# Supplementary material for: Cross-cohort transcriptomic meta-analysis identifies conserved melanogenesis signatures and developmental background in black-boned chicken breast muscle
Source: Anim Biosci. 2026 Jun 24;39(10):260430. doi: 10.5713/ab.260430 (PMC13662945; doi:10.5713/ab.260430)
Supplement: Supplementary file 2 [file ab-260430-Supplementary-10.pdf]

PRJNA729271	stage_1W_vs_E10	ordinary_muscle_development	ENSGALG00010024974	CD44	-0.925994997	0.167610383	-5.52468754	3.30072E-08	6.7912E-08	negative
PRJNA729271	stage_1W_vs_E10	ordinary_muscle_development	ENSGALG00010024977	LRP3	1.111235349	0.104909681	10.5923051	3.23525E-26	1.56525E-25	positive
PRJNA729271	stage_1W_vs_E10	ordinary_muscle_development	ENSGALG00010024983	APOF	1.682720124	0.517603537	3.250982659	0.001150069	0.001702088	positive
PRJNA729271	stage_1W_vs_E10	ordinary_muscle_development	ENSGALG00010024984	SHISAL2A	-0.119273666	0.487502823	-0.244662513	0.806717756	0.827205283	negative
PRJNA729271	stage_1W_vs_E10	ordinary_muscle_development	ENSGALG00010024986	PTS	-1.300155161	0.173984226	-7.472833532	7.84861E-14	2.1971E-13	negative
PRJNA729271	stage_1W_vs_E10	ordinary_muscle_development	ENSGALG00010024989	TIMELESS	-1.072762535	0.160958165	-6.664853146	2.6493E-11	6.51759E-11	negative
PRJNA729271	stage_1W_vs_E10	ordinary_muscle_development	ENSGALG00010024994	NDUFB4	2.092085161	0.103571726	20.19938503	9.9124E-91	2.99334E-89	positive
PRJNA729271	stage_1W_vs_E10	ordinary_muscle_development	ENSGALG00010024996	COA7	0.283325127	0.124470042	2.276251548	0.022830958	0.029669602	positive
PRJNA729271	stage_1W_vs_E10	ordinary_muscle_development	ENSGALG00010024997	HES6	0.151380083	0.25749194	0.587902221	0.556597926	0.592616934	positive
PRJNA729271	stage_1W_vs_E10	ordinary_muscle_development	ENSGALG00010025003	ENSGALG00010025003	9.049048031	0.243183556	37.21077265	4.5694E-303	1.6983E-300	positive
PRJNA729271	stage_1W_vs_E10	ordinary_muscle_development	ENSGALG00010025005	HIP1R	-0.817919793	0.125709928	-6.506405684	7.69702E-11	1.83773E-10	negative
PRJNA729271	stage_1W_vs_E10	ordinary_muscle_development	ENSGALG00010025006	ENSGALG00010025006	-0.793705239	0.357903188	-2.217653448	0.026578471	0.034246147	negative
PRJNA729271	stage_1W_vs_E10	ordinary_muscle_development	ENSGALG00010025007	ZYG11B	0.521070448	0.101388959	5.139321433	2.75732E-07	5.34753E-07	positive
PRJNA729271	stage_1W_vs_E10	ordinary_muscle_development	ENSGALG00010025008	ENSGALG00010025008	-1.465774007	0.603232942	-2.429863996	0.01510449	0.020003616	negative
PRJNA729271	stage_1W_vs_E10	ordinary_muscle_development	ENSGALG00010025012	ENSGALG00010025012	1.848415994	0.823527824	2.244509463	0.024799641	0.032089876	positive
PRJNA729271	stage_1W_vs_E10	ordinary_muscle_development	ENSGALG00010025026	DENR	0.020933233	0.116053236	0.180376127	0.856857295	0.872752898	positive
PRJNA729271	stage_1W_vs_E10	ordinary_muscle_development	ENSGALG00010025028	HES6	-1.304144758	0.136645125	-9.544026941	1.37391E-21	5.512E-21	negative
PRJNA729271	stage_1W_vs_E10	ordinary_muscle_development	ENSGALG00010025031	ECHDC2	0.77350222	0.121277793	6.377937812	1.79488E-10	4.20372E-10	positive
PRJNA729271	stage_1W_vs_E10	ordinary_muscle_development	ENSGALG00010025032	FSTL1	-1.067244406	0.181287052	-5.887041527	3.9317E-09	8.55192E-09	negative
PRJNA729271	stage_1W_vs_E10	ordinary_muscle_development	ENSGALG00010025039	CEBPA	-0.752491263	0.310041966	-2.427062612	0.015221629	0.020153225	negative
PRJNA729271	stage_1W_vs_E10	ordinary_muscle_development	ENSGALG00010025042	BCO2	-2.325844881	0.679631093	-3.422216704	0.000621128	0.00094127	negative
PRJNA729271	stage_1W_vs_E10	ordinary_muscle_development	ENSGALG00010025044	TMEM9	1.212633733	0.212235269	5.713629683	1.10592E-08	2.33984E-08	positive
PRJNA729271	stage_1W_vs_E10	ordinary_muscle_development	ENSGALG00010025047	PER2	0.92656942	0.293029457	3.162035066	0.001566707	0.00229226	positive
PRJNA729271	stage_1W_vs_E10	ordinary_muscle_development	ENSGALG00010025048	ESYT2	1.239039938	0.08213926	15.08462508	2.04433E-51	2.35179E-50	positive
PRJNA729271	stage_1W_vs_E10	ordinary_muscle_development	ENSGALG00010025055	ALS2	1.365179685	0.153983942	8.865727624	7.60061E-19	2.69948E-18	positive
PRJNA729271	stage_1W_vs_E10	ordinary_muscle_development	ENSGALG00010025056	RBMS2	0.006851283	0.175987795	0.038930441	0.968945846	0.973243021	positive
PRJNA729271	stage_1W_vs_E10	ordinary_muscle_development	ENSGALG00010025057	SCP2	0.219019457	0.06668451	3.28441277	0.001021951	0.001520236	positive
PRJNA729271	stage_1W_vs_E10	ordinary_muscle_development	ENSGALG00010025060	GPR156	-0.242910233	0.174508906	-1.391964683	0.163933093	0.19124428	negative
PRJNA729271	stage_1W_vs_E10	ordinary_muscle_development	ENSGALG00010025064	GPN3	0.289914774	0.082657785	3.507410398	0.000452491	0.000692008	positive
PRJNA729271	stage_1W_vs_E10	ordinary_muscle_development	ENSGALG00010025067	SLC1A2	-3.114317615	0.345203631	-9.021682659	1.85222E-19	6.79351E-19	negative
PRJNA729271	stage_1W_vs_E10	ordinary_muscle_development	ENSGALG00010025069	PODN	3.945636957	0.19704556	20.02398304	3.40391E-89	9.84823E-88	positive
PRJNA729271	stage_1W_vs_E10	ordinary_muscle_development	ENSGALG00010025071	CDK15	0.337224255	0.326576575	1.032603929	0.301789289	0.337065476	positive
PRJNA729271	stage_1W_vs_E10	ordinary_muscle_development	ENSGALG00010025072	ENSGALG00010025072	-1.121946666	0.755990437	-1.48407521	0.137788969	0.162536711	negative
PRJNA729271	stage_1W_vs_E10	ordinary_muscle_development	ENSGALG00010025073	IL18	1.423959579	0.200120898	7.115496635	1.11511E-12	2.94792E-12	positive
PRJNA729271	stage_1W_vs_E10	ordinary_muscle_development	ENSGALG00010025080	BAZ2A	1.885436682	0.202195181	9.324834926	1.11156E-20	4.31036E-20	positive
PRJNA729271	stage_1W_vs_E10	ordinary_muscle_development	ENSGALG00010025092	COL1A1	2.537889728	0.220971152	11.48516312	1.5664E-30	9.03138E-30	positive
PRJNA729271	stage_1W_vs_E10	ordinary_muscle_development	ENSGALG00010025097	LMBR1	-0.175331779	0.121540606	-1.442577796	0.149139442	0.175014266	negative
PRJNA729271	stage_1W_vs_E10	ordinary_muscle_development	ENSGALG00010025098	ATP5B	1.711986759	0.13818908	12.38872678	3.00781E-35	2.06041E-34	positive
PRJNA729271	stage_1W_vs_E10	ordinary_muscle_development	ENSGALG00010025102	ARPC3	-1.0405651	0.087093475	-11.94768152	6.67618E-33	4.20196E-32	negative
PRJNA729271	stage_1W_vs_E10	ordinary_muscle_development	ENSGALG00010025103	GSK3B	0.106716415	0.100245832	1.06454715	0.287080907	0.321828132	positive
PRJNA729271	stage_1W_vs_E10	ordinary_muscle_development	ENSGALG00010025113	SDHD	0.925686726	0.12016485	7.703473375	1.32417E-14	3.8596E-14	positive
PRJNA729271	stage_1W_vs_E10	ordinary_muscle_development	ENSGALG00010025114	CEBPG	1.116740455	0.096693519	11.54927929	7.44421E-31	4.3492E-30	positive
PRJNA729271	stage_1W_vs_E10	ordinary_muscle_development	ENSGALG00010025115	MGAT4F	-0.178558958	0.259533762	-0.687998959	0.491453437	0.529558249	negative
PRJNA729271	stage_1W_vs_E10	ordinary_muscle_development	ENSGALG00010025116	ANAPC7	-0.479171482	0.070752418	-6.772510312	1.26567E-11	3.16276E-11	negative
PRJNA729271	stage_1W_vs_E10	ordinary_muscle_development	ENSGALG00010025121	DYNC2I1	-0.648168843	0.157335833	-4.119651768	3.79445E-05	6.30163E-05	negative
PRJNA729271	stage_1W_vs_E10	ordinary_muscle_development	ENSGALG00010025125	PIK3CB	0.519880572	0.132613493	3.920269051	8.84502E-05	0.000142898	positive
PRJNA729271	stage_1W_vs_E10	ordinary_muscle_development	ENSGALG00010025126	ENSGALG00010025126	-0.066446278	0.132674922	-0.500820177	0.616497684	0.650705158	negative
PRJNA729271	stage_1W_vs_E10	ordinary_muscle_development	ENSGALG00010025127	PAMR1	-0.249106626	0.157947645	-1.577146824	0.114761745	0.136877438	negative
PRJNA729271	stage_1W_vs_E10	ordinary_muscle_development	ENSGALG00010025130	ATP2A2	-2.457496799	0.126271292	-19.46203893	2.30433E-84	6.07295E-83	negative
PRJNA729271	stage_1W_vs_E10	ordinary_muscle_development	ENSGALG00010025131	SUMO1	-0.106482157	0.080868011	-1.316740154	0.187925739	0.217240895	negative
PRJNA729271	stage_1W_vs_E10	ordinary_muscle_development	ENSGALG00010025138	CFAP91	-0.805704363	0.279144366	-2.88633575	0.003897561	0.005492966	negative
PRJNA729271	stage_1W_vs_E10	ordinary_muscle_development	ENSGALG00010025139	COPB2	-0.691063413	0.046979546	-14.70987859	5.57073E-49	5.87683E-48	negative
PRJNA729271	stage_1W_vs_E10	ordinary_muscle_development	ENSGALG00010025144	PIH1D2	-0.113682097	0.161606566	-0.703449739	0.481775518	0.5200578	negative
PRJNA729271	stage_1W_vs_E10	ordinary_muscle_development	ENSGALG00010025145	CPT2	-0.615672721	0.095794917	-6.426987334	1.30158E-10	3.0712E-10	negative
PRJNA729271	stage_1W_vs_E10	ordinary_muscle_development	ENSGALG00010025147	COX17	1.992345937	0.113579768	17.54138064	6.92249E-69	1.27987E-67	positive
PRJNA729271	stage_1W_vs_E10	ordinary_muscle_development	ENSGALG00010025149	IFT81	-1.505435099	0.143995806	-10.45471488	1.39417E-25	6.58045E-25	negative
PRJNA729271	stage_1W_vs_E10	ordinary_muscle_development	ENSGALG00010025154	TMEM183A	1.306017291	0.121189596	10.77664528	4.43776E-27	2.22118E-26	positive
PRJNA729271	stage_1W_vs_E10	ordinary_muscle_development	ENSGALG00010025155	CZIB	0.435129662	0.09162947	4.748796014	2.04631E-06	3.73474E-06	positive
PRJNA729271	stage_1W_vs_E10	ordinary_muscle_development	ENSGALG00010025156	DLAT	1.336630068	0.115114529	11.61130637	3.61057E-31	2.13787E-30	positive
PRJNA729271	stage_1W_vs_E10	ordinary_muscle_development	ENSGALG00010025157	NOP58	-1.205582606	0.140656603	-8.57110567	1.02496E-17	3.43351E-17	negative
PRJNA729271	stage_1W_vs_E10	ordinary_muscle_development	ENSGALG00010025160	MRPS22	1.184608997	0.129691365	9.134062181	6.59761E-20	2.46921E-19	positive
PRJNA729271	stage_1W_vs_E10	ordinary_muscle_development	ENSGALG00010025161	ENSGALG00010025161	-1.200093853	0.105812759	-11.34167434	8.15687E-30	4.57031E-29	negative
PRJNA729271	stage_1W_vs_E10	ordinary_muscle_development	ENSGALG00010025163	LDLRAD3	-1.838528452	0.1392711	-13.20107654	8.64901E-40	6.88877E-39	negative
PRJNA729271	stage_1W_vs_E10	ordinary_muscle_development	ENSGALG00010025164	C11orf52	1.196396966	0.125516853	9.5317636	1.54634E-21	6.18662E-21	positive
PRJNA729271	stage_1W_vs_E10	ordinary_muscle_development	ENSGALG00010025167	PTGES3	-0.262900769	0.188611537	-1.39387427	0.163355575	0.190631919	negative
PRJNA729271	stage_1W_vs_E10	ordinary_muscle_development	ENSGALG00010025168	POPDC2	2.036615295	0.211352165	9.636122208	5.62737E-22	2.28292E-21	positive
PRJNA729271	stage_1W_vs_E10	ordinary_muscle_development	ENSGALG00010025169	RBP2	5.71953667	2.103810422	2.718655925	0.006554775	0.009037522	positive
PRJNA729271	stage_1W_vs_E10	ordinary_muscle_development	ENSGALG00010025170	COMMD9	0.029955928	0.09650209	0.310417401	0.756243564	0.78080707	positive
PRJNA729271	stage_1W_vs_E10	ordinary_muscle_development	ENSGALG00010025172	RBP1	6.365109327	0.900892301	7.065338796	1.60225E-12	4.19594E-12	positive
PRJNA729271	stage_1W_vs_E10	ordinary_muscle_development	ENSGALG00010025182	PLA1A	3.084040877	0.193997987	15.89728289	6.61732E-57	8.93092E-56	positive
PRJNA729271	stage_1W_vs_E10	ordinary_muscle_development	ENSGALG00010025186	TMEM120B	-1.046374782	0.209356276	-4.998057863	5.79106E-07	1.09972E-06	negative
PRJNA729271	stage_1W_vs_E10	ordinary_muscle_development	ENSGALG00010025188	PPFIA4	-2.578018713	0.356476801	-7.231939663	4.76144E-13	1.2788E-12	negative
PRJNA729271	stage_1W_vs_E10	ordinary_muscle_development	ENSGALG00010025190	PRR5L	0.322415886	0.241101931	1.337259656	0.181137895	0.209795748	positive
PRJNA729271	stage_1W_vs_E10	ordinary_muscle_development	ENSGALG00010025192	CRYAB	1.360902284	0.182080696	7.474171133	7.7692E-14	2.17571E-13	positive
PRJNA729271	stage_1W_vs_E10	ordinary_muscle_development	ENSGALG00010025195	P2RX7	3.984446528	0.404491285	9.850512678	6.81952E-23	2.87352E-22	positive
PRJNA729271	stage_1W_vs_E10	ordinary_muscle_development	ENSGALG00010025200	PEPD	-0.497999512	0.081037427	-6.145302603	7.98112E-10	1.80309E-09	negative
PRJNA729271	stage_1W_vs_E10	ordinary_muscle_development	ENSGALG00010025204	TRAF6	1.207315647	0.116746972	10.34130157	4.5827E-25	2.11549E-24	positive
PRJNA729271	stage_1W_vs_E10	ordinary_muscle_development	ENSGALG00010025207	BMPR2	0.295174449	0.09751057	3.027102078	0.002469106	0.003546694	positive
PRJNA729271	stage_1W_vs_E10	ordinary_muscle_development	ENSGALG00010025210	HSPB2	0.544234232	0.296397932	1.836160695	0.066333894	0.081559911	positive
PRJNA729271	stage_1W_vs_E10	ordinary_muscle_development	ENSGALG00010025212	P2RX4	0.901935219	0.180085185	5.00838101	5.48898E-07	1.04345E-06	positive
PRJNA729271	stage_1W_vs_E10	ordinary_muscle_development	ENSGALG00010025214	LRP8	-3.693111445	0.275099432	-13.42464219	4.33674E-41	3.60442E-40	negative
PRJNA729271	stage_1W_vs_E10	ordinary_muscle_development	ENSGALG00010025215	MYOG	-1.636392482	0.21514379	-7.606040969	2.8262E-14	8.09599E-14	negative
PRJNA729271	stage_1W_vs_E10	ordinary_muscle_development	ENSGALG00010025216	DPP6	-2.496999954	0.267895191	-9.320809182	1.15456E-20	4.47231E-20	negative
PRJNA729271	stage_1W_vs_E10	ordinary_muscle_development	ENSGALG00010025219	NACA	-1.073203868	0.125781524	-8.53228546	1.43484E-17	4.77239E-17	negative
PRJNA729271	stage_1W_vs_E10	ordinary_muscle_development	ENSGALG00010025222	FAM117B	-2.144317165	0.163553574	-13.1107937	2.85594E-39	2.23525E-38	negative
PRJNA729271	stage_1W_vs_E10	ordinary_muscle_development	ENSGALG00010025225	DMRTB1	1.880049972	0.680620037	2.762260689	0.005740263	0.007961449	positive
PRJNA729271	stage_1W_vs_E10	ordinary_muscle_development	ENSGALG00010025228	FDXACB1	-0.141405709	0.11119412	-1.271701328	0.203479259	0.23385917	negative
PRJNA729271	stage_1W_vs_E10	ordinary_muscle_development	ENSGALG00010025229	ENSGALG00010025229	-1.681067667	0.167363353	-10.04441911	9.72188E-24	4.24709E-23	negative
PRJNA729271	stage_1W_vs_E10	ordinary_muscle_development	ENSGALG00010025230	CHST8	-3.746623829	0.431450906	-8.683777871	3.82836E-18	1.31125E-17	negative
PRJNA729271	stage_1W_vs_E10	ordinary_muscle_development	ENSGALG00010025232	PRIM1	-1.149814512	0.152474207	-7.541042732	4.66228E-14	1.32121E-13	negative
PRJNA729271	stage_1W_vs_E10	ordinary_muscle_development	ENSGALG00010025233	WDR12	-1.192099537	0.077709327	-15.34049483	4.10057E-53	5.02433E-52	negative
PRJNA729271	stage_1W_vs_E10	ordinary_muscle_development	ENSGALG00010025236	ENSGALG00010025236	1.392715518	0.247217614	5.633561032	1.76526E-08	3.69174E-08	positive
PRJNA729271	stage_1W_vs_E10	ordinary_muscle_development	ENSGALG00010025238	IGSF9B	-0.631976282	0.32490327	-1.945121335	0.051760377	0.064533516	negative
PRJNA729271	stage_1W_vs_E10	ordinary_muscle_development	ENSGALG00010025239	RAG2	-3.500027658	0.803111575	-4.358083941	1.31206E-05	2.25588E-05	negative
PRJNA729271	stage_1W_vs_E10	ordinary_muscle_development	ENSGALG00010025242	CLSTN2	-4.535848212	0.317219998	-14.2987461	2.22788E-46	2.17315E-45	negative
PRJNA729271	stage_1W_vs_E10	ordinary_muscle_development	ENSGALG00010025243	PAXIP1	-0.413844807	0.089432764	-4.627440651	3.70212E-06	6.62988E-06	negative
PRJNA729271	stage_1W_vs_E10	ordinary_muscle_development	ENSGALG00010025245	ENSGALG00010025245	1.231810322	0.068116114	18.08397813	4.26195E-73	8.66437E-72	positive
PRJNA729271	stage_1W_vs_E10	ordinary_muscle_development	ENSGALG00010025247	SGCA	3.950718964	0.139676098	28.28486064	5.3065E-176	6.1046E-174	positive
PRJNA729271	stage_1W_vs_E10	ordinary_muscle_development	ENSGALG00010025248	C11orf74	-0.384782405	0.216084849	-1.780700532	0.074961386	0.091523357	negative
PRJNA729271	stage_1W_vs_E10	ordinary_muscle_development	ENSGALG00010025250	GLIS1	-5.76078047	0.576149193	-9.998765141	1.54309E-23	6.68473E-23	negative

PRJNA729271	stage_1W_vs_E10	ordinary_muscle_development	ENSGALG00010025252	PPP1R9B	-0.648995609	0.146765934	-4.421977151	9.78018E-06	1.69838E-05	negative
PRJNA729271	stage_1W_vs_E10	ordinary_muscle_development	ENSGALG00010025256	ANAPC5	-0.875009822	0.122690948	-7.131820505	9.905E-13	2.62473E-12	negative
PRJNA729271	stage_1W_vs_E10	ordinary_muscle_development	ENSGALG00010025257	LPCAT2	0.383222432	0.165366148	2.317417668	0.020480991	0.02673318	positive
PRJNA729271	stage_1W_vs_E10	ordinary_muscle_development	ENSGALG00010025259	SHH	-6.727375874	1.053579774	-6.385255336	1.71112E-10	4.01402E-10	negative
PRJNA729271	stage_1W_vs_E10	ordinary_muscle_development	ENSGALG00010025262	CARF	-0.744759344	0.114760775	-6.489668109	8.60257E-11	2.04988E-10	negative
PRJNA729271	stage_1W_vs_E10	ordinary_muscle_development	ENSGALG00010025263	SAMD14	-2.274927014	0.859797612	-2.64588664	0.008147712	0.011120629	negative
PRJNA729271	stage_1W_vs_E10	ordinary_muscle_development	ENSGALG00010025268	ZBTB39	-0.985327362	0.111248672	-8.856980912	8.22106E-19	2.91498E-18	negative
PRJNA729271	stage_1W_vs_E10	ordinary_muscle_development	ENSGALG00010025270	NDC1	-2.06766818	0.123590803	-16.7299518	7.93024E-63	1.24269E-61	negative
PRJNA729271	stage_1W_vs_E10	ordinary_muscle_development	ENSGALG00010025271	RNF34	1.11664994	0.117445998	9.507773413	1.94787E-21	7.77012E-21	positive
PRJNA729271	stage_1W_vs_E10	ordinary_muscle_development	ENSGALG00010025272	NCAPD3	-2.329185058	0.18727855	-12.43700926	1.6455E-35	1.13783E-34	negative
PRJNA729271	stage_1W_vs_E10	ordinary_muscle_development	ENSGALG00010025274	PDK2	3.891813557	0.269088683	14.46294031	2.07731E-47	2.08522E-46	positive
PRJNA729271	stage_1W_vs_E10	ordinary_muscle_development	ENSGALG00010025280	HSPB11	-0.264507643	0.154311769	-1.714111922	0.086508173	0.104879221	negative
PRJNA729271	stage_1W_vs_E10	ordinary_muscle_development	ENSGALG00010025286	ITGA3	-0.113330364	0.175233671	-0.646738514	0.517801193	0.55497843	negative
PRJNA729271	stage_1W_vs_E10	ordinary_muscle_development	ENSGALG00010025289	KDM2B	-0.854737423	0.074536061	-11.46743485	1.92275E-30	1.10202E-29	negative
PRJNA729271	stage_1W_vs_E10	ordinary_muscle_development	ENSGALG00010025291	DUS2	0.090056726	0.092189968	0.976860366	0.328638286	0.36511167	positive
PRJNA729271	stage_1W_vs_E10	ordinary_muscle_development	ENSGALG00010025292	ATP1A1	0.734909919	0.127518965	5.763142143	8.25622E-09	1.75939E-08	positive
PRJNA729271	stage_1W_vs_E10	ordinary_muscle_development	ENSGALG00010025297	SPSB4	-1.00382984	0.147049055	-6.826496392	8.70135E-12	2.19579E-11	negative
PRJNA729271	stage_1W_vs_E10	ordinary_muscle_development	ENSGALG00010025298	ENSGALG00010025298	-4.468088985	0.350922469	-12.73241066	3.90591E-37	2.8379E-36	negative
PRJNA729271	stage_1W_vs_E10	ordinary_muscle_development	ENSGALG00010025301	MORN3	-1.501280447	0.316347477	-4.745669107	2.07818E-06	3.78813E-06	negative
PRJNA729271	stage_1W_vs_E10	ordinary_muscle_development	ENSGALG00010025303	TMCC2	2.632613562	0.188055293	13.99914629	1.57754E-44	1.45461E-43	positive
PRJNA729271	stage_1W_vs_E10	ordinary_muscle_development	ENSGALG00010025304	DDX28	0.054230756	0.092127188	0.588650946	0.556095451	0.592125436	positive
PRJNA729271	stage_1W_vs_E10	ordinary_muscle_development	ENSGALG00010025305	GPR182	2.577964382	0.401262708	6.424629876	1.32191E-10	3.11866E-10	positive
PRJNA729271	stage_1W_vs_E10	ordinary_muscle_development	ENSGALG00010025311	ENSGALG00010025311	1.920752551	0.582943756	3.294919163	0.0009845	0.001467537	positive
PRJNA729271	stage_1W_vs_E10	ordinary_muscle_development	ENSGALG00010025312	PLEKHM3	0.246795657	0.114343385	2.158372836	0.030898856	0.039526866	positive
PRJNA729271	stage_1W_vs_E10	ordinary_muscle_development	ENSGALG00010025313	DIO1	0.196878168	0.864011762	0.227865148	0.819751077	0.83867108	positive
PRJNA729271	stage_1W_vs_E10	ordinary_muscle_development	ENSGALG00010025315	LRRC42	0.116723367	0.119236768	0.978920926	0.327619052	0.364063029	positive
PRJNA729271	stage_1W_vs_E10	ordinary_muscle_development	ENSGALG00010025316	SLC25A36	-0.346258606	0.110283722	-3.139707282	0.001691167	0.002467882	negative
PRJNA729271	stage_1W_vs_E10	ordinary_muscle_development	ENSGALG00010025318	NUAK2	-2.981610638	0.495138128	-6.021775476	1.72514E-09	3.83821E-09	negative
PRJNA729271	stage_1W_vs_E10	ordinary_muscle_development	ENSGALG00010025319	LDLRAD1	0.958501312	0.345086952	2.777564627	0.005476795	0.007617169	positive
PRJNA729271	stage_1W_vs_E10	ordinary_muscle_development	ENSGALG00010025320	JAM3	-1.804605416	0.145071861	-12.43938969	1.5972E-35	1.1056E-34	negative
PRJNA729271	stage_1W_vs_E10	ordinary_muscle_development	ENSGALG00010025323	KLHDC8A	-1.948720278	0.186646438	-10.44070435	1.61607E-25	7.5981E-25	negative
PRJNA729271	stage_1W_vs_E10	ordinary_muscle_development	ENSGALG00010025325	DPEP2	0.426174776	0.246677337	1.727660841	0.084049048	0.102051512	positive
PRJNA729271	stage_1W_vs_E10	ordinary_muscle_development	ENSGALG00010025327	TMEM59	1.471376384	0.070846641	20.76847052	8.34742E-96	2.88773E-94	positive
PRJNA729271	stage_1W_vs_E10	ordinary_muscle_development	ENSGALG00010025329	MYO1A	-0.631111006	0.207461908	-3.042057272	0.002349672	0.003381167	negative
PRJNA729271	stage_1W_vs_E10	ordinary_muscle_development	ENSGALG00010025330	USH1C	-2.672134519	0.409758691	-6.521239391	6.97288E-11	1.66813E-10	negative
PRJNA729271	stage_1W_vs_E10	ordinary_muscle_development	ENSGALG00010025331	ENSGALG00010025331	0.766616175	0.351317488	2.18211789	0.029100833	0.037328901	positive
PRJNA729271	stage_1W_vs_E10	ordinary_muscle_development	ENSGALG00010025333	FZD5	1.137759957	0.19564134	5.815539573	6.04386E-09	1.30056E-08	positive
PRJNA729271	stage_1W_vs_E10	ordinary_muscle_development	ENSGALG00010025334	RHOF	1.395140421	0.223102479	6.253361354	4.01711E-10	9.23082E-10	positive
PRJNA729271	stage_1W_vs_E10	ordinary_muscle_development	ENSGALG00010025336	ADAM10	-0.291196021	0.063581969	-4.579852188	4.65305E-06	8.2705E-06	negative
PRJNA729271	stage_1W_vs_E10	ordinary_muscle_development	ENSGALG00010025337	NEMP1	-0.012135727	0.145134858	-0.083616901	0.933361029	0.941742177	negative
PRJNA729271	stage_1W_vs_E10	ordinary_muscle_development	ENSGALG00010025338	FADD	-1.236764467	0.09934902	-12.44868311	1.42172E-35	9.87435E-35	negative
PRJNA729271	stage_1W_vs_E10	ordinary_muscle_development	ENSGALG00010025340	METTL21A	1.154020694	0.116111531	9.938898304	2.81928E-23	1.20904E-22	positive
PRJNA729271	stage_1W_vs_E10	ordinary_muscle_development	ENSGALG00010025341	VPS26B	-1.062558807	0.133032595	-7.987206508	1.38031E-15	4.21567E-15	negative
PRJNA729271	stage_1W_vs_E10	ordinary_muscle_development	ENSGALG00010025342	TCEANC2	-0.896713673	0.121694565	-7.368559732	1.72481E-13	4.73327E-13	negative
PRJNA729271	stage_1W_vs_E10	ordinary_muscle_development	ENSGALG00010025343	NAB2	1.541332587	0.288497709	5.342616377	9.16145E-08	1.83723E-07	positive
PRJNA729271	stage_1W_vs_E10	ordinary_muscle_development	ENSGALG00010025344	ENSGALG00010025344	1.367815938	0.531981043	2.571174208	0.010135433	0.013696914	positive
PRJNA729271	stage_1W_vs_E10	ordinary_muscle_development	ENSGALG00010025345	CCM2	-0.519301376	0.090531615	-5.736132906	9.68627E-09	2.05597E-08	negative
PRJNA729271	stage_1W_vs_E10	ordinary_muscle_development	ENSGALG00010025346	PXYLP1	-3.199281119	0.134317054	-23.818875	2.1291E-125	1.3022E-123	negative
PRJNA729271	stage_1W_vs_E10	ordinary_muscle_development	ENSGALG00010025348	HIPK1	0.084429263	0.093540781	0.902593087	0.366741897	0.404468066	positive
PRJNA729271	stage_1W_vs_E10	ordinary_muscle_development	ENSGALG00010025349	SETD1A	-0.019632387	0.127245553	-0.154287413	0.877383122	0.891279581	negative
PRJNA729271	stage_1W_vs_E10	ordinary_muscle_development	ENSGALG00010025351	CREB1	-0.718755593	0.067654371	-10.6239343	2.30631E-26	1.12559E-25	negative
PRJNA729271	stage_1W_vs_E10	ordinary_muscle_development	ENSGALG00010025353	SLC12A4	-0.508983265	0.078027595	-6.523118742	6.88603E-11	1.6479E-10	negative
PRJNA729271	stage_1W_vs_E10	ordinary_muscle_development	ENSGALG00010025354	ENSGALG00010025354	-4.259685645	1.705941377	-2.496970706	0.01252593	0.016746298	negative
PRJNA729271	stage_1W_vs_E10	ordinary_muscle_development	ENSGALG00010025355	STAT6	1.489383433	0.139964469	10.64115373	1.9174E-26	9.37994E-26	positive
PRJNA729271	stage_1W_vs_E10	ordinary_muscle_development	ENSGALG00010025357	OLFML3	-1.352804223	0.121478075	-11.13620068	8.36106E-29	4.48367E-28	negative
PRJNA729271	stage_1W_vs_E10	ordinary_muscle_development	ENSGALG00010025358	INCENP	-2.438082549	0.206077539	-11.83089898	2.70232E-32	1.6654E-31	negative
PRJNA729271	stage_1W_vs_E10	ordinary_muscle_development	ENSGALG00010025359	CDCP2	1.301792041	0.381634577	3.411095638	0.000647024	0.000979285	positive
PRJNA729271	stage_1W_vs_E10	ordinary_muscle_development	ENSGALG00010025360	THYN1	0.544308873	0.111211589	4.894353885	9.86292E-07	1.84628E-06	positive
PRJNA729271	stage_1W_vs_E10	ordinary_muscle_development	ENSGALG00010025362	G6PC2	-1.331872158	0.257822696	-5.16584528	2.39355E-07	4.66449E-07	negative
PRJNA729271	stage_1W_vs_E10	ordinary_muscle_development	ENSGALG00010025364	EPHA4	-4.171922672	0.193680025	-21.5402836	6.5306E-103	2.5793E-101	negative
PRJNA729271	stage_1W_vs_E10	ordinary_muscle_development	ENSGALG00010025365	SIKE1	0.602256807	0.091102221	6.61078072	3.82298E-11	9.32427E-11	positive
PRJNA729271	stage_1W_vs_E10	ordinary_muscle_development	ENSGALG00010025368	LRP1	0.501842553	0.138816357	3.615154322	0.000300169	0.000466689	positive
PRJNA729271	stage_1W_vs_E10	ordinary_muscle_development	ENSGALG00010025370	HPD	2.748288115	0.490616332	5.601705315	2.12253E-08	4.41724E-08	positive
PRJNA729271	stage_1W_vs_E10	ordinary_muscle_development	ENSGALG00010025373	CYB5RL	-0.672302643	0.090930661	-7.393574754	1.42933E-13	3.93657E-13	negative
PRJNA729271	stage_1W_vs_E10	ordinary_muscle_development	ENSGALG00010025377	MYO1G	2.067459228	0.286849865	7.207461053	5.70047E-13	1.52563E-12	positive
PRJNA729271	stage_1W_vs_E10	ordinary_muscle_development	ENSGALG00010025378	PAX3	-2.068578279	0.269983355	-7.661873362	1.8324E-14	5.30153E-14	negative
PRJNA729271	stage_1W_vs_E10	ordinary_muscle_development	ENSGALG00010025379	ACAD8	1.812963549	0.18516612	9.79101115	1.23059E-22	5.11981E-22	positive
PRJNA729271	stage_1W_vs_E10	ordinary_muscle_development	ENSGALG00010025380	SPC25	-2.272766268	0.226472499	-10.03550665	1.06412E-23	4.63612E-23	negative
PRJNA729271	stage_1W_vs_E10	ordinary_muscle_development	ENSGALG00010025381	MRPL37	1.725891401	0.121069656	14.25535888	4.15163E-46	4.00918E-45	positive
PRJNA729271	stage_1W_vs_E10	ordinary_muscle_development	ENSGALG00010025382	MAB21L3	-0.974765601	0.329906925	-2.954668507	0.003130052	0.00445154	negative
PRJNA729271	stage_1W_vs_E10	ordinary_muscle_development	ENSGALG00010025386	TPCN2	-1.035311395	0.101150776	-10.2353282	1.37727E-24	6.22889E-24	negative
PRJNA729271	stage_1W_vs_E10	ordinary_muscle_development	ENSGALG00010025387	PSMD9	-0.179287034	0.069394114	-2.583605781	0.009777348	0.013230271	negative
PRJNA729271	stage_1W_vs_E10	ordinary_muscle_development	ENSGALG00010025389	SNED1	-1.593350058	0.151536954	-10.51459734	7.39962E-26	3.52937E-25	negative
PRJNA729271	stage_1W_vs_E10	ordinary_muscle_development	ENSGALG00010025390	BCL7A	-1.66473228	0.09001715	-18.49350135	2.32928E-76	5.07712E-75	negative
PRJNA729271	stage_1W_vs_E10	ordinary_muscle_development	ENSGALG00010025392	SLC6A2	-1.061383156	0.249069898	-4.261386724	2.03162E-05	3.43903E-05	negative
PRJNA729271	stage_1W_vs_E10	ordinary_muscle_development	ENSGALG00010025398	TSPAN2	0.364348581	0.143810077	2.533539979	0.011291686	0.01516614	positive
PRJNA729271	stage_1W_vs_E10	ordinary_muscle_development	ENSGALG00010025400	SLC22A15	-1.244549065	0.163918772	-7.592474312	3.13854E-14	8.9571E-14	negative
PRJNA729271	stage_1W_vs_E10	ordinary_muscle_development	ENSGALG00010025403	ENSGALG00010025403	0.971010251	0.127648741	7.606892507	2.80765E-14	8.04444E-14	positive
PRJNA729271	stage_1W_vs_E10	ordinary_muscle_development	ENSGALG00010025404	CERS6	-2.443797609	0.292201062	-8.363411109	6.09304E-17	1.97052E-16	negative
PRJNA729271	stage_1W_vs_E10	ordinary_muscle_development	ENSGALG00010025405	TAMALIN	3.278022565	0.16176505	20.26409643	2.66813E-91	8.22863E-90	positive
PRJNA729271	stage_1W_vs_E10	ordinary_muscle_development	ENSGALG00010025406	ENSGALG00010025406	0.424042647	0.147824169	2.868561	0.004123437	0.005796084	positive
PRJNA729271	stage_1W_vs_E10	ordinary_muscle_development	ENSGALG00010025407	MLXIP	1.485002911	0.128053295	11.59675678	4.27992E-31	2.52185E-30	positive
PRJNA729271	stage_1W_vs_E10	ordinary_muscle_development	ENSGALG00010025413	NECAB3	1.140496562	0.121051684	9.421567056	4.44405E-21	1.74523E-20	positive
PRJNA729271	stage_1W_vs_E10	ordinary_muscle_development	ENSGALG00010025414	MMP2	-0.778964456	0.221463957	-3.517341902	0.000435892	0.00066768	negative
PRJNA729271	stage_1W_vs_E10	ordinary_muscle_development	ENSGALG00010025415	ENSGALG00010025415	3.634343912	0.278822514	13.03461425	7.77593E-39	5.99214E-38	positive
PRJNA729271	stage_1W_vs_E10	ordinary_muscle_development	ENSGALG00010025416	ENSGALG00010025416	0.81722086	0.644094992	1.26878934	0.204516208	0.234864715	positive
PRJNA729271	stage_1W_vs_E10	ordinary_muscle_development	ENSGALG00010025417	B3GAT1	-1.885258461	0.199074594	-9.470110813	2.79547E-21	1.10772E-20	negative
PRJNA729271	stage_1W_vs_E10	ordinary_muscle_development	ENSGALG00010025418	CASQ2	4.84397206	0.209409482	23.13157936	2.2282E-118	1.1874E-116	positive
PRJNA729271	stage_1W_vs_E10	ordinary_muscle_development	ENSGALG00010025419	CCND1	-1.53864599	0.134888463	-11.40680199	3.86686E-30	2.19718E-29	negative
PRJNA729271	stage_1W_vs_E10	ordinary_muscle_development	ENSGALG00010025420	STK39	-2.066291817	0.212654251	-9.716672982	2.5601E-22	1.04975E-21	negative
PRJNA729271	stage_1W_vs_E10	ordinary_muscle_development	ENSGALG00010025421	NR4A1	9.475491548	0.396522353	23.89648773	3.3314E-126	2.0636E-124	positive
PRJNA729271	stage_1W_vs_E10	ordinary_muscle_development	ENSGALG00010025422	SSBP3	-0.660038057	0.157388439	-4.193688318	2.74455E-05	4.60443E-05	negative
PRJNA729271	stage_1W_vs_E10	ordinary_muscle_development	ENSGALG00010025423	NGF	-0.979537886	0.354461793	-2.763451253	0.005719363	0.0079355	negative
PRJNA729271	stage_1W_vs_E10	ordinary_muscle_development	ENSGALG00010025424	IRX6	-4.136874649	0.430828717	-9.602133011	7.83068E-22	3.16084E-21	negative
PRJNA729271	stage_1W_vs_E10	ordinary_muscle_development	ENSGALG00010025425	ENSGALG00010025425	0.174760235	0.139882505	1.2493359	0.211542242	0.242549027	positive
PRJNA729271	stage_1W_vs_E10	ordinary_muscle_development	ENSGALG00010025426	ENSGALG00010025426	0.547595939	0.153301643	3.572016113	0.000354244	0.000547183	positive
PRJNA729271	stage_1W_vs_E10	ordinary_muscle_development	ENSGALG00010025427	SLC35G2	-0.721048741	0.133441173	-5.403495244	6.53547E-08	1.32344E-07	negative
PRJNA729271	stage_1W_vs_E10	ordinary_muscle_development	ENSGALG00010025428	VANGL1	-0.369342715	0.111695361	-3.306696989	0.000944029	0.001410546	negative
PRJNA729271	stage_1W_vs_E10	ordinary_muscle_development	ENSGALG00010025429	SCN3B	-4.264155429	0.279674416	-15.2468556	1.72754E-52	2.07463E-51	negative
PRJNA729271	stage_1W_vs_E10	ordinary_muscle_development	ENSGALG00010025430	IRX5	-4.554977719	0.898327121	-5.070511189	3.96749E-07	7.61604E-07	negative
PRJNA729271	stage_1W_vs_E10	ordinary_muscle_development	ENSGALG00010025432	IRX3	-5.198324153	0.880117697	-5.906396579	3.49672E-09	7.62753E-09	negative
PRJNA729271	stage_1W_vs_E10	ordinary_muscle_development	ENSGALG00010025433	CHPF2	0.72882668	0.12161311	5.992994336	2.06012E-09	4.559E-09	positive
PRJNA729271	stage_1W_vs_E10	ordinary_muscle_development	ENSGALG00010025434	SYT6	-0.950348559	0.243953752	-3.895609523	9.79521E-05	0.000157681	negative
PRJNA729271	stage_1W_vs_E10	ordinary_muscle_development	ENSGALG00010025435	SCLY	-0.10822901	0.10032238	-1.078812232	0.280671441	0.315081516	negative
PRJNA729271	stage_1W_vs_E10	ordinary_muscle_development	ENSGALG00010025438	EXOSC6	0.599103177	0.118994054	5.034732056	4.78518E-07	9.13608E-07	positive
PRJNA729271	stage_1W_vs_E10	ordinary_muscle_development	ENSGALG00010025442	ABCF2	2.688492496	0.146843436	18.30856439	7.07095E-75	1.48541E-73	positive
PRJNA729271	stage_1W_vs_E10	ordinary_muscle_development	ENSGALG00010025445	B3GNT4	0.396758867	0.303963835	1.305283133	0.191796455	0.221397596	positive
PRJNA729271	stage_1W_vs_E10	ordinary_muscle_development	ENSGALG00010025448	CHD1L	-0.347854649	0.10896909	-3.192232287	0.001411777	0.002073955	negative
PRJNA729271	stage_1W_vs_E10	ordinary_muscle_development	ENSGALG00010025449	ENSGALG00010025449	0.30855371	0.11690733	2.639301653	0.008307702	0.01132833	positive
PRJNA729271	stage_1W_vs_E10	ordinary_muscle_development	ENSGALG00010025451	LRRC43	0.750339449	0.36505389	2.055421045	0.039838355	0.050353149	positive
PRJNA729271	stage_1W_vs_E10	ordinary_muscle_development	ENSGALG00010025452	TRIM33	0.212932241	0.126227873	1.686887652	0.091624957	0.110804584	positive
PRJNA729271	stage_1W_vs_E10	ordinary_muscle_development	ENSGALG00010025458	BCAS2	0.255645063	0.11576029	2.208400336	0.027216378	0.035032537	positive
PRJNA729271	stage_1W_vs_E10	ordinary_muscle_development	ENSGALG00010025459	DIABLO	0.072834877	0.077983268	0.933980821	0.350313786	0.38779581	positive
PRJNA729271	stage_1W_vs_E10	ordinary_muscle_development	ENSGALG00010025464	VPS33A	0.129665	0.08964747	1.446387729	0.148068482	0.173799712	positive
PRJNA729271	stage_1W_vs_E10	ordinary_muscle_development	ENSGALG00010025465	ENSGALG00010025465	-0.366802765	0.084069715	-4.36307851	1.28245E-05	2.20747E-05	negative
PRJNA729271	stage_1W_vs_E10	ordinary_muscle_development	ENSGALG00010025470	ORAOV1	0.336462598	0.123981395	2.713815234	0.006651325	0.009163669	positive
PRJNA729271	stage_1W_vs_E10	ordinary_muscle_development	ENSGALG00010025472	ZCCHC8	0.309447369	0.097392669	3.177316866	0.001486445	0.002179007	positive
PRJNA729271	stage_1W_vs_E10	ordinary_muscle_development	ENSGALG00010025473	PARS2	0.958456613	0.078368012	12.23020187	2.14405E-34	1.41909E-33	positive
PRJNA729271	stage_1W_vs_E10	ordinary_muscle_development	ENSGALG00010025477	ENSGALG00010025477	0.874273492	0.070318351	12.43307727	1.7285E-35	1.19364E-34	positive
PRJNA729271	stage_1W_vs_E10	ordinary_muscle_development	ENSGALG00010025480	FGF19	-3.163061797	0.604547354	-5.232115858	1.67581E-07	3.29617E-07	negative
PRJNA729271	stage_1W_vs_E10	ordinary_muscle_development	ENSGALG00010025481	TMEM39A	-0.340342233	0.091536004	-3.718124209	0.000200708	0.000315743	negative
PRJNA729271	stage_1W_vs_E10	ordinary_muscle_development	ENSGALG00010025483	FGF4	-2.195013868	0.368384219	-5.958490492	2.54578E-09	5.60127E-09	negative
PRJNA729271	stage_1W_vs_E10	ordinary_muscle_development	ENSGALG00010025485	DENND2C	1.434224166	0.159653625	8.98334857	2.62649E-19	9.55836E-19	positive
PRJNA729271	stage_1W_vs_E10	ordinary_muscle_development	ENSGALG00010025486	B3GALT1	-2.909132991	0.218637132	-13.30575901	2.14306E-40	1.73831E-39	negative
PRJNA729271	stage_1W_vs_E10	ordinary_muscle_development	ENSGALG00010025487	FGF3	-6.392605432	0.932916964	-6.852276978	7.26836E-12	1.84187E-11	negative
PRJNA729271	stage_1W_vs_E10	ordinary_muscle_development	ENSGALG00010025488	SLC4A8	-2.066004038	0.192608315	-10.72645298	7.64756E-27	3.79368E-26	negative
PRJNA729271	stage_1W_vs_E10	ordinary_muscle_development	ENSGALG00010025491	ZMYND11	-0.523981725	0.064518292	-8.12144443	4.60668E-16	1.43909E-15	negative
PRJNA729271	stage_1W_vs_E10	ordinary_muscle_development	ENSGALG00010025496	ATG101	1.429861585	0.109925525	13.00754842	1.10841E-38	8.46939E-38	positive
PRJNA729271	stage_1W_vs_E10	ordinary_muscle_development	ENSGALG00010025499	STAC3	5.122903926	0.158473839	32.32649607	2.9689E-229	6.0611E-227	positive
PRJNA729271	stage_1W_vs_E10	ordinary_muscle_development	ENSGALG00010025504	AMPD1	6.734639011	0.197694973	34.0658081	2.368E-254	5.7206E-252	positive
PRJNA729271	stage_1W_vs_E10	ordinary_muscle_development	ENSGALG00010025505	ENSGALG00010025505	-2.890045925	0.299274669	-9.656834429	4.59854E-22	1.87288E-21	negative

PRJNA729271	stage_1W_vs_E10	ordinary_muscle_development	ENSGALG00010025506	R3HDM2	0.797109134	0.176909306	4.505750175	6.61389E-06	1.16063E-05	positive
PRJNA729271	stage_1W_vs_E10	ordinary_muscle_development	ENSGALG00010025508	ENSGALG00010025508	-0.150998433	0.133712241	-1.129279053	0.258780134	0.292340273	negative
PRJNA729271	stage_1W_vs_E10	ordinary_muscle_development	ENSGALG00010025512	NRAS	-0.226308089	0.077962882	-2.902767086	0.003698816	0.00522506	negative
PRJNA729271	stage_1W_vs_E10	ordinary_muscle_development	ENSGALG00010025513	RSRC2	-0.008824217	0.087748042	-0.100563119	0.919897275	0.929581079	negative
PRJNA729271	stage_1W_vs_E10	ordinary_muscle_development	ENSGALG00010025520	ACOT11	0.842000528	0.296127768	2.843369036	0.004463935	0.006258317	positive
PRJNA729271	stage_1W_vs_E10	ordinary_muscle_development	ENSGALG00010025522	SUMO3	-0.415505465	0.10616342	-3.913828936	9.0844E-05	0.000146619	negative
PRJNA729271	stage_1W_vs_E10	ordinary_muscle_development	ENSGALG00010025523	B4GALT4	-0.821618631	0.132092352	-6.220031832	4.97054E-10	1.13533E-09	negative
PRJNA729271	stage_1W_vs_E10	ordinary_muscle_development	ENSGALG00010025527	KNTC1	-2.810690502	0.241132118	-11.65622615	2.13288E-31	1.27331E-30	negative
PRJNA729271	stage_1W_vs_E10	ordinary_muscle_development	ENSGALG00010025531	ENSGALG00010025531	0.146017187	0.095568397	1.527881512	0.126541959	0.150038912	positive
PRJNA729271	stage_1W_vs_E10	ordinary_muscle_development	ENSGALG00010025533	INHBC	-1.438898811	0.62631868	-2.297390861	0.021596483	0.028125877	negative
PRJNA729271	stage_1W_vs_E10	ordinary_muscle_development	ENSGALG00010025538	ANO1	3.966348665	0.208680305	19.00681839	1.49765E-80	3.63625E-79	positive
PRJNA729271	stage_1W_vs_E10	ordinary_muscle_development	ENSGALG00010025541	TTC21B	-0.542123682	0.12873696	-4.211095887	2.54135E-05	4.27094E-05	negative
PRJNA729271	stage_1W_vs_E10	ordinary_muscle_development	ENSGALG00010025544	CSDE1	0.665156354	0.085969289	7.7371392	1.01679E-14	2.98166E-14	positive
PRJNA729271	stage_1W_vs_E10	ordinary_muscle_development	ENSGALG00010025548	ENSGALG00010025548	-0.937905128	0.198753338	-4.718940268	2.37076E-06	4.30629E-06	negative
PRJNA729271	stage_1W_vs_E10	ordinary_muscle_development	ENSGALG00010025549	DHCR24	-0.705956543	0.245243722	-2.878591699	0.003994551	0.005623746	negative
PRJNA729271	stage_1W_vs_E10	ordinary_muscle_development	ENSGALG00010025553	UBE2G2	-0.300024657	0.096985198	-3.093509769	0.00197804	0.00286717	negative
PRJNA729271	stage_1W_vs_E10	ordinary_muscle_development	ENSGALG00010025554	DIP2C	-0.272521895	0.071727695	-3.79939571	0.000145049	0.000230612	negative
PRJNA729271	stage_1W_vs_E10	ordinary_muscle_development	ENSGALG00010025557	VPS29	-0.751358818	0.078404164	-9.583149435	9.41299E-22	3.78583E-21	negative
PRJNA729271	stage_1W_vs_E10	ordinary_muscle_development	ENSGALG00010025558	GALNT3	-3.320004189	0.246512088	-13.46791637	2.41588E-41	2.03121E-40	negative
PRJNA729271	stage_1W_vs_E10	ordinary_muscle_development	ENSGALG00010025559	SLC6A17	-5.617759333	1.199739244	-4.682483597	2.8342E-06	5.11858E-06	negative
PRJNA729271	stage_1W_vs_E10	ordinary_muscle_development	ENSGALG00010025562	GRAMD1B	-0.23165572	0.185480122	-1.248951735	0.211682728	0.242671713	negative
PRJNA729271	stage_1W_vs_E10	ordinary_muscle_development	ENSGALG00010025563	TSPEAR	0.154609502	0.235532523	0.656425281	0.511550528	0.549050345	positive
PRJNA729271	stage_1W_vs_E10	ordinary_muscle_development	ENSGALG00010025565	ENSGALG00010025565	1.957590381	0.242967064	8.057019542	7.81772E-16	2.4177E-15	positive
PRJNA729271	stage_1W_vs_E10	ordinary_muscle_development	ENSGALG00010025568	MYL3	-4.549619968	0.966240468	-4.708579406	2.49449E-06	4.52763E-06	negative
PRJNA729271	stage_1W_vs_E10	ordinary_muscle_development	ENSGALG00010025569	PPTC7	1.268948712	0.192934243	6.577104683	4.79697E-11	1.16255E-10	positive
PRJNA729271	stage_1W_vs_E10	ordinary_muscle_development	ENSGALG00010025571	SRSF3	-2.09983225	0.230720289	-9.10120326	8.93365E-20	3.32631E-19	negative
PRJNA729271	stage_1W_vs_E10	ordinary_muscle_development	ENSGALG00010025572	ENSGALG00010025572	-0.374204292	0.112678417	-3.3209935	0.000896976	0.001343564	negative
PRJNA729271	stage_1W_vs_E10	ordinary_muscle_development	ENSGALG00010025573	CLMP	-2.486084921	0.16983307	-14.63840299	1.59777E-48	1.66258E-47	negative
PRJNA729271	stage_1W_vs_E10	ordinary_muscle_development	ENSGALG00010025574	ARHGAP31	-1.025882381	0.100296647	-10.22848133	1.47819E-24	6.67697E-24	negative
PRJNA729271	stage_1W_vs_E10	ordinary_muscle_development	ENSGALG00010025575	TRPM2	-1.143307635	0.490536859	-2.330727271	0.019767745	0.025851089	negative
PRJNA729271	stage_1W_vs_E10	ordinary_muscle_development	ENSGALG00010025577	PTH2R	-3.3722521	0.20421877	-16.51293901	2.96105E-61	4.48489E-60	negative
PRJNA729271	stage_1W_vs_E10	ordinary_muscle_development	ENSGALG00010025578	ORMDL2	0.747631826	0.144627848	5.169349045	2.34911E-07	4.58097E-07	positive
PRJNA729271	stage_1W_vs_E10	ordinary_muscle_development	ENSGALG00010025579	CSRNP3	-2.481200564	0.268342955	-9.246378642	2.32227E-20	8.86058E-20	negative
PRJNA729271	stage_1W_vs_E10	ordinary_muscle_development	ENSGALG00010025580	RASSF7	-1.156293981	0.173609035	-6.660332994	2.73208E-11	6.71895E-11	negative
PRJNA729271	stage_1W_vs_E10	ordinary_muscle_development	ENSGALG00010025582	DMAP1	-0.183042237	0.1144347	-1.599534382	0.109701915	0.131133866	negative
PRJNA729271	stage_1W_vs_E10	ordinary_muscle_development	ENSGALG00010025585	SARNP	-1.215156217	0.097561205	-12.45532188	1.30823E-35	9.0992E-35	negative
PRJNA729271	stage_1W_vs_E10	ordinary_muscle_development	ENSGALG00010025586	TCTN1	0.720512638	0.124769143	5.774766273	7.706E-09	1.64529E-08	positive
PRJNA729271	stage_1W_vs_E10	ordinary_muscle_development	ENSGALG00010025587	LRRC56	-4.218810511	0.390876515	-10.79320539	3.70632E-27	1.86409E-26	negative
PRJNA729271	stage_1W_vs_E10	ordinary_muscle_development	ENSGALG00010025588	ENSGALG00010025588	-0.104083569	0.310963756	-0.334712862	0.737841698	0.763568906	negative
PRJNA729271	stage_1W_vs_E10	ordinary_muscle_development	ENSGALG00010025590	HRAS	0.145683571	0.114053963	1.277321429	0.201488791	0.231847268	positive
PRJNA729271	stage_1W_vs_E10	ordinary_muscle_development	ENSGALG00010025591	OTOS	1.005023084	0.346119449	2.903688555	0.003687948	0.005210723	positive
PRJNA729271	stage_1W_vs_E10	ordinary_muscle_development	ENSGALG00010025592	HSPA8	-0.940275668	0.208238792	-4.515372279	6.32056E-06	1.11091E-05	negative
PRJNA729271	stage_1W_vs_E10	ordinary_muscle_development	ENSGALG00010025594	UPK1B	-8.05852909	0.923876576	-8.722516943	2.72085E-18	9.38793E-18	negative
PRJNA729271	stage_1W_vs_E10	ordinary_muscle_development	ENSGALG00010025595	ERI3	-0.081347377	0.093313203	-0.871767057	0.38333547	0.421262141	negative
PRJNA729271	stage_1W_vs_E10	ordinary_muscle_development	ENSGALG00010025597	SCN2A	-5.975472864	0.895071129	-6.67597543	2.45593E-11	6.05112E-11	negative
PRJNA729271	stage_1W_vs_E10	ordinary_muscle_development	ENSGALG00010025598	ENSGALG00010025598	2.433607725	0.399472813	6.092048437	1.11475E-09	2.50167E-09	positive
PRJNA729271	stage_1W_vs_E10	ordinary_muscle_development	ENSGALG00010025599	COPS9	0.748586533	0.143012229	5.234423231	1.65501E-07	3.25588E-07	positive
PRJNA729271	stage_1W_vs_E10	ordinary_muscle_development	ENSGALG00010025600	ENSGALG00010025600	-1.783979518	0.115176871	-15.48904306	4.11368E-54	5.18502E-53	negative
PRJNA729271	stage_1W_vs_E10	ordinary_muscle_development	ENSGALG00010025604	HVCN1	-0.565703354	0.138768409	-4.076600429	4.56989E-05	7.54191E-05	negative
PRJNA729271	stage_1W_vs_E10	ordinary_muscle_development	ENSGALG00010025606	CCDC12	-1.457871406	0.134699966	-10.82310153	2.67567E-27	1.35418E-26	negative
PRJNA729271	stage_1W_vs_E10	ordinary_muscle_development	ENSGALG00010025608	ENSGALG00010025608	-1.083585169	0.493141671	-2.197310089	0.027998308	0.035987893	negative
PRJNA729271	stage_1W_vs_E10	ordinary_muscle_development	ENSGALG00010025610	RNH1	-1.064580583	0.134208253	-7.932303394	2.15118E-15	6.51376E-15	negative
PRJNA729271	stage_1W_vs_E10	ordinary_muscle_development	ENSGALG00010025612	TMEM53	0.310232412	0.149657454	2.072949956	0.038176937	0.048355007	positive
PRJNA729271	stage_1W_vs_E10	ordinary_muscle_development	ENSGALG00010025613	CDKN1A	3.037350567	0.273522685	11.1045655	1.19196E-28	6.35412E-28	positive
PRJNA729271	stage_1W_vs_E10	ordinary_muscle_development	ENSGALG00010025618	ADIPOQ	7.47059074	0.221697084	33.69728914	6.3341E-249	1.4809E-246	positive
PRJNA729271	stage_1W_vs_E10	ordinary_muscle_development	ENSGALG00010025621	PPP1CC	-0.750739162	0.117980551	-6.363245074	1.97535E-10	4.61223E-10	negative
PRJNA729271	stage_1W_vs_E10	ordinary_muscle_development	ENSGALG00010025623	ENSGALG00010025623	0.79843845	0.412911393	1.933679872	0.0531525	0.066155374	positive
PRJNA729271	stage_1W_vs_E10	ordinary_muscle_development	ENSGALG00010025624	ARMH1	1.029044735	0.35053535	2.935637547	0.00332863	0.004724223	positive
PRJNA729271	stage_1W_vs_E10	ordinary_muscle_development	ENSGALG00010025628	ENSGALG00010025628	-4.461373655	0.331177282	-13.47125512	2.30907E-41	1.94254E-40	negative
PRJNA729271	stage_1W_vs_E10	ordinary_muscle_development	ENSGALG00010025629	ST6GAL1	2.919252546	0.143518455	20.34060735	5.62298E-92	1.77185E-90	positive
PRJNA729271	stage_1W_vs_E10	ordinary_muscle_development	ENSGALG00010025635	CHKA	-1.013830497	0.164152435	-6.176152648	6.56825E-10	1.4911E-09	negative
PRJNA729271	stage_1W_vs_E10	ordinary_muscle_development	ENSGALG00010025637	C6orf89	0.06141357	0.12831391	0.478619739	0.632209173	0.665109012	positive
PRJNA729271	stage_1W_vs_E10	ordinary_muscle_development	ENSGALG00010025639	MYL2	-5.7098874	0.456017979	-12.52118922	5.71721E-36	4.02873E-35	negative
PRJNA729271	stage_1W_vs_E10	ordinary_muscle_development	ENSGALG00010025640	SLC38A11	-6.293963003	0.982305832	-6.407335475	1.48085E-10	3.48343E-10	negative
PRJNA729271	stage_1W_vs_E10	ordinary_muscle_development	ENSGALG00010025642	RPS8	0.629794522	0.117534608	5.358375157	8.39737E-08	1.68821E-07	positive
PRJNA729271	stage_1W_vs_E10	ordinary_muscle_development	ENSGALG00010025644	ENSGALG00010025644	3.091785896	0.282923886	10.92797764	8.47154E-28	4.3746E-27	positive
PRJNA729271	stage_1W_vs_E10	ordinary_muscle_development	ENSGALG00010025645	NBEAL2	0.781519201	0.187787163	4.161728564	3.15848E-05	5.28052E-05	positive
PRJNA729271	stage_1W_vs_E10	ordinary_muscle_development	ENSGALG00010025647	JHY	-2.440794281	0.351135355	-6.951149317	3.62322E-12	9.32007E-12	negative
PRJNA729271	stage_1W_vs_E10	ordinary_muscle_development	ENSGALG00010025648	ENSGALG00010025648	1.646248697	0.5098677	3.228776201	0.001243211	0.001833013	positive
PRJNA729271	stage_1W_vs_E10	ordinary_muscle_development	ENSGALG00010025650	PPP1R1A	6.947241719	0.455687629	15.24562282	1.76046E-52	2.11241E-51	positive
PRJNA729271	stage_1W_vs_E10	ordinary_muscle_development	ENSGALG00010025652	KMT5B	-0.358089692	0.077272342	-4.634124988	3.5845E-06	6.43117E-06	negative
PRJNA729271	stage_1W_vs_E10	ordinary_muscle_development	ENSGALG00010025653	IGSF11	-1.121002199	0.203228278	-5.515975485	3.46851E-08	7.12528E-08	negative
PRJNA729271	stage_1W_vs_E10	ordinary_muscle_development	ENSGALG00010025654	CRTAM	2.857321427	0.717631572	3.981599387	6.84531E-05	0.000111561	positive
PRJNA729271	stage_1W_vs_E10	ordinary_muscle_development	ENSGALG00010025658	PDE1B	0.422963839	0.360120673	1.174505856	0.240192468	0.272595507	positive
PRJNA729271	stage_1W_vs_E10	ordinary_muscle_development	ENSGALG00010025659	COBLL1	1.372932166	0.130304229	10.53635925	5.87286E-26	2.80948E-25	positive
PRJNA729271	stage_1W_vs_E10	ordinary_muscle_development	ENSGALG00010025660	KIF2C	-2.892102997	0.204437997	-14.14660208	1.96002E-45	1.86054E-44	negative
PRJNA729271	stage_1W_vs_E10	ordinary_muscle_development	ENSGALG00010025661	SNRPE	-0.995211776	0.131046816	-7.594322434	3.09407E-14	8.83367E-14	negative
PRJNA729271	stage_1W_vs_E10	ordinary_muscle_development	ENSGALG00010025664	GPR55	1.358117471	0.688821864	1.971652676	0.048649269	0.060816831	positive
PRJNA729271	stage_1W_vs_E10	ordinary_muscle_development	ENSGALG00010025666	CUX2	-3.550331961	0.421025555	-8.432580679	3.38125E-17	1.10535E-16	negative
PRJNA729271	stage_1W_vs_E10	ordinary_muscle_development	ENSGALG00010025674	GRB14	2.422444688	0.434875943	5.570426989	2.54116E-08	5.26427E-08	positive
PRJNA729271	stage_1W_vs_E10	ordinary_muscle_development	ENSGALG00010025679	ENSGALG00010025679	-0.162119575	1.345717102	-0.12047077	0.904110233	0.915478717	negative
PRJNA729271	stage_1W_vs_E10	ordinary_muscle_development	ENSGALG00010025685	NCKAP1L	1.240923427	0.195100874	6.36041963	2.01203E-10	4.6941E-10	positive
PRJNA729271	stage_1W_vs_E10	ordinary_muscle_development	ENSGALG00010025688	BEST4	2.25008911	0.492141028	4.572041308	4.82996E-06	8.57293E-06	positive
PRJNA729271	stage_1W_vs_E10	ordinary_muscle_development	ENSGALG00010025691	ENSGALG00010025691	-4.061863995	0.186708959	-21.7550567	6.187E-105	2.5477E-103	negative
PRJNA729271	stage_1W_vs_E10	ordinary_muscle_development	ENSGALG00010025692	ENSGALG00010025692	0.197773558	1.253922065	0.157723963	0.874674317	0.888777022	positive
PRJNA729271	stage_1W_vs_E10	ordinary_muscle_development	ENSGALG00010025696	MTCH1	-0.093188757	0.105956308	-0.879501739	0.379129291	0.416892662	negative
PRJNA729271	stage_1W_vs_E10	ordinary_muscle_development	ENSGALG00010025701	ENSGALG00010025701	1.134916909	3.255211182	0.348646169	0.727354962	0.75409557	positive
PRJNA729271	stage_1W_vs_E10	ordinary_muscle_development	ENSGALG00010025702	GTSF2	-0.400267777	0.188989699	-2.11793436	0.034180627	0.043502344	negative
PRJNA729271	stage_1W_vs_E10	ordinary_muscle_development	ENSGALG00010025703	UBASH3B	-2.11757762	0.144231832	-14.68176331	8.43676E-49	8.84243E-48	negative
PRJNA729271	stage_1W_vs_E10	ordinary_muscle_development	ENSGALG00010025705	ACSL3	-1.032092245	0.08456677	-12.20446565	2.94234E-34	1.9386E-33	negative
PRJNA729271	stage_1W_vs_E10	ordinary_muscle_development	ENSGALG00010025706	ENSGALG00010025706	1.142859933	3.056422803	0.373920758	0.708463272	0.736036061	positive
PRJNA729271	stage_1W_vs_E10	ordinary_muscle_development	ENSGALG00010025708	HOXC8	-2.94098516	0.256412256	-11.46975268	1.87194E-30	1.07418E-29	negative
PRJNA729271	stage_1W_vs_E10	ordinary_muscle_development	ENSGALG00010025710	ENSGALG00010025710	0.169943611	2.061446255	0.082439021	0.934297608	0.942359183	positive
PRJNA729271	stage_1W_vs_E10	ordinary_muscle_development	ENSGALG00010025719	HOXC11	-6.840999646	2.679982774	-2.552628215	0.010691355	0.014410563	negative
PRJNA729271	stage_1W_vs_E10	ordinary_muscle_development	ENSGALG00010025720	PHETA1	-2.736364072	0.28367869	-9.645997984	5.11116E-22	2.07641E-21	negative
PRJNA729271	stage_1W_vs_E10	ordinary_muscle_development	ENSGALG00010025723	SETD2	0.394570413	0.122072956	3.232250841	0.001228192	0.001811974	positive
PRJNA729271	stage_1W_vs_E10	ordinary_muscle_development	ENSGALG00010025727	GLI1	-3.132205088	0.248290055	-12.61510487	1.74325E-36	1.24292E-35	negative
PRJNA729271	stage_1W_vs_E10	ordinary_muscle_development	ENSGALG00010025734	PLK3	0.727696028	0.178211454	4.08332916	4.43951E-05	7.33759E-05	positive
PRJNA729271	stage_1W_vs_E10	ordinary_muscle_development	ENSGALG00010025736	KIF9	0.841044548	0.331768057	2.535037746	0.011243521	0.015104248	positive
PRJNA729271	stage_1W_vs_E10	ordinary_muscle_development	ENSGALG00010025737	ENSGALG00010025737	-0.79079621	0.152862208	-5.17326174	2.30042E-07	4.49086E-07	negative
PRJNA729271	stage_1W_vs_E10	ordinary_muscle_development	ENSGALG00010025738	SOX13	0.353468017	0.153866221	2.297242463	0.021604942	0.028134367	positive
PRJNA729271	stage_1W_vs_E10	ordinary_muscle_development	ENSGALG00010025743	SH2B3	0.11621926	0.110297687	1.053687187	0.292026157	0.327017857	positive
PRJNA729271	stage_1W_vs_E10	ordinary_muscle_development	ENSGALG00010025745	COX14	1.50098602	0.225950067	6.642998793	3.07364E-11	7.52957E-11	positive
PRJNA729271	stage_1W_vs_E10	ordinary_muscle_development	ENSGALG00010025748	ETNK2	0.298141887	0.202756165	1.470445481	0.141441141	0.166546656	positive
PRJNA729271	stage_1W_vs_E10	ordinary_muscle_development	ENSGALG00010025753	SORL1	-1.054450805	0.174170215	-6.054139638	1.4117E-09	3.15343E-09	negative
PRJNA729271	stage_1W_vs_E10	ordinary_muscle_development	ENSGALG00010025755	ENSGALG00010025755	0.168451276	0.261852693	0.643305494	0.520025892	0.557074518	positive
PRJNA729271	stage_1W_vs_E10	ordinary_muscle_development	ENSGALG00010025782	ENSGALG00010025782	1.134841474	4.564438882	0.248626721	0.803649537	0.824584132	positive
PRJNA729271	stage_1W_vs_E10	ordinary_muscle_development	ENSGALG00010025784	ENSGALG00010025784	0.65184097	2.459378008	0.265043018	0.790976323	0.813134879	positive
PRJNA729271	stage_1W_vs_E10	ordinary_muscle_development	ENSGALG00010025787	KCNE4	2.134271979	0.290464051	7.347800773	2.01495E-13	5.51069E-13	positive
PRJNA729271	stage_1W_vs_E10	ordinary_muscle_development	ENSGALG00010025790	LRP5	0.08991129	0.078162122	1.150317925	0.250012951	0.282965388	positive
PRJNA729271	stage_1W_vs_E10	ordinary_muscle_development	ENSGALG00010025791	FIGN	0.521447961	0.236730077	2.202711068	0.02761512	0.03552371	positive
PRJNA729271	stage_1W_vs_E10	ordinary_muscle_development	ENSGALG00010025794	ENSGALG00010025794	1.13486128	4.091796531	0.27735037	0.781511087	0.804145893	positive
PRJNA729271	stage_1W_vs_E10	ordinary_muscle_development	ENSGALG00010025799	SC5D	1.181329942	0.265580945	4.44809752	8.66342E-06	1.51024E-05	positive
PRJNA729271	stage_1W_vs_E10	ordinary_muscle_development	ENSGALG00010025800	ARL2BP	-1.016170193	0.140218887	-7.247027924	4.26016E-13	1.14736E-12	negative
PRJNA729271	stage_1W_vs_E10	ordinary_muscle_development	ENSGALG00010025801	PTPN7	1.169938564	0.373987372	3.1282836	0.001758304	0.002561727	positive
PRJNA729271	stage_1W_vs_E10	ordinary_muscle_development	ENSGALG00010025804	ENSGALG00010025804	0.651231342	2.257954404	0.288416516	0.773027927	0.796264909	positive
PRJNA729271	stage_1W_vs_E10	ordinary_muscle_development	ENSGALG00010025805	ATXN2	0.14323916	0.09399949	1.523829131	0.1275514	0.151087485	positive
PRJNA729271	stage_1W_vs_E10	ordinary_muscle_development	ENSGALG00010025809	ENSGALG00010025809	1.542858193	0.19182012	8.043255261	8.74828E-16	2.69743E-15	positive
PRJNA729271	stage_1W_vs_E10	ordinary_muscle_development	ENSGALG00010025822	KLF7	-0.354900622	0.100105058	-3.545281621	0.000392194	0.000603359	negative
PRJNA729271	stage_1W_vs_E10	ordinary_muscle_development	ENSGALG00010025829	ENSGALG00010025829	-0.512245965	0.259010559	-1.977703018	0.04796222	0.06000452	negative
PRJNA729271	stage_1W_vs_E10	ordinary_muscle_development	ENSGALG00010025830	TBCEL	-0.088319144	0.193647176	-0.456082791	0.648330445	0.679848788	negative
PRJNA729271	stage_1W_vs_E10	ordinary_muscle_development	ENSGALG00010025831	BRAP	0.122157436	0.070015847	1.744711254	0.081035143	0.098548905	positive
PRJNA729271	stage_1W_vs_E10	ordinary_muscle_development	ENSGALG00010025832	ENKD1	1.491364998	0.144829508	10.29738356	7.24038E-25	3.30757E-24	positive
PRJNA729271	stage_1W_vs_E10	ordinary_muscle_development	ENSGALG00010025835	PPP6R3	0.287010537	0.119210927	2.407585814	0.016058388	0.021193014	positive
PRJNA729271	stage_1W_vs_E10	ordinary_muscle_development	ENSGALG00010025836	ENSGALG00010025836	0.458818729	0.113934212	4.027049643	5.64811E-05	9.25496E-05	positive
PRJNA729271	stage_1W_vs_E10	ordinary_muscle_development	ENSGALG00010025837	ENSGALG00010025837	2.866592025	0.175423658	16.34096595	5.04473E-60	7.3862E-59	positive
PRJNA729271	stage_1W_vs_E10	ordinary_muscle_development	ENSGALG00010025846	GRIK4	0.618010336	0.212176603	2.912716703	0.003582995	0.005068857	positive
PRJNA729271	stage_1W_vs_E10	ordinary_muscle_development	ENSGALG00010025847	DEF6	-0.539049563	0.128766169	-4.186266994	2.8358E-05	4.75256E-05	negative
PRJNA729271	stage_1W_vs_E10	ordinary_muscle_development	ENSGALG00010025849	OBSCN	4.698021219	0.229073026	20.50883643	1.79538E-93	5.78312E-92	positive
PRJNA729271	stage_1W_vs_E10	ordinary_muscle_development	ENSGALG00010025852	BTBD19	-0.426964791	0.25181488	-1.695550285	0.08997108	0.108886265	negative
PRJNA729271	stage_1W_vs_E10	ordinary_muscle_development	ENSGALG00010025858	ENSGALG00010025858	1.134937222	2.976788379	0.381262313	0.703008615	0.731104046	positive

PRJNA729271	stage_1W_vs_E10	ordinary_muscle_development	ENSGALG00010025862	PTCH2	-3.61828497	0.314284944	-11.51275314	1.13787E-30	6.61323E-30	negative
PRJNA729271	stage_1W_vs_E10	ordinary_muscle_development	ENSGALG00010025865	LGR6	-3.715677986	0.683354647	-5.437407946	5.40613E-08	1.10059E-07	negative
PRJNA729271	stage_1W_vs_E10	ordinary_muscle_development	ENSGALG00010025869	FASTKD2	0.381016112	0.092035235	4.139893934	3.47466E-05	5.79177E-05	positive
PRJNA729271	stage_1W_vs_E10	ordinary_muscle_development	ENSGALG00010025870	ENSGALG00010025870	2.589589453	0.161677538	16.01700201	9.72263E-58	1.33963E-56	positive
PRJNA729271	stage_1W_vs_E10	ordinary_muscle_development	ENSGALG00010025871	ENSGALG00010025871	-0.43298949	1.597227315	-0.271088208	0.786323191	0.80869552	negative
PRJNA729271	stage_1W_vs_E10	ordinary_muscle_development	ENSGALG00010025874	MDH1B	-1.630524702	0.306771133	-5.31511777	1.06588E-07	2.12488E-07	negative
PRJNA729271	stage_1W_vs_E10	ordinary_muscle_development	ENSGALG00010025876	ENSGALG00010025876	1.134998972	1.784677425	0.635968695	0.524796826	0.561562823	positive
PRJNA729271	stage_1W_vs_E10	ordinary_muscle_development	ENSGALG00010025877	ENSGALG00010025877	1.134927216	3.503381349	0.323951949	0.745974413	0.771358191	positive
PRJNA729271	stage_1W_vs_E10	ordinary_muscle_development	ENSGALG00010025881	GAL	1.267823657	0.59328675	2.136949219	0.032602122	0.041588292	positive
PRJNA729271	stage_1W_vs_E10	ordinary_muscle_development	ENSGALG00010025882	LDHD	3.764351935	0.170877287	22.0295629	1.5001E-107	6.4716E-106	positive
PRJNA729271	stage_1W_vs_E10	ordinary_muscle_development	ENSGALG00010025886	ALDH2	0.951874793	0.109729734	8.674720677	4.14567E-18	1.41792E-17	positive
PRJNA729271	stage_1W_vs_E10	ordinary_muscle_development	ENSGALG00010025887	ZNF76	-0.469796611	0.117240754	-4.007110122	6.14662E-05	0.000100548	negative
PRJNA729271	stage_1W_vs_E10	ordinary_muscle_development	ENSGALG00010025889	TESMIN	1.776784769	0.562391491	3.159337932	0.00158128	0.002312648	positive
PRJNA729271	stage_1W_vs_E10	ordinary_muscle_development	ENSGALG00010025890	SCG2	-0.177438482	0.517416973	-0.342931314	0.731650134	0.758114854	negative
PRJNA729271	stage_1W_vs_E10	ordinary_muscle_development	ENSGALG00010025891	NUP93	-2.044955601	0.111465983	-18.34600604	3.55289E-75	7.51812E-74	negative
PRJNA729271	stage_1W_vs_E10	ordinary_muscle_development	ENSGALG00010025892	ZC3H11A	0.443306108	0.086024068	5.153279993	2.55969E-07	4.97623E-07	positive
PRJNA729271	stage_1W_vs_E10	ordinary_muscle_development	ENSGALG00010025894	DYTN	-1.668964114	0.600012012	-2.78155117	0.00540998	0.007527855	negative
PRJNA729271	stage_1W_vs_E10	ordinary_muscle_development	ENSGALG00010025897	MAPKAPK5	0.372023409	0.120932709	3.076284418	0.002095978	0.003030665	positive
PRJNA729271	stage_1W_vs_E10	ordinary_muscle_development	ENSGALG00010025898	ENSGALG00010025898	1.134995304	2.00257413	0.566768185	0.570871689	0.606566862	positive
PRJNA729271	stage_1W_vs_E10	ordinary_muscle_development	ENSGALG00010025899	ENSGALG00010025899	1.425078184	0.162442155	8.77283478	1.74224E-18	6.07498E-18	positive
PRJNA729271	stage_1W_vs_E10	ordinary_muscle_development	ENSGALG00010025900	FAM237A	0.104630096	0.592913509	0.176467721	0.859926505	0.875448427	positive
PRJNA729271	stage_1W_vs_E10	ordinary_muscle_development	ENSGALG00010025902	ENSGALG00010025902	1.13500581	1.873306446	0.605883683	0.544591995	0.580772585	positive
PRJNA729271	stage_1W_vs_E10	ordinary_muscle_development	ENSGALG00010025904	RPL10A	0.444241788	0.138821445	3.200094821	0.001373824	0.002019632	positive
PRJNA729271	stage_1W_vs_E10	ordinary_muscle_development	ENSGALG00010025905	CPT1A	0.925954334	0.174899325	5.294213311	1.1953E-07	2.37536E-07	positive
PRJNA729271	stage_1W_vs_E10	ordinary_muscle_development	ENSGALG00010025907	ARHGEF12	-0.178162652	0.071937121	-2.476644183	0.013262405	0.017690122	negative
PRJNA729271	stage_1W_vs_E10	ordinary_muscle_development	ENSGALG00010025910	HEATR3	0.132522696	0.112810888	1.174733204	0.240101471	0.272513572	positive
PRJNA729271	stage_1W_vs_E10	ordinary_muscle_development	ENSGALG00010025913	EIF2B3	-0.081720209	0.077615007	-1.052891854	0.29239056	0.327350055	negative
PRJNA729271	stage_1W_vs_E10	ordinary_muscle_development	ENSGALG00010025914	ENSGALG00010025914	-5.663095789	1.762371208	-3.213338804	0.001312014	0.001931314	negative
PRJNA729271	stage_1W_vs_E10	ordinary_muscle_development	ENSGALG00010025917	SCUBE3	-5.698212814	0.495016851	-11.51114916	1.15923E-30	6.732E-30	negative
PRJNA729271	stage_1W_vs_E10	ordinary_muscle_development	ENSGALG00010025919	RGS9BP	-1.007609735	0.330773657	-3.046221222	0.002317373	0.003335351	negative
PRJNA729271	stage_1W_vs_E10	ordinary_muscle_development	ENSGALG00010025920	GTSF1	0.391695714	0.26124315	1.499353047	0.133782067	0.158015895	positive
PRJNA729271	stage_1W_vs_E10	ordinary_muscle_development	ENSGALG00010025924	TMEM116	1.638506851	0.172665485	9.489486846	2.32174E-21	9.2303E-21	positive
PRJNA729271	stage_1W_vs_E10	ordinary_muscle_development	ENSGALG00010025925	KLHL18	0.488599133	0.091581561	5.335125611	9.54784E-08	1.91181E-07	positive
PRJNA729271	stage_1W_vs_E10	ordinary_muscle_development	ENSGALG00010025926	ENSGALG00010025926	0.359036937	0.42826082	0.838360457	0.401828291	0.439983462	positive
PRJNA729271	stage_1W_vs_E10	ordinary_muscle_development	ENSGALG00010025927	ENSGALG00010025927	-3.988646994	1.185881673	-3.363444336	0.000769764	0.001158281	negative
PRJNA729271	stage_1W_vs_E10	ordinary_muscle_development	ENSGALG00010025929	NELFE	0.386486908	0.089867072	4.300650957	1.70297E-05	2.90202E-05	positive
PRJNA729271	stage_1W_vs_E10	ordinary_muscle_development	ENSGALG00010025931	TMEM136	-2.202389679	0.211936269	-10.39175452	2.70334E-25	1.25673E-24	negative
PRJNA729271	stage_1W_vs_E10	ordinary_muscle_development	ENSGALG00010025932	AP1S3	-0.061893517	0.312006893	-0.198372274	0.842753808	0.859958919	negative
PRJNA729271	stage_1W_vs_E10	ordinary_muscle_development	ENSGALG00010025933	SKIC2	0.687998297	0.101640419	6.768943945	1.29726E-11	3.23868E-11	positive
PRJNA729271	stage_1W_vs_E10	ordinary_muscle_development	ENSGALG00010025935	CATH3	-0.549063783	0.379140797	-1.44817911	0.147566965	0.1732391	negative
PRJNA729271	stage_1W_vs_E10	ordinary_muscle_development	ENSGALG00010025936	GOSR2	-0.948439524	0.06982272	-13.58353733	5.01449E-42	4.29581E-41	negative
PRJNA729271	stage_1W_vs_E10	ordinary_muscle_development	ENSGALG00010025938	ERP29	-1.192592764	0.090559838	-13.16911322	1.32141E-39	1.04437E-38	negative
PRJNA729271	stage_1W_vs_E10	ordinary_muscle_development	ENSGALG00010025939	TMEM136-1	-3.853646135	0.883160553	-4.363471764	1.28014E-05	2.20376E-05	negative
PRJNA729271	stage_1W_vs_E10	ordinary_muscle_development	ENSGALG00010025940	HECTD3	0.815069234	0.087798459	9.283411584	1.64137E-20	6.31414E-20	positive
PRJNA729271	stage_1W_vs_E10	ordinary_muscle_development	ENSGALG00010025941	ENSGALG00010025941	-2.886511855	0.714308083	-4.040990045	5.3226E-05	8.73838E-05	negative
PRJNA729271	stage_1W_vs_E10	ordinary_muscle_development	ENSGALG00010025942	ENSGALG00010025942	-1.284622086	1.318310545	-0.974445734	0.329835271	0.366301123	negative
PRJNA729271	stage_1W_vs_E10	ordinary_muscle_development	ENSGALG00010025943	CATH1	-0.016143697	0.755126946	-0.021378785	0.982943497	0.985595323	negative
PRJNA729271	stage_1W_vs_E10	ordinary_muscle_development	ENSGALG00010025946	KRT5	-10.35503788	1.273035171	-8.134133381	4.14896E-16	1.2989E-15	negative
PRJNA729271	stage_1W_vs_E10	ordinary_muscle_development	ENSGALG00010025948	WNT9B	-8.72449705	0.923232858	-9.449942101	3.39018E-21	1.33825E-20	negative
PRJNA729271	stage_1W_vs_E10	ordinary_muscle_development	ENSGALG00010025949	GPATCH1	0.185683636	0.12233126	1.517875608	0.12904576	0.152720305	positive
PRJNA729271	stage_1W_vs_E10	ordinary_muscle_development	ENSGALG00010025951	TBRG4	0.497464557	0.11364655	4.37729574	1.20161E-05	2.07279E-05	positive
PRJNA729271	stage_1W_vs_E10	ordinary_muscle_development	ENSGALG00010025953	POU2F3	-3.716656055	1.082342719	-3.433899439	0.000594965	0.000902755	negative
PRJNA729271	stage_1W_vs_E10	ordinary_muscle_development	ENSGALG00010025954	TUBA1A	-2.738019454	0.158930665	-17.22776066	1.64393E-66	2.80669E-65	negative
PRJNA729271	stage_1W_vs_E10	ordinary_muscle_development	ENSGALG00010025956	ENSGALG00010025956	1.342073622	0.504408244	2.660689309	0.007798088	0.010667543	positive
PRJNA729271	stage_1W_vs_E10	ordinary_muscle_development	ENSGALG00010025957	ENSGALG00010025957	-0.760646632	0.116429101	-6.533131531	6.44085E-11	1.54468E-10	negative
PRJNA729271	stage_1W_vs_E10	ordinary_muscle_development	ENSGALG00010025958	HOXC9	-5.103378307	0.522615609	-9.765070584	1.59001E-22	6.58681E-22	negative
PRJNA729271	stage_1W_vs_E10	ordinary_muscle_development	ENSGALG00010025959	UROD	-0.450283081	0.105296699	-4.276326665	1.90002E-05	3.22417E-05	negative
PRJNA729271	stage_1W_vs_E10	ordinary_muscle_development	ENSGALG00010025960	CMKLR2	1.704451002	0.158393179	10.76088639	5.26612E-27	2.63034E-26	positive
PRJNA729271	stage_1W_vs_E10	ordinary_muscle_development	ENSGALG00010025962	ENSGALG00010025962	0.556875068	0.188538206	2.953645735	0.003140443	0.004464566	positive
PRJNA729271	stage_1W_vs_E10	ordinary_muscle_development	ENSGALG00010025963	OAF	-1.01474219	0.246841712	-4.11090241	3.94116E-05	6.53778E-05	negative
PRJNA729271	stage_1W_vs_E10	ordinary_muscle_development	ENSGALG00010025964	HOXC10	-8.770861242	1.094367161	-8.014550831	1.1054E-15	3.39322E-15	negative
PRJNA729271	stage_1W_vs_E10	ordinary_muscle_development	ENSGALG00010025968	FADS2	-0.228938293	0.156787129	-1.460185502	0.144239099	0.169648307	negative
PRJNA729271	stage_1W_vs_E10	ordinary_muscle_development	ENSGALG00010025970	KLHL12	-0.012821855	0.11069435	-0.115831165	0.907786351	0.918815946	negative
PRJNA729271	stage_1W_vs_E10	ordinary_muscle_development	ENSGALG00010025971	TRAFD1	0.057663292	0.199160123	0.289532315	0.772174055	0.795441896	positive
PRJNA729271	stage_1W_vs_E10	ordinary_muscle_development	ENSGALG00010025972	WNT3	-7.505636581	1.318245216	-5.693657362	1.24347E-08	2.62172E-08	negative
PRJNA729271	stage_1W_vs_E10	ordinary_muscle_development	ENSGALG00010025974	EEF1B2	1.089052886	0.095515401	11.40185634	4.09297E-30	2.32384E-29	positive
PRJNA729271	stage_1W_vs_E10	ordinary_muscle_development	ENSGALG00010025976	NAA25	-0.004117629	0.098781907	-0.041684043	0.966750575	0.971374572	negative
PRJNA729271	stage_1W_vs_E10	ordinary_muscle_development	ENSGALG00010025978	TRIM29	-7.774908415	0.920447238	-8.446881141	2.99188E-17	9.80717E-17	negative
PRJNA729271	stage_1W_vs_E10	ordinary_muscle_development	ENSGALG00010025980	HECTD4	-0.524606326	0.064909404	-8.08213135	6.36445E-16	1.97713E-15	negative
PRJNA729271	stage_1W_vs_E10	ordinary_muscle_development	ENSGALG00010025982	SERPINE2	-1.753183872	0.199453864	-8.789921806	1.49664E-18	5.23626E-18	negative
PRJNA729271	stage_1W_vs_E10	ordinary_muscle_development	ENSGALG00010025983	RNF26	-1.143051841	0.085063319	-13.43765856	3.63766E-41	3.03907E-40	negative
PRJNA729271	stage_1W_vs_E10	ordinary_muscle_development	ENSGALG00010025984	NDUFS1	1.720944182	0.116875329	14.72461466	4.48016E-49	4.7436E-48	positive
PRJNA729271	stage_1W_vs_E10	ordinary_muscle_development	ENSGALG00010025985	CHCHD10	3.481932834	0.317931519	10.9518328	6.51165E-28	3.37939E-27	positive
PRJNA729271	stage_1W_vs_E10	ordinary_muscle_development	ENSGALG00010025986	RAB3IL1	-0.635218493	0.108638222	-5.847099509	5.00218E-09	1.08154E-08	negative
PRJNA729271	stage_1W_vs_E10	ordinary_muscle_development	ENSGALG00010025988	NSF	-0.588569275	0.060524913	-9.724413471	2.37269E-22	9.7511E-22	negative
PRJNA729271	stage_1W_vs_E10	ordinary_muscle_development	ENSGALG00010025990	FAM124B	1.16528067	0.239465309	4.866177375	1.13777E-06	2.11925E-06	positive
PRJNA729271	stage_1W_vs_E10	ordinary_muscle_development	ENSGALG00010025992	MMP11	-3.077923441	0.425372188	-7.235836111	4.6267E-13	1.24354E-12	negative
PRJNA729271	stage_1W_vs_E10	ordinary_muscle_development	ENSGALG00010025994	INO80D	0.624526788	0.108504133	5.755788038	8.62385E-09	1.83612E-08	positive
PRJNA729271	stage_1W_vs_E10	ordinary_muscle_development	ENSGALG00010025996	CUL3	1.692260516	0.096491157	17.53798563	7.34864E-69	1.35692E-67	positive
PRJNA729271	stage_1W_vs_E10	ordinary_muscle_development	ENSGALG00010025997	CCDC43	0.124322977	0.149224392	0.833127712	0.404772706	0.443006673	positive
PRJNA729271	stage_1W_vs_E10	ordinary_muscle_development	ENSGALG00010025998	SMARCB1	-0.685015101	0.143734433	-4.765838533	1.8807E-06	3.44462E-06	negative
PRJNA729271	stage_1W_vs_E10	ordinary_muscle_development	ENSGALG00010025999	DBF4B	-1.652298209	0.167988768	-9.83576799	7.89644E-23	3.31669E-22	negative
PRJNA729271	stage_1W_vs_E10	ordinary_muscle_development	ENSGALG00010026001	ANKS1A	0.724790676	0.092344526	7.848767055	4.20147E-15	1.25697E-14	positive
PRJNA729271	stage_1W_vs_E10	ordinary_muscle_development	ENSGALG00010026002	FTH1	1.253152252	0.141852816	8.834172556	1.00842E-18	3.56079E-18	positive
PRJNA729271	stage_1W_vs_E10	ordinary_muscle_development	ENSGALG00010026003	NECTIN1	-1.164170895	0.156844369	-7.422458987	1.14966E-13	3.18507E-13	negative
PRJNA729271	stage_1W_vs_E10	ordinary_muscle_development	ENSGALG00010026005	DERL3	-0.011858144	0.117679745	-0.10076623	0.919736035	0.929547749	negative
PRJNA729271	stage_1W_vs_E10	ordinary_muscle_development	ENSGALG00010026006	ITGA2B	-0.808437801	0.32453052	-2.491099454	0.012734847	0.017019325	negative
PRJNA729271	stage_1W_vs_E10	ordinary_muscle_development	ENSGALG00010026008	NRP2	-0.705980683	0.101058391	-6.985868991	2.83098E-12	7.32377E-12	negative
PRJNA729271	stage_1W_vs_E10	ordinary_muscle_development	ENSGALG00010026009	ENSGALG00010026009	-1.826539088	0.311079565	-5.871613875	4.31573E-09	9.36054E-09	negative
PRJNA729271	stage_1W_vs_E10	ordinary_muscle_development	ENSGALG00010026010	ZSWIM5	-0.534899251	0.129079893	-4.143939367	3.4139E-05	5.69507E-05	negative
PRJNA729271	stage_1W_vs_E10	ordinary_muscle_development	ENSGALG00010026011	GJC2	-1.286825926	0.165574362	-7.771891214	7.73228E-15	2.27943E-14	negative
PRJNA729271	stage_1W_vs_E10	ordinary_muscle_development	ENSGALG00010026013	RABIF	-0.741898126	0.114191888	-6.49694248	8.19688E-11	1.95578E-10	negative
PRJNA729271	stage_1W_vs_E10	ordinary_muscle_development	ENSGALG00010026016	SLC2A11	-0.048698642	0.37966858	-0.12826619	0.897938321	0.9098648	negative
PRJNA729271	stage_1W_vs_E10	ordinary_muscle_development	ENSGALG00010026017	PLEKHM1	1.446666965	0.142723639	10.13614126	3.81888E-24	1.68919E-23	positive
PRJNA729271	stage_1W_vs_E10	ordinary_muscle_development	ENSGALG00010026023	THY1	-0.663523616	0.301271429	-2.202411359	0.027636264	0.035547755	negative
PRJNA729271	stage_1W_vs_E10	ordinary_muscle_development	ENSGALG00010026030	IBA57	0.915099891	0.082758046	11.05753375	2.01564E-28	1.0663E-27	positive
PRJNA729271	stage_1W_vs_E10	ordinary_muscle_development	ENSGALG00010026036	TUB5A	-1.635183439	0.207360531	-7.885702421	3.12771E-15	9.3961E-15	negative
PRJNA729271	stage_1W_vs_E10	ordinary_muscle_development	ENSGALG00010026040	GUK1	-0.845025138	0.123108513	-6.864067464	6.6927E-12	1.69985E-11	negative
PRJNA729271	stage_1W_vs_E10	ordinary_muscle_development	ENSGALG00010026051	TAF11	0.03437948	0.128120183	0.268337734	0.788439363	0.810756851	positive
PRJNA729271	stage_1W_vs_E10	ordinary_muscle_development	ENSGALG00010026061	MPEG1	2.779197384	0.342540845	8.113477329	4.91914E-16	1.53472E-15	positive
PRJNA729271	stage_1W_vs_E10	ordinary_muscle_development	ENSGALG00010026062	ITGA4	-2.472091617	0.162105674	-15.24987714	1.64943E-52	1.98741E-51	negative
PRJNA729271	stage_1W_vs_E10	ordinary_muscle_development	ENSGALG00010026064	ENSGALG00010026064	0.861623898	0.148073739	5.8188839	5.92419E-09	1.27538E-08	positive
PRJNA729271	stage_1W_vs_E10	ordinary_muscle_development	ENSGALG00010026066	SELENOP2	1.329037925	0.570936668	2.327820227	0.019921652	0.026047658	positive
PRJNA729271	stage_1W_vs_E10	ordinary_muscle_development	ENSGALG00010026068	BLTP3A	0.541424541	0.111420432	4.859293139	1.17806E-06	2.19175E-06	positive
PRJNA729271	stage_1W_vs_E10	ordinary_muscle_development	ENSGALG00010026073	HPDL	-0.290603988	0.234255317	-1.240543832	0.214774312	0.245865871	negative
PRJNA729271	stage_1W_vs_E10	ordinary_muscle_development	ENSGALG00010026076	C1orf35	0.247901212	0.134727956	1.840013163	0.065766305	0.080903216	positive
PRJNA729271	stage_1W_vs_E10	ordinary_muscle_development	ENSGALG00010026078	UBE2E3	0.141993235	0.10697505	1.327349086	0.184393233	0.213378566	positive
PRJNA729271	stage_1W_vs_E10	ordinary_muscle_development	ENSGALG00010026081	KPNA1	0.317336268	0.108083007	2.936042196	0.003324291	0.004718989	positive
PRJNA729271	stage_1W_vs_E10	ordinary_muscle_development	ENSGALG00010026083	MUTYH	-1.643692971	0.1295055	-12.69207074	6.54326E-37	4.7116E-36	negative
PRJNA729271	stage_1W_vs_E10	ordinary_muscle_development	ENSGALG00010026088	SNRPC	-0.147445367	0.081777018	-1.803017166	0.071385519	0.087385617	negative
PRJNA729271	stage_1W_vs_E10	ordinary_muscle_development	ENSGALG00010026090	USP2	5.075894549	0.233777007	21.71254827	1.5616E-104	6.3581E-103	positive
PRJNA729271	stage_1W_vs_E10	ordinary_muscle_development	ENSGALG00010026091	DTX4	0.799866817	0.162449617	4.92378396	8.48865E-07	1.59651E-06	positive
PRJNA729271	stage_1W_vs_E10	ordinary_muscle_development	ENSGALG00010026093	TOE1	-0.746443556	0.129064348	-5.783499216	7.31625E-09	1.56461E-08	negative
PRJNA729271	stage_1W_vs_E10	ordinary_muscle_development	ENSGALG00010026094	C6orf106	1.535194057	0.077668252	19.76604364	5.83785E-87	1.63674E-85	positive
PRJNA729271	stage_1W_vs_E10	ordinary_muscle_development	ENSGALG00010026095	MIF	-1.959943405	0.202463747	-9.680465909	3.65049E-22	1.48969E-21	negative
PRJNA729271	stage_1W_vs_E10	ordinary_muscle_development	ENSGALG00010026097	ARF1	-0.646708638	0.056091651	-11.52949906	9.36873E-31	5.45599E-30	negative
PRJNA729271	stage_1W_vs_E10	ordinary_muscle_development	ENSGALG00010026098	STAT3	1.149950192	0.108059422	10.64183182	1.9035E-26	9.31506E-26	positive
PRJNA729271	stage_1W_vs_E10	ordinary_muscle_development	ENSGALG00010026102	IPO9	-1.342191439	0.060389005	-22.22575856	1.9358E-109	8.6605E-108	negative
PRJNA729271	stage_1W_vs_E10	ordinary_muscle_development	ENSGALG00010026104	DDX51	-0.594196737	0.105103991	-5.653417437	1.57289E-08	3.29799E-08	negative
PRJNA729271	stage_1W_vs_E10	ordinary_muscle_development	ENSGALG00010026105	CNTF	2.674373443	0.184061574	14.52977599	7.84766E-48	7.9882E-47	positive
PRJNA729271	stage_1W_vs_E10	ordinary_muscle_development	ENSGALG00010026106	TESK2	0.688963305	0.118835104	5.797641284	6.72541E-09	1.44187E-08	positive
PRJNA729271	stage_1W_vs_E10	ordinary_muscle_development	ENSGALG00010026111	ENSGALG00010026111	-3.244716316	0.165431925	-19.61360432	1.18341E-85	3.24263E-84	negative
PRJNA729271	stage_1W_vs_E10	ordinary_muscle_development	ENSGALG00010026112	ZFP91	0.744677717	0.120711679	6.169061055	6.86967E-10	1.55733E-09	positive
PRJNA729271	stage_1W_vs_E10	ordinary_muscle_development	ENSGALG00010026113	MFRP	2.259949126	0.488055107	4.630520396	3.64748E-06	6.5401E-06	positive
PRJNA729271	stage_1W_vs_E10	ordinary_muscle_development	ENSGALG00010026114	CWC22	-0.399413041	0.083610284	-4.777080327	1.77859E-06	3.26213E-06	negative
PRJNA729271	stage_1W_vs_E10	ordinary_muscle_development	ENSGALG00010026115	MMACHC	1.341759575	0.173116114	7.750633633	9.14351E-15	2.68671E-14	positive
PRJNA729271	stage_1W_vs_E10	ordinary_muscle_development	ENSGALG00010026116	DLX3	-4.786944707	0.785691134	-6.092654605	1.11054E-09	2.4926E-09	negative
PRJNA729271	stage_1W_vs_E10	ordinary_muscle_development	ENSGALG00010026117	LZTR1	-0.070223784	0.072226538	-0.972271216	0.33091564	0.36747278	negative
PRJNA729271	stage_1W_vs_E10	ordinary_muscle_development	ENSGALG00010026120	C1QTNF5	0.942008136	0.194184515	4.851098127	1.2278E-06	2.2799E-06	positive
PRJNA729271	stage_1W_vs_E10	ordinary_muscle_development	ENSGALG00010026121	ENSGALG00010026121	1.202597389	0.258203349	4.657559229	3.1998E-06	5.75877E-06	positive
PRJNA729271	stage_1W_vs_E10	ordinary_muscle_development	ENSGALG00010026124	PACSIN1	-2.557983828	0.409385659	-6.248347417	4.14818E-10	9.51541E-10	negative

PRJNA729271	stage_1W_vs_E10	ordinary_muscle_development	ENSGALG00010026127	ENSGALG00010026127	0.023511743	0.697178169	0.033724153	0.973097119	0.976871164	positive
PRJNA729271	stage_1W_vs_E10	ordinary_muscle_development	ENSGALG00010026128	PRDX1	-1.485463572	0.142189906	-10.44703957	1.5117E-25	7.12359E-25	negative
PRJNA729271	stage_1W_vs_E10	ordinary_muscle_development	ENSGALG00010026132	DDTL	-0.862373431	0.186306647	-4.628785098	3.67817E-06	6.59025E-06	negative
PRJNA729271	stage_1W_vs_E10	ordinary_muscle_development	ENSGALG00010026137	LPXN	1.588153456	0.339209443	4.681925836	2.84192E-06	5.13189E-06	positive
PRJNA729271	stage_1W_vs_E10	ordinary_muscle_development	ENSGALG00010026141	WNT3A	-8.074065472	0.966025017	-8.358029378	6.37746E-17	2.06112E-16	negative
PRJNA729271	stage_1W_vs_E10	ordinary_muscle_development	ENSGALG00010026143	RNPEP	-0.243742702	0.141826627	-1.7185962	0.085687924	0.103945641	negative
PRJNA729271	stage_1W_vs_E10	ordinary_muscle_development	ENSGALG00010026148	RPS10	0.874710923	0.134063025	6.524624698	6.8172E-11	1.63196E-10	positive
PRJNA729271	stage_1W_vs_E10	ordinary_muscle_development	ENSGALG00010026149	LASP1	-1.259432985	0.129578616	-9.719450815	2.49121E-22	1.02353E-21	negative
PRJNA729271	stage_1W_vs_E10	ordinary_muscle_development	ENSGALG00010026154	ZNF385B	-2.230572712	0.185254822	-12.04056492	2.17461E-33	1.39289E-32	negative
PRJNA729271	stage_1W_vs_E10	ordinary_muscle_development	ENSGALG00010026162	MED1	-0.347623962	0.152217198	-2.283736437	0.022387025	0.029105743	negative
PRJNA729271	stage_1W_vs_E10	ordinary_muscle_development	ENSGALG00010026163	CTNND1	-0.65221947	0.082312356	-7.923712849	2.30522E-15	6.96999E-15	negative
PRJNA729271	stage_1W_vs_E10	ordinary_muscle_development	ENSGALG00010026166	WNT9A	-3.48332496	0.255072708	-13.65620409	1.85399E-42	1.6092E-41	negative
PRJNA729271	stage_1W_vs_E10	ordinary_muscle_development	ENSGALG00010026167	CABIN1	-1.058040377	0.061004385	-17.34367732	2.20188E-67	3.88276E-66	negative
PRJNA729271	stage_1W_vs_E10	ordinary_muscle_development	ENSGALG00010026173	CDK12	0.323833244	0.162184659	1.996694666	0.045858362	0.057531327	positive
PRJNA729271	stage_1W_vs_E10	ordinary_muscle_development	ENSGALG00010026183	SESTD1	0.107158873	0.168447144	0.636157254	0.524673931	0.561484433	positive
PRJNA729271	stage_1W_vs_E10	ordinary_muscle_development	ENSGALG00010026184	NUDT3	0.40532389	0.104305942	3.8859137	0.000101946	0.000163771	positive
PRJNA729271	stage_1W_vs_E10	ordinary_muscle_development	ENSGALG00010026192	SMIM29	3.490363814	0.118044699	29.56815375	3.8376E-192	5.1987E-190	positive
PRJNA729271	stage_1W_vs_E10	ordinary_muscle_development	ENSGALG00010026199	TBX6	-1.804163149	0.483114061	-3.734445537	0.000188129	0.000296567	negative
PRJNA729271	stage_1W_vs_E10	ordinary_muscle_development	ENSGALG00010026203	TMX2	0.812748973	0.102865516	7.90108295	2.76491E-15	8.33035E-15	positive
PRJNA729271	stage_1W_vs_E10	ordinary_muscle_development	ENSGALG00010026205	ENSGALG00010026205	4.981110426	1.304600508	3.818111671	0.000134477	0.000214179	positive
PRJNA729271	stage_1W_vs_E10	ordinary_muscle_development	ENSGALG00010026209	MED19	2.029141916	0.170173733	11.92394313	8.88038E-33	5.55551E-32	positive
PRJNA729271	stage_1W_vs_E10	ordinary_muscle_development	ENSGALG00010026213	AKR1A1	-1.091005836	0.124004822	-8.798092003	1.39162E-18	4.87706E-18	negative
PRJNA729271	stage_1W_vs_E10	ordinary_muscle_development	ENSGALG00010026218	FTSJ3	-0.64491958	0.167850884	-3.842217361	0.000121928	0.000194834	negative
PRJNA729271	stage_1W_vs_E10	ordinary_muscle_development	ENSGALG00010026220	ENSGALG00010026220	-0.025059745	0.097081248	-0.258131669	0.796305291	0.817917035	negative
PRJNA729271	stage_1W_vs_E10	ordinary_muscle_development	ENSGALG00010026221	CRKL	0.432510499	0.146197559	2.958397538	0.00309243	0.00440149	positive
PRJNA729271	stage_1W_vs_E10	ordinary_muscle_development	ENSGALG00010026229	CRHR2	2.499134821	0.209598669	11.92342887	8.93538E-33	5.58751E-32	positive
PRJNA729271	stage_1W_vs_E10	ordinary_muscle_development	ENSGALG00010026232	PSMC5	0.676841604	0.130872664	5.17175692	2.31903E-07	4.52353E-07	positive
PRJNA729271	stage_1W_vs_E10	ordinary_muscle_development	ENSGALG00010026234	ZDHHC5	0.775881989	0.159890618	4.852579852	1.21866E-06	2.26409E-06	positive
PRJNA729271	stage_1W_vs_E10	ordinary_muscle_development	ENSGALG00010026237	KLHL22	2.678563901	0.136916763	19.56344747	3.16909E-85	8.61838E-84	positive
PRJNA729271	stage_1W_vs_E10	ordinary_muscle_development	ENSGALG00010026239	GIP	-3.408738295	1.104893263	-3.085129043	0.002034639	0.002945968	negative
PRJNA729271	stage_1W_vs_E10	ordinary_muscle_development	ENSGALG00010026242	CCDC141	1.212487229	0.253595923	4.781177918	1.74271E-06	3.19835E-06	positive
PRJNA729271	stage_1W_vs_E10	ordinary_muscle_development	ENSGALG00010026244	CLP1	-0.645305463	0.130382733	-4.949316878	7.44744E-07	1.40561E-06	negative
PRJNA729271	stage_1W_vs_E10	ordinary_muscle_development	ENSGALG00010026249	YPEL4	-0.781748422	0.631896173	-1.237146948	0.216032533	0.247208618	negative
PRJNA729271	stage_1W_vs_E10	ordinary_muscle_development	ENSGALG00010026252	ENO2	0.478602766	0.334109879	1.432471162	0.152009025	0.178179753	positive
PRJNA729271	stage_1W_vs_E10	ordinary_muscle_development	ENSGALG00010026254	ENSGALG00010026254	-1.920046559	0.644872834	-2.977403384	0.002907013	0.004148582	negative
PRJNA729271	stage_1W_vs_E10	ordinary_muscle_development	ENSGALG00010026259	MED15	-0.199790043	0.089729276	-2.226587035	0.025974886	0.033517847	negative
PRJNA729271	stage_1W_vs_E10	ordinary_muscle_development	ENSGALG00010026260	ENSGALG00010026260	19.29113953	3.1686546	6.088116872	1.14246E-09	2.56268E-09	positive
PRJNA729271	stage_1W_vs_E10	ordinary_muscle_development	ENSGALG00010026261	IFT70A	-0.320761785	0.106922709	-2.999940671	0.002700322	0.003865031	negative
PRJNA729271	stage_1W_vs_E10	ordinary_muscle_development	ENSGALG00010026262	NAV1	-0.583734189	0.162476616	-3.592727385	0.000327235	0.00050703	negative
PRJNA729271	stage_1W_vs_E10	ordinary_muscle_development	ENSGALG00010026264	FZD7	-1.742291934	0.142036624	-12.26649779	1.37054E-34	9.16752E-34	negative
PRJNA729271	stage_1W_vs_E10	ordinary_muscle_development	ENSGALG00010026266	ENSGALG00010026266	-1.478264571	0.351047487	-4.211010264	2.54231E-05	4.27206E-05	negative
PRJNA729271	stage_1W_vs_E10	ordinary_muscle_development	ENSGALG00010026268	GARS1	0.659802766	0.150571742	4.381982672	1.17604E-05	2.0301E-05	positive
PRJNA729271	stage_1W_vs_E10	ordinary_muscle_development	ENSGALG00010026272	LRRC58	0.553154324	0.096517646	5.731121175	9.9769E-09	2.11611E-08	positive
PRJNA729271	stage_1W_vs_E10	ordinary_muscle_development	ENSGALG00010026277	SMARCD2	0.410920627	0.124264635	3.306818774	0.000943619	0.001410078	positive
PRJNA729271	stage_1W_vs_E10	ordinary_muscle_development	ENSGALG00010026287	NASP	-1.582265936	0.091187323	-17.35181922	1.91096E-67	3.37385E-66	negative
PRJNA729271	stage_1W_vs_E10	ordinary_muscle_development	ENSGALG00010026291	SERPING1	1.202468104	0.171215566	7.02312374	2.16962E-12	5.65013E-12	positive
PRJNA729271	stage_1W_vs_E10	ordinary_muscle_development	ENSGALG00010026295	SMPD4	-0.551152707	0.13291593	-4.146626431	3.3741E-05	5.63257E-05	negative
PRJNA729271	stage_1W_vs_E10	ordinary_muscle_development	ENSGALG00010026298	SNF8	0.195832067	0.115562133	1.694604129	0.090150543	0.109085243	positive
PRJNA729271	stage_1W_vs_E10	ordinary_muscle_development	ENSGALG00010026300	HMGA1	-1.178967317	0.109738394	-10.74343509	6.36318E-27	3.16413E-26	negative
PRJNA729271	stage_1W_vs_E10	ordinary_muscle_development	ENSGALG00010026305	ATP5MC1	1.71826842	0.166987417	10.28980774	7.83326E-25	3.57391E-24	positive
PRJNA729271	stage_1W_vs_E10	ordinary_muscle_development	ENSGALG00010026315	SYT2	-4.373672204	0.562065726	-7.78142485	7.17122E-15	2.11704E-14	negative
PRJNA729271	stage_1W_vs_E10	ordinary_muscle_development	ENSGALG00010026316	ENSGALG00010026316	1.104866391	0.348712428	3.168417022	0.001532715	0.002243885	positive
PRJNA729271	stage_1W_vs_E10	ordinary_muscle_development	ENSGALG00010026320	ENSGALG00010026320	0.56640695	0.174927864	3.237945837	0.001203937	0.001777818	positive
PRJNA729271	stage_1W_vs_E10	ordinary_muscle_development	ENSGALG00010026321	GPBP1L1	0.403843739	0.047493662	8.503108085	1.84581E-17	6.11263E-17	positive
PRJNA729271	stage_1W_vs_E10	ordinary_muscle_development	ENSGALG00010026325	TMEM69	0.868455657	0.137146193	6.332335136	2.41478E-10	5.60664E-10	positive
PRJNA729271	stage_1W_vs_E10	ordinary_muscle_development	ENSGALG00010026329	ENSGALG00010026329	2.517520344	0.270770369	9.297621266	1.43623E-20	5.53968E-20	positive
PRJNA729271	stage_1W_vs_E10	ordinary_muscle_development	ENSGALG00010026334	ENSGALG00010026334	0.97266468	0.127038011	7.656485439	1.9109E-14	5.51544E-14	positive
PRJNA729271	stage_1W_vs_E10	ordinary_muscle_development	ENSGALG00010026335	IPP	-1.142803055	0.106685569	-10.71188042	8.95246E-27	4.42887E-26	negative
PRJNA729271	stage_1W_vs_E10	ordinary_muscle_development	ENSGALG00010026344	TIMM10	0.857423825	0.171337952	5.004284302	5.607E-07	1.06518E-06	positive
PRJNA729271	stage_1W_vs_E10	ordinary_muscle_development	ENSGALG00010026346	FZD2	1.114894442	0.162873477	6.845156511	7.63923E-12	1.93273E-11	positive
PRJNA729271	stage_1W_vs_E10	ordinary_muscle_development	ENSGALG00010026354	ENSGALG00010026354	3.973144514	0.242059166	16.41393951	1.52014E-60	2.26226E-59	positive
PRJNA729271	stage_1W_vs_E10	ordinary_muscle_development	ENSGALG00010026355	PPP1R12B	1.273547858	0.13738898	9.26965075	1.86756E-20	7.15768E-20	positive
PRJNA729271	stage_1W_vs_E10	ordinary_muscle_development	ENSGALG00010026360	CTDSPL	1.257194928	0.126566504	9.933077756	2.98885E-23	1.281E-22	positive
PRJNA729271	stage_1W_vs_E10	ordinary_muscle_development	ENSGALG00010026362	ENSGALG00010026362	-0.383085912	0.394618762	-0.970774705	0.331660481	0.368271693	negative
PRJNA729271	stage_1W_vs_E10	ordinary_muscle_development	ENSGALG00010026367	RBP	2.492793246	0.544835758	4.575311386	4.75512E-06	8.44572E-06	positive
PRJNA729271	stage_1W_vs_E10	ordinary_muscle_development	ENSGALG00010026377	ENSGALG00010026377	-5.036810723	0.284624376	-17.69634348	4.47431E-70	8.4889E-69	negative
PRJNA729271	stage_1W_vs_E10	ordinary_muscle_development	ENSGALG00010026388	SNRPD3	-0.414028187	0.080293919	-5.156407777	2.51732E-07	4.89714E-07	negative
PRJNA729271	stage_1W_vs_E10	ordinary_muscle_development	ENSGALG00010026393	SLC43A3	0.483242726	0.120249008	4.0186837	5.85242E-05	9.58323E-05	positive
PRJNA729271	stage_1W_vs_E10	ordinary_muscle_development	ENSGALG00010026402	TFEB	2.67964236	0.164202449	16.31913759	7.21476E-60	1.05209E-58	positive
PRJNA729271	stage_1W_vs_E10	ordinary_muscle_development	ENSGALG00010026403	GUCD1	2.193637697	0.115762908	18.94940041	4.46643E-80	1.06482E-78	positive
PRJNA729271	stage_1W_vs_E10	ordinary_muscle_development	ENSGALG00010026409	VILL	-3.54756073	0.273726211	-12.96025219	2.05571E-38	1.55926E-37	negative
PRJNA729271	stage_1W_vs_E10	ordinary_muscle_development	ENSGALG00010026410	ENSGALG00010026410	0.640402443	0.319184138	2.006373017	0.044816465	0.05631195	positive
PRJNA729271	stage_1W_vs_E10	ordinary_muscle_development	ENSGALG00010026416	ENSGALG00010026416	4.123397816	1.413666825	2.916810201	0.003536309	0.005005742	positive
PRJNA729271	stage_1W_vs_E10	ordinary_muscle_development	ENSGALG00010026422	P2RX3	-3.099893002	0.390549092	-7.937268485	2.06682E-15	6.26225E-15	negative
PRJNA729271	stage_1W_vs_E10	ordinary_muscle_development	ENSGALG00010026427	PGC	7.535809921	1.052433357	7.160367797	8.04609E-13	2.13879E-12	positive
PRJNA729271	stage_1W_vs_E10	ordinary_muscle_development	ENSGALG00010026433	VPS11	0.100726893	0.071694488	1.404946125	0.160037299	0.186985382	positive
PRJNA729271	stage_1W_vs_E10	ordinary_muscle_development	ENSGALG00010026434	ENSGALG00010026434	0.355271239	0.143427732	2.477005216	0.013248997	0.017673864	positive
PRJNA729271	stage_1W_vs_E10	ordinary_muscle_development	ENSGALG00010026436	PLCD1	-0.993868985	0.077555652	-12.81491375	1.35289E-37	9.96448E-37	negative
PRJNA729271	stage_1W_vs_E10	ordinary_muscle_development	ENSGALG00010026438	TOMM6	1.384822388	0.120580494	11.48463026	1.57608E-30	9.08363E-30	positive
PRJNA729271	stage_1W_vs_E10	ordinary_muscle_development	ENSGALG00010026443	UPB1	0.508860363	0.364741912	1.395124458	0.162978312	0.190222291	positive
PRJNA729271	stage_1W_vs_E10	ordinary_muscle_development	ENSGALG00010026448	ADORA2A	-0.86863479	0.117252918	-7.408214692	1.28011E-13	3.53702E-13	negative
PRJNA729271	stage_1W_vs_E10	ordinary_muscle_development	ENSGALG00010026457	SSRP1	-1.185592276	0.099265362	-11.94366541	7.0066E-33	4.4061E-32	negative
PRJNA729271	stage_1W_vs_E10	ordinary_muscle_development	ENSGALG00010026458	DLEC1	1.06986955	0.18433728	5.803869688	6.48016E-09	1.39135E-08	positive
PRJNA729271	stage_1W_vs_E10	ordinary_muscle_development	ENSGALG00010026480	USP49	-0.003309609	0.213824655	-0.015478145	0.98765072	0.989680643	negative
PRJNA729271	stage_1W_vs_E10	ordinary_muscle_development	ENSGALG00010026481	DIXDC1	-1.735944429	0.139531599	-12.44122791	1.56086E-35	1.08097E-34	negative
PRJNA729271	stage_1W_vs_E10	ordinary_muscle_development	ENSGALG00010026484	TNKS1BP1	-0.57079502	0.112824315	-5.05914901	4.21132E-07	8.06701E-07	negative
PRJNA729271	stage_1W_vs_E10	ordinary_muscle_development	ENSGALG00010026485	ACAA1	0.212329647	0.105661572	2.00952571	0.044481411	0.055905493	positive
PRJNA729271	stage_1W_vs_E10	ordinary_muscle_development	ENSGALG00010026492	SPECC1L	-0.989872501	0.07586367	-13.04804403	6.51999E-39	5.05116E-38	negative
PRJNA729271	stage_1W_vs_E10	ordinary_muscle_development	ENSGALG00010026498	ENSGALG00010026498	-1.327040585	0.088924565	-14.92321705	2.32775E-50	2.58549E-49	negative
PRJNA729271	stage_1W_vs_E10	ordinary_muscle_development	ENSGALG00010026507	APLNR	-0.538947835	0.289656558	-1.860644338	0.062794419	0.077424728	negative
PRJNA729271	stage_1W_vs_E10	ordinary_muscle_development	ENSGALG00010026508	CSRP1	2.140672775	0.148502696	14.41504314	4.16179E-47	4.14321E-46	positive
PRJNA729271	stage_1W_vs_E10	ordinary_muscle_development	ENSGALG00010026516	ENSGALG00010026516	-0.004397437	0.167869109	-0.026195627	0.979101304	0.982354357	negative
PRJNA729271	stage_1W_vs_E10	ordinary_muscle_development	ENSGALG00010026522	MED20	0.404752736	0.128722942	3.144371391	0.001664441	0.002430838	positive
PRJNA729271	stage_1W_vs_E10	ordinary_muscle_development	ENSGALG00010026536	BCR	-0.673493746	0.098130603	-6.863238613	6.73166E-12	1.70945E-11	negative
PRJNA729271	stage_1W_vs_E10	ordinary_muscle_development	ENSGALG00010026537	FJX1	-3.125729022	0.184643409	-16.92846248	2.77535E-64	4.5174E-63	negative
PRJNA729271	stage_1W_vs_E10	ordinary_muscle_development	ENSGALG00010026539	ENSGALG00010026539	-0.258427854	0.389615825	-0.663288904	0.507145542	0.544725797	negative
PRJNA729271	stage_1W_vs_E10	ordinary_muscle_development	ENSGALG00010026550	MYD88	0.146507482	0.105885666	1.38363848	0.16646918	0.193984304	positive
PRJNA729271	stage_1W_vs_E10	ordinary_muscle_development	ENSGALG00010026553	PLPP3	-0.335141372	0.128071351	-2.616833275	0.008874968	0.012067792	negative
PRJNA729271	stage_1W_vs_E10	ordinary_muscle_development	ENSGALG00010026559	RAB36	-2.196658314	0.520897669	-4.217063054	2.47505E-05	4.16386E-05	negative
PRJNA729271	stage_1W_vs_E10	ordinary_muscle_development	ENSGALG00010026560	TNNI1	-5.82548833	0.592227271	-9.836575608	7.83317E-23	3.29107E-22	negative
PRJNA729271	stage_1W_vs_E10	ordinary_muscle_development	ENSGALG00010026565	RSPH14	-2.888894297	0.556592864	-5.190318603	2.09935E-07	4.10606E-07	negative
PRJNA729271	stage_1W_vs_E10	ordinary_muscle_development	ENSGALG00010026566	ENSGALG00010026566	-0.854397322	0.126449094	-6.756848137	1.41026E-11	3.51474E-11	negative
PRJNA729271	stage_1W_vs_E10	ordinary_muscle_development	ENSGALG00010026568	BYSL	0.682557048	0.110270329	6.189852308	6.02206E-10	1.3699E-09	positive
PRJNA729271	stage_1W_vs_E10	ordinary_muscle_development	ENSGALG00010026569	GNAZ	-1.085690984	0.207088599	-5.24264005	1.58295E-07	3.11878E-07	negative
PRJNA729271	stage_1W_vs_E10	ordinary_muscle_development	ENSGALG00010026581	DNER	-5.372025017	0.301794169	-17.80029426	7.02985E-71	1.35864E-69	negative
PRJNA729271	stage_1W_vs_E10	ordinary_muscle_development	ENSGALG00010026584	ENSGALG00010026584	-0.903867994	0.111716916	-8.090699497	5.9323E-16	1.84407E-15	negative
PRJNA729271	stage_1W_vs_E10	ordinary_muscle_development	ENSGALG00010026588	CCND3	1.295145218	0.142645199	9.079486925	1.09088E-19	4.04613E-19	positive
PRJNA729271	stage_1W_vs_E10	ordinary_muscle_development	ENSGALG00010026595	PRKAA2	2.563664998	0.26127098	9.812283763	9.96865E-23	4.16294E-22	positive
PRJNA729271	stage_1W_vs_E10	ordinary_muscle_development	ENSGALG00010026601	ENSGALG00010026601	0.412296457	0.496825409	0.829861858	0.406616892	0.4446691	positive
PRJNA729271	stage_1W_vs_E10	ordinary_muscle_development	ENSGALG00010026612	MFF	0.164236871	0.081654825	2.011355378	0.044287933	0.055686467	positive
PRJNA729271	stage_1W_vs_E10	ordinary_muscle_development	ENSGALG00010026625	ENSGALG00010026625	4.196652708	0.632616754	6.633799505	3.27154E-11	8.00354E-11	positive
PRJNA729271	stage_1W_vs_E10	ordinary_muscle_development	ENSGALG00010026628	FYB2	-1.769473338	0.798396648	-2.216283526	0.026672091	0.034362452	negative
PRJNA729271	stage_1W_vs_E10	ordinary_muscle_development	ENSGALG00010026630	XIRP1	0.155715424	0.243919876	0.638387599	0.523221401	0.560086289	positive
PRJNA729271	stage_1W_vs_E10	ordinary_muscle_development	ENSGALG00010026633	SLC5A1	-0.047213285	0.514657938	-0.091737213	0.92690683	0.935751115	negative
PRJNA729271	stage_1W_vs_E10	ordinary_muscle_development	ENSGALG00010026635	C8A	4.413031921	2.536422101	1.739864954	0.081882734	0.099546274	positive
PRJNA729271	stage_1W_vs_E10	ordinary_muscle_development	ENSGALG00010026638	LAD1	-3.639438289	0.507670157	-7.168903345	7.56009E-13	2.01255E-12	negative
PRJNA729271	stage_1W_vs_E10	ordinary_muscle_development	ENSGALG00010026643	YWHAH	-0.795811957	0.112134487	-7.096942045	1.27548E-12	3.36207E-12	negative
PRJNA729271	stage_1W_vs_E10	ordinary_muscle_development	ENSGALG00010026659	LIPH	-0.713839159	0.270143367	-2.642445624	0.008230968	0.011231091	negative
PRJNA729271	stage_1W_vs_E10	ordinary_muscle_development	ENSGALG00010026666	ENSGALG00010026666	-2.43448165	0.578700697	-4.206806145	2.59005E-05	4.34976E-05	negative
PRJNA729271	stage_1W_vs_E10	ordinary_muscle_development	ENSGALG00010026671	TAF8	0.396070417	0.155378721	2.549064732	0.010801225	0.014550535	positive
PRJNA729271	stage_1W_vs_E10	ordinary_muscle_development	ENSGALG00010026673	DEPDC5	0.324500576	0.121985363	2.66015995	0.007810355	0.010683316	positive
PRJNA729271	stage_1W_vs_E10	ordinary_muscle_development	ENSGALG00010026681	PLXDC1	2.064645902	0.258297525	7.993285655	1.31389E-15	4.02045E-15	positive
PRJNA729271	stage_1W_vs_E10	ordinary_muscle_development	ENSGALG00010026686	ENSGALG00010026686	0.785007101	0.401928731	1.95310024	0.050807719	0.063411218	positive
PRJNA729271	stage_1W_vs_E10	ordinary_muscle_development	ENSGALG00010026690	KRT14	-6.096377841	0.469939703	-12.97268096	1.74807E-38	1.32731E-37	negative
PRJNA729271	stage_1W_vs_E10	ordinary_muscle_development	ENSGALG00010026692	NMNAT3	4.505537276	0.499109782	9.027146802	1.76206E-19	6.46937E-19	positive
PRJNA729271	stage_1W_vs_E10	ordinary_muscle_development	ENSGALG00010026696	CSRNP1	-1.287305399	0.151598842	-8.4915253	2.03943E-17	6.73535E-17	negative
PRJNA729271	stage_1W_vs_E10	ordinary_muscle_development	ENSGALG00010026697	CHN1	-1.961499309	0.119249733	-16.44866832	8.57326E-61	1.28643E-59	negative
PRJNA729271	stage_1W_vs_E10	ordinary_muscle_development	ENSGALG00010026700	C8B	0.215756915	1.024068818	0.210685953	0.833132337	0.8513065	positive
PRJNA729271	stage_1W_vs_E10	ordinary_muscle_development	ENSGALG00010026704	WDFY1	-0.449328507	0.070547258	-6.369184531	1.90036E-10	4.44285E-10	negative
PRJNA729271	stage_1W_vs_E10	ordinary_muscle_development	ENSGALG00010026706	KRT24	-8.591642606	0.653625138	-13.14460248	1.82763E-39	1.4382E-38	negative
PRJNA729271	stage_1W_vs_E10	ordinary_muscle_development	ENSGALG00010026707	GORASP1	0.132342197	0.08148483	1.62413295	0.104347437	0.125135774	positive

PRJNA729271	stage_1W_vs_E10	ordinary_muscle_development	ENSGALG00010026715	WDR48	-0.636570651	0.069151098	-9.205503147	3.40074E-20	1.28628E-19	negative
PRJNA729271	stage_1W_vs_E10	ordinary_muscle_development	ENSGALG00010026719	ENSGALG00010026719	-5.238630434	1.081229553	-4.845067747	1.26568E-06	2.34605E-06	negative
PRJNA729271	stage_1W_vs_E10	ordinary_muscle_development	ENSGALG00010026724	OLA1	1.641548667	0.097529961	16.8312245	1.44091E-63	2.3104E-62	positive
PRJNA729271	stage_1W_vs_E10	ordinary_muscle_development	ENSGALG00010026731	ENSGALG00010026731	-2.908327634	0.554804133	-5.242079969	1.58777E-07	3.12742E-07	negative
PRJNA729271	stage_1W_vs_E10	ordinary_muscle_development	ENSGALG00010026737	SCN11A	-7.41334819	1.151887948	-6.435824075	1.22805E-10	2.90243E-10	negative
PRJNA729271	stage_1W_vs_E10	ordinary_muscle_development	ENSGALG00010026740	DAB1	-4.800352339	0.173272375	-27.70408351	6.2344E-169	6.5962E-167	negative
PRJNA729271	stage_1W_vs_E10	ordinary_muscle_development	ENSGALG00010026743	TNNT2	-5.927866111	0.590225743	-10.04338794	9.82408E-24	4.28787E-23	negative
PRJNA729271	stage_1W_vs_E10	ordinary_muscle_development	ENSGALG00010026746	HOXD10	-3.956579968	0.497260325	-7.956757796	1.76606E-15	5.37118E-15	negative
PRJNA729271	stage_1W_vs_E10	ordinary_muscle_development	ENSGALG00010026755	ENSGALG00010026755	-1.683143386	0.230535164	-7.30102668	2.8558E-13	7.75184E-13	negative
PRJNA729271	stage_1W_vs_E10	ordinary_muscle_development	ENSGALG00010026759	OMA1	-0.522951903	0.111663611	-4.683279543	2.82321E-06	5.10064E-06	negative
PRJNA729271	stage_1W_vs_E10	ordinary_muscle_development	ENSGALG00010026767	ENSGALG00010026767	21.54756802	2.505792354	8.599103588	8.03411E-18	2.70761E-17	positive
PRJNA729271	stage_1W_vs_E10	ordinary_muscle_development	ENSGALG00010026770	ENSGALG00010026770	-1.93863406	0.644033922	-3.010142777	0.002611249	0.003741973	negative
PRJNA729271	stage_1W_vs_E10	ordinary_muscle_development	ENSGALG00010026775	BTG2	3.217130265	0.365956242	8.791024434	1.48202E-18	5.18887E-18	positive
PRJNA729271	stage_1W_vs_E10	ordinary_muscle_development	ENSGALG00010026780	MYSM1	0.40544099	0.078784163	5.14622452	2.65781E-07	5.16171E-07	positive
PRJNA729271	stage_1W_vs_E10	ordinary_muscle_development	ENSGALG00010026782	HOXB9	-5.805459469	0.771204438	-7.527782757	5.16092E-14	1.45937E-13	negative
PRJNA729271	stage_1W_vs_E10	ordinary_muscle_development	ENSGALG00010026786	ENSGALG00010026786	2.973321011	0.571637915	5.201406225	1.97786E-07	3.87473E-07	positive
PRJNA729271	stage_1W_vs_E10	ordinary_muscle_development	ENSGALG00010026796	DCAKD	-0.06780972	0.22528471	-0.300995659	0.763417804	0.787317046	negative
PRJNA729271	stage_1W_vs_E10	ordinary_muscle_development	ENSGALG00010026801	FMOD	2.721806348	0.490245146	5.551929211	2.82534E-08	5.83131E-08	positive
PRJNA729271	stage_1W_vs_E10	ordinary_muscle_development	ENSGALG00010026822	PRELP	-0.787878495	0.201432901	-3.911369448	9.17743E-05	0.000148054	negative
PRJNA729271	stage_1W_vs_E10	ordinary_muscle_development	ENSGALG00010026825	ADAM23	-0.447330524	0.135820591	-3.29353982	0.000989343	0.001474302	negative
PRJNA729271	stage_1W_vs_E10	ordinary_muscle_development	ENSGALG00010026827	FAM117A	1.689568076	0.128221802	13.17691731	1.19161E-39	9.44364E-39	positive
PRJNA729271	stage_1W_vs_E10	ordinary_muscle_development	ENSGALG00010026834	ENSGALG00010026834	0.158935923	2.099079829	0.07571695	0.93964429	0.947158831	positive
PRJNA729271	stage_1W_vs_E10	ordinary_muscle_development	ENSGALG00010026836	ENSGALG00010026836	2.845357384	0.593479223	4.794367311	1.63189E-06	2.99876E-06	positive
PRJNA729271	stage_1W_vs_E10	ordinary_muscle_development	ENSGALG00010026839	FGGY	-0.231818765	0.119898183	-1.933463537	0.053179121	0.066182824	negative
PRJNA729271	stage_1W_vs_E10	ordinary_muscle_development	ENSGALG00010026846	MICAL1	-0.284812995	0.22851024	-1.246390511	0.212621063	0.24359329	negative
PRJNA729271	stage_1W_vs_E10	ordinary_muscle_development	ENSGALG00010026851	ENSGALG00010026851	1.13501618	1.37856128	0.82333386	0.410318163	0.448297413	positive
PRJNA729271	stage_1W_vs_E10	ordinary_muscle_development	ENSGALG00010026854	SMPD2	-0.451305909	0.155684016	-2.898858348	0.003745241	0.005287034	negative
PRJNA729271	stage_1W_vs_E10	ordinary_muscle_development	ENSGALG00010026856	UBE2Z	-0.117710115	0.077976054	-1.509567474	0.131153826	0.15506319	negative
PRJNA729271	stage_1W_vs_E10	ordinary_muscle_development	ENSGALG00010026874	TEAD3	0.953341717	0.160199785	5.950955032	2.66582E-09	5.85915E-09	positive
PRJNA729271	stage_1W_vs_E10	ordinary_muscle_development	ENSGALG00010026885	TULP1	4.485200976	0.625290859	7.172983436	7.33807E-13	1.95489E-12	positive
PRJNA729271	stage_1W_vs_E10	ordinary_muscle_development	ENSGALG00010026886	JUN	1.719602767	0.192774382	8.920286749	4.65082E-19	1.67571E-18	positive
PRJNA729271	stage_1W_vs_E10	ordinary_muscle_development	ENSGALG00010026889	ENSGALG00010026889	-0.45622597	0.288147504	-1.583307036	0.1133515	0.135306761	negative
PRJNA729271	stage_1W_vs_E10	ordinary_muscle_development	ENSGALG00010026893	FKBP5	3.580741926	0.386086699	9.274450361	1.78537E-20	6.85535E-20	positive
PRJNA729271	stage_1W_vs_E10	ordinary_muscle_development	ENSGALG00010026894	IP6K1	-0.331292824	0.067315689	-4.921480104	8.58921E-07	1.61479E-06	negative
PRJNA729271	stage_1W_vs_E10	ordinary_muscle_development	ENSGALG00010026897	ENSGALG00010026897	-0.458964614	0.227239864	-2.019736356	0.043410742	0.0546309	negative
PRJNA729271	stage_1W_vs_E10	ordinary_muscle_development	ENSGALG00010026898	PNPLA2	4.10800807	0.271536851	15.12873136	1.04694E-51	1.21989E-50	positive
PRJNA729271	stage_1W_vs_E10	ordinary_muscle_development	ENSGALG00010026899	HOOK1	-0.18910732	0.167466545	-1.129224468	0.258803155	0.292343495	negative
PRJNA729271	stage_1W_vs_E10	ordinary_muscle_development	ENSGALG00010026901	FAM212A	-0.71120797	0.176077844	-4.03916787	5.36412E-05	8.80254E-05	negative
PRJNA729271	stage_1W_vs_E10	ordinary_muscle_development	ENSGALG00010026907	ENSGALG00010026907	-0.865956814	0.135833729	-6.375123616	1.82815E-10	4.28026E-10	negative
PRJNA729271	stage_1W_vs_E10	ordinary_muscle_development	ENSGALG00010026909	ENSGALG00010026909	-0.250113526	0.158238464	-1.580611431	0.11396691	0.135962993	negative
PRJNA729271	stage_1W_vs_E10	ordinary_muscle_development	ENSGALG00010026916	ENSGALG00010026916	0.960513358	0.214033008	4.487687985	7.20003E-06	1.26105E-05	positive
PRJNA729271	stage_1W_vs_E10	ordinary_muscle_development	ENSGALG00010026917	AMT	-1.523617873	0.14750485	-10.32927308	5.19528E-25	2.39141E-24	negative
PRJNA729271	stage_1W_vs_E10	ordinary_muscle_development	ENSGALG00010026919	NICN1	-0.50593486	0.173067124	-2.923344703	0.003462929	0.004907622	negative
PRJNA729271	stage_1W_vs_E10	ordinary_muscle_development	ENSGALG00010026920	GATA2	1.686622324	0.202849671	8.314641673	9.20299E-17	2.95781E-16	positive
PRJNA729271	stage_1W_vs_E10	ordinary_muscle_development	ENSGALG00010026924	TPH1	-0.140799583	0.355134582	-0.396468242	0.691759631	0.720489751	negative
PRJNA729271	stage_1W_vs_E10	ordinary_muscle_development	ENSGALG00010026942	CDK18	-1.503934593	0.188893464	-7.961813823	1.69535E-15	5.16155E-15	negative
PRJNA729271	stage_1W_vs_E10	ordinary_muscle_development	ENSGALG00010026943	EEFSEC	-0.202476847	0.071213773	-2.84322595	0.00446594	0.006260522	negative
PRJNA729271	stage_1W_vs_E10	ordinary_muscle_development	ENSGALG00010026950	LHFPL5	0.425023476	0.2732577	1.555394323	0.119852185	0.142550047	positive
PRJNA729271	stage_1W_vs_E10	ordinary_muscle_development	ENSGALG00010026951	RUVBL1	-0.833948765	0.106445941	-7.834481624	4.70781E-15	1.40353E-14	negative
PRJNA729271	stage_1W_vs_E10	ordinary_muscle_development	ENSGALG00010026954	MFSD4A	1.332945187	0.175292194	7.604133162	2.8682E-14	8.21144E-14	positive
PRJNA729271	stage_1W_vs_E10	ordinary_muscle_development	ENSGALG00010026958	CLTCL1	0.102055914	0.084215563	1.211841504	0.225573058	0.257111069	positive
PRJNA729271	stage_1W_vs_E10	ordinary_muscle_development	ENSGALG00010026960	SEC61A1	-2.114445694	0.102972068	-20.53416758	1.06626E-93	3.45759E-92	negative
PRJNA729271	stage_1W_vs_E10	ordinary_muscle_development	ENSGALG00010026964	SRPK1	-0.371073736	0.060029414	-6.181531915	6.34825E-10	1.44251E-09	negative
PRJNA729271	stage_1W_vs_E10	ordinary_muscle_development	ENSGALG00010026972	TNNT3	6.291084658	0.158348509	39.72935842	0	0	positive
PRJNA729271	stage_1W_vs_E10	ordinary_muscle_development	ENSGALG00010026973	MGLL	1.841497765	0.124234189	14.82279376	1.04352E-49	1.13133E-48	positive
PRJNA729271	stage_1W_vs_E10	ordinary_muscle_development	ENSGALG00010026977	ELK4	-0.005602367	0.099144131	-0.056507295	0.954937684	0.960770579	negative
PRJNA729271	stage_1W_vs_E10	ordinary_muscle_development	ENSGALG00010026980	LIMK2	-1.026604281	0.118942033	-8.631131114	6.07482E-18	2.05831E-17	negative
PRJNA729271	stage_1W_vs_E10	ordinary_muscle_development	ENSGALG00010026982	ABTB1	1.853211417	0.084637946	21.89575138	2.8516E-106	1.2016E-104	positive
PRJNA729271	stage_1W_vs_E10	ordinary_muscle_development	ENSGALG00010026984	PGR2/3	-1.826543616	1.20114717	-1.520665961	0.128343685	0.151939049	negative
PRJNA729271	stage_1W_vs_E10	ordinary_muscle_development	ENSGALG00010026999	MLEC	-0.827373123	0.083110844	-9.955056211	2.39687E-23	1.03002E-22	negative
PRJNA729271	stage_1W_vs_E10	ordinary_muscle_development	ENSGALG00010027001	SLC26A8	-0.403778277	0.265281951	-1.522072176	0.127990997	0.151558744	negative
PRJNA729271	stage_1W_vs_E10	ordinary_muscle_development	ENSGALG00010027003	SEPTIN2L	-0.702654111	0.082551144	-8.511742857	1.71338E-17	5.68448E-17	negative
PRJNA729271	stage_1W_vs_E10	ordinary_muscle_development	ENSGALG00010027012	RILPL1	2.205662521	0.192002384	11.48768301	1.52138E-30	8.77881E-30	positive
PRJNA729271	stage_1W_vs_E10	ordinary_muscle_development	ENSGALG00010027015	ENSGALG00010027015	1.136459469	0.548559999	2.071714072	0.038292114	0.048492416	positive
PRJNA729271	stage_1W_vs_E10	ordinary_muscle_development	ENSGALG00010027025	ORAI1	2.390242506	0.126944093	18.82909599	4.36142E-79	1.00828E-77	positive
PRJNA729271	stage_1W_vs_E10	ordinary_muscle_development	ENSGALG00010027026	SLC45A3	-0.125387645	0.225944152	-0.554949725	0.578929049	0.614497405	negative
PRJNA729271	stage_1W_vs_E10	ordinary_muscle_development	ENSGALG00010027027	ARHGEF3	0.036649283	0.103173046	0.355221483	0.722423672	0.749358174	positive
PRJNA729271	stage_1W_vs_E10	ordinary_muscle_development	ENSGALG00010027036	NUCKS1	-1.625547497	0.066173886	-24.56478821	3.0061E-133	1.9897E-131	negative
PRJNA729271	stage_1W_vs_E10	ordinary_muscle_development	ENSGALG00010027040	CLIP1	1.428296327	0.107925313	13.23411797	5.57473E-40	4.46194E-39	positive
PRJNA729271	stage_1W_vs_E10	ordinary_muscle_development	ENSGALG00010027041	MAPK14	0.522450478	0.073888312	7.07081351	1.54028E-12	4.03719E-12	positive
PRJNA729271	stage_1W_vs_E10	ordinary_muscle_development	ENSGALG00010027043	IL17RD	-2.871868771	0.177409578	-16.18778873	6.14992E-59	8.73952E-58	negative
PRJNA729271	stage_1W_vs_E10	ordinary_muscle_development	ENSGALG00010027045	MAPK13	-0.021834067	0.201999889	-0.108089498	0.913924698	0.924447907	negative
PRJNA729271	stage_1W_vs_E10	ordinary_muscle_development	ENSGALG00010027046	HESX1	-0.689821476	0.322589715	-2.13838645	0.032485391	0.041446681	negative
PRJNA729271	stage_1W_vs_E10	ordinary_muscle_development	ENSGALG00010027050	GSTT1	2.012770307	0.152072399	13.23560571	5.46543E-40	4.3793E-39	positive
PRJNA729271	stage_1W_vs_E10	ordinary_muscle_development	ENSGALG00010027052	APPL1	0.212578585	0.084899678	2.503879742	0.012283978	0.016434951	positive
PRJNA729271	stage_1W_vs_E10	ordinary_muscle_development	ENSGALG00010027059	ASB14	7.399945563	0.210896852	35.08798495	1.028E-269	2.9801E-267	positive
PRJNA729271	stage_1W_vs_E10	ordinary_muscle_development	ENSGALG00010027063	RAB7L1	-0.032411753	0.089949562	-0.360332525	0.718598479	0.745657166	negative
PRJNA729271	stage_1W_vs_E10	ordinary_muscle_development	ENSGALG00010027073	DNAH12	-1.672403348	0.322644895	-5.183417972	2.17856E-07	4.25755E-07	negative
PRJNA729271	stage_1W_vs_E10	ordinary_muscle_development	ENSGALG00010027074	BRPF3	0.449406515	0.079594866	5.646174632	1.64057E-08	3.43593E-08	positive
PRJNA729271	stage_1W_vs_E10	ordinary_muscle_development	ENSGALG00010027075	SLC41A1	2.090966051	0.093903998	22.26706106	7.7095E-110	3.4597E-108	positive
PRJNA729271	stage_1W_vs_E10	ordinary_muscle_development	ENSGALG00010027076	VDHAP	1.942909125	0.171469543	11.33092844	9.2222E-30	5.15923E-29	positive
PRJNA729271	stage_1W_vs_E10	ordinary_muscle_development	ENSGALG00010027077	ARF4	-0.645764305	0.104713678	-6.166952726	6.96186E-10	1.57749E-09	negative
PRJNA729271	stage_1W_vs_E10	ordinary_muscle_development	ENSGALG00010027078	PDE12	-0.393158929	0.121010401	-3.248968079	0.001158245	0.001713313	negative
PRJNA729271	stage_1W_vs_E10	ordinary_muscle_development	ENSGALG00010027079	PM20D1	0.818385187	0.094412625	8.668175322	4.391E-18	1.49759E-17	positive
PRJNA729271	stage_1W_vs_E10	ordinary_muscle_development	ENSGALG00010027080	TBC1D22B	0.811953435	0.155704945	5.214692676	1.84122E-07	3.6129E-07	positive
PRJNA729271	stage_1W_vs_E10	ordinary_muscle_development	ENSGALG00010027081	PDE4B	1.696353346	0.2907385	5.834636099	5.39081E-09	1.16262E-08	positive
PRJNA729271	stage_1W_vs_E10	ordinary_muscle_development	ENSGALG00010027082	SLC26A9	-3.320782049	0.658092411	-5.046072551	4.50984E-07	8.62631E-07	negative
PRJNA729271	stage_1W_vs_E10	ordinary_muscle_development	ENSGALG00010027083	DENND6A	-0.816245044	0.08863098	-9.209477761	3.27715E-20	1.24118E-19	negative
PRJNA729271	stage_1W_vs_E10	ordinary_muscle_development	ENSGALG00010027084	OSBPL9	0.151122756	0.094385731	1.601118676	0.109350637	0.130757093	positive
PRJNA729271	stage_1W_vs_E10	ordinary_muscle_development	ENSGALG00010027085	PIM1	0.254110527	0.151826387	1.673691463	0.094191252	0.113708853	positive
PRJNA729271	stage_1W_vs_E10	ordinary_muscle_development	ENSGALG00010027086	CACNA1S	5.773446585	0.201095739	28.70993991	2.8672E-181	3.522E-179	positive
PRJNA729271	stage_1W_vs_E10	ordinary_muscle_development	ENSGALG00010027087	RAB7B	-0.316604557	0.352513506	-0.898134546	0.369113824	0.406867291	negative
PRJNA729271	stage_1W_vs_E10	ordinary_muscle_development	ENSGALG00010027088	AGBL4	-0.31904136	0.27125527	-1.176166495	0.23952835	0.271905665	negative
PRJNA729271	stage_1W_vs_E10	ordinary_muscle_development	ENSGALG00010027092	ENSGALG00010027092	-1.554741645	0.200508132	-7.754007929	8.90368E-15	2.61729E-14	negative
PRJNA729271	stage_1W_vs_E10	ordinary_muscle_development	ENSGALG00010027093	SLMAP	0.60919708	0.076263881	7.988015712	1.37128E-15	4.18986E-15	positive
PRJNA729271	stage_1W_vs_E10	ordinary_muscle_development	ENSGALG00010027095	SCN5A	-4.074851065	0.432467003	-9.422339824	4.41146E-21	1.7329E-20	negative
PRJNA729271	stage_1W_vs_E10	ordinary_muscle_development	ENSGALG00010027096	YIPF1	1.65194964	0.109052255	15.14823911	7.78239E-52	9.1193E-51	positive
PRJNA729271	stage_1W_vs_E10	ordinary_muscle_development	ENSGALG00010027097	ENSGALG00010027097	-1.034593744	0.150388638	-6.87946747	6.00767E-12	1.52828E-11	negative
PRJNA729271	stage_1W_vs_E10	ordinary_muscle_development	ENSGALG00010027098	EXOG	0.300983827	0.146026216	2.061162957	0.039287496	0.049705181	positive
PRJNA729271	stage_1W_vs_E10	ordinary_muscle_development	ENSGALG00010027099	GNA11	-0.739057053	0.086300749	-8.563738586	1.09265E-17	3.65605E-17	negative
PRJNA729271	stage_1W_vs_E10	ordinary_muscle_development	ENSGALG00010027100	MIER2	-0.451501307	0.091553967	-4.931531868	8.15873E-07	1.53645E-06	negative
PRJNA729271	stage_1W_vs_E10	ordinary_muscle_development	ENSGALG00010027101	RNF220	-0.270192548	0.080286867	-3.365339287	0.000764496	0.001150833	negative
PRJNA729271	stage_1W_vs_E10	ordinary_muscle_development	ENSGALG00010027102	DUS3L	-0.656383836	0.143957556	-4.559565015	5.12597E-06	9.07639E-06	negative
PRJNA729271	stage_1W_vs_E10	ordinary_muscle_development	ENSGALG00010027103	ACVR2B	-1.507669269	0.187205	-8.053573731	8.04109E-16	2.48414E-15	negative
PRJNA729271	stage_1W_vs_E10	ordinary_muscle_development	ENSGALG00010027105	CHERP	-0.285477838	0.128582922	-2.220184715	0.026406231	0.03403809	negative
PRJNA729271	stage_1W_vs_E10	ordinary_muscle_development	ENSGALG00010027106	CYP2J23	0.387420441	0.147548299	2.625719473	0.008646605	0.011771629	positive
PRJNA729271	stage_1W_vs_E10	ordinary_muscle_development	ENSGALG00010027107	KIF21B	-1.782457101	0.209700159	-8.500027429	1.89546E-17	6.26989E-17	negative
PRJNA729271	stage_1W_vs_E10	ordinary_muscle_development	ENSGALG00010027108	ENSGALG00010027108	-0.276388923	0.145726054	-1.896633555	0.057876319	0.071720718	negative
PRJNA729271	stage_1W_vs_E10	ordinary_muscle_development	ENSGALG00010027109	C19orf44	0.693861905	0.122197114	5.678218422	1.36105E-08	2.86209E-08	positive
PRJNA729271	stage_1W_vs_E10	ordinary_muscle_development	ENSGALG00010027110	IKBKE	-0.656342117	0.226034057	-2.903731083	0.003687447	0.005210523	negative
PRJNA729271	stage_1W_vs_E10	ordinary_muscle_development	ENSGALG00010027111	CALR3	-1.557974354	0.707966868	-2.20063173	0.027762105	0.035700117	negative
PRJNA729271	stage_1W_vs_E10	ordinary_muscle_development	ENSGALG00010027112	XYLB	-1.615288916	0.192327236	-8.398648833	4.51645E-17	1.46686E-16	negative
PRJNA729271	stage_1W_vs_E10	ordinary_muscle_development	ENSGALG00010027113	PIAS4	-0.012821545	0.132947331	-0.096440786	0.9231705	0.932368757	negative
PRJNA729271	stage_1W_vs_E10	ordinary_muscle_development	ENSGALG00010027114	FLNB	-1.003673538	0.113783848	-8.820878826	1.13565E-18	3.99835E-18	negative
PRJNA729271	stage_1W_vs_E10	ordinary_muscle_development	ENSGALG00010027115	C9ORF152	-1.614018596	0.683952591	-2.359839874	0.018282825	0.023974086	negative
PRJNA729271	stage_1W_vs_E10	ordinary_muscle_development	ENSGALG00010027116	CCDC94	0.524887089	0.101637081	5.164326695	2.41306E-07	4.70125E-07	positive
PRJNA729271	stage_1W_vs_E10	ordinary_muscle_development	ENSGALG00010027117	RASSF5	1.950602879	0.234839856	8.306098081	9.89005E-17	3.173E-16	positive
PRJNA729271	stage_1W_vs_E10	ordinary_muscle_development	ENSGALG00010027118	ABHD6	1.074803323	0.160368103	6.70210164	2.05443E-11	5.08174E-11	positive
PRJNA729271	stage_1W_vs_E10	ordinary_muscle_development	ENSGALG00010027119	NUB1	1.634653686	0.097596177	16.74915702	5.74344E-63	9.02941E-62	positive
PRJNA729271	stage_1W_vs_E10	ordinary_muscle_development	ENSGALG00010027121	ELF3	-0.087573621	0.885521188	-0.098895004	0.921221636	0.930659856	negative
PRJNA729271	stage_1W_vs_E10	ordinary_muscle_development	ENSGALG00010027122	DNASE1L3	4.638787547	0.24792504	18.71044389	4.06971E-78	9.20288E-77	positive
PRJNA729271	stage_1W_vs_E10	ordinary_muscle_development	ENSGALG00010027123	WDR86	-2.945100909	0.2317853	-12.70615913	5.46536E-37	3.94524E-36	negative
PRJNA729271	stage_1W_vs_E10	ordinary_muscle_development	ENSGALG00010027124	IP6K2	0.514014725	0.17384625	2.956720227	0.003109301	0.004423331	positive
PRJNA729271	stage_1W_vs_E10	ordinary_muscle_development	ENSGALG00010027125	CRYGN	1.668432831	0.400933466	4.161370835	3.16343E-05	5.28819E-05	positive
PRJNA729271	stage_1W_vs_E10	ordinary_muscle_development	ENSGALG00010027126	ENSGALG00010027126	6.992396385	0.536355942	13.03685825	7.55048E-39	5.8246E-38	positive
PRJNA729271	stage_1W_vs_E10	ordinary_muscle_development	ENSGALG00010027127	S1PR4	1.450167673	0.646720481	2.242340727	0.024939359	0.032262027	positive
PRJNA729271	stage_1W_vs_E10	ordinary_muscle_development	ENSGALG00010027128	EIF2D	0.53749288	0.089958381	5.974906101	2.30223E-09	5.07927E-09	positive
PRJNA729271	stage_1W_vs_E10	ordinary_muscle_development	ENSGALG00010027129	NCLN	1.020078234	0.1284716	7.940106886	2.02007E-15	6.12188E-15	positive
PRJNA729271	stage_1W_vs_E10	ordinary_muscle_development	ENSGALG00010027130	NCKIPSD	0.037108805	0.145570302	0.254920161	0.798784764	0.819998949	positive
PRJNA729271	stage_1W_vs_E10	ordinary_muscle_development	ENSGALG00010027131	UBA52	0.834953921	0.152202285	5.485817264	4.11562E-08	8.43193E-08	positive
PRJNA729271	stage_1W_vs_E10	ordinary_muscle_development	ENSGALG00010027133	CELF5	-3.759646644	0.370926248	-10.13583337	3.83093E-24	1.694E-23	negative

PRJNA729271	stage_1W_vs_E10	ordinary_muscle_development	ENSGALG00010027134	DYRK3	0.015794971	0.148843628	0.106117888	0.915488818	0.925771622	positive
PRJNA729271	stage_1W_vs_E10	ordinary_muscle_development	ENSGALG00010027135	PRKAG2	0.198923534	0.139218608	1.428857372	0.153045232	0.179278377	positive
PRJNA729271	stage_1W_vs_E10	ordinary_muscle_development	ENSGALG00010027136	MAPKAPK2	1.876645118	0.143855316	13.04536508	6.75327E-39	5.2263E-38	positive
PRJNA729271	stage_1W_vs_E10	ordinary_muscle_development	ENSGALG00010027137	FKBP8	1.320834283	0.089010131	14.83914546	8.17912E-50	8.92744E-49	positive
PRJNA729271	stage_1W_vs_E10	ordinary_muscle_development	ENSGALG00010027139	HSD11B1L	0.354009095	0.139486291	2.53794902	0.011150423	0.014987516	positive
PRJNA729271	stage_1W_vs_E10	ordinary_muscle_development	ENSGALG00010027141	PIGR	7.006012893	1.394404362	5.024376775	5.0507E-07	9.62149E-07	positive
PRJNA729271	stage_1W_vs_E10	ordinary_muscle_development	ENSGALG00010027143	GALNT11	-0.987375966	0.121540595	-8.123836855	4.51673E-16	1.4119E-15	negative
PRJNA729271	stage_1W_vs_E10	ordinary_muscle_development	ENSGALG00010027144	ENSGALG00010027144	-4.076063158	0.965538781	-4.221542665	2.42636E-05	4.08432E-05	negative
PRJNA729271	stage_1W_vs_E10	ordinary_muscle_development	ENSGALG00010027145	CELSR3	-1.463433022	0.176030658	-8.313512208	9.29105E-17	2.98479E-16	negative
PRJNA729271	stage_1W_vs_E10	ordinary_muscle_development	ENSGALG00010027146	KXD1	1.620787769	0.121138552	13.37961985	7.95532E-41	6.5444E-40	positive
PRJNA729271	stage_1W_vs_E10	ordinary_muscle_development	ENSGALG00010027147	KMT2C	0.825924549	0.103513229	7.978927493	1.4761E-15	4.50255E-15	positive
PRJNA729271	stage_1W_vs_E10	ordinary_muscle_development	ENSGALG00010027148	ELL	1.744475923	0.165056928	10.56893489	4.15183E-26	2.00136E-25	positive
PRJNA729271	stage_1W_vs_E10	ordinary_muscle_development	ENSGALG00010027149	C1orf116	1.098864077	0.232591524	4.724437323	2.30753E-06	4.19249E-06	positive
PRJNA729271	stage_1W_vs_E10	ordinary_muscle_development	ENSGALG00010027150	YOD1	-0.613900242	0.090863738	-6.756273268	1.41586E-11	3.5281E-11	negative
PRJNA729271	stage_1W_vs_E10	ordinary_muscle_development	ENSGALG00010027151	MICOS13	1.717862739	0.143114358	12.00342689	3.40884E-33	2.17096E-32	positive
PRJNA729271	stage_1W_vs_E10	ordinary_muscle_development	ENSGALG00010027152	C4BPS	2.539370315	0.436272033	5.8206122	5.86325E-09	1.26244E-08	positive
PRJNA729271	stage_1W_vs_E10	ordinary_muscle_development	ENSGALG00010027153	ENSGALG00010027153	3.178282849	0.22798783	13.9405812	3.59005E-44	3.28107E-43	positive
PRJNA729271	stage_1W_vs_E10	ordinary_muscle_development	ENSGALG00010027154	PLPP2	0.371652792	0.13716514	2.709528022	0.006737901	0.009277656	positive
PRJNA729271	stage_1W_vs_E10	ordinary_muscle_development	ENSGALG00010027155	ENSGALG00010027155	0.78392731	0.294585191	2.661122599	0.007788059	0.01065483	positive
PRJNA729271	stage_1W_vs_E10	ordinary_muscle_development	ENSGALG00010027156	ISYNA1	-1.419633969	0.149313107	-9.507765211	1.94802E-21	7.77012E-21	negative
PRJNA729271	stage_1W_vs_E10	ordinary_muscle_development	ENSGALG00010027157	ENSGALG00010027157	0.642130043	0.254279984	2.525287411	0.01156037	0.015509771	positive
PRJNA729271	stage_1W_vs_E10	ordinary_muscle_development	ENSGALG00010027158	XRCC2	-0.326659432	0.135680816	-2.407557986	0.016059612	0.021193014	negative
PRJNA729271	stage_1W_vs_E10	ordinary_muscle_development	ENSGALG00010027159	TMUB1	1.449192147	0.163115915	8.884431318	6.42486E-19	2.29663E-18	positive
PRJNA729271	stage_1W_vs_E10	ordinary_muscle_development	ENSGALG00010027160	FASTK	2.067440444	0.16576596	12.47204458	1.06069E-35	7.41269E-35	positive
PRJNA729271	stage_1W_vs_E10	ordinary_muscle_development	ENSGALG00010027161	C4BPG	0.735844029	0.203626198	3.613700184	0.000301858	0.000469115	positive
PRJNA729271	stage_1W_vs_E10	ordinary_muscle_development	ENSGALG00010027162	SLC4A2	0.807225651	0.129501961	6.233308307	4.56686E-10	1.0456E-09	positive
PRJNA729271	stage_1W_vs_E10	ordinary_muscle_development	ENSGALG00010027163	ENSGALG00010027163	-0.59852975	0.079296768	-7.547971592	4.42089E-14	1.25427E-13	negative
PRJNA729271	stage_1W_vs_E10	ordinary_muscle_development	ENSGALG00010027164	ENSGALG00010027164	1.135239465	0.464699022	2.442956433	0.014567496	0.019329536	positive
PRJNA729271	stage_1W_vs_E10	ordinary_muscle_development	ENSGALG00010027165	ENSGALG00010027165	-3.382186552	0.230081018	-14.69998081	6.4478E-49	6.78728E-48	negative
PRJNA729271	stage_1W_vs_E10	ordinary_muscle_development	ENSGALG00010027166	CDK5	0.586994838	0.098174536	5.979094612	2.24381E-09	4.95491E-09	positive
PRJNA729271	stage_1W_vs_E10	ordinary_muscle_development	ENSGALG00010027167	ENSGALG00010027167	-2.112146024	0.482902688	-4.373854357	1.22072E-05	2.10496E-05	negative
PRJNA729271	stage_1W_vs_E10	ordinary_muscle_development	ENSGALG00010027168	SSBP4	-0.95840471	0.183030026	-5.236325044	1.63805E-07	3.22297E-07	negative
PRJNA729271	stage_1W_vs_E10	ordinary_muscle_development	ENSGALG00010027169	ABCB8	0.824041593	0.111069722	7.419137987	1.17885E-13	3.26225E-13	positive
PRJNA729271	stage_1W_vs_E10	ordinary_muscle_development	ENSGALG00010027170	TLE2	-0.731462931	0.177546948	-4.119828239	3.79155E-05	6.29753E-05	negative
PRJNA729271	stage_1W_vs_E10	ordinary_muscle_development	ENSGALG00010027171	ATG9B	6.060066445	0.273397099	22.16580377	7.3447E-109	3.2657E-107	positive
PRJNA729271	stage_1W_vs_E10	ordinary_muscle_development	ENSGALG00010027172	ENSGALG00010027172	-1.159006208	0.505660746	-2.292062848	0.021902017	0.028493065	negative
PRJNA729271	stage_1W_vs_E10	ordinary_muscle_development	ENSGALG00010027173	ENSGALG00010027173	-0.878439343	0.09781692	-8.980443683	2.69677E-19	9.80183E-19	negative
PRJNA729271	stage_1W_vs_E10	ordinary_muscle_development	ENSGALG00010027174	SHISA5	-0.610246167	0.133226059	-4.580531536	4.63796E-06	8.24469E-06	negative
PRJNA729271	stage_1W_vs_E10	ordinary_muscle_development	ENSGALG00010027175	NOS3	1.063538094	0.163972675	6.486069068	8.81048E-11	2.09881E-10	positive
PRJNA729271	stage_1W_vs_E10	ordinary_muscle_development	ENSGALG00010027176	ENSGALG00010027176	0.617511057	0.170532368	3.621078302	0.000293378	0.000456376	positive
PRJNA729271	stage_1W_vs_E10	ordinary_muscle_development	ENSGALG00010027178	C4BPM	0.894324494	0.080893702	11.05555153	2.06066E-28	1.08933E-27	positive
PRJNA729271	stage_1W_vs_E10	ordinary_muscle_development	ENSGALG00010027183	CD34	-0.029660269	0.157901865	-0.187839892	0.851002163	0.867581682	negative
PRJNA729271	stage_1W_vs_E10	ordinary_muscle_development	ENSGALG00010027184	MPND	1.300574281	0.31567012	4.120042402	3.78803E-05	6.29312E-05	positive
PRJNA729271	stage_1W_vs_E10	ordinary_muscle_development	ENSGALG00010027185	ATRIP	-1.277893216	0.085369348	-14.96899346	1.1708E-50	1.31454E-49	negative
PRJNA729271	stage_1W_vs_E10	ordinary_muscle_development	ENSGALG00010027186	PLXNA2	-0.082115892	0.124537436	-0.659367135	0.509660041	0.547264412	negative
PRJNA729271	stage_1W_vs_E10	ordinary_muscle_development	ENSGALG00010027187	ENSGALG00010027187	-0.168383443	0.13540832	-1.243523606	0.213674948	0.244684655	negative
PRJNA729271	stage_1W_vs_E10	ordinary_muscle_development	ENSGALG00010027188	KCNH2	2.226492314	0.253274438	8.790829151	1.4846E-18	5.19664E-18	positive
PRJNA729271	stage_1W_vs_E10	ordinary_muscle_development	ENSGALG00010027189	ENSGALG00010027189	-1.764044936	0.32203136	-5.477866921	4.30484E-08	8.80963E-08	negative
PRJNA729271	stage_1W_vs_E10	ordinary_muscle_development	ENSGALG00010027190	IPPK	0.231224755	0.114440709	2.02047642	0.043333994	0.054543786	positive
PRJNA729271	stage_1W_vs_E10	ordinary_muscle_development	ENSGALG00010027192	SH3GL1	-1.653553931	0.117921269	-14.02252489	1.13503E-44	1.05194E-43	negative
PRJNA729271	stage_1W_vs_E10	ordinary_muscle_development	ENSGALG00010027193	CAMK1G	-4.848676471	0.915202355	-5.297928315	1.17124E-07	2.32914E-07	negative
PRJNA729271	stage_1W_vs_E10	ordinary_muscle_development	ENSGALG00010027194	ENSGALG00010027194	-1.788421717	0.266784669	-6.703615036	2.03325E-11	5.03279E-11	negative
PRJNA729271	stage_1W_vs_E10	ordinary_muscle_development	ENSGALG00010027195	CENPP	-2.565806651	0.213675576	-12.00795478	3.22728E-33	2.05895E-32	negative
PRJNA729271	stage_1W_vs_E10	ordinary_muscle_development	ENSGALG00010027196	ENSGALG00010027196	0.331558339	0.333297202	0.994782846	0.319841891	0.3560759	positive
PRJNA729271	stage_1W_vs_E10	ordinary_muscle_development	ENSGALG00010027197	ENSGALG00010027197	-2.286558514	0.587673787	-3.890863546	9.98881E-05	0.000160572	negative
PRJNA729271	stage_1W_vs_E10	ordinary_muscle_development	ENSGALG00010027198	ECM2	0.222897642	0.203421352	1.095743583	0.273191004	0.307398199	positive
PRJNA729271	stage_1W_vs_E10	ordinary_muscle_development	ENSGALG00010027199	LAMB3	-2.185576829	0.376939997	-5.798208852	6.70269E-09	1.43728E-08	negative
PRJNA729271	stage_1W_vs_E10	ordinary_muscle_development	ENSGALG00010027200	G0S2	4.005636165	0.317815084	12.60366913	2.01546E-36	1.43417E-35	positive
PRJNA729271	stage_1W_vs_E10	ordinary_muscle_development	ENSGALG00010027201	ASPN	0.598697712	0.316120528	1.893890649	0.058239526	0.072115319	positive
PRJNA729271	stage_1W_vs_E10	ordinary_muscle_development	ENSGALG00010027202	CHAF1A	-1.980462997	0.089427563	-22.14600212	1.14E-108	5.0378E-107	negative
PRJNA729271	stage_1W_vs_E10	ordinary_muscle_development	ENSGALG00010027203	ENSGALG00010027203	-1.043913437	0.308416083	-3.384756805	0.000712413	0.001074774	negative
PRJNA729271	stage_1W_vs_E10	ordinary_muscle_development	ENSGALG00010027204	OMD	-3.405503657	0.843002435	-4.039731697	5.35124E-05	8.7824E-05	negative
PRJNA729271	stage_1W_vs_E10	ordinary_muscle_development	ENSGALG00010027205	OGN	0.358954899	0.22192743	1.617442686	0.105782758	0.126783618	positive
PRJNA729271	stage_1W_vs_E10	ordinary_muscle_development	ENSGALG00010027206	ENSGALG00010027206	6.629532995	0.970987088	6.8276222	8.63336E-12	2.17939E-11	positive
PRJNA729271	stage_1W_vs_E10	ordinary_muscle_development	ENSGALG00010027207	NOL8	-0.369238589	0.12431567	-2.970169311	0.002976357	0.004243365	negative
PRJNA729271	stage_1W_vs_E10	ordinary_muscle_development	ENSGALG00010027208	UBXN6	1.124552117	0.093318392	12.05070181	1.92306E-33	1.23339E-32	positive
PRJNA729271	stage_1W_vs_E10	ordinary_muscle_development	ENSGALG00010027209	TRAF3IP3	1.298258787	0.247577612	5.243845653	1.57264E-07	3.10099E-07	positive
PRJNA729271	stage_1W_vs_E10	ordinary_muscle_development	ENSGALG00010027210	ENSGALG00010027210	-0.968669908	0.269043499	-3.600421171	0.000317702	0.000493051	negative
PRJNA729271	stage_1W_vs_E10	ordinary_muscle_development	ENSGALG00010027211	HDGFRP2	-0.331349821	0.101619136	-3.260702981	0.001111364	0.001646486	negative
PRJNA729271	stage_1W_vs_E10	ordinary_muscle_development	ENSGALG00010027212	IRF6	-4.485212097	0.590402243	-7.596875092	3.03367E-14	8.66942E-14	negative
PRJNA729271	stage_1W_vs_E10	ordinary_muscle_development	ENSGALG00010027213	KCNC4	0.617298488	0.341939585	1.805285245	0.071030057	0.086994565	positive
PRJNA729271	stage_1W_vs_E10	ordinary_muscle_development	ENSGALG00010027214	PLIN4	1.349099823	0.266173631	5.068495386	4.00973E-07	7.69509E-07	positive
PRJNA729271	stage_1W_vs_E10	ordinary_muscle_development	ENSGALG00010027215	ENSGALG00010027215	-2.517833559	0.206451767	-12.19574717	3.27486E-34	2.1528E-33	negative
PRJNA729271	stage_1W_vs_E10	ordinary_muscle_development	ENSGALG00010027216	ENSGALG00010027216	1.339542162	0.58928361	2.273170572	0.023015903	0.029893863	positive
PRJNA729271	stage_1W_vs_E10	ordinary_muscle_development	ENSGALG00010027217	SEMA6B	0.994315241	0.2202629	4.514220232	6.35502E-06	1.11629E-05	positive
PRJNA729271	stage_1W_vs_E10	ordinary_muscle_development	ENSGALG00010027218	IARS1	0.563741172	0.164295625	3.431261006	0.000600782	0.000911391	positive
PRJNA729271	stage_1W_vs_E10	ordinary_muscle_development	ENSGALG00010027219	ENSGALG00010027219	0.787572718	0.137412479	5.731449739	9.95759E-09	2.11233E-08	positive
PRJNA729271	stage_1W_vs_E10	ordinary_muscle_development	ENSGALG00010027220	ANKRD24	-1.553755161	0.636931024	-2.439440226	0.014710036	0.019502604	negative
PRJNA729271	stage_1W_vs_E10	ordinary_muscle_development	ENSGALG00010027221	LRRC61	-1.302482589	0.281454943	-4.627677073	3.6979E-06	6.62314E-06	negative
PRJNA729271	stage_1W_vs_E10	ordinary_muscle_development	ENSGALG00010027222	GUCA1B	0.532115268	0.983430826	0.541080526	0.588452081	0.623509716	positive
PRJNA729271	stage_1W_vs_E10	ordinary_muscle_development	ENSGALG00010027223	SIRT6	-0.389486811	0.0838601	-4.644483004	3.40929E-06	6.12666E-06	negative
PRJNA729271	stage_1W_vs_E10	ordinary_muscle_development	ENSGALG00010027224	RARRES2	0.67721468	0.121776107	5.561145748	2.68009E-08	5.54021E-08	positive
PRJNA729271	stage_1W_vs_E10	ordinary_muscle_development	ENSGALG00010027225	CREB3L3	0.194509747	0.715917043	0.271693137	0.785857979	0.80827442	positive
PRJNA729271	stage_1W_vs_E10	ordinary_muscle_development	ENSGALG00010027227	ENSGALG00010027227	1.561663452	0.182931022	8.536897893	1.37873E-17	4.59103E-17	positive
PRJNA729271	stage_1W_vs_E10	ordinary_muscle_development	ENSGALG00010027229	MDM4	0.227768656	0.081313418	2.801120188	0.005092555	0.007106632	positive
PRJNA729271	stage_1W_vs_E10	ordinary_muscle_development	ENSGALG00010027230	MAP2K2	-0.214866964	0.081632091	-2.632138418	0.008484929	0.011560207	negative
PRJNA729271	stage_1W_vs_E10	ordinary_muscle_development	ENSGALG00010027231	ENSGALG00010027231	-1.143050072	0.159492741	-7.166784307	7.67799E-13	2.04319E-12	negative
PRJNA729271	stage_1W_vs_E10	ordinary_muscle_development	ENSGALG00010027232	ARRDC2	2.64213608	0.327098484	8.07749411	6.61112E-16	2.05112E-15	positive
PRJNA729271	stage_1W_vs_E10	ordinary_muscle_development	ENSGALG00010027233	ENSGALG00010027233	1.169033922	0.263434729	4.437660623	9.09419E-06	1.58305E-05	positive
PRJNA729271	stage_1W_vs_E10	ordinary_muscle_development	ENSGALG00010027234	SLC6A11	-3.537784743	0.47953536	-7.377526327	1.61257E-13	4.43198E-13	negative
PRJNA729271	stage_1W_vs_E10	ordinary_muscle_development	ENSGALG00010027236	PEX11G	0.422425708	0.281169978	1.502385532	0.132997571	0.157191763	positive
PRJNA729271	stage_1W_vs_E10	ordinary_muscle_development	ENSGALG00010027237	CTC-575C13.4	-0.2530449	0.170548849	-1.483709215	0.13788608	0.162624795	negative
PRJNA729271	stage_1W_vs_E10	ordinary_muscle_development	ENSGALG00010027239	ENSGALG00010027239	-0.463028253	0.085129346	-5.439114391	5.35461E-08	1.09056E-07	negative
PRJNA729271	stage_1W_vs_E10	ordinary_muscle_development	ENSGALG00010027240	SLC6A1	0.221261309	0.328285053	0.67399142	0.500316769	0.538108745	positive
PRJNA729271	stage_1W_vs_E10	ordinary_muscle_development	ENSGALG00010027241	LRRN2	-1.853949547	0.272320041	-6.807980567	9.89782E-12	2.48992E-11	negative
PRJNA729271	stage_1W_vs_E10	ordinary_muscle_development	ENSGALG00010027242	ENSGALG00010027242	0.45405476	0.189777708	2.392561088	0.016731243	0.022043207	positive
PRJNA729271	stage_1W_vs_E10	ordinary_muscle_development	ENSGALG00010027243	HRH1	0.381817115	0.199302076	1.915770892	0.055394264	0.068774292	positive
PRJNA729271	stage_1W_vs_E10	ordinary_muscle_development	ENSGALG00010027244	ARHGEF18	0.170215642	0.090604163	1.878673511	0.060289087	0.074506805	positive
PRJNA729271	stage_1W_vs_E10	ordinary_muscle_development	ENSGALG00010027245	LRIG2	-0.042636636	0.066473072	-0.641412153	0.521254939	0.558267352	negative
PRJNA729271	stage_1W_vs_E10	ordinary_muscle_development	ENSGALG00010027246	ZNF767	-0.762040586	0.100725565	-7.565513158	3.86336E-14	1.09889E-13	negative
PRJNA729271	stage_1W_vs_E10	ordinary_muscle_development	ENSGALG00010027247	ATG7	-0.174502826	0.080863811	-2.157984186	0.030929061	0.039562014	negative
PRJNA729271	stage_1W_vs_E10	ordinary_muscle_development	ENSGALG00010027248	ENSGALG00010027248	-1.63430297	0.72664079	-2.249120875	0.024504807	0.031731032	negative
PRJNA729271	stage_1W_vs_E10	ordinary_muscle_development	ENSGALG00010027249	C2CD4C	2.609012099	1.23998627	2.104065312	0.03537275	0.044960366	positive
PRJNA729271	stage_1W_vs_E10	ordinary_muscle_development	ENSGALG00010027250	ENSGALG00010027250	-0.182866347	0.1726624	-1.059097677	0.289555298	0.324426377	negative
PRJNA729271	stage_1W_vs_E10	ordinary_muscle_development	ENSGALG00010027251	ENSGALG00010027251	0.861332356	0.196618624	4.380726192	1.18284E-05	2.0416E-05	positive
PRJNA729271	stage_1W_vs_E10	ordinary_muscle_development	ENSGALG00010027252	MAGI3	-0.757347638	0.073176083	-10.34966087	4.19971E-25	1.94488E-24	negative
PRJNA729271	stage_1W_vs_E10	ordinary_muscle_development	ENSGALG00010027253	VGLL4	-1.255667333	0.088790519	-14.14190779	2.09526E-45	1.98502E-44	negative
PRJNA729271	stage_1W_vs_E10	ordinary_muscle_development	ENSGALG00010027254	INSR	1.185505321	0.132930773	8.918215811	4.7386E-19	1.70649E-18	positive
PRJNA729271	stage_1W_vs_E10	ordinary_muscle_development	ENSGALG00010027255	ENSGALG00010027255	0.098868493	0.179448671	0.550956952	0.581663185	0.617083207	positive
PRJNA729271	stage_1W_vs_E10	ordinary_muscle_development	ENSGALG00010027256	PHTF1	-1.91647113	0.094454877	-20.28980608	1.58218E-91	4.93198E-90	negative
PRJNA729271	stage_1W_vs_E10	ordinary_muscle_development	ENSGALG00010027257	TAFA3	1.987981095	0.266503406	7.459496024	8.68541E-14	2.42339E-13	positive
PRJNA729271	stage_1W_vs_E10	ordinary_muscle_development	ENSGALG00010027258	ENSGALG00010027258	-1.218302712	0.174568212	-6.978949353	2.97396E-12	7.68542E-12	negative
PRJNA729271	stage_1W_vs_E10	ordinary_muscle_development	ENSGALG00010027259	ENSGALG00010027259	-0.45581492	0.085970657	-5.301982529	1.14552E-07	2.27862E-07	negative
PRJNA729271	stage_1W_vs_E10	ordinary_muscle_development	ENSGALG00010027260	LEMD2	0.28089858	0.100722963	2.788823651	0.005289986	0.007371501	positive
PRJNA729271	stage_1W_vs_E10	ordinary_muscle_development	ENSGALG00010027261	ENSGALG00010027261	0.207724733	0.109778565	1.892215783	0.058462236	0.072372544	positive
PRJNA729271	stage_1W_vs_E10	ordinary_muscle_development	ENSGALG00010027262	USE1	-0.234226544	0.135271784	-1.731525503	0.08335808	0.101254955	negative
PRJNA729271	stage_1W_vs_E10	ordinary_muscle_development	ENSGALG00010027263	TAMM41	-1.204169812	0.128075262	-9.402048397	5.3511E-21	2.09294E-20	negative
PRJNA729271	stage_1W_vs_E10	ordinary_muscle_development	ENSGALG00010027264	ENSGALG00010027264	1.171465019	0.093636555	12.51076591	6.51926E-36	4.58944E-35	positive
PRJNA729271	stage_1W_vs_E10	ordinary_muscle_development	ENSGALG00010027265	IP6K3	4.974040871	0.207641185	23.95498213	8.1991E-127	5.1672E-125	positive
PRJNA729271	stage_1W_vs_E10	ordinary_muscle_development	ENSGALG00010027267	MYO9B	-0.716200292	0.0961225	-7.450912071	9.26971E-14	2.58145E-13	negative
PRJNA729271	stage_1W_vs_E10	ordinary_muscle_development	ENSGALG00010027268	UQCRC1	1.570053773	0.126564404	12.40517657	2.44972E-35	1.68368E-34	positive
PRJNA729271	stage_1W_vs_E10	ordinary_muscle_development	ENSGALG00010027269	CDC25A	-1.008233087	0.099447358	-10.13835966	3.73315E-24	1.65278E-23	negative
PRJNA729271	stage_1W_vs_E10	ordinary_muscle_development	ENSGALG00010027270	ENSGALG00010027270	-4.43783879	0.489272377	-9.070282721	1.18709E-19	4.39182E-19	negative
PRJNA729271	stage_1W_vs_E10	ordinary_muscle_development	ENSGALG00010027271	INKA2	-1.009192237	0.147721765	-6.831709833	8.39084E-12	2.11965E-11	negative
PRJNA729271	stage_1W_vs_E10	ordinary_muscle_development	ENSGALG00010027272	DDX20	0.133042473	0.101971052	1.304708256	0.191992209	0.221570627	positive
PRJNA729271	stage_1W_vs_E10	ordinary_muscle_development	ENSGALG00010027273	HAUS8	-1.975261533	0.137433163	-14.3725247	7.69732E-47	7.6055E-46	negative
PRJNA729271	stage_1W_vs_E10	ordinary_muscle_development	ENSGALG00010027274	COPG1	-0.583821598	0.060876386	-9.590280151	8.78462E-22	3.53801E-21	negative
PRJNA729271	stage_1W_vs_E10	ordinary_muscle_development	ENSGALG00010027275	CPAMD8	-0.42050484	0.227571046	-1.847795871	0.064631878	0.079554949	negative
PRJNA729271	stage_1W_vs_E10	ordinary_muscle_development	ENSGALG00010027276	ENSGALG00010027276	-0.153746999	0.138733611	-1.108217387	0.267767944	0.301600462	negative
PRJNA729271	stage_1W_vs_E10	ordinary_muscle_development	ENSGALG00010027277	KCND3	2.49854758	0.382397247	6.533905773	6.40763E-11	1.53722E-10	positive
PRJNA729271	stage_1W_vs_E10	ordinary_muscle_development	ENSGALG00010027278	H1-10	-0.695356909	0.12931845	-5.377089721	7.56995E-08	1.52716E-07	negative

PRJNA729271	stage_1W_vs_E10	ordinary_muscle_development	ENSGALG00010027279	F2RL3	0.020627722	0.381787352	0.054029348	0.956911782	0.962355948	positive
PRJNA729271	stage_1W_vs_E10	ordinary_muscle_development	ENSGALG00010027280	HMCES	-0.609118132	0.126292509	-4.823074111	1.41362E-06	2.61225E-06	negative
PRJNA729271	stage_1W_vs_E10	ordinary_muscle_development	ENSGALG00010027281	ENSGALG00010027281	-1.214893017	0.278357674	-4.364503411	1.27412E-05	2.19443E-05	negative
PRJNA729271	stage_1W_vs_E10	ordinary_muscle_development	ENSGALG00010027282	ENSGALG00010027282	2.321043828	0.128392584	18.07770947	4.7751E-73	9.68042E-72	positive
PRJNA729271	stage_1W_vs_E10	ordinary_muscle_development	ENSGALG00010027283	RAB7A	0.578715124	0.062686844	9.23184339	2.66014E-20	1.01151E-19	positive
PRJNA729271	stage_1W_vs_E10	ordinary_muscle_development	ENSGALG00010027284	CTTNBP2NL	1.313214901	0.089781817	14.62673568	1.89671E-48	1.96659E-47	positive
PRJNA729271	stage_1W_vs_E10	ordinary_muscle_development	ENSGALG00010027285	PTPRS	-1.309085775	0.112331043	-11.65382018	2.19398E-31	1.30818E-30	negative
PRJNA729271	stage_1W_vs_E10	ordinary_muscle_development	ENSGALG00010027286	RPN1	-0.20565703	0.062966704	-3.266123465	0.001090307	0.001618433	negative
PRJNA729271	stage_1W_vs_E10	ordinary_muscle_development	ENSGALG00010027287	DHX30	-0.314351176	0.089528889	-3.511170312	0.000446138	0.000682798	negative
PRJNA729271	stage_1W_vs_E10	ordinary_muscle_development	ENSGALG00010027288	DAG1	1.78866899	0.167717317	10.66478419	1.48744E-26	7.30862E-26	positive
PRJNA729271	stage_1W_vs_E10	ordinary_muscle_development	ENSGALG00010027289	SMARCC1	-0.961198789	0.139577621	-6.886482112	5.71889E-12	1.45686E-11	negative
PRJNA729271	stage_1W_vs_E10	ordinary_muscle_development	ENSGALG00010027290	CSPG5	-4.681285598	0.403269945	-11.60831761	3.739E-31	2.21211E-30	negative
PRJNA729271	stage_1W_vs_E10	ordinary_muscle_development	ENSGALG00010027291	MST1	1.400990674	0.274996716	5.094572378	3.49529E-07	6.73548E-07	positive
PRJNA729271	stage_1W_vs_E10	ordinary_muscle_development	ENSGALG00010027292	KDM4B	0.151862194	0.060703303	2.501712203	0.012359435	0.016531329	positive
PRJNA729271	stage_1W_vs_E10	ordinary_muscle_development	ENSGALG00010027293	ELP6	-0.066110495	0.150208794	-0.440123999	0.659847301	0.690277614	negative
PRJNA729271	stage_1W_vs_E10	ordinary_muscle_development	ENSGALG00010027295	ENSGALG00010027295	3.4115479	0.231716855	14.72291647	4.59412E-49	4.85363E-48	positive
PRJNA729271	stage_1W_vs_E10	ordinary_muscle_development	ENSGALG00010027297	ENSGALG00010027297	1.582614983	2.089282393	0.757492136	0.448755075	0.486915549	positive
PRJNA729271	stage_1W_vs_E10	ordinary_muscle_development	ENSGALG00010027299	PGPEP1	-0.237459362	0.163860445	-1.449156094	0.147293996	0.172946656	negative
PRJNA729271	stage_1W_vs_E10	ordinary_muscle_development	ENSGALG00010027301	SCAP	-0.459407923	0.06514408	-7.052182209	1.76133E-12	4.6001E-12	negative
PRJNA729271	stage_1W_vs_E10	ordinary_muscle_development	ENSGALG00010027303	APEH	0.828806307	0.138477695	5.985124949	2.16224E-09	4.77842E-09	positive
PRJNA729271	stage_1W_vs_E10	ordinary_muscle_development	ENSGALG00010027304	PTPN23	1.055756218	0.136949355	7.70909961	1.26708E-14	3.69545E-14	positive
PRJNA729271	stage_1W_vs_E10	ordinary_muscle_development	ENSGALG00010027305	LSM4	-1.061877997	0.130915979	-8.111141254	5.01465E-16	1.5625E-15	negative
PRJNA729271	stage_1W_vs_E10	ordinary_muscle_development	ENSGALG00010027306	ENSGALG00010027306	3.582923722	0.315462172	11.35769685	6.79115E-30	3.82877E-29	positive
PRJNA729271	stage_1W_vs_E10	ordinary_muscle_development	ENSGALG00010027307	JUND	0.910899793	0.176120077	5.172038364	2.31554E-07	4.51794E-07	positive
PRJNA729271	stage_1W_vs_E10	ordinary_muscle_development	ENSGALG00010027309	ENSGALG00010027309	3.557337697	0.662220931	5.371829143	7.79419E-08	1.57065E-07	positive
PRJNA729271	stage_1W_vs_E10	ordinary_muscle_development	ENSGALG00010027310	ARHGEF2	-0.14304099	0.133427245	-1.072052335	0.283696526	0.318231013	negative
PRJNA729271	stage_1W_vs_E10	ordinary_muscle_development	ENSGALG00010027311	NFASC	2.003750184	0.240362052	8.336383266	7.65974E-17	2.47003E-16	positive
PRJNA729271	stage_1W_vs_E10	ordinary_muscle_development	ENSGALG00010027312	ENSGALG00010027312	1.694522807	0.676012404	2.506644547	0.012188321	0.016314499	positive
PRJNA729271	stage_1W_vs_E10	ordinary_muscle_development	ENSGALG00010027313	BSN	-1.155562861	0.249631732	-4.629070389	3.67311E-06	6.58199E-06	negative
PRJNA729271	stage_1W_vs_E10	ordinary_muscle_development	ENSGALG00010027314	ENSGALG00010027314	4.344162843	0.348182518	12.47668283	1.00069E-35	7.00052E-35	positive
PRJNA729271	stage_1W_vs_E10	ordinary_muscle_development	ENSGALG00010027315	RAB3A	1.086588254	0.222775751	4.877497875	1.0744E-06	2.00508E-06	positive
PRJNA729271	stage_1W_vs_E10	ordinary_muscle_development	ENSGALG00010027316	TRAIP	-3.422611845	0.223591307	-15.30744593	6.81895E-53	8.30594E-52	negative
PRJNA729271	stage_1W_vs_E10	ordinary_muscle_development	ENSGALG00010027317	MPV17L2	1.086009285	0.145459155	7.466077206	8.26209E-14	2.30838E-13	positive
PRJNA729271	stage_1W_vs_E10	ordinary_muscle_development	ENSGALG00010027319	MST1R	2.022233839	0.323632054	6.248558552	4.14258E-10	9.50407E-10	positive
PRJNA729271	stage_1W_vs_E10	ordinary_muscle_development	ENSGALG00010027320	MON1A	1.278898908	0.160355192	7.975413141	1.51872E-15	4.63157E-15	positive
PRJNA729271	stage_1W_vs_E10	ordinary_muscle_development	ENSGALG00010027321	IFI30	2.329751475	0.203594122	11.44311759	2.5457E-30	1.45218E-29	positive
PRJNA729271	stage_1W_vs_E10	ordinary_muscle_development	ENSGALG00010027322	CRABP2	-6.866190495	0.226839493	-30.26893776	2.9397E-201	4.3929E-199	negative
PRJNA729271	stage_1W_vs_E10	ordinary_muscle_development	ENSGALG00010027323	ISG20L2	-0.303252882	0.130494268	-2.323878943	0.020131983	0.026298945	negative
PRJNA729271	stage_1W_vs_E10	ordinary_muscle_development	ENSGALG00010027324	PIK3R2	-0.921986323	0.09799566	-9.408440325	5.03553E-21	1.9727E-20	negative
PRJNA729271	stage_1W_vs_E10	ordinary_muscle_development	ENSGALG00010027325	METTL25B	0.82851778	0.287453889	2.882263253	0.003948297	0.005562306	positive
PRJNA729271	stage_1W_vs_E10	ordinary_muscle_development	ENSGALG00010027326	MRPL24	0.561872496	0.117746626	4.771877677	1.82516E-06	3.34417E-06	positive
PRJNA729271	stage_1W_vs_E10	ordinary_muscle_development	ENSGALG00010027327	RBM6	-0.419088358	0.089361227	-4.689823239	2.73441E-06	4.94637E-06	negative
PRJNA729271	stage_1W_vs_E10	ordinary_muscle_development	ENSGALG00010027328	RBM15	-0.03090639	0.131216935	-0.235536591	0.813792282	0.833398271	negative
PRJNA729271	stage_1W_vs_E10	ordinary_muscle_development	ENSGALG00010027329	MINDY4	-1.507039271	0.123676886	-12.18529446	3.72306E-34	2.44521E-33	negative
PRJNA729271	stage_1W_vs_E10	ordinary_muscle_development	ENSGALG00010027330	ENSGALG00010027330	-0.124144501	0.20077051	-0.618340317	0.536351031	0.572700419	negative
PRJNA729271	stage_1W_vs_E10	ordinary_muscle_development	ENSGALG00010027331	SLC16A4	0.880564905	0.163290669	5.392622302	6.94368E-08	1.40296E-07	positive
PRJNA729271	stage_1W_vs_E10	ordinary_muscle_development	ENSGALG00010027332	HDGF	1.275289862	0.17647586	7.226426656	4.95868E-13	1.33079E-12	positive
PRJNA729271	stage_1W_vs_E10	ordinary_muscle_development	ENSGALG00010027333	PROK1	-0.043712749	0.293749663	-0.148809529	0.881703932	0.894899322	negative
PRJNA729271	stage_1W_vs_E10	ordinary_muscle_development	ENSGALG00010027334	PRCC	-0.138724486	0.114649576	-1.209986901	0.226283919	0.257840218	negative
PRJNA729271	stage_1W_vs_E10	ordinary_muscle_development	ENSGALG00010027335	MAST3	-0.142382749	0.178314851	-0.798490694	0.424585791	0.46256077	negative
PRJNA729271	stage_1W_vs_E10	ordinary_muscle_development	ENSGALG00010027336	RAD54L2	-0.165342455	0.138618444	-1.19278828	0.232952301	0.264793257	negative
PRJNA729271	stage_1W_vs_E10	ordinary_muscle_development	ENSGALG00010027337	AQP1	-1.026404012	0.265965176	-3.859166928	0.000113774	0.000182227	negative
PRJNA729271	stage_1W_vs_E10	ordinary_muscle_development	ENSGALG00010027339	LAMTOR5	0.771230709	0.107554007	7.170636698	7.46498E-13	1.9876E-12	positive
PRJNA729271	stage_1W_vs_E10	ordinary_muscle_development	ENSGALG00010027340	ENSGALG00010027340	3.18814361	0.74024196	4.306893936	1.65563E-05	2.823E-05	positive
PRJNA729271	stage_1W_vs_E10	ordinary_muscle_development	ENSGALG00010027341	ENSGALG00010027341	1.286661868	0.649776537	1.980160553	0.047685491	0.059684068	positive
PRJNA729271	stage_1W_vs_E10	ordinary_muscle_development	ENSGALG00010027346	SIN3B	0.514951915	0.088643215	5.809264871	6.27478E-09	1.34885E-08	positive
PRJNA729271	stage_1W_vs_E10	ordinary_muscle_development	ENSGALG00010027348	ENSGALG00010027348	0.774301926	0.206230249	3.754550711	0.000173653	0.000274492	positive
PRJNA729271	stage_1W_vs_E10	ordinary_muscle_development	ENSGALG00010027350	INSRR	2.058847057	0.336862887	6.111825133	9.84981E-10	2.21663E-09	positive
PRJNA729271	stage_1W_vs_E10	ordinary_muscle_development	ENSGALG00010027351	TMEM38A	4.497656746	0.147821804	30.42620663	2.4734E-203	3.8496E-201	positive
PRJNA729271	stage_1W_vs_E10	ordinary_muscle_development	ENSGALG00010027354	ENSGALG00010027354	3.394457527	0.833605113	4.072021004	4.6607E-05	7.68127E-05	positive
PRJNA729271	stage_1W_vs_E10	ordinary_muscle_development	ENSGALG00010027355	ADCYAP1R1	-2.008484583	0.277571435	-7.235919594	4.62385E-13	1.243E-12	negative
PRJNA729271	stage_1W_vs_E10	ordinary_muscle_development	ENSGALG00010027356	ENSGALG00010027356	0.199827433	0.227787893	0.877252211	0.380349663	0.418164567	positive
PRJNA729271	stage_1W_vs_E10	ordinary_muscle_development	ENSGALG00010027358	RNF126	0.375392532	0.099690889	3.765565084	0.000166173	0.000262985	positive
PRJNA729271	stage_1W_vs_E10	ordinary_muscle_development	ENSGALG00010027359	ENSGALG00010027359	1.628909754	0.116402777	13.99373615	1.70229E-44	1.56764E-43	positive
PRJNA729271	stage_1W_vs_E10	ordinary_muscle_development	ENSGALG00010027360	SMIM7	0.821271287	0.113494352	7.236230463	4.61327E-13	1.24039E-12	positive
PRJNA729271	stage_1W_vs_E10	ordinary_muscle_development	ENSGALG00010027361	FANCD2	0.762613634	0.192203502	3.967740583	7.25573E-05	0.000118064	positive
PRJNA729271	stage_1W_vs_E10	ordinary_muscle_development	ENSGALG00010027362	ENSGALG00010027362	0.536694478	0.144396695	3.716805823	0.000201757	0.000317257	positive
PRJNA729271	stage_1W_vs_E10	ordinary_muscle_development	ENSGALG00010027363	VIPR1	-0.691204958	0.261706801	-2.64114251	0.008262696	0.0112712	negative
PRJNA729271	stage_1W_vs_E10	ordinary_muscle_development	ENSGALG00010027364	FSTL3	1.368625407	0.236101786	5.796760079	6.76083E-09	1.44903E-08	positive
PRJNA729271	stage_1W_vs_E10	ordinary_muscle_development	ENSGALG00010027365	PLEKHA6	1.229937167	0.170847223	7.199046868	6.06349E-13	1.62099E-12	positive
PRJNA729271	stage_1W_vs_E10	ordinary_muscle_development	ENSGALG00010027366	USP4	0.912354565	0.082158337	11.10483248	1.1884E-28	6.33769E-28	positive
PRJNA729271	stage_1W_vs_E10	ordinary_muscle_development	ENSGALG00010027367	SEC22C	-0.549696135	0.09576954	-5.739780494	9.47993E-09	2.01365E-08	negative
PRJNA729271	stage_1W_vs_E10	ordinary_muscle_development	ENSGALG00010027368	EMC3	1.066147798	0.088383014	12.06281334	1.66013E-33	1.06807E-32	positive
PRJNA729271	stage_1W_vs_E10	ordinary_muscle_development	ENSGALG00010027369	ENSGALG00010027369	0.960858069	0.101443569	9.471848016	2.74936E-21	1.09004E-20	positive
PRJNA729271	stage_1W_vs_E10	ordinary_muscle_development	ENSGALG00010027370	NUP210L	-1.510072463	0.371683784	-4.062788121	4.849E-05	7.98164E-05	negative
PRJNA729271	stage_1W_vs_E10	ordinary_muscle_development	ENSGALG00010027372	PIK3C2B	-0.000767187	0.172860911	-0.004438177	0.996458859	0.997009123	negative
PRJNA729271	stage_1W_vs_E10	ordinary_muscle_development	ENSGALG00010027373	MLN	5.58567186	1.06620212	5.238848952	1.61581E-07	3.18136E-07	positive
PRJNA729271	stage_1W_vs_E10	ordinary_muscle_development	ENSGALG00010027374	NKTR	0.440038947	0.118307124	3.719462786	0.000199647	0.000314109	positive
PRJNA729271	stage_1W_vs_E10	ordinary_muscle_development	ENSGALG00010027375	FUT7	1.214384771	0.431595249	2.813712096	0.004897307	0.006844048	positive
PRJNA729271	stage_1W_vs_E10	ordinary_muscle_development	ENSGALG00010027376	DSTYK	0.295181893	0.080025736	3.688587026	0.000225503	0.000353637	positive
PRJNA729271	stage_1W_vs_E10	ordinary_muscle_development	ENSGALG00010027378	ZBTB47	0.047713559	0.164781063	0.289557296	0.77215494	0.795441896	positive
PRJNA729271	stage_1W_vs_E10	ordinary_muscle_development	ENSGALG00010027379	OPTC	-0.95280962	0.65342837	-1.45816996	0.144793702	0.170245353	negative
PRJNA729271	stage_1W_vs_E10	ordinary_muscle_development	ENSGALG00010027380	GPX1	-0.206815788	0.132900953	-1.556164831	0.119668902	0.142355415	negative
PRJNA729271	stage_1W_vs_E10	ordinary_muscle_development	ENSGALG00010027381	KLHL40	0.864484862	0.197223662	4.383271523	1.1691E-05	2.01932E-05	positive
PRJNA729271	stage_1W_vs_E10	ordinary_muscle_development	ENSGALG00010027382	PALM	-0.515392853	0.203787807	-2.529066191	0.011436646	0.015352304	negative
PRJNA729271	stage_1W_vs_E10	ordinary_muscle_development	ENSGALG00010027383	ASS1	2.59356134	0.283042609	9.163148081	5.0405E-20	1.89427E-19	positive
PRJNA729271	stage_1W_vs_E10	ordinary_muscle_development	ENSGALG00010027384	HHATL	1.732609361	0.218598387	7.925993339	2.2633E-15	6.84467E-15	positive
PRJNA729271	stage_1W_vs_E10	ordinary_muscle_development	ENSGALG00010027385	CNTN2	1.672925936	0.61118032	2.737205177	0.006196363	0.008562086	positive
PRJNA729271	stage_1W_vs_E10	ordinary_muscle_development	ENSGALG00010027386	RHOA	-0.456631025	0.066118577	-6.906243978	4.97654E-12	1.2703E-11	negative
PRJNA729271	stage_1W_vs_E10	ordinary_muscle_development	ENSGALG00010027387	ENSGALG00010027387	-0.420163096	0.259925005	-1.61647817	0.10599097	0.127001662	negative
PRJNA729271	stage_1W_vs_E10	ordinary_muscle_development	ENSGALG00010027388	URM1	0.644265922	0.14404786	4.472582402	7.72806E-06	1.35059E-05	positive
PRJNA729271	stage_1W_vs_E10	ordinary_muscle_development	ENSGALG00010027391	TPM3	-1.650284978	0.11499824	-14.35052374	1.05736E-46	1.04049E-45	negative
PRJNA729271	stage_1W_vs_E10	ordinary_muscle_development	ENSGALG00010027392	RBBP5	-0.226033502	0.127204373	-1.77693185	0.075579453	0.092223603	negative
PRJNA729271	stage_1W_vs_E10	ordinary_muscle_development	ENSGALG00010027393	TEX264	1.214515325	0.188830758	6.431766412	1.26129E-10	2.9776E-10	positive
PRJNA729271	stage_1W_vs_E10	ordinary_muscle_development	ENSGALG00010027394	SLC27A4	-0.43272401	0.08714256	-4.965702299	6.84529E-07	1.29466E-06	negative
PRJNA729271	stage_1W_vs_E10	ordinary_muscle_development	ENSGALG00010027395	BCL2L15	0.442419163	0.262574703	1.684926836	0.092002688	0.111233545	positive
PRJNA729271	stage_1W_vs_E10	ordinary_muscle_development	ENSGALG00010027397	RFX5	0.804495629	0.125069753	6.432375613	1.25625E-10	2.96617E-10	positive
PRJNA729271	stage_1W_vs_E10	ordinary_muscle_development	ENSGALG00010027399	MAPKAP1	0.623088431	0.110610764	5.633162696	1.76934E-08	3.69921E-08	positive
PRJNA729271	stage_1W_vs_E10	ordinary_muscle_development	ENSGALG00010027401	ENSGALG00010027401	0.624474656	0.32186879	1.940152868	0.052361115	0.06523761	positive
PRJNA729271	stage_1W_vs_E10	ordinary_muscle_development	ENSGALG00010027402	AP4B1	0.524624355	0.085952122	6.103681267	1.03653E-09	2.33082E-09	positive
PRJNA729271	stage_1W_vs_E10	ordinary_muscle_development	ENSGALG00010027403	ACKR2	0.366215008	0.37480545	0.977080263	0.328529418	0.365018696	positive
PRJNA729271	stage_1W_vs_E10	ordinary_muscle_development	ENSGALG00010027405	DCLRE1B	-1.526362489	0.180160174	-8.472252524	2.40693E-17	7.92918E-17	negative
PRJNA729271	stage_1W_vs_E10	ordinary_muscle_development	ENSGALG00010027407	ENSGALG00010027407	-1.408378635	1.157147701	-1.217112244	0.223561513	0.25507904	negative
PRJNA729271	stage_1W_vs_E10	ordinary_muscle_development	ENSGALG00010027410	ALAS1	0.835444617	0.087087854	9.593124444	8.54571E-22	3.44275E-21	positive
PRJNA729271	stage_1W_vs_E10	ordinary_muscle_development	ENSGALG00010027412	ENSGALG00010027412	0.818187794	0.286623742	2.854570906	0.004309501	0.006047654	positive
PRJNA729271	stage_1W_vs_E10	ordinary_muscle_development	ENSGALG00010027414	PBX3	-1.571100644	0.075161713	-20.90293835	5.03484E-97	1.77567E-95	negative
PRJNA729271	stage_1W_vs_E10	ordinary_muscle_development	ENSGALG00010027417	WNT2B	-1.942937426	0.367157053	-5.291842847	1.2109E-07	2.40537E-07	negative
PRJNA729271	stage_1W_vs_E10	ordinary_muscle_development	ENSGALG00010027418	ENSGALG00010027418	2.092885554	0.900342471	2.324543849	0.020096364	0.026261882	positive
PRJNA729271	stage_1W_vs_E10	ordinary_muscle_development	ENSGALG00010027419	PTBP1	-2.07289986	0.085452986	-24.25778141	5.4732E-130	3.5736E-128	negative
PRJNA729271	stage_1W_vs_E10	ordinary_muscle_development	ENSGALG00010027421	PLPPR3	-3.138817499	0.275332613	-11.40009336	4.17671E-30	2.37045E-29	negative
PRJNA729271	stage_1W_vs_E10	ordinary_muscle_development	ENSGALG00010027423	PEX11B	1.322404189	0.208181349	6.35217418	2.12293E-10	4.94565E-10	positive
PRJNA729271	stage_1W_vs_E10	ordinary_muscle_development	ENSGALG00010027425	CFD	3.760649681	0.238332164	15.77902715	4.33823E-56	5.75848E-55	positive
PRJNA729271	stage_1W_vs_E10	ordinary_muscle_development	ENSGALG00010027427	UBAC1	3.036785583	0.115547676	26.28166725	3.1075E-152	2.7134E-150	positive
PRJNA729271	stage_1W_vs_E10	ordinary_muscle_development	ENSGALG00010027438	ST7L	0.486594105	0.083024452	5.860852932	4.60496E-09	9.97144E-09	positive
PRJNA729271	stage_1W_vs_E10	ordinary_muscle_development	ENSGALG00010027440	TWF2	1.26369138	0.190173743	6.644930877	3.03359E-11	7.43397E-11	positive
PRJNA729271	stage_1W_vs_E10	ordinary_muscle_development	ENSGALG00010027443	MED16	0.198102512	0.155885555	1.270820201	0.20379262	0.234163617	positive
PRJNA729271	stage_1W_vs_E10	ordinary_muscle_development	ENSGALG00010027444	SELENBP1	0.466798566	0.122021206	3.825552794	0.000130479	0.000207994	positive
PRJNA729271	stage_1W_vs_E10	ordinary_muscle_development	ENSGALG00010027445	PPM1M	0.557195615	0.163295157	3.41219927	0.00064441	0.00097543	positive
PRJNA729271	stage_1W_vs_E10	ordinary_muscle_development	ENSGALG00010027448	ADAMTSL5	3.999473292	0.312037419	12.81728746	1.31211E-37	9.67397E-37	positive
PRJNA729271	stage_1W_vs_E10	ordinary_muscle_development	ENSGALG00010027450	CAMSAP1	1.068301518	0.142542163	7.494635212	6.6483E-14	1.87157E-13	positive
PRJNA729271	stage_1W_vs_E10	ordinary_muscle_development	ENSGALG00010027455	R3HDM4	1.318260375	0.146846825	8.977111837	2.77967E-19	1.0093E-18	positive
PRJNA729271	stage_1W_vs_E10	ordinary_muscle_development	ENSGALG00010027457	CAPZA1	-0.309896193	0.128152667	-2.418179818	0.015598366	0.020629409	negative
PRJNA729271	stage_1W_vs_E10	ordinary_muscle_development	ENSGALG00010027460	WDR82	-0.489456713	0.073835215	-6.629041646	3.37873E-11	8.2602E-11	negative
PRJNA729271	stage_1W_vs_E10	ordinary_muscle_development	ENSGALG00010027467	GLYCTK	0.211448768	0.135013018	1.566136145	0.117316748	0.139693934	positive
PRJNA729271	stage_1W_vs_E10	ordinary_muscle_development	ENSGALG00010027468	BSG	0.990921086	0.115157899	8.60489027	7.63898E-18	2.57625E-17	positive
PRJNA729271	stage_1W_vs_E10	ordinary_muscle_development	ENSGALG00010027471	ENSGALG00010027471	1.902521345	0.12893605	14.75554237	2.83404E-49	3.02722E-48	positive
PRJNA729271	stage_1W_vs_E10	ordinary_muscle_development	ENSGALG00010027473	DNAH1	-3.141802027	0.444773314	-7.063827634	1.61978E-12	4.23956E-12	negative
PRJNA729271	stage_1W_vs_E10	ordinary_muscle_development	ENSGALG00010027475	ARID3A	-0.739408259	0.170046764	-4.34826421	1.37219E-05	2.35523E-05	negative
PRJNA729271	stage_1W_vs_E10	ordinary_muscle_development	ENSGALG00010027479	HCN2	-0.318658417	0.276213454	-1.153667257	0.248636613	0.281473579	negative
PRJNA729271	stage_1W_vs_E10	ordinary_muscle_development	ENSGALG00010027487	MPZ	4.912518197	0.533651334	9.205482843	3.40139E-20	1.28628E-19	positive
PRJNA729271	stage_1W_vs_E10	ordinary_muscle_development	ENSGALG00010027490	ATP2B4	0.213187559	0.067858578	3.141644965	0.001680016	0.002452597	positive

PRJNA729271	stage_1W_vs_E10	ordinary_muscle_development	ENSGALG00010027492	PCP4L1	6.221958146	0.483216672	12.87612473	6.1339E-38	4.55719E-37	positive
PRJNA729271	stage_1W_vs_E10	ordinary_muscle_development	ENSGALG00010027497	KCNT1	2.563263813	0.341380253	7.508529836	5.97951E-14	1.68821E-13	positive
PRJNA729271	stage_1W_vs_E10	ordinary_muscle_development	ENSGALG00010027499	MOV10	2.370037061	0.399956935	5.925730633	3.10912E-09	6.80252E-09	positive
PRJNA729271	stage_1W_vs_E10	ordinary_muscle_development	ENSGALG00010027501	BAP1	-0.543038129	0.067487749	-8.046469764	8.52164E-16	2.62811E-15	negative
PRJNA729271	stage_1W_vs_E10	ordinary_muscle_development	ENSGALG00010027502	THOP1	0.591499587	0.138821214	4.260873183	2.0363E-05	3.44613E-05	positive
PRJNA729271	stage_1W_vs_E10	ordinary_muscle_development	ENSGALG00010027510	RHOC	2.796850288	0.181640013	15.39776529	1.69424E-53	2.09539E-52	positive
PRJNA729271	stage_1W_vs_E10	ordinary_muscle_development	ENSGALG00010027511	POLRMT	0.552768113	0.081115032	6.814619891	9.45133E-12	2.38214E-11	positive
PRJNA729271	stage_1W_vs_E10	ordinary_muscle_development	ENSGALG00010027513	SEMA3G	1.443971247	0.181565703	7.952885497	1.82217E-15	5.53601E-15	positive
PRJNA729271	stage_1W_vs_E10	ordinary_muscle_development	ENSGALG00010027516	PPM1J	3.813583495	0.21295514	17.90791944	1.02288E-71	2.01998E-70	positive
PRJNA729271	stage_1W_vs_E10	ordinary_muscle_development	ENSGALG00010027520	FANCE	-1.156090016	0.173712545	-6.655190137	2.82934E-11	6.94871E-11	negative
PRJNA729271	stage_1W_vs_E10	ordinary_muscle_development	ENSGALG00010027522	PSMD5	0.525285827	0.104398376	5.031551728	4.86526E-07	9.27676E-07	positive
PRJNA729271	stage_1W_vs_E10	ordinary_muscle_development	ENSGALG00010027524	ENSGALG00010027524	1.985496948	0.110816772	17.91693542	8.69902E-72	1.72493E-70	positive
PRJNA729271	stage_1W_vs_E10	ordinary_muscle_development	ENSGALG00010027525	ENSGALG00010027525	5.954543542	0.263835267	22.56917205	8.7061E-113	4.1375E-111	positive
PRJNA729271	stage_1W_vs_E10	ordinary_muscle_development	ENSGALG00010027527	ENSGALG00010027527	-1.434462445	0.523065381	-2.74241519	0.00609892	0.008433872	negative
PRJNA729271	stage_1W_vs_E10	ordinary_muscle_development	ENSGALG00010027528	ENSGALG00010027528	6.515448496	2.331562664	2.794455665			positive
PRJNA729271	stage_1W_vs_E10	ordinary_muscle_development	ENSGALG00010027529	ACY1	3.121224861	0.139572424	22.36276172	9.0722E-111	4.1353E-109	positive
PRJNA729271	stage_1W_vs_E10	ordinary_muscle_development	ENSGALG00010027530	PHF19	0.488319064	0.139032221	3.512272622	0.000444292	0.000680187	positive
PRJNA729271	stage_1W_vs_E10	ordinary_muscle_development	ENSGALG00010027531	UFC1	0.67384621	0.167103797	4.03250089	5.51864E-05	9.04895E-05	positive
PRJNA729271	stage_1W_vs_E10	ordinary_muscle_development	ENSGALG00010027533	WDR18	-1.123934551	0.096374944	-11.66210334	1.99063E-31	1.19085E-30	negative
PRJNA729271	stage_1W_vs_E10	ordinary_muscle_development	ENSGALG00010027534	KCNN3	0.611121121	0.191639571	3.188908836	0.001428109	0.002097097	positive
PRJNA729271	stage_1W_vs_E10	ordinary_muscle_development	ENSGALG00010027536	USP21	1.105989726	0.303205916	3.647652202	0.000264648	0.000413191	positive
PRJNA729271	stage_1W_vs_E10	ordinary_muscle_development	ENSGALG00010027537	SGTA	-0.095136942	0.067601014	-1.407330095	0.159329529	0.18617344	negative
PRJNA729271	stage_1W_vs_E10	ordinary_muscle_development	ENSGALG00010027538	ABHD14A	1.435906969	0.13042736	11.00924661	3.44876E-28	1.81122E-27	positive
PRJNA729271	stage_1W_vs_E10	ordinary_muscle_development	ENSGALG00010027539	DDR2L	0.045486142	0.108028042	0.421058655	0.673712251	0.703614027	positive
PRJNA729271	stage_1W_vs_E10	ordinary_muscle_development	ENSGALG00010027540	PPOX	-0.161819834	0.115243994	-1.404149829	0.160274238	0.187247125	negative
PRJNA729271	stage_1W_vs_E10	ordinary_muscle_development	ENSGALG00010027543	TUBB4B	-0.189234757	0.152070878	-1.244385247	0.21335781	0.244379412	negative
PRJNA729271	stage_1W_vs_E10	ordinary_muscle_development	ENSGALG00010027545	PPARD	0.65031731	0.201920707	3.220656855	0.001278972	0.001884397	positive
PRJNA729271	stage_1W_vs_E10	ordinary_muscle_development	ENSGALG00010027547	B4GALT3	1.867559105	0.221237421	8.441425043	3.13492E-17	1.02668E-16	positive
PRJNA729271	stage_1W_vs_E10	ordinary_muscle_development	ENSGALG00010027548	PCBP4	0.06787233	0.147564592	0.459949975	0.645552128	0.67732267	positive
PRJNA729271	stage_1W_vs_E10	ordinary_muscle_development	ENSGALG00010027553	FGF22	0.971393287	0.293145043	3.313695083	0.000920719	0.001376993	positive
PRJNA729271	stage_1W_vs_E10	ordinary_muscle_development	ENSGALG00010027554	ENSGALG00010027554	0.673166677	0.145354029	4.631221311	3.63515E-06	6.52042E-06	positive
PRJNA729271	stage_1W_vs_E10	ordinary_muscle_development	ENSGALG00010027555	ENSGALG00010027555	0.929159216	0.349644106	2.657442814	0.007873595	0.010765754	positive
PRJNA729271	stage_1W_vs_E10	ordinary_muscle_development	ENSGALG00010027557	C6orf132	-2.355289196	0.847906513	-2.777769907	0.005473336	0.00761309	negative
PRJNA729271	stage_1W_vs_E10	ordinary_muscle_development	ENSGALG00010027559	ENSGALG00010027559	0.351160014	0.282129512	1.244676642	0.213250635	0.244275957	positive
PRJNA729271	stage_1W_vs_E10	ordinary_muscle_development	ENSGALG00010027561	ADAMTS4	1.449716333	0.338606079	4.281424416	1.85701E-05	3.15413E-05	positive
PRJNA729271	stage_1W_vs_E10	ordinary_muscle_development	ENSGALG00010027562	SLC39A3	0.358208098	0.146781918	2.440410257	0.014670591	0.019453866	positive
PRJNA729271	stage_1W_vs_E10	ordinary_muscle_development	ENSGALG00010027563	WDR77	-0.390035011	0.122495665	-3.184071967	0.001452189	0.002130946	negative
PRJNA729271	stage_1W_vs_E10	ordinary_muscle_development	ENSGALG00010027567	PARP3	1.762312968	0.128063609	13.76123148	4.36036E-43	3.84917E-42	positive
PRJNA729271	stage_1W_vs_E10	ordinary_muscle_development	ENSGALG00010027568	NELFB	-0.14192436	0.075973034	-1.868088624	0.061749719	0.076181988	negative
PRJNA729271	stage_1W_vs_E10	ordinary_muscle_development	ENSGALG00010027569	ATP5PB	1.542148503	0.125284262	12.30919577	8.08245E-35	5.45924E-34	positive
PRJNA729271	stage_1W_vs_E10	ordinary_muscle_development	ENSGALG00010027570	NDUFS2	1.367868246	0.137326952	9.960668478	2.26534E-23	9.74078E-23	positive
PRJNA729271	stage_1W_vs_E10	ordinary_muscle_development	ENSGALG00010027571	RBM10	0.209332264	0.101699558	2.058339965	0.03955751	0.050016235	positive
PRJNA729271	stage_1W_vs_E10	ordinary_muscle_development	ENSGALG00010027574	GNG7	-0.492569938	0.09589366	-5.136626719	2.79714E-07	5.42184E-07	negative
PRJNA729271	stage_1W_vs_E10	ordinary_muscle_development	ENSGALG00010027575	FCER1G	1.541125516	0.337921115	4.560607338	5.10059E-06	9.03716E-06	positive
PRJNA729271	stage_1W_vs_E10	ordinary_muscle_development	ENSGALG00010027576	GADD45B	2.413897442	0.416834472	5.791021627	6.99596E-09	1.4981E-08	positive
PRJNA729271	stage_1W_vs_E10	ordinary_muscle_development	ENSGALG00010027578	ENSGALG00010027578	1.542324178	0.240336244	6.417359911	1.38658E-10	3.26751E-10	positive
PRJNA729271	stage_1W_vs_E10	ordinary_muscle_development	ENSGALG00010027579	C26H6ORF125	0.234807623	0.158022755	1.485910197	0.137302876	0.161976495	positive
PRJNA729271	stage_1W_vs_E10	ordinary_muscle_development	ENSGALG00010027583	GLE1	-1.08301384	0.07400211	-14.63490491	1.68211E-48	1.74782E-47	negative
PRJNA729271	stage_1W_vs_E10	ordinary_muscle_development	ENSGALG00010027586	HJV	4.974812865	0.288397258	17.24986185	1.12167E-66	1.91956E-65	positive
PRJNA729271	stage_1W_vs_E10	ordinary_muscle_development	ENSGALG00010027590	SEMA3F	-1.372389898	0.237956663	-5.767394283	8.05066E-09	1.71735E-08	negative
PRJNA729271	stage_1W_vs_E10	ordinary_muscle_development	ENSGALG00010027592	MAP1S	0.199328412	0.169065716	1.178999601	0.238398335	0.270644099	positive
PRJNA729271	stage_1W_vs_E10	ordinary_muscle_development	ENSGALG00010027593	ENSGALG00010027593	4.586864718	0.422014252	10.8689806	1.62001E-27	8.27123E-27	positive
PRJNA729271	stage_1W_vs_E10	ordinary_muscle_development	ENSGALG00010027594	ITPR3	-0.268140729	0.139501827	-1.922130594	0.054589327	0.067840371	negative
PRJNA729271	stage_1W_vs_E10	ordinary_muscle_development	ENSGALG00010027595	GNAT1	-0.274069224	0.455634071	-0.601511698	0.547499222	0.583615328	negative
PRJNA729271	stage_1W_vs_E10	ordinary_muscle_development	ENSGALG00010027597	EXOSC2	-0.950949808	0.117071684	-8.122799409	4.55552E-16	1.42372E-15	negative
PRJNA729271	stage_1W_vs_E10	ordinary_muscle_development	ENSGALG00010027600	KIRREL1	-1.04819883	0.142033956	-7.379917154	1.58388E-13	4.35642E-13	negative
PRJNA729271	stage_1W_vs_E10	ordinary_muscle_development	ENSGALG00010027605	FCHO1	0.30886557	0.323230849	0.955557214	0.339295987	0.376317647	positive
PRJNA729271	stage_1W_vs_E10	ordinary_muscle_development	ENSGALG00010027607	ABL1	-1.207456037	0.079897955	-15.11247737	1.34005E-51	1.55393E-50	negative
PRJNA729271	stage_1W_vs_E10	ordinary_muscle_development	ENSGALG00010027608	GNAI2	-0.400187526	0.144531879	-2.768853001	0.005625401	0.007810363	negative
PRJNA729271	stage_1W_vs_E10	ordinary_muscle_development	ENSGALG00010027609	RPL36	0.486335308	0.186800189	2.603505438	0.00922758	0.012524935	positive
PRJNA729271	stage_1W_vs_E10	ordinary_muscle_development	ENSGALG00010027612	BAK1	-1.083399686	0.164542517	-6.58431452	4.56989E-11	1.10937E-10	negative
PRJNA729271	stage_1W_vs_E10	ordinary_muscle_development	ENSGALG00010027614	LONP1	0.056544543	0.07926743	0.713338927	0.475636055	0.514004669	positive
PRJNA729271	stage_1W_vs_E10	ordinary_muscle_development	ENSGALG00010027616	SLC38A3	4.63248931	0.266821557	17.36175054	1.60747E-67	2.84496E-66	positive
PRJNA729271	stage_1W_vs_E10	ordinary_muscle_development	ENSGALG00010027618	PPP1R26	0.362712837	0.07861725	4.613654623	3.9565E-06	7.06622E-06	positive
PRJNA729271	stage_1W_vs_E10	ordinary_muscle_development	ENSGALG00010027619	JAK3	1.740846185	0.349946946	4.974600304	6.53824E-07	1.23771E-06	positive
PRJNA729271	stage_1W_vs_E10	ordinary_muscle_development	ENSGALG00010027621	TSPO2	-3.037749563	0.403094511	-7.53607276	4.84336E-14	1.37092E-13	negative
PRJNA729271	stage_1W_vs_E10	ordinary_muscle_development	ENSGALG00010027622	FIBCD1	-4.069389731	1.222693808	-3.32821652	0.000874039	0.001309884	negative
PRJNA729271	stage_1W_vs_E10	ordinary_muscle_development	ENSGALG00010027623	APOBEC2	2.659477683	0.182662263	14.5595354	5.08019E-48	5.19671E-47	positive
PRJNA729271	stage_1W_vs_E10	ordinary_muscle_development	ENSGALG00010027624	ENSGALG00010027624	3.099136495	0.402581999	7.698149703	1.38051E-14	4.01897E-14	positive
PRJNA729271	stage_1W_vs_E10	ordinary_muscle_development	ENSGALG00010027626	SEMA3B	0.610321701	0.213127822	2.863641614	0.004188016	0.005883435	positive
PRJNA729271	stage_1W_vs_E10	ordinary_muscle_development	ENSGALG00010027627	OARD1	1.636066511	0.093012105	17.58982355	2.94794E-69	5.45727E-68	positive
PRJNA729271	stage_1W_vs_E10	ordinary_muscle_development	ENSGALG00010027628	PIERCE1	-0.506584292	0.249432359	-2.030948572	0.042260212	0.05324772	negative
PRJNA729271	stage_1W_vs_E10	ordinary_muscle_development	ENSGALG00010027632	LAMC3	-1.54412054	0.406614819	-3.797501883	0.000146162	0.000232347	negative
PRJNA729271	stage_1W_vs_E10	ordinary_muscle_development	ENSGALG00010027634	OASL	4.545942461	0.712042558	6.384369035	1.72106E-10	4.03668E-10	positive
PRJNA729271	stage_1W_vs_E10	ordinary_muscle_development	ENSGALG00010027635	CD48	1.621888779	0.228732727	7.090759584	1.33378E-12	3.51192E-12	positive
PRJNA729271	stage_1W_vs_E10	ordinary_muscle_development	ENSGALG00010027636	SLC1A6	0.425103867	0.600116397	0.708369025	0.478716123	0.517063353	positive
PRJNA729271	stage_1W_vs_E10	ordinary_muscle_development	ENSGALG00010027638	MRPS2	1.574816722	0.13978691	11.26583829	1.93502E-29	1.06728E-28	positive
PRJNA729271	stage_1W_vs_E10	ordinary_muscle_development	ENSGALG00010027641	HYAL3	1.240023677	0.163326797	7.592285517	3.14311E-14	8.9684E-14	positive
PRJNA729271	stage_1W_vs_E10	ordinary_muscle_development	ENSGALG00010027642	DIPK1B	-0.70732907	0.168395594	-4.20040129	2.66442E-05	4.47207E-05	negative
PRJNA729271	stage_1W_vs_E10	ordinary_muscle_development	ENSGALG00010027644	NAA80	2.41393884	0.139382486	17.31881032	3.393E-67	5.95418E-66	positive
PRJNA729271	stage_1W_vs_E10	ordinary_muscle_development	ENSGALG00010027645	SLAMF1	4.144475176	0.802779522	5.162656824	2.43469E-07	4.74276E-07	positive
PRJNA729271	stage_1W_vs_E10	ordinary_muscle_development	ENSGALG00010027648	NFYA	-0.21628469	0.072694783	-2.975243632	0.002927559	0.00417626	negative
PRJNA729271	stage_1W_vs_E10	ordinary_muscle_development	ENSGALG00010027649	FBLN2	-1.127399536	0.191695965	-5.881185536	4.07338E-09	8.84815E-09	negative
PRJNA729271	stage_1W_vs_E10	ordinary_muscle_development	ENSGALG00010027650	CCDC124	1.162244482	0.190695189	6.094776098	1.09591E-09	2.46091E-09	positive
PRJNA729271	stage_1W_vs_E10	ordinary_muscle_development	ENSGALG00010027653	WNT7A	-8.594880843	0.92553053	-9.286436873	1.5954E-20	6.14381E-20	negative
PRJNA729271	stage_1W_vs_E10	ordinary_muscle_development	ENSGALG00010027654	AIF1L	0.279166787	0.091533849	3.049874888	0.002289367	0.003296352	positive
PRJNA729271	stage_1W_vs_E10	ordinary_muscle_development	ENSGALG00010027663	COPA	-0.490025181	0.070686027	-6.932419307	4.13704E-12	1.06023E-11	negative
PRJNA729271	stage_1W_vs_E10	ordinary_muscle_development	ENSGALG00010027664	TREMB1	1.302923436	0.325450585	4.00344475	6.24268E-05	0.00010205	positive
PRJNA729271	stage_1W_vs_E10	ordinary_muscle_development	ENSGALG00010027665	TRAF1	0.237204681	0.130347212	1.819790982	0.068790842	0.084394689	positive
PRJNA729271	stage_1W_vs_E10	ordinary_muscle_development	ENSGALG00010027667	PTPDC1	-0.279114747	0.153557979	-1.817650565	0.069117558	0.084773989	negative
PRJNA729271	stage_1W_vs_E10	ordinary_muscle_development	ENSGALG00010027668	RANBP3	-0.539499464	0.054393083	-9.918530663	3.45809E-23	1.47687E-22	negative
PRJNA729271	stage_1W_vs_E10	ordinary_muscle_development	ENSGALG00010027671	BARX1	-4.4016941	0.726737349	-6.056788061	1.38866E-09	3.1034E-09	negative
PRJNA729271	stage_1W_vs_E10	ordinary_muscle_development	ENSGALG00010027672	CNTRL	-0.041800857	0.173884943	-0.240393769	0.810025008	0.830126732	negative
PRJNA729271	stage_1W_vs_E10	ordinary_muscle_development	ENSGALG00010027674	TREM2	0.10624499	0.287867564	0.369075933	0.712071127	0.739413353	positive
PRJNA729271	stage_1W_vs_E10	ordinary_muscle_development	ENSGALG00010027685	ENSGALG00010027685	1.771050819	0.307741608	5.754993055	8.66454E-09	1.84424E-08	positive
PRJNA729271	stage_1W_vs_E10	ordinary_muscle_development	ENSGALG00010027692	PHF2	0.603738042	0.112560283	5.363686257	8.15405E-08	1.64088E-07	positive
PRJNA729271	stage_1W_vs_E10	ordinary_muscle_development	ENSGALG00010027693	RAB14	-0.450952022	0.060125838	-7.500136963	6.37512E-14	1.79606E-13	negative
PRJNA729271	stage_1W_vs_E10	ordinary_muscle_development	ENSGALG00010027695	TREMB2	0.714553944	0.308121485	2.319065783	0.020391478	0.026618739	positive
PRJNA729271	stage_1W_vs_E10	ordinary_muscle_development	ENSGALG00010027697	CATH2	1.6714426	0.926354462	1.804322933	0.071180697	0.087149607	positive
PRJNA729271	stage_1W_vs_E10	ordinary_muscle_development	ENSGALG00010027698	FAM120A	0.234825534	0.066440998	3.534346873	0.000408784	0.000627816	positive
PRJNA729271	stage_1W_vs_E10	ordinary_muscle_development	ENSGALG00010027699	VANGL2	-2.083157527	0.129087241	-16.13759432	1.38849E-58	1.95399E-57	negative
PRJNA729271	stage_1W_vs_E10	ordinary_muscle_development	ENSGALG00010027700	TCF3	-1.358400699	0.09635835	-14.09738444	3.94108E-45	3.69987E-44	negative
PRJNA729271	stage_1W_vs_E10	ordinary_muscle_development	ENSGALG00010027702	FOXP4	-3.734453287	0.261849533	-14.26182907	3.78405E-46	3.66154E-45	negative
PRJNA729271	stage_1W_vs_E10	ordinary_muscle_development	ENSGALG00010027703	NCSTN	-0.083465143	0.101284914	-0.8240629	0.409903802	0.447912221	negative
PRJNA729271	stage_1W_vs_E10	ordinary_muscle_development	ENSGALG00010027704	MDFI	-3.959321814	0.195612185	-20.24067067	4.29288E-91	1.31001E-89	negative
PRJNA729271	stage_1W_vs_E10	ordinary_muscle_development	ENSGALG00010027705	S100A11	0.008371442	0.230136398	0.036376002	0.970982549	0.975018501	positive
PRJNA729271	stage_1W_vs_E10	ordinary_muscle_development	ENSGALG00010027706	GSN	3.24180207	0.171904585	18.85814778	2.51891E-79	5.87002E-78	positive
PRJNA729271	stage_1W_vs_E10	ordinary_muscle_development	ENSGALG00010027707	HIGD1C	1.325659885	0.211482847	6.268403822	3.64768E-10	8.40056E-10	positive
PRJNA729271	stage_1W_vs_E10	ordinary_muscle_development	ENSGALG00010027709	PSMA5	1.831736662	0.106463936	17.20523141	2.42606E-66	4.11776E-65	positive
PRJNA729271	stage_1W_vs_E10	ordinary_muscle_development	ENSGALG00010027710	ENSGALG00010027710	5.601125114	0.280712109	19.9532722	1.40392E-88	4.03768E-87	positive
PRJNA729271	stage_1W_vs_E10	ordinary_muscle_development	ENSGALG00010027711	WNK2	1.298769741	0.186008225	6.98232425	2.90336E-12	7.50566E-12	positive
PRJNA729271	stage_1W_vs_E10	ordinary_muscle_development	ENSGALG00010027712	STOM	1.570786076	0.157166046	9.994436584	1.61201E-23	6.97705E-23	positive
PRJNA729271	stage_1W_vs_E10	ordinary_muscle_development	ENSGALG00010027713	ENSGALG00010027713	1.455998993	0.139318099	10.45089618	1.45147E-25	6.84643E-25	positive
PRJNA729271	stage_1W_vs_E10	ordinary_muscle_development	ENSGALG00010027714	SYPL2	8.173892704	0.162008154	50.45358846	0	0	positive
PRJNA729271	stage_1W_vs_E10	ordinary_muscle_development	ENSGALG00010027715	NINJ1	-0.629944009	0.125126924	-5.034440135	4.79247E-07	9.14881E-07	negative
PRJNA729271	stage_1W_vs_E10	ordinary_muscle_development	ENSGALG00010027717	ATXN7L2	2.064117967	0.224874236	9.17898824	4.35161E-20	1.63963E-19	positive
PRJNA729271	stage_1W_vs_E10	ordinary_muscle_development	ENSGALG00010027718	ENSGALG00010027718	2.263803615	0.471475381	4.801530907	1.57457E-06	2.89628E-06	positive
PRJNA729271	stage_1W_vs_E10	ordinary_muscle_development	ENSGALG00010027719	GPR61	-5.632286433	1.118336045	-5.036309487	4.74593E-07	9.06354E-07	negative
PRJNA729271	stage_1W_vs_E10	ordinary_muscle_development	ENSGALG00010027720	DAB2IP	0.352858364	0.087813568	4.018267028	5.86277E-05	9.59911E-05	positive
PRJNA729271	stage_1W_vs_E10	ordinary_muscle_development	ENSGALG00010027721	SUSD3	-2.142158751	0.211495748	-10.1286138	4.12455E-24	1.82328E-23	negative
PRJNA729271	stage_1W_vs_E10	ordinary_muscle_development	ENSGALG00010027722	GNA13	-0.913668338	0.085559335	-10.67876853	1.27958E-26	6.30438E-26	negative
PRJNA729271	stage_1W_vs_E10	ordinary_muscle_development	ENSGALG00010027723	ENSGALG00010027723	-0.426080365	1.261247666	-0.337824503	0.735495444	0.761554858	negative
PRJNA729271	stage_1W_vs_E10	ordinary_muscle_development	ENSGALG00010027724	TTLL11	-0.53377817	0.113754566	-4.692366972	2.70062E-06	4.8883E-06	negative
PRJNA729271	stage_1W_vs_E10	ordinary_muscle_development	ENSGALG00010027725	BTN1A1	2.28905818	0.256760027	8.915165688	4.87087E-19	1.75281E-18	positive
PRJNA729271	stage_1W_vs_E10	ordinary_muscle_development	ENSGALG00010027727	GSTM2	1.265662928	0.170963185	7.403131426	1.3301E-13	3.67094E-13	positive
PRJNA729271	stage_1W_vs_E10	ordinary_muscle_development	ENSGALG00010027728	CRNN	0.166798045	1.679237626	0.099329626	0.920876557	0.930440937	positive
PRJNA729271	stage_1W_vs_E10	ordinary_muscle_development	ENSGALG00010027729	EPS8L3	1.024785226	0.247301884	4.143863407	3.41503E-05	5.6963E-05	positive
PRJNA729271	stage_1W_vs_E10	ordinary_muscle_development	ENSGALG00010027730	ENSGALG00010027730	-1.894808067	0.146894045	-12.89914828	4.55099E-38	3.40561E-37	negative
PRJNA729271	stage_1W_vs_E10	ordinary_muscle_development	ENSGALG00010027731	OLFML2A	1.822310729	0.102096707	17.84886877	2.9495E-71	5.74638E-70	positive
PRJNA729271	stage_1W_vs_E10	ordinary_muscle_development	ENSGALG00010027732	EEF2	1.313595658	0.117030969	11.22434228	3.09682E-29	1.69262E-28	positive
PRJNA729271	stage_1W_vs_E10	ordinary_muscle_development	ENSGALG00010027733	RPL35	0.296084424	0.154050282	1.921998591	0.054605935	0.067853667	positive

PRJNA729271	stage_1W_vs_E10	ordinary_muscle_development	ENSGALG00010027734	CSF1	0.989364917	0.139997983	7.06699406	1.58326E-12	4.14697E-12	positive
PRJNA729271	stage_1W_vs_E10	ordinary_muscle_development	ENSGALG00010027736	ENSGALG00010027736	1.135005216	1.658492684	0.684359495	0.493748187	0.531675207	positive
PRJNA729271	stage_1W_vs_E10	ordinary_muscle_development	ENSGALG00010027738	ENSGALG00010027738	0.70722129	0.256023957	2.762324665	0.005739138	0.007960651	positive
PRJNA729271	stage_1W_vs_E10	ordinary_muscle_development	ENSGALG00010027739	FGD3	-2.17494365	0.07643377	-28.455271	4.1936E-178	4.9825E-176	negative
PRJNA729271	stage_1W_vs_E10	ordinary_muscle_development	ENSGALG00010027740	MARCHF2	-0.308729227	0.146701698	-2.104469347	0.035337526	0.044919533	negative
PRJNA729271	stage_1W_vs_E10	ordinary_muscle_development	ENSGALG00010027741	RAB11B	-1.101002528	0.062570711	-17.5961327	2.6373E-69	4.88844E-68	negative
PRJNA729271	stage_1W_vs_E10	ordinary_muscle_development	ENSGALG00010027743	C1orf74	2.377556648	0.181497883	13.09963844	3.3083E-39	2.58372E-38	positive
PRJNA729271	stage_1W_vs_E10	ordinary_muscle_development	ENSGALG00010027744	ENSGALG00010027744	1.135017724	1.332846629	0.851574142	0.394450497	0.432558628	positive
PRJNA729271	stage_1W_vs_E10	ordinary_muscle_development	ENSGALG00010027745	BICD2	0.111322111	0.138588583	0.803256002	0.421826783	0.459900655	positive
PRJNA729271	stage_1W_vs_E10	ordinary_muscle_development	ENSGALG00010027746	UQCR11	2.774848504	0.184254237	15.05988976	2.97282E-51	3.39835E-50	positive
PRJNA729271	stage_1W_vs_E10	ordinary_muscle_development	ENSGALG00010027748	ENSGALG00010027748	1.134952076	2.889617951	0.392768904	0.694490174	0.723073917	positive
PRJNA729271	stage_1W_vs_E10	ordinary_muscle_development	ENSGALG00010027751	ANGPTL4	-1.456115986	0.212306534	-6.858554759	6.95607E-12	1.7652E-11	negative
PRJNA729271	stage_1W_vs_E10	ordinary_muscle_development	ENSGALG00010027752	SCAI	-1.305514234	0.086651985	-15.06617808	2.70304E-51	3.09483E-50	negative
PRJNA729271	stage_1W_vs_E10	ordinary_muscle_development	ENSGALG00010027759	ENSGALG00010027759	-3.466525755	1.082332227	-3.202829658	0.001360844	0.002001161	negative
PRJNA729271	stage_1W_vs_E10	ordinary_muscle_development	ENSGALG00010027766	ENSGALG00010027766	-3.117614234	0.940125842	-3.316166937	0.000912613	0.001365434	negative
PRJNA729271	stage_1W_vs_E10	ordinary_muscle_development	ENSGALG00010027767	ENSGALG00010027767	-2.114350509	1.179035241	-1.793288645	0.072926782	0.089174431	negative
PRJNA729271	stage_1W_vs_E10	ordinary_muscle_development	ENSGALG00010027772	ENSGALG00010027772	-2.960169511	1.170293228	-2.529425482	0.011424943	0.015338015	negative
PRJNA729271	stage_1W_vs_E10	ordinary_muscle_development	ENSGALG00010027775	MVB12B	0.818141199	0.087988211	9.29830475	1.42703E-20	5.50712E-20	positive
PRJNA729271	stage_1W_vs_E10	ordinary_muscle_development	ENSGALG00010027776	TKT	-2.165808162	0.1305969	-16.58391712	9.10928E-62	1.38842E-60	negative
PRJNA729271	stage_1W_vs_E10	ordinary_muscle_development	ENSGALG00010027784	PEAR1	1.113455341	0.191621791	5.810692691	6.22149E-09	1.33799E-08	positive
PRJNA729271	stage_1W_vs_E10	ordinary_muscle_development	ENSGALG00010027787	DCP1A	0.06084906	0.137737949	0.441774114	0.658652672	0.689356792	positive
PRJNA729271	stage_1W_vs_E10	ordinary_muscle_development	ENSGALG00010027789	UAP1L1	-1.905284126	0.135747826	-14.03546698	9.45714E-45	8.78726E-44	negative
PRJNA729271	stage_1W_vs_E10	ordinary_muscle_development	ENSGALG00010027801	MBD3	-3.65025301	0.135074223	-27.02405341	7.7105E-161	7.603E-159	negative
PRJNA729271	stage_1W_vs_E10	ordinary_muscle_development	ENSGALG00010027803	SAPCD2	-2.004286132	0.171155979	-11.7102899	1.12893E-31	6.8268E-31	negative
PRJNA729271	stage_1W_vs_E10	ordinary_muscle_development	ENSGALG00010027811	MEX3D	-1.071426991	0.102302707	-10.47310503	1.14814E-25	5.42801E-25	negative
PRJNA729271	stage_1W_vs_E10	ordinary_muscle_development	ENSGALG00010027813	LRRC71	-2.934856898	0.374497145	-7.836793791	4.62196E-15	1.37907E-14	negative
PRJNA729271	stage_1W_vs_E10	ordinary_muscle_development	ENSGALG00010027814	ENSGALG00010027814	-0.121873153	0.133132703	-0.915426112	0.359967975	0.397541775	negative
PRJNA729271	stage_1W_vs_E10	ordinary_muscle_development	ENSGALG00010027816	ZMYND19	-0.248133104	0.11861583	-2.091905465	0.036446973	0.046240602	negative
PRJNA729271	stage_1W_vs_E10	ordinary_muscle_development	ENSGALG00010027821	NMRK2	4.923674858	0.226419611	21.74579678	7.5707E-105	3.1087E-103	positive
PRJNA729271	stage_1W_vs_E10	ordinary_muscle_development	ENSGALG00010027822	DPH7	0.320570051	0.090146359	3.556106492	0.000376392	0.000579725	positive
PRJNA729271	stage_1W_vs_E10	ordinary_muscle_development	ENSGALG00010027826	ARHGEF11	0.889795168	0.164331744	5.41462743	6.14165E-08	1.24665E-07	positive
PRJNA729271	stage_1W_vs_E10	ordinary_muscle_development	ENSGALG00010027830	CACNA1D	-2.710845852	0.382460412	-7.087912288	1.3615E-12	3.58231E-12	negative
PRJNA729271	stage_1W_vs_E10	ordinary_muscle_development	ENSGALG00010027831	ATCAY	-2.437915258	0.216667611	-11.25186755	2.2674E-29	1.2487E-28	negative
PRJNA729271	stage_1W_vs_E10	ordinary_muscle_development	ENSGALG00010027838	ENSGALG00010027838	-0.260078385	0.610795952	-0.425802403	0.670251836	0.700504748	negative
PRJNA729271	stage_1W_vs_E10	ordinary_muscle_development	ENSGALG00010027842	SLAMF8	-2.522390994	0.276072399	-9.13670112	6.43864E-20	2.41033E-19	negative
PRJNA729271	stage_1W_vs_E10	ordinary_muscle_development	ENSGALG00010027847	NRTN	-1.964813712	0.405912752	-4.840482841	1.29524E-06	2.39899E-06	negative
PRJNA729271	stage_1W_vs_E10	ordinary_muscle_development	ENSGALG00010027860	ENSGALG00010027860	2.804078335	0.254659624	11.01108331	3.37916E-28	1.77531E-27	positive
PRJNA729271	stage_1W_vs_E10	ordinary_muscle_development	ENSGALG00010027861	PNPLA7	0.203128557	0.078973079	2.572124074	0.010107666	0.013661938	positive
PRJNA729271	stage_1W_vs_E10	ordinary_muscle_development	ENSGALG00010027863	CHDH	-2.708781724	0.157701336	-17.17665683	3.97149E-66	6.71725E-65	negative
PRJNA729271	stage_1W_vs_E10	ordinary_muscle_development	ENSGALG00010027864	ACTR8	0.137332074	0.08139423	1.687245801	0.091556098	0.11073979	positive
PRJNA729271	stage_1W_vs_E10	ordinary_muscle_development	ENSGALG00010027865	SELENOK	-0.359927407	0.124338529	-2.894737545	0.003794757	0.005353806	negative
PRJNA729271	stage_1W_vs_E10	ordinary_muscle_development	ENSGALG00010027866	KCNA2	-1.079044875	0.140555033	-7.677027637	1.62822E-14	4.72022E-14	negative
PRJNA729271	stage_1W_vs_E10	ordinary_muscle_development	ENSGALG00010027867	UBAP2L	-0.528137877	0.169122555	-3.122811614	0.001791324	0.002609047	negative
PRJNA729271	stage_1W_vs_E10	ordinary_muscle_development	ENSGALG00010027868	CACNA2D3	-3.835992375	0.317381	-12.08639575	1.24635E-33	8.05435E-33	negative
PRJNA729271	stage_1W_vs_E10	ordinary_muscle_development	ENSGALG00010027869	NSMF	-1.201896356	0.167052472	-7.19472356	6.25874E-13	1.67258E-12	negative
PRJNA729271	stage_1W_vs_E10	ordinary_muscle_development	ENSGALG00010027870	HAX1	2.477252325	0.119989116	20.64564197	1.06834E-94	3.5599E-93	positive
PRJNA729271	stage_1W_vs_E10	ordinary_muscle_development	ENSGALG00010027872	KDM5B	0.122597071	0.091479586	1.340157688	0.180194084	0.208819416	positive
PRJNA729271	stage_1W_vs_E10	ordinary_muscle_development	ENSGALG00010027873	RFX2	-0.447984538	0.201656874	-2.221518811	0.026315842	0.033930629	negative
PRJNA729271	stage_1W_vs_E10	ordinary_muscle_development	ENSGALG00010027874	FRS3	-0.191862774	0.134752358	-1.423817559	0.154499291	0.180864742	negative
PRJNA729271	stage_1W_vs_E10	ordinary_muscle_development	ENSGALG00010027875	WNT5A	-1.790976553	0.169742079	-10.55116421	5.01715E-26	2.41206E-25	negative
PRJNA729271	stage_1W_vs_E10	ordinary_muscle_development	ENSGALG00010027876	ANAPC2	-0.280169787	0.110069712	-2.545384945	0.010915733	0.014692502	negative
PRJNA729271	stage_1W_vs_E10	ordinary_muscle_development	ENSGALG00010027877	OAZ1	1.542915142	0.114259426	13.50361364	1.4889E-41	1.26061E-40	positive
PRJNA729271	stage_1W_vs_E10	ordinary_muscle_development	ENSGALG00010027879	ENSGALG00010027879	3.822503242	0.417622895	9.153002112	5.53729E-20	2.07666E-19	positive
PRJNA729271	stage_1W_vs_E10	ordinary_muscle_development	ENSGALG00010027880	AMH	1.347863729	0.353443142	3.813523501	0.000136999	0.000218101	positive
PRJNA729271	stage_1W_vs_E10	ordinary_muscle_development	ENSGALG00010027881	ENSGALG00010027881	-1.843148369	0.326277756	-5.649016317	1.61369E-08	3.38207E-08	negative
PRJNA729271	stage_1W_vs_E10	ordinary_muscle_development	ENSGALG00010027882	CHIA	9.07587484	1.385839486	6.549008695	5.79203E-11	1.39415E-10	positive
PRJNA729271	stage_1W_vs_E10	ordinary_muscle_development	ENSGALG00010027883	ATP8B2	0.209307562	0.117325824	1.783985442	0.074426031	0.090930994	positive
PRJNA729271	stage_1W_vs_E10	ordinary_muscle_development	ENSGALG00010027884	SF3A2	-0.429314587	0.190085958	-2.258528674	0.023912719	0.03101699	negative
PRJNA729271	stage_1W_vs_E10	ordinary_muscle_development	ENSGALG00010027885	PLEKHJ1	-0.943172376	0.075161228	-12.54865574	4.04315E-36	2.85881E-35	negative
PRJNA729271	stage_1W_vs_E10	ordinary_muscle_development	ENSGALG00010027886	IL6R	3.524501147	0.191508363	18.40390204	1.22237E-75	2.61331E-74	positive
PRJNA729271	stage_1W_vs_E10	ordinary_muscle_development	ENSGALG00010027887	ERC2	-3.435254382	0.312898128	-10.97882689	4.83157E-28	2.52192E-27	negative
PRJNA729271	stage_1W_vs_E10	ordinary_muscle_development	ENSGALG00010027888	PFKFB2	-0.113391044	0.145358373	-0.780079208	0.435344254	0.473320954	negative
PRJNA729271	stage_1W_vs_E10	ordinary_muscle_development	ENSGALG00010027889	RBM8A	-1.292386366	0.168059318	-7.690060779	1.47065E-14	4.27196E-14	negative
PRJNA729271	stage_1W_vs_E10	ordinary_muscle_development	ENSGALG00010027890	ENSGALG00010027890	5.194145468	0.824727893	6.298011155	3.01489E-10	6.96871E-10	positive
PRJNA729271	stage_1W_vs_E10	ordinary_muscle_development	ENSGALG00010027891	CCDC66	-0.642632783	0.141843291	-4.530582872	5.88212E-06	1.03724E-05	negative
PRJNA729271	stage_1W_vs_E10	ordinary_muscle_development	ENSGALG00010027892	SHE	0.493299899	0.167457341	2.945824263	0.003220953	0.004575882	positive
PRJNA729271	stage_1W_vs_E10	ordinary_muscle_development	ENSGALG00010027893	DOT1L	0.632453232	0.157907929	4.005202498	6.19644E-05	0.00010134	positive
PRJNA729271	stage_1W_vs_E10	ordinary_muscle_development	ENSGALG00010027894	RSBN1	-0.158854622	0.069099758	-2.298917209	0.021509641	0.028020333	negative
PRJNA729271	stage_1W_vs_E10	ordinary_muscle_development	ENSGALG00010027895	LIX1L	-0.480432207	0.180000936	-2.669053935	0.007606523	0.010413351	negative
PRJNA729271	stage_1W_vs_E10	ordinary_muscle_development	ENSGALG00010027896	TOR4A	0.345269364	0.164289174	2.101595349	0.035588738	0.045207147	positive
PRJNA729271	stage_1W_vs_E10	ordinary_muscle_development	ENSGALG00010027897	AP3D1	-0.074935574	0.098273213	-0.762522888	0.445747988	0.483833839	negative
PRJNA729271	stage_1W_vs_E10	ordinary_muscle_development	ENSGALG00010027898	TASOR	-0.541894472	0.092841188	-5.836789488	5.32163E-09	1.14821E-08	negative
PRJNA729271	stage_1W_vs_E10	ordinary_muscle_development	ENSGALG00010027899	PMVK	-0.467577467	0.178704676	-2.616481436	0.00888412	0.01207797	negative
PRJNA729271	stage_1W_vs_E10	ordinary_muscle_development	ENSGALG00010027901	MOB3A	-0.363584676	0.097954172	-3.711783482	0.000205804	0.000323374	negative
PRJNA729271	stage_1W_vs_E10	ordinary_muscle_development	ENSGALG00010027902	UBE2Q1	0.571057256	0.069806283	8.180599709	2.82435E-16	8.86699E-16	positive
PRJNA729271	stage_1W_vs_E10	ordinary_muscle_development	ENSGALG00010027903	NDUFA8	1.090806457	0.125425939	8.696817179	3.41323E-18	1.17183E-17	positive
PRJNA729271	stage_1W_vs_E10	ordinary_muscle_development	ENSGALG00010027904	MORN5	-2.858857702	0.707499999	-4.040788276	5.32718E-05	8.74491E-05	negative
PRJNA729271	stage_1W_vs_E10	ordinary_muscle_development	ENSGALG00010027905	SYN2	0.293988401	0.270138138	1.088289136	0.276467485	0.310795424	positive
PRJNA729271	stage_1W_vs_E10	ordinary_muscle_development	ENSGALG00010027906	LHX6	2.693280947	0.171466085	15.70736826	1.34654E-55	1.77276E-54	positive
PRJNA729271	stage_1W_vs_E10	ordinary_muscle_development	ENSGALG00010027907	MKNK2	2.778448199	0.188284998	14.75660952	2.78956E-49	2.98191E-48	positive
PRJNA729271	stage_1W_vs_E10	ordinary_muscle_development	ENSGALG00010027908	PBXIP1	1.925209585	0.106043447	18.15491322	1.17413E-73	2.41063E-72	positive
PRJNA729271	stage_1W_vs_E10	ordinary_muscle_development	ENSGALG00010027909	TIMP4	-0.915379018	0.252125821	-3.630643679	0.000282715	0.000440404	negative
PRJNA729271	stage_1W_vs_E10	ordinary_muscle_development	ENSGALG00010027910	CHRNB2	-0.251597241	0.193892568	-1.29761158	0.194420841	0.224194916	negative
PRJNA729271	stage_1W_vs_E10	ordinary_muscle_development	ENSGALG00010027911	PTPN22	1.069916435	0.352502411	3.035203168	0.002403737	0.00345521	positive
PRJNA729271	stage_1W_vs_E10	ordinary_muscle_development	ENSGALG00010027912	RGS3	-1.079530813	0.132090838	-8.172639611	3.01714E-16	9.46407E-16	negative
PRJNA729271	stage_1W_vs_E10	ordinary_muscle_development	ENSGALG00010027913	PYGO2	-0.067810215	0.223206343	-0.303800576	0.761279828	0.785279754	negative
PRJNA729271	stage_1W_vs_E10	ordinary_muscle_development	ENSGALG00010027914	BTBD2	2.205288326	0.124624971	17.69539696	4.55013E-70	8.62145E-69	positive
PRJNA729271	stage_1W_vs_E10	ordinary_muscle_development	ENSGALG00010027915	GSTM3	0.269777031	0.083632798	3.225732465	0.001256507	0.001852052	positive
PRJNA729271	stage_1W_vs_E10	ordinary_muscle_development	ENSGALG00010027916	ADAR	0.138885993	0.128798967	1.078316043	0.28089274	0.315305527	positive
PRJNA729271	stage_1W_vs_E10	ordinary_muscle_development	ENSGALG00010027917	PPARG	-0.387194232	0.453241001	-0.854278918	0.392950465	0.431076742	negative
PRJNA729271	stage_1W_vs_E10	ordinary_muscle_development	ENSGALG00010027918	CSNK1G2	0.714923533	0.100459072	7.116565174	1.1065E-12	2.9257E-12	positive
PRJNA729271	stage_1W_vs_E10	ordinary_muscle_development	ENSGALG00010027919	TSEN2	-0.099944897	0.118884126	-0.840691689	0.400520678	0.438750546	negative
PRJNA729271	stage_1W_vs_E10	ordinary_muscle_development	ENSGALG00010027920	SCAMP4	-0.706607427	0.114476235	-6.172525054	6.72079E-10	1.52454E-09	negative
PRJNA729271	stage_1W_vs_E10	ordinary_muscle_development	ENSGALG00010027921	MKRN2OS	0.996503958	0.231804418	4.29889976	1.71648E-05	2.92435E-05	positive
PRJNA729271	stage_1W_vs_E10	ordinary_muscle_development	ENSGALG00010027922	SHC1	0.371884087	0.113050688	3.289534034	0.001003534	0.00149468	positive
PRJNA729271	stage_1W_vs_E10	ordinary_muscle_development	ENSGALG00010027923	AMPD2	-0.263210648	0.155960975	-1.687669931	0.091474607	0.110650457	negative
PRJNA729271	stage_1W_vs_E10	ordinary_muscle_development	ENSGALG00010027925	MKRN2	-0.212645662	0.069624198	-3.054191894	0.002256677	0.003252837	negative
PRJNA729271	stage_1W_vs_E10	ordinary_muscle_development	ENSGALG00010027926	CKS1B	-2.177639243	0.251817181	-8.647699239	5.25481E-18	1.78506E-17	negative
PRJNA729271	stage_1W_vs_E10	ordinary_muscle_development	ENSGALG00010027927	ENSGALG00010027927	1.913620336	0.195403453	9.793175618	1.20452E-22	5.01423E-22	positive
PRJNA729271	stage_1W_vs_E10	ordinary_muscle_development	ENSGALG00010027928	FLAD1	1.454186798	0.157968722	9.205536276	3.39969E-20	1.28628E-19	positive
PRJNA729271	stage_1W_vs_E10	ordinary_muscle_development	ENSGALG00010027929	CDK5RAP2	-0.769263259	0.137436464	-5.597228242	2.17806E-08	4.5276E-08	negative
PRJNA729271	stage_1W_vs_E10	ordinary_muscle_development	ENSGALG00010027930	RAF1	1.186106972	0.089244593	13.29051918	2.62749E-40	2.12649E-39	positive
PRJNA729271	stage_1W_vs_E10	ordinary_muscle_development	ENSGALG00010027931	ABHD17A	1.429619718	0.171478322	8.337028847	7.61805E-17	2.45713E-16	positive
PRJNA729271	stage_1W_vs_E10	ordinary_muscle_development	ENSGALG00010027932	POLE3	-1.915352772	0.077679866	-24.65700397	3.0956E-134	2.087E-132	negative
PRJNA729271	stage_1W_vs_E10	ordinary_muscle_development	ENSGALG00010027933	ENSGALG00010027933	2.526789037	0.219147544	11.53008145	9.30556E-31	5.42139E-30	positive
PRJNA729271	stage_1W_vs_E10	ordinary_muscle_development	ENSGALG00010027934	CNBP	0.219423221	0.085114242	2.577984769	0.009937838	0.013442419	positive
PRJNA729271	stage_1W_vs_E10	ordinary_muscle_development	ENSGALG00010027935	ISY1	-0.200500643	0.08705348	-2.303189278	0.021268196	0.027715769	negative
PRJNA729271	stage_1W_vs_E10	ordinary_muscle_development	ENSGALG00010027936	ENSGALG00010027936	-0.345273282	0.086240252	-4.003620984	6.23803E-05	0.000101985	negative
PRJNA729271	stage_1W_vs_E10	ordinary_muscle_development	ENSGALG00010027937	HDHD3	0.209925493	0.09344372	2.246544691	0.02466914	0.031932416	positive
PRJNA729271	stage_1W_vs_E10	ordinary_muscle_development	ENSGALG00010027938	ENSGALG00010027938	-0.184554806	0.111775991	-1.651113117	0.098715479	0.11884927	negative
PRJNA729271	stage_1W_vs_E10	ordinary_muscle_development	ENSGALG00010027939	BSPRY	-3.561898626	0.635482663	-5.605028797	2.0822E-08	4.33705E-08	negative
PRJNA729271	stage_1W_vs_E10	ordinary_muscle_development	ENSGALG00010027940	ZBTB7B	1.44100818	0.401963584	3.584922214	0.000337179	0.000521768	positive
PRJNA729271	stage_1W_vs_E10	ordinary_muscle_development	ENSGALG00010027941	KLF2	0.854951789	0.151083461	5.658804639	1.52431E-08	3.1966E-08	positive
PRJNA729271	stage_1W_vs_E10	ordinary_muscle_development	ENSGALG00010027942	KHDC4	0.807549043	0.123018086	6.564474124	5.22168E-11	1.26126E-10	positive
PRJNA729271	stage_1W_vs_E10	ordinary_muscle_development	ENSGALG00010027943	RAB43	-0.795162311	0.095560415	-8.321042859	8.71927E-17	2.80421E-16	negative
PRJNA729271	stage_1W_vs_E10	ordinary_muscle_development	ENSGALG00010027944	WDR31	-3.399307994	0.376990971	-9.016948037	1.93402E-19	7.07563E-19	negative
PRJNA729271	stage_1W_vs_E10	ordinary_muscle_development	ENSGALG00010027946	AP1M1	-0.455851144	0.092921748	-4.905752981	9.30696E-07	1.74475E-06	negative
PRJNA729271	stage_1W_vs_E10	ordinary_muscle_development	ENSGALG00010027947	GP9	0.497978903	0.373605579	1.332900073	0.182564599	0.211346846	positive
PRJNA729271	stage_1W_vs_E10	ordinary_muscle_development	ENSGALG00010027948	PRPF4	0.032865564	0.113465797	0.289651727	0.77208269	0.795441896	positive
PRJNA729271	stage_1W_vs_E10	ordinary_muscle_development	ENSGALG00010027950	CDC26	0.045231549	0.128332174	0.352456809	0.724495704	0.751292405	positive
PRJNA729271	stage_1W_vs_E10	ordinary_muscle_development	ENSGALG00010027951	FAM32A	0.343213147	0.104564131	3.282321984	0.00102956	0.001530926	positive
PRJNA729271	stage_1W_vs_E10	ordinary_muscle_development	ENSGALG00010027952	CIB3	0.579386824	0.474213965	1.221783554	0.22178949	0.253236601	positive
PRJNA729271	stage_1W_vs_E10	ordinary_muscle_development	ENSGALG00010027953	SLC31A1	-1.86229482	0.123081366	-15.13059917	1.01765E-51	1.18766E-50	negative
PRJNA729271	stage_1W_vs_E10	ordinary_muscle_development	ENSGALG00010027954	CFAP92	-0.913309401	0.234822929	-3.88935358	0.000100512	0.000161521	negative
PRJNA729271	stage_1W_vs_E10	ordinary_muscle_development	ENSGALG00010027955	ENSGALG00010027955	0.328054009	0.117056131	2.802535892	0.005070257	0.00707756	positive
PRJNA729271	stage_1W_vs_E10	ordinary_muscle_development	ENSGALG00010027956	RAB8A	-1.033683504	0.067539565	-15.3048588	7.09558E-53	8.63564E-52	negative
PRJNA729271	stage_1W_vs_E10	ordinary_muscle_development	ENSGALG00010027957	ENTREP3	-0.243608932	0.470938453	-0.51728401	0.604957914	0.639409725	negative
PRJNA729271	stage_1W_vs_E10	ordinary_muscle_development	ENSGALG00010027958	ENSGALG00010027958	0.409091761	0.13975572	2.927191543	0.003420382	0.004851118	positive
PRJNA729271	stage_1W_vs_E10	ordinary_muscle_development	ENSGALG00010027959	TPM4	-2.478746595	0.173242484	-14.30796036	1.95153E-46	1.90745E-45	negative
PRJNA729271	stage_1W_vs_E10	ordinary_muscle_development	ENSGALG00010027960	PPP6C	0.503463005	0.119645529	4.207955027	2.57692E-05	4.32821E-05	positive
PRJNA729271	stage_1W_vs_E10	ordinary_muscle_development	ENSGALG00010027961	TINCR	-4.298969422	1.043207045	-4.120916785	3.77368E-05	6.27143E-05	negative

PRJNA729271	stage_1W_vs_E10	ordinary_muscle_development	ENSGALG00010027962	SCAMP3	1.089201189	0.125624524	8.67029107	4.31017E-18	1.47106E-17	positive
PRJNA729271	stage_1W_vs_E10	ordinary_muscle_development	ENSGALG00010027964	RABEPK	-1.181036473	0.1395695	-8.46199543	2.62841E-17	8.64114E-17	negative
PRJNA729271	stage_1W_vs_E10	ordinary_muscle_development	ENSGALG00010027965	ACAD9	1.690910164	0.109276998	15.47361475	5.22867E-54	6.56187E-53	positive
PRJNA729271	stage_1W_vs_E10	ordinary_muscle_development	ENSGALG00010027966	CLK2	0.28299212	0.099560652	2.842409276	0.004477397	0.006274156	positive
PRJNA729271	stage_1W_vs_E10	ordinary_muscle_development	ENSGALG00010027968	FKBP15	0.202019534	0.097036244	2.08189772	0.037351813	0.04735128	positive
PRJNA729271	stage_1W_vs_E10	ordinary_muscle_development	ENSGALG00010027969	HSPA5	-1.207906155	0.129630279	-9.318086542	1.18457E-20	4.58366E-20	negative
PRJNA729271	stage_1W_vs_E10	ordinary_muscle_development	ENSGALG00010027970	GRIN3B	0.064481539	0.177927164	0.362404131	0.717050053	0.744210262	positive
PRJNA729271	stage_1W_vs_E10	ordinary_muscle_development	ENSGALG00010027971	ENSGALG00010027971	0.379286442	0.431227124	0.87955145	0.37910235	0.416892662	positive
PRJNA729271	stage_1W_vs_E10	ordinary_muscle_development	ENSGALG00010027972	TMEM259	0.938396932	0.105130618	8.926009855	4.41651E-19	1.59326E-18	positive
PRJNA729271	stage_1W_vs_E10	ordinary_muscle_development	ENSGALG00010027973	GAPVD1	0.717736861	0.064652336	11.10148373	1.23378E-28	6.56524E-28	positive
PRJNA729271	stage_1W_vs_E10	ordinary_muscle_development	ENSGALG00010027974	ENSGALG00010027974	0.976842485	0.286698681	3.407209558	0.000656307	0.000992506	positive
PRJNA729271	stage_1W_vs_E10	ordinary_muscle_development	ENSGALG00010027975	IQSEC1	-1.148219605	0.119671486	-9.594763457	8.41098E-22	3.39035E-21	negative
PRJNA729271	stage_1W_vs_E10	ordinary_muscle_development	ENSGALG00010027976	LRSAM1	0.265260097	0.097886286	2.709880074	0.006730754	0.009268695	positive
PRJNA729271	stage_1W_vs_E10	ordinary_muscle_development	ENSGALG00010027977	BOLA1	1.790853817	0.155039943	11.55091899	7.30353E-31	4.26873E-30	positive
PRJNA729271	stage_1W_vs_E10	ordinary_muscle_development	ENSGALG00010027979	RPL12	0.604681236	0.119544099	5.0582274	4.23172E-07	8.10501E-07	positive
PRJNA729271	stage_1W_vs_E10	ordinary_muscle_development	ENSGALG00010027980	NUP210	1.190993415	0.142053689	8.384107586	5.1112E-17	1.6578E-16	positive
PRJNA729271	stage_1W_vs_E10	ordinary_muscle_development	ENSGALG00010027981	CNN2	-1.539365482	0.162103271	-9.496202464	2.17684E-21	8.66136E-21	negative
PRJNA729271	stage_1W_vs_E10	ordinary_muscle_development	ENSGALG00010027982	ENTPD2	1.917883179	0.249188256	7.696523132	1.39818E-14	4.06717E-14	positive
PRJNA729271	stage_1W_vs_E10	ordinary_muscle_development	ENSGALG00010027983	POGZ	0.065270745	0.146046674	0.446917026	0.654934971	0.686029167	positive
PRJNA729271	stage_1W_vs_E10	ordinary_muscle_development	ENSGALG00010027984	ARHGAP45	1.661914678	0.168422581	9.867528859	5.75653E-23	2.43339E-22	positive
PRJNA729271	stage_1W_vs_E10	ordinary_muscle_development	ENSGALG00010027985	PSMB4	0.923495856	0.094598234	9.762294866	1.63415E-22	6.76578E-22	positive
PRJNA729271	stage_1W_vs_E10	ordinary_muscle_development	ENSGALG00010027986	CGN	-0.174436566	0.255431491	-0.682909399	0.494664094	0.532542784	negative
PRJNA729271	stage_1W_vs_E10	ordinary_muscle_development	ENSGALG00010027987	TMEM141	0.544756865	0.138467059	3.934198284	8.34749E-05	0.000135116	positive
PRJNA729271	stage_1W_vs_E10	ordinary_muscle_development	ENSGALG00010027988	HDAC11	1.134422045	0.127914243	8.868613991	7.40619E-19	2.63442E-18	positive
PRJNA729271	stage_1W_vs_E10	ordinary_muscle_development	ENSGALG00010027989	ENSGALG00010027989	0.959553487	0.137757874	6.965507352	3.27222E-12	8.43666E-12	positive
PRJNA729271	stage_1W_vs_E10	ordinary_muscle_development	ENSGALG00010027990	ENSGALG00010027990	1.293515713	0.190841509	6.777957908	1.21886E-11	3.05084E-11	positive
PRJNA729271	stage_1W_vs_E10	ordinary_muscle_development	ENSGALG00010027991	ENSGALG00010027991	0.353168935	0.146249526	2.414838155	0.015742203	0.020800659	positive
PRJNA729271	stage_1W_vs_E10	ordinary_muscle_development	ENSGALG00010027993	CLIC3	0.084718267	0.173677116	0.487791766	0.625697345	0.659283622	positive
PRJNA729271	stage_1W_vs_E10	ordinary_muscle_development	ENSGALG00010027994	TUFT1	-1.557281582	0.176416497	-8.82730135	1.07232E-18	3.78273E-18	negative
PRJNA729271	stage_1W_vs_E10	ordinary_muscle_development	ENSGALG00010027995	POLR2E	0.165668481	0.082285726	2.013331954	0.044079719	0.055439091	positive
PRJNA729271	stage_1W_vs_E10	ordinary_muscle_development	ENSGALG00010027996	GPX4	0.800861645	0.121780983	6.576245524	4.82476E-11	1.16889E-10	positive
PRJNA729271	stage_1W_vs_E10	ordinary_muscle_development	ENSGALG00010027998	SNX27	1.073865708	0.091590185	11.72468112	9.52591E-32	5.76767E-31	positive
PRJNA729271	stage_1W_vs_E10	ordinary_muscle_development	ENSGALG00010027999	ENSGALG00010027999	-2.333074153	0.573805678	-4.065965611	4.7834E-05	7.87991E-05	negative
PRJNA729271	stage_1W_vs_E10	ordinary_muscle_development	ENSGALG00010028000	MRPL9	1.437563274	0.165783131	8.671348297	4.27033E-18	1.45849E-17	positive
PRJNA729271	stage_1W_vs_E10	ordinary_muscle_development	ENSGALG00010028001	SBNO2	0.241043146	0.118417753	2.035532179	0.041797354	0.05268742	positive
PRJNA729271	stage_1W_vs_E10	ordinary_muscle_development	ENSGALG00010028002	ABCA2	-0.172835248	0.121170054	-1.426385827	0.153757002	0.180039401	negative
PRJNA729271	stage_1W_vs_E10	ordinary_muscle_development	ENSGALG00010028004	ENSGALG00010028004	-1.153917882	0.300050257	-3.845748683	0.000120185	0.000192198	negative
PRJNA729271	stage_1W_vs_E10	ordinary_muscle_development	ENSGALG00010028005	IER5L	-0.230442659	0.143909795	-1.601299338	0.109310636	0.130720045	negative
PRJNA729271	stage_1W_vs_E10	ordinary_muscle_development	ENSGALG00010028006	ENSGALG00010028006	3.650866413	0.801790809	4.5533902	5.27882E-06	9.33812E-06	positive
PRJNA729271	stage_1W_vs_E10	ordinary_muscle_development	ENSGALG00010028007	STK11	1.379321246	0.119057066	11.5853791	4.88799E-31	2.87313E-30	positive
PRJNA729271	stage_1W_vs_E10	ordinary_muscle_development	ENSGALG00010028008	NTMT1	1.459964371	0.112053304	13.02919521	8.34835E-39	6.4196E-38	positive
PRJNA729271	stage_1W_vs_E10	ordinary_muscle_development	ENSGALG00010028009	RRP9	0.034616148	0.178069853	0.194396453	0.845865467	0.862830398	positive
PRJNA729271	stage_1W_vs_E10	ordinary_muscle_development	ENSGALG00010028010	ASB6	-0.253514561	0.072523286	-3.49562981	0.000472944	0.000722222	negative
PRJNA729271	stage_1W_vs_E10	ordinary_muscle_development	ENSGALG00010028011	ENSGALG00010028011	-0.59607139	0.094181602	-6.328957841	2.46823E-10	5.72797E-10	negative
PRJNA729271	stage_1W_vs_E10	ordinary_muscle_development	ENSGALG00010028012	PRRX2	-4.016961108	0.258704685	-15.52720664	2.27057E-54	2.89208E-53	negative
PRJNA729271	stage_1W_vs_E10	ordinary_muscle_development	ENSGALG00010028013	CBARP	0.773892792	0.214346745	3.610471404	0.000305641	0.00047479	positive
PRJNA729271	stage_1W_vs_E10	ordinary_muscle_development	ENSGALG00010028014	ENSGALG00010028014	1.540085425	0.291099691	5.290577333	1.21931E-07	2.42174E-07	positive
PRJNA729271	stage_1W_vs_E10	ordinary_muscle_development	ENSGALG00010028015	DUSP7	-1.093831885	0.102384219	-10.68359842	1.2147E-26	5.99084E-26	negative
PRJNA729271	stage_1W_vs_E10	ordinary_muscle_development	ENSGALG00010028016	PRF1	3.96256211	1.26820078	3.12455423	0.001780747	0.002594164	positive
PRJNA729271	stage_1W_vs_E10	ordinary_muscle_development	ENSGALG00010028017	ENSGALG00010028017	-1.074915046	1.331875342	-0.807068809	0.419626841	0.457708711	negative
PRJNA729271	stage_1W_vs_E10	ordinary_muscle_development	ENSGALG00010028019	RPL29	0.420378577	0.227134338	1.850792709	0.06419938	0.079042727	positive
PRJNA729271	stage_1W_vs_E10	ordinary_muscle_development	ENSGALG00010028020	ENSGALG00010028020	0.650845753	0.152418564	4.270121264	1.95367E-05	3.31171E-05	positive
PRJNA729271	stage_1W_vs_E10	ordinary_muscle_development	ENSGALG00010028021	ATP5F1D	1.893573833	0.145073271	13.05253422	6.14682E-39	4.76971E-38	positive
PRJNA729271	stage_1W_vs_E10	ordinary_muscle_development	ENSGALG00010028022	ENSGALG00010028022	-0.495169953	0.143498601	-3.450695328	0.000559144	0.000849649	negative
PRJNA729271	stage_1W_vs_E10	ordinary_muscle_development	ENSGALG00010028023	ENSGALG00010028023	0.908028735	0.907296311	1.00080726	0.316919999	0.353039916	positive
PRJNA729271	stage_1W_vs_E10	ordinary_muscle_development	ENSGALG00010028024	RBM3	-1.982174236	0.113044598	-17.53444453	7.82101E-69	1.44048E-67	negative
PRJNA729271	stage_1W_vs_E10	ordinary_muscle_development	ENSGALG00010028025	TNNC1	4.80318514	0.237085031	20.25933532	2.93903E-91	9.04486E-90	positive
PRJNA729271	stage_1W_vs_E10	ordinary_muscle_development	ENSGALG00010028026	ANKMY1	0.898591654	0.166243081	5.405287549	6.47045E-08	1.31082E-07	positive
PRJNA729271	stage_1W_vs_E10	ordinary_muscle_development	ENSGALG00010028027	ENSGALG00010028027	1.580676722	0.229917345	6.874978128	6.19994E-12	1.57663E-11	positive
PRJNA729271	stage_1W_vs_E10	ordinary_muscle_development	ENSGALG00010028028	FAM174C	-0.261766	0.164736075	-1.589002293	0.112059875	0.133831086	negative
PRJNA729271	stage_1W_vs_E10	ordinary_muscle_development	ENSGALG00010028029	EFNA2	-6.421177255	0.312513295	-20.54689304	8.20494E-94	2.67862E-92	negative
PRJNA729271	stage_1W_vs_E10	ordinary_muscle_development	ENSGALG00010028030	PFDN2	0.498611045	0.134882717	3.696626636	0.000218483	0.000342814	positive
PRJNA729271	stage_1W_vs_E10	ordinary_muscle_development	ENSGALG00010028031	NISCH	0.827006732	0.08085138	10.2287275	1.47444E-24	6.6621E-24	positive
PRJNA729271	stage_1W_vs_E10	ordinary_muscle_development	ENSGALG00010028032	ENSGALG00010028032	-3.389618795	0.276792284	-12.246074	1.76327E-34	1.17457E-33	negative
PRJNA729271	stage_1W_vs_E10	ordinary_muscle_development	ENSGALG00010028033	ENSGALG00010028033	2.570081524	0.296584204	8.66560487	4.49122E-18	1.53033E-17	positive
PRJNA729271	stage_1W_vs_E10	ordinary_muscle_development	ENSGALG00010028034	MUM1L1	-0.069174914	0.07522154	-0.919615763	0.357773587	0.395359315	negative
PRJNA729271	stage_1W_vs_E10	ordinary_muscle_development	ENSGALG00010028035	ENSGALG00010028035	1.71590815	1.028933368	1.66765721	0.095383794	0.1150144	positive
PRJNA729271	stage_1W_vs_E10	ordinary_muscle_development	ENSGALG00010028036	NDUFS7	2.616663175	0.121810878	21.48135883	2.326E-102	9.0878E-101	positive
PRJNA729271	stage_1W_vs_E10	ordinary_muscle_development	ENSGALG00010028037	ENSGALG00010028037	-0.313336382	0.2841514	-1.102709267	0.270153441	0.304169188	negative
PRJNA729271	stage_1W_vs_E10	ordinary_muscle_development	ENSGALG00010028039	STAB1	1.861630847	0.162379792	11.46467071	1.98513E-30	1.13688E-29	positive
PRJNA729271	stage_1W_vs_E10	ordinary_muscle_development	ENSGALG00010028042	DPP7	0.975132697	0.191221709	5.099487405	3.40575E-07	6.56728E-07	positive
PRJNA729271	stage_1W_vs_E10	ordinary_muscle_development	ENSGALG00010028043	NT5DC2	1.845347736	0.115613946	15.96129018	2.37765E-57	3.25133E-56	positive
PRJNA729271	stage_1W_vs_E10	ordinary_muscle_development	ENSGALG00010028044	GAMT	4.796541973	0.153263838	31.2959797	5.2927E-215	8.9206E-213	positive
PRJNA729271	stage_1W_vs_E10	ordinary_muscle_development	ENSGALG00010028045	ENSGALG00010028045	-0.334833335	0.576864237	-0.580436978	0.561619975	0.597437365	negative
PRJNA729271	stage_1W_vs_E10	ordinary_muscle_development	ENSGALG00010028046	SMIM4	3.160421012	0.202923175	15.57447054	1.08548E-54	1.39239E-53	positive
PRJNA729271	stage_1W_vs_E10	ordinary_muscle_development	ENSGALG00010028047	ENSGALG00010028047	1.936931027	0.2870252	6.748296068	1.49591E-11	3.72373E-11	positive
PRJNA729271	stage_1W_vs_E10	ordinary_muscle_development	ENSGALG00010028048	S100A10	1.382667373	0.218532144	6.327066335	2.49866E-10	5.79675E-10	positive
PRJNA729271	stage_1W_vs_E10	ordinary_muscle_development	ENSGALG00010028049	MAN1B1	-0.204648785	0.060984644	-3.355742896	0.000791521	0.001190156	negative
PRJNA729271	stage_1W_vs_E10	ordinary_muscle_development	ENSGALG00010028050	PBRM1	-0.805942682	0.087909115	-9.167908008	4.82287E-20	1.81437E-19	negative
PRJNA729271	stage_1W_vs_E10	ordinary_muscle_development	ENSGALG00010028051	GNL3	-0.063612145	0.09239806	-0.688457583	0.491164673	0.529325794	negative
PRJNA729271	stage_1W_vs_E10	ordinary_muscle_development	ENSGALG00010028052	ENSGALG00010028052	-4.036262912	0.22273578	-18.12130461	2.16402E-73	4.41175E-72	negative
PRJNA729271	stage_1W_vs_E10	ordinary_muscle_development	ENSGALG00010028053	ORM1	2.837656338	2.151974511	1.318629158	0.187293121	0.216544132	positive
PRJNA729271	stage_1W_vs_E10	ordinary_muscle_development	ENSGALG00010028054	RPS15	0.115073857	0.140787525	0.817358339	0.413723677	0.451779772	positive
PRJNA729271	stage_1W_vs_E10	ordinary_muscle_development	ENSGALG00010028055	ENSGALG00010028055	-0.232972319	0.118080733	-1.972991802	0.048496496	0.060636308	negative
PRJNA729271	stage_1W_vs_E10	ordinary_muscle_development	ENSGALG00010028056	SLC25A25	1.963644334	0.236199022	8.313515953	9.29075E-17	2.98479E-16	positive
PRJNA729271	stage_1W_vs_E10	ordinary_muscle_development	ENSGALG00010028057	APC2	-1.12519704	0.388663738	-2.895039928	0.003791103	0.005349172	negative
PRJNA729271	stage_1W_vs_E10	ordinary_muscle_development	ENSGALG00010028058	GLT8D1	-1.107124125	0.11604651	-9.540348295	1.42353E-21	5.7095E-21	negative
PRJNA729271	stage_1W_vs_E10	ordinary_muscle_development	ENSGALG00010028059	GZMM	2.145137978	0.580154793	3.697526942	0.00021771	0.000341638	positive
PRJNA729271	stage_1W_vs_E10	ordinary_muscle_development	ENSGALG00010028060	ENSGALG00010028060	1.38734321	0.140079249	9.903988066	3.99999E-23	1.70129E-22	positive
PRJNA729271	stage_1W_vs_E10	ordinary_muscle_development	ENSGALG00010028061	NAIF1	-0.658812397	0.095698521	-6.884248434	5.80934E-12	1.47938E-11	negative
PRJNA729271	stage_1W_vs_E10	ordinary_muscle_development	ENSGALG00010028062	ENSGALG00010028062	0.806138249	0.488495365	1.650247489	0.098892328	0.119027177	positive
PRJNA729271	stage_1W_vs_E10	ordinary_muscle_development	ENSGALG00010028064	SPCS1	-0.381581838	0.08810851	-4.330817063	1.48557E-05	2.54201E-05	negative
PRJNA729271	stage_1W_vs_E10	ordinary_muscle_development	ENSGALG00010028065	REEP6	4.405716644	0.760343446	5.79437709	6.85752E-09	1.46911E-08	positive
PRJNA729271	stage_1W_vs_E10	ordinary_muscle_development	ENSGALG00010028066	ENSGALG00010028066	2.509201157	0.181844743	13.79859059	2.59884E-43	2.31247E-42	positive
PRJNA729271	stage_1W_vs_E10	ordinary_muscle_development	ENSGALG00010028067	FAM102A	-0.994778213	0.114782049	-8.666670639	4.4494E-18	1.51644E-17	negative
PRJNA729271	stage_1W_vs_E10	ordinary_muscle_development	ENSGALG00010028068	POLE4	-0.397679458	0.110788068	-3.589551333	0.000331248	0.000513083	negative
PRJNA729271	stage_1W_vs_E10	ordinary_muscle_development	ENSGALG00010028069	NEK4	-1.284418943	0.116518714	-11.02328459	2.95091E-28	1.55314E-27	negative
PRJNA729271	stage_1W_vs_E10	ordinary_muscle_development	ENSGALG00010028070	MEX3A	-3.907918994	0.164413491	-23.76884635	7.0159E-125	4.2373E-123	negative
PRJNA729271	stage_1W_vs_E10	ordinary_muscle_development	ENSGALG00010028071	DPM2	-0.991917996	0.11704077	-8.474978383	2.35123E-17	7.75099E-17	negative
PRJNA729271	stage_1W_vs_E10	ordinary_muscle_development	ENSGALG00010028072	ENSGALG00010028072	-7.467363633	0.991157905	-7.533979799	4.92167E-14	1.39254E-13	negative
PRJNA729271	stage_1W_vs_E10	ordinary_muscle_development	ENSGALG00010028073	ST6GALNAC4	3.586596621	0.120096763	29.86422387	5.7384E-196	7.9217E-194	positive
PRJNA729271	stage_1W_vs_E10	ordinary_muscle_development	ENSGALG00010028074	ENSGALG00010028074	0.544196949	0.466157638	1.167409702	0.243044955	0.275595449	positive
PRJNA729271	stage_1W_vs_E10	ordinary_muscle_development	ENSGALG00010028075	RAB25	-0.769332602	0.522849356	-1.471423067	0.141176739	0.166248829	negative
PRJNA729271	stage_1W_vs_E10	ordinary_muscle_development	ENSGALG00010028076	ST6GALNAC6	2.000679089	0.152143062	13.1499857	1.70207E-39	1.34011E-38	positive
PRJNA729271	stage_1W_vs_E10	ordinary_muscle_development	ENSGALG00010028078	LAMTOR2	1.388648698	0.13029422	10.657792	1.60359E-26	7.86868E-26	positive
PRJNA729271	stage_1W_vs_E10	ordinary_muscle_development	ENSGALG00010028079	ITIH3	6.561760391	1.529528238	4.290005091	1.78629E-05	3.03935E-05	positive
PRJNA729271	stage_1W_vs_E10	ordinary_muscle_development	ENSGALG00010028080	MUSTN1	2.221538723	0.405236327	5.482081881	4.2035E-08	8.60589E-08	positive
PRJNA729271	stage_1W_vs_E10	ordinary_muscle_development	ENSGALG00010028081	UBQLN4	0.265016514	0.161801551	1.637910844	0.101440294	0.121871285	positive
PRJNA729271	stage_1W_vs_E10	ordinary_muscle_development	ENSGALG00010028082	AK1	7.263546077	0.137101816	52.97921123	0	0	positive
PRJNA729271	stage_1W_vs_E10	ordinary_muscle_development	ENSGALG00010028083	ELAVL1	-0.193379	0.049276767	-3.92434434	8.69663E-05	0.000140579	negative
PRJNA729271	stage_1W_vs_E10	ordinary_muscle_development	ENSGALG00010028084	ENG	2.040733214	0.191868761	10.63608895	2.0245E-26	9.89717E-26	positive
PRJNA729271	stage_1W_vs_E10	ordinary_muscle_development	ENSGALG00010028085	ENSGALG00010028085	-0.905971317	0.290374029	-3.120014968	0.001808419	0.002631566	negative
PRJNA729271	stage_1W_vs_E10	ordinary_muscle_development	ENSGALG00010028086	MATK	0.28559297	0.275579314	1.03636749	0.300045068	0.335401655	positive
PRJNA729271	stage_1W_vs_E10	ordinary_muscle_development	ENSGALG00010028087	STIMATE	-1.08555282	0.090797154	-11.95580229	6.0546E-33	3.81737E-32	negative
PRJNA729271	stage_1W_vs_E10	ordinary_muscle_development	ENSGALG00010028088	NAXE	1.534631087	0.114229227	13.43466227	3.78793E-41	3.15915E-40	positive
PRJNA729271	stage_1W_vs_E10	ordinary_muscle_development	ENSGALG00010028091	FPGS	-0.53542684	0.099088584	-5.4035169	6.53468E-08	1.32344E-07	negative
PRJNA729271	stage_1W_vs_E10	ordinary_muscle_development	ENSGALG00010028093	SFMBT1	-0.6839423	0.144040016	-4.748279802	2.05154E-06	3.74334E-06	negative
PRJNA729271	stage_1W_vs_E10	ordinary_muscle_development	ENSGALG00010028095	CDK9	-0.080288981	0.093690074	-0.856963581	0.391465012	0.429512176	negative
PRJNA729271	stage_1W_vs_E10	ordinary_muscle_development	ENSGALG00010028096	TOR2A	-0.957087391	0.15463871	-6.189183769	6.04765E-10	1.3755E-09	negative
PRJNA729271	stage_1W_vs_E10	ordinary_muscle_development	ENSGALG00010028097	TTC24	2.677530567	0.216099675	12.39025728	2.95095E-35	2.02241E-34	positive
PRJNA729271	stage_1W_vs_E10	ordinary_muscle_development	ENSGALG00010028098	RFT1	-0.574179709	0.120872428	-4.750295137	2.0312E-06	3.70856E-06	negative
PRJNA729271	stage_1W_vs_E10	ordinary_muscle_development	ENSGALG00010028099	PCASP2	2.17972586	0.252872898	8.619847685	6.70432E-18	2.26683E-17	positive
PRJNA729271	stage_1W_vs_E10	ordinary_muscle_development	ENSGALG00010028100	ENSGALG00010028100	1.544843724	0.459052836	3.36528522	0.000764646	0.001150939	positive
PRJNA729271	stage_1W_vs_E10	ordinary_muscle_development	ENSGALG00010028101	MRPL54	1.379360926	0.126531284	10.90134301	1.13568E-27	5.83336E-27	positive
PRJNA729271	stage_1W_vs_E10	ordinary_muscle_development	ENSGALG00010028102	CERCAM	-0.807733243	0.180988982	-4.462886262	8.0863E-06	1.4115E-05	negative
PRJNA729271	stage_1W_vs_E10	ordinary_muscle_development	ENSGALG00010028103	APBA3	0.585498852	0.130547787	4.484938945	7.29348E-06	1.27665E-05	positive
PRJNA729271	stage_1W_vs_E10	ordinary_muscle_development	ENSGALG00010028104	GOLGA1	0.377208495	0.115139182	3.276108875	0.00105248	0.001563404	positive
PRJNA729271	stage_1W_vs_E10	ordinary_muscle_development	ENSGALG00010028105	PRKCD	-0.941226827	0.143852608	-6.542994526	6.0299E-11	1.44948E-10	negative
PRJNA729271	stage_1W_vs_E10	ordinary_muscle_development	ENSGALG00010028106	ARPC5L	0.925920105	0.10176035	9.09026294	9.11453E-20	3.39104E-19	positive
PRJNA729271	stage_1W_vs_E10	ordinary_muscle_development	ENSGALG00010028107	NOTCH1	-0.586140702	0.124715942	-4.699805748	2.60409E-06	4.71888E-06	negative
PRJNA729271	stage_1W_vs_E10	ordinary_muscle_development	ENSGALG00010028108	IQGAP3	-1.617261945	0.211293435	-7.654104076	1.94665E-14	5.61413E-14	negative

PRJNA729271	stage_1W_vs_E10	ordinary_muscle_development	ENSGALG00010028109	TJP3	-0.466022549	0.221209836	-2.106699042	0.035143675	0.044684875	negative
PRJNA729271	stage_1W_vs_E10	ordinary_muscle_development	ENSGALG00010028110	COQ4	0.809313184	0.101080933	8.006586009	1.17937E-15	3.61721E-15	positive
PRJNA729271	stage_1W_vs_E10	ordinary_muscle_development	ENSGALG00010028111	TRUB2	-0.440769117	0.090200593	-4.886543473	1.02622E-06	1.91738E-06	negative
PRJNA729271	stage_1W_vs_E10	ordinary_muscle_development	ENSGALG00010028112	SWI5	0.144343223	0.081685643	1.767057435	0.07721861	0.094128648	positive
PRJNA729271	stage_1W_vs_E10	ordinary_muscle_development	ENSGALG00010028113	PIP5K1C	-0.922235622	0.098964432	-9.318859334	1.17598E-20	4.55161E-20	negative
PRJNA729271	stage_1W_vs_E10	ordinary_muscle_development	ENSGALG00010028114	PFKFB4	-0.682492903	0.091948246	-7.422576647	1.14863E-13	3.18284E-13	negative
PRJNA729271	stage_1W_vs_E10	ordinary_muscle_development	ENSGALG00010028115	CACTIN	0.759017368	0.17505483	4.335883613	1.45176E-05	2.48738E-05	positive
PRJNA729271	stage_1W_vs_E10	ordinary_muscle_development	ENSGALG00010028116	HYAL1	1.952251035	0.146842759	13.29484036	2.48002E-40	2.00938E-39	positive
PRJNA729271	stage_1W_vs_E10	ordinary_muscle_development	ENSGALG00010028117	ENSGALG00010028117	0.840236422	0.228624567	3.675179938	0.000237682	0.000372212	positive
PRJNA729271	stage_1W_vs_E10	ordinary_muscle_development	ENSGALG00010028118	ENSGALG00010028118	0.079505388	0.07758285	1.024780462	0.305466787	0.341041445	positive
PRJNA729271	stage_1W_vs_E10	ordinary_muscle_development	ENSGALG00010028119	HYAL2	0.563035489	0.127267415	4.424034946	9.68743E-06	1.68247E-05	positive
PRJNA729271	stage_1W_vs_E10	ordinary_muscle_development	ENSGALG00010028120	TBXA2R	0.248357604	0.238724951	1.040350426	0.298177124	0.333467897	positive
PRJNA729271	stage_1W_vs_E10	ordinary_muscle_development	ENSGALG00010028121	TUSC2	0.608061065	0.095642153	6.357668113	2.04839E-10	4.77662E-10	positive
PRJNA729271	stage_1W_vs_E10	ordinary_muscle_development	ENSGALG00010028122	SEC16A	-0.071367952	0.088823307	-0.803482261	0.421696046	0.459792702	negative
PRJNA729271	stage_1W_vs_E10	ordinary_muscle_development	ENSGALG00010028123	RASSF1	0.183873428	0.10683756	1.721056039	0.085240656	0.103420382	positive
PRJNA729271	stage_1W_vs_E10	ordinary_muscle_development	ENSGALG00010028124	SUGP2	-0.978106614	0.090215539	-10.84188628	2.17931E-27	1.10683E-26	negative
PRJNA729271	stage_1W_vs_E10	ordinary_muscle_development	ENSGALG00010028125	HMG20B	-1.267973826	0.123741784	-10.24693349	1.22158E-24	5.54029E-24	negative
PRJNA729271	stage_1W_vs_E10	ordinary_muscle_development	ENSGALG00010028126	ZMYND10	1.484197498	0.079503113	18.66841989	8.94606E-78	2.00732E-76	positive
PRJNA729271	stage_1W_vs_E10	ordinary_muscle_development	ENSGALG00010028127	MFSD12	-0.282299378	0.175436868	-1.609122311	0.1075896	0.128768165	negative
PRJNA729271	stage_1W_vs_E10	ordinary_muscle_development	ENSGALG00010028128	DNM1	-2.498279661	0.144782497	-17.25539839	1.01916E-66	1.75033E-65	negative
PRJNA729271	stage_1W_vs_E10	ordinary_muscle_development	ENSGALG00010028129	CYB561D2	1.1838099	0.104029728	11.37953469	5.28811E-30	2.99068E-29	positive
PRJNA729271	stage_1W_vs_E10	ordinary_muscle_development	ENSGALG00010028130	TMEM115	0.894732853	0.126559798	7.069645109	1.5533E-12	4.06998E-12	positive
PRJNA729271	stage_1W_vs_E10	ordinary_muscle_development	ENSGALG00010028131	TMEM161A	1.800827742	0.202057156	8.912467036	4.99094E-19	1.79468E-18	positive
PRJNA729271	stage_1W_vs_E10	ordinary_muscle_development	ENSGALG00010028132	CACNA2D2	-0.132943382	0.13209279	-1.006439354	0.314204271	0.35019791	negative
PRJNA729271	stage_1W_vs_E10	ordinary_muscle_development	ENSGALG00010028133	ENSGALG00010028133	1.607951785	0.293127161	5.485509367	4.1228E-08	8.44544E-08	positive
PRJNA729271	stage_1W_vs_E10	ordinary_muscle_development	ENSGALG00010028134	INPP5E	-1.15337895	0.073911066	-15.60495629	6.73558E-55	8.67072E-54	negative
PRJNA729271	stage_1W_vs_E10	ordinary_muscle_development	ENSGALG00010028136	C3orf18	-1.093718703	0.163515698	-6.688768811	2.25056E-11	5.55549E-11	negative
PRJNA729271	stage_1W_vs_E10	ordinary_muscle_development	ENSGALG00010028137	NUP214	-0.779324966	0.0674453	-11.55491875	6.97134E-31	4.07951E-30	negative
PRJNA729271	stage_1W_vs_E10	ordinary_muscle_development	ENSGALG00010028138	ENSGALG00010028138	-0.378261013	0.507697428	-0.745052056	0.456240251	0.494445042	negative
PRJNA729271	stage_1W_vs_E10	ordinary_muscle_development	ENSGALG00010028139	FZR1	-1.021533972	0.088787468	-11.50538462	1.23936E-30	7.18291E-30	negative
PRJNA729271	stage_1W_vs_E10	ordinary_muscle_development	ENSGALG00010028140	HEMK1	-0.577837505	0.112191731	-5.150446478	2.59867E-07	5.04997E-07	negative
PRJNA729271	stage_1W_vs_E10	ordinary_muscle_development	ENSGALG00010028141	CISH	3.046288464	0.186180233	16.36204025	3.56965E-60	5.25301E-59	positive
PRJNA729271	stage_1W_vs_E10	ordinary_muscle_development	ENSGALG00010028142	PMPCA	0.420683008	0.121928466	3.450244421	0.000560079	0.00085098	positive
PRJNA729271	stage_1W_vs_E10	ordinary_muscle_development	ENSGALG00010028144	FAM78A	1.518883804	0.184888842	8.215118815	2.11954E-16	6.69779E-16	positive
PRJNA729271	stage_1W_vs_E10	ordinary_muscle_development	ENSGALG00010028145	ENSGALG00010028145	0.148589339	0.139059401	1.068531418	0.285280866	0.319909187	positive
PRJNA729271	stage_1W_vs_E10	ordinary_muscle_development	ENSGALG00010028146	MAPKAPK3	0.390834232	0.106123042	3.682840441	0.00023065	0.000361473	positive
PRJNA729271	stage_1W_vs_E10	ordinary_muscle_development	ENSGALG00010028147	SLC25A42	1.28464	0.199245031	6.44753846	1.13681E-10	2.69426E-10	positive
PRJNA729271	stage_1W_vs_E10	ordinary_muscle_development	ENSGALG00010028148	ENSGALG00010028148	6.929762193	0.25498629	27.17699921	1.2149E-162	1.2144E-160	positive
PRJNA729271	stage_1W_vs_E10	ordinary_muscle_development	ENSGALG00010028149	SNAPC4	0.071834306	0.089318819	0.804246043	0.421254892	0.459380805	positive
PRJNA729271	stage_1W_vs_E10	ordinary_muscle_development	ENSGALG00010028150	ARMC6	0.048420588	0.081635804	0.593129315	0.553094613	0.589276473	positive
PRJNA729271	stage_1W_vs_E10	ordinary_muscle_development	ENSGALG00010028151	DOCK3	-1.600641532	0.266870494	-5.997821304	1.99982E-09	4.4303E-09	negative
PRJNA729271	stage_1W_vs_E10	ordinary_muscle_development	ENSGALG00010028152	ENSGALG00010028152	-0.565172515	0.373936069	-1.511414817	0.130682797	0.154544108	negative
PRJNA729271	stage_1W_vs_E10	ordinary_muscle_development	ENSGALG00010028153	MANF	-0.096483487	0.14749129	-0.654163967	0.513006174	0.550286723	negative
PRJNA729271	stage_1W_vs_E10	ordinary_muscle_development	ENSGALG00010028154	ENSGALG00010028154	-1.058022352	0.135022734	-7.835883017	4.65559E-15	1.38825E-14	negative
PRJNA729271	stage_1W_vs_E10	ordinary_muscle_development	ENSGALG00010028155	CIZ1	-0.436817918	0.0789299	-5.534251522	3.1256E-08	6.43911E-08	negative
PRJNA729271	stage_1W_vs_E10	ordinary_muscle_development	ENSGALG00010028156	DOHH	0.839897666	0.095916034	8.756592972	2.01241E-18	6.9801E-18	positive
PRJNA729271	stage_1W_vs_E10	ordinary_muscle_development	ENSGALG00010028157	PRRC2B	-0.332982747	0.145148037	-2.294090607	0.021785295	0.028353942	negative
PRJNA729271	stage_1W_vs_E10	ordinary_muscle_development	ENSGALG00010028158	DDX49	-0.009709691	0.09798959	-0.099089001	0.921067605	0.930569104	negative
PRJNA729271	stage_1W_vs_E10	ordinary_muscle_development	ENSGALG00010028159	DCAF1	0.238975747	0.090169637	2.650290662	0.008042255	0.010983933	positive
PRJNA729271	stage_1W_vs_E10	ordinary_muscle_development	ENSGALG00010028160	ENSGALG00010028160	5.441832955	0.5488835	9.9143679	3.60531E-23	1.53703E-22	positive
PRJNA729271	stage_1W_vs_E10	ordinary_muscle_development	ENSGALG00010028161	ENSGALG00010028161	1.925861021	0.590490745	3.261458433	0.001108407	0.001642441	positive
PRJNA729271	stage_1W_vs_E10	ordinary_muscle_development	ENSGALG00010028162	CARD9	0.317538052	0.178111003	1.782809859	0.07461726	0.091149266	positive
PRJNA729271	stage_1W_vs_E10	ordinary_muscle_development	ENSGALG00010028163	GBP	2.207257091	0.350115664	6.304365433	2.89377E-10	6.6973E-10	positive
PRJNA729271	stage_1W_vs_E10	ordinary_muscle_development	ENSGALG00010028164	BBLN	-0.127092063	0.141602262	-0.897528478	0.369436984	0.407099672	negative
PRJNA729271	stage_1W_vs_E10	ordinary_muscle_development	ENSGALG00010028165	NFIC	1.550587516	0.156170388	9.928818986	3.11928E-23	1.33453E-22	positive
PRJNA729271	stage_1W_vs_E10	ordinary_muscle_development	ENSGALG00010028168	PTGES2	0.99873412	0.137599722	7.25825683	3.92111E-13	1.05762E-12	positive
PRJNA729271	stage_1W_vs_E10	ordinary_muscle_development	ENSGALG00010028169	DNLZ	1.757322584	0.141629007	12.40792842	2.36698E-35	1.62758E-34	positive
PRJNA729271	stage_1W_vs_E10	ordinary_muscle_development	ENSGALG00010028170	COPE	-1.407072211	0.095370267	-14.75378284	2.90892E-49	3.10492E-48	negative
PRJNA729271	stage_1W_vs_E10	ordinary_muscle_development	ENSGALG00010028171	RNF123	2.383951114	0.157725203	15.11458581	1.29785E-51	1.50619E-50	positive
PRJNA729271	stage_1W_vs_E10	ordinary_muscle_development	ENSGALG00010028172	CERS1	0.220361341	0.210687866	1.045913773	0.295600845	0.330739811	positive
PRJNA729271	stage_1W_vs_E10	ordinary_muscle_development	ENSGALG00010028173	GDF3	0.3072451	0.227827338	1.348587498	0.177469499	0.20579363	positive
PRJNA729271	stage_1W_vs_E10	ordinary_muscle_development	ENSGALG00010028174	GMPPB	-0.63306122	0.155278252	-4.076947105	4.56309E-05	7.53239E-05	negative
PRJNA729271	stage_1W_vs_E10	ordinary_muscle_development	ENSGALG00010028175	POMT1	0.138532932	0.093823409	1.476528441	0.139802086	0.164750507	positive
PRJNA729271	stage_1W_vs_E10	ordinary_muscle_development	ENSGALG00010028176	UPF1	-0.214430357	0.095100415	-2.254778348	0.024147245	0.031301584	negative
PRJNA729271	stage_1W_vs_E10	ordinary_muscle_development	ENSGALG00010028177	ENSGALG00010028177	5.147500792	0.388252833	13.25811523	4.04946E-40	3.25551E-39	positive
PRJNA729271	stage_1W_vs_E10	ordinary_muscle_development	ENSGALG00010028178	ENSGALG00010028178	-2.578577693	0.168769981	-15.27865133	1.06114E-52	1.28391E-51	negative
PRJNA729271	stage_1W_vs_E10	ordinary_muscle_development	ENSGALG00010028179	MCM2	-2.983448294	0.145888277	-20.45022634	5.97971E-93	1.91761E-91	negative
PRJNA729271	stage_1W_vs_E10	ordinary_muscle_development	ENSGALG00010028180	ODF2	-1.145475258	0.106008275	-10.80552681	3.24091E-27	1.63171E-26	negative
PRJNA729271	stage_1W_vs_E10	ordinary_muscle_development	ENSGALG00010028181	BGLAP	-0.462220934	0.278895966	-1.657323842	0.09745402	0.117393502	negative
PRJNA729271	stage_1W_vs_E10	ordinary_muscle_development	ENSGALG00010028182	SMG5	0.368963602	0.146618166	2.516493088	0.011852922	0.015886094	positive
PRJNA729271	stage_1W_vs_E10	ordinary_muscle_development	ENSGALG00010028183	CRTC1	0.26698306	0.098133486	2.720611202	0.006516135	0.008985956	positive
PRJNA729271	stage_1W_vs_E10	ordinary_muscle_development	ENSGALG00010028184	TMEM79	1.487584807	0.247430059	6.012142636	1.83087E-09	4.06595E-09	positive
PRJNA729271	stage_1W_vs_E10	ordinary_muscle_development	ENSGALG00010028185	GLMP	0.434157509	0.087174173	4.980345591	6.34708E-07	1.20231E-06	positive
PRJNA729271	stage_1W_vs_E10	ordinary_muscle_development	ENSGALG00010028186	CCT3	0.193494317	0.132204228	1.463601576	0.143302842	0.168574476	positive
PRJNA729271	stage_1W_vs_E10	ordinary_muscle_development	ENSGALG00010028187	TPRA1	0.427273922	0.070141241	6.091621942	1.11772E-09	2.50796E-09	positive
PRJNA729271	stage_1W_vs_E10	ordinary_muscle_development	ENSGALG00010028188	ENSGALG00010028188	0.860406905	0.10809086	7.960033846	1.71992E-15	5.23525E-15	positive
PRJNA729271	stage_1W_vs_E10	ordinary_muscle_development	ENSGALG00010028189	GPSM1	-0.414679003	0.108497536	-3.822013078	0.000132367	0.000210934	negative
PRJNA729271	stage_1W_vs_E10	ordinary_muscle_development	ENSGALG00010028190	ILF2	-0.795297465	0.080920961	-9.828077366	8.52303E-23	3.57366E-22	negative
PRJNA729271	stage_1W_vs_E10	ordinary_muscle_development	ENSGALG00010028191	KLHL26	0.64814914	0.099166346	6.535978855	6.31948E-11	1.51657E-10	positive
PRJNA729271	stage_1W_vs_E10	ordinary_muscle_development	ENSGALG00010028192	ENSGALG00010028192	1.772330994	0.327442858	5.412642094	6.21015E-08	1.25964E-07	positive
PRJNA729271	stage_1W_vs_E10	ordinary_muscle_development	ENSGALG00010028193	BRINP1	-5.711228749	0.292384256	-19.53329781	5.72221E-85	1.53884E-83	negative
PRJNA729271	stage_1W_vs_E10	ordinary_muscle_development	ENSGALG00010028194	ENSGALG00010028194	1.834204962	0.181406434	10.11102486	4.93651E-24	2.1769E-23	positive
PRJNA729271	stage_1W_vs_E10	ordinary_muscle_development	ENSGALG00010028195	ENSGALG00010028195	1.472394378	0.303559695	4.850427777	1.23195E-06	2.28674E-06	positive
PRJNA729271	stage_1W_vs_E10	ordinary_muscle_development	ENSGALG00010028196	ENSGALG00010028196	-1.744694592	0.099553586	-17.52518082	9.20485E-69	1.68891E-67	negative
PRJNA729271	stage_1W_vs_E10	ordinary_muscle_development	ENSGALG00010028197	INTS3	0.155816598	0.153721153	1.013631473	0.310758634	0.346655872	positive
PRJNA729271	stage_1W_vs_E10	ordinary_muscle_development	ENSGALG00010028198	UCK1	-0.187283787	0.092394	-2.02701244	0.04266114	0.053724868	negative
PRJNA729271	stage_1W_vs_E10	ordinary_muscle_development	ENSGALG00010028199	DPP9	0.237301533	0.106476856	2.228667723	0.025836021	0.033356474	positive
PRJNA729271	stage_1W_vs_E10	ordinary_muscle_development	ENSGALG00010028201	ENSGALG00010028201	3.525387447	0.159882799	22.04982315	9.5898E-108	4.1494E-106	positive
PRJNA729271	stage_1W_vs_E10	ordinary_muscle_development	ENSGALG00010028202	QSOX2	-0.524276454	0.067689626	-7.745299914	9.53565E-15	2.79966E-14	negative
PRJNA729271	stage_1W_vs_E10	ordinary_muscle_development	ENSGALG00010028205	PRRT1B	-0.23907362	0.448372293	-0.533203376	0.593892805	0.628355928	negative
PRJNA729271	stage_1W_vs_E10	ordinary_muscle_development	ENSGALG00010028206	RBM15B	0.013233127	0.094436863	0.140126714	0.888559874	0.900907066	positive
PRJNA729271	stage_1W_vs_E10	ordinary_muscle_development	ENSGALG00010028207	ENSGALG00010028207	0.378471543	0.27785862	1.362101138	0.173165969	0.201119422	positive
PRJNA729271	stage_1W_vs_E10	ordinary_muscle_development	ENSGALG00010028211	GATAD2B	0.092758983	0.147187621	0.630209131	0.528557766	0.565086651	positive
PRJNA729271	stage_1W_vs_E10	ordinary_muscle_development	ENSGALG00010028216	NKX2-3	-0.432519637	1.582958047	-0.27323506	0.784672531	0.807226993	negative
PRJNA729271	stage_1W_vs_E10	ordinary_muscle_development	ENSGALG00010028221	TNFAIP8L1	-2.041157749	0.193685572	-10.53851214	5.73998E-26	2.74772E-25	negative
PRJNA729271	stage_1W_vs_E10	ordinary_muscle_development	ENSGALG00010028232	C9orf69	1.164382491	0.108220259	10.75937631	5.35312E-27	2.67103E-26	positive
PRJNA729271	stage_1W_vs_E10	ordinary_muscle_development	ENSGALG00010028239	EDMPN2	1.135008081	1.64549537	0.689766803	0.490340848	0.528516552	positive
PRJNA729271	stage_1W_vs_E10	ordinary_muscle_development	ENSGALG00010028242	ENSGALG00010028242	0.193864295	0.280078985	0.692177226	0.488826039	0.527079777	positive
PRJNA729271	stage_1W_vs_E10	ordinary_muscle_development	ENSGALG00010028245	JAC1	-0.272155029	1.48470997	-0.183305181	0.85455857	0.870839881	negative
PRJNA729271	stage_1W_vs_E10	ordinary_muscle_development	ENSGALG00010028247	MVP	-0.061743953	0.093825479	-0.658072344	0.510491646	0.548076173	negative
PRJNA729271	stage_1W_vs_E10	ordinary_muscle_development	ENSGALG00010028262	C1orf43	2.048426298	0.141727259	14.45329797	2.38964E-47	2.39377E-46	positive
PRJNA729271	stage_1W_vs_E10	ordinary_muscle_development	ENSGALG00010028270	RAPGEF1	0.989728951	0.181226525	5.461280838	4.72712E-08	9.65402E-08	positive
PRJNA729271	stage_1W_vs_E10	ordinary_muscle_development	ENSGALG00010028279	EDBETA	0.355873384	1.629315883	0.218418901	0.82710274	0.845714886	positive
PRJNA729271	stage_1W_vs_E10	ordinary_muscle_development	ENSGALG00010028283	ENSGALG00010028283	0.502920027	0.151764422	3.313820331	0.000920306	0.001376518	positive
PRJNA729271	stage_1W_vs_E10	ordinary_muscle_development	ENSGALG00010028286	KCTD12A	-0.302442997	1.447828087	-0.208894274	0.834530774	0.852588354	negative
PRJNA729271	stage_1W_vs_E10	ordinary_muscle_development	ENSGALG00010028292	ENSGALG00010028292	-1.645411633	0.568701858	-2.893276345	0.003812457	0.005377208	negative
PRJNA729271	stage_1W_vs_E10	ordinary_muscle_development	ENSGALG00010028301	FBN3	-4.276979064	0.186073011	-22.98548855	6.5115E-117	3.3234E-115	negative
PRJNA729271	stage_1W_vs_E10	ordinary_muscle_development	ENSGALG00010028311	ENSGALG00010028311	-3.262403842	0.276761186	-11.78779397	4.51205E-32	2.76192E-31	negative
PRJNA729271	stage_1W_vs_E10	ordinary_muscle_development	ENSGALG00010028314	MEF2D	2.491341363	0.213655173	11.66057123	2.02678E-31	1.21197E-30	positive
PRJNA729271	stage_1W_vs_E10	ordinary_muscle_development	ENSGALG00010028316	TIMM44	-0.283456413	0.081034062	-3.497990906	0.000468777	0.00071616	negative
PRJNA729271	stage_1W_vs_E10	ordinary_muscle_development	ENSGALG00010028318	NACC2	0.859382374	0.195053702	4.405875736	1.05357E-05	1.82565E-05	positive
PRJNA729271	stage_1W_vs_E10	ordinary_muscle_development	ENSGALG00010028321	RHBG	0.653664499	0.190007901	3.4401964	0.000581292	0.000882286	positive
PRJNA729271	stage_1W_vs_E10	ordinary_muscle_development	ENSGALG00010028322	STRBP	0.071723082	0.109192357	0.656850754	0.511276886	0.548797279	positive
PRJNA729271	stage_1W_vs_E10	ordinary_muscle_development	ENSGALG00010028326	CRB2	-0.880854728	0.243734932	-3.613986399	0.000301525	0.000468697	negative
PRJNA729271	stage_1W_vs_E10	ordinary_muscle_development	ENSGALG00010028328	HNRNPM	-1.180743245	0.165978027	-7.113852759	1.12848E-12	2.98272E-12	negative
PRJNA729271	stage_1W_vs_E10	ordinary_muscle_development	ENSGALG00010028329	HAPLN2	0.775092672	0.605368163	1.280365766	0.200416528	0.230705017	positive
PRJNA729271	stage_1W_vs_E10	ordinary_muscle_development	ENSGALG00010028337	ENSGALG00010028337	4.051768941	0.196895033	20.57831975	4.29306E-94	1.41427E-92	positive
PRJNA729271	stage_1W_vs_E10	ordinary_muscle_development	ENSGALG00010028339	BCAN	-3.490732417	0.490754972	-7.112984313	1.1356E-12	3.00101E-12	negative
PRJNA729271	stage_1W_vs_E10	ordinary_muscle_development	ENSGALG00010028344	LSM7	-2.248118294	0.098894665	-22.73245281	2.1404E-114	1.0307E-112	negative
PRJNA729271	stage_1W_vs_E10	ordinary_muscle_development	ENSGALG00010028346	DENND1A	-0.352458356	0.074554733	-4.72751148	2.27288E-06	4.13109E-06	negative
PRJNA729271	stage_1W_vs_E10	ordinary_muscle_development	ENSGALG00010028348	NES	-5.488482797	0.28001369	-19.60076597	1.52314E-85	4.16564E-84	negative
PRJNA729271	stage_1W_vs_E10	ordinary_muscle_development	ENSGALG00010028353	ENSGALG00010028353	1.13501261	1.620473986	0.700420136	0.483664965	0.521903049	positive
PRJNA729271	stage_1W_vs_E10	ordinary_muscle_development	ENSGALG00010028355	RBM18	-0.059598976	0.074263411	-0.802534868	0.422243627	0.46026518	negative
PRJNA729271	stage_1W_vs_E10	ordinary_muscle_development	ENSGALG00010028357	SPPL2C	-0.603911232	0.093614995	-6.451009573	1.11107E-10	2.63585E-10	negative
PRJNA729271	stage_1W_vs_E10	ordinary_muscle_development	ENSGALG00010028360	ENSGALG00010028360	-6.471284703	0.989478289	-6.540097724	6.14786E-11	1.47685E-10	negative
PRJNA729271	stage_1W_vs_E10	ordinary_muscle_development	ENSGALG00010028362	TMPRSS9	-3.832607586	0.525590444	-7.292003933	3.05378E-13	8.28149E-13	negative
PRJNA729271	stage_1W_vs_E10	ordinary_muscle_development	ENSGALG00010028364	MHM2	7.283141837	0.67245436	10.83068572	2.46304E-27	1.24919E-26	positive
PRJNA729271	stage_1W_vs_E10	ordinary_muscle_development	ENSGALG00010028366	MRRF	0.375997551	0.086276116	4.358072273	1.31213E-05	2.25588E-05	positive
PRJNA729271	stage_1W_vs_E10	ordinary_muscle_development	ENSGALG00010028368	TIMM13	0.524939056	0.120971315	4.339368027	1.42893E-05	2.45001E-05	positive
PRJNA729271	stage_1W_vs_E10	ordinary_muscle_development	ENSGALG00010028370	S100A12	1.02189468	0.676770923	1.509956537	0.131054515	0.154958414	positive
PRJNA729271	stage_1W_vs_E10	ordinary_muscle_development	ENSGALG00010028372	PTGS1	-0.152017782	0.164693973	-0.923031845	0.35599061	0.39353904	negative

PRJNA729271	stage_1W_vs_E10	ordinary_muscle_development	ENSGALG00010028374	FDPS	1.528491659	0.129499108	11.80310573	3.76167E-32	2.30844E-31	positive
PRJNA729271	stage_1W_vs_E10	ordinary_muscle_development	ENSGALG00010028375	LMNB2	-2.146507543	0.15287496	-14.04093609	8.75485E-45	8.14516E-44	negative
PRJNA729271	stage_1W_vs_E10	ordinary_muscle_development	ENSGALG00010028377	PDCL	-0.153846266	0.059971563	-2.56532025	0.010308062	0.01391484	negative
PRJNA729271	stage_1W_vs_E10	ordinary_muscle_development	ENSGALG00010028380	RUSC1	1.079279822	0.141329344	7.636629401	2.22982E-14	6.41549E-14	positive
PRJNA729271	stage_1W_vs_E10	ordinary_muscle_development	ENSGALG00010028384	ENSGALG00010028384	-1.442580042	1.16546189	-1.237775387	0.215799357	0.246961289	negative
PRJNA729271	stage_1W_vs_E10	ordinary_muscle_development	ENSGALG00010028386	NCAN	-7.862099443	0.883233916	-8.901491777	5.5101E-19	1.97793E-18	negative
PRJNA729271	stage_1W_vs_E10	ordinary_muscle_development	ENSGALG00010028391	MED26	0.691226629	0.112677223	6.134572835	8.53883E-10	1.92669E-09	positive
PRJNA729271	stage_1W_vs_E10	ordinary_muscle_development	ENSGALG00010028392	ASH1L	0.888510306	0.120802027	7.355094321	1.90792E-13	5.22489E-13	positive
PRJNA729271	stage_1W_vs_E10	ordinary_muscle_development	ENSGALG00010028394	ZBTB26	-0.086150374	0.103875353	-0.829363001	0.406899031	0.444930707	negative
PRJNA729271	stage_1W_vs_E10	ordinary_muscle_development	ENSGALG00010028399	SLC35E1	0.241731739	0.079195834	3.052328986	0.002270731	0.003271143	positive
PRJNA729271	stage_1W_vs_E10	ordinary_muscle_development	ENSGALG00010028405	S100A6	-0.29458264	0.280351382	-1.050762215	0.293367814	0.328393425	negative
PRJNA729271	stage_1W_vs_E10	ordinary_muscle_development	ENSGALG00010028411	LHX2	-5.815822287	0.98243113	-5.919826957	3.22281E-09	7.04487E-09	negative
PRJNA729271	stage_1W_vs_E10	ordinary_muscle_development	ENSGALG00010028412	ENSGALG00010028412	0.98148128	0.317401981	3.092234257	0.00198656	0.002878368	positive
PRJNA729271	stage_1W_vs_E10	ordinary_muscle_development	ENSGALG00010028414	KBTBD12	7.084830245	0.350514841	20.21263988	7.57843E-91	2.2981E-89	positive
PRJNA729271	stage_1W_vs_E10	ordinary_muscle_development	ENSGALG00010028415	FSD1	0.691026127	0.167444327	4.126900796	3.67685E-05	6.11683E-05	positive
PRJNA729271	stage_1W_vs_E10	ordinary_muscle_development	ENSGALG00010028418	ENSGALG00010028418	1.924814299	0.124066287	15.51440243	2.77203E-54	3.52153E-53	positive
PRJNA729271	stage_1W_vs_E10	ordinary_muscle_development	ENSGALG00010028419	ENSGALG00010028419	4.886084918	0.360887089	13.53909591	9.19132E-42	7.82774E-41	positive
PRJNA729271	stage_1W_vs_E10	ordinary_muscle_development	ENSGALG00010028420	ENSGALG00010028420	4.810986306	0.826335622	5.822072989	5.81222E-09	1.25183E-08	positive
PRJNA729271	stage_1W_vs_E10	ordinary_muscle_development	ENSGALG00010028421	ZBTB7A	1.508746814	0.188770114	7.992508876	1.3222E-15	4.04502E-15	positive
PRJNA729271	stage_1W_vs_E10	ordinary_muscle_development	ENSGALG00010028422	RXRA	2.389126367	0.207784955	11.49807196	1.34895E-30	7.80872E-30	positive
PRJNA729271	stage_1W_vs_E10	ordinary_muscle_development	ENSGALG00010028425	DAP3	1.481828751	0.128892075	11.49666299	1.37115E-30	7.93404E-30	positive
PRJNA729271	stage_1W_vs_E10	ordinary_muscle_development	ENSGALG00010028426	GPR21	-0.530753418	0.156120931	-3.399630119	0.000674771	0.001019789	negative
PRJNA729271	stage_1W_vs_E10	ordinary_muscle_development	ENSGALG00010028427	ENSGALG00010028427	0.595341674	0.205929077	2.891003454	0.003840139	0.005415197	positive
PRJNA729271	stage_1W_vs_E10	ordinary_muscle_development	ENSGALG00010028428	RABGAP1	0.295337536	0.067392709	4.382336623	1.17413E-05	2.02735E-05	positive
PRJNA729271	stage_1W_vs_E10	ordinary_muscle_development	ENSGALG00010028429	REXO1	-0.144269423	0.088704661	-1.626401826	0.103864203	0.124638763	negative
PRJNA729271	stage_1W_vs_E10	ordinary_muscle_development	ENSGALG00010028430	NR2C2AP	-0.005541468	0.116547007	-0.047547063	0.962077222	0.96721524	negative
PRJNA729271	stage_1W_vs_E10	ordinary_muscle_development	ENSGALG00010028431	ENSGALG00010028431	-0.577768152	0.273894354	-2.109456234	0.03490522	0.044393363	negative
PRJNA729271	stage_1W_vs_E10	ordinary_muscle_development	ENSGALG00010028432	RFXANK	1.042526753	0.133483891	7.810131598	5.71283E-15	1.69583E-14	positive
PRJNA729271	stage_1W_vs_E10	ordinary_muscle_development	ENSGALG00010028433	MSTO1	-0.461924843	0.094485119	-4.88886344	1.0142E-06	1.89614E-06	negative
PRJNA729271	stage_1W_vs_E10	ordinary_muscle_development	ENSGALG00010028434	COL5A1	-0.392789596	0.195932679	-2.004717122	0.044993297	0.056519442	negative
PRJNA729271	stage_1W_vs_E10	ordinary_muscle_development	ENSGALG00010028435	ATP8B3	1.772734939	0.331809015	5.342636446	9.16044E-08	1.83723E-07	positive
PRJNA729271	stage_1W_vs_E10	ordinary_muscle_development	ENSGALG00010028436	S100A1	3.945678873	0.184360754	21.40194585	1.2815E-101	4.94013E-100	positive
PRJNA729271	stage_1W_vs_E10	ordinary_muscle_development	ENSGALG00010028437	ENSGALG00010028437	0.036472564	0.067532621	0.540073284	0.589146494	0.624154249	positive
PRJNA729271	stage_1W_vs_E10	ordinary_muscle_development	ENSGALG00010028438	MED27	-0.186372324	0.071797868	-2.595791903	0.00943732	0.012791655	negative
PRJNA729271	stage_1W_vs_E10	ordinary_muscle_development	ENSGALG00010028439	BORCS8	-0.137385714	0.12067351	-1.13849107	0.254915486	0.28824401	negative
PRJNA729271	stage_1W_vs_E10	ordinary_muscle_development	ENSGALG00010028440	PSMB7	0.267280775	0.078325983	3.41241522	0.0006439	0.00097476	positive
PRJNA729271	stage_1W_vs_E10	ordinary_muscle_development	ENSGALG00010028442	CHTOP	-0.534492156	0.090879595	-5.88132193	4.07003E-09	8.84218E-09	negative
PRJNA729271	stage_1W_vs_E10	ordinary_muscle_development	ENSGALG00010028443	ENSGALG00010028443	0.309436484	0.348032587	0.889102042	0.373948241	0.411570217	positive
PRJNA729271	stage_1W_vs_E10	ordinary_muscle_development	ENSGALG00010028444	MEF2B	1.614672657	0.312197791	5.171954135	2.31658E-07	4.51937E-07	positive
PRJNA729271	stage_1W_vs_E10	ordinary_muscle_development	ENSGALG00010028445	SYT11	-2.820555727	0.205472083	-13.72719685	6.97815E-43	6.12278E-42	negative
PRJNA729271	stage_1W_vs_E10	ordinary_muscle_development	ENSGALG00010028446	ENSGALG00010028446	-0.749399834	0.222605815	-3.366488137	0.000761319	0.001146288	negative
PRJNA729271	stage_1W_vs_E10	ordinary_muscle_development	ENSGALG00010028447	OLFM1	-3.876774907	0.165296333	-23.45348403	1.218E-121	6.9508E-120	negative
PRJNA729271	stage_1W_vs_E10	ordinary_muscle_development	ENSGALG00010028448	RIT1	-0.394943653	0.058045055	-6.804087877	1.01691E-11	2.5564E-11	negative
PRJNA729271	stage_1W_vs_E10	ordinary_muscle_development	ENSGALG00010028449	EFCAB12	-0.774990457	0.336252372	-2.304788078	0.021178445	0.027601291	negative
PRJNA729271	stage_1W_vs_E10	ordinary_muscle_development	ENSGALG00010028450	PTGDS	9.679208911	0.432694199	22.36962947	7.778E-111	3.5565E-109	positive
PRJNA729271	stage_1W_vs_E10	ordinary_muscle_development	ENSGALG00010028451	TMEM59L	-1.454509256	0.330831056	-4.396531789	1.09994E-05	1.90418E-05	negative
PRJNA729271	stage_1W_vs_E10	ordinary_muscle_development	ENSGALG00010028452	GOLPH3L	-0.580325931	0.673065732	-0.862212862	0.388570393	0.426529939	negative
PRJNA729271	stage_1W_vs_E10	ordinary_muscle_development	ENSGALG00010028453	ENSGALG00010028453	0.135035795	0.095015676	1.421194904	0.155260106	0.181696692	positive
PRJNA729271	stage_1W_vs_E10	ordinary_muscle_development	ENSGALG00010028454	C8G	1.960810568	0.223524953	8.772222271	1.75174E-18	6.10519E-18	positive
PRJNA729271	stage_1W_vs_E10	ordinary_muscle_development	ENSGALG00010028455	ENSA	0.861201304	0.069643085	12.36592693	3.99575E-35	2.72557E-34	positive
PRJNA729271	stage_1W_vs_E10	ordinary_muscle_development	ENSGALG00010028456	FBXW5	0.575260888	0.092991269	6.186181735	6.16389E-10	1.40172E-09	positive
PRJNA729271	stage_1W_vs_E10	ordinary_muscle_development	ENSGALG00010028457	KANK3	1.109747038	0.104611391	10.60828107	2.7272E-26	1.32431E-25	positive
PRJNA729271	stage_1W_vs_E10	ordinary_muscle_development	ENSGALG00010028458	TRAF2	0.473903127	0.108693761	4.359984619	1.30072E-05	2.23679E-05	positive
PRJNA729271	stage_1W_vs_E10	ordinary_muscle_development	ENSGALG00010028459	MCL1	2.382160476	0.209837342	11.35241448	7.21426E-30	4.06415E-29	positive
PRJNA729271	stage_1W_vs_E10	ordinary_muscle_development	ENSGALG00010028460	CRLF1	-1.226399327	0.249899478	-4.907570582	9.22115E-07	1.72956E-06	negative
PRJNA729271	stage_1W_vs_E10	ordinary_muscle_development	ENSGALG00010028461	ENSGALG00010028461	2.113153399	0.31861198	6.632372709	3.30333E-11	8.07859E-11	positive
PRJNA729271	stage_1W_vs_E10	ordinary_muscle_development	ENSGALG00010028462	EDF1	1.06074235	0.105351578	10.06859483	7.60569E-24	3.33166E-23	positive
PRJNA729271	stage_1W_vs_E10	ordinary_muscle_development	ENSGALG00010028463	RPS28	2.98513525	0.169206176	17.64199936	1.17238E-69	2.19841E-68	positive
PRJNA729271	stage_1W_vs_E10	ordinary_muscle_development	ENSGALG00010028464	ENSGALG00010028464	0.713017507	0.110865357	6.431382425	1.26449E-10	2.98465E-10	positive
PRJNA729271	stage_1W_vs_E10	ordinary_muscle_development	ENSGALG00010028465	ENSGALG00010028465	1.271907783	0.132076042	9.630117365	5.96613E-22	2.41764E-21	positive
PRJNA729271	stage_1W_vs_E10	ordinary_muscle_development	ENSGALG00010028466	MAMDC4	1.342321898	0.638350557	2.10279741	0.035483482	0.04508925	positive
PRJNA729271	stage_1W_vs_E10	ordinary_muscle_development	ENSGALG00010028467	ENSGALG00010028467	1.177440794	0.241452411	4.876492181	1.07989E-06	2.0148E-06	positive
PRJNA729271	stage_1W_vs_E10	ordinary_muscle_development	ENSGALG00010028468	PHPT1	-0.415774571	0.102838946	-4.042968034	5.27788E-05	8.66858E-05	negative
PRJNA729271	stage_1W_vs_E10	ordinary_muscle_development	ENSGALG00010028470	AJM1	1.923803803	0.439488668	4.377368394	1.20121E-05	2.07255E-05	positive
PRJNA729271	stage_1W_vs_E10	ordinary_muscle_development	ENSGALG00010028471	TPGS1	0.083364923	0.16839832	0.495046049	0.620567615	0.654571939	positive
PRJNA729271	stage_1W_vs_E10	ordinary_muscle_development	ENSGALG00010028472	RPRD2	0.65085546	0.138684705	4.693058673	2.6915E-06	4.8724E-06	positive
PRJNA729271	stage_1W_vs_E10	ordinary_muscle_development	ENSGALG00010028473	CDC34	2.244114275	0.137167362	16.36040994	3.66651E-60	5.39007E-59	positive
PRJNA729271	stage_1W_vs_E10	ordinary_muscle_development	ENSGALG00010028474	PEX19	1.246558244	0.118656902	10.50556872	8.14305E-26	3.87758E-25	positive
PRJNA729271	stage_1W_vs_E10	ordinary_muscle_development	ENSGALG00010028475	AMBP	6.859360255	1.371034514	5.003054397			positive
PRJNA729271	stage_1W_vs_E10	ordinary_muscle_development	ENSGALG00010028476	ENSGALG00010028476	2.645567915	0.154580652	17.11448279	1.15739E-65	1.93499E-64	positive
PRJNA729271	stage_1W_vs_E10	ordinary_muscle_development	ENSGALG00010028477	NRARP	-0.266206224	0.215497199	-1.235311758	0.216714499	0.247852033	negative
PRJNA729271	stage_1W_vs_E10	ordinary_muscle_development	ENSGALG00010028478	PEA15	0.173306645	0.161362057	1.074023528	0.282812137	0.317337198	positive
PRJNA729271	stage_1W_vs_E10	ordinary_muscle_development	ENSGALG00010028479	CASQ1	9.770596722	0.314436831	31.07332146	5.5246E-212	9.2045E-210	positive
PRJNA729271	stage_1W_vs_E10	ordinary_muscle_development	ENSGALG00010028480	F11R	0.458192091	0.215822253	2.123006709	0.03375329	0.042984883	positive
PRJNA729271	stage_1W_vs_E10	ordinary_muscle_development	ENSGALG00010028481	ENSGALG00010028481	0.765455745	0.144804836	5.286120047	1.24938E-07	2.47909E-07	positive
PRJNA729271	stage_1W_vs_E10	ordinary_muscle_development	ENSGALG00010028482	CD320	0.681164694	0.176204484	3.865762549	0.000110743	0.00017747	positive
PRJNA729271	stage_1W_vs_E10	ordinary_muscle_development	ENSGALG00010028483	EXD3	0.441779038	0.091889345	4.807728674	1.52655E-06	2.81267E-06	positive
PRJNA729271	stage_1W_vs_E10	ordinary_muscle_development	ENSGALG00010028484	NOXA1	0.075292976	0.496340724	0.151696149	0.879426595	0.893105058	positive
PRJNA729271	stage_1W_vs_E10	ordinary_muscle_development	ENSGALG00010028485	USF1	0.126139006	0.18376369	0.68641964	0.492448523	0.530393917	positive
PRJNA729271	stage_1W_vs_E10	ordinary_muscle_development	ENSGALG00010028486	ARHGAP30	1.677696925	0.225246322	7.448276661	9.45674E-14	2.63151E-13	positive
PRJNA729271	stage_1W_vs_E10	ordinary_muscle_development	ENSGALG00010028488	PTGES	1.312920769	0.13039139	10.06907561	7.56861E-24	3.31641E-23	positive
PRJNA729271	stage_1W_vs_E10	ordinary_muscle_development	ENSGALG00010028489	ENSGALG00010028489	2.65637462	0.789744376	3.363587891	0.000769363	0.001157799	positive
PRJNA729271	stage_1W_vs_E10	ordinary_muscle_development	ENSGALG00010028490	NECTIN4	-2.242889691	0.366041825	-6.127413697	8.9319E-10	2.01412E-09	negative
PRJNA729271	stage_1W_vs_E10	ordinary_muscle_development	ENSGALG00010028491	TOR1B	-0.175403523	0.098560508	-1.779653194	0.075132735	0.091709389	negative
PRJNA729271	stage_1W_vs_E10	ordinary_muscle_development	ENSGALG00010028492	ENSGALG00010028492	1.922742465	0.253197141	7.593855351	3.10525E-14	8.86384E-14	positive
PRJNA729271	stage_1W_vs_E10	ordinary_muscle_development	ENSGALG00010028493	TOR1A	-0.286780518	0.087060303	-3.294044572	0.000987568	0.001471808	negative
PRJNA729271	stage_1W_vs_E10	ordinary_muscle_development	ENSGALG00010028494	ENSGALG00010028494	-0.445753976	0.192610338	-2.314278555	0.020652447	0.026944845	negative
PRJNA729271	stage_1W_vs_E10	ordinary_muscle_development	ENSGALG00010028495	LINGO3	-5.487543169	0.634029342	-8.655030303	4.92781E-18	1.67437E-17	negative
PRJNA729271	stage_1W_vs_E10	ordinary_muscle_development	ENSGALG00010028496	ENSGALG00010028496	1.362341628	0.142535356	9.557920671	1.20146E-21	4.82549E-21	positive
PRJNA729271	stage_1W_vs_E10	ordinary_muscle_development	ENSGALG00010028498	C9orf78	0.287038576	0.104157276	2.755818768	0.005854543	0.008114515	positive
PRJNA729271	stage_1W_vs_E10	ordinary_muscle_development	ENSGALG00010028500	VPS45	0.8493108	0.069207313	12.27198055	1.28082E-34	8.58715E-34	positive
PRJNA729271	stage_1W_vs_E10	ordinary_muscle_development	ENSGALG00010028501	USP20	0.686616849	0.087533535	7.844043385	4.36266E-15	1.30385E-14	positive
PRJNA729271	stage_1W_vs_E10	ordinary_muscle_development	ENSGALG00010028502	PLEKHO1	-0.836832757	0.151587	-5.520478397	3.38078E-08	6.95394E-08	negative
PRJNA729271	stage_1W_vs_E10	ordinary_muscle_development	ENSGALG00010028503	NDOR1	0.085077157	0.126873066	0.670569097	0.502495075	0.540251158	positive
PRJNA729271	stage_1W_vs_E10	ordinary_muscle_development	ENSGALG00010028506	SHD	-8.157283113	0.866274576	-9.41650989	4.66331E-21	1.82886E-20	negative
PRJNA729271	stage_1W_vs_E10	ordinary_muscle_development	ENSGALG00010028507	ANP32E	-1.56428816	0.057391807	-27.25629727	1.3995E-163	1.4185E-161	negative
PRJNA729271	stage_1W_vs_E10	ordinary_muscle_development	ENSGALG00010028510	CILP2	0.856184821	0.584625044	1.464502469	0.143056709	0.168325919	positive
PRJNA729271	stage_1W_vs_E10	ordinary_muscle_development	ENSGALG00010028511	YJEFN3	0.995256301	0.40672647	2.446991714	0.014405415	0.019124976	positive
PRJNA729271	stage_1W_vs_E10	ordinary_muscle_development	ENSGALG00010028512	NDUFA13	1.428001296	0.122248601	11.68112592	1.59173E-31	9.57747E-31	positive
PRJNA729271	stage_1W_vs_E10	ordinary_muscle_development	ENSGALG00010028513	ENSGALG00010028513	-1.354281983	1.315732802	-1.029298639	0.303339363	0.338744535	negative
PRJNA729271	stage_1W_vs_E10	ordinary_muscle_development	ENSGALG00010028514	FNBP1	1.351011958	0.058654771	23.03328324	2.1636E-117	1.1322E-115	positive
PRJNA729271	stage_1W_vs_E10	ordinary_muscle_development	ENSGALG00010028515	ENSGALG00010028515	1.134934967	3.388812944	0.334906348	0.737695733	0.763505866	positive
PRJNA729271	stage_1W_vs_E10	ordinary_muscle_development	ENSGALG00010028516	GATAD2A	-0.300211228	0.063205255	-4.749782724	2.03635E-06	3.7175E-06	negative
PRJNA729271	stage_1W_vs_E10	ordinary_muscle_development	ENSGALG00010028517	RNF208	-1.665230551	0.335958783	-4.956651343	7.17185E-07	1.35465E-06	negative
PRJNA729271	stage_1W_vs_E10	ordinary_muscle_development	ENSGALG00010028518	MAU2	0.754824239	0.11482362	6.573771462	4.90565E-11	1.18809E-10	positive
PRJNA729271	stage_1W_vs_E10	ordinary_muscle_development	ENSGALG00010028519	ENSGALG00010028519	1.134989551	2.100649177	0.54030419	0.58898727	0.624031173	positive
PRJNA729271	stage_1W_vs_E10	ordinary_muscle_development	ENSGALG00010028523	SUGP1	-0.536521383	0.116110215	-4.620793983	3.82274E-06	6.83576E-06	negative
PRJNA729271	stage_1W_vs_E10	ordinary_muscle_development	ENSGALG00010028528	UHRF1	-2.871452117	0.185678896	-15.46461221	6.01343E-54	7.53368E-53	negative
PRJNA729271	stage_1W_vs_E10	ordinary_muscle_development	ENSGALG00010028534	ENSGALG00010028534	0.654364453	4.345747042	0.150575827	0.880310332	0.893877286	positive
PRJNA729271	stage_1W_vs_E10	ordinary_muscle_development	ENSGALG00010028535	TICAM1	0.555446602	0.105950826	5.242494322	1.5842E-07	3.12082E-07	positive
PRJNA729271	stage_1W_vs_E10	ordinary_muscle_development	ENSGALG00010028536	GPR107	-0.195312362	0.064247062	-3.040020155	0.002365623	0.003402769	negative
PRJNA729271	stage_1W_vs_E10	ordinary_muscle_development	ENSGALG00010028537	ENSGALG00010028537	0.653873015	2.123141423	0.30797431	0.75810188	0.782614255	positive
PRJNA729271	stage_1W_vs_E10	ordinary_muscle_development	ENSGALG00010028538	FEM1A	2.046556701	0.116915956	17.50451156	1.32358E-68	2.42238E-67	positive
PRJNA729271	stage_1W_vs_E10	ordinary_muscle_development	ENSGALG00010028539	ENSGALG00010028539	1.134925525	3.347138525	0.339073366	0.734554462	0.760797923	positive
PRJNA729271	stage_1W_vs_E10	ordinary_muscle_development	ENSGALG00010028541	ENSGALG00010028541	-0.1826862	0.309375488	-0.590499916	0.554855542	0.590892005	negative
PRJNA729271	stage_1W_vs_E10	ordinary_muscle_development	ENSGALG00010028542	ENSGALG00010028542	1.134986501	1.996452615	0.568501597	0.569694421	0.605360358	positive
PRJNA729271	stage_1W_vs_E10	ordinary_muscle_development	ENSGALG00010028543	ANP32B	-1.490060481	0.093977979	-15.85542166	1.28959E-56	1.7324E-55	negative
PRJNA729271	stage_1W_vs_E10	ordinary_muscle_development	ENSGALG00010028544	ZNF414	0.943167348	0.165272165	5.706752527	1.15152E-08	2.4342E-08	positive
PRJNA729271	stage_1W_vs_E10	ordinary_muscle_development	ENSGALG00010028545	ENSGALG00010028545	1.134996338	1.547225564	0.73356876	0.463211606	0.501475258	positive
PRJNA729271	stage_1W_vs_E10	ordinary_muscle_development	ENSGALG00010028546	ENSGALG00010028546	1.135000728	1.730374514	0.655927789	0.511870588	0.549345887	positive
PRJNA729271	stage_1W_vs_E10	ordinary_muscle_development	ENSGALG00010028547	ZNF618	-0.13260083	0.105256284	-1.25979015	0.207745074	0.238402727	negative
PRJNA729271	stage_1W_vs_E10	ordinary_muscle_development	ENSGALG00010028548	MYO1F	1.181562968	0.249729761	4.731366278	2.23014E-06	4.05747E-06	positive
PRJNA729271	stage_1W_vs_E10	ordinary_muscle_development	ENSGALG00010028549	ADAMTS10	-1.203375869	0.179336743	-6.710146766	1.94429E-11	4.81422E-11	negative
PRJNA729271	stage_1W_vs_E10	ordinary_muscle_development	ENSGALG00010028550	ZAP70	0.700470316	0.604992922	1.157815721	0.246939247	0.279661253	positive
PRJNA729271	stage_1W_vs_E10	ordinary_muscle_development	ENSGALG00010028551	NCS1	-2.378844035	0.228175294	-10.42551099	1.89637E-25	8.88714E-25	negative
PRJNA729271	stage_1W_vs_E10	ordinary_muscle_development	ENSGALG00010028552	ENSGALG00010028552	-4.937921702	0.90397491	-5.462454373	4.69596E-08	9.59515E-08	negative
PRJNA729271	stage_1W_vs_E10	ordinary_muscle_development	ENSGALG00010028553	NFILZ	3.632756586	0.970984039	3.741314419	0.00018306	0.000288922	positive
PRJNA729271	stage_1W_vs_E10	ordinary_muscle_development	ENSGALG00010028554	ENSGALG00010028554	-6.021792226	1.119473167	-5.379130475	7.48465E-08	1.51016E-07	negative
PRJNA729271	stage_1W_vs_E10	ordinary_muscle_development	ENSGALG00010028555	ENSGALG00010028555	-1.346874758	1.425833583	-0.944622692	0.344851538	0.38212851	negative
PRJNA729271	stage_1W_vs_E10	ordinary_muscle_development	ENSGALG00010028556	SPTAN1	-0.173615825	0.093392349	-1.858994086	0.063027977	0.077673059	negative
PRJNA729271	stage_1W_vs_E10	ordinary_muscle_development	ENSGALG00010028558	ENSGALG00010028558	0.220788013	1.589021421	0.138945901	0.889492899	0.901685403	positive

PRJNA729271	stage_1W_vs_E10	ordinary_muscle_development	ENSGALG00010028559	DYNC2I2	-0.744453691	0.126216934	-5.898207691	3.67471E-09	8.00255E-09	negative
PRJNA729271	stage_1W_vs_E10	ordinary_muscle_development	ENSGALG00010028560	ENSGALG00010028560	1.134898448	3.504971005	0.323796815	0.746091868	0.771424611	positive
PRJNA729271	stage_1W_vs_E10	ordinary_muscle_development	ENSGALG00010028561	SET	-1.342626241	0.051853242	-25.89281166	8.0245E-148	6.4981E-146	negative
PRJNA729271	stage_1W_vs_E10	ordinary_muscle_development	ENSGALG00010028562	ACSBG2	1.791415295	0.199359401	8.985858145	2.56723E-19	9.3474E-19	positive
PRJNA729271	stage_1W_vs_E10	ordinary_muscle_development	ENSGALG00010028563	ENSGALG00010028563	1.13497364	2.300871649	0.493279858	0.621814858	0.655744371	positive
PRJNA729271	stage_1W_vs_E10	ordinary_muscle_development	ENSGALG00010028565	PKN1	0.171762144	0.121008524	1.419421862	0.155776062	0.182241649	positive
PRJNA729271	stage_1W_vs_E10	ordinary_muscle_development	ENSGALG00010028566	ZDHHC12	0.706888872	0.124320834	5.686004907	1.30046E-08	2.73626E-08	positive
PRJNA729271	stage_1W_vs_E10	ordinary_muscle_development	ENSGALG00010028567	ENSGALG00010028567	-3.289287356	1.200756272	-2.739346387	0.006156148	0.008508139	negative
PRJNA729271	stage_1W_vs_E10	ordinary_muscle_development	ENSGALG00010028568	MLLT1	-1.593815665	0.068263428	-23.34801684	1.4436E-120	8.142E-119	negative
PRJNA729271	stage_1W_vs_E10	ordinary_muscle_development	ENSGALG00010028569	ZER1	0.775900193	0.068472248	11.33160097	9.15165E-30	5.12372E-29	positive
PRJNA729271	stage_1W_vs_E10	ordinary_muscle_development	ENSGALG00010028572	TBC1D13	-0.417803467	0.069497994	-6.011734215	1.83549E-09	4.07434E-09	negative
PRJNA729271	stage_1W_vs_E10	ordinary_muscle_development	ENSGALG00010028573	KCNN1	1.032764994	0.26302192	3.926535835	8.61781E-05	0.000139321	positive
PRJNA729271	stage_1W_vs_E10	ordinary_muscle_development	ENSGALG00010028574	ENDOG	0.275740604	0.11864088	2.324161826	0.020116822	0.026281509	positive
PRJNA729271	stage_1W_vs_E10	ordinary_muscle_development	ENSGALG00010028575	SETDB1	-0.053720539	0.119232704	-0.450552047	0.652312435	0.683628714	negative
PRJNA729271	stage_1W_vs_E10	ordinary_muscle_development	ENSGALG00010028577	CERS2	0.158946696	0.13258396	1.198838047	0.230590925	0.262314822	positive
PRJNA729271	stage_1W_vs_E10	ordinary_muscle_development	ENSGALG00010028578	SPOUT1	-0.380002016	0.124170005	-3.060336648	0.002210883	0.003189366	negative
PRJNA729271	stage_1W_vs_E10	ordinary_muscle_development	ENSGALG00010028579	OCLN	1.226136397	0.270827184	4.527375648	5.97207E-06	1.05246E-05	positive
PRJNA729271	stage_1W_vs_E10	ordinary_muscle_development	ENSGALG00010028580	PRUNE1	1.702514354	0.150593754	11.30534505	1.23468E-29	6.86749E-29	positive
PRJNA729271	stage_1W_vs_E10	ordinary_muscle_development	ENSGALG00010028581	NR2F6	0.562053604	0.123899699	4.536359722	5.72335E-06	1.00961E-05	positive
PRJNA729271	stage_1W_vs_E10	ordinary_muscle_development	ENSGALG00010028582	KYAT1	2.151576676	0.206069259	10.4410366	1.61042E-25	7.57401E-25	positive
PRJNA729271	stage_1W_vs_E10	ordinary_muscle_development	ENSGALG00010028583	USHBP1	1.491683655	0.197184699	7.564905715	3.88146E-14	1.10382E-13	positive
PRJNA729271	stage_1W_vs_E10	ordinary_muscle_development	ENSGALG00010028584	LRRC8A	0.225101166	0.075884819	2.966353065	0.003013543	0.004295114	positive
PRJNA729271	stage_1W_vs_E10	ordinary_muscle_development	ENSGALG00010028585	MINDY1	1.618012765	0.12081243	13.3927673	6.66503E-41	5.49856E-40	positive
PRJNA729271	stage_1W_vs_E10	ordinary_muscle_development	ENSGALG00010028586	BABAM1	-0.091156291	0.121624516	-0.749489442	0.45356226	0.491763405	negative
PRJNA729271	stage_1W_vs_E10	ordinary_muscle_development	ENSGALG00010028587	ANKLE1	-1.00458809	0.194803595	-5.156927874	2.51034E-07	4.88553E-07	negative
PRJNA729271	stage_1W_vs_E10	ordinary_muscle_development	ENSGALG00010028588	ABHD8	-0.623504588	0.630997783	-0.988124847	0.323091508	0.359527976	negative
PRJNA729271	stage_1W_vs_E10	ordinary_muscle_development	ENSGALG00010028589	PHYHD1	-0.18990133	0.194355331	-0.977083206	0.328527961	0.365018696	negative
PRJNA729271	stage_1W_vs_E10	ordinary_muscle_development	ENSGALG00010028590	ENSGALG00010028590	2.335248278	0.227473507	10.26602309	1.00253E-24	4.55965E-24	positive
PRJNA729271	stage_1W_vs_E10	ordinary_muscle_development	ENSGALG00010028591	GABPB2	-1.364205836	0.129140603	-10.56372517	4.38891E-26	2.11423E-25	negative
PRJNA729271	stage_1W_vs_E10	ordinary_muscle_development	ENSGALG00010028592	SEMA6C	3.23197984	0.158230365	20.42578763	9.86548E-93	3.1542E-91	positive
PRJNA729271	stage_1W_vs_E10	ordinary_muscle_development	ENSGALG00010028593	DOLK	-0.308232683	0.12103747	-2.546588937	0.010878149	0.014645994	negative
PRJNA729271	stage_1W_vs_E10	ordinary_muscle_development	ENSGALG00010028594	ENSGALG00010028594	1.873806727	0.141481177	13.2442122	4.87369E-40	3.91164E-39	positive
PRJNA729271	stage_1W_vs_E10	ordinary_muscle_development	ENSGALG00010028595	SCNM1	1.208140194	0.111279111	10.85684618	1.85029E-27	9.42376E-27	positive
PRJNA729271	stage_1W_vs_E10	ordinary_muscle_development	ENSGALG00010028596	DDA1	0.048685453	0.088680198	0.549000273	0.583005263	0.618461745	positive
PRJNA729271	stage_1W_vs_E10	ordinary_muscle_development	ENSGALG00010028597	NUP188	-1.124034762	0.057901916	-19.41273855	6.02298E-84	1.57587E-82	negative
PRJNA729271	stage_1W_vs_E10	ordinary_muscle_development	ENSGALG00010028598	TMOD4	9.199172342	0.250354455	36.74459217	1.4184E-295	4.8953E-293	positive
PRJNA729271	stage_1W_vs_E10	ordinary_muscle_development	ENSGALG00010028599	VPS72	1.677945194	0.117611446	14.26685288	3.52114E-46	3.41397E-45	positive
PRJNA729271	stage_1W_vs_E10	ordinary_muscle_development	ENSGALG00010028600	PSMD4	1.697070355	0.131783821	12.87768359	6.01128E-38	4.47297E-37	positive
PRJNA729271	stage_1W_vs_E10	ordinary_muscle_development	ENSGALG00010028601	ANO8	7.079602691	0.221516624	31.95969023	3.9627E-224	7.2707E-222	positive
PRJNA729271	stage_1W_vs_E10	ordinary_muscle_development	ENSGALG00010028602	ZNF687	0.753829807	0.168879302	4.463719356	8.05491E-06	1.40653E-05	positive
PRJNA729271	stage_1W_vs_E10	ordinary_muscle_development	ENSGALG00010028603	SH3GLB2	-0.534315022	0.339682723	-1.572982628	0.115722841	0.137955636	negative
PRJNA729271	stage_1W_vs_E10	ordinary_muscle_development	ENSGALG00010028604	PI4KB	-0.046866208	0.140601516	-0.333326478	0.738887854	0.76446677	negative
PRJNA729271	stage_1W_vs_E10	ordinary_muscle_development	ENSGALG00010028605	GTPBP3	2.740026333	0.21152584	12.95362462	2.24117E-38	1.6955E-37	positive
PRJNA729271	stage_1W_vs_E10	ordinary_muscle_development	ENSGALG00010028606	PRPF3	0.805649018	0.135425715	5.949010652	2.69768E-09	5.92827E-09	positive
PRJNA729271	stage_1W_vs_E10	ordinary_muscle_development	ENSGALG00010028607	ENSGALG00010028607	1.564507947	0.23868219	6.554774563	5.5726E-11	1.34378E-10	positive
PRJNA729271	stage_1W_vs_E10	ordinary_muscle_development	ENSGALG00010028608	MIGA2	0.076291209	0.160177087	0.4762929	0.633865723	0.666561495	positive
PRJNA729271	stage_1W_vs_E10	ordinary_muscle_development	ENSGALG00010028609	MRPS21	0.83325861	0.144855972	5.752324851	8.80244E-09	1.87276E-08	positive
PRJNA729271	stage_1W_vs_E10	ordinary_muscle_development	ENSGALG00010028610	ENSGALG00010028610	3.165312821	0.529465863	5.978313318	2.2546E-09	4.97797E-09	positive
PRJNA729271	stage_1W_vs_E10	ordinary_muscle_development	ENSGALG00010028612	DIRAS1	-0.443085311	0.609996814	-0.726373155	0.467610044	0.50593473	negative
PRJNA729271	stage_1W_vs_E10	ordinary_muscle_development	ENSGALG00010028613	ENSGALG00010028613	2.519188499	0.362506169	6.949367246	3.66928E-12	9.4352E-12	positive
PRJNA729271	stage_1W_vs_E10	ordinary_muscle_development	ENSGALG00010028618	DOLPP1	-2.163771383	0.115352905	-18.75784042	1.6705E-78	3.81923E-77	negative
PRJNA729271	stage_1W_vs_E10	ordinary_muscle_development	ENSGALG00010028626	CRAT	2.702952094	0.172895794	15.63341726	4.31053E-55	5.58365E-54	positive
PRJNA729271	stage_1W_vs_E10	ordinary_muscle_development	ENSGALG00010028642	APH1A	0.633706857	0.138530183	4.574503853	4.7735E-06	8.47524E-06	positive
PRJNA729271	stage_1W_vs_E10	ordinary_muscle_development	ENSGALG00010028649	ENSGALG00010028649	0.402142552	0.173072327	2.323552001	0.020149518	0.026319479	positive
PRJNA729271	stage_1W_vs_E10	ordinary_muscle_development	ENSGALG00010028658	PTPA	-0.492958169	0.082315288	-5.988658759	2.11579E-09	4.6779E-09	negative
PRJNA729271	stage_1W_vs_E10	ordinary_muscle_development	ENSGALG00010028662	FCRL4	0.290442823	0.226810497	1.280552823	0.20035078	0.230665969	positive
PRJNA729271	stage_1W_vs_E10	ordinary_muscle_development	ENSGALG00010028669	ZBTB34	-0.049320848	0.111210676	-0.443490228	0.657411186	0.688225851	negative
PRJNA729271	stage_1W_vs_E10	ordinary_muscle_development	ENSGALG00010028671	CADM3	3.104824978	0.212391618	14.61839697	2.14389E-48	2.21811E-47	positive
PRJNA729271	stage_1W_vs_E10	ordinary_muscle_development	ENSGALG00010028675	ZBTB43	-0.181466474	0.071808666	-2.527083207	0.011501425	0.015436403	negative
PRJNA729271	stage_1W_vs_E10	ordinary_muscle_development	ENSGALG00010028678	ENSGALG00010028678	0.731450983	0.371807419	1.967284526	0.049150418	0.061427428	positive
PRJNA729271	stage_1W_vs_E10	ordinary_muscle_development	ENSGALG00010028686	TAGLN2	0.521441794	0.119404923	4.367004167	1.25962E-05	2.17024E-05	positive
PRJNA729271	stage_1W_vs_E10	ordinary_muscle_development	ENSGALG00010028689	CFAP45	0.532110981	0.629014115	0.84594442	0.397583751	0.435763816	positive
PRJNA729271	stage_1W_vs_E10	ordinary_muscle_development	ENSGALG00010028692	RALGPS1	0.315021011	0.111285439	2.830747804	0.004643932	0.006502492	positive
PRJNA729271	stage_1W_vs_E10	ordinary_muscle_development	ENSGALG00010028695	VSIG8	1.485402453	0.918952752	1.616407863	0.10600616	0.127009364	positive
PRJNA729271	stage_1W_vs_E10	ordinary_muscle_development	ENSGALG00010028700	ANGPTL2	1.369963028	0.197677302	6.930300118	4.19949E-12	1.07604E-11	positive
PRJNA729271	stage_1W_vs_E10	ordinary_muscle_development	ENSGALG00010028707	ENSGALG00010028707	-4.206334982	1.23336907	-3.410443058	0.000648574	0.000981426	negative
PRJNA729271	stage_1W_vs_E10	ordinary_muscle_development	ENSGALG00010028712	FBXW2	-0.047889489	0.085220082	-0.561950749	0.574149575	0.609781513	negative
PRJNA729271	stage_1W_vs_E10	ordinary_muscle_development	ENSGALG00010028726	SLC2A8	-0.187775574	0.113263798	-1.657860473	0.097345632	0.117272684	negative
PRJNA729271	stage_1W_vs_E10	ordinary_muscle_development	ENSGALG00010028727	ENSGALG00010028727	-0.900164323	0.183767984	-4.898374038	9.6633E-07	1.80968E-06	negative
PRJNA729271	stage_1W_vs_E10	ordinary_muscle_development	ENSGALG00010028734	ENSGALG00010028734	3.3699968	0.354625964	9.502961278	2.04005E-21	8.1327E-21	positive
PRJNA729271	stage_1W_vs_E10	ordinary_muscle_development	ENSGALG00010028735	ENSGALG00010028735	-0.914883148	0.359646352	-2.543841035	0.010964097	0.014751027	negative
PRJNA729271	stage_1W_vs_E10	ordinary_muscle_development	ENSGALG00010028738	MIDN	0.035639046	0.183734059	0.193970819	0.846198731	0.863048874	positive
PRJNA729271	stage_1W_vs_E10	ordinary_muscle_development	ENSGALG00010028739	GARNL3	-0.252679017	0.077805852	-3.247557998	0.001163999	0.00172165	negative
PRJNA729271	stage_1W_vs_E10	ordinary_muscle_development	ENSGALG00010028740	DAZAP1	-0.055587098	0.105891359	-0.524944612	0.599621695	0.634185806	negative
PRJNA729271	stage_1W_vs_E10	ordinary_muscle_development	ENSGALG00010028742	ENSGALG00010028742	0.109035477	0.144567645	0.754217699	0.450718511	0.488789827	positive
PRJNA729271	stage_1W_vs_E10	ordinary_muscle_development	ENSGALG00010028743	ARRDC1	1.323410346	0.186399596	7.099856313	1.24887E-12	3.29493E-12	positive
PRJNA729271	stage_1W_vs_E10	ordinary_muscle_development	ENSGALG00010028744	HOMER3	-0.071488542	0.208592517	-0.342718632	0.731810145	0.758226451	negative
PRJNA729271	stage_1W_vs_E10	ordinary_muscle_development	ENSGALG00010028745	ENSGALG00010028745	0.09705824	0.140751018	0.689573981	0.490462135	0.528607974	positive
PRJNA729271	stage_1W_vs_E10	ordinary_muscle_development	ENSGALG00010028746	MYDGF	-1.123640753	0.126909037	-8.85390656	8.45081E-19	2.99424E-18	negative
PRJNA729271	stage_1W_vs_E10	ordinary_muscle_development	ENSGALG00010028747	ENSGALG00010028747	-4.324044796	0.192513891	-22.46094956	1.0003E-111	4.6771E-110	negative
PRJNA729271	stage_1W_vs_E10	ordinary_muscle_development	ENSGALG00010028748	PIAS3	1.224385415	0.197319789	6.205081718	5.46685E-10	1.24594E-09	positive
PRJNA729271	stage_1W_vs_E10	ordinary_muscle_development	ENSGALG00010028750	OTUD7B	0.399418827	0.120505566	3.314525967	0.000917987	0.00137319	positive
PRJNA729271	stage_1W_vs_E10	ordinary_muscle_development	ENSGALG00010028751	EHMT1	-0.80840402	0.095967971	-8.423685675	3.64822E-17	1.18994E-16	negative
PRJNA729271	stage_1W_vs_E10	ordinary_muscle_development	ENSGALG00010028752	MTMR11	2.359291804	0.131450326	17.94816246	4.96046E-72	9.89022E-71	positive
PRJNA729271	stage_1W_vs_E10	ordinary_muscle_development	ENSGALG00010028753	SV2A	2.026940649	0.646123694	3.137078343	0.001706405	0.002489116	positive
PRJNA729271	stage_1W_vs_E10	ordinary_muscle_development	ENSGALG00010028756	ENSGALG00010028756	-5.303312274	0.97297999	-5.450587192	5.02038E-08	1.02421E-07	negative
PRJNA729271	stage_1W_vs_E10	ordinary_muscle_development	ENSGALG00010028757	MAT2A	-3.513594953	1.377129337	-2.551390678	0.010729398	0.01445915	negative
PRJNA729271	stage_1W_vs_E10	ordinary_muscle_development	ENSGALG00010028760	SSR2	0.061506791	0.097416485	0.631379699	0.527792287	0.56447644	positive
PRJNA729271	stage_1W_vs_E10	ordinary_muscle_development	ENSGALG00010028761	CTSS	2.603796505	0.23672018	10.99946995	3.84385E-28	2.01361E-27	positive
PRJNA729271	stage_1W_vs_E10	ordinary_muscle_development	ENSGALG00010028762	CACNA1B	-4.305635282	0.89687939	-4.800684829	1.58124E-06	2.9079E-06	negative
PRJNA729271	stage_1W_vs_E10	ordinary_muscle_development	ENSGALG00010028763	CTSK	0.277975069	0.217874194	1.275851278	0.202008097	0.232352592	positive
PRJNA729271	stage_1W_vs_E10	ordinary_muscle_development	ENSGALG00010028764	ARNT	0.662514772	0.125368656	5.284532765	1.26026E-07	2.5E-07	positive
PRJNA729271	stage_1W_vs_E10	ordinary_muscle_development	ENSGALG00010028768	NTNG2	-0.001594386	0.345467851	-0.00461515	0.996317656	0.996936658	negative
PRJNA729271	stage_1W_vs_E10	ordinary_muscle_development	ENSGALG00010028771	SETX	-0.044472816	0.072358906	-0.614614273	0.538809486	0.575028973	negative
PRJNA729271	stage_1W_vs_E10	ordinary_muscle_development	ENSGALG00010028772	TTF1	0.069392671	0.072540151	0.956610511	0.338763881	0.37578499	positive
PRJNA729271	stage_1W_vs_E10	ordinary_muscle_development	ENSGALG00010028782	MLLT11	0.776622925	0.251702698	3.085477155	0.002032258	0.00294311	positive
PRJNA729271	stage_1W_vs_E10	ordinary_muscle_development	ENSGALG00010028783	DDX31	-0.247613789	0.074845397	-3.308336919	0.000938518	0.00140289	negative
PRJNA729271	stage_1W_vs_E10	ordinary_muscle_development	ENSGALG00010028786	GTF3C4	-0.620497601	0.068969989	-8.996631825	2.32749E-19	8.483E-19	negative
PRJNA729271	stage_1W_vs_E10	ordinary_muscle_development	ENSGALG00010028800	ENSGALG00010028800	-1.06155685	0.450785999	-2.354902001	0.018527591	0.024275281	negative
PRJNA729271	stage_1W_vs_E10	ordinary_muscle_development	ENSGALG00010028806	SPACA9	0.253187187	0.438354266	0.577585771	0.563543816	0.599220042	positive
PRJNA729271	stage_1W_vs_E10	ordinary_muscle_development	ENSGALG00010028818	TSC1	0.155167413	0.117618782	1.319240091	0.187088859	0.216325225	positive
PRJNA729271	stage_1W_vs_E10	ordinary_muscle_development	ENSGALG00010028823	GFI1B	-0.422797989	0.343487738	-1.23089689	0.218361431	0.249637112	negative
PRJNA729271	stage_1W_vs_E10	ordinary_muscle_development	ENSGALG00010028826	CEL	6.688114359	2.727377213	2.452214651	0.014197994	0.018870339	positive
PRJNA729271	stage_1W_vs_E10	ordinary_muscle_development	ENSGALG00010028829	GTF3C5	0.115764394	0.112573617	1.02834392	0.303788078	0.33919336	positive
PRJNA729271	stage_1W_vs_E10	ordinary_muscle_development	ENSGALG00010028833	GBGT1	2.655083892	0.166299553	15.96567067	2.21646E-57	3.03951E-56	positive
PRJNA729271	stage_1W_vs_E10	ordinary_muscle_development	ENSGALG00010028849	ENSGALG00010028849	6.250633617	0.325390177	19.20965678	3.07291E-82	7.7599E-81	positive
PRJNA729271	stage_1W_vs_E10	ordinary_muscle_development	ENSGALG00010028853	ENSGALG00010028853	0.653660354	1.440009812	0.453927708	0.64988086	0.681277433	positive
PRJNA729271	stage_1W_vs_E10	ordinary_muscle_development	ENSGALG00010028854	RPL7A	0.664109809	0.140332166	4.732413301	2.21866E-06	4.03709E-06	positive
PRJNA729271	stage_1W_vs_E10	ordinary_muscle_development	ENSGALG00010028855	SURF6	0.00401122	0.168756903	0.023769223	0.98103669	0.983955633	positive
PRJNA729271	stage_1W_vs_E10	ordinary_muscle_development	ENSGALG00010028856	ETV3	-0.168220655	0.110188147	-1.526667429	0.126843731	0.150350799	negative
PRJNA729271	stage_1W_vs_E10	ordinary_muscle_development	ENSGALG00010028857	MED22	0.065831972	0.079464707	0.82844289	0.407419725	0.44536568	positive
PRJNA729271	stage_1W_vs_E10	ordinary_muscle_development	ENSGALG00010028858	ENSGALG00010028858	0.313862634	0.458675988	0.684279627	0.493798609	0.531690004	positive
PRJNA729271	stage_1W_vs_E10	ordinary_muscle_development	ENSGALG00010028859	SURF1	0.954653207	0.108611183	8.789640068	1.5004E-18	5.24814E-18	positive
PRJNA729271	stage_1W_vs_E10	ordinary_muscle_development	ENSGALG00010028860	SURF2	1.161424923	0.170899206	6.795964419	1.0759E-11	2.70047E-11	positive
PRJNA729271	stage_1W_vs_E10	ordinary_muscle_development	ENSGALG00010028861	SURF4	-0.515464186	0.074858509	-6.885846335	5.7445E-12	1.46313E-11	negative
PRJNA729271	stage_1W_vs_E10	ordinary_muscle_development	ENSGALG00010028862	ENSGALG00010028862	-0.315786993	1.406930892	-0.224450963	0.82240642	0.841268952	negative
PRJNA729271	stage_1W_vs_E10	ordinary_muscle_development	ENSGALG00010028865	STKLD1	1.093480681	0.16751475	6.527668048	6.68015E-11	1.59942E-10	positive
PRJNA729271	stage_1W_vs_E10	ordinary_muscle_development	ENSGALG00010028866	HORMAD1	-0.953259183	0.499756903	-1.907445753	0.056462889	0.070059029	negative
PRJNA729271	stage_1W_vs_E10	ordinary_muscle_development	ENSGALG00010028867	REXO4	-0.171115049	0.117586634	-1.45522534	0.145606888	0.171129815	negative
PRJNA729271	stage_1W_vs_E10	ordinary_muscle_development	ENSGALG00010028868	ENSGALG00010028868	0.646019708	4.241825273	0.152297576	0.878952237	0.892748418	positive
PRJNA729271	stage_1W_vs_E10	ordinary_muscle_development	ENSGALG00010028869	ADAMTS13	2.859190859	0.16419661	17.41321497	6.55001E-68	1.17358E-66	positive
PRJNA729271	stage_1W_vs_E10	ordinary_muscle_development	ENSGALG00010028870	CACFD1	-2.598921869	0.231156258	-11.24313868	2.50321E-29	1.37492E-28	negative
PRJNA729271	stage_1W_vs_E10	ordinary_muscle_development	ENSGALG00010028871	PIP5K1A	-0.252764623	0.108845299	-2.322237397	0.020220156	0.02640461	negative
PRJNA729271	stage_1W_vs_E10	ordinary_muscle_development	ENSGALG00010028872	SLC2A6	1.923274428	0.336917613	5.708441336	1.14015E-08	2.41087E-08	positive
PRJNA729271	stage_1W_vs_E10	ordinary_muscle_development	ENSGALG00010028873	TMEM8C	-0.758328612	0.279403445	-2.714099002	0.00664563	0.009157563	negative
PRJNA729271	stage_1W_vs_E10	ordinary_muscle_development	ENSGALG00010028874	CRPL1	2.35381346	0.497517482	4.731117086	2.23288E-06	4.06194E-06	positive
PRJNA729271	stage_1W_vs_E10	ordinary_muscle_development	ENSGALG00010028875	DUSP23	1.768577129	0.119048731	14.85590918	6.36979E-50	6.97357E-49	positive
PRJNA729271	stage_1W_vs_E10	ordinary_muscle_development	ENSGALG00010028876	ADAMTSL2	4.272244892	0.194259558	21.99245652	3.4007E-107	1.4584E-105	positive
PRJNA729271	stage_1W_vs_E10	ordinary_muscle_development	ENSGALG00010028877	FAM163B	-5.741714827	0.947885156	-6.057395022	1.38344E-09	3.0922E-09	negative
PRJNA729271	stage_1W_vs_E10	ordinary_muscle_development	ENSGALG00010028878	IGSF9	1.249393399	0.155449041	8.037318165	9.1826E-16	2.82894E-15	positive
PRJNA729271	stage_1W_vs_E10	ordinary_muscle_development	ENSGALG00010028881	SARDH	-1.596970758	0.23213341	-6.879538624	6.00467E-12	1.52778E-11	negative

PRJNA729271	stage_1W_vs_E10	ordinary_muscle_development	ENSGALG00010028884	VAV2	-1.724333188	0.131353986	-13.1273762	2.2947E-39	1.7989E-38	negative
PRJNA729271	stage_1W_vs_E10	ordinary_muscle_development	ENSGALG00010028885	BRD3	-0.697088816	0.142324687	-4.89787705	9.68776E-07	1.81403E-06	negative
PRJNA729271	stage_1W_vs_E10	ordinary_muscle_development	ENSGALG00010028886	ENSGALG00010028886	0.17694171	1.859824927	0.095138907	0.924204495	0.933348022	positive
PRJNA729271	stage_1W_vs_E10	ordinary_muscle_development	ENSGALG00010028887	SF3B4	0.08655408	0.174678793	0.495504226	0.62024424	0.654326074	positive
PRJNA729271	stage_1W_vs_E10	ordinary_muscle_development	ENSGALG00010028888	WDR5B	-1.219875052	0.082848666	-14.72413632	4.51197E-49	4.77032E-48	negative
PRJNA729271	stage_1W_vs_E10	ordinary_muscle_development	ENSGALG00010028889	FUBP3	-1.065487873	0.083616957	-12.74248563	3.43281E-37	2.50043E-36	negative
PRJNA729271	stage_1W_vs_E10	ordinary_muscle_development	ENSGALG00010028890	ENSGALG00010028890	-6.584357651	0.550293354	-11.96517749	5.40818E-33	3.41278E-32	negative
PRJNA729271	stage_1W_vs_E10	ordinary_muscle_development	ENSGALG00010028891	SLC31A2	2.274110883	0.221217163	10.27999298	8.67339E-25	3.95224E-24	positive
PRJNA729271	stage_1W_vs_E10	ordinary_muscle_development	ENSGALG00010028892	NIBAN2	0.054745985	0.141014004	0.388230835	0.697845216	0.726397998	positive
PRJNA729271	stage_1W_vs_E10	ordinary_muscle_development	ENSGALG00010028893	STXBP1	1.344535838	0.197736492	6.799634318	1.04885E-11	2.63394E-11	positive
PRJNA729271	stage_1W_vs_E10	ordinary_muscle_development	ENSGALG00010028894	AKNA	-1.712412417	0.163369711	-10.48182313	1.04706E-25	4.95821E-25	negative
PRJNA729271	stage_1W_vs_E10	ordinary_muscle_development	ENSGALG00010028895	WHRN	-6.057448549	0.936368514	-6.469086114	9.85974E-11	2.34444E-10	negative
PRJNA729271	stage_1W_vs_E10	ordinary_muscle_development	ENSGALG00010028896	ATP6V1G1	0.863504272	0.098761087	8.743365404	2.26267E-18	7.82756E-18	positive
PRJNA729271	stage_1W_vs_E10	ordinary_muscle_development	ENSGALG00010028897	TMEM268	-0.263209773	0.284544868	-0.925020278	0.354955359	0.392574236	negative
PRJNA729271	stage_1W_vs_E10	ordinary_muscle_development	ENSGALG00010028898	TNFSF15	-2.482631004	0.265449068	-9.352570038	8.55434E-21	3.32605E-20	negative
PRJNA729271	stage_1W_vs_E10	ordinary_muscle_development	ENSGALG00010028899	TNFSF8	1.563751296	0.579729384	2.697381464	0.006988716	0.009611142	positive
PRJNA729271	stage_1W_vs_E10	ordinary_muscle_development	ENSGALG00010028900	TNC	-4.40837494	0.550986999	-8.000869262	1.23544E-15	3.78518E-15	negative
PRJNA729271	stage_1W_vs_E10	ordinary_muscle_development	ENSGALG00010028901	PAPPA	-1.636222617	0.118919851	-13.75903686	4.49475E-43	3.96539E-42	negative
PRJNA729271	stage_1W_vs_E10	ordinary_muscle_development	ENSGALG00010028902	ENSGALG00010028902	-2.791950685	0.649280393	-4.300069303	1.70745E-05	2.9093E-05	negative
PRJNA729271	stage_1W_vs_E10	ordinary_muscle_development	ENSGALG00010028903	ASTN2	-4.009798016	0.251210264	-15.96191953	2.35379E-57	3.22174E-56	negative
PRJNA729271	stage_1W_vs_E10	ordinary_muscle_development	ENSGALG00010028904	TRIM32	-1.024210192	0.087106385	-11.75815291	6.41217E-32	3.90195E-31	negative
PRJNA729271	stage_1W_vs_E10	ordinary_muscle_development	ENSGALG00010028905	COLGALT1	0.200692716	0.08622254	2.327613141	0.019932655	0.026059695	positive
PRJNA729271	stage_1W_vs_E10	ordinary_muscle_development	ENSGALG00010028909	ENSGALG00010028909	-1.385434952	0.141818715	-9.769055912	1.5287E-22	6.34005E-22	negative
PRJNA729271	stage_1W_vs_E10	ordinary_muscle_development	ENSGALG00010028911	LMX1B	-6.702999661	0.79882499	-8.391074067	4.81721E-17	1.56349E-16	negative
PRJNA729271	stage_1W_vs_E10	ordinary_muscle_development	ENSGALG00010028912	EXFABP	-0.713421173	0.501465024	-1.422673844	0.154830726	0.181208831	negative
PRJNA729271	stage_1W_vs_E10	ordinary_muscle_development	ENSGALG00010028914	TLR4	1.219192156	0.229695456	5.307863619	1.10918E-07	2.20814E-07	positive
PRJNA729271	stage_1W_vs_E10	ordinary_muscle_development	ENSGALG00010028915	ADGRD2	0.622106749	0.487504812	1.276103811	0.201918824	0.232275903	positive
PRJNA729271	stage_1W_vs_E10	ordinary_muscle_development	ENSGALG00010028917	NR6A1	-0.218414481	0.129713508	-1.683822182	0.092216039	0.111482192	negative
PRJNA729271	stage_1W_vs_E10	ordinary_muscle_development	ENSGALG00010028918	AGPAT2	2.044511644	0.183789536	11.12420049	9.56596E-29	5.11844E-28	positive
PRJNA729271	stage_1W_vs_E10	ordinary_muscle_development	ENSGALG00010028919	EGFL7	1.483785463	0.168685423	8.79616884	1.41567E-18	4.96013E-18	positive
PRJNA729271	stage_1W_vs_E10	ordinary_muscle_development	ENSGALG00010028921	MRPL41	0.425129201	0.120170251	3.537724159	0.000403591	0.000620169	positive
PRJNA729271	stage_1W_vs_E10	ordinary_muscle_development	ENSGALG00010028923	ENSGALG00010028923	0.903922925	0.18994309	4.758914493	1.94637E-06	3.5604E-06	positive
PRJNA729271	stage_1W_vs_E10	ordinary_muscle_development	ENSGALG00010028924	TMEM203	0.780100535	0.119288902	6.539590223	6.16876E-11	1.48138E-10	positive
PRJNA729271	stage_1W_vs_E10	ordinary_muscle_development	ENSGALG00010028925	ENSGALG00010028925	1.538242542	0.329033816	4.675028725	2.93912E-06	5.30213E-06	positive
PRJNA729271	stage_1W_vs_E10	ordinary_muscle_development	ENSGALG00010028926	ENSGALG00010028926	-1.019005693	0.639642802	-1.59308553	0.111141005	0.132777456	negative
PRJNA729271	stage_1W_vs_E10	ordinary_muscle_development	ENSGALG00010029140	NPDC1	0.896733076	0.190453177	4.708417524	2.49647E-06	4.53066E-06	positive
PRJNA729271	stage_1W_vs_E10	ordinary_muscle_development	ENSGALG00010029141	C5	-0.997811194	0.573046614	-1.74123914	0.081641669	0.099261533	negative
PRJNA729271	stage_1W_vs_E10	ordinary_muscle_development	ENSGALG00010029142	ENSGALG00010029142	0.959166752	0.181825296	5.275210722	1.32604E-07	2.62652E-07	positive
PRJNA729271	stage_1W_vs_E10	ordinary_muscle_development	ENSGALG00010029143	SH2D3C	-0.099873358	0.110265866	-0.905750453	0.365067951	0.402713847	negative
PRJNA729271	stage_1W_vs_E10	ordinary_muscle_development	ENSGALG00010029144	ENSGALG00010029144	6.206588419	0.218216318	28.4423662	6.0566E-178	7.1374E-176	positive
PRJNA729271	stage_1W_vs_E10	ordinary_muscle_development	ENSGALG00010029145	RC3H2	1.121987459	0.133364307	8.412951576	3.9982E-17	1.30263E-16	positive
PRJNA729271	stage_1W_vs_E10	ordinary_muscle_development	ENSGALG00010029146	NEK6	-1.811874695	0.059550404	-30.425901	2.4965E-203	3.8496E-201	negative
PRJNA729271	stage_1W_vs_E10	ordinary_muscle_development	ENSGALG00010029147	RABL6	-0.625176482	0.104218185	-5.998727376	1.9887E-09	4.407E-09	negative
PRJNA729271	stage_1W_vs_E10	ordinary_muscle_development	ENSGALG00010029148	RALGDS	-0.142357524	0.119185537	-1.194419451	0.232313932	0.26412977	negative
PRJNA729271	stage_1W_vs_E10	ordinary_muscle_development	ENSGALG00010029149	ENSGALG00010029149	-0.84598206	0.219534183	-3.853532284	0.000116426	0.00018631	negative
PRJNA729271	stage_1W_vs_E10	ordinary_muscle_development	ENSGALG00010029150	NARF	-0.021176197	0.123292008	-0.171756442	0.863629011	0.878690665	negative
PRJNA729271	stage_1W_vs_E10	ordinary_muscle_development	ENSGALG00010029151	CYBC1	-1.728196608	0.11758303	-14.69767025	6.67158E-49	7.01775E-48	negative
PRJNA729271	stage_1W_vs_E10	ordinary_muscle_development	ENSGALG00010029152	HEXD	-0.202001507	0.130359546	-1.549572037	0.121244269	0.144134805	negative
PRJNA729271	stage_1W_vs_E10	ordinary_muscle_development	ENSGALG00010029153	OGFOD3	-1.403930467	0.08402885	-16.70771956	1.15156E-62	1.78905E-61	negative
PRJNA729271	stage_1W_vs_E10	ordinary_muscle_development	ENSGALG00010029154	UTS2R	-1.199072702	0.146460896	-8.186981875	2.67858E-16	8.42763E-16	negative
PRJNA729271	stage_1W_vs_E10	ordinary_muscle_development	ENSGALG00010029155	ENSGALG00010029155	-1.086605454	0.583727827	-1.861493324	0.062674542	0.077290071	negative
PRJNA729271	stage_1W_vs_E10	ordinary_muscle_development	ENSGALG00010029156	ENSGALG00010029156	-6.66173681	1.276113028	-5.220334455	1.786E-07	3.5074E-07	negative
PRJNA729271	stage_1W_vs_E10	ordinary_muscle_development	ENSGALG00010029158	CD7	0.354623058	0.420642199	0.843051549	0.399199614	0.437435622	positive
PRJNA729271	stage_1W_vs_E10	ordinary_muscle_development	ENSGALG00010029159	SSC4D	-5.348505414	1.067842225	-5.008703801	5.47978E-07	1.04183E-06	negative
PRJNA729271	stage_1W_vs_E10	ordinary_muscle_development	ENSGALG00010029160	DUS1L	1.113789753	0.091200749	12.2125067	2.66548E-34	1.75859E-33	positive
PRJNA729271	stage_1W_vs_E10	ordinary_muscle_development	ENSGALG00010029162	ELN	1.152580324	0.310910554	3.707112253	0.000209636	0.000329146	positive
PRJNA729271	stage_1W_vs_E10	ordinary_muscle_development	ENSGALG00010029163	ANKFY1	-1.224125279	0.095039402	-12.88018704	5.81944E-38	4.33467E-37	negative
PRJNA729271	stage_1W_vs_E10	ordinary_muscle_development	ENSGALG00010029164	CYB5D2	0.527417276	0.08251488	6.391783809	1.63962E-10	3.85128E-10	positive
PRJNA729271	stage_1W_vs_E10	ordinary_muscle_development	ENSGALG00010029165	RAC3	-0.243926939	0.120721636	-2.020573491	0.043323935	0.054540598	negative
PRJNA729271	stage_1W_vs_E10	ordinary_muscle_development	ENSGALG00010029166	ZZEF1	1.498284427	0.123492487	12.1325958	7.09637E-34	4.63133E-33	positive
PRJNA729271	stage_1W_vs_E10	ordinary_muscle_development	ENSGALG00010029167	ENSGALG00010029167	1.327859958	0.126489178	10.49781476	8.84029E-26	4.19856E-25	positive
PRJNA729271	stage_1W_vs_E10	ordinary_muscle_development	ENSGALG00010029168	RFNG	0.861055716	0.079816685	10.78791623	3.9259E-27	1.97111E-26	positive
PRJNA729271	stage_1W_vs_E10	ordinary_muscle_development	ENSGALG00010029169	SBDS	1.217946984	0.077567264	15.70181703	1.46971E-55	1.92686E-54	positive
PRJNA729271	stage_1W_vs_E10	ordinary_muscle_development	ENSGALG00010029170	DCXR	-0.547112989	0.14196566	-3.853840354	0.000116279	0.000186096	negative
PRJNA729271	stage_1W_vs_E10	ordinary_muscle_development	ENSGALG00010029171	ENSGALG00010029171	0.133767703	0.146751163	0.911527379	0.362017566	0.39962262	positive
PRJNA729271	stage_1W_vs_E10	ordinary_muscle_development	ENSGALG00010029172	LRRC45	-0.260554122	0.075364025	-3.457274489	0.000545669	0.000829519	negative
PRJNA729271	stage_1W_vs_E10	ordinary_muscle_development	ENSGALG00010029173	ZNHIT3	0.378676562	0.134722336	2.810786002	0.004942064	0.006903933	positive
PRJNA729271	stage_1W_vs_E10	ordinary_muscle_development	ENSGALG00010029174	CALN1	-10.56803953	0.879843946	-12.01126585	3.10062E-33	1.97989E-32	negative
PRJNA729271	stage_1W_vs_E10	ordinary_muscle_development	ENSGALG00010029175	CENPX	-1.548209722	0.135112716	-11.45865291	2.12799E-30	1.21821E-29	negative
PRJNA729271	stage_1W_vs_E10	ordinary_muscle_development	ENSGALG00010029176	ENSGALG00010029176	1.52893143	0.460469093	3.320377962	0.000898957	0.001346252	positive
PRJNA729271	stage_1W_vs_E10	ordinary_muscle_development	ENSGALG00010029177	ASPSCR1	0.80613177	0.080015637	10.07467787	7.14947E-24	3.13465E-23	positive
PRJNA729271	stage_1W_vs_E10	ordinary_muscle_development	ENSGALG00010029178	NOTUM	0.635468579	0.205463932	3.092847356	0.001982461	0.002873002	positive
PRJNA729271	stage_1W_vs_E10	ordinary_muscle_development	ENSGALG00010029179	PIGW	0.330022212	0.150072394	2.199086746	0.027871755	0.035831582	positive
PRJNA729271	stage_1W_vs_E10	ordinary_muscle_development	ENSGALG00010029180	CA4	4.847097393	0.431863948	11.22366759	3.12055E-29	1.70431E-28	positive
PRJNA729271	stage_1W_vs_E10	ordinary_muscle_development	ENSGALG00010029181	MYO19	0.27896527	0.103613435	2.6923658	0.007094708	0.009746734	positive
PRJNA729271	stage_1W_vs_E10	ordinary_muscle_development	ENSGALG00010029182	HEATR6	-0.429234075	0.068613606	-6.255815686	3.95444E-10	9.09257E-10	negative
PRJNA729271	stage_1W_vs_E10	ordinary_muscle_development	ENSGALG00010029183	ENSGALG00010029183	1.317227932	0.138997093	9.476658115	2.62557E-21	1.0421E-20	positive
PRJNA729271	stage_1W_vs_E10	ordinary_muscle_development	ENSGALG00010029184	DYNLL2	0.091521502	0.096021085	0.953139632	0.340519328	0.377558912	positive
PRJNA729271	stage_1W_vs_E10	ordinary_muscle_development	ENSGALG00010029186	ZNF302	0.570765445	0.139882506	4.080320404	4.49737E-05	7.42729E-05	positive
PRJNA729271	stage_1W_vs_E10	ordinary_muscle_development	ENSGALG00010029187	SRSF1	-1.486388168	0.052896493	-28.09993776	9.8133E-174	1.1027E-171	negative
PRJNA729271	stage_1W_vs_E10	ordinary_muscle_development	ENSGALG00010029188	ENSGALG00010029188	1.317680604	0.246529288	5.344925206	9.04544E-08	1.81522E-07	positive
PRJNA729271	stage_1W_vs_E10	ordinary_muscle_development	ENSGALG00010029189	ENSGALG00010029189	0.264973229	0.359427448	0.737209222	0.460995126	0.499292687	positive
PRJNA729271	stage_1W_vs_E10	ordinary_muscle_development	ENSGALG00010029190	DHRS7C	6.241026814	0.162925311	38.30606042	4.8616E-321	2.0134E-318	positive
PRJNA729271	stage_1W_vs_E10	ordinary_muscle_development	ENSGALG00010029192	DTX2	0.566326628	0.10107616	5.602969351	2.1071E-08	4.38703E-08	positive
PRJNA729271	stage_1W_vs_E10	ordinary_muscle_development	ENSGALG00010029194	DHRS11	-1.796683696	0.13777406	-13.0407981	7.17025E-39	5.53718E-38	negative
PRJNA729271	stage_1W_vs_E10	ordinary_muscle_development	ENSGALG00010029196	MRM1	0.126810172	0.096968286	1.307748923	0.190958484	0.220518102	positive
PRJNA729271	stage_1W_vs_E10	ordinary_muscle_development	ENSGALG00010029197	LHX1	1.449291908	0.650127013	2.229244252	0.025797658	0.03330991	positive
PRJNA729271	stage_1W_vs_E10	ordinary_muscle_development	ENSGALG00010029198	PPM1D	0.354876261	0.174741674	2.030862203	0.042268975	0.053254132	positive
PRJNA729271	stage_1W_vs_E10	ordinary_muscle_development	ENSGALG00010029199	APPBP2	-0.12705013	0.090043819	-1.410981128	0.158250179	0.184986802	negative
PRJNA729271	stage_1W_vs_E10	ordinary_muscle_development	ENSGALG00010029200	GAS7	0.730277081	0.124971264	5.843559994	5.10969E-09	1.10396E-08	positive
PRJNA729271	stage_1W_vs_E10	ordinary_muscle_development	ENSGALG00010029201	PIGS	-0.547681686	0.111343529	-4.918846132	8.70559E-07	1.6354E-06	negative
PRJNA729271	stage_1W_vs_E10	ordinary_muscle_development	ENSGALG00010029202	PSMD12	1.041205989	0.142504388	7.306483719	2.74224E-13	7.44777E-13	positive
PRJNA729271	stage_1W_vs_E10	ordinary_muscle_development	ENSGALG00010029203	ALDOC	-1.657364217	0.173414886	-9.557219996	1.20962E-21	4.85692E-21	negative
PRJNA729271	stage_1W_vs_E10	ordinary_muscle_development	ENSGALG00010029204	HELZ	0.171629879	0.098292685	1.746110393	0.080791771	0.098269424	positive
PRJNA729271	stage_1W_vs_E10	ordinary_muscle_development	ENSGALG00010029205	CACNG1	5.863519666	0.113934303	51.46404115	0	0	positive
PRJNA729271	stage_1W_vs_E10	ordinary_muscle_development	ENSGALG00010029206	CACNG4	-2.688742446	0.373348197	-7.20170197	5.94656E-13	1.59032E-12	negative
PRJNA729271	stage_1W_vs_E10	ordinary_muscle_development	ENSGALG00010029207	SPAG5	-1.92546844	0.222908616	-8.637927383	5.7242E-18	1.94178E-17	negative
PRJNA729271	stage_1W_vs_E10	ordinary_muscle_development	ENSGALG00010029208	CACNG5	-2.055808105	0.313831689	-6.550670869	5.72792E-11	1.37963E-10	negative
PRJNA729271	stage_1W_vs_E10	ordinary_muscle_development	ENSGALG00010029209	PRKCA	0.588045054	0.096512036	6.092971196	1.10834E-09	2.48844E-09	positive
PRJNA729271	stage_1W_vs_E10	ordinary_muscle_development	ENSGALG00010029210	RSKR	-1.180737826	0.439992244	-2.683542361	0.007284675	0.009992558	negative
PRJNA729271	stage_1W_vs_E10	ordinary_muscle_development	ENSGALG00010029211	SDF2	-0.544862719	0.167924145	-3.244695514	0.001175763	0.001738307	negative
PRJNA729271	stage_1W_vs_E10	ordinary_muscle_development	ENSGALG00010029212	SUPT6H	0.154989477	0.113640723	1.363855084	0.17261318	0.200519221	positive
PRJNA729271	stage_1W_vs_E10	ordinary_muscle_development	ENSGALG00010029213	APOH	4.212561611	1.517240907	2.776461927	0.005495407	0.007640123	positive
PRJNA729271	stage_1W_vs_E10	ordinary_muscle_development	ENSGALG00010029214	ENSGALG00010029214	9.41353013	0.37465319	25.12598416	2.587E-139	1.8473E-137	positive
PRJNA729271	stage_1W_vs_E10	ordinary_muscle_development	ENSGALG00010029215	ENSGALG00010029215	8.392843363	0.236576086	35.47629647	1.1407E-275	3.518E-273	positive
PRJNA729271	stage_1W_vs_E10	ordinary_muscle_development	ENSGALG00010029216	RAB34	-0.528823148	0.132692471	-3.985328976	6.73867E-05	0.00010991	negative
PRJNA729271	stage_1W_vs_E10	ordinary_muscle_development	ENSGALG00010029217	CEP112	-0.850912031	0.093586886	-9.092214363	9.70421E-20	3.60766E-19	negative
PRJNA729271	stage_1W_vs_E10	ordinary_muscle_development	ENSGALG00010029218	RPL23A	1.132947156	0.150212353	7.542303519	4.61742E-14	1.30901E-13	positive
PRJNA729271	stage_1W_vs_E10	ordinary_muscle_development	ENSGALG00010029219	TLCD1	0.379404144	0.203082087	1.868230476	0.061729953	0.076164084	positive
PRJNA729271	stage_1W_vs_E10	ordinary_muscle_development	ENSGALG00010029220	NEK8	-0.06025375	0.129938845	-0.463708527	0.642856587	0.674792254	negative
PRJNA729271	stage_1W_vs_E10	ordinary_muscle_development	ENSGALG00010029221	AXIN2	-1.009047087	0.127677749	-7.903077031	2.72101E-15	8.20663E-15	negative
PRJNA729271	stage_1W_vs_E10	ordinary_muscle_development	ENSGALG00010029222	TRAF4	-0.259362894	0.233144647	-1.112454855	0.26594263	0.299754154	negative
PRJNA729271	stage_1W_vs_E10	ordinary_muscle_development	ENSGALG00010029223	NME2	0.62265504	0.145779926	4.271198749	1.94425E-05	3.29613E-05	positive
PRJNA729271	stage_1W_vs_E10	ordinary_muscle_development	ENSGALG00010029224	FAM222B	-0.048832656	0.161670594	-0.302050326	0.762613699	0.786543729	negative
PRJNA729271	stage_1W_vs_E10	ordinary_muscle_development	ENSGALG00010029226	MBTD1	-0.655678319	0.076646055	-8.554625743	1.18252E-17	3.9449E-17	negative
PRJNA729271	stage_1W_vs_E10	ordinary_muscle_development	ENSGALG00010029227	UTP18	-0.795891789	0.081497151	-9.765884804	1.57729E-22	6.53784E-22	negative
PRJNA729271	stage_1W_vs_E10	ordinary_muscle_development	ENSGALG00010029228	ERAL1	0.326007698	0.118435126	2.752626757	0.005911925	0.00818935	positive
PRJNA729271	stage_1W_vs_E10	ordinary_muscle_development	ENSGALG00010029229	CBX4	0.873130124	0.22052565	3.959313226	7.51656E-05	0.000122144	positive
PRJNA729271	stage_1W_vs_E10	ordinary_muscle_development	ENSGALG00010029230	PPM1E	1.296321273	0.164316038	7.889195046	3.04142E-15	9.13876E-15	positive
PRJNA729271	stage_1W_vs_E10	ordinary_muscle_development	ENSGALG00010029231	CBX8	-0.506825114	0.110110221	-4.602888907	4.16671E-06	7.43157E-06	negative
PRJNA729271	stage_1W_vs_E10	ordinary_muscle_development	ENSGALG00010029232	CA10	-5.427107228	0.457688211	-11.85765134	1.96407E-32	1.21611E-31	negative
PRJNA729271	stage_1W_vs_E10	ordinary_muscle_development	ENSGALG00010029233	CBX2	-3.25844996	0.175186457	-18.59989647	3.21929E-77	7.08097E-76	negative
PRJNA729271	stage_1W_vs_E10	ordinary_muscle_development	ENSGALG00010029234	TOM1L1	-2.04172072	0.240447565	-8.491334562	2.04278E-17	6.74488E-17	negative
PRJNA729271	stage_1W_vs_E10	ordinary_muscle_development	ENSGALG00010029235	TRIM37	-0.381264301	0.075274093	-5.065013541	4.08371E-07	7.83293E-07	negative
PRJNA729271	stage_1W_vs_E10	ordinary_muscle_development	ENSGALG00010029237	COX11	0.175794508	0.1043103	1.685303443	0.091930042	0.111154985	positive
PRJNA729271	stage_1W_vs_E10	ordinary_muscle_development	ENSGALG00010029238	CAMKK1	-0.03691187	0.24796797	-0.148857411	0.881666148	0.894899322	negative
PRJNA729271	stage_1W_vs_E10	ordinary_muscle_development	ENSGALG00010029239	RBFOX3	-1.729715334	0.505245167	-3.423516831	0.000618164	0.000936975	negative
PRJNA729271	stage_1W_vs_E10	ordinary_muscle_development	ENSGALG00010029240	MIS12	-2.721852726	0.257687892	-10.56259457	4.44211E-26	2.13915E-25	negative
PRJNA729271	stage_1W_vs_E10	ordinary_muscle_development	ENSGALG00010029241	STXBP4	-2.071503656	0.100236493	-20.6661626	6.98544E-95	2.33843E-93	negative
PRJNA729271	stage_1W_vs_E10	ordinary_muscle_development	ENSGALG00010029242	DERL2	-0.199093073	0.098984028	-2.011365631	0.044286851	0.055686467	negative
PRJNA729271	stage_1W_vs_E10	ordinary_muscle_development	ENSGALG00010029243	NME1	0.047224263	0.33821024	0.139629904	0.888952412	0.901200532	positive

PRJNA729271	stage_1W_vs_E10	ordinary_muscle_development	ENSGALG00010029244	HLF	1.879227303	0.18341032	10.24602817	1.23307E-24	5.59065E-24	positive
PRJNA729271	stage_1W_vs_E10	ordinary_muscle_development	ENSGALG00010029245	SKA2	-1.331062004	0.115844074	-11.49011733	1.47911E-30	8.54171E-30	negative
PRJNA729271	stage_1W_vs_E10	ordinary_muscle_development	ENSGALG00010029246	DHX33	-0.943333734	0.135510346	-6.961341043	3.37049E-12	8.68537E-12	negative
PRJNA729271	stage_1W_vs_E10	ordinary_muscle_development	ENSGALG00010029247	CCDC92B	8.420950373	0.52484433	16.04466293	6.22962E-58	8.61626E-57	positive
PRJNA729271	stage_1W_vs_E10	ordinary_muscle_development	ENSGALG00010029248	C1QBP	0.078755169	0.081357352	0.96801539	0.333036684	0.369743163	positive
PRJNA729271	stage_1W_vs_E10	ordinary_muscle_development	ENSGALG00010029249	PRR11	-1.494466277	0.280482126	-5.328205034	9.91881E-08	1.98335E-07	negative
PRJNA729271	stage_1W_vs_E10	ordinary_muscle_development	ENSGALG00010029250	SPAG9	0.947203375	0.087148097	10.86889333	1.62156E-27	8.27622E-27	positive
PRJNA729271	stage_1W_vs_E10	ordinary_muscle_development	ENSGALG00010029251	ENGASE	-0.681301141	0.193371887	-3.523268831	0.000426259	0.000653753	negative
PRJNA729271	stage_1W_vs_E10	ordinary_muscle_development	ENSGALG00010029252	RPAIN	1.743120828	0.136478709	12.77210815	2.3471E-37	1.71824E-36	positive
PRJNA729271	stage_1W_vs_E10	ordinary_muscle_development	ENSGALG00010029253	NUP88	-0.121794929	0.085553675	-1.42360839	0.154559866	0.180921041	negative
PRJNA729271	stage_1W_vs_E10	ordinary_muscle_development	ENSGALG00010029254	MMD	-0.168954704	0.119068381	-1.418972043	0.155907167	0.182380307	negative
PRJNA729271	stage_1W_vs_E10	ordinary_muscle_development	ENSGALG00010029255	RAP1GAP2	0.285200955	0.193307332	1.475375778	0.140111544	0.165074927	positive
PRJNA729271	stage_1W_vs_E10	ordinary_muscle_development	ENSGALG00010029256	RABEP1	0.239147866	0.096724945	2.472452865	0.013418941	0.017890687	positive
PRJNA729271	stage_1W_vs_E10	ordinary_muscle_development	ENSGALG00010029257	YPEL2	0.582724797	0.161587115	3.606257812	0.000310645	0.000482408	positive
PRJNA729271	stage_1W_vs_E10	ordinary_muscle_development	ENSGALG00010029258	TMEM100	0.800860974	0.234215146	3.419338958	0.000627735	0.000950686	positive
PRJNA729271	stage_1W_vs_E10	ordinary_muscle_development	ENSGALG00010029259	PTRH2	0.142686788	0.107726434	1.324529021	0.185327411	0.214408239	positive
PRJNA729271	stage_1W_vs_E10	ordinary_muscle_development	ENSGALG00010029260	DHX40	0.380135856	0.10220178	3.71946413	0.000199646	0.000314109	positive
PRJNA729271	stage_1W_vs_E10	ordinary_muscle_development	ENSGALG00010029261	IFT22	-1.919929545	0.106078522	-18.09913559	3.23708E-73	6.5901E-72	negative
PRJNA729271	stage_1W_vs_E10	ordinary_muscle_development	ENSGALG00010029262	CLTC	-0.630527817	0.065294681	-9.656648984	4.60687E-22	1.87575E-21	negative
PRJNA729271	stage_1W_vs_E10	ordinary_muscle_development	ENSGALG00010029263	TOB1	3.070503151	0.18827645	16.30848226	8.59007E-60	1.24888E-58	positive
PRJNA729271	stage_1W_vs_E10	ordinary_muscle_development	ENSGALG00010029264	COL26A1	-6.356858722	0.18387087	-34.572408	6.5661E-262	1.7305E-259	negative
PRJNA729271	stage_1W_vs_E10	ordinary_muscle_development	ENSGALG00010029266	CANT1	0.691278392	0.110180694	6.27404282	3.51792E-10	8.10558E-10	positive
PRJNA729271	stage_1W_vs_E10	ordinary_muscle_development	ENSGALG00010029267	ENSGALG00010029267	4.672223572	0.272861355	17.12306814	9.98701E-66	1.67548E-64	positive
PRJNA729271	stage_1W_vs_E10	ordinary_muscle_development	ENSGALG00010029268	PCTP	1.785900741	0.178448471	10.00793525	1.40657E-23	6.0988E-23	positive
PRJNA729271	stage_1W_vs_E10	ordinary_muscle_development	ENSGALG00010029269	WSCD1	0.903594197	0.154005395	5.867289228	4.42978E-09	9.60215E-09	positive
PRJNA729271	stage_1W_vs_E10	ordinary_muscle_development	ENSGALG00010029270	TIMP2	0.927018229	0.149458589	6.202508921	5.557E-10	1.26609E-09	positive
PRJNA729271	stage_1W_vs_E10	ordinary_muscle_development	ENSGALG00010029271	MYL10	-5.866823622	0.801782564	-7.317225251	2.53151E-13	6.89093E-13	negative
PRJNA729271	stage_1W_vs_E10	ordinary_muscle_development	ENSGALG00010029273	SLC13A5	-3.607603327	0.499434379	-7.223378041	5.07118E-13	1.35972E-12	negative
PRJNA729271	stage_1W_vs_E10	ordinary_muscle_development	ENSGALG00010029274	VMP1	0.033648376	0.147024922	0.228861715	0.818976398	0.837937664	positive
PRJNA729271	stage_1W_vs_E10	ordinary_muscle_development	ENSGALG00010029275	PCYT2	0.571847825	0.070881904	8.067613719	7.16854E-16	2.22168E-15	positive
PRJNA729271	stage_1W_vs_E10	ordinary_muscle_development	ENSGALG00010029276	TUBD1	0.571138654	0.172480075	3.311331207	0.000928532	0.001388249	positive
PRJNA729271	stage_1W_vs_E10	ordinary_muscle_development	ENSGALG00010029277	WFIKKN2	0.60687356	0.76328329	0.795082989	0.426565229	0.464542674	positive
PRJNA729271	stage_1W_vs_E10	ordinary_muscle_development	ENSGALG00010029278	MED31	-0.942216449	0.072235505	-13.04367494	6.9047E-39	5.33779E-38	negative
PRJNA729271	stage_1W_vs_E10	ordinary_muscle_development	ENSGALG00010029279	ENSGALG00010029279	1.020902262	0.158440329	6.443449512	1.16788E-10	2.76383E-10	positive
PRJNA729271	stage_1W_vs_E10	ordinary_muscle_development	ENSGALG00010029280	USP36	1.991428539	0.181611075	10.96534745	5.60847E-28	2.91902E-27	positive
PRJNA729271	stage_1W_vs_E10	ordinary_muscle_development	ENSGALG00010029281	KIAA0753	-0.973842488	0.135634967	-7.179877788	6.97738E-13	1.86051E-12	negative
PRJNA729271	stage_1W_vs_E10	ordinary_muscle_development	ENSGALG00010029282	LUC7L3	-0.347353216	0.141285863	-2.458513609	0.013951349	0.018576594	negative
PRJNA729271	stage_1W_vs_E10	ordinary_muscle_development	ENSGALG00010029283	RPS6KB1	-0.018338783	0.077668371	-0.236116493	0.813342276	0.833231769	negative
PRJNA729271	stage_1W_vs_E10	ordinary_muscle_development	ENSGALG00010029284	CYTH1	0.78236773	0.093260038	8.389099424	4.8988E-17	1.58962E-16	positive
PRJNA729271	stage_1W_vs_E10	ordinary_muscle_development	ENSGALG00010029285	CUX1	0.262860486	0.069719913	3.770235431	0.000163094	0.000258252	positive
PRJNA729271	stage_1W_vs_E10	ordinary_muscle_development	ENSGALG00010029286	MAFG	2.386425984	0.181909848	13.11872895	2.57213E-39	2.0153E-38	positive
PRJNA729271	stage_1W_vs_E10	ordinary_muscle_development	ENSGALG00010029287	ANKRD40	0.982330576	0.122951033	7.989608132	1.35368E-15	4.13871E-15	positive
PRJNA729271	stage_1W_vs_E10	ordinary_muscle_development	ENSGALG00010029288	PYCR1	-1.651475397	0.294245588	-5.612574894	1.99338E-08	4.15801E-08	negative
PRJNA729271	stage_1W_vs_E10	ordinary_muscle_development	ENSGALG00010029289	PITPNM3	-0.751663086	0.223228899	-3.36723018	0.000759273	0.001143326	negative
PRJNA729271	stage_1W_vs_E10	ordinary_muscle_development	ENSGALG00010029290	ENSGALG00010029290	-2.03585278	0.199687602	-10.19518866	2.0834E-24	9.33503E-24	negative
PRJNA729271	stage_1W_vs_E10	ordinary_muscle_development	ENSGALG00010029291	RNFT1	-0.052990927	0.113818827	-0.465572598	0.641521461	0.673732327	negative
PRJNA729271	stage_1W_vs_E10	ordinary_muscle_development	ENSGALG00010029292	ENSGALG00010029292	-0.798689015	0.253980404	-3.14468755	0.001662643	0.002428457	negative
PRJNA729271	stage_1W_vs_E10	ordinary_muscle_development	ENSGALG00010029293	DNAH17	-2.068838518	0.150310508	-13.76376508	4.21017E-43	3.72112E-42	negative
PRJNA729271	stage_1W_vs_E10	ordinary_muscle_development	ENSGALG00010029294	PGS1	-0.790324528	0.077505651	-10.19699229	2.04508E-24	9.16617E-24	negative
PRJNA729271	stage_1W_vs_E10	ordinary_muscle_development	ENSGALG00010029295	SOCS3	2.044462439	0.249819882	8.183745907	2.75154E-16	8.64591E-16	positive
PRJNA729271	stage_1W_vs_E10	ordinary_muscle_development	ENSGALG00010029296	FBXO39	-1.331265457	0.161513411	-8.242445312	1.68726E-16	5.36452E-16	negative
PRJNA729271	stage_1W_vs_E10	ordinary_muscle_development	ENSGALG00010029297	SH2B2	4.708570356	0.251228602	18.74217472	2.24271E-78	5.11939E-77	positive
PRJNA729271	stage_1W_vs_E10	ordinary_muscle_development	ENSGALG00010029298	ENDOV	0.452221048	0.145796918	3.101718843	0.001924006	0.002793316	positive
PRJNA729271	stage_1W_vs_E10	ordinary_muscle_development	ENSGALG00010029299	PRKRIP1	-0.000926563	0.091248378	-0.010154296	0.991898184	0.993347321	negative
PRJNA729271	stage_1W_vs_E10	ordinary_muscle_development	ENSGALG00010029300	TEKT1	1.811150293	0.343991587	5.265100541	1.40113E-07	2.77109E-07	positive
PRJNA729271	stage_1W_vs_E10	ordinary_muscle_development	ENSGALG00010029301	RNF213	3.725677331	0.464326383	8.023832955	1.02496E-15	3.15162E-15	positive
PRJNA729271	stage_1W_vs_E10	ordinary_muscle_development	ENSGALG00010029303	SLC26A11	0.099813777	0.152509696	0.65447496	0.512805854	0.550153272	positive
PRJNA729271	stage_1W_vs_E10	ordinary_muscle_development	ENSGALG00010029304	ORAI3	0.071657783	0.117200271	0.611413118	0.54092612	0.577202894	positive
PRJNA729271	stage_1W_vs_E10	ordinary_muscle_development	ENSGALG00010029305	SMTNL2	2.800526769	0.189892159	14.74798533	3.16987E-49	3.37847E-48	positive
PRJNA729271	stage_1W_vs_E10	ordinary_muscle_development	ENSGALG00010029306	CEP95	-1.658110332	0.163097597	-10.16636885	2.80159E-24	1.24644E-23	negative
PRJNA729271	stage_1W_vs_E10	ordinary_muscle_development	ENSGALG00010029307	SGSH	0.556066951	0.164989798	3.37031112	0.000750834	0.001131206	positive
PRJNA729271	stage_1W_vs_E10	ordinary_muscle_development	ENSGALG00010029308	GAA	0.076671246	0.139054002	0.551377488	0.58137493	0.616867697	positive
PRJNA729271	stage_1W_vs_E10	ordinary_muscle_development	ENSGALG00010029309	ALKBH4	1.890987822	0.167022501	11.32175494	1.02401E-29	5.72207E-29	positive
PRJNA729271	stage_1W_vs_E10	ordinary_muscle_development	ENSGALG00010029310	LRWD1	-1.132436423	0.092579179	-12.23208543	2.0949E-34	1.38719E-33	negative
PRJNA729271	stage_1W_vs_E10	ordinary_muscle_development	ENSGALG00010029311	ENSGALG00010029311	0.874649581	0.165933912	5.271071897	1.35629E-07	2.68425E-07	positive
PRJNA729271	stage_1W_vs_E10	ordinary_muscle_development	ENSGALG00010029312	SMURF2	0.349169954	0.094198099	3.706762209	0.000209926	0.000329565	positive
PRJNA729271	stage_1W_vs_E10	ordinary_muscle_development	ENSGALG00010029313	CCDC40	-1.658409394	0.26153932	-6.340956287	2.28343E-10	5.31189E-10	negative
PRJNA729271	stage_1W_vs_E10	ordinary_muscle_development	ENSGALG00010029314	MED13	0.282206844	0.126012726	2.239510674	0.025122708	0.032487612	positive
PRJNA729271	stage_1W_vs_E10	ordinary_muscle_development	ENSGALG00010029315	TBC1D16	-0.484016256	0.096924835	-4.993727945	5.92248E-07	1.12394E-06	negative
PRJNA729271	stage_1W_vs_E10	ordinary_muscle_development	ENSGALG00010029316	INTS2	-0.671915958	0.095342791	-7.047370324	1.82331E-12	4.75938E-12	negative
PRJNA729271	stage_1W_vs_E10	ordinary_muscle_development	ENSGALG00010029317	KPNA2	-3.128501257	0.205865177	-15.19684537	3.71065E-52	4.39068E-51	negative
PRJNA729271	stage_1W_vs_E10	ordinary_muscle_development	ENSGALG00010029318	C17orf58	2.048830435	0.206885904	9.903190082	4.03204E-23	1.71391E-22	positive
PRJNA729271	stage_1W_vs_E10	ordinary_muscle_development	ENSGALG00010029319	BRIP1	-1.695322653	0.129587974	-13.08240727	4.15083E-39	3.22781E-38	negative
PRJNA729271	stage_1W_vs_E10	ordinary_muscle_development	ENSGALG00010029320	TBX4	-6.198049289	0.999500096	-6.201149266	5.60523E-10	1.27688E-09	negative
PRJNA729271	stage_1W_vs_E10	ordinary_muscle_development	ENSGALG00010029321	TBX2	-1.221295325	0.174332359	-7.005557254	2.46005E-12	6.38238E-12	negative
PRJNA729271	stage_1W_vs_E10	ordinary_muscle_development	ENSGALG00010029322	BPTF	-0.411956546	0.097195671	-4.238424835	2.25094E-05	3.79962E-05	negative
PRJNA729271	stage_1W_vs_E10	ordinary_muscle_development	ENSGALG00010029323	NOL11	0.793482114	0.090597441	8.75832809	1.98168E-18	6.8768E-18	positive
PRJNA729271	stage_1W_vs_E10	ordinary_muscle_development	ENSGALG00010029324	BCAS3	1.055895734	0.126203734	8.366596633	5.93063E-17	1.91971E-16	positive
PRJNA729271	stage_1W_vs_E10	ordinary_muscle_development	ENSGALG00010029325	LRRC75A	-1.993780801	0.177764131	-11.21587795	3.40788E-29	1.85634E-28	negative
PRJNA729271	stage_1W_vs_E10	ordinary_muscle_development	ENSGALG00010029326	NCBP3	-0.560115339	0.08172496	-6.853663017	7.19825E-12	1.82506E-11	negative
PRJNA729271	stage_1W_vs_E10	ordinary_muscle_development	ENSGALG00010029327	CRK	-0.641223439	0.055095239	-11.63845464	2.62733E-31	1.56271E-30	negative
PRJNA729271	stage_1W_vs_E10	ordinary_muscle_development	ENSGALG00010029328	ENSGALG00010029328	-0.343662492	0.083080489	-4.136500577	3.52642E-05	5.87467E-05	negative
PRJNA729271	stage_1W_vs_E10	ordinary_muscle_development	ENSGALG00010029329	ENSGALG00010029329	1.132824934	0.178625819	6.341887981	2.26966E-10	5.2807E-10	positive
PRJNA729271	stage_1W_vs_E10	ordinary_muscle_development	ENSGALG00010029330	AFMID	0.814094175	0.127468803	6.386615033	1.69598E-10	3.97915E-10	positive
PRJNA729271	stage_1W_vs_E10	ordinary_muscle_development	ENSGALG00010029331	TK1	-2.450234262	0.278670495	-8.792585891	1.46156E-18	5.11847E-18	negative
PRJNA729271	stage_1W_vs_E10	ordinary_muscle_development	ENSGALG00010029332	YWHAE	-0.898106717	0.148853898	-6.033478002	1.60468E-09	3.57458E-09	negative
PRJNA729271	stage_1W_vs_E10	ordinary_muscle_development	ENSGALG00010029333	ENSGALG00010029333	-1.016279566	0.175730082	-5.783184939	7.32994E-09	1.5673E-08	negative
PRJNA729271	stage_1W_vs_E10	ordinary_muscle_development	ENSGALG00010029335	RNF222	-0.267759922	1.507705825	-0.177594274	0.859041622	0.874670434	negative
PRJNA729271	stage_1W_vs_E10	ordinary_muscle_development	ENSGALG00010029336	SYNGR2	2.737198167	0.109987242	24.88650598	1.0416E-136	7.2586E-135	positive
PRJNA729271	stage_1W_vs_E10	ordinary_muscle_development	ENSGALG00010029337	MYO1C	0.996393166	0.133892582	7.441735415	9.93711E-14	2.76147E-13	positive
PRJNA729271	stage_1W_vs_E10	ordinary_muscle_development	ENSGALG00010029338	INPP5K	-0.034635603	0.120559967	-0.287289421	0.773890722	0.796927111	negative
PRJNA729271	stage_1W_vs_E10	ordinary_muscle_development	ENSGALG00010029339	PITPNA	1.156046422	0.126694684	9.124663974	7.19591E-20	2.68757E-19	positive
PRJNA729271	stage_1W_vs_E10	ordinary_muscle_development	ENSGALG00010029340	NDEL1	0.907806032	0.103702554	8.753940893	2.06029E-18	7.14448E-18	positive
PRJNA729271	stage_1W_vs_E10	ordinary_muscle_development	ENSGALG00010029341	SLC43A2	3.108728066	0.326130984	9.532145723	1.54065E-21	6.16559E-21	positive
PRJNA729271	stage_1W_vs_E10	ordinary_muscle_development	ENSGALG00010029342	TMC6	-0.589387878	0.131873543	-4.469341343	7.84608E-06	1.37056E-05	negative
PRJNA729271	stage_1W_vs_E10	ordinary_muscle_development	ENSGALG00010029343	SCARF1	1.966080555	0.129970807	15.12709358	1.07332E-51	1.24962E-50	positive
PRJNA729271	stage_1W_vs_E10	ordinary_muscle_development	ENSGALG00010029344	ENSGALG00010029344	1.237134636	0.165185686	7.489357356	6.92117E-14	1.94423E-13	positive
PRJNA729271	stage_1W_vs_E10	ordinary_muscle_development	ENSGALG00010029345	RILP	3.36195929	0.241689875	13.91021985	5.49104E-44	4.99326E-43	positive
PRJNA729271	stage_1W_vs_E10	ordinary_muscle_development	ENSGALG00010029346	ARL16	-1.350682656	0.13744776	-9.826880106	8.62494E-23	3.61325E-22	negative
PRJNA729271	stage_1W_vs_E10	ordinary_muscle_development	ENSGALG00010029347	PRPF8	-0.308104079	0.069980224	-4.402730658	1.06897E-05	1.85188E-05	negative
PRJNA729271	stage_1W_vs_E10	ordinary_muscle_development	ENSGALG00010029348	HGS	0.894430465	0.123109673	7.265314252	3.72172E-13	1.00496E-12	positive
PRJNA729271	stage_1W_vs_E10	ordinary_muscle_development	ENSGALG00010029349	MYH10	-2.734217057	0.107718578	-25.38296649	3.8891E-142	2.8615E-140	negative
PRJNA729271	stage_1W_vs_E10	ordinary_muscle_development	ENSGALG00010029350	MRPL12	1.097001785	0.121347383	9.040176697	1.56419E-19	5.75893E-19	positive
PRJNA729271	stage_1W_vs_E10	ordinary_muscle_development	ENSGALG00010029351	ENSGALG00010029351	-0.679817215	0.195563179	-3.476202519	0.000508568	0.000775152	negative
PRJNA729271	stage_1W_vs_E10	ordinary_muscle_development	ENSGALG00010029352	AATF	-0.932548284	0.112580098	-8.28342046	1.19687E-16	3.82718E-16	negative
PRJNA729271	stage_1W_vs_E10	ordinary_muscle_development	ENSGALG00010029353	GCGR	3.05361477	0.893335151	3.418218534	0.000630325	0.000954509	positive
PRJNA729271	stage_1W_vs_E10	ordinary_muscle_development	ENSGALG00010029354	TLCD2	-0.388962531	0.28243053	-1.377197183	0.168451292	0.19610485	negative
PRJNA729271	stage_1W_vs_E10	ordinary_muscle_development	ENSGALG00010029355	MCRIP1	1.524369288	0.17230416	8.846967394	8.99297E-19	3.18167E-18	positive
PRJNA729271	stage_1W_vs_E10	ordinary_muscle_development	ENSGALG00010029357	ARHGDIA	-0.872327071	0.097624469	-8.93553715	4.05205E-19	1.4636E-18	negative
PRJNA729271	stage_1W_vs_E10	ordinary_muscle_development	ENSGALG00010029359	ENSGALG00010029359	-3.120397255	0.533431445	-5.849668748	4.92553E-09	1.06529E-08	negative
PRJNA729271	stage_1W_vs_E10	ordinary_muscle_development	ENSGALG00010029360	ACACA	-0.927624306	0.086444788	-10.73082984	7.29382E-27	3.62068E-26	negative
PRJNA729271	stage_1W_vs_E10	ordinary_muscle_development	ENSGALG00010029361	ALYREF	-1.716758719	0.135814985	-12.64042196	1.26374E-36	9.03695E-36	negative
PRJNA729271	stage_1W_vs_E10	ordinary_muscle_development	ENSGALG00010029362	TADA2A	-0.361014765	0.113281457	-3.186883134	0.001438149	0.00211077	negative
PRJNA729271	stage_1W_vs_E10	ordinary_muscle_development	ENSGALG00010029363	PIK3R6	2.154495114	0.191154398	11.27096807	1.82552E-29	1.00842E-28	positive
PRJNA729271	stage_1W_vs_E10	ordinary_muscle_development	ENSGALG00010029364	ANAPC11	0.354255051	0.128175782	2.763822025	0.005712869	0.007927248	positive
PRJNA729271	stage_1W_vs_E10	ordinary_muscle_development	ENSGALG00010029365	PIK3R5	0.858820166	0.191410487	4.486797885	7.23016E-06	1.26617E-05	positive
PRJNA729271	stage_1W_vs_E10	ordinary_muscle_development	ENSGALG00010029366	MGAT5B	1.491037093	0.344598215	4.326885719	1.51232E-05	2.58534E-05	positive
PRJNA729271	stage_1W_vs_E10	ordinary_muscle_development	ENSGALG00010029367	DUSP14	-0.803178532	0.118943219	-6.752621454	1.45197E-11	3.61621E-11	negative
PRJNA729271	stage_1W_vs_E10	ordinary_muscle_development	ENSGALG00010029368	DDX52	-0.074386643	0.0908238	-0.819021483	0.412774157	0.450878779	negative
PRJNA729271	stage_1W_vs_E10	ordinary_muscle_development	ENSGALG00010029369	MFSD11	-0.595360557	0.085796031	-6.93925522	3.94172E-12	1.01142E-11	negative
PRJNA729271	stage_1W_vs_E10	ordinary_muscle_development	ENSGALG00010029370	SRSF2	-1.134163183	0.126637301	-8.955996185	3.36682E-19	1.21944E-18	negative
PRJNA729271	stage_1W_vs_E10	ordinary_muscle_development	ENSGALG00010029371	AUTS2	-2.052554155	0.17687922	-11.60426957	3.92021E-31	2.31366E-30	negative
PRJNA729271	stage_1W_vs_E10	ordinary_muscle_development	ENSGALG00010029372	METTL23	-0.381344677	0.147758869	-2.580858127	0.009855507	0.013333543	negative
PRJNA729271	stage_1W_vs_E10	ordinary_muscle_development	ENSGALG00010029373	GATSL2	1.2262894	0.16907251	7.253038364	4.07525E-13	1.09817E-12	positive
PRJNA729271	stage_1W_vs_E10	ordinary_muscle_development	ENSGALG00010029374	RCC1L	-0.56176011	0.119510969	-4.700489957	2.59538E-06	4.70427E-06	negative
PRJNA729271	stage_1W_vs_E10	ordinary_muscle_development	ENSGALG00010029375	JMJD6	0.562292678	0.160493889	3.503514559	0.000459162	0.000701914	positive
PRJNA729271	stage_1W_vs_E10	ordinary_muscle_development	ENSGALG00010029376	ENSGALG00010029376	-0.758953064	0.208277096	-3.643958357	0.000268477	0.000418944	negative
PRJNA729271	stage_1W_vs_E10	ordinary_muscle_development	ENSGALG00010029377	NCF1C	1.067926228	0.299021493	3.571402906	0.000355074	0.000548349	positive
PRJNA729271	stage_1W_vs_E10	ordinary_muscle_development	ENSGALG00010029378	ST6GALNAC1	-2.750103389	0.368547244	-7.462010467	8.52121E-14	2.37849E-13	negative
PRJNA729271	stage_1W_vs_E10	ordinary_muscle_development	ENSGALG00010029379	GTF2I	0.595381348	0.112115062	5.310449241	1.09355E-07	2.17824E-07	positive
PRJNA729271	stage_1W_vs_E10	ordinary_muscle_development	ENSGALG00010029380	RGS9	-0.237392811	0.15047603	-1.577612141	0.114654741	0.136772321	negative

PRJNA729271	stage_1W_vs_E10	ordinary_muscle_development	ENSGALG00010029382	GTF2IRD1	0.739411874	0.206087827	3.587848365	0.000333418	0.000516225	positive
PRJNA729271	stage_1W_vs_E10	ordinary_muscle_development	ENSGALG00010029383	GNA13	-0.002685823	0.079094154	-0.033957282	0.972911215	0.976752186	negative
PRJNA729271	stage_1W_vs_E10	ordinary_muscle_development	ENSGALG00010029384	WDR81	-0.044423792	0.075041837	-0.591987005	0.553859291	0.59000444	negative
PRJNA729271	stage_1W_vs_E10	ordinary_muscle_development	ENSGALG00010029385	CLIP2	-1.101623377	0.123398615	-8.927356072	4.36311E-19	1.57439E-18	negative
PRJNA729271	stage_1W_vs_E10	ordinary_muscle_development	ENSGALG00010029386	SLC16A6	-0.666691315	0.239032203	-2.789127607	0.005285024	0.007365294	negative
PRJNA729271	stage_1W_vs_E10	ordinary_muscle_development	ENSGALG00010029387	SERPINF1	1.648289495	0.175962378	9.367283561	7.44232E-21	2.89835E-20	positive
PRJNA729271	stage_1W_vs_E10	ordinary_muscle_development	ENSGALG00010029388	ST6GALNAC2	1.566595889	0.322878789	4.851962843	1.22246E-06	2.27057E-06	positive
PRJNA729271	stage_1W_vs_E10	ordinary_muscle_development	ENSGALG00010029389	SMYD4	0.53104486	0.094727497	5.606026544	2.07024E-08	4.31276E-08	positive
PRJNA729271	stage_1W_vs_E10	ordinary_muscle_development	ENSGALG00010029390	RPA1	-1.446826105	0.081580246	-17.7350055	2.25074E-70	4.30971E-69	negative
PRJNA729271	stage_1W_vs_E10	ordinary_muscle_development	ENSGALG00010029391	ARSG	-0.156522943	0.143548059	-1.090387038	0.275542689	0.309803854	negative
PRJNA729271	stage_1W_vs_E10	ordinary_muscle_development	ENSGALG00010029392	RTN4RL1	2.233679522	0.525833702	4.247882006	2.15801E-05	3.64658E-05	positive
PRJNA729271	stage_1W_vs_E10	ordinary_muscle_development	ENSGALG00010029394	CYGB	-1.073400458	0.143296911	-7.490743853	6.84844E-14	1.92455E-13	negative
PRJNA729271	stage_1W_vs_E10	ordinary_muscle_development	ENSGALG00010029395	DPH1	0.066800251	0.116520025	0.573294173	0.56644553	0.602128774	positive
PRJNA729271	stage_1W_vs_E10	ordinary_muscle_development	ENSGALG00010029396	WIPI1	2.680105021	0.153984095	17.40507699	7.55044E-68	1.34949E-66	positive
PRJNA729271	stage_1W_vs_E10	ordinary_muscle_development	ENSGALG00010029397	OVCA2	-0.307760411	0.15483303	-1.987692229	0.046845739	0.058693836	negative
PRJNA729271	stage_1W_vs_E10	ordinary_muscle_development	ENSGALG00010029398	RHBDF2	0.773207341	0.122702687	6.301470308	2.94835E-10	6.8171E-10	positive
PRJNA729271	stage_1W_vs_E10	ordinary_muscle_development	ENSGALG00010029399	PRKAR1A	-0.986038979	0.054040599	-18.2462631	2.21556E-74	4.60094E-73	negative
PRJNA729271	stage_1W_vs_E10	ordinary_muscle_development	ENSGALG00010029400	AANAT	3.21200581	0.615313842	5.220109786	1.78817E-07	3.51118E-07	positive
PRJNA729271	stage_1W_vs_E10	ordinary_muscle_development	ENSGALG00010029401	HIC1	-1.200950408	0.202540523	-5.929432747	3.03983E-09	6.65494E-09	negative
PRJNA729271	stage_1W_vs_E10	ordinary_muscle_development	ENSGALG00010029402	SMG6	0.25326294	0.088672583	2.856158366	0.004288012	0.006020412	positive
PRJNA729271	stage_1W_vs_E10	ordinary_muscle_development	ENSGALG00010029403	FAM20A	1.885243634	0.157539837	11.96677408	5.30514E-33	3.34922E-32	positive
PRJNA729271	stage_1W_vs_E10	ordinary_muscle_development	ENSGALG00010029404	SRR	-0.233043388	0.192068097	-1.213337308	0.225000884	0.256499239	negative
PRJNA729271	stage_1W_vs_E10	ordinary_muscle_development	ENSGALG00010029405	POLDIP2	0.661384319	0.079059814	8.365619452	5.97999E-17	1.93525E-16	positive
PRJNA729271	stage_1W_vs_E10	ordinary_muscle_development	ENSGALG00010029406	TMEM199	0.428034193	0.101860366	4.202166273	2.64373E-05	4.43785E-05	positive
PRJNA729271	stage_1W_vs_E10	ordinary_muscle_development	ENSGALG00010029407	UBE2O	0.780179637	0.188413917	4.14077499	3.46134E-05	5.77222E-05	positive
PRJNA729271	stage_1W_vs_E10	ordinary_muscle_development	ENSGALG00010029408	KIF12	2.041065028	0.468088809	4.360422611	1.29811E-05	2.23258E-05	positive
PRJNA729271	stage_1W_vs_E10	ordinary_muscle_development	ENSGALG00010029409	ENSGALG00010029409	3.37177829	0.188872007	17.85218653	2.77938E-71	5.42954E-70	positive
PRJNA729271	stage_1W_vs_E10	ordinary_muscle_development	ENSGALG00010029410	VTN	3.526718423	0.880939697	4.003359634	6.24493E-05	0.000102075	positive
PRJNA729271	stage_1W_vs_E10	ordinary_muscle_development	ENSGALG00010029411	SARM1	-3.841633524	0.293634905	-13.08302747	4.1171E-39	3.20329E-38	negative
PRJNA729271	stage_1W_vs_E10	ordinary_muscle_development	ENSGALG00010029412	SLC46A1	0.189479744	0.10998679	1.72275002	0.08493374	0.103065263	positive
PRJNA729271	stage_1W_vs_E10	ordinary_muscle_development	ENSGALG00010029413	B3GNTL1	-0.190741948	0.222598982	-0.856885986	0.391507899	0.429526717	negative
PRJNA729271	stage_1W_vs_E10	ordinary_muscle_development	ENSGALG00010029416	UNC119	-4.400134781	0.512409685	-8.587142105	8.91593E-18	2.99644E-17	negative
PRJNA729271	stage_1W_vs_E10	ordinary_muscle_development	ENSGALG00010029417	ENSGALG00010029417	2.607917788	0.210442626	12.39253587	2.86828E-35	1.96761E-34	positive
PRJNA729271	stage_1W_vs_E10	ordinary_muscle_development	ENSGALG00010029418	ABCA5	1.081066366	0.131661241	8.210968963	2.19411E-16	6.92436E-16	positive
PRJNA729271	stage_1W_vs_E10	ordinary_muscle_development	ENSGALG00010029419	RFC2	-0.529336788	0.133036498	-3.978883953	6.92395E-05	0.000112792	negative
PRJNA729271	stage_1W_vs_E10	ordinary_muscle_development	ENSGALG00010029420	LAT2	0.351977722	0.320171977	1.099339564	0.271619981	0.305701656	positive
PRJNA729271	stage_1W_vs_E10	ordinary_muscle_development	ENSGALG00010029421	TBCD	-0.466965638	0.093761635	-4.98034871	6.34698E-07	1.20231E-06	negative
PRJNA729271	stage_1W_vs_E10	ordinary_muscle_development	ENSGALG00010029422	EIF4H	-0.673303856	0.087438305	-7.700330598	1.35715E-14	3.95334E-14	negative
PRJNA729271	stage_1W_vs_E10	ordinary_muscle_development	ENSGALG00010029423	MYBBP1A	-0.559400862	0.120816509	-4.630169079	3.65367E-06	6.5504E-06	negative
PRJNA729271	stage_1W_vs_E10	ordinary_muscle_development	ENSGALG00010029424	MAP2K6	0.388130554	0.217950675	1.780818317	0.074942136	0.091507562	positive
PRJNA729271	stage_1W_vs_E10	ordinary_muscle_development	ENSGALG00010029425	SPNS2	2.233288271	0.163507256	13.65864936	1.79278E-42	1.557E-41	positive
PRJNA729271	stage_1W_vs_E10	ordinary_muscle_development	ENSGALG00010029426	ZNF750	-4.895583018	0.98431428	-4.973597474	6.57217E-07	1.24398E-06	negative
PRJNA729271	stage_1W_vs_E10	ordinary_muscle_development	ENSGALG00010029427	SPNS3	-1.237961013	0.327389862	-3.781305271	0.000156008	0.000247438	negative
PRJNA729271	stage_1W_vs_E10	ordinary_muscle_development	ENSGALG00010029428	FN3K	0.351314047	0.26148592	1.3435295	0.17910058	0.207601992	positive
PRJNA729271	stage_1W_vs_E10	ordinary_muscle_development	ENSGALG00010029429	UBE2G1	1.797556427	0.089443253	20.09717185	7.81206E-90	2.29222E-88	positive
PRJNA729271	stage_1W_vs_E10	ordinary_muscle_development	ENSGALG00010029430	FN3KRP	-1.024947312	0.098881965	-10.36536152	3.56408E-25	1.65211E-24	negative
PRJNA729271	stage_1W_vs_E10	ordinary_muscle_development	ENSGALG00010029432	ENSGALG00010029432	6.701079723	5.433544473	1.233279631			positive
PRJNA729271	stage_1W_vs_E10	ordinary_muscle_development	ENSGALG00010029436	WDR45B	-0.303710091	0.080245827	-3.784746237	0.000153865	0.0002442	negative
PRJNA729271	stage_1W_vs_E10	ordinary_muscle_development	ENSGALG00010029437	ENSGALG00010029437	0.412280659	2.869422561	0.143680706	0.885752608	0.898662851	positive
PRJNA729271	stage_1W_vs_E10	ordinary_muscle_development	ENSGALG00010029438	ENSGALG00010029438	-0.811997689	0.269917127	-3.00832221	0.002626945	0.003763721	negative
PRJNA729271	stage_1W_vs_E10	ordinary_muscle_development	ENSGALG00010029439	ENSGALG00010029439	-1.266323824	0.211540972	-5.986187038	2.14817E-09	4.74806E-09	negative
PRJNA729271	stage_1W_vs_E10	ordinary_muscle_development	ENSGALG00010029440	FOXK2	1.149133742	0.181332603	6.337160115	2.34039E-10	5.4374E-10	positive
PRJNA729271	stage_1W_vs_E10	ordinary_muscle_development	ENSGALG00010029442	ENSGALG00010029442	-0.847012757	0.283953945	-2.982923017	0.002855098	0.004077305	negative
PRJNA729271	stage_1W_vs_E10	ordinary_muscle_development	ENSGALG00010029443	ENSGALG00010029443	-0.61910951	0.317114035	-1.952324531	0.050899687	0.063509595	negative
PRJNA729271	stage_1W_vs_E10	ordinary_muscle_development	ENSGALG00010029445	SPHK1	0.072938257	0.13769636	0.529703598	0.596317453	0.630829184	positive
PRJNA729271	stage_1W_vs_E10	ordinary_muscle_development	ENSGALG00010029446	PRPSAP1	0.199933912	0.080593678	2.480764208	0.013110106	0.017496639	positive
PRJNA729271	stage_1W_vs_E10	ordinary_muscle_development	ENSGALG00010029449	UBALD2	2.290019658	0.180774062	12.66785532	8.91177E-37	6.40119E-36	positive
PRJNA729271	stage_1W_vs_E10	ordinary_muscle_development	ENSGALG00010029451	ENSGALG00010029451	-1.657294023	0.22304116	-7.430440284	1.08237E-13	3.00324E-13	negative
PRJNA729271	stage_1W_vs_E10	ordinary_muscle_development	ENSGALG00010029452	ENSGALG00010029452	0.396858518	0.933903812	0.424945817	0.670876174	0.700958015	positive
PRJNA729271	stage_1W_vs_E10	ordinary_muscle_development	ENSGALG00010029453	ENSGALG00010029453	-0.697001262	1.520185935	-0.458497376	0.646595147	0.678274473	negative
PRJNA729271	stage_1W_vs_E10	ordinary_muscle_development	ENSGALG00010029454	ENSGALG00010029454	-0.930222548	0.291654105	-3.18947182	0.00142533	0.002093653	negative
PRJNA729271	stage_1W_vs_E10	ordinary_muscle_development	ENSGALG00010029455	ENSGALG00010029455	-0.933172037	0.257070136	-3.630028949	0.000283389	0.000441359	negative
PRJNA729271	stage_1W_vs_E10	ordinary_muscle_development	ENSGALG00010029456	RNF157	-2.635009551	0.519615263	-5.071078047	3.95568E-07	7.5954E-07	negative
PRJNA729271	stage_1W_vs_E10	ordinary_muscle_development	ENSGALG00010029457	ENSGALG00010029457	-0.908962922	0.191181367	-4.754453519	1.98984E-06	3.63579E-06	negative
PRJNA729271	stage_1W_vs_E10	ordinary_muscle_development	ENSGALG00010029460	ENSGALG00010029460	-1.523619145	0.285158526	-5.343060104	9.13904E-08	1.83325E-07	negative
PRJNA729271	stage_1W_vs_E10	ordinary_muscle_development	ENSGALG00010029461	ENSGALG00010029461	-0.83020771	0.203766509	-4.074308936	4.61512E-05	7.60962E-05	negative
PRJNA729271	stage_1W_vs_E10	ordinary_muscle_development	ENSGALG00010029462	ENSGALG00010029462	-2.229861279	1.822901366	-1.223248455	0.221235869	0.252723928	negative
PRJNA729271	stage_1W_vs_E10	ordinary_muscle_development	ENSGALG00010029463	ENSGALG00010029463	-0.374963806	0.445605714	-0.841469923	0.400084728	0.438372373	negative
PRJNA729271	stage_1W_vs_E10	ordinary_muscle_development	ENSGALG00010029464	ENSGALG00010029464	0.574319507	0.521176393	1.101967617	0.27047575	0.304484778	positive
PRJNA729271	stage_1W_vs_E10	ordinary_muscle_development	ENSGALG00010029465	ENSGALG00010029465	-5.339064745	2.502127883	-2.133809699	0.032858362	0.041900409	negative
PRJNA729271	stage_1W_vs_E10	ordinary_muscle_development	ENSGALG00010029466	SLC39A11	-1.634175838	0.128357397	-12.73145044	3.95424E-37	2.87158E-36	negative
PRJNA729271	stage_1W_vs_E10	ordinary_muscle_development	ENSGALG00010029468	ENSGALG00010029468	-2.109204942	0.24257769	-8.694966736	3.46931E-18	1.19052E-17	negative
PRJNA729271	stage_1W_vs_E10	ordinary_muscle_development	ENSGALG00010029469	ENSGALG00010029469	-2.551023187	0.413030857	-6.176350124	6.56004E-10	1.48947E-09	negative
PRJNA729271	stage_1W_vs_E10	ordinary_muscle_development	ENSGALG00010029470	SSTR2	-1.339654238	0.921703498	-1.453454652	0.146097562	0.171653089	negative
PRJNA729271	stage_1W_vs_E10	ordinary_muscle_development	ENSGALG00010029472	ENSGALG00010029472	-2.14360565	0.86071813	-2.490485067	0.012756886	0.017040923	negative
PRJNA729271	stage_1W_vs_E10	ordinary_muscle_development	ENSGALG00010029473	COG1	1.579166138	0.099684404	15.84165709	1.60534E-56	2.15457E-55	positive
PRJNA729271	stage_1W_vs_E10	ordinary_muscle_development	ENSGALG00010029474	ENSGALG00010029474	0.06130815	1.043882653	0.058730883	0.95316646	0.959200829	positive
PRJNA729271	stage_1W_vs_E10	ordinary_muscle_development	ENSGALG00010029477	FAM104A	-0.314482586	0.059381457	-5.295972925	1.18384E-07	2.35356E-07	negative
PRJNA729271	stage_1W_vs_E10	ordinary_muscle_development	ENSGALG00010029479	ENSGALG00010029479	-1.187208816	0.551327505	-2.153364026	0.031290081	0.039995566	negative
PRJNA729271	stage_1W_vs_E10	ordinary_muscle_development	ENSGALG00010029480	ENSGALG00010029480	0.944104737	0.1146207	8.236773454	1.76917E-16	5.61632E-16	positive
PRJNA729271	stage_1W_vs_E10	ordinary_muscle_development	ENSGALG00010029481	ENSGALG00010029481	-1.07398831	0.278215365	-3.860276768	0.000113259	0.000181442	negative
PRJNA729271	stage_1W_vs_E10	ordinary_muscle_development	ENSGALG00010029482	DDX25	-0.273215847	0.18636691	-1.466010505	0.142645425	0.167855612	negative
PRJNA729271	stage_1W_vs_E10	ordinary_muscle_development	ENSGALG00010029484	ENSGALG00010029484	-2.975787578	0.719226866	-4.137481116	3.51139E-05	5.8503E-05	negative
PRJNA729271	stage_1W_vs_E10	ordinary_muscle_development	ENSGALG00010029487	CDC42EP4	-2.098458571	0.148015825	-14.17725824	1.26701E-45	1.20983E-44	negative
PRJNA729271	stage_1W_vs_E10	ordinary_muscle_development	ENSGALG00010029489	ENSGALG00010029489	-0.948627348	0.918245084	-1.033087315	0.301563039	0.336864693	negative
PRJNA729271	stage_1W_vs_E10	ordinary_muscle_development	ENSGALG00010029490	ENSGALG00010029490	-0.970180407	0.611755295	-1.585896215	0.112762855	0.134626273	negative
PRJNA729271	stage_1W_vs_E10	ordinary_muscle_development	ENSGALG00010029491	ENSGALG00010029491	0.040267556	0.199010658	0.202338688	0.839651956	0.857276545	positive
PRJNA729271	stage_1W_vs_E10	ordinary_muscle_development	ENSGALG00010029492	SDK2	-2.330629784	0.23540268	-9.90060855	4.1375E-23	1.75822E-22	negative
PRJNA729271	stage_1W_vs_E10	ordinary_muscle_development	ENSGALG00010029494	MYH7	-4.968582936	0.535545029	-9.277619378	1.73307E-20	6.65631E-20	negative
PRJNA729271	stage_1W_vs_E10	ordinary_muscle_development	ENSGALG00010029495	RPL38	0.291672554	0.188628893	1.546277186	0.122037639	0.145018493	positive
PRJNA729271	stage_1W_vs_E10	ordinary_muscle_development	ENSGALG00010029496	TTYH2	-1.107930905	0.148064426	-7.482762304	7.27764E-14	2.0412E-13	negative
PRJNA729271	stage_1W_vs_E10	ordinary_muscle_development	ENSGALG00010029497	ENSGALG00010029497	-0.505907417	0.588065937	-0.860290293	0.389629042	0.427594865	negative
PRJNA729271	stage_1W_vs_E10	ordinary_muscle_development	ENSGALG00010029498	ENSGALG00010029498	0.204642845	0.148771986	1.375546899	0.168961956	0.196632625	positive
PRJNA729271	stage_1W_vs_E10	ordinary_muscle_development	ENSGALG00010029501	ENSGALG00010029501	-0.779224315	0.392572866	-1.984916386	0.047153775	0.059057285	negative
PRJNA729271	stage_1W_vs_E10	ordinary_muscle_development	ENSGALG00010029502	BTBD17	-0.840430708	0.572111975	-1.468996882	0.141833635	0.166968126	negative
PRJNA729271	stage_1W_vs_E10	ordinary_muscle_development	ENSGALG00010029504	GPR142	-3.012719614	0.626031341	-4.812410202	1.49121E-06	2.75071E-06	negative
PRJNA729271	stage_1W_vs_E10	ordinary_muscle_development	ENSGALG00010029505	VEZF1	-0.04590857	0.110457288	-0.415622819	0.677686026	0.707305512	negative
PRJNA729271	stage_1W_vs_E10	ordinary_muscle_development	ENSGALG00010029506	OXLD1	-1.055575697	0.162799755	-6.483889952	8.93874E-11	2.12824E-10	negative
PRJNA729271	stage_1W_vs_E10	ordinary_muscle_development	ENSGALG00010029507	VPS37D	-0.211196825	0.173648733	-1.216230157	0.223897259	0.255442012	negative
PRJNA729271	stage_1W_vs_E10	ordinary_muscle_development	ENSGALG00010029508	ENSGALG00010029508	-0.944488355	0.146495813	-6.447203739	1.13933E-10	2.69978E-10	negative
PRJNA729271	stage_1W_vs_E10	ordinary_muscle_development	ENSGALG00010029509	DNAJC30	1.396076263	0.167757933	8.321968656	8.65142E-17	2.78363E-16	positive
PRJNA729271	stage_1W_vs_E10	ordinary_muscle_development	ENSGALG00010029510	PDE6G	-0.830838495	0.312674229	-2.65720171	0.007879228	0.010772441	negative
PRJNA729271	stage_1W_vs_E10	ordinary_muscle_development	ENSGALG00010029511	CUEDC1	-0.107952032	0.074035259	-1.458116485	0.144808438	0.17024887	negative
PRJNA729271	stage_1W_vs_E10	ordinary_muscle_development	ENSGALG00010029512	TSPAN10	-1.717975663	0.731386232	-2.34893082	0.018827404	0.024654732	negative
PRJNA729271	stage_1W_vs_E10	ordinary_muscle_development	ENSGALG00010029513	WBSCR22	0.072740171	0.084728023	0.858513726	0.390608855	0.42860525	positive
PRJNA729271	stage_1W_vs_E10	ordinary_muscle_development	ENSGALG00010029514	MRPS23	0.762800902	0.096870852	7.874410964	3.42352E-15	1.02635E-14	positive
PRJNA729271	stage_1W_vs_E10	ordinary_muscle_development	ENSGALG00010029515	NPLOC4	1.421572267	0.15219922	9.340207336	9.61457E-21	3.73327E-20	positive
PRJNA729271	stage_1W_vs_E10	ordinary_muscle_development	ENSGALG00010029516	ENSGALG00010029516	3.699595981	0.226666501	16.3217589	6.91152E-60	1.00889E-58	positive
PRJNA729271	stage_1W_vs_E10	ordinary_muscle_development	ENSGALG00010029517	MSI2	0.807073641	0.111523981	7.236772175	4.59489E-13	1.23568E-12	positive
PRJNA729271	stage_1W_vs_E10	ordinary_muscle_development	ENSGALG00010029518	FAAP100	-0.557022052	0.188135304	-2.960752391	0.003068886	0.004369695	negative
PRJNA729271	stage_1W_vs_E10	ordinary_muscle_development	ENSGALG00010029519	STX1A	-0.883851773	0.118691966	-7.446601514	9.57753E-14	2.66359E-13	negative
PRJNA729271	stage_1W_vs_E10	ordinary_muscle_development	ENSGALG00010029520	MKS1	-0.401394782	0.111451873	-3.601507736	0.000316377	0.000491099	negative
PRJNA729271	stage_1W_vs_E10	ordinary_muscle_development	ENSGALG00010029521	ACTG1	-0.357653975	0.09839215	-3.63498487	0.000277997	0.000433333	negative
PRJNA729271	stage_1W_vs_E10	ordinary_muscle_development	ENSGALG00010029522	AKAP1	1.615403598	0.111903185	14.43572497	3.08386E-47	3.08067E-46	positive
PRJNA729271	stage_1W_vs_E10	ordinary_muscle_development	ENSGALG00010029523	FSCN2	2.593954262	0.667941607	3.88350454	0.000102962	0.000165348	positive
PRJNA729271	stage_1W_vs_E10	ordinary_muscle_development	ENSGALG00010029524	ENSGALG00010029524	0.8062461	0.148117993	5.443269137	5.23115E-08	1.06601E-07	positive
PRJNA729271	stage_1W_vs_E10	ordinary_muscle_development	ENSGALG00010029525	SLC38A10	-1.457539217	0.13893203	-10.49102368	9.49936E-26	4.50567E-25	negative
PRJNA729271	stage_1W_vs_E10	ordinary_muscle_development	ENSGALG00010029526	BCL7B	1.52340266	0.111283634	13.6893684	1.17526E-42	1.025E-41	positive
PRJNA729271	stage_1W_vs_E10	ordinary_muscle_development	ENSGALG00010029527	NDUFAF8	1.799502305	0.15206171	11.83402651	2.60347E-32	1.60516E-31	positive
PRJNA729271	stage_1W_vs_E10	ordinary_muscle_development	ENSGALG00010029528	TBL2	-0.366471524	0.097526253	-3.757670493	0.000171503	0.000271182	negative
PRJNA729271	stage_1W_vs_E10	ordinary_muscle_development	ENSGALG00010029529	TEPSIN	1.430763695	0.125095055	11.43741209	2.71875E-30	1.54967E-29	positive
PRJNA729271	stage_1W_vs_E10	ordinary_muscle_development	ENSGALG00010029530	ENSGALG00010029530	4.343334103	0.355801483	12.20718381	2.84569E-34	1.87663E-33	positive
PRJNA729271	stage_1W_vs_E10	ordinary_muscle_development	ENSGALG00010029531	CEP131	-1.010531204	0.17256749	-5.855860812	4.74546E-09	1.02711E-08	negative
PRJNA729271	stage_1W_vs_E10	ordinary_muscle_development	ENSGALG00010029532	EVI2A	2.395910888	0.45565389	5.258181568	1.45487E-07	2.87542E-07	positive
PRJNA729271	stage_1W_vs_E10	ordinary_muscle_development	ENSGALG00010029533	MLXIPL	0.308464712	0.719542102	0.428695849	0.668144582	0.698453463	positive
PRJNA729271	stage_1W_vs_E10	ordinary_muscle_development	ENSGALG00010029534	NF1	-0.638258841	0.091179221	-7.000047126	2.55876E-12	6.63138E-12	negative
PRJNA729271	stage_1W_vs_E10	ordinary_muscle_development	ENSGALG00010029536	BAZ1B	-0.755847804	0.077770068	-9.719006586	2.50211E-22	1.02742E-21	negative
PRJNA729271	stage_1W_vs_E10	ordinary_muscle_development	ENSGALG00010029537	MYH1B	5.963971513	0.206074164	28.94089874	3.6532E-184	4.728E-182	positive
PRJNA729271	stage_1W_vs_E10	ordinary_muscle_development	ENSGALG00010029538	FZD9	0.710614184	0.172827958	4.111685354	3.92781E-05	6.51639E-05	positive
PRJNA729271	stage_1W_vs_E10	ordinary_muscle_development	ENSGALG00010029539	FKBP6	-2.157909485	0.420417374	-5.132779037	2.85495E-07	5.5302E-07	negative
PRJNA729271	stage_1W_vs_E10	ordinary_muscle_development	ENSGALG00010029541	ENSGALG00010029541	1.003395708	0.228368118	4.393764396	1.11405E-05	1.92721E-05	positive

PRJNA729271	stage_1W_vs_E10	ordinary_muscle_development	ENSGALG00010029542	AATK	2.022004672	0.140485623	14.39296509	5.72863E-47	5.67965E-46	positive
PRJNA729271	stage_1W_vs_E10	ordinary_muscle_development	ENSGALG00010029543	NSUN5	0.52502984	0.13300382	3.947479414	7.89783E-05	0.000128167	positive
PRJNA729271	stage_1W_vs_E10	ordinary_muscle_development	ENSGALG00010029544	ENSGALG00010029544	-0.742359732	0.178406282	-4.161062727	3.1677E-05	5.29472E-05	negative
PRJNA729271	stage_1W_vs_E10	ordinary_muscle_development	ENSGALG00010029545	OMG	2.034181509	0.307735699	6.610157717	3.83911E-11	9.36202E-11	positive
PRJNA729271	stage_1W_vs_E10	ordinary_muscle_development	ENSGALG00010029546	WSB1	0.076177445	0.165392485	0.460585893	0.645095735	0.676946548	positive
PRJNA729271	stage_1W_vs_E10	ordinary_muscle_development	ENSGALG00010029547	HIP1	-0.673961587	0.101230026	-6.657724121	2.781E-11	6.83347E-11	negative
PRJNA729271	stage_1W_vs_E10	ordinary_muscle_development	ENSGALG00010029548	BAIAP2	1.002458012	0.221512564	4.525513109	6.02491E-06	1.06139E-05	positive
PRJNA729271	stage_1W_vs_E10	ordinary_muscle_development	ENSGALG00010029549	KSR1	1.164106506	0.125057068	9.308602269	1.29526E-20	5.0066E-20	positive
PRJNA729271	stage_1W_vs_E10	ordinary_muscle_development	ENSGALG00010029550	CHMP6	1.109453566	0.239363158	4.635022268	3.56899E-06	6.40412E-06	positive
PRJNA729271	stage_1W_vs_E10	ordinary_muscle_development	ENSGALG00010029552	NOS2	-0.427846898	0.132263182	-3.234814795	0.001217217	0.001797062	negative
PRJNA729271	stage_1W_vs_E10	ordinary_muscle_development	ENSGALG00010029553	MYH1C	4.524859174	0.194792262	23.22915256	2.3113E-119	1.269E-117	positive
PRJNA729271	stage_1W_vs_E10	ordinary_muscle_development	ENSGALG00010029554	ENSGALG00010029554	7.373671573	0.365776809	20.1589368	2.24658E-90	6.68668E-89	positive
PRJNA729271	stage_1W_vs_E10	ordinary_muscle_development	ENSGALG00010029555	LYRM9	0.795478038	0.156747174	5.074911508	3.87676E-07	7.44978E-07	positive
PRJNA729271	stage_1W_vs_E10	ordinary_muscle_development	ENSGALG00010029556	NPTX1	2.542383452	0.558966528	4.548364395	5.40645E-06	9.55572E-06	positive
PRJNA729271	stage_1W_vs_E10	ordinary_muscle_development	ENSGALG00010029557	ENSGALG00010029557	3.934007005	0.551944765	7.127537492	1.0218E-12	2.70571E-12	positive
PRJNA729271	stage_1W_vs_E10	ordinary_muscle_development	ENSGALG00010029558	ENSGALG00010029558	-1.040632228	0.755615913	-1.377197343	0.168451243	0.19610485	negative
PRJNA729271	stage_1W_vs_E10	ordinary_muscle_development	ENSGALG00010029560	ENSGALG00010029560	4.150380461	0.167467985	24.78312774	1.363E-135	9.3635E-134	positive
PRJNA729271	stage_1W_vs_E10	ordinary_muscle_development	ENSGALG00010029561	SCO1	0.049597615	0.104305922	0.475501432	0.634429613	0.667057685	positive
PRJNA729271	stage_1W_vs_E10	ordinary_muscle_development	ENSGALG00010029562	ENSGALG00010029562	3.219430559	0.696840335	4.620040483	3.83665E-06	6.85978E-06	positive
PRJNA729271	stage_1W_vs_E10	ordinary_muscle_development	ENSGALG00010029563	ADPRM	0.969617401	0.125003663	7.756711931	8.71596E-15	2.56367E-14	positive
PRJNA729271	stage_1W_vs_E10	ordinary_muscle_development	ENSGALG00010029564	NLK	0.40822435	0.088067771	4.63534329	3.56346E-06	6.39499E-06	positive
PRJNA729271	stage_1W_vs_E10	ordinary_muscle_development	ENSGALG00010029566	TMEM97	-0.637608034	0.146844225	-4.342070891	1.41146E-05	2.42091E-05	negative
PRJNA729271	stage_1W_vs_E10	ordinary_muscle_development	ENSGALG00010029567	IFT20	-1.164482507	0.121796791	-9.560863621	1.16778E-21	4.69152E-21	negative
PRJNA729271	stage_1W_vs_E10	ordinary_muscle_development	ENSGALG00010029568	ENSGALG00010029568	-2.100700548	1.109744641	-1.892958497	0.058363389	0.072256348	negative
PRJNA729271	stage_1W_vs_E10	ordinary_muscle_development	ENSGALG00010029569	TMEM220	1.166506815	0.201823599	5.779833574	7.47746E-09	1.5972E-08	positive
PRJNA729271	stage_1W_vs_E10	ordinary_muscle_development	ENSGALG00010029570	TNFAIP1	-0.952847377	0.080287053	-11.86800785	1.73551E-32	1.07643E-31	negative
PRJNA729271	stage_1W_vs_E10	ordinary_muscle_development	ENSGALG00010029571	EPX	-3.728664469	1.078849481	-3.456148921	0.000547952	0.000832904	negative
PRJNA729271	stage_1W_vs_E10	ordinary_muscle_development	ENSGALG00010029572	TSPOAP1	0.406633002	0.116832532	3.480477535	0.000500521	0.000763448	positive
PRJNA729271	stage_1W_vs_E10	ordinary_muscle_development	ENSGALG00010029573	MAP2K4	-0.201200835	0.060689335	-3.31525852	0.000915584	0.001369738	negative
PRJNA729271	stage_1W_vs_E10	ordinary_muscle_development	ENSGALG00010029574	SGSM2	-0.364172644	0.133677082	-2.724271333	0.006444354	0.008892889	negative
PRJNA729271	stage_1W_vs_E10	ordinary_muscle_development	ENSGALG00010029575	ANKFN1	-3.56915468	0.73977026	-4.82467987	1.40228E-06	2.59228E-06	negative
PRJNA729271	stage_1W_vs_E10	ordinary_muscle_development	ENSGALG00010029576	ATP2A3	1.44487025	0.130710096	11.05400649	2.09645E-28	1.10784E-27	positive
PRJNA729271	stage_1W_vs_E10	ordinary_muscle_development	ENSGALG00010029577	NOG	-5.905830725	0.531373151	-11.11428139	1.06908E-28	5.70556E-28	negative
PRJNA729271	stage_1W_vs_E10	ordinary_muscle_development	ENSGALG00010029578	C17orf67	-4.822481195	0.663755845	-7.26544441	3.71814E-13	1.00418E-12	negative
PRJNA729271	stage_1W_vs_E10	ordinary_muscle_development	ENSGALG00010029579	GALNT17	-0.523389437	0.185046433	-2.828422193	0.004677807	0.006548659	negative
PRJNA729271	stage_1W_vs_E10	ordinary_muscle_development	ENSGALG00010029580	P2RX1	1.243325415	0.348497476	3.567674091	0.000360164	0.000555617	positive
PRJNA729271	stage_1W_vs_E10	ordinary_muscle_development	ENSGALG00010029581	DGKE	0.116849634	0.105171699	1.111036858	0.266552484	0.300371473	positive
PRJNA729271	stage_1W_vs_E10	ordinary_muscle_development	ENSGALG00010029582	SCPEP1	-0.53441202	0.150755813	-3.54488501	0.000392784	0.000604059	negative
PRJNA729271	stage_1W_vs_E10	ordinary_muscle_development	ENSGALG00010029583	LIMK1	-1.521920208	0.216188742	-7.039775484	1.9255E-12	5.0189E-12	negative
PRJNA729271	stage_1W_vs_E10	ordinary_muscle_development	ENSGALG00010029584	TRIM25	0.632194157	0.233252175	2.710346249	0.0067213	0.009257435	positive
PRJNA729271	stage_1W_vs_E10	ordinary_muscle_development	ENSGALG00010029585	ENSGALG00010029585	0.838461401	0.352677787	2.377414826	0.017434469	0.02292178	positive
PRJNA729271	stage_1W_vs_E10	ordinary_muscle_development	ENSGALG00010029586	COIL	0.258275954	0.187465196	1.377727489	0.168287441	0.195961315	positive
PRJNA729271	stage_1W_vs_E10	ordinary_muscle_development	ENSGALG00010029588	RAB11FIP4	-3.963563354	0.165196772	-23.99298314	3.2917E-127	2.1019E-125	negative
PRJNA729271	stage_1W_vs_E10	ordinary_muscle_development	ENSGALG00010029590	UTP6	0.237052343	0.097783068	2.424267791	0.01533929	0.02030338	positive
PRJNA729271	stage_1W_vs_E10	ordinary_muscle_development	ENSGALG00010029591	SUZ12	-0.995630678	0.080700205	-12.33739962	5.69661E-35	3.86394E-34	negative
PRJNA729271	stage_1W_vs_E10	ordinary_muscle_development	ENSGALG00010029592	MNT	0.692120978	0.166053052	4.168071417	3.07188E-05	5.1387E-05	positive
PRJNA729271	stage_1W_vs_E10	ordinary_muscle_development	ENSGALG00010029593	PAFAH1B1	0.435419945	0.07892603	5.516810414	3.45207E-08	7.09454E-08	positive
PRJNA729271	stage_1W_vs_E10	ordinary_muscle_development	ENSGALG00010029594	CRLF3	-1.129848492	0.069357736	-16.29015829	1.15929E-59	1.67703E-58	negative
PRJNA729271	stage_1W_vs_E10	ordinary_muscle_development	ENSGALG00010029595	CLUH	1.546473822	0.134780366	11.47402896	1.78167E-30	1.0244E-29	positive
PRJNA729271	stage_1W_vs_E10	ordinary_muscle_development	ENSGALG00010029596	ATAD5	-1.708288135	0.131819034	-12.9593435	2.0802E-38	1.57619E-37	negative
PRJNA729271	stage_1W_vs_E10	ordinary_muscle_development	ENSGALG00010029597	ADAP2	0.971763673	0.209962657	4.628269089	3.68735E-06	6.60505E-06	positive
PRJNA729271	stage_1W_vs_E10	ordinary_muscle_development	ENSGALG00010029598	SUPT4H1	0.123813718	0.149443236	0.828499977	0.407387408	0.44536394	positive
PRJNA729271	stage_1W_vs_E10	ordinary_muscle_development	ENSGALG00010029599	RNF43	-3.34874215	0.444975712	-7.525674008	5.24491E-14	1.48283E-13	negative
PRJNA729271	stage_1W_vs_E10	ordinary_muscle_development	ENSGALG00010029600	RHOT1	1.447997586	0.10845045	13.35169734	1.1578E-40	9.47375E-40	positive
PRJNA729271	stage_1W_vs_E10	ordinary_muscle_development	ENSGALG00010029601	HSF5	-3.402686103	0.572506599	-5.943487999	2.7902E-09	6.1288E-09	negative
PRJNA729271	stage_1W_vs_E10	ordinary_muscle_development	ENSGALG00010029602	MTMR4	-0.925757516	0.088359742	-10.47714147	1.10019E-25	5.20641E-25	negative
PRJNA729271	stage_1W_vs_E10	ordinary_muscle_development	ENSGALG00010029603	RHBDL3	-0.3898284	0.442710486	-0.880549281	0.37856182	0.416331835	negative
PRJNA729271	stage_1W_vs_E10	ordinary_muscle_development	ENSGALG00010029604	ENSGALG00010029604	-0.010089911	0.121423191	-0.083097067	0.933774358	0.941962511	negative
PRJNA729271	stage_1W_vs_E10	ordinary_muscle_development	ENSGALG00010029605	C17orf75	-1.097828254	0.095410973	-11.50631023	1.22613E-30	7.1091E-30	negative
PRJNA729271	stage_1W_vs_E10	ordinary_muscle_development	ENSGALG00010029606	ABHD11	1.64389276	0.199423387	8.243229576	1.67624E-16	5.33297E-16	positive
PRJNA729271	stage_1W_vs_E10	ordinary_muscle_development	ENSGALG00010029607	ZNF207	-0.488569439	0.065179039	-7.495806158	6.58921E-14	1.85566E-13	negative
PRJNA729271	stage_1W_vs_E10	ordinary_muscle_development	ENSGALG00010029608	ENSGALG00010029608	-2.523811645	0.338399425	-7.458084918	8.77891E-14	2.44806E-13	negative
PRJNA729271	stage_1W_vs_E10	ordinary_muscle_development	ENSGALG00010029609	METTL27	-0.569511414	0.148766398	-3.828226143	0.00012907	0.000205794	negative
PRJNA729271	stage_1W_vs_E10	ordinary_muscle_development	ENSGALG00010029610	ERN1	-0.045101869	0.080127732	-0.562874647	0.573520246	0.609157761	negative
PRJNA729271	stage_1W_vs_E10	ordinary_muscle_development	ENSGALG00010029611	YWHAG	1.051923299	0.130560977	8.056950298	7.82214E-16	2.41805E-15	positive
PRJNA729271	stage_1W_vs_E10	ordinary_muscle_development	ENSGALG00010029612	HSPB1	2.300212891	0.195649196	11.75682262	6.51398E-32	3.96224E-31	positive
PRJNA729271	stage_1W_vs_E10	ordinary_muscle_development	ENSGALG00010029614	MDH2	1.931843922	0.128452466	15.03936808	4.05401E-51	4.61248E-50	positive
PRJNA729271	stage_1W_vs_E10	ordinary_muscle_development	ENSGALG00010029615	ENSGALG00010029615	-2.069612717	0.361796157	-5.720383368	1.06284E-08	2.25166E-08	negative
PRJNA729271	stage_1W_vs_E10	ordinary_muscle_development	ENSGALG00010029616	TMEM120A	1.086352193	0.135145294	8.038401914	9.10177E-16	2.80464E-15	positive
PRJNA729271	stage_1W_vs_E10	ordinary_muscle_development	ENSGALG00010029617	POR	-0.187619283	0.095193121	-1.970933213	0.048731516	0.060914394	negative
PRJNA729271	stage_1W_vs_E10	ordinary_muscle_development	ENSGALG00010029618	TEX2	1.070186779	0.112686145	9.497057316	2.15905E-21	8.59529E-21	positive
PRJNA729271	stage_1W_vs_E10	ordinary_muscle_development	ENSGALG00010029619	ENSGALG00010029619	0.604026008	0.103076385	5.859984382	4.62911E-09	1.00222E-08	positive
PRJNA729271	stage_1W_vs_E10	ordinary_muscle_development	ENSGALG00010029620	ENSGALG00010029620	0.239668747	0.412090417	0.581592624	0.560841112	0.596652618	positive
PRJNA729271	stage_1W_vs_E10	ordinary_muscle_development	ENSGALG00010029621	TAF15	-1.856581098	0.122654205	-15.13670971	9.27388E-52	1.08407E-50	negative
PRJNA729271	stage_1W_vs_E10	ordinary_muscle_development	ENSGALG00010029622	MMP28	-0.241724634	0.158903886	-1.521200267	0.128209589	0.151805096	negative
PRJNA729271	stage_1W_vs_E10	ordinary_muscle_development	ENSGALG00010029623	ENSGALG00010029623	0.673763816	0.388977705	1.732139934	0.083248651	0.101130505	positive
PRJNA729271	stage_1W_vs_E10	ordinary_muscle_development	ENSGALG00010029624	RASL10B	-2.55435786	0.366598634	-6.967723342	3.22111E-12	8.31078E-12	negative
PRJNA729271	stage_1W_vs_E10	ordinary_muscle_development	ENSGALG00010029625	PECAM1	2.460800537	0.174305892	14.11771288	2.95425E-45	2.78064E-44	positive
PRJNA729271	stage_1W_vs_E10	ordinary_muscle_development	ENSGALG00010029626	AP2B1	-0.835451179	0.109685794	-7.616767386	2.60107E-14	7.47177E-14	negative
PRJNA729271	stage_1W_vs_E10	ordinary_muscle_development	ENSGALG00010029627	PEX12	0.032442227	0.14599766	0.222210593	0.824149948	0.842814555	positive
PRJNA729271	stage_1W_vs_E10	ordinary_muscle_development	ENSGALG00010029628	UNC45B	1.521878114	0.160540254	9.479729089	2.54944E-21	1.01244E-20	positive
PRJNA729271	stage_1W_vs_E10	ordinary_muscle_development	ENSGALG00010029629	ENSGALG00010029629	1.601622173	0.259983294	6.160481109	7.25243E-10	1.64179E-09	positive
PRJNA729271	stage_1W_vs_E10	ordinary_muscle_development	ENSGALG00010029630	RAD51D	0.122288368	0.12091657	1.011344996	0.311851337	0.347767744	positive
PRJNA729271	stage_1W_vs_E10	ordinary_muscle_development	ENSGALG00010029631	LIG3	-0.156247517	0.086878564	-1.798458799	0.072104332	0.088198506	negative
PRJNA729271	stage_1W_vs_E10	ordinary_muscle_development	ENSGALG00010029632	TMEM132E	1.981997512	0.225239774	8.79950052	1.37426E-18	4.8174E-18	positive
PRJNA729271	stage_1W_vs_E10	ordinary_muscle_development	ENSGALG00010029633	POLG2	-0.136948472	0.176832014	-0.774455196	0.438661653	0.476641729	negative
PRJNA729271	stage_1W_vs_E10	ordinary_muscle_development	ENSGALG00010029634	DDX5	0.017599092	0.091134883	0.193110382	0.846872522	0.863675312	positive
PRJNA729271	stage_1W_vs_E10	ordinary_muscle_development	ENSGALG00010029635	ENSGALG00010029635	2.965348974	0.574909658	5.157939045	2.49683E-07	4.85988E-07	positive
PRJNA729271	stage_1W_vs_E10	ordinary_muscle_development	ENSGALG00010029636	SPATA20	1.136322159	0.074617629	15.22860185	2.28424E-52	2.72511E-51	positive
PRJNA729271	stage_1W_vs_E10	ordinary_muscle_development	ENSGALG00010029638	EPN3	2.780792091	0.194917215	14.26652892	3.53753E-46	3.42757E-45	positive
PRJNA729271	stage_1W_vs_E10	ordinary_muscle_development	ENSGALG00010029639	ENSGALG00010029639	-1.926466641	0.791682067	-2.433384209	0.014958419	0.019822845	negative
PRJNA729271	stage_1W_vs_E10	ordinary_muscle_development	ENSGALG00010029641	ENSGALG00010029641	-1.104869151	0.72772205	-1.518257075	0.128949604	0.152618969	negative
PRJNA729271	stage_1W_vs_E10	ordinary_muscle_development	ENSGALG00010029642	NAT9	1.707914238	0.143893324	11.86930838	1.70874E-32	1.06074E-31	positive
PRJNA729271	stage_1W_vs_E10	ordinary_muscle_development	ENSGALG00010029644	MYCBPAP	-0.087898764	0.17023742	-0.516330453	0.605623631	0.640020015	negative
PRJNA729271	stage_1W_vs_E10	ordinary_muscle_development	ENSGALG00010029645	ENSGALG00010029645	-1.589206939	0.387278521	-4.103524602	4.06903E-05	6.74218E-05	negative
PRJNA729271	stage_1W_vs_E10	ordinary_muscle_development	ENSGALG00010029646	MRPS17	0.461776663	0.108373031	4.260992415	2.03521E-05	3.4447E-05	positive
PRJNA729271	stage_1W_vs_E10	ordinary_muscle_development	ENSGALG00010029647	RSAD1	0.92828034	0.151878008	6.112012882	9.83822E-10	2.21436E-09	positive
PRJNA729271	stage_1W_vs_E10	ordinary_muscle_development	ENSGALG00010029648	NIPSNAP2	1.362089383	0.121851637	11.17826088	5.2101E-29	2.81477E-28	positive
PRJNA729271	stage_1W_vs_E10	ordinary_muscle_development	ENSGALG00010029649	PSPH	4.807759435	0.257297659	18.68559342	6.48537E-78	1.45971E-76	positive
PRJNA729271	stage_1W_vs_E10	ordinary_muscle_development	ENSGALG00010029650	ACSF2	-0.084079321	0.135056901	-0.62254739	0.533581992	0.570079677	negative
PRJNA729271	stage_1W_vs_E10	ordinary_muscle_development	ENSGALG00010029651	ENSGALG00010029651	2.87062662	0.275530618	10.41853947	2.04063E-25	9.54774E-25	positive
PRJNA729271	stage_1W_vs_E10	ordinary_muscle_development	ENSGALG00010029652	LRRC59	-0.889288271	0.102697942	-8.659260882	4.74833E-18	1.61515E-17	negative
PRJNA729271	stage_1W_vs_E10	ordinary_muscle_development	ENSGALG00010029653	CCT6A	-0.349492226	0.090280304	-3.87119017	0.000108305	0.000173698	negative
PRJNA729271	stage_1W_vs_E10	ordinary_muscle_development	ENSGALG00010029654	EME1	-2.733608149	0.139934544	-19.53490587	5.54481E-85	1.4939E-83	negative
PRJNA729271	stage_1W_vs_E10	ordinary_muscle_development	ENSGALG00010029655	PHKG1	6.086570672	0.128214532	47.47176909	0	0	positive
PRJNA729271	stage_1W_vs_E10	ordinary_muscle_development	ENSGALG00010029656	MRPL27	0.568484728	0.113063496	5.028013003	4.95588E-07	9.44459E-07	positive
PRJNA729271	stage_1W_vs_E10	ordinary_muscle_development	ENSGALG00010029657	CHCHD2	2.76070867	0.146709024	18.81757915	5.42052E-79	1.24913E-77	positive
PRJNA729271	stage_1W_vs_E10	ordinary_muscle_development	ENSGALG00010029658	XYLT2	0.910040579	0.12389662	7.345160673	2.05512E-13	5.61738E-13	positive
PRJNA729271	stage_1W_vs_E10	ordinary_muscle_development	ENSGALG00010029659	VKORC1L1	-1.134172234	0.147008756	-7.714997817	1.20984E-14	3.53419E-14	negative
PRJNA729271	stage_1W_vs_E10	ordinary_muscle_development	ENSGALG00010029660	CD300LG	1.250267703	0.306493662	4.079261203	4.51791E-05	7.45951E-05	positive
PRJNA729271	stage_1W_vs_E10	ordinary_muscle_development	ENSGALG00010029661	KRABZFP	4.382717545	0.578915694	7.570562675	3.71611E-14	1.05825E-13	positive
PRJNA729271	stage_1W_vs_E10	ordinary_muscle_development	ENSGALG00010029663	GUSB	-0.148271842	0.154387177	-0.960389616	0.336859162	0.373815155	negative
PRJNA729271	stage_1W_vs_E10	ordinary_muscle_development	ENSGALG00010029664	RAB37	2.16164102	0.37817607	5.715964572	1.09083E-08	2.30894E-08	positive
PRJNA729271	stage_1W_vs_E10	ordinary_muscle_development	ENSGALG00010029665	ASL1	0.80728988	0.191573011	4.214006328	2.5088E-05	4.21918E-05	positive
PRJNA729271	stage_1W_vs_E10	ordinary_muscle_development	ENSGALG00010029666	SLC9A3R1	-0.252241164	0.200284027	-1.259417281	0.207879651	0.238500517	negative
PRJNA729271	stage_1W_vs_E10	ordinary_muscle_development	ENSGALG00010029667	TMEM104	-0.018899743	0.093713492	-0.2016758	0.840170183	0.857684823	negative
PRJNA729271	stage_1W_vs_E10	ordinary_muscle_development	ENSGALG00010029668	CRCP	-0.652875056	0.182646603	-3.574526129	0.000350863	0.000542134	negative
PRJNA729271	stage_1W_vs_E10	ordinary_muscle_development	ENSGALG00010029669	HID1	0.452578684	0.07459437	6.067196313	1.30162E-09	2.91383E-09	positive
PRJNA729271	stage_1W_vs_E10	ordinary_muscle_development	ENSGALG00010029670	TPST1	-0.398610474	0.170777672	-2.334090102	0.019591004	0.025626894	negative
PRJNA729271	stage_1W_vs_E10	ordinary_muscle_development	ENSGALG00010029671	GRIN2C	2.197493852	0.205238085	10.70704712	9.43225E-27	4.66304E-26	positive
PRJNA729271	stage_1W_vs_E10	ordinary_muscle_development	ENSGALG00010029672	KCTD7	-1.919725852	0.248688436	-7.719401371	1.16877E-14	3.41767E-14	negative
PRJNA729271	stage_1W_vs_E10	ordinary_muscle_development	ENSGALG00010029673	TMEM248	-0.965406049	0.09122356	-10.58285879	3.57869E-26	1.72911E-25	negative
PRJNA729271	stage_1W_vs_E10	ordinary_muscle_development	ENSGALG00010029674	FDXR	-0.805576794	0.114980081	-7.006229111	2.44827E-12	6.3541E-12	negative
PRJNA729271	stage_1W_vs_E10	ordinary_muscle_development	ENSGALG00010029675	DHRS13	0.305845952	0.223818168	1.366492965	0.171784287	0.199648272	positive
PRJNA729271	stage_1W_vs_E10	ordinary_muscle_development	ENSGALG00010029676	FADS6	-0.182427613	1.04846142	-0.173995542	0.861868966	0.877124068	negative
PRJNA729271	stage_1W_vs_E10	ordinary_muscle_development	ENSGALG00010029677	PHF12	0.279402893	0.100305559	2.785517519	0.005344235	0.007442088	positive
PRJNA729271	stage_1W_vs_E10	ordinary_muscle_development	ENSGALG00010029682	CDR2L	-2.468813252	0.149536091	-16.50981536	3.11836E-61	4.71823E-60	negative
PRJNA729271	stage_1W_vs_E10	ordinary_muscle_development	ENSGALG00010029683	MRPL58	0.607034314	0.109705523	5.533306776	3.14249E-08	6.47115E-08	positive
PRJNA729271	stage_1W_vs_E10	ordinary_muscle_development	ENSGALG00010029684	ENSGALG00010029684	-1.142759647	0.698125892	-1.63689624	0.101652152	0.122115692	negative
PRJNA729271	stage_1W_vs_E10	ordinary_muscle_development	ENSGALG00010029685	ATP5PD	1.153621964	0.114964839	10.03456338	1.07434E-23	4.67783E-23	positive
PRJNA729271	stage_1W_vs_E10	ordinary_muscle_development	ENSGALG00010029686	KCTD2	-0.185997205	0.06002352	-3.098738685	0.001943464	0.002819588	negative

PRJNA729271	stage_1W_vs_E10	ordinary_muscle_development	ENSGALG00010029687	SLC16A5	-0.566308646	0.445203247	-1.272022722	0.203365046	0.233764976	negative
PRJNA729271	stage_1W_vs_E10	ordinary_muscle_development	ENSGALG00010029689	ENSGALG00010029689	2.317468611	0.175571162	13.19959716	8.82055E-40	7.02108E-39	positive
PRJNA729271	stage_1W_vs_E10	ordinary_muscle_development	ENSGALG00010029690	ENSGALG00010029690	2.122012554	0.160203716	13.2457137	4.77719E-40	3.83631E-39	positive
PRJNA729271	stage_1W_vs_E10	ordinary_muscle_development	ENSGALG00010029691	NUFIP2	1.366232112	0.139851481	9.769164424	1.52706E-22	6.33508E-22	positive
PRJNA729271	stage_1W_vs_E10	ordinary_muscle_development	ENSGALG00010029692	CRYBA1	-0.123954852	0.505291273	-0.24531366	0.806213576	0.826805276	negative
PRJNA729271	stage_1W_vs_E10	ordinary_muscle_development	ENSGALG00010029693	HN1	-1.256730103	0.127083287	-9.88902738	4.64519E-23	1.96797E-22	negative
PRJNA729271	stage_1W_vs_E10	ordinary_muscle_development	ENSGALG00010029694	ARMC7	0.125526592	0.092038823	1.363843951	0.172616684	0.200519221	positive
PRJNA729271	stage_1W_vs_E10	ordinary_muscle_development	ENSGALG00010029695	TAOK1	0.227724131	0.086486052	2.633073499	0.008461603	0.011529511	positive
PRJNA729271	stage_1W_vs_E10	ordinary_muscle_development	ENSGALG00010029696	SUMO2	-0.63038886	0.108558863	-5.806885258	6.36457E-09	1.36734E-08	negative
PRJNA729271	stage_1W_vs_E10	ordinary_muscle_development	ENSGALG00010029697	GIT1	1.912439943	0.16432644	11.63805378	2.6397E-31	1.56942E-30	positive
PRJNA729271	stage_1W_vs_E10	ordinary_muscle_development	ENSGALG00010029698	NUP85	-0.700644035	0.086275726	-8.120986869	4.62408E-16	1.44422E-15	negative
PRJNA729271	stage_1W_vs_E10	ordinary_muscle_development	ENSGALG00010029699	ANKRD13B	-0.713207605	0.174795191	-4.080247283	4.49878E-05	7.42878E-05	negative
PRJNA729271	stage_1W_vs_E10	ordinary_muscle_development	ENSGALG00010029700	GGA3	0.026519218	0.080474031	0.329537592	0.741749385	0.767193834	positive
PRJNA729271	stage_1W_vs_E10	ordinary_muscle_development	ENSGALG00010029701	MRPS7	0.305344712	0.066011283	4.625644249	3.73436E-06	6.68431E-06	positive
PRJNA729271	stage_1W_vs_E10	ordinary_muscle_development	ENSGALG00010029702	CORO6	5.638632725	0.169529618	33.2604579	1.4412E-242	3.2642E-240	positive
PRJNA729271	stage_1W_vs_E10	ordinary_muscle_development	ENSGALG00010029703	MIF4GD	-0.388864348	0.115889043	-3.355488473	0.00079225	0.001191127	negative
PRJNA729271	stage_1W_vs_E10	ordinary_muscle_development	ENSGALG00010029704	SLC25A19	0.90276215	0.100178355	9.011548929	2.03166E-19	7.42536E-19	positive
PRJNA729271	stage_1W_vs_E10	ordinary_muscle_development	ENSGALG00010029705	GRB2	-1.057037895	0.068739435	-15.37745969	2.31868E-53	2.85793E-52	negative
PRJNA729271	stage_1W_vs_E10	ordinary_muscle_development	ENSGALG00010029706	SSH2	2.242020393	0.216164355	10.37183208	3.33077E-25	1.54643E-24	positive
PRJNA729271	stage_1W_vs_E10	ordinary_muscle_development	ENSGALG00010029707	NSRP1	-0.276742215	0.225972788	-1.224670532	0.220699381	0.252130953	negative
PRJNA729271	stage_1W_vs_E10	ordinary_muscle_development	ENSGALG00010029708	TMEM94	-0.030002029	0.085030355	-0.35283904	0.724209114	0.751048946	negative
PRJNA729271	stage_1W_vs_E10	ordinary_muscle_development	ENSGALG00010029709	SLC6A4	1.333161942	0.195921861	6.8045594	1.01359E-11	2.54892E-11	positive
PRJNA729271	stage_1W_vs_E10	ordinary_muscle_development	ENSGALG00010029710	BLMH	0.054313209	0.082809742	0.655879463	0.511901683	0.549345887	positive
PRJNA729271	stage_1W_vs_E10	ordinary_muscle_development	ENSGALG00010029711	CASKIN2	0.95094801	0.111108859	8.558705533	1.14142E-17	3.81394E-17	positive
PRJNA729271	stage_1W_vs_E10	ordinary_muscle_development	ENSGALG00010029713	CPD	0.584804728	0.116460592	5.021481672	5.12744E-07	9.76511E-07	positive
PRJNA729271	stage_1W_vs_E10	ordinary_muscle_development	ENSGALG00010029716	GOSR1	0.170498003	0.063185357	2.698378419	0.006967818	0.009583829	positive
PRJNA729271	stage_1W_vs_E10	ordinary_muscle_development	ENSGALG00010029717	TRARG1	-1.346299103	0.493184934	-2.729805818	0.006337164	0.008749971	negative
PRJNA729271	stage_1W_vs_E10	ordinary_muscle_development	ENSGALG00010029718	ABR	-0.008561577	0.112285328	-0.076248407	0.939221471	0.946798472	negative
PRJNA729271	stage_1W_vs_E10	ordinary_muscle_development	ENSGALG00010029720	TIMM22	0.453787093	0.106870561	4.246137467	2.17487E-05	3.67293E-05	positive
PRJNA729271	stage_1W_vs_E10	ordinary_muscle_development	ENSGALG00010029721	EXOC7	0.509030282	0.104958751	4.84981268	1.23578E-06	2.29326E-06	positive
PRJNA729271	stage_1W_vs_E10	ordinary_muscle_development	ENSGALG00010029722	SPECC1	0.273688194	0.14236062	1.922499315	0.05454296	0.067792849	positive
PRJNA729271	stage_1W_vs_E10	ordinary_muscle_development	ENSGALG00010029723	ADORA2B	1.040145645	0.180253138	5.770471769	7.90499E-09	1.68653E-08	positive
PRJNA729271	stage_1W_vs_E10	ordinary_muscle_development	ENSGALG00010029724	CACNA1G	-1.888344505	0.216513356	-8.721607497	2.7428E-18	9.46141E-18	negative
PRJNA729271	stage_1W_vs_E10	ordinary_muscle_development	ENSGALG00010029725	TSEN54	-0.402367099	0.069627818	-5.778826791	7.52233E-09	1.60654E-08	negative
PRJNA729271	stage_1W_vs_E10	ordinary_muscle_development	ENSGALG00010029726	SEPTIN9	-1.715928116	0.125893135	-13.63003722	2.65445E-42	2.29025E-41	negative
PRJNA729271	stage_1W_vs_E10	ordinary_muscle_development	ENSGALG00010029727	ZSWIM7	0.530179865	0.135660202	3.908415918	9.30071E-05	0.000149943	positive
PRJNA729271	stage_1W_vs_E10	ordinary_muscle_development	ENSGALG00010029728	TNRC6C	0.080392057	0.103853997	0.774087273	0.438879183	0.476842348	positive
PRJNA729271	stage_1W_vs_E10	ordinary_muscle_development	ENSGALG00010029729	RASL10A	-0.965816052	1.119494816	-0.862724899	0.388288739	0.426323458	negative
PRJNA729271	stage_1W_vs_E10	ordinary_muscle_development	ENSGALG00010029730	TTC19	1.609226207	0.131471469	12.24011734	1.89758E-34	1.26056E-33	positive
PRJNA729271	stage_1W_vs_E10	ordinary_muscle_development	ENSGALG00010029731	NCOR1	0.612549877	0.184627223	3.317765752	0.000907405	0.001357981	positive
PRJNA729271	stage_1W_vs_E10	ordinary_muscle_development	ENSGALG00010029732	PIGL	0.931784533	0.177571487	5.247376984	1.5428E-07	3.0434E-07	positive
PRJNA729271	stage_1W_vs_E10	ordinary_muscle_development	ENSGALG00010029733	ENSGALG00010029733	3.421110488	0.16398584	20.86223105	1.18035E-96	4.13265E-95	positive
PRJNA729271	stage_1W_vs_E10	ordinary_muscle_development	ENSGALG00010029734	SEC14L1	-0.80337849	0.085010014	-9.450398289	3.37544E-21	1.3328E-20	negative
PRJNA729271	stage_1W_vs_E10	ordinary_muscle_development	ENSGALG00010029735	SRP68	-0.442355678	0.114809758	-3.852944971	0.000116706	0.000186737	negative
PRJNA729271	stage_1W_vs_E10	ordinary_muscle_development	ENSGALG00010029736	ENSGALG00010029736	2.210342547	0.133053965	16.61237642	5.67017E-62	8.68807E-61	positive
PRJNA729271	stage_1W_vs_E10	ordinary_muscle_development	ENSGALG00010029737	ENSGALG00010029737	-6.214514779	0.584238353	-10.63695108	2.00586E-26	9.80935E-26	negative
PRJNA729271	stage_1W_vs_E10	ordinary_muscle_development	ENSGALG00010029738	TRPV2	0.658645265	0.167001379	3.943951063	8.015E-05	0.000130011	positive
PRJNA729271	stage_1W_vs_E10	ordinary_muscle_development	ENSGALG00010029739	P2RX5	1.496394819	0.203500984	7.353255934	1.93436E-13	5.29629E-13	positive
PRJNA729271	stage_1W_vs_E10	ordinary_muscle_development	ENSGALG00010029740	HASPIN	-2.090724708	0.21750258	-9.612413356	7.08681E-22	2.86457E-21	negative
PRJNA729271	stage_1W_vs_E10	ordinary_muscle_development	ENSGALG00010029741	EMC6	1.739513681	0.161510056	10.77031193	4.75388E-27	2.37694E-26	positive
PRJNA729271	stage_1W_vs_E10	ordinary_muscle_development	ENSGALG00010029742	TAX1BP3	-1.590734156	0.119722442	-13.28685023	2.75952E-40	2.23086E-39	negative
PRJNA729271	stage_1W_vs_E10	ordinary_muscle_development	ENSGALG00010029743	MYOCD	3.664105179	0.253994415	14.42592815	3.55456E-47	3.546E-46	positive
PRJNA729271	stage_1W_vs_E10	ordinary_muscle_development	ENSGALG00010029744	CTNS	1.119765207	0.08850425	12.65210662	1.08915E-36	7.79614E-36	positive
PRJNA729271	stage_1W_vs_E10	ordinary_muscle_development	ENSGALG00010029745	SHPK	0.720886936	0.168861852	4.269092905	1.9627E-05	3.32623E-05	positive
PRJNA729271	stage_1W_vs_E10	ordinary_muscle_development	ENSGALG00010029746	TRPV1	-0.290647187	0.331822632	-0.875911281	0.381078266	0.418876884	negative
PRJNA729271	stage_1W_vs_E10	ordinary_muscle_development	ENSGALG00010029747	TRPV3	-0.302368537	0.347700935	-0.869622444	0.384506768	0.422453241	negative
PRJNA729271	stage_1W_vs_E10	ordinary_muscle_development	ENSGALG00010029749	ASPA	3.240456348	0.28311737	11.44562888	2.47305E-30	1.41129E-29	positive
PRJNA729271	stage_1W_vs_E10	ordinary_muscle_development	ENSGALG00010029750	TEN1	-2.894634666	0.267844315	-10.80715365	3.18396E-27	1.60416E-26	negative
PRJNA729271	stage_1W_vs_E10	ordinary_muscle_development	ENSGALG00010029752	ENSGALG00010029752	0.814060735	0.169929383	4.790582536	1.66298E-06	3.05512E-06	positive
PRJNA729271	stage_1W_vs_E10	ordinary_muscle_development	ENSGALG00010029753	AKAP10	0.01735994	0.074904614	0.231760629	0.816723938	0.835971793	positive
PRJNA729271	stage_1W_vs_E10	ordinary_muscle_development	ENSGALG00010029754	NLE1	-0.962474101	0.180812603	-5.323047651	1.02043E-07	2.03875E-07	negative
PRJNA729271	stage_1W_vs_E10	ordinary_muscle_development	ENSGALG00010029755	ACOX1	0.442808169	0.119358828	3.7098904	0.000207349	0.000325661	positive
PRJNA729271	stage_1W_vs_E10	ordinary_muscle_development	ENSGALG00010029756	ELAC2	0.155529663	0.082541098	1.884269369	0.059528548	0.073617124	positive
PRJNA729271	stage_1W_vs_E10	ordinary_muscle_development	ENSGALG00010029757	ULK2	0.411397022	0.209323789	1.96536201	0.049372353	0.061678209	positive
PRJNA729271	stage_1W_vs_E10	ordinary_muscle_development	ENSGALG00010029758	DNAH9	0.80702374	0.305789089	2.639151523	0.008311382	0.011332282	positive
PRJNA729271	stage_1W_vs_E10	ordinary_muscle_development	ENSGALG00010029759	ALDH3A2	0.422490167	0.107794778	3.919393635	8.8772E-05	0.000143355	positive
PRJNA729271	stage_1W_vs_E10	ordinary_muscle_development	ENSGALG00010029760	SHISA6	-6.602960115	0.863937532	-7.642867537	2.12437E-14	6.11694E-14	negative
PRJNA729271	stage_1W_vs_E10	ordinary_muscle_development	ENSGALG00010029761	MRPL38	0.787773025	0.117614448	6.69792735	2.11396E-11	5.22721E-11	positive
PRJNA729271	stage_1W_vs_E10	ordinary_muscle_development	ENSGALG00010029762	SLC47A2	4.843711513	0.464889834	10.41905235	2.02965E-25	9.50253E-25	positive
PRJNA729271	stage_1W_vs_E10	ordinary_muscle_development	ENSGALG00010029763	GEMIN4	-0.204644631	0.097564728	-2.097526787	0.035946968	0.045630205	negative
PRJNA729271	stage_1W_vs_E10	ordinary_muscle_development	ENSGALG00010029764	NXN	-1.152907964	0.126332718	-9.125965033	7.10999E-20	2.65617E-19	negative
PRJNA729271	stage_1W_vs_E10	ordinary_muscle_development	ENSGALG00010029765	KCNJ16	-1.621757407	0.311401525	-5.207930201	1.90959E-07	3.74401E-07	negative
PRJNA729271	stage_1W_vs_E10	ordinary_muscle_development	ENSGALG00010029766	ENSGALG00010029766	0.9776704	0.144758773	6.753790335	1.44032E-11	3.5878E-11	positive
PRJNA729271	stage_1W_vs_E10	ordinary_muscle_development	ENSGALG00010029767	GLOD4	-0.187988891	0.087122216	-2.157760658	0.030946445	0.039580757	negative
PRJNA729271	stage_1W_vs_E10	ordinary_muscle_development	ENSGALG00010029768	KCNJ2	0.754137956	0.175538419	4.296141899	1.73796E-05	2.95991E-05	positive
PRJNA729271	stage_1W_vs_E10	ordinary_muscle_development	ENSGALG00010029770	TLCD3A	-1.232029729	0.233751769	-5.270675537	1.35923E-07	2.68969E-07	negative
PRJNA729271	stage_1W_vs_E10	ordinary_muscle_development	ENSGALG00010029771	SOX9	-4.604163777	0.271251788	-16.97376376	1.2843E-64	2.11065E-63	negative
PRJNA729271	stage_1W_vs_E10	ordinary_muscle_development	ENSGALG00010029772	VPS53	0.020769296	0.069255963	0.299891812	0.76425968	0.788129202	positive
PRJNA729271	stage_1W_vs_E10	ordinary_muscle_development	ENSGALG00010029773	TRIM65	1.183415937	0.132357379	8.941065041	3.85437E-19	1.3929E-18	positive
PRJNA729271	stage_1W_vs_E10	ordinary_muscle_development	ENSGALG00010029774	RFLNB	-0.677274743	0.125658242	-5.389815521	7.053E-08	1.42426E-07	negative
PRJNA729271	stage_1W_vs_E10	ordinary_muscle_development	ENSGALG00010029775	ENSGALG00010029775	-0.068436097	0.142362379	-0.480717569	0.630717243	0.663828524	negative
PRJNA729271	stage_1W_vs_E10	ordinary_muscle_development	ENSGALG00010029776	WBP2	1.084238419	0.136396998	7.949136965	1.87815E-15	5.70371E-15	positive
PRJNA729271	stage_1W_vs_E10	ordinary_muscle_development	ENSGALG00010029777	ENSGALG00010029777	-1.071759861	0.144707307	-7.406397669	1.29776E-13	3.58374E-13	negative
PRJNA729271	stage_1W_vs_E10	ordinary_muscle_development	ENSGALG00010029778	DOC2B	-1.021882641	0.357368521	-2.85946462	0.004243568	0.005960321	negative
PRJNA729271	stage_1W_vs_E10	ordinary_muscle_development	ENSGALG00010029780	TEX14	-0.051759858	0.260053844	-0.199035158	0.842235243	0.859490274	negative
PRJNA729271	stage_1W_vs_E10	ordinary_muscle_development	ENSGALG00010029781	UNC13D	0.59360998	0.256468944	2.314549167	0.020637618	0.02692792	positive
PRJNA729271	stage_1W_vs_E10	ordinary_muscle_development	ENSGALG00010029782	UNK	0.059807298	0.121886759	0.490679206	0.623653356	0.657338825	positive
PRJNA729271	stage_1W_vs_E10	ordinary_muscle_development	ENSGALG00010029783	RAD51C	-0.49790626	0.123572962	-4.029429227	5.59553E-05	9.17294E-05	negative
PRJNA729271	stage_1W_vs_E10	ordinary_muscle_development	ENSGALG00010029784	CLDN3	-4.958331925	0.776116481	-6.388644034	1.67363E-10	3.92863E-10	negative
PRJNA729271	stage_1W_vs_E10	ordinary_muscle_development	ENSGALG00010029785	ENSGALG00010029785	2.469110697	0.244787864	10.08673657	6.3237E-24	2.78101E-23	positive
PRJNA729271	stage_1W_vs_E10	ordinary_muscle_development	ENSGALG00010029787	H3-3B	-1.269924828	0.064367791	-19.72919712	1.21078E-86	3.36857E-85	negative
PRJNA729271	stage_1W_vs_E10	ordinary_muscle_development	ENSGALG00010029791	GALK1	-0.201415729	0.112825312	-1.785199836	0.074228909	0.090705449	negative
PRJNA729271	stage_1W_vs_E10	ordinary_muscle_development	ENSGALG00010029804	ITGB4	0.663077977	0.227507316	2.914534742	0.003562192	0.005040411	positive
PRJNA729271	stage_1W_vs_E10	ordinary_muscle_development	ENSGALG00010029812	SAP30BP	-0.044452469	0.115980132	-0.383276582	0.701514701	0.729758546	negative
PRJNA729271	stage_1W_vs_E10	ordinary_muscle_development	ENSGALG00010029835	RECQL5	-0.555862951	0.11059663	-5.026038766	5.00715E-07	9.53977E-07	negative
PRJNA729271	stage_1W_vs_E10	ordinary_muscle_development	ENSGALG00010029841	SMIM5	-0.370984191	0.136829625	-2.711285599	0.006702287	0.009233002	negative
PRJNA729271	stage_1W_vs_E10	ordinary_muscle_development	ENSGALG00010029859	CSNK1D	-0.693527167	0.053682401	-12.91907883	3.51322E-38	2.64014E-37	negative
PRJNA729271	stage_1W_vs_E10	ordinary_muscle_development	ENSGALG00010029864	SLC16A3	2.678221749	0.337878928	7.926572295	2.25278E-15	6.81427E-15	positive
PRJNA729271	stage_1W_vs_E10	ordinary_muscle_development	ENSGALG00010029879	ENSGALG00010029879	-0.920221436	0.345987402	-2.659696367	0.007821112	0.010696011	negative
PRJNA729271	stage_1W_vs_E10	ordinary_muscle_development	ENSGALG00010029895	FASN	-2.738436926	0.169164995	-16.18796443	6.13239E-59	8.72316E-58	negative
PRJNA729271	stage_1W_vs_E10	ordinary_muscle_development	ENSGALG00010029907	NTN1	-0.50860113	0.273394021	-1.860322798	0.06283987	0.077467589	negative
PRJNA729271	stage_1W_vs_E10	ordinary_muscle_development	ENSGALG00010029921	STX8	0.348499131	0.104091718	3.348000564	0.000813968	0.00122264	positive
PRJNA729271	stage_1W_vs_E10	ordinary_muscle_development	ENSGALG00010029925	GGNBP2	1.328792337	0.123944187	10.72089278	8.12156E-27	4.02606E-26	positive
PRJNA729271	stage_1W_vs_E10	ordinary_muscle_development	ENSGALG00010029926	BLTP2	0.502056633	0.13220771	3.797483783	0.000146172	0.000232347	positive
PRJNA729271	stage_1W_vs_E10	ordinary_muscle_development	ENSGALG00010029927	ENSGALG00010029927	-1.479069067	0.749825047	-1.972552228	0.0485466	0.060693718	negative
PRJNA729271	stage_1W_vs_E10	ordinary_muscle_development	ENSGALG00010029928	USP43	-0.11732963	0.137768813	-0.851641441	0.394413131	0.432550378	negative
PRJNA729271	stage_1W_vs_E10	ordinary_muscle_development	ENSGALG00010029929	GDPD1	0.114619008	0.094427087	1.21383611	0.224810314	0.256322308	positive
PRJNA729271	stage_1W_vs_E10	ordinary_muscle_development	ENSGALG00010029930	ARHGAP44	-0.539689721	0.262620634	-2.055016448	0.039877416	0.050389953	negative
PRJNA729271	stage_1W_vs_E10	ordinary_muscle_development	ENSGALG00010029932	RASA4B	2.489880369	0.264543092	9.412002984	4.86771E-21	1.90747E-20	positive
PRJNA729271	stage_1W_vs_E10	ordinary_muscle_development	ENSGALG00010029933	TVP23B	-0.072155783	0.094625371	-0.762541612	0.445736817	0.483833839	negative
PRJNA729271	stage_1W_vs_E10	ordinary_muscle_development	ENSGALG00010029934	TEKT3	1.863522526	0.481024885	3.874066781	0.000107034	0.000171717	positive
PRJNA729271	stage_1W_vs_E10	ordinary_muscle_development	ENSGALG00010029935	SYNRG	-0.24014388	0.074086209	-3.241411384	0.001189394	0.001757597	negative
PRJNA729271	stage_1W_vs_E10	ordinary_muscle_development	ENSGALG00010029936	SERPINF2	2.434631264	0.501704733	4.852717352			positive
PRJNA729271	stage_1W_vs_E10	ordinary_muscle_development	ENSGALG00010029937	PMP22	2.133976868	0.123917824	17.22090337	1.85077E-66	3.1561E-65	positive
PRJNA729271	stage_1W_vs_E10	ordinary_muscle_development	ENSGALG00010029938	ENSGALG00010029938	-1.429200964	0.315856463	-4.524843185	6.04403E-06	1.0645E-05	negative
PRJNA729271	stage_1W_vs_E10	ordinary_muscle_development	ENSGALG00010029939	ENSGALG00010029939	-0.540001556	1.583979208	-0.340914548	0.733167912	0.759416099	negative
PRJNA729271	stage_1W_vs_E10	ordinary_muscle_development	ENSGALG00010029941	COX10	1.450026314	0.172116897	8.424659855	3.618E-17	1.18061E-16	positive
PRJNA729271	stage_1W_vs_E10	ordinary_muscle_development	ENSGALG00010029942	ENSGALG00010029942	-1.41062066	1.508556594	-0.935079709	0.349747223	0.387316525	negative
PRJNA729271	stage_1W_vs_E10	ordinary_muscle_development	ENSGALG00010029943	HS3ST3A1	-1.280183943	0.195574662	-6.545755614	5.91953E-11	1.42365E-10	negative
PRJNA729271	stage_1W_vs_E10	ordinary_muscle_development	ENSGALG00010029945	CCL4	3.07402729	0.699421492	4.395099845	1.10722E-05	1.91586E-05	positive
PRJNA729271	stage_1W_vs_E10	ordinary_muscle_development	ENSGALG00010029946	METRNL	-1.118443505	0.137443933	-8.137452687	4.03681E-16	1.26434E-15	negative
PRJNA729271	stage_1W_vs_E10	ordinary_muscle_development	ENSGALG00010029947	CCL5	6.330640266	0.991757214	6.383256079	1.73362E-10	4.06483E-10	positive
PRJNA729271	stage_1W_vs_E10	ordinary_muscle_development	ENSGALG00010029948	MYADML2	0.943717238	0.219593972	4.297555304	1.72692E-05	2.94145E-05	positive
PRJNA729271	stage_1W_vs_E10	ordinary_muscle_development	ENSGALG00010029950	RFFL	0.039128175	0.104776749	0.373443301	0.708818537	0.736299606	positive
PRJNA729271	stage_1W_vs_E10	ordinary_muscle_development	ENSGALG00010029956	CCL15	5.907885503	0.181474848	32.55484473	1.788E-232	3.7562E-230	positive
PRJNA729271	stage_1W_vs_E10	ordinary_muscle_development	ENSGALG00010029962	ENSGALG00010029962	-0.617502122	0.767308332	-0.804763999	0.42095588	0.459089269	negative
PRJNA729271	stage_1W_vs_E10	ordinary_muscle_development	ENSGALG00010029966	RABGEF1	0.315011569	0.080755841	3.9007899	9.58793E-05	0.00015447	positive
PRJNA729271	stage_1W_vs_E10	ordinary_muscle_development	ENSGALG00010030004	FLOT2	0.601102281	0.07060224	8.513926484	1.68141E-17	5.5835E-17	positive
PRJNA729271	stage_1W_vs_E10	ordinary_muscle_development	ENSGALG00010030014	ENSGALG00010030014	0.551183062	0.304598972	1.809536839	0.070367642	0.086234272	positive
PRJNA729271	stage_1W_vs_E10	ordinary_muscle_development	ENSGALG00010030102	GPS1	0.399999504	0.083033814	4.817308547	1.45508E-06	2.6858E-06	positive
PRJNA729271	stage_1W_vs_E10	ordinary_muscle_development	ENSGALG00010030103	C1QTNF1	2.12274383	0.209715666	10.12200888	4.41264E-24	1.94944E-23	positive

PRJNA729271	stage_1W_vs_E10	ordinary_muscle_development	ENSGALG00010030104	P4HB	-1.674031552	0.110279645	-15.17987797	4.80675E-52	5.66454E-51	negative
PRJNA729271	stage_1W_vs_E10	ordinary_muscle_development	ENSGALG00010030105	RAB40B	1.656726907	0.191131467	8.667996597	4.3979E-18	1.49924E-17	positive
PRJNA729271	stage_1W_vs_E10	ordinary_muscle_development	ENSGALG00010030106	GPRC5C	-1.458717482	0.19528874	-7.469542198	8.04743E-14	2.25014E-13	negative
PRJNA729271	stage_1W_vs_E10	ordinary_muscle_development	ENSGALG00010030107	RPTOR	0.888133221	0.138119938	6.430159408	1.2747E-10	3.00827E-10	positive
PRJNA729271	stage_1W_vs_E10	ordinary_muscle_development	ENSGALG00010030108	LLGL2	0.380578015	0.119403141	3.187336725	0.001435895	0.002107676	positive
PRJNA729271	stage_1W_vs_E10	ordinary_muscle_development	ENSGALG00010030109	FBF1	0.000955212	0.089951562	0.010619181	0.991527279	0.993103089	positive
PRJNA729271	stage_3W_vs_E10	ordinary_muscle_development	ENSGALG00010000007	ND1	-0.057198191	0.243641297	-0.23476394	0.814391958	0.835961435	negative
PRJNA729271	stage_3W_vs_E10	ordinary_muscle_development	ENSGALG00010000011	ND2	0.008758924	0.253305729	0.034578467	0.972415872	0.976525431	positive
PRJNA729271	stage_3W_vs_E10	ordinary_muscle_development	ENSGALG00010000017	COX1	1.420964809	0.272423827	5.21600781	1.8282E-07	3.6331E-07	positive
PRJNA729271	stage_3W_vs_E10	ordinary_muscle_development	ENSGALG00010000020	COX2	2.099250929	0.238991355	8.783794418	1.5805E-18	5.69602E-18	positive
PRJNA729271	stage_3W_vs_E10	ordinary_muscle_development	ENSGALG00010000022	ATP8	-0.1703767	0.279568364	-0.609427682	0.542240994	0.579758295	negative
PRJNA729271	stage_3W_vs_E10	ordinary_muscle_development	ENSGALG00010000023	ATP6	0.375952703	0.198895091	1.890206045	0.058730409	0.073085274	positive
PRJNA729271	stage_3W_vs_E10	ordinary_muscle_development	ENSGALG00010000024	COX3	1.813392898	0.22149688	8.186990709	2.67839E-16	8.6659E-16	positive
PRJNA729271	stage_3W_vs_E10	ordinary_muscle_development	ENSGALG00010000026	ND3	1.099779059	0.318022665	3.458178232	0.000543841	0.000834531	positive
PRJNA729271	stage_3W_vs_E10	ordinary_muscle_development	ENSGALG00010000028	ND4L	0.659135042	0.280503987	2.349824149	0.018782282	0.024770192	positive
PRJNA729271	stage_3W_vs_E10	ordinary_muscle_development	ENSGALG00010000029	ND4	0.712180669	0.255689737	2.785331464	0.005347303	0.007477248	positive
PRJNA729271	stage_3W_vs_E10	ordinary_muscle_development	ENSGALG00010000033	ND5	-0.195733189	0.232922648	-0.840335582	0.400720256	0.439800115	negative
PRJNA729271	stage_3W_vs_E10	ordinary_muscle_development	ENSGALG00010000034	CYTB	0.687135607	0.219330412	3.132878846	0.001731009	0.002538031	positive
PRJNA729271	stage_3W_vs_E10	ordinary_muscle_development	ENSGALG00010000037	ND6	-0.770438924	0.19185758	-4.015681439	5.92743E-05	9.86997E-05	negative
PRJNA729271	stage_3W_vs_E10	ordinary_muscle_development	ENSGALG00010000039	PDIA3	-1.010446156	0.065942375	-15.32316903	5.35421E-53	6.66747E-52	negative
PRJNA729271	stage_3W_vs_E10	ordinary_muscle_development	ENSGALG00010000042	CKMT1B	3.38983684	0.452146583	7.497207684	6.51916E-14	1.86897E-13	positive
PRJNA729271	stage_3W_vs_E10	ordinary_muscle_development	ENSGALG00010000043	PPIP5K1	0.485806181	0.122700499	3.959284465	7.51747E-05	0.000123994	positive
PRJNA729271	stage_3W_vs_E10	ordinary_muscle_development	ENSGALG00010000044	MAP1A	-0.224207858	0.132720902	-1.689318375	0.091158434	0.110860097	negative
PRJNA729271	stage_3W_vs_E10	ordinary_muscle_development	ENSGALG00010000045	TP53BP1	-1.484796003	0.074604446	-19.90224551	3.891E-88	1.22343E-86	negative
PRJNA729271	stage_3W_vs_E10	ordinary_muscle_development	ENSGALG00010000046	TUBGCP4	-0.426132463	0.061753919	-6.900492605	5.18225E-12	1.34137E-11	negative
PRJNA729271	stage_3W_vs_E10	ordinary_muscle_development	ENSGALG00010000047	IQGAP1	-0.786227709	0.097263685	-8.083466177	6.29514E-16	1.99449E-15	negative
PRJNA729271	stage_3W_vs_E10	ordinary_muscle_development	ENSGALG00010000048	MAN2A2	2.378673603	0.147011683	16.18016709	6.96053E-59	1.01809E-57	positive
PRJNA729271	stage_3W_vs_E10	ordinary_muscle_development	ENSGALG00010000049	FES	1.997959304	0.187547586	10.6530793	1.68691E-26	8.45788E-26	positive
PRJNA729271	stage_3W_vs_E10	ordinary_muscle_development	ENSGALG00010000050	CIB1	0.758320177	0.129874492	5.838869264	5.25563E-09	1.15111E-08	positive
PRJNA729271	stage_3W_vs_E10	ordinary_muscle_development	ENSGALG00010000051	GDPGP1	0.208583922	0.187288336	1.113704815	0.265405843	0.301730015	positive
PRJNA729271	stage_3W_vs_E10	ordinary_muscle_development	ENSGALG00010000052	TTLL13	0.404843021	0.133882442	3.023869404	0.002495641	0.003604097	positive
PRJNA729271	stage_3W_vs_E10	ordinary_muscle_development	ENSGALG00010000053	HDDC3	0.701972726	0.116306352	6.035549331	1.58423E-09	3.56741E-09	positive
PRJNA729271	stage_3W_vs_E10	ordinary_muscle_development	ENSGALG00010000054	VPS33B	0.237472601	0.099480797	2.387119997	0.016980948	0.022501036	positive
PRJNA729271	stage_3W_vs_E10	ordinary_muscle_development	ENSGALG00010000055	PRC1	-2.656368888	0.217255311	-12.22694568	2.23173E-34	1.5053E-33	negative
PRJNA729271	stage_3W_vs_E10	ordinary_muscle_development	ENSGALG00010000056	UNC45A	0.10082475	0.081897875	1.231103365	0.218284207	0.251093531	positive
PRJNA729271	stage_3W_vs_E10	ordinary_muscle_development	ENSGALG00010000058	FURIN	1.478353084	0.171865002	8.601827398	7.84567E-18	2.74428E-17	positive
PRJNA729271	stage_3W_vs_E10	ordinary_muscle_development	ENSGALG00010000059	MFAP1	-0.245982805	0.110747401	-2.22111583	0.026343117	0.034169439	negative
PRJNA729271	stage_3W_vs_E10	ordinary_muscle_development	ENSGALG00010000060	HYPK	0.098152463	0.246107643	0.398819238	0.6900264	0.720600337	positive
PRJNA729271	stage_3W_vs_E10	ordinary_muscle_development	ENSGALG00010000061	SERINC4	3.567403064	0.328815934	10.84924023	2.0109E-27	1.04736E-26	positive
PRJNA729271	stage_3W_vs_E10	ordinary_muscle_development	ENSGALG00010000063	SERF2	0.498663403	0.127410498	3.913832919	9.08425E-05	0.00014877	positive
PRJNA729271	stage_3W_vs_E10	ordinary_muscle_development	ENSGALG00010000069	ENSGALG00010000069	1.250769244	0.234824789	5.326393561	1.00182E-07	2.02756E-07	positive
PRJNA729271	stage_3W_vs_E10	ordinary_muscle_development	ENSGALG00010000076	ENSGALG00010000076	0.642706554	0.198146867	3.243586757	0.001180349	0.001758211	positive
PRJNA729271	stage_3W_vs_E10	ordinary_muscle_development	ENSGALG00010000078	ENSGALG00010000078	1.812258469	0.465948907	3.889393114	0.000100495	0.000164022	positive
PRJNA729271	stage_3W_vs_E10	ordinary_muscle_development	ENSGALG00010000081	ENSGALG00010000081	8.162315151	0.298820919	27.31507276	2.8089E-164	3.3102E-162	positive
PRJNA729271	stage_3W_vs_E10	ordinary_muscle_development	ENSGALG00010000084	ENSGALG00010000084	1.813621035	0.261041464	6.947635853	3.71458E-12	9.68571E-12	positive
PRJNA729271	stage_3W_vs_E10	ordinary_muscle_development	ENSGALG00010000085	ENSGALG00010000085	-0.803434122	0.153202843	-5.244250726	1.56919E-07	3.12995E-07	negative
PRJNA729271	stage_3W_vs_E10	ordinary_muscle_development	ENSGALG00010000089	ENSGALG00010000089	1.922580371	0.287052524	6.697660574	2.11782E-11	5.31933E-11	positive
PRJNA729271	stage_3W_vs_E10	ordinary_muscle_development	ENSGALG00010000091	ENSGALG00010000091	2.280126641	0.910157744	2.505199408	0.012238237	0.016478704	positive
PRJNA729271	stage_3W_vs_E10	ordinary_muscle_development	ENSGALG00010000092	INPPL1	1.501653765	0.170305844	8.817394201	1.17154E-18	4.23902E-18	positive
PRJNA729271	stage_3W_vs_E10	ordinary_muscle_development	ENSGALG00010000093	ENSGALG00010000093	0.866156645	0.329653176	2.62747854	0.008602027	0.011781761	positive
PRJNA729271	stage_3W_vs_E10	ordinary_muscle_development	ENSGALG00010000098	ENSGALG00010000098	1.078007881	0.192438826	5.601821125	2.12111E-08	4.48513E-08	positive
PRJNA729271	stage_3W_vs_E10	ordinary_muscle_development	ENSGALG00010000105	CACNA1F	0.144426314	0.297381338	0.485660313	0.627208027	0.661030054	positive
PRJNA729271	stage_3W_vs_E10	ordinary_muscle_development	ENSGALG00010000107	ENSGALG00010000107	-0.782638291	0.547259674	-1.430104076	0.152687151	0.179905726	negative
PRJNA729271	stage_3W_vs_E10	ordinary_muscle_development	ENSGALG00010000121	ENSGALG00010000121	-0.147793168	0.138703381	-1.065534	0.286634348	0.323756322	negative
PRJNA729271	stage_3W_vs_E10	ordinary_muscle_development	ENSGALG00010000123	ENSGALG00010000123	-0.006331205	0.255018983	-0.024826405	0.980193429	0.983177895	negative
PRJNA729271	stage_3W_vs_E10	ordinary_muscle_development	ENSGALG00010000124	ENSGALG00010000124	1.882479863	0.181491635	10.3722679	3.31561E-25	1.567E-24	positive
PRJNA729271	stage_3W_vs_E10	ordinary_muscle_development	ENSGALG00010000126	ENSGALG00010000126	0.448852104	0.203667179	2.203850938	0.027534829	0.035629115	positive
PRJNA729271	stage_3W_vs_E10	ordinary_muscle_development	ENSGALG00010000127	ENSGALG00010000127	0.875349619	0.208438679	4.199554627	2.67441E-05	4.56602E-05	positive
PRJNA729271	stage_3W_vs_E10	ordinary_muscle_development	ENSGALG00010000133	USP39	0.049963097	0.140721401	0.355049742	0.722552327	0.750834897	positive
PRJNA729271	stage_3W_vs_E10	ordinary_muscle_development	ENSGALG00010000134	ENSGALG00010000134	0.801316834	0.126735372	6.322756014	2.56939E-10	6.06964E-10	positive
PRJNA729271	stage_3W_vs_E10	ordinary_muscle_development	ENSGALG00010000137	ENSGALG00010000137	1.925935613	0.538278514	3.57795372	0.000346295	0.000541074	positive
PRJNA729271	stage_3W_vs_E10	ordinary_muscle_development	ENSGALG00010000140	SEPHS3	-0.852785657	0.128201421	-6.651920497	2.89293E-11	7.22236E-11	negative
PRJNA729271	stage_3W_vs_E10	ordinary_muscle_development	ENSGALG00010000141	ENSGALG00010000141	-0.782727614	0.14001087	-5.590477465	2.26446E-08	4.78196E-08	negative
PRJNA729271	stage_3W_vs_E10	ordinary_muscle_development	ENSGALG00010000143	SCARF2	-1.851063836	0.16086088	-11.50723429	1.21307E-30	7.14191E-30	negative
PRJNA729271	stage_3W_vs_E10	ordinary_muscle_development	ENSGALG00010000145	ENSGALG00010000145	0.646016168	0.280020669	2.3070303	0.021053131	0.027639266	positive
PRJNA729271	stage_3W_vs_E10	ordinary_muscle_development	ENSGALG00010000146	ACADM	1.295639504	0.164447347	7.878749814	3.30673E-15	1.01035E-14	positive
PRJNA729271	stage_3W_vs_E10	ordinary_muscle_development	ENSGALG00010000147	ENSGALG00010000147	0.086633419	0.109713096	0.78963608	0.429740331	0.468246718	positive
PRJNA729271	stage_3W_vs_E10	ordinary_muscle_development	ENSGALG00010000148	ENSGALG00010000148	0.61145451	0.299033211	2.044771241	0.040877416	0.051879708	positive
PRJNA729271	stage_3W_vs_E10	ordinary_muscle_development	ENSGALG00010000149	ENSGALG00010000149	-1.036251911	0.142335754	-7.280334598	3.32993E-13	9.18678E-13	negative
PRJNA729271	stage_3W_vs_E10	ordinary_muscle_development	ENSGALG00010000150	B3GAT3	0.499988712	0.125648281	3.979272211	6.91266E-05	0.000114435	positive
PRJNA729271	stage_3W_vs_E10	ordinary_muscle_development	ENSGALG00010000151	TBX5	-4.521286177	0.255727515	-17.68009275	5.96979E-70	1.15376E-68	negative
PRJNA729271	stage_3W_vs_E10	ordinary_muscle_development	ENSGALG00010000153	NF2	0.905284132	0.093196278	9.713736978	2.63495E-22	1.10354E-21	positive
PRJNA729271	stage_3W_vs_E10	ordinary_muscle_development	ENSGALG00010000154	ENSGALG00010000154	-20.88668602	4.021492466	-5.193764801	2.06083E-07	4.07918E-07	negative
PRJNA729271	stage_3W_vs_E10	ordinary_muscle_development	ENSGALG00010000155	MAZ	-1.320290487	0.169754203	-7.777660063	7.38783E-15	2.21895E-14	negative
PRJNA729271	stage_3W_vs_E10	ordinary_muscle_development	ENSGALG00010000156	ENSGALG00010000156	1.912457785	0.48778226	3.920720252	8.82847E-05	0.000144728	positive
PRJNA729271	stage_3W_vs_E10	ordinary_muscle_development	ENSGALG00010000157	ENSGALG00010000157	-18.82335359	5.370802047	-3.504756538			negative
PRJNA729271	stage_3W_vs_E10	ordinary_muscle_development	ENSGALG00010000161	CDC42EP2	0.911857711	0.322444518	2.827952283	0.004684679	0.006591382	positive
PRJNA729271	stage_3W_vs_E10	ordinary_muscle_development	ENSGALG00010000166	PFDN6	0.388771505	0.129424742	3.003842232	0.002665935	0.003837792	positive
PRJNA729271	stage_3W_vs_E10	ordinary_muscle_development	ENSGALG00010000171	DPF2	-0.345474768	0.202364932	-1.707186937	0.087787312	0.106957812	negative
PRJNA729271	stage_3W_vs_E10	ordinary_muscle_development	ENSGALG00010000172	ENSGALG00010000172	0.737537215	1.119832558	0.658613835	0.510143777	0.548230579	positive
PRJNA729271	stage_3W_vs_E10	ordinary_muscle_development	ENSGALG00010000173	NIPSNAP1	-0.863973509	0.129239798	-6.685042264	2.30858E-11	5.79444E-11	negative
PRJNA729271	stage_3W_vs_E10	ordinary_muscle_development	ENSGALG00010000174	ENSGALG00010000174	0.541694903	0.09774916	5.541683479	2.99577E-08	6.26334E-08	positive
PRJNA729271	stage_3W_vs_E10	ordinary_muscle_development	ENSGALG00010000175	ENSGALG00010000175	-1.782617259	0.975285376	-1.827790411	0.067581013	0.083440101	negative
PRJNA729271	stage_3W_vs_E10	ordinary_muscle_development	ENSGALG00010000176	ENSGALG00010000176	-0.49165018	0.514848794	-0.954940919	0.339607575	0.37813887	negative
PRJNA729271	stage_3W_vs_E10	ordinary_muscle_development	ENSGALG00010000177	ENSGALG00010000177	0.252848596	0.210829731	1.199302369	0.230410394	0.264158303	positive
PRJNA729271	stage_3W_vs_E10	ordinary_muscle_development	ENSGALG00010000184	ENSGALG00010000184	2.802252703	3.466239067	0.808441844	0.418836272	0.457431567	positive
PRJNA729271	stage_3W_vs_E10	ordinary_muscle_development	ENSGALG00010000186	THOC5	-0.470266221	0.080016254	-5.87713368	4.17431E-09	9.1788E-09	negative
PRJNA729271	stage_3W_vs_E10	ordinary_muscle_development	ENSGALG00010000188	ENSGALG00010000188	-1.301394468	1.361344382	-0.955962712	0.339091074	0.377650796	negative
PRJNA729271	stage_3W_vs_E10	ordinary_muscle_development	ENSGALG00010000189	ENSGALG00010000189	1.276567689	0.580660085	2.198476737	0.027915151	0.036111567	positive
PRJNA729271	stage_3W_vs_E10	ordinary_muscle_development	ENSGALG00010000191	NEFH	1.265995216	0.444319745	2.84928867	0.00438171	0.006181303	positive
PRJNA729271	stage_3W_vs_E10	ordinary_muscle_development	ENSGALG00010000192	ENSGALG00010000192	0.251651997	0.721297162	0.348888101	0.727173318	0.755203643	positive
PRJNA729271	stage_3W_vs_E10	ordinary_muscle_development	ENSGALG00010000196	KSR2	-4.846682945	1.016376219	-4.768591447	1.85518E-06	3.44932E-06	negative
PRJNA729271	stage_3W_vs_E10	ordinary_muscle_development	ENSGALG00010000197	ENSGALG00010000197	-0.709042924	0.522272458	-1.357611172	0.174587096	0.20383729	negative
PRJNA729271	stage_3W_vs_E10	ordinary_muscle_development	ENSGALG00010000200	GAL3ST1	-3.815490587	0.324416779	-11.76107659	6.19394E-32	3.83189E-31	negative
PRJNA729271	stage_3W_vs_E10	ordinary_muscle_development	ENSGALG00010000202	ENSGALG00010000202	-0.137510476	0.295377363	-0.465541688	0.64154359	0.67474624	negative
PRJNA729271	stage_3W_vs_E10	ordinary_muscle_development	ENSGALG00010000205	RNF215	-0.092416786	0.116097938	-0.796024352	0.426017882	0.464435108	negative
PRJNA729271	stage_3W_vs_E10	ordinary_muscle_development	ENSGALG00010000210	CCDC157	-1.801181175	0.580663082	-3.10193851	0.001922579	0.002801627	negative
PRJNA729271	stage_3W_vs_E10	ordinary_muscle_development	ENSGALG00010000215	SF3A1	-0.698084219	0.090660061	-7.700019321	1.36046E-14	4.03021E-14	negative
PRJNA729271	stage_3W_vs_E10	ordinary_muscle_development	ENSGALG00010000221	TBC1D10A	-0.089225505	0.088078062	-1.013027577	0.311046989	0.348964869	negative
PRJNA729271	stage_3W_vs_E10	ordinary_muscle_development	ENSGALG00010000223	CASTOR1	0.686376939	0.156068583	4.397918702	1.09294E-05	1.92493E-05	positive
PRJNA729271	stage_3W_vs_E10	ordinary_muscle_development	ENSGALG00010000224	MYBPC2	10.51180808	0.251766302	41.75224398	0	0	positive
PRJNA729271	stage_3W_vs_E10	ordinary_muscle_development	ENSGALG00010000225	LIF	3.608177291	0.455852009	7.91523832	2.4678E-15	7.58498E-15	positive
PRJNA729271	stage_3W_vs_E10	ordinary_muscle_development	ENSGALG00010000226	ENSGALG00010000226	0.980783743	0.427412713	2.294699511	0.021750351	0.028510701	positive
PRJNA729271	stage_3W_vs_E10	ordinary_muscle_development	ENSGALG00010000227	ENSGALG00010000227	-0.179223489	0.290409727	-0.617140103	0.537142319	0.574858086	negative
PRJNA729271	stage_3W_vs_E10	ordinary_muscle_development	ENSGALG00010000231	ENSGALG00010000231	3.140251855	0.443502951	7.080565864	1.43567E-12	3.84091E-12	positive
PRJNA729271	stage_3W_vs_E10	ordinary_muscle_development	ENSGALG00010000232	ENSGALG00010000232	0.674490969	0.161723036	4.17065489	3.03726E-05	5.16544E-05	positive
PRJNA729271	stage_3W_vs_E10	ordinary_muscle_development	ENSGALG00010000233	HORMAD2	-1.533538305	0.59631394	-2.571696221	0.010120165	0.013742907	negative
PRJNA729271	stage_3W_vs_E10	ordinary_muscle_development	ENSGALG00010000234	ENSGALG00010000234	-3.396068649	4.6114594	-0.73644119			negative
PRJNA729271	stage_3W_vs_E10	ordinary_muscle_development	ENSGALG00010000235	ENSGALG00010000235	-2.945364754	0.261068012	-11.28198254	1.61074E-29	9.09534E-29	negative
PRJNA729271	stage_3W_vs_E10	ordinary_muscle_development	ENSGALG00010000239	ENSGALG00010000239	-1.921608735	4.644206113	-0.41376474	0.679046409	0.710005349	negative
PRJNA729271	stage_3W_vs_E10	ordinary_muscle_development	ENSGALG00010000242	ENSGALG00010000242	2.179943153	0.19483621	11.1885935	4.63731E-29	2.57244E-28	positive
PRJNA729271	stage_3W_vs_E10	ordinary_muscle_development	ENSGALG00010000246	ENSGALG00010000246	-0.835231265	4.281009707	-0.195101465	0.845313516	0.863258004	negative
PRJNA729271	stage_3W_vs_E10	ordinary_muscle_development	ENSGALG00010000247	ENSGALG00010000247	3.104489153	0.224829222	13.80821016	2.27412E-43	2.05379E-42	positive
PRJNA729271	stage_3W_vs_E10	ordinary_muscle_development	ENSGALG00010000249	ENSGALG00010000249	-22.13773396	4.458072001	-4.965764114			negative
PRJNA729271	stage_3W_vs_E10	ordinary_muscle_development	ENSGALG00010000254	ENSGALG00010000254	-3.360887701	0.723652617	-4.644338488	3.41168E-06	6.24713E-06	negative
PRJNA729271	stage_3W_vs_E10	ordinary_muscle_development	ENSGALG00010000255	ENSGALG00010000255	1.260308838	0.144224911	8.738496206	2.36234E-18	8.46738E-18	positive
PRJNA729271	stage_3W_vs_E10	ordinary_muscle_development	ENSGALG00010000256	ENSGALG00010000256	-1.592227027	0.44703159	-3.561777428	0.000368353	0.000574115	negative
PRJNA729271	stage_3W_vs_E10	ordinary_muscle_development	ENSGALG00010000262	HNRNPKL	-6.357779544	3.532467851	-1.799812429	0.071890261	0.088459196	negative
PRJNA729271	stage_3W_vs_E10	ordinary_muscle_development	ENSGALG00010000263	MTMR3	0.782799807	0.091565656	8.549054725	1.24101E-17	4.29933E-17	positive
PRJNA729271	stage_3W_vs_E10	ordinary_muscle_development	ENSGALG00010000267	ENSGALG00010000267	0.08362685	0.123166661	0.678973103	0.497154904	0.53534361	positive
PRJNA729271	stage_3W_vs_E10	ordinary_muscle_development	ENSGALG00010000269	ENSGALG00010000269	-3.618883868	0.699028255	-5.177020874	2.25457E-07	4.45414E-07	negative
PRJNA729271	stage_3W_vs_E10	ordinary_muscle_development	ENSGALG00010000271	RTN4R	-3.636203541	0.456419972	-7.966793224	1.62845E-15	5.04259E-15	negative
PRJNA729271	stage_3W_vs_E10	ordinary_muscle_development	ENSGALG00010000272	ENSGALG00010000272	0.797194806	0.349140416	2.28330714	0.022412283	0.029343875	positive
PRJNA729271	stage_3W_vs_E10	ordinary_muscle_development	ENSGALG00010000277	ENSGALG00010000277	3.968208291	0.567738653	6.989498196	2.75871E-12	7.25331E-12	positive
PRJNA729271	stage_3W_vs_E10	ordinary_muscle_development	ENSGALG00010000279	ENSGALG00010000279	-1.240427527	0.138889904	-8.931012921	4.22126E-19	1.5601E-18	negative
PRJNA729271	stage_3W_vs_E10	ordinary_muscle_development	ENSGALG00010000281	OSBP2	1.80010112	0.20388003	8.829217466	1.05411E-18	3.82364E-18	positive
PRJNA729271	stage_3W_vs_E10	ordinary_muscle_development	ENSGALG00010000282	ENSGALG00010000282	-21.58307506	4.991011543	-4.324388929	1.52955E-05	2.66477E-05	negative
PRJNA729271	stage_3W_vs_E10	ordinary_muscle_development	ENSGALG00010000284	ENSGALG00010000284	-0.947142053	0.155125987	-6.105631123	1.02395E-09	2.33184E-09	negative
PRJNA729271	stage_3W_vs_E10	ordinary_muscle_development	ENSGALG00010000285	MYLPF	6.086076484	0.145689245	41.77437042	0	0	positive
PRJNA729271	stage_3W_vs_E10	ordinary_muscle_development	ENSGALG00010000286	TCN2	2.013895968	0.137419856	14.6550581	1.2505E-48	1.36081E-47	positive
PRJNA729271	stage_3W_vs_E10	ordinary_muscle_development	ENSGALG00010000287	IQCD	-2.894832466	0.258044757	-11.21833475	3.31453E-29	1.85284E-28	negative

PRJNA729271	stage_3W_vs_E10	ordinary_muscle_development	ENSGALG00010000288	VARS2	0.279137043	0.155008755	1.800782431	0.071737179	0.088278327	positive
PRJNA729271	stage_3W_vs_E10	ordinary_muscle_development	ENSGALG00010000289	QPRT	-3.418204564	0.479179341	-7.133455626	9.78799E-13	2.6435E-12	negative
PRJNA729271	stage_3W_vs_E10	ordinary_muscle_development	ENSGALG00010000292	ABHD16A	0.240561052	0.13983357	1.720338336	0.085370959	0.104171399	positive
PRJNA729271	stage_3W_vs_E10	ordinary_muscle_development	ENSGALG00010000293	ENSGALG00010000293	2.571053159	0.350716269	7.330863688	2.28674E-13	6.36084E-13	positive
PRJNA729271	stage_3W_vs_E10	ordinary_muscle_development	ENSGALG00010000294	DUSP21	-1.116636385	0.308571983	-3.618722523	0.000296061	0.000465193	negative
PRJNA729271	stage_3W_vs_E10	ordinary_muscle_development	ENSGALG00010000298	SLC35E4	1.400268288	0.21287941	6.577753509	4.77609E-11	1.17517E-10	positive
PRJNA729271	stage_3W_vs_E10	ordinary_muscle_development	ENSGALG00010000301	UQCR10	2.516286587	0.154551072	16.28126255	1.34074E-59	1.98712E-58	positive
PRJNA729271	stage_3W_vs_E10	ordinary_muscle_development	ENSGALG00010000302	ENSGALG00010000302	0.781515753	0.231090936	3.381853767	0.000719985	0.001092908	positive
PRJNA729271	stage_3W_vs_E10	ordinary_muscle_development	ENSGALG00010000303	ENSGALG00010000303	-0.523181126	0.11335643	-4.615363487	3.92408E-06	7.15106E-06	negative
PRJNA729271	stage_3W_vs_E10	ordinary_muscle_development	ENSGALG00010000306	ENSGALG00010000306	1.670083366	0.207143593	8.062442794	7.47847E-16	2.36115E-15	positive
PRJNA729271	stage_3W_vs_E10	ordinary_muscle_development	ENSGALG00010000307	ZMAT5	1.196370434	0.111586194	10.72149155	8.06914E-27	4.09102E-26	positive
PRJNA729271	stage_3W_vs_E10	ordinary_muscle_development	ENSGALG00010000308	ENSGALG00010000308	0.344850044	0.33946092	1.015875534	0.309688661	0.347629299	positive
PRJNA729271	stage_3W_vs_E10	ordinary_muscle_development	ENSGALG00010000309	ENSGALG00010000309	-3.397056527	4.320124886	-0.786332946	0.431672456	0.470245923	negative
PRJNA729271	stage_3W_vs_E10	ordinary_muscle_development	ENSGALG00010000312	CABP7	-3.245705281	0.632357393	-5.1327071	2.85604E-07	5.60573E-07	negative
PRJNA729271	stage_3W_vs_E10	ordinary_muscle_development	ENSGALG00010000313	ENSGALG00010000313	-6.9980282	3.871946573	-1.807366932	0.070705086	0.087082184	negative
PRJNA729271	stage_3W_vs_E10	ordinary_muscle_development	ENSGALG00010000318	ENSGALG00010000318	-3.834758105	4.763979614	-0.804948471	0.420849416	0.459284165	negative
PRJNA729271	stage_3W_vs_E10	ordinary_muscle_development	ENSGALG00010000323	AP1B1	0.610458714	0.144764723	4.216902436	2.47681E-05	4.24316E-05	positive
PRJNA729271	stage_3W_vs_E10	ordinary_muscle_development	ENSGALG00010000324	ENSGALG00010000324	-0.477823274	0.178715833	-2.673648256	0.00750311	0.010337191	negative
PRJNA729271	stage_3W_vs_E10	ordinary_muscle_development	ENSGALG00010000326	ENSGALG00010000326	-3.513672767	1.841763764	-1.907776033	0.05642017	0.070379549	negative
PRJNA729271	stage_3W_vs_E10	ordinary_muscle_development	ENSGALG00010000331	ENSGALG00010000331	-0.175471771	0.169824658	-1.033252607	0.3014857	0.339130469	negative
PRJNA729271	stage_3W_vs_E10	ordinary_muscle_development	ENSGALG00010000338	ENSGALG00010000338	-0.989734432	0.194903871	-5.078064511	3.81299E-07	7.4197E-07	negative
PRJNA729271	stage_3W_vs_E10	ordinary_muscle_development	ENSGALG00010000340	NAA10	0.192772835	0.132628901	1.453475319	0.146091827	0.172653978	positive
PRJNA729271	stage_3W_vs_E10	ordinary_muscle_development	ENSGALG00010000341	EWSR1	-1.123315926	0.148226985	-7.578349682	3.49978E-14	1.0156E-13	negative
PRJNA729271	stage_3W_vs_E10	ordinary_muscle_development	ENSGALG00010000346	ENSGALG00010000346	-0.756572359	0.115552359	-6.547441942	5.8531E-11	1.43094E-10	negative
PRJNA729271	stage_3W_vs_E10	ordinary_muscle_development	ENSGALG00010000349	CHIR-IG1-5	2.30835254	0.440004362	5.246203762	1.55265E-07	3.09825E-07	positive
PRJNA729271	stage_3W_vs_E10	ordinary_muscle_development	ENSGALG00010000350	ENSGALG00010000350	0.007996171	0.165808911	0.048225216	0.961536754	0.966676081	positive
PRJNA729271	stage_3W_vs_E10	ordinary_muscle_development	ENSGALG00010000353	ENSGALG00010000353	0.817045506	0.110506595	7.393635707	1.42867E-13	4.0211E-13	positive
PRJNA729271	stage_3W_vs_E10	ordinary_muscle_development	ENSGALG00010000356	ENSGALG00010000356	0.281138258	0.125027308	2.248614831	0.024537012	0.031969797	positive
PRJNA729271	stage_3W_vs_E10	ordinary_muscle_development	ENSGALG00010000366	EMID1	-0.148741351	0.21597698	-0.688690765	0.491017889	0.529434742	negative
PRJNA729271	stage_3W_vs_E10	ordinary_muscle_development	ENSGALG00010000367	HSD17B10	0.77069084	0.138792145	5.552841926	2.81062E-08	5.89239E-08	positive
PRJNA729271	stage_3W_vs_E10	ordinary_muscle_development	ENSGALG00010000373	ENSGALG00010000373	2.845094005	0.46332859	6.140553521	8.22344E-10	1.88427E-09	positive
PRJNA729271	stage_3W_vs_E10	ordinary_muscle_development	ENSGALG00010000376	ASCC2	-0.507657136	0.084778132	-5.988067038	2.1235E-09	4.74196E-09	negative
PRJNA729271	stage_3W_vs_E10	ordinary_muscle_development	ENSGALG00010000377	ENSGALG00010000377	0.157378049	0.251977888	0.624570871	0.532252743	0.570130321	positive
PRJNA729271	stage_3W_vs_E10	ordinary_muscle_development	ENSGALG00010000381	ENSGALG00010000381	-1.295619106	0.314197267	-4.123584902	3.73021E-05	6.30326E-05	negative
PRJNA729271	stage_3W_vs_E10	ordinary_muscle_development	ENSGALG00010000382	ENSGALG00010000382	-0.799526748	0.54598376	-1.464378259	0.143090625	0.169397142	negative
PRJNA729271	stage_3W_vs_E10	ordinary_muscle_development	ENSGALG00010000384	ENSGALG00010000384	-0.325001702	0.147916965	-2.197190178	0.028006867	0.03622698	negative
PRJNA729271	stage_3W_vs_E10	ordinary_muscle_development	ENSGALG00010000386	ENSGALG00010000386	0.431659685	0.360472234	1.197483867	0.231118012	0.264910295	positive
PRJNA729271	stage_3W_vs_E10	ordinary_muscle_development	ENSGALG00010000387	DTX1	-1.211241723	0.456075976	-2.655789355	0.007912302	0.010877164	negative
PRJNA729271	stage_3W_vs_E10	ordinary_muscle_development	ENSGALG00010000388	ENSGALG00010000388	-0.149028726	0.184046538	-0.809733927	0.418093114	0.456723166	negative
PRJNA729271	stage_3W_vs_E10	ordinary_muscle_development	ENSGALG00010000391	ENSGALG00010000391	3.898972782	0.626775795	6.2206818	4.94999E-10	1.15095E-09	positive
PRJNA729271	stage_3W_vs_E10	ordinary_muscle_development	ENSGALG00010000402	PFKFB1	9.830169974	0.318292335	30.88409271	1.9535E-209	3.8789E-207	positive
PRJNA729271	stage_3W_vs_E10	ordinary_muscle_development	ENSGALG00010000405	ENSGALG00010000405	0.740249505	0.447365614	1.654685748	0.09798826	0.118608753	positive
PRJNA729271	stage_3W_vs_E10	ordinary_muscle_development	ENSGALG00010000406	NSMCE3	-0.358663892	0.098509181	-3.640918436	0.000271667	0.000427745	negative
PRJNA729271	stage_3W_vs_E10	ordinary_muscle_development	ENSGALG00010000407	WSB2	0.650981991	0.085128086	7.647088299	2.05581E-14	6.04197E-14	positive
PRJNA729271	stage_3W_vs_E10	ordinary_muscle_development	ENSGALG00010000408	ENSGALG00010000408	9.467036356	0.349953885	27.05223963	3.5946E-161	3.9774E-159	positive
PRJNA729271	stage_3W_vs_E10	ordinary_muscle_development	ENSGALG00010000411	ENSGALG00010000411	2.885678538	0.928471394	3.107988632	0.001883653	0.002747389	positive
PRJNA729271	stage_3W_vs_E10	ordinary_muscle_development	ENSGALG00010000412	RFC5	-1.000347928	0.107400727	-9.314163491	1.22917E-20	4.82449E-20	negative
PRJNA729271	stage_3W_vs_E10	ordinary_muscle_development	ENSGALG00010000422	ITIH6	-2.426548795	0.596431864	-4.068442585	4.73284E-05	7.93552E-05	negative
PRJNA729271	stage_3W_vs_E10	ordinary_muscle_development	ENSGALG00010000423	ENSGALG00010000423	2.789417086	0.19968154	13.9693288	2.39877E-44	2.24034E-43	positive
PRJNA729271	stage_3W_vs_E10	ordinary_muscle_development	ENSGALG00010000428	GNL3L	-0.79446261	0.149460806	-5.315524722	1.0635E-07	2.1497E-07	negative
PRJNA729271	stage_3W_vs_E10	ordinary_muscle_development	ENSGALG00010000431	AIFM3	-3.255606378	1.153951592	-2.821267721	0.004783426	0.006723142	negative
PRJNA729271	stage_3W_vs_E10	ordinary_muscle_development	ENSGALG00010000432	ENSGALG00010000432	0.93460836	0.182304363	5.126637365	2.94963E-07	5.78472E-07	positive
PRJNA729271	stage_3W_vs_E10	ordinary_muscle_development	ENSGALG00010000434	TP53	0.039159497	0.219819901	0.178143548	0.858610243	0.874784246	positive
PRJNA729271	stage_3W_vs_E10	ordinary_muscle_development	ENSGALG00010000435	P2RX6	0.31810373	0.293575127	1.083551363	0.278563765	0.315427059	positive
PRJNA729271	stage_3W_vs_E10	ordinary_muscle_development	ENSGALG00010000436	ENSGALG00010000436	1.476673291	0.378308994	3.903352327	9.48694E-05	0.000155084	positive
PRJNA729271	stage_3W_vs_E10	ordinary_muscle_development	ENSGALG00010000440	ENSGALG00010000440	-1.195323598	0.458897017	-2.604775263	0.009193454	0.012551485	negative
PRJNA729271	stage_3W_vs_E10	ordinary_muscle_development	ENSGALG00010000441	SLC7A4	-1.485368065	0.213722672	-6.949978928	3.65341E-12	9.53306E-12	negative
PRJNA729271	stage_3W_vs_E10	ordinary_muscle_development	ENSGALG00010000442	DGCR6	-0.680566512	0.130424854	-5.218073767	1.80793E-07	3.5943E-07	negative
PRJNA729271	stage_3W_vs_E10	ordinary_muscle_development	ENSGALG00010000443	TBX3	-1.818111894	0.15910072	-11.42742719	3.05012E-30	1.76705E-29	negative
PRJNA729271	stage_3W_vs_E10	ordinary_muscle_development	ENSGALG00010000444	RBM19	-0.32496738	0.118736339	-2.73688225	0.006202449	0.008610717	negative
PRJNA729271	stage_3W_vs_E10	ordinary_muscle_development	ENSGALG00010000449	PLBD2	1.187054186	0.107610334	11.03104271	2.70708E-28	1.46196E-27	positive
PRJNA729271	stage_3W_vs_E10	ordinary_muscle_development	ENSGALG00010000450	ENSGALG00010000450	0.481675599	0.189206004	2.545773333	0.010903596	0.014762528	positive
PRJNA729271	stage_3W_vs_E10	ordinary_muscle_development	ENSGALG00010000452	SLC8B1	2.235202433	0.223389132	10.00586921	1.43625E-23	6.3201E-23	positive
PRJNA729271	stage_3W_vs_E10	ordinary_muscle_development	ENSGALG00010000453	ACHE	-1.576453488	0.253612909	-6.215982836	5.10044E-10	1.1846E-09	negative
PRJNA729271	stage_3W_vs_E10	ordinary_muscle_development	ENSGALG00010000457	TPCN1	0.01809686	0.080607895	0.224504814	0.822364522	0.843428412	positive
PRJNA729271	stage_3W_vs_E10	ordinary_muscle_development	ENSGALG00010000458	ENSGALG00010000458	0.293259622	0.200060214	1.465856784	0.142687308	0.168961075	positive
PRJNA729271	stage_3W_vs_E10	ordinary_muscle_development	ENSGALG00010000462	ENSGALG00010000462	-0.316297632	0.224198573	-1.41079235	0.158305851	0.186177956	negative
PRJNA729271	stage_3W_vs_E10	ordinary_muscle_development	ENSGALG00010000465	ENSGALG00010000465	1.414442034	0.191749679	7.37650273	1.62502E-13	4.5569E-13	positive
PRJNA729271	stage_3W_vs_E10	ordinary_muscle_development	ENSGALG00010000466	FBXO21	-0.149717724	0.080605785	-1.857406699	0.063253314	0.078404035	negative
PRJNA729271	stage_3W_vs_E10	ordinary_muscle_development	ENSGALG00010000467	RNASEK	1.396121968	0.198807807	7.022470558	2.17979E-12	5.76991E-12	positive
PRJNA729271	stage_3W_vs_E10	ordinary_muscle_development	ENSGALG00010000468	TESC	-2.512343329	0.303019085	-8.291039905	1.12263E-16	3.70418E-16	negative
PRJNA729271	stage_3W_vs_E10	ordinary_muscle_development	ENSGALG00010000469	ENSGALG00010000469	6.621917906	0.386075556	17.15187042	6.08616E-66	1.06416E-64	positive
PRJNA729271	stage_3W_vs_E10	ordinary_muscle_development	ENSGALG00010000470	FBXW8	0.122057592	0.108646143	1.123441561	0.261250007	0.297145233	positive
PRJNA729271	stage_3W_vs_E10	ordinary_muscle_development	ENSGALG00010000471	ENSGALG00010000471	-0.289847294	0.20508408	-1.413309575	0.15756472	0.185351454	negative
PRJNA729271	stage_3W_vs_E10	ordinary_muscle_development	ENSGALG00010000475	RNFT2	-0.76047742	0.111062852	-6.847270742	7.52722E-12	1.93212E-11	negative
PRJNA729271	stage_3W_vs_E10	ordinary_muscle_development	ENSGALG00010000476	ENSGALG00010000476	7.885823993	0.214036622	36.84333984	3.7387E-297	1.5939E-294	positive
PRJNA729271	stage_3W_vs_E10	ordinary_muscle_development	ENSGALG00010000479	C12orf49	0.28546054	0.114268907	2.498147115	0.012484437	0.016793051	positive
PRJNA729271	stage_3W_vs_E10	ordinary_muscle_development	ENSGALG00010000483	ENSGALG00010000483	0.878842694	0.247014397	3.557860202	0.000373888	0.000582256	positive
PRJNA729271	stage_3W_vs_E10	ordinary_muscle_development	ENSGALG00010000485	MED13L	0.772087906	0.125982952	6.128510973	8.87053E-10	2.02837E-09	positive
PRJNA729271	stage_3W_vs_E10	ordinary_muscle_development	ENSGALG00010000491	ENSGALG00010000491	1.021545578	0.155909783	6.5521583	5.67114E-11	1.38763E-10	positive
PRJNA729271	stage_3W_vs_E10	ordinary_muscle_development	ENSGALG00010000495	ENSGALG00010000495	0.783680517	0.560690533	1.397705991	0.162201374	0.190434871	positive
PRJNA729271	stage_3W_vs_E10	ordinary_muscle_development	ENSGALG00010000501	ENSGALG00010000501	0.693985151	0.162009384	4.28361084	1.83884E-05	3.18638E-05	positive
PRJNA729271	stage_3W_vs_E10	ordinary_muscle_development	ENSGALG00010000507	TRPT1	1.532487718	0.289862856	5.286940649	1.24379E-07	2.50086E-07	positive
PRJNA729271	stage_3W_vs_E10	ordinary_muscle_development	ENSGALG00010000509	ENSGALG00010000509	-4.759811778	1.158226018	-4.109570761	3.96395E-05	6.68888E-05	negative
PRJNA729271	stage_3W_vs_E10	ordinary_muscle_development	ENSGALG00010000511	FERMT3	1.067122292	0.155599663	6.858127273	6.97691E-12	1.79436E-11	positive
PRJNA729271	stage_3W_vs_E10	ordinary_muscle_development	ENSGALG00010000525	ENSGALG00010000525	-3.432831127	0.576614127	-5.953428757	2.62582E-09	5.83583E-09	negative
PRJNA729271	stage_3W_vs_E10	ordinary_muscle_development	ENSGALG00010000526	POLD1	-2.288517302	0.17238584	-13.27555273	3.20895E-40	2.61313E-39	negative
PRJNA729271	stage_3W_vs_E10	ordinary_muscle_development	ENSGALG00010000529	STIP1	-0.161481673	0.19287634	-0.837229041	0.402463837	0.441579995	negative
PRJNA729271	stage_3W_vs_E10	ordinary_muscle_development	ENSGALG00010000532	ENSGALG00010000532	2.301869549	0.470398782	4.893442836	9.90871E-07	1.87796E-06	positive
PRJNA729271	stage_3W_vs_E10	ordinary_muscle_development	ENSGALG00010000546	FLRT1	-4.17609218	0.756378879	-5.52116445	3.3676E-08	7.01544E-08	negative
PRJNA729271	stage_3W_vs_E10	ordinary_muscle_development	ENSGALG00010000555	OTUB1	-0.884109452	0.102820892	-8.598539003	8.07373E-18	2.82065E-17	negative
PRJNA729271	stage_3W_vs_E10	ordinary_muscle_development	ENSGALG00010000561	ENSGALG00010000561	-0.679643222	0.277811502	-2.446418582	0.014428338	0.019271909	negative
PRJNA729271	stage_3W_vs_E10	ordinary_muscle_development	ENSGALG00010000564	NAA40	-0.923175428	0.090250808	-10.22899906	1.47031E-24	6.76362E-24	negative
PRJNA729271	stage_3W_vs_E10	ordinary_muscle_development	ENSGALG00010000569	SIPA1	0.958866924	0.226943044	4.225143493	2.38789E-05	4.09517E-05	positive
PRJNA729271	stage_3W_vs_E10	ordinary_muscle_development	ENSGALG00010000574	ENSGALG00010000574	3.939586112	0.171312979	22.99642524	5.0615E-117	2.8547E-115	positive
PRJNA729271	stage_3W_vs_E10	ordinary_muscle_development	ENSGALG00010000575	ENSGALG00010000575	1.539923978	0.157884434	9.753488312	1.78236E-22	7.50591E-22	positive
PRJNA729271	stage_3W_vs_E10	ordinary_muscle_development	ENSGALG00010000576	NOS1	3.210405239	0.428511722	7.491989305	6.78375E-14	1.94329E-13	positive
PRJNA729271	stage_3W_vs_E10	ordinary_muscle_development	ENSGALG00010000577	ENSGALG00010000577	0.126098776	0.26914697	0.468512706	0.639417987	0.672741795	positive
PRJNA729271	stage_3W_vs_E10	ordinary_muscle_development	ENSGALG00010000578	ENSGALG00010000578	1.734746276	0.366709742	4.730570467	2.2389E-06	4.14239E-06	positive
PRJNA729271	stage_3W_vs_E10	ordinary_muscle_development	ENSGALG00010000579	ENSGALG00010000579	-2.284548756	0.940476409	-2.429139884	0.015134692	0.020172631	negative
PRJNA729271	stage_3W_vs_E10	ordinary_muscle_development	ENSGALG00010000580	ENSGALG00010000580	0.350203743	0.498033311	0.703173333	0.481947736	0.520476265	positive
PRJNA729271	stage_3W_vs_E10	ordinary_muscle_development	ENSGALG00010000581	ENSGALG00010000581	-0.438141153	0.131350067	-3.335675141	0.000850925	0.001282404	negative
PRJNA729271	stage_3W_vs_E10	ordinary_muscle_development	ENSGALG00010000583	CCS	0.350028652	0.139636224	2.506718117	0.012185785	0.016412651	positive
PRJNA729271	stage_3W_vs_E10	ordinary_muscle_development	ENSGALG00010000584	RBM14	0.2554987	0.212295593	1.203504491	0.228781155	0.262439287	positive
PRJNA729271	stage_3W_vs_E10	ordinary_muscle_development	ENSGALG00010000585	SPTBN2	-2.812457727	0.561096357	-5.012432698	5.37462E-07	1.03473E-06	negative
PRJNA729271	stage_3W_vs_E10	ordinary_muscle_development	ENSGALG00010000586	ENSGALG00010000586	3.692505863	0.254650114	14.50031108	1.20602E-47	1.2631E-46	positive
PRJNA729271	stage_3W_vs_E10	ordinary_muscle_development	ENSGALG00010000589	ENSGALG00010000589	1.453194175	0.315200256	4.610383872	4.01926E-06	7.31532E-06	positive
PRJNA729271	stage_3W_vs_E10	ordinary_muscle_development	ENSGALG00010000590	DNAH5	1.706083602	0.426554365	3.999686185	6.34265E-05	0.000105372	positive
PRJNA729271	stage_3W_vs_E10	ordinary_muscle_development	ENSGALG00010000591	SBK2	4.993134637	0.303498007	16.45195199	8.12083E-61	1.23776E-59	positive
PRJNA729271	stage_3W_vs_E10	ordinary_muscle_development	ENSGALG00010000592	CCT5	-0.871876814	0.119146498	-7.31768727	2.52281E-13	7.00942E-13	negative
PRJNA729271	stage_3W_vs_E10	ordinary_muscle_development	ENSGALG00010000593	ATPSCKMT	-0.809232812	0.146438524	-5.526092364	3.27442E-08	6.82524E-08	negative
PRJNA729271	stage_3W_vs_E10	ordinary_muscle_development	ENSGALG00010000595	MYO10	-2.285222167	0.141549828	-16.1442949	1.24564E-58	1.8095E-57	negative
PRJNA729271	stage_3W_vs_E10	ordinary_muscle_development	ENSGALG00010000596	ENSGALG00010000596	-2.932490268	0.811751571	-3.612546466	0.000303205	0.000475953	negative
PRJNA729271	stage_3W_vs_E10	ordinary_muscle_development	ENSGALG00010000597	CDH9	-4.714486746	0.974709565	-4.836811822	1.31938E-06	2.47758E-06	negative
PRJNA729271	stage_3W_vs_E10	ordinary_muscle_development	ENSGALG00010000598	ENSGALG00010000598	-2.681110852	0.553631337	-4.842772932	1.2804E-06	2.40687E-06	negative
PRJNA729271	stage_3W_vs_E10	ordinary_muscle_development	ENSGALG00010000599	RETREG1	5.467374252	0.374595908	14.59539237	3.00479E-48	3.22865E-47	positive
PRJNA729271	stage_3W_vs_E10	ordinary_muscle_development	ENSGALG00010000601	ENSGALG00010000601	-2.553376944	0.365963956	-6.977126851	3.01278E-12	7.9084E-12	negative
PRJNA729271	stage_3W_vs_E10	ordinary_muscle_development	ENSGALG00010000603	ZNF622	-0.150412559	0.108887374	-1.38135904	0.167168593	0.195917591	negative
PRJNA729271	stage_3W_vs_E10	ordinary_muscle_development	ENSGALG00010000604	ENSGALG00010000604	-0.697441371	0.134800837	-5.17386527	2.293E-07	4.52574E-07	negative
PRJNA729271	stage_3W_vs_E10	ordinary_muscle_development	ENSGALG00010000605	MARCHF11	-1.273717511	0.651016205	-1.956506614	0.050405504	0.063224973	negative
PRJNA729271	stage_3W_vs_E10	ordinary_muscle_development	ENSGALG00010000606	UBE2QL1	0.653491947	0.153998982	4.243482264	2.20078E-05	3.79133E-05	positive
PRJNA729271	stage_3W_vs_E10	ordinary_muscle_development	ENSGALG00010000607	ENSGALG00010000607	-2.01282626	0.295413253	-6.813594981	9.51895E-12	2.43604E-11	negative
PRJNA729271	stage_3W_vs_E10	ordinary_muscle_development	ENSGALG00010000608	ENSGALG00010000608	-3.424181073	1.197924323	-2.858428541	0.00425745	0.006012445	negative
PRJNA729271	stage_3W_vs_E10	ordinary_muscle_development	ENSGALG00010000610	ENSGALG00010000610	-0.292013927	0.079677592	-3.66494418	0.000247393	0.000390669	negative
PRJNA729271	stage_3W_vs_E10	ordinary_muscle_development	ENSGALG00010000611	C7orf57	1.592395856	0.318230905	5.003900728	5.61817E-07	1.08062E-06	positive
PRJNA729271	stage_3W_vs_E10	ordinary_muscle_development	ENSGALG00010000612	ENSGALG00010000612	-0.217033226	0.387543805	-0.560022436	0.575464135	0.611625798	negative
PRJNA729271	stage_3W_vs_E10	ordinary_muscle_development	ENSGALG00010000613	ENSGALG00010000613	-0.265497638	0.177394262	-1.496652907	0.134483596	0.159821245	negative
PRJNA729271	stage_3W_vs_E10	ordinary_muscle_development	ENSGALG00010000614	KAT8	1.302956208	0.183141956	7.114460492	1.12351E-12	3.02084E-12	positive
PRJNA729271	stage_3W_vs_E10	ordinary_muscle_development	ENSGALG00010000615	ENSGALG00010000615	-2.343457594	0.663593676	-3.53146463	0.000413265	0.00064074	negative
PRJNA729271	stage_3W_vs_E10	ordinary_muscle_development	ENSGALG00010000616	PSENEN	1.11034585	0.496438324	2.236623961	0.025310928	0.032895355	positive
PRJNA729271	stage_3W_vs_E10	ordinary_muscle_development	ENSGALG00010000619	ENSGALG00010000619	-0.16523785	0.314764056	-0.524957812	0.599612519	0.635242177	negative

PRJNA729271	stage_3W_vs_E10	ordinary_muscle_development	ENSGALG00010000621	UPP1	-0.576691636	0.238895554	-2.413990666	0.015778867	0.020984923	negative
PRJNA729271	stage_3W_vs_E10	ordinary_muscle_development	ENSGALG00010000622	ENSGALG00010000622	-0.523603302	0.454709615	-1.151511392	0.249521911	0.284766562	negative
PRJNA729271	stage_3W_vs_E10	ordinary_muscle_development	ENSGALG00010000623	ENSGALG00010000623	-0.053860459	0.250418324	-0.215081941	0.829703433	0.849752792	negative
PRJNA729271	stage_3W_vs_E10	ordinary_muscle_development	ENSGALG00010000625	ENSGALG00010000625	3.350835314	0.354104615	9.462839996	2.99691E-21	1.20667E-20	positive
PRJNA729271	stage_3W_vs_E10	ordinary_muscle_development	ENSGALG00010000626	ABCA13	-1.190653623	0.696598327	-1.709239855	0.087406529	0.106520736	negative
PRJNA729271	stage_3W_vs_E10	ordinary_muscle_development	ENSGALG00010000627	ENSGALG00010000627	0.847442301	0.228095624	3.715293991	0.000202968	0.000323121	positive
PRJNA729271	stage_3W_vs_E10	ordinary_muscle_development	ENSGALG00010000630	ENSGALG00010000630	0.530919797	0.369192627	1.438056337	0.15041807	0.177390768	positive
PRJNA729271	stage_3W_vs_E10	ordinary_muscle_development	ENSGALG00010000632	ENSGALG00010000632	-0.527823083	0.417456011	-1.264380122	0.206093624	0.238128902	negative
PRJNA729271	stage_3W_vs_E10	ordinary_muscle_development	ENSGALG00010000633	VWC2	-5.832674152	0.897193957	-6.5010181	7.97782E-11	1.93699E-10	negative
PRJNA729271	stage_3W_vs_E10	ordinary_muscle_development	ENSGALG00010000634	ENSGALG00010000634	0.986110679	0.187969658	5.246116253	1.55339E-07	3.09929E-07	positive
PRJNA729271	stage_3W_vs_E10	ordinary_muscle_development	ENSGALG00010000635	ENSGALG00010000635	-0.793595034	0.133550538	-5.94228257	2.8108E-09	6.23548E-09	negative
PRJNA729271	stage_3W_vs_E10	ordinary_muscle_development	ENSGALG00010000636	ENSGALG00010000636	0.242000312	0.145146854	1.667279079	0.095458925	0.115730772	positive
PRJNA729271	stage_3W_vs_E10	ordinary_muscle_development	ENSGALG00010000639	SCN1B	4.475955588	0.266387072	16.8024505	2.34166E-63	3.83964E-62	positive
PRJNA729271	stage_3W_vs_E10	ordinary_muscle_development	ENSGALG00010000641	ENSGALG00010000641	0.347582947	0.417021628	0.833489018	0.404568989	0.443520458	positive
PRJNA729271	stage_3W_vs_E10	ordinary_muscle_development	ENSGALG00010000643	VKORC1	-0.141975872	0.215975357	-0.657370702	0.510942585	0.548966924	negative
PRJNA729271	stage_3W_vs_E10	ordinary_muscle_development	ENSGALG00010000645	ENSGALG00010000645	-2.920184501	0.615735088	-4.742598819	2.10994E-06	3.91194E-06	negative
PRJNA729271	stage_3W_vs_E10	ordinary_muscle_development	ENSGALG00010000646	ENSGALG00010000646	-0.774160674	0.52784733	-1.466637471	0.142474701	0.168750677	negative
PRJNA729271	stage_3W_vs_E10	ordinary_muscle_development	ENSGALG00010000649	PAPD7	-0.368709229	0.084357613	-4.370787855	1.23799E-05	2.17248E-05	negative
PRJNA729271	stage_3W_vs_E10	ordinary_muscle_development	ENSGALG00010000650	BCKDK	1.232473357	0.168864488	7.298594117	2.9079E-13	8.04543E-13	positive
PRJNA729271	stage_3W_vs_E10	ordinary_muscle_development	ENSGALG00010000651	ENSGALG00010000651	-4.880916891	0.652671466	-7.478367206	7.52517E-14	2.15143E-13	negative
PRJNA729271	stage_3W_vs_E10	ordinary_muscle_development	ENSGALG00010000652	SRD5A1	-0.072349098	0.228923792	-0.316040099	0.751972072	0.77817057	negative
PRJNA729271	stage_3W_vs_E10	ordinary_muscle_development	ENSGALG00010000653	NSUN2	-0.665712673	0.092375801	-7.206569972	5.73788E-13	1.56424E-12	negative
PRJNA729271	stage_3W_vs_E10	ordinary_muscle_development	ENSGALG00010000654	ENSGALG00010000654	-1.735688499	0.181769285	-9.548854753	1.31138E-21	5.35449E-21	negative
PRJNA729271	stage_3W_vs_E10	ordinary_muscle_development	ENSGALG00010000655	ENSGALG00010000655	-3.935634581	1.064802378	-3.696117386	0.000218922	0.000347375	negative
PRJNA729271	stage_3W_vs_E10	ordinary_muscle_development	ENSGALG00010000656	ENSGALG00010000656	0.834610611	0.41801111	1.996623034	0.045866149	0.057886794	positive
PRJNA729271	stage_3W_vs_E10	ordinary_muscle_development	ENSGALG00010000659	ENSGALG00010000659	1.348723923	0.553664481	2.435995027	0.014850889	0.019820506	positive
PRJNA729271	stage_3W_vs_E10	ordinary_muscle_development	ENSGALG00010000660	ENSGALG00010000660	1.568277434	0.176456284	8.887625886	6.24285E-19	2.28684E-18	positive
PRJNA729271	stage_3W_vs_E10	ordinary_muscle_development	ENSGALG00010000661	ENSGALG00010000661	-2.623695876	0.792211833	-3.311861507	0.000926774	0.001391361	negative
PRJNA729271	stage_3W_vs_E10	ordinary_muscle_development	ENSGALG00010000670	ENSGALG00010000670	-3.947399981	0.762410531	-5.177525519	2.24848E-07	4.44332E-07	negative
PRJNA729271	stage_3W_vs_E10	ordinary_muscle_development	ENSGALG00010000674	GRB10	1.763417955	0.16896491	10.43659273	1.6876E-25	8.05987E-25	positive
PRJNA729271	stage_3W_vs_E10	ordinary_muscle_development	ENSGALG00010000684	COBL	1.548070122	0.135240942	11.44675641	2.4411E-30	1.42046E-29	positive
PRJNA729271	stage_3W_vs_E10	ordinary_muscle_development	ENSGALG00010000686	ENSGALG00010000686	-0.220641898	0.133438568	-1.653509183	0.098227279	0.118858369	negative
PRJNA729271	stage_3W_vs_E10	ordinary_muscle_development	ENSGALG00010000687	IKZF1	1.859566067	0.281601749	6.603531663	4.01476E-11	9.93072E-11	positive
PRJNA729271	stage_3W_vs_E10	ordinary_muscle_development	ENSGALG00010000688	ENSGALG00010000688	1.234070865	0.284925853	4.331200031	1.48299E-05	2.58644E-05	positive
PRJNA729271	stage_3W_vs_E10	ordinary_muscle_development	ENSGALG00010000695	FIGNL1	-2.662096555	0.157554137	-16.89639259	4.78287E-64	8.00551E-63	negative
PRJNA729271	stage_3W_vs_E10	ordinary_muscle_development	ENSGALG00010000697	ENSGALG00010000697	0.744901988	0.248094849	3.002488731	0.002677819	0.003854134	positive
PRJNA729271	stage_3W_vs_E10	ordinary_muscle_development	ENSGALG00010000702	DDC	-1.856577192	0.441395502	-4.206153404	2.59754E-05	4.44054E-05	negative
PRJNA729271	stage_3W_vs_E10	ordinary_muscle_development	ENSGALG00010000704	ENSGALG00010000704	-0.443449201	0.094961855	-4.669761341	3.0155E-06	5.53707E-06	negative
PRJNA729271	stage_3W_vs_E10	ordinary_muscle_development	ENSGALG00010000705	ENSGALG00010000705	3.449525468	1.042133176	3.310062042	0.000932753	0.001400047	positive
PRJNA729271	stage_3W_vs_E10	ordinary_muscle_development	ENSGALG00010000710	ENSGALG00010000710	0.813052172	0.506109506	1.606474808	0.108169626	0.130182558	positive
PRJNA729271	stage_3W_vs_E10	ordinary_muscle_development	ENSGALG00010000711	SPRY2	-0.34697641	0.111135225	-3.122110106	0.001795598	0.002624238	negative
PRJNA729271	stage_3W_vs_E10	ordinary_muscle_development	ENSGALG00010000714	FBXL7	-1.92287442	0.099765239	-19.273992	8.88138E-83	2.40178E-81	negative
PRJNA729271	stage_3W_vs_E10	ordinary_muscle_development	ENSGALG00010000715	PCDH20	-2.796674643	0.290656329	-9.621929293	6.46078E-22	2.66806E-21	negative
PRJNA729271	stage_3W_vs_E10	ordinary_muscle_development	ENSGALG00010000717	NDFIP2	0.737265716	0.067070179	10.99245183	4.15483E-28	2.2223E-27	positive
PRJNA729271	stage_3W_vs_E10	ordinary_muscle_development	ENSGALG00010000718	EEF1G	-0.146717221	0.150972906	-0.971811599	0.331144284	0.369453233	negative
PRJNA729271	stage_3W_vs_E10	ordinary_muscle_development	ENSGALG00010000720	RBM26	-0.142011644	0.051285025	-2.76906647	0.005621716	0.007838282	negative
PRJNA729271	stage_3W_vs_E10	ordinary_muscle_development	ENSGALG00010000721	ENSGALG00010000721	-1.651009133	0.156890047	-10.52335162	6.74313E-26	3.27663E-25	negative
PRJNA729271	stage_3W_vs_E10	ordinary_muscle_development	ENSGALG00010000722	ANKH	0.814663559	0.169782664	4.798272908	1.6004E-06	2.98632E-06	positive
PRJNA729271	stage_3W_vs_E10	ordinary_muscle_development	ENSGALG00010000724	OBI1	-1.701708799	0.147843975	-11.51016672	1.17252E-30	6.90598E-30	negative
PRJNA729271	stage_3W_vs_E10	ordinary_muscle_development	ENSGALG00010000725	ENSGALG00010000725	-3.374059637	0.874719823	-3.857303275	0.000114645	0.000186444	negative
PRJNA729271	stage_3W_vs_E10	ordinary_muscle_development	ENSGALG00010000727	ENSGALG00010000727	-0.253655015	0.14870269	-1.705786327	0.08804787	0.107248225	negative
PRJNA729271	stage_3W_vs_E10	ordinary_muscle_development	ENSGALG00010000728	OTULIN	-0.583154969	0.284793241	-2.047643291	0.040594964	0.051530257	negative
PRJNA729271	stage_3W_vs_E10	ordinary_muscle_development	ENSGALG00010000729	EDNRB	0.060256962	0.156112271	0.385984791	0.699507933	0.728926491	positive
PRJNA729271	stage_3W_vs_E10	ordinary_muscle_development	ENSGALG00010000730	INTS5	1.347325381	0.222230255	6.062745061	1.33818E-09	3.02603E-09	positive
PRJNA729271	stage_3W_vs_E10	ordinary_muscle_development	ENSGALG00010000731	ENSGALG00010000731	-2.27557509	0.459627698	-4.950909393	7.38675E-07	1.40957E-06	negative
PRJNA729271	stage_3W_vs_E10	ordinary_muscle_development	ENSGALG00010000732	ENSGALG00010000732	0.071709081	0.143985429	0.498030125	0.618462809	0.652969511	positive
PRJNA729271	stage_3W_vs_E10	ordinary_muscle_development	ENSGALG00010000733	ENSGALG00010000733	-2.292254507	0.507354332	-4.518054468	6.24104E-06	1.12057E-05	negative
PRJNA729271	stage_3W_vs_E10	ordinary_muscle_development	ENSGALG00010000734	SLAIN1	-2.158356675	0.388358402	-5.55764125	2.73445E-08	5.73684E-08	negative
PRJNA729271	stage_3W_vs_E10	ordinary_muscle_development	ENSGALG00010000735	ENSGALG00010000735	1.398489356	0.536588946	2.60625823	0.009153742	0.012501978	positive
PRJNA729271	stage_3W_vs_E10	ordinary_muscle_development	ENSGALG00010000737	ENSGALG00010000737	-3.616225169	1.07616883	-3.360276815	0.000778644	0.001177511	negative
PRJNA729271	stage_3W_vs_E10	ordinary_muscle_development	ENSGALG00010000741	FAM105A	-0.136832615	0.193642662	-0.706624322	0.47979997	0.518427176	negative
PRJNA729271	stage_3W_vs_E10	ordinary_muscle_development	ENSGALG00010000753	TRIO	-0.190244751	0.068381036	-2.782127367	0.005400384	0.00754565	negative
PRJNA729271	stage_3W_vs_E10	ordinary_muscle_development	ENSGALG00010000757	SCEL	-4.333405887	0.507804873	-8.533604383	1.41857E-17	4.89577E-17	negative
PRJNA729271	stage_3W_vs_E10	ordinary_muscle_development	ENSGALG00010000760	CDH10	-2.760800517	0.370094826	-7.459711195	8.67123E-14	2.46692E-13	negative
PRJNA729271	stage_3W_vs_E10	ordinary_muscle_development	ENSGALG00010000762	ENSGALG00010000762	0.625251552	0.300696422	2.079344833	0.037585667	0.047945459	positive
PRJNA729271	stage_3W_vs_E10	ordinary_muscle_development	ENSGALG00010000768	ENSGALG00010000768	-2.56796348	0.408676645	-6.283607125	3.30806E-10	7.76272E-10	negative
PRJNA729271	stage_3W_vs_E10	ordinary_muscle_development	ENSGALG00010000770	ENSGALG00010000770	-1.985562734	0.616900474	-3.218611134	0.00128813	0.001910905	negative
PRJNA729271	stage_3W_vs_E10	ordinary_muscle_development	ENSGALG00010000777	MYCBP2	0.139581184	0.082520103	1.691480966	0.09074498	0.110385069	positive
PRJNA729271	stage_3W_vs_E10	ordinary_muscle_development	ENSGALG00010000783	DAP	-1.410323873	0.163287645	-8.637051975	5.76821E-18	2.03085E-17	negative
PRJNA729271	stage_3W_vs_E10	ordinary_muscle_development	ENSGALG00010000784	FBXL3	0.618339806	0.0953315	6.486206638	8.80244E-11	2.13185E-10	positive
PRJNA729271	stage_3W_vs_E10	ordinary_muscle_development	ENSGALG00010000785	CLN5	0.176650902	0.093139079	1.896635691	0.057876037	0.072077769	positive
PRJNA729271	stage_3W_vs_E10	ordinary_muscle_development	ENSGALG00010000786	ANKRD33B	1.471597629	0.316003859	4.656897657	3.2101E-06	5.8862E-06	positive
PRJNA729271	stage_3W_vs_E10	ordinary_muscle_development	ENSGALG00010000787	SNRNP48	0.113634559	0.105111194	1.081089033	0.279657511	0.316591349	positive
PRJNA729271	stage_3W_vs_E10	ordinary_muscle_development	ENSGALG00010000788	GATD3AL1	0.984049809	0.104414396	9.424464896	4.32304E-21	1.72814E-20	positive
PRJNA729271	stage_3W_vs_E10	ordinary_muscle_development	ENSGALG00010000789	ROPN1L	0.939201864	0.225897523	4.157645702	3.21544E-05	5.45567E-05	positive
PRJNA729271	stage_3W_vs_E10	ordinary_muscle_development	ENSGALG00010000790	MARCHF6	1.31893594	0.098868377	13.34032149	1.34877E-40	1.10704E-39	positive
PRJNA729271	stage_3W_vs_E10	ordinary_muscle_development	ENSGALG00010000791	ACOD1	2.394772423	0.494176487	4.845986173	1.25984E-06	2.37038E-06	positive
PRJNA729271	stage_3W_vs_E10	ordinary_muscle_development	ENSGALG00010000792	ENSGALG00010000792	-1.867813082	1.832728758	-1.019143217	0.30813498	0.345992449	negative
PRJNA729271	stage_3W_vs_E10	ordinary_muscle_development	ENSGALG00010000793	ENSGALG00010000793	1.671919435	1.302624949	1.283500241	0.199316875	0.230777067	positive
PRJNA729271	stage_3W_vs_E10	ordinary_muscle_development	ENSGALG00010000795	ENSGALG00010000795	-1.115794913	0.238935008	-4.66986785	3.01394E-06	5.5349E-06	negative
PRJNA729271	stage_3W_vs_E10	ordinary_muscle_development	ENSGALG00010000796	ENSGALG00010000796	-1.073172456	0.204215604	-5.255095283	1.47948E-07	2.95671E-07	negative
PRJNA729271	stage_3W_vs_E10	ordinary_muscle_development	ENSGALG00010000797	ENSGALG00010000797	4.520201386	0.408480052	11.06590483	1.836E-28	9.97111E-28	positive
PRJNA729271	stage_3W_vs_E10	ordinary_muscle_development	ENSGALG00010000798	ENSGALG00010000798	-6.582100216	0.723833292	-9.093392479	9.5996E-20	3.63306E-19	negative
PRJNA729271	stage_3W_vs_E10	ordinary_muscle_development	ENSGALG00010000799	ENSGALG00010000799	-2.461208928	0.300850661	-8.180832717	2.81889E-16	9.1083E-16	negative
PRJNA729271	stage_3W_vs_E10	ordinary_muscle_development	ENSGALG00010000800	LMO7	0.29805993	0.151664187	1.965262437	0.049383871	0.062040146	positive
PRJNA729271	stage_3W_vs_E10	ordinary_muscle_development	ENSGALG00010000802	SEMA5A	-2.86097232	0.12831575	-22.29634566	4.0094E-110	2.0391E-108	negative
PRJNA729271	stage_3W_vs_E10	ordinary_muscle_development	ENSGALG00010000803	MTRR	-0.018108134	0.097997151	-0.18478225	0.85339983	0.870026061	negative
PRJNA729271	stage_3W_vs_E10	ordinary_muscle_development	ENSGALG00010000804	ENSGALG00010000804	-0.342012868	0.465914454	-0.734067951	0.462907325	0.501557907	negative
PRJNA729271	stage_3W_vs_E10	ordinary_muscle_development	ENSGALG00010000805	FASTKD3	0.489925012	0.129574893	3.781018061	0.000156188	0.000251104	positive
PRJNA729271	stage_3W_vs_E10	ordinary_muscle_development	ENSGALG00010000806	CFAP90	1.208790606	0.435125186	2.77802951	0.005468965	0.007635586	positive
PRJNA729271	stage_3W_vs_E10	ordinary_muscle_development	ENSGALG00010000807	ENSGALG00010000807	2.149726984	0.753080093	2.854579483	0.004309385	0.006083418	positive
PRJNA729271	stage_3W_vs_E10	ordinary_muscle_development	ENSGALG00010000808	ADCY2	-2.00942809	0.183758616	-10.93515034	7.82761E-28	4.14395E-27	negative
PRJNA729271	stage_3W_vs_E10	ordinary_muscle_development	ENSGALG00010000810	UCHL3	-0.557693893	0.137658484	-4.05128603	5.09369E-05	8.52772E-05	negative
PRJNA729271	stage_3W_vs_E10	ordinary_muscle_development	ENSGALG00010000811	ENSGALG00010000811	6.826944052	1.045322584	6.530944757	6.53561E-11	1.59377E-10	positive
PRJNA729271	stage_3W_vs_E10	ordinary_muscle_development	ENSGALG00010000812	ENSGALG00010000812	-0.807425171	0.096298311	-8.384624473	5.08879E-17	1.70588E-16	negative
PRJNA729271	stage_3W_vs_E10	ordinary_muscle_development	ENSGALG00010000813	ENSGALG00010000813	-1.457565488	0.456060833	-3.195989183	0.001393523	0.002061135	negative
PRJNA729271	stage_3W_vs_E10	ordinary_muscle_development	ENSGALG00010000814	CDH18	-2.273048876	0.298846948	-7.606063552	2.8257E-14	8.24282E-14	negative
PRJNA729271	stage_3W_vs_E10	ordinary_muscle_development	ENSGALG00010000815	ENSGALG00010000815	-1.72698294	0.175442908	-9.843560846	7.30776E-23	3.13576E-22	negative
PRJNA729271	stage_3W_vs_E10	ordinary_muscle_development	ENSGALG00010000816	TBC1D4	1.598297706	0.103659235	15.41876809	1.22417E-53	1.56338E-52	positive
PRJNA729271	stage_3W_vs_E10	ordinary_muscle_development	ENSGALG00010000817	ENSGALG00010000817	0.954335585	0.115405256	8.269429152	1.34602E-16	4.42316E-16	positive
PRJNA729271	stage_3W_vs_E10	ordinary_muscle_development	ENSGALG00010000818	KLF12	-1.677508421	0.082125415	-20.42617882	9.78677E-93	3.42655E-91	negative
PRJNA729271	stage_3W_vs_E10	ordinary_muscle_development	ENSGALG00010000820	KLF5	0.106308687	0.194260502	0.547248082	0.584208307	0.619964815	positive
PRJNA729271	stage_3W_vs_E10	ordinary_muscle_development	ENSGALG00010000821	ENSGALG00010000821	3.416683709	0.148407248	23.02235062	2.7843E-117	1.5889E-115	positive
PRJNA729271	stage_3W_vs_E10	ordinary_muscle_development	ENSGALG00010000822	BASP1	-3.512500579	0.166053219	-21.15286049	2.59706E-99	1.05152E-97	negative
PRJNA729271	stage_3W_vs_E10	ordinary_muscle_development	ENSGALG00010000823	PIBF1	-0.159527286	0.161338596	-0.988773237	0.322774101	0.360753381	negative
PRJNA729271	stage_3W_vs_E10	ordinary_muscle_development	ENSGALG00010000843	DIS3	-0.883989778	0.084131171	-10.5072801	7.99666E-26	3.87405E-25	negative
PRJNA729271	stage_3W_vs_E10	ordinary_muscle_development	ENSGALG00010000853	ENSGALG00010000853	0.926247147	0.303940853	3.04745854	0.002307854	0.003341558	positive
PRJNA729271	stage_3W_vs_E10	ordinary_muscle_development	ENSGALG00010000857	ENSGALG00010000857	1.502630384	0.779857197	1.926801971			positive
PRJNA729271	stage_3W_vs_E10	ordinary_muscle_development	ENSGALG00010000859	BORA	-3.221344036	0.200863904	-16.03744609	6.99733E-58	9.95351E-57	negative
PRJNA729271	stage_3W_vs_E10	ordinary_muscle_development	ENSGALG00010000864	MZT1	0.062416149	0.128342217	0.486325938	0.626736092	0.660837975	positive
PRJNA729271	stage_3W_vs_E10	ordinary_muscle_development	ENSGALG00010000870	DACH1	-0.229162551	0.147122046	-1.557635694	0.119319632	0.142748272	negative
PRJNA729271	stage_3W_vs_E10	ordinary_muscle_development	ENSGALG00010000901	PCDH9	-1.865267112	0.245145752	-7.60880863	2.76634E-14	8.07453E-14	negative
PRJNA729271	stage_3W_vs_E10	ordinary_muscle_development	ENSGALG00010001066	SLC33A1	-0.624670422	0.08786783	-7.109205059	1.16713E-12	3.13457E-12	negative
PRJNA729271	stage_3W_vs_E10	ordinary_muscle_development	ENSGALG00010001070	C3orf33	-0.388234308	0.184482257	-2.104453367	0.035338918	0.045210735	negative
PRJNA729271	stage_3W_vs_E10	ordinary_muscle_development	ENSGALG00010001082	P2RY1	3.186937691	0.179981315	17.7070475	3.69978E-70	7.18878E-69	positive
PRJNA729271	stage_3W_vs_E10	ordinary_muscle_development	ENSGALG00010001104	MBNL1	2.361151913	0.122535525	19.26912139	9.75785E-83	2.63389E-81	positive
PRJNA729271	stage_3W_vs_E10	ordinary_muscle_development	ENSGALG00010001111	SUCNR1	4.895454346	0.416468074	11.75469298	6.68031E-32	4.1275E-31	positive
PRJNA729271	stage_3W_vs_E10	ordinary_muscle_development	ENSGALG00010001112	TSC22D2	-0.457049013	0.090939061	-5.025882237	5.01123E-07	9.67086E-07	negative
PRJNA729271	stage_3W_vs_E10	ordinary_muscle_development	ENSGALG00010001113	IGSF10	1.519745521	0.297184252	5.113815785	3.15715E-07	6.18E-07	positive
PRJNA729271	stage_3W_vs_E10	ordinary_muscle_development	ENSGALG00010001114	MED12L	0.868445268	0.101481456	8.557674481	1.15167E-17	3.99939E-17	positive
PRJNA729271	stage_3W_vs_E10	ordinary_muscle_development	ENSGALG00010001115	PFN2	-1.099739262	0.095931043	-11.46385184	2.004E-30	1.17082E-29	negative
PRJNA729271	stage_3W_vs_E10	ordinary_muscle_development	ENSGALG00010001116	RNF13	0.475603579	0.070057868	6.788724671	1.13129E-11	2.88597E-11	positive
PRJNA729271	stage_3W_vs_E10	ordinary_muscle_development	ENSGALG00010001117	P2RY13	1.066329554	0.31496566	3.385542264	0.000710378	0.001078664	positive
PRJNA729271	stage_3W_vs_E10	ordinary_muscle_development	ENSGALG00010001118	P2RY12	0.031713473	0.321301757	0.098703079	0.921374024	0.930424724	positive
PRJNA729271	stage_3W_vs_E10	ordinary_muscle_development	ENSGALG00010001119	COMMD2	-0.539642895	0.099126697	-5.443971295	5.21056E-08	1.07344E-07	negative
PRJNA729271	stage_3W_vs_E10	ordinary_muscle_development	ENSGALG00010001120	GPR87	-4.407826803	1.147030109	-3.842817002	0.00012163	0.000197361	negative
PRJNA729271	stage_3W_vs_E10	ordinary_muscle_development	ENSGALG00010001121	WWTR1	0.249675404	0.091856475	2.718103469	0.00656573	0.009095886	positive
PRJNA729271	stage_3W_vs_E10	ordinary_muscle_development	ENSGALG00010001122	P2RY14	3.66562061	0.422591673	8.674143028	4.16676E-18	1.47924E-17	positive
PRJNA729271	stage_3W_vs_E10	ordinary_muscle_development	ENSGALG00010001123	CP	-0.692649751	0.15477839	-4.475106323	7.63733E-06	1.36049E-05	negative
PRJNA729271	stage_3W_vs_E10	ordinary_muscle_development	ENSGALG00010001124	GPR171	2.785299602	0.57959506	4.80559583	1.54291E-06	2.88314E-06	positive
PRJNA729271	stage_3W_vs_E10	ordinary_muscle_development	ENSGALG00010001125	CLRN1	1.740426568	0.265233325	6.561869881	5.31372E-11	1.3037E-10	positive

PRJNA729271	stage_3W_vs_E10	ordinary_muscle_development	ENSGALG00010001126	MINDY4B	4.382748644	1.183985891	3.701689923	0.000214168	0.000340093	positive
PRJNA729271	stage_3W_vs_E10	ordinary_muscle_development	ENSGALG00010001127	SIAH2	0.856068388	0.099313631	8.619847881	6.70431E-18	2.35471E-17	positive
PRJNA729271	stage_3W_vs_E10	ordinary_muscle_development	ENSGALG00010001129	TM4SF4	-0.746775353	1.010674758	-0.738887903	0.459975069	0.498567159	negative
PRJNA729271	stage_3W_vs_E10	ordinary_muscle_development	ENSGALG00010001131	HPS3	0.281768308	0.099879373	2.82108608	0.004786136	0.006726298	positive
PRJNA729271	stage_3W_vs_E10	ordinary_muscle_development	ENSGALG00010001132	SELENOT	0.497771738	0.094334385	5.276673357	1.3155E-07	2.63956E-07	positive
PRJNA729271	stage_3W_vs_E10	ordinary_muscle_development	ENSGALG00010001133	EIF2A	-0.110850605	0.073623686	-1.505637822	0.132160174	0.157214749	negative
PRJNA729271	stage_3W_vs_E10	ordinary_muscle_development	ENSGALG00010001134	ENSGALG00010001134	0.240854367	0.637126773	0.378032093	0.705406753	0.734598095	positive
PRJNA729271	stage_3W_vs_E10	ordinary_muscle_development	ENSGALG00010001135	GYG1	2.394491139	0.159164599	15.04411879	3.77325E-51	4.43938E-50	positive
PRJNA729271	stage_3W_vs_E10	ordinary_muscle_development	ENSGALG00010001136	SERP1	-0.928650204	0.126712176	-7.328815849	2.32195E-13	6.4563E-13	negative
PRJNA729271	stage_3W_vs_E10	ordinary_muscle_development	ENSGALG00010001138	SPTSSB	-7.066786847	0.994755276	-7.104045607	1.21157E-12	3.25156E-12	negative
PRJNA729271	stage_3W_vs_E10	ordinary_muscle_development	ENSGALG00010001140	NMD3	0.845162966	0.120263144	7.027614073	2.10095E-12	5.57038E-12	positive
PRJNA729271	stage_3W_vs_E10	ordinary_muscle_development	ENSGALG00010001141	MTAP	-0.924979254	0.112488476	-8.222880097	1.98673E-16	6.46558E-16	negative
PRJNA729271	stage_3W_vs_E10	ordinary_muscle_development	ENSGALG00010001142	PPM1L	2.195925958	0.145974467	15.04321961	3.82486E-51	4.49282E-50	positive
PRJNA729271	stage_3W_vs_E10	ordinary_muscle_development	ENSGALG00010001143	CDKN2A	3.400544985	0.412692417	8.239901798	1.72352E-16	5.62667E-16	positive
PRJNA729271	stage_3W_vs_E10	ordinary_muscle_development	ENSGALG00010001144	CDKN2B	5.660032436	1.372515579	4.12383839	3.7261E-05	6.29706E-05	positive
PRJNA729271	stage_3W_vs_E10	ordinary_muscle_development	ENSGALG00010001145	TRIM36	-3.553209146	0.226525611	-15.68568398	1.89511E-55	2.53409E-54	negative
PRJNA729271	stage_3W_vs_E10	ordinary_muscle_development	ENSGALG00010001146	DIPK1C	1.502665815	0.241592184	6.219844481	4.97648E-10	1.1568E-09	positive
PRJNA729271	stage_3W_vs_E10	ordinary_muscle_development	ENSGALG00010001147	PGGT1B	0.054459197	0.112034289	0.486094012	0.626900512	0.660867122	positive
PRJNA729271	stage_3W_vs_E10	ordinary_muscle_development	ENSGALG00010001149	CIDEA	2.070331241	0.620552959	3.336268421	0.000849111	0.001279936	positive
PRJNA729271	stage_3W_vs_E10	ordinary_muscle_development	ENSGALG00010001150	ENSGALG00010001150	2.033430948	0.600564269	3.385867349	0.000709537	0.001077529	positive
PRJNA729271	stage_3W_vs_E10	ordinary_muscle_development	ENSGALG00010001151	CCDC112	2.070886535	0.21457656	9.651038013	4.86606E-22	2.01928E-21	positive
PRJNA729271	stage_3W_vs_E10	ordinary_muscle_development	ENSGALG00010001152	FEM1C	2.602886911	0.197388213	13.186638	1.04754E-39	8.39362E-39	positive
PRJNA729271	stage_3W_vs_E10	ordinary_muscle_development	ENSGALG00010001153	TSHZ1	-2.50326787	0.119557787	-20.93772333	2.42787E-97	9.31003E-96	negative
PRJNA729271	stage_3W_vs_E10	ordinary_muscle_development	ENSGALG00010001154	PTGR3	-0.779418067	0.086283478	-9.033224991	1.66686E-19	6.2561E-19	negative
PRJNA729271	stage_3W_vs_E10	ordinary_muscle_development	ENSGALG00010001155	CYB5A	-0.280263832	0.094249734	-2.973629944	0.002942998	0.004222385	negative
PRJNA729271	stage_3W_vs_E10	ordinary_muscle_development	ENSGALG00010001156	ALDH7A1	-0.022796498	0.181169273	-0.125829825	0.899866633	0.911500129	negative
PRJNA729271	stage_3W_vs_E10	ordinary_muscle_development	ENSGALG00010001157	TIMM21	1.9934159	0.09059769	22.00294404	2.6988E-107	1.304E-105	positive
PRJNA729271	stage_3W_vs_E10	ordinary_muscle_development	ENSGALG00010001158	FRMPD1	-0.376925933	0.367902628	-1.02452634	0.305586736	0.343317295	negative
PRJNA729271	stage_3W_vs_E10	ordinary_muscle_development	ENSGALG00010001159	RTTN	-1.062815382	0.114621249	-9.272411421	1.81984E-20	7.08341E-20	negative
PRJNA729271	stage_3W_vs_E10	ordinary_muscle_development	ENSGALG00010001160	TRMT10B	-0.439464143	0.128583471	-3.417734326	0.000631447	0.000963455	negative
PRJNA729271	stage_3W_vs_E10	ordinary_muscle_development	ENSGALG00010001161	B3GALNT1	0.75444863	0.09655335	7.813800635	5.54888E-15	1.6788E-14	positive
PRJNA729271	stage_3W_vs_E10	ordinary_muscle_development	ENSGALG00010001162	ENSGALG00010001162	0.946627127	0.404296723	2.341416768	0.019210709	0.025312174	positive
PRJNA729271	stage_3W_vs_E10	ordinary_muscle_development	ENSGALG00010001163	ARL14	-0.692370664	0.499714769	-1.38553172	0.16588994	0.194529139	negative
PRJNA729271	stage_3W_vs_E10	ordinary_muscle_development	ENSGALG00010001164	KPNA4	1.291808109	0.074479951	17.34437373	2.17536E-67	3.95136E-66	positive
PRJNA729271	stage_3W_vs_E10	ordinary_muscle_development	ENSGALG00010001165	ENSGALG00010001165	2.156121946	0.158362107	13.615138	3.2553E-42	2.82211E-41	positive
PRJNA729271	stage_3W_vs_E10	ordinary_muscle_development	ENSGALG00010001166	TRIM59	-0.330038804	0.096514465	-3.419578661	0.000627182	0.000957351	negative
PRJNA729271	stage_3W_vs_E10	ordinary_muscle_development	ENSGALG00010001167	SMC4	-1.908360986	0.15464524	-12.34025043	5.49846E-35	3.77905E-34	negative
PRJNA729271	stage_3W_vs_E10	ordinary_muscle_development	ENSGALG00010001168	DOK6	-2.271645646	0.174495698	-13.0183476	9.62308E-39	7.44723E-38	negative
PRJNA729271	stage_3W_vs_E10	ordinary_muscle_development	ENSGALG00010001169	ENSGALG00010001169	-0.367344812	0.103910434	-3.535206213	0.000407457	0.000632141	negative
PRJNA729271	stage_3W_vs_E10	ordinary_muscle_development	ENSGALG00010001170	DCAF10	0.010237063	0.069421234	0.147462995	0.882766586	0.896622638	positive
PRJNA729271	stage_3W_vs_E10	ordinary_muscle_development	ENSGALG00010001171	POLR1E	-0.106827164	0.127251151	-0.839498607	0.40118957	0.440248529	negative
PRJNA729271	stage_3W_vs_E10	ordinary_muscle_development	ENSGALG00010001172	TMEM200C	-3.223759825	0.297278696	-10.84423428	2.12407E-27	1.10511E-26	negative
PRJNA729271	stage_3W_vs_E10	ordinary_muscle_development	ENSGALG00010001173	IFT80	0.133965424	0.110541774	1.211898625	0.225551189	0.258959563	positive
PRJNA729271	stage_3W_vs_E10	ordinary_muscle_development	ENSGALG00010001174	ENSGALG00010001174	-2.019014612	0.143948536	-14.02594755	1.08158E-44	1.01868E-43	negative
PRJNA729271	stage_3W_vs_E10	ordinary_muscle_development	ENSGALG00010001177	SEH1L	-0.689023661	0.072082831	-9.558776349	1.19157E-21	4.86942E-21	negative
PRJNA729271	stage_3W_vs_E10	ordinary_muscle_development	ENSGALG00010001178	ZBTB5	-0.463786498	0.065178072	-7.115682958	1.1136E-12	2.99474E-12	negative
PRJNA729271	stage_3W_vs_E10	ordinary_muscle_development	ENSGALG00010001179	GRHPR	1.576209588	0.184148614	8.559443115	1.13414E-17	3.9404E-17	positive
PRJNA729271	stage_3W_vs_E10	ordinary_muscle_development	ENSGALG00010001180	SPIRE1	-0.026543951	0.160188266	-0.165704716	0.868389334	0.883381529	negative
PRJNA729271	stage_3W_vs_E10	ordinary_muscle_development	ENSGALG00010001181	MFSD1	0.664715753	0.1045732	6.35646375	2.06451E-10	4.90495E-10	positive
PRJNA729271	stage_3W_vs_E10	ordinary_muscle_development	ENSGALG00010001182	ZCCHC7	-0.134394965	0.083895835	-1.601926541	0.109171855	0.131257965	negative
PRJNA729271	stage_3W_vs_E10	ordinary_muscle_development	ENSGALG00010001183	RARRES1	1.988055729	0.187980534	10.57585958	3.85625E-26	1.89608E-25	positive
PRJNA729271	stage_3W_vs_E10	ordinary_muscle_development	ENSGALG00010001185	PTPN2	-0.550523345	0.067424951	-8.164979478	3.2149E-16	1.03418E-15	negative
PRJNA729271	stage_3W_vs_E10	ordinary_muscle_development	ENSGALG00010001186	ENSGALG00010001186	-0.487714076	0.380611876	-1.281394791	0.200055033	0.231594737	negative
PRJNA729271	stage_3W_vs_E10	ordinary_muscle_development	ENSGALG00010001187	GFM1	0.701587312	0.181780447	3.859531233	0.000113605	0.000184773	positive
PRJNA729271	stage_3W_vs_E10	ordinary_muscle_development	ENSGALG00010001188	PSMG2	0.184544985	0.111284352	1.658319264	0.097253043	0.11779693	positive
PRJNA729271	stage_3W_vs_E10	ordinary_muscle_development	ENSGALG00010001190	LXN	0.686250521	0.420606616	1.631573294	0.102769408	0.124023193	positive
PRJNA729271	stage_3W_vs_E10	ordinary_muscle_development	ENSGALG00010001191	TOMM5	0.185068393	0.151179804	1.224160827	0.220891564	0.253891302	positive
PRJNA729271	stage_3W_vs_E10	ordinary_muscle_development	ENSGALG00010001192	ENSGALG00010001192	3.210869501	0.220560873	14.55774751	5.21479E-48	5.56616E-47	positive
PRJNA729271	stage_3W_vs_E10	ordinary_muscle_development	ENSGALG00010001193	PRELID3A	1.13521472	0.227277975	4.994829437	5.88878E-07	1.13132E-06	positive
PRJNA729271	stage_3W_vs_E10	ordinary_muscle_development	ENSGALG00010001194	POT1	-1.024713133	0.110948779	-9.235911797	2.56095E-20	9.92541E-20	negative
PRJNA729271	stage_3W_vs_E10	ordinary_muscle_development	ENSGALG00010001195	ZNF608	-1.028808249	0.142870274	-7.20099585	5.97744E-13	1.62863E-12	negative
PRJNA729271	stage_3W_vs_E10	ordinary_muscle_development	ENSGALG00010001196	LSMEM1	5.431886247	0.205266296	26.46263093	2.611E-154	2.6653E-152	positive
PRJNA729271	stage_3W_vs_E10	ordinary_muscle_development	ENSGALG00010001197	CEP76	-0.699384805	0.144158039	-4.85151441	1.22522E-06	2.30734E-06	negative
PRJNA729271	stage_3W_vs_E10	ordinary_muscle_development	ENSGALG00010001198	IFRD1	0.851982004	0.176183339	4.835769421	1.32632E-06	2.48996E-06	positive
PRJNA729271	stage_3W_vs_E10	ordinary_muscle_development	ENSGALG00010001199	AFG3L2	0.423005109	0.090124295	4.693574676	2.68472E-06	4.94598E-06	positive
PRJNA729271	stage_3W_vs_E10	ordinary_muscle_development	ENSGALG00010001200	CTTNBP2	1.245963422	0.127445309	9.776455721	1.421E-22	6.01207E-22	positive
PRJNA729271	stage_3W_vs_E10	ordinary_muscle_development	ENSGALG00010001201	TUBB6	-1.068753707	0.165514263	-6.457169851	1.06679E-10	2.56914E-10	negative
PRJNA729271	stage_3W_vs_E10	ordinary_muscle_development	ENSGALG00010001202	CFTR	3.669158038	0.246109517	14.90863938	2.89594E-50	3.31831E-49	positive
PRJNA729271	stage_3W_vs_E10	ordinary_muscle_development	ENSGALG00010001203	TMX3	-0.889813656	0.100573874	-8.847363829	8.96109E-19	3.2595E-18	negative
PRJNA729271	stage_3W_vs_E10	ordinary_muscle_development	ENSGALG00010001204	SNX2	0.353086613	0.081379692	4.338755846	1.43292E-05	2.50122E-05	positive
PRJNA729271	stage_3W_vs_E10	ordinary_muscle_development	ENSGALG00010001205	CEP192	-0.322127958	0.104795958	-3.073858625	0.002113095	0.003070301	negative
PRJNA729271	stage_3W_vs_E10	ordinary_muscle_development	ENSGALG00010001206	RSRC1	0.386622299	0.076106015	5.080049148	3.77337E-07	7.34753E-07	positive
PRJNA729271	stage_3W_vs_E10	ordinary_muscle_development	ENSGALG00010001208	MOV10L1	-1.520129593	0.685066566	-2.218951661	0.026490013	0.034350755	negative
PRJNA729271	stage_3W_vs_E10	ordinary_muscle_development	ENSGALG00010001209	CDH7	-1.862680262	0.248734232	-7.48863656	6.95928E-14	1.99318E-13	negative
PRJNA729271	stage_3W_vs_E10	ordinary_muscle_development	ENSGALG00010001210	PANX2	-5.782722709	0.483520788	-11.9596155	5.78291E-33	3.70899E-32	negative
PRJNA729271	stage_3W_vs_E10	ordinary_muscle_development	ENSGALG00010001213	PPIC	-0.526330284	0.218646344	-2.407221967	0.016074397	0.021362279	negative
PRJNA729271	stage_3W_vs_E10	ordinary_muscle_development	ENSGALG00010001214	TES	-2.241120158	0.173290358	-12.93274587	2.94124E-38	2.23914E-37	negative
PRJNA729271	stage_3W_vs_E10	ordinary_muscle_development	ENSGALG00010001215	PRDM6	-2.724370813	0.685042547	-3.976936655	6.98088E-05	0.000115511	negative
PRJNA729271	stage_3W_vs_E10	ordinary_muscle_development	ENSGALG00010001216	SHOX2	-6.897214947	0.887514687	-7.771381193	7.76349E-15	2.32985E-14	negative
PRJNA729271	stage_3W_vs_E10	ordinary_muscle_development	ENSGALG00010001218	ENSGALG00010001218	-0.64253265	0.11153947	-5.760585494	8.38227E-09	1.81779E-08	negative
PRJNA729271	stage_3W_vs_E10	ordinary_muscle_development	ENSGALG00010001219	TFEC	2.215585977	0.388580811	5.701738011	1.18592E-08	2.54741E-08	positive
PRJNA729271	stage_3W_vs_E10	ordinary_muscle_development	ENSGALG00010001220	PLXNB2	-0.922957683	0.082927652	-11.12967344	8.99645E-29	4.93019E-28	negative
PRJNA729271	stage_3W_vs_E10	ordinary_muscle_development	ENSGALG00010001221	GRAMD2B	-0.622792546	0.155768315	-3.998197877	6.38266E-05	0.000106012	negative
PRJNA729271	stage_3W_vs_E10	ordinary_muscle_development	ENSGALG00010001222	ZNF800	-0.616752544	0.10150164	-6.076281567	1.23001E-09	2.78884E-09	negative
PRJNA729271	stage_3W_vs_E10	ordinary_muscle_development	ENSGALG00010001223	GRM8	-3.554906751	0.622788374	-5.708049311	1.14278E-08	2.45803E-08	negative
PRJNA729271	stage_3W_vs_E10	ordinary_muscle_development	ENSGALG00010001224	DNAJB9	1.918575132	0.188408449	10.18306312	2.36013E-24	1.07579E-23	positive
PRJNA729271	stage_3W_vs_E10	ordinary_muscle_development	ENSGALG00010001225	SOCS6	0.207891919	0.097365066	2.135179775	0.032746328	0.04202001	positive
PRJNA729271	stage_3W_vs_E10	ordinary_muscle_development	ENSGALG00010001226	VEPH1	-2.159875088	0.60419042	-3.574825116	0.000350462	0.000547231	negative
PRJNA729271	stage_3W_vs_E10	ordinary_muscle_development	ENSGALG00010001227	ENSGALG00010001227	-3.494974657	0.983958181	-3.551954466	0.000382381	0.00059502	negative
PRJNA729271	stage_3W_vs_E10	ordinary_muscle_development	ENSGALG00010001228	THAP5	0.006827378	0.110158405	0.061977821	0.950580494	0.956984599	positive
PRJNA729271	stage_3W_vs_E10	ordinary_muscle_development	ENSGALG00010001229	ZNF407	0.335128979	0.10314891	3.248982257	0.001158187	0.001726264	positive
PRJNA729271	stage_3W_vs_E10	ordinary_muscle_development	ENSGALG00010001230	PTX3	-3.084416043	0.584266367	-5.279126466	1.29801E-07	2.60627E-07	negative
PRJNA729271	stage_3W_vs_E10	ordinary_muscle_development	ENSGALG00010001231	CNDP1	1.66679071	0.508710594	3.27650088	0.00105102	0.001571705	positive
PRJNA729271	stage_3W_vs_E10	ordinary_muscle_development	ENSGALG00010001232	TSPAN12	-0.077180289	0.103083169	-0.748718633	0.454026811	0.492687414	negative
PRJNA729271	stage_3W_vs_E10	ordinary_muscle_development	ENSGALG00010001233	CNDP2	-0.444415212	0.096574957	-4.601764551	4.18927E-06	7.60945E-06	negative
PRJNA729271	stage_3W_vs_E10	ordinary_muscle_development	ENSGALG00010001234	AASS	1.489166004	0.098491955	15.11967145	1.20141E-51	1.43802E-50	positive
PRJNA729271	stage_3W_vs_E10	ordinary_muscle_development	ENSGALG00010001235	CCDC102B	-2.090274153	0.32211575	-6.489201942	8.62922E-11	2.0913E-10	negative
PRJNA729271	stage_3W_vs_E10	ordinary_muscle_development	ENSGALG00010001237	MELK	-3.81204928	0.282848783	-13.47734023	2.12635E-41	1.79194E-40	negative
PRJNA729271	stage_3W_vs_E10	ordinary_muscle_development	ENSGALG00010001238	GOLIM4	-0.789801042	0.122422458	-6.4514392	1.10793E-10	2.66502E-10	negative
PRJNA729271	stage_3W_vs_E10	ordinary_muscle_development	ENSGALG00010001239	ENSGALG00010001239	0.465499666	0.246584961	1.887786117	0.05905467	0.073469869	positive
PRJNA729271	stage_3W_vs_E10	ordinary_muscle_development	ENSGALG00010001242	ENSGALG00010001242	-2.126614584	0.656075277	-3.241418565	0.001189364	0.001770003	negative
PRJNA729271	stage_3W_vs_E10	ordinary_muscle_development	ENSGALG00010001244	ENSGALG00010001244	0.05892038	0.159093205	0.370351331	0.711120732	0.739963748	positive
PRJNA729271	stage_3W_vs_E10	ordinary_muscle_development	ENSGALG00010001245	SERPINI1	-0.775268847	0.224372626	-3.455273754	0.000549734	0.00084286	negative
PRJNA729271	stage_3W_vs_E10	ordinary_muscle_development	ENSGALG00010001248	PTPRZ1	-2.052140911	0.25640896	-8.003390012	1.2104E-15	3.77631E-15	negative
PRJNA729271	stage_3W_vs_E10	ordinary_muscle_development	ENSGALG00010001249	PDCD10	0.718660843	0.138703253	5.181283257	2.20365E-07	4.35769E-07	positive
PRJNA729271	stage_3W_vs_E10	ordinary_muscle_development	ENSGALG00010001250	ENSGALG00010001250	-3.447510554	0.746662441	-4.617227767	3.889E-06	7.08892E-06	negative
PRJNA729271	stage_3W_vs_E10	ordinary_muscle_development	ENSGALG00010001251	ZNF277	-0.516771783	0.124434813	-4.152951822	3.28214E-05	5.56233E-05	negative
PRJNA729271	stage_3W_vs_E10	ordinary_muscle_development	ENSGALG00010001252	SNCAIP	-2.23520406	0.124190481	-17.9981915	2.01285E-72	4.17998E-71	negative
PRJNA729271	stage_3W_vs_E10	ordinary_muscle_development	ENSGALG00010001253	ENSGALG00010001253	-1.44332074	0.860792711	-1.676734389	0.093594433	0.113612872	negative
PRJNA729271	stage_3W_vs_E10	ordinary_muscle_development	ENSGALG00010001254	NETO1	-2.600503519	0.431908546	-6.020958704	1.73387E-09	3.89469E-09	negative
PRJNA729271	stage_3W_vs_E10	ordinary_muscle_development	ENSGALG00010001258	ENSGALG00010001258	-1.214854271	0.130576347	-9.30378506	1.35533E-20	5.30386E-20	negative
PRJNA729271	stage_3W_vs_E10	ordinary_muscle_development	ENSGALG00010001259	ENSGALG00010001259	0.444752341	0.171978742	2.586089053	0.009707185	0.013213038	positive
PRJNA729271	stage_3W_vs_E10	ordinary_muscle_development	ENSGALG00010001260	ENSGALG00010001260	-5.117243671	1.10682976	-4.62333401	3.77621E-06	6.89372E-06	negative
PRJNA729271	stage_3W_vs_E10	ordinary_muscle_development	ENSGALG00010001262	ENSGALG00010001262	0.306296673	0.145138359	2.110377128	0.034825885	0.044581931	positive
PRJNA729271	stage_3W_vs_E10	ordinary_muscle_development	ENSGALG00010001264	FAM3C	0.931785699	0.112860404	8.256090407	1.50522E-16	4.93846E-16	positive
PRJNA729271	stage_3W_vs_E10	ordinary_muscle_development	ENSGALG00010001265	OPN1LW	2.179327703	0.572840746	3.80442159	0.000142136	0.000229351	positive
PRJNA729271	stage_3W_vs_E10	ordinary_muscle_development	ENSGALG00010001267	BCHE	-0.572138674	0.250795277	-2.281297641	0.02253084	0.029491108	negative
PRJNA729271	stage_3W_vs_E10	ordinary_muscle_development	ENSGALG00010001268	CBLN2	-3.436659687	0.430171855	-7.989038916	1.35995E-15	4.22923E-15	negative
PRJNA729271	stage_3W_vs_E10	ordinary_muscle_development	ENSGALG00010001270	DRAP1	0.207009519	0.110207395	1.87836324	0.060331491	0.074993993	positive
PRJNA729271	stage_3W_vs_E10	ordinary_muscle_development	ENSGALG00010001271	ENSGALG00010001271	0.257653541	0.186002949	1.385212132	0.165987612	0.194612184	positive
PRJNA729271	stage_3W_vs_E10	ordinary_muscle_development	ENSGALG00010001273	ENSGALG00010001273	-0.653713286	0.568028431	-1.150846068	0.249795569	0.285011553	negative
PRJNA729271	stage_3W_vs_E10	ordinary_muscle_development	ENSGALG00010001274	DLGAP1	-2.088744956	0.462467618	-4.516521534	6.28637E-06	1.12801E-05	negative
PRJNA729271	stage_3W_vs_E10	ordinary_muscle_development	ENSGALG00010001276	ENSGALG00010001276	-3.008633903	1.196945224	-2.51359364			negative
PRJNA729271	stage_3W_vs_E10	ordinary_muscle_development	ENSGALG00010001277	ENSGALG00010001277	0.930725169	0.161589459	5.75981362	8.42069E-09	1.82557E-08	positive
PRJNA729271	stage_3W_vs_E10	ordinary_muscle_development	ENSGALG00010001279	LPIN2	-0.699522229	0.137602086	-5.083660047	3.7023E-07	7.21496E-07	negative
PRJNA729271	stage_3W_vs_E10	ordinary_muscle_development	ENSGALG00010001281	LRRN3	-0.227704301	0.146570709	-1.553545743	0.12029281	0.143805714	negative
PRJNA729271	stage_3W_vs_E10	ordinary_muscle_development	ENSGALG00010001283	ENSGALG00010001283	1.075824066	0.177872842	6.048276125	1.46404E-09	3.30189E-09	positive
PRJNA729271	stage_3W_vs_E10	ordinary_muscle_development	ENSGALG00010001285	ENSGALG00010001285	1.413418282	0.215828907	6.548790428	5.8005E-11	1.41832E-10	positive
PRJNA729271	stage_3W_vs_E10	ordinary_muscle_development	ENSGALG00010001286	SRFBP1	-0.745739691	0.112547824	-6.625980565	3.44951E-11	8.57055E-11	negative
PRJNA729271	stage_3W_vs_E10	ordinary_muscle_development	ENSGALG00010001287	PLPP4	-4.727169741	0.431523822	-10.9545974	6.31586E-28	3.35465E-27	negative
PRJNA729271	stage_3W_vs_E10	ordinary_muscle_development	ENSGALG00010001288	TGIF1	-0.60191435	0.155633465	-3.867512365	0.000109951	0.000179052	negative

PRJNA729271	stage_3W_vs_E10	ordinary_muscle_development	ENSGALG00010001289	ENSGALG00010001289	1.621455972	0.344730812	4.703542346	2.55686E-06	4.71823E-06	positive
PRJNA729271	stage_3W_vs_E10	ordinary_muscle_development	ENSGALG00010001291	ENSGALG00010001291	1.342494888	0.142734545	9.405535877	5.17657E-21	2.06195E-20	positive
PRJNA729271	stage_3W_vs_E10	ordinary_muscle_development	ENSGALG00010001292	LOX	0.5622578	0.142208049	3.95376918	7.69296E-05	0.000126773	positive
PRJNA729271	stage_3W_vs_E10	ordinary_muscle_development	ENSGALG00010001295	ENSGALG00010001295	0.790207715	0.12746732	6.199296543	5.67161E-10	1.3141E-09	positive
PRJNA729271	stage_3W_vs_E10	ordinary_muscle_development	ENSGALG00010001297	MYL12A	0.179990303	0.22281631	0.807796803	0.419207566	0.457699131	positive
PRJNA729271	stage_3W_vs_E10	ordinary_muscle_development	ENSGALG00010001298	DOCK4	0.67127156	0.131652334	5.098820049	3.41777E-07	6.67034E-07	positive
PRJNA729271	stage_3W_vs_E10	ordinary_muscle_development	ENSGALG00010001300	MRPL49	0.356359213	0.180782592	1.971203138	0.048700645	0.061229582	positive
PRJNA729271	stage_3W_vs_E10	ordinary_muscle_development	ENSGALG00010001301	GMPS	-1.125960302	0.077241683	-14.57710737	3.92811E-48	4.21451E-47	negative
PRJNA729271	stage_3W_vs_E10	ordinary_muscle_development	ENSGALG00010001302	ENSGALG00010001302	-2.750367443	0.511241056	-5.379785936	7.45745E-08	1.52308E-07	negative
PRJNA729271	stage_3W_vs_E10	ordinary_muscle_development	ENSGALG00010001304	SYVN1	0.126719205	0.177457171	0.714083316	0.475175662	0.513890265	positive
PRJNA729271	stage_3W_vs_E10	ordinary_muscle_development	ENSGALG00010001305	WNT16	-0.739003287	0.392508767	-1.88276887	0.059731698	0.074286654	negative
PRJNA729271	stage_3W_vs_E10	ordinary_muscle_development	ENSGALG00010001306	CEP120	-0.136433938	0.08544054	-1.596829067	0.110303815	0.132509017	negative
PRJNA729271	stage_3W_vs_E10	ordinary_muscle_development	ENSGALG00010001307	CALU	-1.332354982	0.157222861	-8.474308194	2.36481E-17	8.0559E-17	negative
PRJNA729271	stage_3W_vs_E10	ordinary_muscle_development	ENSGALG00010001308	MGMT	-0.139479294	0.1392704	-1.001499911	0.316585183	0.354519641	negative
PRJNA729271	stage_3W_vs_E10	ordinary_muscle_development	ENSGALG00010001313	EMILIN2	1.369764923	0.176337254	7.767870324	7.98167E-15	2.39335E-14	positive
PRJNA729271	stage_3W_vs_E10	ordinary_muscle_development	ENSGALG00010001320	CPED1	-0.002116086	0.197341417	-0.010722967	0.991444474	0.99274074	negative
PRJNA729271	stage_3W_vs_E10	ordinary_muscle_development	ENSGALG00010001330	ENSGALG00010001330	0.953340929	0.142909335	6.670949298	2.54154E-11	6.35933E-11	positive
PRJNA729271	stage_3W_vs_E10	ordinary_muscle_development	ENSGALG00010001333	PLCH1	-1.564021417	0.301588692	-5.185941845	2.14926E-07	4.25188E-07	negative
PRJNA729271	stage_3W_vs_E10	ordinary_muscle_development	ENSGALG00010001334	EBF3	-1.343616285	0.159524104	-8.422653724	3.68051E-17	1.24241E-16	negative
PRJNA729271	stage_3W_vs_E10	ordinary_muscle_development	ENSGALG00010001343	ENSGALG00010001343	-0.468024487	0.305715693	-1.530914172	0.125790606	0.149994639	negative
PRJNA729271	stage_3W_vs_E10	ordinary_muscle_development	ENSGALG00010001347	MYOM1	2.911155181	0.164789391	17.66591385	7.67596E-70	1.47956E-68	positive
PRJNA729271	stage_3W_vs_E10	ordinary_muscle_development	ENSGALG00010001349	ING3	0.904259298	0.104273818	8.67196883	4.24712E-18	1.50666E-17	positive
PRJNA729271	stage_3W_vs_E10	ordinary_muscle_development	ENSGALG00010001351	CAPN1	0.702417289	0.156651962	4.483935468	7.32788E-06	1.30665E-05	positive
PRJNA729271	stage_3W_vs_E10	ordinary_muscle_development	ENSGALG00010001353	GLRX3	0.314415382	0.078566477	4.001902521	6.28352E-05	0.000104425	positive
PRJNA729271	stage_3W_vs_E10	ordinary_muscle_development	ENSGALG00010001358	TCERG1L	-2.846696438	0.536922225	-5.301878573	1.14617E-07	2.31196E-07	negative
PRJNA729271	stage_3W_vs_E10	ordinary_muscle_development	ENSGALG00010001363	HIBADH	1.860790565	0.210132667	8.855313118	8.34492E-19	3.03995E-18	positive
PRJNA729271	stage_3W_vs_E10	ordinary_muscle_development	ENSGALG00010001364	SMCHD1	0.348721159	0.184023317	1.894983552	0.058094581	0.072337509	positive
PRJNA729271	stage_3W_vs_E10	ordinary_muscle_development	ENSGALG00010001365	ENSGALG00010001365	1.065304191	0.278038	3.83150573	0.000127361	0.000206361	positive
PRJNA729271	stage_3W_vs_E10	ordinary_muscle_development	ENSGALG00010001367	ENSGALG00010001367	0.408172471	0.432315096	0.944155027	0.345090433	0.383595539	positive
PRJNA729271	stage_3W_vs_E10	ordinary_muscle_development	ENSGALG00010001368	WIPF3	1.072394442	0.140869522	7.612678938	2.68472E-14	7.84103E-14	positive
PRJNA729271	stage_3W_vs_E10	ordinary_muscle_development	ENSGALG00010001369	PPP2R2D	0.303867288	0.123916732	2.452189326	0.014198993	0.018979565	positive
PRJNA729271	stage_3W_vs_E10	ordinary_muscle_development	ENSGALG00010001372	ENSGALG00010001372	0.443786144	0.304813007	1.455929155	0.145412205	0.171934898	positive
PRJNA729271	stage_3W_vs_E10	ordinary_muscle_development	ENSGALG00010001373	IMPA2	1.482953481	0.13962148	10.62124163	2.37382E-26	1.17878E-25	positive
PRJNA729271	stage_3W_vs_E10	ordinary_muscle_development	ENSGALG00010001378	POLA2	-2.263024838	0.13837641	-16.35412305	4.06518E-60	6.08728E-59	negative
PRJNA729271	stage_3W_vs_E10	ordinary_muscle_development	ENSGALG00010001380	SCRN1	-3.763844723	0.176918741	-21.27442637	1.95895E-100	8.2784E-99	negative
PRJNA729271	stage_3W_vs_E10	ordinary_muscle_development	ENSGALG00010001381	MDFIC	-0.358625974	0.130615289	-2.745666122	0.006038819	0.008393199	negative
PRJNA729271	stage_3W_vs_E10	ordinary_muscle_development	ENSGALG00010001382	BNIP3	2.452800559	0.192867504	12.71754189	4.72491E-37	3.44332E-36	positive
PRJNA729271	stage_3W_vs_E10	ordinary_muscle_development	ENSGALG00010001385	MME	2.33905821	0.253036984	9.243938063	2.37588E-20	9.21306E-20	positive
PRJNA729271	stage_3W_vs_E10	ordinary_muscle_development	ENSGALG00010001386	FKBP14	-1.78040005	0.185648258	-9.590179136	8.79322E-22	3.61685E-21	negative
PRJNA729271	stage_3W_vs_E10	ordinary_muscle_development	ENSGALG00010001388	MPPE1	0.473059295	0.165549228	2.857514358	0.004269733	0.00602803	positive
PRJNA729271	stage_3W_vs_E10	ordinary_muscle_development	ENSGALG00010001395	GNAL	-0.062419359	0.106497344	-0.586111887	0.557800329	0.594945972	negative
PRJNA729271	stage_3W_vs_E10	ordinary_muscle_development	ENSGALG00010001397	KLHL7	-0.069947542	0.063554858	-1.100585287	0.271077192	0.307670808	negative
PRJNA729271	stage_3W_vs_E10	ordinary_muscle_development	ENSGALG00010001398	WDR11	-0.426023623	0.096904345	-4.396331481	1.10096E-05	1.93811E-05	negative
PRJNA729271	stage_3W_vs_E10	ordinary_muscle_development	ENSGALG00010001400	SNX24	1.752535078	0.158796734	11.03634207	2.55212E-28	1.37931E-27	positive
PRJNA729271	stage_3W_vs_E10	ordinary_muscle_development	ENSGALG00010001402	NUP42	-0.010768398	0.131901543	-0.081639666	0.934933258	0.942606773	negative
PRJNA729271	stage_3W_vs_E10	ordinary_muscle_development	ENSGALG00010001406	EML3	0.611304862	0.160270443	3.814208349	0.00013662	0.000220893	positive
PRJNA729271	stage_3W_vs_E10	ordinary_muscle_development	ENSGALG00010001407	FOXP2	-2.162741341	0.153850169	-14.05745184	6.93382E-45	6.5992E-44	negative
PRJNA729271	stage_3W_vs_E10	ordinary_muscle_development	ENSGALG00010001409	GPNMB	-3.408995589	0.984062559	-3.464206171	0.000531799	0.00081709	negative
PRJNA729271	stage_3W_vs_E10	ordinary_muscle_development	ENSGALG00010001411	FGFR2	-5.769147285	0.374598508	-15.40088165	1.61453E-53	2.05106E-52	negative
PRJNA729271	stage_3W_vs_E10	ordinary_muscle_development	ENSGALG00010001412	ENSGALG00010001412	-3.503118138	0.3005194	-11.65687853	2.1166E-31	1.27887E-30	negative
PRJNA729271	stage_3W_vs_E10	ordinary_muscle_development	ENSGALG00010001414	CCDC126	1.448442005	0.170983266	8.471250066	2.42773E-17	8.26833E-17	positive
PRJNA729271	stage_3W_vs_E10	ordinary_muscle_development	ENSGALG00010001415	MC5R	-0.031938213	0.266298083	-0.119934071	0.904535375	0.915717297	negative
PRJNA729271	stage_3W_vs_E10	ordinary_muscle_development	ENSGALG00010001416	AP4M1	0.223389251	0.093007561	2.401839686	0.016312856	0.021665278	positive
PRJNA729271	stage_3W_vs_E10	ordinary_muscle_development	ENSGALG00010001418	RNMT	-1.080669083	0.067429549	-16.02663972	8.32652E-58	1.17864E-56	negative
PRJNA729271	stage_3W_vs_E10	ordinary_muscle_development	ENSGALG00010001419	KCND2	-4.817307094	0.403856372	-11.92826813	8.43093E-33	5.38117E-32	negative
PRJNA729271	stage_3W_vs_E10	ordinary_muscle_development	ENSGALG00010001420	FAM221A	-5.307281443	0.803926566	-6.601699297	4.06471E-11	1.00508E-10	negative
PRJNA729271	stage_3W_vs_E10	ordinary_muscle_development	ENSGALG00010001422	AP1S1	-1.729158125	0.142410606	-12.14206006	6.32142E-34	4.1878E-33	negative
PRJNA729271	stage_3W_vs_E10	ordinary_muscle_development	ENSGALG00010001423	FAM210A	1.494138975	0.136397794	10.95427521	6.33837E-28	3.36414E-27	positive
PRJNA729271	stage_3W_vs_E10	ordinary_muscle_development	ENSGALG00010001426	ATE1	0.425836315	0.090907857	4.684263047	2.80969E-06	5.16833E-06	positive
PRJNA729271	stage_3W_vs_E10	ordinary_muscle_development	ENSGALG00010001427	ASB11	8.79898233	0.462741402	19.01490184	1.28377E-80	3.28187E-79	positive
PRJNA729271	stage_3W_vs_E10	ordinary_muscle_development	ENSGALG00010001432	NSMCE4A	0.533619485	0.112713143	4.734314642	2.19797E-06	4.07046E-06	positive
PRJNA729271	stage_3W_vs_E10	ordinary_muscle_development	ENSGALG00010001436	ENSGALG00010001436	-1.389598546	1.345141072	-1.033050418	0.301580305	0.339184243	negative
PRJNA729271	stage_3W_vs_E10	ordinary_muscle_development	ENSGALG00010001440	CDCA7L	-1.603412157	0.150782644	-10.63393051	2.07192E-26	1.03169E-25	negative
PRJNA729271	stage_3W_vs_E10	ordinary_muscle_development	ENSGALG00010001441	HMGCLL1	-0.850716858	0.301626411	-2.820432252	0.0047959	0.006738714	negative
PRJNA729271	stage_3W_vs_E10	ordinary_muscle_development	ENSGALG00010001442	LDLRAD4	0.501458953	0.113947418	4.400792598	1.07856E-05	1.90053E-05	positive
PRJNA729271	stage_3W_vs_E10	ordinary_muscle_development	ENSGALG00010001443	ANKRD26	0.015817741	0.144997913	0.109089438	0.913131552	0.923323463	positive
PRJNA729271	stage_3W_vs_E10	ordinary_muscle_development	ENSGALG00010001448	GPR37	-5.54517967	0.365937364	-15.15335744	7.19929E-52	8.65287E-51	negative
PRJNA729271	stage_3W_vs_E10	ordinary_muscle_development	ENSGALG00010001450	GPR149	0.628206329	0.405046594	1.550948305	0.120914073	0.144500741	positive
PRJNA729271	stage_3W_vs_E10	ordinary_muscle_development	ENSGALG00010001453	TAX1BP1	1.073123353	0.073424228	14.61538489	2.24084E-48	2.42758E-47	positive
PRJNA729271	stage_3W_vs_E10	ordinary_muscle_development	ENSGALG00010001454	VEGFD	2.788474323	0.164096041	16.99294088	9.26263E-65	1.57584E-63	positive
PRJNA729271	stage_3W_vs_E10	ordinary_muscle_development	ENSGALG00010001456	LSM8	-0.944255298	0.11512466	-8.202024654	2.36372E-16	7.66661E-16	negative
PRJNA729271	stage_3W_vs_E10	ordinary_muscle_development	ENSGALG00010001457	NDC80	-3.75906419	0.230898824	-16.28013572	1.36566E-59	2.02198E-58	negative
PRJNA729271	stage_3W_vs_E10	ordinary_muscle_development	ENSGALG00010001458	JAZF1	-1.23403387	0.160539865	-7.686775315	1.5089E-14	4.45902E-14	negative
PRJNA729271	stage_3W_vs_E10	ordinary_muscle_development	ENSGALG00010001460	MALSU1	0.182773404	0.106198283	1.721057999	0.0852403	0.104020723	positive
PRJNA729271	stage_3W_vs_E10	ordinary_muscle_development	ENSGALG00010001461	ASB9	-2.977044554	0.232229991	-12.81938022	1.27717E-37	9.46453E-37	negative
PRJNA729271	stage_3W_vs_E10	ordinary_muscle_development	ENSGALG00010001462	DHX36	-0.630273142	0.109203577	-5.771542996	7.85489E-09	1.70495E-08	negative
PRJNA729271	stage_3W_vs_E10	ordinary_muscle_development	ENSGALG00010001463	DSEL	-0.168296053	0.158593171	-1.061180957	0.288607672	0.325807493	negative
PRJNA729271	stage_3W_vs_E10	ordinary_muscle_development	ENSGALG00010001465	TACC2	1.639135519	0.157286881	10.4213111	1.98202E-25	9.43183E-25	positive
PRJNA729271	stage_3W_vs_E10	ordinary_muscle_development	ENSGALG00010001466	PRKX	-0.000614836	0.130239108	-0.004720825	0.996233341	0.996714679	negative
PRJNA729271	stage_3W_vs_E10	ordinary_muscle_development	ENSGALG00010001468	ANOS1	-0.742295344	0.153060975	-4.849670816	1.23667E-06	2.32798E-06	negative
PRJNA729271	stage_3W_vs_E10	ordinary_muscle_development	ENSGALG00010001469	ARHGEF26	-0.429475121	0.128360801	-3.345843262	0.000820328	0.001237835	negative
PRJNA729271	stage_3W_vs_E10	ordinary_muscle_development	ENSGALG00010001470	GPR85	-3.42433182	0.338718066	-10.10968165	5.00466E-24	2.24521E-23	negative
PRJNA729271	stage_3W_vs_E10	ordinary_muscle_development	ENSGALG00010001471	BMP5	-2.35180859	0.370626458	-6.345495689	2.2171E-10	5.25973E-10	negative
PRJNA729271	stage_3W_vs_E10	ordinary_muscle_development	ENSGALG00010001472	ENSGALG00010001472	0.953970966	0.182309274	5.232706741	1.67046E-07	3.32691E-07	positive
PRJNA729271	stage_3W_vs_E10	ordinary_muscle_development	ENSGALG00010001475	BMT2	-0.97862555	0.138034899	-7.089696558	1.34406E-12	3.60115E-12	negative
PRJNA729271	stage_3W_vs_E10	ordinary_muscle_development	ENSGALG00010001476	IGF2BP3	-1.972892995	0.194074237	-10.16566149	2.822E-24	1.28148E-23	negative
PRJNA729271	stage_3W_vs_E10	ordinary_muscle_development	ENSGALG00010001477	NLGN4	-1.716735374	0.231650836	-7.410874932	1.25469E-13	3.54185E-13	negative
PRJNA729271	stage_3W_vs_E10	ordinary_muscle_development	ENSGALG00010001479	TMEM168	-0.026139125	0.154985321	-0.168655489	0.866067632	0.881576567	negative
PRJNA729271	stage_3W_vs_E10	ordinary_muscle_development	ENSGALG00010001480	JAKMIP3	-1.693119952	0.321149068	-5.272068703	1.34895E-07	2.70405E-07	negative
PRJNA729271	stage_3W_vs_E10	ordinary_muscle_development	ENSGALG00010001482	TRA2A	-0.936192282	0.065962412	-14.19281448	1.01502E-45	9.93429E-45	negative
PRJNA729271	stage_3W_vs_E10	ordinary_muscle_development	ENSGALG00010001483	ENSGALG00010001483	-2.404399322	0.33447599	-7.188555805	6.54802E-13	1.78108E-12	negative
PRJNA729271	stage_3W_vs_E10	ordinary_muscle_development	ENSGALG00010001486	WNT2	1.750434257	0.262054666	6.679653072	2.39509E-11	6.00324E-11	positive
PRJNA729271	stage_3W_vs_E10	ordinary_muscle_development	ENSGALG00010001487	CDH19	-0.21138742	0.2354987	-0.897616082	0.369390262	0.408071934	negative
PRJNA729271	stage_3W_vs_E10	ordinary_muscle_development	ENSGALG00010001488	GRPR	-2.916259032	0.406984138	-7.165534874	7.74834E-13	2.10244E-12	negative
PRJNA729271	stage_3W_vs_E10	ordinary_muscle_development	ENSGALG00010001491	PHF3	0.299594492	0.08825663	3.3945834	0.000687331	0.001044763	positive
PRJNA729271	stage_3W_vs_E10	ordinary_muscle_development	ENSGALG00010001492	ENSGALG00010001492	2.383152875	0.275587353	8.647540776	5.26211E-18	1.85627E-17	positive
PRJNA729271	stage_3W_vs_E10	ordinary_muscle_development	ENSGALG00010001495	CCNL1	1.119774863	0.107800482	10.38747545	2.82738E-25	1.33843E-24	positive
PRJNA729271	stage_3W_vs_E10	ordinary_muscle_development	ENSGALG00010001496	PTP4A1	0.928107842	0.190224906	4.879002764	1.06624E-06	2.015E-06	positive
PRJNA729271	stage_3W_vs_E10	ordinary_muscle_development	ENSGALG00010001497	LEKR1	-0.438479795	0.271627884	-1.614266505	0.106469632	0.128275066	negative
PRJNA729271	stage_3W_vs_E10	ordinary_muscle_development	ENSGALG00010001499	TIPARP	1.466008598	0.186465286	7.862099339	3.77748E-15	1.15249E-14	positive
PRJNA729271	stage_3W_vs_E10	ordinary_muscle_development	ENSGALG00010001501	CAPZA2	1.611991476	0.106239496	15.17318453	5.32303E-52	6.42977E-51	positive
PRJNA729271	stage_3W_vs_E10	ordinary_muscle_development	ENSGALG00010001502	LGSN	-0.192093413	1.014971315	-0.189259943	0.849889089	0.866995731	negative
PRJNA729271	stage_3W_vs_E10	ordinary_muscle_development	ENSGALG00010001504	RBMS3	-0.494977297	0.09955458	-4.971918903	6.62934E-07	1.27073E-06	negative
PRJNA729271	stage_3W_vs_E10	ordinary_muscle_development	ENSGALG00010001505	SSR3	-0.229101744	0.096941552	-2.363297662	0.018113115	0.023937782	negative
PRJNA729271	stage_3W_vs_E10	ordinary_muscle_development	ENSGALG00010001506	DPYSL4	-2.567832785	0.287377283	-8.935406299	4.05685E-19	1.50048E-18	negative
PRJNA729271	stage_3W_vs_E10	ordinary_muscle_development	ENSGALG00010001507	METTL4	0.020736967	0.095550825	0.217025514	0.828188461	0.848560949	positive
PRJNA729271	stage_3W_vs_E10	ordinary_muscle_development	ENSGALG00010001508	CD109	-1.619670171	0.291251187	-5.561076637	2.68115E-08	5.62584E-08	negative
PRJNA729271	stage_3W_vs_E10	ordinary_muscle_development	ENSGALG00010001510	AP1S2	0.228977873	0.098018223	2.336074518	0.019487358	0.025662692	positive
PRJNA729271	stage_3W_vs_E10	ordinary_muscle_development	ENSGALG00010001512	PLCL2	2.336928055	0.172882988	13.51739744	1.23467E-41	1.04658E-40	positive
PRJNA729271	stage_3W_vs_E10	ordinary_muscle_development	ENSGALG00010001513	ZRSR2	0.040418	0.093043671	0.434398165	0.66399933	0.696027646	positive
PRJNA729271	stage_3W_vs_E10	ordinary_muscle_development	ENSGALG00010001514	MET	1.168790351	0.151007663	7.739940665	9.94632E-15	2.96528E-14	positive
PRJNA729271	stage_3W_vs_E10	ordinary_muscle_development	ENSGALG00010001516	CLTRN	-1.425812459	0.566324396	-2.517660319	0.011813719	0.015939669	negative
PRJNA729271	stage_3W_vs_E10	ordinary_muscle_development	ENSGALG00010001517	CAV2	2.72766549	0.163919483	16.64027629	3.55972E-62	5.63298E-61	positive
PRJNA729271	stage_3W_vs_E10	ordinary_muscle_development	ENSGALG00010001518	SLC17A5	-0.753420192	0.166107585	-4.535736233	5.74029E-06	1.03271E-05	negative
PRJNA729271	stage_3W_vs_E10	ordinary_muscle_development	ENSGALG00010001520	KCNAB1	-3.235979214	0.2341834	-13.81814091	1.98121E-43	1.7971E-42	negative
PRJNA729271	stage_3W_vs_E10	ordinary_muscle_development	ENSGALG00010001521	TBC1D5	-0.019473684	0.074217985	-0.262384975	0.793024655	0.816166741	negative
PRJNA729271	stage_3W_vs_E10	ordinary_muscle_development	ENSGALG00010001524	ACE2	3.773639984	1.461067343	2.582796749	0.009800304	0.013331025	positive
PRJNA729271	stage_3W_vs_E10	ordinary_muscle_development	ENSGALG00010001525	EEF1A1	-1.29002629	0.176706309	-7.300397469	2.86919E-13	7.94287E-13	negative
PRJNA729271	stage_3W_vs_E10	ordinary_muscle_development	ENSGALG00010001526	PPP1R3A	6.654311694	0.149174919	44.60744312	0	0	positive
PRJNA729271	stage_3W_vs_E10	ordinary_muscle_development	ENSGALG00010001527	STK32C	-0.905908249	0.236236306	-3.834754556	0.00012569	0.000203812	negative
PRJNA729271	stage_3W_vs_E10	ordinary_muscle_development	ENSGALG00010001528	NR1D2	3.127139262	0.180286194	17.34541731	2.13621E-67	3.88512E-66	positive
PRJNA729271	stage_3W_vs_E10	ordinary_muscle_development	ENSGALG00010001531	ENSGALG00010001531	-10.26744423	5.524679758	-1.858468668			negative
PRJNA729271	stage_3W_vs_E10	ordinary_muscle_development	ENSGALG00010001532	ENSGALG00010001532	-0.859542263	0.276893394	-3.104235349	0.001907715	0.002780578	negative
PRJNA729271	stage_3W_vs_E10	ordinary_muscle_development	ENSGALG00010001533	ENSGALG00010001533	0.784069813	0.148382827	5.284100793	1.26324E-07	2.53891E-07	positive
PRJNA729271	stage_3W_vs_E10	ordinary_muscle_development	ENSGALG00010001534	SLITRK3	-3.512500223	0.393488987	-8.92655281	4.3949E-19	1.62262E-18	negative
PRJNA729271	stage_3W_vs_E10	ordinary_muscle_development	ENSGALG00010001535	RAP2B	-0.835465478	0.105961357	-7.884624173	3.15484E-15	9.64754E-15	negative
PRJNA729271	stage_3W_vs_E10	ordinary_muscle_development	ENSGALG00010001540	TRABD	-0.565805367	0.130340039	-4.340994306	1.41839E-05	2.47676E-05	negative
PRJNA729271	stage_3W_vs_E10	ordinary_muscle_development	ENSGALG00010001542	MTO1	0.810711704	0.127662692	6.350419928	2.14728E-10	5.09742E-10	positive
PRJNA729271	stage_3W_vs_E10	ordinary_muscle_development	ENSGALG00010001545	SELENOO	2.276359801	0.1284363	17.72364828	2.75455E-70	5.35935E-69	positive
PRJNA729271	stage_3W_vs_E10	ordinary_muscle_development	ENSGALG00010001546	CGAS	-1.27346103	0.17069007	-7.460662642	8.60884E-14	2.44965E-13	negative
PRJNA729271	stage_3W_vs_E10	ordinary_muscle_development	ENSGALG00010001547	PWWP2B	1.203768116	0.109233262	11.02016081	3.05513E-28	1.64564E-27	positive

PRJNA729271	stage_3W_vs_E10	ordinary_muscle_development	ENSGALG00010001548	AKAP17A	0.280266484	0.095209434	2.94368396	0.00324331	0.004639015	positive
PRJNA729271	stage_3W_vs_E10	ordinary_muscle_development	ENSGALG00010001549	ENSGALG00010001549	-2.403368075	0.569943212	-4.216855333	2.47733E-05	4.24354E-05	negative
PRJNA729271	stage_3W_vs_E10	ordinary_muscle_development	ENSGALG00010001551	FHIP1B	2.036429334	0.159796276	12.74390987	3.37069E-37	2.46634E-36	positive
PRJNA729271	stage_3W_vs_E10	ordinary_muscle_development	ENSGALG00010001552	TUBGCP6	0.075150422	0.108385038	0.69336527	0.488080351	0.526393206	positive
PRJNA729271	stage_3W_vs_E10	ordinary_muscle_development	ENSGALG00010001553	CNGA4	0.063108674	0.411725073	0.153278676	0.878178516	0.892337721	positive
PRJNA729271	stage_3W_vs_E10	ordinary_muscle_development	ENSGALG00010001554	ENSGALG00010001554	0.619345625	0.182525643	3.393197886	0.000690817	0.001049952	positive
PRJNA729271	stage_3W_vs_E10	ordinary_muscle_development	ENSGALG00010001556	EPB41L3	-1.597898336	0.117816525	-13.56259939	6.67288E-42	5.73006E-41	negative
PRJNA729271	stage_3W_vs_E10	ordinary_muscle_development	ENSGALG00010001557	THRB	-2.410428567	0.339038664	-7.109597891	1.16382E-12	3.12688E-12	negative
PRJNA729271	stage_3W_vs_E10	ordinary_muscle_development	ENSGALG00010001558	CCKBR	-2.858720227	0.769651338	-3.714305537	0.000203762	0.000324351	negative
PRJNA729271	stage_3W_vs_E10	ordinary_muscle_development	ENSGALG00010001559	HDAC10	-1.627019286	0.130073537	-12.50845735	6.7115E-36	4.74089E-35	negative
PRJNA729271	stage_3W_vs_E10	ordinary_muscle_development	ENSGALG00010001567	INPP5A	-0.369757469	0.082915784	-4.459434036	8.21764E-06	1.45974E-05	negative
PRJNA729271	stage_3W_vs_E10	ordinary_muscle_development	ENSGALG00010001570	SMPD1	2.290970147	0.162012986	14.14065752	2.13282E-45	2.06653E-44	positive
PRJNA729271	stage_3W_vs_E10	ordinary_muscle_development	ENSGALG00010001585	ENSGALG00010001585	0.044712063	0.137433007	0.325337148	0.744925918	0.771870841	positive
PRJNA729271	stage_3W_vs_E10	ordinary_muscle_development	ENSGALG00010001586	ZBTB14	0.168650167	0.094811466	1.778795064	0.075273367	0.092425875	positive
PRJNA729271	stage_3W_vs_E10	ordinary_muscle_development	ENSGALG00010001588	ENSGALG00010001588	1.367043328	0.578706281	2.362240351	0.018164861	0.023997417	positive
PRJNA729271	stage_3W_vs_E10	ordinary_muscle_development	ENSGALG00010001595	AKAIN1	1.539196208	0.255723625	6.018983228	1.75516E-09	3.94129E-09	positive
PRJNA729271	stage_3W_vs_E10	ordinary_muscle_development	ENSGALG00010001599	TBLX1	-0.443190715	0.075209309	-5.892764095	3.79788E-09	8.36758E-09	negative
PRJNA729271	stage_3W_vs_E10	ordinary_muscle_development	ENSGALG00010001604	ENSGALG00010001604	5.703769149	0.421153771	13.54319856	8.69202E-42	7.42432E-41	positive
PRJNA729271	stage_3W_vs_E10	ordinary_muscle_development	ENSGALG00010001605	APBB1	-0.291416823	0.113712932	-2.56274127	0.010384941	0.01408531	negative
PRJNA729271	stage_3W_vs_E10	ordinary_muscle_development	ENSGALG00010001606	PIGA	-0.398141149	0.109993061	-3.619693326	0.000294952	0.000463551	negative
PRJNA729271	stage_3W_vs_E10	ordinary_muscle_development	ENSGALG00010001608	ENSGALG00010001608	-0.5042293	0.496618025	-1.015326216	0.309950352	0.347896109	negative
PRJNA729271	stage_3W_vs_E10	ordinary_muscle_development	ENSGALG00010001609	ENSGALG00010001609	1.455207251	0.458426916	3.174349499	0.001501727	0.002214174	positive
PRJNA729271	stage_3W_vs_E10	ordinary_muscle_development	ENSGALG00010001611	MAPK12	-1.389733939	0.163432893	-8.503391923	1.8413E-17	6.31259E-17	negative
PRJNA729271	stage_3W_vs_E10	ordinary_muscle_development	ENSGALG00010001612	HPX	4.663739432	0.927048607	5.030738837	4.88593E-07	9.44288E-07	positive
PRJNA729271	stage_3W_vs_E10	ordinary_muscle_development	ENSGALG00010001616	OPNVA	-1.765839862	0.664698616	-2.656602285	0.00789325	0.010853032	negative
PRJNA729271	stage_3W_vs_E10	ordinary_muscle_development	ENSGALG00010001617	TRIM3	-0.440549057	0.093576274	-4.707914061	2.50265E-06	4.61936E-06	negative
PRJNA729271	stage_3W_vs_E10	ordinary_muscle_development	ENSGALG00010001620	MOSPD2	0.347550636	0.143580284	2.420601399	0.015494857	0.020618558	positive
PRJNA729271	stage_3W_vs_E10	ordinary_muscle_development	ENSGALG00010001621	DLGAP2	-1.943985159	0.276497678	-7.030746782	2.05431E-12	5.45071E-12	negative
PRJNA729271	stage_3W_vs_E10	ordinary_muscle_development	ENSGALG00010001627	ARFIP2	-0.16127908	0.119765491	-1.34662396	0.178101373	0.207661923	negative
PRJNA729271	stage_3W_vs_E10	ordinary_muscle_development	ENSGALG00010001628	MAPK11	-1.726322412	0.209972103	-8.221675117	2.0068E-16	6.52943E-16	negative
PRJNA729271	stage_3W_vs_E10	ordinary_muscle_development	ENSGALG00010001632	FANCB	-1.29446915	0.277751569	-4.660528666	3.15398E-06	5.78549E-06	negative
PRJNA729271	stage_3W_vs_E10	ordinary_muscle_development	ENSGALG00010001633	B3GAT2	-2.147911163	0.382871004	-5.610012614	2.02312E-08	4.28416E-08	negative
PRJNA729271	stage_3W_vs_E10	ordinary_muscle_development	ENSGALG00010001635	TIMM10B	0.111256484	0.149507542	0.744152987	0.456783926	0.495256415	positive
PRJNA729271	stage_3W_vs_E10	ordinary_muscle_development	ENSGALG00010001639	ENSGALG00010001639	-0.033659347	0.158885123	-0.211847066	0.832226349	0.851734867	negative
PRJNA729271	stage_3W_vs_E10	ordinary_muscle_development	ENSGALG00010001642	ENSGALG00010001642	1.203366263	0.120486043	9.987598815	1.72715E-23	7.57718E-23	positive
PRJNA729271	stage_3W_vs_E10	ordinary_muscle_development	ENSGALG00010001647	ARL8BL	-0.693949283	0.154672509	-4.48657157	7.23784E-06	1.29139E-05	negative
PRJNA729271	stage_3W_vs_E10	ordinary_muscle_development	ENSGALG00010001649	UBE2E2	-0.775320809	0.230659998	-3.361314552	0.000775724	0.001173341	negative
PRJNA729271	stage_3W_vs_E10	ordinary_muscle_development	ENSGALG00010001654	RPS11	0.72148927	0.169384214	4.259483522	2.049E-05	3.53658E-05	positive
PRJNA729271	stage_3W_vs_E10	ordinary_muscle_development	ENSGALG00010001657	SMAP1	-1.044195721	0.098377899	-10.61412909	2.56172E-26	1.26817E-25	negative
PRJNA729271	stage_3W_vs_E10	ordinary_muscle_development	ENSGALG00010001661	TCTN3	-0.478430955	0.105103031	-4.552018643	5.31336E-06	9.57687E-06	negative
PRJNA729271	stage_3W_vs_E10	ordinary_muscle_development	ENSGALG00010001664	SPAM1	0.907468118	2.0670095	0.439024648	0.660643677	0.692956436	positive
PRJNA729271	stage_3W_vs_E10	ordinary_muscle_development	ENSGALG00010001666	PIEZO2	-3.885222269	0.184796749	-21.02429987	3.93152E-98	1.54437E-96	negative
PRJNA729271	stage_3W_vs_E10	ordinary_muscle_development	ENSGALG00010001668	UBE2E1	0.060477515	0.085293748	0.709049802	0.478293572	0.516915101	positive
PRJNA729271	stage_3W_vs_E10	ordinary_muscle_development	ENSGALG00010001670	SHOX	-1.392795174	0.163490405	-8.519124876	1.60763E-17	5.5272E-17	negative
PRJNA729271	stage_3W_vs_E10	ordinary_muscle_development	ENSGALG00010001672	ENSGALG00010001672	2.293634554	0.543607141	4.219287021	2.45076E-05	4.19903E-05	positive
PRJNA729271	stage_3W_vs_E10	ordinary_muscle_development	ENSGALG00010001674	ENSGALG00010001674	-0.000458546	0.105552378	-0.004344247	0.996533804	0.996946475	negative
PRJNA729271	stage_3W_vs_E10	ordinary_muscle_development	ENSGALG00010001677	SDHAF4	0.717433911	0.106149418	6.758717356	1.39219E-11	3.5304E-11	positive
PRJNA729271	stage_3W_vs_E10	ordinary_muscle_development	ENSGALG00010001678	SYAP1	0.746324678	0.058290823	12.80346784	1.5679E-37	1.15834E-36	positive
PRJNA729271	stage_3W_vs_E10	ordinary_muscle_development	ENSGALG00010001681	NAPG	0.232288101	0.091596421	2.535995379	0.011212821	0.015167026	positive
PRJNA729271	stage_3W_vs_E10	ordinary_muscle_development	ENSGALG00010001686	WASL	0.159599162	0.086222165	1.851022437	0.064166325	0.079494947	positive
PRJNA729271	stage_3W_vs_E10	ordinary_muscle_development	ENSGALG00010001688	ENSGALG00010001688	-0.795300201	0.166767892	-4.768904799	1.8523E-06	3.4444E-06	negative
PRJNA729271	stage_3W_vs_E10	ordinary_muscle_development	ENSGALG00010001689	ILK	0.386805357	0.067093993	5.765126453	8.15966E-09	1.77057E-08	positive
PRJNA729271	stage_3W_vs_E10	ordinary_muscle_development	ENSGALG00010001691	CTPS2	0.919801838	0.133849819	6.8718945	6.33548E-12	1.63229E-11	positive
PRJNA729271	stage_3W_vs_E10	ordinary_muscle_development	ENSGALG00010001692	ALDH18A1	-1.342876354	0.255422546	-5.257469928	1.46051E-07	2.9192E-07	negative
PRJNA729271	stage_3W_vs_E10	ordinary_muscle_development	ENSGALG00010001693	NKIRAS1	1.306700124	0.194924113	6.703635097	2.03298E-11	5.11331E-11	positive
PRJNA729271	stage_3W_vs_E10	ordinary_muscle_development	ENSGALG00010001694	ENSGALG00010001694	-0.489106133	0.217761288	-2.246065573	0.024699808	0.032161671	negative
PRJNA729271	stage_3W_vs_E10	ordinary_muscle_development	ENSGALG00010001695	LMOD2	0.492197644	0.471158206	1.04465472	0.296182579	0.333527539	positive
PRJNA729271	stage_3W_vs_E10	ordinary_muscle_development	ENSGALG00010001696	FAM135A	0.514155586	0.090420514	5.686271431	1.29843E-08	2.78331E-08	positive
PRJNA729271	stage_3W_vs_E10	ordinary_muscle_development	ENSGALG00010001697	TAF10	-0.017011871	0.117501278	-0.144780308	0.88488433	0.898332986	negative
PRJNA729271	stage_3W_vs_E10	ordinary_muscle_development	ENSGALG00010001698	RPL15	0.405751139	0.176434872	2.299721891	0.021463981	0.028155693	positive
PRJNA729271	stage_3W_vs_E10	ordinary_muscle_development	ENSGALG00010001699	ASB15	8.298588753	0.249343311	33.28177816	7.0858E-243	1.8674E-240	positive
PRJNA729271	stage_3W_vs_E10	ordinary_muscle_development	ENSGALG00010001700	ENSGALG00010001700	1.551749344	0.200401108	7.743217407	9.69321E-15	2.8922E-14	positive
PRJNA729271	stage_3W_vs_E10	ordinary_muscle_development	ENSGALG00010001701	NDUFA5	2.594911056	0.168717796	15.38018585	2.22309E-53	2.81675E-52	positive
PRJNA729271	stage_3W_vs_E10	ordinary_muscle_development	ENSGALG00010001702	PLEKHA1	0.418569467	0.083136733	5.03471149	4.78569E-07	9.25531E-07	positive
PRJNA729271	stage_3W_vs_E10	ordinary_muscle_development	ENSGALG00010001704	ENSGALG00010001704	-1.937627946	0.147543605	-13.13257834	2.14236E-39	1.69691E-38	negative
PRJNA729271	stage_3W_vs_E10	ordinary_muscle_development	ENSGALG00010001705	HTRA1	2.172088144	0.154612613	14.04858314	7.85907E-45	7.45044E-44	positive
PRJNA729271	stage_3W_vs_E10	ordinary_muscle_development	ENSGALG00010001706	IQUB	2.870594773	0.34139461	8.408436129	4.15514E-17	1.40002E-16	positive
PRJNA729271	stage_3W_vs_E10	ordinary_muscle_development	ENSGALG00010001707	ENSGALG00010001707	4.383836197	1.047946081	4.183265034	2.87352E-05	4.89617E-05	positive
PRJNA729271	stage_3W_vs_E10	ordinary_muscle_development	ENSGALG00010001708	AADAC	3.270617132	0.250424468	13.06029383	5.55131E-39	4.34483E-38	positive
PRJNA729271	stage_3W_vs_E10	ordinary_muscle_development	ENSGALG00010001710	LCMT2	-0.910899998	0.081685448	-11.15131294	7.05575E-29	3.88281E-28	negative
PRJNA729271	stage_3W_vs_E10	ordinary_muscle_development	ENSGALG00010001711	CREB5	-0.210337753	0.175229533	-1.20035561	0.230001261	0.263713675	negative
PRJNA729271	stage_3W_vs_E10	ordinary_muscle_development	ENSGALG00010001712	ATP6AP1	2.057982931	0.15873722	12.96471574	1.93948E-38	1.48588E-37	positive
PRJNA729271	stage_3W_vs_E10	ordinary_muscle_development	ENSGALG00010001713	PNPLA4	0.893182081	0.219229614	4.074185346	4.61757E-05	7.7539E-05	positive
PRJNA729271	stage_3W_vs_E10	ordinary_muscle_development	ENSGALG00010001716	TRIL	-2.05848057	0.152932284	-13.4600786	2.6863E-41	2.25597E-40	negative
PRJNA729271	stage_3W_vs_E10	ordinary_muscle_development	ENSGALG00010001717	STARD10	0.497502561	0.495942518	1.003145612	0.31579061	0.353684507	positive
PRJNA729271	stage_3W_vs_E10	ordinary_muscle_development	ENSGALG00010001721	TM4SF1	-0.319263343	0.162197319	-1.968363873	0.049026186	0.061617494	negative
PRJNA729271	stage_3W_vs_E10	ordinary_muscle_development	ENSGALG00010001725	MTA2	-0.341499287	0.186525005	-1.830849901	0.06712295	0.082931057	negative
PRJNA729271	stage_3W_vs_E10	ordinary_muscle_development	ENSGALG00010001727	COL9A1	-11.21087153	0.812681782	-13.79490937	2.73496E-43	2.46079E-42	negative
PRJNA729271	stage_3W_vs_E10	ordinary_muscle_development	ENSGALG00010001728	STS	0.738821195	0.324325873	2.278021142	0.022725318	0.029737608	positive
PRJNA729271	stage_3W_vs_E10	ordinary_muscle_development	ENSGALG00010001729	ENSGALG00010001729	3.759087384	0.238846977	15.73847586	8.24047E-56	1.11009E-54	positive
PRJNA729271	stage_3W_vs_E10	ordinary_muscle_development	ENSGALG00010001731	ENSGALG00010001731	1.112174611	0.191238123	5.815653258	6.03975E-09	1.31986E-08	positive
PRJNA729271	stage_3W_vs_E10	ordinary_muscle_development	ENSGALG00010001738	PUDP	1.000407853	0.118617026	8.433931368	3.34243E-17	1.13065E-16	positive
PRJNA729271	stage_3W_vs_E10	ordinary_muscle_development	ENSGALG00010001741	SATB1	0.216293071	0.196716453	1.099516933	0.271542652	0.308150845	positive
PRJNA729271	stage_3W_vs_E10	ordinary_muscle_development	ENSGALG00010001745	CADPS2	-1.355720681	0.114318282	-11.85917649	1.92863E-32	1.21863E-31	negative
PRJNA729271	stage_3W_vs_E10	ordinary_muscle_development	ENSGALG00010001749	VAPA	1.311960015	0.105963139	12.38128685	3.30009E-35	2.28765E-34	positive
PRJNA729271	stage_3W_vs_E10	ordinary_muscle_development	ENSGALG00010001751	AVPR2C	-0.637535192	0.434940787	-1.465797667	0.142703417	0.168966347	negative
PRJNA729271	stage_3W_vs_E10	ordinary_muscle_development	ENSGALG00010001752	ARRB1	2.065785773	0.104848389	19.70259909	2.0483E-86	6.17258E-85	positive
PRJNA729271	stage_3W_vs_E10	ordinary_muscle_development	ENSGALG00010001753	PNPLA8	1.115887068	0.204298333	5.462046847	4.70676E-08	9.7172E-08	positive
PRJNA729271	stage_3W_vs_E10	ordinary_muscle_development	ENSGALG00010001754	APCDD1	-4.190647015	0.202488894	-20.69568817	3.78782E-95	1.40062E-93	negative
PRJNA729271	stage_3W_vs_E10	ordinary_muscle_development	ENSGALG00010001755	CUZD1	2.274665385	1.408914945	1.614480273	0.106423292	0.128240554	positive
PRJNA729271	stage_3W_vs_E10	ordinary_muscle_development	ENSGALG00010001756	ENSGALG00010001756	0.303124608	0.218273027	1.388740571	0.164911652	0.193444558	positive
PRJNA729271	stage_3W_vs_E10	ordinary_muscle_development	ENSGALG00010001758	COL19A1	0.03102221	0.280756715	0.110494987	0.912016825	0.922260631	positive
PRJNA729271	stage_3W_vs_E10	ordinary_muscle_development	ENSGALG00010001759	C10orf88	-0.008282644	0.089168149	-0.092887925	0.925992599	0.934437672	negative
PRJNA729271	stage_3W_vs_E10	ordinary_muscle_development	ENSGALG00010001760	RAB5A	0.564039833	0.095577062	5.901414215	3.60399E-09	7.95126E-09	positive
PRJNA729271	stage_3W_vs_E10	ordinary_muscle_development	ENSGALG00010001761	ELOB	0.283175333	0.149640826	1.892366816	0.058442124	0.072751511	positive
PRJNA729271	stage_3W_vs_E10	ordinary_muscle_development	ENSGALG00010001763	ENSGALG00010001763	0.606513914	0.263328145	2.303262775	0.021264063	0.027906074	positive
PRJNA729271	stage_3W_vs_E10	ordinary_muscle_development	ENSGALG00010001764	ENSGALG00010001764	1.343205599	0.385946758	3.480287296	0.000500876	0.000771705	positive
PRJNA729271	stage_3W_vs_E10	ordinary_muscle_development	ENSGALG00010001767	ENSGALG00010001767	0.516437919	0.17416467	2.965227785	0.003024589	0.00433687	positive
PRJNA729271	stage_3W_vs_E10	ordinary_muscle_development	ENSGALG00010001771	ENSGALG00010001771	0.575736368	0.134955848	4.266109083	1.98912E-05	3.43814E-05	positive
PRJNA729271	stage_3W_vs_E10	ordinary_muscle_development	ENSGALG00010001773	NRCAM	-2.105980046	0.250627852	-8.402817272	4.35893E-17	1.46629E-16	negative
PRJNA729271	stage_3W_vs_E10	ordinary_muscle_development	ENSGALG00010001776	LMBRD1	1.305601233	0.131017468	9.965092842	2.16671E-23	9.48264E-23	positive
PRJNA729271	stage_3W_vs_E10	ordinary_muscle_development	ENSGALG00010001778	KAT2B	3.282632872	0.147012435	22.32894699	1.9343E-110	9.9074E-109	positive
PRJNA729271	stage_3W_vs_E10	ordinary_muscle_development	ENSGALG00010001779	ENSGALG00010001779	1.219777302	0.597662772	2.0409123	0.041259545	0.052346355	positive
PRJNA729271	stage_3W_vs_E10	ordinary_muscle_development	ENSGALG00010001780	ENSGALG00010001780	-0.181274154	0.239314554	-0.757472336	0.448766933	0.487621941	negative
PRJNA729271	stage_3W_vs_E10	ordinary_muscle_development	ENSGALG00010001783	SGO1	-3.5186148	0.255860115	-13.75210356	4.94699E-43	4.40729E-42	negative
PRJNA729271	stage_3W_vs_E10	ordinary_muscle_development	ENSGALG00010001785	CNTN1	0.640631245	0.21273272	3.011437289	0.002600141	0.003747916	positive
PRJNA729271	stage_3W_vs_E10	ordinary_muscle_development	ENSGALG00010001786	P2RY8	0.391408125	0.206591739	1.894597174	0.058145789	0.072395054	positive
PRJNA729271	stage_3W_vs_E10	ordinary_muscle_development	ENSGALG00010001788	HOXA1	-2.019894221	0.44854768	-4.503187316	6.69418E-06	1.19734E-05	negative
PRJNA729271	stage_3W_vs_E10	ordinary_muscle_development	ENSGALG00010001790	HOXA2	-1.828755353	0.257291998	-7.107703968	1.17989E-12	3.16773E-12	negative
PRJNA729271	stage_3W_vs_E10	ordinary_muscle_development	ENSGALG00010001793	IKZF5	0.846909435	0.103018125	8.22097504	2.01855E-16	6.56619E-16	positive
PRJNA729271	stage_3W_vs_E10	ordinary_muscle_development	ENSGALG00010001794	ENSGALG00010001794	-2.927731398	0.722721877	-4.050979348	5.10037E-05	8.53792E-05	negative
PRJNA729271	stage_3W_vs_E10	ordinary_muscle_development	ENSGALG00010001795	ENSGALG00010001795	1.498909357	0.477259182	3.140661121	0.00168567	0.00247556	positive
PRJNA729271	stage_3W_vs_E10	ordinary_muscle_development	ENSGALG00010001796	SLCO2B1	2.683098836	0.15493119	17.3180031	3.44093E-67	6.21898E-66	positive
PRJNA729271	stage_3W_vs_E10	ordinary_muscle_development	ENSGALG00010001798	PSTK	0.881188884	0.127204458	6.927342785	4.28819E-12	1.11333E-11	positive
PRJNA729271	stage_3W_vs_E10	ordinary_muscle_development	ENSGALG00010001800	ENSGALG00010001800	0.803182283	0.18264247	4.397565812	1.09472E-05	1.92782E-05	positive
PRJNA729271	stage_3W_vs_E10	ordinary_muscle_development	ENSGALG00010001801	HOXA3	-1.484048103	0.150885239	-9.835608279	7.90882E-23	3.38666E-22	negative
PRJNA729271	stage_3W_vs_E10	ordinary_muscle_development	ENSGALG00010001803	PPP4R1	-0.711376275	0.09698529	-7.334888383	2.21906E-13	6.17851E-13	negative
PRJNA729271	stage_3W_vs_E10	ordinary_muscle_development	ENSGALG00010001805	ENSGALG00010001805	1.889420952	0.256055146	7.378961067	1.59529E-13	4.47615E-13	positive
PRJNA729271	stage_3W_vs_E10	ordinary_muscle_development	ENSGALG00010001806	ENSGALG00010001806	4.17347193	0.90332528	4.620120817	3.83517E-06	6.99343E-06	positive
PRJNA729271	stage_3W_vs_E10	ordinary_muscle_development	ENSGALG00010001807	ASMTL	0.354369011	0.103232596	3.432724018	0.00059755	0.00091337	positive
PRJNA729271	stage_3W_vs_E10	ordinary_muscle_development	ENSGALG00010001808	HOXA4	-1.216601528	0.153493253	-7.926091242	2.26151E-15	6.9598E-15	negative
PRJNA729271	stage_3W_vs_E10	ordinary_muscle_development	ENSGALG00010001811	ACADSB	0.23537481	0.149072102	1.578932663	0.114351503	0.13714422	positive
PRJNA729271	stage_3W_vs_E10	ordinary_muscle_development	ENSGALG00010001813	HOXA5	-0.423214164	0.103380583	-4.093749078	4.24454E-05	7.14322E-05	negative
PRJNA729271	stage_3W_vs_E10	ordinary_muscle_development	ENSGALG00010001817	SLC25A6	-1.347740692	0.113013445	-11.9254899	8.71697E-33	5.55884E-32	negative
PRJNA729271	stage_3W_vs_E10	ordinary_muscle_development	ENSGALG00010001818	ADGRB3	-2.228608016	0.2737432	-8.141236074	3.91263E-16	1.2514E-15	negative
PRJNA729271	stage_3W_vs_E10	ordinary_muscle_development	ENSGALG00010001819	HOXA6	0.853855495	0.142125532	6.007755826	1.88109E-09	4.21167E-09	positive
PRJNA729271	stage_3W_vs_E10	ordinary_muscle_development	ENSGALG00010001820	PGM2L1	2.569135289	0.153684466	16.7169484	9.86425E-63	1.58869E-61	positive
PRJNA729271	stage_3W_vs_E10	ordinary_muscle_development	ENSGALG00010001822	KCNE3	4.594608372	0.297966116	15.41990223	1.20286E-53	1.53888E-52	positive
PRJNA729271	stage_3W_vs_E10	ordinary_muscle_development	ENSGALG00010001823	RALBP1	-0.440447839	0.076444337	-5.761680419	8.32806E-09	1.8063E-08	negative
PRJNA729271	stage_3W_vs_E10	ordinary_muscle_development	ENSGALG00010001825	LIPT2	2.366012787	0.142489009	16.60487925	6.42493E-62	1.01338E-60	positive
PRJNA729271	stage_3W_vs_E10	ordinary_muscle_development	ENSGALG00010001826	HOXA7	1.891586233	0.132753313	14.24888151	4.55519E-46	4.52553E-45	positive

PRJNA729271	stage_3W_vs_E10	ordinary_muscle_development	ENSGALG00010001827	PDZRN4	-3.473417715	0.210665921	-16.48780066	4.48998E-61	6.91628E-60	negative
PRJNA729271	stage_3W_vs_E10	ordinary_muscle_development	ENSGALG00010001828	ENSGALG00010001828	-2.862284951	1.102700784	-2.595704105	0.009439732	0.012865906	negative
PRJNA729271	stage_3W_vs_E10	ordinary_muscle_development	ENSGALG00010001829	POLD3	-0.146957002	0.121401423	-1.210504775	0.22608526	0.259552181	negative
PRJNA729271	stage_3W_vs_E10	ordinary_muscle_development	ENSGALG00010001831	CSF2RA	2.52305986	0.249417985	10.11578961	4.7021E-24	2.11208E-23	positive
PRJNA729271	stage_3W_vs_E10	ordinary_muscle_development	ENSGALG00010001832	BUB3	-1.171770598	0.086865436	-13.48949193	1.80339E-41	1.52066E-40	negative
PRJNA729271	stage_3W_vs_E10	ordinary_muscle_development	ENSGALG00010001834	ZCRB1	0.561793353	0.083266801	6.746906901	1.5103E-11	3.82323E-11	positive
PRJNA729271	stage_3W_vs_E10	ordinary_muscle_development	ENSGALG00010001835	MECP2	0.146430921	0.191580856	0.764329611	0.444670844	0.483424877	positive
PRJNA729271	stage_3W_vs_E10	ordinary_muscle_development	ENSGALG00010001836	TWSG1	-1.000260989	0.14022786	-7.133111717	9.81248E-13	2.64913E-12	negative
PRJNA729271	stage_3W_vs_E10	ordinary_muscle_development	ENSGALG00010001837	TAB3	1.105303101	0.137832732	8.019162659	1.06468E-15	3.32886E-15	positive
PRJNA729271	stage_3W_vs_E10	ordinary_muscle_development	ENSGALG00010001838	PPHLN1	-0.901094205	0.065797243	-13.69501457	1.08739E-42	9.59321E-42	negative
PRJNA729271	stage_3W_vs_E10	ordinary_muscle_development	ENSGALG00010001839	ENSGALG00010001839	7.143099275	0.598938137	11.92627223	8.63546E-33	5.50929E-32	positive
PRJNA729271	stage_3W_vs_E10	ordinary_muscle_development	ENSGALG00010001840	HOXA9	0.133749088	0.144887772	0.923121981	0.355943641	0.394721374	positive
PRJNA729271	stage_3W_vs_E10	ordinary_muscle_development	ENSGALG00010001842	GPR26	-0.125572783	0.503596458	-0.249351998	0.803088513	0.825527834	negative
PRJNA729271	stage_3W_vs_E10	ordinary_muscle_development	ENSGALG00010001843	PRICKLE1	-2.338872301	0.123175259	-18.98816635	2.1366E-80	5.41433E-79	negative
PRJNA729271	stage_3W_vs_E10	ordinary_muscle_development	ENSGALG00010001844	HOXA10	-1.074850444	0.166807076	-6.443674134	1.16615E-10	2.80229E-10	negative
PRJNA729271	stage_3W_vs_E10	ordinary_muscle_development	ENSGALG00010001845	ANKRD12	1.325203294	0.133546868	9.923132729	3.30226E-23	1.43355E-22	positive
PRJNA729271	stage_3W_vs_E10	ordinary_muscle_development	ENSGALG00010001848	HOXA11	-0.855142827	0.363041339	-2.355497117	0.018497941	0.024421864	negative
PRJNA729271	stage_3W_vs_E10	ordinary_muscle_development	ENSGALG00010001849	NDUFV2	0.790222027	0.094575611	8.355452499	6.51824E-17	2.1765E-16	positive
PRJNA729271	stage_3W_vs_E10	ordinary_muscle_development	ENSGALG00010001850	MYOM2	4.816786681	0.187788139	25.65011136	4.216E-145	3.6814E-143	positive
PRJNA729271	stage_3W_vs_E10	ordinary_muscle_development	ENSGALG00010001852	CPXM2	-1.925780456	0.390207253	-4.935275904	8.00375E-07	1.5255E-06	negative
PRJNA729271	stage_3W_vs_E10	ordinary_muscle_development	ENSGALG00010001853	ENSGALG00010001853	0.973744089	0.186923348	5.209322972	1.89531E-07	3.76233E-07	positive
PRJNA729271	stage_3W_vs_E10	ordinary_muscle_development	ENSGALG00010001854	CHRDL2	0.974036219	0.296325602	3.287047126	0.001012439	0.0015162	positive
PRJNA729271	stage_3W_vs_E10	ordinary_muscle_development	ENSGALG00010001856	CHST15	-0.584822797	0.117251052	-4.987782934	6.10761E-07	1.17274E-06	negative
PRJNA729271	stage_3W_vs_E10	ordinary_muscle_development	ENSGALG00010001857	HNRNPA2B1	-0.507530796	0.187693235	-2.704044163	0.006850118	0.009470857	negative
PRJNA729271	stage_3W_vs_E10	ordinary_muscle_development	ENSGALG00010001858	ADAMTS20	0.81595453	0.160830479	5.073382464	3.90806E-07	7.59857E-07	positive
PRJNA729271	stage_3W_vs_E10	ordinary_muscle_development	ENSGALG00010001860	RNF169	0.339381108	0.12930162	2.624724338	0.008671914	0.011872995	positive
PRJNA729271	stage_3W_vs_E10	ordinary_muscle_development	ENSGALG00010001861	CBX3	-1.127724901	0.074580744	-15.1208588	1.17994E-51	1.41349E-50	negative
PRJNA729271	stage_3W_vs_E10	ordinary_muscle_development	ENSGALG00010001864	ENSGALG00010001864	-0.175935534	0.198059258	-0.888297453	0.374380769	0.413112763	negative
PRJNA729271	stage_3W_vs_E10	ordinary_muscle_development	ENSGALG00010001865	CYBB	2.434110218	0.314774556	7.732868411	1.0515E-14	3.13031E-14	positive
PRJNA729271	stage_3W_vs_E10	ordinary_muscle_development	ENSGALG00010001866	PUS7L	-0.067751659	0.11897677	-0.569452831	0.569048872	0.605428905	negative
PRJNA729271	stage_3W_vs_E10	ordinary_muscle_development	ENSGALG00010001868	ENSGALG00010001868	-0.390285084	0.124740149	-3.128784821	0.001755308	0.002569973	negative
PRJNA729271	stage_3W_vs_E10	ordinary_muscle_development	ENSGALG00010001869	CRLF2	8.107566833	0.91897554	8.822396763	1.12035E-18	4.05989E-18	positive
PRJNA729271	stage_3W_vs_E10	ordinary_muscle_development	ENSGALG00010001870	PLP2	2.033235393	0.163769688	12.41521197	2.16114E-35	1.50604E-34	positive
PRJNA729271	stage_3W_vs_E10	ordinary_muscle_development	ENSGALG00010001871	ENSGALG00010001871	-1.025988897	0.124275724	-8.255746686	1.50955E-16	4.95045E-16	negative
PRJNA729271	stage_3W_vs_E10	ordinary_muscle_development	ENSGALG00010001873	ENSGALG00010001873	-0.278292377	0.212315246	-1.310750795	0.189941971	0.220539	negative
PRJNA729271	stage_3W_vs_E10	ordinary_muscle_development	ENSGALG00010001877	OAT	0.719934345	0.136681898	5.267225255	1.38501E-07	2.77404E-07	positive
PRJNA729271	stage_3W_vs_E10	ordinary_muscle_development	ENSGALG00010001878	XK	-0.372798669	0.19747606	-1.887817029	0.059050518	0.073469869	negative
PRJNA729271	stage_3W_vs_E10	ordinary_muscle_development	ENSGALG00010001879	IRAK4	0.936966206	0.16341365	5.733708331	9.82582E-09	2.12132E-08	positive
PRJNA729271	stage_3W_vs_E10	ordinary_muscle_development	ENSGALG00010001880	MTCL1	2.081742025	0.143996827	14.45686043	2.26915E-47	2.3477E-46	positive
PRJNA729271	stage_3W_vs_E10	ordinary_muscle_development	ENSGALG00010001883	KBTBD11	-1.109580157	0.089016681	-12.46485655	1.1608E-35	8.14514E-35	negative
PRJNA729271	stage_3W_vs_E10	ordinary_muscle_development	ENSGALG00010001885	SMPX	4.348168205	0.371146798	11.71549432	1.0617E-31	6.50713E-31	positive
PRJNA729271	stage_3W_vs_E10	ordinary_muscle_development	ENSGALG00010001886	NEU3	-0.245913316	0.084649308	-2.905083586	0.00367155	0.005220632	negative
PRJNA729271	stage_3W_vs_E10	ordinary_muscle_development	ENSGALG00010001888	SNX10	1.655624781	0.199632692	8.293354986	1.10098E-16	3.63359E-16	positive
PRJNA729271	stage_3W_vs_E10	ordinary_muscle_development	ENSGALG00010001890	KLHL34	7.237973628	0.250681318	28.87320712	2.5912E-183	3.8326E-181	positive
PRJNA729271	stage_3W_vs_E10	ordinary_muscle_development	ENSGALG00010001892	RAB12	1.606621825	0.070361505	22.83381834	2.1164E-115	1.162E-113	positive
PRJNA729271	stage_3W_vs_E10	ordinary_muscle_development	ENSGALG00010001895	P4HA3	-1.719647174	0.240631648	-7.146388226	8.90909E-13	2.41063E-12	negative
PRJNA729271	stage_3W_vs_E10	ordinary_muscle_development	ENSGALG00010001896	ARHGEF10	-0.108353315	0.134101376	-0.807995555	0.419093141	0.457608669	negative
PRJNA729271	stage_3W_vs_E10	ordinary_muscle_development	ENSGALG00010001897	TLR7	3.271708298	0.174970993	18.69857535	5.08451E-78	1.20228E-76	positive
PRJNA729271	stage_3W_vs_E10	ordinary_muscle_development	ENSGALG00010001898	LHPP	0.574153757	0.131345831	4.371313148	1.23502E-05	2.16752E-05	positive
PRJNA729271	stage_3W_vs_E10	ordinary_muscle_development	ENSGALG00010001899	TWF1	-0.444524715	0.112641009	-3.946384338	7.93402E-05	0.000130538	negative
PRJNA729271	stage_3W_vs_E10	ordinary_muscle_development	ENSGALG00010001901	PRPS2	0.967300498	0.181916579	5.317275112	1.05333E-07	2.12972E-07	positive
PRJNA729271	stage_3W_vs_E10	ordinary_muscle_development	ENSGALG00010001902	WDR45	0.372617071	0.141982937	2.624379228	0.008680707	0.011883911	positive
PRJNA729271	stage_3W_vs_E10	ordinary_muscle_development	ENSGALG00010001903	NARS2	-0.43710137	0.118538439	-3.687423024	0.000226537	0.000358947	negative
PRJNA729271	stage_3W_vs_E10	ordinary_muscle_development	ENSGALG00010001906	SKAP2	0.109630338	0.127569733	0.859375777	0.390133229	0.429122868	positive
PRJNA729271	stage_3W_vs_E10	ordinary_muscle_development	ENSGALG00010001908	FAM83B	-4.017208077	0.421201618	-9.537494404	1.46325E-21	5.96954E-21	negative
PRJNA729271	stage_3W_vs_E10	ordinary_muscle_development	ENSGALG00010001910	FAM53B	-0.337653213	0.097497145	-3.463211298	0.000533769	0.000819943	negative
PRJNA729271	stage_3W_vs_E10	ordinary_muscle_development	ENSGALG00010001911	USP35	0.099551953	0.345330981	0.28827982	0.773132553	0.79761967	positive
PRJNA729271	stage_3W_vs_E10	ordinary_muscle_development	ENSGALG00010001912	TMEM117	4.737974817	0.205257651	23.08306068	6.8513E-118	3.9566E-116	positive
PRJNA729271	stage_3W_vs_E10	ordinary_muscle_development	ENSGALG00010001914	ENSGALG00010001914	-0.836995107	0.156279396	-5.355761071	8.5197E-08	1.73421E-07	negative
PRJNA729271	stage_3W_vs_E10	ordinary_muscle_development	ENSGALG00010001915	EEF1AKMT2	-0.121353677	0.166077691	-0.730704262	0.464959812	0.503631182	negative
PRJNA729271	stage_3W_vs_E10	ordinary_muscle_development	ENSGALG00010001918	CNKSR2	-0.819050046	0.14656048	-5.588478183	2.29068E-08	4.8331E-08	negative
PRJNA729271	stage_3W_vs_E10	ordinary_muscle_development	ENSGALG00010001921	ENSGALG00010001921	-0.091349347	0.286440367	-0.318912268	0.749793041	0.776303581	negative
PRJNA729271	stage_3W_vs_E10	ordinary_muscle_development	ENSGALG00010001923	RAPGEF5	2.017347258	0.100874553	19.99857454	5.66691E-89	1.81329E-87	positive
PRJNA729271	stage_3W_vs_E10	ordinary_muscle_development	ENSGALG00010001925	FRMPD4	-3.496733903	0.22850703	-15.30252222	7.35501E-53	9.11204E-52	negative
PRJNA729271	stage_3W_vs_E10	ordinary_muscle_development	ENSGALG00010001928	KCTD21	0.460332072	0.166657974	2.762136498	0.005742447	0.007997383	positive
PRJNA729271	stage_3W_vs_E10	ordinary_muscle_development	ENSGALG00010001929	POLR2I	-0.492795051	0.125788833	-3.917637507	8.9421E-05	0.000146508	negative
PRJNA729271	stage_3W_vs_E10	ordinary_muscle_development	ENSGALG00010001935	ABRAXAS2	0.473184418	0.129151872	3.663782871	0.000248517	0.00039236	positive
PRJNA729271	stage_3W_vs_E10	ordinary_muscle_development	ENSGALG00010001938	ALG8	-1.024530422	0.105689949	-9.693735581	3.20584E-22	1.33993E-21	negative
PRJNA729271	stage_3W_vs_E10	ordinary_muscle_development	ENSGALG00010001940	PTPRM	0.583226233	0.110851042	5.261350915	1.43001E-07	2.86021E-07	positive
PRJNA729271	stage_3W_vs_E10	ordinary_muscle_development	ENSGALG00010001947	TINAG	6.511767551	0.301298181	21.61236928	1.3741E-103	6.1666E-102	positive
PRJNA729271	stage_3W_vs_E10	ordinary_muscle_development	ENSGALG00010001948	EMX2	-2.474063069	0.266088702	-9.297888435	1.43262E-20	5.59878E-20	negative
PRJNA729271	stage_3W_vs_E10	ordinary_muscle_development	ENSGALG00010001952	ENSGALG00010001952	0.220206222	0.072634135	3.031718088	0.002431662	0.003514151	positive
PRJNA729271	stage_3W_vs_E10	ordinary_muscle_development	ENSGALG00010001957	NDUFC2	1.529214871	0.111396843	13.72763201	6.93637E-43	6.1607E-42	positive
PRJNA729271	stage_3W_vs_E10	ordinary_muscle_development	ENSGALG00010001958	RAB11FIP2	-0.342169684	0.112117779	-3.051877109	0.002274152	0.003294736	negative
PRJNA729271	stage_3W_vs_E10	ordinary_muscle_development	ENSGALG00010001966	ARHGAP6	1.092298186	0.141161979	7.737906428	1.01067E-14	3.01186E-14	positive
PRJNA729271	stage_3W_vs_E10	ordinary_muscle_development	ENSGALG00010001967	LAMA1	-4.863372911	0.241021205	-20.17819515	1.52203E-90	5.06004E-89	negative
PRJNA729271	stage_3W_vs_E10	ordinary_muscle_development	ENSGALG00010001969	PLEKHA8	-2.282175839	0.128390504	-17.77526978	1.09868E-70	2.16377E-69	negative
PRJNA729271	stage_3W_vs_E10	ordinary_muscle_development	ENSGALG00010001971	MLIP	4.590703386	0.204388013	22.46072711	1.0053E-111	5.2796E-110	positive
PRJNA729271	stage_3W_vs_E10	ordinary_muscle_development	ENSGALG00010001972	FAM204A	0.483401576	0.157931096	3.06083848	0.002207181	0.003200589	positive
PRJNA729271	stage_3W_vs_E10	ordinary_muscle_development	ENSGALG00010001974	ENSGALG00010001974	0.850666768	0.400763523	2.122615257	0.033786105	0.043308242	positive
PRJNA729271	stage_3W_vs_E10	ordinary_muscle_development	ENSGALG00010001976	THRSP	8.699209846	1.423788944	6.109901249	9.96928E-10	2.27244E-09	positive
PRJNA729271	stage_3W_vs_E10	ordinary_muscle_development	ENSGALG00010001982	DMD	2.570187018	0.121812729	21.09949455	8.03908E-99	3.20128E-97	positive
PRJNA729271	stage_3W_vs_E10	ordinary_muscle_development	ENSGALG00010001987	KCTD14	-2.948739796	0.462620738	-6.373989652	1.84173E-10	4.38644E-10	negative
PRJNA729271	stage_3W_vs_E10	ordinary_muscle_development	ENSGALG00010001991	TOMM7	1.316980419	0.130003652	10.13033404	4.05263E-24	1.82829E-23	positive
PRJNA729271	stage_3W_vs_E10	ordinary_muscle_development	ENSGALG00010001995	LRRC1	-1.250272536	0.139115825	-8.987277619	2.5343E-19	9.44821E-19	negative
PRJNA729271	stage_3W_vs_E10	ordinary_muscle_development	ENSGALG00010001997	INTS4	-0.26365178	0.07761437	-3.396945435	0.000681426	0.001036439	negative
PRJNA729271	stage_3W_vs_E10	ordinary_muscle_development	ENSGALG00010002005	COLQ	-0.452647858	0.217680322	-2.079415606	0.037579167	0.047941386	negative
PRJNA729271	stage_3W_vs_E10	ordinary_muscle_development	ENSGALG00010002006	ENSGALG00010002006	3.599024568	0.400003916	8.997473334	2.30972E-19	8.62648E-19	positive
PRJNA729271	stage_3W_vs_E10	ordinary_muscle_development	ENSGALG00010002012	MID1	-2.165170718	0.141232913	-15.33049674	4.78315E-53	5.98203E-52	negative
PRJNA729271	stage_3W_vs_E10	ordinary_muscle_development	ENSGALG00010002017	ARHGAP28	-1.585462463	0.118710361	-13.35572099	1.09691E-40	9.02366E-40	negative
PRJNA729271	stage_3W_vs_E10	ordinary_muscle_development	ENSGALG00010002022	ERNI	-2.218314693	0.738556497	-3.003581584	0.00266822	0.0038407	negative
PRJNA729271	stage_3W_vs_E10	ordinary_muscle_development	ENSGALG00010002023	CLCN4	0.109705359	0.242839031	0.451761639	0.651440708	0.684397555	positive
PRJNA729271	stage_3W_vs_E10	ordinary_muscle_development	ENSGALG00010002024	KLHL31	4.491776262	0.165774688	27.09567007	1.1074E-161	1.2639E-159	positive
PRJNA729271	stage_3W_vs_E10	ordinary_muscle_development	ENSGALG00010002027	ENSGALG00010002027	6.834631677	0.536316998	12.74364174	3.3823E-37	2.47359E-36	positive
PRJNA729271	stage_3W_vs_E10	ordinary_muscle_development	ENSGALG00010002034	WWC3	-0.544103036	0.099948398	-5.443839499	5.21442E-08	1.07393E-07	negative
PRJNA729271	stage_3W_vs_E10	ordinary_muscle_development	ENSGALG00010002039	AAMDC	1.808105825	0.122386922	14.77368491	2.16543E-49	2.39419E-48	positive
PRJNA729271	stage_3W_vs_E10	ordinary_muscle_development	ENSGALG00010002041	GCLC	0.834055609	0.138247416	6.03306474	1.60879E-09	3.62159E-09	positive
PRJNA729271	stage_3W_vs_E10	ordinary_muscle_development	ENSGALG00010002046	FAM126A	-0.466585021	0.088141602	-5.293584512	1.19942E-07	2.41634E-07	negative
PRJNA729271	stage_3W_vs_E10	ordinary_muscle_development	ENSGALG00010002050	ENSGALG00010002050	1.988904204	1.101027203	1.80640787	0.070854653	0.087244155	positive
PRJNA729271	stage_3W_vs_E10	ordinary_muscle_development	ENSGALG00010002054	RSF1	-0.213835781	0.099985044	-2.13867766	0.032461783	0.041673328	negative
PRJNA729271	stage_3W_vs_E10	ordinary_muscle_development	ENSGALG00010002056	PRLHR	-1.235250564	0.439411419	-2.811148072	0.004936506	0.006926879	negative
PRJNA729271	stage_3W_vs_E10	ordinary_muscle_development	ENSGALG00010002062	GSTA4	-0.177589516	0.36294897	-0.4892961	0.624632081	0.659002986	negative
PRJNA729271	stage_3W_vs_E10	ordinary_muscle_development	ENSGALG00010002065	CLNS1A	-0.423340451	0.062898012	-6.730585538	1.68982E-11	4.26723E-11	negative
PRJNA729271	stage_3W_vs_E10	ordinary_muscle_development	ENSGALG00010002072	NEURL4	0.146140632	0.160993636	0.907741673	0.364014721	0.402839623	positive
PRJNA729271	stage_3W_vs_E10	ordinary_muscle_development	ENSGALG00010002079	ARX	2.102121607	0.190079373	11.05917796	1.97903E-28	1.07398E-27	positive
PRJNA729271	stage_3W_vs_E10	ordinary_muscle_development	ENSGALG00010002084	HACL1	-0.711311255	0.091695003	-7.757361147	8.67147E-15	2.59375E-14	negative
PRJNA729271	stage_3W_vs_E10	ordinary_muscle_development	ENSGALG00010002087	POLA1	-1.577565276	0.074894628	-21.06379739	1.70911E-98	6.76871E-97	negative
PRJNA729271	stage_3W_vs_E10	ordinary_muscle_development	ENSGALG00010002088	OZFL1	-0.128431538	0.136464157	-0.941137524	0.346634388	0.385134559	negative
PRJNA729271	stage_3W_vs_E10	ordinary_muscle_development	ENSGALG00010002090	AQP11	0.165031786	0.29203454	0.565110504	0.571998605	0.608280231	positive
PRJNA729271	stage_3W_vs_E10	ordinary_muscle_development	ENSGALG00010002097	PCYT1B	-1.07684143	0.444775932	-2.421087456	0.015474154	0.020596682	negative
PRJNA729271	stage_3W_vs_E10	ordinary_muscle_development	ENSGALG00010002098	ENSGALG00010002098	0.133695435	0.451055213	0.29640592	0.766920105	0.792338341	positive
PRJNA729271	stage_3W_vs_E10	ordinary_muscle_development	ENSGALG00010002100	BTD	0.619632204	0.11596026	5.34348754	9.11751E-08	1.85173E-07	positive
PRJNA729271	stage_3W_vs_E10	ordinary_muscle_development	ENSGALG00010002102	PAK1	-0.904112696	0.272073473	-3.32304611	0.000890402	0.001339391	negative
PRJNA729271	stage_3W_vs_E10	ordinary_muscle_development	ENSGALG00010002106	ENSGALG00010002106	1.207088649	0.869671855	1.387981735	0.165142607	0.193699797	positive
PRJNA729271	stage_3W_vs_E10	ordinary_muscle_development	ENSGALG00010002113	GDPD4	-7.223868159	0.376667614	-19.17836281	5.61198E-82	1.48171E-80	negative
PRJNA729271	stage_3W_vs_E10	ordinary_muscle_development	ENSGALG00010002118	ENSGALG00010002118	2.346176679	0.262737327	8.92974252	4.27001E-19	1.57732E-18	positive
PRJNA729271	stage_3W_vs_E10	ordinary_muscle_development	ENSGALG00010002122	ENSGALG00010002122	2.938392342	0.507303383	5.792179672	6.94788E-09	1.51352E-08	positive
PRJNA729271	stage_3W_vs_E10	ordinary_muscle_development	ENSGALG00010002126	ENSGALG00010002126	3.373738666	0.295903793	11.40147153	4.11111E-30	2.36846E-29	positive
PRJNA729271	stage_3W_vs_E10	ordinary_muscle_development	ENSGALG00010002134	ANKRD28	0.797110132	0.120621222	6.608373898	3.88564E-11	9.61954E-11	positive
PRJNA729271	stage_3W_vs_E10	ordinary_muscle_development	ENSGALG00010002135	ELOVL5	0.035222054	0.168141646	0.209478463	0.834074746	0.853265117	positive
PRJNA729271	stage_3W_vs_E10	ordinary_muscle_development	ENSGALG00010002136	ACAP1	0.159814221	0.239161669	0.668226735	0.503988872	0.541937589	positive
PRJNA729271	stage_3W_vs_E10	ordinary_muscle_development	ENSGALG00010002137	PDK3	1.535396313	0.131450778	11.68038972	1.60558E-31	9.758E-31	positive
PRJNA729271	stage_3W_vs_E10	ordinary_muscle_development	ENSGALG00010002138	MYO7A	-0.792239203	0.171493336	-4.619650073	3.84388E-06	7.00843E-06	negative
PRJNA729271	stage_3W_vs_E10	ordinary_muscle_development	ENSGALG00010002140	ENSGALG00010002140	-0.549416558	0.462646526	-1.187551464	0.23501017	0.26907365	negative
PRJNA729271	stage_3W_vs_E10	ordinary_muscle_development	ENSGALG00010002142	CLDN34	0.196823549	0.257353764	0.764797631	0.444392059	0.483158033	positive
PRJNA729271	stage_3W_vs_E10	ordinary_muscle_development	ENSGALG00010002145	ENSGALG00010002145	0.069659715	0.470907401	0.147926567	0.882400721	0.896313837	positive
PRJNA729271	stage_3W_vs_E10	ordinary_muscle_development	ENSGALG00010002146	CACUL1	0.736827055	0.087651842	8.406292854	4.23174E-17	1.42483E-16	positive
PRJNA729271	stage_3W_vs_E10	ordinary_muscle_development	ENSGALG00010002148	POLR2A	1.037564937	0.177177797	5.856066364	4.73959E-09	1.0395E-08	positive
PRJNA729271	stage_3W_vs_E10	ordinary_muscle_development	ENSGALG00010002149	ENSGALG00010002149	-3.171465548	0.389137696	-8.149982853	3.63976E-16	1.16557E-15	negative
PRJNA729271	stage_3W_vs_E10	ordinary_muscle_development	ENSGALG00010002150	EIF3A	-0.062863243	0.132473313	-0.474535147	0.635118341	0.668606315	negative
PRJNA729271	stage_3W_vs_E10	ordinary_muscle_development	ENSGALG00010002159	GALNT15	2.144724978	0.188750665	11.3627413	6.41011E-30	3.67106E-29	positive

PRJNA729271	stage_3W_vs_E10	ordinary_muscle_development	ENSGALG00010002160	CAPN5	-0.156852742	0.166472542	-0.942213892	0.346083143	0.38460819	negative
PRJNA729271	stage_3W_vs_E10	ordinary_muscle_development	ENSGALG00010002161	CDKL5	-0.391089462	0.174800521	-2.237347228	0.025263655	0.032848643	negative
PRJNA729271	stage_3W_vs_E10	ordinary_muscle_development	ENSGALG00010002173	GCM1	-0.63772478	0.884879959	-0.720690726	0.47109982	0.509748573	negative
PRJNA729271	stage_3W_vs_E10	ordinary_muscle_development	ENSGALG00010002174	ENSGALG00010002174	-0.46659385	0.463820586	-1.005979174	0.314425588	0.352291791	negative
PRJNA729271	stage_3W_vs_E10	ordinary_muscle_development	ENSGALG00010002177	FBXO9	1.538361101	0.154788365	9.938480212	2.83113E-23	1.2305E-22	positive
PRJNA729271	stage_3W_vs_E10	ordinary_muscle_development	ENSGALG00010002181	FIS1	-0.143173696	0.261289902	-0.547949594	0.583726514	0.619498889	negative
PRJNA729271	stage_3W_vs_E10	ordinary_muscle_development	ENSGALG00010002185	ENSGALG00010002185	-0.12597485	0.33796559	-0.372744604	0.709338536	0.738215256	negative
PRJNA729271	stage_3W_vs_E10	ordinary_muscle_development	ENSGALG00010002186	CILK1	-0.32048985	0.074811263	-4.28397858	1.83581E-05	3.18225E-05	negative
PRJNA729271	stage_3W_vs_E10	ordinary_muscle_development	ENSGALG00010002192	ENSGALG00010002192	0.325582183	0.195578188	1.664716227	0.095969384	0.116320447	positive
PRJNA729271	stage_3W_vs_E10	ordinary_muscle_development	ENSGALG00010002194	SHROOM2	-2.614166692	0.135133148	-19.34511795	2.24091E-83	6.16357E-82	negative
PRJNA729271	stage_3W_vs_E10	ordinary_muscle_development	ENSGALG00010002196	ENSGALG00010002196	-0.89988898	0.101110019	-8.900097032	5.57979E-19	2.04809E-18	negative
PRJNA729271	stage_3W_vs_E10	ordinary_muscle_development	ENSGALG00010002197	GSTA2	-0.938781583	0.189322227	-4.958644321	7.09868E-07	1.35674E-06	negative
PRJNA729271	stage_3W_vs_E10	ordinary_muscle_development	ENSGALG00010002202	SFXN4	0.345473296	0.100750523	3.428997551	0.000605815	0.000925515	positive
PRJNA729271	stage_3W_vs_E10	ordinary_muscle_development	ENSGALG00010002206	DPH3P1	1.270134664	0.108225726	11.73597732	8.33578E-32	5.12631E-31	positive
PRJNA729271	stage_3W_vs_E10	ordinary_muscle_development	ENSGALG00010002210	GPR143	-5.740149746	1.231349375	-4.661674307	3.13647E-06	5.7541E-06	negative
PRJNA729271	stage_3W_vs_E10	ordinary_muscle_development	ENSGALG00010002211	ACER3	-0.442213727	0.174316676	-2.53684121	0.011185768	0.015131844	negative
PRJNA729271	stage_3W_vs_E10	ordinary_muscle_development	ENSGALG00010002215	YLEC18	0.482510469	0.353488132	1.364997648	0.172253789	0.201421319	positive
PRJNA729271	stage_3W_vs_E10	ordinary_muscle_development	ENSGALG00010002225	PRDX3	0.998422408	0.105365209	9.475826242	2.64658E-21	1.06918E-20	positive
PRJNA729271	stage_3W_vs_E10	ordinary_muscle_development	ENSGALG00010002226	OXNAD1	0.259985474	0.132637236	1.960124343	0.049981259	0.062730596	positive
PRJNA729271	stage_3W_vs_E10	ordinary_muscle_development	ENSGALG00010002227	ENSGALG00010002227	0.136837856	1.165885206	0.117368207	0.906568277	0.917454945	positive
PRJNA729271	stage_3W_vs_E10	ordinary_muscle_development	ENSGALG00010002231	TMEM14A	-0.913491015	0.136848648	-6.67519212	2.46908E-11	6.18445E-11	negative
PRJNA729271	stage_3W_vs_E10	ordinary_muscle_development	ENSGALG00010002235	MXRA5	-0.506790763	0.201263433	-2.51804689	0.011800761	0.015926632	negative
PRJNA729271	stage_3W_vs_E10	ordinary_muscle_development	ENSGALG00010002238	ENSGALG00010002238	0.40851691	0.390271192	1.046751382	0.295214259	0.332514623	positive
PRJNA729271	stage_3W_vs_E10	ordinary_muscle_development	ENSGALG00010002239	AGPAT5	1.631527221	0.127216354	12.82482304	1.19058E-37	8.83639E-37	positive
PRJNA729271	stage_3W_vs_E10	ordinary_muscle_development	ENSGALG00010002243	ARSH	0.103364782	0.175792008	0.587994773	0.556535801	0.593684607	positive
PRJNA729271	stage_3W_vs_E10	ordinary_muscle_development	ENSGALG00010002247	RFTN1	-0.557478931	0.18552965	-3.00479697	0.002657582	0.003826527	negative
PRJNA729271	stage_3W_vs_E10	ordinary_muscle_development	ENSGALG00010002251	GRK5	-0.263772504	0.150160451	-1.756604367	0.078985268	0.096721421	negative
PRJNA729271	stage_3W_vs_E10	ordinary_muscle_development	ENSGALG00010002253	ENSGALG00010002253	-0.350831774	0.32177464	-1.090302747	0.275579806	0.312365443	negative
PRJNA729271	stage_3W_vs_E10	ordinary_muscle_development	ENSGALG00010002254	ENSGALG00010002254	4.381920254	0.454926255	9.632155119	5.84897E-22	2.42067E-21	positive
PRJNA729271	stage_3W_vs_E10	ordinary_muscle_development	ENSGALG00010002259	TASL	2.038517025	0.492781721	4.136754545	3.52253E-05	5.95718E-05	positive
PRJNA729271	stage_3W_vs_E10	ordinary_muscle_development	ENSGALG00010002261	NROB1	-3.966361292	0.480485028	-8.254911311	1.52015E-16	4.98407E-16	negative
PRJNA729271	stage_3W_vs_E10	ordinary_muscle_development	ENSGALG00010002269	MCPH1	-2.59452215	0.157496288	-16.47354473	5.68399E-61	8.7277E-60	negative
PRJNA729271	stage_3W_vs_E10	ordinary_muscle_development	ENSGALG00010002270	IMMP2L	-0.201624765	0.250114423	-0.806130102	0.420167836	0.458609397	negative
PRJNA729271	stage_3W_vs_E10	ordinary_muscle_development	ENSGALG00010002271	ENSGALG00010002271	3.903792853	0.668371824	5.840750184	5.19663E-09	1.13836E-08	positive
PRJNA729271	stage_3W_vs_E10	ordinary_muscle_development	ENSGALG00010002274	ENSGALG00010002274	3.445158714	0.779906245	4.417401112	9.98947E-06	1.76346E-05	positive
PRJNA729271	stage_3W_vs_E10	ordinary_muscle_development	ENSGALG00010002275	IL1RAPL1	-4.40251139	0.406325461	-10.83493851	2.35123E-27	1.21805E-26	negative
PRJNA729271	stage_3W_vs_E10	ordinary_muscle_development	ENSGALG00010002277	RGS10	-0.837337623	0.177939146	-4.705752729	2.52931E-06	4.66798E-06	negative
PRJNA729271	stage_3W_vs_E10	ordinary_muscle_development	ENSGALG00010002279	ENSGALG00010002279	-1.317832127	1.081643196	-1.218361223	0.223086734	0.256312794	negative
PRJNA729271	stage_3W_vs_E10	ordinary_muscle_development	ENSGALG00010002280	ST7	2.25449153	0.139950926	16.10915766	2.2E-58	3.16674E-57	positive
PRJNA729271	stage_3W_vs_E10	ordinary_muscle_development	ENSGALG00010002281	TGFBR2	2.203369935	0.154764155	14.23695261	5.40324E-46	5.35704E-45	positive
PRJNA729271	stage_3W_vs_E10	ordinary_muscle_development	ENSGALG00010002282	TSKU	-1.317491558	0.165716754	-7.950261688	1.86118E-15	5.74852E-15	negative
PRJNA729271	stage_3W_vs_E10	ordinary_muscle_development	ENSGALG00010002283	ENSGALG00010002283	-1.384728952	0.923110491	-1.50006848	0.133596665	0.158845349	negative
PRJNA729271	stage_3W_vs_E10	ordinary_muscle_development	ENSGALG00010002285	CAV1	2.671792941	0.157409214	16.97354856	1.28902E-64	2.1802E-63	positive
PRJNA729271	stage_3W_vs_E10	ordinary_muscle_development	ENSGALG00010002288	ANGPT2	-0.629663349	0.178091251	-3.535622031	0.000406816	0.000631282	negative
PRJNA729271	stage_3W_vs_E10	ordinary_muscle_development	ENSGALG00010002289	ENSGALG00010002289	5.974939555	0.937388707	6.374025534	1.8413E-10	4.38613E-10	positive
PRJNA729271	stage_3W_vs_E10	ordinary_muscle_development	ENSGALG00010002291	RNASEH1	0.354494952	0.116201348	3.050695704	0.002283118	0.003307396	positive
PRJNA729271	stage_3W_vs_E10	ordinary_muscle_development	ENSGALG00010002293	GYG2	-2.400234862	0.373594954	-6.424698292	1.32131E-10	3.16622E-10	negative
PRJNA729271	stage_3W_vs_E10	ordinary_muscle_development	ENSGALG00010002294	DAZL	1.691144813	0.818488789	2.066179567	0.038811527	0.049426559	positive
PRJNA729271	stage_3W_vs_E10	ordinary_muscle_development	ENSGALG00010002295	YAF2	0.591296792	0.113355198	5.216318295	1.82514E-07	3.62752E-07	positive
PRJNA729271	stage_3W_vs_E10	ordinary_muscle_development	ENSGALG00010002298	ENSGALG00010002298	-1.736128367	0.461380029	-3.762903159	0.000167952	0.000269359	negative
PRJNA729271	stage_3W_vs_E10	ordinary_muscle_development	ENSGALG00010002303	ENSGALG00010002303	1.966897746	0.805655194	2.441364199	0.01463189	0.019536593	positive
PRJNA729271	stage_3W_vs_E10	ordinary_muscle_development	ENSGALG00010002308	GXYLT1	-0.922462712	0.103469314	-8.915326472	4.86381E-19	1.79209E-18	negative
PRJNA729271	stage_3W_vs_E10	ordinary_muscle_development	ENSGALG00010002309	GADL1	6.225221552	0.325576093	19.12063474	1.70026E-81	4.4486E-80	positive
PRJNA729271	stage_3W_vs_E10	ordinary_muscle_development	ENSGALG00010002310	PRRG1	-0.314110318	0.105468724	-2.978231897	0.002899165	0.004161969	negative
PRJNA729271	stage_3W_vs_E10	ordinary_muscle_development	ENSGALG00010002312	ENSGALG00010002312	0.299279083	0.190463119	1.571323018	0.116107637	0.139100769	positive
PRJNA729271	stage_3W_vs_E10	ordinary_muscle_development	ENSGALG00010002315	ENSGALG00010002315	-0.544408965	0.171829941	-3.16830095	0.001533327	0.002259615	negative
PRJNA729271	stage_3W_vs_E10	ordinary_muscle_development	ENSGALG00010002324	SNTG2	-4.371204938	0.505785616	-8.642406591	5.50411E-18	1.93928E-17	negative
PRJNA729271	stage_3W_vs_E10	ordinary_muscle_development	ENSGALG00010002328	ENSGALG00010002328	0.213311335	0.256309546	0.832241087	0.405272877	0.444157746	positive
PRJNA729271	stage_3W_vs_E10	ordinary_muscle_development	ENSGALG00010002329	STT3B	0.043718981	0.072471369	0.603258655	0.546336633	0.583585601	positive
PRJNA729271	stage_3W_vs_E10	ordinary_muscle_development	ENSGALG00010002330	KHDRBS2	-3.199463038	0.304189195	-10.51800355	7.13698E-26	3.46452E-25	negative
PRJNA729271	stage_3W_vs_E10	ordinary_muscle_development	ENSGALG00010002331	ENSGALG00010002331	-3.504118048	0.364048811	-9.625407214	6.2459E-22	2.5808E-21	negative
PRJNA729271	stage_3W_vs_E10	ordinary_muscle_development	ENSGALG00010002332	ENSGALG00010002332	-2.924818079	0.640655357	-4.565353351	4.98654E-06	9.00796E-06	negative
PRJNA729271	stage_3W_vs_E10	ordinary_muscle_development	ENSGALG00010002333	ENSGALG00010002333	2.15801063	0.69793986	3.091972182	0.001988315	0.002894509	positive
PRJNA729271	stage_3W_vs_E10	ordinary_muscle_development	ENSGALG00010002334	OSBPL10	-1.126128118	0.169329023	-6.650532175	2.92035E-11	7.28705E-11	negative
PRJNA729271	stage_3W_vs_E10	ordinary_muscle_development	ENSGALG00010002335	TMEM47	0.459531621	0.134190289	3.424477456	0.000615983	0.000940454	positive
PRJNA729271	stage_3W_vs_E10	ordinary_muscle_development	ENSGALG00010002336	PRIM2	-2.303194248	0.123815504	-18.60182427	3.10557E-77	7.22557E-76	negative
PRJNA729271	stage_3W_vs_E10	ordinary_muscle_development	ENSGALG00010002338	CD99	0.394961608	0.148268316	2.663830133	0.007725656	0.01063369	positive
PRJNA729271	stage_3W_vs_E10	ordinary_muscle_development	ENSGALG00010002339	ZNFY4	0.031672758	0.311144681	0.101794311	0.918919941	0.928205195	positive
PRJNA729271	stage_3W_vs_E10	ordinary_muscle_development	ENSGALG00010002340	RAB23	-0.561041824	0.135896795	-4.128440436	3.65232E-05	6.17381E-05	negative
PRJNA729271	stage_3W_vs_E10	ordinary_muscle_development	ENSGALG00010002341	FAM49A	-0.109424514	0.110432157	-0.990875453	0.3217464	0.359826716	negative
PRJNA729271	stage_3W_vs_E10	ordinary_muscle_development	ENSGALG00010002342	BAG2	3.221055917	0.123325924	26.11823871	2.263E-150	2.1723E-148	positive
PRJNA729271	stage_3W_vs_E10	ordinary_muscle_development	ENSGALG00010002343	GPD1L	0.090225028	0.081696118	1.104397982	0.269420536	0.305934247	positive
PRJNA729271	stage_3W_vs_E10	ordinary_muscle_development	ENSGALG00010002345	SCML2	-2.188504691	0.112713785	-19.41647776	5.60016E-84	1.5701E-82	negative
PRJNA729271	stage_3W_vs_E10	ordinary_muscle_development	ENSGALG00010002346	FBXO15	-1.09827961	0.378878114	-2.898767622	0.003746324	0.005323299	negative
PRJNA729271	stage_3W_vs_E10	ordinary_muscle_development	ENSGALG00010002347	ZNF451	-0.407194397	0.062259052	-6.54032436	6.13856E-11	1.49947E-10	negative
PRJNA729271	stage_3W_vs_E10	ordinary_muscle_development	ENSGALG00010002348	THAP12	-0.141158848	0.153117858	-0.9218967	0.35658246	0.395278584	negative
PRJNA729271	stage_3W_vs_E10	ordinary_muscle_development	ENSGALG00010002349	ZBED1	0.783538424	0.129285051	6.060549281	1.35657E-09	3.06668E-09	positive
PRJNA729271	stage_3W_vs_E10	ordinary_muscle_development	ENSGALG00010002350	BEND6	0.810371691	0.246849193	3.282861415	0.001027592	0.001537939	positive
PRJNA729271	stage_3W_vs_E10	ordinary_muscle_development	ENSGALG00010002352	ENSGALG00010002352	0.405508939	0.106713766	3.799968405	0.000144715	0.000233347	positive
PRJNA729271	stage_3W_vs_E10	ordinary_muscle_development	ENSGALG00010002353	CMTM8	0.3266569	0.138849899	2.352590121	0.018643172	0.024600161	positive
PRJNA729271	stage_3W_vs_E10	ordinary_muscle_development	ENSGALG00010002355	UFM1	-0.576117714	0.100680208	-5.722253904	1.0512E-08	2.26676E-08	negative
PRJNA729271	stage_3W_vs_E10	ordinary_muscle_development	ENSGALG00010002356	WNT11	-4.941475213	0.332812758	-14.84761356	7.20896E-50	8.10658E-49	negative
PRJNA729271	stage_3W_vs_E10	ordinary_muscle_development	ENSGALG00010002357	RAB31	-0.774644159	0.142354586	-5.441652271	5.27886E-08	1.08658E-07	negative
PRJNA729271	stage_3W_vs_E10	ordinary_muscle_development	ENSGALG00010002358	FREM2	-3.606675377	0.235018359	-15.34635586	3.74649E-53	4.70584E-52	negative
PRJNA729271	stage_3W_vs_E10	ordinary_muscle_development	ENSGALG00010002359	CMTM6	0.150993081	0.115700878	1.305029685	0.19188274	0.222596263	positive
PRJNA729271	stage_3W_vs_E10	ordinary_muscle_development	ENSGALG00010002360	DHRSX	1.34375236	0.162542043	8.267106389	1.37249E-16	4.50709E-16	positive
PRJNA729271	stage_3W_vs_E10	ordinary_muscle_development	ENSGALG00010002361	ENSGALG00010002361	0.460722979	0.09899357	4.654069725	3.25447E-06	5.96453E-06	positive
PRJNA729271	stage_3W_vs_E10	ordinary_muscle_development	ENSGALG00010002362	TRPC4	-0.763208287	0.345779546	-2.207210623	0.027299348	0.035343341	negative
PRJNA729271	stage_3W_vs_E10	ordinary_muscle_development	ENSGALG00010002364	VSNL1	-0.820299156	0.396822681	-2.067168023	0.038718324	0.049320864	negative
PRJNA729271	stage_3W_vs_E10	ordinary_muscle_development	ENSGALG00010002365	RAI2	1.200160757	0.138116182	8.689501397	3.64034E-18	1.29584E-17	positive
PRJNA729271	stage_3W_vs_E10	ordinary_muscle_development	ENSGALG00010002366	ENSGALG00010002366	1.590496119	0.756704408	2.101872413	0.035564454	0.045471179	positive
PRJNA729271	stage_3W_vs_E10	ordinary_muscle_development	ENSGALG00010002367	STK31	-5.428891304	0.87271331	-6.220704144	4.94929E-10	1.15095E-09	negative
PRJNA729271	stage_3W_vs_E10	ordinary_muscle_development	ENSGALG00010002368	UVRAG	1.15509747	0.094899963	12.17173789	4.39621E-34	2.9325E-33	positive
PRJNA729271	stage_3W_vs_E10	ordinary_muscle_development	ENSGALG00010002369	GEMIN8	-0.556388176	0.120580486	-4.614247272	3.94523E-06	7.18779E-06	negative
PRJNA729271	stage_3W_vs_E10	ordinary_muscle_development	ENSGALG00010002370	ENSGALG00010002370	-0.154707953	0.156802089	-0.986644721	0.323816838	0.361863008	negative
PRJNA729271	stage_3W_vs_E10	ordinary_muscle_development	ENSGALG00010002371	BAG3	2.569929313	0.154703632	16.61195207	5.71043E-62	9.01664E-61	positive
PRJNA729271	stage_3W_vs_E10	ordinary_muscle_development	ENSGALG00010002372	SMC6	-0.327443744	0.127003916	-2.578217696	0.009931141	0.013496333	negative
PRJNA729271	stage_3W_vs_E10	ordinary_muscle_development	ENSGALG00010002373	POSTN	0.243815022	0.255606955	0.953866932	0.340151004	0.378598541	positive
PRJNA729271	stage_3W_vs_E10	ordinary_muscle_development	ENSGALG00010002374	DST	1.177180434	0.114211965	10.30697991	6.55282E-25	3.0551E-24	positive
PRJNA729271	stage_3W_vs_E10	ordinary_muscle_development	ENSGALG00010002375	NPY	-3.678868572	0.531953948	-6.915765148	4.65345E-12	1.20622E-11	negative
PRJNA729271	stage_3W_vs_E10	ordinary_muscle_development	ENSGALG00010002376	GEN1	-1.70789556	0.193516077	-8.825600345	1.08874E-18	3.9473E-18	negative
PRJNA729271	stage_3W_vs_E10	ordinary_muscle_development	ENSGALG00010002377	ART1A	5.568201415	0.242232367	22.98702478	6.2852E-117	3.5312E-115	positive
PRJNA729271	stage_3W_vs_E10	ordinary_muscle_development	ENSGALG00010002378	ENSGALG00010002378	0.387510093	0.447528661	0.865888884	0.3865511	0.42553795	positive
PRJNA729271	stage_3W_vs_E10	ordinary_muscle_development	ENSGALG00010002379	NHS	-1.766132319	0.135675699	-13.01730765	9.75501E-39	7.5453E-38	negative
PRJNA729271	stage_3W_vs_E10	ordinary_muscle_development	ENSGALG00010002380	CMPK2	4.40065519	0.741731095	5.932952279	2.97535E-09	6.58903E-09	positive
PRJNA729271	stage_3W_vs_E10	ordinary_muscle_development	ENSGALG00010002381	TDRD3	0.772342397	0.096239082	8.025246932	1.01322E-15	3.17343E-15	positive
PRJNA729271	stage_3W_vs_E10	ordinary_muscle_development	ENSGALG00010002382	ART1D	2.321993198	0.529861356	4.382265606	1.17451E-05	2.06381E-05	positive
PRJNA729271	stage_3W_vs_E10	ordinary_muscle_development	ENSGALG00010002383	INPP5F	0.364121666	0.123868315	2.939586823	0.003286502	0.004698939	positive
PRJNA729271	stage_3W_vs_E10	ordinary_muscle_development	ENSGALG00010002384	ENSGALG00010002384	-0.911830499	0.308185201	-2.958709552	0.003089301	0.004426156	negative
PRJNA729271	stage_3W_vs_E10	ordinary_muscle_development	ENSGALG00010002385	PALS2	-2.543529997	0.128012199	-19.86943441	7.48413E-88	2.32795E-86	negative
PRJNA729271	stage_3W_vs_E10	ordinary_muscle_development	ENSGALG00010002386	MHCY2B2	-2.125604598	0.440729297	-4.822925571	1.41468E-06	2.65172E-06	negative
PRJNA729271	stage_3W_vs_E10	ordinary_muscle_development	ENSGALG00010002387	MCMBP	-0.01155714	0.108771439	-0.106251606	0.915382727	0.92534156	negative
PRJNA729271	stage_3W_vs_E10	ordinary_muscle_development	ENSGALG00010002388	ENSGALG00010002388	3.963314171	0.727223438	5.44992634	5.03907E-08	1.03899E-07	positive
PRJNA729271	stage_3W_vs_E10	ordinary_muscle_development	ENSGALG00010002389	COL21A1	-0.980878139	0.322586864	-3.040663615	0.002360574	0.0034138	negative
PRJNA729271	stage_3W_vs_E10	ordinary_muscle_development	ENSGALG00010002391	REPS2	-0.561817532	0.161376025	-3.481418825	0.000498765	0.000768697	negative
PRJNA729271	stage_3W_vs_E10	ordinary_muscle_development	ENSGALG00010002392	DFNA5	-1.670734322	0.207848889	-8.038216271	9.11556E-16	2.86368E-15	negative
PRJNA729271	stage_3W_vs_E10	ordinary_muscle_development	ENSGALG00010002393	GPM6B	-1.054880003	0.107324749	-9.8288607	8.45701E-23	3.61606E-22	negative
PRJNA729271	stage_3W_vs_E10	ordinary_muscle_development	ENSGALG00010002395	CLN8	0.071130713	0.090834825	0.783077559	0.433581569	0.472183684	positive
PRJNA729271	stage_3W_vs_E10	ordinary_muscle_development	ENSGALG00010002396	SEC23IP	-0.094630372	0.088243978	-1.072372006	0.283552976	0.320675696	negative
PRJNA729271	stage_3W_vs_E10	ordinary_muscle_development	ENSGALG00010002398	ENSGALG00010002398	-1.265977611	0.398251906	-3.178836291	0.001478676	0.002181517	negative
PRJNA729271	stage_3W_vs_E10	ordinary_muscle_development	ENSGALG00010002399	CSMD1	-2.263140121	0.399153232	-5.669852923	1.4292E-08	3.0555E-08	negative
PRJNA729271	stage_3W_vs_E10	ordinary_muscle_development	ENSGALG00010002400	RBBP7	-2.093613802	0.159600459	-13.11784326	2.60236E-39	2.05677E-38	negative
PRJNA729271	stage_3W_vs_E10	ordinary_muscle_development	ENSGALG00010002401	ZRANB1	0.02044564	0.161676974	0.126459813	0.899367959	0.911122348	positive
PRJNA729271	stage_3W_vs_E10	ordinary_muscle_development	ENSGALG00010002402	TXLNG	-0.093913273	0.126779002	-0.740763624	0.458836775	0.497370554	negative
PRJNA729271	stage_3W_vs_E10	ordinary_muscle_development	ENSGALG00010002403	ZNFY3	-0.473967233	0.266699714	-1.777156888	0.075542431	0.092748393	negative
PRJNA729271	stage_3W_vs_E10	ordinary_muscle_development	ENSGALG00010002404	OSBPL3	0.075947333	0.252352619	0.300957181	0.763447146	0.788918969	positive
PRJNA729271	stage_3W_vs_E10	ordinary_muscle_development	ENSGALG00010002405	KCNQ5	0.800841495	0.197908578	4.046522407	5.19842E-05	8.69401E-05	positive
PRJNA729271	stage_3W_vs_E10	ordinary_muscle_development	ENSGALG00010002406	DIAPH3	-2.928105854	0.25383318	-11.53555204	8.73252E-31	5.17066E-30	negative
PRJNA729271	stage_3W_vs_E10	ordinary_muscle_development	ENSGALG00010002408	OFD1	-0.164106406	0.115446734	-1.421490243	0.155174288	0.182702567	negative
PRJNA729271	stage_3W_vs_E10	ordinary_muscle_development	ENSGALG00010002409	CYCS	1.628163171	0.166704956	9.766735248	1.56411E-22	6.60793E-22	positive
PRJNA729271	stage_3W_vs_E10	ordinary_muscle_development	ENSGALG00010002410	RNF121	0.432495081	0.104234459	4.149252401	3.33563E-05	5.65034E-05	positive

PRJNA729271	stage_3W_vs_E10	ordinary_muscle_development	ENSGALG00010002411	ENSGALG00010002411	-1.639585926	0.19369873	-8.464618859	2.56992E-17	8.74435E-17	negative
PRJNA729271	stage_3W_vs_E10	ordinary_muscle_development	ENSGALG00010002413	TSSC1	-0.577522813	0.084423946	-6.840746504	7.87816E-12	2.02077E-11	negative
PRJNA729271	stage_3W_vs_E10	ordinary_muscle_development	ENSGALG00010002414	ENSGALG00010002414	2.373224332	0.356769723	6.651977949	2.8918E-11	7.22078E-11	positive
PRJNA729271	stage_3W_vs_E10	ordinary_muscle_development	ENSGALG00010002415	PCDH17	-6.0226235	0.53609283	-11.23429221	2.76702E-29	1.55037E-28	negative
PRJNA729271	stage_3W_vs_E10	ordinary_muscle_development	ENSGALG00010002416	SH3BGRL2	-1.599205637	0.281350756	-5.684028218	1.31559E-08	2.81842E-08	negative
PRJNA729271	stage_3W_vs_E10	ordinary_muscle_development	ENSGALG00010002418	TRAPPC2	-0.251741311	0.083270558	-3.023173089	0.002501391	0.003611473	negative
PRJNA729271	stage_3W_vs_E10	ordinary_muscle_development	ENSGALG00010002419	CTBP2	-1.81190212	0.116234965	-15.58827091	8.74692E-55	1.14635E-53	negative
PRJNA729271	stage_3W_vs_E10	ordinary_muscle_development	ENSGALG00010002420	TMEM272	-2.569184735	0.925273255	-2.77667675	0.005491777	0.007665219	negative
PRJNA729271	stage_3W_vs_E10	ordinary_muscle_development	ENSGALG00010002421	ENSGALG00010002421	-0.169033038	0.186086403	-0.908357813	0.363689207	0.402510121	negative
PRJNA729271	stage_3W_vs_E10	ordinary_muscle_development	ENSGALG00010002422	TRAPPC12	0.238881726	0.103072786	2.317602307	0.020470945	0.026901754	positive
PRJNA729271	stage_3W_vs_E10	ordinary_muscle_development	ENSGALG00010002423	ENSGALG00010002423	3.353155925	0.565404827	5.930539966	3.0194E-09	6.68289E-09	positive
PRJNA729271	stage_3W_vs_E10	ordinary_muscle_development	ENSGALG00010002424	CASK	-0.122552484	0.056658662	-2.162996437	0.030541454	0.039340476	negative
PRJNA729271	stage_3W_vs_E10	ordinary_muscle_development	ENSGALG00010002425	FDX2	0.546252907	0.133516145	4.091287283	4.28985E-05	7.2153E-05	positive
PRJNA729271	stage_3W_vs_E10	ordinary_muscle_development	ENSGALG00010002427	CYB5R4	0.231319644	0.111911918	2.066979533	0.038736083	0.049339149	positive
PRJNA729271	stage_3W_vs_E10	ordinary_muscle_development	ENSGALG00010002428	IBSP	-3.188354543	0.717825781	-4.441682968	8.9258E-06	1.58146E-05	negative
PRJNA729271	stage_3W_vs_E10	ordinary_muscle_development	ENSGALG00010002429	ENSGALG00010002429	0.173589154	0.546954852	0.317373825	0.750959966	0.777234181	positive
PRJNA729271	stage_3W_vs_E10	ordinary_muscle_development	ENSGALG00010002430	NUMA1	1.498665185	0.174198482	8.603204624	7.75206E-18	2.7135E-17	positive
PRJNA729271	stage_3W_vs_E10	ordinary_muscle_development	ENSGALG00010002431	BCKDHB	1.14407456	0.147437018	7.759751073	8.50962E-15	2.54744E-14	positive
PRJNA729271	stage_3W_vs_E10	ordinary_muscle_development	ENSGALG00010002432	RAVER1	-0.783261889	0.185116754	-4.231177744	2.32471E-05	3.99297E-05	negative
PRJNA729271	stage_3W_vs_E10	ordinary_muscle_development	ENSGALG00010002433	GRHL1	-2.164333297	0.332407687	-6.511080767	7.4612E-11	1.8146E-10	negative
PRJNA729271	stage_3W_vs_E10	ordinary_muscle_development	ENSGALG00010002434	GPR34	3.558226509	0.245648422	14.48503713	1.50641E-47	1.5709E-46	positive
PRJNA729271	stage_3W_vs_E10	ordinary_muscle_development	ENSGALG00010002435	ATP7B	-3.104083913	0.754160091	-4.115948258	3.85591E-05	6.51187E-05	negative
PRJNA729271	stage_3W_vs_E10	ordinary_muscle_development	ENSGALG00010002436	ENSGALG00010002436	4.912865272	0.873533963	5.624126229	1.86449E-08	3.95983E-08	positive
PRJNA729271	stage_3W_vs_E10	ordinary_muscle_development	ENSGALG00010002437	TTK	-3.390003346	0.199740569	-16.97203209	1.32275E-64	2.23204E-63	negative
PRJNA729271	stage_3W_vs_E10	ordinary_muscle_development	ENSGALG00010002438	RARB	-1.846715896	0.094176994	-19.60899181	1.29575E-85	3.82523E-84	negative
PRJNA729271	stage_3W_vs_E10	ordinary_muscle_development	ENSGALG00010002439	GPR82	4.702028193	0.847955807	5.545133549	2.9373E-08	6.14641E-08	positive
PRJNA729271	stage_3W_vs_E10	ordinary_muscle_development	ENSGALG00010002440	LRRC51	0.640044224	0.265995612	2.406220991	0.016118511	0.021416978	positive
PRJNA729271	stage_3W_vs_E10	ordinary_muscle_development	ENSGALG00010002441	USO1	-0.483483424	0.068232213	-7.085852916	1.38191E-12	3.69843E-12	negative
PRJNA729271	stage_3W_vs_E10	ordinary_muscle_development	ENSGALG00010002442	ENSGALG00010002442	0.224589134	0.452297421	0.496551878	0.619505095	0.654022313	positive
PRJNA729271	stage_3W_vs_E10	ordinary_muscle_development	ENSGALG00010002443	TYK2	0.399476579	0.13796607	2.895469723	0.003785915	0.005376393	positive
PRJNA729271	stage_3W_vs_E10	ordinary_muscle_development	ENSGALG00010002444	KLF11	0.682733758	0.182266689	3.745795578	0.000179823	0.00028757	positive
PRJNA729271	stage_3W_vs_E10	ordinary_muscle_development	ENSGALG00010002445	EDRF1	0.73718953	0.111125367	6.633854609	3.27032E-11	8.1393E-11	positive
PRJNA729271	stage_3W_vs_E10	ordinary_muscle_development	ENSGALG00010002446	RIMS1	-5.602292125	0.361179485	-15.51110279	2.91823E-54	3.77677E-53	negative
PRJNA729271	stage_3W_vs_E10	ordinary_muscle_development	ENSGALG00010002447	YLEC12	-0.604534198	0.575370298	-1.050687184	0.293402284	0.330704985	negative
PRJNA729271	stage_3W_vs_E10	ordinary_muscle_development	ENSGALG00010002448	RAB9A	0.36689837	0.13578	2.702153267	0.0068892	0.009521258	positive
PRJNA729271	stage_3W_vs_E10	ordinary_muscle_development	ENSGALG00010002449	ALG11	-0.7006714	0.140998189	-4.969364541	6.71727E-07	1.28656E-06	negative
PRJNA729271	stage_3W_vs_E10	ordinary_muscle_development	ENSGALG00010002453	TOP2B	-0.964982391	0.063258618	-15.25456008	1.53526E-52	1.88271E-51	negative
PRJNA729271	stage_3W_vs_E10	ordinary_muscle_development	ENSGALG00010002454	NEK5	-4.451178354	0.870750333	-5.1118882	3.18955E-07	6.24004E-07	negative
PRJNA729271	stage_3W_vs_E10	ordinary_muscle_development	ENSGALG00010002455	NYX	2.036344803	0.35418447	5.749390429	8.95658E-09	1.93827E-08	positive
PRJNA729271	stage_3W_vs_E10	ordinary_muscle_development	ENSGALG00010002456	ELOVL4	-3.340614186	0.599955406	-5.568104152	2.57526E-08	5.41148E-08	negative
PRJNA729271	stage_3W_vs_E10	ordinary_muscle_development	ENSGALG00010002457	ENSGALG00010002457	2.258087966	0.441044839	5.11986031	3.05762E-07	5.98922E-07	positive
PRJNA729271	stage_3W_vs_E10	ordinary_muscle_development	ENSGALG00010002458	ENSGALG00010002458	-0.114974649	0.198051494	-0.580529064	0.561557893	0.598469352	negative
PRJNA729271	stage_3W_vs_E10	ordinary_muscle_development	ENSGALG00010002459	G3BP2	-0.705940838	0.074229548	-9.510240254	1.90223E-21	7.72347E-21	negative
PRJNA729271	stage_3W_vs_E10	ordinary_muscle_development	ENSGALG00010002460	LAMTOR1	1.190713397	0.180267627	6.60525364	3.96837E-11	9.81764E-11	positive
PRJNA729271	stage_3W_vs_E10	ordinary_muscle_development	ENSGALG00010002461	TOMT	-0.157172602	0.342058276	-0.459490717	0.645881817	0.679049607	negative
PRJNA729271	stage_3W_vs_E10	ordinary_muscle_development	ENSGALG00010002462	RRM2	-4.245573437	0.278278331	-15.25657218	1.48866E-52	1.8271E-51	negative
PRJNA729271	stage_3W_vs_E10	ordinary_muscle_development	ENSGALG00010002463	ASRGL1	0.412271006	0.100246039	4.112591485	3.91242E-05	6.60424E-05	positive
PRJNA729271	stage_3W_vs_E10	ordinary_muscle_development	ENSGALG00010002464	NGLY1	0.788752365	0.082851024	9.520128191	1.72966E-21	7.03659E-21	positive
PRJNA729271	stage_3W_vs_E10	ordinary_muscle_development	ENSGALG00010002466	UROS	0.90720731	0.176092779	5.151871151	2.579E-07	5.07709E-07	positive
PRJNA729271	stage_3W_vs_E10	ordinary_muscle_development	ENSGALG00010002467	NEK3	-1.708042871	0.225172884	-7.585473186	3.31276E-14	9.63258E-14	negative
PRJNA729271	stage_3W_vs_E10	ordinary_muscle_development	ENSGALG00010002468	ENSGALG00010002468	0.07650895	0.553878594	0.138133069	0.890135251	0.90296805	positive
PRJNA729271	stage_3W_vs_E10	ordinary_muscle_development	ENSGALG00010002469	MEPE	-3.260494155	1.378464823	-2.365308204	0.018015072	0.023814726	negative
PRJNA729271	stage_3W_vs_E10	ordinary_muscle_development	ENSGALG00010002470	OXSM	0.449476641	0.160601911	2.798700451	0.005130871	0.007187087	positive
PRJNA729271	stage_3W_vs_E10	ordinary_muscle_development	ENSGALG00010002471	OGFRL1	-0.255181986	0.153121934	-1.666527976	0.095608301	0.115902176	negative
PRJNA729271	stage_3W_vs_E10	ordinary_muscle_development	ENSGALG00010002472	CDC37	0.103511925	0.214480224	0.48261757	0.629367301	0.663008883	positive
PRJNA729271	stage_3W_vs_E10	ordinary_muscle_development	ENSGALG00010002473	ENSGALG00010002473	2.734511735	0.627929875	4.354804324	1.33186E-05	2.33043E-05	positive
PRJNA729271	stage_3W_vs_E10	ordinary_muscle_development	ENSGALG00010002474	HPCAL1	-0.213222695	0.14162674	-1.505525686	0.132188978	0.15723611	negative
PRJNA729271	stage_3W_vs_E10	ordinary_muscle_development	ENSGALG00010002475	ENSGALG00010002475	-0.221337973	0.192082648	-1.152305924	0.249195383	0.284438702	negative
PRJNA729271	stage_3W_vs_E10	ordinary_muscle_development	ENSGALG00010002476	DDX3X	0.701891047	0.144954406	4.8421505	1.28441E-06	2.41379E-06	positive
PRJNA729271	stage_3W_vs_E10	ordinary_muscle_development	ENSGALG00010002478	PPME1	-1.336070484	0.057904558	-23.07366681	8.5134E-118	4.8969E-116	negative
PRJNA729271	stage_3W_vs_E10	ordinary_muscle_development	ENSGALG00010002481	CKAP2	-3.715562696	0.247797049	-14.99437832	7.99074E-51	9.31824E-50	negative
PRJNA729271	stage_3W_vs_E10	ordinary_muscle_development	ENSGALG00010002483	COQ2	1.886615594	0.135326454	13.94121797	3.55816E-44	3.29767E-43	positive
PRJNA729271	stage_3W_vs_E10	ordinary_muscle_development	ENSGALG00010002487	BCCIP	-0.459603188	0.118569782	-3.876225301	0.000106089	0.000172938	negative
PRJNA729271	stage_3W_vs_E10	ordinary_muscle_development	ENSGALG00010002492	RPL8	0.354369938	0.129572581	2.734914558	0.006239647	0.008659041	positive
PRJNA729271	stage_3W_vs_E10	ordinary_muscle_development	ENSGALG00010002500	OSR1	-2.949599068	0.203120339	-14.52143635	8.86333E-48	9.35037E-47	negative
PRJNA729271	stage_3W_vs_E10	ordinary_muscle_development	ENSGALG00010002501	TCEANC	0.695572432	0.092506672	7.519159589	5.51295E-14	1.58709E-13	positive
PRJNA729271	stage_3W_vs_E10	ordinary_muscle_development	ENSGALG00010002504	ENSGALG00010002504	0.757766838	0.065593647	11.55244247	7.17519E-31	4.26247E-30	positive
PRJNA729271	stage_3W_vs_E10	ordinary_muscle_development	ENSGALG00010002507	C2CD3	-0.701300135	0.111793591	-6.273169397	3.53772E-10	8.28956E-10	negative
PRJNA729271	stage_3W_vs_E10	ordinary_muscle_development	ENSGALG00010002511	VPS36	-0.142054921	0.077253533	-1.838814552	0.065942467	0.081562937	negative
PRJNA729271	stage_3W_vs_E10	ordinary_muscle_development	ENSGALG00010002512	DAXX	0.706666272	0.230180035	3.070058932	0.002140165	0.003108075	positive
PRJNA729271	stage_3W_vs_E10	ordinary_muscle_development	ENSGALG00010002518	MAST1	0.390992512	0.360100524	1.085787122	0.277573185	0.314428206	positive
PRJNA729271	stage_3W_vs_E10	ordinary_muscle_development	ENSGALG00010002520	TTC32	1.384844228	0.162599076	8.516925562	1.63845E-17	5.62913E-17	positive
PRJNA729271	stage_3W_vs_E10	ordinary_muscle_development	ENSGALG00010002522	HPSE	2.168887359	0.28394147	7.638501556	2.19764E-14	6.44313E-14	positive
PRJNA729271	stage_3W_vs_E10	ordinary_muscle_development	ENSGALG00010002523	DNAJB13	5.432601533	0.41460765	13.10299396	3.16521E-39	2.49346E-38	positive
PRJNA729271	stage_3W_vs_E10	ordinary_muscle_development	ENSGALG00010002526	ZBTB22	-0.516651641	0.10956985	-4.715271967	2.41388E-06	4.45893E-06	negative
PRJNA729271	stage_3W_vs_E10	ordinary_muscle_development	ENSGALG00010002527	LRRC3B	-2.38140272	0.195889744	-12.15685248	5.27523E-34	3.50754E-33	negative
PRJNA729271	stage_3W_vs_E10	ordinary_muscle_development	ENSGALG00010002528	MED14	-0.293313757	0.116969889	-2.50760054	0.0121554	0.016373248	negative
PRJNA729271	stage_3W_vs_E10	ordinary_muscle_development	ENSGALG00010002530	STAG3	-0.342283066	0.122461551	-2.795024742	0.005189574	0.007266507	negative
PRJNA729271	stage_3W_vs_E10	ordinary_muscle_development	ENSGALG00010002533	LPAR2	-0.733709943	0.144305404	-5.084424591	3.68742E-07	7.18693E-07	negative
PRJNA729271	stage_3W_vs_E10	ordinary_muscle_development	ENSGALG00010002536	RGL2	1.628961458	0.141568105	11.50655694	1.22263E-30	7.19237E-30	positive
PRJNA729271	stage_3W_vs_E10	ordinary_muscle_development	ENSGALG00010002539	SNAP91	0.075249309	0.192947488	0.389998907	0.696537355	0.726300911	positive
PRJNA729271	stage_3W_vs_E10	ordinary_muscle_development	ENSGALG00010002543	UCP3	11.37981759	0.502860899	22.63015002	2.1886E-113	1.1793E-111	positive
PRJNA729271	stage_3W_vs_E10	ordinary_muscle_development	ENSGALG00010002544	THSD1	0.309769592	0.126170714	2.4551624	0.014082095	0.018828519	positive
PRJNA729271	stage_3W_vs_E10	ordinary_muscle_development	ENSGALG00010002546	ATP6AP2	0.731925266	0.06251717	11.70758802	1.16548E-31	7.12511E-31	positive
PRJNA729271	stage_3W_vs_E10	ordinary_muscle_development	ENSGALG00010002547	PBX4	-1.131253996	0.106313234	-10.64076365	1.92545E-26	9.61397E-26	negative
PRJNA729271	stage_3W_vs_E10	ordinary_muscle_development	ENSGALG00010002551	WDR35	-0.932958646	0.111252324	-8.385969918	5.03092E-17	1.68726E-16	negative
PRJNA729271	stage_3W_vs_E10	ordinary_muscle_development	ENSGALG00010002553	ENSGALG00010002553	0.31618339	0.223126261	1.41706041	0.156465266	0.184132827	positive
PRJNA729271	stage_3W_vs_E10	ordinary_muscle_development	ENSGALG00010002554	EGFL6	-4.143087039	0.293801522	-14.10165272	3.70979E-45	3.56587E-44	negative
PRJNA729271	stage_3W_vs_E10	ordinary_muscle_development	ENSGALG00010002556	ENSGALG00010002556	4.806856221	0.229467357	20.94788684	1.96144E-97	7.54139E-96	positive
PRJNA729271	stage_3W_vs_E10	ordinary_muscle_development	ENSGALG00010002557	ENSGALG00010002557	0.751875488	0.407932606	1.843136533	0.065309079	0.080834693	positive
PRJNA729271	stage_3W_vs_E10	ordinary_muscle_development	ENSGALG00010002559	NEK10	-2.155107797	0.61255558	-3.518224089	0.000434445	0.000672787	negative
PRJNA729271	stage_3W_vs_E10	ordinary_muscle_development	ENSGALG00010002561	PAAF1	-0.703764152	0.084312226	-8.347118666	6.99484E-17	2.33135E-16	negative
PRJNA729271	stage_3W_vs_E10	ordinary_muscle_development	ENSGALG00010002565	MRPS18C	0.912151505	0.113832599	8.013095673	1.11857E-15	3.49356E-15	positive
PRJNA729271	stage_3W_vs_E10	ordinary_muscle_development	ENSGALG00010002566	TMSB4X	-1.073657124	0.11571139	-9.278750549	1.71478E-20	6.67984E-20	negative
PRJNA729271	stage_3W_vs_E10	ordinary_muscle_development	ENSGALG00010002567	COA4	0.159709456	0.227326879	0.70255421	0.482333607	0.520815378	positive
PRJNA729271	stage_3W_vs_E10	ordinary_muscle_development	ENSGALG00010002569	GTPBP6	0.260260149	0.116057839	2.242503829	0.024928828	0.032436568	positive
PRJNA729271	stage_3W_vs_E10	ordinary_muscle_development	ENSGALG00010002570	ADAM12	-3.062640843	0.270871838	-11.30660489	1.21708E-29	6.90474E-29	negative
PRJNA729271	stage_3W_vs_E10	ordinary_muscle_development	ENSGALG00010002571	BCOR	0.437031312	0.141439855	3.08987387	0.002002415	0.002913865	positive
PRJNA729271	stage_3W_vs_E10	ordinary_muscle_development	ENSGALG00010002573	MVB12A	1.372879541	0.093846675	14.628963	1.83564E-48	1.99607E-47	positive
PRJNA729271	stage_3W_vs_E10	ordinary_muscle_development	ENSGALG00010002575	BGV	1.161952194	0.3973404	2.924324316	0.003452049	0.004920587	positive
PRJNA729271	stage_3W_vs_E10	ordinary_muscle_development	ENSGALG00010002576	HELQ	-0.314760788	0.10656654	-2.953654949	0.003140349	0.004497072	negative
PRJNA729271	stage_3W_vs_E10	ordinary_muscle_development	ENSGALG00010002577	MRPL48	0.149853843	0.103042814	1.454287176	0.145866704	0.172430099	positive
PRJNA729271	stage_3W_vs_E10	ordinary_muscle_development	ENSGALG00010002579	MATN3	-0.91766411	0.572230371	-1.603662015	0.108788573	0.130838572	negative
PRJNA729271	stage_3W_vs_E10	ordinary_muscle_development	ENSGALG00010002581	ATP13A1	0.08323077	0.077050686	1.080208042	0.280049548	0.316985647	positive
PRJNA729271	stage_3W_vs_E10	ordinary_muscle_development	ENSGALG00010002582	ENSGALG00010002582	-4.257532778	0.601452493	-7.078751565	1.45459E-12	3.89009E-12	negative
PRJNA729271	stage_3W_vs_E10	ordinary_muscle_development	ENSGALG00010002584	ENSGALG00010002584	1.755507724	0.435867028	4.027622212	5.63438E-05	9.40252E-05	positive
PRJNA729271	stage_3W_vs_E10	ordinary_muscle_development	ENSGALG00010002586	GPR63	-0.463297521	0.147576401	-3.139374029	0.001693092	0.002485453	negative
PRJNA729271	stage_3W_vs_E10	ordinary_muscle_development	ENSGALG00010002587	DOCK1	-0.456105038	0.066743553	-6.833694321	8.27553E-12	2.12157E-11	negative
PRJNA729271	stage_3W_vs_E10	ordinary_muscle_development	ENSGALG00010002588	FAM175A	-0.92036579	0.08823389	-10.43097832	1.79035E-25	8.53657E-25	negative
PRJNA729271	stage_3W_vs_E10	ordinary_muscle_development	ENSGALG00010002589	SLC4A7	-0.03708241	0.144766512	-0.256153234	0.797832518	0.820415887	negative
PRJNA729271	stage_3W_vs_E10	ordinary_muscle_development	ENSGALG00010002590	ATP6V1C2	-1.8075954	0.262315997	-6.890907997	5.54374E-12	1.43187E-11	negative
PRJNA729271	stage_3W_vs_E10	ordinary_muscle_development	ENSGALG00010002591	ENSGALG00010002591	0.914781437	0.211459307	4.326040077	1.51814E-05	2.64647E-05	positive
PRJNA729271	stage_3W_vs_E10	ordinary_muscle_development	ENSGALG00010002592	RPS6KA3	1.413315889	0.100958809	13.99893583	1.58222E-44	1.48442E-43	positive
PRJNA729271	stage_3W_vs_E10	ordinary_muscle_development	ENSGALG00010002597	ENSGALG00010002597	-0.379037951	0.11697411	-3.240357639	0.001193799	0.00177642	negative
PRJNA729271	stage_3W_vs_E10	ordinary_muscle_development	ENSGALG00010002598	NMT2	0.460064525	0.101932873	4.513406819	6.37945E-06	1.14415E-05	positive
PRJNA729271	stage_3W_vs_E10	ordinary_muscle_development	ENSGALG00010002599	KIFC1	-1.635223826	0.220057094	-7.430907107	1.07855E-13	3.05464E-13	negative
PRJNA729271	stage_3W_vs_E10	ordinary_muscle_development	ENSGALG00010002600	TRPA1	-1.943552452	0.578685125	-3.35856646	0.000783479	0.001184452	negative
PRJNA729271	stage_3W_vs_E10	ordinary_muscle_development	ENSGALG00010002601	PLCXD1	2.274634974	0.186656427	12.18621296	3.68135E-34	2.46017E-33	positive
PRJNA729271	stage_3W_vs_E10	ordinary_muscle_development	ENSGALG00010002603	INSYN2A	0.130611189	0.256864964	0.508481917	0.611115418	0.646059221	positive
PRJNA729271	stage_3W_vs_E10	ordinary_muscle_development	ENSGALG00010002604	BLEC3	0.037325147	0.38852431	0.096069013	0.923465762	0.932277213	positive
PRJNA729271	stage_3W_vs_E10	ordinary_muscle_development	ENSGALG00010002605	EOMES	3.346471976	0.68295516	4.899987839	9.58426E-07	1.81885E-06	positive
PRJNA729271	stage_3W_vs_E10	ordinary_muscle_development	ENSGALG00010002608	RPP38	-0.246169567	0.128134008	-1.92118838	0.054707963	0.068343697	negative
PRJNA729271	stage_3W_vs_E10	ordinary_muscle_development	ENSGALG00010002609	PDIA6	-1.819526147	0.125745147	-14.46995128	1.87605E-47	1.94795E-46	negative
PRJNA729271	stage_3W_vs_E10	ordinary_muscle_development	ENSGALG00010002611	NPS	-0.239306485	0.409335409	-0.584622	0.558801911	0.595838878	negative
PRJNA729271	stage_3W_vs_E10	ordinary_muscle_development	ENSGALG00010002612	JPH1	4.470592164	0.214335116	20.85795479	1.29074E-96	4.88494E-95	positive
PRJNA729271	stage_3W_vs_E10	ordinary_muscle_development	ENSGALG00010002613	ENSGALG00010002613	0.265979653	0.103420963	2.571815679	0.010116674	0.013740529	positive
PRJNA729271	stage_3W_vs_E10	ordinary_muscle_development	ENSGALG00010002614	GPAT3	0.746295192	0.144320819	5.171084787	2.32739E-07	4.59236E-07	positive
PRJNA729271	stage_3W_vs_E10	ordinary_muscle_development	ENSGALG00010002616	ZNFB3	-0.619796021	0.169426588	-3.65819809	0.000253995	0.00040079	negative
PRJNA729271	stage_3W_vs_E10	ordinary_muscle_development	ENSGALG00010002619	RAB6A	-0.941236653	0.06882393	-13.67600861	1.41235E-42	1.24073E-41	negative
PRJNA729271	stage_3W_vs_E10	ordinary_muscle_development	ENSGALG00010002621	SLC25A15	-0.712812483	0.163060276	-4.371466193	1.23415E-05	2.16626E-05	negative
PRJNA729271	stage_3W_vs_E10	ordinary_muscle_development	ENSGALG00010002622	AZI2	0.573048148	0.079612624	7.197955785	6.1122E-13	1.66378E-12	positive

PRJNA729271	stage_3W_vs_E10	ordinary_muscle_development	ENSGALG00010002623	MID1IP1	1.874686252	0.135646499	13.82038066	1.92052E-43	1.74423E-42	positive
PRJNA729271	stage_3W_vs_E10	ordinary_muscle_development	ENSGALG00010002625	FHL5	1.115865983	0.218429978	5.108575275	3.24597E-07	6.347E-07	positive
PRJNA729271	stage_3W_vs_E10	ordinary_muscle_development	ENSGALG00010002626	CHD7	0.511122669	0.135896564	3.761115474	0.000169157	0.000271232	positive
PRJNA729271	stage_3W_vs_E10	ordinary_muscle_development	ENSGALG00010002627	MRPS31	1.487770046	0.146077339	10.18481067	2.31811E-24	1.05697E-23	positive
PRJNA729271	stage_3W_vs_E10	ordinary_muscle_development	ENSGALG00010002628	TRIM7.2	-0.264587527	0.192255645	-1.37622761	0.168751177	0.197676443	negative
PRJNA729271	stage_3W_vs_E10	ordinary_muscle_development	ENSGALG00010002632	HPGD	3.088767152	0.27215106	11.34945845	7.46235E-30	4.2703E-29	positive
PRJNA729271	stage_3W_vs_E10	ordinary_muscle_development	ENSGALG00010002633	ENSGALG00010002633	-1.446781868	0.135410361	-10.68442517	1.20393E-26	6.05724E-26	negative
PRJNA729271	stage_3W_vs_E10	ordinary_muscle_development	ENSGALG00010002634	RGN	1.012531496	0.183226615	5.526115818	3.27398E-08	6.82524E-08	positive
PRJNA729271	stage_3W_vs_E10	ordinary_muscle_development	ENSGALG00010002638	FABP9	3.820248971	0.507457671	7.528212079	5.14398E-14	1.48353E-13	positive
PRJNA729271	stage_3W_vs_E10	ordinary_muscle_development	ENSGALG00010002644	KCNF1	-1.273914947	0.506259546	-2.516327756	0.011858485	0.015998579	negative
PRJNA729271	stage_3W_vs_E10	ordinary_muscle_development	ENSGALG00010002646	FOXO1	1.383755609	0.169250079	8.175804809	2.93897E-16	9.48573E-16	positive
PRJNA729271	stage_3W_vs_E10	ordinary_muscle_development	ENSGALG00010002647	FAM168A	-0.501927827	0.098500899	-5.095667492	3.47515E-07	6.77958E-07	negative
PRJNA729271	stage_3W_vs_E10	ordinary_muscle_development	ENSGALG00010002649	UFL1	0.644236345	0.078825532	8.172939971	3.00964E-16	9.70516E-16	positive
PRJNA729271	stage_3W_vs_E10	ordinary_muscle_development	ENSGALG00010002653	EIF1AX	-0.164934101	0.063543369	-2.595614671	0.009442189	0.012868045	negative
PRJNA729271	stage_3W_vs_E10	ordinary_muscle_development	ENSGALG00010002655	FABP4	8.3049836	0.9462292	8.776925931	1.68004E-18	6.04871E-18	positive
PRJNA729271	stage_3W_vs_E10	ordinary_muscle_development	ENSGALG00010002656	GLRA3	2.387987255	0.548240565	4.355728866	1.32625E-05	2.32118E-05	positive
PRJNA729271	stage_3W_vs_E10	ordinary_muscle_development	ENSGALG00010002658	FBXL12	-0.457560239	0.147105049	-3.110431926	0.00186814	0.002726133	negative
PRJNA729271	stage_3W_vs_E10	ordinary_muscle_development	ENSGALG00010002661	ACBD7	-2.293249255	0.213745907	-10.72885692	7.45122E-27	3.78435E-26	negative
PRJNA729271	stage_3W_vs_E10	ordinary_muscle_development	ENSGALG00010002664	ZNFB1	-0.057899525	0.139934285	-0.413762248	0.679048234	0.710005349	negative
PRJNA729271	stage_3W_vs_E10	ordinary_muscle_development	ENSGALG00010002667	ENSGALG00010002667	1.171296102	1.613934334	0.725739627			positive
PRJNA729271	stage_3W_vs_E10	ordinary_muscle_development	ENSGALG00010002669	IL4I1	7.221166013	1.093921201	6.601175667	4.0791E-11	1.00847E-10	positive
PRJNA729271	stage_3W_vs_E10	ordinary_muscle_development	ENSGALG00010002673	SLC66A3	-0.459629301	0.150844767	-3.047035112	0.002311107	0.003345935	negative
PRJNA729271	stage_3W_vs_E10	ordinary_muscle_development	ENSGALG00010002678	CCNI	0.732094317	0.172942215	4.233173001	2.30417E-05	3.95998E-05	positive
PRJNA729271	stage_3W_vs_E10	ordinary_muscle_development	ENSGALG00010002681	PLEKHB1	0.15567394	0.157662335	0.987388268	0.323452334	0.361483545	positive
PRJNA729271	stage_3W_vs_E10	ordinary_muscle_development	ENSGALG00010002682	UBL5	-0.038155119	0.166192929	-0.229583288	0.818415594	0.839675399	negative
PRJNA729271	stage_3W_vs_E10	ordinary_muscle_development	ENSGALG00010002685	CA8	1.443636378	0.177677329	8.125045462	4.47195E-16	1.42809E-15	positive
PRJNA729271	stage_3W_vs_E10	ordinary_muscle_development	ENSGALG00010002689	COG6	-0.47669843	0.061509721	-7.749968942	9.1915E-15	2.74646E-14	negative
PRJNA729271	stage_3W_vs_E10	ordinary_muscle_development	ENSGALG00010002693	MSC	-1.701545833	0.169495031	-10.03891276	1.02801E-23	4.55409E-23	negative
PRJNA729271	stage_3W_vs_E10	ordinary_muscle_development	ENSGALG00010002700	LHFPL6	-1.316327377	0.171032691	-7.696349585	1.40008E-14	4.14591E-14	negative
PRJNA729271	stage_3W_vs_E10	ordinary_muscle_development	ENSGALG00010002701	TMEM196	-2.158731168	0.561708395	-3.843152762	0.000121464	0.000197114	negative
PRJNA729271	stage_3W_vs_E10	ordinary_muscle_development	ENSGALG00010002703	PIN1	0.355834775	0.096031693	3.705388939	0.000211067	0.000335315	positive
PRJNA729271	stage_3W_vs_E10	ordinary_muscle_development	ENSGALG00010002704	KCNB2	0.474266327	0.25560065	1.855497345	0.063525238	0.07872121	positive
PRJNA729271	stage_3W_vs_E10	ordinary_muscle_development	ENSGALG00010002705	JADE3	-1.591150649	0.056651778	-28.08650854	1.4317E-173	1.853E-171	negative
PRJNA729271	stage_3W_vs_E10	ordinary_muscle_development	ENSGALG00010002706	ROCK2	1.585865264	0.144059475	11.00840652	3.48106E-28	1.87228E-27	positive
PRJNA729271	stage_3W_vs_E10	ordinary_muscle_development	ENSGALG00010002707	RELT	-4.521784856	0.299994857	-15.07287458	2.4425E-51	2.89486E-50	negative
PRJNA729271	stage_3W_vs_E10	ordinary_muscle_development	ENSGALG00010002712	PTPRE	1.170888189	0.124401836	9.412145599	4.86111E-21	1.93896E-20	positive
PRJNA729271	stage_3W_vs_E10	ordinary_muscle_development	ENSGALG00010002713	OLFM2	0.501584276	0.175220036	2.862596578	0.004201852	0.005937978	positive
PRJNA729271	stage_3W_vs_E10	ordinary_muscle_development	ENSGALG00010002716	NHLRC3	-0.54960345	0.109921038	-4.99998418	5.7335E-07	1.10178E-06	negative
PRJNA729271	stage_3W_vs_E10	ordinary_muscle_development	ENSGALG00010002722	TRIM7.1	2.224677677	0.149518489	14.87894698	4.51551E-50	5.13351E-49	positive
PRJNA729271	stage_3W_vs_E10	ordinary_muscle_development	ENSGALG00010002725	TSPAN7	-0.029343273	0.179031995	-0.163899605	0.869810178	0.884640649	negative
PRJNA729271	stage_3W_vs_E10	ordinary_muscle_development	ENSGALG00010002728	RP2	-0.337803522	0.111766185	-3.022412574	0.002507685	0.003619506	negative
PRJNA729271	stage_3W_vs_E10	ordinary_muscle_development	ENSGALG00010002730	E2F6	0.902080678	0.100023272	9.018707968	1.90321E-19	7.12106E-19	positive
PRJNA729271	stage_3W_vs_E10	ordinary_muscle_development	ENSGALG00010002733	RDH8	0.611059258	0.373073668	1.637905084	0.101441495	0.122522663	positive
PRJNA729271	stage_3W_vs_E10	ordinary_muscle_development	ENSGALG00010002739	HEP21	-2.576018051	1.015077512	-2.537755018	0.011156605	0.015095211	negative
PRJNA729271	stage_3W_vs_E10	ordinary_muscle_development	ENSGALG00010002741	PDK4	8.38375639	0.574094923	14.60343238	2.67054E-48	2.87803E-47	positive
PRJNA729271	stage_3W_vs_E10	ordinary_muscle_development	ENSGALG00010002742	PROSER1	-0.050850231	0.121701336	-0.417828041	0.676072847	0.707251438	negative
PRJNA729271	stage_3W_vs_E10	ordinary_muscle_development	ENSGALG00010002747	FUT9	-0.114774978	0.521244839	-0.220193984	0.825720081	0.84639082	negative
PRJNA729271	stage_3W_vs_E10	ordinary_muscle_development	ENSGALG00010002750	ENSGALG00010002750	-3.052701365	0.235535218	-12.96070028	2.04373E-38	1.56492E-37	negative
PRJNA729271	stage_3W_vs_E10	ordinary_muscle_development	ENSGALG00010002752	NDUFA4	2.120272617	0.154108192	13.75833812	4.5384E-43	4.05324E-42	positive
PRJNA729271	stage_3W_vs_E10	ordinary_muscle_development	ENSGALG00010002753	SLC9A7	0.023496537	0.153543428	0.153028605	0.878375717	0.892415619	positive
PRJNA729271	stage_3W_vs_E10	ordinary_muscle_development	ENSGALG00010002758	GREB1	-5.790367013	0.488927372	-11.8430003	2.33932E-32	1.47364E-31	negative
PRJNA729271	stage_3W_vs_E10	ordinary_muscle_development	ENSGALG00010002760	RAB2A	0.986680314	0.092417011	10.67639285	1.31274E-26	6.59783E-26	positive
PRJNA729271	stage_3W_vs_E10	ordinary_muscle_development	ENSGALG00010002762	PPAN	-1.034211003	0.148444216	-6.967000997	3.23768E-12	8.48648E-12	negative
PRJNA729271	stage_3W_vs_E10	ordinary_muscle_development	ENSGALG00010002763	SEPTIN11	-2.335518634	0.116249162	-20.09062764	8.91283E-90	2.92288E-88	negative
PRJNA729271	stage_3W_vs_E10	ordinary_muscle_development	ENSGALG00010002765	MAP7D2	-4.479147247	0.44635636	-10.03491301	1.07054E-23	4.73644E-23	negative
PRJNA729271	stage_3W_vs_E10	ordinary_muscle_development	ENSGALG00010002766	NANOS1	-0.992715632	0.330320117	-3.00531388	0.002653069	0.003820409	negative
PRJNA729271	stage_3W_vs_E10	ordinary_muscle_development	ENSGALG00010002768	TRIM39.2	4.675485359	0.919426261	5.085220596	3.67199E-07	7.15878E-07	positive
PRJNA729271	stage_3W_vs_E10	ordinary_muscle_development	ENSGALG00010002774	TBX18	-0.497092387	0.117443817	-4.232597351	2.31008E-05	3.96878E-05	negative
PRJNA729271	stage_3W_vs_E10	ordinary_muscle_development	ENSGALG00010002779	FBXO8	1.082282907	0.083231955	13.00321375	1.17308E-38	9.03496E-38	positive
PRJNA729271	stage_3W_vs_E10	ordinary_muscle_development	ENSGALG00010002781	EIF3G	0.736789082	0.137640542	5.352994621	8.65104E-08	1.75971E-07	positive
PRJNA729271	stage_3W_vs_E10	ordinary_muscle_development	ENSGALG00010002782	GSTA3	0.143625469	0.146874999	0.977875542	0.328135883	0.366350584	positive
PRJNA729271	stage_3W_vs_E10	ordinary_muscle_development	ENSGALG00010002784	TRIM27.2	0.687188766	0.519200094	1.323552853	0.185651592	0.215990034	positive
PRJNA729271	stage_3W_vs_E10	ordinary_muscle_development	ENSGALG00010002787	CEP44	-0.275741558	0.129584006	-2.127898087	0.033345534	0.042754845	negative
PRJNA729271	stage_3W_vs_E10	ordinary_muscle_development	ENSGALG00010002788	ENSGALG00010002788	6.448019956	0.629780751	10.23851546	1.33265E-24	6.14012E-24	positive
PRJNA729271	stage_3W_vs_E10	ordinary_muscle_development	ENSGALG00010002790	BCLAF3	1.247870194	0.100812773	12.37809607	3.43392E-35	2.37815E-34	positive
PRJNA729271	stage_3W_vs_E10	ordinary_muscle_development	ENSGALG00010002792	ENSGALG00010002792	-0.43050378	0.230586749	-1.866992715	0.061902605	0.076834926	negative
PRJNA729271	stage_3W_vs_E10	ordinary_muscle_development	ENSGALG00010002796	PRDM8	3.174133144	0.702749726	4.516733376	6.28009E-06	1.12717E-05	positive
PRJNA729271	stage_3W_vs_E10	ordinary_muscle_development	ENSGALG00010002800	TUBGCP5	-0.14058801	0.075571446	-1.860332402	0.062838512	0.077909865	negative
PRJNA729271	stage_3W_vs_E10	ordinary_muscle_development	ENSGALG00010002801	FGF5	-2.68151133	0.471473574	-5.687511401	1.28904E-08	2.76369E-08	negative
PRJNA729271	stage_3W_vs_E10	ordinary_muscle_development	ENSGALG00010002805	FAM171A1	-1.230051368	0.100335132	-12.25942836	1.49551E-34	1.01534E-33	negative
PRJNA729271	stage_3W_vs_E10	ordinary_muscle_development	ENSGALG00010002807	P2RY6	2.457505112	0.215949458	11.3800013	5.25989E-30	3.02069E-29	positive
PRJNA729271	stage_3W_vs_E10	ordinary_muscle_development	ENSGALG00010002809	SH3KBP1	-0.662382907	0.110405594	-5.999541193	1.97876E-09	4.42284E-09	negative
PRJNA729271	stage_3W_vs_E10	ordinary_muscle_development	ENSGALG00010002810	TRIM27.1	0.614997698	0.309461209	1.987317569	0.046887216	0.05907113	positive
PRJNA729271	stage_3W_vs_E10	ordinary_muscle_development	ENSGALG00010002812	TRIM13	-0.767901948	0.103161918	-7.443657112	9.79355E-14	2.77749E-13	negative
PRJNA729271	stage_3W_vs_E10	ordinary_muscle_development	ENSGALG00010002814	TERF1	-2.94098368	0.158436725	-18.56251243	6.46111E-77	1.49846E-75	negative
PRJNA729271	stage_3W_vs_E10	ordinary_muscle_development	ENSGALG00010002817	SHFM1	0.89013214	0.091245512	9.755352574	1.74992E-22	7.37355E-22	positive
PRJNA729271	stage_3W_vs_E10	ordinary_muscle_development	ENSGALG00010002819	BMP3	-2.422560959	0.351078116	-6.900347384	5.18755E-12	1.34226E-11	negative
PRJNA729271	stage_3W_vs_E10	ordinary_muscle_development	ENSGALG00010002824	NXPH1	-3.887363801	0.649323162	-5.986793677	2.14018E-09	4.77775E-09	negative
PRJNA729271	stage_3W_vs_E10	ordinary_muscle_development	ENSGALG00010002825	TRIM41	0.774784354	0.120236881	6.44381612	1.16506E-10	2.80013E-10	positive
PRJNA729271	stage_3W_vs_E10	ordinary_muscle_development	ENSGALG00010002827	SBSPON	0.245182339	0.33882869	0.723617411	0.469300649	0.507991406	positive
PRJNA729271	stage_3W_vs_E10	ordinary_muscle_development	ENSGALG00010002832	LPIN1	3.13466971	0.200916096	15.60188443	7.06767E-55	9.30481E-54	positive
PRJNA729271	stage_3W_vs_E10	ordinary_muscle_development	ENSGALG00010002834	MRPL53	0.400093435	0.112136607	3.567911006	0.000359839	0.000561268	positive
PRJNA729271	stage_3W_vs_E10	ordinary_muscle_development	ENSGALG00010002835	CYFIP1	-0.676579626	0.084516384	-8.005307289	1.19169E-15	3.71953E-15	negative
PRJNA729271	stage_3W_vs_E10	ordinary_muscle_development	ENSGALG00010002837	DNMT1	-0.729929185	0.130671757	-5.585975141	2.32392E-08	4.90039E-08	negative
PRJNA729271	stage_3W_vs_E10	ordinary_muscle_development	ENSGALG00010002838	ENSGALG00010002838	1.293547522	0.257314815	5.027100829	4.97951E-07	9.61603E-07	positive
PRJNA729271	stage_3W_vs_E10	ordinary_muscle_development	ENSGALG00010002840	SRPX	0.405746672	0.169950254	2.38744375	0.016965999	0.022483283	positive
PRJNA729271	stage_3W_vs_E10	ordinary_muscle_development	ENSGALG00010002848	TRIB2	-1.633947416	0.107767089	-15.16184058	6.32711E-52	7.62356E-51	negative
PRJNA729271	stage_3W_vs_E10	ordinary_muscle_development	ENSGALG00010002853	CEP162	-0.327417197	0.168640858	-1.941505759	0.052196962	0.065347639	negative
PRJNA729271	stage_3W_vs_E10	ordinary_muscle_development	ENSGALG00010002855	RACK1	0.205700746	0.12529426	1.64174118	0.100643653	0.121619821	positive
PRJNA729271	stage_3W_vs_E10	ordinary_muscle_development	ENSGALG00010002857	DCAF15	-0.829361604	0.149878935	-5.533543492	3.13825E-08	6.54894E-08	negative
PRJNA729271	stage_3W_vs_E10	ordinary_muscle_development	ENSGALG00010002860	NIPA2	-0.031723255	0.108665649	-0.291934528	0.770336683	0.795301298	negative
PRJNA729271	stage_3W_vs_E10	ordinary_muscle_development	ENSGALG00010002864	RNASEH2B	-0.97930413	0.094475035	-10.36574509	3.54981E-25	1.6755E-24	negative
PRJNA729271	stage_3W_vs_E10	ordinary_muscle_development	ENSGALG00010002866	ENSGALG00010002866	2.505502467	0.252466005	9.92411817	3.2698E-23	1.41989E-22	positive
PRJNA729271	stage_3W_vs_E10	ordinary_muscle_development	ENSGALG00010002868	PPP1R9A	0.649921749	0.067294929	9.657811632	4.5549E-22	1.8945E-21	positive
PRJNA729271	stage_3W_vs_E10	ordinary_muscle_development	ENSGALG00010002869	ENSGALG00010002869	0.369658595	0.304662978	1.213336118	0.225001339	0.258348734	positive
PRJNA729271	stage_3W_vs_E10	ordinary_muscle_development	ENSGALG00010002870	FABP5	4.076333239	0.216122725	18.86119679	2.37776E-79	5.81209E-78	positive
PRJNA729271	stage_3W_vs_E10	ordinary_muscle_development	ENSGALG00010002878	P2RY2	5.389941412	0.250257602	21.53757319	6.924E-103	3.0599E-101	positive
PRJNA729271	stage_3W_vs_E10	ordinary_muscle_development	ENSGALG00010002880	RASGEF1B	-0.155097478	0.207592711	-0.747123909	0.454988769	0.493546241	negative
PRJNA729271	stage_3W_vs_E10	ordinary_muscle_development	ENSGALG00010002881	ENSGALG00010002881	5.369268241	0.312444193	17.18472724	3.45568E-66	6.08629E-65	positive
PRJNA729271	stage_3W_vs_E10	ordinary_muscle_development	ENSGALG00010002883	MAP3K15	5.19111438	0.412631153	12.58051977	2.70246E-36	1.93252E-35	positive
PRJNA729271	stage_3W_vs_E10	ordinary_muscle_development	ENSGALG00010002884	ADHFE1	3.774645359	0.25222829	14.96519425	1.23961E-50	1.43631E-49	positive
PRJNA729271	stage_3W_vs_E10	ordinary_muscle_development	ENSGALG00010002886	HERC2	0.580677185	0.105764878	5.490264717	4.01332E-08	8.33305E-08	positive
PRJNA729271	stage_3W_vs_E10	ordinary_muscle_development	ENSGALG00010002888	ENSGALG00010002888	-4.52164866	0.435318715	-10.3869843	2.84197E-25	1.3449E-24	negative
PRJNA729271	stage_3W_vs_E10	ordinary_muscle_development	ENSGALG00010002890	VXN	0.568526052	0.16419094	3.462590877	0.000535001	0.000821662	positive
PRJNA729271	stage_3W_vs_E10	ordinary_muscle_development	ENSGALG00010002894	FCHSD2	-0.585957534	0.072518562	-8.080104096	6.47115E-16	2.04892E-15	negative
PRJNA729271	stage_3W_vs_E10	ordinary_muscle_development	ENSGALG00010002896	OCA2	-2.124789377	0.274661168	-7.736038522	1.02562E-14	3.05516E-14	negative
PRJNA729271	stage_3W_vs_E10	ordinary_muscle_development	ENSGALG00010002904	PON1	3.669928847	0.178660665	20.54133651	9.19959E-94	3.3337E-92	positive
PRJNA729271	stage_3W_vs_E10	ordinary_muscle_development	ENSGALG00010002907	GABRG3	-2.296136694	0.383597237	-5.985800916	2.15328E-09	4.8055E-09	negative
PRJNA729271	stage_3W_vs_E10	ordinary_muscle_development	ENSGALG00010002908	S1PR2	-0.346064115	0.183048839	-1.890556188	0.058683613	0.073033311	negative
PRJNA729271	stage_3W_vs_E10	ordinary_muscle_development	ENSGALG00010002911	MYBL1	-1.135381998	0.238672605	-4.757068776	1.96424E-06	3.64554E-06	negative
PRJNA729271	stage_3W_vs_E10	ordinary_muscle_development	ENSGALG00010002912	RPS3	-0.198406612	0.192295221	-1.031781292	0.302174585	0.339799892	negative
PRJNA729271	stage_3W_vs_E10	ordinary_muscle_development	ENSGALG00010002913	NXNL1	3.971873787	0.561959321	7.067902672	1.57293E-12	4.19882E-12	positive
PRJNA729271	stage_3W_vs_E10	ordinary_muscle_development	ENSGALG00010002914	SLC27A1	2.456516893	0.304574262	8.065411945	7.29893E-16	2.30497E-15	positive
PRJNA729271	stage_3W_vs_E10	ordinary_muscle_development	ENSGALG00010002916	SDCBP	-0.805403072	0.12277066	-6.560224323	5.37269E-11	1.31705E-10	negative
PRJNA729271	stage_3W_vs_E10	ordinary_muscle_development	ENSGALG00010002917	PGLS	-0.248352666	0.09667543	-2.56893263	0.010201229	0.0138478	negative
PRJNA729271	stage_3W_vs_E10	ordinary_muscle_development	ENSGALG00010002918	LRATD1	-4.817326007	0.417103078	-11.54948562	7.42636E-31	4.40626E-30	negative
PRJNA729271	stage_3W_vs_E10	ordinary_muscle_development	ENSGALG00010002919	ENSGALG00010002919	1.3265168	0.202928076	6.536881563	6.28148E-11	1.53309E-10	positive
PRJNA729271	stage_3W_vs_E10	ordinary_muscle_development	ENSGALG00010002920	VCPIP1	1.42315553	0.101242688	14.05687223	6.99083E-45	6.64909E-44	positive
PRJNA729271	stage_3W_vs_E10	ordinary_muscle_development	ENSGALG00010002922	TMEM106B	-0.020164492	0.091331147	-0.220784397	0.825260313	0.845979366	negative
PRJNA729271	stage_3W_vs_E10	ordinary_muscle_development	ENSGALG00010002923	SERPINH1	-0.895694466	0.158578516	-5.64827121	1.62069E-08	3.45318E-08	negative
PRJNA729271	stage_3W_vs_E10	ordinary_muscle_development	ENSGALG00010002925	INTS6	0.2605648	0.121614812	2.14254164	0.032149922	0.041302235	positive
PRJNA729271	stage_3W_vs_E10	ordinary_muscle_development	ENSGALG00010002926	GALNTL6	-2.480918999	0.371193199	-6.683632689	2.33091E-11	5.84845E-11	negative
PRJNA729271	stage_3W_vs_E10	ordinary_muscle_development	ENSGALG00010002927	GABRA5	-3.826594703	0.585291509	-6.537929635	6.23763E-11	1.52264E-10	negative
PRJNA729271	stage_3W_vs_E10	ordinary_muscle_development	ENSGALG00010002928	ASB4	11.63802215	0.545539498	21.33305139	5.6031E-101	2.38873E-99	positive
PRJNA729271	stage_3W_vs_E10	ordinary_muscle_development	ENSGALG00010002929	PDHA2	0.628746457	0.112884068	5.569842295	2.5497E-08	5.35932E-08	positive
PRJNA729271	stage_3W_vs_E10	ordinary_muscle_development	ENSGALG00010002930	SERAF	-5.000996181	0.293329911	-17.04904955	3.55263E-65	6.10859E-64	negative
PRJNA729271	stage_3W_vs_E10	ordinary_muscle_development	ENSGALG00010002934	VWDE	-6.58056315	0.909799856	-7.232978888	4.72513E-13	1.29325E-12	negative
PRJNA729271	stage_3W_vs_E10	ordinary_muscle_development	ENSGALG00010002935	SYTL5	-3.226294667	0.659262027	-4.893797204	9.89088E-07	1.87483E-06	negative
PRJNA729271	stage_3W_vs_E10	ordinary_muscle_development	ENSGALG00010002937	GALNT7	-0.218416131	0.108450629	-2.013968311	0.044012861	0.055630137	negative
PRJNA729271	stage_3W_vs_E10	ordinary_muscle_development	ENSGALG00010002939	HMGB2	-3.194853956	0.173072045	-18.45967649	4.35945E-76	9.87347E-75	negative
PRJNA729271	stage_3W_vs_E10	ordinary_muscle_development	ENSGALG00010002940	BG1	2.171870431	0.404406475	5.3705135	7.85128E-08	1.60175E-07	positive

PRJNA729271	stage_3W_vs_E10	ordinary_muscle_development	ENSGALG00010002942	GABRB3	-4.618955202	0.650217354	-7.103709516	1.21452E-12	3.25888E-12	negative
PRJNA729271	stage_3W_vs_E10	ordinary_muscle_development	ENSGALG00010002943	SAP30	0.160888851	0.147227119	1.092793581	0.274484441	0.311269908	positive
PRJNA729271	stage_3W_vs_E10	ordinary_muscle_development	ENSGALG00010002946	GDPD5	-1.166048899	0.120351957	-9.688657564	3.36928E-22	1.40702E-21	negative
PRJNA729271	stage_3W_vs_E10	ordinary_muscle_development	ENSGALG00010002947	ENSGALG00010002947	-1.827489974	0.743068196	-2.459383921	0.01391757	0.018618844	negative
PRJNA729271	stage_3W_vs_E10	ordinary_muscle_development	ENSGALG00010002948	DLX6	-5.405856545	1.603059143	-3.37221279	0.000745668	0.001129529	negative
PRJNA729271	stage_3W_vs_E10	ordinary_muscle_development	ENSGALG00010002949	ENSGALG00010002949	-0.051467212	1.1831108	-0.043501599	0.96530169	0.970187768	negative
PRJNA729271	stage_3W_vs_E10	ordinary_muscle_development	ENSGALG00010002951	ENSGALG00010002951	-6.129514798	4.350978426	-1.408766994	0.158904078	0.186802953	negative
PRJNA729271	stage_3W_vs_E10	ordinary_muscle_development	ENSGALG00010002952	NBAS	-0.03603706	0.064564654	-0.558154626	0.576738801	0.612845754	negative
PRJNA729271	stage_3W_vs_E10	ordinary_muscle_development	ENSGALG00010002953	WDFY2	-0.384839808	0.13278453	-2.898227751	0.00375278	0.005330381	negative
PRJNA729271	stage_3W_vs_E10	ordinary_muscle_development	ENSGALG00010002954	CHST7	0.018270938	0.125606035	0.145462262	0.88434591	0.897912157	positive
PRJNA729271	stage_3W_vs_E10	ordinary_muscle_development	ENSGALG00010002955	BLEC1	0.651037718	0.464652895	1.401127002	0.161176098	0.189307799	positive
PRJNA729271	stage_3W_vs_E10	ordinary_muscle_development	ENSGALG00010002956	SCRG1	0.066038838	0.389889389	0.169378393	0.865499018	0.881059644	positive
PRJNA729271	stage_3W_vs_E10	ordinary_muscle_development	ENSGALG00010002957	ADGRG2	-1.541593653	0.294088358	-5.241940437	1.58897E-07	3.16853E-07	negative
PRJNA729271	stage_3W_vs_E10	ordinary_muscle_development	ENSGALG00010002958	ENSGALG00010002958	0.443602458	0.182110756	2.435893785	0.014855046	0.019823595	positive
PRJNA729271	stage_3W_vs_E10	ordinary_muscle_development	ENSGALG00010002959	DLX5	-6.51779525	0.852709076	-7.643633022	2.11177E-14	6.20014E-14	negative
PRJNA729271	stage_3W_vs_E10	ordinary_muscle_development	ENSGALG00010002960	BLEC2	2.197097462	0.689207486	3.187860707	0.001433296	0.002117369	positive
PRJNA729271	stage_3W_vs_E10	ordinary_muscle_development	ENSGALG00010002965	PRSS35	0.027336018	0.211566891	0.129207449	0.897193502	0.909046541	positive
PRJNA729271	stage_3W_vs_E10	ordinary_muscle_development	ENSGALG00010002967	OLFM4	-1.106599618	0.825219291	-1.340976429	0.179928106	0.20966781	negative
PRJNA729271	stage_3W_vs_E10	ordinary_muscle_development	ENSGALG00010002972	ME1	3.216507199	0.150058256	21.43505656	6.2957E-102	2.72408E-100	positive
PRJNA729271	stage_3W_vs_E10	ordinary_muscle_development	ENSGALG00010002978	DYNLT3	1.595144464	0.101053833	15.78509612	3.94049E-56	5.33309E-55	positive
PRJNA729271	stage_3W_vs_E10	ordinary_muscle_development	ENSGALG00010002981	KLHL35	-5.729560331	0.397078245	-14.429298	3.38509E-47	3.47499E-46	negative
PRJNA729271	stage_3W_vs_E10	ordinary_muscle_development	ENSGALG00010002982	HAND2	-2.921788001	0.303128687	-9.638771013	5.48406E-22	2.27248E-21	negative
PRJNA729271	stage_3W_vs_E10	ordinary_muscle_development	ENSGALG00010002983	NSMAF	1.143979788	0.111166621	10.29067701	7.76286E-25	3.6065E-24	positive
PRJNA729271	stage_3W_vs_E10	ordinary_muscle_development	ENSGALG00010002985	ENSGALG00010002985	-0.218134222	0.220132391	-0.990922875	0.321723242	0.359826716	negative
PRJNA729271	stage_3W_vs_E10	ordinary_muscle_development	ENSGALG00010002987	PCDH8	-2.122005797	0.381252778	-5.56587629	2.60838E-08	5.47711E-08	negative
PRJNA729271	stage_3W_vs_E10	ordinary_muscle_development	ENSGALG00010002991	BLB1	5.839540784	0.306868376	19.02946422	9.72418E-81	2.49473E-79	positive
PRJNA729271	stage_3W_vs_E10	ordinary_muscle_development	ENSGALG00010002992	ENSGALG00010002992	0.696558101	0.237691188	2.930517138	0.003383983	0.004827838	positive
PRJNA729271	stage_3W_vs_E10	ordinary_muscle_development	ENSGALG00010002994	DUSP4	-4.854339027	0.229487993	-21.15291071	2.5943E-99	1.05152E-97	negative
PRJNA729271	stage_3W_vs_E10	ordinary_muscle_development	ENSGALG00010002995	RWDD2A	-0.88253888	0.128481716	-6.868984233	6.46607E-12	1.66505E-11	negative
PRJNA729271	stage_3W_vs_E10	ordinary_muscle_development	ENSGALG00010002998	LECT1	-7.367783696	0.297854425	-24.73619015	4.3656E-135	3.2787E-133	negative
PRJNA729271	stage_3W_vs_E10	ordinary_muscle_development	ENSGALG00010002999	CMTM7	0.213098633	0.200920484	1.060611788	0.288866363	0.326048741	positive
PRJNA729271	stage_3W_vs_E10	ordinary_muscle_development	ENSGALG00010003001	MAP6	-3.241374998	0.348757048	-9.294077408	1.48488E-20	5.79989E-20	negative
PRJNA729271	stage_3W_vs_E10	ordinary_muscle_development	ENSGALG00010003002	PHKA2	-0.826211088	0.187510698	-4.406207736	1.05196E-05	1.85433E-05	negative
PRJNA729271	stage_3W_vs_E10	ordinary_muscle_development	ENSGALG00010003006	CYP7B1	-1.141867355	0.201708038	-5.660990827	1.50501E-08	3.21331E-08	negative
PRJNA729271	stage_3W_vs_E10	ordinary_muscle_development	ENSGALG00010003009	TNKS	-0.414864072	0.058294122	-7.116739406	1.1051E-12	2.97243E-12	negative
PRJNA729271	stage_3W_vs_E10	ordinary_muscle_development	ENSGALG00010003017	PGM3	-1.386816902	0.155699903	-8.906986272	5.24385E-19	1.92967E-18	negative
PRJNA729271	stage_3W_vs_E10	ordinary_muscle_development	ENSGALG00010003018	MOGAT2	-2.48163549	1.116974976	-2.22174672	0.026300427	0.034120173	negative
PRJNA729271	stage_3W_vs_E10	ordinary_muscle_development	ENSGALG00010003019	TAPBP	3.319294941	0.251570457	13.19429548	9.46355E-40	7.60389E-39	positive
PRJNA729271	stage_3W_vs_E10	ordinary_muscle_development	ENSGALG00010003022	BLB2	6.482374265	0.265644852	24.40240876	1.6124E-131	1.1346E-129	positive
PRJNA729271	stage_3W_vs_E10	ordinary_muscle_development	ENSGALG00010003024	SUGT1	0.997125123	0.071348802	13.97535898	2.204E-44	2.06109E-43	positive
PRJNA729271	stage_3W_vs_E10	ordinary_muscle_development	ENSGALG00010003025	MFHAS1	1.17026365	0.122775703	9.531720229	1.54698E-21	6.30582E-21	positive
PRJNA729271	stage_3W_vs_E10	ordinary_muscle_development	ENSGALG00010003027	SCIN	-1.430061089	0.255118417	-5.605479624	2.07679E-08	4.39461E-08	negative
PRJNA729271	stage_3W_vs_E10	ordinary_muscle_development	ENSGALG00010003030	PPEF1	-0.918000626	0.678996284	-1.351996539	0.176376423	0.205744105	negative
PRJNA729271	stage_3W_vs_E10	ordinary_muscle_development	ENSGALG00010003034	ERI1	-0.636974835	0.062694216	-10.16002554	2.98998E-24	1.35606E-23	negative
PRJNA729271	stage_3W_vs_E10	ordinary_muscle_development	ENSGALG00010003035	DGAT2	3.304932911	0.45610072	7.246059398	4.29072E-13	1.17792E-12	positive
PRJNA729271	stage_3W_vs_E10	ordinary_muscle_development	ENSGALG00010003042	TOX	-3.997311264	0.323446916	-12.35847697	4.38384E-35	3.02589E-34	negative
PRJNA729271	stage_3W_vs_E10	ordinary_muscle_development	ENSGALG00010003052	ENSGALG00010003052	0.421195494	0.093497548	4.504882758	6.64096E-06	1.18811E-05	positive
PRJNA729271	stage_3W_vs_E10	ordinary_muscle_development	ENSGALG00010003057	DDX1	-0.23001952	0.089701	-2.564291578	0.010338665	0.014027797	negative
PRJNA729271	stage_3W_vs_E10	ordinary_muscle_development	ENSGALG00010003061	ENSGALG00010003061	-0.76227888	0.320257103	-2.38020913	0.017302815	0.022906595	negative
PRJNA729271	stage_3W_vs_E10	ordinary_muscle_development	ENSGALG00010003063	GGH	0.171323449	0.088319538	1.939813692	0.052402336	0.065593425	positive
PRJNA729271	stage_3W_vs_E10	ordinary_muscle_development	ENSGALG00010003064	VAV1	1.922653491	0.250816618	7.665574581	1.78032E-14	5.24721E-14	positive
PRJNA729271	stage_3W_vs_E10	ordinary_muscle_development	ENSGALG00010003068	BRD2	0.161766587	0.126357455	1.280229857	0.200464308	0.232012947	positive
PRJNA729271	stage_3W_vs_E10	ordinary_muscle_development	ENSGALG00010003072	ELF1	1.091256043	0.073604481	14.8259457	9.95677E-50	1.11361E-48	positive
PRJNA729271	stage_3W_vs_E10	ordinary_muscle_development	ENSGALG00010003074	TTPA	2.656960074	0.348045296	7.633949092	2.2767E-14	6.67221E-14	positive
PRJNA729271	stage_3W_vs_E10	ordinary_muscle_development	ENSGALG00010003077	EIF2S3	0.458803495	0.081725355	5.613967603	1.97739E-08	4.1904E-08	positive
PRJNA729271	stage_3W_vs_E10	ordinary_muscle_development	ENSGALG00010003081	DOP1A	0.461465735	0.103524197	4.457563996	8.28963E-06	1.47199E-05	positive
PRJNA729271	stage_3W_vs_E10	ordinary_muscle_development	ENSGALG00010003087	TWISTNB	0.425800118	0.178121175	2.390508141	0.016825076	0.022310811	positive
PRJNA729271	stage_3W_vs_E10	ordinary_muscle_development	ENSGALG00010003090	DMA	2.781723626	0.184704706	15.06038308	2.95072E-51	3.48296E-50	positive
PRJNA729271	stage_3W_vs_E10	ordinary_muscle_development	ENSGALG00010003092	ART1F	5.378580761	1.349373411	3.98598395	6.72011E-05	0.000111412	positive
PRJNA729271	stage_3W_vs_E10	ordinary_muscle_development	ENSGALG00010003097	TFPI2	1.877029911	0.135402944	13.86254872	1.06813E-43	9.76818E-43	positive
PRJNA729271	stage_3W_vs_E10	ordinary_muscle_development	ENSGALG00010003101	KLHL15	-0.269517245	0.072425557	-3.721300303	0.0001982	0.000315842	negative
PRJNA729271	stage_3W_vs_E10	ordinary_muscle_development	ENSGALG00010003103	YTHDF3	0.31065939	0.086336832	3.598225484	0.000320396	0.000501906	positive
PRJNA729271	stage_3W_vs_E10	ordinary_muscle_development	ENSGALG00010003107	GNG11	0.216945534	0.181243828	1.196981639	0.231313711	0.265092682	positive
PRJNA729271	stage_3W_vs_E10	ordinary_muscle_development	ENSGALG00010003108	PPP1R3B	-0.59606746	0.183072011	-3.255918018	0.001130264	0.001686726	negative
PRJNA729271	stage_3W_vs_E10	ordinary_muscle_development	ENSGALG00010003109	ART5	5.499287844	0.558318975	9.849724061	6.87324E-23	2.95631E-22	positive
PRJNA729271	stage_3W_vs_E10	ordinary_muscle_development	ENSGALG00010003110	BF1	6.064817075	0.256265764	23.6661229	8.0547E-124	5.0984E-122	positive
PRJNA729271	stage_3W_vs_E10	ordinary_muscle_development	ENSGALG00010003113	WBP4	-0.229400162	0.11726614	-1.956235306	0.050437441	0.063259558	negative
PRJNA729271	stage_3W_vs_E10	ordinary_muscle_development	ENSGALG00010003115	UBE3D	-0.213456779	0.154893856	-1.378084217	0.168177288	0.197067889	negative
PRJNA729271	stage_3W_vs_E10	ordinary_muscle_development	ENSGALG00010003117	ID2	-2.22472932	0.127147882	-17.4971795	1.50542E-68	2.80476E-67	negative
PRJNA729271	stage_3W_vs_E10	ordinary_muscle_development	ENSGALG00010003121	ARMC1	0.169367596	0.082204367	2.0603236	0.039367615	0.050077541	positive
PRJNA729271	stage_3W_vs_E10	ordinary_muscle_development	ENSGALG00010003125	APOO	1.122687239	0.105763293	10.61509342	2.53541E-26	1.25643E-25	positive
PRJNA729271	stage_3W_vs_E10	ordinary_muscle_development	ENSGALG00010003126	ENSGALG00010003126	-2.223334322	0.501995775	-4.428990105	9.46754E-06	1.67438E-05	negative
PRJNA729271	stage_3W_vs_E10	ordinary_muscle_development	ENSGALG00010003131	TAP1	3.687280504	0.346480481	10.64210166	1.89799E-26	9.48669E-26	positive
PRJNA729271	stage_3W_vs_E10	ordinary_muscle_development	ENSGALG00010003132	TPBG	-4.511232397	0.175483526	-25.70744097	9.6512E-146	8.5301E-144	negative
PRJNA729271	stage_3W_vs_E10	ordinary_muscle_development	ENSGALG00010003133	SAT1	2.589572676	0.167018564	15.50469966	3.22421E-54	4.16532E-53	positive
PRJNA729271	stage_3W_vs_E10	ordinary_muscle_development	ENSGALG00010003136	NUP98	-0.113477077	0.104919536	-1.081562896	0.279446799	0.316377517	negative
PRJNA729271	stage_3W_vs_E10	ordinary_muscle_development	ENSGALG00010003152	ENSGALG00010003152	1.360791559	0.126867491	10.72608554	7.67802E-27	3.89681E-26	positive
PRJNA729271	stage_3W_vs_E10	ordinary_muscle_development	ENSGALG00010003154	MTFR1	-0.788249483	0.081837007	-9.631944203	5.86099E-22	2.42382E-21	negative
PRJNA729271	stage_3W_vs_E10	ordinary_muscle_development	ENSGALG00010003159	TAP2	4.659810916	0.263696218	17.67113292	6.99773E-70	1.35063E-68	positive
PRJNA729271	stage_3W_vs_E10	ordinary_muscle_development	ENSGALG00010003160	KIDINS220	0.035252925	0.15146334	0.232748898	0.815956397	0.837448699	positive
PRJNA729271	stage_3W_vs_E10	ordinary_muscle_development	ENSGALG00010003164	C3	4.201013936	0.4586876	9.158769354	5.24925E-20	2.00654E-19	positive
PRJNA729271	stage_3W_vs_E10	ordinary_muscle_development	ENSGALG00010003165	BMP2K	0.457556455	0.109576106	4.175695507	2.97077E-05	5.05592E-05	positive
PRJNA729271	stage_3W_vs_E10	ordinary_muscle_development	ENSGALG00010003166	PGAP2	0.781646769	0.109388014	7.145634517	8.95811E-13	2.42344E-12	positive
PRJNA729271	stage_3W_vs_E10	ordinary_muscle_development	ENSGALG00010003169	MTRF1	0.586966821	0.108097436	5.429979111	5.63606E-08	1.15748E-07	positive
PRJNA729271	stage_3W_vs_E10	ordinary_muscle_development	ENSGALG00010003170	C4	1.151271713	0.406316072	2.833438771	0.004605013	0.006482438	positive
PRJNA729271	stage_3W_vs_E10	ordinary_muscle_development	ENSGALG00010003171	PAQR3	-0.701294504	0.106127058	-6.608065036	3.89376E-11	9.63798E-11	negative
PRJNA729271	stage_3W_vs_E10	ordinary_muscle_development	ENSGALG00010003176	PRAG1	-2.330008353	0.270861983	-8.602197798	7.82039E-18	2.7361E-17	negative
PRJNA729271	stage_3W_vs_E10	ordinary_muscle_development	ENSGALG00010003178	CENPA	-3.030628391	0.296451109	-10.22302937	1.56376E-24	7.18438E-24	negative
PRJNA729271	stage_3W_vs_E10	ordinary_muscle_development	ENSGALG00010003180	IBTK	0.212088838	0.126869258	1.671711824	0.094581161	0.114762593	positive
PRJNA729271	stage_3W_vs_E10	ordinary_muscle_development	ENSGALG00010003183	NAA16	1.331828617	0.087376585	15.24239726	1.84958E-52	2.26241E-51	positive
PRJNA729271	stage_3W_vs_E10	ordinary_muscle_development	ENSGALG00010003184	BET1	-0.035379156	0.080596782	-0.438964869	0.660686992	0.692956436	negative
PRJNA729271	stage_3W_vs_E10	ordinary_muscle_development	ENSGALG00010003186	CYP21A1	-2.127817982	0.282031863	-7.544601376	4.53673E-14	1.31257E-13	negative
PRJNA729271	stage_3W_vs_E10	ordinary_muscle_development	ENSGALG00010003187	RHOG	0.885901981	0.244935122	3.616884228	0.000298171	0.000468406	positive
PRJNA729271	stage_3W_vs_E10	ordinary_muscle_development	ENSGALG00010003189	ANTXR2	0.467854552	0.126786709	3.690091464	0.000224173	0.000355436	positive
PRJNA729271	stage_3W_vs_E10	ordinary_muscle_development	ENSGALG00010003191	CC2D1A	1.524786375	0.159736278	9.545648596	1.35259E-21	5.52118E-21	positive
PRJNA729271	stage_3W_vs_E10	ordinary_muscle_development	ENSGALG00010003192	RGCC	3.179108931	0.307718007	10.33124112	5.08977E-25	2.38681E-24	positive
PRJNA729271	stage_3W_vs_E10	ordinary_muscle_development	ENSGALG00010003193	ACOT9	0.466834512	0.136672671	3.415712212	0.000636154	0.000970433	positive
PRJNA729271	stage_3W_vs_E10	ordinary_muscle_development	ENSGALG00010003198	PRKG2	-0.827682218	0.332044888	-2.492681708	0.012678244	0.017042673	negative
PRJNA729271	stage_3W_vs_E10	ordinary_muscle_development	ENSGALG00010003201	MBOAT2	-0.916443475	0.129281036	-7.088769594	1.3531E-12	3.62468E-12	negative
PRJNA729271	stage_3W_vs_E10	ordinary_muscle_development	ENSGALG00010003202	FAM46A	1.080360605	0.102620078	10.52777024	6.43405E-26	3.12958E-25	positive
PRJNA729271	stage_3W_vs_E10	ordinary_muscle_development	ENSGALG00010003204	PKD2	0.555664017	0.142966946	3.886660749	0.000101633	0.000165766	positive
PRJNA729271	stage_3W_vs_E10	ordinary_muscle_development	ENSGALG00010003205	ENSGALG00010003205	6.685139033	0.263931511	25.32906742	1.5286E-141	1.259E-139	positive
PRJNA729271	stage_3W_vs_E10	ordinary_muscle_development	ENSGALG00010003206	ENSGALG00010003206	-4.680561902	0.968005664	-4.835262927	1.3297E-06	2.49598E-06	negative
PRJNA729271	stage_3W_vs_E10	ordinary_muscle_development	ENSGALG00010003210	EYA1	-0.71873623	0.202005165	-3.558009179	0.000373676	0.000582037	negative
PRJNA729271	stage_3W_vs_E10	ordinary_muscle_development	ENSGALG00010003212	LTB4R	3.319774201	0.706355775	4.699861342	2.60338E-06	4.80102E-06	positive
PRJNA729271	stage_3W_vs_E10	ordinary_muscle_development	ENSGALG00010003214	PRDX4	-1.072148126	0.095266899	-11.25415164	2.20942E-29	1.24274E-28	negative
PRJNA729271	stage_3W_vs_E10	ordinary_muscle_development	ENSGALG00010003218	MAP3K7	0.104303657	0.06064954	1.719776543	0.085473068	0.104287216	positive
PRJNA729271	stage_3W_vs_E10	ordinary_muscle_development	ENSGALG00010003220	SPP1	-0.805193611	0.941885568	-0.854874136	0.39262083	0.431564339	negative
PRJNA729271	stage_3W_vs_E10	ordinary_muscle_development	ENSGALG00010003222	ITGA8	-2.411069286	0.3101988	-7.772658315	7.68559E-15	2.30695E-14	negative
PRJNA729271	stage_3W_vs_E10	ordinary_muscle_development	ENSGALG00010003223	AREG	-2.404242827	1.017484901	-2.362927278	0.018131227	0.023957351	negative
PRJNA729271	stage_3W_vs_E10	ordinary_muscle_development	ENSGALG00010003229	STIM1	0.92123742	0.105582699	8.725268687	2.65549E-18	9.5017E-18	positive
PRJNA729271	stage_3W_vs_E10	ordinary_muscle_development	ENSGALG00010003239	RRM1	-2.54283009	0.120109729	-21.17089189	1.77173E-99	7.23414E-98	negative
PRJNA729271	stage_3W_vs_E10	ordinary_muscle_development	ENSGALG00010003240	PLACL2	5.165544859	0.860295944	6.004381276	1.92063E-09	4.29755E-09	positive
PRJNA729271	stage_3W_vs_E10	ordinary_muscle_development	ENSGALG00010003242	ASAP2	-1.309818126	0.125793515	-10.41244554	2.1756E-25	1.03225E-24	negative
PRJNA729271	stage_3W_vs_E10	ordinary_muscle_development	ENSGALG00010003243	VPS50	-0.021148903	0.091908002	-0.230109486	0.818006694	0.839315285	negative
PRJNA729271	stage_3W_vs_E10	ordinary_muscle_development	ENSGALG00010003245	ENSGALG00010003245	-0.044391969	0.423371933	-0.104853357	0.91649217	0.92626928	negative
PRJNA729271	stage_3W_vs_E10	ordinary_muscle_development	ENSGALG00010003246	VWA8	1.225694088	0.108835827	11.26186223	2.02435E-29	1.13998E-28	positive
PRJNA729271	stage_3W_vs_E10	ordinary_muscle_development	ENSGALG00010003247	ENSGALG00010003247	0.374973343	0.215572241	1.739432415	0.081958731	0.100176389	positive
PRJNA729271	stage_3W_vs_E10	ordinary_muscle_development	ENSGALG00010003248	MRI1	0.237624412	0.095971404	2.475991843	0.013286661	0.017809336	positive
PRJNA729271	stage_3W_vs_E10	ordinary_muscle_development	ENSGALG00010003249	YJU2B	2.029621169	0.316039419	6.422050685	1.34451E-10	3.22019E-10	positive
PRJNA729271	stage_3W_vs_E10	ordinary_muscle_development	ENSGALG00010003250	CD1G	2.411810039	0.686248041	3.514487322	0.000440604	0.000682105	positive
PRJNA729271	stage_3W_vs_E10	ordinary_muscle_development	ENSGALG00010003251	ELOF1	0.004453579	0.154482631	0.028828994	0.977000977	0.980518532	positive
PRJNA729271	stage_3W_vs_E10	ordinary_muscle_development	ENSGALG00010003252	PHEX	-3.282145349	0.198924912	-16.49941836	3.70444E-61	5.7184E-60	negative
PRJNA729271	stage_3W_vs_E10	ordinary_muscle_development	ENSGALG00010003253	ITGB1BP1	0.122890717	0.11339526	1.083737689	0.27848112	0.315361516	positive
PRJNA729271	stage_3W_vs_E10	ordinary_muscle_development	ENSGALG00010003254	DGKH	0.180707457	0.169539188	1.065874268	0.286480483	0.323607746	positive
PRJNA729271	stage_3W_vs_E10	ordinary_muscle_development	ENSGALG00010003255	CALCR	-0.678846916	0.326568781	-2.078725692	0.03764257	0.04801382	negative
PRJNA729271	stage_3W_vs_E10	ordinary_muscle_development	ENSGALG00010003256	ENSGALG00010003256	0.866119489	1.335727105	0.64842548	0.516709795	0.55466996	positive
PRJNA729271	stage_3W_vs_E10	ordinary_muscle_development	ENSGALG00010003257	ENSGALG00010003257	3.035535853	0.792270555	3.83143843	0.000127396	0.000206394	positive
PRJNA729271	stage_3W_vs_E10	ordinary_muscle_development	ENSGALG00010003259	VEGFC	1.202393667	0.139811279	8.600119234	7.96333E-18	2.78342E-17	positive
PRJNA729271	stage_3W_vs_E10	ordinary_muscle_development	ENSGALG00010003260	ACP5	1.651843773	0.264321576	6.24937169	4.12107E-10	9.6238E-10	positive
PRJNA729271	stage_3W_vs_E10	ordinary_muscle_development	ENSGALG00010003261	PEX2	-1.002305908	0.17352238	-5.776234206	7.6391E-09	1.6596E-08	negative

PRJNA729271	stage_3W_vs_E10	ordinary_muscle_development	ENSGALG00010003262	ENSGALG00010003262	-2.577701544	0.616817372	-4.179035257	2.92748E-05	4.98343E-05	negative
PRJNA729271	stage_3W_vs_E10	ordinary_muscle_development	ENSGALG00010003263	ENSGALG00010003263	1.413528172	0.763994095	1.850182063	0.064287313	0.079631226	positive
PRJNA729271	stage_3W_vs_E10	ordinary_muscle_development	ENSGALG00010003264	ENSGALG00010003264	-0.67381104	0.391914391	-1.719281186	0.085563184	0.104388381	negative
PRJNA729271	stage_3W_vs_E10	ordinary_muscle_development	ENSGALG00010003268	SMS	-0.542763438	0.063887227	-8.495648714	1.9683E-17	6.73844E-17	negative
PRJNA729271	stage_3W_vs_E10	ordinary_muscle_development	ENSGALG00010003269	ENSGALG00010003269	0.496055204	0.427503434	1.160353728	0.245904818	0.280837562	positive
PRJNA729271	stage_3W_vs_E10	ordinary_muscle_development	ENSGALG00010003271	AKAP11	0.317016566	0.083771331	3.784308577	0.000154136	0.00024786	positive
PRJNA729271	stage_3W_vs_E10	ordinary_muscle_development	ENSGALG00010003272	CPSF3	-0.18864909	0.066509731	-2.836413379	0.004562335	0.006425481	negative
PRJNA729271	stage_3W_vs_E10	ordinary_muscle_development	ENSGALG00010003273	NFIX	0.816441963	0.198702102	4.108874308	3.97592E-05	6.70752E-05	positive
PRJNA729271	stage_3W_vs_E10	ordinary_muscle_development	ENSGALG00010003274	BACH2	-0.167961682	0.147986261	-1.134981592	0.256383031	0.292022005	negative
PRJNA729271	stage_3W_vs_E10	ordinary_muscle_development	ENSGALG00010003275	MBTPS2	-0.20279448	0.129317311	-1.568192823	0.116836139	0.139938839	negative
PRJNA729271	stage_3W_vs_E10	ordinary_muscle_development	ENSGALG00010003276	TNFSF11	0.671123394	0.352439231	1.904224431	0.056880961	0.070905533	positive
PRJNA729271	stage_3W_vs_E10	ordinary_muscle_development	ENSGALG00010003278	ENSGALG00010003278	0.794110922	0.49879591	1.592055802	0.111372167	0.133748099	positive
PRJNA729271	stage_3W_vs_E10	ordinary_muscle_development	ENSGALG00010003279	ENSGALG00010003279	1.379430456	0.372785136	3.70033653	0.000215314	0.0003418	positive
PRJNA729271	stage_3W_vs_E10	ordinary_muscle_development	ENSGALG00010003280	ENSGALG00010003280	-0.100777815	0.166320981	-0.605923643	0.544565459	0.582045557	negative
PRJNA729271	stage_3W_vs_E10	ordinary_muscle_development	ENSGALG00010003287	ENSGALG00010003287	-6.659005011	1.970620842	-3.379140659	0.000727128	0.001103289	negative
PRJNA729271	stage_3W_vs_E10	ordinary_muscle_development	ENSGALG00010003292	GADD45GIP1	1.029956354	0.123568675	8.33509267	7.74375E-17	2.57444E-16	positive
PRJNA729271	stage_3W_vs_E10	ordinary_muscle_development	ENSGALG00010003294	ENSGALG00010003294	-23.93606849	4.49957563	-5.319627996	1.0398E-07	2.10324E-07	negative
PRJNA729271	stage_3W_vs_E10	ordinary_muscle_development	ENSGALG00010003295	IAH1	0.23679846	0.13332251	1.776132625	0.07571106	0.092939686	positive
PRJNA729271	stage_3W_vs_E10	ordinary_muscle_development	ENSGALG00010003296	ENSGALG00010003296	2.241587358	0.566029286	3.960196782	7.4888E-05	0.000123605	positive
PRJNA729271	stage_3W_vs_E10	ordinary_muscle_development	ENSGALG00010003298	IL7	7.704230871	0.970896586	7.935171449	2.10205E-15	6.47454E-15	positive
PRJNA729271	stage_3W_vs_E10	ordinary_muscle_development	ENSGALG00010003299	RAD23A	2.28265085	0.120197027	18.99090933	2.02786E-80	5.14779E-79	positive
PRJNA729271	stage_3W_vs_E10	ordinary_muscle_development	ENSGALG00010003300	CASP8AP2	0.020552218	0.111028525	0.18510755	0.85314468	0.869827118	positive
PRJNA729271	stage_3W_vs_E10	ordinary_muscle_development	ENSGALG00010003303	MTHFD2L	-0.209859567	0.195304297	-1.074526114	0.282586947	0.31963307	negative
PRJNA729271	stage_3W_vs_E10	ordinary_muscle_development	ENSGALG00010003304	ENSGALG00010003304	-0.391355347	0.65866359	-0.594165752	0.552401257	0.589447602	negative
PRJNA729271	stage_3W_vs_E10	ordinary_muscle_development	ENSGALG00010003305	CALR	-0.752844121	0.078252251	-9.620734353	6.53628E-22	2.69847E-21	negative
PRJNA729271	stage_3W_vs_E10	ordinary_muscle_development	ENSGALG00010003307	COL1A2	-0.064027986	0.220388476	-0.290523292	0.771415934	0.796093991	negative
PRJNA729271	stage_3W_vs_E10	ordinary_muscle_development	ENSGALG00010003308	MDN1	-1.279014462	0.099650702	-12.83497694	1.04434E-37	7.77889E-37	negative
PRJNA729271	stage_3W_vs_E10	ordinary_muscle_development	ENSGALG00010003311	ENSGALG00010003311	-0.496067794	1.182878665	-0.419373355	0.674943294	0.706222699	negative
PRJNA729271	stage_3W_vs_E10	ordinary_muscle_development	ENSGALG00010003315	EPGN	-0.572704723	0.350968484	-1.631783905	0.102725017	0.123979944	negative
PRJNA729271	stage_3W_vs_E10	ordinary_muscle_development	ENSGALG00010003317	LYRM2	0.196887386	0.178062023	1.105723625	0.268846161	0.305353793	positive
PRJNA729271	stage_3W_vs_E10	ordinary_muscle_development	ENSGALG00010003320	OR51E2	0.539883058	0.480477728	1.123638051	0.261166607	0.297073685	positive
PRJNA729271	stage_3W_vs_E10	ordinary_muscle_development	ENSGALG00010003323	STMN2	-2.730867056	0.499011668	-5.472551502	4.43602E-08	9.17655E-08	negative
PRJNA729271	stage_3W_vs_E10	ordinary_muscle_development	ENSGALG00010003324	ENSGALG00010003324	-1.031203908	0.587088699	-1.756470377	0.079008125	0.096741238	negative
PRJNA729271	stage_3W_vs_E10	ordinary_muscle_development	ENSGALG00010003330	HEY1	0.286634271	0.12094073	2.370039196	0.0177862	0.023522898	positive
PRJNA729271	stage_3W_vs_E10	ordinary_muscle_development	ENSGALG00010003331	EPSTI1	4.387633709	0.611499644	7.175202397	7.22003E-13	1.96129E-12	positive
PRJNA729271	stage_3W_vs_E10	ordinary_muscle_development	ENSGALG00010003333	ENSGALG00010003333	20.80457064	2.685995804	7.745570791	9.51534E-15	2.8403E-14	positive
PRJNA729271	stage_3W_vs_E10	ordinary_muscle_development	ENSGALG00010003334	TAC1	-4.675619134	1.071363045	-4.364178097	1.27602E-05	2.23677E-05	negative
PRJNA729271	stage_3W_vs_E10	ordinary_muscle_development	ENSGALG00010003339	ENSGALG00010003339	-1.114781351	0.489207972	-2.278747313	0.02268209	0.029683722	negative
PRJNA729271	stage_3W_vs_E10	ordinary_muscle_development	ENSGALG00010003344	ZFAND1	0.483062566	0.189865452	2.544236255	0.010951698	0.014824885	positive
PRJNA729271	stage_3W_vs_E10	ordinary_muscle_development	ENSGALG00010003346	ENSGALG00010003346	-1.748740198	5.47463579	-0.319425851	0.74940361	0.77601124	negative
PRJNA729271	stage_3W_vs_E10	ordinary_muscle_development	ENSGALG00010003347	GPM6A	-1.15487681	0.360670476	-3.202027575	0.001364639	0.002020475	negative
PRJNA729271	stage_3W_vs_E10	ordinary_muscle_development	ENSGALG00010003348	ADAM17	-0.233252291	0.083534961	-2.792271511	0.005233942	0.007324446	negative
PRJNA729271	stage_3W_vs_E10	ordinary_muscle_development	ENSGALG00010003351	ENSGALG00010003351	-0.78731018	1.596653057	-0.493100349	0.621941684	0.65640343	negative
PRJNA729271	stage_3W_vs_E10	ordinary_muscle_development	ENSGALG00010003352	FARSA	0.141772704	0.101547203	1.396126142	0.162676513	0.190977246	positive
PRJNA729271	stage_3W_vs_E10	ordinary_muscle_development	ENSGALG00010003356	ENSGALG00010003356	5.049329615	1.108152149	4.556530995	5.20054E-06	9.38635E-06	positive
PRJNA729271	stage_3W_vs_E10	ordinary_muscle_development	ENSGALG00010003358	DNAJC15	0.980319158	0.132872349	7.377901895	1.60803E-13	4.51015E-13	positive
PRJNA729271	stage_3W_vs_E10	ordinary_muscle_development	ENSGALG00010003363	HBE1	-7.474891461	0.526330474	-14.20189753	8.91649E-46	8.75641E-45	negative
PRJNA729271	stage_3W_vs_E10	ordinary_muscle_development	ENSGALG00010003364	ANKRD6	-2.860473149	0.168539818	-16.9720911	1.32142E-64	2.23204E-63	negative
PRJNA729271	stage_3W_vs_E10	ordinary_muscle_development	ENSGALG00010003366	CASD1	0.290931189	0.108330806	2.685581311	0.007240376	0.00999136	positive
PRJNA729271	stage_3W_vs_E10	ordinary_muscle_development	ENSGALG00010003371	ENSGALG00010003371	-2.802826265	0.379562504	-7.384360241	1.53188E-13	4.30072E-13	negative
PRJNA729271	stage_3W_vs_E10	ordinary_muscle_development	ENSGALG00010003372	ENOX1	-1.338220812	0.142781586	-9.372502796	7.08326E-21	2.80831E-20	negative
PRJNA729271	stage_3W_vs_E10	ordinary_muscle_development	ENSGALG00010003374	SYCE2	-0.160876309	0.444303607	-0.362086435	0.71728744	0.745898662	negative
PRJNA729271	stage_3W_vs_E10	ordinary_muscle_development	ENSGALG00010003375	YWHAQ	-1.274344311	0.086231237	-14.77822146	2.02442E-49	2.23999E-48	negative
PRJNA729271	stage_3W_vs_E10	ordinary_muscle_development	ENSGALG00010003379	ENSGALG00010003379	-1.84763701	0.464539875	-3.977348576	6.9688E-05	0.000115327	negative
PRJNA729271	stage_3W_vs_E10	ordinary_muscle_development	ENSGALG00010003382	ASNS	2.392115091	0.599242897	3.991895612	6.55472E-05	0.000108807	positive
PRJNA729271	stage_3W_vs_E10	ordinary_muscle_development	ENSGALG00010003383	UBE2J1	0.808841316	0.085458011	9.464780506	2.94178E-21	1.18513E-20	positive
PRJNA729271	stage_3W_vs_E10	ordinary_muscle_development	ENSGALG00010003388	PXDN	-0.80492711	0.186195786	-4.323014642	1.53912E-05	2.68078E-05	negative
PRJNA729271	stage_3W_vs_E10	ordinary_muscle_development	ENSGALG00010003389	ENSGALG00010003389	-3.337525206	0.412321704	-8.094468893	5.75147E-16	1.82704E-15	negative
PRJNA729271	stage_3W_vs_E10	ordinary_muscle_development	ENSGALG00010003395	ENSGALG00010003395	0.740309887	1.00515243	0.736515045	0.461417315	0.500093015	positive
PRJNA729271	stage_3W_vs_E10	ordinary_muscle_development	ENSGALG00010003397	SPATA4	1.562308633	0.410344899	3.807306086	0.000140489	0.000226845	positive
PRJNA729271	stage_3W_vs_E10	ordinary_muscle_development	ENSGALG00010003400	PKIA	1.130067069	0.114525844	9.867354188	5.76656E-23	2.48769E-22	positive
PRJNA729271	stage_3W_vs_E10	ordinary_muscle_development	ENSGALG00010003401	ENSGALG00010003401	0.683295994	1.723791421	0.396391342	0.691816352	0.721949462	positive
PRJNA729271	stage_3W_vs_E10	ordinary_muscle_development	ENSGALG00010003403	GCDH	0.073524893	0.149010288	0.493421588	0.621714732	0.656211683	positive
PRJNA729271	stage_3W_vs_E10	ordinary_muscle_development	ENSGALG00010003405	GABRR2	-2.233767716	0.293490349	-7.611043174	2.71892E-14	7.93772E-14	negative
PRJNA729271	stage_3W_vs_E10	ordinary_muscle_development	ENSGALG00010003406	C1GALT1	-0.933750466	0.106209369	-8.791601692	1.47443E-18	5.31902E-18	negative
PRJNA729271	stage_3W_vs_E10	ordinary_muscle_development	ENSGALG00010003407	CHMP4C	0.690348911	0.209584749	3.293889055	0.000988115	0.001481304	positive
PRJNA729271	stage_3W_vs_E10	ordinary_muscle_development	ENSGALG00010003408	ENSGALG00010003408	-4.258569842	3.709337005	-1.148067656	0.250940635	0.286182888	negative
PRJNA729271	stage_3W_vs_E10	ordinary_muscle_development	ENSGALG00010003412	ASB5	1.961547239	0.146473247	13.39184653	6.74819E-41	5.59583E-40	positive
PRJNA729271	stage_3W_vs_E10	ordinary_muscle_development	ENSGALG00010003419	ENSGALG00010003419	-4.145231145	1.065220243	-3.891431067	9.96547E-05	0.000162723	negative
PRJNA729271	stage_3W_vs_E10	ordinary_muscle_development	ENSGALG00010003420	ENSGALG00010003420	0.197711631	0.631600579	0.313032694	0.754255823	0.78014401	positive
PRJNA729271	stage_3W_vs_E10	ordinary_muscle_development	ENSGALG00010003428	SNX16	1.204105606	0.153254394	7.856907562	3.93733E-15	1.19974E-14	positive
PRJNA729271	stage_3W_vs_E10	ordinary_muscle_development	ENSGALG00010003431	PM20D2	4.849732557	0.285114409	17.00977716	6.95016E-65	1.1866E-63	positive
PRJNA729271	stage_3W_vs_E10	ordinary_muscle_development	ENSGALG00010003432	LACC1	2.341242305	0.305547693	7.662444705	1.82427E-14	5.37454E-14	positive
PRJNA729271	stage_3W_vs_E10	ordinary_muscle_development	ENSGALG00010003437	PNRC1	1.686152192	0.178935562	9.423236892	4.37392E-21	1.74752E-20	positive
PRJNA729271	stage_3W_vs_E10	ordinary_muscle_development	ENSGALG00010003438	KCNS3	-1.278707936	0.176919463	-7.227627261	4.91505E-13	1.34397E-12	negative
PRJNA729271	stage_3W_vs_E10	ordinary_muscle_development	ENSGALG00010003439	GMIP	0.842010231	0.228398618	3.686581984	0.000227286	0.000360056	positive
PRJNA729271	stage_3W_vs_E10	ordinary_muscle_development	ENSGALG00010003442	SPCS3	0.620126189	0.089154397	6.955643368	3.50957E-12	9.16928E-12	positive
PRJNA729271	stage_3W_vs_E10	ordinary_muscle_development	ENSGALG00010003447	ZC2HC1A	-0.936812179	0.126471062	-7.407324367	1.28873E-13	3.63498E-13	negative
PRJNA729271	stage_3W_vs_E10	ordinary_muscle_development	ENSGALG00010003449	BORCS6	1.625540932	0.109011449	14.91165326	2.76813E-50	3.17688E-49	positive
PRJNA729271	stage_3W_vs_E10	ordinary_muscle_development	ENSGALG00010003454	BPNT2	-1.014611951	0.120208092	-8.44046302	3.16083E-17	1.07039E-16	negative
PRJNA729271	stage_3W_vs_E10	ordinary_muscle_development	ENSGALG00010003456	CDS1	-3.882842811	0.411827758	-9.428317397	4.1672E-21	1.66768E-20	negative
PRJNA729271	stage_3W_vs_E10	ordinary_muscle_development	ENSGALG00010003466	TSC22D1	-0.145569188	0.100512693	-1.44826672	0.147542471	0.174197941	negative
PRJNA729271	stage_3W_vs_E10	ordinary_muscle_development	ENSGALG00010003468	ILVBL	0.837698978	0.135403601	6.186681661	6.14439E-10	1.41956E-09	positive
PRJNA729271	stage_3W_vs_E10	ordinary_muscle_development	ENSGALG00010003470	METTL3	0.849995733	0.186563583	4.556064582	5.21209E-06	9.40603E-06	positive
PRJNA729271	stage_3W_vs_E10	ordinary_muscle_development	ENSGALG00010003471	ADI1	1.037728726	0.133150317	7.79366318	6.50939E-15	1.96121E-14	positive
PRJNA729271	stage_3W_vs_E10	ordinary_muscle_development	ENSGALG00010003472	FAM110B	-2.90111318	0.132730604	-21.85715353	6.6461E-106	3.0877E-104	negative
PRJNA729271	stage_3W_vs_E10	ordinary_muscle_development	ENSGALG00010003473	ENSGALG00010003473	-0.719236814	0.257335884	-2.794934012	0.005191031	0.007267845	negative
PRJNA729271	stage_3W_vs_E10	ordinary_muscle_development	ENSGALG00010003474	RPS7	0.327255479	0.166102867	1.970197658	0.048815722	0.061363619	positive
PRJNA729271	stage_3W_vs_E10	ordinary_muscle_development	ENSGALG00010003475	ENSGALG00010003475	6.328656056	2.083163558	3.03800248	0.00238152	0.003443405	positive
PRJNA729271	stage_3W_vs_E10	ordinary_muscle_development	ENSGALG00010003478	COLEC11	1.204071807	0.178076801	6.761530977	1.36541E-11	3.46371E-11	positive
PRJNA729271	stage_3W_vs_E10	ordinary_muscle_development	ENSGALG00010003479	SYDE1	0.350325634	0.201411569	1.739352092	0.08197285	0.100185199	positive
PRJNA729271	stage_3W_vs_E10	ordinary_muscle_development	ENSGALG00010003485	RNGTT	-1.087840449	0.103695667	-10.49070299	9.53165E-26	4.60078E-25	negative
PRJNA729271	stage_3W_vs_E10	ordinary_muscle_development	ENSGALG00010003489	SGCE	-0.403775973	0.119299185	-3.384566058	0.000712909	0.001082393	negative
PRJNA729271	stage_3W_vs_E10	ordinary_muscle_development	ENSGALG00010003503	NUFIP1	-0.208461407	0.085871234	-2.42760464	0.015198902	0.020250952	negative
PRJNA729271	stage_3W_vs_E10	ordinary_muscle_development	ENSGALG00010003506	DYNC1I1	-2.893061043	0.18770584	-15.4127386	1.34394E-53	1.71181E-52	negative
PRJNA729271	stage_3W_vs_E10	ordinary_muscle_development	ENSGALG00010003507	WDFY3	0.547735674	0.097431305	5.621762682	1.89019E-08	4.01205E-08	positive
PRJNA729271	stage_3W_vs_E10	ordinary_muscle_development	ENSGALG00010003509	GPALPP1	-0.307219439	0.072168078	-4.256999074	2.07189E-05	3.5744E-05	negative
PRJNA729271	stage_3W_vs_E10	ordinary_muscle_development	ENSGALG00010003511	UBXN2B	-0.871736728	0.143244799	-6.085643161	1.16025E-09	2.63271E-09	negative
PRJNA729271	stage_3W_vs_E10	ordinary_muscle_development	ENSGALG00010003512	ENSGALG00010003512	0.232038945	0.439980885	0.527384151	0.597926847	0.633548951	positive
PRJNA729271	stage_3W_vs_E10	ordinary_muscle_development	ENSGALG00010003517	GTF2F2	-0.629925242	0.087768623	-7.177112015	7.11994E-13	1.93446E-12	negative
PRJNA729271	stage_3W_vs_E10	ordinary_muscle_development	ENSGALG00010003519	KCTD4	-2.081530213	0.678820108	-3.066394451	0.002166573	0.003143906	negative
PRJNA729271	stage_3W_vs_E10	ordinary_muscle_development	ENSGALG00010003520	ENSGALG00010003520	5.355960699	1.633989951	3.277841882	0.00104604	0.001565065	positive
PRJNA729271	stage_3W_vs_E10	ordinary_muscle_development	ENSGALG00010003522	ENSGALG00010003522	2.314962289	0.640890559	3.612102343	0.000303725	0.000476615	positive
PRJNA729271	stage_3W_vs_E10	ordinary_muscle_development	ENSGALG00010003525	NOL10	-0.78794999	0.067423763	-11.68653231	1.49361E-31	9.09279E-31	negative
PRJNA729271	stage_3W_vs_E10	ordinary_muscle_development	ENSGALG00010003528	GDAP1	-1.553056821	0.207173132	-7.496420066	6.55844E-14	1.87986E-13	negative
PRJNA729271	stage_3W_vs_E10	ordinary_muscle_development	ENSGALG00010003531	PPM1K	-0.624327721	0.150738494	-4.141793533	3.446E-05	5.83049E-05	negative
PRJNA729271	stage_3W_vs_E10	ordinary_muscle_development	ENSGALG00010003539	ENSGALG00010003539	0.61952708	0.212391004	2.916917703	0.00353509	0.005034994	positive
PRJNA729271	stage_3W_vs_E10	ordinary_muscle_development	ENSGALG00010003541	CNR1	-0.987277316	0.219325459	-4.501425977	6.74991E-06	1.20656E-05	negative
PRJNA729271	stage_3W_vs_E10	ordinary_muscle_development	ENSGALG00010003543	SOX11	-3.733675533	0.083456019	-44.7382415	0	0	negative
PRJNA729271	stage_3W_vs_E10	ordinary_muscle_development	ENSGALG00010003552	PI15	-3.683859199	0.389767801	-9.451420031	3.34265E-21	1.34439E-20	negative
PRJNA729271	stage_3W_vs_E10	ordinary_muscle_development	ENSGALG00010003556	ENSGALG00010003556	7.294112785	1.832848069	3.979660349	6.90138E-05	0.000114261	positive
PRJNA729271	stage_3W_vs_E10	ordinary_muscle_development	ENSGALG00010003558	TPT1	2.680839363	0.184626937	14.52030458	9.0109E-48	9.49912E-47	positive
PRJNA729271	stage_3W_vs_E10	ordinary_muscle_development	ENSGALG00010003560	ADGRL3	-3.071894018	0.142172155	-21.60686126	1.5482E-103	6.9264E-102	negative
PRJNA729271	stage_3W_vs_E10	ordinary_muscle_development	ENSGALG00010003571	RPL7	0.288225096	0.157445897	1.83062945	0.06715587	0.082964658	positive
PRJNA729271	stage_3W_vs_E10	ordinary_muscle_development	ENSGALG00010003577	ENSGALG00010003577	5.808098938	1.567175712	3.706093001	0.000210481	0.000334458	positive
PRJNA729271	stage_3W_vs_E10	ordinary_muscle_development	ENSGALG00010003582	RDH10	-1.128502281	0.14167909	-7.965199971	1.64957E-15	5.10472E-15	negative
PRJNA729271	stage_3W_vs_E10	ordinary_muscle_development	ENSGALG00010003583	RSAD2	4.430420555	0.799809504	5.539344725	3.03605E-08	6.34481E-08	positive
PRJNA729271	stage_3W_vs_E10	ordinary_muscle_development	ENSGALG00010003585	PNPLA6	1.0398289	0.142286904	7.307973317	2.71202E-13	7.52045E-13	positive
PRJNA729271	stage_3W_vs_E10	ordinary_muscle_development	ENSGALG00010003589	SLC25A30	3.481408697	0.387408706	8.986397684	2.55466E-19	9.51924E-19	positive
PRJNA729271	stage_3W_vs_E10	ordinary_muscle_development	ENSGALG00010003595	SNX13	0.49697723	0.09416161	5.27917728	1.3066E-07	2.62279E-07	positive
PRJNA729271	stage_3W_vs_E10	ordinary_muscle_development	ENSGALG00010003596	CRISPLD1	-3.413436283	0.156305138	-21.83828582	1.0045E-105	4.652E-104	negative
PRJNA729271	stage_3W_vs_E10	ordinary_muscle_development	ENSGALG00010003597	RNF144A	-0.88762883	0.145613054	-6.095805326	1.08888E-09	2.47387E-09	negative
PRJNA729271	stage_3W_vs_E10	ordinary_muscle_development	ENSGALG00010003604	ODC	-1.59194165	0.216503268	-7.352968231	1.93853E-13	5.41824E-13	negative
PRJNA729271	stage_3W_vs_E10	ordinary_muscle_development	ENSGALG00010003617	COG3	-0.119075055	0.083006718	-1.434523115	0.151423037	0.178517847	negative
PRJNA729271	stage_3W_vs_E10	ordinary_muscle_development	ENSGALG00010003619	HNF4G	-0.768351319	0.527157105	-1.457537634	0.144968031	0.171479648	negative
PRJNA729271	stage_3W_vs_E10	ordinary_muscle_development	ENSGALG00010003627	MCOLN1	0.352164902	0.120028583	2.934008658	0.003346149	0.004778564	positive
PRJNA729271	stage_3W_vs_E10	ordinary_muscle_development	ENSGALG00010003632	TECRL	-0.745805223	0.377349502	-1.976430919	0.048105993	0.060523944	negative
PRJNA729271	stage_3W_vs_E10	ordinary_muscle_development	ENSGALG00010003634	CYP4F11	1.402451122	0.302181092	4.641094886	3.46568E-06	6.342E-06	positive
PRJNA729271	stage_3W_vs_E10	ordinary_muscle_development	ENSGALG00010003639	ENSGALG00010003639	-0.561902946	0.784795109	-0.715986809	0.473999494	0.512694774	negative
PRJNA729271	stage_3W_vs_E10	ordinary_muscle_development	ENSGALG00010003641	ENSGALG00010003641	5.569318275	2.451349918	2.271939324	0.023090175	0.030187796	positive
PRJNA729271	stage_3W_vs_E10	ordinary_muscle_development	ENSGALG00010003650	SPACA1	-1.417883771	0.857260712	-1.653970316	0.098133544	0.118764776	negative

PRJNA729271	stage_3W_vs_E10	ordinary_muscle_development	ENSGALG00010003652	MYCN	-1.412153387	0.143622361	-9.832406165	8.16444E-23	3.49405E-22	negative
PRJNA729271	stage_3W_vs_E10	ordinary_muscle_development	ENSGALG00010003660	IGFBP7	3.397936313	0.164767702	20.62258725	1.72104E-94	6.28376E-93	positive
PRJNA729271	stage_3W_vs_E10	ordinary_muscle_development	ENSGALG00010003665	RDH14	1.867356046	0.091106149	20.49648745	2.31406E-93	8.30255E-92	positive
PRJNA729271	stage_3W_vs_E10	ordinary_muscle_development	ENSGALG00010003666	SLC25A13	-0.480644972	0.077245621	-6.222294083	4.89938E-10	1.13991E-09	negative
PRJNA729271	stage_3W_vs_E10	ordinary_muscle_development	ENSGALG00010003669	ENSGALG00010003669	4.86088976	1.499203189	3.242315515	0.001185627	0.001765348	positive
PRJNA729271	stage_3W_vs_E10	ordinary_muscle_development	ENSGALG00010003671	ENSGALG00010003671	0.022134094	0.406526591	0.054446854	0.95657915	0.962154936	positive
PRJNA729271	stage_3W_vs_E10	ordinary_muscle_development	ENSGALG00010003674	AKIRIN2	0.410977591	0.132024612	3.112886197	0.001852674	0.002704382	positive
PRJNA729271	stage_3W_vs_E10	ordinary_muscle_development	ENSGALG00010003676	POLR2B	0.436942164	0.077815478	5.615106115	1.96442E-08	4.16351E-08	positive
PRJNA729271	stage_3W_vs_E10	ordinary_muscle_development	ENSGALG00010003678	ZNFY1	-0.535468764	0.445880393	-1.20092467	0.229780423	0.26348131	negative
PRJNA729271	stage_3W_vs_E10	ordinary_muscle_development	ENSGALG00010003680	TRAPPC5	0.57870545	0.1063032	5.443913746	5.21225E-08	1.07363E-07	positive
PRJNA729271	stage_3W_vs_E10	ordinary_muscle_development	ENSGALG00010003681	NT5C1B	0.260970815	0.128646667	2.028585907	0.042500485	0.053826521	positive
PRJNA729271	stage_3W_vs_E10	ordinary_muscle_development	ENSGALG00010003683	ENSGALG00010003683	5.487502485	1.565438244	3.50540975	0.000455905	0.000704815	positive
PRJNA729271	stage_3W_vs_E10	ordinary_muscle_development	ENSGALG00010003691	ERICH1	0.031090619	0.14895798	0.208720736	0.834666252	0.853749722	positive
PRJNA729271	stage_3W_vs_E10	ordinary_muscle_development	ENSGALG00010003692	ZNFY2	-0.273398959	0.258825721	-1.056305216	0.290828794	0.328033878	negative
PRJNA729271	stage_3W_vs_E10	ordinary_muscle_development	ENSGALG00010003695	NOA1	0.767855025	0.100379188	7.649544064	2.01693E-14	5.9289E-14	positive
PRJNA729271	stage_3W_vs_E10	ordinary_muscle_development	ENSGALG00010003698	MACC1	-5.950654838	1.308299137	-4.548390096	5.40579E-06	9.73619E-06	negative
PRJNA729271	stage_3W_vs_E10	ordinary_muscle_development	ENSGALG00010003700	REST	-0.656135092	0.067674408	-9.695468507	3.15188E-22	1.31851E-21	negative
PRJNA729271	stage_3W_vs_E10	ordinary_muscle_development	ENSGALG00010003701	SARAF	1.458377945	0.081488735	17.89668169	1.25161E-71	2.51624E-70	positive
PRJNA729271	stage_3W_vs_E10	ordinary_muscle_development	ENSGALG00010003703	TDRP	-1.044128331	0.230338667	-4.533013687	5.8148E-06	1.04586E-05	negative
PRJNA729271	stage_3W_vs_E10	ordinary_muscle_development	ENSGALG00010003713	SPERT	1.014744636	0.255402455	3.973120135	7.09372E-05	0.000117298	positive
PRJNA729271	stage_3W_vs_E10	ordinary_muscle_development	ENSGALG00010003714	ORC3	-0.205389645	0.107274926	-1.914609984	0.055542261	0.069340576	negative
PRJNA729271	stage_3W_vs_E10	ordinary_muscle_development	ENSGALG00010003716	YLEC8	0.097348279	0.6852524	0.142061931	0.887031088	0.900256928	positive
PRJNA729271	stage_3W_vs_E10	ordinary_muscle_development	ENSGALG00010003717	LEPROTL1	0.25341396	0.096895545	2.615331385	0.008914092	0.012190751	positive
PRJNA729271	stage_3W_vs_E10	ordinary_muscle_development	ENSGALG00010003719	RPGR	-0.702385965	0.114494545	-6.134667487	8.53375E-10	1.95413E-09	negative
PRJNA729271	stage_3W_vs_E10	ordinary_muscle_development	ENSGALG00010003720	ENSGALG00010003720	4.096177351	2.644141482	1.549152108	0.121345159	0.14496811	positive
PRJNA729271	stage_3W_vs_E10	ordinary_muscle_development	ENSGALG00010003722	MHCY11	3.272258815	0.652269877	5.016725334	5.25596E-07	1.01243E-06	positive
PRJNA729271	stage_3W_vs_E10	ordinary_muscle_development	ENSGALG00010003724	ENSGALG00010003724	-0.103364477	0.217942716	-0.474273602	0.635304814	0.66875405	negative
PRJNA729271	stage_3W_vs_E10	ordinary_muscle_development	ENSGALG00010003726	ENPEP	-2.91249814	0.477605413	-6.098126315	1.07319E-09	2.43937E-09	negative
PRJNA729271	stage_3W_vs_E10	ordinary_muscle_development	ENSGALG00010003727	FBX025	0.879232167	0.15228353	5.773652403	7.75714E-09	1.68424E-08	positive
PRJNA729271	stage_3W_vs_E10	ordinary_muscle_development	ENSGALG00010003729	RARS2	-0.676633479	0.103994561	-6.506431404	7.6957E-11	1.87038E-10	negative
PRJNA729271	stage_3W_vs_E10	ordinary_muscle_development	ENSGALG00010003731	ENSGALG00010003731	1.613930112	0.579790338	2.783644375	0.005375193	0.007513349	positive
PRJNA729271	stage_3W_vs_E10	ordinary_muscle_development	ENSGALG00010003733	STAU2	-0.038995798	0.070827616	-0.550573352	0.58192618	0.617859653	negative
PRJNA729271	stage_3W_vs_E10	ordinary_muscle_development	ENSGALG00010003734	ZC3H13	-0.502015416	0.141982067	-3.53576637	0.000406594	0.000631004	negative
PRJNA729271	stage_3W_vs_E10	ordinary_muscle_development	ENSGALG00010003736	ENSGALG00010003736	5.266801629	3.018784651	1.744676165	0.081041254	0.0990884	positive
PRJNA729271	stage_3W_vs_E10	ordinary_muscle_development	ENSGALG00010003737	SRPT2	-0.441443331	0.056594655	-7.800088704	6.18637E-15	1.86582E-14	negative
PRJNA729271	stage_3W_vs_E10	ordinary_muscle_development	ENSGALG00010003740	PITX2	-3.518905987	0.360493653	-9.761353515	1.64939E-22	6.96212E-22	negative
PRJNA729271	stage_3W_vs_E10	ordinary_muscle_development	ENSGALG00010003742	ZFHX4	-1.403106827	0.166475641	-8.428301099	3.5072E-17	1.18501E-16	negative
PRJNA729271	stage_3W_vs_E10	ordinary_muscle_development	ENSGALG00010003743	ARL9	0.002447344	0.17636718	0.013876416	0.988928577	0.990568705	positive
PRJNA729271	stage_3W_vs_E10	ordinary_muscle_development	ENSGALG00010003746	HOPX	4.085229661	0.406485869	10.05011484	9.17604E-24	4.08247E-23	positive
PRJNA729271	stage_3W_vs_E10	ordinary_muscle_development	ENSGALG00010003749	MHCY15	1.483426538	0.952125767	1.558015326	0.119229615	0.142652354	positive
PRJNA729271	stage_3W_vs_E10	ordinary_muscle_development	ENSGALG00010003753	SLCO5A1	0.782350917	0.159678793	4.899529266	9.60665E-07	1.82286E-06	positive
PRJNA729271	stage_3W_vs_E10	ordinary_muscle_development	ENSGALG00010003754	FAM110C	-0.367395393	0.252290294	-1.456240694	0.145326094	0.171847098	negative
PRJNA729271	stage_3W_vs_E10	ordinary_muscle_development	ENSGALG00010003755	SLC35A1	-0.654865161	0.090533255	-7.233421109	4.70976E-13	1.28929E-12	negative
PRJNA729271	stage_3W_vs_E10	ordinary_muscle_development	ENSGALG00010003756	MHCY7	1.077845824	0.373519824	2.885645568	0.003906118	0.005538411	positive
PRJNA729271	stage_3W_vs_E10	ordinary_muscle_development	ENSGALG00010003761	UGT8	2.107344305	0.329535412	6.394894846	1.60658E-10	3.83457E-10	positive
PRJNA729271	stage_3W_vs_E10	ordinary_muscle_development	ENSGALG00010003762	EVI5L	-0.379187206	0.099863067	-3.797071516	0.000146416	0.000235941	negative
PRJNA729271	stage_3W_vs_E10	ordinary_muscle_development	ENSGALG00010003764	HDAC9	0.440219539	0.193299133	2.277400477	0.022762322	0.029783341	positive
PRJNA729271	stage_3W_vs_E10	ordinary_muscle_development	ENSGALG00010003765	ENSGALG00010003765	0.846432192	0.873007116	0.969559327	0.332266195	0.370647876	positive
PRJNA729271	stage_3W_vs_E10	ordinary_muscle_development	ENSGALG00010003767	ENSGALG00010003767	-0.33703243	0.414437785	-0.813228047	0.416087322	0.454909167	negative
PRJNA729271	stage_3W_vs_E10	ordinary_muscle_development	ENSGALG00010003768	ARSJ	-2.010700911	0.134348643	-14.96629121	1.21934E-50	1.41508E-49	negative
PRJNA729271	stage_3W_vs_E10	ordinary_muscle_development	ENSGALG00010003769	C6orf163	0.35949657	0.491372078	0.731617823	0.464401867	0.503064424	positive
PRJNA729271	stage_3W_vs_E10	ordinary_muscle_development	ENSGALG00010003772	ENSGALG00010003772	2.391174497	0.440446693	5.428975939	5.66783E-08	1.16384E-07	positive
PRJNA729271	stage_3W_vs_E10	ordinary_muscle_development	ENSGALG00010003775	NCOA2	0.386397262	0.114364899	3.378635098	0.000728466	0.001105088	positive
PRJNA729271	stage_3W_vs_E10	ordinary_muscle_development	ENSGALG00010003777	ENSGALG00010003777	10.32996621	0.798901641	12.93021027	3.03986E-38	2.3118E-37	positive
PRJNA729271	stage_3W_vs_E10	ordinary_muscle_development	ENSGALG00010003778	LCA5	0.365707181	0.122515981	2.984975325	0.002836012	0.004075748	positive
PRJNA729271	stage_3W_vs_E10	ordinary_muscle_development	ENSGALG00010003785	ENSGALG00010003785	5.86883715	1.042086715	5.631812654	1.78325E-08	3.79118E-08	positive
PRJNA729271	stage_3W_vs_E10	ordinary_muscle_development	ENSGALG00010003786	ENSGALG00010003786	-1.036921962	0.617751233	-1.678542925	0.093241158	0.113240937	negative
PRJNA729271	stage_3W_vs_E10	ordinary_muscle_development	ENSGALG00010003787	SMIM8	1.571572187	0.187374239	8.387343926	4.97249E-17	1.66804E-16	positive
PRJNA729271	stage_3W_vs_E10	ordinary_muscle_development	ENSGALG00010003788	NUDT9	0.216056369	0.126556289	1.707195838	0.087785658	0.106957812	positive
PRJNA729271	stage_3W_vs_E10	ordinary_muscle_development	ENSGALG00010003790	ELOVL6	-1.081199208	0.182430579	-5.926633647	3.09208E-09	6.83539E-09	negative
PRJNA729271	stage_3W_vs_E10	ordinary_muscle_development	ENSGALG00010003791	ITGB8	-2.148979708	0.177088104	-12.13508788	6.88361E-34	4.54983E-33	negative
PRJNA729271	stage_3W_vs_E10	ordinary_muscle_development	ENSGALG00010003793	TRAM1L1	-0.0686775	0.08483195	-0.809571163	0.418186688	0.45679096	negative
PRJNA729271	stage_3W_vs_E10	ordinary_muscle_development	ENSGALG00010003795	LCP1	2.077264998	0.200451616	10.36292466	3.65609E-25	1.7251E-24	positive
PRJNA729271	stage_3W_vs_E10	ordinary_muscle_development	ENSGALG00010003796	ENSGALG00010003796	3.175280012	0.301083009	10.54619464	5.28963E-26	2.58333E-25	positive
PRJNA729271	stage_3W_vs_E10	ordinary_muscle_development	ENSGALG00010003797	ENSGALG00010003797	0.478206116	0.1731197	2.762285964	0.005739818	0.007995259	positive
PRJNA729271	stage_3W_vs_E10	ordinary_muscle_development	ENSGALG00010003803	LACTB2	1.233119169	0.132190332	9.328361243	1.0752E-20	4.22704E-20	positive
PRJNA729271	stage_3W_vs_E10	ordinary_muscle_development	ENSGALG00010003804	PSME1	3.132929618	0.253203816	12.37315325	3.65198E-35	2.52796E-34	positive
PRJNA729271	stage_3W_vs_E10	ordinary_muscle_development	ENSGALG00010003806	EGF	4.118370859	0.315382459	13.05833833	5.69575E-39	4.45307E-38	positive
PRJNA729271	stage_3W_vs_E10	ordinary_muscle_development	ENSGALG00010003808	TWIST1	-3.077216471	0.167364317	-18.38633545	1.69026E-75	3.77508E-74	negative
PRJNA729271	stage_3W_vs_E10	ordinary_muscle_development	ENSGALG00010003810	HMGN2P46	0.419057821	0.091022328	4.603901381	4.14649E-06	7.53458E-06	positive
PRJNA729271	stage_3W_vs_E10	ordinary_muscle_development	ENSGALG00010003812	BG8	1.031380289	0.399652703	2.580691391	0.009860268	0.01340756	positive
PRJNA729271	stage_3W_vs_E10	ordinary_muscle_development	ENSGALG00010003813	LRIT3	-2.925515699	0.685958191	-4.264860069	2.00028E-05	3.45619E-05	negative
PRJNA729271	stage_3W_vs_E10	ordinary_muscle_development	ENSGALG00010003814	TPP1	1.533317733	0.13214593	11.60321573	3.96879E-31	2.37619E-30	positive
PRJNA729271	stage_3W_vs_E10	ordinary_muscle_development	ENSGALG00010003815	RASAL3	5.327537608	0.562796949	9.466180691	2.90263E-21	1.17001E-20	positive
PRJNA729271	stage_3W_vs_E10	ordinary_muscle_development	ENSGALG00010003816	KLHL8	-1.17427957	0.183012786	-6.416379955	1.39553E-10	3.33964E-10	negative
PRJNA729271	stage_3W_vs_E10	ordinary_muscle_development	ENSGALG00010003817	BF2	5.528536803	0.281829066	19.61663103	1.11502E-85	3.30517E-84	positive
PRJNA729271	stage_3W_vs_E10	ordinary_muscle_development	ENSGALG00010003818	GAB2	0.255290909	0.1612883	1.582823486	0.113461702	0.13614465	positive
PRJNA729271	stage_3W_vs_E10	ordinary_muscle_development	ENSGALG00010003819	PHIP	-0.04017812	0.094116851	-0.426896136	0.66945498	0.701087345	negative
PRJNA729271	stage_3W_vs_E10	ordinary_muscle_development	ENSGALG00010003820	SH3YL1	-0.613708657	0.300321221	-2.043507467	0.041002229	0.052024449	negative
PRJNA729271	stage_3W_vs_E10	ordinary_muscle_development	ENSGALG00010003821	RUBCNL	1.138033784	0.155619243	7.312937415	2.61365E-13	7.25346E-13	positive
PRJNA729271	stage_3W_vs_E10	ordinary_muscle_development	ENSGALG00010003822	ENSGALG00010003822	-0.845856392	0.167892814	-5.038073816	4.7024E-07	9.10031E-07	negative
PRJNA729271	stage_3W_vs_E10	ordinary_muscle_development	ENSGALG00010003823	PTPN13	0.091970023	0.148918914	0.617584566	0.536849221	0.574586831	positive
PRJNA729271	stage_3W_vs_E10	ordinary_muscle_development	ENSGALG00010003824	SP4	0.305756575	0.158410643	1.930151721	0.053588042	0.067002386	positive
PRJNA729271	stage_3W_vs_E10	ordinary_muscle_development	ENSGALG00010003826	ENSGALG00010003826	-0.175976809	0.106649166	-1.650053316	0.098932032	0.119621271	negative
PRJNA729271	stage_3W_vs_E10	ordinary_muscle_development	ENSGALG00010003827	RRH	-0.628521685	0.752412186	-0.835342245	0.403525038	0.442710825	negative
PRJNA729271	stage_3W_vs_E10	ordinary_muscle_development	ENSGALG00010003829	ENSGALG00010003829	-0.304070417	0.13242439	-2.296181359	0.021665514	0.028407203	negative
PRJNA729271	stage_3W_vs_E10	ordinary_muscle_development	ENSGALG00010003830	DMB2	2.242962875	0.228630001	9.810448608	1.01516E-22	4.32788E-22	positive
PRJNA729271	stage_3W_vs_E10	ordinary_muscle_development	ENSGALG00010003831	IRAK1BP1	0.173681084	0.165535266	1.049208958	0.294081957	0.331342243	positive
PRJNA729271	stage_3W_vs_E10	ordinary_muscle_development	ENSGALG00010003832	ART1B	5.925850485	0.933768901	6.346163893	2.2075E-10	5.2378E-10	positive
PRJNA729271	stage_3W_vs_E10	ordinary_muscle_development	ENSGALG00010003833	ACP1	0.459386476	0.157430058	2.918035356	0.003522444	0.005017475	positive
PRJNA729271	stage_3W_vs_E10	ordinary_muscle_development	ENSGALG00010003834	MAPK10	-1.071897634	0.433917568	-2.47027941	0.013500756	0.018094633	negative
PRJNA729271	stage_3W_vs_E10	ordinary_muscle_development	ENSGALG00010003835	ENSGALG00010003835	1.896147313	0.164660094	11.51552429	1.10187E-30	6.49782E-30	positive
PRJNA729271	stage_3W_vs_E10	ordinary_muscle_development	ENSGALG00010003836	CD1F	3.586425048	0.332485053	10.78672565	3.97708E-27	2.04135E-26	positive
PRJNA729271	stage_3W_vs_E10	ordinary_muscle_development	ENSGALG00010003837	ALKAL2	0.99200659	0.243738133	4.06996877	4.70194E-05	7.88645E-05	positive
PRJNA729271	stage_3W_vs_E10	ordinary_muscle_development	ENSGALG00010003838	GAR1	-2.126882365	0.187146697	-11.36478706	6.2617E-30	3.5889E-29	negative
PRJNA729271	stage_3W_vs_E10	ordinary_muscle_development	ENSGALG00010003839	ENSGALG00010003839	3.48615793	0.727344725	4.792992661	1.64312E-06	3.06446E-06	positive
PRJNA729271	stage_3W_vs_E10	ordinary_muscle_development	ENSGALG00010003841	UBE2W	0.832183789	0.141573028	5.878123813	4.14943E-09	9.12685E-09	positive
PRJNA729271	stage_3W_vs_E10	ordinary_muscle_development	ENSGALG00010003842	ENSGALG00010003842	-0.506492646	0.145197489	-3.488301677	0.000486099	0.000749974	negative
PRJNA729271	stage_3W_vs_E10	ordinary_muscle_development	ENSGALG00010003843	LRCH1	0.403801026	0.108258619	3.729966532	0.000191505	0.000305611	positive
PRJNA729271	stage_3W_vs_E10	ordinary_muscle_development	ENSGALG00010003844	TMEM18	-0.973525526	0.113538683	-8.574395123	9.96089E-18	3.47075E-17	negative
PRJNA729271	stage_3W_vs_E10	ordinary_muscle_development	ENSGALG00010003845	CFI	0.110764951	0.884758117	0.125192354			positive
PRJNA729271	stage_3W_vs_E10	ordinary_muscle_development	ENSGALG00010003848	ESD	0.309987691	0.123822736	2.503479573	0.012297878	0.016555934	positive
PRJNA729271	stage_3W_vs_E10	ordinary_muscle_development	ENSGALG00010003850	PLA2G12A	0.023727003	0.098966875	0.239746919	0.810526459	0.832642171	positive
PRJNA729271	stage_3W_vs_E10	ordinary_muscle_development	ENSGALG00010003851	PHF14	-1.370507061	0.064614155	-21.21063208	7.61826E-100	3.16409E-98	negative
PRJNA729271	stage_3W_vs_E10	ordinary_muscle_development	ENSGALG00010003852	TCEB1	-0.018535656	0.10725943	-0.172811437	0.862799647	0.878370799	negative
PRJNA729271	stage_3W_vs_E10	ordinary_muscle_development	ENSGALG00010003853	ENSGALG00010003853	0.514174617	0.22871373	2.248114346	0.0245689	0.032008467	positive
PRJNA729271	stage_3W_vs_E10	ordinary_muscle_development	ENSGALG00010003858	SUCLA2	2.144982515	0.124160357	17.27590484	7.14441E-67	1.2785E-65	positive
PRJNA729271	stage_3W_vs_E10	ordinary_muscle_development	ENSGALG00010003860	TMEM70	0.736749655	0.113527217	6.489630172	8.60473E-11	2.08606E-10	positive
PRJNA729271	stage_3W_vs_E10	ordinary_muscle_development	ENSGALG00010003861	NUDT15	-0.656210012	0.104042057	-6.307161068	2.842E-10	6.69399E-10	negative
PRJNA729271	stage_3W_vs_E10	ordinary_muscle_development	ENSGALG00010003862	PCK2	-0.895263786	0.14603793	-6.130351124	8.76853E-10	2.006E-09	negative
PRJNA729271	stage_3W_vs_E10	ordinary_muscle_development	ENSGALG00010003863	HTR1B	-3.561448101	0.326450205	-10.90962126	1.03687E-27	5.46527E-27	negative
PRJNA729271	stage_3W_vs_E10	ordinary_muscle_development	ENSGALG00010003865	AKAP8L	-1.037634946	0.190930614	-5.434617979	5.4914E-08	1.12889E-07	negative
PRJNA729271	stage_3W_vs_E10	ordinary_muscle_development	ENSGALG00010003868	HBBA	-3.269040905	0.39900529	-8.192976355	2.54844E-16	8.25468E-16	negative
PRJNA729271	stage_3W_vs_E10	ordinary_muscle_development	ENSGALG00010003869	LY96	3.132112914	0.270035246	11.59890406	4.17389E-31	2.49693E-30	positive
PRJNA729271	stage_3W_vs_E10	ordinary_muscle_development	ENSGALG00010003870	MED4	-0.871617102	0.096100775	-9.069823895	1.1921E-19	4.494E-19	negative
PRJNA729271	stage_3W_vs_E10	ordinary_muscle_development	ENSGALG00010003871	ENSGALG00010003871	-0.810148209	0.205503003	-3.942269446	8.07142E-05	0.000132678	negative
PRJNA729271	stage_3W_vs_E10	ordinary_muscle_development	ENSGALG00010003874	YES1	-0.615572275	0.097855383	-6.290632715	3.16175E-10	7.43021E-10	negative
PRJNA729271	stage_3W_vs_E10	ordinary_muscle_development	ENSGALG00010003875	AKAP8	-0.885152969	0.111789238	-7.91805174	2.41261E-15	7.41849E-15	negative
PRJNA729271	stage_3W_vs_E10	ordinary_muscle_development	ENSGALG00010003878	ARHGAP24	0.35986053	0.089159936	4.036123687	5.43416E-05	9.07676E-05	positive
PRJNA729271	stage_3W_vs_E10	ordinary_muscle_development	ENSGALG00010003879	CASP6	-1.155434021	0.119171753	-9.695535995	3.14979E-22	1.31802E-21	negative
PRJNA729271	stage_3W_vs_E10	ordinary_muscle_development	ENSGALG00010003882	ENSGALG00010003882	-0.406010386	0.121969766	-3.32877891	0.000872276	0.001313625	negative
PRJNA729271	stage_3W_vs_E10	ordinary_muscle_development	ENSGALG00010003884	TYMS	-3.790862026	0.198990837	-19.0504351	6.51584E-81	1.67757E-79	negative
PRJNA729271	stage_3W_vs_E10	ordinary_muscle_development	ENSGALG00010003887	GIPC1	-1.064735119	0.145583124	-7.313588872	2.601E-13	7.21975E-13	negative
PRJNA729271	stage_3W_vs_E10	ordinary_muscle_development	ENSGALG00010003889	ITM2B	0.169157316	0.087739963	1.927939226	0.053862686	0.067334164	positive
PRJNA729271	stage_3W_vs_E10	ordinary_muscle_development	ENSGALG00010003892	OPRK1	-4.915327501	2.563370122	-1.917525471	0.055171208	0.068886783	negative
PRJNA729271	stage_3W_vs_E10	ordinary_muscle_development	ENSGALG00010003894	COPS4	0.296619538	0.124440115	2.383632793	0.017142698	0.022702916	positive
PRJNA729271	stage_3W_vs_E10	ordinary_muscle_development	ENSGALG00010003895	THSD7A	0.506713565	0.16090492	3.149148985	0.001637467	0.002409164	positive
PRJNA729271	stage_3W_vs_E10	ordinary_muscle_development	ENSGALG00010003896	MCUB	-0.788488734	0.133911112	-5.888150164	3.90542E-09	8.60059E-09	negative
PRJNA729271	stage_3W_vs_E10	ordinary_muscle_development	ENSGALG00010003898	NDST4	-3.541394416	0.541486743	-6.54013134	6.14648E-11	1.50115E-10	negative
PRJNA729271	stage_3W_vs_E10	ordinary_muscle_development	ENSGALG00010003899	LIN54	0.22544718	0.165560409	1.361721566	0.173285772	0.202513688	positive
PRJNA729271	stage_3W_vs_E10	ordinary_muscle_development	ENSGALG00010003900	RB1	-0.705766927	0.088239359	-7.998323394	1.26125E-15	3.92988E-15	negative

PRJNA729271	stage_3W_vs_E10	ordinary_muscle_development	ENSGALG00010003902	ENSGALG00010003902	1.046983089	0.1244651	8.411860778	4.03557E-17	1.36005E-16	positive
PRJNA729271	stage_3W_vs_E10	ordinary_muscle_development	ENSGALG00010003903	COL28A1	7.912607723	0.277065522	28.55861553	2.1957E-179	3.09E-177	positive
PRJNA729271	stage_3W_vs_E10	ordinary_muscle_development	ENSGALG00010003909	DNAJB1	-0.338029095	0.157320414	-2.148666451	0.03166085	0.040706406	negative
PRJNA729271	stage_3W_vs_E10	ordinary_muscle_development	ENSGALG00010003910	THAP9	1.137575822	0.147529434	7.710839725	1.24993E-14	3.70884E-14	positive
PRJNA729271	stage_3W_vs_E10	ordinary_muscle_development	ENSGALG00010003913	PSMB5	0.919178087	0.116716651	7.875295263	3.39939E-15	1.03823E-14	positive
PRJNA729271	stage_3W_vs_E10	ordinary_muscle_development	ENSGALG00010003914	CAMK2D	0.704145676	0.105856603	6.651882421	2.89368E-11	7.22298E-11	positive
PRJNA729271	stage_3W_vs_E10	ordinary_muscle_development	ENSGALG00010003915	MIOS	-0.002105004	0.092489909	-0.02275928	0.981842289	0.984559252	negative
PRJNA729271	stage_3W_vs_E10	ordinary_muscle_development	ENSGALG00010003918	HTR1E	-2.716079368	0.740259234	-3.669092182	0.000243413	0.000384595	negative
PRJNA729271	stage_3W_vs_E10	ordinary_muscle_development	ENSGALG00010003920	LPAR6	1.249790407	0.152253414	8.208619929	2.23745E-16	7.26683E-16	positive
PRJNA729271	stage_3W_vs_E10	ordinary_muscle_development	ENSGALG00010003921	COLEC12	0.240752266	0.168255743	1.430871015	0.152467185	0.17967576	positive
PRJNA729271	stage_3W_vs_E10	ordinary_muscle_development	ENSGALG00010003923	ENSGALG00010003923	-0.799267662	0.120478312	-6.634120662	3.26443E-11	8.12743E-11	negative
PRJNA729271	stage_3W_vs_E10	ordinary_muscle_development	ENSGALG00010003925	SEC31A	-1.169157264	0.086157162	-13.57005316	6.02783E-42	5.18536E-41	negative
PRJNA729271	stage_3W_vs_E10	ordinary_muscle_development	ENSGALG00010003928	ENSGALG00010003928	0.980739182	0.155333047	6.313783189	2.72295E-10	6.42089E-10	positive
PRJNA729271	stage_3W_vs_E10	ordinary_muscle_development	ENSGALG00010003929	RCBTB2	0.477101672	0.097219092	4.907489497	9.22496E-07	1.75227E-06	positive
PRJNA729271	stage_3W_vs_E10	ordinary_muscle_development	ENSGALG00010003933	TECR	1.721109801	0.20678013	8.32338096	8.54891E-17	2.83627E-16	positive
PRJNA729271	stage_3W_vs_E10	ordinary_muscle_development	ENSGALG00010003937	ENSGALG00010003937	0.962020352	0.206641362	4.655507222	3.23184E-06	5.92382E-06	positive
PRJNA729271	stage_3W_vs_E10	ordinary_muscle_development	ENSGALG00010003948	SCD5	-3.193047773	0.14768804	-21.62021902	1.1593E-103	5.2185E-102	negative
PRJNA729271	stage_3W_vs_E10	ordinary_muscle_development	ENSGALG00010003958	CYSLTR2	3.822627922	0.361712028	10.56815264	4.1866E-26	2.05502E-25	positive
PRJNA729271	stage_3W_vs_E10	ordinary_muscle_development	ENSGALG00010003960	ASPH	1.628410287	0.19595504	8.310122012	9.56037E-17	3.16604E-16	positive
PRJNA729271	stage_3W_vs_E10	ordinary_muscle_development	ENSGALG00010003962	UMAD1	1.357277574	0.203262801	6.677451898	2.43132E-11	6.09197E-11	positive
PRJNA729271	stage_3W_vs_E10	ordinary_muscle_development	ENSGALG00010003965	SNTG1	-2.99552782	0.52722247	-5.681715009	1.33351E-08	2.85555E-08	negative
PRJNA729271	stage_3W_vs_E10	ordinary_muscle_development	ENSGALG00010003968	DSN1	0.198364511	0.172621746	1.149128172	0.250503133	0.28577388	positive
PRJNA729271	stage_3W_vs_E10	ordinary_muscle_development	ENSGALG00010003972	PENK	-2.801891315	0.241296867	-11.61180149	3.58972E-31	2.1519E-30	negative
PRJNA729271	stage_3W_vs_E10	ordinary_muscle_development	ENSGALG00010003977	MYO6	-1.708278079	0.121305318	-14.08246655	4.86806E-45	4.65759E-44	negative
PRJNA729271	stage_3W_vs_E10	ordinary_muscle_development	ENSGALG00010003978	ANK2	-0.517873471	0.120219513	-4.307732214	1.64937E-05	2.86628E-05	negative
PRJNA729271	stage_3W_vs_E10	ordinary_muscle_development	ENSGALG00010003980	CHCHD7	0.632825126	0.11854545	5.338248972	9.38485E-08	1.90416E-07	positive
PRJNA729271	stage_3W_vs_E10	ordinary_muscle_development	ENSGALG00010003981	TMEM150C	0.798637832	0.162937477	4.901498699	9.51083E-07	1.80562E-06	positive
PRJNA729271	stage_3W_vs_E10	ordinary_muscle_development	ENSGALG00010003982	GLCCI1	-0.587027856	0.121471338	-4.832645023	1.34731E-06	2.52838E-06	negative
PRJNA729271	stage_3W_vs_E10	ordinary_muscle_development	ENSGALG00010003985	PRMT5	0.090151787	0.134162688	0.671958713	0.501609981	0.539659814	positive
PRJNA729271	stage_3W_vs_E10	ordinary_muscle_development	ENSGALG00010003986	FNDC3A	0.635988713	0.063328084	10.0427594	9.8869E-24	4.3893E-23	positive
PRJNA729271	stage_3W_vs_E10	ordinary_muscle_development	ENSGALG00010003988	ENOPH1	-0.818100691	0.089562844	-9.134376028	6.5785E-20	2.50671E-19	negative
PRJNA729271	stage_3W_vs_E10	ordinary_muscle_development	ENSGALG00010003990	ICA1	-0.486910061	0.169776747	-2.867943156	0.004131498	0.005842542	negative
PRJNA729271	stage_3W_vs_E10	ordinary_muscle_development	ENSGALG00010003993	ENSGALG00010003993	0.587705819	0.134205817	4.379138197	1.1915E-05	2.09291E-05	positive
PRJNA729271	stage_3W_vs_E10	ordinary_muscle_development	ENSGALG00010003994	SDR16C5	1.664206167	0.083818881	19.85478876	1.00183E-87	3.10289E-86	positive
PRJNA729271	stage_3W_vs_E10	ordinary_muscle_development	ENSGALG00010003995	MLNR	-3.495394147	0.293973587	-11.89016394	1.33145E-32	8.44242E-32	negative
PRJNA729271	stage_3W_vs_E10	ordinary_muscle_development	ENSGALG00010004000	ENSGALG00010004000	1.438691885	0.175204387	8.211506062	2.18431E-16	7.09742E-16	positive
PRJNA729271	stage_3W_vs_E10	ordinary_muscle_development	ENSGALG00010004001	LARP7	-0.460702311	0.113227392	-4.068823837	4.72511E-05	7.92346E-05	negative
PRJNA729271	stage_3W_vs_E10	ordinary_muscle_development	ENSGALG00010004002	CDADC1	1.243166368	0.121384814	10.2415313	1.29175E-24	5.95546E-24	positive
PRJNA729271	stage_3W_vs_E10	ordinary_muscle_development	ENSGALG00010004003	HNRNPDL	-0.004707398	0.06804047	-0.069185262	0.944842154	0.952001044	negative
PRJNA729271	stage_3W_vs_E10	ordinary_muscle_development	ENSGALG00010004006	NKAIN3	-1.819706784	0.331489085	-5.489492317	4.03091E-08	8.36598E-08	negative
PRJNA729271	stage_3W_vs_E10	ordinary_muscle_development	ENSGALG00010004007	ENSGALG00010004007	-1.609533112	0.164462143	-9.786648067	1.28485E-22	5.44876E-22	negative
PRJNA729271	stage_3W_vs_E10	ordinary_muscle_development	ENSGALG00010004016	ENSGALG00010004016	-4.257036405	0.872675778	-4.878142044	1.0709E-06	2.02302E-06	negative
PRJNA729271	stage_3W_vs_E10	ordinary_muscle_development	ENSGALG00010004018	HNRNPD	-0.478241904	0.161390036	-2.963267838	0.003043916	0.00436415	negative
PRJNA729271	stage_3W_vs_E10	ordinary_muscle_development	ENSGALG00010004029	ARL4A	0.106113259	0.194588155	0.545322294	0.585531873	0.621187478	positive
PRJNA729271	stage_3W_vs_E10	ordinary_muscle_development	ENSGALG00010004034	NEDD8	0.598908182	0.111771019	5.358349485	8.39857E-08	1.71027E-07	positive
PRJNA729271	stage_3W_vs_E10	ordinary_muscle_development	ENSGALG00010004036	ADCYAP1	-4.144351927	0.933877831	-4.437788101	9.0888E-06	1.60936E-05	negative
PRJNA729271	stage_3W_vs_E10	ordinary_muscle_development	ENSGALG00010004038	SENP6	0.312706111	0.087501162	3.573736657	0.000351923	0.000549276	positive
PRJNA729271	stage_3W_vs_E10	ordinary_muscle_development	ENSGALG00010004039	ALPK1	4.825867943	0.218788187	22.05726009	8.1364E-108	4.0115E-106	positive
PRJNA729271	stage_3W_vs_E10	ordinary_muscle_development	ENSGALG00010004040	LGALS1B	-2.57918175	0.225929567	-11.4158664	3.48415E-30	2.01447E-29	negative
PRJNA729271	stage_3W_vs_E10	ordinary_muscle_development	ENSGALG00010004042	PLAG1	-1.349797733	0.124494466	-10.84223078	2.17111E-27	1.12878E-26	negative
PRJNA729271	stage_3W_vs_E10	ordinary_muscle_development	ENSGALG00010004043	CAB39L	0.4018688	0.20116217	1.99773546	0.045745348	0.05773936	positive
PRJNA729271	stage_3W_vs_E10	ordinary_muscle_development	ENSGALG00010004044	RNF138	-0.142325374	0.13300943	-1.070039725	0.284601428	0.321760994	negative
PRJNA729271	stage_3W_vs_E10	ordinary_muscle_development	ENSGALG00010004046	ENSGALG00010004046	-0.237351832	0.12199424	-1.945598678	0.051702966	0.064740367	negative
PRJNA729271	stage_3W_vs_E10	ordinary_muscle_development	ENSGALG00010004047	PDE7A	-1.049074223	0.118876029	-8.824943388	1.09515E-18	3.96955E-18	negative
PRJNA729271	stage_3W_vs_E10	ordinary_muscle_development	ENSGALG00010004049	SETDB2	0.111871142	0.138011125	0.810595099	0.417598232	0.456354522	positive
PRJNA729271	stage_3W_vs_E10	ordinary_muscle_development	ENSGALG00010004055	FILIP1	2.058049694	0.17526252	11.74266863	7.70172E-32	4.7404E-31	positive
PRJNA729271	stage_3W_vs_E10	ordinary_muscle_development	ENSGALG00010004056	SHROOM3	-3.07826329	0.189538424	-16.24084036	2.59347E-59	3.83204E-58	negative
PRJNA729271	stage_3W_vs_E10	ordinary_muscle_development	ENSGALG00010004057	ENSGALG00010004057	-0.955395839	0.440166946	-2.170530631	0.029966671	0.038644741	negative
PRJNA729271	stage_3W_vs_E10	ordinary_muscle_development	ENSGALG00010004058	TMEM30A	-0.109384161	0.061924361	-1.76641566	0.07732614	0.094825922	negative
PRJNA729271	stage_3W_vs_E10	ordinary_muscle_development	ENSGALG00010004060	ETV1	-1.788906416	0.093863299	-19.05863563	5.57087E-81	1.43939E-79	negative
PRJNA729271	stage_3W_vs_E10	ordinary_muscle_development	ENSGALG00010004063	ENSGALG00010004063	2.58610224	0.403586257	6.407805513	1.47629E-10	3.52709E-10	positive
PRJNA729271	stage_3W_vs_E10	ordinary_muscle_development	ENSGALG00010004065	TIFA	2.101660925	0.248816275	8.446637699	2.99812E-17	1.01679E-16	positive
PRJNA729271	stage_3W_vs_E10	ordinary_muscle_development	ENSGALG00010004070	ARL11	-0.303903942	0.402844728	-0.754394733	0.450612233	0.489406887	negative
PRJNA729271	stage_3W_vs_E10	ordinary_muscle_development	ENSGALG00010004072	SGK3	0.276072937	0.134588257	2.051240893	0.040243493	0.051110964	positive
PRJNA729271	stage_3W_vs_E10	ordinary_muscle_development	ENSGALG00010004076	COX7A2	-1.37453339	0.137517324	-9.995347132	1.59727E-23	7.018E-23	negative
PRJNA729271	stage_3W_vs_E10	ordinary_muscle_development	ENSGALG00010004079	AP1AR	-0.274737925	0.149622224	-1.836210682	0.066326503	0.081995963	negative
PRJNA729271	stage_3W_vs_E10	ordinary_muscle_development	ENSGALG00010004081	RPS20	0.74504129	0.167930654	4.436600904	9.13905E-06	1.61806E-05	positive
PRJNA729271	stage_3W_vs_E10	ordinary_muscle_development	ENSGALG00010004082	FAM241A	1.06803889	0.121841028	8.765839453	1.8539E-18	6.6598E-18	positive
PRJNA729271	stage_3W_vs_E10	ordinary_muscle_development	ENSGALG00010004083	PHF11	-0.163467658	0.183778802	-0.889480486	0.373744906	0.412536736	negative
PRJNA729271	stage_3W_vs_E10	ordinary_muscle_development	ENSGALG00010004085	RB1CC1	1.689439546	0.140458188	12.02806025	2.53036E-33	1.64547E-32	positive
PRJNA729271	stage_3W_vs_E10	ordinary_muscle_development	ENSGALG00010004086	UTP3	-0.430803772	0.257998694	-1.669790513	0.094960818	0.115194331	negative
PRJNA729271	stage_3W_vs_E10	ordinary_muscle_development	ENSGALG00010004087	NCOA7	-0.224163388	0.109280637	-2.051263558	0.040241287	0.051110964	negative
PRJNA729271	stage_3W_vs_E10	ordinary_muscle_development	ENSGALG00010004094	DGKB	-2.140093463	0.20324066	-10.52984899	6.29354E-26	3.06226E-25	negative
PRJNA729271	stage_3W_vs_E10	ordinary_muscle_development	ENSGALG00010004095	ANKRD50	-1.221400663	0.113883288	-10.72502107	7.76694E-27	3.93964E-26	negative
PRJNA729271	stage_3W_vs_E10	ordinary_muscle_development	ENSGALG00010004096	COL12A1	-3.711677936	0.414300092	-8.958911697	3.27899E-19	1.21526E-18	negative
PRJNA729271	stage_3W_vs_E10	ordinary_muscle_development	ENSGALG00010004097	ENSGALG00010004097	-0.943548363	0.118874005	-7.937381791	2.06494E-15	6.36294E-15	negative
PRJNA729271	stage_3W_vs_E10	ordinary_muscle_development	ENSGALG00010004101	ARID1B	0.382636172	0.16634491	2.300257768	0.021433621	0.028120957	positive
PRJNA729271	stage_3W_vs_E10	ordinary_muscle_development	ENSGALG00010004102	PCMTD1	3.217985198	0.187485562	17.16390936	4.94688E-66	8.681E-65	positive
PRJNA729271	stage_3W_vs_E10	ordinary_muscle_development	ENSGALG00010004104	SCARB2	1.296275346	0.093960735	13.79592602	2.69668E-43	2.42785E-42	positive
PRJNA729271	stage_3W_vs_E10	ordinary_muscle_development	ENSGALG00010004108	PERPB	1.550840297	0.240780992	6.440875119	1.18787E-10	2.85257E-10	positive
PRJNA729271	stage_3W_vs_E10	ordinary_muscle_development	ENSGALG00010004109	AGMO	-0.580429256	0.504528267	-1.150439516	0.249962892	0.285180018	negative
PRJNA729271	stage_3W_vs_E10	ordinary_muscle_development	ENSGALG00010004113	ECHDC1	0.134026579	0.191299707	0.700610477	0.483546138	0.521930246	positive
PRJNA729271	stage_3W_vs_E10	ordinary_muscle_development	ENSGALG00010004116	SPRY1	-0.534361324	0.095823855	-5.576495789	2.45412E-08	5.16366E-08	negative
PRJNA729271	stage_3W_vs_E10	ordinary_muscle_development	ENSGALG00010004117	STBD1	5.553481096	0.189264241	29.34247421	2.9808E-189	4.9099E-187	positive
PRJNA729271	stage_3W_vs_E10	ordinary_muscle_development	ENSGALG00010004122	TRIM55	1.67012718	0.194821255	8.572612769	1.01163E-17	3.52321E-17	positive
PRJNA729271	stage_3W_vs_E10	ordinary_muscle_development	ENSGALG00010004123	NDUFAF4	0.210871642	0.109866634	1.919341971	0.05494107	0.068609372	positive
PRJNA729271	stage_3W_vs_E10	ordinary_muscle_development	ENSGALG00010004125	MANEA	0.329028952	0.092576353	3.554136038	0.000379223	0.000590233	positive
PRJNA729271	stage_3W_vs_E10	ordinary_muscle_development	ENSGALG00010004127	EPHA7	-4.594835443	0.202471051	-22.69378962	5.1595E-114	2.7906E-112	negative
PRJNA729271	stage_3W_vs_E10	ordinary_muscle_development	ENSGALG00010004131	NUDT6	0.970830385	0.157954195	6.146277953	7.93222E-10	1.81955E-09	positive
PRJNA729271	stage_3W_vs_E10	ordinary_muscle_development	ENSGALG00010004133	CRH	4.461399448	0.956034725	4.666566317	3.06275E-06	5.6217E-06	positive
PRJNA729271	stage_3W_vs_E10	ordinary_muscle_development	ENSGALG00010004135	NUP54	-0.620897289	0.065358434	-9.499880156	2.10132E-21	8.50562E-21	negative
PRJNA729271	stage_3W_vs_E10	ordinary_muscle_development	ENSGALG00010004137	CROT	-0.753299265	0.0845891	-8.905393983	5.31968E-19	1.95658E-18	negative
PRJNA729271	stage_3W_vs_E10	ordinary_muscle_development	ENSGALG00010004139	PPEF2	1.176729739	0.617846737	1.904565758	0.056836541	0.070861204	positive
PRJNA729271	stage_3W_vs_E10	ordinary_muscle_development	ENSGALG00010004141	KPNA3	0.67116142	0.140884789	4.763902635	1.89884E-06	3.52823E-06	positive
PRJNA729271	stage_3W_vs_E10	ordinary_muscle_development	ENSGALG00010004142	RBBP8	-1.469421516	0.115393075	-12.73405287	3.82459E-37	2.79141E-36	negative
PRJNA729271	stage_3W_vs_E10	ordinary_muscle_development	ENSGALG00010004143	TCF24	1.44456119	0.407293625	3.546731652	0.000390042	0.000606485	positive
PRJNA729271	stage_3W_vs_E10	ordinary_muscle_development	ENSGALG00010004146	NAAA	2.810800901	0.170590958	16.47684573	5.38206E-61	8.28162E-60	positive
PRJNA729271	stage_3W_vs_E10	ordinary_muscle_development	ENSGALG00010004147	SPRYD7	0.969471064	0.090706207	10.68803438	1.15799E-26	5.83423E-26	positive
PRJNA729271	stage_3W_vs_E10	ordinary_muscle_development	ENSGALG00010004148	ENSGALG00010004148	0.53288098	0.153423895	3.473259352	0.000514178	0.000791106	positive
PRJNA729271	stage_3W_vs_E10	ordinary_muscle_development	ENSGALG00010004149	SDAD1	-0.723214904	0.121937875	-5.931011224	3.01075E-09	6.66577E-09	negative
PRJNA729271	stage_3W_vs_E10	ordinary_muscle_development	ENSGALG00010004154	COPS5	1.305976838	0.129004202	10.12352167	4.34495E-24	1.95651E-23	positive
PRJNA729271	stage_3W_vs_E10	ordinary_muscle_development	ENSGALG00010004158	JCHAIN	6.487634781	0.469141751	13.82873036	1.71012E-43	1.5551E-42	positive
PRJNA729271	stage_3W_vs_E10	ordinary_muscle_development	ENSGALG00010004172	ST8SIA6	-1.111867685	0.129595796	-8.579504269	9.52829E-18	3.32161E-17	negative
PRJNA729271	stage_3W_vs_E10	ordinary_muscle_development	ENSGALG00010004177	CSPP1	0.24271282	0.12726726	1.907111228	0.056506186	0.070480781	positive
PRJNA729271	stage_3W_vs_E10	ordinary_muscle_development	ENSGALG00010004181	BBS12	-1.425871733	0.13335187	-10.69255147	1.10294E-26	5.56075E-26	negative
PRJNA729271	stage_3W_vs_E10	ordinary_muscle_development	ENSGALG00010004187	ARFGEF1	1.067794509	0.093913296	11.37000352	5.89852E-30	3.38208E-29	positive
PRJNA729271	stage_3W_vs_E10	ordinary_muscle_development	ENSGALG00010004188	BMI1	-0.531080117	0.116643846	-4.553005886	5.28848E-06	9.53439E-06	negative
PRJNA729271	stage_3W_vs_E10	ordinary_muscle_development	ENSGALG00010004191	CABLES1	0.244332421	0.127139248	1.921770227	0.054634676	0.068258027	positive
PRJNA729271	stage_3W_vs_E10	ordinary_muscle_development	ENSGALG00010004200	ABCB1	3.458049675	0.181165564	19.08778689	3.19E-81	8.30146E-80	positive
PRJNA729271	stage_3W_vs_E10	ordinary_muscle_development	ENSGALG00010004201	CPA6	-8.86641787	0.788232324	-11.24848297	2.35609E-29	1.32319E-28	negative
PRJNA729271	stage_3W_vs_E10	ordinary_muscle_development	ENSGALG00010004210	SYNCRIP	-0.850874034	0.070692372	-12.03629199	2.2902E-33	1.49064E-32	negative
PRJNA729271	stage_3W_vs_E10	ordinary_muscle_development	ENSGALG00010004211	RNF146	0.422916847	0.074768569	5.656345308	1.5463E-08	3.29855E-08	positive
PRJNA729271	stage_3W_vs_E10	ordinary_muscle_development	ENSGALG00010004212	RSPO3	-2.560105606	0.389262117	-6.576816741	4.80627E-11	1.1824E-10	negative
PRJNA729271	stage_3W_vs_E10	ordinary_muscle_development	ENSGALG00010004214	REPS1	-0.19394882	0.126223229	-1.536554102	0.124402539	0.148400527	negative
PRJNA729271	stage_3W_vs_E10	ordinary_muscle_development	ENSGALG00010004216	SNX14	0.443078587	0.073363445	6.039500842	1.54592E-09	3.48276E-09	positive
PRJNA729271	stage_3W_vs_E10	ordinary_muscle_development	ENSGALG00010004217	CCDC28A	0.065523144	0.112213083	0.583917155	0.559276051	0.596244253	positive
PRJNA729271	stage_3W_vs_E10	ordinary_muscle_development	ENSGALG00010004219	FGF2	-0.230140551	0.182428053	-1.261541454	0.207113835	0.239231415	negative
PRJNA729271	stage_3W_vs_E10	ordinary_muscle_development	ENSGALG00010004222	SPAG6	-2.42558716	0.256136105	-9.469915066	2.80071E-21	1.13019E-20	negative
PRJNA729271	stage_3W_vs_E10	ordinary_muscle_development	ENSGALG00010004224	NT5E	0.754501058	0.258136377	2.922877688	0.003468127	0.004942047	positive
PRJNA729271	stage_3W_vs_E10	ordinary_muscle_development	ENSGALG00010004225	CETN4	-1.631353046	0.12785309	-12.75959031	2.75647E-37	2.02099E-36	negative
PRJNA729271	stage_3W_vs_E10	ordinary_muscle_development	ENSGALG00010004226	RUNDC3B	0.51602277	0.193709668	2.663897856	0.007724101	0.010632559	positive
PRJNA729271	stage_3W_vs_E10	ordinary_muscle_development	ENSGALG00010004230	TAF1B	0.257953477	0.115319567	2.236857832	0.025295633	0.032884323	positive
PRJNA729271	stage_3W_vs_E10	ordinary_muscle_development	ENSGALG00010004231	ARMC3	-0.26355202	0.387760436	-0.679677439	0.496708724	0.534982386	negative
PRJNA729271	stage_3W_vs_E10	ordinary_muscle_development	ENSGALG00010004233	NHSL1	-3.127970952	0.126300915	-24.76601967	2.0839E-135	1.5815E-133	negative
PRJNA729271	stage_3W_vs_E10	ordinary_muscle_development	ENSGALG00010004235	ADAD1	-1.354349138	0.429534865	-3.153059853	0.001615687	0.00237881	negative
PRJNA729271	stage_3W_vs_E10	ordinary_muscle_development	ENSGALG00010004236	DBF4	-1.372891293	0.125984546	-10.89729916	1.18729E-27	6.24222E-27	negative
PRJNA729271	stage_3W_vs_E10	ordinary_muscle_development	ENSGALG00010004237	RUFY3	0.658545282	0.090945166	7.24112467	4.44979E-13	1.22066E-12	positive
PRJNA729271	stage_3W_vs_E10	ordinary_muscle_development	ENSGALG00010004238	CCNC	-0.808542582	0.124713756	-6.48318686	8.98052E-11	2.1728E-10	negative
PRJNA729271	stage_3W_vs_E10	ordinary_muscle_development	ENSGALG00010004240	ENSGALG00010004240	1.213269865	0.173624676	6.98788844	2.79054E-12	7.33434E-12	positive
PRJNA729271	stage_3W_vs_E10	ordinary_muscle_development	ENSGALG00010004241	PREX2	0.523532463	0.114449101	4.574369384	4.77656E-06	8.64481E-06	positive
PRJNA729271	stage_3W_vs_E10	ordinary_muscle_development	ENSGALG00010004242	ADAM22	-2.342095161	0.17865074	-13.10991023	2.8894E-39	2.27867E-38	negative

PRJNA729271	stage_3W_vs_E10	ordinary_muscle_development	ENSGALG00010004243	BLTP1	0.566021209	0.112712064	5.021833408	5.11806E-07	9.87044E-07	positive
PRJNA729271	stage_3W_vs_E10	ordinary_muscle_development	ENSGALG00010004244	TMEM241	-0.011807521	0.081512003	-0.144856219	0.884824394	0.898332986	negative
PRJNA729271	stage_3W_vs_E10	ordinary_muscle_development	ENSGALG00010004245	ARFGEF3	-1.248616727	0.227529098	-5.487723288	4.07147E-08	8.44654E-08	negative
PRJNA729271	stage_3W_vs_E10	ordinary_muscle_development	ENSGALG00010004246	C8orf34	-3.525174972	0.350856872	-10.04733056	9.43896E-24	4.19815E-23	negative
PRJNA729271	stage_3W_vs_E10	ordinary_muscle_development	ENSGALG00010004247	MEOX2	-0.904312581	0.104589785	-8.646280152	5.32054E-18	1.87643E-17	negative
PRJNA729271	stage_3W_vs_E10	ordinary_muscle_development	ENSGALG00010004248	GRSF1	-0.068918946	0.159420324	-0.432309659	0.665516368	0.697365701	negative
PRJNA729271	stage_3W_vs_E10	ordinary_muscle_development	ENSGALG00010004249	USP45	1.564776948	0.100965437	15.49814461	3.57055E-54	4.60455E-53	positive
PRJNA729271	stage_3W_vs_E10	ordinary_muscle_development	ENSGALG00010004250	MOB1B	-0.849987156	0.088894969	-9.561701481	1.15836E-21	4.73772E-21	negative
PRJNA729271	stage_3W_vs_E10	ordinary_muscle_development	ENSGALG00010004251	CCN2	-0.153800878	0.266043463	-0.578104329	0.563193684	0.599948002	negative
PRJNA729271	stage_3W_vs_E10	ordinary_muscle_development	ENSGALG00010004255	RPS12	0.180015447	0.151084036	1.191492178	0.233460425	0.267426021	positive
PRJNA729271	stage_3W_vs_E10	ordinary_muscle_development	ENSGALG00010004256	ENSGALG00010004256	-3.976068583	0.545061314	-7.294718008	2.99285E-13	8.27258E-13	negative
PRJNA729271	stage_3W_vs_E10	ordinary_muscle_development	ENSGALG00010004257	ENSGALG00010004257	-2.693459139	0.656385282	-4.103472782	4.06994E-05	6.85815E-05	negative
PRJNA729271	stage_3W_vs_E10	ordinary_muscle_development	ENSGALG00010004265	PIP4K2A	0.463288782	0.092605126	5.002841643	5.64913E-07	1.086E-06	positive
PRJNA729271	stage_3W_vs_E10	ordinary_muscle_development	ENSGALG00010004275	ADARB2	-5.07812021	0.975742235	-5.204366512	1.94659E-07	3.86149E-07	negative
PRJNA729271	stage_3W_vs_E10	ordinary_muscle_development	ENSGALG00010004281	VIM	-0.432122875	0.171619486	-2.517912655	0.011805259	0.015929737	negative
PRJNA729271	stage_3W_vs_E10	ordinary_muscle_development	ENSGALG00010004283	DCK	-2.13580245	0.132757969	-16.08794165	3.09973E-58	4.44417E-57	negative
PRJNA729271	stage_3W_vs_E10	ordinary_muscle_development	ENSGALG00010004285	SLC18B1	0.297623493	0.117177339	2.539940711	0.011087126	0.015005406	positive
PRJNA729271	stage_3W_vs_E10	ordinary_muscle_development	ENSGALG00010004296	ENSGALG00010004296	-8.251350841	1.006555331	-8.197612776	2.45208E-16	7.94787E-16	negative
PRJNA729271	stage_3W_vs_E10	ordinary_muscle_development	ENSGALG00010004297	SULF1	-3.078187026	0.185537264	-16.59066731	8.14108E-62	1.27711E-60	negative
PRJNA729271	stage_3W_vs_E10	ordinary_muscle_development	ENSGALG00010004300	VNN2	2.90030013	0.245392514	11.81902448	3.11278E-32	1.9507E-31	positive
PRJNA729271	stage_3W_vs_E10	ordinary_muscle_development	ENSGALG00010004302	CRPPA	0.331329093	0.127548423	2.597672994	0.009385783	0.012794783	positive
PRJNA729271	stage_3W_vs_E10	ordinary_muscle_development	ENSGALG00010004306	PNISR	0.348251926	0.143856141	2.420834608	0.015484921	0.02060912	positive
PRJNA729271	stage_3W_vs_E10	ordinary_muscle_development	ENSGALG00010004307	NPC2	1.426228989	0.140408811	10.15768868	3.0625E-24	1.38809E-23	positive
PRJNA729271	stage_3W_vs_E10	ordinary_muscle_development	ENSGALG00010004308	DSG2	-2.657384612	0.473800661	-5.608655346	2.03905E-08	4.31663E-08	negative
PRJNA729271	stage_3W_vs_E10	ordinary_muscle_development	ENSGALG00010004309	MRPS28	-0.061724784	0.136494376	-0.452214851	0.651114211	0.684104123	negative
PRJNA729271	stage_3W_vs_E10	ordinary_muscle_development	ENSGALG00010004312	PDLIM5	6.150604205	0.181459462	33.89519703	7.8406E-252	2.273E-249	positive
PRJNA729271	stage_3W_vs_E10	ordinary_muscle_development	ENSGALG00010004319	TAAR1	7.055631718	0.813999793	8.667854436	4.40339E-18	1.5598E-17	positive
PRJNA729271	stage_3W_vs_E10	ordinary_muscle_development	ENSGALG00010004323	TRDMT1	-0.187739067	0.158933823	-1.181240488	0.237507201	0.271739434	negative
PRJNA729271	stage_3W_vs_E10	ordinary_muscle_development	ENSGALG00010004325	LTBP2	-0.42266546	0.113880485	-3.711482783	0.000206049	0.000327846	negative
PRJNA729271	stage_3W_vs_E10	ordinary_muscle_development	ENSGALG00010004326	ENSGALG00010004326	-0.645213994	0.954343994	-0.67608116	0.498989118	0.537141028	negative
PRJNA729271	stage_3W_vs_E10	ordinary_muscle_development	ENSGALG00010004328	ENSGALG00010004328	-1.552745005	0.707445442	-2.194861842	0.02817351	0.03642628	negative
PRJNA729271	stage_3W_vs_E10	ordinary_muscle_development	ENSGALG00010004329	COQ3	1.295189312	0.119739428	10.81673211	2.86823E-27	1.48059E-26	positive
PRJNA729271	stage_3W_vs_E10	ordinary_muscle_development	ENSGALG00010004330	STX7	0.435336328	0.103009574	4.22617347	2.37699E-05	4.07841E-05	positive
PRJNA729271	stage_3W_vs_E10	ordinary_muscle_development	ENSGALG00010004332	ENSGALG00010004332	-2.424221921	0.927841171	-2.612755283	0.008981559	0.012279541	negative
PRJNA729271	stage_3W_vs_E10	ordinary_muscle_development	ENSGALG00010004333	DIO2	-0.31556854	0.312210487	-1.010755736	0.312133353	0.350048198	negative
PRJNA729271	stage_3W_vs_E10	ordinary_muscle_development	ENSGALG00010004334	ENSGALG00010004334	0.22072671	0.23174137	0.952470031	0.340858658	0.379269689	positive
PRJNA729271	stage_3W_vs_E10	ordinary_muscle_development	ENSGALG00010004336	ENSGALG00010004336	-3.180140043	0.517776771	-6.141913314	8.15334E-10	1.86909E-09	negative
PRJNA729271	stage_3W_vs_E10	ordinary_muscle_development	ENSGALG00010004337	BMPR1B	-2.762203286	0.251153469	-10.99806941	3.90401E-28	2.09355E-27	negative
PRJNA729271	stage_3W_vs_E10	ordinary_muscle_development	ENSGALG00010004338	ENSGALG00010004338	1.542222287	0.16459564	9.36976389	7.2695E-21	2.88136E-20	positive
PRJNA729271	stage_3W_vs_E10	ordinary_muscle_development	ENSGALG00010004339	B4GALT6	-3.980507722	0.142780832	-27.87844604	4.8707E-171	6.1393E-169	negative
PRJNA729271	stage_3W_vs_E10	ordinary_muscle_development	ENSGALG00010004340	SLC4A4	0.638959816	0.170479022	3.748026041	0.000178232	0.00028512	positive
PRJNA729271	stage_3W_vs_E10	ordinary_muscle_development	ENSGALG00010004341	ENSGALG00010004341	-1.891824651	0.172591759	-10.96126875	5.8671E-28	3.11972E-27	negative
PRJNA729271	stage_3W_vs_E10	ordinary_muscle_development	ENSGALG00010004342	ZNF438	0.053538259	0.130598381	0.409945808	0.681845701	0.712673308	positive
PRJNA729271	stage_3W_vs_E10	ordinary_muscle_development	ENSGALG00010004344	SOSTDC1	-5.485145349	0.392861325	-13.96203953	2.65719E-44	2.47851E-43	negative
PRJNA729271	stage_3W_vs_E10	ordinary_muscle_development	ENSGALG00010004345	FAXC	-2.654283872	0.194690658	-13.63333969	2.53699E-42	2.20863E-41	negative
PRJNA729271	stage_3W_vs_E10	ordinary_muscle_development	ENSGALG00010004346	CEP128	-0.213018486	0.103173768	-2.064657417	0.038955426	0.049596742	negative
PRJNA729271	stage_3W_vs_E10	ordinary_muscle_development	ENSGALG00010004350	MOXD1	1.63878082	0.239003704	6.856717257	7.04609E-12	1.81151E-11	positive
PRJNA729271	stage_3W_vs_E10	ordinary_muscle_development	ENSGALG00010004352	CHMP2A	0.677209611	0.055978179	12.09774285	1.08555E-33	7.14256E-33	positive
PRJNA729271	stage_3W_vs_E10	ordinary_muscle_development	ENSGALG00010004355	ANKMY2	0.209105278	0.123193703	1.697369853	0.089626758	0.109098074	positive
PRJNA729271	stage_3W_vs_E10	ordinary_muscle_development	ENSGALG00010004356	TRAPPC8	0.368316934	0.077250547	4.767822963	1.86227E-06	3.46161E-06	positive
PRJNA729271	stage_3W_vs_E10	ordinary_muscle_development	ENSGALG00010004358	GC	4.790816382	2.121023265	2.258728822	0.023900258	0.031168173	positive
PRJNA729271	stage_3W_vs_E10	ordinary_muscle_development	ENSGALG00010004362	FBXL4	2.232737368	0.120356723	18.55099832	8.00513E-77	1.84768E-75	positive
PRJNA729271	stage_3W_vs_E10	ordinary_muscle_development	ENSGALG00010004364	TSHR	1.383375321	0.45044185	3.071151852	0.002132347	0.003097031	positive
PRJNA729271	stage_3W_vs_E10	ordinary_muscle_development	ENSGALG00010004365	TPD52	-1.083446504	0.155634593	-6.961476128	3.36726E-12	8.81495E-12	negative
PRJNA729271	stage_3W_vs_E10	ordinary_muscle_development	ENSGALG00010004366	UNC5C	-6.043132801	0.407497902	-14.82985008	9.39425E-50	1.0515E-48	negative
PRJNA729271	stage_3W_vs_E10	ordinary_muscle_development	ENSGALG00010004367	TSPAN13	0.797150467	0.177796421	4.483501201	7.34282E-06	1.30915E-05	positive
PRJNA729271	stage_3W_vs_E10	ordinary_muscle_development	ENSGALG00010004368	GJD4	-7.019682702	0.428745326	-16.37261626	3.00034E-60	4.52549E-59	negative
PRJNA729271	stage_3W_vs_E10	ordinary_muscle_development	ENSGALG00010004369	UBE2M	0.502052913	0.23468583	2.139255327	0.032414996	0.041627408	positive
PRJNA729271	stage_3W_vs_E10	ordinary_muscle_development	ENSGALG00010004370	ATP6V1H	0.499794517	0.073839639	6.768647875	1.29991E-11	3.30334E-11	positive
PRJNA729271	stage_3W_vs_E10	ordinary_muscle_development	ENSGALG00010004372	SYNJ2	-0.564594639	0.189189221	-2.984285447	0.002842415	0.004083735	negative
PRJNA729271	stage_3W_vs_E10	ordinary_muscle_development	ENSGALG00010004373	MMS22L	-1.779773075	0.129745368	-13.71743052	7.98452E-43	7.06567E-42	negative
PRJNA729271	stage_3W_vs_E10	ordinary_muscle_development	ENSGALG00010004374	ILDR1	-3.727154161	0.687582277	-5.420666425	5.93773E-08	1.21788E-07	negative
PRJNA729271	stage_3W_vs_E10	ordinary_muscle_development	ENSGALG00010004375	ENSGALG00010004375	0.03908132	0.215405457	0.181431428	0.856028952	0.87239961	positive
PRJNA729271	stage_3W_vs_E10	ordinary_muscle_development	ENSGALG00010004377	AGR2	-4.3470275	0.799352204	-5.438187922	5.38252E-08	1.10729E-07	negative
PRJNA729271	stage_3W_vs_E10	ordinary_muscle_development	ENSGALG00010004378	TRIM28	-0.417836446	0.156180155	-2.675349149	0.007465146	0.01028782	negative
PRJNA729271	stage_3W_vs_E10	ordinary_muscle_development	ENSGALG00010004379	ZBTB10	-0.456710337	0.060565045	-7.540823815	4.67012E-14	1.35036E-13	negative
PRJNA729271	stage_3W_vs_E10	ordinary_muscle_development	ENSGALG00010004380	ISCA2	0.340782505	0.084358203	4.039707981	5.35178E-05	8.94328E-05	positive
PRJNA729271	stage_3W_vs_E10	ordinary_muscle_development	ENSGALG00010004381	ENSGALG00010004381	-0.619693092	0.488701154	-1.268040985	0.204783315	0.23672814	negative
PRJNA729271	stage_3W_vs_E10	ordinary_muscle_development	ENSGALG00010004383	ENSGALG00010004383	-0.49411546	0.484318789	-1.020227732	0.307620468	0.345468249	negative
PRJNA729271	stage_3W_vs_E10	ordinary_muscle_development	ENSGALG00010004384	ENSGALG00010004384	2.47321058	0.511120943	4.838797183	1.30627E-06	2.45392E-06	positive
PRJNA729271	stage_3W_vs_E10	ordinary_muscle_development	ENSGALG00010004385	ADAMTS3	-3.841751913	0.208863456	-18.39360506	1.47816E-75	3.31159E-74	negative
PRJNA729271	stage_3W_vs_E10	ordinary_muscle_development	ENSGALG00010004386	CFAP44	-0.430129138	0.381709131	-1.126850533	0.259805681	0.295664862	negative
PRJNA729271	stage_3W_vs_E10	ordinary_muscle_development	ENSGALG00010004389	ENSGALG00010004389	-0.168894508	0.135057956	-1.250533573	0.211104699	0.243569419	negative
PRJNA729271	stage_3W_vs_E10	ordinary_muscle_development	ENSGALG00010004390	PNLDC1	-0.773682559	0.559712504	-1.382285643	0.166884013	0.195599884	negative
PRJNA729271	stage_3W_vs_E10	ordinary_muscle_development	ENSGALG00010004393	ZBTB45	2.153938655	0.206612125	10.4250351	1.90589E-25	9.08148E-25	positive
PRJNA729271	stage_3W_vs_E10	ordinary_muscle_development	ENSGALG00010004394	CCNY	-1.004805569	0.088889391	-11.30399874	1.25376E-29	7.11004E-29	negative
PRJNA729271	stage_3W_vs_E10	ordinary_muscle_development	ENSGALG00010004395	BOC	-2.167072995	0.192837463	-11.23782151	2.65862E-29	1.49078E-28	negative
PRJNA729271	stage_3W_vs_E10	ordinary_muscle_development	ENSGALG00010004397	ENSGALG00010004397	0.255113927	0.170135696	1.499473264	0.1337509	0.159002648	positive
PRJNA729271	stage_3W_vs_E10	ordinary_muscle_development	ENSGALG00010004398	COX18	-0.989415245	0.127328727	-7.770557922	7.81412E-15	2.34407E-14	negative
PRJNA729271	stage_3W_vs_E10	ordinary_muscle_development	ENSGALG00010004399	POU3F2	-2.012041558	0.693261472	-2.902283826	0.003704527	0.005266489	negative
PRJNA729271	stage_3W_vs_E10	ordinary_muscle_development	ENSGALG00010004404	RGS20	-1.026929382	0.416488521	-2.465684717	0.013675164	0.01831484	negative
PRJNA729271	stage_3W_vs_E10	ordinary_muscle_development	ENSGALG00010004406	ENSGALG00010004406	-1.286492875	0.150122363	-8.569628444	1.03819E-17	3.61398E-17	negative
PRJNA729271	stage_3W_vs_E10	ordinary_muscle_development	ENSGALG00010004408	SERAC1	0.144236173	0.109097821	1.322081157	0.186141128	0.216490062	positive
PRJNA729271	stage_3W_vs_E10	ordinary_muscle_development	ENSGALG00010004416	STXBP6	0.103822654	0.21233568	0.48895529	0.624873351	0.65920955	positive
PRJNA729271	stage_3W_vs_E10	ordinary_muscle_development	ENSGALG00010004420	ENSGALG00010004420	2.492705913	0.276714714	9.008215985	2.09436E-19	7.82616E-19	positive
PRJNA729271	stage_3W_vs_E10	ordinary_muscle_development	ENSGALG00010004421	RAP1GDS1	-1.138507828	0.075490866	-15.08139841	2.14673E-51	2.55057E-50	negative
PRJNA729271	stage_3W_vs_E10	ordinary_muscle_development	ENSGALG00010004424	MRPL18	1.005023448	0.144838074	6.938945107	3.95039E-12	1.02839E-11	positive
PRJNA729271	stage_3W_vs_E10	ordinary_muscle_development	ENSGALG00010004425	CREM	3.494161014	0.240493462	14.52913101	7.9219E-48	8.37549E-47	positive
PRJNA729271	stage_3W_vs_E10	ordinary_muscle_development	ENSGALG00010004429	ENSGALG00010004429	1.00796785	0.345996229	2.913233629	0.003577069	0.005091781	positive
PRJNA729271	stage_3W_vs_E10	ordinary_muscle_development	ENSGALG00010004430	TCEA1	-0.817570906	0.094435409	-8.657461343	4.82387E-18	1.70541E-17	negative
PRJNA729271	stage_3W_vs_E10	ordinary_muscle_development	ENSGALG00010004433	AHR1A	-1.139318938	0.159411514	-7.147030397	8.86752E-13	2.39983E-12	negative
PRJNA729271	stage_3W_vs_E10	ordinary_muscle_development	ENSGALG00010004434	TCP1	-0.714978399	0.086653192	-8.25103357	1.5703E-16	5.14386E-16	negative
PRJNA729271	stage_3W_vs_E10	ordinary_muscle_development	ENSGALG00010004436	NOVA1	0.32202561	0.151053692	2.131861904	0.033018201	0.042357626	positive
PRJNA729271	stage_3W_vs_E10	ordinary_muscle_development	ENSGALG00010004440	GTF2H5	-0.131759106	0.104515636	-1.260664053	0.207429911	0.239558326	negative
PRJNA729271	stage_3W_vs_E10	ordinary_muscle_development	ENSGALG00010004443	ANKRD17	-0.566617079	0.094020474	-6.026528616	1.67519E-09	3.76696E-09	negative
PRJNA729271	stage_3W_vs_E10	ordinary_muscle_development	ENSGALG00010004445	EPHA6	-4.696886523	0.641040069	-7.326978067	2.354E-13	6.54291E-13	negative
PRJNA729271	stage_3W_vs_E10	ordinary_muscle_development	ENSGALG00010004446	ACAT2	-1.971855645	0.128807892	-15.30850024	6.70932E-53	8.33347E-52	negative
PRJNA729271	stage_3W_vs_E10	ordinary_muscle_development	ENSGALG00010004451	CLDND1	-0.270647471	0.084982768	-3.184733544	0.001448873	0.002139509	negative
PRJNA729271	stage_3W_vs_E10	ordinary_muscle_development	ENSGALG00010004454	SRI	0.059789152	0.163643314	0.36536263	0.714840733	0.743461033	positive
PRJNA729271	stage_3W_vs_E10	ordinary_muscle_development	ENSGALG00010004457	AGA	-0.151225431	0.12708376	-1.189966615	0.234059514	0.268048721	negative
PRJNA729271	stage_3W_vs_E10	ordinary_muscle_development	ENSGALG00010004460	ENSGALG00010004460	-0.407629902	0.602958901	-0.676049232	0.499009388	0.537141028	negative
PRJNA729271	stage_3W_vs_E10	ordinary_muscle_development	ENSGALG00010004468	LYPLA1	0.897990896	0.122157907	7.35106649	1.96632E-13	5.49167E-13	positive
PRJNA729271	stage_3W_vs_E10	ordinary_muscle_development	ENSGALG00010004471	C1H3ORF52	0.554696659	0.257114577	2.157390941	0.030975216	0.039870861	positive
PRJNA729271	stage_3W_vs_E10	ordinary_muscle_development	ENSGALG00010004472	NEIL3	-2.331161844	0.23087016	-10.09728517	5.67926E-24	2.5392E-23	negative
PRJNA729271	stage_3W_vs_E10	ordinary_muscle_development	ENSGALG00010004473	JDP2	0.14709497	0.135990706	1.081654582	0.279406041	0.316356083	positive
PRJNA729271	stage_3W_vs_E10	ordinary_muscle_development	ENSGALG00010004477	TULP4	-0.804293988	0.126799834	-6.343020817	2.25303E-10	5.34058E-10	negative
PRJNA729271	stage_3W_vs_E10	ordinary_muscle_development	ENSGALG00010004481	ZNF704	-0.086464196	0.079778843	-1.083798571	0.278454119	0.315361516	negative
PRJNA729271	stage_3W_vs_E10	ordinary_muscle_development	ENSGALG00010004483	STEAP4	2.767339199	0.583839172	4.739899843	2.13824E-06	3.9634E-06	positive
PRJNA729271	stage_3W_vs_E10	ordinary_muscle_development	ENSGALG00010004487	ABHD10	-0.320537262	0.100369014	-3.193587848	0.001405166	0.002077083	negative
PRJNA729271	stage_3W_vs_E10	ordinary_muscle_development	ENSGALG00010004489	LIG1	-3.191856343	0.270221823	-11.81198582	3.38471E-32	2.11654E-31	negative
PRJNA729271	stage_3W_vs_E10	ordinary_muscle_development	ENSGALG00010004494	WTAP	-0.416806222	0.057581607	-7.238530541	4.53572E-13	1.24353E-12	negative
PRJNA729271	stage_3W_vs_E10	ordinary_muscle_development	ENSGALG00010004495	MRPL15	0.475293158	0.113467404	4.188807899	2.80424E-05	4.7798E-05	positive
PRJNA729271	stage_3W_vs_E10	ordinary_muscle_development	ENSGALG00010004501	TAGLN3	-2.737669019	0.351352091	-7.79181081	6.60556E-15	1.98977E-14	negative
PRJNA729271	stage_3W_vs_E10	ordinary_muscle_development	ENSGALG00010004503	ENSGALG00010004503	-0.388879674	0.186472887	-2.08544888	0.037028574	0.047276418	negative
PRJNA729271	stage_3W_vs_E10	ordinary_muscle_development	ENSGALG00010004504	ZNF804B	-2.862473611	0.736154867	-3.888412258	0.000100902	0.000164667	negative
PRJNA729271	stage_3W_vs_E10	ordinary_muscle_development	ENSGALG00010004505	ENSGALG00010004505	1.418686339	0.130452468	10.87512071	1.51455E-27	7.91971E-27	positive
PRJNA729271	stage_3W_vs_E10	ordinary_muscle_development	ENSGALG00010004507	CUL2	0.745277055	0.089822245	8.297243726	1.06555E-16	3.51824E-16	positive
PRJNA729271	stage_3W_vs_E10	ordinary_muscle_development	ENSGALG00010004515	ENSGALG00010004515	0.210138715	0.251853431	0.834369078	0.404073037	0.443096846	positive
PRJNA729271	stage_3W_vs_E10	ordinary_muscle_development	ENSGALG00010004516	ENSGALG00010004516	-0.352081227	0.292826614	-1.202353918	0.229226435	0.26292927	negative
PRJNA729271	stage_3W_vs_E10	ordinary_muscle_development	ENSGALG00010004522	TSPAN5	-1.172730978	0.064172987	-18.27452687	1.3203E-74	2.8909E-73	negative
PRJNA729271	stage_3W_vs_E10	ordinary_muscle_development	ENSGALG00010004533	PAG1	-0.482756318	0.114869023	-4.202667573	2.63788E-05	4.50525E-05	negative
PRJNA729271	stage_3W_vs_E10	ordinary_muscle_development	ENSGALG00010004534	CUBN	1.45729663	0.213589386	6.822888808	8.92277E-12	2.28589E-11	positive
PRJNA729271	stage_3W_vs_E10	ordinary_muscle_development	ENSGALG00010004542	ERMARD	0.017716878	0.128921832	0.137423414	0.890696125	0.903410562	positive
PRJNA729271	stage_3W_vs_E10	ordinary_muscle_development	ENSGALG00010004545	TMPRSS7	-0.055007432	0.600545353	-0.091595799	0.927019189	0.935278287	negative
PRJNA729271	stage_3W_vs_E10	ordinary_muscle_development	ENSGALG00010004553	BATF	0.709891261	0.342279388	2.074011131	0.038078277	0.048539673	positive
PRJNA729271	stage_3W_vs_E10	ordinary_muscle_development	ENSGALG00010004565	ENSGALG00010004565	3.238486614	0.380105245	8.519973498	1.5959E-17	5.49075E-17	positive
PRJNA729271	stage_3W_vs_E10	ordinary_muscle_development	ENSGALG00010004568	RSU1	0.414994207	0.06688001	6.205055952	5.46774E-10	1.26769E-09	positive
PRJNA729271	stage_3W_vs_E10	ordinary_muscle_development	ENSGALG00010004570	ENSGALG00010004570	-0.557270808	0.610610185	-0.912645779	0.361428869	0.400283577	negative
PRJNA729271	stage_3W_vs_E10	ordinary_muscle_development	ENSGALG00010004576	STEAP2	0.892494518	0.210030686	4.249352962	2.14389E-05	3.69421E-05	positive
PRJNA729271	stage_3W_vs_E10	ordinary_muscle_development	ENSGALG00010004577	ZPLD1	-3.847483071	0.435216621	-8.84038634	9.53882E-19	3.46442E-18	negative
PRJNA729271	stage_3W_vs_E10	ordinary_muscle_development	ENSGALG00010004583	XKR4	0.76326052	0.109255804	6.985995203	2.82844E-12	7.42856E-12	positive

PRJNA729271	stage_3W_vs_E10	ordinary_muscle_development	ENSGALG00010004584	EIF4E	0.123856459	0.066483932	1.862953257	0.062468843	0.077478042	positive
PRJNA729271	stage_3W_vs_E10	ordinary_muscle_development	ENSGALG00010004586	ENSGALG00010004586	-4.890608664	0.428728642	-11.40723568	3.84763E-30	2.22108E-29	negative
PRJNA729271	stage_3W_vs_E10	ordinary_muscle_development	ENSGALG00010004588	MINDY3	-0.873036223	0.154010674	-5.668673461	1.43907E-08	3.0757E-08	negative
PRJNA729271	stage_3W_vs_E10	ordinary_muscle_development	ENSGALG00010004591	RRS1	0.123229974	0.161913594	0.761084791	0.446606424	0.485419925	positive
PRJNA729271	stage_3W_vs_E10	ordinary_muscle_development	ENSGALG00010004602	DYNLT2	-0.033746543	0.328367905	-0.102770529	0.918145096	0.927681108	negative
PRJNA729271	stage_3W_vs_E10	ordinary_muscle_development	ENSGALG00010004609	CFAP69	-2.518227825	0.626774029	-4.017760322	5.87539E-05	9.78669E-05	negative
PRJNA729271	stage_3W_vs_E10	ordinary_muscle_development	ENSGALG00010004610	TGS1	-0.685998001	0.123749577	-5.543437154	2.96591E-08	6.2027E-08	negative
PRJNA729271	stage_3W_vs_E10	ordinary_muscle_development	ENSGALG00010004622	ENSGALG00010004622	1.555415198	0.224633461	6.924236439	4.38333E-12	1.13763E-11	positive
PRJNA729271	stage_3W_vs_E10	ordinary_muscle_development	ENSGALG00010004623	MBIP	-0.591279956	0.126739643	-4.665311842	3.08149E-06	5.65539E-06	negative
PRJNA729271	stage_3W_vs_E10	ordinary_muscle_development	ENSGALG00010004624	PHF10	0.237661351	0.075941709	3.12952335	0.001750902	0.002564085	positive
PRJNA729271	stage_3W_vs_E10	ordinary_muscle_development	ENSGALG00010004630	ENSGALG00010004630	2.005412283	0.308317254	6.504379034	7.8015E-11	1.89545E-10	positive
PRJNA729271	stage_3W_vs_E10	ordinary_muscle_development	ENSGALG00010004639	HBS1L	1.296418662	0.078993719	16.41166766	1.57811E-60	2.39525E-59	positive
PRJNA729271	stage_3W_vs_E10	ordinary_muscle_development	ENSGALG00010004641	METAP1	-0.491798343	0.081084546	-6.06525367	1.31746E-09	2.98104E-09	negative
PRJNA729271	stage_3W_vs_E10	ordinary_muscle_development	ENSGALG00010004650	GTPBP10	0.077209316	0.127429095	0.605900214	0.544581017	0.582045557	positive
PRJNA729271	stage_3W_vs_E10	ordinary_muscle_development	ENSGALG00010004666	C6orf120	0.240620911	0.116776983	2.060516588	0.039349182	0.05006288	positive
PRJNA729271	stage_3W_vs_E10	ordinary_muscle_development	ENSGALG00010004669	ELMOD1	-2.893546088	0.36769869	-7.869340221	3.56516E-15	1.08863E-14	negative
PRJNA729271	stage_3W_vs_E10	ordinary_muscle_development	ENSGALG00010004673	RAB18	0.352688777	0.084851023	4.156564839	3.23069E-05	5.47961E-05	positive
PRJNA729271	stage_3W_vs_E10	ordinary_muscle_development	ENSGALG00010004689	BBX	0.374612874	0.083701604	4.4755758	7.62057E-06	1.35767E-05	positive
PRJNA729271	stage_3W_vs_E10	ordinary_muscle_development	ENSGALG00010004705	ENSGALG00010004705	4.293226472	0.226452653	18.95860534	3.74955E-80	9.40308E-79	positive
PRJNA729271	stage_3W_vs_E10	ordinary_muscle_development	ENSGALG00010004707	OTUD1	3.216181982	0.296467167	10.84835808	2.0304E-27	1.05676E-26	positive
PRJNA729271	stage_3W_vs_E10	ordinary_muscle_development	ENSGALG00010004715	WDR27	0.33158879	0.311038691	1.066069269	0.28639233	0.323533382	positive
PRJNA729271	stage_3W_vs_E10	ordinary_muscle_development	ENSGALG00010004716	ADH6	-0.018727577	0.989851914	-0.018919574	0.984905264	0.987425772	negative
PRJNA729271	stage_3W_vs_E10	ordinary_muscle_development	ENSGALG00010004717	ALKBH8	0.180150609	0.174384331	1.033066497	0.301572781	0.339184243	positive
PRJNA729271	stage_3W_vs_E10	ordinary_muscle_development	ENSGALG00010004721	LAMA2	2.76006798	0.158848167	17.37551045	1.26476E-67	2.31473E-66	positive
PRJNA729271	stage_3W_vs_E10	ordinary_muscle_development	ENSGALG00010004728	ENSGALG00010004728	-0.830962771	0.217599061	-3.818779208	0.000134114	0.000216962	negative
PRJNA729271	stage_3W_vs_E10	ordinary_muscle_development	ENSGALG00010004729	ENSGALG00010004729	8.425452635	1.085809505	7.75960479	8.51944E-15	2.54985E-14	positive
PRJNA729271	stage_3W_vs_E10	ordinary_muscle_development	ENSGALG00010004730	CLDN12	-0.158258184	0.063048154	-2.510116054	0.012069149	0.01627218	negative
PRJNA729271	stage_3W_vs_E10	ordinary_muscle_development	ENSGALG00010004735	ENSGALG00010004735	2.987515334	0.164379556	18.17449448	8.21824E-74	1.77004E-72	positive
PRJNA729271	stage_3W_vs_E10	ordinary_muscle_development	ENSGALG00010004736	CD47	0.306819458	0.101107631	3.034582601	0.002408688	0.003481992	positive
PRJNA729271	stage_3W_vs_E10	ordinary_muscle_development	ENSGALG00010004739	ENSGALG00010004739	-0.571397356	0.734574033	-0.777862176	0.436650268	0.475311328	negative
PRJNA729271	stage_3W_vs_E10	ordinary_muscle_development	ENSGALG00010004746	LYN	0.681148036	0.141246909	4.822392515	1.41846E-06	2.65848E-06	positive
PRJNA729271	stage_3W_vs_E10	ordinary_muscle_development	ENSGALG00010004751	PTER	0.928234512	0.12483858	7.435477966	1.04191E-13	2.95373E-13	positive
PRJNA729271	stage_3W_vs_E10	ordinary_muscle_development	ENSGALG00010004755	PAX9	-3.085248299	0.677558422	-4.553479376	5.27658E-06	9.51531E-06	negative
PRJNA729271	stage_3W_vs_E10	ordinary_muscle_development	ENSGALG00010004757	TRMT10A	0.678513662	0.073764616	9.19836232	3.63446E-20	1.3985E-19	positive
PRJNA729271	stage_3W_vs_E10	ordinary_muscle_development	ENSGALG00010004758	FLT3	5.355073352	0.768687256	6.966517667	3.24882E-12	8.51413E-12	positive
PRJNA729271	stage_3W_vs_E10	ordinary_muscle_development	ENSGALG00010004760	THBS2	-2.882895632	0.240895757	-11.96739898	5.26535E-33	3.38153E-32	negative
PRJNA729271	stage_3W_vs_E10	ordinary_muscle_development	ENSGALG00010004762	IFT57	-0.975863436	0.111105457	-8.783217839	1.58863E-18	5.72389E-18	negative
PRJNA729271	stage_3W_vs_E10	ordinary_muscle_development	ENSGALG00010004765	ENSGALG00010004765	3.672568119	0.198747237	18.47858705	3.07119E-76	6.97758E-75	positive
PRJNA729271	stage_3W_vs_E10	ordinary_muscle_development	ENSGALG00010004769	HTR2A	-0.134889734	0.366586526	-0.367961516	0.71290193	0.74160424	negative
PRJNA729271	stage_3W_vs_E10	ordinary_muscle_development	ENSGALG00010004777	CDK14	-1.304119114	0.124693639	-10.45858574	1.33838E-25	6.41742E-25	negative
PRJNA729271	stage_3W_vs_E10	ordinary_muscle_development	ENSGALG00010004778	ENSGALG00010004778	-3.036698389	0.493284922	-6.156073804	7.45705E-10	1.71381E-09	negative
PRJNA729271	stage_3W_vs_E10	ordinary_muscle_development	ENSGALG00010004780	ENSGALG00010004780	0.654776805	0.256958919	2.548176994	0.010828751	0.014662564	positive
PRJNA729271	stage_3W_vs_E10	ordinary_muscle_development	ENSGALG00010004781	ENSGALG00010004781	-1.10122992	0.083362082	-13.21020177	7.66188E-40	6.16994E-39	negative
PRJNA729271	stage_3W_vs_E10	ordinary_muscle_development	ENSGALG00010004787	RIOK3	0.308299884	0.114713598	2.687561794	0.007197578	0.009934193	positive
PRJNA729271	stage_3W_vs_E10	ordinary_muscle_development	ENSGALG00010004791	FZD1	-1.133883836	0.118615249	-9.559342895	1.18507E-21	4.8442E-21	negative
PRJNA729271	stage_3W_vs_E10	ordinary_muscle_development	ENSGALG00010004798	PTPRK	-2.767051803	0.116016291	-23.8505453	9.9953E-126	6.6156E-124	negative
PRJNA729271	stage_3W_vs_E10	ordinary_muscle_development	ENSGALG00010004800	ZC3H12C	-0.098818749	0.278439723	-0.354901764	0.722663187	0.750896265	negative
PRJNA729271	stage_3W_vs_E10	ordinary_muscle_development	ENSGALG00010004803	RMC1	1.882218452	0.139230651	13.51870758	1.21288E-41	1.02871E-40	positive
PRJNA729271	stage_3W_vs_E10	ordinary_muscle_development	ENSGALG00010004808	THEMIS	3.717962916	0.676221227	5.498145825	3.83805E-08	7.97943E-08	positive
PRJNA729271	stage_3W_vs_E10	ordinary_muscle_development	ENSGALG00010004824	ATM	-0.188452198	0.111393763	-1.69176615	0.09069057	0.110331498	negative
PRJNA729271	stage_3W_vs_E10	ordinary_muscle_development	ENSGALG00010004832	C6orf58	5.220055583	1.844102341	2.830675645	0.00464498	0.006537429	positive
PRJNA729271	stage_3W_vs_E10	ordinary_muscle_development	ENSGALG00010004835	HHLA2	-0.517232069	0.410527359	-1.259921067	0.207697838	0.239848643	negative
PRJNA729271	stage_3W_vs_E10	ordinary_muscle_development	ENSGALG00010004837	CENPC	-2.299252855	0.142922477	-16.08741255	3.12633E-58	4.47787E-57	negative
PRJNA729271	stage_3W_vs_E10	ordinary_muscle_development	ENSGALG00010004841	AKAP9	0.167553992	0.194585811	0.861080213	0.389193866	0.428219606	positive
PRJNA729271	stage_3W_vs_E10	ordinary_muscle_development	ENSGALG00010004846	TTLL2	0.009942935	0.357645096	0.027801122	0.977820771	0.981137483	positive
PRJNA729271	stage_3W_vs_E10	ordinary_muscle_development	ENSGALG00010004851	MTCL3	-5.070439066	0.626978089	-8.087107273	6.10985E-16	1.93706E-15	negative
PRJNA729271	stage_3W_vs_E10	ordinary_muscle_development	ENSGALG00010004852	ENSGALG00010004852	5.254159187	0.221700454	23.69936138	3.6608E-124	2.3376E-122	positive
PRJNA729271	stage_3W_vs_E10	ordinary_muscle_development	ENSGALG00010004855	ZEB1	0.082775706	0.092001132	0.899724859	0.368266691	0.406936883	positive
PRJNA729271	stage_3W_vs_E10	ordinary_muscle_development	ENSGALG00010004859	NPC1	1.478015636	0.191273596	7.727232969	1.09909E-14	3.26864E-14	positive
PRJNA729271	stage_3W_vs_E10	ordinary_muscle_development	ENSGALG00010004866	SLC25A21	-1.353305719	0.311051421	-4.350745979	1.35675E-05	2.3717E-05	negative
PRJNA729271	stage_3W_vs_E10	ordinary_muscle_development	ENSGALG00010004868	NPAT	-0.230371208	0.114150714	-2.018131995	0.043577516	0.055118333	negative
PRJNA729271	stage_3W_vs_E10	ordinary_muscle_development	ENSGALG00010004871	C11orf87	0.010035859	0.312589948	0.032105508	0.974387911	0.978166962	positive
PRJNA729271	stage_3W_vs_E10	ordinary_muscle_development	ENSGALG00010004872	KIAA0408	-0.763921438	0.254511127	-3.001524718	0.002686312	0.003865207	negative
PRJNA729271	stage_3W_vs_E10	ordinary_muscle_development	ENSGALG00010004876	ENSGALG00010004876	-0.702009958	0.682150107	-1.029113608	0.303426292	0.341128062	negative
PRJNA729271	stage_3W_vs_E10	ordinary_muscle_development	ENSGALG00010004879	ENSGALG00010004879	-2.815788378	0.763594569	-3.687543747	0.000226429	0.000358816	negative
PRJNA729271	stage_3W_vs_E10	ordinary_muscle_development	ENSGALG00010004880	STAP1	3.742153366	0.56899548	6.576771695	4.80772E-11	1.18255E-10	positive
PRJNA729271	stage_3W_vs_E10	ordinary_muscle_development	ENSGALG00010004881	MIPOL1	-1.667655512	0.15775476	-10.5711898	4.05319E-26	1.99089E-25	negative
PRJNA729271	stage_3W_vs_E10	ordinary_muscle_development	ENSGALG00010004883	KRIT1	-0.015360817	0.073863166	-0.207963148	0.835257742	0.854173908	negative
PRJNA729271	stage_3W_vs_E10	ordinary_muscle_development	ENSGALG00010004887	MYH15	-4.332254994	0.720546943	-6.012453507	1.82736E-09	4.09645E-09	negative
PRJNA729271	stage_3W_vs_E10	ordinary_muscle_development	ENSGALG00010004890	ACAT1	-0.686864517	0.098924301	-6.943334526	3.82951E-12	9.9818E-12	negative
PRJNA729271	stage_3W_vs_E10	ordinary_muscle_development	ENSGALG00010004891	ANKIB1	-0.50896118	0.077631671	-6.556102356	5.52323E-11	1.35281E-10	negative
PRJNA729271	stage_3W_vs_E10	ordinary_muscle_development	ENSGALG00010004892	HEY2	-3.731566864	0.430451566	-8.668958738	4.3609E-18	1.54664E-17	negative
PRJNA729271	stage_3W_vs_E10	ordinary_muscle_development	ENSGALG00010004896	UBA6	0.226285109	0.081821649	2.765589699	0.005681998	0.007919284	positive
PRJNA729271	stage_3W_vs_E10	ordinary_muscle_development	ENSGALG00010004899	GATAD1	-0.098163744	0.106672919	-0.920231159	0.357451975	0.396151569	negative
PRJNA729271	stage_3W_vs_E10	ordinary_muscle_development	ENSGALG00010004901	CUL5	1.001812238	0.092167126	10.86951805	1.61049E-27	8.41229E-27	positive
PRJNA729271	stage_3W_vs_E10	ordinary_muscle_development	ENSGALG00010004902	ANKRD29	3.047233247	0.298731629	10.20057119	1.9711E-24	9.01865E-24	positive
PRJNA729271	stage_3W_vs_E10	ordinary_muscle_development	ENSGALG00010004903	ENSGALG00010004903	1.814425559	0.499904061	3.629547548	0.000283918	0.00044684	positive
PRJNA729271	stage_3W_vs_E10	ordinary_muscle_development	ENSGALG00010004907	ENSGALG00010004907	-1.251072566	0.11915411	-10.4996174	8.67309E-26	4.19474E-25	negative
PRJNA729271	stage_3W_vs_E10	ordinary_muscle_development	ENSGALG00010004917	ENSGALG00010004917	-0.602351682	0.093996043	-6.408266344	1.47184E-10	3.51702E-10	negative
PRJNA729271	stage_3W_vs_E10	ordinary_muscle_development	ENSGALG00010004921	RRAGD	3.167987233	0.132530737	23.90379241	2.7969E-126	1.8597E-124	positive
PRJNA729271	stage_3W_vs_E10	ordinary_muscle_development	ENSGALG00010004924	ENSGALG00010004924	0.585297224	0.356643659	1.641126118	0.100771238	0.121753696	positive
PRJNA729271	stage_3W_vs_E10	ordinary_muscle_development	ENSGALG00010004927	KIF25	-3.464897047	0.28049967	-12.35258869	4.71688E-35	3.25113E-34	negative
PRJNA729271	stage_3W_vs_E10	ordinary_muscle_development	ENSGALG00010004928	HDDC2	-0.478328351	0.122504193	-3.904587576	9.43862E-05	0.000154312	negative
PRJNA729271	stage_3W_vs_E10	ordinary_muscle_development	ENSGALG00010004929	ENSGALG00010004929	2.573660867	0.157761749	16.31359239	7.90065E-60	1.17456E-58	positive
PRJNA729271	stage_3W_vs_E10	ordinary_muscle_development	ENSGALG00010004933	SPTLC2	-0.741985844	0.091589796	-8.101184591	5.44266E-16	1.73083E-15	negative
PRJNA729271	stage_3W_vs_E10	ordinary_muscle_development	ENSGALG00010004934	CIP2A	-3.021163427	0.168202369	-17.96147962	3.90265E-72	8.04678E-71	negative
PRJNA729271	stage_3W_vs_E10	ordinary_muscle_development	ENSGALG00010004935	SVIL	-0.376393404	0.069169733	-5.441591136	5.28068E-08	1.0868E-07	negative
PRJNA729271	stage_3W_vs_E10	ordinary_muscle_development	ENSGALG00010004939	DDX10	-0.315709511	0.10971713	-2.87748606	0.004008576	0.005673695	negative
PRJNA729271	stage_3W_vs_E10	ordinary_muscle_development	ENSGALG00010004942	GABRR1	-2.973742597	0.757330609	-3.926610862	8.61512E-05	0.000141407	negative
PRJNA729271	stage_3W_vs_E10	ordinary_muscle_development	ENSGALG00010004944	ALKBH1	0.058434964	0.097669137	0.598295081	0.549643058	0.586780942	positive
PRJNA729271	stage_3W_vs_E10	ordinary_muscle_development	ENSGALG00010004946	SH2D1B	3.09272013	0.424326356	7.288541198	3.13329E-13	8.64922E-13	positive
PRJNA729271	stage_3W_vs_E10	ordinary_muscle_development	ENSGALG00010004949	RAB39A	-0.55229163	0.32777737	-1.684959613	0.091996364	0.111804083	negative
PRJNA729271	stage_3W_vs_E10	ordinary_muscle_development	ENSGALG00010004950	SLIRP	0.14923558	0.110336224	1.3525529	0.176198508	0.205569654	positive
PRJNA729271	stage_3W_vs_E10	ordinary_muscle_development	ENSGALG00010004951	AFDN	-1.016110641	0.063217338	-16.07329054	3.92677E-58	5.61326E-57	negative
PRJNA729271	stage_3W_vs_E10	ordinary_muscle_development	ENSGALG00010004952	PEX1	1.520556869	0.1200839	12.66245409	9.54676E-37	6.89832E-36	positive
PRJNA729271	stage_3W_vs_E10	ordinary_muscle_development	ENSGALG00010004957	ENSGALG00010004957	-0.210968439	0.145183855	-1.453112248	0.146192591	0.172731783	negative
PRJNA729271	stage_3W_vs_E10	ordinary_muscle_development	ENSGALG00010004958	TPD52L1	-3.838676496	0.461708769	-8.314064515	9.24788E-17	3.06466E-16	negative
PRJNA729271	stage_3W_vs_E10	ordinary_muscle_development	ENSGALG00010004962	ENSGALG00010004962	-0.600846049	0.094924654	-6.329715442	2.45614E-10	5.81063E-10	negative
PRJNA729271	stage_3W_vs_E10	ordinary_muscle_development	ENSGALG00010004963	ENSGALG00010004963	0.436854084	0.24255781	1.801030794	0.071698027	0.088237638	positive
PRJNA729271	stage_3W_vs_E10	ordinary_muscle_development	ENSGALG00010004965	JCAD	0.341807	0.100753285	3.392514693	0.000692542	0.001052464	positive
PRJNA729271	stage_3W_vs_E10	ordinary_muscle_development	ENSGALG00010004966	LAMA3	1.74509138	0.210882041	8.275201507	1.28239E-16	4.21979E-16	positive
PRJNA729271	stage_3W_vs_E10	ordinary_muscle_development	ENSGALG00010004967	SLC35F2	-3.435446759	0.344254255	-9.979387923	1.87621E-23	8.22862E-23	negative
PRJNA729271	stage_3W_vs_E10	ordinary_muscle_development	ENSGALG00010004968	TRAT1	7.346269398	0.928937962	7.908245437	2.61042E-15	8.01483E-15	positive
PRJNA729271	stage_3W_vs_E10	ordinary_muscle_development	ENSGALG00010004969	YTHDC1	0.037707295	0.077594958	0.485950329	0.627002384	0.660926445	positive
PRJNA729271	stage_3W_vs_E10	ordinary_muscle_development	ENSGALG00010004970	RBM48	0.4593677	0.166592097	2.757439929	0.005825592	0.008108503	positive
PRJNA729271	stage_3W_vs_E10	ordinary_muscle_development	ENSGALG00010004973	SLN	10.1500082	0.19488561	52.08187622	0	0	positive
PRJNA729271	stage_3W_vs_E10	ordinary_muscle_development	ENSGALG00010004974	MTPAP	-0.247962258	0.061551521	-4.028531799	5.61263E-05	9.3673E-05	negative
PRJNA729271	stage_3W_vs_E10	ordinary_muscle_development	ENSGALG00010004975	SNW1	-0.030163864	0.0694008	-0.4346328	0.663828983	0.695899407	negative
PRJNA729271	stage_3W_vs_E10	ordinary_muscle_development	ENSGALG00010004976	ENSGALG00010004976	2.395172139	0.22614471	10.59132508	3.26931E-26	1.61241E-25	positive
PRJNA729271	stage_3W_vs_E10	ordinary_muscle_development	ENSGALG00010004977	RNF217	-0.047582386	0.135867479	-0.350211739	0.726179798	0.754333967	negative
PRJNA729271	stage_3W_vs_E10	ordinary_muscle_development	ENSGALG00010004980	FNDC1	-0.005376253	0.377610211	-0.014237572	0.988640445	0.990396288	negative
PRJNA729271	stage_3W_vs_E10	ordinary_muscle_development	ENSGALG00010004981	ENSGALG00010004981	0.49449219	0.199229452	2.482023536	0.013063865	0.017528531	positive
PRJNA729271	stage_3W_vs_E10	ordinary_muscle_development	ENSGALG00010004983	CLXN	-0.91139952	0.271272025	-3.359725425	0.0007802	0.001179618	negative
PRJNA729271	stage_3W_vs_E10	ordinary_muscle_development	ENSGALG00010004987	MAP3K8	1.412674379	0.266008682	5.310632609	1.09245E-07	2.20668E-07	positive
PRJNA729271	stage_3W_vs_E10	ordinary_muscle_development	ENSGALG00010004988	HJURP	-2.752752491	0.222565474	-12.36828174	3.88034E-35	2.68475E-34	negative
PRJNA729271	stage_3W_vs_E10	ordinary_muscle_development	ENSGALG00010004990	ENSGALG00010004990	1.076975755	2.470779645	0.435884988	0.662920178	0.695047232	positive
PRJNA729271	stage_3W_vs_E10	ordinary_muscle_development	ENSGALG00010004991	FAM133B	-0.222866393	0.063090272	-3.532500123	0.00041165	0.000638441	negative
PRJNA729271	stage_3W_vs_E10	ordinary_muscle_development	ENSGALG00010004993	SULT1B	3.142091445	0.24075247	13.05112857	6.2613E-39	4.88206E-38	positive
PRJNA729271	stage_3W_vs_E10	ordinary_muscle_development	ENSGALG00010004995	PIT54	8.344614397	2.199268662	3.794267859	0.00014808	0.00238514	positive
PRJNA729271	stage_3W_vs_E10	ordinary_muscle_development	ENSGALG00010004996	GPR158	0.072687181	0.216274311	0.33608791	0.736804575	0.764439361	positive
PRJNA729271	stage_3W_vs_E10	ordinary_muscle_development	ENSGALG00010004999	ADCK1	-0.355285339	0.189582547	-1.874040329	0.060924867	0.075692633	negative
PRJNA729271	stage_3W_vs_E10	ordinary_muscle_development	ENSGALG00010005003	EXPH5	0.836167743	0.415071079	2.014516992	0.043955283	0.055562207	positive
PRJNA729271	stage_3W_vs_E10	ordinary_muscle_development	ENSGALG00010005004	CDK6	-0.765230331	0.097980257	-7.810046176	5.7167E-15	1.72777E-14	negative
PRJNA729271	stage_3W_vs_E10	ordinary_muscle_development	ENSGALG00010005010	NKAIN2	-5.0660822	0.354732731	-14.28140611	2.85777E-46	2.85678E-45	negative
PRJNA729271	stage_3W_vs_E10	ordinary_muscle_development	ENSGALG00010005012	ENSGALG00010005012	9.992376734	1.059648352	9.429898806	4.10485E-21	1.64455E-20	positive
PRJNA729271	stage_3W_vs_E10	ordinary_muscle_development	ENSGALG00010005014	SULT1B1	0.900569331	0.152222303	5.916145769	3.29573E-09	7.27671E-09	positive
PRJNA729271	stage_3W_vs_E10	ordinary_muscle_development	ENSGALG00010005015	NRXN3	-3.225107597	0.438880778	-7.348482226	2.0047E-13	5.59564E-13	negative
PRJNA729271	stage_3W_vs_E10	ordinary_muscle_development	ENSGALG00010005016	STX11	0.386893973	0.171222407	2.259598963	0.023846152	0.03110321	positive
PRJNA729271	stage_3W_vs_E10	ordinary_muscle_development	ENSGALG00010005017	ENSGALG00010005017	0.697291672	0.171166717	4.073757349	4.62607E-05	7.76727E-05	positive
PRJNA729271	stage_3W_vs_E10	ordinary_muscle_development	ENSGALG00010005023	POGLUT3	0.367523871	0.180415644	2.03709536	0.041640487	0.052774229	positive
PRJNA729271	stage_3W_vs_E10	ordinary_muscle_development	ENSGALG00010005025	THOC1	-0.850343857	0.058434772	-14.55201806	5.67052E-48	6.03925E-47	negative

PRJNA729271	stage_3W_vs_E10	ordinary_muscle_development	ENSGALG00010005029	FAT4	-2.598792045	0.197962948	-13.12766893	2.28585E-39	1.80958E-38	negative
PRJNA729271	stage_3W_vs_E10	ordinary_muscle_development	ENSGALG00010005033	USP14	0.62056857	0.134301118	4.620725276	3.82401E-06	6.97483E-06	positive
PRJNA729271	stage_3W_vs_E10	ordinary_muscle_development	ENSGALG00010005035	NECTIN3	-0.478329727	0.0984536	-4.858427984	1.18321E-06	2.23026E-06	negative
PRJNA729271	stage_3W_vs_E10	ordinary_muscle_development	ENSGALG00010005037	THNSL1	0.267491286	0.141824361	1.886074333	0.059284939	0.073743687	positive
PRJNA729271	stage_3W_vs_E10	ordinary_muscle_development	ENSGALG00010005042	SLC46A3	2.323705605	0.197865797	11.74384679	7.59515E-32	4.67679E-31	positive
PRJNA729271	stage_3W_vs_E10	ordinary_muscle_development	ENSGALG00010005045	UTRN	-0.00120844	0.099377806	-0.012160059	0.990297916	0.991666203	negative
PRJNA729271	stage_3W_vs_E10	ordinary_muscle_development	ENSGALG00010005048	ROCK1	0.068472075	0.114449984	0.598270727	0.549659306	0.586780942	positive
PRJNA729271	stage_3W_vs_E10	ordinary_muscle_development	ENSGALG00010005057	FLVCR2	-1.83913637	0.295292957	-6.228175537	4.71898E-10	1.099E-09	negative
PRJNA729271	stage_3W_vs_E10	ordinary_muscle_development	ENSGALG00010005060	POMP	0.783109471	0.138114241	5.670012496	1.42787E-08	3.0531E-08	positive
PRJNA729271	stage_3W_vs_E10	ordinary_muscle_development	ENSGALG00010005065	ENKUR	-0.838175346	0.407757928	-2.055570937	0.039823892	0.050617968	negative
PRJNA729271	stage_3W_vs_E10	ordinary_muscle_development	ENSGALG00010005068	PLCXD2	2.427937927	0.258195974	9.403469364	5.2793E-21	2.10114E-20	positive
PRJNA729271	stage_3W_vs_E10	ordinary_muscle_development	ENSGALG00010005070	ALB	2.308072721	1.406632577	1.640849757	0.100828607	0.121812857	positive
PRJNA729271	stage_3W_vs_E10	ordinary_muscle_development	ENSGALG00010005075	TRDN	6.949749899	0.1670012	41.61496971	0	0	positive
PRJNA729271	stage_3W_vs_E10	ordinary_muscle_development	ENSGALG00010005077	ERG28	-0.542457099	0.104156069	-5.208118012	1.90766E-07	3.7858E-07	negative
PRJNA729271	stage_3W_vs_E10	ordinary_muscle_development	ENSGALG00010005081	GREB1L	0.445905877	0.179475758	2.484490841	0.012973685	0.017420431	positive
PRJNA729271	stage_3W_vs_E10	ordinary_muscle_development	ENSGALG00010005082	ENSGALG00010005082	-0.436389187	0.08625483	-5.059301464	4.20795E-07	8.16851E-07	negative
PRJNA729271	stage_3W_vs_E10	ordinary_muscle_development	ENSGALG00010005087	ENPP1	1.823755993	0.214116303	8.517595184	1.629E-17	5.59801E-17	positive
PRJNA729271	stage_3W_vs_E10	ordinary_muscle_development	ENSGALG00010005092	ESCO1	2.080243444	0.139574494	14.90418049	3.09588E-50	3.54181E-49	positive
PRJNA729271	stage_3W_vs_E10	ordinary_muscle_development	ENSGALG00010005104	ENPP3	0.077362803	0.199684784	0.387424629	0.698441874	0.72802495	positive
PRJNA729271	stage_3W_vs_E10	ordinary_muscle_development	ENSGALG00010005107	TTLL5	-0.592296381	0.073909444	-8.013811913	1.11207E-15	3.47401E-15	negative
PRJNA729271	stage_3W_vs_E10	ordinary_muscle_development	ENSGALG00010005109	ABHD3	-0.376007395	0.377336929	-0.996476532	0.319018664	0.357079192	negative
PRJNA729271	stage_3W_vs_E10	ordinary_muscle_development	ENSGALG00010005111	RASSF6	-1.626368454	0.42906773	-3.790470223	0.000150362	0.000242006	negative
PRJNA729271	stage_3W_vs_E10	ordinary_muscle_development	ENSGALG00010005114	PHLDB2	-1.258215989	0.100588542	-12.50854192	6.70436E-36	4.73816E-35	negative
PRJNA729271	stage_3W_vs_E10	ordinary_muscle_development	ENSGALG00010005118	PRTFDC1	-1.10472395	0.138583832	-7.971521169	1.56733E-15	4.8606E-15	negative
PRJNA729271	stage_3W_vs_E10	ordinary_muscle_development	ENSGALG00010005119	MIB1	-1.556288868	0.0574181	-27.10449968	8.7145E-162	1.0025E-159	negative
PRJNA729271	stage_3W_vs_E10	ordinary_muscle_development	ENSGALG00010005122	IL8L1	1.633038627	0.933578551	1.749224663	0.080252193	0.098189882	positive
PRJNA729271	stage_3W_vs_E10	ordinary_muscle_development	ENSGALG00010005123	MTIF3	2.275762033	0.102850182	22.12696166	1.7391E-108	8.6625E-107	positive
PRJNA729271	stage_3W_vs_E10	ordinary_muscle_development	ENSGALG00010005126	MED23	-0.789720618	0.058528882	-13.49283631	1.72341E-41	1.45406E-40	negative
PRJNA729271	stage_3W_vs_E10	ordinary_muscle_development	ENSGALG00010005127	GATA6	-2.161759107	0.330282722	-6.545177699	5.94247E-11	1.4523E-10	negative
PRJNA729271	stage_3W_vs_E10	ordinary_muscle_development	ENSGALG00010005131	IL8L2	3.436488904	0.64948933	5.29106291	1.21608E-07	2.4482E-07	positive
PRJNA729271	stage_3W_vs_E10	ordinary_muscle_development	ENSGALG00010005132	LNX2	-0.766915008	0.09732733	-7.879749775	3.28037E-15	1.00272E-14	negative
PRJNA729271	stage_3W_vs_E10	ordinary_muscle_development	ENSGALG00010005138	ENSGALG00010005138	-0.744084462	0.721701191	-1.031014597	0.302533975	0.340176046	negative
PRJNA729271	stage_3W_vs_E10	ordinary_muscle_development	ENSGALG00010005140	DCT	-2.396377837	0.966576361	-2.479243167	0.013166151	0.017657602	negative
PRJNA729271	stage_3W_vs_E10	ordinary_muscle_development	ENSGALG00010005141	CCDC170	-1.169750006	0.316469941	-3.69624364	0.000218813	0.00034724	negative
PRJNA729271	stage_3W_vs_E10	ordinary_muscle_development	ENSGALG00010005143	GTF3A	-0.003569879	0.134647191	-0.026512835	0.978848296	0.982032534	negative
PRJNA729271	stage_3W_vs_E10	ordinary_muscle_development	ENSGALG00010005145	AKAP7	1.740660583	0.174688215	9.964384712	2.18221E-23	9.54181E-23	positive
PRJNA729271	stage_3W_vs_E10	ordinary_muscle_development	ENSGALG00010005146	TGFB3	-0.252396996	0.204688702	-1.233077321	0.217546918	0.250324885	negative
PRJNA729271	stage_3W_vs_E10	ordinary_muscle_development	ENSGALG00010005148	TTC39C	-1.14722295	0.087844379	-13.05971951	5.59335E-39	4.37537E-38	negative
PRJNA729271	stage_3W_vs_E10	ordinary_muscle_development	ENSGALG00010005150	ALCAM	-1.2349875	0.186638016	-6.617020074	3.66512E-11	9.08445E-11	negative
PRJNA729271	stage_3W_vs_E10	ordinary_muscle_development	ENSGALG00010005154	CWF19L2	-0.452303044	0.149646014	-3.022486423	0.002507073	0.003619164	negative
PRJNA729271	stage_3W_vs_E10	ordinary_muscle_development	ENSGALG00010005157	IMPA1	0.020116585	0.108527787	0.185358842	0.852947588	0.869719304	positive
PRJNA729271	stage_3W_vs_E10	ordinary_muscle_development	ENSGALG00010005158	IFT43	-1.729375145	0.202332631	-8.547188518	1.26123E-17	4.36626E-17	negative
PRJNA729271	stage_3W_vs_E10	ordinary_muscle_development	ENSGALG00010005159	ARHGAP21	0.415275284	0.098213473	4.22829244	2.35472E-05	4.04164E-05	positive
PRJNA729271	stage_3W_vs_E10	ordinary_muscle_development	ENSGALG00010005163	GPC6	-3.38633883	0.390656259	-8.668333727	4.3849E-18	1.55363E-17	negative
PRJNA729271	stage_3W_vs_E10	ordinary_muscle_development	ENSGALG00010005165	METTL21C	5.900941612	0.55798195	10.57550627	3.87081E-26	1.90259E-25	positive
PRJNA729271	stage_3W_vs_E10	ordinary_muscle_development	ENSGALG00010005166	CBLB	0.020706727	0.124055735	0.166914711	0.867437158	0.882722663	positive
PRJNA729271	stage_3W_vs_E10	ordinary_muscle_development	ENSGALG00010005167	GUCY1A2	0.189324649	0.178096156	1.063047362	0.287760474	0.324927013	positive
PRJNA729271	stage_3W_vs_E10	ordinary_muscle_development	ENSGALG00010005170	GPATCH2L	0.026700379	0.060726082	0.439685521	0.660164891	0.6926093	positive
PRJNA729271	stage_3W_vs_E10	ordinary_muscle_development	ENSGALG00010005172	OSBPL1A	1.347503539	0.080222732	16.79702882	2.56579E-63	4.19764E-62	positive
PRJNA729271	stage_3W_vs_E10	ordinary_muscle_development	ENSGALG00010005174	TPP2	2.335270129	0.18492902	12.62792679	1.4813E-36	1.06611E-35	positive
PRJNA729271	stage_3W_vs_E10	ordinary_muscle_development	ENSGALG00010005176	BAMBI	-3.531637162	0.314929912	-11.21404168	3.47935E-29	1.94272E-28	negative
PRJNA729271	stage_3W_vs_E10	ordinary_muscle_development	ENSGALG00010005177	AASDHPPT	0.894592302	0.126938086	7.047469627	1.82201E-12	4.84765E-12	positive
PRJNA729271	stage_3W_vs_E10	ordinary_muscle_development	ENSGALG00010005178	EPHA5	-0.690140048	0.200495039	-3.442180173	0.000577046	0.000882587	negative
PRJNA729271	stage_3W_vs_E10	ordinary_muscle_development	ENSGALG00010005180	WAC	1.08947179	0.115287554	9.450038204	3.38707E-21	1.36187E-20	positive
PRJNA729271	stage_3W_vs_E10	ordinary_muscle_development	ENSGALG00010005181	IMPACT	1.417151803	0.129735929	10.92335648	8.91395E-28	4.71045E-27	positive
PRJNA729271	stage_3W_vs_E10	ordinary_muscle_development	ENSGALG00010005182	NEPRO	-0.731374359	0.088389409	-8.27445694	1.29042E-16	4.24431E-16	negative
PRJNA729271	stage_3W_vs_E10	ordinary_muscle_development	ENSGALG00010005184	FGF14	-3.184023143	0.332610693	-9.572822543	1.04026E-21	4.2631E-21	negative
PRJNA729271	stage_3W_vs_E10	ordinary_muscle_development	ENSGALG00010005186	ESR1	0.425552884	0.19199604	2.216466981	0.026659537	0.034558217	positive
PRJNA729271	stage_3W_vs_E10	ordinary_muscle_development	ENSGALG00010005187	KBTBD3	1.314978185	0.252439899	5.209074271	1.89785E-07	3.76686E-07	positive
PRJNA729271	stage_3W_vs_E10	ordinary_muscle_development	ENSGALG00010005190	ENSGALG00010005190	3.284635614	0.273921333	11.99116397	3.95311E-33	2.55122E-32	positive
PRJNA729271	stage_3W_vs_E10	ordinary_muscle_development	ENSGALG00010005191	MSANTD4	-0.322398826	0.104922825	-3.072723454	0.00212115	0.003081694	negative
PRJNA729271	stage_3W_vs_E10	ordinary_muscle_development	ENSGALG00010005193	EPB41L2	0.739471624	0.098325225	7.520670569	5.4496E-14	1.56979E-13	positive
PRJNA729271	stage_3W_vs_E10	ordinary_muscle_development	ENSGALG00010005194	ENSGALG00010005194	2.019683384	1.114356285	1.812421586	0.06992108	0.086160508	positive
PRJNA729271	stage_3W_vs_E10	ordinary_muscle_development	ENSGALG00010005198	TRPC3	0.375532156	0.487325203	0.770598677	0.440944846	0.479482036	positive
PRJNA729271	stage_3W_vs_E10	ordinary_muscle_development	ENSGALG00010005203	ENSGALG00010005203	0.333613441	0.105784964	3.153694326	0.001612179	0.002373995	positive
PRJNA729271	stage_3W_vs_E10	ordinary_muscle_development	ENSGALG00010005204	ITGBL1	1.00189777	0.310611075	3.225570016	0.001257221	0.001867345	positive
PRJNA729271	stage_3W_vs_E10	ordinary_muscle_development	ENSGALG00010005208	ENSGALG00010005208	0.380937141	0.109486828	3.479296543	0.000502732	0.0007744	positive
PRJNA729271	stage_3W_vs_E10	ordinary_muscle_development	ENSGALG00010005211	ESRRB	2.332783498	0.339851023	6.864135585	6.68951E-12	1.72106E-11	positive
PRJNA729271	stage_3W_vs_E10	ordinary_muscle_development	ENSGALG00010005212	TAF13	0.517742391	0.100530431	5.150106168	2.60339E-07	5.12302E-07	positive
PRJNA729271	stage_3W_vs_E10	ordinary_muscle_development	ENSGALG00010005213	ENSGALG00010005213	0.37871082	0.15914593	2.379645025	0.017329323	0.022937497	positive
PRJNA729271	stage_3W_vs_E10	ordinary_muscle_development	ENSGALG00010005214	BBS7	-0.227506463	0.117889555	-1.929827148	0.053628259	0.067046887	negative
PRJNA729271	stage_3W_vs_E10	ordinary_muscle_development	ENSGALG00010005215	VASH1	-1.594880939	0.14246892	-11.19458853	4.3341E-29	2.40885E-28	negative
PRJNA729271	stage_3W_vs_E10	ordinary_muscle_development	ENSGALG00010005216	ENSGALG00010005216	0.133805533	0.164206245	0.814862632	0.415150945	0.454022484	positive
PRJNA729271	stage_3W_vs_E10	ordinary_muscle_development	ENSGALG00010005217	CCNA2	-2.298537755	0.145311121	-15.81804431	2.33638E-56	3.18288E-55	negative
PRJNA729271	stage_3W_vs_E10	ordinary_muscle_development	ENSGALG00010005218	GTPBP8	0.438204705	0.117228734	3.738031546	0.000185467	0.000296236	positive
PRJNA729271	stage_3W_vs_E10	ordinary_muscle_development	ENSGALG00010005219	NALCN	-0.473955676	0.183398963	-2.584287662	0.009758037	0.013276023	negative
PRJNA729271	stage_3W_vs_E10	ordinary_muscle_development	ENSGALG00010005220	ANGEL1	0.067551611	0.085690912	0.788317099	0.430511248	0.4690162	positive
PRJNA729271	stage_3W_vs_E10	ordinary_muscle_development	ENSGALG00010005221	ENSGALG00010005221	1.209678077	0.302310935	4.001436721	6.2959E-05	0.000104619	positive
PRJNA729271	stage_3W_vs_E10	ordinary_muscle_development	ENSGALG00010005222	ABCB1	3.681315558	0.993261121	3.706291809	0.000210316	0.000334269	positive
PRJNA729271	stage_3W_vs_E10	ordinary_muscle_development	ENSGALG00010005223	GRIA4	-3.336121041	0.467374271	-7.138007481	9.46935E-13	2.55888E-12	negative
PRJNA729271	stage_3W_vs_E10	ordinary_muscle_development	ENSGALG00010005224	ENSGALG00010005224	2.388453202	0.381789397	6.2559443	3.95118E-10	9.23449E-10	positive
PRJNA729271	stage_3W_vs_E10	ordinary_muscle_development	ENSGALG00010005226	MYCT1	1.197692622	0.167012624	7.17127	7.43052E-13	2.01809E-12	positive
PRJNA729271	stage_3W_vs_E10	ordinary_muscle_development	ENSGALG00010005227	EXOSC9	-0.40428789	0.104505404	-3.868583577	0.000109469	0.000178288	negative
PRJNA729271	stage_3W_vs_E10	ordinary_muscle_development	ENSGALG00010005228	ZNF521	-1.746872898	0.132316012	-13.2022789	8.51204E-40	6.84315E-39	negative
PRJNA729271	stage_3W_vs_E10	ordinary_muscle_development	ENSGALG00010005229	CIPC	-0.07724566	0.072933556	-1.059123727	0.289543435	0.326762075	negative
PRJNA729271	stage_3W_vs_E10	ordinary_muscle_development	ENSGALG00010005231	ENSGALG00010005231	2.412382518	0.419888229	5.745296843	9.17599E-09	1.98398E-08	positive
PRJNA729271	stage_3W_vs_E10	ordinary_muscle_development	ENSGALG00010005232	GATA3	-2.445122717	0.463512837	-5.275199569	1.32612E-07	2.66013E-07	negative
PRJNA729271	stage_3W_vs_E10	ordinary_muscle_development	ENSGALG00010005236	TMTC4	-1.651834288	0.09190471	-17.97333656	3.15176E-72	6.5078E-71	negative
PRJNA729271	stage_3W_vs_E10	ordinary_muscle_development	ENSGALG00010005237	RAP2A	-2.277834219	0.113277338	-20.10847231	6.22105E-90	2.04941E-88	negative
PRJNA729271	stage_3W_vs_E10	ordinary_muscle_development	ENSGALG00010005238	TMEM63C	-1.107303993	0.170258421	-6.503666525	7.83856E-11	1.90414E-10	negative
PRJNA729271	stage_3W_vs_E10	ordinary_muscle_development	ENSGALG00010005239	ENSGALG00010005239	-1.980191329	0.198657939	-9.967843921	2.10754E-23	9.23483E-23	negative
PRJNA729271	stage_3W_vs_E10	ordinary_muscle_development	ENSGALG00010005240	VIP	-5.491418909	0.438128161	-12.53381865	4.87573E-36	3.461E-35	negative
PRJNA729271	stage_3W_vs_E10	ordinary_muscle_development	ENSGALG00010005241	C4orf54	3.241887128	0.187457583	17.29397695	5.22219E-67	9.40318E-66	positive
PRJNA729271	stage_3W_vs_E10	ordinary_muscle_development	ENSGALG00010005242	CCDC80	-0.298056718	0.233971431	-1.27390219	0.202698082	0.234504645	negative
PRJNA729271	stage_3W_vs_E10	ordinary_muscle_development	ENSGALG00010005243	NGB	-2.839518807	0.556132406	-5.105832311	3.29342E-07	6.43804E-07	negative
PRJNA729271	stage_3W_vs_E10	ordinary_muscle_development	ENSGALG00010005244	FBXO5	-1.531511726	0.098357069	-15.57093705	1.14714E-54	1.49531E-53	negative
PRJNA729271	stage_3W_vs_E10	ordinary_muscle_development	ENSGALG00010005245	KIAA1217	0.743796783	0.136162244	5.462577308	4.69271E-08	9.69096E-08	positive
PRJNA729271	stage_3W_vs_E10	ordinary_muscle_development	ENSGALG00010005246	ENSGALG00010005246	-0.287523494	0.196119956	-1.466059341	0.142632121	0.168909526	negative
PRJNA729271	stage_3W_vs_E10	ordinary_muscle_development	ENSGALG00010005247	THAP7	1.387917989	0.11674665	11.8882896	1.36167E-32	8.63023E-32	positive
PRJNA729271	stage_3W_vs_E10	ordinary_muscle_development	ENSGALG00010005248	TMEM200A	-1.978376404	0.241486912	-8.192478795	2.559E-16	8.28703E-16	negative
PRJNA729271	stage_3W_vs_E10	ordinary_muscle_development	ENSGALG00010005249	PDGFD	-0.619195004	0.228915123	-2.704910872	0.006832272	0.00945069	negative
PRJNA729271	stage_3W_vs_E10	ordinary_muscle_development	ENSGALG00010005250	SLC35A5	-0.405311569	0.090251694	-4.490902618	7.0922E-06	1.26572E-05	negative
PRJNA729271	stage_3W_vs_E10	ordinary_muscle_development	ENSGALG00010005251	MTRF1L	-0.057248069	0.104037291	-0.550264894	0.582137699	0.618038962	negative
PRJNA729271	stage_3W_vs_E10	ordinary_muscle_development	ENSGALG00010005252	KLHL14	-3.294088754	0.241702505	-13.62869101	2.70387E-42	2.34967E-41	negative
PRJNA729271	stage_3W_vs_E10	ordinary_muscle_development	ENSGALG00010005253	POMT2	-0.616820565	0.096804286	-6.371831165	1.86784E-10	4.44791E-10	negative
PRJNA729271	stage_3W_vs_E10	ordinary_muscle_development	ENSGALG00010005254	ENSGALG00010005254	-0.557574557	0.744205227	-0.749221501	0.453723712	0.492527912	negative
PRJNA729271	stage_3W_vs_E10	ordinary_muscle_development	ENSGALG00010005255	BZW2	0.896513712	0.092870706	9.653353025	4.75742E-22	1.9759E-21	positive
PRJNA729271	stage_3W_vs_E10	ordinary_muscle_development	ENSGALG00010005256	OPRM1	-6.10597583	0.731650139	-8.345485776	7.09218E-17	2.36161E-16	negative
PRJNA729271	stage_3W_vs_E10	ordinary_muscle_development	ENSGALG00010005258	ITIH5	2.984094406	0.278838143	10.70188737	9.9726E-27	5.03844E-26	positive
PRJNA729271	stage_3W_vs_E10	ordinary_muscle_development	ENSGALG00010005259	SLC27A5	1.863374338	0.670235796	2.78017729	0.005432923	0.007588188	positive
PRJNA729271	stage_3W_vs_E10	ordinary_muscle_development	ENSGALG00010005260	ANXA5	-0.139382004	0.149991016	-0.929269019	0.352749687	0.391359105	negative
PRJNA729271	stage_3W_vs_E10	ordinary_muscle_development	ENSGALG00010005261	ATG3	0.094176665	0.109541986	0.859731218	0.389937222	0.428939822	positive
PRJNA729271	stage_3W_vs_E10	ordinary_muscle_development	ENSGALG00010005262	ENSGALG00010005262	-0.199947217	0.124841465	-1.601609028	0.109242093	0.131312352	negative
PRJNA729271	stage_3W_vs_E10	ordinary_muscle_development	ENSGALG00010005263	ENSGALG00010005263	2.92928915	0.3145128	9.313735881	1.23413E-20	4.84134E-20	positive
PRJNA729271	stage_3W_vs_E10	ordinary_muscle_development	ENSGALG00010005264	ITIH2	0.400751627	1.075748102	0.372532962			positive
PRJNA729271	stage_3W_vs_E10	ordinary_muscle_development	ENSGALG00010005265	DYNC2H1	-0.997820362	0.09544354	-10.45456153	1.39643E-25	6.68691E-25	negative
PRJNA729271	stage_3W_vs_E10	ordinary_muscle_development	ENSGALG00010005266	SLC19A2	-0.52055473	0.273499956	-1.903308275	0.057000332	0.071042116	negative
PRJNA729271	stage_3W_vs_E10	ordinary_muscle_development	ENSGALG00010005267	CYP51A1	-1.219118052	0.182284639	-6.687991149	2.26255E-11	5.68087E-11	negative
PRJNA729271	stage_3W_vs_E10	ordinary_muscle_development	ENSGALG00010005268	MTTP	2.668782237	0.902566753	2.956880726	0.003107683	0.004451612	positive
PRJNA729271	stage_3W_vs_E10	ordinary_muscle_development	ENSGALG00010005269	KIN	0.27558083	0.105689568	2.607455359	0.009121796	0.01246187	positive
PRJNA729271	stage_3W_vs_E10	ordinary_muscle_development	ENSGALG00010005270	GSTZ1	0.250798992	0.213901821	1.172495823	0.240998048	0.275515948	positive
PRJNA729271	stage_3W_vs_E10	ordinary_muscle_development	ENSGALG00010005272	MBNL2	2.194326739	0.092564933	23.70581031	3.141E-124	2.0146E-122	positive
PRJNA729271	stage_3W_vs_E10	ordinary_muscle_development	ENSGALG00010005273	ENSGALG00010005273	3.447909258	0.628691011	5.48426683	4.15188E-08	8.60842E-08	positive
PRJNA729271	stage_3W_vs_E10	ordinary_muscle_development	ENSGALG00010005275	L3MBTL3	0.341406945	0.171616666	1.989357756	0.046661729	0.058814066	positive
PRJNA729271	stage_3W_vs_E10	ordinary_muscle_development	ENSGALG00010005277	ENSGALG00010005277	1.727856323	0.199590563	8.657004084	4.84325E-18	1.71143E-17	positive
PRJNA729271	stage_3W_vs_E10	ordinary_muscle_development	ENSGALG00010005278	DAPP1	-0.106493129	0.130912999	-0.813464894	0.415951566	0.454795048	negative
PRJNA729271	stage_3W_vs_E10	ordinary_muscle_development	ENSGALG00010005279	CCDC181	0.737945253	0.308107825	2.39508767	0.016616392	0.02204417	positive
PRJNA729271	stage_3W_vs_E10	ordinary_muscle_development	ENSGALG00010005280	TMEM244	-2.408948633	0.630059122	-3.823369186	0.00013164	0.000213103	negative
PRJNA729271	stage_3W_vs_E10	ordinary_muscle_development	ENSGALG00010005281	TMED8	-1.102820922	0.069104737	-15.95868767	2.47889E-57	3.46495E-56	negative
PRJNA729271	stage_3W_vs_E10	ordinary_muscle_development	ENSGALG00010005282	FLT1	1.816545138	0.125161949	14.51355748	9.94274E-48	1.0451E-46	positive
PRJNA729271	stage_3W_vs_E10	ordinary_muscle_development	ENSGALG00010005283	ATP5C1	1.11232247	0.132061658	8.422751044	3.67745E-17	1.24167E-16	positive

PRJNA729271	stage_3W_vs_E10	ordinary_muscle_development	ENSGALG00010005284	ENSGALG00010005284	0.752920245	0.212239089	3.547509782	0.000388891	0.000604761	positive
PRJNA729271	stage_3W_vs_E10	ordinary_muscle_development	ENSGALG00010005285	MPP7	0.835746806	0.127545464	6.552540379	5.65664E-11	1.38432E-10	positive
PRJNA729271	stage_3W_vs_E10	ordinary_muscle_development	ENSGALG00010005286	ENSGALG00010005286	2.284790476	0.590032528	3.872312741	0.000107807	0.000175699	positive
PRJNA729271	stage_3W_vs_E10	ordinary_muscle_development	ENSGALG00010005287	ENSGALG00010005287	-0.105987888	0.385953493	-0.274613107	0.783613495	0.807512983	negative
PRJNA729271	stage_3W_vs_E10	ordinary_muscle_development	ENSGALG00010005288	ENSGALG00010005288	-0.482510993	0.095074802	-5.075067014	3.87359E-07	7.53358E-07	negative
PRJNA729271	stage_3W_vs_E10	ordinary_muscle_development	ENSGALG00010005291	IFNGR1	0.342019621	0.096056265	3.560617529	0.000369984	0.000576595	positive
PRJNA729271	stage_3W_vs_E10	ordinary_muscle_development	ENSGALG00010005292	TAF3	0.108896746	0.098618639	1.104220735	0.269497397	0.305997554	positive
PRJNA729271	stage_3W_vs_E10	ordinary_muscle_development	ENSGALG00010005293	BLZF1	0.062058117	0.096154297	0.645401393	0.518667101	0.556564971	positive
PRJNA729271	stage_3W_vs_E10	ordinary_muscle_development	ENSGALG00010005295	ENSGALG00010005295	2.720666225	0.547960456	4.965077676	6.86736E-07	1.31444E-06	positive
PRJNA729271	stage_3W_vs_E10	ordinary_muscle_development	ENSGALG00010005296	PAN3	0.960907733	0.10079942	9.532869655	1.52994E-21	6.23813E-21	positive
PRJNA729271	stage_3W_vs_E10	ordinary_muscle_development	ENSGALG00010005297	IL22RA2	0.976212629	0.541953202	1.801285841	0.071657838	0.088195667	positive
PRJNA729271	stage_3W_vs_E10	ordinary_muscle_development	ENSGALG00010005299	ODAD2	-4.024513571	0.659614352	-6.101312929	1.05201E-09	2.39385E-09	negative
PRJNA729271	stage_3W_vs_E10	ordinary_muscle_development	ENSGALG00010005300	QRFPR	-1.049629133	0.84085487	-1.248288106	0.211925568	0.244438698	negative
PRJNA729271	stage_3W_vs_E10	ordinary_muscle_development	ENSGALG00010005301	ENSGALG00010005301	1.950538263	0.349168104	5.586244108	2.32033E-08	4.89352E-08	positive
PRJNA729271	stage_3W_vs_E10	ordinary_muscle_development	ENSGALG00010005302	PSPC1	-1.262577995	0.080473154	-15.68943101	1.7865E-55	2.39107E-54	negative
PRJNA729271	stage_3W_vs_E10	ordinary_muscle_development	ENSGALG00010005303	ENSGALG00010005303	2.957160945	0.276509783	10.69459789	1.07887E-26	5.44315E-26	positive
PRJNA729271	stage_3W_vs_E10	ordinary_muscle_development	ENSGALG00010005304	ENSGALG00010005304	1.509585931	0.206812887	7.299283669	2.89304E-13	8.00584E-13	positive
PRJNA729271	stage_3W_vs_E10	ordinary_muscle_development	ENSGALG00010005305	SCAF8	-0.038120452	0.086390402	-0.441257949	0.659026265	0.691715113	negative
PRJNA729271	stage_3W_vs_E10	ordinary_muscle_development	ENSGALG00010005307	TIAM2	-2.122382479	0.157801622	-13.44968737	3.09177E-41	2.58599E-40	negative
PRJNA729271	stage_3W_vs_E10	ordinary_muscle_development	ENSGALG00010005308	ENSGALG00010005308	2.197910142	0.548238367	4.009041094	6.09658E-05	0.00010147	positive
PRJNA729271	stage_3W_vs_E10	ordinary_muscle_development	ENSGALG00010005309	ASXL3	-2.781427017	0.146573839	-18.9762855	2.67878E-80	6.76463E-79	negative
PRJNA729271	stage_3W_vs_E10	ordinary_muscle_development	ENSGALG00010005310	LAMTOR3	0.59793054	0.076175157	7.849416605	4.17977E-15	1.27174E-14	positive
PRJNA729271	stage_3W_vs_E10	ordinary_muscle_development	ENSGALG00010005311	YME1L1	0.601613766	0.078087539	7.704350443	1.3151E-14	3.89985E-14	positive
PRJNA729271	stage_3W_vs_E10	ordinary_muscle_development	ENSGALG00010005313	MPHOSPH8	-0.05282675	0.141290664	-0.373887057	0.708488347	0.737489306	negative
PRJNA729271	stage_3W_vs_E10	ordinary_muscle_development	ENSGALG00010005314	IL20RA	-0.487427578	0.630729002	-0.772800326	0.43964056	0.478279039	negative
PRJNA729271	stage_3W_vs_E10	ordinary_muscle_development	ENSGALG00010005315	ENSGALG00010005315	2.838465748	0.493834909	5.747802951	9.04105E-09	1.95539E-08	positive
PRJNA729271	stage_3W_vs_E10	ordinary_muscle_development	ENSGALG00010005316	PARP4	0.679567646	0.107588391	6.316365904	2.67786E-10	6.3166E-10	positive
PRJNA729271	stage_3W_vs_E10	ordinary_muscle_development	ENSGALG00010005317	CHCHD3	1.222788449	0.109358519	11.181465	5.02535E-29	2.77918E-28	positive
PRJNA729271	stage_3W_vs_E10	ordinary_muscle_development	ENSGALG00010005318	SLC35D3	-2.215405696	0.429967874	-5.152491219	2.57049E-07	5.06102E-07	negative
PRJNA729271	stage_3W_vs_E10	ordinary_muscle_development	ENSGALG00010005319	NOL4	-4.561646291	1.015353771	-4.492666913	7.03368E-06	1.25558E-05	negative
PRJNA729271	stage_3W_vs_E10	ordinary_muscle_development	ENSGALG00010005321	HS6ST3	-3.42242257	0.301748053	-11.34198723	8.12775E-30	4.64374E-29	negative
PRJNA729271	stage_3W_vs_E10	ordinary_muscle_development	ENSGALG00010005322	TFB1M	0.563692184	0.121954391	4.622155707	3.79773E-06	6.92864E-06	positive
PRJNA729271	stage_3W_vs_E10	ordinary_muscle_development	ENSGALG00010005323	DNAJB14	0.846095105	0.062960629	13.4384792	3.59754E-41	3.00382E-40	positive
PRJNA729271	stage_3W_vs_E10	ordinary_muscle_development	ENSGALG00010005324	CENPJ	-3.071035416	0.208992976	-14.69444323	6.99712E-49	7.63155E-48	negative
PRJNA729271	stage_3W_vs_E10	ordinary_muscle_development	ENSGALG00010005326	MASTL	-2.88688394	0.196418231	-14.69763739	6.67482E-49	7.28551E-48	negative
PRJNA729271	stage_3W_vs_E10	ordinary_muscle_development	ENSGALG00010005329	VIPAS39	0.333445259	0.117025151	2.849346959	0.004380908	0.006180772	positive
PRJNA729271	stage_3W_vs_E10	ordinary_muscle_development	ENSGALG00010005331	ENSGALG00010005331	0.690499781	0.852978384	0.809516154	0.418218315	0.456791084	positive
PRJNA729271	stage_3W_vs_E10	ordinary_muscle_development	ENSGALG00010005332	ENSGALG00010005332	2.574024548	0.68082358	3.780751171	0.000156356	0.000251317	positive
PRJNA729271	stage_3W_vs_E10	ordinary_muscle_development	ENSGALG00010005333	PLXNA4	-1.382141278	0.159005314	-8.692421903	3.54794E-18	1.26357E-17	negative
PRJNA729271	stage_3W_vs_E10	ordinary_muscle_development	ENSGALG00010005334	NME7	0.18060094	0.131701683	1.371288021	0.170285189	0.199247966	positive
PRJNA729271	stage_3W_vs_E10	ordinary_muscle_development	ENSGALG00010005335	AHSA1	0.553630018	0.133632997	4.142914041	3.42921E-05	5.8041E-05	positive
PRJNA729271	stage_3W_vs_E10	ordinary_muscle_development	ENSGALG00010005336	PEX7	0.462472471	0.125736106	3.678119871	0.00023496	0.000371699	positive
PRJNA729271	stage_3W_vs_E10	ordinary_muscle_development	ENSGALG00010005337	DCTN1	0.049834613	0.154120311	0.323348771	0.746431122	0.773209399	positive
PRJNA729271	stage_3W_vs_E10	ordinary_muscle_development	ENSGALG00010005338	PCGF1	0.852701592	0.115652384	7.372970313	1.66868E-13	4.67843E-13	positive
PRJNA729271	stage_3W_vs_E10	ordinary_muscle_development	ENSGALG00010005341	UGGT2	-0.480928415	0.119349421	-4.029583149	5.58758E-05	9.32873E-05	negative
PRJNA729271	stage_3W_vs_E10	ordinary_muscle_development	ENSGALG00010005342	MAP3K5	0.647128919	0.145097688	4.459953357	8.19775E-06	1.45638E-05	positive
PRJNA729271	stage_3W_vs_E10	ordinary_muscle_development	ENSGALG00010005343	RNF17	-4.605520461	1.784097237	-2.581429064	0.009839221	0.013381451	negative
PRJNA729271	stage_3W_vs_E10	ordinary_muscle_development	ENSGALG00010005344	ENSGALG00010005344	1.556932658	0.695969073	2.237071614	0.02528166	0.032869106	positive
PRJNA729271	stage_3W_vs_E10	ordinary_muscle_development	ENSGALG00010005345	NOX3	1.56117899	0.499995541	3.122385829	0.001793917	0.002622839	positive
PRJNA729271	stage_3W_vs_E10	ordinary_muscle_development	ENSGALG00010005346	FBXO30	2.428156539	0.21995863	11.03915104	2.47359E-28	1.33786E-27	positive
PRJNA729271	stage_3W_vs_E10	ordinary_muscle_development	ENSGALG00010005347	DOK1	0.183424079	0.148588929	1.234439744	0.217039087	0.249780196	positive
PRJNA729271	stage_3W_vs_E10	ordinary_muscle_development	ENSGALG00010005349	ACBD5	1.28483809	0.103338827	12.43325601	1.72464E-35	1.20475E-34	positive
PRJNA729271	stage_3W_vs_E10	ordinary_muscle_development	ENSGALG00010005350	ARHGAP20	-1.049288755	0.278263092	-3.770851349	0.000162692	0.000261182	negative
PRJNA729271	stage_3W_vs_E10	ordinary_muscle_development	ENSGALG00010005351	DTNA	2.043898824	0.16315252	12.52753455	5.2778E-36	3.74091E-35	positive
PRJNA729271	stage_3W_vs_E10	ordinary_muscle_development	ENSGALG00010005352	ENSGALG00010005352	4.169586962	0.585235719	7.124628295	1.04362E-12	2.81385E-12	positive
PRJNA729271	stage_3W_vs_E10	ordinary_muscle_development	ENSGALG00010005353	ENSGALG00010005353	0.14264098	0.103707772	1.375412637	0.169003553	0.197908103	positive
PRJNA729271	stage_3W_vs_E10	ordinary_muscle_development	ENSGALG00010005354	M1AP	-0.089731587	0.342176242	-0.262237925	0.793138017	0.816225456	negative
PRJNA729271	stage_3W_vs_E10	ordinary_muscle_development	ENSGALG00010005355	FDX1	0.276802314	0.103180227	2.682706962	0.007302895	0.010072839	positive
PRJNA729271	stage_3W_vs_E10	ordinary_muscle_development	ENSGALG00010005356	DNAJC3	0.843157804	0.084053908	10.03115532	1.11208E-23	4.913E-23	positive
PRJNA729271	stage_3W_vs_E10	ordinary_muscle_development	ENSGALG00010005357	TNIP3	0.698979449	0.439760418	1.589455122	0.111957678	0.134428972	positive
PRJNA729271	stage_3W_vs_E10	ordinary_muscle_development	ENSGALG00010005358	SHPRH	0.267539002	0.08946717	2.990359494	0.002786493	0.004006171	positive
PRJNA729271	stage_3W_vs_E10	ordinary_muscle_development	ENSGALG00010005359	MEIS2	-2.51372086	0.134339918	-18.71164508	3.97901E-78	9.45504E-77	negative
PRJNA729271	stage_3W_vs_E10	ordinary_muscle_development	ENSGALG00010005360	CAMK1D	-0.217696569	0.151238649	-1.439424184	0.150030377	0.176962349	negative
PRJNA729271	stage_3W_vs_E10	ordinary_muscle_development	ENSGALG00010005361	ENSGALG00010005361	2.682862255	0.413611509	6.486430369	8.78939E-11	2.12905E-10	positive
PRJNA729271	stage_3W_vs_E10	ordinary_muscle_development	ENSGALG00010005362	ATP1B1	1.694327758	0.174153415	9.728937871	2.2695E-22	9.52416E-22	positive
PRJNA729271	stage_3W_vs_E10	ordinary_muscle_development	ENSGALG00010005363	RDX	0.78317318	0.085387335	9.172006413	4.64295E-20	1.77806E-19	positive
PRJNA729271	stage_3W_vs_E10	ordinary_muscle_development	ENSGALG00010005364	MAP7	-4.752620676	0.43125042	-11.02055894	3.04164E-28	1.63898E-27	negative
PRJNA729271	stage_3W_vs_E10	ordinary_muscle_development	ENSGALG00010005366	AUP1	1.144795795	0.113366886	10.09814979	5.62942E-24	2.51769E-23	positive
PRJNA729271	stage_3W_vs_E10	ordinary_muscle_development	ENSGALG00010005367	H2AZ1	-0.957290973	0.095400812	-10.03441117	1.07599E-23	4.75794E-23	negative
PRJNA729271	stage_3W_vs_E10	ordinary_muscle_development	ENSGALG00010005368	CCDC3	-0.877329404	0.22676989	-3.868809051	0.000109368	0.000178143	negative
PRJNA729271	stage_3W_vs_E10	ordinary_muscle_development	ENSGALG00010005369	DPT	3.54523457	0.312835162	11.33259622	9.04823E-30	5.16355E-29	positive
PRJNA729271	stage_3W_vs_E10	ordinary_muscle_development	ENSGALG00010005371	GRM1	-2.597030989	1.299085843	-1.999121923	0.045595164	0.057564837	negative
PRJNA729271	stage_3W_vs_E10	ordinary_muscle_development	ENSGALG00010005372	CDIN1	-1.025065962	0.110212669	-9.300799746	1.39393E-20	5.45198E-20	negative
PRJNA729271	stage_3W_vs_E10	ordinary_muscle_development	ENSGALG00010005376	HTRA2	0.503729886	0.094646637	5.32221644	1.0251E-07	2.07411E-07	positive
PRJNA729271	stage_3W_vs_E10	ordinary_muscle_development	ENSGALG00010005377	EMCN	2.605493044	0.140425095	18.55432631	7.52448E-77	1.73951E-75	positive
PRJNA729271	stage_3W_vs_E10	ordinary_muscle_development	ENSGALG00010005378	ENSGALG00010005378	-6.801502189	1.026744467	-6.624337807	3.48808E-11	8.66343E-11	negative
PRJNA729271	stage_3W_vs_E10	ordinary_muscle_development	ENSGALG00010005379	DZIP1	-0.531697988	0.167825422	-3.168161181	0.001534064	0.002260472	negative
PRJNA729271	stage_3W_vs_E10	ordinary_muscle_development	ENSGALG00010005380	XCL1	8.519750756	1.013811343	8.403684582	4.32684E-17	1.45584E-16	positive
PRJNA729271	stage_3W_vs_E10	ordinary_muscle_development	ENSGALG00010005381	FGF9	-4.735184231	0.299146389	-15.82898676	1.96358E-56	2.67752E-55	negative
PRJNA729271	stage_3W_vs_E10	ordinary_muscle_development	ENSGALG00010005382	RAB32	-0.969887344	0.128668333	-7.537886925	4.77648E-14	1.38056E-13	negative
PRJNA729271	stage_3W_vs_E10	ordinary_muscle_development	ENSGALG00010005383	OPTN	3.466140464	0.138199022	25.08078866	8.0597E-139	6.3839E-137	positive
PRJNA729271	stage_3W_vs_E10	ordinary_muscle_development	ENSGALG00010005384	MAPRE2	0.469709926	0.172746826	2.719065442	0.006546665	0.009070342	positive
PRJNA729271	stage_3W_vs_E10	ordinary_muscle_development	ENSGALG00010005385	PDSS1	0.291499376	0.099210567	2.938188788	0.003301359	0.004719251	positive
PRJNA729271	stage_3W_vs_E10	ordinary_muscle_development	ENSGALG00010005386	CPOX	-0.811981746	0.141486891	-5.738918577	9.5283E-09	2.05862E-08	negative
PRJNA729271	stage_3W_vs_E10	ordinary_muscle_development	ENSGALG00010005387	CLDN10	-2.706026843	0.551228186	-4.909086495	9.15016E-07	1.73829E-06	negative
PRJNA729271	stage_3W_vs_E10	ordinary_muscle_development	ENSGALG00010005388	ENSGALG00010005388	4.005213379	0.455746148	8.788255031	1.51901E-18	5.47712E-18	positive
PRJNA729271	stage_3W_vs_E10	ordinary_muscle_development	ENSGALG00010005389	MCM10	-2.566609797	0.189647594	-13.53357429	9.90862E-42	8.43863E-41	negative
PRJNA729271	stage_3W_vs_E10	ordinary_muscle_development	ENSGALG00010005391	ADGB	0.332903167	0.384718055	0.865317242	0.38686469	0.425850826	positive
PRJNA729271	stage_3W_vs_E10	ordinary_muscle_development	ENSGALG00010005392	ENSGALG00010005392	0.423207869	0.107184459	3.948406995	7.8673E-05	0.000129469	positive
PRJNA729271	stage_3W_vs_E10	ordinary_muscle_development	ENSGALG00010005394	PHYH	2.798081597	0.135224375	20.6921392	4.07717E-95	1.50378E-93	positive
PRJNA729271	stage_3W_vs_E10	ordinary_muscle_development	ENSGALG00010005395	ENSGALG00010005395	-2.865514408	0.390410142	-7.33975402	2.13987E-13	5.96603E-13	negative
PRJNA729271	stage_3W_vs_E10	ordinary_muscle_development	ENSGALG00010005396	MICU2	0.590371368	0.122524262	4.818403802	1.44711E-06	2.71041E-06	positive
PRJNA729271	stage_3W_vs_E10	ordinary_muscle_development	ENSGALG00010005398	SEPHS1	-1.190777479	0.086967419	-13.69222519	1.12995E-42	9.96268E-42	negative
PRJNA729271	stage_3W_vs_E10	ordinary_muscle_development	ENSGALG00010005399	NDNF	-5.807642792	0.27792802	-20.89621184	5.79667E-97	2.20532E-95	negative
PRJNA729271	stage_3W_vs_E10	ordinary_muscle_development	ENSGALG00010005401	BCLAF1	-1.248849431	0.087048196	-14.34664339	1.1182E-46	1.12871E-45	negative
PRJNA729271	stage_3W_vs_E10	ordinary_muscle_development	ENSGALG00010005402	ABCC4	1.898311027	0.168406273	11.27221088	1.79993E-29	1.01517E-28	positive
PRJNA729271	stage_3W_vs_E10	ordinary_muscle_development	ENSGALG00010005403	ENSGALG00010005403	-0.784809295	0.144991538	-5.412793771	6.20489E-08	1.2707E-07	negative
PRJNA729271	stage_3W_vs_E10	ordinary_muscle_development	ENSGALG00010005404	BEND7	1.6948806	0.186235698	9.10072889	8.97276E-20	3.40027E-19	positive
PRJNA729271	stage_3W_vs_E10	ordinary_muscle_development	ENSGALG00010005405	CEBPD	6.281383609	0.473915822	13.25421798	4.2654E-40	3.45982E-39	positive
PRJNA729271	stage_3W_vs_E10	ordinary_muscle_development	ENSGALG00010005407	DPH6	-0.441972871	0.121107026	-3.649440365	0.000262812	0.000414252	negative
PRJNA729271	stage_3W_vs_E10	ordinary_muscle_development	ENSGALG00010005408	APBB1IP	0.573829323	0.203744102	2.816421762	0.004856187	0.006818796	positive
PRJNA729271	stage_3W_vs_E10	ordinary_muscle_development	ENSGALG00010005409	PRKCQ	2.946679605	0.217704113	13.53525005	9.68523E-42	8.25323E-41	positive
PRJNA729271	stage_3W_vs_E10	ordinary_muscle_development	ENSGALG00010005411	STXBP5	1.248195442	0.095017687	13.13645362	2.03545E-39	1.61399E-38	positive
PRJNA729271	stage_3W_vs_E10	ordinary_muscle_development	ENSGALG00010005412	MTFR2	-2.546287174	0.171660131	-14.83330558	8.92286E-50	1.00029E-48	negative
PRJNA729271	stage_3W_vs_E10	ordinary_muscle_development	ENSGALG00010005413	GAD2	-5.505563708	0.753420821	-7.307421766	2.72317E-13	7.54838E-13	negative
PRJNA729271	stage_3W_vs_E10	ordinary_muscle_development	ENSGALG00010005414	ENSGALG00010005414	-2.229641587	0.247442332	-9.010752394	2.04648E-19	7.65317E-19	negative
PRJNA729271	stage_3W_vs_E10	ordinary_muscle_development	ENSGALG00010005415	PRKDC	-0.848391828	0.088080564	-9.631998115	5.85791E-22	2.42324E-21	negative
PRJNA729271	stage_3W_vs_E10	ordinary_muscle_development	ENSGALG00010005416	PPP3CA	1.403055055	0.162071629	8.65707653	4.84318E-18	1.71143E-17	positive
PRJNA729271	stage_3W_vs_E10	ordinary_muscle_development	ENSGALG00010005417	LOXL3	0.838783905	0.164131042	5.110452573	3.21388E-07	6.2868E-07	positive
PRJNA729271	stage_3W_vs_E10	ordinary_muscle_development	ENSGALG00010005420	DHRS4	-0.381761553	0.119690771	-3.189565495	0.001424868	0.002105778	negative
PRJNA729271	stage_3W_vs_E10	ordinary_muscle_development	ENSGALG00010005421	ENSGALG00010005421	2.688484409	0.321608026	8.359506573	6.29811E-17	2.10494E-16	positive
PRJNA729271	stage_3W_vs_E10	ordinary_muscle_development	ENSGALG00010005423	ST3GAL6	1.694653657	0.149653398	11.32385685	9.99746E-30	5.69403E-29	positive
PRJNA729271	stage_3W_vs_E10	ordinary_muscle_development	ENSGALG00010005424	ENSGALG00010005424	1.18768253	0.132991699	8.930501242	4.24083E-19	1.56693E-18	positive
PRJNA729271	stage_3W_vs_E10	ordinary_muscle_development	ENSGALG00010005425	ELAPOR2	-0.631694029	0.250876525	-2.517947934	0.011804077	0.015929624	negative
PRJNA729271	stage_3W_vs_E10	ordinary_muscle_development	ENSGALG00010005426	ENSGALG00010005426	3.648554904	0.87384826	4.17527284	2.97629E-05	5.06413E-05	positive
PRJNA729271	stage_3W_vs_E10	ordinary_muscle_development	ENSGALG00010005427	ENSGALG00010005427	1.14587273	0.137431873	8.337750971	7.57168E-17	2.51839E-16	positive
PRJNA729271	stage_3W_vs_E10	ordinary_muscle_development	ENSGALG00010005428	ZNF770	0.373520061	0.102043769	3.660390671	0.000251831	0.000397462	positive
PRJNA729271	stage_3W_vs_E10	ordinary_muscle_development	ENSGALG00010005429	ZDHHC20	-1.752387234	0.118825565	-14.74756068	3.18987E-49	3.50282E-48	negative
PRJNA729271	stage_3W_vs_E10	ordinary_muscle_development	ENSGALG00010005430	MCM4	-2.391882012	0.137159437	-17.43869813	4.19522E-68	7.74646E-67	negative
PRJNA729271	stage_3W_vs_E10	ordinary_muscle_development	ENSGALG00010005431	GRM3	-7.209699724	1.035005469	-6.965856647	3.26411E-12	8.55266E-12	negative
PRJNA729271	stage_3W_vs_E10	ordinary_muscle_development	ENSGALG00010005433	GPC5	-1.448206092	0.159780913	-9.063698964	1.261E-19	4.74635E-19	negative
PRJNA729271	stage_3W_vs_E10	ordinary_muscle_development	ENSGALG00010005434	PRDM5	-1.528474094	0.222306189	-6.875535498	6.17574E-12	1.59227E-11	negative
PRJNA729271	stage_3W_vs_E10	ordinary_muscle_development	ENSGALG00010005435	MAD2L1	-3.280440532	0.178261735	-18.40238193	1.25715E-75	2.83397E-74	negative
PRJNA729271	stage_3W_vs_E10	ordinary_muscle_development	ENSGALG00010005436	AQR	-0.801517319	0.055113204	-14.54310867	6.45915E-48	6.86403E-47	negative
PRJNA729271	stage_3W_vs_E10	ordinary_muscle_development	ENSGALG00010005437	PDE7B	3.110118846	0.157738234	19.71696253	1.54216E-86	4.66673E-85	positive
PRJNA729271	stage_3W_vs_E10	ordinary_muscle_development	ENSGALG00010005438	GPR180	-0.521098846	0.139280706	-3.741357018	0.000183029	0.000292536	negative
PRJNA729271	stage_3W_vs_E10	ordinary_muscle_development	ENSGALG00010005439	TBX19	0.091598327	0.552263968	0.165859683	0.868267376	0.883381457	positive
PRJNA729271	stage_3W_vs_E10	ordinary_muscle_development	ENSGALG00010005440	ENSGALG00010005440	4.072241879	0.469766211	8.668656417	4.37249E-18	1.55024E-17	positive
PRJNA729271	stage_3W_vs_E10	ordinary_muscle_development	ENSGALG00010005441	UBE2V2	0.092302915	0.095327188	0.968274808	0.332907143	0.371277144	positive
PRJNA729271	stage_3W_vs_E10	ordinary_muscle_development	ENSGALG00010005442	SASH1	0.431444394	0.097504512	4.424865939	9.65022E-06	1.70502E-05	positive
PRJNA729271	stage_3W_vs_E10	ordinary_muscle_development	ENSGALG00010005443	BANK1	0.696409101	0.427000699	1.630931993	0.10290467	0.124165753	positive

PRJNA729271	stage_3W_vs_E10	ordinary_muscle_development	ENSGALG00010005444	ACTC1	-1.074807796	0.42658418	-2.519567875	0.011749898	0.015863895	negative
PRJNA729271	stage_3W_vs_E10	ordinary_muscle_development	ENSGALG00010005445	SFT2D2	-1.363244491	0.105590495	-12.91067435	3.91854E-38	2.95984E-37	negative
PRJNA729271	stage_3W_vs_E10	ordinary_muscle_development	ENSGALG00010005446	ENSGALG00010005446	3.124813713	0.369700673	8.452280291	2.85668E-17	9.69505E-17	positive
PRJNA729271	stage_3W_vs_E10	ordinary_muscle_development	ENSGALG00010005447	MYO3A	-4.54279065	0.510936197	-8.891111402	6.05007E-19	2.21734E-18	negative
PRJNA729271	stage_3W_vs_E10	ordinary_muscle_development	ENSGALG00010005448	ENSGALG00010005448	0.780110122	0.147345235	5.294437394	1.19383E-07	2.40542E-07	positive
PRJNA729271	stage_3W_vs_E10	ordinary_muscle_development	ENSGALG00010005449	TIPRL	0.215554426	0.08575398	2.513637578	0.011949316	0.016115123	positive
PRJNA729271	stage_3W_vs_E10	ordinary_muscle_development	ENSGALG00010005450	ENSGALG00010005450	3.179271699	0.417689762	7.61156242	2.70802E-14	7.90749E-14	positive
PRJNA729271	stage_3W_vs_E10	ordinary_muscle_development	ENSGALG00010005453	UST	0.265063394	0.114104009	2.322998083	0.020179255	0.026544905	positive
PRJNA729271	stage_3W_vs_E10	ordinary_muscle_development	ENSGALG00010005454	GPR161	-1.468077074	0.098372723	-14.92361943	2.31375E-50	2.66173E-49	negative
PRJNA729271	stage_3W_vs_E10	ordinary_muscle_development	ENSGALG00010005455	ENSGALG00010005455	2.352114037	0.680762604	3.455116396	0.000550055	0.000843263	positive
PRJNA729271	stage_3W_vs_E10	ordinary_muscle_development	ENSGALG00010005456	RPL7L1	-0.625360199	0.070423364	-8.880010355	6.68541E-19	2.44525E-18	negative
PRJNA729271	stage_3W_vs_E10	ordinary_muscle_development	ENSGALG00010005457	SEMA3D	-0.375708234	0.160003474	-2.348125481	0.018868164	0.024876284	negative
PRJNA729271	stage_3W_vs_E10	ordinary_muscle_development	ENSGALG00010005458	PRKD1	0.634922317	0.174250347	3.643736318	0.000268709	0.000423178	positive
PRJNA729271	stage_3W_vs_E10	ordinary_muscle_development	ENSGALG00010005459	ZC3H12D	4.866419168	0.503234618	9.670279012	4.03277E-22	1.68022E-21	positive
PRJNA729271	stage_3W_vs_E10	ordinary_muscle_development	ENSGALG00010005460	TGDS	-1.021427002	0.101967969	-10.01713589	1.28163E-23	5.65173E-23	negative
PRJNA729271	stage_3W_vs_E10	ordinary_muscle_development	ENSGALG00010005462	AHI1	1.112716117	0.149375674	7.449111923	9.39706E-14	2.6687E-13	positive
PRJNA729271	stage_3W_vs_E10	ordinary_muscle_development	ENSGALG00010005463	SKA3	-3.822547757	0.265782742	-14.38222709	6.69064E-47	6.82003E-46	negative
PRJNA729271	stage_3W_vs_E10	ordinary_muscle_development	ENSGALG00010005464	SLC39A8	-4.277577855	0.338603957	-12.63298247	1.38912E-36	1.00076E-35	negative
PRJNA729271	stage_3W_vs_E10	ordinary_muscle_development	ENSGALG00010005465	ENSGALG00010005465	-1.016434252	0.091041487	-11.16451723	6.08207E-29	3.35335E-28	negative
PRJNA729271	stage_3W_vs_E10	ordinary_muscle_development	ENSGALG00010005466	LATS2	0.512646577	0.089181803	5.748331609	9.01283E-09	1.94958E-08	positive
PRJNA729271	stage_3W_vs_E10	ordinary_muscle_development	ENSGALG00010005467	ENSGALG00010005467	3.292936189	0.251524842	13.09189247	3.66364E-39	2.88298E-38	positive
PRJNA729271	stage_3W_vs_E10	ordinary_muscle_development	ENSGALG00010005468	PPIL4	-0.433865708	0.104633174	-4.146540642	3.37536E-05	5.71564E-05	negative
PRJNA729271	stage_3W_vs_E10	ordinary_muscle_development	ENSGALG00010005470	SEMA3A	-1.880341534	0.141638444	-13.27564384	3.20504E-40	2.61142E-39	negative
PRJNA729271	stage_3W_vs_E10	ordinary_muscle_development	ENSGALG00010005472	PFKP	-0.898254552	0.114742467	-7.82844032	4.93959E-15	1.49821E-14	negative
PRJNA729271	stage_3W_vs_E10	ordinary_muscle_development	ENSGALG00010005473	ENSGALG00010005473	3.478951631	0.641312219	5.424739354	5.80392E-08	1.19111E-07	positive
PRJNA729271	stage_3W_vs_E10	ordinary_muscle_development	ENSGALG00010005474	COL8A1	1.602536998	0.343861789	4.66041023	3.1558E-06	5.78809E-06	positive
PRJNA729271	stage_3W_vs_E10	ordinary_muscle_development	ENSGALG00010005475	TMEM181	-0.407935638	0.092082288	-4.430120555	9.41804E-06	1.66603E-05	negative
PRJNA729271	stage_3W_vs_E10	ordinary_muscle_development	ENSGALG00010005476	NFKB1	1.102024479	0.114747112	9.603940852	7.69449E-22	3.16941E-21	positive
PRJNA729271	stage_3W_vs_E10	ordinary_muscle_development	ENSGALG00010005477	TMEM161B	0.366945756	0.161650735	2.269991269	0.023208113	0.030333778	positive
PRJNA729271	stage_3W_vs_E10	ordinary_muscle_development	ENSGALG00010005479	ENSGALG00010005479	0.725571424	0.607162165	1.195020812	0.232078899	0.26592756	positive
PRJNA729271	stage_3W_vs_E10	ordinary_muscle_development	ENSGALG00010005481	ENSGALG00010005481	-2.91061913	0.578564365	-5.030761152	4.88536E-07	9.44288E-07	negative
PRJNA729271	stage_3W_vs_E10	ordinary_muscle_development	ENSGALG00010005482	XPO4	-1.357508503	0.075738262	-17.9236817	7.70555E-72	1.57313E-70	negative
PRJNA729271	stage_3W_vs_E10	ordinary_muscle_development	ENSGALG00010005484	GINM1	1.730610716	0.122192693	14.16296399	1.55303E-45	1.51285E-44	positive
PRJNA729271	stage_3W_vs_E10	ordinary_muscle_development	ENSGALG00010005485	PITRM1	-0.380668998	0.096330816	-3.951684576	7.7603E-05	0.00012781	negative
PRJNA729271	stage_3W_vs_E10	ordinary_muscle_development	ENSGALG00010005486	IRF5	2.583157676	0.312448859	8.267457553	1.36846E-16	4.49486E-16	positive
PRJNA729271	stage_3W_vs_E10	ordinary_muscle_development	ENSGALG00010005487	ENSGALG00010005487	-1.743442433	0.212427946	-8.207217846	2.26373E-16	7.35052E-16	negative
PRJNA729271	stage_3W_vs_E10	ordinary_muscle_development	ENSGALG00010005489	G2E3	-1.654397119	0.087020629	-19.01155097	1.36846E-80	3.49222E-79	negative
PRJNA729271	stage_3W_vs_E10	ordinary_muscle_development	ENSGALG00010005490	DCBLD2	-0.773290566	0.115214233	-6.711762457	1.92288E-11	4.84227E-11	negative
PRJNA729271	stage_3W_vs_E10	ordinary_muscle_development	ENSGALG00010005491	GGACT	0.525635228	0.170774672	3.077946046	0.002084326	0.003029411	positive
PRJNA729271	stage_3W_vs_E10	ordinary_muscle_development	ENSGALG00010005492	TNPO3	0.487661013	0.096501765	5.053389565	4.34037E-07	8.41993E-07	positive
PRJNA729271	stage_3W_vs_E10	ordinary_muscle_development	ENSGALG00010005493	KLF6	0.815968073	0.181042424	4.507054508	6.57338E-06	1.17689E-05	positive
PRJNA729271	stage_3W_vs_E10	ordinary_muscle_development	ENSGALG00010005494	PPDPFL	1.798128308	0.436392211	4.120440887	3.78148E-05	6.38916E-05	positive
PRJNA729271	stage_3W_vs_E10	ordinary_muscle_development	ENSGALG00010005495	TXN	-1.099832991	0.146585186	-7.503029585	6.23595E-14	1.79026E-13	negative
PRJNA729271	stage_3W_vs_E10	ordinary_muscle_development	ENSGALG00010005497	MKX	-2.648373262	0.204268417	-12.96516272	1.92821E-38	1.47802E-37	negative
PRJNA729271	stage_3W_vs_E10	ordinary_muscle_development	ENSGALG00010005498	SMO	0.201197379	0.137009078	1.468496702	0.141969351	0.168193359	positive
PRJNA729271	stage_3W_vs_E10	ordinary_muscle_development	ENSGALG00010005499	PDE5A	-0.699459392	0.112822346	-6.199652942	5.65878E-10	1.31134E-09	negative
PRJNA729271	stage_3W_vs_E10	ordinary_muscle_development	ENSGALG00010005500	KATNA1	-0.325745661	0.131188759	-2.483030282	0.013027001	0.017485543	negative
PRJNA729271	stage_3W_vs_E10	ordinary_muscle_development	ENSGALG00010005502	SCFD1	-0.55966927	0.072054814	-7.767271096	8.01951E-15	2.40419E-14	negative
PRJNA729271	stage_3W_vs_E10	ordinary_muscle_development	ENSGALG00010005503	DYNLT1	-1.405593821	0.148238657	-9.481965454	2.49538E-21	1.00866E-20	negative
PRJNA729271	stage_3W_vs_E10	ordinary_muscle_development	ENSGALG00010005504	LATS1	-0.307816275	0.079673744	-3.863459411	0.000111792	0.000181969	negative
PRJNA729271	stage_3W_vs_E10	ordinary_muscle_development	ENSGALG00010005505	FABP2	1.290826288	0.958190859	1.347149449	0.177932105	0.207508718	positive
PRJNA729271	stage_3W_vs_E10	ordinary_muscle_development	ENSGALG00010005507	USP53	1.657390236	0.119135746	13.91177961	5.37258E-44	4.94135E-43	positive
PRJNA729271	stage_3W_vs_E10	ordinary_muscle_development	ENSGALG00010005508	SS18	-1.380198229	0.065285057	-21.14110486	3.33186E-99	1.34154E-97	negative
PRJNA729271	stage_3W_vs_E10	ordinary_muscle_development	ENSGALG00010005509	COCH	-0.012512099	0.455123717	-0.027491644	0.978067604	0.981317223	negative
PRJNA729271	stage_3W_vs_E10	ordinary_muscle_development	ENSGALG00010005510	MYOZ2	-0.119726989	0.317916786	-0.376598514	0.706471992	0.735548881	negative
PRJNA729271	stage_3W_vs_E10	ordinary_muscle_development	ENSGALG00010005511	AHCYL2	0.709649458	0.098190742	7.227254238	4.92857E-13	1.34741E-12	positive
PRJNA729271	stage_3W_vs_E10	ordinary_muscle_development	ENSGALG00010005512	SMARCAD1	-2.0124786	0.112331871	-17.91547302	8.93068E-72	1.81557E-70	negative
PRJNA729271	stage_3W_vs_E10	ordinary_muscle_development	ENSGALG00010005513	TMEM30CP	0.150761337	0.260349897	0.579072006	0.562540588	0.599340377	positive
PRJNA729271	stage_3W_vs_E10	ordinary_muscle_development	ENSGALG00010005514	EEF1AKMT1	0.455241601	0.143585114	3.170534794	0.001521586	0.002242769	positive
PRJNA729271	stage_3W_vs_E10	ordinary_muscle_development	ENSGALG00010005515	PCCA	-0.223010797	0.09719416	-2.294487609	0.021762506	0.028524055	negative
PRJNA729271	stage_3W_vs_E10	ordinary_muscle_development	ENSGALG00010005516	LARP4B	0.834930418	0.131739265	6.337749177	2.33146E-10	5.52198E-10	positive
PRJNA729271	stage_3W_vs_E10	ordinary_muscle_development	ENSGALG00010005518	HPGDS	0.410219456	0.650783329	0.630347211	0.528467441	0.566368618	positive
PRJNA729271	stage_3W_vs_E10	ordinary_muscle_development	ENSGALG00010005519	PTBP3	-0.25813506	0.249939964	-1.032788258	0.301703	0.33929591	negative
PRJNA729271	stage_3W_vs_E10	ordinary_muscle_development	ENSGALG00010005521	SYTL3	3.084386674	0.380847873	8.098736768	5.55328E-16	1.76524E-15	positive
PRJNA729271	stage_3W_vs_E10	ordinary_muscle_development	ENSGALG00010005522	SYNPO2	1.970064512	0.178103384	11.06135364	1.9316E-28	1.04864E-27	positive
PRJNA729271	stage_3W_vs_E10	ordinary_muscle_development	ENSGALG00010005523	ENSGALG00010005523	4.436762337	0.594218425	7.466551274	8.23239E-14	2.34437E-13	positive
PRJNA729271	stage_3W_vs_E10	ordinary_muscle_development	ENSGALG00010005524	STRIP2	1.728749369	0.27436628	6.300881324	2.95958E-10	6.9664E-10	positive
PRJNA729271	stage_3W_vs_E10	ordinary_muscle_development	ENSGALG00010005526	STRN3	0.852411853	0.069556224	12.25500464	1.5794E-34	1.07179E-33	positive
PRJNA729271	stage_3W_vs_E10	ordinary_muscle_development	ENSGALG00010005527	NUP43	-1.148954434	0.096853763	-11.86277542	1.84748E-32	1.16889E-31	negative
PRJNA729271	stage_3W_vs_E10	ordinary_muscle_development	ENSGALG00010005529	ENSGALG00010005529	2.951981951	0.361347404	8.169373621	3.09995E-16	9.98749E-16	positive
PRJNA729271	stage_3W_vs_E10	ordinary_muscle_development	ENSGALG00010005530	IL17D	-0.046419921	0.211231327	-0.219758698	0.826059088	0.846618573	negative
PRJNA729271	stage_3W_vs_E10	ordinary_muscle_development	ENSGALG00010005531	SUSD1	0.613674779	0.25350583	2.420752134	0.015488434	0.020611903	positive
PRJNA729271	stage_3W_vs_E10	ordinary_muscle_development	ENSGALG00010005532	GTPBP4	0.434313115	0.097164047	4.469895274	7.82579E-06	1.39321E-05	positive
PRJNA729271	stage_3W_vs_E10	ordinary_muscle_development	ENSGALG00010005533	OPN4-1	-1.332980549	0.729502211	-1.827246758	0.067662676	0.083533813	negative
PRJNA729271	stage_3W_vs_E10	ordinary_muscle_development	ENSGALG00010005534	ENSGALG00010005534	-0.778400126	0.163136566	-4.771463238	1.82892E-06	3.40224E-06	negative
PRJNA729271	stage_3W_vs_E10	ordinary_muscle_development	ENSGALG00010005535	AP4S1	1.330656717	0.151088349	8.807143166	1.28376E-18	4.63926E-18	positive
PRJNA729271	stage_3W_vs_E10	ordinary_muscle_development	ENSGALG00010005536	TAF4B	-2.040706464	0.213563146	-9.555517876	1.22967E-21	5.02229E-21	negative
PRJNA729271	stage_3W_vs_E10	ordinary_muscle_development	ENSGALG00010005537	GRIN3A	-4.634742002	0.389645833	-11.89475572	1.26021E-32	7.99421E-32	negative
PRJNA729271	stage_3W_vs_E10	ordinary_muscle_development	ENSGALG00010005538	ENSGALG00010005538	2.679869412	0.314441726	8.522626574	1.55975E-17	5.37276E-17	positive
PRJNA729271	stage_3W_vs_E10	ordinary_muscle_development	ENSGALG00010005539	IFT88	-0.822869656	0.132907878	-6.191278287	5.96782E-10	1.37986E-09	negative
PRJNA729271	stage_3W_vs_E10	ordinary_muscle_development	ENSGALG00010005540	PCMT1	1.133804118	0.137161662	8.266188244	1.3831E-16	4.54089E-16	positive
PRJNA729271	stage_3W_vs_E10	ordinary_muscle_development	ENSGALG00010005542	KCTD1	-0.620758424	0.137196403	-4.524596941	6.05107E-06	1.08714E-05	negative
PRJNA729271	stage_3W_vs_E10	ordinary_muscle_development	ENSGALG00010005543	SEC24D	-1.235612371	0.202603788	-6.098663708	1.06959E-09	2.43195E-09	negative
PRJNA729271	stage_3W_vs_E10	ordinary_muscle_development	ENSGALG00010005544	AQP4	7.016099511	0.308804604	22.72019073	2.8298E-114	1.542E-112	positive
PRJNA729271	stage_3W_vs_E10	ordinary_muscle_development	ENSGALG00010005545	RPL34	1.085029165	0.16689163	6.501399547	7.95762E-11	1.93241E-10	positive
PRJNA729271	stage_3W_vs_E10	ordinary_muscle_development	ENSGALG00010005546	NRF1	1.47510003	0.151896457	9.711220794	2.70083E-22	1.13081E-21	positive
PRJNA729271	stage_3W_vs_E10	ordinary_muscle_development	ENSGALG00010005547	LRP11	-1.303701667	0.222048365	-5.871250929	4.32519E-09	9.50047E-09	negative
PRJNA729271	stage_3W_vs_E10	ordinary_muscle_development	ENSGALG00010005548	EZR	-0.289083973	0.177708455	-1.626731679	0.103794098	0.12518684	negative
PRJNA729271	stage_3W_vs_E10	ordinary_muscle_development	ENSGALG00010005549	RNF20	-0.954101825	0.114185534	-8.355715401	6.50373E-17	2.17216E-16	negative
PRJNA729271	stage_3W_vs_E10	ordinary_muscle_development	ENSGALG00010005550	ENSGALG00010005550	2.550819746	0.543826453	4.690503253	2.72534E-06	5.01699E-06	positive
PRJNA729271	stage_3W_vs_E10	ordinary_muscle_development	ENSGALG00010005551	METTL14	-1.458274757	0.083874347	-17.38642158	1.04562E-67	1.91852E-66	negative
PRJNA729271	stage_3W_vs_E10	ordinary_muscle_development	ENSGALG00010005552	WDR37	0.294029065	0.085851642	3.424850826	0.000615137	0.000939262	positive
PRJNA729271	stage_3W_vs_E10	ordinary_muscle_development	ENSGALG00010005553	PPP1R14C	0.21393033	0.164940925	1.297011831	0.194627116	0.225653499	positive
PRJNA729271	stage_3W_vs_E10	ordinary_muscle_development	ENSGALG00010005554	CRYL1	1.939347985	0.161827187	11.98406781	4.30662E-33	2.77565E-32	positive
PRJNA729271	stage_3W_vs_E10	ordinary_muscle_development	ENSGALG00010005555	PRSS12	-4.861498748	0.194947567	-24.93746817	2.9204E-137	2.2517E-135	negative
PRJNA729271	stage_3W_vs_E10	ordinary_muscle_development	ENSGALG00010005556	IYD	-2.900214679	0.984673666	-2.945356192	0.003225831	0.004614469	negative
PRJNA729271	stage_3W_vs_E10	ordinary_muscle_development	ENSGALG00010005557	CMSS1	-0.35511761	0.167359229	-2.121888415	0.033847109	0.04337644	negative
PRJNA729271	stage_3W_vs_E10	ordinary_muscle_development	ENSGALG00010005558	TAGAP	2.793155915	0.20478242	13.63962745	2.32747E-42	2.02988E-41	positive
PRJNA729271	stage_3W_vs_E10	ordinary_muscle_development	ENSGALG00010005559	FILIP1L	0.504215557	0.130817416	3.854345779	0.00011604	0.000188564	positive
PRJNA729271	stage_3W_vs_E10	ordinary_muscle_development	ENSGALG00010005560	RSPH3	-1.18510939	0.381449774	-3.106855661	0.001890887	0.002757108	negative
PRJNA729271	stage_3W_vs_E10	ordinary_muscle_development	ENSGALG00010005561	CLYBL	0.516825191	0.201716973	2.562130417	0.010403225	0.014108789	positive
PRJNA729271	stage_3W_vs_E10	ordinary_muscle_development	ENSGALG00010005562	HECTD1	0.79477342	0.085358201	9.311037631	1.2659E-20	4.96325E-20	positive
PRJNA729271	stage_3W_vs_E10	ordinary_muscle_development	ENSGALG00010005564	TM9SF2	0.292195647	0.057845329	5.051326523	4.38752E-07	8.51026E-07	positive
PRJNA729271	stage_3W_vs_E10	ordinary_muscle_development	ENSGALG00010005565	PGAP4	-1.140022026	0.178005411	-6.404423424	1.50939E-10	3.60497E-10	negative
PRJNA729271	stage_3W_vs_E10	ordinary_muscle_development	ENSGALG00010005567	NDST3	-1.67916508	0.243722122	-6.889670358	5.59218E-12	1.44335E-11	negative
PRJNA729271	stage_3W_vs_E10	ordinary_muscle_development	ENSGALG00010005568	PLEKHG1	0.192573885	0.132376467	1.454744102	0.145740118	0.172294512	positive
PRJNA729271	stage_3W_vs_E10	ordinary_muscle_development	ENSGALG00010005572	ENSGALG00010005572	1.529069115	0.15228609	10.0407668	1.00887E-23	4.47614E-23	positive
PRJNA729271	stage_3W_vs_E10	ordinary_muscle_development	ENSGALG00010005573	CHST9	0.364932914	0.282728643	1.290753245	0.196789255	0.227996183	positive
PRJNA729271	stage_3W_vs_E10	ordinary_muscle_development	ENSGALG00010005575	ENSGALG00010005575	3.399913459	0.43767421	7.768137539	7.96486E-15	2.3888E-14	positive
PRJNA729271	stage_3W_vs_E10	ordinary_muscle_development	ENSGALG00010005578	HEATR5A	0.372494886	0.120280021	3.0968974	0.001955575	0.002848278	positive
PRJNA729271	stage_3W_vs_E10	ordinary_muscle_development	ENSGALG00010005581	GR42L5	2.895702451	0.744888728	3.88742955	0.000101311	0.000165261	positive
PRJNA729271	stage_3W_vs_E10	ordinary_muscle_development	ENSGALG00010005583	ALDOB	-1.912810091	1.012704289	-1.888814051			negative
PRJNA729271	stage_3W_vs_E10	ordinary_muscle_development	ENSGALG00010005587	LEF1	-6.289920346	0.234039458	-26.87546965	4.2512E-159	4.6332E-157	negative
PRJNA729271	stage_3W_vs_E10	ordinary_muscle_development	ENSGALG00010005588	PLAGL1	-0.235098753	0.127455247	-1.84455924	0.065101681	0.080584873	negative
PRJNA729271	stage_3W_vs_E10	ordinary_muscle_development	ENSGALG00010005590	TBC1D23	-0.042063589	0.056574162	-0.743512362	0.45717154	0.495639601	negative
PRJNA729271	stage_3W_vs_E10	ordinary_muscle_development	ENSGALG00010005593	ENSGALG00010005593	3.644287185	1.055274492	3.453402135	0.000553563	0.000848192	positive
PRJNA729271	stage_3W_vs_E10	ordinary_muscle_development	ENSGALG00010005594	MRPL50	0.727953041	0.150119251	4.849165149	1.23982E-06	2.33362E-06	positive
PRJNA729271	stage_3W_vs_E10	ordinary_muscle_development	ENSGALG00010005596	PARD3	-0.307507442	0.054204462	-5.673102002	1.40235E-08	2.99897E-08	negative
PRJNA729271	stage_3W_vs_E10	ordinary_muscle_development	ENSGALG00010005597	ENSGALG00010005597	2.428840042	0.467166353	5.199090269	2.00266E-07	3.96999E-07	positive
PRJNA729271	stage_3W_vs_E10	ordinary_muscle_development	ENSGALG00010005601	TMEM215	-4.157255913	0.491960117	-8.450392158	2.90326E-17	9.85083E-17	negative
PRJNA729271	stage_3W_vs_E10	ordinary_muscle_development	ENSGALG00010005609	GJB6	-1.231414837	0.542701964	-2.269044371	0.023265628	0.030403369	negative
PRJNA729271	stage_3W_vs_E10	ordinary_muscle_development	ENSGALG00010005610	MTHFD1L	-0.752550636	0.190746348	-3.945295117	7.97018E-05	0.000131103	negative
PRJNA729271	stage_3W_vs_E10	ordinary_muscle_development	ENSGALG00010005617	DTD2	1.033591068	0.208705182	4.95239773	7.33046E-07	1.39938E-06	positive
PRJNA729271	stage_3W_vs_E10	ordinary_muscle_development	ENSGALG00010005618	PHACTR2	-0.809099913	0.06875954	-11.7670932	5.76778E-32	3.57282E-31	negative
PRJNA729271	stage_3W_vs_E10	ordinary_muscle_development	ENSGALG00010005619	CELF2	0.615052238	0.076707203	8.018181008	1.07323E-15	3.35484E-15	positive
PRJNA729271	stage_3W_vs_E10	ordinary_muscle_development	ENSGALG00010005621	CDH2	-3.188615067	0.145516569	-21.91238489	1.9794E-106	9.3763E-105	negative
PRJNA729271	stage_3W_vs_E10	ordinary_muscle_development	ENSGALG00010005629	NRP1	0.599843467	0.17322471	3.462805427	0.000534575	0.000821094	positive
PRJNA729271	stage_3W_vs_E10	ordinary_muscle_development	ENSGALG00010005630	NIT2	-0.549940031	0.16593383	-3.314212844	0.000919015	0.001380428	negative
PRJNA729271	stage_3W_vs_E10	ordinary_muscle_development	ENSGALG00010005632	ITGB1	0.107895241	0.093458974	1.154466352	0.248309026	0.283449309	positive
PRJNA729271	stage_3W_vs_E10	ordinary_muscle_development	ENSGALG00010005634	HADH	1.056814127	0.086638184	12.19801803	3.18482E-34	2.13425E-33	positive
PRJNA729271	stage_3W_vs_E10	ordinary_muscle_development	ENSGALG00010005636	ENSGALG00010005636	3.394386865	0.587444995	5.778220759	7.54947E-09	1.64112E-08	positive

PRJNA729271	stage_3W_vs_E10	ordinary_muscle_development	ENSGALG00010005637	TOPORS	-0.114025766	0.071288538	-1.599496481	0.10971033	0.131839764	negative
PRJNA729271	stage_3W_vs_E10	ordinary_muscle_development	ENSGALG00010005643	ENSGALG00010005643	-2.326040232	1.184635514	-1.963507091	0.049587281	0.062263309	negative
PRJNA729271	stage_3W_vs_E10	ordinary_muscle_development	ENSGALG00010005645	FUCA2	0.323114426	0.15695896	2.058591784	0.03953336	0.050275141	positive
PRJNA729271	stage_3W_vs_E10	ordinary_muscle_development	ENSGALG00010005649	TOMM70	-0.661575956	0.079283812	-8.344401455	7.15755E-17	2.38283E-16	negative
PRJNA729271	stage_3W_vs_E10	ordinary_muscle_development	ENSGALG00010005651	USP6NL	0.035452734	0.121293288	0.292289335	0.770065412	0.795077865	positive
PRJNA729271	stage_3W_vs_E10	ordinary_muscle_development	ENSGALG00010005656	CYP2U1	1.501315969	0.130545118	11.50036088	1.31365E-30	7.70905E-30	positive
PRJNA729271	stage_3W_vs_E10	ordinary_muscle_development	ENSGALG00010005660	UBAC2	1.432970785	0.100357593	14.27864837	2.97312E-46	2.96596E-45	positive
PRJNA729271	stage_3W_vs_E10	ordinary_muscle_development	ENSGALG00010005668	LNP1	-1.493542857	0.37498974	-3.982889922	6.80823E-05	0.000112796	negative
PRJNA729271	stage_3W_vs_E10	ordinary_muscle_development	ENSGALG00010005670	SGMS2	0.288144638	0.20607767	1.398233195	0.162043052	0.190279815	positive
PRJNA729271	stage_3W_vs_E10	ordinary_muscle_development	ENSGALG00010005675	GJA3	-0.187854282	0.285482975	-0.658022714	0.510523536	0.54855735	negative
PRJNA729271	stage_3W_vs_E10	ordinary_muscle_development	ENSGALG00010005679	TMEM45A	-2.035116024	0.206780551	-9.841912161	7.42853E-23	3.1857E-22	negative
PRJNA729271	stage_3W_vs_E10	ordinary_muscle_development	ENSGALG00010005680	ENSGALG00010005680	3.260714655	0.438681285	7.432992388	1.06168E-13	3.00744E-13	positive
PRJNA729271	stage_3W_vs_E10	ordinary_muscle_development	ENSGALG00010005681	EPC1	0.240528405	0.066253172	3.630443618	0.000282935	0.00044534	positive
PRJNA729271	stage_3W_vs_E10	ordinary_muscle_development	ENSGALG00010005688	ENSGALG00010005688	2.238084317	0.499023207	4.484930328	7.29378E-06	1.30105E-05	positive
PRJNA729271	stage_3W_vs_E10	ordinary_muscle_development	ENSGALG00010005692	SEC24B	0.797725439	0.143783126	5.548115858	2.88765E-08	6.04512E-08	positive
PRJNA729271	stage_3W_vs_E10	ordinary_muscle_development	ENSGALG00010005694	ENSGALG00010005694	0.141631937	0.072552536	1.95212938	0.050922846	0.063802113	positive
PRJNA729271	stage_3W_vs_E10	ordinary_muscle_development	ENSGALG00010005695	KIF5B	-0.239437231	0.083690588	-2.860981583	0.004223316	0.005967147	negative
PRJNA729271	stage_3W_vs_E10	ordinary_muscle_development	ENSGALG00010005696	NUBPL	-1.092929206	0.141658111	-7.715260368	1.20736E-14	3.58546E-14	negative
PRJNA729271	stage_3W_vs_E10	ordinary_muscle_development	ENSGALG00010005699	PRPF18	0.661623347	0.092443583	7.157049995	8.24316E-13	2.23378E-12	positive
PRJNA729271	stage_3W_vs_E10	ordinary_muscle_development	ENSGALG00010005704	AKAP12	-1.532718264	0.093806983	-16.33906361	5.20461E-60	7.7614E-59	negative
PRJNA729271	stage_3W_vs_E10	ordinary_muscle_development	ENSGALG00010005705	ECHDC3	1.421674843	0.1228841	11.56923347	5.90075E-31	3.51547E-30	positive
PRJNA729271	stage_3W_vs_E10	ordinary_muscle_development	ENSGALG00010005712	ENSGALG00010005712	0.504995877	0.108424989	4.657559852	3.19979E-06	5.86804E-06	positive
PRJNA729271	stage_3W_vs_E10	ordinary_muscle_development	ENSGALG00010005714	ARHGAP5	-0.872325707	0.068353158	-12.76203958	2.67114E-37	1.95942E-36	negative
PRJNA729271	stage_3W_vs_E10	ordinary_muscle_development	ENSGALG00010005716	ZBTB2	-0.420747834	0.11223695	-3.748746151	0.000177721	0.000284334	negative
PRJNA729271	stage_3W_vs_E10	ordinary_muscle_development	ENSGALG00010005720	COL25A1	-4.78457427	0.240529012	-19.89188014	4.78471E-88	1.49794E-86	negative
PRJNA729271	stage_3W_vs_E10	ordinary_muscle_development	ENSGALG00010005724	PROSER2	0.507778692	0.118562761	4.282783952	1.84569E-05	3.19748E-05	positive
PRJNA729271	stage_3W_vs_E10	ordinary_muscle_development	ENSGALG00010005738	ELAVL2	-4.248227259	0.500719949	-8.484238073	2.17137E-17	7.40738E-17	negative
PRJNA729271	stage_3W_vs_E10	ordinary_muscle_development	ENSGALG00010005740	ENSGALG00010005740	2.507991351	0.319461031	7.850695739	4.13736E-15	1.25963E-14	positive
PRJNA729271	stage_3W_vs_E10	ordinary_muscle_development	ENSGALG00010005742	GPR183	-1.119071493	0.210894328	-5.306313854	1.11864E-07	2.25769E-07	negative
PRJNA729271	stage_3W_vs_E10	ordinary_muscle_development	ENSGALG00010005743	ETNPPL	5.206102801	0.223348879	23.30928559	3.5692E-120	2.129E-118	positive
PRJNA729271	stage_3W_vs_E10	ordinary_muscle_development	ENSGALG00010005748	AIG1	0.708485508	0.178904338	3.960136	7.49071E-05	0.000123623	positive
PRJNA729271	stage_3W_vs_E10	ordinary_muscle_development	ENSGALG00010005752	RMND1	0.506284722	0.09793423	5.169640118	2.34545E-07	4.62675E-07	positive
PRJNA729271	stage_3W_vs_E10	ordinary_muscle_development	ENSGALG00010005755	ZMYM2	0.773299519	0.097021003	7.970434168	1.58118E-15	4.9025E-15	positive
PRJNA729271	stage_3W_vs_E10	ordinary_muscle_development	ENSGALG00010005756	TET2	0.198659899	0.164212265	1.209775034	0.226365229	0.259853013	positive
PRJNA729271	stage_3W_vs_E10	ordinary_muscle_development	ENSGALG00010005757	ARHGAP12	0.750022832	0.133178034	5.631730771	1.7841E-08	3.79242E-08	positive
PRJNA729271	stage_3W_vs_E10	ordinary_muscle_development	ENSGALG00010005758	HIVEP2	1.411264944	0.105095654	13.42838535	4.12303E-41	3.43665E-40	positive
PRJNA729271	stage_3W_vs_E10	ordinary_muscle_development	ENSGALG00010005760	GPR18	-1.924400921	0.346039229	-5.561221848	2.67892E-08	5.62252E-08	negative
PRJNA729271	stage_3W_vs_E10	ordinary_muscle_development	ENSGALG00010005763	AKAP6	2.083205953	0.142520812	14.61685435	2.19301E-48	2.37932E-47	positive
PRJNA729271	stage_3W_vs_E10	ordinary_muscle_development	ENSGALG00010005768	CXXC4	-3.970406232	0.715028362	-5.552795444	2.81137E-08	5.8931E-08	negative
PRJNA729271	stage_3W_vs_E10	ordinary_muscle_development	ENSGALG00010005769	TFG	-0.532236493	0.105913162	-5.025215805	5.02867E-07	9.70321E-07	negative
PRJNA729271	stage_3W_vs_E10	ordinary_muscle_development	ENSGALG00010005772	SVEP1	2.077476301	0.14073242	14.76188861	2.57955E-49	2.84772E-48	positive
PRJNA729271	stage_3W_vs_E10	ordinary_muscle_development	ENSGALG00010005777	ENSGALG00010005777	1.456053615	0.935512514	1.556423452	0.119607432	0.143080773	positive
PRJNA729271	stage_3W_vs_E10	ordinary_muscle_development	ENSGALG00010005779	ARMT1	0.523706524	0.111023754	4.717067338	2.39269E-06	4.4209E-06	positive
PRJNA729271	stage_3W_vs_E10	ordinary_muscle_development	ENSGALG00010005784	RASL11A	1.352623992	0.429889424	3.146446312	0.001652676	0.002430061	positive
PRJNA729271	stage_3W_vs_E10	ordinary_muscle_development	ENSGALG00010005785	RPL21	0.489934188	0.139803165	3.50445705	0.000457539	0.000707115	positive
PRJNA729271	stage_3W_vs_E10	ordinary_muscle_development	ENSGALG00010005789	RNASET2	0.83357809	0.154604132	5.391693478	6.97968E-08	1.42735E-07	positive
PRJNA729271	stage_3W_vs_E10	ordinary_muscle_development	ENSGALG00010005790	MUSK	-0.436649963	0.154961953	-2.817788193	0.00483557	0.006791162	negative
PRJNA729271	stage_3W_vs_E10	ordinary_muscle_development	ENSGALG00010005792	ADGRG6	-0.876719547	0.160134916	-5.474880609	4.37807E-08	9.06055E-08	negative
PRJNA729271	stage_3W_vs_E10	ordinary_muscle_development	ENSGALG00010005799	UFSP2	-0.200514119	0.097633354	-2.053746083	0.040000274	0.050824331	negative
PRJNA729271	stage_3W_vs_E10	ordinary_muscle_development	ENSGALG00010005800	FRMD4A	-1.167520016	0.109930188	-10.6205587	2.39126E-26	1.18662E-25	negative
PRJNA729271	stage_3W_vs_E10	ordinary_muscle_development	ENSGALG00010005814	UPF2	-0.374100741	0.124350324	-3.008442032	0.002625909	0.003783178	negative
PRJNA729271	stage_3W_vs_E10	ordinary_muscle_development	ENSGALG00010005817	VTA1	0.177891538	0.069582607	2.556551788	0.010571533	0.014329005	positive
PRJNA729271	stage_3W_vs_E10	ordinary_muscle_development	ENSGALG00010005821	DOCK9	1.258107793	0.153910855	8.174262888	2.9768E-16	9.60355E-16	positive
PRJNA729271	stage_3W_vs_E10	ordinary_muscle_development	ENSGALG00010005824	NMBR	-5.915919677	1.332630883	-4.439278536	9.02609E-06	1.59865E-05	negative
PRJNA729271	stage_3W_vs_E10	ordinary_muscle_development	ENSGALG00010005826	LRP2BP	-0.139005214	0.206562355	-0.672945531	0.500981944	0.539064159	negative
PRJNA729271	stage_3W_vs_E10	ordinary_muscle_development	ENSGALG00010005828	LPAR1	-2.032034527	0.155873216	-13.03645732	7.59027E-39	5.88978E-38	negative
PRJNA729271	stage_3W_vs_E10	ordinary_muscle_development	ENSGALG00010005829	NPAS3	-0.172445498	0.135543946	-1.272247887	0.203285058	0.235146191	negative
PRJNA729271	stage_3W_vs_E10	ordinary_muscle_development	ENSGALG00010005830	USP12	0.072248395	0.183775666	0.39313363	0.694220787	0.724145819	positive
PRJNA729271	stage_3W_vs_E10	ordinary_muscle_development	ENSGALG00010005831	SEC61A2	0.655264188	0.104855905	6.249187292	4.12594E-10	9.63362E-10	positive
PRJNA729271	stage_3W_vs_E10	ordinary_muscle_development	ENSGALG00010005836	NEBL	0.096886956	0.245925378	0.393968923	0.693603984	0.723652752	positive
PRJNA729271	stage_3W_vs_E10	ordinary_muscle_development	ENSGALG00010005839	RPS6KA2	-0.698431053	0.187468324	-3.725595006	0.000194855	0.000310786	negative
PRJNA729271	stage_3W_vs_E10	ordinary_muscle_development	ENSGALG00010005841	ENSGALG00010005841	2.162074696	0.305856709	7.068913756	1.56151E-12	4.16911E-12	positive
PRJNA729271	stage_3W_vs_E10	ordinary_muscle_development	ENSGALG00010005843	GPR12	-0.662358933	0.439701391	-1.506383527	0.131968747	0.156999917	negative
PRJNA729271	stage_3W_vs_E10	ordinary_muscle_development	ENSGALG00010005848	WASF3	1.307876958	0.186868865	6.998902443	2.57975E-12	6.79512E-12	positive
PRJNA729271	stage_3W_vs_E10	ordinary_muscle_development	ENSGALG00010005849	SNX25	0.107410906	0.097649456	1.099964198	0.271347721	0.307953744	positive
PRJNA729271	stage_3W_vs_E10	ordinary_muscle_development	ENSGALG00010005851	SLC15A1	0.537433181	0.896294998	0.599616402	0.548761914	0.585994972	positive
PRJNA729271	stage_3W_vs_E10	ordinary_muscle_development	ENSGALG00010005853	COMMD3	-1.013950427	0.142948709	-7.093106593	1.31134E-12	3.51478E-12	negative
PRJNA729271	stage_3W_vs_E10	ordinary_muscle_development	ENSGALG00010005858	CFAP97	-1.001790655	0.11336132	-8.837147058	9.81939E-19	3.56454E-18	negative
PRJNA729271	stage_3W_vs_E10	ordinary_muscle_development	ENSGALG00010005859	ENSGALG00010005859	1.81045904	0.517645554	3.497487859	0.000469662	0.000725309	positive
PRJNA729271	stage_3W_vs_E10	ordinary_muscle_development	ENSGALG00010005860	ABI3BP	0.594036762	0.212375814	2.797101755	0.005156329	0.007222049	positive
PRJNA729271	stage_3W_vs_E10	ordinary_muscle_development	ENSGALG00010005861	MPC1L	1.308121726	0.131786435	9.926072647	3.20636E-23	1.39276E-22	positive
PRJNA729271	stage_3W_vs_E10	ordinary_muscle_development	ENSGALG00010005866	DNAJC1	1.486322093	0.14749476	10.07711794	6.97418E-24	3.11336E-23	positive
PRJNA729271	stage_3W_vs_E10	ordinary_muscle_development	ENSGALG00010005871	EGLN3	1.223722265	0.196092031	6.240550705	4.36033E-10	1.01694E-09	positive
PRJNA729271	stage_3W_vs_E10	ordinary_muscle_development	ENSGALG00010005872	STK24	0.437334427	0.135044866	3.238438013	0.001201861	0.001787501	positive
PRJNA729271	stage_3W_vs_E10	ordinary_muscle_development	ENSGALG00010005875	CDK8	-0.917503018	0.152460158	-6.017985494	1.76601E-09	3.96443E-09	negative
PRJNA729271	stage_3W_vs_E10	ordinary_muscle_development	ENSGALG00010005879	FAM107B	-2.208279224	0.124423931	-17.7480265	1.78518E-70	3.48735E-69	negative
PRJNA729271	stage_3W_vs_E10	ordinary_muscle_development	ENSGALG00010005880	SLC25A4	5.803118921	0.179714799	32.29071255	9.4442E-229	2.1061E-226	positive
PRJNA729271	stage_3W_vs_E10	ordinary_muscle_development	ENSGALG00010005881	CORO2A	0.262553681	0.268691805	0.977155524	0.328492163	0.366691868	positive
PRJNA729271	stage_3W_vs_E10	ordinary_muscle_development	ENSGALG00010005884	MYB	-2.739948822	0.303454951	-9.029178177	1.72966E-19	6.48342E-19	negative
PRJNA729271	stage_3W_vs_E10	ordinary_muscle_development	ENSGALG00010005885	SLITRK5	0.902011564	0.367038781	2.457537491	0.013989321	0.018711378	positive
PRJNA729271	stage_3W_vs_E10	ordinary_muscle_development	ENSGALG00010005887	CCNH	0.302952678	0.083765862	3.616660444	0.000298428	0.00046876	positive
PRJNA729271	stage_3W_vs_E10	ordinary_muscle_development	ENSGALG00010005888	IMPG2	0.319758482	0.227390253	1.406210152	0.15966173	0.187664126	positive
PRJNA729271	stage_3W_vs_E10	ordinary_muscle_development	ENSGALG00010005890	SNX32	-0.660609656	0.089425093	-7.387296259	1.49844E-13	4.20929E-13	negative
PRJNA729271	stage_3W_vs_E10	ordinary_muscle_development	ENSGALG00010005891	RNF6	0.106418854	0.117357958	0.906788565	0.364518618	0.403366468	positive
PRJNA729271	stage_3W_vs_E10	ordinary_muscle_development	ENSGALG00010005892	ENSGALG00010005892	0.713300439	0.860191772	0.829234203	0.406971895	0.445817534	positive
PRJNA729271	stage_3W_vs_E10	ordinary_muscle_development	ENSGALG00010005893	SFT2D1	0.230196182	0.066637765	3.454440331	0.000551436	0.000845201	positive
PRJNA729271	stage_3W_vs_E10	ordinary_muscle_development	ENSGALG00010005895	MLLT10	0.68833036	0.098937639	6.95721431	3.47067E-12	9.07419E-12	positive
PRJNA729271	stage_3W_vs_E10	ordinary_muscle_development	ENSGALG00010005899	ENSGALG00010005899	-0.610811074	0.088491333	-6.902496057	5.10966E-12	1.32305E-11	negative
PRJNA729271	stage_3W_vs_E10	ordinary_muscle_development	ENSGALG00010005900	SPTSSA	-0.053100067	0.086965583	-0.610587139	0.541472939	0.579107965	negative
PRJNA729271	stage_3W_vs_E10	ordinary_muscle_development	ENSGALG00010005901	ENSGALG00010005901	0.492638959	0.247140177	1.993358442	0.046222211	0.05830064	positive
PRJNA729271	stage_3W_vs_E10	ordinary_muscle_development	ENSGALG00010005902	EAPP	-0.61328018	0.102143688	-6.004092772	1.92405E-09	4.30387E-09	negative
PRJNA729271	stage_3W_vs_E10	ordinary_muscle_development	ENSGALG00010005903	PRR18	-2.063696643	0.286007531	-7.215532519	5.37233E-13	1.46679E-12	negative
PRJNA729271	stage_3W_vs_E10	ordinary_muscle_development	ENSGALG00010005905	CITED2	0.008204633	0.201787699	0.040659729	0.967567166	0.971992936	positive
PRJNA729271	stage_3W_vs_E10	ordinary_muscle_development	ENSGALG00010005909	BRMS1L	-0.856520301	0.102776747	-8.333794649	7.82917E-17	2.60224E-16	negative
PRJNA729271	stage_3W_vs_E10	ordinary_muscle_development	ENSGALG00010005910	ENSGALG00010005910	3.056222261	0.418426678	7.304080789	2.79169E-13	7.73127E-13	positive
PRJNA729271	stage_3W_vs_E10	ordinary_muscle_development	ENSGALG00010005911	TBXT	-3.143587073	0.562922711	-5.584402643	2.34505E-08	4.94206E-08	negative
PRJNA729271	stage_3W_vs_E10	ordinary_muscle_development	ENSGALG00010005913	NFKBIA	2.360614429	0.142510329	16.56451459	1.25791E-61	1.96269E-60	positive
PRJNA729271	stage_3W_vs_E10	ordinary_muscle_development	ENSGALG00010005915	PAPSS1	-2.383253798	0.101189301	-23.55242868	1.1854E-122	7.4065E-121	negative
PRJNA729271	stage_3W_vs_E10	ordinary_muscle_development	ENSGALG00010005926	NUDT5	-0.116838718	0.233814123	-0.4997077	0.61728091	0.651911606	negative
PRJNA729271	stage_3W_vs_E10	ordinary_muscle_development	ENSGALG00010005934	SLITRK6	-6.183502498	0.336071178	-18.39938355	1.32868E-75	2.98592E-74	negative
PRJNA729271	stage_3W_vs_E10	ordinary_muscle_development	ENSGALG00010005935	ATP8A2	-3.453162728	0.2478503	-13.9324533	4.02296E-44	3.71656E-43	negative
PRJNA729271	stage_3W_vs_E10	ordinary_muscle_development	ENSGALG00010005941	TXLNB	4.587574043	0.181426234	25.28616695	4.5348E-141	3.6928E-139	positive
PRJNA729271	stage_3W_vs_E10	ordinary_muscle_development	ENSGALG00010005943	PROX2	-1.906304277	0.830716317	-2.294771678	0.021746213	0.028507855	negative
PRJNA729271	stage_3W_vs_E10	ordinary_muscle_development	ENSGALG00010005949	SLITRK1	0.286377091	0.359694662	0.796167197	0.425934861	0.464379527	positive
PRJNA729271	stage_3W_vs_E10	ordinary_muscle_development	ENSGALG00010005956	TXNL4B	0.330855933	0.11126251	2.973651527	0.002942791	0.004222385	positive
PRJNA729271	stage_3W_vs_E10	ordinary_muscle_development	ENSGALG00010005963	UGCG	1.178585299	0.103236985	11.41630883	3.46646E-30	2.00504E-29	positive
PRJNA729271	stage_3W_vs_E10	ordinary_muscle_development	ENSGALG00010005964	HSPA14	-0.563694838	0.114526608	-4.921955251	8.56838E-07	1.63033E-06	negative
PRJNA729271	stage_3W_vs_E10	ordinary_muscle_development	ENSGALG00010005966	PLXDC2	1.903047183	0.152927564	12.44410844	1.50556E-35	1.05375E-34	positive
PRJNA729271	stage_3W_vs_E10	ordinary_muscle_development	ENSGALG00010005967	GJD2	-2.777779328	0.282834759	-9.821209177	9.12425E-23	3.89791E-22	negative
PRJNA729271	stage_3W_vs_E10	ordinary_muscle_development	ENSGALG00010005969	HECA	0.536431869	0.065977159	8.130569381	4.27279E-16	1.36509E-15	positive
PRJNA729271	stage_3W_vs_E10	ordinary_muscle_development	ENSGALG00010005970	SHISA2	-3.994492899	0.124709488	-32.03038493	4.1182E-225	9.0445E-223	negative
PRJNA729271	stage_3W_vs_E10	ordinary_muscle_development	ENSGALG00010005975	ABRACL	-0.452781252	0.105664336	-4.285090596	1.82665E-05	3.16676E-05	negative
PRJNA729271	stage_3W_vs_E10	ordinary_muscle_development	ENSGALG00010005978	VPS16	0.164705182	0.114544828	1.43791025	0.150459521	0.177425216	positive
PRJNA729271	stage_3W_vs_E10	ordinary_muscle_development	ENSGALG00010005979	SUV39H2	0.213424821	0.092064797	2.318202279	0.02043833	0.026863765	positive
PRJNA729271	stage_3W_vs_E10	ordinary_muscle_development	ENSGALG00010005981	AREL1	-0.027321822	0.099894521	-0.273506711	0.784463735	0.808159334	negative
PRJNA729271	stage_3W_vs_E10	ordinary_muscle_development	ENSGALG00010005984	IDH3B	1.703418652	0.117558163	14.49000742	1.40128E-47	1.46442E-46	positive
PRJNA729271	stage_3W_vs_E10	ordinary_muscle_development	ENSGALG00010005985	ENSGALG00010005985	-2.877285578	0.321291792	-8.955365964	3.38611E-19	1.254E-18	negative
PRJNA729271	stage_3W_vs_E10	ordinary_muscle_development	ENSGALG00010005988	NUP58	-0.361723542	0.089610987	-4.036598138	5.42318E-05	9.05947E-05	negative
PRJNA729271	stage_3W_vs_E10	ordinary_muscle_development	ENSGALG00010005989	FCF1	-0.18892178	0.100140781	-1.886561885	0.059219277	0.073668334	negative
PRJNA729271	stage_3W_vs_E10	ordinary_muscle_development	ENSGALG00010005990	SLC2A12	2.246329936	0.150426382	14.9330849	2.00759E-50	2.31688E-49	positive
PRJNA729271	stage_3W_vs_E10	ordinary_muscle_development	ENSGALG00010005991	FARP1	-1.192999063	0.090780812	-13.14153323	1.90331E-39	1.51252E-38	negative
PRJNA729271	stage_3W_vs_E10	ordinary_muscle_development	ENSGALG00010005997	ENSGALG00010005997	0.809682889	0.234216322	3.456987461	0.00054625	0.000837784	positive
PRJNA729271	stage_3W_vs_E10	ordinary_muscle_development	ENSGALG00010005998	YLPM1	0.171667053	0.133144096	1.289332819	0.197282406	0.228531006	positive
PRJNA729271	stage_3W_vs_E10	ordinary_muscle_development	ENSGALG00010005999	PDE10A	1.026836947	0.292956911	3.505078418	0.000456473	0.000705542	positive
PRJNA729271	stage_3W_vs_E10	ordinary_muscle_development	ENSGALG00010006001	NOP56	-1.125498075	0.140780083	-7.994725196	1.29863E-15	4.04056E-15	negative
PRJNA729271	stage_3W_vs_E10	ordinary_muscle_development	ENSGALG00010006004	DCLRE1C	-0.959341695	0.148570115	-6.457164643	1.06683E-10	2.56914E-10	negative
PRJNA729271	stage_3W_vs_E10	ordinary_muscle_development	ENSGALG00010006009	MALRD1	1.455910846	0.602669387	2.415770367	0.01570196	0.020886475	positive
PRJNA729271	stage_3W_vs_E10	ordinary_muscle_development	ENSGALG00010006010	IPO5	0.064678771	0.080722302	0.801250324	0.422986743	0.461408251	positive
PRJNA729271	stage_3W_vs_E10	ordinary_muscle_development	ENSGALG00010006013	TRMT10C	0.101817036	0.10552632	0.96484968	0.334620109	0.372871961	positive
PRJNA729271	stage_3W_vs_E10	ordinary_muscle_development	ENSGALG00010006015	CDC123	-0.56588356	0.112179921	-5.044428229	4.5488E-07	8.81245E-07	negative
PRJNA729271	stage_3W_vs_E10	ordinary_muscle_development	ENSGALG00010006016	MTMR6	0.412856799	0.07630785	5.410410571	6.28804E-08	1.28736E-07	positive

PRJNA729271	stage_3W_vs_E10	ordinary_muscle_development	ENSGALG00010006019	SLC10A2	-2.932459656	1.094543029	-2.67916343	0.007380636	0.010175225	negative
PRJNA729271	stage_3W_vs_E10	ordinary_muscle_development	ENSGALG00010006021	ENSGALG00010006021	0.346993743	0.125570072	2.763347485	0.005721182	0.007970832	positive
PRJNA729271	stage_3W_vs_E10	ordinary_muscle_development	ENSGALG00010006022	AFG2A	-0.88653318	0.092039101	-9.632136467	5.85003E-22	2.42067E-21	negative
PRJNA729271	stage_3W_vs_E10	ordinary_muscle_development	ENSGALG00010006024	AMER2	-1.532333565	0.364706218	-4.201555908	2.65087E-05	4.5269E-05	negative
PRJNA729271	stage_3W_vs_E10	ordinary_muscle_development	ENSGALG00010006025	TNFAIP3	1.210546057	0.148512246	8.151153143	3.6047E-16	1.15492E-15	positive
PRJNA729271	stage_3W_vs_E10	ordinary_muscle_development	ENSGALG00010006028	ARL5B	-0.1074155	0.128052217	-0.838841391	0.40155832	0.440619822	negative
PRJNA729271	stage_3W_vs_E10	ordinary_muscle_development	ENSGALG00010006029	HSDL2	-0.413811632	0.135242195	-3.059781976	0.002214982	0.003211579	negative
PRJNA729271	stage_3W_vs_E10	ordinary_muscle_development	ENSGALG00010006031	ERCC5	0.667853148	0.096388708	6.928748862	4.24579E-12	1.10311E-11	positive
PRJNA729271	stage_3W_vs_E10	ordinary_muscle_development	ENSGALG00010006033	NSUN6	-0.488310397	0.175351002	-2.784759667	0.005356741	0.007489001	negative
PRJNA729271	stage_3W_vs_E10	ordinary_muscle_development	ENSGALG00010006035	ADH1C	1.172140314	0.695554944	1.685187238	0.091952453	0.111769459	positive
PRJNA729271	stage_3W_vs_E10	ordinary_muscle_development	ENSGALG00010006037	METTL21EP	6.936042838	0.675853636	10.26264042	1.03828E-24	4.79449E-24	positive
PRJNA729271	stage_3W_vs_E10	ordinary_muscle_development	ENSGALG00010006038	ENSGALG00010006038	0.748492755	0.193058826	3.877019091	0.000105744	0.000172395	positive
PRJNA729271	stage_3W_vs_E10	ordinary_muscle_development	ENSGALG00010006039	PCNP	-1.125098349	0.126384774	-8.902166852	5.47669E-19	2.01228E-18	negative
PRJNA729271	stage_3W_vs_E10	ordinary_muscle_development	ENSGALG00010006041	QKI	-0.136131806	0.085940761	-1.5840191	0.113189373	0.135840367	negative
PRJNA729271	stage_3W_vs_E10	ordinary_muscle_development	ENSGALG00010006047	ZBTB11	0.161571771	0.134948762	1.197282357	0.231196519	0.264979326	positive
PRJNA729271	stage_3W_vs_E10	ordinary_muscle_development	ENSGALG00010006049	AIMP1	-0.244172801	0.124362558	-1.963394811	0.049600316	0.062274282	negative
PRJNA729271	stage_3W_vs_E10	ordinary_muscle_development	ENSGALG00010006052	RALGAPA1	-0.445298476	0.081704788	-5.450090381	5.03442E-08	1.03818E-07	negative
PRJNA729271	stage_3W_vs_E10	ordinary_muscle_development	ENSGALG00010006053	TMEM243	-0.741745199	0.238386013	-3.111529869	0.001861206	0.002716563	negative
PRJNA729271	stage_3W_vs_E10	ordinary_muscle_development	ENSGALG00010006054	ENSGALG00010006054	4.559795454	2.165982731	2.105185507	0.035275163	0.045141122	positive
PRJNA729271	stage_3W_vs_E10	ordinary_muscle_development	ENSGALG00010006055	ENSGALG00010006055	1.977989828	0.577116919	3.427364134	0.000609471	0.000930905	positive
PRJNA729271	stage_3W_vs_E10	ordinary_muscle_development	ENSGALG00010006059	BIVM	-0.236859215	0.055575637	-4.261925351	2.02673E-05	3.49982E-05	negative
PRJNA729271	stage_3W_vs_E10	ordinary_muscle_development	ENSGALG00010006060	ENSGALG00010006060	0.982420765	0.352980594	2.783214661	0.005382318	0.007521132	positive
PRJNA729271	stage_3W_vs_E10	ordinary_muscle_development	ENSGALG00010006061	RPL24	0.282501383	0.159031577	1.776385475	0.075669403	0.092896418	positive
PRJNA729271	stage_3W_vs_E10	ordinary_muscle_development	ENSGALG00010006062	ENSGALG00010006062	1.725177159	0.644712791	2.6758848	0.007453226	0.010272369	positive
PRJNA729271	stage_3W_vs_E10	ordinary_muscle_development	ENSGALG00010006063	POGLUT2	-1.350399619	0.102388819	-13.18893633	1.01609E-39	8.15063E-39	negative
PRJNA729271	stage_3W_vs_E10	ordinary_muscle_development	ENSGALG00010006064	DMTF1	0.018144438	0.081902455	0.221537164	0.824674203	0.845617754	positive
PRJNA729271	stage_3W_vs_E10	ordinary_muscle_development	ENSGALG00010006065	WDR54	0.495542938	0.158168607	3.133004386	0.001730269	0.002537459	positive
PRJNA729271	stage_3W_vs_E10	ordinary_muscle_development	ENSGALG00010006066	ARHGAP18	-0.04873052	0.120756282	-0.403544392	0.686547785	0.717381065	negative
PRJNA729271	stage_3W_vs_E10	ordinary_muscle_development	ENSGALG00010006068	PACRG	-0.233424108	0.279574675	-0.834925795	0.40375949	0.442900999	negative
PRJNA729271	stage_3W_vs_E10	ordinary_muscle_development	ENSGALG00010006069	TTR	3.118305034	1.399255154	2.228546399	0.025844101	0.033566293	positive
PRJNA729271	stage_3W_vs_E10	ordinary_muscle_development	ENSGALG00010006070	TMEM242	1.003174836	0.113447592	8.842627833	9.34932E-19	3.3973E-18	positive
PRJNA729271	stage_3W_vs_E10	ordinary_muscle_development	ENSGALG00010006071	TEX30	0.116492876	0.239133377	0.487146033	0.626154847	0.660369216	positive
PRJNA729271	stage_3W_vs_E10	ordinary_muscle_development	ENSGALG00010006072	ENSGALG00010006072	0.693554065	0.233016684	2.97641376	0.002916411	0.004185068	positive
PRJNA729271	stage_3W_vs_E10	ordinary_muscle_development	ENSGALG00010006073	CEP97	0.049843615	0.10451979	0.476882083	0.633446091	0.666991217	positive
PRJNA729271	stage_3W_vs_E10	ordinary_muscle_development	ENSGALG00010006075	SOX21	-4.497288318	0.417026308	-10.78418371	4.08857E-27	2.09709E-26	negative
PRJNA729271	stage_3W_vs_E10	ordinary_muscle_development	ENSGALG00010006076	PODXL	2.302974902	0.16368778	14.06931475	5.86348E-45	5.60258E-44	positive
PRJNA729271	stage_3W_vs_E10	ordinary_muscle_development	ENSGALG00010006077	TBCK	-0.886582922	0.101541398	-8.731245956	2.51882E-18	9.01712E-18	negative
PRJNA729271	stage_3W_vs_E10	ordinary_muscle_development	ENSGALG00010006078	SPATA13	-2.142427527	0.169044614	-12.67374022	8.26763E-37	5.98E-36	negative
PRJNA729271	stage_3W_vs_E10	ordinary_muscle_development	ENSGALG00010006079	ENSGALG00010006079	-0.830314803	0.827693035	-1.003167561	0.315780022	0.353684507	negative
PRJNA729271	stage_3W_vs_E10	ordinary_muscle_development	ENSGALG00010006080	ENSGALG00010006080	-0.834388514	0.208286205	-4.005971075	6.17632E-05	0.000102717	negative
PRJNA729271	stage_3W_vs_E10	ordinary_muscle_development	ENSGALG00010006081	ZDHHC14	0.309393877	0.078617977	3.935408755	8.30552E-05	0.000136433	positive
PRJNA729271	stage_3W_vs_E10	ordinary_muscle_development	ENSGALG00010006083	CACNB2	-1.433397768	0.180280754	-7.950919544	1.85132E-15	5.72051E-15	negative
PRJNA729271	stage_3W_vs_E10	ordinary_muscle_development	ENSGALG00010006084	TMEM68	-1.560711102	0.153445558	-10.17110641	2.6686E-24	1.21334E-23	negative
PRJNA729271	stage_3W_vs_E10	ordinary_muscle_development	ENSGALG00010006085	DLST	0.623629672	0.090004285	6.928888722	4.24159E-12	1.10228E-11	positive
PRJNA729271	stage_3W_vs_E10	ordinary_muscle_development	ENSGALG00010006086	ENSGALG00010006086	0.382736855	0.291390099	1.313486133	0.189019191	0.219537914	positive
PRJNA729271	stage_3W_vs_E10	ordinary_muscle_development	ENSGALG00010006087	ENSGALG00010006087	-0.405353917	0.589173862	-0.688003904	0.491450323	0.529830601	negative
PRJNA729271	stage_3W_vs_E10	ordinary_muscle_development	ENSGALG00010006091	RTKN	-1.304606955	0.10347021	-12.60852717	1.89501E-36	1.35847E-35	negative
PRJNA729271	stage_3W_vs_E10	ordinary_muscle_development	ENSGALG00010006096	EDIL3	-0.904168302	0.237064661	-3.814015539	0.000136727	0.000221041	negative
PRJNA729271	stage_3W_vs_E10	ordinary_muscle_development	ENSGALG00010006100	RPS6KL1	-1.841082076	0.209226425	-8.799472021	1.37461E-18	4.96265E-18	negative
PRJNA729271	stage_3W_vs_E10	ordinary_muscle_development	ENSGALG00010006101	ENSGALG00010006101	1.057385934	0.113669375	9.302293894	1.37448E-20	5.37734E-20	positive
PRJNA729271	stage_3W_vs_E10	ordinary_muscle_development	ENSGALG00010006103	NXPE3	-0.389330451	0.119295123	-3.263590686	0.0011001	0.001643065	negative
PRJNA729271	stage_3W_vs_E10	ordinary_muscle_development	ENSGALG00010006106	SHOC1	1.356750961	0.375507206	3.613115645	0.00030254	0.000474961	positive
PRJNA729271	stage_3W_vs_E10	ordinary_muscle_development	ENSGALG00010006107	PGF	3.14663535	0.189681545	16.58904322	8.36422E-62	1.3107E-60	positive
PRJNA729271	stage_3W_vs_E10	ordinary_muscle_development	ENSGALG00010006108	MKLN1	0.677313372	0.060867956	11.12758527	9.20966E-29	5.04513E-28	positive
PRJNA729271	stage_3W_vs_E10	ordinary_muscle_development	ENSGALG00010006111	WBP1	0.047050187	0.084558109	0.556424303	0.577920829	0.613966756	positive
PRJNA729271	stage_3W_vs_E10	ordinary_muscle_development	ENSGALG00010006113	EIF2B2	0.397922652	0.168044529	2.367959575	0.01788649	0.023653378	positive
PRJNA729271	stage_3W_vs_E10	ordinary_muscle_development	ENSGALG00010006118	NPNT	-4.393049484	0.21429481	-20.50002745	2.15172E-93	7.73925E-92	negative
PRJNA729271	stage_3W_vs_E10	ordinary_muscle_development	ENSGALG00010006120	MOGS	0.618930606	0.134344696	4.607034188	4.08453E-06	7.42758E-06	positive
PRJNA729271	stage_3W_vs_E10	ordinary_muscle_development	ENSGALG00010006121	ENSGALG00010006121	-0.321056809	0.324448649	-0.989545833	0.322396157	0.360442136	negative
PRJNA729271	stage_3W_vs_E10	ordinary_muscle_development	ENSGALG00010006123	MLH3	0.362979202	0.102906381	3.527275942	0.000419859	0.000650754	positive
PRJNA729271	stage_3W_vs_E10	ordinary_muscle_development	ENSGALG00010006126	ENSGALG00010006126	6.456363792	0.281060575	22.97143169	8.9997E-117	5.0367E-115	positive
PRJNA729271	stage_3W_vs_E10	ordinary_muscle_development	ENSGALG00010006128	ACYP1	1.811406811	0.11747692	15.41925695	1.21494E-53	1.55296E-52	positive
PRJNA729271	stage_3W_vs_E10	ordinary_muscle_development	ENSGALG00010006131	ENSGALG00010006131	0.817552017	0.126465074	6.464646663	1.01536E-10	2.44763E-10	positive
PRJNA729271	stage_3W_vs_E10	ordinary_muscle_development	ENSGALG00010006132	MIPEP	-0.862533583	0.134556547	-6.410194095	1.45334E-10	3.47341E-10	negative
PRJNA729271	stage_3W_vs_E10	ordinary_muscle_development	ENSGALG00010006134	K123	4.675016642	0.706156066	6.620373123	3.58293E-11	8.88987E-11	positive
PRJNA729271	stage_3W_vs_E10	ordinary_muscle_development	ENSGALG00010006135	ZC2HC1C	-0.470996258	0.149658037	-3.147149788	0.001648705	0.002424713	negative
PRJNA729271	stage_3W_vs_E10	ordinary_muscle_development	ENSGALG00010006139	GAREM1	0.04571467	0.130564322	0.350131411	0.726240079	0.754342525	positive
PRJNA729271	stage_3W_vs_E10	ordinary_muscle_development	ENSGALG00010006146	CCDC142	0.992506496	0.17991355	5.516574468	3.45671E-08	7.1959E-08	positive
PRJNA729271	stage_3W_vs_E10	ordinary_muscle_development	ENSGALG00010006147	ENSGALG00010006147	3.620253081	0.215300657	16.81487242	1.89901E-63	3.11734E-62	positive
PRJNA729271	stage_3W_vs_E10	ordinary_muscle_development	ENSGALG00010006151	ENSGALG00010006151	-0.878274636	0.16452909	-5.338111542	9.39197E-08	1.90534E-07	negative
PRJNA729271	stage_3W_vs_E10	ordinary_muscle_development	ENSGALG00010006155	TNFRSF19	-2.477227622	0.196596816	-12.60054802	2.09683E-36	1.50165E-35	negative
PRJNA729271	stage_3W_vs_E10	ordinary_muscle_development	ENSGALG00010006156	NFKBIZ	1.974596494	0.195181576	10.11671561	4.65784E-24	2.0935E-23	positive
PRJNA729271	stage_3W_vs_E10	ordinary_muscle_development	ENSGALG00010006157	SNX9	0.841640962	0.073268187	11.48712696	1.5312E-30	8.98209E-30	positive
PRJNA729271	stage_3W_vs_E10	ordinary_muscle_development	ENSGALG00010006163	ENSGALG00010006163	2.351419918	0.481826649	4.880219731	1.05968E-06	2.00287E-06	positive
PRJNA729271	stage_3W_vs_E10	ordinary_muscle_development	ENSGALG00010006172	ENSGALG00010006172	1.295266592	0.190445231	6.801255079	1.03712E-11	2.64945E-11	positive
PRJNA729271	stage_3W_vs_E10	ordinary_muscle_development	ENSGALG00010006173	TBPL1	0.176104543	0.081399294	2.163465226	0.030505416	0.039297547	positive
PRJNA729271	stage_3W_vs_E10	ordinary_muscle_development	ENSGALG00010006175	IL2RA	7.112469235	0.491919809	14.45859488	2.21269E-47	2.2942E-46	positive
PRJNA729271	stage_3W_vs_E10	ordinary_muscle_development	ENSGALG00010006176	MMR1L3	4.093369773	0.213084829	19.21004787	3.04985E-82	8.09662E-81	positive
PRJNA729271	stage_3W_vs_E10	ordinary_muscle_development	ENSGALG00010006177	NEK9	-0.23970438	0.113809746	-2.106184991	0.035188286	0.045033923	negative
PRJNA729271	stage_3W_vs_E10	ordinary_muscle_development	ENSGALG00010006178	RPS23	0.225245553	0.140874731	1.598906712	0.109841332	0.131986248	positive
PRJNA729271	stage_3W_vs_E10	ordinary_muscle_development	ENSGALG00010006180	CD200L	4.979339583	1.075047913	4.63173736			positive
PRJNA729271	stage_3W_vs_E10	ordinary_muscle_development	ENSGALG00010006183	PRKN	2.021910556	0.137081771	14.74966763	3.09184E-49	3.40032E-48	positive
PRJNA729271	stage_3W_vs_E10	ordinary_muscle_development	ENSGALG00010006184	LBX2	-2.751010215	0.310562819	-8.858144138	8.13575E-19	2.96748E-18	negative
PRJNA729271	stage_3W_vs_E10	ordinary_muscle_development	ENSGALG00010006187	TCF21	-3.640330567	0.683517455	-5.325877984	1.00467E-07	2.03303E-07	negative
PRJNA729271	stage_3W_vs_E10	ordinary_muscle_development	ENSGALG00010006188	TMED10	0.032707212	0.074821115	0.437138798	0.662010692	0.694194095	positive
PRJNA729271	stage_3W_vs_E10	ordinary_muscle_development	ENSGALG00010006193	RBM17	-0.713978905	0.099810052	-7.153376767	8.46688E-13	2.29311E-12	negative
PRJNA729271	stage_3W_vs_E10	ordinary_muscle_development	ENSGALG00010006197	ENSGALG00010006197	-2.291283181	0.597315725	-3.835966618	0.000125071	0.000202832	negative
PRJNA729271	stage_3W_vs_E10	ordinary_muscle_development	ENSGALG00010006198	ENSGALG00010006198	1.827461957	0.680044111	2.687269731	0.007203875	0.009941938	positive
PRJNA729271	stage_3W_vs_E10	ordinary_muscle_development	ENSGALG00010006200	GSTCD	-1.57642849	0.103612498	-15.21465578	2.82702E-52	3.4493E-51	negative
PRJNA729271	stage_3W_vs_E10	ordinary_muscle_development	ENSGALG00010006201	SSTR1	-2.731859068	0.378127176	-7.224709677	5.02173E-13	1.3721E-12	negative
PRJNA729271	stage_3W_vs_E10	ordinary_muscle_development	ENSGALG00010006202	SACS	-0.561634758	0.200291652	-2.804084711	0.005045965	0.007072938	negative
PRJNA729271	stage_3W_vs_E10	ordinary_muscle_development	ENSGALG00010006203	MMR1L4	3.446836116	0.174052554	19.8034216	2.78144E-87	8.52368E-86	positive
PRJNA729271	stage_3W_vs_E10	ordinary_muscle_development	ENSGALG00010006205	CGGBP1	-0.612695719	0.074319824	-8.244041622	1.66489E-16	5.44017E-16	negative
PRJNA729271	stage_3W_vs_E10	ordinary_muscle_development	ENSGALG00010006207	ENSGALG00010006207	-0.154330353	0.206614035	-0.746949997	0.455093745	0.493602563	negative
PRJNA729271	stage_3W_vs_E10	ordinary_muscle_development	ENSGALG00010006212	FOS	3.888006212	0.366211456	10.61683393	2.48859E-26	1.2345E-25	positive
PRJNA729271	stage_3W_vs_E10	ordinary_muscle_development	ENSGALG00010006219	CLEC14A	1.942540747	0.182678703	10.63364648	2.07824E-26	1.03448E-25	positive
PRJNA729271	stage_3W_vs_E10	ordinary_muscle_development	ENSGALG00010006220	SGCG	1.385892985	0.170978299	8.105666024	5.24574E-16	1.66967E-15	positive
PRJNA729271	stage_3W_vs_E10	ordinary_muscle_development	ENSGALG00010006223	ATG10	-0.411810151	0.113672417	-3.622779933	0.000291454	0.000458202	negative
PRJNA729271	stage_3W_vs_E10	ordinary_muscle_development	ENSGALG00010006227	AGPAT4	-1.351934415	0.147034057	-9.194702492	3.76034E-20	1.44578E-19	negative
PRJNA729271	stage_3W_vs_E10	ordinary_muscle_development	ENSGALG00010006229	INTS12	0.154258155	0.132515229	1.164078691	0.244392123	0.279241985	positive
PRJNA729271	stage_3W_vs_E10	ordinary_muscle_development	ENSGALG00010006232	ZNF654	-0.028542611	0.103778808	-0.275033133	0.783290783	0.807237818	negative
PRJNA729271	stage_3W_vs_E10	ordinary_muscle_development	ENSGALG00010006236	C3orf38	0.396060807	0.08363603	4.735528569	2.18485E-06	4.04772E-06	positive
PRJNA729271	stage_3W_vs_E10	ordinary_muscle_development	ENSGALG00010006237	ENSGALG00010006237	2.677081159	0.292935547	9.138806088	6.31456E-20	2.4074E-19	positive
PRJNA729271	stage_3W_vs_E10	ordinary_muscle_development	ENSGALG00010006239	EYA4	1.52942868	0.162258004	9.425905923	4.26409E-21	1.70552E-20	positive
PRJNA729271	stage_3W_vs_E10	ordinary_muscle_development	ENSGALG00010006241	MRC1	2.311177752	0.244065614	9.469493526	2.81204E-21	1.13413E-20	positive
PRJNA729271	stage_3W_vs_E10	ordinary_muscle_development	ENSGALG00010006242	ENSGALG00010006242	1.664198733	0.52784493	3.1528175	0.001617029	0.002380543	positive
PRJNA729271	stage_3W_vs_E10	ordinary_muscle_development	ENSGALG00010006244	EPHA3	-2.157827029	0.162492005	-13.27958894	3.04059E-40	2.47881E-39	negative
PRJNA729271	stage_3W_vs_E10	ordinary_muscle_development	ENSGALG00010006245	PFKFB3	3.929065213	0.508436145	7.727745665	1.09468E-14	3.25618E-14	positive
PRJNA729271	stage_3W_vs_E10	ordinary_muscle_development	ENSGALG00010006246	SUPT20H	-0.146361046	0.09970368	-1.467960322	0.142115001	0.168352153	negative
PRJNA729271	stage_3W_vs_E10	ordinary_muscle_development	ENSGALG00010006249	C9orf72	0.665603479	0.104297324	6.381788677	1.75031E-10	4.17146E-10	positive
PRJNA729271	stage_3W_vs_E10	ordinary_muscle_development	ENSGALG00010006251	SEMA3E	-2.104540496	0.368579867	-5.70986286	1.13067E-08	2.43342E-08	negative
PRJNA729271	stage_3W_vs_E10	ordinary_muscle_development	ENSGALG00010006252	COX7C	1.463744834	0.136534457	10.72069911	8.13858E-27	4.12478E-26	positive
PRJNA729271	stage_3W_vs_E10	ordinary_muscle_development	ENSGALG00010006254	PROS1	1.239721236	0.137260783	9.031867694	1.68766E-19	6.33092E-19	positive
PRJNA729271	stage_3W_vs_E10	ordinary_muscle_development	ENSGALG00010006259	PPA2	0.622671029	0.086970796	7.159541562	8.09473E-13	2.19437E-12	positive
PRJNA729271	stage_3W_vs_E10	ordinary_muscle_development	ENSGALG00010006260	MAP3K4	0.759411249	0.15928389	4.76765885	1.86379E-06	3.46399E-06	positive
PRJNA729271	stage_3W_vs_E10	ordinary_muscle_development	ENSGALG00010006261	PCLO	-2.677663099	0.538738923	-4.97024251	6.68692E-07	1.28125E-06	negative
PRJNA729271	stage_3W_vs_E10	ordinary_muscle_development	ENSGALG00010006263	EXOSC8	-0.649390639	0.1497072	-4.337738179	1.43956E-05	2.51236E-05	negative
PRJNA729271	stage_3W_vs_E10	ordinary_muscle_development	ENSGALG00010006266	CENPW	-3.093100521	0.22993974	-13.45178748	3.00519E-41	2.51648E-40	negative
PRJNA729271	stage_3W_vs_E10	ordinary_muscle_development	ENSGALG00010006269	ENSGALG00010006269	2.503832299	1.242461001	2.015220033	0.043881599	0.055483582	positive
PRJNA729271	stage_3W_vs_E10	ordinary_muscle_development	ENSGALG00010006270	STAM	-0.110029649	0.092974979	-1.183432897	0.236637626	0.270851421	negative
PRJNA729271	stage_3W_vs_E10	ordinary_muscle_development	ENSGALG00010006273	ARL13B	-0.236596913	0.098534005	-2.401170171	0.016342735	0.021700984	negative
PRJNA729271	stage_3W_vs_E10	ordinary_muscle_development	ENSGALG00010006277	GNG10	-0.831414938	0.158636094	-5.241019965	1.59691E-07	3.18307E-07	negative
PRJNA729271	stage_3W_vs_E10	ordinary_muscle_development	ENSGALG00010006279	HACD1	3.658638647	0.141328391	25.88749945	9.2096E-148	8.3433E-146	positive
PRJNA729271	stage_3W_vs_E10	ordinary_muscle_development	ENSGALG00010006281	TRMT11	0.511695606	0.080778386	6.33456089	2.38018E-10	5.63553E-10	positive
PRJNA729271	stage_3W_vs_E10	ordinary_muscle_development	ENSGALG00010006283	DNAJC25	0.788801723	0.10627739	7.422102906	1.15275E-13	3.26096E-13	positive
PRJNA729271	stage_3W_vs_E10	ordinary_muscle_development	ENSGALG00010006285	FZD8	-1.556078802	0.156516389	-9.941954416	2.73409E-23	1.18904E-22	negative
PRJNA729271	stage_3W_vs_E10	ordinary_muscle_development	ENSGALG00010006287	ECPAS	1.590136874	0.122351185	12.99649758	1.28076E-38	9.85382E-38	positive
PRJNA729271	stage_3W_vs_E10	ordinary_muscle_development	ENSGALG00010006288	ARF5	-3.652471423	0.194949011	-18.73552165	2.54139E-78	6.08883E-77	negative
PRJNA729271	stage_3W_vs_E10	ordinary_muscle_development	ENSGALG00010006289	SKIDA1	-1.988526359	0.152796441	-13.01421909	1.01575E-38	7.84409E-38	negative
PRJNA729271	stage_3W_vs_E10	ordinary_muscle_development	ENSGALG00010006291	ENSGALG00010006291	0.263658011	0.207583042	1.270132711	0.204037361	0.23590345	positive
PRJNA729271	stage_3W_vs_E10	ordinary_muscle_development	ENSGALG00010006293	SLC22A3	0.539947742	0.356955257	1.512648241	0.130369034	0.155236929	positive

PRJNA729271	stage_3W_vs_E10	ordinary_muscle_development	ENSGALG00010006300	GCC1	-0.091013756	0.120058611	-0.758077706	0.448404465	0.487264617	negative
PRJNA729271	stage_3W_vs_E10	ordinary_muscle_development	ENSGALG00010006301	ENSGALG00010006301	0.712981332	0.145408032	4.903314645	9.42329E-07	1.78924E-06	positive
PRJNA729271	stage_3W_vs_E10	ordinary_muscle_development	ENSGALG00010006310	ALG5	-0.287910724	0.110685084	-2.601170042	0.009290639	0.01267104	negative
PRJNA729271	stage_3W_vs_E10	ordinary_muscle_development	ENSGALG00010006311	ABCD4	-0.115930776	0.114310826	-1.014171451	0.310500949	0.348460146	negative
PRJNA729271	stage_3W_vs_E10	ordinary_muscle_development	ENSGALG00010006323	ANAPC10	0.28280214	0.104952967	2.694560691	0.007048149	0.009734437	positive
PRJNA729271	stage_3W_vs_E10	ordinary_muscle_development	ENSGALG00010006327	DENND6B	-0.432663433	0.113342728	-3.817302084	0.000134919	0.000218191	negative
PRJNA729271	stage_3W_vs_E10	ordinary_muscle_development	ENSGALG00010006328	SYNDIG1L	-1.940387817	0.445775925	-4.352832235	1.3439E-05	2.35037E-05	negative
PRJNA729271	stage_3W_vs_E10	ordinary_muscle_development	ENSGALG00010006330	STX19	-0.148614813	0.41936535	-0.354380288	0.723053905	0.751248395	negative
PRJNA729271	stage_3W_vs_E10	ordinary_muscle_development	ENSGALG00010006331	SMAD9	-0.878470178	0.154654073	-5.680226583	1.34516E-08	2.88008E-08	negative
PRJNA729271	stage_3W_vs_E10	ordinary_muscle_development	ENSGALG00010006336	ALDH8A1	1.447466001	0.873595946	1.656905584	0.097538565	0.118123611	positive
PRJNA729271	stage_3W_vs_E10	ordinary_muscle_development	ENSGALG00010006339	ENSGALG00010006339	2.763227334	0.725495071	3.808747221	0.000139673	0.000225627	positive
PRJNA729271	stage_3W_vs_E10	ordinary_muscle_development	ENSGALG00010006341	HHIP	-1.043004054	0.220541278	-4.72929178	2.25304E-06	4.16767E-06	negative
PRJNA729271	stage_3W_vs_E10	ordinary_muscle_development	ENSGALG00010006342	RFXAP	0.117550818	0.084608065	1.389357128	0.16472418	0.193255928	positive
PRJNA729271	stage_3W_vs_E10	ordinary_muscle_development	ENSGALG00010006343	SEC23A	-0.091224892	0.094813495	-0.962150926	0.335973796	0.374294072	negative
PRJNA729271	stage_3W_vs_E10	ordinary_muscle_development	ENSGALG00010006344	HINT3	0.48432687	0.10519339	4.604156883	4.1414E-06	7.52817E-06	positive
PRJNA729271	stage_3W_vs_E10	ordinary_muscle_development	ENSGALG00010006345	SMC2	-3.735025188	0.250892534	-14.88695229	4.00619E-50	4.57242E-49	negative
PRJNA729271	stage_3W_vs_E10	ordinary_muscle_development	ENSGALG00010006346	NSUN3	-0.134324816	0.132615418	-1.012889884	0.311112761	0.348984636	negative
PRJNA729271	stage_3W_vs_E10	ordinary_muscle_development	ENSGALG00010006347	PPP6R2	1.545023908	0.135538702	11.39913456	4.22296E-30	2.43097E-29	positive
PRJNA729271	stage_3W_vs_E10	ordinary_muscle_development	ENSGALG00010006351	IGF2R	1.516082875	0.173898886	8.718186228	2.82694E-18	1.01102E-17	positive
PRJNA729271	stage_3W_vs_E10	ordinary_muscle_development	ENSGALG00010006352	GEMIN2	1.115516925	0.096514411	11.55803488	6.72297E-31	4.00039E-30	positive
PRJNA729271	stage_3W_vs_E10	ordinary_muscle_development	ENSGALG00010006353	ABCE1	-0.563218278	0.080825053	-6.96836261	3.20651E-12	8.40781E-12	negative
PRJNA729271	stage_3W_vs_E10	ordinary_muscle_development	ENSGALG00010006355	PTGR1	-1.192983279	0.152725721	-7.811279395	5.66104E-15	1.71201E-14	negative
PRJNA729271	stage_3W_vs_E10	ordinary_muscle_development	ENSGALG00010006359	SGK1	1.789261773	0.163738613	10.92754933	8.51161E-28	4.49948E-27	positive
PRJNA729271	stage_3W_vs_E10	ordinary_muscle_development	ENSGALG00010006361	SERTM1	-1.974746544	0.264178621	-7.47504298	7.71786E-14	2.20565E-13	negative
PRJNA729271	stage_3W_vs_E10	ordinary_muscle_development	ENSGALG00010006363	TRAPPC6A	0.063543294	0.099760111	0.636960935	0.524150289	0.56207431	positive
PRJNA729271	stage_3W_vs_E10	ordinary_muscle_development	ENSGALG00010006372	SBF1	-0.009497471	0.113607611	-0.083598894	0.933375347	0.941232479	negative
PRJNA729271	stage_3W_vs_E10	ordinary_muscle_development	ENSGALG00010006378	CAAP1	-1.270561535	0.104606627	-12.14609023	6.01751E-34	3.99011E-33	negative
PRJNA729271	stage_3W_vs_E10	ordinary_muscle_development	ENSGALG00010006381	SPART	1.416887402	0.156497289	9.053750444	1.3814E-19	5.19145E-19	positive
PRJNA729271	stage_3W_vs_E10	ordinary_muscle_development	ENSGALG00010006382	PNN	-0.355893477	0.196633366	-1.809934316	0.070305974	0.086605345	negative
PRJNA729271	stage_3W_vs_E10	ordinary_muscle_development	ENSGALG00010006387	OTUD4	-0.515961581	0.136260486	-3.786582581	0.000152733	0.00024574	negative
PRJNA729271	stage_3W_vs_E10	ordinary_muscle_development	ENSGALG00010006404	SMAD1	-0.124131097	0.109453027	-1.134103833	0.256750996	0.292395167	negative
PRJNA729271	stage_3W_vs_E10	ordinary_muscle_development	ENSGALG00010006408	ENSGALG00010006408	-0.85813843	0.67952069	-1.262858427	0.206640063	0.238741249	negative
PRJNA729271	stage_3W_vs_E10	ordinary_muscle_development	ENSGALG00010006411	RGS17	-1.841738202	0.408282481	-4.510941044	6.45407E-06	1.15667E-05	negative
PRJNA729271	stage_3W_vs_E10	ordinary_muscle_development	ENSGALG00010006421	LMF2	-1.390098639	0.114025945	-12.19107321	3.46824E-34	2.32096E-33	negative
PRJNA729271	stage_3W_vs_E10	ordinary_muscle_development	ENSGALG00010006434	MMAA	0.119983496	0.116661213	1.028478038	0.303725016	0.341384469	positive
PRJNA729271	stage_3W_vs_E10	ordinary_muscle_development	ENSGALG00010006446	LSAMP	-1.959551538	0.186946013	-10.48191134	1.04608E-25	5.04254E-25	negative
PRJNA729271	stage_3W_vs_E10	ordinary_muscle_development	ENSGALG00010006454	SOD2	1.707222674	0.105256972	16.21956855	3.66762E-59	5.40266E-58	positive
PRJNA729271	stage_3W_vs_E10	ordinary_muscle_development	ENSGALG00010006457	NCAPH2	-3.49345456	0.219203382	-15.93704682	3.50532E-57	4.89025E-56	negative
PRJNA729271	stage_3W_vs_E10	ordinary_muscle_development	ENSGALG00010006463	SCO2	-0.016131618	0.135912072	-0.118691572	0.905519714	0.916628475	negative
PRJNA729271	stage_3W_vs_E10	ordinary_muscle_development	ENSGALG00010006465	PLAA	0.363564556	0.115785422	3.139985573	0.001689562	0.002480773	positive
PRJNA729271	stage_3W_vs_E10	ordinary_muscle_development	ENSGALG00010006472	DCLK1	-2.534668616	0.325599443	-7.784622099	6.99219E-15	2.10361E-14	negative
PRJNA729271	stage_3W_vs_E10	ordinary_muscle_development	ENSGALG00010006478	ENSGALG00010006478	0.748711789	0.131559712	5.691041553	1.26267E-08	2.70825E-08	positive
PRJNA729271	stage_3W_vs_E10	ordinary_muscle_development	ENSGALG00010006480	IFT74	-0.130208862	0.117500245	-1.108158219	0.267793491	0.30427739	negative
PRJNA729271	stage_3W_vs_E10	ordinary_muscle_development	ENSGALG00010006486	CEP43	-0.032635197	0.112257175	-0.290718144	0.771266894	0.796034864	negative
PRJNA729271	stage_3W_vs_E10	ordinary_muscle_development	ENSGALG00010006492	GAP43	-2.966140837	0.468717123	-6.32821097	2.4802E-10	5.86659E-10	negative
PRJNA729271	stage_3W_vs_E10	ordinary_muscle_development	ENSGALG00010006496	UBE2H	1.086717498	0.113690252	9.558581125	1.19382E-21	4.87724E-21	positive
PRJNA729271	stage_3W_vs_E10	ordinary_muscle_development	ENSGALG00010006499	CCR6	2.656484859	0.670746142	3.960492191	7.47954E-05	0.000123467	positive
PRJNA729271	stage_3W_vs_E10	ordinary_muscle_development	ENSGALG00010006503	LRRC19	6.403066142	0.989095497	6.473658166	9.56582E-11	2.31133E-10	positive
PRJNA729271	stage_3W_vs_E10	ordinary_muscle_development	ENSGALG00010006506	ZNF827	-0.33623569	0.105680323	-3.181630045	0.001464488	0.002161245	negative
PRJNA729271	stage_3W_vs_E10	ordinary_muscle_development	ENSGALG00010006515	FBXO33	0.213710872	0.102833497	2.078222351	0.037688884	0.048064436	positive
PRJNA729271	stage_3W_vs_E10	ordinary_muscle_development	ENSGALG00010006516	NBEA	-1.075943327	0.127380702	-8.446674476	2.99718E-17	1.01671E-16	negative
PRJNA729271	stage_3W_vs_E10	ordinary_muscle_development	ENSGALG00010006517	ZBTB20	3.972177363	0.144756759	27.44035851	9.0554E-166	1.0848E-163	positive
PRJNA729271	stage_3W_vs_E10	ordinary_muscle_development	ENSGALG00010006521	ZNF410	-0.516971	0.103851226	-4.977996124	6.42459E-07	1.23229E-06	negative
PRJNA729271	stage_3W_vs_E10	ordinary_muscle_development	ENSGALG00010006524	TAB2	-0.041959743	0.091666307	-0.457744445	0.647136053	0.680220238	negative
PRJNA729271	stage_3W_vs_E10	ordinary_muscle_development	ENSGALG00010006526	LSM6	-0.62758161	0.160153871	-3.918616547	8.90587E-05	0.000145947	negative
PRJNA729271	stage_3W_vs_E10	ordinary_muscle_development	ENSGALG00010006528	ZC3HC1	-0.304450571	0.081488839	-3.736101456	0.000186895	0.000298419	negative
PRJNA729271	stage_3W_vs_E10	ordinary_muscle_development	ENSGALG00010006531	FAM161B	-0.95401513	0.142805957	-6.680499533	2.38129E-11	5.97073E-11	negative
PRJNA729271	stage_3W_vs_E10	ordinary_muscle_development	ENSGALG00010006532	KLHDC10	0.14511728	0.103757973	1.398613285	0.16192898	0.190161271	positive
PRJNA729271	stage_3W_vs_E10	ordinary_muscle_development	ENSGALG00010006537	DRD3	-5.643636229	0.753976501	-7.485161968	7.1459E-14	2.04541E-13	negative
PRJNA729271	stage_3W_vs_E10	ordinary_muscle_development	ENSGALG00010006538	TEK	1.647677164	0.104671515	15.7414094	7.86717E-56	1.06079E-54	positive
PRJNA729271	stage_3W_vs_E10	ordinary_muscle_development	ENSGALG00010006542	RFC3	-2.181613018	0.142725097	-15.2854198	9.56447E-53	1.18089E-51	negative
PRJNA729271	stage_3W_vs_E10	ordinary_muscle_development	ENSGALG00010006544	MOB3B	-3.228355278	0.16708433	-19.32171187	3.52756E-83	9.66578E-82	negative
PRJNA729271	stage_3W_vs_E10	ordinary_muscle_development	ENSGALG00010006546	SMOC2	-0.081551336	0.208299257	-0.39151045	0.695419966	0.725240118	negative
PRJNA729271	stage_3W_vs_E10	ordinary_muscle_development	ENSGALG00010006548	TMEM209	-0.98044718	0.081606894	-12.01426905	2.99002E-33	1.93916E-32	negative
PRJNA729271	stage_3W_vs_E10	ordinary_muscle_development	ENSGALG00010006549	ZDHHC23	-0.147593419	0.181301394	-0.814077684	0.415600449	0.454479707	negative
PRJNA729271	stage_3W_vs_E10	ordinary_muscle_development	ENSGALG00010006552	CPA2	7.571042827	1.637612586	4.623219736	3.77829E-06	6.89665E-06	positive
PRJNA729271	stage_3W_vs_E10	ordinary_muscle_development	ENSGALG00010006557	COQ6	0.219635417	0.109093838	2.013270606	0.044086169	0.055717937	positive
PRJNA729271	stage_3W_vs_E10	ordinary_muscle_development	ENSGALG00010006558	USP10	-0.752528449	0.088940714	-8.461012002	2.65067E-17	9.01278E-17	negative
PRJNA729271	stage_3W_vs_E10	ordinary_muscle_development	ENSGALG00010006564	SLC10A7	-1.107669109	0.144293568	-7.67649679	1.63498E-14	4.82669E-14	negative
PRJNA729271	stage_3W_vs_E10	ordinary_muscle_development	ENSGALG00010006566	CPA5	8.86741647	2.799917437	3.167027839	0.001540056	0.00226907	positive
PRJNA729271	stage_3W_vs_E10	ordinary_muscle_development	ENSGALG00010006568	ENTPD5	0.049459161	0.198998689	0.248540135	0.80371652	0.826056227	positive
PRJNA729271	stage_3W_vs_E10	ordinary_muscle_development	ENSGALG00010006570	ENSGALG00010006570	1.203543339	0.487266403	2.4699904	0.013511668	0.018107584	positive
PRJNA729271	stage_3W_vs_E10	ordinary_muscle_development	ENSGALG00010006574	CPA1	9.164181656	2.55132351	3.591932431	0.000328235	0.000513853	positive
PRJNA729271	stage_3W_vs_E10	ordinary_muscle_development	ENSGALG00010006575	SSBP2	-1.516135768	0.11775173	-12.87569845	6.16786E-38	4.6251E-37	negative
PRJNA729271	stage_3W_vs_E10	ordinary_muscle_development	ENSGALG00010006576	CCDC191	-0.230751504	0.113867524	-2.026490922	0.042714502	0.054078671	negative
PRJNA729271	stage_3W_vs_E10	ordinary_muscle_development	ENSGALG00010006580	ALDH6A1	0.587341199	0.138905024	4.228365402	2.35395E-05	4.0408E-05	positive
PRJNA729271	stage_3W_vs_E10	ordinary_muscle_development	ENSGALG00010006581	CEP41	-1.258330813	0.106151887	-11.85405979	2.05012E-32	1.29371E-31	negative
PRJNA729271	stage_3W_vs_E10	ordinary_muscle_development	ENSGALG00010006585	TTC29	-0.652366945	0.188130393	-3.467631863	0.000525066	0.000807087	negative
PRJNA729271	stage_3W_vs_E10	ordinary_muscle_development	ENSGALG00010006588	ACOT12	-2.18712385	0.774562855	-2.823688013	0.004747457	0.006675176	negative
PRJNA729271	stage_3W_vs_E10	ordinary_muscle_development	ENSGALG00010006592	STARD13	-0.453997185	0.114665839	-3.959306362	7.51678E-05	0.000123994	negative
PRJNA729271	stage_3W_vs_E10	ordinary_muscle_development	ENSGALG00010006598	ENSGALG00010006598	0.647180416	0.15141351	4.274258054	1.91775E-05	3.31795E-05	positive
PRJNA729271	stage_3W_vs_E10	ordinary_muscle_development	ENSGALG00010006602	EDNRA	-0.911253061	0.11854255	-7.687139027	1.50462E-14	4.44818E-14	negative
PRJNA729271	stage_3W_vs_E10	ordinary_muscle_development	ENSGALG00010006603	MEST	-0.675994021	0.224564932	-3.01023858	0.002610426	0.003762366	negative
PRJNA729271	stage_3W_vs_E10	ordinary_muscle_development	ENSGALG00010006606	SULT	-3.262493538	1.256388349	-2.596723809			negative
PRJNA729271	stage_3W_vs_E10	ordinary_muscle_development	ENSGALG00010006608	KL	3.493641905	0.407785479	8.567352413	1.05891E-17	3.68346E-17	positive
PRJNA729271	stage_3W_vs_E10	ordinary_muscle_development	ENSGALG00010006614	TMEM184C	-0.219983777	0.054902197	-4.006830109	6.15391E-05	0.000102365	negative
PRJNA729271	stage_3W_vs_E10	ordinary_muscle_development	ENSGALG00010006615	GRAMD1C	-0.008847182	0.100882912	-0.087697524	0.930117087	0.938207876	negative
PRJNA729271	stage_3W_vs_E10	ordinary_muscle_development	ENSGALG00010006616	COPG2	-0.759359931	0.06897845	-11.00865458	3.47149E-28	1.86783E-27	negative
PRJNA729271	stage_3W_vs_E10	ordinary_muscle_development	ENSGALG00010006619	LIN52	-0.285576264	0.101991624	-2.799997238	0.005110304	0.00715897	negative
PRJNA729271	stage_3W_vs_E10	ordinary_muscle_development	ENSGALG00010006621	DACT2	-1.341673849	0.209559567	-6.402350736	1.53003E-10	3.65366E-10	negative
PRJNA729271	stage_3W_vs_E10	ordinary_muscle_development	ENSGALG00010006623	ENSGALG00010006623	0.514894645	0.185890946	2.769874797	0.005607784	0.007821114	positive
PRJNA729271	stage_3W_vs_E10	ordinary_muscle_development	ENSGALG00010006627	SF3B5	-0.427006485	0.110476302	-3.865140997	0.000111025	0.00018076	negative
PRJNA729271	stage_3W_vs_E10	ordinary_muscle_development	ENSGALG00010006633	FBXO18	0.396057471	0.09292511	4.262114615	2.02502E-05	3.49727E-05	positive
PRJNA729271	stage_3W_vs_E10	ordinary_muscle_development	ENSGALG00010006634	PRMT9	0.760618836	0.095133026	7.995318395	1.29239E-15	4.02333E-15	positive
PRJNA729271	stage_3W_vs_E10	ordinary_muscle_development	ENSGALG00010006635	ENSGALG00010006635	-2.810305318	0.483904474	-5.807562167	6.3389E-09	1.38315E-08	negative
PRJNA729271	stage_3W_vs_E10	ordinary_muscle_development	ENSGALG00010006641	ENSGALG00010006641	-3.222742023	0.501079931	-6.431592695	1.26274E-10	3.02986E-10	negative
PRJNA729271	stage_3W_vs_E10	ordinary_muscle_development	ENSGALG00010006651	ENSGALG00010006651	0.453269896	0.112373946	4.0335853	5.49322E-05	9.17436E-05	positive
PRJNA729271	stage_3W_vs_E10	ordinary_muscle_development	ENSGALG00010006653	CFL2	0.573406417	0.104666757	5.478400532	4.29188E-08	8.88979E-08	positive
PRJNA729271	stage_3W_vs_E10	ordinary_muscle_development	ENSGALG00010006657	PDS5B	-1.217788249	0.088587022	-13.74680205	5.32309E-43	4.73363E-42	negative
PRJNA729271	stage_3W_vs_E10	ordinary_muscle_development	ENSGALG00010006664	ENSGALG00010006664	3.549313607	1.335907047	2.656856713	0.007887296	0.010845874	positive
PRJNA729271	stage_3W_vs_E10	ordinary_muscle_development	ENSGALG00010006670	ANKRD16	0.400150808	0.182010649	2.19850218	0.027913339	0.036111567	positive
PRJNA729271	stage_3W_vs_E10	ordinary_muscle_development	ENSGALG00010006673	BAZ1A	-0.973375876	0.082414259	-11.81077022	3.43401E-32	2.14644E-31	negative
PRJNA729271	stage_3W_vs_E10	ordinary_muscle_development	ENSGALG00010006678	ENSGALG00010006678	-3.951614937	1.29086866	-3.061206038	0.002204473	0.003196983	negative
PRJNA729271	stage_3W_vs_E10	ordinary_muscle_development	ENSGALG00010006681	CRISPLD2	-1.391808899	0.210147842	-6.622998781	3.51984E-11	8.73931E-11	negative
PRJNA729271	stage_3W_vs_E10	ordinary_muscle_development	ENSGALG00010006682	ARHGAP10	-1.012120184	0.121818598	-8.308420873	9.6984E-17	3.20955E-16	negative
PRJNA729271	stage_3W_vs_E10	ordinary_muscle_development	ENSGALG00010006685	ENSGALG00010006685	0.05078485	0.11500797	0.441576789	0.658795484	0.6915392	positive
PRJNA729271	stage_3W_vs_E10	ordinary_muscle_development	ENSGALG00010006694	ENSGALG00010006694	-3.209011371	0.724958485	-4.426476048	9.5785E-06	1.69359E-05	negative
PRJNA729271	stage_3W_vs_E10	ordinary_muscle_development	ENSGALG00010006701	LINGO2	-6.314637062	0.758630992	-8.323726723	8.524E-17	2.82865E-16	negative
PRJNA729271	stage_3W_vs_E10	ordinary_muscle_development	ENSGALG00010006715	IRF8	1.353066327	0.161002085	8.404029841	4.31413E-17	1.45224E-16	positive
PRJNA729271	stage_3W_vs_E10	ordinary_muscle_development	ENSGALG00010006716	ENSGALG00010006716	1.246728011	0.096795311	12.88004552	5.83012E-38	4.37863E-37	positive
PRJNA729271	stage_3W_vs_E10	ordinary_muscle_development	ENSGALG00010006720	SRP54	0.179836539	0.090970209	1.976872874	0.048056002	0.060466297	positive
PRJNA729271	stage_3W_vs_E10	ordinary_muscle_development	ENSGALG00010006728	ENSGALG00010006728	0.36444659	0.136252159	2.674794964	0.007477497	0.010302881	positive
PRJNA729271	stage_3W_vs_E10	ordinary_muscle_development	ENSGALG00010006730	HAPLN1	-8.867575702	0.540119514	-16.41780286	1.42639E-60	2.16724E-59	negative
PRJNA729271	stage_3W_vs_E10	ordinary_muscle_development	ENSGALG00010006735	NR3C2	-0.084217622	0.112596316	-0.74796072	0.45448385	0.493094567	negative
PRJNA729271	stage_3W_vs_E10	ordinary_muscle_development	ENSGALG00010006739	VCAN	-2.990229288	0.253755441	-11.78390215	4.72537E-32	2.94093E-31	negative
PRJNA729271	stage_3W_vs_E10	ordinary_muscle_development	ENSGALG00010006740	BRCA2	-1.800302575	0.154643797	-11.6416087	2.53194E-31	1.52411E-30	negative
PRJNA729271	stage_3W_vs_E10	ordinary_muscle_development	ENSGALG00010006748	FAM177A1	1.159166751	0.076829196	15.08758151	1.95477E-51	2.3244E-50	positive
PRJNA729271	stage_3W_vs_E10	ordinary_muscle_development	ENSGALG00010006752	ZAR1L	-0.513150879	0.438096426	-1.171319483	0.241470383	0.276012397	negative
PRJNA729271	stage_3W_vs_E10	ordinary_muscle_development	ENSGALG00010006760	TASOR2	0.152195127	0.102751438	1.481197048	0.138554073	0.164389071	positive
PRJNA729271	stage_3W_vs_E10	ordinary_muscle_development	ENSGALG00010006761	PPP2R3C	-0.174026276	0.094496933	-1.841607655	0.06553256	0.081083608	negative
PRJNA729271	stage_3W_vs_E10	ordinary_muscle_development	ENSGALG00010006767	ENSGALG00010006767	7.717906117	0.330010745	23.38683279	5.8185E-121	3.5141E-119	positive
PRJNA729271	stage_3W_vs_E10	ordinary_muscle_development	ENSGALG00010006794	PERPA	-3.916974099	0.565745864	-6.923557635	4.4044E-12	1.14268E-11	negative
PRJNA729271	stage_3W_vs_E10	ordinary_muscle_development	ENSGALG00010006803	COX4I1	1.721928113	0.103816125	16.58632622	8.75123E-62	1.36986E-60	positive
PRJNA729271	stage_3W_vs_E10	ordinary_muscle_development	ENSGALG00010006804	PSMA6	0.928676487	0.101811626	9.121517067	7.408E-20	2.81687E-19	positive
PRJNA729271	stage_3W_vs_E10	ordinary_muscle_development	ENSGALG00010006814	ENSGALG00010006814	0.072044045	0.377871505	0.190657524	0.848793919	0.866122341	positive
PRJNA729271	stage_3W_vs_E10	ordinary_muscle_development	ENSGALG00010006816	ENSGALG00010006816	6.442252773	0.50189725	12.83580011	1.0333E-37	7.7006E-37	positive
PRJNA729271	stage_3W_vs_E10	ordinary_muscle_development	ENSGALG00010006819	EMC8	0.652454749	0.13151631	4.961017766	7.01248E-07	1.34088E-06	positive
PRJNA729271	stage_3W_vs_E10	ordinary_muscle_development	ENSGALG00010006821	XRCC4	-0.918217785	0.128300963	-7.15674897	8.26128E-13	2.23827E-12	negative
PRJNA729271	stage_3W_vs_E10	ordinary_muscle_development	ENSGALG00010006828	GTF2A1	-0.014784656	0.106032324	-0.139435362	0.88910613	0.901987217	negative
PRJNA729271	stage_3W_vs_E10	ordinary_muscle_development	ENSGALG00010006833	LTV1	-0.540602417	0.08711654	-6.205508369	5.45204E-10	1.26444E-09	negative
PRJNA729271	stage_3W_vs_E10	ordinary_muscle_development	ENSGALG00010006835	FRY	1.954278681	0.15213595	12.84560735	9.10346E-38	6.80179E-37	positive

PRJNA729271	stage_3W_vs_E10	ordinary_muscle_development	ENSGALG00010006838	ASB13	1.555875905	0.120485599	12.91337657	3.7834E-38	2.86223E-37	positive
PRJNA729271	stage_3W_vs_E10	ordinary_muscle_development	ENSGALG00010006840	ENSGALG00010006840	-4.067284495	0.307136945	-13.24257653	4.98102E-40	4.02901E-39	negative
PRJNA729271	stage_3W_vs_E10	ordinary_muscle_development	ENSGALG00010006843	STON2	1.583161322	0.192030199	8.244335166	1.66081E-16	5.42928E-16	positive
PRJNA729271	stage_3W_vs_E10	ordinary_muscle_development	ENSGALG00010006845	CALML3	-8.418376151	0.824742822	-10.20727423	1.8396E-24	8.43029E-24	negative
PRJNA729271	stage_3W_vs_E10	ordinary_muscle_development	ENSGALG00010006847	SEL1L	0.154368042	0.086645954	1.781595514	0.074815217	0.091878892	positive
PRJNA729271	stage_3W_vs_E10	ordinary_muscle_development	ENSGALG00010006850	IRF2BPL	-0.984044072	0.160295968	-6.138919669	8.30846E-10	1.90315E-09	negative
PRJNA729271	stage_3W_vs_E10	ordinary_muscle_development	ENSGALG00010006851	TMEM167A	-0.195975769	0.10449803	-1.875401573	0.060737499	0.075472786	negative
PRJNA729271	stage_3W_vs_E10	ordinary_muscle_development	ENSGALG00010006853	NET1	-0.798434104	0.084044758	-9.500105941	2.09677E-21	8.48957E-21	negative
PRJNA729271	stage_3W_vs_E10	ordinary_muscle_development	ENSGALG00010006855	DCLK2	0.342100683	0.122793018	2.78599458	0.005336376	0.007462689	positive
PRJNA729271	stage_3W_vs_E10	ordinary_muscle_development	ENSGALG00010006865	EFCAB2	-1.289179271	0.366363228	-3.518855532	0.000433413	0.000671259	negative
PRJNA729271	stage_3W_vs_E10	ordinary_muscle_development	ENSGALG00010006877	GSE1	0.758171094	0.241256077	3.142598946	0.001674551	0.002459481	positive
PRJNA729271	stage_3W_vs_E10	ordinary_muscle_development	ENSGALG00010006883	LRBA	-0.409996531	0.070715629	-5.79782064	6.71822E-09	1.46393E-08	negative
PRJNA729271	stage_3W_vs_E10	ordinary_muscle_development	ENSGALG00010006885	RHOU	-0.762094124	0.153327932	-4.970354142	6.68307E-07	1.28069E-06	negative
PRJNA729271	stage_3W_vs_E10	ordinary_muscle_development	ENSGALG00010006901	TUBAL3	-3.6211372	0.263297202	-13.75304095	4.8833E-43	4.3559E-42	negative
PRJNA729271	stage_3W_vs_E10	ordinary_muscle_development	ENSGALG00010006903	B3GLCT	-1.134474171	0.146432334	-7.747429402	9.37714E-15	2.79962E-14	negative
PRJNA729271	stage_3W_vs_E10	ordinary_muscle_development	ENSGALG00010006912	ENSGALG00010006912	-0.947524146	0.182839429	-5.1822747	2.19196E-07	4.33517E-07	negative
PRJNA729271	stage_3W_vs_E10	ordinary_muscle_development	ENSGALG00010006913	HSPH1	-1.029835255	0.185723365	-5.544995683	2.93961E-08	6.15036E-08	negative
PRJNA729271	stage_3W_vs_E10	ordinary_muscle_development	ENSGALG00010006922	SMYD3	-0.407421371	0.15906338	-2.561377553	0.010425799	0.014135436	negative
PRJNA729271	stage_3W_vs_E10	ordinary_muscle_development	ENSGALG00010006923	ENSGALG00010006923	0.235138981	0.225480518	1.042835019	0.297024709	0.33442389	positive
PRJNA729271	stage_3W_vs_E10	ordinary_muscle_development	ENSGALG00010006926	ID4	-3.034416519	0.230480125	-13.16563207	1.38375E-39	1.10388E-38	negative
PRJNA729271	stage_3W_vs_E10	ordinary_muscle_development	ENSGALG00010006927	UCN3	-4.671558123	1.046782566	-4.462777919	8.09039E-06	1.43872E-05	negative
PRJNA729271	stage_3W_vs_E10	ordinary_muscle_development	ENSGALG00010006939	FBXO31	1.383314544	0.126987866	10.89328132	1.24087E-27	6.51683E-27	positive
PRJNA729271	stage_3W_vs_E10	ordinary_muscle_development	ENSGALG00010006942	ALOX5AP	0.617528792	0.182891871	3.376469321	0.000734226	0.00111301	positive
PRJNA729271	stage_3W_vs_E10	ordinary_muscle_development	ENSGALG00010006944	MAB21L2	-5.8479319	0.552359848	-10.58717776	3.4174E-26	1.6843E-25	negative
PRJNA729271	stage_3W_vs_E10	ordinary_muscle_development	ENSGALG00010006946	PLPPR1	-7.16027822	0.563306955	-12.71114825	5.12759E-37	3.73115E-36	negative
PRJNA729271	stage_3W_vs_E10	ordinary_muscle_development	ENSGALG00010006948	ENSGALG00010006948	-9.75503673	0.534319221	-18.25694518	1.82204E-74	3.98349E-73	negative
PRJNA729271	stage_3W_vs_E10	ordinary_muscle_development	ENSGALG00010006953	MAP1LC3B	1.49164838	0.127788388	11.6728007	1.75547E-31	1.06511E-30	positive
PRJNA729271	stage_3W_vs_E10	ordinary_muscle_development	ENSGALG00010006957	PALM2AKAP2	0.394578785	0.091726281	4.301698269	1.69494E-05	2.94335E-05	positive
PRJNA729271	stage_3W_vs_E10	ordinary_muscle_development	ENSGALG00010006958	KIF26B	-5.386829415	0.254382824	-21.17607366	1.58724E-99	6.49916E-98	negative
PRJNA729271	stage_3W_vs_E10	ordinary_muscle_development	ENSGALG00010006961	ZCCHC14	0.186264672	0.117892943	1.5799476	0.114118867	0.136887866	positive
PRJNA729271	stage_3W_vs_E10	ordinary_muscle_development	ENSGALG00010006963	ENSGALG00010006963	2.406512378	0.413976656	5.813159616	6.13046E-09	1.33928E-08	positive
PRJNA729271	stage_3W_vs_E10	ordinary_muscle_development	ENSGALG00010006966	PGBD5	-3.464220029	0.284259468	-12.18682373	3.65387E-34	2.44294E-33	negative
PRJNA729271	stage_3W_vs_E10	ordinary_muscle_development	ENSGALG00010006983	GTPBP2	0.735993982	0.124171852	5.927220801	3.08105E-09	6.81412E-09	positive
PRJNA729271	stage_3W_vs_E10	ordinary_muscle_development	ENSGALG00010006988	USPL1	0.256058278	0.10397974	2.46257857	0.013794195	0.018462313	positive
PRJNA729271	stage_3W_vs_E10	ordinary_muscle_development	ENSGALG00010006992	SLC35B4	-1.744260581	0.105275103	-16.56859529	1.1754E-61	1.8379E-60	negative
PRJNA729271	stage_3W_vs_E10	ordinary_muscle_development	ENSGALG00010006998	POLH	0.874010159	0.120416945	7.258199081	3.92278E-13	1.07916E-12	positive
PRJNA729271	stage_3W_vs_E10	ordinary_muscle_development	ENSGALG00010007000	LRGUK	-2.289614415	0.574255002	-3.987103996	6.68847E-05	0.000110913	negative
PRJNA729271	stage_3W_vs_E10	ordinary_muscle_development	ENSGALG00010007001	SNX30	-1.02790659	0.093560317	-10.98656595	4.4348E-28	2.36942E-27	negative
PRJNA729271	stage_3W_vs_E10	ordinary_muscle_development	ENSGALG00010007002	EXOC4	-0.208755145	0.120009966	-1.739481744	0.081950061	0.100174239	negative
PRJNA729271	stage_3W_vs_E10	ordinary_muscle_development	ENSGALG00010007004	ENSGALG00010007004	-0.067616285	0.597407514	-0.113182849	0.909885593	0.920490764	negative
PRJNA729271	stage_3W_vs_E10	ordinary_muscle_development	ENSGALG00010007007	ACSL1	1.695360741	0.216895746	7.816477598	5.4322E-15	1.64384E-14	positive
PRJNA729271	stage_3W_vs_E10	ordinary_muscle_development	ENSGALG00010007010	ENSGALG00010007010	1.034151748	0.99974783	1.034412597	0.300943318	0.338572912	positive
PRJNA729271	stage_3W_vs_E10	ordinary_muscle_development	ENSGALG00010007011	HMGB1	-1.931837842	0.101983305	-18.94268708	5.07402E-80	1.26807E-78	negative
PRJNA729271	stage_3W_vs_E10	ordinary_muscle_development	ENSGALG00010007014	ENSGALG00010007014	0.370849095	0.566139286	0.655049214	0.512436068	0.550408359	positive
PRJNA729271	stage_3W_vs_E10	ordinary_muscle_development	ENSGALG00010007017	SRD5A2	1.637380835	0.338236854	4.840929708	1.29233E-06	2.42804E-06	positive
PRJNA729271	stage_3W_vs_E10	ordinary_muscle_development	ENSGALG00010007021	SULT6B1	0.563460503	0.234743109	2.400328192	0.016380378	0.021744993	positive
PRJNA729271	stage_3W_vs_E10	ordinary_muscle_development	ENSGALG00010007025	ENSGALG00010007025	0.949272869	0.773007199	1.22802591	0.219437242	0.252339772	positive
PRJNA729271	stage_3W_vs_E10	ordinary_muscle_development	ENSGALG00010007028	KATNAL1	-0.17931608	0.138286148	-1.296703122	0.194733355	0.225758616	negative
PRJNA729271	stage_3W_vs_E10	ordinary_muscle_development	ENSGALG00010007033	CEBPZOS	1.327720847	0.185350765	7.163287671	7.87648E-13	2.13641E-12	positive
PRJNA729271	stage_3W_vs_E10	ordinary_muscle_development	ENSGALG00010007038	CENPU	-2.324250201	0.207018246	-11.2272722	2.99585E-29	1.67599E-28	negative
PRJNA729271	stage_3W_vs_E10	ordinary_muscle_development	ENSGALG00010007041	NAE1	-0.263557755	0.079549669	-3.313121965	0.000922607	0.001385536	negative
PRJNA729271	stage_3W_vs_E10	ordinary_muscle_development	ENSGALG00010007049	ENSGALG00010007049	-0.634883242	0.526118336	-1.206730878	0.227535804	0.261114043	negative
PRJNA729271	stage_3W_vs_E10	ordinary_muscle_development	ENSGALG00010007051	E2F3	-1.898874362	0.156391379	-12.14180971	6.34079E-34	4.19871E-33	negative
PRJNA729271	stage_3W_vs_E10	ordinary_muscle_development	ENSGALG00010007066	INIP	-0.559464201	0.153183838	-3.652240389	0.000259962	0.000409938	negative
PRJNA729271	stage_3W_vs_E10	ordinary_muscle_development	ENSGALG00010007067	CASP3	-1.26572654	0.107970843	-11.72285502	9.73354E-32	5.96817E-31	negative
PRJNA729271	stage_3W_vs_E10	ordinary_muscle_development	ENSGALG00010007068	NCAM2	-5.104358348	0.31982048	-15.96007343	2.42446E-57	3.39214E-56	negative
PRJNA729271	stage_3W_vs_E10	ordinary_muscle_development	ENSGALG00010007070	UBL3	1.251461623	0.051975467	24.07792944	4.2578E-128	2.9111E-126	positive
PRJNA729271	stage_3W_vs_E10	ordinary_muscle_development	ENSGALG00010007085	PRIMPOL	-0.390294359	0.166888535	-2.338652919	0.019353404	0.025495554	negative
PRJNA729271	stage_3W_vs_E10	ordinary_muscle_development	ENSGALG00010007090	CEBPZ	0.144627394	0.092641349	1.561153807	0.118487465	0.141787815	positive
PRJNA729271	stage_3W_vs_E10	ordinary_muscle_development	ENSGALG00010007092	DYNC1LI2	0.32614913	0.061180612	5.330922939	9.77149E-08	1.97956E-07	positive
PRJNA729271	stage_3W_vs_E10	ordinary_muscle_development	ENSGALG00010007097	ZCCHC9	-0.135153684	0.168242723	-0.803325584	0.421786574	0.460202965	negative
PRJNA729271	stage_3W_vs_E10	ordinary_muscle_development	ENSGALG00010007098	NDUFAF7	0.831664523	0.126716888	6.56317038	5.26756E-11	1.29259E-10	positive
PRJNA729271	stage_3W_vs_E10	ordinary_muscle_development	ENSGALG00010007102	CMTM3	-1.244516517	0.165618958	-7.514336097	5.72005E-14	1.64508E-13	negative
PRJNA729271	stage_3W_vs_E10	ordinary_muscle_development	ENSGALG00010007106	SLC7A1	-2.184946725	0.242541882	-9.008533733	2.0883E-19	7.80554E-19	negative
PRJNA729271	stage_3W_vs_E10	ordinary_muscle_development	ENSGALG00010007108	MBOAT1	0.37905936	0.190140612	1.99357389	0.046198641	0.058278145	positive
PRJNA729271	stage_3W_vs_E10	ordinary_muscle_development	ENSGALG00010007110	ARL6	-0.069333476	0.102770822	-0.674641638	0.499903472	0.538023525	negative
PRJNA729271	stage_3W_vs_E10	ordinary_muscle_development	ENSGALG00010007114	CKLF	0.139677422	0.146538083	0.953181722	0.340498005	0.378955666	positive
PRJNA729271	stage_3W_vs_E10	ordinary_muscle_development	ENSGALG00010007115	ENSGALG00010007115	0.982403862	0.277974946	3.534145342	0.000409096	0.000634616	positive
PRJNA729271	stage_3W_vs_E10	ordinary_muscle_development	ENSGALG00010007118	ENSGALG00010007118	-0.951414537	0.130255416	-7.304222501	2.78875E-13	7.72461E-13	negative
PRJNA729271	stage_3W_vs_E10	ordinary_muscle_development	ENSGALG00010007123	ENSGALG00010007123	2.536583528	0.243406384	10.42118733	1.9846E-25	9.44102E-25	positive
PRJNA729271	stage_3W_vs_E10	ordinary_muscle_development	ENSGALG00010007126	ENSGALG00010007126	2.346079765	1.075320344	2.181749631	0.029128016	0.037606715	positive
PRJNA729271	stage_3W_vs_E10	ordinary_muscle_development	ENSGALG00010007130	IRF2	1.829501937	0.121679699	15.03539175	4.30494E-51	5.04447E-50	positive
PRJNA729271	stage_3W_vs_E10	ordinary_muscle_development	ENSGALG00010007133	BLVRA	0.83499302	0.188490142	4.429902871	9.42755E-06	1.66751E-05	positive
PRJNA729271	stage_3W_vs_E10	ordinary_muscle_development	ENSGALG00010007135	TK2	-0.89572814	0.118094181	-7.584862657	3.32839E-14	9.67611E-14	negative
PRJNA729271	stage_3W_vs_E10	ordinary_muscle_development	ENSGALG00010007137	ENSGALG00010007137	1.421142631	0.359438449	3.953785789	7.69243E-05	0.000126773	positive
PRJNA729271	stage_3W_vs_E10	ordinary_muscle_development	ENSGALG00010007141	PRKD3	-0.061490756	0.082504674	-0.745300274	0.456090215	0.494541268	negative
PRJNA729271	stage_3W_vs_E10	ordinary_muscle_development	ENSGALG00010007143	CKMT2	10.85548045	0.316780733	34.26812087	2.3431E-257	7.0756E-255	positive
PRJNA729271	stage_3W_vs_E10	ordinary_muscle_development	ENSGALG00010007146	ENSGALG00010007146	1.805870572	0.141175264	12.79169258	1.82456E-37	1.34385E-36	positive
PRJNA729271	stage_3W_vs_E10	ordinary_muscle_development	ENSGALG00010007155	FOXL1	-2.460718699	0.772795337	-3.184179018	0.001451652	0.002143394	negative
PRJNA729271	stage_3W_vs_E10	ordinary_muscle_development	ENSGALG00010007160	ENSGALG00010007160	1.431961966	0.293869399	4.87278353	1.10037E-06	2.07788E-06	positive
PRJNA729271	stage_3W_vs_E10	ordinary_muscle_development	ENSGALG00010007162	ENSGALG00010007162	1.90442049	0.411932522	4.623137019	3.7798E-06	6.89854E-06	positive
PRJNA729271	stage_3W_vs_E10	ordinary_muscle_development	ENSGALG00010007165	ENSGALG00010007165	0.626434054	0.325489093	1.924593076	0.054280286	0.067821115	positive
PRJNA729271	stage_3W_vs_E10	ordinary_muscle_development	ENSGALG00010007166	RASGRF2	1.506199845	0.184489627	8.164143797	3.23723E-16	1.04113E-15	positive
PRJNA729271	stage_3W_vs_E10	ordinary_muscle_development	ENSGALG00010007168	MINA	0.000121626	0.107074185	0.001135905	0.999093679	0.99916261	positive
PRJNA729271	stage_3W_vs_E10	ordinary_muscle_development	ENSGALG00010007169	QPCT	0.466641571	0.218216508	2.138433868	0.032481546	0.041695006	positive
PRJNA729271	stage_3W_vs_E10	ordinary_muscle_development	ENSGALG00010007175	FOXC2	-4.460090364	0.541616629	-8.234773691	1.79897E-16	5.86904E-16	negative
PRJNA729271	stage_3W_vs_E10	ordinary_muscle_development	ENSGALG00010007181	GJB2	1.312178084	0.585873639	2.239694698	0.02511075	0.032661551	positive
PRJNA729271	stage_3W_vs_E10	ordinary_muscle_development	ENSGALG00010007182	ENSGALG00010007182	1.072065166	0.293690159	3.650327162	0.000261906	0.000412869	positive
PRJNA729271	stage_3W_vs_E10	ordinary_muscle_development	ENSGALG00010007183	VOPP1	1.247460211	0.31545543	3.954473729	7.67033E-05	0.000126443	positive
PRJNA729271	stage_3W_vs_E10	ordinary_muscle_development	ENSGALG00010007184	VGLL3	0.328927825	0.093258136	3.52706839	0.000420188	0.000651195	positive
PRJNA729271	stage_3W_vs_E10	ordinary_muscle_development	ENSGALG00010007187	ENPP6	-1.084531844	0.280686543	-3.863854084	0.000111612	0.000181695	negative
PRJNA729271	stage_3W_vs_E10	ordinary_muscle_development	ENSGALG00010007188	MAB21L1	-1.673005294	0.216569947	-7.725011316	1.11843E-14	3.3248E-14	negative
PRJNA729271	stage_3W_vs_E10	ordinary_muscle_development	ENSGALG00010007190	ENSGALG00010007190	0.201968546	0.148943478	1.356007993	0.175096625	0.204382797	positive
PRJNA729271	stage_3W_vs_E10	ordinary_muscle_development	ENSGALG00010007201	ENSGALG00010007201	3.213677109	0.252903767	12.70711443	5.39901E-37	3.92078E-36	positive
PRJNA729271	stage_3W_vs_E10	ordinary_muscle_development	ENSGALG00010007203	SLC30A6	0.342535261	0.104520883	3.277194471	0.001048441	0.001568334	positive
PRJNA729271	stage_3W_vs_E10	ordinary_muscle_development	ENSGALG00010007204	LANCL2	-1.102253095	0.087651135	-12.57545713	2.88129E-36	2.05837E-35	negative
PRJNA729271	stage_3W_vs_E10	ordinary_muscle_development	ENSGALG00010007207	EPM2A	1.62778356	0.102929184	15.814597	2.46784E-56	3.35881E-55	positive
PRJNA729271	stage_3W_vs_E10	ordinary_muscle_development	ENSGALG00010007214	MTHFSD	1.166699079	0.138644603	8.415034199	3.9278E-17	1.32496E-16	positive
PRJNA729271	stage_3W_vs_E10	ordinary_muscle_development	ENSGALG00010007217	MSH3	0.057391988	0.143248718	0.400645737	0.688680967	0.71940261	positive
PRJNA729271	stage_3W_vs_E10	ordinary_muscle_development	ENSGALG00010007223	FOXF1	-0.65091366	0.473224856	-1.375484935	0.168981153	0.19789786	negative
PRJNA729271	stage_3W_vs_E10	ordinary_muscle_development	ENSGALG00010007224	DHFR	-3.27510876	0.219749394	-14.90383522	3.11192E-50	3.55736E-49	negative
PRJNA729271	stage_3W_vs_E10	ordinary_muscle_development	ENSGALG00010007226	ENSGALG00010007226	0.016195712	1.521665537	0.010643411	0.991507947	0.99274074	positive
PRJNA729271	stage_3W_vs_E10	ordinary_muscle_development	ENSGALG00010007230	ENSGALG00010007230	-1.008681176	0.349816912	-2.883454579	0.003933394	0.005575261	negative
PRJNA729271	stage_3W_vs_E10	ordinary_muscle_development	ENSGALG00010007243	BEAN1	-1.848418907	0.190886778	-9.683226039	3.54976E-22	1.48111E-21	negative
PRJNA729271	stage_3W_vs_E10	ordinary_muscle_development	ENSGALG00010007251	SPAST	-0.342428682	0.063515866	-5.391230642	6.99768E-08	1.43083E-07	negative
PRJNA729271	stage_3W_vs_E10	ordinary_muscle_development	ENSGALG00010007252	NFATC1	1.196244142	0.152918178	7.822745059	5.16837E-15	1.56498E-14	positive
PRJNA729271	stage_3W_vs_E10	ordinary_muscle_development	ENSGALG00010007259	ANKRD34B	-1.244512037	0.096992841	-12.83096794	1.09981E-37	8.1711E-37	negative
PRJNA729271	stage_3W_vs_E10	ordinary_muscle_development	ENSGALG00010007265	CD200R1A	-0.067707826	0.287384987	-0.235599733	0.81374328	0.835505501	negative
PRJNA729271	stage_3W_vs_E10	ordinary_muscle_development	ENSGALG00010007270	CDH5	0.339873885	0.108431075	3.13446939	0.001721652	0.002525334	positive
PRJNA729271	stage_3W_vs_E10	ordinary_muscle_development	ENSGALG00010007271	CADM2	-3.259590049	0.317210373	-10.27579905	9.05906E-25	4.20061E-24	negative
PRJNA729271	stage_3W_vs_E10	ordinary_muscle_development	ENSGALG00010007275	DPY30	0.594822951	0.097435023	6.104816684	1.02919E-09	2.34303E-09	positive
PRJNA729271	stage_3W_vs_E10	ordinary_muscle_development	ENSGALG00010007277	FAM151B	-0.681092502	0.18971708	-3.590043138	0.000330623	0.00051748	negative
PRJNA729271	stage_3W_vs_E10	ordinary_muscle_development	ENSGALG00010007283	STOX2	-1.130656054	0.123108118	-9.184252624	4.14395E-20	1.58948E-19	negative
PRJNA729271	stage_3W_vs_E10	ordinary_muscle_development	ENSGALG00010007284	ENSGALG00010007284	0.175074306	0.081841886	2.139177312	0.032421311	0.041628745	positive
PRJNA729271	stage_3W_vs_E10	ordinary_muscle_development	ENSGALG00010007287	ENSGALG00010007287	0.625544422	0.620468836	1.008180242	0.313367941	0.351253849	positive
PRJNA729271	stage_3W_vs_E10	ordinary_muscle_development	ENSGALG00010007294	TRAPPC11	0.801050015	0.063885641	12.53881162	4.57809E-36	3.25291E-35	positive
PRJNA729271	stage_3W_vs_E10	ordinary_muscle_development	ENSGALG00010007300	CCDC125	1.359533077	0.238414829	5.702384722	1.18143E-08	2.53851E-08	positive
PRJNA729271	stage_3W_vs_E10	ordinary_muscle_development	ENSGALG00010007302	CDH11	-3.177486424	0.175356842	-18.12011659	2.21126E-73	4.70664E-72	negative
PRJNA729271	stage_3W_vs_E10	ordinary_muscle_development	ENSGALG00010007304	F5	2.204962645	0.35475043	6.215531988	5.11511E-10	1.18782E-09	positive
PRJNA729271	stage_3W_vs_E10	ordinary_muscle_development	ENSGALG00010007318	RMDN2	1.318860456	0.123178078	10.70694139	9.44303E-27	4.77421E-26	positive
PRJNA729271	stage_3W_vs_E10	ordinary_muscle_development	ENSGALG00010007319	ATP9B	0.342139215	0.101368926	3.375188323	0.000737652	0.001117853	positive
PRJNA729271	stage_3W_vs_E10	ordinary_muscle_development	ENSGALG00010007320	ENSGALG00010007320	1.710266835	0.302768039	5.648769401	1.616E-08	3.44369E-08	positive
PRJNA729271	stage_3W_vs_E10	ordinary_muscle_development	ENSGALG00010007321	CD200	1.633705374	0.150746542	10.83743182	2.28804E-27	1.18659E-26	positive
PRJNA729271	stage_3W_vs_E10	ordinary_muscle_development	ENSGALG00010007322	AK6	-0.14199149	0.108127846	-1.313181525	0.189121788	0.219639477	negative
PRJNA729271	stage_3W_vs_E10	ordinary_muscle_development	ENSGALG00010007324	ENSGALG00010007324	0.016158826	0.140741168	0.114812366	0.908593847	0.919312287	positive
PRJNA729271	stage_3W_vs_E10	ordinary_muscle_development	ENSGALG00010007326	RWDD4	1.412832322	0.115422083	12.24057208	1.88698E-34	1.27752E-33	positive
PRJNA729271	stage_3W_vs_E10	ordinary_muscle_development	ENSGALG00010007328	NOVA1	0.378798023	0.135120469	2.803409624	0.00505654	0.007086392	positive
PRJNA729271	stage_3W_vs_E10	ordinary_muscle_development	ENSGALG00010007330	MMP13	-0.549460602	0.708195332	-0.775860243	0.437831509	0.476489806	negative
PRJNA729271	stage_3W_vs_E10	ordinary_muscle_development	ENSGALG00010007333	QTRT2	-1.608440196	0.075150458	-21.40293258	1.2546E-101	5.39641E-100	negative
PRJNA729271	stage_3W_vs_E10	ordinary_muscle_development	ENSGALG00010007337	PRORP	-0.651724511	0.100216656	-6.50315561	7.86524E-11	1.9103E-10	negative
PRJNA729271	stage_3W_vs_E10	ordinary_muscle_development	ENSGALG00010007339	ENSGALG00010007339	0.881295961	0.223656965	3.940391302	8.13488E-05	0.000133691	positive

PRJNA729271	stage_3W_vs_E10	ordinary_muscle_development	ENSGALG00010007344	RAD17	0.385325929	0.107124663	3.596986136	0.000321926	0.000504248	positive
PRJNA729271	stage_3W_vs_E10	ordinary_muscle_development	ENSGALG00010007353	ING2	-0.847505366	0.126091848	-6.721333543	1.80069E-11	4.54088E-11	negative
PRJNA729271	stage_3W_vs_E10	ordinary_muscle_development	ENSGALG00010007357	NOX4	1.071885652	0.117078052	9.15530822	5.42029E-20	2.07028E-19	positive
PRJNA729271	stage_3W_vs_E10	ordinary_muscle_development	ENSGALG00010007360	ROBO1	-3.755359344	0.18785734	-19.99048507	6.66448E-89	2.12311E-87	negative
PRJNA729271	stage_3W_vs_E10	ordinary_muscle_development	ENSGALG00010007365	MEMO1	2.136725726	0.086359726	24.7421551	3.7657E-135	2.8429E-133	positive
PRJNA729271	stage_3W_vs_E10	ordinary_muscle_development	ENSGALG00010007369	ENSGALG00010007369	0.341874815	0.211108386	1.619427923	0.105355223	0.126995755	positive
PRJNA729271	stage_3W_vs_E10	ordinary_muscle_development	ENSGALG00010007374	CMIP	-0.245311505	0.134462942	-1.824380016	0.068094641	0.084017009	negative
PRJNA729271	stage_3W_vs_E10	ordinary_muscle_development	ENSGALG00010007375	CHORDC1	-0.180503997	0.074536297	-2.421692565	0.015448414	0.020564309	negative
PRJNA729271	stage_3W_vs_E10	ordinary_muscle_development	ENSGALG00010007376	CDKN2AIP	-0.807409337	0.061435567	-13.14237629	1.88222E-39	1.49658E-38	negative
PRJNA729271	stage_3W_vs_E10	ordinary_muscle_development	ENSGALG00010007381	EGFR	-0.488664628	0.098267188	-4.972815851	6.59873E-07	1.26503E-06	negative
PRJNA729271	stage_3W_vs_E10	ordinary_muscle_development	ENSGALG00010007382	EIF2AK2	0.864313724	0.256808774	3.365592661	0.000763794	0.001156019	positive
PRJNA729271	stage_3W_vs_E10	ordinary_muscle_development	ENSGALG00010007387	ENSGALG00010007387	-1.991102975	0.515753719	-3.860569304	0.000113123	0.000184031	negative
PRJNA729271	stage_3W_vs_E10	ordinary_muscle_development	ENSGALG00010007388	CYP1B1	-4.25417292	0.21020209	-20.23849015	4.48706E-91	1.51256E-89	negative
PRJNA729271	stage_3W_vs_E10	ordinary_muscle_development	ENSGALG00010007391	NANP	0.223872758	0.138842164	1.612426305	0.106869204	0.128724374	positive
PRJNA729271	stage_3W_vs_E10	ordinary_muscle_development	ENSGALG00010007392	NAALAD2	2.302242701	0.199746869	11.52580117	9.77982E-31	5.78134E-30	positive
PRJNA729271	stage_3W_vs_E10	ordinary_muscle_development	ENSGALG00010007393	PLCG2	1.003228324	0.126892567	7.906123632	2.65527E-15	8.1491E-15	positive
PRJNA729271	stage_3W_vs_E10	ordinary_muscle_development	ENSGALG00010007399	WWC2	-0.911124229	0.10999651	-8.283210367	1.19898E-16	3.94894E-16	negative
PRJNA729271	stage_3W_vs_E10	ordinary_muscle_development	ENSGALG00010007401	SEC61G	-0.037308333	0.220108213	-0.169499959	0.865403405	0.881024185	negative
PRJNA729271	stage_3W_vs_E10	ordinary_muscle_development	ENSGALG00010007403	SDR42E1	-1.719374427	0.249509925	-6.891006143	5.53991E-12	1.43114E-11	negative
PRJNA729271	stage_3W_vs_E10	ordinary_muscle_development	ENSGALG00010007404	FOLH1	1.869205176	0.17000345	10.9951014	4.0346E-28	2.15958E-27	positive
PRJNA729271	stage_3W_vs_E10	ordinary_muscle_development	ENSGALG00010007408	HCLS1	2.47169898	0.243423621	10.1538995	3.18382E-24	1.44262E-23	positive
PRJNA729271	stage_3W_vs_E10	ordinary_muscle_development	ENSGALG00010007411	ENSGALG00010007411	1.402668027	0.576478304	2.433167073	0.014967393	0.019960655	positive
PRJNA729271	stage_3W_vs_E10	ordinary_muscle_development	ENSGALG00010007413	GPATCH11	-0.002305463	0.097455309	-0.023656623	0.981126506	0.983909555	negative
PRJNA729271	stage_3W_vs_E10	ordinary_muscle_development	ENSGALG00010007414	ROBO2	-4.51901851	0.237736599	-19.00850998	1.45013E-80	3.69414E-79	negative
PRJNA729271	stage_3W_vs_E10	ordinary_muscle_development	ENSGALG00010007416	DCTD	-3.144662184	0.254539605	-12.35431392	4.61678E-35	3.18365E-34	negative
PRJNA729271	stage_3W_vs_E10	ordinary_muscle_development	ENSGALG00010007418	NME6	0.612626017	0.145761066	4.202946868	2.63462E-05	4.50022E-05	positive
PRJNA729271	stage_3W_vs_E10	ordinary_muscle_development	ENSGALG00010007419	RABL2A	-0.907574701	0.159171615	-5.701862723	1.18505E-08	2.54592E-08	negative
PRJNA729271	stage_3W_vs_E10	ordinary_muscle_development	ENSGALG00010007423	HEATR5B	-0.541623969	0.066236728	-8.177094227	2.90771E-16	9.39109E-16	negative
PRJNA729271	stage_3W_vs_E10	ordinary_muscle_development	ENSGALG00010007425	ARSA	-0.243876077	0.130763321	-1.865018988	0.062178743	0.077138041	negative
PRJNA729271	stage_3W_vs_E10	ordinary_muscle_development	ENSGALG00010007429	C21orf91	0.64999534	0.208625009	3.115615631	0.001835614	0.002680558	positive
PRJNA729271	stage_3W_vs_E10	ordinary_muscle_development	ENSGALG00010007430	MEF2C	0.426022693	0.14698206	2.898467293	0.003749914	0.005327355	positive
PRJNA729271	stage_3W_vs_E10	ordinary_muscle_development	ENSGALG00010007432	NAT	2.23501458	0.185352414	12.05818973	1.756E-33	1.14706E-32	positive
PRJNA729271	stage_3W_vs_E10	ordinary_muscle_development	ENSGALG00010007433	RNF144B	3.508155512	0.266956247	13.14131269	1.90886E-39	1.51611E-38	positive
PRJNA729271	stage_3W_vs_E10	ordinary_muscle_development	ENSGALG00010007434	ENSGALG00010007434	0.631647187	0.250541854	2.521124425	0.011698048	0.015796833	positive
PRJNA729271	stage_3W_vs_E10	ordinary_muscle_development	ENSGALG00010007436	PNAT3	2.166208345	0.260347998	8.320434036	8.76418E-17	2.90702E-16	positive
PRJNA729271	stage_3W_vs_E10	ordinary_muscle_development	ENSGALG00010007437	TUSC1	0.277608664	0.138419114	2.005565964	0.044902576	0.056725017	positive
PRJNA729271	stage_3W_vs_E10	ordinary_muscle_development	ENSGALG00010007439	HSD17B2	-5.822483176	0.379495518	-15.34269286	3.96404E-53	4.97478E-52	negative
PRJNA729271	stage_3W_vs_E10	ordinary_muscle_development	ENSGALG00010007441	STRN	1.584397183	0.135086732	11.72874183	9.08001E-32	5.57453E-31	positive
PRJNA729271	stage_3W_vs_E10	ordinary_muscle_development	ENSGALG00010007455	TRPC6	1.000402902	0.208520245	4.797629608	1.60554E-06	2.99554E-06	positive
PRJNA729271	stage_3W_vs_E10	ordinary_muscle_development	ENSGALG00010007457	SHANK3	-0.011412969	0.18061642	-0.063188989	0.949616013	0.956080024	negative
PRJNA729271	stage_3W_vs_E10	ordinary_muscle_development	ENSGALG00010007465	PNAT10	1.91255325	0.193543714	9.881763734	4.9946E-23	2.15981E-22	positive
PRJNA729271	stage_3W_vs_E10	ordinary_muscle_development	ENSGALG00010007467	CHODL	-2.864651815	0.333004549	-8.602440484	7.80386E-18	2.73098E-17	negative
PRJNA729271	stage_3W_vs_E10	ordinary_muscle_development	ENSGALG00010007479	NFX1	-0.438145335	0.097211202	-4.507148638	6.57046E-06	1.17666E-05	negative
PRJNA729271	stage_3W_vs_E10	ordinary_muscle_development	ENSGALG00010007480	TENM3	-0.826894167	0.106374628	-7.773415322	7.63977E-15	2.29367E-14	negative
PRJNA729271	stage_3W_vs_E10	ordinary_muscle_development	ENSGALG00010007485	CTSC	1.316020712	0.155354241	8.471096134	2.43095E-17	8.27732E-17	positive
PRJNA729271	stage_3W_vs_E10	ordinary_muscle_development	ENSGALG00010007500	MPHOSPH6	1.016278218	0.116176861	8.747681838	2.1778E-18	7.81368E-18	positive
PRJNA729271	stage_3W_vs_E10	ordinary_muscle_development	ENSGALG00010007506	RAB38	-0.597739477	0.558888715	-1.069514308	0.284837985	0.322003322	negative
PRJNA729271	stage_3W_vs_E10	ordinary_muscle_development	ENSGALG00010007513	GALNT12	-0.464936853	0.129752886	-3.583248637	0.000339347	0.000530791	negative
PRJNA729271	stage_3W_vs_E10	ordinary_muscle_development	ENSGALG00010007514	VIT	-4.009628908	0.372369098	-10.76788844	4.88066E-27	2.49806E-26	negative
PRJNA729271	stage_3W_vs_E10	ordinary_muscle_development	ENSGALG00010007521	SLC12A7	-0.600361565	0.13712665	-4.378153803	1.19689E-05	2.10213E-05	negative
PRJNA729271	stage_3W_vs_E10	ordinary_muscle_development	ENSGALG00010007529	CDH13	2.103397243	0.134284689	15.66371609	2.67783E-55	3.55776E-54	positive
PRJNA729271	stage_3W_vs_E10	ordinary_muscle_development	ENSGALG00010007532	DCUN1D5	1.512281935	0.265733761	5.690966521	1.26322E-08	2.70904E-08	positive
PRJNA729271	stage_3W_vs_E10	ordinary_muscle_development	ENSGALG00010007536	GABBR2	-1.422734029	0.214605805	-6.629522577	3.36774E-11	8.37315E-11	negative
PRJNA729271	stage_3W_vs_E10	ordinary_muscle_development	ENSGALG00010007539	RPS3A	0.036122609	0.17535346	0.205998835	0.836791821	0.855682359	positive
PRJNA729271	stage_3W_vs_E10	ordinary_muscle_development	ENSGALG00010007542	MRPL36	1.184020649	0.109645192	10.79865548	3.49281E-27	1.79724E-26	positive
PRJNA729271	stage_3W_vs_E10	ordinary_muscle_development	ENSGALG00010007544	MLYCD	0.946839127	0.100909498	9.383052603	6.40898E-21	2.54515E-20	positive
PRJNA729271	stage_3W_vs_E10	ordinary_muscle_development	ENSGALG00010007547	ENSGALG00010007547	2.399963704	0.542991549	4.419891451	9.87505E-06	1.74368E-05	positive
PRJNA729271	stage_3W_vs_E10	ordinary_muscle_development	ENSGALG00010007548	HSBP1	-0.442938834	0.087220832	-5.078360544	3.80706E-07	7.40914E-07	negative
PRJNA729271	stage_3W_vs_E10	ordinary_muscle_development	ENSGALG00010007549	SH3D19	0.185014513	0.15807067	1.170454412	0.24181815	0.276388116	positive
PRJNA729271	stage_3W_vs_E10	ordinary_muscle_development	ENSGALG00010007551	FEZ2	-0.243030278	0.111676327	-2.176202294	0.029540137	0.038115034	negative
PRJNA729271	stage_3W_vs_E10	ordinary_muscle_development	ENSGALG00010007555	NDUFS6	1.945324529	0.129354424	15.0387166	4.0941E-51	4.8013E-50	positive
PRJNA729271	stage_3W_vs_E10	ordinary_muscle_development	ENSGALG00010007564	IRX4	-9.004447202	0.947294196	-9.505439011	1.99206E-21	8.08142E-21	negative
PRJNA729271	stage_3W_vs_E10	ordinary_muscle_development	ENSGALG00010007566	FHIP1A	-0.189525219	0.176077509	-1.076373811	0.28176011	0.318797345	negative
PRJNA729271	stage_3W_vs_E10	ordinary_muscle_development	ENSGALG00010007567	TRIP13	-2.947991955	0.146732651	-20.09090642	8.86293E-90	2.91311E-88	negative
PRJNA729271	stage_3W_vs_E10	ordinary_muscle_development	ENSGALG00010007569	GPR89A	-0.199920539	0.082288214	-2.429516086	0.015118994	0.020153561	negative
PRJNA729271	stage_3W_vs_E10	ordinary_muscle_development	ENSGALG00010007574	ENSGALG00010007574	0.311691972	0.188262164	1.655627264	0.097797325	0.118407303	positive
PRJNA729271	stage_3W_vs_E10	ordinary_muscle_development	ENSGALG00010007575	MMP27	-2.869922873	0.342827528	-8.371331461	5.69708E-17	1.90714E-16	negative
PRJNA729271	stage_3W_vs_E10	ordinary_muscle_development	ENSGALG00010007577	WFDC1	0.166335579	0.119446625	1.392551522	0.163755451	0.192150511	positive
PRJNA729271	stage_3W_vs_E10	ordinary_muscle_development	ENSGALG00010007579	GATB	0.453014997	0.178840863	2.533062015	0.011307095	0.015290264	positive
PRJNA729271	stage_3W_vs_E10	ordinary_muscle_development	ENSGALG00010007580	BRD9	-0.611257039	0.101783363	-6.005471055	1.90777E-09	4.2701E-09	negative
PRJNA729271	stage_3W_vs_E10	ordinary_muscle_development	ENSGALG00010007581	LPCAT1	-0.89362676	0.117128594	-7.629450104	2.35757E-14	6.90085E-14	negative
PRJNA729271	stage_3W_vs_E10	ordinary_muscle_development	ENSGALG00010007584	ELP2	-0.493461131	0.115727491	-4.263992293	2.00806E-05	3.46924E-05	negative
PRJNA729271	stage_3W_vs_E10	ordinary_muscle_development	ENSGALG00010007585	KCNG4	-0.13627877	0.591550602	-0.230375506	0.817799992	0.8391626	negative
PRJNA729271	stage_3W_vs_E10	ordinary_muscle_development	ENSGALG00010007586	MMP7	2.798869667	1.500844999	1.864862573	0.06220067	0.07715864	positive
PRJNA729271	stage_3W_vs_E10	ordinary_muscle_development	ENSGALG00010007588	TTC27	-1.073323599	0.103949685	-10.32541468	5.40846E-25	2.52971E-24	negative
PRJNA729271	stage_3W_vs_E10	ordinary_muscle_development	ENSGALG00010007590	FBXW7	-1.19751971	0.141252058	-8.477892109	2.2931E-17	7.81532E-17	negative
PRJNA729271	stage_3W_vs_E10	ordinary_muscle_development	ENSGALG00010007593	SEC61B	-1.279878585	0.119223198	-10.73514727	6.96079E-27	3.54272E-26	negative
PRJNA729271	stage_3W_vs_E10	ordinary_muscle_development	ENSGALG00010007595	ATP2C2	0.498686019	0.16191932	3.0798426	0.0020711	0.003010791	positive
PRJNA729271	stage_3W_vs_E10	ordinary_muscle_development	ENSGALG00010007596	ENSGALG00010007596	-3.333940505	0.516732345	-6.45196791	1.10407E-10	2.65618E-10	negative
PRJNA729271	stage_3W_vs_E10	ordinary_muscle_development	ENSGALG00010007597	SMKR1	-1.527719965	0.728623042	-2.096722005	0.036018191	0.046043186	negative
PRJNA729271	stage_3W_vs_E10	ordinary_muscle_development	ENSGALG00010007601	TMEM154	0.132507696	0.311317339	0.425635449	0.670373505	0.701991546	positive
PRJNA729271	stage_3W_vs_E10	ordinary_muscle_development	ENSGALG00010007603	ALG2	-0.129205653	0.10491774	-1.231494816	0.218137854	0.250945095	negative
PRJNA729271	stage_3W_vs_E10	ordinary_muscle_development	ENSGALG00010007604	ENSGALG00010007604	-5.035504666	0.659950697	-7.630122502	2.34531E-14	6.86634E-14	negative
PRJNA729271	stage_3W_vs_E10	ordinary_muscle_development	ENSGALG00010007610	TLDC1	-0.146847865	0.158312644	-0.927581402	0.353624749	0.392239878	negative
PRJNA729271	stage_3W_vs_E10	ordinary_muscle_development	ENSGALG00010007614	BIRC6	0.117783104	0.088007488	1.338330482	0.180788729	0.210586036	positive
PRJNA729271	stage_3W_vs_E10	ordinary_muscle_development	ENSGALG00010007624	TPPP	1.984449545	0.247057926	8.032324954	9.56426E-16	3.00074E-15	positive
PRJNA729271	stage_3W_vs_E10	ordinary_muscle_development	ENSGALG00010007626	DLK2	-2.90071791	0.374652377	-7.742424942	9.75384E-15	2.90909E-14	negative
PRJNA729271	stage_3W_vs_E10	ordinary_muscle_development	ENSGALG00010007633	ENSGALG00010007633	0.445634044	0.102643364	4.34157678	1.41464E-05	2.4711E-05	positive
PRJNA729271	stage_3W_vs_E10	ordinary_muscle_development	ENSGALG00010007640	TMEM123	-0.91831316	0.218282453	-4.206994872	2.58789E-05	4.42561E-05	negative
PRJNA729271	stage_3W_vs_E10	ordinary_muscle_development	ENSGALG00010007642	ZNF318	0.644995182	0.106655311	6.047473648	1.47135E-09	3.31734E-09	positive
PRJNA729271	stage_3W_vs_E10	ordinary_muscle_development	ENSGALG00010007643	COTL1	-0.475751704	0.164028718	-2.900417122	0.003726664	0.00529744	negative
PRJNA729271	stage_3W_vs_E10	ordinary_muscle_development	ENSGALG00010007650	KLHL36	-0.221612717	0.088319869	-2.509205666	0.012100301	0.016309639	negative
PRJNA729271	stage_3W_vs_E10	ordinary_muscle_development	ENSGALG00010007651	CTNNAL1	-0.659511872	0.136900116	-4.817467579	1.45392E-06	2.72246E-06	negative
PRJNA729271	stage_3W_vs_E10	ordinary_muscle_development	ENSGALG00010007658	BIRC2	1.561062203	0.118655011	13.15631083	1.56545E-39	1.24745E-38	positive
PRJNA729271	stage_3W_vs_E10	ordinary_muscle_development	ENSGALG00010007660	FAM98A	0.830219509	0.096692684	8.586166744	8.99191E-18	3.13689E-17	positive
PRJNA729271	stage_3W_vs_E10	ordinary_muscle_development	ENSGALG00010007663	ARFIP1	-0.306700193	0.110344535	-2.779477862	0.005444636	0.007603816	negative
PRJNA729271	stage_3W_vs_E10	ordinary_muscle_development	ENSGALG00010007671	FAM206A	0.683025137	0.099855162	6.840158524	7.91056E-12	2.02873E-11	positive
PRJNA729271	stage_3W_vs_E10	ordinary_muscle_development	ENSGALG00010007673	FHDC1	-2.279864921	0.341305007	-6.679846102	2.39193E-11	5.99638E-11	negative
PRJNA729271	stage_3W_vs_E10	ordinary_muscle_development	ENSGALG00010007678	CDH8	0.789359262	0.282694578	2.792268837	0.005233985	0.007324446	positive
PRJNA729271	stage_3W_vs_E10	ordinary_muscle_development	ENSGALG00010007679	TMEM135	1.445921826	0.141250339	10.23659009	1.35943E-24	6.25953E-24	positive
PRJNA729271	stage_3W_vs_E10	ordinary_muscle_development	ENSGALG00010007680	CHMP2B	0.100512013	0.125722333	0.799476199	0.424014342	0.462459586	positive
PRJNA729271	stage_3W_vs_E10	ordinary_muscle_development	ENSGALG00010007695	RASGRP3	1.017657743	0.142821027	7.125405568	1.03775E-12	2.79854E-12	positive
PRJNA729271	stage_3W_vs_E10	ordinary_muscle_development	ENSGALG00010007698	ADAMTS18	-1.594207423	0.400426368	-3.981274839	6.85466E-05	0.000113501	negative
PRJNA729271	stage_3W_vs_E10	ordinary_muscle_development	ENSGALG00010007699	SLC39A6	-1.922988425	0.108289325	-17.75787619	1.49796E-70	2.93023E-69	negative
PRJNA729271	stage_3W_vs_E10	ordinary_muscle_development	ENSGALG00010007707	ENSGALG00010007707	4.2748487	0.71785423	5.955037275	2.60013E-09	5.78049E-09	positive
PRJNA729271	stage_3W_vs_E10	ordinary_muscle_development	ENSGALG00010007713	SCLT1	-0.234811755	0.212677323	-1.10407519	0.269560523	0.306045255	negative
PRJNA729271	stage_3W_vs_E10	ordinary_muscle_development	ENSGALG00010007719	LTBP1	-2.43867198	0.191185366	-12.75553682	2.90368E-37	2.12785E-36	negative
PRJNA729271	stage_3W_vs_E10	ordinary_muscle_development	ENSGALG00010007724	RBM11	1.929743599	0.327960912	5.884065846	4.00308E-09	8.81031E-09	positive
PRJNA729271	stage_3W_vs_E10	ordinary_muscle_development	ENSGALG00010007730	COG2	-0.30758575	0.064166297	-4.793571735	1.63838E-06	3.05602E-06	negative
PRJNA729271	stage_3W_vs_E10	ordinary_muscle_development	ENSGALG00010007731	ENSGALG00010007731	0.333639978	0.267922141	1.245287069	0.213026247	0.245649598	positive
PRJNA729271	stage_3W_vs_E10	ordinary_muscle_development	ENSGALG00010007741	ENSGALG00010007741	0.207585023	0.204868561	1.013259538	0.310936209	0.348894593	positive
PRJNA729271	stage_3W_vs_E10	ordinary_muscle_development	ENSGALG00010007748	CSGALNACT1	-2.70072246	0.270152429	-9.997031944	1.57033E-23	6.90384E-23	negative
PRJNA729271	stage_3W_vs_E10	ordinary_muscle_development	ENSGALG00010007751	HSPA13	-0.708579868	0.098288676	-7.209170966	5.62935E-13	1.53552E-12	negative
PRJNA729271	stage_3W_vs_E10	ordinary_muscle_development	ENSGALG00010007753	PRSS23	1.462983884	0.227638841	6.426776197	1.30338E-10	3.1248E-10	positive
PRJNA729271	stage_3W_vs_E10	ordinary_muscle_development	ENSGALG00010007758	DEK	-0.094600429	0.07660577	-1.234899525	0.2168679	0.249603002	negative
PRJNA729271	stage_3W_vs_E10	ordinary_muscle_development	ENSGALG00010007759	AGT	-2.458691906	1.308411919	-1.879142089			negative
PRJNA729271	stage_3W_vs_E10	ordinary_muscle_development	ENSGALG00010007761	ENSGALG00010007761	1.889774653	0.114366684	16.52382135	2.47226E-61	3.83266E-60	positive
PRJNA729271	stage_3W_vs_E10	ordinary_muscle_development	ENSGALG00010007762	SH2D4A	-0.459045982	0.326450844	-1.406171834	0.159673106	0.187664126	negative
PRJNA729271	stage_3W_vs_E10	ordinary_muscle_development	ENSGALG00010007763	FZD4	0.527835887	0.099765675	5.290756424	1.21812E-07	2.45128E-07	positive
PRJNA729271	stage_3W_vs_E10	ordinary_muscle_development	ENSGALG00010007765	NUDT7	0.762808329	0.095108983	8.020360488	1.05435E-15	3.29869E-15	positive
PRJNA729271	stage_3W_vs_E10	ordinary_muscle_development	ENSGALG00010007766	ENSGALG00010007766	2.048976081	0.126265732	16.22749137	3.22368E-59	4.75837E-58	positive
PRJNA729271	stage_3W_vs_E10	ordinary_muscle_development	ENSGALG00010007767	VAT1L	-2.315605225	0.326073762	-7.101476707	1.23431E-12	3.31075E-12	negative
PRJNA729271	stage_3W_vs_E10	ordinary_muscle_development	ENSGALG00010007770	ENSGALG00010007770	-1.165305205	0.439913975	-2.648938819	0.008074495	0.011090666	negative
PRJNA729271	stage_3W_vs_E10	ordinary_muscle_development	ENSGALG00010007771	CAPN9	-2.751939048	0.570666477	-4.822324702	1.41895E-06	2.65904E-06	negative
PRJNA729271	stage_3W_vs_E10	ordinary_muscle_development	ENSGALG00010007772	CLEC3A	-4.655960947	0.756726906	-6.152762521	7.61448E-10	1.74916E-09	negative
PRJNA729271	stage_3W_vs_E10	ordinary_muscle_development	ENSGALG00010007774	ENSGALG00010007774	0.583713573	0.278628447	2.094953254	0.036175148	0.046227521	positive
PRJNA729271	stage_3W_vs_E10	ordinary_muscle_development	ENSGALG00010007777	KIF13A	0.610141665	0.124574976	4.89778672	9.69222E-07	1.83838E-06	positive
PRJNA729271	stage_3W_vs_E10	ordinary_muscle_development	ENSGALG00010007780	SIRT5	0.753956613	0.142306107	5.298132521	1.16993E-07	2.35923E-07	positive
PRJNA729271	stage_3W_vs_E10	ordinary_muscle_development	ENSGALG00010007781	MBP	4.024631768	0.234960427	17.12897708	9.02272E-66	1.57193E-64	positive
PRJNA729271	stage_3W_vs_E10	ordinary_muscle_development	ENSGALG00010007783	NOL7	-0.290838452	0.109596792	-2.653713179	0.007961147	0.010942236	negative
PRJNA729271	stage_3W_vs_E10	ordinary_muscle_development	ENSGALG00010007784	INTS10	-0.372741018	0.094276023	-3.953720202	7.69454E-05	0.000126785	negative

PRJNA729271	stage_3W_vs_E10	ordinary_muscle_development	ENSGALG00010007786	SAMSN1	2.28226272	0.2237962	10.19795118	2.02499E-24	9.26231E-24	positive
PRJNA729271	stage_3W_vs_E10	ordinary_muscle_development	ENSGALG00010007787	C1orf198	-2.307098722	0.120325454	-19.17382106	6.1241E-82	1.61398E-80	negative
PRJNA729271	stage_3W_vs_E10	ordinary_muscle_development	ENSGALG00010007788	PALM2AKAP2	-0.678412824	0.171690327	-3.951374748	7.77035E-05	0.000127961	negative
PRJNA729271	stage_3W_vs_E10	ordinary_muscle_development	ENSGALG00010007789	WWOX	-1.011581213	0.114795168	-8.812053949	1.22873E-18	4.44374E-18	negative
PRJNA729271	stage_3W_vs_E10	ordinary_muscle_development	ENSGALG00010007792	ENSGALG00010007792	1.378417657	0.222274294	6.201426307	5.59537E-10	1.29706E-09	positive
PRJNA729271	stage_3W_vs_E10	ordinary_muscle_development	ENSGALG00010007795	ZNF236	0.213489319	0.115861663	1.842622605	0.065384131	0.080920678	positive
PRJNA729271	stage_3W_vs_E10	ordinary_muscle_development	ENSGALG00010007796	MCUR1	-0.416774776	0.101868387	-4.09130631	4.2895E-05	7.2153E-05	negative
PRJNA729271	stage_3W_vs_E10	ordinary_muscle_development	ENSGALG00010007798	HGSNAT	1.024301599	0.096710079	10.59146691	3.26436E-26	1.61051E-25	positive
PRJNA729271	stage_3W_vs_E10	ordinary_muscle_development	ENSGALG00010007800	MAF	-0.150866308	0.114804561	-1.314114228	0.188807767	0.219309927	negative
PRJNA729271	stage_3W_vs_E10	ordinary_muscle_development	ENSGALG00010007801	TTC13	0.169394253	0.160437421	1.055827574	0.291046998	0.328254453	positive
PRJNA729271	stage_3W_vs_E10	ordinary_muscle_development	ENSGALG00010007802	ENSGALG00010007802	4.355254626	0.71948304	6.053311039	1.41899E-09	3.20477E-09	positive
PRJNA729271	stage_3W_vs_E10	ordinary_muscle_development	ENSGALG00010007803	ME3	0.402175295	0.1776334	2.264074742	0.023569518	0.030770077	positive
PRJNA729271	stage_3W_vs_E10	ordinary_muscle_development	ENSGALG00010007805	ENSGALG00010007805	9.983990516	1.516797605	6.582282623	4.6328E-11	1.14147E-10	positive
PRJNA729271	stage_3W_vs_E10	ordinary_muscle_development	ENSGALG00010007806	DYNLRB2	-1.544279915	0.418863907	-3.68682975	0.000227065	0.000359745	negative
PRJNA729271	stage_3W_vs_E10	ordinary_muscle_development	ENSGALG00010007809	ARV1	-0.254479668	0.119397244	-2.131369698	0.033058697	0.042405824	negative
PRJNA729271	stage_3W_vs_E10	ordinary_muscle_development	ENSGALG00010007811	PRCP	-0.128544243	0.183298615	-0.701283218	0.483126285	0.5215159	negative
PRJNA729271	stage_3W_vs_E10	ordinary_muscle_development	ENSGALG00010007812	POMK	0.03716593	0.121325278	0.306332951	0.759351153	0.7850781	positive
PRJNA729271	stage_3W_vs_E10	ordinary_muscle_development	ENSGALG00010007817	CDYL2	-1.640704215	0.14083258	-11.65003304	2.2937E-31	1.38242E-30	negative
PRJNA729271	stage_3W_vs_E10	ordinary_muscle_development	ENSGALG00010007818	ENSGALG00010007818	0.612803334	0.221863512	2.762073525	0.005743554	0.007998157	positive
PRJNA729271	stage_3W_vs_E10	ordinary_muscle_development	ENSGALG00010007819	NRIP1	1.77885646	0.16643696	10.6878692	1.16005E-26	5.8426E-26	positive
PRJNA729271	stage_3W_vs_E10	ordinary_muscle_development	ENSGALG00010007821	FAM89A	-2.33865272	0.127113983	-18.39807594	1.36113E-75	3.05411E-74	negative
PRJNA729271	stage_3W_vs_E10	ordinary_muscle_development	ENSGALG00010007826	ENSGALG00010007826	9.781006644	1.373365153	7.121927204	1.06428E-12	2.86743E-12	positive
PRJNA729271	stage_3W_vs_E10	ordinary_muscle_development	ENSGALG00010007830	SMIM19	1.187997669	0.130074387	9.133217514	6.64931E-20	2.53236E-19	positive
PRJNA729271	stage_3W_vs_E10	ordinary_muscle_development	ENSGALG00010007835	GUCY1A1	1.228239724	0.186351254	6.59099253	4.36896E-11	1.07737E-10	positive
PRJNA729271	stage_3W_vs_E10	ordinary_muscle_development	ENSGALG00010007845	TFAP2B	-7.726054232	1.094265546	-7.060493001	1.65913E-12	4.4216E-12	negative
PRJNA729271	stage_3W_vs_E10	ordinary_muscle_development	ENSGALG00010007846	ENSGALG00010007846	0.334842712	0.148442017	2.255713828	0.024088559	0.031402434	positive
PRJNA729271	stage_3W_vs_E10	ordinary_muscle_development	ENSGALG00010007847	CMC2	0.596862607	0.114149012	5.228802217	1.70612E-07	3.3956E-07	positive
PRJNA729271	stage_3W_vs_E10	ordinary_muscle_development	ENSGALG00010007849	CXADR	-3.111905734	0.315276759	-9.870393687	5.59446E-23	2.4156E-22	negative
PRJNA729271	stage_3W_vs_E10	ordinary_muscle_development	ENSGALG00010007850	ENSGALG00010007850	-1.703427624	0.285315881	-5.970321802	2.36786E-09	5.27384E-09	negative
PRJNA729271	stage_3W_vs_E10	ordinary_muscle_development	ENSGALG00010007851	AvBD7	2.822905185	0.904155374	3.122146112	0.001795378	0.002624181	positive
PRJNA729271	stage_3W_vs_E10	ordinary_muscle_development	ENSGALG00010007852	SLC20A2	1.616629801	0.207342062	7.796921586	6.34356E-15	1.91204E-14	positive
PRJNA729271	stage_3W_vs_E10	ordinary_muscle_development	ENSGALG00010007853	C1orf131	0.835724429	0.109348826	7.64273798	2.1265E-14	6.23961E-14	positive
PRJNA729271	stage_3W_vs_E10	ordinary_muscle_development	ENSGALG00010007854	FAM181B	0.822785059	0.117354807	7.011089511	2.36469E-12	6.24453E-12	positive
PRJNA729271	stage_3W_vs_E10	ordinary_muscle_development	ENSGALG00010007855	RNF182	-1.791684136	0.225496843	-7.945495438	1.93416E-15	5.97012E-15	negative
PRJNA729271	stage_3W_vs_E10	ordinary_muscle_development	ENSGALG00010007856	PDCD2	1.33257055	0.127944418	10.41523011	2.11286E-25	1.00347E-24	positive
PRJNA729271	stage_3W_vs_E10	ordinary_muscle_development	ENSGALG00010007857	ENSGALG00010007857	-0.399260544	0.33940763	-1.176345223	0.239456951	0.273818911	negative
PRJNA729271	stage_3W_vs_E10	ordinary_muscle_development	ENSGALG00010007859	CENPN	-2.348456559	0.16541754	-14.19714356	9.54234E-46	9.35833E-45	negative
PRJNA729271	stage_3W_vs_E10	ordinary_muscle_development	ENSGALG00010007860	DEFB4A	2.324318952	0.896767063	2.591887066	0.00954511	0.013002196	positive
PRJNA729271	stage_3W_vs_E10	ordinary_muscle_development	ENSGALG00010007861	INTU	-2.527568222	0.182401355	-13.85717895	1.1511E-43	1.05071E-42	negative
PRJNA729271	stage_3W_vs_E10	ordinary_muscle_development	ENSGALG00010007862	ATMIN	0.602075734	0.085616642	7.032227864	2.03261E-12	5.39512E-12	positive
PRJNA729271	stage_3W_vs_E10	ordinary_muscle_development	ENSGALG00010007863	AvBD1	1.737443262	0.836204156	2.07777401	0.037730179	0.048112865	positive
PRJNA729271	stage_3W_vs_E10	ordinary_muscle_development	ENSGALG00010007864	TYR	-3.225435076	1.061502114	-3.038557374	0.002377138	0.003437412	negative
PRJNA729271	stage_3W_vs_E10	ordinary_muscle_development	ENSGALG00010007865	GABRR3	0.709848697	0.425182894	1.66951377	0.095015604	0.115231859	positive
PRJNA729271	stage_3W_vs_E10	ordinary_muscle_development	ENSGALG00010007866	TBP	-0.405152071	0.086041424	-4.708802467	2.49176E-06	4.60045E-06	negative
PRJNA729271	stage_3W_vs_E10	ordinary_muscle_development	ENSGALG00010007867	ZNF516	-1.537315897	0.107475772	-14.3038368	2.07071E-46	2.07716E-45	negative
PRJNA729271	stage_3W_vs_E10	ordinary_muscle_development	ENSGALG00010007868	INHBA	-1.969396349	0.29955247	-6.574462054	4.88294E-11	1.20024E-10	negative
PRJNA729271	stage_3W_vs_E10	ordinary_muscle_development	ENSGALG00010007869	GCSH	-1.060963131	0.124387769	-8.529481128	1.47006E-17	5.07103E-17	negative
PRJNA729271	stage_3W_vs_E10	ordinary_muscle_development	ENSGALG00010007870	AvBD3	-2.552589422	0.792590058	-3.220567047	0.001279373	0.00189869	negative
PRJNA729271	stage_3W_vs_E10	ordinary_muscle_development	ENSGALG00010007871	HSPA4L	-0.943030973	0.088733081	-10.62772709	2.21444E-26	1.10114E-25	negative
PRJNA729271	stage_3W_vs_E10	ordinary_muscle_development	ENSGALG00010007872	KLHL2	-0.714262666	0.077711847	-9.191168338	3.88597E-20	1.49251E-19	negative
PRJNA729271	stage_3W_vs_E10	ordinary_muscle_development	ENSGALG00010007873	ENSGALG00010007873	-0.286171582	0.767563906	-0.372830952	0.709274264	0.738201369	negative
PRJNA729271	stage_3W_vs_E10	ordinary_muscle_development	ENSGALG00010007874	ENSGALG00010007874	-0.81162073	0.595796649	-1.362244537	0.173120725	0.202353431	negative
PRJNA729271	stage_3W_vs_E10	ordinary_muscle_development	ENSGALG00010007875	AvBD4	3.546402646	1.427584025	2.484198886	0.012984327	0.017433106	positive
PRJNA729271	stage_3W_vs_E10	ordinary_muscle_development	ENSGALG00010007877	MSMO1	-0.387344863	0.187377857	-2.067185897	0.038716641	0.049320864	negative
PRJNA729271	stage_3W_vs_E10	ordinary_muscle_development	ENSGALG00010007878	PSMB1	0.585938022	0.12201601	4.802140471	1.56979E-06	2.93147E-06	positive
PRJNA729271	stage_3W_vs_E10	ordinary_muscle_development	ENSGALG00010007879	PLK4	-3.235885724	0.20342146	-15.9072977	5.63957E-57	7.83754E-56	negative
PRJNA729271	stage_3W_vs_E10	ordinary_muscle_development	ENSGALG00010007880	BCO1	1.966943446	0.281326897	6.991665106	2.71643E-12	7.14473E-12	positive
PRJNA729271	stage_3W_vs_E10	ordinary_muscle_development	ENSGALG00010007881	AvBD14	-2.945271336	0.880685471	-3.344294228	0.000824922	0.00124438	negative
PRJNA729271	stage_3W_vs_E10	ordinary_muscle_development	ENSGALG00010007882	CPE	-0.919730937	0.227457323	-4.043531884	5.2652E-05	8.80266E-05	negative
PRJNA729271	stage_3W_vs_E10	ordinary_muscle_development	ENSGALG00010007883	DHTKD1	0.255366722	0.120759936	2.114664268	0.034458572	0.044127308	positive
PRJNA729271	stage_3W_vs_E10	ordinary_muscle_development	ENSGALG00010007884	GLI3	-1.88184745	0.114716394	-16.40434634	1.78033E-60	2.69653E-59	negative
PRJNA729271	stage_3W_vs_E10	ordinary_muscle_development	ENSGALG00010007885	USP38	0.972810296	0.103546261	9.394934071	5.72537E-21	2.27617E-20	positive
PRJNA729271	stage_3W_vs_E10	ordinary_muscle_development	ENSGALG00010007886	GRM5	-2.286450991	0.8372413	-2.730934308	0.006315506	0.00876012	negative
PRJNA729271	stage_3W_vs_E10	ordinary_muscle_development	ENSGALG00010007887	CDT1	-1.903624403	0.129581121	-14.69059993	7.40552E-49	8.07091E-48	negative
PRJNA729271	stage_3W_vs_E10	ordinary_muscle_development	ENSGALG00010007889	DCAF6	2.370431498	0.096986015	24.44096188	6.279E-132	4.4614E-130	positive
PRJNA729271	stage_3W_vs_E10	ordinary_muscle_development	ENSGALG00010007890	ENSGALG00010007890	-1.730305581	0.126418295	-13.68714542	1.21177E-42	1.06711E-41	negative
PRJNA729271	stage_3W_vs_E10	ordinary_muscle_development	ENSGALG00010007891	GDI1	-0.902843553	0.055998003	-16.12278113	1.76485E-58	2.55559E-57	negative
PRJNA729271	stage_3W_vs_E10	ordinary_muscle_development	ENSGALG00010007892	ENSGALG00010007892	-0.653162469	0.475097208	-1.374797534	0.169194223	0.198115378	negative
PRJNA729271	stage_3W_vs_E10	ordinary_muscle_development	ENSGALG00010007893	MFSD8	0.558985446	0.148339935	3.768273509	0.000164381	0.000263748	positive
PRJNA729271	stage_3W_vs_E10	ordinary_muscle_development	ENSGALG00010007894	CD88	2.12772174	0.259079483	8.212621544	2.16411E-16	7.03493E-16	positive
PRJNA729271	stage_3W_vs_E10	ordinary_muscle_development	ENSGALG00010007895	GOLGB1	-0.966590275	0.112274421	-8.60917619	7.35875E-18	2.57957E-17	negative
PRJNA729271	stage_3W_vs_E10	ordinary_muscle_development	ENSGALG00010007896	TIMMDC1	0.600134457	0.105011065	5.714963995	1.09727E-08	2.36329E-08	positive
PRJNA729271	stage_3W_vs_E10	ordinary_muscle_development	ENSGALG00010007897	EFHC1	-0.968304213	0.384418205	-2.518882302	0.0117728	0.015891855	negative
PRJNA729271	stage_3W_vs_E10	ordinary_muscle_development	ENSGALG00010007898	GAB1	0.184288526	0.08954299	2.058101102	0.039580429	0.050330584	positive
PRJNA729271	stage_3W_vs_E10	ordinary_muscle_development	ENSGALG00010007899	APRT	-0.715316415	0.152899059	-4.678357204	2.89182E-06	5.31739E-06	negative
PRJNA729271	stage_3W_vs_E10	ordinary_muscle_development	ENSGALG00010007900	FHOD3	0.034594042	0.200263518	0.172742607	0.862853752	0.878737099	positive
PRJNA729271	stage_3W_vs_E10	ordinary_muscle_development	ENSGALG00010007901	FSTL5	-2.010683238	0.326973376	-6.149379081	7.77869E-10	1.78575E-09	negative
PRJNA729271	stage_3W_vs_E10	ordinary_muscle_development	ENSGALG00010007902	C7orf25	2.228227884	0.141784361	15.71561117	1.18235E-55	1.58687E-54	positive
PRJNA729271	stage_3W_vs_E10	ordinary_muscle_development	ENSGALG00010007903	PSMA2	0.106915979	0.10345193	1.03348463	0.30137716	0.339034686	positive
PRJNA729271	stage_3W_vs_E10	ordinary_muscle_development	ENSGALG00010007904	ENSGALG00010007904	0.033573315	0.123354475	0.272169415	0.785491758	0.809103399	positive
PRJNA729271	stage_3W_vs_E10	ordinary_muscle_development	ENSGALG00010007905	MRPL32	-0.178819199	0.157702662	-1.133900956	0.256836096	0.292446128	negative
PRJNA729271	stage_3W_vs_E10	ordinary_muscle_development	ENSGALG00010007906	NAF1	-0.732313217	0.111642985	-6.559419879	5.40175E-11	1.32395E-10	negative
PRJNA729271	stage_3W_vs_E10	ordinary_muscle_development	ENSGALG00010007907	IRX2	-6.962170113	0.882316202	-7.890788013	3.00284E-15	9.19827E-15	negative
PRJNA729271	stage_3W_vs_E10	ordinary_muscle_development	ENSGALG00010007908	IRX1	-3.994380236	0.61799473	-6.463453553	1.0234E-10	2.46661E-10	negative
PRJNA729271	stage_3W_vs_E10	ordinary_muscle_development	ENSGALG00010007909	GALNS	-0.355703568	0.108054521	-3.29188974	0.000995166	0.001490949	negative
PRJNA729271	stage_3W_vs_E10	ordinary_muscle_development	ENSGALG00010007910	ANKS6	0.135872695	0.147431921	0.921596178	0.356739253	0.395422151	positive
PRJNA729271	stage_3W_vs_E10	ordinary_muscle_development	ENSGALG00010007911	IFI6	5.93315813	0.800602129	7.410869785	1.25474E-13	3.54185E-13	positive
PRJNA729271	stage_3W_vs_E10	ordinary_muscle_development	ENSGALG00010007912	TRAPPC2L	0.019478842	0.1127032	0.172833086	0.862782629	0.878737099	positive
PRJNA729271	stage_3W_vs_E10	ordinary_muscle_development	ENSGALG00010007914	PAQR8	-0.128581054	0.125841007	-1.021773878	0.306887936	0.344699003	negative
PRJNA729271	stage_3W_vs_E10	ordinary_muscle_development	ENSGALG00010007915	HECW1	-0.740095289	0.142433249	-5.196085131	2.03529E-07	4.02971E-07	negative
PRJNA729271	stage_3W_vs_E10	ordinary_muscle_development	ENSGALG00010007916	MCM3	-4.0443904	0.209553979	-19.29999338	5.37161E-83	1.46632E-81	negative
PRJNA729271	stage_3W_vs_E10	ordinary_muscle_development	ENSGALG00010007917	POGLUT1	-2.704954732	0.083210665	-32.50730831	8.4064E-232	2.0308E-229	negative
PRJNA729271	stage_3W_vs_E10	ordinary_muscle_development	ENSGALG00010007918	NPY1R	-1.422460224	0.684452539	-2.078245231	0.037686778	0.048064436	negative
PRJNA729271	stage_3W_vs_E10	ordinary_muscle_development	ENSGALG00010007920	FAT3	-3.880095809	0.185224112	-20.94811393	1.95211E-97	7.52548E-96	negative
PRJNA729271	stage_3W_vs_E10	ordinary_muscle_development	ENSGALG00010007921	FAM120B	-0.428189486	0.101404411	-4.222592321	2.41508E-05	4.14084E-05	negative
PRJNA729271	stage_3W_vs_E10	ordinary_muscle_development	ENSGALG00010007922	IL17A	-1.765617454	0.618821529	-2.853193321	0.004328228	0.006109423	negative
PRJNA729271	stage_3W_vs_E10	ordinary_muscle_development	ENSGALG00010007923	YAP1	-2.17094417	0.092110113	-23.56900995	8.0152E-123	5.0294E-121	negative
PRJNA729271	stage_3W_vs_E10	ordinary_muscle_development	ENSGALG00010007924	ABHD18	0.124380223	0.115698194	1.075040317	0.282356678	0.319447397	positive
PRJNA729271	stage_3W_vs_E10	ordinary_muscle_development	ENSGALG00010007925	STK17A	-0.676293669	0.239282526	-2.826339557	0.004708332	0.006622092	negative
PRJNA729271	stage_3W_vs_E10	ordinary_muscle_development	ENSGALG00010007926	EIF3E	0.792569873	0.131338751	6.03454706	1.59409E-09	3.58906E-09	positive
PRJNA729271	stage_3W_vs_E10	ordinary_muscle_development	ENSGALG00010007927	DLL1	-0.25738806	0.166305098	-1.547685932	0.121697931	0.145365596	negative
PRJNA729271	stage_3W_vs_E10	ordinary_muscle_development	ENSGALG00010007928	C11orf70	-2.723712297	0.707122262	-3.85182654	0.00011724	0.000190472	negative
PRJNA729271	stage_3W_vs_E10	ordinary_muscle_development	ENSGALG00010007929	LRRC14B	7.837672649	0.329465604	23.78904669	4.3362E-125	2.844E-123	positive
PRJNA729271	stage_3W_vs_E10	ordinary_muscle_development	ENSGALG00010007930	CBFA2T3	0.424243696	0.196954826	2.15401524	0.031238977	0.040185389	positive
PRJNA729271	stage_3W_vs_E10	ordinary_muscle_development	ENSGALG00010007931	GBE1	1.847539629	0.188923524	9.779299	1.38164E-22	5.84898E-22	positive
PRJNA729271	stage_3W_vs_E10	ordinary_muscle_development	ENSGALG00010007932	NPY5R	0.587890288	0.180036805	3.265389478	0.001093137	0.00163317	positive
PRJNA729271	stage_3W_vs_E10	ordinary_muscle_development	ENSGALG00010007933	CEP126	0.523972278	0.446321916	1.173978376	0.240403687	0.274858136	positive
PRJNA729271	stage_3W_vs_E10	ordinary_muscle_development	ENSGALG00010007934	PKHD1	-1.447669773	0.362683179	-3.991554769	6.56415E-05	0.000108952	negative
PRJNA729271	stage_3W_vs_E10	ordinary_muscle_development	ENSGALG00010007935	LARP1B	2.035154561	0.122153135	16.66068225	2.53121E-62	4.02301E-61	positive
PRJNA729271	stage_3W_vs_E10	ordinary_muscle_development	ENSGALG00010007936	ENSGALG00010007936	1.712144441	0.139154764	12.30388663	8.63185E-35	5.88794E-34	positive
PRJNA729271	stage_3W_vs_E10	ordinary_muscle_development	ENSGALG00010007937	ANGPTL5	3.515759413	0.401359089	8.759635719	1.95882E-18	7.03149E-18	positive
PRJNA729271	stage_3W_vs_E10	ordinary_muscle_development	ENSGALG00010007938	BCL9	-1.130301169	0.106842319	-10.57915233	3.72311E-26	1.8331E-25	negative
PRJNA729271	stage_3W_vs_E10	ordinary_muscle_development	ENSGALG00010007940	GLIPR2	-1.208608305	0.271721926	-4.44796017	8.66896E-06	1.53746E-05	negative
PRJNA729271	stage_3W_vs_E10	ordinary_muscle_development	ENSGALG00010007941	MRPL19	-0.672994612	0.074209875	-9.068801281	1.20334E-19	4.53519E-19	negative
PRJNA729271	stage_3W_vs_E10	ordinary_muscle_development	ENSGALG00010007942	PGRMC2	-0.724276245	0.086944603	-8.330318596	8.06251E-17	2.67734E-16	negative
PRJNA729271	stage_3W_vs_E10	ordinary_muscle_development	ENSGALG00010007943	BLOC1S4	0.201825569	0.100485297	2.008508464	0.044589288	0.056334152	positive
PRJNA729271	stage_3W_vs_E10	ordinary_muscle_development	ENSGALG00010007944	ACSF3	-0.637947089	0.1433488	-4.450313413	8.57451E-06	1.52108E-05	negative
PRJNA729271	stage_3W_vs_E10	ordinary_muscle_development	ENSGALG00010007945	ENSGALG00010007945	-1.693239076	0.124892404	-13.55758253	7.14524E-42	6.11756E-41	negative
PRJNA729271	stage_3W_vs_E10	ordinary_muscle_development	ENSGALG00010007946	ENSGALG00010007946	-0.303895592	0.156150237	-1.946174393	0.051633795	0.064664926	negative
PRJNA729271	stage_3W_vs_E10	ordinary_muscle_development	ENSGALG00010007948	ENSGALG00010007948	-3.165040079	0.501651035	-6.309246578	2.80397E-10	6.60871E-10	negative
PRJNA729271	stage_3W_vs_E10	ordinary_muscle_development	ENSGALG00010007950	ARC	-1.058415981	0.568338267	-1.862299341	0.062560908	0.077578952	negative
PRJNA729271	stage_3W_vs_E10	ordinary_muscle_development	ENSGALG00010007951	TGFBR1	-0.514575816	0.114030535	-4.512614237	6.40334E-06	1.1478E-05	negative
PRJNA729271	stage_3W_vs_E10	ordinary_muscle_development	ENSGALG00010007952	EMC2	-0.346788023	0.109622438	-3.163476651	0.001558969	0.002296468	negative
PRJNA729271	stage_3W_vs_E10	ordinary_muscle_development	ENSGALG00010007953	SCGN	-3.283025053	0.635304307	-5.167641736	2.37066E-07	4.6752E-07	negative
PRJNA729271	stage_3W_vs_E10	ordinary_muscle_development	ENSGALG00010007954	PSCA	0.30679188	0.595916433	0.514823661	0.60667625	0.642068651	positive
PRJNA729271	stage_3W_vs_E10	ordinary_muscle_development	ENSGALG00010007955	TMA16	0.131872841	0.121519081	1.08520275	0.277831867	0.31469664	positive
PRJNA729271	stage_3W_vs_E10	ordinary_muscle_development	ENSGALG00010007956	HS1BP3	0.28408338	0.131563277	2.159290853	0.030827609	0.039691272	positive
PRJNA729271	stage_3W_vs_E10	ordinary_muscle_development	ENSGALG00010007957	CDH15	0.247127894	0.16322387	1.514042606	0.130015034	0.154840844	positive
PRJNA729271	stage_3W_vs_E10	ordinary_muscle_development	ENSGALG00010007958	ENSGALG00010007958	3.167753848	0.478003479	6.627051862	3.42457E-11	8.51006E-11	positive
PRJNA729271	stage_3W_vs_E10	ordinary_muscle_development	ENSGALG00010007960	TSNAX	-0.562566954	0.062559932	-8.992448261	2.41783E-19	9.0233E-19	negative
PRJNA729271	stage_3W_vs_E10	ordinary_muscle_development	ENSGALG00010007962	ADCY8	-2.306971306	0.491488795	-4.693843138	2.6812E-06	4.94012E-06	negative

PRJNA729271	stage_3W_vs_E10	ordinary_muscle_development	ENSGALG00010007963	LDAH	-0.108917752	0.084806671	-1.284306418	0.199034762	0.230468835	negative
PRJNA729271	stage_3W_vs_E10	ordinary_muscle_development	ENSGALG00010007964	RECQL4	-3.779810456	0.189173485	-19.98065668	8.11494E-89	2.57388E-87	negative
PRJNA729271	stage_3W_vs_E10	ordinary_muscle_development	ENSGALG00010007965	EGLN1	1.89803723	0.157416878	12.05739339	1.77306E-33	1.15716E-32	positive
PRJNA729271	stage_3W_vs_E10	ordinary_muscle_development	ENSGALG00010007966	TMEM74	-1.214394818	0.217909935	-5.572920837	2.50503E-08	5.2685E-08	negative
PRJNA729271	stage_3W_vs_E10	ordinary_muscle_development	ENSGALG00010007968	SLC22A31	-0.641903423	0.222276669	-2.88785785	0.003878751	0.005500685	negative
PRJNA729271	stage_3W_vs_E10	ordinary_muscle_development	ENSGALG00010007970	SPRTN	1.222388793	0.105511688	11.5853401	4.89022E-31	2.92183E-30	positive
PRJNA729271	stage_3W_vs_E10	ordinary_muscle_development	ENSGALG00010007972	EXOC8	-0.270948046	0.112484395	-2.408761199	0.016006768	0.021280207	negative
PRJNA729271	stage_3W_vs_E10	ordinary_muscle_development	ENSGALG00010007973	CARMIL1	-0.205108221	0.134320442	-1.527006742	0.126759335	0.151100046	negative
PRJNA729271	stage_3W_vs_E10	ordinary_muscle_development	ENSGALG00010007975	ANKRD11	0.381267123	0.176195346	2.163888726	0.03047289	0.039266066	positive
PRJNA729271	stage_3W_vs_E10	ordinary_muscle_development	ENSGALG00010007976	ENSGALG00010007976	0.075591078	0.371218857	0.203629412	0.838643107	0.857212597	positive
PRJNA729271	stage_3W_vs_E10	ordinary_muscle_development	ENSGALG00010007977	APOB	1.523696117	1.378832765	1.105062307	0.269132592	0.305655169	positive
PRJNA729271	stage_3W_vs_E10	ordinary_muscle_development	ENSGALG00010007978	JADE1	1.305524583	0.115799209	11.27403712	1.76297E-29	9.94715E-29	positive
PRJNA729271	stage_3W_vs_E10	ordinary_muscle_development	ENSGALG00010007979	RAB30	-0.470646477	0.089803148	-5.240868347	1.59823E-07	3.18525E-07	negative
PRJNA729271	stage_3W_vs_E10	ordinary_muscle_development	ENSGALG00010007980	MARCHF1	1.012276239	0.221701466	4.565942913	4.97254E-06	8.98379E-06	positive
PRJNA729271	stage_3W_vs_E10	ordinary_muscle_development	ENSGALG00010007981	TDRD15	-4.000017433	0.897433653	-4.45717343	8.30474E-06	1.47449E-05	negative
PRJNA729271	stage_3W_vs_E10	ordinary_muscle_development	ENSGALG00010007982	RECK	-0.608761813	0.146575463	-4.153231385	3.27813E-05	5.55618E-05	negative
PRJNA729271	stage_3W_vs_E10	ordinary_muscle_development	ENSGALG00010007983	RCHY1	0.238546161	0.087137348	2.737588031	0.006189155	0.008594731	positive
PRJNA729271	stage_3W_vs_E10	ordinary_muscle_development	ENSGALG00010007984	EIF3H	0.420174555	0.122039092	3.442950525	0.000575405	0.000880263	positive
PRJNA729271	stage_3W_vs_E10	ordinary_muscle_development	ENSGALG00010007985	APELA	-7.200209513	0.708462907	-10.16314254	2.89589E-24	1.3138E-23	negative
PRJNA729271	stage_3W_vs_E10	ordinary_muscle_development	ENSGALG00010007986	MYC	0.19744289	0.188146424	1.049410799	0.29398909	0.33126336	positive
PRJNA729271	stage_3W_vs_E10	ordinary_muscle_development	ENSGALG00010007987	ENSGALG00010007987	0.74800167	0.295111066	2.5346446	0.011256146	0.015224208	positive
PRJNA729271	stage_3W_vs_E10	ordinary_muscle_development	ENSGALG00010007988	TARBP1	0.098186922	0.123189565	0.797039277	0.425428221	0.463896943	positive
PRJNA729271	stage_3W_vs_E10	ordinary_muscle_development	ENSGALG00010007989	SPG7	0.496338664	0.097246625	5.103916626	3.32695E-07	6.50096E-07	positive
PRJNA729271	stage_3W_vs_E10	ordinary_muscle_development	ENSGALG00010007990	TRHR	-4.238295614	1.154799176	-3.670158155	0.0002424	0.000383078	negative
PRJNA729271	stage_3W_vs_E10	ordinary_muscle_development	ENSGALG00010007991	PCF11	0.659387896	0.109734859	6.008919147	1.86764E-09	4.18221E-09	positive
PRJNA729271	stage_3W_vs_E10	ordinary_muscle_development	ENSGALG00010007992	LAPTM4A	0.334089131	0.098997647	3.374717906	0.000738914	0.001119531	positive
PRJNA729271	stage_3W_vs_E10	ordinary_muscle_development	ENSGALG00010007993	LRRC14	0.771752493	0.171247417	4.506651871	6.58586E-06	1.17883E-05	positive
PRJNA729271	stage_3W_vs_E10	ordinary_muscle_development	ENSGALG00010007994	FAM65B	1.557188255	0.165976746	9.381966426	6.47536E-21	2.57081E-20	positive
PRJNA729271	stage_3W_vs_E10	ordinary_muscle_development	ENSGALG00010007995	KIAA0319	-5.092867217	0.874830276	-5.821548883	5.83047E-09	1.27605E-08	negative
PRJNA729271	stage_3W_vs_E10	ordinary_muscle_development	ENSGALG00010007996	NUDCD1	-1.065277804	0.076039741	-14.00948747	1.36386E-44	1.28204E-43	negative
PRJNA729271	stage_3W_vs_E10	ordinary_muscle_development	ENSGALG00010007997	TMEM192	-0.369604238	0.138040887	-2.677498287	0.007417423	0.010223997	negative
PRJNA729271	stage_3W_vs_E10	ordinary_muscle_development	ENSGALG00010007998	RPL13	-0.046419965	0.144826833	-0.320520476	0.74857381	0.775207358	negative
PRJNA729271	stage_3W_vs_E10	ordinary_muscle_development	ENSGALG00010007999	SDC1	-1.625805906	0.208478903	-7.798419344	6.26874E-15	1.89027E-14	negative
PRJNA729271	stage_3W_vs_E10	ordinary_muscle_development	ENSGALG00010008000	C8orf82	-0.655206937	0.174716175	-3.750121798	0.000176749	0.000282841	negative
PRJNA729271	stage_3W_vs_E10	ordinary_muscle_development	ENSGALG00010008001	ALDH5A1	0.269751979	0.136394481	1.977733827	0.047958742	0.060349159	positive
PRJNA729271	stage_3W_vs_E10	ordinary_muscle_development	ENSGALG00010008002	GJA8	-1.165017063	0.173122341	-6.729443798	1.70313E-11	4.3001E-11	negative
PRJNA729271	stage_3W_vs_E10	ordinary_muscle_development	ENSGALG00010008004	ENY2	-0.867597863	0.171094386	-5.070872769	3.95995E-07	7.69637E-07	negative
PRJNA729271	stage_3W_vs_E10	ordinary_muscle_development	ENSGALG00010008005	ANKRD42	-0.367961924	0.344228235	-1.068947537	0.28509331	0.322216572	negative
PRJNA729271	stage_3W_vs_E10	ordinary_muscle_development	ENSGALG00010008006	CPNE7	-0.789496827	0.600577396	-1.314563006	0.18865681	0.219152145	negative
PRJNA729271	stage_3W_vs_E10	ordinary_muscle_development	ENSGALG00010008007	CBR4	0.776995449	0.121164481	6.412732842	1.42934E-10	3.41716E-10	positive
PRJNA729271	stage_3W_vs_E10	ordinary_muscle_development	ENSGALG00010008008	BTC	4.140861502	0.197163503	21.00217051	6.26566E-98	2.448E-96	positive
PRJNA729271	stage_3W_vs_E10	ordinary_muscle_development	ENSGALG00010008009	GPLD1	-0.980165982	1.305472651	-0.750813111	0.452765138	0.491560982	negative
PRJNA729271	stage_3W_vs_E10	ordinary_muscle_development	ENSGALG00010008010	CERK	-0.395589165	0.092389021	-4.281776796	1.85407E-05	3.21084E-05	negative
PRJNA729271	stage_3W_vs_E10	ordinary_muscle_development	ENSGALG00010008011	ADNP2	-0.436971567	0.100981375	-4.327249142	1.50983E-05	2.63231E-05	negative
PRJNA729271	stage_3W_vs_E10	ordinary_muscle_development	ENSGALG00010008012	CCDC90B	-0.133963539	0.079490441	-1.685278601	0.091934832	0.111757413	negative
PRJNA729271	stage_3W_vs_E10	ordinary_muscle_development	ENSGALG00010008013	ENSGALG00010008013	-0.835841846	0.244578278	-3.417481935	0.000632033	0.000964248	negative
PRJNA729271	stage_3W_vs_E10	ordinary_muscle_development	ENSGALG00010008015	ARHGAP39	-1.550690252	0.167115004	-9.279180297	1.70787E-20	6.65474E-20	negative
PRJNA729271	stage_3W_vs_E10	ordinary_muscle_development	ENSGALG00010008016	GSR	-0.774979362	0.143797014	-5.389398157	7.0694E-08	1.44529E-07	negative
PRJNA729271	stage_3W_vs_E10	ordinary_muscle_development	ENSGALG00010008017	GMNN	-1.988948868	0.136798433	-14.53926641	6.83209E-48	7.23912E-47	negative
PRJNA729271	stage_3W_vs_E10	ordinary_muscle_development	ENSGALG00010008018	ENSGALG00010008018	0.490299302	0.206846963	2.370348088	0.017771345	0.023507542	positive
PRJNA729271	stage_3W_vs_E10	ordinary_muscle_development	ENSGALG00010008019	PPP2CB	-0.729296608	0.100322241	-7.269540604	3.60712E-13	9.93827E-13	negative
PRJNA729271	stage_3W_vs_E10	ordinary_muscle_development	ENSGALG00010008020	COA6	1.304845981	0.130193652	10.02234719	1.21581E-23	5.36799E-23	positive
PRJNA729271	stage_3W_vs_E10	ordinary_muscle_development	ENSGALG00010008022	SLC35F3	-0.505435834	0.387826947	-1.303250941	0.192489104	0.223263948	negative
PRJNA729271	stage_3W_vs_E10	ordinary_muscle_development	ENSGALG00010008023	ATAD2	-0.584328691	0.077429188	-7.546620394	4.46698E-14	1.29317E-13	negative
PRJNA729271	stage_3W_vs_E10	ordinary_muscle_development	ENSGALG00010008024	MRS2	0.742357714	0.094427565	7.861663182	3.79066E-15	1.15626E-14	positive
PRJNA729271	stage_3W_vs_E10	ordinary_muscle_development	ENSGALG00010008025	TBC1D22A	-0.181515432	0.097219134	-1.867075185	0.061891089	0.076829197	negative
PRJNA729271	stage_3W_vs_E10	ordinary_muscle_development	ENSGALG00010008026	PURG	0.741909528	0.122179202	6.07230624	1.26086E-09	2.85699E-09	positive
PRJNA729271	stage_3W_vs_E10	ordinary_muscle_development	ENSGALG00010008027	C6orf62	0.610101225	0.107139956	5.69443229	1.23783E-08	2.65616E-08	positive
PRJNA729271	stage_3W_vs_E10	ordinary_muscle_development	ENSGALG00010008028	KCNK1	-3.04743861	0.169809484	-17.94622143	5.13686E-72	1.05615E-70	negative
PRJNA729271	stage_3W_vs_E10	ordinary_muscle_development	ENSGALG00010008029	LY6E	0.272658018	0.314428533	0.867154182	0.385857537	0.424806699	positive
PRJNA729271	stage_3W_vs_E10	ordinary_muscle_development	ENSGALG00010008030	GJA5	-2.778764651	0.153013525	-18.16025515	1.06528E-73	2.28084E-72	negative
PRJNA729271	stage_3W_vs_E10	ordinary_muscle_development	ENSGALG00010008031	MAP3K21	-1.96071756	0.182542004	-10.7411857	6.52016E-27	3.32312E-26	negative
PRJNA729271	stage_3W_vs_E10	ordinary_muscle_development	ENSGALG00010008032	PUM2	-0.652057643	0.054511287	-11.96188312	5.62712E-33	3.61227E-32	negative
PRJNA729271	stage_3W_vs_E10	ordinary_muscle_development	ENSGALG00010008033	ACOT13	0.326766164	0.120015831	2.722692166	0.006475236	0.008974809	positive
PRJNA729271	stage_3W_vs_E10	ordinary_muscle_development	ENSGALG00010008034	DPEP1	-2.1364439	0.425419572	-5.021968995	5.11444E-07	9.86479E-07	negative
PRJNA729271	stage_3W_vs_E10	ordinary_muscle_development	ENSGALG00010008035	TDP2	-0.692960827	0.105725257	-6.554354639	5.5883E-11	1.36828E-10	negative
PRJNA729271	stage_3W_vs_E10	ordinary_muscle_development	ENSGALG00010008036	SPOCK3	-4.947782309	0.198750268	-24.89446859	8.5406E-137	6.55E-135	negative
PRJNA729271	stage_3W_vs_E10	ordinary_muscle_development	ENSGALG00010008037	ENSGALG00010008037	2.691526025	0.897410914	2.999212495	0.002706785	0.003892732	positive
PRJNA729271	stage_3W_vs_E10	ordinary_muscle_development	ENSGALG00010008038	WRN	0.194526842	0.074569336	2.608670721	0.009089466	0.012423556	positive
PRJNA729271	stage_3W_vs_E10	ordinary_muscle_development	ENSGALG00010008039	CHMP1A	-0.290091463	0.090560029	-3.203305776	0.001358596	0.002011939	negative
PRJNA729271	stage_3W_vs_E10	ordinary_muscle_development	ENSGALG00010008040	ENSGALG00010008040	0.454426035	0.271027383	1.676679418	0.093605187	0.113616412	positive
PRJNA729271	stage_3W_vs_E10	ordinary_muscle_development	ENSGALG00010008041	ENSGALG00010008041	-2.60739175	0.237553915	-10.97599992	4.98512E-28	2.65757E-27	negative
PRJNA729271	stage_3W_vs_E10	ordinary_muscle_development	ENSGALG00010008042	ENSGALG00010008042	1.456865754	0.791673701	1.840235128	0.065733725	0.081325567	positive
PRJNA729271	stage_3W_vs_E10	ordinary_muscle_development	ENSGALG00010008043	IL17REL	-4.344285106	0.40757548	-10.65884804	1.58549E-26	7.95489E-26	negative
PRJNA729271	stage_3W_vs_E10	ordinary_muscle_development	ENSGALG00010008045	DCDC2	-1.833890077	0.844924112	-2.170479042	0.029970575	0.038646337	negative
PRJNA729271	stage_3W_vs_E10	ordinary_muscle_development	ENSGALG00010008046	RHOB	-1.074385106	0.169265593	-6.347333133	2.19079E-10	5.19901E-10	negative
PRJNA729271	stage_3W_vs_E10	ordinary_muscle_development	ENSGALG00010008047	ASAP1	-1.878791856	0.132129707	-14.21929939	6.95457E-46	6.86693E-45	negative
PRJNA729271	stage_3W_vs_E10	ordinary_muscle_development	ENSGALG00010008048	ENSGALG00010008048	1.843572691	0.769781368	2.394930258	0.016623527	0.022051617	positive
PRJNA729271	stage_3W_vs_E10	ordinary_muscle_development	ENSGALG00010008050	CDK10	-0.766562198	0.157719775	-4.860279544	1.1722E-06	2.21007E-06	negative
PRJNA729271	stage_3W_vs_E10	ordinary_muscle_development	ENSGALG00010008051	ENSGALG00010008051	-0.145998156	0.078229516	-1.866279678	0.062002247	0.076938718	negative
PRJNA729271	stage_3W_vs_E10	ordinary_muscle_development	ENSGALG00010008052	RBFA	0.621639935	0.121583345	5.11287079	3.17299E-07	6.2085E-07	positive
PRJNA729271	stage_3W_vs_E10	ordinary_muscle_development	ENSGALG00010008054	CRISP3	-4.215963782	0.787628104	-5.352734061	8.66351E-08	1.762E-07	negative
PRJNA729271	stage_3W_vs_E10	ordinary_muscle_development	ENSGALG00010008056	GTF2E2	-0.53169537	0.070963515	-7.492517387	6.7565E-14	1.93587E-13	negative
PRJNA729271	stage_3W_vs_E10	ordinary_muscle_development	ENSGALG00010008057	BAG1	-0.687896146	0.133419517	-5.155888437	2.52431E-07	4.97213E-07	negative
PRJNA729271	stage_3W_vs_E10	ordinary_muscle_development	ENSGALG00010008058	USP25	3.30136042	0.137977108	23.92687074	1.609E-126	1.0797E-124	positive
PRJNA729271	stage_3W_vs_E10	ordinary_muscle_development	ENSGALG00010008059	ENSGALG00010008059	0.800060083	0.092193653	8.678038635	4.02652E-18	1.4305E-17	positive
PRJNA729271	stage_3W_vs_E10	ordinary_muscle_development	ENSGALG00010008062	VPS9D1	0.275159454	0.137450467	2.001880818	0.045297551	0.05719906	positive
PRJNA729271	stage_3W_vs_E10	ordinary_muscle_development	ENSGALG00010008063	KCNV1	-1.90675736	0.332598311	-5.732913539	9.872E-09	2.13097E-08	negative
PRJNA729271	stage_3W_vs_E10	ordinary_muscle_development	ENSGALG00010008064	DDX60	1.469183343	0.630707555	2.329420873	0.01983678	0.026113353	positive
PRJNA729271	stage_3W_vs_E10	ordinary_muscle_development	ENSGALG00010008065	CHMP5	0.282138784	0.085895213	3.284685787	0.001020962	0.001528806	positive
PRJNA729271	stage_3W_vs_E10	ordinary_muscle_development	ENSGALG00010008066	PCNX2	-3.967066037	0.191135174	-20.75529035	1.09817E-95	4.09202E-94	negative
PRJNA729271	stage_3W_vs_E10	ordinary_muscle_development	ENSGALG00010008067	MPC2	2.058035595	0.144836533	14.209368	8.01455E-46	7.89174E-45	positive
PRJNA729271	stage_3W_vs_E10	ordinary_muscle_development	ENSGALG00010008069	DLG2	-2.987269124	0.225846892	-13.22696581	6.1313E-40	4.95115E-39	negative
PRJNA729271	stage_3W_vs_E10	ordinary_muscle_development	ENSGALG00010008071	SMIM18	-0.568728345	0.327290441	-1.737687003	0.08226598	0.100518029	negative
PRJNA729271	stage_3W_vs_E10	ordinary_muscle_development	ENSGALG00010008072	NRSN1	1.690422627	0.197539151	8.557405534	1.15436E-17	4.00777E-17	positive
PRJNA729271	stage_3W_vs_E10	ordinary_muscle_development	ENSGALG00010008073	ZNF276	0.134769256	0.083173249	1.62034377	0.105158451	0.126769108	positive
PRJNA729271	stage_3W_vs_E10	ordinary_muscle_development	ENSGALG00010008074	RHAG	-2.724032821	0.440427869	-6.184969231	6.21146E-10	1.43459E-09	negative
PRJNA729271	stage_3W_vs_E10	ordinary_muscle_development	ENSGALG00010008075	SDHA	1.281779842	0.136458438	9.393188583	5.82108E-21	2.31359E-20	positive
PRJNA729271	stage_3W_vs_E10	ordinary_muscle_development	ENSGALG00010008076	ENSGALG00010008076	4.430615073	1.75599579	2.523135362	0.011631362	0.015711172	positive
PRJNA729271	stage_3W_vs_E10	ordinary_muscle_development	ENSGALG00010008077	ENSGALG00010008077	-0.50120413	1.561947361	-0.320884136	0.748298196	0.774977304	negative
PRJNA729271	stage_3W_vs_E10	ordinary_muscle_development	ENSGALG00010008078	GUCY1B1	0.411829697	0.141359753	2.913344774	0.003575796	0.005090469	positive
PRJNA729271	stage_3W_vs_E10	ordinary_muscle_development	ENSGALG00010008079	CCDC127	0.751080821	0.107932949	6.958772371	3.43251E-12	8.97766E-12	positive
PRJNA729271	stage_3W_vs_E10	ordinary_muscle_development	ENSGALG00010008081	TOP1MT	-0.569007258	0.141385191	-4.024518071	5.70921E-05	9.52192E-05	negative
PRJNA729271	stage_3W_vs_E10	ordinary_muscle_development	ENSGALG00010008083	FANCA	-2.95303572	0.275341063	-10.72501023	7.76785E-27	3.93964E-26	negative
PRJNA729271	stage_3W_vs_E10	ordinary_muscle_development	ENSGALG00010008084	CYP2AC1	2.209012262	1.355875706	1.629214427			positive
PRJNA729271	stage_3W_vs_E10	ordinary_muscle_development	ENSGALG00010008086	RHPN1	1.26990443	0.208520306	6.090075615	1.12857E-09	2.56245E-09	positive
PRJNA729271	stage_3W_vs_E10	ordinary_muscle_development	ENSGALG00010008087	CPNE8	-0.241276795	0.18243052	-1.322568149	0.185979032	0.216353617	negative
PRJNA729271	stage_3W_vs_E10	ordinary_muscle_development	ENSGALG00010008088	SLC35A4	0.346119748	0.070915077	4.880763913	1.05676E-06	1.99839E-06	positive
PRJNA729271	stage_3W_vs_E10	ordinary_muscle_development	ENSGALG00010008091	ENSGALG00010008091	2.26084976	0.270875031	8.346467918	7.03347E-17	2.34368E-16	positive
PRJNA729271	stage_3W_vs_E10	ordinary_muscle_development	ENSGALG00010008092	SPIRE2	2.432859751	0.621769834	3.912797981	9.12329E-05	0.000149359	positive
PRJNA729271	stage_3W_vs_E10	ordinary_muscle_development	ENSGALG00010008093	EFR3A	0.214963196	0.092308472	2.328748285	0.019872405	0.026157874	positive
PRJNA729271	stage_3W_vs_E10	ordinary_muscle_development	ENSGALG00010008094	PIM3	1.157801824	0.141277781	8.195215229	2.50145E-16	8.10609E-16	positive
PRJNA729271	stage_3W_vs_E10	ordinary_muscle_development	ENSGALG00010008096	CPSF1	-1.651226762	0.156332223	-10.56229314	4.4564E-26	2.18523E-25	negative
PRJNA729271	stage_3W_vs_E10	ordinary_muscle_development	ENSGALG00010008098	ENSGALG00010008098	0.366929884	0.166367532	2.205537825	0.027416377	0.035485345	positive
PRJNA729271	stage_3W_vs_E10	ordinary_muscle_development	ENSGALG00010008099	PANK3	-0.146171755	0.07677241	-1.903962058	0.056915126	0.07094202	negative
PRJNA729271	stage_3W_vs_E10	ordinary_muscle_development	ENSGALG00010008100	ENSGALG00010008100	1.653057278	0.23456168	7.047431093	1.82251E-12	4.8481E-12	positive
PRJNA729271	stage_3W_vs_E10	ordinary_muscle_development	ENSGALG00010008101	CENPQ	-3.304136632	0.215638378	-15.32258152	5.40283E-53	6.72224E-52	negative
PRJNA729271	stage_3W_vs_E10	ordinary_muscle_development	ENSGALG00010008103	RBPMS	0.434423221	0.107286114	4.049202696	5.13924E-05	8.59803E-05	positive
PRJNA729271	stage_3W_vs_E10	ordinary_muscle_development	ENSGALG00010008104	TCF25	0.453656397	0.094252778	4.81318862	1.48541E-06	2.77927E-06	positive
PRJNA729271	stage_3W_vs_E10	ordinary_muscle_development	ENSGALG00010008105	MMUT	0.171587568	0.096225841	1.783175558	0.07455773	0.091585957	positive
PRJNA729271	stage_3W_vs_E10	ordinary_muscle_development	ENSGALG00010008106	ENSGALG00010008106	-0.16848134	0.380597964	-0.442675359	0.658000568	0.690788602	negative
PRJNA729271	stage_3W_vs_E10	ordinary_muscle_development	ENSGALG00010008107	TUBB3	-2.752833877	0.21059117	-13.07193402	4.76387E-39	3.73256E-38	negative
PRJNA729271	stage_3W_vs_E10	ordinary_muscle_development	ENSGALG00010008108	ENSGALG00010008108	0.805909179	0.339311658	2.375129648	0.017542787	0.023213684	positive
PRJNA729271	stage_3W_vs_E10	ordinary_muscle_development	ENSGALG00010008110	DEF8	0.578542211	0.152024271	3.805591086	0.000141466	0.000228346	positive
PRJNA729271	stage_3W_vs_E10	ordinary_muscle_development	ENSGALG00010008111	SYTL2	-0.23961723	0.258541102	-0.92680517	0.3540277	0.392656785	negative
PRJNA729271	stage_3W_vs_E10	ordinary_muscle_development	ENSGALG00010008113	DBNDD1	-1.944556937	0.190883245	-10.18715361	2.26293E-24	1.03278E-23	negative
PRJNA729271	stage_3W_vs_E10	ordinary_muscle_development	ENSGALG00010008115	ENSGALG00010008115	-0.365585974	0.202435097	-1.805941651	0.070927454	0.087326378	negative
PRJNA729271	stage_3W_vs_E10	ordinary_muscle_development	ENSGALG00010008116	URAH	-0.469850181	0.441487744	-1.064242862	0.287218695	0.324365795	negative
PRJNA729271	stage_3W_vs_E10	ordinary_muscle_development	ENSGALG00010008117	ENSGALG00010008117	2.039500417	0.178163383	11.44736019	2.42416E-30	1.41117E-29	positive
PRJNA729271	stage_3W_vs_E10	ordinary_muscle_development	ENSGALG00010008118	ENSGALG00010008118	0.270455703	0.613327742	0.440964405	0.659238766	0.691837961	positive
PRJNA729271	stage_3W_vs_E10	ordinary_muscle_development	ENSGALG00010008119	CCDC83	-0.931493746	0.53942488	-1.726827552	0.084198639	0.10279283	negative

PRJNA729271	stage_3W_vs_E10	ordinary_muscle_development	ENSGALG00010008120	CDC5L	-0.209566299	0.064601858	-3.24396706	0.001178774	0.001756046	negative
PRJNA729271	stage_3W_vs_E10	ordinary_muscle_development	ENSGALG00010008121	MPZL1	-0.851257267	0.135683416	-6.273849041	3.5223E-10	8.25477E-10	negative
PRJNA729271	stage_3W_vs_E10	ordinary_muscle_development	ENSGALG00010008123	ENSGALG00010008123	-1.424031496	0.167705291	-8.491273508	2.04385E-17	6.98882E-17	negative
PRJNA729271	stage_3W_vs_E10	ordinary_muscle_development	ENSGALG00010008124	ENSGALG00010008124	0.045526814	0.212603202	0.214139835	0.830438013	0.85017674	positive
PRJNA729271	stage_3W_vs_E10	ordinary_muscle_development	ENSGALG00010008126	CDH3	-3.684296949	0.315533638	-11.67639994	1.68272E-31	1.0214E-30	negative
PRJNA729271	stage_3W_vs_E10	ordinary_muscle_development	ENSGALG00010008127	TBC1D2	-0.387354958	0.13396019	-2.891567689	0.00383325	0.005438818	negative
PRJNA729271	stage_3W_vs_E10	ordinary_muscle_development	ENSGALG00010008128	RARS	-0.655227943	0.070463012	-9.298892042	1.41917E-20	5.54919E-20	negative
PRJNA729271	stage_3W_vs_E10	ordinary_muscle_development	ENSGALG00010008129	TANGO6	0.491877026	0.117001247	4.204032323	2.62201E-05	4.47974E-05	positive
PRJNA729271	stage_3W_vs_E10	ordinary_muscle_development	ENSGALG00010008130	RCSD1	0.94873779	0.120056913	7.90240037	2.73583E-15	8.39277E-15	positive
PRJNA729271	stage_3W_vs_E10	ordinary_muscle_development	ENSGALG00010008131	OC90	-2.93083242	0.809317859	-3.621361356	0.000293057	0.000460622	negative
PRJNA729271	stage_3W_vs_E10	ordinary_muscle_development	ENSGALG00010008132	TDO2	0.746626901	0.651203649	1.146533657	0.251574409	0.286837966	positive
PRJNA729271	stage_3W_vs_E10	ordinary_muscle_development	ENSGALG00010008133	CREG1	3.352750806	0.167074398	20.06741219	1.42219E-89	4.6015E-88	positive
PRJNA729271	stage_3W_vs_E10	ordinary_muscle_development	ENSGALG00010008134	SLC2A13	-0.520564637	0.078001815	-6.673750346	2.49348E-11	6.24338E-11	negative
PRJNA729271	stage_3W_vs_E10	ordinary_muscle_development	ENSGALG00010008135	WWC1	-0.007301428	0.130704466	-0.055862117	0.95545165	0.961220965	negative
PRJNA729271	stage_3W_vs_E10	ordinary_muscle_development	ENSGALG00010008136	ELP1	0.299021773	0.113127811	2.643220705	0.008212149	0.011265862	positive
PRJNA729271	stage_3W_vs_E10	ordinary_muscle_development	ENSGALG00010008137	HHLA1	-3.239906573	0.937180225	-3.457079531	0.000546064	0.000837587	negative
PRJNA729271	stage_3W_vs_E10	ordinary_muscle_development	ENSGALG00010008139	ENSGALG00010008139	0.750640656	0.413115948	1.817021733	0.069213786	0.08532521	positive
PRJNA729271	stage_3W_vs_E10	ordinary_muscle_development	ENSGALG00010008141	APTX	0.563259081	0.153187331	3.676929933	0.000236058	0.000373299	positive
PRJNA729271	stage_3W_vs_E10	ordinary_muscle_development	ENSGALG00010008142	CD247	3.307538712	0.320208666	10.32932291	5.19258E-25	2.43266E-24	positive
PRJNA729271	stage_3W_vs_E10	ordinary_muscle_development	ENSGALG00010008143	DCTN6	0.83014851	0.114819416	7.230035966	4.82866E-13	1.32084E-12	positive
PRJNA729271	stage_3W_vs_E10	ordinary_muscle_development	ENSGALG00010008144	NTPCR	0.519723418	0.132420907	3.924783696	8.68077E-05	0.000142387	positive
PRJNA729271	stage_3W_vs_E10	ordinary_muscle_development	ENSGALG00010008145	ENSGALG00010008145	1.828860389	0.206637332	8.850580711	8.70651E-19	3.17008E-18	positive
PRJNA729271	stage_3W_vs_E10	ordinary_muscle_development	ENSGALG00010008146	SOX4	-2.263043637	0.12005255	-18.8504421	2.91397E-79	7.09882E-78	negative
PRJNA729271	stage_3W_vs_E10	ordinary_muscle_development	ENSGALG00010008147	CDKAL1	0.379147007	0.112311869	3.375840953	0.000735904	0.001115322	positive
PRJNA729271	stage_3W_vs_E10	ordinary_muscle_development	ENSGALG00010008148	ABCD2	1.936843475	0.13809241	14.02570548	1.08528E-44	1.0215E-43	positive
PRJNA729271	stage_3W_vs_E10	ordinary_muscle_development	ENSGALG00010008149	DNAJA1	1.192925309	0.281498651	4.237765633	2.25755E-05	3.8836E-05	positive
PRJNA729271	stage_3W_vs_E10	ordinary_muscle_development	ENSGALG00010008150	TERT	-2.764892654	0.626656193	-4.41213649	1.02356E-05	1.80624E-05	negative
PRJNA729271	stage_3W_vs_E10	ordinary_muscle_development	ENSGALG00010008151	SIPA1L2	-0.848350403	0.122001913	-6.953582823	3.56124E-12	9.29925E-12	negative
PRJNA729271	stage_3W_vs_E10	ordinary_muscle_development	ENSGALG00010008152	ENSGALG00010008152	-3.299572514	0.16876246	-19.5515787	3.99951E-85	1.15946E-83	negative
PRJNA729271	stage_3W_vs_E10	ordinary_muscle_development	ENSGALG00010008153	SMU1	-0.403071411	0.061781341	-6.52416089	6.83833E-11	1.66535E-10	negative
PRJNA729271	stage_3W_vs_E10	ordinary_muscle_development	ENSGALG00010008154	KCNQ3	-0.169247242	0.225990827	-0.748912001	0.453910246	0.49265661	negative
PRJNA729271	stage_3W_vs_E10	ordinary_muscle_development	ENSGALG00010008155	MAP10	0.747234531	0.221298985	3.376583636	0.000733921	0.00111278	positive
PRJNA729271	stage_3W_vs_E10	ordinary_muscle_development	ENSGALG00010008156	HAS3	-2.816005366	1.024786614	-2.74789437	0.005997933	0.008339572	negative
PRJNA729271	stage_3W_vs_E10	ordinary_muscle_development	ENSGALG00010008157	CTSO	-0.796395182	0.156146984	-5.100291795	3.3913E-07	6.62135E-07	negative
PRJNA729271	stage_3W_vs_E10	ordinary_muscle_development	ENSGALG00010008158	CLPTM1L	-0.438445138	0.075343053	-5.819317416	5.90884E-09	1.29281E-08	negative
PRJNA729271	stage_3W_vs_E10	ordinary_muscle_development	ENSGALG00010008159	SUPT3H	-1.990821039	0.137006573	-14.53084328	7.72634E-48	8.17469E-47	negative
PRJNA729271	stage_3W_vs_E10	ordinary_muscle_development	ENSGALG00010008160	TXNL4A	0.13945128	0.090780458	1.536137664	0.124504621	0.148510079	positive
PRJNA729271	stage_3W_vs_E10	ordinary_muscle_development	ENSGALG00010008161	TMEFF1	-2.96964677	0.366567403	-8.101229801	5.44064E-16	1.73077E-15	negative
PRJNA729271	stage_3W_vs_E10	ordinary_muscle_development	ENSGALG00010008162	HSBP1L1	2.005535335	0.191106146	10.49435289	9.17039E-26	4.42935E-25	positive
PRJNA729271	stage_3W_vs_E10	ordinary_muscle_development	ENSGALG00010008163	CHTF8	-3.329584431	0.246097948	-13.52950909	1.04721E-41	8.91324E-41	negative
PRJNA729271	stage_3W_vs_E10	ordinary_muscle_development	ENSGALG00010008164	TG	0.655319408	0.291536821	2.247810093	0.024588302	0.032027987	positive
PRJNA729271	stage_3W_vs_E10	ordinary_muscle_development	ENSGALG00010008165	MRPL1	0.644196517	0.084303324	7.641413061	2.14851E-14	6.30162E-14	positive
PRJNA729271	stage_3W_vs_E10	ordinary_muscle_development	ENSGALG00010008166	RUNX2	-3.416081966	0.405553169	-8.423265375	3.66134E-17	1.23651E-16	negative
PRJNA729271	stage_3W_vs_E10	ordinary_muscle_development	ENSGALG00010008167	UTP4	0.470837985	0.173671606	2.711082112	0.006706402	0.009283668	positive
PRJNA729271	stage_3W_vs_E10	ordinary_muscle_development	ENSGALG00010008168	CAVIN4	5.289758462	0.124857526	42.36635646	0	0	positive
PRJNA729271	stage_3W_vs_E10	ordinary_muscle_development	ENSGALG00010008169	HTR4	-2.157365881	0.415256273	-5.195263797	2.0443E-07	4.04699E-07	negative
PRJNA729271	stage_3W_vs_E10	ordinary_muscle_development	ENSGALG00010008170	PICALM	-0.284634035	0.070824252	-4.018878075	5.84759E-05	9.74151E-05	negative
PRJNA729271	stage_3W_vs_E10	ordinary_muscle_development	ENSGALG00010008171	KIF21A	0.077795327	0.179924257	0.432378205	0.665466556	0.697363919	positive
PRJNA729271	stage_3W_vs_E10	ordinary_muscle_development	ENSGALG00010008172	SNTB2	-0.58912516	0.133967115	-4.397535619	1.09487E-05	1.92786E-05	negative
PRJNA729271	stage_3W_vs_E10	ordinary_muscle_development	ENSGALG00010008174	SLC26A2	-0.804582742	0.123981306	-6.489548839	8.60938E-11	2.08684E-10	negative
PRJNA729271	stage_3W_vs_E10	ordinary_muscle_development	ENSGALG00010008175	ENSGALG00010008175	-1.156556452	0.451440196	-2.561926167	0.010409344	0.014114448	negative
PRJNA729271	stage_3W_vs_E10	ordinary_muscle_development	ENSGALG00010008176	SLA	3.152664455	0.2920789	10.79387951	3.67923E-27	1.89182E-26	positive
PRJNA729271	stage_3W_vs_E10	ordinary_muscle_development	ENSGALG00010008177	NREP	-1.296497676	0.184634729	-7.021959978	2.18777E-12	5.78999E-12	negative
PRJNA729271	stage_3W_vs_E10	ordinary_muscle_development	ENSGALG00010008178	PGR	0.666965337	0.281756623	2.367168269	0.017924781	0.023701852	positive
PRJNA729271	stage_3W_vs_E10	ordinary_muscle_development	ENSGALG00010008179	SEMA6A	-1.36153713	0.09778357	-13.92398672	4.52919E-44	4.17891E-43	negative
PRJNA729271	stage_3W_vs_E10	ordinary_muscle_development	ENSGALG00010008180	POU2F1	0.505968727	0.158408993	3.194065674	0.001402842	0.00207386	positive
PRJNA729271	stage_3W_vs_E10	ordinary_muscle_development	ENSGALG00010008181	DISC1	0.201990242	0.129839549	1.555691189	0.119781543	0.143253586	positive
PRJNA729271	stage_3W_vs_E10	ordinary_muscle_development	ENSGALG00010008182	CLIC5	5.787972964	0.284950887	20.31217736	1.00355E-91	3.39872E-90	positive
PRJNA729271	stage_3W_vs_E10	ordinary_muscle_development	ENSGALG00010008183	ALG10	-0.945032018	0.088362925	-10.69489291	1.07544E-26	5.42774E-26	negative
PRJNA729271	stage_3W_vs_E10	ordinary_muscle_development	ENSGALG00010008184	SLC66A2	-0.206560813	0.085852981	-2.405983009	0.016129015	0.021427007	negative
PRJNA729271	stage_3W_vs_E10	ordinary_muscle_development	ENSGALG00010008185	PDGFC	-1.847640791	0.091326429	-20.23117311	5.20498E-91	1.75049E-89	negative
PRJNA729271	stage_3W_vs_E10	ordinary_muscle_development	ENSGALG00010008186	NOP53	0.519825629	0.12134693	4.283797134	1.8373E-05	3.18409E-05	positive
PRJNA729271	stage_3W_vs_E10	ordinary_muscle_development	ENSGALG00010008187	ENSGALG00010008187	3.937355718	0.374293488	10.5194342	7.02944E-26	3.4146E-25	positive
PRJNA729271	stage_3W_vs_E10	ordinary_muscle_development	ENSGALG00010008188	VPS4A	1.139664242	0.112616647	10.11985595	4.51079E-24	2.02993E-23	positive
PRJNA729271	stage_3W_vs_E10	ordinary_muscle_development	ENSGALG00010008189	EXOC3	0.513069037	0.113847867	4.506619686	6.58685E-06	1.17887E-05	positive
PRJNA729271	stage_3W_vs_E10	ordinary_muscle_development	ENSGALG00010008190	TSNARE1	-0.533657017	0.170458426	-3.13071655	0.001743804	0.002555143	negative
PRJNA729271	stage_3W_vs_E10	ordinary_muscle_development	ENSGALG00010008192	HNRNPU	-0.67565979	0.082939885	-8.146379672	3.74982E-16	1.19986E-15	negative
PRJNA729271	stage_3W_vs_E10	ordinary_muscle_development	ENSGALG00010008193	CNOT6L	1.714564325	0.129508765	13.23898289	5.22517E-40	4.22414E-39	positive
PRJNA729271	stage_3W_vs_E10	ordinary_muscle_development	ENSGALG00010008194	PDF	1.168585179	0.162289896	7.200603433	5.99467E-13	1.63301E-12	positive
PRJNA729271	stage_3W_vs_E10	ordinary_muscle_development	ENSGALG00010008195	ENSGALG00010008195	3.424272919	0.413716048	8.276867513	1.26458E-16	4.16214E-16	positive
PRJNA729271	stage_3W_vs_E10	ordinary_muscle_development	ENSGALG00010008196	PDCD6	0.337461393	0.146720341	2.300031414	0.02144644	0.02813523	positive
PRJNA729271	stage_3W_vs_E10	ordinary_muscle_development	ENSGALG00010008197	STARD4	-0.25832106	0.157851945	-1.636476893	0.101739818	0.122852271	negative
PRJNA729271	stage_3W_vs_E10	ordinary_muscle_development	ENSGALG00010008198	GLRB	-3.192419529	0.385367467	-8.284091937	1.19014E-16	3.92069E-16	negative
PRJNA729271	stage_3W_vs_E10	ordinary_muscle_development	ENSGALG00010008199	TPRKB	0.722918783	0.111803779	6.465960203	1.00658E-10	2.42808E-10	positive
PRJNA729271	stage_3W_vs_E10	ordinary_muscle_development	ENSGALG00010008200	COG8	0.064454748	0.127242301	0.506551258	0.612469716	0.647349316	positive
PRJNA729271	stage_3W_vs_E10	ordinary_muscle_development	ENSGALG00010008201	ENPP4	2.169053283	0.240306878	9.026180607	1.77768E-19	6.65826E-19	positive
PRJNA729271	stage_3W_vs_E10	ordinary_muscle_development	ENSGALG00010008202	KCNG2	0.668230688	0.196116019	3.407323341	0.000656034	0.000999601	positive
PRJNA729271	stage_3W_vs_E10	ordinary_muscle_development	ENSGALG00010008203	CXCL13	2.271329336	1.342514986	1.691846542	0.090675237	0.110328008	positive
PRJNA729271	stage_3W_vs_E10	ordinary_muscle_development	ENSGALG00010008204	ENSGALG00010008204	0.495882763	0.153273391	3.235282777	0.001215223	0.001806446	positive
PRJNA729271	stage_3W_vs_E10	ordinary_muscle_development	ENSGALG00010008205	ENSGALG00010008205	-0.30833467	0.173912538	-1.772929503	0.076240389	0.093549855	negative
PRJNA729271	stage_3W_vs_E10	ordinary_muscle_development	ENSGALG00010008206	CXCL13L2	-1.345972418	0.782964474	-1.719072144	0.085601236	0.104426016	negative
PRJNA729271	stage_3W_vs_E10	ordinary_muscle_development	ENSGALG00010008207	CAMK4	-0.485513898	0.157176676	-3.088969127	0.002008523	0.00292246	negative
PRJNA729271	stage_3W_vs_E10	ordinary_muscle_development	ENSGALG00010008208	ILDR2	1.298737863	0.152957522	8.490840102	2.05149E-17	7.01328E-17	positive
PRJNA729271	stage_3W_vs_E10	ordinary_muscle_development	ENSGALG00010008209	PPARGC1B	2.349089643	0.235051726	9.993926377	1.62033E-23	7.11504E-23	positive
PRJNA729271	stage_3W_vs_E10	ordinary_muscle_development	ENSGALG00010008210	CTDP1	0.215130508	0.085106163	2.52778999	0.011478299	0.015515988	positive
PRJNA729271	stage_3W_vs_E10	ordinary_muscle_development	ENSGALG00010008211	ADGRB1	-0.905975046	0.238704758	-3.795379093	0.000147418	0.00023753	negative
PRJNA729271	stage_3W_vs_E10	ordinary_muscle_development	ENSGALG00010008212	GTSE1	-3.695031807	0.236814121	-15.60308903	6.93555E-55	9.13916E-54	negative
PRJNA729271	stage_3W_vs_E10	ordinary_muscle_development	ENSGALG00010008213	ISCA1	1.230620107	0.116791959	10.53685649	5.8419E-26	2.84729E-25	positive
PRJNA729271	stage_3W_vs_E10	ordinary_muscle_development	ENSGALG00010008214	CCNG2	-0.484696128	0.135971694	-3.564684049	0.000364295	0.000567974	negative
PRJNA729271	stage_3W_vs_E10	ordinary_muscle_development	ENSGALG00010008215	SELENOW	2.823825229	0.127757486	22.10301179	2.9567E-108	1.4677E-106	positive
PRJNA729271	stage_3W_vs_E10	ordinary_muscle_development	ENSGALG00010008217	ARHGEF37	3.85440127	0.161694106	23.83761139	1.3613E-125	8.9694E-124	positive
PRJNA729271	stage_3W_vs_E10	ordinary_muscle_development	ENSGALG00010008218	ARHGAP42	-0.414057174	0.102471699	-4.040697869	5.32924E-05	8.90664E-05	negative
PRJNA729271	stage_3W_vs_E10	ordinary_muscle_development	ENSGALG00010008219	NIP7	-0.319669325	0.119307737	-2.67936794	0.007376129	0.010169979	negative
PRJNA729271	stage_3W_vs_E10	ordinary_muscle_development	ENSGALG00010008221	ENSGALG00010008221	2.353749025	0.519220358	4.533237166	5.80865E-06	1.04488E-05	positive
PRJNA729271	stage_3W_vs_E10	ordinary_muscle_development	ENSGALG00010008222	STYXL2	3.0855525	0.274804678	11.22816583	2.96571E-29	1.65977E-28	positive
PRJNA729271	stage_3W_vs_E10	ordinary_muscle_development	ENSGALG00010008223	ENSGALG00010008223	-0.872552218	0.151627869	-5.754563599	8.68659E-09	1.88097E-08	negative
PRJNA729271	stage_3W_vs_E10	ordinary_muscle_development	ENSGALG00010008224	ENSGALG00010008224	-0.260205376	0.212277965	-1.225776663	0.220282732	0.253271849	negative
PRJNA729271	stage_3W_vs_E10	ordinary_muscle_development	ENSGALG00010008226	COX20	-0.308261321	0.164785462	-1.870682748	0.061389067	0.07623668	negative
PRJNA729271	stage_3W_vs_E10	ordinary_muscle_development	ENSGALG00010008227	DUSP1	3.183596964	0.176863698	18.00028492	1.93819E-72	4.03651E-71	positive
PRJNA729271	stage_3W_vs_E10	ordinary_muscle_development	ENSGALG00010008228	TMED6	2.401275295	0.685745115	3.501702371	0.000462296	0.000714238	positive
PRJNA729271	stage_3W_vs_E10	ordinary_muscle_development	ENSGALG00010008229	PIGY	0.800547919	0.122366974	6.542189403	6.06247E-11	1.48113E-10	positive
PRJNA729271	stage_3W_vs_E10	ordinary_muscle_development	ENSGALG00010008230	TRMU	-0.095242034	0.115157752	-0.827057077	0.408204711	0.447062932	negative
PRJNA729271	stage_3W_vs_E10	ordinary_muscle_development	ENSGALG00010008231	ZCCHC6	0.133754284	0.110048935	1.215407349	0.224210767	0.257481783	positive
PRJNA729271	stage_3W_vs_E10	ordinary_muscle_development	ENSGALG00010008232	PYURF	1.103571736	0.127248166	8.672594428	4.22384E-18	1.49877E-17	positive
PRJNA729271	stage_3W_vs_E10	ordinary_muscle_development	ENSGALG00010008233	CYRIB	-0.443668551	0.071102113	-6.239878593	4.37911E-10	1.02116E-09	negative
PRJNA729271	stage_3W_vs_E10	ordinary_muscle_development	ENSGALG00010008234	COMMD10	-1.209665359	0.120510314	-10.03785746	1.03906E-23	4.60026E-23	negative
PRJNA729271	stage_3W_vs_E10	ordinary_muscle_development	ENSGALG00010008236	ENSGALG00010008236	0.516088989	0.726571938	0.710306802	0.477513903	0.516187935	positive
PRJNA729271	stage_3W_vs_E10	ordinary_muscle_development	ENSGALG00010008237	GRIA2	-5.115611084	0.507031048	-10.08934484	6.15791E-24	2.75236E-23	negative
PRJNA729271	stage_3W_vs_E10	ordinary_muscle_development	ENSGALG00010008238	NTAQ1	-0.35719112	0.163530566	-2.184246835	0.028944115	0.037392598	negative
PRJNA729271	stage_3W_vs_E10	ordinary_muscle_development	ENSGALG00010008240	RCAN2	-0.626992272	0.139270025	-4.501990073	6.73201E-06	1.20381E-05	negative
PRJNA729271	stage_3W_vs_E10	ordinary_muscle_development	ENSGALG00010008241	HERC3	0.239814096	0.083320902	2.878198496	0.003999534	0.005662101	positive
PRJNA729271	stage_3W_vs_E10	ordinary_muscle_development	ENSGALG00010008242	CNTN5	-5.937405486	1.349622269	-4.399309068			negative
PRJNA729271	stage_3W_vs_E10	ordinary_muscle_development	ENSGALG00010008243	YTHDC2	0.254859219	0.103197503	2.469625836	0.013525444	0.01812437	positive
PRJNA729271	stage_3W_vs_E10	ordinary_muscle_development	ENSGALG00010008245	FBXO32	5.066681832	0.49181522	10.30200292	6.90093E-25	3.2112E-24	positive
PRJNA729271	stage_3W_vs_E10	ordinary_muscle_development	ENSGALG00010008246	TERF2	-0.863123528	0.059733416	-14.44959258	2.52173E-47	2.60531E-46	negative
PRJNA729271	stage_3W_vs_E10	ordinary_muscle_development	ENSGALG00010008247	GASK1B	0.238104206	0.161312907	1.476039399	0.139933316	0.165889704	positive
PRJNA729271	stage_3W_vs_E10	ordinary_muscle_development	ENSGALG00010008248	TPK1	-0.811519706	0.164571102	-4.931119108	8.17599E-07	1.55751E-06	negative
PRJNA729271	stage_3W_vs_E10	ordinary_muscle_development	ENSGALG00010008250	KLHL38	6.666457328	0.592433449	11.2526687	2.24689E-29	1.26333E-28	positive
PRJNA729271	stage_3W_vs_E10	ordinary_muscle_development	ENSGALG00010008251	AHRR	-3.540364527	0.327527885	-10.80935301	3.10855E-27	1.60236E-26	negative
PRJNA729271	stage_3W_vs_E10	ordinary_muscle_development	ENSGALG00010008252	ZFP92	-2.262479397	0.131159988	-17.24976819	1.12349E-66	1.99327E-65	negative
PRJNA729271	stage_3W_vs_E10	ordinary_muscle_development	ENSGALG00010008253	ENSGALG00010008253	-0.497611053	0.727083581	-0.684393192	0.493726914	0.532087109	negative
PRJNA729271	stage_3W_vs_E10	ordinary_muscle_development	ENSGALG00010008255	MSANTD3	-2.388831634	0.134000716	-17.82700645	4.36158E-71	8.64857E-70	negative
PRJNA729271	stage_3W_vs_E10	ordinary_muscle_development	ENSGALG00010008257	DESI2	-0.667902005	0.059843918	-11.16073327	6.34659E-29	3.49654E-28	negative
PRJNA729271	stage_3W_vs_E10	ordinary_muscle_development	ENSGALG00010008258	CCDC82	1.054532921	0.130562618	8.076836493	6.64686E-16	2.10363E-15	positive
PRJNA729271	stage_3W_vs_E10	ordinary_muscle_development	ENSGALG00010008259	MCC	-0.734305194	0.109337672	-6.715939537	1.86858E-11	4.70636E-11	negative
PRJNA729271	stage_3W_vs_E10	ordinary_muscle_development	ENSGALG00010008261	CRX	0.00698814	0.387446206	0.018036413	0.985609804	0.987961707	positive
PRJNA729271	stage_3W_vs_E10	ordinary_muscle_development	ENSGALG00010008262	CYB5B	-1.051538152	0.072456909	-14.51260017	1.00825E-47	1.05903E-46	negative
PRJNA729271	stage_3W_vs_E10	ordinary_muscle_development	ENSGALG00010008263	C1orf101	-1.66838302	0.554884594	-3.006720746	0.002640822	0.003803529	negative
PRJNA729271	stage_3W_vs_E10	ordinary_muscle_development	ENSGALG00010008264	CNTNAP2	-3.690377657	0.344517569	-10.71172559	8.96745E-27	4.54011E-26	negative
PRJNA729271	stage_3W_vs_E10	ordinary_muscle_development	ENSGALG00010008265	ENSGALG00010008265	-1.336274421	0.684325826	-1.952687406	0.050856647	0.06374121	negative
PRJNA729271	stage_3W_vs_E10	ordinary_muscle_development	ENSGALG00010008266	ECSCR	0.948823833	0.18688717	5.076987532	3.83466E-07	7.45986E-07	positive

PRJNA729271	stage_3W_vs_E10	ordinary_muscle_development	ENSGALG00010008267	MAML2	-0.743721755	0.119587694	-6.219049214	5.00176E-10	1.16243E-09	negative
PRJNA729271	stage_3W_vs_E10	ordinary_muscle_development	ENSGALG00010008268	ENSGALG00010008268	1.730022738	0.235325626	7.351612182	1.9583E-13	5.47034E-13	positive
PRJNA729271	stage_3W_vs_E10	ordinary_muscle_development	ENSGALG00010008269	ENSGALG00010008269	0.860389083	0.306264335	2.809302247	0.004964901	0.006963352	positive
PRJNA729271	stage_3W_vs_E10	ordinary_muscle_development	ENSGALG00010008270	ENSGALG00010008270	0.830425187	0.162535457	5.10919403	3.23536E-07	6.32796E-07	positive
PRJNA729271	stage_3W_vs_E10	ordinary_muscle_development	ENSGALG00010008272	DCP2	-1.051833534	0.085304208	-12.33038268	6.21509E-35	4.25745E-34	negative
PRJNA729271	stage_3W_vs_E10	ordinary_muscle_development	ENSGALG00010008273	TMEM144	0.464909099	0.119469947	3.891431374	9.96546E-05	0.000162723	positive
PRJNA729271	stage_3W_vs_E10	ordinary_muscle_development	ENSGALG00010008274	NFAT5	-0.698748296	0.110214136	-6.339915387	2.29891E-10	5.44578E-10	negative
PRJNA729271	stage_3W_vs_E10	ordinary_muscle_development	ENSGALG00010008275	CELSR1	-2.347125248	0.176257813	-13.31643239	1.85775E-40	1.52222E-39	negative
PRJNA729271	stage_3W_vs_E10	ordinary_muscle_development	ENSGALG00010008276	DNAJC18	1.130491129	0.09255502	12.21426054	2.60862E-34	1.75543E-33	positive
PRJNA729271	stage_3W_vs_E10	ordinary_muscle_development	ENSGALG00010008277	MZB1	6.619017845	1.027443195	6.442222673	1.17736E-10	2.82782E-10	positive
PRJNA729271	stage_3W_vs_E10	ordinary_muscle_development	ENSGALG00010008278	CYP39A1	0.970046703	0.159483527	6.082425697	1.18378E-09	2.68569E-09	positive
PRJNA729271	stage_3W_vs_E10	ordinary_muscle_development	ENSGALG00010008279	MAPK15	-0.473067942	0.169765572	-2.78659528	0.005326496	0.007450309	negative
PRJNA729271	stage_3W_vs_E10	ordinary_muscle_development	ENSGALG00010008282	FAM83H	-2.921997401	0.296663601	-9.849531209	6.88644E-23	2.96111E-22	negative
PRJNA729271	stage_3W_vs_E10	ordinary_muscle_development	ENSGALG00010008283	NQO1	-0.820210067	0.197346981	-4.156182492	3.23609E-05	5.48815E-05	negative
PRJNA729271	stage_3W_vs_E10	ordinary_muscle_development	ENSGALG00010008284	BCKDHA	1.833177702	0.128366075	14.28085807	2.88033E-46	2.87537E-45	positive
PRJNA729271	stage_3W_vs_E10	ordinary_muscle_development	ENSGALG00010008285	ADSS2	-1.259112634	0.089406837	-14.08295695	4.83439E-45	4.62843E-44	negative
PRJNA729271	stage_3W_vs_E10	ordinary_muscle_development	ENSGALG00010008289	MTMR2	-0.174680408	0.069174362	-2.525218924	0.011562623	0.015624147	negative
PRJNA729271	stage_3W_vs_E10	ordinary_muscle_development	ENSGALG00010008290	FAM13A	0.793725859	0.163417046	4.857056705	1.19143E-06	2.24546E-06	positive
PRJNA729271	stage_3W_vs_E10	ordinary_muscle_development	ENSGALG00010008291	PTGFRN	-0.651271742	0.161164651	-4.04103342	5.32162E-05	8.89493E-05	negative
PRJNA729271	stage_3W_vs_E10	ordinary_muscle_development	ENSGALG00010008292	REEP5	0.865887131	0.113348215	7.639177487	2.18614E-14	6.4107E-14	positive
PRJNA729271	stage_3W_vs_E10	ordinary_muscle_development	ENSGALG00010008293	PLA2G7	1.152861223	0.112722536	10.2274245	1.49441E-24	6.87011E-24	positive
PRJNA729271	stage_3W_vs_E10	ordinary_muscle_development	ENSGALG00010008294	NOB1	0.826143302	0.153654957	5.37661342	7.58999E-08	1.54975E-07	positive
PRJNA729271	stage_3W_vs_E10	ordinary_muscle_development	ENSGALG00010008295	DMAC2	0.155863241	0.13276349	1.173991746	0.240398332	0.274858136	positive
PRJNA729271	stage_3W_vs_E10	ordinary_muscle_development	ENSGALG00010008296	PALLD	-0.589905185	0.15315778	-3.851617504	0.00011734	0.000190614	negative
PRJNA729271	stage_3W_vs_E10	ordinary_muscle_development	ENSGALG00010008297	ENSGALG00010008297	-0.05651456	0.289632913	-0.195124784	0.845295261	0.863258004	negative
PRJNA729271	stage_3W_vs_E10	ordinary_muscle_development	ENSGALG00010008298	ENSGALG00010008298	-1.426890453	0.132135116	-10.79872255	3.49026E-27	1.79656E-26	negative
PRJNA729271	stage_3W_vs_E10	ordinary_muscle_development	ENSGALG00010008299	ANKRD66	1.495066626	0.257012488	5.817097195	5.98783E-09	1.30871E-08	positive
PRJNA729271	stage_3W_vs_E10	ordinary_muscle_development	ENSGALG00010008300	CUL1	0.197312415	0.084930937	2.32321015	0.020167866	0.02653233	positive
PRJNA729271	stage_3W_vs_E10	ordinary_muscle_development	ENSGALG00010008301	FAM91A1	-0.624749278	0.105655985	-5.913051501	3.35827E-09	7.41367E-09	negative
PRJNA729271	stage_3W_vs_E10	ordinary_muscle_development	ENSGALG00010008302	ENSGALG00010008302	0.490892553	0.144564033	3.395675551	0.000684595	0.001040822	positive
PRJNA729271	stage_3W_vs_E10	ordinary_muscle_development	ENSGALG00010008303	TEX10	-1.352301482	0.072311437	-18.70107319	4.85183E-78	1.14914E-76	negative
PRJNA729271	stage_3W_vs_E10	ordinary_muscle_development	ENSGALG00010008306	ENSGALG00010008306	0.305718124	0.301778697	1.013054024	0.311034357	0.348964869	positive
PRJNA729271	stage_3W_vs_E10	ordinary_muscle_development	ENSGALG00010008307	ENSGALG00010008307	0.888206976	0.950737587	0.934229369	0.350185589	0.388692865	positive
PRJNA729271	stage_3W_vs_E10	ordinary_muscle_development	ENSGALG00010008308	WWP2	-2.151157446	0.126476923	-17.00829995	7.12762E-65	1.21547E-63	negative
PRJNA729271	stage_3W_vs_E10	ordinary_muscle_development	ENSGALG00010008309	ENSGALG00010008309	0.185169693	0.281940556	0.65676856	0.511329743	0.549301462	positive
PRJNA729271	stage_3W_vs_E10	ordinary_muscle_development	ENSGALG00010008310	EXOSC5	0.197332853	0.181445134	1.087562106	0.276788467	0.313661858	positive
PRJNA729271	stage_3W_vs_E10	ordinary_muscle_development	ENSGALG00010008311	SRP19	-0.249347285	0.114755799	-2.172851287	0.029791512	0.038425695	negative
PRJNA729271	stage_3W_vs_E10	ordinary_muscle_development	ENSGALG00010008312	ZBTB18	1.520861351	0.217033435	7.007497947	2.42618E-12	6.40339E-12	positive
PRJNA729271	stage_3W_vs_E10	ordinary_muscle_development	ENSGALG00010008313	PSMD7	1.390626656	0.177726404	7.824536048	5.09533E-15	1.54415E-14	positive
PRJNA729271	stage_3W_vs_E10	ordinary_muscle_development	ENSGALG00010008314	INVS	0.036528574	0.105705018	0.345570857	0.729665251	0.757466004	positive
PRJNA729271	stage_3W_vs_E10	ordinary_muscle_development	ENSGALG00010008316	ADGRF5	3.160445187	0.133705686	23.63732826	1.5935E-123	1.0042E-121	positive
PRJNA729271	stage_3W_vs_E10	ordinary_muscle_development	ENSGALG00010008317	CEP57	-0.076204582	0.11065871	-0.68864513	0.491046614	0.529434742	negative
PRJNA729271	stage_3W_vs_E10	ordinary_muscle_development	ENSGALG00010008318	ADPRH	2.181763458	0.132992886	16.40511399	1.75796E-60	2.66545E-59	positive
PRJNA729271	stage_3W_vs_E10	ordinary_muscle_development	ENSGALG00010008319	ERP44	-0.025960412	0.064817192	-0.40051739	0.688775478	0.719449489	negative
PRJNA729271	stage_3W_vs_E10	ordinary_muscle_development	ENSGALG00010008320	GPRIN3	0.57286707	0.114501923	5.00312185	5.64093E-07	1.08456E-06	positive
PRJNA729271	stage_3W_vs_E10	ordinary_muscle_development	ENSGALG00010008321	ZFHX3	0.021885133	0.102009427	0.214540295	0.830125747	0.850065186	positive
PRJNA729271	stage_3W_vs_E10	ordinary_muscle_development	ENSGALG00010008323	AKT3	0.412034556	0.10424722	3.952475246	7.73469E-05	0.000127403	positive
PRJNA729271	stage_3W_vs_E10	ordinary_muscle_development	ENSGALG00010008324	SCRIB	-0.157229887	0.104075358	-1.510731169	0.130856958	0.155766741	negative
PRJNA729271	stage_3W_vs_E10	ordinary_muscle_development	ENSGALG00010008325	AADAT	0.005940771	0.133067797	0.044644691	0.96439052	0.969339199	positive
PRJNA729271	stage_3W_vs_E10	ordinary_muscle_development	ENSGALG00010008327	TGFB1	0.820505262	0.127225799	6.449205025	1.12438E-10	2.70415E-10	positive
PRJNA729271	stage_3W_vs_E10	ordinary_muscle_development	ENSGALG00010008328	ENSGALG00010008328	0.355716854	0.440680858	0.807198332	0.419552227	0.458040937	positive
PRJNA729271	stage_3W_vs_E10	ordinary_muscle_development	ENSGALG00010008330	EZH2	-2.513829633	0.08234335	-30.52862959	1.0868E-204	1.994E-202	negative
PRJNA729271	stage_3W_vs_E10	ordinary_muscle_development	ENSGALG00010008331	CCDC97	0.443530793	0.264324291	1.677979693	0.093351063	0.113364918	positive
PRJNA729271	stage_3W_vs_E10	ordinary_muscle_development	ENSGALG00010008332	DHX38	-0.442753534	0.093786245	-4.720879206	2.34827E-06	4.3405E-06	negative
PRJNA729271	stage_3W_vs_E10	ordinary_muscle_development	ENSGALG00010008333	APC	-0.471048483	0.092206063	-5.108649768	3.24469E-07	6.34536E-07	negative
PRJNA729271	stage_3W_vs_E10	ordinary_muscle_development	ENSGALG00010008334	STX17	0.935911008	0.132783795	7.048382728	1.81009E-12	4.81683E-12	positive
PRJNA729271	stage_3W_vs_E10	ordinary_muscle_development	ENSGALG00010008335	HNRNPUL1	-0.862170419	0.191521762	-4.501683826	6.74172E-06	1.20539E-05	negative
PRJNA729271	stage_3W_vs_E10	ordinary_muscle_development	ENSGALG00010008336	PAIP2B	0.533885915	0.109067211	4.895017562	9.8297E-07	1.86421E-06	positive
PRJNA729271	stage_3W_vs_E10	ordinary_muscle_development	ENSGALG00010008339	FAM76B	-1.016334106	0.114700773	-8.860743315	7.94827E-19	2.89983E-18	negative
PRJNA729271	stage_3W_vs_E10	ordinary_muscle_development	ENSGALG00010008340	PDIA4	-0.845570021	0.105766101	-7.994716735	1.29872E-15	4.04056E-15	negative
PRJNA729271	stage_3W_vs_E10	ordinary_muscle_development	ENSGALG00010008341	DHODH	-1.68243968	0.111298823	-15.1164194	1.26222E-51	1.50956E-50	negative
PRJNA729271	stage_3W_vs_E10	ordinary_muscle_development	ENSGALG00010008342	PLD3	1.142007618	0.146822521	7.778150177	7.35927E-15	2.21083E-14	positive
PRJNA729271	stage_3W_vs_E10	ordinary_muscle_development	ENSGALG00010008343	TNFRSF21	1.908032217	0.223067667	8.553602769	1.19305E-17	4.13714E-17	positive
PRJNA729271	stage_3W_vs_E10	ordinary_muscle_development	ENSGALG00010008344	MFAP3L	1.275182642	0.285340733	4.468982143	7.85927E-06	1.39882E-05	positive
PRJNA729271	stage_3W_vs_E10	ordinary_muscle_development	ENSGALG00010008346	NR4A3	3.775194442	0.522914518	7.21952502	5.21695E-13	1.42517E-12	positive
PRJNA729271	stage_3W_vs_E10	ordinary_muscle_development	ENSGALG00010008347	PUF60	-0.697889384	0.055269913	-12.62693105	1.50016E-36	1.07915E-35	negative
PRJNA729271	stage_3W_vs_E10	ordinary_muscle_development	ENSGALG00010008348	ENSGALG00010008348	-1.318961253	0.103096015	-12.7935231	1.78208E-37	1.31323E-36	negative
PRJNA729271	stage_3W_vs_E10	ordinary_muscle_development	ENSGALG00010008350	LRRC6	-0.485675545	0.179299982	-2.708731693	0.006754094	0.009345226	negative
PRJNA729271	stage_3W_vs_E10	ordinary_muscle_development	ENSGALG00010008351	IST1	-0.33097839	0.068351884	-4.842271683	1.28363E-06	2.41263E-06	negative
PRJNA729271	stage_3W_vs_E10	ordinary_muscle_development	ENSGALG00010008352	ENSGALG00010008352	0.636099423	0.152085167	4.182521124	2.88294E-05	4.91106E-05	positive
PRJNA729271	stage_3W_vs_E10	ordinary_muscle_development	ENSGALG00010008354	TMEM71	2.933309017	0.216330132	13.55941026	6.96942E-42	5.97055E-41	positive
PRJNA729271	stage_3W_vs_E10	ordinary_muscle_development	ENSGALG00010008355	MAFA	4.52274979	0.177386357	25.49660458	2.1498E-143	1.833E-141	positive
PRJNA729271	stage_3W_vs_E10	ordinary_muscle_development	ENSGALG00010008356	ENSGALG00010008356	-0.79638974	0.130581254	-6.098806035	1.06864E-09	2.43017E-09	negative
PRJNA729271	stage_3W_vs_E10	ordinary_muscle_development	ENSGALG00010008358	HPF1	-1.922889483	0.117568455	-16.35548829	3.97509E-60	5.9647E-59	negative
PRJNA729271	stage_3W_vs_E10	ordinary_muscle_development	ENSGALG00010008360	ZC3H3	-0.118440123	0.062432563	-1.897088904	0.057816205	0.072009443	negative
PRJNA729271	stage_3W_vs_E10	ordinary_muscle_development	ENSGALG00010008361	MATR3	-1.07209432	0.056982976	-18.81429151	5.76743E-79	1.39331E-77	negative
PRJNA729271	stage_3W_vs_E10	ordinary_muscle_development	ENSGALG00010008362	RAMP3	0.14999516	0.263734048	0.568736427	0.569535023	0.605901663	positive
PRJNA729271	stage_3W_vs_E10	ordinary_muscle_development	ENSGALG00010008364	ENSGALG00010008364	-3.527232846	0.652839851	-5.402906762	6.55695E-08	1.34166E-07	negative
PRJNA729271	stage_3W_vs_E10	ordinary_muscle_development	ENSGALG00010008366	RPRD1A	-0.323881429	0.10776093	-3.005555247	0.002650964	0.003817757	negative
PRJNA729271	stage_3W_vs_E10	ordinary_muscle_development	ENSGALG00010008367	ENSGALG00010008367	-2.310690045	0.644532469	-3.585063836	0.000336996	0.00052717	negative
PRJNA729271	stage_3W_vs_E10	ordinary_muscle_development	ENSGALG00010008368	SNCA	0.450969137	0.324660642	1.389047755	0.164818229	0.193350617	positive
PRJNA729271	stage_3W_vs_E10	ordinary_muscle_development	ENSGALG00010008372	CLCN3	-0.134349584	0.069293217	-1.938856197	0.052518851	0.065733593	negative
PRJNA729271	stage_3W_vs_E10	ordinary_muscle_development	ENSGALG00010008373	MACIR	-0.638257118	0.165469222	-3.857255798	0.000114667	0.00018646	negative
PRJNA729271	stage_3W_vs_E10	ordinary_muscle_development	ENSGALG00010008374	ZNF821	-0.748957564	0.08749387	-8.560114673	1.12755E-17	3.91846E-17	negative
PRJNA729271	stage_3W_vs_E10	ordinary_muscle_development	ENSGALG00010008375	ENSGALG00010008375	-0.959728333	0.098998923	-9.694331034	3.18719E-22	1.33252E-21	negative
PRJNA729271	stage_3W_vs_E10	ordinary_muscle_development	ENSGALG00010008376	MMRN1	1.683768231	0.257944701	6.527632566	6.68174E-11	1.62858E-10	positive
PRJNA729271	stage_3W_vs_E10	ordinary_muscle_development	ENSGALG00010008377	AKT2	0.425110347	0.116571497	3.646777799	0.000265549	0.000418294	positive
PRJNA729271	stage_3W_vs_E10	ordinary_muscle_development	ENSGALG00010008380	SIL1	0.950352609	0.170485605	5.57438623	2.48404E-08	5.2251E-08	positive
PRJNA729271	stage_3W_vs_E10	ordinary_muscle_development	ENSGALG00010008381	C18orf21	1.227128315	0.112007526	10.95576659	6.23482E-28	3.31282E-27	positive
PRJNA729271	stage_3W_vs_E10	ordinary_muscle_development	ENSGALG00010008382	ENSGALG00010008382	0.313342047	0.130195774	2.406699066	0.016097428	0.021390926	positive
PRJNA729271	stage_3W_vs_E10	ordinary_muscle_development	ENSGALG00010008383	PLD5	-4.380775212	0.455584501	-9.61572486	6.86243E-22	2.83231E-21	negative
PRJNA729271	stage_3W_vs_E10	ordinary_muscle_development	ENSGALG00010008385	CTNNA1	-1.024009834	0.091593216	-11.17997467	5.11046E-29	2.8241E-28	negative
PRJNA729271	stage_3W_vs_E10	ordinary_muscle_development	ENSGALG00010008386	SESN3	-1.598364371	0.228371511	-6.998965705	2.57859E-12	6.79329E-12	negative
PRJNA729271	stage_3W_vs_E10	ordinary_muscle_development	ENSGALG00010008387	PHF20L1	0.476805841	0.112115349	4.252815015	2.111E-05	3.6384E-05	positive
PRJNA729271	stage_3W_vs_E10	ordinary_muscle_development	ENSGALG00010008388	ADCY1	-0.781027773	0.405605386	-1.92558531	0.054156174	0.067677709	negative
PRJNA729271	stage_3W_vs_E10	ordinary_muscle_development	ENSGALG00010008389	GALNT1	-0.717375073	0.088638529	-8.093264677	5.80865E-16	1.84398E-15	negative
PRJNA729271	stage_3W_vs_E10	ordinary_muscle_development	ENSGALG00010008391	ENSGALG00010008391	-0.330734854	0.072520134	-4.560593518	5.10092E-06	9.20885E-06	negative
PRJNA729271	stage_3W_vs_E10	ordinary_muscle_development	ENSGALG00010008392	CD36	3.015647816	0.369325356	8.165287771	3.2067E-16	1.03177E-15	positive
PRJNA729271	stage_3W_vs_E10	ordinary_muscle_development	ENSGALG00010008394	CD2AP	0.97825654	0.110584554	8.846231264	9.05246E-19	3.29191E-18	positive
PRJNA729271	stage_3W_vs_E10	ordinary_muscle_development	ENSGALG00010008395	ENDOD1	0.31870715	0.146639778	2.17340175	0.029750093	0.038379103	positive
PRJNA729271	stage_3W_vs_E10	ordinary_muscle_development	ENSGALG00010008396	LRRTM2	-3.166515325	0.473870489	-6.682237863	2.35321E-11	5.90236E-11	negative
PRJNA729271	stage_3W_vs_E10	ordinary_muscle_development	ENSGALG00010008397	ATXN1L	-0.756539284	0.073132886	-10.34472074	4.42204E-25	2.07906E-24	negative
PRJNA729271	stage_3W_vs_E10	ordinary_muscle_development	ENSGALG00010008399	INO80C	-0.382290067	0.112008933	-3.413031945	0.000642444	0.000979513	negative
PRJNA729271	stage_3W_vs_E10	ordinary_muscle_development	ENSGALG00010008400	MAP1LC3C	-2.38092493	0.2683967	-8.870917286	7.25458E-19	2.65075E-18	negative
PRJNA729271	stage_3W_vs_E10	ordinary_muscle_development	ENSGALG00010008401	PSMD8	0.583948714	0.156009558	3.743031642	0.000181813	0.000290657	positive
PRJNA729271	stage_3W_vs_E10	ordinary_muscle_development	ENSGALG00010008403	GNAT3	-1.306186708	0.456475305	-2.861461934	0.004216921	0.005958693	negative
PRJNA729271	stage_3W_vs_E10	ordinary_muscle_development	ENSGALG00010008405	LRATD2	0.330440917	0.144445734	2.287647469	0.022158059	0.029021511	positive
PRJNA729271	stage_3W_vs_E10	ordinary_muscle_development	ENSGALG00010008407	GNAI1	-0.659151633	0.194427418	-3.390219545	0.000698367	0.001061093	negative
PRJNA729271	stage_3W_vs_E10	ordinary_muscle_development	ENSGALG00010008408	SMARCA4	-0.828020736	0.154124893	-5.372401029	7.76951E-08	1.58551E-07	negative
PRJNA729271	stage_3W_vs_E10	ordinary_muscle_development	ENSGALG00010008409	CWC15	0.521002296	0.084550988	6.161989447	7.18366E-10	1.65413E-09	positive
PRJNA729271	stage_3W_vs_E10	ordinary_muscle_development	ENSGALG00010008410	NAPRT	-0.658304659	0.133018569	-4.948968143	7.4608E-07	1.42332E-06	negative
PRJNA729271	stage_3W_vs_E10	ordinary_muscle_development	ENSGALG00010008411	ENSGALG00010008411	0.21911643	0.404045161	0.54230678	0.587607186	0.623115528	positive
PRJNA729271	stage_3W_vs_E10	ordinary_muscle_development	ENSGALG00010008412	NEK1	-1.181235194	0.105551356	-11.19109449	4.50834E-29	2.50281E-28	negative
PRJNA729271	stage_3W_vs_E10	ordinary_muscle_development	ENSGALG00010008415	ENSGALG00010008415	0.748151242	0.359027831	2.083825199	0.03717607	0.04745484	positive
PRJNA729271	stage_3W_vs_E10	ordinary_muscle_development	ENSGALG00010008416	MSX2	-5.012145021	0.464784266	-10.78380958	4.10524E-27	2.1049E-26	negative
PRJNA729271	stage_3W_vs_E10	ordinary_muscle_development	ENSGALG00010008417	IGFBP1	-0.783611305	0.917036338	-0.854504094	0.392825742	0.431756834	negative
PRJNA729271	stage_3W_vs_E10	ordinary_muscle_development	ENSGALG00010008418	TMEM245	1.751099917	0.088332096	19.82405039	1.84633E-87	5.67004E-86	positive
PRJNA729271	stage_3W_vs_E10	ordinary_muscle_development	ENSGALG00010008422	EXO1	-4.492397716	0.246433047	-18.2296886	3.00025E-74	6.52981E-73	negative
PRJNA729271	stage_3W_vs_E10	ordinary_muscle_development	ENSGALG00010008424	PPIP5K2	-0.547719783	0.120930679	-4.529204544	5.92062E-06	1.0641E-05	negative
PRJNA729271	stage_3W_vs_E10	ordinary_muscle_development	ENSGALG00010008425	MAGI2	-0.535315267	0.107965052	-4.958227273	7.11393E-07	1.3593E-06	negative
PRJNA729271	stage_3W_vs_E10	ordinary_muscle_development	ENSGALG00010008426	AP1G1	-0.58641292	0.056074515	-10.45774404	1.35032E-25	6.47253E-25	negative
PRJNA729271	stage_3W_vs_E10	ordinary_muscle_development	ENSGALG00010008429	IGFBP3	1.451979843	0.118527692	12.25013172	1.67723E-34	1.13765E-33	positive
PRJNA729271	stage_3W_vs_E10	ordinary_muscle_development	ENSGALG00010008430	ENSGALG00010008430	0.376270286	0.389852945	0.965159533	0.334464912	0.372784998	positive
PRJNA729271	stage_3W_vs_E10	ordinary_muscle_development	ENSGALG00010008431	SH3RF1	-0.804274951	0.127519173	-6.307090409	2.8433E-10	6.69595E-10	negative
PRJNA729271	stage_3W_vs_E10	ordinary_muscle_development	ENSGALG00010008433	EBAG9	1.285089062	0.09451107	13.59723321	4.1587E-42	3.59454E-41	positive
PRJNA729271	stage_3W_vs_E10	ordinary_muscle_development	ENSGALG00010008434	PTCHD4	-2.620651266	0.561033109	-4.671116955	2.99566E-06	5.50204E-06	negative
PRJNA729271	stage_3W_vs_E10	ordinary_muscle_development	ENSGALG00010008437	SHKBP1	0.249020194	0.160169129	1.554732769	0.120009727	0.143502804	positive
PRJNA729271	stage_3W_vs_E10	ordinary_muscle_development	ENSGALG00010008438	SYBU	0.330463695	0.262912838	1.256932515	0.208778073	0.241019287	positive
PRJNA729271	stage_3W_vs_E10	ordinary_muscle_development	ENSGALG00010008440	ENSGALG00010008440	0.501582912	0.312698615	1.604045838	0.108703949	0.130749626	positive
PRJNA729271	stage_3W_vs_E10	ordinary_muscle_development	ENSGALG00010008441	PHLPP2	0.07922333	0.086482403	0.916063002	0.359633852	0.398386907	positive
PRJNA729271	stage_3W_vs_E10	ordinary_muscle_development	ENSGALG00010008443	RSFR	5.150149589	0.439056504	11.73003824	8.94183E-32	5.49202E-31	positive

PRJNA729271	stage_3W_vs_E10	ordinary_muscle_development	ENSGALG00010008444	SLC7A11	-3.289704176	0.469112691	-7.012609635	2.33913E-12	6.18041E-12	negative
PRJNA729271	stage_3W_vs_E10	ordinary_muscle_development	ENSGALG00010008447	FRRS1L	-0.123309016	0.155471243	-0.793130705	0.427701675	0.46616556	negative
PRJNA729271	stage_3W_vs_E10	ordinary_muscle_development	ENSGALG00010008448	TNS3	-0.99734908	0.081170233	-12.2871285	1.06212E-34	7.23131E-34	negative
PRJNA729271	stage_3W_vs_E10	ordinary_muscle_development	ENSGALG00010008449	SRA1	0.005998358	0.109526713	0.05476616	0.95632476	0.962032577	positive
PRJNA729271	stage_3W_vs_E10	ordinary_muscle_development	ENSGALG00010008450	GIN1	-0.47760289	0.177180687	-2.695569702	0.007026837	0.009705928	negative
PRJNA729271	stage_3W_vs_E10	ordinary_muscle_development	ENSGALG00010008451	OPN3	1.200516415	0.305750363	3.926459495	8.62054E-05	0.00014148	positive
PRJNA729271	stage_3W_vs_E10	ordinary_muscle_development	ENSGALG00010008452	ENSGALG00010008452	0.728373928	0.40746878	1.787557633	0.073847406	0.090751857	positive
PRJNA729271	stage_3W_vs_E10	ordinary_muscle_development	ENSGALG00010008453	AMOTL1	1.111995816	0.120070242	9.261210727	2.02127E-20	7.85899E-20	positive
PRJNA729271	stage_3W_vs_E10	ordinary_muscle_development	ENSGALG00010008455	FKBP1B	1.973922095	0.150231332	13.13921716	1.96246E-39	1.55783E-38	positive
PRJNA729271	stage_3W_vs_E10	ordinary_muscle_development	ENSGALG00010008456	NHLRC1	0.373255642	0.128044538	2.91504541	0.003556368	0.005063807	positive
PRJNA729271	stage_3W_vs_E10	ordinary_muscle_development	ENSGALG00010008457	MARVELD3	0.491515029	0.119517891	4.112480776	3.9143E-05	6.60664E-05	positive
PRJNA729271	stage_3W_vs_E10	ordinary_muscle_development	ENSGALG00010008459	EPB41L4B	-2.598396373	0.238277898	-10.90489884	1.09215E-27	5.75242E-27	negative
PRJNA729271	stage_3W_vs_E10	ordinary_muscle_development	ENSGALG00010008464	SF3B6	-0.102079128	0.10992074	-0.928661217	0.353064686	0.391678603	negative
PRJNA729271	stage_3W_vs_E10	ordinary_muscle_development	ENSGALG00010008466	C11orf97	-1.033283256	0.352313775	-2.932849433	0.003358667	0.004795969	negative
PRJNA729271	stage_3W_vs_E10	ordinary_muscle_development	ENSGALG00010008468	EPPK1	-2.796378938	0.303486343	-9.21418377	3.13655E-20	1.21012E-19	negative
PRJNA729271	stage_3W_vs_E10	ordinary_muscle_development	ENSGALG00010008469	NOCT	2.476749741	0.275809802	8.979919223	2.70966E-19	1.00786E-18	positive
PRJNA729271	stage_3W_vs_E10	ordinary_muscle_development	ENSGALG00010008473	CSMD3	-5.944456415	0.661401663	-8.987664754	2.52539E-19	9.41742E-19	negative
PRJNA729271	stage_3W_vs_E10	ordinary_muscle_development	ENSGALG00010008474	ENSGALG00010008474	0.928470937	0.140259391	6.619670385	3.60001E-11	8.92765E-11	positive
PRJNA729271	stage_3W_vs_E10	ordinary_muscle_development	ENSGALG00010008476	RNF166	0.150746864	0.134529507	1.120548702	0.262480011	0.298497392	positive
PRJNA729271	stage_3W_vs_E10	ordinary_muscle_development	ENSGALG00010008486	CTU2	-0.703534912	0.138855783	-5.066659067	4.04858E-07	7.86652E-07	negative
PRJNA729271	stage_3W_vs_E10	ordinary_muscle_development	ENSGALG00010008488	CCSER1	-0.890712047	0.154950357	-5.748370419	9.01077E-09	1.94949E-08	negative
PRJNA729271	stage_3W_vs_E10	ordinary_muscle_development	ENSGALG00010008492	ANKRD49	0.702330672	0.122610113	5.728162669	1.01524E-08	2.18987E-08	positive
PRJNA729271	stage_3W_vs_E10	ordinary_muscle_development	ENSGALG00010008497	PAM	-0.51400331	0.129429114	-3.971311351	7.14781E-05	0.000118179	negative
PRJNA729271	stage_3W_vs_E10	ordinary_muscle_development	ENSGALG00010008498	ENSGALG00010008498	2.203475507	0.147707731	14.91780754	2.52432E-50	2.90167E-49	positive
PRJNA729271	stage_3W_vs_E10	ordinary_muscle_development	ENSGALG00010008500	ENSGALG00010008500	0.856282443	0.151028611	5.669670369	1.43073E-08	3.0583E-08	positive
PRJNA729271	stage_3W_vs_E10	ordinary_muscle_development	ENSGALG00010008501	ENSGALG00010008501	-1.342131068	0.416936608	-3.21902909	0.001286254	0.001908317	negative
PRJNA729271	stage_3W_vs_E10	ordinary_muscle_development	ENSGALG00010008504	RNF139	1.364266802	0.087037467	15.67447736	2.26077E-55	3.01748E-54	positive
PRJNA729271	stage_3W_vs_E10	ordinary_muscle_development	ENSGALG00010008505	PTPN3	-0.289784213	0.270537591	-1.071142135	0.284105525	0.321250455	negative
PRJNA729271	stage_3W_vs_E10	ordinary_muscle_development	ENSGALG00010008509	MGARP	0.894700758	0.359452973	2.489062064	0.01280806	0.017209199	positive
PRJNA729271	stage_3W_vs_E10	ordinary_muscle_development	ENSGALG00010008514	PIEZO1	1.750664821	0.166221489	10.5321209	6.14344E-26	2.99024E-25	positive
PRJNA729271	stage_3W_vs_E10	ordinary_muscle_development	ENSGALG00010008515	TAFA5	-1.411557351	0.22006817	-6.414182264	1.41581E-10	3.38593E-10	negative
PRJNA729271	stage_3W_vs_E10	ordinary_muscle_development	ENSGALG00010008516	MRE11	-1.235467571	0.079164654	-15.60630301	6.59494E-55	8.70616E-54	negative
PRJNA729271	stage_3W_vs_E10	ordinary_muscle_development	ENSGALG00010008522	NUDT12	0.842273522	0.149135429	5.647709118	1.626E-08	3.46346E-08	positive
PRJNA729271	stage_3W_vs_E10	ordinary_muscle_development	ENSGALG00010008527	CHUK	0.154355306	0.055063127	2.803242669	0.005059159	0.007089376	positive
PRJNA729271	stage_3W_vs_E10	ordinary_muscle_development	ENSGALG00010008529	GPR83	0.296275573	0.482818774	0.613637227	0.539455078	0.577173021	positive
PRJNA729271	stage_3W_vs_E10	ordinary_muscle_development	ENSGALG00010008533	ENSGALG00010008533	-0.023297188	0.560798883	-0.041542858	0.966863127	0.971487663	negative
PRJNA729271	stage_3W_vs_E10	ordinary_muscle_development	ENSGALG00010008534	RAB33B	1.022809615	0.071770928	14.25102947	4.41721E-46	4.39146E-45	positive
PRJNA729271	stage_3W_vs_E10	ordinary_muscle_development	ENSGALG00010008542	MOCOS	2.500190497	0.154367913	16.19630949	5.35456E-59	7.84776E-58	positive
PRJNA729271	stage_3W_vs_E10	ordinary_muscle_development	ENSGALG00010008561	EEF1D	1.277527959	0.114701269	11.1378712	8.20573E-29	4.50197E-28	positive
PRJNA729271	stage_3W_vs_E10	ordinary_muscle_development	ENSGALG00010008580	PANX1	-1.925770623	0.253908096	-7.584518378	3.33724E-14	9.69989E-14	negative
PRJNA729271	stage_3W_vs_E10	ordinary_muscle_development	ENSGALG00010008588	PYCR3	-2.163813568	0.185258394	-11.67997586	1.61341E-31	9.80152E-31	negative
PRJNA729271	stage_3W_vs_E10	ordinary_muscle_development	ENSGALG00010008592	KIAA0513	1.429551708	0.138896038	10.29224256	7.63765E-25	3.54946E-24	positive
PRJNA729271	stage_3W_vs_E10	ordinary_muscle_development	ENSGALG00010008596	ENSGALG00010008596	-0.058148221	0.15565474	-0.373571797	0.70872292	0.737680506	negative
PRJNA729271	stage_3W_vs_E10	ordinary_muscle_development	ENSGALG00010008618	EIF4EBP3	0.072331355	0.116748008	0.619551081	0.535553382	0.573284563	positive
PRJNA729271	stage_3W_vs_E10	ordinary_muscle_development	ENSGALG00010008623	TRPS1	-2.14064191	0.133045402	-16.08955953	3.01978E-58	4.33813E-57	negative
PRJNA729271	stage_3W_vs_E10	ordinary_muscle_development	ENSGALG00010008636	AGTPBP1	5.132681033	0.238382477	21.53128495	7.9304E-103	3.494E-101	positive
PRJNA729271	stage_3W_vs_E10	ordinary_muscle_development	ENSGALG00010008637	RGS7	-6.041158497	1.027882535	-5.877282898	4.1705E-09	9.17181E-09	negative
PRJNA729271	stage_3W_vs_E10	ordinary_muscle_development	ENSGALG00010008653	ENSGALG00010008653	-0.873702109	0.156878808	-5.569280663	2.55793E-08	5.37585E-08	negative
PRJNA729271	stage_3W_vs_E10	ordinary_muscle_development	ENSGALG00010008656	CCDC6	0.188509186	0.071513347	2.636000048	0.008388972	0.011501906	positive
PRJNA729271	stage_3W_vs_E10	ordinary_muscle_development	ENSGALG00010008658	TSTA3	-1.420094838	0.119329507	-11.90061768	1.17474E-32	7.45857E-32	negative
PRJNA729271	stage_3W_vs_E10	ordinary_muscle_development	ENSGALG00010008666	SLC16A9	1.855277864	0.188228964	9.856495111	6.42532E-23	2.76611E-22	positive
PRJNA729271	stage_3W_vs_E10	ordinary_muscle_development	ENSGALG00010008668	ENSGALG00010008668	0.328771099	0.305226932	1.077136599	0.281419244	0.31843653	positive
PRJNA729271	stage_3W_vs_E10	ordinary_muscle_development	ENSGALG00010008674	RXFP1	2.540065614	0.267332305	9.501528856	2.06831E-21	8.37903E-21	positive
PRJNA729271	stage_3W_vs_E10	ordinary_muscle_development	ENSGALG00010008686	ENSGALG00010008686	-1.15478222	0.406009744	-2.844222919	0.004451989	0.00627678	negative
PRJNA729271	stage_3W_vs_E10	ordinary_muscle_development	ENSGALG00010008688	HBEGF	1.4360806	0.233198013	6.158202554	7.35752E-10	1.69228E-09	positive
PRJNA729271	stage_3W_vs_E10	ordinary_muscle_development	ENSGALG00010008690	YIF1B	-0.589707039	0.124623082	-4.731924696	2.22401E-06	4.11606E-06	negative
PRJNA729271	stage_3W_vs_E10	ordinary_muscle_development	ENSGALG00010008693	C4orf46	-0.703320947	0.105778048	-6.649025605	2.9504E-11	7.35822E-11	negative
PRJNA729271	stage_3W_vs_E10	ordinary_muscle_development	ENSGALG00010008696	PFN4	-0.008779418	0.209433955	-0.041919747	0.966562675	0.9712531	negative
PRJNA729271	stage_3W_vs_E10	ordinary_muscle_development	ENSGALG00010008699	NAA35	0.701414012	0.099737382	7.03260903	2.02707E-12	5.38335E-12	positive
PRJNA729271	stage_3W_vs_E10	ordinary_muscle_development	ENSGALG00010008700	ENSGALG00010008700	0.337596286	0.25626434	1.317375198	0.18771289	0.218160547	positive
PRJNA729271	stage_3W_vs_E10	ordinary_muscle_development	ENSGALG00010008708	ZC3H4	0.823043821	0.245669559	3.350206774	0.000807513	0.001219283	positive
PRJNA729271	stage_3W_vs_E10	ordinary_muscle_development	ENSGALG00010008716	TATDN1	-0.047111553	0.110700114	-0.425578177	0.670415245	0.701991546	negative
PRJNA729271	stage_3W_vs_E10	ordinary_muscle_development	ENSGALG00010008729	TMEM254	-0.59474416	0.119278893	-4.986164324	6.15898E-07	1.18213E-06	negative
PRJNA729271	stage_3W_vs_E10	ordinary_muscle_development	ENSGALG00010008731	NDUFB9	0.866342439	0.087501793	9.900853548	4.12737E-23	1.78906E-22	positive
PRJNA729271	stage_3W_vs_E10	ordinary_muscle_development	ENSGALG00010008739	VSTM5	-0.910069487	0.254065072	-3.582033066	0.000340931	0.000533152	negative
PRJNA729271	stage_3W_vs_E10	ordinary_muscle_development	ENSGALG00010008745	GRAMD4	1.617467084	0.151959293	10.6440814	1.85808E-26	9.2936E-26	positive
PRJNA729271	stage_3W_vs_E10	ordinary_muscle_development	ENSGALG00010008750	PFDN1	0.66742282	0.165777097	4.026025506	5.67275E-05	9.46329E-05	positive
PRJNA729271	stage_3W_vs_E10	ordinary_muscle_development	ENSGALG00010008752	GOLM1	-1.032161806	0.173186556	-5.959826372	2.52506E-09	5.61534E-09	negative
PRJNA729271	stage_3W_vs_E10	ordinary_muscle_development	ENSGALG00010008756	ITSN2	1.310839639	0.072663476	18.03986958	9.47673E-73	1.9908E-71	positive
PRJNA729271	stage_3W_vs_E10	ordinary_muscle_development	ENSGALG00010008762	SAE1	-0.580975728	0.102309167	-5.678628268	1.35779E-08	2.90583E-08	negative
PRJNA729271	stage_3W_vs_E10	ordinary_muscle_development	ENSGALG00010008764	ETFDH	-0.120827301	0.124177965	-0.973017238	0.330544735	0.368841103	negative
PRJNA729271	stage_3W_vs_E10	ordinary_muscle_development	ENSGALG00010008768	PRXL2C	3.149289794	0.143774849	21.90431644	2.363E-106	1.1121E-104	positive
PRJNA729271	stage_3W_vs_E10	ordinary_muscle_development	ENSGALG00010008775	MED17	0.043687104	0.089797693	0.486505866	0.626608548	0.660751557	positive
PRJNA729271	stage_3W_vs_E10	ordinary_muscle_development	ENSGALG00010008780	KARS1	0.269564541	0.057247776	4.70873381	2.4926E-06	4.60141E-06	positive
PRJNA729271	stage_3W_vs_E10	ordinary_muscle_development	ENSGALG00010008782	CCDC9	0.243229012	0.282200557	0.861901249	0.388741862	0.427754747	positive
PRJNA729271	stage_3W_vs_E10	ordinary_muscle_development	ENSGALG00010008790	GPN1	0.438752321	0.094710279	4.632573433	3.61148E-06	6.60298E-06	positive
PRJNA729271	stage_3W_vs_E10	ordinary_muscle_development	ENSGALG00010008793	C11orf54	0.823074357	0.113744049	7.23619711	4.61441E-13	1.2639E-12	positive
PRJNA729271	stage_3W_vs_E10	ordinary_muscle_development	ENSGALG00010008799	ENSGALG00010008799	0.538483544	0.172364017	3.124106486	0.001783459	0.002608601	positive
PRJNA729271	stage_3W_vs_E10	ordinary_muscle_development	ENSGALG00010008807	BRD1	0.035597157	0.061970881	0.574417476	0.56568533	0.602257433	positive
PRJNA729271	stage_3W_vs_E10	ordinary_muscle_development	ENSGALG00010008811	TERF2IP	0.97735124	0.094119675	10.38413322	2.92817E-25	1.38479E-24	positive
PRJNA729271	stage_3W_vs_E10	ordinary_muscle_development	ENSGALG00010008814	ENSGALG00010008814	0.97546466	0.229963575	4.241822474	2.21712E-05	3.81676E-05	positive
PRJNA729271	stage_3W_vs_E10	ordinary_muscle_development	ENSGALG00010008821	ENSGALG00010008821	0.120744904	0.137553861	0.877800913	0.380051769	0.418892129	positive
PRJNA729271	stage_3W_vs_E10	ordinary_muscle_development	ENSGALG00010008824	GREM2	-0.981937913	0.307151978	-3.196912219	0.001389072	0.00205497	negative
PRJNA729271	stage_3W_vs_E10	ordinary_muscle_development	ENSGALG00010008825	PPID	0.310935274	0.108687555	2.860817634	0.0042255	0.005969652	positive
PRJNA729271	stage_3W_vs_E10	ordinary_muscle_development	ENSGALG00010008830	DHX34	0.034296025	0.19880929	0.172507156	0.863038836	0.878863842	positive
PRJNA729271	stage_3W_vs_E10	ordinary_muscle_development	ENSGALG00010008832	MTSS1	0.087886601	0.124532095	0.705734542	0.480353236	0.51890894	positive
PRJNA729271	stage_3W_vs_E10	ordinary_muscle_development	ENSGALG00010008837	ENSGALG00010008837	-0.82329324	0.09910736	-8.307084741	9.80819E-17	3.24514E-16	negative
PRJNA729271	stage_3W_vs_E10	ordinary_muscle_development	ENSGALG00010008839	ZNF512	-0.412034858	0.074492087	-5.531256767	3.17945E-08	6.633E-08	negative
PRJNA729271	stage_3W_vs_E10	ordinary_muscle_development	ENSGALG00010008846	SLC35D2	0.997605396	0.280245749	3.559752112	0.000371205	0.000578312	positive
PRJNA729271	stage_3W_vs_E10	ordinary_muscle_development	ENSGALG00010008850	CYSTM1	2.672277602	0.123142725	21.70065343	2.0227E-104	9.1908E-103	positive
PRJNA729271	stage_3W_vs_E10	ordinary_muscle_development	ENSGALG00010008852	KHDRBS3	0.587488563	0.083262501	7.055860177	1.71536E-12	4.56726E-12	positive
PRJNA729271	stage_3W_vs_E10	ordinary_muscle_development	ENSGALG00010008859	ENSGALG00010008859	-0.906217788	0.213785245	-4.238916425	2.24601E-05	3.86467E-05	negative
PRJNA729271	stage_3W_vs_E10	ordinary_muscle_development	ENSGALG00010008860	SQLE	-2.198604622	0.247392328	-8.887117231	6.27148E-19	2.29616E-18	negative
PRJNA729271	stage_3W_vs_E10	ordinary_muscle_development	ENSGALG00010008862	ZNF367	-1.149249688	0.14917584	-7.703993399	1.31879E-14	3.90916E-14	negative
PRJNA729271	stage_3W_vs_E10	ordinary_muscle_development	ENSGALG00010008864	ADAT1	0.516684702	0.13774225	3.75109816	0.000176062	0.000281807	positive
PRJNA729271	stage_3W_vs_E10	ordinary_muscle_development	ENSGALG00010008866	WASHC5	-0.234595178	0.073256115	-3.202397187	0.001362889	0.00201809	negative
PRJNA729271	stage_3W_vs_E10	ordinary_muscle_development	ENSGALG00010008871	PRR7	-1.680947604	0.197825615	-8.49711806	1.94356E-17	6.65687E-17	negative
PRJNA729271	stage_3W_vs_E10	ordinary_muscle_development	ENSGALG00010008877	FNDC4	0.242697862	0.254255381	0.95454366	0.33980852	0.378333551	positive
PRJNA729271	stage_3W_vs_E10	ordinary_muscle_development	ENSGALG00010008879	HABP4	-1.193137366	0.173706708	-6.868689067	6.47946E-12	1.6682E-11	negative
PRJNA729271	stage_3W_vs_E10	ordinary_muscle_development	ENSGALG00010008880	FMN2	-1.494244791	0.296258507	-5.043719446	4.56569E-07	8.844E-07	negative
PRJNA729271	stage_3W_vs_E10	ordinary_muscle_development	ENSGALG00010008884	FAM135B	-0.01484617	0.508140672	-0.029216654	0.976691799	0.980276113	negative
PRJNA729271	stage_3W_vs_E10	ordinary_muscle_development	ENSGALG00010008888	FBXO46	1.39689669	0.141800228	9.851159721	6.77576E-23	2.91611E-22	positive
PRJNA729271	stage_3W_vs_E10	ordinary_muscle_development	ENSGALG00010008892	ENSGALG00010008892	-0.472747282	0.127113058	-3.719108725	0.000199927	0.000318525	negative
PRJNA729271	stage_3W_vs_E10	ordinary_muscle_development	ENSGALG00010008893	ENSGALG00010008893	4.78399627	0.138146341	34.62991668	8.9618E-263	3.021E-260	positive
PRJNA729271	stage_3W_vs_E10	ordinary_muscle_development	ENSGALG00010008897	NSMCE2	0.118932719	0.16357228	0.72709581	0.467167266	0.505898592	positive
PRJNA729271	stage_3W_vs_E10	ordinary_muscle_development	ENSGALG00010008903	ENSGALG00010008903	0.077502076	0.545837955	0.141987334	0.88709001	0.900256928	positive
PRJNA729271	stage_3W_vs_E10	ordinary_muscle_development	ENSGALG00010008905	GABARAPL2	0.894983319	0.093181148	9.604768129	7.63295E-22	3.14496E-21	positive
PRJNA729271	stage_3W_vs_E10	ordinary_muscle_development	ENSGALG00010008906	DBN1	-3.934359645	0.215509274	-18.25610362	1.85034E-74	4.03925E-73	negative
PRJNA729271	stage_3W_vs_E10	ordinary_muscle_development	ENSGALG00010008910	TRIB1	2.115683864	0.260956872	8.107408135	5.17109E-16	1.64664E-15	positive
PRJNA729271	stage_3W_vs_E10	ordinary_muscle_development	ENSGALG00010008918	PLAC9	-0.070416471	0.245946592	-0.286307978	0.774642248	0.799006575	negative
PRJNA729271	stage_3W_vs_E10	ordinary_muscle_development	ENSGALG00010008921	OPA3	1.132353095	0.125255882	9.040318701	1.56216E-19	5.8677E-19	positive
PRJNA729271	stage_3W_vs_E10	ordinary_muscle_development	ENSGALG00010008922	ENSGALG00010008922	2.639268103	1.321093261	1.997790907	0.045739334	0.057736797	positive
PRJNA729271	stage_3W_vs_E10	ordinary_muscle_development	ENSGALG00010008928	CFC1	-5.31911924	0.892224732	-5.961636174	2.49725E-09	5.55433E-09	negative
PRJNA729271	stage_3W_vs_E10	ordinary_muscle_development	ENSGALG00010008931	FNIP2	-0.068596758	0.132237808	-0.518737866	0.603943552	0.639363263	negative
PRJNA729271	stage_3W_vs_E10	ordinary_muscle_development	ENSGALG00010008932	VASP	1.447016002	0.119770152	12.08160784	1.32111E-33	8.66492E-33	positive
PRJNA729271	stage_3W_vs_E10	ordinary_muscle_development	ENSGALG00010008936	IFT172	-0.533720315	0.094937359	-5.621815462	1.88961E-08	4.01141E-08	negative
PRJNA729271	stage_3W_vs_E10	ordinary_muscle_development	ENSGALG00010008938	ZBED4	0.025933493	0.090403389	0.286864164	0.774216331	0.79862409	positive
PRJNA729271	stage_3W_vs_E10	ordinary_muscle_development	ENSGALG00010008939	RANBP9	1.350265808	0.102431309	13.18215911	1.11163E-39	8.89735E-39	positive
PRJNA729271	stage_3W_vs_E10	ordinary_muscle_development	ENSGALG00010008943	ENSGALG00010008943	3.191301968	0.458593513	6.958890333	3.42963E-12	8.97176E-12	positive
PRJNA729271	stage_3W_vs_E10	ordinary_muscle_development	ENSGALG00010008945	FER1L6	0.968232955	0.55416294	1.747199038	0.080602818	0.098577274	positive
PRJNA729271	stage_3W_vs_E10	ordinary_muscle_development	ENSGALG00010008946	COL22A1	-3.240348765	0.623258462	-5.199094956	2.00315E-07	3.97042E-07	negative
PRJNA729271	stage_3W_vs_E10	ordinary_muscle_development	ENSGALG00010008955	KRTCAP3	-2.59246458	0.4311228	-6.013285728	1.818E-09	4.07672E-09	negative
PRJNA729271	stage_3W_vs_E10	ordinary_muscle_development	ENSGALG00010008956	LVRN	0.461259605	0.483288031	0.954419674	0.339871252	0.378374331	positive
PRJNA729271	stage_3W_vs_E10	ordinary_muscle_development	ENSGALG00010008960	LRRC2	9.73661318	0.397403462	24.50057464	1.4565E-132	1.0609E-130	positive
PRJNA729271	stage_3W_vs_E10	ordinary_muscle_development	ENSGALG00010008966	NRBP1	-0.137559758	0.116749856	-1.178243487	0.23869955	0.273017437	negative
PRJNA729271	stage_3W_vs_E10	ordinary_muscle_development	ENSGALG00010008969	PPP1R37	0.175271424	0.187956896	0.932508608	0.35107374	0.38958918	positive
PRJNA729271	stage_3W_vs_E10	ordinary_muscle_development	ENSGALG00010008971	ANXA11	0.916821949	0.103511889	8.85716562	8.20746E-19	2.99062E-18	positive
PRJNA729271	stage_3W_vs_E10	ordinary_muscle_development	ENSGALG00010008973	KCNK9	-4.298996491	0.932279014	-4.611276697	4.00203E-06	7.28488E-06	negative
PRJNA729271	stage_3W_vs_E10	ordinary_muscle_development	ENSGALG00010008974	SMCO4	0.099123333	0.099622944	0.994984979	0.31974357	0.357835319	positive
PRJNA729271	stage_3W_vs_E10	ordinary_muscle_development	ENSGALG00010008980	ENSGALG00010008980	-0.318985666	0.257982911	-1.236460447	0.216287459	0.249073387	negative
PRJNA729271	stage_3W_vs_E10	ordinary_muscle_development	ENSGALG00010008983	ENSGALG00010008983	-0.491888428	0.347518854	-1.415429475	0.156942613	0.184664597	negative

PRJNA729271	stage_3W_vs_E10	ordinary_muscle_development	ENSGALG00010008988	PPM1G	-0.848030046	0.080791793	-10.49648753	8.96543E-26	4.3318E-25	negative
PRJNA729271	stage_3W_vs_E10	ordinary_muscle_development	ENSGALG00010008991	ENSGALG00010008991	0.043559539	0.131213324	0.331974965	0.739908163	0.767220032	positive
PRJNA729271	stage_3W_vs_E10	ordinary_muscle_development	ENSGALG00010008995	ECD	-0.811321182	0.100563607	-8.067741467	7.16105E-16	2.26241E-15	negative
PRJNA729271	stage_3W_vs_E10	ordinary_muscle_development	ENSGALG00010008996	CHRM3	-1.315107182	0.242791861	-5.41660325	6.07419E-08	1.24516E-07	negative
PRJNA729271	stage_3W_vs_E10	ordinary_muscle_development	ENSGALG00010008997	CHST6	-1.653024311	0.133234667	-12.40686341	2.39867E-35	1.67034E-34	negative
PRJNA729271	stage_3W_vs_E10	ordinary_muscle_development	ENSGALG00010008999	TRAPPC9	-0.741328597	0.065592476	-11.30203716	1.28209E-29	7.265E-29	negative
PRJNA729271	stage_3W_vs_E10	ordinary_muscle_development	ENSGALG00010009000	FAM149B1	-0.607951175	0.103462981	-5.876026089	4.20232E-09	9.23899E-09	negative
PRJNA729271	stage_3W_vs_E10	ordinary_muscle_development	ENSGALG00010009002	ENSGALG00010009002	1.021557742	0.096945426	10.53745167	5.80506E-26	2.83124E-25	positive
PRJNA729271	stage_3W_vs_E10	ordinary_muscle_development	ENSGALG00010009003	ALG12	-1.580534716	0.136774031	-11.55580999	6.89939E-31	4.10199E-30	negative
PRJNA729271	stage_3W_vs_E10	ordinary_muscle_development	ENSGALG00010009007	TMEM65	-0.739405505	0.100312868	-7.370993603	1.69361E-13	4.74741E-13	negative
PRJNA729271	stage_3W_vs_E10	ordinary_muscle_development	ENSGALG00010009011	ACP6	-0.3978242	0.120671858	-3.296743791	0.000978127	0.001466482	negative
PRJNA729271	stage_3W_vs_E10	ordinary_muscle_development	ENSGALG00010009012	DEUP1	-2.802435014	0.931271725	-3.009255988	0.002618884	0.003774181	negative
PRJNA729271	stage_3W_vs_E10	ordinary_muscle_development	ENSGALG00010009013	LIPG	-0.744617772	0.225253554	-3.305687113	0.000947438	0.001421795	negative
PRJNA729271	stage_3W_vs_E10	ordinary_muscle_development	ENSGALG00010009015	TMEM231	-0.019520177	0.091254672	-0.213908793	0.830618183	0.850269108	negative
PRJNA729271	stage_3W_vs_E10	ordinary_muscle_development	ENSGALG00010009016	DNAJC9	-1.480730646	0.111607852	-13.26726233	3.58438E-40	2.91558E-39	negative
PRJNA729271	stage_3W_vs_E10	ordinary_muscle_development	ENSGALG00010009019	ZNF513	0.872108175	0.133458399	6.534681845	6.37449E-11	1.555E-10	positive
PRJNA729271	stage_3W_vs_E10	ordinary_muscle_development	ENSGALG00010009020	ARHGAP35	0.020336279	0.18283482	0.111227605	0.911435862	0.921866082	positive
PRJNA729271	stage_3W_vs_E10	ordinary_muscle_development	ENSGALG00010009021	HNF4beta	-0.938055283	0.464191479	-2.020836927	0.043296649	0.054777422	negative
PRJNA729271	stage_3W_vs_E10	ordinary_muscle_development	ENSGALG00010009024	PMAIP1	1.410219761	0.252160403	5.592550401	2.23758E-08	4.72657E-08	positive
PRJNA729271	stage_3W_vs_E10	ordinary_muscle_development	ENSGALG00010009025	UTP23	0.186798183	0.095944017	1.946949772	0.051540757	0.064556261	positive
PRJNA729271	stage_3W_vs_E10	ordinary_muscle_development	ENSGALG00010009026	RAPGEF2	-0.227737227	0.137052887	-1.661674059	0.096578145	0.117028943	negative
PRJNA729271	stage_3W_vs_E10	ordinary_muscle_development	ENSGALG00010009028	PDLIM7	-1.698924937	0.116719016	-14.55568246	5.37469E-48	5.7326E-47	negative
PRJNA729271	stage_3W_vs_E10	ordinary_muscle_development	ENSGALG00010009029	IGSF3	-0.347198497	0.162955228	-2.130637367	0.033119029	0.042479455	negative
PRJNA729271	stage_3W_vs_E10	ordinary_muscle_development	ENSGALG00010009030	ZDHHC7	-0.439349912	0.090686947	-4.844687451	1.26811E-06	2.38563E-06	negative
PRJNA729271	stage_3W_vs_E10	ordinary_muscle_development	ENSGALG00010009031	SPIN1	-0.853109706	0.079668377	-10.70826017	9.30949E-27	4.70834E-26	negative
PRJNA729271	stage_3W_vs_E10	ordinary_muscle_development	ENSGALG00010009034	SNX17	1.630292997	0.118816466	13.72110322	7.59016E-43	6.73313E-42	positive
PRJNA729271	stage_3W_vs_E10	ordinary_muscle_development	ENSGALG00010009035	ENSGALG00010009035	-1.416161507	0.081890473	-17.29336094	5.2783E-67	9.49243E-66	negative
PRJNA729271	stage_3W_vs_E10	ordinary_muscle_development	ENSGALG00010009037	ENSGALG00010009037	2.589208994	0.179673013	14.41067276	4.4337E-47	4.5386E-46	positive
PRJNA729271	stage_3W_vs_E10	ordinary_muscle_development	ENSGALG00010009038	RAD21	-1.016198759	0.124438539	-8.166270393	3.1807E-16	1.02395E-15	negative
PRJNA729271	stage_3W_vs_E10	ordinary_muscle_development	ENSGALG00010009039	SLC36A4	-0.461773417	0.171960043	-2.685352993	0.007245324	0.009997237	negative
PRJNA729271	stage_3W_vs_E10	ordinary_muscle_development	ENSGALG00010009040	CRELD2	-1.611613109	0.144411178	-11.15989172	6.40696E-29	3.52845E-28	negative
PRJNA729271	stage_3W_vs_E10	ordinary_muscle_development	ENSGALG00010009043	TFAM	-0.605220909	0.083595472	-7.239876709	4.49093E-13	1.23171E-12	negative
PRJNA729271	stage_3W_vs_E10	ordinary_muscle_development	ENSGALG00010009047	SUPT5H	0.067587629	0.110208777	0.613269021	0.539698473	0.577380572	positive
PRJNA729271	stage_3W_vs_E10	ordinary_muscle_development	ENSGALG00010009050	PIK3CG	2.2225952	0.091120784	24.39174793	2.0923E-131	1.4651E-129	positive
PRJNA729271	stage_3W_vs_E10	ordinary_muscle_development	ENSGALG00010009051	TROJANZ	3.939811282	0.236002716	16.6939235	1.45113E-62	2.31653E-61	positive
PRJNA729271	stage_3W_vs_E10	ordinary_muscle_development	ENSGALG00010009054	RYR2	-1.449822435	0.497643406	-2.913376159	0.003575436	0.005090458	negative
PRJNA729271	stage_3W_vs_E10	ordinary_muscle_development	ENSGALG00010009057	NXNL2	1.61521452	0.359520149	4.492695403	7.03273E-06	1.25557E-05	positive
PRJNA729271	stage_3W_vs_E10	ordinary_muscle_development	ENSGALG00010009059	MED29	1.002696791	0.125813398	7.969713921	1.59042E-15	4.9301E-15	positive
PRJNA729271	stage_3W_vs_E10	ordinary_muscle_development	ENSGALG00010009065	EIF2B4	0.543708472	0.094365371	5.761737263	8.32525E-09	1.80596E-08	positive
PRJNA729271	stage_3W_vs_E10	ordinary_muscle_development	ENSGALG00010009067	ENSGALG00010009067	-2.123478626	1.623662025	-1.307832907	0.19092999	0.221597422	negative
PRJNA729271	stage_3W_vs_E10	ordinary_muscle_development	ENSGALG00010009068	AGO2	-0.405556561	0.082285244	-4.928666908	8.27926E-07	1.57698E-06	negative
PRJNA729271	stage_3W_vs_E10	ordinary_muscle_development	ENSGALG00010009069	GRID2	-3.54062823	0.34010272	-10.41046727	2.22129E-25	1.05358E-24	negative
PRJNA729271	stage_3W_vs_E10	ordinary_muscle_development	ENSGALG00010009070	ENSGALG00010009070	0.120027767	1.252662335	0.095818134	0.923665015	0.932413426	positive
PRJNA729271	stage_3W_vs_E10	ordinary_muscle_development	ENSGALG00010009073	PRKAR2B	1.148660916	0.403581308	2.846169761	0.00442486	0.006239745	positive
PRJNA729271	stage_3W_vs_E10	ordinary_muscle_development	ENSGALG00010009076	TMEM214	0.120937931	0.115774182	1.044601908	0.296206997	0.333529125	positive
PRJNA729271	stage_3W_vs_E10	ordinary_muscle_development	ENSGALG00010009078	PTTG2	-1.988331171	0.171277324	-11.60884073	3.7162E-31	2.2268E-30	negative
PRJNA729271	stage_3W_vs_E10	ordinary_muscle_development	ENSGALG00010009079	KCTD15	-1.293937663	0.098961334	-13.07518411	4.56457E-39	3.58028E-38	negative
PRJNA729271	stage_3W_vs_E10	ordinary_muscle_development	ENSGALG00010009080	TLL1	-0.073656506	0.168360688	-0.437492308	0.661754353	0.693975499	negative
PRJNA729271	stage_3W_vs_E10	ordinary_muscle_development	ENSGALG00010009081	HBP1	1.518571867	0.121690255	12.47899323	9.7208E-36	6.83663E-35	positive
PRJNA729271	stage_3W_vs_E10	ordinary_muscle_development	ENSGALG00010009083	MED30	0.620292934	0.1267483	4.893895502	9.88593E-07	1.87438E-06	positive
PRJNA729271	stage_3W_vs_E10	ordinary_muscle_development	ENSGALG00010009085	PAF1	-0.182578619	0.088813661	-2.055749284	0.039806689	0.05060054	negative
PRJNA729271	stage_3W_vs_E10	ordinary_muscle_development	ENSGALG00010009086	UBE2D1	2.111060144	0.170416246	12.38766958	3.04772E-35	2.11777E-34	positive
PRJNA729271	stage_3W_vs_E10	ordinary_muscle_development	ENSGALG00010009088	ENSGALG00010009088	-0.842612153	0.145785756	-5.77979754	7.47906E-09	1.62605E-08	negative
PRJNA729271	stage_3W_vs_E10	ordinary_muscle_development	ENSGALG00010009089	AGBL5	-0.677704303	0.15922396	-4.256296003	2.07841E-05	3.58505E-05	negative
PRJNA729271	stage_3W_vs_E10	ordinary_muscle_development	ENSGALG00010009091	EXT1	-0.573023696	0.112437898	-5.096357229	3.46251E-07	6.75584E-07	negative
PRJNA729271	stage_3W_vs_E10	ordinary_muscle_development	ENSGALG00010009092	EED	-0.856533108	0.114713778	-7.466697746	8.22324E-14	2.34222E-13	negative
PRJNA729271	stage_3W_vs_E10	ordinary_muscle_development	ENSGALG00010009093	SLU7	0.58823542	0.131495022	4.473442482	7.69703E-06	1.37079E-05	positive
PRJNA729271	stage_3W_vs_E10	ordinary_muscle_development	ENSGALG00010009094	DARS	0.133044286	0.095176828	1.397864254	0.162153835	0.190394478	positive
PRJNA729271	stage_3W_vs_E10	ordinary_muscle_development	ENSGALG00010009096	ENSGALG00010009096	1.501349685	1.350773696	1.111473883	0.266364426	0.30274792	positive
PRJNA729271	stage_3W_vs_E10	ordinary_muscle_development	ENSGALG00010009097	C1QTNF2	0.110416655	0.18176191	0.607479615	0.543532667	0.581053618	positive
PRJNA729271	stage_3W_vs_E10	ordinary_muscle_development	ENSGALG00010009098	CAD	-1.803961765	0.198217527	-9.100919539	8.95702E-20	3.39519E-19	negative
PRJNA729271	stage_3W_vs_E10	ordinary_muscle_development	ENSGALG00010009099	CXCR4	0.035402996	0.18999551	0.186335963	0.852181307	0.869028285	positive
PRJNA729271	stage_3W_vs_E10	ordinary_muscle_development	ENSGALG00010009100	ENSGALG00010009100	-0.266644277	0.15412258	-1.730079246	0.083616118	0.102098865	negative
PRJNA729271	stage_3W_vs_E10	ordinary_muscle_development	ENSGALG00010009101	HIKESHI	0.434056968	0.09774384	4.440760332	8.96416E-06	1.58807E-05	positive
PRJNA729271	stage_3W_vs_E10	ordinary_muscle_development	ENSGALG00010009102	HTRA3	1.735535682	0.216409284	8.019691426	1.06011E-15	3.31528E-15	positive
PRJNA729271	stage_3W_vs_E10	ordinary_muscle_development	ENSGALG00010009103	AP3S1	-1.175398908	0.123812975	-9.493341946	2.23744E-21	9.04648E-21	negative
PRJNA729271	stage_3W_vs_E10	ordinary_muscle_development	ENSGALG00010009104	MTR	0.678475588	0.12238695	5.543692274	2.96159E-08	6.19455E-08	positive
PRJNA729271	stage_3W_vs_E10	ordinary_muscle_development	ENSGALG00010009106	THSD7B	-1.674381622	0.238951867	-7.007192045	2.43148E-12	6.41623E-12	negative
PRJNA729271	stage_3W_vs_E10	ordinary_muscle_development	ENSGALG00010009109	ENSGALG00010009109	-0.295834309	0.217784876	-1.35837857	0.174343591	0.203618592	negative
PRJNA729271	stage_3W_vs_E10	ordinary_muscle_development	ENSGALG00010009111	LRFN1	0.604295973	0.243940854	2.477223327	0.013240902	0.017752926	positive
PRJNA729271	stage_3W_vs_E10	ordinary_muscle_development	ENSGALG00010009112	ENSGALG00010009112	7.496619583	0.264726924	28.31831187	2.0566E-176	2.7602E-174	positive
PRJNA729271	stage_3W_vs_E10	ordinary_muscle_development	ENSGALG00010009113	RPLP1	0.614015651	0.176357025	3.481662566	0.000498311	0.00076808	positive
PRJNA729271	stage_3W_vs_E10	ordinary_muscle_development	ENSGALG00010009114	SETD7	1.424741575	0.149910296	9.503960815	2.02055E-21	8.19242E-21	positive
PRJNA729271	stage_3W_vs_E10	ordinary_muscle_development	ENSGALG00010009115	COG5	0.137180122	0.114615084	1.196876693	0.23135462	0.265118603	positive
PRJNA729271	stage_3W_vs_E10	ordinary_muscle_development	ENSGALG00010009116	S1PR3	-1.347132172	0.185271265	-7.271133867	3.56482E-13	9.82734E-13	negative
PRJNA729271	stage_3W_vs_E10	ordinary_muscle_development	ENSGALG00010009117	ERGIC1	0.189961687	0.0668836	2.840183333	0.004508761	0.006352498	positive
PRJNA729271	stage_3W_vs_E10	ordinary_muscle_development	ENSGALG00010009118	ACTN2	4.094098504	0.143906461	28.44971983	4.9121E-178	6.7811E-176	positive
PRJNA729271	stage_3W_vs_E10	ordinary_muscle_development	ENSGALG00010009119	CISD1	1.139522212	0.153003692	7.447677854	9.49974E-14	2.6968E-13	positive
PRJNA729271	stage_3W_vs_E10	ordinary_muscle_development	ENSGALG00010009120	ATG12	0.12744823	0.089038165	1.431388779	0.152318823	0.179530113	positive
PRJNA729271	stage_3W_vs_E10	ordinary_muscle_development	ENSGALG00010009121	SYCN	1.886831081	0.735862521	2.564108141	0.010344131	0.014033899	positive
PRJNA729271	stage_3W_vs_E10	ordinary_muscle_development	ENSGALG00010009123	ENSGALG00010009123	-2.300326692	0.13756387	-16.7218812	9.0807E-63	1.46575E-61	negative
PRJNA729271	stage_3W_vs_E10	ordinary_muscle_development	ENSGALG00010009125	CD72L1	7.619652321	0.928619835	8.205351674	2.29917E-16	7.46393E-16	positive
PRJNA729271	stage_3W_vs_E10	ordinary_muscle_development	ENSGALG00010009126	JPH3	1.840471712	0.135397553	13.59309435	4.40074E-42	3.79695E-41	positive
PRJNA729271	stage_3W_vs_E10	ordinary_muscle_development	ENSGALG00010009127	SCOC	0.230406305	0.07420813	3.104866076	0.001903652	0.002775441	positive
PRJNA729271	stage_3W_vs_E10	ordinary_muscle_development	ENSGALG00010009129	ENSGALG00010009129	-0.748472706	0.452707923	-1.653323628	0.098265017	0.118894108	negative
PRJNA729271	stage_3W_vs_E10	ordinary_muscle_development	ENSGALG00010009130	HEATR1	-0.889850779	0.073338966	-12.13339687	7.02728E-34	4.64268E-33	negative
PRJNA729271	stage_3W_vs_E10	ordinary_muscle_development	ENSGALG00010009131	CDKL2	-1.761599866	0.304013842	-5.794472558	6.85362E-09	1.49321E-08	negative
PRJNA729271	stage_3W_vs_E10	ordinary_muscle_development	ENSGALG00010009133	BOD1L1	0.299623396	0.129143826	2.320075271	0.020336806	0.026740022	positive
PRJNA729271	stage_3W_vs_E10	ordinary_muscle_development	ENSGALG00010009135	TENM4	-2.766884003	0.186670358	-14.82229975	1.05123E-49	1.17483E-48	negative
PRJNA729271	stage_3W_vs_E10	ordinary_muscle_development	ENSGALG00010009136	KHK	-1.623153616	0.458586478	-3.539471167	0.00040093	0.000622347	negative
PRJNA729271	stage_3W_vs_E10	ordinary_muscle_development	ENSGALG00010009137	IPMK	0.137885486	0.091951721	1.499542201	0.133733029	0.158994444	positive
PRJNA729271	stage_3W_vs_E10	ordinary_muscle_development	ENSGALG00010009138	ENSGALG00010009138	0.208303	0.106331739	1.958991765	0.050113751	0.062886228	positive
PRJNA729271	stage_3W_vs_E10	ordinary_muscle_development	ENSGALG00010009139	CKS2	-3.718251215	0.314900601	-11.80769805	3.5618E-32	2.2244E-31	negative
PRJNA729271	stage_3W_vs_E10	ordinary_muscle_development	ENSGALG00010009141	MAML3	-0.186812681	0.118422707	-1.577507267	0.114678851	0.137514059	negative
PRJNA729271	stage_3W_vs_E10	ordinary_muscle_development	ENSGALG00010009142	LGALS8	-0.182760542	0.134009403	-1.363788944	0.172634001	0.201833348	negative
PRJNA729271	stage_3W_vs_E10	ordinary_muscle_development	ENSGALG00010009143	MCCC2	0.715131272	0.135057648	5.295007588	1.19011E-07	2.39926E-07	positive
PRJNA729271	stage_3W_vs_E10	ordinary_muscle_development	ENSGALG00010009144	CREBZF	0.439569198	0.154909542	2.837586321	0.004545605	0.006403163	positive
PRJNA729271	stage_3W_vs_E10	ordinary_muscle_development	ENSGALG00010009147	HNMT	2.415232908	0.226924452	10.64333476	1.87303E-26	9.36516E-26	positive
PRJNA729271	stage_3W_vs_E10	ordinary_muscle_development	ENSGALG00010009149	FUT4	-1.541727907	0.216842031	-7.109912688	1.16116E-12	3.12033E-12	negative
PRJNA729271	stage_3W_vs_E10	ordinary_muscle_development	ENSGALG00010009150	ENSGALG00010009150	-1.157533224	0.428240353	-2.702998948	0.006871696	0.009498878	negative
PRJNA729271	stage_3W_vs_E10	ordinary_muscle_development	ENSGALG00010009151	CCNJL	0.681965179	0.360810882	1.890090387	0.058745873	0.073098242	positive
PRJNA729271	stage_3W_vs_E10	ordinary_muscle_development	ENSGALG00010009152	RPP25L	0.630089749	0.122633871	5.137974899	2.77715E-07	5.45828E-07	positive
PRJNA729271	stage_3W_vs_E10	ordinary_muscle_development	ENSGALG00010009153	SECISBP2	0.253123767	0.124202538	2.037991914	0.041550742	0.052669698	positive
PRJNA729271	stage_3W_vs_E10	ordinary_muscle_development	ENSGALG00010009155	PAK4	-0.429005896	0.221871428	-1.93357883	0.053164932	0.066490569	negative
PRJNA729271	stage_3W_vs_E10	ordinary_muscle_development	ENSGALG00010009157	ABHD1	1.129452343	0.136693015	8.262692445	1.42423E-16	4.67486E-16	positive
PRJNA729271	stage_3W_vs_E10	ordinary_muscle_development	ENSGALG00010009159	PTK2	0.088459338	0.098934401	0.894121123	0.371257089	0.409946789	positive
PRJNA729271	stage_3W_vs_E10	ordinary_muscle_development	ENSGALG00010009160	ENSGALG00010009160	-3.628177197	0.395250773	-9.179431002	4.33375E-20	1.66098E-19	negative
PRJNA729271	stage_3W_vs_E10	ordinary_muscle_development	ENSGALG00010009163	SAMD12	-0.512044896	0.178365558	-2.870761045	0.004094849	0.005791846	negative
PRJNA729271	stage_3W_vs_E10	ordinary_muscle_development	ENSGALG00010009164	TMEM128	0.677748451	0.100085669	6.771683258	1.27293E-11	3.23646E-11	positive
PRJNA729271	stage_3W_vs_E10	ordinary_muscle_development	ENSGALG00010009166	CLGN	-0.681157736	0.231924772	-2.936977065	0.003314286	0.004735861	negative
PRJNA729271	stage_3W_vs_E10	ordinary_muscle_development	ENSGALG00010009167	DCTN3	-0.245155911	0.110889209	-2.21081847	0.02704841	0.035030978	negative
PRJNA729271	stage_3W_vs_E10	ordinary_muscle_development	ENSGALG00010009168	MRPS12	0.683387671	0.174881037	3.907728839	9.31678E-05	0.00015244	positive
PRJNA729271	stage_3W_vs_E10	ordinary_muscle_development	ENSGALG00010009170	RPL26L1	0.502359988	0.191282071	2.626278493	0.008632416	0.011820033	positive
PRJNA729271	stage_3W_vs_E10	ordinary_muscle_development	ENSGALG00010009172	SPOPL	0.62941319	0.131887055	4.772365195	1.82075E-06	3.3879E-06	positive
PRJNA729271	stage_3W_vs_E10	ordinary_muscle_development	ENSGALG00010009173	LYAR	-0.20190097	0.142115698	-1.420680283	0.155409727	0.182950052	negative
PRJNA729271	stage_3W_vs_E10	ordinary_muscle_development	ENSGALG00010009178	ENSGALG00010009178	0.757386964	0.251697165	3.009119963	0.002620056	0.003775496	positive
PRJNA729271	stage_3W_vs_E10	ordinary_muscle_development	ENSGALG00010009180	NFKBIB	0.752048041	0.146495468	5.133592548	2.84263E-07	5.58017E-07	positive
PRJNA729271	stage_3W_vs_E10	ordinary_muscle_development	ENSGALG00010009181	ARID3C	-5.043518902	0.839139824	-6.010343875	1.8513E-09	4.14755E-09	negative
PRJNA729271	stage_3W_vs_E10	ordinary_muscle_development	ENSGALG00010009186	PREB	-0.432450471	0.098961202	-4.369899146	1.24304E-05	2.18055E-05	negative
PRJNA729271	stage_3W_vs_E10	ordinary_muscle_development	ENSGALG00010009188	ENSGALG00010009188	-1.445942552	0.517881107	-2.792035727	0.005237757	0.007329017	negative
PRJNA729271	stage_3W_vs_E10	ordinary_muscle_development	ENSGALG00010009189	ENSGALG00010009189	-1.728680493	0.260187904	-6.643969489	3.05346E-11	7.61002E-11	negative
PRJNA729271	stage_3W_vs_E10	ordinary_muscle_development	ENSGALG00010009190	ZBTB49	0.225941292	0.138229029	1.634543003	0.102144884	0.123320848	positive
PRJNA729271	stage_3W_vs_E10	ordinary_muscle_development	ENSGALG00010009192	LSM14A	-0.374363647	0.059587991	-6.282535083	3.33096E-10	7.8114E-10	negative
PRJNA729271	stage_3W_vs_E10	ordinary_muscle_development	ENSGALG00010009193	EDARADD	-1.371949795	0.220752066	-6.214889941	5.13606E-10	1.19249E-09	negative
PRJNA729271	stage_3W_vs_E10	ordinary_muscle_development	ENSGALG00010009195	ENSGALG00010009195	3.412596217	0.618562059	5.516982765	3.44869E-08	7.18024E-08	positive
PRJNA729271	stage_3W_vs_E10	ordinary_muscle_development	ENSGALG00010009197	FH	0.800638744	0.102489647	7.811898705	5.63328E-15	1.70397E-14	positive
PRJNA729271	stage_3W_vs_E10	ordinary_muscle_development	ENSGALG00010009198	KCNN2	-0.231078764	0.178951224	-1.291294684	0.196601513	0.227815088	negative
PRJNA729271	stage_3W_vs_E10	ordinary_muscle_development	ENSGALG00010009199	SEMA4D	-2.313211515	0.153160932	-15.10314335	1.54394E-51	1.84193E-50	negative
PRJNA729271	stage_3W_vs_E10	ordinary_muscle_development	ENSGALG00010009200	ADGRA3	-2.208218846	0.094844411	-23.28254046	6.6628E-120	3.9419E-118	negative
PRJNA729271	stage_3W_vs_E10	ordinary_muscle_development	ENSGALG00010009202	FABP6	1.631110218	0.282600072	5.771796887	7.84306E-09	1.70264E-08	positive
PRJNA729271	stage_3W_vs_E10	ordinary_muscle_development	ENSGALG00010009205	SIRT2	0.566394078	0.097517054	5.808154098	6.31654E-09	1.37889E-08	positive

PRJNA729271	stage_3W_vs_E10	ordinary_muscle_development	ENSGALG00010009208	SLC5A6	0.167283734	0.190880081	0.876381301	0.38082278	0.419646206	positive
PRJNA729271	stage_3W_vs_E10	ordinary_muscle_development	ENSGALG00010009209	SLC6A19	5.604773773	1.696721139	3.303296955	0.000955551	0.001433377	positive
PRJNA729271	stage_3W_vs_E10	ordinary_muscle_development	ENSGALG00010009210	PCDH15	-3.799602753	0.349220878	-10.88022793	1.43204E-27	7.5018E-27	negative
PRJNA729271	stage_3W_vs_E10	ordinary_muscle_development	ENSGALG00010009212	NXPH2	-2.376422643	0.300663794	-7.903920226	2.70266E-15	8.29277E-15	negative
PRJNA729271	stage_3W_vs_E10	ordinary_muscle_development	ENSGALG00010009213	DUS4L	-0.871691548	0.094510827	-9.223192426	2.88384E-20	1.115E-19	negative
PRJNA729271	stage_3W_vs_E10	ordinary_muscle_development	ENSGALG00010009214	PCDH10	-5.425745208	0.299558959	-18.11244516	2.54203E-73	5.39482E-72	negative
PRJNA729271	stage_3W_vs_E10	ordinary_muscle_development	ENSGALG00010009216	NSG1	-2.801822501	0.217512001	-12.88123177	5.74119E-38	4.31408E-37	negative
PRJNA729271	stage_3W_vs_E10	ordinary_muscle_development	ENSGALG00010009219	TNFRSF11B	1.141595151	0.180485615	6.325130943	2.53018E-10	5.98091E-10	positive
PRJNA729271	stage_3W_vs_E10	ordinary_muscle_development	ENSGALG00010009220	GADD45G	1.582831324	0.189197141	8.366042493	5.95857E-17	1.99192E-16	positive
PRJNA729271	stage_3W_vs_E10	ordinary_muscle_development	ENSGALG00010009223	ENSGALG00010009223	0.06472317	0.10880994	0.594827736	0.551958625	0.589018646	positive
PRJNA729271	stage_3W_vs_E10	ordinary_muscle_development	ENSGALG00010009224	ENSGALG00010009224	5.145654778	0.446803592	11.51659224	1.08831E-30	6.42043E-30	positive
PRJNA729271	stage_3W_vs_E10	ordinary_muscle_development	ENSGALG00010009225	HNRNPL	-0.754571503	0.12557359	-6.00899842	1.86673E-09	4.18082E-09	negative
PRJNA729271	stage_3W_vs_E10	ordinary_muscle_development	ENSGALG00010009227	ENSGALG00010009227	0.05699897	0.17349877	0.328526653	0.742513495	0.769591213	positive
PRJNA729271	stage_3W_vs_E10	ordinary_muscle_development	ENSGALG00010009228	ATP6V0E1	1.288682784	0.098874483	13.03352232	7.88804E-39	6.11429E-38	positive
PRJNA729271	stage_3W_vs_E10	ordinary_muscle_development	ENSGALG00010009232	ECH1	-0.036126064	0.165452547	-0.218346982	0.827158771	0.847565841	negative
PRJNA729271	stage_3W_vs_E10	ordinary_muscle_development	ENSGALG00010009234	PCDH18	-1.262429413	0.128752037	-9.805121858	1.07018E-22	4.55706E-22	negative
PRJNA729271	stage_3W_vs_E10	ordinary_muscle_development	ENSGALG00010009235	KLHDC4	0.793747232	0.130183795	6.097127792	1.07991E-09	2.45388E-09	positive
PRJNA729271	stage_3W_vs_E10	ordinary_muscle_development	ENSGALG00010009236	DTWD2	-0.143629038	0.116180184	-1.236261069	0.216361537	0.24911911	negative
PRJNA729271	stage_3W_vs_E10	ordinary_muscle_development	ENSGALG00010009237	DENND3	-0.746410653	0.178014752	-4.192970789	2.75325E-05	4.6962E-05	negative
PRJNA729271	stage_3W_vs_E10	ordinary_muscle_development	ENSGALG00010009239	STX18	0.609260049	0.081427854	7.482206882	7.30848E-14	2.09071E-13	positive
PRJNA729271	stage_3W_vs_E10	ordinary_muscle_development	ENSGALG00010009243	BCAP29	0.377404571	0.099296655	3.800778278	0.000144242	0.000232672	positive
PRJNA729271	stage_3W_vs_E10	ordinary_muscle_development	ENSGALG00010009246	RUSC2	2.172632369	0.141778503	15.3241311	5.27553E-53	6.57513E-52	positive
PRJNA729271	stage_3W_vs_E10	ordinary_muscle_development	ENSGALG00010009250	ERO1B	-0.454107202	0.108901758	-4.169879429	3.04761E-05	5.18183E-05	negative
PRJNA729271	stage_3W_vs_E10	ordinary_muscle_development	ENSGALG00010009252	SIRT1	-0.09121318	0.096460979	-0.945596662	0.344354347	0.382836038	negative
PRJNA729271	stage_3W_vs_E10	ordinary_muscle_development	ENSGALG00010009253	SLC45A4	-0.222759087	0.14057084	-1.584674937	0.113040209	0.135683822	negative
PRJNA729271	stage_3W_vs_E10	ordinary_muscle_development	ENSGALG00010009254	SLC26A4	-0.099182261	0.729662935	-0.135928873	0.89187751	0.904292425	negative
PRJNA729271	stage_3W_vs_E10	ordinary_muscle_development	ENSGALG00010009256	CREBRF	2.14873829	0.172277734	12.47252465	1.05432E-35	7.40424E-35	positive
PRJNA729271	stage_3W_vs_E10	ordinary_muscle_development	ENSGALG00010009257	ENSGALG00010009257	1.851241947	0.187960045	9.849124858	6.91434E-23	2.97222E-22	positive
PRJNA729271	stage_3W_vs_E10	ordinary_muscle_development	ENSGALG00010009258	DNAJC12	-0.367689456	0.168256896	-2.185286097	0.028867876	0.037299922	negative
PRJNA729271	stage_3W_vs_E10	ordinary_muscle_development	ENSGALG00010009259	MGAT4D	1.826197646	0.803063994	2.274037511	0.022963731	0.03003332	positive
PRJNA729271	stage_3W_vs_E10	ordinary_muscle_development	ENSGALG00010009263	COLEC10	-0.143501782	0.220922096	-0.649558304	0.515977574	0.553924969	negative
PRJNA729271	stage_3W_vs_E10	ordinary_muscle_development	ENSGALG00010009266	PCDH7	-3.080654289	0.257064323	-11.98398226	4.31106E-33	2.77728E-32	negative
PRJNA729271	stage_3W_vs_E10	ordinary_muscle_development	ENSGALG00010009268	PWWP2A	-0.131895501	0.133822616	-0.985599479	0.324329694	0.362352313	negative
PRJNA729271	stage_3W_vs_E10	ordinary_muscle_development	ENSGALG00010009269	ENSGALG00010009269	-5.920511324	0.585374229	-10.11406213	4.78579E-24	2.14901E-23	negative
PRJNA729271	stage_3W_vs_E10	ordinary_muscle_development	ENSGALG00010009273	MAL2	-0.466143107	0.487450531	-0.956288029	0.338926736	0.377496776	negative
PRJNA729271	stage_3W_vs_E10	ordinary_muscle_development	ENSGALG00010009275	CBLL1	-0.034433959	0.143477568	-0.239995418	0.810333809	0.832503265	negative
PRJNA729271	stage_3W_vs_E10	ordinary_muscle_development	ENSGALG00010009276	ENSGALG00010009276	-0.537812982	0.329228913	-1.633553314	0.102352677	0.123561427	negative
PRJNA729271	stage_3W_vs_E10	ordinary_muscle_development	ENSGALG00010009277	ACTN4	-0.639578753	0.128675038	-4.970495934	6.67819E-07	1.27992E-06	negative
PRJNA729271	stage_3W_vs_E10	ordinary_muscle_development	ENSGALG00010009279	ELMOD2	-2.272395686	0.11428596	-19.88341953	5.66389E-88	1.76936E-86	negative
PRJNA729271	stage_3W_vs_E10	ordinary_muscle_development	ENSGALG00010009280	HSD17B4	0.166209327	0.083160964	1.998645999	0.04564667	0.057624846	positive
PRJNA729271	stage_3W_vs_E10	ordinary_muscle_development	ENSGALG00010009284	CCN3	-3.866723714	0.682304134	-5.667155633	1.45187E-08	3.10214E-08	negative
PRJNA729271	stage_3W_vs_E10	ordinary_muscle_development	ENSGALG00010009286	ENSGALG00010009286	-0.062986897	0.18565408	-0.3392702	0.734406189	0.762060113	negative
PRJNA729271	stage_3W_vs_E10	ordinary_muscle_development	ENSGALG00010009289	GPR137B	0.184465698	0.14583395	1.264902295	0.205906354	0.23793149	positive
PRJNA729271	stage_3W_vs_E10	ordinary_muscle_development	ENSGALG00010009294	ACOX3	0.898021705	0.139085064	6.456636509	1.07056E-10	2.57726E-10	positive
PRJNA729271	stage_3W_vs_E10	ordinary_muscle_development	ENSGALG00010009297	CTNNA3	4.878127686	0.204046864	23.90689859	2.5964E-126	1.7343E-124	positive
PRJNA729271	stage_3W_vs_E10	ordinary_muscle_development	ENSGALG00010009299	MAP4K1	3.331215002	0.29190865	11.41184068	3.64924E-30	2.10908E-29	positive
PRJNA729271	stage_3W_vs_E10	ordinary_muscle_development	ENSGALG00010009303	TTC1	0.498877921	0.095980381	5.197707218	2.01762E-07	3.99746E-07	positive
PRJNA729271	stage_3W_vs_E10	ordinary_muscle_development	ENSGALG00010009306	TBC1D9	-1.48611414	0.108821531	-13.65643479	1.84813E-42	1.61767E-41	negative
PRJNA729271	stage_3W_vs_E10	ordinary_muscle_development	ENSGALG00010009307	DIRAS2	3.635984598	0.310549464	11.70823017	1.15669E-31	7.07733E-31	positive
PRJNA729271	stage_3W_vs_E10	ordinary_muscle_development	ENSGALG00010009309	TRIM54	2.583785889	0.148943473	17.34742613	2.06282E-67	3.75635E-66	positive
PRJNA729271	stage_3W_vs_E10	ordinary_muscle_development	ENSGALG00010009311	BNIP1	-0.045854176	0.111543517	-0.411087773	0.681008175	0.711951929	negative
PRJNA729271	stage_3W_vs_E10	ordinary_muscle_development	ENSGALG00010009313	UNC13B	-1.355272618	0.199892158	-6.780018932	1.2016E-11	3.05941E-11	negative
PRJNA729271	stage_3W_vs_E10	ordinary_muscle_development	ENSGALG00010009317	LRRTM3	-3.231810387	0.523656917	-6.171617874	6.75947E-10	1.55818E-09	negative
PRJNA729271	stage_3W_vs_E10	ordinary_muscle_development	ENSGALG00010009320	GPR20	-1.803206736	0.227925618	-7.911382446	2.54546E-15	7.82036E-15	negative
PRJNA729271	stage_3W_vs_E10	ordinary_muscle_development	ENSGALG00010009322	ENSGALG00010009322	2.635714172	0.165042484	15.96991332	2.07073E-57	2.90283E-56	positive
PRJNA729271	stage_3W_vs_E10	ordinary_muscle_development	ENSGALG00010009323	RNF150	0.304196646	0.107557451	2.828224747	0.004680693	0.006586414	positive
PRJNA729271	stage_3W_vs_E10	ordinary_muscle_development	ENSGALG00010009326	DLD	0.557200421	0.131799904	4.227623872	2.36172E-05	4.0527E-05	positive
PRJNA729271	stage_3W_vs_E10	ordinary_muscle_development	ENSGALG00010009327	NID1	1.896950034	0.163069396	11.63277767	2.80809E-31	1.68753E-30	positive
PRJNA729271	stage_3W_vs_E10	ordinary_muscle_development	ENSGALG00010009328	ENSGALG00010009328	-1.5141488	0.138099659	-10.96417482	5.68165E-28	3.02222E-27	negative
PRJNA729271	stage_3W_vs_E10	ordinary_muscle_development	ENSGALG00010009330	EIF3K	0.442442939	0.141482783	3.127185716	0.001764884	0.002582996	positive
PRJNA729271	stage_3W_vs_E10	ordinary_muscle_development	ENSGALG00010009332	ENSGALG00010009332	0.605731168	0.162887045	3.718719119	0.000200236	0.000318947	positive
PRJNA729271	stage_3W_vs_E10	ordinary_muscle_development	ENSGALG00010009333	ADRA1B	2.570132411	0.31156499	8.249105297	1.59585E-16	5.22281E-16	positive
PRJNA729271	stage_3W_vs_E10	ordinary_muscle_development	ENSGALG00010009334	SS18L2	1.060973259	0.162814521	6.516453507	7.19891E-11	1.75198E-10	positive
PRJNA729271	stage_3W_vs_E10	ordinary_muscle_development	ENSGALG00010009335	PTP4A3	3.293796542	0.195035617	16.8881797	5.4973E-64	9.18012E-63	positive
PRJNA729271	stage_3W_vs_E10	ordinary_muscle_development	ENSGALG00010009336	LAMB1	0.377412843	0.109994743	3.431189808	0.00060094	0.000918455	positive
PRJNA729271	stage_3W_vs_E10	ordinary_muscle_development	ENSGALG00010009337	ZNF330	0.2415591	0.099559879	2.426269517	0.015254937	0.020319822	positive
PRJNA729271	stage_3W_vs_E10	ordinary_muscle_development	ENSGALG00010009338	KYNU	0.017850951	0.573166762	0.031144429	0.975154358	0.978800804	positive
PRJNA729271	stage_3W_vs_E10	ordinary_muscle_development	ENSGALG00010009340	SYK	1.784908916	0.137142652	13.0149803	1.00568E-38	7.77045E-38	positive
PRJNA729271	stage_3W_vs_E10	ordinary_muscle_development	ENSGALG00010009344	ENPP2	-0.635450238	0.257851739	-2.464401601	0.013724224	0.018377148	negative
PRJNA729271	stage_3W_vs_E10	ordinary_muscle_development	ENSGALG00010009345	ZFAT	0.479372129	0.124483213	3.850897786	0.000117686	0.000191153	positive
PRJNA729271	stage_3W_vs_E10	ordinary_muscle_development	ENSGALG00010009346	STC2	-0.600497816	0.307203993	-1.954720089	0.050616118	0.063467182	negative
PRJNA729271	stage_3W_vs_E10	ordinary_muscle_development	ENSGALG00010009347	TESK1	2.527930385	0.141527081	17.86181386	2.33913E-71	4.6702E-70	positive
PRJNA729271	stage_3W_vs_E10	ordinary_muscle_development	ENSGALG00010009348	ERGIC2	0.256640934	0.082000869	3.129734327	0.001749645	0.002562504	positive
PRJNA729271	stage_3W_vs_E10	ordinary_muscle_development	ENSGALG00010009351	GTF3C2	0.470060317	0.121675792	3.863219713	0.000111902	0.000182119	positive
PRJNA729271	stage_3W_vs_E10	ordinary_muscle_development	ENSGALG00010009352	HSF1	1.427773217	0.177845548	8.028163969	9.89422E-16	3.10023E-15	positive
PRJNA729271	stage_3W_vs_E10	ordinary_muscle_development	ENSGALG00010009355	GARRE1	0.102105364	0.072291396	1.412413785	0.15782816	0.185631222	positive
PRJNA729271	stage_3W_vs_E10	ordinary_muscle_development	ENSGALG00010009357	LYST	0.17472068	0.116607128	1.498370496	0.134037018	0.159316652	positive
PRJNA729271	stage_3W_vs_E10	ordinary_muscle_development	ENSGALG00010009359	REEP3	-0.890424819	0.110902518	-8.028896341	9.83534E-16	3.08445E-15	negative
PRJNA729271	stage_3W_vs_E10	ordinary_muscle_development	ENSGALG00010009360	TNFAIP8	0.270256169	0.12796078	2.112023459	0.034684437	0.04440478	positive
PRJNA729271	stage_3W_vs_E10	ordinary_muscle_development	ENSGALG00010009361	LRRK2	1.260331965	0.08707186	14.47461853	1.75296E-47	1.82406E-46	positive
PRJNA729271	stage_3W_vs_E10	ordinary_muscle_development	ENSGALG00010009362	IL15	5.768493906	0.308267833	18.71260408	3.90804E-78	9.30166E-77	positive
PRJNA729271	stage_3W_vs_E10	ordinary_muscle_development	ENSGALG00010009363	TAF2	-0.280283139	0.077339367	-3.624068174	0.000290005	0.000456023	negative
PRJNA729271	stage_3W_vs_E10	ordinary_muscle_development	ENSGALG00010009365	CA5B	1.234227319	0.159981553	7.714810226	1.21162E-14	3.5974E-14	positive
PRJNA729271	stage_3W_vs_E10	ordinary_muscle_development	ENSGALG00010009367	DMXL1	0.709215586	0.119132379	5.953172381	2.62994E-09	5.84409E-09	positive
PRJNA729271	stage_3W_vs_E10	ordinary_muscle_development	ENSGALG00010009368	DSCC1	-2.299011285	0.198321014	-11.59237362	4.50473E-31	2.69373E-30	negative
PRJNA729271	stage_3W_vs_E10	ordinary_muscle_development	ENSGALG00010009370	ENSGALG00010009370	0.567137279	0.456700761	1.241813737	0.214305293	0.24702626	positive
PRJNA729271	stage_3W_vs_E10	ordinary_muscle_development	ENSGALG00010009371	PHTF2	2.325060363	0.154465397	15.05230565	3.33409E-51	3.92588E-50	positive
PRJNA729271	stage_3W_vs_E10	ordinary_muscle_development	ENSGALG00010009372	RAB28	-0.166351647	0.097777895	-1.701321622	0.08888261	0.108210434	negative
PRJNA729271	stage_3W_vs_E10	ordinary_muscle_development	ENSGALG00010009373	ARHGAP15	1.026035986	0.215290063	4.765830685	1.88077E-06	3.4951E-06	positive
PRJNA729271	stage_3W_vs_E10	ordinary_muscle_development	ENSGALG00010009376	CDO1	-0.187678717	0.250110351	-0.750383646	0.453023678	0.491804839	negative
PRJNA729271	stage_3W_vs_E10	ordinary_muscle_development	ENSGALG00010009378	ENSGALG00010009378	-0.927205067	0.08394002	-11.046043	2.29092E-28	1.24091E-27	negative
PRJNA729271	stage_3W_vs_E10	ordinary_muscle_development	ENSGALG00010009379	RYR1	4.207862455	0.219475689	19.17233967	6.30102E-82	1.65759E-80	positive
PRJNA729271	stage_3W_vs_E10	ordinary_muscle_development	ENSGALG00010009381	INPP4B	-1.162223067	0.116074947	-10.01269526	1.3405E-23	5.90593E-23	negative
PRJNA729271	stage_3W_vs_E10	ordinary_muscle_development	ENSGALG00010009383	ASXL2	0.699679857	0.111355693	6.283287677	3.31487E-10	7.77744E-10	positive
PRJNA729271	stage_3W_vs_E10	ordinary_muscle_development	ENSGALG00010009385	GPI	3.213925343	0.177945013	18.06133981	6.42442E-73	1.35549E-71	positive
PRJNA729271	stage_3W_vs_E10	ordinary_muscle_development	ENSGALG00010009386	TMEM60	0.566120925	0.110677689	5.115041062	3.13673E-07	6.14085E-07	positive
PRJNA729271	stage_3W_vs_E10	ordinary_muscle_development	ENSGALG00010009387	DEPTOR	3.122511109	0.13816275	22.60023848	4.3104E-113	2.297E-111	positive
PRJNA729271	stage_3W_vs_E10	ordinary_muscle_development	ENSGALG00010009388	TMED7	-0.076503207	0.073556659	-1.04005821	0.298312858	0.335822062	negative
PRJNA729271	stage_3W_vs_E10	ordinary_muscle_development	ENSGALG00010009389	CPEB4	2.021698691	0.125226906	16.14428364	1.24587E-58	1.8095E-57	positive
PRJNA729271	stage_3W_vs_E10	ordinary_muscle_development	ENSGALG00010009391	RSBN1L	0.179743778	0.107079292	1.678604475	0.093229154	0.113235846	positive
PRJNA729271	stage_3W_vs_E10	ordinary_muscle_development	ENSGALG00010009392	MAPRE3	1.509148108	0.105137066	14.35410142	1.00419E-46	1.01646E-45	positive
PRJNA729271	stage_3W_vs_E10	ordinary_muscle_development	ENSGALG00010009393	MARVELD2	-0.339913574	0.131750763	-2.579974232	0.009880769	0.013432915	negative
PRJNA729271	stage_3W_vs_E10	ordinary_muscle_development	ENSGALG00010009394	BANP	-0.235209335	0.095485088	-2.463309621	0.013766098	0.018429814	negative
PRJNA729271	stage_3W_vs_E10	ordinary_muscle_development	ENSGALG00010009396	AUH	0.663505092	0.111460721	5.952815353	2.63569E-09	5.85596E-09	positive
PRJNA729271	stage_3W_vs_E10	ordinary_muscle_development	ENSGALG00010009398	WTIP	-0.123168451	0.138957664	-0.88637393	0.375416063	0.414160578	negative
PRJNA729271	stage_3W_vs_E10	ordinary_muscle_development	ENSGALG00010009400	CNTFR	-0.616088724	0.284532815	-2.165264219	0.030367457	0.039137218	negative
PRJNA729271	stage_3W_vs_E10	ordinary_muscle_development	ENSGALG00010009404	GNG4	-1.173671875	0.405400292	-2.895093804	0.003790452	0.005382309	negative
PRJNA729271	stage_3W_vs_E10	ordinary_muscle_development	ENSGALG00010009407	PTPN12	-0.144614655	0.087534895	-1.652080056	0.098518232	0.11917064	negative
PRJNA729271	stage_3W_vs_E10	ordinary_muscle_development	ENSGALG00010009408	ENSGALG00010009408	-0.199198196	0.091228034	-2.183519552	0.028997571	0.03745498	negative
PRJNA729271	stage_3W_vs_E10	ordinary_muscle_development	ENSGALG00010009410	JMJD1C	0.103171947	0.108150451	0.953966873	0.340100411	0.378598541	positive
PRJNA729271	stage_3W_vs_E10	ordinary_muscle_development	ENSGALG00010009411	SMARCA5	-1.671650116	0.097729711	-17.1048303	1.36599E-65	2.37126E-64	negative
PRJNA729271	stage_3W_vs_E10	ordinary_muscle_development	ENSGALG00010009415	HMP19	-4.734446712	0.459534312	-10.30270556	6.85069E-25	3.18885E-24	negative
PRJNA729271	stage_3W_vs_E10	ordinary_muscle_development	ENSGALG00010009416	COL14A1	-4.569308474	0.474084092	-9.638181399	5.51564E-22	2.28492E-21	negative
PRJNA729271	stage_3W_vs_E10	ordinary_muscle_development	ENSGALG00010009424	ENSGALG00010009424	0.371384068	1.258870355	0.295013753	0.767983373	0.793154186	positive
PRJNA729271	stage_3W_vs_E10	ordinary_muscle_development	ENSGALG00010009431	SMN	0.10491701	0.149537063	0.701612083	0.482921114	0.521345064	positive
PRJNA729271	stage_3W_vs_E10	ordinary_muscle_development	ENSGALG00010009432	BOP1	-0.405326311	0.182962372	-2.215353389	0.026735818	0.034650901	negative
PRJNA729271	stage_3W_vs_E10	ordinary_muscle_development	ENSGALG00010009435	GTDC1	-0.52086119	0.090459852	-5.757926647	8.51533E-09	1.84526E-08	negative
PRJNA729271	stage_3W_vs_E10	ordinary_muscle_development	ENSGALG00010009437	DPYSL5	-4.937671071	0.257004145	-19.21241803	2.91373E-82	7.74946E-81	negative
PRJNA729271	stage_3W_vs_E10	ordinary_muscle_development	ENSGALG00010009439	B3GALNT2	1.139662102	0.097485814	11.69054307	1.42473E-31	8.68436E-31	positive
PRJNA729271	stage_3W_vs_E10	ordinary_muscle_development	ENSGALG00010009440	DOK3	-0.23085822	0.217599181	-1.060933309	0.28872021	0.325909154	negative
PRJNA729271	stage_3W_vs_E10	ordinary_muscle_development	ENSGALG00010009441	ZNF518B	-0.838405234	0.098655969	-8.498271713	1.92434E-17	6.59261E-17	negative
PRJNA729271	stage_3W_vs_E10	ordinary_muscle_development	ENSGALG00010009444	NFIL3	2.598100866	0.221199281	11.74552131	7.44619E-32	4.58897E-31	positive
PRJNA729271	stage_3W_vs_E10	ordinary_muscle_development	ENSGALG00010009446	ENSGALG00010009446	1.695458649	0.249617497	6.792226795	1.10416E-11	2.81923E-11	positive
PRJNA729271	stage_3W_vs_E10	ordinary_muscle_development	ENSGALG00010009447	DDX41	-0.531165684	0.106511679	-4.986924317	6.13481E-07	1.1778E-06	negative
PRJNA729271	stage_3W_vs_E10	ordinary_muscle_development	ENSGALG00010009451	ROR2	-1.804950446	0.141071442	-12.79458423	1.75791E-37	1.29608E-36	negative
PRJNA729271	stage_3W_vs_E10	ordinary_muscle_development	ENSGALG00010009452	TBCE	-0.067757408	0.085596043	-0.7915951	0.428596805	0.467035836	negative
PRJNA729271	stage_3W_vs_E10	ordinary_muscle_development	ENSGALG00010009453	GSAP	-0.069216226	0.114462355	-0.604707339	0.545373471	0.58274553	negative
PRJNA729271	stage_3W_vs_E10	ordinary_muscle_development	ENSGALG00010009457	BDP1	-0.051287645	0.129731035	-0.395338282	0.692593251	0.722708169	negative
PRJNA729271	stage_3W_vs_E10	ordinary_muscle_development	ENSGALG00010009458	SCX	-4.005177833	0.463846437	-8.634706479	5.8878E-18	2.07195E-17	negative
PRJNA729271	stage_3W_vs_E10	ordinary_muscle_development	ENSGALG00010009461	ZEB2	0.10271896	0.125581157	0.817948824	0.41338641	0.452263266	positive
PRJNA729271	stage_3W_vs_E10	ordinary_muscle_development	ENSGALG00010009462	NRBF2	2.361350504	0.119331305	19.78818967	3.76318E-87	1.14836E-85	positive

PRJNA729271	stage_3W_vs_E10	ordinary_muscle_development	ENSGALG00010009463	MRPL13	0.246477378	0.108942783	2.262447966	0.02366974	0.030889789	positive
PRJNA729271	stage_3W_vs_E10	ordinary_muscle_development	ENSGALG00010009464	SPTLC1	0.290280706	0.147726948	1.964981415	0.049416389	0.062075618	positive
PRJNA729271	stage_3W_vs_E10	ordinary_muscle_development	ENSGALG00010009465	GGPS1	0.788433559	0.098136798	8.034025759	9.43254E-16	2.96069E-15	positive
PRJNA729271	stage_3W_vs_E10	ordinary_muscle_development	ENSGALG00010009466	FAM193B	-0.731239191	0.112240076	-6.514956292	7.27108E-11	1.76925E-10	negative
PRJNA729271	stage_3W_vs_E10	ordinary_muscle_development	ENSGALG00010009467	EGR2	1.371257207	0.304226634	4.507354241	6.5641E-06	1.17567E-05	positive
PRJNA729271	stage_3W_vs_E10	ordinary_muscle_development	ENSGALG00010009469	DNMT3A	0.491497227	0.210706766	2.332612457	0.019668495	0.025898876	positive
PRJNA729271	stage_3W_vs_E10	ordinary_muscle_development	ENSGALG00010009470	ZNF469	-1.449783935	0.212282357	-6.829507447	8.52067E-12	2.18403E-11	negative
PRJNA729271	stage_3W_vs_E10	ordinary_muscle_development	ENSGALG00010009472	ADO	0.058878986	0.084607015	0.695911395	0.486484321	0.52474998	positive
PRJNA729271	stage_3W_vs_E10	ordinary_muscle_development	ENSGALG00010009474	ACVR2A	-0.915730463	0.096097216	-9.529209125	1.58486E-21	6.45477E-21	negative
PRJNA729271	stage_3W_vs_E10	ordinary_muscle_development	ENSGALG00010009477	ZNF365	1.507642958	0.242749928	6.21068344	5.27547E-10	1.22407E-09	positive
PRJNA729271	stage_3W_vs_E10	ordinary_muscle_development	ENSGALG00010009479	MTBP	-3.267700711	0.15179321	-21.52731802	8.6391E-103	3.7946E-101	negative
PRJNA729271	stage_3W_vs_E10	ordinary_muscle_development	ENSGALG00010009482	ARID4B	0.239630552	0.107853713	2.221810885	0.026296089	0.034117598	positive
PRJNA729271	stage_3W_vs_E10	ordinary_muscle_development	ENSGALG00010009484	RTKN2	-1.881382057	0.13637178	-13.79597783	2.69474E-43	2.42761E-42	negative
PRJNA729271	stage_3W_vs_E10	ordinary_muscle_development	ENSGALG00010009486	B4GALT1	-0.033608369	0.128317098	-0.261916532	0.793385794	0.816422482	negative
PRJNA729271	stage_3W_vs_E10	ordinary_muscle_development	ENSGALG00010009489	MROH1	-0.344351982	0.138713275	-2.482473157	0.01304739	0.017508046	negative
PRJNA729271	stage_3W_vs_E10	ordinary_muscle_development	ENSGALG00010009490	SNTB1	0.141864812	0.159897923	0.887221103	0.374959873	0.413688793	positive
PRJNA729271	stage_3W_vs_E10	ordinary_muscle_development	ENSGALG00010009491	RBM34	0.0325388	0.102087568	0.318734203	0.749928076	0.776332486	positive
PRJNA729271	stage_3W_vs_E10	ordinary_muscle_development	ENSGALG00010009492	CDC42SE2	-1.201343622	0.12588866	-9.542905796	1.38885E-21	5.66763E-21	negative
PRJNA729271	stage_3W_vs_E10	ordinary_muscle_development	ENSGALG00010009493	ENSGALG00010009493	0.78498602	0.392330389	2.000829003	0.045410821	0.057337095	positive
PRJNA729271	stage_3W_vs_E10	ordinary_muscle_development	ENSGALG00010009494	UBA2	-0.617801987	0.074792709	-8.260190051	1.45441E-16	4.77284E-16	negative
PRJNA729271	stage_3W_vs_E10	ordinary_muscle_development	ENSGALG00010009497	HS3ST1	3.102999286	0.186178112	16.66683185	2.28383E-62	3.63382E-61	positive
PRJNA729271	stage_3W_vs_E10	ordinary_muscle_development	ENSGALG00010009498	ORC4	-1.890450153	0.118210033	-15.9922987	1.44596E-57	2.03487E-56	negative
PRJNA729271	stage_3W_vs_E10	ordinary_muscle_development	ENSGALG00010009499	MXD3	-2.17217468	0.254533513	-8.533943753	1.41442E-17	4.88258E-17	negative
PRJNA729271	stage_3W_vs_E10	ordinary_muscle_development	ENSGALG00010009500	CCDC146	1.14537612	0.566605109	2.021471571	0.043230972	0.05470388	positive
PRJNA729271	stage_3W_vs_E10	ordinary_muscle_development	ENSGALG00010009501	HGH1	-0.453373846	0.112868255	-4.016841114	5.89835E-05	9.8238E-05	negative
PRJNA729271	stage_3W_vs_E10	ordinary_muscle_development	ENSGALG00010009502	ARID5B	-1.189138391	0.097201786	-12.23370925	2.05343E-34	1.38827E-33	negative
PRJNA729271	stage_3W_vs_E10	ordinary_muscle_development	ENSGALG00010009503	IL11RA	-2.193966004	0.136441339	-16.07992134	3.52827E-58	5.04859E-57	negative
PRJNA729271	stage_3W_vs_E10	ordinary_muscle_development	ENSGALG00010009506	HAS2	-2.000473341	0.2593911	-7.712189594	1.23677E-14	3.67056E-14	negative
PRJNA729271	stage_3W_vs_E10	ordinary_muscle_development	ENSGALG00010009508	TOMM20	1.34959412	0.089850726	15.020403	5.39785E-51	6.30983E-50	positive
PRJNA729271	stage_3W_vs_E10	ordinary_muscle_development	ENSGALG00010009511	ZFPM1	-1.355026164	0.264537132	-5.12225317	3.01906E-07	5.91529E-07	negative
PRJNA729271	stage_3W_vs_E10	ordinary_muscle_development	ENSGALG00010009514	PRELID1	-1.074227062	0.091608578	-11.72627149	9.34867E-32	5.73704E-31	negative
PRJNA729271	stage_3W_vs_E10	ordinary_muscle_development	ENSGALG00010009515	MBD5	0.602911586	0.095826251	6.291716325	3.13975E-10	7.37971E-10	positive
PRJNA729271	stage_3W_vs_E10	ordinary_muscle_development	ENSGALG00010009518	FGL2	7.587401526	0.288162284	26.33030742	8.6281E-153	8.6251E-151	positive
PRJNA729271	stage_3W_vs_E10	ordinary_muscle_development	ENSGALG00010009520	SPINK4	0.563136246	0.590344384	0.953911414	0.340128485	0.378598541	positive
PRJNA729271	stage_3W_vs_E10	ordinary_muscle_development	ENSGALG00010009522	TAF5L	0.583641907	0.080652641	7.236488448	4.60451E-13	1.26143E-12	positive
PRJNA729271	stage_3W_vs_E10	ordinary_muscle_development	ENSGALG00010009524	FAM185A	1.862904552	0.159836937	11.65503157	2.163E-31	1.30582E-30	positive
PRJNA729271	stage_3W_vs_E10	ordinary_muscle_development	ENSGALG00010009525	ENSGALG00010009525	0.35318828	0.156749618	2.253200256	0.024246525	0.03160552	positive
PRJNA729271	stage_3W_vs_E10	ordinary_muscle_development	ENSGALG00010009526	ENSGALG00010009526	-1.145770569	0.257547417	-4.448775222	8.63613E-06	1.53183E-05	negative
PRJNA729271	stage_3W_vs_E10	ordinary_muscle_development	ENSGALG00010009527	ENSGALG00010009527	-2.036481686	0.536833861	-3.793504534	0.000148536	0.000239199	negative
PRJNA729271	stage_3W_vs_E10	ordinary_muscle_development	ENSGALG00010009530	KIF3C	-1.796329494	0.161598838	-11.11598029	1.04892E-28	5.7266E-28	negative
PRJNA729271	stage_3W_vs_E10	ordinary_muscle_development	ENSGALG00010009531	FBXL13	-1.740116637	0.499308123	-3.485055735	0.000492035	0.000758889	negative
PRJNA729271	stage_3W_vs_E10	ordinary_muscle_development	ENSGALG00010009534	XPA	0.44145349	0.131020531	3.369345922	0.000753468	0.001140986	positive
PRJNA729271	stage_3W_vs_E10	ordinary_muscle_development	ENSGALG00010009538	CRIM1	1.56637006	0.073909924	21.19295987	1.10902E-99	4.57984E-98	positive
PRJNA729271	stage_3W_vs_E10	ordinary_muscle_development	ENSGALG00010009540	RAB10	0.45261129	0.089989195	5.029618143	4.91458E-07	9.49697E-07	positive
PRJNA729271	stage_3W_vs_E10	ordinary_muscle_development	ENSGALG00010009541	PMPCB	-0.017986684	0.092399981	-0.194661123	0.845658249	0.863530562	negative
PRJNA729271	stage_3W_vs_E10	ordinary_muscle_development	ENSGALG00010009542	WDR1	0.049133958	0.143106957	0.343337311	0.731344715	0.759100726	positive
PRJNA729271	stage_3W_vs_E10	ordinary_muscle_development	ENSGALG00010009543	MAF1	2.003576324	0.129544717	15.46629123	5.85866E-54	7.52181E-53	positive
PRJNA729271	stage_3W_vs_E10	ordinary_muscle_development	ENSGALG00010009546	GAREM2	-2.321009007	0.620047821	-3.743274195	0.000181638	0.000290408	negative
PRJNA729271	stage_3W_vs_E10	ordinary_muscle_development	ENSGALG00010009548	PIK3C3	-0.030307192	0.081814457	-0.370438102	0.711056089	0.739949602	negative
PRJNA729271	stage_3W_vs_E10	ordinary_muscle_development	ENSGALG00010009551	HADHA	0.604399664	0.130162209	4.643434295	3.42665E-06	6.27217E-06	positive
PRJNA729271	stage_3W_vs_E10	ordinary_muscle_development	ENSGALG00010009554	ABCB10	0.685037746	0.1014191	6.754524036	1.43305E-11	3.63211E-11	positive
PRJNA729271	stage_3W_vs_E10	ordinary_muscle_development	ENSGALG00010009555	EPC2	0.029251866	0.076918985	0.380294491	0.703726825	0.733006634	positive
PRJNA729271	stage_3W_vs_E10	ordinary_muscle_development	ENSGALG00010009557	CIDEC	3.475617351	1.055597918	3.292557982	0.000992804	0.001487872	positive
PRJNA729271	stage_3W_vs_E10	ordinary_muscle_development	ENSGALG00010009558	TMEM26	-3.986333287	0.204316123	-19.51061532	8.92019E-85	2.56036E-83	negative
PRJNA729271	stage_3W_vs_E10	ordinary_muscle_development	ENSGALG00010009560	RAB24	1.420123799	0.141354741	10.04652399	9.51651E-24	4.23135E-23	positive
PRJNA729271	stage_3W_vs_E10	ordinary_muscle_development	ENSGALG00010009562	HADHB	0.84450945	0.189542462	4.455515874	8.36917E-06	1.48556E-05	positive
PRJNA729271	stage_3W_vs_E10	ordinary_muscle_development	ENSGALG00010009565	TMEM8B	0.260053108	0.229327931	1.133979217	0.256803266	0.292431718	positive
PRJNA729271	stage_3W_vs_E10	ordinary_muscle_development	ENSGALG00010009567	NSD1	-0.258708463	0.106741931	-2.423681692	0.015364066	0.020455785	negative
PRJNA729271	stage_3W_vs_E10	ordinary_muscle_development	ENSGALG00010009569	ZC3H18	-0.221429657	0.143650372	-1.541448544	0.123207657	0.147071975	negative
PRJNA729271	stage_3W_vs_E10	ordinary_muscle_development	ENSGALG00010009570	SHARPIN	2.298615847	0.134625021	17.07420975	2.30931E-65	3.99445E-64	positive
PRJNA729271	stage_3W_vs_E10	ordinary_muscle_development	ENSGALG00010009571	DNAJC2	0.684716684	0.184863969	3.703894746	0.000212314	0.00033726	positive
PRJNA729271	stage_3W_vs_E10	ordinary_muscle_development	ENSGALG00010009574	TAF1C	-0.402044204	0.145822022	-2.757088392	0.005831858	0.008116446	negative
PRJNA729271	stage_3W_vs_E10	ordinary_muscle_development	ENSGALG00010009575	RHOBTB1	-3.559144529	0.255813813	-13.91302716	5.27967E-44	4.86206E-43	negative
PRJNA729271	stage_3W_vs_E10	ordinary_muscle_development	ENSGALG00010009578	KIF5C	-2.050344064	0.159220459	-12.87739076	6.03412E-38	4.5295E-37	negative
PRJNA729271	stage_3W_vs_E10	ordinary_muscle_development	ENSGALG00010009582	TIGD5	-0.910891747	0.138907317	-6.557550501	5.46988E-11	1.34019E-10	negative
PRJNA729271	stage_3W_vs_E10	ordinary_muscle_development	ENSGALG00010009583	LYPD6B	-2.821346206	0.310685528	-9.081035163	1.07547E-19	4.06068E-19	negative
PRJNA729271	stage_3W_vs_E10	ordinary_muscle_development	ENSGALG00010009591	LYPD6	-3.189426254	0.533309544	-5.980440978	2.22534E-09	4.96023E-09	negative
PRJNA729271	stage_3W_vs_E10	ordinary_muscle_development	ENSGALG00010009593	NCBP1	-1.011735245	0.063181337	-16.01319766	1.03359E-57	1.45879E-56	negative
PRJNA729271	stage_3W_vs_E10	ordinary_muscle_development	ENSGALG00010009594	HINT2	1.047886761	0.161870099	6.473627712	9.56775E-11	2.31141E-10	positive
PRJNA729271	stage_3W_vs_E10	ordinary_muscle_development	ENSGALG00010009595	ENSGALG00010009595	1.45000467	0.185422011	7.820024512	5.28131E-15	1.59884E-14	positive
PRJNA729271	stage_3W_vs_E10	ordinary_muscle_development	ENSGALG00010009596	URB2	-0.860465539	0.09818744	-8.763499054	1.89281E-18	6.79791E-18	negative
PRJNA729271	stage_3W_vs_E10	ordinary_muscle_development	ENSGALG00010009597	CDK1	-3.726548567	0.261417266	-14.25517382	4.16265E-46	4.14122E-45	negative
PRJNA729271	stage_3W_vs_E10	ordinary_muscle_development	ENSGALG00010009600	LRRC17	-1.71669696	0.217507843	-7.892574982	2.96014E-15	9.06939E-15	negative
PRJNA729271	stage_3W_vs_E10	ordinary_muscle_development	ENSGALG00010009601	ZHX2	0.210577608	0.136248678	1.545538728	0.122216009	0.145912285	positive
PRJNA729271	stage_3W_vs_E10	ordinary_muscle_development	ENSGALG00010009602	TSTD2	0.073021494	0.080762749	0.904148197	0.365916825	0.404604804	positive
PRJNA729271	stage_3W_vs_E10	ordinary_muscle_development	ENSGALG00010009603	HINT1	1.621427976	0.176994113	9.160914714	5.14593E-20	1.96808E-19	positive
PRJNA729271	stage_3W_vs_E10	ordinary_muscle_development	ENSGALG00010009605	ENSGALG00010009605	-0.453954147	0.176040038	-2.578698311	0.009917336	0.013478836	negative
PRJNA729271	stage_3W_vs_E10	ordinary_muscle_development	ENSGALG00010009606	CHSY3	-0.569420286	0.215194701	-2.646070201	0.008143291	0.011177747	negative
PRJNA729271	stage_3W_vs_E10	ordinary_muscle_development	ENSGALG00010009608	AFAP1	-1.543818993	0.107235398	-14.39654281	5.43973E-47	5.55665E-46	negative
PRJNA729271	stage_3W_vs_E10	ordinary_muscle_development	ENSGALG00010009609	MMADHC	-0.455111986	0.072358998	-6.289639172	3.18205E-10	7.4767E-10	negative
PRJNA729271	stage_3W_vs_E10	ordinary_muscle_development	ENSGALG00010009612	DERL1	-0.150233911	0.063484149	-2.366479102	0.017958188	0.023741694	negative
PRJNA729271	stage_3W_vs_E10	ordinary_muscle_development	ENSGALG00010009615	ENSGALG00010009615	2.425666369	0.395165727	6.138352104	8.33819E-10	1.90965E-09	positive
PRJNA729271	stage_3W_vs_E10	ordinary_muscle_development	ENSGALG00010009616	ARMC10	-0.195940959	0.176842819	-1.107994994	0.267863978	0.304333623	negative
PRJNA729271	stage_3W_vs_E10	ordinary_muscle_development	ENSGALG00010009617	SLC25A46	1.130273369	0.096896633	11.66473314	1.93007E-31	1.16909E-30	positive
PRJNA729271	stage_3W_vs_E10	ordinary_muscle_development	ENSGALG00010009619	ENSGALG00010009619	-0.396308048	0.191414529	-2.070417797	0.038413237	0.048945136	negative
PRJNA729271	stage_3W_vs_E10	ordinary_muscle_development	ENSGALG00010009620	TMOD1	-0.67368063	0.232592641	-2.896397016	0.003774745	0.005361055	negative
PRJNA729271	stage_3W_vs_E10	ordinary_muscle_development	ENSGALG00010009623	XPO5	-1.086825929	0.111809903	-9.720301145	2.4705E-22	1.03557E-21	negative
PRJNA729271	stage_3W_vs_E10	ordinary_muscle_development	ENSGALG00010009629	ENSGALG00010009629	0.218120224	0.570860154	0.382090469	0.702394261	0.73172379	positive
PRJNA729271	stage_3W_vs_E10	ordinary_muscle_development	ENSGALG00010009634	TBC1D31	-0.727761807	0.105054621	-6.927461194	4.2846E-12	1.1126E-11	negative
PRJNA729271	stage_3W_vs_E10	ordinary_muscle_development	ENSGALG00010009635	TDRD7	1.101649895	0.138259433	7.967990841	1.61275E-15	4.99717E-15	positive
PRJNA729271	stage_3W_vs_E10	ordinary_muscle_development	ENSGALG00010009637	CYBA	0.656991436	0.135632798	4.84389798	1.27316E-06	2.39389E-06	positive
PRJNA729271	stage_3W_vs_E10	ordinary_muscle_development	ENSGALG00010009638	RND3	-0.991654181	0.150284946	-6.598493117	4.15358E-11	1.02636E-10	negative
PRJNA729271	stage_3W_vs_E10	ordinary_muscle_development	ENSGALG00010009640	NPR2	-0.258556422	0.188392071	-1.372437919	0.169927153	0.19886114	negative
PRJNA729271	stage_3W_vs_E10	ordinary_muscle_development	ENSGALG00010009643	PSMC2	0.699535444	0.108527967	6.445669828	1.15091E-10	2.76703E-10	positive
PRJNA729271	stage_3W_vs_E10	ordinary_muscle_development	ENSGALG00010009644	SLC27A6	0.413236698	0.214601445	1.925600723	0.054154248	0.067677709	positive
PRJNA729271	stage_3W_vs_E10	ordinary_muscle_development	ENSGALG00010009646	MVD	0.151375054	0.168369856	0.899062676	0.368619277	0.407251252	positive
PRJNA729271	stage_3W_vs_E10	ordinary_muscle_development	ENSGALG00010009647	GALNT2	-1.856682381	0.079096782	-23.47355136	7.5997E-122	4.6677E-120	negative
PRJNA729271	stage_3W_vs_E10	ordinary_muscle_development	ENSGALG00010009649	TENM2	-1.890594273	0.315983581	-5.983204147	2.1879E-09	4.88128E-09	negative
PRJNA729271	stage_3W_vs_E10	ordinary_muscle_development	ENSGALG00010009650	KLHL29	-0.318063157	0.192735168	-1.650260097	0.09888975	0.119600077	negative
PRJNA729271	stage_3W_vs_E10	ordinary_muscle_development	ENSGALG00010009652	CIAO2B	0.392436727	0.080308939	4.886588379	1.02598E-06	1.94299E-06	positive
PRJNA729271	stage_3W_vs_E10	ordinary_muscle_development	ENSGALG00010009653	ABLIM2	3.592014842	0.183014929	19.62689531	9.11149E-86	2.71193E-84	positive
PRJNA729271	stage_3W_vs_E10	ordinary_muscle_development	ENSGALG00010009654	NDUFA12	1.925815602	0.139437344	13.81133305	2.17764E-43	1.96789E-42	positive
PRJNA729271	stage_3W_vs_E10	ordinary_muscle_development	ENSGALG00010009657	MSMP	0.006310039	0.85490887	0.00738095	0.994110908	0.995003287	positive
PRJNA729271	stage_3W_vs_E10	ordinary_muscle_development	ENSGALG00010009658	NAPEPLD	1.661417175	0.122309639	13.58369775	5.00352E-42	4.30933E-41	positive
PRJNA729271	stage_3W_vs_E10	ordinary_muscle_development	ENSGALG00010009659	APOLD1	-0.367103265	0.257064408	-1.42805948	0.153274739	0.18055403	negative
PRJNA729271	stage_3W_vs_E10	ordinary_muscle_development	ENSGALG00010009663	FER	-0.08898513	0.122391322	-0.727054246	0.467192726	0.505898592	negative
PRJNA729271	stage_3W_vs_E10	ordinary_muscle_development	ENSGALG00010009665	DGKQ	0.040984503	0.171918031	0.238395606	0.811574275	0.833600419	positive
PRJNA729271	stage_3W_vs_E10	ordinary_muscle_development	ENSGALG00010009666	RRAD	0.963370158	0.364886192	2.640193518	0.00828587	0.011364845	positive
PRJNA729271	stage_3W_vs_E10	ordinary_muscle_development	ENSGALG00010009668	TFAP2A	-7.951648299	0.965742506	-8.233714733	1.81496E-16	5.91984E-16	negative
PRJNA729271	stage_3W_vs_E10	ordinary_muscle_development	ENSGALG00010009671	MINAR2	3.342814696	0.806120273	4.146793979	3.37163E-05	5.70999E-05	positive
PRJNA729271	stage_3W_vs_E10	ordinary_muscle_development	ENSGALG00010009672	DDX47	-0.565897792	0.120586739	-4.692869175	2.694E-06	4.96182E-06	negative
PRJNA729271	stage_3W_vs_E10	ordinary_muscle_development	ENSGALG00010009676	ACO1	-0.532027146	0.096030101	-5.540212264	3.02105E-08	6.31528E-08	negative
PRJNA729271	stage_3W_vs_E10	ordinary_muscle_development	ENSGALG00010009677	SLC26A5	3.064777076	0.16054528	19.0897987	3.0695E-81	8.00223E-80	positive
PRJNA729271	stage_3W_vs_E10	ordinary_muscle_development	ENSGALG00010009678	JARID2	-0.474649041	0.100723126	-4.712413714	2.448E-06	4.5208E-06	negative
PRJNA729271	stage_3W_vs_E10	ordinary_muscle_development	ENSGALG00010009679	ANK3	1.858700889	0.102845848	18.07268772	5.23025E-73	1.10675E-71	positive
PRJNA729271	stage_3W_vs_E10	ordinary_muscle_development	ENSGALG00010009683	ADAMTS19	2.163391622	0.238403907	9.074480574	1.14221E-19	4.30818E-19	positive
PRJNA729271	stage_3W_vs_E10	ordinary_muscle_development	ENSGALG00010009686	MAT2B	1.106327474	0.086751002	12.75290719	3.00332E-37	2.19976E-36	positive
PRJNA729271	stage_3W_vs_E10	ordinary_muscle_development	ENSGALG00010009689	ENSGALG00010009689	-2.243926344	0.539580484	-4.158649934	3.20134E-05	5.43302E-05	negative
PRJNA729271	stage_3W_vs_E10	ordinary_muscle_development	ENSGALG00010009690	RBM43	0.17496178	0.18579194	0.941708129	0.346342092	0.384839297	positive
PRJNA729271	stage_3W_vs_E10	ordinary_muscle_development	ENSGALG00010009695	ATAD2B	0.308035952	0.076162061	4.044480268	5.24393E-05	8.76812E-05	positive
PRJNA729271	stage_3W_vs_E10	ordinary_muscle_development	ENSGALG00010009697	ENSGALG00010009697	-0.965453098	0.282733775	-3.414707343	0.000638506	0.000973815	negative
PRJNA729271	stage_3W_vs_E10	ordinary_muscle_development	ENSGALG00010009699	POLR1C	-0.814678782	0.110259563	-7.388735807	1.48231E-13	4.1664E-13	negative
PRJNA729271	stage_3W_vs_E10	ordinary_muscle_development	ENSGALG00010009700	SH3TC1	0.877858738	0.206264507	4.255985428	2.0813E-05	3.58935E-05	positive
PRJNA729271	stage_3W_vs_E10	ordinary_muscle_development	ENSGALG00010009702	ENSGALG00010009702	-0.940237237	0.124935003	-7.525811164	5.2394E-14	1.51074E-13	negative
PRJNA729271	stage_3W_vs_E10	ordinary_muscle_development	ENSGALG00010009704	RGP1	0.339243542	0.130135499	2.606848594	0.009137976	0.012482797	positive
PRJNA729271	stage_3W_vs_E10	ordinary_muscle_development	ENSGALG00010009705	DTNBP1	-0.405979271	0.072179647	-5.624567174	1.85974E-08	3.95031E-08	negative
PRJNA729271	stage_3W_vs_E10	ordinary_muscle_development	ENSGALG00010009710	ENSGALG00010009710	-0.935951108	0.112736552	-8.302108689	1.0228E-16	3.38248E-16	negative
PRJNA729271	stage_3W_vs_E10	ordinary_muscle_development	ENSGALG00010009712	UBXN2A	3.149706583	0.177920733	17.70286427	3.98513E-70	7.73287E-69	positive
PRJNA729271	stage_3W_vs_E10	ordinary_muscle_development	ENSGALG00010009715	NUDCD2	-0.361798946	0.093100867	-3.88609643	0.000101869	0.000166133	negative
PRJNA729271	stage_3W_vs_E10	ordinary_muscle_development	ENSGALG00010009717	EDN1	2.917134348	0.24850756	11.73861408	8.07997E-32	4.9711E-31	positive
PRJNA729271	stage_3W_vs_E10	ordinary_muscle_development	ENSGALG00010009718	ENSGALG00010009718	-1.058614563	0.185192118	-5.71630465	1.08865E-08	2.34508E-08	negative

PRJNA729271	stage_3W_vs_E10	ordinary_muscle_development	ENSGALG00010009719	YIPF3	0.159297456	0.081819623	1.946934614	0.051542574	0.064556261	positive
PRJNA729271	stage_3W_vs_E10	ordinary_muscle_development	ENSGALG00010009720	RELN	-0.982751211	0.169070735	-5.812663015	6.14868E-09	1.34305E-08	negative
PRJNA729271	stage_3W_vs_E10	ordinary_muscle_development	ENSGALG00010009721	ZHX1	1.028243796	0.155944749	6.59364168	4.29167E-11	1.05904E-10	positive
PRJNA729271	stage_3W_vs_E10	ordinary_muscle_development	ENSGALG00010009722	GBA2	0.234084027	0.096489613	2.426002344	0.015266172	0.020331051	positive
PRJNA729271	stage_3W_vs_E10	ordinary_muscle_development	ENSGALG00010009724	ISOC1	0.781396235	0.142499238	5.483511683	4.16965E-08	8.64279E-08	positive
PRJNA729271	stage_3W_vs_E10	ordinary_muscle_development	ENSGALG00010009726	PRR16	-0.066372699	0.145794834	-0.455247265	0.648931361	0.681909531	negative
PRJNA729271	stage_3W_vs_E10	ordinary_muscle_development	ENSGALG00010009727	CDH20	2.810911761	0.213647853	13.15675171	1.55634E-39	1.24088E-38	positive
PRJNA729271	stage_3W_vs_E10	ordinary_muscle_development	ENSGALG00010009728	CCDC149	-1.216086257	0.103943274	-11.6995185	1.28179E-31	7.82295E-31	negative
PRJNA729271	stage_3W_vs_E10	ordinary_muscle_development	ENSGALG00010009730	C8orf76	0.005818858	0.114397177	0.050865398	0.959432778	0.964757414	positive
PRJNA729271	stage_3W_vs_E10	ordinary_muscle_development	ENSGALG00010009731	NMI	1.086832743	0.241681732	4.496958607	6.89324E-06	1.23127E-05	positive
PRJNA729271	stage_3W_vs_E10	ordinary_muscle_development	ENSGALG00010009732	HMMR	-3.334727446	0.24840693	-13.42445418	4.34776E-41	3.62188E-40	negative
PRJNA729271	stage_3W_vs_E10	ordinary_muscle_development	ENSGALG00010009736	WISP1	-1.526932116	0.331288475	-4.609071046	4.04472E-06	7.35888E-06	negative
PRJNA729271	stage_3W_vs_E10	ordinary_muscle_development	ENSGALG00010009738	ENSGALG00010009738	-0.910301346	0.26008712	-3.49998626	0.000465282	0.000718775	negative
PRJNA729271	stage_3W_vs_E10	ordinary_muscle_development	ENSGALG00010009740	LRRC73	-2.58361983	0.370929261	-6.965262928	3.27791E-12	8.58726E-12	negative
PRJNA729271	stage_3W_vs_E10	ordinary_muscle_development	ENSGALG00010009741	C2H6orf52	0.193225541	0.069769135	2.769498854	0.00561426	0.007829392	positive
PRJNA729271	stage_3W_vs_E10	ordinary_muscle_development	ENSGALG00010009743	CCNG1	1.762128056	0.172669398	10.20521339	1.87908E-24	8.60575E-24	positive
PRJNA729271	stage_3W_vs_E10	ordinary_muscle_development	ENSGALG00010009744	DAPK1	-0.029763121	0.102539879	-0.29025898	0.771618118	0.796227281	negative
PRJNA729271	stage_3W_vs_E10	ordinary_muscle_development	ENSGALG00010009749	NDRG1	1.459380555	0.166932605	8.742333817	2.28344E-18	8.1866E-18	positive
PRJNA729271	stage_3W_vs_E10	ordinary_muscle_development	ENSGALG00010009751	ENSGALG00010009751	-0.093028096	0.211730551	-0.439370208	0.66039331	0.692748663	negative
PRJNA729271	stage_3W_vs_E10	ordinary_muscle_development	ENSGALG00010009761	PDP2	0.82794082	0.11583356	7.147676529	8.8259E-13	2.38901E-12	positive
PRJNA729271	stage_3W_vs_E10	ordinary_muscle_development	ENSGALG00010009762	MAN2A1	0.213829197	0.102614909	2.083802438	0.037178141	0.04745484	positive
PRJNA729271	stage_3W_vs_E10	ordinary_muscle_development	ENSGALG00010009765	STX10	-0.613239078	0.098557823	-6.222124851	4.90466E-10	1.14096E-09	negative
PRJNA729271	stage_3W_vs_E10	ordinary_muscle_development	ENSGALG00010009769	TMCC3	2.1986266	0.224088319	9.811428849	1.00535E-22	4.2873E-22	positive
PRJNA729271	stage_3W_vs_E10	ordinary_muscle_development	ENSGALG00010009772	ENSGALG00010009772	-0.965550942	0.594011001	-1.625476533	0.10406106	0.125477503	negative
PRJNA729271	stage_3W_vs_E10	ordinary_muscle_development	ENSGALG00010009774	PIAS2	-0.934907016	0.062327363	-14.99994509	7.34801E-51	8.57564E-50	negative
PRJNA729271	stage_3W_vs_E10	ordinary_muscle_development	ENSGALG00010009775	KIAA1614	-0.165238011	0.137447482	-1.202190159	0.229289861	0.262960403	negative
PRJNA729271	stage_3W_vs_E10	ordinary_muscle_development	ENSGALG00010009777	ST3GAL1	-2.058320487	0.191807687	-10.73116786	7.26719E-27	3.69218E-26	negative
PRJNA729271	stage_3W_vs_E10	ordinary_muscle_development	ENSGALG00010009778	ENSGALG00010009778	-0.019584819	0.111655072	-0.17540465	0.860761687	0.876914581	negative
PRJNA729271	stage_3W_vs_E10	ordinary_muscle_development	ENSGALG00010009779	PPP1R3E	2.784934687	0.343869067	8.098822948	5.54935E-16	1.76437E-15	positive
PRJNA729271	stage_3W_vs_E10	ordinary_muscle_development	ENSGALG00010009780	MFSD2B	-1.440322154	0.261393157	-5.51017543	3.58476E-08	7.45818E-08	negative
PRJNA729271	stage_3W_vs_E10	ordinary_muscle_development	ENSGALG00010009784	ENSGALG00010009784	-1.046371815	0.180248991	-5.805146574	6.43097E-09	1.40239E-08	negative
PRJNA729271	stage_3W_vs_E10	ordinary_muscle_development	ENSGALG00010009786	DIPK2B	0.435806699	0.160448639	2.716175721	0.006604086	0.009148148	positive
PRJNA729271	stage_3W_vs_E10	ordinary_muscle_development	ENSGALG00010009788	PAK1IP1	0.686414987	0.091481352	7.503332297	6.22156E-14	1.78648E-13	positive
PRJNA729271	stage_3W_vs_E10	ordinary_muscle_development	ENSGALG00010009791	GABRG2	-1.402218561	0.338029156	-4.148217793	3.35074E-05	5.67526E-05	negative
PRJNA729271	stage_3W_vs_E10	ordinary_muscle_development	ENSGALG00010009793	TNFAIP6	-0.464796024	0.288828637	-1.609244947	0.107562792	0.129516753	negative
PRJNA729271	stage_3W_vs_E10	ordinary_muscle_development	ENSGALG00010009795	NDUFA6	1.053373306	0.11458556	9.192897458	3.82399E-20	1.46948E-19	positive
PRJNA729271	stage_3W_vs_E10	ordinary_muscle_development	ENSGALG00010009796	ENSGALG00010009796	-1.063400742	0.198119897	-5.367460615	7.98529E-08	1.62863E-07	negative
PRJNA729271	stage_3W_vs_E10	ordinary_muscle_development	ENSGALG00010009798	YIPF4	0.906770633	0.077107219	11.75986686	6.28333E-32	3.88553E-31	positive
PRJNA729271	stage_3W_vs_E10	ordinary_muscle_development	ENSGALG00010009801	ENSGALG00010009801	-1.728215451	0.652707788	-2.647762877	0.008102634	0.011125099	negative
PRJNA729271	stage_3W_vs_E10	ordinary_muscle_development	ENSGALG00010009804	XPR1	0.778408138	0.117060638	6.64961467	2.93861E-11	7.3301E-11	positive
PRJNA729271	stage_3W_vs_E10	ordinary_muscle_development	ENSGALG00010009806	WDCP	-0.486779408	0.173708731	-2.802273701	0.00507438	0.007110019	negative
PRJNA729271	stage_3W_vs_E10	ordinary_muscle_development	ENSGALG00010009809	SMDT1	1.51825586	0.089404837	16.98180902	1.11981E-64	1.90065E-63	positive
PRJNA729271	stage_3W_vs_E10	ordinary_muscle_development	ENSGALG00010009811	ENSGALG00010009811	0.239573777	0.086985364	2.754184924	0.005883851	0.008188021	positive
PRJNA729271	stage_3W_vs_E10	ordinary_muscle_development	ENSGALG00010009822	ENSGALG00010009822	-0.923928151	0.204917541	-4.508780192	6.52014E-06	1.16808E-05	negative
PRJNA729271	stage_3W_vs_E10	ordinary_muscle_development	ENSGALG00010009830	RNASEL	-0.994763073	0.197125566	-5.046342245	4.50348E-07	8.72817E-07	negative
PRJNA729271	stage_3W_vs_E10	ordinary_muscle_development	ENSGALG00010009831	N6AMT1	-0.423906589	0.12065638	-3.513337534	0.000442515	0.000684918	negative
PRJNA729271	stage_3W_vs_E10	ordinary_muscle_development	ENSGALG00010009833	SOD3	4.161360516	0.31097768	13.38154079	7.75235E-41	6.42116E-40	positive
PRJNA729271	stage_3W_vs_E10	ordinary_muscle_development	ENSGALG00010009840	ENSGALG00010009840	-0.243626643	0.178934081	-1.361544101	0.173341807	0.202562841	negative
PRJNA729271	stage_3W_vs_E10	ordinary_muscle_development	ENSGALG00010009841	GABRA1	-0.808659779	0.244000999	-3.314165848	0.00091917	0.001380517	negative
PRJNA729271	stage_3W_vs_E10	ordinary_muscle_development	ENSGALG00010009842	PHETA2	-0.370785449	0.090287328	-4.106727466	4.01304E-05	6.76699E-05	negative
PRJNA729271	stage_3W_vs_E10	ordinary_muscle_development	ENSGALG00010009847	ENSGALG00010009847	-0.631363205	0.118388616	-5.332972271	9.66181E-08	1.95871E-07	negative
PRJNA729271	stage_3W_vs_E10	ordinary_muscle_development	ENSGALG00010009850	RIF1	-2.052436137	0.106371998	-19.29489125	5.92896E-83	1.61239E-81	negative
PRJNA729271	stage_3W_vs_E10	ordinary_muscle_development	ENSGALG00010009852	ENSGALG00010009852	-0.786582279	0.256376696	-3.068072451	0.002154444	0.003128184	negative
PRJNA729271	stage_3W_vs_E10	ordinary_muscle_development	ENSGALG00010009857	ORC5	-1.277423468	0.140146471	-9.114917097	7.87306E-20	2.99292E-19	negative
PRJNA729271	stage_3W_vs_E10	ordinary_muscle_development	ENSGALG00010009858	ST8SIA5	-1.639929438	0.252441149	-6.496284172	8.23281E-11	1.9969E-10	negative
PRJNA729271	stage_3W_vs_E10	ordinary_muscle_development	ENSGALG00010009866	NAGA	0.741980564	0.114507206	6.479771772	9.18615E-11	2.22144E-10	positive
PRJNA729271	stage_3W_vs_E10	ordinary_muscle_development	ENSGALG00010009868	LTN1	0.792682559	0.094716471	8.369004343	5.81071E-17	1.94428E-16	positive
PRJNA729271	stage_3W_vs_E10	ordinary_muscle_development	ENSGALG00010009876	SELENOI	-0.789537616	0.110997289	-7.113125246	1.13444E-12	3.04909E-12	negative
PRJNA729271	stage_3W_vs_E10	ordinary_muscle_development	ENSGALG00010009883	ENSGALG00010009883	-0.75400924	0.204109292	-3.694144614	0.000220628	0.000350006	negative
PRJNA729271	stage_3W_vs_E10	ordinary_muscle_development	ENSGALG00010009884	GFOD1	0.898883943	0.114280702	7.865579493	3.67393E-15	1.12113E-14	positive
PRJNA729271	stage_3W_vs_E10	ordinary_muscle_development	ENSGALG00010009910	ENSGALG00010009910	-0.075483374	0.177767741	-0.424617949	0.671115206	0.702622963	negative
PRJNA729271	stage_3W_vs_E10	ordinary_muscle_development	ENSGALG00010009914	RWDD2B	-0.265649365	0.123439624	-2.1520591	0.0313927	0.040379553	negative
PRJNA729271	stage_3W_vs_E10	ordinary_muscle_development	ENSGALG00010009941	CACNA1E	-3.304324575	0.499123395	-6.620255847	3.58578E-11	8.8954E-11	negative
PRJNA729271	stage_3W_vs_E10	ordinary_muscle_development	ENSGALG00010009943	DHX15	-0.631053848	0.0689662	-9.150190172	5.68334E-20	2.16847E-19	negative
PRJNA729271	stage_3W_vs_E10	ordinary_muscle_development	ENSGALG00010009948	WBP2NL	0.712229252	0.064882861	10.97715543	4.92178E-28	2.62477E-27	positive
PRJNA729271	stage_3W_vs_E10	ordinary_muscle_development	ENSGALG00010009972	PTK2B	2.282433493	0.258938644	8.814572662	1.20142E-18	4.34606E-18	positive
PRJNA729271	stage_3W_vs_E10	ordinary_muscle_development	ENSGALG00010009985	USP16	-0.733090382	0.116057567	-6.31660994	2.67363E-10	6.30767E-10	negative
PRJNA729271	stage_3W_vs_E10	ordinary_muscle_development	ENSGALG00010009989	TBC1D7	-0.851729362	0.135929028	-6.265985799	3.70474E-10	8.66972E-10	negative
PRJNA729271	stage_3W_vs_E10	ordinary_muscle_development	ENSGALG00010009991	LHFPL3	-3.971172041	0.577712177	-6.873962844	6.24425E-12	1.60907E-11	negative
PRJNA729271	stage_3W_vs_E10	ordinary_muscle_development	ENSGALG00010009993	ENSGALG00010009993	-0.67411064	0.370641014	-1.818769683	0.068946576	0.085017492	negative
PRJNA729271	stage_3W_vs_E10	ordinary_muscle_development	ENSGALG00010009995	SEPTIN3	0.273606968	0.371756377	0.735984597	0.461740072	0.500405409	positive
PRJNA729271	stage_3W_vs_E10	ordinary_muscle_development	ENSGALG00010009999	GABRB2	-2.345786797	0.235948056	-9.941962804	2.73386E-23	1.18904E-22	negative
PRJNA729271	stage_3W_vs_E10	ordinary_muscle_development	ENSGALG00010010006	PPARGC1A	2.505206262	0.265542788	9.434284708	3.93671E-21	1.58024E-20	positive
PRJNA729271	stage_3W_vs_E10	ordinary_muscle_development	ENSGALG00010010007	CTIF	0.248644689	0.095319177	2.60854843	0.009092714	0.012426824	positive
PRJNA729271	stage_3W_vs_E10	ordinary_muscle_development	ENSGALG00010010009	ELF2	-0.08126483	0.08820229	-0.921346039	0.356869792	0.395536598	negative
PRJNA729271	stage_3W_vs_E10	ordinary_muscle_development	ENSGALG00010010011	EPB41L4A	0.69987747	0.122752638	5.701526937	1.18739E-08	2.55019E-08	positive
PRJNA729271	stage_3W_vs_E10	ordinary_muscle_development	ENSGALG00010010014	GLUL	3.10590939	0.392384872	7.915466709	2.46327E-15	7.57267E-15	positive
PRJNA729271	stage_3W_vs_E10	ordinary_muscle_development	ENSGALG00010010017	CCDC59	-0.513836252	0.106063406	-4.84461392	1.26858E-06	2.3862E-06	negative
PRJNA729271	stage_3W_vs_E10	ordinary_muscle_development	ENSGALG00010010022	KMT2E	0.446584507	0.101237159	4.411270645	1.02766E-05	1.81326E-05	positive
PRJNA729271	stage_3W_vs_E10	ordinary_muscle_development	ENSGALG00010010035	ATP10B	-0.511164778	0.29889579	-1.710177246	0.087233102	0.106318323	negative
PRJNA729271	stage_3W_vs_E10	ordinary_muscle_development	ENSGALG00010010037	NAA15	0.475769439	0.138173701	3.443270567	0.000574724	0.000879314	positive
PRJNA729271	stage_3W_vs_E10	ordinary_muscle_development	ENSGALG00010010042	METTL25	-0.661735378	0.115302076	-5.739145373	9.51555E-09	2.05648E-08	negative
PRJNA729271	stage_3W_vs_E10	ordinary_muscle_development	ENSGALG00010010049	ZBTB7C	-0.797731109	0.308399994	-2.586676797	0.009690644	0.013194241	negative
PRJNA729271	stage_3W_vs_E10	ordinary_muscle_development	ENSGALG00010010052	ZNF346	-0.42874115	0.114226768	-3.753421002	0.000174438	0.000279297	negative
PRJNA729271	stage_3W_vs_E10	ordinary_muscle_development	ENSGALG00010010053	CHRNA2	-5.720268378	0.884210364	-6.469352328	9.84239E-11	2.37538E-10	negative
PRJNA729271	stage_3W_vs_E10	ordinary_muscle_development	ENSGALG00010010059	ENSGALG00010010059	-1.264912118	0.132056254	-9.578585516	9.8383E-22	4.03984E-21	negative
PRJNA729271	stage_3W_vs_E10	ordinary_muscle_development	ENSGALG00010010061	CYP2D6	1.008921051	0.203334324	4.961882627	6.98132E-07	1.33572E-06	positive
PRJNA729271	stage_3W_vs_E10	ordinary_muscle_development	ENSGALG00010010062	RGS7BP	-0.75508698	0.174846056	-4.318581719	1.57035E-05	2.73322E-05	negative
PRJNA729271	stage_3W_vs_E10	ordinary_muscle_development	ENSGALG00010010064	ENSGALG00010010064	0.126342231	0.088126725	1.433642635	0.15167427	0.178799491	positive
PRJNA729271	stage_3W_vs_E10	ordinary_muscle_development	ENSGALG00010010071	GLT8D2	-1.504930139	0.26945487	-5.58509164	2.33577E-08	4.92322E-08	negative
PRJNA729271	stage_3W_vs_E10	ordinary_muscle_development	ENSGALG00010010077	CCT8	-0.64340454	0.115328366	-5.578892363	2.42055E-08	5.09599E-08	negative
PRJNA729271	stage_3W_vs_E10	ordinary_muscle_development	ENSGALG00010010080	CDC42EP3	2.603032507	0.117901345	22.07805611	5.1371E-108	2.5414E-106	positive
PRJNA729271	stage_3W_vs_E10	ordinary_muscle_development	ENSGALG00010010084	HK3	0.766170207	0.218671787	3.503745114	0.000458764	0.000708933	positive
PRJNA729271	stage_3W_vs_E10	ordinary_muscle_development	ENSGALG00010010085	TDG	-1.096643022	0.103690187	-10.57615047	3.8443E-26	1.89084E-25	negative
PRJNA729271	stage_3W_vs_E10	ordinary_muscle_development	ENSGALG00010010094	SLC2A9	2.055819382	0.241318107	8.519126079	1.60762E-17	5.5272E-17	positive
PRJNA729271	stage_3W_vs_E10	ordinary_muscle_development	ENSGALG00010010095	C12orf73	0.895648743	0.097788432	9.159045987	5.23581E-20	2.00193E-19	positive
PRJNA729271	stage_3W_vs_E10	ordinary_muscle_development	ENSGALG00010010100	IRF2BP2	1.050324946	0.162252497	6.473397716	9.58233E-11	2.31416E-10	positive
PRJNA729271	stage_3W_vs_E10	ordinary_muscle_development	ENSGALG00010010106	C7orf73	-0.538874853	0.206653593	-2.607623929	0.009117306	0.012456909	negative
PRJNA729271	stage_3W_vs_E10	ordinary_muscle_development	ENSGALG00010010107	MGST2	0.851181615	0.302837624	2.810686477	0.004943593	0.006934809	positive
PRJNA729271	stage_3W_vs_E10	ordinary_muscle_development	ENSGALG00010010111	CNOT4	0.661565728	0.134651349	4.913175635	8.96129E-07	1.70353E-06	positive
PRJNA729271	stage_3W_vs_E10	ordinary_muscle_development	ENSGALG00010010115	NEB	4.513465132	0.171691462	26.28823283	2.6143E-152	2.5604E-150	positive
PRJNA729271	stage_3W_vs_E10	ordinary_muscle_development	ENSGALG00010010116	DRD5	-6.047361318	0.919845244	-6.574324712	4.88745E-11	1.20115E-10	negative
PRJNA729271	stage_3W_vs_E10	ordinary_muscle_development	ENSGALG00010010119	SRPK2	-1.162828984	0.125676747	-9.252538863	2.19222E-20	8.50999E-20	negative
PRJNA729271	stage_3W_vs_E10	ordinary_muscle_development	ENSGALG00010010122	THBS4	-5.012622808	0.251224011	-19.95280143	1.41721E-88	4.47547E-87	negative
PRJNA729271	stage_3W_vs_E10	ordinary_muscle_development	ENSGALG00010010124	ENSGALG00010010124	0.351275171	0.147395044	2.383222396	0.017161823	0.022726167	positive
PRJNA729271	stage_3W_vs_E10	ordinary_muscle_development	ENSGALG00010010125	AHCTF1	-0.01743051	0.066625581	-0.26161888	0.793615287	0.816542701	negative
PRJNA729271	stage_3W_vs_E10	ordinary_muscle_development	ENSGALG00010010127	FAM13C	1.845936224	0.331621756	5.566390598	2.6007E-08	5.46177E-08	positive
PRJNA729271	stage_3W_vs_E10	ordinary_muscle_development	ENSGALG00010010136	EPHX2	-0.139730298	0.11866159	-1.177552883	0.238974903	0.273310811	negative
PRJNA729271	stage_3W_vs_E10	ordinary_muscle_development	ENSGALG00010010142	ENSGALG00010010142	1.033906476	0.288813461	3.579841713	0.000343802	0.000537469	positive
PRJNA729271	stage_3W_vs_E10	ordinary_muscle_development	ENSGALG00010010146	PHACTR1	-4.570487922	0.363589543	-12.57046031	3.06932E-36	2.19161E-35	negative
PRJNA729271	stage_3W_vs_E10	ordinary_muscle_development	ENSGALG00010010150	NTRK2	-0.167784571	0.298329758	-0.562413124	0.573834579	0.610072776	negative
PRJNA729271	stage_3W_vs_E10	ordinary_muscle_development	ENSGALG00010010153	ENSGALG00010010153	3.354453615	0.433761736	7.733401402	1.0471E-14	3.11787E-14	positive
PRJNA729271	stage_3W_vs_E10	ordinary_muscle_development	ENSGALG00010010154	IQSEC3	-6.112289807	0.759415607	-8.048675518	8.36948E-16	2.63386E-15	negative
PRJNA729271	stage_3W_vs_E10	ordinary_muscle_development	ENSGALG00010010155	BRINP3	-4.119111396	0.519362847	-7.931085984	2.17238E-15	6.68832E-15	negative
PRJNA729271	stage_3W_vs_E10	ordinary_muscle_development	ENSGALG00010010156	ENSGALG00010010156	-0.125379113	0.097741876	-1.282757383	0.199577089	0.231059896	negative
PRJNA729271	stage_3W_vs_E10	ordinary_muscle_development	ENSGALG00010010161	SEPSECS	-0.427504225	0.06501341	-6.575631483	4.84471E-11	1.19125E-10	negative
PRJNA729271	stage_3W_vs_E10	ordinary_muscle_development	ENSGALG00010010164	ENSGALG00010010164	1.548678708	0.388884888	3.982357653	6.8235E-05	0.000113023	positive
PRJNA729271	stage_3W_vs_E10	ordinary_muscle_development	ENSGALG00010010167	PUS7	-0.585054155	0.069528549	-8.414588823	3.94275E-17	1.32938E-16	negative
PRJNA729271	stage_3W_vs_E10	ordinary_muscle_development	ENSGALG00010010168	SNCB	-3.918510154	0.373660159	-10.48682891	9.93055E-26	4.79013E-25	negative
PRJNA729271	stage_3W_vs_E10	ordinary_muscle_development	ENSGALG00010010171	ENSGALG00010010171	3.084763591	0.377758218	8.165973491	3.18853E-16	1.02615E-15	positive
PRJNA729271	stage_3W_vs_E10	ordinary_muscle_development	ENSGALG00010010178	LGI2	-4.03634112	0.211046628	-19.12535231	1.5532E-81	4.07119E-80	negative
PRJNA729271	stage_3W_vs_E10	ordinary_muscle_development	ENSGALG00010010179	CDHR2	1.246264364	0.835223161	1.492133387	0.135664181	0.161118583	positive
PRJNA729271	stage_3W_vs_E10	ordinary_muscle_development	ENSGALG00010010183	NKX3-2	-4.631201961	0.442687118	-10.46156929	1.2969E-25	6.22674E-25	negative
PRJNA729271	stage_3W_vs_E10	ordinary_muscle_development	ENSGALG00010010185	PHYHIPL	-2.124357212	0.357422798	-5.943541437	2.78929E-09	6.18965E-09	negative
PRJNA729271	stage_3W_vs_E10	ordinary_muscle_development	ENSGALG00010010186	TNPO1	0.633193526	0.072068101	8.786044269	1.54919E-18	5.58454E-18	positive
PRJNA729271	stage_3W_vs_E10	ordinary_muscle_development	ENSGALG00010010188	DSP	-5.460220421	0.356981129	-15.29554358	8.18744E-53	1.0126E-51	negative
PRJNA729271	stage_3W_vs_E10	ordinary_muscle_development	ENSGALG00010010190	RNF44	-0.200008536	0.184555533	-1.083730911	0.278484126	0.315361516	negative
PRJNA729271	stage_3W_vs_E10	ordinary_muscle_development	ENSGALG00010010191	RINT1	-0.288049213	0.093215349	-3.090147874	0.002000569	0.00291147	negative
PRJNA729271	stage_3W_vs_E10	ordinary_muscle_development	ENSGALG00010010195	ENSGALG00010010195	3.066646702	0.281770991	10.88347203	1.38197E-27	7.24995E-27	positive
PRJNA729271	stage_3W_vs_E10	ordinary_muscle_development	ENSGALG00010010196	EFCAB10	-0.739187216	0.360317977	-2.051485809	0.04021966	0.051089648	negative
PRJNA729271	stage_3W_vs_E10	ordinary_muscle_development	ENSGALG00010010197	SCCPDH	-0.421800732	0.1215046	-3.471479545	0.000517599	0.0007962	negative

PRJNA729271	stage_3W_vs_E10	ordinary_muscle_development	ENSGALG00010010201	RIOK1	-0.316941113	0.098414539	-3.220470424	0.001279804	0.001899136	negative
PRJNA729271	stage_3W_vs_E10	ordinary_muscle_development	ENSGALG00010010205	ARL5A	0.870752697	0.082404173	10.56685194	4.24506E-26	2.08301E-25	positive
PRJNA729271	stage_3W_vs_E10	ordinary_muscle_development	ENSGALG00010010206	SCARA5	1.757754605	0.158043464	11.12196962	9.80824E-29	5.3629E-28	positive
PRJNA729271	stage_3W_vs_E10	ordinary_muscle_development	ENSGALG00010010214	FAF2	-1.212244109	0.079757401	-15.19914262	3.5828E-52	4.35311E-51	negative
PRJNA729271	stage_3W_vs_E10	ordinary_muscle_development	ENSGALG00010010215	ENSGALG00010010215	0.707066645	0.123358933	5.731783068	9.93803E-09	2.14427E-08	positive
PRJNA729271	stage_3W_vs_E10	ordinary_muscle_development	ENSGALG00010010216	ATXN7L1	0.649193586	0.128144053	5.066123395	4.05999E-07	7.88762E-07	positive
PRJNA729271	stage_3W_vs_E10	ordinary_muscle_development	ENSGALG00010010218	PBK	-3.726762874	0.232465899	-16.03143895	7.70771E-58	1.09425E-56	negative
PRJNA729271	stage_3W_vs_E10	ordinary_muscle_development	ENSGALG00010010220	LHX9	-8.095464375	0.902676048	-8.968294211	3.01142E-19	1.11823E-18	negative
PRJNA729271	stage_3W_vs_E10	ordinary_muscle_development	ENSGALG00010010222	PAH	-0.787603112	1.074838429	-0.732764191	0.463702265	0.50234412	negative
PRJNA729271	stage_3W_vs_E10	ordinary_muscle_development	ENSGALG00010010223	ENSGALG00010010223	2.469040092	0.194290751	12.70796512	5.34061E-37	3.88031E-36	positive
PRJNA729271	stage_3W_vs_E10	ordinary_muscle_development	ENSGALG00010010226	BICC1	0.029837647	0.164006257	0.181929933	0.855637714	0.872062204	positive
PRJNA729271	stage_3W_vs_E10	ordinary_muscle_development	ENSGALG00010010231	C1orf53	-0.541088864	0.241024879	-2.244950253	0.024771326	0.032246105	negative
PRJNA729271	stage_3W_vs_E10	ordinary_muscle_development	ENSGALG00010010234	ENSGALG00010010234	1.15539867	0.268692952	4.300070628	1.70744E-05	2.96434E-05	positive
PRJNA729271	stage_3W_vs_E10	ordinary_muscle_development	ENSGALG00010010243	ESCO2	-0.791002105	0.111982023	-7.063652554	1.62182E-12	4.32615E-12	negative
PRJNA729271	stage_3W_vs_E10	ordinary_muscle_development	ENSGALG00010010246	CLTB	0.574364698	0.066518105	8.634712255	5.8875E-18	2.07195E-17	positive
PRJNA729271	stage_3W_vs_E10	ordinary_muscle_development	ENSGALG00010010252	WDR7	1.159904443	0.095328075	12.16750096	4.63043E-34	3.08589E-33	positive
PRJNA729271	stage_3W_vs_E10	ordinary_muscle_development	ENSGALG00010010253	IL10RB	1.174317133	0.121590267	9.657986277	4.54714E-22	1.89181E-21	positive
PRJNA729271	stage_3W_vs_E10	ordinary_muscle_development	ENSGALG00010010254	WDR41	1.750685569	0.110826721	15.79660167	3.28346E-56	4.45218E-55	positive
PRJNA729271	stage_3W_vs_E10	ordinary_muscle_development	ENSGALG00010010255	CACNB4	0.239040171	0.175862184	1.359247143	0.174068287	0.203329827	positive
PRJNA729271	stage_3W_vs_E10	ordinary_muscle_development	ENSGALG00010010257	ENSGALG00010010257	1.028304089	0.135007072	7.616668312	2.60307E-14	7.6133E-14	positive
PRJNA729271	stage_3W_vs_E10	ordinary_muscle_development	ENSGALG00010010259	IGF1	0.487504229	0.396013065	1.231030671	0.218311393	0.251104876	positive
PRJNA729271	stage_3W_vs_E10	ordinary_muscle_development	ENSGALG00010010262	HSPA9	0.18264427	0.145667912	1.253840111	0.209900127	0.242256735	positive
PRJNA729271	stage_3W_vs_E10	ordinary_muscle_development	ENSGALG00010010263	RAB4A	-0.627721181	0.137397667	-4.568645138	4.90887E-06	8.87651E-06	negative
PRJNA729271	stage_3W_vs_E10	ordinary_muscle_development	ENSGALG00010010266	EFNA5	-2.142103403	0.233348427	-9.179849337	4.31695E-20	1.6554E-19	negative
PRJNA729271	stage_3W_vs_E10	ordinary_muscle_development	ENSGALG00010010267	CCSER2	1.130789218	0.106797851	10.58812705	3.38293E-26	1.66787E-25	positive
PRJNA729271	stage_3W_vs_E10	ordinary_muscle_development	ENSGALG00010010270	SSR1	-0.744096243	0.077763675	-9.568686688	1.08272E-21	4.43335E-21	negative
PRJNA729271	stage_3W_vs_E10	ordinary_muscle_development	ENSGALG00010010271	HUNK	-2.389197954	0.115583737	-20.67071038	6.35737E-95	2.32702E-93	negative
PRJNA729271	stage_3W_vs_E10	ordinary_muscle_development	ENSGALG00010010274	MIS18A	-2.434474302	0.103850567	-23.4420897	1.5918E-121	9.7356E-120	negative
PRJNA729271	stage_3W_vs_E10	ordinary_muscle_development	ENSGALG00010010275	PDE8B	-3.995986498	0.977434664	-4.088238984	4.3466E-05	7.30905E-05	negative
PRJNA729271	stage_3W_vs_E10	ordinary_muscle_development	ENSGALG00010010279	CCSAP	1.030860636	0.240137345	4.292796001	1.76437E-05	3.06062E-05	positive
PRJNA729271	stage_3W_vs_E10	ordinary_muscle_development	ENSGALG00010010280	HSP90B1	-1.26470741	0.135588009	-9.327575614	1.0832E-20	4.25732E-20	negative
PRJNA729271	stage_3W_vs_E10	ordinary_muscle_development	ENSGALG00010010284	CCDC25	0.752595278	0.094094965	7.998252345	1.26198E-15	3.9313E-15	positive
PRJNA729271	stage_3W_vs_E10	ordinary_muscle_development	ENSGALG00010010285	ENSGALG00010010285	4.339967744	0.567885146	7.642333623	2.1332E-14	6.25798E-14	positive
PRJNA729271	stage_3W_vs_E10	ordinary_muscle_development	ENSGALG00010010287	STAM2	0.279188843	0.096943166	2.87992289	0.003977724	0.005633879	positive
PRJNA729271	stage_3W_vs_E10	ordinary_muscle_development	ENSGALG00010010288	ENSGALG00010010288	1.354366373	0.163197347	8.298948486	1.05037E-16	3.46971E-16	positive
PRJNA729271	stage_3W_vs_E10	ordinary_muscle_development	ENSGALG00010010290	ENSGALG00010010290	0.602906332	0.166438966	3.622386913	0.000291897	0.000458849	positive
PRJNA729271	stage_3W_vs_E10	ordinary_muscle_development	ENSGALG00010010291	TCF20	-0.644991252	0.117634182	-5.483025791	4.18112E-08	8.66533E-08	negative
PRJNA729271	stage_3W_vs_E10	ordinary_muscle_development	ENSGALG00010010293	ENSGALG00010010293	3.002055319	0.476218583	6.303944078	2.90165E-10	6.83227E-10	positive
PRJNA729271	stage_3W_vs_E10	ordinary_muscle_development	ENSGALG00010010295	ENSGALG00010010295	8.2779553	0.181412506	45.63056585	0	0	positive
PRJNA729271	stage_3W_vs_E10	ordinary_muscle_development	ENSGALG00010010298	KCNIP4	-3.421758937	0.468181115	-7.308622302	2.69895E-13	7.4859E-13	negative
PRJNA729271	stage_3W_vs_E10	ordinary_muscle_development	ENSGALG00010010300	DENND1B	1.891967483	0.090783001	20.84054804	1.85701E-96	7.00972E-95	positive
PRJNA729271	stage_3W_vs_E10	ordinary_muscle_development	ENSGALG00010010301	ENSGALG00010010301	-2.056984885	0.560024607	-3.673025899	0.000239695	0.000378927	negative
PRJNA729271	stage_3W_vs_E10	ordinary_muscle_development	ENSGALG00010010302	WDR36	-0.274283079	0.104090186	-2.635052248	0.008412433	0.011532982	negative
PRJNA729271	stage_3W_vs_E10	ordinary_muscle_development	ENSGALG00010010303	ETF1	1.160174825	0.18448026	6.288883289	3.19758E-10	7.51076E-10	positive
PRJNA729271	stage_3W_vs_E10	ordinary_muscle_development	ENSGALG00010010305	URB1	-0.902701478	0.102599623	-8.798292383	1.38914E-18	5.01383E-18	negative
PRJNA729271	stage_3W_vs_E10	ordinary_muscle_development	ENSGALG00010010308	PACRGL	-0.911838251	0.223855363	-4.073336635	4.63444E-05	7.78042E-05	negative
PRJNA729271	stage_3W_vs_E10	ordinary_muscle_development	ENSGALG00010010310	NT5DC3	1.607106705	0.117988613	13.6208628	3.00991E-42	2.61249E-41	positive
PRJNA729271	stage_3W_vs_E10	ordinary_muscle_development	ENSGALG00010010311	CTSV	1.430513281	0.189348968	7.554904011	4.19169E-14	1.21468E-13	positive
PRJNA729271	stage_3W_vs_E10	ordinary_muscle_development	ENSGALG00010010313	GRID1	-3.664463466	0.234904064	-15.59983	7.29882E-55	9.5917E-54	negative
PRJNA729271	stage_3W_vs_E10	ordinary_muscle_development	ENSGALG00010010314	CRB1	-0.995174952	0.285716098	-3.483090239	0.000495661	0.000764158	negative
PRJNA729271	stage_3W_vs_E10	ordinary_muscle_development	ENSGALG00010010317	ENSGALG00010010317	-0.196889203	0.107917845	-1.824436014	0.068086182	0.084013723	negative
PRJNA729271	stage_3W_vs_E10	ordinary_muscle_development	ENSGALG00010010320	SYPL1	2.305734098	0.569206676	4.050785411	5.1046E-05	8.54402E-05	positive
PRJNA729271	stage_3W_vs_E10	ordinary_muscle_development	ENSGALG00010010321	NUP133	-1.130911638	0.073169343	-15.45608575	6.86451E-54	8.8054E-53	negative
PRJNA729271	stage_3W_vs_E10	ordinary_muscle_development	ENSGALG00010010322	ENSGALG00010010322	0.294316529	0.067013957	4.391869127	1.1238E-05	1.97736E-05	positive
PRJNA729271	stage_3W_vs_E10	ordinary_muscle_development	ENSGALG00010010323	FBP2	10.43085165	0.241137031	43.2569465	0	0	positive
PRJNA729271	stage_3W_vs_E10	ordinary_muscle_development	ENSGALG00010010327	FBP1	0.346425411	0.57713345	0.600251833	0.548338414	0.585586481	positive
PRJNA729271	stage_3W_vs_E10	ordinary_muscle_development	ENSGALG00010010330	ALPK2	1.205454883	0.153881279	7.833668196	4.73838E-15	1.43869E-14	positive
PRJNA729271	stage_3W_vs_E10	ordinary_muscle_development	ENSGALG00010010333	WAPL	0.411473274	0.063032597	6.527944134	6.66786E-11	1.62547E-10	positive
PRJNA729271	stage_3W_vs_E10	ordinary_muscle_development	ENSGALG00010010334	ZBTB41	0.40674628	0.086676562	4.692690493	2.69635E-06	4.96552E-06	positive
PRJNA729271	stage_3W_vs_E10	ordinary_muscle_development	ENSGALG00010010336	FMNL2	-1.149801811	0.089371403	-12.86543319	7.04459E-38	5.27434E-37	negative
PRJNA729271	stage_3W_vs_E10	ordinary_muscle_development	ENSGALG00010010337	SCARA3	-1.037391664	0.15615835	-6.643203281	3.06938E-11	7.64839E-11	negative
PRJNA729271	stage_3W_vs_E10	ordinary_muscle_development	ENSGALG00010010338	CNST	1.689206733	0.155029187	10.89605617	1.20362E-27	6.32347E-27	positive
PRJNA729271	stage_3W_vs_E10	ordinary_muscle_development	ENSGALG00010010345	ENSGALG00010010345	-0.418771837	0.139192263	-3.008585589	0.002624669	0.003781767	negative
PRJNA729271	stage_3W_vs_E10	ordinary_muscle_development	ENSGALG00010010346	TFB2M	0.70340764	0.139113706	5.056350376	4.27356E-07	8.29476E-07	positive
PRJNA729271	stage_3W_vs_E10	ordinary_muscle_development	ENSGALG00010010347	FBXW11	0.153280034	0.083835051	1.828352595	0.067496651	0.083350141	positive
PRJNA729271	stage_3W_vs_E10	ordinary_muscle_development	ENSGALG00010010349	RREB1	0.43403621	0.082398383	5.267533106	1.38269E-07	2.76978E-07	positive
PRJNA729271	stage_3W_vs_E10	ordinary_muscle_development	ENSGALG00010010351	NAMPTP1	4.089664794	0.212504588	19.24506586	1.55266E-82	4.1371E-81	positive
PRJNA729271	stage_3W_vs_E10	ordinary_muscle_development	ENSGALG00010010352	MALT1	0.086940315	0.115161324	0.754943688	0.450282771	0.489085708	positive
PRJNA729271	stage_3W_vs_E10	ordinary_muscle_development	ENSGALG00010010356	STAB2	1.08791651	0.279204341	3.896488517	9.75974E-05	0.000159436	positive
PRJNA729271	stage_3W_vs_E10	ordinary_muscle_development	ENSGALG00010010358	ASPM	-3.082152934	0.182627221	-16.87674445	6.67244E-64	1.10914E-62	negative
PRJNA729271	stage_3W_vs_E10	ordinary_muscle_development	ENSGALG00010010361	PIGM	-0.789992444	0.165146529	-4.783584905	1.72196E-06	3.20779E-06	negative
PRJNA729271	stage_3W_vs_E10	ordinary_muscle_development	ENSGALG00010010366	CLU	3.394591547	0.23508873	14.43961836	2.91451E-47	3.00469E-46	positive
PRJNA729271	stage_3W_vs_E10	ordinary_muscle_development	ENSGALG00010010368	ENSGALG00010010368	-0.295468493	0.080148424	-3.686516551	0.000227345	0.000360109	negative
PRJNA729271	stage_3W_vs_E10	ordinary_muscle_development	ENSGALG00010010369	ENSGALG00010010369	-0.055780263	0.243838851	-0.228758718	0.819056454	0.840213963	negative
PRJNA729271	stage_3W_vs_E10	ordinary_muscle_development	ENSGALG00010010371	SOCS1L	-2.479895563	0.214690542	-11.55102384	7.29462E-31	4.33165E-30	negative
PRJNA729271	stage_3W_vs_E10	ordinary_muscle_development	ENSGALG00010010374	CMTM4	-0.281469258	0.112205219	-2.508521983	0.012123742	0.016336678	negative
PRJNA729271	stage_3W_vs_E10	ordinary_muscle_development	ENSGALG00010010379	SLC35F6	1.228737464	0.186882163	6.574931732	4.86755E-11	1.19666E-10	positive
PRJNA729271	stage_3W_vs_E10	ordinary_muscle_development	ENSGALG00010010381	MAST4	1.332641683	0.131534151	10.1315261	4.00352E-24	1.80669E-23	positive
PRJNA729271	stage_3W_vs_E10	ordinary_muscle_development	ENSGALG00010010382	SEC11C	0.893010089	0.150674224	5.926760819	3.08968E-09	6.83219E-09	positive
PRJNA729271	stage_3W_vs_E10	ordinary_muscle_development	ENSGALG00010010383	SLIT2	-1.14656893	0.160868099	-7.127385324	1.02293E-12	2.76013E-12	negative
PRJNA729271	stage_3W_vs_E10	ordinary_muscle_development	ENSGALG00010010386	PRPF40A	0.754725494	0.146193067	5.162525891	2.4364E-07	4.80157E-07	positive
PRJNA729271	stage_3W_vs_E10	ordinary_muscle_development	ENSGALG00010010390	KCNK3	0.194679838	0.442425632	0.440028389	0.659916546	0.692398866	positive
PRJNA729271	stage_3W_vs_E10	ordinary_muscle_development	ENSGALG00010010393	FANCC	-2.56615327	0.149707372	-17.14112831	7.3216E-66	1.27863E-64	negative
PRJNA729271	stage_3W_vs_E10	ordinary_muscle_development	ENSGALG00010010394	MRPL42	0.39677336	0.123310984	3.217664374	0.001292389	0.001916831	positive
PRJNA729271	stage_3W_vs_E10	ordinary_muscle_development	ENSGALG00010010395	GRP	-0.80968939	0.888762304	-0.911030301	0.362279408	0.401133605	negative
PRJNA729271	stage_3W_vs_E10	ordinary_muscle_development	ENSGALG00010010397	PICK1	-0.514244119	0.092556237	-5.556017982	2.75998E-08	5.78874E-08	negative
PRJNA729271	stage_3W_vs_E10	ordinary_muscle_development	ENSGALG00010010399	FGF18	0.466966772	0.18092378	2.581013791	0.009851065	0.013396302	positive
PRJNA729271	stage_3W_vs_E10	ordinary_muscle_development	ENSGALG00010010403	ENSGALG00010010403	-0.891312614	0.446921913	-1.994336343	0.04611531	0.058180992	negative
PRJNA729271	stage_3W_vs_E10	ordinary_muscle_development	ENSGALG00010010404	COP1	0.102393255	0.116401287	0.87965742	0.379044923	0.417909497	positive
PRJNA729271	stage_3W_vs_E10	ordinary_muscle_development	ENSGALG00010010405	EVA1C	2.93781679	0.227432701	12.9173016	3.5953E-38	2.72562E-37	positive
PRJNA729271	stage_3W_vs_E10	ordinary_muscle_development	ENSGALG00010010406	PTCH1	-1.221935624	0.094900178	-12.8760098	6.14303E-38	4.60887E-37	negative
PRJNA729271	stage_3W_vs_E10	ordinary_muscle_development	ENSGALG00010010407	SOX10	-2.295290761	0.310436523	-7.393752326	1.42742E-13	4.01835E-13	negative
PRJNA729271	stage_3W_vs_E10	ordinary_muscle_development	ENSGALG00010010408	GAS8	-0.42101145	0.117507988	-3.582832607	0.000339888	0.00053158	negative
PRJNA729271	stage_3W_vs_E10	ordinary_muscle_development	ENSGALG00010010410	CACNA2D1	2.369953615	0.158085371	14.99160614	8.33138E-51	9.69987E-50	positive
PRJNA729271	stage_3W_vs_E10	ordinary_muscle_development	ENSGALG00010010411	CD180	2.339720721	0.42020898	5.567993144	2.5769E-08	5.41414E-08	positive
PRJNA729271	stage_3W_vs_E10	ordinary_muscle_development	ENSGALG00010010416	SLC35B3	-0.415644533	0.127160794	-3.268653168	0.001080607	0.001614783	negative
PRJNA729271	stage_3W_vs_E10	ordinary_muscle_development	ENSGALG00010010417	OTOF	-0.160651749	0.674599831	-0.238143773	0.811769585	0.833682877	negative
PRJNA729271	stage_3W_vs_E10	ordinary_muscle_development	ENSGALG00010010418	BRINP2	-5.902541414	0.615673508	-9.587129113	9.057E-22	3.72429E-21	negative
PRJNA729271	stage_3W_vs_E10	ordinary_muscle_development	ENSGALG00010010420	NPM1	-1.202563897	0.112362873	-10.70250225	9.90663E-27	5.00685E-26	negative
PRJNA729271	stage_3W_vs_E10	ordinary_muscle_development	ENSGALG00010010421	POLR2F	0.821873921	0.090226293	9.109028981	8.31226E-20	3.15491E-19	positive
PRJNA729271	stage_3W_vs_E10	ordinary_muscle_development	ENSGALG00010010422	CDH1	-3.872841389	0.910325636	-4.254347277	2.0966E-05	3.61487E-05	negative
PRJNA729271	stage_3W_vs_E10	ordinary_muscle_development	ENSGALG00010010423	C21orf59	0.430964553	0.09652157	4.464955916	8.00852E-06	1.42451E-05	positive
PRJNA729271	stage_3W_vs_E10	ordinary_muscle_development	ENSGALG00010010425	BLOC1S5	0.733248708	0.078477379	9.343440273	9.32537E-21	3.68013E-20	positive
PRJNA729271	stage_3W_vs_E10	ordinary_muscle_development	ENSGALG00010010430	ASTN1	-5.783984637	0.698051717	-8.285897018	1.17222E-16	3.86431E-16	negative
PRJNA729271	stage_3W_vs_E10	ordinary_muscle_development	ENSGALG00010010431	SOCS2	-0.303685317	0.244336342	-1.242898683	0.213905171	0.246643501	negative
PRJNA729271	stage_3W_vs_E10	ordinary_muscle_development	ENSGALG00010010433	ENSGALG00010010433	-0.977416068	1.616439262	-0.604672313	0.545396748	0.58274553	negative
PRJNA729271	stage_3W_vs_E10	ordinary_muscle_development	ENSGALG00010010434	PROM1	-2.295404559	0.151481518	-15.1530338	7.23483E-52	8.68839E-51	negative
PRJNA729271	stage_3W_vs_E10	ordinary_muscle_development	ENSGALG00010010435	GAS1	-1.074258338	0.165873039	-6.476389065	9.39436E-11	2.27104E-10	negative
PRJNA729271	stage_3W_vs_E10	ordinary_muscle_development	ENSGALG00010010438	LDB3	3.719451153	0.185206044	20.08277413	1.04399E-89	3.40824E-88	positive
PRJNA729271	stage_3W_vs_E10	ordinary_muscle_development	ENSGALG00010010439	HGF	1.174912553	0.239293562	4.909921283	9.1113E-07	1.73113E-06	positive
PRJNA729271	stage_3W_vs_E10	ordinary_muscle_development	ENSGALG00010010444	TXNDC5	-2.012464371	0.140235133	-14.35064326	1.05554E-46	1.0662E-45	negative
PRJNA729271	stage_3W_vs_E10	ordinary_muscle_development	ENSGALG00010010445	LMAN1L	-0.884136676	0.083342563	-10.60846516	2.72183E-26	1.3456E-25	negative
PRJNA729271	stage_3W_vs_E10	ordinary_muscle_development	ENSGALG00010010446	C22orf23	-0.260961601	0.604548546	-0.431663599	0.665985928	0.69775685	negative
PRJNA729271	stage_3W_vs_E10	ordinary_muscle_development	ENSGALG00010010447	BTF3	0.01883236	0.096537519	0.195078138	0.845331777	0.863258004	positive
PRJNA729271	stage_3W_vs_E10	ordinary_muscle_development	ENSGALG00010010448	GALNT13	-1.552545773	0.378105719	-4.106115556	4.02368E-05	6.78414E-05	negative
PRJNA729271	stage_3W_vs_E10	ordinary_muscle_development	ENSGALG00010010449	PAPPA2	-3.909062955	0.778975207	-5.018212287	5.21545E-07	1.00516E-06	negative
PRJNA729271	stage_3W_vs_E10	ordinary_muscle_development	ENSGALG00010010451	RANBP17	-2.133675016	0.513526676	-4.154944847	3.25366E-05	5.51729E-05	negative
PRJNA729271	stage_3W_vs_E10	ordinary_muscle_development	ENSGALG00010010453	DRC1	-0.698960008	0.412175973	-1.695780574	0.089927443	0.109436511	negative
PRJNA729271	stage_3W_vs_E10	ordinary_muscle_development	ENSGALG00010010456	BMP6	0.144487168	0.17517827	0.824800749	0.409484722	0.448306616	positive
PRJNA729271	stage_3W_vs_E10	ordinary_muscle_development	ENSGALG00010010457	AGGF1	-0.32285428	0.090679113	-3.560404033	0.000370285	0.000577002	negative
PRJNA729271	stage_3W_vs_E10	ordinary_muscle_development	ENSGALG00010010460	SEMA3C	0.508175769	0.142109116	3.575954751	0.000348952	0.000544991	positive
PRJNA729271	stage_3W_vs_E10	ordinary_muscle_development	ENSGALG00010010461	SLC7A5	1.743325863	0.261291593	6.671955434	2.52417E-11	6.31697E-11	positive
PRJNA729271	stage_3W_vs_E10	ordinary_muscle_development	ENSGALG00010010463	ACAA2	-0.071643286	0.12865458	-0.556865418	0.577619384	0.613691488	negative
PRJNA729271	stage_3W_vs_E10	ordinary_muscle_development	ENSGALG00010010465	BMPR1A	-0.749190809	0.097920529	-7.651008585	1.99409E-14	5.86414E-14	negative
PRJNA729271	stage_3W_vs_E10	ordinary_muscle_development	ENSGALG00010010466	CRADD	1.272637946	0.12285488	10.35887177	3.81435E-25	1.79743E-24	positive
PRJNA729271	stage_3W_vs_E10	ordinary_muscle_development	ENSGALG00010010467	FGFBP2	-8.356980268	0.547318936	-15.26894049	1.23158E-52	1.51671E-51	negative
PRJNA729271	stage_3W_vs_E10	ordinary_muscle_development	ENSGALG00010010468	ENSGALG00010010468	3.323222628	1.339235603	2.481432409	0.013085553	0.017556006	positive
PRJNA729271	stage_3W_vs_E10	ordinary_muscle_development	ENSGALG00010010471	KCNJ3	-1.692688892	0.1727887	-9.796293923	1.16792E-22	4.96453E-22	negative
PRJNA729271	stage_3W_vs_E10	ordinary_muscle_development	ENSGALG00010010472	MMRN2	1.587838698	0.136411053	11.64010293	2.57704E-31	1.55061E-30	positive
PRJNA729271	stage_3W_vs_E10	ordinary_muscle_development	ENSGALG00010010473	TRIM35	0.84510116	0.177124819	4.77121819	1.83115E-06	3.40594E-06	positive
PRJNA729271	stage_3W_vs_E10	ordinary_muscle_development	ENSGALG00010010475	CCDC71L	0.587681364	0.107900072	5.446533573	5.13609E-08	1.0587E-07	positive

PRJNA729271	stage_3W_vs_E10	ordinary_muscle_development	ENSGALG00010010478	ENSGALG00010010478	0.828398235	0.209191498	3.95999953	7.49499E-05	0.000123679	positive
PRJNA729271	stage_3W_vs_E10	ordinary_muscle_development	ENSGALG00010010483	FGFBP1	-4.92907048	1.091126541	-4.517414155	6.25994E-06	1.12383E-05	negative
PRJNA729271	stage_3W_vs_E10	ordinary_muscle_development	ENSGALG00010010486	ENSGALG00010010486	-3.186016886	0.48047948	-6.630911451	3.3362E-11	8.29615E-11	negative
PRJNA729271	stage_3W_vs_E10	ordinary_muscle_development	ENSGALG00010010494	ENSGALG00010010494	0.552453778	0.264053987	2.092200097	0.03642062	0.046520698	positive
PRJNA729271	stage_3W_vs_E10	ordinary_muscle_development	ENSGALG00010010496	MRPL17	0.564194782	0.142291819	3.965054269	7.33793E-05	0.000121225	positive
PRJNA729271	stage_3W_vs_E10	ordinary_muscle_development	ENSGALG00010010499	NR4A2	2.934281011	0.455295687	6.444781036	1.15767E-10	2.78283E-10	positive
PRJNA729271	stage_3W_vs_E10	ordinary_muscle_development	ENSGALG00010010505	CD38	2.947016161	0.193075059	15.26357765	1.33712E-52	1.64529E-51	positive
PRJNA729271	stage_3W_vs_E10	ordinary_muscle_development	ENSGALG00010010511	GABRP	-3.671526225	0.605561645	-6.063009863	1.33597E-09	3.022E-09	negative
PRJNA729271	stage_3W_vs_E10	ordinary_muscle_development	ENSGALG00010010512	PLXNC1	0.377460218	0.198797425	1.898717844	0.057601584	0.071754466	positive
PRJNA729271	stage_3W_vs_E10	ordinary_muscle_development	ENSGALG00010010515	RXRG	-1.111347733	0.383441955	-2.898346718	0.003751356	0.005328882	negative
PRJNA729271	stage_3W_vs_E10	ordinary_muscle_development	ENSGALG00010010517	STMN4	-7.030344815	0.49646414	-14.16083106	1.6009E-45	1.55843E-44	negative
PRJNA729271	stage_3W_vs_E10	ordinary_muscle_development	ENSGALG00010010521	SNCG	-0.447122201	0.390686727	-1.144451986	0.252436239	0.287707445	negative
PRJNA729271	stage_3W_vs_E10	ordinary_muscle_development	ENSGALG00010010526	ENSGALG00010010526	0.391724381	0.254509171	1.539136603	0.123770951	0.147720044	positive
PRJNA729271	stage_3W_vs_E10	ordinary_muscle_development	ENSGALG00010010528	GPD2	2.030240064	0.12456144	16.29910556	1.00147E-59	1.4858E-58	positive
PRJNA729271	stage_3W_vs_E10	ordinary_muscle_development	ENSGALG00010010534	SYNJ1	0.697065115	0.12042858	5.788203417	7.11432E-09	1.54931E-08	positive
PRJNA729271	stage_3W_vs_E10	ordinary_muscle_development	ENSGALG00010010535	LMX1A	-0.982685055	0.419160777	-2.344410804	0.019057168	0.025116717	negative
PRJNA729271	stage_3W_vs_E10	ordinary_muscle_development	ENSGALG00010010537	ENSGALG00010010537	0.100477697	0.217141456	0.462729221	0.64355847	0.676705116	positive
PRJNA729271	stage_3W_vs_E10	ordinary_muscle_development	ENSGALG00010010544	ENSGALG00010010544	-1.151674894	0.17301717	-6.656419688	2.80578E-11	7.01083E-11	negative
PRJNA729271	stage_3W_vs_E10	ordinary_muscle_development	ENSGALG00010010546	MICALL1	-0.67940958	0.169026366	-4.019547929	5.83099E-05	9.7172E-05	negative
PRJNA729271	stage_3W_vs_E10	ordinary_muscle_development	ENSGALG00010010547	BST1	1.258910843	0.164358631	7.659535936	1.86606E-14	5.49544E-14	positive
PRJNA729271	stage_3W_vs_E10	ordinary_muscle_development	ENSGALG00010010567	ERMN	-0.223958563	0.491729656	-0.455450592	0.648785105	0.681819623	negative
PRJNA729271	stage_3W_vs_E10	ordinary_muscle_development	ENSGALG00010010572	AQP3	3.785988759	0.146059904	25.92079449	3.8825E-148	3.5618E-146	positive
PRJNA729271	stage_3W_vs_E10	ordinary_muscle_development	ENSGALG00010010573	CEP83	-0.770896112	0.157543747	-4.893219362	9.91997E-07	1.87985E-06	negative
PRJNA729271	stage_3W_vs_E10	ordinary_muscle_development	ENSGALG00010010575	PAXBP1	0.510240874	0.122750803	4.156721283	3.22847E-05	5.47651E-05	positive
PRJNA729271	stage_3W_vs_E10	ordinary_muscle_development	ENSGALG00010010577	PBX1	-0.630413943	0.145861472	-4.32200453	1.54618E-05	2.69244E-05	negative
PRJNA729271	stage_3W_vs_E10	ordinary_muscle_development	ENSGALG00010010578	GLUD2	0.752889999	0.111309723	6.763919466	1.34308E-11	3.40825E-11	positive
PRJNA729271	stage_3W_vs_E10	ordinary_muscle_development	ENSGALG00010010585	FBXL5	1.227367773	0.163231372	7.519190456	5.51164E-14	1.58703E-13	positive
PRJNA729271	stage_3W_vs_E10	ordinary_muscle_development	ENSGALG00010010586	CYTIP	1.173129284	0.245157002	4.785216311	1.70803E-06	3.18307E-06	positive
PRJNA729271	stage_3W_vs_E10	ordinary_muscle_development	ENSGALG00010010588	SLC25A3	0.703011472	0.101001007	6.960440241	3.39211E-12	8.87521E-12	positive
PRJNA729271	stage_3W_vs_E10	ordinary_muscle_development	ENSGALG00010010593	EIF3L	0.425878272	0.103757473	4.104555168	4.05094E-05	6.82851E-05	positive
PRJNA729271	stage_3W_vs_E10	ordinary_muscle_development	ENSGALG00010010596	SHLD2	0.680750189	0.132961594	5.119900925	3.05696E-07	5.98874E-07	positive
PRJNA729271	stage_3W_vs_E10	ordinary_muscle_development	ENSGALG00010010599	F2RL1	-1.617288705	0.202319769	-7.993725528	1.30921E-15	4.07232E-15	negative
PRJNA729271	stage_3W_vs_E10	ordinary_muscle_development	ENSGALG00010010604	HIVEP1	0.215942301	0.10000768	2.159257185	0.03083022	0.039691272	positive
PRJNA729271	stage_3W_vs_E10	ordinary_muscle_development	ENSGALG00010010605	NUF2	-3.517422041	0.271984889	-12.93241714	2.95384E-38	2.24755E-37	negative
PRJNA729271	stage_3W_vs_E10	ordinary_muscle_development	ENSGALG00010010614	IKBIP	-0.258239429	0.14231504	-1.814561761	0.069591283	0.085783285	negative
PRJNA729271	stage_3W_vs_E10	ordinary_muscle_development	ENSGALG00010010618	APAF1	-0.077992155	0.135921223	-0.573804097	0.566100376	0.602645781	negative
PRJNA729271	stage_3W_vs_E10	ordinary_muscle_development	ENSGALG00010010619	LRRC52	-1.513295138	0.287716892	-5.259667327	1.44316E-07	2.88573E-07	negative
PRJNA729271	stage_3W_vs_E10	ordinary_muscle_development	ENSGALG00010010620	ANKRD54	0.527559619	0.110490817	4.774691984	1.79983E-06	3.34983E-06	positive
PRJNA729271	stage_3W_vs_E10	ordinary_muscle_development	ENSGALG00010010623	SMAD2	-0.337763912	0.077202606	-4.3750325	1.21414E-05	2.13166E-05	negative
PRJNA729271	stage_3W_vs_E10	ordinary_muscle_development	ENSGALG00010010625	FAM169A	-7.336310995	0.520653034	-14.09059491	4.33891E-45	4.16507E-44	negative
PRJNA729271	stage_3W_vs_E10	ordinary_muscle_development	ENSGALG00010010626	ADTRP	2.008333309	0.57442913	3.496224695	0.000471891	0.000728596	positive
PRJNA729271	stage_3W_vs_E10	ordinary_muscle_development	ENSGALG00010010629	ENSGALG00010010629	-1.11639795	0.349063801	-3.198263316	0.00138258	0.002045574	negative
PRJNA729271	stage_3W_vs_E10	ordinary_muscle_development	ENSGALG00010010633	EPCIP	1.106651731	0.327695237	3.377076031	0.000732608	0.001110906	positive
PRJNA729271	stage_3W_vs_E10	ordinary_muscle_development	ENSGALG00010010635	ALDH9A1	-0.455469954	0.076665939	-5.940968797	2.83343E-09	6.28374E-09	negative
PRJNA729271	stage_3W_vs_E10	ordinary_muscle_development	ENSGALG00010010638	ACVR1C	1.714076627	0.432879998	3.959703926	7.50427E-05	0.000123818	positive
PRJNA729271	stage_3W_vs_E10	ordinary_muscle_development	ENSGALG00010010642	CC2D2A	-0.857659294	0.120388004	-7.12412588	1.04743E-12	2.82332E-12	negative
PRJNA729271	stage_3W_vs_E10	ordinary_muscle_development	ENSGALG00010010645	FAM219A	-0.076818839	0.086409202	-0.889012252	0.373996495	0.41277193	negative
PRJNA729271	stage_3W_vs_E10	ordinary_muscle_development	ENSGALG00010010646	MCM3AP	-0.468208658	0.05809903	-8.058803391	7.70449E-16	2.43145E-15	negative
PRJNA729271	stage_3W_vs_E10	ordinary_muscle_development	ENSGALG00010010648	CENPM	-3.191533215	0.286518533	-11.13901144	8.10136E-29	4.44808E-28	negative
PRJNA729271	stage_3W_vs_E10	ordinary_muscle_development	ENSGALG00010010651	NUAK1	0.874333218	0.133599654	6.544427272	5.97238E-11	1.45937E-10	positive
PRJNA729271	stage_3W_vs_E10	ordinary_muscle_development	ENSGALG00010010655	C1QTNF7	0.995635183	0.248559342	4.005623666	6.18541E-05	0.000102854	positive
PRJNA729271	stage_3W_vs_E10	ordinary_muscle_development	ENSGALG00010010657	TMEM170B	-1.446499585	0.180092461	-8.031982983	9.59097E-16	3.00846E-15	negative
PRJNA729271	stage_3W_vs_E10	ordinary_muscle_development	ENSGALG00010010659	ADAMTS1	-0.284422256	0.152336662	-1.867063723	0.06189269	0.076829197	negative
PRJNA729271	stage_3W_vs_E10	ordinary_muscle_development	ENSGALG00010010661	CPEB2	-0.011787338	0.183128277	-0.064366564	0.948678353	0.955401426	negative
PRJNA729271	stage_3W_vs_E10	ordinary_muscle_development	ENSGALG00010010662	MGST3	-0.909971126	0.152890671	-5.951776648	2.65247E-09	5.89235E-09	negative
PRJNA729271	stage_3W_vs_E10	ordinary_muscle_development	ENSGALG00010010663	EFR3B	-1.249244485	0.172375241	-7.247238525	4.25355E-13	1.16815E-12	negative
PRJNA729271	stage_3W_vs_E10	ordinary_muscle_development	ENSGALG00010010666	C12orf75	-0.265192087	0.379677714	-0.698466296	0.484885628	0.523220218	negative
PRJNA729271	stage_3W_vs_E10	ordinary_muscle_development	ENSGALG00010010671	SIGMAR1	-0.67012388	0.141445141	-4.737694601	2.16163E-06	4.00522E-06	negative
PRJNA729271	stage_3W_vs_E10	ordinary_muscle_development	ENSGALG00010010678	KCNIP1	-3.249404933	0.421045022	-7.717476194	1.18656E-14	3.52441E-14	negative
PRJNA729271	stage_3W_vs_E10	ordinary_muscle_development	ENSGALG00010010680	ENSGALG00010010680	-0.974858926	0.624417718	-1.561228801	0.118469776	0.141778352	negative
PRJNA729271	stage_3W_vs_E10	ordinary_muscle_development	ENSGALG00010010682	SMIM13	-0.714292922	0.105657561	-6.760452508	1.37561E-11	3.48898E-11	negative
PRJNA729271	stage_3W_vs_E10	ordinary_muscle_development	ENSGALG00010010685	STIM2	0.355935974	0.107825874	3.301025638	0.000963321	0.001444732	positive
PRJNA729271	stage_3W_vs_E10	ordinary_muscle_development	ENSGALG00010010688	IQGAP2	-0.463274926	0.169355637	-2.735515244	0.00622827	0.00864408	negative
PRJNA729271	stage_3W_vs_E10	ordinary_muscle_development	ENSGALG00010010689	POFUT2	-0.649164586	0.150095383	-4.325013698	1.52522E-05	2.65818E-05	negative
PRJNA729271	stage_3W_vs_E10	ordinary_muscle_development	ENSGALG00010010690	MTPN	-0.4894699	0.135270687	-3.618447657	0.000296375	0.000465636	negative
PRJNA729271	stage_3W_vs_E10	ordinary_muscle_development	ENSGALG00010010693	KCNMB1	1.108732851	0.330654508	3.353146033	0.000798985	0.001207265	positive
PRJNA729271	stage_3W_vs_E10	ordinary_muscle_development	ENSGALG00010010697	NEDD9	-0.592464376	0.139117256	-4.258741099	2.05581E-05	3.5475E-05	negative
PRJNA729271	stage_3W_vs_E10	ordinary_muscle_development	ENSGALG00010010698	GPRIN2	-2.322584889	0.20234989	-11.47806352	1.70046E-30	9.95486E-30	negative
PRJNA729271	stage_3W_vs_E10	ordinary_muscle_development	ENSGALG00010010699	DNAJC27	-0.783547892	0.15353244	-5.103467992	3.33485E-07	6.51552E-07	negative
PRJNA729271	stage_3W_vs_E10	ordinary_muscle_development	ENSGALG00010010706	SREBF2	0.583491038	0.13849505	4.213082263	2.51909E-05	4.31101E-05	positive
PRJNA729271	stage_3W_vs_E10	ordinary_muscle_development	ENSGALG00010010710	UCK2	-0.205698616	0.099802623	-2.061054214	0.039297868	0.050001984	negative
PRJNA729271	stage_3W_vs_E10	ordinary_muscle_development	ENSGALG00010010711	ENSGALG00010010711	-1.172222172	0.428238212	-2.737313342	0.006194326	0.008601088	negative
PRJNA729271	stage_3W_vs_E10	ordinary_muscle_development	ENSGALG00010010712	ADAMTS5	0.496741893	0.145268522	3.419473723	0.000627424	0.000957619	positive
PRJNA729271	stage_3W_vs_E10	ordinary_muscle_development	ENSGALG00010010713	NSA2	0.676543527	0.090545724	7.471844059	7.90787E-14	2.25861E-13	positive
PRJNA729271	stage_3W_vs_E10	ordinary_muscle_development	ENSGALG00010010719	SH3BP4	-0.465143496	0.165627364	-2.808373473	0.004979244	0.006982793	negative
PRJNA729271	stage_3W_vs_E10	ordinary_muscle_development	ENSGALG00010010722	TBC1D19	-1.324078338	0.169628166	-7.805769342	5.91397E-15	1.78552E-14	negative
PRJNA729271	stage_3W_vs_E10	ordinary_muscle_development	ENSGALG00010010724	ADCY3	-0.682023275	0.15422615	-4.422228488	9.76881E-06	1.72513E-05	negative
PRJNA729271	stage_3W_vs_E10	ordinary_muscle_development	ENSGALG00010010728	ELOVL2	-0.338550242	0.95201702	-0.355613645	0.722129924	0.750503567	negative
PRJNA729271	stage_3W_vs_E10	ordinary_muscle_development	ENSGALG00010010732	CCKAR	-5.856002043	1.165005816	-5.026586101	4.99288E-07	9.6393E-07	negative
PRJNA729271	stage_3W_vs_E10	ordinary_muscle_development	ENSGALG00010010733	GFM2	-0.183063271	0.126551893	-1.446547077	0.148023818	0.174723554	negative
PRJNA729271	stage_3W_vs_E10	ordinary_muscle_development	ENSGALG00010010734	AMN1	2.231189175	0.140509973	15.87922291	8.82651E-57	1.21501E-55	positive
PRJNA729271	stage_3W_vs_E10	ordinary_muscle_development	ENSGALG00010010740	CCDC134	1.000027927	0.095428317	10.47936255	1.07466E-25	5.17484E-25	positive
PRJNA729271	stage_3W_vs_E10	ordinary_muscle_development	ENSGALG00010010742	LCP2	2.975189594	0.258448628	11.51172525	1.15151E-30	6.78504E-30	positive
PRJNA729271	stage_3W_vs_E10	ordinary_muscle_development	ENSGALG00010010743	KLHL20	-0.22870353	0.084450047	-2.708151607	0.006765911	0.009360683	negative
PRJNA729271	stage_3W_vs_E10	ordinary_muscle_development	ENSGALG00010010745	ATVR1	-0.258097032	0.063649813	-4.054953478	5.01443E-05	8.39794E-05	negative
PRJNA729271	stage_3W_vs_E10	ordinary_muscle_development	ENSGALG00010010748	SF3B1	0.083263916	0.071733026	1.160747293	0.245744683	0.280698911	positive
PRJNA729271	stage_3W_vs_E10	ordinary_muscle_development	ENSGALG00010010749	NCOA4	-0.066506732	0.098269369	-0.676779885	0.498545622	0.536761405	negative
PRJNA729271	stage_3W_vs_E10	ordinary_muscle_development	ENSGALG00010010751	SLC30A5	-0.070731839	0.078400843	-0.90218212	0.366960131	0.405603714	negative
PRJNA729271	stage_3W_vs_E10	ordinary_muscle_development	ENSGALG00010010752	ENSGALG00010010752	-0.640158903	0.257753823	-2.483605852	0.013005967	0.017460544	negative
PRJNA729271	stage_3W_vs_E10	ordinary_muscle_development	ENSGALG00010010753	ENSGALG00010010753	-2.378999336	1.164811279	-2.042390367			negative
PRJNA729271	stage_3W_vs_E10	ordinary_muscle_development	ENSGALG00010010754	DNAI1	-2.68344149	0.300565095	-8.927987769	4.33828E-19	1.60212E-18	negative
PRJNA729271	stage_3W_vs_E10	ordinary_muscle_development	ENSGALG00010010756	CENPL	-3.821042211	0.221445232	-17.2550214	1.02584E-66	1.82224E-65	negative
PRJNA729271	stage_3W_vs_E10	ordinary_muscle_development	ENSGALG00010010759	ENSGALG00010010759	0.061800328	0.270817883	0.228198845	0.819491659	0.84060092	positive
PRJNA729271	stage_3W_vs_E10	ordinary_muscle_development	ENSGALG00010010760	FOXI1	3.645440554	0.63734781	5.719703582	1.0671E-08	2.30036E-08	positive
PRJNA729271	stage_3W_vs_E10	ordinary_muscle_development	ENSGALG00010010761	MEI1	0.797144358	0.259784313	3.068485346	0.002151469	0.003124178	positive
PRJNA729271	stage_3W_vs_E10	ordinary_muscle_development	ENSGALG00010010762	MRPL39	0.053806388	0.110956101	0.484934019	0.62772315	0.661445701	positive
PRJNA729271	stage_3W_vs_E10	ordinary_muscle_development	ENSGALG00010010765	ENSGALG00010010765	3.145691686	0.797843942	3.94274058	8.05558E-05	0.000132447	positive
PRJNA729271	stage_3W_vs_E10	ordinary_muscle_development	ENSGALG00010010766	CENPO	-3.752907629	0.274381683	-13.6776901	1.38007E-42	1.21311E-41	negative
PRJNA729271	stage_3W_vs_E10	ordinary_muscle_development	ENSGALG00010010768	ENSGALG00010010768	0.863540601	0.0913587	9.452198868	3.31786E-21	1.33479E-20	positive
PRJNA729271	stage_3W_vs_E10	ordinary_muscle_development	ENSGALG00010010769	HEXB	0.26773136	0.119645972	2.237696393	0.025240861	0.03282195	positive
PRJNA729271	stage_3W_vs_E10	ordinary_muscle_development	ENSGALG00010010771	ENSGALG00010010771	-0.376039673	0.071861905	-5.232809682	1.66953E-07	3.32552E-07	negative
PRJNA729271	stage_3W_vs_E10	ordinary_muscle_development	ENSGALG00010010774	KPTN	-0.536260403	0.137482356	-3.900576176	9.5964E-05	0.000156821	negative
PRJNA729271	stage_3W_vs_E10	ordinary_muscle_development	ENSGALG00010010776	GCAT	0.830207497	0.19391834	4.281222184	1.8587E-05	3.21847E-05	positive
PRJNA729271	stage_3W_vs_E10	ordinary_muscle_development	ENSGALG00010010778	UPP2	3.139115654	0.364760605	8.605961321	7.56798E-18	2.64993E-17	positive
PRJNA729271	stage_3W_vs_E10	ordinary_muscle_development	ENSGALG00010010779	DOCK2	1.94072895	0.185319165	10.47235966	1.15722E-25	5.56717E-25	positive
PRJNA729271	stage_3W_vs_E10	ordinary_muscle_development	ENSGALG00010010780	PARG	-0.991095385	0.049988291	-19.82655062	1.75682E-87	5.40661E-86	negative
PRJNA729271	stage_3W_vs_E10	ordinary_muscle_development	ENSGALG00010010781	NAPA	0.637863195	0.097335115	6.55326906	5.6291E-11	1.37781E-10	positive
PRJNA729271	stage_3W_vs_E10	ordinary_muscle_development	ENSGALG00010010783	F2RL2	-0.553668744	0.146857229	-3.770115698	0.000163172	0.000261837	negative
PRJNA729271	stage_3W_vs_E10	ordinary_muscle_development	ENSGALG00010010784	CCDC148	0.839461112	0.298953558	2.807998395	0.004985048	0.006990255	positive
PRJNA729271	stage_3W_vs_E10	ordinary_muscle_development	ENSGALG00010010786	NABP1	0.322527854	0.154194987	2.091688323	0.036466406	0.046575077	positive
PRJNA729271	stage_3W_vs_E10	ordinary_muscle_development	ENSGALG00010010789	ENSGALG00010010789	-1.006278736	0.289884584	-3.471308205	0.000517929	0.000796539	negative
PRJNA729271	stage_3W_vs_E10	ordinary_muscle_development	ENSGALG00010010791	H-10	0.019037002	0.294146492	0.064719459	0.948397369	0.955184816	positive
PRJNA729271	stage_3W_vs_E10	ordinary_muscle_development	ENSGALG00010010792	XRCC6	-1.186950853	0.149320326	-7.949023969	1.87987E-15	5.805E-15	negative
PRJNA729271	stage_3W_vs_E10	ordinary_muscle_development	ENSGALG00010010796	CAVIN2	2.356633844	0.145972322	16.14438822	1.24376E-58	1.8095E-57	positive
PRJNA729271	stage_3W_vs_E10	ordinary_muscle_development	ENSGALG00010010799	DARS2	-0.610401779	0.107690363	-5.668118851	1.44374E-08	3.08521E-08	negative
PRJNA729271	stage_3W_vs_E10	ordinary_muscle_development	ENSGALG00010010800	BICRA	0.519772853	0.190584145	2.727261777	0.006386235	0.008853145	positive
PRJNA729271	stage_3W_vs_E10	ordinary_muscle_development	ENSGALG00010010805	PTRHD1	-0.400809659	0.163458254	-2.452061306	0.014204046	0.018984569	negative
PRJNA729271	stage_3W_vs_E10	ordinary_muscle_development	ENSGALG00010010806	INSYN2B	2.392208984	0.74677346	3.203393144	0.001358184	0.002011534	positive
PRJNA729271	stage_3W_vs_E10	ordinary_muscle_development	ENSGALG00010010809	CENPH	-3.123823006	0.166922621	-18.71419819	3.79286E-78	9.04236E-77	negative
PRJNA729271	stage_3W_vs_E10	ordinary_muscle_development	ENSGALG00010010810	GALT	0.493130785	0.11183918	4.409284698	1.03713E-05	1.82929E-05	positive
PRJNA729271	stage_3W_vs_E10	ordinary_muscle_development	ENSGALG00010010813	EHD2	2.011877313	0.148592975	13.53951837	9.13861E-42	7.7966E-41	positive
PRJNA729271	stage_3W_vs_E10	ordinary_muscle_development	ENSGALG00010010816	AP2S1	-1.155436452	0.113316953	-10.19650121	2.05544E-24	9.39862E-24	negative
PRJNA729271	stage_3W_vs_E10	ordinary_muscle_development	ENSGALG00010010821	RGS8	-1.78621111	0.426487406	-4.188191929	2.81186E-05	4.79222E-05	negative
PRJNA729271	stage_3W_vs_E10	ordinary_muscle_development	ENSGALG00010010822	OGDHL	-1.226939271	0.368523991	-3.329333508	0.000870541	0.001311421	negative
PRJNA729271	stage_3W_vs_E10	ordinary_muscle_development	ENSGALG00010010823	TMEFF2	-2.512197527	0.185238813	-13.56193927	6.73321E-42	5.77844E-41	negative
PRJNA729271	stage_3W_vs_E10	ordinary_muscle_development	ENSGALG00010010824	PKP4	-0.251708254	0.109810893	-2.292197506	0.021894249	0.028686354	negative
PRJNA729271	stage_3W_vs_E10	ordinary_muscle_development	ENSGALG00010010825	MAK	4.029931046	0.365320645	11.03121627	2.70186E-28	1.45969E-27	positive
PRJNA729271	stage_3W_vs_E10	ordinary_muscle_development	ENSGALG00010010826	NCOA1	-0.180270835	0.162875757	-1.10679967	0.268380551	0.304872734	negative
PRJNA729271	stage_3W_vs_E10	ordinary_muscle_development	ENSGALG00010010828	SPDL1	-1.32539469	0.126460345	-10.48071382	1.05941E-25	5.10511E-25	negative
PRJNA729271	stage_3W_vs_E10	ordinary_muscle_development	ENSGALG00010010829	LAMB4	-4.363577926	0.289395264	-15.07826307	2.25113E-51	2.67242E-50	negative
PRJNA729271	stage_3W_vs_E10	ordinary_muscle_development	ENSGALG00010010831	DESI1	-0.331141324	0.093105783	-3.556613918	0.000375666	0.000584822	negative

PRJNA729271	stage_3W_vs_E10	ordinary_muscle_development	ENSGALG00010010834	KGD4	1.610875169	0.124348757	12.95449354	2.21594E-38	1.69409E-37	positive
PRJNA729271	stage_3W_vs_E10	ordinary_muscle_development	ENSGALG00010010836	DIP2A	-0.360089575	0.106005408	-3.396898157	0.000681543	0.001036509	negative
PRJNA729271	stage_3W_vs_E10	ordinary_muscle_development	ENSGALG00010010837	RBPJ	-0.387537294	0.123069148	-3.148939439	0.001638641	0.002410403	negative
PRJNA729271	stage_3W_vs_E10	ordinary_muscle_development	ENSGALG00010010838	JAM2	1.705627681	0.173221567	9.846508801	7.09663E-23	3.04697E-22	positive
PRJNA729271	stage_3W_vs_E10	ordinary_muscle_development	ENSGALG00010010840	RGS16	3.108600298	0.541008858	5.745932349	9.14159E-09	1.97684E-08	positive
PRJNA729271	stage_3W_vs_E10	ordinary_muscle_development	ENSGALG00010010841	PMM1	0.653062893	0.132933143	4.912716849	8.9823E-07	1.7073E-06	positive
PRJNA729271	stage_3W_vs_E10	ordinary_muscle_development	ENSGALG00010010843	ZNF395	-0.076441239	0.180611314	-0.423236159	0.672122967	0.703525593	negative
PRJNA729271	stage_3W_vs_E10	ordinary_muscle_development	ENSGALG00010010844	CDC14B	0.83968969	0.174473872	4.812695914	1.48908E-06	2.78577E-06	positive
PRJNA729271	stage_3W_vs_E10	ordinary_muscle_development	ENSGALG00010010845	FKRP	0.952584573	0.251014271	3.794941896	0.000147678	0.000237897	positive
PRJNA729271	stage_3W_vs_E10	ordinary_muscle_development	ENSGALG00010010848	RGR	0.990210258	0.572166277	1.73063373	0.083517112	0.101986566	positive
PRJNA729271	stage_3W_vs_E10	ordinary_muscle_development	ENSGALG00010010849	ACBD6	-0.378597601	0.109451746	-3.459036652	0.000542111	0.000832052	negative
PRJNA729271	stage_3W_vs_E10	ordinary_muscle_development	ENSGALG00010010850	ENSGALG00010010850	3.944593184	0.105670036	37.32934467	5.4859E-305	2.4096E-302	positive
PRJNA729271	stage_3W_vs_E10	ordinary_muscle_development	ENSGALG00010010851	PNOC	4.957872919	0.753973253	6.575661536	4.84373E-11	1.19121E-10	positive
PRJNA729271	stage_3W_vs_E10	ordinary_muscle_development	ENSGALG00010010853	SMIM20	0.838003883	0.145602981	5.755403333	8.64352E-09	1.87192E-08	positive
PRJNA729271	stage_3W_vs_E10	ordinary_muscle_development	ENSGALG00010010854	LRIT1	1.608669119	0.559440588	2.875495904	0.004033934	0.005708471	positive
PRJNA729271	stage_3W_vs_E10	ordinary_muscle_development	ENSGALG00010010855	ATP5PF	1.151705392	0.148441559	7.758645231	8.58414E-15	2.56816E-14	positive
PRJNA729271	stage_3W_vs_E10	ordinary_muscle_development	ENSGALG00010010856	CSDC2	-5.82522192	0.912451064	-6.384147214	1.72355E-10	4.1097E-10	negative
PRJNA729271	stage_3W_vs_E10	ordinary_muscle_development	ENSGALG00010010857	SEL1L3	0.608157743	0.060317476	10.08261263	6.59488E-24	2.94585E-23	positive
PRJNA729271	stage_3W_vs_E10	ordinary_muscle_development	ENSGALG00010010859	ENSGALG00010010859	-2.476426464	0.285311684	-8.679723268	3.96733E-18	1.41051E-17	negative
PRJNA729271	stage_3W_vs_E10	ordinary_muscle_development	ENSGALG00010010861	ENSGALG00010010861	0.926349606	0.236850049	3.911122715	9.18681E-05	0.000150347	positive
PRJNA729271	stage_3W_vs_E10	ordinary_muscle_development	ENSGALG00010010862	STRN4	0.21965615	0.152979951	1.435849262	0.151045239	0.178086931	positive
PRJNA729271	stage_3W_vs_E10	ordinary_muscle_development	ENSGALG00010010865	ENSGALG00010010865	1.671404576	0.156430485	10.68464744	1.20105E-26	6.04484E-26	positive
PRJNA729271	stage_3W_vs_E10	ordinary_muscle_development	ENSGALG00010010867	QSOX1	-0.833807092	0.164084111	-5.081583379	3.74302E-07	7.29038E-07	negative
PRJNA729271	stage_3W_vs_E10	ordinary_muscle_development	ENSGALG00010010868	SLC39A10	-2.28601326	0.110905516	-20.61225934	2.13052E-94	7.75925E-93	negative
PRJNA729271	stage_3W_vs_E10	ordinary_muscle_development	ENSGALG00010010869	ENSGALG00010010869	5.951250119	0.173419342	34.31710701	4.3612E-258	1.345E-255	positive
PRJNA729271	stage_3W_vs_E10	ordinary_muscle_development	ENSGALG00010010870	NOL6	-1.25315018	0.124042016	-10.10262671	5.3782E-24	2.40682E-23	negative
PRJNA729271	stage_3W_vs_E10	ordinary_muscle_development	ENSGALG00010010871	GCNT2	-1.623942499	0.93384703	-1.738981275	0.082038057	0.10025644	negative
PRJNA729271	stage_3W_vs_E10	ordinary_muscle_development	ENSGALG00010010874	ENSGALG00010010874	3.154840007	0.259570965	12.15405584	5.45888E-34	3.62633E-33	positive
PRJNA729271	stage_3W_vs_E10	ordinary_muscle_development	ENSGALG00010010876	ENSGALG00010010876	0.587822121	0.157919901	3.722280206	0.000197432	0.000314653	positive
PRJNA729271	stage_3W_vs_E10	ordinary_muscle_development	ENSGALG00010010880	ELP3	0.232245168	0.106008871	2.190808799	0.028465632	0.036794126	positive
PRJNA729271	stage_3W_vs_E10	ordinary_muscle_development	ENSGALG00010010882	CEP350	0.766130748	0.098275037	7.795781825	6.40109E-15	1.92898E-14	positive
PRJNA729271	stage_3W_vs_E10	ordinary_muscle_development	ENSGALG00010010884	DNAH7	-2.713739113	0.311655997	-8.707482403	3.10698E-18	1.10925E-17	negative
PRJNA729271	stage_3W_vs_E10	ordinary_muscle_development	ENSGALG00010010885	SNRPD2	-1.044695551	0.27556662	-3.791081636	0.000149993	0.000241437	negative
PRJNA729271	stage_3W_vs_E10	ordinary_muscle_development	ENSGALG00010010887	GABPA	0.120671182	0.066111793	1.825259549	0.067961871	0.083867472	positive
PRJNA729271	stage_3W_vs_E10	ordinary_muscle_development	ENSGALG00010010888	GNPAT	0.996944886	0.118588384	8.406766761	4.21468E-17	1.41942E-16	positive
PRJNA729271	stage_3W_vs_E10	ordinary_muscle_development	ENSGALG00010010889	CCL19	0.455333612	0.652634261	0.697685732	0.485373753	0.523668966	positive
PRJNA729271	stage_3W_vs_E10	ordinary_muscle_development	ENSGALG00010010890	NOL12	-0.503603365	0.135361347	-3.720437012	0.000198878	0.000316889	negative
PRJNA729271	stage_3W_vs_E10	ordinary_muscle_development	ENSGALG00010010891	POLR3H	-0.713676994	0.10793524	-6.612085139	3.78944E-11	9.38618E-11	negative
PRJNA729271	stage_3W_vs_E10	ordinary_muscle_development	ENSGALG00010010893	CCL21	-0.822279023	0.492279702	-1.670349235	0.094850287	0.115069879	negative
PRJNA729271	stage_3W_vs_E10	ordinary_muscle_development	ENSGALG00010010894	SPAG1	0.451007901	0.139685198	3.228745116	0.001243347	0.001847305	positive
PRJNA729271	stage_3W_vs_E10	ordinary_muscle_development	ENSGALG00010010896	LY86	4.158504206	0.292287391	14.22744988	6.18981E-46	6.12432E-45	positive
PRJNA729271	stage_3W_vs_E10	ordinary_muscle_development	ENSGALG00010010897	CEP170	-0.149568078	0.090639538	-1.650141654	0.098913967	0.119618236	negative
PRJNA729271	stage_3W_vs_E10	ordinary_muscle_development	ENSGALG00010010898	PHF24	-4.766324983	3.569493153	-1.335294614	0.181779941	0.211672577	negative
PRJNA729271	stage_3W_vs_E10	ordinary_muscle_development	ENSGALG00010010900	SLC34A2	-0.581522665	1.015198807	-0.572816537	0.566768921	0.603268873	negative
PRJNA729271	stage_3W_vs_E10	ordinary_muscle_development	ENSGALG00010010904	TOR1AIP1	-0.808665756	0.082580554	-9.792447681	1.21323E-22	5.15106E-22	negative
PRJNA729271	stage_3W_vs_E10	ordinary_muscle_development	ENSGALG00010010905	ENC1	-0.326800013	0.14822061	-2.204821669	0.027466611	0.035544016	negative
PRJNA729271	stage_3W_vs_E10	ordinary_muscle_development	ENSGALG00010010906	ATP6V1C1	0.951295249	0.138738449	6.85675279	7.04434E-12	1.81138E-11	positive
PRJNA729271	stage_3W_vs_E10	ordinary_muscle_development	ENSGALG00010010908	STK17B	0.98119054	0.122512949	8.008872115	1.15765E-15	3.61408E-15	positive
PRJNA729271	stage_3W_vs_E10	ordinary_muscle_development	ENSGALG00010010909	SLIT3	-2.447699259	0.329423467	-7.430251638	1.08391E-13	3.06862E-13	negative
PRJNA729271	stage_3W_vs_E10	ordinary_muscle_development	ENSGALG00010010911	ENSGALG00010010911	-1.173690173	0.350120442	-3.352246918	0.000801585	0.001210688	negative
PRJNA729271	stage_3W_vs_E10	ordinary_muscle_development	ENSGALG00010010912	BAALC	-1.366286887	0.360906981	-3.785703682	0.000153274	0.000246528	negative
PRJNA729271	stage_3W_vs_E10	ordinary_muscle_development	ENSGALG00010010913	IFT56	-2.101909043	0.149886695	-14.02331972	1.12239E-44	1.05575E-43	negative
PRJNA729271	stage_3W_vs_E10	ordinary_muscle_development	ENSGALG00010010915	APP	-1.58049603	0.110379059	-14.3188033	1.66974E-46	1.68075E-45	negative
PRJNA729271	stage_3W_vs_E10	ordinary_muscle_development	ENSGALG00010010916	ENSGALG00010010916	-3.829678907	0.887629978	-4.314499288	1.59965E-05	2.78254E-05	negative
PRJNA729271	stage_3W_vs_E10	ordinary_muscle_development	ENSGALG00010010917	TANC1	0.29578985	0.068563085	4.314126919	1.60235E-05	2.7869E-05	positive
PRJNA729271	stage_3W_vs_E10	ordinary_muscle_development	ENSGALG00010010918	TOR1AIP2	1.001588662	0.118810083	8.430165511	3.45177E-17	1.16655E-16	positive
PRJNA729271	stage_3W_vs_E10	ordinary_muscle_development	ENSGALG00010010919	CDHR1	-3.119780729	0.655734158	-4.757691349	1.9582E-06	3.63479E-06	negative
PRJNA729271	stage_3W_vs_E10	ordinary_muscle_development	ENSGALG00010010920	UBN2	0.789459955	0.113410027	6.961112509	3.37596E-12	8.83614E-12	positive
PRJNA729271	stage_3W_vs_E10	ordinary_muscle_development	ENSGALG00010010921	ENSGALG00010010921	-0.361950442	0.130533448	-2.772855895	0.005556672	0.007752812	negative
PRJNA729271	stage_3W_vs_E10	ordinary_muscle_development	ENSGALG00010010922	DYM	1.222143637	0.101212955	12.07497244	1.43212E-33	9.38454E-33	positive
PRJNA729271	stage_3W_vs_E10	ordinary_muscle_development	ENSGALG00010010923	F13A1	3.368416731	0.230750217	14.59767525	2.90586E-48	3.12931E-47	positive
PRJNA729271	stage_3W_vs_E10	ordinary_muscle_development	ENSGALG00010010924	ENSGALG00010010924	-2.071447574	0.227322656	-9.112367444	8.06035E-20	3.06011E-19	negative
PRJNA729271	stage_3W_vs_E10	ordinary_muscle_development	ENSGALG00010010928	HECW2	-0.417295986	0.116702295	-3.575730773	0.000349251	0.000545399	negative
PRJNA729271	stage_3W_vs_E10	ordinary_muscle_development	ENSGALG00010010929	FMC1	0.074342231	0.149201261	0.498268115	0.618295078	0.652887532	positive
PRJNA729271	stage_3W_vs_E10	ordinary_muscle_development	ENSGALG00010010930	FAM163A	-5.276316342	0.747482069	-7.058786507	1.67963E-12	4.47459E-12	negative
PRJNA729271	stage_3W_vs_E10	ordinary_muscle_development	ENSGALG00010010931	CYYR1	-2.983410703	0.199893005	-14.92503806	2.26506E-50	2.60779E-49	negative
PRJNA729271	stage_3W_vs_E10	ordinary_muscle_development	ENSGALG00010010932	GHITM	0.884526526	0.08817447	10.03154912	1.10765E-23	4.89493E-23	positive
PRJNA729271	stage_3W_vs_E10	ordinary_muscle_development	ENSGALG00010010933	ANAPC4	-0.768519806	0.111400992	-6.898680101	5.24879E-12	1.35714E-11	negative
PRJNA729271	stage_3W_vs_E10	ordinary_muscle_development	ENSGALG00010010934	ENSGALG00010010934	-0.173501735	0.119785604	-1.448435619	0.147495259	0.174156385	negative
PRJNA729271	stage_3W_vs_E10	ordinary_muscle_development	ENSGALG00010010935	LGALS1A	0.582741977	0.227874164	2.557297275	0.010548902	0.014299667	positive
PRJNA729271	stage_3W_vs_E10	ordinary_muscle_development	ENSGALG00010010937	CDK7	0.420500502	0.114737668	3.664886256	0.000247449	0.000390715	positive
PRJNA729271	stage_3W_vs_E10	ordinary_muscle_development	ENSGALG00010010938	FZD6	0.002122636	0.120774923	0.017575135	0.985977793	0.988227639	positive
PRJNA729271	stage_3W_vs_E10	ordinary_muscle_development	ENSGALG00010010939	ZCCHC4	-0.064335776	0.1382178	-0.465466643	0.641597319	0.67474624	negative
PRJNA729271	stage_3W_vs_E10	ordinary_muscle_development	ENSGALG00010010940	NRG3	-3.809386221	0.413290583	-9.217210314	3.0493E-20	1.17803E-19	negative
PRJNA729271	stage_3W_vs_E10	ordinary_muscle_development	ENSGALG00010010941	RIPK4	-5.921240361	0.805993778	-7.346508773	2.03451E-13	5.67774E-13	negative
PRJNA729271	stage_3W_vs_E10	ordinary_muscle_development	ENSGALG00010010942	NRN1	4.175175471	0.336521927	12.40684525	2.39921E-35	1.67034E-34	positive
PRJNA729271	stage_3W_vs_E10	ordinary_muscle_development	ENSGALG00010010943	WDSUB1	0.016631367	0.103724628	0.16034154	0.872612038	0.887179034	positive
PRJNA729271	stage_3W_vs_E10	ordinary_muscle_development	ENSGALG00010010945	PI4K2B	-0.52233863	0.098783528	-5.287709829	1.23857E-07	2.49099E-07	negative
PRJNA729271	stage_3W_vs_E10	ordinary_muscle_development	ENSGALG00010010946	SH2D4B	-0.256638432	0.342599185	-0.74909236	0.45380154	0.492575507	negative
PRJNA729271	stage_3W_vs_E10	ordinary_muscle_development	ENSGALG00010010947	LUC7L2	-0.381076659	0.070370171	-5.415315251	6.11808E-08	1.25363E-07	negative
PRJNA729271	stage_3W_vs_E10	ordinary_muscle_development	ENSGALG00010010948	PRDM15	-0.36647927	0.084751853	-4.324144655	1.53125E-05	2.6674E-05	negative
PRJNA729271	stage_3W_vs_E10	ordinary_muscle_development	ENSGALG00010010949	FZD3	-3.037496592	0.237086148	-12.81178431	1.40858E-37	1.0433E-36	negative
PRJNA729271	stage_3W_vs_E10	ordinary_muscle_development	ENSGALG00010010951	ENSGALG00010010951	-0.905793668	0.133544978	-6.782686093	1.17962E-11	3.00555E-11	negative
PRJNA729271	stage_3W_vs_E10	ordinary_muscle_development	ENSGALG00010010952	ENSGALG00010010952	1.795162405	0.475299415	3.776908505	0.000158787	0.000255083	positive
PRJNA729271	stage_3W_vs_E10	ordinary_muscle_development	ENSGALG00010010953	EXTL3	-0.3108264	0.110751771	-2.80651404	0.005008073	0.007021184	negative
PRJNA729271	stage_3W_vs_E10	ordinary_muscle_development	ENSGALG00010010954	MTERF2	0.996184487	0.225070987	4.426090179	9.59564E-06	1.69621E-05	positive
PRJNA729271	stage_3W_vs_E10	ordinary_muscle_development	ENSGALG00010010955	GTF3C3	0.166087692	0.081916158	2.027532736	0.04260796	0.053957923	positive
PRJNA729271	stage_3W_vs_E10	ordinary_muscle_development	ENSGALG00010010956	AXDND1	2.849790268	0.571552398	4.986052508	6.16254E-07	1.18266E-06	positive
PRJNA729271	stage_3W_vs_E10	ordinary_muscle_development	ENSGALG00010010957	ZMAT2	0.114407025	0.075300043	1.519348744	0.128674736	0.153307603	positive
PRJNA729271	stage_3W_vs_E10	ordinary_muscle_development	ENSGALG00010010958	LCORL	0.310042814	0.160270048	1.934502536	0.053051371	0.066359995	positive
PRJNA729271	stage_3W_vs_E10	ordinary_muscle_development	ENSGALG00010010959	TMEM263	-1.47625241	0.090483322	-16.31518803	7.69688E-60	1.14544E-58	negative
PRJNA729271	stage_3W_vs_E10	ordinary_muscle_development	ENSGALG00010010960	POLDIP3	-0.397217564	0.070184895	-5.659587607	1.51737E-08	3.23874E-08	negative
PRJNA729271	stage_3W_vs_E10	ordinary_muscle_development	ENSGALG00010010961	C2orf66	0.875786935	0.365188433	2.398178187	0.016476848	0.021869051	positive
PRJNA729271	stage_3W_vs_E10	ordinary_muscle_development	ENSGALG00010010962	ANKDD1B	0.891953451	0.265817883	3.355505808	0.0007922	0.001197387	positive
PRJNA729271	stage_3W_vs_E10	ordinary_muscle_development	ENSGALG00010010963	TSPAN14	-0.313402518	0.074022707	-4.23386998	2.29704E-05	3.94825E-05	negative
PRJNA729271	stage_3W_vs_E10	ordinary_muscle_development	ENSGALG00010010964	INTS9	-0.766376616	0.092876441	-8.251571716	1.56325E-16	5.1219E-16	negative
PRJNA729271	stage_3W_vs_E10	ordinary_muscle_development	ENSGALG00010010965	BAZ2B	0.245936713	0.131989001	1.863312172	0.062418359	0.077422053	positive
PRJNA729271	stage_3W_vs_E10	ordinary_muscle_development	ENSGALG00010010966	ENSGALG00010010966	2.258461948	0.532419092	4.241887609	2.21648E-05	3.81611E-05	positive
PRJNA729271	stage_3W_vs_E10	ordinary_muscle_development	ENSGALG00010010967	PRXL2A	-0.127410641	0.39046009	-0.326309	0.74419058	0.771219164	negative
PRJNA729271	stage_3W_vs_E10	ordinary_muscle_development	ENSGALG00010010968	RRP7A	-0.187256829	0.246711704	-0.759010723	0.447846142	0.486730886	negative
PRJNA729271	stage_3W_vs_E10	ordinary_muscle_development	ENSGALG00010010970	TPM2	-0.536224425	0.352465434	-1.521353227	0.12817122	0.152757921	negative
PRJNA729271	stage_3W_vs_E10	ordinary_muscle_development	ENSGALG00010010971	LCORL	0.290430882	0.124276731	2.336969118	0.01944079	0.025603693	positive
PRJNA729271	stage_3W_vs_E10	ordinary_muscle_development	ENSGALG00010010972	EXOSC3	-0.917870137	0.169634997	-5.41085361	6.2725E-08	1.28436E-07	negative
PRJNA729271	stage_3W_vs_E10	ordinary_muscle_development	ENSGALG00010010973	PGAP1	-0.346279149	0.112907166	-3.066936853	0.002162645	0.003139464	negative
PRJNA729271	stage_3W_vs_E10	ordinary_muscle_development	ENSGALG00010010974	NCAPG	-2.851092414	0.194465496	-14.66117366	1.1428E-48	1.24455E-47	negative
PRJNA729271	stage_3W_vs_E10	ordinary_muscle_development	ENSGALG00010010975	ATP8B1	-4.393587844	0.958407026	-4.584260886	4.55595E-06	8.25481E-06	negative
PRJNA729271	stage_3W_vs_E10	ordinary_muscle_development	ENSGALG00010010976	DYDC2	-2.417585923	1.013475892	-2.385439991	0.017058705	0.022595808	negative
PRJNA729271	stage_3W_vs_E10	ordinary_muscle_development	ENSGALG00010010977	FARS2	-0.260868136	0.082443371	-3.164209953	0.001555046	0.002290923	negative
PRJNA729271	stage_3W_vs_E10	ordinary_muscle_development	ENSGALG00010010978	SERHL	-0.005535626	0.134763443	-0.041076614	0.967234818	0.971726413	negative
PRJNA729271	stage_3W_vs_E10	ordinary_muscle_development	ENSGALG00010010979	ENSGALG00010010979	-2.781439456	0.456108135	-6.098201814	1.07268E-09	2.4386E-09	negative
PRJNA729271	stage_3W_vs_E10	ordinary_muscle_development	ENSGALG00010010980	POLK	0.930695973	0.124722889	7.462110456	8.51475E-14	2.42335E-13	positive
PRJNA729271	stage_3W_vs_E10	ordinary_muscle_development	ENSGALG00010010981	RIC8B	-0.850000675	0.064798288	-13.11764094	2.60932E-39	2.06115E-38	negative
PRJNA729271	stage_3W_vs_E10	ordinary_muscle_development	ENSGALG00010010982	ZBTB21	0.583873442	0.119585249	4.88248714	1.04756E-06	1.98204E-06	positive
PRJNA729271	stage_3W_vs_E10	ordinary_muscle_development	ENSGALG00010010983	MED28	0.447933051	0.088298834	5.072921477	3.91754E-07	7.61598E-07	positive
PRJNA729271	stage_3W_vs_E10	ordinary_muscle_development	ENSGALG00010010984	MAT1A	-2.634198523	0.390921029	-6.738441596	1.60094E-11	4.04844E-11	negative
PRJNA729271	stage_3W_vs_E10	ordinary_muscle_development	ENSGALG00010010985	CERT1	1.336196986	0.10772735	12.40350745	2.5013E-35	1.74058E-34	positive
PRJNA729271	stage_3W_vs_E10	ordinary_muscle_development	ENSGALG00010010986	NARS	-0.292188436	0.130116869	-2.245584585	0.024730629	0.03219891	negative
PRJNA729271	stage_3W_vs_E10	ordinary_muscle_development	ENSGALG00010010987	MARCHF7	-0.269391128	0.081031685	-3.324515921	0.000885722	0.00133249	negative
PRJNA729271	stage_3W_vs_E10	ordinary_muscle_development	ENSGALG00010010988	ANKRD44	-0.634776155	0.086148671	-7.368380145	1.72714E-13	4.83952E-13	negative
PRJNA729271	stage_3W_vs_E10	ordinary_muscle_development	ENSGALG00010010989	TRIQK	-1.904422992	0.182427901	-10.43931869	1.63983E-25	7.83951E-25	negative
PRJNA729271	stage_3W_vs_E10	ordinary_muscle_development	ENSGALG00010010990	MED18	0.50345984	0.119287472	4.220559209	2.43697E-05	4.17738E-05	positive
PRJNA729271	stage_3W_vs_E10	ordinary_muscle_development	ENSGALG00010010991	C2CD2	-0.586660747	0.162884631	-3.601694911	0.000316149	0.000495522	negative
PRJNA729271	stage_3W_vs_E10	ordinary_muscle_development	ENSGALG00010010992	PCDHA6	0.607921609	0.294397644	2.064967641	0.038926062	0.04956371	positive
PRJNA729271	stage_3W_vs_E10	ordinary_muscle_development	ENSGALG00010010993	ENSGALG00010010993	-1.234688228	0.42033037	-2.937423316	0.00330952	0.00473045	negative
PRJNA729271	stage_3W_vs_E10	ordinary_muscle_development	ENSGALG00010010994	NAA20	0.105648128	0.149656606	0.705936949	0.480227348	0.518845852	positive
PRJNA729271	stage_3W_vs_E10	ordinary_muscle_development	ENSGALG00010010995	HEBP1	-0.493919622	0.183553648	-2.690873362	0.007126524	0.009839871	negative
PRJNA729271	stage_3W_vs_E10	ordinary_muscle_development	ENSGALG00010010996	RFX4	-0.80528212	0.733754289	-1.097481993	0.272430748	0.309086075	negative
PRJNA729271	stage_3W_vs_E10	ordinary_muscle_development	ENSGALG00010010997	LAP3	-1.369928204	0.150302766	-9.114457715	7.90649E-20	3.00484E-19	negative
PRJNA729271	stage_3W_vs_E10	ordinary_muscle_development	ENSGALG00010010998	SOAT1	0.050894286	0.166237586	0.306153902	0.759487469	0.785163032	positive
PRJNA729271	stage_3W_vs_E10	ordinary_muscle_development	ENSGALG00010011001	ANGPT1	-2.14925834	0.25407995	-8.458984418	2.69716E-17	9.1687E-17	negative
PRJNA729271	stage_3W_vs_E10	ordinary_muscle_development	ENSGALG00010011002	RPRM	-1.903376128	0.688969702	-2.762641265	0.005733574	0.007987329	negative

PRJNA729271	stage_3W_vs_E10	ordinary_muscle_development	ENSGALG00010011003	FECH	-0.592518332	0.112351098	-5.273809901	1.3362E-07	2.67962E-07	negative
PRJNA729271	stage_3W_vs_E10	ordinary_muscle_development	ENSGALG00010011004	UMODL1	-1.21223157	0.412107187	-2.941544355	0.003265801	0.004670263	negative
PRJNA729271	stage_3W_vs_E10	ordinary_muscle_development	ENSGALG00010011006	TMEM64	-0.637713267	0.101249542	-6.298431125	3.00673E-10	7.07395E-10	negative
PRJNA729271	stage_3W_vs_E10	ordinary_muscle_development	ENSGALG00010011007	ENSGALG00010011007	-1.08966157	0.183832485	-5.927470149	3.07637E-09	6.80586E-09	negative
PRJNA729271	stage_3W_vs_E10	ordinary_muscle_development	ENSGALG00010011009	SFTPA1	0.394735447	1.248154542	0.316255266	0.751808762	0.778057119	positive
PRJNA729271	stage_3W_vs_E10	ordinary_muscle_development	ENSGALG00010011013	ENSGALG00010011013	5.282969933	0.327289983	16.14155703	1.30215E-58	1.88935E-57	positive
PRJNA729271	stage_3W_vs_E10	ordinary_muscle_development	ENSGALG00010011015	LYRM4	0.283557555	0.182966844	1.549775627	0.121195379	0.144809853	positive
PRJNA729271	stage_3W_vs_E10	ordinary_muscle_development	ENSGALG00010011018	QDPR	-0.924032567	0.105274976	-8.77732392	1.6741E-18	6.02885E-18	negative
PRJNA729271	stage_3W_vs_E10	ordinary_muscle_development	ENSGALG00010011019	NECAB1	-2.620417544	0.228445457	-11.47064852	1.85266E-30	1.08327E-29	negative
PRJNA729271	stage_3W_vs_E10	ordinary_muscle_development	ENSGALG00010011020	SFTPA2	-0.848460328	0.919809836	-0.922430153	0.356304248	0.39509104	negative
PRJNA729271	stage_3W_vs_E10	ordinary_muscle_development	ENSGALG00010011021	PPP1R3G	1.998945434	0.18374302	10.87902785	1.45102E-27	7.59847E-27	positive
PRJNA729271	stage_3W_vs_E10	ordinary_muscle_development	ENSGALG00010011022	C5orf22	-0.018919632	0.194362591	-0.097341942	0.92245485	0.931451274	negative
PRJNA729271	stage_3W_vs_E10	ordinary_muscle_development	ENSGALG00010011025	POLR3B	-0.349771715	0.09129689	-3.831146019	0.000127548	0.000206617	negative
PRJNA729271	stage_3W_vs_E10	ordinary_muscle_development	ENSGALG00010011027	FAM234B	-0.150966187	0.098100763	-1.538889014	0.123831394	0.147780014	negative
PRJNA729271	stage_3W_vs_E10	ordinary_muscle_development	ENSGALG00010011028	HMBOX1	-1.190268142	0.087249383	-13.64213817	2.24869E-42	1.96473E-41	negative
PRJNA729271	stage_3W_vs_E10	ordinary_muscle_development	ENSGALG00010011029	HMGCR	-0.816392661	0.125766515	-6.491335631	8.50787E-11	2.06327E-10	negative
PRJNA729271	stage_3W_vs_E10	ordinary_muscle_development	ENSGALG00010011033	DROSHA	-1.057093742	0.101931928	-10.37058514	3.37452E-25	1.59423E-24	negative
PRJNA729271	stage_3W_vs_E10	ordinary_muscle_development	ENSGALG00010011034	FAM180A	2.481304964	0.385144964	6.442522161	1.17504E-10	2.82271E-10	positive
PRJNA729271	stage_3W_vs_E10	ordinary_muscle_development	ENSGALG00010011038	GSG1	-2.774307465	0.518949465	-5.34600699	8.99157E-08	1.82667E-07	negative
PRJNA729271	stage_3W_vs_E10	ordinary_muscle_development	ENSGALG00010011040	CDH6	-3.027306023	0.226602754	-13.35952881	1.04222E-40	8.58349E-40	negative
PRJNA729271	stage_3W_vs_E10	ordinary_muscle_development	ENSGALG00010011042	SLC13A4	0.181051944	0.523536737	0.345824717	0.729474449	0.75732217	positive
PRJNA729271	stage_3W_vs_E10	ordinary_muscle_development	ENSGALG00010011044	MBL2	-0.403318713	0.64251394	-0.627719786	0.530187521	0.568002078	negative
PRJNA729271	stage_3W_vs_E10	ordinary_muscle_development	ENSGALG00010011045	ENSGALG00010011045	-2.408288945	0.426621507	-5.645024706	1.65158E-08	3.51536E-08	negative
PRJNA729271	stage_3W_vs_E10	ordinary_muscle_development	ENSGALG00010011049	ABL2	-0.738287949	0.130490389	-5.657795599	1.5333E-08	3.27129E-08	negative
PRJNA729271	stage_3W_vs_E10	ordinary_muscle_development	ENSGALG00010011050	SYT10	2.319697665	0.412664523	5.621267488	1.89562E-08	4.0218E-08	positive
PRJNA729271	stage_3W_vs_E10	ordinary_muscle_development	ENSGALG00010011055	TOR3A	-0.931381657	0.138351963	-6.731972813	1.67378E-11	4.2282E-11	negative
PRJNA729271	stage_3W_vs_E10	ordinary_muscle_development	ENSGALG00010011056	ENSGALG00010011056	-1.051623355	0.53262552	-1.974414136	0.048334672	0.060795824	negative
PRJNA729271	stage_3W_vs_E10	ordinary_muscle_development	ENSGALG00010011059	ENSGALG00010011059	-1.678809499	0.20847874	-8.052665211	8.10103E-16	2.55215E-15	negative
PRJNA729271	stage_3W_vs_E10	ordinary_muscle_development	ENSGALG00010011061	KIF13B	0.74999991	0.154615722	4.850735105	1.23005E-06	2.31613E-06	positive
PRJNA729271	stage_3W_vs_E10	ordinary_muscle_development	ENSGALG00010011062	TMEM55A	-0.231857375	0.121226322	-1.912599264	0.055799374	0.069653111	negative
PRJNA729271	stage_3W_vs_E10	ordinary_muscle_development	ENSGALG00010011064	MYLIP	1.361521774	0.096151787	14.16012964	1.61696E-45	1.573E-44	positive
PRJNA729271	stage_3W_vs_E10	ordinary_muscle_development	ENSGALG00010011067	TMTC2	-0.560741022	0.102987389	-5.444754223	5.1877E-08	1.06903E-07	negative
PRJNA729271	stage_3W_vs_E10	ordinary_muscle_development	ENSGALG00010011069	ENSGALG00010011069	-1.838857583	0.55343824	-3.32260666	0.000891806	0.001341225	negative
PRJNA729271	stage_3W_vs_E10	ordinary_muscle_development	ENSGALG00010011076	PKP2	-2.73459811	0.30686161	-8.911502838	5.03454E-19	1.85406E-18	negative
PRJNA729271	stage_3W_vs_E10	ordinary_muscle_development	ENSGALG00010011077	RASGEF1A	0.344610667	0.146110907	2.358555388	0.018346222	0.024228178	positive
PRJNA729271	stage_3W_vs_E10	ordinary_muscle_development	ENSGALG00010011080	EMP1	0.36877967	0.255139665	1.445403131	0.148344684	0.175073783	positive
PRJNA729271	stage_3W_vs_E10	ordinary_muscle_development	ENSGALG00010011085	UGT1A1	3.023433208	1.324305188	2.283033575	0.022428391	0.029362312	positive
PRJNA729271	stage_3W_vs_E10	ordinary_muscle_development	ENSGALG00010011086	OTUD6A	0.565809135	0.113139024	5.001007756	5.70314E-07	1.09609E-06	positive
PRJNA729271	stage_3W_vs_E10	ordinary_muscle_development	ENSGALG00010011090	GRIN2B	-3.406106295	0.490215107	-6.948187128	3.7001E-12	9.64969E-12	negative
PRJNA729271	stage_3W_vs_E10	ordinary_muscle_development	ENSGALG00010011091	YARS2	0.424036237	0.099408478	4.265594278	1.99371E-05	3.44525E-05	positive
PRJNA729271	stage_3W_vs_E10	ordinary_muscle_development	ENSGALG00010011094	CSGALNACT2	-0.496418712	0.090934571	-5.45907577	4.78619E-08	9.87979E-08	negative
PRJNA729271	stage_3W_vs_E10	ordinary_muscle_development	ENSGALG00010011095	TNN	-2.761778539	0.478238404	-5.774899127	7.69992E-09	1.67232E-08	negative
PRJNA729271	stage_3W_vs_E10	ordinary_muscle_development	ENSGALG00010011096	LDB2	-0.61714203	0.114941417	-5.369187604	7.90921E-08	1.61334E-07	negative
PRJNA729271	stage_3W_vs_E10	ordinary_muscle_development	ENSGALG00010011099	CCBE1	-0.388327242	0.198893451	-1.952438556	0.050886159	0.063767172	negative
PRJNA729271	stage_3W_vs_E10	ordinary_muscle_development	ENSGALG00010011101	ENSGALG00010011101	-1.217168143	0.835756504	-1.45636694	0.14529121	0.171825104	negative
PRJNA729271	stage_3W_vs_E10	ordinary_muscle_development	ENSGALG00010011104	ABCG1	-0.081943826	0.252558653	-0.324454635	0.745593862	0.772507722	negative
PRJNA729271	stage_3W_vs_E10	ordinary_muscle_development	ENSGALG00010011106	USP40	0.428762323	0.115357682	3.71680771	0.000201756	0.000321333	positive
PRJNA729271	stage_3W_vs_E10	ordinary_muscle_development	ENSGALG00010011107	ENSGALG00010011107	0.77954597	0.191314176	4.074690044	4.60757E-05	7.738E-05	positive
PRJNA729271	stage_3W_vs_E10	ordinary_muscle_development	ENSGALG00010011109	ENSGALG00010011109	-1.512818449	1.255794234	-1.204670645	0.228330473	0.261943031	negative
PRJNA729271	stage_3W_vs_E10	ordinary_muscle_development	ENSGALG00010011111	MRPS14	0.988793011	0.116478541	8.489057326	2.0832E-17	7.11666E-17	positive
PRJNA729271	stage_3W_vs_E10	ordinary_muscle_development	ENSGALG00010011116	TAPT1	1.009508083	0.127286242	7.931007031	2.17376E-15	6.69115E-15	positive
PRJNA729271	stage_3W_vs_E10	ordinary_muscle_development	ENSGALG00010011117	SLC6A15	-6.960330309	0.591760427	-11.76207464	6.12115E-32	3.78847E-31	negative
PRJNA729271	stage_3W_vs_E10	ordinary_muscle_development	ENSGALG00010011118	GMPR2	2.64777494	0.135680241	19.51481606	8.21643E-85	2.36304E-83	positive
PRJNA729271	stage_3W_vs_E10	ordinary_muscle_development	ENSGALG00010011119	ENSGALG00010011119	-1.323465888	0.296837007	-4.458560953	8.25117E-06	1.46552E-05	negative
PRJNA729271	stage_3W_vs_E10	ordinary_muscle_development	ENSGALG00010011120	ENSGALG00010011120	-3.704326776	3.111103183	-1.190679498	0.233779429	0.267770275	negative
PRJNA729271	stage_3W_vs_E10	ordinary_muscle_development	ENSGALG00010011121	NUP35	-0.428068359	0.104414378	-4.099707043	4.13673E-05	6.96665E-05	negative
PRJNA729271	stage_3W_vs_E10	ordinary_muscle_development	ENSGALG00010011124	CACYBP	0.601548406	0.067342312	8.932696053	4.15751E-19	1.53732E-18	positive
PRJNA729271	stage_3W_vs_E10	ordinary_muscle_development	ENSGALG00010011126	ATXN1	0.562604874	0.14177971	3.968162126	7.24291E-05	0.00011971	positive
PRJNA729271	stage_3W_vs_E10	ordinary_muscle_development	ENSGALG00010011128	GCNT4	1.562190227	0.236235648	6.612847135	3.76998E-11	9.33957E-11	positive
PRJNA729271	stage_3W_vs_E10	ordinary_muscle_development	ENSGALG00010011129	ENSGALG00010011129	0.467248918	0.116585887	4.007765692	6.12959E-05	0.000101984	positive
PRJNA729271	stage_3W_vs_E10	ordinary_muscle_development	ENSGALG00010011130	DNM1L	0.148513195	0.115157244	1.289655691	0.19717023	0.228419316	positive
PRJNA729271	stage_3W_vs_E10	ordinary_muscle_development	ENSGALG00010011131	RET	0.28493247	0.495214474	0.575371854	0.565039835	0.601649336	positive
PRJNA729271	stage_3W_vs_E10	ordinary_muscle_development	ENSGALG00010011133	CDC37L1	0.875164753	0.118467798	7.387364072	1.49768E-13	4.20796E-13	positive
PRJNA729271	stage_3W_vs_E10	ordinary_muscle_development	ENSGALG00010011136	ENSGALG00010011136	-0.990344724	0.39991451	-2.476391077	0.013271811	0.017792722	negative
PRJNA729271	stage_3W_vs_E10	ordinary_muscle_development	ENSGALG00010011139	UBASH3A	6.148878692	0.564982064	10.88331662	1.38433E-27	7.25707E-27	positive
PRJNA729271	stage_3W_vs_E10	ordinary_muscle_development	ENSGALG00010011141	MSX1	-5.291951405	0.897849724	-5.894027988	3.76893E-09	8.30506E-09	negative
PRJNA729271	stage_3W_vs_E10	ordinary_muscle_development	ENSGALG00010011142	DUSP19	-0.044564146	0.150737909	-0.295639938	0.76750507	0.792716687	negative
PRJNA729271	stage_3W_vs_E10	ordinary_muscle_development	ENSGALG00010011143	AK3	2.634690054	0.162724793	16.1910795	5.82968E-59	8.53548E-58	positive
PRJNA729271	stage_3W_vs_E10	ordinary_muscle_development	ENSGALG00010011144	ATF7IP	-0.179682244	0.062497251	-2.87504238	0.004039733	0.005716119	negative
PRJNA729271	stage_3W_vs_E10	ordinary_muscle_development	ENSGALG00010011146	BMS1	-0.790511136	0.129109503	-6.122795899	9.19474E-10	2.09919E-09	negative
PRJNA729271	stage_3W_vs_E10	ordinary_muscle_development	ENSGALG00010011148	ENSGALG00010011148	-0.125130519	0.183778875	-0.680875424	0.49595032	0.534284644	negative
PRJNA729271	stage_3W_vs_E10	ordinary_muscle_development	ENSGALG00010011150	FGD4	2.404474938	0.162378559	14.80783518	1.30372E-49	1.45365E-48	positive
PRJNA729271	stage_3W_vs_E10	ordinary_muscle_development	ENSGALG00010011152	SLC18A3	-0.874052105	0.644099774	-1.357013524	0.174776913	0.204042474	negative
PRJNA729271	stage_3W_vs_E10	ordinary_muscle_development	ENSGALG00010011154	SLC26A7	-7.216803467	0.943769855	-7.646783198	2.06069E-14	6.05509E-14	negative
PRJNA729271	stage_3W_vs_E10	ordinary_muscle_development	ENSGALG00010011157	ENSGALG00010011157	-5.56265615	1.574356484	-3.533288811	0.000410424	0.000636607	negative
PRJNA729271	stage_3W_vs_E10	ordinary_muscle_development	ENSGALG00010011161	ENSGALG00010011161	-0.105862697	0.094647645	-1.118492658	0.263356641	0.299470817	negative
PRJNA729271	stage_3W_vs_E10	ordinary_muscle_development	ENSGALG00010011166	SOX7	0.893112199	0.141384615	6.316898061	2.66865E-10	6.29695E-10	positive
PRJNA729271	stage_3W_vs_E10	ordinary_muscle_development	ENSGALG00010011170	RBM24	1.148024855	0.14277381	8.04086444	8.92069E-16	2.80428E-15	positive
PRJNA729271	stage_3W_vs_E10	ordinary_muscle_development	ENSGALG00010011175	GPR52	-0.067806242	0.189422428	-0.357963111	0.72037092	0.748782825	negative
PRJNA729271	stage_3W_vs_E10	ordinary_muscle_development	ENSGALG00010011178	RSPO2	-3.118071165	0.321842316	-9.688195136	3.38457E-22	1.413E-21	negative
PRJNA729271	stage_3W_vs_E10	ordinary_muscle_development	ENSGALG00010011181	ENSGALG00010011181	-0.686898319	0.291294263	-2.358090795	0.0183692	0.024254103	negative
PRJNA729271	stage_3W_vs_E10	ordinary_muscle_development	ENSGALG00010011184	FAM8A1	0.839783717	0.135602404	6.192985442	5.90352E-10	1.36543E-09	positive
PRJNA729271	stage_3W_vs_E10	ordinary_muscle_development	ENSGALG00010011187	STK32B	-6.896986812	0.77542339	-8.894478679	5.86942E-19	2.15222E-18	negative
PRJNA729271	stage_3W_vs_E10	ordinary_muscle_development	ENSGALG00010011190	ENSGALG00010011190	-0.745458351	0.21534221	-3.461738184	0.000536699	0.000824182	negative
PRJNA729271	stage_3W_vs_E10	ordinary_muscle_development	ENSGALG00010011192	RPL17	0.334856013	0.123500174	2.711380903	0.006700361	0.009276192	positive
PRJNA729271	stage_3W_vs_E10	ordinary_muscle_development	ENSGALG00010011193	FAM20B	-0.96350781	0.078975875	-12.20002704	3.10721E-34	2.0861E-33	negative
PRJNA729271	stage_3W_vs_E10	ordinary_muscle_development	ENSGALG00010011198	PINX1	-0.667132983	0.066656757	-10.00848246	1.39882E-23	6.15913E-23	negative
PRJNA729271	stage_3W_vs_E10	ordinary_muscle_development	ENSGALG00010011199	ENSGALG00010011199	-4.912169146	1.718897299	-2.857744409	0.004266639	0.006024248	negative
PRJNA729271	stage_3W_vs_E10	ordinary_muscle_development	ENSGALG00010011200	PCDHB4	-1.596939184	0.621056619	-2.571326244	0.010130984	0.01375631	negative
PRJNA729271	stage_3W_vs_E10	ordinary_muscle_development	ENSGALG00010011203	ENSGALG00010011203	-0.284386574	0.104189665	-2.729508462	0.006342882	0.00879641	negative
PRJNA729271	stage_3W_vs_E10	ordinary_muscle_development	ENSGALG00010011205	CYTL1	-9.484887271	0.966382976	-9.814832741	9.7199E-23	4.1487E-22	negative
PRJNA729271	stage_3W_vs_E10	ordinary_muscle_development	ENSGALG00010011210	NUP153	-0.49854114	0.098277816	-5.0727739	3.92058E-07	7.62087E-07	negative
PRJNA729271	stage_3W_vs_E10	ordinary_muscle_development	ENSGALG00010011211	SLC37A1	0.57000315	0.150199285	3.794979125	0.000147656	0.000237887	positive
PRJNA729271	stage_3W_vs_E10	ordinary_muscle_development	ENSGALG00010011212	NCKAP1	-0.637311426	0.063264688	-10.07373062	7.21869E-24	3.21953E-23	negative
PRJNA729271	stage_3W_vs_E10	ordinary_muscle_development	ENSGALG00010011213	XKR6	-0.177979239	0.186994399	-0.951789141	0.34120393	0.379595592	negative
PRJNA729271	stage_3W_vs_E10	ordinary_muscle_development	ENSGALG00010011218	ENSGALG00010011218	-1.005284115	0.594915803	-1.689792252	0.091067707	0.110759054	negative
PRJNA729271	stage_3W_vs_E10	ordinary_muscle_development	ENSGALG00010011222	RCL1	-0.243358662	0.089826122	-2.709219277	0.006744175	0.009332393	negative
PRJNA729271	stage_3W_vs_E10	ordinary_muscle_development	ENSGALG00010011231	PLBD1	1.377603536	0.204906393	6.723087134	1.77914E-11	4.4881E-11	positive
PRJNA729271	stage_3W_vs_E10	ordinary_muscle_development	ENSGALG00010011235	ENSGALG00010011235	-0.528930538	0.092494087	-5.71853355	1.07447E-08	2.31556E-08	negative
PRJNA729271	stage_3W_vs_E10	ordinary_muscle_development	ENSGALG00010011237	ENSGALG00010011237	-0.570781074	0.261867665	-2.179654651	0.02928307	0.037790074	negative
PRJNA729271	stage_3W_vs_E10	ordinary_muscle_development	ENSGALG00010011239	RALGPS2	-2.202666821	0.077958032	-28.25452045	1.2525E-175	1.6504E-173	negative
PRJNA729271	stage_3W_vs_E10	ordinary_muscle_development	ENSGALG00010011243	ALX1	-7.201297367	0.72986494	-9.866616372	5.80912E-23	2.5053E-22	negative
PRJNA729271	stage_3W_vs_E10	ordinary_muscle_development	ENSGALG00010011244	PDE9A	-0.915693798	0.171030281	-5.353986412	8.60373E-08	1.75033E-07	negative
PRJNA729271	stage_3W_vs_E10	ordinary_muscle_development	ENSGALG00010011246	DNAJB5	2.111181853	0.232902591	9.06465595	1.24998E-19	4.70732E-19	positive
PRJNA729271	stage_3W_vs_E10	ordinary_muscle_development	ENSGALG00010011247	ENSGALG00010011247	-0.451187234	0.096516023	-4.674739164	2.94327E-06	5.41062E-06	negative
PRJNA729271	stage_3W_vs_E10	ordinary_muscle_development	ENSGALG00010011250	ENSGALG00010011250	0.054887758	0.099230929	0.553131549	0.580173335	0.616224261	positive
PRJNA729271	stage_3W_vs_E10	ordinary_muscle_development	ENSGALG00010011251	FRZB	0.020553541	0.175460641	0.117140464	0.906748745	0.917573517	positive
PRJNA729271	stage_3W_vs_E10	ordinary_muscle_development	ENSGALG00010011253	KDM4C	0.872420594	0.108257758	8.058735072	7.7088E-16	2.43228E-15	positive
PRJNA729271	stage_3W_vs_E10	ordinary_muscle_development	ENSGALG00010011262	ENSGALG00010011262	-1.459864552	0.208467178	-7.002850849	2.50806E-12	6.61348E-12	negative
PRJNA729271	stage_3W_vs_E10	ordinary_muscle_development	ENSGALG00010011267	EVC2	-1.091735979	0.165672251	-6.589733495	4.40617E-11	1.08618E-10	negative
PRJNA729271	stage_3W_vs_E10	ordinary_muscle_development	ENSGALG00010011271	ANGPTL1	-2.864641149	0.162350396	-17.64480545	1.11558E-69	2.14176E-68	negative
PRJNA729271	stage_3W_vs_E10	ordinary_muscle_development	ENSGALG00010011273	RUNX1T1	-1.332802181	0.099477389	-13.39804147	6.20798E-41	5.15081E-40	negative
PRJNA729271	stage_3W_vs_E10	ordinary_muscle_development	ENSGALG00010011274	ENSGALG00010011274	-1.344141933	0.550748312	-2.440573857	0.014663947	0.019577592	negative
PRJNA729271	stage_3W_vs_E10	ordinary_muscle_development	ENSGALG00010011275	WDR4	-1.003158801	0.104543062	-9.595651619	8.33885E-22	3.4319E-21	negative
PRJNA729271	stage_3W_vs_E10	ordinary_muscle_development	ENSGALG00010011278	ENSGALG00010011278	0.359063262	0.52412696	0.685069248	0.493300225	0.531706332	positive
PRJNA729271	stage_3W_vs_E10	ordinary_muscle_development	ENSGALG00010011280	FAM199X	-0.21036352	0.081440789	-2.583024097	0.009793848	0.013323494	negative
PRJNA729271	stage_3W_vs_E10	ordinary_muscle_development	ENSGALG00010011281	ENSGALG00010011281	-1.300503852	0.635166183	-2.047501719	0.040608848	0.051543367	negative
PRJNA729271	stage_3W_vs_E10	ordinary_muscle_development	ENSGALG00010011282	CRY1	1.183531603	0.12682882	9.331724438	1.04162E-20	4.09723E-20	positive
PRJNA729271	stage_3W_vs_E10	ordinary_muscle_development	ENSGALG00010011283	LONRF3	2.348304447	0.139616557	16.81967023	1.75131E-63	2.88141E-62	positive
PRJNA729271	stage_3W_vs_E10	ordinary_muscle_development	ENSGALG00010011285	ENSGALG00010011285	2.604936799	0.255505297	10.19523598	2.08238E-24	9.5158E-24	positive
PRJNA729271	stage_3W_vs_E10	ordinary_muscle_development	ENSGALG00010011287	ENSGALG00010011287	0.464062676	0.116787188	3.973575227	7.08018E-05	0.000117087	positive
PRJNA729271	stage_3W_vs_E10	ordinary_muscle_development	ENSGALG00010011288	ENSGALG00010011288	-0.745076973	0.302460881	-2.46338294	0.013763283	0.018427747	negative
PRJNA729271	stage_3W_vs_E10	ordinary_muscle_development	ENSGALG00010011293	NDUFV3	1.568754374	0.167316981	9.375942375	6.85605E-21	2.71897E-20	positive
PRJNA729271	stage_3W_vs_E10	ordinary_muscle_development	ENSGALG00010011295	FAM167A	-1.066116677	0.383383524	-2.78080984	0.005422349	0.007574879	negative
PRJNA729271	stage_3W_vs_E10	ordinary_muscle_development	ENSGALG00010011297	OSR2	-0.637233499	0.185311665	-3.438712295	0.000584488	0.000893687	negative
PRJNA729271	stage_3W_vs_E10	ordinary_muscle_development	ENSGALG00010011300	EVC	-0.997496003	0.162358653	-6.143780974	8.05799E-10	1.84753E-09	negative
PRJNA729271	stage_3W_vs_E10	ordinary_muscle_development	ENSGALG00010011304	COMMD5	-0.650657989	0.092917885	-7.002505365	2.51425E-12	6.62861E-12	negative
PRJNA729271	stage_3W_vs_E10	ordinary_muscle_development	ENSGALG00010011306	ECI2	1.017499859	0.137762452	7.385901176	1.51424E-13	4.25202E-13	positive
PRJNA729271	stage_3W_vs_E10	ordinary_muscle_development	ENSGALG00010011307	ENSGALG00010011307	-2.779610565	0.480711414	-5.78228535	7.36926E-09	1.60266E-08	negative
PRJNA729271	stage_3W_vs_E10	ordinary_muscle_development	ENSGALG00010011308	SPIC	4.527370107	1.409642136	3.211715932	0.001319448	0.001955962	positive
PRJNA729271	stage_3W_vs_E10	ordinary_muscle_development	ENSGALG00010011311	DOCK8	1.062627133	0.189695119	5.601763183	2.12182E-08	4.48597E-08	positive

PRJNA729271	stage_3W_vs_E10	ordinary_muscle_development	ENSGALG00010011312	VPS13B	-0.364889989	0.053060457	-6.876872303	6.11809E-12	1.57769E-11	negative
PRJNA729271	stage_3W_vs_E10	ordinary_muscle_development	ENSGALG00010011313	RASSF9	-0.757429145	0.251864707	-3.007285747	0.002635919	0.003797221	negative
PRJNA729271	stage_3W_vs_E10	ordinary_muscle_development	ENSGALG00010011316	PARPBP	-3.242865288	0.23321792	-13.90487186	5.91727E-44	5.43542E-43	negative
PRJNA729271	stage_3W_vs_E10	ordinary_muscle_development	ENSGALG00010011317	BLK	2.403187608	0.354219571	6.784457446	1.16524E-11	2.97099E-11	positive
PRJNA729271	stage_3W_vs_E10	ordinary_muscle_development	ENSGALG00010011318	PRP4K	-0.103333982	0.097715657	-1.057496671	0.290284973	0.327445968	negative
PRJNA729271	stage_3W_vs_E10	ordinary_muscle_development	ENSGALG00010011321	MAP2	-3.257764971	0.1582482	-20.58642667	3.63187E-94	1.3194E-92	negative
PRJNA729271	stage_3W_vs_E10	ordinary_muscle_development	ENSGALG00010011322	GATA4	-2.592983913	1.251317358	-2.072203263	0.038246489	0.04874124	negative
PRJNA729271	stage_3W_vs_E10	ordinary_muscle_development	ENSGALG00010011323	GUCY2C	0.112704826	0.801413418	0.140632567	0.888160219	0.901153744	positive
PRJNA729271	stage_3W_vs_E10	ordinary_muscle_development	ENSGALG00010011326	COX6C	1.151793248	0.135487885	8.501079264	1.87836E-17	6.4366E-17	positive
PRJNA729271	stage_3W_vs_E10	ordinary_muscle_development	ENSGALG00010011329	PCDHGC5	-0.955647487	0.117185625	-8.154988999	3.49212E-16	1.12037E-15	negative
PRJNA729271	stage_3W_vs_E10	ordinary_muscle_development	ENSGALG00010011333	ENSGALG00010011333	-1.812958021	0.133013441	-13.6298859	2.65996E-42	2.3129E-41	negative
PRJNA729271	stage_3W_vs_E10	ordinary_muscle_development	ENSGALG00010011335	VCP	0.605943331	0.173652249	3.489406763	0.000484094	0.000747119	positive
PRJNA729271	stage_3W_vs_E10	ordinary_muscle_development	ENSGALG00010011339	ENSGALG00010011339	-0.50345975	0.303267844	-1.660115836	0.096891152	0.117388603	negative
PRJNA729271	stage_3W_vs_E10	ordinary_muscle_development	ENSGALG00010011340	PKNOX1	-0.388718732	0.109008506	-3.565948641	0.000362542	0.000565302	negative
PRJNA729271	stage_3W_vs_E10	ordinary_muscle_development	ENSGALG00010011342	RASAL2	0.142034541	0.066916934	2.122550045	0.033791575	0.043311423	positive
PRJNA729271	stage_3W_vs_E10	ordinary_muscle_development	ENSGALG00010011343	PXDC1	-0.725862704	0.141434327	-5.132153684	2.86445E-07	5.62148E-07	negative
PRJNA729271	stage_3W_vs_E10	ordinary_muscle_development	ENSGALG00010011345	CRMP1	-2.948329151	0.234376516	-12.57945634	2.73908E-36	1.95774E-35	negative
PRJNA729271	stage_3W_vs_E10	ordinary_muscle_development	ENSGALG00010011347	RGS22	-1.943262034	0.42647401	-4.556577863	5.19938E-06	9.38543E-06	negative
PRJNA729271	stage_3W_vs_E10	ordinary_muscle_development	ENSGALG00010011348	SLC22A23	-0.109130552	0.163536703	-0.667315348	0.504570725	0.542523007	negative
PRJNA729271	stage_3W_vs_E10	ordinary_muscle_development	ENSGALG00010011349	ENSGALG00010011349	-0.534169982	0.090745201	-5.886481901	3.94503E-09	8.68518E-09	negative
PRJNA729271	stage_3W_vs_E10	ordinary_muscle_development	ENSGALG00010011351	KANK1	-2.128036401	0.111620832	-19.06486768	4.94527E-81	1.28003E-79	negative
PRJNA729271	stage_3W_vs_E10	ordinary_muscle_development	ENSGALG00010011352	NTS	-0.864064935	0.549283278	-1.573077079	0.115700971	0.138670877	negative
PRJNA729271	stage_3W_vs_E10	ordinary_muscle_development	ENSGALG00010011353	ENSGALG00010011353	-3.884573941	0.219579634	-17.69095733	4.92319E-70	9.54032E-69	negative
PRJNA729271	stage_3W_vs_E10	ordinary_muscle_development	ENSGALG00010011357	UNC80	-0.715562192	0.42175775	-1.696618954	0.089768724	0.109261706	negative
PRJNA729271	stage_3W_vs_E10	ordinary_muscle_development	ENSGALG00010011359	RNF19A	-0.088482365	0.08673721	-1.020120028	0.307671539	0.345498835	negative
PRJNA729271	stage_3W_vs_E10	ordinary_muscle_development	ENSGALG00010011360	TCF4	0.630672899	0.171099431	3.686002316	0.000227804	0.000360798	positive
PRJNA729271	stage_3W_vs_E10	ordinary_muscle_development	ENSGALG00010011361	NUP37	-1.229861963	0.091257749	-13.47679494	2.14212E-41	1.80418E-40	negative
PRJNA729271	stage_3W_vs_E10	ordinary_muscle_development	ENSGALG00010011363	FDFT1	0.882880382	0.146207327	6.03855087	1.55504E-09	3.50223E-09	positive
PRJNA729271	stage_3W_vs_E10	ordinary_muscle_development	ENSGALG00010011365	PSMG4	0.053945924	0.17888864	0.301561487	0.762986371	0.788499033	positive
PRJNA729271	stage_3W_vs_E10	ordinary_muscle_development	ENSGALG00010011367	IER3IP1	0.701858318	0.112222533	6.25416572	3.99646E-10	9.33732E-10	positive
PRJNA729271	stage_3W_vs_E10	ordinary_muscle_development	ENSGALG00010011368	ENSGALG00010011368	-2.679979316	0.300483535	-8.918889071	4.70989E-19	1.73803E-18	negative
PRJNA729271	stage_3W_vs_E10	ordinary_muscle_development	ENSGALG00010011370	MGAT4C	-5.535214555	1.574826278	-3.514809622	0.000440069	0.000681351	negative
PRJNA729271	stage_3W_vs_E10	ordinary_muscle_development	ENSGALG00010011371	ENSGALG00010011371	1.300065413	0.191640872	6.783862953	1.17004E-11	2.9822E-11	positive
PRJNA729271	stage_3W_vs_E10	ordinary_muscle_development	ENSGALG00010011372	VMA21	-0.10670348	0.092383971	-1.154999924	0.248090457	0.283244422	negative
PRJNA729271	stage_3W_vs_E10	ordinary_muscle_development	ENSGALG00010011374	ENSGALG00010011374	-0.50982709	0.092943367	-5.485352082	4.12647E-08	8.55818E-08	negative
PRJNA729271	stage_3W_vs_E10	ordinary_muscle_development	ENSGALG00010011375	C12orf50	0.665406064	0.136589563	4.871573283	1.10713E-06	2.09038E-06	positive
PRJNA729271	stage_3W_vs_E10	ordinary_muscle_development	ENSGALG00010011377	SEC16B	1.453005641	0.300359743	4.837551224	1.31449E-06	2.46902E-06	positive
PRJNA729271	stage_3W_vs_E10	ordinary_muscle_development	ENSGALG00010011378	TUBB2B	-3.442119314	0.195028656	-17.6493003	1.03025E-69	1.98056E-68	negative
PRJNA729271	stage_3W_vs_E10	ordinary_muscle_development	ENSGALG00010011379	CELF4	-5.517156316	0.917081813	-6.015991418	1.78789E-09	4.01168E-09	negative
PRJNA729271	stage_3W_vs_E10	ordinary_muscle_development	ENSGALG00010011381	RLIG1	-0.076457485	0.118313198	-0.646229554	0.518130702	0.556030542	negative
PRJNA729271	stage_3W_vs_E10	ordinary_muscle_development	ENSGALG00010011382	ENSGALG00010011382	-0.121202328	0.197519288	-0.613622746	0.539464649	0.577173021	negative
PRJNA729271	stage_3W_vs_E10	ordinary_muscle_development	ENSGALG00010011384	PUM3	-0.950423478	0.081552493	-11.65413158	2.18598E-31	1.31914E-30	negative
PRJNA729271	stage_3W_vs_E10	ordinary_muscle_development	ENSGALG00010011386	CTSB	1.977834819	0.126020916	15.69449647	1.64946E-55	2.2097E-54	positive
PRJNA729271	stage_3W_vs_E10	ordinary_muscle_development	ENSGALG00010011388	GPR50	-0.160002006	0.165824061	-0.964890168	0.334599827	0.372871961	negative
PRJNA729271	stage_3W_vs_E10	ordinary_muscle_development	ENSGALG00010011389	RC3H1	0.629076156	0.085716788	7.339007534	2.15184E-13	5.99824E-13	positive
PRJNA729271	stage_3W_vs_E10	ordinary_muscle_development	ENSGALG00010011394	SERPINC1	2.068353129	0.916077123	2.257837333	0.023955803	0.031237798	positive
PRJNA729271	stage_3W_vs_E10	ordinary_muscle_development	ENSGALG00010011398	ENSGALG00010011398	0.272614795	0.187591704	1.453234811	0.14615857	0.172718773	positive
PRJNA729271	stage_3W_vs_E10	ordinary_muscle_development	ENSGALG00010011399	JAKMIP1	-2.401930033	0.261017129	-9.202193151	3.50717E-20	1.35024E-19	negative
PRJNA729271	stage_3W_vs_E10	ordinary_muscle_development	ENSGALG00010011400	BPHL	1.011838501	0.198608155	5.094647309	3.49391E-07	6.81527E-07	positive
PRJNA729271	stage_3W_vs_E10	ordinary_muscle_development	ENSGALG00010011401	ANKRD46	-0.362131679	0.06813036	-5.315276137	1.06496E-07	2.15234E-07	negative
PRJNA729271	stage_3W_vs_E10	ordinary_muscle_development	ENSGALG00010011403	SV2C	-6.658192188	0.802227415	-8.299631829	1.04435E-16	3.4506E-16	negative
PRJNA729271	stage_3W_vs_E10	ordinary_muscle_development	ENSGALG00010011406	UBE2R2	-0.359697126	0.115972937	-3.101560901	0.001925033	0.00280492	negative
PRJNA729271	stage_3W_vs_E10	ordinary_muscle_development	ENSGALG00010011407	CEP290	-0.564282732	0.180241828	-3.130698002	0.001743914	0.002555143	negative
PRJNA729271	stage_3W_vs_E10	ordinary_muscle_development	ENSGALG00010011411	ENSGALG00010011411	-0.651665975	0.225534364	-2.889430962	0.003859397	0.005474845	negative
PRJNA729271	stage_3W_vs_E10	ordinary_muscle_development	ENSGALG00010011412	KANSL1L	0.564846688	0.12028009	4.696094649	2.65183E-06	4.88912E-06	positive
PRJNA729271	stage_3W_vs_E10	ordinary_muscle_development	ENSGALG00010011413	ZBTB37	0.505130147	0.104039006	4.855199651	1.20266E-06	2.26631E-06	positive
PRJNA729271	stage_3W_vs_E10	ordinary_muscle_development	ENSGALG00010011414	PABPC1	0.000577037	0.114190037	0.005053306	0.995968062	0.996578443	positive
PRJNA729271	stage_3W_vs_E10	ordinary_muscle_development	ENSGALG00010011416	ENSGALG00010011416	0.222903973	0.438214903	0.508663607	0.610988036	0.645971669	positive
PRJNA729271	stage_3W_vs_E10	ordinary_muscle_development	ENSGALG00010011417	WFS1	-0.07433203	0.145163884	-0.512055944	0.608611857	0.643788139	negative
PRJNA729271	stage_3W_vs_E10	ordinary_muscle_development	ENSGALG00010011418	RIPK1	-0.476853582	0.086146145	-5.535402434	3.10515E-08	6.48266E-08	negative
PRJNA729271	stage_3W_vs_E10	ordinary_muscle_development	ENSGALG00010011419	RNF180	-0.098239891	0.150188461	-0.654110775	0.51304044	0.55097586	negative
PRJNA729271	stage_3W_vs_E10	ordinary_muscle_development	ENSGALG00010011420	HMGB3	-2.123834329	0.100108941	-21.21523111	6.90865E-100	2.87761E-98	negative
PRJNA729271	stage_3W_vs_E10	ordinary_muscle_development	ENSGALG00010011421	ENSGALG00010011421	1.053715652	0.116788983	9.022389111	1.84031E-19	6.88751E-19	positive
PRJNA729271	stage_3W_vs_E10	ordinary_muscle_development	ENSGALG00010011422	KATNAL2	-1.786667447	0.239881943	-7.448111456	9.46858E-14	2.68848E-13	negative
PRJNA729271	stage_3W_vs_E10	ordinary_muscle_development	ENSGALG00010011423	GDF6	-0.925182828	0.365903365	-2.528489533	0.01145545	0.015486546	negative
PRJNA729271	stage_3W_vs_E10	ordinary_muscle_development	ENSGALG00010011424	ACADL	0.766629891	0.097301675	7.878897184	3.30283E-15	1.00937E-14	positive
PRJNA729271	stage_3W_vs_E10	ordinary_muscle_development	ENSGALG00010011425	WASHC3	0.035638671	0.079514298	0.448204565	0.654005568	0.686992587	positive
PRJNA729271	stage_3W_vs_E10	ordinary_muscle_development	ENSGALG00010011426	NQO2	0.60191252	0.127014973	4.738909959	2.14871E-06	3.98179E-06	positive
PRJNA729271	stage_3W_vs_E10	ordinary_muscle_development	ENSGALG00010011427	TMTC3	-0.76781233	0.088071907	-8.718016387	2.83118E-18	1.01228E-17	negative
PRJNA729271	stage_3W_vs_E10	ordinary_muscle_development	ENSGALG00010011428	UQCRB	1.73303764	0.162866958	10.64081791	1.92433E-26	9.61169E-26	positive
PRJNA729271	stage_3W_vs_E10	ordinary_muscle_development	ENSGALG00010011429	UBAP2	0.005107345	0.107356816	0.047573551	0.962056111	0.967126939	positive
PRJNA729271	stage_3W_vs_E10	ordinary_muscle_development	ENSGALG00010011430	ENSGALG00010011430	-0.419766185	0.33857165	-1.239814925	0.215043856	0.247789711	negative
PRJNA729271	stage_3W_vs_E10	ordinary_muscle_development	ENSGALG00010011431	DRAM1	4.017765251	0.399984911	10.04479203	9.68518E-24	4.30238E-23	positive
PRJNA729271	stage_3W_vs_E10	ordinary_muscle_development	ENSGALG00010011433	RNASE6	6.225039307	0.329774007	18.87668276	1.77383E-79	4.35053E-78	positive
PRJNA729271	stage_3W_vs_E10	ordinary_muscle_development	ENSGALG00010011434	NPL	1.147673803	0.179583751	6.39074415	1.6508E-10	3.93818E-10	positive
PRJNA729271	stage_3W_vs_E10	ordinary_muscle_development	ENSGALG00010011436	UBAP2L2	-2.862893751	2.312470566	-1.238023867			negative
PRJNA729271	stage_3W_vs_E10	ordinary_muscle_development	ENSGALG00010011437	DMRT1	-2.500963032	0.598610576	-4.177946616	2.94153E-05	5.00674E-05	negative
PRJNA729271	stage_3W_vs_E10	ordinary_muscle_development	ENSGALG00010011438	ENSGALG00010011438	4.196352452	0.440803914	9.519771297	1.73561E-21	7.05683E-21	positive
PRJNA729271	stage_3W_vs_E10	ordinary_muscle_development	ENSGALG00010011439	ENSGALG00010011439	-0.72321982	0.334839712	-2.159898586	0.030780521	0.03963433	negative
PRJNA729271	stage_3W_vs_E10	ordinary_muscle_development	ENSGALG00010011440	FUNDC1	0.272939441	0.11127724	2.452787651	0.014175399	0.018949775	positive
PRJNA729271	stage_3W_vs_E10	ordinary_muscle_development	ENSGALG00010011441	CD99L2	1.086407318	0.079644252	13.64074991	2.29192E-42	2.00128E-41	positive
PRJNA729271	stage_3W_vs_E10	ordinary_muscle_development	ENSGALG00010011442	ENSGALG00010011442	-3.118528718	2.106416131	-1.480490333	0.138742439	0.164599087	negative
PRJNA729271	stage_3W_vs_E10	ordinary_muscle_development	ENSGALG00010011443	ENSGALG00010011443	-1.549380953	0.17301935	-8.954957644	3.39866E-19	1.25833E-18	negative
PRJNA729271	stage_3W_vs_E10	ordinary_muscle_development	ENSGALG00010011445	MTERF3	-0.022858558	0.080788303	-0.282943908	0.777219836	0.801437114	negative
PRJNA729271	stage_3W_vs_E10	ordinary_muscle_development	ENSGALG00010011446	ENSGALG00010011446	-0.53996862	0.229835856	-2.349366327	0.018805395	0.024796161	negative
PRJNA729271	stage_3W_vs_E10	ordinary_muscle_development	ENSGALG00010011447	LAMC1	1.356030923	0.13034867	10.40310516	2.39983E-25	1.1379E-24	positive
PRJNA729271	stage_3W_vs_E10	ordinary_muscle_development	ENSGALG00010011448	AvBD10	5.195381866	2.626532444	1.978038337	0.047924382	0.060311158	positive
PRJNA729271	stage_3W_vs_E10	ordinary_muscle_development	ENSGALG00010011449	DUSP22	-1.03074007	0.145268571	-7.095409974	1.28969E-12	3.45801E-12	negative
PRJNA729271	stage_3W_vs_E10	ordinary_muscle_development	ENSGALG00010011450	PTDSS1	-0.915278307	0.069468079	-13.17552347	1.21383E-39	9.70994E-39	negative
PRJNA729271	stage_3W_vs_E10	ordinary_muscle_development	ENSGALG00010011451	CRHBP	-9.978451143	0.945446261	-10.55422349	4.85638E-26	2.37574E-25	negative
PRJNA729271	stage_3W_vs_E10	ordinary_muscle_development	ENSGALG00010011452	KITLG	1.006351539	0.18867763	5.33370882	9.62268E-08	1.95105E-07	positive
PRJNA729271	stage_3W_vs_E10	ordinary_muscle_development	ENSGALG00010011453	GNPTAB	0.319689907	0.124346388	2.570962552	0.010141629	0.013769475	positive
PRJNA729271	stage_3W_vs_E10	ordinary_muscle_development	ENSGALG00010011456	KIAA1328	-0.826544858	0.098033079	-8.431285276	3.41889E-17	1.15598E-16	negative
PRJNA729271	stage_3W_vs_E10	ordinary_muscle_development	ENSGALG00010011457	ENSGALG00010011457	1.529302697	0.257333972	5.942871384	2.80072E-09	6.21406E-09	positive
PRJNA729271	stage_3W_vs_E10	ordinary_muscle_development	ENSGALG00010011458	TPGS2	-0.935979384	0.130106841	-7.193929074	6.29529E-13	1.7133E-12	negative
PRJNA729271	stage_3W_vs_E10	ordinary_muscle_development	ENSGALG00010011459	ENSGALG00010011459	-0.079518303	0.280790772	-0.283194146	0.777028015	0.80129632	negative
PRJNA729271	stage_3W_vs_E10	ordinary_muscle_development	ENSGALG00010011460	LAMC2	-2.320809488	0.678764307	-3.41916843	0.000628128	0.000958593	negative
PRJNA729271	stage_3W_vs_E10	ordinary_muscle_development	ENSGALG00010011461	IRF4	1.067044646	0.352101029	3.030507035	0.002441435	0.003527572	positive
PRJNA729271	stage_3W_vs_E10	ordinary_muscle_development	ENSGALG00010011463	IMP3	-0.643714648	0.130926681	-4.916604028	8.80584E-07	1.67463E-06	negative
PRJNA729271	stage_3W_vs_E10	ordinary_muscle_development	ENSGALG00010011464	FANCG	-1.979796625	0.164954091	-12.00210684	3.46366E-33	2.24534E-32	negative
PRJNA729271	stage_3W_vs_E10	ordinary_muscle_development	ENSGALG00010011465	PPP2R2C	-1.435204727	0.341371766	-4.204227965	2.61975E-05	4.47639E-05	negative
PRJNA729271	stage_3W_vs_E10	ordinary_muscle_development	ENSGALG00010011466	MTMR1	-0.038627047	0.06633333	-0.582317314	0.560352965	0.597273052	negative
PRJNA729271	stage_3W_vs_E10	ordinary_muscle_development	ENSGALG00010011467	ENSGALG00010011467	-2.415948538	4.011473588	-0.602259615			negative
PRJNA729271	stage_3W_vs_E10	ordinary_muscle_development	ENSGALG00010011468	EFHC2	-1.324049512	0.290846808	-4.552394849	5.30387E-06	9.56094E-06	negative
PRJNA729271	stage_3W_vs_E10	ordinary_muscle_development	ENSGALG00010011469	CPS1	1.02174336	0.332598702	3.072000445	0.002126294	0.003088859	positive
PRJNA729271	stage_3W_vs_E10	ordinary_muscle_development	ENSGALG00010011471	NMNAT2	-3.36100006	0.449936768	-7.46993867	8.02322E-14	2.2893E-13	negative
PRJNA729271	stage_3W_vs_E10	ordinary_muscle_development	ENSGALG00010011475	NDP	-3.708358466	0.355757411	-10.42384037	1.92999E-25	9.19331E-25	negative
PRJNA729271	stage_3W_vs_E10	ordinary_muscle_development	ENSGALG00010011477	SDC2	0.348171737	0.167250773	2.081734696	0.03736671	0.047678737	positive
PRJNA729271	stage_3W_vs_E10	ordinary_muscle_development	ENSGALG00010011478	HDAC3	-0.703724453	0.077288701	-9.105140103	8.61552E-20	3.26659E-19	negative
PRJNA729271	stage_3W_vs_E10	ordinary_muscle_development	ENSGALG00010011480	BLTP3B	-0.005355458	0.064228316	-0.083381578	0.933548136	0.941293581	negative
PRJNA729271	stage_3W_vs_E10	ordinary_muscle_development	ENSGALG00010011482	DMRT2	-1.106581379	0.164355877	-6.732837283	1.66386E-11	4.20388E-11	negative
PRJNA729271	stage_3W_vs_E10	ordinary_muscle_development	ENSGALG00010011484	CHPT1	0.987704498	0.078125269	12.64257401	1.22962E-36	8.87172E-36	positive
PRJNA729271	stage_3W_vs_E10	ordinary_muscle_development	ENSGALG00010011485	MTM1	1.204517955	0.07592504	15.86456798	1.11482E-56	1.53169E-55	positive
PRJNA729271	stage_3W_vs_E10	ordinary_muscle_development	ENSGALG00010011487	MAN2B2	-0.583963447	0.15869083	-3.679881489	0.000233342	0.000369408	negative
PRJNA729271	stage_3W_vs_E10	ordinary_muscle_development	ENSGALG00010011493	ENSGALG00010011493	1.181240714	0.260675354	4.531462976	5.85766E-06	1.0533E-05	positive
PRJNA729271	stage_3W_vs_E10	ordinary_muscle_development	ENSGALG00010011496	ACTR6	-0.865128301	0.086990087	-9.94513658	2.64811E-23	1.15234E-22	negative
PRJNA729271	stage_3W_vs_E10	ordinary_muscle_development	ENSGALG00010011497	CPQ	0.711829681	0.248827666	2.860733663	0.00422662	0.005970651	positive
PRJNA729271	stage_3W_vs_E10	ordinary_muscle_development	ENSGALG00010011499	SMG7	-0.122182985	0.147175929	-0.830183209	0.406435207	0.44526327	negative
PRJNA729271	stage_3W_vs_E10	ordinary_muscle_development	ENSGALG00010011503	NCF2	1.574279978	0.368590608	4.271080007	1.94529E-05	3.36479E-05	positive
PRJNA729271	stage_3W_vs_E10	ordinary_muscle_development	ENSGALG00010011505	MYL1	6.238360495	0.152229511	40.97996801	0	0	positive
PRJNA729271	stage_3W_vs_E10	ordinary_muscle_development	ENSGALG00010011509	EXOC2	0.406018628	0.066916875	6.067507281	1.29911E-09	2.94044E-09	positive
PRJNA729271	stage_3W_vs_E10	ordinary_muscle_development	ENSGALG00010011510	ENSGALG00010011510	0.762190757	0.263288264	2.894890744	0.003792905	0.005385264	positive
PRJNA729271	stage_3W_vs_E10	ordinary_muscle_development	ENSGALG00010011515	SERINC5	-0.35342953	0.086113439	-4.104231984	4.0566E-05	6.83647E-05	negative
PRJNA729271	stage_3W_vs_E10	ordinary_muscle_development	ENSGALG00010011523	PIGO	0.067235472	0.120245303	0.559152589	0.576057587	0.612211652	positive
PRJNA729271	stage_3W_vs_E10	ordinary_muscle_development	ENSGALG00010011525	ENSGALG00010011525	-2.380679306	0.252613678	-9.424190029	4.33438E-21	1.7322E-20	negative
PRJNA729271	stage_3W_vs_E10	ordinary_muscle_development	ENSGALG00010011529	SCYL2	0.450633559	0.081884465	5.503285159	3.72779E-08	7.75352E-08	positive
PRJNA729271	stage_3W_vs_E10	ordinary_muscle_development	ENSGALG00010011530	ARPC5	-1.055178625	0.119956846	-8.796318505	1.41378E-18	5.1015E-18	negative
PRJNA729271	stage_3W_vs_E10	ordinary_muscle_development	ENSGALG00010011531	MTDH	0.426364935	0.071824232	5.936226882	2.91656E-09	6.4602E-09	positive
PRJNA729271	stage_3W_vs_E10	ordinary_muscle_development	ENSGALG00010011533	ENSGALG00010011533	-0.121762741	0.272854008	-0.446256011	0.655412328	0.688270788	negative
PRJNA729271	stage_3W_vs_E10	ordinary_muscle_development	ENSGALG00010011536	FCHSD1	-0.666904234	0.17522508	-3.805986194	0.00014124	0.000228033	negative
PRJNA729271	stage_3W_vs_E10	ordinary_muscle_development	ENSGALG00010011538	ENSGALG00010011538	3.484651328	0.252781557	13.78522772	3.12778E-43	2.80876E-42	positive

PRJNA729271	stage_3W_vs_E10	ordinary_muscle_development	ENSGALG00010011539	LANCL1	-0.09136275	0.106532157	-0.857607252	0.391109369	0.43009865	negative
PRJNA729271	stage_3W_vs_E10	ordinary_muscle_development	ENSGALG00010011544	STOML2	-0.234311581	0.092056815	-2.545293152	0.010918603	0.014781466	negative
PRJNA729271	stage_3W_vs_E10	ordinary_muscle_development	ENSGALG00010011545	USP12-like	-0.375404882	0.076400692	-4.913631964	8.94045E-07	1.69979E-06	negative
PRJNA729271	stage_3W_vs_E10	ordinary_muscle_development	ENSGALG00010011546	MAOA	2.219379228	0.125792249	17.64321125	1.14751E-69	2.20014E-68	positive
PRJNA729271	stage_3W_vs_E10	ordinary_muscle_development	ENSGALG00010011549	KIAA0232	1.037343671	0.143709634	7.218330752	5.26296E-13	1.43747E-12	positive
PRJNA729271	stage_3W_vs_E10	ordinary_muscle_development	ENSGALG00010011551	GMDS	-1.714731413	0.156910107	-10.92811325	8.45889E-28	4.47324E-27	negative
PRJNA729271	stage_3W_vs_E10	ordinary_muscle_development	ENSGALG00010011555	ENSGALG00010011555	-4.147789528	4.289209579	-0.967028878	0.333529597	0.371906185	negative
PRJNA729271	stage_3W_vs_E10	ordinary_muscle_development	ENSGALG00010011556	LAPTM4B	-0.787291446	0.144879385	-5.43411643	5.50686E-08	1.13174E-07	negative
PRJNA729271	stage_3W_vs_E10	ordinary_muscle_development	ENSGALG00010011558	MYBPC1	0.971841317	0.559892352	1.735764585			positive
PRJNA729271	stage_3W_vs_E10	ordinary_muscle_development	ENSGALG00010011559	MTX3	0.432171518	0.126020618	3.42937152	0.000604981	0.000924436	positive
PRJNA729271	stage_3W_vs_E10	ordinary_muscle_development	ENSGALG00010011562	ENSGALG00010011562	-9.019800581	0.892338086	-10.10805291	5.08855E-24	2.28213E-23	negative
PRJNA729271	stage_3W_vs_E10	ordinary_muscle_development	ENSGALG00010011564	ARAP3	0.967488015	0.121866815	7.938896367	2.03988E-15	6.28707E-15	positive
PRJNA729271	stage_3W_vs_E10	ordinary_muscle_development	ENSGALG00010011570	RGL1	-1.156922639	0.137064217	-8.440734329	3.1535E-17	1.06824E-16	negative
PRJNA729271	stage_3W_vs_E10	ordinary_muscle_development	ENSGALG00010011571	ATOSB	0.930450336	0.114543032	8.123150916	4.54234E-16	1.44993E-15	positive
PRJNA729271	stage_3W_vs_E10	ordinary_muscle_development	ENSGALG00010011572	PTCD2	1.045370562	0.101154073	10.33443865	4.92286E-25	2.31003E-24	positive
PRJNA729271	stage_3W_vs_E10	ordinary_muscle_development	ENSGALG00010011573	CMAS	0.172995506	0.177841911	0.972748802	0.330678164	0.368961588	positive
PRJNA729271	stage_3W_vs_E10	ordinary_muscle_development	ENSGALG00010011574	GAS2L3	-1.866176347	0.160791809	-11.6061655	3.83428E-31	2.2966E-30	negative
PRJNA729271	stage_3W_vs_E10	ordinary_muscle_development	ENSGALG00010011575	ENSGALG00010011575	-3.19451656	0.522845302	-6.109869498	9.97126E-10	2.27254E-09	negative
PRJNA729271	stage_3W_vs_E10	ordinary_muscle_development	ENSGALG00010011577	ENSGALG00010011577	0.296493083	0.160222875	1.850504068	0.064240932	0.079580575	positive
PRJNA729271	stage_3W_vs_E10	ordinary_muscle_development	ENSGALG00010011578	MYLK4	3.324581251	0.328479948	10.12110867	4.45342E-24	2.00473E-23	positive
PRJNA729271	stage_3W_vs_E10	ordinary_muscle_development	ENSGALG00010011584	ENSGALG00010011584	-4.351597912	1.141622303	-3.81176673	0.000137977	0.000222988	negative
PRJNA729271	stage_3W_vs_E10	ordinary_muscle_development	ENSGALG00010011586	ENSGALG00010011586	0.422189473	0.252847509	1.669739502	0.094970915	0.115196938	positive
PRJNA729271	stage_3W_vs_E10	ordinary_muscle_development	ENSGALG00010011588	COLGALT2	-0.210960086	0.198532461	-1.062597444	0.287964546	0.325132116	negative
PRJNA729271	stage_3W_vs_E10	ordinary_muscle_development	ENSGALG00010011589	RERG	-2.023635612	0.225566858	-8.97133395	2.92945E-19	1.08822E-18	negative
PRJNA729271	stage_3W_vs_E10	ordinary_muscle_development	ENSGALG00010011593	WRNIP1	-0.838024663	0.102426944	-8.181681765	2.79909E-16	9.04837E-16	negative
PRJNA729271	stage_3W_vs_E10	ordinary_muscle_development	ENSGALG00010011595	ENSGALG00010011595	0.547910272	0.162710271	3.367398188	0.00075881	0.001148836	positive
PRJNA729271	stage_3W_vs_E10	ordinary_muscle_development	ENSGALG00010011597	TBC1D14	-0.264273629	0.130379168	-2.026962083	0.042666291	0.054022352	negative
PRJNA729271	stage_3W_vs_E10	ordinary_muscle_development	ENSGALG00010011601	ANO4	-1.593831955	0.509389442	-3.128906535	0.001754581	0.002569214	negative
PRJNA729271	stage_3W_vs_E10	ordinary_muscle_development	ENSGALG00010011603	ENSGALG00010011603	0.606391814	0.276563484	2.192595366	0.028336545	0.036630538	positive
PRJNA729271	stage_3W_vs_E10	ordinary_muscle_development	ENSGALG00010011605	APPL2	-0.761719378	0.142131732	-5.359249265	8.35685E-08	1.7025E-07	negative
PRJNA729271	stage_3W_vs_E10	ordinary_muscle_development	ENSGALG00010011607	TSEN15	0.35614712	0.156961381	2.269011129	0.023267649	0.030403369	positive
PRJNA729271	stage_3W_vs_E10	ordinary_muscle_development	ENSGALG00010011609	ENSGALG00010011609	-0.094514422	0.853053712	-0.110795393	0.911778598	0.922148394	negative
PRJNA729271	stage_3W_vs_E10	ordinary_muscle_development	ENSGALG00010011612	ENSGALG00010011612	-1.246757096	0.493395823	-2.526890256	0.011507746	0.015552893	negative
PRJNA729271	stage_3W_vs_E10	ordinary_muscle_development	ENSGALG00010011617	MAMLD1	0.907822082	0.097122404	9.347195347	9.00026E-21	3.55474E-20	positive
PRJNA729271	stage_3W_vs_E10	ordinary_muscle_development	ENSGALG00010011619	PCDH1	-0.493069867	0.161184363	-3.059042809	0.002220454	0.00321887	negative
PRJNA729271	stage_3W_vs_E10	ordinary_muscle_development	ENSGALG00010011621	WASHC4	0.648643573	0.087682425	7.397646381	1.38619E-13	3.90456E-13	positive
PRJNA729271	stage_3W_vs_E10	ordinary_muscle_development	ENSGALG00010011622	BACH1	0.634989334	0.159949679	3.969931899	7.18932E-05	0.000118838	positive
PRJNA729271	stage_3W_vs_E10	ordinary_muscle_development	ENSGALG00010011625	ERBB4	1.80591187	0.216506747	8.341134372	7.35812E-17	2.44848E-16	positive
PRJNA729271	stage_3W_vs_E10	ordinary_muscle_development	ENSGALG00010011626	SERPINB1	1.923787557	0.564665562	3.40695039	0.000656931	0.001000863	positive
PRJNA729271	stage_3W_vs_E10	ordinary_muscle_development	ENSGALG00010011628	MATN2	1.622341406	0.191844021	8.456564857	2.75369E-17	9.35648E-17	positive
PRJNA729271	stage_3W_vs_E10	ordinary_muscle_development	ENSGALG00010011630	DELE1	0.616170426	0.100729291	6.117092858	9.52978E-10	2.17397E-09	positive
PRJNA729271	stage_3W_vs_E10	ordinary_muscle_development	ENSGALG00010011633	TADA2B	-0.332607241	0.087282663	-3.810690815	0.000138579	0.000223911	negative
PRJNA729271	stage_3W_vs_E10	ordinary_muscle_development	ENSGALG00010011634	AVD	9.683435701	0.882605093	10.97142513	5.24393E-28	2.79451E-27	positive
PRJNA729271	stage_3W_vs_E10	ordinary_muscle_development	ENSGALG00010011635	PCDH12	-1.239278868	0.200311658	-6.186753581	6.14159E-10	1.41913E-09	negative
PRJNA729271	stage_3W_vs_E10	ordinary_muscle_development	ENSGALG00010011636	GRIK1	-0.630717843	0.400464583	-1.574965353	0.115264442	0.138159108	negative
PRJNA729271	stage_3W_vs_E10	ordinary_muscle_development	ENSGALG00010011637	CECR2	-0.414616714	0.156416092	-2.650729265	0.008031819	0.011036233	negative
PRJNA729271	stage_3W_vs_E10	ordinary_muscle_development	ENSGALG00010011638	GRPEL1	0.307360478	0.081484848	3.771995468	0.000161947	0.000260016	positive
PRJNA729271	stage_3W_vs_E10	ordinary_muscle_development	ENSGALG00010011639	ENSGALG00010011639	-0.4862629	0.146377138	-3.321986664	0.00089379	0.001344069	negative
PRJNA729271	stage_3W_vs_E10	ordinary_muscle_development	ENSGALG00010011641	ENSGALG00010011641	-3.937422055	0.861945871	-4.568061856	4.92255E-06	8.89902E-06	negative
PRJNA729271	stage_3W_vs_E10	ordinary_muscle_development	ENSGALG00010011643	C1orf21	0.896632775	0.080026852	11.20414896	3.89075E-29	2.16826E-28	positive
PRJNA729271	stage_3W_vs_E10	ordinary_muscle_development	ENSGALG00010011647	FBXO38	0.404819656	0.082632232	4.899052666	9.62998E-07	1.82705E-06	positive
PRJNA729271	stage_3W_vs_E10	ordinary_muscle_development	ENSGALG00010011648	ENSGALG00010011648	0.444704653	0.220349867	2.018175278	0.04357301	0.055117443	positive
PRJNA729271	stage_3W_vs_E10	ordinary_muscle_development	ENSGALG00010011649	MRPS27	0.417292766	0.136720372	3.052162319	0.002271992	0.003291936	positive
PRJNA729271	stage_3W_vs_E10	ordinary_muscle_development	ENSGALG00010011650	RPL30	0.5336432	0.155321838	3.435725499	0.000590969	0.000903502	positive
PRJNA729271	stage_3W_vs_E10	ordinary_muscle_development	ENSGALG00010011651	SERPINB6	0.111523159	0.127887169	0.872043374	0.383184716	0.42212057	positive
PRJNA729271	stage_3W_vs_E10	ordinary_muscle_development	ENSGALG00010011653	NR2C1	-0.112756573	0.088761479	-1.270332299	0.203966288	0.235840088	negative
PRJNA729271	stage_3W_vs_E10	ordinary_muscle_development	ENSGALG00010011654	CLDN8	-3.738096842	1.364434345	-2.739667802	0.006150131	0.008542995	negative
PRJNA729271	stage_3W_vs_E10	ordinary_muscle_development	ENSGALG00010011655	SORCS2	-0.880645695	0.185081988	-4.758138289	1.95387E-06	3.62721E-06	negative
PRJNA729271	stage_3W_vs_E10	ordinary_muscle_development	ENSGALG00010011657	ALDH1L2	0.608987449	0.142284585	4.280066236	1.86838E-05	3.23485E-05	positive
PRJNA729271	stage_3W_vs_E10	ordinary_muscle_development	ENSGALG00010011658	TENM1	-2.281400626	0.322216276	-7.080339493	1.43802E-12	3.84648E-12	negative
PRJNA729271	stage_3W_vs_E10	ordinary_muscle_development	ENSGALG00010011659	CD14	1.276018111	0.152306346	8.377970721	5.3848E-17	1.80344E-16	positive
PRJNA729271	stage_3W_vs_E10	ordinary_muscle_development	ENSGALG00010011660	ENSGALG00010011660	-0.367716315	0.085844813	-4.283500702	1.83976E-05	3.18757E-05	negative
PRJNA729271	stage_3W_vs_E10	ordinary_muscle_development	ENSGALG00010011662	RIDA	-1.182511835	0.171334608	-6.901768689	5.1359E-12	1.32961E-11	negative
PRJNA729271	stage_3W_vs_E10	ordinary_muscle_development	ENSGALG00010011663	UTP20	-0.617388065	0.137275124	-4.497450427	6.87732E-06	1.22858E-05	negative
PRJNA729271	stage_3W_vs_E10	ordinary_muscle_development	ENSGALG00010011664	SERPINB10B	5.462322496	0.535160838	10.20688007	1.84709E-24	8.46192E-24	positive
PRJNA729271	stage_3W_vs_E10	ordinary_muscle_development	ENSGALG00010011667	ENSGALG00010011667	-0.468813025	0.410884965	-1.140983644	0.253876729	0.289212763	negative
PRJNA729271	stage_3W_vs_E10	ordinary_muscle_development	ENSGALG00010011669	ENSGALG00010011669	-9.163400521	0.970237157	-9.444495561	3.5712E-21	1.43472E-20	negative
PRJNA729271	stage_3W_vs_E10	ordinary_muscle_development	ENSGALG00010011670	TMCO6	-0.036793129	0.171476925	-0.214566066	0.830105653	0.850065186	negative
PRJNA729271	stage_3W_vs_E10	ordinary_muscle_development	ENSGALG00010011671	SLC41A2	-1.407410593	0.19460578	-7.232110957	4.75543E-13	1.3013E-12	negative
PRJNA729271	stage_3W_vs_E10	ordinary_muscle_development	ENSGALG00010011673	POP1	-1.425193899	0.125967987	-11.31393725	1.11951E-29	6.36363E-29	negative
PRJNA729271	stage_3W_vs_E10	ordinary_muscle_development	ENSGALG00010011674	ENSGALG00010011674	-0.6896582	0.495490328	-1.391870156	0.163961721	0.192376974	negative
PRJNA729271	stage_3W_vs_E10	ordinary_muscle_development	ENSGALG00010011677	EDEM3	-0.290205655	0.080002198	-3.627471041	0.000286211	0.000450399	negative
PRJNA729271	stage_3W_vs_E10	ordinary_muscle_development	ENSGALG00010011679	OVAL	-8.019757279	1.214507677	-6.603298961	4.02107E-11	9.94463E-11	negative
PRJNA729271	stage_3W_vs_E10	ordinary_muscle_development	ENSGALG00010011681	MAP1B	-2.164663164	0.358941533	-6.030684576	1.63267E-09	3.67362E-09	negative
PRJNA729271	stage_3W_vs_E10	ordinary_muscle_development	ENSGALG00010011682	USP15	1.719002946	0.122341096	14.05090357	7.60574E-45	7.215E-44	positive
PRJNA729271	stage_3W_vs_E10	ordinary_muscle_development	ENSGALG00010011683	NIPAL2	-1.832552901	0.291963725	-6.27664584	3.45955E-10	8.11033E-10	negative
PRJNA729271	stage_3W_vs_E10	ordinary_muscle_development	ENSGALG00010011685	NDUFA2	1.341955393	0.111272646	12.06006547	1.71646E-33	1.12174E-32	positive
PRJNA729271	stage_3W_vs_E10	ordinary_muscle_development	ENSGALG00010011688	PDE3A	-0.18830762	0.1481746	-1.270849528	0.203782185	0.235646013	negative
PRJNA729271	stage_3W_vs_E10	ordinary_muscle_development	ENSGALG00010011692	ARL1	-0.682596076	0.08963595	-7.615204322	2.63275E-14	7.69233E-14	negative
PRJNA729271	stage_3W_vs_E10	ordinary_muscle_development	ENSGALG00010011696	ZFC3H1	0.525735643	0.108947154	4.825602339	1.39581E-06	2.61737E-06	positive
PRJNA729271	stage_3W_vs_E10	ordinary_muscle_development	ENSGALG00010011699	TIAM1	-1.306412635	0.144727375	-9.026714105	1.76904E-19	6.62932E-19	negative
PRJNA729271	stage_3W_vs_E10	ordinary_muscle_development	ENSGALG00010011702	NUP205	-0.590659088	0.083839334	-7.045130981	1.85287E-12	4.92525E-12	negative
PRJNA729271	stage_3W_vs_E10	ordinary_muscle_development	ENSGALG00010011703	YWHAZ	-1.233099847	0.09443052	-13.05827656	5.70037E-39	4.45428E-38	negative
PRJNA729271	stage_3W_vs_E10	ordinary_muscle_development	ENSGALG00010011708	IK	0.303535209	0.091478557	3.318102299	0.000906313	0.001362195	positive
PRJNA729271	stage_3W_vs_E10	ordinary_muscle_development	ENSGALG00010011712	DCAF12L2	-0.004861117	0.0760854	-0.063890278	0.949057593	0.955584177	negative
PRJNA729271	stage_3W_vs_E10	ordinary_muscle_development	ENSGALG00010011713	AVPR1A	-4.84650991	0.991925434	-4.88596193	1.02925E-06	1.94892E-06	negative
PRJNA729271	stage_3W_vs_E10	ordinary_muscle_development	ENSGALG00010011715	SLCO1B3	-0.962579322	1.838626849	-0.523531636	0.600604342	0.636199937	negative
PRJNA729271	stage_3W_vs_E10	ordinary_muscle_development	ENSGALG00010011720	IKZF2	0.793014112	0.172012197	4.610220248	4.02243E-06	7.32016E-06	positive
PRJNA729271	stage_3W_vs_E10	ordinary_muscle_development	ENSGALG00010011723	SPRY3	1.889029641	0.155905278	12.11652146	8.63468E-34	5.69684E-33	positive
PRJNA729271	stage_3W_vs_E10	ordinary_muscle_development	ENSGALG00010011724	ARHGEF28	-0.262291415	0.15068847	-1.740620327	0.081750152	0.099946732	negative
PRJNA729271	stage_3W_vs_E10	ordinary_muscle_development	ENSGALG00010011726	WDR55	-0.375351351	0.086023515	-4.363357509	1.28081E-05	2.24464E-05	negative
PRJNA729271	stage_3W_vs_E10	ordinary_muscle_development	ENSGALG00010011729	FAM129A	-1.104447338	0.23263159	-4.747624089	2.0582E-06	3.81895E-06	negative
PRJNA729271	stage_3W_vs_E10	ordinary_muscle_development	ENSGALG00010011734	ENSGALG00010011734	-1.445613399	0.295635575	-4.889849267	1.00913E-06	1.91182E-06	negative
PRJNA729271	stage_3W_vs_E10	ordinary_muscle_development	ENSGALG00010011738	FGD6	0.831775871	0.116920318	7.114040435	1.12694E-12	3.02949E-12	positive
PRJNA729271	stage_3W_vs_E10	ordinary_muscle_development	ENSGALG00010011745	AFAP1L1	-1.017200512	0.193329331	-5.261490894	1.42892E-07	2.85843E-07	negative
PRJNA729271	stage_3W_vs_E10	ordinary_muscle_development	ENSGALG00010011746	SOD1	0.20775852	0.142471723	1.45824389	0.14477333	0.171277295	positive
PRJNA729271	stage_3W_vs_E10	ordinary_muscle_development	ENSGALG00010011747	UTP15	-0.521840642	0.116579804	-4.47625253	7.59646E-06	1.35354E-05	negative
PRJNA729271	stage_3W_vs_E10	ordinary_muscle_development	ENSGALG00010011751	ZNF706	1.768020666	0.109289533	16.17740156	7.28027E-59	1.06379E-57	positive
PRJNA729271	stage_3W_vs_E10	ordinary_muscle_development	ENSGALG00010011752	RNF2	-2.545925032	0.1302199	-19.55096754	4.04771E-85	1.17109E-83	negative
PRJNA729271	stage_3W_vs_E10	ordinary_muscle_development	ENSGALG00010011758	SERPINB11	0.756031261	0.468504023	1.613713487	0.106589587	0.128408915	positive
PRJNA729271	stage_3W_vs_E10	ordinary_muscle_development	ENSGALG00010011759	CREB3	0.464400428	0.130216758	3.566364534	0.000361968	0.000564467	positive
PRJNA729271	stage_3W_vs_E10	ordinary_muscle_development	ENSGALG00010011764	PCYOX1L	-0.847568777	0.133986996	-6.325753998	2.51999E-10	5.9578E-10	negative
PRJNA729271	stage_3W_vs_E10	ordinary_muscle_development	ENSGALG00010011770	CHST11	-3.565066178	0.304862962	-11.69399575	1.36796E-31	8.34184E-31	negative
PRJNA729271	stage_3W_vs_E10	ordinary_muscle_development	ENSGALG00010011772	GRHL2	-3.724973219	0.483406908	-7.705668152	1.3016E-14	3.86139E-14	negative
PRJNA729271	stage_3W_vs_E10	ordinary_muscle_development	ENSGALG00010011774	ANKRA2	-0.186821766	0.092702547	-2.015281898	0.043875121	0.05548023	negative
PRJNA729271	stage_3W_vs_E10	ordinary_muscle_development	ENSGALG00010011775	VEZT	0.334502838	0.079848762	4.189205045	2.79933E-05	4.772E-05	positive
PRJNA729271	stage_3W_vs_E10	ordinary_muscle_development	ENSGALG00010011776	IL17RA	-0.552471204	0.137424601	-4.020176895	5.81545E-05	9.69464E-05	negative
PRJNA729271	stage_3W_vs_E10	ordinary_muscle_development	ENSGALG00010011780	SERPINB5	-6.305936077	0.955944242	-6.596552186	4.20829E-11	1.03899E-10	negative
PRJNA729271	stage_3W_vs_E10	ordinary_muscle_development	ENSGALG00010011784	GRPEL2	0.034664842	0.134044514	0.258606943	0.795938525	0.818526352	positive
PRJNA729271	stage_3W_vs_E10	ordinary_muscle_development	ENSGALG00010011789	TXNRD1	-0.432849163	0.134378849	-3.221110808	0.001276948	0.001895479	negative
PRJNA729271	stage_3W_vs_E10	ordinary_muscle_development	ENSGALG00010011790	AMY2A	3.924442278	1.107386185	3.543878667	0.000394287	0.000612582	positive
PRJNA729271	stage_3W_vs_E10	ordinary_muscle_development	ENSGALG00010011792	HDHD5	1.477410463	0.140867615	10.48793552	9.81495E-26	4.73594E-25	positive
PRJNA729271	stage_3W_vs_E10	ordinary_muscle_development	ENSGALG00010011793	TLN1	0.071135564	0.11411062	0.623391264	0.533027437	0.570917956	positive
PRJNA729271	stage_3W_vs_E10	ordinary_muscle_development	ENSGALG00010011796	METAP2	0.549305063	0.089163736	6.160633095	7.24547E-10	1.66756E-09	positive
PRJNA729271	stage_3W_vs_E10	ordinary_muscle_development	ENSGALG00010011797	SCAF4	-0.015798807	0.156423829	-0.101000004	0.919550457	0.928712645	negative
PRJNA729271	stage_3W_vs_E10	ordinary_muscle_development	ENSGALG00010011802	VPS4B	0.191040827	0.092558039	2.064011183	0.039016656	0.049665973	positive
PRJNA729271	stage_3W_vs_E10	ordinary_muscle_development	ENSGALG00010011808	SMARCA2	0.374129362	0.122652523	3.050319331	0.002285982	0.003311213	positive
PRJNA729271	stage_3W_vs_E10	ordinary_muscle_development	ENSGALG00010011810	FGFR4	-0.84721523	0.126474944	-6.698680395	2.1031E-11	5.28418E-11	negative
PRJNA729271	stage_3W_vs_E10	ordinary_muscle_development	ENSGALG00010011814	ENSGALG00010011814	4.008524445	0.524221795	7.646619205	2.06332E-14	6.06158E-14	positive
PRJNA729271	stage_3W_vs_E10	ordinary_muscle_development	ENSGALG00010011817	KDSR	0.594088319	0.089553944	6.633859873	3.2702E-11	8.1393E-11	positive
PRJNA729271	stage_3W_vs_E10	ordinary_muscle_development	ENSGALG00010011819	USP44	0.833124074	0.138451614	6.017438522	1.77199E-09	3.97661E-09	positive
PRJNA729271	stage_3W_vs_E10	ordinary_muscle_development	ENSGALG00010011823	ENSGALG00010011823	-4.779093409	0.774366026	-6.171620716	6.75935E-10	1.55818E-09	negative
PRJNA729271	stage_3W_vs_E10	ordinary_muscle_development	ENSGALG00010011825	TAFA2	-5.814300953	0.462544103	-12.57026284	3.07699E-36	2.19601E-35	negative
PRJNA729271	stage_3W_vs_E10	ordinary_muscle_development	ENSGALG00010011837	VLDLR	2.0998299	0.134368622	15.62738289	4.7387E-55	6.2671E-54	positive
PRJNA729271	stage_3W_vs_E10	ordinary_muscle_development	ENSGALG00010011839	CBS	-1.63180072	0.46045738	-3.543869189	0.000394301	0.000612582	negative
PRJNA729271	stage_3W_vs_E10	ordinary_muscle_development	ENSGALG00010011841	KCNMB4	-3.879203099	0.278297339	-13.93905923	3.66742E-44	3.39243E-43	negative
PRJNA729271	stage_3W_vs_E10	ordinary_muscle_development	ENSGALG00010011843	BCL2	0.513016441	0.112017281	4.579797285	4.65427E-06	8.42874E-06	positive
PRJNA729271	stage_3W_vs_E10	ordinary_muscle_development	ENSGALG00010011846	UBAP1	0.598265799	0.104724518	5.712757698	1.1116E-08	2.39308E-08	positive
PRJNA729271	stage_3W_vs_E10	ordinary_muscle_development	ENSGALG00010011850	SPAG16	-1.587554297	0.357533249	-4.440298349	8.98342E-06	1.59128E-05	negative
PRJNA729271	stage_3W_vs_E10	ordinary_muscle_development	ENSGALG00010011856	NFYB	0.060533406	0.128718371	0.470277908	0.638156479	0.671560778	positive
PRJNA729271	stage_3W_vs_E10	ordinary_muscle_development	ENSGALG00010011857	ENSGALG00010011857	0.543504066	0.244380116	2.224010999	0.026147704	0.03393419	positive

PRJNA729271	stage_3W_vs_E10	ordinary_muscle_development	ENSGALG00010011859	NTN4	2.574465966	0.198828723	12.94815925	2.40656E-38	1.83692E-37	positive
PRJNA729271	stage_3W_vs_E10	ordinary_muscle_development	ENSGALG00010011860	CECR1	0.679700517	0.155710267	4.365161837	1.27029E-05	2.227E-05	positive
PRJNA729271	stage_3W_vs_E10	ordinary_muscle_development	ENSGALG00010011861	ENSGALG00010011861	-3.202100201	0.601132248	-5.326781602	9.99682E-08	2.02352E-07	negative
PRJNA729271	stage_3W_vs_E10	ordinary_muscle_development	ENSGALG00010011866	KLF8	0.535120928	0.187161532	2.859139497	0.004247919	0.006000155	positive
PRJNA729271	stage_3W_vs_E10	ordinary_muscle_development	ENSGALG00010011867	ENSGALG00010011867	0.282122552	0.274278859	1.028597511	0.303668848	0.341347805	positive
PRJNA729271	stage_3W_vs_E10	ordinary_muscle_development	ENSGALG00010011868	SNRPF	-1.534383125	0.125366958	-12.23913503	1.92069E-34	1.29913E-33	negative
PRJNA729271	stage_3W_vs_E10	ordinary_muscle_development	ENSGALG00010011870	AvBD6	3.104379197	1.393288281	2.228095391	0.025874155	0.033597229	positive
PRJNA729271	stage_3W_vs_E10	ordinary_muscle_development	ENSGALG00010011871	CCT2	-1.078356457	0.09801658	-11.00177598	3.74679E-28	2.01371E-27	negative
PRJNA729271	stage_3W_vs_E10	ordinary_muscle_development	ENSGALG00010011873	RFX3	-0.172558289	0.192759806	-0.895198499	0.370680987	0.409373022	negative
PRJNA729271	stage_3W_vs_E10	ordinary_muscle_development	ENSGALG00010011874	KCNS2	-5.473725769	0.787020648	-6.954996391	3.52571E-12	9.2098E-12	negative
PRJNA729271	stage_3W_vs_E10	ordinary_muscle_development	ENSGALG00010011875	PSD2	-2.145603683	0.29646	-7.237413751	4.57321E-13	1.2531E-12	negative
PRJNA729271	stage_3W_vs_E10	ordinary_muscle_development	ENSGALG00010011876	AMDHD1	-0.619778116	0.463044248	-1.338485724	0.180738151	0.210544041	negative
PRJNA729271	stage_3W_vs_E10	ordinary_muscle_development	ENSGALG00010011880	U2AF1	-0.236251665	0.156366307	-1.510886004	0.130817497	0.155732558	negative
PRJNA729271	stage_3W_vs_E10	ordinary_muscle_development	ENSGALG00010011881	RIOK2	-0.678228974	0.096594554	-7.021399724	2.19656E-12	5.81219E-12	negative
PRJNA729271	stage_3W_vs_E10	ordinary_muscle_development	ENSGALG00010011882	ENSGALG00010011882	0.232182157	0.286103462	0.811532147	0.417060139	0.455835223	positive
PRJNA729271	stage_3W_vs_E10	ordinary_muscle_development	ENSGALG00010011883	HCFC2	0.988845407	0.10668212	9.269082848	1.87753E-20	7.30404E-20	positive
PRJNA729271	stage_3W_vs_E10	ordinary_muscle_development	ENSGALG00010011885	NCALD	-0.94306742	0.212783625	-4.43204885	9.33419E-06	1.6514E-05	negative
PRJNA729271	stage_3W_vs_E10	ordinary_muscle_development	ENSGALG00010011886	NTNG1	-4.769279504	0.22351458	-21.33766619	5.0766E-101	2.17067E-99	negative
PRJNA729271	stage_3W_vs_E10	ordinary_muscle_development	ENSGALG00010011887	PGM5	2.213270407	0.132246988	16.73588519	7.1782E-63	1.16125E-61	positive
PRJNA729271	stage_3W_vs_E10	ordinary_muscle_development	ENSGALG00010011888	CAND1	-1.31306341	0.070433108	-18.64270147	1.44743E-77	3.40041E-76	negative
PRJNA729271	stage_3W_vs_E10	ordinary_muscle_development	ENSGALG00010011891	HAL	1.098279849	0.538892385	2.038031858	0.041546747	0.052669241	positive
PRJNA729271	stage_3W_vs_E10	ordinary_muscle_development	ENSGALG00010011893	LIX1	-2.496957268	0.450909239	-5.537605023	3.06636E-08	6.40629E-08	negative
PRJNA729271	stage_3W_vs_E10	ordinary_muscle_development	ENSGALG00010011894	VWC2L	-1.860639648	0.349047628	-5.330618229	9.7879E-08	1.98261E-07	negative
PRJNA729271	stage_3W_vs_E10	ordinary_muscle_development	ENSGALG00010011895	PHF21B	-2.818031629	0.895833232	-3.145710081	0.001656842	0.002435692	negative
PRJNA729271	stage_3W_vs_E10	ordinary_muscle_development	ENSGALG00010011896	SIK1	4.726406319	0.298113024	15.85441069	1.31051E-56	1.79885E-55	positive
PRJNA729271	stage_3W_vs_E10	ordinary_muscle_development	ENSGALG00010011898	ENSGALG00010011898	1.313258749	0.109734796	11.9675691	5.25456E-33	3.3761E-32	positive
PRJNA729271	stage_3W_vs_E10	ordinary_muscle_development	ENSGALG00010011899	PHLPP1	-0.119388402	0.085226941	-1.400829372	0.161265103	0.189396991	negative
PRJNA729271	stage_3W_vs_E10	ordinary_muscle_development	ENSGALG00010011902	TP53I3	-1.306399453	0.255826837	-5.106577045	3.28047E-07	6.4136E-07	negative
PRJNA729271	stage_3W_vs_E10	ordinary_muscle_development	ENSGALG00010011903	LTA4H	-0.001985976	0.115688169	-0.01716663	0.986303684	0.98834925	negative
PRJNA729271	stage_3W_vs_E10	ordinary_muscle_development	ENSGALG00010011904	KIF24	-3.573749898	0.338957092	-10.54336961	5.45101E-26	2.66035E-25	negative
PRJNA729271	stage_3W_vs_E10	ordinary_muscle_development	ENSGALG00010011906	ENSGALG00010011906	-5.343610919	1.014519298	-5.267135807	1.38569E-07	2.77501E-07	negative
PRJNA729271	stage_3W_vs_E10	ordinary_muscle_development	ENSGALG00010011907	BIK	-1.262002061	0.253092414	-4.986329073	6.15373E-07	1.18128E-06	negative
PRJNA729271	stage_3W_vs_E10	ordinary_muscle_development	ENSGALG00010011908	ENSGALG00010011908	-1.630064441	0.225221312	-7.237611858	4.56654E-13	1.2515E-12	negative
PRJNA729271	stage_3W_vs_E10	ordinary_muscle_development	ENSGALG00010011909	C8orf48	-1.49060048	0.213769416	-6.97293611	3.10394E-12	8.14474E-12	negative
PRJNA729271	stage_3W_vs_E10	ordinary_muscle_development	ENSGALG00010011911	NUDT2	-0.229329419	0.139160234	-1.64795224	0.099362472	0.120121688	negative
PRJNA729271	stage_3W_vs_E10	ordinary_muscle_development	ENSGALG00010011912	TCP11X2	1.811330327	0.125289538	14.45715549	2.25944E-47	2.33933E-46	positive
PRJNA729271	stage_3W_vs_E10	ordinary_muscle_development	ENSGALG00010011913	CXXC5	0.495333567	0.111455298	4.444235289	8.8205E-06	1.56357E-05	positive
PRJNA729271	stage_3W_vs_E10	ordinary_muscle_development	ENSGALG00010011915	RRM2B	0.546276814	0.121391343	4.500129926	6.79119E-06	1.21379E-05	positive
PRJNA729271	stage_3W_vs_E10	ordinary_muscle_development	ENSGALG00010011916	MCAT	0.181530673	0.097457755	1.862660118	0.0625101	0.077522579	positive
PRJNA729271	stage_3W_vs_E10	ordinary_muscle_development	ENSGALG00010011917	HSF2BP	-0.136133764	0.176636148	-0.770701614	0.440883815	0.47945164	negative
PRJNA729271	stage_3W_vs_E10	ordinary_muscle_development	ENSGALG00010011921	MEGF10	0.319535878	0.167331131	1.909602086	0.056184466	0.070109662	positive
PRJNA729271	stage_3W_vs_E10	ordinary_muscle_development	ENSGALG00010011922	PIK3R1	0.43400519	0.173060219	2.50782757	0.012147593	0.016364253	positive
PRJNA729271	stage_3W_vs_E10	ordinary_muscle_development	ENSGALG00010011923	BARD1	-3.310920719	0.179330076	-18.4627185	4.1207E-76	9.34734E-75	negative
PRJNA729271	stage_3W_vs_E10	ordinary_muscle_development	ENSGALG00010011925	RRP1B	-1.060283011	0.446977526	-2.372117051	0.017686487	0.023399564	negative
PRJNA729271	stage_3W_vs_E10	ordinary_muscle_development	ENSGALG00010011926	DYRK2	0.868303933	0.194866022	4.455902186	8.35411E-06	1.48307E-05	positive
PRJNA729271	stage_3W_vs_E10	ordinary_muscle_development	ENSGALG00010011927	ENSGALG00010011927	-1.715131718	0.521240043	-3.290483415	0.001000154	0.001498267	negative
PRJNA729271	stage_3W_vs_E10	ordinary_muscle_development	ENSGALG00010011929	ELK3	-1.381233335	0.136294778	-10.13416183	3.89701E-24	1.75973E-23	negative
PRJNA729271	stage_3W_vs_E10	ordinary_muscle_development	ENSGALG00010011930	TSPO	0.105524517	0.150968558	0.698983405	0.484562402	0.522910364	positive
PRJNA729271	stage_3W_vs_E10	ordinary_muscle_development	ENSGALG00010011931	ENSGALG00010011931	-2.898087477	1.3047324	-2.221212164			negative
PRJNA729271	stage_3W_vs_E10	ordinary_muscle_development	ENSGALG00010011932	VAV3	0.894658269	0.165061362	5.420155603	5.95472E-08	1.22119E-07	positive
PRJNA729271	stage_3W_vs_E10	ordinary_muscle_development	ENSGALG00010011935	ST8SIA4	0.149280791	0.124950104	1.194723227	0.232195184	0.266039775	positive
PRJNA729271	stage_3W_vs_E10	ordinary_muscle_development	ENSGALG00010011936	CKAP4	-1.860856819	0.175676473	-10.59252148	3.22778E-26	1.59301E-25	negative
PRJNA729271	stage_3W_vs_E10	ordinary_muscle_development	ENSGALG00010011937	SLC1A1	-1.794953941	0.336404906	-5.335694908	9.51793E-08	1.93035E-07	negative
PRJNA729271	stage_3W_vs_E10	ordinary_muscle_development	ENSGALG00010011941	ENSGALG00010011941	-2.870920379	0.243255864	-11.80206029	3.8087E-32	2.3745E-31	negative
PRJNA729271	stage_3W_vs_E10	ordinary_muscle_development	ENSGALG00010011943	TTLL12	-0.59923923	0.05516462	-10.86274551	1.73454E-27	9.04722E-27	negative
PRJNA729271	stage_3W_vs_E10	ordinary_muscle_development	ENSGALG00010011945	ENSGALG00010011945	0.824377101	0.455793427	1.808663866	0.07050324	0.086840964	positive
PRJNA729271	stage_3W_vs_E10	ordinary_muscle_development	ENSGALG00010011947	THAP1	1.143989481	0.111440096	10.26551056	1.00786E-24	4.66145E-24	positive
PRJNA729271	stage_3W_vs_E10	ordinary_muscle_development	ENSGALG00010011949	ABCA12	-3.701931215	0.482769302	-7.668116419	1.7454E-14	5.14534E-14	negative
PRJNA729271	stage_3W_vs_E10	ordinary_muscle_development	ENSGALG00010011950	PDXK	0.402131685	0.165079809	2.435983466	0.014851363	0.019820506	positive
PRJNA729271	stage_3W_vs_E10	ordinary_muscle_development	ENSGALG00010011952	UBE2D2	1.187829291	0.173619462	6.841567628	7.83312E-12	2.00958E-11	positive
PRJNA729271	stage_3W_vs_E10	ordinary_muscle_development	ENSGALG00010011953	CDK17	0.278877911	0.058295929	4.783831654	1.71985E-06	3.20427E-06	positive
PRJNA729271	stage_3W_vs_E10	ordinary_muscle_development	ENSGALG00010011954	ENSGALG00010011954	1.987449467	0.435120544	4.567583618	4.93379E-06	8.91823E-06	positive
PRJNA729271	stage_3W_vs_E10	ordinary_muscle_development	ENSGALG00010011957	ENSGALG00010011957	3.275025505	0.210011693	15.5944912	7.93531E-55	1.04093E-53	positive
PRJNA729271	stage_3W_vs_E10	ordinary_muscle_development	ENSGALG00010011960	CFAP54	-0.44440013	0.290852222	-1.527924132	0.126531376	0.150840718	negative
PRJNA729271	stage_3W_vs_E10	ordinary_muscle_development	ENSGALG00010011961	AGPAT3	0.464266204	0.103205934	4.498444856	6.84523E-06	1.22315E-05	positive
PRJNA729271	stage_3W_vs_E10	ordinary_muscle_development	ENSGALG00010011962	CPSF6	-1.104116632	0.060190263	-18.34377476	3.70178E-75	8.17947E-74	negative
PRJNA729271	stage_3W_vs_E10	ordinary_muscle_development	ENSGALG00010011963	ZCCHC2	0.048631297	0.100772256	0.482586173	0.629389599	0.663008883	positive
PRJNA729271	stage_3W_vs_E10	ordinary_muscle_development	ENSGALG00010011964	ENSGALG00010011964	-4.119406131	4.384389365	-0.939562112	0.347442218	0.385972943	negative
PRJNA729271	stage_3W_vs_E10	ordinary_muscle_development	ENSGALG00010011965	ATIC	-0.401162867	0.147028875	-2.728463143	0.00636302	0.008822649	negative
PRJNA729271	stage_3W_vs_E10	ordinary_muscle_development	ENSGALG00010011966	SLC25A24	0.10667185	0.137930681	0.773372892	0.439301728	0.477946299	positive
PRJNA729271	stage_3W_vs_E10	ordinary_muscle_development	ENSGALG00010011967	MTMR9	0.116640881	0.137399752	0.848916239	0.395927902	0.434803768	positive
PRJNA729271	stage_3W_vs_E10	ordinary_muscle_development	ENSGALG00010011968	ENSGALG00010011968	5.645014362	0.224105889	25.18904963	5.2806E-140	4.2761E-138	positive
PRJNA729271	stage_3W_vs_E10	ordinary_muscle_development	ENSGALG00010011969	TMEM173	1.79159272	0.293386772	6.106589978	1.01782E-09	2.31825E-09	positive
PRJNA729271	stage_3W_vs_E10	ordinary_muscle_development	ENSGALG00010011971	RNF170	-0.353896331	0.09015659	-3.925351799	8.66031E-05	0.000142084	negative
PRJNA729271	stage_3W_vs_E10	ordinary_muscle_development	ENSGALG00010011972	IPO11	-0.021055508	0.081081016	-0.259684808	0.795106912	0.817845209	negative
PRJNA729271	stage_3W_vs_E10	ordinary_muscle_development	ENSGALG00010011973	TRAPPC10	0.303623877	0.0496056	6.120758151	9.31312E-10	2.12521E-09	positive
PRJNA729271	stage_3W_vs_E10	ordinary_muscle_development	ENSGALG00010011976	TNFRSF11A	0.385368688	0.089368734	4.312119788	1.61697E-05	2.81132E-05	positive
PRJNA729271	stage_3W_vs_E10	ordinary_muscle_development	ENSGALG00010011977	GATD3A	1.247746642	0.118230647	10.55349582	4.89415E-26	2.39341E-25	positive
PRJNA729271	stage_3W_vs_E10	ordinary_muscle_development	ENSGALG00010011978	ENSGALG00010011978	-2.503861581	5.478910529	-0.456999903	0.647671115	0.680733291	negative
PRJNA729271	stage_3W_vs_E10	ordinary_muscle_development	ENSGALG00010011979	NEDD1	0.144134048	0.076942506	1.873269467	0.061031184	0.075818222	positive
PRJNA729271	stage_3W_vs_E10	ordinary_muscle_development	ENSGALG00010011980	AvBD9	17.22107747	2.34779115	7.335012516	2.21701E-13	6.17397E-13	positive
PRJNA729271	stage_3W_vs_E10	ordinary_muscle_development	ENSGALG00010011981	SCUBE1	-3.996092445	0.349921298	-11.41997492	3.3233E-30	1.923E-29	negative
PRJNA729271	stage_3W_vs_E10	ordinary_muscle_development	ENSGALG00010011982	STK3	-0.96814451	0.105754156	-9.154671016	5.45237E-20	2.08198E-19	negative
PRJNA729271	stage_3W_vs_E10	ordinary_muscle_development	ENSGALG00010011983	LYZ	2.001528549	0.529612402	3.779232779	0.000157312	0.000252811	positive
PRJNA729271	stage_3W_vs_E10	ordinary_muscle_development	ENSGALG00010011984	FN1	-0.093398074	0.240242831	-0.388765292	0.697449778	0.727147704	negative
PRJNA729271	stage_3W_vs_E10	ordinary_muscle_development	ENSGALG00010011987	H2BC32	-3.07349242	0.376056579	-8.17295213	3.00933E-16	9.70516E-16	negative
PRJNA729271	stage_3W_vs_E10	ordinary_muscle_development	ENSGALG00010011988	HINTW	-5.62304107	3.177965552	-1.769383896	0.076829831	0.094233238	negative
PRJNA729271	stage_3W_vs_E10	ordinary_muscle_development	ENSGALG00010011989	H1-010	-1.29645329	0.137081312	-9.457549482	3.15244E-21	1.26894E-20	negative
PRJNA729271	stage_3W_vs_E10	ordinary_muscle_development	ENSGALG00010011991	CSNK1A1	-0.125777002	0.056390078	-2.230481066	0.025715523	0.033403218	negative
PRJNA729271	stage_3W_vs_E10	ordinary_muscle_development	ENSGALG00010011992	YEATS4	-1.346301168	0.086314669	-15.59759409	7.55895E-55	9.92455E-54	negative
PRJNA729271	stage_3W_vs_E10	ordinary_muscle_development	ENSGALG00010011993	RELCH	-0.107103094	0.051593741	-2.075893143	0.037903835	0.048325806	negative
PRJNA729271	stage_3W_vs_E10	ordinary_muscle_development	ENSGALG00010011996	LRRC70	1.704014454	0.217615522	7.830390207	4.86358E-15	1.47546E-14	positive
PRJNA729271	stage_3W_vs_E10	ordinary_muscle_development	ENSGALG00010011997	DENND5B	-0.917765044	0.135594689	-6.768443906	1.30175E-11	3.30626E-11	negative
PRJNA729271	stage_3W_vs_E10	ordinary_muscle_development	ENSGALG00010011998	MPPED1	-0.98571887	0.076432414	-12.89660777	4.7035E-38	3.54536E-37	negative
PRJNA729271	stage_3W_vs_E10	ordinary_muscle_development	ENSGALG00010012001	EEIG2	0.667373391	0.252311527	2.645037262	0.008168192	0.011209804	positive
PRJNA729271	stage_3W_vs_E10	ordinary_muscle_development	ENSGALG00010012004	LGR5	-3.881368878	0.20990878	-18.49074093	2.45163E-76	5.5787E-75	negative
PRJNA729271	stage_3W_vs_E10	ordinary_muscle_development	ENSGALG00010012007	MREG	-0.939275909	0.214910275	-4.370549103	1.23934E-05	2.17459E-05	negative
PRJNA729271	stage_3W_vs_E10	ordinary_muscle_development	ENSGALG00010012008	ZNF366	4.363774476	0.175685145	24.83860818	3.4334E-136	2.6193E-134	positive
PRJNA729271	stage_3W_vs_E10	ordinary_muscle_development	ENSGALG00010012010	HENMT1	-0.940747776	0.168006639	-5.599467875	2.15011E-08	4.54312E-08	negative
PRJNA729271	stage_3W_vs_E10	ordinary_muscle_development	ENSGALG00010012011	HOOK3	0.903639317	0.068152507	13.25907665	3.99788E-40	3.24828E-39	positive
PRJNA729271	stage_3W_vs_E10	ordinary_muscle_development	ENSGALG00010012013	FOXD1	-6.350316235	1.054387768	-6.022752185	1.71476E-09	3.85236E-09	negative
PRJNA729271	stage_3W_vs_E10	ordinary_muscle_development	ENSGALG00010012015	ENSGALG00010012015	1.284019561	0.215212249	5.966294055	2.42703E-09	5.40147E-09	positive
PRJNA729271	stage_3W_vs_E10	ordinary_muscle_development	ENSGALG00010012016	WDR91	-0.516386943	0.064321786	-8.028181088	9.89284E-16	3.10023E-15	negative
PRJNA729271	stage_3W_vs_E10	ordinary_muscle_development	ENSGALG00010012017	N4BP3	-0.696586088	0.131264144	-5.306750693	1.11597E-07	2.2526E-07	negative
PRJNA729271	stage_3W_vs_E10	ordinary_muscle_development	ENSGALG00010012019	ENSGALG00010012019	7.538209345	0.300588062	25.07820601	8.5999E-139	6.7747E-137	positive
PRJNA729271	stage_3W_vs_E10	ordinary_muscle_development	ENSGALG00010012021	ENSGALG00010012021	0.176535648	0.130162203	1.356274279	0.175011916	0.204300372	positive
PRJNA729271	stage_3W_vs_E10	ordinary_muscle_development	ENSGALG00010012024	UBR5	0.22553613	0.075756562	2.977116744	0.002909732	0.004176724	positive
PRJNA729271	stage_3W_vs_E10	ordinary_muscle_development	ENSGALG00010012026	SYT1	-3.748260558	0.291843458	-12.84339414	9.36756E-38	6.99551E-37	negative
PRJNA729271	stage_3W_vs_E10	ordinary_muscle_development	ENSGALG00010012028	TMEM140	5.523803872	0.169555909	32.57806772	8.3872E-233	2.0961E-230	positive
PRJNA729271	stage_3W_vs_E10	ordinary_muscle_development	ENSGALG00010012031	GRK6	-0.224362178	0.108454548	-2.068720788	0.038572295	0.049143484	negative
PRJNA729271	stage_3W_vs_E10	ordinary_muscle_development	ENSGALG00010012034	ARHGEF9	0.884518248	0.1508275	5.864436188	4.50661E-09	9.88999E-09	positive
PRJNA729271	stage_3W_vs_E10	ordinary_muscle_development	ENSGALG00010012035	ENSGALG00010012035	-1.021901181	0.559712391	-1.825761226	0.067886236	0.083781268	negative
PRJNA729271	stage_3W_vs_E10	ordinary_muscle_development	ENSGALG00010012039	ODF1	-1.406737843	0.706768039	-1.990381236	0.046548955	0.058687232	negative
PRJNA729271	stage_3W_vs_E10	ordinary_muscle_development	ENSGALG00010012040	B4GALT7	0.221006405	0.1503128	1.470309947	0.141477828	0.167652152	positive
PRJNA729271	stage_3W_vs_E10	ordinary_muscle_development	ENSGALG00010012041	ICOSLG	0.460035874	0.150389305	3.058966681	0.002221019	0.003219366	positive
PRJNA729271	stage_3W_vs_E10	ordinary_muscle_development	ENSGALG00010012042	FNDC7	-3.994711844	0.429088958	-9.309752137	1.28131E-20	5.02233E-20	negative
PRJNA729271	stage_3W_vs_E10	ordinary_muscle_development	ENSGALG00010012043	NUP50	-0.043425136	0.095545921	-0.454494928	0.649472642	0.682428847	negative
PRJNA729271	stage_3W_vs_E10	ordinary_muscle_development	ENSGALG00010012044	ENSGALG00010012044	2.796830862	0.608952453	4.592855889	4.37221E-06	7.93281E-06	positive
PRJNA729271	stage_3W_vs_E10	ordinary_muscle_development	ENSGALG00010012045	ENSGALG00010012045	0.663893937	0.300682043	2.207960045	0.027247059	0.035278795	positive
PRJNA729271	stage_3W_vs_E10	ordinary_muscle_development	ENSGALG00010012046	LOXHD1	-0.349223095	0.275163773	-1.269146339	0.204388876	0.236291016	negative
PRJNA729271	stage_3W_vs_E10	ordinary_muscle_development	ENSGALG00010012047	LMAN2	0.046016506	0.090668382	0.507525394	0.611786223	0.646674054	positive
PRJNA729271	stage_3W_vs_E10	ordinary_muscle_development	ENSGALG00010012048	PDE6H	-2.411898726	0.608988887	-3.960497105	7.47939E-05	0.000123467	negative
PRJNA729271	stage_3W_vs_E10	ordinary_muscle_development	ENSGALG00010012049	IFNAR1	1.142185855	0.144399753	7.909887866	2.57621E-15	7.91315E-15	positive
PRJNA729271	stage_3W_vs_E10	ordinary_muscle_development	ENSGALG00010012051	ARK2C	-2.396636355	0.21050935	-11.38494017	4.97023E-30	2.8566E-29	negative
PRJNA729271	stage_3W_vs_E10	ordinary_muscle_development	ENSGALG00010012052	KLF10	2.009595146	0.122916289	16.34929881	4.40014E-60	6.58205E-59	positive
PRJNA729271	stage_3W_vs_E10	ordinary_muscle_development	ENSGALG00010012054	FAM60A	-2.095775829	0.091966467	-22.78847828	5.9653E-115	3.2629E-113	negative
PRJNA729271	stage_3W_vs_E10	ordinary_muscle_development	ENSGALG00010012055	ENSGALG00010012055	0.770142016	0.156446389	4.922721594	8.53488E-07	1.62438E-06	positive
PRJNA729271	stage_3W_vs_E10	ordinary_muscle_development	ENSGALG00010012058	ENSGALG00010012058	-1.997602072	0.313667765	-6.368528406	1.9085E-10	4.541E-10	negative
PRJNA729271	stage_3W_vs_E10	ordinary_muscle_development	ENSGALG00010012059	ARHGDIB	-1.166570958	0.14684512	-7.944226945	1.95406E-15	6.02896E-15	negative
PRJNA729271	stage_3W_vs_E10	ordinary_muscle_development	ENSGALG00010012060	ENSGALG00010012060	1.71236063	0.123922654	13.81797898	1.98567E-43	1.80002E-42	positive
PRJNA729271	stage_3W_vs_E10	ordinary_muscle_development	ENSGALG00010012061	AZIN1	0.975026148	0.120416608	8.097106894	5.62816E-16	1.78865E-15	positive

PRJNA729271	stage_3W_vs_E10	ordinary_muscle_development	ENSGALG00010012062	HMGA2	-6.0433143	0.175307628	-34.4726261	2.0634E-260	6.7975E-258	negative
PRJNA729271	stage_3W_vs_E10	ordinary_muscle_development	ENSGALG00010012063	PTPRD	-1.130366021	0.196983065	-5.738391874	9.55798E-09	2.06461E-08	negative
PRJNA729271	stage_3W_vs_E10	ordinary_muscle_development	ENSGALG00010012064	RGS14	-0.386718645	0.172033471	-2.24792677	0.02458086	0.03202117	negative
PRJNA729271	stage_3W_vs_E10	ordinary_muscle_development	ENSGALG00010012066	STXBP3	-0.545225123	0.121982906	-4.469684637	7.8335E-06	1.39441E-05	negative
PRJNA729271	stage_3W_vs_E10	ordinary_muscle_development	ENSGALG00010012067	ENSGALG00010012067	0.1894206	0.083413882	2.27085222	0.023155926	0.030271026	positive
PRJNA729271	stage_3W_vs_E10	ordinary_muscle_development	ENSGALG00010012069	ERP27	-0.890337942	0.603035751	-1.476426464	0.139829443	0.165793683	negative
PRJNA729271	stage_3W_vs_E10	ordinary_muscle_development	ENSGALG00010012070	DNAJC28	0.619090238	0.161468341	3.834127701	0.000126011	0.000204309	positive
PRJNA729271	stage_3W_vs_E10	ordinary_muscle_development	ENSGALG00010012071	ENSGALG00010012071	-0.839068131	0.151781201	-5.528142634	3.23639E-08	6.74888E-08	negative
PRJNA729271	stage_3W_vs_E10	ordinary_muscle_development	ENSGALG00010012072	CAPRIN2	-0.117570538	0.150558333	-0.780896915	0.434863127	0.47350819	negative
PRJNA729271	stage_3W_vs_E10	ordinary_muscle_development	ENSGALG00010012074	KIAA0930	-0.438224666	0.088377367	-4.958562149	7.10168E-07	1.35714E-06	negative
PRJNA729271	stage_3W_vs_E10	ordinary_muscle_development	ENSGALG00010012075	PIGN	-0.359559491	0.096532635	-3.724745442	0.000195512	0.0003118	negative
PRJNA729271	stage_3W_vs_E10	ordinary_muscle_development	ENSGALG00010012077	IFNGR2	0.901347873	0.068025227	13.25020019	4.50001E-40	3.644E-39	positive
PRJNA729271	stage_3W_vs_E10	ordinary_muscle_development	ENSGALG00010012078	TMBIM4	-0.07073964	0.082581463	-0.856604339	0.391663587	0.430642774	negative
PRJNA729271	stage_3W_vs_E10	ordinary_muscle_development	ENSGALG00010012079	ENSGALG00010012079	-1.213833953	0.37632781	-3.225469714	0.001257661	0.001867808	negative
PRJNA729271	stage_3W_vs_E10	ordinary_muscle_development	ENSGALG00010012081	CHIC1	0.256469877	0.124197723	2.065012711	0.038921798	0.049562633	positive
PRJNA729271	stage_3W_vs_E10	ordinary_muscle_development	ENSGALG00010012082	HAUS1	-2.622052318	0.21469794	-12.21275025	2.65751E-34	1.7875E-33	negative
PRJNA729271	stage_3W_vs_E10	ordinary_muscle_development	ENSGALG00010012083	MGP	4.219341246	0.277659603	15.19609333	3.75348E-52	4.55668E-51	positive
PRJNA729271	stage_3W_vs_E10	ordinary_muscle_development	ENSGALG00010012084	TMEM50B	0.745749061	0.105381614	7.076652514	1.47678E-12	3.94725E-12	positive
PRJNA729271	stage_3W_vs_E10	ordinary_muscle_development	ENSGALG00010012085	IPO8	-0.432570685	0.0968306	-4.467293225	7.92155E-06	1.40956E-05	negative
PRJNA729271	stage_3W_vs_E10	ordinary_muscle_development	ENSGALG00010012086	CIBAR1	-1.810823206	0.134629254	-13.45044373	3.0603E-41	2.56115E-40	negative
PRJNA729271	stage_3W_vs_E10	ordinary_muscle_development	ENSGALG00010012087	TMEM171	0.660132802	0.168707073	3.912893455	9.11968E-05	0.000149316	positive
PRJNA729271	stage_3W_vs_E10	ordinary_muscle_development	ENSGALG00010012088	ATP5F1AZ	0.520965815	0.169893134	3.066432424	0.002166298	0.003143821	positive
PRJNA729271	stage_3W_vs_E10	ordinary_muscle_development	ENSGALG00010012089	ENSGALG00010012089	-2.110167123	0.288446499	-7.315627432	2.56182E-13	7.11506E-13	negative
PRJNA729271	stage_3W_vs_E10	ordinary_muscle_development	ENSGALG00010012090	HELB	0.472343728	0.223156687	2.11664608	0.034289896	0.043915183	positive
PRJNA729271	stage_3W_vs_E10	ordinary_muscle_development	ENSGALG00010012091	ENSGALG00010012091	-0.643625612	0.444693913	-1.447345226	0.147800258	0.174485087	negative
PRJNA729271	stage_3W_vs_E10	ordinary_muscle_development	ENSGALG00010012092	OC3	9.038242096	0.697742516	12.95354931	2.24337E-38	1.71416E-37	positive
PRJNA729271	stage_3W_vs_E10	ordinary_muscle_development	ENSGALG00010012093	ENSGALG00010012093	-1.76259038	0.0694395	-25.38310859	3.8751E-142	3.2468E-140	negative
PRJNA729271	stage_3W_vs_E10	ordinary_muscle_development	ENSGALG00010012094	ENSGALG00010012094	0.257319558	0.336929079	0.763720242	0.445033975	0.483783371	positive
PRJNA729271	stage_3W_vs_E10	ordinary_muscle_development	ENSGALG00010012095	RNF38	0.015561093	0.086906672	0.17905522	0.857894343	0.874116301	positive
PRJNA729271	stage_3W_vs_E10	ordinary_muscle_development	ENSGALG00010012096	ENSGALG00010012096	-2.62148718	0.169192545	-15.4941057	3.80214E-54	4.8945E-53	negative
PRJNA729271	stage_3W_vs_E10	ordinary_muscle_development	ENSGALG00010012097	GART	-0.078198122	0.192162249	-0.406938003	0.684053524	0.714929398	negative
PRJNA729271	stage_3W_vs_E10	ordinary_muscle_development	ENSGALG00010012098	ART4	1.976981328	0.529104772	3.736464749	0.000186626	0.000298021	positive
PRJNA729271	stage_3W_vs_E10	ordinary_muscle_development	ENSGALG00010012099	UPK3A	-4.490041747	0.525899073	-8.537839249	1.36755E-17	4.72417E-17	negative
PRJNA729271	stage_3W_vs_E10	ordinary_muscle_development	ENSGALG00010012100	UBE2F	1.310853171	0.11101128	11.8082881	3.53689E-32	2.2098E-31	positive
PRJNA729271	stage_3W_vs_E10	ordinary_muscle_development	ENSGALG00010012101	ENSGALG00010012101	-2.156411174	0.138420263	-15.57872478	1.01561E-54	1.32624E-53	negative
PRJNA729271	stage_3W_vs_E10	ordinary_muscle_development	ENSGALG00010012102	SON	0.339296969	0.104852219	3.235954106	0.001212369	0.001802758	positive
PRJNA729271	stage_3W_vs_E10	ordinary_muscle_development	ENSGALG00010012103	ENSGALG00010012103	0.949285769	0.207940773	4.565173796	4.99081E-06	9.01342E-06	positive
PRJNA729271	stage_3W_vs_E10	ordinary_muscle_development	ENSGALG00010012105	DONSON	-2.734399616	0.218921222	-12.49033597	8.42969E-36	5.94301E-35	negative
PRJNA729271	stage_3W_vs_E10	ordinary_muscle_development	ENSGALG00010012106	TMTC1	-0.553492918	0.139251896	-3.974760363	7.04502E-05	0.000116533	negative
PRJNA729271	stage_3W_vs_E10	ordinary_muscle_development	ENSGALG00010012107	CLCC1	0.15531482	0.084896893	1.829452337	0.067331873	0.083167917	positive
PRJNA729271	stage_3W_vs_E10	ordinary_muscle_development	ENSGALG00010012108	PSTPIP2	0.906607854	0.333416778	2.719142872	0.006545133	0.009069086	positive
PRJNA729271	stage_3W_vs_E10	ordinary_muscle_development	ENSGALG00010012110	ENSGALG00010012110	0.911637352	0.112664935	8.091580184	5.88956E-16	1.86926E-15	positive
PRJNA729271	stage_3W_vs_E10	ordinary_muscle_development	ENSGALG00010012111	DRD1	-0.521732461	0.33536797	-1.555701523	0.119779084	0.143253586	negative
PRJNA729271	stage_3W_vs_E10	ordinary_muscle_development	ENSGALG00010012112	MBD2	1.927539318	0.189592228	10.16676338	2.79027E-24	1.26747E-23	positive
PRJNA729271	stage_3W_vs_E10	ordinary_muscle_development	ENSGALG00010012113	WDR47	-0.679720409	0.095902123	-7.087647123	1.36411E-12	3.65284E-12	negative
PRJNA729271	stage_3W_vs_E10	ordinary_muscle_development	ENSGALG00010012114	TAL2	2.029759336	0.303698904	6.683459534	2.33366E-11	5.85435E-11	positive
PRJNA729271	stage_3W_vs_E10	ordinary_muscle_development	ENSGALG00010012116	RBM12B	-0.493792698	0.078004533	-6.330307697	2.44673E-10	5.78931E-10	negative
PRJNA729271	stage_3W_vs_E10	ordinary_muscle_development	ENSGALG00010012118	ENSGALG00010012118	1.05021644	0.718069366	1.462555695	0.143588994	0.169973252	positive
PRJNA729271	stage_3W_vs_E10	ordinary_muscle_development	ENSGALG00010012119	EPG5	0.596565523	0.122393365	4.874165549	1.09269E-06	2.06366E-06	positive
PRJNA729271	stage_3W_vs_E10	ordinary_muscle_development	ENSGALG00010012120	ENSGALG00010012120	-0.069076191	0.188297301	-0.36684642	0.71373358	0.742416092	negative
PRJNA729271	stage_3W_vs_E10	ordinary_muscle_development	ENSGALG00010012121	SREK1	-0.314623892	0.100363591	-3.134840935	0.001719473	0.002522393	negative
PRJNA729271	stage_3W_vs_E10	ordinary_muscle_development	ENSGALG00010012122	GRIP1	-4.270838645	0.287301301	-14.86536478	5.53128E-50	6.2491E-49	negative
PRJNA729271	stage_3W_vs_E10	ordinary_muscle_development	ENSGALG00010012123	ENSGALG00010012123	-1.751605074	0.210582012	-8.317923546	8.95177E-17	2.96789E-16	negative
PRJNA729271	stage_3W_vs_E10	ordinary_muscle_development	ENSGALG00010012124	ERAP1	2.989868525	0.146262654	20.44177685	7.11029E-93	2.49549E-91	positive
PRJNA729271	stage_3W_vs_E10	ordinary_muscle_development	ENSGALG00010012125	RNF152	-0.321248431	0.149019022	-2.155754526	0.031102838	0.040024471	negative
PRJNA729271	stage_3W_vs_E10	ordinary_muscle_development	ENSGALG00010012127	ENSGALG00010012127	-0.599358185	0.116832125	-5.130080336	2.89619E-07	5.68298E-07	negative
PRJNA729271	stage_3W_vs_E10	ordinary_muscle_development	ENSGALG00010012128	ENSGALG00010012128	2.878765495	0.756100679	3.807383823	0.000140445	0.000226799	positive
PRJNA729271	stage_3W_vs_E10	ordinary_muscle_development	ENSGALG00010012129	CRYZL1	-0.65979439	0.097946763	-6.736255172	1.62521E-11	4.10909E-11	negative
PRJNA729271	stage_3W_vs_E10	ordinary_muscle_development	ENSGALG00010012130	TMEM67	-1.64338105	0.170276274	-9.651262672	4.85541E-22	2.01544E-21	negative
PRJNA729271	stage_3W_vs_E10	ordinary_muscle_development	ENSGALG00010012131	SLC14A1	1.299554782	0.241310694	5.385400709	7.22834E-08	1.47757E-07	positive
PRJNA729271	stage_3W_vs_E10	ordinary_muscle_development	ENSGALG00010012132	MC4R	-3.342544352	1.013679694	-3.297436429	0.000975718	0.001463021	negative
PRJNA729271	stage_3W_vs_E10	ordinary_muscle_development	ENSGALG00010012133	RAB3IP	-2.459358213	0.238531701	-10.31040404	6.32349E-25	2.95008E-24	negative
PRJNA729271	stage_3W_vs_E10	ordinary_muscle_development	ENSGALG00010012135	CAMSAP2	-0.328963938	0.092194033	-3.568169528	0.000359484	0.000560775	negative
PRJNA729271	stage_3W_vs_E10	ordinary_muscle_development	ENSGALG00010012136	ENSGALG00010012136	0.504879743	0.178588874	2.827050373	0.004697894	0.006609334	positive
PRJNA729271	stage_3W_vs_E10	ordinary_muscle_development	ENSGALG00010012137	ENSGALG00010012137	0.628781991	0.939679396	0.669145236	0.503402836	0.541347586	positive
PRJNA729271	stage_3W_vs_E10	ordinary_muscle_development	ENSGALG00010012138	DDX11	-2.628057041	0.181972894	-14.44202474	2.81451E-47	2.90572E-46	negative
PRJNA729271	stage_3W_vs_E10	ordinary_muscle_development	ENSGALG00010012140	GLOD5	-0.697911008	0.736098219	-0.948122126	0.343067285	0.381522196	negative
PRJNA729271	stage_3W_vs_E10	ordinary_muscle_development	ENSGALG00010012141	ENSGALG00010012141	0.299956038	0.093882922	3.195001103	0.001398303	0.002067571	positive
PRJNA729271	stage_3W_vs_E10	ordinary_muscle_development	ENSGALG00010012144	H1-01	-0.746231522	0.157684876	-4.732422912	2.21856E-06	4.10702E-06	negative
PRJNA729271	stage_3W_vs_E10	ordinary_muscle_development	ENSGALG00010012145	ENSGALG00010012145	7.545340119	0.867013362	8.702680314	3.24136E-18	1.15581E-17	positive
PRJNA729271	stage_3W_vs_E10	ordinary_muscle_development	ENSGALG00010012147	ARFGAP3	-0.348109383	0.122781249	-2.835199891	0.004579702	0.006447434	negative
PRJNA729271	stage_3W_vs_E10	ordinary_muscle_development	ENSGALG00010012148	SETBP1	-0.509432594	0.109586248	-4.64869089	3.34048E-06	6.11986E-06	negative
PRJNA729271	stage_3W_vs_E10	ordinary_muscle_development	ENSGALG00010012149	SFXN1	0.069563315	0.172832858	0.402488949	0.68732422	0.718140602	positive
PRJNA729271	stage_3W_vs_E10	ordinary_muscle_development	ENSGALG00010012151	MYRFL	-0.189485992	0.493955259	-0.383609626	0.701267805	0.730602805	negative
PRJNA729271	stage_3W_vs_E10	ordinary_muscle_development	ENSGALG00010012154	CWC27	-0.065856176	0.113498761	-0.58023696	0.561754832	0.598591215	negative
PRJNA729271	stage_3W_vs_E10	ordinary_muscle_development	ENSGALG00010012156	ENSGALG00010012156	-3.398687903	0.659830359	-5.150851056	2.59307E-07	5.1041E-07	negative
PRJNA729271	stage_3W_vs_E10	ordinary_muscle_development	ENSGALG00010012157	H2BC32	-2.730722097	0.199198471	-13.70854947	9.02439E-43	7.981E-42	negative
PRJNA729271	stage_3W_vs_E10	ordinary_muscle_development	ENSGALG00010012159	WASHC1	-0.198337381	0.102473342	-1.935502219	0.052928697	0.06621226	negative
PRJNA729271	stage_3W_vs_E10	ordinary_muscle_development	ENSGALG00010012160	SYT4	-4.635592611	0.441444609	-10.50096098	8.55052E-26	4.13822E-25	negative
PRJNA729271	stage_3W_vs_E10	ordinary_muscle_development	ENSGALG00010012163	PDP1	1.214707224	0.220832818	5.500573841	3.78557E-08	7.87145E-08	positive
PRJNA729271	stage_3W_vs_E10	ordinary_muscle_development	ENSGALG00010012168	ENSGALG00010012168	-3.124506244	0.593093966	-5.268147075	1.37808E-07	2.7613E-07	negative
PRJNA729271	stage_3W_vs_E10	ordinary_muscle_development	ENSGALG00010012169	H3C33	-3.348927906	0.229115018	-14.61679785	2.19483E-48	2.37951E-47	negative
PRJNA729271	stage_3W_vs_E10	ordinary_muscle_development	ENSGALG00010012171	CDH17	-0.513541504	0.396630361	-1.294760953	0.195402706	0.226516492	negative
PRJNA729271	stage_3W_vs_E10	ordinary_muscle_development	ENSGALG00010012172	CNOT2	-0.40480934	0.089242827	-4.536043431	5.73194E-06	1.03134E-05	negative
PRJNA729271	stage_3W_vs_E10	ordinary_muscle_development	ENSGALG00010012175	PACSIN2	0.563566059	0.154474567	3.648277308	0.000264005	0.000415996	positive
PRJNA729271	stage_3W_vs_E10	ordinary_muscle_development	ENSGALG00010012179	ENSGALG00010012179	-1.940520335	0.180424346	-10.7553131	5.59437E-27	2.85697E-26	negative
PRJNA729271	stage_3W_vs_E10	ordinary_muscle_development	ENSGALG00010012180	DDX59	-0.113767916	0.106019184	-1.073088019	0.283231626	0.320337267	negative
PRJNA729271	stage_3W_vs_E10	ordinary_muscle_development	ENSGALG00010012186	KIF20A	-3.643363325	0.249877391	-14.58060416	3.73199E-48	4.00705E-47	negative
PRJNA729271	stage_3W_vs_E10	ordinary_muscle_development	ENSGALG00010012187	ENSGALG00010012187	-2.867537352	0.863932374	-3.319168767	0.000902858	0.001357284	negative
PRJNA729271	stage_3W_vs_E10	ordinary_muscle_development	ENSGALG00010012188	ADAMTS6	-0.582840379	0.183073664	-3.183638581	0.001454365	0.00214718	negative
PRJNA729271	stage_3W_vs_E10	ordinary_muscle_development	ENSGALG00010012190	CAST	0.806667365	0.132407902	6.092290223	1.11307E-09	2.52803E-09	positive
PRJNA729271	stage_3W_vs_E10	ordinary_muscle_development	ENSGALG00010012191	BEST3	5.3649407	0.373299048	14.37169671	7.78991E-47	7.92385E-46	positive
PRJNA729271	stage_3W_vs_E10	ordinary_muscle_development	ENSGALG00010012193	TTLL1	-0.456185671	0.213229785	-2.139408762	0.032402578	0.041615751	negative
PRJNA729271	stage_3W_vs_E10	ordinary_muscle_development	ENSGALG00010012194	LRRFIP1	1.709539991	0.159868207	10.6934332	1.0925E-26	5.51004E-26	positive
PRJNA729271	stage_3W_vs_E10	ordinary_muscle_development	ENSGALG00010012195	ITSN1	-0.083252409	0.072099565	-1.154686704	0.248218746	0.28336857	negative
PRJNA729271	stage_3W_vs_E10	ordinary_muscle_development	ENSGALG00010012196	CENPK	-3.708614562	0.268155822	-13.83007288	1.6785E-43	1.5273E-42	negative
PRJNA729271	stage_3W_vs_E10	ordinary_muscle_development	ENSGALG00010012198	TMEM5	1.081844098	0.098126041	11.02504583	2.89371E-28	1.56101E-27	positive
PRJNA729271	stage_3W_vs_E10	ordinary_muscle_development	ENSGALG00010012199	ENSGALG00010012199	1.092583499	0.284612691	3.838843224	0.000123615	0.000200538	positive
PRJNA729271	stage_3W_vs_E10	ordinary_muscle_development	ENSGALG00010012200	GEM	1.229708796	0.238835802	5.148762387	2.62211E-07	5.15915E-07	positive
PRJNA729271	stage_3W_vs_E10	ordinary_muscle_development	ENSGALG00010012205	SLC6A13	-5.835917354	0.571541382	-10.21083955	1.77324E-24	8.13131E-24	negative
PRJNA729271	stage_3W_vs_E10	ordinary_muscle_development	ENSGALG00010012206	RIBC2	-4.251942958	0.858832984	-4.950837981	7.38946E-07	1.4099E-06	negative
PRJNA729271	stage_3W_vs_E10	ordinary_muscle_development	ENSGALG00010012207	GRIA3	-3.07123084	0.276160871	-11.12116581	9.89702E-29	5.4094E-28	negative
PRJNA729271	stage_3W_vs_E10	ordinary_muscle_development	ENSGALG00010012209	KIF14	-2.02199068	0.23143478	-8.73676237	2.39886E-18	8.59405E-18	negative
PRJNA729271	stage_3W_vs_E10	ordinary_muscle_development	ENSGALG00010012210	PPWD1	-1.226870945	0.076778567	-15.97934163	1.78016E-57	2.49791E-56	negative
PRJNA729271	stage_3W_vs_E10	ordinary_muscle_development	ENSGALG00010012211	ENSGALG00010012211	-6.069805143	4.076154142	-1.489100984	0.136460779	0.162008048	negative
PRJNA729271	stage_3W_vs_E10	ordinary_muscle_development	ENSGALG00010012212	RAD54B	-3.443719475	0.204458032	-16.84316064	1.17774E-63	1.94879E-62	negative
PRJNA729271	stage_3W_vs_E10	ordinary_muscle_development	ENSGALG00010012215	RAB17	4.64736841	0.768328902	6.04867056	1.46046E-09	3.29484E-09	positive
PRJNA729271	stage_3W_vs_E10	ordinary_muscle_development	ENSGALG00010012216	BRD8	-0.186803035	0.057936147	-3.224291665	0.001262847	0.001875317	negative
PRJNA729271	stage_3W_vs_E10	ordinary_muscle_development	ENSGALG00010012219	KDM5A	-0.699259134	0.077714023	-8.997850107	2.30181E-19	8.59915E-19	negative
PRJNA729271	stage_3W_vs_E10	ordinary_muscle_development	ENSGALG00010012226	SLCO4C1	1.704023833	0.493356082	3.453943093	0.000552454	0.000846672	positive
PRJNA729271	stage_3W_vs_E10	ordinary_muscle_development	ENSGALG00010012228	NHP2	-0.944564835	0.114702439	-8.23491503	1.79685E-16	5.86343E-16	negative
PRJNA729271	stage_3W_vs_E10	ordinary_muscle_development	ENSGALG00010012229	FSBP	0.569980314	0.201823237	2.824156037	0.00474053	0.006666083	positive
PRJNA729271	stage_3W_vs_E10	ordinary_muscle_development	ENSGALG00010012230	PTPRQ	-1.617060081	0.424281105	-3.811294117	0.000138241	0.00022339	negative
PRJNA729271	stage_3W_vs_E10	ordinary_muscle_development	ENSGALG00010012232	PCSK1	-0.092745158	0.439612273	-0.210970357	0.832910403	0.852254432	negative
PRJNA729271	stage_3W_vs_E10	ordinary_muscle_development	ENSGALG00010012234	C1GALT1C1	-0.13741798	0.062763917	-2.189442388	0.028564702	0.03691889	negative
PRJNA729271	stage_3W_vs_E10	ordinary_muscle_development	ENSGALG00010012236	PRLH	-2.173058841	0.642671117	-3.381292211	0.000721458	0.001095029	negative
PRJNA729271	stage_3W_vs_E10	ordinary_muscle_development	ENSGALG00010012240	TRIM14	-0.090780327	0.228566634	-0.397172264	0.691240434	0.72155625	negative
PRJNA729271	stage_3W_vs_E10	ordinary_muscle_development	ENSGALG00010012242	CCDC77	-2.514755849	0.148734403	-16.90769449	3.94856E-64	6.61669E-63	negative
PRJNA729271	stage_3W_vs_E10	ordinary_muscle_development	ENSGALG00010012243	ENSGALG00010012243	-4.288488192	1.680890327	-2.55131454	0.010731591	0.014540513	negative
PRJNA729271	stage_3W_vs_E10	ordinary_muscle_development	ENSGALG00010012244	ENSGALG00010012244	-1.444084701	0.085771188	-16.83647776	1.31855E-63	2.17929E-62	negative
PRJNA729271	stage_3W_vs_E10	ordinary_muscle_development	ENSGALG00010012245	ENSGALG00010012245	-0.591741232	0.079187062	-7.472700949	7.85653E-14	2.24483E-13	negative
PRJNA729271	stage_3W_vs_E10	ordinary_muscle_development	ENSGALG00010012247	TRIM23	1.263550821	0.125372643	10.07836153	6.88649E-24	3.07516E-23	positive
PRJNA729271	stage_3W_vs_E10	ordinary_muscle_development	ENSGALG00010012248	H2AC39	-2.855309771	0.210066976	-13.59237816	4.44402E-42	3.83201E-41	negative
PRJNA729271	stage_3W_vs_E10	ordinary_muscle_development	ENSGALG00010012250	NANS	-0.655259537	0.111159657	-5.894760327	3.75226E-09	8.26957E-09	negative
PRJNA729271	stage_3W_vs_E10	ordinary_muscle_development	ENSGALG00010012253	MYF6	2.269152501	0.289190373	7.846569977	4.2757E-15	1.30011E-14	positive
PRJNA729271	stage_3W_vs_E10	ordinary_muscle_development	ENSGALG00010012259	IL1RAPL2	-1.816181822	0.23138586	-7.84914782	4.18874E-15	1.2742E-14	negative
PRJNA729271	stage_3W_vs_E10	ordinary_muscle_development	ENSGALG00010012260	ENSGALG00010012260	2.222872883	0.30225825	7.354217415	1.92049E-13	5.36885E-13	positive
PRJNA729271	stage_3W_vs_E10	ordinary_muscle_development	ENSGALG00010012263	SHLD3	0.708007018	0.126089287	5.615124271	1.96421E-08	4.16351E-08	positive
PRJNA729271	stage_3W_vs_E10	ordinary_muscle_development	ENSGALG00010012264	MYF5	1.486932517	0.1551442	9.584196606	9.318E-22	3.82944E-21	positive
PRJNA729271	stage_3W_vs_E10	ordinary_muscle_development	ENSGALG00010012266	H3C33	-3.13200601	0.203528026	-15.38857363	1.9529E-53	2.47658E-52	negative
PRJNA729271	stage_3W_vs_E10	ordinary_muscle_development	ENSGALG00010012268	ENSGALG00010012268	-0.907991589	0.168927131	-5.375048894	7.65619E-08	1.56305E-07	negative

PRJNA729271	stage_3W_vs_E10	ordinary_muscle_development	ENSGALG00010012271	ENSGALG00010012271	-0.242580559	0.300079753	-0.808386959	0.418867857	0.457431597	negative
PRJNA729271	stage_3W_vs_E10	ordinary_muscle_development	ENSGALG00010012274	B4GALNT3	-0.017828123	0.277678063	-0.064204289	0.948807562	0.955403577	negative
PRJNA729271	stage_3W_vs_E10	ordinary_muscle_development	ENSGALG00010012275	VIRMA	-0.102670513	0.073647748	-1.394075387	0.163294841	0.191653864	negative
PRJNA729271	stage_3W_vs_E10	ordinary_muscle_development	ENSGALG00010012277	H3C33	-2.996877785	0.21050761	-14.2364344	5.44344E-46	5.39321E-45	negative
PRJNA729271	stage_3W_vs_E10	ordinary_muscle_development	ENSGALG00010012282	ENSGALG00010012282	-2.885963739	1.548921186	-1.8632089			negative
PRJNA729271	stage_3W_vs_E10	ordinary_muscle_development	ENSGALG00010012283	CUPIN1A	1.176932491	0.746822191	1.57592062	0.115044098	0.137929214	positive
PRJNA729271	stage_3W_vs_E10	ordinary_muscle_development	ENSGALG00010012285	FBLN1	-2.459678941	0.199347219	-12.33866696	5.60766E-35	3.85045E-34	negative
PRJNA729271	stage_3W_vs_E10	ordinary_muscle_development	ENSGALG00010012286	ENSGALG00010012286	-6.041929925	2.716502518	-2.224157675	0.026137838	0.033924423	negative
PRJNA729271	stage_3W_vs_E10	ordinary_muscle_development	ENSGALG00010012288	MCTS1	1.005158837	0.113016524	8.893910419	5.89953E-19	2.16271E-18	positive
PRJNA729271	stage_3W_vs_E10	ordinary_muscle_development	ENSGALG00010012290	ENSGALG00010012290	0.34919839	0.094956561	3.677454033	0.000235574	0.000372614	positive
PRJNA729271	stage_3W_vs_E10	ordinary_muscle_development	ENSGALG00010012291	NR5A2	-1.947816503	0.205754125	-9.466719102	2.88771E-21	1.16432E-20	negative
PRJNA729271	stage_3W_vs_E10	ordinary_muscle_development	ENSGALG00010012293	SRGAP1	-1.930713025	0.107726318	-17.92238941	7.88667E-72	1.60784E-70	negative
PRJNA729271	stage_3W_vs_E10	ordinary_muscle_development	ENSGALG00010012294	ENSGALG00010012294	0.611342731	0.48838114	1.251773833	0.210652288	0.243066781	positive
PRJNA729271	stage_3W_vs_E10	ordinary_muscle_development	ENSGALG00010012295	CUL4B	-0.263472887	0.065555925	-4.019055287	5.8432E-05	9.7353E-05	negative
PRJNA729271	stage_3W_vs_E10	ordinary_muscle_development	ENSGALG00010012297	ABLIM3	-0.681261012	0.198057227	-3.439718014	0.000582321	0.000890561	negative
PRJNA729271	stage_3W_vs_E10	ordinary_muscle_development	ENSGALG00010012300	NINJ2	-2.689109007	0.226709697	-11.86146441	1.87664E-32	1.18682E-31	negative
PRJNA729271	stage_3W_vs_E10	ordinary_muscle_development	ENSGALG00010012303	CA9	1.724083238	0.211070473	8.16828244	3.12811E-16	1.0076E-15	positive
PRJNA729271	stage_3W_vs_E10	ordinary_muscle_development	ENSGALG00010012304	ESRP1	-5.795772451	0.79320273	-7.306798413	2.73583E-13	7.57947E-13	negative
PRJNA729271	stage_3W_vs_E10	ordinary_muscle_development	ENSGALG00010012307	ENSGALG00010012307	-2.298593386	0.173297146	-13.26388483	3.74957E-40	3.04824E-39	negative
PRJNA729271	stage_3W_vs_E10	ordinary_muscle_development	ENSGALG00010012308	TRABD2A	1.576877514	0.34500565	4.57058461	4.86366E-06	8.79804E-06	positive
PRJNA729271	stage_3W_vs_E10	ordinary_muscle_development	ENSGALG00010012309	TRAPPC13	-0.021368925	0.124764305	-0.171274347	0.86400805	0.879727219	negative
PRJNA729271	stage_3W_vs_E10	ordinary_muscle_development	ENSGALG00010012311	ENSGALG00010012311	-4.454150537	0.554052349	-8.039223268	9.04097E-16	2.84148E-15	negative
PRJNA729271	stage_3W_vs_E10	ordinary_muscle_development	ENSGALG00010012312	SH3TC2	1.496203795	0.176228552	8.490132711	2.06401E-17	7.05277E-17	positive
PRJNA729271	stage_3W_vs_E10	ordinary_muscle_development	ENSGALG00010012314	ADRB2	5.14770943	0.261548662	19.68165079	3.09747E-86	9.27641E-85	positive
PRJNA729271	stage_3W_vs_E10	ordinary_muscle_development	ENSGALG00010012315	ATP6V1E1	0.563527359	0.100567506	5.60347352	2.10098E-08	4.44515E-08	positive
PRJNA729271	stage_3W_vs_E10	ordinary_muscle_development	ENSGALG00010012316	ENSGALG00010012316	-2.90859687	0.986921927	-2.94713978	0.003207282	0.004588388	negative
PRJNA729271	stage_3W_vs_E10	ordinary_muscle_development	ENSGALG00010012317	ENSGALG00010012317	-4.427975472	1.151720799	-3.844660507	0.000120719	0.000195993	negative
PRJNA729271	stage_3W_vs_E10	ordinary_muscle_development	ENSGALG00010012318	POLR1D	-0.596515767	0.141194119	-4.224791883	2.39162E-05	4.10109E-05	negative
PRJNA729271	stage_3W_vs_E10	ordinary_muscle_development	ENSGALG00010012320	DPY19L4	-0.647439257	0.061697926	-10.49369562	9.23443E-26	4.4588E-25	negative
PRJNA729271	stage_3W_vs_E10	ordinary_muscle_development	ENSGALG00010012322	ENSGALG00010012322	-2.149708085	0.275493413	-7.803119738	6.03952E-15	1.82229E-14	negative
PRJNA729271	stage_3W_vs_E10	ordinary_muscle_development	ENSGALG00010012323	SMAD7B	-2.300209727	3.603715603	-0.638288361	0.523285987	0.561272042	negative
PRJNA729271	stage_3W_vs_E10	ordinary_muscle_development	ENSGALG00010012324	ENSGALG00010012324	-2.404852352	0.164613212	-14.60910892	2.45711E-48	2.65591E-47	negative
PRJNA729271	stage_3W_vs_E10	ordinary_muscle_development	ENSGALG00010012325	MLPH	-2.783159358	0.580991675	-4.790360139	1.66482E-06	3.10374E-06	negative
PRJNA729271	stage_3W_vs_E10	ordinary_muscle_development	ENSGALG00010012326	SGTB	0.158392664	0.115943917	1.366114472	0.171903037	0.201059833	positive
PRJNA729271	stage_3W_vs_E10	ordinary_muscle_development	ENSGALG00010012327	UBTD2	-0.865235613	0.088342952	-9.794053617	1.1941E-22	5.07135E-22	negative
PRJNA729271	stage_3W_vs_E10	ordinary_muscle_development	ENSGALG00010012329	ATXN10	-0.28320376	0.107300504	-2.63935163	0.008306477	0.011389877	negative
PRJNA729271	stage_3W_vs_E10	ordinary_muscle_development	ENSGALG00010012331	ENSGALG00010012331	-1.030018968	0.266028386	-3.871838583	0.000108017	0.000175982	negative
PRJNA729271	stage_3W_vs_E10	ordinary_muscle_development	ENSGALG00010012334	ARHGEF39	-2.62167171	0.24532526	-10.68651354	1.17713E-26	5.92654E-26	negative
PRJNA729271	stage_3W_vs_E10	ordinary_muscle_development	ENSGALG00010012336	ENSGALG00010012336	-1.14965582	4.134899613	-0.278037178	0.780983819	0.804917553	negative
PRJNA729271	stage_3W_vs_E10	ordinary_muscle_development	ENSGALG00010012337	ACSS3	0.480618567	0.154601219	3.108763111	0.001878723	0.002741025	positive
PRJNA729271	stage_3W_vs_E10	ordinary_muscle_development	ENSGALG00010012338	H3C33	-3.174947081	0.22635983	-14.02610652	1.07916E-44	1.01706E-43	negative
PRJNA729271	stage_3W_vs_E10	ordinary_muscle_development	ENSGALG00010012339	WNK1	0.525837942	0.15490427	3.394599406	0.000687291	0.001044763	positive
PRJNA729271	stage_3W_vs_E10	ordinary_muscle_development	ENSGALG00010012340	ENHO	-1.318033338	0.164851098	-7.995296095	1.29263E-15	4.02333E-15	negative
PRJNA729271	stage_3W_vs_E10	ordinary_muscle_development	ENSGALG00010012341	GOPC	-0.371241281	0.080747584	-4.597552792	4.27482E-06	7.76194E-06	negative
PRJNA729271	stage_3W_vs_E10	ordinary_muscle_development	ENSGALG00010012342	ENSGALG00010012342	-0.382972043	1.626488097	-0.235459481	0.813852125	0.835525643	negative
PRJNA729271	stage_3W_vs_E10	ordinary_muscle_development	ENSGALG00010012343	BCL2L13	1.737180026	0.170160265	10.20908155	1.80566E-24	8.27736E-24	positive
PRJNA729271	stage_3W_vs_E10	ordinary_muscle_development	ENSGALG00010012345	H2BC32	-2.399387285	0.165453596	-14.50187448	1.17886E-47	1.23555E-46	negative
PRJNA729271	stage_3W_vs_E10	ordinary_muscle_development	ENSGALG00010012346	NLN	-0.67460727	0.140450158	-4.803179149	1.56166E-06	2.91742E-06	negative
PRJNA729271	stage_3W_vs_E10	ordinary_muscle_development	ENSGALG00010012347	ENSGALG00010012347	-1.989638521	0.494946507	-4.019906176	5.82213E-05	9.70467E-05	negative
PRJNA729271	stage_3W_vs_E10	ordinary_muscle_development	ENSGALG00010012350	H2AC32	-2.557100867	0.192446712	-13.28731907	2.74229E-40	2.23814E-39	negative
PRJNA729271	stage_3W_vs_E10	ordinary_muscle_development	ENSGALG00010012352	PTPRC	3.32368316	0.192776893	17.24108687	1.30559E-66	2.31351E-65	positive
PRJNA729271	stage_3W_vs_E10	ordinary_muscle_development	ENSGALG00010012353	PIGG	0.087869757	0.152409854	0.576535927	0.564252993	0.600855652	positive
PRJNA729271	stage_3W_vs_E10	ordinary_muscle_development	ENSGALG00010012354	DCBLD1	-0.158463751	0.150918535	-1.049995289	0.293720279	0.330986196	negative
PRJNA729271	stage_3W_vs_E10	ordinary_muscle_development	ENSGALG00010012358	STK10	0.984029213	0.148598716	6.62205731	3.54234E-11	8.79366E-11	positive
PRJNA729271	stage_3W_vs_E10	ordinary_muscle_development	ENSGALG00010012360	ROS1	1.945397909	0.379221456	5.129978477	2.89775E-07	5.68529E-07	positive
PRJNA729271	stage_3W_vs_E10	ordinary_muscle_development	ENSGALG00010012361	WIF1	-7.260481816	0.486279375	-14.93068015	2.08132E-50	2.40006E-49	negative
PRJNA729271	stage_3W_vs_E10	ordinary_muscle_development	ENSGALG00010012362	COL6A3	2.617793708	0.292465369	8.950781823	3.52973E-19	1.30652E-18	positive
PRJNA729271	stage_3W_vs_E10	ordinary_muscle_development	ENSGALG00010012366	INTS8	-0.712075446	0.068495901	-10.39588414	2.58875E-25	1.22707E-24	negative
PRJNA729271	stage_3W_vs_E10	ordinary_muscle_development	ENSGALG00010012367	RAD52	-2.936157114	0.166889791	-17.59338961	2.76813E-69	5.2177E-68	negative
PRJNA729271	stage_3W_vs_E10	ordinary_muscle_development	ENSGALG00010012371	ATP6V1G3	1.2939258	0.429646753	3.011603811	0.002598715	0.003746234	positive
PRJNA729271	stage_3W_vs_E10	ordinary_muscle_development	ENSGALG00010012372	LEMD3	-0.016253205	0.073477156	-0.221200795	0.82493609	0.845706813	negative
PRJNA729271	stage_3W_vs_E10	ordinary_muscle_development	ENSGALG00010012373	WNT7B	-9.229689837	0.964819024	-9.566239483	1.10865E-21	4.53695E-21	negative
PRJNA729271	stage_3W_vs_E10	ordinary_muscle_development	ENSGALG00010012376	VGLL2	1.249299495	0.186269902	6.706931609	1.98759E-11	5.00177E-11	positive
PRJNA729271	stage_3W_vs_E10	ordinary_muscle_development	ENSGALG00010012378	RPS19BP1	-0.037044612	0.091258148	-0.405932104	0.684792489	0.715650117	negative
PRJNA729271	stage_3W_vs_E10	ordinary_muscle_development	ENSGALG00010012382	SH3PXD2B	-1.664660783	0.134475967	-12.37887198	3.40089E-35	2.3564E-34	negative
PRJNA729271	stage_3W_vs_E10	ordinary_muscle_development	ENSGALG00010012384	NRK	0.153155134	0.121912562	1.25627032	0.209017979	0.241269693	positive
PRJNA729271	stage_3W_vs_E10	ordinary_muscle_development	ENSGALG00010012387	ERBIN	-0.198000151	0.089058998	-2.22324701	0.026199149	0.03399791	negative
PRJNA729271	stage_3W_vs_E10	ordinary_muscle_development	ENSGALG00010012390	MMP16	-1.708636859	0.17496873	-9.765384106	1.5851E-22	6.6927E-22	negative
PRJNA729271	stage_3W_vs_E10	ordinary_muscle_development	ENSGALG00010012391	NEURL1B	-1.360464392	0.159285336	-8.541052347	1.33005E-17	4.59901E-17	negative
PRJNA729271	stage_3W_vs_E10	ordinary_muscle_development	ENSGALG00010012393	ENSGALG00010012393	-3.194393528	1.626743801	-1.963673398	0.049567979	0.062244465	negative
PRJNA729271	stage_3W_vs_E10	ordinary_muscle_development	ENSGALG00010012398	ENSGALG00010012398	-5.533617668	0.927856302	-5.963873559	2.46327E-09	5.48129E-09	negative
PRJNA729271	stage_3W_vs_E10	ordinary_muscle_development	ENSGALG00010012399	ERC1	-0.803991403	0.085848013	-9.36528845	7.58428E-21	3.00285E-20	negative
PRJNA729271	stage_3W_vs_E10	ordinary_muscle_development	ENSGALG00010012401	RIPK2	0.603365927	0.130779799	4.613601896	3.9575E-06	7.20925E-06	positive
PRJNA729271	stage_3W_vs_E10	ordinary_muscle_development	ENSGALG00010012404	VAMP7	0.327397385	0.051905624	6.307551299	2.83485E-10	6.67822E-10	positive
PRJNA729271	stage_3W_vs_E10	ordinary_muscle_development	ENSGALG00010012405	MSRB3	2.492482218	0.125560487	19.85084853	1.08356E-87	3.34886E-86	positive
PRJNA729271	stage_3W_vs_E10	ordinary_muscle_development	ENSGALG00010012406	ENSGALG00010012406	4.278384751	0.238857566	17.91186616	9.52866E-72	1.92902E-70	positive
PRJNA729271	stage_3W_vs_E10	ordinary_muscle_development	ENSGALG00010012407	ATF4	1.062388506	0.128785044	8.249315881	1.59304E-16	5.21479E-16	positive
PRJNA729271	stage_3W_vs_E10	ordinary_muscle_development	ENSGALG00010012410	CACNA1I	-3.233105614	0.442442534	-7.307402358	2.72356E-13	7.54838E-13	negative
PRJNA729271	stage_3W_vs_E10	ordinary_muscle_development	ENSGALG00010012412	PURA	1.39940752	0.130596185	10.71553142	8.60616E-27	4.36023E-26	positive
PRJNA729271	stage_3W_vs_E10	ordinary_muscle_development	ENSGALG00010012413	NEK7	0.384936077	0.072553438	5.305552571	1.12332E-07	2.26682E-07	positive
PRJNA729271	stage_3W_vs_E10	ordinary_muscle_development	ENSGALG00010012414	ENSGALG00010012414	7.685520411	0.203317309	37.80062035	1.1099E-312	5.1898E-310	positive
PRJNA729271	stage_3W_vs_E10	ordinary_muscle_development	ENSGALG00010012415	BID	-2.055320944	0.115735223	-17.7588196	1.473E-70	2.88529E-69	negative
PRJNA729271	stage_3W_vs_E10	ordinary_muscle_development	ENSGALG00010012418	ENSGALG00010012418	-0.131871963	0.808414182	-0.163124257	0.870420601	0.885199369	negative
PRJNA729271	stage_3W_vs_E10	ordinary_muscle_development	ENSGALG00010012419	HACE1	0.568692216	0.09621581	5.910590136	3.40884E-09	7.52416E-09	positive
PRJNA729271	stage_3W_vs_E10	ordinary_muscle_development	ENSGALG00010012426	MIEF1	-0.717019973	0.082299118	-8.712365192	2.97599E-18	1.06354E-17	negative
PRJNA729271	stage_3W_vs_E10	ordinary_muscle_development	ENSGALG00010012427	C7	5.513372834	0.447337028	12.32487472	6.65468E-35	4.55643E-34	positive
PRJNA729271	stage_3W_vs_E10	ordinary_muscle_development	ENSGALG00010012428	CCNE2	-3.730251452	0.244585145	-15.25134101	1.61286E-52	1.9762E-51	negative
PRJNA729271	stage_3W_vs_E10	ordinary_muscle_development	ENSGALG00010012429	CXCR7	1.205641205	0.101027592	11.93378141	7.8906E-33	5.04073E-32	positive
PRJNA729271	stage_3W_vs_E10	ordinary_muscle_development	ENSGALG00010012431	LAMP2	0.587834935	0.079436937	7.400020184	1.36164E-13	3.83763E-13	positive
PRJNA729271	stage_3W_vs_E10	ordinary_muscle_development	ENSGALG00010012433	ENSGALG00010012433	0.1385532	0.73748581	0.18787236	0.85097671	0.867983071	positive
PRJNA729271	stage_3W_vs_E10	ordinary_muscle_development	ENSGALG00010012436	ENSGALG00010012436	5.228569343	0.199621621	26.19239997	3.2437E-151	3.1345E-149	positive
PRJNA729271	stage_3W_vs_E10	ordinary_muscle_development	ENSGALG00010012437	ENSGALG00010012437	-3.488401217	3.834440382	-0.909754976	0.362951744	0.401755291	negative
PRJNA729271	stage_3W_vs_E10	ordinary_muscle_development	ENSGALG00010012439	TENT2	0.57984757	0.101159285	5.732025211	9.92385E-09	2.14152E-08	positive
PRJNA729271	stage_3W_vs_E10	ordinary_muscle_development	ENSGALG00010012441	FST	-0.03191465	0.23929916	-0.133367163	0.893903013	0.906029241	negative
PRJNA729271	stage_3W_vs_E10	ordinary_muscle_development	ENSGALG00010012442	CPLX1	-6.803306553	0.530692991	-12.81966536	1.27249E-37	9.43462E-37	negative
PRJNA729271	stage_3W_vs_E10	ordinary_muscle_development	ENSGALG00010012445	SOBP	-0.535682925	0.16486217	-3.249277416	0.001156986	0.001724829	negative
PRJNA729271	stage_3W_vs_E10	ordinary_muscle_development	ENSGALG00010012447	COPS8	0.489462309	0.116507582	4.201119811	2.65598E-05	4.5351E-05	positive
PRJNA729271	stage_3W_vs_E10	ordinary_muscle_development	ENSGALG00010012449	SUB1	-1.384531192	0.109685805	-12.62270169	1.58296E-36	1.13702E-35	negative
PRJNA729271	stage_3W_vs_E10	ordinary_muscle_development	ENSGALG00010012450	PPFIA2	-2.959539531	0.290655175	-10.18230463	2.3786E-24	1.08353E-23	negative
PRJNA729271	stage_3W_vs_E10	ordinary_muscle_development	ENSGALG00010012454	IQCA1	2.207018638	0.413241189	5.340751841	9.25619E-08	1.87911E-07	positive
PRJNA729271	stage_3W_vs_E10	ordinary_muscle_development	ENSGALG00010012456	ENSGALG00010012456	-1.762871395	0.093960083	-18.76191821	1.54714E-78	3.71288E-77	negative
PRJNA729271	stage_3W_vs_E10	ordinary_muscle_development	ENSGALG00010012458	MRPS30	0.268836765	0.108037707	2.488360524	0.012833356	0.017241451	positive
PRJNA729271	stage_3W_vs_E10	ordinary_muscle_development	ENSGALG00010012464	ENSGALG00010012464	0.923382354	0.236679206	3.901408866	9.56345E-05	0.0001563	positive
PRJNA729271	stage_3W_vs_E10	ordinary_muscle_development	ENSGALG00010012466	PDSS2	0.359245302	0.121111327	2.966240327	0.003014648	0.004323471	positive
PRJNA729271	stage_3W_vs_E10	ordinary_muscle_development	ENSGALG00010012469	CETN2	-0.705538288	0.130604454	-5.40209976	6.58653E-08	1.34733E-07	negative
PRJNA729271	stage_3W_vs_E10	ordinary_muscle_development	ENSGALG00010012470	CHD1	0.308332903	0.105435284	2.924380639	0.003451424	0.00492018	positive
PRJNA729271	stage_3W_vs_E10	ordinary_muscle_development	ENSGALG00010012474	ASB18	2.285498417	0.933857195	2.44737464	0.014390117	0.019222629	positive
PRJNA729271	stage_3W_vs_E10	ordinary_muscle_development	ENSGALG00010012477	ENSGALG00010012477	-0.749995939	0.260919211	-2.874437406	0.00404748	0.005726522	negative
PRJNA729271	stage_3W_vs_E10	ordinary_muscle_development	ENSGALG00010012483	ENSGALG00010012483	2.027407596	0.251824905	8.050862161	8.22128E-16	2.58947E-15	positive
PRJNA729271	stage_3W_vs_E10	ordinary_muscle_development	ENSGALG00010012485	C6	4.147874808	1.238575145	3.34890848	0.000811306	0.001224732	positive
PRJNA729271	stage_3W_vs_E10	ordinary_muscle_development	ENSGALG00010012486	RADX	1.432082745	0.106761431	13.41385868	5.01596E-41	4.17373E-40	positive
PRJNA729271	stage_3W_vs_E10	ordinary_muscle_development	ENSGALG00010012489	GBX2	-4.439039712	1.068441194	-4.15468791	3.25732E-05	5.52285E-05	negative
PRJNA729271	stage_3W_vs_E10	ordinary_muscle_development	ENSGALG00010012497	PLCXD3	-0.166401698	0.411166601	-0.40470626	0.68569344	0.716540005	negative
PRJNA729271	stage_3W_vs_E10	ordinary_muscle_development	ENSGALG00010012498	KCNT2	0.947188578	0.213645181	4.433465678	9.27303E-06	1.64118E-05	positive
PRJNA729271	stage_3W_vs_E10	ordinary_muscle_development	ENSGALG00010012506	TP53INP1	-0.516493487	0.12626	-4.090713505	4.30048E-05	7.23233E-05	negative
PRJNA729271	stage_3W_vs_E10	ordinary_muscle_development	ENSGALG00010012508	MDM1	-0.699444708	0.06884584	-10.15957845	3.00372E-24	1.36187E-23	negative
PRJNA729271	stage_3W_vs_E10	ordinary_muscle_development	ENSGALG00010012509	MICAL3	0.795489085	0.09331009	8.525220418	1.52519E-17	5.25747E-17	positive
PRJNA729271	stage_3W_vs_E10	ordinary_muscle_development	ENSGALG00010012511	OXCT1	-0.855746536	0.122245081	-7.000253362	2.555E-12	6.73236E-12	negative
PRJNA729271	stage_3W_vs_E10	ordinary_muscle_development	ENSGALG00010012517	MGAT3	0.846006365	0.175277967	4.826655505	1.38845E-06	2.60424E-06	positive
PRJNA729271	stage_3W_vs_E10	ordinary_muscle_development	ENSGALG00010012519	ENSGALG00010012519	-2.712865636	1.72150911	-1.575864816	0.115056961	0.137933227	negative
PRJNA729271	stage_3W_vs_E10	ordinary_muscle_development	ENSGALG00010012521	HOMER1	0.958441438	0.132114376	7.25463395	4.0275E-13	1.10712E-12	positive
PRJNA729271	stage_3W_vs_E10	ordinary_muscle_development	ENSGALG00010012529	BEND3	-1.203173598	0.126322382	-9.524627184	1.65635E-21	6.74027E-21	negative
PRJNA729271	stage_3W_vs_E10	ordinary_muscle_development	ENSGALG00010012532	FAM174A	1.087121406	0.150325775	7.231769843	4.7674E-13	1.30433E-12	positive
PRJNA729271	stage_3W_vs_E10	ordinary_muscle_development	ENSGALG00010012535	JMY	-0.318014483	0.103767949	-3.064669651	0.002179105	0.003161459	negative
PRJNA729271	stage_3W_vs_E10	ordinary_muscle_development	ENSGALG00010012536	FBXO4	0.224159725	0.134294396	1.669166643	0.095084359	0.11529595	positive
PRJNA729271	stage_3W_vs_E10	ordinary_muscle_development	ENSGALG00010012538	RAP1B	-0.53982409	0.088045121	-6.131220939	8.72072E-10	1.99537E-09	negative
PRJNA729271	stage_3W_vs_E10	ordinary_muscle_development	ENSGALG00010012547	ENSGALG00010012547	-2.402556053	0.488483969	-4.918392831	8.72577E-07	1.66006E-06	negative
PRJNA729271	stage_3W_vs_E10	ordinary_muscle_development	ENSGALG00010012550	TAB1	-0.164618146	0.096447868	-1.706809589	0.08785745	0.107034269	negative
PRJNA729271	stage_3W_vs_E10	ordinary_muscle_development	ENSGALG00010012551	PCGF3	-0.399250974	0.156306565	-2.554281553	0.010640717	0.014421431	negative
PRJNA729271	stage_3W_vs_E10	ordinary_muscle_development	ENSGALG00010012553	ENSGALG00010012553	0.46187171	0.22390019	2.062846442	0.03912722	0.049793596	positive
PRJNA729271	stage_3W_vs_E10	ordinary_muscle_development	ENSGALG00010012555	ENSGALG00010012555	-1.523711919	0.213138257	-7.148936752	8.74526E-13	2.36762E-12	negative

PRJNA729271	stage_3W_vs_E10	ordinary_muscle_development	ENSGALG00010012561	OSGIN2	-0.966120035	0.128215495	-7.535126961	4.8786E-14	1.40867E-13	negative
PRJNA729271	stage_3W_vs_E10	ordinary_muscle_development	ENSGALG00010012565	SYNGR1	1.039541511	0.122017092	8.519638451	1.60052E-17	5.50535E-17	positive
PRJNA729271	stage_3W_vs_E10	ordinary_muscle_development	ENSGALG00010012569	C6orf203	1.406764361	0.12065369	11.65952203	2.05191E-31	1.24082E-30	positive
PRJNA729271	stage_3W_vs_E10	ordinary_muscle_development	ENSGALG00010012571	AGAP1	0.035663182	0.082274199	0.43346738	0.664675259	0.696635421	positive
PRJNA729271	stage_3W_vs_E10	ordinary_muscle_development	ENSGALG00010012574	PHAX	-0.308182928	0.105181316	-2.930015892	0.003389447	0.004835157	negative
PRJNA729271	stage_3W_vs_E10	ordinary_muscle_development	ENSGALG00010012578	NDUFS4	0.733572669	0.152842228	4.79954185	1.59029E-06	2.96861E-06	positive
PRJNA729271	stage_3W_vs_E10	ordinary_muscle_development	ENSGALG00010012582	CD24	-3.283266048	0.227400163	-14.43827484	2.97188E-47	3.06165E-46	negative
PRJNA729271	stage_3W_vs_E10	ordinary_muscle_development	ENSGALG00010012583	NDUFAF6	0.125088915	0.102774462	1.217120605	0.223558332	0.256793567	positive
PRJNA729271	stage_3W_vs_E10	ordinary_muscle_development	ENSGALG00010012586	CDC73	0.633920081	0.133616456	4.744326404	2.09201E-06	3.8797E-06	positive
PRJNA729271	stage_3W_vs_E10	ordinary_muscle_development	ENSGALG00010012587	MBLAC2	1.179143955	0.144938595	8.135472484	4.10335E-16	1.31211E-15	positive
PRJNA729271	stage_3W_vs_E10	ordinary_muscle_development	ENSGALG00010012588	PEX26	0.645190339	0.104378487	6.181257819	6.35929E-10	1.4678E-09	positive
PRJNA729271	stage_3W_vs_E10	ordinary_muscle_development	ENSGALG00010012593	TUBA8B	5.5163596	0.955007743	5.776245943	7.63857E-09	1.6596E-08	positive
PRJNA729271	stage_3W_vs_E10	ordinary_muscle_development	ENSGALG00010012599	DMGDH	-3.32006435	0.199953743	-16.60416203	6.50218E-62	1.02445E-60	negative
PRJNA729271	stage_3W_vs_E10	ordinary_muscle_development	ENSGALG00010012600	NUP107	-1.11273684	0.100327661	-11.09102742	1.38686E-28	7.54598E-28	negative
PRJNA729271	stage_3W_vs_E10	ordinary_muscle_development	ENSGALG00010012601	TUBA8A	4.045791154	0.237243396	17.05333523	3.30145E-65	5.6902E-64	positive
PRJNA729271	stage_3W_vs_E10	ordinary_muscle_development	ENSGALG00010012604	ENSGALG00010012604	-4.111363641	2.165199249	-1.898838475			negative
PRJNA729271	stage_3W_vs_E10	ordinary_muscle_development	ENSGALG00010012606	ATP5PO	0.406378536	0.114236655	3.557339254	0.00037463	0.000583335	positive
PRJNA729271	stage_3W_vs_E10	ordinary_muscle_development	ENSGALG00010012607	LMNB1	-3.603050653	0.165813293	-21.72956455	1.0782E-104	4.9301E-103	negative
PRJNA729271	stage_3W_vs_E10	ordinary_muscle_development	ENSGALG00010012610	RPL3	-1.244884553	0.155051335	-8.028854126	9.83873E-16	3.08484E-15	negative
PRJNA729271	stage_3W_vs_E10	ordinary_muscle_development	ENSGALG00010012614	LHFPL2	-1.246991173	0.145470452	-8.572126909	1.01591E-17	3.53726E-17	negative
PRJNA729271	stage_3W_vs_E10	ordinary_muscle_development	ENSGALG00010012618	B3GALT2	-0.186913371	0.329032257	-0.56807005	0.569987404	0.606338428	negative
PRJNA729271	stage_3W_vs_E10	ordinary_muscle_development	ENSGALG00010012624	PDGFB	0.488315279	0.18438287	2.648376609	0.008087937	0.011107025	positive
PRJNA729271	stage_3W_vs_E10	ordinary_muscle_development	ENSGALG00010012629	NUS1	0.2255688	0.110098813	2.048785026	0.040483141	0.051392812	positive
PRJNA729271	stage_3W_vs_E10	ordinary_muscle_development	ENSGALG00010012631	GLRX2	0.573898623	0.154832762	3.70657098	0.000210084	0.000333937	positive
PRJNA729271	stage_3W_vs_E10	ordinary_muscle_development	ENSGALG00010012633	USP18	1.778826872	0.541645321	3.284117493	0.001023023	0.001531575	positive
PRJNA729271	stage_3W_vs_E10	ordinary_muscle_development	ENSGALG00010012640	PRRC1	-0.603688159	0.074072473	-8.149966284	3.64026E-16	1.16557E-15	negative
PRJNA729271	stage_3W_vs_E10	ordinary_muscle_development	ENSGALG00010012648	MRPS6	-0.251051485	0.131956856	-1.902527031	0.057102288	0.07116307	negative
PRJNA729271	stage_3W_vs_E10	ordinary_muscle_development	ENSGALG00010012650	DSE	-0.90919823	0.105570016	-8.612277082	7.16235E-18	2.51254E-17	negative
PRJNA729271	stage_3W_vs_E10	ordinary_muscle_development	ENSGALG00010012653	CBX7	2.522018525	0.249554413	10.10608666	5.19168E-24	2.32766E-23	positive
PRJNA729271	stage_3W_vs_E10	ordinary_muscle_development	ENSGALG00010012654	TENT5B	2.787768293	0.308641028	9.032396977	1.67952E-19	6.302E-19	positive
PRJNA729271	stage_3W_vs_E10	ordinary_muscle_development	ENSGALG00010012662	SLC35E3	-1.704849948	0.108946943	-15.64844221	3.40452E-55	4.50671E-54	negative
PRJNA729271	stage_3W_vs_E10	ordinary_muscle_development	ENSGALG00010012667	SCAMP1	-0.489997064	0.089199904	-5.493246512	3.94611E-08	8.19586E-08	negative
PRJNA729271	stage_3W_vs_E10	ordinary_muscle_development	ENSGALG00010012669	ERCC8	0.0881022	0.148468039	0.593408529	0.552907782	0.589944667	positive
PRJNA729271	stage_3W_vs_E10	ordinary_muscle_development	ENSGALG00010012670	ENSGALG00010012670	-0.402700825	0.218220495	-1.845384982	0.064981556	0.080443049	negative
PRJNA729271	stage_3W_vs_E10	ordinary_muscle_development	ENSGALG00010012671	NBN	0.276956248	0.183734634	1.507370943	0.131715602	0.156748845	positive
PRJNA729271	stage_3W_vs_E10	ordinary_muscle_development	ENSGALG00010012672	RO60	-0.825839912	0.090943122	-9.080839695	1.0774E-19	4.06692E-19	negative
PRJNA729271	stage_3W_vs_E10	ordinary_muscle_development	ENSGALG00010012673	ENSGALG00010012673	-2.082445113	0.340390912	-6.117804671	9.48733E-10	2.16463E-09	negative
PRJNA729271	stage_3W_vs_E10	ordinary_muscle_development	ENSGALG00010012674	ENSGALG00010012674	-0.264960377	0.141115657	-1.877611477	0.060434335	0.07510895	negative
PRJNA729271	stage_3W_vs_E10	ordinary_muscle_development	ENSGALG00010012676	ENSGALG00010012676	0.58748482	0.208876047	2.812600241	0.00491427	0.006897681	positive
PRJNA729271	stage_3W_vs_E10	ordinary_muscle_development	ENSGALG00010012680	TRPM8	-0.824109026	0.31654965	-2.603411586	0.009230107	0.012595594	negative
PRJNA729271	stage_3W_vs_E10	ordinary_muscle_development	ENSGALG00010012682	ENSGALG00010012682	-1.178826684	0.112115399	-10.51440475	7.41475E-26	3.59695E-25	negative
PRJNA729271	stage_3W_vs_E10	ordinary_muscle_development	ENSGALG00010012683	MDM2	1.017784418	0.085836514	11.85724314	1.97367E-32	1.24601E-31	positive
PRJNA729271	stage_3W_vs_E10	ordinary_muscle_development	ENSGALG00010012685	NPTXR	-0.046133017	0.263777722	-0.174893529	0.861163295	0.877262067	negative
PRJNA729271	stage_3W_vs_E10	ordinary_muscle_development	ENSGALG00010012688	NDUFAF2	0.849280879	0.112040427	7.580128875	3.45212E-14	1.00257E-13	positive
PRJNA729271	stage_3W_vs_E10	ordinary_muscle_development	ENSGALG00010012690	NT5DC1	1.881707558	0.334963889	5.61764304	1.9358E-08	4.10465E-08	positive
PRJNA729271	stage_3W_vs_E10	ordinary_muscle_development	ENSGALG00010012693	UCHL5	-0.201809954	0.077496927	-2.604102679	0.009211516	0.012573775	negative
PRJNA729271	stage_3W_vs_E10	ordinary_muscle_development	ENSGALG00010012696	DNAL4	-1.253973725	0.095461767	-13.13587384	2.0511E-39	1.62552E-38	negative
PRJNA729271	stage_3W_vs_E10	ordinary_muscle_development	ENSGALG00010012699	RNF128	0.086240655	0.143878722	0.59939826	0.548907337	0.586064515	positive
PRJNA729271	stage_3W_vs_E10	ordinary_muscle_development	ENSGALG00010012702	PTPRO	-2.018805025	0.334669554	-6.032233889	1.61708E-09	3.6397E-09	negative
PRJNA729271	stage_3W_vs_E10	ordinary_muscle_development	ENSGALG00010012704	SMIM15	0.197978093	0.067096436	2.950649906	0.003171061	0.004538362	positive
PRJNA729271	stage_3W_vs_E10	ordinary_muscle_development	ENSGALG00010012705	CPM	1.682303257	0.184737999	9.106427819	8.51391E-20	3.22891E-19	positive
PRJNA729271	stage_3W_vs_E10	ordinary_muscle_development	ENSGALG00010012706	TBC1D8B	1.159366918	0.103451501	11.20686413	3.77326E-29	2.1044E-28	positive
PRJNA729271	stage_3W_vs_E10	ordinary_muscle_development	ENSGALG00010012708	SPP2	-2.001045176	1.206698216	-1.658281373	0.097260687	0.11779693	negative
PRJNA729271	stage_3W_vs_E10	ordinary_muscle_development	ENSGALG00010012709	PPP1R8	-1.396063572	0.058800219	-23.74248916	1.3137E-124	8.5008E-123	negative
PRJNA729271	stage_3W_vs_E10	ordinary_muscle_development	ENSGALG00010012711	ELL2	-0.114388521	0.125589652	-0.910811669	0.362394613	0.401230516	negative
PRJNA729271	stage_3W_vs_E10	ordinary_muscle_development	ENSGALG00010012712	EEF1E1	0.990720118	0.132663631	7.467910442	8.14783E-14	2.32394E-13	positive
PRJNA729271	stage_3W_vs_E10	ordinary_muscle_development	ENSGALG00010012714	RGS2	0.524497024	0.172946264	3.032716704	0.00242363	0.003502893	positive
PRJNA729271	stage_3W_vs_E10	ordinary_muscle_development	ENSGALG00010012719	COL10A1	-4.6714252	0.790983361	-5.90584509	3.50844E-09	7.74163E-09	negative
PRJNA729271	stage_3W_vs_E10	ordinary_muscle_development	ENSGALG00010012720	ACO2	0.984980528	0.119903207	8.214797165	2.12523E-16	6.9101E-16	positive
PRJNA729271	stage_3W_vs_E10	ordinary_muscle_development	ENSGALG00010012722	ENSGALG00010012722	0.082252589	0.098842325	0.832159594	0.405318868	0.444174566	positive
PRJNA729271	stage_3W_vs_E10	ordinary_muscle_development	ENSGALG00010012725	ENSGALG00010012725	1.771022181	0.255539812	6.930513765	4.19315E-12	1.09002E-11	positive
PRJNA729271	stage_3W_vs_E10	ordinary_muscle_development	ENSGALG00010012726	IL7R	6.614627185	0.462746701	14.29427195	2.37577E-46	2.38152E-45	positive
PRJNA729271	stage_3W_vs_E10	ordinary_muscle_development	ENSGALG00010012730	CAP2	1.960991534	0.167775304	11.68820137	1.46455E-31	8.91962E-31	positive
PRJNA729271	stage_3W_vs_E10	ordinary_muscle_development	ENSGALG00010012732	MGST1	-0.900320081	0.175200248	-5.138805988	2.7649E-07	5.43493E-07	negative
PRJNA729271	stage_3W_vs_E10	ordinary_muscle_development	ENSGALG00010012733	ENSGALG00010012733	-4.429067129	1.265917764	-3.498700511	0.000467531	0.000722173	negative
PRJNA729271	stage_3W_vs_E10	ordinary_muscle_development	ENSGALG00010012734	TMEM19	-0.420336396	0.145249326	-2.893895675	0.003804946	0.005400243	negative
PRJNA729271	stage_3W_vs_E10	ordinary_muscle_development	ENSGALG00010012738	RAB21	0.833928638	0.068365277	12.19813157	3.18038E-34	2.13227E-33	positive
PRJNA729271	stage_3W_vs_E10	ordinary_muscle_development	ENSGALG00010012741	ENSGALG00010012741	0.242631821	0.151064016	1.606152325	0.108240446	0.130246161	positive
PRJNA729271	stage_3W_vs_E10	ordinary_muscle_development	ENSGALG00010012742	ENSGALG00010012742	-2.805169535	0.174822422	-16.04582239	6.11436E-58	8.70606E-57	negative
PRJNA729271	stage_3W_vs_E10	ordinary_muscle_development	ENSGALG00010012743	ENSGALG00010012743	1.530966421	0.500100342	3.061318485	0.002203646	0.003196102	positive
PRJNA729271	stage_3W_vs_E10	ordinary_muscle_development	ENSGALG00010012744	ARL4C	-1.912446441	0.232393125	-8.229358926	1.88218E-16	6.13083E-16	negative
PRJNA729271	stage_3W_vs_E10	ordinary_muscle_development	ENSGALG00010012745	MARCHF3	0.010948688	0.302850364	0.036152138	0.971161049	0.975400458	positive
PRJNA729271	stage_3W_vs_E10	ordinary_muscle_development	ENSGALG00010012747	TOB2	1.445183125	0.145199981	9.953053128	2.44563E-23	1.06551E-22	positive
PRJNA729271	stage_3W_vs_E10	ordinary_muscle_development	ENSGALG00010012748	ENSGALG00010012748	0.519278053	0.21149266	2.455300585	0.014076683	0.018824259	positive
PRJNA729271	stage_3W_vs_E10	ordinary_muscle_development	ENSGALG00010012749	RGS4	2.528549927	0.176620633	14.31627712	1.73154E-46	1.74054E-45	positive
PRJNA729271	stage_3W_vs_E10	ordinary_muscle_development	ENSGALG00010012751	AP3B1	-0.250697357	0.074909225	-3.346682012	0.00081785	0.001234354	negative
PRJNA729271	stage_3W_vs_E10	ordinary_muscle_development	ENSGALG00010012753	GTPBP1	-0.814640691	0.053680293	-15.17578694	5.11605E-52	6.18491E-51	negative
PRJNA729271	stage_3W_vs_E10	ordinary_muscle_development	ENSGALG00010012755	CLVS2	-2.826658676	0.406778784	-6.948884227	3.68187E-12	9.60386E-12	negative
PRJNA729271	stage_3W_vs_E10	ordinary_muscle_development	ENSGALG00010012756	RPA2	-1.726834042	0.105586429	-16.35469693	4.02707E-60	6.03644E-59	negative
PRJNA729271	stage_3W_vs_E10	ordinary_muscle_development	ENSGALG00010012758	NAB1	-0.510380877	0.128969891	-3.957364574	7.57812E-05	0.00012498	negative
PRJNA729271	stage_3W_vs_E10	ordinary_muscle_development	ENSGALG00010012759	ATP1B4	7.172503105	0.197604693	36.29723049	1.7892E-288	7.2039E-286	positive
PRJNA729271	stage_3W_vs_E10	ordinary_muscle_development	ENSGALG00010012760	PLEKHF2	0.826328943	0.105170323	7.857054369	3.93272E-15	1.19859E-14	positive
PRJNA729271	stage_3W_vs_E10	ordinary_muscle_development	ENSGALG00010012761	SMPDL3A	2.214964069	0.224585279	9.86246328	6.05455E-23	2.61037E-22	positive
PRJNA729271	stage_3W_vs_E10	ordinary_muscle_development	ENSGALG00010012763	SMPDL3B	-0.163389955	0.13288248	-1.22958237	0.218853535	0.251708482	negative
PRJNA729271	stage_3W_vs_E10	ordinary_muscle_development	ENSGALG00010012765	PHF5A	0.041209055	0.106936719	0.385359262	0.699971261	0.72935687	positive
PRJNA729271	stage_3W_vs_E10	ordinary_muscle_development	ENSGALG00010012767	JOSD1	-0.725415065	0.071299901	-10.17413848	2.58679E-24	1.17688E-23	negative
PRJNA729271	stage_3W_vs_E10	ordinary_muscle_development	ENSGALG00010012768	EPS8	-1.031214817	0.116906418	-8.820857224	1.13587E-18	4.11404E-18	negative
PRJNA729271	stage_3W_vs_E10	ordinary_muscle_development	ENSGALG00010012769	NEMP2	-0.934199192	0.083454492	-11.19411518	4.35731E-29	2.41989E-28	negative
PRJNA729271	stage_3W_vs_E10	ordinary_muscle_development	ENSGALG00010012771	ITGA2	-1.78320254	0.191453562	-9.314021216	1.23082E-20	4.82966E-20	negative
PRJNA729271	stage_3W_vs_E10	ordinary_muscle_development	ENSGALG00010012772	TOMM22	-0.51421663	0.074445282	-6.907309863	4.9393E-12	1.2794E-11	negative
PRJNA729271	stage_3W_vs_E10	ordinary_muscle_development	ENSGALG00010012773	XKR8	-0.276243452	0.123962155	-2.2284499	0.025850529	0.033569559	negative
PRJNA729271	stage_3W_vs_E10	ordinary_muscle_development	ENSGALG00010012775	KCNE2	3.191486053	0.145391027	21.95105242	8.462E-107	4.0347E-105	positive
PRJNA729271	stage_3W_vs_E10	ordinary_muscle_development	ENSGALG00010012778	TBC1D15	0.655269728	0.151340053	4.329783925	1.49256E-05	2.6025E-05	positive
PRJNA729271	stage_3W_vs_E10	ordinary_muscle_development	ENSGALG00010012780	FABP7	-2.941010881	0.252857551	-11.63109772	2.86391E-31	1.72036E-30	negative
PRJNA729271	stage_3W_vs_E10	ordinary_muscle_development	ENSGALG00010012781	SPIN1L	-6.380749909	3.267766094	-1.952633611	0.050863025	0.063743693	negative
PRJNA729271	stage_3W_vs_E10	ordinary_muscle_development	ENSGALG00010012786	TEF	0.925312331	0.087816317	10.53690662	5.83879E-26	2.84673E-25	positive
PRJNA729271	stage_3W_vs_E10	ordinary_muscle_development	ENSGALG00010012788	RGS1	4.00420679	0.62922811	6.363680717	1.96975E-10	4.68443E-10	positive
PRJNA729271	stage_3W_vs_E10	ordinary_muscle_development	ENSGALG00010012790	PITX1	-5.543374748	0.550749724	-10.06514304	7.87727E-24	3.50787E-23	negative
PRJNA729271	stage_3W_vs_E10	ordinary_muscle_development	ENSGALG00010012791	CFAP418	0.094024785	0.190245599	0.494228435	0.62114486	0.655657934	positive
PRJNA729271	stage_3W_vs_E10	ordinary_muscle_development	ENSGALG00010012792	CBY1	0.008691065	0.143065508	0.06074885	0.951559226	0.957836874	positive
PRJNA729271	stage_3W_vs_E10	ordinary_muscle_development	ENSGALG00010012795	TBCA	-0.443375487	0.136392955	-3.250721324	0.001151126	0.0017168	negative
PRJNA729271	stage_3W_vs_E10	ordinary_muscle_development	ENSGALG00010012796	MOCS2	1.329985649	0.120149162	11.06945424	1.76472E-28	9.59116E-28	positive
PRJNA729271	stage_3W_vs_E10	ordinary_muscle_development	ENSGALG00010012797	ZC3H7B	-0.066009921	0.070468821	-0.936725197	0.348899932	0.38738449	negative
PRJNA729271	stage_3W_vs_E10	ordinary_muscle_development	ENSGALG00010012798	MFSD6	0.521504399	0.095462183	5.46294232	4.68307E-08	9.67242E-08	positive
PRJNA729271	stage_3W_vs_E10	ordinary_muscle_development	ENSGALG00010012801	DMC1	3.5828115	0.417868263	8.574021566	9.99327E-18	3.48119E-17	positive
PRJNA729271	stage_3W_vs_E10	ordinary_muscle_development	ENSGALG00010012803	NUP155	-0.998473094	0.090425773	-11.04190828	2.39884E-28	1.29792E-27	negative
PRJNA729271	stage_3W_vs_E10	ordinary_muscle_development	ENSGALG00010012804	ENSGALG00010012804	0.727717588	0.15929626	4.568328159	4.9163E-06	8.88883E-06	positive
PRJNA729271	stage_3W_vs_E10	ordinary_muscle_development	ENSGALG00010012808	EYA3	-0.231456536	0.092617314	-2.499063372	0.012452204	0.016755913	negative
PRJNA729271	stage_3W_vs_E10	ordinary_muscle_development	ENSGALG00010012809	RHOBTB3	4.773652456	0.238460206	20.01865438	3.78814E-89	1.2148E-87	positive
PRJNA729271	stage_3W_vs_E10	ordinary_muscle_development	ENSGALG00010012812	DECR1	0.354639741	0.23987763	1.478419398	0.139295556	0.165201202	positive
PRJNA729271	stage_3W_vs_E10	ordinary_muscle_development	ENSGALG00010012818	RGS18	1.998689453	0.191805213	10.42041256	2.00083E-25	9.51512E-25	positive
PRJNA729271	stage_3W_vs_E10	ordinary_muscle_development	ENSGALG00010012819	RANGAP1	-0.079605159	0.067306369	-1.18272847	0.236916777	0.271149521	negative
PRJNA729271	stage_3W_vs_E10	ordinary_muscle_development	ENSGALG00010012821	ZBTB33	0.009392452	0.051826303	0.181229438	0.856187489	0.872499835	positive
PRJNA729271	stage_3W_vs_E10	ordinary_muscle_development	ENSGALG00010012824	HIBCH	1.09464757	0.11720414	9.339666379	9.66381E-21	3.80955E-20	positive
PRJNA729271	stage_3W_vs_E10	ordinary_muscle_development	ENSGALG00010012827	ENSGALG00010012827	-0.062519907	0.18847561	-0.331713515	0.740105594	0.767369856	negative
PRJNA729271	stage_3W_vs_E10	ordinary_muscle_development	ENSGALG00010012829	RGS5	4.824628411	0.18048416	26.73158911	2.0216E-157	2.1389E-155	positive
PRJNA729271	stage_3W_vs_E10	ordinary_muscle_development	ENSGALG00010012830	ARRDC3	1.516663792	0.148979439	10.18035644	2.42671E-24	1.10509E-23	positive
PRJNA729271	stage_3W_vs_E10	ordinary_muscle_development	ENSGALG00010012831	ENSGALG00010012831	-1.416705747	0.383871666	-3.690571283	0.000223751	0.000354805	negative
PRJNA729271	stage_3W_vs_E10	ordinary_muscle_development	ENSGALG00010012832	CHADL	-2.843392747	0.355973939	-7.987643023	1.37543E-15	4.27646E-15	negative
PRJNA729271	stage_3W_vs_E10	ordinary_muscle_development	ENSGALG00010012833	JAK2	0.908367277	0.105211115	8.633757724	5.93687E-18	2.0882E-17	positive
PRJNA729271	stage_3W_vs_E10	ordinary_muscle_development	ENSGALG00010012834	TPH2	-5.19705514	0.920291029	-5.64718657	1.63095E-08	3.47349E-08	negative
PRJNA729271	stage_3W_vs_E10	ordinary_muscle_development	ENSGALG00010012836	ENSGALG00010012836	-1.923633417	1.153348378	-1.66786849			negative
PRJNA729271	stage_3W_vs_E10	ordinary_muscle_development	ENSGALG00010012837	PKIB	1.968594374	0.261291163	7.53410238	4.91705E-14	1.41949E-13	positive
PRJNA729271	stage_3W_vs_E10	ordinary_muscle_development	ENSGALG00010012838	C2orf88	-0.148076482	0.234540336	-0.631347619	0.527813258	0.565793016	negative
PRJNA729271	stage_3W_vs_E10	ordinary_muscle_development	ENSGALG00010012839	TGFBI	-1.116463542	0.18004264	-6.201106261	5.60676E-10	1.29949E-09	negative
PRJNA729271	stage_3W_vs_E10	ordinary_muscle_development	ENSGALG00010012841	L3MBTL2	-0.921354502	0.094795234	-9.719417977	2.49202E-22	1.04428E-21	negative
PRJNA729271	stage_3W_vs_E10	ordinary_muscle_development	ENSGALG00010012842	DDX17	-0.182912781	0.049113589	-3.724280504	0.000195873	0.000312307	negative
PRJNA729271	stage_3W_vs_E10	ordinary_muscle_development	ENSGALG00010012843	ENSGALG00010012843	0.234074305	0.157484538	1.486331976	0.137191333	0.162812213	positive
PRJNA729271	stage_3W_vs_E10	ordinary_muscle_development	ENSGALG00010012845	HDHD2	-0.006673967	0.102328466	-0.065221023	0.947998023	0.954848957	negative
PRJNA729271	stage_3W_vs_E10	ordinary_muscle_development	ENSGALG00010012847	RLN3	0.545175986	0.694576834	0.784903785	0.432509983	0.471087481	positive
PRJNA729271	stage_3W_vs_E10	ordinary_muscle_development	ENSGALG00010012848	SERINC1	1.106538256	0.092413881	11.97372344	4.87889E-33	3.13751E-32	positive

PRJNA729271	stage_3W_vs_E10	ordinary_muscle_development	ENSGALG00010012849	TRHDE	-4.294427417	0.38039152	-11.28949305	1.47887E-29	8.367E-29	negative
PRJNA729271	stage_3W_vs_E10	ordinary_muscle_development	ENSGALG00010012850	CALB1	3.018802026	1.092886906	2.76222728	0.00574085	0.007995928	positive
PRJNA729271	stage_3W_vs_E10	ordinary_muscle_development	ENSGALG00010012851	ENSGALG00010012851	-0.719515616	0.255972669	-2.810907978	0.004940191	0.006930708	negative
PRJNA729271	stage_3W_vs_E10	ordinary_muscle_development	ENSGALG00010012852	MSTN	0.297430184	0.419255532	0.70942459	0.478061033	0.516702309	positive
PRJNA729271	stage_3W_vs_E10	ordinary_muscle_development	ENSGALG00010012854	LECT2	2.676242027	0.747765067	3.578987766	0.000344928	0.00053917	positive
PRJNA729271	stage_3W_vs_E10	ordinary_muscle_development	ENSGALG00010012856	AVR2	2.589237911	1.050634886	2.464450729	0.013722343	0.018376327	positive
PRJNA729271	stage_3W_vs_E10	ordinary_muscle_development	ENSGALG00010012857	NSDHL	-2.478257733	0.229550512	-10.79613248	3.59009E-27	1.84664E-26	negative
PRJNA729271	stage_3W_vs_E10	ordinary_muscle_development	ENSGALG00010012859	FBXL21	1.152021094	0.110864006	10.39129953	2.71627E-25	1.28668E-24	positive
PRJNA729271	stage_3W_vs_E10	ordinary_muscle_development	ENSGALG00010012860	ENSGALG00010012860	1.31751473	0.256450475	5.137501624	2.78415E-07	5.47055E-07	positive
PRJNA729271	stage_3W_vs_E10	ordinary_muscle_development	ENSGALG00010012861	WDR70	-0.314383555	0.082640909	-3.804212226	0.000142256	0.00022952	negative
PRJNA729271	stage_3W_vs_E10	ordinary_muscle_development	ENSGALG00010012863	KCNE1	1.01365324	0.423057707	2.396016488	0.016574345	0.021992414	positive
PRJNA729271	stage_3W_vs_E10	ordinary_muscle_development	ENSGALG00010012865	EP300	0.124258305	0.123363143	1.007256317	0.313811616	0.351658243	positive
PRJNA729271	stage_3W_vs_E10	ordinary_muscle_development	ENSGALG00010012867	A4GALT	0.714736629	0.102073362	7.002185597	2.52E-12	6.64134E-12	positive
PRJNA729271	stage_3W_vs_E10	ordinary_muscle_development	ENSGALG00010012868	CTHRC1	-4.168908712	0.598658407	-6.963752059	3.31328E-12	8.67677E-12	negative
PRJNA729271	stage_3W_vs_E10	ordinary_muscle_development	ENSGALG00010012869	ENSGALG00010012869	0.74629962	0.216015411	3.454844333	0.000550611	0.000844025	positive
PRJNA729271	stage_3W_vs_E10	ordinary_muscle_development	ENSGALG00010012870	KCNC2	-2.76267868	0.217592784	-12.69655467	6.17906E-37	4.48276E-36	negative
PRJNA729271	stage_3W_vs_E10	ordinary_muscle_development	ENSGALG00010012871	ENSGALG00010012871	1.580098926	0.34902306	4.52720496	5.9769E-06	1.07408E-05	positive
PRJNA729271	stage_3W_vs_E10	ordinary_muscle_development	ENSGALG00010012873	PTAFR	2.808176026	0.220594247	12.73005103	4.02576E-37	2.93528E-36	positive
PRJNA729271	stage_3W_vs_E10	ordinary_muscle_development	ENSGALG00010012875	HSF2	-0.643453927	0.133677288	-4.813487291	1.48319E-06	2.77548E-06	negative
PRJNA729271	stage_3W_vs_E10	ordinary_muscle_development	ENSGALG00010012876	KDELR3	-1.545465363	0.164118946	-9.416739491	4.65313E-21	1.85805E-20	negative
PRJNA729271	stage_3W_vs_E10	ordinary_muscle_development	ENSGALG00010012877	SLC25A32	-0.425116293	0.095317058	-4.460023246	8.19508E-06	1.45609E-05	negative
PRJNA729271	stage_3W_vs_E10	ordinary_muscle_development	ENSGALG00010012878	GDNF	0.546251096	0.314649258	1.736063512	0.082552605	0.100851244	positive
PRJNA729271	stage_3W_vs_E10	ordinary_muscle_development	ENSGALG00010012879	RCAN1	0.007153828	0.173572627	0.041215184	0.967124349	0.971682778	positive
PRJNA729271	stage_3W_vs_E10	ordinary_muscle_development	ENSGALG00010012880	CLK4	1.53524277	0.138861829	11.05590194	2.05263E-28	1.11351E-27	positive
PRJNA729271	stage_3W_vs_E10	ordinary_muscle_development	ENSGALG00010012882	ENSGALG00010012882	0.605841966	0.606161822	0.999472326	0.317565939	0.355535513	positive
PRJNA729271	stage_3W_vs_E10	ordinary_muscle_development	ENSGALG00010012883	DCAF13	-0.541927082	0.066819177	-8.110352577	5.04731E-16	1.60828E-15	negative
PRJNA729271	stage_3W_vs_E10	ordinary_muscle_development	ENSGALG00010012884	ENSGALG00010012884	-0.302182394	0.214329985	-1.409893222	0.158571214	0.186459784	negative
PRJNA729271	stage_3W_vs_E10	ordinary_muscle_development	ENSGALG00010012885	PMS1	0.379176936	0.129175001	2.935373965	0.003331459	0.0047588	positive
PRJNA729271	stage_3W_vs_E10	ordinary_muscle_development	ENSGALG00010012887	KCNJ4	-0.502849495	0.462522276	-1.087189787	0.276952943	0.31379918	negative
PRJNA729271	stage_3W_vs_E10	ordinary_muscle_development	ENSGALG00010012888	ENSGALG00010012888	-3.677811803	3.667265771	-1.00287572	0.315920829	0.353803014	negative
PRJNA729271	stage_3W_vs_E10	ordinary_muscle_development	ENSGALG00010012889	RIC1	0.800827751	0.104685916	7.649813675	2.01271E-14	5.91769E-14	positive
PRJNA729271	stage_3W_vs_E10	ordinary_muscle_development	ENSGALG00010012890	MFAP3	-0.307864622	0.069988818	-4.398768708	1.08867E-05	1.91787E-05	negative
PRJNA729271	stage_3W_vs_E10	ordinary_muscle_development	ENSGALG00010012891	CAPS2	-3.576411002	0.357043057	-10.0167499	1.28665E-23	5.67212E-23	negative
PRJNA729271	stage_3W_vs_E10	ordinary_muscle_development	ENSGALG00010012892	ORMDL1	0.222342958	0.161566637	1.37616876	0.168769391	0.197681804	positive
PRJNA729271	stage_3W_vs_E10	ordinary_muscle_development	ENSGALG00010012893	ENSGALG00010012893	2.142888766	0.187031456	11.45737091	2.15971E-30	1.25875E-29	positive
PRJNA729271	stage_3W_vs_E10	ordinary_muscle_development	ENSGALG00010012894	CLIC6	-1.475628335	0.704019133	-2.096006011	0.036081658	0.046120249	negative
PRJNA729271	stage_3W_vs_E10	ordinary_muscle_development	ENSGALG00010012896	STK38L	0.51948784	0.098933885	5.250858564	1.51392E-07	3.02429E-07	positive
PRJNA729271	stage_3W_vs_E10	ordinary_muscle_development	ENSGALG00010012897	ENSGALG00010012897	0.733143678	0.297981229	2.460368662	0.013879436	0.018571257	positive
PRJNA729271	stage_3W_vs_E10	ordinary_muscle_development	ENSGALG00010012898	FAM114A2	1.212963309	0.114249418	10.61680077	2.48947E-26	1.23452E-25	positive
PRJNA729271	stage_3W_vs_E10	ordinary_muscle_development	ENSGALG00010012899	ENSGALG00010012899	1.13718108	0.129536784	8.778827465	1.65188E-18	5.95029E-18	positive
PRJNA729271	stage_3W_vs_E10	ordinary_muscle_development	ENSGALG00010012900	RBX1	0.970546894	0.171709062	5.652275326	1.58338E-08	3.37615E-08	positive
PRJNA729271	stage_3W_vs_E10	ordinary_muscle_development	ENSGALG00010012903	RGMB	-0.079899186	0.092618568	-0.862669199	0.388319372	0.427322297	negative
PRJNA729271	stage_3W_vs_E10	ordinary_muscle_development	ENSGALG00010012904	ERMP1	-0.217079794	0.135497434	-1.602095233	0.109134552	0.131224001	negative
PRJNA729271	stage_3W_vs_E10	ordinary_muscle_development	ENSGALG00010012905	NKAIN1	-4.147692939	0.33578961	-12.35205859	4.74807E-35	3.27107E-34	negative
PRJNA729271	stage_3W_vs_E10	ordinary_muscle_development	ENSGALG00010012908	STRAP	-0.402929277	0.083035729	-4.85248075	1.21927E-06	2.29642E-06	negative
PRJNA729271	stage_3W_vs_E10	ordinary_muscle_development	ENSGALG00010012910	XPNPEP3	0.152717925	0.099317605	1.53767225	0.124128772	0.148098325	positive
PRJNA729271	stage_3W_vs_E10	ordinary_muscle_development	ENSGALG00010012911	CSNK1E	-1.025905283	0.085697795	-11.97119813	5.0297E-33	3.23306E-32	negative
PRJNA729271	stage_3W_vs_E10	ordinary_muscle_development	ENSGALG00010012912	NKAPL	0.054930567	0.09973498	0.550765313	0.581794565	0.617810419	positive
PRJNA729271	stage_3W_vs_E10	ordinary_muscle_development	ENSGALG00010012913	EGFLAM	-1.786691634	0.156185771	-11.43952885	2.65322E-30	1.54019E-29	negative
PRJNA729271	stage_3W_vs_E10	ordinary_muscle_development	ENSGALG00010012915	ENSGALG00010012915	5.533923179	0.278390595	19.87826914	6.27619E-88	1.95642E-86	positive
PRJNA729271	stage_3W_vs_E10	ordinary_muscle_development	ENSGALG00010012917	RUNX1	0.675359162	0.248976602	2.712540685	0.006676958	0.009244676	positive
PRJNA729271	stage_3W_vs_E10	ordinary_muscle_development	ENSGALG00010012918	GLRX	1.66557831	0.276885563	6.015403229	1.79439E-09	4.02565E-09	positive
PRJNA729271	stage_3W_vs_E10	ordinary_muscle_development	ENSGALG00010012921	EBF1	-0.340823663	0.140531997	-2.425238876	0.015298317	0.020370118	negative
PRJNA729271	stage_3W_vs_E10	ordinary_muscle_development	ENSGALG00010012922	SNRNP40	-1.095382266	0.129704618	-8.445206397	3.03509E-17	1.02885E-16	negative
PRJNA729271	stage_3W_vs_E10	ordinary_muscle_development	ENSGALG00010012923	MLANA	-3.366751797	0.86589634	-3.888169568	0.000101003	0.000164814	negative
PRJNA729271	stage_3W_vs_E10	ordinary_muscle_development	ENSGALG00010012924	GLIPR1	0.329659122	0.200738411	1.642232401	0.10054185	0.12150693	positive
PRJNA729271	stage_3W_vs_E10	ordinary_muscle_development	ENSGALG00010012925	SETD4	-1.434009202	0.194350996	-7.378450508	1.60142E-13	4.49248E-13	negative
PRJNA729271	stage_3W_vs_E10	ordinary_muscle_development	ENSGALG00010012926	OSGEPL1	0.604182051	0.164258394	3.678241546	0.000234848	0.000371598	positive
PRJNA729271	stage_3W_vs_E10	ordinary_muscle_development	ENSGALG00010012927	ENSGALG00010012927	-4.951935041	3.739202879	-1.324329062			negative
PRJNA729271	stage_3W_vs_E10	ordinary_muscle_development	ENSGALG00010012930	IL6ST	0.635709566	0.090864222	6.996258281	2.62889E-12	6.91825E-12	positive
PRJNA729271	stage_3W_vs_E10	ordinary_muscle_development	ENSGALG00010012932	ATF6	0.471495775	0.118237884	3.98768786	6.67204E-05	0.000110679	positive
PRJNA729271	stage_3W_vs_E10	ordinary_muscle_development	ENSGALG00010012933	ZCCHC17	-0.582323061	0.147482487	-3.948421758	7.86681E-05	0.000129469	negative
PRJNA729271	stage_3W_vs_E10	ordinary_muscle_development	ENSGALG00010012934	NDUFA1	1.597718583	0.151423753	10.55130755	5.0095E-26	2.44758E-25	positive
PRJNA729271	stage_3W_vs_E10	ordinary_muscle_development	ENSGALG00010012938	UPF3B	0.306677195	0.150288796	2.040585883	0.041292006	0.052382954	positive
PRJNA729271	stage_3W_vs_E10	ordinary_muscle_development	ENSGALG00010012939	GJA1	-3.24959469	0.207390057	-15.66899948	2.46428E-55	3.28609E-54	negative
PRJNA729271	stage_3W_vs_E10	ordinary_muscle_development	ENSGALG00010012940	FBN2	-4.001274015	0.224980818	-17.78495627	9.24355E-71	1.82293E-69	negative
PRJNA729271	stage_3W_vs_E10	ordinary_muscle_development	ENSGALG00010012943	ASNSD1	-0.09571574	0.122241765	-0.783003583	0.433625008	0.472195514	negative
PRJNA729271	stage_3W_vs_E10	ordinary_muscle_development	ENSGALG00010012944	NCK1	-0.481257377	0.052807343	-9.113455681	7.97988E-20	3.03035E-19	negative
PRJNA729271	stage_3W_vs_E10	ordinary_muscle_development	ENSGALG00010012945	CLINT1	0.023588949	0.06283134	0.375432846	0.707338581	0.736398242	positive
PRJNA729271	stage_3W_vs_E10	ordinary_muscle_development	ENSGALG00010012948	ST13P5	-0.808235186	0.095353196	-8.476225422	2.32618E-17	7.92618E-17	negative
PRJNA729271	stage_3W_vs_E10	ordinary_muscle_development	ENSGALG00010012949	ENSGALG00010012949	-0.223923126	0.170999679	-1.30949442	0.190366927	0.221014706	negative
PRJNA729271	stage_3W_vs_E10	ordinary_muscle_development	ENSGALG00010012952	BRD10	1.166488671	0.186363467	6.259213177	3.86925E-10	9.04738E-10	positive
PRJNA729271	stage_3W_vs_E10	ordinary_muscle_development	ENSGALG00010012954	RIMS2	-0.353241131	0.333659541	-1.058687335	0.289742198	0.326960931	negative
PRJNA729271	stage_3W_vs_E10	ordinary_muscle_development	ENSGALG00010012955	RPL39L	0.410304924	0.1551714	2.644204559	0.008188315	0.011235293	positive
PRJNA729271	stage_3W_vs_E10	ordinary_muscle_development	ENSGALG00010012956	FABP3	3.578546016	0.168202331	21.27524622	1.925E-100	8.15873E-99	positive
PRJNA729271	stage_3W_vs_E10	ordinary_muscle_development	ENSGALG00010012957	DERA	0.198191171	0.227528689	0.871060136	0.383721319	0.422647456	positive
PRJNA729271	stage_3W_vs_E10	ordinary_muscle_development	ENSGALG00010012958	OLFML2B	0.606871076	0.205054856	2.959554768	0.003080839	0.004415778	positive
PRJNA729271	stage_3W_vs_E10	ordinary_muscle_development	ENSGALG00010012959	TBC1D32	0.028211091	0.146529351	0.192528601	0.847328166	0.864809306	positive
PRJNA729271	stage_3W_vs_E10	ordinary_muscle_development	ENSGALG00010012962	SLC25A17	-1.058134206	0.210159432	-5.034911799	4.78069E-07	9.24688E-07	negative
PRJNA729271	stage_3W_vs_E10	ordinary_muscle_development	ENSGALG00010012963	UIMC1	-0.221604447	0.091046901	-2.433959255	0.014934676	0.019924095	negative
PRJNA729271	stage_3W_vs_E10	ordinary_muscle_development	ENSGALG00010012964	CBR3	-0.371466478	0.072511319	-5.122875766	3.00911E-07	5.89658E-07	negative
PRJNA729271	stage_3W_vs_E10	ordinary_muscle_development	ENSGALG00010012965	ENSGALG00010012965	-0.620680741	0.085366653	-7.270763452	3.57461E-13	9.85245E-13	negative
PRJNA729271	stage_3W_vs_E10	ordinary_muscle_development	ENSGALG00010012966	GLIPR1L	1.699944791	0.26452068	6.426509987	1.30567E-10	3.12976E-10	positive
PRJNA729271	stage_3W_vs_E10	ordinary_muscle_development	ENSGALG00010012967	ECRG4	-2.04377534	0.210147189	-9.725446957	2.34872E-22	9.85373E-22	negative
PRJNA729271	stage_3W_vs_E10	ordinary_muscle_development	ENSGALG00010012968	LSM11	-1.165917681	0.078599808	-14.83359448	8.88453E-50	9.96759E-49	negative
PRJNA729271	stage_3W_vs_E10	ordinary_muscle_development	ENSGALG00010012969	SLC12A2	-0.151650173	0.142466919	-1.064458852	0.287120885	0.324280601	negative
PRJNA729271	stage_3W_vs_E10	ordinary_muscle_development	ENSGALG00010012970	UNC5A	-2.162391641	0.208528931	-10.36974404	3.40435E-25	1.60737E-24	negative
PRJNA729271	stage_3W_vs_E10	ordinary_muscle_development	ENSGALG00010012971	ENSGALG00010012971	-1.072631005	0.609198559	-1.760724788	0.078284995	0.095920626	negative
PRJNA729271	stage_3W_vs_E10	ordinary_muscle_development	ENSGALG00010012973	GLDC	-5.42757944	0.360547129	-15.05373085	3.26301E-51	3.84532E-50	negative
PRJNA729271	stage_3W_vs_E10	ordinary_muscle_development	ENSGALG00010012974	SLC49A3	-0.804789051	0.216829236	-3.711626095	0.000205932	0.000327696	negative
PRJNA729271	stage_3W_vs_E10	ordinary_muscle_development	ENSGALG00010012975	KRR1	-0.29964554	0.092885463	-3.225968101	0.001255473	0.00186494	negative
PRJNA729271	stage_3W_vs_E10	ordinary_muscle_development	ENSGALG00010012976	SLC9A2	2.716757087	0.229772388	11.82368826	2.94467E-32	1.84695E-31	positive
PRJNA729271	stage_3W_vs_E10	ordinary_muscle_development	ENSGALG00010012977	NKRF	-0.376682046	0.099277233	-3.794244012	0.000148094	0.000238514	negative
PRJNA729271	stage_3W_vs_E10	ordinary_muscle_development	ENSGALG00010012978	HSD17B7	-0.241676383	0.210346915	-1.148941895	0.250579941	0.285827612	negative
PRJNA729271	stage_3W_vs_E10	ordinary_muscle_development	ENSGALG00010012979	HTR1A	0.517889531	0.549231072	0.9429356	0.345713844	0.384229579	positive
PRJNA729271	stage_3W_vs_E10	ordinary_muscle_development	ENSGALG00010012980	KICS2	-0.255642777	0.07684522	-3.326723199	0.000878736	0.001322255	negative
PRJNA729271	stage_3W_vs_E10	ordinary_muscle_development	ENSGALG00010012982	DDR2	-1.281011774	0.244497358	-5.239368569	1.61127E-07	3.21077E-07	negative
PRJNA729271	stage_3W_vs_E10	ordinary_muscle_development	ENSGALG00010012985	ARNTL2	0.098367808	0.170199494	0.577955937	0.563293868	0.600010627	positive
PRJNA729271	stage_3W_vs_E10	ordinary_muscle_development	ENSGALG00010012986	TMEM184B	0.506874108	0.140229874	3.614594347	0.000300818	0.000472309	positive
PRJNA729271	stage_3W_vs_E10	ordinary_muscle_development	ENSGALG00010012988	RPL18A	0.195061671	0.134906571	1.445901926	0.148204712	0.174922832	positive
PRJNA729271	stage_3W_vs_E10	ordinary_muscle_development	ENSGALG00010012991	MFSD9	0.210742901	0.101150817	2.083452297	0.037210016	0.047491342	positive
PRJNA729271	stage_3W_vs_E10	ordinary_muscle_development	ENSGALG00010012992	HARS1	-0.407194359	0.127253224	-3.199874585	0.001374874	0.002034589	negative
PRJNA729271	stage_3W_vs_E10	ordinary_muscle_development	ENSGALG00010012993	DOP1B	0.064449576	0.098696924	0.653004912	0.513753112	0.551700352	positive
PRJNA729271	stage_3W_vs_E10	ordinary_muscle_development	ENSGALG00010012995	SERINC2	0.349718564	0.151348313	2.310686898	0.020850155	0.027380232	positive
PRJNA729271	stage_3W_vs_E10	ordinary_muscle_development	ENSGALG00010012997	UBE2A	0.804041976	0.057318762	14.02755298	1.05737E-44	9.97179E-44	positive
PRJNA729271	stage_3W_vs_E10	ordinary_muscle_development	ENSGALG00010012998	MAFF	0.041114361	0.200117367	0.205451238	0.837219592	0.855938636	positive
PRJNA729271	stage_3W_vs_E10	ordinary_muscle_development	ENSGALG00010013001	ADGRV1	-0.953771896	0.317812571	-3.001051508	0.002690491	0.003870066	negative
PRJNA729271	stage_3W_vs_E10	ordinary_muscle_development	ENSGALG00010013004	UAP1	-0.274170631	0.153655858	-1.784316164	0.074372305	0.091365926	negative
PRJNA729271	stage_3W_vs_E10	ordinary_muscle_development	ENSGALG00010013005	SLC40A1	0.80652726	0.090669232	8.895269606	5.82776E-19	2.13749E-18	positive
PRJNA729271	stage_3W_vs_E10	ordinary_muscle_development	ENSGALG00010013006	GRIK2	-6.164462944	1.174255433	-5.249678025	1.52365E-07	3.04248E-07	negative
PRJNA729271	stage_3W_vs_E10	ordinary_muscle_development	ENSGALG00010013008	STEEP1	-0.490576	0.110302869	-4.44753618	8.68608E-06	1.54031E-05	negative
PRJNA729271	stage_3W_vs_E10	ordinary_muscle_development	ENSGALG00010013012	TMEM182	2.251004678	0.151455815	14.86245129	5.7772E-50	6.51677E-49	positive
PRJNA729271	stage_3W_vs_E10	ordinary_muscle_development	ENSGALG00010013013	DCSTAMP	5.053372922	1.068241518	4.730552815	2.23909E-06	4.14239E-06	positive
PRJNA729271	stage_3W_vs_E10	ordinary_muscle_development	ENSGALG00010013015	PLA2G6	0.776406735	0.149017506	5.21017132	1.88666E-07	3.74568E-07	positive
PRJNA729271	stage_3W_vs_E10	ordinary_muscle_development	ENSGALG00010013016	QRSL1	1.008305563	0.121616698	8.290848047	1.12444E-16	3.70932E-16	positive
PRJNA729271	stage_3W_vs_E10	ordinary_muscle_development	ENSGALG00010013017	SCMH1	0.800572912	0.158050881	5.065285999	4.07788E-07	7.92131E-07	positive
PRJNA729271	stage_3W_vs_E10	ordinary_muscle_development	ENSGALG00010013018	ID1	-0.504108739	0.19338169	-2.606806979	0.009139086	0.012483137	negative
PRJNA729271	stage_3W_vs_E10	ordinary_muscle_development	ENSGALG00010013019	DPYS	-0.783400894	0.964111614	-0.812562449	0.416468968	0.455267665	negative
PRJNA729271	stage_3W_vs_E10	ordinary_muscle_development	ENSGALG00010013021	SLC25A5	-1.575855195	0.164654948	-9.57065193	1.06234E-21	4.35111E-21	negative
PRJNA729271	stage_3W_vs_E10	ordinary_muscle_development	ENSGALG00010013027	UHMK1	1.115100741	0.125256971	8.902504417	5.46005E-19	2.00668E-18	positive
PRJNA729271	stage_3W_vs_E10	ordinary_muscle_development	ENSGALG00010013029	MYLK2	8.910225106	0.253733953	35.11640836	3.7872E-270	1.3389E-267	positive
PRJNA729271	stage_3W_vs_E10	ordinary_muscle_development	ENSGALG00010013032	BAIAP2L2	1.661333702	0.387137197	4.291330601	1.77606E-05	3.08052E-05	positive
PRJNA729271	stage_3W_vs_E10	ordinary_muscle_development	ENSGALG00010013033	MAP3K1	-0.020772252	0.1253397	-0.16572763	0.868371301	0.883381529	negative
PRJNA729271	stage_3W_vs_E10	ordinary_muscle_development	ENSGALG00010013036	ENSGALG00010013036	1.659604996	0.400827379	4.140448189	3.46628E-05	5.86343E-05	positive
PRJNA729271	stage_3W_vs_E10	ordinary_muscle_development	ENSGALG00010013037	MORC4	0.960497152	0.089446717	10.73820465	6.73412E-27	3.42976E-26	positive
PRJNA729271	stage_3W_vs_E10	ordinary_muscle_development	ENSGALG00010013040	SLC25A43	2.082171568	0.203751034	10.2191951	1.62686E-24	7.47191E-24	positive
PRJNA729271	stage_3W_vs_E10	ordinary_muscle_development	ENSGALG00010013041	LRP12	0.278937673	0.074915227	3.723377521	0.000196575	0.000313357	positive
PRJNA729271	stage_3W_vs_E10	ordinary_muscle_development	ENSGALG00010013042	SLC25A48	-2.95034599	0.354961595	-8.311732959	9.43145E-17	3.12406E-16	negative
PRJNA729271	stage_3W_vs_E10	ordinary_muscle_development	ENSGALG00010013044	ENSGALG00010013044	0.175184656	0.126694971	1.382727782	0.16674835	0.195456682	positive
PRJNA729271	stage_3W_vs_E10	ordinary_muscle_development	ENSGALG00010013045	FSD1L	1.265288329	0.144242913	8.771927207	1.75634E-18	6.31559E-18	positive
PRJNA729271	stage_3W_vs_E10	ordinary_muscle_development	ENSGALG00010013046	SLC12A5	-0.649418767	0.375764161	-1.728261592	0.083941336	0.102487337	negative

PRJNA729271	stage_3W_vs_E10	ordinary_muscle_development	ENSGALG00010013047	CTPS1	0.124066542	0.100181946	1.238412175	0.215563268	0.248331163	positive
PRJNA729271	stage_3W_vs_E10	ordinary_muscle_development	ENSGALG00010013048	WDR75	-1.288350389	0.088392464	-14.57534198	4.03099E-48	4.32169E-47	negative
PRJNA729271	stage_3W_vs_E10	ordinary_muscle_development	ENSGALG00010013051	XPOT	0.612927628	0.078467934	7.81118598	5.66523E-15	1.71292E-14	positive
PRJNA729271	stage_3W_vs_E10	ordinary_muscle_development	ENSGALG00010013053	MRTFA	0.509746546	0.174681661	2.918145739	0.003521197	0.005016192	positive
PRJNA729271	stage_3W_vs_E10	ordinary_muscle_development	ENSGALG00010013056	ENSGALG00010013056	2.691224622	0.55282728	4.868111107	1.1267E-06	2.12595E-06	positive
PRJNA729271	stage_3W_vs_E10	ordinary_muscle_development	ENSGALG00010013058	ENSGALG00010013058	0.505737041	0.275620448	1.834903924	0.066519926	0.082228068	positive
PRJNA729271	stage_3W_vs_E10	ordinary_muscle_development	ENSGALG00010013062	ENSGALG00010013062	0.148930927	0.104733307	1.422001563	0.155025797	0.182542561	positive
PRJNA729271	stage_3W_vs_E10	ordinary_muscle_development	ENSGALG00010013063	PARP12	1.569573957	0.216956633	7.234505505	4.67228E-13	1.27927E-12	positive
PRJNA729271	stage_3W_vs_E10	ordinary_muscle_development	ENSGALG00010013065	FKTN	-0.077267573	0.103196696	-0.74874076	0.454013472	0.492687414	negative
PRJNA729271	stage_3W_vs_E10	ordinary_muscle_development	ENSGALG00010013071	ENSGALG00010013071	-1.15539603	0.225971161	-5.113024263	3.17042E-07	6.20429E-07	negative
PRJNA729271	stage_3W_vs_E10	ordinary_muscle_development	ENSGALG00010013076	RTN4IP1	-0.139624109	0.081919688	-1.704402356	0.088305944	0.107553538	negative
PRJNA729271	stage_3W_vs_E10	ordinary_muscle_development	ENSGALG00010013078	COL5A2	-1.229778511	0.242034838	-5.08099793	3.75457E-07	7.31191E-07	negative
PRJNA729271	stage_3W_vs_E10	ordinary_muscle_development	ENSGALG00010013079	ENSGALG00010013079	-1.42827472	0.207427524	-6.885656703	5.75216E-12	1.48385E-11	negative
PRJNA729271	stage_3W_vs_E10	ordinary_muscle_development	ENSGALG00010013080	ARHGEF7	-0.14368392	0.099587464	-1.442791226	0.149079291	0.175897788	negative
PRJNA729271	stage_3W_vs_E10	ordinary_muscle_development	ENSGALG00010013081	NCOA5	-1.026603873	0.10233722	-10.03157863	1.10732E-23	4.89493E-23	negative
PRJNA729271	stage_3W_vs_E10	ordinary_muscle_development	ENSGALG00010013082	CHAF1B	-2.255286242	0.116563128	-19.34819588	2.11103E-83	5.82104E-82	negative
PRJNA729271	stage_3W_vs_E10	ordinary_muscle_development	ENSGALG00010013084	SGSM3	0.793991349	0.115174765	6.893796123	5.43229E-12	1.40408E-11	positive
PRJNA729271	stage_3W_vs_E10	ordinary_muscle_development	ENSGALG00010013085	LYSMD3	-0.067261288	0.089275386	-0.75341358	0.451201425	0.489936674	negative
PRJNA729271	stage_3W_vs_E10	ordinary_muscle_development	ENSGALG00010013086	CXCL14	-3.639631857	0.518277923	-7.022548518	2.17857E-12	5.76775E-12	negative
PRJNA729271	stage_3W_vs_E10	ordinary_muscle_development	ENSGALG00010013087	GPN2	-0.472994186	0.093127145	-5.079015238	3.79396E-07	7.38564E-07	negative
PRJNA729271	stage_3W_vs_E10	ordinary_muscle_development	ENSGALG00010013089	TBK1	0.458800279	0.076117236	6.02754778	1.66466E-09	3.74503E-09	positive
PRJNA729271	stage_3W_vs_E10	ordinary_muscle_development	ENSGALG00010013093	GPATCH3	0.259003615	0.158592088	1.633143356	0.10243885	0.123655157	positive
PRJNA729271	stage_3W_vs_E10	ordinary_muscle_development	ENSGALG00010013095	ADSL	2.89561169	0.127567043	22.6987443	4.6097E-114	2.5026E-112	positive
PRJNA729271	stage_3W_vs_E10	ordinary_muscle_development	ENSGALG00010013096	CLDN14	-1.233050383	0.431333856	-2.858691396	0.004253924	0.006008051	negative
PRJNA729271	stage_3W_vs_E10	ordinary_muscle_development	ENSGALG00010013097	ENSGALG00010013097	-0.356935903	0.420732229	-0.848368341	0.396232868	0.435105714	negative
PRJNA729271	stage_3W_vs_E10	ordinary_muscle_development	ENSGALG00010013099	SIM2	0.432647814	0.202117749	2.140573087	0.032308481	0.041502254	positive
PRJNA729271	stage_3W_vs_E10	ordinary_muscle_development	ENSGALG00010013100	ZFPM2	-1.616764252	0.176433505	-9.163589725	5.0199E-20	1.92089E-19	negative
PRJNA729271	stage_3W_vs_E10	ordinary_muscle_development	ENSGALG00010013103	AIRIM	0.038458858	0.146973598	0.261671886	0.793574418	0.816542701	positive
PRJNA729271	stage_3W_vs_E10	ordinary_muscle_development	ENSGALG00010013104	HLCS	-0.306100221	0.109522701	-2.794856404	0.005192277	0.007268887	negative
PRJNA729271	stage_3W_vs_E10	ordinary_muscle_development	ENSGALG00010013106	SETD9	0.560336743	0.178852962	3.132946402	0.00173061	0.002537703	positive
PRJNA729271	stage_3W_vs_E10	ordinary_muscle_development	ENSGALG00010013109	ENSGALG00010013109	2.065724441	1.469777571	1.405467386	0.159882341	0.187894805	positive
PRJNA729271	stage_3W_vs_E10	ordinary_muscle_development	ENSGALG00010013110	ANKRD10	1.537344231	0.154264473	9.965640172	2.15481E-23	9.4334E-23	positive
PRJNA729271	stage_3W_vs_E10	ordinary_muscle_development	ENSGALG00010013111	RFESD	2.740035061	0.271938571	10.07593387	7.0587E-24	3.14915E-23	positive
PRJNA729271	stage_3W_vs_E10	ordinary_muscle_development	ENSGALG00010013112	CD40	0.472994012	0.161597162	2.926994549	0.003422549	0.004879977	positive
PRJNA729271	stage_3W_vs_E10	ordinary_muscle_development	ENSGALG00010013114	PIGP	0.274447971	0.121032201	2.267561596	0.023355941	0.030513237	positive
PRJNA729271	stage_3W_vs_E10	ordinary_muscle_development	ENSGALG00010013115	ENSGALG00010013115	-2.830442485	0.385397174	-7.344222203	2.06959E-13	5.77342E-13	negative
PRJNA729271	stage_3W_vs_E10	ordinary_muscle_development	ENSGALG00010013116	TARS	-0.528909451	0.168141004	-3.14563039	0.001657293	0.002436108	negative
PRJNA729271	stage_3W_vs_E10	ordinary_muscle_development	ENSGALG00010013117	ARSK	0.132855502	0.131492138	1.010368402	0.312318821	0.350229097	positive
PRJNA729271	stage_3W_vs_E10	ordinary_muscle_development	ENSGALG00010013119	RNF114	0.659393564	0.078668679	8.381907143	5.20769E-17	1.74533E-16	positive
PRJNA729271	stage_3W_vs_E10	ordinary_muscle_development	ENSGALG00010013121	PCBD2	-0.161879572	0.165238906	-0.97966984	0.327249118	0.365473147	negative
PRJNA729271	stage_3W_vs_E10	ordinary_muscle_development	ENSGALG00010013123	PPFIBP1	0.11328246	0.108222357	1.04675654	0.295211879	0.332514623	positive
PRJNA729271	stage_3W_vs_E10	ordinary_muscle_development	ENSGALG00010013125	POU3F3	-6.860035716	0.849907729	-8.071506449	6.94361E-16	2.19467E-15	negative
PRJNA729271	stage_3W_vs_E10	ordinary_muscle_development	ENSGALG00010013129	RASSF3	-0.401735532	0.111328367	-3.608563951	0.000307897	0.000482952	negative
PRJNA729271	stage_3W_vs_E10	ordinary_muscle_development	ENSGALG00010013131	SPATA2	0.204456347	0.116890021	1.749134312	0.080267805	0.098200695	positive
PRJNA729271	stage_3W_vs_E10	ordinary_muscle_development	ENSGALG00010013132	SKIC3	-0.514810039	0.089680382	-5.740497856	9.43986E-09	2.04073E-08	negative
PRJNA729271	stage_3W_vs_E10	ordinary_muscle_development	ENSGALG00010013133	TXNDC15	-0.159107657	0.120059754	-1.325237234	0.185092479	0.215356838	negative
PRJNA729271	stage_3W_vs_E10	ordinary_muscle_development	ENSGALG00010013134	ENSGALG00010013134	-3.810158089	5.428609441	-0.701866312	0.482762539	0.521239703	negative
PRJNA729271	stage_3W_vs_E10	ordinary_muscle_development	ENSGALG00010013135	TTC3	-0.340293368	0.085018038	-4.002602006	6.26496E-05	0.000104129	negative
PRJNA729271	stage_3W_vs_E10	ordinary_muscle_development	ENSGALG00010013137	TMEM121	-6.748967207	0.395893394	-17.04743575	3.65207E-65	6.27213E-64	negative
PRJNA729271	stage_3W_vs_E10	ordinary_muscle_development	ENSGALG00010013139	TMEM185A	0.885399465	0.081940037	10.80545595	3.24342E-27	1.6701E-26	positive
PRJNA729271	stage_3W_vs_E10	ordinary_muscle_development	ENSGALG00010013143	ENSGALG00010013143	0.672820018	0.413803147	1.625942248	0.103961942	0.125368415	positive
PRJNA729271	stage_3W_vs_E10	ordinary_muscle_development	ENSGALG00010013144	TEDC1	-1.82494378	0.174703332	-10.44595862	1.52903E-25	7.31943E-25	negative
PRJNA729271	stage_3W_vs_E10	ordinary_muscle_development	ENSGALG00010013145	ENSGALG00010013145	-0.231725085	0.285949967	-0.810369338	0.417727935	0.45642744	negative
PRJNA729271	stage_3W_vs_E10	ordinary_muscle_development	ENSGALG00010013146	TNRC6B	0.681470465	0.119375432	5.708632444	1.13888E-08	2.45035E-08	positive
PRJNA729271	stage_3W_vs_E10	ordinary_muscle_development	ENSGALG00010013149	MCTP1	0.796362478	0.196962992	4.043208669	5.27247E-05	8.81379E-05	positive
PRJNA729271	stage_3W_vs_E10	ordinary_muscle_development	ENSGALG00010013150	GNS	1.061499938	0.103029467	10.30287715	6.83848E-25	3.18419E-24	positive
PRJNA729271	stage_3W_vs_E10	ordinary_muscle_development	ENSGALG00010013152	TBXAS1	0.934487178	0.219646891	4.254497636	2.09519E-05	3.61287E-05	positive
PRJNA729271	stage_3W_vs_E10	ordinary_muscle_development	ENSGALG00010013154	ENSGALG00010013154	-1.296208223	0.415925283	-3.116444888	0.001830459	0.002673569	negative
PRJNA729271	stage_3W_vs_E10	ordinary_muscle_development	ENSGALG00010013156	ABCC9	2.121041381	0.129611533	16.36460371	3.42248E-60	5.14615E-59	positive
PRJNA729271	stage_3W_vs_E10	ordinary_muscle_development	ENSGALG00010013158	CRIP1	3.598103647	0.184434775	19.50881362	9.24017E-85	2.64696E-83	positive
PRJNA729271	stage_3W_vs_E10	ordinary_muscle_development	ENSGALG00010013159	ENSGALG00010013159	-1.522107834	1.454768673	-1.04628857	0.295427821	0.332729314	negative
PRJNA729271	stage_3W_vs_E10	ordinary_muscle_development	ENSGALG00010013160	CRYBG1	-5.200844828	0.619097158	-8.400692467	4.43853E-17	1.49238E-16	negative
PRJNA729271	stage_3W_vs_E10	ordinary_muscle_development	ENSGALG00010013162	IDS	0.269280997	0.110600603	2.434715459	0.014903504	0.0198846	positive
PRJNA729271	stage_3W_vs_E10	ordinary_muscle_development	ENSGALG00010013163	ENSGALG00010013163	-0.817304748	0.488457787	-1.673235168	0.09428101	0.114407973	negative
PRJNA729271	stage_3W_vs_E10	ordinary_muscle_development	ENSGALG00010013164	FAM83F	-1.934997066	0.45096087	-4.290831415	1.78005E-05	3.08709E-05	negative
PRJNA729271	stage_3W_vs_E10	ordinary_muscle_development	ENSGALG00010013165	NUDC	-0.34992996	0.081163263	-4.311432862	1.622E-05	2.81973E-05	negative
PRJNA729271	stage_3W_vs_E10	ordinary_muscle_development	ENSGALG00010013168	VPS26C	-0.247846888	0.138267116	-1.792522291	0.073049342	0.089832036	negative
PRJNA729271	stage_3W_vs_E10	ordinary_muscle_development	ENSGALG00010013170	COL3A1	2.415211786	0.234906245	10.28159889	8.53006E-25	3.95785E-24	positive
PRJNA729271	stage_3W_vs_E10	ordinary_muscle_development	ENSGALG00010013171	SLC9A8	0.654083145	0.135810795	4.816135156	1.46365E-06	2.73963E-06	positive
PRJNA729271	stage_3W_vs_E10	ordinary_muscle_development	ENSGALG00010013172	TNFSF13B	3.890179057	0.409109273	9.508899744	1.9269E-21	7.81925E-21	positive
PRJNA729271	stage_3W_vs_E10	ordinary_muscle_development	ENSGALG00010013173	KCNJ8	0.495314126	0.155315079	3.189092312	0.001427203	0.002109013	positive
PRJNA729271	stage_3W_vs_E10	ordinary_muscle_development	ENSGALG00010013174	ADAMTS12	0.318445987	0.165327034	1.926157994	0.054084649	0.067599982	positive
PRJNA729271	stage_3W_vs_E10	ordinary_muscle_development	ENSGALG00010013175	HIPK2	2.58616317	0.162828043	15.88278731	8.3389E-57	1.15007E-55	positive
PRJNA729271	stage_3W_vs_E10	ordinary_muscle_development	ENSGALG00010013177	OXR1	0.901971675	0.069130459	13.04738445	6.57667E-39	5.12245E-38	positive
PRJNA729271	stage_3W_vs_E10	ordinary_muscle_development	ENSGALG00010013179	TBC1D30	0.762992113	0.182405164	4.182952381	2.87748E-05	4.90233E-05	positive
PRJNA729271	stage_3W_vs_E10	ordinary_muscle_development	ENSGALG00010013180	C5orf24	-0.276654643	0.059576046	-4.643722794	3.42187E-06	6.2642E-06	negative
PRJNA729271	stage_3W_vs_E10	ordinary_muscle_development	ENSGALG00010013181	DYRK1A	0.472605911	0.079187344	5.968200024	2.39885E-09	5.33958E-09	positive
PRJNA729271	stage_3W_vs_E10	ordinary_muscle_development	ENSGALG00010013183	CLTA	-1.121390767	0.137731989	-8.141832402	3.8934E-16	1.24553E-15	negative
PRJNA729271	stage_3W_vs_E10	ordinary_muscle_development	ENSGALG00010013184	LDHB	-1.67275272	0.114833096	-14.56681726	4.56677E-48	4.88887E-47	negative
PRJNA729271	stage_3W_vs_E10	ordinary_muscle_development	ENSGALG00010013185	ENSGALG00010013185	-0.958354425	0.324734266	-2.951195865	0.003165461	0.004531242	negative
PRJNA729271	stage_3W_vs_E10	ordinary_muscle_development	ENSGALG00010013186	DDX46	-0.729310573	0.0804928	-9.060569026	1.29772E-19	4.88202E-19	negative
PRJNA729271	stage_3W_vs_E10	ordinary_muscle_development	ENSGALG00010013187	KDF1	-3.583671704	0.27531648	-13.01655355	9.85181E-39	7.6161E-38	negative
PRJNA729271	stage_3W_vs_E10	ordinary_muscle_development	ENSGALG00010013188	ENSGALG00010013188	1.021180719	0.362312677	2.81850673	0.00482476	0.006777294	positive
PRJNA729271	stage_3W_vs_E10	ordinary_muscle_development	ENSGALG00010013190	SLC45A2	-3.452451166	0.571576129	-6.040229796	1.53895E-09	3.4676E-09	negative
PRJNA729271	stage_3W_vs_E10	ordinary_muscle_development	ENSGALG00010013191	ABRA	6.079445417	0.418836933	14.51506525	9.72651E-48	1.02386E-46	positive
PRJNA729271	stage_3W_vs_E10	ordinary_muscle_development	ENSGALG00010013192	B4GALT5	0.258727446	0.082407699	3.139602861	0.00169177	0.002483765	positive
PRJNA729271	stage_3W_vs_E10	ordinary_muscle_development	ENSGALG00010013194	GRAP2	-0.329447566	0.33911743	-0.971485205	0.331306717	0.369606008	negative
PRJNA729271	stage_3W_vs_E10	ordinary_muscle_development	ENSGALG00010013196	MRPS9	-0.092550889	0.114199989	-0.810428171	0.417694132	0.456424911	negative
PRJNA729271	stage_3W_vs_E10	ordinary_muscle_development	ENSGALG00010013198	CAMLG	-0.592402997	0.086847182	-6.821211523	9.02759E-12	2.31192E-11	negative
PRJNA729271	stage_3W_vs_E10	ordinary_muscle_development	ENSGALG00010013199	RALYL	-4.005433472	0.693302701	-5.777322755	7.58986E-09	1.64965E-08	negative
PRJNA729271	stage_3W_vs_E10	ordinary_muscle_development	ENSGALG00010013202	ENSGALG00010013202	-1.300466852	0.745146615	-1.745249628	0.080941426	0.098974689	negative
PRJNA729271	stage_3W_vs_E10	ordinary_muscle_development	ENSGALG00010013203	AMACR	0.633210485	0.119708863	5.289587314	1.22593E-07	2.46666E-07	positive
PRJNA729271	stage_3W_vs_E10	ordinary_muscle_development	ENSGALG00010013204	ENSGALG00010013204	0.606035005	0.349119235	1.735896922	0.082582062	0.100878728	positive
PRJNA729271	stage_3W_vs_E10	ordinary_muscle_development	ENSGALG00010013205	KCNJ6	-1.380988131	0.395403333	-3.492606197	0.000478331	0.000738402	negative
PRJNA729271	stage_3W_vs_E10	ordinary_muscle_development	ENSGALG00010013206	AFF2	-0.67783852	0.112623378	-6.018630674	1.75899E-09	3.94927E-09	negative
PRJNA729271	stage_3W_vs_E10	ordinary_muscle_development	ENSGALG00010013207	C1QTNF3	-2.970295798	0.199733058	-14.87132792	5.05996E-50	5.73898E-49	negative
PRJNA729271	stage_3W_vs_E10	ordinary_muscle_development	ENSGALG00010013208	ENSGALG00010013208	0.699266772	0.295487067	2.366488589	0.017957728	0.023741694	positive
PRJNA729271	stage_3W_vs_E10	ordinary_muscle_development	ENSGALG00010013209	KIAA1549	-1.263253928	0.104674099	-12.06844801	1.55031E-33	1.01452E-32	negative
PRJNA729271	stage_3W_vs_E10	ordinary_muscle_development	ENSGALG00010013212	ENSGALG00010013212	0.380067287	0.153492619	2.476127442	0.013281616	0.017804219	positive
PRJNA729271	stage_3W_vs_E10	ordinary_muscle_development	ENSGALG00010013213	SH2D1A	3.493025318	0.352720945	9.903084476	4.0363E-23	1.75064E-22	positive
PRJNA729271	stage_3W_vs_E10	ordinary_muscle_development	ENSGALG00010013214	RAI14	0.627711622	0.169146227	3.711058959	0.000206394	0.000328323	positive
PRJNA729271	stage_3W_vs_E10	ordinary_muscle_development	ENSGALG00010013215	SF3A3	-0.943197344	0.082555956	-11.42494601	3.13851E-30	1.81752E-29	negative
PRJNA729271	stage_3W_vs_E10	ordinary_muscle_development	ENSGALG00010013217	TGFBRAP1	0.233674111	0.083234465	2.807420108	0.004994007	0.00700214	positive
PRJNA729271	stage_3W_vs_E10	ordinary_muscle_development	ENSGALG00010013220	CSTF1	-0.002935483	0.086720391	-0.033849972	0.972996787	0.976987881	negative
PRJNA729271	stage_3W_vs_E10	ordinary_muscle_development	ENSGALG00010013221	C2orf49	0.263971528	0.117807521	2.240701834	0.025045395	0.032582391	positive
PRJNA729271	stage_3W_vs_E10	ordinary_muscle_development	ENSGALG00010013224	ATP6V0A4	0.653102734	0.722138748	0.904400623	0.365783008	0.404487695	positive
PRJNA729271	stage_3W_vs_E10	ordinary_muscle_development	ENSGALG00010013225	GNE	-1.084747838	0.097165259	-11.16394733	6.1212E-29	3.37364E-28	negative
PRJNA729271	stage_3W_vs_E10	ordinary_muscle_development	ENSGALG00010013226	DUSP6	-1.20744786	0.114885393	-10.51002067	7.76765E-26	3.76562E-25	negative
PRJNA729271	stage_3W_vs_E10	ordinary_muscle_development	ENSGALG00010013227	CITED4	1.897200716	0.161142278	11.773451	5.34906E-32	3.31912E-31	positive
PRJNA729271	stage_3W_vs_E10	ordinary_muscle_development	ENSGALG00010013228	RTFDC1	0.725335925	0.084518811	8.581946574	9.3281E-18	3.2526E-17	positive
PRJNA729271	stage_3W_vs_E10	ordinary_muscle_development	ENSGALG00010013230	ENSGALG00010013230	4.840375709	0.340419847	14.2188411	7.00026E-46	6.90734E-45	positive
PRJNA729271	stage_3W_vs_E10	ordinary_muscle_development	ENSGALG00010013232	FHL2	0.061447608	0.421581494	0.145754992	0.884114807	0.897740394	positive
PRJNA729271	stage_3W_vs_E10	ordinary_muscle_development	ENSGALG00010013233	LRRCC1	-0.979939729	0.131213431	-7.468288306	8.12447E-14	2.31774E-13	negative
PRJNA729271	stage_3W_vs_E10	ordinary_muscle_development	ENSGALG00010013235	GCNT7	0.833500455	0.247637408	3.365809963	0.000763193	0.00115523	positive
PRJNA729271	stage_3W_vs_E10	ordinary_muscle_development	ENSGALG00010013236	MON2	0.099186486	0.082706165	1.199263515	0.230425497	0.264158303	positive
PRJNA729271	stage_3W_vs_E10	ordinary_muscle_development	ENSGALG00010013238	RAD1	0.119665278	0.131221261	0.911935131	0.361802867	0.400667168	positive
PRJNA729271	stage_3W_vs_E10	ordinary_muscle_development	ENSGALG00010013241	SEC24A	0.48930786	0.135521411	3.610557602	0.000305539	0.000479359	positive
PRJNA729271	stage_3W_vs_E10	ordinary_muscle_development	ENSGALG00010013242	SULT4A1	-0.165189281	0.159005312	-1.038891591	0.298855158	0.336406423	negative
PRJNA729271	stage_3W_vs_E10	ordinary_muscle_development	ENSGALG00010013244	TRIM24	0.074090939	0.147887277	0.500996034	0.616373914	0.651048596	positive
PRJNA729271	stage_3W_vs_E10	ordinary_muscle_development	ENSGALG00010013246	LNPEP	2.685998066	0.174721543	15.37302169	2.48313E-53	3.13801E-52	positive
PRJNA729271	stage_3W_vs_E10	ordinary_muscle_development	ENSGALG00010013247	ATG5	0.207054616	0.092046965	2.249445336	0.024484177	0.031903825	positive
PRJNA729271	stage_3W_vs_E10	ordinary_muscle_development	ENSGALG00010013248	ENSGALG00010013248	-1.103608915	0.691662924	-1.595587789	0.110580856	0.13283082	negative
PRJNA729271	stage_3W_vs_E10	ordinary_muscle_development	ENSGALG00010013249	AURKA	-3.859539384	0.234420405	-16.46417849	6.63574E-61	1.01676E-59	negative
PRJNA729271	stage_3W_vs_E10	ordinary_muscle_development	ENSGALG00010013250	E2F5	0.211453721	0.093467826	2.262315612	0.023677911	0.03089767	positive
PRJNA729271	stage_3W_vs_E10	ordinary_muscle_development	ENSGALG00010013251	MTA1	-0.884404927	0.082996606	-10.65591677	1.63624E-26	8.2067E-26	negative
PRJNA729271	stage_3W_vs_E10	ordinary_muscle_development	ENSGALG00010013252	PPM1H	-2.327170124	0.193982037	-11.9968331	3.69153E-33	2.38878E-32	negative
PRJNA729271	stage_3W_vs_E10	ordinary_muscle_development	ENSGALG00010013253	SHB	0.870165778	0.098343525	8.848226437	8.89211E-19	3.23522E-18	positive
PRJNA729271	stage_3W_vs_E10	ordinary_muscle_development	ENSGALG00010013254	SAR1B	2.232479516	0.141462446	15.78142879	4.17626E-56	5.64692E-55	positive
PRJNA729271	stage_3W_vs_E10	ordinary_muscle_development	ENSGALG00010013256	BRIX1	-0.495290662	0.117792432	-4.20477489	2.61342E-05	4.46716E-05	negative
PRJNA729271	stage_3W_vs_E10	ordinary_muscle_development	ENSGALG00010013257	STX12	-0.414910983	0.089938804	-4.613258825	3.96404E-06	7.21935E-06	negative
PRJNA729271	stage_3W_vs_E10	ordinary_muscle_development	ENSGALG00010013258	RBIS	1.223529941	0.144268077	8.480947186	2.23368E-17	7.61816E-17	positive

PRJNA729271	stage_3W_vs_E10	ordinary_muscle_development	ENSGALG00010013259	PTPRB	2.658825251	0.139093971	19.11531628	1.88275E-81	4.91721E-80	positive
PRJNA729271	stage_3W_vs_E10	ordinary_muscle_development	ENSGALG00010013260	FAM210B	-0.258318595	0.08242399	-3.134021956	0.00172428	0.002528932	negative
PRJNA729271	stage_3W_vs_E10	ordinary_muscle_development	ENSGALG00010013261	JADE2	2.202010622	0.197116328	11.1711224	5.64628E-29	3.11664E-28	positive
PRJNA729271	stage_3W_vs_E10	ordinary_muscle_development	ENSGALG00010013262	CA13	-1.483132744	0.263767631	-5.622876237	1.87804E-08	3.98743E-08	negative
PRJNA729271	stage_3W_vs_E10	ordinary_muscle_development	ENSGALG00010013263	ATP11A	-0.548868681	0.113298229	-4.844459482	1.26957E-06	2.38775E-06	negative
PRJNA729271	stage_3W_vs_E10	ordinary_muscle_development	ENSGALG00010013264	DNAJC21	0.39679024	0.121587549	3.263411766	0.001100795	0.001643932	positive
PRJNA729271	stage_3W_vs_E10	ordinary_muscle_development	ENSGALG00010013265	PACS2	0.822552642	0.122147524	6.734091834	1.64957E-11	4.16923E-11	positive
PRJNA729271	stage_3W_vs_E10	ordinary_muscle_development	ENSGALG00010013266	KIAA1755	1.579105675	0.188953978	8.357091441	6.42835E-17	2.14747E-16	positive
PRJNA729271	stage_3W_vs_E10	ordinary_muscle_development	ENSGALG00010013267	PNPLA3	0.105245672	0.17936035	0.586783376	0.557349203	0.594508551	positive
PRJNA729271	stage_3W_vs_E10	ordinary_muscle_development	ENSGALG00010013268	PTPRR	2.115025041	0.256655567	8.240713664	1.71186E-16	5.59114E-16	positive
PRJNA729271	stage_3W_vs_E10	ordinary_muscle_development	ENSGALG00010013269	INPP5B	1.313693443	0.100165246	13.11526204	2.6925E-39	2.12454E-38	positive
PRJNA729271	stage_3W_vs_E10	ordinary_muscle_development	ENSGALG00010013271	GULP1	0.473171129	0.139176241	3.3979817	0.000674356	0.001026225	positive
PRJNA729271	stage_3W_vs_E10	ordinary_muscle_development	ENSGALG00010013273	STAG2	1.087893719	0.11618506	9.363456207	7.71701E-21	3.05456E-20	positive
PRJNA729271	stage_3W_vs_E10	ordinary_muscle_development	ENSGALG00010013274	MTF1	1.354443917	0.119002514	11.3816412	5.1619E-30	2.96559E-29	positive
PRJNA729271	stage_3W_vs_E10	ordinary_muscle_development	ENSGALG00010013275	MAPRE1	-1.4971224	0.076533952	-19.56154563	3.28953E-85	9.57466E-84	negative
PRJNA729271	stage_3W_vs_E10	ordinary_muscle_development	ENSGALG00010013276	POC1B	-1.591100719	0.110504165	-14.39855885	5.28337E-47	5.40074E-46	negative
PRJNA729271	stage_3W_vs_E10	ordinary_muscle_development	ENSGALG00010013277	YRDC	-0.957781724	0.094018446	-10.18716819	2.26259E-24	1.03278E-23	negative
PRJNA729271	stage_3W_vs_E10	ordinary_muscle_development	ENSGALG00010013278	SAMM50	0.977278213	0.10270314	9.515563152	1.80731E-21	7.34427E-21	positive
PRJNA729271	stage_3W_vs_E10	ordinary_muscle_development	ENSGALG00010013280	FRS2	-0.268062256	0.111829	-2.397072814	0.016526639	0.021933129	negative
PRJNA729271	stage_3W_vs_E10	ordinary_muscle_development	ENSGALG00010013281	BIRC8	1.89392367	0.138615406	13.66315427	1.68523E-42	1.47687E-41	positive
PRJNA729271	stage_3W_vs_E10	ordinary_muscle_development	ENSGALG00010013282	SEC22B	-1.16308836	0.090363839	-12.87117029	6.54036E-38	4.90189E-37	negative
PRJNA729271	stage_3W_vs_E10	ordinary_muscle_development	ENSGALG00010013283	PAWR	-0.732573313	0.279968752	-2.616625274	0.008880377	0.012148081	negative
PRJNA729271	stage_3W_vs_E10	ordinary_muscle_development	ENSGALG00010013284	MANEAL	-0.650545122	0.305523364	-2.129281092	0.033231011	0.042615545	negative
PRJNA729271	stage_3W_vs_E10	ordinary_muscle_development	ENSGALG00010013285	ATP2B1	-0.464939314	0.10793398	-4.307626889	1.65015E-05	2.8673E-05	negative
PRJNA729271	stage_3W_vs_E10	ordinary_muscle_development	ENSGALG00010013286	RNF14	1.447872659	0.118769712	12.19058827	3.48894E-34	2.33374E-33	positive
PRJNA729271	stage_3W_vs_E10	ordinary_muscle_development	ENSGALG00010013288	CA3A	5.785759273	0.775703706	7.458723253	8.73649E-14	2.485E-13	positive
PRJNA729271	stage_3W_vs_E10	ordinary_muscle_development	ENSGALG00010013289	TM9SF4	-1.218366067	0.07533768	-16.17206788	7.93883E-59	1.15885E-57	negative
PRJNA729271	stage_3W_vs_E10	ordinary_muscle_development	ENSGALG00010013290	AKR1D1	-3.095115575	0.338635166	-9.139970928	6.24691E-20	2.38224E-19	negative
PRJNA729271	stage_3W_vs_E10	ordinary_muscle_development	ENSGALG00010013292	PRDM1	-2.156365568	0.293279729	-7.352589877	1.94403E-13	5.43256E-13	negative
PRJNA729271	stage_3W_vs_E10	ordinary_muscle_development	ENSGALG00010013293	TUBGCP3	-0.79822436	0.057783404	-13.81407641	2.09626E-43	1.89671E-42	negative
PRJNA729271	stage_3W_vs_E10	ordinary_muscle_development	ENSGALG00010013295	CA3B	-0.226570069	0.400955607	-0.565075199	0.572022617	0.608280231	negative
PRJNA729271	stage_3W_vs_E10	ordinary_muscle_development	ENSGALG00010013296	AGXT2	-0.054734911	0.404710212	-0.1352447	0.892418406	0.904777562	negative
PRJNA729271	stage_3W_vs_E10	ordinary_muscle_development	ENSGALG00010013297	FCHO2	0.929557317	0.123706091	7.514240483	5.72423E-14	1.64596E-13	positive
PRJNA729271	stage_3W_vs_E10	ordinary_muscle_development	ENSGALG00010013298	EPYC	-7.887907304	0.439683392	-17.93997099	5.74851E-72	1.17857E-70	negative
PRJNA729271	stage_3W_vs_E10	ordinary_muscle_development	ENSGALG00010013299	PLAGL2	-1.297491243	0.112966828	-11.4855951	1.55859E-30	9.13165E-30	negative
PRJNA729271	stage_3W_vs_E10	ordinary_muscle_development	ENSGALG00010013300	GNPDA1	-1.138932	0.09901433	-11.50269865	1.27854E-30	7.50607E-30	negative
PRJNA729271	stage_3W_vs_E10	ordinary_muscle_development	ENSGALG00010013301	PARVB	-2.451986345	0.141690343	-17.30524676	4.29437E-67	7.7518E-66	negative
PRJNA729271	stage_3W_vs_E10	ordinary_muscle_development	ENSGALG00010013302	ENSGALG00010013302	2.196109162	0.582405775	3.770754441	0.000162755	0.000261229	positive
PRJNA729271	stage_3W_vs_E10	ordinary_muscle_development	ENSGALG00010013303	TFPI	0.50209277	0.232255893	2.161808526	0.030632938	0.03945481	positive
PRJNA729271	stage_3W_vs_E10	ordinary_muscle_development	ENSGALG00010013304	KCNJ15	0.819770456	0.23313132	3.516346312	0.00043753	0.000677491	positive
PRJNA729271	stage_3W_vs_E10	ordinary_muscle_development	ENSGALG00010013305	THOC2	-0.326584396	0.091034249	-3.58748931	0.000333877	0.000522461	negative
PRJNA729271	stage_3W_vs_E10	ordinary_muscle_development	ENSGALG00010013306	ARSB	-0.129129286	0.150079507	-0.860405853	0.389565361	0.428595818	negative
PRJNA729271	stage_3W_vs_E10	ordinary_muscle_development	ENSGALG00010013307	POFUT1	-1.094965269	0.089533635	-12.22965273	2.15859E-34	1.45665E-33	negative
PRJNA729271	stage_3W_vs_E10	ordinary_muscle_development	ENSGALG00010013309	ENSGALG00010013309	-0.834268481	0.290553543	-2.871307208	0.00408778	0.005782412	negative
PRJNA729271	stage_3W_vs_E10	ordinary_muscle_development	ENSGALG00010013310	MYO16	-2.453941874	0.300903056	-8.155257401	3.48437E-16	1.11813E-15	negative
PRJNA729271	stage_3W_vs_E10	ordinary_muscle_development	ENSGALG00010013311	RSPO1	-3.707004582	0.263570859	-14.0645464	6.27237E-45	5.98933E-44	negative
PRJNA729271	stage_3W_vs_E10	ordinary_muscle_development	ENSGALG00010013312	ENSGALG00010013312	2.303354068	0.560047018	4.112786949	3.90911E-05	6.59941E-05	positive
PRJNA729271	stage_3W_vs_E10	ordinary_muscle_development	ENSGALG00010013313	GAK	-0.03554954	0.093964436	-0.378329729	0.705185663	0.73442062	negative
PRJNA729271	stage_3W_vs_E10	ordinary_muscle_development	ENSGALG00010013314	KIT	0.208039668	0.205377551	1.012962065	0.311078281	0.348972965	positive
PRJNA729271	stage_3W_vs_E10	ordinary_muscle_development	ENSGALG00010013316	KDM7A	1.86059788	0.129506234	14.36685956	8.35341E-47	8.47324E-46	positive
PRJNA729271	stage_3W_vs_E10	ordinary_muscle_development	ENSGALG00010013317	PREP	-0.622709494	0.109665226	-5.67827668	1.36059E-08	2.91095E-08	negative
PRJNA729271	stage_3W_vs_E10	ordinary_muscle_development	ENSGALG00010013318	KERA	-6.595389642	0.494070296	-13.3490916	1.19902E-40	9.8525E-40	negative
PRJNA729271	stage_3W_vs_E10	ordinary_muscle_development	ENSGALG00010013319	ENSGALG00010013319	-4.107385512	1.592282575	-2.579558162			negative
PRJNA729271	stage_3W_vs_E10	ordinary_muscle_development	ENSGALG00010013320	CALCRL	0.502330253	0.123563734	4.06535344	4.79597E-05	8.03951E-05	positive
PRJNA729271	stage_3W_vs_E10	ordinary_muscle_development	ENSGALG00010013322	IRS2	1.526755374	0.511694644	2.983723581	0.002847639	0.004090835	positive
PRJNA729271	stage_3W_vs_E10	ordinary_muscle_development	ENSGALG00010013323	NOTCH2	-1.412939083	0.108952175	-12.96843395	1.84767E-38	1.41703E-37	negative
PRJNA729271	stage_3W_vs_E10	ordinary_muscle_development	ENSGALG00010013324	PARVG	0.975901792	0.148029918	6.592598353	4.32195E-11	1.06596E-10	positive
PRJNA729271	stage_3W_vs_E10	ordinary_muscle_development	ENSGALG00010013325	CA2	2.839741439	0.316904112	8.960885441	3.22082E-19	1.19462E-18	positive
PRJNA729271	stage_3W_vs_E10	ordinary_muscle_development	ENSGALG00010013326	FGF20	-5.195265105	0.852736229	-6.092464386	1.11186E-09	2.52568E-09	negative
PRJNA729271	stage_3W_vs_E10	ordinary_muscle_development	ENSGALG00010013327	SLF1	-1.473086202	0.100052616	-14.72311527	4.58063E-49	5.01216E-48	negative
PRJNA729271	stage_3W_vs_E10	ordinary_muscle_development	ENSGALG00010013328	SLC37A3	-0.461429685	0.078903988	-5.847989381	4.9755E-09	1.09074E-08	negative
PRJNA729271	stage_3W_vs_E10	ordinary_muscle_development	ENSGALG00010013329	LIFR	-0.638663365	0.122831893	-5.199491333	1.99835E-07	3.96198E-07	negative
PRJNA729271	stage_3W_vs_E10	ordinary_muscle_development	ENSGALG00010013330	ERG	-0.323510996	0.128997526	-2.507885267	0.01214561	0.016363102	negative
PRJNA729271	stage_3W_vs_E10	ordinary_muscle_development	ENSGALG00010013331	KIF3B	-0.962321115	0.151251368	-6.362396109	1.9863E-10	4.72152E-10	negative
PRJNA729271	stage_3W_vs_E10	ordinary_muscle_development	ENSGALG00010013332	LUM	-0.912057003	0.320730618	-2.843685486	0.004459504	0.006286765	negative
PRJNA729271	stage_3W_vs_E10	ordinary_muscle_development	ENSGALG00010013333	PPP1R12A	0.020736786	0.098110884	0.211360708	0.832605815	0.852002915	positive
PRJNA729271	stage_3W_vs_E10	ordinary_muscle_development	ENSGALG00010013334	SH3BGRL3	0.555028837	0.120988738	4.587442142	4.4871E-06	8.13311E-06	positive
PRJNA729271	stage_3W_vs_E10	ordinary_muscle_development	ENSGALG00010013335	POPDC3	3.843234413	0.175693855	21.87460912	4.5338E-106	2.1131E-104	positive
PRJNA729271	stage_3W_vs_E10	ordinary_muscle_development	ENSGALG00010013336	MTNR1A	1.470373155	0.678522351	2.16702243	0.030233144	0.038971047	positive
PRJNA729271	stage_3W_vs_E10	ordinary_muscle_development	ENSGALG00010013337	RAB19	0.562063686	0.254188565	2.211207599	0.027021464	0.034999207	positive
PRJNA729271	stage_3W_vs_E10	ordinary_muscle_development	ENSGALG00010013339	ATP6V0D2	3.890547313	0.53616051	7.256310831	3.9779E-13	1.0939E-12	positive
PRJNA729271	stage_3W_vs_E10	ordinary_muscle_development	ENSGALG00010013340	LIN28B	-6.886302832	0.667007023	-10.32418339	5.4783E-25	2.56072E-24	negative
PRJNA729271	stage_3W_vs_E10	ordinary_muscle_development	ENSGALG00010013343	SHISAL1	-6.614483281	0.520166234	-12.71609507	4.81319E-37	3.50589E-36	negative
PRJNA729271	stage_3W_vs_E10	ordinary_muscle_development	ENSGALG00010013344	OSMR	1.348559174	0.123113897	10.95375267	6.37506E-28	3.38237E-27	positive
PRJNA729271	stage_3W_vs_E10	ordinary_muscle_development	ENSGALG00010013345	DCN	0.273852781	0.213779845	1.281003741	0.200192352	0.231735198	positive
PRJNA729271	stage_3W_vs_E10	ordinary_muscle_development	ENSGALG00010013346	HMGCS2	-0.500079394	0.706488191	-0.707838292	0.479045684	0.517650752	negative
PRJNA729271	stage_3W_vs_E10	ordinary_muscle_development	ENSGALG00010013347	ENSGALG00010013347	0.032259362	0.315436722	0.102268865	0.918543252	0.927954031	positive
PRJNA729271	stage_3W_vs_E10	ordinary_muscle_development	ENSGALG00010013348	MKRN4P	0.462925106	0.069976439	6.615442438	3.70442E-11	9.17873E-11	positive
PRJNA729271	stage_3W_vs_E10	ordinary_muscle_development	ENSGALG00010013349	ETS2	-0.253079447	0.101426304	-2.495205269	0.012588428	0.01693136	negative
PRJNA729271	stage_3W_vs_E10	ordinary_muscle_development	ENSGALG00010013351	EDAR	-4.529692106	0.42911401	-10.55591755	4.76957E-26	2.33485E-25	negative
PRJNA729271	stage_3W_vs_E10	ordinary_muscle_development	ENSGALG00010013352	ASXL1	-0.637037736	0.089176363	-7.14357163	9.09365E-13	2.45965E-12	negative
PRJNA729271	stage_3W_vs_E10	ordinary_muscle_development	ENSGALG00010013353	NDFIP1	-0.11878578	0.084731085	-1.401915008	0.160940628	0.189092519	negative
PRJNA729271	stage_3W_vs_E10	ordinary_muscle_development	ENSGALG00010013354	WWP1	4.115046371	0.14535495	28.3103284	2.5789E-176	3.4295E-174	positive
PRJNA729271	stage_3W_vs_E10	ordinary_muscle_development	ENSGALG00010013355	FAM171B	-2.087910299	0.150772979	-13.8480404	1.3073E-43	1.19103E-42	negative
PRJNA729271	stage_3W_vs_E10	ordinary_muscle_development	ENSGALG00010013356	AMER1	-0.944527395	0.065945324	-14.32288646	1.57446E-46	1.58594E-45	negative
PRJNA729271	stage_3W_vs_E10	ordinary_muscle_development	ENSGALG00010013357	RICTOR	0.080610319	0.10990525	0.733452853	0.463282274	0.501926643	positive
PRJNA729271	stage_3W_vs_E10	ordinary_muscle_development	ENSGALG00010013358	RPS16	0.415970307	0.174149272	2.388584815	0.016913404	0.022415634	positive
PRJNA729271	stage_3W_vs_E10	ordinary_muscle_development	ENSGALG00010013359	BTG1	0.736754189	0.132481036	5.561204919	2.67918E-08	5.62252E-08	positive
PRJNA729271	stage_3W_vs_E10	ordinary_muscle_development	ENSGALG00010013360	PRR5	-1.116550299	0.115902205	-9.633555263	5.76979E-22	2.38883E-21	negative
PRJNA729271	stage_3W_vs_E10	ordinary_muscle_development	ENSGALG00010013361	ENSGALG00010013361	0.080260073	0.230936101	0.347542342	0.728183914	0.756144841	positive
PRJNA729271	stage_3W_vs_E10	ordinary_muscle_development	ENSGALG00010013362	ENSGALG00010013362	2.278097896	0.367031357	6.206820887	5.40672E-10	1.25433E-09	positive
PRJNA729271	stage_3W_vs_E10	ordinary_muscle_development	ENSGALG00010013363	HS3ST5	2.381888223	0.228223473	10.43664874	1.6866E-25	8.05777E-25	positive
PRJNA729271	stage_3W_vs_E10	ordinary_muscle_development	ENSGALG00010013364	OTOGL	2.901016806	0.357430334	8.116313939	4.80557E-16	1.53193E-15	positive
PRJNA729271	stage_3W_vs_E10	ordinary_muscle_development	ENSGALG00010013365	CEP85	2.095033879	0.227162107	9.222637984	2.89879E-20	1.12048E-19	positive
PRJNA729271	stage_3W_vs_E10	ordinary_muscle_development	ENSGALG00010013367	NOL4L	-0.625699804	0.186000338	-3.363971329	0.000768295	0.001162346	negative
PRJNA729271	stage_3W_vs_E10	ordinary_muscle_development	ENSGALG00010013369	MTMR8	-0.487210337	0.062768034	-7.762077366	8.35494E-15	2.50372E-14	negative
PRJNA729271	stage_3W_vs_E10	ordinary_muscle_development	ENSGALG00010013370	CNKSR1	2.970157151	0.234935795	12.64242069	1.23202E-36	8.88461E-36	positive
PRJNA729271	stage_3W_vs_E10	ordinary_muscle_development	ENSGALG00010013371	FAT1	-1.236590057	0.156647613	-7.894088071	2.92446E-15	8.96194E-15	negative
PRJNA729271	stage_3W_vs_E10	ordinary_muscle_development	ENSGALG00010013372	SPRY4	-2.732144465	0.309696008	-8.82202029	1.12413E-18	4.07254E-18	negative
PRJNA729271	stage_3W_vs_E10	ordinary_muscle_development	ENSGALG00010013373	ENSGALG00010013373	0.61423702	0.563324591	1.090378494	0.275546451	0.312352062	positive
PRJNA729271	stage_3W_vs_E10	ordinary_muscle_development	ENSGALG00010013374	RMDN1	0.09160594	0.167531795	0.54679734	0.584517971	0.62020262	positive
PRJNA729271	stage_3W_vs_E10	ordinary_muscle_development	ENSGALG00010013375	PSMG1	-0.102684289	0.08810378	-1.165492427	0.243819724	0.278609925	negative
PRJNA729271	stage_3W_vs_E10	ordinary_muscle_development	ENSGALG00010013376	ARHGAP8	3.002272462	0.372808157	8.053129754	8.07033E-16	2.54303E-15	positive
PRJNA729271	stage_3W_vs_E10	ordinary_muscle_development	ENSGALG00010013378	SH3RF3	-0.893100009	0.146139596	-6.111280134	9.88351E-10	2.25325E-09	negative
PRJNA729271	stage_3W_vs_E10	ordinary_muscle_development	ENSGALG00010013379	ZNF593	0.772759282	0.131697188	5.867697654	4.41888E-09	9.70333E-09	positive
PRJNA729271	stage_3W_vs_E10	ordinary_muscle_development	ENSGALG00010013380	PHGDH	-1.113169262	0.610436965	-1.82356136	0.068218413	0.084162558	negative
PRJNA729271	stage_3W_vs_E10	ordinary_muscle_development	ENSGALG00010013381	DENND2A	-1.561007098	0.089817657	-17.37973523	1.17495E-67	2.15308E-66	negative
PRJNA729271	stage_3W_vs_E10	ordinary_muscle_development	ENSGALG00010013382	ASB12	1.625022047	0.220895772	7.356510398	1.8878E-13	5.27849E-13	positive
PRJNA729271	stage_3W_vs_E10	ordinary_muscle_development	ENSGALG00010013383	SEPTIN10	0.942871986	0.13824984	6.820058432	9.10035E-12	2.32973E-11	positive
PRJNA729271	stage_3W_vs_E10	ordinary_muscle_development	ENSGALG00010013385	PGRMC1	0.295312697	0.155400336	1.900334989	0.057389172	0.071496008	positive
PRJNA729271	stage_3W_vs_E10	ordinary_muscle_development	ENSGALG00010013386	ITGAV	0.148175251	0.121841233	1.21613388	0.223933926	0.257204617	positive
PRJNA729271	stage_3W_vs_E10	ordinary_muscle_development	ENSGALG00010013387	NDST1	-0.808131314	0.093895656	-8.606695397	7.51969E-18	2.63407E-17	negative
PRJNA729271	stage_3W_vs_E10	ordinary_muscle_development	ENSGALG00010013388	SOWAHC	0.443276461	0.210853129	2.102299657	0.035527035	0.045427344	positive
PRJNA729271	stage_3W_vs_E10	ordinary_muscle_development	ENSGALG00010013389	NAP1L1	-1.139563418	0.062174656	-18.32842344	4.90929E-75	1.08146E-73	negative
PRJNA729271	stage_3W_vs_E10	ordinary_muscle_development	ENSGALG00010013390	SLC16A2	-3.145084901	0.13666344	-23.01335965	3.4258E-117	1.9474E-115	negative
PRJNA729271	stage_3W_vs_E10	ordinary_muscle_development	ENSGALG00010013391	HMGN1	0.317056735	0.087411711	3.627165399	0.00028655	0.000450835	positive
PRJNA729271	stage_3W_vs_E10	ordinary_muscle_development	ENSGALG00010013392	HDAC2	-1.145257653	0.069350621	-16.51402168	2.90839E-61	4.50397E-60	negative
PRJNA729271	stage_3W_vs_E10	ordinary_muscle_development	ENSGALG00010013393	ADCK2	-1.000327546	0.165074197	-6.059866176	1.36235E-09	3.07925E-09	negative
PRJNA729271	stage_3W_vs_E10	ordinary_muscle_development	ENSGALG00010013394	DNMT3B	-7.480440992	0.349005263	-21.4336051	6.4951E-102	2.80198E-100	negative
PRJNA729271	stage_3W_vs_E10	ordinary_muscle_development	ENSGALG00010013396	FYB1	1.619111831	0.186050066	8.702559819	3.24481E-18	1.15675E-17	positive
PRJNA729271	stage_3W_vs_E10	ordinary_muscle_development	ENSGALG00010013397	MATN1	-8.845844293	0.476995675	-18.54491509	8.96421E-77	2.05921E-75	negative
PRJNA729271	stage_3W_vs_E10	ordinary_muscle_development	ENSGALG00010013398	CHAMP1	0.980679182	0.155296873	6.314867529	2.70393E-10	6.37707E-10	positive
PRJNA729271	stage_3W_vs_E10	ordinary_muscle_development	ENSGALG00010013399	FGF1	4.323682388	0.321348723	13.45479874	2.88524E-41	2.41883E-40	positive
PRJNA729271	stage_3W_vs_E10	ordinary_muscle_development	ENSGALG00010013400	BBS10	0.371582852	0.163937508	2.266612789	0.02341389	0.030583431	positive
PRJNA729271	stage_3W_vs_E10	ordinary_muscle_development	ENSGALG00010013402	MARCKS	-2.043084123	0.155172351	-13.16654749	1.36708E-39	1.09118E-38	negative
PRJNA729271	stage_3W_vs_E10	ordinary_muscle_development	ENSGALG00010013403	CPNE3	1.008093383	0.102908168	9.796048252	1.17077E-22	4.97516E-22	positive
PRJNA729271	stage_3W_vs_E10	ordinary_muscle_development	ENSGALG00010013404	ENSGALG00010013404	-0.007183192	0.227169172	-0.031620454	0.974774732	0.978487516	negative
PRJNA729271	stage_3W_vs_E10	ordinary_muscle_development	ENSGALG00010013405	RPS14	0.016017269	0.175365318	0.091336584	0.92722515	0.935420973	positive
PRJNA729271	stage_3W_vs_E10	ordinary_muscle_development	ENSGALG00010013406	ENSGALG00010013406	0.810222196	0.268986821	3.012126	0.002594249	0.003740167	positive
PRJNA729271	stage_3W_vs_E10	ordinary_muscle_development	ENSGALG00010013407	ZC3H15	1.619592274	0.11240778	14.4081866	4.5962E-47	4.70162E-46	positive
PRJNA729271	stage_3W_vs_E10	ordinary_muscle_development	ENSGALG00010013408	RLIM	-0.182477514	0.065264792	-2.795956424	0.005174638	0.007246993	negative

PRJNA729271	stage_3W_vs_E10	ordinary_muscle_development	ENSGALG00010013409	DAB2	-0.346746727	0.147865538	-2.345013801	0.019026375	0.025078413	negative
PRJNA729271	stage_3W_vs_E10	ordinary_muscle_development	ENSGALG00010013410	KIAA0825	-0.537542864	0.103022656	-5.217715042	1.81144E-07	3.60077E-07	negative
PRJNA729271	stage_3W_vs_E10	ordinary_muscle_development	ENSGALG00010013411	ENSGALG00010013411	0.918220029	0.44789076	2.050098171	0.040354852	0.051238926	positive
PRJNA729271	stage_3W_vs_E10	ordinary_muscle_development	ENSGALG00010013412	COMMD7	-1.220079407	0.103570718	-11.78015788	4.94005E-32	3.0719E-31	negative
PRJNA729271	stage_3W_vs_E10	ordinary_muscle_development	ENSGALG00010013413	WRB	-0.194626846	0.083698318	-2.325337597	0.020053915	0.02638721	negative
PRJNA729271	stage_3W_vs_E10	ordinary_muscle_development	ENSGALG00010013414	LAPTM5	2.360129606	0.172887832	13.65121872	1.9853E-42	1.73669E-41	positive
PRJNA729271	stage_3W_vs_E10	ordinary_muscle_development	ENSGALG00010013416	NDUFB2	0.855867914	0.110418272	7.751143878	9.10684E-15	2.72229E-14	positive
PRJNA729271	stage_3W_vs_E10	ordinary_muscle_development	ENSGALG00010013417	AKR1B1	-0.148212404	0.088293907	-1.678625491	0.093225055	0.113235846	negative
PRJNA729271	stage_3W_vs_E10	ordinary_muscle_development	ENSGALG00010013418	HCN1	-1.775846368	0.802083252	-2.214042449	0.026825859	0.034758275	negative
PRJNA729271	stage_3W_vs_E10	ordinary_muscle_development	ENSGALG00010013419	ENSGALG00010013419	2.145619767	0.335784788	6.389865904	1.66031E-10	3.96022E-10	positive
PRJNA729271	stage_3W_vs_E10	ordinary_muscle_development	ENSGALG00010013420	ENSGALG00010013420	-3.678149491	4.240851426	-0.867313924			negative
PRJNA729271	stage_3W_vs_E10	ordinary_muscle_development	ENSGALG00010013423	ZNF804A	-2.499050847	0.472616719	-5.28769031	1.2387E-07	2.49099E-07	negative
PRJNA729271	stage_3W_vs_E10	ordinary_muscle_development	ENSGALG00010013425	ENSGALG00010013425	0.695419281	0.220816339	3.149310795	0.00163656	0.002408075	positive
PRJNA729271	stage_3W_vs_E10	ordinary_muscle_development	ENSGALG00010013427	NEXMIF	-3.675025684	0.218769506	-16.79861945	2.4979E-63	4.0912E-62	negative
PRJNA729271	stage_3W_vs_E10	ordinary_muscle_development	ENSGALG00010013428	BPGM	3.9287182	0.152786243	25.71382161	8.189E-146	7.2821E-144	positive
PRJNA729271	stage_3W_vs_E10	ordinary_muscle_development	ENSGALG00010013429	PDE4DIP	2.122023548	0.187525899	11.3158959	1.09478E-29	6.22797E-29	positive
PRJNA729271	stage_3W_vs_E10	ordinary_muscle_development	ENSGALG00010013431	TMEM175	-0.39675876	0.087429171	-4.538059285	5.67743E-06	1.02178E-05	negative
PRJNA729271	stage_3W_vs_E10	ordinary_muscle_development	ENSGALG00010013434	CORIN	-5.171545486	0.445195228	-11.61635427	3.40352E-31	2.04197E-30	negative
PRJNA729271	stage_3W_vs_E10	ordinary_muscle_development	ENSGALG00010013435	SDC3	-2.756433888	0.181629101	-15.17616872	5.08636E-52	6.15416E-51	negative
PRJNA729271	stage_3W_vs_E10	ordinary_muscle_development	ENSGALG00010013436	NPAS2	0.721261221	0.205359219	3.512193041	0.000444425	0.000687727	positive
PRJNA729271	stage_3W_vs_E10	ordinary_muscle_development	ENSGALG00010013437	ENSGALG00010013437	0.567971956	0.124038211	4.579007972	4.67186E-06	8.45849E-06	positive
PRJNA729271	stage_3W_vs_E10	ordinary_muscle_development	ENSGALG00010013438	RPL37	0.795157301	0.18748652	4.241143857	2.22384E-05	3.82787E-05	positive
PRJNA729271	stage_3W_vs_E10	ordinary_muscle_development	ENSGALG00010013440	EEA1	0.163044478	0.09824665	1.65954237	0.09700655	0.117518591	positive
PRJNA729271	stage_3W_vs_E10	ordinary_muscle_development	ENSGALG00010013442	ENSGALG00010013442	1.325785404	0.103854018	12.76585564	2.5434E-37	1.86666E-36	positive
PRJNA729271	stage_3W_vs_E10	ordinary_muscle_development	ENSGALG00010013444	OSBPL8	-1.099471017	0.103290458	-10.64445873	1.85057E-26	9.25922E-26	negative
PRJNA729271	stage_3W_vs_E10	ordinary_muscle_development	ENSGALG00010013446	ATP5I	1.706311251	0.145217432	11.75004421	7.05819E-32	4.35355E-31	positive
PRJNA729271	stage_3W_vs_E10	ordinary_muscle_development	ENSGALG00010013449	LCA5L	-0.39158889	0.222066148	-1.763388491	0.077834989	0.095401503	negative
PRJNA729271	stage_3W_vs_E10	ordinary_muscle_development	ENSGALG00010013450	CD74	5.573235423	0.190010988	29.3311217	4.1605E-189	6.776E-187	positive
PRJNA729271	stage_3W_vs_E10	ordinary_muscle_development	ENSGALG00010013451	PRKAB2	4.108594433	0.166926669	24.61316968	9.1305E-134	6.787E-132	positive
PRJNA729271	stage_3W_vs_E10	ordinary_muscle_development	ENSGALG00010013452	LSS	-0.949214609	0.225604526	-4.207427146	2.58295E-05	4.41768E-05	negative
PRJNA729271	stage_3W_vs_E10	ordinary_muscle_development	ENSGALG00010013453	PUM1	-0.175192286	0.089321584	-1.96136565	0.049836384	0.062554415	negative
PRJNA729271	stage_3W_vs_E10	ordinary_muscle_development	ENSGALG00010013457	SH3BGR	3.371504218	0.147501898	22.85736153	1.2347E-115	6.8049E-114	positive
PRJNA729271	stage_3W_vs_E10	ordinary_muscle_development	ENSGALG00010013458	LAMA4	0.420910688	0.143713714	2.928813656	0.003402583	0.004852464	positive
PRJNA729271	stage_3W_vs_E10	ordinary_muscle_development	ENSGALG00010013459	ATP10D	-0.785541267	0.144813812	-5.424491329	5.81198E-08	1.19259E-07	negative
PRJNA729271	stage_3W_vs_E10	ordinary_muscle_development	ENSGALG00010013461	S100B	3.104620886	0.574326869	5.405668883	6.4567E-08	1.32152E-07	positive
PRJNA729271	stage_3W_vs_E10	ordinary_muscle_development	ENSGALG00010013463	ENSGALG00010013463	2.149937618	0.208653401	10.30387048	6.76821E-25	3.15248E-24	positive
PRJNA729271	stage_3W_vs_E10	ordinary_muscle_development	ENSGALG00010013464	ABCB7	-0.126401359	0.07453127	-1.695950687	0.089895219	0.109406482	negative
PRJNA729271	stage_3W_vs_E10	ordinary_muscle_development	ENSGALG00010013465	CALD1	-0.416358287	0.157098648	-2.650298341	0.008042072	0.011049273	negative
PRJNA729271	stage_3W_vs_E10	ordinary_muscle_development	ENSGALG00010013466	BRAF	0.216936015	0.061417465	3.532155146	0.000412187	0.000639206	positive
PRJNA729271	stage_3W_vs_E10	ordinary_muscle_development	ENSGALG00010013468	DEPDC1B	-3.483990781	0.283289038	-12.29836075	9.24309E-35	6.30191E-34	negative
PRJNA729271	stage_3W_vs_E10	ordinary_muscle_development	ENSGALG00010013470	RPL31	0.136996279	0.146345207	0.93611729	0.349212802	0.387702172	positive
PRJNA729271	stage_3W_vs_E10	ordinary_muscle_development	ENSGALG00010013472	ENSGALG00010013472	1.082487803	0.229683525	4.712953637	2.44152E-06	4.5094E-06	positive
PRJNA729271	stage_3W_vs_E10	ordinary_muscle_development	ENSGALG00010013476	ZDHHC17	-0.586934631	0.130256957	-4.505975299	6.60688E-06	1.18216E-05	negative
PRJNA729271	stage_3W_vs_E10	ordinary_muscle_development	ENSGALG00010013477	RFC1	-0.402891567	0.089876613	-4.482718625	7.36981E-06	1.31364E-05	negative
PRJNA729271	stage_3W_vs_E10	ordinary_muscle_development	ENSGALG00010013480	TBC1D8	1.225111483	0.152275106	8.045382575	8.59764E-16	2.7039E-15	positive
PRJNA729271	stage_3W_vs_E10	ordinary_muscle_development	ENSGALG00010013482	ENSGALG00010013482	-0.522528349	0.235035882	-2.223185436	0.026203298	0.034000252	negative
PRJNA729271	stage_3W_vs_E10	ordinary_muscle_development	ENSGALG00010013483	ENSGALG00010013483	-1.077764555	0.209776112	-5.13768963	2.78137E-07	5.46582E-07	negative
PRJNA729271	stage_3W_vs_E10	ordinary_muscle_development	ENSGALG00010013484	TCOF1	-0.578482833	0.102237645	-5.658217533	1.52953E-08	3.26374E-08	negative
PRJNA729271	stage_3W_vs_E10	ordinary_muscle_development	ENSGALG00010013486	PLPP6	1.733733202	0.160275934	10.81717737	2.85433E-27	1.47394E-26	positive
PRJNA729271	stage_3W_vs_E10	ordinary_muscle_development	ENSGALG00010013487	ENSGALG00010013487	-0.097221415	0.092868106	-1.046876257	0.295156654	0.332501414	negative
PRJNA729271	stage_3W_vs_E10	ordinary_muscle_development	ENSGALG00010013488	UPRT	-0.757671497	0.120807229	-6.271739727	3.57036E-10	8.3647E-10	negative
PRJNA729271	stage_3W_vs_E10	ordinary_muscle_development	ENSGALG00010013491	CNOT11	1.199087486	0.110197273	10.88128095	1.4156E-27	7.41831E-27	positive
PRJNA729271	stage_3W_vs_E10	ordinary_muscle_development	ENSGALG00010013492	B3GALT5	-2.715883226	0.63728374	-4.261654666	2.02919E-05	3.50323E-05	negative
PRJNA729271	stage_3W_vs_E10	ordinary_muscle_development	ENSGALG00010013493	ENSGALG00010013493	0.334588512	0.127228417	2.629825318	0.008542875	0.011706275	positive
PRJNA729271	stage_3W_vs_E10	ordinary_muscle_development	ENSGALG00010013494	ASB1	-0.445509598	0.145045862	-3.071508496	0.002129801	0.003093643	negative
PRJNA729271	stage_3W_vs_E10	ordinary_muscle_development	ENSGALG00010013497	FAM172A	-0.519394866	0.108026916	-4.808013474	1.52437E-06	2.84923E-06	negative
PRJNA729271	stage_3W_vs_E10	ordinary_muscle_development	ENSGALG00010013498	KDR	0.667763545	0.125257303	5.331134611	9.7601E-08	1.97753E-07	positive
PRJNA729271	stage_3W_vs_E10	ordinary_muscle_development	ENSGALG00010013501	AKR1B10	-1.164340419	0.135547495	-8.589907303	8.70395E-18	3.0379E-17	negative
PRJNA729271	stage_3W_vs_E10	ordinary_muscle_development	ENSGALG00010013502	PAFAH2	1.186078011	0.206012825	5.757301812	8.5469E-09	1.85128E-08	positive
PRJNA729271	stage_3W_vs_E10	ordinary_muscle_development	ENSGALG00010013504	IGSF5	-1.469394178	0.804711001	-1.825989922	0.06785178	0.083745874	negative
PRJNA729271	stage_3W_vs_E10	ordinary_muscle_development	ENSGALG00010013506	RNF149	-0.255640698	0.104349176	-2.449858344	0.014291243	0.01909759	negative
PRJNA729271	stage_3W_vs_E10	ordinary_muscle_development	ENSGALG00010013508	TUBE1	0.12596624	0.192251991	0.655214229	0.512329835	0.550335034	positive
PRJNA729271	stage_3W_vs_E10	ordinary_muscle_development	ENSGALG00010013509	ENSGALG00010013509	0.646114704	0.192015495	3.364909193	0.000765689	0.001158644	positive
PRJNA729271	stage_3W_vs_E10	ordinary_muscle_development	ENSGALG00010013510	MRPS33	0.089190238	0.087782923	1.016031757	0.309614264	0.347572704	positive
PRJNA729271	stage_3W_vs_E10	ordinary_muscle_development	ENSGALG00010013511	FAM78B	1.197362453	0.226308834	5.290833901	1.2176E-07	2.45058E-07	positive
PRJNA729271	stage_3W_vs_E10	ordinary_muscle_development	ENSGALG00010013512	ENSGALG00010013512	-4.246883791	3.485153042	-1.218564505			negative
PRJNA729271	stage_3W_vs_E10	ordinary_muscle_development	ENSGALG00010013514	LMBRD2	0.759668541	0.135401483	5.61048906	2.01756E-08	4.27301E-08	positive
PRJNA729271	stage_3W_vs_E10	ordinary_muscle_development	ENSGALG00010013516	CSRP2	-1.765063698	0.135292446	-13.04628423	6.67231E-39	5.19416E-38	negative
PRJNA729271	stage_3W_vs_E10	ordinary_muscle_development	ENSGALG00010013517	STMN1	-3.537678066	0.218084198	-16.2216158	3.54738E-59	5.23086E-58	negative
PRJNA729271	stage_3W_vs_E10	ordinary_muscle_development	ENSGALG00010013520	CREG2	-0.736523002	0.192143165	-3.833199077	0.000126488	0.000205037	negative
PRJNA729271	stage_3W_vs_E10	ordinary_muscle_development	ENSGALG00010013523	TMEM178B	-1.977512857	0.19439899	-10.17244411	2.63219E-24	1.19717E-23	negative
PRJNA729271	stage_3W_vs_E10	ordinary_muscle_development	ENSGALG00010013524	ZDHHC15	0.579158796	0.121151125	4.780465682	1.7489E-06	3.25755E-06	positive
PRJNA729271	stage_3W_vs_E10	ordinary_muscle_development	ENSGALG00010013526	SIRPA	0.31138823	0.235766684	1.32074738	0.18658561	0.216937388	positive
PRJNA729271	stage_3W_vs_E10	ordinary_muscle_development	ENSGALG00010013527	ADARB1	0.209763207	0.099662134	2.104743283	0.035313661	0.045182409	positive
PRJNA729271	stage_3W_vs_E10	ordinary_muscle_development	ENSGALG00010013528	PRDX6	0.610484828	0.131397958	4.64607544	3.38309E-06	6.19635E-06	positive
PRJNA729271	stage_3W_vs_E10	ordinary_muscle_development	ENSGALG00010013531	ARSI	-4.777938171	0.272118967	-17.55826955	5.1419E-69	9.64189E-68	negative
PRJNA729271	stage_3W_vs_E10	ordinary_muscle_development	ENSGALG00010013532	WDR19	-0.484256269	0.075494682	-6.414442137	1.41339E-10	3.38128E-10	negative
PRJNA729271	stage_3W_vs_E10	ordinary_muscle_development	ENSGALG00010013533	SKP2	-0.919201104	0.081652023	-11.25754237	2.12605E-29	1.19678E-28	negative
PRJNA729271	stage_3W_vs_E10	ordinary_muscle_development	ENSGALG00010013537	LMO3	-2.940554701	0.450617619	-6.525609696	6.77255E-11	1.65016E-10	negative
PRJNA729271	stage_3W_vs_E10	ordinary_muscle_development	ENSGALG00010013538	E2F7	-2.02462593	0.268120318	-7.551184271	4.31318E-14	1.24939E-13	negative
PRJNA729271	stage_3W_vs_E10	ordinary_muscle_development	ENSGALG00010013539	NSFL1C	0.306654032	0.091773434	3.341424849	0.000833496	0.001257051	positive
PRJNA729271	stage_3W_vs_E10	ordinary_muscle_development	ENSGALG00010013544	ENSGALG00010013544	-0.832761673	0.289897962	-2.872602715	0.004071057	0.005759318	negative
PRJNA729271	stage_3W_vs_E10	ordinary_muscle_development	ENSGALG00010013546	IDUA	0.036691699	0.13169153	0.278618523	0.780537596	0.804514858	positive
PRJNA729271	stage_3W_vs_E10	ordinary_muscle_development	ENSGALG00010013549	PCP4	-4.744084927	0.419344996	-11.31308344	1.13046E-29	6.42083E-29	negative
PRJNA729271	stage_3W_vs_E10	ordinary_muscle_development	ENSGALG00010013550	KLHL5	-0.45038233	0.12501569	-3.602606429	0.000315042	0.000494	negative
PRJNA729271	stage_3W_vs_E10	ordinary_muscle_development	ENSGALG00010013551	UBE2N	-0.441015314	0.111766816	-3.945852009	7.95167E-05	0.000130813	negative
PRJNA729271	stage_3W_vs_E10	ordinary_muscle_development	ENSGALG00010013552	FGF16	0.655443115	0.403769047	1.62331194	0.104522738	0.126023714	positive
PRJNA729271	stage_3W_vs_E10	ordinary_muscle_development	ENSGALG00010013553	TADA1	0.491084267	0.110490141	4.444598081	8.80563E-06	1.56113E-05	positive
PRJNA729271	stage_3W_vs_E10	ordinary_muscle_development	ENSGALG00010013554	ENSGALG00010013554	-4.561551489	5.466207082	-0.834500307	0.403999114	0.443063264	negative
PRJNA729271	stage_3W_vs_E10	ordinary_muscle_development	ENSGALG00010013556	RASL11B	-3.105135599	0.174748844	-17.76913385	1.22569E-70	2.40736E-69	negative
PRJNA729271	stage_3W_vs_E10	ordinary_muscle_development	ENSGALG00010013558	SNAPC3	-0.42002167	0.086105756	-4.877974382	1.07181E-06	2.02448E-06	negative
PRJNA729271	stage_3W_vs_E10	ordinary_muscle_development	ENSGALG00010013561	AGK	0.279071594	0.107917159	2.585979806	0.009710262	0.013214745	positive
PRJNA729271	stage_3W_vs_E10	ordinary_muscle_development	ENSGALG00010013562	RERGL	0.707930292	0.158102009	4.477680558	7.54584E-06	1.34469E-05	positive
PRJNA729271	stage_3W_vs_E10	ordinary_muscle_development	ENSGALG00010013563	FKBP1A	0.551299834	0.098926858	5.57280241	2.50674E-08	5.27131E-08	positive
PRJNA729271	stage_3W_vs_E10	ordinary_muscle_development	ENSGALG00010013565	ENSGALG00010013565	-0.683740961	5.324803546	-0.128406796	0.897827054	0.909624879	negative
PRJNA729271	stage_3W_vs_E10	ordinary_muscle_development	ENSGALG00010013569	ENSGALG00010013569	-4.791799359	0.592009884	-8.094120536	5.76796E-16	1.83187E-15	negative
PRJNA729271	stage_3W_vs_E10	ordinary_muscle_development	ENSGALG00010013572	DENND11	0.260702562	0.103893724	2.509319627	0.012096398	0.016305894	positive
PRJNA729271	stage_3W_vs_E10	ordinary_muscle_development	ENSGALG00010013573	ENSGALG00010013573	0.184772526	0.465769002	0.396704214	0.691585592	0.721864561	positive
PRJNA729271	stage_3W_vs_E10	ordinary_muscle_development	ENSGALG00010013574	NR2F1	-2.215202294	0.164638398	-13.45495539	2.87913E-41	2.4151E-40	negative
PRJNA729271	stage_3W_vs_E10	ordinary_muscle_development	ENSGALG00010013576	CAMK2A	-0.538940291	0.187195877	-2.879017962	0.003989156	0.005648414	negative
PRJNA729271	stage_3W_vs_E10	ordinary_muscle_development	ENSGALG00010013579	H2AC32	-2.813447597	0.177678577	-15.8344784	1.79945E-56	2.45603E-55	negative
PRJNA729271	stage_3W_vs_E10	ordinary_muscle_development	ENSGALG00010013580	RANBP3L	-1.023690874	0.315634144	-3.243283069	0.001181608	0.001759906	negative
PRJNA729271	stage_3W_vs_E10	ordinary_muscle_development	ENSGALG00010013582	MAP4K4	-1.416719	0.107567791	-13.17047585	1.29777E-39	1.037E-38	negative
PRJNA729271	stage_3W_vs_E10	ordinary_muscle_development	ENSGALG00010013583	FASLG	1.342558824	0.321845079	4.171444316	3.02675E-05	5.14878E-05	positive
PRJNA729271	stage_3W_vs_E10	ordinary_muscle_development	ENSGALG00010013587	SLC26A1	0.305528975	0.466986452	0.654256615	0.512946492	0.550915783	positive
PRJNA729271	stage_3W_vs_E10	ordinary_muscle_development	ENSGALG00010013589	DSCAM	-5.396443156	0.888973852	-6.070418318	1.27577E-09	2.88988E-09	negative
PRJNA729271	stage_3W_vs_E10	ordinary_muscle_development	ENSGALG00010013592	H3C33	-3.133338431	0.184812859	-16.95411485	1.79443E-64	3.02094E-63	negative
PRJNA729271	stage_3W_vs_E10	ordinary_muscle_development	ENSGALG00010013596	SDCBP2	-0.385168521	0.123382449	-3.12174482	0.001797827	0.002627231	negative
PRJNA729271	stage_3W_vs_E10	ordinary_muscle_development	ENSGALG00010013598	PAQR7	2.114040344	0.224830843	9.402803976	5.3128E-21	2.11389E-20	positive
PRJNA729271	stage_3W_vs_E10	ordinary_muscle_development	ENSGALG00010013604	FYN	-0.760806112	0.11180364	-6.804842038	1.0116E-11	2.58747E-11	negative
PRJNA729271	stage_3W_vs_E10	ordinary_muscle_development	ENSGALG00010013609	PIGC	1.967776854	0.161022179	12.22053301	2.41496E-34	1.62738E-33	positive
PRJNA729271	stage_3W_vs_E10	ordinary_muscle_development	ENSGALG00010013612	TWIST2	-4.371220029	0.291788308	-14.98079229	9.80413E-51	1.13962E-49	negative
PRJNA729271	stage_3W_vs_E10	ordinary_muscle_development	ENSGALG00010013614	ATRX	-0.851907249	0.086800512	-9.814541762	9.74799E-23	4.15947E-22	negative
PRJNA729271	stage_3W_vs_E10	ordinary_muscle_development	ENSGALG00010013617	PIK3C2G	1.018413867	0.158452396	6.427254455	1.29929E-10	3.11551E-10	positive
PRJNA729271	stage_3W_vs_E10	ordinary_muscle_development	ENSGALG00010013619	IL1R2	0.936419641	0.196003736	4.777560152	1.77435E-06	3.30369E-06	positive
PRJNA729271	stage_3W_vs_E10	ordinary_muscle_development	ENSGALG00010013621	PDGFRA	-0.217783432	0.164688048	-1.322399739	0.186035076	0.216401446	negative
PRJNA729271	stage_3W_vs_E10	ordinary_muscle_development	ENSGALG00010013624	SST1	-8.043386331	1.740043611	-4.622519964	3.79106E-06	6.91735E-06	negative
PRJNA729271	stage_3W_vs_E10	ordinary_muscle_development	ENSGALG00010013625	SSBP1	-0.340029186	0.11242683	-3.024448755	0.002490867	0.00359756	negative
PRJNA729271	stage_3W_vs_E10	ordinary_muscle_development	ENSGALG00010013627	MYCBP	0.350595525	0.147209862	2.38160351	0.017237446	0.022824223	positive
PRJNA729271	stage_3W_vs_E10	ordinary_muscle_development	ENSGALG00010013632	SLC1A3	-9.662092634	0.969739135	-9.963599783	2.19951E-23	9.61458E-23	negative
PRJNA729271	stage_3W_vs_E10	ordinary_muscle_development	ENSGALG00010013635	BCL6	0.527544816	0.160445671	3.287996572	0.001009031	0.001511408	positive
PRJNA729271	stage_3W_vs_E10	ordinary_muscle_development	ENSGALG00010013638	COMMD8	-0.566327517	0.117649132	-4.813699065	1.48162E-06	2.77289E-06	negative
PRJNA729271	stage_3W_vs_E10	ordinary_muscle_development	ENSGALG00010013639	ENSGALG00010013639	1.015684777	0.450931543	2.252414567	0.024296086	0.031667275	positive
PRJNA729271	stage_3W_vs_E10	ordinary_muscle_development	ENSGALG00010013640	SNPH	-2.4916867	0.653929757	-3.810327752	0.000138783	0.000224215	negative
PRJNA729271	stage_3W_vs_E10	ordinary_muscle_development	ENSGALG00010013645	RRAGC	-0.272050513	0.121489102	-2.239299737	0.02513642	0.032692006	negative
PRJNA729271	stage_3W_vs_E10	ordinary_muscle_development	ENSGALG00010013646	CLDN1	-0.44278808	0.336669027	-1.315202897	0.188441722	0.218919833	negative
PRJNA729271	stage_3W_vs_E10	ordinary_muscle_development	ENSGALG00010013648	NAV3	-1.011723456	0.118947366	-8.505639847	1.80597E-17	6.19293E-17	negative
PRJNA729271	stage_3W_vs_E10	ordinary_muscle_development	ENSGALG00010013650	BACE2	-0.810927694	0.16089248	-5.040183957	4.65085E-07	9.00414E-07	negative
PRJNA729271	stage_3W_vs_E10	ordinary_muscle_development	ENSGALG00010013651	ENSGALG00010013651	-0.00645272	0.159721357	-0.04039986	0.967774342	0.972133686	negative
PRJNA729271	stage_3W_vs_E10	ordinary_muscle_development	ENSGALG00010013654	SLC6A7	-5.353370319	0.815113646	-6.567636731	5.11201E-11	1.25506E-10	negative
PRJNA729271	stage_3W_vs_E10	ordinary_muscle_development	ENSGALG00010013657	MAGT1	-0.84823505	0.104867797	-8.088613247	6.03479E-16	1.9141E-15	negative

PRJNA729271	stage_3W_vs_E10	ordinary_muscle_development	ENSGALG00010013661	GABRA4	-1.996887087	0.392958964	-5.081668236	3.74134E-07	7.2881E-07	negative
PRJNA729271	stage_3W_vs_E10	ordinary_muscle_development	ENSGALG00010013663	OPA1	0.214954745	0.106355841	2.021090176	0.043270431	0.054749031	positive
PRJNA729271	stage_3W_vs_E10	ordinary_muscle_development	ENSGALG00010013664	REV3L	0.220426931	0.096236116	2.290480338	0.021993487	0.02881117	positive
PRJNA729271	stage_3W_vs_E10	ordinary_muscle_development	ENSGALG00010013666	COX7B	1.20674051	0.143359263	8.417597056	3.84285E-17	1.29691E-16	positive
PRJNA729271	stage_3W_vs_E10	ordinary_muscle_development	ENSGALG00010013667	SOWAHA	-1.496251813	0.489986423	-3.05365974	0.002260683	0.003275878	negative
PRJNA729271	stage_3W_vs_E10	ordinary_muscle_development	ENSGALG00010013669	NPR3	-0.293438653	0.255887235	-1.146749869	0.251485013	0.286758596	negative
PRJNA729271	stage_3W_vs_E10	ordinary_muscle_development	ENSGALG00010013672	ENSGALG00010013672	-4.414870773	0.499370139	-8.840878591	9.49688E-19	3.45006E-18	negative
PRJNA729271	stage_3W_vs_E10	ordinary_muscle_development	ENSGALG00010013675	CHRNA6	1.30696304	0.370657856	3.526063237	0.000421786	0.000653602	positive
PRJNA729271	stage_3W_vs_E10	ordinary_muscle_development	ENSGALG00010013677	GABRG1	-3.864253892	0.480720163	-8.038468517	9.09682E-16	2.85841E-15	negative
PRJNA729271	stage_3W_vs_E10	ordinary_muscle_development	ENSGALG00010013678	ATP7A	0.377195247	0.101684537	3.709465145	0.000207698	0.000330324	positive
PRJNA729271	stage_3W_vs_E10	ordinary_muscle_development	ENSGALG00010013679	MFSD4B	1.417275849	0.213727158	6.631238912	3.32881E-11	8.27919E-11	positive
PRJNA729271	stage_3W_vs_E10	ordinary_muscle_development	ENSGALG00010013686	UQCRQ	2.684267947	0.137876786	19.46859957	2.02739E-84	5.72845E-83	positive
PRJNA729271	stage_3W_vs_E10	ordinary_muscle_development	ENSGALG00010013690	CREB3L2	-1.113096074	0.102084551	-10.90366823	1.10702E-27	5.82866E-27	negative
PRJNA729271	stage_3W_vs_E10	ordinary_muscle_development	ENSGALG00010013696	CETN3	-0.334656316	0.109939644	-3.04400037	0.002334549	0.003378185	negative
PRJNA729271	stage_3W_vs_E10	ordinary_muscle_development	ENSGALG00010013697	HDAC4	-0.085411714	0.067403264	-1.267174749	0.205092813	0.237067011	negative
PRJNA729271	stage_3W_vs_E10	ordinary_muscle_development	ENSGALG00010013698	TFAP2E	-5.660106598	0.935179228	-6.052429769	1.42677E-09	3.22135E-09	negative
PRJNA729271	stage_3W_vs_E10	ordinary_muscle_development	ENSGALG00010013709	PSMF1	0.459381871	0.10054926	4.568724544	4.90701E-06	8.87425E-06	positive
PRJNA729271	stage_3W_vs_E10	ordinary_muscle_development	ENSGALG00010013715	IL1R1	0.751349504	0.233453396	3.218413256	0.00128902	0.001912028	positive
PRJNA729271	stage_3W_vs_E10	ordinary_muscle_development	ENSGALG00010013719	NCDN	-0.108881442	0.139710017	-0.779338838	0.435780142	0.474399787	negative
PRJNA729271	stage_3W_vs_E10	ordinary_muscle_development	ENSGALG00010013720	SLC16A10	0.489777963	0.189925296	2.578792679	0.009914627	0.013476418	positive
PRJNA729271	stage_3W_vs_E10	ordinary_muscle_development	ENSGALG00010013732	FNTA	0.434802778	0.07516948	5.784299373	7.28152E-09	1.58477E-08	positive
PRJNA729271	stage_3W_vs_E10	ordinary_muscle_development	ENSGALG00010013737	AFF4	0.710414567	0.084445431	8.412705802	4.00659E-17	1.35059E-16	positive
PRJNA729271	stage_3W_vs_E10	ordinary_muscle_development	ENSGALG00010013740	GNPDA2	-0.925017122	0.11937395	-7.748902678	9.269E-15	2.76848E-14	negative
PRJNA729271	stage_3W_vs_E10	ordinary_muscle_development	ENSGALG00010013742	RSPO4	-4.755423108	1.375038368	-3.458393029	0.000543408	0.000833954	negative
PRJNA729271	stage_3W_vs_E10	ordinary_muscle_development	ENSGALG00010013744	ENSGALG00010013744	0.132388699	0.308085621	0.429713982	0.667403711	0.699141201	positive
PRJNA729271	stage_3W_vs_E10	ordinary_muscle_development	ENSGALG00010013746	ENSGALG00010013746	-0.025696747	0.086808657	-0.296015949	0.767217901	0.792476554	negative
PRJNA729271	stage_3W_vs_E10	ordinary_muscle_development	ENSGALG00010013749	NELL2	-3.038418659	0.46473015	-6.538027843	6.23353E-11	1.5219E-10	negative
PRJNA729271	stage_3W_vs_E10	ordinary_muscle_development	ENSGALG00010013751	FUT10	-0.043082333	0.170601064	-0.252532615	0.800629408	0.823058388	negative
PRJNA729271	stage_3W_vs_E10	ordinary_muscle_development	ENSGALG00010013753	PLEKHA5	0.568896504	0.123801788	4.595220401	4.32292E-06	7.84534E-06	positive
PRJNA729271	stage_3W_vs_E10	ordinary_muscle_development	ENSGALG00010013755	ANGPT4	1.369651706	0.154113316	8.887302821	6.26102E-19	2.29291E-18	positive
PRJNA729271	stage_3W_vs_E10	ordinary_muscle_development	ENSGALG00010013757	DGKI	-1.803140471	0.281110881	-6.414338936	1.41435E-10	3.38301E-10	negative
PRJNA729271	stage_3W_vs_E10	ordinary_muscle_development	ENSGALG00010013758	FAM3B	-3.476354484	0.637396219	-5.453992946	4.92512E-08	1.01622E-07	negative
PRJNA729271	stage_3W_vs_E10	ordinary_muscle_development	ENSGALG00010013760	RPF2	-0.95236365	0.116078464	-8.204481858	2.31587E-16	7.5148E-16	negative
PRJNA729271	stage_3W_vs_E10	ordinary_muscle_development	ENSGALG00010013766	DNM3	-0.069822384	0.107749355	-0.648007443	0.516980136	0.554877977	negative
PRJNA729271	stage_3W_vs_E10	ordinary_muscle_development	ENSGALG00010013767	PGK2	5.152406419	0.171876614	29.97735592	1.9367E-197	3.3822E-195	positive
PRJNA729271	stage_3W_vs_E10	ordinary_muscle_development	ENSGALG00010013768	FAM110A	-1.539562972	0.243368786	-6.32604943	2.51517E-10	5.94738E-10	negative
PRJNA729271	stage_3W_vs_E10	ordinary_muscle_development	ENSGALG00010013771	PELO	0.378117872	0.102622511	3.684550951	0.000229106	0.00036282	positive
PRJNA729271	stage_3W_vs_E10	ordinary_muscle_development	ENSGALG00010013772	KIAA0319L	1.45438092	0.143015937	10.16936259	2.71681E-24	1.23487E-23	positive
PRJNA729271	stage_3W_vs_E10	ordinary_muscle_development	ENSGALG00010013775	DBX2	-2.607076486	0.402829833	-6.471905183	9.67748E-11	2.33636E-10	negative
PRJNA729271	stage_3W_vs_E10	ordinary_muscle_development	ENSGALG00010013777	CPLANE1	-1.152307528	0.09563135	-12.04947467	1.9519E-33	1.2733E-32	negative
PRJNA729271	stage_3W_vs_E10	ordinary_muscle_development	ENSGALG00010013778	ENSGALG00010013778	-5.205581258	0.743837024	-6.998282003	2.5912E-12	6.82403E-12	negative
PRJNA729271	stage_3W_vs_E10	ordinary_muscle_development	ENSGALG00010013779	SLC52A3	-0.403133701	0.242557163	-1.662015234	0.09650972	0.116955806	negative
PRJNA729271	stage_3W_vs_E10	ordinary_muscle_development	ENSGALG00010013783	NDUFA10	0.936691915	0.095621863	9.795792403	1.17373E-22	4.98631E-22	positive
PRJNA729271	stage_3W_vs_E10	ordinary_muscle_development	ENSGALG00010013784	ANO6	1.547433278	0.094425006	16.38796055	2.33136E-60	3.52378E-59	positive
PRJNA729271	stage_3W_vs_E10	ordinary_muscle_development	ENSGALG00010013787	ZCCHC10	-0.251342362	0.103596987	-2.426155131	0.015259746	0.020324361	negative
PRJNA729271	stage_3W_vs_E10	ordinary_muscle_development	ENSGALG00010013791	AEBP2	-0.071382326	0.064719753	-1.102944971	0.270051063	0.306578177	negative
PRJNA729271	stage_3W_vs_E10	ordinary_muscle_development	ENSGALG00010013792	FGF10	0.24305256	0.229646861	1.058375278	0.289884386	0.327054892	positive
PRJNA729271	stage_3W_vs_E10	ordinary_muscle_development	ENSGALG00010013793	METTL13	-1.22281236	0.132751659	-9.211277443	3.22267E-20	1.24302E-19	negative
PRJNA729271	stage_3W_vs_E10	ordinary_muscle_development	ENSGALG00010013794	GTF3C6	-0.925161036	0.179336881	-5.158788486	2.48553E-07	4.89707E-07	negative
PRJNA729271	stage_3W_vs_E10	ordinary_muscle_development	ENSGALG00010013797	IL1RL1	1.407996022	0.315620324	4.461043587	8.15615E-06	1.44935E-05	positive
PRJNA729271	stage_3W_vs_E10	ordinary_muscle_development	ENSGALG00010013802	PYROXD1	0.718408471	0.139711354	5.142090822	2.71698E-07	5.34291E-07	positive
PRJNA729271	stage_3W_vs_E10	ordinary_muscle_development	ENSGALG00010013805	HSPA4	0.402918279	0.145237804	2.774196991	0.005533816	0.007722409	positive
PRJNA729271	stage_3W_vs_E10	ordinary_muscle_development	ENSGALG00010013808	PLAAT1	1.409886655	0.13634884	10.34029078	4.6313E-25	2.17533E-24	positive
PRJNA729271	stage_3W_vs_E10	ordinary_muscle_development	ENSGALG00010013809	MX1	6.979775966	1.212086029	5.758482319	8.48736E-09	1.83948E-08	positive
PRJNA729271	stage_3W_vs_E10	ordinary_muscle_development	ENSGALG00010013814	AMD1	-0.676107866	0.111042086	-6.088753312	1.13793E-09	2.58289E-09	negative
PRJNA729271	stage_3W_vs_E10	ordinary_muscle_development	ENSGALG00010013815	SRXN1	0.40219487	0.250391267	1.606265567	0.108215573	0.130227042	positive
PRJNA729271	stage_3W_vs_E10	ordinary_muscle_development	ENSGALG00010013820	MASP1	-3.763813773	0.505371944	-7.44761124	9.50454E-14	2.69764E-13	negative
PRJNA729271	stage_3W_vs_E10	ordinary_muscle_development	ENSGALG00010013823	GUF1	0.185326485	0.111960275	1.655287866	0.097866119	0.118470803	positive
PRJNA729271	stage_3W_vs_E10	ordinary_muscle_development	ENSGALG00010013828	PTN	-8.226725205	0.277310666	-29.66609737	2.1029E-193	3.5861E-191	negative
PRJNA729271	stage_3W_vs_E10	ordinary_muscle_development	ENSGALG00010013831	TCERG1	0.048231285	0.11291997	0.427128037	0.669286073	0.7009611	positive
PRJNA729271	stage_3W_vs_E10	ordinary_muscle_development	ENSGALG00010013833	TMPRSS2	-2.16012319	1.241386241	-1.740089521	0.0818433	0.100052175	negative
PRJNA729271	stage_3W_vs_E10	ordinary_muscle_development	ENSGALG00010013836	IL18R1	1.307216879	0.251498767	5.197706913	2.01762E-07	3.99746E-07	positive
PRJNA729271	stage_3W_vs_E10	ordinary_muscle_development	ENSGALG00010013837	IFI27L2	2.320122258	0.334702207	6.931900078	4.15225E-12	1.07959E-11	positive
PRJNA729271	stage_3W_vs_E10	ordinary_muscle_development	ENSGALG00010013842	CDK19	0.676978467	0.110824115	6.108584472	1.00519E-09	2.29055E-09	positive
PRJNA729271	stage_3W_vs_E10	ordinary_muscle_development	ENSGALG00010013846	YIPF7	8.234535018	0.319026283	25.8114627	6.5936E-147	5.8996E-145	positive
PRJNA729271	stage_3W_vs_E10	ordinary_muscle_development	ENSGALG00010013848	ITPA	-1.180406346	0.102410013	-11.52627867	9.72575E-31	5.75172E-30	negative
PRJNA729271	stage_3W_vs_E10	ordinary_muscle_development	ENSGALG00010013857	IL18RAP	1.953149141	0.58213868	3.355126893	0.000793286	0.001198778	positive
PRJNA729271	stage_3W_vs_E10	ordinary_muscle_development	ENSGALG00010013859	MYOC	1.236562005	0.247103106	5.004234968	5.60843E-07	1.07903E-06	positive
PRJNA729271	stage_3W_vs_E10	ordinary_muscle_development	ENSGALG00010013861	TCF15	1.72106788	0.156458448	11.00015948	3.81457E-28	2.04786E-27	positive
PRJNA729271	stage_3W_vs_E10	ordinary_muscle_development	ENSGALG00010013866	KCTD8	-2.567419399	0.403772657	-6.358576673	2.03632E-10	4.83876E-10	negative
PRJNA729271	stage_3W_vs_E10	ordinary_muscle_development	ENSGALG00010013867	RECQL	-1.089518788	0.127973622	-8.513620025	1.68586E-17	5.78927E-17	negative
PRJNA729271	stage_3W_vs_E10	ordinary_muscle_development	ENSGALG00010013871	ENSGALG00010013871	0.559811021	0.231135908	2.421999354	0.015435378	0.020548843	positive
PRJNA729271	stage_3W_vs_E10	ordinary_muscle_development	ENSGALG00010013872	GLS2	2.001308136	0.196889405	10.16463093	2.852E-24	1.2947E-23	positive
PRJNA729271	stage_3W_vs_E10	ordinary_muscle_development	ENSGALG00010013878	ARID2	-0.379476546	0.08204849	-4.625027774	3.74548E-06	6.8428E-06	negative
PRJNA729271	stage_3W_vs_E10	ordinary_muscle_development	ENSGALG00010013880	ZMYM4	-0.830345165	0.090566177	-9.168380453	4.80179E-20	1.8384E-19	negative
PRJNA729271	stage_3W_vs_E10	ordinary_muscle_development	ENSGALG00010013889	ENSGALG00010013889	0.30411122	0.420478127	0.723250985	0.4695257	0.50819706	positive
PRJNA729271	stage_3W_vs_E10	ordinary_muscle_development	ENSGALG00010013890	SFPQ	-1.413518166	0.072505957	-19.49520048	1.20581E-84	3.4406E-83	negative
PRJNA729271	stage_3W_vs_E10	ordinary_muscle_development	ENSGALG00010013893	CSNK2A1	-0.134784058	0.078797271	-1.71051682	0.087170346	0.10625077	negative
PRJNA729271	stage_3W_vs_E10	ordinary_muscle_development	ENSGALG00010013897	AMOT	-1.254317525	0.07600558	-16.50296627	3.49304E-61	5.39783E-60	negative
PRJNA729271	stage_3W_vs_E10	ordinary_muscle_development	ENSGALG00010013898	EMB	-1.22213496	0.294825428	-4.145283419	3.39394E-05	5.74575E-05	negative
PRJNA729271	stage_3W_vs_E10	ordinary_muscle_development	ENSGALG00010013899	WNT5B	0.372651026	0.141177371	2.639594605	0.008300525	0.011382792	positive
PRJNA729271	stage_3W_vs_E10	ordinary_muscle_development	ENSGALG00010013901	NFXL1	-0.111485203	0.121205986	-0.919799476	0.357677557	0.396371269	negative
PRJNA729271	stage_3W_vs_E10	ordinary_muscle_development	ENSGALG00010013908	TBC1D20	0.993020613	0.115443645	8.601778072	7.84905E-18	2.7448E-17	positive
PRJNA729271	stage_3W_vs_E10	ordinary_muscle_development	ENSGALG00010013910	ADIPOR2	1.852114335	0.207776562	8.913971403	4.92365E-19	1.81368E-18	positive
PRJNA729271	stage_3W_vs_E10	ordinary_muscle_development	ENSGALG00010013911	TNIK	-0.126821577	0.148188015	-0.855815342	0.392099928	0.431057145	negative
PRJNA729271	stage_3W_vs_E10	ordinary_muscle_development	ENSGALG00010013914	NRG1	-0.308715315	0.230388731	-1.339975763	0.180253225	0.2100129	negative
PRJNA729271	stage_3W_vs_E10	ordinary_muscle_development	ENSGALG00010013915	ENSGALG00010013915	1.230179026	0.19051476	6.457132383	1.06706E-10	2.56926E-10	positive
PRJNA729271	stage_3W_vs_E10	ordinary_muscle_development	ENSGALG00010013917	TRPC5	-2.067680199	0.578507251	-3.574164706	0.000351348	0.000548437	negative
PRJNA729271	stage_3W_vs_E10	ordinary_muscle_development	ENSGALG00010013921	PPP2R2B	0.912308109	0.105537406	8.644405308	5.40862E-18	1.90703E-17	positive
PRJNA729271	stage_3W_vs_E10	ordinary_muscle_development	ENSGALG00010013922	ENSGALG00010013922	-3.186623419	0.954727997	-3.3377291	0.000844661	0.00127336	negative
PRJNA729271	stage_3W_vs_E10	ordinary_muscle_development	ENSGALG00010013923	FMR1NB	1.767009298	0.503557415	3.509052283	0.000449706	0.000695455	positive
PRJNA729271	stage_3W_vs_E10	ordinary_muscle_development	ENSGALG00010013928	CCNO	1.960454983	0.473023776	4.144516789	3.40531E-05	5.76433E-05	positive
PRJNA729271	stage_3W_vs_E10	ordinary_muscle_development	ENSGALG00010013930	SLC22A16	2.61812775	0.303578596	8.624217201	6.45325E-18	2.26763E-17	positive
PRJNA729271	stage_3W_vs_E10	ordinary_muscle_development	ENSGALG00010013934	STAT1	1.501744811	0.282600563	5.31401918	1.07233E-07	2.16694E-07	positive
PRJNA729271	stage_3W_vs_E10	ordinary_muscle_development	ENSGALG00010013936	DLGAP3	-3.943539871	0.527867151	-7.470705209	7.97662E-14	2.2769E-13	negative
PRJNA729271	stage_3W_vs_E10	ordinary_muscle_development	ENSGALG00010013938	ENSGALG00010013938	0.807783967	0.407670848	1.981461197	0.047539577	0.059858076	positive
PRJNA729271	stage_3W_vs_E10	ordinary_muscle_development	ENSGALG00010013940	UPF3A	-0.984421509	0.256407326	-3.839287758	0.000123392	0.000200197	negative
PRJNA729271	stage_3W_vs_E10	ordinary_muscle_development	ENSGALG00010013942	CACNA2D4	0.835085884	0.522302413	1.598855113	0.1098528	0.131989086	positive
PRJNA729271	stage_3W_vs_E10	ordinary_muscle_development	ENSGALG00010013944	METTL24	-3.126868669	0.241638847	-12.94025656	2.66737E-38	2.03492E-37	negative
PRJNA729271	stage_3W_vs_E10	ordinary_muscle_development	ENSGALG00010013945	SCAF11	-0.257314422	0.076942896	-3.344225844	0.000825125	0.001244557	negative
PRJNA729271	stage_3W_vs_E10	ordinary_muscle_development	ENSGALG00010013947	ENSGALG00010013947	-0.561373835	0.189070185	-2.969129354	0.002986449	0.004283876	negative
PRJNA729271	stage_3W_vs_E10	ordinary_muscle_development	ENSGALG00010013949	PRRC2C	-0.37227811	0.126212928	-2.949603626	0.003181818	0.004553308	negative
PRJNA729271	stage_3W_vs_E10	ordinary_muscle_development	ENSGALG00010013953	DHX29	-0.709707149	0.082113785	-8.642972006	5.47693E-18	1.93018E-17	negative
PRJNA729271	stage_3W_vs_E10	ordinary_muscle_development	ENSGALG00010013954	LPL	1.069476463	0.334092249	3.201141204	0.001368844	0.00202608	positive
PRJNA729271	stage_3W_vs_E10	ordinary_muscle_development	ENSGALG00010013958	TUSC3	0.686259403	0.174644636	3.929461669	8.51362E-05	0.000139788	positive
PRJNA729271	stage_3W_vs_E10	ordinary_muscle_development	ENSGALG00010013959	STK32A	-1.423007748	0.457813637	-3.108268586	0.00188187	0.00274534	negative
PRJNA729271	stage_3W_vs_E10	ordinary_muscle_development	ENSGALG00010013964	SLC38A1	-1.482731001	0.161301294	-9.192306926	3.84505E-20	1.47718E-19	negative
PRJNA729271	stage_3W_vs_E10	ordinary_muscle_development	ENSGALG00010013965	SLC2A2	1.529650376	1.023159203	1.495026748			positive
PRJNA729271	stage_3W_vs_E10	ordinary_muscle_development	ENSGALG00010013967	MLLT3	-0.279668389	0.154166967	-1.814061698	0.069668228	0.08587083	negative
PRJNA729271	stage_3W_vs_E10	ordinary_muscle_development	ENSGALG00010013971	ENSGALG00010013971	-1.55794469	0.406468219	-3.832881926	0.000126651	0.000205278	negative
PRJNA729271	stage_3W_vs_E10	ordinary_muscle_development	ENSGALG00010013973	ENSGALG00010013973	1.045957284	0.235390405	4.443500087	8.85071E-06	1.56854E-05	positive
PRJNA729271	stage_3W_vs_E10	ordinary_muscle_development	ENSGALG00010013976	CDC40	-0.159343338	0.075222883	-2.11828278	0.034151126	0.04374519	negative
PRJNA729271	stage_3W_vs_E10	ordinary_muscle_development	ENSGALG00010013977	SLC38A2	0.258635673	0.192701342	1.342158132	0.179544728	0.209271537	positive
PRJNA729271	stage_3W_vs_E10	ordinary_muscle_development	ENSGALG00010013980	FMO4	1.018511138	0.376715272	2.703662989	0.006857981	0.009480823	positive
PRJNA729271	stage_3W_vs_E10	ordinary_muscle_development	ENSGALG00010013981	RLF	0.545360689	0.106894838	5.101843092	3.36362E-07	6.56818E-07	positive
PRJNA729271	stage_3W_vs_E10	ordinary_muscle_development	ENSGALG00010013989	ENSGALG00010013989	1.550213978	0.202801933	7.643980276	2.10608E-14	6.18468E-14	positive
PRJNA729271	stage_3W_vs_E10	ordinary_muscle_development	ENSGALG00010013994	CHRM2	-5.500315376	0.617222076	-8.911404166	5.03903E-19	1.85524E-18	negative
PRJNA729271	stage_3W_vs_E10	ordinary_muscle_development	ENSGALG00010013995	REM1	0.216223872	0.211260425	1.023494446	0.306074124	0.343838211	positive
PRJNA729271	stage_3W_vs_E10	ordinary_muscle_development	ENSGALG00010014000	STAT4	8.164192791	0.923235243	8.843025497	9.3161E-19	3.38608E-18	positive
PRJNA729271	stage_3W_vs_E10	ordinary_muscle_development	ENSGALG00010014001	CDC16	-0.990146168	0.08640376	-11.45952637	2.10664E-30	1.2288E-29	negative
PRJNA729271	stage_3W_vs_E10	ordinary_muscle_development	ENSGALG00010014002	ZDHHC18	-0.052741196	0.269351634	-0.195807969	0.844760472	0.862917762	negative
PRJNA729271	stage_3W_vs_E10	ordinary_muscle_development	ENSGALG00010014010	EIF5A2	-0.068557902	0.10239543	-0.66954064	0.503150665	0.541118692	negative
PRJNA729271	stage_3W_vs_E10	ordinary_muscle_development	ENSGALG00010014011	SFN	-5.061055663	0.569400383	-8.888395263	6.19978E-19	2.27163E-18	negative
PRJNA729271	stage_3W_vs_E10	ordinary_muscle_development	ENSGALG00010014014	PARP8	0.954420173	0.196379541	4.860079451	1.17339E-06	2.21202E-06	positive
PRJNA729271	stage_3W_vs_E10	ordinary_muscle_development	ENSGALG00010014018	DPYSL3	-1.548828566	0.238896246	-6.483268751	8.97564E-11	2.17199E-10	negative
PRJNA729271	stage_3W_vs_E10	ordinary_muscle_development	ENSGALG00010014019	IRAG2	1.371923803	0.222753103	6.158943607	7.32318E-10	1.68491E-09	positive
PRJNA729271	stage_3W_vs_E10	ordinary_muscle_development	ENSGALG00010014020	PDXP	-0.78963053	0.105634343	-7.47513078	7.71271E-14	2.20461E-13	negative
PRJNA729271	stage_3W_vs_E10	ordinary_muscle_development	ENSGALG00010014026	SH3BP1	3.540631166	0.316439434	11.18896949	4.61769E-29	2.56254E-28	positive
PRJNA729271	stage_3W_vs_E10	ordinary_muscle_development	ENSGALG00010014030	WASF1	-3.541623068	0.416196446	-8.509498591	1.74687E-17	5.99311E-17	negative
PRJNA729271	stage_3W_vs_E10	ordinary_muscle_development	ENSGALG00010014031	RPL22L1	0.183660748	0.188379905	0.974948723	0.329585696	0.367827584	positive
PRJNA729271	stage_3W_vs_E10	ordinary_muscle_development	ENSGALG00010014032	PSD3	-0.853736984	0.078251926	-10.91010831	1.03133E-27	5.43804E-27	negative

PRJNA729271	stage_3W_vs_E10	ordinary_muscle_development	ENSGALG00010014033	SLC38A4	3.291991809	0.473718202	6.949261807	3.67203E-12	9.57992E-12	positive
PRJNA729271	stage_3W_vs_E10	ordinary_muscle_development	ENSGALG00010014035	PSIP1	-1.074606588	0.118308533	-9.083086089	1.05539E-19	3.98694E-19	negative
PRJNA729271	stage_3W_vs_E10	ordinary_muscle_development	ENSGALG00010014041	SKIV2L2	0.169940497	0.098441404	1.726311191	0.084291443	0.102897462	positive
PRJNA729271	stage_3W_vs_E10	ordinary_muscle_development	ENSGALG00010014042	SGCZ	-1.989271294	0.366042961	-5.434529562	5.49412E-08	1.12929E-07	negative
PRJNA729271	stage_3W_vs_E10	ordinary_muscle_development	ENSGALG00010014045	GOLT1B	-1.499288976	0.111435195	-13.45435775	2.9025E-41	2.43189E-40	negative
PRJNA729271	stage_3W_vs_E10	ordinary_muscle_development	ENSGALG00010014047	HM13	0.10291322	0.167197322	0.615519548	0.538211664	0.575917471	positive
PRJNA729271	stage_3W_vs_E10	ordinary_muscle_development	ENSGALG00010014056	FMO3	4.734620519	0.253815416	18.65379413	1.17626E-77	2.76784E-76	positive
PRJNA729271	stage_3W_vs_E10	ordinary_muscle_development	ENSGALG00010014062	GGA1	1.049804353	0.103467782	10.14619558	3.44533E-24	1.56014E-23	positive
PRJNA729271	stage_3W_vs_E10	ordinary_muscle_development	ENSGALG00010014065	CHUNK-1	-2.983595879	0.954507932	-3.125794746	0.001773253	0.002594982	negative
PRJNA729271	stage_3W_vs_E10	ordinary_muscle_development	ENSGALG00010014066	BCL2L1	0.087907739	0.116637003	0.753686532	0.451037471	0.489795336	positive
PRJNA729271	stage_3W_vs_E10	ordinary_muscle_development	ENSGALG00010014076	PRRX1	-2.872334312	0.248188793	-11.57318297	5.63525E-31	3.35868E-30	negative
PRJNA729271	stage_3W_vs_E10	ordinary_muscle_development	ENSGALG00010014077	SLITRK2	0.003267694	0.100221843	0.032604608	0.973989895	0.977835125	positive
PRJNA729271	stage_3W_vs_E10	ordinary_muscle_development	ENSGALG00010014080	MYO1B	-0.273759418	0.160789517	-1.702594935	0.088643899	0.10792888	negative
PRJNA729271	stage_3W_vs_E10	ordinary_muscle_development	ENSGALG00010014082	HNRNPR	-1.049325274	0.043247328	-24.26335482	4.7799E-130	3.3151E-128	negative
PRJNA729271	stage_3W_vs_E10	ordinary_muscle_development	ENSGALG00010014083	ENSGALG00010014083	-0.667878131	0.145678381	-4.584607023	4.54841E-06	8.24218E-06	negative
PRJNA729271	stage_3W_vs_E10	ordinary_muscle_development	ENSGALG00010014089	FIG4	-0.06758054	0.114300905	-0.59125114	0.554352162	0.591398733	negative
PRJNA729271	stage_3W_vs_E10	ordinary_muscle_development	ENSGALG00010014091	CHIC2	0.612837067	0.098156266	6.243483885	4.2793E-10	9.98527E-10	positive
PRJNA729271	stage_3W_vs_E10	ordinary_muscle_development	ENSGALG00010014092	TYRP1	-3.520624506	1.097545925	-3.207724091	0.001337898	0.001982501	negative
PRJNA729271	stage_3W_vs_E10	ordinary_muscle_development	ENSGALG00010014093	PLD1	0.198173111	0.093773665	2.113313057	0.034573982	0.04427119	positive
PRJNA729271	stage_3W_vs_E10	ordinary_muscle_development	ENSGALG00010014096	JAKMIP2	-3.082366377	0.161865617	-19.04274943	7.54604E-81	1.93936E-79	negative
PRJNA729271	stage_3W_vs_E10	ordinary_muscle_development	ENSGALG00010014099	TPX2	-2.660704644	0.144762048	-18.37984943	1.90497E-75	4.24808E-74	negative
PRJNA729271	stage_3W_vs_E10	ordinary_muscle_development	ENSGALG00010014102	TRIM2	0.929114901	0.137207032	6.771627438	1.27342E-11	3.23714E-11	positive
PRJNA729271	stage_3W_vs_E10	ordinary_muscle_development	ENSGALG00010014104	ALDH1A1	3.358922984	0.518560042	6.477404176	9.3314E-11	2.25619E-10	positive
PRJNA729271	stage_3W_vs_E10	ordinary_muscle_development	ENSGALG00010014105	AHR1B	-2.962912609	0.660697091	-4.484524982	7.30765E-06	1.30336E-05	negative
PRJNA729271	stage_3W_vs_E10	ordinary_muscle_development	ENSGALG00010014109	AMIGO2	-0.89793339	0.164033481	-5.474086035	4.39775E-08	9.09999E-08	negative
PRJNA729271	stage_3W_vs_E10	ordinary_muscle_development	ENSGALG00010014111	GORAB	-0.121809931	0.081513551	-1.494351922	0.135083661	0.160481736	negative
PRJNA729271	stage_3W_vs_E10	ordinary_muscle_development	ENSGALG00010014114	ANXA1	3.126886261	0.332013289	9.417955143	4.59958E-21	1.83717E-20	positive
PRJNA729271	stage_3W_vs_E10	ordinary_muscle_development	ENSGALG00010014120	SOX5	-2.440551139	0.136863916	-17.83195456	3.99217E-71	7.92693E-70	negative
PRJNA729271	stage_3W_vs_E10	ordinary_muscle_development	ENSGALG00010014122	ABCA1	0.963506325	0.140644448	6.850653088	7.35136E-12	1.88832E-11	positive
PRJNA729271	stage_3W_vs_E10	ordinary_muscle_development	ENSGALG00010014125	PLPP1	3.528089604	0.138605136	25.45424874	6.3349E-143	5.3698E-141	positive
PRJNA729271	stage_3W_vs_E10	ordinary_muscle_development	ENSGALG00010014128	COL4A1	3.001151349	0.144542814	20.76306159	9.34211E-96	3.49005E-94	positive
PRJNA729271	stage_3W_vs_E10	ordinary_muscle_development	ENSGALG00010014131	CDC23	-0.978089145	0.118638414	-8.244287107	1.66148E-16	5.43024E-16	negative
PRJNA729271	stage_3W_vs_E10	ordinary_muscle_development	ENSGALG00010014134	RPAP3	-0.143212601	0.113743631	-1.259082375	0.20800058	0.240159981	negative
PRJNA729271	stage_3W_vs_E10	ordinary_muscle_development	ENSGALG00010014135	ZBTB24	0.665958288	0.152659471	4.362377817	1.28656E-05	2.25417E-05	positive
PRJNA729271	stage_3W_vs_E10	ordinary_muscle_development	ENSGALG00010014142	PPIL6	-0.82935334	0.588723541	-1.408731403	0.158914606	0.186802953	negative
PRJNA729271	stage_3W_vs_E10	ordinary_muscle_development	ENSGALG00010014143	NFATC2	-0.05268084	0.152981852	-0.344360061	0.730575521	0.758356644	negative
PRJNA729271	stage_3W_vs_E10	ordinary_muscle_development	ENSGALG00010014145	NTMT2	6.432545376	0.251384722	25.5884499	2.0512E-144	1.7804E-142	positive
PRJNA729271	stage_3W_vs_E10	ordinary_muscle_development	ENSGALG00010014147	ENSGALG00010014147	-1.589509979	0.361786777	-4.393499379	1.11541E-05	1.9633E-05	negative
PRJNA729271	stage_3W_vs_E10	ordinary_muscle_development	ENSGALG00010014148	KCNG1	-1.848192054	0.389500243	-4.745034408	2.08471E-06	3.86714E-06	negative
PRJNA729271	stage_3W_vs_E10	ordinary_muscle_development	ENSGALG00010014149	LGALS2	4.955522447	0.504595744	9.820777344	9.16342E-23	3.91349E-22	positive
PRJNA729271	stage_3W_vs_E10	ordinary_muscle_development	ENSGALG00010014151	ATP23	0.345703611	0.089655008	3.855931947	0.00011529	0.00018743	positive
PRJNA729271	stage_3W_vs_E10	ordinary_muscle_development	ENSGALG00010014153	FOCAD	0.342614734	0.132507457	2.585626063	0.009720232	0.013226745	positive
PRJNA729271	stage_3W_vs_E10	ordinary_muscle_development	ENSGALG00010014154	DNAI7	-1.850915978	0.485493289	-3.812444	0.000137599	0.000222402	negative
PRJNA729271	stage_3W_vs_E10	ordinary_muscle_development	ENSGALG00010014155	ENSGALG00010014155	-4.909777187	1.43208803	-3.428404599	0.00060714	0.000927442	negative
PRJNA729271	stage_3W_vs_E10	ordinary_muscle_development	ENSGALG00010014156	CD164	0.352425776	0.092213424	3.821848945	0.000132455	0.000214326	positive
PRJNA729271	stage_3W_vs_E10	ordinary_muscle_development	ENSGALG00010014159	CDC42EP1	-0.834493049	0.150380992	-5.549192355	2.86992E-08	6.00976E-08	negative
PRJNA729271	stage_3W_vs_E10	ordinary_muscle_development	ENSGALG00010014161	GFRA3	0.438262219	0.250755549	1.747766784	0.080504419	0.09848186	positive
PRJNA729271	stage_3W_vs_E10	ordinary_muscle_development	ENSGALG00010014162	COL18A1	1.038221306	0.188360788	5.511875985	3.55029E-08	7.38858E-08	positive
PRJNA729271	stage_3W_vs_E10	ordinary_muscle_development	ENSGALG00010014165	LUZP1	-1.617077816	0.120648374	-13.40322929	5.78885E-41	4.80856E-40	negative
PRJNA729271	stage_3W_vs_E10	ordinary_muscle_development	ENSGALG00010014166	FGF12	-2.163157814	0.213264941	-10.14305401	3.55799E-24	1.60914E-23	negative
PRJNA729271	stage_3W_vs_E10	ordinary_muscle_development	ENSGALG00010014167	SLC38A9	0.242433714	0.108310986	2.2383114	0.025200755	0.032772739	positive
PRJNA729271	stage_3W_vs_E10	ordinary_muscle_development	ENSGALG00010014170	CEMIP2	-0.784378048	0.106710444	-7.350527427	1.97426E-13	5.5128E-13	negative
PRJNA729271	stage_3W_vs_E10	ordinary_muscle_development	ENSGALG00010014171	DLC1	0.071624253	0.132809118	0.539302226	0.589678333	0.625266089	positive
PRJNA729271	stage_3W_vs_E10	ordinary_muscle_development	ENSGALG00010014173	MND1	-2.395245574	0.203919642	-11.74602679	7.4018E-32	4.56355E-31	negative
PRJNA729271	stage_3W_vs_E10	ordinary_muscle_development	ENSGALG00010014176	ABHD17B	-0.202648573	0.089665023	-2.260062687	0.023817361	0.031068452	negative
PRJNA729271	stage_3W_vs_E10	ordinary_muscle_development	ENSGALG00010014178	LRTM2	-1.927200511	0.462653306	-4.165539259	3.10617E-05	5.27707E-05	negative
PRJNA729271	stage_3W_vs_E10	ordinary_muscle_development	ENSGALG00010014179	CTBPL	-0.743735533	0.042651398	-17.43754158	4.28097E-68	7.89474E-67	negative
PRJNA729271	stage_3W_vs_E10	ordinary_muscle_development	ENSGALG00010014180	SLC19A1	-0.449214496	0.144288107	-3.113316176	0.001849977	0.002700717	negative
PRJNA729271	stage_3W_vs_E10	ordinary_muscle_development	ENSGALG00010014184	SLC44A1	0.733844369	0.132300556	5.546797296	2.9095E-08	6.08999E-08	positive
PRJNA729271	stage_3W_vs_E10	ordinary_muscle_development	ENSGALG00010014186	MOCS3	-0.462940209	0.108822659	-4.254079185	2.09911E-05	3.61877E-05	negative
PRJNA729271	stage_3W_vs_E10	ordinary_muscle_development	ENSGALG00010014189	DDX4	-2.508836442	0.596399689	-4.206636067	2.592E-05	4.43212E-05	negative
PRJNA729271	stage_3W_vs_E10	ordinary_muscle_development	ENSGALG00010014190	HACD4	2.189795283	0.219747649	9.965045333	2.16775E-23	9.48431E-23	positive
PRJNA729271	stage_3W_vs_E10	ordinary_muscle_development	ENSGALG00010014192	KDM1A	-2.008679104	0.062778137	-31.99647519	1.2207E-224	2.641E-222	negative
PRJNA729271	stage_3W_vs_E10	ordinary_muscle_development	ENSGALG00010014193	CARD10	-1.826987272	0.362322076	-5.042439839	4.59633E-07	8.90217E-07	negative
PRJNA729271	stage_3W_vs_E10	ordinary_muscle_development	ENSGALG00010014194	LRIG3	-1.849677759	0.093822488	-19.71465256	1.61421E-86	4.87458E-85	negative
PRJNA729271	stage_3W_vs_E10	ordinary_muscle_development	ENSGALG00010014197	SH3GL2	-2.932444889	0.364096572	-8.054030482	8.01113E-16	2.52492E-15	negative
PRJNA729271	stage_3W_vs_E10	ordinary_muscle_development	ENSGALG00010014198	KDM3B	-0.61692442	0.045780666	-13.47565403	2.17549E-41	1.83123E-40	negative
PRJNA729271	stage_3W_vs_E10	ordinary_muscle_development	ENSGALG00010014199	LNX1	-1.614330426	0.260105102	-6.206454299	5.41934E-10	1.25705E-09	negative
PRJNA729271	stage_3W_vs_E10	ordinary_muscle_development	ENSGALG00010014200	DCP1B	0.056308913	0.089396486	0.629878373	0.528774164	0.566655442	positive
PRJNA729271	stage_3W_vs_E10	ordinary_muscle_development	ENSGALG00010014203	TMEM38B	1.543170049	0.168995633	9.131419695	6.76068E-20	2.57343E-19	positive
PRJNA729271	stage_3W_vs_E10	ordinary_muscle_development	ENSGALG00010014204	KIFAP3	-0.859639315	0.084778628	-10.13981162	3.67808E-24	1.6619E-23	negative
PRJNA729271	stage_3W_vs_E10	ordinary_muscle_development	ENSGALG00010014205	DPM1	-0.162788418	0.085406685	-1.906038355	0.05664523	0.070635978	negative
PRJNA729271	stage_3W_vs_E10	ordinary_muscle_development	ENSGALG00010014209	CCDC50	-0.711555872	0.107640725	-6.610470833	3.831E-11	9.48749E-11	negative
PRJNA729271	stage_3W_vs_E10	ordinary_muscle_development	ENSGALG00010014210	ENSGALG00010014210	0.835679599	0.247262755	3.379722914	0.000725589	0.00110107	positive
PRJNA729271	stage_3W_vs_E10	ordinary_muscle_development	ENSGALG00010014213	ETFRF1	0.991970324	0.150928584	6.572448353	4.94946E-11	1.21577E-10	positive
PRJNA729271	stage_3W_vs_E10	ordinary_muscle_development	ENSGALG00010014214	REEP2	0.387998632	0.132460823	2.929157641	0.00339882	0.004848051	positive
PRJNA729271	stage_3W_vs_E10	ordinary_muscle_development	ENSGALG00010014217	RASA3	-0.263213075	0.109922224	-2.394539217	0.016641264	0.022073126	negative
PRJNA729271	stage_3W_vs_E10	ordinary_muscle_development	ENSGALG00010014218	IL31RA	1.672260802	0.165946886	10.07708458	6.97655E-24	3.11346E-23	positive
PRJNA729271	stage_3W_vs_E10	ordinary_muscle_development	ENSGALG00010014220	C9orf85	-0.488306121	0.132388117	-3.68844375	0.00022563	0.000357667	negative
PRJNA729271	stage_3W_vs_E10	ordinary_muscle_development	ENSGALG00010014221	SCYL3	1.808661113	0.130679053	13.8404823	1.45229E-43	1.32229E-42	positive
PRJNA729271	stage_3W_vs_E10	ordinary_muscle_development	ENSGALG00010014222	TBX22	-1.739081786	0.414280471	-4.197836753	2.69477E-05	4.59862E-05	negative
PRJNA729271	stage_3W_vs_E10	ordinary_muscle_development	ENSGALG00010014224	NMUR2	-6.519787199	0.967166025	-6.741125136	1.57165E-11	3.97644E-11	negative
PRJNA729271	stage_3W_vs_E10	ordinary_muscle_development	ENSGALG00010014225	ZMPSTE24	-0.357327958	0.076176357	-4.69079875	2.72141E-06	5.01039E-06	negative
PRJNA729271	stage_3W_vs_E10	ordinary_muscle_development	ENSGALG00010014226	SLC16A7	1.430294877	0.258384502	5.535528894	3.10291E-08	6.47891E-08	positive
PRJNA729271	stage_3W_vs_E10	ordinary_muscle_development	ENSGALG00010014234	GLRA1	-1.040962009	0.751767501	-1.384686099	0.166148471	0.194785029	negative
PRJNA729271	stage_3W_vs_E10	ordinary_muscle_development	ENSGALG00010014236	WDR44	-0.155436942	0.082188363	-1.891228099	0.058593902	0.072927924	negative
PRJNA729271	stage_3W_vs_E10	ordinary_muscle_development	ENSGALG00010014238	PHLDA1	-2.542161294	0.122027414	-20.83270645	2.18744E-96	8.23558E-95	negative
PRJNA729271	stage_3W_vs_E10	ordinary_muscle_development	ENSGALG00010014240	PIGV	0.437882646	0.129202876	3.389109138	0.000701201	0.001065287	positive
PRJNA729271	stage_3W_vs_E10	ordinary_muscle_development	ENSGALG00010014241	ENSGALG00010014241	-3.125769526	0.882128492	-3.543440161	0.000394943	0.000613382	negative
PRJNA729271	stage_3W_vs_E10	ordinary_muscle_development	ENSGALG00010014243	ENSGALG00010014243	0.692020266	0.143928638	4.808079017	1.52388E-06	2.84867E-06	positive
PRJNA729271	stage_3W_vs_E10	ordinary_muscle_development	ENSGALG00010014244	SESN1	4.511075876	0.369801114	12.1986541	3.16004E-34	2.11961E-33	positive
PRJNA729271	stage_3W_vs_E10	ordinary_muscle_development	ENSGALG00010014246	KRAS	-1.408887867	0.115201142	-12.22980821	2.15447E-34	1.45454E-33	negative
PRJNA729271	stage_3W_vs_E10	ordinary_muscle_development	ENSGALG00010014249	ENSGALG00010014249	0.556941569	0.100753332	5.527773207	3.24321E-08	6.76213E-08	positive
PRJNA729271	stage_3W_vs_E10	ordinary_muscle_development	ENSGALG00010014250	ENSGALG00010014250	5.149927835	0.253048891	20.35151315	4.50162E-92	1.53171E-90	positive
PRJNA729271	stage_3W_vs_E10	ordinary_muscle_development	ENSGALG00010014257	NOP16	-0.342717743	0.124670325	-2.748992131	0.005977882	0.008313288	negative
PRJNA729271	stage_3W_vs_E10	ordinary_muscle_development	ENSGALG00010014258	ENSGALG00010014258	-2.245449825	0.630086933	-3.563714317	0.000365644	0.000570016	negative
PRJNA729271	stage_3W_vs_E10	ordinary_muscle_development	ENSGALG00010014260	ADNP	-0.69868752	0.075706456	-9.228902724	2.73418E-20	1.05826E-19	negative
PRJNA729271	stage_3W_vs_E10	ordinary_muscle_development	ENSGALG00010014263	ZNF131	-0.116226335	0.119060309	-0.976197159	0.328966771	0.367193388	negative
PRJNA729271	stage_3W_vs_E10	ordinary_muscle_development	ENSGALG00010014265	ZNF462	-1.05169916	0.139938263	-7.515450988	5.67151E-14	1.63177E-13	negative
PRJNA729271	stage_3W_vs_E10	ordinary_muscle_development	ENSGALG00010014267	SCFD2	-0.809931531	0.075688777	-10.70081404	1.00888E-26	5.09537E-26	negative
PRJNA729271	stage_3W_vs_E10	ordinary_muscle_development	ENSGALG00010014272	PARD6B	-1.217241968	0.255278111	-4.768297468	1.85789E-06	3.45391E-06	negative
PRJNA729271	stage_3W_vs_E10	ordinary_muscle_development	ENSGALG00010014274	FIRRM	-1.020927023	0.151456729	-6.740417506	1.57606E-11	3.98692E-11	negative
PRJNA729271	stage_3W_vs_E10	ordinary_muscle_development	ENSGALG00010014278	G3BP1	-0.484539831	0.139482112	-3.473849269	0.000513049	0.000789537	negative
PRJNA729271	stage_3W_vs_E10	ordinary_muscle_development	ENSGALG00010014284	TMEM131L	-0.877928342	0.085198161	-10.30454568	6.72085E-25	3.13243E-24	negative
PRJNA729271	stage_3W_vs_E10	ordinary_muscle_development	ENSGALG00010014288	ARMC2	-1.801739662	0.250474875	-7.193294972	6.32461E-13	1.72095E-12	negative
PRJNA729271	stage_3W_vs_E10	ordinary_muscle_development	ENSGALG00010014291	OSTN	-2.644363382	0.486811706	-5.432004509	5.57245E-08	1.1449E-07	negative
PRJNA729271	stage_3W_vs_E10	ordinary_muscle_development	ENSGALG00010014292	NIM1K	3.159158731	0.444980561	7.099543239	1.2517E-12	3.35678E-12	positive
PRJNA729271	stage_3W_vs_E10	ordinary_muscle_development	ENSGALG00010014294	TMPO	-0.32565129	0.102089909	-3.189847969	0.001423477	0.002103935	negative
PRJNA729271	stage_3W_vs_E10	ordinary_muscle_development	ENSGALG00010014295	CACNA1C	-1.659990331	0.179746187	-9.235190803	2.57826E-20	9.98981E-20	negative
PRJNA729271	stage_3W_vs_E10	ordinary_muscle_development	ENSGALG00010014298	METTL18	-0.636417063	0.225126091	-2.826936055	0.004699571	0.006610411	negative
PRJNA729271	stage_3W_vs_E10	ordinary_muscle_development	ENSGALG00010014300	ELFN2	-0.194656229	0.438184759	-0.444233225	0.656873973	0.689705776	negative
PRJNA729271	stage_3W_vs_E10	ordinary_muscle_development	ENSGALG00010014301	ARID1A	-0.129927617	0.11948616	-1.087386332	0.276866109	0.313725317	negative
PRJNA729271	stage_3W_vs_E10	ordinary_muscle_development	ENSGALG00010014302	FOXO3	1.44712934	0.233443025	6.199068654	5.67983E-10	1.31558E-09	positive
PRJNA729271	stage_3W_vs_E10	ordinary_muscle_development	ENSGALG00010014303	GABRA2	0.280043499	0.362998058	0.77147382	0.440426134	0.478989857	positive
PRJNA729271	stage_3W_vs_E10	ordinary_muscle_development	ENSGALG00010014305	ARL10	-1.115803013	0.291130307	-3.832658383	0.000126766	0.000205419	negative
PRJNA729271	stage_3W_vs_E10	ordinary_muscle_development	ENSGALG00010014306	ENSGALG00010014306	-0.042838528	0.311453682	-0.137543817	0.890600961	0.903377252	negative
PRJNA729271	stage_3W_vs_E10	ordinary_muscle_development	ENSGALG00010014308	ENSGALG00010014308	2.253463383	0.198747359	11.3383312	8.4745E-30	4.83804E-29	positive
PRJNA729271	stage_3W_vs_E10	ordinary_muscle_development	ENSGALG00010014310	COL6A1	1.545061001	0.23773655	6.499046955	8.08304E-11	1.96156E-10	positive
PRJNA729271	stage_3W_vs_E10	ordinary_muscle_development	ENSGALG00010014312	KIAA1191	-0.215176278	0.083348578	-2.581643079	0.009833122	0.013374412	negative
PRJNA729271	stage_3W_vs_E10	ordinary_muscle_development	ENSGALG00010014314	ENSGALG00010014314	0.014418606	0.16716327	0.08625463	0.931264004	0.939299404	positive
PRJNA729271	stage_3W_vs_E10	ordinary_muscle_development	ENSGALG00010014317	RAD23B	1.206794806	0.146171341	8.256028838	1.50599E-16	4.93989E-16	positive
PRJNA729271	stage_3W_vs_E10	ordinary_muscle_development	ENSGALG00010014318	ENSGALG00010014318	2.153961782	0.464237032	4.639788802	3.48765E-06	6.3798E-06	positive
PRJNA729271	stage_3W_vs_E10	ordinary_muscle_development	ENSGALG00010014319	ENSGALG00010014319	0.144776702	0.115589479	1.252507611	0.210384957	0.242777641	positive
PRJNA729271	stage_3W_vs_E10	ordinary_muscle_development	ENSGALG00010014320	UTS2B	-3.842133578	1.118605126	-3.434754131	0.000593092	0.000906651	negative
PRJNA729271	stage_3W_vs_E10	ordinary_muscle_development	ENSGALG00010014323	KLF4	0.378071252	0.177128432	2.134447009	0.032806207	0.042093119	positive
PRJNA729271	stage_3W_vs_E10	ordinary_muscle_development	ENSGALG00010014324	COL4A2	2.811613217	0.142221762	19.76921937	5.48176E-87	1.6623E-85	positive
PRJNA729271	stage_3W_vs_E10	ordinary_muscle_development	ENSGALG00010014328	RASSF8	-1.123020036	0.092163724	-12.18505492	3.73402E-34	2.49422E-33	negative
PRJNA729271	stage_3W_vs_E10	ordinary_muscle_development	ENSGALG00010014329	IER5	1.030287626	0.154347794	6.675104321	2.47056E-11	6.18708E-11	positive
PRJNA729271	stage_3W_vs_E10	ordinary_muscle_development	ENSGALG00010014334	HMGCS1	0.131376898	0.158091709	0.83101698	0.405964027	0.444814315	positive
PRJNA729271	stage_3W_vs_E10	ordinary_muscle_development	ENSGALG00010014338	BHLHE41	1.165099159	0.223564069	5.211477689	1.87342E-07	3.72143E-07	positive
PRJNA729271	stage_3W_vs_E10	ordinary_muscle_development	ENSGALG00010014340	EFNB1	-2.621130326	0.138789061	-18.88571266	1.49506E-79	3.67928E-78	negative

PRJNA729271	stage_3W_vs_E10	ordinary_muscle_development	ENSGALG00010014344	LSG1	-0.754314226	0.088151487	-8.557022164	1.1582E-17	4.01919E-17	negative
PRJNA729271	stage_3W_vs_E10	ordinary_muscle_development	ENSGALG00010014346	AFG1L	0.719837782	0.159481022	4.513626582	6.37284E-06	1.14325E-05	positive
PRJNA729271	stage_3W_vs_E10	ordinary_muscle_development	ENSGALG00010014347	KIAA0040	2.46424716	0.331500836	7.433607678	1.05675E-13	2.99406E-13	positive
PRJNA729271	stage_3W_vs_E10	ordinary_muscle_development	ENSGALG00010014350	PTGER4	-0.653746787	0.174801586	-3.739936247	0.000184067	0.00029413	negative
PRJNA729271	stage_3W_vs_E10	ordinary_muscle_development	ENSGALG00010014355	RAB20	1.048640053	0.16527961	6.344642601	2.22942E-10	5.28549E-10	positive
PRJNA729271	stage_3W_vs_E10	ordinary_muscle_development	ENSGALG00010014359	TMEM271	0.292881969	0.387145223	0.756517069	0.449339243	0.488170613	positive
PRJNA729271	stage_3W_vs_E10	ordinary_muscle_development	ENSGALG00010014361	SSPN	2.206727955	0.149097795	14.80054055	1.4531E-49	1.61648E-48	positive
PRJNA729271	stage_3W_vs_E10	ordinary_muscle_development	ENSGALG00010014362	NAXD	1.904683246	0.106380945	17.90436484	1.09032E-71	2.19808E-70	positive
PRJNA729271	stage_3W_vs_E10	ordinary_muscle_development	ENSGALG00010014367	OPHN1	-0.217841051	0.109566051	-1.988216699	0.046787729	0.058952376	negative
PRJNA729271	stage_3W_vs_E10	ordinary_muscle_development	ENSGALG00010014371	TTC33	-0.410882734	0.101928042	-4.03110591	5.5515E-05	9.26956E-05	negative
PRJNA729271	stage_3W_vs_E10	ordinary_muscle_development	ENSGALG00010014372	CCDC171	-0.821268919	0.126910761	-6.471231539	9.72073E-11	2.34641E-10	negative
PRJNA729271	stage_3W_vs_E10	ordinary_muscle_development	ENSGALG00010014375	H2BC35	-2.584025159	0.247157246	-10.45498443	1.39022E-25	6.65935E-25	negative
PRJNA729271	stage_3W_vs_E10	ordinary_muscle_development	ENSGALG00010014379	CARS2	-0.358072161	0.08973012	-3.990545901	6.59214E-05	0.000109391	negative
PRJNA729271	stage_3W_vs_E10	ordinary_muscle_development	ENSGALG00010014380	SNX3	0.642001926	0.117362815	5.470232859	4.49445E-08	9.29079E-08	positive
PRJNA729271	stage_3W_vs_E10	ordinary_muscle_development	ENSGALG00010014388	MFNG	2.895541793	0.219255177	13.2062642	8.07328E-40	6.49402E-39	positive
PRJNA729271	stage_3W_vs_E10	ordinary_muscle_development	ENSGALG00010014389	COL6A2	1.555416818	0.264477847	5.881085445	4.07585E-09	8.96773E-09	positive
PRJNA729271	stage_3W_vs_E10	ordinary_muscle_development	ENSGALG00010014390	SPARC	1.028811359	0.159053605	6.468330962	9.90913E-11	2.39109E-10	positive
PRJNA729271	stage_3W_vs_E10	ordinary_muscle_development	ENSGALG00010014393	ATP8A1	1.622316763	0.143805422	11.28133237	1.62269E-29	9.15925E-29	positive
PRJNA729271	stage_3W_vs_E10	ordinary_muscle_development	ENSGALG00010014396	ING1	0.174048088	0.117694134	1.478817011	0.139189228	0.165094952	positive
PRJNA729271	stage_3W_vs_E10	ordinary_muscle_development	ENSGALG00010014397	PRKAA1	-0.234989751	0.076400538	-3.075760428	0.002099665	0.003051398	negative
PRJNA729271	stage_3W_vs_E10	ordinary_muscle_development	ENSGALG00010014399	GAS6	2.756329052	0.189848707	14.51855581	9.24373E-48	9.73749E-47	positive
PRJNA729271	stage_3W_vs_E10	ordinary_muscle_development	ENSGALG00010014400	RIPOR3	-0.398307343	0.100853678	-3.949358602	7.83609E-05	0.000129014	negative
PRJNA729271	stage_3W_vs_E10	ordinary_muscle_development	ENSGALG00010014403	H2BC37	-1.221579031	0.188662887	-6.474930233	9.48558E-11	2.29232E-10	negative
PRJNA729271	stage_3W_vs_E10	ordinary_muscle_development	ENSGALG00010014407	YIPF6	-0.48926656	0.090928852	-5.380762511	7.4171E-08	1.51509E-07	negative
PRJNA729271	stage_3W_vs_E10	ordinary_muscle_development	ENSGALG00010014408	ENSGALG00010014408	-1.562065885	0.378746903	-4.124300085	3.71864E-05	6.28517E-05	negative
PRJNA729271	stage_3W_vs_E10	ordinary_muscle_development	ENSGALG00010014411	ATP13A3	-0.589671135	0.081133089	-7.267948751	3.64987E-13	1.00522E-12	negative
PRJNA729271	stage_3W_vs_E10	ordinary_muscle_development	ENSGALG00010014414	TMEM255B	1.138496427	0.500090653	2.276580098	0.022811312	0.029844749	positive
PRJNA729271	stage_3W_vs_E10	ordinary_muscle_development	ENSGALG00010014416	H2BC32	-3.027968718	0.270106545	-11.21027526	3.63063E-29	2.02641E-28	negative
PRJNA729271	stage_3W_vs_E10	ordinary_muscle_development	ENSGALG00010014418	RAC2	2.940743927	0.291495417	10.08847398	6.21278E-24	2.77603E-23	positive
PRJNA729271	stage_3W_vs_E10	ordinary_muscle_development	ENSGALG00010014427	TMEM267	-0.811168559	0.136460436	-5.944349754	2.77556E-09	6.16107E-09	negative
PRJNA729271	stage_3W_vs_E10	ordinary_muscle_development	ENSGALG00010014431	ENSGALG00010014431	-1.440082488	0.158259282	-9.099513612	9.07373E-20	3.43763E-19	negative
PRJNA729271	stage_3W_vs_E10	ordinary_muscle_development	ENSGALG00010014439	ENSGALG00010014439	-1.309802997	0.841825965	-1.555907102			negative
PRJNA729271	stage_3W_vs_E10	ordinary_muscle_development	ENSGALG00010014442	SHISA3	-5.746118505	0.997838986	-5.758562838	8.48331E-09	1.83887E-08	negative
PRJNA729271	stage_3W_vs_E10	ordinary_muscle_development	ENSGALG00010014449	ENSGALG00010014449	-1.318227866	0.154975448	-8.506043283	1.7997E-17	6.17289E-17	negative
PRJNA729271	stage_3W_vs_E10	ordinary_muscle_development	ENSGALG00010014453	NR2E1	1.830864045	0.334790025	5.468693528	4.53365E-08	9.37049E-08	positive
PRJNA729271	stage_3W_vs_E10	ordinary_muscle_development	ENSGALG00010014455	C4orf19	-2.311197419	0.264303604	-8.744479385	2.24046E-18	8.03451E-18	negative
PRJNA729271	stage_3W_vs_E10	ordinary_muscle_development	ENSGALG00010014465	SIMC1	2.025072247	0.147356443	13.74267872	5.63516E-43	5.00807E-42	positive
PRJNA729271	stage_3W_vs_E10	ordinary_muscle_development	ENSGALG00010014469	TMCO3	-0.496006264	0.127002882	-3.905472523	9.40414E-05	0.000153783	negative
PRJNA729271	stage_3W_vs_E10	ordinary_muscle_development	ENSGALG00010014470	RPS6KA1	-0.870258862	0.129325252	-6.729226086	1.70568E-11	4.30578E-11	negative
PRJNA729271	stage_3W_vs_E10	ordinary_muscle_development	ENSGALG00010014474	PTPN1	-0.185127524	0.077566817	-2.386684558	0.017001072	0.022523584	negative
PRJNA729271	stage_3W_vs_E10	ordinary_muscle_development	ENSGALG00010014476	C5orf34	0.140328669	0.177019694	0.792729139	0.427935649	0.466385507	positive
PRJNA729271	stage_3W_vs_E10	ordinary_muscle_development	ENSGALG00010014477	ITPR2	0.482795124	0.09796974	4.928002532	8.30745E-07	1.58193E-06	positive
PRJNA729271	stage_3W_vs_E10	ordinary_muscle_development	ENSGALG00010014482	THOC3	-1.601896874	0.087828154	-18.23899053	2.53089E-74	5.51659E-73	negative
PRJNA729271	stage_3W_vs_E10	ordinary_muscle_development	ENSGALG00010014487	OSTM1	0.437824129	0.08302256	5.27355612	1.33805E-07	2.68295E-07	positive
PRJNA729271	stage_3W_vs_E10	ordinary_muscle_development	ENSGALG00010014488	ENSGALG00010014488	-1.03287721	0.409975465	-2.519362365	0.011756759	0.01587168	negative
PRJNA729271	stage_3W_vs_E10	ordinary_muscle_development	ENSGALG00010014492	CPLX2	-2.26216999	0.349788215	-6.467256167	9.97984E-11	2.40775E-10	negative
PRJNA729271	stage_3W_vs_E10	ordinary_muscle_development	ENSGALG00010014494	KLHL9	-1.165017663	0.068238986	-17.07261112	2.37343E-65	4.10046E-64	negative
PRJNA729271	stage_3W_vs_E10	ordinary_muscle_development	ENSGALG00010014504	SEC63	-0.551916024	0.045937422	-12.01451899	2.98099E-33	1.93418E-32	negative
PRJNA729271	stage_3W_vs_E10	ordinary_muscle_development	ENSGALG00010014505	CYTH4	2.982518456	0.208970357	14.27244753	3.2497E-46	3.23742E-45	positive
PRJNA729271	stage_3W_vs_E10	ordinary_muscle_development	ENSGALG00010014512	HMGN4	-1.402401676	0.111212187	-12.6101439	1.85654E-36	1.33154E-35	negative
PRJNA729271	stage_3W_vs_E10	ordinary_muscle_development	ENSGALG00010014515	ETNK1	-1.970165657	0.113729593	-17.32324548	3.14131E-67	5.69878E-66	negative
PRJNA729271	stage_3W_vs_E10	ordinary_muscle_development	ENSGALG00010014517	DCUN1D2	2.01251986	0.126571978	15.90020073	6.3162E-57	8.76109E-56	positive
PRJNA729271	stage_3W_vs_E10	ordinary_muscle_development	ENSGALG00010014522	FAT2	-6.276099712	0.543461629	-11.54837688	7.52277E-31	4.45982E-30	negative
PRJNA729271	stage_3W_vs_E10	ordinary_muscle_development	ENSGALG00010014537	GKAP1	2.692451773	0.11969679	22.49393463	4.7587E-112	2.5083E-110	positive
PRJNA729271	stage_3W_vs_E10	ordinary_muscle_development	ENSGALG00010014539	PAIP1	-0.250399657	0.085154852	-2.940521319	0.003276605	0.00468525	negative
PRJNA729271	stage_3W_vs_E10	ordinary_muscle_development	ENSGALG00010014547	DHDDS	0.123215917	0.106593901	1.155937778	0.247706609	0.282828459	positive
PRJNA729271	stage_3W_vs_E10	ordinary_muscle_development	ENSGALG00010014550	SCML4	-6.787126018	0.984979851	-6.890624224	5.55481E-12	1.43422E-11	negative
PRJNA729271	stage_3W_vs_E10	ordinary_muscle_development	ENSGALG00010014555	VGLL1	-6.665375096	0.874120304	-7.625237701	2.43585E-14	7.12854E-14	negative
PRJNA729271	stage_3W_vs_E10	ordinary_muscle_development	ENSGALG00010014567	UTP11	0.044916327	0.115743783	0.388066866	0.697966551	0.727581817	positive
PRJNA729271	stage_3W_vs_E10	ordinary_muscle_development	ENSGALG00010014570	KIF27	-0.686695116	0.162908738	-4.215213534	2.49542E-05	4.27201E-05	negative
PRJNA729271	stage_3W_vs_E10	ordinary_muscle_development	ENSGALG00010014572	IL1RAP	0.221140405	0.124660187	1.773945712	0.07607213	0.093367107	positive
PRJNA729271	stage_3W_vs_E10	ordinary_muscle_development	ENSGALG00010014573	CEBPB	4.91319848	0.347186997	14.15144728	1.82953E-45	1.77622E-44	positive
PRJNA729271	stage_3W_vs_E10	ordinary_muscle_development	ENSGALG00010014576	HRH2	0.458706276	0.267023753	1.71784821	0.085824305	0.10468933	positive
PRJNA729271	stage_3W_vs_E10	ordinary_muscle_development	ENSGALG00010014578	ENSGALG00010014578	6.143890204	0.301071409	20.40675408	1.45642E-92	5.06253E-91	positive
PRJNA729271	stage_3W_vs_E10	ordinary_muscle_development	ENSGALG00010014579	NNT	0.289745952	0.144664901	2.00287665	0.04519053	0.057073864	positive
PRJNA729271	stage_3W_vs_E10	ordinary_muscle_development	ENSGALG00010014592	QNG1	0.043503036	0.092585126	0.469870681	0.638447412	0.671769399	positive
PRJNA729271	stage_3W_vs_E10	ordinary_muscle_development	ENSGALG00010014599	SSTR3	-0.095789944	0.367109966	-0.260929838	0.794146615	0.816973398	negative
PRJNA729271	stage_3W_vs_E10	ordinary_muscle_development	ENSGALG00010014602	FRK	-3.058228011	1.30591905	-2.341820507	0.019189941	0.025287109	negative
PRJNA729271	stage_3W_vs_E10	ordinary_muscle_development	ENSGALG00010014603	ENSGALG00010014603	4.863146256	0.221470599	21.95842824	7.1945E-107	3.4417E-105	positive
PRJNA729271	stage_3W_vs_E10	ordinary_muscle_development	ENSGALG00010014611	TMEM189	0.619267953	0.103412126	5.988349512	2.11981E-09	4.73446E-09	positive
PRJNA729271	stage_3W_vs_E10	ordinary_muscle_development	ENSGALG00010014614	GHR	0.899239002	0.090307992	9.95746866	2.33943E-23	1.02047E-22	positive
PRJNA729271	stage_3W_vs_E10	ordinary_muscle_development	ENSGALG00010014617	GRTP1	1.528871348	0.202544078	7.548338931	4.40844E-14	1.27648E-13	positive
PRJNA729271	stage_3W_vs_E10	ordinary_muscle_development	ENSGALG00010014624	GPRC6A	-0.104098428	0.448045262	-0.232339088	0.816274655	0.837656717	negative
PRJNA729271	stage_3W_vs_E10	ordinary_muscle_development	ENSGALG00010014626	C2CD5	-0.286929997	0.091404286	-3.139130663	0.001694499	0.002487267	negative
PRJNA729271	stage_3W_vs_E10	ordinary_muscle_development	ENSGALG00010014632	NWD2	-1.237888867	0.170673425	-7.252967871	4.07737E-13	1.12062E-12	negative
PRJNA729271	stage_3W_vs_E10	ordinary_muscle_development	ENSGALG00010014634	CCDC152	4.111344171	0.441788501	9.306136679	1.32567E-20	5.19199E-20	positive
PRJNA729271	stage_3W_vs_E10	ordinary_muscle_development	ENSGALG00010014641	DOCK11	0.251754744	0.096269255	2.615110554	0.008919858	0.012197485	positive
PRJNA729271	stage_3W_vs_E10	ordinary_muscle_development	ENSGALG00010014645	SLC36A1	0.022798969	0.118883947	0.191775004	0.847918452	0.865350838	positive
PRJNA729271	stage_3W_vs_E10	ordinary_muscle_development	ENSGALG00010014648	C1QTNF6	-0.063883599	0.274881617	-0.232404044	0.816224209	0.837656717	negative
PRJNA729271	stage_3W_vs_E10	ordinary_muscle_development	ENSGALG00010014653	FAM162B	2.699422954	0.480409917	5.618999233	1.92067E-08	4.07376E-08	positive
PRJNA729271	stage_3W_vs_E10	ordinary_muscle_development	ENSGALG00010014654	LAMP1	1.506104367	0.104353703	14.43268733	3.22275E-47	3.31304E-46	positive
PRJNA729271	stage_3W_vs_E10	ordinary_muscle_development	ENSGALG00010014655	FHL3	-1.665955217	0.134921438	-12.34759456	5.01897E-35	3.45442E-34	negative
PRJNA729271	stage_3W_vs_E10	ordinary_muscle_development	ENSGALG00010014658	SELENOP1	2.995366649	0.173449593	17.26937833	7.99995E-67	1.42631E-65	positive
PRJNA729271	stage_3W_vs_E10	ordinary_muscle_development	ENSGALG00010014668	GP5	-0.427618949	0.155044112	-2.758047008	0.005814784	0.008094237	negative
PRJNA729271	stage_3W_vs_E10	ordinary_muscle_development	ENSGALG00010014672	ISL1	-5.119373635	1.231354105	-4.157515384	3.21728E-05	5.45815E-05	negative
PRJNA729271	stage_3W_vs_E10	ordinary_muscle_development	ENSGALG00010014676	UBE2V1	-0.336953429	0.057784603	-5.831197426	5.5031E-09	1.20476E-08	negative
PRJNA729271	stage_3W_vs_E10	ordinary_muscle_development	ENSGALG00010014677	ENSGALG00010014677	-1.372847103	0.090157845	-15.22715085	2.3355E-52	2.85439E-51	negative
PRJNA729271	stage_3W_vs_E10	ordinary_muscle_development	ENSGALG00010014682	IL13RA1	0.700943419	0.125655718	5.578285093	2.42901E-08	5.11305E-08	positive
PRJNA729271	stage_3W_vs_E10	ordinary_muscle_development	ENSGALG00010014687	CUL4A	2.233286057	0.114213427	19.55362089	3.84256E-85	1.11619E-83	positive
PRJNA729271	stage_3W_vs_E10	ordinary_muscle_development	ENSGALG00010014690	CSF3R	4.088967796	0.365667786	11.18219311	4.98428E-29	2.75752E-28	positive
PRJNA729271	stage_3W_vs_E10	ordinary_muscle_development	ENSGALG00010014691	PABIR2	0.181302558	0.053913368	3.362849801	0.000771423	0.001166957	positive
PRJNA729271	stage_3W_vs_E10	ordinary_muscle_development	ENSGALG00010014696	SPINK5	-0.851218158	0.635398564	-1.339660185	0.180355847	0.210115577	negative
PRJNA729271	stage_3W_vs_E10	ordinary_muscle_development	ENSGALG00010014697	ENSGALG00010014697	1.220461298	0.666861121	1.830158123	0.067226298	0.083044587	positive
PRJNA729271	stage_3W_vs_E10	ordinary_muscle_development	ENSGALG00010014702	ENSGALG00010014702	0.354533448	0.268639244	1.319738109	0.186922472	0.217311616	positive
PRJNA729271	stage_3W_vs_E10	ordinary_muscle_development	ENSGALG00010014703	PCID2	-0.685506153	0.080288028	-8.538086778	1.36462E-17	4.71519E-17	negative
PRJNA729271	stage_3W_vs_E10	ordinary_muscle_development	ENSGALG00010014705	ARAP2	0.606864412	0.215881987	2.81109332	0.004937346	0.006927387	positive
PRJNA729271	stage_3W_vs_E10	ordinary_muscle_development	ENSGALG00010014707	KPNA5	1.577895121	0.129263445	12.20681621	2.85858E-34	1.92007E-33	positive
PRJNA729271	stage_3W_vs_E10	ordinary_muscle_development	ENSGALG00010014710	NPEPL1	1.467106288	0.099353774	14.76648778	2.40946E-49	2.66198E-48	positive
PRJNA729271	stage_3W_vs_E10	ordinary_muscle_development	ENSGALG00010014713	ST8SIA1	-3.081410754	0.140005783	-22.00916771	2.3527E-107	1.1482E-105	negative
PRJNA729271	stage_3W_vs_E10	ordinary_muscle_development	ENSGALG00010014716	TMEM35A	-2.20011283	0.133710514	-16.45429938	7.81206E-61	1.19196E-59	negative
PRJNA729271	stage_3W_vs_E10	ordinary_muscle_development	ENSGALG00010014717	ELOVL7	1.82108659	0.182233789	9.993133558	1.63335E-23	7.17003E-23	positive
PRJNA729271	stage_3W_vs_E10	ordinary_muscle_development	ENSGALG00010014719	ENSGALG00010014719	1.318944673	1.010010033	1.305872843	0.191595803	0.222281188	positive
PRJNA729271	stage_3W_vs_E10	ordinary_muscle_development	ENSGALG00010014722	KCTD17	-3.662377758	0.457770252	-8.00047129	1.23944E-15	3.86442E-15	negative
PRJNA729271	stage_3W_vs_E10	ordinary_muscle_development	ENSGALG00010014726	PWP2	-1.658974031	0.063020107	-26.32451962	1.0051E-152	9.9782E-151	negative
PRJNA729271	stage_3W_vs_E10	ordinary_muscle_development	ENSGALG00010014728	INTS13	0.740236681	0.111827886	6.619428357	3.60591E-11	8.94075E-11	positive
PRJNA729271	stage_3W_vs_E10	ordinary_muscle_development	ENSGALG00010014729	MRPS15	0.710121436	0.095059331	7.470297018	8.0014E-14	2.28353E-13	positive
PRJNA729271	stage_3W_vs_E10	ordinary_muscle_development	ENSGALG00010014734	ZUP1	-0.33089372	0.095913585	-3.44991506	0.000560763	0.000858498	negative
PRJNA729271	stage_3W_vs_E10	ordinary_muscle_development	ENSGALG00010014735	STX16	0.915071479	0.104390055	8.76588752	1.85311E-18	6.65861E-18	positive
PRJNA729271	stage_3W_vs_E10	ordinary_muscle_development	ENSGALG00010014736	KIF2A	-1.353295166	0.090018155	-15.03358037	4.42432E-51	5.18017E-50	negative
PRJNA729271	stage_3W_vs_E10	ordinary_muscle_development	ENSGALG00010014737	RMI1	-0.204240707	0.114183494	-1.788706052	0.073662168	0.090531891	negative
PRJNA729271	stage_3W_vs_E10	ordinary_muscle_development	ENSGALG00010014738	BRWD1	0.514868809	0.086458335	5.955109005	2.59899E-09	5.77885E-09	positive
PRJNA729271	stage_3W_vs_E10	ordinary_muscle_development	ENSGALG00010014739	TRMT2B	0.763033515	0.17577012	4.341087763	1.41779E-05	2.47601E-05	positive
PRJNA729271	stage_3W_vs_E10	ordinary_muscle_development	ENSGALG00010014740	TRMT9B	-2.957910724	0.508321233	-5.81897928	5.92081E-09	1.29523E-08	negative
PRJNA729271	stage_3W_vs_E10	ordinary_muscle_development	ENSGALG00010014742	PROZ	14.40115518	2.942242462	4.894618771	9.84965E-07	1.86775E-06	positive
PRJNA729271	stage_3W_vs_E10	ordinary_muscle_development	ENSGALG00010014744	IL2RB	5.444675916	0.424521761	12.8254342	1.18123E-37	8.77148E-37	positive
PRJNA729271	stage_3W_vs_E10	ordinary_muscle_development	ENSGALG00010014745	LSM10	1.374720718	0.182390646	7.537232575	4.8005E-14	1.38695E-13	positive
PRJNA729271	stage_3W_vs_E10	ordinary_muscle_development	ENSGALG00010014748	FGFR1OP2	0.244879786	0.107702675	2.273664853	0.022986145	0.030059922	positive
PRJNA729271	stage_3W_vs_E10	ordinary_muscle_development	ENSGALG00010014750	LONRF1	1.121937959	0.180587884	6.212697862	5.20825E-10	1.20887E-09	positive
PRJNA729271	stage_3W_vs_E10	ordinary_muscle_development	ENSGALG00010014753	ENSGALG00010014753	-2.579479757	0.457795429	-5.634568618	1.75497E-08	3.73269E-08	negative
PRJNA729271	stage_3W_vs_E10	ordinary_muscle_development	ENSGALG00010014754	DIMT1	-0.142998525	0.142386595	-1.004297667	0.315235158	0.353144262	negative
PRJNA729271	stage_3W_vs_E10	ordinary_muscle_development	ENSGALG00010014758	FXN	-0.764141538	0.101875544	-7.500735745	6.34606E-14	1.82079E-13	negative
PRJNA729271	stage_3W_vs_E10	ordinary_muscle_development	ENSGALG00010014762	STK40	0.050885667	0.104391316	0.487451151	0.625938652	0.660237285	positive
PRJNA729271	stage_3W_vs_E10	ordinary_muscle_development	ENSGALG00010014764	ENSGALG00010014764	6.679028096	2.289879682	2.916759403	0.003536885	0.005037055	positive
PRJNA729271	stage_3W_vs_E10	ordinary_muscle_development	ENSGALG00010014766	F10	0.263530542	0.215052543	1.225423976	0.220415518	0.25340442	positive
PRJNA729271	stage_3W_vs_E10	ordinary_muscle_development	ENSGALG00010014767	LRRC15	0.137370314	0.252606888	0.54381064	0.586571786	0.622108585	positive
PRJNA729271	stage_3W_vs_E10	ordinary_muscle_development	ENSGALG00010014769	CCDC69	1.017247926	0.322984445	3.149526057	0.001635355	0.002406546	positive
PRJNA729271	stage_3W_vs_E10	ordinary_muscle_development	ENSGALG00010014777	APCDD1L	-1.734294279	0.22384052	-7.747901401	9.34236E-15	2.78981E-14	negative
PRJNA729271	stage_3W_vs_E10	ordinary_muscle_development	ENSGALG00010014780	EVA1B	0.261934443	0.125493796	2.087230222	0.036867329	0.047074694	positive
PRJNA729271	stage_3W_vs_E10	ordinary_muscle_development	ENSGALG00010014783	ENSGALG00010014783	-0.181159513	0.119531208	-1.515583378	0.129624733	0.154401389	negative
PRJNA729271	stage_3W_vs_E10	ordinary_muscle_development	ENSGALG00010014789	F7	0.077350204	0.917387795	0.084315711	0.932805422	0.940723202	positive
PRJNA729271	stage_3W_vs_E10	ordinary_muscle_development	ENSGALG00010014792	ENSGALG00010014792	1.601547902	0.264186721	6.062181684	1.34287E-09	3.03618E-09	positive

PRJNA729271	stage_3W_vs_E10	ordinary_muscle_development	ENSGALG00010014793	TLR2A	1.495647558	0.174915181	8.550701823	1.22342E-17	4.23943E-17	positive
PRJNA729271	stage_3W_vs_E10	ordinary_muscle_development	ENSGALG00010014795	THRAP3	-0.414565761	0.095401806	-4.345470781	1.38977E-05	2.42825E-05	negative
PRJNA729271	stage_3W_vs_E10	ordinary_muscle_development	ENSGALG00010014796	PRLR	-1.047442962	0.249800413	-4.193119416	2.75145E-05	4.69368E-05	negative
PRJNA729271	stage_3W_vs_E10	ordinary_muscle_development	ENSGALG00010014797	ZDHHC9	0.156446052	0.09211387	1.698398434	0.089432588	0.108870862	positive
PRJNA729271	stage_3W_vs_E10	ordinary_muscle_development	ENSGALG00010014801	MPST	-0.735917903	0.087959537	-8.366550425	5.93295E-17	1.98473E-16	negative
PRJNA729271	stage_3W_vs_E10	ordinary_muscle_development	ENSGALG00010014802	ENSGALG00010014802	-2.117024277	0.4471004	-4.735008681	2.19046E-06	4.05759E-06	negative
PRJNA729271	stage_3W_vs_E10	ordinary_muscle_development	ENSGALG00010014805	SPEF2	-1.375464714	0.369853693	-3.718942758	0.000200058	0.000318699	negative
PRJNA729271	stage_3W_vs_E10	ordinary_muscle_development	ENSGALG00010014806	MAP7D1	1.471757617	0.226401411	6.500655661	7.99707E-11	1.94134E-10	positive
PRJNA729271	stage_3W_vs_E10	ordinary_muscle_development	ENSGALG00010014807	HES1	0.293514566	0.224252597	1.308856934	0.190582817	0.221229914	positive
PRJNA729271	stage_3W_vs_E10	ordinary_muscle_development	ENSGALG00010014810	ENTREP1	3.458263492	0.215850872	16.02154055	9.03829E-58	1.27815E-56	positive
PRJNA729271	stage_3W_vs_E10	ordinary_muscle_development	ENSGALG00010014812	RWDD1	-0.398693404	0.083486498	-4.775543525	1.79223E-06	3.33654E-06	negative
PRJNA729271	stage_3W_vs_E10	ordinary_muscle_development	ENSGALG00010014813	SASH3	2.597476953	0.252077782	10.30426772	6.7403E-25	3.14049E-24	positive
PRJNA729271	stage_3W_vs_E10	ordinary_muscle_development	ENSGALG00010014816	TST	0.497247405	0.108477954	4.583856796	4.56477E-06	8.26976E-06	positive
PRJNA729271	stage_3W_vs_E10	ordinary_muscle_development	ENSGALG00010014818	ENSGALG00010014818	-0.146050811	0.469507656	-0.311072267	0.755745684	0.781517706	negative
PRJNA729271	stage_3W_vs_E10	ordinary_muscle_development	ENSGALG00010014821	C3AR1L	2.852439884	0.227634142	12.5308087	5.06436E-36	3.59139E-35	positive
PRJNA729271	stage_3W_vs_E10	ordinary_muscle_development	ENSGALG00010014824	ANXA6	-0.127966517	0.090800949	-1.409308146	0.158744071	0.186632761	negative
PRJNA729271	stage_3W_vs_E10	ordinary_muscle_development	ENSGALG00010014829	ENSGALG00010014829	4.609485491	0.385902567	11.94468729	6.92102E-33	4.43306E-32	positive
PRJNA729271	stage_3W_vs_E10	ordinary_muscle_development	ENSGALG00010014830	TRAPPC3L	-2.69568375	0.552288097	-4.880937619	1.05583E-06	1.9969E-06	negative
PRJNA729271	stage_3W_vs_E10	ordinary_muscle_development	ENSGALG00010014831	VAPB	0.496195398	0.060333849	8.224162777	1.96559E-16	6.39821E-16	positive
PRJNA729271	stage_3W_vs_E10	ordinary_muscle_development	ENSGALG00010014837	APBA1	-0.800371387	0.114025534	-7.019229456	2.23095E-12	5.90211E-12	negative
PRJNA729271	stage_3W_vs_E10	ordinary_muscle_development	ENSGALG00010014839	TM7SF3	0.658716506	0.106562923	6.181479333	6.35037E-10	1.46598E-09	positive
PRJNA729271	stage_3W_vs_E10	ordinary_muscle_development	ENSGALG00010014840	NCF4	2.296407763	0.208201686	11.02972701	2.74698E-28	1.48241E-27	positive
PRJNA729271	stage_3W_vs_E10	ordinary_muscle_development	ENSGALG00010014847	PAICS	-1.628752883	0.092373054	-17.63233763	1.39095E-69	2.65637E-68	negative
PRJNA729271	stage_3W_vs_E10	ordinary_muscle_development	ENSGALG00010014851	CALHM4	0.154471823	0.459851462	0.335916781	0.736933622	0.764518527	positive
PRJNA729271	stage_3W_vs_E10	ordinary_muscle_development	ENSGALG00010014856	MMGT1	0.907198982	0.070889601	12.79734927	1.69644E-37	1.25139E-36	positive
PRJNA729271	stage_3W_vs_E10	ordinary_muscle_development	ENSGALG00010014859	TRAPPC3	0.244986864	0.128355668	1.908656373	0.056306435	0.070255812	positive
PRJNA729271	stage_3W_vs_E10	ordinary_muscle_development	ENSGALG00010014861	MED21	-0.422676303	0.097261707	-4.345762754	1.38793E-05	2.42531E-05	negative
PRJNA729271	stage_3W_vs_E10	ordinary_muscle_development	ENSGALG00010014862	ENSGALG00010014862	-0.655313565	0.334591686	-1.958547066	0.050165854	0.062940713	negative
PRJNA729271	stage_3W_vs_E10	ordinary_muscle_development	ENSGALG00010014865	TLR2	3.505824193	0.372439478	9.413137967	4.81542E-21	1.92179E-20	positive
PRJNA729271	stage_3W_vs_E10	ordinary_muscle_development	ENSGALG00010014866	FAM26E	0.401637985	0.14930104	2.690121816	0.007142594	0.009860181	positive
PRJNA729271	stage_3W_vs_E10	ordinary_muscle_development	ENSGALG00010014867	TP63	0.018423594	0.27136436	0.067892462	0.945871239	0.952971684	positive
PRJNA729271	stage_3W_vs_E10	ordinary_muscle_development	ENSGALG00010014870	ENSGALG00010014870	4.279486356	1.910531722	2.239945198	0.025094481	0.032643319	positive
PRJNA729271	stage_3W_vs_E10	ordinary_muscle_development	ENSGALG00010014873	CALHM6	1.553865077	0.685254669	2.267573132	0.023355237	0.030513237	positive
PRJNA729271	stage_3W_vs_E10	ordinary_muscle_development	ENSGALG00010014876	PCNT	-0.266077473	0.103429725	-2.572543564	0.010095425	0.01371445	negative
PRJNA729271	stage_3W_vs_E10	ordinary_muscle_development	ENSGALG00010014877	ENSGALG00010014877	-0.266781167	0.091085367	-2.928913559	0.00340149	0.004851382	negative
PRJNA729271	stage_3W_vs_E10	ordinary_muscle_development	ENSGALG00010014878	TMCO1	1.136333876	0.128855391	8.818675487	1.15822E-18	4.19394E-18	positive
PRJNA729271	stage_3W_vs_E10	ordinary_muscle_development	ENSGALG00010014880	SFRP2	-3.126645102	0.293843696	-10.64050428	1.93082E-26	9.6373E-26	negative
PRJNA729271	stage_3W_vs_E10	ordinary_muscle_development	ENSGALG00010014881	ENSGALG00010014881	-0.402736688	0.47081356	-0.855405882	0.392326488	0.431273506	negative
PRJNA729271	stage_3W_vs_E10	ordinary_muscle_development	ENSGALG00010014882	XPNPEP2	-0.739968149	0.657639439	-1.125188218	0.260509285	0.296372505	negative
PRJNA729271	stage_3W_vs_E10	ordinary_muscle_development	ENSGALG00010014883	PPAT	-0.982138454	0.122064885	-8.04603595	8.55189E-16	2.6901E-15	negative
PRJNA729271	stage_3W_vs_E10	ordinary_muscle_development	ENSGALG00010014886	RABGAP1L	0.472961308	0.069666549	6.788929753	1.12968E-11	2.88238E-11	positive
PRJNA729271	stage_3W_vs_E10	ordinary_muscle_development	ENSGALG00010014887	MTMR12	-0.105648145	0.131797854	-0.801592301	0.422788832	0.461227074	negative
PRJNA729271	stage_3W_vs_E10	ordinary_muscle_development	ENSGALG00010014889	PVALB	1.307322961	0.417483603	3.131435469	0.00173954	0.002549508	positive
PRJNA729271	stage_3W_vs_E10	ordinary_muscle_development	ENSGALG00010014893	SLC9A6	-0.326300609	0.089521329	-3.644948209	0.000267446	0.000421235	negative
PRJNA729271	stage_3W_vs_E10	ordinary_muscle_development	ENSGALG00010014894	RAB22A	-0.131144109	0.078566517	-1.669211199	0.095075531	0.115294891	negative
PRJNA729271	stage_3W_vs_E10	ordinary_muscle_development	ENSGALG00010014897	COL8A2	-3.829709134	0.25350589	-15.10698285	1.45658E-51	1.73914E-50	negative
PRJNA729271	stage_3W_vs_E10	ordinary_muscle_development	ENSGALG00010014898	ENSGALG00010014898	2.469093982	0.657233096	3.756801046	0.000172099	0.000275735	positive
PRJNA729271	stage_3W_vs_E10	ordinary_muscle_development	ENSGALG00010014900	UBLCP1	-0.159720535	0.083179534	-1.920190317	0.054833864	0.068483273	negative
PRJNA729271	stage_3W_vs_E10	ordinary_muscle_development	ENSGALG00010014902	AASDH	0.202511225	0.126403395	1.602102735	0.109132893	0.131224001	positive
PRJNA729271	stage_3W_vs_E10	ordinary_muscle_development	ENSGALG00010014903	MCF2L	2.00232705	0.153503695	13.04416187	6.86073E-39	5.3351E-38	positive
PRJNA729271	stage_3W_vs_E10	ordinary_muscle_development	ENSGALG00010014906	IFT27	-0.31608999	0.109294115	-2.892104381	0.003826708	0.005430067	negative
PRJNA729271	stage_3W_vs_E10	ordinary_muscle_development	ENSGALG00010014907	ENSGALG00010014907	-0.807628555	0.071313894	-11.32498185	9.86993E-30	5.62582E-29	negative
PRJNA729271	stage_3W_vs_E10	ordinary_muscle_development	ENSGALG00010014908	ENSGALG00010014908	-0.344357461	0.101915488	-3.378853094	0.000727889	0.001104328	negative
PRJNA729271	stage_3W_vs_E10	ordinary_muscle_development	ENSGALG00010014909	FBXL14	-0.214784525	0.097639265	-2.199776132	0.027822782	0.035998503	negative
PRJNA729271	stage_3W_vs_E10	ordinary_muscle_development	ENSGALG00010014912	ENSGALG00010014912	2.994889666	0.312599686	9.580590772	9.64914E-22	3.96441E-21	positive
PRJNA729271	stage_3W_vs_E10	ordinary_muscle_development	ENSGALG00010014914	PTAR1	0.182630733	0.180374303	1.012509712	0.311294405	0.349161372	positive
PRJNA729271	stage_3W_vs_E10	ordinary_muscle_development	ENSGALG00010014916	DYNLT2B	-0.040134166	0.152957287	-0.262388059	0.793022278	0.816166741	negative
PRJNA729271	stage_3W_vs_E10	ordinary_muscle_development	ENSGALG00010014917	EIF3D	0.349629719	0.105018815	3.329210292	0.000870926	0.001311865	positive
PRJNA729271	stage_3W_vs_E10	ordinary_muscle_development	ENSGALG00010014918	PLRG1	-0.384578878	0.084418586	-4.555618596	5.22316E-06	9.42484E-06	negative
PRJNA729271	stage_3W_vs_E10	ordinary_muscle_development	ENSGALG00010014922	MAN1A1	0.559473758	0.164558352	3.399850275	0.000674228	0.001026137	positive
PRJNA729271	stage_3W_vs_E10	ordinary_muscle_development	ENSGALG00010014923	CIMIP1	-2.837641962	0.436197399	-6.505407804	7.74829E-11	1.88284E-10	negative
PRJNA729271	stage_3W_vs_E10	ordinary_muscle_development	ENSGALG00010014928	SUCO	-1.048022037	0.081657927	-12.83429643	1.05356E-37	7.84351E-37	negative
PRJNA729271	stage_3W_vs_E10	ordinary_muscle_development	ENSGALG00010014929	FHL1	1.146076438	0.284931342	4.02228983	5.76351E-05	9.60916E-05	positive
PRJNA729271	stage_3W_vs_E10	ordinary_muscle_development	ENSGALG00010014931	RNF145	-0.34132118	0.108700956	-3.140001647	0.001689469	0.002480773	negative
PRJNA729271	stage_3W_vs_E10	ordinary_muscle_development	ENSGALG00010014934	MAMDC2	1.776218308	0.203828199	8.71429135	2.92583E-18	1.04587E-17	positive
PRJNA729271	stage_3W_vs_E10	ordinary_muscle_development	ENSGALG00010014935	PCYT1A	0.050378018	0.131960082	0.3817671	0.702634124	0.731921065	positive
PRJNA729271	stage_3W_vs_E10	ordinary_muscle_development	ENSGALG00010014936	FOXRED2	-0.802894047	0.172553473	-4.653015872	3.27115E-06	5.99435E-06	negative
PRJNA729271	stage_3W_vs_E10	ordinary_muscle_development	ENSGALG00010014940	VAMP4	-0.015672587	0.084570915	-0.185318877	0.852978933	0.869719304	negative
PRJNA729271	stage_3W_vs_E10	ordinary_muscle_development	ENSGALG00010014942	PMEPA1	-1.896136629	0.147365411	-12.86690418	6.91173E-38	5.17755E-37	negative
PRJNA729271	stage_3W_vs_E10	ordinary_muscle_development	ENSGALG00010014943	APLN	-0.638941749	0.307549406	-2.077525553	0.037753079	0.048137833	negative
PRJNA729271	stage_3W_vs_E10	ordinary_muscle_development	ENSGALG00010014944	FGB	0.27989192	0.919064051	0.304540167			positive
PRJNA729271	stage_3W_vs_E10	ordinary_muscle_development	ENSGALG00010014945	TXN2	0.73753615	0.104364293	7.06693955	1.58388E-12	4.22727E-12	positive
PRJNA729271	stage_3W_vs_E10	ordinary_muscle_development	ENSGALG00010014946	PCK1	3.833097666	1.042102665	3.678234202	0.000234854	0.000371598	positive
PRJNA729271	stage_3W_vs_E10	ordinary_muscle_development	ENSGALG00010014948	RBM38	3.923489155	0.258951911	15.15141999	7.41471E-52	8.89704E-51	positive
PRJNA729271	stage_3W_vs_E10	ordinary_muscle_development	ENSGALG00010014949	AGO3	-0.08178065	0.109237898	-0.748647234	0.454069855	0.492687414	negative
PRJNA729271	stage_3W_vs_E10	ordinary_muscle_development	ENSGALG00010014952	C21orf58	-1.30860607	0.641481237	-2.039975597	0.041352756	0.052446251	negative
PRJNA729271	stage_3W_vs_E10	ordinary_muscle_development	ENSGALG00010014953	ENSGALG00010014953	2.144079264	0.325886029	6.579230379	4.7289E-11	1.16415E-10	positive
PRJNA729271	stage_3W_vs_E10	ordinary_muscle_development	ENSGALG00010014956	FGG	2.266701053	1.532231063	1.479346756	0.139047663	0.164947694	positive
PRJNA729271	stage_3W_vs_E10	ordinary_muscle_development	ENSGALG00010014965	ABHD13	0.148180444	0.099054513	1.495948442	0.134667091	0.160013074	positive
PRJNA729271	stage_3W_vs_E10	ordinary_muscle_development	ENSGALG00010014967	HNRNPH1	-0.816801569	0.107732134	-7.581782166	3.4084E-14	9.90274E-14	negative
PRJNA729271	stage_3W_vs_E10	ordinary_muscle_development	ENSGALG00010014970	SLC51A	1.000098971	0.505065852	1.980135792	0.047688272	0.060029658	positive
PRJNA729271	stage_3W_vs_E10	ordinary_muscle_development	ENSGALG00010014971	SMC5	-0.526374334	0.125191488	-4.204553706	2.61598E-05	4.47101E-05	negative
PRJNA729271	stage_3W_vs_E10	ordinary_muscle_development	ENSGALG00010014972	FGA	1.054858911	1.667970578	0.632420574	0.527112093	0.565124985	positive
PRJNA729271	stage_3W_vs_E10	ordinary_muscle_development	ENSGALG00010014974	LIG4	0.513908028	0.11125966	4.618996922	3.856E-06	7.02964E-06	positive
PRJNA729271	stage_3W_vs_E10	ordinary_muscle_development	ENSGALG00010014976	ITGB2	3.182672299	0.209446371	15.19564308	3.77936E-52	4.58426E-51	positive
PRJNA729271	stage_3W_vs_E10	ordinary_muscle_development	ENSGALG00010014980	ENSGALG00010014980	0.888534514	0.72739416	1.221530999	0.221885036	0.254992753	positive
PRJNA729271	stage_3W_vs_E10	ordinary_muscle_development	ENSGALG00010014982	ENSGALG00010014982	1.144198019	0.324901636	3.52167515	0.000428829	0.000664232	positive
PRJNA729271	stage_3W_vs_E10	ordinary_muscle_development	ENSGALG00010014984	LPP	0.495444676	0.092572541	5.351961516	8.70059E-08	1.76904E-07	positive
PRJNA729271	stage_3W_vs_E10	ordinary_muscle_development	ENSGALG00010014985	ENSGALG00010014985	0.195648654	0.107631663	1.817761133	0.069100649	0.085192984	positive
PRJNA729271	stage_3W_vs_E10	ordinary_muscle_development	ENSGALG00010014987	KLF9	7.571459602	0.289971231	26.11107172	2.7295E-150	2.6029E-148	positive
PRJNA729271	stage_3W_vs_E10	ordinary_muscle_development	ENSGALG00010014990	AGO1	-1.181972791	0.048369856	-24.43614453	7.0648E-132	4.9954E-130	negative
PRJNA729271	stage_3W_vs_E10	ordinary_muscle_development	ENSGALG00010014992	ARL15	-1.2155723	0.09830517	-12.36529374	4.02736E-35	2.78382E-34	negative
PRJNA729271	stage_3W_vs_E10	ordinary_muscle_development	ENSGALG00010014994	ENSGALG00010014994	0.337649247	0.119033569	2.83658844	0.004559835	0.006422583	positive
PRJNA729271	stage_3W_vs_E10	ordinary_muscle_development	ENSGALG00010014995	LRAT	-0.87844343	0.245343244	-3.580467163	0.00034298	0.000536242	negative
PRJNA729271	stage_3W_vs_E10	ordinary_muscle_development	ENSGALG00010014997	RAE1	-0.301030131	0.089319664	-3.370255955	0.000750984	0.001137462	negative
PRJNA729271	stage_3W_vs_E10	ordinary_muscle_development	ENSGALG00010014998	NALF1	0.336198636	0.469285254	0.716405711	0.47374087	0.512491522	positive
PRJNA729271	stage_3W_vs_E10	ordinary_muscle_development	ENSGALG00010015002	ADAM19	-1.478790407	0.20160577	-7.335059952	2.21622E-13	6.17297E-13	negative
PRJNA729271	stage_3W_vs_E10	ordinary_muscle_development	ENSGALG00010015006	HSPB3	4.208809151	0.329576112	12.77037078	2.40008E-37	1.76327E-36	positive
PRJNA729271	stage_3W_vs_E10	ordinary_muscle_development	ENSGALG00010015009	AGO4	0.47392773	0.104220485	4.54735679	5.43239E-06	9.78288E-06	positive
PRJNA729271	stage_3W_vs_E10	ordinary_muscle_development	ENSGALG00010015013	SNX18	-1.101702859	0.110848884	-9.938781696	2.82258E-23	1.22715E-22	negative
PRJNA729271	stage_3W_vs_E10	ordinary_muscle_development	ENSGALG00010015016	ARGLU1	0.221425174	0.079830265	2.77369959	0.005542283	0.00773348	positive
PRJNA729271	stage_3W_vs_E10	ordinary_muscle_development	ENSGALG00010015017	FAM184A	0.43759897	0.188464031	2.321923011	0.020237082	0.026616142	positive
PRJNA729271	stage_3W_vs_E10	ordinary_muscle_development	ENSGALG00010015018	MAP7D3	-0.217009171	0.06884696	-3.152051621	0.001621276	0.002386312	negative
PRJNA729271	stage_3W_vs_E10	ordinary_muscle_development	ENSGALG00010015020	NIPAL4	-1.891010047	0.86534794	-2.185259777	0.028869805	0.037299922	negative
PRJNA729271	stage_3W_vs_E10	ordinary_muscle_development	ENSGALG00010015021	RBM46	-3.01950474	1.05184664	-2.870670139	0.004096027	0.005792947	negative
PRJNA729271	stage_3W_vs_E10	ordinary_muscle_development	ENSGALG00010015024	CRACD	-2.194283234	0.392367202	-5.592422673	2.23923E-08	4.72936E-08	negative
PRJNA729271	stage_3W_vs_E10	ordinary_muscle_development	ENSGALG00010015025	TRAF3IP1	-0.61403241	0.187450554	-3.275703366	0.001053992	0.001575987	negative
PRJNA729271	stage_3W_vs_E10	ordinary_muscle_development	ENSGALG00010015027	CLSPN	-3.300725324	0.192284413	-17.16584966	4.7843E-66	8.41608E-65	negative
PRJNA729271	stage_3W_vs_E10	ordinary_muscle_development	ENSGALG00010015028	MCM9	-2.705259571	0.275140479	-9.832284888	8.17428E-23	3.49723E-22	negative
PRJNA729271	stage_3W_vs_E10	ordinary_muscle_development	ENSGALG00010015029	TFRC	-1.457253434	0.182720982	-7.975293365	1.52019E-15	4.71745E-15	negative
PRJNA729271	stage_3W_vs_E10	ordinary_muscle_development	ENSGALG00010015031	MYH9	0.193888927	0.109563231	1.769653251	0.076784922	0.094186126	positive
PRJNA729271	stage_3W_vs_E10	ordinary_muscle_development	ENSGALG00010015032	C1orf216	-1.311584307	0.251680661	-5.211303484	1.87518E-07	3.72442E-07	negative
PRJNA729271	stage_3W_vs_E10	ordinary_muscle_development	ENSGALG00010015033	BMP7	-1.362988798	0.324602764	-4.198943902	2.68163E-05	4.57781E-05	negative
PRJNA729271	stage_3W_vs_E10	ordinary_muscle_development	ENSGALG00010015035	SLC35F1	-3.943142072	0.137911654	-28.59179743	8.4973E-180	1.2075E-177	negative
PRJNA729271	stage_3W_vs_E10	ordinary_muscle_development	ENSGALG00010015037	CYFIP2	-1.162103833	0.195687447	-5.938571175	2.87517E-09	6.37046E-09	negative
PRJNA729271	stage_3W_vs_E10	ordinary_muscle_development	ENSGALG00010015038	PCSK5	0.112112189	0.205948376	0.544370347	0.586186647	0.621791105	positive
PRJNA729271	stage_3W_vs_E10	ordinary_muscle_development	ENSGALG00010015039	FAM114A1	-0.533700169	0.127365596	-4.190300868	2.78585E-05	4.75069E-05	negative
PRJNA729271	stage_3W_vs_E10	ordinary_muscle_development	ENSGALG00010015040	ZC4H2	-1.154290265	0.088497783	-13.04315462	6.95199E-39	5.40317E-38	negative
PRJNA729271	stage_3W_vs_E10	ordinary_muscle_development	ENSGALG00010015041	TFAP2C	-7.21758998	0.749733928	-9.626868554	6.15774E-22	2.54509E-21	negative
PRJNA729271	stage_3W_vs_E10	ordinary_muscle_development	ENSGALG00010015042	EFNB2	-0.583896297	0.133730983	-4.366200602	1.26426E-05	2.21697E-05	negative
PRJNA729271	stage_3W_vs_E10	ordinary_muscle_development	ENSGALG00010015043	COQ10B	3.149636319	0.221712583	14.20594299	8.41621E-46	8.27632E-45	positive
PRJNA729271	stage_3W_vs_E10	ordinary_muscle_development	ENSGALG00010015044	ZC3H12B	-0.395777867	0.129476576	-3.056752663	0.002237488	0.003242591	negative
PRJNA729271	stage_3W_vs_E10	ordinary_muscle_development	ENSGALG00010015046	PSMB2	0.150326827	0.093713393	1.60411251	0.108689255	0.130742801	positive
PRJNA729271	stage_3W_vs_E10	ordinary_muscle_development	ENSGALG00010015047	NPY2R	-2.853734193	0.213198323	-13.38535012	7.36495E-41	6.10377E-40	negative
PRJNA729271	stage_3W_vs_E10	ordinary_muscle_development	ENSGALG00010015049	DCK2	-3.171549739	0.252494179	-12.5608826	3.46446E-36	2.4689E-35	negative
PRJNA729271	stage_3W_vs_E10	ordinary_muscle_development	ENSGALG00010015050	HIVEP3	-0.336071822	0.177243254	-1.896105013	0.05794616	0.072158899	negative
PRJNA729271	stage_3W_vs_E10	ordinary_muscle_development	ENSGALG00010015052	ENSGALG00010015052	-0.582541764	0.108764162	-5.35600839	8.50806E-08	1.73208E-07	negative
PRJNA729271	stage_3W_vs_E10	ordinary_muscle_development	ENSGALG00010015054	ENSGALG00010015054	-2.749353771	0.179357923	-15.32886718	4.90466E-53	6.12343E-52	negative
PRJNA729271	stage_3W_vs_E10	ordinary_muscle_development	ENSGALG00010015055	ZNF335	0.820628661	0.181970503	4.509679583	6.49256E-06	1.16343E-05	positive
PRJNA729271	stage_3W_vs_E10	ordinary_muscle_development	ENSGALG00010015056	ENSGALG00010015056	-1.228332857	0.495367198	-2.479641084	0.013151469	0.017641176	negative
PRJNA729271	stage_3W_vs_E10	ordinary_muscle_development	ENSGALG00010015057	LAS1L	-0.595643833	0.113132618	-5.265005299	1.40185E-07	2.80622E-07	negative

PRJNA729271	stage_3W_vs_E10	ordinary_muscle_development	ENSGALG00010015059	ENSGALG00010015059	0.2991993	0.164998295	1.813347835	0.06977819	0.085999053	positive
PRJNA729271	stage_3W_vs_E10	ordinary_muscle_development	ENSGALG00010015061	MMP9	0.447124317	0.46186955	0.968074897	0.333006966	0.371359899	positive
PRJNA729271	stage_3W_vs_E10	ordinary_muscle_development	ENSGALG00010015062	ENSGALG00010015062	0.470201955	0.230592446	2.039103891	0.04143966	0.052542676	positive
PRJNA729271	stage_3W_vs_E10	ordinary_muscle_development	ENSGALG00010015063	EEF1AKMT4	-2.561047935	0.257227094	-9.95636929	2.36544E-23	1.03119E-22	negative
PRJNA729271	stage_3W_vs_E10	ordinary_muscle_development	ENSGALG00010015064	RFK	0.328292915	0.145152851	2.261704904	0.023715642	0.030943027	positive
PRJNA729271	stage_3W_vs_E10	ordinary_muscle_development	ENSGALG00010015065	F11	5.473094302	1.205614473	4.539672029	5.63418E-06	1.01425E-05	positive
PRJNA729271	stage_3W_vs_E10	ordinary_muscle_development	ENSGALG00010015066	RUFY1	-0.275363573	0.070603257	-3.900153968	9.61315E-05	0.000157077	negative
PRJNA729271	stage_3W_vs_E10	ordinary_muscle_development	ENSGALG00010015068	ESM1	-2.325300581	0.332455661	-6.994317909	2.66553E-12	7.01212E-12	negative
PRJNA729271	stage_3W_vs_E10	ordinary_muscle_development	ENSGALG00010015069	HSPD1	-1.417972863	0.102773634	-13.79704896	2.65501E-43	2.3933E-42	negative
PRJNA729271	stage_3W_vs_E10	ordinary_muscle_development	ENSGALG00010015070	EDN2	1.402895015	0.42673598	3.287501126	0.001010808	0.001513914	positive
PRJNA729271	stage_3W_vs_E10	ordinary_muscle_development	ENSGALG00010015071	SNAI1	-2.282104359	0.130398432	-17.50101071	1.40749E-68	2.62569E-67	negative
PRJNA729271	stage_3W_vs_E10	ordinary_muscle_development	ENSGALG00010015072	MAP9	-0.599676025	0.140033468	-4.282376452	1.84908E-05	3.20296E-05	negative
PRJNA729271	stage_3W_vs_E10	ordinary_muscle_development	ENSGALG00010015075	ENSGALG00010015075	3.230611803	0.16697253	19.34816343	2.11236E-83	5.82104E-82	positive
PRJNA729271	stage_3W_vs_E10	ordinary_muscle_development	ENSGALG00010015076	ITK	3.686072525	0.567299359	6.497579223	8.16227E-11	1.98045E-10	positive
PRJNA729271	stage_3W_vs_E10	ordinary_muscle_development	ENSGALG00010015080	ASF1A	-1.139005652	0.07914082	-14.39213864	5.79751E-47	5.91378E-46	negative
PRJNA729271	stage_3W_vs_E10	ordinary_muscle_development	ENSGALG00010015083	CEP135	-2.793574068	0.200206609	-13.95345581	2.99723E-44	2.78671E-43	negative
PRJNA729271	stage_3W_vs_E10	ordinary_muscle_development	ENSGALG00010015084	FOXO6	0.794165137	0.202687449	3.918176183	8.92215E-05	0.000146198	positive
PRJNA729271	stage_3W_vs_E10	ordinary_muscle_development	ENSGALG00010015085	PSMD2	0.419827229	0.163430944	2.568835614	0.010204085	0.01385038	positive
PRJNA729271	stage_3W_vs_E10	ordinary_muscle_development	ENSGALG00010015086	FOXN3	0.303434562	0.096095779	3.157626336	0.001590593	0.002342577	positive
PRJNA729271	stage_3W_vs_E10	ordinary_muscle_development	ENSGALG00010015087	GABRA3	-5.426812999	0.808837834	-6.70939559	1.95432E-11	4.91889E-11	negative
PRJNA729271	stage_3W_vs_E10	ordinary_muscle_development	ENSGALG00010015088	ENSGALG00010015088	-1.303908364	1.085343412	-1.201378614			negative
PRJNA729271	stage_3W_vs_E10	ordinary_muscle_development	ENSGALG00010015090	GZMA	2.634071297	0.501664348	5.250664727	1.51551E-07	3.02706E-07	positive
PRJNA729271	stage_3W_vs_E10	ordinary_muscle_development	ENSGALG00010015091	MSN	-0.6780512	0.121315593	-5.589151246	2.28182E-08	4.81511E-08	negative
PRJNA729271	stage_3W_vs_E10	ordinary_muscle_development	ENSGALG00010015092	HSPE1	-0.219550429	0.122573511	-1.791173536	0.073265451	0.090090153	negative
PRJNA729271	stage_3W_vs_E10	ordinary_muscle_development	ENSGALG00010015094	CDK5RAP1	2.106134222	0.212961318	9.889750128	4.61178E-23	1.99605E-22	positive
PRJNA729271	stage_3W_vs_E10	ordinary_muscle_development	ENSGALG00010015095	RBFOX2	-0.677465724	0.133233142	-5.084813817	3.67987E-07	7.17317E-07	negative
PRJNA729271	stage_3W_vs_E10	ordinary_muscle_development	ENSGALG00010015096	GABRE	-4.80663357	1.209021987	-3.975637847	7.01909E-05	0.000116117	negative
PRJNA729271	stage_3W_vs_E10	ordinary_muscle_development	ENSGALG00010015097	MFSD2A	-1.13899013	0.165928483	-6.86434367	6.67976E-12	1.71916E-11	negative
PRJNA729271	stage_3W_vs_E10	ordinary_muscle_development	ENSGALG00010015098	HAVCR1	2.279107792	0.212759025	10.71215566	8.92588E-27	4.52064E-26	positive
PRJNA729271	stage_3W_vs_E10	ordinary_muscle_development	ENSGALG00010015100	MOB4	0.70492753	0.088739403	7.943793916	1.96089E-15	6.04876E-15	positive
PRJNA729271	stage_3W_vs_E10	ordinary_muscle_development	ENSGALG00010015101	CAP1	-1.080855483	0.099614199	-10.8504158	1.9852E-27	1.03435E-26	negative
PRJNA729271	stage_3W_vs_E10	ordinary_muscle_development	ENSGALG00010015103	ITPK1	-1.036911276	0.09309129	-11.13864979	8.13432E-29	4.46448E-28	negative
PRJNA729271	stage_3W_vs_E10	ordinary_muscle_development	ENSGALG00010015104	CNGA2	1.49596587	0.276915078	5.402255016	6.58083E-08	1.34635E-07	positive
PRJNA729271	stage_3W_vs_E10	ordinary_muscle_development	ENSGALG00010015105	ENSGALG00010015105	8.546559465	0.938432106	9.107275221	8.44769E-20	3.20464E-19	positive
PRJNA729271	stage_3W_vs_E10	ordinary_muscle_development	ENSGALG00010015107	MB	1.219029646	0.562390701	2.167584997	0.030190276	0.038919251	positive
PRJNA729271	stage_3W_vs_E10	ordinary_muscle_development	ENSGALG00010015108	ENSGALG00010015108	0.469113763	0.088320566	5.311489527	1.08733E-07	2.19663E-07	positive
PRJNA729271	stage_3W_vs_E10	ordinary_muscle_development	ENSGALG00010015109	PPT1	1.016539603	0.107389945	9.46587319	2.91118E-21	1.17313E-20	positive
PRJNA729271	stage_3W_vs_E10	ordinary_muscle_development	ENSGALG00010015110	ENSGALG00010015110	3.525938058	0.692406759	5.092292948	3.53759E-07	6.89861E-07	positive
PRJNA729271	stage_3W_vs_E10	ordinary_muscle_development	ENSGALG00010015111	ADAMTS2	1.132919536	0.142317506	7.960507262	1.71335E-15	5.29645E-15	positive
PRJNA729271	stage_3W_vs_E10	ordinary_muscle_development	ENSGALG00010015112	CBLN4	-2.662190995	0.393573908	-6.764145032	1.34099E-11	3.40354E-11	negative
PRJNA729271	stage_3W_vs_E10	ordinary_muscle_development	ENSGALG00010015115	CLDN2	-0.372361561	0.415413002	-0.896364723	0.370058002	0.408716149	negative
PRJNA729271	stage_3W_vs_E10	ordinary_muscle_development	ENSGALG00010015117	RASD2	-0.6235439	0.207859158	-2.999838472	0.002701228	0.003885126	negative
PRJNA729271	stage_3W_vs_E10	ordinary_muscle_development	ENSGALG00010015118	GPX8	-0.671469675	0.185892837	-3.612133132	0.000303689	0.00047661	negative
PRJNA729271	stage_3W_vs_E10	ordinary_muscle_development	ENSGALG00010015119	TLR1A	0.84455186	0.213065794	3.963807812	7.37636E-05	0.000121805	positive
PRJNA729271	stage_3W_vs_E10	ordinary_muscle_development	ENSGALG00010015121	UNC79	-4.491840462	0.297686891	-15.08914432	1.90902E-51	2.27186E-50	negative
PRJNA729271	stage_3W_vs_E10	ordinary_muscle_development	ENSGALG00010015122	ENSGALG00010015122	-3.893047842	0.217421876	-17.90550205	1.06828E-71	2.15664E-70	negative
PRJNA729271	stage_3W_vs_E10	ordinary_muscle_development	ENSGALG00010015123	CEP85L	1.881591081	0.250582598	7.508865716	5.96419E-14	1.7136E-13	positive
PRJNA729271	stage_3W_vs_E10	ordinary_muscle_development	ENSGALG00010015124	UXS1	0.046597778	0.095945857	0.48566743	0.627202979	0.661030054	positive
PRJNA729271	stage_3W_vs_E10	ordinary_muscle_development	ENSGALG00010015126	MCM5	-3.600406676	0.184750219	-19.48796977	1.38883E-84	3.93954E-83	negative
PRJNA729271	stage_3W_vs_E10	ordinary_muscle_development	ENSGALG00010015127	RPL11	0.60535296	0.151559115	3.994170593	6.49211E-05	0.000107793	positive
PRJNA729271	stage_3W_vs_E10	ordinary_muscle_development	ENSGALG00010015128	OCRL	0.341002097	0.106705701	3.195725196	0.001394799	0.002062811	positive
PRJNA729271	stage_3W_vs_E10	ordinary_muscle_development	ENSGALG00010015129	PRRG3	-1.587944424	0.141660326	-11.20952115	3.66169E-29	2.04296E-28	negative
PRJNA729271	stage_3W_vs_E10	ordinary_muscle_development	ENSGALG00010015131	DOK5	1.835689404	0.241452072	7.602707192	2.89999E-14	8.45782E-14	positive
PRJNA729271	stage_3W_vs_E10	ordinary_muscle_development	ENSGALG00010015132	GCNT1	-0.463349248	0.288751123	-1.604666484	0.108567222	0.130608804	negative
PRJNA729271	stage_3W_vs_E10	ordinary_muscle_development	ENSGALG00010015133	RFTN2	-1.450998042	0.128478591	-11.29369519	1.40982E-29	7.98257E-29	negative
PRJNA729271	stage_3W_vs_E10	ordinary_muscle_development	ENSGALG00010015134	PLN	4.16132408	0.521062873	7.986222576	1.39136E-15	4.32507E-15	positive
PRJNA729271	stage_3W_vs_E10	ordinary_muscle_development	ENSGALG00010015136	TCEB3	0.889764847	0.090074898	9.878055602	5.18288E-23	2.23922E-22	positive
PRJNA729271	stage_3W_vs_E10	ordinary_muscle_development	ENSGALG00010015137	AMN	0.330101963	0.480502214	0.686993635	0.492086739	0.530477263	positive
PRJNA729271	stage_3W_vs_E10	ordinary_muscle_development	ENSGALG00010015138	ENSGALG00010015138	0.450582814	0.455486159	0.98923492	0.322548217	0.360528717	positive
PRJNA729271	stage_3W_vs_E10	ordinary_muscle_development	ENSGALG00010015140	ASCC3	0.478041376	0.072644072	6.580597148	4.68563E-11	1.15389E-10	positive
PRJNA729271	stage_3W_vs_E10	ordinary_muscle_development	ENSGALG00010015141	PFDN4	0.951045641	0.135522495	7.017621991	2.25676E-12	5.96712E-12	positive
PRJNA729271	stage_3W_vs_E10	ordinary_muscle_development	ENSGALG00010015142	HNRNPAB	-1.120537682	0.128756763	-8.70274815	3.23942E-18	1.1554E-17	negative
PRJNA729271	stage_3W_vs_E10	ordinary_muscle_development	ENSGALG00010015143	EXOC1	0.273882267	0.079212126	3.457580069	0.00054505	0.000836209	positive
PRJNA729271	stage_3W_vs_E10	ordinary_muscle_development	ENSGALG00010015145	CLIC2	1.613145177	0.12467002	12.93931914	2.70012E-38	2.05882E-37	positive
PRJNA729271	stage_3W_vs_E10	ordinary_muscle_development	ENSGALG00010015146	ENSGALG00010015146	1.033205443	0.127746543	8.087932682	6.06859E-16	1.9244E-15	positive
PRJNA729271	stage_3W_vs_E10	ordinary_muscle_development	ENSGALG00010015148	ENSGALG00010015148	-0.235019655	0.076040198	-3.09072914	0.001996657	0.002906361	negative
PRJNA729271	stage_3W_vs_E10	ordinary_muscle_development	ENSGALG00010015149	ENSGALG00010015149	1.407537998	0.138414122	10.16903464	2.72597E-24	1.23865E-23	positive
PRJNA729271	stage_3W_vs_E10	ordinary_muscle_development	ENSGALG00010015151	ENSGALG00010015151	-4.709551346	0.500461337	-9.410419948	4.94159E-21	1.96943E-20	negative
PRJNA729271	stage_3W_vs_E10	ordinary_muscle_development	ENSGALG00010015152	BOLL	3.62801716	0.273778725	13.25164021	4.41449E-40	3.57874E-39	positive
PRJNA729271	stage_3W_vs_E10	ordinary_muscle_development	ENSGALG00010015153	ERCC6L	-4.136003033	0.260313388	-15.88855289	7.60633E-57	1.05204E-55	negative
PRJNA729271	stage_3W_vs_E10	ordinary_muscle_development	ENSGALG00010015154	CYP24A1	-0.922485513	0.658324397	-1.401262837	0.16113549	0.18927544	negative
PRJNA729271	stage_3W_vs_E10	ordinary_muscle_development	ENSGALG00010015155	HMOX1	-0.242785179	0.178356737	-1.361233583	0.173439885	0.202644779	negative
PRJNA729271	stage_3W_vs_E10	ordinary_muscle_development	ENSGALG00010015156	CNOT6	-0.684200773	0.074253868	-9.21434522	3.13184E-20	1.20863E-19	negative
PRJNA729271	stage_3W_vs_E10	ordinary_muscle_development	ENSGALG00010015157	CDC42BPB	-0.50856139	0.070067524	-7.258161238	3.92388E-13	1.07925E-12	negative
PRJNA729271	stage_3W_vs_E10	ordinary_muscle_development	ENSGALG00010015158	PIN4	0.367748081	0.16440805	2.236800941	0.025299353	0.032886209	positive
PRJNA729271	stage_3W_vs_E10	ordinary_muscle_development	ENSGALG00010015160	SIM1	-7.133288604	0.595670621	-11.97522315	4.79147E-33	3.08266E-32	negative
PRJNA729271	stage_3W_vs_E10	ordinary_muscle_development	ENSGALG00010015161	GFPT2	-0.21631705	0.200534432	-1.078702784	0.280720244	0.317670383	negative
PRJNA729271	stage_3W_vs_E10	ordinary_muscle_development	ENSGALG00010015163	PLK2	-0.984465091	0.209710204	-4.694407208	2.67381E-06	4.92839E-06	negative
PRJNA729271	stage_3W_vs_E10	ordinary_muscle_development	ENSGALG00010015165	KLF3	0.605896285	0.117075056	5.175280753	2.27569E-07	4.49463E-07	positive
PRJNA729271	stage_3W_vs_E10	ordinary_muscle_development	ENSGALG00010015166	YTHDF2	0.383384877	0.180779217	2.12073535	0.03394408	0.043495353	positive
PRJNA729271	stage_3W_vs_E10	ordinary_muscle_development	ENSGALG00010015167	RAB3C	-3.237158135	0.447374576	-7.23590098	4.62449E-13	1.26643E-12	negative
PRJNA729271	stage_3W_vs_E10	ordinary_muscle_development	ENSGALG00010015169	ENSGALG00010015169	-0.555502723	0.613008793	-0.906190465	0.364835049	0.403624182	negative
PRJNA729271	stage_3W_vs_E10	ordinary_muscle_development	ENSGALG00010015170	EXOC1L	-2.896910181	0.989581613	-2.927409061	0.00341799	0.004873956	negative
PRJNA729271	stage_3W_vs_E10	ordinary_muscle_development	ENSGALG00010015171	NHSL2	1.862296009	0.19973694	9.323743557	1.12306E-20	4.4116E-20	positive
PRJNA729271	stage_3W_vs_E10	ordinary_muscle_development	ENSGALG00010015172	PPP4R3A	0.25434641	0.076454675	3.326760739	0.000878618	0.001322214	positive
PRJNA729271	stage_3W_vs_E10	ordinary_muscle_development	ENSGALG00010015173	ENS-1	-0.765294245	0.692591053	-1.104972758	0.269171394	0.305675286	negative
PRJNA729271	stage_3W_vs_E10	ordinary_muscle_development	ENSGALG00010015174	ST6GAL2	-5.000245707	0.222644983	-22.45838034	1.0598E-111	5.5458E-110	negative
PRJNA729271	stage_3W_vs_E10	ordinary_muscle_development	ENSGALG00010015175	MAPK9	0.065526414	0.068992036	0.9497678	0.342230251	0.380649745	positive
PRJNA729271	stage_3W_vs_E10	ordinary_muscle_development	ENSGALG00010015177	TC2N	0.130470525	0.159044248	0.820341051	0.41202171	0.45083828	positive
PRJNA729271	stage_3W_vs_E10	ordinary_muscle_development	ENSGALG00010015180	PITHD1	1.138189931	0.12438722	9.150376819	5.67353E-20	2.16529E-19	positive
PRJNA729271	stage_3W_vs_E10	ordinary_muscle_development	ENSGALG00010015181	VPS13A	1.318885143	0.119992828	10.99136644	4.20511E-28	2.24836E-27	positive
PRJNA729271	stage_3W_vs_E10	ordinary_muscle_development	ENSGALG00010015182	PHYKPL	-0.862475058	0.325835535	-2.646964391	0.00812179	0.011150346	negative
PRJNA729271	stage_3W_vs_E10	ordinary_muscle_development	ENSGALG00010015183	SLC5A7	-5.013660022	0.76270594	-6.573516422	4.91407E-11	1.20748E-10	negative
PRJNA729271	stage_3W_vs_E10	ordinary_muscle_development	ENSGALG00010015186	ENSGALG00010015186	1.113879464	0.336621289	3.308998867	0.000936302	0.001405229	positive
PRJNA729271	stage_3W_vs_E10	ordinary_muscle_development	ENSGALG00010015188	SULT1C3	-0.986578829	1.184939544	-0.832598451	0.405071233	0.443970323	negative
PRJNA729271	stage_3W_vs_E10	ordinary_muscle_development	ENSGALG00010015190	FBLN5	0.66337113	0.192142898	3.452488411	0.000555441	0.00085089	positive
PRJNA729271	stage_3W_vs_E10	ordinary_muscle_development	ENSGALG00010015191	TOM1	5.43653374	0.186647185	29.1273278	1.6186E-186	2.5228E-184	positive
PRJNA729271	stage_3W_vs_E10	ordinary_muscle_development	ENSGALG00010015192	ENSGALG00010015192	1.665601229	0.223041012	7.46769043	8.16146E-14	2.32691E-13	positive
PRJNA729271	stage_3W_vs_E10	ordinary_muscle_development	ENSGALG00010015193	LYPLA2	-1.164282187	0.107867341	-10.7936487	3.68848E-27	1.89591E-26	negative
PRJNA729271	stage_3W_vs_E10	ordinary_muscle_development	ENSGALG00010015194	TMLHE	0.008604601	0.113488646	0.075819051	0.939563059	0.947011094	positive
PRJNA729271	stage_3W_vs_E10	ordinary_muscle_development	ENSGALG00010015196	PDE4D	1.523761815	0.21750943	7.00549773	2.46109E-12	6.49318E-12	positive
PRJNA729271	stage_3W_vs_E10	ordinary_muscle_development	ENSGALG00010015198	NME5	0.99784345	0.217452234	4.588793745	4.45815E-06	8.08266E-06	positive
PRJNA729271	stage_3W_vs_E10	ordinary_muscle_development	ENSGALG00010015199	ENSGALG00010015199	-1.037782321	0.175286672	-5.920486196	3.20991E-09	7.08831E-09	negative
PRJNA729271	stage_3W_vs_E10	ordinary_muscle_development	ENSGALG00010015200	TBC1D1	-0.59839885	0.086203898	-6.941668085	3.87497E-12	1.0093E-11	negative
PRJNA729271	stage_3W_vs_E10	ordinary_muscle_development	ENSGALG00010015201	GCC2	0.381130167	0.108447627	3.514416844	0.000440721	0.000682213	positive
PRJNA729271	stage_3W_vs_E10	ordinary_muscle_development	ENSGALG00010015203	HMGXB4	-0.559407009	0.065975503	-8.479010898	2.27116E-17	7.74418E-17	negative
PRJNA729271	stage_3W_vs_E10	ordinary_muscle_development	ENSGALG00010015204	ENSGALG00010015204	0.216692644	0.139591985	1.552328697	0.120583595	0.144117679	positive
PRJNA729271	stage_3W_vs_E10	ordinary_muscle_development	ENSGALG00010015207	AKR1E2	-0.491204504	0.178917399	-2.745426132	0.006043237	0.008398535	negative
PRJNA729271	stage_3W_vs_E10	ordinary_muscle_development	ENSGALG00010015208	GNA14	1.704674874	0.590954033	2.884615009	0.003918926	0.005555485	positive
PRJNA729271	stage_3W_vs_E10	ordinary_muscle_development	ENSGALG00010015212	GNAQ	0.403922848	0.097234674	4.15410297	3.26566E-05	5.53635E-05	positive
PRJNA729271	stage_3W_vs_E10	ordinary_muscle_development	ENSGALG00010015213	RUBCN	0.665606135	0.117999372	5.640759996	1.69301E-08	3.60197E-08	positive
PRJNA729271	stage_3W_vs_E10	ordinary_muscle_development	ENSGALG00010015218	PLCL1	-1.677401805	0.159545095	-10.51365326	7.4741E-26	3.62452E-25	negative
PRJNA729271	stage_3W_vs_E10	ordinary_muscle_development	ENSGALG00010015227	FYTTD1	-1.101676585	0.076157082	-14.46584554	1.99143E-47	2.06626E-46	negative
PRJNA729271	stage_3W_vs_E10	ordinary_muscle_development	ENSGALG00010015233	ENSGALG00010015233	-0.109212517	0.763411368	-0.143058542	0.886243947	0.899587255	negative
PRJNA729271	stage_3W_vs_E10	ordinary_muscle_development	ENSGALG00010015234	TRIP11	0.463320075	0.143510393	3.228477507	0.00124451	0.001848845	positive
PRJNA729271	stage_3W_vs_E10	ordinary_muscle_development	ENSGALG00010015241	ENSGALG00010015241	-0.144065727	0.169925024	-0.847819373	0.396538572	0.435408424	negative
PRJNA729271	stage_3W_vs_E10	ordinary_muscle_development	ENSGALG00010015248	ENSGALG00010015248	0.731330951	0.133682231	5.470666851	4.48345E-08	9.27071E-08	positive
PRJNA729271	stage_3W_vs_E10	ordinary_muscle_development	ENSGALG00010015250	RORB	-2.564215297	0.554065818	-4.627997638	3.69218E-06	6.74797E-06	negative
PRJNA729271	stage_3W_vs_E10	ordinary_muscle_development	ENSGALG00010015253	ENSGALG00010015253	-0.728501131	0.073955905	-9.850479537	6.82177E-23	2.93504E-22	negative
PRJNA729271	stage_3W_vs_E10	ordinary_muscle_development	ENSGALG00010015260	RASGEF1C	0.318095587	0.14330461	2.219716363	0.026438027	0.03428641	positive
PRJNA729271	stage_3W_vs_E10	ordinary_muscle_development	ENSGALG00010015261	IQCG	0.056115773	0.401171445	0.139879779	0.888754978	0.901694086	positive
PRJNA729271	stage_3W_vs_E10	ordinary_muscle_development	ENSGALG00010015283	ENSGALG00010015283	-3.139036098	0.943481219	-3.327078519	0.000877616	0.001320844	negative
PRJNA729271	stage_3W_vs_E10	ordinary_muscle_development	ENSGALG00010015285	ALG13	0.083388315	0.410194111	0.203289889	0.838908458	0.85742336	positive
PRJNA729271	stage_3W_vs_E10	ordinary_muscle_development	ENSGALG00010015291	ZFP64	0.254016813	0.092002586	2.760974715	0.005762914	0.008022804	positive
PRJNA729271	stage_3W_vs_E10	ordinary_muscle_development	ENSGALG00010015296	RPL35A	0.112740714	0.195392729	0.57699544	0.563942536	0.600569176	positive
PRJNA729271	stage_3W_vs_E10	ordinary_muscle_development	ENSGALG00010015298	ENSGALG00010015298	3.120319699	0.232228985	13.43639211	3.70043E-41	3.08795E-40	positive
PRJNA729271	stage_3W_vs_E10	ordinary_muscle_development	ENSGALG00010015299	GALE	-0.677978941	0.095627117	-7.089818866	1.34288E-12	3.59863E-12	negative
PRJNA729271	stage_3W_vs_E10	ordinary_muscle_development	ENSGALG00010015300	ATXN3	1.607260639	0.126719766	12.68358279	7.29209E-37	5.2823E-36	positive
PRJNA729271	stage_3W_vs_E10	ordinary_muscle_development	ENSGALG00010015306	BCAT1	1.445169852	0.090566106	15.95707177	2.5439E-57	3.55239E-56	positive
PRJNA729271	stage_3W_vs_E10	ordinary_muscle_development	ENSGALG00010015310	PGM2	-1.529778326	0.09444426	-16.1976845	5.23619E-59	7.68982E-58	negative
PRJNA729271	stage_3W_vs_E10	ordinary_muscle_development	ENSGALG00010015314	GMEB1	-0.039384327	0.067862721	-0.580352908	0.561676655	0.598551912	negative

PRJNA729271	stage_3W_vs_E10	ordinary_muscle_development	ENSGALG00010015318	SALL4	-0.75195899	0.185700012	-4.049321163	5.13664E-05	8.59467E-05	negative
PRJNA729271	stage_3W_vs_E10	ordinary_muscle_development	ENSGALG00010015320	TRPM6	-2.835885106	0.427122714	-6.639509005	3.1473E-11	7.83985E-11	negative
PRJNA729271	stage_3W_vs_E10	ordinary_muscle_development	ENSGALG00010015327	LARGE1	-0.133383564	0.126449762	-1.054834446	0.291501048	0.32874097	negative
PRJNA729271	stage_3W_vs_E10	ordinary_muscle_development	ENSGALG00010015328	ENSGALG00010015328	0.732204102	0.399679127	1.831979837	0.066954425	0.082729894	positive
PRJNA729271	stage_3W_vs_E10	ordinary_muscle_development	ENSGALG00010015333	MED7	0.118695096	0.067315148	1.763274687	0.077854172	0.095416947	positive
PRJNA729271	stage_3W_vs_E10	ordinary_muscle_development	ENSGALG00010015339	ENSGALG00010015339	-0.252787697	0.813945021	-0.310570973	0.756126797	0.781856037	negative
PRJNA729271	stage_3W_vs_E10	ordinary_muscle_development	ENSGALG00010015348	CPSF2	-0.69205658	0.061325787	-11.28491974	1.55784E-29	8.80688E-29	negative
PRJNA729271	stage_3W_vs_E10	ordinary_muscle_development	ENSGALG00010015353	RELL1	0.885212189	0.090963578	9.731501368	2.21302E-22	9.2952E-22	positive
PRJNA729271	stage_3W_vs_E10	ordinary_muscle_development	ENSGALG00010015356	ENSGALG00010015356	-0.690886556	0.086783222	-7.961061374	1.7057E-15	5.27391E-15	negative
PRJNA729271	stage_3W_vs_E10	ordinary_muscle_development	ENSGALG00010015362	ATP9A	-1.100103862	0.136264524	-8.073296172	6.84254E-16	2.16367E-15	negative
PRJNA729271	stage_3W_vs_E10	ordinary_muscle_development	ENSGALG00010015364	CLOCK	3.496621296	0.202068092	17.30417337	4.37516E-67	7.8878E-66	positive
PRJNA729271	stage_3W_vs_E10	ordinary_muscle_development	ENSGALG00010015370	ENSGALG00010015370	1.615578729	0.142655698	11.32502068	9.86556E-30	5.62554E-29	positive
PRJNA729271	stage_3W_vs_E10	ordinary_muscle_development	ENSGALG00010015374	LIMS1	-1.253756691	0.151100332	-8.297511163	1.06315E-16	3.51114E-16	negative
PRJNA729271	stage_3W_vs_E10	ordinary_muscle_development	ENSGALG00010015384	TAF12	0.179748146	0.116338895	1.545039133	0.122336798	0.146044464	positive
PRJNA729271	stage_3W_vs_E10	ordinary_muscle_development	ENSGALG00010015386	SLC24A4	0.999175043	0.245458404	4.070649143	4.68823E-05	7.86536E-05	positive
PRJNA729271	stage_3W_vs_E10	ordinary_muscle_development	ENSGALG00010015388	PSMD1	0.573222311	0.12221594	4.690241806	2.72882E-06	5.02277E-06	positive
PRJNA729271	stage_3W_vs_E10	ordinary_muscle_development	ENSGALG00010015390	CCNB3	-3.727337829	0.214699576	-17.3607135	1.63677E-67	2.99179E-66	negative
PRJNA729271	stage_3W_vs_E10	ordinary_muscle_development	ENSGALG00010015391	SYN3	-2.112127693	0.481565166	-4.385964434	1.15473E-05	2.03079E-05	negative
PRJNA729271	stage_3W_vs_E10	ordinary_muscle_development	ENSGALG00010015392	TMEM165	-0.534947759	0.081882977	-6.533076602	6.44322E-11	1.5715E-10	negative
PRJNA729271	stage_3W_vs_E10	ordinary_muscle_development	ENSGALG00010015393	DCX	-2.740686701	0.378330111	-7.244167509	4.35103E-13	1.19425E-12	negative
PRJNA729271	stage_3W_vs_E10	ordinary_muscle_development	ENSGALG00010015397	SRD5A3	-0.057497009	0.082390137	-0.697862758	0.485263027	0.523588475	negative
PRJNA729271	stage_3W_vs_E10	ordinary_muscle_development	ENSGALG00010015404	BEND4	-3.740811565	0.36871324	-10.14558514	3.46694E-24	1.56895E-23	negative
PRJNA729271	stage_3W_vs_E10	ordinary_muscle_development	ENSGALG00010015407	TIMP3	0.584501804	0.175501709	3.330462178	0.000867019	0.001306252	positive
PRJNA729271	stage_3W_vs_E10	ordinary_muscle_development	ENSGALG00010015409	FAM13B	0.251326646	0.132128691	1.902135285	0.057153471	0.071220732	positive
PRJNA729271	stage_3W_vs_E10	ordinary_muscle_development	ENSGALG00010015410	HTR2B	-1.666874443	0.215403325	-7.738387704	1.00685E-14	3.0011E-14	negative
PRJNA729271	stage_3W_vs_E10	ordinary_muscle_development	ENSGALG00010015413	CARNMT1	0.538347831	0.105996239	5.078933309	3.7956E-07	7.38784E-07	positive
PRJNA729271	stage_3W_vs_E10	ordinary_muscle_development	ENSGALG00010015416	CAPN6	2.354271494	0.16228605	14.50692463	1.09522E-47	1.14954E-46	positive
PRJNA729271	stage_3W_vs_E10	ordinary_muscle_development	ENSGALG00010015422	ENSGALG00010015422	-0.930969377	0.511355101	-1.820592726	0.068668789	0.084682159	negative
PRJNA729271	stage_3W_vs_E10	ordinary_muscle_development	ENSGALG00010015430	HMGCL	0.59291258	0.130868165	4.530609707	5.88137E-06	1.05744E-05	positive
PRJNA729271	stage_3W_vs_E10	ordinary_muscle_development	ENSGALG00010015431	RIN3	-0.481710109	0.158602457	-3.037217184	0.002387733	0.003452044	negative
PRJNA729271	stage_3W_vs_E10	ordinary_muscle_development	ENSGALG00010015436	SLC30A9	-0.099534177	0.098608658	-1.009385774	0.312789658	0.350729953	negative
PRJNA729271	stage_3W_vs_E10	ordinary_muscle_development	ENSGALG00010015440	ENSGALG00010015440	0.986247114	0.36542968	2.698869761	0.00695754	0.009612042	positive
PRJNA729271	stage_3W_vs_E10	ordinary_muscle_development	ENSGALG00010015443	PAK3	-0.134333237	0.10170736	-1.320781861	0.18657411	0.216937388	negative
PRJNA729271	stage_3W_vs_E10	ordinary_muscle_development	ENSGALG00010015449	HDX	-0.975485692	0.094161003	-10.35976321	3.77896E-25	1.78192E-24	negative
PRJNA729271	stage_3W_vs_E10	ordinary_muscle_development	ENSGALG00010015466	NMRK1	0.708659666	0.124204551	5.705585337	1.15944E-08	2.49237E-08	positive
PRJNA729271	stage_3W_vs_E10	ordinary_muscle_development	ENSGALG00010015469	TMEM33	-0.150443908	0.073146376	-2.056751348	0.03971015	0.050482251	negative
PRJNA729271	stage_3W_vs_E10	ordinary_muscle_development	ENSGALG00010015472	LGMN	0.836532063	0.133995412	6.242990321	4.29283E-10	1.00152E-09	positive
PRJNA729271	stage_3W_vs_E10	ordinary_muscle_development	ENSGALG00010015477	ARMC9	-1.233877713	0.105551748	-11.68978947	1.43742E-31	8.75808E-31	negative
PRJNA729271	stage_3W_vs_E10	ordinary_muscle_development	ENSGALG00010015478	FBXO7	-0.02067904	0.062634213	-0.330155655	0.741282352	0.768480023	negative
PRJNA729271	stage_3W_vs_E10	ordinary_muscle_development	ENSGALG00010015481	GOLGA5	0.128482879	0.071092655	1.807259537	0.070721822	0.087095396	positive
PRJNA729271	stage_3W_vs_E10	ordinary_muscle_development	ENSGALG00010015483	SGCD	1.21790847	0.162212638	7.50809852	5.99924E-14	1.72298E-13	positive
PRJNA729271	stage_3W_vs_E10	ordinary_muscle_development	ENSGALG00010015485	BCAS1	0.879746332	0.188958122	4.65577412	3.22766E-06	5.91689E-06	positive
PRJNA729271	stage_3W_vs_E10	ordinary_muscle_development	ENSGALG00010015486	MTFR1L	2.3083381	0.118441197	19.48931753	1.35273E-84	3.84467E-83	positive
PRJNA729271	stage_3W_vs_E10	ordinary_muscle_development	ENSGALG00010015487	RPS6KA6	-0.057933302	0.117774376	-0.49190073	0.622789523	0.657202543	negative
PRJNA729271	stage_3W_vs_E10	ordinary_muscle_development	ENSGALG00010015490	RANBP2	0.269627923	0.082870381	3.253610282	0.001139485	0.001700312	positive
PRJNA729271	stage_3W_vs_E10	ordinary_muscle_development	ENSGALG00010015493	OSTF1	-0.002304052	0.141317654	-0.016304063	0.986991816	0.988970439	negative
PRJNA729271	stage_3W_vs_E10	ordinary_muscle_development	ENSGALG00010015494	CHRDL1	-0.675785978	0.240070673	-2.814945994	0.004878543	0.00684886	negative
PRJNA729271	stage_3W_vs_E10	ordinary_muscle_development	ENSGALG00010015498	MID2	0.557620726	0.135459161	4.11652282	3.84631E-05	6.49718E-05	positive
PRJNA729271	stage_3W_vs_E10	ordinary_muscle_development	ENSGALG00010015502	BPIFB6	0.389581987	1.442562783	0.270062414	0.787112238	0.810599779	positive
PRJNA729271	stage_3W_vs_E10	ordinary_muscle_development	ENSGALG00010015505	CHGA	-0.304807272	0.45733396	-0.66648729	0.505099685	0.543000313	negative
PRJNA729271	stage_3W_vs_E10	ordinary_muscle_development	ENSGALG00010015507	AMMECR1	-1.401534722	0.072669064	-19.28653891	6.96851E-83	1.89155E-81	negative
PRJNA729271	stage_3W_vs_E10	ordinary_muscle_development	ENSGALG00010015508	FUCA1	1.035382794	0.103119468	10.04061418	1.01043E-23	4.4817E-23	positive
PRJNA729271	stage_3W_vs_E10	ordinary_muscle_development	ENSGALG00010015511	BPIFB4	-2.698421314	0.492662488	-5.477220978	4.32058E-08	8.94668E-08	negative
PRJNA729271	stage_3W_vs_E10	ordinary_muscle_development	ENSGALG00010015516	SELENON	-0.984443602	0.113278821	-8.690447118	3.61016E-18	1.28541E-17	negative
PRJNA729271	stage_3W_vs_E10	ordinary_muscle_development	ENSGALG00010015520	UBE3A	0.962224047	0.078162321	12.31058692	7.94434E-35	5.43175E-34	positive
PRJNA729271	stage_3W_vs_E10	ordinary_muscle_development	ENSGALG00010015521	DKC1	-1.017731717	0.082191845	-12.38239282	3.25492E-35	2.25742E-34	negative
PRJNA729271	stage_3W_vs_E10	ordinary_muscle_development	ENSGALG00010015523	MRPL22	-0.495927113	0.1455869	-3.406399304	0.000658258	0.001002594	negative
PRJNA729271	stage_3W_vs_E10	ordinary_muscle_development	ENSGALG00010015532	NXT2	-0.561137759	0.111150732	-5.048439635	4.45433E-07	8.63637E-07	negative
PRJNA729271	stage_3W_vs_E10	ordinary_muscle_development	ENSGALG00010015533	PSAT1	-1.125131953	0.277706543	-4.051514022	5.08873E-05	8.5204E-05	negative
PRJNA729271	stage_3W_vs_E10	ordinary_muscle_development	ENSGALG00010015540	ACSL4	1.118549391	0.151518881	7.382244264	1.55643E-13	4.36796E-13	positive
PRJNA729271	stage_3W_vs_E10	ordinary_muscle_development	ENSGALG00010015541	RASEF	-1.993186165	0.363561158	-5.482395794	4.19604E-08	8.69501E-08	negative
PRJNA729271	stage_3W_vs_E10	ordinary_muscle_development	ENSGALG00010015542	CNGA3	-2.273992124	0.32794398	-6.934087115	4.08853E-12	1.06359E-11	negative
PRJNA729271	stage_3W_vs_E10	ordinary_muscle_development	ENSGALG00010015544	RTCB	-0.000382076	0.073210986	-0.005218832	0.995835993	0.996523488	negative
PRJNA729271	stage_3W_vs_E10	ordinary_muscle_development	ENSGALG00010015551	VWA3B	-2.390151099	0.545298773	-4.383195451	1.16951E-05	2.05554E-05	negative
PRJNA729271	stage_3W_vs_E10	ordinary_muscle_development	ENSGALG00010015553	ASCL4	-6.519041726	1.056783178	-6.16875993	6.88276E-10	1.58635E-09	negative
PRJNA729271	stage_3W_vs_E10	ordinary_muscle_development	ENSGALG00010015555	PNRC2	-0.245091106	0.102323515	-2.395256916	0.016608723	0.022036013	negative
PRJNA729271	stage_3W_vs_E10	ordinary_muscle_development	ENSGALG00010015557	IRS4	1.01586606	0.19199189	5.291192554	1.21521E-07	2.4468E-07	positive
PRJNA729271	stage_3W_vs_E10	ordinary_muscle_development	ENSGALG00010015558	MPP1	0.826644406	0.259449878	3.186142973	0.001441833	0.002129764	positive
PRJNA729271	stage_3W_vs_E10	ordinary_muscle_development	ENSGALG00010015562	IRF9	2.077014835	0.269060393	7.719511637	1.16776E-14	3.4693E-14	positive
PRJNA729271	stage_3W_vs_E10	ordinary_muscle_development	ENSGALG00010015563	B3GNT7	-2.53705138	0.322417626	-7.868835873	3.57956E-15	1.09279E-14	negative
PRJNA729271	stage_3W_vs_E10	ordinary_muscle_development	ENSGALG00010015564	PKD2L2	-0.888256011	0.30779277	-2.885889788	0.003903088	0.005534657	negative
PRJNA729271	stage_3W_vs_E10	ordinary_muscle_development	ENSGALG00010015565	ENSGALG00010015565	-0.533652674	0.452900962	-1.178298831	0.238677494	0.273013753	negative
PRJNA729271	stage_3W_vs_E10	ordinary_muscle_development	ENSGALG00010015567	ENSGALG00010015567	4.880520555	0.426264812	11.44950372	2.36496E-30	1.37726E-29	positive
PRJNA729271	stage_3W_vs_E10	ordinary_muscle_development	ENSGALG00010015570	LIMCH1	1.24289121	0.127828975	9.723078892	2.404E-22	1.00798E-21	positive
PRJNA729271	stage_3W_vs_E10	ordinary_muscle_development	ENSGALG00010015571	DUSP15	0.624649022	0.301268985	2.073393058	0.038135715	0.048608616	positive
PRJNA729271	stage_3W_vs_E10	ordinary_muscle_development	ENSGALG00010015573	MAN1C1	1.064413434	0.122973747	8.655615233	4.9026E-18	1.73198E-17	positive
PRJNA729271	stage_3W_vs_E10	ordinary_muscle_development	ENSGALG00010015574	C14orf132	0.292253859	0.255500772	1.143847263	0.252686985	0.287970583	positive
PRJNA729271	stage_3W_vs_E10	ordinary_muscle_development	ENSGALG00010015580	GEMIN5	-0.381322171	0.071581624	-5.327095858	9.97955E-08	2.0203E-07	negative
PRJNA729271	stage_3W_vs_E10	ordinary_muscle_development	ENSGALG00010015583	PRDM4	1.004338972	0.134425614	7.471336318	7.93845E-14	2.26645E-13	positive
PRJNA729271	stage_3W_vs_E10	ordinary_muscle_development	ENSGALG00010015584	TTLL9	2.744500276	0.227042415	12.08805093	1.2215E-33	8.01887E-33	positive
PRJNA729271	stage_3W_vs_E10	ordinary_muscle_development	ENSGALG00010015585	BDKRB1	3.034356758	0.293072769	10.353595	4.03061E-25	1.89687E-24	positive
PRJNA729271	stage_3W_vs_E10	ordinary_muscle_development	ENSGALG00010015588	BDKRB2	0.183246145	0.434681508	0.42156416	0.673343171	0.704675348	positive
PRJNA729271	stage_3W_vs_E10	ordinary_muscle_development	ENSGALG00010015589	FRMD3	0.772058151	0.179500815	4.301140083	1.69922E-05	2.95042E-05	positive
PRJNA729271	stage_3W_vs_E10	ordinary_muscle_development	ENSGALG00010015596	UCHL1	-1.695659985	0.503685616	-3.366504685	0.000761273	0.001152444	negative
PRJNA729271	stage_3W_vs_E10	ordinary_muscle_development	ENSGALG00010015605	LDLRAP1	-0.221287986	0.058181672	-3.803396823	0.000142725	0.000230251	negative
PRJNA729271	stage_3W_vs_E10	ordinary_muscle_development	ENSGALG00010015606	TMEM131	-0.060050317	0.108699698	-0.552442355	0.580645318	0.616680385	negative
PRJNA729271	stage_3W_vs_E10	ordinary_muscle_development	ENSGALG00010015610	ENSGALG00010015610	1.734108213	0.286498661	6.052762019	1.42383E-09	3.21521E-09	positive
PRJNA729271	stage_3W_vs_E10	ordinary_muscle_development	ENSGALG00010015613	PWP1	-0.616320049	0.080913395	-7.617033612	2.59572E-14	7.59333E-14	negative
PRJNA729271	stage_3W_vs_E10	ordinary_muscle_development	ENSGALG00010015614	ATG2B	1.003354633	0.106859275	9.389495037	6.02886E-21	2.39551E-20	positive
PRJNA729271	stage_3W_vs_E10	ordinary_muscle_development	ENSGALG00010015616	NADK2	-0.364783156	0.118233666	-3.085273147	0.002033653	0.002957837	negative
PRJNA729271	stage_3W_vs_E10	ordinary_muscle_development	ENSGALG00010015617	SRSF10	-0.003197044	0.055703026	-0.057394439	0.954230993	0.960259528	negative
PRJNA729271	stage_3W_vs_E10	ordinary_muscle_development	ENSGALG00010015618	NCL	-1.401859033	0.101131984	-13.86167834	1.08116E-43	9.88113E-43	negative
PRJNA729271	stage_3W_vs_E10	ordinary_muscle_development	ENSGALG00010015620	PDRG1	1.84518235	0.142847615	12.91713792	3.60296E-38	2.73E-37	positive
PRJNA729271	stage_3W_vs_E10	ordinary_muscle_development	ENSGALG00010015621	IDNK	1.001248773	0.149731884	6.686944329	2.27879E-11	5.72064E-11	positive
PRJNA729271	stage_3W_vs_E10	ordinary_muscle_development	ENSGALG00010015624	ITGA1	1.500525377	0.129038119	11.62854351	2.95089E-31	1.77188E-30	positive
PRJNA729271	stage_3W_vs_E10	ordinary_muscle_development	ENSGALG00010015626	XKR7	-0.920874799	0.58587625	-1.571790629	0.115999116	0.138993732	negative
PRJNA729271	stage_3W_vs_E10	ordinary_muscle_development	ENSGALG00010015628	UBQLN1	0.729516537	0.118280034	6.167706513	6.92876E-10	1.59619E-09	positive
PRJNA729271	stage_3W_vs_E10	ordinary_muscle_development	ENSGALG00010015631	GSKIP	-0.42218891	0.106510687	-3.963817365	7.37607E-05	0.000121805	negative
PRJNA729271	stage_3W_vs_E10	ordinary_muscle_development	ENSGALG00010015632	COL4A5	0.567437491	0.16167914	3.509651845	0.000448694	0.000694002	positive
PRJNA729271	stage_3W_vs_E10	ordinary_muscle_development	ENSGALG00010015633	CCM2L	0.705212307	0.187381127	3.763518342	0.000167539	0.000268757	positive
PRJNA729271	stage_3W_vs_E10	ordinary_muscle_development	ENSGALG00010015634	ENSGALG00010015634	-1.039856177	1.147989227	-0.905806564	0.365038246	0.403787346	negative
PRJNA729271	stage_3W_vs_E10	ordinary_muscle_development	ENSGALG00010015635	ABTB3	-2.656349892	0.132676276	-20.02128768	3.59314E-89	1.15482E-87	negative
PRJNA729271	stage_3W_vs_E10	ordinary_muscle_development	ENSGALG00010015636	APBB2	0.366330927	0.124212568	2.949225946	0.00318571	0.004558427	positive
PRJNA729271	stage_3W_vs_E10	ordinary_muscle_development	ENSGALG00010015642	ENSGALG00010015642	-1.763691882	0.899081894	-1.961658769	0.049802225	0.062516953	negative
PRJNA729271	stage_3W_vs_E10	ordinary_muscle_development	ENSGALG00010015643	ENSGALG00010015643	-2.140915014	0.121391982	-17.63637915	1.29498E-69	2.47961E-68	negative
PRJNA729271	stage_3W_vs_E10	ordinary_muscle_development	ENSGALG00010015645	MACO1	0.36127834	0.094123096	3.838360162	0.000123859	0.00020091	positive
PRJNA729271	stage_3W_vs_E10	ordinary_muscle_development	ENSGALG00010015647	PDZD2	0.051601667	0.136755888	0.377326839	0.705930729	0.735090942	positive
PRJNA729271	stage_3W_vs_E10	ordinary_muscle_development	ENSGALG00010015650	ENSGALG00010015650	-0.311654599	1.28639052	-0.242270597	0.808570496	0.830750591	negative
PRJNA729271	stage_3W_vs_E10	ordinary_muscle_development	ENSGALG00010015653	HCK	2.422901629	0.515381541	4.701180456	2.58662E-06	4.77132E-06	positive
PRJNA729271	stage_3W_vs_E10	ordinary_muscle_development	ENSGALG00010015655	ENSGALG00010015655	5.586131103	0.361982906	15.43203011	9.96846E-54	1.27644E-52	positive
PRJNA729271	stage_3W_vs_E10	ordinary_muscle_development	ENSGALG00010015657	NMUR1	-3.840542958	1.196859156	-3.208851216	0.001332664	0.001974948	negative
PRJNA729271	stage_3W_vs_E10	ordinary_muscle_development	ENSGALG00010015660	KLHL3	-0.17097354	0.126226793	-1.354494839	0.175578559	0.204879344	negative
PRJNA729271	stage_3W_vs_E10	ordinary_muscle_development	ENSGALG00010015668	ZNF217	0.810544538	0.134661134	6.019142376	1.75344E-09	3.93803E-09	positive
PRJNA729271	stage_3W_vs_E10	ordinary_muscle_development	ENSGALG00010015669	INPP4A	0.435104982	0.130267953	3.34007691	0.000837552	0.001263037	positive
PRJNA729271	stage_3W_vs_E10	ordinary_muscle_development	ENSGALG00010015670	ENSGALG00010015670	-1.957186975	0.132190352	-14.80582316	1.34332E-49	1.49665E-48	negative
PRJNA729271	stage_3W_vs_E10	ordinary_muscle_development	ENSGALG00010015674	RFKL	1.688541251	0.192996567	8.749074026	2.15111E-18	7.7198E-18	positive
PRJNA729271	stage_3W_vs_E10	ordinary_muscle_development	ENSGALG00010015676	TGM2	2.009183316	0.192411759	10.44210255	1.59244E-25	7.61796E-25	positive
PRJNA729271	stage_3W_vs_E10	ordinary_muscle_development	ENSGALG00010015677	GRHL3	-3.359580688	0.277477256	-12.10758943	9.62823E-34	6.33793E-33	negative
PRJNA729271	stage_3W_vs_E10	ordinary_muscle_development	ENSGALG00010015680	ENSGALG00010015680	-0.30680929	0.10396295	-2.95114068	0.003166027	0.004531604	negative
PRJNA729271	stage_3W_vs_E10	ordinary_muscle_development	ENSGALG00010015684	PDE6D	-0.97967322	0.101191377	-9.681390337	3.61762E-22	1.50856E-21	negative
PRJNA729271	stage_3W_vs_E10	ordinary_muscle_development	ENSGALG00010015686	CNOT8	-0.897207589	0.054038213	-16.60320623	6.60657E-62	1.03976E-60	negative
PRJNA729271	stage_3W_vs_E10	ordinary_muscle_development	ENSGALG00010015691	EXOC3L4	-1.62017601	0.837048205	-1.935582683	0.052918833	0.066205635	negative
PRJNA729271	stage_3W_vs_E10	ordinary_muscle_development	ENSGALG00010015693	COA5	0.039522237	0.107774606	0.366711956	0.713833887	0.74246715	positive
PRJNA729271	stage_3W_vs_E10	ordinary_muscle_development	ENSGALG00010015695	ASAH1	1.209208798	0.093655369	12.91125982	3.88886E-38	2.93895E-37	positive
PRJNA729271	stage_3W_vs_E10	ordinary_muscle_development	ENSGALG00010015697	FAXDC2	0.347270144	0.350671385	0.990300776	0.322027127	0.360085104	positive
PRJNA729271	stage_3W_vs_E10	ordinary_muscle_development	ENSGALG00010015698	TNFAIP2	0.152123575	0.200506147	0.758697812	0.448033346	0.486897837	positive
PRJNA729271	stage_3W_vs_E10	ordinary_muscle_development	ENSGALG00010015701	UNC50	-0.201355549	0.086809221	-2.319517998	0.020366966	0.02677725	negative
PRJNA729271	stage_3W_vs_E10	ordinary_muscle_development	ENSGALG00010015703	GK5	0.194451908	0.187224156	1.038604802	0.298988572	0.336530466	positive
PRJNA729271	stage_3W_vs_E10	ordinary_muscle_development	ENSGALG00010015705	NPY6R	-4.807612757	1.15567342	-4.160009802	3.18234E-05	5.40267E-05	negative
PRJNA729271	stage_3W_vs_E10	ordinary_muscle_development	ENSGALG00010015706	ENSGALG00010015706	1.846788744	0.16014201	11.53219411	9.07997E-31	5.3742E-30	positive
PRJNA729271	stage_3W_vs_E10	ordinary_muscle_development	ENSGALG00010015709	HNRNPA0	-2.018179966	0.114593416	-17.61165723	2.00488E-69	3.79383E-68	negative

PRJNA729271	stage_3W_vs_E10	ordinary_muscle_development	ENSGALG00010015711	LBFABP	-0.289238003	0.70032713	-0.413004138	0.679603582	0.710534761	negative
PRJNA729271	stage_3W_vs_E10	ordinary_muscle_development	ENSGALG00010015716	MGAT4A	0.424287492	0.131606185	3.223917561	0.001264498	0.001877576	positive
PRJNA729271	stage_3W_vs_E10	ordinary_muscle_development	ENSGALG00010015719	XRN1	0.392219835	0.099320898	3.949016188	7.8473E-05	0.000129175	positive
PRJNA729271	stage_3W_vs_E10	ordinary_muscle_development	ENSGALG00010015729	RHCE	0.134195235	0.197602012	0.679118767	0.497062613	0.535283995	positive
PRJNA729271	stage_3W_vs_E10	ordinary_muscle_development	ENSGALG00010015731	PHF6	-1.493435339	0.070248983	-21.25917383	2.71148E-100	1.14252E-98	negative
PRJNA729271	stage_3W_vs_E10	ordinary_muscle_development	ENSGALG00010015733	COL4A6	0.251654059	0.152749775	1.647492172	0.099456924	0.120225845	positive
PRJNA729271	stage_3W_vs_E10	ordinary_muscle_development	ENSGALG00010015734	TSHZ2	-1.604460346	0.167578836	-9.57436144	1.02489E-21	4.20247E-21	negative
PRJNA729271	stage_3W_vs_E10	ordinary_muscle_development	ENSGALG00010015738	CRACDL	-3.269898821	0.45083787	-7.252937345	4.07829E-13	1.12066E-12	negative
PRJNA729271	stage_3W_vs_E10	ordinary_muscle_development	ENSGALG00010015742	HPRT1	1.396661005	0.085459545	16.34294928	4.88326E-60	7.2972E-59	positive
PRJNA729271	stage_3W_vs_E10	ordinary_muscle_development	ENSGALG00010015746	PAPOLB	-0.00984357	0.072069306	-0.136584771	0.891359015	0.903956406	negative
PRJNA729271	stage_3W_vs_E10	ordinary_muscle_development	ENSGALG00010015749	ATR	-1.262009233	0.134864035	-9.357641071	8.15367E-21	3.22652E-20	negative
PRJNA729271	stage_3W_vs_E10	ordinary_muscle_development	ENSGALG00010015751	MYOM3	-2.243674868	0.53950604	-4.158757643	3.19983E-05	5.4311E-05	negative
PRJNA729271	stage_3W_vs_E10	ordinary_muscle_development	ENSGALG00010015753	RPRD1B	-0.443004347	0.078616575	-5.634999298	1.75059E-08	3.72392E-08	negative
PRJNA729271	stage_3W_vs_E10	ordinary_muscle_development	ENSGALG00010015756	PCM1	-0.266169024	0.103254518	-2.577795424	0.009943285	0.013511569	negative
PRJNA729271	stage_3W_vs_E10	ordinary_muscle_development	ENSGALG00010015758	PCDH19	0.114666985	0.170292172	0.673354412	0.500721844	0.538824286	positive
PRJNA729271	stage_3W_vs_E10	ordinary_muscle_development	ENSGALG00010015759	EIF5	0.683622006	0.091548386	7.467329941	8.18384E-14	2.33238E-13	positive
PRJNA729271	stage_3W_vs_E10	ordinary_muscle_development	ENSGALG00010015764	TTI1	0.142500201	0.096997443	1.469112967	0.141802151	0.168009006	positive
PRJNA729271	stage_3W_vs_E10	ordinary_muscle_development	ENSGALG00010015765	ENSGALG00010015765	0.688389976	0.370678537	1.857107728	0.063295829	0.078450025	positive
PRJNA729271	stage_3W_vs_E10	ordinary_muscle_development	ENSGALG00010015766	TLE1	-0.779409561	0.101354413	-7.689942028	1.47202E-14	4.35358E-14	negative
PRJNA729271	stage_3W_vs_E10	ordinary_muscle_development	ENSGALG00010015769	TMEM50A	0.14563609	0.099782279	1.459538624	0.144416918	0.17089748	positive
PRJNA729271	stage_3W_vs_E10	ordinary_muscle_development	ENSGALG00010015774	PLS1	-2.055421683	0.374066726	-5.494799558	3.91154E-08	8.12872E-08	negative
PRJNA729271	stage_3W_vs_E10	ordinary_muscle_development	ENSGALG00010015776	SPOCK1	-6.316830842	0.275076492	-22.96390649	1.0701E-116	5.9659E-115	negative
PRJNA729271	stage_3W_vs_E10	ordinary_muscle_development	ENSGALG00010015778	PABIR3	-1.697610323	0.088152002	-19.25776265	1.21517E-82	3.26183E-81	negative
PRJNA729271	stage_3W_vs_E10	ordinary_muscle_development	ENSGALG00010015782	ATG4A	0.525551189	0.081402937	6.456169843	1.07386E-10	2.58479E-10	positive
PRJNA729271	stage_3W_vs_E10	ordinary_muscle_development	ENSGALG00010015785	FGL1	2.231223387	0.190327137	11.72309647	9.70583E-32	5.9537E-31	positive
PRJNA729271	stage_3W_vs_E10	ordinary_muscle_development	ENSGALG00010015788	TRPC1	0.012256991	0.128141185	0.095652237	0.923796776	0.932481495	positive
PRJNA729271	stage_3W_vs_E10	ordinary_muscle_development	ENSGALG00010015792	MARK3	0.337486584	0.081398649	4.146095652	3.38193E-05	5.72609E-05	positive
PRJNA729271	stage_3W_vs_E10	ordinary_muscle_development	ENSGALG00010015793	RSRP1	2.276674838	0.232748025	9.781714939	1.34905E-22	5.71434E-22	positive
PRJNA729271	stage_3W_vs_E10	ordinary_muscle_development	ENSGALG00010015795	PSMD10	-0.417273759	0.134760807	-3.096402938	0.00195884	0.002852746	negative
PRJNA729271	stage_3W_vs_E10	ordinary_muscle_development	ENSGALG00010015796	LARP1	-0.13931289	0.141212268	-0.986549486	0.323863544	0.361887301	negative
PRJNA729271	stage_3W_vs_E10	ordinary_muscle_development	ENSGALG00010015797	PIP5K1B	-0.803928407	0.165041804	-4.871059256	1.11002E-06	2.09528E-06	negative
PRJNA729271	stage_3W_vs_E10	ordinary_muscle_development	ENSGALG00010015798	IL22RA1	-1.832459184	0.286688107	-6.391821418	1.63921E-10	3.91118E-10	negative
PRJNA729271	stage_3W_vs_E10	ordinary_muscle_development	ENSGALG00010015800	BVES	3.236392381	0.181328676	17.84821048	2.98447E-71	5.93415E-70	positive
PRJNA729271	stage_3W_vs_E10	ordinary_muscle_development	ENSGALG00010015801	VSTM2L	-2.722113	0.291937277	-9.324307707	1.1171E-20	4.38938E-20	negative
PRJNA729271	stage_3W_vs_E10	ordinary_muscle_development	ENSGALG00010015802	TRPC7	-2.499592209	0.844757001	-2.958948203	0.00308691	0.004423604	negative
PRJNA729271	stage_3W_vs_E10	ordinary_muscle_development	ENSGALG00010015803	SYF2	0.400435105	0.15975639	2.506535761	0.012192072	0.016419594	positive
PRJNA729271	stage_3W_vs_E10	ordinary_muscle_development	ENSGALG00010015804	CKB	-2.258509427	0.160620263	-14.06117377	6.5786E-45	6.27347E-44	negative
PRJNA729271	stage_3W_vs_E10	ordinary_muscle_development	ENSGALG00010015807	ENSGALG00010015807	0.20363884	0.94095107	0.216418097	0.828661861	0.848985982	positive
PRJNA729271	stage_3W_vs_E10	ordinary_muscle_development	ENSGALG00010015809	PCOLCE2	4.024032689	0.231930757	17.35014686	1.96741E-67	3.58711E-66	positive
PRJNA729271	stage_3W_vs_E10	ordinary_muscle_development	ENSGALG00010015810	LIPT1	-0.074858657	0.104433167	-0.716809222	0.473491821	0.512260333	negative
PRJNA729271	stage_3W_vs_E10	ordinary_muscle_development	ENSGALG00010015811	CTNNBL1	-0.587067877	0.055867548	-10.50820908	7.91829E-26	3.83737E-25	negative
PRJNA729271	stage_3W_vs_E10	ordinary_muscle_development	ENSGALG00010015813	ENSGALG00010015813	1.505072673	0.138383829	10.8760733	1.49881E-27	7.84307E-27	positive
PRJNA729271	stage_3W_vs_E10	ordinary_muscle_development	ENSGALG00010015814	PAQR9	1.575809755	0.414067991	3.805678754	0.000141416	0.000228291	positive
PRJNA729271	stage_3W_vs_E10	ordinary_muscle_development	ENSGALG00010015816	MITD1	-0.427010136	0.215201714	-1.984232037	0.047229979	0.059478587	negative
PRJNA729271	stage_3W_vs_E10	ordinary_muscle_development	ENSGALG00010015820	ENSGALG00010015820	2.138970887	0.513491268	4.165544811	3.1061E-05	5.27707E-05	positive
PRJNA729271	stage_3W_vs_E10	ordinary_muscle_development	ENSGALG00010015822	WFDC2	-0.258055235	0.501224079	-0.514850036	0.606657818	0.642068651	negative
PRJNA729271	stage_3W_vs_E10	ordinary_muscle_development	ENSGALG00010015825	RUNX3	-2.503905547	0.506295329	-4.945543448	7.59317E-07	1.4482E-06	negative
PRJNA729271	stage_3W_vs_E10	ordinary_muscle_development	ENSGALG00010015827	ENSGALG00010015827	1.889836009	0.14761719	12.80227597	1.59215E-37	1.17566E-36	positive
PRJNA729271	stage_3W_vs_E10	ordinary_muscle_development	ENSGALG00010015829	SMAD5	-0.408702243	0.047996326	-8.515281765	1.66186E-17	5.7082E-17	negative
PRJNA729271	stage_3W_vs_E10	ordinary_muscle_development	ENSGALG00010015831	LYG2	3.949151891	0.62322106	6.336679144	2.3477E-10	5.55954E-10	positive
PRJNA729271	stage_3W_vs_E10	ordinary_muscle_development	ENSGALG00010015832	RAB39B	-1.717340506	0.0873247	-19.66614831	4.20538E-86	1.25684E-84	negative
PRJNA729271	stage_3W_vs_E10	ordinary_muscle_development	ENSGALG00010015834	STARD8	-0.116170755	0.133775216	-0.868402666	0.385173936	0.42411852	negative
PRJNA729271	stage_3W_vs_E10	ordinary_muscle_development	ENSGALG00010015837	GDA	3.022639994	0.158612568	19.05674964	5.77531E-81	1.48956E-79	positive
PRJNA729271	stage_3W_vs_E10	ordinary_muscle_development	ENSGALG00010015838	IFNLR1	-0.789206291	1.269691321	-0.621573352	0.534222448	0.572028838	negative
PRJNA729271	stage_3W_vs_E10	ordinary_muscle_development	ENSGALG00010015839	U2SURP	-0.327736839	0.071116272	-4.608464845	4.05653E-06	7.37944E-06	negative
PRJNA729271	stage_3W_vs_E10	ordinary_muscle_development	ENSGALG00010015840	MTUS1	1.885622953	0.145801263	12.93283002	2.93802E-38	2.23786E-37	positive
PRJNA729271	stage_3W_vs_E10	ordinary_muscle_development	ENSGALG00010015843	ENSGALG00010015843	-2.704958992	0.525177059	-5.150565782	2.59702E-07	5.11117E-07	negative
PRJNA729271	stage_3W_vs_E10	ordinary_muscle_development	ENSGALG00010015845	SAP30L	1.54633906	0.12516234	12.35466724	4.59654E-35	3.1712E-34	positive
PRJNA729271	stage_3W_vs_E10	ordinary_muscle_development	ENSGALG00010015848	UBE2B	2.588146099	0.161029499	16.07249674	3.97738E-58	5.68002E-57	positive
PRJNA729271	stage_3W_vs_E10	ordinary_muscle_development	ENSGALG00010015852	ZFAND5	1.998577777	0.285735522	6.994502334	2.66202E-12	7.00418E-12	positive
PRJNA729271	stage_3W_vs_E10	ordinary_muscle_development	ENSGALG00010015855	NIPAL3	-0.902489743	0.081363888	-11.09201843	1.37158E-28	7.46564E-28	negative
PRJNA729271	stage_3W_vs_E10	ordinary_muscle_development	ENSGALG00010015856	TXNDC9	0.923123738	0.132975564	6.942055466	3.86435E-12	1.00672E-11	positive
PRJNA729271	stage_3W_vs_E10	ordinary_muscle_development	ENSGALG00010015858	CHST2	-1.070697192	0.09270081	-11.55003054	7.37942E-31	4.38021E-30	negative
PRJNA729271	stage_3W_vs_E10	ordinary_muscle_development	ENSGALG00010015861	PPP2CA	-0.984229065	0.067728572	-14.53196253	7.60111E-48	8.04807E-47	negative
PRJNA729271	stage_3W_vs_E10	ordinary_muscle_development	ENSGALG00010015862	PDGFRL	0.412899959	0.209224966	1.973473654	0.048441623	0.060925061	positive
PRJNA729271	stage_3W_vs_E10	ordinary_muscle_development	ENSGALG00010015863	ENSGALG00010015863	-2.608283841	0.566208831	-4.606575699	4.09354E-06	7.44303E-06	negative
PRJNA729271	stage_3W_vs_E10	ordinary_muscle_development	ENSGALG00010015865	VRK1	-2.093796725	0.143452468	-14.59575255	2.98896E-48	3.21641E-47	negative
PRJNA729271	stage_3W_vs_E10	ordinary_muscle_development	ENSGALG00010015866	ARHGEF6	0.136520721	0.071490882	1.909624237	0.056181612	0.070109662	positive
PRJNA729271	stage_3W_vs_E10	ordinary_muscle_development	ENSGALG00010015867	PICALML	0.407386258	0.056587472	7.199230583	6.05533E-13	1.64861E-12	positive
PRJNA729271	stage_3W_vs_E10	ordinary_muscle_development	ENSGALG00010015869	SLC7A2	2.691773541	0.193793624	13.88989733	7.29398E-44	6.6873E-43	positive
PRJNA729271	stage_3W_vs_E10	ordinary_muscle_development	ENSGALG00010015870	EIF5B	0.340423299	0.156048297	2.18152524	0.02914459	0.037624762	positive
PRJNA729271	stage_3W_vs_E10	ordinary_muscle_development	ENSGALG00010015872	TMC1	-2.106653505	0.399976322	-5.266945541	1.38712E-07	2.7775E-07	negative
PRJNA729271	stage_3W_vs_E10	ordinary_muscle_development	ENSGALG00010015874	CLIC4	-1.099607526	0.114838448	-9.575255897	1.01605E-21	4.16979E-21	negative
PRJNA729271	stage_3W_vs_E10	ordinary_muscle_development	ENSGALG00010015875	DNTTIP1	1.286968209	0.138975807	9.260375884	2.03714E-20	7.91856E-20	positive
PRJNA729271	stage_3W_vs_E10	ordinary_muscle_development	ENSGALG00010015876	SKP1	-0.437883266	0.077453807	-5.65347638	1.57235E-08	3.35362E-08	negative
PRJNA729271	stage_3W_vs_E10	ordinary_muscle_development	ENSGALG00010015877	REV1	0.254300304	0.103975858	2.445762964	0.0144546	0.019305208	positive
PRJNA729271	stage_3W_vs_E10	ordinary_muscle_development	ENSGALG00010015879	TRMT61A	-1.520845379	0.124157492	-12.24932424	1.69402E-34	1.14849E-33	negative
PRJNA729271	stage_3W_vs_E10	ordinary_muscle_development	ENSGALG00010015881	GAB3	2.08253958	0.174895442	11.90734051	1.08379E-32	6.88713E-32	positive
PRJNA729271	stage_3W_vs_E10	ordinary_muscle_development	ENSGALG00010015886	AFF3	-1.466460067	0.211308142	-6.939912738	3.92342E-12	1.02156E-11	negative
PRJNA729271	stage_3W_vs_E10	ordinary_muscle_development	ENSGALG00010015887	SLC9A9	0.573022297	0.180499236	3.174652204	0.001500162	0.002212315	positive
PRJNA729271	stage_3W_vs_E10	ordinary_muscle_development	ENSGALG00010015889	SH3BP5	1.720730123	0.164949284	10.43187383	1.77356E-25	8.45927E-25	positive
PRJNA729271	stage_3W_vs_E10	ordinary_muscle_development	ENSGALG00010015892	UBE2C	-3.342270148	0.231454406	-14.44029604	2.886E-47	2.97741E-46	negative
PRJNA729271	stage_3W_vs_E10	ordinary_muscle_development	ENSGALG00010015893	TCF7	-1.902155719	0.442476535	-4.298884951	1.7166E-05	2.97917E-05	negative
PRJNA729271	stage_3W_vs_E10	ordinary_muscle_development	ENSGALG00010015894	CEP78	-2.310084191	0.122634543	-18.83714109	3.74664E-79	9.09674E-78	negative
PRJNA729271	stage_3W_vs_E10	ordinary_muscle_development	ENSGALG00010015895	RBMXL1	-1.244320874	0.085377178	-14.57439681	4.08717E-48	4.37868E-47	negative
PRJNA729271	stage_3W_vs_E10	ordinary_muscle_development	ENSGALG00010015896	LONRF2	-0.523803908	0.078933125	-6.636046715	3.22208E-11	8.02474E-11	negative
PRJNA729271	stage_3W_vs_E10	ordinary_muscle_development	ENSGALG00010015898	ENSGALG00010015898	1.618763212	0.217988216	7.425920738	1.11998E-13	3.16949E-13	positive
PRJNA729271	stage_3W_vs_E10	ordinary_muscle_development	ENSGALG00010015899	SMARCA1	-0.790138136	0.116131818	-6.8038041	1.01892E-11	2.60481E-11	negative
PRJNA729271	stage_3W_vs_E10	ordinary_muscle_development	ENSGALG00010015900	ENSGALG00010015900	1.628832806	0.158593876	10.27046472	9.57423E-25	4.4324E-24	positive
PRJNA729271	stage_3W_vs_E10	ordinary_muscle_development	ENSGALG00010015902	ENSGALG00010015902	1.947642287	0.456568043	4.265831389	1.99159E-05	3.44201E-05	positive
PRJNA729271	stage_3W_vs_E10	ordinary_muscle_development	ENSGALG00010015904	MTMR7	1.455066466	0.259783046	5.601083252	2.13016E-08	4.50295E-08	positive
PRJNA729271	stage_3W_vs_E10	ordinary_muscle_development	ENSGALG00010015905	ANPEP	0.485173969	0.217711056	2.22852242	0.025845698	0.033566293	positive
PRJNA729271	stage_3W_vs_E10	ordinary_muscle_development	ENSGALG00010015906	BAG5	1.169235062	0.100283285	11.65932152	2.05675E-31	1.24323E-30	positive
PRJNA729271	stage_3W_vs_E10	ordinary_muscle_development	ENSGALG00010015908	CHRD	0.840955077	0.137339539	6.123182596	9.17245E-10	2.09443E-09	positive
PRJNA729271	stage_3W_vs_E10	ordinary_muscle_development	ENSGALG00010015909	TNNC2	4.376384894	0.106577703	41.0628561	0	0	positive
PRJNA729271	stage_3W_vs_E10	ordinary_muscle_development	ENSGALG00010015910	C5orf15	0.569047516	0.092838443	6.129438406	8.81898E-10	2.0169E-09	positive
PRJNA729271	stage_3W_vs_E10	ordinary_muscle_development	ENSGALG00010015911	TM9SF2L	0.589636116	0.077994722	7.559948957	4.03228E-14	1.16872E-13	positive
PRJNA729271	stage_3W_vs_E10	ordinary_muscle_development	ENSGALG00010015912	ENSGALG00010015912	0.361442193	0.263657552	1.370877449	0.170413162	0.199365519	positive
PRJNA729271	stage_3W_vs_E10	ordinary_muscle_development	ENSGALG00010015913	CHST10	-0.834724677	0.105131404	-7.939822394	2.02471E-15	6.24163E-15	negative
PRJNA729271	stage_3W_vs_E10	ordinary_muscle_development	ENSGALG00010015914	SRRM1	-0.83028304	0.118034359	-7.0342487	2.00338E-12	5.32141E-12	negative
PRJNA729271	stage_3W_vs_E10	ordinary_muscle_development	ENSGALG00010015916	COA8	1.777702031	0.144018413	12.34357461	5.27604E-35	3.6279E-34	positive
PRJNA729271	stage_3W_vs_E10	ordinary_muscle_development	ENSGALG00010015917	TMEM63A	1.428941297	0.105457508	13.54992478	7.93114E-42	6.78241E-41	positive
PRJNA729271	stage_3W_vs_E10	ordinary_muscle_development	ENSGALG00010015918	BICD1	-1.536808688	0.196925992	-7.8039911	5.99794E-15	1.81012E-14	negative
PRJNA729271	stage_3W_vs_E10	ordinary_muscle_development	ENSGALG00010015919	ENSGALG00010015919	1.059527522	0.226734484	4.672987986	2.96849E-06	5.45421E-06	positive
PRJNA729271	stage_3W_vs_E10	ordinary_muscle_development	ENSGALG00010015921	SNX21	2.063632547	0.109248414	18.88935931	1.39529E-79	3.43958E-78	positive
PRJNA729271	stage_3W_vs_E10	ordinary_muscle_development	ENSGALG00010015923	RCAN3	-0.313842672	0.120442513	-2.605746635	0.009167425	0.012518306	negative
PRJNA729271	stage_3W_vs_E10	ordinary_muscle_development	ENSGALG00010015927	GLA	2.324010484	0.16631423	13.97361179	2.25875E-44	2.11093E-43	positive
PRJNA729271	stage_3W_vs_E10	ordinary_muscle_development	ENSGALG00010015928	CAPN7	0.427890451	0.121823588	3.512377687	0.000444116	0.000687323	positive
PRJNA729271	stage_3W_vs_E10	ordinary_muscle_development	ENSGALG00010015929	SLC6A12	-4.548650027	1.054493682	-4.313586801	1.60627E-05	2.79305E-05	negative
PRJNA729271	stage_3W_vs_E10	ordinary_muscle_development	ENSGALG00010015931	RBM47	2.122370888	0.27273698	7.781749602	7.15283E-15	2.15104E-14	positive
PRJNA729271	stage_3W_vs_E10	ordinary_muscle_development	ENSGALG00010015932	ACOT8	2.16304687	0.141282294	15.31010578	6.54574E-53	8.13726E-52	positive
PRJNA729271	stage_3W_vs_E10	ordinary_muscle_development	ENSGALG00010015933	ENSGALG00010015933	-0.122132458	1.033258414	-0.118201272	0.905908182	0.916914957	negative
PRJNA729271	stage_3W_vs_E10	ordinary_muscle_development	ENSGALG00010015934	TMC2	-1.99727994	1.288779615	-1.549745137	0.1212027	0.144809853	negative
PRJNA729271	stage_3W_vs_E10	ordinary_muscle_development	ENSGALG00010015935	EPHX1	0.059087629	0.112143787	0.526891685	0.598268808	0.63386495	positive
PRJNA729271	stage_3W_vs_E10	ordinary_muscle_development	ENSGALG00010015936	RPL36AL	0.330693404	0.13952163	2.370194527	0.017778729	0.023515163	positive
PRJNA729271	stage_3W_vs_E10	ordinary_muscle_development	ENSGALG00010015937	ENSGALG00010015937	0.958889845	0.19181139	4.999128791	5.75899E-07	1.10653E-06	positive
PRJNA729271	stage_3W_vs_E10	ordinary_muscle_development	ENSGALG00010015939	SUN2	0.85650894	0.079443788	10.78132049	4.21787E-27	2.16188E-26	positive
PRJNA729271	stage_3W_vs_E10	ordinary_muscle_development	ENSGALG00010015942	AP3S2	-0.305318839	0.131524599	-2.321382018	0.020266235	0.026652067	negative
PRJNA729271	stage_3W_vs_E10	ordinary_muscle_development	ENSGALG00010015946	TLE4	0.525112804	0.098399775	5.336524431	9.47451E-08	1.92182E-07	positive
PRJNA729271	stage_3W_vs_E10	ordinary_muscle_development	ENSGALG00010015954	CTSA	1.233180575	0.096299273	12.80571018	1.52326E-37	1.12651E-36	positive
PRJNA729271	stage_3W_vs_E10	ordinary_muscle_development	ENSGALG00010015957	ENSGALG00010015957	3.635770489	0.213568242	17.02392854	5.45824E-65	9.32986E-64	positive
PRJNA729271	stage_3W_vs_E10	ordinary_muscle_development	ENSGALG00010015959	TRNAU1AP	1.334202971	0.129025581	10.34060812	4.61599E-25	2.16884E-24	positive
PRJNA729271	stage_3W_vs_E10	ordinary_muscle_development	ENSGALG00010015960	CHRNA9	2.033860165	0.848838517	2.396050749	0.016572796	0.021992371	positive
PRJNA729271	stage_3W_vs_E10	ordinary_muscle_development	ENSGALG00010015963	DIPK2A	-0.370461639	0.108989878	-3.399046271	0.000676213	0.001028942	negative
PRJNA729271	stage_3W_vs_E10	ordinary_muscle_development	ENSGALG00010015964	KLC1	0.004634597	0.087435289	0.053006022	0.95772711	0.963175915	positive
PRJNA729271	stage_3W_vs_E10	ordinary_muscle_development	ENSGALG00010015967	ZSWIM1	-0.944574016	0.225446862	-4.189785598	2.79218E-05	4.76037E-05	negative
PRJNA729271	stage_3W_vs_E10	ordinary_muscle_development	ENSGALG00010015970	THPO	0.881160461	0.376654114	2.339442016	0.019312569	0.025444073	positive
PRJNA729271	stage_3W_vs_E10	ordinary_muscle_development	ENSGALG00010015971	ENSGALG00010015971	-1.068115719	0.158423867	-6.742138915	1.56072E-11	3.94948E-11	negative
PRJNA729271	stage_3W_vs_E10	ordinary_muscle_development	ENSGALG00010015972	FSTL4	-3.720042712	0.318758699	-11.67040374	1.80564E-31	1.09464E-30	negative
PRJNA729271	stage_3W_vs_E10	ordinary_muscle_development	ENSGALG00010015974	VPS37A	2.833056398	0.162799554	17.40211403	7.95135E-68	1.46077E-66	positive
PRJNA729271	stage_3W_vs_E10	ordinary_muscle_development	ENSGALG00010015975	BTK	2.209061999	0.171212552	12.90245352	4.35991E-38	3.28808E-37	positive

PRJNA729271	stage_3W_vs_E10	ordinary_muscle_development	ENSGALG00010015979	ENSGALG00010015979	4.034592829	0.202178141	19.95563327	1.33916E-88	4.23823E-87	positive
PRJNA729271	stage_3W_vs_E10	ordinary_muscle_development	ENSGALG00010015980	EXOSC7	-1.132384251	0.093054681	-12.16901973	4.54508E-34	3.0304E-33	negative
PRJNA729271	stage_3W_vs_E10	ordinary_muscle_development	ENSGALG00010015983	CNOT7	-0.381553431	0.064974729	-5.872335847	4.29697E-09	9.44135E-09	negative
PRJNA729271	stage_3W_vs_E10	ordinary_muscle_development	ENSGALG00010015984	PDCL3	0.78183314	0.107534137	7.270557653	3.58006E-13	9.8656E-13	positive
PRJNA729271	stage_3W_vs_E10	ordinary_muscle_development	ENSGALG00010015985	TIMM8A	-0.562323487	0.111679837	-5.035138871	4.77503E-07	9.23715E-07	negative
PRJNA729271	stage_3W_vs_E10	ordinary_muscle_development	ENSGALG00010015986	NEURL2	3.399973757	0.229437764	14.81871904	1.10879E-49	1.2382E-48	positive
PRJNA729271	stage_3W_vs_E10	ordinary_muscle_development	ENSGALG00010015987	POLR2H	-0.215685281	0.096053679	-2.245466106	0.024738226	0.032205908	negative
PRJNA729271	stage_3W_vs_E10	ordinary_muscle_development	ENSGALG00010015989	ENAH	-1.420415499	0.163383216	-8.693766298	3.50619E-18	1.24901E-17	negative
PRJNA729271	stage_3W_vs_E10	ordinary_muscle_development	ENSGALG00010015992	ATP10A	-0.806133467	0.157640656	-5.113740874	3.15841E-07	6.18162E-07	negative
PRJNA729271	stage_3W_vs_E10	ordinary_muscle_development	ENSGALG00010015994	ENSGALG00010015994	-0.029418327	0.107369965	-0.273990277	0.784092094	0.807891306	negative
PRJNA729271	stage_3W_vs_E10	ordinary_muscle_development	ENSGALG00010016004	XRCC3	-1.187117417	0.172687975	-6.874349068	6.22736E-12	1.60501E-11	negative
PRJNA729271	stage_3W_vs_E10	ordinary_muscle_development	ENSGALG00010016006	PLTP	-0.078210196	0.317256548	-0.246520352	0.805279452	0.827545243	negative
PRJNA729271	stage_3W_vs_E10	ordinary_muscle_development	ENSGALG00010016008	TAF7	-0.668474292	0.095341485	-7.011368571	2.35998E-12	6.23436E-12	negative
PRJNA729271	stage_3W_vs_E10	ordinary_muscle_development	ENSGALG00010016011	ADD1	-0.930626564	0.08101811	-11.48664863	1.5397E-30	9.02829E-30	negative
PRJNA729271	stage_3W_vs_E10	ordinary_muscle_development	ENSGALG00010016015	ZDHHC3	0.854035139	0.082000259	10.41502982	2.11731E-25	1.00526E-24	positive
PRJNA729271	stage_3W_vs_E10	ordinary_muscle_development	ENSGALG00010016016	ZDHHC2	0.314769158	0.276908068	1.136728012	0.255652005	0.291212245	positive
PRJNA729271	stage_3W_vs_E10	ordinary_muscle_development	ENSGALG00010016017	RAPGEF6	0.470356445	0.07150085	6.578333623	4.7575E-11	1.171E-10	positive
PRJNA729271	stage_3W_vs_E10	ordinary_muscle_development	ENSGALG00010016022	EPHB3	-2.304899824	0.170404277	-13.52606789	1.09738E-41	9.31844E-41	negative
PRJNA729271	stage_3W_vs_E10	ordinary_muscle_development	ENSGALG00010016025	PCIF1	0.137406674	0.108042431	1.271784365	0.203449746	0.235317911	positive
PRJNA729271	stage_3W_vs_E10	ordinary_muscle_development	ENSGALG00010016027	COPS2	1.42451156	0.14972934	9.513910618	1.83627E-21	7.45774E-21	positive
PRJNA729271	stage_3W_vs_E10	ordinary_muscle_development	ENSGALG00010016030	TGM4	1.848790569	0.70570473	2.619779193	0.008798672	0.012040856	positive
PRJNA729271	stage_3W_vs_E10	ordinary_muscle_development	ENSGALG00010016040	RPSA2	0.058851857	0.105924168	0.555603671	0.578481821	0.614472666	positive
PRJNA729271	stage_3W_vs_E10	ordinary_muscle_development	ENSGALG00010016041	ENSGALG00010016041	0.775889212	0.089713062	8.648564599	5.21512E-18	1.84059E-17	positive
PRJNA729271	stage_3W_vs_E10	ordinary_muscle_development	ENSGALG00010016043	ADAMTSL1	-0.43862039	0.192874983	-2.274117587	0.022958918	0.030029734	negative
PRJNA729271	stage_3W_vs_E10	ordinary_muscle_development	ENSGALG00010016047	SRP9	0.453928333	0.08859779	5.123472408	2.9996E-07	5.87874E-07	positive
PRJNA729271	stage_3W_vs_E10	ordinary_muscle_development	ENSGALG00010016049	ZFYVE21	0.682214584	0.121810152	5.600638129	2.13564E-08	4.51387E-08	positive
PRJNA729271	stage_3W_vs_E10	ordinary_muscle_development	ENSGALG00010016053	DRP2	2.246288516	0.462762553	4.854084455	1.20944E-06	2.27851E-06	positive
PRJNA729271	stage_3W_vs_E10	ordinary_muscle_development	ENSGALG00010016057	SOHO1	-4.531914145	0.796125665	-5.692460809	1.25221E-08	2.68662E-08	negative
PRJNA729271	stage_3W_vs_E10	ordinary_muscle_development	ENSGALG00010016059	EPDR1	0.392595697	0.200912963	1.954058566	0.050694291	0.063559705	positive
PRJNA729271	stage_3W_vs_E10	ordinary_muscle_development	ENSGALG00010016060	ENSGALG00010016060	2.276379695	1.361871684	1.671508206	0.094621339	0.114801734	positive
PRJNA729271	stage_3W_vs_E10	ordinary_muscle_development	ENSGALG00010016066	C14orf2	1.358278511	0.149442324	9.088981465	9.99712E-20	3.77857E-19	positive
PRJNA729271	stage_3W_vs_E10	ordinary_muscle_development	ENSGALG00010016067	AHSG	2.152820391	0.86163093	2.498541214	0.012470564	0.016775947	positive
PRJNA729271	stage_3W_vs_E10	ordinary_muscle_development	ENSGALG00010016069	GALK2	0.530536028	0.144860523	3.662392042	0.000249871	0.000394454	positive
PRJNA729271	stage_3W_vs_E10	ordinary_muscle_development	ENSGALG00010016073	SAXO1	5.085868498	0.974306247	5.219989619	1.78933E-07	3.55829E-07	positive
PRJNA729271	stage_3W_vs_E10	ordinary_muscle_development	ENSGALG00010016076	RAB3GAP2	0.178870533	0.090537085	1.975660391	0.048193254	0.060628469	positive
PRJNA729271	stage_3W_vs_E10	ordinary_muscle_development	ENSGALG00010016083	CDH22	-2.967039294	0.584497087	-5.076225975	3.85005E-07	7.4888E-07	negative
PRJNA729271	stage_3W_vs_E10	ordinary_muscle_development	ENSGALG00010016086	TDRD9	-2.135374004	0.361871282	-5.900921425	3.61477E-09	7.97384E-09	negative
PRJNA729271	stage_3W_vs_E10	ordinary_muscle_development	ENSGALG00010016087	IARS2	0.320389332	0.090070817	3.557082534	0.000374996	0.000583842	positive
PRJNA729271	stage_3W_vs_E10	ordinary_muscle_development	ENSGALG00010016091	HMX1	-3.159571266	0.908638755	-3.477257876	0.00050657	0.000780063	negative
PRJNA729271	stage_3W_vs_E10	ordinary_muscle_development	ENSGALG00010016094	FGF7	0.578629403	0.184938863	3.12876047	0.001755453	0.002569973	positive
PRJNA729271	stage_3W_vs_E10	ordinary_muscle_development	ENSGALG00010016097	FNIP1	1.319118243	0.109994889	11.99254119	3.88792E-33	2.51026E-32	positive
PRJNA729271	stage_3W_vs_E10	ordinary_muscle_development	ENSGALG00010016100	SFRP4	-1.430576584	0.413453794	-3.460063988	0.000540047	0.00082906	negative
PRJNA729271	stage_3W_vs_E10	ordinary_muscle_development	ENSGALG00010016103	BPNT1	-1.179508809	0.091530461	-12.88651668	5.36111E-38	4.03266E-37	negative
PRJNA729271	stage_3W_vs_E10	ordinary_muscle_development	ENSGALG00010016107	UVSSA	0.25414443	0.090268987	2.815412464	0.004871467	0.006839588	positive
PRJNA729271	stage_3W_vs_E10	ordinary_muscle_development	ENSGALG00010016109	MLH1	-0.178634852	0.101178492	-1.765541752	0.07747276	0.094997686	negative
PRJNA729271	stage_3W_vs_E10	ordinary_muscle_development	ENSGALG00010016111	EPB41	-0.04584281	0.102330311	-0.447988571	0.654161445	0.687106532	negative
PRJNA729271	stage_3W_vs_E10	ordinary_muscle_development	ENSGALG00010016113	DTWD1	0.229822684	0.148216451	1.550588229	0.121000395	0.14459198	positive
PRJNA729271	stage_3W_vs_E10	ordinary_muscle_development	ENSGALG00010016114	HAUS6	-1.583760657	0.152057408	-10.41554425	2.1059E-25	1.00049E-24	negative
PRJNA729271	stage_3W_vs_E10	ordinary_muscle_development	ENSGALG00010016115	RD3L	-1.076435008	0.475494252	-2.263823387	0.02358498	0.030784717	negative
PRJNA729271	stage_3W_vs_E10	ordinary_muscle_development	ENSGALG00010016117	ENSGALG00010016117	0.125527453	0.240331514	0.522309582	0.601454798	0.637007694	positive
PRJNA729271	stage_3W_vs_E10	ordinary_muscle_development	ENSGALG00010016118	EPRS	0.252232585	0.087509658	2.882339983	0.003947336	0.005593024	positive
PRJNA729271	stage_3W_vs_E10	ordinary_muscle_development	ENSGALG00010016119	FETUB	0.347404797	1.485210347	0.233909491			positive
PRJNA729271	stage_3W_vs_E10	ordinary_muscle_development	ENSGALG00010016121	SLC35C2	-0.368919099	0.099391578	-3.711774227	0.000205812	0.00032754	negative
PRJNA729271	stage_3W_vs_E10	ordinary_muscle_development	ENSGALG00010016122	AFG2B	0.311755271	0.131084712	2.37827331	0.017393928	0.023020908	positive
PRJNA729271	stage_3W_vs_E10	ordinary_muscle_development	ENSGALG00010016127	ENSGALG00010016127	-0.240355772	0.174920035	-1.374089434	0.169413919	0.198340583	negative
PRJNA729271	stage_3W_vs_E10	ordinary_muscle_development	ENSGALG00010016129	CENPI	-3.438167714	0.245588041	-13.99973589	1.56451E-44	1.46876E-43	negative
PRJNA729271	stage_3W_vs_E10	ordinary_muscle_development	ENSGALG00010016131	SMYD1	0.26945349	0.217585093	1.238382122	0.215574407	0.248331163	positive
PRJNA729271	stage_3W_vs_E10	ordinary_muscle_development	ENSGALG00010016137	STARD3NL	0.198677026	0.139889711	1.420240446	0.155537693	0.183085825	positive
PRJNA729271	stage_3W_vs_E10	ordinary_muscle_development	ENSGALG00010016139	LURAP1L	-0.165384782	0.192282563	-0.860113258	0.389726613	0.428740685	negative
PRJNA729271	stage_3W_vs_E10	ordinary_muscle_development	ENSGALG00010016141	RRP15	-1.509465081	0.179851657	-8.392833874	4.74562E-17	1.59378E-16	negative
PRJNA729271	stage_3W_vs_E10	ordinary_muscle_development	ENSGALG00010016147	FABP1	3.116486716	0.796333358	3.913545355	9.09508E-05	0.00014893	positive
PRJNA729271	stage_3W_vs_E10	ordinary_muscle_development	ENSGALG00010016150	POLG	0.219491644	0.094864224	2.313745202	0.020681703	0.02717133	positive
PRJNA729271	stage_3W_vs_E10	ordinary_muscle_development	ENSGALG00010016159	ASPG	-1.673845509	0.191676089	-8.732677703	2.48713E-18	8.90587E-18	negative
PRJNA729271	stage_3W_vs_E10	ordinary_muscle_development	ENSGALG00010016165	KK34	4.959024413	0.896762126	5.529921783	3.20374E-08	6.68271E-08	positive
PRJNA729271	stage_3W_vs_E10	ordinary_muscle_development	ENSGALG00010016168	PLIN2	1.270380403	0.229346655	5.539127666	3.03982E-08	6.35176E-08	positive
PRJNA729271	stage_3W_vs_E10	ordinary_muscle_development	ENSGALG00010016169	RHOH	0.151217211	0.120372684	1.25624192	0.209028272	0.241269693	positive
PRJNA729271	stage_3W_vs_E10	ordinary_muscle_development	ENSGALG00010016173	ENSGALG00010016173	-3.34641177	0.470047781	-7.119301276	1.08476E-12	2.91988E-12	negative
PRJNA729271	stage_3W_vs_E10	ordinary_muscle_development	ENSGALG00010016177	ENSGALG00010016177	0.589987849	1.672168971	0.35282789	0.724217474	0.752403403	positive
PRJNA729271	stage_3W_vs_E10	ordinary_muscle_development	ENSGALG00010016181	FANCI	-3.173256833	0.184514055	-17.19791393	2.75264E-66	4.85988E-65	negative
PRJNA729271	stage_3W_vs_E10	ordinary_muscle_development	ENSGALG00010016182	ELMO2	-0.142744093	0.062119759	-2.297885469	0.021568309	0.028287426	negative
PRJNA729271	stage_3W_vs_E10	ordinary_muscle_development	ENSGALG00010016190	THNSL2	0.415041563	0.152829345	2.715719051	0.006613201	0.0091599	positive
PRJNA729271	stage_3W_vs_E10	ordinary_muscle_development	ENSGALG00010016193	TCAIM	0.022152301	0.135040066	0.164042431	0.869697741	0.884588363	positive
PRJNA729271	stage_3W_vs_E10	ordinary_muscle_development	ENSGALG00010016197	SNRPN	-1.69928054	0.116874632	-14.53934456	6.82429E-48	7.23615E-47	negative
PRJNA729271	stage_3W_vs_E10	ordinary_muscle_development	ENSGALG00010016199	RLBP1	-6.61068611	0.722190065	-9.153665266	5.50339E-20	2.10091E-19	negative
PRJNA729271	stage_3W_vs_E10	ordinary_muscle_development	ENSGALG00010016200	MICU3	1.128347966	0.116865268	9.655118103	4.6762E-22	1.94384E-21	positive
PRJNA729271	stage_3W_vs_E10	ordinary_muscle_development	ENSGALG00010016205	CD40LG	8.098713154	1.003066742	8.073952428	6.80584E-16	2.153E-15	positive
PRJNA729271	stage_3W_vs_E10	ordinary_muscle_development	ENSGALG00010016206	KNG1	1.15398367	1.172346963	0.984336299	0.324950188	0.36301757	positive
PRJNA729271	stage_3W_vs_E10	ordinary_muscle_development	ENSGALG00010016209	ADAMTS7	-1.730897883	0.167640473	-10.32505967	5.4285E-25	2.53826E-24	negative
PRJNA729271	stage_3W_vs_E10	ordinary_muscle_development	ENSGALG00010016211	TACC3	-1.840692874	0.161877405	-11.37090672	5.8378E-30	3.34859E-29	negative
PRJNA729271	stage_3W_vs_E10	ordinary_muscle_development	ENSGALG00010016215	SPATA17	-3.016092212	0.382969759	-7.875536224	3.39285E-15	1.03644E-14	negative
PRJNA729271	stage_3W_vs_E10	ordinary_muscle_development	ENSGALG00010016218	N4BP2	0.14051683	0.075595611	1.858796132	0.063056041	0.07817288	positive
PRJNA729271	stage_3W_vs_E10	ordinary_muscle_development	ENSGALG00010016221	TMEM200B	-0.499345468	0.202488058	-2.466048971	0.013661266	0.018299607	negative
PRJNA729271	stage_3W_vs_E10	ordinary_muscle_development	ENSGALG00010016224	MOSPD1	2.390085709	0.115040539	20.77603021	7.13176E-96	2.67811E-94	positive
PRJNA729271	stage_3W_vs_E10	ordinary_muscle_development	ENSGALG00010016225	P4HA2	1.313051909	0.156279107	8.401967075	4.39061E-17	1.47661E-16	positive
PRJNA729271	stage_3W_vs_E10	ordinary_muscle_development	ENSGALG00010016229	ENSGALG00010016229	0.970616147	0.219386173	4.424235743	9.67843E-06	1.70959E-05	positive
PRJNA729271	stage_3W_vs_E10	ordinary_muscle_development	ENSGALG00010016235	DDX27	-0.322634455	0.123596688	-2.610381066	0.009044141	0.012363605	negative
PRJNA729271	stage_3W_vs_E10	ordinary_muscle_development	ENSGALG00010016236	GPATCH2	-0.700908174	0.066314977	-10.56937967	4.13219E-26	2.029E-25	negative
PRJNA729271	stage_3W_vs_E10	ordinary_muscle_development	ENSGALG00010016240	EIF4A2	1.230019206	0.100309719	12.26221369	1.44498E-34	9.81488E-34	positive
PRJNA729271	stage_3W_vs_E10	ordinary_muscle_development	ENSGALG00010016241	TOPAZ1	0.35099114	0.809915617	0.433367542	0.664747777	0.696661053	positive
PRJNA729271	stage_3W_vs_E10	ordinary_muscle_development	ENSGALG00010016242	PDS5A	0.579086822	0.092090591	6.288230057	3.21106E-10	7.5412E-10	positive
PRJNA729271	stage_3W_vs_E10	ordinary_muscle_development	ENSGALG00010016245	FAM96A	-0.17335606	0.104681154	-1.656038861	0.097713949	0.118316239	negative
PRJNA729271	stage_3W_vs_E10	ordinary_muscle_development	ENSGALG00010016247	KIF26A	-2.629064569	0.167612613	-15.68536233	1.90473E-55	2.54462E-54	negative
PRJNA729271	stage_3W_vs_E10	ordinary_muscle_development	ENSGALG00010016251	MPDZ	-0.406052738	0.073100166	-5.554744373	2.78018E-08	5.82942E-08	negative
PRJNA729271	stage_3W_vs_E10	ordinary_muscle_development	ENSGALG00010016253	EDN3	-0.917769767	0.209270178	-4.385573598	1.15681E-05	2.03395E-05	negative
PRJNA729271	stage_3W_vs_E10	ordinary_muscle_development	ENSGALG00010016257	ENSGALG00010016257	-0.963544657	0.113043466	-8.523665217	1.54582E-17	5.32604E-17	negative
PRJNA729271	stage_3W_vs_E10	ordinary_muscle_development	ENSGALG00010016258	CTNNB1	-2.161171217	0.077340461	-27.94360401	7.8843E-172	1.0025E-169	negative
PRJNA729271	stage_3W_vs_E10	ordinary_muscle_development	ENSGALG00010016260	INTS6L	1.181176381	0.144004579	8.202352946	2.35727E-16	7.64741E-16	positive
PRJNA729271	stage_3W_vs_E10	ordinary_muscle_development	ENSGALG00010016262	SPG11	0.392933415	0.060802593	6.462445011	1.03025E-10	2.4827E-10	positive
PRJNA729271	stage_3W_vs_E10	ordinary_muscle_development	ENSGALG00010016263	HGFAC	-1.121511952	1.417484118	-0.791198954			negative
PRJNA729271	stage_3W_vs_E10	ordinary_muscle_development	ENSGALG00010016266	PDLIM4	-0.913506227	0.121283209	-7.532009073	4.99655E-14	1.44187E-13	negative
PRJNA729271	stage_3W_vs_E10	ordinary_muscle_development	ENSGALG00010016270	RFC4	-1.624067004	0.112102947	-14.487282	1.45799E-47	1.52259E-46	negative
PRJNA729271	stage_3W_vs_E10	ordinary_muscle_development	ENSGALG00010016274	ZNF831	3.039942598	0.47114674	6.452220375	1.10223E-10	2.6522E-10	positive
PRJNA729271	stage_3W_vs_E10	ordinary_muscle_development	ENSGALG00010016276	ACTR2	-1.000068183	0.107785705	-9.278300743	1.72203E-20	6.70629E-20	negative
PRJNA729271	stage_3W_vs_E10	ordinary_muscle_development	ENSGALG00010016279	PRELID3B	-0.410157952	0.106190935	-3.862457301	0.000112252	0.000182656	negative
PRJNA729271	stage_3W_vs_E10	ordinary_muscle_development	ENSGALG00010016281	SLC22A4	-1.485635747	0.220080625	-6.750415899	1.47422E-11	3.73254E-11	negative
PRJNA729271	stage_3W_vs_E10	ordinary_muscle_development	ENSGALG00010016286	ULK4	-1.767574073	0.14553711	-12.14517775	6.08502E-34	4.03303E-33	negative
PRJNA729271	stage_3W_vs_E10	ordinary_muscle_development	ENSGALG00010016287	BCL11B	-4.439662203	0.347379103	-12.78045273	2.10839E-37	1.55211E-36	negative
PRJNA729271	stage_3W_vs_E10	ordinary_muscle_development	ENSGALG00010016289	IRF1	4.508930718	0.255980291	17.61436674	1.91116E-69	3.62121E-68	positive
PRJNA729271	stage_3W_vs_E10	ordinary_muscle_development	ENSGALG00010016292	PHKA1	5.782623243	0.147885024	39.10215565	0	0	positive
PRJNA729271	stage_3W_vs_E10	ordinary_muscle_development	ENSGALG00010016295	UBE2K	0.777547209	0.092940021	8.366118318	5.95474E-17	1.99154E-16	positive
PRJNA729271	stage_3W_vs_E10	ordinary_muscle_development	ENSGALG00010016299	SRSF4	-1.080583841	0.110160883	-9.809142911	1.02838E-22	4.38037E-22	negative
PRJNA729271	stage_3W_vs_E10	ordinary_muscle_development	ENSGALG00010016303	ENSGALG00010016303	0.677086072	0.203398588	3.328863182	0.000872012	0.001313364	positive
PRJNA729271	stage_3W_vs_E10	ordinary_muscle_development	ENSGALG00010016304	EIF3J	1.466310662	0.099536489	14.73138829	4.05293E-49	4.44382E-48	positive
PRJNA729271	stage_3W_vs_E10	ordinary_muscle_development	ENSGALG00010016305	DENND4C	1.007309289	0.095945813	10.49873106	8.75491E-26	4.2329E-25	positive
PRJNA729271	stage_3W_vs_E10	ordinary_muscle_development	ENSGALG00010016307	RAD50	-0.453467258	0.128385231	-3.532082722	0.0004123	0.000639313	negative
PRJNA729271	stage_3W_vs_E10	ordinary_muscle_development	ENSGALG00010016308	RPS6	-0.198506322	0.140719587	-1.410651679	0.158347345	0.186211648	negative
PRJNA729271	stage_3W_vs_E10	ordinary_muscle_development	ENSGALG00010016310	HDAC8	-0.4801059	0.092781249	-5.174600505	2.28399E-07	4.50893E-07	negative
PRJNA729271	stage_3W_vs_E10	ordinary_muscle_development	ENSGALG00010016312	MECR	-0.076217462	0.146698894	-0.519550353	0.603377009	0.638810149	negative
PRJNA729271	stage_3W_vs_E10	ordinary_muscle_development	ENSGALG00010016314	ACER2	0.173477893	0.157337339	1.102585659	0.270207141	0.306731342	positive
PRJNA729271	stage_3W_vs_E10	ordinary_muscle_development	ENSGALG00010016315	RPS4Y1	0.054094541	0.13644069	0.39646927	0.691758874	0.721941455	positive
PRJNA729271	stage_3W_vs_E10	ordinary_muscle_development	ENSGALG00010016317	CTDSPL2	-0.809360777	0.057465429	-14.08430764	4.74284E-45	4.54379E-44	negative
PRJNA729271	stage_3W_vs_E10	ordinary_muscle_development	ENSGALG00010016319	RBM41	0.874602735	0.184420249	4.742444174	2.11155E-06	3.91443E-06	positive
PRJNA729271	stage_3W_vs_E10	ordinary_muscle_development	ENSGALG00010016322	EBF4	-1.336482756	0.136176864	-9.814315884	9.76984E-23	4.16756E-22	negative
PRJNA729271	stage_3W_vs_E10	ordinary_muscle_development	ENSGALG00010016324	TUBB1	-1.237357472	0.213022026	-5.808589354	6.30014E-09	1.37552E-08	negative
PRJNA729271	stage_3W_vs_E10	ordinary_muscle_development	ENSGALG00010016328	USH2A	4.571557456	0.609737469	7.497583284	6.50052E-14	1.864E-13	positive
PRJNA729271	stage_3W_vs_E10	ordinary_muscle_development	ENSGALG00010016332	ENSGALG00010016332	-0.643215631	0.237415125	-2.709244543	0.006743661	0.009332393	negative
PRJNA729271	stage_3W_vs_E10	ordinary_muscle_development	ENSGALG00010016335	ABHD4	3.057393029	0.182930202	16.71344039	1.04621E-62	1.67939E-61	positive
PRJNA729271	stage_3W_vs_E10	ordinary_muscle_development	ENSGALG00010016336	SLC24A2	0.244775503	0.349619396	0.70011992	0.483852417	0.522183067	positive
PRJNA729271	stage_3W_vs_E10	ordinary_muscle_development	ENSGALG00010016338	PTPRU	-1.486177057	0.151978323	-9.778875251	1.38744E-22	5.8718E-22	negative
PRJNA729271	stage_3W_vs_E10	ordinary_muscle_development	ENSGALG00010016339	SETD3	0.806079284	0.121813359	6.617330727	3.65743E-11	9.06694E-11	positive
PRJNA729271	stage_3W_vs_E10	ordinary_muscle_development	ENSGALG00010016340	RGS12	-2.08999879	0.102471501	-20.39590291	1.81827E-92	6.26029E-91	negative

PRJNA729271	stage_3W_vs_E10	ordinary_muscle_development	ENSGALG00010016341	SLX4	-1.003695083	0.065281348	-15.37491355	2.41165E-53	3.05034E-52	negative
PRJNA729271	stage_3W_vs_E10	ordinary_muscle_development	ENSGALG00010016344	SMIM14	0.162370643	0.111960917	1.450243949	0.146990503	0.173616961	positive
PRJNA729271	stage_3W_vs_E10	ordinary_muscle_development	ENSGALG00010016345	CTSZ	1.444324433	0.110071101	13.12174057	2.47192E-39	1.95474E-38	positive
PRJNA729271	stage_3W_vs_E10	ordinary_muscle_development	ENSGALG00010016346	TRAP1	0.04837242	0.103924018	0.465459486	0.641602444	0.67474624	positive
PRJNA729271	stage_3W_vs_E10	ordinary_muscle_development	ENSGALG00010016347	ANO10	0.126525225	0.090155183	1.403415991	0.160492827	0.188596962	positive
PRJNA729271	stage_3W_vs_E10	ordinary_muscle_development	ENSGALG00010016350	CASC4	0.26174743	0.062785199	4.168935287	3.06026E-05	5.20273E-05	positive
PRJNA729271	stage_3W_vs_E10	ordinary_muscle_development	ENSGALG00010016353	C1orf232	0.008569899	0.146017382	0.058690952	0.953198266	0.959353484	positive
PRJNA729271	stage_3W_vs_E10	ordinary_muscle_development	ENSGALG00010016355	MCF2L2	1.279591888	0.228017632	5.611811144	2.0022E-08	4.24172E-08	positive
PRJNA729271	stage_3W_vs_E10	ordinary_muscle_development	ENSGALG00010016361	NELFCD	-1.329805278	0.083903979	-15.84913244	1.42535E-56	1.95094E-55	negative
PRJNA729271	stage_3W_vs_E10	ordinary_muscle_development	ENSGALG00010016362	NUP62	-0.545456436	0.17038488	-3.201319491	0.001367998	0.002025033	negative
PRJNA729271	stage_3W_vs_E10	ordinary_muscle_development	ENSGALG00010016366	KIF3A	-0.555775167	0.098698675	-5.631029666	1.79137E-08	3.80732E-08	negative
PRJNA729271	stage_3W_vs_E10	ordinary_muscle_development	ENSGALG00010016368	CCNK	-0.542744857	0.151376926	-3.585386954	0.000336579	0.000526574	negative
PRJNA729271	stage_3W_vs_E10	ordinary_muscle_development	ENSGALG00010016369	MSANTD1	-5.210202948	0.440536358	-11.82695333	2.83237E-32	1.77959E-31	negative
PRJNA729271	stage_3W_vs_E10	ordinary_muscle_development	ENSGALG00010016370	TMEM255A	4.883625299	0.185143343	26.37753663	2.4808E-153	2.4971E-151	positive
PRJNA729271	stage_3W_vs_E10	ordinary_muscle_development	ENSGALG00010016372	DNAAF6	-0.164541253	0.198121789	-0.830505588	0.40625299	0.445097285	negative
PRJNA729271	stage_3W_vs_E10	ordinary_muscle_development	ENSGALG00010016373	SRL	3.285408322	0.13165229	24.95519304	1.8755E-137	1.4537E-135	positive
PRJNA729271	stage_3W_vs_E10	ordinary_muscle_development	ENSGALG00010016374	FRMD5	7.879423724	0.335243868	23.50355805	3.751E-122	2.3335E-120	positive
PRJNA729271	stage_3W_vs_E10	ordinary_muscle_development	ENSGALG00010016375	CCNI2	-1.931930018	0.142211538	-13.58490352	4.9218E-42	4.24147E-41	negative
PRJNA729271	stage_3W_vs_E10	ordinary_muscle_development	ENSGALG00010016377	FRMPD3	3.324713102	0.277969151	11.96072689	5.70602E-33	3.6613E-32	positive
PRJNA729271	stage_3W_vs_E10	ordinary_muscle_development	ENSGALG00010016378	WDR76	-0.849400375	0.173783516	-4.887692424	1.02025E-06	1.93238E-06	negative
PRJNA729271	stage_3W_vs_E10	ordinary_muscle_development	ENSGALG00010016379	TFAP4	-1.302632429	0.15640848	-8.328400263	8.19421E-17	2.72045E-16	negative
PRJNA729271	stage_3W_vs_E10	ordinary_muscle_development	ENSGALG00010016382	UGDH	-0.966344402	0.18615129	-5.191177582	2.08968E-07	4.13515E-07	negative
PRJNA729271	stage_3W_vs_E10	ordinary_muscle_development	ENSGALG00010016383	FAM110D	0.879495871	0.175295543	5.017217538	5.24252E-07	1.00997E-06	positive
PRJNA729271	stage_3W_vs_E10	ordinary_muscle_development	ENSGALG00010016384	GNAS	0.016466309	0.074363113	0.221431136	0.824756751	0.845642576	positive
PRJNA729271	stage_3W_vs_E10	ordinary_muscle_development	ENSGALG00010016385	HTT	0.274691401	0.10656619	2.57765995	0.009947183	0.0135156	positive
PRJNA729271	stage_3W_vs_E10	ordinary_muscle_development	ENSGALG00010016387	CCDC85C	-1.218848256	0.164034021	-7.430460127	1.08221E-13	3.06438E-13	negative
PRJNA729271	stage_3W_vs_E10	ordinary_muscle_development	ENSGALG00010016390	ENSGALG00010016390	3.419283614	0.307807774	11.10850309	1.14055E-28	6.22452E-28	positive
PRJNA729271	stage_3W_vs_E10	ordinary_muscle_development	ENSGALG00010016392	STMN3	-0.380969237	0.332354976	-1.146272102	0.251682582	0.286910501	negative
PRJNA729271	stage_3W_vs_E10	ordinary_muscle_development	ENSGALG00010016393	BLM	-2.33875623	0.128617606	-18.18379532	6.93629E-74	1.49838E-72	negative
PRJNA729271	stage_3W_vs_E10	ordinary_muscle_development	ENSGALG00010016394	PDIK1L	0.350551358	0.096027235	3.650541004	0.000261689	0.000412571	positive
PRJNA729271	stage_3W_vs_E10	ordinary_muscle_development	ENSGALG00010016395	ENSGALG00010016395	-0.750047311	0.161987774	-4.630271123	3.65187E-06	6.67599E-06	negative
PRJNA729271	stage_3W_vs_E10	ordinary_muscle_development	ENSGALG00010016397	LIAS	0.252906654	0.104089058	2.42971411	0.015110737	0.020146259	positive
PRJNA729271	stage_3W_vs_E10	ordinary_muscle_development	ENSGALG00010016398	SEPTIN6	-0.631914377	0.068794082	-9.185592117	4.09269E-20	1.57065E-19	negative
PRJNA729271	stage_3W_vs_E10	ordinary_muscle_development	ENSGALG00010016399	ACSM5	6.710987458	0.327512256	20.49079795	2.60095E-93	9.30885E-92	positive
PRJNA729271	stage_3W_vs_E10	ordinary_muscle_development	ENSGALG00010016400	ENSGALG00010016400	0.569431074	0.086635356	6.572733106	4.94E-11	1.21365E-10	positive
PRJNA729271	stage_3W_vs_E10	ordinary_muscle_development	ENSGALG00010016402	SEPTIN8	-1.126729814	0.078389409	-14.37349553	7.59016E-47	7.7315E-46	negative
PRJNA729271	stage_3W_vs_E10	ordinary_muscle_development	ENSGALG00010016403	GRK4	-0.873687335	0.134745392	-6.48398672	8.93301E-11	2.16239E-10	negative
PRJNA729271	stage_3W_vs_E10	ordinary_muscle_development	ENSGALG00010016404	ESRRG	0.75355587	0.14987588	5.027866181	4.95968E-07	9.58029E-07	positive
PRJNA729271	stage_3W_vs_E10	ordinary_muscle_development	ENSGALG00010016407	RPL9	0.310099705	0.180720545	1.715907313	0.086179008	0.10510431	positive
PRJNA729271	stage_3W_vs_E10	ordinary_muscle_development	ENSGALG00010016409	GMEB2	1.787937554	0.205634016	8.694755781	3.47577E-18	1.23848E-17	positive
PRJNA729271	stage_3W_vs_E10	ordinary_muscle_development	ENSGALG00010016410	TRAK1	1.182661651	0.096696756	12.2306239	2.13294E-34	1.44135E-33	positive
PRJNA729271	stage_3W_vs_E10	ordinary_muscle_development	ENSGALG00010016411	CTSH	1.208481023	0.122806765	9.840508589	7.53291E-23	3.2295E-22	positive
PRJNA729271	stage_3W_vs_E10	ordinary_muscle_development	ENSGALG00010016412	TMSB15B	-3.07147436	0.215645693	-14.24315186	4.94457E-46	4.90901E-45	negative
PRJNA729271	stage_3W_vs_E10	ordinary_muscle_development	ENSGALG00010016415	RASGRF1	3.271946567	0.513813626	6.367963792	1.91554E-10	4.55699E-10	positive
PRJNA729271	stage_3W_vs_E10	ordinary_muscle_development	ENSGALG00010016416	CYP46A1	1.000413633	0.111650798	8.960201402	3.24086E-19	1.20144E-18	positive
PRJNA729271	stage_3W_vs_E10	ordinary_muscle_development	ENSGALG00010016417	NOP14	-0.4784737	0.106442293	-4.495146478	6.95221E-06	1.24134E-05	negative
PRJNA729271	stage_3W_vs_E10	ordinary_muscle_development	ENSGALG00010016418	NFIB	-0.103086032	0.098141583	-1.050380773	0.293543082	0.330812235	negative
PRJNA729271	stage_3W_vs_E10	ordinary_muscle_development	ENSGALG00010016421	ENSGALG00010016421	-5.154886466	0.88609436	-5.817536706	5.97212E-09	1.30547E-08	negative
PRJNA729271	stage_3W_vs_E10	ordinary_muscle_development	ENSGALG00010016422	KCTD3	0.055144963	0.090452954	0.609653532	0.542091342	0.579683807	positive
PRJNA729271	stage_3W_vs_E10	ordinary_muscle_development	ENSGALG00010016423	TRMT44	-0.618610679	0.109815923	-5.633160119	1.76937E-08	3.76222E-08	negative
PRJNA729271	stage_3W_vs_E10	ordinary_muscle_development	ENSGALG00010016425	TRIM63	5.54531197	0.413864535	13.3988576	6.1401E-41	5.09741E-40	positive
PRJNA729271	stage_3W_vs_E10	ordinary_muscle_development	ENSGALG00010016426	HELZ2	2.164547942	0.367606529	5.888219526	3.90378E-09	8.59829E-09	positive
PRJNA729271	stage_3W_vs_E10	ordinary_muscle_development	ENSGALG00010016428	ADRA1D	0.513737016	0.205360106	2.501639803	0.012361962	0.016638909	positive
PRJNA729271	stage_3W_vs_E10	ordinary_muscle_development	ENSGALG00010016429	ENSGALG00010016429	-1.614402231	0.45546904	-3.544482913	0.000393384	0.000611354	negative
PRJNA729271	stage_3W_vs_E10	ordinary_muscle_development	ENSGALG00010016431	B3GNT5	-3.880416367	0.161502135	-24.02702832	1.4515E-127	9.8312E-126	negative
PRJNA729271	stage_3W_vs_E10	ordinary_muscle_development	ENSGALG00010016433	TMED3	-1.127048215	0.148707375	-7.578966496	3.48318E-14	1.01139E-13	negative
PRJNA729271	stage_3W_vs_E10	ordinary_muscle_development	ENSGALG00010016434	GPR139	-2.489128508	0.594942463	-4.183813836	2.86659E-05	4.88493E-05	negative
PRJNA729271	stage_3W_vs_E10	ordinary_muscle_development	ENSGALG00010016436	SLC30A2	2.467185885	0.535031575	4.611290251	4.00177E-06	7.28488E-06	positive
PRJNA729271	stage_3W_vs_E10	ordinary_muscle_development	ENSGALG00010016441	ENSGALG00010016441	-1.874284868	0.67748364	-2.766538933	0.005665482	0.007897025	negative
PRJNA729271	stage_3W_vs_E10	ordinary_muscle_development	ENSGALG00010016445	ZDHHC21	0.255237441	0.135994621	1.87682012	0.060542752	0.075237242	positive
PRJNA729271	stage_3W_vs_E10	ordinary_muscle_development	ENSGALG00010016447	ID3	-1.321166602	0.113197841	-11.67130567	1.7866E-31	1.08355E-30	negative
PRJNA729271	stage_3W_vs_E10	ordinary_muscle_development	ENSGALG00010016449	FMR1	0.363266733	0.106675958	3.405329004	0.000660844	0.001006296	positive
PRJNA729271	stage_3W_vs_E10	ordinary_muscle_development	ENSGALG00010016450	ENSGALG00010016450	-0.37087128	0.269815005	-1.374539125	0.169274373	0.198193217	negative
PRJNA729271	stage_3W_vs_E10	ordinary_muscle_development	ENSGALG00010016451	USP46	0.42495615	0.147477234	2.881503391	0.00395783	0.005607345	positive
PRJNA729271	stage_3W_vs_E10	ordinary_muscle_development	ENSGALG00010016452	GLCE	-0.933496574	0.115511396	-8.081424032	6.40148E-16	2.02774E-15	negative
PRJNA729271	stage_3W_vs_E10	ordinary_muscle_development	ENSGALG00010016453	MYCL	-2.401642125	0.188363333	-12.75005113	3.11541E-37	2.2807E-36	negative
PRJNA729271	stage_3W_vs_E10	ordinary_muscle_development	ENSGALG00010016454	ANKS3	-0.013426678	0.094550884	-0.142004783	0.887076228	0.900256928	negative
PRJNA729271	stage_3W_vs_E10	ordinary_muscle_development	ENSGALG00010016455	RBM27	-0.66240104	0.091878589	-7.209525611	5.61471E-13	1.53181E-12	negative
PRJNA729271	stage_3W_vs_E10	ordinary_muscle_development	ENSGALG00010016456	LAMP3	0.118132296	0.353203685	0.33445941	0.738032916	0.765494605	positive
PRJNA729271	stage_3W_vs_E10	ordinary_muscle_development	ENSGALG00010016457	SMOX	0.306589659	0.178898597	1.713762233	0.086572402	0.10555745	positive
PRJNA729271	stage_3W_vs_E10	ordinary_muscle_development	ENSGALG00010016458	ZC3H12A	1.766123171	0.087991392	20.07154495	1.30873E-89	4.26292E-88	positive
PRJNA729271	stage_3W_vs_E10	ordinary_muscle_development	ENSGALG00010016459	PAQR5	2.91765006	0.272382213	10.71160273	8.97936E-27	4.54372E-26	positive
PRJNA729271	stage_3W_vs_E10	ordinary_muscle_development	ENSGALG00010016460	LBR	-1.536323708	0.069769151	-22.02010048	1.8485E-107	9.0829E-106	negative
PRJNA729271	stage_3W_vs_E10	ordinary_muscle_development	ENSGALG00010016461	HPCAL4	-3.832288246	0.405613869	-9.448119364	3.44973E-21	1.38668E-20	negative
PRJNA729271	stage_3W_vs_E10	ordinary_muscle_development	ENSGALG00010016462	RNF24	-1.352916957	0.159982518	-8.456654964	2.75157E-17	9.35145E-17	negative
PRJNA729271	stage_3W_vs_E10	ordinary_muscle_development	ENSGALG00010016463	FREM1	-2.254245016	0.1446829	-15.58059054	9.86393E-55	1.29041E-53	negative
PRJNA729271	stage_3W_vs_E10	ordinary_muscle_development	ENSGALG00010016464	PANK2	-0.496331539	0.089591483	-5.539941102	3.02573E-08	6.32415E-08	negative
PRJNA729271	stage_3W_vs_E10	ordinary_muscle_development	ENSGALG00010016465	STK35	-1.364945684	0.201659423	-6.768568827	1.30063E-11	3.30457E-11	negative
PRJNA729271	stage_3W_vs_E10	ordinary_muscle_development	ENSGALG00010016466	TMEM164	0.145335465	0.233614126	0.622117624	0.533864527	0.571730057	positive
PRJNA729271	stage_3W_vs_E10	ordinary_muscle_development	ENSGALG00010016468	TTC39B	-0.065020144	0.163363761	-0.398008368	0.690624017	0.721022754	negative
PRJNA729271	stage_3W_vs_E10	ordinary_muscle_development	ENSGALG00010016469	MAVS	0.343782626	0.102930813	3.33993889	0.000837968	0.001263534	positive
PRJNA729271	stage_3W_vs_E10	ordinary_muscle_development	ENSGALG00010016470	TNIP1	1.600213019	0.158065068	10.12376129	4.33432E-24	1.95233E-23	positive
PRJNA729271	stage_3W_vs_E10	ordinary_muscle_development	ENSGALG00010016471	POMGNT2	0.889533647	0.135114368	6.583560734	4.59313E-11	1.13188E-10	positive
PRJNA729271	stage_3W_vs_E10	ordinary_muscle_development	ENSGALG00010016472	TFDP1	-1.427233012	0.122312503	-11.66874172	1.84126E-31	1.11577E-30	negative
PRJNA729271	stage_3W_vs_E10	ordinary_muscle_development	ENSGALG00010016473	RAB9B	-0.456043945	0.159885818	-2.852310175	0.004340273	0.006125231	negative
PRJNA729271	stage_3W_vs_E10	ordinary_muscle_development	ENSGALG00010016474	MAPK8IP3	-0.73153485	0.11148923	-6.561484472	5.32747E-11	1.30641E-10	negative
PRJNA729271	stage_3W_vs_E10	ordinary_muscle_development	ENSGALG00010016475	MCCC1	0.430924534	0.084079114	5.125226898	2.9718E-07	5.82662E-07	positive
PRJNA729271	stage_3W_vs_E10	ordinary_muscle_development	ENSGALG00010016477	CNIH3	-0.35274836	0.205656234	-1.715233002	0.086302516	0.105246086	negative
PRJNA729271	stage_3W_vs_E10	ordinary_muscle_development	ENSGALG00010016479	ENSGALG00010016479	-3.83567973	0.500120195	-7.669515781	1.72647E-14	5.09055E-14	negative
PRJNA729271	stage_3W_vs_E10	ordinary_muscle_development	ENSGALG00010016480	GPX3	4.009250783	0.133251929	30.08775054	7.008E-199	1.2388E-196	positive
PRJNA729271	stage_3W_vs_E10	ordinary_muscle_development	ENSGALG00010016482	EML1	1.510781808	0.140086463	10.7846381	4.06842E-27	2.08749E-26	positive
PRJNA729271	stage_3W_vs_E10	ordinary_muscle_development	ENSGALG00010016483	WDR26	0.279930183	0.102820685	2.722508432	0.006478838	0.008978942	positive
PRJNA729271	stage_3W_vs_E10	ordinary_muscle_development	ENSGALG00010016489	CCK	-5.829287104	0.648390524	-8.990395274	2.46343E-19	9.19109E-19	negative
PRJNA729271	stage_3W_vs_E10	ordinary_muscle_development	ENSGALG00010016491	DNAJC8	-0.226740038	0.053781222	-4.215970335	2.48706E-05	4.25922E-05	negative
PRJNA729271	stage_3W_vs_E10	ordinary_muscle_development	ENSGALG00010016496	PLP1	1.924037093	0.336977775	5.709685434	1.13185E-08	2.4356E-08	positive
PRJNA729271	stage_3W_vs_E10	ordinary_muscle_development	ENSGALG00010016498	KIF23	-3.270331646	0.210079091	-15.56714488	1.2172E-54	1.58379E-53	negative
PRJNA729271	stage_3W_vs_E10	ordinary_muscle_development	ENSGALG00010016500	ENSGALG00010016500	0.541037005	0.106838575	5.06406046	4.10419E-07	7.96923E-07	positive
PRJNA729271	stage_3W_vs_E10	ordinary_muscle_development	ENSGALG00010016502	YIPF5	-1.644326391	0.13357997	-12.30967778	8.03433E-35	5.49069E-34	negative
PRJNA729271	stage_3W_vs_E10	ordinary_muscle_development	ENSGALG00010016504	SNRK	0.733209155	0.084750741	8.651359839	5.08894E-18	1.79693E-17	positive
PRJNA729271	stage_3W_vs_E10	ordinary_muscle_development	ENSGALG00010016505	KCTD16	-4.012152016	0.474176453	-8.461305902	2.644E-17	8.9922E-17	negative
PRJNA729271	stage_3W_vs_E10	ordinary_muscle_development	ENSGALG00010016507	SPATA18	-0.230730957	0.142285495	-1.621605606	0.104887822	0.126453379	negative
PRJNA729271	stage_3W_vs_E10	ordinary_muscle_development	ENSGALG00010016508	CREBBP	0.178151509	0.119733481	1.487900524	0.13677713	0.162360535	positive
PRJNA729271	stage_3W_vs_E10	ordinary_muscle_development	ENSGALG00010016510	THG1L	-0.653727978	0.112565681	-5.807524723	6.34032E-09	1.38325E-08	negative
PRJNA729271	stage_3W_vs_E10	ordinary_muscle_development	ENSGALG00010016514	GASK1A	-2.578015286	0.255959567	-10.07196298	7.34963E-24	3.27592E-23	negative
PRJNA729271	stage_3W_vs_E10	ordinary_muscle_development	ENSGALG00010016518	TMEM42	0.550510278	0.140183941	3.927056657	8.59917E-05	0.000141177	positive
PRJNA729271	stage_3W_vs_E10	ordinary_muscle_development	ENSGALG00010016519	MEX3B	-3.46714312	0.181828538	-19.06820105	4.63994E-81	1.20315E-79	negative
PRJNA729271	stage_3W_vs_E10	ordinary_muscle_development	ENSGALG00010016520	CHMP1B	0.411715559	0.082728641	4.976699178	6.46777E-07	1.24041E-06	positive
PRJNA729271	stage_3W_vs_E10	ordinary_muscle_development	ENSGALG00010016526	EIF1B	1.423687415	0.113689453	12.52259886	5.61655E-36	3.97713E-35	positive
PRJNA729271	stage_3W_vs_E10	ordinary_muscle_development	ENSGALG00010016527	MRPS34	-0.29706037	0.098286332	-3.022397558	0.002507809	0.003619506	negative
PRJNA729271	stage_3W_vs_E10	ordinary_muscle_development	ENSGALG00010016529	DCUN1D1	-0.037779678	0.097579782	-0.387167067	0.698632529	0.728170521	negative
PRJNA729271	stage_3W_vs_E10	ordinary_muscle_development	ENSGALG00010016533	CNIH4	0.379728866	0.109709261	3.461228916	0.000537715	0.000825655	positive
PRJNA729271	stage_3W_vs_E10	ordinary_muscle_development	ENSGALG00010016534	TRIT1	-0.179686418	0.144634591	-1.242347471	0.214108388	0.246838549	negative
PRJNA729271	stage_3W_vs_E10	ordinary_muscle_development	ENSGALG00010016535	BNC2	-1.629951363	0.13259567	-12.29264399	9.92073E-35	6.75756E-34	negative
PRJNA729271	stage_3W_vs_E10	ordinary_muscle_development	ENSGALG00010016541	UBE2J2	0.117446294	0.079047465	1.485769261	0.137340163	0.162975495	positive
PRJNA729271	stage_3W_vs_E10	ordinary_muscle_development	ENSGALG00010016542	ENOX2	-0.033031872	0.076764849	-0.430299452	0.666977826	0.698745561	negative
PRJNA729271	stage_3W_vs_E10	ordinary_muscle_development	ENSGALG00010016543	TCEA3	5.133017715	0.173972095	29.50483359	2.4959E-191	4.2068E-189	positive
PRJNA729271	stage_3W_vs_E10	ordinary_muscle_development	ENSGALG00010016544	ADCY9	1.417786982	0.135500383	10.46334298	1.27284E-25	6.11326E-25	positive
PRJNA729271	stage_3W_vs_E10	ordinary_muscle_development	ENSGALG00010016546	C1QTNF12	-0.584766363	0.406270435	-1.43935249	0.150050678	0.176971894	negative
PRJNA729271	stage_3W_vs_E10	ordinary_muscle_development	ENSGALG00010016547	SGCB	2.171222396	0.143214306	15.16065302	6.44256E-52	7.75622E-51	positive
PRJNA729271	stage_3W_vs_E10	ordinary_muscle_development	ENSGALG00010016548	EVL	0.064965959	0.108788757	0.597175299	0.550390349	0.587518088	positive
PRJNA729271	stage_3W_vs_E10	ordinary_muscle_development	ENSGALG00010016549	PTPRA	-0.841651813	0.103476148	-8.133776015	4.16121E-16	1.32973E-15	negative
PRJNA729271	stage_3W_vs_E10	ordinary_muscle_development	ENSGALG00010016550	FBLIM1	-2.075302712	0.146976399	-14.11997251	2.86103E-45	2.76471E-44	negative
PRJNA729271	stage_3W_vs_E10	ordinary_muscle_development	ENSGALG00010016552	ENSGALG00010016552	3.288226154	0.442792492	7.426110909	1.11837E-13	3.16555E-13	positive
PRJNA729271	stage_3W_vs_E10	ordinary_muscle_development	ENSGALG00010016553	SLC25A14	-0.014269839	0.095052769	-0.150125444	0.880665646	0.894676797	negative
PRJNA729271	stage_3W_vs_E10	ordinary_muscle_development	ENSGALG00010016556	ASAP3	-1.066777496	0.197080257	-5.412908986	6.2009E-08	1.27006E-07	negative
PRJNA729271	stage_3W_vs_E10	ordinary_muscle_development	ENSGALG00010016558	NR2F2	-1.231102892	0.110156556	-11.17593849	5.3482E-29	2.95435E-28	negative
PRJNA729271	stage_3W_vs_E10	ordinary_muscle_development	ENSGALG00010016559	ROGDI	-0.84314307	0.117932029	-7.149398472	8.7159E-13	2.36011E-12	negative
PRJNA729271	stage_3W_vs_E10	ordinary_muscle_development	ENSGALG00010016560	DCUN1D4	1.361590545	0.169138916	8.050131689	8.2705E-16	2.60384E-15	positive
PRJNA729271	stage_3W_vs_E10	ordinary_muscle_development	ENSGALG00010016561	DEGS2	3.825917304	0.546737153	6.997726939	2.60148E-12	6.84862E-12	positive
PRJNA729271	stage_3W_vs_E10	ordinary_muscle_development	ENSGALG00010016562	GLIS2	0.715716854	0.220770615	3.241902698	0.001187346	0.001767544	positive
PRJNA729271	stage_3W_vs_E10	ordinary_muscle_development	ENSGALG00010016564	SPEN	0.850722635	0.165909029	5.12764518	2.93389E-07	5.75463E-07	positive

PRJNA729271	stage_3W_vs_E10	ordinary_muscle_development	ENSGALG00010016565	NVL	-0.493267101	0.097653704	-5.051186791	4.39073E-07	8.51535E-07	negative
PRJNA729271	stage_3W_vs_E10	ordinary_muscle_development	ENSGALG00010016566	CNTLN	-2.470776668	0.216822533	-11.39538698	4.40867E-30	2.53687E-29	negative
PRJNA729271	stage_3W_vs_E10	ordinary_muscle_development	ENSGALG00010016567	E2F2	-2.172984158	0.281130287	-7.729455918	1.08007E-14	3.21405E-14	negative
PRJNA729271	stage_3W_vs_E10	ordinary_muscle_development	ENSGALG00010016568	PAM16	1.828076106	0.133570401	13.68623656	1.22702E-42	1.07988E-41	positive
PRJNA729271	stage_3W_vs_E10	ordinary_muscle_development	ENSGALG00010016569	YY1	0.345917503	0.086752265	3.98741756	6.67964E-05	0.000110792	positive
PRJNA729271	stage_3W_vs_E10	ordinary_muscle_development	ENSGALG00010016572	RAB33A	0.062499958	0.114475962	0.545965779	0.585089461	0.620763559	positive
PRJNA729271	stage_3W_vs_E10	ordinary_muscle_development	ENSGALG00010016573	DEGS1	-0.247287099	0.128425044	-1.925536416	0.054162285	0.06767951	negative
PRJNA729271	stage_3W_vs_E10	ordinary_muscle_development	ENSGALG00010016575	CWH43	-0.497107961	0.402059556	-1.236403795	0.216308506	0.249077836	negative
PRJNA729271	stage_3W_vs_E10	ordinary_muscle_development	ENSGALG00010016576	MRPS26	0.282009833	0.106439239	2.649491262	0.008061306	0.011074649	positive
PRJNA729271	stage_3W_vs_E10	ordinary_muscle_development	ENSGALG00010016577	ZBTB17	1.281230858	0.185136434	6.92046849	4.50152E-12	1.16726E-11	positive
PRJNA729271	stage_3W_vs_E10	ordinary_muscle_development	ENSGALG00010016580	MARS2	-0.43675816	0.110001422	-3.970477406	7.17288E-05	0.00011858	negative
PRJNA729271	stage_3W_vs_E10	ordinary_muscle_development	ENSGALG00010016581	H2AFYMACROH2A1	0.366253476	0.076035058	4.816902728	1.45804E-06	2.72982E-06	positive
PRJNA729271	stage_3W_vs_E10	ordinary_muscle_development	ENSGALG00010016583	VPS41	0.20685323	0.082778226	2.498884535	0.01245849	0.016761259	positive
PRJNA729271	stage_3W_vs_E10	ordinary_muscle_development	ENSGALG00010016584	EHBP1	-0.042306353	0.16070179	-0.263259998	0.79235019	0.815588411	negative
PRJNA729271	stage_3W_vs_E10	ordinary_muscle_development	ENSGALG00010016588	ATP11B	-0.605792207	0.100520307	-6.02656546	1.6748E-09	3.76669E-09	negative
PRJNA729271	stage_3W_vs_E10	ordinary_muscle_development	ENSGALG00010016589	FBXO28	0.149880113	0.077167729	1.942264138	0.052105132	0.065238308	positive
PRJNA729271	stage_3W_vs_E10	ordinary_muscle_development	ENSGALG00010016591	HOMER2	-0.394547009	0.203563874	-1.938197586	0.052599121	0.065817012	negative
PRJNA729271	stage_3W_vs_E10	ordinary_muscle_development	ENSGALG00010016592	TMEM129	0.539421854	0.102706554	5.25206848	1.50401E-07	3.00532E-07	positive
PRJNA729271	stage_3W_vs_E10	ordinary_muscle_development	ENSGALG00010016597	FRYL	0.603925106	0.125349654	4.817923989	1.4506E-06	2.71659E-06	positive
PRJNA729271	stage_3W_vs_E10	ordinary_muscle_development	ENSGALG00010016598	AIFM1	0.159322977	0.088439086	1.801499589	0.071624172	0.088161716	positive
PRJNA729271	stage_3W_vs_E10	ordinary_muscle_development	ENSGALG00010016599	ENSGALG00010016599	-0.369376754	0.284542963	-1.298140535	0.194239047	0.225221582	negative
PRJNA729271	stage_3W_vs_E10	ordinary_muscle_development	ENSGALG00010016600	GNL2	-0.248198968	0.10953943	-2.265841343	0.023461099	0.030639574	negative
PRJNA729271	stage_3W_vs_E10	ordinary_muscle_development	ENSGALG00010016602	HSPB7	4.809498469	0.513406179	9.367823495	7.40436E-21	2.93401E-20	positive
PRJNA729271	stage_3W_vs_E10	ordinary_muscle_development	ENSGALG00010016603	ENSGALG00010016603	0.457312854	0.125160849	3.653801136	0.000258386	0.000407564	positive
PRJNA729271	stage_3W_vs_E10	ordinary_muscle_development	ENSGALG00010016604	TP53BP2	-1.374413387	0.063775931	-21.5506597	5.2198E-103	2.3209E-101	negative
PRJNA729271	stage_3W_vs_E10	ordinary_muscle_development	ENSGALG00010016605	WDPCP	-0.423163078	0.165680018	-2.554098457	0.010646315	0.014427668	negative
PRJNA729271	stage_3W_vs_E10	ordinary_muscle_development	ENSGALG00010016606	VASN	-1.200168265	0.181085443	-6.627635262	3.41107E-11	8.47796E-11	negative
PRJNA729271	stage_3W_vs_E10	ordinary_muscle_development	ENSGALG00010016607	FAM103A1	-0.24080014	0.114457909	-2.103831373	0.035393159	0.045276131	negative
PRJNA729271	stage_3W_vs_E10	ordinary_muscle_development	ENSGALG00010016609	SOX2	-1.884362575	0.575058668	-3.276817964	0.00104984	0.001570265	negative
PRJNA729271	stage_3W_vs_E10	ordinary_muscle_development	ENSGALG00010016611	DNALI1	-0.115641821	0.205942626	-0.561524453	0.574440065	0.610626925	negative
PRJNA729271	stage_3W_vs_E10	ordinary_muscle_development	ENSGALG00010016612	GRIA1	-1.17370691	0.33605544	-3.49259905	0.000478344	0.000738402	negative
PRJNA729271	stage_3W_vs_E10	ordinary_muscle_development	ENSGALG00010016613	SNIP1	-0.454565277	0.077146873	-5.892206091	3.81073E-09	8.39462E-09	negative
PRJNA729271	stage_3W_vs_E10	ordinary_muscle_development	ENSGALG00010016614	DNAJC19	1.191942838	0.138786129	8.588342728	8.82327E-18	3.0788E-17	positive
PRJNA729271	stage_3W_vs_E10	ordinary_muscle_development	ENSGALG00010016615	BCORL1	0.780036192	0.126183669	6.181752342	6.33939E-10	1.46367E-09	positive
PRJNA729271	stage_3W_vs_E10	ordinary_muscle_development	ENSGALG00010016616	SLBP	-3.467963228	0.248330795	-13.96509533	2.54565E-44	2.37599E-43	negative
PRJNA729271	stage_3W_vs_E10	ordinary_muscle_development	ENSGALG00010016618	TRPM3	-2.344826494	0.501898451	-4.671914186	2.98406E-06	5.48212E-06	negative
PRJNA729271	stage_3W_vs_E10	ordinary_muscle_development	ENSGALG00010016619	SLC25A47	0.298625876	0.33419133	0.893577567	0.371547953	0.410205467	positive
PRJNA729271	stage_3W_vs_E10	ordinary_muscle_development	ENSGALG00010016620	ENSGALG00010016620	0.20544955	0.121377442	1.69265018	0.090522073	0.110150894	positive
PRJNA729271	stage_3W_vs_E10	ordinary_muscle_development	ENSGALG00010016622	KIAA1143	-0.393234244	0.133964755	-2.935355987	0.003331652	0.0047588	negative
PRJNA729271	stage_3W_vs_E10	ordinary_muscle_development	ENSGALG00010016623	DNAJA3	1.719430078	0.140978771	12.19637582	3.24969E-34	2.17571E-33	positive
PRJNA729271	stage_3W_vs_E10	ordinary_muscle_development	ENSGALG00010016624	MDH1	0.736981543	0.154714133	4.763504985	1.90259E-06	3.53474E-06	positive
PRJNA729271	stage_3W_vs_E10	ordinary_muscle_development	ENSGALG00010016626	CAPN2	0.490764418	0.229331425	2.139978934	0.032356469	0.041560215	positive
PRJNA729271	stage_3W_vs_E10	ordinary_muscle_development	ENSGALG00010016629	LARS1	-0.512188477	0.071448699	-7.168618613	7.57583E-13	2.0564E-12	negative
PRJNA729271	stage_3W_vs_E10	ordinary_muscle_development	ENSGALG00010016630	ENSGALG00010016630	2.082768299	0.330645192	6.299103529	2.99372E-10	7.04448E-10	positive
PRJNA729271	stage_3W_vs_E10	ordinary_muscle_development	ENSGALG00010016631	FAM53A	1.658574306	0.091189221	18.18827155	6.39245E-74	1.38296E-72	positive
PRJNA729271	stage_3W_vs_E10	ordinary_muscle_development	ENSGALG00010016637	POU6F2	-3.303247063	0.864152668	-3.822527181	0.000132091	0.000213809	negative
PRJNA729271	stage_3W_vs_E10	ordinary_muscle_development	ENSGALG00010016640	OCIAD1	1.474589049	0.103433455	14.2564033	4.08997E-46	4.07171E-45	positive
PRJNA729271	stage_3W_vs_E10	ordinary_muscle_development	ENSGALG00010016643	BTBD1	1.30230973	0.173214423	7.518483187	5.54154E-14	1.59469E-13	positive
PRJNA729271	stage_3W_vs_E10	ordinary_muscle_development	ENSGALG00010016644	NMRAL1	-2.290404915	0.207323409	-11.04749785	2.2541E-28	1.22143E-27	negative
PRJNA729271	stage_3W_vs_E10	ordinary_muscle_development	ENSGALG00010016647	EGR1	1.588073543	0.204676211	7.758955173	8.56319E-15	2.56242E-14	positive
PRJNA729271	stage_3W_vs_E10	ordinary_muscle_development	ENSGALG00010016648	ADRA2C	-1.85158771	0.291960393	-6.341914022	2.26928E-10	5.37734E-10	negative
PRJNA729271	stage_3W_vs_E10	ordinary_muscle_development	ENSGALG00010016651	ENSGALG00010016651	2.70143067	0.179586642	15.04249227	3.86712E-51	4.53878E-50	positive
PRJNA729271	stage_3W_vs_E10	ordinary_muscle_development	ENSGALG00010016652	FXR1	2.434675249	0.098831744	24.63454706	5.389E-134	4.0265E-132	positive
PRJNA729271	stage_3W_vs_E10	ordinary_muscle_development	ENSGALG00010016653	EPHA2	-0.40052207	0.20945199	-1.912238077	0.055845664	0.069704891	negative
PRJNA729271	stage_3W_vs_E10	ordinary_muscle_development	ENSGALG00010016654	SMIM3	0.828870386	0.183312803	4.521617551	6.13689E-06	1.10228E-05	positive
PRJNA729271	stage_3W_vs_E10	ordinary_muscle_development	ENSGALG00010016655	TRANK1	0.697704266	0.323678933	2.155544259	0.031119269	0.040038506	positive
PRJNA729271	stage_3W_vs_E10	ordinary_muscle_development	ENSGALG00010016656	CAPN8	-4.932779651	1.188356961	-4.150924187	3.31135E-05	5.61053E-05	negative
PRJNA729271	stage_3W_vs_E10	ordinary_muscle_development	ENSGALG00010016657	SLC25A29	1.671132244	0.279337256	5.982489649	2.19752E-09	4.90048E-09	positive
PRJNA729271	stage_3W_vs_E10	ordinary_muscle_development	ENSGALG00010016658	ZAR1	-2.320002137	0.581265923	-3.991292188	6.57143E-05	0.00010906	negative
PRJNA729271	stage_3W_vs_E10	ordinary_muscle_development	ENSGALG00010016661	UBE4B	-0.561783648	0.128616473	-4.367898099	1.25448E-05	2.20035E-05	negative
PRJNA729271	stage_3W_vs_E10	ordinary_muscle_development	ENSGALG00010016664	TM6SF1	3.0548265	0.248400623	12.29798246	9.28648E-35	6.32851E-34	positive
PRJNA729271	stage_3W_vs_E10	ordinary_muscle_development	ENSGALG00010016665	ENSGALG00010016665	7.675966294	0.987461333	7.773434807	7.6386E-15	2.29367E-14	positive
PRJNA729271	stage_3W_vs_E10	ordinary_muscle_development	ENSGALG00010016667	WARS1	-0.096094783	0.143173729	-0.671176087	0.502108361	0.540155907	negative
PRJNA729271	stage_3W_vs_E10	ordinary_muscle_development	ENSGALG00010016671	CASS4	-0.588824822	0.24775629	-2.376629148	0.017471644	0.023121654	negative
PRJNA729271	stage_3W_vs_E10	ordinary_muscle_development	ENSGALG00010016672	DCTN4	0.094293625	0.080622191	1.169574084	0.242172412	0.276771198	positive
PRJNA729271	stage_3W_vs_E10	ordinary_muscle_development	ENSGALG00010016673	LRPAP1	0.248187926	0.079295106	3.129927425	0.001748495	0.002561079	positive
PRJNA729271	stage_3W_vs_E10	ordinary_muscle_development	ENSGALG00010016674	SLC10A4	-0.620478169	0.358470927	-1.730902346	0.083469183	0.101936626	negative
PRJNA729271	stage_3W_vs_E10	ordinary_muscle_development	ENSGALG00010016675	SKIL	-0.238243577	0.139660454	-1.70587715	0.088030955	0.107236633	negative
PRJNA729271	stage_3W_vs_E10	ordinary_muscle_development	ENSGALG00010016676	NLRC3	4.025590254	0.407435676	9.880308698	5.06766E-23	2.19074E-22	positive
PRJNA729271	stage_3W_vs_E10	ordinary_muscle_development	ENSGALG00010016677	MEAF6	0.715472355	0.087749972	8.153533725	3.53442E-16	1.13319E-15	positive
PRJNA729271	stage_3W_vs_E10	ordinary_muscle_development	ENSGALG00010016678	NOX1	3.203073832	0.311554818	10.28093179	8.58931E-25	3.98407E-24	positive
PRJNA729271	stage_3W_vs_E10	ordinary_muscle_development	ENSGALG00010016680	BPIFB3	-0.974573688	1.018056798	-0.957288129	0.338421845	0.376963394	negative
PRJNA729271	stage_3W_vs_E10	ordinary_muscle_development	ENSGALG00010016681	SUSD4	2.065168515	0.281568316	7.334520244	2.22517E-13	6.19433E-13	positive
PRJNA729271	stage_3W_vs_E10	ordinary_muscle_development	ENSGALG00010016682	GPR141	1.629638157	0.429132608	3.797516498	0.000146153	0.000235545	positive
PRJNA729271	stage_3W_vs_E10	ordinary_muscle_development	ENSGALG00010016683	RBM22	-0.633623272	0.074206472	-8.538652479	1.35796E-17	4.69329E-17	negative
PRJNA729271	stage_3W_vs_E10	ordinary_muscle_development	ENSGALG00010016691	ENSGALG00010016691	0.9485003	0.301413885	3.146836782	0.001650471	0.002427064	positive
PRJNA729271	stage_3W_vs_E10	ordinary_muscle_development	ENSGALG00010016692	PRKCI	-1.789630615	0.075385494	-23.73972118	1.4031E-124	9.0389E-123	negative
PRJNA729271	stage_3W_vs_E10	ordinary_muscle_development	ENSGALG00010016696	DOK7	0.751842924	0.129451154	5.807927574	6.32509E-09	1.38055E-08	positive
PRJNA729271	stage_3W_vs_E10	ordinary_muscle_development	ENSGALG00010016699	SYNPO	1.978791163	0.144812086	13.66454432	1.65336E-42	1.44981E-41	positive
PRJNA729271	stage_3W_vs_E10	ordinary_muscle_development	ENSGALG00010016700	CLUAP1	-0.763031503	0.125614856	-6.074373112	1.24473E-09	2.82132E-09	negative
PRJNA729271	stage_3W_vs_E10	ordinary_muscle_development	ENSGALG00010016702	WDR25	0.280979513	0.096548696	2.910236221	0.003611557	0.005139864	positive
PRJNA729271	stage_3W_vs_E10	ordinary_muscle_development	ENSGALG00010016704	CSTF2	-0.811367108	0.073897795	-10.97958483	4.79121E-28	2.55796E-27	negative
PRJNA729271	stage_3W_vs_E10	ordinary_muscle_development	ENSGALG00010016706	RTN4	2.198063985	0.125441842	17.52257419	9.63648E-69	1.80233E-67	positive
PRJNA729271	stage_3W_vs_E10	ordinary_muscle_development	ENSGALG00010016707	KIF1B	1.434249206	0.130432797	10.99607796	3.99116E-28	2.13712E-27	positive
PRJNA729271	stage_3W_vs_E10	ordinary_muscle_development	ENSGALG00010016712	ENSGALG00010016712	-0.137612884	0.161684293	-0.851120914	0.394702189	0.433654834	negative
PRJNA729271	stage_3W_vs_E10	ordinary_muscle_development	ENSGALG00010016720	INTS15	-0.370117766	0.100463141	-3.684115	0.000229499	0.000363402	negative
PRJNA729271	stage_3W_vs_E10	ordinary_muscle_development	ENSGALG00010016724	PGD	-1.606092139	0.110873398	-14.48582052	1.48933E-47	1.55421E-46	negative
PRJNA729271	stage_3W_vs_E10	ordinary_muscle_development	ENSGALG00010016729	ELMO1	0.783706757	0.094242291	8.315871215	9.10807E-17	3.01901E-16	positive
PRJNA729271	stage_3W_vs_E10	ordinary_muscle_development	ENSGALG00010016731	C4orf48	-1.821350991	0.276734662	-6.581578813	4.65479E-11	1.14649E-10	negative
PRJNA729271	stage_3W_vs_E10	ordinary_muscle_development	ENSGALG00010016732	ARHGAP26	-0.065337046	0.076898981	-0.849647742	0.395520961	0.43438978	negative
PRJNA729271	stage_3W_vs_E10	ordinary_muscle_development	ENSGALG00010016733	PHC3	0.660765429	0.154790631	4.268768876	1.96555E-05	3.39862E-05	positive
PRJNA729271	stage_3W_vs_E10	ordinary_muscle_development	ENSGALG00010016735	ENSGALG00010016735	-1.020557468	0.342448449	-2.980178388	0.002880806	0.004136022	negative
PRJNA729271	stage_3W_vs_E10	ordinary_muscle_development	ENSGALG00010016736	CENPS	-3.690382375	0.276258322	-13.35844782	1.05746E-40	8.7041E-40	negative
PRJNA729271	stage_3W_vs_E10	ordinary_muscle_development	ENSGALG00010016738	NAA60	1.193147856	0.149436382	7.98431972	1.41299E-15	4.39137E-15	positive
PRJNA729271	stage_3W_vs_E10	ordinary_muscle_development	ENSGALG00010016741	NELFA	-0.32118862	0.089783445	-3.577370196	0.000347068	0.000542166	negative
PRJNA729271	stage_3W_vs_E10	ordinary_muscle_development	ENSGALG00010016742	TRMT12	-0.271681444	0.139605813	-1.946061118	0.051647398	0.064676375	negative
PRJNA729271	stage_3W_vs_E10	ordinary_muscle_development	ENSGALG00010016744	CRYBG2	-7.81486364	1.035850956	-7.544390047	4.54409E-14	1.31444E-13	negative
PRJNA729271	stage_3W_vs_E10	ordinary_muscle_development	ENSGALG00010016745	BEGAIN	0.725678902	0.364735143	1.989605105	0.046634453	0.058784799	positive
PRJNA729271	stage_3W_vs_E10	ordinary_muscle_development	ENSGALG00010016746	HDGFL3	-1.8509961	0.124453905	-14.87294512	4.93919E-50	5.60638E-49	negative
PRJNA729271	stage_3W_vs_E10	ordinary_muscle_development	ENSGALG00010016750	SYTL4	0.482267501	0.126630864	3.808451475	0.00013984	0.000225872	positive
PRJNA729271	stage_3W_vs_E10	ordinary_muscle_development	ENSGALG00010016751	DFFA	-1.159905287	0.090845333	-12.76791275	2.47708E-37	1.81891E-36	negative
PRJNA729271	stage_3W_vs_E10	ordinary_muscle_development	ENSGALG00010016752	ENSGALG00010016752	1.863420519	0.171315227	10.87714472	1.4813E-27	7.75425E-27	positive
PRJNA729271	stage_3W_vs_E10	ordinary_muscle_development	ENSGALG00010016754	ENSGALG00010016754	0.244954123	0.184134021	1.330303447	0.183418312	0.21351176	positive
PRJNA729271	stage_3W_vs_E10	ordinary_muscle_development	ENSGALG00010016756	NUDT16L1	-0.406775204	0.107082773	-3.798698823	0.000145458	0.000234502	negative
PRJNA729271	stage_3W_vs_E10	ordinary_muscle_development	ENSGALG00010016757	SRPX2	-1.032109989	0.280491786	-3.679644249	0.00023356	0.000369711	negative
PRJNA729271	stage_3W_vs_E10	ordinary_muscle_development	ENSGALG00010016758	NR3C1	0.167849796	0.068562907	2.448113743	0.014360631	0.019185009	positive
PRJNA729271	stage_3W_vs_E10	ordinary_muscle_development	ENSGALG00010016760	DLK1	0.186598248	0.262483339	0.710895589	0.477148939	0.515870357	positive
PRJNA729271	stage_3W_vs_E10	ordinary_muscle_development	ENSGALG00010016761	GPR160	3.529637541	0.341810742	10.32629203	5.35924E-25	2.50911E-24	positive
PRJNA729271	stage_3W_vs_E10	ordinary_muscle_development	ENSGALG00010016762	EML6	0.097663046	0.123223268	0.792569842	0.428028485	0.466451612	positive
PRJNA729271	stage_3W_vs_E10	ordinary_muscle_development	ENSGALG00010016763	UBALD1	1.267947177	0.172275472	7.359998287	1.83913E-13	5.14437E-13	positive
PRJNA729271	stage_3W_vs_E10	ordinary_muscle_development	ENSGALG00010016766	AOAH	1.717358362	0.325704534	5.272749318	1.34395E-07	2.69441E-07	positive
PRJNA729271	stage_3W_vs_E10	ordinary_muscle_development	ENSGALG00010016767	SLAIN2	0.809329909	0.092229935	8.775132555	1.70703E-18	6.14169E-18	positive
PRJNA729271	stage_3W_vs_E10	ordinary_muscle_development	ENSGALG00010016768	GALNT10	-0.087840646	0.100706127	-0.872247286	0.383073488	0.422030114	negative
PRJNA729271	stage_3W_vs_E10	ordinary_muscle_development	ENSGALG00010016769	BNC1	-8.64241096	0.474372361	-18.21862249	3.67285E-74	7.9817E-73	negative
PRJNA729271	stage_3W_vs_E10	ordinary_muscle_development	ENSGALG00010016772	TSPAN6	-1.304704946	0.127004175	-10.27292961	9.33267E-25	4.32333E-24	negative
PRJNA729271	stage_3W_vs_E10	ordinary_muscle_development	ENSGALG00010016773	RPS27A	0.159559132	0.161285113	0.989298572	0.322517083	0.360521718	positive
PRJNA729271	stage_3W_vs_E10	ordinary_muscle_development	ENSGALG00010016774	PEX14	-0.063495989	0.118915047	-0.533960926	0.593368568	0.628948987	negative
PRJNA729271	stage_3W_vs_E10	ordinary_muscle_development	ENSGALG00010016777	TNMD	-2.905800725	0.489954101	-5.930761105	3.01534E-09	6.67491E-09	negative
PRJNA729271	stage_3W_vs_E10	ordinary_muscle_development	ENSGALG00010016778	MTIF2	0.30297674	0.107767642	2.811388797	0.004932814	0.006922368	positive
PRJNA729271	stage_3W_vs_E10	ordinary_muscle_development	ENSGALG00010016779	CTNNA2	-2.783410134	0.155116599	-17.94398638	5.34774E-72	1.09795E-70	negative
PRJNA729271	stage_3W_vs_E10	ordinary_muscle_development	ENSGALG00010016780	PRORSD1P	-1.566838015	0.141552275	-11.0689709	1.77426E-28	9.63941E-28	negative
PRJNA729271	stage_3W_vs_E10	ordinary_muscle_development	ENSGALG00010016781	CDIP1	1.491320249	0.143607345	10.38470733	2.91061E-25	1.37693E-24	positive
PRJNA729271	stage_3W_vs_E10	ordinary_muscle_development	ENSGALG00010016783	CLBA1	0.107915424	0.29853684	0.361481096	0.717739835	0.74631556	positive
PRJNA729271	stage_3W_vs_E10	ordinary_muscle_development	ENSGALG00010016785	PDGFRB	0.305660794	0.194418785	1.572177262	0.115909447	0.138897771	positive
PRJNA729271	stage_3W_vs_E10	ordinary_muscle_development	ENSGALG00010016786	DISP1	-0.631871741	0.111787414	-5.652440826	1.58185E-08	3.37339E-08	negative
PRJNA729271	stage_3W_vs_E10	ordinary_muscle_development	ENSGALG00010016787	PPP2R5C	-0.227789665	0.075484963	-3.017682685	0.002547155	0.003674829	negative
PRJNA729271	stage_3W_vs_E10	ordinary_muscle_development	ENSGALG00010016788	ANLN	-3.32848449	0.290197402	-11.46972534	1.87253E-30	1.09445E-29	negative
PRJNA729271	stage_3W_vs_E10	ordinary_muscle_development	ENSGALG00010016789	DISP3	1.284748965	0.111653548	11.50656637	1.22249E-30	7.19237E-30	positive
PRJNA729271	stage_3W_vs_E10	ordinary_muscle_development	ENSGALG00010016791	LRRTM1	-4.792150706	0.631943764	-7.583191696	3.37156E-14	9.79767E-14	negative
PRJNA729271	stage_3W_vs_E10	ordinary_muscle_development	ENSGALG00010016792	HMOX2	-0.902857565	0.122936703	-7.344084747	2.07172E-13	5.77825E-13	negative
PRJNA729271	stage_3W_vs_E10	ordinary_muscle_development	ENSGALG00010016793	SLC9A1	0.411446407	0.155710642	2.6423782	0.008232607	0.011292859	positive

PRJNA729271	stage_3W_vs_E10	ordinary_muscle_development	ENSGALG00010016795	GUCA2B	2.857382587	1.2258565	2.330927467	0.019757185	0.026010935	positive
PRJNA729271	stage_3W_vs_E10	ordinary_muscle_development	ENSGALG00010016796	BROX	0.528282003	0.084085033	6.282711454	3.32718E-10	7.80507E-10	positive
PRJNA729271	stage_3W_vs_E10	ordinary_muscle_development	ENSGALG00010016797	SEC62	-0.238366758	0.109239479	-2.182056893	0.029105334	0.037584126	negative
PRJNA729271	stage_3W_vs_E10	ordinary_muscle_development	ENSGALG00010016799	WDTC1	1.553630784	0.13190802	11.77813736	5.05988E-32	3.14372E-31	positive
PRJNA729271	stage_3W_vs_E10	ordinary_muscle_development	ENSGALG00010016800	RPL14	-0.008939341	0.169054753	-0.052878378	0.957828812	0.963211366	negative
PRJNA729271	stage_3W_vs_E10	ordinary_muscle_development	ENSGALG00010016802	TMEM222	-0.399336103	0.085272636	-4.683051001	2.82636E-06	5.19834E-06	negative
PRJNA729271	stage_3W_vs_E10	ordinary_muscle_development	ENSGALG00010016804	CSF1R	2.87605657	0.1357511	21.18624865	1.2789E-99	5.26637E-98	positive
PRJNA729271	stage_3W_vs_E10	ordinary_muscle_development	ENSGALG00010016805	ENSGALG00010016805	-4.77424246	0.86269549	-5.534099244	3.12832E-08	6.52915E-08	negative
PRJNA729271	stage_3W_vs_E10	ordinary_muscle_development	ENSGALG00010016807	VSIG4	5.304147673	0.229775614	23.08403219	6.6991E-118	3.8841E-116	positive
PRJNA729271	stage_3W_vs_E10	ordinary_muscle_development	ENSGALG00010016808	ENTPD3	-2.766891532	0.619483429	-4.466449627	7.95284E-06	1.41478E-05	negative
PRJNA729271	stage_3W_vs_E10	ordinary_muscle_development	ENSGALG00010016809	SYTL1	1.321052414	0.486899133	2.713195248	0.006663783	0.009227315	positive
PRJNA729271	stage_3W_vs_E10	ordinary_muscle_development	ENSGALG00010016810	SH3GL3	-2.19191943	0.202216761	-10.83945472	2.23801E-27	1.16231E-26	negative
PRJNA729271	stage_3W_vs_E10	ordinary_muscle_development	ENSGALG00010016812	LRRC31	-0.897834921	0.131227441	-6.841822971	7.81917E-12	2.00635E-11	negative
PRJNA729271	stage_3W_vs_E10	ordinary_muscle_development	ENSGALG00010016813	ENSGALG00010016813	-2.409594846	0.551353573	-4.370325984	1.24061E-05	2.17655E-05	negative
PRJNA729271	stage_3W_vs_E10	ordinary_muscle_development	ENSGALG00010016814	HMGXB3	0.4260976	0.099828696	4.26828776	1.96979E-05	3.40555E-05	positive
PRJNA729271	stage_3W_vs_E10	ordinary_muscle_development	ENSGALG00010016816	MAP3K6	3.497569497	0.258976129	13.50537409	1.45373E-41	1.23012E-40	positive
PRJNA729271	stage_3W_vs_E10	ordinary_muscle_development	ENSGALG00010016817	GGCT	-0.522528063	0.099663742	-5.242910311	1.58063E-07	3.15235E-07	negative
PRJNA729271	stage_3W_vs_E10	ordinary_muscle_development	ENSGALG00010016820	EIF2AK3	-0.566709001	0.109055926	-5.196498927	2.03077E-07	4.02186E-07	negative
PRJNA729271	stage_3W_vs_E10	ordinary_muscle_development	ENSGALG00010016823	HSF3	1.163681426	0.078739879	14.77880632	2.00691E-49	2.22232E-48	positive
PRJNA729271	stage_3W_vs_E10	ordinary_muscle_development	ENSGALG00010016825	ENSGALG00010016825	3.673052244	0.290701377	12.63513879	1.35155E-36	9.74181E-36	positive
PRJNA729271	stage_3W_vs_E10	ordinary_muscle_development	ENSGALG00010016828	LACTBL1	4.226888881	0.379229117	11.14600302	7.48956E-29	4.11684E-28	positive
PRJNA729271	stage_3W_vs_E10	ordinary_muscle_development	ENSGALG00010016831	AIDA	-0.754318646	0.067673026	-11.14651864	7.4463E-29	4.09462E-28	negative
PRJNA729271	stage_3W_vs_E10	ordinary_muscle_development	ENSGALG00010016832	DYNC1H1	-0.224836134	0.094192666	-2.386981328	0.016987354	0.022507468	negative
PRJNA729271	stage_3W_vs_E10	ordinary_muscle_development	ENSGALG00010016834	ENSGALG00010016834	1.374554218	0.07421846	18.52038181	1.41431E-76	3.23349E-75	positive
PRJNA729271	stage_3W_vs_E10	ordinary_muscle_development	ENSGALG00010016835	CCDC88A	-1.644470639	0.15211114	-10.81098095	3.05387E-27	1.57474E-26	negative
PRJNA729271	stage_3W_vs_E10	ordinary_muscle_development	ENSGALG00010016836	HEPH	2.15126883	0.148545296	14.48224136	1.56896E-47	1.63377E-46	positive
PRJNA729271	stage_3W_vs_E10	ordinary_muscle_development	ENSGALG00010016840	LRRC34	-0.136541752	0.204710979	-0.666997697	0.504773605	0.54270089	negative
PRJNA729271	stage_3W_vs_E10	ordinary_muscle_development	ENSGALG00010016842	NPY7R	0.08478578	0.431815839	0.196347083	0.844338512	0.862608312	positive
PRJNA729271	stage_3W_vs_E10	ordinary_muscle_development	ENSGALG00010016845	MIA3	1.254728674	0.137456279	9.128201935	6.96464E-20	2.65037E-19	positive
PRJNA729271	stage_3W_vs_E10	ordinary_muscle_development	ENSGALG00010016847	EPHB2	-1.829027701	0.10736947	-17.03489557	4.52549E-65	7.75378E-64	negative
PRJNA729271	stage_3W_vs_E10	ordinary_muscle_development	ENSGALG00010016848	ENSGALG00010016848	-0.107404349	0.172865343	-0.621318001	0.534390412	0.572166422	negative
PRJNA729271	stage_3W_vs_E10	ordinary_muscle_development	ENSGALG00010016849	WASF2	0.835573097	0.152703381	5.471870305	4.45311E-08	9.21059E-08	positive
PRJNA729271	stage_3W_vs_E10	ordinary_muscle_development	ENSGALG00010016851	TEC	2.295919415	0.136994042	16.75926476	4.8458E-63	7.88326E-62	positive
PRJNA729271	stage_3W_vs_E10	ordinary_muscle_development	ENSGALG00010016852	AHDC1	-0.272688553	0.17026017	-1.601599202	0.109244267	0.131312352	negative
PRJNA729271	stage_3W_vs_E10	ordinary_muscle_development	ENSGALG00010016854	TAF1A	0.054299634	0.104177796	0.5212208	0.602212966	0.637717486	positive
PRJNA729271	stage_3W_vs_E10	ordinary_muscle_development	ENSGALG00010016856	NOD1	-0.275591364	0.138738113	-1.986414243	0.046987346	0.059183315	negative
PRJNA729271	stage_3W_vs_E10	ordinary_muscle_development	ENSGALG00010016858	ENSGALG00010016858	-2.65629975	0.193057183	-13.75913455	4.48868E-43	4.01131E-42	negative
PRJNA729271	stage_3W_vs_E10	ordinary_muscle_development	ENSGALG00010016859	RPIA	0.937881667	0.130262361	7.199943704	6.02374E-13	1.64063E-12	positive
PRJNA729271	stage_3W_vs_E10	ordinary_muscle_development	ENSGALG00010016860	VSX1	-1.661510332	0.514419949	-3.2298715	0.001238459	0.001840232	negative
PRJNA729271	stage_3W_vs_E10	ordinary_muscle_development	ENSGALG00010016861	FGR	1.028201735	0.121516894	8.461389174	2.64211E-17	8.98789E-17	positive
PRJNA729271	stage_3W_vs_E10	ordinary_muscle_development	ENSGALG00010016862	TXK	6.011257282	0.506932892	11.8580928	1.95375E-32	1.23397E-31	positive
PRJNA729271	stage_3W_vs_E10	ordinary_muscle_development	ENSGALG00010016863	MYNN	-0.200529082	0.082178664	-2.440159919	0.014680761	0.019598235	negative
PRJNA729271	stage_3W_vs_E10	ordinary_muscle_development	ENSGALG00010016864	ENSGALG00010016864	0.519052511	0.26563335	1.954018617	0.050699016	0.063560131	positive
PRJNA729271	stage_3W_vs_E10	ordinary_muscle_development	ENSGALG00010016865	CFAP36	-1.278888461	0.138774419	-9.215592244	3.09565E-20	1.1953E-19	negative
PRJNA729271	stage_3W_vs_E10	ordinary_muscle_development	ENSGALG00010016866	ZNRF2	0.061645562	0.105337617	0.585218873	0.558400556	0.595454724	positive
PRJNA729271	stage_3W_vs_E10	ordinary_muscle_development	ENSGALG00010016867	FLT4	-0.471825939	0.120543381	-3.914158827	9.07199E-05	0.000148586	negative
PRJNA729271	stage_3W_vs_E10	ordinary_muscle_development	ENSGALG00010016868	NIPAL1	2.730773058	0.465551803	5.865669593	4.47324E-09	9.81823E-09	positive
PRJNA729271	stage_3W_vs_E10	ordinary_muscle_development	ENSGALG00010016869	HSP90AA1	0.314613739	0.156347948	2.012266504	0.044191851	0.055836897	positive
PRJNA729271	stage_3W_vs_E10	ordinary_muscle_development	ENSGALG00010016872	EDA2R	1.798081831	0.280307822	6.41466877	1.41129E-10	3.37681E-10	positive
PRJNA729271	stage_3W_vs_E10	ordinary_muscle_development	ENSGALG00010016873	ENTPD6	0.844441159	0.138840487	6.082095899	1.18622E-09	2.6908E-09	positive
PRJNA729271	stage_3W_vs_E10	ordinary_muscle_development	ENSGALG00010016874	ADAMTSL3	-0.578527179	0.145402323	-3.978802852	6.92631E-05	0.000114648	negative
PRJNA729271	stage_3W_vs_E10	ordinary_muscle_development	ENSGALG00010016875	SESN2	3.058872801	0.217615561	14.05631464	7.04612E-45	6.69289E-44	positive
PRJNA729271	stage_3W_vs_E10	ordinary_muscle_development	ENSGALG00010016876	MTURN	-1.05038239	0.192583319	-5.454171183	4.92018E-08	1.01535E-07	negative
PRJNA729271	stage_3W_vs_E10	ordinary_muscle_development	ENSGALG00010016877	UBE2D3	0.965700305	0.13545879	7.129107722	1.01022E-12	2.72632E-12	positive
PRJNA729271	stage_3W_vs_E10	ordinary_muscle_development	ENSGALG00010016878	SUCLG1	1.366725703	0.12539177	10.89964443	1.15709E-27	6.08782E-27	positive
PRJNA729271	stage_3W_vs_E10	ordinary_muscle_development	ENSGALG00010016879	RNF130	-0.238512357	0.100914211	-2.363516041	0.018102443	0.02392586	negative
PRJNA729271	stage_3W_vs_E10	ordinary_muscle_development	ENSGALG00010016880	AR	1.335974094	0.201534848	6.628997942	3.37973E-11	8.40151E-11	positive
PRJNA729271	stage_3W_vs_E10	ordinary_muscle_development	ENSGALG00010016881	C1QB	3.202088944	0.289972287	11.04274128	2.3767E-28	1.28642E-27	positive
PRJNA729271	stage_3W_vs_E10	ordinary_muscle_development	ENSGALG00010016882	ENSGALG00010016882	-0.745076464	0.04969538	-14.99287181	8.1741E-51	9.5244E-50	negative
PRJNA729271	stage_3W_vs_E10	ordinary_muscle_development	ENSGALG00010016883	TMEM251	0.404709817	0.076804355	5.269360302	1.369E-07	2.74349E-07	positive
PRJNA729271	stage_3W_vs_E10	ordinary_muscle_development	ENSGALG00010016884	SBK1	-1.450151473	0.288532053	-5.02596318	5.00912E-07	9.66807E-07	negative
PRJNA729271	stage_3W_vs_E10	ordinary_muscle_development	ENSGALG00010016885	CDCA4	-0.56480703	0.153550982	-3.678302945	0.000234791	0.000371579	negative
PRJNA729271	stage_3W_vs_E10	ordinary_muscle_development	ENSGALG00010016887	TJP2	0.238750541	0.139003062	1.717591957	0.085871068	0.104737558	positive
PRJNA729271	stage_3W_vs_E10	ordinary_muscle_development	ENSGALG00010016888	ENSGALG00010016888	-0.278969105	0.084960396	-3.28351935	0.001025196	0.001534613	negative
PRJNA729271	stage_3W_vs_E10	ordinary_muscle_development	ENSGALG00010016889	FUNDC2	1.690500493	0.086875909	19.45879499	2.45489E-84	6.92289E-83	positive
PRJNA729271	stage_3W_vs_E10	ordinary_muscle_development	ENSGALG00010016890	UBR7	-0.643725659	0.075571826	-8.518064102	1.62242E-17	5.57672E-17	negative
PRJNA729271	stage_3W_vs_E10	ordinary_muscle_development	ENSGALG00010016891	ENSGALG00010016891	-2.15783518	1.166841741	-1.849295499			negative
PRJNA729271	stage_3W_vs_E10	ordinary_muscle_development	ENSGALG00010016892	MAL	0.04464155	0.463704955	0.096271455	0.923304982	0.932179822	positive
PRJNA729271	stage_3W_vs_E10	ordinary_muscle_development	ENSGALG00010016893	TBC1D9B	0.013643029	0.050829448	0.268407964	0.78838531	0.811795487	positive
PRJNA729271	stage_3W_vs_E10	ordinary_muscle_development	ENSGALG00010016894	BTBD7	0.413442842	0.091618411	4.512661111	6.40193E-06	1.14775E-05	positive
PRJNA729271	stage_3W_vs_E10	ordinary_muscle_development	ENSGALG00010016895	ENSGALG00010016895	0.595498377	0.159303274	3.738142751	0.000185385	0.000296137	positive
PRJNA729271	stage_3W_vs_E10	ordinary_muscle_development	ENSGALG00010016896	SPTBN1	-0.303644638	0.098781409	-3.073904693	0.002112769	0.003070134	negative
PRJNA729271	stage_3W_vs_E10	ordinary_muscle_development	ENSGALG00010016898	ENSGALG00010016898	0.760738389	0.629454918	1.208566916	0.226829273	0.26034447	positive
PRJNA729271	stage_3W_vs_E10	ordinary_muscle_development	ENSGALG00010016899	SAXO2	-1.170929579	0.559188713	-2.093979283	0.036261826	0.046330116	negative
PRJNA729271	stage_3W_vs_E10	ordinary_muscle_development	ENSGALG00010016900	CISD2	-0.339436613	0.090286944	-3.759531528	0.000170232	0.000272864	negative
PRJNA729271	stage_3W_vs_E10	ordinary_muscle_development	ENSGALG00010016901	MECOM	-0.878433386	0.193072183	-4.54976669	5.37054E-06	9.67632E-06	negative
PRJNA729271	stage_3W_vs_E10	ordinary_muscle_development	ENSGALG00010016902	MRNIP	-1.357128549	0.145051482	-9.356185318	8.26675E-21	3.26948E-20	negative
PRJNA729271	stage_3W_vs_E10	ordinary_muscle_development	ENSGALG00010016903	PPM1A	1.645064361	0.147073561	11.18531673	4.81184E-29	2.66823E-28	positive
PRJNA729271	stage_3W_vs_E10	ordinary_muscle_development	ENSGALG00010016904	EAF1	0.751169086	0.069847494	10.75441707	5.64901E-27	2.88318E-26	positive
PRJNA729271	stage_3W_vs_E10	ordinary_muscle_development	ENSGALG00010016905	C1QC	4.512982385	0.370524267	12.17999139	3.97324E-34	2.65279E-33	positive
PRJNA729271	stage_3W_vs_E10	ordinary_muscle_development	ENSGALG00010016907	SQSTM1	4.037084042	0.159264065	25.34836743	9.3667E-142	7.7583E-140	positive
PRJNA729271	stage_3W_vs_E10	ordinary_muscle_development	ENSGALG00010016908	CMC4	1.870574004	0.158083749	11.83280389	2.64167E-32	1.66122E-31	positive
PRJNA729271	stage_3W_vs_E10	ordinary_muscle_development	ENSGALG00010016909	MRPS5	-0.316295006	0.13443113	-2.352840488	0.018630624	0.024585843	negative
PRJNA729271	stage_3W_vs_E10	ordinary_muscle_development	ENSGALG00010016910	NSD2	-1.072125198	0.111114455	-9.648836382	4.97166E-22	2.06252E-21	negative
PRJNA729271	stage_3W_vs_E10	ordinary_muscle_development	ENSGALG00010016911	AXIN1	-0.391803463	0.099934853	-3.920588774	8.83329E-05	0.000144791	negative
PRJNA729271	stage_3W_vs_E10	ordinary_muscle_development	ENSGALG00010016912	CCDC88C	1.143050343	0.159624642	7.160863956	8.01702E-13	2.17371E-12	positive
PRJNA729271	stage_3W_vs_E10	ordinary_muscle_development	ENSGALG00010016913	BRCC3	-0.310892193	0.111392327	-2.790965972	0.0052551	0.007352574	negative
PRJNA729271	stage_3W_vs_E10	ordinary_muscle_development	ENSGALG00010016915	PLOD2	-1.739797378	0.168152695	-10.34653284	4.33916E-25	2.04076E-24	negative
PRJNA729271	stage_3W_vs_E10	ordinary_muscle_development	ENSGALG00010016916	ACYP2	4.410404054	0.157176994	28.060112	3.0068E-173	3.8569E-171	positive
PRJNA729271	stage_3W_vs_E10	ordinary_muscle_development	ENSGALG00010016917	METTL6	1.899907281	0.169408801	11.21492665	3.44472E-29	1.92413E-28	positive
PRJNA729271	stage_3W_vs_E10	ordinary_muscle_development	ENSGALG00010016918	RCOR1	-0.571772021	0.074908396	-7.632949711	2.29442E-14	6.7228E-14	negative
PRJNA729271	stage_3W_vs_E10	ordinary_muscle_development	ENSGALG00010016919	ARHGDIG	0.565013778	0.197055129	2.867287856	0.004140063	0.005851872	positive
PRJNA729271	stage_3W_vs_E10	ordinary_muscle_development	ENSGALG00010016920	C1QA	4.320863695	0.317683906	13.60114131	3.94233E-42	3.40955E-41	positive
PRJNA729271	stage_3W_vs_E10	ordinary_muscle_development	ENSGALG00010016921	EFL1	-0.029090001	0.074503621	-0.390450838	0.696203202	0.726004706	negative
PRJNA729271	stage_3W_vs_E10	ordinary_muscle_development	ENSGALG00010016922	ENSGALG00010016922	-0.21585465	0.194966383	-1.107137792	0.268234358	0.304730545	negative
PRJNA729271	stage_3W_vs_E10	ordinary_muscle_development	ENSGALG00010016923	ENSGALG00010016923	-3.621400371	0.184992807	-19.5758983	2.48222E-85	7.25398E-84	negative
PRJNA729271	stage_3W_vs_E10	ordinary_muscle_development	ENSGALG00010016924	LETM1	0.324828551	0.117087682	2.774233329	0.005533198	0.00772229	positive
PRJNA729271	stage_3W_vs_E10	ordinary_muscle_development	ENSGALG00010016925	PHACTR4	-0.160214153	0.134644688	-1.18990326	0.234084417	0.268056061	negative
PRJNA729271	stage_3W_vs_E10	ordinary_muscle_development	ENSGALG00010016926	ENSGALG00010016926	-0.105792258	0.184581914	-0.573145309	0.566546311	0.603076212	negative
PRJNA729271	stage_3W_vs_E10	ordinary_muscle_development	ENSGALG00010016927	MGAT4B	1.624070819	0.114733463	14.15516254	1.73536E-45	1.68706E-44	positive
PRJNA729271	stage_3W_vs_E10	ordinary_muscle_development	ENSGALG00010016928	RGS11	3.484633101	0.378669832	9.202299219	3.50371E-20	1.34926E-19	positive
PRJNA729271	stage_3W_vs_E10	ordinary_muscle_development	ENSGALG00010016930	C1OH15ORF48	5.485563319	0.383409549	14.30732056	1.96957E-46	1.97707E-45	positive
PRJNA729271	stage_3W_vs_E10	ordinary_muscle_development	ENSGALG00010016931	SLC30A4	0.09142586	0.082427634	1.109165166	0.267358937	0.303807447	positive
PRJNA729271	stage_3W_vs_E10	ordinary_muscle_development	ENSGALG00010016932	OGT	0.701712443	0.092172427	7.613040754	2.67721E-14	7.82068E-14	positive
PRJNA729271	stage_3W_vs_E10	ordinary_muscle_development	ENSGALG00010016934	SLC25A38	-0.361924741	0.14050332	-2.575915936	0.009997495	0.013582687	negative
PRJNA729271	stage_3W_vs_E10	ordinary_muscle_development	ENSGALG00010016935	PRIMA1	-3.151901139	0.26662795	-11.8213456	3.02795E-32	1.89836E-31	negative
PRJNA729271	stage_3W_vs_E10	ordinary_muscle_development	ENSGALG00010016936	RCC1	-1.352777534	0.165823228	-8.157949566	3.4076E-16	1.09446E-15	negative
PRJNA729271	stage_3W_vs_E10	ordinary_muscle_development	ENSGALG00010016937	DHRS7	-0.229991306	0.171862108	-1.338231612	0.180820947	0.21060664	negative
PRJNA729271	stage_3W_vs_E10	ordinary_muscle_development	ENSGALG00010016938	PSME4	1.396125017	0.166251833	8.397651895	4.55495E-17	1.53081E-16	positive
PRJNA729271	stage_3W_vs_E10	ordinary_muscle_development	ENSGALG00010016939	FAM234A	0.128877686	0.079883256	1.613325393	0.106673832	0.128499726	positive
PRJNA729271	stage_3W_vs_E10	ordinary_muscle_development	ENSGALG00010016940	FGFR3	-5.586470929	0.228441933	-24.45466493	4.489E-132	3.2212E-130	negative
PRJNA729271	stage_3W_vs_E10	ordinary_muscle_development	ENSGALG00010016941	SLC8A1	1.75651904	0.169600121	10.35682659	3.89676E-25	1.83567E-24	positive
PRJNA729271	stage_3W_vs_E10	ordinary_muscle_development	ENSGALG00010016942	ZBTB40	1.080588338	0.149722923	7.217253811	5.30479E-13	1.44862E-12	positive
PRJNA729271	stage_3W_vs_E10	ordinary_muscle_development	ENSGALG00010016943	PCNX4	-0.513909798	0.109963935	-4.673439525	2.96197E-06	5.44292E-06	negative
PRJNA729271	stage_3W_vs_E10	ordinary_muscle_development	ENSGALG00010016944	OPRD1	-6.056749868	0.903571754	-6.70311997	2.04016E-11	5.13048E-11	negative
PRJNA729271	stage_3W_vs_E10	ordinary_muscle_development	ENSGALG00010016945	BDH2	0.33410135	0.14558658	2.294863639	0.02174094	0.028503521	positive
PRJNA729271	stage_3W_vs_E10	ordinary_muscle_development	ENSGALG00010016946	TAF1	0.459395818	0.106380171	4.318434676	1.5714E-05	2.73438E-05	positive
PRJNA729271	stage_3W_vs_E10	ordinary_muscle_development	ENSGALG00010016947	CANX	-0.69853222	0.07147166	-9.773555277	1.46229E-22	6.18315E-22	negative
PRJNA729271	stage_3W_vs_E10	ordinary_muscle_development	ENSGALG00010016948	CCR8	2.871680374	0.481614363	5.962613654	2.48235E-09	5.52289E-09	positive
PRJNA729271	stage_3W_vs_E10	ordinary_muscle_development	ENSGALG00010016949	LUC7L	0.022331055	0.094768665	0.235637542	0.813713939	0.835505501	positive
PRJNA729271	stage_3W_vs_E10	ordinary_muscle_development	ENSGALG00010016950	ENSGALG00010016950	3.191819574	0.994439272	3.209667664	0.001328885	0.001969549	positive
PRJNA729271	stage_3W_vs_E10	ordinary_muscle_development	ENSGALG00010016952	GINS1	-3.807967844	0.247991534	-15.35523321	3.26732E-53	4.11467E-52	negative
PRJNA729271	stage_3W_vs_E10	ordinary_muscle_development	ENSGALG00010016953	GJA4	0.754333739	0.163040836	4.626655321	3.71618E-06	6.79098E-06	positive
PRJNA729271	stage_3W_vs_E10	ordinary_muscle_development	ENSGALG00010016955	ENSGALG00010016955	-6.924431329	0.294669897	-23.49894371	4.1814E-122	2.5902E-120	negative
PRJNA729271	stage_3W_vs_E10	ordinary_muscle_development	ENSGALG00010016956	CENPE	-3.476187974	0.25799843	-13.47367878	2.23449E-41	1.8798E-40	negative
PRJNA729271	stage_3W_vs_E10	ordinary_muscle_development	ENSGALG00010016957	WNT4	-2.713476344	0.264161418	-10.27203885	9.41926E-25	4.36205E-24	negative
PRJNA729271	stage_3W_vs_E10	ordinary_muscle_development	ENSGALG00010016958	TLR15	1.968808539	0.49938655	3.942454074	8.06521E-05	0.000132591	positive
PRJNA729271	stage_3W_vs_E10	ordinary_muscle_development	ENSGALG00010016959	PERCC1	-2.039796934	1.353248828	-1.507333235	0.131725263	0.156748845	negative
PRJNA729271	stage_3W_vs_E10	ordinary_muscle_development	ENSGALG00010016960	GJB3	-6.541446616	1.067158386	-6.129780455	8.80004E-10	2.01289E-09	negative
PRJNA729271	stage_3W_vs_E10	ordinary_muscle_development	ENSGALG00010016961	EIF4A3	-0.680298233	0.074784537	-9.096776692	9.30524E-20	3.52258E-19	negative
PRJNA729271	stage_3W_vs_E10	ordinary_muscle_development	ENSGALG00010016962	SQOR	2.434018312	0.135883046	17.91259755	9.40426E-72	1.9065E-70	positive

PRJNA729271	stage_3W_vs_E10	ordinary_muscle_development	ENSGALG00010016963	CD8A	4.752806618	0.656248269	7.242391089	4.40842E-13	1.20954E-12	positive
PRJNA729271	stage_3W_vs_E10	ordinary_muscle_development	ENSGALG00010016964	MAML1	0.369061593	0.084836164	4.35028621	1.3596E-05	2.37639E-05	positive
PRJNA729271	stage_3W_vs_E10	ordinary_muscle_development	ENSGALG00010016965	SLC25A33	0.052881206	0.338746892	0.156108315	0.875947644	0.890258106	positive
PRJNA729271	stage_3W_vs_E10	ordinary_muscle_development	ENSGALG00010016966	KDM3A	0.339907231	0.158223989	2.148266098	0.031692622	0.040743642	positive
PRJNA729271	stage_3W_vs_E10	ordinary_muscle_development	ENSGALG00010016967	ERLEC1	0.235013768	0.074754034	3.143827245	0.001667539	0.002450356	positive
PRJNA729271	stage_3W_vs_E10	ordinary_muscle_development	ENSGALG00010016968	C15orf61	0.906701047	0.146709951	6.18022868	6.40088E-10	1.47717E-09	positive
PRJNA729271	stage_3W_vs_E10	ordinary_muscle_development	ENSGALG00010016969	CHMP3	1.633379752	0.082259813	19.85635137	9.71147E-88	3.0143E-86	positive
PRJNA729271	stage_3W_vs_E10	ordinary_muscle_development	ENSGALG00010016970	SYNE3	1.342967465	0.098790227	13.59413275	4.33873E-42	3.74568E-41	positive
PRJNA729271	stage_3W_vs_E10	ordinary_muscle_development	ENSGALG00010016972	ASB3	-1.618317173	0.115603329	-13.99888036	1.58346E-44	1.48462E-43	negative
PRJNA729271	stage_3W_vs_E10	ordinary_muscle_development	ENSGALG00010016973	PLSCR1	3.250409248	0.120142445	27.05462867	3.3694E-161	3.786E-159	positive
PRJNA729271	stage_3W_vs_E10	ordinary_muscle_development	ENSGALG00010016974	CFAP96	-1.948417102	0.69031084	-2.822521378	0.004764765	0.006698212	negative
PRJNA729271	stage_3W_vs_E10	ordinary_muscle_development	ENSGALG00010016975	MALL	0.932018652	0.222926637	4.180831263	2.90445E-05	4.94538E-05	positive
PRJNA729271	stage_3W_vs_E10	ordinary_muscle_development	ENSGALG00010016976	ENSGALG00010016976	6.930894935	0.900527149	7.696486373	1.39859E-14	4.14232E-14	positive
PRJNA729271	stage_3W_vs_E10	ordinary_muscle_development	ENSGALG00010016977	CLCN7	0.380049034	0.118895971	3.196483707	0.001391137	0.002057815	positive
PRJNA729271	stage_3W_vs_E10	ordinary_muscle_development	ENSGALG00010016978	FAM181A	4.924432425	0.348904516	14.11398305	3.11477E-45	3.0079E-44	positive
PRJNA729271	stage_3W_vs_E10	ordinary_muscle_development	ENSGALG00010016979	PLSCR5	-1.226786775	0.520137503	-2.358581657	0.018344923	0.024228178	negative
PRJNA729271	stage_3W_vs_E10	ordinary_muscle_development	ENSGALG00010016980	NPHP1	-0.374729466	0.101616693	-3.687676271	0.000226311	0.000358669	negative
PRJNA729271	stage_3W_vs_E10	ordinary_muscle_development	ENSGALG00010016981	ENSGALG00010016981	1.811066568	0.444950491	4.070265354	4.69596E-05	7.87733E-05	positive
PRJNA729271	stage_3W_vs_E10	ordinary_muscle_development	ENSGALG00010016982	GLRX5	-0.258063195	0.141164673	-1.828100399	0.067534485	0.083389757	negative
PRJNA729271	stage_3W_vs_E10	ordinary_muscle_development	ENSGALG00010016983	ZFAND2A	0.127345831	0.285160685	0.446575695	0.655181448	0.688078183	positive
PRJNA729271	stage_3W_vs_E10	ordinary_muscle_development	ENSGALG00010016984	RNF103	1.508248068	0.1141199	13.2163459	7.06119E-40	5.69571E-39	positive
PRJNA729271	stage_3W_vs_E10	ordinary_muscle_development	ENSGALG00010016985	ENSGALG00010016985	1.69990564	0.185011708	9.188097646	3.9985E-20	1.53491E-19	positive
PRJNA729271	stage_3W_vs_E10	ordinary_muscle_development	ENSGALG00010016986	GFER	0.411392829	0.08565166	4.803092308	1.56234E-06	2.91831E-06	positive
PRJNA729271	stage_3W_vs_E10	ordinary_muscle_development	ENSGALG00010016987	ASB2	5.844509933	0.317838103	18.38832376	1.6294E-75	3.64478E-74	positive
PRJNA729271	stage_3W_vs_E10	ordinary_muscle_development	ENSGALG00010016988	ECI1	0.653930641	0.110220392	5.932937012	2.97563E-09	6.58903E-09	positive
PRJNA729271	stage_3W_vs_E10	ordinary_muscle_development	ENSGALG00010016989	RHOG2	-0.13548379	0.093207107	-1.453577881	0.146063373	0.172634425	negative
PRJNA729271	stage_3W_vs_E10	ordinary_muscle_development	ENSGALG00010016991	PDLIM3	5.453618948	0.199133189	27.38679058	3.9401E-165	4.6813E-163	positive
PRJNA729271	stage_3W_vs_E10	ordinary_muscle_development	ENSGALG00010016993	ENSGALG00010016993	0.652276758	0.179983175	3.624098522	0.000289971	0.000456019	positive
PRJNA729271	stage_3W_vs_E10	ordinary_muscle_development	ENSGALG00010016994	ATL2	1.218416626	0.115475512	10.55129872	5.00997E-26	2.44758E-25	positive
PRJNA729271	stage_3W_vs_E10	ordinary_muscle_development	ENSGALG00010016998	DNASE1L2	1.024153151	0.341119054	3.002333463	0.002679185	0.003855718	positive
PRJNA729271	stage_3W_vs_E10	ordinary_muscle_development	ENSGALG00010016999	ITGB1BP2	3.064619919	0.172434251	17.7726867	1.15046E-70	2.26268E-69	positive
PRJNA729271	stage_3W_vs_E10	ordinary_muscle_development	ENSGALG00010017001	TPCN3	-0.911210074	0.118897259	-7.663844261	1.80449E-14	5.31735E-14	negative
PRJNA729271	stage_3W_vs_E10	ordinary_muscle_development	ENSGALG00010017003	CPZ	-0.449913827	0.398969882	-1.127688699	0.259451412	0.295284879	negative
PRJNA729271	stage_3W_vs_E10	ordinary_muscle_development	ENSGALG00010017004	CX3CR1	0.629210698	0.405086354	1.553275473	0.120357337	0.143870988	positive
PRJNA729271	stage_3W_vs_E10	ordinary_muscle_development	ENSGALG00010017006	DNAH3	0.226316622	0.245419004	0.922164213	0.356442927	0.395154128	positive
PRJNA729271	stage_3W_vs_E10	ordinary_muscle_development	ENSGALG00010017008	RTN1	-0.543867205	0.167745673	-3.242213014	0.001186053	0.001765802	negative
PRJNA729271	stage_3W_vs_E10	ordinary_muscle_development	ENSGALG00010017010	NONO	-1.700254599	0.055548933	-30.60823129	9.5114E-206	1.7905E-203	negative
PRJNA729271	stage_3W_vs_E10	ordinary_muscle_development	ENSGALG00010017012	SYNGR3	1.75605548	0.178705386	9.826539204	8.65417E-23	3.69927E-22	positive
PRJNA729271	stage_3W_vs_E10	ordinary_muscle_development	ENSGALG00010017016	ZIC1	-6.776282246	1.000494197	-6.772935082	1.26196E-11	3.20913E-11	negative
PRJNA729271	stage_3W_vs_E10	ordinary_muscle_development	ENSGALG00010017017	ENSGALG00010017017	1.380829664	0.260906489	5.292431278	1.20701E-07	2.43129E-07	positive
PRJNA729271	stage_3W_vs_E10	ordinary_muscle_development	ENSGALG00010017018	HP1BP3	-1.344219163	0.084028178	-15.99724283	1.3356E-57	1.8814E-56	negative
PRJNA729271	stage_3W_vs_E10	ordinary_muscle_development	ENSGALG00010017020	GJB5	-3.622173878	0.588376709	-6.156215606	7.45038E-10	1.71282E-09	negative
PRJNA729271	stage_3W_vs_E10	ordinary_muscle_development	ENSGALG00010017022	CCR4	2.591806493	0.526238981	4.925151091	8.42952E-07	1.60454E-06	positive
PRJNA729271	stage_3W_vs_E10	ordinary_muscle_development	ENSGALG00010017024	BUB1	-4.099001503	0.223595931	-18.33218286	4.58145E-75	1.01078E-73	negative
PRJNA729271	stage_3W_vs_E10	ordinary_muscle_development	ENSGALG00010017033	LDAF1	0.6823667	0.110845386	6.156022594	7.45946E-10	1.71409E-09	positive
PRJNA729271	stage_3W_vs_E10	ordinary_muscle_development	ENSGALG00010017034	ZMYM3	-0.103325038	0.091473884	-1.129557787	0.258662607	0.294410246	negative
PRJNA729271	stage_3W_vs_E10	ordinary_muscle_development	ENSGALG00010017035	PIK3CD	1.476360775	0.200817527	7.351572577	1.95625E-13	5.46565E-13	positive
PRJNA729271	stage_3W_vs_E10	ordinary_muscle_development	ENSGALG00010017038	ZNF598	-0.090116898	0.131449513	-0.685562818	0.492988835	0.531410215	negative
PRJNA729271	stage_3W_vs_E10	ordinary_muscle_development	ENSGALG00010017039	MAP2K5	-0.870972078	0.078864109	-11.04396023	2.34466E-28	1.26955E-27	negative
PRJNA729271	stage_3W_vs_E10	ordinary_muscle_development	ENSGALG00010017041	JKAMP	0.353704282	0.088771981	3.984413513	6.7647E-05	0.000112113	positive
PRJNA729271	stage_3W_vs_E10	ordinary_muscle_development	ENSGALG00010017042	HNRNPLL	-1.036679009	0.070505082	-14.70360684	6.11158E-49	6.68079E-48	negative
PRJNA729271	stage_3W_vs_E10	ordinary_muscle_development	ENSGALG00010017045	GLB1	-0.115193863	0.099251416	-1.160626903	0.24579366	0.280732732	negative
PRJNA729271	stage_3W_vs_E10	ordinary_muscle_development	ENSGALG00010017049	TRAF3	0.111288267	0.089623918	1.241725088	0.21433801	0.247044327	positive
PRJNA729271	stage_3W_vs_E10	ordinary_muscle_development	ENSGALG00010017050	L3HYPDH	-1.140494222	0.156815967	-7.272819508	3.52061E-13	9.70728E-13	negative
PRJNA729271	stage_3W_vs_E10	ordinary_muscle_development	ENSGALG00010017054	GALM	-0.571608663	0.151183825	-3.780885037	0.000156272	0.00025121	negative
PRJNA729271	stage_3W_vs_E10	ordinary_muscle_development	ENSGALG00010017055	GPR135	-1.709649628	0.344608189	-4.96114045	7.00805E-07	1.3403E-06	negative
PRJNA729271	stage_3W_vs_E10	ordinary_muscle_development	ENSGALG00010017056	AGTR1	3.914717443	0.202300007	19.3510495	1.99733E-83	5.52507E-82	positive
PRJNA729271	stage_3W_vs_E10	ordinary_muscle_development	ENSGALG00010017057	NDUFAB1	0.97248586	0.121383104	8.011707002	1.13127E-15	3.53248E-15	positive
PRJNA729271	stage_3W_vs_E10	ordinary_muscle_development	ENSGALG00010017060	CLSTN1	-1.682075744	0.102612808	-16.39245409	2.16524E-60	3.27611E-59	negative
PRJNA729271	stage_3W_vs_E10	ordinary_muscle_development	ENSGALG00010017063	TMPPE	0.334454823	0.108028752	3.095979681	0.001961638	0.002856534	positive
PRJNA729271	stage_3W_vs_E10	ordinary_muscle_development	ENSGALG00010017065	VRK2	-0.298650336	0.133845067	-2.231313722	0.025660355	0.033337533	negative
PRJNA729271	stage_3W_vs_E10	ordinary_muscle_development	ENSGALG00010017068	ZP2	-1.297959733	0.920672583	-1.40979514	0.158600182	0.186478718	negative
PRJNA729271	stage_3W_vs_E10	ordinary_muscle_development	ENSGALG00010017070	BLOC1S6	0.328402466	0.104636479	3.138508374	0.001698101	0.002492302	positive
PRJNA729271	stage_3W_vs_E10	ordinary_muscle_development	ENSGALG00010017077	CTNNBIP1	-0.732348174	0.105357326	-6.951089208	3.62477E-12	9.46343E-12	negative
PRJNA729271	stage_3W_vs_E10	ordinary_muscle_development	ENSGALG00010017080	PALB2	-1.087909584	0.103814739	-10.47933651	1.07495E-25	5.17484E-25	negative
PRJNA729271	stage_3W_vs_E10	ordinary_muscle_development	ENSGALG00010017083	ENSGALG00010017083	-0.256836915	0.421273106	-0.609668434	0.542081469	0.579683807	negative
PRJNA729271	stage_3W_vs_E10	ordinary_muscle_development	ENSGALG00010017085	ENSGALG00010017085	-0.790050989	0.120407266	-6.561489343	5.3273E-11	1.30641E-10	negative
PRJNA729271	stage_3W_vs_E10	ordinary_muscle_development	ENSGALG00010017089	CRTAP	-0.63442184	0.15756027	-4.02653436	5.6605E-05	9.44502E-05	negative
PRJNA729271	stage_3W_vs_E10	ordinary_muscle_development	ENSGALG00010017094	SORBS2	0.495679615	0.147737436	3.355138869	0.000793252	0.001198778	positive
PRJNA729271	stage_3W_vs_E10	ordinary_muscle_development	ENSGALG00010017096	DCTN5	-0.514175932	0.091378833	-5.626860362	1.83519E-08	3.89931E-08	negative
PRJNA729271	stage_3W_vs_E10	ordinary_muscle_development	ENSGALG00010017098	DAAM1	-1.513293446	0.125418058	-12.06599329	1.59724E-33	1.04476E-32	negative
PRJNA729271	stage_3W_vs_E10	ordinary_muscle_development	ENSGALG00010017100	SUSD5	-7.666469817	0.612281102	-12.52116028	5.71929E-36	4.04593E-35	negative
PRJNA729271	stage_3W_vs_E10	ordinary_muscle_development	ENSGALG00010017101	CSMD2	-1.955605418	0.251362479	-7.780021195	7.25124E-15	2.18019E-14	negative
PRJNA729271	stage_3W_vs_E10	ordinary_muscle_development	ENSGALG00010017104	FANCL	-3.021033434	0.1835897	-16.45535361	7.67722E-61	1.17262E-59	negative
PRJNA729271	stage_3W_vs_E10	ordinary_muscle_development	ENSGALG00010017105	PLK1	-4.318777362	0.239936592	-17.99966116	1.96014E-72	4.07637E-71	negative
PRJNA729271	stage_3W_vs_E10	ordinary_muscle_development	ENSGALG00010017106	CRYM	-1.086979958	0.119008827	-9.133607852	6.62537E-20	2.52391E-19	negative
PRJNA729271	stage_3W_vs_E10	ordinary_muscle_development	ENSGALG00010017109	ITGB1BP3	0.827078605	0.1274659	6.488626388	8.66225E-11	2.09895E-10	positive
PRJNA729271	stage_3W_vs_E10	ordinary_muscle_development	ENSGALG00010017110	BRF1	-0.154986935	0.071899894	-2.155593387	0.031115429	0.040037119	negative
PRJNA729271	stage_3W_vs_E10	ordinary_muscle_development	ENSGALG00010017111	GJB1	-1.808582686	0.482811447	-3.745939944	0.00017972	0.000287436	negative
PRJNA729271	stage_3W_vs_E10	ordinary_muscle_development	ENSGALG00010017112	LZIC	-0.290135097	0.062975873	-4.607083377	4.08356E-06	7.42676E-06	negative
PRJNA729271	stage_3W_vs_E10	ordinary_muscle_development	ENSGALG00010017115	LGALSL	2.655137678	0.191677039	13.85214263	1.23473E-43	1.12633E-42	positive
PRJNA729271	stage_3W_vs_E10	ordinary_muscle_development	ENSGALG00010017116	ENSGALG00010017116	-3.10339165	0.328963072	-9.433860249	3.95268E-21	1.58622E-20	negative
PRJNA729271	stage_3W_vs_E10	ordinary_muscle_development	ENSGALG00010017119	BTBD6	-1.795344477	0.151980449	-11.81299627	3.34427E-32	2.09215E-31	negative
PRJNA729271	stage_3W_vs_E10	ordinary_muscle_development	ENSGALG00010017120	ERN2	1.275555042	0.141361899	9.023329839	1.82457E-19	6.83036E-19	positive
PRJNA729271	stage_3W_vs_E10	ordinary_muscle_development	ENSGALG00010017122	NCBP2	-1.074564204	0.192994131	-5.567859487	2.57888E-08	5.41672E-08	negative
PRJNA729271	stage_3W_vs_E10	ordinary_muscle_development	ENSGALG00010017127	SOX8	-1.85642362	0.137443326	-13.50682984	1.42528E-41	1.20674E-40	negative
PRJNA729271	stage_3W_vs_E10	ordinary_muscle_development	ENSGALG00010017131	TLR3	3.912141871	0.374684298	10.44116845	1.60819E-25	7.69076E-25	positive
PRJNA729271	stage_3W_vs_E10	ordinary_muscle_development	ENSGALG00010017132	TMEM108	-3.162285302	0.321614871	-9.832522012	8.15505E-23	3.49106E-22	negative
PRJNA729271	stage_3W_vs_E10	ordinary_muscle_development	ENSGALG00010017133	WDR20	0.673238905	0.11228377	5.995870165	2.02399E-09	4.52323E-09	positive
PRJNA729271	stage_3W_vs_E10	ordinary_muscle_development	ENSGALG00010017135	ENSGALG00010017135	0.598038699	0.132929464	4.498917539	6.83003E-06	1.22058E-05	positive
PRJNA729271	stage_3W_vs_E10	ordinary_muscle_development	ENSGALG00010017136	ENSGALG00010017136	-0.252972534	0.418412673	-0.604600555	0.545444438	0.582753529	negative
PRJNA729271	stage_3W_vs_E10	ordinary_muscle_development	ENSGALG00010017139	COL9A2	-9.172423012	0.600944186	-15.26335262	1.34174E-52	1.64957E-51	negative
PRJNA729271	stage_3W_vs_E10	ordinary_muscle_development	ENSGALG00010017140	UQCRC2	0.762292166	0.115064694	6.624900625	3.47482E-11	8.63196E-11	positive
PRJNA729271	stage_3W_vs_E10	ordinary_muscle_development	ENSGALG00010017141	AFTPH	0.720782538	0.075280068	9.574679655	1.02174E-21	4.19073E-21	positive
PRJNA729271	stage_3W_vs_E10	ordinary_muscle_development	ENSGALG00010017142	RBP7	2.632725853	0.694409276	3.791317232	0.00014985	0.000241235	positive
PRJNA729271	stage_3W_vs_E10	ordinary_muscle_development	ENSGALG00010017143	FAM149A	0.406766061	0.269639561	1.508554822	0.131412587	0.15640245	positive
PRJNA729271	stage_3W_vs_E10	ordinary_muscle_development	ENSGALG00010017144	CPB1	3.014805843	0.723197865	4.168715074	3.06322E-05	5.20714E-05	positive
PRJNA729271	stage_3W_vs_E10	ordinary_muscle_development	ENSGALG00010017147	SRSF7L	-1.089467748	0.071237472	-15.29346446	8.45311E-53	1.04457E-51	negative
PRJNA729271	stage_3W_vs_E10	ordinary_muscle_development	ENSGALG00010017148	MOK	-0.228103238	0.469243156	-0.486108823	0.626890012	0.660867122	negative
PRJNA729271	stage_3W_vs_E10	ordinary_muscle_development	ENSGALG00010017152	CYP4V2	3.993350297	0.195282106	20.44913297	6.1149E-93	2.15135E-91	positive
PRJNA729271	stage_3W_vs_E10	ordinary_muscle_development	ENSGALG00010017153	SLC35F4	-2.18063618	0.671439396	-3.247703656	0.001163404	0.001733862	negative
PRJNA729271	stage_3W_vs_E10	ordinary_muscle_development	ENSGALG00010017154	PIGZ	0.584353753	0.186285645	3.136869469	0.001707621	0.002505767	positive
PRJNA729271	stage_3W_vs_E10	ordinary_muscle_development	ENSGALG00010017162	BCL11A	-3.34245065	0.230284323	-14.51445158	9.81394E-48	1.03232E-46	negative
PRJNA729271	stage_3W_vs_E10	ordinary_muscle_development	ENSGALG00010017163	CDV3	-0.434183985	0.124730456	-3.480978109	0.000499586	0.000769881	negative
PRJNA729271	stage_3W_vs_E10	ordinary_muscle_development	ENSGALG00010017167	SMAP2	-0.073062863	0.096559634	-0.756660519	0.449253274	0.4881138	negative
PRJNA729271	stage_3W_vs_E10	ordinary_muscle_development	ENSGALG00010017169	CCDC198	0.382675071	0.242192441	1.580045475	0.114096453	0.136872306	positive
PRJNA729271	stage_3W_vs_E10	ordinary_muscle_development	ENSGALG00010017174	DHX57	-0.742971641	0.098032415	-7.578836465	3.48668E-14	1.012E-13	negative
PRJNA729271	stage_3W_vs_E10	ordinary_muscle_development	ENSGALG00010017180	ENSGALG00010017180	-2.928898381	0.590859938	-4.957009596	7.15864E-07	1.36748E-06	negative
PRJNA729271	stage_3W_vs_E10	ordinary_muscle_development	ENSGALG00010017183	NFYC	0.260151483	0.093444898	2.78400949	0.005369146	0.00750562	positive
PRJNA729271	stage_3W_vs_E10	ordinary_muscle_development	ENSGALG00010017184	CARD11	2.347581483	0.210079123	11.17474908	5.42033E-29	2.99306E-28	positive
PRJNA729271	stage_3W_vs_E10	ordinary_muscle_development	ENSGALG00010017185	NAA30	0.372353353	0.064002238	5.81781766	5.96209E-09	1.30367E-08	positive
PRJNA729271	stage_3W_vs_E10	ordinary_muscle_development	ENSGALG00010017187	ZNF839	0.988001274	0.137938774	7.16260735	7.91568E-13	2.14664E-12	positive
PRJNA729271	stage_3W_vs_E10	ordinary_muscle_development	ENSGALG00010017188	ENSGALG00010017188	1.968628683	0.155147205	12.68877956	6.82408E-37	4.94576E-36	positive
PRJNA729271	stage_3W_vs_E10	ordinary_muscle_development	ENSGALG00010017190	STK26	-0.270377299	0.067864467	-3.984077544	6.77428E-05	0.000112259	negative
PRJNA729271	stage_3W_vs_E10	ordinary_muscle_development	ENSGALG00010017198	TOPBP1	-1.068624392	0.069563416	-15.36187335	2.94928E-53	3.72385E-52	negative
PRJNA729271	stage_3W_vs_E10	ordinary_muscle_development	ENSGALG00010017200	KCNQ4	0.903554514	0.308275844	2.930993562	0.003378798	0.004821864	positive
PRJNA729271	stage_3W_vs_E10	ordinary_muscle_development	ENSGALG00010017201	AP5M1	-1.435531887	0.154168183	-9.311466593	1.26079E-20	4.94458E-20	negative
PRJNA729271	stage_3W_vs_E10	ordinary_muscle_development	ENSGALG00010017207	ENSGALG00010017207	9.141585328	0.280419656	32.59965961	4.147E-233	1.0546E-230	positive
PRJNA729271	stage_3W_vs_E10	ordinary_muscle_development	ENSGALG00010017208	CINP	-0.390158726	0.170935711	-2.282488101	0.022460539	0.029401744	negative
PRJNA729271	stage_3W_vs_E10	ordinary_muscle_development	ENSGALG00010017211	UBFD1	1.773017738	0.127173765	13.94169417	3.53449E-44	3.27783E-43	positive
PRJNA729271	stage_3W_vs_E10	ordinary_muscle_development	ENSGALG00010017212	TINAGL1	2.903078155	0.13524139	21.4659	3.2441E-102	1.41635E-100	positive
PRJNA729271	stage_3W_vs_E10	ordinary_muscle_development	ENSGALG00010017215	FRMD7	2.374800753	0.607965763	3.906142248	9.37813E-05	0.000153375	positive
PRJNA729271	stage_3W_vs_E10	ordinary_muscle_development	ENSGALG00010017219	EARS2	-1.662699387	0.153016437	-10.86614888	1.67106E-27	8.72239E-27	negative
PRJNA729271	stage_3W_vs_E10	ordinary_muscle_development	ENSGALG00010017221	SPPL2A	0.099067078	0.075659864	1.30937426	0.190407606	0.22104423	positive
PRJNA729271	stage_3W_vs_E10	ordinary_muscle_development	ENSGALG00010017223	NOC2L	-1.214024747	0.077037025	-15.75897751	5.95893E-56	8.04983E-55	negative
PRJNA729271	stage_3W_vs_E10	ordinary_muscle_development	ENSGALG00010017226	PEF1	-0.253515426	0.075671694	-3.350201564	0.000807528	0.001219283	negative
PRJNA729271	stage_3W_vs_E10	ordinary_muscle_development	ENSGALG00010017228	RAP2C	-0.639116723	0.072079597	-8.866818791	7.52652E-19	2.74873E-18	negative
PRJNA729271	stage_3W_vs_E10	ordinary_muscle_development	ENSGALG00010017240	MELTF	0.108309724	0.23781776	0.455431605	0.648798763	0.681819623	positive
PRJNA729271	stage_3W_vs_E10	ordinary_muscle_development	ENSGALG00010017246	GGA2	0.633881865	0.129832559	4.882302782	1.04854E-06	1.98363E-06	positive
PRJNA729271	stage_3W_vs_E10	ordinary_muscle_development	ENSGALG00010017248	TECPR2	0.653963597	0.083828103	7.801245329	6.12992E-15	1.84918E-14	positive
PRJNA729271	stage_3W_vs_E10	ordinary_muscle_development	ENSGALG00010017250	COL16A1	-0.744820888	0.170211167	-4.375863828	1.20953E-05	2.12381E-05	negative

PRJNA729271	stage_3W_vs_E10	ordinary_muscle_development	ENSGALG00010017252	EXOC5	-0.397280855	0.088038123	-4.512600231	6.40377E-06	1.1478E-05	negative
PRJNA729271	stage_3W_vs_E10	ordinary_muscle_development	ENSGALG00010017256	CARHSP1	0.303762551	0.128661495	2.360943734	0.018228496	0.02407929	positive
PRJNA729271	stage_3W_vs_E10	ordinary_muscle_development	ENSGALG00010017263	COG7	-0.262305636	0.071694935	-3.658635539	0.000253562	0.00040015	negative
PRJNA729271	stage_3W_vs_E10	ordinary_muscle_development	ENSGALG00010017264	PIAS1	0.725710579	0.08568391	8.469624897	2.46185E-17	8.38057E-17	positive
PRJNA729271	stage_3W_vs_E10	ordinary_muscle_development	ENSGALG00010017266	PMM2	-1.125431188	0.10942393	-10.28505545	8.22947E-25	3.8196E-24	negative
PRJNA729271	stage_3W_vs_E10	ordinary_muscle_development	ENSGALG00010017272	NRXN1	1.42404738	0.134159915	10.61455189	2.55015E-26	1.26331E-25	positive
PRJNA729271	stage_3W_vs_E10	ordinary_muscle_development	ENSGALG00010017278	CCR5	1.768682206	0.227151123	7.786367872	6.8963E-15	2.07519E-14	positive
PRJNA729271	stage_3W_vs_E10	ordinary_muscle_development	ENSGALG00010017280	SOS1	0.956233831	0.155034587	6.167874201	6.92142E-10	1.59475E-09	positive
PRJNA729271	stage_3W_vs_E10	ordinary_muscle_development	ENSGALG00010017283	INF2	0.847389276	0.136905372	6.189598442	6.03177E-10	1.3942E-09	positive
PRJNA729271	stage_3W_vs_E10	ordinary_muscle_development	ENSGALG00010017284	ENSGALG00010017284	1.3565271	0.195382929	6.942915146	3.8409E-12	1.00097E-11	positive
PRJNA729271	stage_3W_vs_E10	ordinary_muscle_development	ENSGALG00010017285	IL21R	5.123075294	0.816342326	6.275645805	3.48186E-10	8.16131E-10	positive
PRJNA729271	stage_3W_vs_E10	ordinary_muscle_development	ENSGALG00010017289	ADGRB2	-1.579096133	0.320460896	-4.92757822	8.32551E-07	1.58516E-06	negative
PRJNA729271	stage_3W_vs_E10	ordinary_muscle_development	ENSGALG00010017290	DACT1	-1.858197179	0.157382806	-11.80686265	3.59735E-32	2.24467E-31	negative
PRJNA729271	stage_3W_vs_E10	ordinary_muscle_development	ENSGALG00010017296	SCNN1G	2.169713546	0.223803713	9.694716476	3.17518E-22	1.32788E-21	positive
PRJNA729271	stage_3W_vs_E10	ordinary_muscle_development	ENSGALG00010017299	USP31	1.27321634	0.110147972	11.55914468	6.63665E-31	3.95065E-30	positive
PRJNA729271	stage_3W_vs_E10	ordinary_muscle_development	ENSGALG00010017300	TRPM7	-0.58119517	0.104682349	-5.55198821	2.82439E-08	5.91868E-08	negative
PRJNA729271	stage_3W_vs_E10	ordinary_muscle_development	ENSGALG00010017301	LHCGR	-2.308647664	0.552078907	-4.181734954	2.89293E-05	4.92634E-05	negative
PRJNA729271	stage_3W_vs_E10	ordinary_muscle_development	ENSGALG00010017304	ANKRD9	5.573766871	0.297554192	18.73193864	2.71835E-78	6.50205E-77	positive
PRJNA729271	stage_3W_vs_E10	ordinary_muscle_development	ENSGALG00010017305	HS3ST2	3.171628989	0.478060228	6.634371162	3.25889E-11	8.11503E-11	positive
PRJNA729271	stage_3W_vs_E10	ordinary_muscle_development	ENSGALG00010017307	TMEM186	0.314421465	0.156940621	2.003442221	0.045129843	0.057002186	positive
PRJNA729271	stage_3W_vs_E10	ordinary_muscle_development	ENSGALG00010017310	SAMD11	-1.503273412	0.179067345	-8.395017044	4.65827E-17	1.56481E-16	negative
PRJNA729271	stage_3W_vs_E10	ordinary_muscle_development	ENSGALG00010017312	PTP4A2	1.715633504	0.154582943	11.09846579	1.27616E-28	6.95409E-28	positive
PRJNA729271	stage_3W_vs_E10	ordinary_muscle_development	ENSGALG00010017313	MAP4K3	0.711363327	0.082323139	8.641110363	5.56693E-18	1.96094E-17	positive
PRJNA729271	stage_3W_vs_E10	ordinary_muscle_development	ENSGALG00010017314	CALML4	0.952102641	0.219755305	4.332558173	1.47387E-05	2.57084E-05	positive
PRJNA729271	stage_3W_vs_E10	ordinary_muscle_development	ENSGALG00010017315	HS3ST4	-2.027890799	0.923703211	-2.195392172	0.028135478	0.036380353	negative
PRJNA729271	stage_3W_vs_E10	ordinary_muscle_development	ENSGALG00010017318	ADSS1	4.1565661	0.123658644	33.61322718	1.0751E-247	2.8858E-245	positive
PRJNA729271	stage_3W_vs_E10	ordinary_muscle_development	ENSGALG00010017322	KHDRBS1	-1.527177595	0.071177713	-21.45583973	4.0277E-102	1.74796E-100	negative
PRJNA729271	stage_3W_vs_E10	ordinary_muscle_development	ENSGALG00010017323	ENSGALG00010017323	0.77172947	0.105240134	7.333033901	2.25E-13	6.26224E-13	positive
PRJNA729271	stage_3W_vs_E10	ordinary_muscle_development	ENSGALG00010017326	STON1	-0.160551181	0.122909709	-1.306253047	0.191466518	0.222148978	negative
PRJNA729271	stage_3W_vs_E10	ordinary_muscle_development	ENSGALG00010017327	CLN6	0.924469634	0.131492501	7.030588265	2.05665E-12	5.45591E-12	positive
PRJNA729271	stage_3W_vs_E10	ordinary_muscle_development	ENSGALG00010017332	FGFR1	-0.548466278	0.159036834	-3.448674522	0.000563345	0.000862269	negative
PRJNA729271	stage_3W_vs_E10	ordinary_muscle_development	ENSGALG00010017334	KDM8	-0.654352185	0.157645439	-4.150784122	3.31338E-05	5.61331E-05	negative
PRJNA729271	stage_3W_vs_E10	ordinary_muscle_development	ENSGALG00010017335	THUMPD2	-0.069323752	0.137443798	-0.504378903	0.613995141	0.648821753	negative
PRJNA729271	stage_3W_vs_E10	ordinary_muscle_development	ENSGALG00010017337	ABAT	1.152430523	0.201592945	5.716621306	1.08663E-08	2.34141E-08	positive
PRJNA729271	stage_3W_vs_E10	ordinary_muscle_development	ENSGALG00010017338	MBNL3	0.141402925	0.074737732	1.891988428	0.058492523	0.072807996	positive
PRJNA729271	stage_3W_vs_E10	ordinary_muscle_development	ENSGALG00010017340	SIVA1	-0.654502059	0.105588381	-6.1986182	5.6961E-10	1.31914E-09	negative
PRJNA729271	stage_3W_vs_E10	ordinary_muscle_development	ENSGALG00010017341	TA3	-0.541437399	0.081035101	-6.681516894	2.36482E-11	5.93045E-11	negative
PRJNA729271	stage_3W_vs_E10	ordinary_muscle_development	ENSGALG00010017343	DLG1	0.299403667	0.099205882	3.01800317	0.002544462	0.00367131	positive
PRJNA729271	stage_3W_vs_E10	ordinary_muscle_development	ENSGALG00010017344	RERE	1.170848336	0.153412763	7.632013873	2.31115E-14	6.77042E-14	positive
PRJNA729271	stage_3W_vs_E10	ordinary_muscle_development	ENSGALG00010017346	GM2A	-1.347788926	0.166574222	-8.091221497	5.90693E-16	1.87436E-15	negative
PRJNA729271	stage_3W_vs_E10	ordinary_muscle_development	ENSGALG00010017347	TMEM39B	-0.996550365	0.122735921	-8.119467881	4.68232E-16	1.49362E-15	negative
PRJNA729271	stage_3W_vs_E10	ordinary_muscle_development	ENSGALG00010017349	ENSGALG00010017349	0.630028009	0.114800564	5.488021912	4.0646E-08	8.43349E-08	positive
PRJNA729271	stage_3W_vs_E10	ordinary_muscle_development	ENSGALG00010017351	CCR2	2.944579479	0.205184328	14.35089854	1.05166E-46	1.06303E-45	positive
PRJNA729271	stage_3W_vs_E10	ordinary_muscle_development	ENSGALG00010017352	GPCPD1	1.766260136	0.238320558	7.411278967	1.25087E-13	3.53232E-13	positive
PRJNA729271	stage_3W_vs_E10	ordinary_muscle_development	ENSGALG00010017354	FEM1B	-0.113023524	0.105171151	-1.074662806	0.282525722	0.319588757	negative
PRJNA729271	stage_3W_vs_E10	ordinary_muscle_development	ENSGALG00010017356	NSMCE1	-0.500690429	0.118478627	-4.22599788	2.37884E-05	4.08084E-05	negative
PRJNA729271	stage_3W_vs_E10	ordinary_muscle_development	ENSGALG00010017359	EHD3	1.041823913	0.095236319	10.93935512	7.47296E-28	3.96053E-27	positive
PRJNA729271	stage_3W_vs_E10	ordinary_muscle_development	ENSGALG00010017361	METTL22	-0.023789655	0.15149973	-0.157027706	0.875223012	0.88958401	negative
PRJNA729271	stage_3W_vs_E10	ordinary_muscle_development	ENSGALG00010017362	ENO1	-0.09455107	0.130175566	-0.726335002	0.467633427	0.506337979	negative
PRJNA729271	stage_3W_vs_E10	ordinary_muscle_development	ENSGALG00010017363	TIMM9	0.486003333	0.14064438	3.455547469	0.000549176	0.000842094	positive
PRJNA729271	stage_3W_vs_E10	ordinary_muscle_development	ENSGALG00010017364	AKT1	-0.297906579	0.073407444	-4.058261195	4.94395E-05	8.28181E-05	negative
PRJNA729271	stage_3W_vs_E10	ordinary_muscle_development	ENSGALG00010017367	TIMD4	0.396952857	0.308727153	1.285772415	0.1985225	0.229894035	positive
PRJNA729271	stage_3W_vs_E10	ordinary_muscle_development	ENSGALG00010017368	ERLIN2	0.018867472	0.05041937	0.374210786	0.7082475	0.737291548	positive
PRJNA729271	stage_3W_vs_E10	ordinary_muscle_development	ENSGALG00010017369	CAPN14	0.52256688	0.489625057	1.067279692	0.285845556	0.322940868	positive
PRJNA729271	stage_3W_vs_E10	ordinary_muscle_development	ENSGALG00010017371	TMEM114	-3.521867824	1.345936528	-2.616667095	0.008879289	0.012147739	negative
PRJNA729271	stage_3W_vs_E10	ordinary_muscle_development	ENSGALG00010017372	ENSGALG00010017372	0.928420402	0.109990846	8.440887881	3.14936E-17	1.06709E-16	positive
PRJNA729271	stage_3W_vs_E10	ordinary_muscle_development	ENSGALG00010017373	RP1-2705.3	-0.160247865	0.106513708	-1.504481146	0.132457524	0.157542612	negative
PRJNA729271	stage_3W_vs_E10	ordinary_muscle_development	ENSGALG00010017375	BDH1A	-0.579843158	0.161465631	-3.591124342	0.000329254	0.000515394	negative
PRJNA729271	stage_3W_vs_E10	ordinary_muscle_development	ENSGALG00010017376	GALNT14	-0.542801289	0.280974646	-1.931851488	0.053377839	0.066745322	negative
PRJNA729271	stage_3W_vs_E10	ordinary_muscle_development	ENSGALG00010017377	USP8	0.511643274	0.075226136	6.801403064	1.03605E-11	2.6472E-11	positive
PRJNA729271	stage_3W_vs_E10	ordinary_muscle_development	ENSGALG00010017378	ENSGALG00010017378	0.669831177	0.53905333	1.24260651	0.21401287	0.246748055	positive
PRJNA729271	stage_3W_vs_E10	ordinary_muscle_development	ENSGALG00010017380	MARCKSL1	-1.859733264	0.135565556	-13.71833173	7.88591E-43	6.98266E-42	negative
PRJNA729271	stage_3W_vs_E10	ordinary_muscle_development	ENSGALG00010017381	CA6	-0.105099787	0.673841124	-0.155971167	0.876055748	0.890305551	negative
PRJNA729271	stage_3W_vs_E10	ordinary_muscle_development	ENSGALG00010017383	ENSGALG00010017383	-0.029692626	0.151490362	-0.196003401	0.844607504	0.862822311	negative
PRJNA729271	stage_3W_vs_E10	ordinary_muscle_development	ENSGALG00010017384	ARID4A	0.078897678	0.092597611	0.852048741	0.39418704	0.433121676	positive
PRJNA729271	stage_3W_vs_E10	ordinary_muscle_development	ENSGALG00010017386	ENSGALG00010017386	1.017389479	0.524807204	1.938596634	0.052550474	0.065761816	positive
PRJNA729271	stage_3W_vs_E10	ordinary_muscle_development	ENSGALG00010017387	HS6ST2	-0.960253079	0.114975655	-8.351794823	6.72334E-17	2.24343E-16	negative
PRJNA729271	stage_3W_vs_E10	ordinary_muscle_development	ENSGALG00010017388	PLPBP	0.41542697	0.082445712	5.038794111	4.68474E-07	9.06855E-07	positive
PRJNA729271	stage_3W_vs_E10	ordinary_muscle_development	ENSGALG00010017390	DDX24	-1.284301859	0.103714306	-12.38307338	3.22743E-35	2.2405E-34	negative
PRJNA729271	stage_3W_vs_E10	ordinary_muscle_development	ENSGALG00010017391	CRAMP1	0.170399286	0.1168734	1.457981764	0.14484557	0.171348774	positive
PRJNA729271	stage_3W_vs_E10	ordinary_muscle_development	ENSGALG00010017393	VDAC1	3.338346517	0.163591933	20.40654735	1.46259E-92	5.07182E-91	positive
PRJNA729271	stage_3W_vs_E10	ordinary_muscle_development	ENSGALG00010017395	BRF2	-0.183133038	0.124491207	-1.471051997	0.141277055	0.167427922	negative
PRJNA729271	stage_3W_vs_E10	ordinary_muscle_development	ENSGALG00010017396	CCR8L	2.986342409	0.273039284	10.93740932	7.63505E-28	4.04348E-27	positive
PRJNA729271	stage_3W_vs_E10	ordinary_muscle_development	ENSGALG00010017398	SLC22A5	-0.034436684	0.18314879	-0.188025722	0.850856487	0.867921519	negative
PRJNA729271	stage_3W_vs_E10	ordinary_muscle_development	ENSGALG00010017399	CPXM1	-0.764436714	0.291417916	-2.623163065	0.008711757	0.011925291	negative
PRJNA729271	stage_3W_vs_E10	ordinary_muscle_development	ENSGALG00010017400	GPC4	-1.424072913	0.119155822	-11.95134979	6.38792E-33	4.09341E-32	negative
PRJNA729271	stage_3W_vs_E10	ordinary_muscle_development	ENSGALG00010017402	SLC2A5	2.275505582	0.226637721	10.04027739	1.01389E-23	4.49565E-23	positive
PRJNA729271	stage_3W_vs_E10	ordinary_muscle_development	ENSGALG00010017403	ITGA11	-0.441018679	0.292676009	-1.506849436	0.131849255	0.156880372	negative
PRJNA729271	stage_3W_vs_E10	ordinary_muscle_development	ENSGALG00010017405	XCR1	6.224272747	0.902013331	6.900422123	5.18482E-12	1.3418E-11	positive
PRJNA729271	stage_3W_vs_E10	ordinary_muscle_development	ENSGALG00010017406	IFT140	-1.099114784	0.125058091	-8.788833835	1.5112E-18	5.45034E-18	negative
PRJNA729271	stage_3W_vs_E10	ordinary_muscle_development	ENSGALG00010017407	EPN2	0.443554377	0.08494209	5.221844402	1.7715E-07	3.52331E-07	positive
PRJNA729271	stage_3W_vs_E10	ordinary_muscle_development	ENSGALG00010017409	GPC3	-1.63087508	0.33785607	-4.827129724	1.38515E-06	2.59839E-06	negative
PRJNA729271	stage_3W_vs_E10	ordinary_muscle_development	ENSGALG00010017411	RAB11FIP1	-0.271526964	0.138696708	-1.957703021	0.050264871	0.063059486	negative
PRJNA729271	stage_3W_vs_E10	ordinary_muscle_development	ENSGALG00010017413	ZIC3	-5.662159044	0.995874432	-5.685615437	1.30342E-08	2.79278E-08	negative
PRJNA729271	stage_3W_vs_E10	ordinary_muscle_development	ENSGALG00010017414	ENSGALG00010017414	-0.156493728	0.198250645	-0.78937311	0.429893968	0.468378913	negative
PRJNA729271	stage_3W_vs_E10	ordinary_muscle_development	ENSGALG00010017415	HDAC1	-1.749031504	0.064962307	-26.92378996	1.1568E-159	1.2702E-157	negative
PRJNA729271	stage_3W_vs_E10	ordinary_muscle_development	ENSGALG00010017418	CHGB	-1.228690299	0.287942593	-4.267136331	1.97998E-05	3.42276E-05	negative
PRJNA729271	stage_3W_vs_E10	ordinary_muscle_development	ENSGALG00010017419	B9D1	-1.041740746	0.102175237	-10.19562838	2.07399E-24	9.48044E-24	negative
PRJNA729271	stage_3W_vs_E10	ordinary_muscle_development	ENSGALG00010017421	GPR157	2.455120699	0.261734648	9.3801899	6.5854E-21	2.61378E-20	positive
PRJNA729271	stage_3W_vs_E10	ordinary_muscle_development	ENSGALG00010017422	ENSGALG00010017422	-0.827044099	0.243504203	-3.39642638	0.000682719	0.001038189	negative
PRJNA729271	stage_3W_vs_E10	ordinary_muscle_development	ENSGALG00010017423	TMEM204	-0.221095	0.133503938	-1.656093466	0.097702892	0.118312734	negative
PRJNA729271	stage_3W_vs_E10	ordinary_muscle_development	ENSGALG00010017424	APOD	3.564991003	0.480661371	7.416845245	1.19943E-13	3.39101E-13	positive
PRJNA729271	stage_3W_vs_E10	ordinary_muscle_development	ENSGALG00010017425	H6PD	0.961319095	0.124781296	7.704031959	1.31839E-14	3.90878E-14	positive
PRJNA729271	stage_3W_vs_E10	ordinary_muscle_development	ENSGALG00010017428	PSMA3	0.287403475	0.10784075	2.665073032	0.00769716	0.010598494	positive
PRJNA729271	stage_3W_vs_E10	ordinary_muscle_development	ENSGALG00010017429	TELO2	-0.25202179	0.103691027	-2.430507212	0.015077706	0.02010407	negative
PRJNA729271	stage_3W_vs_E10	ordinary_muscle_development	ENSGALG00010017430	CACNA1H	-3.771406837	0.233933447	-16.12170846	1.79575E-58	2.59775E-57	negative
PRJNA729271	stage_3W_vs_E10	ordinary_muscle_development	ENSGALG00010017431	FYCO1	3.949635112	0.117140912	33.71695717	3.2623E-249	8.922E-247	positive
PRJNA729271	stage_3W_vs_E10	ordinary_muscle_development	ENSGALG00010017433	RNPS1	-0.784117336	0.07945005	-9.869312056	5.65511E-23	2.44106E-22	negative
PRJNA729271	stage_3W_vs_E10	ordinary_muscle_development	ENSGALG00010017434	FGF13	2.641079641	0.148296012	17.80951223	5.96272E-71	1.17912E-69	positive
PRJNA729271	stage_3W_vs_E10	ordinary_muscle_development	ENSGALG00010017435	ACTR10	-0.824242153	0.090755268	-9.082031001	1.06567E-19	4.02474E-19	negative
PRJNA729271	stage_3W_vs_E10	ordinary_muscle_development	ENSGALG00010017437	DRG2	0.211999162	0.13959789	1.518641599	0.128852734	0.153507058	positive
PRJNA729271	stage_3W_vs_E10	ordinary_muscle_development	ENSGALG00010017438	ADRB3	1.743218266	0.844463869	2.064289936	0.038990235	0.049636699	positive
PRJNA729271	stage_3W_vs_E10	ordinary_muscle_development	ENSGALG00010017439	PPP4R4	-2.241971524	0.278364242	-8.054093117	8.00702E-16	2.52418E-15	negative
PRJNA729271	stage_3W_vs_E10	ordinary_muscle_development	ENSGALG00010017441	SPSB1	0.81639136	0.256575804	3.181871978	0.001463265	0.00215966	positive
PRJNA729271	stage_3W_vs_E10	ordinary_muscle_development	ENSGALG00010017442	LCK	1.737144739	0.24571684	7.069701615	1.55267E-12	4.14704E-12	positive
PRJNA729271	stage_3W_vs_E10	ordinary_muscle_development	ENSGALG00010017445	F9	2.641657865	1.215677778	2.172991818	0.029780933	0.038415469	positive
PRJNA729271	stage_3W_vs_E10	ordinary_muscle_development	ENSGALG00010017447	CAPN13	-0.045859028	0.520842107	-0.08804785	0.929838645	0.937992286	negative
PRJNA729271	stage_3W_vs_E10	ordinary_muscle_development	ENSGALG00010017449	TRMT6	-1.028591213	0.086779233	-11.85296507	2.07709E-32	1.30959E-31	negative
PRJNA729271	stage_3W_vs_E10	ordinary_muscle_development	ENSGALG00010017450	ENSGALG00010017450	2.085572186	0.209664883	9.947169785	2.59457E-23	1.12938E-22	positive
PRJNA729271	stage_3W_vs_E10	ordinary_muscle_development	ENSGALG00010017451	GABPB1	-0.684379788	0.077669459	-8.811440185	1.23548E-18	4.46703E-18	negative
PRJNA729271	stage_3W_vs_E10	ordinary_muscle_development	ENSGALG00010017452	ARMH4	-4.41905179	0.466573447	-9.471288645	2.76412E-21	1.11573E-20	negative
PRJNA729271	stage_3W_vs_E10	ordinary_muscle_development	ENSGALG00010017453	PPP1R2	1.239385884	0.138019619	8.9797805	2.71307E-19	1.00888E-18	positive
PRJNA729271	stage_3W_vs_E10	ordinary_muscle_development	ENSGALG00010017454	ENSGALG00010017454	1.282675809	0.157922788	8.122170498	4.57919E-16	1.46137E-15	positive
PRJNA729271	stage_3W_vs_E10	ordinary_muscle_development	ENSGALG00010017455	CCR9	3.700946769	0.681506766	5.430535621	5.61852E-08	1.15404E-07	positive
PRJNA729271	stage_3W_vs_E10	ordinary_muscle_development	ENSGALG00010017457	ZMAT4	-1.937015983	0.263550567	-7.349693852	1.98661E-13	5.54622E-13	negative
PRJNA729271	stage_3W_vs_E10	ordinary_muscle_development	ENSGALG00010017458	ENSGALG00010017458	0.860022157	0.171863979	5.004086157	5.61277E-07	1.07972E-06	positive
PRJNA729271	stage_3W_vs_E10	ordinary_muscle_development	ENSGALG00010017459	FAM167B	1.807502851	0.286509665	6.308697641	2.81393E-10	6.63111E-10	positive
PRJNA729271	stage_3W_vs_E10	ordinary_muscle_development	ENSGALG00010017462	MCM8	-0.378577941	0.118950496	-3.182651205	0.001459333	0.002154296	negative
PRJNA729271	stage_3W_vs_E10	ordinary_muscle_development	ENSGALG00010017463	PPP1R36	1.160291304	0.325235752	3.567539232	0.000360349	0.000562004	positive
PRJNA729271	stage_3W_vs_E10	ordinary_muscle_development	ENSGALG00010017464	SH3BP2	0.346135568	0.129766448	2.667373372	0.007644669	0.010529217	positive
PRJNA729271	stage_3W_vs_E10	ordinary_muscle_development	ENSGALG00010017465	TMEM234	0.109865161	0.232468873	0.472601598	0.636497442	0.669960818	positive
PRJNA729271	stage_3W_vs_E10	ordinary_muscle_development	ENSGALG00010017466	SPIA4	4.748062183	2.758055333	1.721525354			positive
PRJNA729271	stage_3W_vs_E10	ordinary_muscle_development	ENSGALG00010017467	SFRP1	-5.562028494	0.12663832	-43.92058035	0	0	negative
PRJNA729271	stage_3W_vs_E10	ordinary_muscle_development	ENSGALG00010017468	AMDHD2	0.087203051	0.127789044	0.682398491	0.49498701	0.533326151	positive
PRJNA729271	stage_3W_vs_E10	ordinary_muscle_development	ENSGALG00010017470	HSPA2	2.716176003	0.169908105	15.98614731	1.59601E-57	2.24169E-56	positive
PRJNA729271	stage_3W_vs_E10	ordinary_muscle_development	ENSGALG00010017471	CRLS1	1.111254628	0.088486559	12.55845676	3.57232E-36	2.54451E-35	positive
PRJNA729271	stage_3W_vs_E10	ordinary_muscle_development	ENSGALG00010017472	LZTFL1	-1.376267368	0.171515427	-8.02416079	1.02222E-15	3.20093E-15	negative
PRJNA729271	stage_3W_vs_E10	ordinary_muscle_development	ENSGALG00010017473	EIF3I	0.45281353	0.104442606	4.335525021	1.45413E-05	2.53702E-05	positive
PRJNA729271	stage_3W_vs_E10	ordinary_muscle_development	ENSGALG00010017475	STUM	1.569314256	0.248166153	6.323643405	2.55467E-10	6.03683E-10	positive
PRJNA729271	stage_3W_vs_E10	ordinary_muscle_development	ENSGALG00010017476	ATP6V0C	1.636063909	0.111853614	14.626831	1.89406E-48	2.05651E-47	positive
PRJNA729271	stage_3W_vs_E10	ordinary_muscle_development	ENSGALG00010017477	MCF2	1.712981434	0.182011475	9.411392504	4.89607E-21	1.95183E-20	positive

PRJNA729271	stage_3W_vs_E10	ordinary_muscle_development	ENSGALG00010017479	SSTR5	0.859007675	0.494508721	1.737093075	0.082370743	0.100637553	positive
PRJNA729271	stage_3W_vs_E10	ordinary_muscle_development	ENSGALG00010017480	DCDC2B	0.898530002	0.192973181	4.656242898	3.22032E-06	5.90419E-06	positive
PRJNA729271	stage_3W_vs_E10	ordinary_muscle_development	ENSGALG00010017481	SPIA1	3.003253672	2.575050419	1.16628927			positive
PRJNA729271	stage_3W_vs_E10	ordinary_muscle_development	ENSGALG00010017482	CORO2B	-1.538861488	0.148844829	-10.33869633	4.709E-25	2.21111E-24	negative
PRJNA729271	stage_3W_vs_E10	ordinary_muscle_development	ENSGALG00010017484	IQCC	0.043579483	0.13362602	0.326130219	0.744325835	0.771304187	positive
PRJNA729271	stage_3W_vs_E10	ordinary_muscle_development	ENSGALG00010017485	LBH	-0.648949892	0.107938833	-6.012200372	1.83022E-09	4.10222E-09	negative
PRJNA729271	stage_3W_vs_E10	ordinary_muscle_development	ENSGALG00010017487	C1QTNF8	-0.3968401	0.510337196	-0.777603717	0.436802669	0.475441518	negative
PRJNA729271	stage_3W_vs_E10	ordinary_muscle_development	ENSGALG00010017489	TNFRSF1B	1.245879197	0.177300924	7.026918797	2.11144E-12	5.59615E-12	positive
PRJNA729271	stage_3W_vs_E10	ordinary_muscle_development	ENSGALG00010017491	CCDC28B	1.627238394	0.190295177	8.551127894	1.21891E-17	4.22481E-17	positive
PRJNA729271	stage_3W_vs_E10	ordinary_muscle_development	ENSGALG00010017493	YPEL5	0.291675462	0.134350207	2.171008648	0.029930519	0.038601554	positive
PRJNA729271	stage_3W_vs_E10	ordinary_muscle_development	ENSGALG00010017494	TBC1D24	-1.364767114	0.095940763	-14.22510167	6.40112E-46	6.32908E-45	negative
PRJNA729271	stage_3W_vs_E10	ordinary_muscle_development	ENSGALG00010017495	NT5M	-0.074227131	0.134802878	-0.550634619	0.581884172	0.617859653	negative
PRJNA729271	stage_3W_vs_E10	ordinary_muscle_development	ENSGALG00010017496	PARP1	-1.650610056	0.103910704	-15.88488953	8.06399E-57	1.11428E-55	negative
PRJNA729271	stage_3W_vs_E10	ordinary_muscle_development	ENSGALG00010017499	SNRPD1	-1.394218219	0.098976097	-14.08641335	4.60356E-45	4.41618E-44	negative
PRJNA729271	stage_3W_vs_E10	ordinary_muscle_development	ENSGALG00010017500	ACAP2	0.346071098	0.100463876	3.444731682	0.000571627	0.000874668	positive
PRJNA729271	stage_3W_vs_E10	ordinary_muscle_development	ENSGALG00010017502	NAT8B	1.00026839	0.117348007	8.523948686	1.54204E-17	5.31428E-17	positive
PRJNA729271	stage_3W_vs_E10	ordinary_muscle_development	ENSGALG00010017503	MED9	0.98063139	0.12532867	7.824477773	5.09769E-15	1.54454E-14	positive
PRJNA729271	stage_3W_vs_E10	ordinary_muscle_development	ENSGALG00010017505	TNFRSF8	1.377303731	0.296520721	4.6448819	3.40271E-06	6.2315E-06	positive
PRJNA729271	stage_3W_vs_E10	ordinary_muscle_development	ENSGALG00010017509	RASD1	0.483510261	0.301931748	1.601389267	0.109290728	0.131346415	positive
PRJNA729271	stage_3W_vs_E10	ordinary_muscle_development	ENSGALG00010017510	HDC	5.278206852	0.376029744	14.03667378	9.29751E-45	8.79109E-44	positive
PRJNA729271	stage_3W_vs_E10	ordinary_muscle_development	ENSGALG00010017513	TNIP2	0.868400997	0.142612748	6.089224205	1.13459E-09	2.57571E-09	positive
PRJNA729271	stage_3W_vs_E10	ordinary_muscle_development	ENSGALG00010017514	TXLNA	-0.288028615	0.096769195	-2.976449413	0.002916072	0.004184996	negative
PRJNA729271	stage_3W_vs_E10	ordinary_muscle_development	ENSGALG00010017516	AAK1	-0.48023511	0.080847632	-5.940002153	2.85018E-09	6.31897E-09	negative
PRJNA729271	stage_3W_vs_E10	ordinary_muscle_development	ENSGALG00010017517	LIN9	-0.725049767	0.102912805	-7.045282354	1.85086E-12	4.9208E-12	negative
PRJNA729271	stage_3W_vs_E10	ordinary_muscle_development	ENSGALG00010017518	PEMT	1.035583995	0.147169911	7.036655693	1.96909E-12	5.23129E-12	positive
PRJNA729271	stage_3W_vs_E10	ordinary_muscle_development	ENSGALG00010017519	NTN3	-3.633034561	0.237384983	-15.30439926	7.14587E-53	8.86052E-52	negative
PRJNA729271	stage_3W_vs_E10	ordinary_muscle_development	ENSGALG00010017520	ZBTB1	-1.005087321	0.081490835	-12.33374671	5.96092E-35	4.08721E-34	negative
PRJNA729271	stage_3W_vs_E10	ordinary_muscle_development	ENSGALG00010017521	MIIP	0.753410002	0.114001615	6.608766044	3.87537E-11	9.59573E-11	positive
PRJNA729271	stage_3W_vs_E10	ordinary_muscle_development	ENSGALG00010017522	XXYLT1	-0.660039785	0.107724803	-6.127092049	8.94997E-10	2.04586E-09	negative
PRJNA729271	stage_3W_vs_E10	ordinary_muscle_development	ENSGALG00010017524	ACBD3	-0.310298282	0.059938252	-5.176965798	2.25524E-07	4.45484E-07	negative
PRJNA729271	stage_3W_vs_E10	ordinary_muscle_development	ENSGALG00010017525	ENSGALG00010017525	-3.439909296	0.629113902	-5.467864064	4.55491E-08	9.41175E-08	negative
PRJNA729271	stage_3W_vs_E10	ordinary_muscle_development	ENSGALG00010017526	PLEKHA2	1.76268582	0.170217764	10.35547512	3.95219E-25	1.86057E-24	positive
PRJNA729271	stage_3W_vs_E10	ordinary_muscle_development	ENSGALG00010017528	RAI1	-0.001208904	0.178167631	-0.006785208	0.994586229	0.995410301	negative
PRJNA729271	stage_3W_vs_E10	ordinary_muscle_development	ENSGALG00010017529	MFN2	0.751097882	0.118422168	6.342544611	2.26001E-10	5.35625E-10	positive
PRJNA729271	stage_3W_vs_E10	ordinary_muscle_development	ENSGALG00010017530	TLR1B	1.247800875	0.211216572	5.907684518	3.4695E-09	7.65687E-09	positive
PRJNA729271	stage_3W_vs_E10	ordinary_muscle_development	ENSGALG00010017531	SACM1L	-0.559925947	0.112397785	-4.981645737	6.30458E-07	1.20959E-06	negative
PRJNA729271	stage_3W_vs_E10	ordinary_muscle_development	ENSGALG00010017532	MIXL1	0.566713733	0.320038603	1.770766798	0.076599489	0.093974574	positive
PRJNA729271	stage_3W_vs_E10	ordinary_muscle_development	ENSGALG00010017533	FAM193A	-0.125008192	0.078382772	-1.59484271	0.110747414	0.133019867	negative
PRJNA729271	stage_3W_vs_E10	ordinary_muscle_development	ENSGALG00010017534	ATP11C	-1.107871699	0.084707956	-13.07872074	4.35711E-39	3.42125E-38	negative
PRJNA729271	stage_3W_vs_E10	ordinary_muscle_development	ENSGALG00010017535	LIMD1	-0.414098028	0.090592372	-4.571003278	4.85395E-06	8.78157E-06	negative
PRJNA729271	stage_3W_vs_E10	ordinary_muscle_development	ENSGALG00010017536	GSC	-1.906042302	0.46018488	-4.141905532	3.44432E-05	5.82833E-05	negative
PRJNA729271	stage_3W_vs_E10	ordinary_muscle_development	ENSGALG00010017537	MANBA	0.963321333	0.187370602	5.141261879	2.72899E-07	5.36581E-07	positive
PRJNA729271	stage_3W_vs_E10	ordinary_muscle_development	ENSGALG00010017538	H3-3A	-0.344041154	0.076028212	-4.525177486	6.03448E-06	1.08429E-05	negative
PRJNA729271	stage_3W_vs_E10	ordinary_muscle_development	ENSGALG00010017539	KPNA6	1.190375994	0.138256587	8.609904353	7.31216E-18	2.56385E-17	positive
PRJNA729271	stage_3W_vs_E10	ordinary_muscle_development	ENSGALG00010017540	EIF4G1	0.595166887	0.164594379	3.615961182	0.000299235	0.000469977	positive
PRJNA729271	stage_3W_vs_E10	ordinary_muscle_development	ENSGALG00010017541	SREBF1	2.19249813	0.227094482	9.654563639	4.70157E-22	1.95326E-21	positive
PRJNA729271	stage_3W_vs_E10	ordinary_muscle_development	ENSGALG00010017542	PLOD1	-0.753811895	0.125401619	-6.011181512	1.84176E-09	4.12681E-09	negative
PRJNA729271	stage_3W_vs_E10	ordinary_muscle_development	ENSGALG00010017543	ZBTB25	-0.002290742	0.177823001	-0.012882145	0.989721819	0.991165232	negative
PRJNA729271	stage_3W_vs_E10	ordinary_muscle_development	ENSGALG00010017544	SDE2	0.344337413	0.068433163	5.031733108	4.86066E-07	9.39779E-07	positive
PRJNA729271	stage_3W_vs_E10	ordinary_muscle_development	ENSGALG00010017545	DICER1	-0.351520347	0.12092888	-2.906835379	0.003651053	0.005192505	negative
PRJNA729271	stage_3W_vs_E10	ordinary_muscle_development	ENSGALG00010017546	NFU1	1.107255556	0.108102807	10.24261615	1.27735E-24	5.89092E-24	positive
PRJNA729271	stage_3W_vs_E10	ordinary_muscle_development	ENSGALG00010017547	RNF4	0.138749208	0.065389925	2.121874404	0.033848286	0.04337644	positive
PRJNA729271	stage_3W_vs_E10	ordinary_muscle_development	ENSGALG00010017550	TM2D2	0.866104356	0.074950942	11.55561672	6.91493E-31	4.10955E-30	positive
PRJNA729271	stage_3W_vs_E10	ordinary_muscle_development	ENSGALG00010017551	NALF2	-1.14285461	0.289097665	-3.953178279	7.71199E-05	0.000127043	negative
PRJNA729271	stage_3W_vs_E10	ordinary_muscle_development	ENSGALG00010017552	LARS2	0.043612576	0.102809901	0.424205995	0.671415589	0.702886679	positive
PRJNA729271	stage_3W_vs_E10	ordinary_muscle_development	ENSGALG00010017553	EDA	0.503023	0.109013436	4.614321139	3.94382E-06	7.18614E-06	positive
PRJNA729271	stage_3W_vs_E10	ordinary_muscle_development	ENSGALG00010017554	FAM131A	-3.189029915	0.378379523	-8.42812499	3.51248E-17	1.18652E-16	negative
PRJNA729271	stage_3W_vs_E10	ordinary_muscle_development	ENSGALG00010017555	CELA2A	4.506392869	1.200071632	3.755103238	0.00017327	0.000277458	positive
PRJNA729271	stage_3W_vs_E10	ordinary_muscle_development	ENSGALG00010017556	TOM1L2	-0.364986994	0.115804898	-3.151740567	0.001623004	0.002388612	negative
PRJNA729271	stage_3W_vs_E10	ordinary_muscle_development	ENSGALG00010017558	ENSGALG00010017558	-1.921811305	0.075577316	-25.42841427	1.2236E-142	1.0311E-140	negative
PRJNA729271	stage_3W_vs_E10	ordinary_muscle_development	ENSGALG00010017559	DUSP10	1.959994265	0.149762383	13.08736033	3.88889E-39	3.05857E-38	positive
PRJNA729271	stage_3W_vs_E10	ordinary_muscle_development	ENSGALG00010017561	CFAP99	-1.620876561	0.687149037	-2.358842805	0.018332019	0.024213835	negative
PRJNA729271	stage_3W_vs_E10	ordinary_muscle_development	ENSGALG00010017562	SLC30A10	-3.965157751	0.626118696	-6.332917027	2.40569E-10	5.69407E-10	negative
PRJNA729271	stage_3W_vs_E10	ordinary_muscle_development	ENSGALG00010017563	ALK	0.778150516	0.155151865	5.015411924	5.292E-07	1.0191E-06	positive
PRJNA729271	stage_3W_vs_E10	ordinary_muscle_development	ENSGALG00010017565	ENSGALG00010017565	-1.519515879	0.193348465	-7.858949781	3.87367E-15	1.18084E-14	negative
PRJNA729271	stage_3W_vs_E10	ordinary_muscle_development	ENSGALG00010017568	CDCP1	-1.753124676	0.24027689	-7.296268384	2.95858E-13	8.18215E-13	negative
PRJNA729271	stage_3W_vs_E10	ordinary_muscle_development	ENSGALG00010017569	CLMN	-1.50640969	0.246866032	-6.102134339	1.04661E-09	2.38195E-09	negative
PRJNA729271	stage_3W_vs_E10	ordinary_muscle_development	ENSGALG00010017570	DRC3	-0.724717561	0.387592259	-1.869793689	0.061512473	0.076376846	negative
PRJNA729271	stage_3W_vs_E10	ordinary_muscle_development	ENSGALG00010017571	ADAM9	1.09127944	0.149789651	7.285412802	3.20687E-13	8.84895E-13	positive
PRJNA729271	stage_3W_vs_E10	ordinary_muscle_development	ENSGALG00010017573	ENSGALG00010017573	0.248823395	0.228652764	1.08821512	0.276500151	0.313359632	positive
PRJNA729271	stage_3W_vs_E10	ordinary_muscle_development	ENSGALG00010017574	DYNC1LI1	-0.059650069	0.055718299	-1.070565139	0.284365005	0.321518779	negative
PRJNA729271	stage_3W_vs_E10	ordinary_muscle_development	ENSGALG00010017575	CLCN2	2.571602015	0.713973666	3.601816336	0.000316002	0.000495397	positive
PRJNA729271	stage_3W_vs_E10	ordinary_muscle_development	ENSGALG00010017576	ENSGALG00010017576	-3.195136333	0.301850145	-10.58517407	3.49131E-26	1.72014E-25	negative
PRJNA729271	stage_3W_vs_E10	ordinary_muscle_development	ENSGALG00010017577	CLPX	1.462477956	0.13752436	10.63431929	2.0633E-26	1.02775E-25	positive
PRJNA729271	stage_3W_vs_E10	ordinary_muscle_development	ENSGALG00010017578	BSDC1	0.276880505	0.100271348	2.761312283	0.00575696	0.008015285	positive
PRJNA729271	stage_3W_vs_E10	ordinary_muscle_development	ENSGALG00010017579	P3H2	-0.09820001	0.143793592	-0.682923406	0.494655243	0.533008307	negative
PRJNA729271	stage_3W_vs_E10	ordinary_muscle_development	ENSGALG00010017580	LYPLAL1	0.451918327	0.153889568	2.936640428	0.003317886	0.004740537	positive
PRJNA729271	stage_3W_vs_E10	ordinary_muscle_development	ENSGALG00010017581	EFCAB11	-2.80149813	0.468996312	-5.973390534	2.32373E-09	5.17634E-09	negative
PRJNA729271	stage_3W_vs_E10	ordinary_muscle_development	ENSGALG00010017582	CLCN6	-0.270357344	0.103570983	-2.610358007	0.009044751	0.012363605	negative
PRJNA729271	stage_3W_vs_E10	ordinary_muscle_development	ENSGALG00010017583	MTHFD1	-1.600074778	0.1072723	-14.91601067	2.59321E-50	2.97849E-49	negative
PRJNA729271	stage_3W_vs_E10	ordinary_muscle_development	ENSGALG00010017584	WNT11B	-1.013878653	0.679613343	-1.491846302	0.135739442	0.161181553	negative
PRJNA729271	stage_3W_vs_E10	ordinary_muscle_development	ENSGALG00010017585	ENSGALG00010017585	-0.593907499	0.121638637	-4.882556334	1.04719E-06	1.9816E-06	negative
PRJNA729271	stage_3W_vs_E10	ordinary_muscle_development	ENSGALG00010017586	ZFYVE28	0.596094402	0.158990527	3.749244759	0.000177368	0.0002838	positive
PRJNA729271	stage_3W_vs_E10	ordinary_muscle_development	ENSGALG00010017587	MB21D2	-0.896437511	0.148810673	-6.02401354	1.70144E-09	3.82422E-09	negative
PRJNA729271	stage_3W_vs_E10	ordinary_muscle_development	ENSGALG00010017588	NOX5	-1.222635216	0.491347616	-2.48833041	0.012834443	0.017241451	negative
PRJNA729271	stage_3W_vs_E10	ordinary_muscle_development	ENSGALG00010017589	ATPAF2	0.785404582	0.144741913	5.42624156	5.75531E-08	1.1813E-07	positive
PRJNA729271	stage_3W_vs_E10	ordinary_muscle_development	ENSGALG00010017590	ENSGALG00010017590	-0.778484839	0.193343207	-4.02644009	5.66277E-05	9.44772E-05	negative
PRJNA729271	stage_3W_vs_E10	ordinary_muscle_development	ENSGALG00010017591	ESR2	-1.627931166	0.258338885	-6.301533601	2.94715E-10	6.93827E-10	negative
PRJNA729271	stage_3W_vs_E10	ordinary_muscle_development	ENSGALG00010017592	GID4	0.952720853	0.109830748	8.674445634	4.1557E-18	1.47567E-17	positive
PRJNA729271	stage_3W_vs_E10	ordinary_muscle_development	ENSGALG00010017593	MTHFR	1.018911291	0.13175451	7.73340728	1.04706E-14	3.11787E-14	positive
PRJNA729271	stage_3W_vs_E10	ordinary_muscle_development	ENSGALG00010017594	CEP68	0.001323267	0.102795428	0.012872824	0.989729256	0.991165232	positive
PRJNA729271	stage_3W_vs_E10	ordinary_muscle_development	ENSGALG00010017595	CCNF	-2.666247937	0.129865096	-20.53090498	1.14031E-93	4.12191E-92	negative
PRJNA729271	stage_3W_vs_E10	ordinary_muscle_development	ENSGALG00010017596	TDP1	-2.144376812	0.101070262	-21.21669395	6.69706E-100	2.79752E-98	negative
PRJNA729271	stage_3W_vs_E10	ordinary_muscle_development	ENSGALG00010017597	CNOT10	-0.16763363	0.135694433	-1.235375882	0.216690644	0.24944822	negative
PRJNA729271	stage_3W_vs_E10	ordinary_muscle_development	ENSGALG00010017598	ENSGALG00010017598	0.812131326	0.117276677	6.924917604	4.36229E-12	1.13237E-11	positive
PRJNA729271	stage_3W_vs_E10	ordinary_muscle_development	ENSGALG00010017599	KCNK13	0.733506717	0.220883855	3.320780131	0.000897662	0.001349613	positive
PRJNA729271	stage_3W_vs_E10	ordinary_muscle_development	ENSGALG00010017600	MXD4	0.650138165	0.103197865	6.299918729	2.97802E-10	7.00866E-10	positive
PRJNA729271	stage_3W_vs_E10	ordinary_muscle_development	ENSGALG00010017601	ENSGALG00010017601	1.085539103	0.115004819	9.439074887	3.76085E-21	1.51049E-20	positive
PRJNA729271	stage_3W_vs_E10	ordinary_muscle_development	ENSGALG00010017603	CYP3A5	0.956686061	1.359692206	0.703604872	0.481678876	0.520224671	positive
PRJNA729271	stage_3W_vs_E10	ordinary_muscle_development	ENSGALG00010017604	GATM	2.90085264	0.241219159	12.02579702	2.60066E-33	1.69043E-32	positive
PRJNA729271	stage_3W_vs_E10	ordinary_muscle_development	ENSGALG00010017605	GFPT1	-1.345814228	0.072359151	-18.59908803	3.2682E-77	7.59176E-76	negative
PRJNA729271	stage_3W_vs_E10	ordinary_muscle_development	ENSGALG00010017606	ENSGALG00010017606	1.132928317	0.24455359	4.632638261	3.61035E-06	6.60174E-06	positive
PRJNA729271	stage_3W_vs_E10	ordinary_muscle_development	ENSGALG00010017608	RBBP4	-1.432513312	0.081282156	-17.62395812	1.61313E-69	3.06915E-68	negative
PRJNA729271	stage_3W_vs_E10	ordinary_muscle_development	ENSGALG00010017609	TRIM71	-2.347623889	0.220607654	-10.64162483	1.90773E-26	9.53208E-26	negative
PRJNA729271	stage_3W_vs_E10	ordinary_muscle_development	ENSGALG00010017610	PSMC1	0.449121259	0.118993268	3.774341756	0.000160431	0.000257667	positive
PRJNA729271	stage_3W_vs_E10	ordinary_muscle_development	ENSGALG00010017612	CLDN11	-0.689563261	0.423959079	-1.626485421	0.103846432	0.12523954	negative
PRJNA729271	stage_3W_vs_E10	ordinary_muscle_development	ENSGALG00010017613	HAUS3	-2.098109365	0.123511048	-16.98722017	1.02115E-64	1.73524E-63	negative
PRJNA729271	stage_3W_vs_E10	ordinary_muscle_development	ENSGALG00010017615	AGTRAP	0.551197953	0.134408363	4.100920083	4.11511E-05	6.93184E-05	positive
PRJNA729271	stage_3W_vs_E10	ordinary_muscle_development	ENSGALG00010017616	PDCD7	1.203263676	0.155023122	7.76183358	8.37102E-15	2.50802E-14	positive
PRJNA729271	stage_3W_vs_E10	ordinary_muscle_development	ENSGALG00010017619	POLN	0.182100528	0.332811343	0.547158419	0.5842699	0.619984788	positive
PRJNA729271	stage_3W_vs_E10	ordinary_muscle_development	ENSGALG00010017620	UBAP1L	0.120223618	0.335448158	0.358397014	0.720046226	0.748498999	positive
PRJNA729271	stage_3W_vs_E10	ordinary_muscle_development	ENSGALG00010017621	SYNE2	1.371992876	0.155916161	8.799555269	1.37359E-18	4.9602E-18	positive
PRJNA729271	stage_3W_vs_E10	ordinary_muscle_development	ENSGALG00010017622	RAB1B	0.317530204	0.11250659	2.822325364	0.004767678	0.006701658	positive
PRJNA729271	stage_3W_vs_E10	ordinary_muscle_development	ENSGALG00010017625	YBX1	-0.163290638	0.09126452	-1.789201745	0.073582331	0.090456779	negative
PRJNA729271	stage_3W_vs_E10	ordinary_muscle_development	ENSGALG00010017626	COPS7B	-0.765328913	0.140741547	-5.43783216	5.39327E-08	1.10934E-07	negative
PRJNA729271	stage_3W_vs_E10	ordinary_muscle_development	ENSGALG00010017628	NRDE2	-0.110600747	0.104207114	-1.061355059	0.288528572	0.325743567	negative
PRJNA729271	stage_3W_vs_E10	ordinary_muscle_development	ENSGALG00010017629	ENSGALG00010017629	-0.343205571	0.14256765	-2.407317304	0.016070201	0.021358661	negative
PRJNA729271	stage_3W_vs_E10	ordinary_muscle_development	ENSGALG00010017631	NAT8L	-4.548973299	0.286024622	-15.90413182	5.93199E-57	8.23604E-56	negative
PRJNA729271	stage_3W_vs_E10	ordinary_muscle_development	ENSGALG00010017632	AWAT1	0.507121054	0.609010919	0.832696161	0.40501611	0.443943475	positive
PRJNA729271	stage_3W_vs_E10	ordinary_muscle_development	ENSGALG00010017633	HLX	0.450787348	0.152361577	2.958668171	0.003089716	0.004426313	positive
PRJNA729271	stage_3W_vs_E10	ordinary_muscle_development	ENSGALG00010017634	PPIH	-0.382174937	0.097142413	-3.934171764	8.34841E-05	0.000137122	negative
PRJNA729271	stage_3W_vs_E10	ordinary_muscle_development	ENSGALG00010017636	ZBTB8OS	-0.411629605	0.159177293	-2.585981942	0.009710202	0.013214745	negative
PRJNA729271	stage_3W_vs_E10	ordinary_muscle_development	ENSGALG00010017638	GFRA2	-0.238121118	0.259351808	-0.918139419	0.35854588	0.397272782	negative
PRJNA729271	stage_3W_vs_E10	ordinary_muscle_development	ENSGALG00010017639	ENSGALG00010017639	-0.882633234	0.134130303	-6.580416337	4.69133E-11	1.1551E-10	negative
PRJNA729271	stage_3W_vs_E10	ordinary_muscle_development	ENSGALG00010017641	GPRC5B	1.202762895	0.159222099	7.553994701	4.22107E-14	1.22296E-13	positive
PRJNA729271	stage_3W_vs_E10	ordinary_muscle_development	ENSGALG00010017642	MTARC1	3.142105413	0.121587561	25.84232622	2.9677E-147	2.6719E-145	positive
PRJNA729271	stage_3W_vs_E10	ordinary_muscle_development	ENSGALG00010017643	CALM1	0.730365863	0.060923796	11.98818711	4.09775E-33	2.64339E-32	positive
PRJNA729271	stage_3W_vs_E10	ordinary_muscle_development	ENSGALG00010017644	RBBP6	0.410497497	0.097136499	4.225986103	2.37897E-05	4.08084E-05	positive
PRJNA729271	stage_3W_vs_E10	ordinary_muscle_development	ENSGALG00010017645	SYNC	1.075047668	0.270292852	3.977344052	6.96893E-05	0.000115327	positive
PRJNA729271	stage_3W_vs_E10	ordinary_muscle_development	ENSGALG00010017646	MAEA	-0.403009887	0.086857663	-4.639888661	3.48597E-06	6.37752E-06	negative
PRJNA729271	stage_3W_vs_E10	ordinary_muscle_development	ENSGALG00010017647	ITGA9	-0.37822432	0.167233843	-2.261649393	0.023719074	0.030943027	negative

PRJNA729271	stage_3W_vs_E10	ordinary_muscle_development	ENSGALG00010017648	ENSGALG00010017648	-1.110165674	0.159712561	-6.951022909	3.62647E-12	9.46618E-12	negative
PRJNA729271	stage_3W_vs_E10	ordinary_muscle_development	ENSGALG00010017649	ENSGALG00010017649	-0.609322008	0.11336742	-5.374754109	7.66873E-08	1.56517E-07	negative
PRJNA729271	stage_3W_vs_E10	ordinary_muscle_development	ENSGALG00010017651	PPCS	0.035778937	0.122845762	0.291250888	0.770859446	0.795784322	positive
PRJNA729271	stage_3W_vs_E10	ordinary_muscle_development	ENSGALG00010017652	C1orf115	2.475524819	0.169606857	14.59566473	2.99281E-48	3.21816E-47	positive
PRJNA729271	stage_3W_vs_E10	ordinary_muscle_development	ENSGALG00010017653	ENSGALG00010017653	1.354417159	0.172595599	7.847344686	4.24938E-15	1.29238E-14	positive
PRJNA729271	stage_3W_vs_E10	ordinary_muscle_development	ENSGALG00010017654	KBTBD13	6.035241104	0.312754106	19.29708029	5.6831E-83	1.54843E-81	positive
PRJNA729271	stage_3W_vs_E10	ordinary_muscle_development	ENSGALG00010017655	ENSGALG00010017655	-0.955466524	0.109671065	-8.712111257	2.98267E-18	1.06566E-17	negative
PRJNA729271	stage_3W_vs_E10	ordinary_muscle_development	ENSGALG00010017656	FSD2	7.803916501	0.207845972	37.54663338	1.5989E-308	7.2423E-306	positive
PRJNA729271	stage_3W_vs_E10	ordinary_muscle_development	ENSGALG00010017657	DIS3L2	0.037635536	0.094750566	0.39720645	0.691215226	0.72155625	positive
PRJNA729271	stage_3W_vs_E10	ordinary_muscle_development	ENSGALG00010017658	WHAMM	2.905418958	0.161405319	18.00076336	1.92152E-72	4.00755E-71	positive
PRJNA729271	stage_3W_vs_E10	ordinary_muscle_development	ENSGALG00010017659	TNRC6A	-0.647948656	0.079113861	-8.19007751	2.61058E-16	8.45217E-16	negative
PRJNA729271	stage_3W_vs_E10	ordinary_muscle_development	ENSGALG00010017660	CLIP4	3.664626159	0.246864334	14.84469667	7.52946E-50	8.46043E-49	positive
PRJNA729271	stage_3W_vs_E10	ordinary_muscle_development	ENSGALG00010017661	NHSL3	-0.462685882	0.177036346	-2.613507859	0.008961802	0.012253686	negative
PRJNA729271	stage_3W_vs_E10	ordinary_muscle_development	ENSGALG00010017662	GOLGA4	0.881356903	0.165317575	5.331295863	9.75144E-08	1.97633E-07	positive
PRJNA729271	stage_3W_vs_E10	ordinary_muscle_development	ENSGALG00010017663	ZMYND12	-0.350239312	0.220390908	-1.58917314	0.112021309	0.134494233	negative
PRJNA729271	stage_3W_vs_E10	ordinary_muscle_development	ENSGALG00010017664	ENSGALG00010017664	0.627519093	0.074207734	8.456249256	2.76115E-17	9.37743E-17	positive
PRJNA729271	stage_3W_vs_E10	ordinary_muscle_development	ENSGALG00010017665	CTBP1	-0.020367167	0.062882548	-0.323892203	0.746019647	0.772838392	negative
PRJNA729271	stage_3W_vs_E10	ordinary_muscle_development	ENSGALG00010017667	SLC2A1	-0.797693458	0.112024229	-7.12072257	1.07363E-12	2.89099E-12	negative
PRJNA729271	stage_3W_vs_E10	ordinary_muscle_development	ENSGALG00010017668	ENSGALG00010017668	4.474056444	0.593368734	7.540094699	4.6963E-14	1.35766E-13	positive
PRJNA729271	stage_3W_vs_E10	ordinary_muscle_development	ENSGALG00010017669	ENSGALG00010017669	0.320094886	0.1791271	1.786970741	0.073942218	0.090852966	positive
PRJNA729271	stage_3W_vs_E10	ordinary_muscle_development	ENSGALG00010017670	FAM179A	2.592041647	0.330342305	7.846532535	4.27698E-15	1.30023E-14	positive
PRJNA729271	stage_3W_vs_E10	ordinary_muscle_development	ENSGALG00010017671	ANTXR1	-0.855695545	0.18514854	-4.621670489	3.80662E-06	6.944E-06	negative
PRJNA729271	stage_3W_vs_E10	ordinary_muscle_development	ENSGALG00010017672	SPRED2	-0.948723003	0.079078833	-11.9971801	3.67609E-33	2.37985E-32	negative
PRJNA729271	stage_3W_vs_E10	ordinary_muscle_development	ENSGALG00010017673	WDR89	-0.215006571	0.090991456	-2.362931428	0.018131024	0.023957351	negative
PRJNA729271	stage_3W_vs_E10	ordinary_muscle_development	ENSGALG00010017675	ENSGALG00010017675	0.899222249	0.472059054	1.904893554	0.056793909	0.070815287	positive
PRJNA729271	stage_3W_vs_E10	ordinary_muscle_development	ENSGALG00010017676	SPON2	2.50317044	0.262702133	9.52855011	1.59495E-21	6.49223E-21	positive
PRJNA729271	stage_3W_vs_E10	ordinary_muscle_development	ENSGALG00010017677	WDR43	-0.489124759	0.110203076	-4.438394786	9.06323E-06	1.60503E-05	negative
PRJNA729271	stage_3W_vs_E10	ordinary_muscle_development	ENSGALG00010017678	YARS	-0.338143132	0.137720977	-2.455276889	0.014077611	0.018824259	negative
PRJNA729271	stage_3W_vs_E10	ordinary_muscle_development	ENSGALG00010017679	RASL12	3.247098323	0.178668076	18.17391441	8.30561E-74	1.7862E-72	positive
PRJNA729271	stage_3W_vs_E10	ordinary_muscle_development	ENSGALG00010017681	ENSGALG00010017681	6.574351537	1.23097121	5.340784158	9.25454E-08	1.87904E-07	positive
PRJNA729271	stage_3W_vs_E10	ordinary_muscle_development	ENSGALG00010017684	TRMT61B	0.250991728	0.096220124	2.608515962	0.009093577	0.012426831	positive
PRJNA729271	stage_3W_vs_E10	ordinary_muscle_development	ENSGALG00010017685	PHC2	1.011444897	0.183278633	5.518618742	3.41675E-08	7.11474E-08	positive
PRJNA729271	stage_3W_vs_E10	ordinary_muscle_development	ENSGALG00010017687	TTC7B	0.6527102	0.096853912	6.739120698	1.59348E-11	4.03027E-11	positive
PRJNA729271	stage_3W_vs_E10	ordinary_muscle_development	ENSGALG00010017688	SPDYA	-0.882992233	0.563515843	-1.566934177	0.117130078	0.140244547	negative
PRJNA729271	stage_3W_vs_E10	ordinary_muscle_development	ENSGALG00010017691	LZTS1	-2.946872694	0.285515868	-10.32122214	5.64994E-25	2.63755E-24	negative
PRJNA729271	stage_3W_vs_E10	ordinary_muscle_development	ENSGALG00010017692	EPCAM	-4.735447093	1.002006432	-4.725964766	2.29025E-06	4.23542E-06	negative
PRJNA729271	stage_3W_vs_E10	ordinary_muscle_development	ENSGALG00010017693	MTFMT	-0.080475695	0.11774382	-0.68348126	0.494302788	0.532668122	negative
PRJNA729271	stage_3W_vs_E10	ordinary_muscle_development	ENSGALG00010017694	ATP6V1B2	0.014028048	0.070673667	0.198490452	0.842661355	0.861076936	positive
PRJNA729271	stage_3W_vs_E10	ordinary_muscle_development	ENSGALG00010017695	FNDC5	-1.80769708	0.20858303	-8.666558739	4.45377E-18	1.57688E-17	negative
PRJNA729271	stage_3W_vs_E10	ordinary_muscle_development	ENSGALG00010017698	ENSGALG00010017698	0.358389581	0.811652563	0.44155541	0.658810956	0.6915392	positive
PRJNA729271	stage_3W_vs_E10	ordinary_muscle_development	ENSGALG00010017699	RNF19B	-0.142385706	0.249287178	-0.571171401	0.567883462	0.604366431	negative
PRJNA729271	stage_3W_vs_E10	ordinary_muscle_development	ENSGALG00010017700	VPS35L	-0.133290992	0.078854784	-1.690334897	0.090963902	0.110642088	negative
PRJNA729271	stage_3W_vs_E10	ordinary_muscle_development	ENSGALG00010017701	PPP2R5E	0.196731523	0.099140928	1.984362334	0.047215462	0.059465472	positive
PRJNA729271	stage_3W_vs_E10	ordinary_muscle_development	ENSGALG00010017703	RPS6KA5	0.979759079	0.13981456	7.007561142	2.42508E-12	6.40167E-12	positive
PRJNA729271	stage_3W_vs_E10	ordinary_muscle_development	ENSGALG00010017704	ZNF362	-0.447609641	0.200640596	-2.230902672	0.025687577	0.033369907	negative
PRJNA729271	stage_3W_vs_E10	ordinary_muscle_development	ENSGALG00010017705	MAK16	1.119011609	0.102922683	10.87235166	1.56124E-27	8.15795E-27	positive
PRJNA729271	stage_3W_vs_E10	ordinary_muscle_development	ENSGALG00010017706	FGFRL1	-1.775733281	0.145841988	-12.17573418	4.1861E-34	2.79363E-33	negative
PRJNA729271	stage_3W_vs_E10	ordinary_muscle_development	ENSGALG00010017707	AK2	-0.714148586	0.095867993	-7.449291083	9.38431E-14	2.6656E-13	negative
PRJNA729271	stage_3W_vs_E10	ordinary_muscle_development	ENSGALG00010017708	MEIS1	-1.833079066	0.104899046	-17.47469715	2.23328E-68	4.14487E-67	negative
PRJNA729271	stage_3W_vs_E10	ordinary_muscle_development	ENSGALG00010017710	RAB41	-0.228403571	0.116205706	-1.965510804	0.049355146	0.062020186	negative
PRJNA729271	stage_3W_vs_E10	ordinary_muscle_development	ENSGALG00010017711	LRRFIP2	1.162990765	0.134083347	8.673640603	4.1852E-18	1.48542E-17	positive
PRJNA729271	stage_3W_vs_E10	ordinary_muscle_development	ENSGALG00010017712	MSH2	-2.124907612	0.075269879	-28.23051721	2.4692E-175	3.2244E-173	negative
PRJNA729271	stage_3W_vs_E10	ordinary_muscle_development	ENSGALG00010017713	ALKBH5	2.831973831	0.149066781	18.99802101	1.77097E-80	4.50355E-79	positive
PRJNA729271	stage_3W_vs_E10	ordinary_muscle_development	ENSGALG00010017714	ATAD3A	-1.300512756	0.156093512	-8.331625941	7.97395E-17	2.64915E-16	negative
PRJNA729271	stage_3W_vs_E10	ordinary_muscle_development	ENSGALG00010017715	CILP	2.857176665	0.168589254	16.947561	2.00605E-64	3.37328E-63	positive
PRJNA729271	stage_3W_vs_E10	ordinary_muscle_development	ENSGALG00010017717	LLGL1	-0.338631609	0.150006653	-2.257443941	0.023980349	0.031266992	negative
PRJNA729271	stage_3W_vs_E10	ordinary_muscle_development	ENSGALG00010017718	ATOH8	1.127059467	0.298464367	3.776194389	0.000159243	0.000255787	positive
PRJNA729271	stage_3W_vs_E10	ordinary_muscle_development	ENSGALG00010017719	ENSGALG00010017719	3.80405474	1.770267768	2.148858386	0.031645627	0.040694053	positive
PRJNA729271	stage_3W_vs_E10	ordinary_muscle_development	ENSGALG00010017720	ENSGALG00010017720	-2.574988283	0.417790908	-6.163342078	7.12254E-10	1.64031E-09	negative
PRJNA729271	stage_3W_vs_E10	ordinary_muscle_development	ENSGALG00010017721	VWA1	1.074398886	0.120290058	8.931734734	4.1938E-19	1.55035E-18	positive
PRJNA729271	stage_3W_vs_E10	ordinary_muscle_development	ENSGALG00010017723	KCNK12	-2.547294121	0.407649212	-6.248740456	4.13776E-10	9.65966E-10	negative
PRJNA729271	stage_3W_vs_E10	ordinary_muscle_development	ENSGALG00010017724	FLII	1.640321685	0.127726739	12.8424298	9.48501E-38	7.07956E-37	positive
PRJNA729271	stage_3W_vs_E10	ordinary_muscle_development	ENSGALG00010017725	ENSGALG00010017725	1.307307433	0.392762621	3.328492489	0.000873174	0.00131484	positive
PRJNA729271	stage_3W_vs_E10	ordinary_muscle_development	ENSGALG00010017726	TTI2	0.089634708	0.123867441	0.723634134	0.46929038	0.507991406	positive
PRJNA729271	stage_3W_vs_E10	ordinary_muscle_development	ENSGALG00010017727	AZIN2	0.731690637	0.150530058	4.86076102	1.16935E-06	2.20499E-06	positive
PRJNA729271	stage_3W_vs_E10	ordinary_muscle_development	ENSGALG00010017729	MIEF2	1.86425734	0.13969869	13.3448448	1.26935E-40	1.04245E-39	positive
PRJNA729271	stage_3W_vs_E10	ordinary_muscle_development	ENSGALG00010017730	TARP	7.336380943	0.537868777	13.63972264	2.32443E-42	2.02845E-41	positive
PRJNA729271	stage_3W_vs_E10	ordinary_muscle_development	ENSGALG00010017732	RHOJ	-0.741479853	0.080538959	-9.206474239	3.37013E-20	1.29886E-19	negative
PRJNA729271	stage_3W_vs_E10	ordinary_muscle_development	ENSGALG00010017733	TRIM62	-0.264307704	0.131914719	-2.003625572	0.045110184	0.05698232	negative
PRJNA729271	stage_3W_vs_E10	ordinary_muscle_development	ENSGALG00010017734	TOP3A	-0.199032086	0.118111304	-1.685123095	0.091964825	0.111775124	negative
PRJNA729271	stage_3W_vs_E10	ordinary_muscle_development	ENSGALG00010017736	ENSGALG00010017736	7.733298602	4.318436248	1.790763637	0.073331233	0.090155744	positive
PRJNA729271	stage_3W_vs_E10	ordinary_muscle_development	ENSGALG00010017737	ENSGALG00010017737	0.25595141	0.183601651	1.394058328	0.163299992	0.191653864	positive
PRJNA729271	stage_3W_vs_E10	ordinary_muscle_development	ENSGALG00010017738	NDUFS5	0.487774481	0.111979432	4.355929235	1.32504E-05	2.31933E-05	positive
PRJNA729271	stage_3W_vs_E10	ordinary_muscle_development	ENSGALG00010017739	MRPL20	-0.207737437	0.099225497	-2.093589278	0.036296583	0.04636635	negative
PRJNA729271	stage_3W_vs_E10	ordinary_muscle_development	ENSGALG00010017741	SMCR8	1.817438964	0.138267166	13.1444002	1.83253E-39	1.45788E-38	positive
PRJNA729271	stage_3W_vs_E10	ordinary_muscle_development	ENSGALG00010017742	DGLUCY	-0.271055766	0.242747906	-1.116614232	0.264159304	0.300359987	negative
PRJNA729271	stage_3W_vs_E10	ordinary_muscle_development	ENSGALG00010017743	KCNH5	-4.193268049	0.496944981	-8.43809316	3.22557E-17	1.09138E-16	negative
PRJNA729271	stage_3W_vs_E10	ordinary_muscle_development	ENSGALG00010017744	ETAA1	-0.762487104	0.099190501	-7.687098009	1.5051E-14	4.4487E-14	negative
PRJNA729271	stage_3W_vs_E10	ordinary_muscle_development	ENSGALG00010017745	SHMT1	-1.056816838	0.190027589	-5.561386349	2.6764E-08	5.61831E-08	negative
PRJNA729271	stage_3W_vs_E10	ordinary_muscle_development	ENSGALG00010017746	MSH6	-1.271704219	0.089958489	-14.13656708	2.26045E-45	2.18727E-44	negative
PRJNA729271	stage_3W_vs_E10	ordinary_muscle_development	ENSGALG00010017747	DUSP26	0.753476466	0.12501867	6.026911556	1.67122E-09	3.75922E-09	positive
PRJNA729271	stage_3W_vs_E10	ordinary_muscle_development	ENSGALG00010017748	ENSGALG00010017748	-4.063823071	0.760309256	-5.344960674	9.04367E-08	1.837E-07	negative
PRJNA729271	stage_3W_vs_E10	ordinary_muscle_development	ENSGALG00010017751	ST3GAL5	0.14740915	0.125783212	1.17193024	0.241225065	0.275753732	positive
PRJNA729271	stage_3W_vs_E10	ordinary_muscle_development	ENSGALG00010017753	GNRHR	0.933064113	0.338969138	2.752652109	0.005911468	0.008223294	positive
PRJNA729271	stage_3W_vs_E10	ordinary_muscle_development	ENSGALG00010017754	PDZD11	-0.995941191	0.161298608	-6.174518207	6.63655E-10	1.53058E-09	negative
PRJNA729271	stage_3W_vs_E10	ordinary_muscle_development	ENSGALG00010017755	C1D	-0.585912865	0.139834801	-4.190036107	2.7891E-05	4.75568E-05	negative
PRJNA729271	stage_3W_vs_E10	ordinary_muscle_development	ENSGALG00010017756	EIF2B5	-0.348294244	0.118998657	-2.926875414	0.00342386	0.004881366	negative
PRJNA729271	stage_3W_vs_E10	ordinary_muscle_development	ENSGALG00010017757	ENSGALG00010017757	1.241183352	0.138521408	8.960227641	3.24009E-19	1.20144E-18	positive
PRJNA729271	stage_3W_vs_E10	ordinary_muscle_development	ENSGALG00010017759	ENSGALG00010017759	5.195302759	0.844225859	6.153925165	7.55884E-10	1.73665E-09	positive
PRJNA729271	stage_3W_vs_E10	ordinary_muscle_development	ENSGALG00010017761	PARP16	0.753382029	0.106760716	7.056734479	1.70461E-12	4.53946E-12	positive
PRJNA729271	stage_3W_vs_E10	ordinary_muscle_development	ENSGALG00010017762	FBXO11	-0.370940303	0.055815536	-6.645825397	3.01523E-11	7.51732E-11	negative
PRJNA729271	stage_3W_vs_E10	ordinary_muscle_development	ENSGALG00010017763	POLR1A	-0.423668321	0.08672202	-4.885360386	1.0324E-06	1.95463E-06	negative
PRJNA729271	stage_3W_vs_E10	ordinary_muscle_development	ENSGALG00010017764	IGDCC3	-2.150011062	0.251100286	-8.562360072	1.1058E-17	3.84379E-17	negative
PRJNA729271	stage_3W_vs_E10	ordinary_muscle_development	ENSGALG00010017765	SYT16	-3.647057558	0.312665024	-11.66442446	1.93709E-31	1.17285E-30	negative
PRJNA729271	stage_3W_vs_E10	ordinary_muscle_development	ENSGALG00010017766	DNAAF10	1.108126957	0.153357727	7.225765388	4.98287E-13	1.36174E-12	positive
PRJNA729271	stage_3W_vs_E10	ordinary_muscle_development	ENSGALG00010017767	KIF4B	-4.050703074	0.225298893	-17.97924088	2.83345E-72	5.8589E-71	negative
PRJNA729271	stage_3W_vs_E10	ordinary_muscle_development	ENSGALG00010017768	FOXN2	0.233913473	0.081693196	2.863316475	0.004192316	0.00592508	positive
PRJNA729271	stage_3W_vs_E10	ordinary_muscle_development	ENSGALG00010017770	PTCD3	-0.203347229	0.070331331	-2.891275151	0.00383682	0.00544335	negative
PRJNA729271	stage_3W_vs_E10	ordinary_muscle_development	ENSGALG00010017771	PNO1	0.709978747	0.157182258	4.516914042	6.27474E-06	1.12634E-05	positive
PRJNA729271	stage_3W_vs_E10	ordinary_muscle_development	ENSGALG00010017772	ENSGALG00010017772	3.192125835	0.332135681	9.610909079	7.19112E-22	2.96544E-21	positive
PRJNA729271	stage_3W_vs_E10	ordinary_muscle_development	ENSGALG00010017773	DVL3	-1.750660144	0.085591469	-20.45367555	5.57149E-93	1.97454E-91	negative
PRJNA729271	stage_3W_vs_E10	ordinary_muscle_development	ENSGALG00010017774	ASH2L	0.787745395	0.162884378	4.836224357	1.32329E-06	2.48459E-06	positive
PRJNA729271	stage_3W_vs_E10	ordinary_muscle_development	ENSGALG00010017775	PRPSAP2	-1.064310415	0.086280613	-12.33545265	5.836E-35	4.00534E-34	negative
PRJNA729271	stage_3W_vs_E10	ordinary_muscle_development	ENSGALG00010017776	GPR68	0.774341066	0.362194273	2.13791697	0.032523531	0.041745204	positive
PRJNA729271	stage_3W_vs_E10	ordinary_muscle_development	ENSGALG00010017778	PPP3R1	1.125403064	0.078568299	14.32388234	1.55205E-46	1.56446E-45	positive
PRJNA729271	stage_3W_vs_E10	ordinary_muscle_development	ENSGALG00010017779	SNAPC1	0.430414821	0.123546966	3.483815386	0.00049432	0.000762252	positive
PRJNA729271	stage_3W_vs_E10	ordinary_muscle_development	ENSGALG00010017780	TAF9	0.389966754	0.136741088	2.851862308	0.004346392	0.006133271	positive
PRJNA729271	stage_3W_vs_E10	ordinary_muscle_development	ENSGALG00010017781	CYP26C1	1.421247499	0.298520266	4.760974923	1.9266E-06	3.57751E-06	positive
PRJNA729271	stage_3W_vs_E10	ordinary_muscle_development	ENSGALG00010017782	IGDCC4	-1.241434588	0.118506689	-10.47564995	1.11767E-25	5.3787E-25	negative
PRJNA729271	stage_3W_vs_E10	ordinary_muscle_development	ENSGALG00010017783	STAR	0.836170064	0.379050593	2.205958991	0.027386871	0.035450321	positive
PRJNA729271	stage_3W_vs_E10	ordinary_muscle_development	ENSGALG00010017786	KAZALD1	-2.891336612	0.170637751	-16.94429633	2.12056E-64	3.56171E-63	negative
PRJNA729271	stage_3W_vs_E10	ordinary_muscle_development	ENSGALG00010017787	CNRIP1	2.286585172	0.234372907	9.756183878	1.73564E-22	7.31551E-22	positive
PRJNA729271	stage_3W_vs_E10	ordinary_muscle_development	ENSGALG00010017789	IMMT	1.381112488	0.130094435	10.61623031	2.50473E-26	1.24166E-25	positive
PRJNA729271	stage_3W_vs_E10	ordinary_muscle_development	ENSGALG00010017790	R3HCC1L	0.397932024	0.17503084	2.273496631	0.022996269	0.030070448	positive
PRJNA729271	stage_3W_vs_E10	ordinary_muscle_development	ENSGALG00010017791	CCNL2	0.34853507	0.111505714	3.125714885	0.001773734	0.002595425	positive
PRJNA729271	stage_3W_vs_E10	ordinary_muscle_development	ENSGALG00010017792	LSM1	0.10407397	0.122375686	0.850446467	0.395076911	0.433967856	positive
PRJNA729271	stage_3W_vs_E10	ordinary_muscle_development	ENSGALG00010017793	FAM83G	-4.472633542	0.48812182	-9.162945312	5.04998E-20	1.93189E-19	negative
PRJNA729271	stage_3W_vs_E10	ordinary_muscle_development	ENSGALG00010017795	MACF1	-0.72526869	0.114302552	-6.345166223	2.22185E-10	5.27013E-10	negative
PRJNA729271	stage_3W_vs_E10	ordinary_muscle_development	ENSGALG00010017796	MXI1	1.847828515	0.131921064	14.00707712	1.41093E-44	1.32544E-43	positive
PRJNA729271	stage_3W_vs_E10	ordinary_muscle_development	ENSGALG00010017797	PPP1R21	-0.031831144	0.091638462	-0.347355717	0.728324097	0.756236231	negative
PRJNA729271	stage_3W_vs_E10	ordinary_muscle_development	ENSGALG00010017798	BAG4	0.733404556	0.15685736	4.675614552	2.93075E-06	5.38828E-06	positive
PRJNA729271	stage_3W_vs_E10	ordinary_muscle_development	ENSGALG00010017799	GRAPL	2.054221691	0.306595622	6.700101192	2.08275E-11	5.23396E-11	positive
PRJNA729271	stage_3W_vs_E10	ordinary_muscle_development	ENSGALG00010017801	SMNDC1	-0.342764395	0.071776351	-4.775450286	1.79306E-06	3.33766E-06	negative
PRJNA729271	stage_3W_vs_E10	ordinary_muscle_development	ENSGALG00010017803	ENSGALG00010017803	0.804178652	0.118189263	6.804159983	1.01641E-11	2.59884E-11	positive
PRJNA729271	stage_3W_vs_E10	ordinary_muscle_development	ENSGALG00010017804	DUSP5	-1.046695461	0.255496027	-4.096719135	4.19047E-05	7.05387E-05	negative
PRJNA729271	stage_3W_vs_E10	ordinary_muscle_development	ENSGALG00010017805	DPP8	0.940461518	0.106280041	8.848900626	8.83856E-19	3.21654E-18	positive
PRJNA729271	stage_3W_vs_E10	ordinary_muscle_development	ENSGALG00010017806	ENSGALG00010017806	3.85021108	0.748254341	5.145591371	2.66679E-07	5.24494E-07	positive
PRJNA729271	stage_3W_vs_E10	ordinary_muscle_development	ENSGALG00010017807	MRPL35	0.719978958	0.14658212	4.911778853	9.02538E-07	1.71526E-06	positive
PRJNA729271	stage_3W_vs_E10	ordinary_muscle_development	ENSGALG00010017808	SFRP5	0.507051965	0.378085868	1.341102662	0.179887123	0.209636907	positive
PRJNA729271	stage_3W_vs_E10	ordinary_muscle_development	ENSGALG00010017809	MOSMO	-0.119765513	0.102912514	-1.16376045	0.244521104	0.279345319	negative
PRJNA729271	stage_3W_vs_E10	ordinary_muscle_development	ENSGALG00010017810	HIF1A	-0.769813635	0.078711542	-9.780187456	1.36957E-22	5.79955E-22	negative

PRJNA729271	stage_3W_vs_E10	ordinary_muscle_development	ENSGALG00010017811	HACD3	-0.727991115	0.066122286	-11.00976935	3.4288E-28	1.84554E-27	negative
PRJNA729271	stage_3W_vs_E10	ordinary_muscle_development	ENSGALG00010017812	ENSGALG00010017812	5.058421733	1.725095901	2.932255378	0.003365099	0.004803734	positive
PRJNA729271	stage_3W_vs_E10	ordinary_muscle_development	ENSGALG00010017813	DDHD2	0.131437357	0.160863728	0.81707268	0.413886895	0.452776644	positive
PRJNA729271	stage_3W_vs_E10	ordinary_muscle_development	ENSGALG00010017814	ENSGALG00010017814	-2.767643074	0.260169917	-10.63782895	1.98705E-26	9.90452E-26	negative
PRJNA729271	stage_3W_vs_E10	ordinary_muscle_development	ENSGALG00010017815	VWA9	-0.769794825	0.08611167	-8.939494833	3.90953E-19	1.44637E-18	negative
PRJNA729271	stage_3W_vs_E10	ordinary_muscle_development	ENSGALG00010017816	PRKCH	0.946659762	0.283029305	3.344741151	0.000823594	0.001242506	positive
PRJNA729271	stage_3W_vs_E10	ordinary_muscle_development	ENSGALG00010017817	REEP1	2.683836442	0.116501623	23.03690171	1.9903E-117	1.1403E-115	positive
PRJNA729271	stage_3W_vs_E10	ordinary_muscle_development	ENSGALG00010017818	SLC38A6	-0.587464086	0.138446403	-4.243260014	2.20296E-05	3.79419E-05	negative
PRJNA729271	stage_3W_vs_E10	ordinary_muscle_development	ENSGALG00010017820	FLRT2	-0.425587793	0.134949543	-3.153680879	0.001612253	0.002373995	negative
PRJNA729271	stage_3W_vs_E10	ordinary_muscle_development	ENSGALG00010017821	ENSGALG00010017821	-4.160409704	0.415207717	-10.02006836	1.24417E-23	5.48987E-23	negative
PRJNA729271	stage_3W_vs_E10	ordinary_muscle_development	ENSGALG00010017822	VWA3A	-1.7078869	0.268749447	-6.354941075	2.08507E-10	4.95297E-10	negative
PRJNA729271	stage_3W_vs_E10	ordinary_muscle_development	ENSGALG00010017823	PPIE	-0.166109131	0.089939232	-1.846904043	0.064761049	0.080197471	negative
PRJNA729271	stage_3W_vs_E10	ordinary_muscle_development	ENSGALG00010017824	YAE1	0.031573391	0.123668533	0.255306589	0.798486311	0.821029941	positive
PRJNA729271	stage_3W_vs_E10	ordinary_muscle_development	ENSGALG00010017825	PLPP5	-1.48504777	0.151053712	-9.831256394	8.2582E-23	3.53209E-22	negative
PRJNA729271	stage_3W_vs_E10	ordinary_muscle_development	ENSGALG00010017826	PABPC4	3.591517798	0.139904351	25.67123729	2.4497E-145	2.152E-143	positive
PRJNA729271	stage_3W_vs_E10	ordinary_muscle_development	ENSGALG00010017828	ENSGALG00010017828	0.958711005	0.236100535	4.060604962	4.89457E-05	8.20005E-05	positive
PRJNA729271	stage_3W_vs_E10	ordinary_muscle_development	ENSGALG00010017830	DENND4A	1.204024205	0.127638501	9.433080141	3.9822E-21	1.59718E-20	positive
PRJNA729271	stage_3W_vs_E10	ordinary_muscle_development	ENSGALG00010017831	PLEK	2.152802358	0.180404388	11.93320393	7.94554E-33	5.07359E-32	positive
PRJNA729271	stage_3W_vs_E10	ordinary_muscle_development	ENSGALG00010017832	POU3F1	-3.42374631	0.552449503	-6.197392326	5.74063E-10	1.32882E-09	negative
PRJNA729271	stage_3W_vs_E10	ordinary_muscle_development	ENSGALG00010017834	ENSGALG00010017834	-1.870197329	0.413531675	-4.522500793	6.11133E-06	1.09783E-05	negative
PRJNA729271	stage_3W_vs_E10	ordinary_muscle_development	ENSGALG00010017838	B3GNT2	-0.462254478	0.124634713	-3.708874248	0.000208183	0.00033106	negative
PRJNA729271	stage_3W_vs_E10	ordinary_muscle_development	ENSGALG00010017841	GALC	-1.048210967	0.267244067	-3.922298371	8.77083E-05	0.000143832	negative
PRJNA729271	stage_3W_vs_E10	ordinary_muscle_development	ENSGALG00010017843	NSD3	0.048051686	0.071245958	0.674447885	0.500026608	0.538116095	positive
PRJNA729271	stage_3W_vs_E10	ordinary_muscle_development	ENSGALG00010017848	CYP3A4	0.791609624	0.197698637	4.004122813	6.2248E-05	0.000103473	positive
PRJNA729271	stage_3W_vs_E10	ordinary_muscle_development	ENSGALG00010017849	FBX048	3.10210026	0.173270131	17.9032603	1.11216E-71	2.239E-70	positive
PRJNA729271	stage_3W_vs_E10	ordinary_muscle_development	ENSGALG00010017850	TRMT5	-0.871863116	0.094307182	-9.2449281	2.35399E-20	9.13061E-20	negative
PRJNA729271	stage_3W_vs_E10	ordinary_muscle_development	ENSGALG00010017852	LETM2	0.548116293	0.113642731	4.823153119	1.41306E-06	2.64904E-06	positive
PRJNA729271	stage_3W_vs_E10	ordinary_muscle_development	ENSGALG00010017855	SDR42E2	-1.389167672	0.339097526	-4.096661182	4.19152E-05	7.05481E-05	negative
PRJNA729271	stage_3W_vs_E10	ordinary_muscle_development	ENSGALG00010017856	FASTKD5	0.423854337	0.115399413	3.672933222	0.000239782	0.000379023	positive
PRJNA729271	stage_3W_vs_E10	ordinary_muscle_development	ENSGALG00010017858	GPR65	3.915943367	0.280544552	13.95836538	2.79777E-44	2.60293E-43	positive
PRJNA729271	stage_3W_vs_E10	ordinary_muscle_development	ENSGALG00010017859	EXOC6	2.239870417	0.133632709	16.76139343	4.67537E-63	7.61455E-62	positive
PRJNA729271	stage_3W_vs_E10	ordinary_muscle_development	ENSGALG00010017860	CYSLTR1	0.425192999	0.202921818	2.095353781	0.036139555	0.046190182	positive
PRJNA729271	stage_3W_vs_E10	ordinary_muscle_development	ENSGALG00010017861	PRSS56	-0.261626923	0.556663014	-0.469991569	0.638361041	0.671727281	negative
PRJNA729271	stage_3W_vs_E10	ordinary_muscle_development	ENSGALG00010017865	UBOX5	0.182559656	0.135242558	1.349868408	0.177058197	0.206506161	positive
PRJNA729271	stage_3W_vs_E10	ordinary_muscle_development	ENSGALG00010017866	RALA	-0.539808114	0.095571428	-5.648216502	1.62121E-08	3.45377E-08	negative
PRJNA729271	stage_3W_vs_E10	ordinary_muscle_development	ENSGALG00010017867	APLF	1.209947951	0.108055446	11.19747315	4.19529E-29	2.3326E-28	positive
PRJNA729271	stage_3W_vs_E10	ordinary_muscle_development	ENSGALG00010017868	CHRND	1.407462923	0.240931547	5.841754392	5.16539E-09	1.13169E-08	positive
PRJNA729271	stage_3W_vs_E10	ordinary_muscle_development	ENSGALG00010017870	RAB11A	0.462451462	0.073582113	6.284835324	3.28201E-10	7.7041E-10	positive
PRJNA729271	stage_3W_vs_E10	ordinary_muscle_development	ENSGALG00010017873	HSPG2	1.617760073	0.109631183	14.75638622	2.79881E-49	3.08742E-48	positive
PRJNA729271	stage_3W_vs_E10	ordinary_muscle_development	ENSGALG00010017876	COMMD1	-0.378467664	0.137775663	-2.746984879	0.006014591	0.008361129	negative
PRJNA729271	stage_3W_vs_E10	ordinary_muscle_development	ENSGALG00010017878	CHRNG	0.474304519	0.248725103	1.906942699	0.056528008	0.070501934	positive
PRJNA729271	stage_3W_vs_E10	ordinary_muscle_development	ENSGALG00010017880	CDK13	0.221730311	0.084347233	2.628779905	0.00856918	0.011741211	positive
PRJNA729271	stage_3W_vs_E10	ordinary_muscle_development	ENSGALG00010017881	KCNK10	-1.559022495	0.490183432	-3.180487944	0.001470273	0.002169561	negative
PRJNA729271	stage_3W_vs_E10	ordinary_muscle_development	ENSGALG00010017882	LZTS3	1.946576448	0.200034506	9.731203319	2.21952E-22	9.31978E-22	positive
PRJNA729271	stage_3W_vs_E10	ordinary_muscle_development	ENSGALG00010017886	DDRGK1	0.338012722	0.101730135	3.322641053	0.000891696	0.001341199	positive
PRJNA729271	stage_3W_vs_E10	ordinary_muscle_development	ENSGALG00010017887	PROKR1	6.898174693	0.387304449	17.81072931	5.83446E-71	1.15534E-69	positive
PRJNA729271	stage_3W_vs_E10	ordinary_muscle_development	ENSGALG00010017888	MPLKIP	-0.304385278	0.110568086	-2.752921661	0.005906603	0.008217315	negative
PRJNA729271	stage_3W_vs_E10	ordinary_muscle_development	ENSGALG00010017890	ANAPC1	-0.394888331	0.065247356	-6.052173719	1.42904E-09	3.22597E-09	negative
PRJNA729271	stage_3W_vs_E10	ordinary_muscle_development	ENSGALG00010017891	GOLGA7B	4.475510366	0.322876995	13.86134791	1.08615E-43	9.92047E-43	positive
PRJNA729271	stage_3W_vs_E10	ordinary_muscle_development	ENSGALG00010017894	KAT6A	0.051946011	0.081172233	0.639948036	0.522206383	0.560196975	positive
PRJNA729271	stage_3W_vs_E10	ordinary_muscle_development	ENSGALG00010017895	MNAT1	0.422883566	0.080592621	5.247174738	1.54449E-07	3.08282E-07	positive
PRJNA729271	stage_3W_vs_E10	ordinary_muscle_development	ENSGALG00010017898	NHERF2	0.67955155	0.114690557	5.925087202	3.12132E-09	6.89793E-09	positive
PRJNA729271	stage_3W_vs_E10	ordinary_muscle_development	ENSGALG00010017901	HTR7L	-2.475204279	0.716127262	-3.456374878	0.000547493	0.000839602	negative
PRJNA729271	stage_3W_vs_E10	ordinary_muscle_development	ENSGALG00010017903	ENSGALG00010017903	-4.275939706	0.490818872	-8.711848602	2.98959E-18	1.06787E-17	negative
PRJNA729271	stage_3W_vs_E10	ordinary_muscle_development	ENSGALG00010017904	MEGF11	-5.123890711	0.470515637	-10.88994776	1.28714E-27	6.75737E-27	negative
PRJNA729271	stage_3W_vs_E10	ordinary_muscle_development	ENSGALG00010017905	EIF4E2	-0.05790983	0.087245442	-0.663757654	0.506845432	0.544807159	negative
PRJNA729271	stage_3W_vs_E10	ordinary_muscle_development	ENSGALG00010017909	NTHL1	-0.614417334	0.139517211	-4.403881995	1.06331E-05	1.87411E-05	negative
PRJNA729271	stage_3W_vs_E10	ordinary_muscle_development	ENSGALG00010017915	BFSP1	3.357146214	0.428654838	7.831816921	4.80869E-15	1.45942E-14	positive
PRJNA729271	stage_3W_vs_E10	ordinary_muscle_development	ENSGALG00010017918	SIX4	0.245467141	0.201625058	1.217443624	0.223435474	0.256672784	positive
PRJNA729271	stage_3W_vs_E10	ordinary_muscle_development	ENSGALG00010017920	AP3M2	-0.482753121	0.080872023	-5.969346403	2.38206E-09	5.30383E-09	negative
PRJNA729271	stage_3W_vs_E10	ordinary_muscle_development	ENSGALG00010017921	SPATA7	0.896350663	0.201632054	4.445477029	8.76971E-06	1.55495E-05	positive
PRJNA729271	stage_3W_vs_E10	ordinary_muscle_development	ENSGALG00010017922	MERTK	0.93822586	0.13029082	7.2010128	5.97669E-13	1.62863E-12	positive
PRJNA729271	stage_3W_vs_E10	ordinary_muscle_development	ENSGALG00010017927	PCSK2	0.29794307	0.125694756	2.370369932	0.017770295	0.023507542	positive
PRJNA729271	stage_3W_vs_E10	ordinary_muscle_development	ENSGALG00010017930	TSC2	-0.579831248	0.078720675	-7.365679366	1.76247E-13	4.93376E-13	negative
PRJNA729271	stage_3W_vs_E10	ordinary_muscle_development	ENSGALG00010017933	SIX1	0.905042895	0.195754993	4.623345125	3.77601E-06	6.89372E-06	positive
PRJNA729271	stage_3W_vs_E10	ordinary_muscle_development	ENSGALG00010017937	PTPN21	1.539806871	0.119257186	12.91164848	3.86928E-38	2.92567E-37	positive
PRJNA729271	stage_3W_vs_E10	ordinary_muscle_development	ENSGALG00010017941	DIS3L	0.96865624	0.135675954	7.139483535	9.36822E-13	2.5325E-12	positive
PRJNA729271	stage_3W_vs_E10	ordinary_muscle_development	ENSGALG00010017946	PTGDR	1.350704998	0.248293021	5.439963614	5.32915E-08	1.09647E-07	positive
PRJNA729271	stage_3W_vs_E10	ordinary_muscle_development	ENSGALG00010017948	PLAT	2.029445182	0.156008712	13.00853747	1.09416E-38	8.4361E-38	positive
PRJNA729271	stage_3W_vs_E10	ordinary_muscle_development	ENSGALG00010017949	SFXN3	1.259214257	0.137892502	9.131854431	6.73358E-20	2.56378E-19	positive
PRJNA729271	stage_3W_vs_E10	ordinary_muscle_development	ENSGALG00010017950	FBLN7	0.27334511	0.309534385	0.883084798	0.377190457	0.415959805	positive
PRJNA729271	stage_3W_vs_E10	ordinary_muscle_development	ENSGALG00010017954	PTGER2	1.025237374	0.260013162	3.943021063	8.04616E-05	0.000132308	positive
PRJNA729271	stage_3W_vs_E10	ordinary_muscle_development	ENSGALG00010017955	IKBKB	0.651534123	0.062909649	10.35666435	3.90338E-25	1.83819E-24	positive
PRJNA729271	stage_3W_vs_E10	ordinary_muscle_development	ENSGALG00010017956	TIPIN	-0.69869422	0.080887709	-8.63782928	5.72911E-18	2.01758E-17	negative
PRJNA729271	stage_3W_vs_E10	ordinary_muscle_development	ENSGALG00010017957	THUMPD1	-0.213803937	0.098689966	-2.166420204	0.030279091	0.039026803	negative
PRJNA729271	stage_3W_vs_E10	ordinary_muscle_development	ENSGALG00010017958	CAPN10	-1.131271415	0.092994974	-12.1648662	4.78228E-34	3.18416E-33	negative
PRJNA729271	stage_3W_vs_E10	ordinary_muscle_development	ENSGALG00010017959	PDZD7	1.748976083	0.277975521	6.29183489	3.13735E-10	7.37527E-10	positive
PRJNA729271	stage_3W_vs_E10	ordinary_muscle_development	ENSGALG00010017962	TXNDC16	-0.488035915	0.150655078	-3.239425598	0.001197707	0.001781688	negative
PRJNA729271	stage_3W_vs_E10	ordinary_muscle_development	ENSGALG00010017963	LPAR4	-2.334057384	0.170759293	-13.66869903	1.56162E-42	1.37103E-41	negative
PRJNA729271	stage_3W_vs_E10	ordinary_muscle_development	ENSGALG00010017970	AMPH	3.590203058	0.663140807	5.413937768	6.16536E-08	1.26314E-07	positive
PRJNA729271	stage_3W_vs_E10	ordinary_muscle_development	ENSGALG00010017972	RNPEPL1	1.284322342	0.171690149	7.480466099	7.40595E-14	2.11776E-13	positive
PRJNA729271	stage_3W_vs_E10	ordinary_muscle_development	ENSGALG00010017973	PKD1	1.625938047	0.129017914	12.60242079	2.04762E-36	1.46714E-35	positive
PRJNA729271	stage_3W_vs_E10	ordinary_muscle_development	ENSGALG00010017974	MXRA8	0.43004777	0.183943315	2.337936395	0.019390548	0.025542166	positive
PRJNA729271	stage_3W_vs_E10	ordinary_muscle_development	ENSGALG00010017976	GPR137C	-0.985092884	0.108084649	-9.114086912	7.93357E-20	3.01355E-19	negative
PRJNA729271	stage_3W_vs_E10	ordinary_muscle_development	ENSGALG00010017979	ENSGALG00010017979	-0.855229608	0.172845427	-4.947944667	7.50012E-07	1.43064E-06	negative
PRJNA729271	stage_3W_vs_E10	ordinary_muscle_development	ENSGALG00010017983	SLC4A11	2.421920329	0.202046452	11.9869481	4.15949E-33	2.68202E-32	positive
PRJNA729271	stage_3W_vs_E10	ordinary_muscle_development	ENSGALG00010017985	ABCC5	0.992303865	0.267643522	3.707557935	0.000209268	0.000332712	positive
PRJNA729271	stage_3W_vs_E10	ordinary_muscle_development	ENSGALG00010017987	ERI2	-2.581069963	0.246746997	-10.46039059	1.31313E-25	6.29843E-25	negative
PRJNA729271	stage_3W_vs_E10	ordinary_muscle_development	ENSGALG00010017991	RAB26	-1.124871469	0.246551675	-4.562416666	5.05682E-06	9.13036E-06	negative
PRJNA729271	stage_3W_vs_E10	ordinary_muscle_development	ENSGALG00010017992	ENSGALG00010017992	5.165148478	0.564076782	9.156818086	5.34501E-20	2.0426E-19	positive
PRJNA729271	stage_3W_vs_E10	ordinary_muscle_development	ENSGALG00010017993	REXO5	-1.090928055	0.095745951	-11.39398628	4.48014E-30	2.57697E-29	negative
PRJNA729271	stage_3W_vs_E10	ordinary_muscle_development	ENSGALG00010017996	ZC3H14	0.078179607	0.091722624	0.852348132	0.394020899	0.432971946	positive
PRJNA729271	stage_3W_vs_E10	ordinary_muscle_development	ENSGALG00010017997	ENSGALG00010017997	-0.174335068	0.346722095	-0.502809225	0.615098407	0.649843397	negative
PRJNA729271	stage_3W_vs_E10	ordinary_muscle_development	ENSGALG00010017999	GPR174	4.199617053	0.546892994	7.679047092	1.60276E-14	4.73351E-14	positive
PRJNA729271	stage_3W_vs_E10	ordinary_muscle_development	ENSGALG00010018000	MAP2K1	0.495666826	0.072991311	6.790764837	1.11541E-11	2.84745E-11	positive
PRJNA729271	stage_3W_vs_E10	ordinary_muscle_development	ENSGALG00010018001	TRAF7	-0.214229224	0.085986958	-2.491415309	0.01272353	0.017101962	negative
PRJNA729271	stage_3W_vs_E10	ordinary_muscle_development	ENSGALG00010018002	ZC3H8	0.294233516	0.079490062	3.701513242	0.000214317	0.000340293	positive
PRJNA729271	stage_3W_vs_E10	ordinary_muscle_development	ENSGALG00010018004	CDC42BPA	-0.126002498	0.062217738	-2.025186085	0.04284826	0.054243278	negative
PRJNA729271	stage_3W_vs_E10	ordinary_muscle_development	ENSGALG00010018006	DCUN1D3	0.04745471	0.103336491	0.459225093	0.646072533	0.679200853	positive
PRJNA729271	stage_3W_vs_E10	ordinary_muscle_development	ENSGALG00010018007	DNAAF9	-0.549656777	0.179655502	-3.059504271	0.002217036	0.003214237	negative
PRJNA729271	stage_3W_vs_E10	ordinary_muscle_development	ENSGALG00010018008	ABCF3	2.188877623	0.13170266	16.61984375	5.00629E-62	7.91344E-61	positive
PRJNA729271	stage_3W_vs_E10	ordinary_muscle_development	ENSGALG00010018010	ENSGALG00010018010	-0.469985142	0.119567303	-3.930716271	8.46932E-05	0.000139092	negative
PRJNA729271	stage_3W_vs_E10	ordinary_muscle_development	ENSGALG00010018011	LIPA	0.948381107	0.185322053	5.117475736	3.09652E-07	6.06459E-07	positive
PRJNA729271	stage_3W_vs_E10	ordinary_muscle_development	ENSGALG00010018012	DVL1	1.284861941	0.138017198	9.309433628	1.28516E-20	5.03605E-20	positive
PRJNA729271	stage_3W_vs_E10	ordinary_muscle_development	ENSGALG00010018014	LYRM1	0.326119448	0.095737478	3.406392718	0.000658274	0.001002594	positive
PRJNA729271	stage_3W_vs_E10	ordinary_muscle_development	ENSGALG00010018016	IFIT5	7.732709667	0.856953761	9.023485303	1.82198E-19	6.82244E-19	positive
PRJNA729271	stage_3W_vs_E10	ordinary_muscle_development	ENSGALG00010018019	NAPB	0.328749448	0.352067729	0.933767628	0.350423772	0.388927456	positive
PRJNA729271	stage_3W_vs_E10	ordinary_muscle_development	ENSGALG00010018020	SNAPC5	-0.790870084	0.104188704	-7.59074693	3.18067E-14	9.25778E-14	negative
PRJNA729271	stage_3W_vs_E10	ordinary_muscle_development	ENSGALG00010018021	ATRN	1.384221619	0.070687404	19.58229531	2.1893E-85	6.41088E-84	positive
PRJNA729271	stage_3W_vs_E10	ordinary_muscle_development	ENSGALG00010018022	XPO6	-0.566080446	0.117023701	-4.83731452	1.31605E-06	2.47165E-06	negative
PRJNA729271	stage_3W_vs_E10	ordinary_muscle_development	ENSGALG00010018025	CPTP	-0.605112232	0.135602465	-4.462398467	8.10473E-06	1.4411E-05	negative
PRJNA729271	stage_3W_vs_E10	ordinary_muscle_development	ENSGALG00010018026	CCDC39	-4.225357054	1.017740589	-4.151703389	3.3001E-05	5.59211E-05	negative
PRJNA729271	stage_3W_vs_E10	ordinary_muscle_development	ENSGALG00010018027	EML5	-1.283849162	0.325106839	-3.949006939	7.84761E-05	0.000129175	negative
PRJNA729271	stage_3W_vs_E10	ordinary_muscle_development	ENSGALG00010018030	POLB	0.840318361	0.100382805	8.371138474	5.70642E-17	1.90983E-16	positive
PRJNA729271	stage_3W_vs_E10	ordinary_muscle_development	ENSGALG00010018032	ERO1A	0.385602949	0.122561054	3.146211104	0.001654006	0.002431769	positive
PRJNA729271	stage_3W_vs_E10	ordinary_muscle_development	ENSGALG00010018033	COQ8A	2.106049518	0.213202752	9.878153526	5.17782E-23	2.2377E-22	positive
PRJNA729271	stage_3W_vs_E10	ordinary_muscle_development	ENSGALG00010018035	ITM2A	1.38406913	0.08632538	16.03316581	7.49644E-58	1.0653E-56	positive
PRJNA729271	stage_3W_vs_E10	ordinary_muscle_development	ENSGALG00010018036	RPL4	-0.045375367	0.107056144	-0.423846455	0.671677797	0.703110397	negative
PRJNA729271	stage_3W_vs_E10	ordinary_muscle_development	ENSGALG00010018037	GZF1	-0.09882709	0.083335801	-1.185889964	0.235665757	0.26980295	negative
PRJNA729271	stage_3W_vs_E10	ordinary_muscle_development	ENSGALG00010018039	TTC14	0.252641173	0.131531647	1.9207634	0.054761543	0.068398842	positive
PRJNA729271	stage_3W_vs_E10	ordinary_muscle_development	ENSGALG00010018042	ZWILCH	-1.83032444	0.141018752	-12.97929824	1.60343E-38	1.23168E-37	negative
PRJNA729271	stage_3W_vs_E10	ordinary_muscle_development	ENSGALG00010018043	PSMC6	0.115704962	0.091042188	1.27089391	0.203766393	0.235646013	positive
PRJNA729271	stage_3W_vs_E10	ordinary_muscle_development	ENSGALG00010018044	INTS11	-0.848857892	0.060381659	-14.05820761	6.86018E-45	6.5334E-44	negative
PRJNA729271	stage_3W_vs_E10	ordinary_muscle_development	ENSGALG00010018045	PSEN2	1.088789849	0.073117844	14.89089102	3.77702E-50	4.31426E-49	positive
PRJNA729271	stage_3W_vs_E10	ordinary_muscle_development	ENSGALG00010018046	CAPN15	-0.680036765	0.104220232	-6.52499764	6.80026E-11	1.65636E-10	negative
PRJNA729271	stage_3W_vs_E10	ordinary_muscle_development	ENSGALG00010018048	GFRA4	-4.669671751	0.175495326	-26.60852482	5.4089E-156	5.6002E-154	negative
PRJNA729271	stage_3W_vs_E10	ordinary_muscle_development	ENSGALG00010018049	SUGCT	-1.249014954	0.175469007	-7.118151393	1.09384E-12	2.94379E-12	negative
PRJNA729271	stage_3W_vs_E10	ordinary_muscle_development	ENSGALG00010018051	GNPNAT1	-0.651141333	0.134731737	-4.832872685	1.34577E-06	2.52582E-06	negative
PRJNA729271	stage_3W_vs_E10	ordinary_muscle_development	ENSGALG00010018052	PPP1RL	-0.515182863	0.13170778	-3.91155985	9.17019E-05	0.000150093	negative
PRJNA729271	stage_3W_vs_E10	ordinary_muscle_development	ENSGALG00010018053	DIAPH2	2.13327372	0.091190211	23.39367025	4.9571E-121	3.0064E-119	positive
PRJNA729271	stage_3W_vs_E10	ordinary_muscle_development	ENSGALG00010018054	SMAD6	-1.081585364	0.160831581	-6.72495638	1.75645E-11	4.43164E-11	negative

PRJNA729271	stage_3W_vs_E10	ordinary_muscle_development	ENSGALG00010018055	PEX5L	-0.525674442	0.506494854	-1.037867289	0.299331844	0.336838373	negative
PRJNA729271	stage_3W_vs_E10	ordinary_muscle_development	ENSGALG00010018057	SSTR4	-0.426445448	0.178804892	-2.3849764	0.017080217	0.022622236	negative
PRJNA729271	stage_3W_vs_E10	ordinary_muscle_development	ENSGALG00010018058	ITPKB	-0.296041314	0.09254803	-3.198785691	0.001380077	0.00204208	negative
PRJNA729271	stage_3W_vs_E10	ordinary_muscle_development	ENSGALG00010018060	CASKIN1	-3.689043642	0.413650056	-8.918271827	4.73621E-19	1.74685E-18	negative
PRJNA729271	stage_3W_vs_E10	ordinary_muscle_development	ENSGALG00010018061	NDUFAF5	0.830081664	0.134971059	6.150071509	7.7448E-10	1.77825E-09	positive
PRJNA729271	stage_3W_vs_E10	ordinary_muscle_development	ENSGALG00010018062	GOLGA7	-0.965282913	0.094606366	-10.20314963	1.91945E-24	8.78787E-24	negative
PRJNA729271	stage_3W_vs_E10	ordinary_muscle_development	ENSGALG00010018063	DECR2	-0.03134291	0.098748975	-0.317399847	0.750940223	0.777234181	negative
PRJNA729271	stage_3W_vs_E10	ordinary_muscle_development	ENSGALG00010018064	TTC8	-1.494482514	0.161875144	-9.232316193	2.64842E-20	1.02562E-19	negative
PRJNA729271	stage_3W_vs_E10	ordinary_muscle_development	ENSGALG00010018067	ENSGALG00010018067	-0.386394053	0.178843961	-2.160509369	0.030733259	0.039576989	negative
PRJNA729271	stage_3W_vs_E10	ordinary_muscle_development	ENSGALG00010018068	ADAM33	-0.326996077	0.138852581	-2.354987386	0.018523334	0.024453163	negative
PRJNA729271	stage_3W_vs_E10	ordinary_muscle_development	ENSGALG00010018069	ENSGALG00010018069	-0.94703996	0.379920621	-2.492731136	0.012676479	0.017041882	negative
PRJNA729271	stage_3W_vs_E10	ordinary_muscle_development	ENSGALG00010018071	GPAT4	-0.065606463	0.079427291	-0.825993971	0.408807512	0.447659204	negative
PRJNA729271	stage_3W_vs_E10	ordinary_muscle_development	ENSGALG00010018075	MLST8	1.377541803	0.102431994	13.44835482	3.14798E-41	2.63149E-40	positive
PRJNA729271	stage_3W_vs_E10	ordinary_muscle_development	ENSGALG00010018076	FOXA2	-5.933503628	1.564284352	-3.793110646	0.000148772	0.000239552	negative
PRJNA729271	stage_3W_vs_E10	ordinary_muscle_development	ENSGALG00010018078	NKX6-3	5.99384741	0.691744932	8.664823018	4.52215E-18	1.6007E-17	positive
PRJNA729271	stage_3W_vs_E10	ordinary_muscle_development	ENSGALG00010018080	BRICD5	2.097500316	0.319150544	6.572134556	4.9599E-11	1.21813E-10	positive
PRJNA729271	stage_3W_vs_E10	ordinary_muscle_development	ENSGALG00010018081	DIO3	-4.889068486	0.269038067	-18.17240413	8.53743E-74	1.83333E-72	negative
PRJNA729271	stage_3W_vs_E10	ordinary_muscle_development	ENSGALG00010018082	RAB11FIP3	-0.288874356	0.177086551	-1.631260846	0.102835291	0.12409237	negative
PRJNA729271	stage_3W_vs_E10	ordinary_muscle_development	ENSGALG00010018083	PAX1	-4.675735161	0.906353194	-5.15884447	2.48479E-07	4.89627E-07	negative
PRJNA729271	stage_3W_vs_E10	ordinary_muscle_development	ENSGALG00010018085	PGP	2.161555644	0.159408763	13.55982946	6.9297E-42	5.94004E-41	positive
PRJNA729271	stage_3W_vs_E10	ordinary_muscle_development	ENSGALG00010018087	ENSGALG00010018087	-1.013952007	0.056598222	-17.91490906	9.02165E-72	1.8315E-70	negative
PRJNA729271	stage_3W_vs_E10	ordinary_muscle_development	ENSGALG00010018089	TASP1	0.621249697	0.090717788	6.848157499	7.48072E-12	1.92053E-11	positive
PRJNA729271	stage_3W_vs_E10	ordinary_muscle_development	ENSGALG00010018092	ENSGALG00010018092	-4.504558315	0.220122633	-20.46385808	4.5215E-93	1.60635E-91	negative
PRJNA729271	stage_3W_vs_E10	ordinary_muscle_development	ENSGALG00010018095	E4F1	-0.178533216	0.06968546	-2.56198662	0.010407533	0.014113312	negative
PRJNA729271	stage_3W_vs_E10	ordinary_muscle_development	ENSGALG00010018099	SMAD3	-2.013681286	0.13132589	-15.33346764	4.56929E-53	5.72444E-52	negative
PRJNA729271	stage_3W_vs_E10	ordinary_muscle_development	ENSGALG00010018102	ZDHHC4	0.835393814	0.16580162	5.038514189	4.6916E-07	9.08061E-07	positive
PRJNA729271	stage_3W_vs_E10	ordinary_muscle_development	ENSGALG00010018103	BTRC	-0.257230291	0.10765219	-2.389457097	0.016873294	0.022365982	negative
PRJNA729271	stage_3W_vs_E10	ordinary_muscle_development	ENSGALG00010018111	NKX2-2	-0.966329142	0.83953769	-1.151025325	0.249721817	0.284949834	negative
PRJNA729271	stage_3W_vs_E10	ordinary_muscle_development	ENSGALG00010018115	KLHL4	-1.565146483	0.196060312	-7.982984748	1.42837E-15	4.43819E-15	negative
PRJNA729271	stage_3W_vs_E10	ordinary_muscle_development	ENSGALG00010018119	GRID2IP	-4.124354473	0.941105977	-4.382454871	1.17349E-05	2.06229E-05	negative
PRJNA729271	stage_3W_vs_E10	ordinary_muscle_development	ENSGALG00010018121	FERMT2	-0.367024209	0.159977391	-2.294225492	0.02177755	0.028541192	negative
PRJNA729271	stage_3W_vs_E10	ordinary_muscle_development	ENSGALG00010018123	USP13	3.921129426	0.140701721	27.86838288	6.4501E-171	8.0599E-169	positive
PRJNA729271	stage_3W_vs_E10	ordinary_muscle_development	ENSGALG00010018128	SEPTIN12	-0.383184771	0.19620473	-1.952984374	0.050821447	0.06370811	negative
PRJNA729271	stage_3W_vs_E10	ordinary_muscle_development	ENSGALG00010018137	NDUFB5	1.77240441	0.127405271	13.91154693	5.39009E-44	4.9543E-43	positive
PRJNA729271	stage_3W_vs_E10	ordinary_muscle_development	ENSGALG00010018140	CHPF	-1.400010186	0.107224286	-13.05683854	5.80905E-39	4.53431E-38	negative
PRJNA729271	stage_3W_vs_E10	ordinary_muscle_development	ENSGALG00010018142	DDHD1	-1.063962157	0.143520558	-7.413308365	1.23187E-13	3.48001E-13	negative
PRJNA729271	stage_3W_vs_E10	ordinary_muscle_development	ENSGALG00010018143	MRPL47	0.603757669	0.123077424	4.905511114	9.31844E-07	1.76956E-06	positive
PRJNA729271	stage_3W_vs_E10	ordinary_muscle_development	ENSGALG00010018145	ENSGALG00010018145	-2.113466701	0.37310394	-5.66455209	1.47409E-08	3.14868E-08	negative
PRJNA729271	stage_3W_vs_E10	ordinary_muscle_development	ENSGALG00010018147	ISM1	-0.291668953	0.286683137	-1.017391381	0.308967281	0.346873266	negative
PRJNA729271	stage_3W_vs_E10	ordinary_muscle_development	ENSGALG00010018148	XRN2	-0.550683358	0.055307111	-9.956827433	2.35457E-23	1.02676E-22	negative
PRJNA729271	stage_3W_vs_E10	ordinary_muscle_development	ENSGALG00010018149	ENSGALG00010018149	1.941954987	0.291413628	6.663912742	2.66632E-11	6.6658E-11	positive
PRJNA729271	stage_3W_vs_E10	ordinary_muscle_development	ENSGALG00010018150	AAGAB	-1.834350304	0.081794774	-22.42625296	2.1826E-111	1.138E-109	negative
PRJNA729271	stage_3W_vs_E10	ordinary_muscle_development	ENSGALG00010018154	SLC16A12	-2.199873488	0.268123802	-8.204693027	2.31181E-16	7.50328E-16	negative
PRJNA729271	stage_3W_vs_E10	ordinary_muscle_development	ENSGALG00010018157	FBXL2	-0.859183686	0.153889645	-5.583115638	2.36248E-08	4.97661E-08	negative
PRJNA729271	stage_3W_vs_E10	ordinary_muscle_development	ENSGALG00010018160	ENSGALG00010018160	0.854234161	0.164216837	5.201867084	1.97296E-07	3.91326E-07	positive
PRJNA729271	stage_3W_vs_E10	ordinary_muscle_development	ENSGALG00010018162	ENSGALG00010018162	-0.58517623	0.363780181	-1.608598435	0.107704177	0.129676222	negative
PRJNA729271	stage_3W_vs_E10	ordinary_muscle_development	ENSGALG00010018163	CCT7	-0.639895757	0.08103916	-7.89613013	2.87696E-15	8.81827E-15	negative
PRJNA729271	stage_3W_vs_E10	ordinary_muscle_development	ENSGALG00010018165	DACH2	0.90036868	0.181828311	4.951751868	7.35483E-07	1.40385E-06	positive
PRJNA729271	stage_3W_vs_E10	ordinary_muscle_development	ENSGALG00010018166	MSS51	6.392360713	0.235977465	27.0888609	1.3321E-161	1.5085E-159	positive
PRJNA729271	stage_3W_vs_E10	ordinary_muscle_development	ENSGALG00010018168	ENSGALG00010018168	-8.705368458	0.855453739	-10.17631704	2.52955E-24	1.1512E-23	negative
PRJNA729271	stage_3W_vs_E10	ordinary_muscle_development	ENSGALG00010018172	ACTL6A	-1.894125736	0.094570751	-20.02866342	3.09863E-89	9.98104E-88	negative
PRJNA729271	stage_3W_vs_E10	ordinary_muscle_development	ENSGALG00010018174	BMP4	0.949712243	0.203471467	4.667545078	3.0482E-06	5.5957E-06	positive
PRJNA729271	stage_3W_vs_E10	ordinary_muscle_development	ENSGALG00010018177	KIF20B	-3.307016156	0.191600134	-17.25998873	9.41293E-67	1.67412E-65	negative
PRJNA729271	stage_3W_vs_E10	ordinary_muscle_development	ENSGALG00010018179	PRADC1	0.913897753	0.115881466	7.886487698	3.1081E-15	9.50864E-15	positive
PRJNA729271	stage_3W_vs_E10	ordinary_muscle_development	ENSGALG00010018183	CYP17A1	-0.774600629	0.379924661	-2.038826927	0.041467304	0.052573128	negative
PRJNA729271	stage_3W_vs_E10	ordinary_muscle_development	ENSGALG00010018185	GNB4	-0.733289015	0.069678482	-10.52389486	6.70435E-26	3.25887E-25	negative
PRJNA729271	stage_3W_vs_E10	ordinary_muscle_development	ENSGALG00010018186	SPTLC3	-4.243663285	0.670935131	-6.324997888	2.53236E-10	5.98509E-10	negative
PRJNA729271	stage_3W_vs_E10	ordinary_muscle_development	ENSGALG00010018188	UBP1	-0.140904993	0.052094075	-2.704818013	0.006834182	0.009452136	negative
PRJNA729271	stage_3W_vs_E10	ordinary_muscle_development	ENSGALG00010018189	MIS18BP1	-1.561648073	0.121950893	-12.80554848	1.52643E-37	1.12828E-36	negative
PRJNA729271	stage_3W_vs_E10	ordinary_muscle_development	ENSGALG00010018192	IQCH	-2.04944898	0.230102805	-8.906666664	5.25899E-19	1.93475E-18	negative
PRJNA729271	stage_3W_vs_E10	ordinary_muscle_development	ENSGALG00010018193	ANK1	4.34632558	0.246552569	17.62839298	1.49146E-69	2.84457E-68	positive
PRJNA729271	stage_3W_vs_E10	ordinary_muscle_development	ENSGALG00010018198	BTBD3	-0.470211136	0.102444415	-4.589914804	4.43427E-06	8.04138E-06	negative
PRJNA729271	stage_3W_vs_E10	ordinary_muscle_development	ENSGALG00010018199	BORCS7	-0.858324068	0.121090765	-7.088270235	1.35799E-12	3.6371E-12	negative
PRJNA729271	stage_3W_vs_E10	ordinary_muscle_development	ENSGALG00010018206	KIZ	-0.004127328	0.125385179	-0.032917189	0.973740625	0.977652585	negative
PRJNA729271	stage_3W_vs_E10	ordinary_muscle_development	ENSGALG00010018209	GMPPA	0.130049833	0.131319233	0.990333482	0.322011146	0.360085104	positive
PRJNA729271	stage_3W_vs_E10	ordinary_muscle_development	ENSGALG00010018215	CFAP70	3.386050087	0.263878445	12.83185557	1.08729E-37	8.0863E-37	positive
PRJNA729271	stage_3W_vs_E10	ordinary_muscle_development	ENSGALG00010018216	FANCM	-0.513486748	0.086642323	-5.926511761	3.09437E-09	6.83942E-09	negative
PRJNA729271	stage_3W_vs_E10	ordinary_muscle_development	ENSGALG00010018217	ENSGALG00010018217	-3.449888714	0.688394717	-5.011497951	5.4008E-07	1.0395E-06	negative
PRJNA729271	stage_3W_vs_E10	ordinary_muscle_development	ENSGALG00010018219	SLC24A5	-3.818584474	0.672471421	-5.678433841	1.35934E-08	2.90871E-08	negative
PRJNA729271	stage_3W_vs_E10	ordinary_muscle_development	ENSGALG00010018226	JAG1	-0.297930792	0.102464378	-2.907652376	0.003641529	0.005180993	negative
PRJNA729271	stage_3W_vs_E10	ordinary_muscle_development	ENSGALG00010018229	AS3MT	0.645545263	0.189041148	3.414839943	0.000638195	0.000973444	positive
PRJNA729271	stage_3W_vs_E10	ordinary_muscle_development	ENSGALG00010018231	SMYD5	-0.93696945	0.080755775	-11.60250706	4.0018E-31	2.39497E-30	negative
PRJNA729271	stage_3W_vs_E10	ordinary_muscle_development	ENSGALG00010018233	RASSF2	0.661659355	0.183568677	3.604424053	0.000312846	0.000490609	positive
PRJNA729271	stage_3W_vs_E10	ordinary_muscle_development	ENSGALG00010018240	SOUL	-1.378491285	0.738463071	-1.866703075	0.061943064	0.076871979	negative
PRJNA729271	stage_3W_vs_E10	ordinary_muscle_development	ENSGALG00010018241	RALGAPA2	0.53434831	0.090976192	5.873496121	4.26699E-09	9.37832E-09	positive
PRJNA729271	stage_3W_vs_E10	ordinary_muscle_development	ENSGALG00010018244	CHM	0.260378109	0.081747635	3.185145456	0.001446813	0.002136901	positive
PRJNA729271	stage_3W_vs_E10	ordinary_muscle_development	ENSGALG00010018251	USP48	0.166514361	0.166444596	1.000419151	0.317107706	0.355064343	positive
PRJNA729271	stage_3W_vs_E10	ordinary_muscle_development	ENSGALG00010018255	MFN1	1.000364571	0.11271532	8.875142866	6.98437E-19	2.5533E-18	positive
PRJNA729271	stage_3W_vs_E10	ordinary_muscle_development	ENSGALG00010018258	OSCP1	-0.82208159	0.283172024	-2.903117262	0.003694683	0.005253009	negative
PRJNA729271	stage_3W_vs_E10	ordinary_muscle_development	ENSGALG00010018259	WDR90	-0.630575535	0.13067681	-4.825458572	1.39681E-06	2.61892E-06	negative
PRJNA729271	stage_3W_vs_E10	ordinary_muscle_development	ENSGALG00010018261	PRNP	0.76295947	0.194627497	3.920101122	8.85118E-05	0.000145068	positive
PRJNA729271	stage_3W_vs_E10	ordinary_muscle_development	ENSGALG00010018263	SLX4IP	-1.664360269	0.118526157	-14.04213481	8.608E-45	8.14977E-44	negative
PRJNA729271	stage_3W_vs_E10	ordinary_muscle_development	ENSGALG00010018264	CFAP61	-1.444059237	0.360477233	-4.005965164	6.17648E-05	0.000102717	negative
PRJNA729271	stage_3W_vs_E10	ordinary_muscle_development	ENSGALG00010018265	PDCD4	0.018996152	0.065329662	0.290773773	0.771224345	0.796034864	positive
PRJNA729271	stage_3W_vs_E10	ordinary_muscle_development	ENSGALG00010018267	ADPRHL2	-0.635467356	0.077353063	-8.215154368	2.11892E-16	6.89111E-16	negative
PRJNA729271	stage_3W_vs_E10	ordinary_muscle_development	ENSGALG00010018273	PROM2	-6.04998479	1.077725316	-5.6136612	1.9809E-08	4.19721E-08	negative
PRJNA729271	stage_3W_vs_E10	ordinary_muscle_development	ENSGALG00010018274	ZNF711	0.004402974	0.097336518	0.045234557	0.96392035	0.968933805	positive
PRJNA729271	stage_3W_vs_E10	ordinary_muscle_development	ENSGALG00010018275	ZNF639	-0.057658646	0.083503334	-0.690495134	0.489882869	0.528258606	negative
PRJNA729271	stage_3W_vs_E10	ordinary_muscle_development	ENSGALG00010018276	ARHGAP17	-0.112939104	0.072200025	-1.564252966	0.117758173	0.1409733	negative
PRJNA729271	stage_3W_vs_E10	ordinary_muscle_development	ENSGALG00010018278	PUSL1	1.123159745	0.145293785	7.73026693	1.07321E-14	3.1943E-14	positive
PRJNA729271	stage_3W_vs_E10	ordinary_muscle_development	ENSGALG00010018283	POF1B	0.319242987	0.178627405	1.787200492	0.07390509	0.090815045	positive
PRJNA729271	stage_3W_vs_E10	ordinary_muscle_development	ENSGALG00010018284	ENSGALG00010018284	1.307670402	0.16705411	7.827825395	4.96381E-15	1.50492E-14	positive
PRJNA729271	stage_3W_vs_E10	ordinary_muscle_development	ENSGALG00010018285	BBIP1	-0.446013477	0.192837487	-2.312898203	0.020728237	0.02722753	negative
PRJNA729271	stage_3W_vs_E10	ordinary_muscle_development	ENSGALG00010018287	MKKS	0.305428208	0.102479715	2.980377224	0.002878936	0.004134157	positive
PRJNA729271	stage_3W_vs_E10	ordinary_muscle_development	ENSGALG00010018288	CRNKL1	-0.189894188	0.061654123	-3.079991704	0.002070064	0.003009586	negative
PRJNA729271	stage_3W_vs_E10	ordinary_muscle_development	ENSGALG00010018289	HEYL	-1.923246012	0.219212724	-8.773423264	1.73315E-18	6.23375E-18	negative
PRJNA729271	stage_3W_vs_E10	ordinary_muscle_development	ENSGALG00010018290	RHOT2	-0.178739825	0.134401763	-1.32989197	0.183553868	0.213652398	negative
PRJNA729271	stage_3W_vs_E10	ordinary_muscle_development	ENSGALG00010018294	FKBP3	0.579740365	0.177794337	3.260735833	0.001111235	0.001658842	positive
PRJNA729271	stage_3W_vs_E10	ordinary_muscle_development	ENSGALG00010018296	PIK3CA	0.901565996	0.096384262	9.353871489	8.44969E-21	3.34092E-20	positive
PRJNA729271	stage_3W_vs_E10	ordinary_muscle_development	ENSGALG00010018298	ENSGALG00010018298	0.198604723	0.197649185	1.004834514	0.314976542	0.352881819	positive
PRJNA729271	stage_3W_vs_E10	ordinary_muscle_development	ENSGALG00010018299	SHOC2	0.356764035	0.082606185	4.318853805	1.56842E-05	2.73018E-05	positive
PRJNA729271	stage_3W_vs_E10	ordinary_muscle_development	ENSGALG00010018301	ENSGALG00010018301	-0.216476799	0.310677745	-0.696788882	0.485934925	0.524235393	negative
PRJNA729271	stage_3W_vs_E10	ordinary_muscle_development	ENSGALG00010018302	RAB11FIP5	1.002506671	0.100462691	9.978895212	1.88554E-23	8.26708E-23	positive
PRJNA729271	stage_3W_vs_E10	ordinary_muscle_development	ENSGALG00010018306	DES	4.397472941	0.150905689	29.1405379	1.101E-186	1.7347E-184	positive
PRJNA729271	stage_3W_vs_E10	ordinary_muscle_development	ENSGALG00010018308	ACAP3	-1.033786436	0.177942295	-5.809672387	6.25952E-09	1.36685E-08	negative
PRJNA729271	stage_3W_vs_E10	ordinary_muscle_development	ENSGALG00010018309	DPYSL2	-1.423078086	0.12433843	-11.44519905	2.48533E-30	1.44562E-29	negative
PRJNA729271	stage_3W_vs_E10	ordinary_muscle_development	ENSGALG00010018310	APOOL	-0.033945712	0.112261862	-0.302379736	0.7623626	0.787966764	negative
PRJNA729271	stage_3W_vs_E10	ordinary_muscle_development	ENSGALG00010018311	NKX2-3	2.719580813	0.723460753	3.759126946	0.000170507	0.000273275	positive
PRJNA729271	stage_3W_vs_E10	ordinary_muscle_development	ENSGALG00010018312	PRPF39	-0.387824642	0.07914193	-4.900368784	9.56569E-07	1.81557E-06	negative
PRJNA729271	stage_3W_vs_E10	ordinary_muscle_development	ENSGALG00010018313	MYEF2	-1.739000735	0.082395583	-21.10550915	7.07883E-99	2.82666E-97	negative
PRJNA729271	stage_3W_vs_E10	ordinary_muscle_development	ENSGALG00010018314	SLC24A3	-1.570579825	0.13176426	-11.91961932	9.35349E-33	5.95689E-32	negative
PRJNA729271	stage_3W_vs_E10	ordinary_muscle_development	ENSGALG00010018315	CLASP2	0.63740959	0.11727207	5.435306055	5.47025E-08	1.1247E-07	positive
PRJNA729271	stage_3W_vs_E10	ordinary_muscle_development	ENSGALG00010018317	ZMAT3	-0.627356748	0.138217249	-4.538917916	5.65436E-06	1.01776E-05	negative
PRJNA729271	stage_3W_vs_E10	ordinary_muscle_development	ENSGALG00010018319	STUB1	0.953736331	0.201495043	4.733299222	2.20899E-06	4.09037E-06	positive
PRJNA729271	stage_3W_vs_E10	ordinary_muscle_development	ENSGALG00010018322	NT5C1A	4.310632858	0.240717084	17.90746545	1.03126E-71	2.08481E-70	positive
PRJNA729271	stage_3W_vs_E10	ordinary_muscle_development	ENSGALG00010018323	GOT1	2.121914311	0.142366401	14.90460036	3.07648E-50	3.5224E-49	positive
PRJNA729271	stage_3W_vs_E10	ordinary_muscle_development	ENSGALG00010018325	ENSGALG00010018325	0.717273288	0.195142798	3.675632902	0.00023726	0.000375119	positive
PRJNA729271	stage_3W_vs_E10	ordinary_muscle_development	ENSGALG00010018326	LCMT1	-1.05822884	0.112245124	-9.427837913	4.18629E-21	1.67486E-20	negative
PRJNA729271	stage_3W_vs_E10	ordinary_muscle_development	ENSGALG00010018327	SNAP25	-1.266154687	0.557718253	-2.27024072	0.023192982	0.030316734	negative
PRJNA729271	stage_3W_vs_E10	ordinary_muscle_development	ENSGALG00010018328	ENSGALG00010018328	3.319886955	0.54192731	6.126074281	9.00738E-10	2.05804E-09	positive
PRJNA729271	stage_3W_vs_E10	ordinary_muscle_development	ENSGALG00010018329	ENSGALG00010018329	2.639397659	0.220346022	11.97842211	4.61015E-33	2.96732E-32	positive
PRJNA729271	stage_3W_vs_E10	ordinary_muscle_development	ENSGALG00010018333	ENSGALG00010018333	1.453725195	0.207607223	7.002286219	2.51819E-12	6.63778E-12	positive
PRJNA729271	stage_3W_vs_E10	ordinary_muscle_development	ENSGALG00010018336	JMJD8	-0.396632986	0.149576395	-2.651708412	0.008008567	0.01100637	negative
PRJNA729271	stage_3W_vs_E10	ordinary_muscle_development	ENSGALG00010018337	TMEM151B	-2.927613693	0.351150318	-8.337209287	7.60644E-17	2.52937E-16	negative
PRJNA729271	stage_3W_vs_E10	ordinary_muscle_development	ENSGALG00010018338	PDCD6IP	0.301322064	0.084692085	3.557853879	0.000373897	0.000582256	positive
PRJNA729271	stage_3W_vs_E10	ordinary_muscle_development	ENSGALG00010018339	FAM76A	0.286969977	0.115563743	2.483218083	0.013020135	0.017477945	positive
PRJNA729271	stage_3W_vs_E10	ordinary_muscle_development	ENSGALG00010018340	DNPEP	1.384434067	0.096881127	14.29002849	2.52506E-46	2.52593E-45	positive
PRJNA729271	stage_3W_vs_E10	ordinary_muscle_development	ENSGALG00010018342	BNIP3L	-0.520870115	0.10627375	-4.9012114	9.52475E-07	1.80803E-06	negative
PRJNA729271	stage_3W_vs_E10	ordinary_muscle_development	ENSGALG00010018344	SFXN5	-1.093748492	0.197536395	-5.536946703	3.0779E-08	6.42948E-08	negative
PRJNA729271	stage_3W_vs_E10	ordinary_muscle_development	ENSGALG00010018345	WDR24	0.585561423	0.170887069	3.426598784	0.000611191	0.000933434	positive
PRJNA729271	stage_3W_vs_E10	ordinary_muscle_development	ENSGALG00010018347	CTXN2	-5.993365689	0.96375169	-6.218786178	5.01015E-10	1.16419E-09	negative

PRJNA729271	stage_3W_vs_E10	ordinary_muscle_development	ENSGALG00010018348	TOGARAM1	0.560439547	0.107261638	5.224976586	1.74177E-07	3.46514E-07	positive
PRJNA729271	stage_3W_vs_E10	ordinary_muscle_development	ENSGALG00010018349	KCNMB2	0.987400094	0.191259834	5.162610844	2.43529E-07	4.80005E-07	positive
PRJNA729271	stage_3W_vs_E10	ordinary_muscle_development	ENSGALG00010018350	CNNM1	-1.298707431	0.262771009	-4.942354308	7.71848E-07	1.47152E-06	negative
PRJNA729271	stage_3W_vs_E10	ordinary_muscle_development	ENSGALG00010018351	RIN2	0.86768737	0.086584832	10.02123981	1.22951E-23	5.42683E-23	positive
PRJNA729271	stage_3W_vs_E10	ordinary_muscle_development	ENSGALG00010018354	AARS2	-0.072582057	0.118686306	-0.611545335	0.540838615	0.578472235	negative
PRJNA729271	stage_3W_vs_E10	ordinary_muscle_development	ENSGALG00010018355	PRKCB	0.1460128	0.152796475	0.955603197	0.339272746	0.377824097	positive
PRJNA729271	stage_3W_vs_E10	ordinary_muscle_development	ENSGALG00010018356	ADRA1A	-2.234875688	0.444581587	-5.026919144	4.98422E-07	9.62386E-07	negative
PRJNA729271	stage_3W_vs_E10	ordinary_muscle_development	ENSGALG00010018359	CACNG3	-4.233242455	0.762555578	-5.55138875	2.83409E-08	5.93729E-08	negative
PRJNA729271	stage_3W_vs_E10	ordinary_muscle_development	ENSGALG00010018361	ASIC4	-2.767803544	0.325139964	-8.512652547	1.69999E-17	5.83641E-17	negative
PRJNA729271	stage_3W_vs_E10	ordinary_muscle_development	ENSGALG00010018363	TCTE1	0.129865711	0.389087536	0.333769907	0.738553193	0.765979431	positive
PRJNA729271	stage_3W_vs_E10	ordinary_muscle_development	ENSGALG00010018365	HPSE2	-1.970858365	0.262115906	-7.519033845	5.51825E-14	1.5883E-13	negative
PRJNA729271	stage_3W_vs_E10	ordinary_muscle_development	ENSGALG00010018367	PAK5	-1.222267918	0.222863739	-5.484373211	4.14938E-08	8.60447E-08	negative
PRJNA729271	stage_3W_vs_E10	ordinary_muscle_development	ENSGALG00010018370	ZBTB8A	-0.112164132	0.111328913	-1.007502266	0.31369347	0.351580209	negative
PRJNA729271	stage_3W_vs_E10	ordinary_muscle_development	ENSGALG00010018371	ENSGALG00010018371	0.60450002	0.136329239	4.434118633	9.24497E-06	1.63641E-05	positive
PRJNA729271	stage_3W_vs_E10	ordinary_muscle_development	ENSGALG00010018372	NLGN3	-1.644422657	0.478194296	-3.438816961	0.000584262	0.000893436	negative
PRJNA729271	stage_3W_vs_E10	ordinary_muscle_development	ENSGALG00010018374	HPS1	-0.374710299	0.134620554	-2.783455331	0.005378327	0.007516279	negative
PRJNA729271	stage_3W_vs_E10	ordinary_muscle_development	ENSGALG00010018376	PYGB	-0.574037223	0.143149352	-4.010058125	6.07038E-05	0.000101045	negative
PRJNA729271	stage_3W_vs_E10	ordinary_muscle_development	ENSGALG00010018377	RAP1GAP	0.341500562	0.321033467	1.063753776	0.287440256	0.324590723	positive
PRJNA729271	stage_3W_vs_E10	ordinary_muscle_development	ENSGALG00010018378	ARPP21	0.328770919	0.145218832	2.263968899	0.023576028	0.030775803	positive
PRJNA729271	stage_3W_vs_E10	ordinary_muscle_development	ENSGALG00010018379	SRBD1	0.931019092	0.125924331	7.393480525	1.43034E-13	4.02501E-13	positive
PRJNA729271	stage_3W_vs_E10	ordinary_muscle_development	ENSGALG00010018380	SLC20A1	-1.468313283	0.143048017	-10.26447843	1.0187E-24	4.70556E-24	negative
PRJNA729271	stage_3W_vs_E10	ordinary_muscle_development	ENSGALG00010018381	HAPSTR1	0.303974004	0.067480002	4.504653147	6.64814E-06	1.18925E-05	positive
PRJNA729271	stage_3W_vs_E10	ordinary_muscle_development	ENSGALG00010018383	METRN	-2.328494263	0.169232257	-13.75916335	4.48689E-43	4.01131E-42	negative
PRJNA729271	stage_3W_vs_E10	ordinary_muscle_development	ENSGALG00010018384	LAMP5	-0.228355501	0.240200764	-0.950685988	0.341763803	0.380160105	negative
PRJNA729271	stage_3W_vs_E10	ordinary_muscle_development	ENSGALG00010018385	SPR	-1.392789161	0.144100219	-9.665420169	4.22881E-22	1.76089E-21	negative
PRJNA729271	stage_3W_vs_E10	ordinary_muscle_development	ENSGALG00010018387	NT5DC4	-2.589132911	0.354954458	-7.294267895	3.00287E-13	8.2987E-13	negative
PRJNA729271	stage_3W_vs_E10	ordinary_muscle_development	ENSGALG00010018388	SMC3	-1.074917888	0.066158668	-16.24757459	2.32377E-59	3.43705E-58	negative
PRJNA729271	stage_3W_vs_E10	ordinary_muscle_development	ENSGALG00010018391	STAC	-1.852815401	0.22503625	-8.233408617	1.8196E-16	5.93366E-16	negative
PRJNA729271	stage_3W_vs_E10	ordinary_muscle_development	ENSGALG00010018393	PRKCE	-0.054538645	0.076652794	-0.711502374	0.47677298	0.515502339	negative
PRJNA729271	stage_3W_vs_E10	ordinary_muscle_development	ENSGALG00010018394	KLHL28	-0.040613414	0.097695578	-0.415713941	0.677619339	0.708715803	negative
PRJNA729271	stage_3W_vs_E10	ordinary_muscle_development	ENSGALG00010018395	MED12	-0.059000903	0.079020978	-0.746648604	0.455275701	0.493731953	negative
PRJNA729271	stage_3W_vs_E10	ordinary_muscle_development	ENSGALG00010018396	TBL1XR1	0.021872077	0.074602374	0.293182044	0.769383008	0.794429883	positive
PRJNA729271	stage_3W_vs_E10	ordinary_muscle_development	ENSGALG00010018397	FAM173A	2.537661392	0.15892482	15.96768456	2.14606E-57	3.00552E-56	positive
PRJNA729271	stage_3W_vs_E10	ordinary_muscle_development	ENSGALG00010018398	LRFN5	-0.824500879	0.195370375	-4.220193966	2.44092E-05	4.18267E-05	negative
PRJNA729271	stage_3W_vs_E10	ordinary_muscle_development	ENSGALG00010018399	ABHD12	0.155116186	0.162836976	0.952585769	0.34079999	0.379233522	positive
PRJNA729271	stage_3W_vs_E10	ordinary_muscle_development	ENSGALG00010018401	ENSGALG00010018401	-0.785023498	0.10308541	-7.61527261	2.63136E-14	7.68982E-14	negative
PRJNA729271	stage_3W_vs_E10	ordinary_muscle_development	ENSGALG00010018404	IL2RG	3.210688677	0.253322122	12.67433202	8.20547E-37	5.93801E-36	positive
PRJNA729271	stage_3W_vs_E10	ordinary_muscle_development	ENSGALG00010018405	DCLK3	-1.520085897	0.192245515	-7.907003171	2.63659E-15	8.09347E-15	negative
PRJNA729271	stage_3W_vs_E10	ordinary_muscle_development	ENSGALG00010018406	TMEM260	-0.682653227	0.128368697	-5.317910383	1.04966E-07	2.12289E-07	negative
PRJNA729271	stage_3W_vs_E10	ordinary_muscle_development	ENSGALG00010018410	EXOC6B	0.320905604	0.099173804	3.235790007	0.001213066	0.001803482	positive
PRJNA729271	stage_3W_vs_E10	ordinary_muscle_development	ENSGALG00010018413	SLC15A2	-1.866981109	0.361091999	-5.170375175	2.33624E-07	4.60921E-07	negative
PRJNA729271	stage_3W_vs_E10	ordinary_muscle_development	ENSGALG00010018414	EPAS1	1.547316021	0.137716587	11.23550946	2.72915E-29	1.52974E-28	positive
PRJNA729271	stage_3W_vs_E10	ordinary_muscle_development	ENSGALG00010018415	PELI2	-1.899282085	0.131367321	-14.45779721	2.23848E-47	2.31928E-46	negative
PRJNA729271	stage_3W_vs_E10	ordinary_muscle_development	ENSGALG00010018416	DUT	-2.387927069	0.135493319	-17.62394696	1.61345E-69	3.06915E-68	negative
PRJNA729271	stage_3W_vs_E10	ordinary_muscle_development	ENSGALG00010018418	PLCB4	-0.407860744	0.11908288	-3.425015794	0.000614764	0.000938791	negative
PRJNA729271	stage_3W_vs_E10	ordinary_muscle_development	ENSGALG00010018419	USP7	0.629994162	0.133565434	4.716745522	2.39647E-06	4.42733E-06	positive
PRJNA729271	stage_3W_vs_E10	ordinary_muscle_development	ENSGALG00010018420	RHOQ	2.757168187	0.131479751	20.97028763	1.22521E-97	4.76124E-96	positive
PRJNA729271	stage_3W_vs_E10	ordinary_muscle_development	ENSGALG00010018422	ENSGALG00010018422	0.521464556	0.21429547	2.433390476	0.01495816	0.019952013	positive
PRJNA729271	stage_3W_vs_E10	ordinary_muscle_development	ENSGALG00010018425	CYP26B1	-2.748195003	0.234926962	-11.69808258	1.30366E-31	7.95309E-31	negative
PRJNA729271	stage_3W_vs_E10	ordinary_muscle_development	ENSGALG00010018426	ENSGALG00010018426	2.99630617	0.348100915	8.607579121	7.46196E-18	2.61448E-17	positive
PRJNA729271	stage_3W_vs_E10	ordinary_muscle_development	ENSGALG00010018427	PIGF	-0.08456191	0.142828217	-0.592053252	0.55381493	0.590869086	negative
PRJNA729271	stage_3W_vs_E10	ordinary_muscle_development	ENSGALG00010018428	PPP2R5D	-0.529107304	0.064840065	-8.160190833	3.34496E-16	1.07482E-15	negative
PRJNA729271	stage_3W_vs_E10	ordinary_muscle_development	ENSGALG00010018430	LOXL4	-1.751345405	0.267206898	-6.554267187	5.59158E-11	1.36886E-10	negative
PRJNA729271	stage_3W_vs_E10	ordinary_muscle_development	ENSGALG00010018434	HSPBAP1	1.938704041	0.158710142	12.2153759	2.57309E-34	1.73233E-33	positive
PRJNA729271	stage_3W_vs_E10	ordinary_muscle_development	ENSGALG00010018436	FOXO4	0.879882854	0.145281215	6.056411714	1.39191E-09	3.14559E-09	positive
PRJNA729271	stage_3W_vs_E10	ordinary_muscle_development	ENSGALG00010018438	PPP2R2A	0.847757889	0.098181017	8.634641572	5.89115E-18	2.07263E-17	positive
PRJNA729271	stage_3W_vs_E10	ordinary_muscle_development	ENSGALG00010018440	MEA1	0.905132587	0.100765794	8.982538157	2.64591E-19	9.84912E-19	positive
PRJNA729271	stage_3W_vs_E10	ordinary_muscle_development	ENSGALG00010018441	CRIPT	0.90282617	0.093175943	9.689477141	3.34235E-22	1.39618E-21	positive
PRJNA729271	stage_3W_vs_E10	ordinary_muscle_development	ENSGALG00010018443	PLCB1	1.649834368	0.146589937	11.25475873	2.19426E-29	1.2347E-28	positive
PRJNA729271	stage_3W_vs_E10	ordinary_muscle_development	ENSGALG00010018444	NUBP2	-0.385090027	0.098194157	-3.921720389	8.7919E-05	0.000144161	negative
PRJNA729271	stage_3W_vs_E10	ordinary_muscle_development	ENSGALG00010018446	FBN1	1.8916484	0.232071921	8.151130027	3.60539E-16	1.15492E-15	positive
PRJNA729271	stage_3W_vs_E10	ordinary_muscle_development	ENSGALG00010018448	KLHDC3	2.295585657	0.164546105	13.95101787	3.10147E-44	2.88178E-43	positive
PRJNA729271	stage_3W_vs_E10	ordinary_muscle_development	ENSGALG00010018451	PIK3R4	0.011370511	0.111397427	0.102071581	0.918699858	0.92804756	positive
PRJNA729271	stage_3W_vs_E10	ordinary_muscle_development	ENSGALG00010018452	SPSB3	0.466724351	0.119439579	3.907618843	9.32102E-05	0.000152492	positive
PRJNA729271	stage_3W_vs_E10	ordinary_muscle_development	ENSGALG00010018453	TMX4	0.098607016	0.092256986	1.068829804	0.285146367	0.32225141	positive
PRJNA729271	stage_3W_vs_E10	ordinary_muscle_development	ENSGALG00010018455	RBM20	4.267312699	0.246343259	17.32262829	3.17518E-67	5.75304E-66	positive
PRJNA729271	stage_3W_vs_E10	ordinary_muscle_development	ENSGALG00010018458	SEMA5B	-2.190739873	0.109334781	-20.03698955	2.62151E-89	8.46299E-88	negative
PRJNA729271	stage_3W_vs_E10	ordinary_muscle_development	ENSGALG00010018459	SNX12	0.427014952	0.102365374	4.171478441	3.0263E-05	5.14861E-05	positive
PRJNA729271	stage_3W_vs_E10	ordinary_muscle_development	ENSGALG00010018462	KTN1	0.686313651	0.11634345	5.899031261	3.65642E-09	8.06203E-09	positive
PRJNA729271	stage_3W_vs_E10	ordinary_muscle_development	ENSGALG00010018464	ENSGALG00010018464	-3.953965657	1.179339516	-3.352694966	0.000800289	0.001208982	negative
PRJNA729271	stage_3W_vs_E10	ordinary_muscle_development	ENSGALG00010018465	NAALADL2	-2.91502675	0.207610927	-14.04081562	8.76975E-45	8.29749E-44	negative
PRJNA729271	stage_3W_vs_E10	ordinary_muscle_development	ENSGALG00010018466	DYSF	1.904336392	0.182542393	10.43229664	1.76568E-25	8.42447E-25	positive
PRJNA729271	stage_3W_vs_E10	ordinary_muscle_development	ENSGALG00010018467	TLX1	-6.387222332	1.166590458	-5.475119642	4.37216E-08	9.04962E-08	negative
PRJNA729271	stage_3W_vs_E10	ordinary_muscle_development	ENSGALG00010018469	KLHDC7A	-0.730240493	0.209278729	-3.489320189	0.000484251	0.000747281	negative
PRJNA729271	stage_3W_vs_E10	ordinary_muscle_development	ENSGALG00010018470	IQCB1	1.294035334	0.157308099	8.226120234	1.93375E-16	6.29598E-16	positive
PRJNA729271	stage_3W_vs_E10	ordinary_muscle_development	ENSGALG00010018471	HHEX	1.486973181	0.179780242	8.271060062	1.32773E-16	4.36405E-16	positive
PRJNA729271	stage_3W_vs_E10	ordinary_muscle_development	ENSGALG00010018472	LMF1	-0.009942239	0.141455833	-0.070285111	0.943966733	0.951185109	negative
PRJNA729271	stage_3W_vs_E10	ordinary_muscle_development	ENSGALG00010018476	ENSGALG00010018476	-0.445241306	0.376475849	-1.182655692	0.236945631	0.271161134	negative
PRJNA729271	stage_3W_vs_E10	ordinary_muscle_development	ENSGALG00010018480	EME2	-0.079993405	0.244649367	-0.326971642	0.743689334	0.770754819	negative
PRJNA729271	stage_3W_vs_E10	ordinary_muscle_development	ENSGALG00010018482	TWNK	-0.080439466	0.127819067	-0.629322902	0.529137681	0.567003082	negative
PRJNA729271	stage_3W_vs_E10	ordinary_muscle_development	ENSGALG00010018484	NLGN1	-3.260026383	0.275886297	-11.81655783	3.20551E-32	2.00795E-31	negative
PRJNA729271	stage_3W_vs_E10	ordinary_muscle_development	ENSGALG00010018485	ATP2C1	-0.00427705	0.066622466	-0.064198321	0.948812314	0.955403577	negative
PRJNA729271	stage_3W_vs_E10	ordinary_muscle_development	ENSGALG00010018486	ENSGALG00010018486	0.634178819	0.100328039	6.321052667	2.59787E-10	6.13493E-10	positive
PRJNA729271	stage_3W_vs_E10	ordinary_muscle_development	ENSGALG00010018487	ENSGALG00010018487	-2.037881312	0.132787078	-15.34698515	3.71033E-53	4.66446E-52	negative
PRJNA729271	stage_3W_vs_E10	ordinary_muscle_development	ENSGALG00010018488	ENSGALG00010018488	1.024833491	0.15377541	6.664482236	2.656E-11	6.64115E-11	positive
PRJNA729271	stage_3W_vs_E10	ordinary_muscle_development	ENSGALG00010018489	SLC7A3	-1.675262236	0.208952926	-8.017414571	1.07994E-15	3.37511E-15	negative
PRJNA729271	stage_3W_vs_E10	ordinary_muscle_development	ENSGALG00010018490	NME3	-0.291001738	0.120724496	-2.410461404	0.015932357	0.021183225	negative
PRJNA729271	stage_3W_vs_E10	ordinary_muscle_development	ENSGALG00010018491	TIA1	0.151381686	0.120174091	1.259686547	0.20778246	0.239927249	positive
PRJNA729271	stage_3W_vs_E10	ordinary_muscle_development	ENSGALG00010018492	ENSGALG00010018492	0.235134342	0.173878335	1.352292352	0.17628181	0.205650289	positive
PRJNA729271	stage_3W_vs_E10	ordinary_muscle_development	ENSGALG00010018494	HAO1	0.818389109	0.284369235	2.87791015	0.004003191	0.005666627	positive
PRJNA729271	stage_3W_vs_E10	ordinary_muscle_development	ENSGALG00010018495	ENSGALG00010018495	-0.260328843	0.685715466	-0.379645577	0.704208524	0.73345567	negative
PRJNA729271	stage_3W_vs_E10	ordinary_muscle_development	ENSGALG00010018496	GNG13	-1.797052554	0.528749942	-3.398681322	0.000677116	0.0010301	negative
PRJNA729271	stage_3W_vs_E10	ordinary_muscle_development	ENSGALG00010018497	ASTE1	-1.114501571	0.113614439	-9.809506402	1.02469E-22	4.36591E-22	negative
PRJNA729271	stage_3W_vs_E10	ordinary_muscle_development	ENSGALG00010018498	ENSGALG00010018498	2.484088927	0.383126689	6.483727183	8.9484E-11	2.16575E-10	positive
PRJNA729271	stage_3W_vs_E10	ordinary_muscle_development	ENSGALG00010018501	ATG14	0.640465846	0.134493352	4.762063234	1.91624E-06	3.55872E-06	positive
PRJNA729271	stage_3W_vs_E10	ordinary_muscle_development	ENSGALG00010018502	ARHGEF10L	0.997534269	0.121994832	8.176856811	2.91344E-16	9.40751E-16	positive
PRJNA729271	stage_3W_vs_E10	ordinary_muscle_development	ENSGALG00010018503	BMP2	-3.114700469	0.192735171	-16.16051941	9.57515E-59	1.3963E-57	negative
PRJNA729271	stage_3W_vs_E10	ordinary_muscle_development	ENSGALG00010018504	SLCO3A1	-0.647278314	0.182657711	-3.543668151	0.000394602	0.000612984	negative
PRJNA729271	stage_3W_vs_E10	ordinary_muscle_development	ENSGALG00010018505	CHTF18	-1.737999626	0.146332145	-11.87708704	1.55699E-32	9.86389E-32	negative
PRJNA729271	stage_3W_vs_E10	ordinary_muscle_development	ENSGALG00010018506	ENSGALG00010018506	0.448087008	0.144670488	3.097293811	0.001952962	0.002845044	positive
PRJNA729271	stage_3W_vs_E10	ordinary_muscle_development	ENSGALG00010018508	ENSGALG00010018508	-0.309127338	0.101479207	-3.046213576	0.002317432	0.003354086	negative
PRJNA729271	stage_3W_vs_E10	ordinary_muscle_development	ENSGALG00010018509	ENSGALG00010018509	-1.409614852	0.112915663	-12.48378499	9.15299E-36	6.44355E-35	negative
PRJNA729271	stage_3W_vs_E10	ordinary_muscle_development	ENSGALG00010018510	ENSGALG00010018510	0.316593581	0.126532877	2.502065776	0.012347098	0.016620653	positive
PRJNA729271	stage_3W_vs_E10	ordinary_muscle_development	ENSGALG00010018511	SOCS5	-0.145491605	0.100811458	-1.443205049	0.148962718	0.175788862	negative
PRJNA729271	stage_3W_vs_E10	ordinary_muscle_development	ENSGALG00010018512	FERMT1	0.312470611	0.169246374	1.846247001	0.064856349	0.080308627	positive
PRJNA729271	stage_3W_vs_E10	ordinary_muscle_development	ENSGALG00010018513	RPUSD1	0.170243159	0.153951693	1.105821938	0.268803597	0.305329374	positive
PRJNA729271	stage_3W_vs_E10	ordinary_muscle_development	ENSGALG00010018514	EAF2	-0.214013598	0.24229242	-0.883286393	0.377081554	0.415871347	negative
PRJNA729271	stage_3W_vs_E10	ordinary_muscle_development	ENSGALG00010018515	SEMA4G	-0.394872722	0.179782892	-2.19638652	0.02806429	0.036294779	negative
PRJNA729271	stage_3W_vs_E10	ordinary_muscle_development	ENSGALG00010018516	LRRN4	0.518203085	0.155700797	3.328198012	0.000874097	0.001315957	positive
PRJNA729271	stage_3W_vs_E10	ordinary_muscle_development	ENSGALG00010018517	ECT2	-3.381847099	0.205435512	-16.46184276	6.89684E-61	1.05565E-59	negative
PRJNA729271	stage_3W_vs_E10	ordinary_muscle_development	ENSGALG00010018518	ENSGALG00010018518	0.403591604	0.790889794	0.510300685	0.609840823	0.644899885	positive
PRJNA729271	stage_3W_vs_E10	ordinary_muscle_development	ENSGALG00010018519	OGDH	2.140296609	0.13616804	15.718054	1.13765E-55	1.52828E-54	positive
PRJNA729271	stage_3W_vs_E10	ordinary_muscle_development	ENSGALG00010018520	ENSGALG00010018520	0.245736283	0.124850852	1.968238731	0.049040576	0.061630236	positive
PRJNA729271	stage_3W_vs_E10	ordinary_muscle_development	ENSGALG00010018521	ENSGALG00010018521	0.532412833	0.158930028	3.349982636	0.000808166	0.00122012	positive
PRJNA729271	stage_3W_vs_E10	ordinary_muscle_development	ENSGALG00010018522	NEK11	-0.539304622	0.26376683	-2.044626391	0.040891706	0.0518933	negative
PRJNA729271	stage_3W_vs_E10	ordinary_muscle_development	ENSGALG00010018523	TEX11	0.258707344	0.165538621	1.562821672	0.118094545	0.141340965	positive
PRJNA729271	stage_3W_vs_E10	ordinary_muscle_development	ENSGALG00010018524	SEC22A	-0.150219341	0.116643855	-1.287846167	0.197799518	0.229093409	negative
PRJNA729271	stage_3W_vs_E10	ordinary_muscle_development	ENSGALG00010018525	ST8SIA2	-1.931624912	0.291197118	-6.633392971	3.28057E-11	8.16341E-11	negative
PRJNA729271	stage_3W_vs_E10	ordinary_muscle_development	ENSGALG00010018526	ENSGALG00010018526	0.620168423	0.100544382	6.168106201	6.91127E-10	1.59267E-09	positive
PRJNA729271	stage_3W_vs_E10	ordinary_muscle_development	ENSGALG00010018527	CCT4	-0.902632168	0.081519831	-11.07254716	1.70485E-28	9.26923E-28	negative
PRJNA729271	stage_3W_vs_E10	ordinary_muscle_development	ENSGALG00010018528	ENSGALG00010018528	-0.881388341	0.786061959	-1.121270825	0.2621726	0.298171192	negative
PRJNA729271	stage_3W_vs_E10	ordinary_muscle_development	ENSGALG00010018529	UBR2	0.727019919	0.092619864	7.849503174	4.17689E-15	1.27113E-14	positive
PRJNA729271	stage_3W_vs_E10	ordinary_muscle_development	ENSGALG00010018530	FAM161A	-0.807229123	0.228039592	-3.539863911	0.000400333	0.000621488	negative
PRJNA729271	stage_3W_vs_E10	ordinary_muscle_development	ENSGALG00010018531	ADCY5	-0.054044275	0.135750495	-0.398114755	0.690545599	0.721022754	negative
PRJNA729271	stage_3W_vs_E10	ordinary_muscle_development	ENSGALG00010018532	MFSD13A	-0.93488967	0.132970184	-7.030821812	2.05321E-12	5.44878E-12	negative
PRJNA729271	stage_3W_vs_E10	ordinary_muscle_development	ENSGALG00010018533	PCYOX1	1.388406729	0.172807506	8.034412168	9.40286E-16	2.95201E-15	positive
PRJNA729271	stage_3W_vs_E10	ordinary_muscle_development	ENSGALG00010018534	NCEH1	2.24290808	0.194797596	11.51404395	1.12096E-30	6.60768E-30	positive
PRJNA729271	stage_3W_vs_E10	ordinary_muscle_development	ENSGALG00010018535	FAM174B	0.124758681	0.10270895	1.214681687	0.22448752	0.257779181	positive
PRJNA729271	stage_3W_vs_E10	ordinary_muscle_development	ENSGALG00010018536	ENSGALG00010018536	1.455483494	0.237619003	6.1252824	9.05229E-10	2.06797E-09	positive
PRJNA729271	stage_3W_vs_E10	ordinary_muscle_development	ENSGALG00010018537	ENSGALG00010018537	-0.461160794	0.678661497	-0.679515187	0.496811488	0.535053312	negative
PRJNA729271	stage_3W_vs_E10	ordinary_muscle_development	ENSGALG00010018539	CHD2	-0.161178466	0.088477187	-1.821695186	0.068501249	0.084489925	negative

PRJNA729271	stage_3W_vs_E10	ordinary_muscle_development	ENSGALG00010018540	TNFSF10	1.188690874	0.108752525	10.930237	8.26323E-28	4.37297E-27	positive
PRJNA729271	stage_3W_vs_E10	ordinary_muscle_development	ENSGALG00010018542	ZMIZ2	2.28509184	0.262314598	8.711264463	3.00504E-18	1.07312E-17	positive
PRJNA729271	stage_3W_vs_E10	ordinary_muscle_development	ENSGALG00010018543	MRPL3	-0.640544816	0.077672875	-8.246698975	1.6283E-16	5.32661E-16	negative
PRJNA729271	stage_3W_vs_E10	ordinary_muscle_development	ENSGALG00010018544	HACD2	-1.019145155	0.102378662	-9.954663735	2.40635E-23	1.04871E-22	negative
PRJNA729271	stage_3W_vs_E10	ordinary_muscle_development	ENSGALG00010018546	SNRPGP15	-0.841390869	0.136187329	-6.178187603	6.48416E-10	1.49615E-09	negative
PRJNA729271	stage_3W_vs_E10	ordinary_muscle_development	ENSGALG00010018548	RGMA	-1.857925227	0.125535722	-14.79997247	1.46543E-49	1.62894E-48	negative
PRJNA729271	stage_3W_vs_E10	ordinary_muscle_development	ENSGALG00010018549	GHSR	-5.050209876	0.902854749	-5.593601718	2.22407E-08	4.69871E-08	negative
PRJNA729271	stage_3W_vs_E10	ordinary_muscle_development	ENSGALG00010018551	FBXO34	1.535075512	0.111140477	13.8120292	2.1567E-43	1.95018E-42	positive
PRJNA729271	stage_3W_vs_E10	ordinary_muscle_development	ENSGALG00010018552	DTD1	1.035062614	0.102453425	10.10276241	5.37076E-24	2.40424E-23	positive
PRJNA729271	stage_3W_vs_E10	ordinary_muscle_development	ENSGALG00010018553	ENSGALG00010018553	-1.343327257	0.513632525	-2.615346946	0.008913686	0.012190751	negative
PRJNA729271	stage_3W_vs_E10	ordinary_muscle_development	ENSGALG00010018554	PPIA	-1.672907463	0.123827618	-13.50997049	1.36576E-41	1.15703E-40	negative
PRJNA729271	stage_3W_vs_E10	ordinary_muscle_development	ENSGALG00010018555	UBN1	-0.569678759	0.075335906	-7.561849197	3.97379E-14	1.152E-13	negative
PRJNA729271	stage_3W_vs_E10	ordinary_muscle_development	ENSGALG00010018556	MRPL2	2.08057113	0.125704788	16.55124807	1.56818E-61	2.44416E-60	positive
PRJNA729271	stage_3W_vs_E10	ordinary_muscle_development	ENSGALG00010018557	TBCC	0.224241176	0.102224079	2.193623829	0.028262464	0.036538032	positive
PRJNA729271	stage_3W_vs_E10	ordinary_muscle_development	ENSGALG00010018558	ENSGALG00010018558	0.198854284	0.242155019	0.821185888	0.411540394	0.450345614	positive
PRJNA729271	stage_3W_vs_E10	ordinary_muscle_development	ENSGALG00010018559	DLG3	-2.465321175	0.139426043	-17.68192747	5.77865E-70	1.11831E-68	negative
PRJNA729271	stage_3W_vs_E10	ordinary_muscle_development	ENSGALG00010018560	ENSGALG00010018560	1.086225625	0.080058821	13.56784438	6.21222E-42	5.34082E-41	positive
PRJNA729271	stage_3W_vs_E10	ordinary_muscle_development	ENSGALG00010018561	ACTR1A	0.199506817	0.059110883	3.375128365	0.000737813	0.00111798	positive
PRJNA729271	stage_3W_vs_E10	ordinary_muscle_development	ENSGALG00010018562	ENSGALG00010018562	-8.453419202	1.124116245	-7.52005786	5.4752E-14	1.57685E-13	negative
PRJNA729271	stage_3W_vs_E10	ordinary_muscle_development	ENSGALG00010018564	DLGAP5	-2.853371372	0.155477506	-18.35230993	3.16372E-75	7.00124E-74	negative
PRJNA729271	stage_3W_vs_E10	ordinary_muscle_development	ENSGALG00010018566	ENSGALG00010018566	1.729055913	0.141633975	12.20791772	2.82015E-34	1.89513E-33	positive
PRJNA729271	stage_3W_vs_E10	ordinary_muscle_development	ENSGALG00010018568	FAM136A	-1.467785656	0.19741174	-7.435148755	1.0445E-13	2.96052E-13	negative
PRJNA729271	stage_3W_vs_E10	ordinary_muscle_development	ENSGALG00010018573	SEC23B	-0.654595417	0.079530473	-8.230749753	1.86045E-16	6.06415E-16	negative
PRJNA729271	stage_3W_vs_E10	ordinary_muscle_development	ENSGALG00010018574	ENSGALG00010018574	-0.837998032	0.226067907	-3.706842093	0.00020986	0.000333617	negative
PRJNA729271	stage_3W_vs_E10	ordinary_muscle_development	ENSGALG00010018576	PTK7	-2.589651571	0.116276893	-22.27142051	6.9948E-110	3.5451E-108	negative
PRJNA729271	stage_3W_vs_E10	ordinary_muscle_development	ENSGALG00010018577	NARFL	0.840043806	0.080669606	10.41338671	2.15419E-25	1.02243E-24	positive
PRJNA729271	stage_3W_vs_E10	ordinary_muscle_development	ENSGALG00010018579	FNDC3B	0.359657795	0.10149503	3.543600051	0.000394704	0.000613076	positive
PRJNA729271	stage_3W_vs_E10	ordinary_muscle_development	ENSGALG00010018581	SUFU	-1.451159944	0.093185724	-15.57277107	1.11471E-54	1.45435E-53	negative
PRJNA729271	stage_3W_vs_E10	ordinary_muscle_development	ENSGALG00010018582	RCC2	-2.479301395	0.112764409	-21.98655949	3.8726E-107	1.8649E-105	negative
PRJNA729271	stage_3W_vs_E10	ordinary_muscle_development	ENSGALG00010018583	PPL	2.521952052	0.212999655	11.84016963	2.41963E-32	1.52357E-31	positive
PRJNA729271	stage_3W_vs_E10	ordinary_muscle_development	ENSGALG00010018585	GDPD2	-3.12590471	0.745405733	-4.193561402	2.74609E-05	4.68509E-05	negative
PRJNA729271	stage_3W_vs_E10	ordinary_muscle_development	ENSGALG00010018587	SRF	0.527549774	0.173470689	3.041146468	0.002356792	0.00340867	positive
PRJNA729271	stage_3W_vs_E10	ordinary_muscle_development	ENSGALG00010018588	POLR3F	0.820001697	0.101802792	8.054805577	7.96052E-16	2.51007E-15	positive
PRJNA729271	stage_3W_vs_E10	ordinary_muscle_development	ENSGALG00010018590	TGFA	-0.399720628	0.50920757	-0.784985637	0.43246199	0.471070605	negative
PRJNA729271	stage_3W_vs_E10	ordinary_muscle_development	ENSGALG00010018593	LGALS3	0.848272079	0.326525865	2.597871014	0.009380372	0.012788609	positive
PRJNA729271	stage_3W_vs_E10	ordinary_muscle_development	ENSGALG00010018594	BICRAL	0.128931366	0.104457755	1.234291947	0.217094136	0.249823714	positive
PRJNA729271	stage_3W_vs_E10	ordinary_muscle_development	ENSGALG00010018598	ENSGALG00010018598	0.359690918	0.078094443	4.605845248	4.10794E-06	7.46828E-06	positive
PRJNA729271	stage_3W_vs_E10	ordinary_muscle_development	ENSGALG00010018601	CPNE4	-4.722807321	0.425779999	-11.09213053	1.36986E-28	7.45908E-28	negative
PRJNA729271	stage_3W_vs_E10	ordinary_muscle_development	ENSGALG00010018602	MCTP2	-0.515967868	0.221635467	-2.328002263	0.019911983	0.026205212	negative
PRJNA729271	stage_3W_vs_E10	ordinary_muscle_development	ENSGALG00010018604	FAM43A	0.698025998	0.124054251	5.626780163	1.83604E-08	3.90055E-08	positive
PRJNA729271	stage_3W_vs_E10	ordinary_muscle_development	ENSGALG00010018606	DZANK1	-0.747996231	0.249191107	-3.001697127	0.002684791	0.003863402	negative
PRJNA729271	stage_3W_vs_E10	ordinary_muscle_development	ENSGALG00010018607	HAGHL	0.401011934	0.116359314	3.446324324	0.000568268	0.00086962	positive
PRJNA729271	stage_3W_vs_E10	ordinary_muscle_development	ENSGALG00010018608	TRIM8	-0.129815691	0.127184649	-1.020686785	0.307402858	0.345250614	negative
PRJNA729271	stage_3W_vs_E10	ordinary_muscle_development	ENSGALG00010018609	ENSGALG00010018609	0.46573189	0.12444022	3.742615445	0.000182115	0.000291107	positive
PRJNA729271	stage_3W_vs_E10	ordinary_muscle_development	ENSGALG00010018611	NUDCD3	0.909003212	0.11218368	8.102811532	5.37034E-16	1.70896E-15	positive
PRJNA729271	stage_3W_vs_E10	ordinary_muscle_development	ENSGALG00010018613	PADI3	0.609903012	0.408695312	1.492317123	0.13561603	0.161074595	positive
PRJNA729271	stage_3W_vs_E10	ordinary_muscle_development	ENSGALG00010018615	HTR2C	-1.163857578	0.299213383	-3.889724336	0.000100358	0.000163817	negative
PRJNA729271	stage_3W_vs_E10	ordinary_muscle_development	ENSGALG00010018616	ENSGALG00010018616	-1.286014662	0.850132456	-1.512722696	0.130350112	0.15522715	negative
PRJNA729271	stage_3W_vs_E10	ordinary_muscle_development	ENSGALG00010018618	FAHD1	0.609761874	0.170249764	3.5815725	0.000341532	0.000534036	positive
PRJNA729271	stage_3W_vs_E10	ordinary_muscle_development	ENSGALG00010018619	MAPK1IP1L	-0.583267644	0.077389708	-7.536759842	4.81792E-14	1.39171E-13	negative
PRJNA729271	stage_3W_vs_E10	ordinary_muscle_development	ENSGALG00010018623	ARRDC4	1.752882805	0.131044669	13.37622363	8.32719E-41	6.88942E-40	positive
PRJNA729271	stage_3W_vs_E10	ordinary_muscle_development	ENSGALG00010018624	ENSGALG00010018624	1.494056201	0.386102509	3.869584288	0.000109021	0.000177597	positive
PRJNA729271	stage_3W_vs_E10	ordinary_muscle_development	ENSGALG00010018627	MYLK	-0.876107552	0.180133702	-4.86365151	1.1524E-06	2.17387E-06	negative
PRJNA729271	stage_3W_vs_E10	ordinary_muscle_development	ENSGALG00010018628	XDH	3.837633718	0.565721279	6.783612109	1.17208E-11	2.98686E-11	positive
PRJNA729271	stage_3W_vs_E10	ordinary_muscle_development	ENSGALG00010018631	KAT14	-0.340553451	0.052496206	-6.487201235	8.74455E-11	2.11854E-10	negative
PRJNA729271	stage_3W_vs_E10	ordinary_muscle_development	ENSGALG00010018633	ENSGALG00010018633	1.502469458	0.179892267	8.352051376	6.70875E-17	2.23908E-16	positive
PRJNA729271	stage_3W_vs_E10	ordinary_muscle_development	ENSGALG00010018638	SEC14L5	1.447221013	0.15951479	9.072644691	1.16163E-19	4.38027E-19	positive
PRJNA729271	stage_3W_vs_E10	ordinary_muscle_development	ENSGALG00010018641	ENSGALG00010018641	-3.839787448	0.750591608	-5.115681291	3.12611E-07	6.12088E-07	negative
PRJNA729271	stage_3W_vs_E10	ordinary_muscle_development	ENSGALG00010018644	ESF1	-0.285447538	0.096710516	-2.951566676	0.003161663	0.004526252	negative
PRJNA729271	stage_3W_vs_E10	ordinary_muscle_development	ENSGALG00010018649	HAGH	3.213335421	0.104777754	30.66810754	1.516E-206	2.8913E-204	positive
PRJNA729271	stage_3W_vs_E10	ordinary_muscle_development	ENSGALG00010018654	ENSGALG00010018654	2.031662481	0.21580642	9.414281918	4.76327E-21	1.9015E-20	positive
PRJNA729271	stage_3W_vs_E10	ordinary_muscle_development	ENSGALG00010018655	BIRC5	-3.404846974	0.215399451	-15.80712933	2.77842E-56	3.77089E-55	negative
PRJNA729271	stage_3W_vs_E10	ordinary_muscle_development	ENSGALG00010018662	PADI1	1.651574678	0.83110559	1.987201984	0.046900018	0.059078453	positive
PRJNA729271	stage_3W_vs_E10	ordinary_muscle_development	ENSGALG00010018665	IL13RA2	-0.861970927	0.331689838	-2.598725763	0.009357049	0.012759213	negative
PRJNA729271	stage_3W_vs_E10	ordinary_muscle_development	ENSGALG00010018668	OVOL2	-3.346940672	0.791573497	-4.228212144	2.35556E-05	4.0426E-05	negative
PRJNA729271	stage_3W_vs_E10	ordinary_muscle_development	ENSGALG00010018670	MRAS	1.309028778	0.213057299	6.144022208	8.04576E-10	1.84501E-09	positive
PRJNA729271	stage_3W_vs_E10	ordinary_muscle_development	ENSGALG00010018672	PKDCC	-1.733285727	0.099265363	-17.46113313	2.83255E-68	5.23697E-67	negative
PRJNA729271	stage_3W_vs_E10	ordinary_muscle_development	ENSGALG00010018673	NAGPA	-0.301887536	0.092432161	-3.266044334	0.001090612	0.001629566	negative
PRJNA729271	stage_3W_vs_E10	ordinary_muscle_development	ENSGALG00010018684	PADI2	2.681884746	1.079269427	2.48490755	0.012958508	0.017403278	positive
PRJNA729271	stage_3W_vs_E10	ordinary_muscle_development	ENSGALG00010018685	ARL3	-2.186411875	0.105722344	-20.68069806	5.16875E-95	1.89674E-93	negative
PRJNA729271	stage_3W_vs_E10	ordinary_muscle_development	ENSGALG00010018693	ENSGALG00010018693	0.824123973	0.163923575	5.027489027	4.96944E-07	9.59787E-07	positive
PRJNA729271	stage_3W_vs_E10	ordinary_muscle_development	ENSGALG00010018694	MGME1	-1.781388538	0.107020222	-16.6453452	3.27075E-62	5.18136E-61	negative
PRJNA729271	stage_3W_vs_E10	ordinary_muscle_development	ENSGALG00010018698	MRPS16	1.434122529	0.113071011	12.68337943	7.31104E-37	5.29338E-36	positive
PRJNA729271	stage_3W_vs_E10	ordinary_muscle_development	ENSGALG00010018701	SFXN2	-2.862857946	0.142509278	-20.08892327	9.22407E-90	3.01812E-88	negative
PRJNA729271	stage_3W_vs_E10	ordinary_muscle_development	ENSGALG00010018704	PGPEP1L	5.492353697	0.416939809	13.17301341	1.25487E-39	1.00327E-38	positive
PRJNA729271	stage_3W_vs_E10	ordinary_muscle_development	ENSGALG00010018707	SLC45A1	0.395083141	0.09936588	3.976044289	7.00711E-05	0.000115932	positive
PRJNA729271	stage_3W_vs_E10	ordinary_muscle_development	ENSGALG00010018709	SNX5	-0.237095779	0.113704686	-2.085189159	0.037052134	0.047302332	negative
PRJNA729271	stage_3W_vs_E10	ordinary_muscle_development	ENSGALG00010018713	ENSGALG00010018713	-0.155921323	0.135433165	-1.15127874	0.24961758	0.284853317	negative
PRJNA729271	stage_3W_vs_E10	ordinary_muscle_development	ENSGALG00010018715	DNAJC13	-0.1931006	0.103256211	-1.870111236	0.061468372	0.076328627	negative
PRJNA729271	stage_3W_vs_E10	ordinary_muscle_development	ENSGALG00010018717	MEIOB	-5.659349565	0.986228426	-5.738376035	9.55887E-09	2.06461E-08	negative
PRJNA729271	stage_3W_vs_E10	ordinary_muscle_development	ENSGALG00010018719	ERRFI1	-0.291895391	0.086073922	-3.391217519	0.000695829	0.001057347	negative
PRJNA729271	stage_3W_vs_E10	ordinary_muscle_development	ENSGALG00010018721	LRCH2	0.18219627	0.188819031	0.964925356	0.3345822	0.372871961	positive
PRJNA729271	stage_3W_vs_E10	ordinary_muscle_development	ENSGALG00010018724	RRBP1	-1.069605919	0.141908093	-7.537314461	4.79748E-14	1.38635E-13	negative
PRJNA729271	stage_3W_vs_E10	ordinary_muscle_development	ENSGALG00010018726	EBF2	-0.938582033	0.156256353	-6.00668077	1.8936E-09	4.23903E-09	negative
PRJNA729271	stage_3W_vs_E10	ordinary_muscle_development	ENSGALG00010018727	PARK7	2.393862199	0.131664334	18.18155397	7.22569E-74	1.55858E-72	positive
PRJNA729271	stage_3W_vs_E10	ordinary_muscle_development	ENSGALG00010018729	HS3ST6	-6.833996582	0.675538974	-10.11636166	4.67471E-24	2.10043E-23	negative
PRJNA729271	stage_3W_vs_E10	ordinary_muscle_development	ENSGALG00010018730	IGF1R	-0.405387829	0.087586709	-4.628417185	3.68471E-06	6.73517E-06	negative
PRJNA729271	stage_3W_vs_E10	ordinary_muscle_development	ENSGALG00010018732	ENSGALG00010018732	4.146134701	0.218355173	18.9880306	2.14213E-80	5.41887E-79	positive
PRJNA729271	stage_3W_vs_E10	ordinary_muscle_development	ENSGALG00010018736	EEF2KMT	0.118943077	0.131817351	0.902332475	0.366880279	0.405546377	positive
PRJNA729271	stage_3W_vs_E10	ordinary_muscle_development	ENSGALG00010018747	FLRT3	-3.384442359	0.128630698	-26.31131155	1.4235E-152	1.4037E-150	negative
PRJNA729271	stage_3W_vs_E10	ordinary_muscle_development	ENSGALG00010018750	SOCS4	0.168013098	0.125980563	1.333643016	0.182320878	0.212268364	positive
PRJNA729271	stage_3W_vs_E10	ordinary_muscle_development	ENSGALG00010018752	PLS3	-1.154695317	0.141090633	-8.184067864	2.74419E-16	8.87485E-16	negative
PRJNA729271	stage_3W_vs_E10	ordinary_muscle_development	ENSGALG00010018753	SYNM	2.441996205	0.153595498	15.89887875	6.4509E-57	8.93937E-56	positive
PRJNA729271	stage_3W_vs_E10	ordinary_muscle_development	ENSGALG00010018756	EML4	-2.767475842	0.064118556	-43.16185558	0	0	negative
PRJNA729271	stage_3W_vs_E10	ordinary_muscle_development	ENSGALG00010018758	ENSGALG00010018758	-1.209746236	0.135985469	-8.89614342	5.78209E-19	2.12181E-18	negative
PRJNA729271	stage_3W_vs_E10	ordinary_muscle_development	ENSGALG00010018764	TMEM177	-0.306251511	0.092665354	-3.304919227	0.000950038	0.001425401	negative
PRJNA729271	stage_3W_vs_E10	ordinary_muscle_development	ENSGALG00010018767	NDUFB6	1.647498528	0.168979602	9.749688783	1.85035E-22	7.78542E-22	positive
PRJNA729271	stage_3W_vs_E10	ordinary_muscle_development	ENSGALG00010018774	MSRB1	1.577992054	0.122246916	12.90823614	4.0446E-38	3.05187E-37	positive
PRJNA729271	stage_3W_vs_E10	ordinary_muscle_development	ENSGALG00010018780	WDHD1	-3.717653608	0.215064652	-17.28621402	5.975E-67	1.07188E-65	negative
PRJNA729271	stage_3W_vs_E10	ordinary_muscle_development	ENSGALG00010018783	ENSGALG00010018783	-0.118050411	0.17865185	-0.660784711	0.508750389	0.546814244	negative
PRJNA729271	stage_3W_vs_E10	ordinary_muscle_development	ENSGALG00010018786	DSTN	-1.370037176	0.114929656	-11.92065847	9.23755E-33	5.88564E-32	negative
PRJNA729271	stage_3W_vs_E10	ordinary_muscle_development	ENSGALG00010018796	PER3	1.161469798	0.309286149	3.755324326	0.000173117	0.000277244	positive
PRJNA729271	stage_3W_vs_E10	ordinary_muscle_development	ENSGALG00010018799	ENSGALG00010018799	-0.616065836	0.306112473	-2.012547313	0.044162274	0.055804391	negative
PRJNA729271	stage_3W_vs_E10	ordinary_muscle_development	ENSGALG00010018806	GCH1	1.115158541	0.30973158	3.600403103	0.000317724	0.000497936	positive
PRJNA729271	stage_3W_vs_E10	ordinary_muscle_development	ENSGALG00010018807	RD3	-2.719076342	0.252534084	-10.76716576	4.91911E-27	2.51686E-26	negative
PRJNA729271	stage_3W_vs_E10	ordinary_muscle_development	ENSGALG00010018808	MATCAP2	-1.221554991	0.091365709	-13.36995037	9.06013E-41	7.47448E-40	negative
PRJNA729271	stage_3W_vs_E10	ordinary_muscle_development	ENSGALG00010018811	VAMP3	1.26954994	0.122927885	10.32759928	5.28672E-25	2.47596E-24	positive
PRJNA729271	stage_3W_vs_E10	ordinary_muscle_development	ENSGALG00010018815	ENSGALG00010018815	2.04271524	0.170222845	12.00024148	3.54261E-33	2.29549E-32	positive
PRJNA729271	stage_3W_vs_E10	ordinary_muscle_development	ENSGALG00010018819	LRRC28	0.178627647	0.078467433	2.276455859	0.02281874	0.029851772	positive
PRJNA729271	stage_3W_vs_E10	ordinary_muscle_development	ENSGALG00010018822	TRAF5	1.316997773	0.193468313	6.807304781	9.94441E-12	2.54402E-11	positive
PRJNA729271	stage_3W_vs_E10	ordinary_muscle_development	ENSGALG00010018823	RAB40C	-0.657261288	0.077225761	-8.510907187	1.72578E-17	5.92355E-17	negative
PRJNA729271	stage_3W_vs_E10	ordinary_muscle_development	ENSGALG00010018829	PTPN4	2.250561791	0.137988876	16.30973345	8.41592E-60	1.24988E-58	positive
PRJNA729271	stage_3W_vs_E10	ordinary_muscle_development	ENSGALG00010018831	WBP1L	1.385445744	0.24016168	5.768804353	7.98359E-09	1.73263E-08	positive
PRJNA729271	stage_3W_vs_E10	ordinary_muscle_development	ENSGALG00010018832	EEPD1	1.18497479	0.184251089	6.431304145	1.26514E-10	3.03511E-10	positive
PRJNA729271	stage_3W_vs_E10	ordinary_muscle_development	ENSGALG00010018834	SAMD4A	1.065026935	0.171638628	6.205053879	5.46782E-10	1.26769E-09	positive
PRJNA729271	stage_3W_vs_E10	ordinary_muscle_development	ENSGALG00010018835	CAMK2B	0.504529557	0.179374102	2.812722418	0.004912404	0.006895728	positive
PRJNA729271	stage_3W_vs_E10	ordinary_muscle_development	ENSGALG00010018837	RCOR3	-1.030015979	0.095084258	-10.83266563	2.41035E-27	1.24734E-26	negative
PRJNA729271	stage_3W_vs_E10	ordinary_muscle_development	ENSGALG00010018838	WFIKKN1	-6.562469965	0.427264926	-15.3592527	3.07094E-53	3.87409E-52	negative
PRJNA729271	stage_3W_vs_E10	ordinary_muscle_development	ENSGALG00010018839	RPL3L	11.40234531	0.249391779	45.72061426	0	0	positive
PRJNA729271	stage_3W_vs_E10	ordinary_muscle_development	ENSGALG00010018843	MCRIP2	2.384338384	0.235811462	10.11120647	4.92737E-24	2.21121E-23	positive
PRJNA729271	stage_3W_vs_E10	ordinary_muscle_development	ENSGALG00010018847	CGRRF1	0.034487835	0.156695086	0.220095194	0.82579018	0.846409827	positive
PRJNA729271	stage_3W_vs_E10	ordinary_muscle_development	ENSGALG00010018848	MEF2A	1.146238167	0.114638308	9.998735908	1.54355E-23	6.78814E-23	positive
PRJNA729271	stage_3W_vs_E10	ordinary_muscle_development	ENSGALG00010018849	KCNH1	-3.919838072	0.568179122	-6.898947745	5.23891E-12	1.35483E-11	negative
PRJNA729271	stage_3W_vs_E10	ordinary_muscle_development	ENSGALG00010018852	NDUFB10	2.354940058	0.115661568	20.36060977	3.73901E-92	1.27522E-90	positive
PRJNA729271	stage_3W_vs_E10	ordinary_muscle_development	ENSGALG00010018854	LYSMD4	0.470946164	0.171412044	2.747450846	0.006006051	0.008350059	positive
PRJNA729271	stage_3W_vs_E10	ordinary_muscle_development	ENSGALG00010018855	SYNDIG1	-3.510258627	0.840711156	-4.175344412	2.97536E-05	5.06313E-05	negative
PRJNA729271	stage_3W_vs_E10	ordinary_muscle_development	ENSGALG00010018857	ENSGALG00010018857	0.578245478	0.081972671	7.054125117	1.7369E-12	4.62376E-12	positive
PRJNA729271	stage_3W_vs_E10	ordinary_muscle_development	ENSGALG00010018858	EPB41L5	-1.357115969	0.118431908	-11.45904002	2.1185E-30	1.23522E-29	negative
PRJNA729271	stage_3W_vs_E10	ordinary_muscle_development	ENSGALG00010018860	AGTR2	1.298907513	0.214962652	6.042479935	1.51763E-09	3.42063E-09	positive
PRJNA729271	stage_3W_vs_E10	ordinary_muscle_development	ENSGALG00010018864	ABCA3	1.205263508	0.118848645	10.14116325	3.62754E-24	1.63958E-23	positive
PRJNA729271	stage_3W_vs_E10	ordinary_muscle_development	ENSGALG00010018865	ENSGALG00010018865	-2.877554742	0.235396645	-12.22428102	2.30613E-34	1.55476E-33	negative

PRJNA729271	stage_3W_vs_E10	ordinary_muscle_development	ENSGALG00010018867	RPS2	-0.046808129	0.155938191	-0.300171039	0.764046694	0.789482238	negative
PRJNA729271	stage_3W_vs_E10	ordinary_muscle_development	ENSGALG00010018872	KCTD9	0.134888563	0.101040454	1.334995617	0.181877781	0.211769494	positive
PRJNA729271	stage_3W_vs_E10	ordinary_muscle_development	ENSGALG00010018874	ENSGALG00010018874	-0.532592705	0.116449046	-4.573611583	4.79388E-06	8.67507E-06	negative
PRJNA729271	stage_3W_vs_E10	ordinary_muscle_development	ENSGALG00010018876	GMFB	-1.209557934	0.098273178	-12.30811861	8.19104E-35	5.59251E-34	negative
PRJNA729271	stage_3W_vs_E10	ordinary_muscle_development	ENSGALG00010018877	CST7	5.336241013	0.790336299	6.751861228	1.4596E-11	3.69812E-11	positive
PRJNA729271	stage_3W_vs_E10	ordinary_muscle_development	ENSGALG00010018880	TMEM17	-0.22539139	0.255853351	-0.880939762	0.378350425	0.417175522	negative
PRJNA729271	stage_3W_vs_E10	ordinary_muscle_development	ENSGALG00010018881	ENSGALG00010018881	0.643158779	0.639020739	1.006475597	0.314186845	0.352051508	positive
PRJNA729271	stage_3W_vs_E10	ordinary_muscle_development	ENSGALG00010018882	HHAT	-0.824550409	0.162381562	-5.077857354	3.81715E-07	7.42679E-07	negative
PRJNA729271	stage_3W_vs_E10	ordinary_muscle_development	ENSGALG00010018883	TBL3	-1.530967847	0.076921888	-19.90288968	3.84131E-88	1.21043E-86	negative
PRJNA729271	stage_3W_vs_E10	ordinary_muscle_development	ENSGALG00010018885	EFEMP1	0.935095163	0.207365138	4.509413546	6.50071E-06	1.16474E-05	positive
PRJNA729271	stage_3W_vs_E10	ordinary_muscle_development	ENSGALG00010018887	ADAMTS17	-3.972236659	0.209815183	-18.93207446	6.20693E-80	1.54853E-78	negative
PRJNA729271	stage_3W_vs_E10	ordinary_muscle_development	ENSGALG00010018891	GNRH1	-2.720567927	0.371303919	-7.327064934	2.35248E-13	6.53993E-13	negative
PRJNA729271	stage_3W_vs_E10	ordinary_muscle_development	ENSGALG00010018892	APMAP	-0.623040322	0.124001657	-5.024451566	5.04873E-07	9.73934E-07	negative
PRJNA729271	stage_3W_vs_E10	ordinary_muscle_development	ENSGALG00010018894	SERTAD4	-1.021564	0.092914063	-10.9947188	4.05174E-28	2.16796E-27	negative
PRJNA729271	stage_3W_vs_E10	ordinary_muscle_development	ENSGALG00010018895	SEPTIN7	-1.113410356	0.089917911	-12.38252032	3.24975E-35	2.25491E-34	negative
PRJNA729271	stage_3W_vs_E10	ordinary_muscle_development	ENSGALG00010018897	CCDC85A	-0.677933501	0.16759343	-4.045107856	5.2299E-05	8.74567E-05	negative
PRJNA729271	stage_3W_vs_E10	ordinary_muscle_development	ENSGALG00010018899	ACSS1	0.243314872	0.098590146	2.467943112	0.013589191	0.018206427	positive
PRJNA729271	stage_3W_vs_E10	ordinary_muscle_development	ENSGALG00010018901	TCF7L2	-0.751848055	0.148354835	-5.067903962	4.0222E-07	7.81631E-07	negative
PRJNA729271	stage_3W_vs_E10	ordinary_muscle_development	ENSGALG00010018902	NOXO1	0.814624031	0.497741798	1.63663979	0.101705757	0.122821373	positive
PRJNA729271	stage_3W_vs_E10	ordinary_muscle_development	ENSGALG00010018903	CAMTA1	-0.259815469	0.096861466	-2.682340867	0.007310892	0.01008291	negative
PRJNA729271	stage_3W_vs_E10	ordinary_muscle_development	ENSGALG00010018904	UGP2	2.724260829	0.271178352	10.04601144	9.56612E-24	4.2521E-23	positive
PRJNA729271	stage_3W_vs_E10	ordinary_muscle_development	ENSGALG00010018908	ENSGALG00010018908	-0.397618226	0.242222618	-1.641540451	0.100685277	0.121659978	negative
PRJNA729271	stage_3W_vs_E10	ordinary_muscle_development	ENSGALG00010018909	TTBK1	0.070327886	0.131528443	0.534697163	0.592859284	0.628501084	positive
PRJNA729271	stage_3W_vs_E10	ordinary_muscle_development	ENSGALG00010018911	CNIH1	0.628845245	0.093388265	6.733664468	1.65442E-11	4.18077E-11	positive
PRJNA729271	stage_3W_vs_E10	ordinary_muscle_development	ENSGALG00010018913	ENSGALG00010018913	1.378106701	0.217193084	6.345076338	2.22315E-10	5.27148E-10	positive
PRJNA729271	stage_3W_vs_E10	ordinary_muscle_development	ENSGALG00010018915	DOCK5	-1.001849726	0.19084208	-5.249626944	1.52408E-07	3.0429E-07	negative
PRJNA729271	stage_3W_vs_E10	ordinary_muscle_development	ENSGALG00010018916	CERS3	-0.526049904	0.319690386	-1.645498042	0.099867146	0.1207016	negative
PRJNA729271	stage_3W_vs_E10	ordinary_muscle_development	ENSGALG00010018917	DNAJC11	-0.000524676	0.105406082	-0.004977661	0.996028417	0.996578443	negative
PRJNA729271	stage_3W_vs_E10	ordinary_muscle_development	ENSGALG00010018919	RALB	0.057866249	0.107578174	0.537899523	0.590646418	0.626246787	positive
PRJNA729271	stage_3W_vs_E10	ordinary_muscle_development	ENSGALG00010018920	ACAD11	-0.420242316	0.116251046	-3.614955137	0.0003004	0.000471703	negative
PRJNA729271	stage_3W_vs_E10	ordinary_muscle_development	ENSGALG00010018922	EEF2K	1.786199238	0.114035828	15.66349165	2.6873E-55	3.56707E-54	positive
PRJNA729271	stage_3W_vs_E10	ordinary_muscle_development	ENSGALG00010018923	VPS54	-0.295537837	0.092503938	-3.194867627	0.00139895	0.002068317	negative
PRJNA729271	stage_3W_vs_E10	ordinary_muscle_development	ENSGALG00010018925	LINS1	-0.097618353	0.127476452	-0.765775574	0.44380985	0.482561232	negative
PRJNA729271	stage_3W_vs_E10	ordinary_muscle_development	ENSGALG00010018926	SLC22A7	0.93395159	0.96671126	0.96611225	0.333988015	0.372310719	positive
PRJNA729271	stage_3W_vs_E10	ordinary_muscle_development	ENSGALG00010018932	CDKN3	-2.60186783	0.238925492	-10.88987119	1.28822E-27	6.76061E-27	negative
PRJNA729271	stage_3W_vs_E10	ordinary_muscle_development	ENSGALG00010018933	PHF13	-0.011702861	0.088190958	-0.132699107	0.894431349	0.906501357	negative
PRJNA729271	stage_3W_vs_E10	ordinary_muscle_development	ENSGALG00010018936	SYT14	0.923015127	0.220696165	4.182288927	2.88589E-05	4.91524E-05	positive
PRJNA729271	stage_3W_vs_E10	ordinary_muscle_development	ENSGALG00010018937	INHBB	-3.909088326	0.41269091	-9.472193921	2.74027E-21	1.10641E-20	negative
PRJNA729271	stage_3W_vs_E10	ordinary_muscle_development	ENSGALG00010018941	KLHL21	1.945873963	0.143626652	13.54813981	8.12635E-42	6.94525E-41	positive
PRJNA729271	stage_3W_vs_E10	ordinary_muscle_development	ENSGALG00010018943	ACKR4	4.202953297	0.342668947	12.26534627	1.39017E-34	9.44701E-34	positive
PRJNA729271	stage_3W_vs_E10	ordinary_muscle_development	ENSGALG00010018944	NEFL	-5.141069762	0.255589332	-20.11457096	5.5013E-90	1.82058E-88	negative
PRJNA729271	stage_3W_vs_E10	ordinary_muscle_development	ENSGALG00010018945	HABP2	-1.906947578	0.710577309	-2.683659545	0.007282122	0.010047055	negative
PRJNA729271	stage_3W_vs_E10	ordinary_muscle_development	ENSGALG00010018947	ENSGALG00010018947	1.041204531	0.211149512	4.931124504	8.17576E-07	1.55751E-06	positive
PRJNA729271	stage_3W_vs_E10	ordinary_muscle_development	ENSGALG00010018948	POLR3E	1.425493404	0.107194293	13.2982211	2.37041E-40	1.93681E-39	positive
PRJNA729271	stage_3W_vs_E10	ordinary_muscle_development	ENSGALG00010018950	TTL	0.06125506	0.097033229	0.631279209	0.527857979	0.565799113	positive
PRJNA729271	stage_3W_vs_E10	ordinary_muscle_development	ENSGALG00010018951	YKT6	-1.025459725	0.092244534	-11.11675328	1.03988E-28	5.67936E-28	negative
PRJNA729271	stage_3W_vs_E10	ordinary_muscle_development	ENSGALG00010018955	ZBTB48	1.173981625	0.140712474	8.343124017	7.23532E-17	2.40817E-16	positive
PRJNA729271	stage_3W_vs_E10	ordinary_muscle_development	ENSGALG00010018958	PELI3	-0.58809334	0.118359518	-4.968703399	6.74021E-07	1.29078E-06	negative
PRJNA729271	stage_3W_vs_E10	ordinary_muscle_development	ENSGALG00010018959	UBA5	-0.621061593	0.117644029	-5.279159512	1.29778E-07	2.60616E-07	negative
PRJNA729271	stage_3W_vs_E10	ordinary_muscle_development	ENSGALG00010018960	RBFOX1	-0.472261361	0.219913687	-2.147485077	0.031754684	0.040819807	negative
PRJNA729271	stage_3W_vs_E10	ordinary_muscle_development	ENSGALG00010018961	ASB7	-0.262671642	0.099511602	-2.639608205	0.008300192	0.011382792	negative
PRJNA729271	stage_3W_vs_E10	ordinary_muscle_development	ENSGALG00010018968	GLI2	-2.268812748	0.199259541	-11.38621889	4.89786E-30	2.81612E-29	negative
PRJNA729271	stage_3W_vs_E10	ordinary_muscle_development	ENSGALG00010018969	DIEXF	-0.591835406	0.090057365	-6.571760191	4.97239E-11	1.22099E-10	negative
PRJNA729271	stage_3W_vs_E10	ordinary_muscle_development	ENSGALG00010018971	POLR1B	-0.181813781	0.09940019	-1.829108988	0.067383283	0.083217235	negative
PRJNA729271	stage_3W_vs_E10	ordinary_muscle_development	ENSGALG00010018973	NEFM	-7.608825108	0.891026963	-8.539388169	1.34934E-17	4.66462E-17	negative
PRJNA729271	stage_3W_vs_E10	ordinary_muscle_development	ENSGALG00010018974	TAS1R1	1.497567223	0.27384813	5.4686049	4.53592E-08	9.37384E-08	positive
PRJNA729271	stage_3W_vs_E10	ordinary_muscle_development	ENSGALG00010018977	NPHP3	-0.190545215	0.11134857	-1.711249774	0.087035014	0.106094738	negative
PRJNA729271	stage_3W_vs_E10	ordinary_muscle_development	ENSGALG00010018983	MDGA2	-4.379158969	0.266619891	-16.4247272	1.27255E-60	1.93756E-59	negative
PRJNA729271	stage_3W_vs_E10	ordinary_muscle_development	ENSGALG00010018985	NOL9	-0.536470879	0.08966851	-5.98282361	2.19302E-09	4.89194E-09	negative
PRJNA729271	stage_3W_vs_E10	ordinary_muscle_development	ENSGALG00010018986	CTDSP1	1.846564431	0.191249874	9.6552452	4.67041E-22	1.94198E-21	positive
PRJNA729271	stage_3W_vs_E10	ordinary_muscle_development	ENSGALG00010018987	MRPS10	0.393826168	0.106777304	3.688294722	0.000225762	0.000357837	positive
PRJNA729271	stage_3W_vs_E10	ordinary_muscle_development	ENSGALG00010018990	GCK	-1.185267662	0.409531115	-2.894206615	0.003801179	0.005395427	negative
PRJNA729271	stage_3W_vs_E10	ordinary_muscle_development	ENSGALG00010018991	RYBP	-2.611529875	0.13485751	-19.36510518	1.52044E-83	4.23009E-82	negative
PRJNA729271	stage_3W_vs_E10	ordinary_muscle_development	ENSGALG00010018993	ENSGALG00010018993	0.152819764	0.484971033	0.315111117	0.752677288	0.778789157	positive
PRJNA729271	stage_3W_vs_E10	ordinary_muscle_development	ENSGALG00010018998	RPS29	1.739979638	0.439936079	3.955073753	7.6511E-05	0.000126141	positive
PRJNA729271	stage_3W_vs_E10	ordinary_muscle_development	ENSGALG00010018999	THUMPD3	-0.753740757	0.072274676	-10.42883619	1.83117E-25	8.72833E-25	negative
PRJNA729271	stage_3W_vs_E10	ordinary_muscle_development	ENSGALG00010019000	TRERF1	0.162939136	0.119353308	1.365183242	0.172195464	0.20138561	positive
PRJNA729271	stage_3W_vs_E10	ordinary_muscle_development	ENSGALG00010019001	ALDH1A3	0.195491036	0.159946138	1.222230426	0.221620501	0.254708941	positive
PRJNA729271	stage_3W_vs_E10	ordinary_muscle_development	ENSGALG00010019004	ECE2	0.054674721	0.200228154	0.273062106	0.784805475	0.808453938	positive
PRJNA729271	stage_3W_vs_E10	ordinary_muscle_development	ENSGALG00010019005	ENSGALG00010019005	-1.343180751	0.219617571	-6.115998572	9.59542E-10	2.18791E-09	negative
PRJNA729271	stage_3W_vs_E10	ordinary_muscle_development	ENSGALG00010019006	ENSGALG00010019006	-1.050098776	0.08019614	-13.09413117	3.55721E-39	2.80075E-38	negative
PRJNA729271	stage_3W_vs_E10	ordinary_muscle_development	ENSGALG00010019008	HERPUD2	0.21059693	0.044800879	4.700732116	2.5923E-06	4.7812E-06	positive
PRJNA729271	stage_3W_vs_E10	ordinary_muscle_development	ENSGALG00010019009	NRAP	4.458009685	0.218343205	20.41744185	1.17034E-92	4.07792E-91	positive
PRJNA729271	stage_3W_vs_E10	ordinary_muscle_development	ENSGALG00010019011	VIL1	2.443673503	0.388650581	6.287584853	3.22443E-10	7.57137E-10	positive
PRJNA729271	stage_3W_vs_E10	ordinary_muscle_development	ENSGALG00010019012	ADAM28	-0.524701009	0.365182509	-1.436818568	0.150769553	0.17777635	negative
PRJNA729271	stage_3W_vs_E10	ordinary_muscle_development	ENSGALG00010019013	LRRK1	-0.159179794	0.101623633	-1.566365913	0.117262978	0.140392076	negative
PRJNA729271	stage_3W_vs_E10	ordinary_muscle_development	ENSGALG00010019015	GSG1L	-5.17550442	1.073981452	-4.818988643	1.44288E-06	2.70318E-06	negative
PRJNA729271	stage_3W_vs_E10	ordinary_muscle_development	ENSGALG00010019016	PLEKHG5	0.502495452	0.144518295	3.477036943	0.000506988	0.000780541	positive
PRJNA729271	stage_3W_vs_E10	ordinary_muscle_development	ENSGALG00010019017	IRAK2	0.865269808	0.081972828	10.55556861	4.78732E-26	2.34275E-25	positive
PRJNA729271	stage_3W_vs_E10	ordinary_muscle_development	ENSGALG00010019018	SH3BGRL	-1.509727749	0.11046176	-13.66742436	1.58921E-42	1.39441E-41	negative
PRJNA729271	stage_3W_vs_E10	ordinary_muscle_development	ENSGALG00010019019	TBX20	-0.531995658	0.31996808	-1.66265228	0.09638206	0.116810866	negative
PRJNA729271	stage_3W_vs_E10	ordinary_muscle_development	ENSGALG00010019020	KCNK2	1.271148688	0.441648175	2.878193004	0.003999604	0.005662101	positive
PRJNA729271	stage_3W_vs_E10	ordinary_muscle_development	ENSGALG00010019021	LRR1	-1.415763244	0.153942531	-9.196699801	3.69112E-20	1.41992E-19	negative
PRJNA729271	stage_3W_vs_E10	ordinary_muscle_development	ENSGALG00010019023	AP2M1	-0.097608193	0.071548997	-1.364214692	0.172500005	0.201692956	negative
PRJNA729271	stage_3W_vs_E10	ordinary_muscle_development	ENSGALG00010019024	CNPY3	-0.584163035	0.10353305	-5.642285565	1.67807E-08	3.57071E-08	negative
PRJNA729271	stage_3W_vs_E10	ordinary_muscle_development	ENSGALG00010019025	DNAAF2	-0.607211882	0.244294753	-2.485570704	0.012934389	0.017372495	negative
PRJNA729271	stage_3W_vs_E10	ordinary_muscle_development	ENSGALG00010019026	CHSY1	-2.357727394	0.12713565	-18.54497452	8.95431E-77	2.05921E-75	negative
PRJNA729271	stage_3W_vs_E10	ordinary_muscle_development	ENSGALG00010019027	GNLY	3.235042223	0.311355717	10.39018088	2.74831E-25	1.30143E-24	positive
PRJNA729271	stage_3W_vs_E10	ordinary_muscle_development	ENSGALG00010019028	TNFRSF25	7.114776391	0.701361263	10.14423917	3.51507E-24	1.59023E-23	positive
PRJNA729271	stage_3W_vs_E10	ordinary_muscle_development	ENSGALG00010019029	CENPF	-3.257711079	0.261651706	-12.45056309	1.38862E-35	9.72839E-35	negative
PRJNA729271	stage_3W_vs_E10	ordinary_muscle_development	ENSGALG00010019030	CDR2	-2.49386719	0.154719247	-16.11866168	1.88651E-58	2.7236E-57	negative
PRJNA729271	stage_3W_vs_E10	ordinary_muscle_development	ENSGALG00010019032	GNMT	-1.809172683	0.191886282	-9.428358604	4.16556E-21	1.66749E-20	negative
PRJNA729271	stage_3W_vs_E10	ordinary_muscle_development	ENSGALG00010019033	CYP2AB4	-2.390812554	1.092394078	-2.188598969	0.028626001	0.036994818	negative
PRJNA729271	stage_3W_vs_E10	ordinary_muscle_development	ENSGALG00010019034	CASP7	1.133943077	0.166490108	6.810873565	9.70079E-12	2.48213E-11	positive
PRJNA729271	stage_3W_vs_E10	ordinary_muscle_development	ENSGALG00010019035	ENSGALG00010019035	-1.69539371	0.516726548	-3.281026915	0.001034299	0.001547658	negative
PRJNA729271	stage_3W_vs_E10	ordinary_muscle_development	ENSGALG00010019036	REL	0.496585918	0.109842877	4.520875006	6.15845E-06	1.10602E-05	positive
PRJNA729271	stage_3W_vs_E10	ordinary_muscle_development	ENSGALG00010019038	PLEKHS1	-1.272675765	0.465953415	-2.731336922	0.006307795	0.0087511	negative
PRJNA729271	stage_3W_vs_E10	ordinary_muscle_development	ENSGALG00010019040	HMGN5	-2.878836604	0.083728565	-34.38296829	4.5317E-259	1.428E-256	negative
PRJNA729271	stage_3W_vs_E10	ordinary_muscle_development	ENSGALG00010019041	KATNIP	-0.365023633	0.089472157	-4.079745562	4.5085E-05	7.5769E-05	negative
PRJNA729271	stage_3W_vs_E10	ordinary_muscle_development	ENSGALG00010019042	PEX6	0.682969288	0.10552719	6.471974564	9.67304E-11	2.33568E-10	positive
PRJNA729271	stage_3W_vs_E10	ordinary_muscle_development	ENSGALG00010019043	METTL9	-1.008070497	0.103592378	-9.731126182	2.2212E-22	9.32415E-22	negative
PRJNA729271	stage_3W_vs_E10	ordinary_muscle_development	ENSGALG00010019045	LBX1	0.903894071	0.265369779	3.406168076	0.000658816	0.001003314	positive
PRJNA729271	stage_3W_vs_E10	ordinary_muscle_development	ENSGALG00010019046	SELENOS	0.866159797	0.080558924	10.75187898	5.80665E-27	2.9626E-26	positive
PRJNA729271	stage_3W_vs_E10	ordinary_muscle_development	ENSGALG00010019047	GHRL	-0.050658849	0.41568367	-0.121868749	0.903002966	0.914392611	negative
PRJNA729271	stage_3W_vs_E10	ordinary_muscle_development	ENSGALG00010019049	POLD2	-0.511123372	0.162561227	-3.144189929	0.001665473	0.002447636	negative
PRJNA729271	stage_3W_vs_E10	ordinary_muscle_development	ENSGALG00010019050	TNS1	0.886834273	0.133696525	6.633188664	3.28511E-11	8.17332E-11	positive
PRJNA729271	stage_3W_vs_E10	ordinary_muscle_development	ENSGALG00010019051	IGSF6	3.086047837	0.24907333	12.39011757	2.9561E-35	2.05509E-34	positive
PRJNA729271	stage_3W_vs_E10	ordinary_muscle_development	ENSGALG00010019059	BRWD3	0.605337191	0.077381372	7.822776628	5.16707E-15	1.56491E-14	positive
PRJNA729271	stage_3W_vs_E10	ordinary_muscle_development	ENSGALG00010019062	PUS10	-0.955158804	0.09971	-9.579368221	9.76404E-22	4.01048E-21	negative
PRJNA729271	stage_3W_vs_E10	ordinary_muscle_development	ENSGALG00010019064	ENSGALG00010019064	-1.28799613	0.172376183	-7.472007477	7.89805E-14	2.25625E-13	negative
PRJNA729271	stage_3W_vs_E10	ordinary_muscle_development	ENSGALG00010019065	CAND2	3.95455352	0.120801311	32.73601501	4.8016E-235	1.2429E-232	positive
PRJNA729271	stage_3W_vs_E10	ordinary_muscle_development	ENSGALG00010019067	GTF3C1	-0.570326054	0.058195902	-9.800106744	1.12467E-22	4.78347E-22	negative
PRJNA729271	stage_3W_vs_E10	ordinary_muscle_development	ENSGALG00010019068	SNRPA1	0.68802628	0.163026177	4.220342353	2.43932E-05	4.18041E-05	positive
PRJNA729271	stage_3W_vs_E10	ordinary_muscle_development	ENSGALG00010019072	AEBP1	0.928737433	0.211497413	4.391247239	1.12702E-05	1.98279E-05	positive
PRJNA729271	stage_3W_vs_E10	ordinary_muscle_development	ENSGALG00010019076	ESPN	-0.085750138	0.396849815	-0.216077053	0.828927687	0.849138291	negative
PRJNA729271	stage_3W_vs_E10	ordinary_muscle_development	ENSGALG00010019079	CAPG	0.776590069	0.246722334	3.14762777	0.001646012	0.002420998	positive
PRJNA729271	stage_3W_vs_E10	ordinary_muscle_development	ENSGALG00010019081	PCSK6	1.36905832	0.135126917	10.13164771	3.99854E-24	1.80501E-23	positive
PRJNA729271	stage_3W_vs_E10	ordinary_muscle_development	ENSGALG00010019082	POLR3K	1.623561167	0.17529741	9.261752169	2.01105E-20	7.82133E-20	positive
PRJNA729271	stage_3W_vs_E10	ordinary_muscle_development	ENSGALG00010019083	OTOA	-0.772287861	0.233941994	-3.301193801	0.000962744	0.001444016	negative
PRJNA729271	stage_3W_vs_E10	ordinary_muscle_development	ENSGALG00010019087	PEX13	0.737251576	0.086438316	8.529221911	1.47335E-17	5.08119E-17	positive
PRJNA729271	stage_3W_vs_E10	ordinary_muscle_development	ENSGALG00010019093	RPL32	0.788629393	0.151738108	5.197306079	2.02197E-07	4.00499E-07	positive
PRJNA729271	stage_3W_vs_E10	ordinary_muscle_development	ENSGALG00010019094	SNRNP25	0.342322434	0.16782358	2.039775534	0.041372688	0.052462349	positive
PRJNA729271	stage_3W_vs_E10	ordinary_muscle_development	ENSGALG00010019097	STC1	-3.098048135	0.281606141	-11.00135147	3.76447E-28	2.02171E-27	negative
PRJNA729271	stage_3W_vs_E10	ordinary_muscle_development	ENSGALG00010019101	POLE2	-1.938388377	0.122773182	-15.78836962	3.74125E-56	5.06817E-55	negative
PRJNA729271	stage_3W_vs_E10	ordinary_muscle_development	ENSGALG00010019104	POLL	0.006398317	0.120622929	0.053043955	0.957696886	0.963175915	positive
PRJNA729271	stage_3W_vs_E10	ordinary_muscle_development	ENSGALG00010019108	KDELR2	-0.598416146	0.099680672	-6.003331759	1.93309E-09	4.32343E-09	negative
PRJNA729271	stage_3W_vs_E10	ordinary_muscle_development	ENSGALG00010019110	MBD4	1.167569803	0.106523304	10.96069839	5.9042E-28	3.1383E-27	positive
PRJNA729271	stage_3W_vs_E10	ordinary_muscle_development	ENSGALG00010019113	KLHDC1	1.367142799	0.133179371	10.26542467	1.00876E-24	4.66411E-24	positive
PRJNA729271	stage_3W_vs_E10	ordinary_muscle_development	ENSGALG00010019114	TENT5D	-0.481453164	0.336368036	-1.431328523	0.152336083	0.179535858	negative
PRJNA729271	stage_3W_vs_E10	ordinary_muscle_development	ENSGALG00010019115	DBNL	-1.044098259	0.103106316	-10.12642387	4.21794E-24	1.90109E-23	negative
PRJNA729271	stage_3W_vs_E10	ordinary_muscle_development	ENSGALG00010019120	DAGLB	1.564686751	0.077956592	20.07125649	1.31634E-89	4.27812E-88	positive

PRJNA729271	stage_3W_vs_E10	ordinary_muscle_development	ENSGALG00010019123	PLEKHO2	1.717952803	0.117608982	14.60732655	2.52223E-48	2.72427E-47	positive
PRJNA729271	stage_3W_vs_E10	ordinary_muscle_development	ENSGALG00010019124	BMPER	-1.598507033	0.250955467	-6.369684045	1.89418E-10	4.50766E-10	negative
PRJNA729271	stage_3W_vs_E10	ordinary_muscle_development	ENSGALG00010019125	ENSGALG00010019125	2.247615596	0.19700417	11.40897475	3.77148E-30	2.17799E-29	positive
PRJNA729271	stage_3W_vs_E10	ordinary_muscle_development	ENSGALG00010019127	TMBIM1	1.502019711	0.121802129	12.3316376	6.11904E-35	4.19364E-34	positive
PRJNA729271	stage_3W_vs_E10	ordinary_muscle_development	ENSGALG00010019130	RPS6KC1	-0.803324682	0.083811798	-9.584864013	9.25795E-22	3.80584E-21	negative
PRJNA729271	stage_3W_vs_E10	ordinary_muscle_development	ENSGALG00010019132	ENSGALG00010019132	-1.082279122	0.153969986	-7.029156484	2.07786E-12	5.51016E-12	negative
PRJNA729271	stage_3W_vs_E10	ordinary_muscle_development	ENSGALG00010019135	KIAA1841	-1.239331219	0.196399217	-6.310265575	2.78557E-10	6.56641E-10	negative
PRJNA729271	stage_3W_vs_E10	ordinary_muscle_development	ENSGALG00010019139	DPCD	-1.553447085	0.136863801	-11.35031381	7.3897E-30	4.2304E-29	negative
PRJNA729271	stage_3W_vs_E10	ordinary_muscle_development	ENSGALG00010019141	ACOT7	-0.018640707	0.151321999	-0.123185702	0.901960048	0.913556767	negative
PRJNA729271	stage_3W_vs_E10	ordinary_muscle_development	ENSGALG00010019144	IFT122	-1.179997022	0.074241073	-15.89412682	6.95914E-57	9.63445E-56	negative
PRJNA729271	stage_3W_vs_E10	ordinary_muscle_development	ENSGALG00010019145	RAC1	-0.649253512	0.077383166	-8.39011304	4.85675E-17	1.63073E-16	negative
PRJNA729271	stage_3W_vs_E10	ordinary_muscle_development	ENSGALG00010019146	KLHDC2	1.437592047	0.085134426	16.88614253	5.6904E-64	9.49163E-63	positive
PRJNA729271	stage_3W_vs_E10	ordinary_muscle_development	ENSGALG00010019148	ENSGALG00010019148	0.554865292	0.215877388	2.570279819	0.01016164	0.013795352	positive
PRJNA729271	stage_3W_vs_E10	ordinary_muscle_development	ENSGALG00010019149	COX5B	1.510615545	0.180048069	8.390068037	4.85861E-17	1.63098E-16	positive
PRJNA729271	stage_3W_vs_E10	ordinary_muscle_development	ENSGALG00010019158	ENSGALG00010019158	2.602276158	0.251609175	10.34253284	4.52419E-25	2.1264E-24	positive
PRJNA729271	stage_3W_vs_E10	ordinary_muscle_development	ENSGALG00010019166	PNPT1	-0.386549526	0.109508316	-3.529864597	0.000415772	0.000644558	negative
PRJNA729271	stage_3W_vs_E10	ordinary_muscle_development	ENSGALG00010019169	GPR153	-3.167675358	0.261363491	-12.11980811	8.29531E-34	5.47793E-33	negative
PRJNA729271	stage_3W_vs_E10	ordinary_muscle_development	ENSGALG00010019172	NEMF	0.279890862	0.086616647	3.231374929	0.001231962	0.001830954	positive
PRJNA729271	stage_3W_vs_E10	ordinary_muscle_development	ENSGALG00010019173	RHO	-2.036433145	0.809454566	-2.515809078	0.01187595	0.01601916	negative
PRJNA729271	stage_3W_vs_E10	ordinary_muscle_development	ENSGALG00010019174	ENSGALG00010019174	0.024868364	0.444860688	0.055901465	0.955420304	0.961220965	positive
PRJNA729271	stage_3W_vs_E10	ordinary_muscle_development	ENSGALG00010019183	ANGEL2	0.604228172	0.077944904	7.75199073	9.0463E-15	2.70475E-14	positive
PRJNA729271	stage_3W_vs_E10	ordinary_muscle_development	ENSGALG00010019184	PNKD	-0.736970483	0.189460485	-3.889837421	0.000100311	0.000163759	negative
PRJNA729271	stage_3W_vs_E10	ordinary_muscle_development	ENSGALG00010019185	ANKDD1A	0.376745648	0.208506296	1.806879001	0.070781147	0.087161051	positive
PRJNA729271	stage_3W_vs_E10	ordinary_muscle_development	ENSGALG00010019188	ENSGALG00010019188	1.006856114	0.372798078	2.700808219	0.006917121	0.009558935	positive
PRJNA729271	stage_3W_vs_E10	ordinary_muscle_development	ENSGALG00010019189	ICMT	-0.106310579	0.094066921	-1.130159016	0.258409226	0.294144945	negative
PRJNA729271	stage_3W_vs_E10	ordinary_muscle_development	ENSGALG00010019193	ENSGALG00010019193	3.606186104	1.042396041	3.459516307	0.000541147	0.00083066	positive
PRJNA729271	stage_3W_vs_E10	ordinary_muscle_development	ENSGALG00010019194	ENSGALG00010019194	0.331878269	0.188012517	1.765192413	0.077531433	0.09505355	positive
PRJNA729271	stage_3W_vs_E10	ordinary_muscle_development	ENSGALG00010019204	ARF6	-0.331974477	0.230102596	-1.442723737	0.14909831	0.175905909	negative
PRJNA729271	stage_3W_vs_E10	ordinary_muscle_development	ENSGALG00010019205	ENSGALG00010019205	-0.18993134	0.259974987	-0.730575438	0.465038519	0.503678797	negative
PRJNA729271	stage_3W_vs_E10	ordinary_muscle_development	ENSGALG00010019206	RNF207	1.232648529	0.282379635	4.365217512	1.26996E-05	2.2267E-05	positive
PRJNA729271	stage_3W_vs_E10	ordinary_muscle_development	ENSGALG00010019208	PLXND1	0.286219922	0.158122767	1.810112024	0.070278417	0.086578757	positive
PRJNA729271	stage_3W_vs_E10	ordinary_muscle_development	ENSGALG00010019209	VCPKMT	0.202480292	0.085078354	2.379927233	0.017316057	0.022922032	positive
PRJNA729271	stage_3W_vs_E10	ordinary_muscle_development	ENSGALG00010019212	RPL22	0.055674079	0.113887021	0.48885359	0.624945355	0.659237531	positive
PRJNA729271	stage_3W_vs_E10	ordinary_muscle_development	ENSGALG00010019213	RHBDF1	-0.450417203	0.111091533	-4.054469235	5.02483E-05	8.41438E-05	negative
PRJNA729271	stage_3W_vs_E10	ordinary_muscle_development	ENSGALG00010019215	ENSGALG00010019215	-0.039947239	0.101576646	-0.393271883	0.694118683	0.724091422	negative
PRJNA729271	stage_3W_vs_E10	ordinary_muscle_development	ENSGALG00010019218	RMND5A	1.387952172	0.13726691	10.11133835	4.92074E-24	2.20892E-23	positive
PRJNA729271	stage_3W_vs_E10	ordinary_muscle_development	ENSGALG00010019221	VASH2	-2.719959427	0.232786596	-11.68434726	1.53252E-31	9.32183E-31	negative
PRJNA729271	stage_3W_vs_E10	ordinary_muscle_development	ENSGALG00010019223	AAMP	-0.318456766	0.126173818	-2.523952832	0.01160435	0.015676146	negative
PRJNA729271	stage_3W_vs_E10	ordinary_muscle_development	ENSGALG00010019225	PROK2	-7.165032572	0.908203034	-7.889240955	3.0403E-15	9.30514E-15	negative
PRJNA729271	stage_3W_vs_E10	ordinary_muscle_development	ENSGALG00010019227	CLCN5	0.561726331	0.07173383	7.830703185	4.85149E-15	1.4721E-14	positive
PRJNA729271	stage_3W_vs_E10	ordinary_muscle_development	ENSGALG00010019230	SPG21	-0.125960153	0.097539668	-1.291373606	0.196574158	0.227801601	negative
PRJNA729271	stage_3W_vs_E10	ordinary_muscle_development	ENSGALG00010019231	ENSGALG00010019231	3.740726912	0.311123158	12.02329949	2.6805E-33	1.74154E-32	positive
PRJNA729271	stage_3W_vs_E10	ordinary_muscle_development	ENSGALG00010019232	SOS2	0.989389047	0.093072245	10.63033393	2.1534E-26	1.07116E-25	positive
PRJNA729271	stage_3W_vs_E10	ordinary_muscle_development	ENSGALG00010019233	ARPC2	-0.405546072	0.053448755	-7.587568113	3.25965E-14	9.48007E-14	negative
PRJNA729271	stage_3W_vs_E10	ordinary_muscle_development	ENSGALG00010019236	MPG	0.073313256	0.208004343	0.352460219	0.724493148	0.752635862	positive
PRJNA729271	stage_3W_vs_E10	ordinary_muscle_development	ENSGALG00010019237	SORCS3	-2.817092711	0.347406243	-8.10892945	5.10677E-16	1.62651E-15	negative
PRJNA729271	stage_3W_vs_E10	ordinary_muscle_development	ENSGALG00010019239	CYTH3	0.274491702	0.081104759	3.384409316	0.000713316	0.001082898	positive
PRJNA729271	stage_3W_vs_E10	ordinary_muscle_development	ENSGALG00010019240	ENSGALG00010019240	0.121138103	0.206784846	0.585817119	0.557998419	0.595113463	positive
PRJNA729271	stage_3W_vs_E10	ordinary_muscle_development	ENSGALG00010019243	SERTAD2	0.305111451	0.155278131	1.964935108	0.049421749	0.062076971	positive
PRJNA729271	stage_3W_vs_E10	ordinary_muscle_development	ENSGALG00010019246	IL17RC	1.051134332	0.134789652	7.798331102	6.27312E-15	1.8912E-14	positive
PRJNA729271	stage_3W_vs_E10	ordinary_muscle_development	ENSGALG00010019247	HBZ	-8.202865306	0.831217499	-9.868494492	5.70139E-23	2.4603E-22	negative
PRJNA729271	stage_3W_vs_E10	ordinary_muscle_development	ENSGALG00010019248	RMI2	-0.487069739	0.168921805	-2.883403598	0.00393403	0.005575261	negative
PRJNA729271	stage_3W_vs_E10	ordinary_muscle_development	ENSGALG00010019250	CXCR1	0.607395405	0.243840341	2.490955362	0.012740012	0.017120942	positive
PRJNA729271	stage_3W_vs_E10	ordinary_muscle_development	ENSGALG00010019252	TATDN3	0.15878629	0.116637172	1.361369514	0.173396945	0.202610941	positive
PRJNA729271	stage_3W_vs_E10	ordinary_muscle_development	ENSGALG00010019253	HBAD	-4.054560976	0.427054596	-9.494245028	2.21813E-21	8.97092E-21	negative
PRJNA729271	stage_3W_vs_E10	ordinary_muscle_development	ENSGALG00010019255	ENSGALG00010019255	2.616329325	0.198318313	13.19257552	9.682E-40	7.7751E-39	positive
PRJNA729271	stage_3W_vs_E10	ordinary_muscle_development	ENSGALG00010019256	BBS9	-0.929724286	0.10486099	-8.866255112	7.5647E-19	2.76128E-18	negative
PRJNA729271	stage_3W_vs_E10	ordinary_muscle_development	ENSGALG00010019257	SLC1A4	-1.047481066	0.158737706	-6.598816938	4.14452E-11	1.02429E-10	negative
PRJNA729271	stage_3W_vs_E10	ordinary_muscle_development	ENSGALG00010019259	L2HGDH	0.28428038	0.108515068	2.619731858	0.008799893	0.01204139	positive
PRJNA729271	stage_3W_vs_E10	ordinary_muscle_development	ENSGALG00010019260	TRAIL-LIKE	-0.420146413	0.176441759	-2.381218687	0.017255465	0.022845996	negative
PRJNA729271	stage_3W_vs_E10	ordinary_muscle_development	ENSGALG00010019262	TMC3	-5.235237461	0.616453267	-8.492513128	2.02216E-17	6.91791E-17	negative
PRJNA729271	stage_3W_vs_E10	ordinary_muscle_development	ENSGALG00010019263	SNRPB2	-0.73443925	0.102494193	-7.16566693	7.74088E-13	2.10081E-12	negative
PRJNA729271	stage_3W_vs_E10	ordinary_muscle_development	ENSGALG00010019264	HK2	0.827546721	0.214558951	3.856966667	0.000114803	0.000186659	positive
PRJNA729271	stage_3W_vs_E10	ordinary_muscle_development	ENSGALG00010019265	RUFY4	4.946423784	0.659476372	7.500532235	6.35592E-14	1.82326E-13	positive
PRJNA729271	stage_3W_vs_E10	ordinary_muscle_development	ENSGALG00010019266	ATP5S	0.013422358	0.157178257	0.085395771	0.931946755	0.939922642	positive
PRJNA729271	stage_3W_vs_E10	ordinary_muscle_development	ENSGALG00010019267	STARD5	0.081261749	0.18352275	0.442788421	0.657918779	0.690752767	positive
PRJNA729271	stage_3W_vs_E10	ordinary_muscle_development	ENSGALG00010019268	NSL1	-1.312249656	0.095192722	-13.78518894	3.12946E-43	2.80876E-42	negative
PRJNA729271	stage_3W_vs_E10	ordinary_muscle_development	ENSGALG00010019269	SOCS1	0.283870036	0.255190754	1.112383703	0.265973209	0.302351318	positive
PRJNA729271	stage_3W_vs_E10	ordinary_muscle_development	ENSGALG00010019270	ENSGALG00010019270	0.049789883	0.19673763	0.25307758	0.800208263	0.822683792	positive
PRJNA729271	stage_3W_vs_E10	ordinary_muscle_development	ENSGALG00010019271	CDKL1	-0.376382111	0.19202298	-1.960088895	0.049985401	0.062730596	negative
PRJNA729271	stage_3W_vs_E10	ordinary_muscle_development	ENSGALG00010019274	ENSGALG00010019274	-3.701309397	0.985613764	-3.755334524	0.00017311	0.000277244	negative
PRJNA729271	stage_3W_vs_E10	ordinary_muscle_development	ENSGALG00010019275	CTAGE1	2.728971848	0.57762254	4.724489888	2.30694E-06	4.26573E-06	positive
PRJNA729271	stage_3W_vs_E10	ordinary_muscle_development	ENSGALG00010019277	IGFBP5	-0.317738985	0.132976724	-2.389433096	0.016874397	0.022365982	negative
PRJNA729271	stage_3W_vs_E10	ordinary_muscle_development	ENSGALG00010019278	JPT2	-2.13078471	0.07392632	-28.82308651	1.102E-182	1.6135E-180	negative
PRJNA729271	stage_3W_vs_E10	ordinary_muscle_development	ENSGALG00010019280	TLNRD1	-0.292205346	0.116902321	-2.499568379	0.01243447	0.016733603	negative
PRJNA729271	stage_3W_vs_E10	ordinary_muscle_development	ENSGALG00010019281	ENSGALG00010019281	0.792914108	1.124302206	0.70524998	0.480654683	0.519164108	positive
PRJNA729271	stage_3W_vs_E10	ordinary_muscle_development	ENSGALG00010019282	IGFBP2	-3.205485586	0.159377797	-20.11249782	5.73613E-90	1.89397E-88	negative
PRJNA729271	stage_3W_vs_E10	ordinary_muscle_development	ENSGALG00010019283	NT5C3A	2.770699408	0.255400257	10.84845976	2.02814E-27	1.05596E-26	positive
PRJNA729271	stage_3W_vs_E10	ordinary_muscle_development	ENSGALG00010019284	CHD5	1.150279468	0.131699834	8.734099622	2.45605E-18	8.79673E-18	positive
PRJNA729271	stage_3W_vs_E10	ordinary_muscle_development	ENSGALG00010019285	BATF3	-0.55949739	0.274683959	-2.036876828	0.041662387	0.052797368	negative
PRJNA729271	stage_3W_vs_E10	ordinary_muscle_development	ENSGALG00010019287	AHSA2	-0.630400116	0.097500677	-6.465597307	1.00899E-10	2.4331E-10	negative
PRJNA729271	stage_3W_vs_E10	ordinary_muscle_development	ENSGALG00010019288	RPL37A	0.636991785	0.15670215	4.06498435	4.80357E-05	8.05038E-05	positive
PRJNA729271	stage_3W_vs_E10	ordinary_muscle_development	ENSGALG00010019289	FKBP9	-2.061178571	0.166728121	-12.36251307	4.16916E-35	2.88045E-34	negative
PRJNA729271	stage_3W_vs_E10	ordinary_muscle_development	ENSGALG00010019291	ADD2	-0.996059073	0.277037561	-3.595393602	0.000323901	0.000507233	negative
PRJNA729271	stage_3W_vs_E10	ordinary_muscle_development	ENSGALG00010019292	ENSGALG00010019292	-0.648757235	0.096744334	-6.705893832	2.00177E-11	5.03657E-11	negative
PRJNA729271	stage_3W_vs_E10	ordinary_muscle_development	ENSGALG00010019294	SMARCAL1	-0.316563544	0.097333986	-3.25234335	0.001144577	0.001707207	negative
PRJNA729271	stage_3W_vs_E10	ordinary_muscle_development	ENSGALG00010019295	MAP4K5	0.267608645	0.130055333	2.057652214	0.039623531	0.050376553	positive
PRJNA729271	stage_3W_vs_E10	ordinary_muscle_development	ENSGALG00010019296	ENSGALG00010019296	0.390127766	0.101182272	3.855692893	0.000115402	0.000187592	positive
PRJNA729271	stage_3W_vs_E10	ordinary_muscle_development	ENSGALG00010019297	SLC25A26	1.535884997	0.145389037	10.56396705	4.37761E-26	2.14733E-25	positive
PRJNA729271	stage_3W_vs_E10	ordinary_muscle_development	ENSGALG00010019298	PGAP6	0.265058424	0.093484082	2.835332153	0.004577806	0.006445825	positive
PRJNA729271	stage_3W_vs_E10	ordinary_muscle_development	ENSGALG00010019300	MESDC2	-0.366310133	0.066169928	-5.53590042	3.09633E-08	6.46706E-08	negative
PRJNA729271	stage_3W_vs_E10	ordinary_muscle_development	ENSGALG00010019302	USP34	-0.138775573	0.094653283	-1.466146426	0.1426084	0.168895233	negative
PRJNA729271	stage_3W_vs_E10	ordinary_muscle_development	ENSGALG00010019305	SNRNP27	0.546127205	0.118114481	4.623710829	3.76935E-06	6.88294E-06	positive
PRJNA729271	stage_3W_vs_E10	ordinary_muscle_development	ENSGALG00010019307	ATF3	3.012467654	0.486244388	6.195377733	5.81454E-10	1.34528E-09	positive
PRJNA729271	stage_3W_vs_E10	ordinary_muscle_development	ENSGALG00010019310	CRELD1	1.154187488	0.096649213	11.9420268	7.14603E-33	4.57113E-32	positive
PRJNA729271	stage_3W_vs_E10	ordinary_muscle_development	ENSGALG00010019312	KBTBD2	0.317680236	0.072036736	4.409975425	1.03382E-05	1.82369E-05	positive
PRJNA729271	stage_3W_vs_E10	ordinary_muscle_development	ENSGALG00010019313	MRPL28	-0.023474835	0.099448857	-0.236049316	0.813394403	0.835292374	negative
PRJNA729271	stage_3W_vs_E10	ordinary_muscle_development	ENSGALG00010019314	GKN2	-4.317679086	1.307013348	-3.303469772	0.000954963	0.001432642	negative
PRJNA729271	stage_3W_vs_E10	ordinary_muscle_development	ENSGALG00010019316	CLEC16A	0.295645716	0.08775091	3.369147011	0.000754012	0.001141691	positive
PRJNA729271	stage_3W_vs_E10	ordinary_muscle_development	ENSGALG00010019318	NOG2	0.490827256	0.276836721	1.772984648	0.076231251	0.093546561	positive
PRJNA729271	stage_3W_vs_E10	ordinary_muscle_development	ENSGALG00010019319	ACTR3	-0.973284071	0.066839074	-14.56160322	4.92882E-48	5.26867E-47	negative
PRJNA729271	stage_3W_vs_E10	ordinary_muscle_development	ENSGALG00010019322	EGR3	1.198160545	0.314612821	3.808365277	0.000139889	0.000225926	positive
PRJNA729271	stage_3W_vs_E10	ordinary_muscle_development	ENSGALG00010019323	LRIG1	-0.694154246	0.119939058	-5.787557937	7.14171E-09	1.55481E-08	negative
PRJNA729271	stage_3W_vs_E10	ordinary_muscle_development	ENSGALG00010019325	SLITRK4	-2.536564048	0.210130164	-12.07139425	1.49578E-33	9.79285E-33	negative
PRJNA729271	stage_3W_vs_E10	ordinary_muscle_development	ENSGALG00010019331	CEMIP	-1.010517099	0.320890559	-3.149101992	0.00163773	0.002409307	negative
PRJNA729271	stage_3W_vs_E10	ordinary_muscle_development	ENSGALG00010019334	ATL1	-1.355638042	0.131310366	-10.32392252	5.49321E-25	2.56603E-24	negative
PRJNA729271	stage_3W_vs_E10	ordinary_muscle_development	ENSGALG00010019335	HIPK3	1.300688373	0.115175157	11.29313305	1.41887E-29	8.03067E-29	positive
PRJNA729271	stage_3W_vs_E10	ordinary_muscle_development	ENSGALG00010019336	DEXI	1.399657196	0.23353355	5.993388097	2.05514E-09	4.59214E-09	positive
PRJNA729271	stage_3W_vs_E10	ordinary_muscle_development	ENSGALG00010019337	XPO1	-0.77858856	0.084204001	-9.246455633	2.3206E-20	9.00593E-20	negative
PRJNA729271	stage_3W_vs_E10	ordinary_muscle_development	ENSGALG00010019344	DNAJC24	-0.243095855	0.085206686	-2.853013834	0.004330674	0.00611228	negative
PRJNA729271	stage_3W_vs_E10	ordinary_muscle_development	ENSGALG00010019345	ENSGALG00010019345	0.280230206	0.185732742	1.50878194	0.131354518	0.156346176	positive
PRJNA729271	stage_3W_vs_E10	ordinary_muscle_development	ENSGALG00010019346	POU3F4	-0.863665814	0.5350073	-1.614306596	0.10646094	0.128275066	negative
PRJNA729271	stage_3W_vs_E10	ordinary_muscle_development	ENSGALG00010019350	SAV1	0.231919646	0.088168539	2.630412727	0.008528126	0.011687169	positive
PRJNA729271	stage_3W_vs_E10	ordinary_muscle_development	ENSGALG00010019355	ENTPD4	0.769454825	0.096622798	7.96349147	1.67252E-15	5.17352E-15	positive
PRJNA729271	stage_3W_vs_E10	ordinary_muscle_development	ENSGALG00010019356	AVL9	-0.109250728	0.095312573	-1.146236261	0.251697408	0.286910501	negative
PRJNA729271	stage_3W_vs_E10	ordinary_muscle_development	ENSGALG00010019365	ENSGALG00010019365	0.263642471	0.17676316	1.491501232	0.135829948	0.161275811	positive
PRJNA729271	stage_3W_vs_E10	ordinary_muscle_development	ENSGALG00010019371	TMEM87A	1.17795641	0.076472494	15.40366148	1.54658E-53	1.96647E-52	positive
PRJNA729271	stage_3W_vs_E10	ordinary_muscle_development	ENSGALG00010019373	DENND5A	0.144393873	0.061406863	2.351428934	0.018701462	0.024665849	positive
PRJNA729271	stage_3W_vs_E10	ordinary_muscle_development	ENSGALG00010019376	ENSGALG00010019376	4.833078955	0.31473716	15.35592097	3.23286E-53	4.07481E-52	positive
PRJNA729271	stage_3W_vs_E10	ordinary_muscle_development	ENSGALG00010019388	GPR75	-0.390019067	0.136008947	-2.867598601	0.004135999	0.005847197	negative
PRJNA729271	stage_3W_vs_E10	ordinary_muscle_development	ENSGALG00010019390	TVP23A	-0.599872729	0.218720525	-2.742644885	0.006094656	0.00846837	negative
PRJNA729271	stage_3W_vs_E10	ordinary_muscle_development	ENSGALG00010019391	GANC	3.617783113	0.130740025	27.67158039	1.5351E-168	1.8699E-166	positive
PRJNA729271	stage_3W_vs_E10	ordinary_muscle_development	ENSGALG00010019392	NIN	-2.123402313	0.112252243	-18.91634633	8.36566E-80	2.07282E-78	negative
PRJNA729271	stage_3W_vs_E10	ordinary_muscle_development	ENSGALG00010019398	SLC35F5	1.131359951	0.120032701	9.425431074	4.28342E-21	1.71278E-20	positive
PRJNA729271	stage_3W_vs_E10	ordinary_muscle_development	ENSGALG00010019399	CD93	0.37738128	0.152008099	2.482639299	0.013041307	0.017501504	positive
PRJNA729271	stage_3W_vs_E10	ordinary_muscle_development	ENSGALG00010019403	PACC1	-0.253245501	0.105421926	-2.402209012	0.016296394	0.021645398	negative
PRJNA729271	stage_3W_vs_E10	ordinary_muscle_development	ENSGALG00010019411	LSM5	-0.431489611	0.156966852	-2.748921855	0.005979164	0.008314273	negative
PRJNA729271	stage_3W_vs_E10	ordinary_muscle_development	ENSGALG00010019416	GPR39	-0.454633982	0.534268839	-0.850946095	0.394799298	0.43369578	negative
PRJNA729271	stage_3W_vs_E10	ordinary_muscle_development	ENSGALG00010019418	ZNF703	-0.504579796	0.239554035	-2.106329777	0.035175716	0.045021811	negative
PRJNA729271	stage_3W_vs_E10	ordinary_muscle_development	ENSGALG00010019422	IMMP1L	1.256333959	0.138090319	9.097914794	9.20827E-20	3.48769E-19	positive
PRJNA729271	stage_3W_vs_E10	ordinary_muscle_development	ENSGALG00010019423	LYPD1	-1.492767107	0.182899035	-8.161700292	3.30341E-16	1.06194E-15	negative

PRJNA729271	stage_3W_vs_E10	ordinary_muscle_development	ENSGALG00010019426	GRM7	-3.118548069	0.271503846	-11.48620217	1.54767E-30	9.07139E-30	negative
PRJNA729271	stage_3W_vs_E10	ordinary_muscle_development	ENSGALG00010019430	ENSGALG00010019430	0.612188369	0.402476374	1.521054177	0.128246242	0.152834768	positive
PRJNA729271	stage_3W_vs_E10	ordinary_muscle_development	ENSGALG00010019441	LMCD1	1.925055435	0.138208457	13.92863705	4.24377E-44	3.91805E-43	positive
PRJNA729271	stage_3W_vs_E10	ordinary_muscle_development	ENSGALG00010019443	NCKAP5	-0.59322338	0.134025197	-4.426207846	9.59041E-06	1.69549E-05	negative
PRJNA729271	stage_3W_vs_E10	ordinary_muscle_development	ENSGALG00010019451	CAV3	4.141066956	0.142798236	28.99942665	6.69E-185	1.0208E-182	positive
PRJNA729271	stage_3W_vs_E10	ordinary_muscle_development	ENSGALG00010019458	ELP4	0.111231621	0.100203699	1.110055037	0.266975311	0.303395306	positive
PRJNA729271	stage_3W_vs_E10	ordinary_muscle_development	ENSGALG00010019461	MGAT5	0.299033342	0.070187201	4.260511	2.0396E-05	3.52078E-05	positive
PRJNA729271	stage_3W_vs_E10	ordinary_muscle_development	ENSGALG00010019470	TGFB2	-1.495794559	0.200593539	-7.456843139	8.86201E-14	2.51971E-13	negative
PRJNA729271	stage_3W_vs_E10	ordinary_muscle_development	ENSGALG00010019471	NUBP1	0.502068572	0.092239189	5.443115621	5.23566E-08	1.078E-07	positive
PRJNA729271	stage_3W_vs_E10	ordinary_muscle_development	ENSGALG00010019472	CAPN3	8.110127469	0.2555924	31.73070664	5.8613E-221	1.2313E-218	positive
PRJNA729271	stage_3W_vs_E10	ordinary_muscle_development	ENSGALG00010019473	OXTR	0.396884456	0.446450289	0.888977936	0.374014938	0.41277193	positive
PRJNA729271	stage_3W_vs_E10	ordinary_muscle_development	ENSGALG00010019474	TMEM163	-1.103390858	0.555223524	-1.987291262	0.046890129	0.05907113	negative
PRJNA729271	stage_3W_vs_E10	ordinary_muscle_development	ENSGALG00010019477	NCAPH	-3.065605892	0.18283985	-16.76661785	4.28198E-63	6.9817E-62	negative
PRJNA729271	stage_3W_vs_E10	ordinary_muscle_development	ENSGALG00010019481	PYGL	0.173555408	0.09831196	1.765353965	0.077504295	0.095028317	positive
PRJNA729271	stage_3W_vs_E10	ordinary_muscle_development	ENSGALG00010019483	SCUBE2	-2.816578417	0.263899367	-10.67292602	1.36266E-26	6.84639E-26	negative
PRJNA729271	stage_3W_vs_E10	ordinary_muscle_development	ENSGALG00010019488	TEKT5	-2.854840784	1.264696627	-2.257332488	0.023987307	0.031273252	negative
PRJNA729271	stage_3W_vs_E10	ordinary_muscle_development	ENSGALG00010019495	EMP2	0.462397308	0.196814855	2.349402479	0.018803569	0.024796009	positive
PRJNA729271	stage_3W_vs_E10	ordinary_muscle_development	ENSGALG00010019498	ENSGALG00010019498	-1.084987278	0.296817865	-3.655397489	0.000256784	0.000405102	negative
PRJNA729271	stage_3W_vs_E10	ordinary_muscle_development	ENSGALG00010019509	ZNF106	1.133668826	0.222180252	5.102473399	3.35243E-07	6.5481E-07	positive
PRJNA729271	stage_3W_vs_E10	ordinary_muscle_development	ENSGALG00010019515	ENSGALG00010019515	-0.334905986	0.109245412	-3.065629757	0.002172121	0.003151641	negative
PRJNA729271	stage_3W_vs_E10	ordinary_muscle_development	ENSGALG00010019520	CD6	4.197566777	0.989473554	4.242222302	2.21317E-05	3.81116E-05	positive
PRJNA729271	stage_3W_vs_E10	ordinary_muscle_development	ENSGALG00010019524	HHIPL2	1.353648452	0.35867024	3.774075186	0.000160602	0.000257914	positive
PRJNA729271	stage_3W_vs_E10	ordinary_muscle_development	ENSGALG00010019529	SNAP23	0.0036806	0.067334547	0.054661388	0.956408231	0.962049779	positive
PRJNA729271	stage_3W_vs_E10	ordinary_muscle_development	ENSGALG00010019534	PPP2R5A	0.956090538	0.1044169	9.156473088	5.36212E-20	2.0486E-19	positive
PRJNA729271	stage_3W_vs_E10	ordinary_muscle_development	ENSGALG00010019540	UNC5D	-1.591066449	0.38811011	-4.099523327	4.14002E-05	6.97056E-05	negative
PRJNA729271	stage_3W_vs_E10	ordinary_muscle_development	ENSGALG00010019546	HSD17B12	-0.871582503	0.077796102	-11.20342123	3.92285E-29	2.18531E-28	negative
PRJNA729271	stage_3W_vs_E10	ordinary_muscle_development	ENSGALG00010019557	TRIM9	-1.537671981	0.353040025	-4.355517423	1.32753E-05	2.32314E-05	negative
PRJNA729271	stage_3W_vs_E10	ordinary_muscle_development	ENSGALG00010019572	NFKBIE	0.310515211	0.183941598	1.688118484	0.091388487	0.111130547	positive
PRJNA729271	stage_3W_vs_E10	ordinary_muscle_development	ENSGALG00010019577	MXD1	-0.186230342	0.18958434	-0.982308675	0.325947802	0.364103991	negative
PRJNA729271	stage_3W_vs_E10	ordinary_muscle_development	ENSGALG00010019580	CD5	3.390174603	0.441903583	7.671751794	1.69663E-14	5.00665E-14	positive
PRJNA729271	stage_3W_vs_E10	ordinary_muscle_development	ENSGALG00010019581	SLC35B2	-0.292071142	0.133729903	-2.18403764	0.028959482	0.037409116	negative
PRJNA729271	stage_3W_vs_E10	ordinary_muscle_development	ENSGALG00010019584	RAD18	-1.278282992	0.117942927	-10.83814875	2.27018E-27	1.17859E-26	negative
PRJNA729271	stage_3W_vs_E10	ordinary_muscle_development	ENSGALG00010019585	PDE1C	-0.801119516	0.211518718	-3.787463936	0.000152193	0.000244924	negative
PRJNA729271	stage_3W_vs_E10	ordinary_muscle_development	ENSGALG00010019586	GLYR1	-0.695328487	0.090653032	-7.670217667	1.71705E-14	5.06587E-14	negative
PRJNA729271	stage_3W_vs_E10	ordinary_muscle_development	ENSGALG00010019587	CCNT2	-0.096657137	0.127897004	-0.755741998	0.449803899	0.488602182	negative
PRJNA729271	stage_3W_vs_E10	ordinary_muscle_development	ENSGALG00010019588	CHMP7	1.019856357	0.09254572	11.02002723	3.05966E-28	1.64747E-27	positive
PRJNA729271	stage_3W_vs_E10	ordinary_muscle_development	ENSGALG00010019591	ENSGALG00010019591	-1.971976509	0.227301053	-8.675615385	4.1132E-18	1.46094E-17	negative
PRJNA729271	stage_3W_vs_E10	ordinary_muscle_development	ENSGALG00010019592	ALKBH3	0.214041135	0.132263164	1.6182974	0.105598521	0.127278443	positive
PRJNA729271	stage_3W_vs_E10	ordinary_muscle_development	ENSGALG00010019594	TMX1	-0.130479685	0.325268214	-0.401144899	0.688313446	0.719070515	negative
PRJNA729271	stage_3W_vs_E10	ordinary_muscle_development	ENSGALG00010019595	GRIN2A	-2.617334065	0.234882406	-11.14316781	7.73193E-29	4.24685E-28	negative
PRJNA729271	stage_3W_vs_E10	ordinary_muscle_development	ENSGALG00010019598	KCNAB2	-0.918793833	0.135428257	-6.784358421	1.16604E-11	2.97251E-11	negative
PRJNA729271	stage_3W_vs_E10	ordinary_muscle_development	ENSGALG00010019600	MAP3K19	-1.859957544	0.260642986	-7.136035279	9.60614E-13	2.59487E-12	negative
PRJNA729271	stage_3W_vs_E10	ordinary_muscle_development	ENSGALG00010019602	SLC29A1	1.598754011	0.19378449	8.250164981	1.58176E-16	5.17904E-16	positive
PRJNA729271	stage_3W_vs_E10	ordinary_muscle_development	ENSGALG00010019610	CORO7	0.228600554	0.100986975	2.263663732	0.023594805	0.030794768	positive
PRJNA729271	stage_3W_vs_E10	ordinary_muscle_development	ENSGALG00010019613	FRMD6	-0.718787553	0.124919147	-5.754022286	8.71447E-09	1.88672E-08	negative
PRJNA729271	stage_3W_vs_E10	ordinary_muscle_development	ENSGALG00010019614	MICALL2	0.361517681	0.157135322	2.300677382	0.021409873	0.028092343	positive
PRJNA729271	stage_3W_vs_E10	ordinary_muscle_development	ENSGALG00010019617	ANO3	-1.337123569	0.145076788	-9.216660966	3.06496E-20	1.18376E-19	negative
PRJNA729271	stage_3W_vs_E10	ordinary_muscle_development	ENSGALG00010019618	NPHP4	-0.410554189	0.105606048	-3.887601101	0.00010124	0.000165163	negative
PRJNA729271	stage_3W_vs_E10	ordinary_muscle_development	ENSGALG00010019621	ENSGALG00010019621	0.326055078	0.151188478	2.156613267	0.03103581	0.039941762	positive
PRJNA729271	stage_3W_vs_E10	ordinary_muscle_development	ENSGALG00010019622	C2orf42	1.079759525	0.141024425	7.656542634	1.91005E-14	5.62157E-14	positive
PRJNA729271	stage_3W_vs_E10	ordinary_muscle_development	ENSGALG00010019624	SRGAP3	0.149192479	0.130034949	1.147326009	0.251246908	0.286509632	positive
PRJNA729271	stage_3W_vs_E10	ordinary_muscle_development	ENSGALG00010019625	INTS1	-0.475044149	0.076732747	-6.190891967	5.98247E-10	1.38303E-09	negative
PRJNA729271	stage_3W_vs_E10	ordinary_muscle_development	ENSGALG00010019630	AJAP1	-0.850171427	0.375274785	-2.265463763	0.023484235	0.030667026	negative
PRJNA729271	stage_3W_vs_E10	ordinary_muscle_development	ENSGALG00010019634	GNG2	-2.541327187	0.296983809	-8.557123698	1.15718E-17	4.01661E-17	negative
PRJNA729271	stage_3W_vs_E10	ordinary_muscle_development	ENSGALG00010019635	LRRC57	1.077058199	0.075756279	14.21741167	7.1447E-46	7.04506E-45	positive
PRJNA729271	stage_3W_vs_E10	ordinary_muscle_development	ENSGALG00010019640	MGRN1	-0.505873518	0.117272897	-4.313643918	1.60586E-05	2.79267E-05	negative
PRJNA729271	stage_3W_vs_E10	ordinary_muscle_development	ENSGALG00010019641	CAPN11	0.8885388	0.081722232	10.87266923	1.55581E-27	8.13253E-27	positive
PRJNA729271	stage_3W_vs_E10	ordinary_muscle_development	ENSGALG00010019643	RAB3GAP1	-0.35517513	0.070846048	-5.013337219	5.3494E-07	1.03002E-06	negative
PRJNA729271	stage_3W_vs_E10	ordinary_muscle_development	ENSGALG00010019645	ENSGALG00010019645	3.131422326	0.161396772	19.40201331	7.42075E-84	2.07652E-82	positive
PRJNA729271	stage_3W_vs_E10	ordinary_muscle_development	ENSGALG00010019646	VPS37C	1.394275655	0.125310541	11.12656317	9.31584E-29	5.10137E-28	positive
PRJNA729271	stage_3W_vs_E10	ordinary_muscle_development	ENSGALG00010019648	PRDM16	-2.348611666	0.204915674	-11.46135686	2.06258E-30	1.20407E-29	negative
PRJNA729271	stage_3W_vs_E10	ordinary_muscle_development	ENSGALG00010019649	TMEM63B	-0.271886769	0.076904182	-3.535396412	0.000407164	0.000631753	negative
PRJNA729271	stage_3W_vs_E10	ordinary_muscle_development	ENSGALG00010019650	KBTBD8	0.839924472	0.086651759	9.69310352	3.22575E-22	1.34786E-21	positive
PRJNA729271	stage_3W_vs_E10	ordinary_muscle_development	ENSGALG00010019652	TNFRSF10B	1.264678356	0.316273142	3.998690331	6.36939E-05	0.000105804	positive
PRJNA729271	stage_3W_vs_E10	ordinary_muscle_development	ENSGALG00010019655	RTRAF	0.817207141	0.098222881	8.319926416	8.80179E-17	2.91883E-16	positive
PRJNA729271	stage_3W_vs_E10	ordinary_muscle_development	ENSGALG00010019657	PPP1R17	-2.250771174	0.274457763	-8.200792524	2.38807E-16	7.74388E-16	negative
PRJNA729271	stage_3W_vs_E10	ordinary_muscle_development	ENSGALG00010019659	MRPL14	1.463391533	0.124602906	11.74444146	7.54191E-32	4.64599E-31	positive
PRJNA729271	stage_3W_vs_E10	ordinary_muscle_development	ENSGALG00010019660	ENSGALG00010019660	0.058082914	0.544588666	0.10665465	0.915062961	0.92508283	positive
PRJNA729271	stage_3W_vs_E10	ordinary_muscle_development	ENSGALG00010019667	ITPRID1	1.214686216	0.599908475	2.024785893	0.042889354	0.054290559	positive
PRJNA729271	stage_3W_vs_E10	ordinary_muscle_development	ENSGALG00010019670	ENSGALG00010019670	-0.934761226	0.122421823	-7.635576726	2.24812E-14	6.58978E-14	negative
PRJNA729271	stage_3W_vs_E10	ordinary_muscle_development	ENSGALG00010019673	NID2	-0.587222197	0.133011208	-4.414832457	1.01088E-05	1.78409E-05	negative
PRJNA729271	stage_3W_vs_E10	ordinary_muscle_development	ENSGALG00010019675	MAFK	1.912101228	0.136980473	13.95893292	2.77558E-44	2.58395E-43	positive
PRJNA729271	stage_3W_vs_E10	ordinary_muscle_development	ENSGALG00010019679	RHOBTB2	-2.208018113	0.177789904	-12.4192548	2.05466E-35	1.43253E-34	negative
PRJNA729271	stage_3W_vs_E10	ordinary_muscle_development	ENSGALG00010019680	CCDC34	-0.047083016	0.200446885	-0.234890237	0.814293927	0.835920005	negative
PRJNA729271	stage_3W_vs_E10	ordinary_muscle_development	ENSGALG00010019682	TMEM158	-1.665542365	0.37233956	-4.473181323	7.70644E-06	1.37229E-05	negative
PRJNA729271	stage_3W_vs_E10	ordinary_muscle_development	ENSGALG00010019683	TMEM184A	0.737174769	0.284331214	2.592662128	0.009523628	0.012975373	positive
PRJNA729271	stage_3W_vs_E10	ordinary_muscle_development	ENSGALG00010019684	PIGQ	-0.271293948	0.129766829	-2.090626317	0.036561575	0.046692513	negative
PRJNA729271	stage_3W_vs_E10	ordinary_muscle_development	ENSGALG00010019690	ENSGALG00010019690	-0.374899735	0.091220115	-4.109836255	3.9594E-05	6.68197E-05	negative
PRJNA729271	stage_3W_vs_E10	ordinary_muscle_development	ENSGALG00010019692	MMEL1	-0.918217334	0.304533475	-3.015160601	0.002568432	0.003704789	negative
PRJNA729271	stage_3W_vs_E10	ordinary_muscle_development	ENSGALG00010019693	HAUS2	-0.155890588	0.117939867	-1.321780258	0.186241334	0.216578714	negative
PRJNA729271	stage_3W_vs_E10	ordinary_muscle_development	ENSGALG00010019695	PGA5	10.6448579	2.385118113	4.463031764	8.08081E-06	1.43719E-05	positive
PRJNA729271	stage_3W_vs_E10	ordinary_muscle_development	ENSGALG00010019697	EIF4EBP1	2.794117836	0.145168538	19.2474063	1.48409E-82	3.96168E-81	positive
PRJNA729271	stage_3W_vs_E10	ordinary_muscle_development	ENSGALG00010019700	HES5B	-2.91498274	0.268986141	-10.83692538	2.30074E-27	1.19232E-26	negative
PRJNA729271	stage_3W_vs_E10	ordinary_muscle_development	ENSGALG00010019706	NPRL3	0.403795506	0.098811059	4.086541623	4.37851E-05	7.36099E-05	positive
PRJNA729271	stage_3W_vs_E10	ordinary_muscle_development	ENSGALG00010019713	NUDT14	0.117857898	0.114539069	1.028975517	0.30349118	0.34117455	positive
PRJNA729271	stage_3W_vs_E10	ordinary_muscle_development	ENSGALG00010019716	VEGFA	0.322576995	0.130723932	2.467620048	0.013601461	0.01822118	positive
PRJNA729271	stage_3W_vs_E10	ordinary_muscle_development	ENSGALG00010019717	ZRANB3	-0.969063362	0.14274034	-6.788994362	1.12918E-11	2.88159E-11	negative
PRJNA729271	stage_3W_vs_E10	ordinary_muscle_development	ENSGALG00010019724	HES5A	-5.269065594	0.371131441	-14.1973032	9.52063E-46	9.34336E-45	negative
PRJNA729271	stage_3W_vs_E10	ordinary_muscle_development	ENSGALG00010019726	PEBP4	4.694094087	0.223988917	20.95681406	1.62614E-97	6.30237E-96	positive
PRJNA729271	stage_3W_vs_E10	ordinary_muscle_development	ENSGALG00010019731	PANK4	1.241552061	0.133442024	9.304055968	1.35188E-20	5.29179E-20	positive
PRJNA729271	stage_3W_vs_E10	ordinary_muscle_development	ENSGALG00010019732	HBA1	-2.983537247	0.403714291	-7.390219547	1.46587E-13	4.12177E-13	negative
PRJNA729271	stage_3W_vs_E10	ordinary_muscle_development	ENSGALG00010019735	ENSGALG00010019735	-0.32223385	0.17150148	-1.878898359	0.060258373	0.074915955	negative
PRJNA729271	stage_3W_vs_E10	ordinary_muscle_development	ENSGALG00010019738	ADRA2A	-2.022518273	0.35800378	-5.649432723	1.60978E-08	3.43093E-08	negative
PRJNA729271	stage_3W_vs_E10	ordinary_muscle_development	ENSGALG00010019743	KCNU1	3.860461273	0.430745244	8.962284155	3.18021E-19	1.18046E-18	positive
PRJNA729271	stage_3W_vs_E10	ordinary_muscle_development	ENSGALG00010019744	KBP	-0.452716728	0.463876399	-0.975942576	0.329092922	0.367305914	negative
PRJNA729271	stage_3W_vs_E10	ordinary_muscle_development	ENSGALG00010019752	GPAM	-0.173907422	0.167627918	-1.037460967	0.299521078	0.337025153	negative
PRJNA729271	stage_3W_vs_E10	ordinary_muscle_development	ENSGALG00010019761	MRPS18A	0.878067681	0.086619543	10.13706208	3.78306E-24	1.7088E-23	positive
PRJNA729271	stage_3W_vs_E10	ordinary_muscle_development	ENSGALG00010019767	SUCLG2	-0.935722727	0.07848891	-11.92171899	9.1207E-33	5.81375E-32	negative
PRJNA729271	stage_3W_vs_E10	ordinary_muscle_development	ENSGALG00010019771	VWCE	-3.869838098	0.459895362	-8.414605611	3.94219E-17	1.32938E-16	negative
PRJNA729271	stage_3W_vs_E10	ordinary_muscle_development	ENSGALG00010019772	ENSGALG00010019772	-0.136982864	0.166082358	-0.824788774	0.409491521	0.448306616	negative
PRJNA729271	stage_3W_vs_E10	ordinary_muscle_development	ENSGALG00010019778	ABHD17C	0.108771029	0.132085702	0.823488292	0.410230373	0.449013762	positive
PRJNA729271	stage_3W_vs_E10	ordinary_muscle_development	ENSGALG00010019785	ACSL5	1.742269706	0.213297718	8.168252915	3.12887E-16	1.00762E-15	positive
PRJNA729271	stage_3W_vs_E10	ordinary_muscle_development	ENSGALG00010019786	R3HDM1	0.637693768	0.087188411	7.313973983	2.59355E-13	7.20045E-13	positive
PRJNA729271	stage_3W_vs_E10	ordinary_muscle_development	ENSGALG00010019789	ARNT2	-2.513257324	0.176843522	-14.21175792	7.74564E-46	7.63243E-45	negative
PRJNA729271	stage_3W_vs_E10	ordinary_muscle_development	ENSGALG00010019794	RSPH9	-0.42227831	0.471072583	-0.896418779	0.370029141	0.408715416	negative
PRJNA729271	stage_3W_vs_E10	ordinary_muscle_development	ENSGALG00010019799	DOK2	1.194348804	0.264660746	4.51275387	6.39913E-06	1.14754E-05	positive
PRJNA729271	stage_3W_vs_E10	ordinary_muscle_development	ENSGALG00010019800	EXT2	-0.501509571	0.060532186	-8.285006748	1.18102E-16	3.89155E-16	negative
PRJNA729271	stage_3W_vs_E10	ordinary_muscle_development	ENSGALG00010019801	FIBIN	-4.99627616	0.189220869	-26.40446678	1.2175E-153	1.2341E-151	negative
PRJNA729271	stage_3W_vs_E10	ordinary_muscle_development	ENSGALG00010019803	FAH	-1.715731699	0.501283385	-3.422678173	0.000620074	0.000946601	negative
PRJNA729271	stage_3W_vs_E10	ordinary_muscle_development	ENSGALG00010019804	ELFN1	-2.9559403	0.319700312	-9.245972524	2.33111E-20	9.04428E-20	negative
PRJNA729271	stage_3W_vs_E10	ordinary_muscle_development	ENSGALG00010019807	ZDHHC6	1.156338479	0.07783015	14.85720489	6.2478E-50	7.04214E-49	positive
PRJNA729271	stage_3W_vs_E10	ordinary_muscle_development	ENSGALG00010019810	ZFAND6	0.80173979	0.124599986	6.434509462	1.23873E-10	2.97274E-10	positive
PRJNA729271	stage_3W_vs_E10	ordinary_muscle_development	ENSGALG00010019811	UBXN4	1.049222078	0.110411782	9.502809001	2.04303E-21	8.27894E-21	positive
PRJNA729271	stage_3W_vs_E10	ordinary_muscle_development	ENSGALG00010019813	ENSGALG00010019813	4.013539867	0.216753544	18.5166055	1.51706E-76	3.46295E-75	positive
PRJNA729271	stage_3W_vs_E10	ordinary_muscle_development	ENSGALG00010019816	ENSGALG00010019816	4.689900915	0.687670394	6.819983757	9.10508E-12	2.33053E-11	positive
PRJNA729271	stage_3W_vs_E10	ordinary_muscle_development	ENSGALG00010019820	BBOX1	0.403157953	0.153940914	2.618913594	0.008821029	0.012069172	positive
PRJNA729271	stage_3W_vs_E10	ordinary_muscle_development	ENSGALG00010019823	LCT	-1.146096314	0.212957449	-5.381808983	7.3741E-08	1.50673E-07	negative
PRJNA729271	stage_3W_vs_E10	ordinary_muscle_development	ENSGALG00010019825	BCL2A1	3.400032997	0.274941022	12.36640851	3.97187E-35	2.74677E-34	positive
PRJNA729271	stage_3W_vs_E10	ordinary_muscle_development	ENSGALG00010019830	JAG2	-0.381494309	0.098528971	-3.871899851	0.00010799	0.000175958	negative
PRJNA729271	stage_3W_vs_E10	ordinary_muscle_development	ENSGALG00010019832	MAD2L1BP	-2.785781268	0.147159609	-18.93033886	6.41484E-80	1.59765E-78	negative
PRJNA729271	stage_3W_vs_E10	ordinary_muscle_development	ENSGALG00010019833	MTHFS	-0.754344979	0.131185096	-5.750233835	8.91201E-09	1.92892E-08	negative
PRJNA729271	stage_3W_vs_E10	ordinary_muscle_development	ENSGALG00010019834	PLCH2	-1.894098255	0.248190553	-7.63162913	2.31805E-14	6.78929E-14	negative
PRJNA729271	stage_3W_vs_E10	ordinary_muscle_development	ENSGALG00010019836	MINAR1	1.525908627	0.485123566	3.145401985	0.001658588	0.002437764	positive
PRJNA729271	stage_3W_vs_E10	ordinary_muscle_development	ENSGALG00010019838	ALX4	-4.029076745	0.222012197	-18.14799725	1.33168E-73	2.84702E-72	negative
PRJNA729271	stage_3W_vs_E10	ordinary_muscle_development	ENSGALG00010019843	MCM6	-2.666958441	0.112656089	-23.67345143	6.7698E-124	4.3039E-122	negative
PRJNA729271	stage_3W_vs_E10	ordinary_muscle_development	ENSGALG00010019846	ENSGALG00010019846	0.241063919	0.071895912	3.352957231	0.000799531	0.001207963	positive
PRJNA729271	stage_3W_vs_E10	ordinary_muscle_development	ENSGALG00010019848	MAD1L1	-0.939397988	0.127554504	-7.36467906	1.77574E-13	4.96993E-13	negative
PRJNA729271	stage_3W_vs_E10	ordinary_muscle_development	ENSGALG00010019854	CCDC14	-1.020376329	0.150800816	-6.766384671	1.3204E-11	3.35247E-11	negative
PRJNA729271	stage_3W_vs_E10	ordinary_muscle_development	ENSGALG00010019855	TTC7A	0.741238906	0.124810868	5.93889715	2.86946E-09	6.35878E-09	positive
PRJNA729271	stage_3W_vs_E10	ordinary_muscle_development	ENSGALG00010019859	BIN3	-0.630314415	0.137514355	-4.583626303	4.56981E-06	8.27785E-06	negative
PRJNA729271	stage_3W_vs_E10	ordinary_muscle_development	ENSGALG00010019865	ENSGALG00010019865	0.383035376	0.135094678	2.835310634	0.004578115	0.006445825	positive

PRJNA729271	stage_3W_vs_E10	ordinary_muscle_development	ENSGALG00010019867	PEX11A	-0.752178592	0.377670188	-1.99162819	0.046411868	0.058524578	negative
PRJNA729271	stage_3W_vs_E10	ordinary_muscle_development	ENSGALG00010019871	TAFA1	-3.533401798	0.640110991	-5.519983017	3.39032E-08	7.06074E-08	negative
PRJNA729271	stage_3W_vs_E10	ordinary_muscle_development	ENSGALG00010019872	VTI1A	-0.011049473	0.109083584	-0.101293635	0.919317369	0.928541932	negative
PRJNA729271	stage_3W_vs_E10	ordinary_muscle_development	ENSGALG00010019873	LGR4	-2.033230918	0.099417568	-20.4514248	5.83459E-93	2.06274E-91	negative
PRJNA729271	stage_3W_vs_E10	ordinary_muscle_development	ENSGALG00010019875	PLEKHH1	-1.935979816	0.271260352	-7.136980408	9.54034E-13	2.57758E-12	negative
PRJNA729271	stage_3W_vs_E10	ordinary_muscle_development	ENSGALG00010019876	MRM2	0.777353235	0.223327432	3.480778101	0.00049996	0.000770375	positive
PRJNA729271	stage_3W_vs_E10	ordinary_muscle_development	ENSGALG00010019878	MCFD2	0.275124158	0.149977609	1.83443489	0.066589465	0.082307008	positive
PRJNA729271	stage_3W_vs_E10	ordinary_muscle_development	ENSGALG00010019891	PIGH	-1.070066744	0.105897173	-10.10477158	5.2618E-24	2.35692E-23	negative
PRJNA729271	stage_3W_vs_E10	ordinary_muscle_development	ENSGALG00010019896	DDB1	-0.153572376	0.090991163	-1.687772431	0.091454922	0.111202004	negative
PRJNA729271	stage_3W_vs_E10	ordinary_muscle_development	ENSGALG00010019898	NUDT1	-1.643433019	0.189157183	-8.688187193	3.68269E-18	1.3106E-17	negative
PRJNA729271	stage_3W_vs_E10	ordinary_muscle_development	ENSGALG00010019899	ENSGALG00010019899	-0.047138991	0.449433442	-0.104885367	0.91646677	0.92626928	negative
PRJNA729271	stage_3W_vs_E10	ordinary_muscle_development	ENSGALG00010019905	ENSGALG00010019905	-0.396519046	0.124686277	-3.180133821	0.001472071	0.002171993	negative
PRJNA729271	stage_3W_vs_E10	ordinary_muscle_development	ENSGALG00010019906	ENSGALG00010019906	1.562940571	0.232587466	6.71979705	1.81978E-11	4.58663E-11	positive
PRJNA729271	stage_3W_vs_E10	ordinary_muscle_development	ENSGALG00010019910	ENSGALG00010019910	-0.396727121	0.256630506	-1.545907881	0.122126817	0.14581781	negative
PRJNA729271	stage_3W_vs_E10	ordinary_muscle_development	ENSGALG00010019912	ARG2	-5.484007354	0.230975585	-23.74280101	1.304E-124	8.4758E-123	negative
PRJNA729271	stage_3W_vs_E10	ordinary_muscle_development	ENSGALG00010019916	NOC3L	-0.02525936	0.113464276	-0.222619493	0.823831664	0.844873353	negative
PRJNA729271	stage_3W_vs_E10	ordinary_muscle_development	ENSGALG00010019919	LIN7C	0.154896195	0.107023412	1.447311306	0.147809754	0.174485087	positive
PRJNA729271	stage_3W_vs_E10	ordinary_muscle_development	ENSGALG00010019921	CALM2	0.549725817	0.112633413	4.880663762	1.05729E-06	1.99875E-06	positive
PRJNA729271	stage_3W_vs_E10	ordinary_muscle_development	ENSGALG00010019925	RDH11	-1.602750462	0.1439267	-11.13588	8.39121E-29	4.60199E-28	negative
PRJNA729271	stage_3W_vs_E10	ordinary_muscle_development	ENSGALG00010019926	KIF7	-1.164172248	0.102087468	-11.40367441	4.00837E-30	2.31111E-29	negative
PRJNA729271	stage_3W_vs_E10	ordinary_muscle_development	ENSGALG00010019931	ZFYVE26	1.049554463	0.134004461	7.832235263	4.79272E-15	1.45488E-14	positive
PRJNA729271	stage_3W_vs_E10	ordinary_muscle_development	ENSGALG00010019932	KALRN	-0.068165938	0.116635123	-0.584437479	0.558926017	0.595927372	negative
PRJNA729271	stage_3W_vs_E10	ordinary_muscle_development	ENSGALG00010019934	TKFC	0.148433551	0.151268771	0.981257067	0.326465989	0.364654736	positive
PRJNA729271	stage_3W_vs_E10	ordinary_muscle_development	ENSGALG00010019935	ENSGALG00010019935	3.343791574	1.264514173	2.644329059	0.008185304	0.011232224	positive
PRJNA729271	stage_3W_vs_E10	ordinary_muscle_development	ENSGALG00010019938	TAFA4	-4.51689546	0.509422623	-8.866695861	7.53483E-19	2.75107E-18	negative
PRJNA729271	stage_3W_vs_E10	ordinary_muscle_development	ENSGALG00010019947	ENSGALG00010019947	-0.353028559	0.286563398	-1.231938768	0.217971958	0.250774151	negative
PRJNA729271	stage_3W_vs_E10	ordinary_muscle_development	ENSGALG00010019948	TICRR	-2.263712842	0.133351657	-16.97551344	1.24659E-64	2.11091E-63	negative
PRJNA729271	stage_3W_vs_E10	ordinary_muscle_development	ENSGALG00010019954	CYB561A3	1.591589906	0.123772373	12.85900775	7.6553E-38	5.72863E-37	positive
PRJNA729271	stage_3W_vs_E10	ordinary_muscle_development	ENSGALG00010019956	SLC29A3	-0.937391842	0.177546516	-5.279697183	1.29398E-07	2.59889E-07	negative
PRJNA729271	stage_3W_vs_E10	ordinary_muscle_development	ENSGALG00010019961	HTATSF1	-0.46873105	0.069778274	-6.717435443	1.84951E-11	4.65993E-11	negative
PRJNA729271	stage_3W_vs_E10	ordinary_muscle_development	ENSGALG00010019962	SNX8	0.910636661	0.141653361	6.428627292	1.28761E-10	3.08802E-10	positive
PRJNA729271	stage_3W_vs_E10	ordinary_muscle_development	ENSGALG00010019967	CEP170B	-0.404241927	0.107491481	-3.760688053	0.000169447	0.000271666	negative
PRJNA729271	stage_3W_vs_E10	ordinary_muscle_development	ENSGALG00010019968	RHCG	1.070460309	0.906345483	1.181073143	0.237573668	0.271794026	positive
PRJNA729271	stage_3W_vs_E10	ordinary_muscle_development	ENSGALG00010019972	PPP1CB	-0.064930622	0.091495375	-0.709660157	0.477914905	0.51658289	negative
PRJNA729271	stage_3W_vs_E10	ordinary_muscle_development	ENSGALG00010019975	ENSGALG00010019975	-0.164151818	0.198852474	-0.825495475	0.409090351	0.447935083	negative
PRJNA729271	stage_3W_vs_E10	ordinary_muscle_development	ENSGALG00010019977	ZMIZ1	-0.304576594	0.088466796	-3.442835161	0.00057565	0.000880545	negative
PRJNA729271	stage_3W_vs_E10	ordinary_muscle_development	ENSGALG00010019979	ENSGALG00010019979	-3.651247108	0.564736083	-6.465404318	1.01028E-10	2.4358E-10	negative
PRJNA729271	stage_3W_vs_E10	ordinary_muscle_development	ENSGALG00010019982	PLCE1	-0.567794616	0.113103425	-5.020136378	5.16348E-07	9.95672E-07	negative
PRJNA729271	stage_3W_vs_E10	ordinary_muscle_development	ENSGALG00010019985	UMPS	-1.705477353	0.087700026	-19.44671442	3.10713E-84	8.74522E-83	negative
PRJNA729271	stage_3W_vs_E10	ordinary_muscle_development	ENSGALG00010019989	ENSGALG00010019989	1.222799469	0.276294073	4.425717339	9.61223E-06	1.69852E-05	positive
PRJNA729271	stage_3W_vs_E10	ordinary_muscle_development	ENSGALG00010019990	MARCHF8	0.792613144	0.106292528	7.456903705	8.85794E-14	2.51905E-13	positive
PRJNA729271	stage_3W_vs_E10	ordinary_muscle_development	ENSGALG00010019991	EIF3B	0.316381181	0.075162326	4.209305352	2.56157E-05	4.38163E-05	positive
PRJNA729271	stage_3W_vs_E10	ordinary_muscle_development	ENSGALG00010019992	CHAC2	-0.219185847	0.139785826	-1.568011961	0.116878341	0.13996632	negative
PRJNA729271	stage_3W_vs_E10	ordinary_muscle_development	ENSGALG00010019994	PLD4	3.450844358	0.579595484	5.953884142	2.61852E-09	5.8205E-09	positive
PRJNA729271	stage_3W_vs_E10	ordinary_muscle_development	ENSGALG00010019996	ITGB5	0.271560236	0.124445222	2.182166836	0.029097222	0.037576999	positive
PRJNA729271	stage_3W_vs_E10	ordinary_muscle_development	ENSGALG00010019997	FSHR	0.223767576	0.423592469	0.52826146	0.597317877	0.632949969	positive
PRJNA729271	stage_3W_vs_E10	ordinary_muscle_development	ENSGALG00010019998	PLEK2	-5.321457831	0.752145643	-7.075036437	1.49409E-12	3.99279E-12	negative
PRJNA729271	stage_3W_vs_E10	ordinary_muscle_development	ENSGALG00010019999	ERLIN1	-0.834302297	0.064383095	-12.9584062	2.10577E-38	1.61072E-37	negative
PRJNA729271	stage_3W_vs_E10	ordinary_muscle_development	ENSGALG00010020003	ENSGALG00010020003	1.199109262	0.186957471	6.413807675	1.41929E-10	3.39371E-10	positive
PRJNA729271	stage_3W_vs_E10	ordinary_muscle_development	ENSGALG00010020005	EOGT	0.538233021	0.163472238	3.292504135	0.000992994	0.001488003	positive
PRJNA729271	stage_3W_vs_E10	ordinary_muscle_development	ENSGALG00010020006	PLB1	-1.600802207	0.389731856	-4.10744511	4.0006E-05	6.74758E-05	negative
PRJNA729271	stage_3W_vs_E10	ordinary_muscle_development	ENSGALG00010020008	EIF2S1	-0.316232136	0.078961734	-4.004878284	6.20494E-05	0.000103155	negative
PRJNA729271	stage_3W_vs_E10	ordinary_muscle_development	ENSGALG00010020009	MACROD2	-0.744701749	0.117832342	-6.320011441	2.61544E-10	6.1744E-10	negative
PRJNA729271	stage_3W_vs_E10	ordinary_muscle_development	ENSGALG00010020010	SORBS3	2.592911237	0.205486424	12.61840653	1.6717E-36	1.20016E-35	positive
PRJNA729271	stage_3W_vs_E10	ordinary_muscle_development	ENSGALG00010020011	TLE3	-0.869415774	0.204127247	-4.25918533	2.05173E-05	3.54088E-05	negative
PRJNA729271	stage_3W_vs_E10	ordinary_muscle_development	ENSGALG00010020013	ADAM8	1.374826317	0.516944705	2.659522972	0.007825139	0.010764487	positive
PRJNA729271	stage_3W_vs_E10	ordinary_muscle_development	ENSGALG00010020015	ATP6V1D	0.926487981	0.078114232	11.86068091	1.89428E-32	1.19745E-31	positive
PRJNA729271	stage_3W_vs_E10	ordinary_muscle_development	ENSGALG00010020017	ENSGALG00010020017	4.55848183	0.28334713	16.08797603	3.09801E-58	4.44417E-57	positive
PRJNA729271	stage_3W_vs_E10	ordinary_muscle_development	ENSGALG00010020018	ENSGALG00010020018	1.344112359	0.204845644	6.561586257	5.32384E-11	1.30596E-10	positive
PRJNA729271	stage_3W_vs_E10	ordinary_muscle_development	ENSGALG00010020019	PEX10	1.777723976	0.096753641	18.37371662	2.13293E-75	4.74913E-74	positive
PRJNA729271	stage_3W_vs_E10	ordinary_muscle_development	ENSGALG00010020023	FOSL2	0.954267954	0.208361301	4.579871357	4.65262E-06	8.42681E-06	positive
PRJNA729271	stage_3W_vs_E10	ordinary_muscle_development	ENSGALG00010020025	LOXL2	-0.724359066	0.140170602	-5.167696041	2.36997E-07	4.67448E-07	negative
PRJNA729271	stage_3W_vs_E10	ordinary_muscle_development	ENSGALG00010020027	RER1	0.104501687	0.070179176	1.489069732	0.136469007	0.162008048	positive
PRJNA729271	stage_3W_vs_E10	ordinary_muscle_development	ENSGALG00010020030	MRPL33	0.879106419	0.134268892	6.547357349	5.85641E-11	1.43151E-10	positive
PRJNA729271	stage_3W_vs_E10	ordinary_muscle_development	ENSGALG00010020032	CHST12	-0.288061978	0.12405408	-2.322067738	0.020229289	0.026608306	negative
PRJNA729271	stage_3W_vs_E10	ordinary_muscle_development	ENSGALG00010020036	TMF1	0.502387226	0.083483523	6.017800985	1.76802E-09	3.96833E-09	positive
PRJNA729271	stage_3W_vs_E10	ordinary_muscle_development	ENSGALG00010020037	MPP5	-0.224861791	0.089525427	-2.511708678	0.012014823	0.016200452	negative
PRJNA729271	stage_3W_vs_E10	ordinary_muscle_development	ENSGALG00010020038	SKI	-0.59110647	0.091195219	-6.481770393	9.06525E-11	2.19294E-10	negative
PRJNA729271	stage_3W_vs_E10	ordinary_muscle_development	ENSGALG00010020041	CDH23	-0.367138999	0.153595748	-2.390294026	0.016834889	0.022321782	negative
PRJNA729271	stage_3W_vs_E10	ordinary_muscle_development	ENSGALG00010020046	BDNF	-2.55484648	0.252856624	-10.10393337	5.30699E-24	2.37642E-23	negative
PRJNA729271	stage_3W_vs_E10	ordinary_muscle_development	ENSGALG00010020048	ENSGALG00010020048	-1.009880557	0.268337222	-3.763475483	0.000167568	0.000268773	negative
PRJNA729271	stage_3W_vs_E10	ordinary_muscle_development	ENSGALG00010020053	LFNG	-0.436839964	0.13592577	-3.213812678	0.001309851	0.001942133	negative
PRJNA729271	stage_3W_vs_E10	ordinary_muscle_development	ENSGALG00010020055	R3HCC1	-0.852977919	0.084707746	-10.06965662	7.52403E-24	3.35159E-23	negative
PRJNA729271	stage_3W_vs_E10	ordinary_muscle_development	ENSGALG00010020056	UACA	-1.939871625	0.16613096	-11.67676169	1.67558E-31	1.01749E-30	negative
PRJNA729271	stage_3W_vs_E10	ordinary_muscle_development	ENSGALG00010020057	RBKS	0.54026152	0.090262604	5.985441349	2.15804E-09	4.81539E-09	positive
PRJNA729271	stage_3W_vs_E10	ordinary_muscle_development	ENSGALG00010020059	PRKCZ	0.079418256	0.139396848	0.569727771	0.568862352	0.605309063	positive
PRJNA729271	stage_3W_vs_E10	ordinary_muscle_development	ENSGALG00010020061	UBA3	0.233371287	0.072227629	3.231052869	0.001233351	0.00183283	positive
PRJNA729271	stage_3W_vs_E10	ordinary_muscle_development	ENSGALG00010020063	ENSGALG00010020063	1.689500742	0.128449823	13.15300175	1.6355E-39	1.30256E-38	positive
PRJNA729271	stage_3W_vs_E10	ordinary_muscle_development	ENSGALG00010020064	TMEM138	0.031458204	0.132000867	0.238318161	0.811634336	0.83360304	positive
PRJNA729271	stage_3W_vs_E10	ordinary_muscle_development	ENSGALG00010020065	TTYH3	-2.188441545	0.112901774	-19.38358867	1.06178E-83	2.95971E-82	negative
PRJNA729271	stage_3W_vs_E10	ordinary_muscle_development	ENSGALG00010020066	ENSGALG00010020066	2.682777989	0.376411407	7.127249437	1.02394E-12	2.76234E-12	positive
PRJNA729271	stage_3W_vs_E10	ordinary_muscle_development	ENSGALG00010020069	TMEM216	-0.845782927	0.205824703	-4.109239139	3.96965E-05	6.69771E-05	negative
PRJNA729271	stage_3W_vs_E10	ordinary_muscle_development	ENSGALG00010020070	ARL6IP5	0.993058528	0.114345136	8.684746581	3.79588E-18	1.35021E-17	positive
PRJNA729271	stage_3W_vs_E10	ordinary_muscle_development	ENSGALG00010020071	ENSGALG00010020071	0.342628293	0.143614431	2.385751143	0.01704428	0.022578765	positive
PRJNA729271	stage_3W_vs_E10	ordinary_muscle_development	ENSGALG00010020074	ENSGALG00010020074	-0.471094132	0.207989549	-2.264989438	0.023513328	0.030702251	negative
PRJNA729271	stage_3W_vs_E10	ordinary_muscle_development	ENSGALG00010020075	CPSF7	-0.747689421	0.146670101	-5.09776306	3.43691E-07	6.70678E-07	negative
PRJNA729271	stage_3W_vs_E10	ordinary_muscle_development	ENSGALG00010020080	SLC4A1AP	0.109494494	0.102397263	1.069310754	0.284929666	0.322081846	positive
PRJNA729271	stage_3W_vs_E10	ordinary_muscle_development	ENSGALG00010020083	CFAP74	-2.514856774	0.483577479	-5.200525011	1.98726E-07	3.94055E-07	negative
PRJNA729271	stage_3W_vs_E10	ordinary_muscle_development	ENSGALG00010020084	ENSGALG00010020084	-0.512823613	0.087705957	-5.847078474	5.00282E-09	1.09623E-08	negative
PRJNA729271	stage_3W_vs_E10	ordinary_muscle_development	ENSGALG00010020087	ENSGALG00010020087	1.997182229	0.485220265	4.116032187	3.85451E-05	6.51026E-05	positive
PRJNA729271	stage_3W_vs_E10	ordinary_muscle_development	ENSGALG00010020088	LMOD3	4.080098777	0.194005748	21.03081383	3.42721E-98	1.35361E-96	positive
PRJNA729271	stage_3W_vs_E10	ordinary_muscle_development	ENSGALG00010020090	MORF4L2	-0.560587815	0.053558265	-10.46687774	1.22621E-25	5.89125E-25	negative
PRJNA729271	stage_3W_vs_E10	ordinary_muscle_development	ENSGALG00010020091	SUPT7L	-0.232913892	0.08384735	-2.777832486	0.005472282	0.007639481	negative
PRJNA729271	stage_3W_vs_E10	ordinary_muscle_development	ENSGALG00010020092	SDHAF2	1.761297252	0.136376746	12.91493822	3.70741E-38	2.80621E-37	positive
PRJNA729271	stage_3W_vs_E10	ordinary_muscle_development	ENSGALG00010020094	CLEC3B	3.692455272	0.227661849	16.21903401	3.69968E-59	5.44435E-58	positive
PRJNA729271	stage_3W_vs_E10	ordinary_muscle_development	ENSGALG00010020095	LRFN2	-0.284538144	0.318337372	-0.893825762	0.371415123	0.410090052	negative
PRJNA729271	stage_3W_vs_E10	ordinary_muscle_development	ENSGALG00010020096	GPHN	1.754661775	0.115559535	15.18405008	4.5105E-52	5.46196E-51	positive
PRJNA729271	stage_3W_vs_E10	ordinary_muscle_development	ENSGALG00010020097	RETSAT	0.812818182	0.228774493	3.552923106	0.000380976	0.000592897	positive
PRJNA729271	stage_3W_vs_E10	ordinary_muscle_development	ENSGALG00010020098	GABRD	1.668411315	0.330655242	5.045773061	4.51691E-07	8.75303E-07	positive
PRJNA729271	stage_3W_vs_E10	ordinary_muscle_development	ENSGALG00010020100	MOCS1	2.595722868	0.15698978	16.53434298	2.0763E-61	3.22572E-60	positive
PRJNA729271	stage_3W_vs_E10	ordinary_muscle_development	ENSGALG00010020101	KIF15	-4.096289428	0.212156315	-19.30788355	4.61084E-83	1.26102E-81	negative
PRJNA729271	stage_3W_vs_E10	ordinary_muscle_development	ENSGALG00010020102	TMEM127	0.49162937	0.202223842	2.431114775	0.015052445	0.020072235	positive
PRJNA729271	stage_3W_vs_E10	ordinary_muscle_development	ENSGALG00010020103	PSAP	1.271939736	0.104997903	12.11395369	8.90938E-34	5.87274E-33	positive
PRJNA729271	stage_3W_vs_E10	ordinary_muscle_development	ENSGALG00010020104	BRAT1	0.328838988	0.112457507	2.924117712	0.003454341	0.00492337	positive
PRJNA729271	stage_3W_vs_E10	ordinary_muscle_development	ENSGALG00010020106	ENSGALG00010020106	2.459642606	0.161763862	15.20514269	3.26918E-52	3.98208E-51	positive
PRJNA729271	stage_3W_vs_E10	ordinary_muscle_development	ENSGALG00010020108	RPAP1	-0.878113235	0.117839167	-7.451794324	9.20792E-14	2.61652E-13	negative
PRJNA729271	stage_3W_vs_E10	ordinary_muscle_development	ENSGALG00010020109	WDFY4	2.104049041	0.359053251	5.859991613	4.62891E-09	1.01538E-08	positive
PRJNA729271	stage_3W_vs_E10	ordinary_muscle_development	ENSGALG00010020111	ADRA2B	1.516287069	0.120635504	12.56916097	3.12018E-36	2.22464E-35	positive
PRJNA729271	stage_3W_vs_E10	ordinary_muscle_development	ENSGALG00010020113	CIAO1	0.020094421	0.104193561	0.192856652	0.847071235	0.864607953	positive
PRJNA729271	stage_3W_vs_E10	ordinary_muscle_development	ENSGALG00010020116	GNA12	-0.013106511	0.071746218	-0.182678771	0.855050074	0.871565663	negative
PRJNA729271	stage_3W_vs_E10	ordinary_muscle_development	ENSGALG00010020117	AKAP13	-0.614818492	0.102602826	-5.992217936	2.06998E-09	4.6246E-09	negative
PRJNA729271	stage_3W_vs_E10	ordinary_muscle_development	ENSGALG00010020118	GNB1	-0.919699868	0.093491039	-9.837305012	7.7766E-23	3.33201E-22	negative
PRJNA729271	stage_3W_vs_E10	ordinary_muscle_development	ENSGALG00010020120	DAAM2	-1.015815936	0.103053814	-9.857140606	6.38416E-23	2.75038E-22	negative
PRJNA729271	stage_3W_vs_E10	ordinary_muscle_development	ENSGALG00010020121	ENSGALG00010020121	-2.735722095	0.282989726	-9.667213495	4.15538E-22	1.73081E-21	negative
PRJNA729271	stage_3W_vs_E10	ordinary_muscle_development	ENSGALG00010020123	ENSGALG00010020123	-0.662052151	0.157389107	-4.206467408	2.59393E-05	4.4349E-05	negative
PRJNA729271	stage_3W_vs_E10	ordinary_muscle_development	ENSGALG00010020125	NAV2	0.093893157	0.130220736	0.721030769	0.470890584	0.50956021	positive
PRJNA729271	stage_3W_vs_E10	ordinary_muscle_development	ENSGALG00010020126	NADK	0.138623581	0.104486547	1.326712234	0.184603892	0.214805605	positive
PRJNA729271	stage_3W_vs_E10	ordinary_muscle_development	ENSGALG00010020127	FRMD4B	-1.2288909	0.141216631	-8.702168385	3.25602E-18	1.16046E-17	negative
PRJNA729271	stage_3W_vs_E10	ordinary_muscle_development	ENSGALG00010020128	KIF6	-0.287559023	0.524611073	-0.548137541	0.583597465	0.619407282	negative
PRJNA729271	stage_3W_vs_E10	ordinary_muscle_development	ENSGALG00010020129	MGAT2	-0.520301669	0.117308506	-4.43532773	9.19323E-06	1.62745E-05	negative
PRJNA729271	stage_3W_vs_E10	ordinary_muscle_development	ENSGALG00010020130	PDXDC1	-0.099960463	0.073592343	-1.358299787	0.174368578	0.203631368	negative
PRJNA729271	stage_3W_vs_E10	ordinary_muscle_development	ENSGALG00010020131	SNRNP200	-0.846571526	0.124945793	-6.775510458	1.23968E-11	3.15414E-11	negative
PRJNA729271	stage_3W_vs_E10	ordinary_muscle_development	ENSGALG00010020132	SHC4	-0.418432278	0.230929483	-1.811948276	0.069994189	0.086243265	negative
PRJNA729271	stage_3W_vs_E10	ordinary_muscle_development	ENSGALG00010020133	ENSGALG00010020133	2.464818002	0.366752561	6.720656559	1.80908E-11	4.56044E-11	positive
PRJNA729271	stage_3W_vs_E10	ordinary_muscle_development	ENSGALG00010020136	KCNK17	1.048841653	0.282204044	3.716607448	0.000201916	0.000321552	positive
PRJNA729271	stage_3W_vs_E10	ordinary_muscle_development	ENSGALG00010020137	NTAN1	-0.276337285	0.082876361	-3.334331796	0.000855046	0.00128848	negative
PRJNA729271	stage_3W_vs_E10	ordinary_muscle_development	ENSGALG00010020138	ENSGALG00010020138	0.501098799	0.084946317	5.899005618	3.65699E-09	8.06206E-09	positive
PRJNA729271	stage_3W_vs_E10	ordinary_muscle_development	ENSGALG00010020140	SECISBP2L	0.685815105	0.094486919	7.258307398	3.91964E-13	1.0785E-12	positive
PRJNA729271	stage_3W_vs_E10	ordinary_muscle_development	ENSGALG00010020141	THOC7	0.214017335	0.127471133	1.678947465	0.093162283	0.113173591	positive
PRJNA729271	stage_3W_vs_E10	ordinary_muscle_development	ENSGALG00010020142	SLC35E2B	-1.335413354	0.070443039	-18.95735021	3.8401E-80	9.61351E-79	negative
PRJNA729271	stage_3W_vs_E10	ordinary_muscle_development	ENSGALG00010020143	KCNK5	-0.8117445	0.180103727	-4.507094412	6.57214E-06	1.17681E-05	negative

PRJNA729271	stage_3W_vs_E10	ordinary_muscle_development	ENSGALG00010020147	ABHD2	1.364857165	0.146091723	9.342467417	9.41148E-21	3.7131E-20	positive
PRJNA729271	stage_3W_vs_E10	ordinary_muscle_development	ENSGALG00010020149	FAHD2A	1.357519291	0.17206143	7.88973619	3.02826E-15	9.27221E-15	positive
PRJNA729271	stage_3W_vs_E10	ordinary_muscle_development	ENSGALG00010020152	ATXN7	0.805399916	0.10723436	7.510651604	5.88338E-14	1.69071E-13	positive
PRJNA729271	stage_3W_vs_E10	ordinary_muscle_development	ENSGALG00010020153	AXL	-0.692232004	0.121740192	-5.686141869	1.29941E-08	2.78501E-08	negative
PRJNA729271	stage_3W_vs_E10	ordinary_muscle_development	ENSGALG00010020155	MFGE8	2.022520216	0.134198975	15.07105562	2.51069E-51	2.97081E-50	positive
PRJNA729271	stage_3W_vs_E10	ordinary_muscle_development	ENSGALG00010020160	SYT7	-3.856963909	0.353391275	-10.91414584	9.86523E-28	5.20745E-27	negative
PRJNA729271	stage_3W_vs_E10	ordinary_muscle_development	ENSGALG00010020163	CDC2L1	-0.177686592	0.095769523	-1.855356343	0.063545357	0.078739097	negative
PRJNA729271	stage_3W_vs_E10	ordinary_muscle_development	ENSGALG00010020164	C10orf71	4.18924798	0.147853575	28.33376183	1.3269E-176	1.7975E-174	positive
PRJNA729271	stage_3W_vs_E10	ordinary_muscle_development	ENSGALG00010020166	HAPLN3	-0.15623694	0.17422686	-0.896744282	0.369855386	0.408554627	negative
PRJNA729271	stage_3W_vs_E10	ordinary_muscle_development	ENSGALG00010020168	COX7A2L	1.724741628	0.114425035	15.07311426	2.43366E-51	2.88673E-50	positive
PRJNA729271	stage_3W_vs_E10	ordinary_muscle_development	ENSGALG00010020170	SORCS1	-1.911439255	0.309999275	-6.165947498	7.00623E-10	1.61378E-09	negative
PRJNA729271	stage_3W_vs_E10	ordinary_muscle_development	ENSGALG00010020171	RRN3	-0.194185091	0.076040677	-2.553700197	0.010658499	0.014442829	negative
PRJNA729271	stage_3W_vs_E10	ordinary_muscle_development	ENSGALG00010020175	ACAN	-11.2841594	0.616857515	-18.29297549	9.41337E-75	2.06738E-73	negative
PRJNA729271	stage_3W_vs_E10	ordinary_muscle_development	ENSGALG00010020176	ENSGALG00010020176	0.632108204	0.242728176	2.604181412	0.0092094	0.012572071	positive
PRJNA729271	stage_3W_vs_E10	ordinary_muscle_development	ENSGALG00010020177	KCNG3	-3.841983039	0.297138253	-12.92995095	3.05013E-38	2.31839E-37	negative
PRJNA729271	stage_3W_vs_E10	ordinary_muscle_development	ENSGALG00010020179	PSMD6	0.982561678	0.134465478	7.307166832	2.72834E-13	7.56017E-13	positive
PRJNA729271	stage_3W_vs_E10	ordinary_muscle_development	ENSGALG00010020180	TCF7L1	-1.265515593	0.21980287	-5.757502587	8.53675E-09	1.84935E-08	negative
PRJNA729271	stage_3W_vs_E10	ordinary_muscle_development	ENSGALG00010020181	LEUTX	2.343182671	0.21580514	10.85786311	1.82981E-27	9.54068E-27	positive
PRJNA729271	stage_3W_vs_E10	ordinary_muscle_development	ENSGALG00010020183	XPNPEP1	-0.715936377	0.069035465	-10.37055926	3.37543E-25	1.59423E-24	negative
PRJNA729271	stage_3W_vs_E10	ordinary_muscle_development	ENSGALG00010020184	RSL1D1	-1.02757034	0.070669257	-14.54055676	6.70451E-48	7.11434E-47	negative
PRJNA729271	stage_3W_vs_E10	ordinary_muscle_development	ENSGALG00010020186	MGA	0.01292512	0.094902747	0.136193321	0.891668455	0.904206958	positive
PRJNA729271	stage_3W_vs_E10	ordinary_muscle_development	ENSGALG00010020187	MMP23B	-0.750376646	0.208339147	-3.601707394	0.000316134	0.000495522	negative
PRJNA729271	stage_3W_vs_E10	ordinary_muscle_development	ENSGALG00010020193	VSTM4	-2.378722669	0.182808884	-13.0120737	1.04468E-38	8.05889E-38	negative
PRJNA729271	stage_3W_vs_E10	ordinary_muscle_development	ENSGALG00010020194	PRICKLE2	-1.628619304	0.157608478	-10.33332294	4.98047E-25	2.33631E-24	negative
PRJNA729271	stage_3W_vs_E10	ordinary_muscle_development	ENSGALG00010020199	DET1	-0.165917287	0.084125532	-1.972258384	0.048580118	0.061083345	negative
PRJNA729271	stage_3W_vs_E10	ordinary_muscle_development	ENSGALG00010020202	MAPKBP1	-0.050127271	0.091329104	-0.548864145	0.583098686	0.619001033	negative
PRJNA729271	stage_3W_vs_E10	ordinary_muscle_development	ENSGALG00010020204	GSPT2	-0.142058474	0.086665119	-1.639165515	0.101178794	0.122215551	negative
PRJNA729271	stage_3W_vs_E10	ordinary_muscle_development	ENSGALG00010020206	MRPS11	0.832991575	0.080695078	10.32270618	5.56326E-25	2.59792E-24	positive
PRJNA729271	stage_3W_vs_E10	ordinary_muscle_development	ENSGALG00010020211	ADD3	-0.664917582	0.083451461	-7.967716491	1.61633E-15	5.00721E-15	negative
PRJNA729271	stage_3W_vs_E10	ordinary_muscle_development	ENSGALG00010020212	JMJD7	-0.002402769	0.139779589	-0.017189698	0.98628528	0.98834925	negative
PRJNA729271	stage_3W_vs_E10	ordinary_muscle_development	ENSGALG00010020215	PRMT3	-0.140338549	0.079938424	-1.755583131	0.079159615	0.096910356	negative
PRJNA729271	stage_3W_vs_E10	ordinary_muscle_development	ENSGALG00010020224	MRPL46	-0.027280937	0.087371962	-0.312239033	0.75485887	0.780656344	negative
PRJNA729271	stage_3W_vs_E10	ordinary_muscle_development	ENSGALG00010020226	TC1M	1.695974447	0.197988469	8.566026371	1.07117E-17	3.72431E-17	positive
PRJNA729271	stage_3W_vs_E10	ordinary_muscle_development	ENSGALG00010020228	TM9SF3	-0.601056676	0.083139371	-7.229507128	4.8475E-13	1.32575E-12	negative
PRJNA729271	stage_3W_vs_E10	ordinary_muscle_development	ENSGALG00010020230	ENSGALG00010020230	0.38377437	0.216947254	1.76897547	0.076897968	0.094308829	positive
PRJNA729271	stage_3W_vs_E10	ordinary_muscle_development	ENSGALG00010020236	SLC35G1	2.455126111	0.285735356	8.592307735	8.52397E-18	2.97579E-17	positive
PRJNA729271	stage_3W_vs_E10	ordinary_muscle_development	ENSGALG00010020237	MIB2	0.150588926	0.075478508	1.995123247	0.046029439	0.058077709	positive
PRJNA729271	stage_3W_vs_E10	ordinary_muscle_development	ENSGALG00010020240	SV2B	-1.593624064	0.385178724	-4.137362647	3.51321E-05	5.94211E-05	negative
PRJNA729271	stage_3W_vs_E10	ordinary_muscle_development	ENSGALG00010020244	SLC25A37	-2.074210568	0.217678526	-9.528779001	1.59144E-21	6.47975E-21	negative
PRJNA729271	stage_3W_vs_E10	ordinary_muscle_development	ENSGALG00010020246	ADAMTS9	-1.151534593	0.130198904	-8.844426191	9.19998E-19	3.34471E-18	negative
PRJNA729271	stage_3W_vs_E10	ordinary_muscle_development	ENSGALG00010020248	NOLC1	-0.627030852	0.080784871	-7.761736147	8.37746E-15	2.50943E-14	negative
PRJNA729271	stage_3W_vs_E10	ordinary_muscle_development	ENSGALG00010020250	ENSGALG00010020250	0.311068973	0.244491109	1.272312004	0.203262285	0.235138613	positive
PRJNA729271	stage_3W_vs_E10	ordinary_muscle_development	ENSGALG00010020252	DMTN	0.908726378	0.188588469	4.818568077	1.44592E-06	2.70853E-06	positive
PRJNA729271	stage_3W_vs_E10	ordinary_muscle_development	ENSGALG00010020254	ENSGALG00010020254	-1.555082773	0.13546137	-11.47989849	1.66476E-30	9.74977E-30	negative
PRJNA729271	stage_3W_vs_E10	ordinary_muscle_development	ENSGALG00010020257	ZNF710	0.862591887	0.160978882	5.358416423	8.39546E-08	1.70988E-07	positive
PRJNA729271	stage_3W_vs_E10	ordinary_muscle_development	ENSGALG00010020259	ENSGALG00010020259	-1.136994895	0.198903042	-5.716327324	1.08851E-08	2.34508E-08	negative
PRJNA729271	stage_3W_vs_E10	ordinary_muscle_development	ENSGALG00010020261	SSUT2	0.886478409	0.090565833	9.788221261	1.26502E-22	5.3678E-22	positive
PRJNA729271	stage_3W_vs_E10	ordinary_muscle_development	ENSGALG00010020265	ENSGALG00010020265	-1.181561997	0.228737993	-5.165569474	2.39708E-07	4.72666E-07	negative
PRJNA729271	stage_3W_vs_E10	ordinary_muscle_development	ENSGALG00010020268	CPLANE2	0.176662831	0.098243422	1.798215363	0.072142886	0.08876251	positive
PRJNA729271	stage_3W_vs_E10	ordinary_muscle_development	ENSGALG00010020269	ENSGALG00010020269	-3.522342465	0.9581467	-3.676203724	0.00023673	0.000374322	negative
PRJNA729271	stage_3W_vs_E10	ordinary_muscle_development	ENSGALG00010020272	ENSGALG00010020272	-1.234047019	0.093104735	-13.25439597	4.25529E-40	3.45549E-39	negative
PRJNA729271	stage_3W_vs_E10	ordinary_muscle_development	ENSGALG00010020275	ELOVL3	-2.95025375	0.656782556	-4.491979461	7.05642E-06	1.25949E-05	negative
PRJNA729271	stage_3W_vs_E10	ordinary_muscle_development	ENSGALG00010020277	IDH2	0.931471356	0.124733231	7.467708062	8.16036E-14	2.32691E-13	positive
PRJNA729271	stage_3W_vs_E10	ordinary_muscle_development	ENSGALG00010020282	MAGI1	-0.956494842	0.138072581	-6.927478522	4.28408E-12	1.1126E-11	negative
PRJNA729271	stage_3W_vs_E10	ordinary_muscle_development	ENSGALG00010020283	FBXO42	-0.001787553	0.222676689	-0.00802757	0.993594994	0.994555586	negative
PRJNA729271	stage_3W_vs_E10	ordinary_muscle_development	ENSGALG00010020284	HEG1	1.17239207	0.117346018	9.990897775	1.67062E-23	7.3314E-23	positive
PRJNA729271	stage_3W_vs_E10	ordinary_muscle_development	ENSGALG00010020285	ADAMTS14	-3.974109646	0.275405904	-14.43000891	3.35038E-47	3.4418E-46	negative
PRJNA729271	stage_3W_vs_E10	ordinary_muscle_development	ENSGALG00010020287	GALNT16	-0.170139949	0.161720646	-1.05206078	0.292771665	0.330045519	negative
PRJNA729271	stage_3W_vs_E10	ordinary_muscle_development	ENSGALG00010020290	NELL1	-3.652521523	0.580225136	-6.295007395	3.07385E-10	7.22834E-10	negative
PRJNA729271	stage_3W_vs_E10	ordinary_muscle_development	ENSGALG00010020291	SNX29	0.411967711	0.144971545	2.841714281	0.004487169	0.006325149	positive
PRJNA729271	stage_3W_vs_E10	ordinary_muscle_development	ENSGALG00010020292	NPM2	-0.383615034	0.237495118	-1.615254396	0.106255615	0.128049147	negative
PRJNA729271	stage_3W_vs_E10	ordinary_muscle_development	ENSGALG00010020293	SEMA4B	-0.480947804	0.113615607	-4.23311389	2.30478E-05	3.96014E-05	negative
PRJNA729271	stage_3W_vs_E10	ordinary_muscle_development	ENSGALG00010020295	LGI1	-0.134088371	0.14751262	-0.908995929	0.363352275	0.402167931	negative
PRJNA729271	stage_3W_vs_E10	ordinary_muscle_development	ENSGALG00010020298	ENSGALG00010020298	-0.168606751	0.17449704	-0.966244186	0.333922007	0.372265767	negative
PRJNA729271	stage_3W_vs_E10	ordinary_muscle_development	ENSGALG00010020300	EHD4	0.809764133	0.128791596	6.28739885	3.22829E-10	7.57922E-10	positive
PRJNA729271	stage_3W_vs_E10	ordinary_muscle_development	ENSGALG00010020302	HAAO	2.123954683	1.459147661	1.45561326	0.14549956	0.172024154	positive
PRJNA729271	stage_3W_vs_E10	ordinary_muscle_development	ENSGALG00010020303	EXD2	-0.580795234	0.074653618	-7.779867245	7.26007E-15	2.18239E-14	negative
PRJNA729271	stage_3W_vs_E10	ordinary_muscle_development	ENSGALG00010020304	ZFP36L2	1.017540626	0.155226428	6.555202225	5.55665E-11	1.36076E-10	positive
PRJNA729271	stage_3W_vs_E10	ordinary_muscle_development	ENSGALG00010020305	XPO7	-0.122336376	0.138282845	-0.884682234	0.37632804	0.415103489	negative
PRJNA729271	stage_3W_vs_E10	ordinary_muscle_development	ENSGALG00010020307	SHQ1	-1.235318338	0.228569864	-5.404554729	6.49696E-08	1.32957E-07	negative
PRJNA729271	stage_3W_vs_E10	ordinary_muscle_development	ENSGALG00010020309	GXYLT2	-0.962572425	0.15977414	-6.024582102	1.69547E-09	3.81198E-09	negative
PRJNA729271	stage_3W_vs_E10	ordinary_muscle_development	ENSGALG00010020311	SGPL1	-0.49003097	0.134550825	-3.641976696	0.000270553	0.000426036	negative
PRJNA729271	stage_3W_vs_E10	ordinary_muscle_development	ENSGALG00010020313	FRA10AC1	0.180809877	0.114557424	1.578333993	0.1144889	0.137297643	positive
PRJNA729271	stage_3W_vs_E10	ordinary_muscle_development	ENSGALG00010020314	THADA	-0.319634165	0.079515183	-4.019787803	5.82506E-05	9.70843E-05	negative
PRJNA729271	stage_3W_vs_E10	ordinary_muscle_development	ENSGALG00010020315	ANO5	0.413488726	0.108383786	3.815042268	0.00013616	0.000220173	positive
PRJNA729271	stage_3W_vs_E10	ordinary_muscle_development	ENSGALG00010020316	PDPK1	0.625473268	0.112158941	5.576668807	2.45168E-08	5.15928E-08	positive
PRJNA729271	stage_3W_vs_E10	ordinary_muscle_development	ENSGALG00010020319	NTRK3	-1.662767184	0.1669764	-9.958096983	2.3247E-23	1.01434E-22	negative
PRJNA729271	stage_3W_vs_E10	ordinary_muscle_development	ENSGALG00010020321	SZRD1	1.080669468	0.169421428	6.378587878	1.78728E-10	4.25816E-10	positive
PRJNA729271	stage_3W_vs_E10	ordinary_muscle_development	ENSGALG00010020323	ARHGAP25	1.081605065	0.153478351	7.047280999	1.82448E-12	4.85244E-12	positive
PRJNA729271	stage_3W_vs_E10	ordinary_muscle_development	ENSGALG00010020324	PLEKHH2	0.877644406	0.184271141	4.762788143	1.90936E-06	3.54649E-06	positive
PRJNA729271	stage_3W_vs_E10	ordinary_muscle_development	ENSGALG00010020329	PPP4R2	-0.071933003	0.105832484	-0.679687374	0.496702432	0.534982386	negative
PRJNA729271	stage_3W_vs_E10	ordinary_muscle_development	ENSGALG00010020334	CABP4	0.400643732	0.483566922	0.828517653	0.407377401	0.446228023	positive
PRJNA729271	stage_3W_vs_E10	ordinary_muscle_development	ENSGALG00010020336	OSBP	0.321523246	0.134466465	2.39110358	0.016797813	0.022278736	positive
PRJNA729271	stage_3W_vs_E10	ordinary_muscle_development	ENSGALG00010020338	NECAP2	-0.600759912	0.168168235	-3.572374489	0.000353759	0.000552023	negative
PRJNA729271	stage_3W_vs_E10	ordinary_muscle_development	ENSGALG00010020340	DCAF5	0.728039801	0.096429752	7.549949941	4.35426E-14	1.26104E-13	positive
PRJNA729271	stage_3W_vs_E10	ordinary_muscle_development	ENSGALG00010020341	DYNC2LI1	-1.291706757	0.130705784	-9.882552415	4.95543E-23	2.14351E-22	negative
PRJNA729271	stage_3W_vs_E10	ordinary_muscle_development	ENSGALG00010020342	CPPED1	-1.039351781	0.107670306	-9.653095802	4.76937E-22	1.98029E-21	negative
PRJNA729271	stage_3W_vs_E10	ordinary_muscle_development	ENSGALG00010020343	PATL1	0.789381859	0.17517839	4.506160041	6.60113E-06	1.18128E-05	positive
PRJNA729271	stage_3W_vs_E10	ordinary_muscle_development	ENSGALG00010020345	AGBL1	-1.102814625	0.456425772	-2.416197096	0.015683569	0.020863926	negative
PRJNA729271	stage_3W_vs_E10	ordinary_muscle_development	ENSGALG00010020346	PCBD1	-2.291466275	0.296166747	-7.737081561	1.01725E-14	3.03083E-14	negative
PRJNA729271	stage_3W_vs_E10	ordinary_muscle_development	ENSGALG00010020350	PDZRN3	0.296199145	0.108034693	2.74170394	0.00611214	0.00849185	positive
PRJNA729271	stage_3W_vs_E10	ordinary_muscle_development	ENSGALG00010020351	CROCC	-0.540075794	0.151093796	-3.574440574	0.000350978	0.000547918	negative
PRJNA729271	stage_3W_vs_E10	ordinary_muscle_development	ENSGALG00010020352	ABCG5	-0.79962668	0.644468439	-1.240753825	0.214696704	0.247418407	negative
PRJNA729271	stage_3W_vs_E10	ordinary_muscle_development	ENSGALG00010020356	STX3	-0.529552676	0.402176077	-1.316718487	0.187933004	0.218381345	negative
PRJNA729271	stage_3W_vs_E10	ordinary_muscle_development	ENSGALG00010020357	RBP4	0.058791499	0.738988832	0.079556681	0.936589849	0.944211286	positive
PRJNA729271	stage_3W_vs_E10	ordinary_muscle_development	ENSGALG00010020362	CDS2	-0.524102969	0.127301697	-4.117014791	3.83811E-05	6.48409E-05	negative
PRJNA729271	stage_3W_vs_E10	ordinary_muscle_development	ENSGALG00010020364	MRPL16	1.511183293	0.109929366	13.74685719	5.31904E-43	4.73293E-42	positive
PRJNA729271	stage_3W_vs_E10	ordinary_muscle_development	ENSGALG00010020365	UNC5B	-0.862916873	0.141380081	-6.10352511	1.03754E-09	2.36168E-09	negative
PRJNA729271	stage_3W_vs_E10	ordinary_muscle_development	ENSGALG00010020369	CNTN3	-4.720164775	0.367794365	-12.83370607	1.06162E-37	7.89946E-37	negative
PRJNA729271	stage_3W_vs_E10	ordinary_muscle_development	ENSGALG00010020370	CEP55	-2.2037883	0.230157502	-9.57513129	1.01728E-21	4.17364E-21	negative
PRJNA729271	stage_3W_vs_E10	ordinary_muscle_development	ENSGALG00010020373	MS4A4A	-1.041203476	0.216878713	-4.800856028	1.57989E-06	2.94971E-06	negative
PRJNA729271	stage_3W_vs_E10	ordinary_muscle_development	ENSGALG00010020374	PCNA	-2.09089595	0.096684041	-21.62607112	1.0212E-103	4.6113E-102	negative
PRJNA729271	stage_3W_vs_E10	ordinary_muscle_development	ENSGALG00010020375	LRPPRC	0.060725662	0.101748448	0.596821508	0.550626557	0.587683671	positive
PRJNA729271	stage_3W_vs_E10	ordinary_muscle_development	ENSGALG00010020377	TMEM230	-0.095039755	0.097541976	-0.974347243	0.329884155	0.368132329	negative
PRJNA729271	stage_3W_vs_E10	ordinary_muscle_development	ENSGALG00010020379	COL13A1	-2.467091687	0.201718372	-12.23037675	2.13944E-34	1.44507E-33	negative
PRJNA729271	stage_3W_vs_E10	ordinary_muscle_development	ENSGALG00010020382	MFAP2	-4.287343147	0.159646073	-26.85529969	7.3143E-159	7.8534E-157	negative
PRJNA729271	stage_3W_vs_E10	ordinary_muscle_development	ENSGALG00010020383	BAAT	-0.46033035	0.105608574	-4.358834996	1.30757E-05	2.28958E-05	negative
PRJNA729271	stage_3W_vs_E10	ordinary_muscle_development	ENSGALG00010020385	SLC12A8	-4.22124464	0.580207664	-7.27540311	3.45388E-13	9.52691E-13	negative
PRJNA729271	stage_3W_vs_E10	ordinary_muscle_development	ENSGALG00010020386	PPM1B	0.928946768	0.071867204	12.92587881	3.21601E-38	2.44191E-37	positive
PRJNA729271	stage_3W_vs_E10	ordinary_muscle_development	ENSGALG00010020388	MYOF	1.885309308	0.165918218	11.36288305	6.39971E-30	3.66656E-29	positive
PRJNA729271	stage_3W_vs_E10	ordinary_muscle_development	ENSGALG00010020389	ACTN1	-1.182702965	0.157420258	-7.513029011	5.77747E-14	1.66094E-13	negative
PRJNA729271	stage_3W_vs_E10	ordinary_muscle_development	ENSGALG00010020390	KLHL25	0.692516673	0.222299382	3.11524336	0.001837932	0.002683673	positive
PRJNA729271	stage_3W_vs_E10	ordinary_muscle_development	ENSGALG00010020392	SLC23A2	-0.391017609	0.082213331	-4.756133882	1.97336E-06	3.66199E-06	negative
PRJNA729271	stage_3W_vs_E10	ordinary_muscle_development	ENSGALG00010020393	SH3A9	-1.822285831	0.312451195	-5.832225511	5.46929E-09	1.19754E-08	negative
PRJNA729271	stage_3W_vs_E10	ordinary_muscle_development	ENSGALG00010020396	CHL1	-0.559306821	0.194246982	-2.879359132	0.003984843	0.005643409	negative
PRJNA729271	stage_3W_vs_E10	ordinary_muscle_development	ENSGALG00010020399	ATP13A2	0.289774549	0.130875142	2.214129782	0.026819852	0.034753641	positive
PRJNA729271	stage_3W_vs_E10	ordinary_muscle_development	ENSGALG00010020402	SLC3A1	3.169662358	0.309880507	10.22866004	1.47547E-24	6.78518E-24	positive
PRJNA729271	stage_3W_vs_E10	ordinary_muscle_development	ENSGALG00010020405	UBE2I	-0.618939417	0.081666573	-7.578858709	3.48608E-14	1.012E-13	negative
PRJNA729271	stage_3W_vs_E10	ordinary_muscle_development	ENSGALG00010020413	CYP26A1	0.316704775	0.318561364	0.994171957	0.320139158	0.358222719	positive
PRJNA729271	stage_3W_vs_E10	ordinary_muscle_development	ENSGALG00010020415	TSR3	0.205130234	0.091711623	2.236687433	0.025306776	0.032892909	positive
PRJNA729271	stage_3W_vs_E10	ordinary_muscle_development	ENSGALG00010020416	ENSGALG00010020416	-5.762329737	1.103478365	-5.22196893	1.77031E-07	3.52142E-07	negative
PRJNA729271	stage_3W_vs_E10	ordinary_muscle_development	ENSGALG00010020417	ENSGALG00010020417	4.543951953	0.747329627	6.080251323	1.19994E-09	2.72151E-09	positive
PRJNA729271	stage_3W_vs_E10	ordinary_muscle_development	ENSGALG00010020422	ERCC4	0.054597461	0.08815185	0.619356947	0.535681237	0.573379082	positive
PRJNA729271	stage_3W_vs_E10	ordinary_muscle_development	ENSGALG00010020428	SEMA4C	-0.301343014	0.151507594	-1.988963098	0.046705276	0.058858718	negative
PRJNA729271	stage_3W_vs_E10	ordinary_muscle_development	ENSGALG00010020429	ZNF148	0.033926423	0.101890204	0.332970411	0.739156619	0.766550418	positive
PRJNA729271	stage_3W_vs_E10	ordinary_muscle_development	ENSGALG00010020433	GNPTG	-0.191105664	0.071500692	-2.672780613	0.007522543	0.010362978	negative
PRJNA729271	stage_3W_vs_E10	ordinary_muscle_development	ENSGALG00010020436	CNTN4	-3.751476904	0.273460161	-13.71855003	7.8622E-43	6.96593E-42	negative
PRJNA729271	stage_3W_vs_E10	ordinary_muscle_development	ENSGALG00010020437	SNX4	-1.114776547	0.085033653	-13.10982773	2.89255E-39	2.27991E-38	negative
PRJNA729271	stage_3W_vs_E10	ordinary_muscle_development	ENSGALG00010020438	HTR7	-0.142561688	0.284669929	-0.500796444	0.616514389	0.651149524	negative
PRJNA729271	stage_3W_vs_E10	ordinary_muscle_development	ENSGALG00010020440	PAX7	-0.566602169	0.187396235	-3.023551506	0.002498265	0.003607526	negative
PRJNA729271	stage_3W_vs_E10	ordinary_muscle_development	ENSGALG00010020443	PREPL	0.346908404	0.135311981	2.563767085	0.0103543	0.014045067	positive

PRJNA729271	stage_3W_vs_E10	ordinary_muscle_development	ENSGALG00010020444	ENSGALG00010020444	-3.09422523	1.199661159	-2.579249321	0.009901529	0.013459877	negative
PRJNA729271	stage_3W_vs_E10	ordinary_muscle_development	ENSGALG00010020449	PRPF19	-0.598432226	0.09770217	-6.125065839	9.06461E-10	2.07046E-09	negative
PRJNA729271	stage_3W_vs_E10	ordinary_muscle_development	ENSGALG00010020450	ANKRD39	0.806343613	0.180213028	4.474391348	7.66293E-06	1.36488E-05	positive
PRJNA729271	stage_3W_vs_E10	ordinary_muscle_development	ENSGALG00010020453	ENSGALG00010020453	-0.945859054	0.144162826	-6.56104685	5.34313E-11	1.31003E-10	negative
PRJNA729271	stage_3W_vs_E10	ordinary_muscle_development	ENSGALG00010020455	ENSGALG00010020455	0.393461774	0.671748263	0.585728011	0.558058308	0.595133547	positive
PRJNA729271	stage_3W_vs_E10	ordinary_muscle_development	ENSGALG00010020457	IL5RA	3.380863788	0.929907479	3.635699104	0.000277228	0.000436405	positive
PRJNA729271	stage_3W_vs_E10	ordinary_muscle_development	ENSGALG00010020460	MAPK8	0.350599991	0.140810175	2.489876825	0.012778737	0.017171391	positive
PRJNA729271	stage_3W_vs_E10	ordinary_muscle_development	ENSGALG00010020462	CNNM4	0.62501056	0.18097158	3.453639293	0.000553077	0.000847536	positive
PRJNA729271	stage_3W_vs_E10	ordinary_muscle_development	ENSGALG00010020467	ALDH4A1	0.770497914	0.130882725	5.886933603	3.93427E-09	8.6628E-09	positive
PRJNA729271	stage_3W_vs_E10	ordinary_muscle_development	ENSGALG00010020468	ENSGALG00010020468	0.03072696	0.457334464	0.067187065	0.946432783	0.953404906	positive
PRJNA729271	stage_3W_vs_E10	ordinary_muscle_development	ENSGALG00010020473	TMEM109	2.995709532	0.159030871	18.83728307	3.73661E-79	9.0876E-78	positive
PRJNA729271	stage_3W_vs_E10	ordinary_muscle_development	ENSGALG00010020476	TMEM132A	-1.340774081	0.114085299	-11.75238259	6.86553E-32	4.23652E-31	negative
PRJNA729271	stage_3W_vs_E10	ordinary_muscle_development	ENSGALG00010020478	ENSGALG00010020478	2.21305024	0.680857855	3.250385118	0.001152488	0.0017183	positive
PRJNA729271	stage_3W_vs_E10	ordinary_muscle_development	ENSGALG00010020481	RPP30	-0.360542052	0.122933894	-2.932812424	0.003359068	0.004796068	negative
PRJNA729271	stage_3W_vs_E10	ordinary_muscle_development	ENSGALG00010020484	TRNT1	0.523104078	0.123796177	4.225526921	2.38382E-05	4.08869E-05	positive
PRJNA729271	stage_3W_vs_E10	ordinary_muscle_development	ENSGALG00010020485	LMAN2L	-0.603899005	0.132280658	-4.565285778	4.98814E-06	9.00974E-06	negative
PRJNA729271	stage_3W_vs_E10	ordinary_muscle_development	ENSGALG00010020486	MRTFB	1.610003297	0.111595447	14.42714144	3.49259E-47	3.58028E-46	positive
PRJNA729271	stage_3W_vs_E10	ordinary_muscle_development	ENSGALG00010020489	IFFO2	-1.547948741	0.189411222	-8.172423621	3.02255E-16	9.74463E-16	negative
PRJNA729271	stage_3W_vs_E10	ordinary_muscle_development	ENSGALG00010020493	DEPDC7	-1.501177868	0.162142517	-9.258385148	2.07547E-20	8.06541E-20	negative
PRJNA729271	stage_3W_vs_E10	ordinary_muscle_development	ENSGALG00010020497	OSBPL11	-0.399546339	0.069856094	-5.719563148	1.06798E-08	2.30192E-08	negative
PRJNA729271	stage_3W_vs_E10	ordinary_muscle_development	ENSGALG00010020498	FRMPD2	-1.141849822	0.579382335	-1.970805381	0.048746141	0.061281467	negative
PRJNA729271	stage_3W_vs_E10	ordinary_muscle_development	ENSGALG00010020507	ANKRD1	7.290074003	0.585290642	12.45547678	1.30569E-35	9.15624E-35	positive
PRJNA729271	stage_3W_vs_E10	ordinary_muscle_development	ENSGALG00010020509	CRBN	0.894910509	0.102598826	8.722424464	2.72307E-18	9.7411E-18	positive
PRJNA729271	stage_3W_vs_E10	ordinary_muscle_development	ENSGALG00010020514	CAMKMT	-1.123094583	0.139222055	-8.0669301	7.20878E-16	2.27699E-15	negative
PRJNA729271	stage_3W_vs_E10	ordinary_muscle_development	ENSGALG00010020517	UBR4	0.516628879	0.094111995	5.489511485	4.03047E-08	8.36598E-08	positive
PRJNA729271	stage_3W_vs_E10	ordinary_muscle_development	ENSGALG00010020520	ARHGAP22	-0.880631898	0.2028477	-4.341345244	1.41613E-05	2.47341E-05	negative
PRJNA729271	stage_3W_vs_E10	ordinary_muscle_development	ENSGALG00010020521	ENSGALG00010020521	-1.067416657	0.942940455	-1.132008549	0.25763084	0.293304982	negative
PRJNA729271	stage_3W_vs_E10	ordinary_muscle_development	ENSGALG00010020524	ENSGALG00010020524	0.459027467	0.404540601	1.134688252	0.256505961	0.292139067	positive
PRJNA729271	stage_3W_vs_E10	ordinary_muscle_development	ENSGALG00010020525	ENSGALG00010020525	0.985607805	0.722797687	1.363601216	0.172693109	0.20188617	positive
PRJNA729271	stage_3W_vs_E10	ordinary_muscle_development	ENSGALG00010020530	SRSF5A	-0.503547617	0.069373872	-7.258462024	3.91516E-13	1.07747E-12	negative
PRJNA729271	stage_3W_vs_E10	ordinary_muscle_development	ENSGALG00010020533	ENSGALG00010020533	-0.081583289	0.097737505	-0.834718352	0.403876306	0.442962096	negative
PRJNA729271	stage_3W_vs_E10	ordinary_muscle_development	ENSGALG00010020535	LMLN	-0.409295054	0.149571789	-2.736445538	0.006210687	0.008621329	negative
PRJNA729271	stage_3W_vs_E10	ordinary_muscle_development	ENSGALG00010020537	CCDC167	-0.689426138	0.098930991	-6.968758062	3.19751E-12	8.38573E-12	negative
PRJNA729271	stage_3W_vs_E10	ordinary_muscle_development	ENSGALG00010020540	PCGF5	1.866964085	0.16139924	11.567366	6.03057E-31	3.59134E-30	positive
PRJNA729271	stage_3W_vs_E10	ordinary_muscle_development	ENSGALG00010020544	BMERB1	-0.179204239	0.163448624	-1.096394909	0.272905991	0.309576799	negative
PRJNA729271	stage_3W_vs_E10	ordinary_muscle_development	ENSGALG00010020545	STYX	2.056978674	0.140777444	14.6115643	2.37013E-48	2.56572E-47	positive
PRJNA729271	stage_3W_vs_E10	ordinary_muscle_development	ENSGALG00010020546	LRRC20	3.085567564	0.236424681	13.05095369	6.27569E-39	4.89065E-38	positive
PRJNA729271	stage_3W_vs_E10	ordinary_muscle_development	ENSGALG00010020548	CMTR1	0.042161828	0.185044299	0.227847212	0.819765022	0.840821822	positive
PRJNA729271	stage_3W_vs_E10	ordinary_muscle_development	ENSGALG00010020550	HECTD2	1.569526519	0.212256256	7.394488844	1.41953E-13	3.99769E-13	positive
PRJNA729271	stage_3W_vs_E10	ordinary_muscle_development	ENSGALG00010020554	ZFP36L1	0.688883599	0.21073831	3.268905398	0.001079644	0.001613677	positive
PRJNA729271	stage_3W_vs_E10	ordinary_muscle_development	ENSGALG00010020556	EMC1	-0.176171991	0.085916194	-2.050509709	0.040314717	0.051192451	negative
PRJNA729271	stage_3W_vs_E10	ordinary_muscle_development	ENSGALG00010020557	ENSGALG00010020557	3.216957301	0.256411779	12.54605898	4.17791E-36	2.97294E-35	positive
PRJNA729271	stage_3W_vs_E10	ordinary_muscle_development	ENSGALG00010020558	EIF4EBP2	0.440631839	0.100117865	4.401131006	1.07688E-05	1.8978E-05	positive
PRJNA729271	stage_3W_vs_E10	ordinary_muscle_development	ENSGALG00010020562	LRRN1	-6.09459014	0.251213464	-24.26060313	5.1105E-130	3.5274E-128	negative
PRJNA729271	stage_3W_vs_E10	ordinary_muscle_development	ENSGALG00010020568	PLA2G4F	1.475312204	0.384368138	3.838279137	0.0001239	0.000200954	positive
PRJNA729271	stage_3W_vs_E10	ordinary_muscle_development	ENSGALG00010020569	DTX3L	3.452772587	0.250298229	13.7946345	2.74541E-43	2.46865E-42	positive
PRJNA729271	stage_3W_vs_E10	ordinary_muscle_development	ENSGALG00010020571	MRTO4	-0.710889436	0.082492932	-8.617579979	6.8384E-18	2.40064E-17	negative
PRJNA729271	stage_3W_vs_E10	ordinary_muscle_development	ENSGALG00010020572	PPP1R3C	3.416291642	0.271778054	12.57015273	3.08128E-36	2.19799E-35	positive
PRJNA729271	stage_3W_vs_E10	ordinary_muscle_development	ENSGALG00010020574	DTL	-3.53097005	0.19403388	-18.19769848	5.3822E-74	1.16789E-72	negative
PRJNA729271	stage_3W_vs_E10	ordinary_muscle_development	ENSGALG00010020576	AKR7L	0.701428993	0.104015612	6.743497245	1.54619E-11	3.91339E-11	positive
PRJNA729271	stage_3W_vs_E10	ordinary_muscle_development	ENSGALG00010020578	TNKS2	0.365512621	0.062820558	5.818359955	5.94278E-09	1.29984E-08	positive
PRJNA729271	stage_3W_vs_E10	ordinary_muscle_development	ENSGALG00010020580	ENSGALG00010020580	-0.60407235	0.116624419	-5.179638685	2.22316E-07	4.39448E-07	negative
PRJNA729271	stage_3W_vs_E10	ordinary_muscle_development	ENSGALG00010020582	KANSL3	1.152949065	0.147701535	7.805938266	5.90605E-15	1.7835E-14	positive
PRJNA729271	stage_3W_vs_E10	ordinary_muscle_development	ENSGALG00010020584	PARP9	3.291855113	0.287631804	11.44468404	2.50014E-30	1.45307E-29	positive
PRJNA729271	stage_3W_vs_E10	ordinary_muscle_development	ENSGALG00010020586	C11orf16	0.840570333	0.097079682	8.658560885	4.77757E-18	1.68987E-17	positive
PRJNA729271	stage_3W_vs_E10	ordinary_muscle_development	ENSGALG00010020588	PALD1	-1.045530775	0.110017661	-9.503299434	2.03343E-21	8.24234E-21	negative
PRJNA729271	stage_3W_vs_E10	ordinary_muscle_development	ENSGALG00010020590	INTS7	-0.950195929	0.079218394	-11.99463757	3.79073E-33	2.45078E-32	negative
PRJNA729271	stage_3W_vs_E10	ordinary_muscle_development	ENSGALG00010020594	PQLC2	0.118797628	0.148927563	0.797687315	0.425051967	0.463521537	positive
PRJNA729271	stage_3W_vs_E10	ordinary_muscle_development	ENSGALG00010020595	ZFYVE27	0.800686358	0.13069832	6.126217657	8.99927E-10	2.05651E-09	positive
PRJNA729271	stage_3W_vs_E10	ordinary_muscle_development	ENSGALG00010020596	SUMF1	0.536795856	0.120321117	4.461360301	8.1441E-06	1.44756E-05	positive
PRJNA729271	stage_3W_vs_E10	ordinary_muscle_development	ENSGALG00010020597	ARID5A	2.017732107	0.237771241	8.486022519	2.1383E-17	7.29973E-17	positive
PRJNA729271	stage_3W_vs_E10	ordinary_muscle_development	ENSGALG00010020599	PARN	-1.473483804	0.06838038	-21.54834198	5.4877E-103	2.4325E-101	negative
PRJNA729271	stage_3W_vs_E10	ordinary_muscle_development	ENSGALG00010020601	AKIP1	-0.758512058	0.168687432	-4.496553463	6.90638E-06	1.23346E-05	negative
PRJNA729271	stage_3W_vs_E10	ordinary_muscle_development	ENSGALG00010020602	DENND2B	-0.93822519	0.097430776	-9.629659386	5.99278E-22	2.47762E-21	negative
PRJNA729271	stage_3W_vs_E10	ordinary_muscle_development	ENSGALG00010020603	VPS39	0.497311633	0.092678319	5.36599753	8.0503E-08	1.6412E-07	positive
PRJNA729271	stage_3W_vs_E10	ordinary_muscle_development	ENSGALG00010020605	STAMBP	-0.261080514	0.089057446	-2.931596679	0.003372244	0.004813459	negative
PRJNA729271	stage_3W_vs_E10	ordinary_muscle_development	ENSGALG00010020607	ENSGALG00010020607	4.023675334	0.238457655	16.87375202	7.01927E-64	1.16546E-62	positive
PRJNA729271	stage_3W_vs_E10	ordinary_muscle_development	ENSGALG00010020608	CAPZB	0.974250598	0.099385748	9.802719389	1.09595E-22	4.66542E-22	positive
PRJNA729271	stage_3W_vs_E10	ordinary_muscle_development	ENSGALG00010020609	AVPI1	-0.318036384	0.234057781	-1.35879432	0.174211774	0.203481036	negative
PRJNA729271	stage_3W_vs_E10	ordinary_muscle_development	ENSGALG00010020611	LPGAT1	-0.276200642	0.099739253	-2.769227093	0.005618946	0.007835172	negative
PRJNA729271	stage_3W_vs_E10	ordinary_muscle_development	ENSGALG00010020612	RPL27A	0.536888262	0.19278104	2.784964028	0.005353366	0.007485005	positive
PRJNA729271	stage_3W_vs_E10	ordinary_muscle_development	ENSGALG00010020613	ACTG2	-0.973318593	0.27587783	-3.52807833	0.000418588	0.000648854	negative
PRJNA729271	stage_3W_vs_E10	ordinary_muscle_development	ENSGALG00010020614	FAIM	-0.550805518	0.110923205	-4.965647336	6.84723E-07	1.31076E-06	negative
PRJNA729271	stage_3W_vs_E10	ordinary_muscle_development	ENSGALG00010020616	PI4K2A	1.064760539	0.13023239	8.175850398	2.93786E-16	9.48426E-16	positive
PRJNA729271	stage_3W_vs_E10	ordinary_muscle_development	ENSGALG00010020617	DGUOK	-0.387073749	0.114228534	-3.388590721	0.000702528	0.001067191	negative
PRJNA729271	stage_3W_vs_E10	ordinary_muscle_development	ENSGALG00010020618	ENSGALG00010020618	-0.06499968	0.316119489	-0.205617439	0.837089754	0.855912474	negative
PRJNA729271	stage_3W_vs_E10	ordinary_muscle_development	ENSGALG00010020619	QARS	-0.051014099	0.099547108	-0.51246189	0.608327786	0.643581582	negative
PRJNA729271	stage_3W_vs_E10	ordinary_muscle_development	ENSGALG00010020620	MARF1	0.322729786	0.103259398	3.125427725	0.001775467	0.002597698	positive
PRJNA729271	stage_3W_vs_E10	ordinary_muscle_development	ENSGALG00010020622	ABCG2	1.75826995	0.116352616	15.11156351	1.35877E-51	1.62369E-50	positive
PRJNA729271	stage_3W_vs_E10	ordinary_muscle_development	ENSGALG00010020623	NEK2	-2.69996877	0.232073758	-11.63409768	2.76499E-31	1.66301E-30	negative
PRJNA729271	stage_3W_vs_E10	ordinary_muscle_development	ENSGALG00010020624	MINOS1	0.974669485	0.135702796	7.182383241	6.85066E-13	1.86235E-12	positive
PRJNA729271	stage_3W_vs_E10	ordinary_muscle_development	ENSGALG00010020625	SLC30A1	-0.356731021	0.076514206	-4.662284811	3.12718E-06	5.73851E-06	negative
PRJNA729271	stage_3W_vs_E10	ordinary_muscle_development	ENSGALG00010020626	MORN4	-0.56794518	0.119013649	-4.772101216	1.82314E-06	3.39191E-06	negative
PRJNA729271	stage_3W_vs_E10	ordinary_muscle_development	ENSGALG00010020629	TRIM66	-3.405933024	0.253587675	-13.43098803	3.98065E-41	3.31988E-40	negative
PRJNA729271	stage_3W_vs_E10	ordinary_muscle_development	ENSGALG00010020630	QRICH1	-0.059380486	0.12614274	-0.47074042	0.637826116	0.671261857	negative
PRJNA729271	stage_3W_vs_E10	ordinary_muscle_development	ENSGALG00010020632	BLOC1S2	0.136579435	0.111528375	1.224616019	0.220719929	0.253734267	positive
PRJNA729271	stage_3W_vs_E10	ordinary_muscle_development	ENSGALG00010020633	HOGA1	0.214725366	0.35547899	0.604045168	0.545813613	0.583104976	positive
PRJNA729271	stage_3W_vs_E10	ordinary_muscle_development	ENSGALG00010020635	NDE1	-0.523861044	0.134093528	-3.90668402	9.35714E-05	0.000153066	negative
PRJNA729271	stage_3W_vs_E10	ordinary_muscle_development	ENSGALG00010020636	NBL1	-0.837917715	0.178722684	-4.688368	2.75392E-06	5.0664E-06	negative
PRJNA729271	stage_3W_vs_E10	ordinary_muscle_development	ENSGALG00010020638	IMPDH2	1.288981862	0.111338181	11.57717729	5.37881E-31	3.20979E-30	positive
PRJNA729271	stage_3W_vs_E10	ordinary_muscle_development	ENSGALG00010020640	PTPN14	-0.622911959	0.092911071	-6.704388927	2.02251E-11	5.08786E-11	negative
PRJNA729271	stage_3W_vs_E10	ordinary_muscle_development	ENSGALG00010020642	ANKRD2	3.923610773	0.629203623	6.235836269	4.49371E-10	1.04721E-09	positive
PRJNA729271	stage_3W_vs_E10	ordinary_muscle_development	ENSGALG00010020646	CEP70	-0.281162616	0.115831789	-2.427335534	0.015210181	0.020263932	negative
PRJNA729271	stage_3W_vs_E10	ordinary_muscle_development	ENSGALG00010020648	TET3	-0.669249606	0.150970263	-4.432989595	9.29354E-06	1.64461E-05	negative
PRJNA729271	stage_3W_vs_E10	ordinary_muscle_development	ENSGALG00010020649	MYH11	3.336425744	0.212096116	15.7307253	9.31381E-56	1.25352E-54	positive
PRJNA729271	stage_3W_vs_E10	ordinary_muscle_development	ENSGALG00010020651	LMO1	-1.069056381	0.192576185	-5.551342598	2.83484E-08	5.938E-08	negative
PRJNA729271	stage_3W_vs_E10	ordinary_muscle_development	ENSGALG00010020653	NDUFAF3	1.15353371	0.117440588	9.822274646	9.0283E-23	3.85805E-22	positive
PRJNA729271	stage_3W_vs_E10	ordinary_muscle_development	ENSGALG00010020654	SAYSD1	0.176405271	0.148230511	1.190073959	0.234017324	0.26802158	positive
PRJNA729271	stage_3W_vs_E10	ordinary_muscle_development	ENSGALG00010020655	RIC3	-1.703473312	0.14003358	-12.16474874	4.78917E-34	3.18728E-33	negative
PRJNA729271	stage_3W_vs_E10	ordinary_muscle_development	ENSGALG00010020657	DALRD3	0.657774584	0.092362932	7.121629498	1.06658E-12	2.8731E-12	positive
PRJNA729271	stage_3W_vs_E10	ordinary_muscle_development	ENSGALG00010020659	RAD51B	-0.921768251	0.136449394	-6.755385454	1.42456E-11	3.61123E-11	negative
PRJNA729271	stage_3W_vs_E10	ordinary_muscle_development	ENSGALG00010020662	BFAR	-0.191975254	0.087401963	-2.19646387	0.028058758	0.036290863	negative
PRJNA729271	stage_3W_vs_E10	ordinary_muscle_development	ENSGALG00010020664	ESYT3	1.710751242	0.432318301	3.957156659	7.58472E-05	0.000125075	positive
PRJNA729271	stage_3W_vs_E10	ordinary_muscle_development	ENSGALG00010020666	TMCO4	1.061245088	0.204964015	5.177714181	2.24621E-07	4.43943E-07	positive
PRJNA729271	stage_3W_vs_E10	ordinary_muscle_development	ENSGALG00010020667	UBTD1	-0.226073243	0.158004331	-1.430804095	0.152486369	0.179683759	negative
PRJNA729271	stage_3W_vs_E10	ordinary_muscle_development	ENSGALG00010020670	ENSGALG00010020670	-0.370599289	0.135070475	-2.743747581	0.006074222	0.008440788	negative
PRJNA729271	stage_3W_vs_E10	ordinary_muscle_development	ENSGALG00010020673	MMS19	-0.202897488	0.128261354	-1.581906647	0.113670883	0.13638436	negative
PRJNA729271	stage_3W_vs_E10	ordinary_muscle_development	ENSGALG00010020674	TRPV4	-8.789368279	0.94523584	-9.298598198	1.42309E-20	5.56304E-20	negative
PRJNA729271	stage_3W_vs_E10	ordinary_muscle_development	ENSGALG00010020675	SMYD2	-0.816387326	0.179277643	-4.553759809	5.26955E-06	9.50381E-06	negative
PRJNA729271	stage_3W_vs_E10	ordinary_muscle_development	ENSGALG00010020676	CYP2C23a	6.398481094	1.378212527	4.642593916	3.44062E-06	6.29695E-06	positive
PRJNA729271	stage_3W_vs_E10	ordinary_muscle_development	ENSGALG00010020677	CYR61	1.113058283	0.232891461	4.779300532	1.75906E-06	3.27564E-06	positive
PRJNA729271	stage_3W_vs_E10	ordinary_muscle_development	ENSGALG00010020679	OTUD3	0.671013284	0.107132437	6.263399802	3.76673E-10	8.81336E-10	positive
PRJNA729271	stage_3W_vs_E10	ordinary_muscle_development	ENSGALG00010020680	CFAP221	0.485234823	0.50353987	0.963647274	0.335222797	0.373514831	positive
PRJNA729271	stage_3W_vs_E10	ordinary_muscle_development	ENSGALG00010020681	BCL10	-0.091962276	0.087349961	-1.052802705	0.292431425	0.329713247	negative
PRJNA729271	stage_3W_vs_E10	ordinary_muscle_development	ENSGALG00010020682	ZDHHC16	0.493710992	0.168360705	2.932459765	0.003362885	0.004801046	positive
PRJNA729271	stage_3W_vs_E10	ordinary_muscle_development	ENSGALG00010020684	P4HTM	1.332524516	0.12008065	11.09691294	1.29852E-28	7.07329E-28	positive
PRJNA729271	stage_3W_vs_E10	ordinary_muscle_development	ENSGALG00010020685	RPL6	0.143802485	0.1160427	1.239220433	0.215263876	0.248012867	positive
PRJNA729271	stage_3W_vs_E10	ordinary_muscle_development	ENSGALG00010020686	C1orf52	0.092309773	0.12277395	0.751867747	0.452130596	0.490908838	positive
PRJNA729271	stage_3W_vs_E10	ordinary_muscle_development	ENSGALG00010020691	ENSGALG00010020691	1.184202024	0.163276657	7.252733169	4.08444E-13	1.12214E-12	positive
PRJNA729271	stage_3W_vs_E10	ordinary_muscle_development	ENSGALG00010020692	TUB	-0.882707618	0.154757434	-5.703813979	1.17156E-08	2.51768E-08	negative
PRJNA729271	stage_3W_vs_E10	ordinary_muscle_development	ENSGALG00010020693	PTGFR	-0.362143138	0.189817489	-1.907849167	0.056410714	0.070379549	negative
PRJNA729271	stage_3W_vs_E10	ordinary_muscle_development	ENSGALG00010020696	MOB1A	-0.825801977	0.1015073	-8.135394983	4.10597E-16	1.31266E-15	negative
PRJNA729271	stage_3W_vs_E10	ordinary_muscle_development	ENSGALG00010020699	PLA2G10	0.462513767	0.410717043	1.126112916	0.260117728	0.295996739	positive
PRJNA729271	stage_3W_vs_E10	ordinary_muscle_development	ENSGALG00010020700	MTHFD2	1.717779777	0.131301721	13.08269054	4.13539E-39	3.25068E-38	positive
PRJNA729271	stage_3W_vs_E10	ordinary_muscle_development	ENSGALG00010020701	PTPN11	-0.763262839	0.085363774	-8.941296763	3.8463E-19	1.42334E-18	negative
PRJNA729271	stage_3W_vs_E10	ordinary_muscle_development	ENSGALG00010020704	PROX1	0.639951067	0.233798423	2.737191549	0.00619662	0.008603449	positive
PRJNA729271	stage_3W_vs_E10	ordinary_muscle_development	ENSGALG00010020705	EXOSC1	1.535611578	0.173113762	8.870534398	7.27957E-19	2.65921E-18	positive
PRJNA729271	stage_3W_vs_E10	ordinary_muscle_development	ENSGALG00010020707	SLC4A5	-2.043141724	0.484536336	-4.216694539	2.47909E-05	4.24607E-05	negative
PRJNA729271	stage_3W_vs_E10	ordinary_muscle_development	ENSGALG00010020708	FAM214A	0.362574242	0.133826876	2.709278236	0.006742977	0.009332393	positive
PRJNA729271	stage_3W_vs_E10	ordinary_muscle_development	ENSGALG00010020712	ZC3H7A	-0.756986248	0.071070566	-10.651192	1.72146E-26	8.62517E-26	negative
PRJNA729271	stage_3W_vs_E10	ordinary_muscle_development	ENSGALG00010020713	SCTR	-4.772716313	1.330124724	-3.588172016	0.000333005	0.000521151	negative

PRJNA729271	stage_3W_vs_E10	ordinary_muscle_development	ENSGALG00010020714	PGAM1	4.43445703	0.14444457	30.70006047	5.6811E-207	1.098E-204	positive
PRJNA729271	stage_3W_vs_E10	ordinary_muscle_development	ENSGALG00010020715	ENSGALG00010020715	-1.133665969	0.881161682	-1.286558405	0.198248249	0.229594789	negative
PRJNA729271	stage_3W_vs_E10	ordinary_muscle_development	ENSGALG00010020716	RRAS	0.720082827	0.179293383	4.01622646	5.91374E-05	9.84831E-05	positive
PRJNA729271	stage_3W_vs_E10	ordinary_muscle_development	ENSGALG00010020719	RPH3A	-2.129399472	0.670094573	-3.177759615	0.001484177	0.002189411	negative
PRJNA729271	stage_3W_vs_E10	ordinary_muscle_development	ENSGALG00010020720	ENSGALG00010020720	-6.191448294	0.601526862	-10.29288746	7.58665E-25	3.52689E-24	negative
PRJNA729271	stage_3W_vs_E10	ordinary_muscle_development	ENSGALG00010020721	PTDSS2	0.336727206	0.169951776	1.981310309	0.047556485	0.059874164	positive
PRJNA729271	stage_3W_vs_E10	ordinary_muscle_development	ENSGALG00010020723	RPS24	0.307383685	0.190111086	1.616863549	0.105907738	0.127640531	positive
PRJNA729271	stage_3W_vs_E10	ordinary_muscle_development	ENSGALG00010020724	TACR1	-4.034491281	0.300544857	-13.42392388	4.37899E-41	3.64581E-40	negative
PRJNA729271	stage_3W_vs_E10	ordinary_muscle_development	ENSGALG00010020725	FOPNL	-0.41947397	0.110444179	-3.798063181	0.000145831	0.000235078	negative
PRJNA729271	stage_3W_vs_E10	ordinary_muscle_development	ENSGALG00010020728	TMEM37	1.598923438	0.207906695	7.690581758	1.46467E-14	4.33275E-14	positive
PRJNA729271	stage_3W_vs_E10	ordinary_muscle_development	ENSGALG00010020730	RRP12	-0.496656448	0.462126062	-1.074720706	0.28249979	0.319584364	negative
PRJNA729271	stage_3W_vs_E10	ordinary_muscle_development	ENSGALG00010020732	ARIH2	1.603071326	0.142753744	11.22962721	2.91707E-29	1.63381E-28	positive
PRJNA729271	stage_3W_vs_E10	ordinary_muscle_development	ENSGALG00010020733	TMEM229B	-1.213300053	0.108848825	-11.14665275	7.43509E-29	4.09001E-28	negative
PRJNA729271	stage_3W_vs_E10	ordinary_muscle_development	ENSGALG00010020736	LRRTM4	-0.926666687	0.233660408	-3.965869503	7.31289E-05	0.000120826	negative
PRJNA729271	stage_3W_vs_E10	ordinary_muscle_development	ENSGALG00010020737	POLR3A	-0.150003893	0.115931169	-1.293904782	0.195698313	0.226822888	negative
PRJNA729271	stage_3W_vs_E10	ordinary_muscle_development	ENSGALG00010020739	PKP3	-4.91995717	0.786006765	-6.259433617	3.86378E-10	9.03752E-10	negative
PRJNA729271	stage_3W_vs_E10	ordinary_muscle_development	ENSGALG00010020742	THAP10	-1.152248324	0.205126465	-5.617258241	1.94011E-08	4.1132E-08	negative
PRJNA729271	stage_3W_vs_E10	ordinary_muscle_development	ENSGALG00010020743	PURB	0.60004166	0.169430546	3.541519957	0.000397829	0.000617732	positive
PRJNA729271	stage_3W_vs_E10	ordinary_muscle_development	ENSGALG00010020744	CHST3	-1.775436955	0.11929378	-14.88289625	4.25666E-50	4.84684E-49	negative
PRJNA729271	stage_3W_vs_E10	ordinary_muscle_development	ENSGALG00010020745	ABCC1	1.033192567	0.166309344	6.212474568	5.21566E-10	1.21039E-09	positive
PRJNA729271	stage_3W_vs_E10	ordinary_muscle_development	ENSGALG00010020747	DBI	-0.047357978	0.120889784	-0.391745081	0.695246577	0.725136016	negative
PRJNA729271	stage_3W_vs_E10	ordinary_muscle_development	ENSGALG00010020750	AIFM2	1.486894332	0.284071506	5.234225533	1.65678E-07	3.30058E-07	positive
PRJNA729271	stage_3W_vs_E10	ordinary_muscle_development	ENSGALG00010020753	SYDE2	1.82173759	0.094862937	19.20389189	3.43373E-82	9.08247E-81	positive
PRJNA729271	stage_3W_vs_E10	ordinary_muscle_development	ENSGALG00010020754	SLC25A20	-0.643282845	0.115116223	-5.588116325	2.29546E-08	4.84199E-08	negative
PRJNA729271	stage_3W_vs_E10	ordinary_muscle_development	ENSGALG00010020756	MACROH2A2	-4.816699854	0.173079852	-27.82935047	1.9154E-170	2.373E-168	negative
PRJNA729271	stage_3W_vs_E10	ordinary_muscle_development	ENSGALG00010020759	ARHGAP19	-3.656917553	0.181363258	-20.16349729	2.04876E-90	6.79562E-89	negative
PRJNA729271	stage_3W_vs_E10	ordinary_muscle_development	ENSGALG00010020760	TXNDC11	-0.388975555	0.095556214	-4.070646364	4.68829E-05	7.86536E-05	negative
PRJNA729271	stage_3W_vs_E10	ordinary_muscle_development	ENSGALG00010020762	TTBK2	0.114714836	0.14122012	0.812312266	0.416612474	0.455380274	positive
PRJNA729271	stage_3W_vs_E10	ordinary_muscle_development	ENSGALG00010020764	PRKAR2A	0.016969664	0.098848836	0.171672881	0.863694707	0.879469953	positive
PRJNA729271	stage_3W_vs_E10	ordinary_muscle_development	ENSGALG00010020765	GLT1D1	-2.644023497	0.211487049	-12.50205868	7.27428E-36	5.13343E-35	negative
PRJNA729271	stage_3W_vs_E10	ordinary_muscle_development	ENSGALG00010020766	C2orf76	0.280153649	0.12046509	2.325600296	0.020039884	0.026371141	positive
PRJNA729271	stage_3W_vs_E10	ordinary_muscle_development	ENSGALG00010020767	UBXN10	-0.264817463	0.174679647	-1.516017847	0.12951484	0.154283169	negative
PRJNA729271	stage_3W_vs_E10	ordinary_muscle_development	ENSGALG00010020768	DNAH8	0.362374982	0.598427609	0.605545227	0.544816783	0.582211683	positive
PRJNA729271	stage_3W_vs_E10	ordinary_muscle_development	ENSGALG00010020770	B4GALNT4	-5.305702058	0.209744827	-25.29598531	3.5363E-141	2.8959E-139	negative
PRJNA729271	stage_3W_vs_E10	ordinary_muscle_development	ENSGALG00010020771	LINGO1	3.115797187	0.199988351	15.57989337	9.9721E-55	1.30339E-53	positive
PRJNA729271	stage_3W_vs_E10	ordinary_muscle_development	ENSGALG00010020773	ZCCHC24	0.948139254	0.089450772	10.59956479	2.9937E-26	1.47899E-25	positive
PRJNA729271	stage_3W_vs_E10	ordinary_muscle_development	ENSGALG00010020775	UBR1	0.980223266	0.080915066	12.11422441	8.88001E-34	5.85604E-33	positive
PRJNA729271	stage_3W_vs_E10	ordinary_muscle_development	ENSGALG00010020776	MCOLN3	0.057889826	0.437780518	0.132234815	0.894798565	0.906810123	positive
PRJNA729271	stage_3W_vs_E10	ordinary_muscle_development	ENSGALG00010020778	SLIT1	-1.734412595	0.231708568	-7.485319196	7.13735E-14	2.04337E-13	negative
PRJNA729271	stage_3W_vs_E10	ordinary_muscle_development	ENSGALG00010020779	SLC15A4	0.281635905	0.065124446	4.324580409	1.52822E-05	2.66277E-05	positive
PRJNA729271	stage_3W_vs_E10	ordinary_muscle_development	ENSGALG00010020781	SNN	-0.015102781	0.136591844	-0.110568686	0.91195838	0.922260631	negative
PRJNA729271	stage_3W_vs_E10	ordinary_muscle_development	ENSGALG00010020782	PPIF	-0.090321574	0.157908227	-0.571987765	0.567330263	0.603822026	negative
PRJNA729271	stage_3W_vs_E10	ordinary_muscle_development	ENSGALG00010020786	DDX19	-0.302148432	0.131326721	-2.300738415	0.021406421	0.028090356	negative
PRJNA729271	stage_3W_vs_E10	ordinary_muscle_development	ENSGALG00010020787	TMEM132C	-4.529305981	0.312396768	-14.49856861	1.23703E-47	1.29464E-46	negative
PRJNA729271	stage_3W_vs_E10	ordinary_muscle_development	ENSGALG00010020790	PRPH2L	2.744574889	0.302689395	9.06729781	1.22005E-19	4.59699E-19	positive
PRJNA729271	stage_3W_vs_E10	ordinary_muscle_development	ENSGALG00010020794	STEAP3	-0.077702599	0.142580024	-0.544975354	0.585770469	0.621395122	negative
PRJNA729271	stage_3W_vs_E10	ordinary_muscle_development	ENSGALG00010020797	DGCR8	-0.895520801	0.066598872	-13.44648611	3.22854E-41	2.69727E-40	negative
PRJNA729271	stage_3W_vs_E10	ordinary_muscle_development	ENSGALG00010020798	C1QL2	-1.270857401	0.568447428	-2.235663913	0.025373795	0.032974104	negative
PRJNA729271	stage_3W_vs_E10	ordinary_muscle_development	ENSGALG00010020803	ABCC6	2.206863912	0.156378887	14.11228811	3.19055E-45	3.07494E-44	positive
PRJNA729271	stage_3W_vs_E10	ordinary_muscle_development	ENSGALG00010020806	HTD2	0.417096356	0.172862361	2.41288129	0.015826973	0.021046971	positive
PRJNA729271	stage_3W_vs_E10	ordinary_muscle_development	ENSGALG00010020809	ENSGALG00010020809	0.194333231	0.221675409	0.876656692	0.380673136	0.419545097	positive
PRJNA729271	stage_3W_vs_E10	ordinary_muscle_development	ENSGALG00010020810	IFITM5	0.710947326	0.242543952	2.931210277	0.003376441	0.004818976	positive
PRJNA729271	stage_3W_vs_E10	ordinary_muscle_development	ENSGALG00010020811	MCOLN2	1.336110978	0.637819866	2.094809285	0.036187949	0.046239803	positive
PRJNA729271	stage_3W_vs_E10	ordinary_muscle_development	ENSGALG00010020813	NPY4R	0.539707988	0.544554001	0.991100949	0.321636289	0.359786851	positive
PRJNA729271	stage_3W_vs_E10	ordinary_muscle_development	ENSGALG00010020816	TRMT2A	0.239570627	0.089839883	2.666640022	0.007661368	0.010551215	positive
PRJNA729271	stage_3W_vs_E10	ordinary_muscle_development	ENSGALG00010020818	TMEM62	0.625982013	0.195350892	3.204398033	0.001353452	0.002004935	positive
PRJNA729271	stage_3W_vs_E10	ordinary_muscle_development	ENSGALG00010020820	VWA5B1	-0.040605376	0.440828887	-0.092111422	0.926609513	0.934930036	negative
PRJNA729271	stage_3W_vs_E10	ordinary_muscle_development	ENSGALG00010020827	ENSGALG00010020827	-0.585094877	0.092097125	-6.353019992	2.11128E-10	5.0136E-10	negative
PRJNA729271	stage_3W_vs_E10	ordinary_muscle_development	ENSGALG00010020828	CAMK2N1	-0.288992535	0.221595096	-1.3041468	0.192183535	0.222927363	negative
PRJNA729271	stage_3W_vs_E10	ordinary_muscle_development	ENSGALG00010020831	MUL1	0.890613198	0.143668545	6.199082759	5.67932E-10	1.31558E-09	positive
PRJNA729271	stage_3W_vs_E10	ordinary_muscle_development	ENSGALG00010020833	RANBP1	-2.387189165	0.084005754	-28.41697205	1.2478E-177	1.7063E-175	negative
PRJNA729271	stage_3W_vs_E10	ordinary_muscle_development	ENSGALG00010020835	PTGR2	-0.450863173	0.115519841	-3.902906813	9.50443E-05	0.000155353	negative
PRJNA729271	stage_3W_vs_E10	ordinary_muscle_development	ENSGALG00010020839	PGGHG	-1.413926223	0.149142805	-9.480351526	2.53428E-21	1.0241E-20	negative
PRJNA729271	stage_3W_vs_E10	ordinary_muscle_development	ENSGALG00010020840	LPAR3	-2.715143896	0.295931957	-9.174892523	4.52025E-20	1.73153E-19	negative
PRJNA729271	stage_3W_vs_E10	ordinary_muscle_development	ENSGALG00010020843	HMG20A	-1.415148213	0.084258944	-16.79522846	2.64484E-63	4.32209E-62	negative
PRJNA729271	stage_3W_vs_E10	ordinary_muscle_development	ENSGALG00010020845	RAB35	0.056665109	0.092521578	0.612452898	0.540238152	0.577872631	positive
PRJNA729271	stage_3W_vs_E10	ordinary_muscle_development	ENSGALG00010020847	MIDEAS	0.035887942	0.09017061	0.398000433	0.690629867	0.721022754	positive
PRJNA729271	stage_3W_vs_E10	ordinary_muscle_development	ENSGALG00010020854	ENSGALG00010020854	12.05893292	0.380983462	31.65211652	7.0914E-220	1.4684E-217	positive
PRJNA729271	stage_3W_vs_E10	ordinary_muscle_development	ENSGALG00010020855	DNAL1	0.473633032	0.098597571	4.803698784	1.55761E-06	2.91023E-06	positive
PRJNA729271	stage_3W_vs_E10	ordinary_muscle_development	ENSGALG00010020856	VTG2	-4.329927722	0.986898223	-4.387410599	1.14708E-05	2.01759E-05	negative
PRJNA729271	stage_3W_vs_E10	ordinary_muscle_development	ENSGALG00010020861	CDA	1.740384358	0.173411642	10.03614483	1.05726E-23	4.67937E-23	positive
PRJNA729271	stage_3W_vs_E10	ordinary_muscle_development	ENSGALG00010020865	BICDL1	-2.013604961	0.836845915	-2.406183654	0.016120159	0.021417204	negative
PRJNA729271	stage_3W_vs_E10	ordinary_muscle_development	ENSGALG00010020866	LCOR	-0.788845567	0.100016074	-7.887187916	3.09072E-15	9.45745E-15	negative
PRJNA729271	stage_3W_vs_E10	ordinary_muscle_development	ENSGALG00010020870	RIOX1	-0.123162891	0.147823604	-0.833174727	0.404746194	0.443681168	negative
PRJNA729271	stage_3W_vs_E10	ordinary_muscle_development	ENSGALG00010020873	PINK1	2.788607455	0.136223233	20.47086526	3.91606E-93	1.39467E-91	positive
PRJNA729271	stage_3W_vs_E10	ordinary_muscle_development	ENSGALG00010020875	ENSGALG00010020875	0.76859546	0.172518452	4.4551493	8.38348E-06	1.48792E-05	positive
PRJNA729271	stage_3W_vs_E10	ordinary_muscle_development	ENSGALG00010020878	XYLT1	-0.696605185	0.125111378	-5.567880346	2.57857E-08	5.41672E-08	negative
PRJNA729271	stage_3W_vs_E10	ordinary_muscle_development	ENSGALG00010020880	PXK	2.488449792	0.16342604	15.22676433	2.34934E-52	2.86889E-51	positive
PRJNA729271	stage_3W_vs_E10	ordinary_muscle_development	ENSGALG00010020886	GBF1	-0.435644963	0.086431793	-5.040332345	4.64724E-07	8.99836E-07	negative
PRJNA729271	stage_3W_vs_E10	ordinary_muscle_development	ENSGALG00010020887	ARPP19	-0.941375715	0.071254051	-13.2115395	7.52691E-40	6.06462E-39	negative
PRJNA729271	stage_3W_vs_E10	ordinary_muscle_development	ENSGALG00010020889	PPRC1	0.144791534	0.281617595	0.514142359	0.607152464	0.642478827	positive
PRJNA729271	stage_3W_vs_E10	ordinary_muscle_development	ENSGALG00010020890	RPS15A	0.216411191	0.188360818	1.148918298	0.250589672	0.285827612	positive
PRJNA729271	stage_3W_vs_E10	ordinary_muscle_development	ENSGALG00010020894	PSMD13	0.644888854	0.120754139	5.34051139	9.26848E-08	1.88134E-07	positive
PRJNA729271	stage_3W_vs_E10	ordinary_muscle_development	ENSGALG00010020896	MARCO	4.856992589	0.390548895	12.43632397	1.65968E-35	1.15993E-34	positive
PRJNA729271	stage_3W_vs_E10	ordinary_muscle_development	ENSGALG00010020898	ENSGALG00010020898	0.060966757	0.129460889	0.470927998	0.637692154	0.671169603	positive
PRJNA729271	stage_3W_vs_E10	ordinary_muscle_development	ENSGALG00010020900	PDHB	0.425908112	0.116101364	3.668416084	0.000244058	0.000385529	positive
PRJNA729271	stage_3W_vs_E10	ordinary_muscle_development	ENSGALG00010020903	AGMAT	1.846494099	0.377772049	4.887852613	1.01942E-06	1.93106E-06	positive
PRJNA729271	stage_3W_vs_E10	ordinary_muscle_development	ENSGALG00010020905	KCTD6	-0.537590051	0.088488365	-6.075262531	1.23785E-09	2.80617E-09	negative
PRJNA729271	stage_3W_vs_E10	ordinary_muscle_development	ENSGALG00010020906	SIRT3	1.755177683	0.108368478	16.19638587	5.34792E-59	7.84596E-58	positive
PRJNA729271	stage_3W_vs_E10	ordinary_muscle_development	ENSGALG00010020912	PDE8A	1.141471165	0.14373701	7.941386598	1.99933E-15	6.16603E-15	positive
PRJNA729271	stage_3W_vs_E10	ordinary_muscle_development	ENSGALG00010020913	TMEM132B	-3.541908582	0.282456217	-12.53967296	4.52861E-36	3.21933E-35	negative
PRJNA729271	stage_3W_vs_E10	ordinary_muscle_development	ENSGALG00010020915	EN1	-4.414520475	0.392319439	-11.25236234	2.25471E-29	1.26724E-28	negative
PRJNA729271	stage_3W_vs_E10	ordinary_muscle_development	ENSGALG00010020916	ARL6IP1	-0.347345866	0.097894984	-3.54814774	0.000387951	0.000603363	negative
PRJNA729271	stage_3W_vs_E10	ordinary_muscle_development	ENSGALG00010020918	ENSGALG00010020918	-3.157796167	0.364278218	-8.66863844	4.37318E-18	1.55024E-17	negative
PRJNA729271	stage_3W_vs_E10	ordinary_muscle_development	ENSGALG00010020920	ACOX2	1.919734052	0.205636696	9.335561635	1.00457E-20	3.95472E-20	positive
PRJNA729271	stage_3W_vs_E10	ordinary_muscle_development	ENSGALG00010020921	LDB1	-0.543260702	0.152839033	-3.554463101	0.000378752	0.000589562	negative
PRJNA729271	stage_3W_vs_E10	ordinary_muscle_development	ENSGALG00010020922	INSIG2	0.349060838	0.08013328	4.356003377	1.32459E-05	2.31883E-05	positive
PRJNA729271	stage_3W_vs_E10	ordinary_muscle_development	ENSGALG00010020924	DNAJC16	0.983468803	0.119365616	8.239129803	1.73468E-16	5.66182E-16	positive
PRJNA729271	stage_3W_vs_E10	ordinary_muscle_development	ENSGALG00010020925	RIC8A	0.085456808	0.103822544	0.823104542	0.410448546	0.449218641	positive
PRJNA729271	stage_3W_vs_E10	ordinary_muscle_development	ENSGALG00010020927	ENSGALG00010020927	-0.628922526	0.118268224	-5.317764162	1.0505E-07	2.1243E-07	negative
PRJNA729271	stage_3W_vs_E10	ordinary_muscle_development	ENSGALG00010020928	AACS	-1.079449382	0.151651713	-7.117950458	1.09544E-12	2.94753E-12	negative
PRJNA729271	stage_3W_vs_E10	ordinary_muscle_development	ENSGALG00010020932	CASP9	0.018450809	0.120573572	0.153025315	0.878378312	0.892415619	positive
PRJNA729271	stage_3W_vs_E10	ordinary_muscle_development	ENSGALG00010020934	ABCD3	0.114781273	0.153676353	0.746902634	0.455122336	0.493602563	positive
PRJNA729271	stage_3W_vs_E10	ordinary_muscle_development	ENSGALG00010020935	BRI3BP	-0.047870341	0.065065536	-0.735724992	0.461898077	0.500539221	negative
PRJNA729271	stage_3W_vs_E10	ordinary_muscle_development	ENSGALG00010020936	BET1L	-0.295398029	0.126394314	-2.337114855	0.019433213	0.02559604	negative
PRJNA729271	stage_3W_vs_E10	ordinary_muscle_development	ENSGALG00010020938	SMG1	0.147687054	0.080000015	1.846087833	0.064879453	0.080330373	positive
PRJNA729271	stage_3W_vs_E10	ordinary_muscle_development	ENSGALG00010020939	CTRC	9.273531567	2.111168429	4.392606217	1.12E-05	1.97091E-05	positive
PRJNA729271	stage_3W_vs_E10	ordinary_muscle_development	ENSGALG00010020940	ENSGALG00010020940	-1.239469017	0.134636905	-9.206012398	3.38465E-20	1.30411E-19	negative
PRJNA729271	stage_3W_vs_E10	ordinary_muscle_development	ENSGALG00010020941	ENSGALG00010020941	2.591513872	0.184026261	14.08230466	4.87922E-45	4.66519E-44	positive
PRJNA729271	stage_3W_vs_E10	ordinary_muscle_development	ENSGALG00010020943	FAM107A	0.926759837	0.367008883	2.525170042	0.011564232	0.015624864	positive
PRJNA729271	stage_3W_vs_E10	ordinary_muscle_development	ENSGALG00010020944	MYO5A	-1.171375584	0.156514858	-7.484117474	7.20295E-14	2.06134E-13	negative
PRJNA729271	stage_3W_vs_E10	ordinary_muscle_development	ENSGALG00010020945	NUMB	0.388914232	0.086264019	4.508417729	6.53129E-06	1.16993E-05	positive
PRJNA729271	stage_3W_vs_E10	ordinary_muscle_development	ENSGALG00010020946	CTBS	0.719375561	0.215499288	3.338180679	0.000843289	0.001271424	positive
PRJNA729271	stage_3W_vs_E10	ordinary_muscle_development	ENSGALG00010020949	HPS6	0.716023956	0.109571123	6.53478706	6.37001E-11	1.55417E-10	positive
PRJNA729271	stage_3W_vs_E10	ordinary_muscle_development	ENSGALG00010020950	EFHD2	-2.155878656	0.280796548	-7.677724915	1.61939E-14	4.78163E-14	negative
PRJNA729271	stage_3W_vs_E10	ordinary_muscle_development	ENSGALG00010020951	ENSGALG00010020951	-0.516412646	0.24941573	-2.070489481	0.03840653	0.048940893	negative
PRJNA729271	stage_3W_vs_E10	ordinary_muscle_development	ENSGALG00010020952	GNG5	-0.881359901	0.099195439	-8.885084957	6.38719E-19	2.33735E-18	negative
PRJNA729271	stage_3W_vs_E10	ordinary_muscle_development	ENSGALG00010020954	PLIN1	10.25859554	1.405808315	7.297293258	2.93614E-13	8.12202E-13	positive
PRJNA729271	stage_3W_vs_E10	ordinary_muscle_development	ENSGALG00010020955	FHAD1	-1.228085329	0.980007064	-1.253139262	0.21015503	0.242531621	negative
PRJNA729271	stage_3W_vs_E10	ordinary_muscle_development	ENSGALG00010020957	RPF1	-0.051482809	0.101367472	-0.507882934	0.611535444	0.646456115	negative
PRJNA729271	stage_3W_vs_E10	ordinary_muscle_development	ENSGALG00010020959	ENSGALG00010020959	-2.744635178	0.386395686	-7.103172422	1.21925E-12	3.27097E-12	negative
PRJNA729271	stage_3W_vs_E10	ordinary_muscle_development	ENSGALG00010020960	CLEC19A	1.39889362	0.378159295	3.699217864	0.000216265	0.000343234	positive
PRJNA729271	stage_3W_vs_E10	ordinary_muscle_development	ENSGALG00010020962	MYO5C	-3.843873617	0.382895539	-10.03896162	1.0275E-23	4.55323E-23	negative
PRJNA729271	stage_3W_vs_E10	ordinary_muscle_development	ENSGALG00010020963	CCDC93	1.43067467	0.098696679	14.49567177	1.29034E-47	1.34946E-46	positive
PRJNA729271	stage_3W_vs_E10	ordinary_muscle_development	ENSGALG00010020965	DHX37	-0.887029035	0.094578394	-9.378770276	6.67466E-21	2.64776E-20	negative
PRJNA729271	stage_3W_vs_E10	ordinary_muscle_development	ENSGALG00010020966	PAPLN	1.189239022	0.247125667	4.812284524	1.49215E-06	2.79116E-06	positive
PRJNA729271	stage_3W_vs_E10	ordinary_muscle_development	ENSGALG00010020967	VPS51	0.003295056	0.07594517	0.043387302	0.9653928	0.970212066	positive
PRJNA729271	stage_3W_vs_E10	ordinary_muscle_development	ENSGALG00010020969	RBP3	-1.256304382	0.339195481	-3.703776881	0.000212413	0.00033738	negative
PRJNA729271	stage_3W_vs_E10	ordinary_muscle_development	ENSGALG00010020970	ARMH3	0.448032992	0.078635104	5.697620672	1.21491E-08	2.60852E-08	positive
PRJNA729271	stage_3W_vs_E10	ordinary_muscle_development	ENSGALG00010020971	TMEM51	-0.825223806	0.285098708	-2.894519624	0.003797392	0.005391106	negative
PRJNA729271	stage_3W_vs_E10	ordinary_muscle_development	ENSGALG00010020973	GNB5	-1.42343224	0.166540989	-8.547038473	1.26287E-17	4.3709E-17	negative

PRJNA729271	stage_3W_vs_E10	ordinary_muscle_development	ENSGALG00010020975	HCCS	0.806787796	0.130180767	6.197442345	5.7388E-10	1.32861E-09	positive
PRJNA729271	stage_3W_vs_E10	ordinary_muscle_development	ENSGALG00010020976	GDF10	-1.645531626	0.312942289	-5.258259064	1.45426E-07	2.90751E-07	negative
PRJNA729271	stage_3W_vs_E10	ordinary_muscle_development	ENSGALG00010020978	BCL2L10	1.92196173	0.11529917	16.66934572	2.18978E-62	3.48801E-61	positive
PRJNA729271	stage_3W_vs_E10	ordinary_muscle_development	ENSGALG00010020979	ENSGALG00010020979	0.432107575	0.247263053	1.747562244	0.080539858	0.098516898	positive
PRJNA729271	stage_3W_vs_E10	ordinary_muscle_development	ENSGALG00010020981	CIT	-3.000282287	0.24037357	-12.48174784	9.39024E-36	6.60736E-35	negative
PRJNA729271	stage_3W_vs_E10	ordinary_muscle_development	ENSGALG00010020982	TLCD4	0.208261278	0.151764059	1.372270091	0.169979373	0.198906193	positive
PRJNA729271	stage_3W_vs_E10	ordinary_muscle_development	ENSGALG00010020984	PSEN1	-1.140143781	0.060290878	-18.91071776	9.30812E-80	2.30241E-78	negative
PRJNA729271	stage_3W_vs_E10	ordinary_muscle_development	ENSGALG00010020986	CFAP20DC	-3.336402304	0.377416526	-8.840106554	9.56274E-19	3.47224E-18	negative
PRJNA729271	stage_3W_vs_E10	ordinary_muscle_development	ENSGALG00010020987	NADSYN1	0.034083847	0.092337245	0.369123494	0.712035678	0.740862619	positive
PRJNA729271	stage_3W_vs_E10	ordinary_muscle_development	ENSGALG00010020989	MAPK4	0.940699529	0.152794665	6.1566255	7.43113E-10	1.70866E-09	positive
PRJNA729271	stage_3W_vs_E10	ordinary_muscle_development	ENSGALG00010020990	UBB	-1.124729383	0.106700515	-10.54099305	5.59054E-26	2.72753E-25	negative
PRJNA729271	stage_3W_vs_E10	ordinary_muscle_development	ENSGALG00010020991	ALG14	0.934562785	0.136395749	6.851846884	7.29026E-12	1.87296E-11	positive
PRJNA729271	stage_3W_vs_E10	ordinary_muscle_development	ENSGALG00010020992	KCNH7	3.560459746	0.428922464	8.300940244	1.03291E-16	3.41436E-16	positive
PRJNA729271	stage_3W_vs_E10	ordinary_muscle_development	ENSGALG00010020994	DHCR7	-1.745105718	0.14668327	-11.89710128	1.2253E-32	7.77613E-32	negative
PRJNA729271	stage_3W_vs_E10	ordinary_muscle_development	ENSGALG00010020998	SYT17	-0.570670873	0.298651563	-1.910824998	0.056027074	0.0699253	negative
PRJNA729271	stage_3W_vs_E10	ordinary_muscle_development	ENSGALG00010020999	IFIH1	2.949004838	0.33480045	8.808246348	1.27119E-18	4.59498E-18	positive
PRJNA729271	stage_3W_vs_E10	ordinary_muscle_development	ENSGALG00010021000	NFKB2	1.337558594	0.113973576	11.73569029	8.36411E-32	5.14155E-31	positive
PRJNA729271	stage_3W_vs_E10	ordinary_muscle_development	ENSGALG00010021002	SLC44A3	0.034328948	0.158801567	0.216175121	0.828851246	0.849119995	positive
PRJNA729271	stage_3W_vs_E10	ordinary_muscle_development	ENSGALG00010021003	KAZN	-1.022997414	0.122787008	-8.331479296	7.98384E-17	2.65183E-16	negative
PRJNA729271	stage_3W_vs_E10	ordinary_muscle_development	ENSGALG00010021004	PSD	0.211610944	0.17091035	1.238140023	0.215664146	0.248414796	positive
PRJNA729271	stage_3W_vs_E10	ordinary_muscle_development	ENSGALG00010021010	LEO1	-0.612773183	0.112555011	-5.444210598	5.20356E-08	1.07215E-07	negative
PRJNA729271	stage_3W_vs_E10	ordinary_muscle_development	ENSGALG00010021011	SCARB1	0.645527966	0.105864923	6.0976568	1.07635E-09	2.44616E-09	positive
PRJNA729271	stage_3W_vs_E10	ordinary_muscle_development	ENSGALG00010021012	KCNIP2	-1.420739268	0.722853623	-1.96545915	0.049361119	0.062020436	negative
PRJNA729271	stage_3W_vs_E10	ordinary_muscle_development	ENSGALG00010021013	FAP	0.383735958	0.296645721	1.293583323	0.195809386	0.226933481	positive
PRJNA729271	stage_3W_vs_E10	ordinary_muscle_development	ENSGALG00010021014	FBXL15	0.624978439	0.090133942	6.933885541	4.09436E-12	1.06492E-11	positive
PRJNA729271	stage_3W_vs_E10	ordinary_muscle_development	ENSGALG00010021015	RBM25	-0.337568243	0.135394462	-2.493220461	0.012659022	0.017019991	negative
PRJNA729271	stage_3W_vs_E10	ordinary_muscle_development	ENSGALG00010021016	FHIT	-0.634876918	0.218396493	-2.906992277	0.003649222	0.005190411	negative
PRJNA729271	stage_3W_vs_E10	ordinary_muscle_development	ENSGALG00010021017	COQ7	0.202656123	0.15696535	1.291088271	0.196673071	0.227879789	positive
PRJNA729271	stage_3W_vs_E10	ordinary_muscle_development	ENSGALG00010021018	F3	-0.086362709	0.222255567	-0.388573885	0.697591389	0.727243036	negative
PRJNA729271	stage_3W_vs_E10	ordinary_muscle_development	ENSGALG00010021019	VSIG10	-1.054938149	0.096816006	-10.89631962	1.20014E-27	6.30748E-27	negative
PRJNA729271	stage_3W_vs_E10	ordinary_muscle_development	ENSGALG00010021020	ZDHHC13	0.168522926	0.118403828	1.423289509	0.154652247	0.182132298	positive
PRJNA729271	stage_3W_vs_E10	ordinary_muscle_development	ENSGALG00010021021	GCG	2.113551739	0.344127006	6.141778194	8.16028E-10	1.87039E-09	positive
PRJNA729271	stage_3W_vs_E10	ordinary_muscle_development	ENSGALG00010021022	ZFYVE1	1.579314757	0.077525667	20.37150809	2.99317E-92	1.02567E-90	positive
PRJNA729271	stage_3W_vs_E10	ordinary_muscle_development	ENSGALG00010021023	MGEA5	0.555336835	0.09718798	5.714048549	1.1032E-08	2.3757E-08	positive
PRJNA729271	stage_3W_vs_E10	ordinary_muscle_development	ENSGALG00010021024	ENSGALG00010021024	0.581245736	0.186076533	3.123691771	0.001785974	0.002612017	positive
PRJNA729271	stage_3W_vs_E10	ordinary_muscle_development	ENSGALG00010021025	CUEDC2	0.727667311	0.114911229	6.332429954	2.4133E-10	5.71114E-10	positive
PRJNA729271	stage_3W_vs_E10	ordinary_muscle_development	ENSGALG00010021026	ADGRA2	-1.00296216	0.135645914	-7.393972495	1.42506E-13	4.01248E-13	negative
PRJNA729271	stage_3W_vs_E10	ordinary_muscle_development	ENSGALG00010021027	PLA2G4A	0.566435446	0.166452788	3.402979626	0.000666553	0.001014669	positive
PRJNA729271	stage_3W_vs_E10	ordinary_muscle_development	ENSGALG00010021028	ENSGALG00010021028	0.65051588	0.322866339	2.014814808	0.043924057	0.055527578	positive
PRJNA729271	stage_3W_vs_E10	ordinary_muscle_development	ENSGALG00010021029	PRDM2	-0.76632496	0.075866184	-10.10100841	5.4677E-24	2.44612E-23	negative
PRJNA729271	stage_3W_vs_E10	ordinary_muscle_development	ENSGALG00010021030	CSRP3	1.778985808	0.548798807	3.241599261	0.00118861	0.001769063	positive
PRJNA729271	stage_3W_vs_E10	ordinary_muscle_development	ENSGALG00010021031	DDX54	-0.313284285	0.110291179	-2.84051986	0.004504007	0.006346416	negative
PRJNA729271	stage_3W_vs_E10	ordinary_muscle_development	ENSGALG00010021032	TMC7	0.071232224	0.118368391	0.601784174	0.547317811	0.584582689	positive
PRJNA729271	stage_3W_vs_E10	ordinary_muscle_development	ENSGALG00010021033	RNF122	-2.480764097	0.235098339	-10.5520273	4.97127E-26	2.43031E-25	negative
PRJNA729271	stage_3W_vs_E10	ordinary_muscle_development	ENSGALG00010021034	NPM3	-0.457751104	0.278102629	-1.645979062	0.09976807	0.120591909	negative
PRJNA729271	stage_3W_vs_E10	ordinary_muscle_development	ENSGALG00010021036	E2F8	-2.876710721	0.208192702	-13.81753873	1.99785E-43	1.80992E-42	negative
PRJNA729271	stage_3W_vs_E10	ordinary_muscle_development	ENSGALG00010021038	PTGS2	0.434464913	0.29928497	1.45167635	0.146591618	0.173174043	positive
PRJNA729271	stage_3W_vs_E10	ordinary_muscle_development	ENSGALG00010021040	FGF8	-4.486660893	0.638450428	-7.027422486	2.10384E-12	5.57701E-12	negative
PRJNA729271	stage_3W_vs_E10	ordinary_muscle_development	ENSGALG00010021041	GLTP	-0.173003371	0.184175546	-0.939339533	0.347556448	0.386070251	negative
PRJNA729271	stage_3W_vs_E10	ordinary_muscle_development	ENSGALG00010021042	LRRC38	0.30729695	0.097748994	3.143735152	0.001668063	0.002450449	positive
PRJNA729271	stage_3W_vs_E10	ordinary_muscle_development	ENSGALG00010021043	PTPRG	-0.770830412	0.0909204	-8.478079886	2.28941E-17	7.80455E-17	negative
PRJNA729271	stage_3W_vs_E10	ordinary_muscle_development	ENSGALG00010021044	EIF4G2	0.150295314	0.068021615	2.209522881	0.027138293	0.035141107	positive
PRJNA729271	stage_3W_vs_E10	ordinary_muscle_development	ENSGALG00010021045	DPP4	2.595859759	0.258963526	10.02403622	1.1952E-23	5.27862E-23	positive
PRJNA729271	stage_3W_vs_E10	ordinary_muscle_development	ENSGALG00010021046	ENSGALG00010021046	-1.680023638	0.252396233	-6.656294414	2.80817E-11	7.0156E-11	negative
PRJNA729271	stage_3W_vs_E10	ordinary_muscle_development	ENSGALG00010021047	TMC5	-0.272619003	0.483773395	-0.563526241	0.573076596	0.609311616	negative
PRJNA729271	stage_3W_vs_E10	ordinary_muscle_development	ENSGALG00010021048	API5	-0.388928365	0.075761015	-5.133621341	2.8422E-07	5.58007E-07	negative
PRJNA729271	stage_3W_vs_E10	ordinary_muscle_development	ENSGALG00010021050	CTR9	-0.163227709	0.09466465	-1.724273103	0.084658553	0.103328201	negative
PRJNA729271	stage_3W_vs_E10	ordinary_muscle_development	ENSGALG00010021051	GDE1	0.630237815	0.134971325	4.66942007	3.02051E-06	5.54558E-06	positive
PRJNA729271	stage_3W_vs_E10	ordinary_muscle_development	ENSGALG00010021052	CEP15	-1.738226084	0.152029916	-11.43344764	2.84581E-30	1.65066E-29	negative
PRJNA729271	stage_3W_vs_E10	ordinary_muscle_development	ENSGALG00010021053	ENSGALG00010021053	-4.183645859	0.532584434	-7.85536639	3.98605E-15	1.21407E-14	negative
PRJNA729271	stage_3W_vs_E10	ordinary_muscle_development	ENSGALG00010021055	IRAG1	0.056212883	0.221752729	0.253493533	0.799886858	0.82241169	positive
PRJNA729271	stage_3W_vs_E10	ordinary_muscle_development	ENSGALG00010021057	ZDHHC8	-0.762247062	0.081699384	-9.329899726	1.05971E-20	4.16726E-20	negative
PRJNA729271	stage_3W_vs_E10	ordinary_muscle_development	ENSGALG00010021058	CCP110	-0.435029648	0.09524784	-4.567343991	4.93943E-06	8.92731E-06	negative
PRJNA729271	stage_3W_vs_E10	ordinary_muscle_development	ENSGALG00010021059	ENSGALG00010021059	-2.63357695	1.385081825	-1.90138727	0.057251306	0.071336515	negative
PRJNA729271	stage_3W_vs_E10	ordinary_muscle_development	ENSGALG00010021062	MYO9A	-1.857273554	0.102628823	-18.09699747	3.36519E-73	7.13135E-72	negative
PRJNA729271	stage_3W_vs_E10	ordinary_muscle_development	ENSGALG00010021064	LYVE1	3.123293978	0.284219226	10.98903131	4.31533E-28	2.30644E-27	positive
PRJNA729271	stage_3W_vs_E10	ordinary_muscle_development	ENSGALG00010021065	CNNM2	-0.628158038	0.113152172	-5.551444795	2.83318E-08	5.93625E-08	negative
PRJNA729271	stage_3W_vs_E10	ordinary_muscle_development	ENSGALG00010021067	SLC4A10	-3.207368359	0.31363034	-10.22658827	1.50736E-24	6.92747E-24	negative
PRJNA729271	stage_3W_vs_E10	ordinary_muscle_development	ENSGALG00010021069	PGAM5	-0.210693692	0.071696871	-2.938673481	0.003296201	0.004712342	negative
PRJNA729271	stage_3W_vs_E10	ordinary_muscle_development	ENSGALG00010021070	RNF141	0.514368015	0.085588378	6.009788067	1.85766E-09	4.16115E-09	positive
PRJNA729271	stage_3W_vs_E10	ordinary_muscle_development	ENSGALG00010021071	HIF1AN	1.10902346	0.095213237	11.64778658	2.35496E-31	1.41875E-30	positive
PRJNA729271	stage_3W_vs_E10	ordinary_muscle_development	ENSGALG00010021072	ENSGALG00010021072	0.852465724	0.08975332	9.497873982	2.14219E-21	8.66622E-21	positive
PRJNA729271	stage_3W_vs_E10	ordinary_muscle_development	ENSGALG00010021073	NDUFB8	0.637183141	0.107510269	5.926718896	3.09047E-09	6.83289E-09	positive
PRJNA729271	stage_3W_vs_E10	ordinary_muscle_development	ENSGALG00010021074	TTC17	0.567169454	0.069084406	8.209804293	2.21549E-16	7.19713E-16	positive
PRJNA729271	stage_3W_vs_E10	ordinary_muscle_development	ENSGALG00010021075	DHRS3	-1.741896544	0.134206877	-12.97918991	1.6057E-38	1.23276E-37	negative
PRJNA729271	stage_3W_vs_E10	ordinary_muscle_development	ENSGALG00010021077	SEC31B	0.253942717	0.079250754	3.204294002	0.001353941	0.002005455	positive
PRJNA729271	stage_3W_vs_E10	ordinary_muscle_development	ENSGALG00010021078	ENSGALG00010021078	-2.604025873	0.259277094	-10.04340892	9.82199E-24	4.36182E-23	negative
PRJNA729271	stage_3W_vs_E10	ordinary_muscle_development	ENSGALG00010021080	TTLL7	2.78885966	0.173128757	16.10858706	2.2204E-58	3.19292E-57	positive
PRJNA729271	stage_3W_vs_E10	ordinary_muscle_development	ENSGALG00010021081	KCNH6	-1.182138575	0.221671988	-5.332827955	9.66949E-08	1.96E-07	negative
PRJNA729271	stage_3W_vs_E10	ordinary_muscle_development	ENSGALG00010021082	SENP8	0.496604122	0.163164181	3.04358542	0.002337771	0.00338251	positive
PRJNA729271	stage_3W_vs_E10	ordinary_muscle_development	ENSGALG00010021083	SCD	-0.715116528	0.562003241	-1.272442001			negative
PRJNA729271	stage_3W_vs_E10	ordinary_muscle_development	ENSGALG00010021084	ENSGALG00010021084	-1.861426778	0.655054427	-2.84163682	0.004488259	0.006326071	negative
PRJNA729271	stage_3W_vs_E10	ordinary_muscle_development	ENSGALG00010021085	AMPD3	1.862012601	0.184040859	10.11738705	4.62601E-24	2.07984E-23	positive
PRJNA729271	stage_3W_vs_E10	ordinary_muscle_development	ENSGALG00010021086	PSMD14	0.13415682	0.10919134	1.228639745	0.219206907	0.2520949	positive
PRJNA729271	stage_3W_vs_E10	ordinary_muscle_development	ENSGALG00010021087	THBS1	-0.914961935	0.356203047	-2.56865275	0.010209471	0.013856393	negative
PRJNA729271	stage_3W_vs_E10	ordinary_muscle_development	ENSGALG00010021088	ANKLE2	-0.289819689	0.087335405	-3.31846733	0.000905129	0.001360556	negative
PRJNA729271	stage_3W_vs_E10	ordinary_muscle_development	ENSGALG00010021090	ADM	3.335801849	0.255073967	13.07778244	4.41122E-39	3.46187E-38	positive
PRJNA729271	stage_3W_vs_E10	ordinary_muscle_development	ENSGALG00010021091	TANK	0.400388422	0.081528879	4.911001184	9.06125E-07	1.72185E-06	positive
PRJNA729271	stage_3W_vs_E10	ordinary_muscle_development	ENSGALG00010021092	VPS13D	1.043271614	0.094662344	11.02097804	3.02751E-28	1.63198E-27	positive
PRJNA729271	stage_3W_vs_E10	ordinary_muscle_development	ENSGALG00010021093	GRAMD2	2.646647024	0.219493069	12.05799818	1.76009E-33	1.14921E-32	positive
PRJNA729271	stage_3W_vs_E10	ordinary_muscle_development	ENSGALG00010021094	ENSGALG00010021094	3.444373562	0.313714078	10.97934011	4.8042E-28	2.56395E-27	positive
PRJNA729271	stage_3W_vs_E10	ordinary_muscle_development	ENSGALG00010021095	NT5C2	-0.170222981	0.106040664	-1.605261362	0.108436298	0.130471001	negative
PRJNA729271	stage_3W_vs_E10	ordinary_muscle_development	ENSGALG00010021096	ZFAND4	-0.874750541	0.10440836	-8.378165702	5.37589E-17	1.80128E-16	negative
PRJNA729271	stage_3W_vs_E10	ordinary_muscle_development	ENSGALG00010021097	KATNBL1	-0.036766568	0.129305285	-0.284339254	0.776150407	0.800505205	negative
PRJNA729271	stage_3W_vs_E10	ordinary_muscle_development	ENSGALG00010021098	SDK1	-1.0474626	0.14273465	-7.338530611	2.15952E-13	6.01849E-13	negative
PRJNA729271	stage_3W_vs_E10	ordinary_muscle_development	ENSGALG00010021099	HMCN1	-2.362144674	0.264856418	-8.918585745	4.7228E-19	1.74235E-18	negative
PRJNA729271	stage_3W_vs_E10	ordinary_muscle_development	ENSGALG00010021100	EMC7	1.046010527	0.085204333	12.27649456	1.21134E-34	8.23564E-34	positive
PRJNA729271	stage_3W_vs_E10	ordinary_muscle_development	ENSGALG00010021101	TMEM240	-3.677294083	0.555443799	-6.620461133	3.5808E-11	8.8861E-11	negative
PRJNA729271	stage_3W_vs_E10	ordinary_muscle_development	ENSGALG00010021102	GOLGA3	1.323962277	0.111961304	11.82517736	2.89291E-32	1.81606E-31	positive
PRJNA729271	stage_3W_vs_E10	ordinary_muscle_development	ENSGALG00010021103	RBMS1	-0.2076384	0.074319463	-2.793863036	0.005208253	0.007290549	negative
PRJNA729271	stage_3W_vs_E10	ordinary_muscle_development	ENSGALG00010021104	INA	-1.794315657	0.369689728	-4.853571844	1.21257E-06	2.28411E-06	negative
PRJNA729271	stage_3W_vs_E10	ordinary_muscle_development	ENSGALG00010021105	ENSGALG00010021105	2.761641863	0.407688298	6.773905157	1.25352E-11	3.18824E-11	positive
PRJNA729271	stage_3W_vs_E10	ordinary_muscle_development	ENSGALG00010021106	ITGB6	-2.593456209	0.213620825	-12.14046528	6.44585E-34	4.26438E-33	negative
PRJNA729271	stage_3W_vs_E10	ordinary_muscle_development	ENSGALG00010021108	PCGF6	0.344388559	0.105360225	3.268677138	0.001080515	0.001614783	positive
PRJNA729271	stage_3W_vs_E10	ordinary_muscle_development	ENSGALG00010021110	PKLR	4.979437236	0.168775126	29.50338327	2.6052E-191	4.3405E-189	positive
PRJNA729271	stage_3W_vs_E10	ordinary_muscle_development	ENSGALG00010021111	AVEN	0.596612128	0.078339837	7.615692764	2.62281E-14	7.66639E-14	positive
PRJNA729271	stage_3W_vs_E10	ordinary_muscle_development	ENSGALG00010021112	TAF5	-1.023812958	0.072255055	-14.16943028	1.41645E-45	1.38352E-44	negative
PRJNA729271	stage_3W_vs_E10	ordinary_muscle_development	ENSGALG00010021113	SYP	-1.718157858	0.312244928	-5.502596525	3.74238E-08	7.78276E-08	negative
PRJNA729271	stage_3W_vs_E10	ordinary_muscle_development	ENSGALG00010021114	SBF2	-0.206948498	0.098393486	-2.10327437	0.035441793	0.045330343	negative
PRJNA729271	stage_3W_vs_E10	ordinary_muscle_development	ENSGALG00010021115	ALPL	-1.039251907	0.246542335	-4.21530812	2.49437E-05	4.27072E-05	negative
PRJNA729271	stage_3W_vs_E10	ordinary_muscle_development	ENSGALG00010021116	FAM21C	0.623188304	0.06381182	9.766032427	1.575E-22	6.65197E-22	positive
PRJNA729271	stage_3W_vs_E10	ordinary_muscle_development	ENSGALG00010021118	USMG5	1.328581533	0.179709013	7.392959918	1.43596E-13	4.04002E-13	positive
PRJNA729271	stage_3W_vs_E10	ordinary_muscle_development	ENSGALG00010021120	PDCD11	-1.591492026	0.088469773	-17.98910497	2.37157E-72	4.91788E-71	negative
PRJNA729271	stage_3W_vs_E10	ordinary_muscle_development	ENSGALG00010021121	PLA2R1	1.574666579	0.091340111	17.23959571	1.3397E-66	2.37106E-65	positive
PRJNA729271	stage_3W_vs_E10	ordinary_muscle_development	ENSGALG00010021122	CYP1A1	1.888688814	0.952032678	1.983848725	0.047272706	0.059527224	positive
PRJNA729271	stage_3W_vs_E10	ordinary_muscle_development	ENSGALG00010021123	RYR3	6.993806588	0.216124971	32.36001169	1.0031E-229	2.3453E-227	positive
PRJNA729271	stage_3W_vs_E10	ordinary_muscle_development	ENSGALG00010021124	CALHM2	0.392414975	0.131101725	2.993209841	0.002760599	0.003969336	positive
PRJNA729271	stage_3W_vs_E10	ordinary_muscle_development	ENSGALG00010021125	LY75	2.428858475	0.123953177	19.59496748	1.70695E-85	5.02497E-84	positive
PRJNA729271	stage_3W_vs_E10	ordinary_muscle_development	ENSGALG00010021126	ECE1	0.745646609	0.103997447	7.16985493	7.50773E-13	2.03829E-12	positive
PRJNA729271	stage_3W_vs_E10	ordinary_muscle_development	ENSGALG00010021127	SWAP70	-0.341279781	0.07778602	-4.387417987	1.14704E-05	2.01759E-05	negative
PRJNA729271	stage_3W_vs_E10	ordinary_muscle_development	ENSGALG00010021129	TXNRD3	-0.133902633	0.085613848	-1.564030068	0.117810507	0.141024305	negative
PRJNA729271	stage_3W_vs_E10	ordinary_muscle_development	ENSGALG00010021130	ENSGALG00010021130	3.980904761	1.102894039	3.609507912	0.000306778	0.00048125	positive
PRJNA729271	stage_3W_vs_E10	ordinary_muscle_development	ENSGALG00010021131	EDC3	-0.822251668	0.116921429	-7.032514715	2.02844E-12	5.38601E-12	negative
PRJNA729271	stage_3W_vs_E10	ordinary_muscle_development	ENSGALG00010021133	ENSGALG00010021133	-0.892869107	0.103864813	-8.596454219	8.22169E-18	2.87165E-17	negative
PRJNA729271	stage_3W_vs_E10	ordinary_muscle_development	ENSGALG00010021134	CHFR	-0.678881179	0.093628872	-7.250767439	4.14417E-13	1.13833E-12	negative
PRJNA729271	stage_3W_vs_E10	ordinary_muscle_development	ENSGALG00010021135	CLK3	0.844054449	0.105510383	7.99972876	1.24694E-15	3.88695E-15	positive
PRJNA729271	stage_3W_vs_E10	ordinary_muscle_development	ENSGALG00010021136	WEE1	-2.271314086	0.150701071	-15.07165191	2.48813E-51	2.94653E-50	negative
PRJNA729271	stage_3W_vs_E10	ordinary_muscle_development	ENSGALG00010021137	PLAU	0.605928748	0.190401468	3.182374347	0.001460729	0.002156137	positive
PRJNA729271	stage_3W_vs_E10	ordinary_muscle_development	ENSGALG00010021138	DDX18	-0.38148618	0.09952083	-3.833229473	0.000126472	0.000205034	negative
PRJNA729271	stage_3W_vs_E10	ordinary_muscle_development	ENSGALG00010021139	ARHGAP29	1.013391441	0.095500446	10.6113791	2.63826E-26	1.30562E-25	positive
PRJNA729271	stage_3W_vs_E10	ordinary_muscle_development	ENSGALG00010021140	SLC11A1	0.363513605	0.435748038	0.834228896	0.404152011	0.443096846	positive

PRJNA729271	stage_3W_vs_E10	ordinary_muscle_development	ENSGALG00010021141	SGSM1	-1.000054291	0.161873655	-6.177992897	6.49216E-10	1.49775E-09	negative
PRJNA729271	stage_3W_vs_E10	ordinary_muscle_development	ENSGALG00010021142	WNT6	-4.768324714	0.543387232	-8.775187257	1.7062E-18	6.14138E-18	negative
PRJNA729271	stage_3W_vs_E10	ordinary_muscle_development	ENSGALG00010021143	ABCA4	-0.984160121	0.356008142	-2.764431497	0.005702208	0.007945924	negative
PRJNA729271	stage_3W_vs_E10	ordinary_muscle_development	ENSGALG00010021144	FOXK1	0.160656841	0.109020928	1.473633036	0.140580422	0.166643213	positive
PRJNA729271	stage_3W_vs_E10	ordinary_muscle_development	ENSGALG00010021145	EIF4G3	0.165549857	0.157452691	1.051426025	0.293062968	0.330348217	positive
PRJNA729271	stage_3W_vs_E10	ordinary_muscle_development	ENSGALG00010021146	AP5Z1	-0.080109793	0.085997069	-0.931540961	0.351573806	0.390114241	negative
PRJNA729271	stage_3W_vs_E10	ordinary_muscle_development	ENSGALG00010021147	ARID3B	-0.529348542	0.085918815	-6.161031693	7.22725E-10	1.66381E-09	negative
PRJNA729271	stage_3W_vs_E10	ordinary_muscle_development	ENSGALG00010021148	GPATCH8	-0.103339698	0.105520176	-0.979335913	0.327414031	0.365600985	negative
PRJNA729271	stage_3W_vs_E10	ordinary_muscle_development	ENSGALG00010021149	ENSGALG00010021149	-3.313092712	1.345538149	-2.462280771	0.013805654	0.018475945	negative
PRJNA729271	stage_3W_vs_E10	ordinary_muscle_development	ENSGALG00010021150	ENSGALG00010021150	0.788537092	0.253063986	3.115959339	0.001833476	0.002677706	positive
PRJNA729271	stage_3W_vs_E10	ordinary_muscle_development	ENSGALG00010021151	WNT10A	-6.759566263	0.963663626	-7.014445787	2.30862E-12	6.10264E-12	negative
PRJNA729271	stage_3W_vs_E10	ordinary_muscle_development	ENSGALG00010021152	STK11IP	1.088975314	0.125892825	8.650018899	5.14909E-18	1.81773E-17	positive
PRJNA729271	stage_3W_vs_E10	ordinary_muscle_development	ENSGALG00010021153	PTPRF	-3.367387724	0.1627749	-20.68738929	4.49923E-95	1.65524E-93	negative
PRJNA729271	stage_3W_vs_E10	ordinary_muscle_development	ENSGALG00010021154	CHST13	-2.665889195	0.214335193	-12.43794429	1.62636E-35	1.1372E-34	negative
PRJNA729271	stage_3W_vs_E10	ordinary_muscle_development	ENSGALG00010021155	RADIL	-2.757417373	0.235393148	-11.7140936	1.07939E-31	6.61277E-31	negative
PRJNA729271	stage_3W_vs_E10	ordinary_muscle_development	ENSGALG00010021156	DDOST	-1.118237084	0.081035617	-13.79932838	2.57238E-43	2.32026E-42	negative
PRJNA729271	stage_3W_vs_E10	ordinary_muscle_development	ENSGALG00010021157	ENSGALG00010021157	8.626871078	0.803892853	10.73136929	7.25136E-27	3.68543E-26	positive
PRJNA729271	stage_3W_vs_E10	ordinary_muscle_development	ENSGALG00010021158	ACTG1L	-2.187668538	0.129002876	-16.95829279	1.6713E-64	2.81692E-63	negative
PRJNA729271	stage_3W_vs_E10	ordinary_muscle_development	ENSGALG00010021159	MMD2	1.783797295	0.238220496	7.488009337	6.99261E-14	2.00233E-13	positive
PRJNA729271	stage_3W_vs_E10	ordinary_muscle_development	ENSGALG00010021160	ZNF143	0.41877046	0.164155223	2.551063872	0.010739464	0.014549821	positive
PRJNA729271	stage_3W_vs_E10	ordinary_muscle_development	ENSGALG00010021161	ENSGALG00010021161	-2.026157852	0.112198499	-18.05868945	6.74039E-73	1.42009E-71	negative
PRJNA729271	stage_3W_vs_E10	ordinary_muscle_development	ENSGALG00010021162	WIPI2	-0.10368583	0.094822958	-1.093467586	0.274188553	0.310958695	negative
PRJNA729271	stage_3W_vs_E10	ordinary_muscle_development	ENSGALG00010021163	INHA	-1.786355945	0.377297182	-4.734612473	2.19474E-06	4.06501E-06	negative
PRJNA729271	stage_3W_vs_E10	ordinary_muscle_development	ENSGALG00010021164	SLC41A3	0.996052026	0.212201083	4.693906414	2.68037E-06	4.93922E-06	positive
PRJNA729271	stage_3W_vs_E10	ordinary_muscle_development	ENSGALG00010021165	PTRF	3.308937327	0.105586284	31.33870413	1.3867E-215	2.7918E-213	positive
PRJNA729271	stage_3W_vs_E10	ordinary_muscle_development	ENSGALG00010021166	NEURL1	0.429324378	0.298084898	1.440275509	0.149789468	0.176692573	positive
PRJNA729271	stage_3W_vs_E10	ordinary_muscle_development	ENSGALG00010021167	KIAA1671	0.342194537	0.126087393	2.71394727	0.006648675	0.009208154	positive
PRJNA729271	stage_3W_vs_E10	ordinary_muscle_development	ENSGALG00010021168	IVNS1ABP	0.572427093	0.105683915	5.41640698	6.08086E-08	1.24635E-07	positive
PRJNA729271	stage_3W_vs_E10	ordinary_muscle_development	ENSGALG00010021169	CAMK2G	2.092499033	0.140066238	14.93935341	1.8274E-50	2.11061E-49	positive
PRJNA729271	stage_3W_vs_E10	ordinary_muscle_development	ENSGALG00010021170	UBL7	-0.253643045	0.138112562	-1.836495104	0.066284466	0.081950983	negative
PRJNA729271	stage_3W_vs_E10	ordinary_muscle_development	ENSGALG00010021171	ENSGALG00010021171	-0.796739685	0.214039334	-3.722398451	0.000197339	0.000314541	negative
PRJNA729271	stage_3W_vs_E10	ordinary_muscle_development	ENSGALG00010021172	SLC29A4	-1.540241279	0.287793079	-5.351905217	8.7033E-08	1.76935E-07	negative
PRJNA729271	stage_3W_vs_E10	ordinary_muscle_development	ENSGALG00010021173	KLF15	2.684998758	0.287185155	9.349364724	8.81757E-21	3.48353E-20	positive
PRJNA729271	stage_3W_vs_E10	ordinary_muscle_development	ENSGALG00010021175	IPO7	0.447753518	0.097579603	4.588597444	4.46234E-06	8.08925E-06	positive
PRJNA729271	stage_3W_vs_E10	ordinary_muscle_development	ENSGALG00010021176	ENSGALG00010021176	-0.032943311	0.118023728	-0.279124473	0.780149303	0.804171821	negative
PRJNA729271	stage_3W_vs_E10	ordinary_muscle_development	ENSGALG00010021177	OBSL1	0.22770542	0.188419741	1.208500862	0.226854664	0.260352998	positive
PRJNA729271	stage_3W_vs_E10	ordinary_muscle_development	ENSGALG00010021178	NDST2	-0.593545729	0.097810819	-6.068303447	1.29268E-09	2.92681E-09	negative
PRJNA729271	stage_3W_vs_E10	ordinary_muscle_development	ENSGALG00010021180	TMEM198	-0.812589288	0.146560134	-5.544408749	2.94949E-08	6.17014E-08	negative
PRJNA729271	stage_3W_vs_E10	ordinary_muscle_development	ENSGALG00010021181	DAGLA	0.048473993	0.128576093	0.377006267	0.706168947	0.735286179	positive
PRJNA729271	stage_3W_vs_E10	ordinary_muscle_development	ENSGALG00010021182	ZSWIM8	0.12950545	0.069374618	1.866755491	0.061935741	0.076869472	positive
PRJNA729271	stage_3W_vs_E10	ordinary_muscle_development	ENSGALG00010021183	CFAP100	4.262263603	0.450346357	9.46441231	2.95216E-21	1.18899E-20	positive
PRJNA729271	stage_3W_vs_E10	ordinary_muscle_development	ENSGALG00010021184	DRAXIN	-0.863666862	0.436421603	-1.978973672	0.047818971	0.060183727	negative
PRJNA729271	stage_3W_vs_E10	ordinary_muscle_development	ENSGALG00010021186	SWT1	0.592680829	0.089821213	6.598450504	4.15477E-11	1.02648E-10	positive
PRJNA729271	stage_3W_vs_E10	ordinary_muscle_development	ENSGALG00010021187	SEMA7A	2.079759344	0.278012933	7.480800699	7.38711E-14	2.11279E-13	positive
PRJNA729271	stage_3W_vs_E10	ordinary_muscle_development	ENSGALG00010021188	TNRC18	-0.885521972	0.133702151	-6.623094427	3.51756E-11	8.73515E-11	negative
PRJNA729271	stage_3W_vs_E10	ordinary_muscle_development	ENSGALG00010021189	ENSGALG00010021189	4.079396765	0.359530117	11.34646744	7.72198E-30	4.41713E-29	positive
PRJNA729271	stage_3W_vs_E10	ordinary_muscle_development	ENSGALG00010021190	ENSGALG00010021190	0.518822527	0.102245651	5.074274763	3.88977E-07	7.56401E-07	positive
PRJNA729271	stage_3W_vs_E10	ordinary_muscle_development	ENSGALG00010021191	MYRF	-1.49980631	0.215621179	-6.955746741	3.50699E-12	9.16421E-12	negative
PRJNA729271	stage_3W_vs_E10	ordinary_muscle_development	ENSGALG00010021192	ENSGALG00010021192	-2.861688527	0.180605433	-15.84497477	1.52282E-56	2.08042E-55	negative
PRJNA729271	stage_3W_vs_E10	ordinary_muscle_development	ENSGALG00010021193	USP37	-0.124584396	0.157052152	-0.79326768	0.427621883	0.46611364	negative
PRJNA729271	stage_3W_vs_E10	ordinary_muscle_development	ENSGALG00010021194	CYP11A1	-0.112250382	0.366912471	-0.305932316	0.75965618	0.785281439	negative
PRJNA729271	stage_3W_vs_E10	ordinary_muscle_development	ENSGALG00010021195	GCLM	-0.363305743	0.090493937	-4.014697082	5.95222E-05	9.91011E-05	negative
PRJNA729271	stage_3W_vs_E10	ordinary_muscle_development	ENSGALG00010021196	ATP6V0A1	2.072649844	0.120029624	17.26781912	8.21902E-67	1.46357E-65	positive
PRJNA729271	stage_3W_vs_E10	ordinary_muscle_development	ENSGALG00010021197	CHCHD1	0.574609168	0.104911325	5.47709381	4.32368E-08	8.95183E-08	positive
PRJNA729271	stage_3W_vs_E10	ordinary_muscle_development	ENSGALG00010021199	TMEM258	-0.588453943	0.136539302	-4.309777003	1.63419E-05	2.84058E-05	negative
PRJNA729271	stage_3W_vs_E10	ordinary_muscle_development	ENSGALG00010021200	FTO	-0.37124805	0.164148448	-2.261660427	0.023718392	0.030943027	negative
PRJNA729271	stage_3W_vs_E10	ordinary_muscle_development	ENSGALG00010021201	TRMT1L	-0.298871411	0.089206256	-3.350341368	0.00080712	0.001218921	negative
PRJNA729271	stage_3W_vs_E10	ordinary_muscle_development	ENSGALG00010021202	UROC1	-3.078727912	0.690091738	-4.461331359	8.1452E-06	1.44758E-05	negative
PRJNA729271	stage_3W_vs_E10	ordinary_muscle_development	ENSGALG00010021203	FUT11	-0.18901155	0.082664246	-2.286496993	0.0222252	0.029106819	negative
PRJNA729271	stage_3W_vs_E10	ordinary_muscle_development	ENSGALG00010021204	NAGLU	0.166641277	0.095837059	1.738797901	0.082070318	0.10028741	positive
PRJNA729271	stage_3W_vs_E10	ordinary_muscle_development	ENSGALG00010021206	HSD17B1	0.120970092	0.430496181	0.281001545	0.778709215	0.802858672	positive
PRJNA729271	stage_3W_vs_E10	ordinary_muscle_development	ENSGALG00010021207	MAD2L2	-0.180895876	0.122090439	-1.48165473	0.138432188	0.164257905	negative
PRJNA729271	stage_3W_vs_E10	ordinary_muscle_development	ENSGALG00010021210	ENSGALG00010021210	-0.742886966	0.064167915	-11.57723391	5.37526E-31	3.20899E-30	negative
PRJNA729271	stage_3W_vs_E10	ordinary_muscle_development	ENSGALG00010021211	FEN1	-2.982568008	0.159435133	-18.70709392	4.33371E-78	1.0281E-76	negative
PRJNA729271	stage_3W_vs_E10	ordinary_muscle_development	ENSGALG00010021212	MYO18B	3.417625772	0.161073915	21.21774818	6.5486E-100	2.74341E-98	positive
PRJNA729271	stage_3W_vs_E10	ordinary_muscle_development	ENSGALG00010021213	FBXO44	0.858725472	0.192642049	4.457622184	8.28738E-06	1.47177E-05	positive
PRJNA729271	stage_3W_vs_E10	ordinary_muscle_development	ENSGALG00010021214	FBXL18	-0.14610646	0.115804695	-1.261662664	0.207070197	0.239200072	negative
PRJNA729271	stage_3W_vs_E10	ordinary_muscle_development	ENSGALG00010021215	SH3PXD2A	-0.533331749	0.111737163	-4.773091903	1.81419E-06	3.37613E-06	negative
PRJNA729271	stage_3W_vs_E10	ordinary_muscle_development	ENSGALG00010021217	FBXO2	-1.434342162	0.210289667	-6.820792395	9.05397E-12	2.31827E-11	negative
PRJNA729271	stage_3W_vs_E10	ordinary_muscle_development	ENSGALG00010021218	DPF3	0.162513077	0.174720786	0.930130186	0.352303683	0.390894205	positive
PRJNA729271	stage_3W_vs_E10	ordinary_muscle_development	ENSGALG00010021219	STRA6	-8.129132976	0.749860458	-10.84086098	2.20387E-27	1.14499E-26	negative
PRJNA729271	stage_3W_vs_E10	ordinary_muscle_development	ENSGALG00010021220	SYNPO2L	2.855459639	0.205850534	13.87151925	9.4259E-44	8.63643E-43	positive
PRJNA729271	stage_3W_vs_E10	ordinary_muscle_development	ENSGALG00010021222	ENSGALG00010021222	5.174562448	0.302349588	17.11450139	1.15702E-65	2.01333E-64	positive
PRJNA729271	stage_3W_vs_E10	ordinary_muscle_development	ENSGALG00010021223	DFFB	0.292715234	0.110542004	2.648000056	0.008096951	0.01111835	positive
PRJNA729271	stage_3W_vs_E10	ordinary_muscle_development	ENSGALG00010021224	MYOZ1	7.900363516	0.140957719	56.04775383	0	0	positive
PRJNA729271	stage_3W_vs_E10	ordinary_muscle_development	ENSGALG00010021227	CHCHD4	-1.000228789	0.097600469	-10.2481965	1.20572E-24	5.56413E-24	negative
PRJNA729271	stage_3W_vs_E10	ordinary_muscle_development	ENSGALG00010021228	ESRP2	-7.308849245	0.996935888	-7.331313212	2.27908E-13	6.34075E-13	negative
PRJNA729271	stage_3W_vs_E10	ordinary_muscle_development	ENSGALG00010021229	GLO1	2.296121457	0.178217075	12.88384659	5.5499E-38	4.1725E-37	positive
PRJNA729271	stage_3W_vs_E10	ordinary_muscle_development	ENSGALG00010021230	C1orf174	0.196754001	0.0584613	3.365542714	0.000763932	0.001156108	positive
PRJNA729271	stage_3W_vs_E10	ordinary_muscle_development	ENSGALG00010021231	STN1	-1.347801623	0.119025046	-11.32368078	1.00176E-29	5.70324E-29	negative
PRJNA729271	stage_3W_vs_E10	ordinary_muscle_development	ENSGALG00010021232	ACTB	-1.328037548	0.109387234	-12.14069959	6.42741E-34	4.25413E-33	negative
PRJNA729271	stage_3W_vs_E10	ordinary_muscle_development	ENSGALG00010021233	COASY	0.576112644	0.061591359	9.353790052	8.4562E-21	3.34259E-20	positive
PRJNA729271	stage_3W_vs_E10	ordinary_muscle_development	ENSGALG00010021234	ISLR2	-1.366080861	0.339475081	-4.024097608	5.71942E-05	9.53785E-05	negative
PRJNA729271	stage_3W_vs_E10	ordinary_muscle_development	ENSGALG00010021235	XRCC5	-1.537779376	0.140030147	-10.98177361	4.67651E-28	2.49764E-27	negative
PRJNA729271	stage_3W_vs_E10	ordinary_muscle_development	ENSGALG00010021236	BTBD9	0.227659036	0.073799654	3.084825241	0.002036718	0.002961997	positive
PRJNA729271	stage_3W_vs_E10	ordinary_muscle_development	ENSGALG00010021237	FSCN1	-4.754685514	0.225376522	-21.0966319	8.54073E-99	3.39172E-97	negative
PRJNA729271	stage_3W_vs_E10	ordinary_muscle_development	ENSGALG00010021238	NDUFS8	1.887494052	0.146800023	12.85758692	7.7973E-38	5.83188E-37	positive
PRJNA729271	stage_3W_vs_E10	ordinary_muscle_development	ENSGALG00010021239	USP54	-0.028129662	0.070462278	-0.3992159	0.689734128	0.720398918	negative
PRJNA729271	stage_3W_vs_E10	ordinary_muscle_development	ENSGALG00010021240	SLK	-0.025089584	0.07865983	-0.318963103	0.749754492	0.776303581	negative
PRJNA729271	stage_3W_vs_E10	ordinary_muscle_development	ENSGALG00010021241	TMEM43	-0.420925409	0.171806694	-2.449994226	0.014285851	0.019092145	negative
PRJNA729271	stage_3W_vs_E10	ordinary_muscle_development	ENSGALG00010021242	BCAR3	3.867701938	0.223933328	17.27166729	7.68886E-67	1.37254E-65	positive
PRJNA729271	stage_3W_vs_E10	ordinary_muscle_development	ENSGALG00010021243	DYL1	-1.230568533	0.095176848	-12.92928433	3.07669E-38	2.33735E-37	negative
PRJNA729271	stage_3W_vs_E10	ordinary_muscle_development	ENSGALG00010021244	PPP3CB	0.649107019	0.087192437	7.444533523	9.72876E-14	2.75965E-13	positive
PRJNA729271	stage_3W_vs_E10	ordinary_muscle_development	ENSGALG00010021245	ZFAND3	0.854729991	0.070352172	12.14930502	5.78553E-34	3.8398E-33	positive
PRJNA729271	stage_3W_vs_E10	ordinary_muscle_development	ENSGALG00010021246	PECR	0.323836153	0.115418493	2.805756218	0.005019865	0.007037036	positive
PRJNA729271	stage_3W_vs_E10	ordinary_muscle_development	ENSGALG00010021247	GNAO1	-2.285446096	0.229286245	-9.967654603	2.11156E-23	9.24965E-23	negative
PRJNA729271	stage_3W_vs_E10	ordinary_muscle_development	ENSGALG00010021248	ANXA7	0.576557323	0.168138749	3.429056809	0.000605683	0.000925411	positive
PRJNA729271	stage_3W_vs_E10	ordinary_muscle_development	ENSGALG00010021249	ENSGALG00010021249	0.1580412	0.350429013	0.450993479	0.651994249	0.684929456	positive
PRJNA729271	stage_3W_vs_E10	ordinary_muscle_development	ENSGALG00010021250	ISLR	0.38817006	0.145734283	2.663546633	0.007732169	0.010640634	positive
PRJNA729271	stage_3W_vs_E10	ordinary_muscle_development	ENSGALG00010021251	RNF216	-0.116788993	0.084847436	-1.376458711	0.168679662	0.19760864	negative
PRJNA729271	stage_3W_vs_E10	ordinary_muscle_development	ENSGALG00010021252	XPC	0.570558298	0.131442816	4.34073397	1.42008E-05	2.4794E-05	positive
PRJNA729271	stage_3W_vs_E10	ordinary_muscle_development	ENSGALG00010021253	TMEM169	-2.075034094	0.11746791	-17.66468898	7.8444E-70	1.51002E-68	negative
PRJNA729271	stage_3W_vs_E10	ordinary_muscle_development	ENSGALG00010021254	FNBP1L	-0.49822676	0.108387745	-4.59670748	4.2922E-06	7.79153E-06	negative
PRJNA729271	stage_3W_vs_E10	ordinary_muscle_development	ENSGALG00010021255	EIF3F	0.770907173	0.091796412	8.398009832	4.54109E-17	1.52651E-16	positive
PRJNA729271	stage_3W_vs_E10	ordinary_muscle_development	ENSGALG00010021256	ENSGALG00010021256	0.563546773	0.290324108	1.941095337	0.052246715	0.065404277	positive
PRJNA729271	stage_3W_vs_E10	ordinary_muscle_development	ENSGALG00010021257	RGS6	-1.316298611	0.328435059	-4.007798588	6.12897E-05	0.000101984	negative
PRJNA729271	stage_3W_vs_E10	ordinary_muscle_development	ENSGALG00010021258	LSM3	-1.097443294	0.113567929	-9.663320467	4.31642E-22	1.79685E-21	negative
PRJNA729271	stage_3W_vs_E10	ordinary_muscle_development	ENSGALG00010021259	CEP104	-0.858267408	0.13474027	-6.369791336	1.89286E-10	4.50524E-10	negative
PRJNA729271	stage_3W_vs_E10	ordinary_muscle_development	ENSGALG00010021260	KDM4A	0.101577944	0.067655618	1.501397017	0.133252905	0.158462618	positive
PRJNA729271	stage_3W_vs_E10	ordinary_muscle_development	ENSGALG00010021261	JUP	0.023474985	0.128540331	0.182627385	0.855090396	0.871565663	positive
PRJNA729271	stage_3W_vs_E10	ordinary_muscle_development	ENSGALG00010021262	MDGA1	-3.130970151	0.410611361	-7.62514252	2.43765E-14	7.13236E-14	negative
PRJNA729271	stage_3W_vs_E10	ordinary_muscle_development	ENSGALG00010021263	STOML1	0.116506695	0.24368054	0.478112431	0.632570185	0.666117314	positive
PRJNA729271	stage_3W_vs_E10	ordinary_muscle_development	ENSGALG00010021264	CBFB	-1.481974545	0.170255588	-8.704410616	3.19229E-18	1.13915E-17	negative
PRJNA729271	stage_3W_vs_E10	ordinary_muscle_development	ENSGALG00010021265	SIPA1L1	-0.880142547	0.090226826	-9.754776777	1.75987E-22	7.41336E-22	negative
PRJNA729271	stage_3W_vs_E10	ordinary_muscle_development	ENSGALG00010021266	COQ5	-0.009619365	0.099592335	-0.096587404	0.92305406	0.931991404	negative
PRJNA729271	stage_3W_vs_E10	ordinary_muscle_development	ENSGALG00010021267	LRRC47	0.117170955	0.078757583	1.487741871	0.136818981	0.162396915	positive
PRJNA729271	stage_3W_vs_E10	ordinary_muscle_development	ENSGALG00010021269	NUDT13	-0.02372371	0.174454615	-0.135987864	0.891830875	0.904292425	negative
PRJNA729271	stage_3W_vs_E10	ordinary_muscle_development	ENSGALG00010021271	ENSGALG00010021271	-0.433267573	0.46205393	-0.937699139	0.348399046	0.386917268	negative
PRJNA729271	stage_3W_vs_E10	ordinary_muscle_development	ENSGALG00010021272	ENSGALG00010021272	0.73499085	0.357762357	2.054410801	0.039935949	0.050751497	positive
PRJNA729271	stage_3W_vs_E10	ordinary_muscle_development	ENSGALG00010021275	MAP3K2	0.341098186	0.091105588	3.743987536	0.000181123	0.000289616	positive
PRJNA729271	stage_3W_vs_E10	ordinary_muscle_development	ENSGALG00010021277	ENSGALG00010021277	-0.301523353	0.24320519	-1.239789962	0.215053092	0.247789711	negative
PRJNA729271	stage_3W_vs_E10	ordinary_muscle_development	ENSGALG00010021287	COL17A1	-7.24443059	0.498812049	-14.52336728	8.61711E-48	9.09723E-47	negative
PRJNA729271	stage_3W_vs_E10	ordinary_muscle_development	ENSGALG00010021290	ERCC3	-0.058011	0.068169706	-0.850979175	0.394780921	0.43369578	negative
PRJNA729271	stage_3W_vs_E10	ordinary_muscle_development	ENSGALG00010021293	PCNX1	0.199649612	0.068837402	2.900307187	0.003727971	0.005298778	positive
PRJNA729271	stage_3W_vs_E10	ordinary_muscle_development	ENSGALG00010021296	GATC	0.145614531	0.121994863	1.193611996	0.232629779	0.266516649	positive
PRJNA729271	stage_3W_vs_E10	ordinary_muscle_development	ENSGALG00010021298	RPGRIP1L	-1.240370738	0.146717457	-8.45414554	2.81138E-17	9.54356E-17	negative
PRJNA729271	stage_3W_vs_E10	ordinary_muscle_development	ENSGALG00010021299	CYP27C1	-5.060032412	0.194636734	-25.99731467	5.3108E-149	4.9346E-147	negative
PRJNA729271	stage_3W_vs_E10	ordinary_muscle_development	ENSGALG00010021301	SMURF1	-0.015443687	0.080632961	-0.1915307	0.848109831	0.865485215	negative
PRJNA729271	stage_3W_vs_E10	ordinary_muscle_development	ENSGALG00010021302	P4HA1	-0.609406323	0.060161221	-10.12955375	4.08509E-24	1.84236E-23	negative
PRJNA729271	stage_3W_vs_E10	ordinary_muscle_development	ENSGALG00010021307	MAP3K9	-0.340770577	0.134728794	-2.529307708	0.011428778	0.01545193	negative
PRJNA729271	stage_3W_vs_E10	ordinary_muscle_development	ENSGALG00010021309	ST3GAL3	0.156850786	0.126765248	1.237332694	0.215963595	0.248739953	positive

PRJNA729271	stage_3W_vs_E10	ordinary_muscle_development	ENSGALG00010021311	TRIAP1	0.220820138	0.120491758	1.832657624	0.066853503	0.082619279	positive
PRJNA729271	stage_3W_vs_E10	ordinary_muscle_development	ENSGALG00010021313	KPNA7	4.530353088	1.212460346	3.736495881	0.000186603	0.000298017	positive
PRJNA729271	stage_3W_vs_E10	ordinary_muscle_development	ENSGALG00010021315	COX6A1	-0.291363639	0.135706509	-2.147013001	0.031792247	0.040860845	negative
PRJNA729271	stage_3W_vs_E10	ordinary_muscle_development	ENSGALG00010021318	TP73	-1.78940918	0.268352737	-6.668123447	2.59095E-11	6.48072E-11	negative
PRJNA729271	stage_3W_vs_E10	ordinary_muscle_development	ENSGALG00010021320	SLC6A6	1.380652184	0.19508723	7.077101788	1.472E-12	3.93521E-12	positive
PRJNA729271	stage_3W_vs_E10	ordinary_muscle_development	ENSGALG00010021325	ARPC1A	0.357584197	0.063469225	5.633977666	1.761E-08	3.74496E-08	positive
PRJNA729271	stage_3W_vs_E10	ordinary_muscle_development	ENSGALG00010021327	IGSF1	3.868606013	0.414561199	9.331809203	1.04079E-20	4.09507E-20	positive
PRJNA729271	stage_3W_vs_E10	ordinary_muscle_development	ENSGALG00010021329	PSMC3IP	-2.375735453	0.170433183	-13.93939495	3.65021E-44	3.37866E-43	negative
PRJNA729271	stage_3W_vs_E10	ordinary_muscle_development	ENSGALG00010021330	COMMD4	-0.545668968	0.131215956	-4.158556498	3.20265E-05	5.43461E-05	negative
PRJNA729271	stage_3W_vs_E10	ordinary_muscle_development	ENSGALG00010021332	OIT3	0.924629268	0.127206942	7.268701319	3.6296E-13	9.9983E-13	positive
PRJNA729271	stage_3W_vs_E10	ordinary_muscle_development	ENSGALG00010021335	NFATC3	0.217842823	0.101832559	2.139225652	0.032417398	0.041627408	positive
PRJNA729271	stage_3W_vs_E10	ordinary_muscle_development	ENSGALG00010021338	PDIA5	-0.84031041	0.158313373	-5.307892781	1.109E-07	2.23916E-07	negative
PRJNA729271	stage_3W_vs_E10	ordinary_muscle_development	ENSGALG00010021341	L3MBTL1	-0.67769589	0.157034156	-4.315595468	1.59173E-05	2.76943E-05	negative
PRJNA729271	stage_3W_vs_E10	ordinary_muscle_development	ENSGALG00010021342	SFR1	-3.208644536	0.192616737	-16.65818137	2.63928E-62	4.19019E-61	negative
PRJNA729271	stage_3W_vs_E10	ordinary_muscle_development	ENSGALG00010021345	NEIL1	0.845440612	0.150433785	5.620018207	1.90937E-08	4.0504E-08	positive
PRJNA729271	stage_3W_vs_E10	ordinary_muscle_development	ENSGALG00010021346	RPS21	0.690197288	0.278457336	2.47864645	0.013188196	0.01768553	positive
PRJNA729271	stage_3W_vs_E10	ordinary_muscle_development	ENSGALG00010021348	SLC4A3	-0.083192494	0.116820326	-0.712140577	0.47637773	0.515113405	negative
PRJNA729271	stage_3W_vs_E10	ordinary_muscle_development	ENSGALG00010021350	CABLES2	-0.552469729	0.136471596	-4.048239659	5.16043E-05	8.63148E-05	negative
PRJNA729271	stage_3W_vs_E10	ordinary_muscle_development	ENSGALG00010021352	CFAP43	-0.829786079	0.228789067	-3.626860707	0.000286888	0.000451318	negative
PRJNA729271	stage_3W_vs_E10	ordinary_muscle_development	ENSGALG00010021353	IPO13	1.046075415	0.178510586	5.860018925	4.62814E-09	1.01536E-08	positive
PRJNA729271	stage_3W_vs_E10	ordinary_muscle_development	ENSGALG00010021354	ENSGALG00010021354	-0.402317736	0.113278712	-3.551574082	0.000382934	0.000595817	negative
PRJNA729271	stage_3W_vs_E10	ordinary_muscle_development	ENSGALG00010021358	MMP15	0.374728309	0.129260748	2.899010843	0.003743419	0.005319693	positive
PRJNA729271	stage_3W_vs_E10	ordinary_muscle_development	ENSGALG00010021360	PTPRN	-0.44551038	0.267511605	-1.665387115	0.095835548	0.116167944	negative
PRJNA729271	stage_3W_vs_E10	ordinary_muscle_development	ENSGALG00010021361	WRAP73	-0.851479523	0.093113059	-9.144576824	5.98638E-20	2.28349E-19	negative
PRJNA729271	stage_3W_vs_E10	ordinary_muscle_development	ENSGALG00010021362	ARPC1B	0.988212894	0.120102655	8.228068681	1.90256E-16	6.19582E-16	positive
PRJNA729271	stage_3W_vs_E10	ordinary_muscle_development	ENSGALG00010021363	ENSGALG00010021363	0.123056986	0.073751665	1.668531632	0.095210238	0.115438929	positive
PRJNA729271	stage_3W_vs_E10	ordinary_muscle_development	ENSGALG00010021367	GRIP2	-0.953029485	0.181256688	-5.257899701	1.4571E-07	2.91279E-07	negative
PRJNA729271	stage_3W_vs_E10	ordinary_muscle_development	ENSGALG00010021372	CD86	4.583394774	0.556689493	8.233305695	1.82117E-16	5.93743E-16	positive
PRJNA729271	stage_3W_vs_E10	ordinary_muscle_development	ENSGALG00010021373	CEP250	-0.59872251	0.153890447	-3.890576188	0.000100006	0.000163279	negative
PRJNA729271	stage_3W_vs_E10	ordinary_muscle_development	ENSGALG00010021375	TUBG1	-1.008258927	0.118097375	-8.53752191	1.37131E-17	4.73603E-17	negative
PRJNA729271	stage_3W_vs_E10	ordinary_muscle_development	ENSGALG00010021378	KIF11	-3.559647296	0.188972564	-18.83684712	3.7675E-79	9.1321E-78	negative
PRJNA729271	stage_3W_vs_E10	ordinary_muscle_development	ENSGALG00010021381	DNAJB2	2.199687036	0.146954765	14.96846345	1.18016E-50	1.37071E-49	positive
PRJNA729271	stage_3W_vs_E10	ordinary_muscle_development	ENSGALG00010021382	TPRG1L	0.331505296	0.086825772	3.818051797	0.00013451	0.000217578	positive
PRJNA729271	stage_3W_vs_E10	ordinary_muscle_development	ENSGALG00010021383	MSI1	-1.176154585	0.11999326	-9.801838731	1.10555E-22	4.7049E-22	negative
PRJNA729271	stage_3W_vs_E10	ordinary_muscle_development	ENSGALG00010021384	ARTN	0.195460331	0.419071892	0.46641241	0.64092033	0.67417563	positive
PRJNA729271	stage_3W_vs_E10	ordinary_muscle_development	ENSGALG00010021385	GDF5	-8.028630492	0.682273796	-11.76746131	5.74267E-32	3.55879E-31	negative
PRJNA729271	stage_3W_vs_E10	ordinary_muscle_development	ENSGALG00010021387	SLC2A14	1.402321412	0.093057362	15.06943004	2.57323E-51	3.04233E-50	positive
PRJNA729271	stage_3W_vs_E10	ordinary_muscle_development	ENSGALG00010021388	DPH2	-1.128822747	0.154371135	-7.312395201	2.62422E-13	7.2814E-13	negative
PRJNA729271	stage_3W_vs_E10	ordinary_muscle_development	ENSGALG00010021390	GSTO2	-1.074236898	0.215203811	-4.991718746	5.98444E-07	1.14954E-06	negative
PRJNA729271	stage_3W_vs_E10	ordinary_muscle_development	ENSGALG00010021391	ENSGALG00010021391	1.617095467	0.365370734	4.425903112	9.60396E-06	1.69747E-05	positive
PRJNA729271	stage_3W_vs_E10	ordinary_muscle_development	ENSGALG00010021393	CCDC174	0.108696161	0.113300824	0.95935897	0.337377937	0.37582948	positive
PRJNA729271	stage_3W_vs_E10	ordinary_muscle_development	ENSGALG00010021394	MAN2C1	-0.452402396	0.098103111	-4.611498997	3.99776E-06	7.27892E-06	negative
PRJNA729271	stage_3W_vs_E10	ordinary_muscle_development	ENSGALG00010021396	MED6	0.158281231	0.078624848	2.013119699	0.044102038	0.055733134	positive
PRJNA729271	stage_3W_vs_E10	ordinary_muscle_development	ENSGALG00010021397	ENSGALG00010021397	6.17191421	1.687341813	3.657773525	0.000254416	0.00040141	positive
PRJNA729271	stage_3W_vs_E10	ordinary_muscle_development	ENSGALG00010021400	B4GALT2	0.109020661	0.092073317	1.184063578	0.236387896	0.270586951	positive
PRJNA729271	stage_3W_vs_E10	ordinary_muscle_development	ENSGALG00010021401	PDAP1	-0.490463378	0.094736508	-5.177131676	2.25323E-07	4.4521E-07	negative
PRJNA729271	stage_3W_vs_E10	ordinary_muscle_development	ENSGALG00010021402	ENSGALG00010021402	0.22548816	0.087865549	2.566286352	0.010279393	0.013949986	positive
PRJNA729271	stage_3W_vs_E10	ordinary_muscle_development	ENSGALG00010021404	UQCC1	0.164535928	0.093955363	1.751213794	0.07990909	0.097811372	positive
PRJNA729271	stage_3W_vs_E10	ordinary_muscle_development	ENSGALG00010021405	IDE	1.333690369	0.113864707	11.71293899	1.0942E-31	6.69781E-31	positive
PRJNA729271	stage_3W_vs_E10	ordinary_muscle_development	ENSGALG00010021407	SIN3A	-0.573322474	0.112304105	-5.105089192	3.30639E-07	6.46252E-07	negative
PRJNA729271	stage_3W_vs_E10	ordinary_muscle_development	ENSGALG00010021408	TTC9	-0.00010166	0.217032398	-0.000468409	0.999626264	0.999626264	negative
PRJNA729271	stage_3W_vs_E10	ordinary_muscle_development	ENSGALG00010021409	MEGF6	-2.723290792	0.281307178	-9.680843596	3.63702E-22	1.51621E-21	negative
PRJNA729271	stage_3W_vs_E10	ordinary_muscle_development	ENSGALG00010021411	MCU	1.02302918	0.128952754	7.933364329	2.13288E-15	6.5681E-15	positive
PRJNA729271	stage_3W_vs_E10	ordinary_muscle_development	ENSGALG00010021412	ZNF319	0.286618946	0.123902287	2.313265977	0.02070802	0.027203439	positive
PRJNA729271	stage_3W_vs_E10	ordinary_muscle_development	ENSGALG00010021413	SYNJ2BP	1.296237561	0.149550089	8.667581355	4.41396E-18	1.56317E-17	positive
PRJNA729271	stage_3W_vs_E10	ordinary_muscle_development	ENSGALG00010021414	BUD31	0.641810004	0.111098504	5.776945524	7.60689E-09	1.6531E-08	positive
PRJNA729271	stage_3W_vs_E10	ordinary_muscle_development	ENSGALG00010021415	SIRT4	0.739935026	0.149007375	4.965761102	6.84322E-07	1.31016E-06	positive
PRJNA729271	stage_3W_vs_E10	ordinary_muscle_development	ENSGALG00010021416	PLEKHH3	1.102317906	0.122054797	9.031336176	1.69588E-19	6.35846E-19	positive
PRJNA729271	stage_3W_vs_E10	ordinary_muscle_development	ENSGALG00010021417	STK16	-1.540429122	0.166424279	-9.256036051	2.12163E-20	8.24256E-20	negative
PRJNA729271	stage_3W_vs_E10	ordinary_muscle_development	ENSGALG00010021419	MANSC1	-1.372725757	0.201176733	-6.823481694	8.88601E-12	2.27727E-11	negative
PRJNA729271	stage_3W_vs_E10	ordinary_muscle_development	ENSGALG00010021420	PLXNB3	-1.971294195	0.192490885	-10.24097422	1.29921E-24	5.98795E-24	negative
PRJNA729271	stage_3W_vs_E10	ordinary_muscle_development	ENSGALG00010021421	GLB1L	-0.274648306	0.168503224	-1.629929081	0.103116487	0.124390269	negative
PRJNA729271	stage_3W_vs_E10	ordinary_muscle_development	ENSGALG00010021424	PTCD1	0.087564788	0.107765863	0.812546626	0.416478043	0.455267665	positive
PRJNA729271	stage_3W_vs_E10	ordinary_muscle_development	ENSGALG00010021425	PTPN9	-1.447031932	0.126435362	-11.44483556	2.49577E-30	1.45111E-29	negative
PRJNA729271	stage_3W_vs_E10	ordinary_muscle_development	ENSGALG00010021426	SLC38A7	-0.451362555	0.146170284	-3.087922818	0.002015608	0.002932474	negative
PRJNA729271	stage_3W_vs_E10	ordinary_muscle_development	ENSGALG00010021427	RPLP0	0.342556041	0.135397964	2.529994028	0.011406447	0.015423176	positive
PRJNA729271	stage_3W_vs_E10	ordinary_muscle_development	ENSGALG00010021429	MED8	0.854388641	0.11387645	7.502768504	6.24838E-14	1.79347E-13	positive
PRJNA729271	stage_3W_vs_E10	ordinary_muscle_development	ENSGALG00010021431	CPSF4	-0.488063766	0.106928029	-4.564413769	5.00892E-06	9.04501E-06	negative
PRJNA729271	stage_3W_vs_E10	ordinary_muscle_development	ENSGALG00010021432	SYT9	-0.07635603	0.293178267	-0.260442326	0.794522598	0.817302183	negative
PRJNA729271	stage_3W_vs_E10	ordinary_muscle_development	ENSGALG00010021433	ENSGALG00010021433	4.525621668	0.271024926	16.69817508	1.35135E-62	2.15963E-61	positive
PRJNA729271	stage_3W_vs_E10	ordinary_muscle_development	ENSGALG00010021434	ANKZF1	0.262591839	0.177571255	1.478796999	0.139194578	0.165094952	positive
PRJNA729271	stage_3W_vs_E10	ordinary_muscle_development	ENSGALG00010021435	GCN1	0.460454837	0.088982266	5.174680951	2.28301E-07	4.50834E-07	positive
PRJNA729271	stage_3W_vs_E10	ordinary_muscle_development	ENSGALG00010021438	GOT2	1.455291084	0.110015842	13.22801389	6.04641E-40	4.88532E-39	positive
PRJNA729271	stage_3W_vs_E10	ordinary_muscle_development	ENSGALG00010021439	ITPRIP	0.255670435	0.186255921	1.372683525	0.169850753	0.198787781	positive
PRJNA729271	stage_3W_vs_E10	ordinary_muscle_development	ENSGALG00010021441	ENSGALG00010021441	0.276446203	0.319904579	0.864152065	0.387504361	0.426490183	positive
PRJNA729271	stage_3W_vs_E10	ordinary_muscle_development	ENSGALG00010021443	SNUPN	-0.035474382	0.134701614	-0.263355286	0.792276751	0.815570734	negative
PRJNA729271	stage_3W_vs_E10	ordinary_muscle_development	ENSGALG00010021444	ATG9A	2.145336831	0.245825471	8.727073006	2.61348E-18	9.35369E-18	positive
PRJNA729271	stage_3W_vs_E10	ordinary_muscle_development	ENSGALG00010021446	HOXB8	-1.248672052	0.613279959	-2.03605553	0.04174478	0.052892533	negative
PRJNA729271	stage_3W_vs_E10	ordinary_muscle_development	ENSGALG00010021447	OCSTAMP	0.963704832	0.655920525	1.469240245	0.141767638	0.167981845	positive
PRJNA729271	stage_3W_vs_E10	ordinary_muscle_development	ENSGALG00010021448	SZT2	0.306940023	0.100397831	3.057237598	0.002233871	0.003237673	positive
PRJNA729271	stage_3W_vs_E10	ordinary_muscle_development	ENSGALG00010021450	OLFML1	3.665647549	0.231242003	15.85199702	1.36183E-56	1.86753E-55	positive
PRJNA729271	stage_3W_vs_E10	ordinary_muscle_development	ENSGALG00010021452	SLC25A1	-0.921446642	0.118895036	-7.750085051	9.1831E-15	2.74452E-14	negative
PRJNA729271	stage_3W_vs_E10	ordinary_muscle_development	ENSGALG00010021454	ATP5J2	1.149212019	0.174189417	6.597484737	4.18192E-11	1.03283E-10	positive
PRJNA729271	stage_3W_vs_E10	ordinary_muscle_development	ENSGALG00010021455	CFAP58	0.320811533	0.503615046	0.637017372	0.524113528	0.56207431	positive
PRJNA729271	stage_3W_vs_E10	ordinary_muscle_development	ENSGALG00010021457	USB1	0.789407686	0.112806089	6.997917346	2.59795E-12	6.84057E-12	positive
PRJNA729271	stage_3W_vs_E10	ordinary_muscle_development	ENSGALG00010021458	USP30	0.140744613	0.080404585	1.750455061	0.080039822	0.097963119	positive
PRJNA729271	stage_3W_vs_E10	ordinary_muscle_development	ENSGALG00010021459	ARHGEF16	-0.116604725	0.127590734	-0.913896493	0.360771237	0.399585778	negative
PRJNA729271	stage_3W_vs_E10	ordinary_muscle_development	ENSGALG00010021461	SNX33	0.75787148	0.109193033	6.940657845	3.90278E-12	1.01636E-11	positive
PRJNA729271	stage_3W_vs_E10	ordinary_muscle_development	ENSGALG00010021462	SETD5	-0.781130488	0.095475685	-8.18145991	2.80425E-16	9.06302E-16	negative
PRJNA729271	stage_3W_vs_E10	ordinary_muscle_development	ENSGALG00010021464	ENSGALG00010021464	1.900513178	0.406651406	4.673568444	2.96011E-06	5.44019E-06	positive
PRJNA729271	stage_3W_vs_E10	ordinary_muscle_development	ENSGALG00010021465	ABCB6	1.849486286	0.211492822	8.74491281	2.23188E-18	8.00571E-18	positive
PRJNA729271	stage_3W_vs_E10	ordinary_muscle_development	ENSGALG00010021466	HES4	-0.947097769	0.181958947	-5.205007963	1.93988E-07	3.8487E-07	negative
PRJNA729271	stage_3W_vs_E10	ordinary_muscle_development	ENSGALG00010021467	LHFPL4	0.929275176	0.242711781	3.828718876	0.000128812	0.000208618	positive
PRJNA729271	stage_3W_vs_E10	ordinary_muscle_development	ENSGALG00010021469	CSPG4	-0.852062075	0.162408564	-5.246410995	1.55091E-07	3.09519E-07	negative
PRJNA729271	stage_3W_vs_E10	ordinary_muscle_development	ENSGALG00010021470	ENSGALG00010021470	-0.319959017	0.554273833	-0.577258023	0.563765166	0.600424399	negative
PRJNA729271	stage_3W_vs_E10	ordinary_muscle_development	ENSGALG00010021473	SLC8A3	2.147177672	0.131231432	16.36176357	3.58591E-60	5.3863E-59	positive
PRJNA729271	stage_3W_vs_E10	ordinary_muscle_development	ENSGALG00010021474	PPFIBP2	0.357193305	0.181013551	1.973295941	0.048461855	0.060945219	positive
PRJNA729271	stage_3W_vs_E10	ordinary_muscle_development	ENSGALG00010021476	ALKBH2	0.107435784	0.120257743	0.893379343	0.371654061	0.410291365	positive
PRJNA729271	stage_3W_vs_E10	ordinary_muscle_development	ENSGALG00010021477	ZFAND2B	0.568673254	0.117842117	4.825721626	1.39497E-06	2.61614E-06	positive
PRJNA729271	stage_3W_vs_E10	ordinary_muscle_development	ENSGALG00010021478	SLC13A3	-0.348558617	0.195732127	-1.780794101	0.074946093	0.092031822	negative
PRJNA729271	stage_3W_vs_E10	ordinary_muscle_development	ENSGALG00010021479	SKAP1	1.319098166	0.41552163	3.17455957	0.001500641	0.002212796	positive
PRJNA729271	stage_3W_vs_E10	ordinary_muscle_development	ENSGALG00010021480	ENSGALG00010021480	0.085665878	0.0750081	1.142088353	0.253417296	0.288717407	positive
PRJNA729271	stage_3W_vs_E10	ordinary_muscle_development	ENSGALG00010021481	MICU1	0.127153164	0.175270662	0.725467471	0.468165294	0.506876004	positive
PRJNA729271	stage_3W_vs_E10	ordinary_muscle_development	ENSGALG00010021483	UNG	-2.161362405	0.158767975	-13.61333987	3.33641E-42	2.89069E-41	negative
PRJNA729271	stage_3W_vs_E10	ordinary_muscle_development	ENSGALG00010021484	MARCHF5	0.976759249	0.070703799	13.8148058	2.07513E-43	1.87876E-42	positive
PRJNA729271	stage_3W_vs_E10	ordinary_muscle_development	ENSGALG00010021485	SMOC1	-3.736167614	0.234812426	-15.91128577	5.29155E-57	7.36094E-56	negative
PRJNA729271	stage_3W_vs_E10	ordinary_muscle_development	ENSGALG00010021487	KLHL17	-2.281672844	0.165410775	-13.79397955	2.77045E-43	2.48963E-42	negative
PRJNA729271	stage_3W_vs_E10	ordinary_muscle_development	ENSGALG00010021488	HYI	-0.428341194	0.133795542	-3.201460888	0.001367326	0.002024246	negative
PRJNA729271	stage_3W_vs_E10	ordinary_muscle_development	ENSGALG00010021489	CNPPD1	0.953004304	0.085211413	11.18399837	4.88389E-29	2.70715E-28	positive
PRJNA729271	stage_3W_vs_E10	ordinary_muscle_development	ENSGALG00010021490	ACACB	5.78319795	0.328983351	17.57899885	3.56821E-69	6.70833E-68	positive
PRJNA729271	stage_3W_vs_E10	ordinary_muscle_development	ENSGALG00010021492	PARP6	0.885512436	0.154413851	5.734669722	9.77025E-09	2.10964E-08	positive
PRJNA729271	stage_3W_vs_E10	ordinary_muscle_development	ENSGALG00010021493	UNC93B1	0.659039155	0.130367016	5.055259933	4.29805E-07	8.34006E-07	positive
PRJNA729271	stage_3W_vs_E10	ordinary_muscle_development	ENSGALG00010021494	SLC2A10	-1.383495396	0.244873578	-5.649835341	1.60602E-08	3.42341E-08	negative
PRJNA729271	stage_3W_vs_E10	ordinary_muscle_development	ENSGALG00010021496	MTMR14	0.778769319	0.115254351	6.756962442	1.40915E-11	3.57278E-11	positive
PRJNA729271	stage_3W_vs_E10	ordinary_muscle_development	ENSGALG00010021498	B3GALT6	-1.742500902	0.115708139	-15.05944975	2.99267E-51	3.5296E-50	negative
PRJNA729271	stage_3W_vs_E10	ordinary_muscle_development	ENSGALG00010021501	SLC23A3	4.094690849	0.554620038	7.382875782	1.54907E-13	4.34812E-13	positive
PRJNA729271	stage_3W_vs_E10	ordinary_muscle_development	ENSGALG00010021502	DNAJB12	1.107852989	0.145752814	7.600902939	2.94072E-14	8.57315E-14	positive
PRJNA729271	stage_3W_vs_E10	ordinary_muscle_development	ENSGALG00010021503	KCNS1	-2.784574485	0.665803884	-4.182274318	2.88607E-05	4.91524E-05	negative
PRJNA729271	stage_3W_vs_E10	ordinary_muscle_development	ENSGALG00010021504	ODR4	-0.753095834	0.07874653	-9.563543078	1.13793E-21	4.65545E-21	negative
PRJNA729271	stage_3W_vs_E10	ordinary_muscle_development	ENSGALG00010021505	IHH	-5.119868422	0.59165285	-8.653500819	4.99433E-18	1.76396E-17	negative
PRJNA729271	stage_3W_vs_E10	ordinary_muscle_development	ENSGALG00010021506	MAX	0.343382127	0.253499364	1.354568003	0.175555234	0.204868619	positive
PRJNA729271	stage_3W_vs_E10	ordinary_muscle_development	ENSGALG00010021509	DDIT4	2.529839516	0.277762051	9.107937912	8.39626E-20	3.18596E-19	positive
PRJNA729271	stage_3W_vs_E10	ordinary_muscle_development	ENSGALG00010021510	NTSR1	1.08800267	0.391530475	2.778845431	0.005455248	0.007617167	positive
PRJNA729271	stage_3W_vs_E10	ordinary_muscle_development	ENSGALG00010021512	CPNE9	1.640612293	0.41061941	3.99545723	6.45695E-05	0.000107221	positive
PRJNA729271	stage_3W_vs_E10	ordinary_muscle_development	ENSGALG00010021513	SDF4	0.078123236	0.097147545	0.804170976	0.421298239	0.459739364	positive
PRJNA729271	stage_3W_vs_E10	ordinary_muscle_development	ENSGALG00010021514	TPR	-0.5090319	0.079296231	-6.419370665	1.36839E-10	3.27685E-10	negative
PRJNA729271	stage_3W_vs_E10	ordinary_muscle_development	ENSGALG00010021516	SRSF5	-0.926682923	0.152721034	-6.067814631	1.29662E-09	2.93528E-09	negative
PRJNA729271	stage_3W_vs_E10	ordinary_muscle_development	ENSGALG00010021518	CPEB3	2.244053514	0.145912734	15.379422	2.24947E-53	2.84769E-52	positive
PRJNA729271	stage_3W_vs_E10	ordinary_muscle_development	ENSGALG00010021519	FNTB	0.795981057	0.099211038	8.023109834	1.03101E-15	3.22706E-15	positive
PRJNA729271	stage_3W_vs_E10	ordinary_muscle_development	ENSGALG00010021521	ENSGALG00010021521	1.288616897	0.260601923	4.944771261	7.62333E-07	1.45376E-06	positive
PRJNA729271	stage_3W_vs_E10	ordinary_muscle_development	ENSGALG00010021523	CELF6	-1.439060565	0.165353633	-8.70292679	3.23433E-18	1.15387E-17	negative

PRJNA729271	stage_3W_vs_E10	ordinary_muscle_development	ENSGALG00010021525	ENSGALG00010021525	5.58024715	0.722325586	7.725390399	1.11511E-14	3.3156E-14	positive
PRJNA729271	stage_3W_vs_E10	ordinary_muscle_development	ENSGALG00010021526	KCNJ12	3.111784568	0.213949671	14.54447	6.33193E-48	6.73378E-47	positive
PRJNA729271	stage_3W_vs_E10	ordinary_muscle_development	ENSGALG00010021528	BRPF1	0.25927303	0.166789192	1.554495391	0.120066295	0.143558603	positive
PRJNA729271	stage_3W_vs_E10	ordinary_muscle_development	ENSGALG00010021529	BTAF1	0.451555346	0.075418706	5.987312274	2.13337E-09	4.76328E-09	positive
PRJNA729271	stage_3W_vs_E10	ordinary_muscle_development	ENSGALG00010021530	MLF2	-0.485165535	0.060840468	-7.97438865	1.53137E-15	4.75111E-15	negative
PRJNA729271	stage_3W_vs_E10	ordinary_muscle_development	ENSGALG00010021531	RAB15	-0.835073972	0.263129829	-3.173619565	0.001505509	0.002219523	negative
PRJNA729271	stage_3W_vs_E10	ordinary_muscle_development	ENSGALG00010021532	DPP10	-2.078545056	0.185857473	-11.18354307	4.90902E-29	2.71692E-28	negative
PRJNA729271	stage_3W_vs_E10	ordinary_muscle_development	ENSGALG00010021533	SSX2IP	-4.502084832	0.341401593	-13.18706452	1.04163E-39	8.35089E-39	negative
PRJNA729271	stage_3W_vs_E10	ordinary_muscle_development	ENSGALG00010021534	KIFC3	0.848911647	0.169705829	5.00225392	5.66639E-07	1.08917E-06	positive
PRJNA729271	stage_3W_vs_E10	ordinary_muscle_development	ENSGALG00010021535	SUSD6	0.419610786	0.085758934	4.892910453	9.93556E-07	1.88256E-06	positive
PRJNA729271	stage_3W_vs_E10	ordinary_muscle_development	ENSGALG00010021538	TNFRSF18	3.161649932	0.39910376	7.92187459	2.33956E-15	7.19693E-15	positive
PRJNA729271	stage_3W_vs_E10	ordinary_muscle_development	ENSGALG00010021539	HEXA	-0.294318239	0.119642018	-2.459990585	0.013894066	0.018589116	negative
PRJNA729271	stage_3W_vs_E10	ordinary_muscle_development	ENSGALG00010021540	STK4	-0.85407679	0.077611368	-11.00453214	3.63397E-28	1.9538E-27	negative
PRJNA729271	stage_3W_vs_E10	ordinary_muscle_development	ENSGALG00010021541	CYP27A1	3.251531032	0.275483211	11.80300979	3.76596E-32	2.34886E-31	positive
PRJNA729271	stage_3W_vs_E10	ordinary_muscle_development	ENSGALG00010021543	PLEKHD1	0.667136121	0.261779715	2.548463772	0.010819852	0.014654622	positive
PRJNA729271	stage_3W_vs_E10	ordinary_muscle_development	ENSGALG00010021545	CYB5R2	-1.528363062	0.143061437	-10.68326372	1.21909E-26	6.1314E-26	negative
PRJNA729271	stage_3W_vs_E10	ordinary_muscle_development	ENSGALG00010021546	OGG1	-1.400649353	0.185655845	-7.544332116	4.54611E-14	1.31476E-13	negative
PRJNA729271	stage_3W_vs_E10	ordinary_muscle_development	ENSGALG00010021549	ANAPC16	0.723162573	0.090133403	8.023247181	1.02986E-15	3.22415E-15	positive
PRJNA729271	stage_3W_vs_E10	ordinary_muscle_development	ENSGALG00010021550	MAP2K3	1.206727966	0.171732294	7.026796978	2.11329E-12	5.60001E-12	positive
PRJNA729271	stage_3W_vs_E10	ordinary_muscle_development	ENSGALG00010021552	ENSGALG00010021552	0.578052347	0.17329169	3.335718786	0.000850792	0.001282336	positive
PRJNA729271	stage_3W_vs_E10	ordinary_muscle_development	ENSGALG00010021553	SLC39A9	-0.862794062	0.087209717	-9.893324836	4.44998E-23	1.92775E-22	negative
PRJNA729271	stage_3W_vs_E10	ordinary_muscle_development	ENSGALG00010021554	SNX11	0.632864462	0.147286396	4.296829033	1.73259E-05	3.00657E-05	positive
PRJNA729271	stage_3W_vs_E10	ordinary_muscle_development	ENSGALG00010021556	CAMK1	-0.189630822	0.142912566	-1.32690096	0.184541447	0.214750182	negative
PRJNA729271	stage_3W_vs_E10	ordinary_muscle_development	ENSGALG00010021558	CHURC1	2.12298837	0.179384482	11.83484964	2.57805E-32	1.62262E-31	positive
PRJNA729271	stage_3W_vs_E10	ordinary_muscle_development	ENSGALG00010021559	MYO1H	-0.115764444	0.588230066	-0.196801304	0.843983031	0.862305916	negative
PRJNA729271	stage_3W_vs_E10	ordinary_muscle_development	ENSGALG00010021561	TGIF2	-1.940880419	0.100290663	-19.35255348	1.93989E-83	5.37643E-82	negative
PRJNA729271	stage_3W_vs_E10	ordinary_muscle_development	ENSGALG00010021562	LRP6	-0.1480824	0.082431861	-1.796421895	0.072427444	0.089097426	negative
PRJNA729271	stage_3W_vs_E10	ordinary_muscle_development	ENSGALG00010021564	PRKAG3	11.08744105	0.629710101	17.60721485	2.16856E-69	4.09821E-68	positive
PRJNA729271	stage_3W_vs_E10	ordinary_muscle_development	ENSGALG00010021565	CBX1	-1.977376224	0.106106414	-18.63578407	1.64723E-77	3.85728E-76	negative
PRJNA729271	stage_3W_vs_E10	ordinary_muscle_development	ENSGALG00010021566	ENSGALG00010021566	-0.148766461	0.096952324	-1.53442903	0.12492414	0.148998224	negative
PRJNA729271	stage_3W_vs_E10	ordinary_muscle_development	ENSGALG00010021568	TADA3	0.786720565	0.141200833	5.571642521	2.52349E-08	5.30577E-08	positive
PRJNA729271	stage_3W_vs_E10	ordinary_muscle_development	ENSGALG00010021570	KATNB1	-0.983649578	0.106912402	-9.200518889	3.56225E-20	1.37108E-19	negative
PRJNA729271	stage_3W_vs_E10	ordinary_muscle_development	ENSGALG00010021572	MYL9	-0.220218497	0.11308223	-1.947419123	0.051484507	0.064494679	negative
PRJNA729271	stage_3W_vs_E10	ordinary_muscle_development	ENSGALG00010021573	NATD1	1.014303925	0.195782409	5.180771502	2.2097E-07	4.36906E-07	positive
PRJNA729271	stage_3W_vs_E10	ordinary_muscle_development	ENSGALG00010021574	TTLL4	1.787805082	0.225243234	7.937219911	2.06763E-15	6.36989E-15	positive
PRJNA729271	stage_3W_vs_E10	ordinary_muscle_development	ENSGALG00010021575	ADGRL2	-0.520947269	0.083089946	-6.269678738	3.61794E-10	8.47342E-10	negative
PRJNA729271	stage_3W_vs_E10	ordinary_muscle_development	ENSGALG00010021576	ERH	-1.558355581	0.122773831	-12.69289691	6.47459E-37	4.69481E-36	negative
PRJNA729271	stage_3W_vs_E10	ordinary_muscle_development	ENSGALG00010021577	ARIH1	0.444242355	0.076538298	5.804183849	6.46803E-09	1.41005E-08	positive
PRJNA729271	stage_3W_vs_E10	ordinary_muscle_development	ENSGALG00010021578	ENSGALG00010021578	6.315305	0.932915722	6.76942713	1.29293E-11	3.28618E-11	positive
PRJNA729271	stage_3W_vs_E10	ordinary_muscle_development	ENSGALG00010021579	C20H20ORF24	0.458540186	0.111843265	4.099846202	4.13425E-05	6.96327E-05	positive
PRJNA729271	stage_3W_vs_E10	ordinary_muscle_development	ENSGALG00010021580	KCTD10	-1.371389051	0.11071819	-12.38630302	3.10008E-35	2.15313E-34	negative
PRJNA729271	stage_3W_vs_E10	ordinary_muscle_development	ENSGALG00010021582	STK36	-0.137986975	0.145827621	-0.946233469	0.344029519	0.382504247	negative
PRJNA729271	stage_3W_vs_E10	ordinary_muscle_development	ENSGALG00010021583	NFE2L1	2.091675237	0.220786676	9.473738539	2.70003E-21	1.09047E-20	positive
PRJNA729271	stage_3W_vs_E10	ordinary_muscle_development	ENSGALG00010021584	OVCH2	0.70093022	0.367728288	1.906109054	0.056636058	0.070630617	positive
PRJNA729271	stage_3W_vs_E10	ordinary_muscle_development	ENSGALG00010021586	BBS4	-0.452795044	0.123924847	-3.653787408	0.0002584	0.000407564	negative
PRJNA729271	stage_3W_vs_E10	ordinary_muscle_development	ENSGALG00010021588	RNF25	0.734333605	0.138133418	5.316118407	1.06004E-07	2.143E-07	positive
PRJNA729271	stage_3W_vs_E10	ordinary_muscle_development	ENSGALG00010021593	DCAF7	-1.134196287	0.057349242	-19.77700574	4.6977E-87	1.43053E-85	negative
PRJNA729271	stage_3W_vs_E10	ordinary_muscle_development	ENSGALG00010021594	ADPGK	-1.728547011	0.091961881	-18.79634247	8.09068E-79	1.94808E-77	negative
PRJNA729271	stage_3W_vs_E10	ordinary_muscle_development	ENSGALG00010021595	UBE3B	0.342318115	0.084072187	4.071716559	4.66679E-05	7.83202E-05	positive
PRJNA729271	stage_3W_vs_E10	ordinary_muscle_development	ENSGALG00010021597	GGT7	-0.995672297	0.188779993	-5.274246934	1.33302E-07	2.67361E-07	negative
PRJNA729271	stage_3W_vs_E10	ordinary_muscle_development	ENSGALG00010021599	ARPC4	-0.12471572	0.103360265	-1.206611849	0.227581661	0.261145993	negative
PRJNA729271	stage_3W_vs_E10	ordinary_muscle_development	ENSGALG00010021600	FMN1	0.01594605	0.130482958	0.122207913	0.902734359	0.91421327	positive
PRJNA729271	stage_3W_vs_E10	ordinary_muscle_development	ENSGALG00010021601	LPAR5	1.429937859	0.354150041	4.037661142	5.39868E-05	9.01957E-05	positive
PRJNA729271	stage_3W_vs_E10	ordinary_muscle_development	ENSGALG00010021602	PLEKHG4	0.305032022	0.097838237	3.117717882	0.001822572	0.002662854	positive
PRJNA729271	stage_3W_vs_E10	ordinary_muscle_development	ENSGALG00010021604	SPTB	1.697170407	0.189370028	8.962191234	3.18289E-19	1.18116E-18	positive
PRJNA729271	stage_3W_vs_E10	ordinary_muscle_development	ENSGALG00010021605	MAP3K3	-0.165068353	0.112703084	-1.464630312	0.143021807	0.169329502	negative
PRJNA729271	stage_3W_vs_E10	ordinary_muscle_development	ENSGALG00010021606	RNF8	-0.027004615	0.094090548	-0.287006672	0.774107211	0.79856836	negative
PRJNA729271	stage_3W_vs_E10	ordinary_muscle_development	ENSGALG00010021607	SLC9A5	2.010030308	0.184361041	10.90268473	1.11906E-27	5.88989E-27	positive
PRJNA729271	stage_3W_vs_E10	ordinary_muscle_development	ENSGALG00010021609	ENSGALG00010021609	0.923001054	0.204533502	4.512713284	6.40035E-06	1.14761E-05	positive
PRJNA729271	stage_3W_vs_E10	ordinary_muscle_development	ENSGALG00010021610	TMEM11	0.088283233	0.090157523	0.979210946	0.327475761	0.365641747	positive
PRJNA729271	stage_3W_vs_E10	ordinary_muscle_development	ENSGALG00010021611	GREM1	-1.211708612	0.601375257	-2.014896018	0.043915545	0.05552166	negative
PRJNA729271	stage_3W_vs_E10	ordinary_muscle_development	ENSGALG00010021612	BCS1L	-0.103662687	0.132813824	-0.780511271	0.435089996	0.473719634	negative
PRJNA729271	stage_3W_vs_E10	ordinary_muscle_development	ENSGALG00010021613	ADGRL4	2.819924717	0.149397844	18.87527047	1.82189E-79	4.46086E-78	positive
PRJNA729271	stage_3W_vs_E10	ordinary_muscle_development	ENSGALG00010021615	FLVCR1	-1.475481016	0.178176745	-8.280996597	1.22149E-16	4.02122E-16	negative
PRJNA729271	stage_3W_vs_E10	ordinary_muscle_development	ENSGALG00010021617	SORBS1	0.747808758	0.111456696	6.709410755	1.95412E-11	4.91889E-11	positive
PRJNA729271	stage_3W_vs_E10	ordinary_muscle_development	ENSGALG00010021618	NEO1	-2.381040204	0.108196976	-22.00653195	2.4935E-107	1.2088E-105	negative
PRJNA729271	stage_3W_vs_E10	ordinary_muscle_development	ENSGALG00010021619	FHOD1	4.032660281	0.178690558	22.56784203	8.9719E-113	4.7637E-111	positive
PRJNA729271	stage_3W_vs_E10	ordinary_muscle_development	ENSGALG00010021621	ZNF518A	0.218784845	0.136955391	1.597489836	0.110156561	0.132343088	positive
PRJNA729271	stage_3W_vs_E10	ordinary_muscle_development	ENSGALG00010021622	PTHLH	-1.909856447	0.261250192	-7.310449918	2.66249E-13	7.38619E-13	negative
PRJNA729271	stage_3W_vs_E10	ordinary_muscle_development	ENSGALG00010021623	FAR1	1.415907211	0.129427471	10.93977341	7.43856E-28	3.94374E-27	positive
PRJNA729271	stage_3W_vs_E10	ordinary_muscle_development	ENSGALG00010021624	HSD11B2	1.516517874	0.360821413	4.202959749	2.63447E-05	4.50022E-05	positive
PRJNA729271	stage_3W_vs_E10	ordinary_muscle_development	ENSGALG00010021625	HCN4	-3.01360891	1.026489641	-2.935839575	0.003326463	0.004752324	negative
PRJNA729271	stage_3W_vs_E10	ordinary_muscle_development	ENSGALG00010021626	ZNF142	1.0527915	0.212444542	4.955606256	7.21051E-07	1.37721E-06	positive
PRJNA729271	stage_3W_vs_E10	ordinary_muscle_development	ENSGALG00010021627	NCOA6	0.287414976	0.103862433	2.767265976	0.005652861	0.007880949	positive
PRJNA729271	stage_3W_vs_E10	ordinary_muscle_development	ENSGALG00010021628	REC114	-0.533087433	0.563247074	-0.946453977	0.343917086	0.382408573	negative
PRJNA729271	stage_3W_vs_E10	ordinary_muscle_development	ENSGALG00010021629	DHRS7B	1.588467661	0.100879841	15.74613571	7.30085E-56	9.85343E-55	positive
PRJNA729271	stage_3W_vs_E10	ordinary_muscle_development	ENSGALG00010021630	VTG1	-1.121316106	0.272966269	-4.107892563	3.99286E-05	6.7353E-05	negative
PRJNA729271	stage_3W_vs_E10	ordinary_muscle_development	ENSGALG00010021632	LIMD2	-3.874286678	0.168855902	-22.94433678	1.6784E-116	9.3211E-115	negative
PRJNA729271	stage_3W_vs_E10	ordinary_muscle_development	ENSGALG00010021633	CCNJ	-1.052251633	0.107374851	-9.799796021	1.12813E-22	4.7968E-22	negative
PRJNA729271	stage_3W_vs_E10	ordinary_muscle_development	ENSGALG00010021634	PLCD4	5.119490118	0.241649952	21.18556233	1.29767E-99	5.32854E-98	positive
PRJNA729271	stage_3W_vs_E10	ordinary_muscle_development	ENSGALG00010021635	FOXJ2	0.524011498	0.11383066	4.603430192	4.15589E-06	7.55071E-06	positive
PRJNA729271	stage_3W_vs_E10	ordinary_muscle_development	ENSGALG00010021636	C1orf159	-0.306849587	0.198315217	-1.547282112	0.121795233	0.145469834	negative
PRJNA729271	stage_3W_vs_E10	ordinary_muscle_development	ENSGALG00010021637	ATP6V0D1	0.220887195	0.103076814	2.142937751	0.032118097	0.041268666	positive
PRJNA729271	stage_3W_vs_E10	ordinary_muscle_development	ENSGALG00010021638	SRC	-1.03149343	0.175683439	-5.87131854	4.32343E-09	9.49804E-09	negative
PRJNA729271	stage_3W_vs_E10	ordinary_muscle_development	ENSGALG00010021639	SCG5	0.554667712	0.344874643	1.608316887	0.107765794	0.129739633	positive
PRJNA729271	stage_3W_vs_E10	ordinary_muscle_development	ENSGALG00010021640	PLEKHG3	-0.091678948	0.108782304	-0.842774463	0.399354592	0.43840085	negative
PRJNA729271	stage_3W_vs_E10	ordinary_muscle_development	ENSGALG00010021642	NPTN	-0.283796671	0.090503292	-3.135760755	0.00171409	0.002515005	negative
PRJNA729271	stage_3W_vs_E10	ordinary_muscle_development	ENSGALG00010021643	ENSGALG00010021643	0.455781599	0.12854747	3.545628696	0.000391678	0.000608833	positive
PRJNA729271	stage_3W_vs_E10	ordinary_muscle_development	ENSGALG00010021644	TARDBP	-0.659369313	0.154175288	-4.276750979	1.89641E-05	3.2822E-05	negative
PRJNA729271	stage_3W_vs_E10	ordinary_muscle_development	ENSGALG00010021645	ASCC1	-0.023286221	0.138249084	-0.168436711	0.866239729	0.881689831	negative
PRJNA729271	stage_3W_vs_E10	ordinary_muscle_development	ENSGALG00010021646	RPUSD3	0.320425248	0.16568463	1.93394673	0.053119678	0.066439704	positive
PRJNA729271	stage_3W_vs_E10	ordinary_muscle_development	ENSGALG00010021647	ARHGAP11B	-2.04066299	0.138840182	-14.69792799	6.64624E-49	7.25978E-48	negative
PRJNA729271	stage_3W_vs_E10	ordinary_muscle_development	ENSGALG00010021648	BRE	-0.099143965	0.067283491	-1.473525879	0.140609291	0.166663805	negative
PRJNA729271	stage_3W_vs_E10	ordinary_muscle_development	ENSGALG00010021649	PXN	0.398333704	0.096348503	4.134300918	3.56037E-05	6.01977E-05	positive
PRJNA729271	stage_3W_vs_E10	ordinary_muscle_development	ENSGALG00010021650	CD276	-1.507508761	0.111172917	-13.56003609	6.91021E-42	5.92683E-41	negative
PRJNA729271	stage_3W_vs_E10	ordinary_muscle_development	ENSGALG00010021651	RNF113B	-0.029457503	0.155308459	-0.18967095	0.849566986	0.866789151	negative
PRJNA729271	stage_3W_vs_E10	ordinary_muscle_development	ENSGALG00010021652	BORCS5	-0.169836972	0.096387541	-1.762022046	0.078065573	0.095659859	negative
PRJNA729271	stage_3W_vs_E10	ordinary_muscle_development	ENSGALG00010021655	USP22	-0.512328279	0.09324321	-5.494537131	3.91737E-08	8.13965E-08	negative
PRJNA729271	stage_3W_vs_E10	ordinary_muscle_development	ENSGALG00010021656	SETD6	-0.064693554	0.078502264	-0.824097941	0.40988391	0.448702309	negative
PRJNA729271	stage_3W_vs_E10	ordinary_muscle_development	ENSGALG00010021657	JAGN1	1.055512133	0.141408024	7.464301578	8.37426E-14	2.38384E-13	positive
PRJNA729271	stage_3W_vs_E10	ordinary_muscle_development	ENSGALG00010021658	STRADA	-0.412019349	0.090464537	-4.554484712	5.25141E-06	9.47346E-06	negative
PRJNA729271	stage_3W_vs_E10	ordinary_muscle_development	ENSGALG00010021659	SPOCK2	-1.876286242	0.152441216	-12.30826082	8.17662E-35	5.5853E-34	negative
PRJNA729271	stage_3W_vs_E10	ordinary_muscle_development	ENSGALG00010021660	RNF223	2.294377593	0.452968414	5.065204371	4.07962E-07	7.92364E-07	positive
PRJNA729271	stage_3W_vs_E10	ordinary_muscle_development	ENSGALG00010021661	DUSP16	0.323581219	0.120588004	2.683361599	0.007288614	0.010055054	positive
PRJNA729271	stage_3W_vs_E10	ordinary_muscle_development	ENSGALG00010021662	RASGRP1	1.684773892	0.15924539	10.57973418	3.70006E-26	1.82237E-25	positive
PRJNA729271	stage_3W_vs_E10	ordinary_muscle_development	ENSGALG00010021663	CNOT9	0.703750105	0.13958306	5.041801678	4.61169E-07	8.93072E-07	positive
PRJNA729271	stage_3W_vs_E10	ordinary_muscle_development	ENSGALG00010021664	TYSND1	-0.483843885	0.166505219	-2.905878198	0.00366224	0.005207904	negative
PRJNA729271	stage_3W_vs_E10	ordinary_muscle_development	ENSGALG00010021665	CNOT1	0.098676274	0.091436064	1.079183317	0.280506015	0.31745274	positive
PRJNA729271	stage_3W_vs_E10	ordinary_muscle_development	ENSGALG00010021666	CCDC47	0.971256664	0.091998012	10.55736579	4.69657E-26	2.30144E-25	positive
PRJNA729271	stage_3W_vs_E10	ordinary_muscle_development	ENSGALG00010021667	TBX15	-0.192587592	0.191804825	-1.00408106	0.315339544	0.353233902	negative
PRJNA729271	stage_3W_vs_E10	ordinary_muscle_development	ENSGALG00010021668	PYGO1	-1.080617359	0.132489956	-8.156220967	3.4567E-16	1.10974E-15	negative
PRJNA729271	stage_3W_vs_E10	ordinary_muscle_development	ENSGALG00010021669	ENSGALG00010021669	-0.696489172	0.074218657	-9.384286893	6.33436E-21	2.51621E-20	negative
PRJNA729271	stage_3W_vs_E10	ordinary_muscle_development	ENSGALG00010021670	BLCAP	1.912517292	0.108523547	17.62306287	1.63887E-69	3.11342E-68	positive
PRJNA729271	stage_3W_vs_E10	ordinary_muscle_development	ENSGALG00010021671	TNFRSF13B	2.77384055	0.734408301	3.77697331	0.000158746	0.000255045	positive
PRJNA729271	stage_3W_vs_E10	ordinary_muscle_development	ENSGALG00010021674	ING4	-1.293518251	0.125896545	-10.2744539	9.18632E-25	4.25826E-24	negative
PRJNA729271	stage_3W_vs_E10	ordinary_muscle_development	ENSGALG00010021675	DDX42	-0.704652977	0.059678599	-11.80746517	3.57167E-32	2.2296E-31	negative
PRJNA729271	stage_3W_vs_E10	ordinary_muscle_development	ENSGALG00010021676	TP53INP2	2.593098015	0.275720895	9.404793242	5.21326E-21	2.07542E-20	positive
PRJNA729271	stage_3W_vs_E10	ordinary_muscle_development	ENSGALG00010021677	ENSGALG00010021677	-0.566031754	0.153892027	-3.678109684	0.000234969	0.000371699	negative
PRJNA729271	stage_3W_vs_E10	ordinary_muscle_development	ENSGALG00010021678	DNAAF4	0.729903689	0.157833684	4.624511505	3.75482E-06	6.85813E-06	positive
PRJNA729271	stage_3W_vs_E10	ordinary_muscle_development	ENSGALG00010021679	MASP2	-3.636726519	1.231013046	-2.954255059	0.003134248	0.004488779	negative
PRJNA729271	stage_3W_vs_E10	ordinary_muscle_development	ENSGALG00010021680	PIGT	0.693648696	0.117650496	5.895841643	3.72776E-09	8.21684E-09	positive
PRJNA729271	stage_3W_vs_E10	ordinary_muscle_development	ENSGALG00010021681	BTBD10	-0.449516059	0.080915581	-5.555370824	2.77023E-08	5.80939E-08	negative
PRJNA729271	stage_3W_vs_E10	ordinary_muscle_development	ENSGALG00010021682	MYL4	-7.186030537	0.32999384	-21.77625659	3.8964E-105	1.793E-103	negative
PRJNA729271	stage_3W_vs_E10	ordinary_muscle_development	ENSGALG00010021683	HFM1	-0.985764863	0.431298226	-2.285575973	0.022279077	0.029174742	negative
PRJNA729271	stage_3W_vs_E10	ordinary_muscle_development	ENSGALG00010021684	BIN1	2.292557107	0.121193736	18.91646536	8.34679E-80	2.07169E-78	positive
PRJNA729271	stage_3W_vs_E10	ordinary_muscle_development	ENSGALG00010021685	DBNDD2	1.858381499	0.138592689	13.40894319	5.35971E-41	4.4572E-40	positive
PRJNA729271	stage_3W_vs_E10	ordinary_muscle_development	ENSGALG00010021686	CCPG1	2.808992311	0.164836661	17.04106536	4.07245E-65	6.98582E-64	positive
PRJNA729271	stage_3W_vs_E10	ordinary_muscle_development	ENSGALG00010021687	ZNF384	-0.355269734	0.107137847	-3.316005902	0.000913139	0.001372028	negative
PRJNA729271	stage_3W_vs_E10	ordinary_muscle_development	ENSGALG00010021688	ITPR1	-0.118696118	0.117083043	-1.013777191	0.310689081	0.348644285	negative
PRJNA729271	stage_3W_vs_E10	ordinary_muscle_development	ENSGALG00010021689	MPRIP	-0.192488944	0.109616898	-1.756015249	0.079085805	0.096828173	negative
PRJNA729271	stage_3W_vs_E10	ordinary_muscle_development	ENSGALG00010021691	PIGB	-0.923974037	0.150498172	-6.139436954	8.28145E-10	1.89726E-09	negative

PRJNA729271	stage_3W_vs_E10	ordinary_muscle_development	ENSGALG00010021693	PPA1	0.018431928	0.091150294	0.202214688	0.839748891	0.858221827	positive
PRJNA729271	stage_3W_vs_E10	ordinary_muscle_development	ENSGALG00010021694	ENSGALG00010021694	3.063328057	1.113097208	2.75207595	0.005921879	0.008236195	positive
PRJNA729271	stage_3W_vs_E10	ordinary_muscle_development	ENSGALG00010021695	FNBP4	0.152608497	0.070805721	2.15531307	0.031137344	0.040058205	positive
PRJNA729271	stage_3W_vs_E10	ordinary_muscle_development	ENSGALG00010021697	PIANP	-2.060823746	0.256969112	-8.01973331	1.05975E-15	3.31486E-15	negative
PRJNA729271	stage_3W_vs_E10	ordinary_muscle_development	ENSGALG00010021698	GYPC	-0.396207654	0.135194857	-2.930641463	0.003382629	0.004826382	negative
PRJNA729271	stage_3W_vs_E10	ordinary_muscle_development	ENSGALG00010021699	SYS1	-0.110303023	0.083852519	-1.315440772	0.18836181	0.218844537	negative
PRJNA729271	stage_3W_vs_E10	ordinary_muscle_development	ENSGALG00010021700	FLCN	0.652033837	0.077879318	8.372361934	5.64747E-17	1.89097E-16	positive
PRJNA729271	stage_3W_vs_E10	ordinary_muscle_development	ENSGALG00010021701	DR1	-0.34293705	0.076713364	-4.470369052	7.80847E-06	1.39029E-05	negative
PRJNA729271	stage_3W_vs_E10	ordinary_muscle_development	ENSGALG00010021702	PLD6	-0.940398204	0.353050241	-2.66363847	0.007730059	0.01063874	negative
PRJNA729271	stage_3W_vs_E10	ordinary_muscle_development	ENSGALG00010021703	CD82	-0.355275754	0.173820838	-2.043919228	0.040961527	0.051977356	negative
PRJNA729271	stage_3W_vs_E10	ordinary_muscle_development	ENSGALG00010021704	COPS7A	1.7605739	0.101828646	17.28957384	5.63674E-67	1.01245E-65	positive
PRJNA729271	stage_3W_vs_E10	ordinary_muscle_development	ENSGALG00010021705	CCDC18	-0.408837791	0.190812864	-2.142611259	0.032144326	0.041298707	negative
PRJNA729271	stage_3W_vs_E10	ordinary_muscle_development	ENSGALG00010021706	ENSGALG00010021706	2.125072026	0.15890453	13.37326272	8.66548E-41	7.15704E-40	positive
PRJNA729271	stage_3W_vs_E10	ordinary_muscle_development	ENSGALG00010021707	DLX2	-4.768620609	1.134199094	-4.204394654	2.61782E-05	4.47362E-05	negative
PRJNA729271	stage_3W_vs_E10	ordinary_muscle_development	ENSGALG00010021708	CNTNAP5	-6.643693878	0.470393313	-14.12369965	2.71363E-45	2.62402E-44	negative
PRJNA729271	stage_3W_vs_E10	ordinary_muscle_development	ENSGALG00010021710	ENSGALG00010021710	-0.798260663	0.32215157	-2.477903993	0.01321567	0.017720734	negative
PRJNA729271	stage_3W_vs_E10	ordinary_muscle_development	ENSGALG00010021711	ENSGALG00010021711	0.834680393	0.220086206	3.792515701	0.000149129	0.0002401	positive
PRJNA729271	stage_3W_vs_E10	ordinary_muscle_development	ENSGALG00010021713	RAB27A	0.484423924	0.079889332	6.063687233	1.33036E-09	3.00976E-09	positive
PRJNA729271	stage_3W_vs_E10	ordinary_muscle_development	ENSGALG00010021714	TSPAN18	0.052106476	0.118549914	0.439531964	0.660276127	0.692675867	positive
PRJNA729271	stage_3W_vs_E10	ordinary_muscle_development	ENSGALG00010021715	MMAB	-0.464185148	0.133606838	-3.474261917	0.000512261	0.000788407	negative
PRJNA729271	stage_3W_vs_E10	ordinary_muscle_development	ENSGALG00010021716	TFCP2L1	-0.044269972	0.387956903	-0.114110541	0.909150167	0.919810963	negative
PRJNA729271	stage_3W_vs_E10	ordinary_muscle_development	ENSGALG00010021717	SDC4	1.204919712	0.197500917	6.10083096	1.05518E-09	2.40033E-09	positive
PRJNA729271	stage_3W_vs_E10	ordinary_muscle_development	ENSGALG00010021718	SST7	-3.819827432	0.490558491	-7.786691089	6.87869E-15	2.07075E-14	negative
PRJNA729271	stage_3W_vs_E10	ordinary_muscle_development	ENSGALG00010021719	BHLHE40	3.82488125	0.213853213	17.88554496	1.52851E-71	3.0602E-70	positive
PRJNA729271	stage_3W_vs_E10	ordinary_muscle_development	ENSGALG00010021720	CACNB1	1.309403234	0.260941792	5.017989735	5.2215E-07	1.00619E-06	positive
PRJNA729271	stage_3W_vs_E10	ordinary_muscle_development	ENSGALG00010021721	TP53I11	-1.073282269	0.14373359	-7.467163851	8.19417E-14	2.33486E-13	negative
PRJNA729271	stage_3W_vs_E10	ordinary_muscle_development	ENSGALG00010021722	AARS1	0.601530425	0.158826812	3.787335502	0.000152271	0.000245024	positive
PRJNA729271	stage_3W_vs_E10	ordinary_muscle_development	ENSGALG00010021723	COPS3	1.349723796	0.105984664	12.73508581	3.77431E-37	2.7561E-36	positive
PRJNA729271	stage_3W_vs_E10	ordinary_muscle_development	ENSGALG00010021725	SH2D6	1.176086087	0.211416676	5.562882307	2.65355E-08	5.57115E-08	positive
PRJNA729271	stage_3W_vs_E10	ordinary_muscle_development	ENSGALG00010021726	ARNTL	0.058221599	0.146815849	0.396562085	0.691690416	0.721921989	positive
PRJNA729271	stage_3W_vs_E10	ordinary_muscle_development	ENSGALG00010021727	MVK	-1.117043924	0.104861063	-10.65260917	1.69545E-26	8.49778E-26	negative
PRJNA729271	stage_3W_vs_E10	ordinary_muscle_development	ENSGALG00010021728	TMED5	0.223361087	0.064730713	3.450619905	0.000559301	0.00085635	positive
PRJNA729271	stage_3W_vs_E10	ordinary_muscle_development	ENSGALG00010021729	RBPJL	-3.587378061	0.653638975	-5.488317248	4.05781E-08	8.42061E-08	negative
PRJNA729271	stage_3W_vs_E10	ordinary_muscle_development	ENSGALG00010021730	ARL8B	0.2196769	0.072110467	3.046394094	0.002316041	0.003352557	positive
PRJNA729271	stage_3W_vs_E10	ordinary_muscle_development	ENSGALG00010021731	SRM	-0.76061642	0.119963908	-6.340377127	2.29203E-10	5.43037E-10	negative
PRJNA729271	stage_3W_vs_E10	ordinary_muscle_development	ENSGALG00010021732	CASR	-1.801706984	0.300074178	-6.004205349	1.92271E-09	4.30155E-09	negative
PRJNA729271	stage_3W_vs_E10	ordinary_muscle_development	ENSGALG00010021733	ENSGALG00010021733	1.733461944	0.595224425	2.912282952	0.003587975	0.005106804	positive
PRJNA729271	stage_3W_vs_E10	ordinary_muscle_development	ENSGALG00010021734	PARD3B	0.291655214	0.063819395	4.570009053	4.87703E-06	8.82113E-06	positive
PRJNA729271	stage_3W_vs_E10	ordinary_muscle_development	ENSGALG00010021735	PIGBOS1	1.079856056	0.144924674	7.451153938	9.25273E-14	2.62874E-13	positive
PRJNA729271	stage_3W_vs_E10	ordinary_muscle_development	ENSGALG00010021736	EDEM1	0.57062023	0.082496869	6.916871386	4.61727E-12	1.19705E-11	positive
PRJNA729271	stage_3W_vs_E10	ordinary_muscle_development	ENSGALG00010021738	PRDM11	-0.438796875	0.07344409	-5.974570282	2.30697E-09	5.1406E-09	negative
PRJNA729271	stage_3W_vs_E10	ordinary_muscle_development	ENSGALG00010021739	EXOSC10	0.582516307	0.109955433	5.297749194	1.17239E-07	2.36386E-07	positive
PRJNA729271	stage_3W_vs_E10	ordinary_muscle_development	ENSGALG00010021740	PDLIM1	1.073619596	0.158290568	6.782587298	1.18042E-11	3.00708E-11	positive
PRJNA729271	stage_3W_vs_E10	ordinary_muscle_development	ENSGALG00010021741	RPL19	0.342288934	0.146021895	2.344093222	0.019073403	0.025135829	positive
PRJNA729271	stage_3W_vs_E10	ordinary_muscle_development	ENSGALG00010021742	MATN4	-8.264551842	0.512501336	-16.12591277	1.67762E-58	2.43171E-57	negative
PRJNA729271	stage_3W_vs_E10	ordinary_muscle_development	ENSGALG00010021743	SYT13	-6.175620164	0.597833035	-10.33000821	5.15562E-25	2.4169E-24	negative
PRJNA729271	stage_3W_vs_E10	ordinary_muscle_development	ENSGALG00010021744	LITAF	2.927170376	0.342466419	8.547320887	1.25979E-17	4.3623E-17	positive
PRJNA729271	stage_3W_vs_E10	ordinary_muscle_development	ENSGALG00010021745	RSL24D1	0.272766386	0.125488733	2.173632484	0.029732747	0.038360139	positive
PRJNA729271	stage_3W_vs_E10	ordinary_muscle_development	ENSGALG00010021746	MTOR	0.277344955	0.110695905	2.505467165	0.012228975	0.016467763	positive
PRJNA729271	stage_3W_vs_E10	ordinary_muscle_development	ENSGALG00010021747	AKTIP	1.177089532	0.114780061	10.25517432	1.12173E-24	5.1782E-24	positive
PRJNA729271	stage_3W_vs_E10	ordinary_muscle_development	ENSGALG00010021748	CDCA7	-3.140675202	0.180400406	-17.4094686	6.993E-68	1.28797E-66	negative
PRJNA729271	stage_3W_vs_E10	ordinary_muscle_development	ENSGALG00010021749	CLASP1	0.014760862	0.091071918	0.16207918	0.871243501	0.885911929	positive
PRJNA729271	stage_3W_vs_E10	ordinary_muscle_development	ENSGALG00010021750	HELLS	-3.4605676	0.154677482	-22.37279504	7.2452E-111	3.7373E-109	negative
PRJNA729271	stage_3W_vs_E10	ordinary_muscle_development	ENSGALG00010021751	MTCH2	-0.90111653	0.093781538	-9.608677276	7.34869E-22	3.02956E-21	negative
PRJNA729271	stage_3W_vs_E10	ordinary_muscle_development	ENSGALG00010021752	CSTB	0.238057845	0.109110768	2.18179975	0.029124315	0.037605287	positive
PRJNA729271	stage_3W_vs_E10	ordinary_muscle_development	ENSGALG00010021753	MTF2	-1.461534985	0.09791353	-14.92679296	2.20625E-50	2.54209E-49	negative
PRJNA729271	stage_3W_vs_E10	ordinary_muscle_development	ENSGALG00010021754	SLC17A9	-1.809157387	0.484977925	-3.730391205	0.000191183	0.00030513	negative
PRJNA729271	stage_3W_vs_E10	ordinary_muscle_development	ENSGALG00010021755	ENSGALG00010021755	2.765953471	0.153566807	18.01140179	1.58562E-72	3.31653E-71	positive
PRJNA729271	stage_3W_vs_E10	ordinary_muscle_development	ENSGALG00010021756	USP42	-1.717347152	0.129441879	-13.26732249	3.5815E-40	2.91487E-39	negative
PRJNA729271	stage_3W_vs_E10	ordinary_muscle_development	ENSGALG00010021757	ZP4	-0.82521488	0.414968208	-1.988621931	0.046742949	0.058901073	negative
PRJNA729271	stage_3W_vs_E10	ordinary_muscle_development	ENSGALG00010021758	RASSF10	-5.578484725	0.53419914	-10.4427063	1.58234E-25	7.57215E-25	negative
PRJNA729271	stage_3W_vs_E10	ordinary_muscle_development	ENSGALG00010021759	NIFK	-0.40576819	0.093543994	-4.337725753	1.43965E-05	2.51236E-05	negative
PRJNA729271	stage_3W_vs_E10	ordinary_muscle_development	ENSGALG00010021760	CSTA	2.059967926	0.392290486	5.251128942	1.5117E-07	3.02027E-07	positive
PRJNA729271	stage_3W_vs_E10	ordinary_muscle_development	ENSGALG00010021761	MAFB	0.177465725	0.16967356	1.04592445	0.295595915	0.332892766	positive
PRJNA729271	stage_3W_vs_E10	ordinary_muscle_development	ENSGALG00010021762	LAMB2	2.817565584	0.198740474	14.17711014	1.26968E-45	1.241E-44	positive
PRJNA729271	stage_3W_vs_E10	ordinary_muscle_development	ENSGALG00010021764	FAM222A	-2.155217316	0.885488368	-2.433930691	0.014935855	0.019924095	negative
PRJNA729271	stage_3W_vs_E10	ordinary_muscle_development	ENSGALG00010021765	EIF2AK1	0.010455923	0.104972946	0.099605884	0.920657223	0.929765655	positive
PRJNA729271	stage_3W_vs_E10	ordinary_muscle_development	ENSGALG00010021766	AGRN	-1.583040891	0.150728663	-10.50258694	8.40447E-26	4.0689E-25	negative
PRJNA729271	stage_3W_vs_E10	ordinary_muscle_development	ENSGALG00010021767	CCDC58	0.56389281	0.212183964	2.65756563	0.007870726	0.010825143	positive
PRJNA729271	stage_3W_vs_E10	ordinary_muscle_development	ENSGALG00010021768	DIPK1A	1.093877853	0.149080912	7.337477606	2.17657E-13	6.06369E-13	positive
PRJNA729271	stage_3W_vs_E10	ordinary_muscle_development	ENSGALG00010021769	ENSGALG00010021769	-0.76699053	0.178555895	-4.295520622	1.74284E-05	3.02399E-05	negative
PRJNA729271	stage_3W_vs_E10	ordinary_muscle_development	ENSGALG00010021770	RALGAPB	-0.023475454	0.097999439	-0.239546826	0.810681591	0.832742518	negative
PRJNA729271	stage_3W_vs_E10	ordinary_muscle_development	ENSGALG00010021771	ANKRD61	0.779667003	0.14364764	5.427635307	5.71056E-08	1.17228E-07	positive
PRJNA729271	stage_3W_vs_E10	ordinary_muscle_development	ENSGALG00010021772	TSN	-0.6281184	0.083345761	-7.536296902	4.83505E-14	1.39637E-13	negative
PRJNA729271	stage_3W_vs_E10	ordinary_muscle_development	ENSGALG00010021773	ENSGALG00010021773	-1.380031497	0.938110814	-1.47107514	0.141270797	0.167427922	negative
PRJNA729271	stage_3W_vs_E10	ordinary_muscle_development	ENSGALG00010021774	FBXL20	0.754654069	0.072180439	10.45510505	1.38845E-25	6.65308E-25	positive
PRJNA729271	stage_3W_vs_E10	ordinary_muscle_development	ENSGALG00010021775	CCDC36	1.829815202	0.324058975	5.646549989	1.637E-08	3.48586E-08	positive
PRJNA729271	stage_3W_vs_E10	ordinary_muscle_development	ENSGALG00010021776	PMS2	-0.661906618	0.094593456	-6.997382744	2.60788E-12	6.86422E-12	negative
PRJNA729271	stage_3W_vs_E10	ordinary_muscle_development	ENSGALG00010021778	RBL2	0.497279862	0.10380502	4.790518418	1.66351E-06	3.10169E-06	positive
PRJNA729271	stage_3W_vs_E10	ordinary_muscle_development	ENSGALG00010021779	UNC13C	0.59842784	0.246307756	2.429593974	0.015115746	0.020151084	positive
PRJNA729271	stage_3W_vs_E10	ordinary_muscle_development	ENSGALG00010021781	RAPGEF4	-0.545840727	0.268370634	-2.033906309	0.041961043	0.053157255	negative
PRJNA729271	stage_3W_vs_E10	ordinary_muscle_development	ENSGALG00010021782	ADAM11	-1.792854118	0.341754945	-5.246022466	1.55418E-07	3.10044E-07	negative
PRJNA729271	stage_3W_vs_E10	ordinary_muscle_development	ENSGALG00010021783	GIT2	-0.847343115	0.048511424	-17.46687795	2.56132E-68	4.74762E-67	negative
PRJNA729271	stage_3W_vs_E10	ordinary_muscle_development	ENSGALG00010021784	ANGPTL7	3.292972786	0.850410107	3.872217368	0.00010785	0.000175748	positive
PRJNA729271	stage_3W_vs_E10	ordinary_muscle_development	ENSGALG00010021785	CCDC71	-0.220291723	0.090160536	-2.443327574	0.014552522	0.019432409	negative
PRJNA729271	stage_3W_vs_E10	ordinary_muscle_development	ENSGALG00010021786	FAM162A	-0.027703321	0.173451616	-0.159717858	0.873103333	0.887616272	negative
PRJNA729271	stage_3W_vs_E10	ordinary_muscle_development	ENSGALG00010021788	RPL5	0.344894406	0.123907733	2.783477659	0.005377957	0.007516279	positive
PRJNA729271	stage_3W_vs_E10	ordinary_muscle_development	ENSGALG00010021789	CHD9	0.223256492	0.090779783	2.459319534	0.013920066	0.018620465	positive
PRJNA729271	stage_3W_vs_E10	ordinary_muscle_development	ENSGALG00010021790	PHF21A	0.478519659	0.127970242	3.739304174	0.00018453	0.000294805	positive
PRJNA729271	stage_3W_vs_E10	ordinary_muscle_development	ENSGALG00010021791	PLEKHM2	1.934798923	0.184012396	10.51450321	7.40701E-26	3.5944E-25	positive
PRJNA729271	stage_3W_vs_E10	ordinary_muscle_development	ENSGALG00010021792	KLHDC8B	3.727716341	0.258272779	14.43325294	3.19643E-47	3.28831E-46	positive
PRJNA729271	stage_3W_vs_E10	ordinary_muscle_development	ENSGALG00010021793	ENSGALG00010021793	0.010763673	0.159732775	0.067385501	0.946274811	0.953312023	positive
PRJNA729271	stage_3W_vs_E10	ordinary_muscle_development	ENSGALG00010021794	ENSGALG00010021794	-1.216413583	0.199165213	-6.10756047	1.01165E-09	2.30456E-09	negative
PRJNA729271	stage_3W_vs_E10	ordinary_muscle_development	ENSGALG00010021795	NDRG3	-0.310219954	0.106654441	-2.908645447	0.003629983	0.005165073	negative
PRJNA729271	stage_3W_vs_E10	ordinary_muscle_development	ENSGALG00010021797	SLC25A34	-0.162760008	0.179815012	-0.9051525	0.365384602	0.404139617	negative
PRJNA729271	stage_3W_vs_E10	ordinary_muscle_development	ENSGALG00010021798	RANBP10	0.526175584	0.181460423	2.899671323	0.003735541	0.005309018	positive
PRJNA729271	stage_3W_vs_E10	ordinary_muscle_development	ENSGALG00010021799	ENSGALG00010021799	0.005466239	0.211334277	0.02586537	0.979364721	0.982482638	positive
PRJNA729271	stage_3W_vs_E10	ordinary_muscle_development	ENSGALG00010021800	TMEM82	0.027622446	0.351470935	0.078590983	0.93735796	0.944919927	positive
PRJNA729271	stage_3W_vs_E10	ordinary_muscle_development	ENSGALG00010021801	VAMP1	2.700049167	0.242287014	11.14401105	7.65904E-29	4.20841E-28	positive
PRJNA729271	stage_3W_vs_E10	ordinary_muscle_development	ENSGALG00010021803	TLL2	-3.515838951	0.173988519	-20.20730437	8.44352E-91	2.82653E-89	negative
PRJNA729271	stage_3W_vs_E10	ordinary_muscle_development	ENSGALG00010021805	FUT8	-0.518315219	0.108826103	-4.762783983	1.9094E-06	3.54649E-06	negative
PRJNA729271	stage_3W_vs_E10	ordinary_muscle_development	ENSGALG00010021807	CDC42	-0.215875237	0.082983373	-2.601427594	0.009283666	0.012663913	negative
PRJNA729271	stage_3W_vs_E10	ordinary_muscle_development	ENSGALG00010021810	ANKRD13A	-1.135486688	0.09717287	-11.68522331	1.5168E-31	9.23009E-31	negative
PRJNA729271	stage_3W_vs_E10	ordinary_muscle_development	ENSGALG00010021812	TEAD1	-0.335047522	0.083484853	-4.013273186	5.98825E-05	9.96896E-05	negative
PRJNA729271	stage_3W_vs_E10	ordinary_muscle_development	ENSGALG00010021817	EVI5	0.274494483	0.100512697	2.730943374	0.006315332	0.00876012	positive
PRJNA729271	stage_3W_vs_E10	ordinary_muscle_development	ENSGALG00010021821	PPP1R19L	-1.393329325	0.120375368	-11.57487078	5.52544E-31	3.29594E-30	negative
PRJNA729271	stage_3W_vs_E10	ordinary_muscle_development	ENSGALG00010021823	ENSGALG00010021823	0.261954501	0.252328221	1.038149834	0.299200304	0.336716491	positive
PRJNA729271	stage_3W_vs_E10	ordinary_muscle_development	ENSGALG00010021826	FBXO40	6.748922948	0.207501766	32.52465303	4.7802E-232	1.1744E-229	positive
PRJNA729271	stage_3W_vs_E10	ordinary_muscle_development	ENSGALG00010021827	CCZ1	-0.173910909	0.095522607	-1.820625659	0.06866378	0.084682159	negative
PRJNA729271	stage_3W_vs_E10	ordinary_muscle_development	ENSGALG00010021829	ERCC6	-0.02115012	0.096711945	-0.218691908	0.826890053	0.847410302	negative
PRJNA729271	stage_3W_vs_E10	ordinary_muscle_development	ENSGALG00010021831	ENSGALG00010021831	1.482034648	0.356165598	4.161083095	3.16742E-05	5.37797E-05	positive
PRJNA729271	stage_3W_vs_E10	ordinary_muscle_development	ENSGALG00010021835	NUDT8	-0.046008287	0.17419161	-0.264124589	0.791683917	0.81501835	negative
PRJNA729271	stage_3W_vs_E10	ordinary_muscle_development	ENSGALG00010021837	ENSGALG00010021837	0.504360305	0.32409179	1.556226723	0.119654189	0.143124894	positive
PRJNA729271	stage_3W_vs_E10	ordinary_muscle_development	ENSGALG00010021839	C1orf50	0.025490555	0.121126162	0.210446321	0.833319343	0.852612683	positive
PRJNA729271	stage_3W_vs_E10	ordinary_muscle_development	ENSGALG00010021840	ENSGALG00010021840	0.383500834	0.219205632	1.749502655	0.08020417	0.098147695	positive
PRJNA729271	stage_3W_vs_E10	ordinary_muscle_development	ENSGALG00010021841	CENPT	-2.010689122	0.095960152	-20.95337584	1.74791E-97	6.75624E-96	negative
PRJNA729271	stage_3W_vs_E10	ordinary_muscle_development	ENSGALG00010021842	ENSGALG00010021842	-2.544677632	0.190324214	-13.37022536	9.02669E-41	7.45114E-40	negative
PRJNA729271	stage_3W_vs_E10	ordinary_muscle_development	ENSGALG00010021843	ENSGALG00010021843	2.605467191	0.375810739	6.932923725	4.12231E-12	1.07199E-11	positive
PRJNA729271	stage_3W_vs_E10	ordinary_muscle_development	ENSGALG00010021847	P3H1	-1.428397184	0.151964989	-9.399514959	5.48152E-21	2.18042E-20	negative
PRJNA729271	stage_3W_vs_E10	ordinary_muscle_development	ENSGALG00010021848	LARGE2	1.287938723	0.136944742	9.404805949	5.21263E-21	2.07542E-20	positive
PRJNA729271	stage_3W_vs_E10	ordinary_muscle_development	ENSGALG00010021851	ENSGALG00010021851	1.180182833	0.216808792	5.443427011	5.22652E-08	1.07627E-07	positive
PRJNA729271	stage_3W_vs_E10	ordinary_muscle_development	ENSGALG00010021853	ENSGALG00010021853	-0.262565145	0.618279488	-0.424670639	0.671076791	0.702622963	negative
PRJNA729271	stage_3W_vs_E10	ordinary_muscle_development	ENSGALG00010021854	TAPBPL	1.699727681	0.152888921	11.11740255	1.03234E-28	5.64031E-28	positive
PRJNA729271	stage_3W_vs_E10	ordinary_muscle_development	ENSGALG00010021860	ENSGALG00010021860	1.513761746	0.257264282	5.884072734	4.00292E-09	8.81031E-09	positive
PRJNA729271	stage_3W_vs_E10	ordinary_muscle_development	ENSGALG00010021862	PDK1	-0.49119581	0.106704852	-4.603312779	4.15823E-06	7.55402E-06	negative
PRJNA729271	stage_3W_vs_E10	ordinary_muscle_development	ENSGALG00010021863	CLDN19	1.267339764	0.299159309	4.236337383	2.27195E-05	3.90791E-05	positive
PRJNA729271	stage_3W_vs_E10	ordinary_muscle_development	ENSGALG00010021864	GFI1	3.663562931	0.448389962	8.170483818	3.07155E-16	9.89821E-16	positive
PRJNA729271	stage_3W_vs_E10	ordinary_muscle_development	ENSGALG00010021866	SCNN1A	1.52404755	0.365606488	4.168546244	3.06549E-05	5.21039E-05	positive
PRJNA729271	stage_3W_vs_E10	ordinary_muscle_development	ENSGALG00010021867	CXCL12	-0.259176496	0.182352172	-1.421296459	0.155230592	0.182754015	negative
PRJNA729271	stage_3W_vs_E10	ordinary_muscle_development	ENSGALG00010021869	GJC3	0.002271422	0.121930729	0.018628794	0.985137232	0.987590026	positive
PRJNA729271	stage_3W_vs_E10	ordinary_muscle_development	ENSGALG00010021870	ENSGALG00010021870	-0.332384086	0.169114763	-1.965435077	0.049363903	0.062020436	negative

PRJNA729271	stage_3W_vs_E10	ordinary_muscle_development	ENSGALG00010021871	ENSGALG00010021871	4.899351141	0.250047175	19.59370723	1.74973E-85	5.13409E-84	positive
PRJNA729271	stage_3W_vs_E10	ordinary_muscle_development	ENSGALG00010021874	TMCC1	0.706115004	0.078502857	8.994768174	2.36731E-19	8.83931E-19	positive
PRJNA729271	stage_3W_vs_E10	ordinary_muscle_development	ENSGALG00010021877	PLEKHN1	0.809051234	0.147606165	5.481147974	4.22575E-08	8.75532E-08	positive
PRJNA729271	stage_3W_vs_E10	ordinary_muscle_development	ENSGALG00010021879	CREBL2	1.188152444	0.154228268	7.703856488	1.3202E-14	3.91256E-14	positive
PRJNA729271	stage_3W_vs_E10	ordinary_muscle_development	ENSGALG00010021881	PHF20	-0.58487541	0.049099188	-11.91211983	1.02342E-32	6.51491E-32	negative
PRJNA729271	stage_3W_vs_E10	ordinary_muscle_development	ENSGALG00010021887	NUTF2	-0.732118667	0.110579931	-6.620719177	3.57455E-11	8.87212E-11	negative
PRJNA729271	stage_3W_vs_E10	ordinary_muscle_development	ENSGALG00010021892	NDUFV1	1.049329827	0.121832403	8.612896078	7.12377E-18	2.49961E-17	positive
PRJNA729271	stage_3W_vs_E10	ordinary_muscle_development	ENSGALG00010021897	ENSGALG00010021897	0.277682002	0.20890359	1.329235187	0.18377039	0.213870076	positive
PRJNA729271	stage_3W_vs_E10	ordinary_muscle_development	ENSGALG00010021902	LMTK2	1.016744576	0.118947009	8.547878442	1.25372E-17	4.34232E-17	positive
PRJNA729271	stage_3W_vs_E10	ordinary_muscle_development	ENSGALG00010021903	MORC2	-0.543347603	0.07910003	-6.869120072	6.45991E-12	1.66376E-11	negative
PRJNA729271	stage_3W_vs_E10	ordinary_muscle_development	ENSGALG00010021905	RBSN	-0.154461649	0.073220473	-2.109541794	0.034897842	0.044670101	negative
PRJNA729271	stage_3W_vs_E10	ordinary_muscle_development	ENSGALG00010021908	EDC4	0.154755264	0.108359023	1.42817146	0.153242513	0.18053074	positive
PRJNA729271	stage_3W_vs_E10	ordinary_muscle_development	ENSGALG00010021913	ENSGALG00010021913	1.453184172	0.175658453	8.272782486	1.30868E-16	4.30242E-16	positive
PRJNA729271	stage_3W_vs_E10	ordinary_muscle_development	ENSGALG00010021916	MRPS25	0.684878566	0.148148062	4.622933	3.78352E-06	6.90446E-06	positive
PRJNA729271	stage_3W_vs_E10	ordinary_muscle_development	ENSGALG00010021919	PARVA	0.086771782	0.12476291	0.695493407	0.486746141	0.524993327	positive
PRJNA729271	stage_3W_vs_E10	ordinary_muscle_development	ENSGALG00010021922	MRPL51	-0.25477937	0.138453949	-1.8401741	0.065742681	0.081329706	negative
PRJNA729271	stage_3W_vs_E10	ordinary_muscle_development	ENSGALG00010021924	ENSGALG00010021924	0.577659127	0.467660763	1.235209734	0.216752458	0.249489945	positive
PRJNA729271	stage_3W_vs_E10	ordinary_muscle_development	ENSGALG00010021928	ENSGALG00010021928	6.135502343	0.86860991	7.063587773	1.62258E-12	4.32737E-12	positive
PRJNA729271	stage_3W_vs_E10	ordinary_muscle_development	ENSGALG00010021931	ITGA6	0.381412923	0.121808668	3.131246145	0.001740662	0.002550894	positive
PRJNA729271	stage_3W_vs_E10	ordinary_muscle_development	ENSGALG00010021932	RPAP2	1.040725894	0.161691776	6.436480089	1.22276E-10	2.9349E-10	positive
PRJNA729271	stage_3W_vs_E10	ordinary_muscle_development	ENSGALG00010021933	PRR14L	-0.533997387	0.08984001	-5.943870512	2.7837E-09	6.17818E-09	negative
PRJNA729271	stage_3W_vs_E10	ordinary_muscle_development	ENSGALG00010021934	EFTUD2	-1.124485509	0.067361647	-16.69326021	1.46734E-62	2.33984E-61	negative
PRJNA729271	stage_3W_vs_E10	ordinary_muscle_development	ENSGALG00010021935	CETP	6.259999713	0.295996367	21.14890729	2.82408E-99	1.14025E-97	positive
PRJNA729271	stage_3W_vs_E10	ordinary_muscle_development	ENSGALG00010021938	CABP2	0.03297795	0.163684777	0.201472307	0.840329281	0.858754437	positive
PRJNA729271	stage_3W_vs_E10	ordinary_muscle_development	ENSGALG00010021943	OCM3	-3.418875	0.568897055	-6.009654945			negative
PRJNA729271	stage_3W_vs_E10	ordinary_muscle_development	ENSGALG00010021945	C1QTNF4	-2.657552047	0.221589919	-11.993109	3.86135E-33	2.49422E-32	negative
PRJNA729271	stage_3W_vs_E10	ordinary_muscle_development	ENSGALG00010021947	CDK2AP2	1.235959598	0.246602409	5.011952653	5.38805E-07	1.03718E-06	positive
PRJNA729271	stage_3W_vs_E10	ordinary_muscle_development	ENSGALG00010021949	ENSGALG00010021949	-1.249579365	0.247527779	-5.048238907	4.45901E-07	8.64314E-07	negative
PRJNA729271	stage_3W_vs_E10	ordinary_muscle_development	ENSGALG00010021951	PSKH1	-0.102568231	0.143716875	-0.713682584	0.475423478	0.514119913	negative
PRJNA729271	stage_3W_vs_E10	ordinary_muscle_development	ENSGALG00010021953	PITPNM1	0.099615224	0.178394761	0.558397697	0.576572845	0.612714325	positive
PRJNA729271	stage_3W_vs_E10	ordinary_muscle_development	ENSGALG00010021958	BHLHA15	0.084720877	0.393526315	0.215286435	0.829544005	0.849649545	positive
PRJNA729271	stage_3W_vs_E10	ordinary_muscle_development	ENSGALG00010021960	CDKN1B	0.568188518	0.144758819	3.925070133	8.67045E-05	0.000142234	positive
PRJNA729271	stage_3W_vs_E10	ordinary_muscle_development	ENSGALG00010021965	PEX16	0.305909832	0.101933246	3.001080049	0.002690238	0.003870066	positive
PRJNA729271	stage_3W_vs_E10	ordinary_muscle_development	ENSGALG00010021966	ADGRA1	-0.448767343	0.159151217	-2.819754386	0.004806042	0.00675231	negative
PRJNA729271	stage_3W_vs_E10	ordinary_muscle_development	ENSGALG00010021969	CCDC103	-1.391809084	0.288022795	-4.832287964	1.34973E-06	2.5326E-06	negative
PRJNA729271	stage_3W_vs_E10	ordinary_muscle_development	ENSGALG00010021970	ERGIC3	-0.482014481	0.091041108	-5.294470757	1.19362E-07	2.40532E-07	negative
PRJNA729271	stage_3W_vs_E10	ordinary_muscle_development	ENSGALG00010021971	ICOS	2.006668853	0.363374592	5.522314708	3.34563E-08	6.97166E-08	positive
PRJNA729271	stage_3W_vs_E10	ordinary_muscle_development	ENSGALG00010021972	PISD	0.079781361	0.183708008	0.434283526	0.664082566	0.696064559	positive
PRJNA729271	stage_3W_vs_E10	ordinary_muscle_development	ENSGALG00010021974	GFAP	3.603638776	0.813731383	4.428536063	9.48749E-06	1.6777E-05	positive
PRJNA729271	stage_3W_vs_E10	ordinary_muscle_development	ENSGALG00010021978	NR2C2	0.147652775	0.07398918	1.995599558	0.045977528	0.05801726	positive
PRJNA729271	stage_3W_vs_E10	ordinary_muscle_development	ENSGALG00010021980	GLMN	0.100042719	0.132277252	0.756310839	0.449462851	0.488268308	positive
PRJNA729271	stage_3W_vs_E10	ordinary_muscle_development	ENSGALG00010021983	CTLA4	-1.297786389	0.52325716	-2.480207607	0.013130591	0.0176148	negative
PRJNA729271	stage_3W_vs_E10	ordinary_muscle_development	ENSGALG00010021988	FAM180B	4.171568618	0.262613671	15.88481134	8.07405E-57	1.1146E-55	positive
PRJNA729271	stage_3W_vs_E10	ordinary_muscle_development	ENSGALG00010021989	NCAPD2	-2.22567532	0.15300269	-14.54664174	6.13413E-48	6.52821E-47	negative
PRJNA729271	stage_3W_vs_E10	ordinary_muscle_development	ENSGALG00010021994	ENSGALG00010021994	-4.722194297	0.400429876	-11.79281213	4.25105E-32	2.648E-31	negative
PRJNA729271	stage_3W_vs_E10	ordinary_muscle_development	ENSGALG00010021995	CD28	4.287210522	0.467779336	9.165027587	4.95343E-20	1.89596E-19	positive
PRJNA729271	stage_3W_vs_E10	ordinary_muscle_development	ENSGALG00010021999	ANKRD22	-3.066669921	0.539731898	-5.681839318	1.33254E-08	2.85389E-08	negative
PRJNA729271	stage_3W_vs_E10	ordinary_muscle_development	ENSGALG00010022002	KIF18B	-2.957399517	0.242418324	-12.19957083	3.12466E-34	2.09685E-33	negative
PRJNA729271	stage_3W_vs_E10	ordinary_muscle_development	ENSGALG00010022005	NLRC5	4.432208942	0.181087705	24.47548238	2.6952E-132	1.9534E-130	positive
PRJNA729271	stage_3W_vs_E10	ordinary_muscle_development	ENSGALG00010022006	SFI1	-0.054335528	0.10816878	-0.50232172	0.615441233	0.650158201	negative
PRJNA729271	stage_3W_vs_E10	ordinary_muscle_development	ENSGALG00010022012	ENSGALG00010022012	-0.337519322	2.998349023	-0.11256839	0.910372747	0.920919322	negative
PRJNA729271	stage_3W_vs_E10	ordinary_muscle_development	ENSGALG00010022017	CNP1	2.746050454	0.58092473	4.727033144	2.27824E-06	4.21374E-06	positive
PRJNA729271	stage_3W_vs_E10	ordinary_muscle_development	ENSGALG00010022019	BTBD8	-0.001360074	0.154441807	-0.008806387	0.99297361	0.994070895	negative
PRJNA729271	stage_3W_vs_E10	ordinary_muscle_development	ENSGALG00010022021	TECPR1	1.558976737	0.077216568	20.18966622	1.20677E-90	4.02118E-89	positive
PRJNA729271	stage_3W_vs_E10	ordinary_muscle_development	ENSGALG00010022022	STAMBPL1	-1.133325395	0.251478241	-4.506653904	6.58579E-06	1.17883E-05	negative
PRJNA729271	stage_3W_vs_E10	ordinary_muscle_development	ENSGALG00010022024	MICAL2	2.530486659	0.183072933	13.82228724	1.87031E-43	1.6997E-42	positive
PRJNA729271	stage_3W_vs_E10	ordinary_muscle_development	ENSGALG00010022027	EIF4ENIF1	-0.513373652	0.067543293	-7.600660712	2.94623E-14	8.58748E-14	negative
PRJNA729271	stage_3W_vs_E10	ordinary_muscle_development	ENSGALG00010022028	FGD5	0.343109268	0.115262676	2.976759516	0.002913124	0.00418118	positive
PRJNA729271	stage_3W_vs_E10	ordinary_muscle_development	ENSGALG00010022029	ENSGALG00010022029	0.032550147	0.652632261	0.049875174	0.960221861	0.965483898	positive
PRJNA729271	stage_3W_vs_E10	ordinary_muscle_development	ENSGALG00010022030	ZNF341	0.110449415	0.156469198	0.705885991	0.48025904	0.518845852	positive
PRJNA729271	stage_3W_vs_E10	ordinary_muscle_development	ENSGALG00010022032	KBTBD4	0.20718097	0.081966361	2.527634093	0.011483396	0.015521431	positive
PRJNA729271	stage_3W_vs_E10	ordinary_muscle_development	ENSGALG00010022033	RAPH1	-0.770932725	0.11772095	-6.548815006	5.79954E-11	1.41832E-10	negative
PRJNA729271	stage_3W_vs_E10	ordinary_muscle_development	ENSGALG00010022034	ENSGALG00010022034	2.020841947	0.104839452	19.27558678	8.61182E-83	2.33324E-81	positive
PRJNA729271	stage_3W_vs_E10	ordinary_muscle_development	ENSGALG00010022037	DRG1	0.786665868	0.093201943	8.440444923	3.16132E-17	1.07039E-16	positive
PRJNA729271	stage_3W_vs_E10	ordinary_muscle_development	ENSGALG00010022038	GAPDH	5.374698623	0.137180683	39.17970464	0	0	positive
PRJNA729271	stage_3W_vs_E10	ordinary_muscle_development	ENSGALG00010022041	MAPK8IP1	-0.151692105	0.151954791	-0.998271293	0.318147824	0.356159462	negative
PRJNA729271	stage_3W_vs_E10	ordinary_muscle_development	ENSGALG00010022042	AIP	0.143476002	0.085396215	1.680121337	0.09293371	0.112914848	positive
PRJNA729271	stage_3W_vs_E10	ordinary_muscle_development	ENSGALG00010022044	PXMP4	0.78144893	0.058398859	13.38123616	7.78419E-41	6.44385E-40	positive
PRJNA729271	stage_3W_vs_E10	ordinary_muscle_development	ENSGALG00010022046	CRY2	1.154847018	0.103262284	11.18362844	4.90429E-29	2.71535E-28	positive
PRJNA729271	stage_3W_vs_E10	ordinary_muscle_development	ENSGALG00010022049	EPHX4	-1.412251887	0.613111492	-2.303417738	0.021255351	0.027897167	negative
PRJNA729271	stage_3W_vs_E10	ordinary_muscle_development	ENSGALG00010022050	ENSGALG00010022050	0.592843989	0.197820184	2.996883222	0.002727552	0.003922209	positive
PRJNA729271	stage_3W_vs_E10	ordinary_muscle_development	ENSGALG00010022051	BAIAP2L1	-0.286606544	0.114250936	-2.50857065	0.012122072	0.016335946	negative
PRJNA729271	stage_3W_vs_E10	ordinary_muscle_development	ENSGALG00010022052	IFFO1	0.341424405	0.10479683	3.257964999	0.001122142	0.001674779	positive
PRJNA729271	stage_3W_vs_E10	ordinary_muscle_development	ENSGALG00010022054	ENSGALG00010022054	2.321459714	0.418833965	5.542673013	2.97889E-08	6.22894E-08	positive
PRJNA729271	stage_3W_vs_E10	ordinary_muscle_development	ENSGALG00010022055	E2F1	-1.862037824	0.166188791	-11.20435269	3.88181E-29	2.16411E-28	negative
PRJNA729271	stage_3W_vs_E10	ordinary_muscle_development	ENSGALG00010022058	NLRP3	-0.767575748	0.129579627	-5.923583568	3.15001E-09	6.95814E-09	negative
PRJNA729271	stage_3W_vs_E10	ordinary_muscle_development	ENSGALG00010022059	NPTX2	-1.592561803	0.250991587	-6.345080418	2.22309E-10	5.27148E-10	negative
PRJNA729271	stage_3W_vs_E10	ordinary_muscle_development	ENSGALG00010022062	MITF	1.694445594	0.174996335	9.682749056	3.56985E-22	1.48907E-21	positive
PRJNA729271	stage_3W_vs_E10	ordinary_muscle_development	ENSGALG00010022065	PABPC1L	-0.330903884	0.258967661	-1.277780721	0.201326754	0.23295532	negative
PRJNA729271	stage_3W_vs_E10	ordinary_muscle_development	ENSGALG00010022066	DAD1	-0.550244096	0.139488495	-3.944727457	7.98908E-05	0.000131384	negative
PRJNA729271	stage_3W_vs_E10	ordinary_muscle_development	ENSGALG00010022067	TMEM130	-0.988280544	0.239364499	-4.128768247	3.64712E-05	6.16573E-05	negative
PRJNA729271	stage_3W_vs_E10	ordinary_muscle_development	ENSGALG00010022069	NOP2	-1.102777145	0.136027512	-8.107015487	5.18783E-16	1.6516E-15	negative
PRJNA729271	stage_3W_vs_E10	ordinary_muscle_development	ENSGALG00010022071	ACTA2	-0.613972605	0.19823928	-3.097128906	0.001954049	0.002846341	negative
PRJNA729271	stage_3W_vs_E10	ordinary_muscle_development	ENSGALG00010022072	SMTN	0.098084668	0.312873538	0.313496208	0.753903701	0.779946767	positive
PRJNA729271	stage_3W_vs_E10	ordinary_muscle_development	ENSGALG00010022073	PTPMT1	0.957784133	0.132318512	7.238474166	4.53761E-13	1.24381E-12	positive
PRJNA729271	stage_3W_vs_E10	ordinary_muscle_development	ENSGALG00010022076	YWHAB	-1.368197288	0.07009083	-19.52034647	7.37366E-85	2.12487E-83	negative
PRJNA729271	stage_3W_vs_E10	ordinary_muscle_development	ENSGALG00010022079	ABI2	-0.551185353	0.076475416	-7.20735343	5.70498E-13	1.55556E-12	negative
PRJNA729271	stage_3W_vs_E10	ordinary_muscle_development	ENSGALG00010022081	FAS	1.71399843	0.172119862	9.958167602	2.32305E-23	1.01393E-22	positive
PRJNA729271	stage_3W_vs_E10	ordinary_muscle_development	ENSGALG00010022082	TGFBR3	1.612198555	0.144362526	11.16770812	5.86753E-29	3.2363E-28	positive
PRJNA729271	stage_3W_vs_E10	ordinary_muscle_development	ENSGALG00010022084	SELENOM	-1.5564149	0.203262691	-7.65715977	1.9009E-14	5.59577E-14	negative
PRJNA729271	stage_3W_vs_E10	ordinary_muscle_development	ENSGALG00010022085	MLLT6	0.511123479	0.177440696	2.880531307	0.003970055	0.005624116	positive
PRJNA729271	stage_3W_vs_E10	ordinary_muscle_development	ENSGALG00010022087	SLC35C1	-0.277692227	0.074821743	-3.711384079	0.000206129	0.000327938	negative
PRJNA729271	stage_3W_vs_E10	ordinary_muscle_development	ENSGALG00010022088	DKK3	-1.675376747	0.270565194	-6.192136983	5.93539E-10	1.37258E-09	negative
PRJNA729271	stage_3W_vs_E10	ordinary_muscle_development	ENSGALG00010022090	TRRAP	-1.085219155	0.091109287	-11.91118043	1.03502E-32	6.58584E-32	negative
PRJNA729271	stage_3W_vs_E10	ordinary_muscle_development	ENSGALG00010022092	CTRL	5.886791064	1.777199698	3.312397065	0.000925002	0.001388844	positive
PRJNA729271	stage_3W_vs_E10	ordinary_muscle_development	ENSGALG00010022093	CHD4	-1.144437272	0.136598596	-8.378104178	5.3787E-17	1.80181E-16	negative
PRJNA729271	stage_3W_vs_E10	ordinary_muscle_development	ENSGALG00010022097	INPP5J	-0.908682739	0.294034889	-3.09039088	0.001998932	0.002909381	negative
PRJNA729271	stage_3W_vs_E10	ordinary_muscle_development	ENSGALG00010022098	CDC7	-3.434391698	0.18214248	-18.85552288	2.64709E-79	6.45952E-78	negative
PRJNA729271	stage_3W_vs_E10	ordinary_muscle_development	ENSGALG00010022100	ENTPD1	0.416638692	0.165582803	2.516195431	0.011862938	0.016003098	positive
PRJNA729271	stage_3W_vs_E10	ordinary_muscle_development	ENSGALG00010022101	ENSGALG00010022101	1.223958158	0.181945871	6.727045531	1.73143E-11	4.37002E-11	positive
PRJNA729271	stage_3W_vs_E10	ordinary_muscle_development	ENSGALG00010022104	EXOC3L1	0.046862119	0.142483529	0.328894989	0.742235062	0.769357639	positive
PRJNA729271	stage_3W_vs_E10	ordinary_muscle_development	ENSGALG00010022105	DLG5	-1.441759656	0.085351352	-16.89205413	5.14791E-64	8.60657E-63	negative
PRJNA729271	stage_3W_vs_E10	ordinary_muscle_development	ENSGALG00010022108	USP47	0.761606013	0.08796589	8.657969775	4.80241E-18	1.69824E-17	positive
PRJNA729271	stage_3W_vs_E10	ordinary_muscle_development	ENSGALG00010022111	PCGF2	-2.303838273	0.204905023	-11.24344459	2.49455E-29	1.39932E-28	negative
PRJNA729271	stage_3W_vs_E10	ordinary_muscle_development	ENSGALG00010022115	E2F4	-0.089688797	0.185089254	-0.484570526	0.627981026	0.66166933	negative
PRJNA729271	stage_3W_vs_E10	ordinary_muscle_development	ENSGALG00010022116	CYP20A1	-0.66552503	0.102807887	-6.473482211	9.57697E-11	2.31325E-10	negative
PRJNA729271	stage_3W_vs_E10	ordinary_muscle_development	ENSGALG00010022117	FAM20C	0.054255596	0.121330331	0.44717257	0.654750465	0.687675385	positive
PRJNA729271	stage_3W_vs_E10	ordinary_muscle_development	ENSGALG00010022120	FOXL3	-6.771829066	0.934810889	-7.244063096	4.35439E-13	1.19494E-12	negative
PRJNA729271	stage_3W_vs_E10	ordinary_muscle_development	ENSGALG00010022122	PSMB3	0.069512365	0.157851141	0.440366565	0.659671637	0.692192006	positive
PRJNA729271	stage_3W_vs_E10	ordinary_muscle_development	ENSGALG00010022124	ENSGALG00010022124	0.497555799	0.15515522	3.206826029	0.001342081	0.001988497	positive
PRJNA729271	stage_3W_vs_E10	ordinary_muscle_development	ENSGALG00010022125	CBFA2T2	0.404961635	0.186640791	2.169738105	0.030026692	0.038715255	positive
PRJNA729271	stage_3W_vs_E10	ordinary_muscle_development	ENSGALG00010022129	ELMO3	-0.089564872	0.247905631	-0.361286155	0.717885543	0.746413524	negative
PRJNA729271	stage_3W_vs_E10	ordinary_muscle_development	ENSGALG00010022134	CH25H	0.336658321	0.233720576	1.440430818	0.14974555	0.176655143	positive
PRJNA729271	stage_3W_vs_E10	ordinary_muscle_development	ENSGALG00010022135	CHST1	-4.677197384	0.279450617	-16.73711596	7.03134E-63	1.13876E-61	negative
PRJNA729271	stage_3W_vs_E10	ordinary_muscle_development	ENSGALG00010022137	PDDC1	-0.938031717	0.129813334	-7.226004364	4.97412E-13	1.3596E-12	negative
PRJNA729271	stage_3W_vs_E10	ordinary_muscle_development	ENSGALG00010022138	SPAG17	0.821795951	0.209115467	3.929866886	8.49929E-05	0.000139569	positive
PRJNA729271	stage_3W_vs_E10	ordinary_muscle_development	ENSGALG00010022142	TMEM208	1.392925579	0.104195883	13.36833604	9.2589E-41	7.63411E-40	positive
PRJNA729271	stage_3W_vs_E10	ordinary_muscle_development	ENSGALG00010022143	MATCAP1	-0.728882226	0.199763291	-3.648729571	0.00026354	0.00041531	negative
PRJNA729271	stage_3W_vs_E10	ordinary_muscle_development	ENSGALG00010022144	SNTA1	3.443012246	0.186605777	18.45072694	5.14484E-76	1.16341E-74	positive
PRJNA729271	stage_3W_vs_E10	ordinary_muscle_development	ENSGALG00010022148	TALDO1	-0.107166589	0.085776893	-1.249364312	0.211531855	0.244003998	negative
PRJNA729271	stage_3W_vs_E10	ordinary_muscle_development	ENSGALG00010022149	NBEAL1	0.164970001	0.106251292	1.55263996	0.120509174	0.144040609	positive
PRJNA729271	stage_3W_vs_E10	ordinary_muscle_development	ENSGALG00010022150	CPNE2	-1.21373656	0.133518448	-9.090403435	9.86722E-20	3.73142E-19	negative
PRJNA729271	stage_3W_vs_E10	ordinary_muscle_development	ENSGALG00010022151	PIP4K2B	-0.380474478	0.154849762	-2.457055614	0.0140081	0.018734767	negative
PRJNA729271	stage_3W_vs_E10	ordinary_muscle_development	ENSGALG00010022152	ZNF644	-0.253817058	0.100531605	-2.524748892	0.011578099	0.015642142	negative
PRJNA729271	stage_3W_vs_E10	ordinary_muscle_development	ENSGALG00010022154	CPN1	1.717892823	0.193943531	8.857695929	8.16852E-19	2.97793E-18	positive
PRJNA729271	stage_3W_vs_E10	ordinary_muscle_development	ENSGALG00010022157	EPS8L2	0.561390444	0.136938295	4.099586935	4.13888E-05	6.96946E-05	positive
PRJNA729271	stage_3W_vs_E10	ordinary_muscle_development	ENSGALG00010022158	CWC25	-0.340801275	0.079682174	-4.277007734	1.89422E-05	3.27881E-05	negative
PRJNA729271	stage_3W_vs_E10	ordinary_muscle_development	ENSGALG00010022159	KIAA2013	0.330455095	0.089465127	3.693674894	0.000221036	0.000350615	positive
PRJNA729271	stage_3W_vs_E10	ordinary_muscle_development	ENSGALG00010022161	CELF1	0.092964107	0.094285651	0.985983613	0.324141154	0.362169585	positive
PRJNA729271	stage_3W_vs_E10	ordinary_muscle_development	ENSGALG00010022162	POLE	-2.895446801	0.164518225	-17.59955047	2.48287E-69	4.6861E-68	negative
PRJNA729271	stage_3W_vs_E10	ordinary_muscle_development	ENSGALG00010022164	PDGFA	-0.808047825	0.109186368	-7.400629242	1.35541E-13	3.82081E-13	negative

PRJNA729271	stage_3W_vs_E10	ordinary_muscle_development	ENSGALG00010022165	FAM192A	0.068995988	0.088956386	0.775615901	0.437975809	0.476611062	positive
PRJNA729271	stage_3W_vs_E10	ordinary_muscle_development	ENSGALG00010022166	SDHB	1.270635001	0.107837796	11.78283543	4.78557E-32	2.97712E-31	positive
PRJNA729271	stage_3W_vs_E10	ordinary_muscle_development	ENSGALG00010022167	WDR3	-0.874405615	0.072641538	-12.03726742	2.26329E-33	1.47378E-32	negative
PRJNA729271	stage_3W_vs_E10	ordinary_muscle_development	ENSGALG00010022169	TOX2	-1.093064016	0.253112701	-4.318487415	1.57102E-05	2.73406E-05	negative
PRJNA729271	stage_3W_vs_E10	ordinary_muscle_development	ENSGALG00010022170	RSPRY1	-0.774575619	0.142068605	-5.452123778	4.97718E-08	1.02667E-07	negative
PRJNA729271	stage_3W_vs_E10	ordinary_muscle_development	ENSGALG00010022171	FOXP1	-0.585638428	0.094834635	-6.175364407	6.6011E-10	1.52264E-09	negative
PRJNA729271	stage_3W_vs_E10	ordinary_muscle_development	ENSGALG00010022172	GIPC2	1.198482623	0.181770485	6.593384089	4.29912E-11	1.06069E-10	positive
PRJNA729271	stage_3W_vs_E10	ordinary_muscle_development	ENSGALG00010022173	RPL23	0.375820531	0.104037627	3.612351994	0.000303432	0.000476259	positive
PRJNA729271	stage_3W_vs_E10	ordinary_muscle_development	ENSGALG00010022174	KCNMA1	1.1674881	0.125047191	9.336380035	9.96841E-21	3.92748E-20	positive
PRJNA729271	stage_3W_vs_E10	ordinary_muscle_development	ENSGALG00010022176	ARL2BPL	-1.478502704	0.147241829	-10.04132258	1.0032E-23	4.45235E-23	negative
PRJNA729271	stage_3W_vs_E10	ordinary_muscle_development	ENSGALG00010022178	ENSGALG00010022178	0.705381783	0.32600004	2.163747537	0.030483731	0.039273102	positive
PRJNA729271	stage_3W_vs_E10	ordinary_muscle_development	ENSGALG00010022180	PLLP	0.52085647	0.402385699	1.294420927	0.195520066	0.226634415	positive
PRJNA729271	stage_3W_vs_E10	ordinary_muscle_development	ENSGALG00010022181	ENSGALG00010022181	-3.254609529	0.245551284	-13.2542965	4.26094E-40	3.45814E-39	negative
PRJNA729271	stage_3W_vs_E10	ordinary_muscle_development	ENSGALG00010022182	CNTNAP1	-1.215205162	0.304772146	-3.98725795	6.68413E-05	0.000110854	negative
PRJNA729271	stage_3W_vs_E10	ordinary_muscle_development	ENSGALG00010022184	PRKAR1B	-1.340376884	0.094695184	-14.15464678	1.74814E-45	1.69834E-44	negative
PRJNA729271	stage_3W_vs_E10	ordinary_muscle_development	ENSGALG00010022186	DNAJB4	1.432172399	0.112432439	12.73807109	3.63265E-37	2.654E-36	positive
PRJNA729271	stage_3W_vs_E10	ordinary_muscle_development	ENSGALG00010022190	ACVR1B	1.097992177	0.16342848	6.718487367	1.83621E-11	4.62723E-11	positive
PRJNA729271	stage_3W_vs_E10	ordinary_muscle_development	ENSGALG00010022193	GDAP2	-0.457128899	0.082052489	-5.57117649	2.53025E-08	5.31921E-08	negative
PRJNA729271	stage_3W_vs_E10	ordinary_muscle_development	ENSGALG00010022194	ENSGALG00010022194	-1.953951105	0.094146098	-20.75445651	1.11738E-95	4.15295E-94	negative
PRJNA729271	stage_3W_vs_E10	ordinary_muscle_development	ENSGALG00010022196	PLXNA1	-0.569502921	0.108884044	-5.230361581	1.69179E-07	3.36847E-07	negative
PRJNA729271	stage_3W_vs_E10	ordinary_muscle_development	ENSGALG00010022199	RBM39	-0.251294011	0.061590717	-4.08006308	4.50235E-05	7.56743E-05	negative
PRJNA729271	stage_3W_vs_E10	ordinary_muscle_development	ENSGALG00010022200	DNMBP	0.158906708	0.1095767	1.450187015	0.147006375	0.17362156	positive
PRJNA729271	stage_3W_vs_E10	ordinary_muscle_development	ENSGALG00010022202	CX3CL1	2.349438698	0.159257293	14.75247164	2.966E-49	3.26688E-48	positive
PRJNA729271	stage_3W_vs_E10	ordinary_muscle_development	ENSGALG00010022203	DNAAF5	0.025566748	0.13175394	0.194049212	0.846137349	0.863898068	positive
PRJNA729271	stage_3W_vs_E10	ordinary_muscle_development	ENSGALG00010022204	ENSGALG00010022204	0.93416712	0.328857804	2.840641482	0.00450229	0.00634523	positive
PRJNA729271	stage_3W_vs_E10	ordinary_muscle_development	ENSGALG00010022205	GALNT18	-0.771867413	0.108813534	-7.093487196	1.30774E-12	3.50577E-12	negative
PRJNA729271	stage_3W_vs_E10	ordinary_muscle_development	ENSGALG00010022207	ENSGALG00010022207	4.070950457	0.305443692	13.32798995	1.59128E-40	1.30461E-39	positive
PRJNA729271	stage_3W_vs_E10	ordinary_muscle_development	ENSGALG00010022208	CCL17	2.802046666	0.686722136	4.080320874	4.49736E-05	7.55992E-05	positive
PRJNA729271	stage_3W_vs_E10	ordinary_muscle_development	ENSGALG00010022209	ZFYVE19	-0.692142415	0.121728329	-5.685960038	1.3008E-08	2.78756E-08	negative
PRJNA729271	stage_3W_vs_E10	ordinary_muscle_development	ENSGALG00010022210	EZH1	0.93325699	0.082867919	11.26198172	2.02161E-29	1.13887E-28	positive
PRJNA729271	stage_3W_vs_E10	ordinary_muscle_development	ENSGALG00010022211	ENSGALG00010022211	-0.313895489	0.516928595	-0.607231815	0.543697081	0.581186519	negative
PRJNA729271	stage_3W_vs_E10	ordinary_muscle_development	ENSGALG00010022213	IDH1	-0.61350879	0.080405008	-7.630231029	2.34333E-14	6.86194E-14	negative
PRJNA729271	stage_3W_vs_E10	ordinary_muscle_development	ENSGALG00010022214	ENSGALG00010022214	-1.437207548	0.26266702	-5.471594976	4.46003E-08	9.22359E-08	negative
PRJNA729271	stage_3W_vs_E10	ordinary_muscle_development	ENSGALG00010022215	ENSGALG00010022215	1.395216248	0.129098646	10.80736543	3.17662E-27	1.63629E-26	positive
PRJNA729271	stage_3W_vs_E10	ordinary_muscle_development	ENSGALG00010022216	ROMO1	0.175876214	0.144690339	1.21553529	0.224161998	0.257446174	positive
PRJNA729271	stage_3W_vs_E10	ordinary_muscle_development	ENSGALG00010022218	ENSGALG00010022218	4.774213975	0.246652567	19.35602793	1.81341E-83	5.0355E-82	positive
PRJNA729271	stage_3W_vs_E10	ordinary_muscle_development	ENSGALG00010022219	EIF4E3	0.895250099	0.136528257	6.557251338	5.48086E-11	1.34266E-10	positive
PRJNA729271	stage_3W_vs_E10	ordinary_muscle_development	ENSGALG00010022221	CUTC	0.465911949	0.082447223	5.651032625	1.59487E-08	3.40015E-08	positive
PRJNA729271	stage_3W_vs_E10	ordinary_muscle_development	ENSGALG00010022222	ENSGALG00010022222	-0.263498213	0.109757578	-2.400729127	0.016362443	0.021725164	negative
PRJNA729271	stage_3W_vs_E10	ordinary_muscle_development	ENSGALG00010022223	DOK4	-1.962265865	0.180615912	-10.86430225	1.70522E-27	8.89745E-27	negative
PRJNA729271	stage_3W_vs_E10	ordinary_muscle_development	ENSGALG00010022224	NHEJ1	1.183427993	0.141729572	8.34990167	6.83198E-17	2.27811E-16	positive
PRJNA729271	stage_3W_vs_E10	ordinary_muscle_development	ENSGALG00010022225	ENSGALG00010022225	0.519343592	0.51558257	1.007294703	0.313793175	0.351658243	positive
PRJNA729271	stage_3W_vs_E10	ordinary_muscle_development	ENSGALG00010022226	FBRSL1	-0.064603078	0.133032499	-0.48561877	0.627237486	0.661030054	negative
PRJNA729271	stage_3W_vs_E10	ordinary_muscle_development	ENSGALG00010022227	TMEM80	0.362618374	0.15820516	2.292076785	0.021901213	0.028692885	positive
PRJNA729271	stage_3W_vs_E10	ordinary_muscle_development	ENSGALG00010022228	FUBP1	-1.937309435	0.055856055	-34.68396441	1.3748E-263	4.7446E-261	negative
PRJNA729271	stage_3W_vs_E10	ordinary_muscle_development	ENSGALG00010022229	RAPSN	0.434759332	0.178674386	2.43324934	0.014963992	0.019957956	positive
PRJNA729271	stage_3W_vs_E10	ordinary_muscle_development	ENSGALG00010022230	NFS1	-0.440969114	0.087414361	-5.044584281	4.54508E-07	8.80644E-07	negative
PRJNA729271	stage_3W_vs_E10	ordinary_muscle_development	ENSGALG00010022231	ZNF503	-2.945678312	0.213903507	-13.77106131	3.80581E-43	3.40736E-42	negative
PRJNA729271	stage_3W_vs_E10	ordinary_muscle_development	ENSGALG00010022232	GPR27	-4.06992205	0.211297207	-19.26159892	1.1284E-82	3.03453E-81	negative
PRJNA729271	stage_3W_vs_E10	ordinary_muscle_development	ENSGALG00010022233	FAM46C	3.157750838	0.164027769	19.25131864	1.37615E-82	3.68032E-81	positive
PRJNA729271	stage_3W_vs_E10	ordinary_muscle_development	ENSGALG00010022234	COX15	0.239588241	0.094534441	2.534401631	0.011263955	0.015233348	positive
PRJNA729271	stage_3W_vs_E10	ordinary_muscle_development	ENSGALG00010022235	RAMP2	1.142940166	0.112965047	10.11764431	4.61386E-24	2.07502E-23	positive
PRJNA729271	stage_3W_vs_E10	ordinary_muscle_development	ENSGALG00010022236	SHF	0.888533241	0.181916603	4.88428888	1.03803E-06	1.96477E-06	positive
PRJNA729271	stage_3W_vs_E10	ordinary_muscle_development	ENSGALG00010022237	SUN1	0.873720833	0.090876656	9.614359451	6.95408E-22	2.86933E-21	positive
PRJNA729271	stage_3W_vs_E10	ordinary_muscle_development	ENSGALG00010022238	COMTD1	-0.004255376	0.165769452	-0.025670447	0.979520196	0.982570605	negative
PRJNA729271	stage_3W_vs_E10	ordinary_muscle_development	ENSGALG00010022239	DEAF1	0.441253337	0.114219557	3.863203024	0.00011191	0.000182119	positive
PRJNA729271	stage_3W_vs_E10	ordinary_muscle_development	ENSGALG00010022240	ENSGALG00010022240	-1.097095502	0.134519713	-8.155648541	3.47311E-16	1.11476E-15	negative
PRJNA729271	stage_3W_vs_E10	ordinary_muscle_development	ENSGALG00010022241	POLR2C	0.399434788	0.094374228	4.232456227	2.31153E-05	3.9708E-05	positive
PRJNA729271	stage_3W_vs_E10	ordinary_muscle_development	ENSGALG00010022242	USP19	0.566860856	0.126028277	4.497886278	6.86324E-06	1.22621E-05	positive
PRJNA729271	stage_3W_vs_E10	ordinary_muscle_development	ENSGALG00010022243	ENSGALG00010022243	2.370219656	0.558913075	4.240766163	2.22758E-05	3.83386E-05	positive
PRJNA729271	stage_3W_vs_E10	ordinary_muscle_development	ENSGALG00010022244	ENTPD7	-1.15329026	0.174831825	-6.596569372	4.20781E-11	1.03899E-10	negative
PRJNA729271	stage_3W_vs_E10	ordinary_muscle_development	ENSGALG00010022245	PIKFYVE	1.031744204	0.099810737	10.33700619	4.79277E-25	2.24971E-24	positive
PRJNA729271	stage_3W_vs_E10	ordinary_muscle_development	ENSGALG00010022246	RHOV	-4.892570878	0.497366898	-9.836945118	7.80446E-23	3.34296E-22	negative
PRJNA729271	stage_3W_vs_E10	ordinary_muscle_development	ENSGALG00010022247	GALNT9	-1.958640622	0.212674319	-9.20957751	3.27411E-20	1.26252E-19	negative
PRJNA729271	stage_3W_vs_E10	ordinary_muscle_development	ENSGALG00010022248	FIGNL2	-3.247659894	0.240101319	-13.52620599	1.09533E-41	9.30641E-41	negative
PRJNA729271	stage_3W_vs_E10	ordinary_muscle_development	ENSGALG00010022250	SLC25A28	2.050691006	0.126909618	16.15867287	9.86629E-59	1.43731E-57	positive
PRJNA729271	stage_3W_vs_E10	ordinary_muscle_development	ENSGALG00010022251	WNK4	-0.595699413	0.153027547	-3.892759355	9.91105E-05	0.000161889	negative
PRJNA729271	stage_3W_vs_E10	ordinary_muscle_development	ENSGALG00010022252	COQ9	3.999956251	0.131461757	30.426767	2.4315E-203	4.4056E-201	positive
PRJNA729271	stage_3W_vs_E10	ordinary_muscle_development	ENSGALG00010022253	ALPK3	3.807154191	0.217254152	17.52396513	9.4037E-69	1.76107E-67	positive
PRJNA729271	stage_3W_vs_E10	ordinary_muscle_development	ENSGALG00010022255	NOC4L	-2.539733937	0.094565098	-26.85699046	6.9892E-159	7.5603E-157	negative
PRJNA729271	stage_3W_vs_E10	ordinary_muscle_development	ENSGALG00010022256	PSMC3	0.329151518	0.091999167	3.577766278	0.000346543	0.000541404	positive
PRJNA729271	stage_3W_vs_E10	ordinary_muscle_development	ENSGALG00010022257	KCNK18	-1.13379378	1.156296039	-0.980539361	0.32681995	0.365021974	negative
PRJNA729271	stage_3W_vs_E10	ordinary_muscle_development	ENSGALG00010022258	ENSGALG00010022258	5.070874757	0.674058958	7.522894982	5.35765E-14	1.54453E-13	positive
PRJNA729271	stage_3W_vs_E10	ordinary_muscle_development	ENSGALG00010022259	GET4	0.522263102	0.084974733	6.146098739	7.94119E-10	1.82132E-09	positive
PRJNA729271	stage_3W_vs_E10	ordinary_muscle_development	ENSGALG00010022264	SCN8A	-1.114895761	0.257069949	-4.336935392	1.44483E-05	2.52111E-05	negative
PRJNA729271	stage_3W_vs_E10	ordinary_muscle_development	ENSGALG00010022266	COA3	-0.075583724	0.146873963	-0.514616221	0.606821228	0.642175199	negative
PRJNA729271	stage_3W_vs_E10	ordinary_muscle_development	ENSGALG00010022269	CNTD1	-0.401806566	0.389737345	-1.030967576	0.302556025	0.340176046	negative
PRJNA729271	stage_3W_vs_E10	ordinary_muscle_development	ENSGALG00010022271	SLC18A2	-0.451133386	0.221999008	-2.032141451	0.042139338	0.053373795	negative
PRJNA729271	stage_3W_vs_E10	ordinary_muscle_development	ENSGALG00010022274	CCDC91	0.90091089	0.174170578	5.172577956	2.30886E-07	4.55643E-07	positive
PRJNA729271	stage_3W_vs_E10	ordinary_muscle_development	ENSGALG00010022275	VDAC2	1.93539807	0.12402475	15.6049343	6.73791E-55	8.8868E-54	positive
PRJNA729271	stage_3W_vs_E10	ordinary_muscle_development	ENSGALG00010022276	BECN1	0.742914491	0.088040422	8.438334086	3.21892E-17	1.08938E-16	positive
PRJNA729271	stage_3W_vs_E10	ordinary_muscle_development	ENSGALG00010022279	EP400	0.148464257	0.149137454	0.995486064	0.319499919	0.35759025	positive
PRJNA729271	stage_3W_vs_E10	ordinary_muscle_development	ENSGALG00010022280	PDZD8	0.1029701	0.06749218	1.525659718	0.127094631	0.151487269	positive
PRJNA729271	stage_3W_vs_E10	ordinary_muscle_development	ENSGALG00010022282	TMT1A	2.497555639	0.222943428	11.20264301	3.95747E-29	2.20375E-28	positive
PRJNA729271	stage_3W_vs_E10	ordinary_muscle_development	ENSGALG00010022290	NEXN	1.741169128	0.220067262	7.911986131	2.53315E-15	7.78418E-15	positive
PRJNA729271	stage_3W_vs_E10	ordinary_muscle_development	ENSGALG00010022293	DNAJC10	-0.552528326	0.063643355	-8.681634152	3.90123E-18	1.38735E-17	negative
PRJNA729271	stage_3W_vs_E10	ordinary_muscle_development	ENSGALG00010022294	ENSGALG00010022294	1.460287575	0.637644262	2.290128936	0.022013843	0.02883523	positive
PRJNA729271	stage_3W_vs_E10	ordinary_muscle_development	ENSGALG00010022296	CIAPIN1	-0.065530124	0.097424897	-0.672621944	0.501187838	0.539245673	negative
PRJNA729271	stage_3W_vs_E10	ordinary_muscle_development	ENSGALG00010022297	VPS18	-0.397513542	0.057820454	-6.874964073	6.20055E-12	1.59838E-11	negative
PRJNA729271	stage_3W_vs_E10	ordinary_muscle_development	ENSGALG00010022299	MRGBP	-0.092141113	0.079979872	-1.152053774	0.249298977	0.284534541	negative
PRJNA729271	stage_3W_vs_E10	ordinary_muscle_development	ENSGALG00010022302	ENSGALG00010022302	-0.207570533	0.151547233	-1.369675511	0.170788216	0.199788168	negative
PRJNA729271	stage_3W_vs_E10	ordinary_muscle_development	ENSGALG00010022303	DUSP13A	6.049581821	0.202881104	29.81836007	2.2586E-195	3.8975E-193	positive
PRJNA729271	stage_3W_vs_E10	ordinary_muscle_development	ENSGALG00010022304	ADAP1	-1.430435738	0.190899254	-7.493144739	6.72427E-14	1.92701E-13	negative
PRJNA729271	stage_3W_vs_E10	ordinary_muscle_development	ENSGALG00010022307	ENSGALG00010022307	8.852662734	0.634172839	13.95938487	2.75804E-44	2.56927E-43	positive
PRJNA729271	stage_3W_vs_E10	ordinary_muscle_development	ENSGALG00010022308	OGFR	0.169357344	0.181236037	0.934457333	0.350068035	0.388621902	positive
PRJNA729271	stage_3W_vs_E10	ordinary_muscle_development	ENSGALG00010022309	WSCD2	-2.079925071	0.404195665	-5.145837151	2.6633E-07	5.23878E-07	negative
PRJNA729271	stage_3W_vs_E10	ordinary_muscle_development	ENSGALG00010022310	DCLRE1A	-2.120882895	0.10395069	-20.40277849	1.57979E-92	5.45216E-91	negative
PRJNA729271	stage_3W_vs_E10	ordinary_muscle_development	ENSGALG00010022311	DLL4	1.036710287	0.141274281	7.338280387	2.16356E-13	6.02859E-13	positive
PRJNA729271	stage_3W_vs_E10	ordinary_muscle_development	ENSGALG00010022312	ZNF592	-0.1509335	0.055627558	-2.713286449	0.006661949	0.009225657	negative
PRJNA729271	stage_3W_vs_E10	ordinary_muscle_development	ENSGALG00010022313	ENSGALG00010022313	0.400998967	0.37555713	1.067744252	0.285635891	0.322729148	positive
PRJNA729271	stage_3W_vs_E10	ordinary_muscle_development	ENSGALG00010022314	DUSP13B	6.886617328	0.551393379	12.48948135	8.52073E-36	6.00428E-35	positive
PRJNA729271	stage_3W_vs_E10	ordinary_muscle_development	ENSGALG00010022316	INO80	0.240914331	0.069153022	3.483786008	0.000494375	0.000762255	positive
PRJNA729271	stage_3W_vs_E10	ordinary_muscle_development	ENSGALG00010022317	PSME3	-1.484020789	0.074271759	-19.98095648	8.06636E-89	2.56408E-87	negative
PRJNA729271	stage_3W_vs_E10	ordinary_muscle_development	ENSGALG00010022320	COL9A3	-9.622822114	0.575780867	-16.71264655	1.06024E-62	1.70002E-61	negative
PRJNA729271	stage_3W_vs_E10	ordinary_muscle_development	ENSGALG00010022322	ENSGALG00010022322	2.434308254	0.373051592	6.525393021	6.78235E-11	1.65227E-10	positive
PRJNA729271	stage_3W_vs_E10	ordinary_muscle_development	ENSGALG00010022323	DUSP29	6.234319583	0.381501353	16.34153991	4.99746E-60	7.46017E-59	positive
PRJNA729271	stage_3W_vs_E10	ordinary_muscle_development	ENSGALG00010022325	MIGA1	0.438194157	0.105365685	4.158793773	3.19933E-05	5.43087E-05	positive
PRJNA729271	stage_3W_vs_E10	ordinary_muscle_development	ENSGALG00010022326	COX19	1.075175078	0.094112612	11.42434636	3.16024E-30	1.82938E-29	positive
PRJNA729271	stage_3W_vs_E10	ordinary_muscle_development	ENSGALG00010022328	ENSGALG00010022328	-0.547500238	0.23252567	-2.354579764	0.018543663	0.024475542	negative
PRJNA729271	stage_3W_vs_E10	ordinary_muscle_development	ENSGALG00010022332	KAT6B	-1.117552157	0.062008723	-18.02249921	1.29748E-72	2.71776E-71	negative
PRJNA729271	stage_3W_vs_E10	ordinary_muscle_development	ENSGALG00010022333	ENSGALG00010022333	1.068889566	0.132680144	8.05613812	7.87427E-16	2.48395E-15	positive
PRJNA729271	stage_3W_vs_E10	ordinary_muscle_development	ENSGALG00010022334	PDE1A	-1.533622554	0.122383063	-12.53133007	5.03118E-36	3.5696E-35	negative
PRJNA729271	stage_3W_vs_E10	ordinary_muscle_development	ENSGALG00010022340	GFOD2	-0.593170464	0.094798181	-6.257192441	3.9197E-10	9.16239E-10	negative
PRJNA729271	stage_3W_vs_E10	ordinary_muscle_development	ENSGALG00010022343	GATA5	-3.045902811	0.614959152	-4.953016473	7.30718E-07	1.39512E-06	negative
PRJNA729271	stage_3W_vs_E10	ordinary_muscle_development	ENSGALG00010022345	USP33	0.171624922	0.122389655	1.402282911	0.160830781	0.188978776	positive
PRJNA729271	stage_3W_vs_E10	ordinary_muscle_development	ENSGALG00010022347	SEC11A	-1.341304222	0.104999677	-12.77436523	2.28E-37	1.67589E-36	negative
PRJNA729271	stage_3W_vs_E10	ordinary_muscle_development	ENSGALG00010022348	G6PC1	2.161342288	0.626239494	3.45130307	0.000557887	0.000854456	positive
PRJNA729271	stage_3W_vs_E10	ordinary_muscle_development	ENSGALG00010022353	SLC39A13	0.37318034	0.095058663	3.925789896	8.64456E-05	0.000141858	positive
PRJNA729271	stage_3W_vs_E10	ordinary_muscle_development	ENSGALG00010022354	FAR2	-0.253966427	0.136956805	-1.854354208	0.063688501	0.078909721	negative
PRJNA729271	stage_3W_vs_E10	ordinary_muscle_development	ENSGALG00010022356	TCFL5	-1.165453773	0.233635177	-4.988348881	6.08975E-07	1.16946E-06	negative
PRJNA729271	stage_3W_vs_E10	ordinary_muscle_development	ENSGALG00010022357	ENSGALG00010022357	-5.110786089	0.788046833	-6.485383704	8.85062E-11	2.14281E-10	negative
PRJNA729271	stage_3W_vs_E10	ordinary_muscle_development	ENSGALG00010022359	ZZZ3	-0.255821767	0.079100193	-3.234148472	0.00122006	0.001813451	negative
PRJNA729271	stage_3W_vs_E10	ordinary_muscle_development	ENSGALG00010022363	KIF18A	-2.831170112	0.20763213	-13.63551064	2.46261E-42	2.14516E-41	negative
PRJNA729271	stage_3W_vs_E10	ordinary_muscle_development	ENSGALG00010022365	PLCG1	-2.002994258	0.112052681	-17.87546923	1.83128E-71	3.6613E-70	negative
PRJNA729271	stage_3W_vs_E10	ordinary_muscle_development	ENSGALG00010022366	AARSD1	0.932511212	0.112353269	8.2998138	1.04275E-16	3.44611E-16	positive
PRJNA729271	stage_3W_vs_E10	ordinary_muscle_development	ENSGALG00010022373	AK5	-4.480997598	0.252617081	-17.73830015	2.1226E-70	4.14092E-69	negative
PRJNA729271	stage_3W_vs_E10	ordinary_muscle_development	ENSGALG00010022375	NMB	-2.126352141	0.443264392	-4.797028995	1.61036E-06	3.00415E-06	negative
PRJNA729271	stage_3W_vs_E10	ordinary_muscle_development	ENSGALG00010022377	ZHX3	1.164485484	0.089579568	12.99945406	1.2322E-38	9.48524E-38	positive
PRJNA729271	stage_3W_vs_E10	ordinary_muscle_development	ENSGALG00010022379	METTL15	-1.053228801	0.124035666	-8.491338321	2.04271E-17	6.98657E-17	negative
PRJNA729271	stage_3W_vs_E10	ordinary_muscle_development	ENSGALG00010022382	CALB2	-4.873873535	0.96911305	-5.029210507	4.92503E-07	9.51591E-07	negative
PRJNA729271	stage_3W_vs_E10	ordinary_muscle_development	ENSGALG00010022385	ENSGALG00010022385	2.657209013	0.627597869	4.233935687	2.29637E-05	3.94756E-05	positive

PRJNA729271	stage_3W_vs_E10	ordinary_muscle_development	ENSGALG00010022386	SPI1	2.836683707	0.243484736	11.65035541	2.28504E-31	1.37777E-30	positive
PRJNA729271	stage_3W_vs_E10	ordinary_muscle_development	ENSGALG00010022388	CYP2W1	-0.007546721	0.435316741	-0.01733616	0.986168439	0.98834925	negative
PRJNA729271	stage_3W_vs_E10	ordinary_muscle_development	ENSGALG00010022391	ENSGALG00010022391	0.681541515	0.179470321	3.79751655	0.000146153	0.000235545	positive
PRJNA729271	stage_3W_vs_E10	ordinary_muscle_development	ENSGALG00010022392	EMILIN3	-4.566878337	0.196015713	-23.29853188	4.5878E-120	2.7254E-118	negative
PRJNA729271	stage_3W_vs_E10	ordinary_muscle_development	ENSGALG00010022395	PTGES3L	1.581380892	0.133414039	11.85318202	2.07172E-32	1.30677E-31	positive
PRJNA729271	stage_3W_vs_E10	ordinary_muscle_development	ENSGALG00010022396	PPP1R1C	-2.237362651	0.348372794	-6.422323133	1.3421E-10	3.21497E-10	negative
PRJNA729271	stage_3W_vs_E10	ordinary_muscle_development	ENSGALG00010022397	ATF1	-0.321462113	0.047602415	-6.753063086	1.44756E-11	3.66825E-11	negative
PRJNA729271	stage_3W_vs_E10	ordinary_muscle_development	ENSGALG00010022400	NHLRC2	0.089660298	0.071891245	1.24716574	0.212336732	0.24487397	positive
PRJNA729271	stage_3W_vs_E10	ordinary_muscle_development	ENSGALG00010022401	LCAT	3.302066357	0.436285855	7.568584496	3.77313E-14	1.09427E-13	positive
PRJNA729271	stage_3W_vs_E10	ordinary_muscle_development	ENSGALG00010022406	ITPRID2	0.74189641	0.10435749	7.109182218	1.16733E-12	3.13457E-12	positive
PRJNA729271	stage_3W_vs_E10	ordinary_muscle_development	ENSGALG00010022407	RUNDC1	0.690153535	0.095119543	7.255643915	3.99755E-13	1.0991E-12	positive
PRJNA729271	stage_3W_vs_E10	ordinary_muscle_development	ENSGALG00010022408	RAD51AP1	-0.148663179	0.136902288	-1.085907192	0.277520055	0.31439259	negative
PRJNA729271	stage_3W_vs_E10	ordinary_muscle_development	ENSGALG00010022409	CARNS1	8.795922348	0.338395967	25.99298812	5.9439E-149	5.4877E-147	positive
PRJNA729271	stage_3W_vs_E10	ordinary_muscle_development	ENSGALG00010022411	ADRB1	0.8401224	0.290421825	2.892766061	0.003818656	0.005419172	positive
PRJNA729271	stage_3W_vs_E10	ordinary_muscle_development	ENSGALG00010022412	CHAC1	7.254437342	0.650370842	11.154309	6.82208E-29	3.75564E-28	positive
PRJNA729271	stage_3W_vs_E10	ordinary_muscle_development	ENSGALG00010022415	DIP2B	-0.410299366	0.109481665	-3.747653696	0.000178496	0.000285512	negative
PRJNA729271	stage_3W_vs_E10	ordinary_muscle_development	ENSGALG00010022416	CCDC186	-0.066850762	0.086624868	-0.771727146	0.440276049	0.478898494	negative
PRJNA729271	stage_3W_vs_E10	ordinary_muscle_development	ENSGALG00010022419	DUOX2	2.174449889	0.705840314	3.080654145	0.002065464	0.0030032	positive
PRJNA729271	stage_3W_vs_E10	ordinary_muscle_development	ENSGALG00010022421	CMKLR1	1.61459887	0.175718585	9.188549227	3.98175E-20	1.52889E-19	positive
PRJNA729271	stage_3W_vs_E10	ordinary_muscle_development	ENSGALG00010022422	RPS6KB2	1.908298886	0.153362246	12.44308122	1.52505E-35	1.06688E-34	positive
PRJNA729271	stage_3W_vs_E10	ordinary_muscle_development	ENSGALG00010022425	VWA2	-3.911882525	0.497172064	-7.868266968	3.59588E-15	1.09754E-14	negative
PRJNA729271	stage_3W_vs_E10	ordinary_muscle_development	ENSGALG00010022426	EXD1	-0.812188304	0.234917815	-3.457329554	0.000545557	0.000836898	negative
PRJNA729271	stage_3W_vs_E10	ordinary_muscle_development	ENSGALG00010022427	RPL27	0.303207541	0.138665475	2.18661163	0.028770888	0.037178748	positive
PRJNA729271	stage_3W_vs_E10	ordinary_muscle_development	ENSGALG00010022429	ADK	-1.301179919	0.131407893	-9.90183989	4.08686E-23	1.77203E-22	negative
PRJNA729271	stage_3W_vs_E10	ordinary_muscle_development	ENSGALG00010022431	LARP4	-0.416504146	0.132970651	-3.132301309	0.001734418	0.002542515	negative
PRJNA729271	stage_3W_vs_E10	ordinary_muscle_development	ENSGALG00010022433	LNPK	1.440317846	0.106926121	13.47021512	2.34183E-41	1.96896E-40	positive
PRJNA729271	stage_3W_vs_E10	ordinary_muscle_development	ENSGALG00010022435	PIGK	0.379602245	0.163761992	2.318011888	0.020448675	0.026874925	positive
PRJNA729271	stage_3W_vs_E10	ordinary_muscle_development	ENSGALG00010022436	AFAP1L2	-0.415893651	0.156113505	-2.664046602	0.007720687	0.010628868	negative
PRJNA729271	stage_3W_vs_E10	ordinary_muscle_development	ENSGALG00010022438	RPN2	-0.947759916	0.10142325	-9.344602127	9.22356E-21	3.64095E-20	negative
PRJNA729271	stage_3W_vs_E10	ordinary_muscle_development	ENSGALG00010022439	IFI35	1.854967719	0.203520121	9.1144193	7.90929E-20	3.00512E-19	positive
PRJNA729271	stage_3W_vs_E10	ordinary_muscle_development	ENSGALG00010022440	SORD	1.171628298	0.12319726	9.510181443	1.9033E-21	7.72567E-21	positive
PRJNA729271	stage_3W_vs_E10	ordinary_muscle_development	ENSGALG00010022441	FICD	-0.110808702	0.120149517	-0.922256743	0.356394671	0.395130852	negative
PRJNA729271	stage_3W_vs_E10	ordinary_muscle_development	ENSGALG00010022448	FERRY3	-0.315740888	0.121049247	-2.608367212	0.00909753	0.012431061	negative
PRJNA729271	stage_3W_vs_E10	ordinary_muscle_development	ENSGALG00010022449	ENSGALG00010022449	-0.492700743	0.102418077	-4.810681473	1.50417E-06	2.81291E-06	negative
PRJNA729271	stage_3W_vs_E10	ordinary_muscle_development	ENSGALG00010022451	CHP1	0.200056818	0.120974519	1.653710381	0.098186372	0.11881879	positive
PRJNA729271	stage_3W_vs_E10	ordinary_muscle_development	ENSGALG00010022452	AP3M1	-0.355589365	0.08154307	-4.360755161	1.29614E-05	2.27013E-05	negative
PRJNA729271	stage_3W_vs_E10	ordinary_muscle_development	ENSGALG00010022453	ENSGALG00010022453	2.825272146	0.233275529	12.11130954	9.20133E-34	6.06242E-33	positive
PRJNA729271	stage_3W_vs_E10	ordinary_muscle_development	ENSGALG00010022454	ABLIM1	2.223163436	0.140293359	15.84653367	1.48552E-56	2.03138E-55	positive
PRJNA729271	stage_3W_vs_E10	ordinary_muscle_development	ENSGALG00010022455	C7orf50	-1.106070614	0.154093927	-7.177898783	7.07909E-13	1.92372E-12	negative
PRJNA729271	stage_3W_vs_E10	ordinary_muscle_development	ENSGALG00010022456	ATP5G3	0.031404501	0.1582843	0.198405657	0.842727692	0.861084019	positive
PRJNA729271	stage_3W_vs_E10	ordinary_muscle_development	ENSGALG00010022459	FGF6	3.043794472	0.734935145	4.141582413	3.44918E-05	5.83518E-05	positive
PRJNA729271	stage_3W_vs_E10	ordinary_muscle_development	ENSGALG00010022460	VAT1	-1.18419983	0.119257367	-9.929783471	3.08926E-23	1.34229E-22	negative
PRJNA729271	stage_3W_vs_E10	ordinary_muscle_development	ENSGALG00010022462	ITGA5	-0.205244724	0.130529187	-1.572404829	0.115856695	0.138846035	negative
PRJNA729271	stage_3W_vs_E10	ordinary_muscle_development	ENSGALG00010022463	DUSP12	0.255182126	0.115637095	2.206749719	0.02733155	0.035381871	positive
PRJNA729271	stage_3W_vs_E10	ordinary_muscle_development	ENSGALG00010022466	NEDD4	0.370606617	0.137274565	2.699747169	0.006939219	0.009587644	positive
PRJNA729271	stage_3W_vs_E10	ordinary_muscle_development	ENSGALG00010022467	SART3	-0.887173347	0.116850476	-7.592381101	3.1408E-14	9.14725E-14	negative
PRJNA729271	stage_3W_vs_E10	ordinary_muscle_development	ENSGALG00010022468	MYBPC3	-0.381554328	0.429320099	-0.888740893	0.374142349	0.412881107	negative
PRJNA729271	stage_3W_vs_E10	ordinary_muscle_development	ENSGALG00010022470	ENSGALG00010022470	0.349461968	0.169126641	2.066273928	0.038802622	0.049419559	positive
PRJNA729271	stage_3W_vs_E10	ordinary_muscle_development	ENSGALG00010022471	ST6GALNAC5	-2.495336803	0.164555119	-15.16413967	6.10942E-52	7.36739E-51	negative
PRJNA729271	stage_3W_vs_E10	ordinary_muscle_development	ENSGALG00010022472	QSER1	0.225239566	0.087578144	2.571869614	0.010115098	0.013739888	positive
PRJNA729271	stage_3W_vs_E10	ordinary_muscle_development	ENSGALG00010022473	ZNF385A	1.300420957	0.426586693	3.048433013	0.002300382	0.003331197	positive
PRJNA729271	stage_3W_vs_E10	ordinary_muscle_development	ENSGALG00010022474	VCL	-0.393349309	0.119795711	-3.28350078	0.001025264	0.001534613	negative
PRJNA729271	stage_3W_vs_E10	ordinary_muscle_development	ENSGALG00010022476	OIP5	-1.241300947	0.114570705	-10.83436593	2.36599E-27	1.22526E-26	negative
PRJNA729271	stage_3W_vs_E10	ordinary_muscle_development	ENSGALG00010022478	ATP6V1A	0.042541612	0.096444468	0.441099558	0.659140923	0.691785365	positive
PRJNA729271	stage_3W_vs_E10	ordinary_muscle_development	ENSGALG00010022479	ENSGALG00010022479	1.655866401	0.640121139	2.586801623	0.009687135	0.013190701	positive
PRJNA729271	stage_3W_vs_E10	ordinary_muscle_development	ENSGALG00010022480	PIF1	-3.753801797	0.283944638	-13.22018906	6.70951E-40	5.41505E-39	negative
PRJNA729271	stage_3W_vs_E10	ordinary_muscle_development	ENSGALG00010022481	CORO1B	-0.931814906	0.151327396	-6.157608822	7.38515E-10	1.69836E-09	negative
PRJNA729271	stage_3W_vs_E10	ordinary_muscle_development	ENSGALG00010022482	PRTG	0.389208922	0.292799046	1.329269772	0.183758983	0.213870076	positive
PRJNA729271	stage_3W_vs_E10	ordinary_muscle_development	ENSGALG00010022483	TOP2A	-4.012281687	0.240171171	-16.70588791	1.18747E-62	1.89982E-61	negative
PRJNA729271	stage_3W_vs_E10	ordinary_muscle_development	ENSGALG00010022485	ATF2	-0.310329629	0.057737719	-5.374816181	7.66608E-08	1.56485E-07	negative
PRJNA729271	stage_3W_vs_E10	ordinary_muscle_development	ENSGALG00010022486	FAM183A	-1.701179868	0.669866702	-2.539579684	0.011098576	0.015019499	negative
PRJNA729271	stage_3W_vs_E10	ordinary_muscle_development	ENSGALG00010022489	NUSAP1	-2.839281563	0.241584647	-11.7527401	6.83654E-32	4.22043E-31	negative
PRJNA729271	stage_3W_vs_E10	ordinary_muscle_development	ENSGALG00010022493	ENSGALG00010022493	-0.26995073	0.163595999	-1.650105946	0.098921269	0.119618236	negative
PRJNA729271	stage_3W_vs_E10	ordinary_muscle_development	ENSGALG00010022494	FHIP2A	0.147374082	0.065450348	2.251692871	0.024341687	0.031723859	positive
PRJNA729271	stage_3W_vs_E10	ordinary_muscle_development	ENSGALG00010022496	TUBGCP2	0.148246092	0.071974076	2.059715113	0.039425784	0.050142734	positive
PRJNA729271	stage_3W_vs_E10	ordinary_muscle_development	ENSGALG00010022497	CORO1C	-0.050305245	0.149207602	-0.337149341	0.736004324	0.763663756	negative
PRJNA729271	stage_3W_vs_E10	ordinary_muscle_development	ENSGALG00010022498	NAA50	0.195097374	0.113863064	1.71343864	0.086631873	0.105621078	positive
PRJNA729271	stage_3W_vs_E10	ordinary_muscle_development	ENSGALG00010022500	ENSGALG00010022500	-0.506720748	0.443553776	-1.14241108	0.253283188	0.288604655	negative
PRJNA729271	stage_3W_vs_E10	ordinary_muscle_development	ENSGALG00010022502	ENSGALG00010022502	-2.348331307	0.574498537	-4.0876193	4.35823E-05	7.32774E-05	negative
PRJNA729271	stage_3W_vs_E10	ordinary_muscle_development	ENSGALG00010022504	RAD9A	1.421991851	0.142665266	9.96733048	2.11846E-23	9.27707E-23	positive
PRJNA729271	stage_3W_vs_E10	ordinary_muscle_development	ENSGALG00010022505	ZNF511	1.361634651	0.118374566	11.50276359	1.27758E-30	7.50346E-30	positive
PRJNA729271	stage_3W_vs_E10	ordinary_muscle_development	ENSGALG00010022506	EBNA1BP2	-1.297091392	0.087153064	-14.88291204	4.25566E-50	4.84684E-49	negative
PRJNA729271	stage_3W_vs_E10	ordinary_muscle_development	ENSGALG00010022507	ENSGALG00010022507	-0.588337375	0.22231671	-2.646392959	0.008135525	0.011168144	negative
PRJNA729271	stage_3W_vs_E10	ordinary_muscle_development	ENSGALG00010022508	USF3	0.527654624	0.122007485	4.324772549	1.52689E-05	2.66077E-05	positive
PRJNA729271	stage_3W_vs_E10	ordinary_muscle_development	ENSGALG00010022511	IGFBP4	-0.530258422	0.271608474	-1.952289682	0.050903822	0.063783791	negative
PRJNA729271	stage_3W_vs_E10	ordinary_muscle_development	ENSGALG00010022512	PRAP1	2.203511193	0.840267637	2.622392077	0.008731492	0.011951178	positive
PRJNA729271	stage_3W_vs_E10	ordinary_muscle_development	ENSGALG00010022513	NDUFAF1	0.07258932	0.084981857	0.854174328	0.393008405	0.431892103	positive
PRJNA729271	stage_3W_vs_E10	ordinary_muscle_development	ENSGALG00010022515	CHRNA1	-2.355302057	0.173113253	-13.60555601	3.71135E-42	3.2117E-41	negative
PRJNA729271	stage_3W_vs_E10	ordinary_muscle_development	ENSGALG00010022516	TRUB1	-0.641361636	0.152295989	-4.211283827	2.53923E-05	4.34497E-05	negative
PRJNA729271	stage_3W_vs_E10	ordinary_muscle_development	ENSGALG00010022517	HNRNPA1	-0.616140663	0.181432466	-3.395978005	0.000683839	0.001039782	negative
PRJNA729271	stage_3W_vs_E10	ordinary_muscle_development	ENSGALG00010022518	ECHS1	0.282435725	0.112409149	2.512568844	0.011985572	0.016162514	positive
PRJNA729271	stage_3W_vs_E10	ordinary_muscle_development	ENSGALG00010022520	TMOD2	-3.152987051	0.218141654	-14.45385142	2.37051E-47	2.45083E-46	negative
PRJNA729271	stage_3W_vs_E10	ordinary_muscle_development	ENSGALG00010022521	RTF1	-0.224567717	0.091217394	-2.461895778	0.013820482	0.018494081	negative
PRJNA729271	stage_3W_vs_E10	ordinary_muscle_development	ENSGALG00010022522	TNS4	-2.269355389	0.386228165	-5.875685917	4.21096E-09	9.25658E-09	negative
PRJNA729271	stage_3W_vs_E10	ordinary_muscle_development	ENSGALG00010022524	SOX18	0.211897655	0.231780202	0.914218095	0.360602258	0.39942914	positive
PRJNA729271	stage_3W_vs_E10	ordinary_muscle_development	ENSGALG00010022525	ISCU	0.866070983	0.194110387	4.461744653	8.12951E-06	1.44515E-05	positive
PRJNA729271	stage_3W_vs_E10	ordinary_muscle_development	ENSGALG00010022526	PPP1CA	1.97339492	0.137335501	14.36915364	8.08128E-47	8.2087E-46	positive
PRJNA729271	stage_3W_vs_E10	ordinary_muscle_development	ENSGALG00010022528	SIDT1	-3.738889193	0.516498999	-7.238908888	4.52309E-13	1.2403E-12	negative
PRJNA729271	stage_3W_vs_E10	ordinary_muscle_development	ENSGALG00010022529	CCR7	-0.809377894	0.356115637	-2.272795153	0.023038527	0.030122989	negative
PRJNA729271	stage_3W_vs_E10	ordinary_muscle_development	ENSGALG00010022531	ENSGALG00010022531	0.310976999	0.387057423	0.803438922	0.421721086	0.46016615	positive
PRJNA729271	stage_3W_vs_E10	ordinary_muscle_development	ENSGALG00010022532	CBX5	-3.446992889	0.183091655	-18.82659743	4.5721E-79	1.10639E-77	negative
PRJNA729271	stage_3W_vs_E10	ordinary_muscle_development	ENSGALG00010022533	TMEM119	-0.552508365	0.13269273	-4.163817908	3.1297E-05	5.31641E-05	negative
PRJNA729271	stage_3W_vs_E10	ordinary_muscle_development	ENSGALG00010022534	CFAP57	-1.093892162	0.541023996	-2.021892132	0.043187497	0.054653638	negative
PRJNA729271	stage_3W_vs_E10	ordinary_muscle_development	ENSGALG00010022536	SMUG1	0.224122958	0.099740226	2.247066872	0.024635754	0.032081147	positive
PRJNA729271	stage_3W_vs_E10	ordinary_muscle_development	ENSGALG00010022538	WIPF1	-0.219557658	0.158355926	-1.386482102	0.165599742	0.194204551	negative
PRJNA729271	stage_3W_vs_E10	ordinary_muscle_development	ENSGALG00010022539	LYSMD2	0.876454846	0.101404041	8.643194453	5.46628E-18	1.92689E-17	positive
PRJNA729271	stage_3W_vs_E10	ordinary_muscle_development	ENSGALG00010022540	GPR146	1.69486848	0.237905859	7.124114093	1.04752E-12	2.82332E-12	positive
PRJNA729271	stage_3W_vs_E10	ordinary_muscle_development	ENSGALG00010022541	ITPKA	-1.723112555	0.12725286	-13.54085528	8.9738E-42	7.66049E-41	negative
PRJNA729271	stage_3W_vs_E10	ordinary_muscle_development	ENSGALG00010022542	ENSGALG00010022542	0.141142022	0.745743648	0.189263459	0.849886334	0.866995731	positive
PRJNA729271	stage_3W_vs_E10	ordinary_muscle_development	ENSGALG00010022544	SPICE1	-0.259647335	0.070141919	-3.701742694	0.000214124	0.00034006	negative
PRJNA729271	stage_3W_vs_E10	ordinary_muscle_development	ENSGALG00010022545	CSTF3	-0.700919651	0.067151186	-10.43793399	1.66393E-25	7.95206E-25	negative
PRJNA729271	stage_3W_vs_E10	ordinary_muscle_development	ENSGALG00010022546	MTG1	1.19368419	0.157030547	7.601605003	2.9248E-14	8.52847E-14	positive
PRJNA729271	stage_3W_vs_E10	ordinary_muscle_development	ENSGALG00010022547	SMARCE1	-0.958734678	0.061961952	-15.47295783	5.28231E-54	6.78786E-53	negative
PRJNA729271	stage_3W_vs_E10	ordinary_muscle_development	ENSGALG00010022548	ENSGALG00010022548	-0.373536987	0.116321761	-3.211239111	0.001321639	0.00195901	negative
PRJNA729271	stage_3W_vs_E10	ordinary_muscle_development	ENSGALG00010022550	TRIM45	-1.144787935	0.167785359	-6.822931064	8.92015E-12	2.28562E-11	negative
PRJNA729271	stage_3W_vs_E10	ordinary_muscle_development	ENSGALG00010022551	GPER1	-0.529456746	0.581498701	-0.910503747	0.362556907	0.401379543	negative
PRJNA729271	stage_3W_vs_E10	ordinary_muscle_development	ENSGALG00010022552	GPR155	1.079732328	0.10509	10.2743584	9.19542E-25	4.26111E-24	positive
PRJNA729271	stage_3W_vs_E10	ordinary_muscle_development	ENSGALG00010022553	PRPF6	0.174009118	0.169395559	1.027235422	0.304309623	0.341988524	positive
PRJNA729271	stage_3W_vs_E10	ordinary_muscle_development	ENSGALG00010022554	SCG3	-3.521390152	0.357618846	-9.846768961	7.07829E-23	3.04E-22	negative
PRJNA729271	stage_3W_vs_E10	ordinary_muscle_development	ENSGALG00010022557	WNT1	-0.690639524	0.421882554	-1.637042152	0.101621662	0.122730045	negative
PRJNA729271	stage_3W_vs_E10	ordinary_muscle_development	ENSGALG00010022558	NYAP2	-1.855465423	0.591834981	-3.135106041	0.00171792	0.002520369	negative
PRJNA729271	stage_3W_vs_E10	ordinary_muscle_development	ENSGALG00010022559	SSH1	0.289603896	0.086278516	3.356616574	0.000789025	0.001192711	positive
PRJNA729271	stage_3W_vs_E10	ordinary_muscle_development	ENSGALG00010022560	SAMD10	-4.554805507	1.448819092	-3.143805554	0.001667662	0.002450356	negative
PRJNA729271	stage_3W_vs_E10	ordinary_muscle_development	ENSGALG00010022562	GHRH	-4.05011755	1.144466134	-3.538870596	0.000401843	0.000623698	negative
PRJNA729271	stage_3W_vs_E10	ordinary_muscle_development	ENSGALG00010022565	ARF3	-1.749305323	0.176589376	-9.906062083	3.91785E-23	1.69977E-22	negative
PRJNA729271	stage_3W_vs_E10	ordinary_muscle_development	ENSGALG00010022567	PAOX	0.694550037	0.124031192	5.599801331	2.14598E-08	4.53505E-08	positive
PRJNA729271	stage_3W_vs_E10	ordinary_muscle_development	ENSGALG00010022568	SCRN3	2.858222073	0.166768558	17.13885461	7.61357E-66	1.32802E-64	positive
PRJNA729271	stage_3W_vs_E10	ordinary_muscle_development	ENSGALG00010022569	KIAA1549L	-3.933349896	0.280226507	-14.03632346	9.34357E-45	8.82888E-44	negative
PRJNA729271	stage_3W_vs_E10	ordinary_muscle_development	ENSGALG00010022571	RIPOR1	0.620187467	0.107671411	5.760001327	8.41133E-09	1.82382E-08	positive
PRJNA729271	stage_3W_vs_E10	ordinary_muscle_development	ENSGALG00010022572	ENSGALG00010022572	-4.960689499	0.410490757	-12.0847776	1.27114E-33	8.34093E-33	negative
PRJNA729271	stage_3W_vs_E10	ordinary_muscle_development	ENSGALG00010022573	AP4E1	-0.233319818	0.082137715	-2.840593006	0.004502974	0.006345577	negative
PRJNA729271	stage_3W_vs_E10	ordinary_muscle_development	ENSGALG00010022574	ITPRIPL2	-0.349758341	0.111667255	-3.132147739	0.001735325	0.002543588	negative
PRJNA729271	stage_3W_vs_E10	ordinary_muscle_development	ENSGALG00010022575	TMEM41B	0.424992548	0.109595287	3.877835986	0.00010539	0.000171836	positive
PRJNA729271	stage_3W_vs_E10	ordinary_muscle_development	ENSGALG00010022578	ZNF512B	-0.657575998	0.13581187	-4.841815363	1.28658E-06	2.41755E-06	negative
PRJNA729271	stage_3W_vs_E10	ordinary_muscle_development	ENSGALG00010022579	TNFAIP8L3	-0.514390113	0.147386738	-3.490070543	0.000482893	0.000745345	negative
PRJNA729271	stage_3W_vs_E10	ordinary_muscle_development	ENSGALG00010022581	TMEM125	-2.38069811	0.702925943	-3.38684058	0.000707025	0.00107391	negative
PRJNA729271	stage_3W_vs_E10	ordinary_muscle_development	ENSGALG00010022582	ENSGALG00010022582	0.593701467	0.215588313	2.753866657	0.005889576	0.0081952	positive
PRJNA729271	stage_3W_vs_E10	ordinary_muscle_development	ENSGALG00010022583	FKBP11	-0.221060845	0.120575242	-1.833385046	0.06674533	0.082492629	negative
PRJNA729271	stage_3W_vs_E10	ordinary_muscle_development	ENSGALG00010022585	CIR1	0.172963225	0.09371601	1.845610213	0.064948822	0.080409394	positive
PRJNA729271	stage_3W_vs_E10	ordinary_muscle_development	ENSGALG00010022587	ENSGALG00010022587	-3.214504901	0.984544234	-3.264967474	0.001094766	0.001635268	negative
PRJNA729271	stage_3W_vs_E10	ordinary_muscle_development	ENSGALG00010022588	TCP11L1	0.12177361	0.077228071	1.576805009	0.114840399	0.137696383	positive

PRJNA729271	stage_3W_vs_E10	ordinary_muscle_development	ENSGALG00010022590	ENSGALG00010022590	-1.190667683	0.163118618	-7.299397813	2.89058E-13	8.00058E-13	negative
PRJNA729271	stage_3W_vs_E10	ordinary_muscle_development	ENSGALG00010022592	CCDC65	0.022724614	0.403361742	0.05633805	0.955072508	0.960972928	positive
PRJNA729271	stage_3W_vs_E10	ordinary_muscle_development	ENSGALG00010022593	MANBAL	-2.356582935	0.148398485	-15.880101	8.70381E-57	1.19926E-55	negative
PRJNA729271	stage_3W_vs_E10	ordinary_muscle_development	ENSGALG00010022597	SLC04A1	1.846027196	0.222778228	8.286389613	1.16738E-16	3.84922E-16	positive
PRJNA729271	stage_3W_vs_E10	ordinary_muscle_development	ENSGALG00010022599	RNLS	-1.507196025	0.2814299	-5.355493585	8.53232E-08	1.73653E-07	negative
PRJNA729271	stage_3W_vs_E10	ordinary_muscle_development	ENSGALG00010022603	ATRNL1	-0.459012617	0.16970319	-2.704796637	0.006834622	0.009452136	negative
PRJNA729271	stage_3W_vs_E10	ordinary_muscle_development	ENSGALG00010022608	P3H4	-2.198277924	0.208829418	-10.52666786	6.50982E-26	3.16538E-25	negative
PRJNA729271	stage_3W_vs_E10	ordinary_muscle_development	ENSGALG00010022609	RBBP8NL	-8.052839093	1.061065745	-7.589387491	3.21421E-14	9.35354E-14	negative
PRJNA729271	stage_3W_vs_E10	ordinary_muscle_development	ENSGALG00010022614	DAO	-0.869940191	0.205613417	-4.230950509	2.32706E-05	3.99653E-05	negative
PRJNA729271	stage_3W_vs_E10	ordinary_muscle_development	ENSGALG00010022616	GLDN	2.356541416	0.334154669	7.052247459	1.76051E-12	4.68574E-12	positive
PRJNA729271	stage_3W_vs_E10	ordinary_muscle_development	ENSGALG00010022619	MADD	-0.12780108	0.132163566	-0.966991769	0.333548148	0.371906185	negative
PRJNA729271	stage_3W_vs_E10	ordinary_muscle_development	ENSGALG00010022622	MPPED2	-1.650318811	0.138223185	-11.93952239	7.36448E-33	4.70671E-32	negative
PRJNA729271	stage_3W_vs_E10	ordinary_muscle_development	ENSGALG00010022623	TOP1	-1.256484439	0.126710943	-9.916147815	3.54162E-23	1.537E-22	negative
PRJNA729271	stage_3W_vs_E10	ordinary_muscle_development	ENSGALG00010022625	ENSGALG00010022625	0.411135865	0.122898816	3.345319985	0.000821877	0.001240044	positive
PRJNA729271	stage_3W_vs_E10	ordinary_muscle_development	ENSGALG00010022627	CTCF	-0.644177881	0.098571526	-6.535131453	6.35537E-11	1.55086E-10	negative
PRJNA729271	stage_3W_vs_E10	ordinary_muscle_development	ENSGALG00010022628	SVOP	1.66592186	0.546219665	3.049911908	0.002289085	0.003315376	positive
PRJNA729271	stage_3W_vs_E10	ordinary_muscle_development	ENSGALG00010022630	ENSGALG00010022630	-3.347577443	0.332392046	-10.07117192	7.40899E-24	3.30136E-23	negative
PRJNA729271	stage_3W_vs_E10	ordinary_muscle_development	ENSGALG00010022637	DMXL2	-0.615650942	0.110142404	-5.589590539	2.27606E-08	4.80435E-08	negative
PRJNA729271	stage_3W_vs_E10	ordinary_muscle_development	ENSGALG00010022642	SEC13	-0.339953636	0.068525614	-4.960971722	7.01414E-07	1.34094E-06	negative
PRJNA729271	stage_3W_vs_E10	ordinary_muscle_development	ENSGALG00010022643	SP3	-0.039392943	0.076844688	-0.512630663	0.6082097	0.643503621	negative
PRJNA729271	stage_3W_vs_E10	ordinary_muscle_development	ENSGALG00010022644	ARMC8	0.65434121	0.085154671	7.684149316	1.54017E-14	4.5505E-14	positive
PRJNA729271	stage_3W_vs_E10	ordinary_muscle_development	ENSGALG00010022645	ENSGALG00010022645	-1.93278039	0.758444894	-2.548346498	0.010823491	0.01465818	negative
PRJNA729271	stage_3W_vs_E10	ordinary_muscle_development	ENSGALG00010022647	SVIP	-0.682637255	0.134330097	-5.081789333	3.73896E-07	7.28444E-07	negative
PRJNA729271	stage_3W_vs_E10	ordinary_muscle_development	ENSGALG00010022649	PATZ1	-3.358631023	0.103817441	-32.3513177	1.3294E-229	3.0108E-227	negative
PRJNA729271	stage_3W_vs_E10	ordinary_muscle_development	ENSGALG00010022650	NR1H3	1.420674734	0.098465154	14.42819803	3.4395E-47	3.52835E-46	positive
PRJNA729271	stage_3W_vs_E10	ordinary_muscle_development	ENSGALG00010022652	CARMIL2	-0.072624801	0.256293936	-0.283365273	0.776896846	0.801218056	negative
PRJNA729271	stage_3W_vs_E10	ordinary_muscle_development	ENSGALG00010022655	NRIP3	-7.82748962	0.574011948	-13.63645765	2.43085E-42	2.11877E-41	negative
PRJNA729271	stage_3W_vs_E10	ordinary_muscle_development	ENSGALG00010022656	FKBP10	-2.449899032	0.212500336	-11.52891842	9.43212E-31	5.58035E-30	negative
PRJNA729271	stage_3W_vs_E10	ordinary_muscle_development	ENSGALG00010022657	MRPS35	0.463808046	0.111415527	4.162867213	3.14276E-05	5.33736E-05	positive
PRJNA729271	stage_3W_vs_E10	ordinary_muscle_development	ENSGALG00010022659	TIE1	0.660726803	0.151443908	4.362848341	1.2838E-05	2.2496E-05	positive
PRJNA729271	stage_3W_vs_E10	ordinary_muscle_development	ENSGALG00010022660	METAP1D	0.716892178	0.182043387	3.938029224	8.21536E-05	0.000134983	positive
PRJNA729271	stage_3W_vs_E10	ordinary_muscle_development	ENSGALG00010022661	ENSGALG00010022661	-0.903306763	0.38869185	-2.323966305	0.0201273	0.026481366	negative
PRJNA729271	stage_3W_vs_E10	ordinary_muscle_development	ENSGALG00010022662	GFRA1	-4.753539593	0.172728208	-27.52034334	1.0024E-166	1.2109E-164	negative
PRJNA729271	stage_3W_vs_E10	ordinary_muscle_development	ENSGALG00010022664	TRPC4AP	0.775621888	0.097366179	7.966029865	1.63853E-15	5.07273E-15	positive
PRJNA729271	stage_3W_vs_E10	ordinary_muscle_development	ENSGALG00010022666	ENSGALG00010022666	-0.635599602	0.103616899	-6.134130712	8.56261E-10	1.96043E-09	negative
PRJNA729271	stage_3W_vs_E10	ordinary_muscle_development	ENSGALG00010022667	PTEN	0.787674951	0.041537518	18.96297564	3.45058E-80	8.66832E-79	positive
PRJNA729271	stage_3W_vs_E10	ordinary_muscle_development	ENSGALG00010022669	MANSC4	-6.613007914	1.139271136	-5.804595327	6.45216E-09	1.4068E-08	negative
PRJNA729271	stage_3W_vs_E10	ordinary_muscle_development	ENSGALG00010022671	DBR1	-0.41648418	0.056358618	-7.389893365	1.46947E-13	4.13109E-13	negative
PRJNA729271	stage_3W_vs_E10	ordinary_muscle_development	ENSGALG00010022673	KLHL42	0.36199065	0.148530454	2.437147668	0.014803633	0.019760443	positive
PRJNA729271	stage_3W_vs_E10	ordinary_muscle_development	ENSGALG00010022674	ENSGALG00010022674	2.53282631	0.329763758	7.68072976	1.58185E-14	4.67269E-14	positive
PRJNA729271	stage_3W_vs_E10	ordinary_muscle_development	ENSGALG00010022681	PPP2R3A	0.803661042	0.093160732	8.626607229	6.31987E-18	2.22184E-17	positive
PRJNA729271	stage_3W_vs_E10	ordinary_muscle_development	ENSGALG00010022682	EIF1	1.263161425	0.15552277	8.122035301	4.5843E-16	1.46268E-15	positive
PRJNA729271	stage_3W_vs_E10	ordinary_muscle_development	ENSGALG00010022684	ENSGALG00010022684	-3.05056065	0.539107797	-5.658535578	1.5267E-08	3.25818E-08	negative
PRJNA729271	stage_3W_vs_E10	ordinary_muscle_development	ENSGALG00010022685	ATAD1	0.554083019	0.107859261	5.137092671	2.79021E-07	5.48098E-07	positive
PRJNA729271	stage_3W_vs_E10	ordinary_muscle_development	ENSGALG00010022689	MPL	1.061420625	0.408047014	2.601221399	0.009289248	0.012670335	positive
PRJNA729271	stage_3W_vs_E10	ordinary_muscle_development	ENSGALG00010022691	ASIC2	-4.312243465	0.6300501	-6.844286614	7.68579E-12	1.97248E-11	negative
PRJNA729271	stage_3W_vs_E10	ordinary_muscle_development	ENSGALG00010022695	PIK3IP1	2.695575028	0.202480618	13.31275584	1.95149E-40	1.59722E-39	positive
PRJNA729271	stage_3W_vs_E10	ordinary_muscle_development	ENSGALG00010022698	SEMA6D	0.64278579	0.117003005	5.493754554	3.93477E-08	8.17348E-08	positive
PRJNA729271	stage_3W_vs_E10	ordinary_muscle_development	ENSGALG00010022702	CDC20	-3.396158557	0.237604685	-14.29331479	2.40866E-46	2.41282E-45	negative
PRJNA729271	stage_3W_vs_E10	ordinary_muscle_development	ENSGALG00010022707	SALL1	-4.528328719	0.567703074	-7.976579534	1.50444E-15	4.66957E-15	negative
PRJNA729271	stage_3W_vs_E10	ordinary_muscle_development	ENSGALG00010022708	SPRED1	-0.255380267	0.130585084	-1.955661845	0.050505002	0.063333334	negative
PRJNA729271	stage_3W_vs_E10	ordinary_muscle_development	ENSGALG00010022709	RNF5	-0.538051215	0.08522676	-6.313172262	2.73373E-10	6.44525E-10	negative
PRJNA729271	stage_3W_vs_E10	ordinary_muscle_development	ENSGALG00010022711	LRRC49	-0.233722891	0.191874514	-1.218102845	0.223184893	0.256405249	negative
PRJNA729271	stage_3W_vs_E10	ordinary_muscle_development	ENSGALG00010022712	DDX23	-0.068766677	0.201706278	-0.340924823	0.733160177	0.760930601	negative
PRJNA729271	stage_3W_vs_E10	ordinary_muscle_development	ENSGALG00010022713	PNLIPRP1	8.687787021	2.787425566	3.116778122	0.001828391	0.002670818	positive
PRJNA729271	stage_3W_vs_E10	ordinary_muscle_development	ENSGALG00010022717	ELOVL1	0.871993391	0.170897167	5.102444969	3.35293E-07	6.5482E-07	positive
PRJNA729271	stage_3W_vs_E10	ordinary_muscle_development	ENSGALG00010022718	C12orf43	-0.969026847	0.142413818	-6.804303544	1.01539E-11	2.5967E-11	negative
PRJNA729271	stage_3W_vs_E10	ordinary_muscle_development	ENSGALG00010022719	MSL2	0.041648358	0.097356531	0.427792128	0.668802468	0.700555845	positive
PRJNA729271	stage_3W_vs_E10	ordinary_muscle_development	ENSGALG00010022724	LUZP2	-2.5311677	0.415513356	-6.091663868	1.11743E-09	2.53755E-09	negative
PRJNA729271	stage_3W_vs_E10	ordinary_muscle_development	ENSGALG00010022725	KRT222	-6.159245157	0.91234631	-6.750994759	1.46835E-11	3.71898E-11	negative
PRJNA729271	stage_3W_vs_E10	ordinary_muscle_development	ENSGALG00010022727	ACP2	0.451952958	0.082814458	5.457416117	4.83113E-08	9.97113E-08	positive
PRJNA729271	stage_3W_vs_E10	ordinary_muscle_development	ENSGALG00010022729	ENSGALG00010022729	-0.514525818	0.133115405	-3.86526126	0.00011097	0.000180691	negative
PRJNA729271	stage_3W_vs_E10	ordinary_muscle_development	ENSGALG00010022731	LRRC8B	-0.67757569	0.177112424	-3.825681315	0.000130411	0.000211137	negative
PRJNA729271	stage_3W_vs_E10	ordinary_muscle_development	ENSGALG00010022733	PCCB	0.360376057	0.115129317	3.130184974	0.001746963	0.002559351	positive
PRJNA729271	stage_3W_vs_E10	ordinary_muscle_development	ENSGALG00010022734	ENSGALG00010022734	1.077125584	0.118874875	9.061002862	1.29257E-19	4.8639E-19	positive
PRJNA729271	stage_3W_vs_E10	ordinary_muscle_development	ENSGALG00010022737	CYLD	0.553269551	0.078995808	7.003783716	2.49141E-12	6.57077E-12	positive
PRJNA729271	stage_3W_vs_E10	ordinary_muscle_development	ENSGALG00010022742	IFT46	-0.376569132	0.122963026	-3.062458253	0.002195271	0.003184593	negative
PRJNA729271	stage_3W_vs_E10	ordinary_muscle_development	ENSGALG00010022743	ZNF326	-1.629423814	0.153158632	-10.63879841	1.96649E-26	9.80539E-26	negative
PRJNA729271	stage_3W_vs_E10	ordinary_muscle_development	ENSGALG00010022745	PAPSS2	-0.444989568	0.237801403	-1.871265527	0.061308286	0.076142885	negative
PRJNA729271	stage_3W_vs_E10	ordinary_muscle_development	ENSGALG00010022747	NT5C3B	-0.195281357	0.137258267	-1.422729295	0.154814645	0.18230874	negative
PRJNA729271	stage_3W_vs_E10	ordinary_muscle_development	ENSGALG00010022748	ENSGALG00010022748	-0.556124842	0.326306967	-1.704299627	0.088325125	0.107567861	negative
PRJNA729271	stage_3W_vs_E10	ordinary_muscle_development	ENSGALG00010022749	HSPA12A	-1.353739502	0.131101763	-10.32586799	5.38297E-25	2.51941E-24	negative
PRJNA729271	stage_3W_vs_E10	ordinary_muscle_development	ENSGALG00010022751	SLC25A12	3.433625494	0.190943301	17.98243497	2.67483E-72	5.53882E-71	positive
PRJNA729271	stage_3W_vs_E10	ordinary_muscle_development	ENSGALG00010022754	SNX20	0.194149873	0.183453345	1.058306532	0.289915716	0.327054892	positive
PRJNA729271	stage_3W_vs_E10	ordinary_muscle_development	ENSGALG00010022755	MINPP1	-0.808396617	0.116210858	-6.956291599	3.49346E-12	9.1305E-12	negative
PRJNA729271	stage_3W_vs_E10	ordinary_muscle_development	ENSGALG00010022759	THSD4	-0.748892567	0.121110678	-6.183538746	6.26803E-10	1.44743E-09	negative
PRJNA729271	stage_3W_vs_E10	ordinary_muscle_development	ENSGALG00010022760	ELAVL4	-4.269435585	0.186160592	-22.93415348	2.1209E-116	1.1734E-114	negative
PRJNA729271	stage_3W_vs_E10	ordinary_muscle_development	ENSGALG00010022764	MFAP5	2.564254401	0.222873775	11.50541107	1.23898E-30	7.28263E-30	positive
PRJNA729271	stage_3W_vs_E10	ordinary_muscle_development	ENSGALG00010022767	CACNB3	-0.798234397	0.476743132	-1.674349022	0.094062025	0.114151796	negative
PRJNA729271	stage_3W_vs_E10	ordinary_muscle_development	ENSGALG00010022768	ENSGALG00010022768	0.853487056	0.551921547	1.546391984	0.122009929	0.145702251	positive
PRJNA729271	stage_3W_vs_E10	ordinary_muscle_development	ENSGALG00010022769	FXYD6	0.929267942	0.097673966	9.513977754	1.83508E-21	7.45502E-21	positive
PRJNA729271	stage_3W_vs_E10	ordinary_muscle_development	ENSGALG00010022770	HAT1	-1.316197505	0.092764826	-14.18854063	1.0788E-45	1.05515E-44	negative
PRJNA729271	stage_3W_vs_E10	ordinary_muscle_development	ENSGALG00010022771	SGMS1	1.203551782	0.219473069	5.483824442	4.16228E-08	8.62875E-08	positive
PRJNA729271	stage_3W_vs_E10	ordinary_muscle_development	ENSGALG00010022776	CDH4	-1.376823669	0.181953556	-7.56689619	3.82247E-14	1.10836E-13	negative
PRJNA729271	stage_3W_vs_E10	ordinary_muscle_development	ENSGALG00010022780	DYNC1I2	-0.784844195	0.058028045	-13.52525648	1.10956E-41	9.41632E-41	negative
PRJNA729271	stage_3W_vs_E10	ordinary_muscle_development	ENSGALG00010022782	TMPRSS13	0.410858011	0.231629241	1.773774372	0.076100479	0.093393992	positive
PRJNA729271	stage_3W_vs_E10	ordinary_muscle_development	ENSGALG00010022783	STAG1	-0.091103637	0.055244023	-1.649113022	0.099124482	0.119843971	negative
PRJNA729271	stage_3W_vs_E10	ordinary_muscle_development	ENSGALG00010022784	DMRTA2	-4.837791259	0.993126971	-4.871271652	1.10882E-06	2.0933E-06	negative
PRJNA729271	stage_3W_vs_E10	ordinary_muscle_development	ENSGALG00010022786	KRT40	0.165343819	0.571015855	0.28956082	0.772152244	0.796721724	positive
PRJNA729271	stage_3W_vs_E10	ordinary_muscle_development	ENSGALG00010022787	ENO4	-1.54033989	0.340089282	-4.529222094	5.92012E-06	1.0641E-05	negative
PRJNA729271	stage_3W_vs_E10	ordinary_muscle_development	ENSGALG00010022788	CYBRD1	2.373748943	0.17320738	13.70466395	9.52071E-43	8.40967E-42	positive
PRJNA729271	stage_3W_vs_E10	ordinary_muscle_development	ENSGALG00010022789	ENSGALG00010022789	0.710781111	0.332310618	2.138905811	0.032443297	0.041653286	positive
PRJNA729271	stage_3W_vs_E10	ordinary_muscle_development	ENSGALG00010022792	DDB2	-0.718416523	0.126141871	-5.695305748	1.23151E-08	2.64377E-08	negative
PRJNA729271	stage_3W_vs_E10	ordinary_muscle_development	ENSGALG00010022795	TAF4	0.245697489	0.110818667	2.217112834	0.026615382	0.034504066	positive
PRJNA729271	stage_3W_vs_E10	ordinary_muscle_development	ENSGALG00010022796	RIMKLB	-2.061422535	0.138563953	-14.87704772	4.64551E-50	5.27717E-49	negative
PRJNA729271	stage_3W_vs_E10	ordinary_muscle_development	ENSGALG00010022797	EFHD1	1.094205908	0.131917542	8.294620193	1.08933E-16	3.59594E-16	positive
PRJNA729271	stage_3W_vs_E10	ordinary_muscle_development	ENSGALG00010022799	DCAF17	-0.09946284	0.153576015	-0.647645664	0.517214155	0.555088048	negative
PRJNA729271	stage_3W_vs_E10	ordinary_muscle_development	ENSGALG00010022800	KLHL10	-0.458129435	0.445970945	-1.027262964	0.304296657	0.341988524	negative
PRJNA729271	stage_3W_vs_E10	ordinary_muscle_development	ENSGALG00010022801	LSM14B	-0.405889347	0.103079971	-3.937616054	8.22951E-05	0.0001352	negative
PRJNA729271	stage_3W_vs_E10	ordinary_muscle_development	ENSGALG00010022803	ENSGALG00010022803	0.828260722	0.154659278	5.355389814	8.53722E-08	1.73729E-07	positive
PRJNA729271	stage_3W_vs_E10	ordinary_muscle_development	ENSGALG00010022804	SNX19	-0.124679849	0.114206196	-1.091708271	0.274961349	0.311761948	negative
PRJNA729271	stage_3W_vs_E10	ordinary_muscle_development	ENSGALG00010022805	ENSGALG00010022805	-0.640393408	0.276161867	-2.318905995	0.020400134	0.026818424	negative
PRJNA729271	stage_3W_vs_E10	ordinary_muscle_development	ENSGALG00010022806	SPPL3	-0.512551853	0.063726807	-8.042955238	8.76974E-16	2.75743E-15	negative
PRJNA729271	stage_3W_vs_E10	ordinary_muscle_development	ENSGALG00010022807	ADCY6	-0.4294269	0.148651307	-2.888820222	0.003866901	0.005484416	negative
PRJNA729271	stage_3W_vs_E10	ordinary_muscle_development	ENSGALG00010022808	PSMA7	0.580251214	0.136601758	4.247758036	2.1592E-05	3.72015E-05	positive
PRJNA729271	stage_3W_vs_E10	ordinary_muscle_development	ENSGALG00010022809	MYO1E	-0.391098921	0.155888718	-2.508834039	0.012113038	0.016325289	negative
PRJNA729271	stage_3W_vs_E10	ordinary_muscle_development	ENSGALG00010022811	GIGYF2	-0.014351296	0.09801005	-0.146426779	0.883584488	0.897327623	negative
PRJNA729271	stage_3W_vs_E10	ordinary_muscle_development	ENSGALG00010022813	USP24	1.8029588	0.121849249	14.79663448	1.53997E-49	1.70918E-48	positive
PRJNA729271	stage_3W_vs_E10	ordinary_muscle_development	ENSGALG00010022815	LRRC8D	0.435050895	0.236538997	1.839235391	0.065880571	0.081493334	positive
PRJNA729271	stage_3W_vs_E10	ordinary_muscle_development	ENSGALG00010022816	METTL8	0.570608191	0.137122518	4.161301862	3.16438E-05	5.37345E-05	positive
PRJNA729271	stage_3W_vs_E10	ordinary_muscle_development	ENSGALG00010022817	KRT23	-3.505163224	0.886658068	-3.953229945	7.71033E-05	0.00012703	negative
PRJNA729271	stage_3W_vs_E10	ordinary_muscle_development	ENSGALG00010022819	PACSIN3	2.087276167	0.108331339	19.2675194	1.00646E-82	2.71164E-81	positive
PRJNA729271	stage_3W_vs_E10	ordinary_muscle_development	ENSGALG00010022820	SHTN1	-1.382250449	0.175203202	-7.889413154	3.03611E-15	9.29427E-15	negative
PRJNA729271	stage_3W_vs_E10	ordinary_muscle_development	ENSGALG00010022821	ENSGALG00010022821	1.431876405	1.398546812	1.023831589			positive
PRJNA729271	stage_3W_vs_E10	ordinary_muscle_development	ENSGALG00010022823	IGHMBP2	0.068918576	0.11494924	0.599556604	0.548801776	0.585994972	positive
PRJNA729271	stage_3W_vs_E10	ordinary_muscle_development	ENSGALG00010022824	PUS1	-1.436579098	0.085983904	-16.70753508	1.15513E-62	1.85012E-61	negative
PRJNA729271	stage_3W_vs_E10	ordinary_muscle_development	ENSGALG00010022825	SS18L1	1.8150616	0.258780784	7.013896367	2.31771E-12	6.12493E-12	positive
PRJNA729271	stage_3W_vs_E10	ordinary_muscle_development	ENSGALG00010022826	SNORC	-5.159532927	0.537558299	-9.598089989	8.14395E-22	3.3536E-21	negative
PRJNA729271	stage_3W_vs_E10	ordinary_muscle_development	ENSGALG00010022827	CMTR2	-0.084876756	0.09389577	-0.903946543	0.366023748	0.404692161	negative
PRJNA729271	stage_3W_vs_E10	ordinary_muscle_development	ENSGALG00010022828	PRKG1	-2.198828311	0.173008792	-12.70934435	5.24725E-37	3.81439E-36	negative
PRJNA729271	stage_3W_vs_E10	ordinary_muscle_development	ENSGALG00010022830	MTG2	-0.581120472	0.130540178	-4.451659862	8.52091E-06	1.51213E-05	negative
PRJNA729271	stage_3W_vs_E10	ordinary_muscle_development	ENSGALG00010022831	LRRC8C	0.765686128	0.088806388	8.621971331	6.58112E-18	2.312E-17	positive
PRJNA729271	stage_3W_vs_E10	ordinary_muscle_development	ENSGALG00010022833	HRH3	-0.856334001	0.545234747	-1.570578553	0.116280575	0.13928493	negative
PRJNA729271	stage_3W_vs_E10	ordinary_muscle_development	ENSGALG00010022834	CHRNA7	-4.0137805	0.284593342	-14.10356433	3.61063E-45	3.47286E-44	negative
PRJNA729271	stage_3W_vs_E10	ordinary_muscle_development	ENSGALG00010022837	ITGB3	-1.017172587	0.150667248	-6.75111945	1.46709E-11	3.71643E-11	negative
PRJNA729271	stage_3W_vs_E10	ordinary_muscle_development	ENSGALG00010022838	ARFGAP2	0.495279277	0.07918337	6.254839585	3.97925E-10	9.2986E-10	positive
PRJNA729271	stage_3W_vs_E10	ordinary_muscle_development	ENSGALG00010022839	SYT12	-1.683166051	0.30876091	-5.451357342	4.99868E-08	1.03096E-07	negative
PRJNA729271	stage_3W_vs_E10	ordinary_muscle_development	ENSGALG00010022840	MARVELD1	0.069199013	0.191776188	0.360832144	0.718224932	0.746712838	positive
PRJNA729271	stage_3W_vs_E10	ordinary_muscle_development	ENSGALG00010022841	ENSGALG00010022841	-8.385986527	0.70906416	-11.8268374	2.83628E-32	1.78128E-31	negative
PRJNA729271	stage_3W_vs_E10	ordinary_muscle_development	ENSGALG00010022842	PHC1	-1.195990184	0.143542236	-8.331974024	7.95054E-17	2.64198E-16	negative
PRJNA729271	stage_3W_vs_E10	ordinary_muscle_development	ENSGALG00010022845	HYDIN	2.259750633	0.32133198	7.032448595	2.0294E-12	5.38758E-12	positive

PRJNA729271	stage_3W_vs_E10	ordinary_muscle_development	ENSGALG00010022848	SGIP1	-2.382203767	0.855011171	-2.786166832	0.005333541	0.007459444	negative
PRJNA729271	stage_3W_vs_E10	ordinary_muscle_development	ENSGALG00010022850	ULK1	3.1156092	0.249487001	12.48806224	8.67406E-36	6.10935E-35	positive
PRJNA729271	stage_3W_vs_E10	ordinary_muscle_development	ENSGALG00010022858	HERC4	0.405643455	0.127222302	3.18846183	0.001430319	0.002113402	positive
PRJNA729271	stage_3W_vs_E10	ordinary_muscle_development	ENSGALG00010022860	MOB3C	-1.269502991	0.132136157	-9.607536792	7.43052E-22	3.06242E-21	negative
PRJNA729271	stage_3W_vs_E10	ordinary_muscle_development	ENSGALG00010022862	ACADS	0.244943515	0.123146338	1.989044248	0.046696319	0.058852547	positive
PRJNA729271	stage_3W_vs_E10	ordinary_muscle_development	ENSGALG00010022863	FAM81A	-1.375812788	0.359206214	-3.830147519	0.000128066	0.000207434	negative
PRJNA729271	stage_3W_vs_E10	ordinary_muscle_development	ENSGALG00010022864	CSTPP1	-1.762467731	0.215885438	-8.163902784	3.2437E-16	1.04298E-15	negative
PRJNA729271	stage_3W_vs_E10	ordinary_muscle_development	ENSGALG00010022866	KLHL11	-0.371405368	0.098957228	-3.753190917	0.000174598	0.000279522	negative
PRJNA729271	stage_3W_vs_E10	ordinary_muscle_development	ENSGALG00010022867	ATPAF1	-0.429739931	0.134082104	-3.205050625	0.001350387	0.002000599	negative
PRJNA729271	stage_3W_vs_E10	ordinary_muscle_development	ENSGALG00010022868	VAC14	-0.73582598	0.102703968	-7.164533085	7.80521E-13	2.11747E-12	negative
PRJNA729271	stage_3W_vs_E10	ordinary_muscle_development	ENSGALG00010022872	KRT13	-2.652772312	0.4795605	-5.531673918	3.17189E-08	6.61819E-08	negative
PRJNA729271	stage_3W_vs_E10	ordinary_muscle_development	ENSGALG00010022873	NGEF	-0.049959557	0.371903141	-0.134334861	0.893137788	0.905380253	negative
PRJNA729271	stage_3W_vs_E10	ordinary_muscle_development	ENSGALG00010022874	EFCAB14	0.977084228	0.07609048	12.84108385	9.65137E-38	7.20003E-37	positive
PRJNA729271	stage_3W_vs_E10	ordinary_muscle_development	ENSGALG00010022875	SPON1	-0.511824826	0.244292488	-2.095131252	0.036159326	0.046211377	negative
PRJNA729271	stage_3W_vs_E10	ordinary_muscle_development	ENSGALG00010022877	KYAT3	0.062786207	0.101300634	0.619800733	0.535388986	0.573150912	positive
PRJNA729271	stage_3W_vs_E10	ordinary_muscle_development	ENSGALG00010022879	ENSGALG00010022879	2.224027266	0.277074491	8.026820718	1.00031E-15	3.13367E-15	positive
PRJNA729271	stage_3W_vs_E10	ordinary_muscle_development	ENSGALG00010022881	CCNB1	-2.938909038	0.212887717	-13.80497233	2.37864E-43	2.14685E-42	negative
PRJNA729271	stage_3W_vs_E10	ordinary_muscle_development	ENSGALG00010022885	M6PR	0.323843903	0.099235646	3.263382838	0.001100907	0.001643932	positive
PRJNA729271	stage_3W_vs_E10	ordinary_muscle_development	ENSGALG00010022886	PLA2G15	2.613449033	0.281136556	9.296012828	1.45811E-20	5.69686E-20	positive
PRJNA729271	stage_3W_vs_E10	ordinary_muscle_development	ENSGALG00010022888	OSBPL2	1.115786129	0.090454128	12.33538094	5.8412E-35	4.00701E-34	positive
PRJNA729271	stage_3W_vs_E10	ordinary_muscle_development	ENSGALG00010022890	MYPN	5.380334815	0.166074211	32.39717228	3.0083E-230	7.1484E-228	positive
PRJNA729271	stage_3W_vs_E10	ordinary_muscle_development	ENSGALG00010022891	NTM	-1.376638986	0.228991992	-6.011734175	1.83549E-09	4.1134E-09	negative
PRJNA729271	stage_3W_vs_E10	ordinary_muscle_development	ENSGALG00010022900	MMP17	0.03666346	0.199641861	0.183646152	0.854291055	0.870873398	positive
PRJNA729271	stage_3W_vs_E10	ordinary_muscle_development	ENSGALG00010022911	SMPD3	-4.700440381	0.362079793	-12.98178047	1.5523E-38	1.19303E-37	negative
PRJNA729271	stage_3W_vs_E10	ordinary_muscle_development	ENSGALG00010022917	CYP4B7	-1.243761663	0.485788745	-2.56029329	0.010458386	0.014178293	negative
PRJNA729271	stage_3W_vs_E10	ordinary_muscle_development	ENSGALG00010022919	ACLY	-2.198408517	0.125862343	-17.46676943	2.5662E-68	4.75058E-67	negative
PRJNA729271	stage_3W_vs_E10	ordinary_muscle_development	ENSGALG00010022921	ENSGALG00010022921	0.566676631	0.19896283	2.848153244	0.004397374	0.006202194	positive
PRJNA729271	stage_3W_vs_E10	ordinary_muscle_development	ENSGALG00010022922	ATOH7	0.391456388	0.426962999	0.916839139	0.35922694	0.39799698	positive
PRJNA729271	stage_3W_vs_E10	ordinary_muscle_development	ENSGALG00010022923	CD63	1.794165776	0.126386357	14.19588178	9.71566E-46	9.52187E-45	positive
PRJNA729271	stage_3W_vs_E10	ordinary_muscle_development	ENSGALG00010022928	RNF111	0.162795013	0.0832022	1.956619094	0.050392268	0.063213841	positive
PRJNA729271	stage_3W_vs_E10	ordinary_muscle_development	ENSGALG00010022929	LRP4	-0.694902404	0.117334732	-5.92239307	3.1729E-09	7.00765E-09	negative
PRJNA729271	stage_3W_vs_E10	ordinary_muscle_development	ENSGALG00010022930	GTF2B	-0.068505277	0.117462619	-0.583209173	0.559752497	0.596676897	negative
PRJNA729271	stage_3W_vs_E10	ordinary_muscle_development	ENSGALG00010022931	RDH5	0.963782165	0.121315804	7.944407378	1.95121E-15	6.02147E-15	positive
PRJNA729271	stage_3W_vs_E10	ordinary_muscle_development	ENSGALG00010022932	OPCML	-3.170471776	0.280369828	-11.3081775	1.19547E-29	6.78478E-29	negative
PRJNA729271	stage_3W_vs_E10	ordinary_muscle_development	ENSGALG00010022933	UNC119B	-0.883346714	0.07678502	-11.50415426	1.25716E-30	7.38649E-30	negative
PRJNA729271	stage_3W_vs_E10	ordinary_muscle_development	ENSGALG00010022937	BLOC1S1	0.709274723	0.161305636	4.397085818	1.09714E-05	1.93162E-05	positive
PRJNA729271	stage_3W_vs_E10	ordinary_muscle_development	ENSGALG00010022942	NEU2	-1.47873432	0.499758494	-2.958897825	0.003087414	0.00442389	negative
PRJNA729271	stage_3W_vs_E10	ordinary_muscle_development	ENSGALG00010022945	KCNA4	-5.006724442	0.463905178	-10.79255993	3.73245E-27	1.91783E-26	negative
PRJNA729271	stage_3W_vs_E10	ordinary_muscle_development	ENSGALG00010022946	KRT17	-5.463495928	0.514405616	-10.6209881	2.38028E-26	1.18158E-25	negative
PRJNA729271	stage_3W_vs_E10	ordinary_muscle_development	ENSGALG00010022949	INSC	-3.703109979	0.280351894	-13.20879245	7.80667E-40	6.28305E-39	negative
PRJNA729271	stage_3W_vs_E10	ordinary_muscle_development	ENSGALG00010022950	ITGA7	3.607074661	0.154244319	23.38546203	6.0084E-121	3.6137E-119	positive
PRJNA729271	stage_3W_vs_E10	ordinary_muscle_development	ENSGALG00010022953	ENSGALG00010022953	-1.337455936	1.166613399	-1.146443147	0.251611839	0.286858078	negative
PRJNA729271	stage_3W_vs_E10	ordinary_muscle_development	ENSGALG00010022954	ADRM1	0.767156955	0.143274418	5.35445871	8.58129E-08	1.74601E-07	positive
PRJNA729271	stage_3W_vs_E10	ordinary_muscle_development	ENSGALG00010022956	PBLD	0.857448051	0.121407789	7.062545661	1.6348E-12	4.35835E-12	positive
PRJNA729271	stage_3W_vs_E10	ordinary_muscle_development	ENSGALG00010022957	SLTM	0.082032564	0.142813204	0.574404617	0.565694029	0.602257433	positive
PRJNA729271	stage_3W_vs_E10	ordinary_muscle_development	ENSGALG00010022958	SFSWAP	0.128461755	0.097457032	1.318137357	0.187457671	0.217889898	positive
PRJNA729271	stage_3W_vs_E10	ordinary_muscle_development	ENSGALG00010022959	ADAMTS8	0.056239057	0.264550273	0.212583628	0.831651743	0.851206893	positive
PRJNA729271	stage_3W_vs_E10	ordinary_muscle_development	ENSGALG00010022967	ADAMTS15	-1.387449728	0.163481438	-8.486894575	2.12232E-17	7.24689E-17	negative
PRJNA729271	stage_3W_vs_E10	ordinary_muscle_development	ENSGALG00010022969	TTN	4.299873731	0.575497524	7.471576417	7.92397E-14	2.26277E-13	positive
PRJNA729271	stage_3W_vs_E10	ordinary_muscle_development	ENSGALG00010022971	CKAP5	-1.307589249	0.058572877	-22.32414237	2.1538E-110	1.0993E-108	negative
PRJNA729271	stage_3W_vs_E10	ordinary_muscle_development	ENSGALG00010022972	ENSGALG00010022972	-2.957259925	1.107157547	-2.67103804			negative
PRJNA729271	stage_3W_vs_E10	ordinary_muscle_development	ENSGALG00010022974	INPP5D	2.155691771	0.154079291	13.99079497	1.77418E-44	1.66236E-43	positive
PRJNA729271	stage_3W_vs_E10	ordinary_muscle_development	ENSGALG00010022976	MINDY2	-0.120116607	0.065914992	-1.82229571	0.068410129	0.084391899	negative
PRJNA729271	stage_3W_vs_E10	ordinary_muscle_development	ENSGALG00010022978	ARL14EP	0.009307028	0.086248419	0.107909544	0.914067447	0.924205332	positive
PRJNA729271	stage_3W_vs_E10	ordinary_muscle_development	ENSGALG00010022979	LAMA5	-0.634157747	0.149592016	-4.239248608	2.24269E-05	3.85941E-05	negative
PRJNA729271	stage_3W_vs_E10	ordinary_muscle_development	ENSGALG00010022981	HNRNPH3	-1.830448377	0.129954537	-14.08529796	4.67682E-45	4.4835E-44	negative
PRJNA729271	stage_3W_vs_E10	ordinary_muscle_development	ENSGALG00010022983	PKN2	0.245527751	0.061005766	4.024664698	5.70566E-05	9.51709E-05	positive
PRJNA729271	stage_3W_vs_E10	ordinary_muscle_development	ENSGALG00010022986	ALG9	-0.541445574	0.106407341	-5.088423132	3.61053E-07	7.0399E-07	negative
PRJNA729271	stage_3W_vs_E10	ordinary_muscle_development	ENSGALG00010022987	F2	0.845762456	1.417458237	0.596675396			positive
PRJNA729271	stage_3W_vs_E10	ordinary_muscle_development	ENSGALG00010022990	SLC25A22	-1.393181061	0.101651887	-13.70541263	9.42301E-43	8.32845E-42	negative
PRJNA729271	stage_3W_vs_E10	ordinary_muscle_development	ENSGALG00010022991	TAL1	1.50264079	0.17484626	8.594068822	8.39427E-18	2.93122E-17	positive
PRJNA729271	stage_3W_vs_E10	ordinary_muscle_development	ENSGALG00010022992	SLC7A6	0.321206949	0.106494218	3.016191437	0.002559716	0.003692584	positive
PRJNA729271	stage_3W_vs_E10	ordinary_muscle_development	ENSGALG00010022994	LIPC	-3.245039694	0.904764664	-3.58661188	0.000335002	0.000524164	negative
PRJNA729271	stage_3W_vs_E10	ordinary_muscle_development	ENSGALG00010022997	ARHGAP1	-1.42484224	0.096197624	-14.81161566	1.23242E-49	1.37521E-48	negative
PRJNA729271	stage_3W_vs_E10	ordinary_muscle_development	ENSGALG00010022999	ENSGALG00010022999	2.085952632	0.476503244	4.37762525	1.19979E-05	2.10698E-05	positive
PRJNA729271	stage_3W_vs_E10	ordinary_muscle_development	ENSGALG00010023002	ATG16L1	1.1108014	0.133679711	8.309423985	9.61677E-17	3.18399E-16	positive
PRJNA729271	stage_3W_vs_E10	ordinary_muscle_development	ENSGALG00010023003	ENSGALG00010023003	-4.920702502	0.63249858	-7.77978427	7.26483E-15	2.18337E-14	negative
PRJNA729271	stage_3W_vs_E10	ordinary_muscle_development	ENSGALG00010023005	IRF7	2.454474495	0.347433126	7.064595494	1.61085E-12	4.29845E-12	positive
PRJNA729271	stage_3W_vs_E10	ordinary_muscle_development	ENSGALG00010023007	LMO4	0.036930173	0.142254525	0.259606313	0.795167465	0.817849458	positive
PRJNA729271	stage_3W_vs_E10	ordinary_muscle_development	ENSGALG00010023008	CDHR5	0.638393799	0.846455754	0.754196301	0.450731358	0.489499591	positive
PRJNA729271	stage_3W_vs_E10	ordinary_muscle_development	ENSGALG00010023012	ENSGALG00010023012	1.065694475	0.506857993	2.1025504	0.03550509	0.045403288	positive
PRJNA729271	stage_3W_vs_E10	ordinary_muscle_development	ENSGALG00010023013	ENSGALG00010023013	1.098809415	0.407865435	2.694048869	0.007058981	0.009748469	positive
PRJNA729271	stage_3W_vs_E10	ordinary_muscle_development	ENSGALG00010023015	METTL2B	-0.276726907	0.102317044	-2.70460226	0.006838622	0.009456766	negative
PRJNA729271	stage_3W_vs_E10	ordinary_muscle_development	ENSGALG00010023017	BUB1B	-3.427434062	0.20472874	-16.74134299	6.54937E-63	1.06189E-61	negative
PRJNA729271	stage_3W_vs_E10	ordinary_muscle_development	ENSGALG00010023019	CABP1	0.030818613	0.327290465	0.094162881	0.924979771	0.933610596	positive
PRJNA729271	stage_3W_vs_E10	ordinary_muscle_development	ENSGALG00010023024	PRMT7	-0.021886826	0.112543542	-0.194474292	0.845804523	0.863619087	negative
PRJNA729271	stage_3W_vs_E10	ordinary_muscle_development	ENSGALG00010023026	PHRF1	0.026548138	0.09138785	0.290499642	0.771434025	0.796093991	positive
PRJNA729271	stage_3W_vs_E10	ordinary_muscle_development	ENSGALG00010023028	TMEM61	-1.923886048	0.312921106	-6.148150476	7.83916E-10	1.79906E-09	negative
PRJNA729271	stage_3W_vs_E10	ordinary_muscle_development	ENSGALG00010023030	HS2ST1	-0.839495498	0.085523231	-9.815993766	9.60865E-23	4.10243E-22	negative
PRJNA729271	stage_3W_vs_E10	ordinary_muscle_development	ENSGALG00010023041	CD151	1.346085142	0.11929016	11.28412552	1.57197E-29	8.88332E-29	positive
PRJNA729271	stage_3W_vs_E10	ordinary_muscle_development	ENSGALG00010023042	ENSGALG00010023042	-3.101536491	0.760384689	-4.078904447	4.52484E-05	7.60348E-05	negative
PRJNA729271	stage_3W_vs_E10	ordinary_muscle_development	ENSGALG00010023043	CNP	0.974518545	0.145613479	6.69250231	2.19386E-11	5.50936E-11	positive
PRJNA729271	stage_3W_vs_E10	ordinary_muscle_development	ENSGALG00010023046	SLC7A6OS	-0.569846816	0.074557961	-7.643004336	2.12211E-14	6.22797E-14	negative
PRJNA729271	stage_3W_vs_E10	ordinary_muscle_development	ENSGALG00010023048	ARFGAP1	-0.246919694	0.083600779	-2.953557354	0.003141342	0.00449805	negative
PRJNA729271	stage_3W_vs_E10	ordinary_muscle_development	ENSGALG00010023050	RUFY2	-0.43870832	0.094404449	-4.647114903	3.3661E-06	6.166E-06	negative
PRJNA729271	stage_3W_vs_E10	ordinary_muscle_development	ENSGALG00010023052	RAD54L	-2.23410646	0.139055936	-16.06624296	4.39956E-58	6.27674E-57	negative
PRJNA729271	stage_3W_vs_E10	ordinary_muscle_development	ENSGALG00010023053	MAN1A2	-0.787505549	0.047795195	-16.47666772	5.39792E-61	8.29723E-60	negative
PRJNA729271	stage_3W_vs_E10	ordinary_muscle_development	ENSGALG00010023054	AQP9	1.796898092	0.230983253	7.779343618	7.29018E-15	2.19053E-14	positive
PRJNA729271	stage_3W_vs_E10	ordinary_muscle_development	ENSGALG00010023056	SAG	1.659124303	0.434604963	3.817545691	0.000134786	0.000218001	positive
PRJNA729271	stage_3W_vs_E10	ordinary_muscle_development	ENSGALG00010023057	PAFAH1B2	-1.493680392	0.075479046	-19.78933847	3.67838E-87	1.12485E-85	negative
PRJNA729271	stage_3W_vs_E10	ordinary_muscle_development	ENSGALG00010023058	PPARA	0.706167231	0.204897138	3.44644751	0.000568009	0.000869316	positive
PRJNA729271	stage_3W_vs_E10	ordinary_muscle_development	ENSGALG00010023059	SELENOF	-0.325321693	0.093239753	-3.489087873	0.000484672	0.000747852	negative
PRJNA729271	stage_3W_vs_E10	ordinary_muscle_development	ENSGALG00010023060	ALDH1A2	-1.784326781	0.167986289	-10.62185965	2.35816E-26	1.1714E-25	negative
PRJNA729271	stage_3W_vs_E10	ordinary_muscle_development	ENSGALG00010023062	STX2	0.007600181	0.10348298	0.073443778	0.941452982	0.948850019	positive
PRJNA729271	stage_3W_vs_E10	ordinary_muscle_development	ENSGALG00010023063	ENSGALG00010023063	-0.635901369	0.104451752	-6.087991426	1.14336E-09	2.5948E-09	negative
PRJNA729271	stage_3W_vs_E10	ordinary_muscle_development	ENSGALG00010023064	ATG13	0.476408463	0.068703562	6.934261513	4.08349E-12	1.06247E-11	positive
PRJNA729271	stage_3W_vs_E10	ordinary_muscle_development	ENSGALG00010023065	DNA2	-3.477169268	0.235718367	-14.75137177	3.01474E-49	3.31805E-48	negative
PRJNA729271	stage_3W_vs_E10	ordinary_muscle_development	ENSGALG00010023066	TLR21	1.727017731	0.184775843	9.346555836	9.05483E-21	3.57531E-20	positive
PRJNA729271	stage_3W_vs_E10	ordinary_muscle_development	ENSGALG00010023068	CDPF1	-0.622133498	0.126813261	-4.905902522	9.29987E-07	1.76627E-06	negative
PRJNA729271	stage_3W_vs_E10	ordinary_muscle_development	ENSGALG00010023069	ENSGALG00010023069	-0.314835521	0.288164349	-1.092555419	0.274589045	0.311364172	negative
PRJNA729271	stage_3W_vs_E10	ordinary_muscle_development	ENSGALG00010023070	SIDT2	2.058519978	0.199986559	10.29329163	7.55487E-25	3.51324E-24	positive
PRJNA729271	stage_3W_vs_E10	ordinary_muscle_development	ENSGALG00010023071	POLR2M	0.865116593	0.081128894	10.66348312	1.5084E-26	7.57336E-26	positive
PRJNA729271	stage_3W_vs_E10	ordinary_muscle_development	ENSGALG00010023073	POMGNT1	-0.233947468	0.086475443	-2.705363051	0.006822978	0.009438734	negative
PRJNA729271	stage_3W_vs_E10	ordinary_muscle_development	ENSGALG00010023074	CCNT1	0.065515736	0.167254337	0.391713225	0.695270117	0.725136016	positive
PRJNA729271	stage_3W_vs_E10	ordinary_muscle_development	ENSGALG00010023075	PKDREJ	-2.107773893	0.302762824	-6.961798899	3.35955E-12	8.79636E-12	negative
PRJNA729271	stage_3W_vs_E10	ordinary_muscle_development	ENSGALG00010023076	DNAJC7	1.108621334	0.079961934	13.8643631	1.04146E-43	9.53626E-43	positive
PRJNA729271	stage_3W_vs_E10	ordinary_muscle_development	ENSGALG00010023077	KANSL2	0.005152502	0.152297038	0.033831922	0.97301118	0.976987881	positive
PRJNA729271	stage_3W_vs_E10	ordinary_muscle_development	ENSGALG00010023078	TAGLN	1.624360543	0.197382709	8.229497665	1.88E-16	6.12511E-16	positive
PRJNA729271	stage_3W_vs_E10	ordinary_muscle_development	ENSGALG00010023079	SLC25A16	1.025126873	0.09458678	10.83795081	2.2751E-27	1.1807E-26	positive
PRJNA729271	stage_3W_vs_E10	ordinary_muscle_development	ENSGALG00010023080	MCRS1	0.440261816	0.111728196	3.94047191	8.13215E-05	0.000133661	positive
PRJNA729271	stage_3W_vs_E10	ordinary_muscle_development	ENSGALG00010023081	PRLHR2	-8.093915636	0.799937998	-10.11817873	4.58875E-24	2.06437E-23	negative
PRJNA729271	stage_3W_vs_E10	ordinary_muscle_development	ENSGALG00010023083	HARBI1	0.491553637	0.154699515	3.177473687	0.001485642	0.002191348	positive
PRJNA729271	stage_3W_vs_E10	ordinary_muscle_development	ENSGALG00010023084	ENSGALG00010023084	0.838172654	0.241286379	3.47376697	0.000513207	0.000789695	positive
PRJNA729271	stage_3W_vs_E10	ordinary_muscle_development	ENSGALG00010023086	PCSK7	0.339018941	0.091472511	3.706238479	0.00021036	0.000334302	positive
PRJNA729271	stage_3W_vs_E10	ordinary_muscle_development	ENSGALG00010023087	SH3GLB1	1.720633243	0.117632628	14.62717673	1.88446E-48	2.04762E-47	positive
PRJNA729271	stage_3W_vs_E10	ordinary_muscle_development	ENSGALG00010023089	ENSGALG00010023089	1.050742865	0.759239252	1.383941705	0.166376305	0.195036356	positive
PRJNA729271	stage_3W_vs_E10	ordinary_muscle_development	ENSGALG00010023090	LRRC41	-0.031869389	0.091843542	-0.34699652	0.728593933	0.75646222	negative
PRJNA729271	stage_3W_vs_E10	ordinary_muscle_development	ENSGALG00010023091	ENSGALG00010023091	0.258865896	0.131797713	1.964115228	0.049516731	0.062190886	positive
PRJNA729271	stage_3W_vs_E10	ordinary_muscle_development	ENSGALG00010023093	POP5	-0.559612624	0.131270671	-4.263043846	2.01661E-05	3.48358E-05	negative
PRJNA729271	stage_3W_vs_E10	ordinary_muscle_development	ENSGALG00010023094	CCDC32	-0.278459917	0.102195286	-2.724782407	0.006434388	0.008919045	negative
PRJNA729271	stage_3W_vs_E10	ordinary_muscle_development	ENSGALG00010023096	SPATS2	0.231847689	0.170697868	1.358234241	0.174389369	0.203639241	positive
PRJNA729271	stage_3W_vs_E10	ordinary_muscle_development	ENSGALG00010023097	LURAP1	1.025346585	0.211493354	4.848126737	1.24633E-06	2.34526E-06	positive
PRJNA729271	stage_3W_vs_E10	ordinary_muscle_development	ENSGALG00010023101	CLCA2	0.576402077	1.213787843	0.474878769	0.634873385	0.668396986	positive
PRJNA729271	stage_3W_vs_E10	ordinary_muscle_development	ENSGALG00010023102	RNF10	0.50361446	0.101514601	4.961005176	7.01293E-07	1.34088E-06	positive
PRJNA729271	stage_3W_vs_E10	ordinary_muscle_development	ENSGALG00010023103	DGKD	0.668458964	0.125147674	5.341361486	9.22511E-08	1.87333E-07	positive
PRJNA729271	stage_3W_vs_E10	ordinary_muscle_development	ENSGALG00010023104	ENSGALG00010023104	1.275684344	0.157280695	8.110876819	5.02558E-16	1.60171E-15	positive
PRJNA729271	stage_3W_vs_E10	ordinary_muscle_development	ENSGALG00010023107	ZC3HAV1	1.0551302	0.29150392	3.619608954	0.000295049	0.000463652	positive
PRJNA729271	stage_3W_vs_E10	ordinary_muscle_development	ENSGALG00010023108	TET1	-0.309650703	0.061702995	-5.018406392	5.21019E-07	1.00428E-06	negative
PRJNA729271	stage_3W_vs_E10	ordinary_muscle_development	ENSGALG00010023110	LIMA1	-1.329660827	0.154921887	-8.582782294	9.26055E-18	3.22983E-17	negative
PRJNA729271	stage_3W_vs_E10	ordinary_muscle_development	ENSGALG00010023112	PCMT1L	2.911713035	0.129862043	22.42158655	2.4239E-111	1.2593E-109	positive
PRJNA729271	stage_3W_vs_E10	ordinary_muscle_development	ENSGALG00010023113	MYZAP	0.246724036	0.131615722	1.874578753	0.060850698	0.075606967	positive

PRJNA729271	stage_3W_vs_E10	ordinary_muscle_development	ENSGALG00010023114	UQCRHL	1.695011915	0.16736741	10.12749085	4.17218E-24	1.88105E-23	positive
PRJNA729271	stage_3W_vs_E10	ordinary_muscle_development	ENSGALG00010023115	RPLP2	0.577492575	0.136335192	4.235829113	2.2771E-05	3.91583E-05	positive
PRJNA729271	stage_3W_vs_E10	ordinary_muscle_development	ENSGALG00010023116	PROC	3.616808432	1.018925525	3.549629826	0.000385773	0.000600105	positive
PRJNA729271	stage_3W_vs_E10	ordinary_muscle_development	ENSGALG00010023117	RPUSD2	-0.334508876	0.103845501	-3.221216845	0.001276475	0.001894972	negative
PRJNA729271	stage_3W_vs_E10	ordinary_muscle_development	ENSGALG00010023118	PIDD1	0.854542073	0.135152548	6.32279661	2.56871E-10	6.06903E-10	positive
PRJNA729271	stage_3W_vs_E10	ordinary_muscle_development	ENSGALG00010023119	NDRG4	-1.355684836	0.176218389	-7.69320866	1.4349E-14	4.24641E-14	negative
PRJNA729271	stage_3W_vs_E10	ordinary_muscle_development	ENSGALG00010023120	CHRM4	-0.799424103	0.248215799	-3.22068179	0.001278861	0.001898125	negative
PRJNA729271	stage_3W_vs_E10	ordinary_muscle_development	ENSGALG00010023122	ENSGALG00010023122	2.044878085	0.663279192	3.082982417	0.002049373	0.002980102	positive
PRJNA729271	stage_3W_vs_E10	ordinary_muscle_development	ENSGALG00010023123	RNF214	0.178494323	0.144488467	1.235353425	0.216698999	0.24944822	positive
PRJNA729271	stage_3W_vs_E10	ordinary_muscle_development	ENSGALG00010023124	NSUN4	1.253633879	0.133830133	9.367351398	7.43754E-21	2.94636E-20	positive
PRJNA729271	stage_3W_vs_E10	ordinary_muscle_development	ENSGALG00010023125	COL20A1	0.058844468	0.282866876	0.208028839	0.835206449	0.854173908	positive
PRJNA729271	stage_3W_vs_E10	ordinary_muscle_development	ENSGALG00010023126	CGNL1	0.282169816	0.137410684	2.053478002	0.040026241	0.050852867	positive
PRJNA729271	stage_3W_vs_E10	ordinary_muscle_development	ENSGALG00010023127	CERS5	-1.032098646	0.097992042	-10.53247417	6.12043E-26	2.98003E-25	negative
PRJNA729271	stage_3W_vs_E10	ordinary_muscle_development	ENSGALG00010023128	IWS1	0.231484859	0.137162854	1.687664348	0.091475679	0.111217914	positive
PRJNA729271	stage_3W_vs_E10	ordinary_muscle_development	ENSGALG00010023130	DYNLT5	-1.943604471	0.618238879	-3.143775874	0.001667831	0.002450356	negative
PRJNA729271	stage_3W_vs_E10	ordinary_muscle_development	ENSGALG00010023131	SOX6	0.808530396	0.137047737	5.899626037	3.64326E-09	8.03519E-09	positive
PRJNA729271	stage_3W_vs_E10	ordinary_muscle_development	ENSGALG00010023134	KNL1	-3.709964143	0.219792647	-16.87938239	6.38089E-64	1.06189E-62	negative
PRJNA729271	stage_3W_vs_E10	ordinary_muscle_development	ENSGALG00010023135	GINS3	-1.664292049	0.071657566	-23.22562917	2.5088E-119	1.4783E-117	negative
PRJNA729271	stage_3W_vs_E10	ordinary_muscle_development	ENSGALG00010023136	MDK	-5.860656485	0.415897624	-14.09158442	4.27853E-45	4.10983E-44	negative
PRJNA729271	stage_3W_vs_E10	ordinary_muscle_development	ENSGALG00010023137	ETV6	-0.100062058	0.113888355	-0.878597796	0.379619391	0.418447382	negative
PRJNA729271	stage_3W_vs_E10	ordinary_muscle_development	ENSGALG00010023140	CCAR1	-0.486288722	0.083816248	-5.801843089	6.55899E-09	1.42945E-08	negative
PRJNA729271	stage_3W_vs_E10	ordinary_muscle_development	ENSGALG00010023142	TLK2	1.245999392	0.167935827	7.419497169	1.17566E-13	3.32446E-13	positive
PRJNA729271	stage_3W_vs_E10	ordinary_muscle_development	ENSGALG00010023144	C11orf58	0.017885768	0.106395668	0.168106166	0.866499759	0.881892572	positive
PRJNA729271	stage_3W_vs_E10	ordinary_muscle_development	ENSGALG00010023147	RAD51	-4.167577723	0.204480987	-20.38124804	2.4532E-92	8.42633E-91	negative
PRJNA729271	stage_3W_vs_E10	ordinary_muscle_development	ENSGALG00010023149	ADGRD1	-0.076395099	0.184510984	-0.414040929	0.678844132	0.709896598	negative
PRJNA729271	stage_3W_vs_E10	ordinary_muscle_development	ENSGALG00010023151	ENSGALG00010023151	-0.050708239	0.490978377	-0.103279983	0.917740762	0.927337214	negative
PRJNA729271	stage_3W_vs_E10	ordinary_muscle_development	ENSGALG00010023155	CFAP263	-1.101818651	0.355180972	-3.102133099	0.001921316	0.002800067	negative
PRJNA729271	stage_3W_vs_E10	ordinary_muscle_development	ENSGALG00010023157	STOX1	-3.228653102	0.397104208	-8.13049329	4.27547E-16	1.36564E-15	negative
PRJNA729271	stage_3W_vs_E10	ordinary_muscle_development	ENSGALG00010023158	ENSGALG00010023158	3.635671662	0.446925955	8.13484117	4.12479E-16	1.31839E-15	positive
PRJNA729271	stage_3W_vs_E10	ordinary_muscle_development	ENSGALG00010023159	DAW1	0.351041729	0.489165775	0.717633463	0.472983319	0.511748392	positive
PRJNA729271	stage_3W_vs_E10	ordinary_muscle_development	ENSGALG00010023160	ENSGALG00010023160	-1.299412554	0.11099737	-11.70669675	1.17779E-31	7.19735E-31	negative
PRJNA729271	stage_3W_vs_E10	ordinary_muscle_development	ENSGALG00010023161	ODF2L	-0.766055339	0.409001237	-1.872990276	0.061069728	0.075853102	negative
PRJNA729271	stage_3W_vs_E10	ordinary_muscle_development	ENSGALG00010023162	CHRNA4	-2.689822501	0.287456441	-9.357322076	8.17832E-21	3.23539E-20	negative
PRJNA729271	stage_3W_vs_E10	ordinary_muscle_development	ENSGALG00010023163	CRTAC1	-1.735608854	0.288611763	-6.013645577	1.81397E-09	4.06831E-09	negative
PRJNA729271	stage_3W_vs_E10	ordinary_muscle_development	ENSGALG00010023164	NKIRAS2	0.728567047	0.132807019	5.485907696	4.11352E-08	8.53254E-08	positive
PRJNA729271	stage_3W_vs_E10	ordinary_muscle_development	ENSGALG00010023165	CSNK2A2	0.466849657	0.129420655	3.607226808	0.000309487	0.000485394	positive
PRJNA729271	stage_3W_vs_E10	ordinary_muscle_development	ENSGALG00010023166	KDM2A	-0.208467819	0.113961649	-1.829280468	0.067357603	0.083192609	negative
PRJNA729271	stage_3W_vs_E10	ordinary_muscle_development	ENSGALG00010023167	GPD1	11.53424578	0.209875543	54.95755065	0	0	positive
PRJNA729271	stage_3W_vs_E10	ordinary_muscle_development	ENSGALG00010023168	SPHKAP	-3.719062624	0.410604846	-9.057522481	1.33447E-19	5.01898E-19	negative
PRJNA729271	stage_3W_vs_E10	ordinary_muscle_development	ENSGALG00010023169	RAN	-1.03217341	0.097004461	-10.64047367	1.93145E-26	9.6373E-26	negative
PRJNA729271	stage_3W_vs_E10	ordinary_muscle_development	ENSGALG00010023170	TCF12	-1.358011567	0.064943052	-20.91080597	4.2696E-97	1.63292E-95	negative
PRJNA729271	stage_3W_vs_E10	ordinary_muscle_development	ENSGALG00010023171	ENSGALG00010023171	-0.908616169	0.115001418	-7.900912752	2.76868E-15	8.48997E-15	negative
PRJNA729271	stage_3W_vs_E10	ordinary_muscle_development	ENSGALG00010023172	DHX58	3.62453921	0.322226468	11.24842175	2.35773E-29	1.3236E-28	positive
PRJNA729271	stage_3W_vs_E10	ordinary_muscle_development	ENSGALG00010023174	KCNQ2	-2.807543511	0.372861943	-7.529713239	5.08519E-14	1.46686E-13	negative
PRJNA729271	stage_3W_vs_E10	ordinary_muscle_development	ENSGALG00010023175	GRK2	0.467644953	0.137003115	3.413389204	0.000641602	0.000978332	positive
PRJNA729271	stage_3W_vs_E10	ordinary_muscle_development	ENSGALG00010023177	LZTS2	0.575586326	0.170813871	3.369669712	0.000752583	0.001139765	positive
PRJNA729271	stage_3W_vs_E10	ordinary_muscle_development	ENSGALG00010023178	CFAP20	-0.405837458	0.098664757	-4.113297115	3.90048E-05	6.58561E-05	negative
PRJNA729271	stage_3W_vs_E10	ordinary_muscle_development	ENSGALG00010023179	KAT2A	-0.721659927	0.130885128	-5.513689273	3.51389E-08	7.31387E-08	negative
PRJNA729271	stage_3W_vs_E10	ordinary_muscle_development	ENSGALG00010023180	EEF1A2	6.84114131	0.121645505	56.23834022	0	0	positive
PRJNA729271	stage_3W_vs_E10	ordinary_muscle_development	ENSGALG00010023181	PLEKHA7	0.139909616	0.165108315	0.847380799	0.396782903	0.435610708	positive
PRJNA729271	stage_3W_vs_E10	ordinary_muscle_development	ENSGALG00010023182	EPHB6	-2.234498367	0.144949277	-15.41572625	1.2832E-53	1.63732E-52	negative
PRJNA729271	stage_3W_vs_E10	ordinary_muscle_development	ENSGALG00010023183	ENSGALG00010023183	-1.139129535	0.526433356	-2.163862761	0.030474884	0.039266066	negative
PRJNA729271	stage_3W_vs_E10	ordinary_muscle_development	ENSGALG00010023184	SMARCD1	-1.168246319	0.055570992	-21.02259268	4.07552E-98	1.59661E-96	negative
PRJNA729271	stage_3W_vs_E10	ordinary_muscle_development	ENSGALG00010023185	FBXW4	2.334851838	0.116332511	20.0705016	1.33649E-89	4.33387E-88	positive
PRJNA729271	stage_3W_vs_E10	ordinary_muscle_development	ENSGALG00010023186	MIER1	-0.263465035	0.099249983	-2.654560015	0.007941191	0.010915843	negative
PRJNA729271	stage_3W_vs_E10	ordinary_muscle_development	ENSGALG00010023187	ENSGALG00010023187	4.376026274	0.230011882	19.02521833	1.05447E-80	2.70046E-79	positive
PRJNA729271	stage_3W_vs_E10	ordinary_muscle_development	ENSGALG00010023188	KIF1BP	0.384384101	0.057868166	6.64241029	3.08594E-11	7.68834E-11	positive
PRJNA729271	stage_3W_vs_E10	ordinary_muscle_development	ENSGALG00010023189	RMDN3	-0.445710973	0.085195371	-5.23163368	1.68018E-07	3.34583E-07	negative
PRJNA729271	stage_3W_vs_E10	ordinary_muscle_development	ENSGALG00010023190	SF3B3	-1.193405327	0.056478085	-21.13041408	4.17867E-99	1.67783E-97	negative
PRJNA729271	stage_3W_vs_E10	ordinary_muscle_development	ENSGALG00010023193	PPDPF	4.709625117	0.312609601	15.06551657	2.73023E-51	3.22532E-50	positive
PRJNA729271	stage_3W_vs_E10	ordinary_muscle_development	ENSGALG00010023194	MRC2	-0.261945396	0.193297622	-1.355140296	0.175372862	0.204672274	negative
PRJNA729271	stage_3W_vs_E10	ordinary_muscle_development	ENSGALG00010023195	ENSGALG00010023195	-5.153968867	0.205236775	-25.11230678	3.6496E-139	2.9227E-137	negative
PRJNA729271	stage_3W_vs_E10	ordinary_muscle_development	ENSGALG00010023196	DGKZ	1.011058099	0.127140879	7.952266048	1.8313E-15	5.65986E-15	positive
PRJNA729271	stage_3W_vs_E10	ordinary_muscle_development	ENSGALG00010023198	PTK6	-2.106607693	0.581094838	-3.625239039	0.000288694	0.000454061	negative
PRJNA729271	stage_3W_vs_E10	ordinary_muscle_development	ENSGALG00010023199	RPS13	0.592768228	0.196709258	3.013423127	0.002583185	0.003725327	positive
PRJNA729271	stage_3W_vs_E10	ordinary_muscle_development	ENSGALG00010023200	VPS26A	0.347669974	0.08246839	4.215796778	2.48898E-05	4.26199E-05	positive
PRJNA729271	stage_3W_vs_E10	ordinary_muscle_development	ENSGALG00010023201	ZNF280D	0.001168139	0.117966493	0.009902297	0.992099239	0.993264157	positive
PRJNA729271	stage_3W_vs_E10	ordinary_muscle_development	ENSGALG00010023202	PID1	-0.353811983	0.157241435	-2.250119272	0.024441375	0.031850915	negative
PRJNA729271	stage_3W_vs_E10	ordinary_muscle_development	ENSGALG00010023203	COG4	-0.197751996	0.063948244	-3.0923757	0.001985614	0.002890867	negative
PRJNA729271	stage_3W_vs_E10	ordinary_muscle_development	ENSGALG00010023204	SRGN	5.389011539	0.305844229	17.62011842	1.72643E-69	3.27548E-68	positive
PRJNA729271	stage_3W_vs_E10	ordinary_muscle_development	ENSGALG00010023205	DNAJC17	-1.119475253	0.116698457	-9.592888189	8.56531E-22	3.5241E-21	negative
PRJNA729271	stage_3W_vs_E10	ordinary_muscle_development	ENSGALG00010023206	HSPB9	3.240922619	0.313756493	10.3294201	5.18732E-25	2.43098E-24	positive
PRJNA729271	stage_3W_vs_E10	ordinary_muscle_development	ENSGALG00010023207	MNS1	-0.980667149	0.162060739	-6.051232129	1.43742E-09	3.24438E-09	negative
PRJNA729271	stage_3W_vs_E10	ordinary_muscle_development	ENSGALG00010023208	BACE1	-0.647091059	0.079029487	-8.187969842	2.65669E-16	8.59763E-16	negative
PRJNA729271	stage_3W_vs_E10	ordinary_muscle_development	ENSGALG00010023209	PIK3C2A	-0.489890478	0.106972689	-4.579584592	4.659E-06	8.43626E-06	negative
PRJNA729271	stage_3W_vs_E10	ordinary_muscle_development	ENSGALG00010023210	ZNHIT6	-0.066245022	0.137315728	-0.482428508	0.629501573	0.663054838	negative
PRJNA729271	stage_3W_vs_E10	ordinary_muscle_development	ENSGALG00010023211	TLK1	-0.023873204	0.076245337	-0.313110345	0.75419683	0.780138661	negative
PRJNA729271	stage_3W_vs_E10	ordinary_muscle_development	ENSGALG00010023212	RIMBP2	1.594221723	0.214322804	7.438413885	1.01901E-13	2.8894E-13	positive
PRJNA729271	stage_3W_vs_E10	ordinary_muscle_development	ENSGALG00010023213	FCSK	0.605106957	0.114022747	5.306896869	1.11507E-07	2.25111E-07	positive
PRJNA729271	stage_3W_vs_E10	ordinary_muscle_development	ENSGALG00010023214	SUPV3L1	-0.120889157	0.08920691	-1.355154629	0.175368297	0.204672274	negative
PRJNA729271	stage_3W_vs_E10	ordinary_muscle_development	ENSGALG00010023215	TRIP12	0.382218504	0.056180729	6.803373815	1.02197E-11	2.61214E-11	positive
PRJNA729271	stage_3W_vs_E10	ordinary_muscle_development	ENSGALG00010023216	SLC35D1	-0.122789967	0.12302619	-0.998079897	0.318240617	0.356235829	negative
PRJNA729271	stage_3W_vs_E10	ordinary_muscle_development	ENSGALG00010023217	CREB3L1	-1.13164134	0.20765813	-5.449540261	5.05002E-08	1.0411E-07	negative
PRJNA729271	stage_3W_vs_E10	ordinary_muscle_development	ENSGALG00010023218	SSH3	0.383390333	0.130057226	2.94785875	0.003199832	0.004578183	positive
PRJNA729271	stage_3W_vs_E10	ordinary_muscle_development	ENSGALG00010023219	TEX9	-0.016870548	0.150121852	-0.112379031	0.910522881	0.92100692	negative
PRJNA729271	stage_3W_vs_E10	ordinary_muscle_development	ENSGALG00010023220	ST3GAL2	-1.158251712	0.088301871	-13.11695546	2.63302E-39	2.07874E-38	negative
PRJNA729271	stage_3W_vs_E10	ordinary_muscle_development	ENSGALG00010023221	AMY1A	2.276478379	0.13614012	16.72158345	9.12618E-63	1.47146E-61	positive
PRJNA729271	stage_3W_vs_E10	ordinary_muscle_development	ENSGALG00010023222	ENSGALG00010023222	-1.27216781	0.620917842	-2.048850467	0.040476739	0.051389186	negative
PRJNA729271	stage_3W_vs_E10	ordinary_muscle_development	ENSGALG00010023223	CEP164	-1.721013803	0.103696561	-16.59663335	7.3711E-62	1.15883E-60	negative
PRJNA729271	stage_3W_vs_E10	ordinary_muscle_development	ENSGALG00010023225	PDPR	-0.443458432	0.122796113	-3.611339339	0.00030462	0.000477967	negative
PRJNA729271	stage_3W_vs_E10	ordinary_muscle_development	ENSGALG00010023226	ENSGALG00010023226	0.296321821	0.203468697	1.456350904	0.14529564	0.171825104	positive
PRJNA729271	stage_3W_vs_E10	ordinary_muscle_development	ENSGALG00010023227	RNPC3	-0.173639937	0.124558858	-1.394039252	0.163305752	0.191653864	negative
PRJNA729271	stage_3W_vs_E10	ordinary_muscle_development	ENSGALG00010023228	GORASP2	1.135140104	0.10149784	11.18388437	4.89017E-29	2.70903E-28	positive
PRJNA729271	stage_3W_vs_E10	ordinary_muscle_development	ENSGALG00010023229	HKDC1	-0.081725834	0.399584005	-0.204527291	0.837941468	0.856616234	negative
PRJNA729271	stage_3W_vs_E10	ordinary_muscle_development	ENSGALG00010023230	ENSGALG00010023230	0.952849474	0.083211387	11.45095045	2.32581E-30	1.35501E-29	positive
PRJNA729271	stage_3W_vs_E10	ordinary_muscle_development	ENSGALG00010023231	ASIC1	-3.363576838	0.334197874	-10.06462668	7.91872E-24	3.52524E-23	negative
PRJNA729271	stage_3W_vs_E10	ordinary_muscle_development	ENSGALG00010023233	SEZ6L	-0.976877754	1.078189913	-0.906034959	0.364917349	0.403684422	negative
PRJNA729271	stage_3W_vs_E10	ordinary_muscle_development	ENSGALG00010023234	ENSGALG00010023234	-0.462182299	0.095057018	-4.862158617	1.16113E-06	2.19005E-06	negative
PRJNA729271	stage_3W_vs_E10	ordinary_muscle_development	ENSGALG00010023235	ENSGALG00010023235	0.223040248	0.353619362	0.630735396	0.528213551	0.566138378	positive
PRJNA729271	stage_3W_vs_E10	ordinary_muscle_development	ENSGALG00010023237	GAD1	-4.875203297	0.742465261	-6.566237576	5.16025E-11	1.26669E-10	negative
PRJNA729271	stage_3W_vs_E10	ordinary_muscle_development	ENSGALG00010023238	NUCB2	-0.070682896	0.078109618	-0.904919243	0.365508172	0.404214614	negative
PRJNA729271	stage_3W_vs_E10	ordinary_muscle_development	ENSGALG00010023239	RBPMS2	0.265686863	0.20787039	1.278137128	0.20120108	0.232828489	positive
PRJNA729271	stage_3W_vs_E10	ordinary_muscle_development	ENSGALG00010023240	PIWIL1	-3.087668325	0.476806968	-6.475719803	9.4361E-11	2.28075E-10	negative
PRJNA729271	stage_3W_vs_E10	ordinary_muscle_development	ENSGALG00010023243	HK1	1.462754362	0.114189684	12.80986435	1.44387E-37	1.06889E-36	positive
PRJNA729271	stage_3W_vs_E10	ordinary_muscle_development	ENSGALG00010023244	PPIB	-1.55197144	0.118881897	-13.05473319	5.97189E-39	4.65891E-38	negative
PRJNA729271	stage_3W_vs_E10	ordinary_muscle_development	ENSGALG00010023245	TANC2	-1.576327911	0.149497538	-10.54417303	5.40462E-26	2.6386E-25	negative
PRJNA729271	stage_3W_vs_E10	ordinary_muscle_development	ENSGALG00010023246	SP5	-6.430563656	0.974196043	-6.600892809	4.08689E-11	1.01022E-10	negative
PRJNA729271	stage_3W_vs_E10	ordinary_muscle_development	ENSGALG00010023248	PRDM10	-0.20378706	0.121229024	-1.681008834	0.092761199	0.112714693	negative
PRJNA729271	stage_3W_vs_E10	ordinary_muscle_development	ENSGALG00010023249	IL12RB2	2.296480846	0.419571886	5.473390669	4.41505E-08	9.13448E-08	positive
PRJNA729271	stage_3W_vs_E10	ordinary_muscle_development	ENSGALG00010023250	FZD10	-5.556774422	0.472773306	-11.75357059	6.76966E-32	4.18092E-31	negative
PRJNA729271	stage_3W_vs_E10	ordinary_muscle_development	ENSGALG00010023251	ENSGALG00010023251	10.19949672	0.917024928	11.12237673	9.76358E-29	5.34049E-28	positive
PRJNA729271	stage_3W_vs_E10	ordinary_muscle_development	ENSGALG00010023252	GLG1	-0.266009159	0.079949774	-3.327203409	0.000877223	0.001320526	negative
PRJNA729271	stage_3W_vs_E10	ordinary_muscle_development	ENSGALG00010023254	MYO3B	-1.084248884	0.31201471	-3.474992839	0.000510867	0.000786346	negative
PRJNA729271	stage_3W_vs_E10	ordinary_muscle_development	ENSGALG00010023255	SNX22	2.471387516	0.306624975	8.059968089	7.63144E-16	2.40892E-15	positive
PRJNA729271	stage_3W_vs_E10	ordinary_muscle_development	ENSGALG00010023257	PAK6	2.666956853	0.288235235	9.252709346	2.18873E-20	8.4987E-20	positive
PRJNA729271	stage_3W_vs_E10	ordinary_muscle_development	ENSGALG00010023258	ENSGALG00010023258	-0.690545172	0.367680319	-1.878112961	0.060365714	0.075030099	negative
PRJNA729271	stage_3W_vs_E10	ordinary_muscle_development	ENSGALG00010023259	TSPAN15	1.905147793	0.287936043	6.616565866	3.67639E-11	9.11084E-11	positive
PRJNA729271	stage_3W_vs_E10	ordinary_muscle_development	ENSGALG00010023260	ENSGALG00010023260	-4.863917412	0.3446497	-14.11264079	3.17463E-45	3.06367E-44	negative
PRJNA729271	stage_3W_vs_E10	ordinary_muscle_development	ENSGALG00010023261	SNX1	-0.499113043	0.096191466	-5.188745565	2.11715E-07	4.18894E-07	negative
PRJNA729271	stage_3W_vs_E10	ordinary_muscle_development	ENSGALG00010023262	POLD4	2.501079805	0.205721748	12.1575858	5.2281E-34	3.4778E-33	positive
PRJNA729271	stage_3W_vs_E10	ordinary_muscle_development	ENSGALG00010023263	SRP14	-0.794610832	0.07264018	-10.93899872	7.50239E-28	3.97468E-27	negative
PRJNA729271	stage_3W_vs_E10	ordinary_muscle_development	ENSGALG00010023264	RACGAP1	-3.341130704	0.235136513	-14.20932316	8.01969E-46	7.89174E-45	negative
PRJNA729271	stage_3W_vs_E10	ordinary_muscle_development	ENSGALG00010023265	RAB5C	-0.333527371	0.071112631	-4.690128377	2.73034E-06	5.02492E-06	negative
PRJNA729271	stage_3W_vs_E10	ordinary_muscle_development	ENSGALG00010023266	RFWD3	-1.484530704	0.144289633	-10.28854723	7.93647E-25	3.68479E-24	negative
PRJNA729271	stage_3W_vs_E10	ordinary_muscle_development	ENSGALG00010023267	DSCAML1	-3.363322026	0.844117298	-3.984424955	6.76437E-05	0.000112113	negative
PRJNA729271	stage_3W_vs_E10	ordinary_muscle_development	ENSGALG00010023268	KCNJ11	3.064002567	0.179966277	17.02542624	5.32036E-65	9.10492E-64	positive
PRJNA729271	stage_3W_vs_E10	ordinary_muscle_development	ENSGALG00010023269	OPRL1	-2.822975632	0.58666574	-4.811897884	1.49504E-06	2.7962E-06	negative
PRJNA729271	stage_3W_vs_E10	ordinary_muscle_development	ENSGALG00010023271	PATL2	-0.52315191	0.421140094	-1.242227747	0.214152545	0.246869822	negative
PRJNA729271	stage_3W_vs_E10	ordinary_muscle_development	ENSGALG00010023273	HDAC7	0.384864067	0.113932472	3.378001549	0.000730147	0.00110729	positive
PRJNA729271	stage_3W_vs_E10	ordinary_muscle_development	ENSGALG00010023274	ZBTB38	1.065081568	0.114616938	9.292532063	1.50661E-20	5.88157E-20	positive
PRJNA729271	stage_3W_vs_E10	ordinary_muscle_development	ENSGALG00010023275	TWIST3	-7.458074315	0.571196586	-13.05693083	5.80201E-39	4.53126E-38	negative

PRJNA729271	stage_3W_vs_E10	ordinary_muscle_development	ENSGALG00010023276	ABCC8	1.268775082	0.162152432	7.824582498	5.09344E-15	1.5439E-14	positive
PRJNA729271	stage_3W_vs_E10	ordinary_muscle_development	ENSGALG00010023278	ENSGALG00010023278	-0.876871997	0.307851775	-2.848357771	0.004394549	0.006198812	negative
PRJNA729271	stage_3W_vs_E10	ordinary_muscle_development	ENSGALG00010023279	FA2H	-0.541392301	0.439329055	-1.232316174	0.217831	0.250631874	negative
PRJNA729271	stage_3W_vs_E10	ordinary_muscle_development	ENSGALG00010023281	B2M	4.849562067	0.199030825	24.36588438	3.9347E-131	2.742E-129	positive
PRJNA729271	stage_3W_vs_E10	ordinary_muscle_development	ENSGALG00010023282	SERBP1	-0.605782689	0.087334141	-6.936378829	4.02278E-12	1.04705E-11	negative
PRJNA729271	stage_3W_vs_E10	ordinary_muscle_development	ENSGALG00010023285	SLC48A1	2.173386227	0.110915891	19.59490396	1.70908E-85	5.02497E-84	positive
PRJNA729271	stage_3W_vs_E10	ordinary_muscle_development	ENSGALG00010023286	HCRT	-1.904274262	0.714418626	-2.665487981	0.007687668	0.010586429	negative
PRJNA729271	stage_3W_vs_E10	ordinary_muscle_development	ENSGALG00010023287	RGS19	-0.004782234	0.199671879	-0.023950461	0.980892123	0.983742568	negative
PRJNA729271	stage_3W_vs_E10	ordinary_muscle_development	ENSGALG00010023289	NFRKB	-0.380235252	0.087333765	-4.353817253	1.33787E-05	2.34011E-05	negative
PRJNA729271	stage_3W_vs_E10	ordinary_muscle_development	ENSGALG00010023290	SLC16A14	-4.409035116	0.281752898	-15.64858834	3.39671E-55	4.50048E-54	negative
PRJNA729271	stage_3W_vs_E10	ordinary_muscle_development	ENSGALG00010023293	COL11A1	-5.816834105	0.489860685	-11.87446611	1.60656E-32	1.01735E-31	negative
PRJNA729271	stage_3W_vs_E10	ordinary_muscle_development	ENSGALG00010023295	ENSGALG00010023295	1.206713919	0.501651708	2.405481534	0.016151168	0.021454471	positive
PRJNA729271	stage_3W_vs_E10	ordinary_muscle_development	ENSGALG00010023299	PASK	-1.534552162	0.141976159	-10.80852007	3.1369E-27	1.6164E-26	negative
PRJNA729271	stage_3W_vs_E10	ordinary_muscle_development	ENSGALG00010023304	ENSGALG00010023304	-0.173721852	0.174677539	-0.994528848	0.319965468	0.358056007	negative
PRJNA729271	stage_3W_vs_E10	ordinary_muscle_development	ENSGALG00010023305	PLCB2	1.936795114	0.157153653	12.32421314	6.70952E-35	4.59181E-34	positive
PRJNA729271	stage_3W_vs_E10	ordinary_muscle_development	ENSGALG00010023309	PIK3R3	-1.722120422	0.202353764	-8.510444205	1.73268E-17	5.94584E-17	negative
PRJNA729271	stage_3W_vs_E10	ordinary_muscle_development	ENSGALG00010023311	ETS1	-0.607043768	0.13399099	-4.530481979	5.88493E-06	1.05794E-05	negative
PRJNA729271	stage_3W_vs_E10	ordinary_muscle_development	ENSGALG00010023312	UBR3	2.479252493	0.114076979	21.73315351	9.9714E-105	4.5739E-103	positive
PRJNA729271	stage_3W_vs_E10	ordinary_muscle_development	ENSGALG00010023317	RAPGEF3	0.890419978	0.149003687	5.975825143	2.28928E-09	5.10196E-09	positive
PRJNA729271	stage_3W_vs_E10	ordinary_muscle_development	ENSGALG00010023319	EIF2AK4	0.114884372	0.061456382	1.869364395	0.061572135	0.076444376	positive
PRJNA729271	stage_3W_vs_E10	ordinary_muscle_development	ENSGALG00010023320	ENSGALG00010023320	-1.005946422	0.140547058	-7.157363768	8.22433E-13	2.22909E-12	negative
PRJNA729271	stage_3W_vs_E10	ordinary_muscle_development	ENSGALG00010023322	PRKAB1	-0.100287741	0.09137402	-1.097552027	0.272400151	0.309075552	negative
PRJNA729271	stage_3W_vs_E10	ordinary_muscle_development	ENSGALG00010023324	LAG3	4.594563019	0.957492559	4.798536527	1.59829E-06	2.98316E-06	positive
PRJNA729271	stage_3W_vs_E10	ordinary_muscle_development	ENSGALG00010023328	TCEA2	-1.593337371	0.111607625	-14.27624111	3.07759E-46	3.06807E-45	negative
PRJNA729271	stage_3W_vs_E10	ordinary_muscle_development	ENSGALG00010023330	TSPAN1	1.364252483	1.378661145	0.989548801	0.322394706	0.360442136	positive
PRJNA729271	stage_3W_vs_E10	ordinary_muscle_development	ENSGALG00010023331	CD4	-1.424166557	0.32311213	-4.407654258	1.04496E-05	1.84249E-05	negative
PRJNA729271	stage_3W_vs_E10	ordinary_muscle_development	ENSGALG00010023332	PPP1R7	0.897516801	0.074339758	12.07317348	1.46378E-33	9.58769E-33	positive
PRJNA729271	stage_3W_vs_E10	ordinary_muscle_development	ENSGALG00010023333	ENDOU	-1.627454536	0.591312913	-2.752272951	0.005918317	0.008232032	negative
PRJNA729271	stage_3W_vs_E10	ordinary_muscle_development	ENSGALG00010023334	RPS17	0.241243458	0.151635155	1.590946759	0.111621558	0.134036491	positive
PRJNA729271	stage_3W_vs_E10	ordinary_muscle_development	ENSGALG00010023335	WDR59	0.003002406	0.069606253	0.043134136	0.965594608	0.970347605	positive
PRJNA729271	stage_3W_vs_E10	ordinary_muscle_development	ENSGALG00010023336	GPR162	-1.880918992	0.155484183	-12.09717256	1.09312E-33	7.18907E-33	negative
PRJNA729271	stage_3W_vs_E10	ordinary_muscle_development	ENSGALG00010023337	FLI1	0.498804128	0.116493645	4.281814052	1.85376E-05	3.21069E-05	positive
PRJNA729271	stage_3W_vs_E10	ordinary_muscle_development	ENSGALG00010023338	HDLBP	0.336275763	0.124054749	2.710704486	0.006714043	0.009293359	positive
PRJNA729271	stage_3W_vs_E10	ordinary_muscle_development	ENSGALG00010023340	P3H3	-1.183656454	0.159975073	-7.399005556	1.37208E-13	3.86631E-13	negative
PRJNA729271	stage_3W_vs_E10	ordinary_muscle_development	ENSGALG00010023342	FITM2	-0.337165503	0.13657202	-2.468774374	0.013557667	0.01816587	negative
PRJNA729271	stage_3W_vs_E10	ordinary_muscle_development	ENSGALG00010023343	TMEM233	5.113929611	0.333604792	15.32930501	4.87171E-53	6.08754E-52	positive
PRJNA729271	stage_3W_vs_E10	ordinary_muscle_development	ENSGALG00010023347	STAT5A	2.639033543	0.169162012	15.60062753	7.20821E-55	9.48121E-54	positive
PRJNA729271	stage_3W_vs_E10	ordinary_muscle_development	ENSGALG00010023349	ENSGALG00010023349	3.51807934	0.563299339	6.245488143	4.22479E-10	9.85965E-10	positive
PRJNA729271	stage_3W_vs_E10	ordinary_muscle_development	ENSGALG00010023350	ZNRF1	1.395033303	0.230668442	6.047785687	1.4685E-09	3.31144E-09	positive
PRJNA729271	stage_3W_vs_E10	ordinary_muscle_development	ENSGALG00010023351	GNB3	0.220249527	0.380279303	0.579178317	0.56246886	0.599308007	positive
PRJNA729271	stage_3W_vs_E10	ordinary_muscle_development	ENSGALG00010023354	SEPTIN2	-2.931437906	0.172494395	-16.99439514	9.03576E-65	1.53905E-63	negative
PRJNA729271	stage_3W_vs_E10	ordinary_muscle_development	ENSGALG00010023356	ENSGALG00010023356	0.452891827	0.18660902	2.426955717	0.015226114	0.020283294	positive
PRJNA729271	stage_3W_vs_E10	ordinary_muscle_development	ENSGALG00010023359	CDCA3	-3.409297056	0.281845465	-12.09633462	1.10433E-33	7.25952E-33	negative
PRJNA729271	stage_3W_vs_E10	ordinary_muscle_development	ENSGALG00010023362	GADD45A	0.815518801	0.148801966	5.480564713	4.2397E-08	8.78298E-08	positive
PRJNA729271	stage_3W_vs_E10	ordinary_muscle_development	ENSGALG00010023363	CPEB1	-1.689539453	0.240513495	-7.024717909	2.14499E-12	5.68195E-12	negative
PRJNA729271	stage_3W_vs_E10	ordinary_muscle_development	ENSGALG00010023364	CTRB1	10.14735755	2.210648108	4.590218367	4.42783E-06	8.0307E-06	positive
PRJNA729271	stage_3W_vs_E10	ordinary_muscle_development	ENSGALG00010023366	GDAP1L1	-4.07199866	0.489854703	-8.312666254	9.35754E-17	3.10029E-16	negative
PRJNA729271	stage_3W_vs_E10	ordinary_muscle_development	ENSGALG00010023368	ENSGALG00010023368	-1.896063512	0.233798723	-8.109811249	5.06985E-16	1.61511E-15	negative
PRJNA729271	stage_3W_vs_E10	ordinary_muscle_development	ENSGALG00010023369	ACE	3.355643978	0.476320593	7.044927358	1.85558E-12	4.93155E-12	positive
PRJNA729271	stage_3W_vs_E10	ordinary_muscle_development	ENSGALG00010023374	ENSGALG00010023374	-1.083493754	0.207841328	-5.21308136	1.85729E-07	3.6899E-07	negative
PRJNA729271	stage_3W_vs_E10	ordinary_muscle_development	ENSGALG00010023375	ST14	-0.665427347	0.150351627	-4.425807415	9.60822E-06	1.69801E-05	negative
PRJNA729271	stage_3W_vs_E10	ordinary_muscle_development	ENSGALG00010023377	OSER1	1.498116848	0.149134364	10.04541679	9.624E-24	4.27651E-23	positive
PRJNA729271	stage_3W_vs_E10	ordinary_muscle_development	ENSGALG00010023379	CCDC60	-0.063012468	0.547453552	-0.115101031	0.908365043	0.919144942	negative
PRJNA729271	stage_3W_vs_E10	ordinary_muscle_development	ENSGALG00010023380	USP5	-0.648510741	0.097023663	-6.684047146	2.32432E-11	5.83293E-11	negative
PRJNA729271	stage_3W_vs_E10	ordinary_muscle_development	ENSGALG00010023381	HOXB7	-0.425559066	0.164338077	-2.589534166	0.009610589	0.01309016	negative
PRJNA729271	stage_3W_vs_E10	ordinary_muscle_development	ENSGALG00010023383	HOXB6	0.015770249	0.170757525	0.092354638	0.926416279	0.934800137	positive
PRJNA729271	stage_3W_vs_E10	ordinary_muscle_development	ENSGALG00010023384	AP3B2	-1.949487918	0.23097587	-8.440223303	3.16732E-17	1.07217E-16	negative
PRJNA729271	stage_3W_vs_E10	ordinary_muscle_development	ENSGALG00010023387	ENSGALG00010023387	1.620762583	0.182624713	8.874826161	7.00427E-19	2.55993E-18	positive
PRJNA729271	stage_3W_vs_E10	ordinary_muscle_development	ENSGALG00010023388	FARP2	-0.908906259	0.084674265	-10.73414995	7.03635E-27	3.57992E-26	negative
PRJNA729271	stage_3W_vs_E10	ordinary_muscle_development	ENSGALG00010023389	GNG12	-0.411252145	0.110333761	-3.727346384	0.000193506	0.000308771	negative
PRJNA729271	stage_3W_vs_E10	ordinary_muscle_development	ENSGALG00010023390	HSPB8	2.861008128	0.250726759	11.41086073	3.69059E-30	2.13213E-29	positive
PRJNA729271	stage_3W_vs_E10	ordinary_muscle_development	ENSGALG00010023392	TPI1	4.728805747	0.149507077	31.62931051	1.4603E-219	2.9812E-217	positive
PRJNA729271	stage_3W_vs_E10	ordinary_muscle_development	ENSGALG00010023394	HOXB5	-1.200282756	0.177457838	-6.76376298	1.34453E-11	3.41134E-11	negative
PRJNA729271	stage_3W_vs_E10	ordinary_muscle_development	ENSGALG00010023396	ENSGALG00010023396	-0.223096753	0.147556164	-1.511944646	0.130547945	0.155424432	negative
PRJNA729271	stage_3W_vs_E10	ordinary_muscle_development	ENSGALG00010023397	ENSGALG00010023397	0.389174985	0.24252814	1.60465909	0.10856885	0.130608804	positive
PRJNA729271	stage_3W_vs_E10	ordinary_muscle_development	ENSGALG00010023401	LRRC23	1.459205939	0.368041045	3.964791314	7.34602E-05	0.000121345	positive
PRJNA729271	stage_3W_vs_E10	ordinary_muscle_development	ENSGALG00010023405	DNAJA2	-0.054787254	0.076679697	-0.714494931	0.47492119	0.513653384	negative
PRJNA729271	stage_3W_vs_E10	ordinary_muscle_development	ENSGALG00010023408	ZBTB44	0.279981538	0.126971154	2.205079894	0.027448489	0.035523736	positive
PRJNA729271	stage_3W_vs_E10	ordinary_muscle_development	ENSGALG00010023411	ENSGALG00010023411	3.736172233	0.180437679	20.70616432	3.0478E-95	1.12987E-93	positive
PRJNA729271	stage_3W_vs_E10	ordinary_muscle_development	ENSGALG00010023412	GSS	-0.758192207	0.108025059	-7.018669685	2.23991E-12	5.92363E-12	negative
PRJNA729271	stage_3W_vs_E10	ordinary_muscle_development	ENSGALG00010023414	SUDS3	-0.611620555	0.076407399	-8.004729435	1.1973E-15	3.73623E-15	negative
PRJNA729271	stage_3W_vs_E10	ordinary_muscle_development	ENSGALG00010023415	HOXB4	0.19570206	0.165621904	1.181619428	0.237356741	0.271610165	positive
PRJNA729271	stage_3W_vs_E10	ordinary_muscle_development	ENSGALG00010023421	OLFM3	-0.869383458	0.253469336	-3.429935435	0.000603725	0.000922614	negative
PRJNA729271	stage_3W_vs_E10	ordinary_muscle_development	ENSGALG00010023422	WLS	-2.557928517	0.156282129	-16.36737701	3.27007E-60	4.92719E-59	negative
PRJNA729271	stage_3W_vs_E10	ordinary_muscle_development	ENSGALG00010023426	KCNJ1	0.404319572	0.443749306	0.911144122	0.362219441	0.401097846	positive
PRJNA729271	stage_3W_vs_E10	ordinary_muscle_development	ENSGALG00010023428	HOXB3	-2.088151508	0.198526594	-10.51824576	7.11866E-26	3.45678E-25	negative
PRJNA729271	stage_3W_vs_E10	ordinary_muscle_development	ENSGALG00010023429	BMF	0.978551859	0.146097176	6.697951926	2.11361E-11	5.30966E-11	positive
PRJNA729271	stage_3W_vs_E10	ordinary_muscle_development	ENSGALG00010023431	BCAR1	-0.269399799	0.174255898	-1.546001037	0.122104317	0.145802956	negative
PRJNA729271	stage_3W_vs_E10	ordinary_muscle_development	ENSGALG00010023432	TMOD3	-0.949457747	0.175055727	-5.423745708	5.83629E-08	1.19741E-07	negative
PRJNA729271	stage_3W_vs_E10	ordinary_muscle_development	ENSGALG00010023434	ATN1	-0.934482364	0.191428411	-4.881628387	1.05213E-06	1.99017E-06	negative
PRJNA729271	stage_3W_vs_E10	ordinary_muscle_development	ENSGALG00010023437	KCNJ5	-1.567256922	0.259453558	-6.04060679	1.53536E-09	3.46004E-09	negative
PRJNA729271	stage_3W_vs_E10	ordinary_muscle_development	ENSGALG00010023438	HOXB2	-0.563474533	0.244444542	-2.305122172	0.021159732	0.027774184	negative
PRJNA729271	stage_3W_vs_E10	ordinary_muscle_development	ENSGALG00010023439	ACSS2	0.936285354	0.132657125	7.057934938	1.68995E-12	4.50126E-12	positive
PRJNA729271	stage_3W_vs_E10	ordinary_muscle_development	ENSGALG00010023441	ENSGALG00010023441	2.261838991	0.145285115	15.56827748	1.19584E-54	1.55739E-53	positive
PRJNA729271	stage_3W_vs_E10	ordinary_muscle_development	ENSGALG00010023442	STK25	0.015040849	0.112512815	0.133681211	0.893654663	0.905840863	positive
PRJNA729271	stage_3W_vs_E10	ordinary_muscle_development	ENSGALG00010023443	ENSGALG00010023443	1.07848931	0.179656647	6.003058196	1.93635E-09	4.33006E-09	positive
PRJNA729271	stage_3W_vs_E10	ordinary_muscle_development	ENSGALG00010023447	C12orf57	0.125409778	0.114177649	1.098374148	0.272041146	0.308692376	positive
PRJNA729271	stage_3W_vs_E10	ordinary_muscle_development	ENSGALG00010023448	FAM219B	-0.12417785	0.121420411	-1.022709853	0.306445051	0.344228225	negative
PRJNA729271	stage_3W_vs_E10	ordinary_muscle_development	ENSGALG00010023453	ARHGAP32	-1.787705372	0.185041766	-9.661091178	4.4114E-22	1.83587E-21	negative
PRJNA729271	stage_3W_vs_E10	ordinary_muscle_development	ENSGALG00010023454	HOXB1	0.805148192	0.406477434	1.980794319	0.047614344	0.059941803	positive
PRJNA729271	stage_3W_vs_E10	ordinary_muscle_development	ENSGALG00010023455	CFDP1	-0.753644163	0.09652573	-7.807702315	5.82399E-15	1.75909E-14	negative
PRJNA729271	stage_3W_vs_E10	ordinary_muscle_development	ENSGALG00010023456	MPI	0.623451991	0.14446609	4.315559399	1.59199E-05	2.76955E-05	positive
PRJNA729271	stage_3W_vs_E10	ordinary_muscle_development	ENSGALG00010023469	ENSGALG00010023469	0.783185361	0.213415586	3.669766466	0.000242772	0.000383624	positive
PRJNA729271	stage_3W_vs_E10	ordinary_muscle_development	ENSGALG00010023472	PTPN6	1.895689068	0.194402136	9.751379807	1.81978E-22	7.65903E-22	positive
PRJNA729271	stage_3W_vs_E10	ordinary_muscle_development	ENSGALG00010023473	BOK	-0.697749824	0.201195427	-3.46802029	0.000524308	0.000806007	negative
PRJNA729271	stage_3W_vs_E10	ordinary_muscle_development	ENSGALG00010023475	ENSGALG00010023475	-3.446693446	0.601230599	-5.732731255	9.88262E-09	2.13294E-08	negative
PRJNA729271	stage_3W_vs_E10	ordinary_muscle_development	ENSGALG00010023476	SCAMP2	0.100640266	0.151010723	0.666444504	0.505127024	0.543000313	positive
PRJNA729271	stage_3W_vs_E10	ordinary_muscle_development	ENSGALG00010023478	TAOK3	0.914806655	0.122859787	7.445940455	9.62562E-14	2.73147E-13	positive
PRJNA729271	stage_3W_vs_E10	ordinary_muscle_development	ENSGALG00010023479	MCCC2L	0.913086348	0.156066649	5.850618009	4.8975E-09	1.07397E-08	positive
PRJNA729271	stage_3W_vs_E10	ordinary_muscle_development	ENSGALG00010023482	MTCL2	-0.700302835	0.099721746	-7.02256892	2.17825E-12	5.76775E-12	negative
PRJNA729271	stage_3W_vs_E10	ordinary_muscle_development	ENSGALG00010023485	BARX2	-5.420422243	0.322991303	-16.78194489	3.30822E-63	5.40007E-62	negative
PRJNA729271	stage_3W_vs_E10	ordinary_muscle_development	ENSGALG00010023487	GRN	0.4154292	0.146016481	2.845084319	0.004439967	0.00626044	positive
PRJNA729271	stage_3W_vs_E10	ordinary_muscle_development	ENSGALG00010023489	ETV5	-1.013242587	0.218558176	-4.636031498	3.55162E-06	6.49517E-06	negative
PRJNA729271	stage_3W_vs_E10	ordinary_muscle_development	ENSGALG00010023490	PHB2	0.431561475	0.102832491	4.196742396	2.70782E-05	4.62034E-05	positive
PRJNA729271	stage_3W_vs_E10	ordinary_muscle_development	ENSGALG00010023496	PLEKHA3	0.357543629	0.103548282	3.452917065	0.000554559	0.000849629	positive
PRJNA729271	stage_3W_vs_E10	ordinary_muscle_development	ENSGALG00010023499	PEBP1	-0.24469323	0.139860096	-1.749557146	0.08019476	0.098144465	negative
PRJNA729271	stage_3W_vs_E10	ordinary_muscle_development	ENSGALG00010023501	ENSGALG00010023501	8.654818782	2.963350014	2.920619819	0.003493358	0.004977512	positive
PRJNA729271	stage_3W_vs_E10	ordinary_muscle_development	ENSGALG00010023506	RPE65	-1.287023233	0.600252278	-2.144137189	0.032021896	0.041152352	negative
PRJNA729271	stage_3W_vs_E10	ordinary_muscle_development	ENSGALG00010023510	ASPHD2	-0.945456447	0.256118256	-3.691484006	0.000222949	0.000353572	negative
PRJNA729271	stage_3W_vs_E10	ordinary_muscle_development	ENSGALG00010023513	CDCA9	-1.586101405	0.217037745	-7.30795191	2.71245E-13	7.52045E-13	negative
PRJNA729271	stage_3W_vs_E10	ordinary_muscle_development	ENSGALG00010023514	ULK3	0.607980704	0.105137149	5.782739147	7.3494E-09	1.59882E-08	positive
PRJNA729271	stage_3W_vs_E10	ordinary_muscle_development	ENSGALG00010023518	METTL5	0.626348519	0.118670548	5.278045213	1.30569E-07	2.62133E-07	positive
PRJNA729271	stage_3W_vs_E10	ordinary_muscle_development	ENSGALG00010023519	DGKG	-0.503599086	0.257107679	-1.958708849	0.050146894	0.06292237	negative
PRJNA729271	stage_3W_vs_E10	ordinary_muscle_development	ENSGALG00010023522	EMG1	-1.720461847	0.164889895	-10.43400412	1.73423E-25	8.27712E-25	negative
PRJNA729271	stage_3W_vs_E10	ordinary_muscle_development	ENSGALG00010023526	KNSTRN	-3.210117168	0.194937449	-16.46742162	6.28945E-61	9.64715E-60	negative
PRJNA729271	stage_3W_vs_E10	ordinary_muscle_development	ENSGALG00010023527	TMEM98	-1.075061573	0.229709151	-4.68009903	2.86736E-06	5.27308E-06	negative
PRJNA729271	stage_3W_vs_E10	ordinary_muscle_development	ENSGALG00010023533	FKBP7	-1.076308535	0.163231801	-6.593742917	4.28874E-11	1.05849E-10	negative
PRJNA729271	stage_3W_vs_E10	ordinary_muscle_development	ENSGALG00010023536	ENSGALG00010023536	-0.314965665	0.205794526	-1.530486114	0.125896448	0.150108498	negative
PRJNA729271	stage_3W_vs_E10	ordinary_muscle_development	ENSGALG00010023541	PRSS2	9.238243836	2.479433473	3.72594947	0.000194581	0.000310384	positive
PRJNA729271	stage_3W_vs_E10	ordinary_muscle_development	ENSGALG00010023543	ENSGALG00010023543	-2.886138394	0.913702318	-3.15872942	0.001584585	0.002333967	negative
PRJNA729271	stage_3W_vs_E10	ordinary_muscle_development	ENSGALG00010023544	SSB	-0.850889682	0.102657019	-8.288665422	1.14526E-16	3.77715E-16	negative
PRJNA729271	stage_3W_vs_E10	ordinary_muscle_development	ENSGALG00010023545	PTPRT	-2.716465406	0.146399505	-18.55515436	7.40942E-77	1.71565E-75	negative
PRJNA729271	stage_3W_vs_E10	ordinary_muscle_development	ENSGALG00010023546	SLC11A2	0.528556928	0.155164538	3.406428639	0.000658188	0.001002594	positive
PRJNA729271	stage_3W_vs_E10	ordinary_muscle_development	ENSGALG00010023551	ENSGALG00010023551	0.129680614	0.176471869	0.734851479	0.462429951	0.50107813	positive
PRJNA729271	stage_3W_vs_E10	ordinary_muscle_development	ENSGALG00010023552	SGPP2	0.72675887	0.341196174	2.130032295	0.033168947	0.042539718	positive
PRJNA729271	stage_3W_vs_E10	ordinary_muscle_development	ENSGALG00010023555	HPS4	-0.908719724	0.169351193	-5.365889109	8.05514E-08	1.64195E-07	negative
PRJNA729271	stage_3W_vs_E10	ordinary_muscle_development	ENSGALG00010023556	IVD	-0.159813703	0.121900748	-1.311014955	0.189852711	0.22045302	negative
PRJNA729271	stage_3W_vs_E10	ordinary_muscle_development	ENSGALG00010023559	PJVK	0.593465971	0.716518422	0.828263382	0.407521356	0.446351976	positive
PRJNA729271	stage_3W_vs_E10	ordinary_muscle_development	ENSGALG00010023569	LPCAT3	0.404529899	0.102381916	3.951185058	7.77652E-05	0.000128048	positive
PRJNA729271	stage_3W_vs_E10	ordinary_muscle_development	ENSGALG00010023571	APLP2	0.641879837	0.098638717	6.507382234	7.64717E-11	1.8592E-10	positive
PRJNA729271	stage_3W_vs_E10	ordinary_muscle_development	ENSGALG00010023572	MYBL2	-1.787213468	0.129766025	-13.77258394	3.72642E-43	3.33835E-42	negative

PRJNA729271	stage_3W_vs_E10	ordinary_muscle_development	ENSGALG00010023574	DEPDC1	-3.063454588	0.216259884	-14.16561652	1.49549E-45	1.45875E-44	negative
PRJNA729271	stage_3W_vs_E10	ordinary_muscle_development	ENSGALG00010023578	SRRD	-0.125730043	0.101673673	-1.236603725	0.216234236	0.249031881	negative
PRJNA729271	stage_3W_vs_E10	ordinary_muscle_development	ENSGALG00010023580	RDM1	-1.884962945	0.169816293	-11.10001231	1.25427E-28	6.83738E-28	negative
PRJNA729271	stage_3W_vs_E10	ordinary_muscle_development	ENSGALG00010023585	TFIP11	-0.044981948	0.096222355	-0.467479183	0.640157082	0.673470526	negative
PRJNA729271	stage_3W_vs_E10	ordinary_muscle_development	ENSGALG00010023588	FARSB	0.067916889	0.134302771	0.505699831	0.613067387	0.647933778	positive
PRJNA729271	stage_3W_vs_E10	ordinary_muscle_development	ENSGALG00010023590	S1PR1	1.435637585	0.114820914	12.50327607	7.16371E-36	5.05786E-35	positive
PRJNA729271	stage_3W_vs_E10	ordinary_muscle_development	ENSGALG00010023592	BAHD1	-0.701823916	0.105301946	-6.664871288	2.64897E-11	6.62472E-11	negative
PRJNA729271	stage_3W_vs_E10	ordinary_muscle_development	ENSGALG00010023593	CSK	-0.318266219	0.08185563	-3.888140874	0.000101015	0.000164815	negative
PRJNA729271	stage_3W_vs_E10	ordinary_muscle_development	ENSGALG00010023597	MOGAT1	0.272347538	0.33933791	0.802585062	0.422214605	0.460635308	positive
PRJNA729271	stage_3W_vs_E10	ordinary_muscle_development	ENSGALG00010023598	IL10RA	3.356112898	0.3594763	9.33611728	9.99316E-21	3.93617E-20	positive
PRJNA729271	stage_3W_vs_E10	ordinary_muscle_development	ENSGALG00010023600	CHST14	-2.623457482	0.08638251	-30.37023924	1.3582E-202	2.4305E-200	negative
PRJNA729271	stage_3W_vs_E10	ordinary_muscle_development	ENSGALG00010023601	TPST2	1.246039281	0.104011618	11.97980861	4.53369E-33	2.91941E-32	positive
PRJNA729271	stage_3W_vs_E10	ordinary_muscle_development	ENSGALG00010023603	C1S	3.348294213	0.392922129	8.521521096	1.57471E-17	5.42044E-17	positive
PRJNA729271	stage_3W_vs_E10	ordinary_muscle_development	ENSGALG00010023604	ENSGALG00010023604	0.788967299	0.395758346	1.993558206	0.046200357	0.058278145	positive
PRJNA729271	stage_3W_vs_E10	ordinary_muscle_development	ENSGALG00010023605	INSYN1	3.400480821	0.304463953	11.16874686	5.79933E-29	3.19989E-28	positive
PRJNA729271	stage_3W_vs_E10	ordinary_muscle_development	ENSGALG00010023606	LRRC7	-3.650121106	0.370721109	-9.846002867	7.13243E-23	3.06143E-22	negative
PRJNA729271	stage_3W_vs_E10	ordinary_muscle_development	ENSGALG00010023612	GPT2	-0.069179179	0.18799984	-0.367974671	0.712892121	0.74160424	negative
PRJNA729271	stage_3W_vs_E10	ordinary_muscle_development	ENSGALG00010023613	COPB1	-0.745782824	0.051703168	-14.42431591	3.6386E-47	3.72731E-46	negative
PRJNA729271	stage_3W_vs_E10	ordinary_muscle_development	ENSGALG00010023615	PRSS3	6.622492485	5.087112577	1.301817561	0.192978759	0.22381398	positive
PRJNA729271	stage_3W_vs_E10	ordinary_muscle_development	ENSGALG00010023617	ENSGALG00010023617	0.524734315	0.302452207	1.734933003	0.082752673	0.101078621	positive
PRJNA729271	stage_3W_vs_E10	ordinary_muscle_development	ENSGALG00010023620	IRS1	1.301806581	0.141129014	9.224230704	2.85604E-20	1.10484E-19	positive
PRJNA729271	stage_3W_vs_E10	ordinary_muscle_development	ENSGALG00010023621	CD79B	4.210407756	0.891405103	4.723338177	2.32005E-06	4.28942E-06	positive
PRJNA729271	stage_3W_vs_E10	ordinary_muscle_development	ENSGALG00010023623	OSBPL6	0.548119482	0.217663535	2.518196183	0.01179576	0.015921365	positive
PRJNA729271	stage_3W_vs_E10	ordinary_muscle_development	ENSGALG00010023624	PSMA1	0.55734663	0.070530126	7.902249157	2.73915E-15	8.40119E-15	positive
PRJNA729271	stage_3W_vs_E10	ordinary_muscle_development	ENSGALG00010023626	LOXL1	0.886226685	0.195053615	4.543503004	5.5327E-06	9.96106E-06	positive
PRJNA729271	stage_3W_vs_E10	ordinary_muscle_development	ENSGALG00010023627	IFT52	0.202619367	0.101183475	2.002494635	0.04523156	0.057120705	positive
PRJNA729271	stage_3W_vs_E10	ordinary_muscle_development	ENSGALG00010023631	ENSGALG00010023631	2.445569391	0.463187371	5.279870614	1.29275E-07	2.59679E-07	positive
PRJNA729271	stage_3W_vs_E10	ordinary_muscle_development	ENSGALG00010023635	SCN4A	9.278015672	0.317385792	29.23261184	7.4705E-188	1.1899E-185	positive
PRJNA729271	stage_3W_vs_E10	ordinary_muscle_development	ENSGALG00010023636	PDE3B	0.09928879	0.081307204	1.221156122	0.222026914	0.255135573	positive
PRJNA729271	stage_3W_vs_E10	ordinary_muscle_development	ENSGALG00010023637	RHBDD1	0.55323349	0.104682567	5.284867444	1.25796E-07	2.52865E-07	positive
PRJNA729271	stage_3W_vs_E10	ordinary_muscle_development	ENSGALG00010023638	DCTN2	0.257435823	0.131093932	1.963750868	0.04955899	0.062238569	positive
PRJNA729271	stage_3W_vs_E10	ordinary_muscle_development	ENSGALG00010023639	KLHL23	1.172320482	0.216295245	5.420001166	5.95986E-08	1.22207E-07	positive
PRJNA729271	stage_3W_vs_E10	ordinary_muscle_development	ENSGALG00010023641	LRRC40	-1.834870044	0.113817787	-16.12111855	1.81298E-58	2.62005E-57	negative
PRJNA729271	stage_3W_vs_E10	ordinary_muscle_development	ENSGALG00010023642	PAX6	-1.437521547	0.960783976	-1.496196423	0.134602477	0.15994941	negative
PRJNA729271	stage_3W_vs_E10	ordinary_muscle_development	ENSGALG00010023643	SCN4B	2.952608611	0.15165882	19.46875629	2.02119E-84	5.72211E-83	positive
PRJNA729271	stage_3W_vs_E10	ordinary_muscle_development	ENSGALG00010023646	DPH5	1.112987085	0.141579844	7.861197269	3.80479E-15	1.16008E-14	positive
PRJNA729271	stage_3W_vs_E10	ordinary_muscle_development	ENSGALG00010023648	SCN2B	2.475022911	0.359175905	6.890837814	5.54647E-12	1.43232E-11	positive
PRJNA729271	stage_3W_vs_E10	ordinary_muscle_development	ENSGALG00010023652	SLC30A7	-1.133710111	0.09683246	-11.70795529	1.16044E-31	7.09731E-31	negative
PRJNA729271	stage_3W_vs_E10	ordinary_muscle_development	ENSGALG00010023655	C1R	1.480091328	0.185109474	7.995762173	1.28775E-15	4.00986E-15	positive
PRJNA729271	stage_3W_vs_E10	ordinary_muscle_development	ENSGALG00010023658	ENSGALG00010023658	-1.316750003	0.347763455	-3.786338053	0.000152884	0.000245954	negative
PRJNA729271	stage_3W_vs_E10	ordinary_muscle_development	ENSGALG00010023660	CRYBB1	-1.999093289	0.760907816	-2.627247675	0.008607866	0.011788644	negative
PRJNA729271	stage_3W_vs_E10	ordinary_muscle_development	ENSGALG00010023663	PHOSPHO2	0.870931969	0.089247156	9.75865238	1.69391E-22	7.1445E-22	positive
PRJNA729271	stage_3W_vs_E10	ordinary_muscle_development	ENSGALG00010023664	SLC6A9	-0.994896007	0.32658296	-3.046380638	0.002316144	0.003352557	negative
PRJNA729271	stage_3W_vs_E10	ordinary_muscle_development	ENSGALG00010023665	C16orf87	-0.432357991	0.138215216	-3.128150457	0.001759101	0.002575053	negative
PRJNA729271	stage_3W_vs_E10	ordinary_muscle_development	ENSGALG00010023666	ENSGALG00010023666	1.947547566	0.248591828	7.83431855	4.71392E-15	1.43156E-14	positive
PRJNA729271	stage_3W_vs_E10	ordinary_muscle_development	ENSGALG00010023668	RCN1	-1.304739921	0.161466697	-8.080551233	6.44747E-16	2.04186E-15	negative
PRJNA729271	stage_3W_vs_E10	ordinary_muscle_development	ENSGALG00010023670	EXTL2	0.691448799	0.126227432	5.477801372	4.30643E-08	8.91866E-08	positive
PRJNA729271	stage_3W_vs_E10	ordinary_muscle_development	ENSGALG00010023671	ENSGALG00010023671	5.875511896	0.63245649	9.289985939	1.54309E-20	6.02074E-20	positive
PRJNA729271	stage_3W_vs_E10	ordinary_muscle_development	ENSGALG00010023676	ENSGALG00010023676	0.717736681	0.255264156	2.811740948	0.004927417	0.006915464	positive
PRJNA729271	stage_3W_vs_E10	ordinary_muscle_development	ENSGALG00010023677	MN1	0.483422466	0.161078233	3.00116568	0.002689482	0.003869384	positive
PRJNA729271	stage_3W_vs_E10	ordinary_muscle_development	ENSGALG00010023680	MPZL3	-3.487354975	0.37968232	-9.184928542	4.11801E-20	1.57995E-19	negative
PRJNA729271	stage_3W_vs_E10	ordinary_muscle_development	ENSGALG00010023686	MYLK3	-0.401008191	0.22505116	-1.781853477	0.074773129	0.091842768	negative
PRJNA729271	stage_3W_vs_E10	ordinary_muscle_development	ENSGALG00010023689	WT1	-2.719484472	0.261601627	-10.39551819	2.59871E-25	1.23139E-24	negative
PRJNA729271	stage_3W_vs_E10	ordinary_muscle_development	ENSGALG00010023693	SRSF11	-0.611284417	0.068974311	-8.862494028	7.8244E-19	2.85535E-18	negative
PRJNA729271	stage_3W_vs_E10	ordinary_muscle_development	ENSGALG00010023694	PPCDC	1.98444575	0.15016842	13.21480074	7.20769E-40	5.81065E-39	positive
PRJNA729271	stage_3W_vs_E10	ordinary_muscle_development	ENSGALG00010023695	CLSTN3	-0.734019842	0.130829369	-5.610512755	2.01728E-08	4.27301E-08	negative
PRJNA729271	stage_3W_vs_E10	ordinary_muscle_development	ENSGALG00010023696	LRRC4C	0.525899346	0.529142675	0.993870597	0.320285871	0.35835922	positive
PRJNA729271	stage_3W_vs_E10	ordinary_muscle_development	ENSGALG00010023698	ENSGALG00010023698	-1.975825046	0.150402422	-13.13692305	2.02286E-39	1.60489E-38	negative
PRJNA729271	stage_3W_vs_E10	ordinary_muscle_development	ENSGALG00010023699	PPIG	0.043442454	0.202907573	0.214099716	0.830469298	0.85017674	positive
PRJNA729271	stage_3W_vs_E10	ordinary_muscle_development	ENSGALG00010023702	COL4A4	-0.361805455	0.27363438	-1.322222211	0.186094167	0.216452813	negative
PRJNA729271	stage_3W_vs_E10	ordinary_muscle_development	ENSGALG00010023703	PITPNB	-1.108115378	0.081176225	-13.65073798	1.99844E-42	1.74713E-41	negative
PRJNA729271	stage_3W_vs_E10	ordinary_muscle_development	ENSGALG00010023704	ANKRD13C	-0.330336872	0.068788409	-4.802217053	1.56918E-06	2.93072E-06	negative
PRJNA729271	stage_3W_vs_E10	ordinary_muscle_development	ENSGALG00010023706	CDK5RAP3	-0.654720184	0.129277546	-5.064453981	4.09573E-07	7.95385E-07	negative
PRJNA729271	stage_3W_vs_E10	ordinary_muscle_development	ENSGALG00010023707	MPZL2	-0.180178336	0.268988775	-0.66983589	0.502962412	0.540994372	negative
PRJNA729271	stage_3W_vs_E10	ordinary_muscle_development	ENSGALG00010023710	CDC14A	0.771027508	0.119769178	6.437612082	1.21368E-10	2.91359E-10	positive
PRJNA729271	stage_3W_vs_E10	ordinary_muscle_development	ENSGALG00010023711	ENSGALG00010023711	-0.365497122	0.347924724	-1.050506323	0.293485385	0.330772931	negative
PRJNA729271	stage_3W_vs_E10	ordinary_muscle_development	ENSGALG00010023712	ARHGAP40	-2.050251081	0.165851041	-12.36200307	4.1957E-35	2.89741E-34	negative
PRJNA729271	stage_3W_vs_E10	ordinary_muscle_development	ENSGALG00010023714	EIF3M	0.032695086	0.110422506	0.296090781	0.767160754	0.792473997	positive
PRJNA729271	stage_3W_vs_E10	ordinary_muscle_development	ENSGALG00010023715	CD3E	2.957181169	0.340425051	8.686731967	3.73015E-18	1.32716E-17	positive
PRJNA729271	stage_3W_vs_E10	ordinary_muscle_development	ENSGALG00010023716	FASTKD1	-0.395215386	0.10535999	-3.75109552	0.000176064	0.000281807	negative
PRJNA729271	stage_3W_vs_E10	ordinary_muscle_development	ENSGALG00010023720	SCAMP5	-1.053492315	0.097809413	-10.77086842	4.72523E-27	2.41936E-26	negative
PRJNA729271	stage_3W_vs_E10	ordinary_muscle_development	ENSGALG00010023724	CYP2R1	0.068522297	0.12074945	0.567475029	0.570391488	0.606723755	positive
PRJNA729271	stage_3W_vs_E10	ordinary_muscle_development	ENSGALG00010023726	PNPO	-0.09915025	0.188980905	-0.524657501	0.599821306	0.635416928	negative
PRJNA729271	stage_3W_vs_E10	ordinary_muscle_development	ENSGALG00010023730	SP2	1.118329211	0.181662749	6.156073353	7.45707E-10	1.71381E-09	positive
PRJNA729271	stage_3W_vs_E10	ordinary_muscle_development	ENSGALG00010023731	ACTR5	0.074640738	0.097825747	0.762996864	0.445465266	0.484215901	positive
PRJNA729271	stage_3W_vs_E10	ordinary_muscle_development	ENSGALG00010023732	TM2D1	0.870113917	0.076713047	11.34245022	8.08486E-30	4.62106E-29	positive
PRJNA729271	stage_3W_vs_E10	ordinary_muscle_development	ENSGALG00010023733	ORC6	-1.935341578	0.192861297	-10.0348883	1.07081E-23	4.73644E-23	negative
PRJNA729271	stage_3W_vs_E10	ordinary_muscle_development	ENSGALG00010023736	KIF5A	-1.83608486	0.553405937	-3.317790321	0.000907326	0.001363576	negative
PRJNA729271	stage_3W_vs_E10	ordinary_muscle_development	ENSGALG00010023738	ENSGALG00010023738	1.025693848	0.385431877	2.661154691	0.007787317	0.010715508	positive
PRJNA729271	stage_3W_vs_E10	ordinary_muscle_development	ENSGALG00010023740	PPP1R16B	0.322042373	0.10295436	3.128011015	0.001759936	0.002576014	positive
PRJNA729271	stage_3W_vs_E10	ordinary_muscle_development	ENSGALG00010023743	CD3D	2.841522299	0.218062245	13.03078534	8.17617E-39	6.33424E-38	positive
PRJNA729271	stage_3W_vs_E10	ordinary_muscle_development	ENSGALG00010023744	MT4	1.614050265	0.316213026	5.104313014	3.31998E-07	6.48822E-07	positive
PRJNA729271	stage_3W_vs_E10	ordinary_muscle_development	ENSGALG00010023746	TTC28	-1.740085445	0.090056079	-19.32224305	3.49144E-83	9.58494E-82	negative
PRJNA729271	stage_3W_vs_E10	ordinary_muscle_development	ENSGALG00010023748	ENSGALG00010023748	-1.088529009	0.174156135	-6.25030527	4.09651E-10	9.56799E-10	negative
PRJNA729271	stage_3W_vs_E10	ordinary_muscle_development	ENSGALG00010023749	RPP25	-0.17369031	0.27985123	-0.6206523	0.53482842	0.572593098	negative
PRJNA729271	stage_3W_vs_E10	ordinary_muscle_development	ENSGALG00010023750	ENSGALG00010023750	-2.794580451	0.171117078	-16.3313942	5.902E-60	8.79235E-59	negative
PRJNA729271	stage_3W_vs_E10	ordinary_muscle_development	ENSGALG00010023751	PRRG4	-0.871910611	0.280537688	-3.107998135	0.001883593	0.002747389	negative
PRJNA729271	stage_3W_vs_E10	ordinary_muscle_development	ENSGALG00010023752	C1QL1	-2.440888663	0.351188527	-6.950365623	3.64341E-12	9.50868E-12	negative
PRJNA729271	stage_3W_vs_E10	ordinary_muscle_development	ENSGALG00010023759	CALCA	-1.271252461	0.275917722	-4.607360666	4.07812E-06	7.41779E-06	negative
PRJNA729271	stage_3W_vs_E10	ordinary_muscle_development	ENSGALG00010023760	ZW10	-0.927957845	0.093821954	-9.890625879	4.57161E-23	1.97925E-22	negative
PRJNA729271	stage_3W_vs_E10	ordinary_muscle_development	ENSGALG00010023762	BBS5	2.971421128	0.152075395	19.53913143	5.10434E-85	1.47385E-83	positive
PRJNA729271	stage_3W_vs_E10	ordinary_muscle_development	ENSGALG00010023764	COX5A	1.602906356	0.177061843	9.052805107	1.39342E-19	5.23525E-19	positive
PRJNA729271	stage_3W_vs_E10	ordinary_muscle_development	ENSGALG00010023766	ENSGALG00010023766	-3.967860002	0.337264352	-11.76483662	5.92409E-32	3.66808E-31	negative
PRJNA729271	stage_3W_vs_E10	ordinary_muscle_development	ENSGALG00010023767	ENSGALG00010023767	3.476831952	0.208747126	16.65571169	2.75053E-62	4.36202E-61	positive
PRJNA729271	stage_3W_vs_E10	ordinary_muscle_development	ENSGALG00010023770	SCRN2	0.005054315	0.139299976	0.036283672	0.971056169	0.975362703	positive
PRJNA729271	stage_3W_vs_E10	ordinary_muscle_development	ENSGALG00010023773	OVOA	1.439963113	0.515440307	2.793656403	0.005211582	0.007294504	positive
PRJNA729271	stage_3W_vs_E10	ordinary_muscle_development	ENSGALG00010023775	ENSGALG00010023775	2.024668194	0.13279281	15.24682091	1.72846E-52	2.11605E-51	positive
PRJNA729271	stage_3W_vs_E10	ordinary_muscle_development	ENSGALG00010023777	OAZ2	0.577429424	0.13223255	4.366772204	1.26096E-05	2.21145E-05	positive
PRJNA729271	stage_3W_vs_E10	ordinary_muscle_development	ENSGALG00010023778	FANCF	-3.554808967	0.23912233	-14.86606862	5.47345E-50	6.18858E-49	negative
PRJNA729271	stage_3W_vs_E10	ordinary_muscle_development	ENSGALG00010023781	COL4A3	1.225955511	0.246874968	4.965896383	6.83845E-07	1.30942E-06	positive
PRJNA729271	stage_3W_vs_E10	ordinary_muscle_development	ENSGALG00010023783	VPS35	-0.218882574	0.062981261	-3.475360267	0.000510168	0.000785353	negative
PRJNA729271	stage_3W_vs_E10	ordinary_muscle_development	ENSGALG00010023786	ENSGALG00010023786	-2.970460619	0.448727434	-6.619743738	3.59822E-11	8.92475E-11	negative
PRJNA729271	stage_3W_vs_E10	ordinary_muscle_development	ENSGALG00010023788	RTCA	-0.262841858	0.104930457	-2.504914835	0.012248088	0.016490436	negative
PRJNA729271	stage_3W_vs_E10	ordinary_muscle_development	ENSGALG00010023791	VTI1B	0.492105178	0.080316574	6.127068861	8.95127E-10	2.04586E-09	positive
PRJNA729271	stage_3W_vs_E10	ordinary_muscle_development	ENSGALG00010023792	ZNF609	-0.459235635	0.177091487	-2.59321124	0.009508435	0.012957109	negative
PRJNA729271	stage_3W_vs_E10	ordinary_muscle_development	ENSGALG00010023793	RBM45	-0.191110887	0.065207849	-2.930795736	0.00338095	0.004824461	negative
PRJNA729271	stage_3W_vs_E10	ordinary_muscle_development	ENSGALG00010023794	MT3	2.44808676	0.458877119	5.334950593	9.55706E-08	1.93802E-07	positive
PRJNA729271	stage_3W_vs_E10	ordinary_muscle_development	ENSGALG00010023798	ARCN1	-0.884444056	0.057141368	-15.47817441	4.871E-54	6.26488E-53	negative
PRJNA729271	stage_3W_vs_E10	ordinary_muscle_development	ENSGALG00010023799	NMT1	0.345387874	0.126606663	2.728038671	0.006371214	0.008833165	positive
PRJNA729271	stage_3W_vs_E10	ordinary_muscle_development	ENSGALG00010023800	PEX5	0.451045953	0.072060461	6.259271022	3.86781E-10	9.04549E-10	positive
PRJNA729271	stage_3W_vs_E10	ordinary_muscle_development	ENSGALG00010023802	ENSGALG00010023802	2.722242062	0.271170223	10.03886796	1.02848E-23	4.55477E-23	positive
PRJNA729271	stage_3W_vs_E10	ordinary_muscle_development	ENSGALG00010023805	GALNT6	-0.161224325	0.315807499	-0.510514558	0.609691018	0.644835557	negative
PRJNA729271	stage_3W_vs_E10	ordinary_muscle_development	ENSGALG00010023806	PATJ	-1.815981696	0.176171542	-10.30803091	6.48157E-25	3.02286E-24	negative
PRJNA729271	stage_3W_vs_E10	ordinary_muscle_development	ENSGALG00010023807	MRPL10	1.182800266	0.124520156	9.498865914	2.12189E-21	8.58647E-21	positive
PRJNA729271	stage_3W_vs_E10	ordinary_muscle_development	ENSGALG00010023808	DHX35	-0.388525899	0.116611884	-3.331786492	0.000862904	0.001300187	negative
PRJNA729271	stage_3W_vs_E10	ordinary_muscle_development	ENSGALG00010023809	CDON	-1.687490297	0.100333324	-16.81884175	1.77597E-63	2.91867E-62	negative
PRJNA729271	stage_3W_vs_E10	ordinary_muscle_development	ENSGALG00010023811	MRPL44	0.978990623	0.083935169	11.66365221	1.95474E-31	1.18255E-30	positive
PRJNA729271	stage_3W_vs_E10	ordinary_muscle_development	ENSGALG00010023813	CELA1	7.331689439	5.258869098	1.394157052	0.163270184	0.191653864	positive
PRJNA729271	stage_3W_vs_E10	ordinary_muscle_development	ENSGALG00010023815	PLCD3	0.585927248	0.13712423	4.272966552	1.92889E-05	3.33683E-05	positive
PRJNA729271	stage_3W_vs_E10	ordinary_muscle_development	ENSGALG00010023817	BBS2	0.180159262	0.116484936	1.546631428	0.121952147	0.145645247	positive
PRJNA729271	stage_3W_vs_E10	ordinary_muscle_development	ENSGALG00010023818	ALOX5	2.585626757	0.183216673	14.11239876	3.18555E-45	3.07216E-44	positive
PRJNA729271	stage_3W_vs_E10	ordinary_muscle_development	ENSGALG00010023819	BIN2	2.395206589	0.225654977	10.6144638	2.55256E-26	1.26407E-25	positive
PRJNA729271	stage_3W_vs_E10	ordinary_muscle_development	ENSGALG00010023820	CYP1C1	0.766884186	0.817125709	0.938514329	0.347980157	0.386511293	positive
PRJNA729271	stage_3W_vs_E10	ordinary_muscle_development	ENSGALG00010023821	KANK4	-0.402661065	0.124088223	-3.244957929	0.00117468	0.001750307	negative
PRJNA729271	stage_3W_vs_E10	ordinary_muscle_development	ENSGALG00010023822	CHEK2	-1.440582088	0.130943509	-11.00155402	3.75602E-28	2.01792E-27	negative
PRJNA729271	stage_3W_vs_E10	ordinary_muscle_development	ENSGALG00010023823	MUC2	2.92732926	0.520501533	5.62405502	1.86526E-08	3.96088E-08	positive
PRJNA729271	stage_3W_vs_E10	ordinary_muscle_development	ENSGALG00010023826	DBT	1.209975797	0.126972967	9.529396874	1.582E-21	6.44492E-21	positive
PRJNA729271	stage_3W_vs_E10	ordinary_muscle_development	ENSGALG00010023828	CHEK1	-3.200948021	0.201960211	-15.8493993	1.41931E-56	1.94451E-55	negative
PRJNA729271	stage_3W_vs_E10	ordinary_muscle_development	ENSGALG00010023829	USP1	-1.419796375	0.101513404	-13.98629467	1.89006E-44	1.76865E-43	negative
PRJNA729271	stage_3W_vs_E10	ordinary_muscle_development	ENSGALG00010023830	AGFG1	1.325818172	0.112095311	11.82759704	2.81073E-32	1.76676E-31	positive
PRJNA729271	stage_3W_vs_E10	ordinary_muscle_development	ENSGALG00010023831	SATB2	-4.525681563	0.554811019	-8.157158753	3.42997E-16	1.10141E-15	negative

PRJNA729271	stage_3W_vs_E10	ordinary_muscle_development	ENSGALG00010023832	ENSGALG00010023832	-0.273621028	0.491136515	-0.55711807	0.577446762	0.613553058	negative
PRJNA729271	stage_3W_vs_E10	ordinary_muscle_development	ENSGALG00010023833	PIP4K2C	0.072078546	0.107654229	0.66953752	0.503152655	0.541118692	positive
PRJNA729271	stage_3W_vs_E10	ordinary_muscle_development	ENSGALG00010023835	INAFM2	0.669730906	0.120691817	5.549099537	2.87145E-08	6.01208E-08	positive
PRJNA729271	stage_3W_vs_E10	ordinary_muscle_development	ENSGALG00010023836	NDUFS3	1.578630875	0.14752577	10.70071264	1.00998E-26	5.09917E-26	positive
PRJNA729271	stage_3W_vs_E10	ordinary_muscle_development	ENSGALG00010023837	STT3A	-0.907907523	0.093138231	-9.747957527	1.88217E-22	7.91472E-22	negative
PRJNA729271	stage_3W_vs_E10	ordinary_muscle_development	ENSGALG00010023840	SMAGP	-2.811794124	0.637806667	-4.408536741	1.04071E-05	1.8354E-05	negative
PRJNA729271	stage_3W_vs_E10	ordinary_muscle_development	ENSGALG00010023841	OSBPL7	0.498745869	0.146494905	3.404527071	0.000662787	0.001009149	positive
PRJNA729271	stage_3W_vs_E10	ordinary_muscle_development	ENSGALG00010023847	PIK3AP1	1.174740334	0.182457764	6.438423398	1.20721E-10	2.89854E-10	positive
PRJNA729271	stage_3W_vs_E10	ordinary_muscle_development	ENSGALG00010023850	DAZAP2	1.246286726	0.203745811	6.116870431	9.54309E-10	2.17667E-09	positive
PRJNA729271	stage_3W_vs_E10	ordinary_muscle_development	ENSGALG00010023851	HSCB	0.24336636	0.149268802	1.630389986	0.1030191	0.124283134	positive
PRJNA729271	stage_3W_vs_E10	ordinary_muscle_development	ENSGALG00010023852	LRRC39	3.145393486	0.190081833	16.54757553	1.66681E-61	2.59511E-60	positive
PRJNA729271	stage_3W_vs_E10	ordinary_muscle_development	ENSGALG00010023854	SHCBP1	-3.473390461	0.228431616	-15.2053841	3.25715E-52	3.97076E-51	negative
PRJNA729271	stage_3W_vs_E10	ordinary_muscle_development	ENSGALG00010023857	ACBD4	0.719059313	0.151926837	4.732931497	2.213E-06	4.09726E-06	positive
PRJNA729271	stage_3W_vs_E10	ordinary_muscle_development	ENSGALG00010023859	ENSGALG00010023859	3.049712802	0.765843143	3.982163751	6.82907E-05	0.000113103	positive
PRJNA729271	stage_3W_vs_E10	ordinary_muscle_development	ENSGALG00010023860	TRIP4	0.074719125	0.114498729	0.652576027	0.514029645	0.551956419	positive
PRJNA729271	stage_3W_vs_E10	ordinary_muscle_development	ENSGALG00010023861	ENSGALG00010023861	-0.631303071	0.154794115	-4.078340266	4.53583E-05	7.62106E-05	negative
PRJNA729271	stage_3W_vs_E10	ordinary_muscle_development	ENSGALG00010023862	TRMT13	-0.595246755	0.132714892	-4.485154186	7.28612E-06	1.29984E-05	negative
PRJNA729271	stage_3W_vs_E10	ordinary_muscle_development	ENSGALG00010023863	EYA2	-4.118454762	0.241561062	-17.04933205	3.5355E-65	6.08636E-64	negative
PRJNA729271	stage_3W_vs_E10	ordinary_muscle_development	ENSGALG00010023864	EPHA1	-4.802537056	0.638396583	-7.522811339	5.36108E-14	1.54521E-13	negative
PRJNA729271	stage_3W_vs_E10	ordinary_muscle_development	ENSGALG00010023866	AMBRA1	-0.064819215	0.074728571	-0.867395345	0.385725431	0.424693515	negative
PRJNA729271	stage_3W_vs_E10	ordinary_muscle_development	ENSGALG00010023867	ENSGALG00010023867	1.217081772	0.479128207	2.540200626	0.01107889	0.014995658	positive
PRJNA729271	stage_3W_vs_E10	ordinary_muscle_development	ENSGALG00010023869	DOCK7	-1.130331996	0.047581025	-23.75594044	9.5391E-125	6.2283E-123	negative
PRJNA729271	stage_3W_vs_E10	ordinary_muscle_development	ENSGALG00010023871	UQCRFS1	1.428829735	0.140261221	10.18691924	2.26839E-24	1.03495E-23	positive
PRJNA729271	stage_3W_vs_E10	ordinary_muscle_development	ENSGALG00010023874	HEXIM1	2.403444391	0.223747781	10.74175743	6.4799E-27	3.30493E-26	positive
PRJNA729271	stage_3W_vs_E10	ordinary_muscle_development	ENSGALG00010023878	ENSGALG00010023878	4.258111569	1.42874938	2.980306853	0.002879598	0.004134697	positive
PRJNA729271	stage_3W_vs_E10	ordinary_muscle_development	ENSGALG00010023879	EI24	0.614956862	0.11684179	5.263158534	1.41601E-07	2.83379E-07	positive
PRJNA729271	stage_3W_vs_E10	ordinary_muscle_development	ENSGALG00010023880	ENSGALG00010023880	-2.825426159	0.204990082	-13.78323346	3.21541E-43	2.88412E-42	negative
PRJNA729271	stage_3W_vs_E10	ordinary_muscle_development	ENSGALG00010023881	VSTM2B	-2.180312692	0.242710958	-8.983165447	2.63086E-19	9.79563E-19	negative
PRJNA729271	stage_3W_vs_E10	ordinary_muscle_development	ENSGALG00010023883	TBX21	6.453505375	0.341646586	18.88941862	1.39372E-79	3.43958E-78	positive
PRJNA729271	stage_3W_vs_E10	ordinary_muscle_development	ENSGALG00010023884	C2orf69	-0.634762985	0.126052309	-5.03571087	4.76079E-07	9.21207E-07	negative
PRJNA729271	stage_3W_vs_E10	ordinary_muscle_development	ENSGALG00010023887	RASA2	-0.114641679	0.128128456	-0.894740189	0.37092599	0.409612389	negative
PRJNA729271	stage_3W_vs_E10	ordinary_muscle_development	ENSGALG00010023888	CTH	-0.787290722	0.258836183	-3.041656353	0.002352803	0.003403242	negative
PRJNA729271	stage_3W_vs_E10	ordinary_muscle_development	ENSGALG00010023889	SAMD8	2.431921524	0.189899058	12.80639065	1.50996E-37	1.11725E-36	positive
PRJNA729271	stage_3W_vs_E10	ordinary_muscle_development	ENSGALG00010023890	SASS6	-0.991179741	0.120281541	-8.240497523	1.71496E-16	5.59999E-16	negative
PRJNA729271	stage_3W_vs_E10	ordinary_muscle_development	ENSGALG00010023891	ENSGALG00010023891	4.518694555	0.263265849	17.16399821	4.93932E-66	8.67823E-65	positive
PRJNA729271	stage_3W_vs_E10	ordinary_muscle_development	ENSGALG00010023896	POP4	-0.232282713	0.125625068	-1.849015622	0.064455557	0.079832803	negative
PRJNA729271	stage_3W_vs_E10	ordinary_muscle_development	ENSGALG00010023902	PTGER3	0.19757549	0.234714319	0.841770076	0.399916665	0.43895139	positive
PRJNA729271	stage_3W_vs_E10	ordinary_muscle_development	ENSGALG00010023903	TBKBP1	-0.354460996	0.398024036	-0.890551734	0.373169703	0.411933199	negative
PRJNA729271	stage_3W_vs_E10	ordinary_muscle_development	ENSGALG00010023909	FOXRED1	0.209446855	0.153736337	1.362377033	0.173078928	0.202320892	positive
PRJNA729271	stage_3W_vs_E10	ordinary_muscle_development	ENSGALG00010023910	RNF7	0.782211017	0.100394288	7.791389683	6.62762E-15	1.996E-14	positive
PRJNA729271	stage_3W_vs_E10	ordinary_muscle_development	ENSGALG00010023911	TFCP2	-1.229333046	0.123521771	-9.952359287	2.46274E-23	1.07264E-22	negative
PRJNA729271	stage_3W_vs_E10	ordinary_muscle_development	ENSGALG00010023913	ZMYND8	-0.948593692	0.083736878	-11.32826672	9.50674E-30	5.42307E-29	negative
PRJNA729271	stage_3W_vs_E10	ordinary_muscle_development	ENSGALG00010023914	CCDC117	-0.250981249	0.065297661	-3.843648398	0.000121219	0.000196738	negative
PRJNA729271	stage_3W_vs_E10	ordinary_muscle_development	ENSGALG00010023917	ZRANB2	-0.144318872	0.079607521	-1.812879878	0.06985035	0.086080669	negative
PRJNA729271	stage_3W_vs_E10	ordinary_muscle_development	ENSGALG00010023918	MFSD14A	-0.713523526	0.080553307	-8.857780709	8.16231E-19	2.97642E-18	negative
PRJNA729271	stage_3W_vs_E10	ordinary_muscle_development	ENSGALG00010023920	ENSGALG00010023920	0.587533839	0.381986577	1.538100743	0.124023984	0.14799684	positive
PRJNA729271	stage_3W_vs_E10	ordinary_muscle_development	ENSGALG00010023921	GRK7	1.021649206	0.178975971	5.708303741	1.14108E-08	2.45472E-08	positive
PRJNA729271	stage_3W_vs_E10	ordinary_muscle_development	ENSGALG00010023923	CSNK1G1	-0.98209523	0.098130257	-10.00807766	1.40455E-23	6.1825E-23	negative
PRJNA729271	stage_3W_vs_E10	ordinary_muscle_development	ENSGALG00010023925	NCOA3	-0.453623528	0.077286757	-5.8693565	4.3749E-09	9.6082E-09	negative
PRJNA729271	stage_3W_vs_E10	ordinary_muscle_development	ENSGALG00010023926	ZYX	-0.145239691	0.138459867	-1.048965985	0.294193774	0.331442465	negative
PRJNA729271	stage_3W_vs_E10	ordinary_muscle_development	ENSGALG00010023927	KPNB1	-0.393918341	0.130300178	-3.023160418	0.002501496	0.003611473	negative
PRJNA729271	stage_3W_vs_E10	ordinary_muscle_development	ENSGALG00010023930	SRPRA	0.498320711	0.13113629	3.800021436	0.000144684	0.000233332	positive
PRJNA729271	stage_3W_vs_E10	ordinary_muscle_development	ENSGALG00010023931	CSRNP2	-1.59874338	0.152712073	-10.46900451	1.19897E-25	5.76421E-25	negative
PRJNA729271	stage_3W_vs_E10	ordinary_muscle_development	ENSGALG00010023937	XBP1	-0.556507675	0.110514493	-5.035608092	4.76334E-07	9.21578E-07	negative
PRJNA729271	stage_3W_vs_E10	ordinary_muscle_development	ENSGALG00010023940	ATP1B3	0.073420497	0.071584731	1.025644656	0.30505911	0.342777659	positive
PRJNA729271	stage_3W_vs_E10	ordinary_muscle_development	ENSGALG00010023942	NEGR1	2.156564063	0.220372803	9.785980991	1.29335E-22	5.48161E-22	positive
PRJNA729271	stage_3W_vs_E10	ordinary_muscle_development	ENSGALG00010023945	LETMD1	0.470188909	0.210522569	2.233437065	0.025520136	0.033161307	positive
PRJNA729271	stage_3W_vs_E10	ordinary_muscle_development	ENSGALG00010023946	ZNRF3	-1.0360388	0.129136897	-8.022794592	1.03366E-15	3.23465E-15	negative
PRJNA729271	stage_3W_vs_E10	ordinary_muscle_development	ENSGALG00010023947	ENSGALG00010023947	-8.69400238	0.849244301	-10.23733968	1.34895E-24	6.21321E-24	negative
PRJNA729271	stage_3W_vs_E10	ordinary_muscle_development	ENSGALG00010023948	SULF2	-1.542617901	0.220933135	-6.982284025	2.90419E-12	7.62614E-12	negative
PRJNA729271	stage_3W_vs_E10	ordinary_muscle_development	ENSGALG00010023953	SLC35A3	0.980549783	0.15389846	6.371407369	1.87301E-10	4.45948E-10	positive
PRJNA729271	stage_3W_vs_E10	ordinary_muscle_development	ENSGALG00010023954	PLEKHF1	0.628032476	0.095417635	6.581932963	4.64371E-11	1.14396E-10	positive
PRJNA729271	stage_3W_vs_E10	ordinary_muscle_development	ENSGALG00010023956	KREMEN1	-2.054063003	0.132356714	-15.51914479	2.57459E-54	3.33799E-53	negative
PRJNA729271	stage_3W_vs_E10	ordinary_muscle_development	ENSGALG00010023957	YBX3	2.633004389	0.13880488	18.96910527	3.07089E-80	7.72787E-79	positive
PRJNA729271	stage_3W_vs_E10	ordinary_muscle_development	ENSGALG00010023962	FPGT	-1.363401142	0.118059169	-11.54845616	7.51584E-31	4.45753E-30	negative
PRJNA729271	stage_3W_vs_E10	ordinary_muscle_development	ENSGALG00010023966	PEAK1	0.629915975	0.098067579	6.423284642	1.33365E-10	3.19524E-10	positive
PRJNA729271	stage_3W_vs_E10	ordinary_muscle_development	ENSGALG00010023967	PRPH	-3.113260166	0.377764184	-8.241279341	1.70379E-16	5.56602E-16	negative
PRJNA729271	stage_3W_vs_E10	ordinary_muscle_development	ENSGALG00010023968	TYW5	0.827236273	0.112438989	7.357201281	1.87806E-13	5.25226E-13	positive
PRJNA729271	stage_3W_vs_E10	ordinary_muscle_development	ENSGALG00010023972	ENSGALG00010023972	2.887125948	0.505878219	5.707156067	1.14879E-08	2.47059E-08	positive
PRJNA729271	stage_3W_vs_E10	ordinary_muscle_development	ENSGALG00010023974	PREX1	0.016760808	0.10302245	0.162690834	0.870761864	0.8854843	positive
PRJNA729271	stage_3W_vs_E10	ordinary_muscle_development	ENSGALG00010023978	TFDP2	0.126030788	0.13982114	0.901371475	0.367390843	0.406017861	positive
PRJNA729271	stage_3W_vs_E10	ordinary_muscle_development	ENSGALG00010023979	FAM118B	-0.618065457	0.115976724	-5.329219841	9.86355E-08	1.99738E-07	negative
PRJNA729271	stage_3W_vs_E10	ordinary_muscle_development	ENSGALG00010023981	ENSGALG00010023981	1.536869455	0.363294932	4.230363041	2.33314E-05	4.00651E-05	positive
PRJNA729271	stage_3W_vs_E10	ordinary_muscle_development	ENSGALG00010023984	FMNL1	3.163941211	0.197397746	16.02825402	8.11304E-58	1.14955E-56	positive
PRJNA729271	stage_3W_vs_E10	ordinary_muscle_development	ENSGALG00010023986	SYCE3	-0.537065368	0.625787919	-0.858222653	0.390769529	0.429790146	negative
PRJNA729271	stage_3W_vs_E10	ordinary_muscle_development	ENSGALG00010023987	TNNI3K	-1.593327694	0.459661348	-3.466307755	0.000527659	0.000810987	negative
PRJNA729271	stage_3W_vs_E10	ordinary_muscle_development	ENSGALG00010023989	ENSGALG00010023989	-0.18957106	0.100760121	-1.881409611	0.05991622	0.074509747	negative
PRJNA729271	stage_3W_vs_E10	ordinary_muscle_development	ENSGALG00010023991	TSPAN3	-0.025813788	0.105989614	-0.243550166	0.807579228	0.829790949	negative
PRJNA729271	stage_3W_vs_E10	ordinary_muscle_development	ENSGALG00010023992	FAM131B	-2.072875976	0.146973792	-14.10371159	3.6031E-45	3.46792E-44	negative
PRJNA729271	stage_3W_vs_E10	ordinary_muscle_development	ENSGALG00010023993	C19orf12	-0.322612972	0.175496426	-1.838287991	0.06601998	0.081651844	negative
PRJNA729271	stage_3W_vs_E10	ordinary_muscle_development	ENSGALG00010023996	MYO7B	5.283406096	0.460981744	11.46120462	2.06621E-30	1.2057E-29	positive
PRJNA729271	stage_3W_vs_E10	ordinary_muscle_development	ENSGALG00010023997	ARFGEF2	-0.180844687	0.062851213	-2.877346029	0.004010356	0.00567566	negative
PRJNA729271	stage_3W_vs_E10	ordinary_muscle_development	ENSGALG00010023999	DGCR14	0.057244429	0.092044472	0.621921424	0.533993537	0.571825969	positive
PRJNA729271	stage_3W_vs_E10	ordinary_muscle_development	ENSGALG00010024001	ENSGALG00010024001	5.239726083	0.449208275	11.66435789	1.9386E-31	1.17328E-30	positive
PRJNA729271	stage_3W_vs_E10	ordinary_muscle_development	ENSGALG00010024002	DGCR2	-0.70870672	0.061019138	-11.61449897	3.47821E-31	2.08592E-30	negative
PRJNA729271	stage_3W_vs_E10	ordinary_muscle_development	ENSGALG00010024003	ERICH3	-1.18523899	0.303423263	-3.906223208	9.37499E-05	0.000153341	negative
PRJNA729271	stage_3W_vs_E10	ordinary_muscle_development	ENSGALG00010024004	EIF4B	0.181737779	0.125160943	1.452032676	0.14649252	0.17307108	positive
PRJNA729271	stage_3W_vs_E10	ordinary_muscle_development	ENSGALG00010024005	STYK1	4.526036263	0.249173816	18.16417282	9.91907E-74	2.12688E-72	positive
PRJNA729271	stage_3W_vs_E10	ordinary_muscle_development	ENSGALG00010024008	CCNE1	0.331736899	0.102057612	3.250486568	0.001152077	0.001718041	positive
PRJNA729271	stage_3W_vs_E10	ordinary_muscle_development	ENSGALG00010024009	CSE1L	-0.946854655	0.061460949	-15.40579294	1.49642E-53	1.90436E-52	negative
PRJNA729271	stage_3W_vs_E10	ordinary_muscle_development	ENSGALG00010024010	ENSGALG00010024010	1.668146004	0.088336347	18.88402748	1.54355E-79	3.79215E-78	positive
PRJNA729271	stage_3W_vs_E10	ordinary_muscle_development	ENSGALG00010024011	AGPS	0.6101681	0.0698261	8.738395799	2.36444E-18	8.47281E-18	positive
PRJNA729271	stage_3W_vs_E10	ordinary_muscle_development	ENSGALG00010024013	PTPRJ	0.716591685	0.119440144	5.999588279	1.97818E-09	4.42224E-09	positive
PRJNA729271	stage_3W_vs_E10	ordinary_muscle_development	ENSGALG00010024016	CRYZ	0.158453052	0.103089005	1.537050941	0.124280835	0.148267548	positive
PRJNA729271	stage_3W_vs_E10	ordinary_muscle_development	ENSGALG00010024017	TYW3	-1.823287905	0.137622702	-13.24845302	4.606E-40	3.72775E-39	negative
PRJNA729271	stage_3W_vs_E10	ordinary_muscle_development	ENSGALG00010024018	KRT18	-6.309824314	1.091156351	-5.782694942	7.35133E-09	1.599E-08	negative
PRJNA729271	stage_3W_vs_E10	ordinary_muscle_development	ENSGALG00010024019	MAP3K14	0.991060852	0.106189937	9.332907389	1.03006E-20	4.05395E-20	positive
PRJNA729271	stage_3W_vs_E10	ordinary_muscle_development	ENSGALG00010024020	MAIP1	0.523078165	0.121843121	4.293046325	1.76238E-05	3.05754E-05	positive
PRJNA729271	stage_3W_vs_E10	ordinary_muscle_development	ENSGALG00010024021	IGLL1	13.24081881	1.20722399	10.96798847	5.44707E-28	2.89957E-27	positive
PRJNA729271	stage_3W_vs_E10	ordinary_muscle_development	ENSGALG00010024023	LHX8	-4.357571989	0.532430555	-8.184301129	2.73888E-16	8.85966E-16	negative
PRJNA729271	stage_3W_vs_E10	ordinary_muscle_development	ENSGALG00010024024	AGL	4.846598639	0.122587651	39.53578193	0	0	positive
PRJNA729271	stage_3W_vs_E10	ordinary_muscle_development	ENSGALG00010024025	CLCN1	7.985512678	0.333169302	23.96833268	5.951E-127	4.0121E-125	positive
PRJNA729271	stage_3W_vs_E10	ordinary_muscle_development	ENSGALG00010024026	TIRAP	0.598589363	0.103491138	5.783967343	7.29591E-09	1.58766E-08	positive
PRJNA729271	stage_3W_vs_E10	ordinary_muscle_development	ENSGALG00010024029	SCAPER	0.193486833	0.147009732	1.316149825	0.18812376	0.218585483	positive
PRJNA729271	stage_3W_vs_E10	ordinary_muscle_development	ENSGALG00010024030	STAU1	0.107964267	0.089601432	1.204939087	0.228226818	0.261844841	positive
PRJNA729271	stage_3W_vs_E10	ordinary_muscle_development	ENSGALG00010024031	LIMS2	0.627967305	0.115170886	5.452483074	4.96713E-08	1.02474E-07	positive
PRJNA729271	stage_3W_vs_E10	ordinary_muscle_development	ENSGALG00010024034	LRP2	-1.093912772	0.248185097	-4.407648909	1.04499E-05	1.84249E-05	negative
PRJNA729271	stage_3W_vs_E10	ordinary_muscle_development	ENSGALG00010024035	OSBPL5	-0.745620103	0.137165596	-5.435911969	5.45169E-08	1.12104E-07	negative
PRJNA729271	stage_3W_vs_E10	ordinary_muscle_development	ENSGALG00010024038	NKD1	-3.294354645	0.171116482	-19.25211767	1.35509E-82	3.63069E-81	negative
PRJNA729271	stage_3W_vs_E10	ordinary_muscle_development	ENSGALG00010024039	MRPL45	-0.319816669	0.107250499	-2.981959741	0.002864097	0.004113662	negative
PRJNA729271	stage_3W_vs_E10	ordinary_muscle_development	ENSGALG00010024040	ENSGALG00010024040	-4.891284787	0.912785862	-5.358633378	8.38538E-08	1.70807E-07	negative
PRJNA729271	stage_3W_vs_E10	ordinary_muscle_development	ENSGALG00010024042	DCPS	-0.442983892	0.130833497	-3.385859904	0.000709556	0.001077529	negative
PRJNA729271	stage_3W_vs_E10	ordinary_muscle_development	ENSGALG00010024045	PSTPIP1	2.708425305	0.243477415	11.1239283	9.5952E-29	5.25038E-28	positive
PRJNA729271	stage_3W_vs_E10	ordinary_muscle_development	ENSGALG00010024046	ENSGALG00010024046	-9.072379232	0.986823518	-9.193517447	3.80201E-20	1.46142E-19	negative
PRJNA729271	stage_3W_vs_E10	ordinary_muscle_development	ENSGALG00010024049	RCN2	-0.755094705	0.144067516	-5.241255802	1.59487E-07	3.17944E-07	negative
PRJNA729271	stage_3W_vs_E10	ordinary_muscle_development	ENSGALG00010024051	ENSGALG00010024051	-7.646530927	0.859595747	-8.895496466	5.81587E-19	2.13366E-18	negative
PRJNA729271	stage_3W_vs_E10	ordinary_muscle_development	ENSGALG00010024052	ENSGALG00010024052	0.734933479	0.166111709	4.424332773	9.67408E-06	1.70903E-05	positive
PRJNA729271	stage_3W_vs_E10	ordinary_muscle_development	ENSGALG00010024053	ISL2	-1.578770676	0.602324024	-2.621131838	0.008763836	0.011994317	negative
PRJNA729271	stage_3W_vs_E10	ordinary_muscle_development	ENSGALG00010024054	ENSGALG00010024054	-6.726345172	1.032516126	-6.514518275	7.29233E-11	1.77397E-10	negative
PRJNA729271	stage_3W_vs_E10	ordinary_muscle_development	ENSGALG00010024057	HSD3B1	-2.38216938	0.765103713	-3.113524792	0.00184867	0.00269908	negative
PRJNA729271	stage_3W_vs_E10	ordinary_muscle_development	ENSGALG00010024058	SLC44A5	0.201082315	0.105400587	1.907791227	0.056418205	0.070379549	positive
PRJNA729271	stage_3W_vs_E10	ordinary_muscle_development	ENSGALG00010024060	FRRS1	0.589271298	0.161368313	3.651716288	0.000260494	0.000410731	positive
PRJNA729271	stage_3W_vs_E10	ordinary_muscle_development	ENSGALG00010024061	ENSGALG00010024061	1.038968261	1.285608081	0.808153182	0.419002406	0.457544061	positive
PRJNA729271	stage_3W_vs_E10	ordinary_muscle_development	ENSGALG00010024062	ST3GAL4	-0.162893449	0.133063777	-1.224175753	0.220885934	0.253891302	negative
PRJNA729271	stage_3W_vs_E10	ordinary_muscle_development	ENSGALG00010024063	BRD7	0.323483235	0.103776664	3.117109601	0.001826337	0.002668086	positive
PRJNA729271	stage_3W_vs_E10	ordinary_muscle_development	ENSGALG00010024064	GPR17	-1.740732847	0.223043899	-7.804440538	5.97661E-15	1.80406E-14	negative
PRJNA729271	stage_3W_vs_E10	ordinary_muscle_development	ENSGALG00010024066	ENSGALG00010024066	6.611983837	2.117457042	3.122605893	0.001792576	0.002621143	positive
PRJNA729271	stage_3W_vs_E10	ordinary_muscle_development	ENSGALG00010024068	CASP2	-1.428591961	0.086516203	-16.51242102	2.98658E-61	4.62011E-60	negative
PRJNA729271	stage_3W_vs_E10	ordinary_muscle_development	ENSGALG00010024069	ENSGALG00010024069	6.934680777	2.220319906	3.123280009	0.001788475	0.00261541	positive
PRJNA729271	stage_3W_vs_E10	ordinary_muscle_development	ENSGALG00010024073	RABGGTB	0.591459243	0.124802016	4.739180212	2.14585E-06	3.97699E-06	positive
PRJNA729271	stage_3W_vs_E10	ordinary_muscle_development	ENSGALG00010024074	ADCY7	0.37888782	0.22181547	1.708121713	0.087613761	0.10676431	positive

PRJNA729271	stage_3W_vs_E10	ordinary_muscle_development	ENSGALG00010024075	SPATS2L	-0.586697914	0.137212062	-4.27584795	1.90411E-05	3.29514E-05	negative
PRJNA729271	stage_3W_vs_E10	ordinary_muscle_development	ENSGALG00010024077	SFT2D3	0.385627841	0.115960301	3.325516037	0.00088255	0.001327856	positive
PRJNA729271	stage_3W_vs_E10	ordinary_muscle_development	ENSGALG00010024078	KIRREL3	-2.588443278	0.259756706	-9.964875671	2.17145E-23	9.49765E-23	negative
PRJNA729271	stage_3W_vs_E10	ordinary_muscle_development	ENSGALG00010024080	TENT4B	0.012895906	0.087534286	0.147324054	0.882876247	0.896671189	positive
PRJNA729271	stage_3W_vs_E10	ordinary_muscle_development	ENSGALG00010024081	CARS	-0.210524461	0.098908048	-2.128486665	0.033296754	0.042696077	negative
PRJNA729271	stage_3W_vs_E10	ordinary_muscle_development	ENSGALG00010024083	PALMD	-0.940485815	0.191077538	-4.922011371	8.56592E-07	1.63008E-06	negative
PRJNA729271	stage_3W_vs_E10	ordinary_muscle_development	ENSGALG00010024086	GABARAPL1	0.155119392	0.215031348	0.721380363	0.470675525	0.509365517	positive
PRJNA729271	stage_3W_vs_E10	ordinary_muscle_development	ENSGALG00010024089	ENSGALG00010024089	7.736543816	1.753844582	4.411191217	1.02803E-05	1.8137E-05	positive
PRJNA729271	stage_3W_vs_E10	ordinary_muscle_development	ENSGALG00010024091	ETFA	-0.737306511	0.117116205	-6.295512313	3.06386E-10	7.20602E-10	negative
PRJNA729271	stage_3W_vs_E10	ordinary_muscle_development	ENSGALG00010024095	RBM12	-1.344426354	0.053523259	-25.11854435	3.1196E-139	2.5122E-137	negative
PRJNA729271	stage_3W_vs_E10	ordinary_muscle_development	ENSGALG00010024096	MYO1D	-0.294315294	0.220847809	-1.332661146	0.182643029	0.212626352	negative
PRJNA729271	stage_3W_vs_E10	ordinary_muscle_development	ENSGALG00010024099	ENSGALG00010024099	6.657520685	1.917355121	3.472241847	0.000516131	0.000794027	positive
PRJNA729271	stage_3W_vs_E10	ordinary_muscle_development	ENSGALG00010024101	CNEP1R1	0.20123159	0.089741143	2.242355981	0.024938374	0.032446076	positive
PRJNA729271	stage_3W_vs_E10	ordinary_muscle_development	ENSGALG00010024102	SGF29	0.383663559	0.090006761	4.262608214	2.02055E-05	3.48997E-05	positive
PRJNA729271	stage_3W_vs_E10	ordinary_muscle_development	ENSGALG00010024104	PLPPR5	-1.033224593	0.168092002	-6.146780226	7.90716E-10	1.81438E-09	negative
PRJNA729271	stage_3W_vs_E10	ordinary_muscle_development	ENSGALG00010024106	WDR33	-0.135123891	0.063657517	-2.12266983	0.033781529	0.043306205	negative
PRJNA729271	stage_3W_vs_E10	ordinary_muscle_development	ENSGALG00010024107	NFE2L2	1.616488915	0.062393786	25.90785115	5.4325E-148	4.9524E-146	positive
PRJNA729271	stage_3W_vs_E10	ordinary_muscle_development	ENSGALG00010024110	CPNE1	-0.766755998	0.126307689	-6.070540943	1.2748E-09	2.88813E-09	negative
PRJNA729271	stage_3W_vs_E10	ordinary_muscle_development	ENSGALG00010024112	NAP1L4	0.574170928	0.090410847	6.350686317	2.14356E-10	5.08943E-10	positive
PRJNA729271	stage_3W_vs_E10	ordinary_muscle_development	ENSGALG00010024113	ENSGALG00010024113	1.028419527	0.164312494	6.258924691	3.87641E-10	9.06267E-10	positive
PRJNA729271	stage_3W_vs_E10	ordinary_muscle_development	ENSGALG00010024115	POLR2D	-0.598762062	0.073707742	-8.123462232	4.5307E-16	1.44653E-15	negative
PRJNA729271	stage_3W_vs_E10	ordinary_muscle_development	ENSGALG00010024116	MCAM	0.018810263	0.183660376	0.102418734	0.918424314	0.927898546	positive
PRJNA729271	stage_3W_vs_E10	ordinary_muscle_development	ENSGALG00010024118	TMEM266	-1.254096323	0.138363545	-9.063777075	1.2601E-19	4.74419E-19	negative
PRJNA729271	stage_3W_vs_E10	ordinary_muscle_development	ENSGALG00010024119	AMMECR1L	-0.833822631	0.075535118	-11.03887381	2.48123E-28	1.3415E-27	negative
PRJNA729271	stage_3W_vs_E10	ordinary_muscle_development	ENSGALG00010024121	NRG4	0.962774508	0.167730669	5.740002809	9.4675E-09	2.0464E-08	positive
PRJNA729271	stage_3W_vs_E10	ordinary_muscle_development	ENSGALG00010024122	SOCS7	0.642321089	0.104744768	6.132249862	8.66449E-10	1.98282E-09	positive
PRJNA729271	stage_3W_vs_E10	ordinary_muscle_development	ENSGALG00010024123	PLPPR4	-0.420193272	0.393238297	-1.068546159	0.285274221	0.322370767	negative
PRJNA729271	stage_3W_vs_E10	ordinary_muscle_development	ENSGALG00010024124	DIDO1	-0.170717826	0.066992736	-2.548303541	0.010824824	0.014658616	negative
PRJNA729271	stage_3W_vs_E10	ordinary_muscle_development	ENSGALG00010024125	ENSGALG00010024125	-3.088723541	1.676827863	-1.842003946	0.065474573	0.081018775	negative
PRJNA729271	stage_3W_vs_E10	ordinary_muscle_development	ENSGALG00010024126	CDK5R1	-1.577938568	0.302298839	-5.21979698	1.79119E-07	3.5615E-07	negative
PRJNA729271	stage_3W_vs_E10	ordinary_muscle_development	ENSGALG00010024128	PHLDA2	-2.585390772	0.236709372	-10.9222155	9.02666E-28	4.76828E-27	negative
PRJNA729271	stage_3W_vs_E10	ordinary_muscle_development	ENSGALG00010024129	GID8	0.141603289	0.101043203	1.401413304	0.161090516	0.18924931	positive
PRJNA729271	stage_3W_vs_E10	ordinary_muscle_development	ENSGALG00010024131	ENSGALG00010024131	0.076005447	1.302045091	0.058373897	0.953450806	0.959541029	positive
PRJNA729271	stage_3W_vs_E10	ordinary_muscle_development	ENSGALG00010024132	ENSGALG00010024132	-1.477368776	0.151175498	-9.772541152	1.477E-22	6.24355E-22	negative
PRJNA729271	stage_3W_vs_E10	ordinary_muscle_development	ENSGALG00010024133	PSMD11	0.543549813	0.140832219	3.85955585	0.000113593	0.000184773	positive
PRJNA729271	stage_3W_vs_E10	ordinary_muscle_development	ENSGALG00010024135	FBXO22	0.907710541	0.087148215	10.41571001	2.10223E-25	9.99076E-25	positive
PRJNA729271	stage_3W_vs_E10	ordinary_muscle_development	ENSGALG00010024136	SAP130	-0.439090188	0.176069244	-2.49384945	0.012636613	0.016991438	negative
PRJNA729271	stage_3W_vs_E10	ordinary_muscle_development	ENSGALG00010024137	ENSGALG00010024137	0.754885228	0.735221125	1.026745834			positive
PRJNA729271	stage_3W_vs_E10	ordinary_muscle_development	ENSGALG00010024138	CBL	0.591555076	0.148866479	3.973729208	7.0756E-05	0.000117025	positive
PRJNA729271	stage_3W_vs_E10	ordinary_muscle_development	ENSGALG00010024139	SLC22A18	-2.020571385	0.753642374	-2.681074544	0.007338617	0.010120183	negative
PRJNA729271	stage_3W_vs_E10	ordinary_muscle_development	ENSGALG00010024140	KRT6A	-5.532434983	1.054095903	-5.248511986	1.53333E-07	3.06095E-07	negative
PRJNA729271	stage_3W_vs_E10	ordinary_muscle_development	ENSGALG00010024141	YTHDF1	-0.498519405	0.096339964	-5.174585751	2.28417E-07	4.50893E-07	negative
PRJNA729271	stage_3W_vs_E10	ordinary_muscle_development	ENSGALG00010024142	DNAJC6	-1.307741993	0.258784845	-5.053394806	4.34025E-07	8.41993E-07	negative
PRJNA729271	stage_3W_vs_E10	ordinary_muscle_development	ENSGALG00010024143	UBE2Q2	2.497003379	0.116361913	21.45894065	3.7679E-102	1.64011E-100	positive
PRJNA729271	stage_3W_vs_E10	ordinary_muscle_development	ENSGALG00010024144	HNRNPA3	-1.525795734	0.126849877	-12.02835803	2.52125E-33	1.64028E-32	negative
PRJNA729271	stage_3W_vs_E10	ordinary_muscle_development	ENSGALG00010024145	RAP1GAP3	-0.349549764	0.184131285	-1.898372478	0.057647032	0.07180491	negative
PRJNA729271	stage_3W_vs_E10	ordinary_muscle_development	ENSGALG00010024146	CDKN1C	-2.325893577	0.24471504	-9.504497871	2.01015E-21	8.15254E-21	negative
PRJNA729271	stage_3W_vs_E10	ordinary_muscle_development	ENSGALG00010024147	ENSGALG00010024147	-0.281386691	0.270102672	-1.041776778	0.297515183	0.334950103	negative
PRJNA729271	stage_3W_vs_E10	ordinary_muscle_development	ENSGALG00010024148	UGGT1	-0.421426973	0.058600618	-7.191510757	6.40783E-13	1.74327E-12	negative
PRJNA729271	stage_3W_vs_E10	ordinary_muscle_development	ENSGALG00010024149	TCIRG1	0.839120691	0.152414188	5.505528718	3.68062E-08	7.65651E-08	positive
PRJNA729271	stage_3W_vs_E10	ordinary_muscle_development	ENSGALG00010024150	NHERF4	1.967124115	0.342082131	5.750443939	8.90094E-09	1.92681E-08	positive
PRJNA729271	stage_3W_vs_E10	ordinary_muscle_development	ENSGALG00010024151	LEPROT	-0.483606834	0.148279282	-3.261459235	0.001108404	0.001654785	negative
PRJNA729271	stage_3W_vs_E10	ordinary_muscle_development	ENSGALG00010024152	ZNF423	-1.389658593	0.108121559	-12.85274287	8.30137E-38	6.20569E-37	negative
PRJNA729271	stage_3W_vs_E10	ordinary_muscle_development	ENSGALG00010024153	KCNQ1	2.123404189	0.201119967	10.55789846	4.67E-26	2.2892E-25	positive
PRJNA729271	stage_3W_vs_E10	ordinary_muscle_development	ENSGALG00010024154	VPS29L	0.845796572	0.47401295	1.784332206	0.0743697	0.091365926	positive
PRJNA729271	stage_3W_vs_E10	ordinary_muscle_development	ENSGALG00010024155	SNX7	-0.950482884	0.166058203	-5.723793645	1.04171E-08	2.24663E-08	negative
PRJNA729271	stage_3W_vs_E10	ordinary_muscle_development	ENSGALG00010024157	CHRNB4	-3.689844461	0.425658829	-8.668549106	4.37661E-18	1.55108E-17	negative
PRJNA729271	stage_3W_vs_E10	ordinary_muscle_development	ENSGALG00010024158	ENSGALG00010024158	0.509924577	0.337207018	1.51220037	0.130482897	0.15535975	positive
PRJNA729271	stage_3W_vs_E10	ordinary_muscle_development	ENSGALG00010024159	NLRX1	0.423388778	0.161829526	2.616264103	0.008889777	0.012159792	positive
PRJNA729271	stage_3W_vs_E10	ordinary_muscle_development	ENSGALG00010024162	SRCIN1	-3.653782931	0.573939874	-6.366142336	1.93842E-10	4.61066E-10	negative
PRJNA729271	stage_3W_vs_E10	ordinary_muscle_development	ENSGALG00010024163	HS6ST1	-1.258284542	0.097615969	-12.89015065	5.11438E-38	3.85106E-37	negative
PRJNA729271	stage_3W_vs_E10	ordinary_muscle_development	ENSGALG00010024164	SNAP29	1.635817827	0.112011815	14.60397576	2.64934E-48	2.8573E-47	positive
PRJNA729271	stage_3W_vs_E10	ordinary_muscle_development	ENSGALG00010024165	LEPR	-1.317309042	0.279753399	-4.708822303	2.49152E-06	4.60045E-06	negative
PRJNA729271	stage_3W_vs_E10	ordinary_muscle_development	ENSGALG00010024166	ENSGALG00010024166	6.538284498	0.882653572	7.407531909	1.28672E-13	3.63E-13	positive
PRJNA729271	stage_3W_vs_E10	ordinary_muscle_development	ENSGALG00010024167	CHRNA3	-0.428430152	0.523697786	-0.81808662	0.413307728	0.452211316	negative
PRJNA729271	stage_3W_vs_E10	ordinary_muscle_development	ENSGALG00010024168	BIRC7	-1.610043709	0.368964693	-4.36367962	1.27893E-05	2.2416E-05	negative
PRJNA729271	stage_3W_vs_E10	ordinary_muscle_development	ENSGALG00010024169	FAM168B	-0.681778798	0.072438841	-9.411784962	4.87782E-21	1.94509E-20	negative
PRJNA729271	stage_3W_vs_E10	ordinary_muscle_development	ENSGALG00010024170	NUP160	-1.223471966	0.087732365	-13.94550314	3.35077E-44	3.10943E-43	negative
PRJNA729271	stage_3W_vs_E10	ordinary_muscle_development	ENSGALG00010024171	ENSGALG00010024171	-2.491791409	0.642059026	-3.880938216	0.000104054	0.000169678	negative
PRJNA729271	stage_3W_vs_E10	ordinary_muscle_development	ENSGALG00010024172	ABCG4	-3.360489801	0.423221172	-7.940268635	2.01744E-15	6.22054E-15	negative
PRJNA729271	stage_3W_vs_E10	ordinary_muscle_development	ENSGALG00010024173	CBLN1	-1.946225914	0.429391987	-4.532515677	5.82853E-06	1.0482E-05	negative
PRJNA729271	stage_3W_vs_E10	ordinary_muscle_development	ENSGALG00010024174	PLEKHB2	0.904138728	0.10129156	8.92610135	4.41286E-19	1.62884E-18	positive
PRJNA729271	stage_3W_vs_E10	ordinary_muscle_development	ENSGALG00010024175	DHRS9	5.6227028	0.394977228	14.23551132	5.5158E-46	5.46117E-45	positive
PRJNA729271	stage_3W_vs_E10	ordinary_muscle_development	ENSGALG00010024177	ENSGALG00010024177	1.435064221	0.401041748	3.57834123	0.000345782	0.000540447	positive
PRJNA729271	stage_3W_vs_E10	ordinary_muscle_development	ENSGALG00010024178	N4BP1	1.223017295	0.120334093	10.16351446	2.88486E-24	1.30921E-23	positive
PRJNA729271	stage_3W_vs_E10	ordinary_muscle_development	ENSGALG00010024179	ATG4C	-0.443325267	0.116666069	-3.799950326	0.000144725	0.000233347	negative
PRJNA729271	stage_3W_vs_E10	ordinary_muscle_development	ENSGALG00010024180	HINFP	0.387373319	0.139847014	2.769979184	0.005605988	0.00781936	positive
PRJNA729271	stage_3W_vs_E10	ordinary_muscle_development	ENSGALG00010024181	FOXD3	0.837543062	0.314789039	2.660648742	0.007799027	0.010729584	positive
PRJNA729271	stage_3W_vs_E10	ordinary_muscle_development	ENSGALG00010024183	MTX2	0.514807883	0.106290676	4.843396452	1.27638E-06	2.39963E-06	positive
PRJNA729271	stage_3W_vs_E10	ordinary_muscle_development	ENSGALG00010024184	ENSGALG00010024184	-3.39826794	0.226240985	-15.02056732	5.38449E-51	6.29929E-50	negative
PRJNA729271	stage_3W_vs_E10	ordinary_muscle_development	ENSGALG00010024185	CHRNA5	0.355319982	0.154979116	2.292695884	0.02186552	0.028651302	positive
PRJNA729271	stage_3W_vs_E10	ordinary_muscle_development	ENSGALG00010024186	HAO2	-1.993499072	0.772674701	-2.579997856	0.009880093	0.013432915	negative
PRJNA729271	stage_3W_vs_E10	ordinary_muscle_development	ENSGALG00010024188	TSSC4	-1.441810586	0.08491966	-16.97852517	1.18425E-64	2.00768E-63	negative
PRJNA729271	stage_3W_vs_E10	ordinary_muscle_development	ENSGALG00010024189	KAT7	-0.175555711	0.140442089	-1.250022069	0.211291486	0.243746127	negative
PRJNA729271	stage_3W_vs_E10	ordinary_muscle_development	ENSGALG00010024190	WARS2	-1.431490765	0.114538385	-12.49791296	7.66369E-36	5.4056E-35	negative
PRJNA729271	stage_3W_vs_E10	ordinary_muscle_development	ENSGALG00010024191	DPYD	-0.631595324	0.110684336	-5.70627558	1.15475E-08	2.48266E-08	negative
PRJNA729271	stage_3W_vs_E10	ordinary_muscle_development	ENSGALG00010024192	ALG6	-0.71601092	0.094128123	-7.606769367	2.81032E-14	8.1996E-14	negative
PRJNA729271	stage_3W_vs_E10	ordinary_muscle_development	ENSGALG00010024193	GSTK1	0.25999607	0.12655294	2.054445113	0.03993263	0.050751497	positive
PRJNA729271	stage_3W_vs_E10	ordinary_muscle_development	ENSGALG00010024194	NKAIN4	-0.831257436	0.086836306	-9.57269461	1.04155E-21	4.26718E-21	negative
PRJNA729271	stage_3W_vs_E10	ordinary_muscle_development	ENSGALG00010024195	PSMA4	0.461198812	0.120672176	3.821915112	0.000132419	0.000214292	positive
PRJNA729271	stage_3W_vs_E10	ordinary_muscle_development	ENSGALG00010024196	MRPL21	0.339685295	0.108793444	3.122295643	0.001794466	0.002623113	positive
PRJNA729271	stage_3W_vs_E10	ordinary_muscle_development	ENSGALG00010024197	KRT7	-4.766629332	0.312363416	-15.25988347	1.41501E-52	1.73818E-51	negative
PRJNA729271	stage_3W_vs_E10	ordinary_muscle_development	ENSGALG00010024198	PI4KA	0.102764464	0.064446282	1.594575529	0.110807189	0.133080636	positive
PRJNA729271	stage_3W_vs_E10	ordinary_muscle_development	ENSGALG00010024199	SLC35B1	-0.124165206	0.101707479	-1.220807038	0.222159088	0.255267221	negative
PRJNA729271	stage_3W_vs_E10	ordinary_muscle_development	ENSGALG00010024200	GCHFR	-3.408461337	0.744739944	-4.576713473	4.72338E-06	8.54962E-06	negative
PRJNA729271	stage_3W_vs_E10	ordinary_muscle_development	ENSGALG00010024201	IREB2	-0.184166303	0.069666117	-2.643556302	0.008204012	0.011255765	negative
PRJNA729271	stage_3W_vs_E10	ordinary_muscle_development	ENSGALG00010024203	SPOP	0.179080037	0.072124802	2.482918954	0.013031073	0.017489389	positive
PRJNA729271	stage_3W_vs_E10	ordinary_muscle_development	ENSGALG00010024204	PTBP2	0.276795952	0.107867359	2.566077028	0.010285598	0.013957101	positive
PRJNA729271	stage_3W_vs_E10	ordinary_muscle_development	ENSGALG00010024205	ANKRD13D	0.170632431	0.169084108	1.009157117	0.312899289	0.350825742	positive
PRJNA729271	stage_3W_vs_E10	ordinary_muscle_development	ENSGALG00010024206	KRT80	-2.949546889	0.656081058	-4.495704991	6.93398E-06	1.23824E-05	negative
PRJNA729271	stage_3W_vs_E10	ordinary_muscle_development	ENSGALG00010024207	KCTD18	-0.800047206	0.124860764	-6.407514901	1.47911E-10	3.53323E-10	negative
PRJNA729271	stage_3W_vs_E10	ordinary_muscle_development	ENSGALG00010024208	SERPIND1	2.188793768	0.581734793	3.762528552	0.000168204	0.000269733	positive
PRJNA729271	stage_3W_vs_E10	ordinary_muscle_development	ENSGALG00010024209	RWDD3	-0.403753116	0.144119489	-2.801516428	0.005086305	0.007126038	negative
PRJNA729271	stage_3W_vs_E10	ordinary_muscle_development	ENSGALG00010024210	NXPH3	-4.997449528	1.247172595	-4.007023204	6.14888E-05	0.000102293	negative
PRJNA729271	stage_3W_vs_E10	ordinary_muscle_development	ENSGALG00010024211	TRPM5	-0.010024383	0.757461291	-0.013234185	0.989440956	0.991013451	negative
PRJNA729271	stage_3W_vs_E10	ordinary_muscle_development	ENSGALG00010024212	RIMS4	-0.580238924	0.202339979	-2.86764349	0.004135412	0.005846937	negative
PRJNA729271	stage_3W_vs_E10	ordinary_muscle_development	ENSGALG00010024213	ENSGALG00010024213	-0.664865363	0.191590985	-3.470233032	0.000520007	0.000799565	negative
PRJNA729271	stage_3W_vs_E10	ordinary_muscle_development	ENSGALG00010024214	CRABP1	-10.39832292	0.548130602	-18.97052069	2.98931E-80	7.53566E-79	negative
PRJNA729271	stage_3W_vs_E10	ordinary_muscle_development	ENSGALG00010024215	ENSGALG00010024215	0.874879336	0.156680009	5.583860651	2.35237E-08	4.95605E-08	positive
PRJNA729271	stage_3W_vs_E10	ordinary_muscle_development	ENSGALG00010024218	NGFR	-2.803041697	0.233689394	-11.9947322	3.7864E-33	2.44908E-32	negative
PRJNA729271	stage_3W_vs_E10	ordinary_muscle_development	ENSGALG00010024220	SIAH1	2.40910027	0.200455684	12.01811903	2.85394E-33	1.8534E-32	positive
PRJNA729271	stage_3W_vs_E10	ordinary_muscle_development	ENSGALG00010024222	ENSGALG00010024222	-0.812890748	0.318957794	-2.548584058	0.010816122	0.014650938	negative
PRJNA729271	stage_3W_vs_E10	ordinary_muscle_development	ENSGALG00010024223	ENSGALG00010024223	-2.107971701	0.316715634	-6.655723535	2.8191E-11	7.04167E-11	negative
PRJNA729271	stage_3W_vs_E10	ordinary_muscle_development	ENSGALG00010024225	BCDIN3D	0.380118918	0.176152218	2.15790026	0.030935587	0.039823387	positive
PRJNA729271	stage_3W_vs_E10	ordinary_muscle_development	ENSGALG00010024226	POU2AF1	3.490129711	1.255881706	2.779027431	0.005452192	0.007613634	positive
PRJNA729271	stage_3W_vs_E10	ordinary_muscle_development	ENSGALG00010024227	MUC6	0.649375183	0.476764972	1.362044659	0.173183792	0.202410826	positive
PRJNA729271	stage_3W_vs_E10	ordinary_muscle_development	ENSGALG00010024230	LONP2	0.505438021	0.150405124	3.36051065	0.000777985	0.001176638	positive
PRJNA729271	stage_3W_vs_E10	ordinary_muscle_development	ENSGALG00010024233	FAIM2	-5.206609371	0.340152792	-15.30667835	6.89989E-53	8.56283E-52	negative
PRJNA729271	stage_3W_vs_E10	ordinary_muscle_development	ENSGALG00010024234	ENSGALG00010024234	0.594125171	0.27055114	2.195981032	0.028093301	0.036329057	positive
PRJNA729271	stage_3W_vs_E10	ordinary_muscle_development	ENSGALG00010024238	ATF7	0.754806953	0.208087074	3.627361082	0.000286333	0.000450542	positive
PRJNA729271	stage_3W_vs_E10	ordinary_muscle_development	ENSGALG00010024240	WDR61	-0.684730131	0.126488734	-5.413368546	6.185E-08	1.26698E-07	negative
PRJNA729271	stage_3W_vs_E10	ordinary_muscle_development	ENSGALG00010024242	KMT2A	-0.023750078	0.125203	-0.18969256	0.849550051	0.866789151	negative
PRJNA729271	stage_3W_vs_E10	ordinary_muscle_development	ENSGALG00010024243	MPP3	0.52783922	0.202813922	2.602578827	0.009252554	0.012623849	positive
PRJNA729271	stage_3W_vs_E10	ordinary_muscle_development	ENSGALG00010024245	TARBP2	0.605469366	0.164116877	3.689257185	0.00022491	0.000356564	positive
PRJNA729271	stage_3W_vs_E10	ordinary_muscle_development	ENSGALG00010024250	AP2A2	-0.337579823	0.05367355	-6.289500542	3.18489E-10	7.48217E-10	negative
PRJNA729271	stage_3W_vs_E10	ordinary_muscle_development	ENSGALG00010024251	ENSGALG00010024251	2.966667767	0.206078217	14.39583383	5.4958E-47	5.60998E-46	positive
PRJNA729271	stage_3W_vs_E10	ordinary_muscle_development	ENSGALG00010024254	ENSGALG00010024254	1.517475002	0.317489385	4.779608622	1.75637E-06	3.27105E-06	positive
PRJNA729271	stage_3W_vs_E10	ordinary_muscle_development	ENSGALG00010024255	CFAP97D1	1.390461491	0.670057214	2.075138455	0.037973705	0.048410629	positive
PRJNA729271	stage_3W_vs_E10	ordinary_muscle_development	ENSGALG00010024258	DNAJA4	0.408481426	0.151653578	2.693516579	0.007070263	0.009763119	positive
PRJNA729271	stage_3W_vs_E10	ordinary_muscle_development	ENSGALG00010024259	ENSGALG00010024259	1.481623608	0.134724335	10.99744604	3.93108E-28	2.10729E-27	positive
PRJNA729271	stage_3W_vs_E10	ordinary_muscle_development	ENSGALG00010024263	PHKB	4.430249785	0.131317153	33.73702287	1.6571E-249	4.6192E-247	positive

PRJNA729271	stage_3W_vs_E10	ordinary_muscle_development	ENSGALG00010024264	AMER3	-0.575216317	0.305027673	-1.88578404	0.059324062	0.073786021	negative
PRJNA729271	stage_3W_vs_E10	ordinary_muscle_development	ENSGALG00010024266	ENSGALG00010024266	0.213490982	0.24420998	0.874210719	0.38200351	0.420915308	positive
PRJNA729271	stage_3W_vs_E10	ordinary_muscle_development	ENSGALG00010024267	MAP3K12	-2.170708084	0.217681286	-9.971955462	2.02208E-23	8.86303E-23	negative
PRJNA729271	stage_3W_vs_E10	ordinary_muscle_development	ENSGALG00010024270	RAB18L	0.876761067	0.774280491	1.132355881	0.257484845	0.293161797	positive
PRJNA729271	stage_3W_vs_E10	ordinary_muscle_development	ENSGALG00010024274	ACSBG1	0.202089666	0.313942029	0.643716507	0.519759284	0.557654391	positive
PRJNA729271	stage_3W_vs_E10	ordinary_muscle_development	ENSGALG00010024277	ITGB3BP	-2.560315018	0.19767871	-12.95190065	2.29208E-38	1.75046E-37	negative
PRJNA729271	stage_3W_vs_E10	ordinary_muscle_development	ENSGALG00010024280	ENSGALG00010024280	3.29990972	0.123928221	26.62758885	3.254E-156	3.3933E-154	positive
PRJNA729271	stage_3W_vs_E10	ordinary_muscle_development	ENSGALG00010024282	HIC2	-2.36384116	0.1523629	-15.5145456	2.76586E-54	3.58276E-53	negative
PRJNA729271	stage_3W_vs_E10	ordinary_muscle_development	ENSGALG00010024283	UBE4A	0.222892237	0.11037889	2.019337541	0.043452149	0.054969357	positive
PRJNA729271	stage_3W_vs_E10	ordinary_muscle_development	ENSGALG00010024285	CCND2	-0.783156877	0.097446122	-8.036819333	9.22005E-16	2.89587E-15	negative
PRJNA729271	stage_3W_vs_E10	ordinary_muscle_development	ENSGALG00010024286	ADA	1.345276202	0.179165968	7.508547631	5.97869E-14	1.71742E-13	positive
PRJNA729271	stage_3W_vs_E10	ordinary_muscle_development	ENSGALG00010024288	CHID1	0.328220073	0.133805747	2.452959464	0.01416863	0.018942474	positive
PRJNA729271	stage_3W_vs_E10	ordinary_muscle_development	ENSGALG00010024290	SOST	-0.733320764	0.482555736	-1.519660237	0.12859639	0.153226854	negative
PRJNA729271	stage_3W_vs_E10	ordinary_muscle_development	ENSGALG00010024291	ENSGALG00010024291	2.087914185	0.541578451	3.855238664	0.000115617	0.000187919	positive
PRJNA729271	stage_3W_vs_E10	ordinary_muscle_development	ENSGALG00010024300	MEOX1	-4.159601714	0.292043515	-14.24308878	4.94903E-46	4.91008E-45	negative
PRJNA729271	stage_3W_vs_E10	ordinary_muscle_development	ENSGALG00010024304	PKIG	0.022884522	0.132030143	0.173328013	0.862393606	0.878453641	positive
PRJNA729271	stage_3W_vs_E10	ordinary_muscle_development	ENSGALG00010024305	ENSGALG00010024305	5.477307565	0.720845734	7.598446251	2.99707E-14	8.73042E-14	positive
PRJNA729271	stage_3W_vs_E10	ordinary_muscle_development	ENSGALG00010024309	ABCB11	2.091738035	0.39091498	5.350877151	8.75289E-08	1.77893E-07	positive
PRJNA729271	stage_3W_vs_E10	ordinary_muscle_development	ENSGALG00010024311	KLHL6	1.968639324	0.123108439	15.99109966	1.47406E-57	2.0724E-56	positive
PRJNA729271	stage_3W_vs_E10	ordinary_muscle_development	ENSGALG00010024313	SERINC3	2.412338357	0.106662485	22.61655865	2.9782E-113	1.5989E-111	positive
PRJNA729271	stage_3W_vs_E10	ordinary_muscle_development	ENSGALG00010024314	ITFG1	2.014157569	0.145418716	13.85074511	1.25899E-43	1.14774E-42	positive
PRJNA729271	stage_3W_vs_E10	ordinary_muscle_development	ENSGALG00010024321	UBE2L3	0.159964465	0.082126905	1.94777174	0.051442282	0.064447353	positive
PRJNA729271	stage_3W_vs_E10	ordinary_muscle_development	ENSGALG00010024322	IDH3A	1.622955321	0.158833846	10.21794385	1.64799E-24	7.56418E-24	positive
PRJNA729271	stage_3W_vs_E10	ordinary_muscle_development	ENSGALG00010024323	TTPAL	-1.117203325	0.102375506	-10.91279902	1.00125E-27	5.28327E-27	negative
PRJNA729271	stage_3W_vs_E10	ordinary_muscle_development	ENSGALG00010024325	PCBP2	0.16825361	0.170044704	0.989466922	0.322434746	0.360457477	positive
PRJNA729271	stage_3W_vs_E10	ordinary_muscle_development	ENSGALG00010024326	KLHL24	2.791971783	0.178381423	15.65169592	3.23483E-55	4.28992E-54	positive
PRJNA729271	stage_3W_vs_E10	ordinary_muscle_development	ENSGALG00010024327	PPP1R1B	4.335715947	0.414065834	10.47107874	1.17299E-25	5.64115E-25	positive
PRJNA729271	stage_3W_vs_E10	ordinary_muscle_development	ENSGALG00010024328	YDJC	-2.488998903	0.155562677	-15.9999747	1.27827E-57	1.80239E-56	negative
PRJNA729271	stage_3W_vs_E10	ordinary_muscle_development	ENSGALG00010024330	SDF2L1	-1.473096675	0.102883474	-14.31810779	1.68653E-46	1.69648E-45	negative
PRJNA729271	stage_3W_vs_E10	ordinary_muscle_development	ENSGALG00010024332	EFCAB7	-0.329096949	0.165204537	-1.992057573	0.046364741	0.058470239	negative
PRJNA729271	stage_3W_vs_E10	ordinary_muscle_development	ENSGALG00010024333	ETV4	-4.580911973	0.245856893	-18.63243254	1.7537E-77	4.09997E-76	negative
PRJNA729271	stage_3W_vs_E10	ordinary_muscle_development	ENSGALG00010024334	USP28	1.35569654	0.122903434	11.03058304	2.72095E-28	1.46891E-27	positive
PRJNA729271	stage_3W_vs_E10	ordinary_muscle_development	ENSGALG00010024336	TSPAN4	-0.513500229	0.117150315	-4.383259497	1.16917E-05	2.05519E-05	negative
PRJNA729271	stage_3W_vs_E10	ordinary_muscle_development	ENSGALG00010024338	NDUFA9	0.624574155	0.114073183	5.475205803	4.37003E-08	9.04651E-08	positive
PRJNA729271	stage_3W_vs_E10	ordinary_muscle_development	ENSGALG00010024339	TOP3B	0.049079601	0.086152867	0.569680422	0.568894471	0.605309063	positive
PRJNA729271	stage_3W_vs_E10	ordinary_muscle_development	ENSGALG00010024340	NETO2	-0.582526287	0.373474415	-1.559748844	0.118819245	0.142173101	negative
PRJNA729271	stage_3W_vs_E10	ordinary_muscle_development	ENSGALG00010024342	HNF4A	2.355317798	1.339893364	1.757839737	0.078774782	0.096488124	positive
PRJNA729271	stage_3W_vs_E10	ordinary_muscle_development	ENSGALG00010024343	POLR2L	0.797316884	0.126131261	6.321326528	2.59327E-10	6.12506E-10	positive
PRJNA729271	stage_3W_vs_E10	ordinary_muscle_development	ENSGALG00010024344	YEATS2	-0.013813322	0.133117707	-0.103767729	0.917353676	0.927010704	negative
PRJNA729271	stage_3W_vs_E10	ordinary_muscle_development	ENSGALG00010024345	STARD3	0.382274807	0.091734639	4.167180564	3.0839E-05	5.24046E-05	positive
PRJNA729271	stage_3W_vs_E10	ordinary_muscle_development	ENSGALG00010024346	PPM1F	0.231796751	0.091282427	2.539335971	0.011106311	0.015028564	positive
PRJNA729271	stage_3W_vs_E10	ordinary_muscle_development	ENSGALG00010024347	OTOG	-1.689716959	0.290439837	-5.817786486	5.9632E-09	1.30372E-08	negative
PRJNA729271	stage_3W_vs_E10	ordinary_muscle_development	ENSGALG00010024349	R3HDML	-4.227006453	0.604695803	-6.990302289	2.74295E-12	7.21317E-12	negative
PRJNA729271	stage_3W_vs_E10	ordinary_muscle_development	ENSGALG00010024350	PGM1	7.305923898	0.135813035	53.79398164	0	0	positive
PRJNA729271	stage_3W_vs_E10	ordinary_muscle_development	ENSGALG00010024351	SP1	1.201712181	0.153214736	7.843319856	4.38788E-15	1.33338E-14	positive
PRJNA729271	stage_3W_vs_E10	ordinary_muscle_development	ENSGALG00010024354	C2CD2L	-0.114445625	0.078291257	-1.46179316	0.143797899	0.170206643	negative
PRJNA729271	stage_3W_vs_E10	ordinary_muscle_development	ENSGALG00010024356	PARL	0.444601821	0.117483532	3.784375674	0.000154095	0.00024782	positive
PRJNA729271	stage_3W_vs_E10	ordinary_muscle_development	ENSGALG00010024358	MAPK3	0.527554942	0.061731193	8.546002709	1.27425E-17	4.40923E-17	positive
PRJNA729271	stage_3W_vs_E10	ordinary_muscle_development	ENSGALG00010024360	FTL	-0.840784827	0.241780783	-3.477467545	0.000506174	0.000779536	negative
PRJNA729271	stage_3W_vs_E10	ordinary_muscle_development	ENSGALG00010024361	H2AX	-3.110296225	0.221724217	-14.02776955	1.05415E-44	9.95434E-44	negative
PRJNA729271	stage_3W_vs_E10	ordinary_muscle_development	ENSGALG00010024362	DHX8	-0.099578013	0.057783947	-1.723281619	0.08483761	0.103538028	negative
PRJNA729271	stage_3W_vs_E10	ordinary_muscle_development	ENSGALG00010024363	EDEM2	-0.235741068	0.112792973	-2.090033289	0.036614809	0.046756379	negative
PRJNA729271	stage_3W_vs_E10	ordinary_muscle_development	ENSGALG00010024365	ENSGALG00010024365	0.312810539	0.105488865	2.965341775	0.003023468	0.004335692	positive
PRJNA729271	stage_3W_vs_E10	ordinary_muscle_development	ENSGALG00010024366	PARD6A	0.218686543	0.446851165	0.48939459	0.624562365	0.658977397	positive
PRJNA729271	stage_3W_vs_E10	ordinary_muscle_development	ENSGALG00010024367	ENSGALG00010024367	-2.248570486	0.236670263	-9.500857664	2.08169E-21	8.43086E-21	negative
PRJNA729271	stage_3W_vs_E10	ordinary_muscle_development	ENSGALG00010024368	DPAGT1	0.071512635	0.079290652	0.901904995	0.367107338	0.405735483	positive
PRJNA729271	stage_3W_vs_E10	ordinary_muscle_development	ENSGALG00010024369	MYOD1	1.443106478	0.275436435	5.239344888	1.61148E-07	3.21077E-07	positive
PRJNA729271	stage_3W_vs_E10	ordinary_muscle_development	ENSGALG00010024370	HERPUD1	0.181064104	0.092560526	1.956169781	0.050445157	0.063263761	positive
PRJNA729271	stage_3W_vs_E10	ordinary_muscle_development	ENSGALG00010024371	YPEL1	0.767060284	0.105054211	7.301566303	2.84437E-13	7.87567E-13	positive
PRJNA729271	stage_3W_vs_E10	ordinary_muscle_development	ENSGALG00010024373	TCAP	12.97841122	0.478228224	27.13853045	3.4583E-162	4.0426E-160	positive
PRJNA729271	stage_3W_vs_E10	ordinary_muscle_development	ENSGALG00010024374	ENSGALG00010024374	1.124774932	0.586028912	1.919316451	0.054944297	0.068609372	positive
PRJNA729271	stage_3W_vs_E10	ordinary_muscle_development	ENSGALG00010024377	CIB2	-0.47496176	0.171451156	-2.770245304	0.005601409	0.007813726	negative
PRJNA729271	stage_3W_vs_E10	ordinary_muscle_development	ENSGALG00010024378	ZDHHC1	-0.277831169	0.175651936	-1.581714241	0.11371482	0.136425784	negative
PRJNA729271	stage_3W_vs_E10	ordinary_muscle_development	ENSGALG00010024382	ENSGALG00010024382	0.592953014	0.443855025	1.335915965	0.181576742	0.21145295	positive
PRJNA729271	stage_3W_vs_E10	ordinary_muscle_development	ENSGALG00010024383	DUSP28	1.152315998	0.190442777	6.050720397	1.442E-09	3.2542E-09	positive
PRJNA729271	stage_3W_vs_E10	ordinary_muscle_development	ENSGALG00010024386	TPPP3	2.427826311	0.267468075	9.077069514	1.11537E-19	4.20915E-19	positive
PRJNA729271	stage_3W_vs_E10	ordinary_muscle_development	ENSGALG00010024387	PPIL2	-0.246181554	0.06597504	-3.731434691	0.000190392	0.000303902	negative
PRJNA729271	stage_3W_vs_E10	ordinary_muscle_development	ENSGALG00010024389	AAAS	0.035305873	0.107199478	0.329347429	0.741893099	0.769058175	positive
PRJNA729271	stage_3W_vs_E10	ordinary_muscle_development	ENSGALG00010024391	TBC1D2B	-0.294828586	0.11063936	-2.664771262	0.00770407	0.010607001	negative
PRJNA729271	stage_3W_vs_E10	ordinary_muscle_development	ENSGALG00010024392	CHMP4B	0.289046643	0.084730827	3.411351593	0.000646417	0.000985259	positive
PRJNA729271	stage_3W_vs_E10	ordinary_muscle_development	ENSGALG00010024393	OGFOD1	-1.32430194	0.107277766	-12.34460775	5.20875E-35	3.58333E-34	negative
PRJNA729271	stage_3W_vs_E10	ordinary_muscle_development	ENSGALG00010024396	ENSGALG00010024396	0.6618213	0.325117133	2.035639571	0.041786561	0.052940845	positive
PRJNA729271	stage_3W_vs_E10	ordinary_muscle_development	ENSGALG00010024398	PHB	0.018987805	0.088120953	0.215474351	0.829397508	0.849559527	positive
PRJNA729271	stage_3W_vs_E10	ordinary_muscle_development	ENSGALG00010024399	ROR1	-0.760888566	0.168512858	-4.515314588	6.32228E-06	1.13432E-05	negative
PRJNA729271	stage_3W_vs_E10	ordinary_muscle_development	ENSGALG00010024400	KCNA1	-4.154120372	0.473397255	-8.775125604	1.70713E-18	6.14169E-18	negative
PRJNA729271	stage_3W_vs_E10	ordinary_muscle_development	ENSGALG00010024401	KCNC1	1.631402564	0.221477003	7.366013377	1.75806E-13	4.92237E-13	positive
PRJNA729271	stage_3W_vs_E10	ordinary_muscle_development	ENSGALG00010024402	GPC1	-1.87317057	0.120185846	-15.5856171	9.11786E-55	1.19389E-53	negative
PRJNA729271	stage_3W_vs_E10	ordinary_muscle_development	ENSGALG00010024403	HMBS	-0.818278752	0.077357204	-10.57792562	3.77217E-26	1.85663E-25	negative
PRJNA729271	stage_3W_vs_E10	ordinary_muscle_development	ENSGALG00010024406	NUDT21	-1.248210666	0.066370516	-18.80670412	6.65488E-79	1.60503E-77	negative
PRJNA729271	stage_3W_vs_E10	ordinary_muscle_development	ENSGALG00010024407	HIRA	-0.762494301	0.082049719	-9.29307627	1.49892E-20	5.85314E-20	negative
PRJNA729271	stage_3W_vs_E10	ordinary_muscle_development	ENSGALG00010024408	ENSGALG00010024408	-3.150688583	1.394701643	-2.25904128	0.023880817	0.031145622	negative
PRJNA729271	stage_3W_vs_E10	ordinary_muscle_development	ENSGALG00010024410	MYG1	0.05101528	0.107346837	0.47523785	0.634617452	0.66817607	positive
PRJNA729271	stage_3W_vs_E10	ordinary_muscle_development	ENSGALG00010024412	PIGU	-0.545929635	0.101757728	-5.364994356	8.09518E-08	1.64988E-07	negative
PRJNA729271	stage_3W_vs_E10	ordinary_muscle_development	ENSGALG00010024413	AMFR	1.613679852	0.091888833	17.56121835	4.88161E-69	9.16567E-68	positive
PRJNA729271	stage_3W_vs_E10	ordinary_muscle_development	ENSGALG00010024415	SIK2	0.883557606	0.05957102	14.83200406	9.09758E-50	1.01908E-48	positive
PRJNA729271	stage_3W_vs_E10	ordinary_muscle_development	ENSGALG00010024416	VDR	0.27278481	0.289529403	0.942166172	0.34610757	0.38460819	positive
PRJNA729271	stage_3W_vs_E10	ordinary_muscle_development	ENSGALG00010024419	PGAP3	0.378596601	0.126819391	2.985321065	0.002832808	0.004071951	positive
PRJNA729271	stage_3W_vs_E10	ordinary_muscle_development	ENSGALG00010024420	TMEM106C	0.633758849	0.157303937	4.028881027	5.6043E-05	9.35556E-05	positive
PRJNA729271	stage_3W_vs_E10	ordinary_muscle_development	ENSGALG00010024421	DAPK2	-0.78660393	0.261583595	-3.007084332	0.002637666	0.003799361	negative
PRJNA729271	stage_3W_vs_E10	ordinary_muscle_development	ENSGALG00010024423	SERGEF	-0.389225452	0.120823789	-3.221430602	0.001275523	0.001893753	negative
PRJNA729271	stage_3W_vs_E10	ordinary_muscle_development	ENSGALG00010024424	SAMHD1	1.235072131	0.179171045	6.89325738	5.45291E-12	1.40916E-11	positive
PRJNA729271	stage_3W_vs_E10	ordinary_muscle_development	ENSGALG00010024425	MRPL40	0.935840418	0.088197315	10.61075855	2.65584E-26	1.31387E-25	positive
PRJNA729271	stage_3W_vs_E10	ordinary_muscle_development	ENSGALG00010024426	PHAF1	1.217613286	0.104555377	11.64563045	2.41529E-31	1.45449E-30	positive
PRJNA729271	stage_3W_vs_E10	ordinary_muscle_development	ENSGALG00010024427	C22orf39	0.028376651	0.13634271	0.208127379	0.83512951	0.854163297	positive
PRJNA729271	stage_3W_vs_E10	ordinary_muscle_development	ENSGALG00010024428	SAAL1	-1.463845249	0.064765098	-22.60237832	4.1065E-113	2.1964E-111	negative
PRJNA729271	stage_3W_vs_E10	ordinary_muscle_development	ENSGALG00010024430	ZNF652	-0.405248975	0.065221444	-6.21343154	5.18398E-10	1.20342E-09	negative
PRJNA729271	stage_3W_vs_E10	ordinary_muscle_development	ENSGALG00010024431	FEZ1	-3.432549455	0.161265643	-21.28506355	1.56135E-100	6.63689E-99	negative
PRJNA729271	stage_3W_vs_E10	ordinary_muscle_development	ENSGALG00010024433	ENSGALG00010024433	-1.938365949	0.169599419	-11.42908366	2.9925E-30	1.73436E-29	negative
PRJNA729271	stage_3W_vs_E10	ordinary_muscle_development	ENSGALG00010024434	B3GNT9	-1.352351218	0.15994056	-8.455336257	2.78284E-17	9.44889E-17	negative
PRJNA729271	stage_3W_vs_E10	ordinary_muscle_development	ENSGALG00010024437	HERC1	0.110935795	0.081546505	1.360399191	0.17370365	0.202920246	positive
PRJNA729271	stage_3W_vs_E10	ordinary_muscle_development	ENSGALG00010024438	RBL1	-2.150937147	0.122783032	-17.51819541	1.04075E-68	1.94402E-67	negative
PRJNA729271	stage_3W_vs_E10	ordinary_muscle_development	ENSGALG00010024439	UFD1	1.005806169	0.111943412	8.984951853	2.58848E-19	9.64028E-19	positive
PRJNA729271	stage_3W_vs_E10	ordinary_muscle_development	ENSGALG00010024440	PKNOX2	2.048641899	0.205659226	9.961342059	2.25005E-23	9.8325E-23	positive
PRJNA729271	stage_3W_vs_E10	ordinary_muscle_development	ENSGALG00010024441	SAA	8.661322651	0.787593338	10.99720151	3.94175E-28	2.11223E-27	positive
PRJNA729271	stage_3W_vs_E10	ordinary_muscle_development	ENSGALG00010024446	GRM4	-5.548780033	0.61919109	-8.961337013	3.20765E-19	1.19004E-18	negative
PRJNA729271	stage_3W_vs_E10	ordinary_muscle_development	ENSGALG00010024449	CACHD1	-0.748276635	0.084729203	-8.831389935	1.03383E-18	3.75103E-18	negative
PRJNA729271	stage_3W_vs_E10	ordinary_muscle_development	ENSGALG00010024451	PHOSPHO1	2.248125578	0.267955784	8.389912486	4.86504E-17	1.63276E-16	positive
PRJNA729271	stage_3W_vs_E10	ordinary_muscle_development	ENSGALG00010024452	SLC16A1	0.001375729	0.25216503	0.005455669	0.995647027	0.99640318	positive
PRJNA729271	stage_3W_vs_E10	ordinary_muscle_development	ENSGALG00010024453	HTR3A	2.178056446	0.609538238	3.573289269	0.000352525	0.000550156	positive
PRJNA729271	stage_3W_vs_E10	ordinary_muscle_development	ENSGALG00010024454	HPS5	0.355637843	0.120427095	2.953138114	0.003145612	0.004503718	positive
PRJNA729271	stage_3W_vs_E10	ordinary_muscle_development	ENSGALG00010024457	CHD6	-0.659436706	0.101225891	-6.514506296	7.29291E-11	1.77397E-10	negative
PRJNA729271	stage_3W_vs_E10	ordinary_muscle_development	ENSGALG00010024459	AHCYL1	-0.496309429	0.093278708	-5.320715072	1.0336E-07	2.09101E-07	negative
PRJNA729271	stage_3W_vs_E10	ordinary_muscle_development	ENSGALG00010024460	IMP4	0.060131863	0.155339256	0.387100238	0.698682001	0.728170521	positive
PRJNA729271	stage_3W_vs_E10	ordinary_muscle_development	ENSGALG00010024462	ZBTB32	6.732409415	0.428594793	15.70809895	1.33111E-55	1.78487E-54	positive
PRJNA729271	stage_3W_vs_E10	ordinary_muscle_development	ENSGALG00010024463	FBXL22	-0.902415621	0.229091879	-3.93909913	8.17881E-05	0.000134397	negative
PRJNA729271	stage_3W_vs_E10	ordinary_muscle_development	ENSGALG00010024465	SENP1	-0.835216152	0.160851435	-5.192469387	2.07523E-07	4.10711E-07	negative
PRJNA729271	stage_3W_vs_E10	ordinary_muscle_development	ENSGALG00010024466	ADGRG3	4.305615422	0.683840399	6.296228517	3.04975E-10	7.17398E-10	positive
PRJNA729271	stage_3W_vs_E10	ordinary_muscle_development	ENSGALG00010024467	ENSGALG00010024467	1.163862879	0.284442294	4.091736376	4.28155E-05	7.20301E-05	positive
PRJNA729271	stage_3W_vs_E10	ordinary_muscle_development	ENSGALG00010024469	GTF2H1	0.441856305	0.127492691	3.465738314	0.000528778	0.00081262	positive
PRJNA729271	stage_3W_vs_E10	ordinary_muscle_development	ENSGALG00010024471	CCDC61	-0.572058331	0.11726408	-4.878376495	1.06963E-06	2.02115E-06	negative
PRJNA729271	stage_3W_vs_E10	ordinary_muscle_development	ENSGALG00010024472	LDHA	7.865910101	0.201250351	39.08519947	0	0	positive
PRJNA729271	stage_3W_vs_E10	ordinary_muscle_development	ENSGALG00010024473	RAVER2	-1.345361134	0.061107761	-22.01620725	2.0143E-107	9.8641E-106	negative
PRJNA729271	stage_3W_vs_E10	ordinary_muscle_development	ENSGALG00010024474	STRIP1	0.351872543	0.103392228	3.403278461	0.000665824	0.001013666	positive
PRJNA729271	stage_3W_vs_E10	ordinary_muscle_development	ENSGALG00010024475	ADGRG1	1.087532705	0.157087931	6.92308248	4.4192E-12	1.14632E-11	positive
PRJNA729271	stage_3W_vs_E10	ordinary_muscle_development	ENSGALG00010024476	KCNB1	-1.593542786	0.192576642	-8.274849784	1.28618E-16	4.23131E-16	negative
PRJNA729271	stage_3W_vs_E10	ordinary_muscle_development	ENSGALG00010024479	ERBB2	0.118871954	0.128730056	0.923420355	0.355788188	0.394579173	positive
PRJNA729271	stage_3W_vs_E10	ordinary_muscle_development	ENSGALG00010024480	CDC45	-3.072724093	0.22032532	-13.9463049	3.31332E-44	3.07666E-43	negative
PRJNA729271	stage_3W_vs_E10	ordinary_muscle_development	ENSGALG00010024482	USP3	-1.300283597	0.059182186	-21.97086124	5.4721E-107	2.6264E-105	negative
PRJNA729271	stage_3W_vs_E10	ordinary_muscle_development	ENSGALG00010024486	GPR37L1	-1.091069806	0.320116421	-3.408353131	0.000653563	0.000995941	negative
PRJNA729271	stage_3W_vs_E10	ordinary_muscle_development	ENSGALG00010024488	ADGRG5	2.523753795	0.419008282	6.02315969	1.71045E-09	3.84327E-09	positive
PRJNA729271	stage_3W_vs_E10	ordinary_muscle_development	ENSGALG00010024490	TGM3	2.215557881	0.840833173	2.634955366	0.008414835	0.011535183	positive

PRJNA729271	stage_3W_vs_E10	ordinary_muscle_development	ENSGALG00010024492	NTF3	-2.601522494	0.255486553	-10.18262006	2.3709E-24	1.08036E-23	negative
PRJNA729271	stage_3W_vs_E10	ordinary_muscle_development	ENSGALG00010024493	JAK1	0.319436916	0.060159254	5.309854979	1.09712E-07	2.2158E-07	positive
PRJNA729271	stage_3W_vs_E10	ordinary_muscle_development	ENSGALG00010024494	DDAH1	-0.432035555	0.172888289	-2.498928973	0.012456928	0.016760713	negative
PRJNA729271	stage_3W_vs_E10	ordinary_muscle_development	ENSGALG00010024495	CLDN5	0.144007962	0.17101897	0.842058411	0.399755259	0.438807458	positive
PRJNA729271	stage_3W_vs_E10	ordinary_muscle_development	ENSGALG00010024496	UEVLD	1.777870103	0.207864322	8.553031527	1.19897E-17	4.15668E-17	positive
PRJNA729271	stage_3W_vs_E10	ordinary_muscle_development	ENSGALG00010024497	ABI3	1.777598328	0.177815974	9.996842761	1.57333E-23	6.91494E-23	positive
PRJNA729271	stage_3W_vs_E10	ordinary_muscle_development	ENSGALG00010024498	CCDC102A	-1.307977522	0.121810478	-10.73780797	6.76311E-27	3.44332E-26	negative
PRJNA729271	stage_3W_vs_E10	ordinary_muscle_development	ENSGALG00010024499	CA12	-0.07183847	0.547459311	-0.13122157	0.895600033	0.907495455	negative
PRJNA729271	stage_3W_vs_E10	ordinary_muscle_development	ENSGALG00010024501	ARL8A	0.367751347	0.08962987	4.102999896	4.07827E-05	6.87139E-05	positive
PRJNA729271	stage_3W_vs_E10	ordinary_muscle_development	ENSGALG00010024502	VPS8	0.480944936	0.072978231	6.590252044	4.39081E-11	1.08258E-10	positive
PRJNA729271	stage_3W_vs_E10	ordinary_muscle_development	ENSGALG00010024504	CASC3	0.683870155	0.186465852	3.667535621	0.000244899	0.000386817	positive
PRJNA729271	stage_3W_vs_E10	ordinary_muscle_development	ENSGALG00010024505	DNASE2B	2.742638497	0.209764421	13.07485077	4.58463E-39	3.59406E-38	positive
PRJNA729271	stage_3W_vs_E10	ordinary_muscle_development	ENSGALG00010024506	RAB8B	0.411958492	0.081108242	5.079119951	3.79187E-07	7.38256E-07	positive
PRJNA729271	stage_3W_vs_E10	ordinary_muscle_development	ENSGALG00010024508	RPS27L	3.421222702	0.188761158	18.12461173	2.03776E-73	4.35013E-72	positive
PRJNA729271	stage_3W_vs_E10	ordinary_muscle_development	ENSGALG00010024509	SEPTIN5	0.225929956	0.10739867	2.103656924	0.035408385	0.045291611	positive
PRJNA729271	stage_3W_vs_E10	ordinary_muscle_development	ENSGALG00010024510	CNN3	-0.863559826	0.101152711	-8.537189177	1.37526E-17	4.74856E-17	negative
PRJNA729271	stage_3W_vs_E10	ordinary_muscle_development	ENSGALG00010024511	ENSGALG00010024511	-0.857511189	0.170835238	-5.019521727	5.18003E-07	9.9873E-07	negative
PRJNA729271	stage_3W_vs_E10	ordinary_muscle_development	ENSGALG00010024512	CCNDBP1	0.251142546	0.115046454	2.182966421	0.029038283	0.037504224	positive
PRJNA729271	stage_3W_vs_E10	ordinary_muscle_development	ENSGALG00010024513	SPTY2D1	0.400403621	0.138863836	2.883426181	0.003933748	0.005575261	positive
PRJNA729271	stage_3W_vs_E10	ordinary_muscle_development	ENSGALG00010024514	FAF1	0.572758705	0.068056125	8.415975853	3.89638E-17	1.31466E-16	positive
PRJNA729271	stage_3W_vs_E10	ordinary_muscle_development	ENSGALG00010024515	C3orf70	-1.318339782	0.157793038	-8.354866569	6.55067E-17	2.18683E-16	negative
PRJNA729271	stage_3W_vs_E10	ordinary_muscle_development	ENSGALG00010024516	EHHADH	-0.101815658	0.241537666	-0.421531181	0.673367247	0.704675348	negative
PRJNA729271	stage_3W_vs_E10	ordinary_muscle_development	ENSGALG00010024517	LACTB	-0.018276005	0.097795101	-0.186880576	0.851754269	0.868653918	negative
PRJNA729271	stage_3W_vs_E10	ordinary_muscle_development	ENSGALG00010024518	ENSGALG00010024518	0.141960324	1.33961853	0.105970708	0.915605593	0.925502306	positive
PRJNA729271	stage_3W_vs_E10	ordinary_muscle_development	ENSGALG00010024519	MIEN1	0.828957666	0.138761167	5.973988881	2.31522E-09	5.15817E-09	positive
PRJNA729271	stage_3W_vs_E10	ordinary_muscle_development	ENSGALG00010024520	MAP3K13	-0.659186363	0.139300526	-4.732116808	2.2219E-06	4.11269E-06	negative
PRJNA729271	stage_3W_vs_E10	ordinary_muscle_development	ENSGALG00010024521	HEPACAM	-1.650357797	0.508945273	-3.242701889	0.00118402	0.001763318	negative
PRJNA729271	stage_3W_vs_E10	ordinary_muscle_development	ENSGALG00010024522	DNTTIP2	0.026982415	0.075239196	0.358621791	0.719878043	0.748377841	positive
PRJNA729271	stage_3W_vs_E10	ordinary_muscle_development	ENSGALG00010024523	TOX3	-2.766782474	0.319515809	-8.659297585	4.7468E-18	1.6794E-17	negative
PRJNA729271	stage_3W_vs_E10	ordinary_muscle_development	ENSGALG00010024524	TMEM41A	0.867530701	0.132458518	6.54945196	5.77486E-11	1.41253E-10	positive
PRJNA729271	stage_3W_vs_E10	ordinary_muscle_development	ENSGALG00010024525	GP1BB	0.305931191	0.190428857	1.606537982	0.108155757	0.130176675	positive
PRJNA729271	stage_3W_vs_E10	ordinary_muscle_development	ENSGALG00010024526	TGM6	1.010440707	1.583567651	0.638078648	0.523422485	0.561376908	positive
PRJNA729271	stage_3W_vs_E10	ordinary_muscle_development	ENSGALG00010024527	ATP6V0B	1.076166767	0.098750741	10.89780949	1.18065E-27	6.20957E-27	positive
PRJNA729271	stage_3W_vs_E10	ordinary_muscle_development	ENSGALG00010024528	TBX1	-1.654454298	0.223624879	-7.398346299	1.37891E-13	3.8848E-13	negative
PRJNA729271	stage_3W_vs_E10	ordinary_muscle_development	ENSGALG00010024529	GRB7	-4.366290868	1.094830521	-3.988097503	6.66053E-05	0.000110513	negative
PRJNA729271	stage_3W_vs_E10	ordinary_muscle_development	ENSGALG00010024530	ENSGALG00010024530	0.838343573	0.529474112	1.583351393	0.113341395	0.136011551	positive
PRJNA729271	stage_3W_vs_E10	ordinary_muscle_development	ENSGALG00010024531	CEP63	-1.088178384	0.131528266	-8.273342418	1.30255E-16	4.28322E-16	negative
PRJNA729271	stage_3W_vs_E10	ordinary_muscle_development	ENSGALG00010024532	TMEM86A	0.387587238	0.214848486	1.804002649	0.071230892	0.087692524	positive
PRJNA729271	stage_3W_vs_E10	ordinary_muscle_development	ENSGALG00010024533	CDKN2C	2.371254415	0.222763638	10.64471041	1.84557E-26	9.23742E-26	positive
PRJNA729271	stage_3W_vs_E10	ordinary_muscle_development	ENSGALG00010024534	VWF	4.437905652	0.125408599	35.38757063	2.6519E-274	1.0116E-271	positive
PRJNA729271	stage_3W_vs_E10	ordinary_muscle_development	ENSGALG00010024535	ENSGALG00010024535	1.849506438	0.151635803	12.19703	3.22369E-34	2.15931E-33	positive
PRJNA729271	stage_3W_vs_E10	ordinary_muscle_development	ENSGALG00010024536	URI1	-0.266156627	0.062030788	-4.290718107	1.78096E-05	3.08829E-05	negative
PRJNA729271	stage_3W_vs_E10	ordinary_muscle_development	ENSGALG00010024537	EPB42	-0.818412239	0.181096072	-4.519215848	6.20691E-06	1.11458E-05	negative
PRJNA729271	stage_3W_vs_E10	ordinary_muscle_development	ENSGALG00010024540	PTPN5	-2.312499081	0.302079655	-7.655262588	1.92918E-14	5.6767E-14	negative
PRJNA729271	stage_3W_vs_E10	ordinary_muscle_development	ENSGALG00010024541	WIPF2	0.612362666	0.202737932	3.020464205	0.002523875	0.003642331	positive
PRJNA729271	stage_3W_vs_E10	ordinary_muscle_development	ENSGALG00010024542	ZNFX1	3.496345311	0.424843224	8.229730679	1.87635E-16	6.11458E-16	positive
PRJNA729271	stage_3W_vs_E10	ordinary_muscle_development	ENSGALG00010024544	ZNF536	-1.678809578	0.248113937	-6.766284857	1.32131E-11	3.35419E-11	negative
PRJNA729271	stage_3W_vs_E10	ordinary_muscle_development	ENSGALG00010024545	PHACTR3	-1.226237027	0.288980873	-4.243315527	2.20241E-05	3.7937E-05	negative
PRJNA729271	stage_3W_vs_E10	ordinary_muscle_development	ENSGALG00010024546	KY	5.348333727	0.401845026	13.30944364	2.03995E-40	1.66774E-39	positive
PRJNA729271	stage_3W_vs_E10	ordinary_muscle_development	ENSGALG00010024547	PFKM	9.506597485	0.197740655	48.07608987	0	0	positive
PRJNA729271	stage_3W_vs_E10	ordinary_muscle_development	ENSGALG00010024548	CDC6	-3.513225953	0.331385401	-10.60163165	2.92826E-26	1.44716E-25	negative
PRJNA729271	stage_3W_vs_E10	ordinary_muscle_development	ENSGALG00010024549	GNB1L	-0.135206428	0.132795218	-1.018157356	0.308603182	0.346491334	negative
PRJNA729271	stage_3W_vs_E10	ordinary_muscle_development	ENSGALG00010024551	RNF11	1.769924933	0.126525161	13.98871903	1.82673E-44	1.71049E-43	positive
PRJNA729271	stage_3W_vs_E10	ordinary_muscle_development	ENSGALG00010024552	CD81	0.869041175	0.066218058	13.12393027	2.4015E-39	1.90009E-38	positive
PRJNA729271	stage_3W_vs_E10	ordinary_muscle_development	ENSGALG00010024553	ASB8	-0.093329943	0.113491043	-0.82235515	0.410874797	0.449651203	negative
PRJNA729271	stage_3W_vs_E10	ordinary_muscle_development	ENSGALG00010024554	ST6GALNAC3	-2.789764988	0.213242615	-13.08258667	4.14105E-39	3.25336E-38	negative
PRJNA729271	stage_3W_vs_E10	ordinary_muscle_development	ENSGALG00010024555	EPHB1	-1.14059948	0.163202948	-6.988841149	2.77166E-12	7.28604E-12	negative
PRJNA729271	stage_3W_vs_E10	ordinary_muscle_development	ENSGALG00010024556	ENSGALG00010024556	-0.843304864	0.213760424	-3.945093515	7.97688E-05	0.000131198	negative
PRJNA729271	stage_3W_vs_E10	ordinary_muscle_development	ENSGALG00010024557	TSPAN32	0.951111895	0.286810042	3.316173623	0.000912591	0.001371347	positive
PRJNA729271	stage_3W_vs_E10	ordinary_muscle_development	ENSGALG00010024558	PFDN5	0.124024336	0.108231106	1.145921365	0.25182769	0.287036437	positive
PRJNA729271	stage_3W_vs_E10	ordinary_muscle_development	ENSGALG00010024559	ENSGALG00010024559	-2.487544121	0.351148497	-7.084023265	1.40028E-12	3.74693E-12	negative
PRJNA729271	stage_3W_vs_E10	ordinary_muscle_development	ENSGALG00010024560	A4GNT	0.065024805	0.475846926	0.136650677	0.891306918	0.903956406	positive
PRJNA729271	stage_3W_vs_E10	ordinary_muscle_development	ENSGALG00010024561	ROBO3	-1.127388237	0.557077419	-2.023755043	0.042995362	0.054419994	negative
PRJNA729271	stage_3W_vs_E10	ordinary_muscle_development	ENSGALG00010024563	ESPL1	-2.21328302	0.170777748	-12.96001991	2.06194E-38	1.57803E-37	negative
PRJNA729271	stage_3W_vs_E10	ordinary_muscle_development	ENSGALG00010024564	PPP1R3D	2.236080821	0.19873822	11.25138797	2.27976E-29	1.28082E-28	positive
PRJNA729271	stage_3W_vs_E10	ordinary_muscle_development	ENSGALG00010024565	FAM217B	0.801231642	0.137958507	5.807772642	6.33094E-09	1.38162E-08	positive
PRJNA729271	stage_3W_vs_E10	ordinary_muscle_development	ENSGALG00010024566	MTSS2	-0.206258437	0.22361955	-0.92236317	0.356339174	0.395099543	negative
PRJNA729271	stage_3W_vs_E10	ordinary_muscle_development	ENSGALG00010024567	IKZF3	2.870608153	0.38860016	7.387048298	1.50124E-13	4.21633E-13	positive
PRJNA729271	stage_3W_vs_E10	ordinary_muscle_development	ENSGALG00010024568	DZIP1L	-0.199329824	0.275163632	-0.724404683	0.468817326	0.507544039	negative
PRJNA729271	stage_3W_vs_E10	ordinary_muscle_development	ENSGALG00010024569	ASCL2	-1.467072739	0.286338636	-5.123558446	2.99823E-07	5.87685E-07	negative
PRJNA729271	stage_3W_vs_E10	ordinary_muscle_development	ENSGALG00010024570	TPM1	5.65635726	0.164137777	34.46103246	3.078E-260	9.9146E-258	positive
PRJNA729271	stage_3W_vs_E10	ordinary_muscle_development	ENSGALG00010024571	KCNA3	-2.91390234	0.393684426	-7.401619548	1.34533E-13	3.79316E-13	negative
PRJNA729271	stage_3W_vs_E10	ordinary_muscle_development	ENSGALG00010024573	ENSGALG00010024573	1.42733842	0.269915366	5.288096189	1.23596E-07	2.48616E-07	positive
PRJNA729271	stage_3W_vs_E10	ordinary_muscle_development	ENSGALG00010024575	TXNRD2	0.453224219	0.124978659	3.626412886	0.000287386	0.000452052	positive
PRJNA729271	stage_3W_vs_E10	ordinary_muscle_development	ENSGALG00010024576	CSAD	-0.011304107	0.156249086	-0.072346709	0.942325993	0.949663858	negative
PRJNA729271	stage_3W_vs_E10	ordinary_muscle_development	ENSGALG00010024578	ENSGALG00010024578	1.92186716	0.586523815	3.276707802	0.00105025	0.001570715	positive
PRJNA729271	stage_3W_vs_E10	ordinary_muscle_development	ENSGALG00010024579	ZNF740	-0.357902218	0.105949236	-3.378053771	0.000730008	0.001107195	negative
PRJNA729271	stage_3W_vs_E10	ordinary_muscle_development	ENSGALG00010024580	IGF2	0.057004519	0.193429379	0.294704555	0.768219583	0.793341612	positive
PRJNA729271	stage_3W_vs_E10	ordinary_muscle_development	ENSGALG00010024581	RARA	0.720969107	0.171971022	4.192387179	2.76034E-05	4.70775E-05	positive
PRJNA729271	stage_3W_vs_E10	ordinary_muscle_development	ENSGALG00010024582	TLN2	1.338947157	0.107344008	12.47342244	1.0425E-35	7.32482E-35	positive
PRJNA729271	stage_3W_vs_E10	ordinary_muscle_development	ENSGALG00010024583	ITGB7	2.994960085	0.483554823	6.193630886	5.87938E-10	1.36006E-09	positive
PRJNA729271	stage_3W_vs_E10	ordinary_muscle_development	ENSGALG00010024585	TBCCD1	-1.379324758	0.130460777	-10.57271607	3.98775E-26	1.95941E-25	negative
PRJNA729271	stage_3W_vs_E10	ordinary_muscle_development	ENSGALG00010024586	DLGAP4	0.310176862	0.095461461	3.249236492	0.001157152	0.0017249	positive
PRJNA729271	stage_3W_vs_E10	ordinary_muscle_development	ENSGALG00010024587	ENSGALG00010024587	3.49607612	0.17817645	19.6214265	1.01466E-85	3.01382E-84	positive
PRJNA729271	stage_3W_vs_E10	ordinary_muscle_development	ENSGALG00010024588	TTC39A	-4.322205913	0.399436902	-10.82074764	2.74529E-27	1.41864E-26	negative
PRJNA729271	stage_3W_vs_E10	ordinary_muscle_development	ENSGALG00010024589	ADORA1	0.529571649	0.483305753	1.095728006	0.273197823	0.309883593	positive
PRJNA729271	stage_3W_vs_E10	ordinary_muscle_development	ENSGALG00010024590	MRPL23	0.167562125	0.104075799	1.610000844	0.107397672	0.129328675	positive
PRJNA729271	stage_3W_vs_E10	ordinary_muscle_development	ENSGALG00010024592	COMT	-0.110205432	0.198190526	-0.556058022	0.578171191	0.614187718	negative
PRJNA729271	stage_3W_vs_E10	ordinary_muscle_development	ENSGALG00010024593	SRSF6	-0.809346535	0.075007549	-10.79020101	3.82951E-27	1.967E-26	negative
PRJNA729271	stage_3W_vs_E10	ordinary_muscle_development	ENSGALG00010024594	TNNI2	6.451275072	0.183197539	35.21485665	1.1846E-271	4.4029E-269	positive
PRJNA729271	stage_3W_vs_E10	ordinary_muscle_development	ENSGALG00010024595	MYBPHL	4.841683203	0.316404455	15.30219667	7.3919E-53	9.14992E-52	positive
PRJNA729271	stage_3W_vs_E10	ordinary_muscle_development	ENSGALG00010024596	EPS15	1.095154509	0.066549374	16.45627056	7.56183E-61	1.15621E-59	positive
PRJNA729271	stage_3W_vs_E10	ordinary_muscle_development	ENSGALG00010024597	DNAJB11	-1.416141487	0.101711357	-13.92314023	4.58317E-44	4.22602E-43	negative
PRJNA729271	stage_3W_vs_E10	ordinary_muscle_development	ENSGALG00010024598	LRRC3C	0.807015431	0.236086555	3.418303225	0.000630129	0.000961545	positive
PRJNA729271	stage_3W_vs_E10	ordinary_muscle_development	ENSGALG00010024599	ENSGALG00010024599	0.115327654	0.21589909	0.534173876	0.593221241	0.62883881	positive
PRJNA729271	stage_3W_vs_E10	ordinary_muscle_development	ENSGALG00010024600	CD9	0.177807414	0.334991498	0.530781872	0.595569944	0.631143905	positive
PRJNA729271	stage_3W_vs_E10	ordinary_muscle_development	ENSGALG00010024601	ENSGALG00010024601	-0.253721073	0.50625792	-0.501169589	0.616251775	0.650967022	negative
PRJNA729271	stage_3W_vs_E10	ordinary_muscle_development	ENSGALG00010024602	PROCR	0.610555917	0.244802646	2.49407401	0.012628621	0.016982267	positive
PRJNA729271	stage_3W_vs_E10	ordinary_muscle_development	ENSGALG00010024603	ENSGALG00010024603	-1.281173329	1.248632204	-1.026061418	0.304862635	0.342583448	negative
PRJNA729271	stage_3W_vs_E10	ordinary_muscle_development	ENSGALG00010024604	MFSD5	1.336520751	0.168165238	7.947663653	1.90062E-15	5.86783E-15	positive
PRJNA729271	stage_3W_vs_E10	ordinary_muscle_development	ENSGALG00010024605	LSP1P1	-0.382454114	0.195847113	-1.952819769	0.050840955	0.063727053	negative
PRJNA729271	stage_3W_vs_E10	ordinary_muscle_development	ENSGALG00010024607	RARG	-0.863188986	0.142529062	-6.056231433	1.39347E-09	3.14813E-09	negative
PRJNA729271	stage_3W_vs_E10	ordinary_muscle_development	ENSGALG00010024608	DENND2D	1.362878189	0.332623651	4.097358039	4.17892E-05	7.03525E-05	positive
PRJNA729271	stage_3W_vs_E10	ordinary_muscle_development	ENSGALG00010024609	PRR33	14.12154389	0.836415753	16.88340258	5.9608E-64	9.93124E-63	positive
PRJNA729271	stage_3W_vs_E10	ordinary_muscle_development	ENSGALG00010024610	PFKL	-1.215078263	0.139587555	-8.704775015	3.18205E-18	1.13578E-17	negative
PRJNA729271	stage_3W_vs_E10	ordinary_muscle_development	ENSGALG00010024611	ENSGALG00010024611	-1.991020735	0.276060621	-7.212259138	5.50311E-13	1.50192E-12	negative
PRJNA729271	stage_3W_vs_E10	ordinary_muscle_development	ENSGALG00010024612	ENSGALG00010024612	-1.679342737	0.603996167	-2.780386415	0.005429425	0.007584033	negative
PRJNA729271	stage_3W_vs_E10	ordinary_muscle_development	ENSGALG00010024613	ENSGALG00010024613	-0.153502941	0.095722254	-1.603628563	0.108795951	0.130838572	negative
PRJNA729271	stage_3W_vs_E10	ordinary_muscle_development	ENSGALG00010024614	ENSGALG00010024614	-5.490308722	0.787299364	-6.973597301	3.08938E-12	8.108E-12	negative
PRJNA729271	stage_3W_vs_E10	ordinary_muscle_development	ENSGALG00010024615	SYT8	-2.901453506	0.684525444	-4.238634999	2.24883E-05	3.86905E-05	negative
PRJNA729271	stage_3W_vs_E10	ordinary_muscle_development	ENSGALG00010024616	ARVCF	-1.343215439	0.099514781	-13.49764756	1.61449E-41	1.36455E-40	negative
PRJNA729271	stage_3W_vs_E10	ordinary_muscle_development	ENSGALG00010024618	VPS13C	-0.034506589	0.122776042	-0.281053111	0.778669664	0.802858672	negative
PRJNA729271	stage_3W_vs_E10	ordinary_muscle_development	ENSGALG00010024619	ENSGALG00010024619	-0.115073067	0.212139078	-0.542441628	0.587514309	0.623062622	negative
PRJNA729271	stage_3W_vs_E10	ordinary_muscle_development	ENSGALG00010024620	MSANTD2	-0.861768047	0.132179072	-6.519701158	7.04476E-11	1.71505E-10	negative
PRJNA729271	stage_3W_vs_E10	ordinary_muscle_development	ENSGALG00010024621	THRA	1.082035276	0.18502102	5.84817484	4.96996E-09	1.08969E-08	positive
PRJNA729271	stage_3W_vs_E10	ordinary_muscle_development	ENSGALG00010024622	PRMT8	-1.092383013	0.213473479	-5.117183738	3.10131E-07	6.07316E-07	negative
PRJNA729271	stage_3W_vs_E10	ordinary_muscle_development	ENSGALG00010024623	NRDC	1.046926793	0.109362687	9.572979837	1.03868E-21	4.25782E-21	positive
PRJNA729271	stage_3W_vs_E10	ordinary_muscle_development	ENSGALG00010024624	CTSD	0.729149198	0.128696329	5.665656547	1.46463E-08	3.12893E-08	positive
PRJNA729271	stage_3W_vs_E10	ordinary_muscle_development	ENSGALG00010024625	SPRYD3	0.606258821	0.078843848	7.689361147	1.47872E-14	4.3725E-14	positive
PRJNA729271	stage_3W_vs_E10	ordinary_muscle_development	ENSGALG00010024626	TSHZ3	-1.981486347	0.100635464	-19.68974228	2.64029E-86	7.92362E-85	negative
PRJNA729271	stage_3W_vs_E10	ordinary_muscle_development	ENSGALG00010024627	CEPT1	0.333287676	0.078696097	4.235123353	2.28426E-05	3.92769E-05	positive
PRJNA729271	stage_3W_vs_E10	ordinary_muscle_development	ENSGALG00010024628	TANGO2	0.30368283	0.111240062	2.729977177	0.006333871	0.008784754	positive
PRJNA729271	stage_3W_vs_E10	ordinary_muscle_development	ENSGALG00010024629	ENSGALG00010024629	0.664315663	0.172809818	3.844200921	0.000120946	0.000196317	positive
PRJNA729271	stage_3W_vs_E10	ordinary_muscle_development	ENSGALG00010024630	CFAP410	-1.233487275	0.100120553	-12.32002065	7.06762E-35	4.8346E-34	negative
PRJNA729271	stage_3W_vs_E10	ordinary_muscle_development	ENSGALG00010024631	EPB41L1	-0.993989134	0.150766946	-6.592884964	4.31361E-11	1.06409E-10	negative
PRJNA729271	stage_3W_vs_E10	ordinary_muscle_development	ENSGALG00010024632	IFITM10	-2.245789674	0.547174448	-4.104339452	4.05472E-05	6.83408E-05	negative
PRJNA729271	stage_3W_vs_E10	ordinary_muscle_development	ENSGALG00010024633	NRGN	3.794247697	0.278657814	13.61615397	3.21034E-42	2.7848E-41	positive
PRJNA729271	stage_3W_vs_E10	ordinary_muscle_development	ENSGALG00010024634	ENSGALG00010024634	1.770477386	0.163761631	10.81130773	3.04301E-27	1.56969E-26	positive
PRJNA729271	stage_3W_vs_E10	ordinary_muscle_development	ENSGALG00010024635	ENSGALG00010024635	-0.987278082	0.192669858	-5.124195818	2.9881E-07	5.8578E-07	negative
PRJNA729271	stage_3W_vs_E10	ordinary_muscle_development	ENSGALG00010024636	PANX3	-1.912387227	0.311385573	-6.14154089	8.17248E-10	1.87289E-09	negative
PRJNA729271	stage_3W_vs_E10	ordinary_muscle_development	ENSGALG00010024637	DUSP8	1.779531229	0.276980867	6.42474423	1.32091E-10	3.16578E-10	positive

PRJNA729271	stage_3W_vs_E10	ordinary_muscle_development	ENSGALG00010024638	ZNF507	0.390851803	0.129195596	3.025271877	0.002484097	0.00358814	positive
PRJNA729271	stage_3W_vs_E10	ordinary_muscle_development	ENSGALG00010024639	AAR2	-0.326010293	0.076102581	-4.283827006	1.83706E-05	3.18404E-05	negative
PRJNA729271	stage_3W_vs_E10	ordinary_muscle_development	ENSGALG00010024640	GSDMA	-0.450048104	0.29336527	-1.534087876	0.125008036	0.149086019	negative
PRJNA729271	stage_3W_vs_E10	ordinary_muscle_development	ENSGALG00010024641	TRA2B	-1.628117693	0.088637916	-18.36818553	2.36176E-75	5.24254E-74	negative
PRJNA729271	stage_3W_vs_E10	ordinary_muscle_development	ENSGALG00010024642	RAB3B	1.029915989	0.22177653	4.643935902	3.41834E-06	6.25853E-06	positive
PRJNA729271	stage_3W_vs_E10	ordinary_muscle_development	ENSGALG00010024643	ETV7	1.971906194	0.383843173	5.137270464	2.78758E-07	5.47654E-07	positive
PRJNA729271	stage_3W_vs_E10	ordinary_muscle_development	ENSGALG00010024644	EIF6	-0.560529619	0.075599263	-7.414485183	1.22098E-13	3.44993E-13	negative
PRJNA729271	stage_3W_vs_E10	ordinary_muscle_development	ENSGALG00010024645	PARP11	0.482457006	0.117908204	4.091801837	4.28034E-05	7.20181E-05	positive
PRJNA729271	stage_3W_vs_E10	ordinary_muscle_development	ENSGALG00010024646	RORA	1.792535805	0.192258091	9.323591	1.12468E-20	4.41675E-20	positive
PRJNA729271	stage_3W_vs_E10	ordinary_muscle_development	ENSGALG00010024647	RPUSD4	-0.468665084	0.135811248	-3.450856191	0.000558811	0.000855691	negative
PRJNA729271	stage_3W_vs_E10	ordinary_muscle_development	ENSGALG00010024648	MMP24	-2.006645395	0.128079216	-15.66722108	2.5342E-55	3.37373E-54	negative
PRJNA729271	stage_3W_vs_E10	ordinary_muscle_development	ENSGALG00010024649	NCOR2	-0.156510745	0.129841253	-1.20540076	0.228048628	0.261661115	negative
PRJNA729271	stage_3W_vs_E10	ordinary_muscle_development	ENSGALG00010024650	PRKAG1	1.185711208	0.120293714	9.856801101	6.40577E-23	2.75852E-22	positive
PRJNA729271	stage_3W_vs_E10	ordinary_muscle_development	ENSGALG00010024651	PSMD3	0.473419414	0.127593694	3.710366875	0.000206959	0.000329186	positive
PRJNA729271	stage_3W_vs_E10	ordinary_muscle_development	ENSGALG00010024652	PUS3	-0.501154923	0.125761676	-3.984957405	6.74922E-05	0.000111882	negative
PRJNA729271	stage_3W_vs_E10	ordinary_muscle_development	ENSGALG00010024653	RFLNA	-3.899637678	0.262368044	-14.8632342	5.71007E-50	6.44606E-49	negative
PRJNA729271	stage_3W_vs_E10	ordinary_muscle_development	ENSGALG00010024654	CCDC92	-1.296664441	0.276530667	-4.689043907	2.74484E-06	5.05033E-06	negative
PRJNA729271	stage_3W_vs_E10	ordinary_muscle_development	ENSGALG00010024655	IGF2BP2	-1.948169726	0.167465617	-11.633252	2.79252E-31	1.67887E-30	negative
PRJNA729271	stage_3W_vs_E10	ordinary_muscle_development	ENSGALG00010024656	HYLS1	-1.069550613	0.188296319	-5.680146161	1.3458E-08	2.88101E-08	negative
PRJNA729271	stage_3W_vs_E10	ordinary_muscle_development	ENSGALG00010024657	DPY19L3	-0.886704736	0.128762606	-6.886352803	5.72409E-12	1.47687E-11	negative
PRJNA729271	stage_3W_vs_E10	ordinary_muscle_development	ENSGALG00010024659	BEND5	0.669961777	0.107438427	6.235774243	4.49549E-10	1.04745E-09	positive
PRJNA729271	stage_3W_vs_E10	ordinary_muscle_development	ENSGALG00010024660	KMT2D	0.686716379	0.1574899	4.360383601	1.29835E-05	2.27371E-05	positive
PRJNA729271	stage_3W_vs_E10	ordinary_muscle_development	ENSGALG00010024662	MOB2	0.201237921	0.071403994	2.818300622	0.004827858	0.006780989	positive
PRJNA729271	stage_3W_vs_E10	ordinary_muscle_development	ENSGALG00010024663	ICE2	-1.016799514	0.12459662	-8.160731088	3.33003E-16	1.07026E-15	negative
PRJNA729271	stage_3W_vs_E10	ordinary_muscle_development	ENSGALG00010024664	DNAH10	-4.296247066	0.270588777	-15.87740304	9.08632E-57	1.24958E-55	negative
PRJNA729271	stage_3W_vs_E10	ordinary_muscle_development	ENSGALG00010024665	MAP1LC3A	3.232430926	0.147553579	21.9068284	2.2362E-106	1.0558E-104	positive
PRJNA729271	stage_3W_vs_E10	ordinary_muscle_development	ENSGALG00010024666	MED24	-1.354454403	0.116513513	-11.62486966	3.08063E-31	1.84902E-30	negative
PRJNA729271	stage_3W_vs_E10	ordinary_muscle_development	ENSGALG00010024667	RBM7	1.113804044	0.135046924	8.24753363	1.61697E-16	5.29074E-16	positive
PRJNA729271	stage_3W_vs_E10	ordinary_muscle_development	ENSGALG00010024668	ENSGALG00010024668	0.531197984	0.185225736	2.867841129	0.00413283	0.005843856	positive
PRJNA729271	stage_3W_vs_E10	ordinary_muscle_development	ENSGALG00010024669	STIL	-1.711495537	0.185552496	-9.223780724	2.86805E-20	1.10919E-19	negative
PRJNA729271	stage_3W_vs_E10	ordinary_muscle_development	ENSGALG00010024670	SENP2	0.740178761	0.093861183	7.885887816	3.12307E-15	9.55242E-15	positive
PRJNA729271	stage_3W_vs_E10	ordinary_muscle_development	ENSGALG00010024671	KCTD20	1.660782402	0.280146997	5.928253444	3.06174E-09	6.77555E-09	positive
PRJNA729271	stage_3W_vs_E10	ordinary_muscle_development	ENSGALG00010024672	DYNLRB1	-0.278672762	0.105343481	-2.64537265	0.0081601	0.011199758	negative
PRJNA729271	stage_3W_vs_E10	ordinary_muscle_development	ENSGALG00010024673	REXO2	1.614487112	0.097279773	16.59632892	7.40857E-62	1.16346E-60	positive
PRJNA729271	stage_3W_vs_E10	ordinary_muscle_development	ENSGALG00010024674	DHH	2.023125275	0.330463548	6.122083021	9.23599E-10	2.10828E-09	positive
PRJNA729271	stage_3W_vs_E10	ordinary_muscle_development	ENSGALG00010024675	NR1D1	3.798618503	0.416246676	9.125926939	7.1125E-20	2.70521E-19	positive
PRJNA729271	stage_3W_vs_E10	ordinary_muscle_development	ENSGALG00010024676	ENSGALG00010024676	0.716582019	0.165489662	4.330071206	1.49061E-05	2.59942E-05	positive
PRJNA729271	stage_3W_vs_E10	ordinary_muscle_development	ENSGALG00010024677	LMBR1L	-3.84111083	0.491915629	-7.808474874	5.78841E-15	1.74907E-14	negative
PRJNA729271	stage_3W_vs_E10	ordinary_muscle_development	ENSGALG00010024678	AMOTL2	0.398288627	0.091543107	4.350831443	1.35622E-05	2.37106E-05	positive
PRJNA729271	stage_3W_vs_E10	ordinary_muscle_development	ENSGALG00010024679	MSL1	0.150971976	0.111203282	1.357621585	0.17458379	0.20383729	positive
PRJNA729271	stage_3W_vs_E10	ordinary_muscle_development	ENSGALG00010024680	BRSK1	-1.622483661	0.27589758	-5.880746262	4.08421E-09	8.98476E-09	negative
PRJNA729271	stage_3W_vs_E10	ordinary_muscle_development	ENSGALG00010024681	ATP6V0A2	1.26055555	0.084922832	14.84354114	7.66032E-50	8.6008E-49	positive
PRJNA729271	stage_3W_vs_E10	ordinary_muscle_development	ENSGALG00010024682	ENSGALG00010024682	-2.676345805	0.17951183	-14.90902192	2.87939E-50	3.30196E-49	negative
PRJNA729271	stage_3W_vs_E10	ordinary_muscle_development	ENSGALG00010024683	SHISA4	2.944436135	0.174661488	16.85795858	9.17021E-64	1.52085E-62	positive
PRJNA729271	stage_3W_vs_E10	ordinary_muscle_development	ENSGALG00010024684	ENSGALG00010024684	-0.758679794	0.32853402	-2.309288375	0.020927584	0.027476932	negative
PRJNA729271	stage_3W_vs_E10	ordinary_muscle_development	ENSGALG00010024685	ENSGALG00010024685	-1.362557198	0.222184087	-6.132559791	8.64762E-10	1.97927E-09	negative
PRJNA729271	stage_3W_vs_E10	ordinary_muscle_development	ENSGALG00010024686	CMPK1	-1.105458381	0.133685375	-8.269104855	1.34969E-16	4.4342E-16	negative
PRJNA729271	stage_3W_vs_E10	ordinary_muscle_development	ENSGALG00010024687	RYK	-0.38639165	0.10734676	-3.599471943	0.000318864	0.000499669	negative
PRJNA729271	stage_3W_vs_E10	ordinary_muscle_development	ENSGALG00010024688	CADM1	-2.485825993	0.132981573	-18.69301083	5.64363E-78	1.33232E-76	negative
PRJNA729271	stage_3W_vs_E10	ordinary_muscle_development	ENSGALG00010024689	FOXD2	-0.212608747	0.311783715	-0.681911007	0.495295225	0.533618573	negative
PRJNA729271	stage_3W_vs_E10	ordinary_muscle_development	ENSGALG00010024690	TUBA1C	-3.702718032	0.650234369	-5.694436053	1.2378E-08	2.65616E-08	negative
PRJNA729271	stage_3W_vs_E10	ordinary_muscle_development	ENSGALG00010024691	TCTN2	-0.878997152	0.151985251	-5.78343717	7.31895E-09	1.59244E-08	negative
PRJNA729271	stage_3W_vs_E10	ordinary_muscle_development	ENSGALG00010024692	DRAM2	1.826584549	0.104517562	17.476341	2.16984E-68	4.03746E-67	positive
PRJNA729271	stage_3W_vs_E10	ordinary_muscle_development	ENSGALG00010024693	ANXA2	0.536175544	0.201495639	2.660978407	0.007791395	0.010720102	positive
PRJNA729271	stage_3W_vs_E10	ordinary_muscle_development	ENSGALG00010024694	GTF2H3	-0.813733339	0.098242255	-8.282926148	1.20185E-16	3.95748E-16	negative
PRJNA729271	stage_3W_vs_E10	ordinary_muscle_development	ENSGALG00010024695	TRABD2B	-0.508021903	0.194998928	-2.605254849	0.009180595	0.012535109	negative
PRJNA729271	stage_3W_vs_E10	ordinary_muscle_development	ENSGALG00010024696	TRADD	-0.04716406	0.114795768	-0.410851908	0.681181128	0.712081383	negative
PRJNA729271	stage_3W_vs_E10	ordinary_muscle_development	ENSGALG00010024697	IGF2BP1	-2.11877646	0.123823582	-17.11125158	1.22342E-65	2.12632E-64	negative
PRJNA729271	stage_3W_vs_E10	ordinary_muscle_development	ENSGALG00010024698	ENSGALG00010024698	-2.515195776	0.409087452	-6.148308292	7.83137E-10	1.79756E-09	negative
PRJNA729271	stage_3W_vs_E10	ordinary_muscle_development	ENSGALG00010024699	FBXL8	-0.666720722	0.13438499	-4.96127375	7.00324E-07	1.33956E-06	negative
PRJNA729271	stage_3W_vs_E10	ordinary_muscle_development	ENSGALG00010024700	TOLLIP	0.807167705	0.083982484	9.611143538	7.17477E-22	2.95954E-21	positive
PRJNA729271	stage_3W_vs_E10	ordinary_muscle_development	ENSGALG00010024701	EIF2B1	-0.094819841	0.100887721	-0.939855117	0.347291883	0.385835505	negative
PRJNA729271	stage_3W_vs_E10	ordinary_muscle_development	ENSGALG00010024702	INAVA	-2.620583612	0.824829916	-3.177119987	0.001487455	0.002193799	negative
PRJNA729271	stage_3W_vs_E10	ordinary_muscle_development	ENSGALG00010024703	HSF4	-0.851549858	0.623819891	-1.365057239	0.17223506	0.201415668	negative
PRJNA729271	stage_3W_vs_E10	ordinary_muscle_development	ENSGALG00010024705	SLCO2A1	2.376975187	0.235221062	10.10528208	5.23447E-24	2.3454E-23	positive
PRJNA729271	stage_3W_vs_E10	ordinary_muscle_development	ENSGALG00010024706	ENSGALG00010024706	0.181512848	1.061505587	0.170995659	0.864227179	0.879888527	positive
PRJNA729271	stage_3W_vs_E10	ordinary_muscle_development	ENSGALG00010024707	ENSGALG00010024707	-1.08455568	0.375965528	-2.884721072	0.003917606	0.005554157	negative
PRJNA729271	stage_3W_vs_E10	ordinary_muscle_development	ENSGALG00010024708	BNIP2	0.844816096	0.062338553	13.55206466	7.70325E-42	6.59142E-41	positive
PRJNA729271	stage_3W_vs_E10	ordinary_muscle_development	ENSGALG00010024709	TEAD4	1.062511194	0.096619426	10.99686922	3.9563E-28	2.11924E-27	positive
PRJNA729271	stage_3W_vs_E10	ordinary_muscle_development	ENSGALG00010024710	HSDL1	-0.528051341	0.127487945	-4.141970766	3.44334E-05	5.82735E-05	negative
PRJNA729271	stage_3W_vs_E10	ordinary_muscle_development	ENSGALG00010024711	DDX55	-0.427241613	0.092102967	-4.638738851	3.50542E-06	6.41148E-06	negative
PRJNA729271	stage_3W_vs_E10	ordinary_muscle_development	ENSGALG00010024712	LRIF1	0.595086137	0.09805075	6.069164582	1.28577E-09	2.91208E-09	positive
PRJNA729271	stage_3W_vs_E10	ordinary_muscle_development	ENSGALG00010024713	RND2	-1.995493219	0.460485622	-4.333453906	1.46788E-05	2.56071E-05	negative
PRJNA729271	stage_3W_vs_E10	ordinary_muscle_development	ENSGALG00010024714	SRPRB	-0.86305636	0.175198683	-4.926157797	8.38623E-07	1.59651E-06	negative
PRJNA729271	stage_3W_vs_E10	ordinary_muscle_development	ENSGALG00010024715	DNAAF1	0.036246664	0.173512126	0.208899888	0.834526392	0.853666905	positive
PRJNA729271	stage_3W_vs_E10	ordinary_muscle_development	ENSGALG00010024716	GTF2A2	-0.255057249	0.077037329	-3.310826729	0.000930208	0.001396371	negative
PRJNA729271	stage_3W_vs_E10	ordinary_muscle_development	ENSGALG00010024717	TF	2.809585626	0.652399707	4.306540296	1.65828E-05	2.88072E-05	positive
PRJNA729271	stage_3W_vs_E10	ordinary_muscle_development	ENSGALG00010024718	ENSGALG00010024718	0.876497317	0.192502457	4.553174712	5.28424E-06	9.52793E-06	positive
PRJNA729271	stage_3W_vs_E10	ordinary_muscle_development	ENSGALG00010024719	PYM1	-0.760075068	0.135653353	-5.603068804	2.1059E-08	4.4549E-08	negative
PRJNA729271	stage_3W_vs_E10	ordinary_muscle_development	ENSGALG00010024720	BUD13	-0.438760332	0.108341881	-4.049775824	5.12667E-05	8.57997E-05	negative
PRJNA729271	stage_3W_vs_E10	ordinary_muscle_development	ENSGALG00010024721	ENSGALG00010024721	-0.734341831	0.859467513	-0.854414878	0.392875155	0.431778404	negative
PRJNA729271	stage_3W_vs_E10	ordinary_muscle_development	ENSGALG00010024722	BRCA1	-3.748373736	0.26500888	-14.14433259	2.02428E-45	1.96399E-44	negative
PRJNA729271	stage_3W_vs_E10	ordinary_muscle_development	ENSGALG00010024723	ITCH	0.57319996	0.100173782	5.722055696	1.05243E-08	2.26907E-08	positive
PRJNA729271	stage_3W_vs_E10	ordinary_muscle_development	ENSGALG00010024724	HOXD3	-1.698039058	0.254493564	-6.672227909	2.51949E-11	6.30634E-11	negative
PRJNA729271	stage_3W_vs_E10	ordinary_muscle_development	ENSGALG00010024726	TMED2	-0.217732679	0.058600216	-3.715561056	0.000202753	0.000322815	negative
PRJNA729271	stage_3W_vs_E10	ordinary_muscle_development	ENSGALG00010024728	SLC5A9	-1.510779408	0.758843168	-1.990898082	0.046492093	0.058620641	negative
PRJNA729271	stage_3W_vs_E10	ordinary_muscle_development	ENSGALG00010024729	FOXM1	-2.960697972	0.17334496	-17.07980421	2.09822E-65	3.63365E-64	negative
PRJNA729271	stage_3W_vs_E10	ordinary_muscle_development	ENSGALG00010024731	AOX1	4.413182286	0.197475385	22.34801211	1.2624E-110	6.4888E-109	positive
PRJNA729271	stage_3W_vs_E10	ordinary_muscle_development	ENSGALG00010024732	AHCY	-1.207320082	0.224950922	-5.367037716	8.00403E-08	1.63222E-07	negative
PRJNA729271	stage_3W_vs_E10	ordinary_muscle_development	ENSGALG00010024733	MBTPS1	-0.249214626	0.088088653	-2.82913426	0.004667411	0.006568362	negative
PRJNA729271	stage_3W_vs_E10	ordinary_muscle_development	ENSGALG00010024734	ZPR1	0.15061372	0.114235718	1.318446833	0.187354112	0.217795962	positive
PRJNA729271	stage_3W_vs_E10	ordinary_muscle_development	ENSGALG00010024735	OTUD7A	1.61761587	0.179511777	9.011196371	2.03821E-19	7.62421E-19	positive
PRJNA729271	stage_3W_vs_E10	ordinary_muscle_development	ENSGALG00010024736	DGKA	-0.064699971	0.146907718	-0.440412334	0.659638494	0.692192006	negative
PRJNA729271	stage_3W_vs_E10	ordinary_muscle_development	ENSGALG00010024737	STK38	-0.816715028	0.062634533	-13.03937287	7.30555E-39	5.67491E-38	negative
PRJNA729271	stage_3W_vs_E10	ordinary_muscle_development	ENSGALG00010024738	NBR1	0.16296142	0.078337176	2.080256516	0.037502011	0.047847166	positive
PRJNA729271	stage_3W_vs_E10	ordinary_muscle_development	ENSGALG00010024739	ASIP	-7.18314256	0.608983592	-11.79529736	4.1274E-32	2.57208E-31	negative
PRJNA729271	stage_3W_vs_E10	ordinary_muscle_development	ENSGALG00010024741	RILPL2	-0.630966908	0.156454934	-4.032898755	5.5093E-05	9.20015E-05	negative
PRJNA729271	stage_3W_vs_E10	ordinary_muscle_development	ENSGALG00010024742	PAK2	-0.17851188	0.069993295	-2.550414021	0.010759506	0.014575611	negative
PRJNA729271	stage_3W_vs_E10	ordinary_muscle_development	ENSGALG00010024744	KLF13	2.983724114	0.129664773	23.01106179	3.6122E-117	2.0453E-115	positive
PRJNA729271	stage_3W_vs_E10	ordinary_muscle_development	ENSGALG00010024745	PMEL	-7.805510465	0.744816663	-10.47977423	1.06999E-25	5.15437E-25	negative
PRJNA729271	stage_3W_vs_E10	ordinary_muscle_development	ENSGALG00010024746	HOXD4	-1.083095916	0.312742667	-3.463217629	0.000533756	0.000819943	negative
PRJNA729271	stage_3W_vs_E10	ordinary_muscle_development	ENSGALG00010024747	TULP3	0.074743828	0.086459863	0.864491629	0.387317877	0.426317308	positive
PRJNA729271	stage_3W_vs_E10	ordinary_muscle_development	ENSGALG00010024748	TRPM1	1.122779733	0.386186078	2.907354242	0.003645002	0.005185425	positive
PRJNA729271	stage_3W_vs_E10	ordinary_muscle_development	ENSGALG00010024749	APOA5	1.329479842	0.692071481	1.921015209	0.05472979	0.068365073	positive
PRJNA729271	stage_3W_vs_E10	ordinary_muscle_development	ENSGALG00010024750	APOA4	5.791487514	1.548745364	3.739470445	0.000184408	0.000294643	positive
PRJNA729271	stage_3W_vs_E10	ordinary_muscle_development	ENSGALG00010024751	MTMR10	1.252820015	0.055884821	22.4178945	2.6335E-111	1.3633E-109	positive
PRJNA729271	stage_3W_vs_E10	ordinary_muscle_development	ENSGALG00010024752	MPP2	-0.530825992	0.223539429	-2.374641446	0.017566004	0.023242285	negative
PRJNA729271	stage_3W_vs_E10	ordinary_muscle_development	ENSGALG00010024753	HOXD8	-1.238857713	0.177292214	-6.987659985	2.79509E-12	7.34496E-12	negative
PRJNA729271	stage_3W_vs_E10	ordinary_muscle_development	ENSGALG00010024754	EIF2S2	1.566125175	0.144514431	10.8371542	2.29499E-27	1.18977E-26	positive
PRJNA729271	stage_3W_vs_E10	ordinary_muscle_development	ENSGALG00010024755	PIGX	0.638570597	0.156596756	4.077802204	4.54634E-05	7.63783E-05	positive
PRJNA729271	stage_3W_vs_E10	ordinary_muscle_development	ENSGALG00010024756	CDK2	-2.89801772	0.171930422	-16.85575879	9.51787E-64	1.5767E-62	negative
PRJNA729271	stage_3W_vs_E10	ordinary_muscle_development	ENSGALG00010024757	PPFIA1	-0.437788198	0.075673967	-5.785188924	7.24309E-09	1.57664E-08	negative
PRJNA729271	stage_3W_vs_E10	ordinary_muscle_development	ENSGALG00010024758	SPATA6	0.027643244	0.105702143	0.261520188	0.793691383	0.816563035	positive
PRJNA729271	stage_3W_vs_E10	ordinary_muscle_development	ENSGALG00010024759	APOC3	1.31614594	0.982115466	1.340113241			positive
PRJNA729271	stage_3W_vs_E10	ordinary_muscle_development	ENSGALG00010024760	ENSGALG00010024760	2.653244634	0.184763885	14.36019077	9.19736E-47	9.32278E-46	positive
PRJNA729271	stage_3W_vs_E10	ordinary_muscle_development	ENSGALG00010024761	NECAB2	0.491819523	0.233903797	2.102657289	0.035495738	0.045395335	positive
PRJNA729271	stage_3W_vs_E10	ordinary_muscle_development	ENSGALG00010024762	FAN1	-0.319642596	0.088948321	-3.593576499	0.00032617	0.000510675	negative
PRJNA729271	stage_3W_vs_E10	ordinary_muscle_development	ENSGALG00010024763	HOXD9	-1.23272883	0.310974754	-3.964080089	7.36795E-05	0.000121694	negative
PRJNA729271	stage_3W_vs_E10	ordinary_muscle_development	ENSGALG00010024764	ENSGALG00010024764	0.788512384	0.257113651	3.066785367	0.002163742	0.003140741	positive
PRJNA729271	stage_3W_vs_E10	ordinary_muscle_development	ENSGALG00010024765	SNRNP35	-0.008875147	0.133487746	-0.066486603	0.946990423	0.95390036	negative
PRJNA729271	stage_3W_vs_E10	ordinary_muscle_development	ENSGALG00010024767	CTTN	-1.201860286	0.069570781	-17.27536001	7.2122E-67	1.28904E-65	negative
PRJNA729271	stage_3W_vs_E10	ordinary_muscle_development	ENSGALG00010024769	RALY	3.621527561	0.203896118	17.76163072	1.40104E-70	2.74805E-69	positive
PRJNA729271	stage_3W_vs_E10	ordinary_muscle_development	ENSGALG00010024770	CDC27	0.392246495	0.07203013	5.445589116	5.16342E-08	1.06418E-07	positive
PRJNA729271	stage_3W_vs_E10	ordinary_muscle_development	ENSGALG00010024771	AOX2	5.061307574	0.263486482	19.20898383	3.113E-82	8.24916E-81	positive
PRJNA729271	stage_3W_vs_E10	ordinary_muscle_development	ENSGALG00010024772	ELAPOR1	-0.647589458	0.236545651	-2.73769336	0.006187174	0.008592803	negative
PRJNA729271	stage_3W_vs_E10	ordinary_muscle_development	ENSGALG00010024773	APOA1	7.162135825	0.264044947	27.12468433	5.0377E-162	5.8417E-160	positive
PRJNA729271	stage_3W_vs_E10	ordinary_muscle_development	ENSGALG00010024775	ITFG2	0.774971752	0.093899926	8.253166806	1.54252E-16	5.05511E-16	positive
PRJNA729271	stage_3W_vs_E10	ordinary_muscle_development	ENSGALG00010024776	MPHOSPH10	-0.487857257	0.107214463	-4.550293316	5.35712E-06	9.65334E-06	negative
PRJNA729271	stage_3W_vs_E10	ordinary_muscle_development	ENSGALG00010024778	RAB5B	-0.067915081	0.140801528	-0.482346191	0.629560039	0.663054838	negative
PRJNA729271	stage_3W_vs_E10	ordinary_muscle_development	ENSGALG00010024779	ENSGALG00010024779	-4.083243234	1.082917986	-3.770593237	0.00016286	0.000261366	negative
PRJNA729271	stage_3W_vs_E10	ordinary_muscle_development	ENSGALG00010024780	HOXD11	-4.079454668	0.862310955	-4.730839431	2.23593E-06	4.1376E-06	negative

PRJNA729271	stage_3W_vs_E10	ordinary_muscle_development	ENSGALG00010024781	CEP19	-0.814322383	0.16106361	-5.055905431	4.28354E-07	8.31301E-07	negative
PRJNA729271	stage_3W_vs_E10	ordinary_muscle_development	ENSGALG00010024782	HOXD12	-5.003157634	2.178365665	-2.296748299	0.021633132	0.028368067	negative
PRJNA729271	stage_3W_vs_E10	ordinary_muscle_development	ENSGALG00010024783	UBE2T	-2.991219735	0.246830558	-12.11851464	8.42726E-34	5.56253E-33	negative
PRJNA729271	stage_3W_vs_E10	ordinary_muscle_development	ENSGALG00010024784	ENSGALG00010024784	0.609622248	0.138081973	4.414930026	1.01043E-05	1.7835E-05	positive
PRJNA729271	stage_3W_vs_E10	ordinary_muscle_development	ENSGALG00010024785	MCEE	0.67755577	0.200665046	3.376551038	0.000734008	0.001112796	positive
PRJNA729271	stage_3W_vs_E10	ordinary_muscle_development	ENSGALG00010024786	PDCD5	1.58313695	0.096690662	16.37321453	2.97099E-60	4.48588E-59	positive
PRJNA729271	stage_3W_vs_E10	ordinary_muscle_development	ENSGALG00010024787	SARS1	0.773050449	0.099222738	7.791061467	6.64486E-15	2.00077E-14	positive
PRJNA729271	stage_3W_vs_E10	ordinary_muscle_development	ENSGALG00010024788	SIK3	0.327596144	0.166403626	1.968683928	0.048989398	0.061576598	positive
PRJNA729271	stage_3W_vs_E10	ordinary_muscle_development	ENSGALG00010024789	SUOX	0.796677678	0.173536245	4.590843116	4.41459E-06	8.0077E-06	positive
PRJNA729271	stage_3W_vs_E10	ordinary_muscle_development	ENSGALG00010024791	SHANK2	-3.122845267	0.464347099	-6.725239103	1.75304E-11	4.42381E-11	negative
PRJNA729271	stage_3W_vs_E10	ordinary_muscle_development	ENSGALG00010024792	BZW1	-0.769339967	0.069124794	-11.12972527	8.99122E-29	4.92919E-28	negative
PRJNA729271	stage_3W_vs_E10	ordinary_muscle_development	ENSGALG00010024793	FKBP4	-1.144467635	0.117168683	-9.76769222	1.54941E-22	6.54773E-22	negative
PRJNA729271	stage_3W_vs_E10	ordinary_muscle_development	ENSGALG00010024794	DDX6	-0.955705144	0.064882538	-14.72977428	4.1509E-49	4.54779E-48	negative
PRJNA729271	stage_3W_vs_E10	ordinary_muscle_development	ENSGALG00010024795	NRROS	1.716017954	0.205115842	8.366091747	5.95608E-17	1.99154E-16	positive
PRJNA729271	stage_3W_vs_E10	ordinary_muscle_development	ENSGALG00010024797	IKZF4	0.673835369	0.23660048	2.847988179	0.004399656	0.006204808	positive
PRJNA729271	stage_3W_vs_E10	ordinary_muscle_development	ENSGALG00010024798	FBXO45	-0.307645633	0.084912185	-3.623103468	0.000291089	0.000457679	negative
PRJNA729271	stage_3W_vs_E10	ordinary_muscle_development	ENSGALG00010024799	ENSGALG00010024799	1.369821012	0.185406066	7.38822113	1.48806E-13	4.18175E-13	positive
PRJNA729271	stage_3W_vs_E10	ordinary_muscle_development	ENSGALG00010024800	ENSGALG00010024800	0.612564687	0.130252986	4.70288402	2.56512E-06	4.73287E-06	positive
PRJNA729271	stage_3W_vs_E10	ordinary_muscle_development	ENSGALG00010024801	CXCR5	1.868627802	1.119198137	1.669613038	0.094995949	0.115217662	positive
PRJNA729271	stage_3W_vs_E10	ordinary_muscle_development	ENSGALG00010024802	CD59	0.805119994	0.16385096	4.913733767	8.93581E-07	1.69913E-06	positive
PRJNA729271	stage_3W_vs_E10	ordinary_muscle_development	ENSGALG00010024803	CLK1	0.004807377	0.13807446	0.034817282	0.97222544	0.976401839	positive
PRJNA729271	stage_3W_vs_E10	ordinary_muscle_development	ENSGALG00010024804	WDR53	0.000194274	0.105606271	0.001839606	0.998532207	0.998670003	positive
PRJNA729271	stage_3W_vs_E10	ordinary_muscle_development	ENSGALG00010024805	ENSGALG00010024805	0.834317885	0.371228515	2.247450971	0.024611221	0.032054959	positive
PRJNA729271	stage_3W_vs_E10	ordinary_muscle_development	ENSGALG00010024806	ANKRD27	0.667026278	0.072990088	9.13858718	6.32735E-20	2.41165E-19	positive
PRJNA729271	stage_3W_vs_E10	ordinary_muscle_development	ENSGALG00010024807	PPIL3	-0.42025105	0.192740702	-2.180395972	0.029228122	0.037722521	negative
PRJNA729271	stage_3W_vs_E10	ordinary_muscle_development	ENSGALG00010024808	CELSR2	-0.81630315	0.250935129	-3.253044538	0.001141756	0.00170335	negative
PRJNA729271	stage_3W_vs_E10	ordinary_muscle_development	ENSGALG00010024809	KANSL1	0.428524067	0.170846227	2.508244249	0.012133277	0.016348006	positive
PRJNA729271	stage_3W_vs_E10	ordinary_muscle_development	ENSGALG00010024810	ADIPOR1	1.222701682	0.11250973	10.86751947	1.64616E-27	8.59547E-27	positive
PRJNA729271	stage_3W_vs_E10	ordinary_muscle_development	ENSGALG00010024811	NIF3L1	-0.512489752	0.137592103	-3.72470323	0.000195545	0.000311818	negative
PRJNA729271	stage_3W_vs_E10	ordinary_muscle_development	ENSGALG00010024812	SBNO1	0.649255311	0.111715667	5.811676427	6.18503E-09	1.35079E-08	positive
PRJNA729271	stage_3W_vs_E10	ordinary_muscle_development	ENSGALG00010024813	RNF168	-0.197179315	0.095697993	-2.060433121	0.039357153	0.050068627	negative
PRJNA729271	stage_3W_vs_E10	ordinary_muscle_development	ENSGALG00010024814	APBA2	-3.857491315	0.177720781	-21.70534752	1.8264E-104	8.3249E-103	negative
PRJNA729271	stage_3W_vs_E10	ordinary_muscle_development	ENSGALG00010024816	NFIA	-0.892278344	0.122891875	-7.260678117	3.85154E-13	1.06016E-12	negative
PRJNA729271	stage_3W_vs_E10	ordinary_muscle_development	ENSGALG00010024817	UCKL1	3.115975612	0.219913716	14.16908262	1.42348E-45	1.38945E-44	positive
PRJNA729271	stage_3W_vs_E10	ordinary_muscle_development	ENSGALG00010024818	FBXO3	0.010310604	0.076511383	0.134759081	0.89280236	0.905103525	positive
PRJNA729271	stage_3W_vs_E10	ordinary_muscle_development	ENSGALG00010024820	NUDT19	0.924670117	0.138641708	6.669494538	2.56686E-11	6.42158E-11	positive
PRJNA729271	stage_3W_vs_E10	ordinary_muscle_development	ENSGALG00010024821	NECAP1	-0.637099136	0.106386399	-5.988539362	2.11734E-09	4.72967E-09	negative
PRJNA729271	stage_3W_vs_E10	ordinary_muscle_development	ENSGALG00010024822	RPS26	0.752835736	0.137113275	5.490611575	4.00544E-08	8.31789E-08	positive
PRJNA729271	stage_3W_vs_E10	ordinary_muscle_development	ENSGALG00010024823	ASB10	12.0336168	0.865475995	13.90404456	5.98609E-44	5.49514E-43	positive
PRJNA729271	stage_3W_vs_E10	ordinary_muscle_development	ENSGALG00010024824	DNAJC5	-1.129709459	0.110057187	-10.26474954	1.01584E-24	4.69386E-24	negative
PRJNA729271	stage_3W_vs_E10	ordinary_muscle_development	ENSGALG00010024825	UBXN7	-0.513719868	0.075699373	-6.786315994	1.15033E-11	2.93402E-11	negative
PRJNA729271	stage_3W_vs_E10	ordinary_muscle_development	ENSGALG00010024826	RBM33	0.429278161	0.11162872	3.845588852	0.000120263	0.000195275	positive
PRJNA729271	stage_3W_vs_E10	ordinary_muscle_development	ENSGALG00010024827	ENSGALG00010024827	0.167065584	0.360997518	0.462788734	0.643515807	0.676705116	positive
PRJNA729271	stage_3W_vs_E10	ordinary_muscle_development	ENSGALG00010024828	TIMM17B	1.137482512	0.132970832	8.554376152	1.18508E-17	4.11049E-17	positive
PRJNA729271	stage_3W_vs_E10	ordinary_muscle_development	ENSGALG00010024829	ERBB3	-3.0723759	0.223420405	-13.75154565	4.9853E-43	4.43869E-42	negative
PRJNA729271	stage_3W_vs_E10	ordinary_muscle_development	ENSGALG00010024830	ORC2	-1.735676743	0.115985383	-14.96461618	1.25043E-50	1.44769E-49	negative
PRJNA729271	stage_3W_vs_E10	ordinary_muscle_development	ENSGALG00010024833	MAPT	2.441949313	0.201042235	12.14644926	5.99115E-34	3.97445E-33	positive
PRJNA729271	stage_3W_vs_E10	ordinary_muscle_development	ENSGALG00010024834	INSIG1	1.311808818	0.259078378	5.063366652	4.11917E-07	7.99723E-07	positive
PRJNA729271	stage_3W_vs_E10	ordinary_muscle_development	ENSGALG00010024835	ENTREP2	-1.772538814	0.150584531	-11.77105512	5.50319E-32	3.41183E-31	negative
PRJNA729271	stage_3W_vs_E10	ordinary_muscle_development	ENSGALG00010024836	TXNDC12	-0.711405614	0.106549185	-6.676781376	2.44247E-11	6.11884E-11	negative
PRJNA729271	stage_3W_vs_E10	ordinary_muscle_development	ENSGALG00010024837	TNFRSF1A	1.254606891	0.155901012	8.047458269	8.45312E-16	2.6596E-15	positive
PRJNA729271	stage_3W_vs_E10	ordinary_muscle_development	ENSGALG00010024838	EN2	-4.97610501	0.914502981	-5.441321805	5.28867E-08	1.08829E-07	negative
PRJNA729271	stage_3W_vs_E10	ordinary_muscle_development	ENSGALG00010024839	PA2G4	-0.746469893	0.099230449	-7.522589114	5.3702E-14	1.54754E-13	negative
PRJNA729271	stage_3W_vs_E10	ordinary_muscle_development	ENSGALG00010024840	BTF3L4	-1.075587158	0.101883937	-10.55698462	4.71567E-26	2.31003E-25	negative
PRJNA729271	stage_3W_vs_E10	ordinary_muscle_development	ENSGALG00010024841	ENSGALG00010024841	-2.552550756	0.225668874	-11.31104486	1.15703E-29	6.56921E-29	negative
PRJNA729271	stage_3W_vs_E10	ordinary_muscle_development	ENSGALG00010024842	ZFYVE9	-0.124181178	0.076062166	-1.63262743	0.102547379	0.123775856	negative
PRJNA729271	stage_3W_vs_E10	ordinary_muscle_development	ENSGALG00010024843	BCL9L	-0.104551409	0.134841267	-0.775366559	0.43812309	0.476735544	negative
PRJNA729271	stage_3W_vs_E10	ordinary_muscle_development	ENSGALG00010024844	TM4SF19	1.828453772	0.381612208	4.791392236	1.65628E-06	3.08861E-06	positive
PRJNA729271	stage_3W_vs_E10	ordinary_muscle_development	ENSGALG00010024845	HYCC2	0.645664841	0.109749668	5.883068758	4.02729E-09	8.86223E-09	positive
PRJNA729271	stage_3W_vs_E10	ordinary_muscle_development	ENSGALG00010024846	LMO2	0.947469035	0.142478671	6.649900826	2.93291E-11	7.31712E-11	positive
PRJNA729271	stage_3W_vs_E10	ordinary_muscle_development	ENSGALG00010024847	PPIL1	0.205927275	0.149329737	1.379010495	0.167891518	0.196748933	positive
PRJNA729271	stage_3W_vs_E10	ordinary_muscle_development	ENSGALG00010024848	ESYT1	-0.186077587	0.086268689	-2.156953929	0.031009254	0.03991113	negative
PRJNA729271	stage_3W_vs_E10	ordinary_muscle_development	ENSGALG00010024849	CENATAC	0.456133645	0.190880412	2.389630447	0.016865333	0.022358058	positive
PRJNA729271	stage_3W_vs_E10	ordinary_muscle_development	ENSGALG00010024850	CDK2AP1	-0.193131055	0.116863634	-1.652618941	0.098408441	0.119047767	negative
PRJNA729271	stage_3W_vs_E10	ordinary_muscle_development	ENSGALG00010024851	CPNE5	-4.829058377	0.988739155	-4.884056984	1.03925E-06	1.96683E-06	negative
PRJNA729271	stage_3W_vs_E10	ordinary_muscle_development	ENSGALG00010024852	CEP89	-0.734070112	0.094466684	-7.770677225	7.80676E-15	2.34235E-14	negative
PRJNA729271	stage_3W_vs_E10	ordinary_muscle_development	ENSGALG00010024853	TJP1	-0.08655402	0.067680105	-1.278869479	0.200943022	0.232548431	negative
PRJNA729271	stage_3W_vs_E10	ordinary_muscle_development	ENSGALG00010024854	C12orf65	1.585584022	0.149145505	10.63112173	2.13529E-26	1.06251E-25	positive
PRJNA729271	stage_3W_vs_E10	ordinary_muscle_development	ENSGALG00010024855	CAPRIN1	-0.168438137	0.071802132	-2.345865394	0.018982962	0.025023466	negative
PRJNA729271	stage_3W_vs_E10	ordinary_muscle_development	ENSGALG00010024856	SORT1	3.07374516	0.131851767	23.31212712	3.34E-120	2.0006E-118	positive
PRJNA729271	stage_3W_vs_E10	ordinary_muscle_development	ENSGALG00010024857	SLC37A4	1.089592838	0.141603604	7.694668819	1.41861E-14	4.19905E-14	positive
PRJNA729271	stage_3W_vs_E10	ordinary_muscle_development	ENSGALG00010024858	SLC25A39	0.408099294	0.121732234	3.352434119	0.000801043	0.001209996	positive
PRJNA729271	stage_3W_vs_E10	ordinary_muscle_development	ENSGALG00010024859	TARSL2	0.242557298	0.103121448	2.352151782	0.018665157	0.024624688	positive
PRJNA729271	stage_3W_vs_E10	ordinary_muscle_development	ENSGALG00010024860	NEU4	-4.678017181	0.900044963	-5.197537203	2.01946E-07	4.00056E-07	negative
PRJNA729271	stage_3W_vs_E10	ordinary_muscle_development	ENSGALG00010024861	ORC1	0.255690679	0.062932724	4.062920883	4.84625E-05	8.12002E-05	positive
PRJNA729271	stage_3W_vs_E10	ordinary_muscle_development	ENSGALG00010024862	METTL16	0.480761702	0.119505867	4.022912975	5.74827E-05	9.58487E-05	positive
PRJNA729271	stage_3W_vs_E10	ordinary_muscle_development	ENSGALG00010024863	LMOD1	0.825387838	0.293802325	2.809330523	0.004964465	0.006963352	positive
PRJNA729271	stage_3W_vs_E10	ordinary_muscle_development	ENSGALG00010024864	MPHOSPH9	-0.924114732	0.130890277	-7.060224447	1.66234E-12	4.42934E-12	negative
PRJNA729271	stage_3W_vs_E10	ordinary_muscle_development	ENSGALG00010024865	SNAP47	1.185180144	0.141937943	8.349988175	6.82698E-17	2.27697E-16	positive
PRJNA729271	stage_3W_vs_E10	ordinary_muscle_development	ENSGALG00010024866	TRAPPC4	0.542318407	0.084888164	6.388622183	1.67387E-10	3.9919E-10	positive
PRJNA729271	stage_3W_vs_E10	ordinary_muscle_development	ENSGALG00010024867	TM2D3	0.428304463	0.105386829	4.064117578	4.82145E-05	8.07942E-05	positive
PRJNA729271	stage_3W_vs_E10	ordinary_muscle_development	ENSGALG00010024868	FAAP24	-2.950383621	0.239779238	-12.30458335	8.55769E-35	5.8401E-34	negative
PRJNA729271	stage_3W_vs_E10	ordinary_muscle_development	ENSGALG00010024869	NDUFB3	2.169040995	0.121440694	17.86090738	2.37743E-71	4.74014E-70	positive
PRJNA729271	stage_3W_vs_E10	ordinary_muscle_development	ENSGALG00010024870	TPD52L2	0.042531488	0.082847394	0.513371467	0.6076915	0.643002285	positive
PRJNA729271	stage_3W_vs_E10	ordinary_muscle_development	ENSGALG00010024871	POLQ	-2.069609862	0.144135744	-14.35875523	9.38985E-47	9.51124E-46	negative
PRJNA729271	stage_3W_vs_E10	ordinary_muscle_development	ENSGALG00010024872	RUNDC3A	-1.148519163	0.205444656	-5.590406616	2.26538E-08	4.78321E-08	negative
PRJNA729271	stage_3W_vs_E10	ordinary_muscle_development	ENSGALG00010024873	HYOU1	-1.185936128	0.108967081	-10.88343483	1.38253E-27	7.25029E-27	negative
PRJNA729271	stage_3W_vs_E10	ordinary_muscle_development	ENSGALG00010024874	JMJD4	0.112430101	0.081612124	1.377615185	0.16832213	0.19722167	positive
PRJNA729271	stage_3W_vs_E10	ordinary_muscle_development	ENSGALG00010024876	CFLAR	0.477197335	0.094904928	5.028161835	4.95204E-07	9.56681E-07	positive
PRJNA729271	stage_3W_vs_E10	ordinary_muscle_development	ENSGALG00010024877	PRPF38A	-0.906198213	0.080395382	-11.27176953	1.80897E-29	1.01988E-28	negative
PRJNA729271	stage_3W_vs_E10	ordinary_muscle_development	ENSGALG00010024878	RHPN2	-0.786514151	0.266031775	-2.956466951	0.003111856	0.004457149	negative
PRJNA729271	stage_3W_vs_E10	ordinary_muscle_development	ENSGALG00010024879	D2HGDH	0.649639231	0.149193468	4.354340959	1.33468E-05	2.3348E-05	positive
PRJNA729271	stage_3W_vs_E10	ordinary_muscle_development	ENSGALG00010024880	NAT10	-1.425084552	0.139692114	-10.20161061	1.95011E-24	8.92545E-24	negative
PRJNA729271	stage_3W_vs_E10	ordinary_muscle_development	ENSGALG00010024881	CASP10	1.044084127	0.286116051	3.649163076	0.000263096	0.000414654	positive
PRJNA729271	stage_3W_vs_E10	ordinary_muscle_development	ENSGALG00010024882	MYL6	-1.74733754	0.164940163	-10.59376631	3.18513E-26	1.57249E-25	negative
PRJNA729271	stage_3W_vs_E10	ordinary_muscle_development	ENSGALG00010024883	ING5	1.652334667	0.122691895	13.46734979	2.43448E-41	2.04567E-40	positive
PRJNA729271	stage_3W_vs_E10	ordinary_muscle_development	ENSGALG00010024884	ZBTB46	1.549084901	0.132891486	11.65676557	2.11941E-31	1.28004E-30	positive
PRJNA729271	stage_3W_vs_E10	ordinary_muscle_development	ENSGALG00010024885	SLC4A1	-1.02251955	0.23781856	-4.29957843	1.71123E-05	2.97058E-05	negative
PRJNA729271	stage_3W_vs_E10	ordinary_muscle_development	ENSGALG00010024886	ABTB2	-2.368459701	0.148667611	-15.93124211	3.8464E-57	5.35577E-56	negative
PRJNA729271	stage_3W_vs_E10	ordinary_muscle_development	ENSGALG00010024887	CASP18	2.799047079	0.248411303	11.26779274	1.89255E-29	1.06658E-28	positive
PRJNA729271	stage_3W_vs_E10	ordinary_muscle_development	ENSGALG00010024888	PITPNM2	0.310463766	0.127870762	2.427949608	0.015184453	0.020235234	positive
PRJNA729271	stage_3W_vs_E10	ordinary_muscle_development	ENSGALG00010024889	SLC2A4RG	-0.50864792	0.519368965	-0.979357555	0.327403342	0.365600985	negative
PRJNA729271	stage_3W_vs_E10	ordinary_muscle_development	ENSGALG00010024890	SMARCC2	-0.053322892	0.146335122	-0.364388882	0.715567636	0.744163645	negative
PRJNA729271	stage_3W_vs_E10	ordinary_muscle_development	ENSGALG00010024891	PKP1	-2.303062335	0.490722945	-4.693202875	2.68961E-06	4.95436E-06	negative
PRJNA729271	stage_3W_vs_E10	ordinary_muscle_development	ENSGALG00010024893	ARL6IP4	-0.20913701	0.140168256	-1.492042612	0.135687975	0.161133639	negative
PRJNA729271	stage_3W_vs_E10	ordinary_muscle_development	ENSGALG00010024894	TUT4	0.989236056	0.121842318	8.118985826	4.70095E-16	1.49924E-15	positive
PRJNA729271	stage_3W_vs_E10	ordinary_muscle_development	ENSGALG00010024895	ENSGALG00010024895	1.195642214	0.411271966	2.907181412	0.003647016	0.005187782	positive
PRJNA729271	stage_3W_vs_E10	ordinary_muscle_development	ENSGALG00010024896	RNF41	0.782359535	0.100052622	7.81948058	5.30417E-15	1.60543E-14	positive
PRJNA729271	stage_3W_vs_E10	ordinary_muscle_development	ENSGALG00010024897	ADAL	0.497731246	0.191984254	2.592562863	0.009526377	0.012977898	positive
PRJNA729271	stage_3W_vs_E10	ordinary_muscle_development	ENSGALG00010024898	NABP2	-0.210793122	0.31240038	-0.674753091	0.499832648	0.537987245	negative
PRJNA729271	stage_3W_vs_E10	ordinary_muscle_development	ENSGALG00010024899	OGFOD2	-0.47882837	0.128013834	-3.740442385	0.000183697	0.00029357	negative
PRJNA729271	stage_3W_vs_E10	ordinary_muscle_development	ENSGALG00010024900	DTYMK	-1.66192352	0.105705474	-15.72220867	1.06544E-55	1.43261E-54	negative
PRJNA729271	stage_3W_vs_E10	ordinary_muscle_development	ENSGALG00010024901	ZGPAT	-0.548763206	0.131566527	-4.170994096	3.03274E-05	5.15836E-05	negative
PRJNA729271	stage_3W_vs_E10	ordinary_muscle_development	ENSGALG00010024902	LARP6	1.027065984	0.116051445	8.850092165	8.7447E-19	3.18319E-18	positive
PRJNA729271	stage_3W_vs_E10	ordinary_muscle_development	ENSGALG00010024903	CASP8	-0.595042602	0.11060754	-5.379765279	7.4583E-08	1.52308E-07	negative
PRJNA729271	stage_3W_vs_E10	ordinary_muscle_development	ENSGALG00010024904	ALS2CL	1.634090019	0.159121811	10.26942824	9.67765E-25	4.47742E-24	positive
PRJNA729271	stage_3W_vs_E10	ordinary_muscle_development	ENSGALG00010024905	GPX7	-1.88081202	0.209504644	-8.977424017	2.7718E-19	1.03045E-18	negative
PRJNA729271	stage_3W_vs_E10	ordinary_muscle_development	ENSGALG00010024907	PHLDB1	0.744197533	0.133304048	5.582707676	2.36803E-08	4.98758E-08	positive
PRJNA729271	stage_3W_vs_E10	ordinary_muscle_development	ENSGALG00010024908	ENSGALG00010024908	1.232477557	0.125754091	9.800695545	1.11813E-22	4.75706E-22	positive
PRJNA729271	stage_3W_vs_E10	ordinary_muscle_development	ENSGALG00010024910	UBTF	-0.990081277	0.123794924	-7.997753456	1.2671E-15	3.94641E-15	negative
PRJNA729271	stage_3W_vs_E10	ordinary_muscle_development	ENSGALG00010024911	CAT	0.70003795	0.153283319	4.566954553	4.94861E-06	8.94279E-06	positive
PRJNA729271	stage_3W_vs_E10	ordinary_muscle_development	ENSGALG00010024915	STXBP5L	-0.144402712	0.17309345	-0.834247116	0.404141746	0.443096846	negative
PRJNA729271	stage_3W_vs_E10	ordinary_muscle_development	ENSGALG00010024917	TRAK2	0.799193178	0.12261239	6.518045828	7.12292E-11	1.73378E-10	positive
PRJNA729271	stage_3W_vs_E10	ordinary_muscle_development	ENSGALG00010024918	ATG4B	-0.528436801	0.075371312	-7.011113202	2.36429E-12	6.24453E-12	negative
PRJNA729271	stage_3W_vs_E10	ordinary_muscle_development	ENSGALG00010024919	ANKRD52	-0.657201537	0.124252294	-5.289250719	1.22818E-07	2.47086E-07	negative
PRJNA729271	stage_3W_vs_E10	ordinary_muscle_development	ENSGALG00010024920	VIPR2	0.960023101	0.298905198	3.211797943	0.001319071	0.001955604	positive
PRJNA729271	stage_3W_vs_E10	ordinary_muscle_development	ENSGALG00010024921	STRADB	-0.534587862	0.109532074	-4.880651338	1.05736E-06	1.99875E-06	negative
PRJNA729271	stage_3W_vs_E10	ordinary_muscle_development	ENSGALG00010024924	MNX1	-3.394208829	0.657323146	-5.16368372	2.42137E-07	4.77325E-07	negative
PRJNA729271	stage_3W_vs_E10	ordinary_muscle_development	ENSGALG00010024925	GTF2E1	-0.643986406	0.083961415	-7.670028021	1.71959E-14	5.07233E-14	negative
PRJNA729271	stage_3W_vs_E10	ordinary_muscle_development	ENSGALG00010024926	ELF5	-4.935198991	0.847965765	-5.820045092	5.88318E-09	1.28739E-08	negative
PRJNA729271	stage_3W_vs_E10	ordinary_muscle_development	ENSGALG00010024927	NOM1	-0.294612251	0.113397635	-2.598045818	0.009375598	0.012783303	negative

PRJNA729271	stage_3W_vs_E10	ordinary_muscle_development	ENSGALG00010024928	THAP4	1.5091555	0.084076329	17.94982631	4.81407E-72	9.91192E-71	positive
PRJNA729271	stage_3W_vs_E10	ordinary_muscle_development	ENSGALG00010024930	TMEM237	-0.168019977	0.100020145	-1.679861358	0.092984294	0.112966837	negative
PRJNA729271	stage_3W_vs_E10	ordinary_muscle_development	ENSGALG00010024931	UBE3C	0.613985306	0.088398827	6.945627333	3.76782E-12	9.82277E-12	positive
PRJNA729271	stage_3W_vs_E10	ordinary_muscle_development	ENSGALG00010024932	ANKK1	-1.414994842	1.757202222	-0.805254412	0.420672883	0.459126078	negative
PRJNA729271	stage_3W_vs_E10	ordinary_muscle_development	ENSGALG00010024933	CS	0.452650893	0.157169898	2.880010099	0.003976624	0.005632871	positive
PRJNA729271	stage_3W_vs_E10	ordinary_muscle_development	ENSGALG00010024934	G6PC3	0.801228299	0.221608488	3.615512681	0.000299754	0.00047074	positive
PRJNA729271	stage_3W_vs_E10	ordinary_muscle_development	ENSGALG00010024935	KLHL30	3.568978415	0.387684034	9.205894754	3.38836E-20	1.30519E-19	positive
PRJNA729271	stage_3W_vs_E10	ordinary_muscle_development	ENSGALG00010024936	EHF	-0.482138887	0.567002911	-0.850328768	0.395142327	0.434006822	negative
PRJNA729271	stage_3W_vs_E10	ordinary_muscle_development	ENSGALG00010024937	ILKAP	-1.027813523	0.127261571	-8.076385642	6.67147E-16	2.11096E-15	negative
PRJNA729271	stage_3W_vs_E10	ordinary_muscle_development	ENSGALG00010024938	ENSGALG00010024938	0.479606605	0.175276882	2.736279875	0.006213815	0.008624845	positive
PRJNA729271	stage_3W_vs_E10	ordinary_muscle_development	ENSGALG00010024939	COQ10A	2.334958936	0.202711147	11.51865093	1.06262E-30	6.27143E-30	positive
PRJNA729271	stage_3W_vs_E10	ordinary_muscle_development	ENSGALG00010024940	RABL3	-0.136693885	0.091165794	-1.499398829	0.133770197	0.159012548	negative
PRJNA729271	stage_3W_vs_E10	ordinary_muscle_development	ENSGALG00010024941	PDHX	0.546591697	0.118185801	4.6248508	3.74868E-06	6.84778E-06	positive
PRJNA729271	stage_3W_vs_E10	ordinary_muscle_development	ENSGALG00010024942	DNAJB6	0.338962339	0.066008333	5.135144706	2.81927E-07	5.53581E-07	positive
PRJNA729271	stage_3W_vs_E10	ordinary_muscle_development	ENSGALG00010024943	ABCB9	0.441735758	0.175240282	2.520743246	0.011710727	0.015812481	positive
PRJNA729271	stage_3W_vs_E10	ordinary_muscle_development	ENSGALG00010024944	ENSGALG00010024944	-0.194020654	0.112484023	-1.724872999	0.084550363	0.103204843	negative
PRJNA729271	stage_3W_vs_E10	ordinary_muscle_development	ENSGALG00010024945	APIP	0.798560732	0.080186383	9.958807193	2.30815E-23	1.00773E-22	positive
PRJNA729271	stage_3W_vs_E10	ordinary_muscle_development	ENSGALG00010024946	KIF1A	-5.113062109	0.346694645	-14.74802735	3.1679E-49	3.48132E-48	negative
PRJNA729271	stage_3W_vs_E10	ordinary_muscle_development	ENSGALG00010024947	MPP4	1.269534143	0.312111212	4.067569803	4.7506E-05	7.96437E-05	positive
PRJNA729271	stage_3W_vs_E10	ordinary_muscle_development	ENSGALG00010024949	PAN2	-0.359971295	0.115007612	-3.129977996	0.001748194	0.002560897	negative
PRJNA729271	stage_3W_vs_E10	ordinary_muscle_development	ENSGALG00010024950	NCAM1	-1.267579783	0.155221588	-8.166259584	3.18098E-16	1.02395E-15	negative
PRJNA729271	stage_3W_vs_E10	ordinary_muscle_development	ENSGALG00010024953	NCAPG2	-3.030674949	0.228094023	-13.28695467	2.75567E-40	2.2478E-39	negative
PRJNA729271	stage_3W_vs_E10	ordinary_muscle_development	ENSGALG00010024956	VPS37B	-0.621871909	0.138781803	-4.480932622	7.43176E-06	1.32452E-05	negative
PRJNA729271	stage_3W_vs_E10	ordinary_muscle_development	ENSGALG00010024960	ARFRP1	-0.697403195	0.102235919	-6.821508528	9.00894E-12	2.30756E-11	negative
PRJNA729271	stage_3W_vs_E10	ordinary_muscle_development	ENSGALG00010024961	LSM12	0.709337595	0.202846843	3.496912171	0.000470677	0.000726799	positive
PRJNA729271	stage_3W_vs_E10	ordinary_muscle_development	ENSGALG00010024963	TNFRSF6B	1.279180033	0.577735015	2.214129313	0.026819885	0.034753641	positive
PRJNA729271	stage_3W_vs_E10	ordinary_muscle_development	ENSGALG00010024964	ITM2C	-1.780692602	0.174389025	-10.21103593	1.76965E-24	8.11744E-24	negative
PRJNA729271	stage_3W_vs_E10	ordinary_muscle_development	ENSGALG00010024965	STAT2	0.961556402	0.218214768	4.406468039	1.0507E-05	1.85233E-05	positive
PRJNA729271	stage_3W_vs_E10	ordinary_muscle_development	ENSGALG00010024966	AGAP3	-0.279795666	0.115344104	-2.425747439	0.015276898	0.020343466	negative
PRJNA729271	stage_3W_vs_E10	ordinary_muscle_development	ENSGALG00010024968	TMEM101	0.974922145	0.131579383	7.409383779	1.26888E-13	3.58107E-13	positive
PRJNA729271	stage_3W_vs_E10	ordinary_muscle_development	ENSGALG00010024970	CAB39	0.629990771	0.077611282	8.117257613	4.76836E-16	1.5204E-15	positive
PRJNA729271	stage_3W_vs_E10	ordinary_muscle_development	ENSGALG00010024973	RTEL1	-1.831628459	0.089431227	-20.48086013	3.18976E-93	1.13881E-91	negative
PRJNA729271	stage_3W_vs_E10	ordinary_muscle_development	ENSGALG00010024974	CD44	0.56029426	0.165766886	3.380013193	0.000724824	0.001100023	positive
PRJNA729271	stage_3W_vs_E10	ordinary_muscle_development	ENSGALG00010024977	LRP3	1.277907301	0.104404684	12.23994228	1.90168E-34	1.28687E-33	positive
PRJNA729271	stage_3W_vs_E10	ordinary_muscle_development	ENSGALG00010024983	APOF	1.207400038	0.533132923	2.264726088	0.023529494	0.030720592	positive
PRJNA729271	stage_3W_vs_E10	ordinary_muscle_development	ENSGALG00010024984	SHISAL2A	-0.215918059	0.499992263	-0.431842799	0.665855671	0.697670807	negative
PRJNA729271	stage_3W_vs_E10	ordinary_muscle_development	ENSGALG00010024986	PTS	-1.341314389	0.17271231	-7.766177128	8.08904E-15	2.42454E-14	negative
PRJNA729271	stage_3W_vs_E10	ordinary_muscle_development	ENSGALG00010024989	TIMELESS	-1.662100015	0.166063946	-10.00879515	1.3944E-23	6.14156E-23	negative
PRJNA729271	stage_3W_vs_E10	ordinary_muscle_development	ENSGALG00010024994	NDUFB4	1.600260429	0.103694439	15.43246137	9.90206E-54	1.26906E-52	positive
PRJNA729271	stage_3W_vs_E10	ordinary_muscle_development	ENSGALG00010024996	COA7	0.326552451	0.124270708	2.627750802	0.008595146	0.011773449	positive
PRJNA729271	stage_3W_vs_E10	ordinary_muscle_development	ENSGALG00010024997	HES6	0.258607749	0.252283994	1.025066016	0.305332039	0.34305774	positive
PRJNA729271	stage_3W_vs_E10	ordinary_muscle_development	ENSGALG00010025003	ENSGALG00010025003	9.681129245	0.243146034	39.81611007	0	0	positive
PRJNA729271	stage_3W_vs_E10	ordinary_muscle_development	ENSGALG00010025005	HIP1R	-0.814631117	0.12482248	-6.526317344	6.74064E-11	1.64266E-10	negative
PRJNA729271	stage_3W_vs_E10	ordinary_muscle_development	ENSGALG00010025006	ENSGALG00010025006	-1.139597177	0.371554863	-3.067103385	0.002161441	0.003138029	negative
PRJNA729271	stage_3W_vs_E10	ordinary_muscle_development	ENSGALG00010025007	ZYG11B	0.463580163	0.101255459	4.578322654	4.68719E-06	8.48518E-06	positive
PRJNA729271	stage_3W_vs_E10	ordinary_muscle_development	ENSGALG00010025008	ENSGALG00010025008	-2.689467858	0.619085798	-4.344257074	1.39748E-05	2.44142E-05	negative
PRJNA729271	stage_3W_vs_E10	ordinary_muscle_development	ENSGALG00010025012	ENSGALG00010025012	3.321942378	0.759868566	4.371732856	1.23264E-05	2.16388E-05	positive
PRJNA729271	stage_3W_vs_E10	ordinary_muscle_development	ENSGALG00010025026	DENR	0.256978784	0.115514102	2.224652917	0.026104547	0.033887284	positive
PRJNA729271	stage_3W_vs_E10	ordinary_muscle_development	ENSGALG00010025028	HES6	-1.077168305	0.135158873	-7.969645538	1.5913E-15	4.93178E-15	negative
PRJNA729271	stage_3W_vs_E10	ordinary_muscle_development	ENSGALG00010025031	ECHDC2	0.435736398	0.122006753	3.571412119	0.000355062	0.000553996	positive
PRJNA729271	stage_3W_vs_E10	ordinary_muscle_development	ENSGALG00010025032	FSTL1	-0.796237275	0.181266408	-4.39263559	1.11985E-05	1.97088E-05	negative
PRJNA729271	stage_3W_vs_E10	ordinary_muscle_development	ENSGALG00010025039	CEBPA	0.306484334	0.308712321	0.992782968	0.320815732	0.358896661	positive
PRJNA729271	stage_3W_vs_E10	ordinary_muscle_development	ENSGALG00010025042	BCO2	-0.444732376	0.510185426	-0.871707331	0.38336806	0.42229045	negative
PRJNA729271	stage_3W_vs_E10	ordinary_muscle_development	ENSGALG00010025044	TMEM9	0.57680812	0.214364026	2.690787863	0.007128351	0.009841456	positive
PRJNA729271	stage_3W_vs_E10	ordinary_muscle_development	ENSGALG00010025047	PER2	1.191939268	0.292899936	4.06944189	4.71259E-05	7.90339E-05	positive
PRJNA729271	stage_3W_vs_E10	ordinary_muscle_development	ENSGALG00010025048	ESYT2	1.154078761	0.082102976	14.05647896	7.02978E-45	6.68175E-44	positive
PRJNA729271	stage_3W_vs_E10	ordinary_muscle_development	ENSGALG00010025055	ALS2	1.07387371	0.154052712	6.970819917	3.15099E-12	8.26522E-12	positive
PRJNA729271	stage_3W_vs_E10	ordinary_muscle_development	ENSGALG00010025056	RBMS2	0.045133374	0.174080995	0.259266523	0.795429607	0.81806103	positive
PRJNA729271	stage_3W_vs_E10	ordinary_muscle_development	ENSGALG00010025057	SCP2	0.239446908	0.066542459	3.598407851	0.000320171	0.000501609	positive
PRJNA729271	stage_3W_vs_E10	ordinary_muscle_development	ENSGALG00010025060	GPR156	-0.829583153	0.185920634	-4.462028427	8.11875E-06	1.44341E-05	negative
PRJNA729271	stage_3W_vs_E10	ordinary_muscle_development	ENSGALG00010025064	GPN3	0.383331415	0.082224785	4.661993524	3.13161E-06	5.74591E-06	positive
PRJNA729271	stage_3W_vs_E10	ordinary_muscle_development	ENSGALG00010025067	SLC1A2	-2.059640846	0.341898071	-6.024137083	1.70014E-09	3.82189E-09	negative
PRJNA729271	stage_3W_vs_E10	ordinary_muscle_development	ENSGALG00010025069	PODN	4.567111701	0.196936617	23.1907695	5.6425E-119	3.2979E-117	positive
PRJNA729271	stage_3W_vs_E10	ordinary_muscle_development	ENSGALG00010025071	CDK15	0.070506463	0.329250831	0.214142096	0.830436249	0.85017674	positive
PRJNA729271	stage_3W_vs_E10	ordinary_muscle_development	ENSGALG00010025072	ENSGALG00010025072	-0.047675451	0.664121688	-0.071787223	0.942771242	0.95004652	negative
PRJNA729271	stage_3W_vs_E10	ordinary_muscle_development	ENSGALG00010025073	IL18	1.867142994	0.199359102	9.365727347	7.55283E-21	2.99121E-20	positive
PRJNA729271	stage_3W_vs_E10	ordinary_muscle_development	ENSGALG00010025080	BAZ2A	1.533535852	0.202395216	7.576937271	3.53808E-14	1.02651E-13	positive
PRJNA729271	stage_3W_vs_E10	ordinary_muscle_development	ENSGALG00010025092	COL1A1	1.72071682	0.22098297	7.786648995	6.88098E-15	2.07101E-14	positive
PRJNA729271	stage_3W_vs_E10	ordinary_muscle_development	ENSGALG00010025097	LMBR1	0.015267444	0.121271338	0.125894906	0.899815115	0.911500129	positive
PRJNA729271	stage_3W_vs_E10	ordinary_muscle_development	ENSGALG00010025098	ATP5B	1.00537488	0.138217113	7.273881329	3.49303E-13	9.63308E-13	positive
PRJNA729271	stage_3W_vs_E10	ordinary_muscle_development	ENSGALG00010025102	ARPC3	-0.498548857	0.085691975	-5.817917672	5.95852E-09	1.30309E-08	negative
PRJNA729271	stage_3W_vs_E10	ordinary_muscle_development	ENSGALG00010025103	GSK3B	0.011766321	0.100220751	0.117404043	0.90653988	0.917454945	positive
PRJNA729271	stage_3W_vs_E10	ordinary_muscle_development	ENSGALG00010025113	SDHD	0.91086773	0.120153602	7.58086079	3.4327E-14	9.97133E-14	positive
PRJNA729271	stage_3W_vs_E10	ordinary_muscle_development	ENSGALG00010025114	CEBPG	0.630220105	0.096966683	6.499346859	8.06695E-11	1.95798E-10	positive
PRJNA729271	stage_3W_vs_E10	ordinary_muscle_development	ENSGALG00010025115	MGAT4F	-0.090536953	0.254009926	-0.356430767	0.721517992	0.74992136	negative
PRJNA729271	stage_3W_vs_E10	ordinary_muscle_development	ENSGALG00010025116	ANAPC7	-0.553579019	0.070605631	-7.840437253	4.4898E-15	1.36378E-14	negative
PRJNA729271	stage_3W_vs_E10	ordinary_muscle_development	ENSGALG00010025121	DYNC2I1	-0.270895806	0.153073929	-1.769705704	0.076776179	0.094183371	negative
PRJNA729271	stage_3W_vs_E10	ordinary_muscle_development	ENSGALG00010025125	PIK3CB	0.809544063	0.131418316	6.160055066	7.27197E-10	1.67339E-09	positive
PRJNA729271	stage_3W_vs_E10	ordinary_muscle_development	ENSGALG00010025126	ENSGALG00010025126	-0.145205711	0.132401435	-1.096707989	0.272769063	0.309445689	negative
PRJNA729271	stage_3W_vs_E10	ordinary_muscle_development	ENSGALG00010025127	PAMR1	0.509073579	0.155910124	3.265173338	0.001093971	0.001634248	positive
PRJNA729271	stage_3W_vs_E10	ordinary_muscle_development	ENSGALG00010025130	ATP2A2	-2.181539649	0.125941123	-17.32190081	3.21558E-67	5.81896E-66	negative
PRJNA729271	stage_3W_vs_E10	ordinary_muscle_development	ENSGALG00010025131	SUMO1	-0.258322163	0.080850209	-3.195071049	0.001397964	0.002067281	negative
PRJNA729271	stage_3W_vs_E10	ordinary_muscle_development	ENSGALG00010025138	CFAP91	-0.820730921	0.276352465	-2.969870098	0.002979257	0.004273984	negative
PRJNA729271	stage_3W_vs_E10	ordinary_muscle_development	ENSGALG00010025139	COPB2	-0.719013076	0.046890042	-15.33402507	4.53023E-53	5.68043E-52	negative
PRJNA729271	stage_3W_vs_E10	ordinary_muscle_development	ENSGALG00010025144	PIH1D2	-0.221832175	0.161263555	-1.375587769	0.168949296	0.197876538	negative
PRJNA729271	stage_3W_vs_E10	ordinary_muscle_development	ENSGALG00010025145	CPT2	-0.5347618	0.095454459	-5.602271548	2.11561E-08	4.47414E-08	negative
PRJNA729271	stage_3W_vs_E10	ordinary_muscle_development	ENSGALG00010025147	COX17	2.471499512	0.113289431	21.81579942	1.6427E-105	7.5833E-104	positive
PRJNA729271	stage_3W_vs_E10	ordinary_muscle_development	ENSGALG00010025149	IFT81	-1.226076551	0.142372978	-8.611722307	7.1971E-18	2.52412E-17	negative
PRJNA729271	stage_3W_vs_E10	ordinary_muscle_development	ENSGALG00010025154	TMEM183A	1.732895572	0.120729236	14.35357029	1.01191E-46	1.02356E-45	positive
PRJNA729271	stage_3W_vs_E10	ordinary_muscle_development	ENSGALG00010025155	CZIB	0.397408633	0.091407435	4.347662023	1.37596E-05	2.4047E-05	positive
PRJNA729271	stage_3W_vs_E10	ordinary_muscle_development	ENSGALG00010025156	DLAT	0.821472854	0.115259156	7.127180888	1.02445E-12	2.76321E-12	positive
PRJNA729271	stage_3W_vs_E10	ordinary_muscle_development	ENSGALG00010025157	NOP58	-1.193563069	0.140515939	-8.494147165	1.99392E-17	6.8229E-17	negative
PRJNA729271	stage_3W_vs_E10	ordinary_muscle_development	ENSGALG00010025160	MRPS22	0.678549527	0.130157853	5.213281511	1.85529E-07	3.68642E-07	positive
PRJNA729271	stage_3W_vs_E10	ordinary_muscle_development	ENSGALG00010025161	ENSGALG00010025161	-1.02298111	0.105057261	-9.737367069	2.08897E-22	8.77668E-22	negative
PRJNA729271	stage_3W_vs_E10	ordinary_muscle_development	ENSGALG00010025163	LDLRAD3	-1.66184773	0.137930064	-12.04848077	1.97558E-33	1.28817E-32	negative
PRJNA729271	stage_3W_vs_E10	ordinary_muscle_development	ENSGALG00010025164	C11orf52	1.479019525	0.124610381	11.86915176	1.71195E-32	1.08361E-31	positive
PRJNA729271	stage_3W_vs_E10	ordinary_muscle_development	ENSGALG00010025167	PTGES3	-0.11250957	0.188459662	-0.596995502	0.550510384	0.587602947	negative
PRJNA729271	stage_3W_vs_E10	ordinary_muscle_development	ENSGALG00010025168	POPDC2	1.495502492	0.211550815	7.069235316	1.5579E-12	4.16023E-12	positive
PRJNA729271	stage_3W_vs_E10	ordinary_muscle_development	ENSGALG00010025169	RBP2	6.312003888	2.095258257	3.012518322	0.002590898	0.003735707	positive
PRJNA729271	stage_3W_vs_E10	ordinary_muscle_development	ENSGALG00010025170	COMMD9	0.166877858	0.095398936	1.7492633	0.080245517	0.098189882	positive
PRJNA729271	stage_3W_vs_E10	ordinary_muscle_development	ENSGALG00010025172	RBP1	6.529473025	0.898849744	7.2642542	3.75102E-13	1.03288E-12	positive
PRJNA729271	stage_3W_vs_E10	ordinary_muscle_development	ENSGALG00010025182	PLA1A	3.670707112	0.191643406	19.15383992	8.99064E-82	2.36086E-80	positive
PRJNA729271	stage_3W_vs_E10	ordinary_muscle_development	ENSGALG00010025186	TMEM120B	-1.224424849	0.208699322	-5.866932574	4.43931E-09	9.74524E-09	negative
PRJNA729271	stage_3W_vs_E10	ordinary_muscle_development	ENSGALG00010025188	PPFIA4	-3.471997933	0.418223213	-8.301781972	1.02561E-16	3.39103E-16	negative
PRJNA729271	stage_3W_vs_E10	ordinary_muscle_development	ENSGALG00010025190	PRR5L	1.041449727	0.238775842	4.361621005	1.29102E-05	2.26171E-05	positive
PRJNA729271	stage_3W_vs_E10	ordinary_muscle_development	ENSGALG00010025192	CRYAB	2.172861781	0.181689641	11.95919464	5.81229E-33	3.72619E-32	positive
PRJNA729271	stage_3W_vs_E10	ordinary_muscle_development	ENSGALG00010025195	P2RX7	4.795611039	0.399823816	11.9943106	3.80573E-33	2.45938E-32	positive
PRJNA729271	stage_3W_vs_E10	ordinary_muscle_development	ENSGALG00010025200	PEPD	-0.223953602	0.080352061	-2.787154428	0.005317313	0.007438183	negative
PRJNA729271	stage_3W_vs_E10	ordinary_muscle_development	ENSGALG00010025204	TRAF6	1.32491868	0.116202256	11.40183272	4.09408E-30	2.35959E-29	positive
PRJNA729271	stage_3W_vs_E10	ordinary_muscle_development	ENSGALG00010025207	BMPR2	-0.086460341	0.097683182	-0.885109797	0.376097416	0.414880673	negative
PRJNA729271	stage_3W_vs_E10	ordinary_muscle_development	ENSGALG00010025210	HSPB2	1.272368097	0.295652897	4.30358745	1.68054E-05	2.91905E-05	positive
PRJNA729271	stage_3W_vs_E10	ordinary_muscle_development	ENSGALG00010025212	P2RX4	1.318848849	0.177715529	7.421123259	1.16131E-13	3.28453E-13	positive
PRJNA729271	stage_3W_vs_E10	ordinary_muscle_development	ENSGALG00010025214	LRP8	-2.759875001	0.231791738	-11.90670137	1.09213E-32	6.93707E-32	negative
PRJNA729271	stage_3W_vs_E10	ordinary_muscle_development	ENSGALG00010025215	MYOG	-1.439812984	0.214341409	-6.717381343	1.85019E-11	4.66085E-11	negative
PRJNA729271	stage_3W_vs_E10	ordinary_muscle_development	ENSGALG00010025216	DPP6	-3.294244377	0.287956404	-11.44008027	2.63641E-30	1.53104E-29	negative
PRJNA729271	stage_3W_vs_E10	ordinary_muscle_development	ENSGALG00010025219	NACA	-1.029469124	0.125718024	-8.188715442	2.64029E-16	8.54645E-16	negative
PRJNA729271	stage_3W_vs_E10	ordinary_muscle_development	ENSGALG00010025222	FAM117B	-1.327325724	0.145908799	-9.0969546	9.29002E-20	3.51773E-19	negative
PRJNA729271	stage_3W_vs_E10	ordinary_muscle_development	ENSGALG00010025225	DMRTB1	2.310304441	0.666670939	3.465434455	0.000529376	0.000813453	positive
PRJNA729271	stage_3W_vs_E10	ordinary_muscle_development	ENSGALG00010025228	FDXACB1	-0.349768045	0.111953729	-3.1242197	0.001782773	0.002607861	negative
PRJNA729271	stage_3W_vs_E10	ordinary_muscle_development	ENSGALG00010025229	ENSGALG00010025229	-1.236716603	0.166366509	-7.433687284	1.05611E-13	2.99284E-13	negative
PRJNA729271	stage_3W_vs_E10	ordinary_muscle_development	ENSGALG00010025230	CHST8	-3.304098327	0.371109252	-8.903303561	5.42087E-19	1.99278E-18	negative
PRJNA729271	stage_3W_vs_E10	ordinary_muscle_development	ENSGALG00010025232	PRIM1	-1.477220492	0.153198808	-9.642506416	5.28808E-22	2.19252E-21	negative
PRJNA729271	stage_3W_vs_E10	ordinary_muscle_development	ENSGALG00010025233	WDR12	-1.02629136	0.076545357	-13.40762394	5.45589E-41	4.53459E-40	negative
PRJNA729271	stage_3W_vs_E10	ordinary_muscle_development	ENSGALG00010025236	ENSGALG00010025236	1.125713619	0.249235747	4.516661966	6.28221E-06	1.12741E-05	positive
PRJNA729271	stage_3W_vs_E10	ordinary_muscle_development	ENSGALG00010025238	IGSF9B	-0.618720533	0.325482362	-1.900934138	0.05731064	0.071404308	negative
PRJNA729271	stage_3W_vs_E10	ordinary_muscle_development	ENSGALG00010025239	RAG2	-1.722367367	0.628860127	-2.738871958	0.006165038	0.008562881	negative
PRJNA729271	stage_3W_vs_E10	ordinary_muscle_development	ENSGALG00010025242	CLSTN2	-4.566685093	0.313520518	-14.56582529	4.63355E-48	4.9567E-47	negative
PRJNA729271	stage_3W_vs_E10	ordinary_muscle_development	ENSGALG00010025243	PAXIP1	-0.696163262	0.090142112	-7.722952619	1.13665E-14	3.37757E-14	negative
PRJNA729271	stage_3W_vs_E10	ordinary_muscle_development	ENSGALG00010025245	ENSGALG00010025245	1.376857932	0.067868373	20.28718062	1.66896E-91	5.63905E-90	positive
PRJNA729271	stage_3W_vs_E10	ordinary_muscle_development	ENSGALG00010025247	SGCA	3.421715945	0.139874084	24.46283013	3.6751E-132	2.6503E-130	positive
PRJNA729271	stage_3W_vs_E10	ordinary_muscle_development	ENSGALG00010025248	C11orf74	0.243925292	0.210955917	1.156285611	0.247564352	0.2826883	positive

PRJNA729271	stage_3W_vs_E10	ordinary_muscle_development	ENSGALG00010025250	GLIS1	-3.60726412	0.450774661	-8.002366672	1.2205E-15	3.80701E-15	negative
PRJNA729271	stage_3W_vs_E10	ordinary_muscle_development	ENSGALG00010025252	PPP1R9B	-0.32988517	0.145536934	-2.266676644	0.023409986	0.030581088	negative
PRJNA729271	stage_3W_vs_E10	ordinary_muscle_development	ENSGALG00010025256	ANAPC5	-0.966175477	0.122445128	-7.890681273	3.00541E-15	9.2042E-15	negative
PRJNA729271	stage_3W_vs_E10	ordinary_muscle_development	ENSGALG00010025257	LPCAT2	0.919085625	0.164414758	5.590043369	2.27013E-08	4.79253E-08	positive
PRJNA729271	stage_3W_vs_E10	ordinary_muscle_development	ENSGALG00010025259	SHH	-4.89489056	0.869319098	-5.630717846	1.79461E-08	3.81365E-08	negative
PRJNA729271	stage_3W_vs_E10	ordinary_muscle_development	ENSGALG00010025262	CARF	-0.548412589	0.112591024	-4.87083756	1.11126E-06	2.09736E-06	negative
PRJNA729271	stage_3W_vs_E10	ordinary_muscle_development	ENSGALG00010025263	SAMD14	-1.892713663	0.819605467	-2.309298486	0.020927024	0.027476932	negative
PRJNA729271	stage_3W_vs_E10	ordinary_muscle_development	ENSGALG00010025268	ZBTB39	-1.37424691	0.11295831	-12.16596556	4.71833E-34	3.14302E-33	negative
PRJNA729271	stage_3W_vs_E10	ordinary_muscle_development	ENSGALG00010025270	NDC1	-2.232994397	0.123800808	-18.0369937	9.98291E-73	2.0941E-71	negative
PRJNA729271	stage_3W_vs_E10	ordinary_muscle_development	ENSGALG00010025271	RNF34	0.919331927	0.11749667	7.82432324	5.10395E-15	1.54612E-14	positive
PRJNA729271	stage_3W_vs_E10	ordinary_muscle_development	ENSGALG00010025272	NCAPD3	-3.303258169	0.193587222	-17.06341018	2.77851E-65	4.79458E-64	negative
PRJNA729271	stage_3W_vs_E10	ordinary_muscle_development	ENSGALG00010025274	PDK2	3.483344387	0.269255026	12.93697071	2.78392E-38	2.1216E-37	positive
PRJNA729271	stage_3W_vs_E10	ordinary_muscle_development	ENSGALG00010025280	HSPB11	-0.137656842	0.15187944	-0.906356006	0.364747451	0.403558072	negative
PRJNA729271	stage_3W_vs_E10	ordinary_muscle_development	ENSGALG00010025286	ITGA3	-0.815417014	0.178374108	-4.571386641	4.84507E-06	8.76661E-06	negative
PRJNA729271	stage_3W_vs_E10	ordinary_muscle_development	ENSGALG00010025289	KDM2B	-1.213893478	0.075334562	-16.11336749	2.0552E-58	2.96419E-57	negative
PRJNA729271	stage_3W_vs_E10	ordinary_muscle_development	ENSGALG00010025291	DUS2	0.24188424	0.091309982	2.64904489	0.008071961	0.011088237	positive
PRJNA729271	stage_3W_vs_E10	ordinary_muscle_development	ENSGALG00010025292	ATP1A1	0.773333494	0.127500601	6.065332134	1.31681E-09	2.98005E-09	positive
PRJNA729271	stage_3W_vs_E10	ordinary_muscle_development	ENSGALG00010025297	SPSB4	-1.1749253	0.147382421	-7.971950074	1.5619E-15	4.8448E-15	negative
PRJNA729271	stage_3W_vs_E10	ordinary_muscle_development	ENSGALG00010025298	ENSGALG00010025298	-4.282811107	0.344489159	-12.43235381	1.74422E-35	1.21784E-34	negative
PRJNA729271	stage_3W_vs_E10	ordinary_muscle_development	ENSGALG00010025301	MORN3	-1.851352009	0.329340497	-5.621391917	1.89425E-08	4.01949E-08	negative
PRJNA729271	stage_3W_vs_E10	ordinary_muscle_development	ENSGALG00010025303	TMCC2	2.637188148	0.188000117	14.02758779	1.05686E-44	9.97179E-44	positive
PRJNA729271	stage_3W_vs_E10	ordinary_muscle_development	ENSGALG00010025304	DDX28	-0.07429847	0.092137623	-0.80638579	0.420020438	0.458483037	negative
PRJNA729271	stage_3W_vs_E10	ordinary_muscle_development	ENSGALG00010025305	GPR182	1.401266239	0.42856153	3.269696746	0.001076628	0.001609336	positive
PRJNA729271	stage_3W_vs_E10	ordinary_muscle_development	ENSGALG00010025311	ENSGALG00010025311	0.433971602	0.673621725	0.644236352	0.519422182	0.557333964	positive
PRJNA729271	stage_3W_vs_E10	ordinary_muscle_development	ENSGALG00010025312	PLEKHM3	0.344747882	0.113732371	3.031220396	0.002435674	0.003519599	positive
PRJNA729271	stage_3W_vs_E10	ordinary_muscle_development	ENSGALG00010025313	DIO1	-0.386009855	0.86877046	-0.444317426	0.656813104	0.689691824	negative
PRJNA729271	stage_3W_vs_E10	ordinary_muscle_development	ENSGALG00010025315	LRRC42	-0.063630759	0.119460748	-0.532649929	0.594275933	0.629864701	negative
PRJNA729271	stage_3W_vs_E10	ordinary_muscle_development	ENSGALG00010025316	SLC25A36	0.05129669	0.109777163	0.467280161	0.640299449	0.673571414	positive
PRJNA729271	stage_3W_vs_E10	ordinary_muscle_development	ENSGALG00010025318	NUAK2	-2.115898147	0.448165065	-4.721247403	2.34403E-06	4.3332E-06	negative
PRJNA729271	stage_3W_vs_E10	ordinary_muscle_development	ENSGALG00010025319	LDLRAD1	0.912058891	0.344045054	2.650986786	0.008025698	0.011028868	positive
PRJNA729271	stage_3W_vs_E10	ordinary_muscle_development	ENSGALG00010025320	JAM3	-1.859116352	0.145008605	-12.82073122	1.25511E-37	9.31057E-37	negative
PRJNA729271	stage_3W_vs_E10	ordinary_muscle_development	ENSGALG00010025323	KLHDC8A	-2.171635516	0.188113977	-11.54425388	7.89234E-31	4.677E-30	negative
PRJNA729271	stage_3W_vs_E10	ordinary_muscle_development	ENSGALG00010025325	DPEP2	1.92916928	0.240289239	8.028529652	9.86478E-16	3.09234E-15	positive
PRJNA729271	stage_3W_vs_E10	ordinary_muscle_development	ENSGALG00010025327	TMEM59	1.773841028	0.070638309	25.1116009	3.715E-139	2.9587E-137	positive
PRJNA729271	stage_3W_vs_E10	ordinary_muscle_development	ENSGALG00010025329	MYO1A	-1.423003591	0.211726166	-6.720962342	1.80528E-11	4.55167E-11	negative
PRJNA729271	stage_3W_vs_E10	ordinary_muscle_development	ENSGALG00010025330	USH1C	-2.782289964	0.404886639	-6.871775196	6.34078E-12	1.63337E-11	negative
PRJNA729271	stage_3W_vs_E10	ordinary_muscle_development	ENSGALG00010025331	ENSGALG00010025331	2.16747704	0.337688458	6.4185701	1.3756E-10	3.29304E-10	positive
PRJNA729271	stage_3W_vs_E10	ordinary_muscle_development	ENSGALG00010025333	FZD5	1.618109114	0.192917183	8.387584205	4.96234E-17	1.66502E-16	positive
PRJNA729271	stage_3W_vs_E10	ordinary_muscle_development	ENSGALG00010025334	RHOF	1.915087461	0.217174109	8.818212599	1.16301E-18	4.21026E-18	positive
PRJNA729271	stage_3W_vs_E10	ordinary_muscle_development	ENSGALG00010025336	ADAM10	-0.158998395	0.063343869	-2.510083428	0.012070264	0.01627218	negative
PRJNA729271	stage_3W_vs_E10	ordinary_muscle_development	ENSGALG00010025337	NEMP1	-0.303670388	0.147295503	-2.061640582	0.039241967	0.04993524	negative
PRJNA729271	stage_3W_vs_E10	ordinary_muscle_development	ENSGALG00010025338	FADD	-0.939241767	0.096959441	-9.686955256	3.42589E-22	1.42984E-21	negative
PRJNA729271	stage_3W_vs_E10	ordinary_muscle_development	ENSGALG00010025340	METTL21A	1.184648426	0.115778175	10.232053	1.42467E-24	6.55573E-24	positive
PRJNA729271	stage_3W_vs_E10	ordinary_muscle_development	ENSGALG00010025341	VPS26B	-1.038940629	0.132251176	-7.855813928	3.97184E-15	1.21E-14	negative
PRJNA729271	stage_3W_vs_E10	ordinary_muscle_development	ENSGALG00010025342	TCEANC2	-0.958226237	0.120977302	-7.92071091	2.36156E-15	7.26308E-15	negative
PRJNA729271	stage_3W_vs_E10	ordinary_muscle_development	ENSGALG00010025343	NAB2	0.931506595	0.292152534	3.188425525	0.001430499	0.002113452	positive
PRJNA729271	stage_3W_vs_E10	ordinary_muscle_development	ENSGALG00010025344	ENSGALG00010025344	1.141615492	0.532461576	2.144033566	0.032030197	0.041159372	positive
PRJNA729271	stage_3W_vs_E10	ordinary_muscle_development	ENSGALG00010025345	CCM2	-0.342437235	0.090101869	-3.800556401	0.000144372	0.000232855	negative
PRJNA729271	stage_3W_vs_E10	ordinary_muscle_development	ENSGALG00010025346	PXYLP1	-2.257121964	0.127994234	-17.63455974	1.33734E-69	2.55735E-68	negative
PRJNA729271	stage_3W_vs_E10	ordinary_muscle_development	ENSGALG00010025348	HIPK1	0.231969226	0.093122581	2.491009417	0.012738074	0.017119925	positive
PRJNA729271	stage_3W_vs_E10	ordinary_muscle_development	ENSGALG00010025349	SETD1A	-0.158738239	0.127274382	-1.247212808	0.212319478	0.244873554	negative
PRJNA729271	stage_3W_vs_E10	ordinary_muscle_development	ENSGALG00010025351	CREB1	-0.663929027	0.067346445	-9.858412397	6.30382E-23	2.71704E-22	negative
PRJNA729271	stage_3W_vs_E10	ordinary_muscle_development	ENSGALG00010025353	SLC12A4	-0.358631882	0.07775591	-4.612278123	3.9828E-06	7.25259E-06	negative
PRJNA729271	stage_3W_vs_E10	ordinary_muscle_development	ENSGALG00010025354	ENSGALG00010025354	-3.898248649	1.705941377	-2.285101177	0.022306896	0.029208533	negative
PRJNA729271	stage_3W_vs_E10	ordinary_muscle_development	ENSGALG00010025355	STAT6	1.451808568	0.13983186	10.38253064	2.97775E-25	1.40778E-24	positive
PRJNA729271	stage_3W_vs_E10	ordinary_muscle_development	ENSGALG00010025357	OLFML3	-1.604411158	0.121637538	-13.19009892	1.00054E-39	8.03035E-39	negative
PRJNA729271	stage_3W_vs_E10	ordinary_muscle_development	ENSGALG00010025358	INCENP	-3.155765215	0.207608344	-15.20057023	3.50556E-52	4.26642E-51	negative
PRJNA729271	stage_3W_vs_E10	ordinary_muscle_development	ENSGALG00010025359	CDCP2	1.112311502	0.384297563	2.894401653	0.003798819	0.005392604	positive
PRJNA729271	stage_3W_vs_E10	ordinary_muscle_development	ENSGALG00010025360	THYN1	0.731906956	0.110936733	6.597516795	4.18101E-11	1.03278E-10	positive
PRJNA729271	stage_3W_vs_E10	ordinary_muscle_development	ENSGALG00010025362	G6PC2	-0.889308396	0.247122471	-3.598654514	0.000319868	0.000501187	negative
PRJNA729271	stage_3W_vs_E10	ordinary_muscle_development	ENSGALG00010025364	EPHA4	-4.055532867	0.191240147	-21.20649319	8.31884E-100	3.44519E-98	negative
PRJNA729271	stage_3W_vs_E10	ordinary_muscle_development	ENSGALG00010025365	SIKE1	0.637888353	0.09101659	7.008484441	2.40913E-12	6.36073E-12	positive
PRJNA729271	stage_3W_vs_E10	ordinary_muscle_development	ENSGALG00010025368	LRP1	0.96133701	0.138743461	6.928881547	4.24181E-12	1.10228E-11	positive
PRJNA729271	stage_3W_vs_E10	ordinary_muscle_development	ENSGALG00010025370	HPD	1.013259612	0.496674931	2.040086078	0.041341753	0.052436884	positive
PRJNA729271	stage_3W_vs_E10	ordinary_muscle_development	ENSGALG00010025373	CYB5RL	-0.734385465	0.09065127	-8.101215386	5.44128E-16	1.73077E-15	negative
PRJNA729271	stage_3W_vs_E10	ordinary_muscle_development	ENSGALG00010025377	MYO1G	3.07848797	0.280645227	10.96932238	5.36731E-28	2.85816E-27	positive
PRJNA729271	stage_3W_vs_E10	ordinary_muscle_development	ENSGALG00010025378	PAX3	-2.918667689	0.28292987	-10.31586975	5.97379E-25	2.78784E-24	negative
PRJNA729271	stage_3W_vs_E10	ordinary_muscle_development	ENSGALG00010025379	ACAD8	1.112165754	0.185921601	5.981907142	2.2054E-09	4.91653E-09	positive
PRJNA729271	stage_3W_vs_E10	ordinary_muscle_development	ENSGALG00010025380	SPC25	-2.372892712	0.226364431	-10.48262179	1.03825E-25	5.00646E-25	negative
PRJNA729271	stage_3W_vs_E10	ordinary_muscle_development	ENSGALG00010025381	MRPL37	0.7089424	0.12215938	5.803421732	6.49751E-09	1.41626E-08	positive
PRJNA729271	stage_3W_vs_E10	ordinary_muscle_development	ENSGALG00010025382	MAB21L3	-0.682758166	0.31594475	-2.161004941	0.030694958	0.039531178	negative
PRJNA729271	stage_3W_vs_E10	ordinary_muscle_development	ENSGALG00010025386	TPCN2	-0.647367755	0.098693256	-6.559391988	5.40276E-11	1.32397E-10	negative
PRJNA729271	stage_3W_vs_E10	ordinary_muscle_development	ENSGALG00010025387	PSMD9	-0.013417655	0.068744913	-0.195180338	0.845251771	0.863258004	negative
PRJNA729271	stage_3W_vs_E10	ordinary_muscle_development	ENSGALG00010025389	SNED1	-2.49396387	0.155572078	-16.03092213	7.77208E-58	1.10231E-56	negative
PRJNA729271	stage_3W_vs_E10	ordinary_muscle_development	ENSGALG00010025390	BCL7A	-1.488460575	0.088992136	-16.72575409	8.50923E-63	1.37504E-61	negative
PRJNA729271	stage_3W_vs_E10	ordinary_muscle_development	ENSGALG00010025392	SLC6A2	-0.071051235	0.239863116	-0.296215759	0.767065315	0.792431882	negative
PRJNA729271	stage_3W_vs_E10	ordinary_muscle_development	ENSGALG00010025398	TSPAN2	0.373540349	0.143500028	2.603068125	0.009239359	0.012607033	positive
PRJNA729271	stage_3W_vs_E10	ordinary_muscle_development	ENSGALG00010025400	SLC22A15	-0.896216513	0.158827338	-5.642709393	1.67395E-08	3.56245E-08	negative
PRJNA729271	stage_3W_vs_E10	ordinary_muscle_development	ENSGALG00010025403	ENSGALG00010025403	0.689808074	0.128104187	5.384742613	7.25484E-08	1.48278E-07	positive
PRJNA729271	stage_3W_vs_E10	ordinary_muscle_development	ENSGALG00010025404	CERS6	-1.739619458	0.284182121	-6.121495081	9.27014E-10	2.11574E-09	negative
PRJNA729271	stage_3W_vs_E10	ordinary_muscle_development	ENSGALG00010025405	TAMALIN	2.378255251	0.162766164	14.61148432	2.37291E-48	2.56682E-47	positive
PRJNA729271	stage_3W_vs_E10	ordinary_muscle_development	ENSGALG00010025406	ENSGALG00010025406	-0.346686909	0.149355702	-2.321216424	0.020275166	0.026661393	negative
PRJNA729271	stage_3W_vs_E10	ordinary_muscle_development	ENSGALG00010025407	MLXIP	1.285936874	0.128093848	10.03902131	1.02688E-23	4.55187E-23	positive
PRJNA729271	stage_3W_vs_E10	ordinary_muscle_development	ENSGALG00010025413	NECAB3	0.763402751	0.12150988	6.282639317	3.32873E-10	7.80743E-10	positive
PRJNA729271	stage_3W_vs_E10	ordinary_muscle_development	ENSGALG00010025414	MMP2	0.010675537	0.221392404	0.04821998	0.961540927	0.966676081	positive
PRJNA729271	stage_3W_vs_E10	ordinary_muscle_development	ENSGALG00010025415	ENSGALG00010025415	4.285530865	0.275790725	15.53906815	1.88707E-54	2.451E-53	positive
PRJNA729271	stage_3W_vs_E10	ordinary_muscle_development	ENSGALG00010025416	ENSGALG00010025416	2.115788642	0.603621316	3.505158924	0.000456335	0.000705404	positive
PRJNA729271	stage_3W_vs_E10	ordinary_muscle_development	ENSGALG00010025417	B3GAT1	-3.40111477	0.235013286	-14.47201059	1.82071E-47	1.89184E-46	negative
PRJNA729271	stage_3W_vs_E10	ordinary_muscle_development	ENSGALG00010025418	CASQ2	4.253874668	0.2094145	20.31318113	9.83251E-92	3.33776E-90	positive
PRJNA729271	stage_3W_vs_E10	ordinary_muscle_development	ENSGALG00010025419	CCND1	-1.439316998	0.134763142	-10.68034606	1.25802E-26	6.325E-26	negative
PRJNA729271	stage_3W_vs_E10	ordinary_muscle_development	ENSGALG00010025420	STK39	-1.463091922	0.208583105	-7.014431591	2.30886E-12	6.10264E-12	negative
PRJNA729271	stage_3W_vs_E10	ordinary_muscle_development	ENSGALG00010025421	NR4A1	7.698181008	0.396902506	19.39564729	8.39889E-84	2.3457E-82	positive
PRJNA729271	stage_3W_vs_E10	ordinary_muscle_development	ENSGALG00010025422	SSBP3	-1.068171599	0.157826242	-6.76802276	1.30554E-11	3.31532E-11	negative
PRJNA729271	stage_3W_vs_E10	ordinary_muscle_development	ENSGALG00010025423	NGF	-0.27739786	0.339829413	-0.816285609	0.414336803	0.45320042	negative
PRJNA729271	stage_3W_vs_E10	ordinary_muscle_development	ENSGALG00010025424	IRX6	-2.881016611	0.393581931	-7.319992056	2.47986E-13	6.8914E-13	negative
PRJNA729271	stage_3W_vs_E10	ordinary_muscle_development	ENSGALG00010025425	ENSGALG00010025425	-0.0202217	0.140123942	-0.144312954	0.88525335	0.898644674	negative
PRJNA729271	stage_3W_vs_E10	ordinary_muscle_development	ENSGALG00010025426	ENSGALG00010025426	0.858565665	0.153135643	5.606569763	2.06376E-08	4.36767E-08	positive
PRJNA729271	stage_3W_vs_E10	ordinary_muscle_development	ENSGALG00010025427	SLC35G2	-0.906388639	0.133649593	-6.781828655	1.18664E-11	3.02238E-11	negative
PRJNA729271	stage_3W_vs_E10	ordinary_muscle_development	ENSGALG00010025428	VANGL1	-0.557675664	0.112383711	-4.962246376	6.96825E-07	1.3334E-06	negative
PRJNA729271	stage_3W_vs_E10	ordinary_muscle_development	ENSGALG00010025429	SCN3B	-3.852665331	0.258824372	-14.8852494	4.10951E-50	4.68665E-49	negative
PRJNA729271	stage_3W_vs_E10	ordinary_muscle_development	ENSGALG00010025430	IRX5	-4.971597933	0.926387524	-5.366650352	8.02123E-08	1.6355E-07	negative
PRJNA729271	stage_3W_vs_E10	ordinary_muscle_development	ENSGALG00010025432	IRX3	-4.131590266	0.706547112	-5.8475793	4.98778E-09	1.09327E-08	negative
PRJNA729271	stage_3W_vs_E10	ordinary_muscle_development	ENSGALG00010025433	CHPF2	0.605002566	0.121608322	4.975009562	6.52444E-07	1.25095E-06	positive
PRJNA729271	stage_3W_vs_E10	ordinary_muscle_development	ENSGALG00010025434	SYT6	-1.386953845	0.246837578	-5.618892621	1.92185E-08	4.07568E-08	negative
PRJNA729271	stage_3W_vs_E10	ordinary_muscle_development	ENSGALG00010025435	SCLY	-0.380576194	0.101311209	-3.756506277	0.000172302	0.00027603	negative
PRJNA729271	stage_3W_vs_E10	ordinary_muscle_development	ENSGALG00010025438	EXOSC6	0.000282192	0.121321002	0.002325994	0.998144127	0.998350754	positive
PRJNA729271	stage_3W_vs_E10	ordinary_muscle_development	ENSGALG00010025442	ABCF2	2.379633901	0.146859711	16.20344944	4.76761E-59	7.00877E-58	positive
PRJNA729271	stage_3W_vs_E10	ordinary_muscle_development	ENSGALG00010025445	B3GNT4	0.944160969	0.299473268	3.152738725	0.001617465	0.002380944	positive
PRJNA729271	stage_3W_vs_E10	ordinary_muscle_development	ENSGALG00010025448	CHD1L	-0.305099367	0.108156034	-2.820918578	0.004788636	0.006729159	negative
PRJNA729271	stage_3W_vs_E10	ordinary_muscle_development	ENSGALG00010025449	ENSGALG00010025449	-0.07507332	0.118723931	-0.632335194	0.527167871	0.565142984	negative
PRJNA729271	stage_3W_vs_E10	ordinary_muscle_development	ENSGALG00010025451	LRRC43	1.083029227	0.345163268	3.137730247	0.001702615	0.002498674	positive
PRJNA729271	stage_3W_vs_E10	ordinary_muscle_development	ENSGALG00010025452	TRIM33	0.100752618	0.126200781	0.798351781	0.424666377	0.463135891	positive
PRJNA729271	stage_3W_vs_E10	ordinary_muscle_development	ENSGALG00010025458	BCAS2	0.046254707	0.11589988	0.399091935	0.689825465	0.720442403	positive
PRJNA729271	stage_3W_vs_E10	ordinary_muscle_development	ENSGALG00010025459	DIABLO	-0.243623694	0.078475543	-3.104453738	0.001906307	0.002779033	negative
PRJNA729271	stage_3W_vs_E10	ordinary_muscle_development	ENSGALG00010025464	VPS33A	-0.036014356	0.089764867	-0.401207708	0.688267206	0.719070515	negative
PRJNA729271	stage_3W_vs_E10	ordinary_muscle_development	ENSGALG00010025465	ENSGALG00010025465	-0.267825546	0.083312807	-3.214698362	0.001305817	0.001936349	negative
PRJNA729271	stage_3W_vs_E10	ordinary_muscle_development	ENSGALG00010025470	ORAOV1	0.506650697	0.123391712	4.106035079	4.02508E-05	6.78572E-05	positive
PRJNA729271	stage_3W_vs_E10	ordinary_muscle_development	ENSGALG00010025472	ZCCHC8	0.175025484	0.097410156	1.796788867	0.072369144	0.089033335	positive
PRJNA729271	stage_3W_vs_E10	ordinary_muscle_development	ENSGALG00010025473	PARS2	0.534127899	0.07878761	6.779338728	1.20727E-11	3.07331E-11	positive
PRJNA729271	stage_3W_vs_E10	ordinary_muscle_development	ENSGALG00010025477	ENSGALG00010025477	0.748665145	0.070326683	10.64553478	1.82931E-26	9.15918E-26	positive
PRJNA729271	stage_3W_vs_E10	ordinary_muscle_development	ENSGALG00010025480	FGF19	-3.149981305	0.585138273	-5.383311005	7.3128E-08	1.49442E-07	negative
PRJNA729271	stage_3W_vs_E10	ordinary_muscle_development	ENSGALG00010025481	TMEM39A	-0.36156493	0.091148506	-3.966767474	7.2854E-05	0.000120399	negative
PRJNA729271	stage_3W_vs_E10	ordinary_muscle_development	ENSGALG00010025483	FGF4	-2.291270471	0.368171591	-6.223376622	4.86567E-10	1.13262E-09	negative
PRJNA729271	stage_3W_vs_E10	ordinary_muscle_development	ENSGALG00010025485	DENND2C	0.719549757	0.160277984	4.489386118	7.14287E-06	1.2746E-05	positive
PRJNA729271	stage_3W_vs_E10	ordinary_muscle_development	ENSGALG00010025486	B3GALT1	-2.924032835	0.216620417	-13.49841753	1.59771E-41	1.35116E-40	negative
PRJNA729271	stage_3W_vs_E10	ordinary_muscle_development	ENSGALG00010025487	FGF3	-6.517550338	0.932916964	-6.986206267	2.82419E-12	7.41874E-12	negative
PRJNA729271	stage_3W_vs_E10	ordinary_muscle_development	ENSGALG00010025488	SLC4A8	-3.060508178	0.212042979	-14.4334332	3.18808E-47	3.28205E-46	negative
PRJNA729271	stage_3W_vs_E10	ordinary_muscle_development	ENSGALG00010025491	ZMYND11	-0.35819182	0.064169191	-5.581990617	2.37781E-08	5.00674E-08	negative
PRJNA729271	stage_3W_vs_E10	ordinary_muscle_development	ENSGALG00010025496	ATG101	0.659438137	0.110909883	5.945711207	2.75259E-09	6.11195E-09	positive
PRJNA729271	stage_3W_vs_E10	ordinary_muscle_development	ENSGALG00010025499	STAC3	4.844436665	0.158518015	30.5607957	4.0643E-205	7.5529E-203	positive
PRJNA729271	stage_3W_vs_E10	ordinary_muscle_development	ENSGALG00010025504	AMPD1	5.48588182	0.197734179	27.74372067	2.0746E-169	2.5484E-167	positive

PRJNA729271	stage_3W_vs_E10	ordinary_muscle_development	ENSGALG00010025505	ENSGALG00010025505	-2.572754754	0.286638897	-8.975595354	2.81823E-19	1.04744E-18	negative
PRJNA729271	stage_3W_vs_E10	ordinary_muscle_development	ENSGALG00010025506	R3HDM2	0.549736534	0.177454788	3.097896308	0.001948996	0.002839552	positive
PRJNA729271	stage_3W_vs_E10	ordinary_muscle_development	ENSGALG00010025508	ENSGALG00010025508	-0.192601553	0.133491123	-1.442804202	0.149075635	0.175897788	negative
PRJNA729271	stage_3W_vs_E10	ordinary_muscle_development	ENSGALG00010025512	NRAS	-0.186535414	0.077669231	-2.40166422	0.016320682	0.021673686	negative
PRJNA729271	stage_3W_vs_E10	ordinary_muscle_development	ENSGALG00010025513	RSRC2	0.0144176	0.087520075	0.164734777	0.869152743	0.88409607	positive
PRJNA729271	stage_3W_vs_E10	ordinary_muscle_development	ENSGALG00010025520	ACOT11	1.204162571	0.295520289	4.074720473	4.60696E-05	7.73789E-05	positive
PRJNA729271	stage_3W_vs_E10	ordinary_muscle_development	ENSGALG00010025522	SUMO3	-0.293333224	0.10546723	-2.781273601	0.005414608	0.007564795	negative
PRJNA729271	stage_3W_vs_E10	ordinary_muscle_development	ENSGALG00010025523	B4GALT4	-1.044993439	0.132540374	-7.884340481	3.16201E-15	9.66744E-15	negative
PRJNA729271	stage_3W_vs_E10	ordinary_muscle_development	ENSGALG00010025527	KNTC1	-3.598251248	0.243179835	-14.79666786	1.53921E-49	1.70918E-48	negative
PRJNA729271	stage_3W_vs_E10	ordinary_muscle_development	ENSGALG00010025531	ENSGALG00010025531	0.079418695	0.095196459	0.83426102	0.404133913	0.443096846	positive
PRJNA729271	stage_3W_vs_E10	ordinary_muscle_development	ENSGALG00010025533	INHBC	-2.589747629	0.676514645	-3.828073272	0.00012915	0.000209142	negative
PRJNA729271	stage_3W_vs_E10	ordinary_muscle_development	ENSGALG00010025538	ANO1	3.593477537	0.208807714	17.20950564	2.25347E-66	3.98342E-65	positive
PRJNA729271	stage_3W_vs_E10	ordinary_muscle_development	ENSGALG00010025541	TTC21B	-0.376313671	0.128127198	-2.937031934	0.0033137	0.00473549	negative
PRJNA729271	stage_3W_vs_E10	ordinary_muscle_development	ENSGALG00010025544	CSDE1	0.730801025	0.08595504	8.502131207	1.86141E-17	6.38004E-17	positive
PRJNA729271	stage_3W_vs_E10	ordinary_muscle_development	ENSGALG00010025548	ENSGALG00010025548	-2.268342658	0.207883239	-10.9116188	1.01434E-27	5.35037E-27	negative
PRJNA729271	stage_3W_vs_E10	ordinary_muscle_development	ENSGALG00010025549	DHCR24	-2.073795506	0.248147294	-8.357115123	6.42706E-17	2.14747E-16	negative
PRJNA729271	stage_3W_vs_E10	ordinary_muscle_development	ENSGALG00010025553	UBE2G2	-0.058785232	0.09645591	-0.609451844	0.542224983	0.579758295	negative
PRJNA729271	stage_3W_vs_E10	ordinary_muscle_development	ENSGALG00010025554	DIP2C	-0.353080513	0.071646819	-4.928069667	8.3046E-07	1.58159E-06	negative
PRJNA729271	stage_3W_vs_E10	ordinary_muscle_development	ENSGALG00010025557	VPS29	-0.498993637	0.077753239	-6.417657261	1.38387E-10	3.31229E-10	negative
PRJNA729271	stage_3W_vs_E10	ordinary_muscle_development	ENSGALG00010025558	GALNT3	-2.82758271	0.234131632	-12.0768932	1.39907E-33	9.17208E-33	negative
PRJNA729271	stage_3W_vs_E10	ordinary_muscle_development	ENSGALG00010025559	SLC6A17	-5.261976907	1.199739244	-4.385933804	1.15489E-05	2.03083E-05	negative
PRJNA729271	stage_3W_vs_E10	ordinary_muscle_development	ENSGALG00010025562	GRAMD1B	0.528956632	0.183099397	2.888904284	0.003865867	0.005483486	positive
PRJNA729271	stage_3W_vs_E10	ordinary_muscle_development	ENSGALG00010025563	TSPEAR	-0.101534738	0.237597999	-0.427338352	0.669132902	0.70085132	negative
PRJNA729271	stage_3W_vs_E10	ordinary_muscle_development	ENSGALG00010025565	ENSGALG00010025565	1.460875701	0.244023596	5.986616553	2.14251E-09	4.78221E-09	positive
PRJNA729271	stage_3W_vs_E10	ordinary_muscle_development	ENSGALG00010025568	MYL3	-4.480892127	0.965546824	-4.6407818	3.47093E-06	6.35082E-06	negative
PRJNA729271	stage_3W_vs_E10	ordinary_muscle_development	ENSGALG00010025569	PPTC7	1.165163674	0.192925141	6.039459999	1.54631E-09	3.4831E-09	positive
PRJNA729271	stage_3W_vs_E10	ordinary_muscle_development	ENSGALG00010025571	SRSF3	-1.522952481	0.229618364	-6.63253781	3.29964E-11	8.20804E-11	negative
PRJNA729271	stage_3W_vs_E10	ordinary_muscle_development	ENSGALG00010025572	ENSGALG00010025572	-0.41569737	0.112504434	-3.694942093	0.000219937	0.000348948	negative
PRJNA729271	stage_3W_vs_E10	ordinary_muscle_development	ENSGALG00010025573	CLMP	-2.118298653	0.169114275	-12.52584178	5.39162E-36	3.81973E-35	negative
PRJNA729271	stage_3W_vs_E10	ordinary_muscle_development	ENSGALG00010025574	ARHGAP31	-1.137947835	0.100301591	-11.34526201	7.82914E-30	4.47666E-29	negative
PRJNA729271	stage_3W_vs_E10	ordinary_muscle_development	ENSGALG00010025575	TRPM2	-0.817388907	0.464346514	-1.760299437	0.07835705	0.096000797	negative
PRJNA729271	stage_3W_vs_E10	ordinary_muscle_development	ENSGALG00010025577	PTH2R	-3.058424526	0.19842186	-15.41374788	1.3231E-53	1.68676E-52	negative
PRJNA729271	stage_3W_vs_E10	ordinary_muscle_development	ENSGALG00010025578	ORMDL2	0.537824151	0.144891401	3.711912132	0.000205699	0.000327398	positive
PRJNA729271	stage_3W_vs_E10	ordinary_muscle_development	ENSGALG00010025579	CSRNP3	-3.019354167	0.282433608	-10.6904918	1.12771E-26	5.68366E-26	negative
PRJNA729271	stage_3W_vs_E10	ordinary_muscle_development	ENSGALG00010025580	RASSF7	-1.301851725	0.171118223	-7.607908171	2.78568E-14	8.12933E-14	negative
PRJNA729271	stage_3W_vs_E10	ordinary_muscle_development	ENSGALG00010025582	DMAP1	-0.424723914	0.114745892	-3.701430246	0.000214388	0.000340367	negative
PRJNA729271	stage_3W_vs_E10	ordinary_muscle_development	ENSGALG00010025585	SARNP	-1.167183034	0.097137874	-12.01573581	2.93743E-33	1.90677E-32	negative
PRJNA729271	stage_3W_vs_E10	ordinary_muscle_development	ENSGALG00010025586	TCTN1	0.658715305	0.124488007	5.291395706	1.21386E-07	2.44442E-07	positive
PRJNA729271	stage_3W_vs_E10	ordinary_muscle_development	ENSGALG00010025587	LRRC56	-3.316854536	0.306268741	-10.82988269	2.48473E-27	1.28538E-26	negative
PRJNA729271	stage_3W_vs_E10	ordinary_muscle_development	ENSGALG00010025588	ENSGALG00010025588	-0.471694508	0.315083458	-1.497046246	0.134381225	0.159712681	negative
PRJNA729271	stage_3W_vs_E10	ordinary_muscle_development	ENSGALG00010025590	HRAS	0.0723503	0.114012618	0.634581518	0.525701385	0.563654233	positive
PRJNA729271	stage_3W_vs_E10	ordinary_muscle_development	ENSGALG00010025591	OTOS	1.357460622	0.343322855	3.953889474	7.68909E-05	0.000126738	positive
PRJNA729271	stage_3W_vs_E10	ordinary_muscle_development	ENSGALG00010025592	HSPA8	-0.366079274	0.208196537	-1.758335073	0.078690513	0.096393053	negative
PRJNA729271	stage_3W_vs_E10	ordinary_muscle_development	ENSGALG00010025594	UPK1B	-5.893634154	0.648363573	-9.090014312	9.9026E-20	3.74382E-19	negative
PRJNA729271	stage_3W_vs_E10	ordinary_muscle_development	ENSGALG00010025595	ERI3	-0.50581249	0.094747572	-5.338527217	9.37046E-08	1.90151E-07	negative
PRJNA729271	stage_3W_vs_E10	ordinary_muscle_development	ENSGALG00010025597	SCN2A	-4.511155277	0.669825321	-6.734823446	1.64129E-11	4.14903E-11	negative
PRJNA729271	stage_3W_vs_E10	ordinary_muscle_development	ENSGALG00010025598	ENSGALG00010025598	2.280644059	0.399812904	5.704278274	1.16837E-08	2.5112E-08	positive
PRJNA729271	stage_3W_vs_E10	ordinary_muscle_development	ENSGALG00010025599	COPS9	0.677388787	0.142778574	4.744330796	2.09197E-06	3.8797E-06	positive
PRJNA729271	stage_3W_vs_E10	ordinary_muscle_development	ENSGALG00010025600	ENSGALG00010025600	-1.617579135	0.113745619	-14.22102362	6.78531E-46	6.70437E-45	negative
PRJNA729271	stage_3W_vs_E10	ordinary_muscle_development	ENSGALG00010025604	HVCN1	-0.287661731	0.135733906	-2.119306368	0.034064584	0.043642049	negative
PRJNA729271	stage_3W_vs_E10	ordinary_muscle_development	ENSGALG00010025606	CCDC12	-0.99017095	0.132616669	-7.466413966	8.24098E-14	2.34636E-13	negative
PRJNA729271	stage_3W_vs_E10	ordinary_muscle_development	ENSGALG00010025608	ENSGALG00010025608	-0.715311796	0.478285569	-1.495574699	0.13476452	0.160115715	negative
PRJNA729271	stage_3W_vs_E10	ordinary_muscle_development	ENSGALG00010025610	RNH1	-0.946594099	0.133998415	-7.064218624	1.61522E-12	4.30934E-12	negative
PRJNA729271	stage_3W_vs_E10	ordinary_muscle_development	ENSGALG00010025612	TMEM53	0.195540544	0.149555386	1.307479111	0.191050045	0.221719007	positive
PRJNA729271	stage_3W_vs_E10	ordinary_muscle_development	ENSGALG00010025613	CDKN1A	3.718978484	0.272826036	13.63131808	2.60826E-42	2.26932E-41	positive
PRJNA729271	stage_3W_vs_E10	ordinary_muscle_development	ENSGALG00010025618	ADIPOQ	7.17581945	0.221767281	32.35743074	1.0906E-229	2.5093E-227	positive
PRJNA729271	stage_3W_vs_E10	ordinary_muscle_development	ENSGALG00010025621	PPP1CC	-0.553039702	0.117610583	-4.702295391	2.57253E-06	4.74594E-06	negative
PRJNA729271	stage_3W_vs_E10	ordinary_muscle_development	ENSGALG00010025623	ENSGALG00010025623	2.400032654	0.388761838	6.173529443	6.67821E-10	1.53994E-09	positive
PRJNA729271	stage_3W_vs_E10	ordinary_muscle_development	ENSGALG00010025624	ARMH1	0.731779542	0.356690967	2.051578568	0.040210637	0.051082663	positive
PRJNA729271	stage_3W_vs_E10	ordinary_muscle_development	ENSGALG00010025628	ENSGALG00010025628	-5.15047867	0.359983761	-14.30753058	1.96363E-46	1.97247E-45	negative
PRJNA729271	stage_3W_vs_E10	ordinary_muscle_development	ENSGALG00010025629	ST6GAL1	3.137284004	0.14333136	21.88832936	3.3559E-106	1.5742E-104	positive
PRJNA729271	stage_3W_vs_E10	ordinary_muscle_development	ENSGALG00010025635	CHKA	-1.018788228	0.163833509	-6.218436228	5.02134E-10	1.1666E-09	negative
PRJNA729271	stage_3W_vs_E10	ordinary_muscle_development	ENSGALG00010025637	C6orf89	0.353408698	0.127528643	2.771210365	0.005584833	0.007791353	positive
PRJNA729271	stage_3W_vs_E10	ordinary_muscle_development	ENSGALG00010025639	MYL2	-6.524391008	0.467996669	-13.94110564	3.56376E-44	3.30075E-43	negative
PRJNA729271	stage_3W_vs_E10	ordinary_muscle_development	ENSGALG00010025640	SLC38A11	-5.951511227	0.93248995	-6.382386454	1.74349E-10	4.15657E-10	negative
PRJNA729271	stage_3W_vs_E10	ordinary_muscle_development	ENSGALG00010025642	RPS8	0.150554034	0.117551211	1.280752726	0.200280534	0.23181876	positive
PRJNA729271	stage_3W_vs_E10	ordinary_muscle_development	ENSGALG00010025644	ENSGALG00010025644	3.832036935	0.282304602	13.57412136	5.70234E-42	4.90828E-41	positive
PRJNA729271	stage_3W_vs_E10	ordinary_muscle_development	ENSGALG00010025645	NBEAL2	1.582727052	0.185546963	8.530061739	1.4627E-17	5.04684E-17	positive
PRJNA729271	stage_3W_vs_E10	ordinary_muscle_development	ENSGALG00010025647	JHY	-2.471955325	0.360042059	-6.865740447	6.61472E-12	1.70272E-11	negative
PRJNA729271	stage_3W_vs_E10	ordinary_muscle_development	ENSGALG00010025648	ENSGALG00010025648	3.687546177	0.4852199	7.599742257	2.96721E-14	8.64691E-14	positive
PRJNA729271	stage_3W_vs_E10	ordinary_muscle_development	ENSGALG00010025650	PPP1R1A	6.061251826	0.455890382	13.295415	2.46104E-40	2.00973E-39	positive
PRJNA729271	stage_3W_vs_E10	ordinary_muscle_development	ENSGALG00010025652	KMT5B	-0.307494025	0.076941343	-3.99647332	6.42931E-05	0.000106775	negative
PRJNA729271	stage_3W_vs_E10	ordinary_muscle_development	ENSGALG00010025653	IGSF11	-2.320246832	0.213969034	-10.84384405	2.13315E-27	1.10944E-26	negative
PRJNA729271	stage_3W_vs_E10	ordinary_muscle_development	ENSGALG00010025654	CRTAM	4.820822346	0.684104542	7.046908842	1.82936E-12	4.86454E-12	positive
PRJNA729271	stage_3W_vs_E10	ordinary_muscle_development	ENSGALG00010025658	PDE1B	0.202795818	0.360959552	0.561824219	0.574235789	0.610454548	positive
PRJNA729271	stage_3W_vs_E10	ordinary_muscle_development	ENSGALG00010025659	COBLL1	1.397787816	0.130141193	10.7405487	6.5653E-27	3.34496E-26	positive
PRJNA729271	stage_3W_vs_E10	ordinary_muscle_development	ENSGALG00010025660	KIF2C	-3.629645413	0.209120021	-17.35675713	1.75352E-67	3.20117E-66	negative
PRJNA729271	stage_3W_vs_E10	ordinary_muscle_development	ENSGALG00010025661	SNRPE	-1.113399142	0.131133378	-8.490585353	2.05599E-17	7.02701E-17	negative
PRJNA729271	stage_3W_vs_E10	ordinary_muscle_development	ENSGALG00010025664	GPR55	2.485396159	0.622513511	3.992517617	6.53755E-05	0.000108535	positive
PRJNA729271	stage_3W_vs_E10	ordinary_muscle_development	ENSGALG00010025666	CUX2	-4.486719984	0.509450755	-8.806974849	1.28569E-18	4.64507E-18	negative
PRJNA729271	stage_3W_vs_E10	ordinary_muscle_development	ENSGALG00010025674	GRB14	2.179486879	0.435487785	5.004702672	5.59483E-07	1.07661E-06	positive
PRJNA729271	stage_3W_vs_E10	ordinary_muscle_development	ENSGALG00010025679	ENSGALG00010025679	0.653798129	1.276559549	0.512156389	0.608541562	0.643760761	positive
PRJNA729271	stage_3W_vs_E10	ordinary_muscle_development	ENSGALG00010025685	NCKAP1L	2.192603197	0.191277295	11.46295591	2.02484E-30	1.18252E-29	positive
PRJNA729271	stage_3W_vs_E10	ordinary_muscle_development	ENSGALG00010025688	BEST4	2.139350939	0.491126641	4.356006701	1.32457E-05	2.31883E-05	positive
PRJNA729271	stage_3W_vs_E10	ordinary_muscle_development	ENSGALG00010025691	ENSGALG00010025691	-3.858073839	0.181649276	-21.23913684	4.15454E-100	1.74551E-98	negative
PRJNA729271	stage_3W_vs_E10	ordinary_muscle_development	ENSGALG00010025692	ENSGALG00010025692	4.709961126	0.957685644	4.91806592	8.74035E-07	1.66262E-06	positive
PRJNA729271	stage_3W_vs_E10	ordinary_muscle_development	ENSGALG00010025696	MTCH1	0.300913793	0.103439179	2.909089152	0.003624835	0.005158254	positive
PRJNA729271	stage_3W_vs_E10	ordinary_muscle_development	ENSGALG00010025701	ENSGALG00010025701	1.009972569	3.255211182	0.310263302	0.756360736	0.782042148	positive
PRJNA729271	stage_3W_vs_E10	ordinary_muscle_development	ENSGALG00010025702	GTSF2	-0.29665413	0.184017714	-1.612095511	0.106941157	0.128800339	negative
PRJNA729271	stage_3W_vs_E10	ordinary_muscle_development	ENSGALG00010025703	UBASH3B	-1.653037539	0.143029085	-11.55735238	6.77661E-31	4.03065E-30	negative
PRJNA729271	stage_3W_vs_E10	ordinary_muscle_development	ENSGALG00010025705	ACSL3	-0.817905087	0.083563994	-9.787769166	1.27068E-22	5.39027E-22	negative
PRJNA729271	stage_3W_vs_E10	ordinary_muscle_development	ENSGALG00010025706	ENSGALG00010025706	1.503885699	3.056422803	0.49204112	0.622690275	0.657145652	positive
PRJNA729271	stage_3W_vs_E10	ordinary_muscle_development	ENSGALG00010025708	HOXC8	-2.817787712	0.250928321	-11.22945272	2.92283E-29	1.6364E-28	negative
PRJNA729271	stage_3W_vs_E10	ordinary_muscle_development	ENSGALG00010025710	ENSGALG00010025710	1.004523474	2.061446255	0.487290645	0.626052377	0.66030919	positive
PRJNA729271	stage_3W_vs_E10	ordinary_muscle_development	ENSGALG00010025719	HOXC11	-6.965944158	2.679982774	-2.599249601	0.009342781	0.012740955	negative
PRJNA729271	stage_3W_vs_E10	ordinary_muscle_development	ENSGALG00010025720	PHETA1	-2.798061317	0.273405928	-10.23409162	1.39498E-24	6.42117E-24	negative
PRJNA729271	stage_3W_vs_E10	ordinary_muscle_development	ENSGALG00010025723	SETD2	0.000424208	0.122213247	0.003471046	0.997230511	0.99750578	positive
PRJNA729271	stage_3W_vs_E10	ordinary_muscle_development	ENSGALG00010025727	GLI1	-3.359562254	0.252011605	-13.33098231	1.52871E-40	1.25403E-39	negative
PRJNA729271	stage_3W_vs_E10	ordinary_muscle_development	ENSGALG00010025734	PLK3	0.207061346	0.181302425	1.142077089	0.253421978	0.288717407	positive
PRJNA729271	stage_3W_vs_E10	ordinary_muscle_development	ENSGALG00010025736	KIF9	1.326006908	0.322871708	4.106915764	4.00977E-05	6.76227E-05	positive
PRJNA729271	stage_3W_vs_E10	ordinary_muscle_development	ENSGALG00010025737	ENSGALG00010025737	-0.737341002	0.152208858	-4.844271294	1.27077E-06	2.3897E-06	negative
PRJNA729271	stage_3W_vs_E10	ordinary_muscle_development	ENSGALG00010025738	SOX13	-0.275147608	0.157078087	-1.751661316	0.079832062	0.09772534	negative
PRJNA729271	stage_3W_vs_E10	ordinary_muscle_development	ENSGALG00010025743	SH2B3	0.38563907	0.10883526	3.543328419	0.00039511	0.000613577	positive
PRJNA729271	stage_3W_vs_E10	ordinary_muscle_development	ENSGALG00010025745	COX14	1.307419777	0.226198305	5.779971589	7.47132E-09	1.62462E-08	positive
PRJNA729271	stage_3W_vs_E10	ordinary_muscle_development	ENSGALG00010025748	ETNK2	-1.651363289	0.231335468	-7.138392149	9.44289E-13	2.5522E-12	negative
PRJNA729271	stage_3W_vs_E10	ordinary_muscle_development	ENSGALG00010025753	SORL1	-1.206144417	0.174283249	-6.920598635	4.49739E-12	1.16639E-11	negative
PRJNA729271	stage_3W_vs_E10	ordinary_muscle_development	ENSGALG00010025755	ENSGALG00010025755	-0.715737736	0.283385154	-2.525671247	0.011547749	0.015605502	negative
PRJNA729271	stage_3W_vs_E10	ordinary_muscle_development	ENSGALG00010025782	ENSGALG00010025782	1.009897773	4.564438882	0.221253433	0.824895106	0.845706813	positive
PRJNA729271	stage_3W_vs_E10	ordinary_muscle_development	ENSGALG00010025784	ENSGALG00010025784	0.526896345	2.459378008	0.214239675	0.830360158	0.85017674	positive
PRJNA729271	stage_3W_vs_E10	ordinary_muscle_development	ENSGALG00010025787	KCNE4	2.959410354	0.289703799	10.21529703	1.6936E-24	7.77103E-24	positive
PRJNA729271	stage_3W_vs_E10	ordinary_muscle_development	ENSGALG00010025790	LRP5	0.137167718	0.077943773	1.759829081	0.078436791	0.096090372	positive
PRJNA729271	stage_3W_vs_E10	ordinary_muscle_development	ENSGALG00010025791	FIGN	0.165124619	0.237278027	0.695911967	0.486483962	0.52474998	positive
PRJNA729271	stage_3W_vs_E10	ordinary_muscle_development	ENSGALG00010025794	ENSGALG00010025794	1.009917323	4.091796531	0.246815137	0.805051295	0.827369436	positive
PRJNA729271	stage_3W_vs_E10	ordinary_muscle_development	ENSGALG00010025799	SC5D	0.372227261	0.271463412	1.371187587	0.170316487	0.199268502	positive
PRJNA729271	stage_3W_vs_E10	ordinary_muscle_development	ENSGALG00010025800	ARL2BP	-1.146016155	0.140045957	-8.183143435	2.76534E-16	8.94123E-16	negative
PRJNA729271	stage_3W_vs_E10	ordinary_muscle_development	ENSGALG00010025801	PTPN7	2.757374136	0.360089171	7.657475863	1.89623E-14	5.58315E-14	positive
PRJNA729271	stage_3W_vs_E10	ordinary_muscle_development	ENSGALG00010025804	ENSGALG00010025804	2.097898315	2.237222647	0.937724423	0.34838605	0.386917268	positive
PRJNA729271	stage_3W_vs_E10	ordinary_muscle_development	ENSGALG00010025805	ATXN2	-0.146466639	0.094153635	-1.555613215	0.119800094	0.143263953	negative
PRJNA729271	stage_3W_vs_E10	ordinary_muscle_development	ENSGALG00010025809	ENSGALG00010025809	1.431615786	0.191724726	7.467037853	8.20202E-14	2.33664E-13	positive
PRJNA729271	stage_3W_vs_E10	ordinary_muscle_development	ENSGALG00010025822	KLF7	-0.20745694	0.098730809	-2.101238136	0.035620067	0.045538267	negative
PRJNA729271	stage_3W_vs_E10	ordinary_muscle_development	ENSGALG00010025829	ENSGALG00010025829	-1.112474696	0.259602441	-4.285301377	1.82492E-05	3.16413E-05	negative
PRJNA729271	stage_3W_vs_E10	ordinary_muscle_development	ENSGALG00010025830	TBCEL	0.280287264	0.191990757	1.459899781	0.144317619	0.170793917	positive
PRJNA729271	stage_3W_vs_E10	ordinary_muscle_development	ENSGALG00010025831	BRAP	0.098670408	0.069735076	1.414932246	0.157088363	0.184821089	positive
PRJNA729271	stage_3W_vs_E10	ordinary_muscle_development	ENSGALG00010025832	ENKD1	0.965073007	0.146318806	6.595686738	4.23292E-11	1.04489E-10	positive
PRJNA729271	stage_3W_vs_E10	ordinary_muscle_development	ENSGALG00010025835	PPP6R3	0.130064096	0.119230516	1.090862476	0.275333401	0.312159378	positive
PRJNA729271	stage_3W_vs_E10	ordinary_muscle_development	ENSGALG00010025836	ENSGALG00010025836	0.521857417	0.113672868	4.590870494	4.41401E-06	8.00765E-06	positive
PRJNA729271	stage_3W_vs_E10	ordinary_muscle_development	ENSGALG00010025837	ENSGALG00010025837	2.484223335	0.176649763	14.06298705	6.41215E-45	6.11877E-44	positive
PRJNA729271	stage_3W_vs_E10	ordinary_muscle_development	ENSGALG00010025846	GRIK4	0.133591604	0.216820153	0.616140161	0.537802018	0.575521613	positive
PRJNA729271	stage_3W_vs_E10	ordinary_muscle_development	ENSGALG00010025847	DEF6	0.15963935	0.125572253	1.271294783	0.203623798	0.235500435	positive
PRJNA729271	stage_3W_vs_E10	ordinary_muscle_development	ENSGALG00010025849	OBSCN	4.270514387	0.229075874	18.64235771	1.45676E-77	3.41679E-76	positive
PRJNA729271	stage_3W_vs_E10	ordinary_muscle_development	ENSGALG00010025852	BTBD19	-0.332146015	0.246653789	-1.3466082	0.178106452	0.207661923	negative

PRJNA729271	stage_3W_vs_E10	ordinary_muscle_development	ENSGALG00010025858	ENSGALG00010025858	1.009992773	2.976788379	0.33928941	0.73439172	0.762060113	positive
PRJNA729271	stage_3W_vs_E10	ordinary_muscle_development	ENSGALG00010025862	PTCH2	-4.175478199	0.335325672	-12.45200873	1.36369E-35	9.55837E-35	negative
PRJNA729271	stage_3W_vs_E10	ordinary_muscle_development	ENSGALG00010025865	LGR6	-3.794062326	0.678952844	-5.588108748	2.29556E-08	4.84199E-08	negative
PRJNA729271	stage_3W_vs_E10	ordinary_muscle_development	ENSGALG00010025869	FASTKD2	0.081676415	0.092454967	0.883418356	0.377010277	0.415824377	positive
PRJNA729271	stage_3W_vs_E10	ordinary_muscle_development	ENSGALG00010025870	ENSGALG00010025870	2.187152417	0.162504184	13.45905296	2.72385E-41	2.28617E-40	positive
PRJNA729271	stage_3W_vs_E10	ordinary_muscle_development	ENSGALG00010025871	ENSGALG00010025871	0.677221693	1.551722732	0.436432153	0.662523215	0.694681279	positive
PRJNA729271	stage_3W_vs_E10	ordinary_muscle_development	ENSGALG00010025874	MDH1B	-2.445033219	0.32970531	-7.415813885	1.2088E-13	3.41618E-13	negative
PRJNA729271	stage_3W_vs_E10	ordinary_muscle_development	ENSGALG00010025876	ENSGALG00010025876	1.010054169	1.784677425	0.565958954	0.571421684	0.607730377	positive
PRJNA729271	stage_3W_vs_E10	ordinary_muscle_development	ENSGALG00010025877	ENSGALG00010025877	1.00998298	3.503381349	0.288288051	0.773126253	0.79761967	positive
PRJNA729271	stage_3W_vs_E10	ordinary_muscle_development	ENSGALG00010025881	GAL	1.301894034	0.596857152	2.181248948	0.029165008	0.037644416	positive
PRJNA729271	stage_3W_vs_E10	ordinary_muscle_development	ENSGALG00010025882	LDHD	2.607946395	0.172730466	15.09835787	1.66017E-51	1.97896E-50	positive
PRJNA729271	stage_3W_vs_E10	ordinary_muscle_development	ENSGALG00010025886	ALDH2	1.136044436	0.109665466	10.3591812	3.80203E-25	1.79221E-24	positive
PRJNA729271	stage_3W_vs_E10	ordinary_muscle_development	ENSGALG00010025887	ZNF76	-0.503255979	0.116438094	-4.322090499	1.54558E-05	2.69172E-05	negative
PRJNA729271	stage_3W_vs_E10	ordinary_muscle_development	ENSGALG00010025889	TESMIN	1.478098879	0.570079166	2.592795822	0.009519927	0.012971549	positive
PRJNA729271	stage_3W_vs_E10	ordinary_muscle_development	ENSGALG00010025890	SCG2	0.647687414	0.511178805	1.267046692	0.205138595	0.237101023	positive
PRJNA729271	stage_3W_vs_E10	ordinary_muscle_development	ENSGALG00010025891	NUP93	-2.065606931	0.110987509	-18.61116576	2.60879E-77	6.07949E-76	negative
PRJNA729271	stage_3W_vs_E10	ordinary_muscle_development	ENSGALG00010025892	ZC3H11A	0.299189186	0.086046021	3.477083341	0.0005069	0.000780489	positive
PRJNA729271	stage_3W_vs_E10	ordinary_muscle_development	ENSGALG00010025894	DYTN	-1.414531457	0.557445669	-2.537523448	0.011163989	0.015103791	negative
PRJNA729271	stage_3W_vs_E10	ordinary_muscle_development	ENSGALG00010025897	MAPKAPK5	0.781874638	0.120150391	6.507466458	7.64288E-11	1.85847E-10	positive
PRJNA729271	stage_3W_vs_E10	ordinary_muscle_development	ENSGALG00010025898	ENSGALG00010025898	1.010050552	2.00257413	0.504376111	0.613997102	0.648821753	positive
PRJNA729271	stage_3W_vs_E10	ordinary_muscle_development	ENSGALG00010025899	ENSGALG00010025899	1.043001987	0.163900133	6.363643325	1.97023E-10	4.6848E-10	positive
PRJNA729271	stage_3W_vs_E10	ordinary_muscle_development	ENSGALG00010025900	FAM237A	1.81547214	0.559905195	3.242463466	0.001185011	0.001764613	positive
PRJNA729271	stage_3W_vs_E10	ordinary_muscle_development	ENSGALG00010025902	ENSGALG00010025902	1.488271267	1.873306446	0.794462257	0.426926373	0.465390523	positive
PRJNA729271	stage_3W_vs_E10	ordinary_muscle_development	ENSGALG00010025904	RPL10A	0.25579527	0.138824872	1.842575229	0.065391053	0.080922336	positive
PRJNA729271	stage_3W_vs_E10	ordinary_muscle_development	ENSGALG00010025905	CPT1A	0.643642839	0.174927438	3.679484735	0.000233706	0.000369902	positive
PRJNA729271	stage_3W_vs_E10	ordinary_muscle_development	ENSGALG00010025907	ARHGEF12	0.113661497	0.071602779	1.58738946	0.112424465	0.134967088	positive
PRJNA729271	stage_3W_vs_E10	ordinary_muscle_development	ENSGALG00010025910	HEATR3	-0.073173127	0.112869201	-0.648300209	0.516790799	0.554715835	negative
PRJNA729271	stage_3W_vs_E10	ordinary_muscle_development	ENSGALG00010025913	EIF2B3	-0.015775864	0.077200523	-0.204349184	0.838080637	0.856698085	negative
PRJNA729271	stage_3W_vs_E10	ordinary_muscle_development	ENSGALG00010025914	ENSGALG00010025914	-5.788040555	1.762371208	-3.284234632	0.001022597	0.001531097	negative
PRJNA729271	stage_3W_vs_E10	ordinary_muscle_development	ENSGALG00010025917	SCUBE3	-4.971498493	0.410491776	-12.11107939	9.22718E-34	6.07669E-33	negative
PRJNA729271	stage_3W_vs_E10	ordinary_muscle_development	ENSGALG00010025919	RGS9BP	-0.980183548	0.325099133	-3.015029718	0.002569541	0.003706019	negative
PRJNA729271	stage_3W_vs_E10	ordinary_muscle_development	ENSGALG00010025920	GTSF1	0.884184489	0.259925667	3.401682097	0.000669725	0.001019391	positive
PRJNA729271	stage_3W_vs_E10	ordinary_muscle_development	ENSGALG00010025924	TMEM116	1.750035275	0.171713119	10.19162241	2.16127E-24	9.87006E-24	positive
PRJNA729271	stage_3W_vs_E10	ordinary_muscle_development	ENSGALG00010025925	KLHL18	0.529473756	0.091180193	5.806894439	6.36422E-09	1.38816E-08	positive
PRJNA729271	stage_3W_vs_E10	ordinary_muscle_development	ENSGALG00010025926	ENSGALG00010025926	0.10069975	0.431663618	0.233282922	0.815541719	0.837082369	positive
PRJNA729271	stage_3W_vs_E10	ordinary_muscle_development	ENSGALG00010025927	ENSGALG00010025927	-3.152586824	1.185881673	-2.658432875	0.007850499	0.010798347	negative
PRJNA729271	stage_3W_vs_E10	ordinary_muscle_development	ENSGALG00010025929	NELFE	0.172609268	0.090154471	1.914594656	0.055544217	0.069340576	positive
PRJNA729271	stage_3W_vs_E10	ordinary_muscle_development	ENSGALG00010025931	TMEM136	-2.477909953	0.217233222	-11.40668045	3.87226E-30	2.23352E-29	negative
PRJNA729271	stage_3W_vs_E10	ordinary_muscle_development	ENSGALG00010025932	AP1S3	-0.273508146	0.312062511	-0.876453071	0.380783778	0.41963513	negative
PRJNA729271	stage_3W_vs_E10	ordinary_muscle_development	ENSGALG00010025933	SKIC2	0.310682678	0.102125902	3.042153567	0.00234892	0.003397964	positive
PRJNA729271	stage_3W_vs_E10	ordinary_muscle_development	ENSGALG00010025935	CATH3	-0.842993006	0.380363049	-2.216285226	0.026671974	0.034571248	negative
PRJNA729271	stage_3W_vs_E10	ordinary_muscle_development	ENSGALG00010025936	GOSR2	-0.594268752	0.068476047	-8.678490865	4.01055E-18	1.42552E-17	negative
PRJNA729271	stage_3W_vs_E10	ordinary_muscle_development	ENSGALG00010025938	ERP29	-1.132980091	0.090319054	-12.54419799	4.27723E-36	3.04212E-35	negative
PRJNA729271	stage_3W_vs_E10	ordinary_muscle_development	ENSGALG00010025939	TMEM136-1	-2.096955696	0.626538332	-3.346891306	0.000817232	0.00123355	negative
PRJNA729271	stage_3W_vs_E10	ordinary_muscle_development	ENSGALG00010025940	HECTD3	0.995524304	0.087316596	11.40131831	4.11835E-30	2.37169E-29	positive
PRJNA729271	stage_3W_vs_E10	ordinary_muscle_development	ENSGALG00010025941	ENSGALG00010025941	-3.040655375	0.718121345	-4.234180473	2.29387E-05	3.94373E-05	negative
PRJNA729271	stage_3W_vs_E10	ordinary_muscle_development	ENSGALG00010025942	ENSGALG00010025942	-1.409566959	1.318310545	-1.069222244	0.284969538	0.322101797	negative
PRJNA729271	stage_3W_vs_E10	ordinary_muscle_development	ENSGALG00010025943	CATH1	0.577032114	0.739780066	0.780004951	0.435387961	0.474008449	positive
PRJNA729271	stage_3W_vs_E10	ordinary_muscle_development	ENSGALG00010025946	KRT5	-9.042955991	1.266480146	-7.140227205	9.31768E-13	2.5193E-12	negative
PRJNA729271	stage_3W_vs_E10	ordinary_muscle_development	ENSGALG00010025948	WNT9B	-7.88790585	0.923232858	-8.543788044	1.29892E-17	4.49245E-17	negative
PRJNA729271	stage_3W_vs_E10	ordinary_muscle_development	ENSGALG00010025949	GPATCH1	0.177346114	0.122045957	1.453109288	0.146193413	0.172731783	positive
PRJNA729271	stage_3W_vs_E10	ordinary_muscle_development	ENSGALG00010025951	TBRG4	-0.120542373	0.1145521	-1.052293007	0.292665139	0.329951092	negative
PRJNA729271	stage_3W_vs_E10	ordinary_muscle_development	ENSGALG00010025953	POU2F3	-3.401780508	1.082342719	-3.142979066	0.001672378	0.002456538	negative
PRJNA729271	stage_3W_vs_E10	ordinary_muscle_development	ENSGALG00010025954	TUBA1A	-2.918852522	0.158952165	-18.36308759	2.59429E-75	5.74987E-74	negative
PRJNA729271	stage_3W_vs_E10	ordinary_muscle_development	ENSGALG00010025956	ENSGALG00010025956	1.081631748	0.508610609	2.126640162	0.033449993	0.042884985	positive
PRJNA729271	stage_3W_vs_E10	ordinary_muscle_development	ENSGALG00010025957	ENSGALG00010025957	-0.467678177	0.115487186	-4.049610975	5.13028E-05	8.58502E-05	negative
PRJNA729271	stage_3W_vs_E10	ordinary_muscle_development	ENSGALG00010025958	HOXC9	-4.524113015	0.44768945	-10.10547159	5.22436E-24	2.34159E-23	negative
PRJNA729271	stage_3W_vs_E10	ordinary_muscle_development	ENSGALG00010025959	UROD	-0.565786159	0.105323062	-5.371911399	7.79064E-08	1.5896E-07	negative
PRJNA729271	stage_3W_vs_E10	ordinary_muscle_development	ENSGALG00010025960	CMKLR2	2.400773902	0.157946489	15.19991944	3.54056E-52	4.30541E-51	positive
PRJNA729271	stage_3W_vs_E10	ordinary_muscle_development	ENSGALG00010025962	ENSGALG00010025962	1.043648392	0.183853364	5.67652596	1.37458E-08	2.94045E-08	positive
PRJNA729271	stage_3W_vs_E10	ordinary_muscle_development	ENSGALG00010025963	OAF	-0.932406536	0.246531443	-3.782099858	0.000155511	0.000250042	negative
PRJNA729271	stage_3W_vs_E10	ordinary_muscle_development	ENSGALG00010025964	HOXC10	-7.987340727	1.041376486	-7.669983753	1.72018E-14	5.07305E-14	negative
PRJNA729271	stage_3W_vs_E10	ordinary_muscle_development	ENSGALG00010025968	FADS2	-0.081086232	0.156452657	-0.518279676	0.604263151	0.639654887	negative
PRJNA729271	stage_3W_vs_E10	ordinary_muscle_development	ENSGALG00010025970	KLHL12	-0.33239925	0.111368625	-2.984675903	0.002838789	0.004079335	negative
PRJNA729271	stage_3W_vs_E10	ordinary_muscle_development	ENSGALG00010025971	TRAFD1	0.335734545	0.198453796	1.691751689	0.090693328	0.110331498	positive
PRJNA729271	stage_3W_vs_E10	ordinary_muscle_development	ENSGALG00010025972	WNT3	-7.142000188	1.318245216	-5.417808538	6.0334E-08	1.23697E-07	negative
PRJNA729271	stage_3W_vs_E10	ordinary_muscle_development	ENSGALG00010025974	EEF1B2	0.999132851	0.095511699	10.46084258	1.30688E-25	6.27261E-25	positive
PRJNA729271	stage_3W_vs_E10	ordinary_muscle_development	ENSGALG00010025976	NAA25	-0.356004395	0.098997388	-3.596098861	0.000323025	0.000505916	negative
PRJNA729271	stage_3W_vs_E10	ordinary_muscle_development	ENSGALG00010025978	TRIM29	-7.48152521	0.839102657	-8.916102393	4.82987E-19	1.78004E-18	negative
PRJNA729271	stage_3W_vs_E10	ordinary_muscle_development	ENSGALG00010025980	HECTD4	-0.490871015	0.06468427	-7.588723098	3.23073E-14	9.39785E-14	negative
PRJNA729271	stage_3W_vs_E10	ordinary_muscle_development	ENSGALG00010025982	SERPINE2	-1.182557372	0.199098517	-5.939558925	2.8579E-09	6.33511E-09	negative
PRJNA729271	stage_3W_vs_E10	ordinary_muscle_development	ENSGALG00010025983	RNF26	-1.236328092	0.084924321	-14.55799796	5.19573E-48	5.54989E-47	negative
PRJNA729271	stage_3W_vs_E10	ordinary_muscle_development	ENSGALG00010025984	NDUFS1	1.309630724	0.116923819	11.20071801	4.04442E-29	2.2513E-28	positive
PRJNA729271	stage_3W_vs_E10	ordinary_muscle_development	ENSGALG00010025985	CHCHD10	3.427093561	0.317895368	10.78057092	4.25238E-27	2.1788E-26	positive
PRJNA729271	stage_3W_vs_E10	ordinary_muscle_development	ENSGALG00010025986	RAB3IL1	-0.280059611	0.108228835	-2.587661693	0.009662983	0.013160288	negative
PRJNA729271	stage_3W_vs_E10	ordinary_muscle_development	ENSGALG00010025988	NSF	-0.376867035	0.059657131	-6.31721683	2.66316E-10	6.285E-10	negative
PRJNA729271	stage_3W_vs_E10	ordinary_muscle_development	ENSGALG00010025990	FAM124B	1.120028972	0.238592414	4.6943193	2.67496E-06	4.92988E-06	positive
PRJNA729271	stage_3W_vs_E10	ordinary_muscle_development	ENSGALG00010025992	MMP11	-2.424922358	0.364235759	-6.65756257	2.78406E-11	6.95775E-11	negative
PRJNA729271	stage_3W_vs_E10	ordinary_muscle_development	ENSGALG00010025994	INO80D	0.506652789	0.10846444	4.671141873	2.9953E-06	5.50204E-06	positive
PRJNA729271	stage_3W_vs_E10	ordinary_muscle_development	ENSGALG00010025996	CUL3	1.971285867	0.096399314	20.44916906	6.11072E-93	2.15135E-91	positive
PRJNA729271	stage_3W_vs_E10	ordinary_muscle_development	ENSGALG00010025997	CCDC43	-0.077719032	0.149441337	-0.520063812	0.6030191	0.638477856	negative
PRJNA729271	stage_3W_vs_E10	ordinary_muscle_development	ENSGALG00010025998	SMARCB1	-1.380383584	0.144997898	-9.520024771	1.73138E-21	7.04162E-21	negative
PRJNA729271	stage_3W_vs_E10	ordinary_muscle_development	ENSGALG00010025999	DBF4B	-1.767211495	0.167722814	-10.53650041	5.86406E-26	2.85713E-25	negative
PRJNA729271	stage_3W_vs_E10	ordinary_muscle_development	ENSGALG00010026001	ANKS1A	0.964059039	0.091836924	10.49751014	8.86886E-26	4.28657E-25	positive
PRJNA729271	stage_3W_vs_E10	ordinary_muscle_development	ENSGALG00010026002	FTH1	1.751312793	0.141823635	12.34852564	4.96123E-35	3.4163E-34	positive
PRJNA729271	stage_3W_vs_E10	ordinary_muscle_development	ENSGALG00010026003	NECTIN1	-1.078530912	0.156500405	-6.891553486	5.51864E-12	1.42589E-11	negative
PRJNA729271	stage_3W_vs_E10	ordinary_muscle_development	ENSGALG00010026005	DERL3	0.295497712	0.114247776	2.586463596	0.009696641	0.013199926	positive
PRJNA729271	stage_3W_vs_E10	ordinary_muscle_development	ENSGALG00010026006	ITGA2B	-0.100398796	0.303009087	-0.331339224	0.740388267	0.767608034	negative
PRJNA729271	stage_3W_vs_E10	ordinary_muscle_development	ENSGALG00010026008	NRP2	-1.077245655	0.101249563	-10.63950914	1.95155E-26	9.73423E-26	negative
PRJNA729271	stage_3W_vs_E10	ordinary_muscle_development	ENSGALG00010026009	ENSGALG00010026009	-1.547012651	0.308440246	-5.015599206	5.28684E-07	1.01824E-06	negative
PRJNA729271	stage_3W_vs_E10	ordinary_muscle_development	ENSGALG00010026010	ZSWIM5	-1.003298076	0.130424812	-7.692539962	1.44242E-14	4.2678E-14	negative
PRJNA729271	stage_3W_vs_E10	ordinary_muscle_development	ENSGALG00010026011	GJC2	-1.596956061	0.165955288	-9.622809132	6.40574E-22	2.64609E-21	negative
PRJNA729271	stage_3W_vs_E10	ordinary_muscle_development	ENSGALG00010026013	RABIF	-1.052935376	0.115351836	-9.128033102	6.97551E-20	2.6538E-19	negative
PRJNA729271	stage_3W_vs_E10	ordinary_muscle_development	ENSGALG00010026016	SLC2A11	-0.534794404	0.381619579	-1.401380941	0.161100189	0.18924931	negative
PRJNA729271	stage_3W_vs_E10	ordinary_muscle_development	ENSGALG00010026017	PLEKHM1	0.764526952	0.145130277	5.267866686	1.38018E-07	2.76513E-07	positive
PRJNA729271	stage_3W_vs_E10	ordinary_muscle_development	ENSGALG00010026023	THY1	0.19054293	0.299626386	0.635935082	0.524818736	0.562749488	positive
PRJNA729271	stage_3W_vs_E10	ordinary_muscle_development	ENSGALG00010026030	IBA57	0.404888305	0.083487062	4.849713187	1.2364E-06	2.32779E-06	positive
PRJNA729271	stage_3W_vs_E10	ordinary_muscle_development	ENSGALG00010026036	TUB5A	-2.279199562	0.20774168	-10.97131574	5.25028E-28	2.79687E-27	negative
PRJNA729271	stage_3W_vs_E10	ordinary_muscle_development	ENSGALG00010026040	GUK1	-0.842531302	0.120912765	-6.968092266	3.21268E-12	8.42245E-12	negative
PRJNA729271	stage_3W_vs_E10	ordinary_muscle_development	ENSGALG00010026051	TAF11	0.204560277	0.12720339	1.608135425	0.107805522	0.129776683	positive
PRJNA729271	stage_3W_vs_E10	ordinary_muscle_development	ENSGALG00010026061	MPEG1	4.217882628	0.341821229	12.33944025	5.55407E-35	3.81546E-34	positive
PRJNA729271	stage_3W_vs_E10	ordinary_muscle_development	ENSGALG00010026062	ITGA4	-1.744246637	0.160882796	-10.84172257	2.18321E-27	1.13466E-26	negative
PRJNA729271	stage_3W_vs_E10	ordinary_muscle_development	ENSGALG00010026064	ENSGALG00010026064	0.590999469	0.148413165	3.982122938	6.83024E-05	0.000113109	positive
PRJNA729271	stage_3W_vs_E10	ordinary_muscle_development	ENSGALG00010026066	SELENOP2	-0.531830528	0.591118191	-0.899702523	0.368278581	0.406936883	negative
PRJNA729271	stage_3W_vs_E10	ordinary_muscle_development	ENSGALG00010026068	BLTP3A	0.421063145	0.111419365	3.779084037	0.000157406	0.000252921	positive
PRJNA729271	stage_3W_vs_E10	ordinary_muscle_development	ENSGALG00010026073	HPDL	-0.849804926	0.243911204	-3.484074987	0.000493841	0.000761595	negative
PRJNA729271	stage_3W_vs_E10	ordinary_muscle_development	ENSGALG00010026076	C1orf35	0.0761497	0.135059458	0.563823522	0.572874242	0.609141148	positive
PRJNA729271	stage_3W_vs_E10	ordinary_muscle_development	ENSGALG00010026078	UBE2E3	-0.041636965	0.107095448	-0.388783709	0.697436153	0.727147704	negative
PRJNA729271	stage_3W_vs_E10	ordinary_muscle_development	ENSGALG00010026081	KPNA1	0.354100665	0.107912103	3.28138044	0.001033003	0.001545879	positive
PRJNA729271	stage_3W_vs_E10	ordinary_muscle_development	ENSGALG00010026083	MUTYH	-1.989863656	0.133836023	-14.86792283	5.32397E-50	6.02427E-49	negative
PRJNA729271	stage_3W_vs_E10	ordinary_muscle_development	ENSGALG00010026088	SNRPC	-0.35659036	0.08192772	-4.352499502	1.34594E-05	2.35365E-05	negative
PRJNA729271	stage_3W_vs_E10	ordinary_muscle_development	ENSGALG00010026090	USP2	4.237806849	0.233929367	18.11575396	2.39371E-73	5.08751E-72	positive
PRJNA729271	stage_3W_vs_E10	ordinary_muscle_development	ENSGALG00010026091	DTX4	0.404486249	0.162653134	2.486802685	0.012889687	0.017314059	positive
PRJNA729271	stage_3W_vs_E10	ordinary_muscle_development	ENSGALG00010026093	TOE1	-1.053847235	0.130452831	-8.078377631	6.56341E-16	2.07767E-15	negative
PRJNA729271	stage_3W_vs_E10	ordinary_muscle_development	ENSGALG00010026094	C6orf106	1.544552821	0.077608738	19.90179018	3.92651E-88	1.23192E-86	positive
PRJNA729271	stage_3W_vs_E10	ordinary_muscle_development	ENSGALG00010026095	MIF	-2.084054554	0.202343639	-10.29958029	7.07694E-25	3.29205E-24	negative
PRJNA729271	stage_3W_vs_E10	ordinary_muscle_development	ENSGALG00010026097	ARF1	-0.513719379	0.055981382	-9.176611161	4.44871E-20	1.70458E-19	negative
PRJNA729271	stage_3W_vs_E10	ordinary_muscle_development	ENSGALG00010026098	STAT3	1.16182366	0.108023447	10.75529153	5.59568E-27	2.85697E-26	positive
PRJNA729271	stage_3W_vs_E10	ordinary_muscle_development	ENSGALG00010026102	IPO9	-1.484267053	0.060436949	-24.55893415	3.4718E-133	2.5545E-131	negative
PRJNA729271	stage_3W_vs_E10	ordinary_muscle_development	ENSGALG00010026104	DDX51	-0.557331044	0.104187095	-5.349328968	8.82809E-08	1.79371E-07	negative
PRJNA729271	stage_3W_vs_E10	ordinary_muscle_development	ENSGALG00010026105	CNTF	2.389001816	0.184970117	12.91560958	3.67522E-38	2.7833E-37	positive
PRJNA729271	stage_3W_vs_E10	ordinary_muscle_development	ENSGALG00010026106	TESK2	0.609077336	0.118762876	5.128516229	2.92035E-07	5.72884E-07	positive
PRJNA729271	stage_3W_vs_E10	ordinary_muscle_development	ENSGALG00010026111	ENSGALG00010026111	-3.622165322	0.167035617	-21.68498778	2.8433E-104	1.2879E-102	negative
PRJNA729271	stage_3W_vs_E10	ordinary_muscle_development	ENSGALG00010026112	ZFP91	0.631160993	0.12070368	5.229012008	1.70418E-07	3.39222E-07	positive
PRJNA729271	stage_3W_vs_E10	ordinary_muscle_development	ENSGALG00010026113	MFRP	1.124671756	0.516036393	2.179442714	0.029298796	0.037807001	positive
PRJNA729271	stage_3W_vs_E10	ordinary_muscle_development	ENSGALG00010026114	CWC22	-0.248182361	0.083091389	-2.986860176	0.002818586	0.00405191	negative
PRJNA729271	stage_3W_vs_E10	ordinary_muscle_development	ENSGALG00010026115	MMACHC	1.126108525	0.173741795	6.481506234	9.08114E-11	2.19641E-10	positive
PRJNA729271	stage_3W_vs_E10	ordinary_muscle_development	ENSGALG00010026116	DLX3	-7.385278918	0.984540594	-7.501243687	6.32151E-14	1.8141E-13	negative
PRJNA729271	stage_3W_vs_E10	ordinary_muscle_development	ENSGALG00010026117	LZTR1	-0.05227437	0.071561345	-0.730483336	0.465094795	0.503702111	negative
PRJNA729271	stage_3W_vs_E10	ordinary_muscle_development	ENSGALG00010026120	C1QTNF5	1.067590618	0.193244199	5.524567482	3.30298E-08	6.88379E-08	positive
PRJNA729271	stage_3W_vs_E10	ordinary_muscle_development	ENSGALG00010026121	ENSGALG00010026121	1.808842342	0.25755788	7.023051836	2.17074E-12	5.7491E-12	positive

PRJNA729271	stage_3W_vs_E10	ordinary_muscle_development	ENSGALG00010026124	PACSIN1	-2.617251024	0.403515383	-6.486124524	8.80724E-11	2.13266E-10	negative
PRJNA729271	stage_3W_vs_E10	ordinary_muscle_development	ENSGALG00010026127	ENSGALG00010026127	0.462295728	0.655515745	0.705239701	0.480661079	0.519164108	positive
PRJNA729271	stage_3W_vs_E10	ordinary_muscle_development	ENSGALG00010026128	PRDX1	-1.309524456	0.142109608	-9.214890321	3.11597E-20	1.20282E-19	negative
PRJNA729271	stage_3W_vs_E10	ordinary_muscle_development	ENSGALG00010026132	DDTL	-0.601439644	0.185871556	-3.235780966	0.001213104	0.001803482	negative
PRJNA729271	stage_3W_vs_E10	ordinary_muscle_development	ENSGALG00010026137	LPXN	2.386387489	0.323926302	7.367069213	1.7442E-13	4.88544E-13	positive
PRJNA729271	stage_3W_vs_E10	ordinary_muscle_development	ENSGALG00010026141	WNT3A	-8.199010373	0.966025017	-8.487368577	2.11369E-17	7.2191E-17	negative
PRJNA729271	stage_3W_vs_E10	ordinary_muscle_development	ENSGALG00010026143	RNPEP	-0.285333137	0.141547324	-2.015814414	0.043819386	0.055414587	negative
PRJNA729271	stage_3W_vs_E10	ordinary_muscle_development	ENSGALG00010026148	RPS10	0.568767819	0.134073608	4.242205672	2.21334E-05	3.81116E-05	positive
PRJNA729271	stage_3W_vs_E10	ordinary_muscle_development	ENSGALG00010026149	LASP1	-1.162070446	0.129386599	-8.981381825	2.67387E-19	9.95065E-19	negative
PRJNA729271	stage_3W_vs_E10	ordinary_muscle_development	ENSGALG00010026154	ZNF385B	-1.953321739	0.181520247	-10.76090282	5.26518E-27	2.69012E-26	negative
PRJNA729271	stage_3W_vs_E10	ordinary_muscle_development	ENSGALG00010026162	MED1	-0.377801281	0.152062637	-2.484510916	0.012972953	0.017420431	negative
PRJNA729271	stage_3W_vs_E10	ordinary_muscle_development	ENSGALG00010026163	CTNND1	-0.758977935	0.082280623	-9.224260879	2.85523E-20	1.10482E-19	negative
PRJNA729271	stage_3W_vs_E10	ordinary_muscle_development	ENSGALG00010026166	WNT9A	-1.951509153	0.244999308	-7.965365989	1.64735E-15	5.09896E-15	negative
PRJNA729271	stage_3W_vs_E10	ordinary_muscle_development	ENSGALG00010026167	CABIN1	-1.07803971	0.060537278	-17.80786547	6.14074E-71	1.21267E-69	negative
PRJNA729271	stage_3W_vs_E10	ordinary_muscle_development	ENSGALG00010026173	CDK12	0.002922798	0.162439392	0.017993162	0.985644308	0.987961707	positive
PRJNA729271	stage_3W_vs_E10	ordinary_muscle_development	ENSGALG00010026183	SESTD1	0.164562225	0.168368868	0.977391053	0.328375591	0.366589971	positive
PRJNA729271	stage_3W_vs_E10	ordinary_muscle_development	ENSGALG00010026184	NUDT3	0.227615203	0.104342119	2.181431672	0.029151503	0.037630336	positive
PRJNA729271	stage_3W_vs_E10	ordinary_muscle_development	ENSGALG00010026192	SMIM29	2.980541146	0.118893934	25.06890841	1.0862E-138	8.5103E-137	positive
PRJNA729271	stage_3W_vs_E10	ordinary_muscle_development	ENSGALG00010026199	TBX6	-1.082273949	0.41181306	-2.628070974	0.00858706	0.011763485	negative
PRJNA729271	stage_3W_vs_E10	ordinary_muscle_development	ENSGALG00010026203	TMX2	0.394160349	0.103603438	3.804510327	0.000142085	0.000229294	positive
PRJNA729271	stage_3W_vs_E10	ordinary_muscle_development	ENSGALG00010026205	ENSGALG00010026205	2.171365131	1.31375	1.652799338	0.098371709	0.119013264	positive
PRJNA729271	stage_3W_vs_E10	ordinary_muscle_development	ENSGALG00010026209	MED19	1.60839795	0.170505268	9.433127587	3.9804E-21	1.5969E-20	positive
PRJNA729271	stage_3W_vs_E10	ordinary_muscle_development	ENSGALG00010026213	AKR1A1	-1.12556489	0.123803449	-9.09154709	9.76397E-20	3.69334E-19	negative
PRJNA729271	stage_3W_vs_E10	ordinary_muscle_development	ENSGALG00010026218	FTSJ3	-0.811397748	0.167921706	-4.832000394	1.35168E-06	2.53593E-06	negative
PRJNA729271	stage_3W_vs_E10	ordinary_muscle_development	ENSGALG00010026220	ENSGALG00010026220	0.058059737	0.096497353	0.60167181	0.547392618	0.584619511	positive
PRJNA729271	stage_3W_vs_E10	ordinary_muscle_development	ENSGALG00010026221	CRKL	0.362307443	0.146117448	2.479563178	0.013154342	0.017643397	positive
PRJNA729271	stage_3W_vs_E10	ordinary_muscle_development	ENSGALG00010026229	CRHR2	1.747727297	0.21045348	8.304577805	1.00175E-16	3.31364E-16	positive
PRJNA729271	stage_3W_vs_E10	ordinary_muscle_development	ENSGALG00010026232	PSMC5	0.692270688	0.130821663	5.291712956	1.21176E-07	2.44052E-07	positive
PRJNA729271	stage_3W_vs_E10	ordinary_muscle_development	ENSGALG00010026234	ZDHHC5	0.422163253	0.160291696	2.633718791	0.00844554	0.011575086	positive
PRJNA729271	stage_3W_vs_E10	ordinary_muscle_development	ENSGALG00010026237	KLHL22	2.557349565	0.136914848	18.67839468	7.42178E-78	1.74925E-76	positive
PRJNA729271	stage_3W_vs_E10	ordinary_muscle_development	ENSGALG00010026239	GIP	-4.748817408	1.165194245	-4.075558586	4.5904E-05	7.71096E-05	negative
PRJNA729271	stage_3W_vs_E10	ordinary_muscle_development	ENSGALG00010026242	CCDC141	0.241699038	0.253876543	0.952033752	0.341079865	0.379486693	positive
PRJNA729271	stage_3W_vs_E10	ordinary_muscle_development	ENSGALG00010026244	CLP1	-0.77454196	0.130371235	-5.941049518	2.83203E-09	6.2816E-09	negative
PRJNA729271	stage_3W_vs_E10	ordinary_muscle_development	ENSGALG00010026249	YPEL4	0.453059646	0.542690482	0.834839859	0.40380788	0.442920562	positive
PRJNA729271	stage_3W_vs_E10	ordinary_muscle_development	ENSGALG00010026252	ENO2	0.94115161	0.320635041	2.935273722	0.003332536	0.004759593	positive
PRJNA729271	stage_3W_vs_E10	ordinary_muscle_development	ENSGALG00010026254	ENSGALG00010026254	-2.538698357	0.729777941	-3.478727176	0.000503801	0.000775964	negative
PRJNA729271	stage_3W_vs_E10	ordinary_muscle_development	ENSGALG00010026259	MED15	-0.14230488	0.088862352	-1.601407981	0.109286586	0.131346415	negative
PRJNA729271	stage_3W_vs_E10	ordinary_muscle_development	ENSGALG00010026260	ENSGALG00010026260	20.97472475	3.155754843	6.646500059	3.00144E-11	7.48425E-11	positive
PRJNA729271	stage_3W_vs_E10	ordinary_muscle_development	ENSGALG00010026261	IFT70A	-0.61789606	0.107726396	-5.735790716	9.70585E-09	2.09604E-08	negative
PRJNA729271	stage_3W_vs_E10	ordinary_muscle_development	ENSGALG00010026262	NAV1	-0.455926433	0.161808057	-2.817699204	0.00483691	0.006792386	negative
PRJNA729271	stage_3W_vs_E10	ordinary_muscle_development	ENSGALG00010026264	FZD7	-2.14131266	0.143077095	-14.96614573	1.22201E-50	1.41704E-49	negative
PRJNA729271	stage_3W_vs_E10	ordinary_muscle_development	ENSGALG00010026266	ENSGALG00010026266	-2.386678599	0.401386605	-5.946084318	2.74633E-09	6.09898E-09	negative
PRJNA729271	stage_3W_vs_E10	ordinary_muscle_development	ENSGALG00010026268	GARS1	0.413533838	0.150606837	2.745783975	0.00603665	0.00839099	positive
PRJNA729271	stage_3W_vs_E10	ordinary_muscle_development	ENSGALG00010026272	LRRC58	0.523019856	0.096276732	5.432463748	5.55812E-08	1.14212E-07	positive
PRJNA729271	stage_3W_vs_E10	ordinary_muscle_development	ENSGALG00010026277	SMARCD2	0.306068203	0.124281253	2.462706124	0.013789289	0.018457451	positive
PRJNA729271	stage_3W_vs_E10	ordinary_muscle_development	ENSGALG00010026287	NASP	-1.910975409	0.092003477	-20.77068688	7.97103E-96	2.98553E-94	negative
PRJNA729271	stage_3W_vs_E10	ordinary_muscle_development	ENSGALG00010026291	SERPING1	1.719140984	0.169347149	10.15157915	3.26044E-24	1.47688E-23	positive
PRJNA729271	stage_3W_vs_E10	ordinary_muscle_development	ENSGALG00010026295	SMPD4	-0.80096518	0.133880751	-5.982676175	2.19501E-09	4.89562E-09	negative
PRJNA729271	stage_3W_vs_E10	ordinary_muscle_development	ENSGALG00010026298	SNF8	0.356877392	0.114865787	3.106907649	0.001890554	0.0027569	positive
PRJNA729271	stage_3W_vs_E10	ordinary_muscle_development	ENSGALG00010026300	HMGA1	-1.41634443	0.11032549	-12.83787114	1.00603E-37	7.50124E-37	negative
PRJNA729271	stage_3W_vs_E10	ordinary_muscle_development	ENSGALG00010026305	ATP5MC1	1.269365522	0.167029582	7.599644967	2.96944E-14	8.65167E-14	positive
PRJNA729271	stage_3W_vs_E10	ordinary_muscle_development	ENSGALG00010026315	SYT2	-3.397768773	0.420299936	-8.08415248	6.2598E-16	1.98373E-15	negative
PRJNA729271	stage_3W_vs_E10	ordinary_muscle_development	ENSGALG00010026316	ENSGALG00010026316	0.964069317	0.348660398	2.765066877	0.005691113	0.007931226	positive
PRJNA729271	stage_3W_vs_E10	ordinary_muscle_development	ENSGALG00010026320	ENSGALG00010026320	0.682188936	0.174682092	3.905316956	9.4102E-05	0.000153864	positive
PRJNA729271	stage_3W_vs_E10	ordinary_muscle_development	ENSGALG00010026321	GPBP1L1	0.543555626	0.047239918	11.50627793	1.22659E-30	7.21274E-30	positive
PRJNA729271	stage_3W_vs_E10	ordinary_muscle_development	ENSGALG00010026325	TMEM69	0.584834897	0.137338035	4.258360749	2.05931E-05	3.55312E-05	positive
PRJNA729271	stage_3W_vs_E10	ordinary_muscle_development	ENSGALG00010026329	ENSGALG00010026329	1.17824171	0.275613434	4.274979238	1.91155E-05	3.30762E-05	positive
PRJNA729271	stage_3W_vs_E10	ordinary_muscle_development	ENSGALG00010026334	ENSGALG00010026334	1.499509839	0.126525465	11.851447	2.11507E-32	1.33295E-31	positive
PRJNA729271	stage_3W_vs_E10	ordinary_muscle_development	ENSGALG00010026335	IPP	-1.16891037	0.105757153	-11.05277835	2.12534E-28	1.15208E-27	negative
PRJNA729271	stage_3W_vs_E10	ordinary_muscle_development	ENSGALG00010026344	TIMM10	0.361590732	0.171782469	2.104933843	0.035297067	0.045165165	positive
PRJNA729271	stage_3W_vs_E10	ordinary_muscle_development	ENSGALG00010026346	FZD2	0.023027585	0.163431281	0.140900722	0.887948372	0.901001866	positive
PRJNA729271	stage_3W_vs_E10	ordinary_muscle_development	ENSGALG00010026354	ENSGALG00010026354	1.897704449	0.244551478	7.75993858	8.49705E-15	2.54473E-14	positive
PRJNA729271	stage_3W_vs_E10	ordinary_muscle_development	ENSGALG00010026355	PPP1R12B	1.080280847	0.137418308	7.861258537	3.80293E-15	1.15976E-14	positive
PRJNA729271	stage_3W_vs_E10	ordinary_muscle_development	ENSGALG00010026360	CTDSPL	1.310925033	0.126531503	10.3604636	3.75139E-25	1.76949E-24	positive
PRJNA729271	stage_3W_vs_E10	ordinary_muscle_development	ENSGALG00010026362	ENSGALG00010026362	-0.195947321	0.38514639	-0.508760631	0.610920018	0.645946871	negative
PRJNA729271	stage_3W_vs_E10	ordinary_muscle_development	ENSGALG00010026367	RBP	2.156907285	0.549690863	3.923855078	8.71432E-05	0.000142922	positive
PRJNA729271	stage_3W_vs_E10	ordinary_muscle_development	ENSGALG00010026377	ENSGALG00010026377	-4.722523483	0.257098052	-18.3685697	2.34511E-75	5.21355E-74	negative
PRJNA729271	stage_3W_vs_E10	ordinary_muscle_development	ENSGALG00010026388	SNRPD3	-0.502627866	0.080215225	-6.265990827	3.70462E-10	8.66972E-10	negative
PRJNA729271	stage_3W_vs_E10	ordinary_muscle_development	ENSGALG00010026393	SLC43A3	0.04815073	0.122238217	0.393908971	0.693648247	0.723652752	positive
PRJNA729271	stage_3W_vs_E10	ordinary_muscle_development	ENSGALG00010026402	TFEB	2.294987381	0.164398632	13.95989345	2.73843E-44	2.55264E-43	positive
PRJNA729271	stage_3W_vs_E10	ordinary_muscle_development	ENSGALG00010026403	GUCD1	2.088462601	0.115751336	18.04266521	9.00922E-73	1.89534E-71	positive
PRJNA729271	stage_3W_vs_E10	ordinary_muscle_development	ENSGALG00010026409	VILL	-3.429084319	0.271865725	-12.61315425	1.78695E-36	1.28227E-35	negative
PRJNA729271	stage_3W_vs_E10	ordinary_muscle_development	ENSGALG00010026410	ENSGALG00010026410	-0.166046545	0.32197847	-0.515706982	0.606059077	0.641509152	negative
PRJNA729271	stage_3W_vs_E10	ordinary_muscle_development	ENSGALG00010026416	ENSGALG00010026416	0.26018934	1.436643539	0.181109185	0.856281876	0.872534677	positive
PRJNA729271	stage_3W_vs_E10	ordinary_muscle_development	ENSGALG00010026422	P2RX3	-2.819132549	0.352455316	-7.998553073	1.2589E-15	3.9234E-15	negative
PRJNA729271	stage_3W_vs_E10	ordinary_muscle_development	ENSGALG00010026427	PGC	3.350553537	1.056756937	3.170599992	0.001521245	0.002242494	positive
PRJNA729271	stage_3W_vs_E10	ordinary_muscle_development	ENSGALG00010026433	VPS11	0.161541804	0.071289032	2.266012033	0.023450647	0.030628683	positive
PRJNA729271	stage_3W_vs_E10	ordinary_muscle_development	ENSGALG00010026434	ENSGALG00010026434	0.139561907	0.144116364	0.968397364	0.332845955	0.371237467	positive
PRJNA729271	stage_3W_vs_E10	ordinary_muscle_development	ENSGALG00010026436	PLCD1	-0.995751824	0.077253035	-12.88948477	5.15873E-38	3.88244E-37	negative
PRJNA729271	stage_3W_vs_E10	ordinary_muscle_development	ENSGALG00010026438	TOMM6	0.771726772	0.120927778	6.381716302	1.75114E-10	4.17274E-10	positive
PRJNA729271	stage_3W_vs_E10	ordinary_muscle_development	ENSGALG00010026443	UPB1	0.571030918	0.361633077	1.579033985	0.114328262	0.137127692	positive
PRJNA729271	stage_3W_vs_E10	ordinary_muscle_development	ENSGALG00010026448	ADORA2A	-0.943138839	0.116841264	-8.071967096	6.91745E-16	2.18688E-15	negative
PRJNA729271	stage_3W_vs_E10	ordinary_muscle_development	ENSGALG00010026457	SSRP1	-1.020283857	0.098826359	-10.32400537	5.48847E-25	2.56465E-24	negative
PRJNA729271	stage_3W_vs_E10	ordinary_muscle_development	ENSGALG00010026458	DLEC1	1.084373698	0.18303195	5.924504966	3.1324E-09	6.9203E-09	positive
PRJNA729271	stage_3W_vs_E10	ordinary_muscle_development	ENSGALG00010026480	USP49	0.087575446	0.213338208	0.410500523	0.68143882	0.712299394	positive
PRJNA729271	stage_3W_vs_E10	ordinary_muscle_development	ENSGALG00010026481	DIXDC1	-1.490338029	0.137703497	-10.82280453	2.68435E-27	1.38765E-26	negative
PRJNA729271	stage_3W_vs_E10	ordinary_muscle_development	ENSGALG00010026484	TNKS1BP1	-0.418955988	0.112249406	-3.732367089	0.000189689	0.000302846	negative
PRJNA729271	stage_3W_vs_E10	ordinary_muscle_development	ENSGALG00010026485	ACAA1	0.478416183	0.105122199	4.551048108	5.33793E-06	9.61996E-06	positive
PRJNA729271	stage_3W_vs_E10	ordinary_muscle_development	ENSGALG00010026492	SPECC1L	-0.944452957	0.075694482	-12.47717051	9.94586E-36	6.99152E-35	negative
PRJNA729271	stage_3W_vs_E10	ordinary_muscle_development	ENSGALG00010026498	ENSGALG00010026498	-1.215679183	0.088613058	-13.71896203	7.81766E-43	6.9307E-42	negative
PRJNA729271	stage_3W_vs_E10	ordinary_muscle_development	ENSGALG00010026507	APLNR	-2.358903908	0.300702842	-7.844634577	4.34216E-15	1.31977E-14	negative
PRJNA729271	stage_3W_vs_E10	ordinary_muscle_development	ENSGALG00010026508	CSRP1	1.863097038	0.148788642	12.52176924	5.67557E-36	4.01697E-35	positive
PRJNA729271	stage_3W_vs_E10	ordinary_muscle_development	ENSGALG00010026516	ENSGALG00010026516	0.345023383	0.167493697	2.059918605	0.039406323	0.050122381	positive
PRJNA729271	stage_3W_vs_E10	ordinary_muscle_development	ENSGALG00010026522	MED20	0.091377437	0.129050654	0.708074183	0.478899191	0.517531035	positive
PRJNA729271	stage_3W_vs_E10	ordinary_muscle_development	ENSGALG00010026536	BCR	-0.371345154	0.097232037	-3.819164616	0.000133904	0.000216647	negative
PRJNA729271	stage_3W_vs_E10	ordinary_muscle_development	ENSGALG00010026537	FJX1	-3.306784655	0.185197337	-17.85546542	2.62087E-71	5.21834E-70	negative
PRJNA729271	stage_3W_vs_E10	ordinary_muscle_development	ENSGALG00010026539	ENSGALG00010026539	0.197158995	0.362548514	0.543814103	0.586569403	0.622108585	positive
PRJNA729271	stage_3W_vs_E10	ordinary_muscle_development	ENSGALG00010026550	MYD88	0.685985527	0.105041655	6.530604695	6.55047E-11	1.59712E-10	positive
PRJNA729271	stage_3W_vs_E10	ordinary_muscle_development	ENSGALG00010026553	PLPP3	-0.415397698	0.128038774	-3.244311746	0.001177348	0.001754102	negative
PRJNA729271	stage_3W_vs_E10	ordinary_muscle_development	ENSGALG00010026559	RAB36	-1.413740224	0.46719458	-3.026020173	0.002477958	0.003579629	negative
PRJNA729271	stage_3W_vs_E10	ordinary_muscle_development	ENSGALG00010026560	TNNI1	-4.810301951	0.589900502	-8.154429323	3.50833E-16	1.12532E-15	negative
PRJNA729271	stage_3W_vs_E10	ordinary_muscle_development	ENSGALG00010026565	RSPH14	-3.266722691	0.587864475	-5.556931623	2.74558E-08	5.75937E-08	negative
PRJNA729271	stage_3W_vs_E10	ordinary_muscle_development	ENSGALG00010026566	ENSGALG00010026566	-0.234542681	0.123904216	-1.89293543	0.058366457	0.072663557	negative
PRJNA729271	stage_3W_vs_E10	ordinary_muscle_development	ENSGALG00010026568	BYSL	0.21450924	0.110757388	1.936748825	0.052776054	0.066032707	positive
PRJNA729271	stage_3W_vs_E10	ordinary_muscle_development	ENSGALG00010026569	GNAZ	-1.508250833	0.209263371	-7.207428743	5.70183E-13	1.55499E-12	negative
PRJNA729271	stage_3W_vs_E10	ordinary_muscle_development	ENSGALG00010026581	DNER	-6.642889318	0.377737327	-17.58600182	3.15358E-69	5.93652E-68	negative
PRJNA729271	stage_3W_vs_E10	ordinary_muscle_development	ENSGALG00010026584	ENSGALG00010026584	-0.570710715	0.111097194	-5.137039863	2.791E-07	5.48178E-07	negative
PRJNA729271	stage_3W_vs_E10	ordinary_muscle_development	ENSGALG00010026588	CCND3	1.117202482	0.142721192	7.827866816	4.96217E-15	1.50474E-14	positive
PRJNA729271	stage_3W_vs_E10	ordinary_muscle_development	ENSGALG00010026595	PRKAA2	2.797311328	0.261148161	10.71158731	8.98086E-27	4.54372E-26	positive
PRJNA729271	stage_3W_vs_E10	ordinary_muscle_development	ENSGALG00010026601	ENSGALG00010026601	0.853154833	0.467115902	1.82643072	0.067785408	0.08367108	positive
PRJNA729271	stage_3W_vs_E10	ordinary_muscle_development	ENSGALG00010026612	MFF	0.262185617	0.080964221	3.238289875	0.001202486	0.001788246	positive
PRJNA729271	stage_3W_vs_E10	ordinary_muscle_development	ENSGALG00010026625	ENSGALG00010026625	4.27248237	0.629288552	6.789385183	1.12612E-11	2.8743E-11	positive
PRJNA729271	stage_3W_vs_E10	ordinary_muscle_development	ENSGALG00010026628	FYB2	-2.806305194	0.872429794	-3.216654465	0.001296947	0.001923393	negative
PRJNA729271	stage_3W_vs_E10	ordinary_muscle_development	ENSGALG00010026630	XIRP1	0.677207297	0.243836091	2.777305421	0.005481165	0.007651144	positive
PRJNA729271	stage_3W_vs_E10	ordinary_muscle_development	ENSGALG00010026633	SLC5A1	0.084835358	0.510553504	0.166163502	0.868028281	0.883200192	positive
PRJNA729271	stage_3W_vs_E10	ordinary_muscle_development	ENSGALG00010026635	C8A	1.489107657	2.609162035	0.570722568	0.568187718	0.604645839	positive
PRJNA729271	stage_3W_vs_E10	ordinary_muscle_development	ENSGALG00010026638	LAD1	-3.438407473	0.485175552	-7.086934734	1.37115E-12	3.67033E-12	negative
PRJNA729271	stage_3W_vs_E10	ordinary_muscle_development	ENSGALG00010026643	YWHAH	-0.642549638	0.111961866	-5.73900435	9.52348E-09	2.05788E-08	negative
PRJNA729271	stage_3W_vs_E10	ordinary_muscle_development	ENSGALG00010026659	LIPH	-0.073205874	0.251592247	-0.290970309	0.771074028	0.795949155	negative
PRJNA729271	stage_3W_vs_E10	ordinary_muscle_development	ENSGALG00010026666	ENSGALG00010026666	-0.762332291	0.467469929	-1.630762201	0.102940506	0.124198655	negative
PRJNA729271	stage_3W_vs_E10	ordinary_muscle_development	ENSGALG00010026671	TAF8	0.313347446	0.155308604	2.017579447	0.043635079	0.055186325	positive
PRJNA729271	stage_3W_vs_E10	ordinary_muscle_development	ENSGALG00010026673	DEPDC5	0.347174617	0.121839939	2.849431969	0.004379737	0.006179723	positive
PRJNA729271	stage_3W_vs_E10	ordinary_muscle_development	ENSGALG00010026681	PLXDC1	2.468372905	0.257776706	9.575624369	1.01244E-21	4.15612E-21	positive
PRJNA729271	stage_3W_vs_E10	ordinary_muscle_development	ENSGALG00010026686	ENSGALG00010026686	1.141921872	0.38877691	2.937216282	0.00331173	0.004733143	positive
PRJNA729271	stage_3W_vs_E10	ordinary_muscle_development	ENSGALG00010026690	KRT14	-4.275556174	0.436890693	-9.786329266	1.2889E-22	5.46436E-22	negative
PRJNA729271	stage_3W_vs_E10	ordinary_muscle_development	ENSGALG00010026692	NMNAT3	5.060354015	0.49679963	10.18590537	2.29216E-24	1.04547E-23	positive
PRJNA729271	stage_3W_vs_E10	ordinary_muscle_development	ENSGALG00010026696	CSRNP1	-1.219604494	0.151406966	-8.055141251	7.93871E-16	2.50373E-15	negative
PRJNA729271	stage_3W_vs_E10	ordinary_muscle_development	ENSGALG00010026697	CHN1	-2.139985378	0.11942961	-17.9183821	8.47575E-72	1.72551E-70	negative
PRJNA729271	stage_3W_vs_E10	ordinary_muscle_development	ENSGALG00010026700	C8B	-2.467132842	1.0658619	-2.314683396	0.020630265	0.027106209	negative
PRJNA729271	stage_3W_vs_E10	ordinary_muscle_development	ENSGALG00010026704	WDFY1	-0.345575177	0.070038007	-4.934109199	8.05174E-07	1.53445E-06	negative
PRJNA729271	stage_3W_vs_E10	ordinary_muscle_development	ENSGALG00010026706	KRT24	-7.330475218	0.588091866	-12.46484715	1.16094E-35	8.14514E-35	negative

PRJNA729271	stage_3W_vs_E10	ordinary_muscle_development	ENSGALG00010026707	GORASP1	0.111733372	0.081343724	1.373595473	0.169567303	0.198504124	positive
PRJNA729271	stage_3W_vs_E10	ordinary_muscle_development	ENSGALG00010026715	WDR48	-0.42368557	0.067755822	-6.253124186	4.02322E-10	9.39832E-10	negative
PRJNA729271	stage_3W_vs_E10	ordinary_muscle_development	ENSGALG00010026719	ENSGALG00010026719	-4.064172065	1.008767149	-4.028850534	5.60502E-05	9.35569E-05	negative
PRJNA729271	stage_3W_vs_E10	ordinary_muscle_development	ENSGALG00010026724	OLA1	1.335829058	0.097582084	13.68928609	1.1766E-42	1.03676E-41	positive
PRJNA729271	stage_3W_vs_E10	ordinary_muscle_development	ENSGALG00010026731	ENSGALG00010026731	-2.379048456	0.515449077	-4.615486883	3.92175E-06	7.14771E-06	negative
PRJNA729271	stage_3W_vs_E10	ordinary_muscle_development	ENSGALG00010026737	SCN11A	-7.05772542	1.151887948	-6.127093725	8.94988E-10	2.04586E-09	negative
PRJNA729271	stage_3W_vs_E10	ordinary_muscle_development	ENSGALG00010026740	DAB1	-5.692149831	0.199738964	-28.49794417	1.2422E-178	1.7313E-176	negative
PRJNA729271	stage_3W_vs_E10	ordinary_muscle_development	ENSGALG00010026743	TNNT2	-3.992193718	0.587255103	-6.798057089	1.06039E-11	2.70797E-11	negative
PRJNA729271	stage_3W_vs_E10	ordinary_muscle_development	ENSGALG00010026746	HOXD10	-3.522358909	0.464139043	-7.58901661	3.22342E-14	9.37847E-14	negative
PRJNA729271	stage_3W_vs_E10	ordinary_muscle_development	ENSGALG00010026755	ENSGALG00010026755	-1.068568272	0.205055357	-5.21112097	1.87703E-07	3.72757E-07	negative
PRJNA729271	stage_3W_vs_E10	ordinary_muscle_development	ENSGALG00010026759	OMA1	-0.440385734	0.110575432	-3.982672528	6.81446E-05	0.000112886	negative
PRJNA729271	stage_3W_vs_E10	ordinary_muscle_development	ENSGALG00010026767	ENSGALG00010026767	18.0240288	2.527262717	7.131838206	9.90373E-13	2.67327E-12	positive
PRJNA729271	stage_3W_vs_E10	ordinary_muscle_development	ENSGALG00010026770	ENSGALG00010026770	-0.765399799	0.583694037	-1.311303098	0.189755382	0.220357656	negative
PRJNA729271	stage_3W_vs_E10	ordinary_muscle_development	ENSGALG00010026775	BTG2	2.687438383	0.365990154	7.342925359	2.08975E-13	5.82742E-13	positive
PRJNA729271	stage_3W_vs_E10	ordinary_muscle_development	ENSGALG00010026780	MYSM1	0.470327271	0.078363142	6.001893973	1.95029E-09	4.36055E-09	positive
PRJNA729271	stage_3W_vs_E10	ordinary_muscle_development	ENSGALG00010026782	HOXB9	-4.871645147	0.634782869	-7.674506332	1.66057E-14	4.90123E-14	negative
PRJNA729271	stage_3W_vs_E10	ordinary_muscle_development	ENSGALG00010026786	ENSGALG00010026786	1.783427217	0.590874645	3.018283542	0.002542109	0.00366828	positive
PRJNA729271	stage_3W_vs_E10	ordinary_muscle_development	ENSGALG00010026796	DCAKD	-0.346597887	0.228757219	-1.515134205	0.129738421	0.15452411	negative
PRJNA729271	stage_3W_vs_E10	ordinary_muscle_development	ENSGALG00010026801	FMOD	1.842883949	0.490672653	3.755831794	0.000172767	0.000276744	positive
PRJNA729271	stage_3W_vs_E10	ordinary_muscle_development	ENSGALG00010026822	PRELP	-0.211309971	0.20049027	-1.053966215	0.291898385	0.329163459	negative
PRJNA729271	stage_3W_vs_E10	ordinary_muscle_development	ENSGALG00010026825	ADAM23	-0.760211303	0.136347822	-5.575529492	2.46778E-08	5.19165E-08	negative
PRJNA729271	stage_3W_vs_E10	ordinary_muscle_development	ENSGALG00010026827	FAM117A	1.478634991	0.12832608	11.52248233	1.0164E-30	6.006E-30	positive
PRJNA729271	stage_3W_vs_E10	ordinary_muscle_development	ENSGALG00010026834	ENSGALG00010026834	0.033991195	2.099079829	0.016193379	0.987080117	0.988990551	positive
PRJNA729271	stage_3W_vs_E10	ordinary_muscle_development	ENSGALG00010026836	ENSGALG00010026836	2.305338339	0.608925989	3.785908927	0.000153148	0.000246352	positive
PRJNA729271	stage_3W_vs_E10	ordinary_muscle_development	ENSGALG00010026839	FGGY	-0.046663896	0.118238935	-0.394657614	0.693095588	0.723180287	negative
PRJNA729271	stage_3W_vs_E10	ordinary_muscle_development	ENSGALG00010026846	MICAL1	0.710196369	0.224070563	3.169521058	0.001526904	0.002250378	positive
PRJNA729271	stage_3W_vs_E10	ordinary_muscle_development	ENSGALG00010026851	ENSGALG00010026851	1.488987607	1.37856128	1.080102588	0.280096499	0.317014036	positive
PRJNA729271	stage_3W_vs_E10	ordinary_muscle_development	ENSGALG00010026854	SMPD2	-0.122834667	0.150907578	-0.813972821	0.415660522	0.454511109	negative
PRJNA729271	stage_3W_vs_E10	ordinary_muscle_development	ENSGALG00010026856	UBE2Z	-0.066377609	0.077364591	-0.857984357	0.390901101	0.429902236	negative
PRJNA729271	stage_3W_vs_E10	ordinary_muscle_development	ENSGALG00010026874	TEAD3	0.559194155	0.160334729	3.487667073	0.000487254	0.000751677	positive
PRJNA729271	stage_3W_vs_E10	ordinary_muscle_development	ENSGALG00010026885	TULP1	4.50301957	0.624358422	7.212234855	5.50409E-13	1.50192E-12	positive
PRJNA729271	stage_3W_vs_E10	ordinary_muscle_development	ENSGALG00010026886	JUN	1.23662946	0.192885882	6.411197359	1.44381E-10	3.45119E-10	positive
PRJNA729271	stage_3W_vs_E10	ordinary_muscle_development	ENSGALG00010026889	ENSGALG00010026889	-0.334635094	0.279126398	-1.198865804	0.23058013	0.264314669	negative
PRJNA729271	stage_3W_vs_E10	ordinary_muscle_development	ENSGALG00010026893	FKBP5	3.642664578	0.386065888	9.435344297	3.89712E-21	1.56479E-20	positive
PRJNA729271	stage_3W_vs_E10	ordinary_muscle_development	ENSGALG00010026894	IP6K1	-0.310520251	0.066736777	-4.652910495	3.27282E-06	5.99666E-06	negative
PRJNA729271	stage_3W_vs_E10	ordinary_muscle_development	ENSGALG00010026897	ENSGALG00010026897	-0.802857033	0.232741888	-3.449559682	0.000561502	0.000859538	negative
PRJNA729271	stage_3W_vs_E10	ordinary_muscle_development	ENSGALG00010026898	PNPLA2	4.167523309	0.271527845	15.34841962	3.6292E-53	4.56643E-52	positive
PRJNA729271	stage_3W_vs_E10	ordinary_muscle_development	ENSGALG00010026899	HOOK1	-0.087290813	0.166841005	-0.523197599	0.600836752	0.636399614	negative
PRJNA729271	stage_3W_vs_E10	ordinary_muscle_development	ENSGALG00010026901	FAM212A	-0.922808984	0.176575631	-5.226140097	1.73085E-07	3.44389E-07	negative
PRJNA729271	stage_3W_vs_E10	ordinary_muscle_development	ENSGALG00010026907	ENSGALG00010026907	-1.013517388	0.135928996	-7.456226516	8.90356E-14	2.53103E-13	negative
PRJNA729271	stage_3W_vs_E10	ordinary_muscle_development	ENSGALG00010026909	ENSGALG00010026909	0.386648421	0.154986091	2.494729801	0.012605308	0.01695249	positive
PRJNA729271	stage_3W_vs_E10	ordinary_muscle_development	ENSGALG00010026916	ENSGALG00010026916	0.889193631	0.213203842	4.170626674	3.03763E-05	5.16547E-05	positive
PRJNA729271	stage_3W_vs_E10	ordinary_muscle_development	ENSGALG00010026917	AMT	-1.828979759	0.149401788	-12.24202056	1.8536E-34	1.25551E-33	negative
PRJNA729271	stage_3W_vs_E10	ordinary_muscle_development	ENSGALG00010026919	NICN1	-0.489850198	0.170841188	-2.867283954	0.004140114	0.005851872	negative
PRJNA729271	stage_3W_vs_E10	ordinary_muscle_development	ENSGALG00010026920	GATA2	1.861468397	0.20058088	9.280388024	1.68862E-20	6.5815E-20	positive
PRJNA729271	stage_3W_vs_E10	ordinary_muscle_development	ENSGALG00010026924	TPH1	0.740201556	0.346965879	2.133355469	0.032895577	0.042204053	positive
PRJNA729271	stage_3W_vs_E10	ordinary_muscle_development	ENSGALG00010026942	CDK18	-1.704495729	0.19026647	-8.958466159	3.29226E-19	1.21956E-18	negative
PRJNA729271	stage_3W_vs_E10	ordinary_muscle_development	ENSGALG00010026943	EEFSEC	-0.388131379	0.071508149	-5.427792289	5.70554E-08	1.17141E-07	negative
PRJNA729271	stage_3W_vs_E10	ordinary_muscle_development	ENSGALG00010026950	LHFPL5	0.241210918	0.273746006	0.881148631	0.378237379	0.417082602	positive
PRJNA729271	stage_3W_vs_E10	ordinary_muscle_development	ENSGALG00010026951	RUVBL1	-0.948884299	0.106419522	-8.916449564	4.81476E-19	1.77492E-18	negative
PRJNA729271	stage_3W_vs_E10	ordinary_muscle_development	ENSGALG00010026954	MFSD4A	0.850685298	0.177599879	4.789897951	1.66866E-06	3.1105E-06	positive
PRJNA729271	stage_3W_vs_E10	ordinary_muscle_development	ENSGALG00010026958	CLTCL1	-0.097725014	0.08424836	-1.159963405	0.246063706	0.280996881	negative
PRJNA729271	stage_3W_vs_E10	ordinary_muscle_development	ENSGALG00010026960	SEC61A1	-1.793885512	0.102655965	-17.47473234	2.2319E-68	4.14487E-67	negative
PRJNA729271	stage_3W_vs_E10	ordinary_muscle_development	ENSGALG00010026964	SRPK1	-0.518119289	0.060054817	-8.627439267	6.27408E-18	2.20628E-17	negative
PRJNA729271	stage_3W_vs_E10	ordinary_muscle_development	ENSGALG00010026972	TNNT3	6.12288027	0.158348666	38.6670783	0	0	positive
PRJNA729271	stage_3W_vs_E10	ordinary_muscle_development	ENSGALG00010026973	MGLL	1.661459392	0.12432116	13.36425266	9.78124E-41	8.06021E-40	positive
PRJNA729271	stage_3W_vs_E10	ordinary_muscle_development	ENSGALG00010026977	ELK4	0.015563575	0.098504348	0.157998859	0.874457698	0.888930804	positive
PRJNA729271	stage_3W_vs_E10	ordinary_muscle_development	ENSGALG00010026980	LIMK2	-1.147229078	0.119012009	-9.639607735	5.43954E-22	2.25468E-21	negative
PRJNA729271	stage_3W_vs_E10	ordinary_muscle_development	ENSGALG00010026982	ABTB1	1.561994297	0.084785535	18.42288661	8.60877E-76	1.94368E-74	positive
PRJNA729271	stage_3W_vs_E10	ordinary_muscle_development	ENSGALG00010026984	PGR2/3	2.182704041	0.926918	2.354797339	0.01853281	0.024463444	positive
PRJNA729271	stage_3W_vs_E10	ordinary_muscle_development	ENSGALG00010026999	MLEC	-0.80886969	0.082887522	-9.758642489	1.69408E-22	7.1445E-22	negative
PRJNA729271	stage_3W_vs_E10	ordinary_muscle_development	ENSGALG00010027001	SLC26A8	0.164390151	0.253062855	0.649602057	0.515949304	0.553924969	positive
PRJNA729271	stage_3W_vs_E10	ordinary_muscle_development	ENSGALG00010027003	SEPTIN2L	-0.952198111	0.082653502	-11.52036009	1.04175E-30	6.15076E-30	negative
PRJNA729271	stage_3W_vs_E10	ordinary_muscle_development	ENSGALG00010027012	RILPL1	1.98261335	0.192011194	10.32550918	5.40314E-25	2.52803E-24	positive
PRJNA729271	stage_3W_vs_E10	ordinary_muscle_development	ENSGALG00010027015	ENSGALG00010027015	1.510079305	0.5237674	2.883110525	0.003937693	0.005579905	positive
PRJNA729271	stage_3W_vs_E10	ordinary_muscle_development	ENSGALG00010027025	ORAI1	2.153587212	0.127319631	16.91480876	3.49951E-64	5.87099E-63	positive
PRJNA729271	stage_3W_vs_E10	ordinary_muscle_development	ENSGALG00010027026	SLC45A3	1.152217319	0.201787003	5.71006705	1.12932E-08	2.43087E-08	positive
PRJNA729271	stage_3W_vs_E10	ordinary_muscle_development	ENSGALG00010027027	ARHGEF3	0.545743929	0.101875461	5.356971405	8.46285E-08	1.72312E-07	positive
PRJNA729271	stage_3W_vs_E10	ordinary_muscle_development	ENSGALG00010027036	NUCKS1	-1.623385851	0.066081777	-24.56631647	2.8952E-133	2.1411E-131	negative
PRJNA729271	stage_3W_vs_E10	ordinary_muscle_development	ENSGALG00010027040	CLIP1	1.3001474	0.10794102	12.04497981	2.06127E-33	1.34344E-32	positive
PRJNA729271	stage_3W_vs_E10	ordinary_muscle_development	ENSGALG00010027041	MAPK14	0.546674396	0.073785949	7.408922723	1.27329E-13	3.59284E-13	positive
PRJNA729271	stage_3W_vs_E10	ordinary_muscle_development	ENSGALG00010027043	IL17RD	-3.043763724	0.177437794	-17.15397636	5.86952E-66	1.02876E-64	negative
PRJNA729271	stage_3W_vs_E10	ordinary_muscle_development	ENSGALG00010027045	MAPK13	0.303178461	0.193989606	1.562859308	0.118085691	0.141340965	positive
PRJNA729271	stage_3W_vs_E10	ordinary_muscle_development	ENSGALG00010027046	HESX1	-0.423088264	0.310914957	-1.360784532	0.173581791	0.202794234	negative
PRJNA729271	stage_3W_vs_E10	ordinary_muscle_development	ENSGALG00010027050	GSTT1	2.27144056	0.15179312	14.96405481	1.26103E-50	1.45879E-49	positive
PRJNA729271	stage_3W_vs_E10	ordinary_muscle_development	ENSGALG00010027052	APPL1	0.288495512	0.084508151	3.413818779	0.000640592	0.000976894	positive
PRJNA729271	stage_3W_vs_E10	ordinary_muscle_development	ENSGALG00010027059	ASB14	7.224438722	0.210919316	34.25214374	4.0524E-257	1.1988E-254	positive
PRJNA729271	stage_3W_vs_E10	ordinary_muscle_development	ENSGALG00010027063	RAB7L1	0.610242054	0.087529625	6.971834453	3.12835E-12	8.20731E-12	positive
PRJNA729271	stage_3W_vs_E10	ordinary_muscle_development	ENSGALG00010027073	DNAH12	-1.322270896	0.312573624	-4.230270237	2.33411E-05	4.00769E-05	negative
PRJNA729271	stage_3W_vs_E10	ordinary_muscle_development	ENSGALG00010027074	BRPF3	-0.132423766	0.080731525	-1.640298073	0.100943206	0.121941143	negative
PRJNA729271	stage_3W_vs_E10	ordinary_muscle_development	ENSGALG00010027075	SLC41A1	1.338724839	0.094661978	14.14216002	2.08776E-45	2.02422E-44	positive
PRJNA729271	stage_3W_vs_E10	ordinary_muscle_development	ENSGALG00010027076	VDHAP	1.903195481	0.171383682	11.1048815	1.18775E-28	6.4772E-28	positive
PRJNA729271	stage_3W_vs_E10	ordinary_muscle_development	ENSGALG00010027077	ARF4	-0.889896974	0.104867818	-8.485891924	2.1407E-17	7.30622E-17	negative
PRJNA729271	stage_3W_vs_E10	ordinary_muscle_development	ENSGALG00010027078	PDE12	-0.763296077	0.122195121	-6.246534793	4.19659E-10	9.79541E-10	negative
PRJNA729271	stage_3W_vs_E10	ordinary_muscle_development	ENSGALG00010027079	PM20D1	0.805336512	0.094119879	8.556497527	1.16348E-17	4.03655E-17	positive
PRJNA729271	stage_3W_vs_E10	ordinary_muscle_development	ENSGALG00010027080	TBC1D22B	0.956710438	0.155352569	6.158317472	7.35219E-10	1.69132E-09	positive
PRJNA729271	stage_3W_vs_E10	ordinary_muscle_development	ENSGALG00010027081	PDE4B	1.11991956	0.291212611	3.845711062	0.000120203	0.000195199	positive
PRJNA729271	stage_3W_vs_E10	ordinary_muscle_development	ENSGALG00010027082	SLC26A9	-5.144929711	0.723987343	-7.106380744	1.19125E-12	3.19764E-12	negative
PRJNA729271	stage_3W_vs_E10	ordinary_muscle_development	ENSGALG00010027083	DENND6A	-0.680373003	0.087801424	-7.748997342	9.2621E-15	2.76698E-14	negative
PRJNA729271	stage_3W_vs_E10	ordinary_muscle_development	ENSGALG00010027084	OSBPL9	0.268630119	0.093990124	2.858067489	0.004262297	0.006018704	positive
PRJNA729271	stage_3W_vs_E10	ordinary_muscle_development	ENSGALG00010027085	PIM1	0.714375867	0.149245191	4.786592196	1.69637E-06	3.16174E-06	positive
PRJNA729271	stage_3W_vs_E10	ordinary_muscle_development	ENSGALG00010027086	CACNA1S	5.241736715	0.20111144	26.06384164	9.3749E-150	8.8583E-148	positive
PRJNA729271	stage_3W_vs_E10	ordinary_muscle_development	ENSGALG00010027087	RAB7B	0.145855401	0.335565604	0.434655396	0.663812579	0.695899407	positive
PRJNA729271	stage_3W_vs_E10	ordinary_muscle_development	ENSGALG00010027088	AGBL4	-0.043956228	0.263606027	-0.166749708	0.867566992	0.882792808	negative
PRJNA729271	stage_3W_vs_E10	ordinary_muscle_development	ENSGALG00010027092	ENSGALG00010027092	-2.483832883	0.20701987	-11.99804096	3.63806E-33	2.35628E-32	negative
PRJNA729271	stage_3W_vs_E10	ordinary_muscle_development	ENSGALG00010027093	SLMAP	0.648957495	0.076115482	8.525959242	1.51549E-17	5.22526E-17	positive
PRJNA729271	stage_3W_vs_E10	ordinary_muscle_development	ENSGALG00010027095	SCN5A	-3.003108284	0.412045724	-7.288288918	3.13916E-13	8.66378E-13	negative
PRJNA729271	stage_3W_vs_E10	ordinary_muscle_development	ENSGALG00010027096	YIPF1	1.285778314	0.109381262	11.75501441	6.65494E-32	4.11358E-31	positive
PRJNA729271	stage_3W_vs_E10	ordinary_muscle_development	ENSGALG00010027097	ENSGALG00010027097	-0.724252585	0.14935876	-4.849080074	1.24035E-06	2.33432E-06	negative
PRJNA729271	stage_3W_vs_E10	ordinary_muscle_development	ENSGALG00010027098	EXOG	0.582122917	0.14533115	4.005493099	6.18882E-05	0.000102899	positive
PRJNA729271	stage_3W_vs_E10	ordinary_muscle_development	ENSGALG00010027099	GNA11	-0.800042358	0.086169517	-9.284517187	1.62442E-20	6.33296E-20	negative
PRJNA729271	stage_3W_vs_E10	ordinary_muscle_development	ENSGALG00010027100	MIER2	-0.663590394	0.092167992	-7.199792245	6.03044E-13	1.64214E-12	negative
PRJNA729271	stage_3W_vs_E10	ordinary_muscle_development	ENSGALG00010027101	RNF220	-0.325899709	0.080015471	-4.072958681	4.64197E-05	7.79216E-05	negative
PRJNA729271	stage_3W_vs_E10	ordinary_muscle_development	ENSGALG00010027102	DUS3L	-1.007441711	0.1455054	-6.923741028	4.3987E-12	1.14141E-11	negative
PRJNA729271	stage_3W_vs_E10	ordinary_muscle_development	ENSGALG00010027103	ACVR2B	-1.55613838	0.186447444	-8.346257494	7.04601E-17	2.34732E-16	negative
PRJNA729271	stage_3W_vs_E10	ordinary_muscle_development	ENSGALG00010027105	CHERP	-0.658944228	0.129131735	-5.102883723	3.34517E-07	6.53479E-07	negative
PRJNA729271	stage_3W_vs_E10	ordinary_muscle_development	ENSGALG00010027106	CYP2J23	0.775486346	0.142987697	5.423448033	5.84602E-08	1.19924E-07	positive
PRJNA729271	stage_3W_vs_E10	ordinary_muscle_development	ENSGALG00010027107	KIF21B	-1.123833264	0.202398891	-5.552566303	2.81506E-08	5.89998E-08	negative
PRJNA729271	stage_3W_vs_E10	ordinary_muscle_development	ENSGALG00010027108	ENSGALG00010027108	0.17772598	0.141225439	1.258455848	0.208226945	0.240402196	positive
PRJNA729271	stage_3W_vs_E10	ordinary_muscle_development	ENSGALG00010027109	C19orf44	0.439090408	0.122783454	3.576136631	0.000348709	0.000544671	positive
PRJNA729271	stage_3W_vs_E10	ordinary_muscle_development	ENSGALG00010027110	IKBKE	0.921228734	0.202294163	4.553906652	5.26587E-06	9.49836E-06	positive
PRJNA729271	stage_3W_vs_E10	ordinary_muscle_development	ENSGALG00010027111	CALR3	-2.901405926	0.893678033	-3.246589734	0.001167966	0.001740482	negative
PRJNA729271	stage_3W_vs_E10	ordinary_muscle_development	ENSGALG00010027112	XYLB	-1.291502831	0.187443416	-6.890094406	5.57554E-12	1.43931E-11	negative
PRJNA729271	stage_3W_vs_E10	ordinary_muscle_development	ENSGALG00010027113	PIAS4	-0.609331516	0.135924562	-4.482865395	7.36474E-06	1.3129E-05	negative
PRJNA729271	stage_3W_vs_E10	ordinary_muscle_development	ENSGALG00010027114	FLNB	-0.823891162	0.113692375	-7.24667034	4.27142E-13	1.17284E-12	negative
PRJNA729271	stage_3W_vs_E10	ordinary_muscle_development	ENSGALG00010027115	C9ORF152	-1.09142575	0.640814305	-1.703185684	0.088533325	0.107803305	negative
PRJNA729271	stage_3W_vs_E10	ordinary_muscle_development	ENSGALG00010027116	CCDC94	0.137895435	0.102603684	1.343961826	0.178960732	0.208607624	positive
PRJNA729271	stage_3W_vs_E10	ordinary_muscle_development	ENSGALG00010027117	RASSF5	3.136404573	0.228514327	13.72519883	7.17321E-43	6.36716E-42	positive
PRJNA729271	stage_3W_vs_E10	ordinary_muscle_development	ENSGALG00010027118	ABHD6	1.206387039	0.159256847	7.575103131	3.58842E-14	1.04091E-13	positive
PRJNA729271	stage_3W_vs_E10	ordinary_muscle_development	ENSGALG00010027119	NUB1	1.897525411	0.097303843	19.50103251	1.07588E-84	3.07592E-83	positive
PRJNA729271	stage_3W_vs_E10	ordinary_muscle_development	ENSGALG00010027121	ELF3	0.186231097	0.874841974	0.212873985	0.831425254	0.851035171	positive
PRJNA729271	stage_3W_vs_E10	ordinary_muscle_development	ENSGALG00010027122	DNASE1L3	5.041454173	0.247603783	20.36097397	3.71132E-92	1.26877E-90	positive
PRJNA729271	stage_3W_vs_E10	ordinary_muscle_development	ENSGALG00010027123	WDR86	-2.577261812	0.227278523	-11.3396628	8.34654E-30	4.76687E-29	negative
PRJNA729271	stage_3W_vs_E10	ordinary_muscle_development	ENSGALG00010027124	IP6K2	0.835944173	0.173439801	4.819794345	1.43706E-06	2.69264E-06	positive
PRJNA729271	stage_3W_vs_E10	ordinary_muscle_development	ENSGALG00010027125	CRYGN	1.182353076	0.407896386	2.898660333	0.003747606	0.005324599	positive
PRJNA729271	stage_3W_vs_E10	ordinary_muscle_development	ENSGALG00010027126	ENSGALG00010027126	7.673085137	0.534878527	14.34547239	1.13723E-46	1.14713E-45	positive
PRJNA729271	stage_3W_vs_E10	ordinary_muscle_development	ENSGALG00010027127	S1PR4	2.60060314	0.603581839	4.308617275	1.64278E-05	2.85517E-05	positive
PRJNA729271	stage_3W_vs_E10	ordinary_muscle_development	ENSGALG00010027128	EIF2D	0.444195779	0.089867909	4.942763018	7.70231E-07	1.46863E-06	positive
PRJNA729271	stage_3W_vs_E10	ordinary_muscle_development	ENSGALG00010027129	NCLN	0.863824714	0.128491628	6.72280932	1.78254E-11	4.49589E-11	positive
PRJNA729271	stage_3W_vs_E10	ordinary_muscle_development	ENSGALG00010027130	NCKIPSD	-0.117771168	0.145440324	-0.809755951	0.418080454	0.456723166	negative
PRJNA729271	stage_3W_vs_E10	ordinary_muscle_development	ENSGALG00010027131	UBA52	0.850645989	0.152185035	5.589550849	2.27658E-08	4.80474E-08	positive

PRJNA729271	stage_3W_vs_E10	ordinary_muscle_development	ENSGALG00010027133	CELF5	-3.966212577	0.377598106	-10.50379362	8.29769E-26	4.01854E-25	negative
PRJNA729271	stage_3W_vs_E10	ordinary_muscle_development	ENSGALG00010027134	DYRK3	-0.206279151	0.148903299	-1.385322901	0.165953754	0.194588227	negative
PRJNA729271	stage_3W_vs_E10	ordinary_muscle_development	ENSGALG00010027135	PRKAG2	0.615761793	0.138599902	4.44728902	8.88251E-06	1.57398E-05	positive
PRJNA729271	stage_3W_vs_E10	ordinary_muscle_development	ENSGALG00010027136	MAPKAPK2	1.576480465	0.143913501	10.95436117	6.33236E-28	3.36218E-27	positive
PRJNA729271	stage_3W_vs_E10	ordinary_muscle_development	ENSGALG00010027137	FKBP8	0.945110995	0.089351357	10.57746664	3.79069E-26	1.86511E-25	positive
PRJNA729271	stage_3W_vs_E10	ordinary_muscle_development	ENSGALG00010027139	HSD11B1L	-0.014876725	0.142244073	-0.104585907	0.916704398	0.926419177	negative
PRJNA729271	stage_3W_vs_E10	ordinary_muscle_development	ENSGALG00010027141	PIGR	7.754470371	1.38905343	5.582557305	2.37007E-08	4.99117E-08	positive
PRJNA729271	stage_3W_vs_E10	ordinary_muscle_development	ENSGALG00010027143	GALNT11	-1.078740445	0.121392717	-8.886368754	6.31386E-19	2.31109E-18	negative
PRJNA729271	stage_3W_vs_E10	ordinary_muscle_development	ENSGALG00010027144	ENSGALG00010027144	-3.01628802	0.754073133	-3.999994014	6.33441E-05	0.000105247	negative
PRJNA729271	stage_3W_vs_E10	ordinary_muscle_development	ENSGALG00010027145	CELSR3	-1.466652975	0.174450931	-8.407252226	4.19728E-17	1.41389E-16	negative
PRJNA729271	stage_3W_vs_E10	ordinary_muscle_development	ENSGALG00010027146	KXD1	1.730514794	0.120433973	14.36899204	8.10016E-47	8.22211E-46	positive
PRJNA729271	stage_3W_vs_E10	ordinary_muscle_development	ENSGALG00010027147	KMT2C	0.75468144	0.103494626	7.291986724	3.05417E-13	8.43564E-13	positive
PRJNA729271	stage_3W_vs_E10	ordinary_muscle_development	ENSGALG00010027148	ELL	0.984539636	0.165612466	5.944840136	2.76727E-09	6.1436E-09	positive
PRJNA729271	stage_3W_vs_E10	ordinary_muscle_development	ENSGALG00010027149	C1orf116	2.127449406	0.223221991	9.530644348	1.5631E-21	6.36973E-21	positive
PRJNA729271	stage_3W_vs_E10	ordinary_muscle_development	ENSGALG00010027150	YOD1	-0.890781887	0.091288784	-9.757845899	1.70743E-22	7.19873E-22	negative
PRJNA729271	stage_3W_vs_E10	ordinary_muscle_development	ENSGALG00010027151	MICOS13	1.302407253	0.143346664	9.085717234	1.03017E-19	3.89269E-19	positive
PRJNA729271	stage_3W_vs_E10	ordinary_muscle_development	ENSGALG00010027152	C4BPS	4.510044552	0.429518099	10.50024333	8.61577E-26	4.16841E-25	positive
PRJNA729271	stage_3W_vs_E10	ordinary_muscle_development	ENSGALG00010027153	ENSGALG00010027153	3.235515207	0.227948843	14.1940409	9.97417E-46	9.76862E-45	positive
PRJNA729271	stage_3W_vs_E10	ordinary_muscle_development	ENSGALG00010027154	PLPP2	0.096486226	0.137524198	0.701594541	0.482932057	0.521345064	positive
PRJNA729271	stage_3W_vs_E10	ordinary_muscle_development	ENSGALG00010027155	ENSGALG00010027155	2.26028332	0.28938954	7.810521829	5.69517E-15	1.72162E-14	positive
PRJNA729271	stage_3W_vs_E10	ordinary_muscle_development	ENSGALG00010027156	ISYNA1	-2.25987115	0.152872325	-14.78273557	1.89318E-49	2.09799E-48	negative
PRJNA729271	stage_3W_vs_E10	ordinary_muscle_development	ENSGALG00010027157	ENSGALG00010027157	1.689771172	0.249423516	6.774706715	1.24659E-11	3.17117E-11	positive
PRJNA729271	stage_3W_vs_E10	ordinary_muscle_development	ENSGALG00010027158	XRCC2	0.188491248	0.132162783	1.426205189	0.153809122	0.181154077	positive
PRJNA729271	stage_3W_vs_E10	ordinary_muscle_development	ENSGALG00010027159	TMUB1	0.579400748	0.164423197	3.523838239	0.000425344	0.000658974	positive
PRJNA729271	stage_3W_vs_E10	ordinary_muscle_development	ENSGALG00010027160	FASTK	1.841251714	0.16580541	11.1048953	1.18756E-28	6.4772E-28	positive
PRJNA729271	stage_3W_vs_E10	ordinary_muscle_development	ENSGALG00010027161	C4BPG	1.217839511	0.202800024	6.005125082	1.91184E-09	4.27855E-09	positive
PRJNA729271	stage_3W_vs_E10	ordinary_muscle_development	ENSGALG00010027162	SLC4A2	0.794340384	0.129232998	6.146575537	7.91736E-10	1.81643E-09	positive
PRJNA729271	stage_3W_vs_E10	ordinary_muscle_development	ENSGALG00010027163	ENSGALG00010027163	-0.790038632	0.079416692	-9.948017341	2.57257E-23	1.12014E-22	negative
PRJNA729271	stage_3W_vs_E10	ordinary_muscle_development	ENSGALG00010027164	ENSGALG00010027164	1.410988901	0.463439489	3.04460223	0.002329882	0.00337177	positive
PRJNA729271	stage_3W_vs_E10	ordinary_muscle_development	ENSGALG00010027165	ENSGALG00010027165	-3.126971899	0.224755475	-13.91277299	5.29847E-44	4.87627E-43	negative
PRJNA729271	stage_3W_vs_E10	ordinary_muscle_development	ENSGALG00010027166	CDK5	0.177270737	0.099082563	1.789121439	0.07359526	0.090465001	positive
PRJNA729271	stage_3W_vs_E10	ordinary_muscle_development	ENSGALG00010027167	ENSGALG00010027167	-2.369418987	0.496683524	-4.770480342	1.83787E-06	3.41801E-06	negative
PRJNA729271	stage_3W_vs_E10	ordinary_muscle_development	ENSGALG00010027168	SSBP4	-1.518748731	0.185844621	-8.172142545	3.0296E-16	9.7652E-16	negative
PRJNA729271	stage_3W_vs_E10	ordinary_muscle_development	ENSGALG00010027169	ABCB8	-0.012024266	0.112209102	-0.107159449	0.914662485	0.924742465	negative
PRJNA729271	stage_3W_vs_E10	ordinary_muscle_development	ENSGALG00010027170	TLE2	-0.817906694	0.176879806	-4.624081799	3.76261E-06	6.8715E-06	negative
PRJNA729271	stage_3W_vs_E10	ordinary_muscle_development	ENSGALG00010027171	ATG9B	4.603221977	0.273561509	16.82700901	1.54723E-63	2.55144E-62	positive
PRJNA729271	stage_3W_vs_E10	ordinary_muscle_development	ENSGALG00010027172	ENSGALG00010027172	-2.014184006	0.551385854	-3.65294828	0.000259246	0.000408854	negative
PRJNA729271	stage_3W_vs_E10	ordinary_muscle_development	ENSGALG00010027173	ENSGALG00010027173	-1.012261066	0.097816928	-10.34852644	4.24976E-25	1.99936E-24	negative
PRJNA729271	stage_3W_vs_E10	ordinary_muscle_development	ENSGALG00010027174	SHISA5	-0.27516962	0.132772341	-2.072492043	0.038219578	0.048711226	negative
PRJNA729271	stage_3W_vs_E10	ordinary_muscle_development	ENSGALG00010027175	NOS3	0.823130422	0.164514153	5.003401864	5.63273E-07	1.08328E-06	positive
PRJNA729271	stage_3W_vs_E10	ordinary_muscle_development	ENSGALG00010027176	ENSGALG00010027176	0.513844877	0.170531185	3.013201817	0.002585069	0.003727674	positive
PRJNA729271	stage_3W_vs_E10	ordinary_muscle_development	ENSGALG00010027178	C4BPM	1.038993642	0.08048439	12.90925655	3.99136E-38	3.01327E-37	positive
PRJNA729271	stage_3W_vs_E10	ordinary_muscle_development	ENSGALG00010027183	CD34	0.084676659	0.157529279	0.537529653	0.590901808	0.626471745	positive
PRJNA729271	stage_3W_vs_E10	ordinary_muscle_development	ENSGALG00010027184	MPND	0.770260192	0.321126031	2.398622712	0.016456861	0.021844524	positive
PRJNA729271	stage_3W_vs_E10	ordinary_muscle_development	ENSGALG00010027185	ATRIP	-1.215466824	0.08397716	-14.47377863	1.7745E-47	1.84515E-46	negative
PRJNA729271	stage_3W_vs_E10	ordinary_muscle_development	ENSGALG00010027186	PLXNA2	-0.74250601	0.125856866	-5.899606706	3.64369E-09	8.03519E-09	negative
PRJNA729271	stage_3W_vs_E10	ordinary_muscle_development	ENSGALG00010027187	ENSGALG00010027187	-0.538219026	0.137401422	-3.917128492	8.961E-05	0.000146785	negative
PRJNA729271	stage_3W_vs_E10	ordinary_muscle_development	ENSGALG00010027188	KCNH2	0.203612868	0.263198724	0.773608871	0.439162124	0.47783028	positive
PRJNA729271	stage_3W_vs_E10	ordinary_muscle_development	ENSGALG00010027189	ENSGALG00010027189	-1.731302031	0.314954921	-5.496983577	3.86343E-08	8.03103E-08	negative
PRJNA729271	stage_3W_vs_E10	ordinary_muscle_development	ENSGALG00010027190	IPPK	0.115672009	0.114435227	1.010807698	0.312108478	0.350047384	positive
PRJNA729271	stage_3W_vs_E10	ordinary_muscle_development	ENSGALG00010027192	SH3GL1	-1.714206337	0.117786731	-14.55347579	5.55094E-48	5.91624E-47	negative
PRJNA729271	stage_3W_vs_E10	ordinary_muscle_development	ENSGALG00010027193	CAMK1G	-3.731589631	0.683168209	-5.462182785	4.70315E-08	9.71114E-08	negative
PRJNA729271	stage_3W_vs_E10	ordinary_muscle_development	ENSGALG00010027194	ENSGALG00010027194	-2.084881858	0.267011616	-7.808206574	5.80075E-15	1.75243E-14	negative
PRJNA729271	stage_3W_vs_E10	ordinary_muscle_development	ENSGALG00010027195	CENPP	-3.087893981	0.224501164	-13.75446756	4.78793E-43	4.27346E-42	negative
PRJNA729271	stage_3W_vs_E10	ordinary_muscle_development	ENSGALG00010027196	ENSGALG00010027196	0.906141126	0.327871438	2.763708637	0.005714854	0.007962781	positive
PRJNA729271	stage_3W_vs_E10	ordinary_muscle_development	ENSGALG00010027197	ENSGALG00010027197	-2.690143253	0.60828962	-4.422471081	9.75784E-06	1.72341E-05	negative
PRJNA729271	stage_3W_vs_E10	ordinary_muscle_development	ENSGALG00010027198	ECM2	0.80981078	0.203124202	3.986776432	6.69771E-05	0.000111054	positive
PRJNA729271	stage_3W_vs_E10	ordinary_muscle_development	ENSGALG00010027199	LAMB3	-0.953341014	0.319621468	-2.982718963	0.002857002	0.004103879	negative
PRJNA729271	stage_3W_vs_E10	ordinary_muscle_development	ENSGALG00010027200	G0S2	4.719364378	0.317381231	14.86970215	5.18435E-50	5.87546E-49	positive
PRJNA729271	stage_3W_vs_E10	ordinary_muscle_development	ENSGALG00010027201	ASPN	0.685018703	0.316021274	2.167634775	0.030186486	0.038917825	positive
PRJNA729271	stage_3W_vs_E10	ordinary_muscle_development	ENSGALG00010027202	CHAF1A	-1.829637112	0.087499254	-20.91031674	4.3136E-97	1.64541E-95	negative
PRJNA729271	stage_3W_vs_E10	ordinary_muscle_development	ENSGALG00010027203	ENSGALG00010027203	-1.308344575	0.310701368	-4.210939211	2.54311E-05	4.35057E-05	negative
PRJNA729271	stage_3W_vs_E10	ordinary_muscle_development	ENSGALG00010027204	OMD	-4.949956829	0.933510218	-5.302520242	1.14215E-07	2.30417E-07	negative
PRJNA729271	stage_3W_vs_E10	ordinary_muscle_development	ENSGALG00010027205	OGN	0.603850155	0.221892951	2.721357987	0.006501431	0.009009393	positive
PRJNA729271	stage_3W_vs_E10	ordinary_muscle_development	ENSGALG00010027206	ENSGALG00010027206	6.165206734	0.973274316	6.334500597	2.38111E-10	5.63682E-10	positive
PRJNA729271	stage_3W_vs_E10	ordinary_muscle_development	ENSGALG00010027207	NOL8	-0.207762163	0.123834093	-1.677746072	0.093396681	0.113401315	negative
PRJNA729271	stage_3W_vs_E10	ordinary_muscle_development	ENSGALG00010027208	UBXN6	0.990336712	0.093339884	10.6100577	2.67733E-26	1.32405E-25	positive
PRJNA729271	stage_3W_vs_E10	ordinary_muscle_development	ENSGALG00010027209	TRAF3IP3	2.212470656	0.235412288	9.398280238	5.54621E-21	2.20555E-20	positive
PRJNA729271	stage_3W_vs_E10	ordinary_muscle_development	ENSGALG00010027210	ENSGALG00010027210	-0.6505776	0.263843488	-2.465770919	0.013671874	0.018312125	negative
PRJNA729271	stage_3W_vs_E10	ordinary_muscle_development	ENSGALG00010027211	HDGFRP2	-0.721331398	0.101921839	-7.077299704	1.4699E-12	3.93032E-12	negative
PRJNA729271	stage_3W_vs_E10	ordinary_muscle_development	ENSGALG00010027212	IRF6	-3.57633407	0.560366628	-6.382132502	1.74639E-10	4.16278E-10	negative
PRJNA729271	stage_3W_vs_E10	ordinary_muscle_development	ENSGALG00010027213	KCNC4	0.029148149	0.349668667	0.08335934	0.933565818	0.941293581	positive
PRJNA729271	stage_3W_vs_E10	ordinary_muscle_development	ENSGALG00010027214	PLIN4	0.620034402	0.266317894	2.328174024	0.019902865	0.026195589	positive
PRJNA729271	stage_3W_vs_E10	ordinary_muscle_development	ENSGALG00010027215	ENSGALG00010027215	-2.325818687	0.200769136	-11.584543	4.93592E-31	2.94793E-30	negative
PRJNA729271	stage_3W_vs_E10	ordinary_muscle_development	ENSGALG00010027216	ENSGALG00010027216	1.774903972	0.567951129	3.125099825	0.001777447	0.002600333	positive
PRJNA729271	stage_3W_vs_E10	ordinary_muscle_development	ENSGALG00010027217	SEMA6B	0.369887303	0.2205915	1.676797623	0.093582063	0.11360737	positive
PRJNA729271	stage_3W_vs_E10	ordinary_muscle_development	ENSGALG00010027218	IARS1	0.137282487	0.164357589	0.835267102	0.403567336	0.442723722	positive
PRJNA729271	stage_3W_vs_E10	ordinary_muscle_development	ENSGALG00010027219	ENSGALG00010027219	0.181618972	0.137786841	1.318115512	0.187464982	0.217889898	positive
PRJNA729271	stage_3W_vs_E10	ordinary_muscle_development	ENSGALG00010027220	ANKRD24	-0.901306074	0.58192509	-1.548835218	0.121421337	0.145047164	negative
PRJNA729271	stage_3W_vs_E10	ordinary_muscle_development	ENSGALG00010027221	LRRC61	-0.714051966	0.274243759	-2.603712729	0.009222002	0.012585718	negative
PRJNA729271	stage_3W_vs_E10	ordinary_muscle_development	ENSGALG00010027222	GUCA1B	0.485815168	0.951948962	0.51033741	0.609815098	0.644899885	positive
PRJNA729271	stage_3W_vs_E10	ordinary_muscle_development	ENSGALG00010027223	SIRT6	-0.41612297	0.083544745	-4.980839566	6.3309E-07	1.21448E-06	negative
PRJNA729271	stage_3W_vs_E10	ordinary_muscle_development	ENSGALG00010027224	RARRES2	0.794240188	0.120993216	6.564336535	5.2265E-11	1.28273E-10	positive
PRJNA729271	stage_3W_vs_E10	ordinary_muscle_development	ENSGALG00010027225	CREB3L3	-0.939754561	0.744604856	-1.26208492	0.206918231	0.239043577	negative
PRJNA729271	stage_3W_vs_E10	ordinary_muscle_development	ENSGALG00010027227	ENSGALG00010027227	1.61830351	0.182769076	8.854361714	8.4164E-19	3.06522E-18	positive
PRJNA729271	stage_3W_vs_E10	ordinary_muscle_development	ENSGALG00010027229	MDM4	0.499357712	0.080111607	6.233275437	4.56782E-10	1.06414E-09	positive
PRJNA729271	stage_3W_vs_E10	ordinary_muscle_development	ENSGALG00010027230	MAP2K2	-0.456424868	0.081822083	-5.578260188	2.42936E-08	5.11305E-08	negative
PRJNA729271	stage_3W_vs_E10	ordinary_muscle_development	ENSGALG00010027231	ENSGALG00010027231	-1.299992913	0.159478228	-8.151538476	3.59323E-16	1.15153E-15	negative
PRJNA729271	stage_3W_vs_E10	ordinary_muscle_development	ENSGALG00010027232	ARRDC2	3.182829114	0.326835981	9.738306977	2.06974E-22	8.69842E-22	positive
PRJNA729271	stage_3W_vs_E10	ordinary_muscle_development	ENSGALG00010027233	ENSGALG00010027233	1.567615158	0.258304572	6.068863386	1.28819E-09	2.91709E-09	positive
PRJNA729271	stage_3W_vs_E10	ordinary_muscle_development	ENSGALG00010027234	SLC6A11	-5.316262703	0.716816405	-7.4164914	1.20264E-13	3.39942E-13	negative
PRJNA729271	stage_3W_vs_E10	ordinary_muscle_development	ENSGALG00010027236	PEX11G	-0.090684174	0.281666964	-0.321955308	0.747486552	0.774247361	negative
PRJNA729271	stage_3W_vs_E10	ordinary_muscle_development	ENSGALG00010027237	CTC-575C13.4	-0.002310639	0.162975656	-0.014177815	0.988688119	0.990396288	negative
PRJNA729271	stage_3W_vs_E10	ordinary_muscle_development	ENSGALG00010027239	ENSGALG00010027239	-0.5256271	0.084816188	-6.197249769	5.74583E-10	1.32981E-09	negative
PRJNA729271	stage_3W_vs_E10	ordinary_muscle_development	ENSGALG00010027240	SLC6A1	0.053826623	0.321498231	0.167424321	0.86703619	0.882376576	positive
PRJNA729271	stage_3W_vs_E10	ordinary_muscle_development	ENSGALG00010027241	LRRN2	-1.148415256	0.262061119	-4.382242062	1.17464E-05	2.06381E-05	negative
PRJNA729271	stage_3W_vs_E10	ordinary_muscle_development	ENSGALG00010027242	ENSGALG00010027242	0.197755031	0.190914826	1.035828571	0.300282126	0.337855268	positive
PRJNA729271	stage_3W_vs_E10	ordinary_muscle_development	ENSGALG00010027243	HRH1	0.651914466	0.198001956	3.292464774	0.000993133	0.001488058	positive
PRJNA729271	stage_3W_vs_E10	ordinary_muscle_development	ENSGALG00010027244	ARHGEF18	0.278883178	0.090181244	3.092474294	0.001984954	0.002890197	positive
PRJNA729271	stage_3W_vs_E10	ordinary_muscle_development	ENSGALG00010027245	LRIG2	-0.21303896	0.066574457	-3.200010453	0.001374226	0.002033838	negative
PRJNA729271	stage_3W_vs_E10	ordinary_muscle_development	ENSGALG00010027246	ZNF767	-0.853485088	0.100149315	-8.522126105	1.56651E-17	5.39347E-17	negative
PRJNA729271	stage_3W_vs_E10	ordinary_muscle_development	ENSGALG00010027247	ATG7	0.027627595	0.079461401	0.347685732	0.728076213	0.756087169	positive
PRJNA729271	stage_3W_vs_E10	ordinary_muscle_development	ENSGALG00010027248	ENSGALG00010027248	-1.109526859	0.647765052	-1.712853845	0.086739431	0.105743318	negative
PRJNA729271	stage_3W_vs_E10	ordinary_muscle_development	ENSGALG00010027249	C2CD4C	3.265116466	1.10342372	2.959077647	0.003085613	0.004422184	positive
PRJNA729271	stage_3W_vs_E10	ordinary_muscle_development	ENSGALG00010027250	ENSGALG00010027250	-0.395027291	0.17359676	-2.275545304	0.022873238	0.029920367	negative
PRJNA729271	stage_3W_vs_E10	ordinary_muscle_development	ENSGALG00010027251	ENSGALG00010027251	0.21701617	0.199803752	1.08614662	0.27741413	0.314297156	positive
PRJNA729271	stage_3W_vs_E10	ordinary_muscle_development	ENSGALG00010027252	MAGI3	-0.678465242	0.072667423	-9.336580487	9.94956E-21	3.92112E-20	negative
PRJNA729271	stage_3W_vs_E10	ordinary_muscle_development	ENSGALG00010027253	VGLL4	-0.980071164	0.088099552	-11.12458742	9.52455E-29	5.21368E-28	negative
PRJNA729271	stage_3W_vs_E10	ordinary_muscle_development	ENSGALG00010027254	INSR	0.834538997	0.133137464	6.268250661	3.65127E-10	8.54872E-10	positive
PRJNA729271	stage_3W_vs_E10	ordinary_muscle_development	ENSGALG00010027255	ENSGALG00010027255	-0.270526687	0.181041314	-1.494281505	0.135102057	0.160490438	negative
PRJNA729271	stage_3W_vs_E10	ordinary_muscle_development	ENSGALG00010027256	PHTF1	-1.623754353	0.090585876	-17.92502783	7.52129E-72	1.53767E-70	negative
PRJNA729271	stage_3W_vs_E10	ordinary_muscle_development	ENSGALG00010027257	TAFA3	1.791452296	0.267276187	6.702625904	2.04707E-11	5.14697E-11	positive
PRJNA729271	stage_3W_vs_E10	ordinary_muscle_development	ENSGALG00010027258	ENSGALG00010027258	-0.886934341	0.169606325	-5.22937066	1.70088E-07	3.38611E-07	negative
PRJNA729271	stage_3W_vs_E10	ordinary_muscle_development	ENSGALG00010027259	ENSGALG00010027259	-0.582971544	0.085965866	-6.781430514	1.18992E-11	3.03019E-11	negative
PRJNA729271	stage_3W_vs_E10	ordinary_muscle_development	ENSGALG00010027260	LEMD2	0.166637979	0.100717389	1.65451052	0.098023828	0.118641899	positive
PRJNA729271	stage_3W_vs_E10	ordinary_muscle_development	ENSGALG00010027261	ENSGALG00010027261	-0.222467992	0.111428422	-1.996510289	0.045878407	0.057897224	negative
PRJNA729271	stage_3W_vs_E10	ordinary_muscle_development	ENSGALG00010027262	USE1	-0.135824868	0.134612271	-1.009008068	0.312970765	0.350878741	negative
PRJNA729271	stage_3W_vs_E10	ordinary_muscle_development	ENSGALG00010027263	TAMM41	-1.372781148	0.127803398	-10.74135097	6.50849E-27	3.31835E-26	negative
PRJNA729271	stage_3W_vs_E10	ordinary_muscle_development	ENSGALG00010027264	ENSGALG00010027264	1.094479625	0.093521348	11.70299237	1.23037E-31	7.5123E-31	positive
PRJNA729271	stage_3W_vs_E10	ordinary_muscle_development	ENSGALG00010027265	IP6K3	4.859900465	0.207638596	23.40557369	3.7501E-121	2.2839E-119	positive
PRJNA729271	stage_3W_vs_E10	ordinary_muscle_development	ENSGALG00010027267	MYO9B	-0.235024959	0.095423881	-2.462957442	0.013779627	0.018446222	negative
PRJNA729271	stage_3W_vs_E10	ordinary_muscle_development	ENSGALG00010027268	UQCRC1	0.936055904	0.126647475	7.391034894	1.4569E-13	4.09737E-13	positive
PRJNA729271	stage_3W_vs_E10	ordinary_muscle_development	ENSGALG00010027269	CDC25A	-1.522613337	0.104706304	-14.54175426	6.58824E-48	6.99608E-47	negative
PRJNA729271	stage_3W_vs_E10	ordinary_muscle_development	ENSGALG00010027270	ENSGALG00010027270	-3.540402428	0.437445633	-8.093354155	5.80438E-16	1.84303E-15	negative
PRJNA729271	stage_3W_vs_E10	ordinary_muscle_development	ENSGALG00010027271	INKA2	-1.454177957	0.149890298	-9.701614964	2.96764E-22	1.24216E-21	negative
PRJNA729271	stage_3W_vs_E10	ordinary_muscle_development	ENSGALG00010027272	DDX20	-0.19744263	0.102455763	-1.927101254	0.053967013	0.067458766	negative
PRJNA729271	stage_3W_vs_E10	ordinary_muscle_development	ENSGALG00010027273	HAUS8	-1.735693422	0.134641794	-12.8911935	5.04568E-38	3.80131E-37	negative
PRJNA729271	stage_3W_vs_E10	ordinary_muscle_development	ENSGALG00010027274	COPG1	-0.814448363	0.060988581	-13.35411234	1.12086E-40	9.21549E-40	negative
PRJNA729271	stage_3W_vs_E10	ordinary_muscle_development	ENSGALG00010027275	CPAMD8	0.049662912	0.227287533	0.218502579	0.827037549	0.847501539	positive
PRJNA729271	stage_3W_vs_E10	ordinary_muscle_development	ENSGALG00010027276	ENSGALG00010027276	0.03080834	0.138511634	0.222424208	0.823983669	0.844969457	positive
PRJNA729271	stage_3W_vs_E10	ordinary_muscle_development	ENSGALG00010027277	KCND3	1.844830425	0.387530189	4.760481839	1.93131E-06	3.58581E-06	positive

PRJNA729271	stage_3W_vs_E10	ordinary_muscle_development	ENSGALG00010027278	H1-10	-1.163933731	0.129432694	-8.992579029	2.41496E-19	9.01489E-19	negative
PRJNA729271	stage_3W_vs_E10	ordinary_muscle_development	ENSGALG00010027279	F2RL3	1.055209385	0.346149405	3.048421777	0.002300468	0.003331197	positive
PRJNA729271	stage_3W_vs_E10	ordinary_muscle_development	ENSGALG00010027280	HMCES	-0.669108808	0.12601844	-5.309610314	1.0986E-07	2.21847E-07	negative
PRJNA729271	stage_3W_vs_E10	ordinary_muscle_development	ENSGALG00010027281	ENSGALG00010027281	-0.93045703	0.265053334	-3.510452091	0.000447345	0.000692025	negative
PRJNA729271	stage_3W_vs_E10	ordinary_muscle_development	ENSGALG00010027282	ENSGALG00010027282	2.361243813	0.128320848	18.40109258	1.28743E-75	2.89771E-74	positive
PRJNA729271	stage_3W_vs_E10	ordinary_muscle_development	ENSGALG00010027283	RAB7A	0.608465889	0.062603313	9.719387996	2.49275E-22	1.04429E-21	positive
PRJNA729271	stage_3W_vs_E10	ordinary_muscle_development	ENSGALG00010027284	CTTNBP2NL	1.211275347	0.089743155	13.49713348	1.6258E-41	1.37331E-40	positive
PRJNA729271	stage_3W_vs_E10	ordinary_muscle_development	ENSGALG00010027285	PTPRS	-1.194623858	0.112208434	-10.6464712	1.81101E-26	9.07067E-26	negative
PRJNA729271	stage_3W_vs_E10	ordinary_muscle_development	ENSGALG00010027286	RPN1	0.020201756	0.062827521	0.321543104	0.747798853	0.774515497	positive
PRJNA729271	stage_3W_vs_E10	ordinary_muscle_development	ENSGALG00010027287	DHX30	-0.658488939	0.089844919	-7.329172836	2.31578E-13	6.44036E-13	negative
PRJNA729271	stage_3W_vs_E10	ordinary_muscle_development	ENSGALG00010027288	DAG1	1.015338159	0.167871182	6.048317205	1.46367E-09	3.30156E-09	positive
PRJNA729271	stage_3W_vs_E10	ordinary_muscle_development	ENSGALG00010027289	SMARCC1	-1.166225816	0.139651168	-8.350992236	6.76919E-17	2.25821E-16	negative
PRJNA729271	stage_3W_vs_E10	ordinary_muscle_development	ENSGALG00010027290	CSPG5	-5.353034355	0.470688903	-11.37276516	5.7148E-30	3.27933E-29	negative
PRJNA729271	stage_3W_vs_E10	ordinary_muscle_development	ENSGALG00010027291	MST1	2.34716202	0.272490513	8.613738484	7.07159E-18	2.48191E-17	positive
PRJNA729271	stage_3W_vs_E10	ordinary_muscle_development	ENSGALG00010027292	KDM4B	0.157848415	0.0606629	2.602058505	0.009266604	0.012641829	positive
PRJNA729271	stage_3W_vs_E10	ordinary_muscle_development	ENSGALG00010027293	ELP6	-0.177332901	0.150083244	-1.181563621	0.237378895	0.271614074	negative
PRJNA729271	stage_3W_vs_E10	ordinary_muscle_development	ENSGALG00010027295	ENSGALG00010027295	2.701973623	0.234526831	11.52095736	1.03455E-30	6.11076E-30	positive
PRJNA729271	stage_3W_vs_E10	ordinary_muscle_development	ENSGALG00010027297	ENSGALG00010027297	-0.258783778	2.135263231	-0.121195258	0.90353638	0.914769842	negative
PRJNA729271	stage_3W_vs_E10	ordinary_muscle_development	ENSGALG00010027299	PGPEP1	-0.367462528	0.163855248	-2.242604564	0.024922326	0.032431018	negative
PRJNA729271	stage_3W_vs_E10	ordinary_muscle_development	ENSGALG00010027301	SCAP	-0.734338865	0.065675468	-11.18132666	5.03319E-29	2.78246E-28	negative
PRJNA729271	stage_3W_vs_E10	ordinary_muscle_development	ENSGALG00010027303	APEH	0.256092522	0.139330771	1.83801841	0.066059693	0.08168702	positive
PRJNA729271	stage_3W_vs_E10	ordinary_muscle_development	ENSGALG00010027304	PTPN23	0.735152941	0.13767771	5.339665679	9.31181E-08	1.88987E-07	positive
PRJNA729271	stage_3W_vs_E10	ordinary_muscle_development	ENSGALG00010027305	LSM4	-0.973065266	0.130541033	-7.454095027	9.04868E-14	2.57178E-13	negative
PRJNA729271	stage_3W_vs_E10	ordinary_muscle_development	ENSGALG00010027306	ENSGALG00010027306	4.419068933	0.313156861	14.11135913	3.23287E-45	3.11365E-44	positive
PRJNA729271	stage_3W_vs_E10	ordinary_muscle_development	ENSGALG00010027307	JUND	0.379235984	0.176272304	2.151421271	0.031442964	0.040440617	positive
PRJNA729271	stage_3W_vs_E10	ordinary_muscle_development	ENSGALG00010027309	ENSGALG00010027309	5.047828394	0.650964718	7.754380932	8.87755E-15	2.65484E-14	positive
PRJNA729271	stage_3W_vs_E10	ordinary_muscle_development	ENSGALG00010027310	ARHGEF2	-0.250731219	0.133386071	-1.879740644	0.060143433	0.074785885	negative
PRJNA729271	stage_3W_vs_E10	ordinary_muscle_development	ENSGALG00010027311	NFASC	2.310303282	0.239534366	9.644976317	5.16231E-22	2.14099E-21	positive
PRJNA729271	stage_3W_vs_E10	ordinary_muscle_development	ENSGALG00010027312	ENSGALG00010027312	3.913802744	0.612310217	6.391862545	1.63877E-10	3.91077E-10	positive
PRJNA729271	stage_3W_vs_E10	ordinary_muscle_development	ENSGALG00010027313	BSN	-0.890246369	0.248793732	-3.5782508	0.000345901	0.000540518	negative
PRJNA729271	stage_3W_vs_E10	ordinary_muscle_development	ENSGALG00010027314	ENSGALG00010027314	2.95972201	0.350477913	8.444817489	3.04521E-17	1.03204E-16	positive
PRJNA729271	stage_3W_vs_E10	ordinary_muscle_development	ENSGALG00010027315	RAB3A	-0.06147146	0.231362714	-0.265693028	0.790475633	0.813832254	negative
PRJNA729271	stage_3W_vs_E10	ordinary_muscle_development	ENSGALG00010027316	TRAIP	-4.358296419	0.238240854	-18.29365679	9.29643E-75	2.04479E-73	negative
PRJNA729271	stage_3W_vs_E10	ordinary_muscle_development	ENSGALG00010027317	MPV17L2	1.020997695	0.145316807	7.026012466	2.12519E-12	5.63054E-12	positive
PRJNA729271	stage_3W_vs_E10	ordinary_muscle_development	ENSGALG00010027319	MST1R	2.333462329	0.318230827	7.332609323	2.25714E-13	6.28091E-13	positive
PRJNA729271	stage_3W_vs_E10	ordinary_muscle_development	ENSGALG00010027320	MON1A	1.26589507	0.160251973	7.899403959	2.8024E-15	8.59155E-15	positive
PRJNA729271	stage_3W_vs_E10	ordinary_muscle_development	ENSGALG00010027321	IFI30	3.208521623	0.202932554	15.81077831	2.62207E-56	3.56538E-55	positive
PRJNA729271	stage_3W_vs_E10	ordinary_muscle_development	ENSGALG00010027322	CRABP2	-6.907586537	0.223731487	-30.87444967	2.6319E-209	5.1553E-207	negative
PRJNA729271	stage_3W_vs_E10	ordinary_muscle_development	ENSGALG00010027323	ISG20L2	-0.40242553	0.130353866	-3.087177559	0.002020669	0.002939541	negative
PRJNA729271	stage_3W_vs_E10	ordinary_muscle_development	ENSGALG00010027324	PIK3R2	-1.125603693	0.098103259	-11.47366254	1.78923E-30	1.04703E-29	negative
PRJNA729271	stage_3W_vs_E10	ordinary_muscle_development	ENSGALG00010027325	METTL25B	-0.122607889	0.292475612	-0.419207222	0.675064695	0.706284324	negative
PRJNA729271	stage_3W_vs_E10	ordinary_muscle_development	ENSGALG00010027326	MRPL24	0.621132594	0.117549942	5.283988953	1.26401E-07	2.54011E-07	positive
PRJNA729271	stage_3W_vs_E10	ordinary_muscle_development	ENSGALG00010027327	RBM6	-0.315028756	0.088837336	-3.54613014	0.000390933	0.000607741	negative
PRJNA729271	stage_3W_vs_E10	ordinary_muscle_development	ENSGALG00010027328	RBM15	-0.360615236	0.131569618	-2.740870141	0.006127672	0.008512613	negative
PRJNA729271	stage_3W_vs_E10	ordinary_muscle_development	ENSGALG00010027329	MINDY4	-1.393252077	0.122141777	-11.40684301	3.86504E-30	2.23024E-29	negative
PRJNA729271	stage_3W_vs_E10	ordinary_muscle_development	ENSGALG00010027330	ENSGALG00010027330	-0.18911615	0.200066069	-0.945268485	0.344521824	0.382992855	negative
PRJNA729271	stage_3W_vs_E10	ordinary_muscle_development	ENSGALG00010027331	SLC16A4	0.659931086	0.163399444	4.038759678	5.37346E-05	8.97848E-05	positive
PRJNA729271	stage_3W_vs_E10	ordinary_muscle_development	ENSGALG00010027332	HDGF	1.007424631	0.17653167	5.70676428	1.15144E-08	2.47591E-08	positive
PRJNA729271	stage_3W_vs_E10	ordinary_muscle_development	ENSGALG00010027333	PROK1	-0.235075036	0.293802515	-0.800112403	0.423645676	0.462092262	negative
PRJNA729271	stage_3W_vs_E10	ordinary_muscle_development	ENSGALG00010027334	PRCC	-0.449854838	0.115063074	-3.909636877	9.2435E-05	0.000151258	negative
PRJNA729271	stage_3W_vs_E10	ordinary_muscle_development	ENSGALG00010027335	MAST3	0.345427552	0.174468544	1.979884417	0.047716518	0.060059997	positive
PRJNA729271	stage_3W_vs_E10	ordinary_muscle_development	ENSGALG00010027336	RAD54L2	-0.219364545	0.138448918	-1.584443905	0.113092737	0.135735632	negative
PRJNA729271	stage_3W_vs_E10	ordinary_muscle_development	ENSGALG00010027337	AQP1	-0.698351186	0.265711112	-2.628234781	0.008582926	0.011758932	negative
PRJNA729271	stage_3W_vs_E10	ordinary_muscle_development	ENSGALG00010027339	LAMTOR5	0.788937174	0.107082707	7.367549799	1.73793E-13	4.86881E-13	positive
PRJNA729271	stage_3W_vs_E10	ordinary_muscle_development	ENSGALG00010027340	ENSGALG00010027340	3.599538326	0.729877976	4.931698781	8.15176E-07	1.5533E-06	positive
PRJNA729271	stage_3W_vs_E10	ordinary_muscle_development	ENSGALG00010027341	ENSGALG00010027341	2.363704618	0.580646888	4.070812511	4.68494E-05	7.86157E-05	positive
PRJNA729271	stage_3W_vs_E10	ordinary_muscle_development	ENSGALG00010027346	SIN3B	0.131240404	0.088908645	1.476126475	0.139909943	0.165875562	positive
PRJNA729271	stage_3W_vs_E10	ordinary_muscle_development	ENSGALG00010027348	ENSGALG00010027348	0.805832988	0.205268234	3.925755926	8.64578E-05	0.000141862	positive
PRJNA729271	stage_3W_vs_E10	ordinary_muscle_development	ENSGALG00010027350	INSRR	2.121718387	0.333508216	6.36181744	1.9938E-10	4.73851E-10	positive
PRJNA729271	stage_3W_vs_E10	ordinary_muscle_development	ENSGALG00010027351	TMEM38A	3.947150536	0.147846339	26.69765487	5.0114E-157	5.2637E-155	positive
PRJNA729271	stage_3W_vs_E10	ordinary_muscle_development	ENSGALG00010027354	ENSGALG00010027354	4.012587859	0.815988408	4.917456941	8.76757E-07	1.66758E-06	positive
PRJNA729271	stage_3W_vs_E10	ordinary_muscle_development	ENSGALG00010027355	ADCYAP1R1	-2.022440996	0.269116757	-7.51510616	5.68648E-14	1.63575E-13	negative
PRJNA729271	stage_3W_vs_E10	ordinary_muscle_development	ENSGALG00010027356	ENSGALG00010027356	0.407433409	0.223592922	1.822210673	0.068423026	0.084400626	positive
PRJNA729271	stage_3W_vs_E10	ordinary_muscle_development	ENSGALG00010027358	RNF126	0.148627303	0.099946796	1.487064203	0.136997855	0.162595915	positive
PRJNA729271	stage_3W_vs_E10	ordinary_muscle_development	ENSGALG00010027359	ENSGALG00010027359	1.074451007	0.11655914	9.218075928	3.02479E-20	1.16887E-19	positive
PRJNA729271	stage_3W_vs_E10	ordinary_muscle_development	ENSGALG00010027360	SMIM7	0.860761479	0.113382988	7.591628134	3.15911E-14	9.19687E-14	positive
PRJNA729271	stage_3W_vs_E10	ordinary_muscle_development	ENSGALG00010027361	FANCD2	0.288931645	0.192451129	1.501324756	0.133271585	0.158471831	positive
PRJNA729271	stage_3W_vs_E10	ordinary_muscle_development	ENSGALG00010027362	ENSGALG00010027362	0.0727652	0.144458365	0.503710537	0.614464802	0.649268648	positive
PRJNA729271	stage_3W_vs_E10	ordinary_muscle_development	ENSGALG00010027363	VIPR1	0.04070341	0.257762863	0.157910297	0.874527484	0.888939403	positive
PRJNA729271	stage_3W_vs_E10	ordinary_muscle_development	ENSGALG00010027364	FSTL3	1.679252167	0.233918007	7.178806746	7.03225E-13	1.91135E-12	positive
PRJNA729271	stage_3W_vs_E10	ordinary_muscle_development	ENSGALG00010027365	PLEKHA6	1.359319999	0.169185791	8.034480857	9.39759E-16	2.951E-15	positive
PRJNA729271	stage_3W_vs_E10	ordinary_muscle_development	ENSGALG00010027366	USP4	0.651591551	0.082243957	7.922667814	2.32468E-15	7.15267E-15	positive
PRJNA729271	stage_3W_vs_E10	ordinary_muscle_development	ENSGALG00010027367	SEC22C	-0.664517139	0.095717158	-6.942508076	3.85199E-12	1.00368E-11	negative
PRJNA729271	stage_3W_vs_E10	ordinary_muscle_development	ENSGALG00010027368	EMC3	1.450582268	0.087769802	16.52712239	2.34054E-61	3.63235E-60	positive
PRJNA729271	stage_3W_vs_E10	ordinary_muscle_development	ENSGALG00010027369	ENSGALG00010027369	0.701573086	0.1016108	6.904512973	5.0376E-12	1.30463E-11	positive
PRJNA729271	stage_3W_vs_E10	ordinary_muscle_development	ENSGALG00010027370	NUP210L	-1.139272047	0.350290903	-3.252359792	0.00114451	0.001707207	negative
PRJNA729271	stage_3W_vs_E10	ordinary_muscle_development	ENSGALG00010027372	PIK3C2B	-0.460532572	0.173391901	-2.656021233	0.007906864	0.010870719	negative
PRJNA729271	stage_3W_vs_E10	ordinary_muscle_development	ENSGALG00010027373	MLN	3.952465099	1.126175046	3.509636547	0.00044872	0.000694002	positive
PRJNA729271	stage_3W_vs_E10	ordinary_muscle_development	ENSGALG00010027374	NKTR	0.049449736	0.118499604	0.417298748	0.676459904	0.707554215	positive
PRJNA729271	stage_3W_vs_E10	ordinary_muscle_development	ENSGALG00010027375	FUT7	2.037843361	0.409539204	4.975942083	6.49311E-07	1.24511E-06	positive
PRJNA729271	stage_3W_vs_E10	ordinary_muscle_development	ENSGALG00010027376	DSTYK	0.445152358	0.079448969	5.60299731	2.10676E-08	4.45609E-08	positive
PRJNA729271	stage_3W_vs_E10	ordinary_muscle_development	ENSGALG00010027378	ZBTB47	0.080141852	0.164632487	0.486792455	0.626405418	0.660585415	positive
PRJNA729271	stage_3W_vs_E10	ordinary_muscle_development	ENSGALG00010027379	OPTC	-1.64166763	0.686818018	-2.390251255	0.01683685	0.02232234	negative
PRJNA729271	stage_3W_vs_E10	ordinary_muscle_development	ENSGALG00010027380	GPX1	0.182427636	0.132486536	1.376952267	0.168527006	0.197445761	positive
PRJNA729271	stage_3W_vs_E10	ordinary_muscle_development	ENSGALG00010027381	KLHL40	1.137763225	0.197054142	5.773861007	7.74754E-09	1.6824E-08	positive
PRJNA729271	stage_3W_vs_E10	ordinary_muscle_development	ENSGALG00010027382	PALM	-0.674098592	0.203926867	-3.305589895	0.000947767	0.001422141	negative
PRJNA729271	stage_3W_vs_E10	ordinary_muscle_development	ENSGALG00010027383	ASS1	3.305796464	0.282224002	11.71337816	1.08854E-31	6.66601E-31	positive
PRJNA729271	stage_3W_vs_E10	ordinary_muscle_development	ENSGALG00010027384	HHATL	1.086115099	0.218803077	4.963893168	6.9094E-07	1.32231E-06	positive
PRJNA729271	stage_3W_vs_E10	ordinary_muscle_development	ENSGALG00010027385	CNTN2	0.517824761	0.67113114	0.771570161	0.440369053	0.478963715	positive
PRJNA729271	stage_3W_vs_E10	ordinary_muscle_development	ENSGALG00010027386	RHOA	-0.350350499	0.066041864	-5.304975932	1.12688E-07	2.27368E-07	negative
PRJNA729271	stage_3W_vs_E10	ordinary_muscle_development	ENSGALG00010027387	ENSGALG00010027387	-0.9685297	0.273510863	-3.541101395	0.00039846	0.000618647	negative
PRJNA729271	stage_3W_vs_E10	ordinary_muscle_development	ENSGALG00010027388	URM1	0.809217614	0.143950732	5.621490107	1.89317E-08	4.0178E-08	positive
PRJNA729271	stage_3W_vs_E10	ordinary_muscle_development	ENSGALG00010027391	TPM3	-1.38742544	0.114764811	-12.08929309	1.20317E-33	7.90572E-33	negative
PRJNA729271	stage_3W_vs_E10	ordinary_muscle_development	ENSGALG00010027392	RBBP5	-0.226341241	0.127043004	-1.781611213	0.074812655	0.091878892	negative
PRJNA729271	stage_3W_vs_E10	ordinary_muscle_development	ENSGALG00010027393	TEX264	1.119029612	0.188770524	5.927989103	3.06667E-09	6.78543E-09	positive
PRJNA729271	stage_3W_vs_E10	ordinary_muscle_development	ENSGALG00010027394	SLC27A4	-0.656197141	0.087355853	-7.511770777	5.83329E-14	1.67665E-13	negative
PRJNA729271	stage_3W_vs_E10	ordinary_muscle_development	ENSGALG00010027395	BCL2L15	0.936022538	0.251389079	3.723401758	0.000196556	0.000313357	positive
PRJNA729271	stage_3W_vs_E10	ordinary_muscle_development	ENSGALG00010027397	RFX5	0.809938255	0.124474402	6.506865996	7.67348E-11	1.86529E-10	positive
PRJNA729271	stage_3W_vs_E10	ordinary_muscle_development	ENSGALG00010027399	MAPKAP1	0.702425642	0.110270877	6.370001419	1.89026E-10	4.49982E-10	positive
PRJNA729271	stage_3W_vs_E10	ordinary_muscle_development	ENSGALG00010027401	ENSGALG00010027401	1.043109977	0.314845583	3.313084359	0.000922731	0.001385579	positive
PRJNA729271	stage_3W_vs_E10	ordinary_muscle_development	ENSGALG00010027402	AP4B1	0.12253092	0.086743641	1.412563715	0.157784044	0.185594394	positive
PRJNA729271	stage_3W_vs_E10	ordinary_muscle_development	ENSGALG00010027403	ACKR2	1.413910461	0.367435659	3.848049109	0.000119062	0.000193368	positive
PRJNA729271	stage_3W_vs_E10	ordinary_muscle_development	ENSGALG00010027405	DCLRE1B	-2.425932221	0.194944476	-12.44422143	1.50343E-35	1.05277E-34	negative
PRJNA729271	stage_3W_vs_E10	ordinary_muscle_development	ENSGALG00010027407	ENSGALG00010027407	-0.638800222	1.157147701	-0.552047264	0.580915972	0.616922632	negative
PRJNA729271	stage_3W_vs_E10	ordinary_muscle_development	ENSGALG00010027410	ALAS1	0.261835012	0.087734797	2.984391835	0.002841426	0.00408272	positive
PRJNA729271	stage_3W_vs_E10	ordinary_muscle_development	ENSGALG00010027412	ENSGALG00010027412	0.577686436	0.289815659	1.993289245	0.046229783	0.058305117	positive
PRJNA729271	stage_3W_vs_E10	ordinary_muscle_development	ENSGALG00010027414	PBX3	-1.554683865	0.074475096	-20.87521796	8.99578E-97	3.41345E-95	negative
PRJNA729271	stage_3W_vs_E10	ordinary_muscle_development	ENSGALG00010027417	WNT2B	-1.181650283	0.362426632	-3.260384803	0.001112612	0.001660725	negative
PRJNA729271	stage_3W_vs_E10	ordinary_muscle_development	ENSGALG00010027418	ENSGALG00010027418	2.69283215	0.866174534	3.108879383	0.001877984	0.002740223	positive
PRJNA729271	stage_3W_vs_E10	ordinary_muscle_development	ENSGALG00010027419	PTBP1	-1.618500838	0.084834684	-19.07829157	3.8256E-81	9.93766E-80	negative
PRJNA729271	stage_3W_vs_E10	ordinary_muscle_development	ENSGALG00010027421	PLPPR3	-2.984327504	0.26658926	-11.1944776	4.33952E-29	2.41094E-28	negative
PRJNA729271	stage_3W_vs_E10	ordinary_muscle_development	ENSGALG00010027423	PEX11B	0.761916746	0.214901389	3.545424956	0.000391981	0.000609239	positive
PRJNA729271	stage_3W_vs_E10	ordinary_muscle_development	ENSGALG00010027425	CFD	4.144155281	0.238088602	17.40593734	7.43785E-68	1.36817E-66	positive
PRJNA729271	stage_3W_vs_E10	ordinary_muscle_development	ENSGALG00010027427	UBAC1	2.824640269	0.115557623	24.4435651	5.8913E-132	4.2066E-130	positive
PRJNA729271	stage_3W_vs_E10	ordinary_muscle_development	ENSGALG00010027438	ST7L	0.481893298	0.082643489	5.830989254	5.50997E-09	1.20609E-08	positive
PRJNA729271	stage_3W_vs_E10	ordinary_muscle_development	ENSGALG00010027440	TWF2	0.699800298	0.190302447	3.677305838	0.00023571	0.00037279	positive
PRJNA729271	stage_3W_vs_E10	ordinary_muscle_development	ENSGALG00010027443	MED16	-0.420668807	0.156803058	-2.682784458	0.007301203	0.010071463	negative
PRJNA729271	stage_3W_vs_E10	ordinary_muscle_development	ENSGALG00010027444	SELENBP1	0.518520756	0.121666672	4.261814241	2.02774E-05	3.50114E-05	positive
PRJNA729271	stage_3W_vs_E10	ordinary_muscle_development	ENSGALG00010027445	PPM1M	0.190222533	0.163605588	1.162689707	0.244955419	0.279819434	positive
PRJNA729271	stage_3W_vs_E10	ordinary_muscle_development	ENSGALG00010027448	ADAMTSL5	2.75305927	0.314004897	8.767567941	1.82567E-18	6.56163E-18	positive
PRJNA729271	stage_3W_vs_E10	ordinary_muscle_development	ENSGALG00010027450	CAMSAP1	0.810892296	0.142675785	5.683461249	1.31996E-08	2.82736E-08	positive
PRJNA729271	stage_3W_vs_E10	ordinary_muscle_development	ENSGALG00010027455	R3HDM4	1.258258791	0.146727837	8.575460666	9.8691E-18	3.43959E-17	positive
PRJNA729271	stage_3W_vs_E10	ordinary_muscle_development	ENSGALG00010027457	CAPZA1	-0.239889312	0.128071038	-1.873095708	0.06105517	0.075841519	negative
PRJNA729271	stage_3W_vs_E10	ordinary_muscle_development	ENSGALG00010027460	WDR82	-0.631424968	0.07371168	-8.56614544	1.07007E-17	3.72136E-17	negative
PRJNA729271	stage_3W_vs_E10	ordinary_muscle_development	ENSGALG00010027467	GLYCTK	0.308610741	0.134274746	2.298352819	0.021541716	0.028255106	positive
PRJNA729271	stage_3W_vs_E10	ordinary_muscle_development	ENSGALG00010027468	BSG	0.744747022	0.11518031	6.465923051	1.00682E-10	2.42827E-10	positive
PRJNA729271	stage_3W_vs_E10	ordinary_muscle_development	ENSGALG00010027471	ENSGALG00010027471	1.37718721	0.129163504	10.66235563	1.5268E-26	7.6631E-26	positive
PRJNA729271	stage_3W_vs_E10	ordinary_muscle_development	ENSGALG00010027473	DNAH1	-2.663319766	0.403130273	-6.606598281	3.93251E-11	9.73225E-11	negative
PRJNA729271	stage_3W_vs_E10	ordinary_muscle_development	ENSGALG00010027475	ARID3A	-0.730731264	0.169077477	-4.321872285	1.54711E-05	2.69373E-05	negative
PRJNA729271	stage_3W_vs_E10	ordinary_muscle_development	ENSGALG00010027479	HCN2	-0.502750506	0.277711192	-1.810335774	0.070243733	0.086543383	negative
PRJNA729271	stage_3W_vs_E10	ordinary_muscle_development	ENSGALG00010027487	MPZ	2.875466814	0.54309925	5.294551255	1.19309E-07	2.40459E-07	positive

PRJNA729271	stage_3W_vs_E10	ordinary_muscle_development	ENSGALG00010027490	ATP2B4	0.103776552	0.067852601	1.52944102	0.12615515	0.150404581	positive
PRJNA729271	stage_3W_vs_E10	ordinary_muscle_development	ENSGALG00010027492	PCP4L1	2.605157551	0.4915414	5.299975854	1.15818E-07	2.33586E-07	positive
PRJNA729271	stage_3W_vs_E10	ordinary_muscle_development	ENSGALG00010027497	KCNT1	3.171430234	0.339525707	9.340766162	9.56395E-21	3.7712E-20	positive
PRJNA729271	stage_3W_vs_E10	ordinary_muscle_development	ENSGALG00010027499	MOV10	2.67640462	0.399442359	6.700352528	2.07917E-11	5.22587E-11	positive
PRJNA729271	stage_3W_vs_E10	ordinary_muscle_development	ENSGALG00010027501	BAP1	-0.706989224	0.067586488	-10.46051131	1.31146E-25	6.29249E-25	negative
PRJNA729271	stage_3W_vs_E10	ordinary_muscle_development	ENSGALG00010027502	THOP1	0.080394563	0.139009849	0.57833717	0.563036504	0.599824646	positive
PRJNA729271	stage_3W_vs_E10	ordinary_muscle_development	ENSGALG00010027510	RHOC	2.696664493	0.181522955	14.85577675	6.38239E-50	7.18824E-49	positive
PRJNA729271	stage_3W_vs_E10	ordinary_muscle_development	ENSGALG00010027511	POLRMT	0.212655912	0.081669884	2.603847361	0.00921838	0.01258196	positive
PRJNA729271	stage_3W_vs_E10	ordinary_muscle_development	ENSGALG00010027513	SEMA3G	0.80195017	0.182325585	4.39845111	1.09026E-05	1.92045E-05	positive
PRJNA729271	stage_3W_vs_E10	ordinary_muscle_development	ENSGALG00010027516	PPM1J	3.198279224	0.213955765	14.94831991	1.59728E-50	1.8463E-49	positive
PRJNA729271	stage_3W_vs_E10	ordinary_muscle_development	ENSGALG00010027520	FANCE	-1.477836308	0.177227357	-8.338646643	7.51456E-17	2.49997E-16	negative
PRJNA729271	stage_3W_vs_E10	ordinary_muscle_development	ENSGALG00010027522	PSMD5	0.450802876	0.104346463	4.320250679	1.55852E-05	2.71328E-05	positive
PRJNA729271	stage_3W_vs_E10	ordinary_muscle_development	ENSGALG00010027524	ENSGALG00010027524	1.6741342	0.110896689	15.09634064	1.71173E-51	2.03875E-50	positive
PRJNA729271	stage_3W_vs_E10	ordinary_muscle_development	ENSGALG00010027525	ENSGALG00010027525	6.120607994	0.263729797	23.20787437	3.7916E-119	2.225E-117	positive
PRJNA729271	stage_3W_vs_E10	ordinary_muscle_development	ENSGALG00010027527	ENSGALG00010027527	-0.863022035	0.491212302	-1.756922681	0.078930989	0.09666312	negative
PRJNA729271	stage_3W_vs_E10	ordinary_muscle_development	ENSGALG00010027528	ENSGALG00010027528	1.729465882	2.41609901	0.715809193			positive
PRJNA729271	stage_3W_vs_E10	ordinary_muscle_development	ENSGALG00010027529	ACY1	2.248984728	0.140036512	16.05998814	4.86644E-58	6.936E-57	positive
PRJNA729271	stage_3W_vs_E10	ordinary_muscle_development	ENSGALG00010027530	PHF19	0.148159402	0.140585337	1.053875217	0.291940051	0.329184834	positive
PRJNA729271	stage_3W_vs_E10	ordinary_muscle_development	ENSGALG00010027531	UFC1	0.37143841	0.167968552	2.211356858	0.027011135	0.034988954	positive
PRJNA729271	stage_3W_vs_E10	ordinary_muscle_development	ENSGALG00010027533	WDR18	-1.384082452	0.097457136	-14.20196108	8.90841E-46	8.7544E-45	negative
PRJNA729271	stage_3W_vs_E10	ordinary_muscle_development	ENSGALG00010027534	KCNN3	0.047767632	0.196094757	0.243594639	0.80754478	0.829790949	positive
PRJNA729271	stage_3W_vs_E10	ordinary_muscle_development	ENSGALG00010027536	USP21	0.99707474	0.302812968	3.292708191	0.000992274	0.001487231	positive
PRJNA729271	stage_3W_vs_E10	ordinary_muscle_development	ENSGALG00010027537	SGTA	-0.217654645	0.067590597	-3.220191174	0.001281051	0.001900792	negative
PRJNA729271	stage_3W_vs_E10	ordinary_muscle_development	ENSGALG00010027538	ABHD14A	1.178037558	0.131355999	8.968281382	3.01177E-19	1.11823E-18	positive
PRJNA729271	stage_3W_vs_E10	ordinary_muscle_development	ENSGALG00010027539	DDR2L	-0.01311968	0.107951458	-0.121533145	0.903268763	0.914562778	negative
PRJNA729271	stage_3W_vs_E10	ordinary_muscle_development	ENSGALG00010027540	PPOX	-0.258043296	0.115052054	-2.242839551	0.024907163	0.032414197	negative
PRJNA729271	stage_3W_vs_E10	ordinary_muscle_development	ENSGALG00010027543	TUBB4B	-0.754555239	0.152210693	-4.95730768	7.14767E-07	1.36557E-06	negative
PRJNA729271	stage_3W_vs_E10	ordinary_muscle_development	ENSGALG00010027545	PPARD	0.434130246	0.202033288	2.14880553	0.031649819	0.040695833	positive
PRJNA729271	stage_3W_vs_E10	ordinary_muscle_development	ENSGALG00010027547	B4GALT3	1.171720151	0.222282181	5.27131841	1.35447E-07	2.71475E-07	positive
PRJNA729271	stage_3W_vs_E10	ordinary_muscle_development	ENSGALG00010027548	PCBP4	-0.131319377	0.147863318	-0.888113288	0.374479816	0.413190601	negative
PRJNA729271	stage_3W_vs_E10	ordinary_muscle_development	ENSGALG00010027553	FGF22	-0.436488685	0.307719763	-1.418461661	0.156056024	0.183666131	negative
PRJNA729271	stage_3W_vs_E10	ordinary_muscle_development	ENSGALG00010027554	ENSGALG00010027554	0.275870109	0.147873623	1.865580247	0.062100115	0.077047092	positive
PRJNA729271	stage_3W_vs_E10	ordinary_muscle_development	ENSGALG00010027555	ENSGALG00010027555	0.97594847	0.345223737	2.827002801	0.004698592	0.006609675	positive
PRJNA729271	stage_3W_vs_E10	ordinary_muscle_development	ENSGALG00010027557	C6orf132	-2.506780033	0.846545865	-2.961186316	0.003064565	0.004392886	negative
PRJNA729271	stage_3W_vs_E10	ordinary_muscle_development	ENSGALG00010027559	ENSGALG00010027559	1.8473843	0.269556279	6.853427067	7.21014E-12	1.8527E-11	positive
PRJNA729271	stage_3W_vs_E10	ordinary_muscle_development	ENSGALG00010027561	ADAMTS4	1.895316759	0.3329765	5.692043605	1.25528E-08	2.6928E-08	positive
PRJNA729271	stage_3W_vs_E10	ordinary_muscle_development	ENSGALG00010027562	SLC39A3	0.216943358	0.146888439	1.476926019	0.13969547	0.165648383	positive
PRJNA729271	stage_3W_vs_E10	ordinary_muscle_development	ENSGALG00010027563	WDR77	-0.712786997	0.122584966	-5.814636334	6.07658E-09	1.32771E-08	negative
PRJNA729271	stage_3W_vs_E10	ordinary_muscle_development	ENSGALG00010027567	PARP3	1.374271712	0.129191999	10.63743669	1.99543E-26	9.94287E-26	positive
PRJNA729271	stage_3W_vs_E10	ordinary_muscle_development	ENSGALG00010027568	NELFB	-0.258994002	0.075953736	-3.409891517	0.000649887	0.000990444	negative
PRJNA729271	stage_3W_vs_E10	ordinary_muscle_development	ENSGALG00010027569	ATP5PB	1.181783719	0.125306436	9.431149442	4.05619E-21	1.6255E-20	positive
PRJNA729271	stage_3W_vs_E10	ordinary_muscle_development	ENSGALG00010027570	NDUFS2	0.64533628	0.137610297	4.689592948	2.73749E-06	5.03744E-06	positive
PRJNA729271	stage_3W_vs_E10	ordinary_muscle_development	ENSGALG00010027571	RBM10	0.079012881	0.101701579	0.776909087	0.43721241	0.47585177	positive
PRJNA729271	stage_3W_vs_E10	ordinary_muscle_development	ENSGALG00010027574	GNG7	-0.765416724	0.096107743	-7.964152554	1.6636E-15	5.14704E-15	negative
PRJNA729271	stage_3W_vs_E10	ordinary_muscle_development	ENSGALG00010027575	FCER1G	2.447917891	0.330221229	7.412963409	1.23508E-13	3.4884E-13	positive
PRJNA729271	stage_3W_vs_E10	ordinary_muscle_development	ENSGALG00010027576	GADD45B	1.848601406	0.417030179	4.432776086	9.30275E-06	1.64604E-05	positive
PRJNA729271	stage_3W_vs_E10	ordinary_muscle_development	ENSGALG00010027578	ENSGALG00010027578	2.376968422	0.237365639	10.01395328	1.32355E-23	5.83306E-23	positive
PRJNA729271	stage_3W_vs_E10	ordinary_muscle_development	ENSGALG00010027579	C26H6ORF125	0.325512229	0.157753596	2.063421923	0.039072559	0.049732766	positive
PRJNA729271	stage_3W_vs_E10	ordinary_muscle_development	ENSGALG00010027583	GLE1	-1.085710363	0.073387572	-14.79419916	1.59673E-49	1.77082E-48	negative
PRJNA729271	stage_3W_vs_E10	ordinary_muscle_development	ENSGALG00010027586	HJV	4.060821497	0.289105696	14.04614838	8.13391E-45	7.70595E-44	positive
PRJNA729271	stage_3W_vs_E10	ordinary_muscle_development	ENSGALG00010027590	SEMA3F	-2.104590405	0.240007819	-8.768841012	1.80515E-18	6.48949E-18	negative
PRJNA729271	stage_3W_vs_E10	ordinary_muscle_development	ENSGALG00010027592	MAP1S	-0.407848771	0.169414972	-2.407395089	0.016066778	0.02135607	negative
PRJNA729271	stage_3W_vs_E10	ordinary_muscle_development	ENSGALG00010027593	ENSGALG00010027593	3.816648082	0.428828265	8.900178444	5.5757E-19	2.04711E-18	positive
PRJNA729271	stage_3W_vs_E10	ordinary_muscle_development	ENSGALG00010027594	ITPR3	-0.740638665	0.140248494	-5.280902823	1.28549E-07	2.58256E-07	negative
PRJNA729271	stage_3W_vs_E10	ordinary_muscle_development	ENSGALG00010027595	GNAT1	0.387436758	0.427343334	0.906617061	0.364609336	0.403436056	positive
PRJNA729271	stage_3W_vs_E10	ordinary_muscle_development	ENSGALG00010027597	EXOSC2	-1.093184762	0.11709683	-9.33573318	1.00295E-20	3.94939E-20	negative
PRJNA729271	stage_3W_vs_E10	ordinary_muscle_development	ENSGALG00010027600	KIRREL1	-0.942860254	0.141760286	-6.651088821	2.90933E-11	7.26079E-11	negative
PRJNA729271	stage_3W_vs_E10	ordinary_muscle_development	ENSGALG00010027605	FCHO1	1.544335605	0.305915704	5.048239049	4.45901E-07	8.64314E-07	positive
PRJNA729271	stage_3W_vs_E10	ordinary_muscle_development	ENSGALG00010027607	ABL1	-1.077611993	0.079604582	-13.5370598	9.4496E-42	8.05718E-41	negative
PRJNA729271	stage_3W_vs_E10	ordinary_muscle_development	ENSGALG00010027608	GNAI2	-0.529720737	0.14451769	-3.665438725	0.000246915	0.000389958	negative
PRJNA729271	stage_3W_vs_E10	ordinary_muscle_development	ENSGALG00010027609	RPL36	0.413276668	0.186792513	2.212490538	0.026932789	0.034893706	positive
PRJNA729271	stage_3W_vs_E10	ordinary_muscle_development	ENSGALG00010027612	BAK1	-0.813977927	0.163790729	-4.969621492	6.70837E-07	1.2852E-06	negative
PRJNA729271	stage_3W_vs_E10	ordinary_muscle_development	ENSGALG00010027614	LONP1	-0.198779489	0.07946058	-2.501611349	0.012362956	0.016638909	negative
PRJNA729271	stage_3W_vs_E10	ordinary_muscle_development	ENSGALG00010027616	SLC38A3	4.052635192	0.267482446	15.15103235	7.45857E-52	8.94227E-51	positive
PRJNA729271	stage_3W_vs_E10	ordinary_muscle_development	ENSGALG00010027618	PPP1R26	0.301561042	0.078439596	3.844500172	0.000120798	0.0001961	positive
PRJNA729271	stage_3W_vs_E10	ordinary_muscle_development	ENSGALG00010027619	JAK3	2.775632795	0.338475324	8.200399252	2.3959E-16	7.76752E-16	positive
PRJNA729271	stage_3W_vs_E10	ordinary_muscle_development	ENSGALG00010027621	TSPO2	-4.045517665	0.413496709	-9.7836756	1.32316E-22	5.60632E-22	negative
PRJNA729271	stage_3W_vs_E10	ordinary_muscle_development	ENSGALG00010027622	FIBCD1	-1.012338344	0.974839788	-1.038466378	0.29905298	0.336576827	negative
PRJNA729271	stage_3W_vs_E10	ordinary_muscle_development	ENSGALG00010027623	APOBEC2	2.532418722	0.182660643	13.86406335	1.04582E-43	9.57021E-43	positive
PRJNA729271	stage_3W_vs_E10	ordinary_muscle_development	ENSGALG00010027624	ENSGALG00010027624	5.222376372	0.392357163	13.31026132	2.01775E-40	1.65052E-39	positive
PRJNA729271	stage_3W_vs_E10	ordinary_muscle_development	ENSGALG00010027626	SEMA3B	0.121811535	0.214816332	0.567049695	0.570680419	0.606986548	positive
PRJNA729271	stage_3W_vs_E10	ordinary_muscle_development	ENSGALG00010027627	OARD1	1.756310348	0.092835796	18.91846057	8.03678E-80	1.99817E-78	positive
PRJNA729271	stage_3W_vs_E10	ordinary_muscle_development	ENSGALG00010027628	PIERCE1	-0.746931042	0.25213714	-2.962399913	0.00305251	0.00437604	negative
PRJNA729271	stage_3W_vs_E10	ordinary_muscle_development	ENSGALG00010027632	LAMC3	-2.343856637	0.443416856	-5.285898825	1.25089E-07	2.51479E-07	negative
PRJNA729271	stage_3W_vs_E10	ordinary_muscle_development	ENSGALG00010027634	OASL	4.805581057	0.711886944	6.750483483	1.47353E-11	3.73146E-11	positive
PRJNA729271	stage_3W_vs_E10	ordinary_muscle_development	ENSGALG00010027635	CD48	2.946264814	0.22553475	13.06346281	5.32494E-39	4.16991E-38	positive
PRJNA729271	stage_3W_vs_E10	ordinary_muscle_development	ENSGALG00010027636	SLC1A6	2.16242287	0.592748026	3.648131714	0.000264154	0.000416186	positive
PRJNA729271	stage_3W_vs_E10	ordinary_muscle_development	ENSGALG00010027638	MRPS2	1.001702254	0.140425204	7.133350876	9.79544E-13	2.64502E-12	positive
PRJNA729271	stage_3W_vs_E10	ordinary_muscle_development	ENSGALG00010027641	HYAL3	1.021267794	0.16359027	6.242839486	4.29698E-10	1.00233E-09	positive
PRJNA729271	stage_3W_vs_E10	ordinary_muscle_development	ENSGALG00010027642	DIPK1B	-0.928359162	0.169098379	-5.490053568	4.01812E-08	8.34183E-08	negative
PRJNA729271	stage_3W_vs_E10	ordinary_muscle_development	ENSGALG00010027644	NAA80	2.374447229	0.138949584	17.08855229	1.80602E-65	3.13137E-64	positive
PRJNA729271	stage_3W_vs_E10	ordinary_muscle_development	ENSGALG00010027645	SLAMF1	5.186709517	0.785142702	6.606072379	3.9465E-11	9.7652E-11	positive
PRJNA729271	stage_3W_vs_E10	ordinary_muscle_development	ENSGALG00010027648	NFYA	-0.41239735	0.07286358	-5.659855695	1.515E-08	3.23416E-08	negative
PRJNA729271	stage_3W_vs_E10	ordinary_muscle_development	ENSGALG00010027649	FBLN2	-1.025364572	0.191662437	-5.34984626	8.8029E-08	1.78884E-07	negative
PRJNA729271	stage_3W_vs_E10	ordinary_muscle_development	ENSGALG00010027650	CCDC124	0.633432568	0.191081353	3.314988917	0.000916468	0.001376743	positive
PRJNA729271	stage_3W_vs_E10	ordinary_muscle_development	ENSGALG00010027653	WNT7A	-6.090174292	0.564593129	-10.78683743	3.97225E-27	2.03959E-26	negative
PRJNA729271	stage_3W_vs_E10	ordinary_muscle_development	ENSGALG00010027654	AIF1L	0.805801122	0.091190972	8.836413373	9.88406E-19	3.58712E-18	positive
PRJNA729271	stage_3W_vs_E10	ordinary_muscle_development	ENSGALG00010027663	COPA	-0.773944397	0.070810811	-10.92974904	8.30779E-28	4.39494E-27	negative
PRJNA729271	stage_3W_vs_E10	ordinary_muscle_development	ENSGALG00010027664	TREMB1	2.206254967	0.302423123	7.295258869	2.98085E-13	8.24098E-13	positive
PRJNA729271	stage_3W_vs_E10	ordinary_muscle_development	ENSGALG00010027665	TRAF1	0.188737379	0.129538009	1.457003864	0.145115314	0.171639859	positive
PRJNA729271	stage_3W_vs_E10	ordinary_muscle_development	ENSGALG00010027667	PTPDC1	-0.267253971	0.152986613	-1.746910829	0.080652807	0.098630089	negative
PRJNA729271	stage_3W_vs_E10	ordinary_muscle_development	ENSGALG00010027668	RANBP3	-0.460035258	0.053985514	-8.521457376	1.57558E-17	5.42213E-17	negative
PRJNA729271	stage_3W_vs_E10	ordinary_muscle_development	ENSGALG00010027671	BARX1	-4.310344124	0.692187074	-6.227137559	4.75034E-10	1.10612E-09	negative
PRJNA729271	stage_3W_vs_E10	ordinary_muscle_development	ENSGALG00010027672	CNTRL	-0.276071953	0.173955844	-1.587023159	0.1125074	0.135055466	negative
PRJNA729271	stage_3W_vs_E10	ordinary_muscle_development	ENSGALG00010027674	TREM2	1.460599159	0.280813034	5.201322521	1.97875E-07	3.92421E-07	positive
PRJNA729271	stage_3W_vs_E10	ordinary_muscle_development	ENSGALG00010027685	ENSGALG00010027685	3.454941405	0.295171873	11.70484628	1.20377E-31	7.353E-31	positive
PRJNA729271	stage_3W_vs_E10	ordinary_muscle_development	ENSGALG00010027692	PHF2	0.384708197	0.112746567	3.412149978	0.000644526	0.000982584	positive
PRJNA729271	stage_3W_vs_E10	ordinary_muscle_development	ENSGALG00010027693	RAB14	-0.266390044	0.059849512	-4.450997771	8.54722E-06	1.51643E-05	negative
PRJNA729271	stage_3W_vs_E10	ordinary_muscle_development	ENSGALG00010027695	TREMB2	2.182715812	0.29475822	7.405105835	1.31046E-13	3.69555E-13	positive
PRJNA729271	stage_3W_vs_E10	ordinary_muscle_development	ENSGALG00010027697	CATH2	1.10289325	0.945895387	1.16597804	0.243623324	0.278407449	positive
PRJNA729271	stage_3W_vs_E10	ordinary_muscle_development	ENSGALG00010027698	FAM120A	-0.041655024	0.066528472	-0.626123267	0.531234092	0.569081233	negative
PRJNA729271	stage_3W_vs_E10	ordinary_muscle_development	ENSGALG00010027699	VANGL2	-2.370747603	0.131185552	-18.07171266	5.32352E-73	1.12485E-71	negative
PRJNA729271	stage_3W_vs_E10	ordinary_muscle_development	ENSGALG00010027700	TCF3	-1.730277502	0.096526469	-17.92542003	7.46844E-72	1.52903E-70	negative
PRJNA729271	stage_3W_vs_E10	ordinary_muscle_development	ENSGALG00010027702	FOXP4	-3.180018006	0.254221364	-12.50885434	6.67804E-36	4.72186E-35	negative
PRJNA729271	stage_3W_vs_E10	ordinary_muscle_development	ENSGALG00010027703	NCSTN	-0.090757251	0.100892697	-0.899542328	0.36836386	0.407000088	negative
PRJNA729271	stage_3W_vs_E10	ordinary_muscle_development	ENSGALG00010027704	MDFI	-5.064467289	0.218678299	-23.15944153	1.1678E-118	6.7981E-117	negative
PRJNA729271	stage_3W_vs_E10	ordinary_muscle_development	ENSGALG00010027705	S100A11	0.146604173	0.22964111	0.63840561	0.52320968	0.561231726	positive
PRJNA729271	stage_3W_vs_E10	ordinary_muscle_development	ENSGALG00010027706	GSN	4.130205047	0.171888125	24.02844898	1.4027E-127	9.5453E-126	positive
PRJNA729271	stage_3W_vs_E10	ordinary_muscle_development	ENSGALG00010027707	HIGD1C	1.096959883	0.21153226	5.18578056	2.15112E-07	4.25498E-07	positive
PRJNA729271	stage_3W_vs_E10	ordinary_muscle_development	ENSGALG00010027709	PSMA5	1.386780676	0.10661428	13.00745715	1.10974E-38	8.55164E-38	positive
PRJNA729271	stage_3W_vs_E10	ordinary_muscle_development	ENSGALG00010027710	ENSGALG00010027710	3.668001878	0.28387075	12.92138014	3.4097E-38	2.58762E-37	positive
PRJNA729271	stage_3W_vs_E10	ordinary_muscle_development	ENSGALG00010027711	WNK2	0.788657428	0.186170121	4.236219138	2.27315E-05	3.9095E-05	positive
PRJNA729271	stage_3W_vs_E10	ordinary_muscle_development	ENSGALG00010027712	STOM	1.279634871	0.157068994	8.146960352	3.73186E-16	1.19438E-15	positive
PRJNA729271	stage_3W_vs_E10	ordinary_muscle_development	ENSGALG00010027713	ENSGALG00010027713	0.898857095	0.139507987	6.443051143	1.17095E-10	2.81335E-10	positive
PRJNA729271	stage_3W_vs_E10	ordinary_muscle_development	ENSGALG00010027714	SYPL2	7.428783847	0.162044835	45.84400279	0	0	positive
PRJNA729271	stage_3W_vs_E10	ordinary_muscle_development	ENSGALG00010027715	NINJ1	0.110915775	0.122545342	0.905099881	0.365412475	0.404139617	positive
PRJNA729271	stage_3W_vs_E10	ordinary_muscle_development	ENSGALG00010027717	ATXN7L2	1.420912407	0.226724175	6.267141156	3.67737E-10	8.60844E-10	positive
PRJNA729271	stage_3W_vs_E10	ordinary_muscle_development	ENSGALG00010027718	ENSGALG00010027718	2.563598943	0.461864036	5.550548959	2.84774E-08	5.96416E-08	positive
PRJNA729271	stage_3W_vs_E10	ordinary_muscle_development	ENSGALG00010027719	GPR61	-5.277157063	1.118336045	-4.718757914	2.37289E-06	4.38544E-06	negative
PRJNA729271	stage_3W_vs_E10	ordinary_muscle_development	ENSGALG00010027720	DAB2IP	0.270076025	0.087734676	3.078327024	0.002081663	0.003025843	positive
PRJNA729271	stage_3W_vs_E10	ordinary_muscle_development	ENSGALG00010027721	SUSD3	-1.630780574	0.192182982	-8.485561824	2.14679E-17	7.32526E-17	negative
PRJNA729271	stage_3W_vs_E10	ordinary_muscle_development	ENSGALG00010027722	GNAI3	-0.759634064	0.085180747	-8.917907963	4.75179E-19	1.75215E-18	negative
PRJNA729271	stage_3W_vs_E10	ordinary_muscle_development	ENSGALG00010027723	ENSGALG00010027723	2.16396025	1.021714736	2.11796911	0.034177684	0.04377534	positive
PRJNA729271	stage_3W_vs_E10	ordinary_muscle_development	ENSGALG00010027724	TTLL11	-0.475006855	0.112547448	-4.220503127	2.43758E-05	4.17792E-05	negative
PRJNA729271	stage_3W_vs_E10	ordinary_muscle_development	ENSGALG00010027725	BTN1A1	3.196009967	0.2490793	12.83129498	1.09518E-37	8.14085E-37	positive
PRJNA729271	stage_3W_vs_E10	ordinary_muscle_development	ENSGALG00010027727	GSTM2	1.210921134	0.170864553	7.087023683	1.37027E-12	3.66865E-12	positive
PRJNA729271	stage_3W_vs_E10	ordinary_muscle_development	ENSGALG00010027728	CRNN	0.524404908	1.679237626	0.312287493	0.754822045	0.780656344	positive
PRJNA729271	stage_3W_vs_E10	ordinary_muscle_development	ENSGALG00010027729	EPS8L3	1.31769128	0.243972993	5.400971893	6.62808E-08	1.35564E-07	positive
PRJNA729271	stage_3W_vs_E10	ordinary_muscle_development	ENSGALG00010027730	ENSGALG00010027730	-2.740748441	0.158581074	-17.28294786	6.3232E-67	1.13294E-65	negative
PRJNA729271	stage_3W_vs_E10	ordinary_muscle_development	ENSGALG00010027731	OLFML2A	1.174046465	0.102674909	11.43459954	2.80829E-30	1.62955E-29	positive
PRJNA729271	stage_3W_vs_E10	ordinary_muscle_development	ENSGALG00010027732	EEF2	0.917662132	0.117035662	7.840876141	4.47414E-15	1.35931E-14	positive

PRJNA729271	stage_3W_vs_E10	ordinary_muscle_development	ENSGALG00010027733	RPL35	-0.004356335	0.154069962	-0.028275046	0.977442783	0.980894014	negative
PRJNA729271	stage_3W_vs_E10	ordinary_muscle_development	ENSGALG00010027734	CSF1	1.105542161	0.139049444	7.950712548	1.85442E-15	5.72885E-15	positive
PRJNA729271	stage_3W_vs_E10	ordinary_muscle_development	ENSGALG00010027736	ENSGALG00010027736	2.348887455	1.640592724	1.431730996	0.152220822	0.179429196	positive
PRJNA729271	stage_3W_vs_E10	ordinary_muscle_development	ENSGALG00010027738	ENSGALG00010027738	1.512913749	0.239834243	6.308164052	2.82365E-10	6.65292E-10	positive
PRJNA729271	stage_3W_vs_E10	ordinary_muscle_development	ENSGALG00010027739	FGD3	-1.994471009	0.075179069	-26.52960498	4.4161E-155	4.5398E-153	negative
PRJNA729271	stage_3W_vs_E10	ordinary_muscle_development	ENSGALG00010027740	MARCHF2	-0.296956482	0.146072049	-2.032945277	0.042058052	0.053275493	negative
PRJNA729271	stage_3W_vs_E10	ordinary_muscle_development	ENSGALG00010027741	RAB11B	-1.219106343	0.062541382	-19.49279498	1.26386E-84	3.59914E-83	negative
PRJNA729271	stage_3W_vs_E10	ordinary_muscle_development	ENSGALG00010027743	C1orf74	2.196025735	0.181654661	12.08901396	1.20727E-33	7.92903E-33	positive
PRJNA729271	stage_3W_vs_E10	ordinary_muscle_development	ENSGALG00010027744	ENSGALG00010027744	2.19312981	1.30245149	1.683847596	0.092211126	0.11205569	positive
PRJNA729271	stage_3W_vs_E10	ordinary_muscle_development	ENSGALG00010027745	BICD2	0.245352302	0.138520668	1.771232449	0.076522055	0.093887523	positive
PRJNA729271	stage_3W_vs_E10	ordinary_muscle_development	ENSGALG00010027746	UQCR11	2.590573339	0.184264334	14.05900577	6.78325E-45	6.46438E-44	positive
PRJNA729271	stage_3W_vs_E10	ordinary_muscle_development	ENSGALG00010027748	ENSGALG00010027748	1.010007594	2.889617951	0.349529803	0.7266916	0.754757434	positive
PRJNA729271	stage_3W_vs_E10	ordinary_muscle_development	ENSGALG00010027751	ANGPTL4	-1.744017632	0.212824007	-8.194647116	2.51329E-16	8.14265E-16	negative
PRJNA729271	stage_3W_vs_E10	ordinary_muscle_development	ENSGALG00010027752	SCAI	-1.144593354	0.085582816	-13.3741025	8.56817E-41	7.0807E-40	negative
PRJNA729271	stage_3W_vs_E10	ordinary_muscle_development	ENSGALG00010027759	ENSGALG00010027759	-2.143614028	1.050537645	-2.040492349	0.041301312	0.052390174	negative
PRJNA729271	stage_3W_vs_E10	ordinary_muscle_development	ENSGALG00010027766	ENSGALG00010027766	-1.415321507	0.685965085	-2.063255898	0.039088322	0.049748461	negative
PRJNA729271	stage_3W_vs_E10	ordinary_muscle_development	ENSGALG00010027767	ENSGALG00010027767	-0.821036915	1.172314853	-0.700355295	0.483705447	0.522063325	negative
PRJNA729271	stage_3W_vs_E10	ordinary_muscle_development	ENSGALG00010027772	ENSGALG00010027772	-1.067701394	1.067282473	-1.000392512	0.317120592	0.355064343	negative
PRJNA729271	stage_3W_vs_E10	ordinary_muscle_development	ENSGALG00010027775	MVB12B	0.757086909	0.087972479	8.605951695	7.56861E-18	2.64993E-17	positive
PRJNA729271	stage_3W_vs_E10	ordinary_muscle_development	ENSGALG00010027776	TKT	-1.6732671	0.128953086	-12.97578173	1.67875E-38	1.28817E-37	negative
PRJNA729271	stage_3W_vs_E10	ordinary_muscle_development	ENSGALG00010027784	PEAR1	0.654914936	0.192694198	3.398726814	0.000677003	0.001030037	positive
PRJNA729271	stage_3W_vs_E10	ordinary_muscle_development	ENSGALG00010027787	DCP1A	-0.041789955	0.137696749	-0.303492675	0.761514429	0.787146235	negative
PRJNA729271	stage_3W_vs_E10	ordinary_muscle_development	ENSGALG00010027789	UAP1L1	-1.61255816	0.13502388	-11.94276275	7.08307E-33	4.53486E-32	negative
PRJNA729271	stage_3W_vs_E10	ordinary_muscle_development	ENSGALG00010027801	MBD3	-3.945709634	0.136121673	-28.98663785	9.6973E-185	1.4642E-182	negative
PRJNA729271	stage_3W_vs_E10	ordinary_muscle_development	ENSGALG00010027803	SAPCD2	-2.119771244	0.171073435	-12.39100184	2.92368E-35	2.03353E-34	negative
PRJNA729271	stage_3W_vs_E10	ordinary_muscle_development	ENSGALG00010027811	MEX3D	-1.11588942	0.101759964	-10.96589835	5.57442E-28	2.96627E-27	negative
PRJNA729271	stage_3W_vs_E10	ordinary_muscle_development	ENSGALG00010027813	LRRC71	-2.890734094	0.361314201	-8.000610225	1.23804E-15	3.86089E-15	negative
PRJNA729271	stage_3W_vs_E10	ordinary_muscle_development	ENSGALG00010027814	ENSGALG00010027814	-0.417369403	0.133777261	-3.119882999	0.001809229	0.002643626	negative
PRJNA729271	stage_3W_vs_E10	ordinary_muscle_development	ENSGALG00010027816	ZMYND19	-0.734915146	0.120151855	-6.116552668	9.56213E-10	2.18066E-09	negative
PRJNA729271	stage_3W_vs_E10	ordinary_muscle_development	ENSGALG00010027821	NMRK2	4.200948857	0.226478693	18.54898051	8.31134E-77	1.91531E-75	positive
PRJNA729271	stage_3W_vs_E10	ordinary_muscle_development	ENSGALG00010027822	DPH7	0.121364576	0.090231139	1.345040944	0.178612013	0.208234627	positive
PRJNA729271	stage_3W_vs_E10	ordinary_muscle_development	ENSGALG00010027826	ARHGEF11	0.575272887	0.164464299	3.497858742	0.000469009	0.000724379	positive
PRJNA729271	stage_3W_vs_E10	ordinary_muscle_development	ENSGALG00010027830	CACNA1D	-2.491759233	0.367609202	-6.778283083	1.21612E-11	3.0953E-11	negative
PRJNA729271	stage_3W_vs_E10	ordinary_muscle_development	ENSGALG00010027831	ATCAY	-3.104874672	0.227851986	-13.6267176	2.77797E-42	2.41262E-41	negative
PRJNA729271	stage_3W_vs_E10	ordinary_muscle_development	ENSGALG00010027838	ENSGALG00010027838	-0.528069042	0.641042879	-0.823765554	0.410072784	0.44887517	negative
PRJNA729271	stage_3W_vs_E10	ordinary_muscle_development	ENSGALG00010027842	SLAMF8	-2.349801065	0.263779181	-8.908212745	5.18618E-19	1.90893E-18	negative
PRJNA729271	stage_3W_vs_E10	ordinary_muscle_development	ENSGALG00010027847	NRTN	-1.381337027	0.367583003	-3.757891453	0.000171351	0.000274598	negative
PRJNA729271	stage_3W_vs_E10	ordinary_muscle_development	ENSGALG00010027860	ENSGALG00010027860	2.164468722	0.257555657	8.403887318	4.31937E-17	1.45366E-16	positive
PRJNA729271	stage_3W_vs_E10	ordinary_muscle_development	ENSGALG00010027861	PNPLA7	-0.092224363	0.079231271	-1.163989441	0.244428291	0.279261297	negative
PRJNA729271	stage_3W_vs_E10	ordinary_muscle_development	ENSGALG00010027863	CHDH	-2.298391011	0.154437418	-14.88234545	4.29186E-50	4.88308E-49	negative
PRJNA729271	stage_3W_vs_E10	ordinary_muscle_development	ENSGALG00010027864	ACTR8	-0.121967987	0.081656068	-1.493679394	0.135259437	0.160664225	negative
PRJNA729271	stage_3W_vs_E10	ordinary_muscle_development	ENSGALG00010027865	SELENOK	0.080149358	0.121559616	0.659341979	0.509676191	0.547768695	positive
PRJNA729271	stage_3W_vs_E10	ordinary_muscle_development	ENSGALG00010027866	KCNA2	-1.115783183	0.140130503	-7.962457546	1.68655E-15	5.21583E-15	negative
PRJNA729271	stage_3W_vs_E10	ordinary_muscle_development	ENSGALG00010027867	UBAP2L	-0.704709025	0.169270266	-4.163218044	3.13793E-05	5.32978E-05	negative
PRJNA729271	stage_3W_vs_E10	ordinary_muscle_development	ENSGALG00010027868	CACNA2D3	-4.18742326	0.334069555	-12.53458508	4.82882E-36	3.42939E-35	negative
PRJNA729271	stage_3W_vs_E10	ordinary_muscle_development	ENSGALG00010027869	NSMF	-1.247457678	0.166688316	-7.483773948	7.22181E-14	2.06633E-13	negative
PRJNA729271	stage_3W_vs_E10	ordinary_muscle_development	ENSGALG00010027870	HAX1	2.066992182	0.120256865	17.18814296	3.25802E-66	5.74513E-65	positive
PRJNA729271	stage_3W_vs_E10	ordinary_muscle_development	ENSGALG00010027872	KDM5B	-0.528457634	0.091931833	-5.748363938	9.01111E-09	1.94949E-08	negative
PRJNA729271	stage_3W_vs_E10	ordinary_muscle_development	ENSGALG00010027873	RFX2	0.364048742	0.198057825	1.838093206	0.066048672	0.08168036	positive
PRJNA729271	stage_3W_vs_E10	ordinary_muscle_development	ENSGALG00010027874	FRS3	-0.482317886	0.135509143	-3.559301425	0.000371843	0.000579243	negative
PRJNA729271	stage_3W_vs_E10	ordinary_muscle_development	ENSGALG00010027875	WNT5A	-1.306760012	0.1691893	-7.72365634	1.13039E-14	3.35966E-14	negative
PRJNA729271	stage_3W_vs_E10	ordinary_muscle_development	ENSGALG00010027876	ANAPC2	-0.619746531	0.110508265	-5.608146436	2.04505E-08	4.32871E-08	negative
PRJNA729271	stage_3W_vs_E10	ordinary_muscle_development	ENSGALG00010027877	OAZ1	1.128228189	0.114287272	9.871862132	5.51315E-23	2.3812E-22	positive
PRJNA729271	stage_3W_vs_E10	ordinary_muscle_development	ENSGALG00010027879	ENSGALG00010027879	3.788574449	0.417394314	9.076727496	1.11888E-19	4.22129E-19	positive
PRJNA729271	stage_3W_vs_E10	ordinary_muscle_development	ENSGALG00010027880	AMH	1.26848217	0.352133443	3.602276906	0.000315442	0.000494574	positive
PRJNA729271	stage_3W_vs_E10	ordinary_muscle_development	ENSGALG00010027881	ENSGALG00010027881	-2.279447178	0.332322743	-6.859136873	6.92778E-12	1.78204E-11	negative
PRJNA729271	stage_3W_vs_E10	ordinary_muscle_development	ENSGALG00010027882	CHIA	5.906117109	1.38932288	4.251075971	2.12746E-05	3.66633E-05	positive
PRJNA729271	stage_3W_vs_E10	ordinary_muscle_development	ENSGALG00010027883	ATP8B2	0.061186722	0.117354555	0.521383438	0.602099687	0.637644112	positive
PRJNA729271	stage_3W_vs_E10	ordinary_muscle_development	ENSGALG00010027884	SF3A2	-0.679062484	0.190604818	-3.562672196	0.000367099	0.000572223	negative
PRJNA729271	stage_3W_vs_E10	ordinary_muscle_development	ENSGALG00010027885	PLEKHJ1	-1.121163648	0.075472311	-14.85529761	6.42818E-50	7.2342E-49	negative
PRJNA729271	stage_3W_vs_E10	ordinary_muscle_development	ENSGALG00010027886	IL6R	2.454386832	0.192113215	12.77573142	2.24032E-37	1.64756E-36	positive
PRJNA729271	stage_3W_vs_E10	ordinary_muscle_development	ENSGALG00010027887	ERC2	-2.82962585	0.295955446	-9.560985926	1.1664E-21	4.76925E-21	negative
PRJNA729271	stage_3W_vs_E10	ordinary_muscle_development	ENSGALG00010027888	PFKFB2	-0.029771058	0.144829339	-0.205559581	0.837134953	0.855912474	negative
PRJNA729271	stage_3W_vs_E10	ordinary_muscle_development	ENSGALG00010027889	RBM8A	-1.336086505	0.167401121	-7.981347426	1.44744E-15	4.49458E-15	negative
PRJNA729271	stage_3W_vs_E10	ordinary_muscle_development	ENSGALG00010027890	ENSGALG00010027890	5.138375364	0.823708022	6.238102853	4.42909E-10	1.03248E-09	positive
PRJNA729271	stage_3W_vs_E10	ordinary_muscle_development	ENSGALG00010027891	CCDC66	-0.680856402	0.141348556	-4.816861391	1.45834E-06	2.73003E-06	negative
PRJNA729271	stage_3W_vs_E10	ordinary_muscle_development	ENSGALG00010027892	SHE	-0.162503398	0.168345357	-0.965297773	0.334395687	0.372736503	negative
PRJNA729271	stage_3W_vs_E10	ordinary_muscle_development	ENSGALG00010027893	DOT1L	0.257325279	0.158173632	1.6268532	0.10376828	0.125166116	positive
PRJNA729271	stage_3W_vs_E10	ordinary_muscle_development	ENSGALG00010027894	RSBN1	-0.126141827	0.068269653	-1.847699844	0.064645777	0.080061563	negative
PRJNA729271	stage_3W_vs_E10	ordinary_muscle_development	ENSGALG00010027895	LIX1L	-0.708614898	0.180459174	-3.926732475	8.61077E-05	0.000141351	negative
PRJNA729271	stage_3W_vs_E10	ordinary_muscle_development	ENSGALG00010027896	TOR4A	0.813375143	0.159701207	5.0931058	3.52245E-07	6.87001E-07	positive
PRJNA729271	stage_3W_vs_E10	ordinary_muscle_development	ENSGALG00010027897	AP3D1	-0.083188251	0.098148632	-0.84757423	0.396675131	0.435525377	negative
PRJNA729271	stage_3W_vs_E10	ordinary_muscle_development	ENSGALG00010027898	TASOR	-0.501225701	0.092549308	-5.415769276	6.10258E-08	1.25063E-07	negative
PRJNA729271	stage_3W_vs_E10	ordinary_muscle_development	ENSGALG00010027899	PMVK	-1.091686742	0.18289491	-5.968929057	2.38816E-09	5.3166E-09	negative
PRJNA729271	stage_3W_vs_E10	ordinary_muscle_development	ENSGALG00010027901	MOB3A	-0.459306046	0.097885781	-4.692265237	2.70197E-06	4.97523E-06	negative
PRJNA729271	stage_3W_vs_E10	ordinary_muscle_development	ENSGALG00010027902	UBE2Q1	0.478196386	0.069761732	6.854709241	7.14577E-12	1.83681E-11	positive
PRJNA729271	stage_3W_vs_E10	ordinary_muscle_development	ENSGALG00010027903	NDUFA8	0.782547259	0.125453212	6.237761831	4.43876E-10	1.03457E-09	positive
PRJNA729271	stage_3W_vs_E10	ordinary_muscle_development	ENSGALG00010027904	MORN5	-2.268785553	0.603297782	-3.760639642	0.000169479	0.000271688	negative
PRJNA729271	stage_3W_vs_E10	ordinary_muscle_development	ENSGALG00010027905	SYN2	0.527602449	0.267427005	1.972883963	0.048508784	0.060998944	positive
PRJNA729271	stage_3W_vs_E10	ordinary_muscle_development	ENSGALG00010027906	LHX6	2.244052298	0.172113593	13.03820492	7.41831E-39	5.75942E-38	positive
PRJNA729271	stage_3W_vs_E10	ordinary_muscle_development	ENSGALG00010027907	MKNK2	2.383473664	0.188337514	12.65533145	1.04533E-36	7.54964E-36	positive
PRJNA729271	stage_3W_vs_E10	ordinary_muscle_development	ENSGALG00010027908	PBXIP1	1.438547271	0.106342846	13.52744753	1.07699E-41	9.16133E-41	positive
PRJNA729271	stage_3W_vs_E10	ordinary_muscle_development	ENSGALG00010027909	TIMP4	0.560267923	0.249685775	2.243892039	0.024839349	0.032331749	positive
PRJNA729271	stage_3W_vs_E10	ordinary_muscle_development	ENSGALG00010027910	CHRNB2	-0.183347333	0.189673905	-0.966645009	0.333721526	0.372070881	negative
PRJNA729271	stage_3W_vs_E10	ordinary_muscle_development	ENSGALG00010027911	PTPN22	2.560095814	0.332437586	7.700981843	1.35025E-14	4.00078E-14	positive
PRJNA729271	stage_3W_vs_E10	ordinary_muscle_development	ENSGALG00010027912	RGS3	-1.29635666	0.132437038	-9.788475204	1.26184E-22	5.35591E-22	negative
PRJNA729271	stage_3W_vs_E10	ordinary_muscle_development	ENSGALG00010027913	PYGO2	-0.417747003	0.224891963	-1.857545275	0.063233616	0.078386322	negative
PRJNA729271	stage_3W_vs_E10	ordinary_muscle_development	ENSGALG00010027914	BTBD2	1.645057143	0.124907544	13.17019844	1.30255E-39	1.04024E-38	positive
PRJNA729271	stage_3W_vs_E10	ordinary_muscle_development	ENSGALG00010027915	GSTM3	0.193608289	0.083559565	2.317009295	0.020503225	0.026941733	positive
PRJNA729271	stage_3W_vs_E10	ordinary_muscle_development	ENSGALG00010027916	ADAR	0.037841762	0.128753072	0.293909584	0.768826994	0.793912323	positive
PRJNA729271	stage_3W_vs_E10	ordinary_muscle_development	ENSGALG00010027917	PPARG	0.758356162	0.451971048	1.677886592	0.093369241	0.113377494	positive
PRJNA729271	stage_3W_vs_E10	ordinary_muscle_development	ENSGALG00010027918	CSNK1G2	0.522690029	0.100541568	5.198745556	2.00638E-07	3.97627E-07	positive
PRJNA729271	stage_3W_vs_E10	ordinary_muscle_development	ENSGALG00010027919	TSEN2	0.03225973	0.117603541	0.274309172	0.783847036	0.807696225	positive
PRJNA729271	stage_3W_vs_E10	ordinary_muscle_development	ENSGALG00010027920	SCAMP4	-0.517387859	0.113852901	-4.544353745	5.51041E-06	9.92215E-06	negative
PRJNA729271	stage_3W_vs_E10	ordinary_muscle_development	ENSGALG00010027921	MKRN2OS	1.06872644	0.231298636	4.620547957	3.82728E-06	6.97992E-06	positive
PRJNA729271	stage_3W_vs_E10	ordinary_muscle_development	ENSGALG00010027922	SHC1	-0.031045316	0.113434671	-0.273684537	0.784327062	0.808075966	negative
PRJNA729271	stage_3W_vs_E10	ordinary_muscle_development	ENSGALG00010027923	AMPD2	-0.356892056	0.155390831	-2.296738192	0.021633709	0.028368067	negative
PRJNA729271	stage_3W_vs_E10	ordinary_muscle_development	ENSGALG00010027925	MKRN2	-0.364235402	0.069693629	-5.226236703	1.72995E-07	3.44256E-07	negative
PRJNA729271	stage_3W_vs_E10	ordinary_muscle_development	ENSGALG00010027926	CKS1B	-3.353381862	0.258145982	-12.99025396	1.38965E-38	1.06859E-37	negative
PRJNA729271	stage_3W_vs_E10	ordinary_muscle_development	ENSGALG00010027927	ENSGALG00010027927	1.229164138	0.195606588	6.283858575	3.30271E-10	7.75143E-10	positive
PRJNA729271	stage_3W_vs_E10	ordinary_muscle_development	ENSGALG00010027928	FLAD1	0.687518816	0.159773508	4.303083929	1.68437E-05	2.92535E-05	positive
PRJNA729271	stage_3W_vs_E10	ordinary_muscle_development	ENSGALG00010027929	CDK5RAP2	-0.57898496	0.137157191	-4.221324133	2.42871E-05	4.16371E-05	negative
PRJNA729271	stage_3W_vs_E10	ordinary_muscle_development	ENSGALG00010027930	RAF1	1.209725184	0.089170301	13.56645842	6.33078E-42	5.43952E-41	positive
PRJNA729271	stage_3W_vs_E10	ordinary_muscle_development	ENSGALG00010027931	ABHD17A	0.767789965	0.1718938	4.46665305	7.94528E-06	1.41361E-05	positive
PRJNA729271	stage_3W_vs_E10	ordinary_muscle_development	ENSGALG00010027932	POLE3	-2.022033471	0.077580468	-26.06369277	9.4114E-150	8.8583E-148	negative
PRJNA729271	stage_3W_vs_E10	ordinary_muscle_development	ENSGALG00010027933	ENSGALG00010027933	2.68213474	0.218372833	12.28236454	1.12657E-34	7.66648E-34	positive
PRJNA729271	stage_3W_vs_E10	ordinary_muscle_development	ENSGALG00010027934	CNBP	0.070804132	0.08511798	0.831835203	0.40550197	0.444341628	positive
PRJNA729271	stage_3W_vs_E10	ordinary_muscle_development	ENSGALG00010027935	ISY1	-0.527440047	0.088166517	-5.982316916	2.19986E-09	4.90493E-09	negative
PRJNA729271	stage_3W_vs_E10	ordinary_muscle_development	ENSGALG00010027936	ENSGALG00010027936	-0.361922532	0.085899579	-4.213321376	2.51643E-05	4.30695E-05	negative
PRJNA729271	stage_3W_vs_E10	ordinary_muscle_development	ENSGALG00010027937	HDHD3	0.085649986	0.093468945	0.916346993	0.359484928	0.398252371	positive
PRJNA729271	stage_3W_vs_E10	ordinary_muscle_development	ENSGALG00010027938	ENSGALG00010027938	-0.068846957	0.109458236	-0.62897923	0.529362654	0.567202223	negative
PRJNA729271	stage_3W_vs_E10	ordinary_muscle_development	ENSGALG00010027939	BSPRY	-2.08767417	0.452882928	-4.609743585	4.03166E-06	7.33604E-06	negative
PRJNA729271	stage_3W_vs_E10	ordinary_muscle_development	ENSGALG00010027940	ZBTB7B	2.084092633	0.393899749	5.290921459	1.21702E-07	2.44975E-07	positive
PRJNA729271	stage_3W_vs_E10	ordinary_muscle_development	ENSGALG00010027941	KLF2	0.583124912	0.151316216	3.853684211	0.000116354	0.000189053	positive
PRJNA729271	stage_3W_vs_E10	ordinary_muscle_development	ENSGALG00010027942	KHDC4	0.635653193	0.123095228	5.163914174	2.41839E-07	4.76802E-07	positive
PRJNA729271	stage_3W_vs_E10	ordinary_muscle_development	ENSGALG00010027943	RAB43	-0.497678705	0.094573346	-5.262356967	1.4222E-07	2.84539E-07	negative
PRJNA729271	stage_3W_vs_E10	ordinary_muscle_development	ENSGALG00010027944	WDR31	-3.017189154	0.338931708	-8.902056315	5.48214E-19	2.01378E-18	negative
PRJNA729271	stage_3W_vs_E10	ordinary_muscle_development	ENSGALG00010027946	AP1M1	-0.349283173	0.092629588	-3.770751648	0.000162757	0.000261229	negative
PRJNA729271	stage_3W_vs_E10	ordinary_muscle_development	ENSGALG00010027947	GP9	1.95061515	0.322421095	6.049899277	1.44936E-09	3.27032E-09	positive
PRJNA729271	stage_3W_vs_E10	ordinary_muscle_development	ENSGALG00010027948	PRPF4	-0.26706751	0.113737839	-2.348097278	0.018869592	0.024876284	negative
PRJNA729271	stage_3W_vs_E10	ordinary_muscle_development	ENSGALG00010027950	CDC26	0.28198123	0.127502168	2.21157988	0.026995707	0.034972095	positive
PRJNA729271	stage_3W_vs_E10	ordinary_muscle_development	ENSGALG00010027951	FAM32A	0.426956553	0.104338807	4.092020654	4.27631E-05	7.19585E-05	positive
PRJNA729271	stage_3W_vs_E10	ordinary_muscle_development	ENSGALG00010027952	CIB3	0.743146719	0.461355048	1.610791344	0.107225208	0.129131721	positive
PRJNA729271	stage_3W_vs_E10	ordinary_muscle_development	ENSGALG00010027953	SLC31A1	-1.662082334	0.12165435	-13.6623338	1.70433E-42	1.4927E-41	negative
PRJNA729271	stage_3W_vs_E10	ordinary_muscle_development	ENSGALG00010027954	CFAP92	-0.415107004	0.228284629	-1.818374746	0.069006876	0.085084609	negative
PRJNA729271	stage_3W_vs_E10	ordinary_muscle_development	ENSGALG00010027955	ENSGALG00010027955	0.230955368	0.116905154	1.975578999	0.048202479	0.060634812	positive
PRJNA729271	stage_3W_vs_E10	ordinary_muscle_development	ENSGALG00010027956	RAB8A	-0.804016237	0.065471418	-12.2804159	1.15403E-34	7.84971E-34	negative
PRJNA729271	stage_3W_vs_E10	ordinary_muscle_development	ENSGALG00010027957	ENTREP3	-0.711440154	0.48157507	-1.477319318	0.139590062	0.165536934	negative
PRJNA729271	stage_3W_vs_E10	ordinary_muscle_development	ENSGALG00010027958	ENSGALG00010027958	0.966430695	0.138751512	6.965190384	3.2796E-12	8.59013E-12	positive
PRJNA729271	stage_3W_vs_E10	ordinary_muscle_development	ENSGALG00010027959	TPM4	-2.712564924	0.173612132	-15.62428207	4.97493E-55	6.57353E-54	negative
PRJNA729271	stage_3W_vs_E10	ordinary_muscle_development	ENSGALG00010027960	PPP6C	-0.002341413	0.120628716	-0.019410083	0.984513967	0.987101746	negative

PRJNA729271	stage_3W_vs_E10	ordinary_muscle_development	ENSGALG00010027961	TINCR	-5.382015546	1.055257061	-5.100193824	3.39306E-07	6.62389E-07	negative
PRJNA729271	stage_3W_vs_E10	ordinary_muscle_development	ENSGALG00010027962	SCAMP3	0.842403682	0.12614843	6.677876864	2.42429E-11	6.07538E-11	positive
PRJNA729271	stage_3W_vs_E10	ordinary_muscle_development	ENSGALG00010027964	RABEPK	-0.971377696	0.138398941	-7.018678689	2.23976E-12	5.92363E-12	negative
PRJNA729271	stage_3W_vs_E10	ordinary_muscle_development	ENSGALG00010027965	ACAD9	0.913671536	0.109430728	8.349314225	6.86604E-17	2.28894E-16	positive
PRJNA729271	stage_3W_vs_E10	ordinary_muscle_development	ENSGALG00010027966	CLK2	0.112356415	0.099779013	1.126052576	0.260143266	0.296002562	positive
PRJNA729271	stage_3W_vs_E10	ordinary_muscle_development	ENSGALG00010027968	FKBP15	0.053270954	0.097064608	0.548819549	0.583129293	0.619001033	positive
PRJNA729271	stage_3W_vs_E10	ordinary_muscle_development	ENSGALG00010027969	HSPA5	-1.153856374	0.129572347	-8.90511286	5.33318E-19	1.96104E-18	negative
PRJNA729271	stage_3W_vs_E10	ordinary_muscle_development	ENSGALG00010027970	GRIN3B	-0.195800087	0.178981214	-1.093970051	0.273968114	0.310733005	negative
PRJNA729271	stage_3W_vs_E10	ordinary_muscle_development	ENSGALG00010027971	ENSGALG00010027971	-0.598413959	0.488190712	-1.225779074	0.220281824	0.253271849	negative
PRJNA729271	stage_3W_vs_E10	ordinary_muscle_development	ENSGALG00010027972	TMEM259	0.343099525	0.105906885	3.23963381	0.001196833	0.00178057	positive
PRJNA729271	stage_3W_vs_E10	ordinary_muscle_development	ENSGALG00010027973	GAPVD1	0.685932643	0.064547843	10.62673224	2.23818E-26	1.11218E-25	positive
PRJNA729271	stage_3W_vs_E10	ordinary_muscle_development	ENSGALG00010027974	ENSGALG00010027974	1.088512418	0.281901531	3.861321415	0.000112775	0.000183486	positive
PRJNA729271	stage_3W_vs_E10	ordinary_muscle_development	ENSGALG00010027975	IQSEC1	-0.592581533	0.118405175	-5.004692869	5.59512E-07	1.07661E-06	negative
PRJNA729271	stage_3W_vs_E10	ordinary_muscle_development	ENSGALG00010027976	LRSAM1	0.170864246	0.097789808	1.747260267	0.080592202	0.098572607	positive
PRJNA729271	stage_3W_vs_E10	ordinary_muscle_development	ENSGALG00010027977	BOLA1	1.172415094	0.155680649	7.53089803	5.03925E-14	1.4539E-13	positive
PRJNA729271	stage_3W_vs_E10	ordinary_muscle_development	ENSGALG00010027979	RPL12	0.419819072	0.119548496	3.511705172	0.000445242	0.000688844	positive
PRJNA729271	stage_3W_vs_E10	ordinary_muscle_development	ENSGALG00010027980	NUP210	1.664401202	0.141187968	11.78854848	4.47182E-32	2.78432E-31	positive
PRJNA729271	stage_3W_vs_E10	ordinary_muscle_development	ENSGALG00010027981	CNN2	-1.124518096	0.158960188	-7.074212167	1.503E-12	4.01585E-12	negative
PRJNA729271	stage_3W_vs_E10	ordinary_muscle_development	ENSGALG00010027982	ENTPD2	1.362620065	0.249410397	5.463365117	4.67192E-08	9.65216E-08	positive
PRJNA729271	stage_3W_vs_E10	ordinary_muscle_development	ENSGALG00010027983	POGZ	-0.582990032	0.146984752	-3.966330015	7.29878E-05	0.000120606	negative
PRJNA729271	stage_3W_vs_E10	ordinary_muscle_development	ENSGALG00010027984	ARHGAP45	2.528781015	0.165457124	15.2836031	9.83493E-53	1.21325E-51	positive
PRJNA729271	stage_3W_vs_E10	ordinary_muscle_development	ENSGALG00010027985	PSMB4	0.776907091	0.094600257	8.212526248	2.16583E-16	7.03894E-16	positive
PRJNA729271	stage_3W_vs_E10	ordinary_muscle_development	ENSGALG00010027986	CGN	-1.200843804	0.281165137	-4.270955558	1.94637E-05	3.36626E-05	negative
PRJNA729271	stage_3W_vs_E10	ordinary_muscle_development	ENSGALG00010027987	TMEM141	0.382637259	0.138547822	2.761770286	0.005748891	0.00800482	positive
PRJNA729271	stage_3W_vs_E10	ordinary_muscle_development	ENSGALG00010027988	HDAC11	0.918551862	0.128098817	7.1706506	7.46422E-13	2.02686E-12	positive
PRJNA729271	stage_3W_vs_E10	ordinary_muscle_development	ENSGALG00010027989	ENSGALG00010027989	0.665481149	0.13861746	4.800846502	1.57996E-06	2.94971E-06	positive
PRJNA729271	stage_3W_vs_E10	ordinary_muscle_development	ENSGALG00010027990	ENSGALG00010027990	0.755560473	0.192705164	3.920810728	8.82516E-05	0.00014469	positive
PRJNA729271	stage_3W_vs_E10	ordinary_muscle_development	ENSGALG00010027991	ENSGALG00010027991	0.324049854	0.145906487	2.220942063	0.026354886	0.034181645	positive
PRJNA729271	stage_3W_vs_E10	ordinary_muscle_development	ENSGALG00010027993	CLIC3	0.013418119	0.173293877	0.077429853	0.938281593	0.945785236	positive
PRJNA729271	stage_3W_vs_E10	ordinary_muscle_development	ENSGALG00010027994	TUFT1	-1.172677465	0.16854934	-6.957472918	3.46431E-12	9.05919E-12	negative
PRJNA729271	stage_3W_vs_E10	ordinary_muscle_development	ENSGALG00010027995	POLR2E	0.168997787	0.082120925	2.057913825	0.039598406	0.050349026	positive
PRJNA729271	stage_3W_vs_E10	ordinary_muscle_development	ENSGALG00010027996	GPX4	0.634154961	0.121800283	5.20651466	1.9242E-07	3.81812E-07	positive
PRJNA729271	stage_3W_vs_E10	ordinary_muscle_development	ENSGALG00010027998	SNX27	0.96443173	0.09156539	10.53271032	6.10509E-26	2.97356E-25	positive
PRJNA729271	stage_3W_vs_E10	ordinary_muscle_development	ENSGALG00010027999	ENSGALG00010027999	-2.051616863	0.54286781	-3.779219957	0.00015732	0.000252811	negative
PRJNA729271	stage_3W_vs_E10	ordinary_muscle_development	ENSGALG00010028000	MRPL9	1.204292733	0.165854399	7.261144357	3.83829E-13	1.05671E-12	positive
PRJNA729271	stage_3W_vs_E10	ordinary_muscle_development	ENSGALG00010028001	SBNO2	0.819796224	0.11609758	7.061268836	1.64989E-12	4.39779E-12	positive
PRJNA729271	stage_3W_vs_E10	ordinary_muscle_development	ENSGALG00010028002	ABCA2	-0.376688467	0.121346836	-3.104229831	0.001907751	0.002780578	negative
PRJNA729271	stage_3W_vs_E10	ordinary_muscle_development	ENSGALG00010028004	ENSGALG00010028004	-0.396389241	0.279766395	-1.416857951	0.156524462	0.184187537	negative
PRJNA729271	stage_3W_vs_E10	ordinary_muscle_development	ENSGALG00010028005	IER5L	-0.134183256	0.14360763	-0.93437414	0.350110932	0.388639758	negative
PRJNA729271	stage_3W_vs_E10	ordinary_muscle_development	ENSGALG00010028006	ENSGALG00010028006	3.541587628	0.796990253	4.44370256	8.84238E-06	1.56726E-05	positive
PRJNA729271	stage_3W_vs_E10	ordinary_muscle_development	ENSGALG00010028007	STK11	1.001316995	0.119415473	8.385152882	5.06599E-17	1.69862E-16	positive
PRJNA729271	stage_3W_vs_E10	ordinary_muscle_development	ENSGALG00010028008	NTMT1	0.883518391	0.11248551	7.854508493	4.01343E-15	1.22216E-14	positive
PRJNA729271	stage_3W_vs_E10	ordinary_muscle_development	ENSGALG00010028009	RRP9	-1.165286009	0.180520961	-6.455128562	1.08127E-10	2.60219E-10	negative
PRJNA729271	stage_3W_vs_E10	ordinary_muscle_development	ENSGALG00010028010	ASB6	-0.471311537	0.073093012	-6.448106644	1.13256E-10	2.72337E-10	negative
PRJNA729271	stage_3W_vs_E10	ordinary_muscle_development	ENSGALG00010028011	ENSGALG00010028011	-0.9012541	0.09574949	-9.412625597	4.83895E-21	1.93065E-20	negative
PRJNA729271	stage_3W_vs_E10	ordinary_muscle_development	ENSGALG00010028012	PRRX2	-3.506399385	0.254414495	-13.78223116	3.26038E-43	2.92264E-42	negative
PRJNA729271	stage_3W_vs_E10	ordinary_muscle_development	ENSGALG00010028013	CBARP	0.607407981	0.214413929	2.832875567	0.004613134	0.006493239	positive
PRJNA729271	stage_3W_vs_E10	ordinary_muscle_development	ENSGALG00010028014	ENSGALG00010028014	1.096905275	0.291946999	3.757206889	0.00017182	0.000275319	positive
PRJNA729271	stage_3W_vs_E10	ordinary_muscle_development	ENSGALG00010028015	DUSP7	-0.681424502	0.101667449	-6.702484513	2.04905E-11	5.15106E-11	negative
PRJNA729271	stage_3W_vs_E10	ordinary_muscle_development	ENSGALG00010028016	PRF1	4.902552302	1.239434433	3.955475314	7.63826E-05	0.000125943	positive
PRJNA729271	stage_3W_vs_E10	ordinary_muscle_development	ENSGALG00010028017	ENSGALG00010028017	-3.091091952	1.340854463	-2.305315034	0.021148936	0.027762528	negative
PRJNA729271	stage_3W_vs_E10	ordinary_muscle_development	ENSGALG00010028019	RPL29	0.899944495	0.227110613	3.9625823	7.41434E-05	0.000122418	positive
PRJNA729271	stage_3W_vs_E10	ordinary_muscle_development	ENSGALG00010028020	ENSGALG00010028020	0.371791145	0.153806115	2.417271538	0.015637347	0.020804345	positive
PRJNA729271	stage_3W_vs_E10	ordinary_muscle_development	ENSGALG00010028021	ATP5F1D	1.569836043	0.145107961	10.81840056	2.8165E-27	1.45492E-26	positive
PRJNA729271	stage_3W_vs_E10	ordinary_muscle_development	ENSGALG00010028022	ENSGALG00010028022	-0.613949502	0.14347714	-4.279075425	1.87671E-05	3.24889E-05	negative
PRJNA729271	stage_3W_vs_E10	ordinary_muscle_development	ENSGALG00010028023	ENSGALG00010028023	1.238836334	0.892836597	1.387528623	0.165280629	0.193846001	positive
PRJNA729271	stage_3W_vs_E10	ordinary_muscle_development	ENSGALG00010028024	RBM3	-2.196769615	0.113065758	-19.42913277	4.37694E-84	1.22953E-82	negative
PRJNA729271	stage_3W_vs_E10	ordinary_muscle_development	ENSGALG00010028025	TNNC1	4.395780206	0.23708712	18.54078031	9.68069E-77	2.22028E-75	positive
PRJNA729271	stage_3W_vs_E10	ordinary_muscle_development	ENSGALG00010028026	ANKMY1	1.018682788	0.164366392	6.197634281	5.73181E-10	1.3272E-09	positive
PRJNA729271	stage_3W_vs_E10	ordinary_muscle_development	ENSGALG00010028027	ENSGALG00010028027	1.459995372	0.229952959	6.34910453	2.16572E-10	5.14035E-10	positive
PRJNA729271	stage_3W_vs_E10	ordinary_muscle_development	ENSGALG00010028028	FAM174C	-0.238210565	0.163232648	-1.459331629	0.144473854	0.170950899	negative
PRJNA729271	stage_3W_vs_E10	ordinary_muscle_development	ENSGALG00010028029	EFNA2	-6.964263926	0.355819032	-19.57248854	2.654E-85	7.7404E-84	negative
PRJNA729271	stage_3W_vs_E10	ordinary_muscle_development	ENSGALG00010028030	PFDN2	0.241608119	0.1350579	1.788922516	0.073627295	0.090496705	positive
PRJNA729271	stage_3W_vs_E10	ordinary_muscle_development	ENSGALG00010028031	NISCH	0.844479337	0.080666373	10.4687902	1.20169E-25	5.77536E-25	positive
PRJNA729271	stage_3W_vs_E10	ordinary_muscle_development	ENSGALG00010028032	ENSGALG00010028032	-4.577258102	0.342198297	-13.37603999	8.34779E-41	6.90252E-40	negative
PRJNA729271	stage_3W_vs_E10	ordinary_muscle_development	ENSGALG00010028033	ENSGALG00010028033	3.069710138	0.290778384	10.55687186	4.72134E-26	2.31202E-25	positive
PRJNA729271	stage_3W_vs_E10	ordinary_muscle_development	ENSGALG00010028034	MUM1L1	-0.399528727	0.076223143	-5.241562225	1.59218E-07	3.17451E-07	negative
PRJNA729271	stage_3W_vs_E10	ordinary_muscle_development	ENSGALG00010028035	ENSGALG00010028035	1.006767938	1.058460841	0.951162196	0.341522046	0.379920342	positive
PRJNA729271	stage_3W_vs_E10	ordinary_muscle_development	ENSGALG00010028036	NDUFS7	2.01645489	0.121949487	16.53516498	2.04817E-61	3.18543E-60	positive
PRJNA729271	stage_3W_vs_E10	ordinary_muscle_development	ENSGALG00010028037	ENSGALG00010028037	0.14633533	0.27548377	0.531194015	0.59528434	0.630887366	positive
PRJNA729271	stage_3W_vs_E10	ordinary_muscle_development	ENSGALG00010028039	STAB1	2.459374852	0.162257807	15.15720505	6.78978E-52	8.16746E-51	positive
PRJNA729271	stage_3W_vs_E10	ordinary_muscle_development	ENSGALG00010028042	DPP7	1.078945667	0.19110905	5.645706831	1.64504E-08	3.50248E-08	positive
PRJNA729271	stage_3W_vs_E10	ordinary_muscle_development	ENSGALG00010028043	NT5DC2	1.931997952	0.115583824	16.71512406	1.01708E-62	1.63625E-61	positive
PRJNA729271	stage_3W_vs_E10	ordinary_muscle_development	ENSGALG00010028044	GAMT	3.377327939	0.153468676	22.00662721	2.4883E-107	1.2088E-105	positive
PRJNA729271	stage_3W_vs_E10	ordinary_muscle_development	ENSGALG00010028045	ENSGALG00010028045	-0.275629752	0.567846063	-0.485395198	0.627396038	0.661149078	negative
PRJNA729271	stage_3W_vs_E10	ordinary_muscle_development	ENSGALG00010028046	SMIM4	2.852845893	0.202968448	14.05561271	7.11633E-45	6.75516E-44	positive
PRJNA729271	stage_3W_vs_E10	ordinary_muscle_development	ENSGALG00010028047	ENSGALG00010028047	1.14332916	0.288757398	3.959480057	7.51131E-05	0.00012392	positive
PRJNA729271	stage_3W_vs_E10	ordinary_muscle_development	ENSGALG00010028048	S100A10	2.027989494	0.218378	9.286601635	1.59293E-20	6.21187E-20	positive
PRJNA729271	stage_3W_vs_E10	ordinary_muscle_development	ENSGALG00010028049	MAN1B1	-0.005636439	0.060360883	-0.093378994	0.925602478	0.934174065	negative
PRJNA729271	stage_3W_vs_E10	ordinary_muscle_development	ENSGALG00010028050	PBRM1	-0.858612903	0.087829897	-9.775861481	1.42936E-22	6.04569E-22	negative
PRJNA729271	stage_3W_vs_E10	ordinary_muscle_development	ENSGALG00010028051	GNL3	-0.566310989	0.092821926	-6.101047623	1.05376E-09	2.39745E-09	negative
PRJNA729271	stage_3W_vs_E10	ordinary_muscle_development	ENSGALG00010028052	ENSGALG00010028052	-4.113794784	0.222337027	-18.50251773	1.9705E-76	4.49095E-75	negative
PRJNA729271	stage_3W_vs_E10	ordinary_muscle_development	ENSGALG00010028053	ORM1	1.335996464	2.179049036	0.613109867	0.539803697	0.577450523	positive
PRJNA729271	stage_3W_vs_E10	ordinary_muscle_development	ENSGALG00010028054	RPS15	-0.068188006	0.14079093	-0.484321014	0.628158065	0.661807759	negative
PRJNA729271	stage_3W_vs_E10	ordinary_muscle_development	ENSGALG00010028055	ENSGALG00010028055	-0.25182827	0.117887368	-2.136176877	0.032664999	0.041923071	negative
PRJNA729271	stage_3W_vs_E10	ordinary_muscle_development	ENSGALG00010028056	SLC25A25	1.462468328	0.236391696	6.186631562	6.14634E-10	1.41978E-09	positive
PRJNA729271	stage_3W_vs_E10	ordinary_muscle_development	ENSGALG00010028057	APC2	-1.270852223	0.389641327	-3.26159505	0.001107873	0.001654163	negative
PRJNA729271	stage_3W_vs_E10	ordinary_muscle_development	ENSGALG00010028058	GLT8D1	-1.144432258	0.115786222	-9.884010725	4.88382E-23	2.11316E-22	negative
PRJNA729271	stage_3W_vs_E10	ordinary_muscle_development	ENSGALG00010028059	GZMM	3.207311532	0.557035607	5.757821387	8.52064E-09	1.84614E-08	positive
PRJNA729271	stage_3W_vs_E10	ordinary_muscle_development	ENSGALG00010028060	ENSGALG00010028060	0.802094151	0.14102742	5.687504963	1.28909E-08	2.76369E-08	positive
PRJNA729271	stage_3W_vs_E10	ordinary_muscle_development	ENSGALG00010028061	NAIF1	-0.880062903	0.095873369	-9.179430269	4.33378E-20	1.66098E-19	negative
PRJNA729271	stage_3W_vs_E10	ordinary_muscle_development	ENSGALG00010028062	ENSGALG00010028062	0.517725811	0.489194687	1.058322636	0.289908377	0.327054892	positive
PRJNA729271	stage_3W_vs_E10	ordinary_muscle_development	ENSGALG00010028064	SPCS1	-0.092657107	0.087470866	-1.059291067	0.289467242	0.326701524	negative
PRJNA729271	stage_3W_vs_E10	ordinary_muscle_development	ENSGALG00010028065	REEP6	2.695915553	0.774744994	3.479745687	0.00050189	0.000773185	positive
PRJNA729271	stage_3W_vs_E10	ordinary_muscle_development	ENSGALG00010028066	ENSGALG00010028066	2.066745755	0.182899335	11.29990851	1.31355E-29	7.44036E-29	positive
PRJNA729271	stage_3W_vs_E10	ordinary_muscle_development	ENSGALG00010028067	FAM102A	-0.550881131	0.1129249	-4.878296402	1.07006E-06	2.02171E-06	negative
PRJNA729271	stage_3W_vs_E10	ordinary_muscle_development	ENSGALG00010028068	POLE4	-0.586749278	0.111100789	-5.281234101	1.28317E-07	2.57825E-07	negative
PRJNA729271	stage_3W_vs_E10	ordinary_muscle_development	ENSGALG00010028069	NEK4	-1.073336691	0.115572772	-9.287106908	1.58539E-20	6.18413E-20	negative
PRJNA729271	stage_3W_vs_E10	ordinary_muscle_development	ENSGALG00010028070	MEX3A	-4.325471865	0.173063168	-24.99360162	7.1755E-138	5.5919E-136	negative
PRJNA729271	stage_3W_vs_E10	ordinary_muscle_development	ENSGALG00010028071	DPM2	-0.814442576	0.115865734	-7.029192723	2.07732E-12	5.50974E-12	negative
PRJNA729271	stage_3W_vs_E10	ordinary_muscle_development	ENSGALG00010028072	ENSGALG00010028072	-7.593279038	0.991157905	-7.661018492	1.84464E-14	5.43347E-14	negative
PRJNA729271	stage_3W_vs_E10	ordinary_muscle_development	ENSGALG00010028073	ST6GALNAC4	3.07580703	0.120535069	25.51794315	1.2464E-143	1.069E-141	positive
PRJNA729271	stage_3W_vs_E10	ordinary_muscle_development	ENSGALG00010028074	ENSGALG00010028074	2.141926925	0.440183509	4.865986299	1.13887E-06	2.14864E-06	positive
PRJNA729271	stage_3W_vs_E10	ordinary_muscle_development	ENSGALG00010028075	RAB25	-0.861809772	0.521896405	-1.651304288	0.098676457	0.119352073	negative
PRJNA729271	stage_3W_vs_E10	ordinary_muscle_development	ENSGALG00010028076	ST6GALNAC6	1.386996926	0.1525482	9.092188077	9.70656E-20	3.67258E-19	positive
PRJNA729271	stage_3W_vs_E10	ordinary_muscle_development	ENSGALG00010028078	LAMTOR2	0.911523837	0.1305561	6.981855623	2.91306E-12	7.64805E-12	positive
PRJNA729271	stage_3W_vs_E10	ordinary_muscle_development	ENSGALG00010028079	ITIH3	4.26799428	1.542416269	2.767083287	0.00565603	0.007884609	positive
PRJNA729271	stage_3W_vs_E10	ordinary_muscle_development	ENSGALG00010028080	MUSTN1	3.9869231	0.404865066	9.847535469	7.02453E-23	3.0178E-22	positive
PRJNA729271	stage_3W_vs_E10	ordinary_muscle_development	ENSGALG00010028081	UBQLN4	-0.338250727	0.162422165	-2.082540438	0.037293133	0.047589045	negative
PRJNA729271	stage_3W_vs_E10	ordinary_muscle_development	ENSGALG00010028082	AK1	6.962460736	0.137104288	50.78222435	0	0	positive
PRJNA729271	stage_3W_vs_E10	ordinary_muscle_development	ENSGALG00010028083	ELAVL1	-0.297008957	0.049245967	-6.031132575	1.62815E-09	3.66402E-09	negative
PRJNA729271	stage_3W_vs_E10	ordinary_muscle_development	ENSGALG00010028084	ENG	1.436319229	0.192346548	7.46735122	8.18252E-14	2.33238E-13	positive
PRJNA729271	stage_3W_vs_E10	ordinary_muscle_development	ENSGALG00010028085	ENSGALG00010028085	-1.096217269	0.290909669	-3.768239372	0.000164403	0.000263754	negative
PRJNA729271	stage_3W_vs_E10	ordinary_muscle_development	ENSGALG00010028086	MATK	1.177160093	0.256148934	4.595608015	4.31489E-06	7.83175E-06	positive
PRJNA729271	stage_3W_vs_E10	ordinary_muscle_development	ENSGALG00010028087	STIMATE	-0.658369585	0.089341274	-7.369153786	1.71714E-13	4.81245E-13	negative
PRJNA729271	stage_3W_vs_E10	ordinary_muscle_development	ENSGALG00010028088	NAXE	0.988264363	0.114577649	8.625280548	6.39357E-18	2.24721E-17	positive
PRJNA729271	stage_3W_vs_E10	ordinary_muscle_development	ENSGALG00010028091	FPGS	-1.064218305	0.1019939	-10.43413676	1.73181E-25	8.26829E-25	negative
PRJNA729271	stage_3W_vs_E10	ordinary_muscle_development	ENSGALG00010028093	SFMBT1	-0.566383143	0.141307685	-4.008155274	6.11949E-05	0.000101839	negative
PRJNA729271	stage_3W_vs_E10	ordinary_muscle_development	ENSGALG00010028095	CDK9	-0.285857761	0.093938207	-3.043040424	0.002342009	0.003388304	negative
PRJNA729271	stage_3W_vs_E10	ordinary_muscle_development	ENSGALG00010028096	TOR2A	-0.998178521	0.153798737	-6.490160728	8.57448E-11	2.07908E-10	negative
PRJNA729271	stage_3W_vs_E10	ordinary_muscle_development	ENSGALG00010028097	TTC24	1.338042324	0.221265987	6.047211954	1.47374E-09	3.32221E-09	positive
PRJNA729271	stage_3W_vs_E10	ordinary_muscle_development	ENSGALG00010028098	RFT1	-0.373981208	0.119389113	-3.132456541	0.001733501	0.002541428	negative
PRJNA729271	stage_3W_vs_E10	ordinary_muscle_development	ENSGALG00010028099	PCASP2	3.227732976	0.243582028	13.25111297	4.44561E-40	3.60196E-39	positive
PRJNA729271	stage_3W_vs_E10	ordinary_muscle_development	ENSGALG00010028100	ENSGALG00010028100	2.02962321	0.442699571	4.584651406	4.54744E-06	8.24146E-06	positive
PRJNA729271	stage_3W_vs_E10	ordinary_muscle_development	ENSGALG00010028101	MRPL54	1.287209066	0.126475406	10.17755445	2.49785E-24	1.13713E-23	positive
PRJNA729271	stage_3W_vs_E10	ordinary_muscle_development	ENSGALG00010028102	CERCAM	-0.952668253	0.181011494	-5.263026294	1.41703E-07	2.83544E-07	negative
PRJNA729271	stage_3W_vs_E10	ordinary_muscle_development	ENSGALG00010028103	APBA3	0.348056907	0.131407972	2.648674211	0.008080819	0.011098301	positive
PRJNA729271	stage_3W_vs_E10	ordinary_muscle_development	ENSGALG00010028104	GOLGA1	0.402607538	0.114789498	3.507355144	0.000452585	0.000699831	positive
PRJNA729271	stage_3W_vs_E10	ordinary_muscle_development	ENSGALG00010028105	PRKCD	-0.353621822	0.140666532	-2.513901621	0.011940374	0.016104561	negative
PRJNA729271	stage_3W_vs_E10	ordinary_muscle_development	ENSGALG00010028106	ARPC5L	0.595685966	0.102582792	5.806880038	6.36477E-09	1.38816E-08	positive
PRJNA729271	stage_3W_vs_E10	ordinary_muscle_development	ENSGALG00010028107	NOTCH1	-0.911066167	0.12493114	-7.292546675	3.0415E-13	8.40224E-13	negative

PRJNA729271	stage_3W_vs_E10	ordinary_muscle_development	ENSGALG00010028108	IQGAP3	-3.143565276	0.219952181	-14.29203955	2.45318E-46	2.45572E-45	negative
PRJNA729271	stage_3W_vs_E10	ordinary_muscle_development	ENSGALG00010028109	TJP3	-0.285246242	0.218301751	-1.30666035	0.191328091	0.222023911	negative
PRJNA729271	stage_3W_vs_E10	ordinary_muscle_development	ENSGALG00010028110	COQ4	0.27378029	0.101816926	2.688946725	0.007167786	0.009894015	positive
PRJNA729271	stage_3W_vs_E10	ordinary_muscle_development	ENSGALG00010028111	TRUB2	-0.586473095	0.090271556	-6.496765073	8.20655E-11	1.99086E-10	negative
PRJNA729271	stage_3W_vs_E10	ordinary_muscle_development	ENSGALG00010028112	SWI5	0.230363684	0.081182083	2.837617315	0.004545164	0.006403163	positive
PRJNA729271	stage_3W_vs_E10	ordinary_muscle_development	ENSGALG00010028113	PIP5K1C	-1.05470716	0.098855562	-10.66917369	1.41882E-26	7.12608E-26	negative
PRJNA729271	stage_3W_vs_E10	ordinary_muscle_development	ENSGALG00010028114	PFKFB4	-0.298148898	0.090961062	-3.277764027	0.001046328	0.001565335	negative
PRJNA729271	stage_3W_vs_E10	ordinary_muscle_development	ENSGALG00010028115	CACTIN	0.473386413	0.175154925	2.702672579	0.006878447	0.009507303	positive
PRJNA729271	stage_3W_vs_E10	ordinary_muscle_development	ENSGALG00010028116	HYAL1	1.333962044	0.147788074	9.026181961	1.77766E-19	6.65826E-19	positive
PRJNA729271	stage_3W_vs_E10	ordinary_muscle_development	ENSGALG00010028117	ENSGALG00010028117	0.710376155	0.228311928	3.111428131	0.001861848	0.002717226	positive
PRJNA729271	stage_3W_vs_E10	ordinary_muscle_development	ENSGALG00010028118	ENSGALG00010028118	0.082987918	0.077454275	1.071443995	0.28396984	0.321122081	positive
PRJNA729271	stage_3W_vs_E10	ordinary_muscle_development	ENSGALG00010028119	HYAL2	0.103839399	0.128169224	0.810174203	0.417840062	0.456515542	positive
PRJNA729271	stage_3W_vs_E10	ordinary_muscle_development	ENSGALG00010028120	TBXA2R	-0.546768411	0.258872984	-2.112110747	0.034676951	0.044399118	negative
PRJNA729271	stage_3W_vs_E10	ordinary_muscle_development	ENSGALG00010028121	TUSC2	0.441602305	0.095722163	4.613375764	3.96181E-06	7.21619E-06	positive
PRJNA729271	stage_3W_vs_E10	ordinary_muscle_development	ENSGALG00010028122	SEC16A	-0.163198843	0.088793494	-1.837959468	0.066068378	0.081690791	negative
PRJNA729271	stage_3W_vs_E10	ordinary_muscle_development	ENSGALG00010028123	RASSF1	0.25735706	0.106012902	2.427601301	0.015199042	0.020250952	positive
PRJNA729271	stage_3W_vs_E10	ordinary_muscle_development	ENSGALG00010028124	SUGP2	-1.061050583	0.09013127	-11.77228044	5.42382E-32	3.36407E-31	negative
PRJNA729271	stage_3W_vs_E10	ordinary_muscle_development	ENSGALG00010028125	HMG20B	-1.169696968	0.122661339	-9.535987298	1.48466E-21	6.05519E-21	negative
PRJNA729271	stage_3W_vs_E10	ordinary_muscle_development	ENSGALG00010028126	ZMYND10	1.056060815	0.080306235	13.15042127	1.69229E-39	1.34705E-38	positive
PRJNA729271	stage_3W_vs_E10	ordinary_muscle_development	ENSGALG00010028127	MFSD12	0.024913231	0.170501797	0.146117115	0.883828936	0.89751299	positive
PRJNA729271	stage_3W_vs_E10	ordinary_muscle_development	ENSGALG00010028128	DNM1	-2.233684473	0.144043037	-15.50706314	3.10772E-54	4.01841E-53	negative
PRJNA729271	stage_3W_vs_E10	ordinary_muscle_development	ENSGALG00010028129	CYB561D2	0.854060824	0.104671628	8.159430005	3.36609E-16	1.08137E-15	positive
PRJNA729271	stage_3W_vs_E10	ordinary_muscle_development	ENSGALG00010028130	TMEM115	0.812106305	0.126519999	6.418797904	1.37355E-10	3.28866E-10	positive
PRJNA729271	stage_3W_vs_E10	ordinary_muscle_development	ENSGALG00010028131	TMEM161A	1.08248384	0.203091078	5.330041335	9.81904E-08	1.98864E-07	positive
PRJNA729271	stage_3W_vs_E10	ordinary_muscle_development	ENSGALG00010028132	CACNA2D2	-0.522704551	0.133618595	-3.91191474	9.15672E-05	0.000149889	negative
PRJNA729271	stage_3W_vs_E10	ordinary_muscle_development	ENSGALG00010028133	ENSGALG00010028133	1.038131596	0.299934489	3.461194472	0.000537784	0.000825673	positive
PRJNA729271	stage_3W_vs_E10	ordinary_muscle_development	ENSGALG00010028134	INPP5E	-1.025357036	0.072515366	-14.13985878	2.15717E-45	2.08872E-44	negative
PRJNA729271	stage_3W_vs_E10	ordinary_muscle_development	ENSGALG00010028136	C3orf18	-1.231842429	0.163530674	-7.532791261	4.9667E-14	1.43354E-13	negative
PRJNA729271	stage_3W_vs_E10	ordinary_muscle_development	ENSGALG00010028137	NUP214	-0.835894647	0.067278004	-12.42448642	1.92458E-35	1.34313E-34	negative
PRJNA729271	stage_3W_vs_E10	ordinary_muscle_development	ENSGALG00010028138	ENSGALG00010028138	-0.67306632	0.514486311	-1.308229793	0.19079538	0.221458923	negative
PRJNA729271	stage_3W_vs_E10	ordinary_muscle_development	ENSGALG00010028139	FZR1	-0.818085884	0.088185458	-9.276879715	1.74514E-20	6.79448E-20	negative
PRJNA729271	stage_3W_vs_E10	ordinary_muscle_development	ENSGALG00010028140	HEMK1	-0.635207617	0.111177368	-5.713461542	1.10701E-08	2.38356E-08	negative
PRJNA729271	stage_3W_vs_E10	ordinary_muscle_development	ENSGALG00010028141	CISH	2.747809849	0.186225994	14.75524333	2.84663E-49	3.13779E-48	positive
PRJNA729271	stage_3W_vs_E10	ordinary_muscle_development	ENSGALG00010028142	PMPCA	-0.086828896	0.122212748	-0.710473313	0.477410674	0.516114836	negative
PRJNA729271	stage_3W_vs_E10	ordinary_muscle_development	ENSGALG00010028144	FAM78A	1.535078978	0.184752928	8.30882085	9.66577E-17	3.19948E-16	positive
PRJNA729271	stage_3W_vs_E10	ordinary_muscle_development	ENSGALG00010028145	ENSGALG00010028145	-0.068845419	0.140222935	-0.490971177	0.623446834	0.657848282	negative
PRJNA729271	stage_3W_vs_E10	ordinary_muscle_development	ENSGALG00010028146	MAPKAPK3	0.421634573	0.105836568	3.983826967	6.78143E-05	0.000112365	positive
PRJNA729271	stage_3W_vs_E10	ordinary_muscle_development	ENSGALG00010028147	SLC25A42	1.118091417	0.199329159	5.609271731	2.0318E-08	4.30192E-08	positive
PRJNA729271	stage_3W_vs_E10	ordinary_muscle_development	ENSGALG00010028148	ENSGALG00010028148	6.688267828	0.25505831	26.22250512	1.472E-151	1.432E-149	positive
PRJNA729271	stage_3W_vs_E10	ordinary_muscle_development	ENSGALG00010028149	SNAPC4	0.00195281	0.089040818	0.021931629	0.982502494	0.985153131	positive
PRJNA729271	stage_3W_vs_E10	ordinary_muscle_development	ENSGALG00010028150	ARMC6	-0.283854438	0.082083497	-3.458118223	0.000543963	0.000834629	negative
PRJNA729271	stage_3W_vs_E10	ordinary_muscle_development	ENSGALG00010028151	DOCK3	-2.028351091	0.278139909	-7.292556834	3.04127E-13	8.40224E-13	negative
PRJNA729271	stage_3W_vs_E10	ordinary_muscle_development	ENSGALG00010028152	ENSGALG00010028152	0.321022717	0.336087519	0.955175956	0.339488723	0.378035572	positive
PRJNA729271	stage_3W_vs_E10	ordinary_muscle_development	ENSGALG00010028153	MANF	-0.712433547	0.148139189	-4.809217289	1.51522E-06	2.83286E-06	negative
PRJNA729271	stage_3W_vs_E10	ordinary_muscle_development	ENSGALG00010028154	ENSGALG00010028154	-1.498907414	0.139223384	-10.76620444	4.97073E-27	2.54147E-26	negative
PRJNA729271	stage_3W_vs_E10	ordinary_muscle_development	ENSGALG00010028155	CIZ1	-0.330777512	0.07854672	-4.21121994	2.53995E-05	4.34568E-05	negative
PRJNA729271	stage_3W_vs_E10	ordinary_muscle_development	ENSGALG00010028156	DOHH	0.496246671	0.096297672	5.153257198	2.56E-07	5.04106E-07	positive
PRJNA729271	stage_3W_vs_E10	ordinary_muscle_development	ENSGALG00010028157	PRRC2B	-0.534459346	0.145180612	-3.68134104	0.00023201	0.000367339	negative
PRJNA729271	stage_3W_vs_E10	ordinary_muscle_development	ENSGALG00010028158	DDX49	-0.349208617	0.098527587	-3.54427249	0.000393698	0.000611777	negative
PRJNA729271	stage_3W_vs_E10	ordinary_muscle_development	ENSGALG00010028159	DCAF1	0.113693278	0.090155272	1.261082959	0.20727896	0.239403069	positive
PRJNA729271	stage_3W_vs_E10	ordinary_muscle_development	ENSGALG00010028160	ENSGALG00010028160	5.112046929	0.549204097	9.308100504	1.30139E-20	5.09828E-20	positive
PRJNA729271	stage_3W_vs_E10	ordinary_muscle_development	ENSGALG00010028161	ENSGALG00010028161	1.943397452	0.590211333	3.292714562	0.000992252	0.001487231	positive
PRJNA729271	stage_3W_vs_E10	ordinary_muscle_development	ENSGALG00010028162	CARD9	0.968255239	0.174600386	5.545550406	2.93031E-08	6.13266E-08	positive
PRJNA729271	stage_3W_vs_E10	ordinary_muscle_development	ENSGALG00010028163	GBP	3.116278616	0.345036059	9.031747641	1.68952E-19	6.33623E-19	positive
PRJNA729271	stage_3W_vs_E10	ordinary_muscle_development	ENSGALG00010028164	BBLN	-0.130976123	0.141153253	-0.927900138	0.353459373	0.392086448	negative
PRJNA729271	stage_3W_vs_E10	ordinary_muscle_development	ENSGALG00010028165	NFIC	1.668336935	0.155462975	10.73141008	7.24816E-27	3.6851E-26	positive
PRJNA729271	stage_3W_vs_E10	ordinary_muscle_development	ENSGALG00010028168	PTGES2	0.402756422	0.137916689	2.920287782	0.003497083	0.004982329	positive
PRJNA729271	stage_3W_vs_E10	ordinary_muscle_development	ENSGALG00010028169	DNLZ	1.666824588	0.141507244	11.77907608	5.00385E-32	3.11024E-31	positive
PRJNA729271	stage_3W_vs_E10	ordinary_muscle_development	ENSGALG00010028170	COPE	-1.292104008	0.094944505	-13.60904462	3.53839E-42	3.06386E-41	negative
PRJNA729271	stage_3W_vs_E10	ordinary_muscle_development	ENSGALG00010028171	RNF123	1.796865359	0.157973962	11.37444007	5.60615E-30	3.21826E-29	positive
PRJNA729271	stage_3W_vs_E10	ordinary_muscle_development	ENSGALG00010028172	CERS1	0.288913044	0.210345898	1.373514037	0.1695926	0.198517705	positive
PRJNA729271	stage_3W_vs_E10	ordinary_muscle_development	ENSGALG00010028173	GDF3	0.488611061	0.227118608	2.151347548	0.031448778	0.040444507	positive
PRJNA729271	stage_3W_vs_E10	ordinary_muscle_development	ENSGALG00010028174	GMPPB	-0.912922243	0.155725412	-5.862384522	4.56267E-09	1.00115E-08	negative
PRJNA729271	stage_3W_vs_E10	ordinary_muscle_development	ENSGALG00010028175	POMT1	-0.012407116	0.093965269	-0.132039385	0.89495314	0.906903367	negative
PRJNA729271	stage_3W_vs_E10	ordinary_muscle_development	ENSGALG00010028176	UPF1	0.030205629	0.094740464	0.318825008	0.749859214	0.776316642	positive
PRJNA729271	stage_3W_vs_E10	ordinary_muscle_development	ENSGALG00010028177	ENSGALG00010028177	6.08121679	0.388191782	15.66549597	2.6039E-55	3.46272E-54	positive
PRJNA729271	stage_3W_vs_E10	ordinary_muscle_development	ENSGALG00010028178	ENSGALG00010028178	-2.790472924	0.169208691	-16.49130964	4.23666E-61	6.53302E-60	negative
PRJNA729271	stage_3W_vs_E10	ordinary_muscle_development	ENSGALG00010028179	MCM2	-3.23918088	0.14621896	-22.15294702	9.7714E-109	4.9179E-107	negative
PRJNA729271	stage_3W_vs_E10	ordinary_muscle_development	ENSGALG00010028180	ODF2	-1.372784439	0.106273345	-12.91748591	3.5867E-38	2.72053E-37	negative
PRJNA729271	stage_3W_vs_E10	ordinary_muscle_development	ENSGALG00010028181	BGLAP	-0.986084121	0.284070349	-3.471267328	0.000518008	0.000796576	negative
PRJNA729271	stage_3W_vs_E10	ordinary_muscle_development	ENSGALG00010028182	SMG5	0.437088075	0.146426085	2.985042408	0.00283539	0.004075258	positive
PRJNA729271	stage_3W_vs_E10	ordinary_muscle_development	ENSGALG00010028183	CRTC1	0.20721558	0.097731418	2.120255537	0.033984501	0.043543299	positive
PRJNA729271	stage_3W_vs_E10	ordinary_muscle_development	ENSGALG00010028184	TMEM79	1.031606746	0.248340779	4.15399658	3.26718E-05	5.53827E-05	positive
PRJNA729271	stage_3W_vs_E10	ordinary_muscle_development	ENSGALG00010028185	GLMP	0.349341892	0.086921278	4.019060723	5.84306E-05	9.7353E-05	positive
PRJNA729271	stage_3W_vs_E10	ordinary_muscle_development	ENSGALG00010028186	CCT3	-0.098618593	0.132261165	-0.745635297	0.455887755	0.494358719	negative
PRJNA729271	stage_3W_vs_E10	ordinary_muscle_development	ENSGALG00010028187	TPRA1	0.62432325	0.069461507	8.988046398	2.51664E-19	9.3872E-19	positive
PRJNA729271	stage_3W_vs_E10	ordinary_muscle_development	ENSGALG00010028188	ENSGALG00010028188	0.399509164	0.109109161	3.661554729	0.000250689	0.000395703	positive
PRJNA729271	stage_3W_vs_E10	ordinary_muscle_development	ENSGALG00010028189	GPSM1	-0.238487419	0.107502651	-2.218432912	0.026525329	0.034390398	negative
PRJNA729271	stage_3W_vs_E10	ordinary_muscle_development	ENSGALG00010028190	ILF2	-1.103044615	0.081134504	-13.59525917	4.27245E-42	3.69065E-41	negative
PRJNA729271	stage_3W_vs_E10	ordinary_muscle_development	ENSGALG00010028191	KLHL26	0.280871606	0.100488987	2.795048641	0.005189191	0.007266507	positive
PRJNA729271	stage_3W_vs_E10	ordinary_muscle_development	ENSGALG00010028192	ENSGALG00010028192	2.313563197	0.311688065	7.422687808	1.14767E-13	3.24722E-13	positive
PRJNA729271	stage_3W_vs_E10	ordinary_muscle_development	ENSGALG00010028193	BRINP1	-6.655268103	0.365775436	-18.19495637	5.65842E-74	1.22599E-72	negative
PRJNA729271	stage_3W_vs_E10	ordinary_muscle_development	ENSGALG00010028194	ENSGALG00010028194	1.497396247	0.181578423	8.246553882	1.63028E-16	5.33187E-16	positive
PRJNA729271	stage_3W_vs_E10	ordinary_muscle_development	ENSGALG00010028195	ENSGALG00010028195	1.173620764	0.306208515	3.832750258	0.000126719	0.000205365	positive
PRJNA729271	stage_3W_vs_E10	ordinary_muscle_development	ENSGALG00010028196	ENSGALG00010028196	-1.450982555	0.097588226	-14.86841821	5.28472E-50	5.98454E-49	negative
PRJNA729271	stage_3W_vs_E10	ordinary_muscle_development	ENSGALG00010028197	INTS3	-0.27186719	0.15421087	-1.762957366	0.077907681	0.095474453	negative
PRJNA729271	stage_3W_vs_E10	ordinary_muscle_development	ENSGALG00010028198	UCK1	-0.223709122	0.092099955	-2.428981869	0.015141289	0.020179569	negative
PRJNA729271	stage_3W_vs_E10	ordinary_muscle_development	ENSGALG00010028199	DPP9	0.127000375	0.106451297	1.193037368	0.232854739	0.266753294	positive
PRJNA729271	stage_3W_vs_E10	ordinary_muscle_development	ENSGALG00010028201	ENSGALG00010028201	3.497416834	0.159854473	21.87875494	4.1399E-106	1.9357E-104	positive
PRJNA729271	stage_3W_vs_E10	ordinary_muscle_development	ENSGALG00010028202	QSOX2	-0.478729323	0.067266326	-7.11692391	1.10362E-12	2.96901E-12	negative
PRJNA729271	stage_3W_vs_E10	ordinary_muscle_development	ENSGALG00010028205	PRRT1B	-0.820439157	0.465164125	-1.76376275	0.077771931	0.095332274	negative
PRJNA729271	stage_3W_vs_E10	ordinary_muscle_development	ENSGALG00010028206	RBM15B	-0.598163967	0.095884194	-6.23840015	4.42069E-10	1.03069E-09	negative
PRJNA729271	stage_3W_vs_E10	ordinary_muscle_development	ENSGALG00010028207	ENSGALG00010028207	1.687786625	0.246449378	6.848410963	7.46748E-12	1.91747E-11	positive
PRJNA729271	stage_3W_vs_E10	ordinary_muscle_development	ENSGALG00010028211	GATAD2B	0.061645573	0.146624438	0.420431781	0.674170055	0.70546455	positive
PRJNA729271	stage_3W_vs_E10	ordinary_muscle_development	ENSGALG00010028216	NKX2-3	0.779274796	1.570831234	0.496090719	0.619830407	0.654318094	positive
PRJNA729271	stage_3W_vs_E10	ordinary_muscle_development	ENSGALG00010028221	TNFAIP8L1	-1.69141376	0.190956939	-8.857566371	8.17801E-19	2.98065E-18	negative
PRJNA729271	stage_3W_vs_E10	ordinary_muscle_development	ENSGALG00010028232	C9orf69	1.316736632	0.107831175	12.21109416	2.71217E-34	1.82342E-33	positive
PRJNA729271	stage_3W_vs_E10	ordinary_muscle_development	ENSGALG00010028239	EDMPN2	1.933090204	1.641856324	1.177380856	0.239043527	0.273367726	positive
PRJNA729271	stage_3W_vs_E10	ordinary_muscle_development	ENSGALG00010028242	ENSGALG00010028242	0.226683125	0.279464111	0.811135013	0.417288141	0.456050034	positive
PRJNA729271	stage_3W_vs_E10	ordinary_muscle_development	ENSGALG00010028245	JAC1	-0.397099888	1.48470997	-0.267459569	0.78911534	0.812489477	negative
PRJNA729271	stage_3W_vs_E10	ordinary_muscle_development	ENSGALG00010028247	MVP	-0.035999801	0.093500234	-0.385023645	0.700219898	0.7295635	negative
PRJNA729271	stage_3W_vs_E10	ordinary_muscle_development	ENSGALG00010028262	C1orf43	2.006266519	0.141645002	14.16404742	1.52927E-45	1.4907E-44	positive
PRJNA729271	stage_3W_vs_E10	ordinary_muscle_development	ENSGALG00010028270	RAPGEF1	0.734693278	0.1814535	4.048934168	5.14514E-05	8.6069E-05	positive
PRJNA729271	stage_3W_vs_E10	ordinary_muscle_development	ENSGALG00010028279	EDBETA	0.23092855	1.629315883	0.141733443	0.887290558	0.900397412	positive
PRJNA729271	stage_3W_vs_E10	ordinary_muscle_development	ENSGALG00010028283	ENSGALG00010028283	0.696206456	0.151451722	4.59688704	4.2885E-06	7.7858E-06	positive
PRJNA729271	stage_3W_vs_E10	ordinary_muscle_development	ENSGALG00010028286	KCTD12A	0.533532767	1.447828087	0.368505606	0.712496266	0.741288643	positive
PRJNA729271	stage_3W_vs_E10	ordinary_muscle_development	ENSGALG00010028292	ENSGALG00010028292	-1.731225979	0.564544748	-3.066587697	0.002165173	0.003142503	negative
PRJNA729271	stage_3W_vs_E10	ordinary_muscle_development	ENSGALG00010028301	FBN3	-4.771397654	0.186532373	-25.57946144	2.5824E-144	2.2281E-142	negative
PRJNA729271	stage_3W_vs_E10	ordinary_muscle_development	ENSGALG00010028311	ENSGALG00010028311	-3.979158528	0.315052653	-12.63013815	1.44025E-36	1.03708E-35	negative
PRJNA729271	stage_3W_vs_E10	ordinary_muscle_development	ENSGALG00010028314	MEF2D	1.99756006	0.213841828	9.341297157	9.5161E-21	3.75336E-20	positive
PRJNA729271	stage_3W_vs_E10	ordinary_muscle_development	ENSGALG00010028316	TIMM44	-0.401846456	0.081013055	-4.960267906	7.0396E-07	1.34563E-06	negative
PRJNA729271	stage_3W_vs_E10	ordinary_muscle_development	ENSGALG00010028318	NACC2	1.054905287	0.1919668	5.495248588	3.9016E-08	8.10923E-08	positive
PRJNA729271	stage_3W_vs_E10	ordinary_muscle_development	ENSGALG00010028321	RHBG	1.047718729	0.185591127	5.645306132	1.64888E-08	3.51013E-08	positive
PRJNA729271	stage_3W_vs_E10	ordinary_muscle_development	ENSGALG00010028322	STRBP	0.26210964	0.108683903	2.411669366	0.015879675	0.021115116	positive
PRJNA729271	stage_3W_vs_E10	ordinary_muscle_development	ENSGALG00010028326	CRB2	-1.810123312	0.260035412	-6.961064631	3.37711E-12	8.83755E-12	negative
PRJNA729271	stage_3W_vs_E10	ordinary_muscle_development	ENSGALG00010028328	HNRNPM	-1.421057734	0.166037703	-8.558644875	1.14202E-17	3.96683E-17	negative
PRJNA729271	stage_3W_vs_E10	ordinary_muscle_development	ENSGALG00010028329	HAPLN2	1.055170358	0.588497995	1.792988875	0.072974703	0.089747864	positive
PRJNA729271	stage_3W_vs_E10	ordinary_muscle_development	ENSGALG00010028337	ENSGALG00010028337	4.01353963	0.196695246	20.40486339	1.51384E-92	5.23703E-91	positive
PRJNA729271	stage_3W_vs_E10	ordinary_muscle_development	ENSGALG00010028339	BCAN	-4.036517315	0.521301662	-7.743150675	9.6983E-15	2.89312E-14	negative
PRJNA729271	stage_3W_vs_E10	ordinary_muscle_development	ENSGALG00010028344	LSM7	-2.053577425	0.097200582	-21.1272132	4.47172E-99	1.79054E-97	negative
PRJNA729271	stage_3W_vs_E10	ordinary_muscle_development	ENSGALG00010028346	DENND1A	-0.177502761	0.0742485	-2.390657866	0.016818217	0.022303756	negative
PRJNA729271	stage_3W_vs_E10	ordinary_muscle_development	ENSGALG00010028348	NES	-5.752736497	0.280373795	-20.51809621	1.48411E-93	5.3513E-92	negative
PRJNA729271	stage_3W_vs_E10	ordinary_muscle_development	ENSGALG00010028353	ENSGALG00010028353	1.010067771	1.620473986	0.623316252	0.53307672	0.570928555	positive
PRJNA729271	stage_3W_vs_E10	ordinary_muscle_development	ENSGALG00010028355	RBM18	-0.096339013	0.074092569	-1.300252019	0.193514607	0.224399538	negative
PRJNA729271	stage_3W_vs_E10	ordinary_muscle_development	ENSGALG00010028357	SPPL2C	-0.715928547	0.093557386	-7.652293184	1.97426E-14	5.80701E-14	negative
PRJNA729271	stage_3W_vs_E10	ordinary_muscle_development	ENSGALG00010028360	ENSGALG00010028360	-4.445122745	0.767860422	-5.788972341	7.08184E-09	1.54247E-08	negative
PRJNA729271	stage_3W_vs_E10	ordinary_muscle_development	ENSGALG00010028362	TMPRSS9	-3.015209139	0.438413038	-6.877553528	6.08891E-12	1.57044E-11	negative
PRJNA729271	stage_3W_vs_E10	ordinary_muscle_development	ENSGALG00010028364	MHM2	9.799601882	0.670888519	14.60690056	2.53804E-48	2.73931E-47	positive
PRJNA729271	stage_3W_vs_E10	ordinary_muscle_development	ENSGALG00010028366	MRRF	-0.027790249	0.087480092	-0.317675125	0.750731382	0.777108575	negative
PRJNA729271	stage_3W_vs_E10	ordinary_muscle_development	ENSGALG00010028368	TIMM13	0.175598504	0.121198357	1.448852179	0.147378868	0.174033132	positive
PRJNA729271	stage_3W_vs_E10	ordinary_muscle_development	ENSGALG00010028370	S100A12	4.044474731	0.657484255	6.151439677	7.67827E-10	1.76325E-09	positive

PRJNA729271	stage_3W_vs_E10	ordinary_muscle_development	ENSGALG00010028372	PTGS1	-0.240349279	0.164491081	-1.461169071	0.143969049	0.17039531	negative
PRJNA729271	stage_3W_vs_E10	ordinary_muscle_development	ENSGALG00010028374	FDPS	0.173742364	0.131447487	1.321762542	0.186247235	0.216578714	positive
PRJNA729271	stage_3W_vs_E10	ordinary_muscle_development	ENSGALG00010028375	LMNB2	-2.562564694	0.152983935	-16.75054761	5.61074E-63	9.10725E-62	negative
PRJNA729271	stage_3W_vs_E10	ordinary_muscle_development	ENSGALG00010028377	PDCL	-0.210372961	0.059670222	-3.525593717	0.000422535	0.000654692	negative
PRJNA729271	stage_3W_vs_E10	ordinary_muscle_development	ENSGALG00010028380	RUSC1	0.507369681	0.142988643	3.548321541	0.000387695	0.00060303	positive
PRJNA729271	stage_3W_vs_E10	ordinary_muscle_development	ENSGALG00010028384	ENSGALG00010028384	-1.567524937	1.16546189	-1.344981719	0.178631138	0.208240176	negative
PRJNA729271	stage_3W_vs_E10	ordinary_muscle_development	ENSGALG00010028386	NCAN	-7.504573835	0.883233916	-8.496700247	1.95056E-17	6.67928E-17	negative
PRJNA729271	stage_3W_vs_E10	ordinary_muscle_development	ENSGALG00010028391	MED26	0.603306805	0.112515974	5.361965821	8.23211E-08	1.67732E-07	positive
PRJNA729271	stage_3W_vs_E10	ordinary_muscle_development	ENSGALG00010028392	ASH1L	0.833200726	0.120790406	6.897904843	5.27751E-12	1.36432E-11	positive
PRJNA729271	stage_3W_vs_E10	ordinary_muscle_development	ENSGALG00010028394	ZBTB26	-0.115306519	0.103746405	-1.111426639	0.266384751	0.30274792	negative
PRJNA729271	stage_3W_vs_E10	ordinary_muscle_development	ENSGALG00010028399	SLC35E1	0.103496133	0.07921441	1.306531648	0.191371824	0.222056883	positive
PRJNA729271	stage_3W_vs_E10	ordinary_muscle_development	ENSGALG00010028405	S100A6	0.891403766	0.27989201	3.184813194	0.001448475	0.002139138	positive
PRJNA729271	stage_3W_vs_E10	ordinary_muscle_development	ENSGALG00010028411	LHX2	-6.43554749	0.98243113	-6.55063474	5.7293E-11	1.40162E-10	negative
PRJNA729271	stage_3W_vs_E10	ordinary_muscle_development	ENSGALG00010028412	ENSGALG00010028412	1.958155733	0.314558383	6.225094739	4.81265E-10	1.12045E-09	positive
PRJNA729271	stage_3W_vs_E10	ordinary_muscle_development	ENSGALG00010028414	KBTBD12	7.555551869	0.350293106	21.56922798	3.4948E-103	1.5587E-101	positive
PRJNA729271	stage_3W_vs_E10	ordinary_muscle_development	ENSGALG00010028415	FSD1	0.384829079	0.167832422	2.292936456	0.021851664	0.028635735	positive
PRJNA729271	stage_3W_vs_E10	ordinary_muscle_development	ENSGALG00010028418	ENSGALG00010028418	1.615501976	0.124134522	13.01412332	1.01703E-38	7.84974E-38	positive
PRJNA729271	stage_3W_vs_E10	ordinary_muscle_development	ENSGALG00010028419	ENSGALG00010028419	4.804092898	0.360826828	13.31412334	1.91608E-40	1.56913E-39	positive
PRJNA729271	stage_3W_vs_E10	ordinary_muscle_development	ENSGALG00010028420	ENSGALG00010028420	4.157147324	0.827278405	5.025088652	5.032E-07	9.70835E-07	positive
PRJNA729271	stage_3W_vs_E10	ordinary_muscle_development	ENSGALG00010028421	ZBTB7A	1.381225524	0.188786238	7.316346474	2.54813E-13	7.07842E-13	positive
PRJNA729271	stage_3W_vs_E10	ordinary_muscle_development	ENSGALG00010028422	RXRA	1.996516669	0.208064146	9.595678587	8.33667E-22	3.4319E-21	positive
PRJNA729271	stage_3W_vs_E10	ordinary_muscle_development	ENSGALG00010028425	DAP3	1.291518114	0.128905762	10.01908751	1.25658E-23	5.54293E-23	positive
PRJNA729271	stage_3W_vs_E10	ordinary_muscle_development	ENSGALG00010028426	GPR21	-0.921234541	0.159838898	-5.763519113	8.23779E-09	1.78726E-08	negative
PRJNA729271	stage_3W_vs_E10	ordinary_muscle_development	ENSGALG00010028427	ENSGALG00010028427	0.578405588	0.205142274	2.819533856	0.004809346	0.006756296	positive
PRJNA729271	stage_3W_vs_E10	ordinary_muscle_development	ENSGALG00010028428	RABGAP1	0.593935768	0.06686769	8.882253423	6.55194E-19	2.39703E-18	positive
PRJNA729271	stage_3W_vs_E10	ordinary_muscle_development	ENSGALG00010028429	REXO1	-0.077054282	0.088289338	-0.872747307	0.382800826	0.42176178	negative
PRJNA729271	stage_3W_vs_E10	ordinary_muscle_development	ENSGALG00010028430	NR2C2AP	0.080131045	0.115784231	0.692072174	0.488892005	0.527229344	positive
PRJNA729271	stage_3W_vs_E10	ordinary_muscle_development	ENSGALG00010028431	ENSGALG00010028431	0.056421852	0.268586192	0.210069818	0.833613182	0.852853125	positive
PRJNA729271	stage_3W_vs_E10	ordinary_muscle_development	ENSGALG00010028432	RFXANK	1.255977035	0.132948806	9.44707271	3.48439E-21	1.40023E-20	positive
PRJNA729271	stage_3W_vs_E10	ordinary_muscle_development	ENSGALG00010028433	MSTO1	-0.704788689	0.095678967	-7.366182011	1.75584E-13	4.9171E-13	negative
PRJNA729271	stage_3W_vs_E10	ordinary_muscle_development	ENSGALG00010028434	COL5A1	-0.728086852	0.195946738	-3.715738576	0.000202611	0.000322624	negative
PRJNA729271	stage_3W_vs_E10	ordinary_muscle_development	ENSGALG00010028435	ATP8B3	1.667546878	0.33136355	5.032378721	4.84431E-07	9.36743E-07	positive
PRJNA729271	stage_3W_vs_E10	ordinary_muscle_development	ENSGALG00010028436	S100A1	3.66280125	0.184579583	19.84402171	1.24121E-87	3.82794E-86	positive
PRJNA729271	stage_3W_vs_E10	ordinary_muscle_development	ENSGALG00010028437	ENSGALG00010028437	0.090454328	0.067160405	1.346840128	0.178031728	0.207608197	positive
PRJNA729271	stage_3W_vs_E10	ordinary_muscle_development	ENSGALG00010028438	MED27	-0.503989223	0.072897921	-6.913629596	4.72408E-12	1.22431E-11	negative
PRJNA729271	stage_3W_vs_E10	ordinary_muscle_development	ENSGALG00010028439	BORCS8	-0.154866069	0.120220549	-1.288183011	0.197682264	0.228975901	negative
PRJNA729271	stage_3W_vs_E10	ordinary_muscle_development	ENSGALG00010028440	PSMB7	-0.085495698	0.078402985	-1.090464829	0.275508438	0.312333397	negative
PRJNA729271	stage_3W_vs_E10	ordinary_muscle_development	ENSGALG00010028442	CHTOP	-0.818984702	0.091131588	-8.986836684	2.54448E-19	9.48374E-19	negative
PRJNA729271	stage_3W_vs_E10	ordinary_muscle_development	ENSGALG00010028443	ENSGALG00010028443	0.543599382	0.332253859	1.636096517	0.101819389	0.122938113	positive
PRJNA729271	stage_3W_vs_E10	ordinary_muscle_development	ENSGALG00010028444	MEF2B	1.165801719	0.314354471	3.708557783	0.000208443	0.000331437	positive
PRJNA729271	stage_3W_vs_E10	ordinary_muscle_development	ENSGALG00010028445	SYT11	-2.58739627	0.204623839	-12.64464731	1.19761E-36	8.64508E-36	negative
PRJNA729271	stage_3W_vs_E10	ordinary_muscle_development	ENSGALG00010028446	ENSGALG00010028446	-0.931458755	0.222871438	-4.179354535	2.92338E-05	4.97702E-05	negative
PRJNA729271	stage_3W_vs_E10	ordinary_muscle_development	ENSGALG00010028447	OLFM1	-4.060792099	0.165634098	-24.51664333	9.817E-133	7.1867E-131	negative
PRJNA729271	stage_3W_vs_E10	ordinary_muscle_development	ENSGALG00010028448	RIT1	-0.318354728	0.057656253	-5.521599312	3.35928E-08	6.9991E-08	negative
PRJNA729271	stage_3W_vs_E10	ordinary_muscle_development	ENSGALG00010028449	EFCAB12	-0.907213082	0.336369552	-2.69707254	0.006995203	0.009663153	negative
PRJNA729271	stage_3W_vs_E10	ordinary_muscle_development	ENSGALG00010028450	PTGDS	9.288787864	0.432720282	21.46603302	3.2348E-102	1.41635E-100	positive
PRJNA729271	stage_3W_vs_E10	ordinary_muscle_development	ENSGALG00010028451	TMEM59L	-1.908573346	0.344804968	-5.535225769	3.10828E-08	6.48826E-08	negative
PRJNA729271	stage_3W_vs_E10	ordinary_muscle_development	ENSGALG00010028452	GOLPH3L	-0.453415944	0.63301606	-0.716278738	0.473819253	0.512538065	negative
PRJNA729271	stage_3W_vs_E10	ordinary_muscle_development	ENSGALG00010028453	ENSGALG00010028453	0.001445688	0.095022861	0.015214109	0.987861366	0.9897049	positive
PRJNA729271	stage_3W_vs_E10	ordinary_muscle_development	ENSGALG00010028454	C8G	1.367325316	0.227360294	6.01391428	1.81096E-09	4.0622E-09	positive
PRJNA729271	stage_3W_vs_E10	ordinary_muscle_development	ENSGALG00010028455	ENSA	0.887895937	0.069487088	12.77785502	2.17999E-37	1.60401E-36	positive
PRJNA729271	stage_3W_vs_E10	ordinary_muscle_development	ENSGALG00010028456	FBXW5	0.211598282	0.093248887	2.269177558	0.02325753	0.030395627	positive
PRJNA729271	stage_3W_vs_E10	ordinary_muscle_development	ENSGALG00010028457	KANK3	0.841152431	0.104707765	8.033333865	9.48591E-16	2.9768E-15	positive
PRJNA729271	stage_3W_vs_E10	ordinary_muscle_development	ENSGALG00010028458	TRAF2	0.377182625	0.108654607	3.471391001	0.000517769	0.000796378	positive
PRJNA729271	stage_3W_vs_E10	ordinary_muscle_development	ENSGALG00010028459	MCL1	2.252146665	0.209834985	10.73294173	7.12899E-27	3.62578E-26	positive
PRJNA729271	stage_3W_vs_E10	ordinary_muscle_development	ENSGALG00010028460	CRLF1	-1.57646401	0.251378053	-6.271287372	3.58075E-10	8.38768E-10	negative
PRJNA729271	stage_3W_vs_E10	ordinary_muscle_development	ENSGALG00010028461	ENSGALG00010028461	2.553749923	0.31571727	8.088724192	6.02929E-16	1.91277E-15	positive
PRJNA729271	stage_3W_vs_E10	ordinary_muscle_development	ENSGALG00010028462	EDF1	1.027683141	0.105309642	9.758680416	1.69344E-22	7.1445E-22	positive
PRJNA729271	stage_3W_vs_E10	ordinary_muscle_development	ENSGALG00010028463	RPS28	2.166277006	0.169216952	12.80177297	1.6025E-37	1.1827E-36	positive
PRJNA729271	stage_3W_vs_E10	ordinary_muscle_development	ENSGALG00010028464	ENSGALG00010028464	-0.107593493	0.112938809	-0.952670693	0.340756947	0.379214737	negative
PRJNA729271	stage_3W_vs_E10	ordinary_muscle_development	ENSGALG00010028465	ENSGALG00010028465	1.078364972	0.132285284	8.151813555	3.58507E-16	1.14917E-15	positive
PRJNA729271	stage_3W_vs_E10	ordinary_muscle_development	ENSGALG00010028466	MAMDC4	0.803036233	0.647154832	1.24087188	0.214653082	0.247387805	positive
PRJNA729271	stage_3W_vs_E10	ordinary_muscle_development	ENSGALG00010028467	ENSGALG00010028467	1.223965036	0.237792832	5.147190631	2.64417E-07	5.20184E-07	positive
PRJNA729271	stage_3W_vs_E10	ordinary_muscle_development	ENSGALG00010028468	PHPT1	-0.852933137	0.104658406	-8.14968588	3.64871E-16	1.16802E-15	negative
PRJNA729271	stage_3W_vs_E10	ordinary_muscle_development	ENSGALG00010028470	AJM1	2.043597185	0.43308758	4.718669574	2.37392E-06	4.38679E-06	positive
PRJNA729271	stage_3W_vs_E10	ordinary_muscle_development	ENSGALG00010028471	TPGS1	-0.056026489	0.168460242	-0.332579891	0.739451425	0.766801288	negative
PRJNA729271	stage_3W_vs_E10	ordinary_muscle_development	ENSGALG00010028472	RPRD2	0.58706045	0.138802731	4.229458922	2.34254E-05	4.02169E-05	positive
PRJNA729271	stage_3W_vs_E10	ordinary_muscle_development	ENSGALG00010028473	CDC34	1.675552324	0.138073603	12.13521116	6.87325E-34	4.54506E-33	positive
PRJNA729271	stage_3W_vs_E10	ordinary_muscle_development	ENSGALG00010028474	PEX19	0.726148605	0.119434511	6.079889296	1.20266E-09	2.72724E-09	positive
PRJNA729271	stage_3W_vs_E10	ordinary_muscle_development	ENSGALG00010028475	AMBP	5.162001054	1.377842736	3.746437035			positive
PRJNA729271	stage_3W_vs_E10	ordinary_muscle_development	ENSGALG00010028476	ENSGALG00010028476	2.130467026	0.154762848	13.76601069	4.08136E-43	3.65181E-42	positive
PRJNA729271	stage_3W_vs_E10	ordinary_muscle_development	ENSGALG00010028477	NRARP	-0.781665775	0.218448466	-3.578261688	0.000345887	0.000540518	negative
PRJNA729271	stage_3W_vs_E10	ordinary_muscle_development	ENSGALG00010028478	PEA15	0.131068582	0.160847481	0.814862505	0.415151018	0.454022484	positive
PRJNA729271	stage_3W_vs_E10	ordinary_muscle_development	ENSGALG00010028479	CASQ1	8.430169426	0.314675647	26.7900281	4.2225E-158	4.5004E-156	positive
PRJNA729271	stage_3W_vs_E10	ordinary_muscle_development	ENSGALG00010028480	F11R	0.235982041	0.216487842	1.090047547	0.2756922	0.312468406	positive
PRJNA729271	stage_3W_vs_E10	ordinary_muscle_development	ENSGALG00010028481	ENSGALG00010028481	0.300064212	0.146288357	2.051183144	0.040249115	0.051113625	positive
PRJNA729271	stage_3W_vs_E10	ordinary_muscle_development	ENSGALG00010028482	CD320	0.08902589	0.178751215	0.498043554	0.618453344	0.652969511	positive
PRJNA729271	stage_3W_vs_E10	ordinary_muscle_development	ENSGALG00010028483	EXD3	0.429135597	0.091355023	4.697449372	2.6343E-06	4.85743E-06	positive
PRJNA729271	stage_3W_vs_E10	ordinary_muscle_development	ENSGALG00010028484	NOXA1	0.71651215	0.471185546	1.520658169	0.128345641	0.15294065	positive
PRJNA729271	stage_3W_vs_E10	ordinary_muscle_development	ENSGALG00010028485	USF1	-0.11237638	0.184541612	-0.608948732	0.542558421	0.580054899	negative
PRJNA729271	stage_3W_vs_E10	ordinary_muscle_development	ENSGALG00010028486	ARHGAP30	2.963309641	0.21907895	13.52621804	1.09515E-41	9.30641E-41	positive
PRJNA729271	stage_3W_vs_E10	ordinary_muscle_development	ENSGALG00010028488	PTGES	1.467904361	0.129709495	11.31686125	1.0828E-29	6.1622E-29	positive
PRJNA729271	stage_3W_vs_E10	ordinary_muscle_development	ENSGALG00010028489	ENSGALG00010028489	2.660758624	0.787652343	3.378087614	0.000729918	0.001107175	positive
PRJNA729271	stage_3W_vs_E10	ordinary_muscle_development	ENSGALG00010028490	NECTIN4	-2.481335643	0.368613688	-6.731534197	1.67883E-11	4.24023E-11	negative
PRJNA729271	stage_3W_vs_E10	ordinary_muscle_development	ENSGALG00010028491	TOR1B	-0.103857694	0.098118723	-1.058490066	0.289832077	0.327036896	negative
PRJNA729271	stage_3W_vs_E10	ordinary_muscle_development	ENSGALG00010028492	ENSGALG00010028492	1.481205728	0.258555013	5.728783638	1.01153E-08	2.1822E-08	positive
PRJNA729271	stage_3W_vs_E10	ordinary_muscle_development	ENSGALG00010028493	TOR1A	-0.438283591	0.087115843	-5.031043434	4.87818E-07	9.4304E-07	negative
PRJNA729271	stage_3W_vs_E10	ordinary_muscle_development	ENSGALG00010028494	ENSGALG00010028494	-1.069961349	0.20080415	-5.328382641	9.90912E-08	2.00632E-07	negative
PRJNA729271	stage_3W_vs_E10	ordinary_muscle_development	ENSGALG00010028495	LINGO3	-4.998731283	0.512753524	-9.74879947	1.86663E-22	7.85164E-22	negative
PRJNA729271	stage_3W_vs_E10	ordinary_muscle_development	ENSGALG00010028496	ENSGALG00010028496	1.753348229	0.139630193	12.55708519	3.63478E-36	2.58773E-35	positive
PRJNA729271	stage_3W_vs_E10	ordinary_muscle_development	ENSGALG00010028498	C9orf78	0.386096152	0.103467172	3.73158117	0.000190282	0.000303759	positive
PRJNA729271	stage_3W_vs_E10	ordinary_muscle_development	ENSGALG00010028500	VPS45	0.423897099	0.06979984	6.073038223	1.25513E-09	2.84444E-09	positive
PRJNA729271	stage_3W_vs_E10	ordinary_muscle_development	ENSGALG00010028501	USP20	0.606438593	0.087454541	6.934329374	4.08153E-12	1.06215E-11	positive
PRJNA729271	stage_3W_vs_E10	ordinary_muscle_development	ENSGALG00010028502	PLEKHO1	-0.723281096	0.151177978	-4.784301951	1.71583E-06	3.19718E-06	negative
PRJNA729271	stage_3W_vs_E10	ordinary_muscle_development	ENSGALG00010028503	NDOR1	-0.274395509	0.127784189	-2.147335375	0.031766591	0.040831493	negative
PRJNA729271	stage_3W_vs_E10	ordinary_muscle_development	ENSGALG00010028506	SHD	-6.086205898	0.51690916	-11.77422722	5.30006E-32	3.29012E-31	negative
PRJNA729271	stage_3W_vs_E10	ordinary_muscle_development	ENSGALG00010028507	ANP32E	-1.196317622	0.057032739	-20.97598043	1.08703E-97	4.23561E-96	negative
PRJNA729271	stage_3W_vs_E10	ordinary_muscle_development	ENSGALG00010028510	CILP2	0.233423682	0.586036877	0.398308863	0.690402528	0.720941189	positive
PRJNA729271	stage_3W_vs_E10	ordinary_muscle_development	ENSGALG00010028511	YJEFN3	-0.146404731	0.451633949	-0.324166798	0.745811757	0.772678252	negative
PRJNA729271	stage_3W_vs_E10	ordinary_muscle_development	ENSGALG00010028512	NDUFA13	1.05359176	0.122381503	8.60907678	7.36513E-18	2.58118E-17	positive
PRJNA729271	stage_3W_vs_E10	ordinary_muscle_development	ENSGALG00010028513	ENSGALG00010028513	-0.976362925	1.315732802	-0.742067784	0.458046268	0.496550793	negative
PRJNA729271	stage_3W_vs_E10	ordinary_muscle_development	ENSGALG00010028514	FNBP1	1.672437567	0.058287308	28.69299723	4.6655E-181	6.6957E-179	positive
PRJNA729271	stage_3W_vs_E10	ordinary_muscle_development	ENSGALG00010028515	ENSGALG00010028515	1.009990682	3.388812944	0.298036716	0.765675141	0.791108501	positive
PRJNA729271	stage_3W_vs_E10	ordinary_muscle_development	ENSGALG00010028516	GATAD2A	-0.720276509	0.063719296	-11.30389937	1.25518E-29	7.11531E-29	negative
PRJNA729271	stage_3W_vs_E10	ordinary_muscle_development	ENSGALG00010028517	RNF208	-2.660893969	0.342908468	-7.75977912	8.50774E-15	2.5474E-14	negative
PRJNA729271	stage_3W_vs_E10	ordinary_muscle_development	ENSGALG00010028518	MAU2	0.414597588	0.115365669	3.593769189	0.000325929	0.000510353	positive
PRJNA729271	stage_3W_vs_E10	ordinary_muscle_development	ENSGALG00010028519	ENSGALG00010028519	1.010044824	2.100649177	0.480825087	0.630640818	0.664133876	positive
PRJNA729271	stage_3W_vs_E10	ordinary_muscle_development	ENSGALG00010028523	SUGP1	-0.336385973	0.114285572	-2.943380925	0.003246487	0.004643101	negative
PRJNA729271	stage_3W_vs_E10	ordinary_muscle_development	ENSGALG00010028528	UHRF1	-3.412562918	0.186655582	-18.28267269	1.13715E-74	2.49364E-73	negative
PRJNA729271	stage_3W_vs_E10	ordinary_muscle_development	ENSGALG00010028534	ENSGALG00010028534	0.52942063	4.345747042	0.121824999	0.903037615	0.914392611	positive
PRJNA729271	stage_3W_vs_E10	ordinary_muscle_development	ENSGALG00010028535	TICAM1	0.590164566	0.105654006	5.585822915	2.32596E-08	4.90397E-08	positive
PRJNA729271	stage_3W_vs_E10	ordinary_muscle_development	ENSGALG00010028536	GPR107	-0.068132863	0.063766226	-1.068478837	0.285304572	0.322379932	negative
PRJNA729271	stage_3W_vs_E10	ordinary_muscle_development	ENSGALG00010028537	ENSGALG00010028537	0.528928294	2.123141423	0.249125324	0.803263841	0.825649509	positive
PRJNA729271	stage_3W_vs_E10	ordinary_muscle_development	ENSGALG00010028538	FEM1A	1.817514699	0.116927753	15.54391196	1.74969E-54	2.2746E-53	positive
PRJNA729271	stage_3W_vs_E10	ordinary_muscle_development	ENSGALG00010028539	ENSGALG00010028539	1.009981222	3.347138525	0.301744674	0.76284671	0.788410913	positive
PRJNA729271	stage_3W_vs_E10	ordinary_muscle_development	ENSGALG00010028541	ENSGALG00010028541	-0.272305804	0.309845574	-0.878843614	0.379486075	0.418332242	negative
PRJNA729271	stage_3W_vs_E10	ordinary_muscle_development	ENSGALG00010028542	ENSGALG00010028542	1.62158371	1.985081662	0.816885139	0.413994071	0.452859713	positive
PRJNA729271	stage_3W_vs_E10	ordinary_muscle_development	ENSGALG00010028543	ANP32B	-1.767332024	0.094082538	-18.78491019	1.00356E-78	2.41237E-77	negative
PRJNA729271	stage_3W_vs_E10	ordinary_muscle_development	ENSGALG00010028544	ZNF414	0.872705122	0.164228443	5.313970621	1.07262E-07	2.16722E-07	positive
PRJNA729271	stage_3W_vs_E10	ordinary_muscle_development	ENSGALG00010028545	ENSGALG00010028545	4.781568909	1.371896064	3.485372569	0.000491452	0.000758072	positive
PRJNA729271	stage_3W_vs_E10	ordinary_muscle_development	ENSGALG00010028546	ENSGALG00010028546	1.977008605	1.730374514	1.142532203	0.253232868	0.288570002	positive
PRJNA729271	stage_3W_vs_E10	ordinary_muscle_development	ENSGALG00010028547	ZNF618	-0.410962593	0.105588454	-3.892116789	9.93734E-05	0.000162301	negative
PRJNA729271	stage_3W_vs_E10	ordinary_muscle_development	ENSGALG00010028548	MYO1F	2.449994137	0.247521453	9.898108234	4.24223E-23	1.8383E-22	positive
PRJNA729271	stage_3W_vs_E10	ordinary_muscle_development	ENSGALG00010028549	ADAMTS10	-1.620334117	0.180552726	-8.97429882	2.85162E-19	1.05958E-18	negative
PRJNA729271	stage_3W_vs_E10	ordinary_muscle_development	ENSGALG00010028550	ZAP70	2.633996333	0.56933546	4.626439978	3.72005E-06	6.79718E-06	positive
PRJNA729271	stage_3W_vs_E10	ordinary_muscle_development	ENSGALG00010028551	NCS1	-2.455612365	0.226675047	-10.83318343	2.39675E-27	1.24075E-26	negative
PRJNA729271	stage_3W_vs_E10	ordinary_muscle_development	ENSGALG00010028552	ENSGALG00010028552	-3.712543168	0.752104604	-4.936205879	7.9657E-07	1.51845E-06	negative
PRJNA729271	stage_3W_vs_E10	ordinary_muscle_development	ENSGALG00010028553	NFILZ	5.831780427	0.917846047	6.353767547	2.10104E-10	4.99011E-10	positive
PRJNA729271	stage_3W_vs_E10	ordinary_muscle_development	ENSGALG00010028554	ENSGALG00010028554	-2.371728729	0.840052332	-2.82331069	0.004753049	0.00668239	negative
PRJNA729271	stage_3W_vs_E10	ordinary_muscle_development	ENSGALG00010028555	ENSGALG00010028555	2.038885597	1.227909427	1.660452759	0.096823404	0.117316329	positive
PRJNA729271	stage_3W_vs_E10	ordinary_muscle_development	ENSGALG00010028556	SPTAN1	-0.398749614	0.093407003	-4.268947746	1.96397E-05	3.3963E-05	negative

PRJNA729271	stage_3W_vs_E10	ordinary_muscle_development	ENSGALG00010028558	ENSGALG00010028558	3.486858048	1.423762184	2.449045273	0.014323544	0.019137227	positive
PRJNA729271	stage_3W_vs_E10	ordinary_muscle_development	ENSGALG00010028559	DYNC2I2	-0.779618376	0.125125748	-6.230679045	4.64418E-10	1.08175E-09	negative
PRJNA729271	stage_3W_vs_E10	ordinary_muscle_development	ENSGALG00010028560	ENSGALG00010028560	2.23879864	3.496215449	0.640349164	0.521945625	0.559958688	positive
PRJNA729271	stage_3W_vs_E10	ordinary_muscle_development	ENSGALG00010028561	SET	-1.142158438	0.051615229	-22.12832252	1.6874E-108	8.434E-107	negative
PRJNA729271	stage_3W_vs_E10	ordinary_muscle_development	ENSGALG00010028562	ACSBG2	1.854373701	0.199265466	9.30604654	1.32679E-20	5.19499E-20	positive
PRJNA729271	stage_3W_vs_E10	ordinary_muscle_development	ENSGALG00010028563	ENSGALG00010028563	2.267698007	2.288276346	0.991007057	0.321682134	0.359810366	positive
PRJNA729271	stage_3W_vs_E10	ordinary_muscle_development	ENSGALG00010028565	PKN1	0.17465653	0.120384961	1.450816856	0.146830865	0.173442538	positive
PRJNA729271	stage_3W_vs_E10	ordinary_muscle_development	ENSGALG00010028566	ZDHHC12	0.875292051	0.123119191	7.109306408	1.16628E-12	3.13291E-12	positive
PRJNA729271	stage_3W_vs_E10	ordinary_muscle_development	ENSGALG00010028567	ENSGALG00010028567	-2.882379704	1.200756272	-2.400470246	0.016374022	0.021738546	negative
PRJNA729271	stage_3W_vs_E10	ordinary_muscle_development	ENSGALG00010028568	MLLT1	-1.517313453	0.067380175	-22.51869271	2.7228E-112	1.4404E-110	negative
PRJNA729271	stage_3W_vs_E10	ordinary_muscle_development	ENSGALG00010028569	ZER1	0.428269433	0.068855418	6.219836394	4.97674E-10	1.1568E-09	positive
PRJNA729271	stage_3W_vs_E10	ordinary_muscle_development	ENSGALG00010028572	TBC1D13	-0.530926323	0.069465272	-7.643046816	2.12141E-14	6.22718E-14	negative
PRJNA729271	stage_3W_vs_E10	ordinary_muscle_development	ENSGALG00010028573	KCNN1	0.319205221	0.264045501	1.208902329	0.226700371	0.260217127	positive
PRJNA729271	stage_3W_vs_E10	ordinary_muscle_development	ENSGALG00010028574	ENDOG	-0.161171557	0.120091284	-1.3420754	0.179571549	0.20928597	negative
PRJNA729271	stage_3W_vs_E10	ordinary_muscle_development	ENSGALG00010028575	SETDB1	-0.1692746	0.119214247	-1.41991922	0.1556312	0.183181018	negative
PRJNA729271	stage_3W_vs_E10	ordinary_muscle_development	ENSGALG00010028577	CERS2	-0.149514275	0.132865026	-1.125309493	0.260457908	0.296337314	negative
PRJNA729271	stage_3W_vs_E10	ordinary_muscle_development	ENSGALG00010028578	SPOUT1	-0.637254246	0.124949757	-5.100083932	3.39503E-07	6.62684E-07	negative
PRJNA729271	stage_3W_vs_E10	ordinary_muscle_development	ENSGALG00010028579	OCLN	0.628195229	0.271826957	2.311011518	0.020832219	0.027359156	positive
PRJNA729271	stage_3W_vs_E10	ordinary_muscle_development	ENSGALG00010028580	PRUNE1	0.918366976	0.151695831	6.054002732	1.4129E-09	3.19153E-09	positive
PRJNA729271	stage_3W_vs_E10	ordinary_muscle_development	ENSGALG00010028581	NR2F6	-0.044164389	0.124310202	-0.355275662	0.722383086	0.75071285	negative
PRJNA729271	stage_3W_vs_E10	ordinary_muscle_development	ENSGALG00010028582	KYAT1	1.650292093	0.206767151	7.981403659	1.44678E-15	4.4935E-15	positive
PRJNA729271	stage_3W_vs_E10	ordinary_muscle_development	ENSGALG00010028583	USHBP1	0.829917477	0.20235832	4.101227348	4.10965E-05	6.92344E-05	positive
PRJNA729271	stage_3W_vs_E10	ordinary_muscle_development	ENSGALG00010028584	LRRC8A	0.204737274	0.075710572	2.704209847	0.006846704	0.009467039	positive
PRJNA729271	stage_3W_vs_E10	ordinary_muscle_development	ENSGALG00010028585	MINDY1	1.096187274	0.121033123	9.056919645	1.34186E-19	5.04548E-19	positive
PRJNA729271	stage_3W_vs_E10	ordinary_muscle_development	ENSGALG00010028586	BABAM1	-0.326169198	0.121747391	-2.679065184	0.007382802	0.010177243	negative
PRJNA729271	stage_3W_vs_E10	ordinary_muscle_development	ENSGALG00010028587	ANKLE1	-1.646628785	0.206295035	-7.981911851	1.44084E-15	4.47599E-15	negative
PRJNA729271	stage_3W_vs_E10	ordinary_muscle_development	ENSGALG00010028588	ABHD8	-3.812465523	0.869489466	-4.38471732	1.16136E-05	2.04172E-05	negative
PRJNA729271	stage_3W_vs_E10	ordinary_muscle_development	ENSGALG00010028589	PHYHD1	0.035544061	0.190151164	0.186925285	0.851719214	0.868653918	positive
PRJNA729271	stage_3W_vs_E10	ordinary_muscle_development	ENSGALG00010028590	ENSGALG00010028590	1.591854093	0.227813438	6.987533778	2.7976E-12	7.35023E-12	positive
PRJNA729271	stage_3W_vs_E10	ordinary_muscle_development	ENSGALG00010028591	GABPB2	-1.189258128	0.126123833	-9.429289433	4.12876E-21	1.65322E-20	negative
PRJNA729271	stage_3W_vs_E10	ordinary_muscle_development	ENSGALG00010028592	SEMA6C	2.685980634	0.160289633	16.75704529	5.03009E-63	8.17389E-62	positive
PRJNA729271	stage_3W_vs_E10	ordinary_muscle_development	ENSGALG00010028593	DOLK	-0.102032638	0.117425018	-0.868917377	0.384892324	0.423840632	negative
PRJNA729271	stage_3W_vs_E10	ordinary_muscle_development	ENSGALG00010028594	ENSGALG00010028594	1.960884844	0.141070167	13.90006754	6.32816E-44	5.80549E-43	positive
PRJNA729271	stage_3W_vs_E10	ordinary_muscle_development	ENSGALG00010028595	SCNM1	0.77263738	0.11180972	6.910288109	4.8367E-12	1.25305E-11	positive
PRJNA729271	stage_3W_vs_E10	ordinary_muscle_development	ENSGALG00010028596	DDA1	0.18908404	0.088528714	2.135849841	0.032691655	0.041953567	positive
PRJNA729271	stage_3W_vs_E10	ordinary_muscle_development	ENSGALG00010028597	NUP188	-1.137792704	0.057552077	-19.769794	5.41968E-87	1.64692E-85	negative
PRJNA729271	stage_3W_vs_E10	ordinary_muscle_development	ENSGALG00010028598	TMOD4	9.198771549	0.25035025	36.74360846	1.4707E-295	6.0907E-293	positive
PRJNA729271	stage_3W_vs_E10	ordinary_muscle_development	ENSGALG00010028599	VPS72	1.481310858	0.117641065	12.59178376	2.34318E-36	1.67725E-35	positive
PRJNA729271	stage_3W_vs_E10	ordinary_muscle_development	ENSGALG00010028600	PSMD4	1.374435858	0.131875861	10.42219437	1.96369E-25	9.3477E-25	positive
PRJNA729271	stage_3W_vs_E10	ordinary_muscle_development	ENSGALG00010028601	ANO8	6.415210764	0.221535107	28.95798709	2.2263E-184	3.3268E-182	positive
PRJNA729271	stage_3W_vs_E10	ordinary_muscle_development	ENSGALG00010028602	ZNF687	0.261856434	0.170250917	1.538061812	0.124033502	0.14799684	positive
PRJNA729271	stage_3W_vs_E10	ordinary_muscle_development	ENSGALG00010028603	SH3GLB2	0.113073659	0.337953622	0.334583361	0.737939398	0.765452381	positive
PRJNA729271	stage_3W_vs_E10	ordinary_muscle_development	ENSGALG00010028604	PI4KB	-0.226113224	0.140641558	-1.607726958	0.107894992	0.129873601	negative
PRJNA729271	stage_3W_vs_E10	ordinary_muscle_development	ENSGALG00010028605	GTPBP3	1.835389422	0.213377308	8.601614861	7.86022E-18	2.74804E-17	positive
PRJNA729271	stage_3W_vs_E10	ordinary_muscle_development	ENSGALG00010028606	PRPF3	0.328431608	0.135762215	2.419168007	0.015556054	0.02069809	positive
PRJNA729271	stage_3W_vs_E10	ordinary_muscle_development	ENSGALG00010028607	ENSGALG00010028607	1.161523865	0.238897869	4.862010158	1.162E-06	2.19141E-06	positive
PRJNA729271	stage_3W_vs_E10	ordinary_muscle_development	ENSGALG00010028608	MIGA2	0.077644449	0.159701614	0.486184501	0.626836359	0.660867122	positive
PRJNA729271	stage_3W_vs_E10	ordinary_muscle_development	ENSGALG00010028609	MRPS21	0.53216297	0.145050451	3.668812932	0.000243679	0.000384973	positive
PRJNA729271	stage_3W_vs_E10	ordinary_muscle_development	ENSGALG00010028610	ENSGALG00010028610	3.800976948	0.529165827	7.182959955	6.82181E-13	1.85485E-12	positive
PRJNA729271	stage_3W_vs_E10	ordinary_muscle_development	ENSGALG00010028612	DIRAS1	-1.25001326	0.665062944	-1.879541284	0.060170622	0.074813276	negative
PRJNA729271	stage_3W_vs_E10	ordinary_muscle_development	ENSGALG00010028613	ENSGALG00010028613	2.056124966	0.363098796	5.662714906	1.48997E-08	3.18212E-08	positive
PRJNA729271	stage_3W_vs_E10	ordinary_muscle_development	ENSGALG00010028618	DOLPP1	-1.810425001	0.110617108	-16.36659141	3.31254E-60	4.98601E-59	negative
PRJNA729271	stage_3W_vs_E10	ordinary_muscle_development	ENSGALG00010028626	CRAT	2.469500236	0.172921293	14.28106505	2.87179E-46	2.86882E-45	positive
PRJNA729271	stage_3W_vs_E10	ordinary_muscle_development	ENSGALG00010028642	APH1A	0.542364838	0.138456557	3.917220327	8.95759E-05	0.000146745	positive
PRJNA729271	stage_3W_vs_E10	ordinary_muscle_development	ENSGALG00010028649	ENSGALG00010028649	0.114460058	0.173856194	0.658360542	0.510306484	0.548364777	positive
PRJNA729271	stage_3W_vs_E10	ordinary_muscle_development	ENSGALG00010028658	PTPA	-0.576433684	0.082291682	-7.004762414	2.47405E-12	6.52619E-12	negative
PRJNA729271	stage_3W_vs_E10	ordinary_muscle_development	ENSGALG00010028662	FCRL4	0.19904963	0.226341956	0.879419939	0.379173625	0.418019599	positive
PRJNA729271	stage_3W_vs_E10	ordinary_muscle_development	ENSGALG00010028669	ZBTB34	0.08016646	0.110922754	0.722723315	0.469849889	0.508509978	positive
PRJNA729271	stage_3W_vs_E10	ordinary_muscle_development	ENSGALG00010028671	CADM3	3.793351258	0.211970749	17.89563548	1.27534E-71	2.56039E-70	positive
PRJNA729271	stage_3W_vs_E10	ordinary_muscle_development	ENSGALG00010028675	ZBTB43	0.013734754	0.070811971	0.193960904	0.846206495	0.863907814	positive
PRJNA729271	stage_3W_vs_E10	ordinary_muscle_development	ENSGALG00010028678	ENSGALG00010028678	1.466892666	0.360190324	4.07254878	4.65015E-05	7.80499E-05	positive
PRJNA729271	stage_3W_vs_E10	ordinary_muscle_development	ENSGALG00010028686	TAGLN2	0.520318213	0.119296	4.361573013	1.29131E-05	2.26193E-05	positive
PRJNA729271	stage_3W_vs_E10	ordinary_muscle_development	ENSGALG00010028689	CFAP45	0.17796496	0.635350083	0.280105355	0.779396677	0.803510301	positive
PRJNA729271	stage_3W_vs_E10	ordinary_muscle_development	ENSGALG00010028692	RALGPS1	0.393975891	0.110663491	3.560125273	0.000370678	0.000577553	positive
PRJNA729271	stage_3W_vs_E10	ordinary_muscle_development	ENSGALG00010028695	VSIG8	0.965597629	0.957782896	1.00815919	0.313378046	0.351253849	positive
PRJNA729271	stage_3W_vs_E10	ordinary_muscle_development	ENSGALG00010028700	ANGPTL2	2.492724363	0.197450925	12.62452612	1.5467E-36	1.11152E-35	positive
PRJNA729271	stage_3W_vs_E10	ordinary_muscle_development	ENSGALG00010028707	ENSGALG00010028707	-4.33127985	1.23336907	-3.511746772	0.000445172	0.00068881	negative
PRJNA729271	stage_3W_vs_E10	ordinary_muscle_development	ENSGALG00010028712	FBXW2	0.013718369	0.084824686	0.161726145	0.871521515	0.886132461	positive
PRJNA729271	stage_3W_vs_E10	ordinary_muscle_development	ENSGALG00010028726	SLC2A8	0.030414923	0.11219083	0.271099899	0.786314199	0.809893009	positive
PRJNA729271	stage_3W_vs_E10	ordinary_muscle_development	ENSGALG00010028727	ENSGALG00010028727	-0.945027546	0.182424223	-5.180384121	2.21429E-07	4.37755E-07	negative
PRJNA729271	stage_3W_vs_E10	ordinary_muscle_development	ENSGALG00010028734	ENSGALG00010028734	1.17108975	0.359970056	3.253297687	0.001140739	0.001702009	positive
PRJNA729271	stage_3W_vs_E10	ordinary_muscle_development	ENSGALG00010028735	ENSGALG00010028735	-1.12846777	0.363170326	-3.107268656	0.001888247	0.002753812	negative
PRJNA729271	stage_3W_vs_E10	ordinary_muscle_development	ENSGALG00010028738	MIDN	-0.936136094	0.186246046	-5.026340761	4.99927E-07	9.65035E-07	negative
PRJNA729271	stage_3W_vs_E10	ordinary_muscle_development	ENSGALG00010028739	GARNL3	-0.404504032	0.077843816	-5.196354103	2.03235E-07	4.02444E-07	negative
PRJNA729271	stage_3W_vs_E10	ordinary_muscle_development	ENSGALG00010028740	DAZAP1	-0.035942254	0.105743849	-0.339899242	0.733932407	0.761677543	negative
PRJNA729271	stage_3W_vs_E10	ordinary_muscle_development	ENSGALG00010028742	ENSGALG00010028742	-0.231866984	0.147873246	-1.568011731	0.116878395	0.13996632	negative
PRJNA729271	stage_3W_vs_E10	ordinary_muscle_development	ENSGALG00010028743	ARRDC1	1.272846542	0.186254807	6.833899	8.26372E-12	2.11892E-11	positive
PRJNA729271	stage_3W_vs_E10	ordinary_muscle_development	ENSGALG00010028744	HOMER3	0.130908306	0.208484137	0.627905356	0.530065942	0.567913803	positive
PRJNA729271	stage_3W_vs_E10	ordinary_muscle_development	ENSGALG00010028745	ENSGALG00010028745	0.312380485	0.139013359	2.247125647	0.024631998	0.032079139	positive
PRJNA729271	stage_3W_vs_E10	ordinary_muscle_development	ENSGALG00010028746	MYDGF	-0.778243892	0.125057587	-6.223084197	4.87476E-10	1.13436E-09	negative
PRJNA729271	stage_3W_vs_E10	ordinary_muscle_development	ENSGALG00010028747	ENSGALG00010028747	-4.533420463	0.192940084	-23.49651959	4.427E-122	2.7306E-120	negative
PRJNA729271	stage_3W_vs_E10	ordinary_muscle_development	ENSGALG00010028748	PIAS3	1.006644274	0.198041266	5.083002616	3.71515E-07	7.23902E-07	positive
PRJNA729271	stage_3W_vs_E10	ordinary_muscle_development	ENSGALG00010028750	OTUD7B	0.311332151	0.120409918	2.585602218	0.009720904	0.013226745	positive
PRJNA729271	stage_3W_vs_E10	ordinary_muscle_development	ENSGALG00010028751	EHMT1	-0.918127514	0.095971445	-9.566673876	1.104E-21	4.51921E-21	negative
PRJNA729271	stage_3W_vs_E10	ordinary_muscle_development	ENSGALG00010028752	MTMR11	2.562417902	0.130103124	19.69528339	2.36669E-86	7.11725E-85	positive
PRJNA729271	stage_3W_vs_E10	ordinary_muscle_development	ENSGALG00010028753	SV2A	2.215072909	0.636039191	3.482604439	0.000496561	0.000765464	positive
PRJNA729271	stage_3W_vs_E10	ordinary_muscle_development	ENSGALG00010028756	ENSGALG00010028756	-4.663872442	0.883663085	-5.277885342	1.30683E-07	2.62289E-07	negative
PRJNA729271	stage_3W_vs_E10	ordinary_muscle_development	ENSGALG00010028757	MAT2A	-2.644131564	1.363928508	-1.938614486	0.052548298	0.065761816	negative
PRJNA729271	stage_3W_vs_E10	ordinary_muscle_development	ENSGALG00010028760	SSR2	0.072698542	0.097307453	0.747101482	0.455002306	0.493546241	positive
PRJNA729271	stage_3W_vs_E10	ordinary_muscle_development	ENSGALG00010028761	CTSS	3.734752705	0.236250441	15.80844753	2.72089E-56	3.69628E-55	positive
PRJNA729271	stage_3W_vs_E10	ordinary_muscle_development	ENSGALG00010028762	CACNA1B	-4.466034287	0.901967149	-4.951437857	7.36671E-07	1.40593E-06	negative
PRJNA729271	stage_3W_vs_E10	ordinary_muscle_development	ENSGALG00010028763	CTSK	0.827742182	0.217520447	3.805353437	0.000141602	0.00022854	positive
PRJNA729271	stage_3W_vs_E10	ordinary_muscle_development	ENSGALG00010028764	ARNT	0.479261144	0.125389975	3.822164763	0.000132285	0.000214099	positive
PRJNA729271	stage_3W_vs_E10	ordinary_muscle_development	ENSGALG00010028768	NTNG2	-0.302029511	0.346845768	-0.870789091	0.383869323	0.422778348	negative
PRJNA729271	stage_3W_vs_E10	ordinary_muscle_development	ENSGALG00010028771	SETX	0.185795174	0.07181839	2.587013913	0.009681168	0.013183816	positive
PRJNA729271	stage_3W_vs_E10	ordinary_muscle_development	ENSGALG00010028772	TTF1	0.159563896	0.071712926	2.225036769	0.02607877	0.033856854	positive
PRJNA729271	stage_3W_vs_E10	ordinary_muscle_development	ENSGALG00010028782	MLLT11	0.766189177	0.250276174	3.061374813	0.002203231	0.00319582	positive
PRJNA729271	stage_3W_vs_E10	ordinary_muscle_development	ENSGALG00010028783	DDX31	-0.413469455	0.075033031	-5.510499189	3.57817E-08	7.44554E-08	negative
PRJNA729271	stage_3W_vs_E10	ordinary_muscle_development	ENSGALG00010028786	GTF3C4	-0.673889501	0.068496702	-9.838276546	7.70189E-23	3.30097E-22	negative
PRJNA729271	stage_3W_vs_E10	ordinary_muscle_development	ENSGALG00010028800	ENSGALG00010028800	-1.137668277	0.451196583	-2.521447016	0.011687327	0.015783827	negative
PRJNA729271	stage_3W_vs_E10	ordinary_muscle_development	ENSGALG00010028806	SPACA9	-1.360520816	0.586791638	-2.318575673	0.020418055	0.02683955	negative
PRJNA729271	stage_3W_vs_E10	ordinary_muscle_development	ENSGALG00010028818	TSC1	0.146897081	0.11749848	1.250204094	0.211225002	0.243688825	positive
PRJNA729271	stage_3W_vs_E10	ordinary_muscle_development	ENSGALG00010028823	GFI1B	0.30027765	0.320500396	0.936902586	0.348808669	0.3873425	positive
PRJNA729271	stage_3W_vs_E10	ordinary_muscle_development	ENSGALG00010028826	CEL	8.180923601	2.723337344	3.00400669	0.002664495	0.0038361	positive
PRJNA729271	stage_3W_vs_E10	ordinary_muscle_development	ENSGALG00010028829	GTF3C5	-0.267314288	0.113550936	-2.354135489	0.018565843	0.024502585	negative
PRJNA729271	stage_3W_vs_E10	ordinary_muscle_development	ENSGALG00010028833	GBGT1	2.54887647	0.166298859	15.3270833	5.0412E-53	6.28848E-52	positive
PRJNA729271	stage_3W_vs_E10	ordinary_muscle_development	ENSGALG00010028849	ENSGALG00010028849	6.094857615	0.325408143	18.72988657	2.82517E-78	6.74644E-77	positive
PRJNA729271	stage_3W_vs_E10	ordinary_muscle_development	ENSGALG00010028853	ENSGALG00010028853	3.479462685	1.275092016	2.728793406	0.006356651	0.008814662	positive
PRJNA729271	stage_3W_vs_E10	ordinary_muscle_development	ENSGALG00010028854	RPL7A	0.418277361	0.14033466	2.980570604	0.002877119	0.004131957	positive
PRJNA729271	stage_3W_vs_E10	ordinary_muscle_development	ENSGALG00010028855	SURF6	-0.375955212	0.169252209	-2.22127211	0.026332537	0.034158772	negative
PRJNA729271	stage_3W_vs_E10	ordinary_muscle_development	ENSGALG00010028856	ETV3	-0.297404002	0.110152788	-2.699922594	0.006935561	0.009583504	negative
PRJNA729271	stage_3W_vs_E10	ordinary_muscle_development	ENSGALG00010028857	MED22	0.245727119	0.078699963	3.122328261	0.001794268	0.002623087	positive
PRJNA729271	stage_3W_vs_E10	ordinary_muscle_development	ENSGALG00010028858	ENSGALG00010028858	-0.124401289	0.45972351	-0.270600233	0.786698517	0.810231278	negative
PRJNA729271	stage_3W_vs_E10	ordinary_muscle_development	ENSGALG00010028859	SURF1	0.675483059	0.109164161	6.187773089	6.10201E-10	1.41021E-09	positive
PRJNA729271	stage_3W_vs_E10	ordinary_muscle_development	ENSGALG00010028860	SURF2	0.787562373	0.171274709	4.598240899	4.26073E-06	7.73732E-06	positive
PRJNA729271	stage_3W_vs_E10	ordinary_muscle_development	ENSGALG00010028861	SURF4	-0.492495659	0.074779987	-6.585928646	4.52051E-11	1.11418E-10	negative
PRJNA729271	stage_3W_vs_E10	ordinary_muscle_development	ENSGALG00010028862	ENSGALG00010028862	0.613280706	1.399767172	0.438130511	0.661291678	0.693540473	positive
PRJNA729271	stage_3W_vs_E10	ordinary_muscle_development	ENSGALG00010028865	STKLD1	1.221126896	0.166251498	7.345057984	2.0567E-13	5.73857E-13	positive
PRJNA729271	stage_3W_vs_E10	ordinary_muscle_development	ENSGALG00010028866	HORMAD1	-0.745260325	0.483960214	-1.539920644	0.123579698	0.14750393	negative
PRJNA729271	stage_3W_vs_E10	ordinary_muscle_development	ENSGALG00010028867	REXO4	-0.053910364	0.116854435	-0.461346328	0.644550152	0.677698712	negative
PRJNA729271	stage_3W_vs_E10	ordinary_muscle_development	ENSGALG00010028868	ENSGALG00010028868	0.521075829	4.241825273	0.12284236	0.902231929	0.913768294	positive
PRJNA729271	stage_3W_vs_E10	ordinary_muscle_development	ENSGALG00010028869	ADAMTS13	3.265081019	0.163276195	19.99728749	5.81503E-89	1.85658E-87	positive
PRJNA729271	stage_3W_vs_E10	ordinary_muscle_development	ENSGALG00010028870	CACFD1	-2.49070122	0.229543105	-10.85069064	1.97924E-27	1.03161E-26	negative
PRJNA729271	stage_3W_vs_E10	ordinary_muscle_development	ENSGALG00010028871	PIP5K1A	-0.771886636	0.109580509	-7.044013945	1.86779E-12	4.96309E-12	negative
PRJNA729271	stage_3W_vs_E10	ordinary_muscle_development	ENSGALG00010028872	SLC2A6	2.979908868	0.328678649	9.066329317	1.23094E-19	4.63682E-19	positive
PRJNA729271	stage_3W_vs_E10	ordinary_muscle_development	ENSGALG00010028873	TMEM8C	-0.777515543	0.278842135	-2.78837179	0.005297371	0.007411002	negative
PRJNA729271	stage_3W_vs_E10	ordinary_muscle_development	ENSGALG00010028874	CRPL1	2.282920631	0.497030508	4.593119728	4.36668E-06	7.92377E-06	positive
PRJNA729271	stage_3W_vs_E10	ordinary_muscle_development	ENSGALG00010028875	DUSP23	1.228973147	0.119724626	10.26499881	1.01322E-24	4.68324E-24	positive
PRJNA729271	stage_3W_vs_E10	ordinary_muscle_development	ENSGALG00010028876	ADAMTSL2	3.821351806	0.194402059	19.65695126	5.04127E-86	1.50356E-84	positive
PRJNA729271	stage_3W_vs_E10	ordinary_muscle_development	ENSGALG00010028877	FAM163B	-3.915871887	0.743531126	-5.266587703	1.38983E-07	2.78254E-07	negative
PRJNA729271	stage_3W_vs_E10	ordinary_muscle_development	ENSGALG00010028878	IGSF9	0.558658331	0.157362181	3.550143536	0.000385021	0.000599	positive

PRJNA729271	stage_3W_vs_E10	ordinary_muscle_development	ENSGALG00010028881	SARDH	-2.252077028	0.233263006	-9.654668622	4.69675E-22	1.95182E-21	negative
PRJNA729271	stage_3W_vs_E10	ordinary_muscle_development	ENSGALG00010028884	VAV2	-1.561446082	0.130759699	-11.94134046	7.20525E-33	4.60698E-32	negative
PRJNA729271	stage_3W_vs_E10	ordinary_muscle_development	ENSGALG00010028885	BRD3	-0.694782871	0.141970625	-4.893849507	9.88825E-07	1.87458E-06	negative
PRJNA729271	stage_3W_vs_E10	ordinary_muscle_development	ENSGALG00010028886	ENSGALG00010028886	0.051996924	1.859824927	0.027957967	0.977695676	0.981079877	positive
PRJNA729271	stage_3W_vs_E10	ordinary_muscle_development	ENSGALG00010028887	SF3B4	-0.415878436	0.17603	-2.362542951	0.018150038	0.02398002	negative
PRJNA729271	stage_3W_vs_E10	ordinary_muscle_development	ENSGALG00010028888	WDR5B	-1.043014971	0.082042711	-12.71307301	5.00291E-37	3.64225E-36	negative
PRJNA729271	stage_3W_vs_E10	ordinary_muscle_development	ENSGALG00010028889	FUBP3	-1.213237543	0.083657847	-14.50237586	1.17028E-47	1.22744E-46	negative
PRJNA729271	stage_3W_vs_E10	ordinary_muscle_development	ENSGALG00010028890	ENSGALG00010028890	-7.440875602	0.676552926	-10.99821657	3.89764E-28	2.09091E-27	negative
PRJNA729271	stage_3W_vs_E10	ordinary_muscle_development	ENSGALG00010028891	SLC31A2	2.53947244	0.217904104	11.65408266	2.18723E-31	1.31935E-30	positive
PRJNA729271	stage_3W_vs_E10	ordinary_muscle_development	ENSGALG00010028892	NIBAN2	-0.072013145	0.1410033	-0.510719571	0.609547435	0.644730741	negative
PRJNA729271	stage_3W_vs_E10	ordinary_muscle_development	ENSGALG00010028893	STXBP1	1.16231532	0.197927474	5.872430424	4.29452E-09	9.4374E-09	positive
PRJNA729271	stage_3W_vs_E10	ordinary_muscle_development	ENSGALG00010028894	AKNA	-1.291684068	0.159770102	-8.084641946	6.23471E-16	1.97621E-15	negative
PRJNA729271	stage_3W_vs_E10	ordinary_muscle_development	ENSGALG00010028895	WHRN	-4.329915666	0.839340031	-5.158714591	2.48651E-07	4.89834E-07	negative
PRJNA729271	stage_3W_vs_E10	ordinary_muscle_development	ENSGALG00010028896	ATP6V1G1	0.871021979	0.098692593	8.825606416	1.08868E-18	3.9473E-18	positive
PRJNA729271	stage_3W_vs_E10	ordinary_muscle_development	ENSGALG00010028897	TMEM268	0.93947009	0.278449646	3.373931712	0.000741027	0.001122616	positive
PRJNA729271	stage_3W_vs_E10	ordinary_muscle_development	ENSGALG00010028898	TNFSF15	-1.406298135	0.251844601	-5.583991599	2.3506E-08	4.95304E-08	negative
PRJNA729271	stage_3W_vs_E10	ordinary_muscle_development	ENSGALG00010028899	TNFSF8	2.961937555	0.530320441	5.585184593	2.33452E-08	4.9213E-08	positive
PRJNA729271	stage_3W_vs_E10	ordinary_muscle_development	ENSGALG00010028900	TNC	-2.407140905	0.549523781	-4.380412619	1.18455E-05	2.08096E-05	negative
PRJNA729271	stage_3W_vs_E10	ordinary_muscle_development	ENSGALG00010028901	PAPPA	-1.850777504	0.119438992	-15.49558878	3.71541E-54	4.78711E-53	negative
PRJNA729271	stage_3W_vs_E10	ordinary_muscle_development	ENSGALG00010028902	ENSGALG00010028902	-1.777452009	0.506841924	-3.506915912	0.000453332	0.000700912	negative
PRJNA729271	stage_3W_vs_E10	ordinary_muscle_development	ENSGALG00010028903	ASTN2	-5.050190034	0.284923801	-17.72470402	2.70332E-70	5.26675E-69	negative
PRJNA729271	stage_3W_vs_E10	ordinary_muscle_development	ENSGALG00010028904	TRIM32	-0.942584491	0.085697274	-10.99900209	3.86384E-28	2.07354E-27	negative
PRJNA729271	stage_3W_vs_E10	ordinary_muscle_development	ENSGALG00010028905	COLGALT1	-0.132637423	0.086598323	-1.531639622	0.125611389	0.149793261	negative
PRJNA729271	stage_3W_vs_E10	ordinary_muscle_development	ENSGALG00010028909	ENSGALG00010028909	-1.159791395	0.137573547	-8.430337252	3.4467E-17	1.16511E-16	negative
PRJNA729271	stage_3W_vs_E10	ordinary_muscle_development	ENSGALG00010028911	LMX1B	-7.681343126	0.927087933	-8.2854526	1.17661E-16	3.87788E-16	negative
PRJNA729271	stage_3W_vs_E10	ordinary_muscle_development	ENSGALG00010028912	EXFABP	2.818606115	0.49658134	5.676020999	1.37864E-08	2.9487E-08	positive
PRJNA729271	stage_3W_vs_E10	ordinary_muscle_development	ENSGALG00010028914	TLR4	2.298630447	0.227032524	10.12467468	4.29404E-24	1.93479E-23	positive
PRJNA729271	stage_3W_vs_E10	ordinary_muscle_development	ENSGALG00010028915	ADGRD2	2.49200707	0.460814978	5.407825676	6.37945E-08	1.30589E-07	positive
PRJNA729271	stage_3W_vs_E10	ordinary_muscle_development	ENSGALG00010028917	NR6A1	-0.555144806	0.13114225	-4.233149923	2.30441E-05	3.95998E-05	negative
PRJNA729271	stage_3W_vs_E10	ordinary_muscle_development	ENSGALG00010028918	AGPAT2	1.613788814	0.184182312	8.761909843	1.9197E-18	6.89275E-18	positive
PRJNA729271	stage_3W_vs_E10	ordinary_muscle_development	ENSGALG00010028919	EGFL7	1.004938381	0.169240276	5.937938686	2.88628E-09	6.3941E-09	positive
PRJNA729271	stage_3W_vs_E10	ordinary_muscle_development	ENSGALG00010028921	MRPL41	-0.085286168	0.121335522	-0.702895299	0.482121001	0.520624593	negative
PRJNA729271	stage_3W_vs_E10	ordinary_muscle_development	ENSGALG00010028923	ENSGALG00010028923	1.546495898	0.183118692	8.445319706	3.03214E-17	1.02809E-16	positive
PRJNA729271	stage_3W_vs_E10	ordinary_muscle_development	ENSGALG00010028924	TMEM203	0.638610791	0.119311426	5.352469677	8.67618E-08	1.76433E-07	positive
PRJNA729271	stage_3W_vs_E10	ordinary_muscle_development	ENSGALG00010028925	ENSGALG00010028925	1.701449019	0.324071935	5.250220194	1.51918E-07	3.03395E-07	positive
PRJNA729271	stage_3W_vs_E10	ordinary_muscle_development	ENSGALG00010028926	ENSGALG00010028926	-2.722191316	0.661768912	-4.1135074	3.89693E-05	6.58037E-05	negative
PRJNA729271	stage_3W_vs_E10	ordinary_muscle_development	ENSGALG00010029140	NPDC1	0.465879274	0.191331788	2.434928761	0.014894722	0.019874712	positive
PRJNA729271	stage_3W_vs_E10	ordinary_muscle_development	ENSGALG00010029141	C5	-1.273692037	0.574096704	-2.218601898	0.02651382	0.034378551	negative
PRJNA729271	stage_3W_vs_E10	ordinary_muscle_development	ENSGALG00010029142	ENSGALG00010029142	0.988262343	0.180897201	5.463115715	4.67849E-08	9.66435E-08	positive
PRJNA729271	stage_3W_vs_E10	ordinary_muscle_development	ENSGALG00010029143	SH2D3C	-0.358730319	0.110731711	-3.239634939	0.001196828	0.00178057	negative
PRJNA729271	stage_3W_vs_E10	ordinary_muscle_development	ENSGALG00010029144	ENSGALG00010029144	5.905877352	0.21830556	27.05326127	3.4965E-161	3.8986E-159	positive
PRJNA729271	stage_3W_vs_E10	ordinary_muscle_development	ENSGALG00010029145	RC3H2	0.943523115	0.133407064	7.072512387	1.52153E-12	4.06462E-12	positive
PRJNA729271	stage_3W_vs_E10	ordinary_muscle_development	ENSGALG00010029146	NEK6	-1.70775258	0.05928266	-28.8069493	1.7554E-182	2.5445E-180	negative
PRJNA729271	stage_3W_vs_E10	ordinary_muscle_development	ENSGALG00010029147	RABL6	-0.798592195	0.104320203	-7.655201666	1.93009E-14	5.67824E-14	negative
PRJNA729271	stage_3W_vs_E10	ordinary_muscle_development	ENSGALG00010029148	RALGDS	0.050139269	0.118854446	0.421854386	0.673131306	0.704530167	positive
PRJNA729271	stage_3W_vs_E10	ordinary_muscle_development	ENSGALG00010029149	ENSGALG00010029149	-1.684171786	0.242095373	-6.95664592	3.48469E-12	9.10922E-12	negative
PRJNA729271	stage_3W_vs_E10	ordinary_muscle_development	ENSGALG00010029150	NARF	-0.000487242	0.123148728	-0.003956536	0.996843149	0.997187125	negative
PRJNA729271	stage_3W_vs_E10	ordinary_muscle_development	ENSGALG00010029151	CYBC1	-0.9734976	0.114607706	-8.49417227	1.99348E-17	6.8229E-17	negative
PRJNA729271	stage_3W_vs_E10	ordinary_muscle_development	ENSGALG00010029152	HEXD	-0.052727377	0.127349124	-0.414038004	0.678846275	0.709896598	negative
PRJNA729271	stage_3W_vs_E10	ordinary_muscle_development	ENSGALG00010029153	OGFOD3	-1.168021855	0.081270464	-14.37203381	7.75208E-47	7.8909E-46	negative
PRJNA729271	stage_3W_vs_E10	ordinary_muscle_development	ENSGALG00010029154	UTS2R	-1.678230797	0.146830683	-11.42970096	2.9713E-30	1.72276E-29	negative
PRJNA729271	stage_3W_vs_E10	ordinary_muscle_development	ENSGALG00010029155	ENSGALG00010029155	-1.713802141	0.586208548	-2.923536593	0.003460795	0.004932084	negative
PRJNA729271	stage_3W_vs_E10	ordinary_muscle_development	ENSGALG00010029156	ENSGALG00010029156	-7.272035184	1.276113028	-5.698582353	1.20808E-08	2.59424E-08	negative
PRJNA729271	stage_3W_vs_E10	ordinary_muscle_development	ENSGALG00010029158	CD7	2.000394013	0.402533462	4.969509872	6.71224E-07	1.28577E-06	positive
PRJNA729271	stage_3W_vs_E10	ordinary_muscle_development	ENSGALG00010029159	SSC4D	-4.991120118	1.067842225	-4.674023933	2.95355E-06	5.42882E-06	negative
PRJNA729271	stage_3W_vs_E10	ordinary_muscle_development	ENSGALG00010029160	DUS1L	0.966762211	0.091226207	10.59741759	3.06322E-26	1.51282E-25	positive
PRJNA729271	stage_3W_vs_E10	ordinary_muscle_development	ENSGALG00010029162	ELN	0.692629806	0.310949563	2.227466731	0.025916097	0.033648676	positive
PRJNA729271	stage_3W_vs_E10	ordinary_muscle_development	ENSGALG00010029163	ANKFY1	-0.742053856	0.094522315	-7.850567959	4.14157E-15	1.26065E-14	negative
PRJNA729271	stage_3W_vs_E10	ordinary_muscle_development	ENSGALG00010029164	CYB5D2	0.578586217	0.081915629	7.063196941	1.62715E-12	4.33876E-12	positive
PRJNA729271	stage_3W_vs_E10	ordinary_muscle_development	ENSGALG00010029165	RAC3	-0.446995878	0.120825216	-3.699524749	0.000216004	0.000342857	negative
PRJNA729271	stage_3W_vs_E10	ordinary_muscle_development	ENSGALG00010029166	ZZEF1	1.688747781	0.123404448	13.68465889	1.25394E-42	1.10291E-41	positive
PRJNA729271	stage_3W_vs_E10	ordinary_muscle_development	ENSGALG00010029167	ENSGALG00010029167	1.366745699	0.126372072	10.81525117	2.91493E-27	1.50416E-26	positive
PRJNA729271	stage_3W_vs_E10	ordinary_muscle_development	ENSGALG00010029168	RFNG	0.643769743	0.079965922	8.050551078	8.24221E-16	2.5955E-15	positive
PRJNA729271	stage_3W_vs_E10	ordinary_muscle_development	ENSGALG00010029169	SBDS	1.383288902	0.077328619	17.88844699	1.45096E-71	2.90895E-70	positive
PRJNA729271	stage_3W_vs_E10	ordinary_muscle_development	ENSGALG00010029170	DCXR	-0.893334661	0.142705514	-6.259986966	3.8501E-10	9.00696E-10	negative
PRJNA729271	stage_3W_vs_E10	ordinary_muscle_development	ENSGALG00010029171	ENSGALG00010029171	-0.041005794	0.14688071	-0.279177534	0.780108584	0.804171821	negative
PRJNA729271	stage_3W_vs_E10	ordinary_muscle_development	ENSGALG00010029172	LRRC45	-0.203507832	0.074424499	-2.734419906	0.00624903	0.008670402	negative
PRJNA729271	stage_3W_vs_E10	ordinary_muscle_development	ENSGALG00010029173	ZNHIT3	0.166799202	0.134982144	1.235713093	0.216565229	0.249333836	positive
PRJNA729271	stage_3W_vs_E10	ordinary_muscle_development	ENSGALG00010029174	CALN1	-9.251509049	0.853624077	-10.83791953	2.27588E-27	1.1807E-26	negative
PRJNA729271	stage_3W_vs_E10	ordinary_muscle_development	ENSGALG00010029175	CENPX	-1.253922443	0.132943886	-9.431967739	4.02467E-21	1.61376E-20	negative
PRJNA729271	stage_3W_vs_E10	ordinary_muscle_development	ENSGALG00010029176	ENSGALG00010029176	1.397546356	0.460591784	3.034240743	0.002411419	0.003485592	positive
PRJNA729271	stage_3W_vs_E10	ordinary_muscle_development	ENSGALG00010029177	ASPSCR1	0.19025956	0.080880893	2.352342463	0.01865559	0.024614307	positive
PRJNA729271	stage_3W_vs_E10	ordinary_muscle_development	ENSGALG00010029178	NOTUM	0.461312089	0.205604424	2.243687559	0.024852511	0.032345977	positive
PRJNA729271	stage_3W_vs_E10	ordinary_muscle_development	ENSGALG00010029179	PIGW	0.301460741	0.148724057	2.026980349	0.042664422	0.054022352	positive
PRJNA729271	stage_3W_vs_E10	ordinary_muscle_development	ENSGALG00010029180	CA4	6.487535276	0.4270764	15.19057312	4.08334E-52	4.94883E-51	positive
PRJNA729271	stage_3W_vs_E10	ordinary_muscle_development	ENSGALG00010029181	MYO19	-0.050473251	0.104644312	-0.482331527	0.629570455	0.663054838	negative
PRJNA729271	stage_3W_vs_E10	ordinary_muscle_development	ENSGALG00010029182	HEATR6	-0.45306524	0.068082285	-6.654671503	2.83933E-11	7.091E-11	negative
PRJNA729271	stage_3W_vs_E10	ordinary_muscle_development	ENSGALG00010029183	ENSGALG00010029183	1.488332289	0.13874525	10.72708643	7.59533E-27	3.85619E-26	positive
PRJNA729271	stage_3W_vs_E10	ordinary_muscle_development	ENSGALG00010029184	DYNLL2	0.519073105	0.095581038	5.430712156	5.61296E-08	1.15306E-07	positive
PRJNA729271	stage_3W_vs_E10	ordinary_muscle_development	ENSGALG00010029186	ZNF302	0.642313701	0.139508872	4.604106474	4.14241E-06	7.52905E-06	positive
PRJNA729271	stage_3W_vs_E10	ordinary_muscle_development	ENSGALG00010029187	SRSF1	-1.537196233	0.052792623	-29.11763316	2.1474E-186	3.3113E-184	negative
PRJNA729271	stage_3W_vs_E10	ordinary_muscle_development	ENSGALG00010029188	ENSGALG00010029188	1.659434719	0.240507527	6.899720523	5.21049E-12	1.34796E-11	positive
PRJNA729271	stage_3W_vs_E10	ordinary_muscle_development	ENSGALG00010029189	ENSGALG00010029189	1.115632485	0.318463146	3.503176107	0.000459745	0.000710373	positive
PRJNA729271	stage_3W_vs_E10	ordinary_muscle_development	ENSGALG00010029190	DHRS7C	6.733332342	0.162750286	41.37216913	0	0	positive
PRJNA729271	stage_3W_vs_E10	ordinary_muscle_development	ENSGALG00010029192	DTX2	0.182046528	0.101506008	1.793455701	0.072900088	0.089663706	positive
PRJNA729271	stage_3W_vs_E10	ordinary_muscle_development	ENSGALG00010029194	DHRS11	-2.283189362	0.143300766	-15.93284826	3.74885E-57	5.22495E-56	negative
PRJNA729271	stage_3W_vs_E10	ordinary_muscle_development	ENSGALG00010029196	MRM1	-0.189732536	0.098188201	-1.932335388	0.053318123	0.066676404	negative
PRJNA729271	stage_3W_vs_E10	ordinary_muscle_development	ENSGALG00010029197	LHX1	1.461643316	0.640969329	2.280363895	0.022586115	0.029560789	positive
PRJNA729271	stage_3W_vs_E10	ordinary_muscle_development	ENSGALG00010029198	PPM1D	-0.105684551	0.175192674	-0.603247546	0.546344022	0.583585601	negative
PRJNA729271	stage_3W_vs_E10	ordinary_muscle_development	ENSGALG00010029199	APPBP2	-0.162135681	0.089962747	-1.802253555	0.071505522	0.088023146	negative
PRJNA729271	stage_3W_vs_E10	ordinary_muscle_development	ENSGALG00010029200	GAS7	1.423268727	0.124378482	11.44304632	2.5478E-30	1.48017E-29	positive
PRJNA729271	stage_3W_vs_E10	ordinary_muscle_development	ENSGALG00010029201	PIGS	-0.96609987	0.113052932	-8.545553432	1.27922E-17	4.42536E-17	negative
PRJNA729271	stage_3W_vs_E10	ordinary_muscle_development	ENSGALG00010029202	PSMD12	1.074747125	0.14241698	7.546481667	4.47174E-14	1.29429E-13	positive
PRJNA729271	stage_3W_vs_E10	ordinary_muscle_development	ENSGALG00010029203	ALDOC	-1.776903934	0.173401841	-10.24731872	1.21672E-24	5.61309E-24	negative
PRJNA729271	stage_3W_vs_E10	ordinary_muscle_development	ENSGALG00010029204	HELZ	0.164497953	0.098101294	1.676817366	0.093578201	0.11360737	positive
PRJNA729271	stage_3W_vs_E10	ordinary_muscle_development	ENSGALG00010029205	CACNG1	5.547534788	0.113945761	48.68574943	0	0	positive
PRJNA729271	stage_3W_vs_E10	ordinary_muscle_development	ENSGALG00010029206	CACNG4	-2.288384792	0.349987798	-6.538470218	6.21513E-11	1.51766E-10	negative
PRJNA729271	stage_3W_vs_E10	ordinary_muscle_development	ENSGALG00010029207	SPAG5	-3.489540535	0.231958461	-15.04381658	3.79051E-51	4.45608E-50	negative
PRJNA729271	stage_3W_vs_E10	ordinary_muscle_development	ENSGALG00010029208	CACNG5	-5.03036083	0.630332181	-7.980491844	1.45751E-15	4.52488E-15	negative
PRJNA729271	stage_3W_vs_E10	ordinary_muscle_development	ENSGALG00010029209	PRKCA	0.439173842	0.096541216	4.549081309	5.38806E-06	9.70668E-06	positive
PRJNA729271	stage_3W_vs_E10	ordinary_muscle_development	ENSGALG00010029210	RSKR	-2.308231485	0.567798525	-4.065229801	4.79852E-05	8.04284E-05	negative
PRJNA729271	stage_3W_vs_E10	ordinary_muscle_development	ENSGALG00010029211	SDF2	-1.178402281	0.169433422	-6.954957702	3.52668E-12	9.21067E-12	negative
PRJNA729271	stage_3W_vs_E10	ordinary_muscle_development	ENSGALG00010029212	SUPT6H	0.107620948	0.113605509	0.947321559	0.343474948	0.381946251	positive
PRJNA729271	stage_3W_vs_E10	ordinary_muscle_development	ENSGALG00010029213	APOH	1.047940987	1.549739293	0.676204696	0.498910692	0.537114563	positive
PRJNA729271	stage_3W_vs_E10	ordinary_muscle_development	ENSGALG00010029214	ENSGALG00010029214	8.294117883	0.374770122	22.13121429	1.5826E-108	7.9375E-107	positive
PRJNA729271	stage_3W_vs_E10	ordinary_muscle_development	ENSGALG00010029215	ENSGALG00010029215	7.993574889	0.23667178	33.77493877	4.6027E-250	1.3082E-247	positive
PRJNA729271	stage_3W_vs_E10	ordinary_muscle_development	ENSGALG00010029216	RAB34	-0.907797976	0.133478012	-6.801104986	1.0382E-11	2.65175E-11	negative
PRJNA729271	stage_3W_vs_E10	ordinary_muscle_development	ENSGALG00010029217	CEP112	-0.624347389	0.091150261	-6.849650067	7.40309E-12	1.90127E-11	negative
PRJNA729271	stage_3W_vs_E10	ordinary_muscle_development	ENSGALG00010029218	RPL23A	0.998199476	0.150212555	6.64524663	3.0271E-11	7.54562E-11	positive
PRJNA729271	stage_3W_vs_E10	ordinary_muscle_development	ENSGALG00010029219	TLCD1	0.319592314	0.202902619	1.575101969	0.11523291	0.138132734	positive
PRJNA729271	stage_3W_vs_E10	ordinary_muscle_development	ENSGALG00010029220	NEK8	-0.076639309	0.128832629	-0.59487499	0.551927036	0.589018646	negative
PRJNA729271	stage_3W_vs_E10	ordinary_muscle_development	ENSGALG00010029221	AXIN2	-1.266255165	0.129083014	-9.809618852	1.02354E-22	4.36233E-22	negative
PRJNA729271	stage_3W_vs_E10	ordinary_muscle_development	ENSGALG00010029222	TRAF4	-0.663336236	0.236603767	-2.803574276	0.005053959	0.007083459	negative
PRJNA729271	stage_3W_vs_E10	ordinary_muscle_development	ENSGALG00010029223	NME2	0.001223361	0.145838958	0.008388434	0.993307076	0.994336055	positive
PRJNA729271	stage_3W_vs_E10	ordinary_muscle_development	ENSGALG00010029224	FAM222B	-0.296057573	0.161604782	-1.831985231	0.066953621	0.082729894	negative
PRJNA729271	stage_3W_vs_E10	ordinary_muscle_development	ENSGALG00010029226	MBTD1	-0.679863497	0.0763811	-8.900938834	5.53763E-19	2.03364E-18	negative
PRJNA729271	stage_3W_vs_E10	ordinary_muscle_development	ENSGALG00010029227	UTP18	-0.743455769	0.080847634	-9.195764098	3.72339E-20	1.43196E-19	negative
PRJNA729271	stage_3W_vs_E10	ordinary_muscle_development	ENSGALG00010029228	ERAL1	0.130600822	0.11861423	1.101055262	0.270872606	0.30746268	positive
PRJNA729271	stage_3W_vs_E10	ordinary_muscle_development	ENSGALG00010029229	CBX4	0.113620192	0.222848351	0.509854308	0.610153535	0.645183505	positive
PRJNA729271	stage_3W_vs_E10	ordinary_muscle_development	ENSGALG00010029230	PPM1E	0.608899874	0.164927038	3.691934824	0.000222555	0.000352985	positive
PRJNA729271	stage_3W_vs_E10	ordinary_muscle_development	ENSGALG00010029231	CBX8	-1.066162646	0.114024368	-9.350305238	8.73951E-21	3.45363E-20	negative
PRJNA729271	stage_3W_vs_E10	ordinary_muscle_development	ENSGALG00010029232	CA10	-5.53396355	0.463378781	-11.9426348	7.09398E-33	4.53983E-32	negative
PRJNA729271	stage_3W_vs_E10	ordinary_muscle_development	ENSGALG00010029233	CBX2	-4.303304552	0.19617051	-21.93655177	1.164E-106	5.5318E-105	negative
PRJNA729271	stage_3W_vs_E10	ordinary_muscle_development	ENSGALG00010029234	TOM1L1	-2.081965777	0.239907151	-8.678214756	4.02029E-18	1.42864E-17	negative
PRJNA729271	stage_3W_vs_E10	ordinary_muscle_development	ENSGALG00010029235	TRIM37	-0.293561103	0.074386118	-3.946450082	7.93184E-05	0.000130517	negative
PRJNA729271	stage_3W_vs_E10	ordinary_muscle_development	ENSGALG00010029237	COX11	-0.188979125	0.105199762	-1.796383578	0.072433534	0.089097426	negative
PRJNA729271	stage_3W_vs_E10	ordinary_muscle_development	ENSGALG00010029238	CAMKK1	-0.077896471	0.246661333	-0.315803333	0.752151788	0.778300983	negative
PRJNA729271	stage_3W_vs_E10	ordinary_muscle_development	ENSGALG00010029239	RBFOX3	-3.056907014	0.669439088	-4.566370666	4.96241E-06	8.96661E-06	negative
PRJNA729271	stage_3W_vs_E10	ordinary_muscle_development	ENSGALG00010029240	MIS12	-3.73694254	0.268561291	-13.91467296	5.15954E-44	4.75445E-43	negative
PRJNA729271	stage_3W_vs_E10	ordinary_muscle_development	ENSGALG00010029241	STXBP4	-2.128116112	0.099628372	-21.36054285	3.1117E-101	1.33446E-99	negative
PRJNA729271	stage_3W_vs_E10	ordinary_muscle_development	ENSGALG00010029242	DERL2	-0.115795066	0.09812401	-1.180089021	0.237964811	0.272220025	negative

PRJNA729271	stage_3W_vs_E10	ordinary_muscle_development	ENSGALG00010029243	NME1	-0.277130737	0.343417682	-0.806978649	0.419678784	0.458144599	negative
PRJNA729271	stage_3W_vs_E10	ordinary_muscle_development	ENSGALG00010029244	HLF	2.0972297	0.182789175	11.47348962	1.79281E-30	1.0487E-29	positive
PRJNA729271	stage_3W_vs_E10	ordinary_muscle_development	ENSGALG00010029245	SKA2	-1.295476534	0.115445009	-11.22158979	3.19475E-29	1.78657E-28	negative
PRJNA729271	stage_3W_vs_E10	ordinary_muscle_development	ENSGALG00010029246	DHX33	-1.344200173	0.136368344	-9.857127671	6.38498E-23	2.75038E-22	negative
PRJNA729271	stage_3W_vs_E10	ordinary_muscle_development	ENSGALG00010029247	CCDC92B	6.658471575	0.527325759	12.62686578	1.5014E-36	1.0795E-35	positive
PRJNA729271	stage_3W_vs_E10	ordinary_muscle_development	ENSGALG00010029248	C1QBP	-0.370118277	0.081564219	-4.537752951	5.68568E-06	1.02314E-05	negative
PRJNA729271	stage_3W_vs_E10	ordinary_muscle_development	ENSGALG00010029249	PRR11	-3.001217888	0.317348114	-9.457178894	3.16363E-21	1.27309E-20	negative
PRJNA729271	stage_3W_vs_E10	ordinary_muscle_development	ENSGALG00010029250	SPAG9	0.71316574	0.08720243	8.178278321	2.87928E-16	9.30135E-16	positive
PRJNA729271	stage_3W_vs_E10	ordinary_muscle_development	ENSGALG00010029251	ENGASE	-0.854408597	0.194197911	-4.399679651	1.08411E-05	1.91007E-05	negative
PRJNA729271	stage_3W_vs_E10	ordinary_muscle_development	ENSGALG00010029252	RPAIN	1.65839813	0.136427415	12.15590089	5.33702E-34	3.547E-33	positive
PRJNA729271	stage_3W_vs_E10	ordinary_muscle_development	ENSGALG00010029253	NUP88	-0.07705019	0.085261597	-0.903691618	0.366158945	0.404810763	negative
PRJNA729271	stage_3W_vs_E10	ordinary_muscle_development	ENSGALG00010029254	MMD	-0.277518198	0.119040661	-2.331289118	0.01973812	0.025988195	negative
PRJNA729271	stage_3W_vs_E10	ordinary_muscle_development	ENSGALG00010029255	RAP1GAP2	0.633350209	0.189272814	3.346229152	0.000819187	0.001236243	positive
PRJNA729271	stage_3W_vs_E10	ordinary_muscle_development	ENSGALG00010029256	RABEP1	0.444366937	0.096518843	4.603939742	4.14572E-06	7.53414E-06	positive
PRJNA729271	stage_3W_vs_E10	ordinary_muscle_development	ENSGALG00010029257	YPEL2	0.414426492	0.161641173	2.563867137	0.010351316	0.014042333	positive
PRJNA729271	stage_3W_vs_E10	ordinary_muscle_development	ENSGALG00010029258	TMEM100	0.389619791	0.234921025	1.658513921	0.09721378	0.117759798	positive
PRJNA729271	stage_3W_vs_E10	ordinary_muscle_development	ENSGALG00010029259	PTRH2	-0.08470677	0.108060714	-0.783881269	0.433109782	0.471705334	negative
PRJNA729271	stage_3W_vs_E10	ordinary_muscle_development	ENSGALG00010029260	DHX40	0.149467736	0.102469212	1.458659962	0.144658721	0.171155674	positive
PRJNA729271	stage_3W_vs_E10	ordinary_muscle_development	ENSGALG00010029261	IFT22	-1.663940565	0.101344402	-16.41867272	1.40609E-60	2.13864E-59	negative
PRJNA729271	stage_3W_vs_E10	ordinary_muscle_development	ENSGALG00010029262	CLTC	-0.558595156	0.065200056	-8.567403047	1.05845E-17	3.68272E-17	negative
PRJNA729271	stage_3W_vs_E10	ordinary_muscle_development	ENSGALG00010029263	TOB1	2.788090202	0.188313496	14.80557827	1.34823E-49	1.50096E-48	positive
PRJNA729271	stage_3W_vs_E10	ordinary_muscle_development	ENSGALG00010029264	COL26A1	-6.687798621	0.184867933	-36.17608803	1.4473E-286	5.67E-284	negative
PRJNA729271	stage_3W_vs_E10	ordinary_muscle_development	ENSGALG00010029266	CANT1	0.196811121	0.1114993	1.765133245	0.077541375	0.095057698	positive
PRJNA729271	stage_3W_vs_E10	ordinary_muscle_development	ENSGALG00010029267	ENSGALG00010029267	4.521813195	0.272954706	16.56616682	1.22383E-61	1.91157E-60	positive
PRJNA729271	stage_3W_vs_E10	ordinary_muscle_development	ENSGALG00010029268	PCTP	1.807763388	0.178256536	10.14135821	3.62031E-24	1.63682E-23	positive
PRJNA729271	stage_3W_vs_E10	ordinary_muscle_development	ENSGALG00010029269	WSCD1	0.506633238	0.154787823	3.273081998	0.001063816	0.001590512	positive
PRJNA729271	stage_3W_vs_E10	ordinary_muscle_development	ENSGALG00010029270	TIMP2	0.9366468	0.149421088	6.268504768	3.64531E-10	8.53616E-10	positive
PRJNA729271	stage_3W_vs_E10	ordinary_muscle_development	ENSGALG00010029271	MYL10	-5.538264281	0.801176154	-6.912667398	4.75624E-12	1.23242E-11	negative
PRJNA729271	stage_3W_vs_E10	ordinary_muscle_development	ENSGALG00010029273	SLC13A5	-1.629639577	0.443985962	-3.670475461	0.0002421	0.000382645	negative
PRJNA729271	stage_3W_vs_E10	ordinary_muscle_development	ENSGALG00010029274	VMP1	0.174143318	0.146582241	1.188024669	0.234823691	0.268881381	positive
PRJNA729271	stage_3W_vs_E10	ordinary_muscle_development	ENSGALG00010029275	PCYT2	0.388255286	0.070972694	5.470488221	4.48798E-08	9.27873E-08	positive
PRJNA729271	stage_3W_vs_E10	ordinary_muscle_development	ENSGALG00010029276	TUBD1	0.26071748	0.173593465	1.501885345	0.133126722	0.158325553	positive
PRJNA729271	stage_3W_vs_E10	ordinary_muscle_development	ENSGALG00010029277	WFIKKN2	2.426443266	0.742022798	3.2700387	0.001075328	0.001607557	positive
PRJNA729271	stage_3W_vs_E10	ordinary_muscle_development	ENSGALG00010029278	MED31	-0.71096803	0.071091597	-10.0007323	1.51274E-23	6.65469E-23	negative
PRJNA729271	stage_3W_vs_E10	ordinary_muscle_development	ENSGALG00010029279	ENSGALG00010029279	1.05662886	0.158360411	6.672304362	2.51818E-11	6.30414E-11	positive
PRJNA729271	stage_3W_vs_E10	ordinary_muscle_development	ENSGALG00010029280	USP36	1.001779114	0.182332392	5.494246532	3.92382E-08	8.1519E-08	positive
PRJNA729271	stage_3W_vs_E10	ordinary_muscle_development	ENSGALG00010029281	KIAA0753	-0.738585264	0.133593328	-5.528608934	3.2278E-08	6.73194E-08	negative
PRJNA729271	stage_3W_vs_E10	ordinary_muscle_development	ENSGALG00010029282	LUC7L3	-0.212326559	0.140910599	-1.506817517	0.131857438	0.156880372	negative
PRJNA729271	stage_3W_vs_E10	ordinary_muscle_development	ENSGALG00010029283	RPS6KB1	0.032358665	0.077519267	0.417427388	0.676365825	0.707506866	positive
PRJNA729271	stage_3W_vs_E10	ordinary_muscle_development	ENSGALG00010029284	CYTH1	0.859130927	0.092845534	9.253336101	2.17593E-20	8.45126E-20	positive
PRJNA729271	stage_3W_vs_E10	ordinary_muscle_development	ENSGALG00010029285	CUX1	0.117607207	0.069730718	1.686591092	0.091682006	0.111459421	positive
PRJNA729271	stage_3W_vs_E10	ordinary_muscle_development	ENSGALG00010029286	MAFG	1.900519873	0.182343455	10.42274798	1.95229E-25	9.29648E-25	positive
PRJNA729271	stage_3W_vs_E10	ordinary_muscle_development	ENSGALG00010029287	ANKRD40	0.903629998	0.122721772	7.36324113	1.79498E-13	5.02282E-13	positive
PRJNA729271	stage_3W_vs_E10	ordinary_muscle_development	ENSGALG00010029288	PYCR1	-2.2830792	0.294934731	-7.740964218	9.86657E-15	2.94211E-14	negative
PRJNA729271	stage_3W_vs_E10	ordinary_muscle_development	ENSGALG00010029289	PITPNM3	-0.245800207	0.220513107	-1.114673911	0.264990185	0.301281099	negative
PRJNA729271	stage_3W_vs_E10	ordinary_muscle_development	ENSGALG00010029290	ENSGALG00010029290	-1.678926081	0.198243719	-8.469000134	2.47508E-17	8.42365E-17	negative
PRJNA729271	stage_3W_vs_E10	ordinary_muscle_development	ENSGALG00010029291	RNFT1	-0.057146576	0.113494795	-0.503517147	0.614600728	0.649364935	negative
PRJNA729271	stage_3W_vs_E10	ordinary_muscle_development	ENSGALG00010029292	ENSGALG00010029292	-0.666514182	0.253054008	-2.633881155	0.008441503	0.011570646	negative
PRJNA729271	stage_3W_vs_E10	ordinary_muscle_development	ENSGALG00010029293	DNAH17	-1.866373538	0.14650064	-12.73969541	3.55781E-37	2.60063E-36	negative
PRJNA729271	stage_3W_vs_E10	ordinary_muscle_development	ENSGALG00010029294	PGS1	-0.917778044	0.077557924	-11.83345291	2.62132E-32	1.64914E-31	negative
PRJNA729271	stage_3W_vs_E10	ordinary_muscle_development	ENSGALG00010029295	SOCS3	2.470193831	0.248012093	9.95997333	2.28124E-23	9.96281E-23	positive
PRJNA729271	stage_3W_vs_E10	ordinary_muscle_development	ENSGALG00010029296	FBXO39	-1.465345593	0.161797985	-9.056636821	1.34535E-19	5.05726E-19	negative
PRJNA729271	stage_3W_vs_E10	ordinary_muscle_development	ENSGALG00010029297	SH2B2	3.653920644	0.251546407	14.52583117	8.31278E-48	8.78235E-47	positive
PRJNA729271	stage_3W_vs_E10	ordinary_muscle_development	ENSGALG00010029298	ENDOV	0.355981915	0.145561019	2.445585475	0.014461716	0.019312934	positive
PRJNA729271	stage_3W_vs_E10	ordinary_muscle_development	ENSGALG00010029299	PRKRIP1	0.102754274	0.09078682	1.131819289	0.257710416	0.293372535	positive
PRJNA729271	stage_3W_vs_E10	ordinary_muscle_development	ENSGALG00010029300	TEKT1	2.43942685	0.342606504	7.12020007	1.07771E-12	2.90144E-12	positive
PRJNA729271	stage_3W_vs_E10	ordinary_muscle_development	ENSGALG00010029301	RNF213	4.513478893	0.464199533	9.723143981	2.40247E-22	1.00763E-21	positive
PRJNA729271	stage_3W_vs_E10	ordinary_muscle_development	ENSGALG00010029303	SLC26A11	0.266757479	0.150290717	1.774943156	0.075907274	0.093172659	positive
PRJNA729271	stage_3W_vs_E10	ordinary_muscle_development	ENSGALG00010029304	ORAI3	0.079077334	0.116785539	0.677115804	0.498332482	0.536571782	positive
PRJNA729271	stage_3W_vs_E10	ordinary_muscle_development	ENSGALG00010029305	SMTNL2	2.624135013	0.189896596	13.81875752	1.96431E-43	1.78289E-42	positive
PRJNA729271	stage_3W_vs_E10	ordinary_muscle_development	ENSGALG00010029306	CEP95	-1.540011756	0.161834367	-9.515974783	1.80017E-21	7.3173E-21	negative
PRJNA729271	stage_3W_vs_E10	ordinary_muscle_development	ENSGALG00010029307	SGSH	0.613399022	0.163555299	3.750407516	0.000176547	0.00028255	positive
PRJNA729271	stage_3W_vs_E10	ordinary_muscle_development	ENSGALG00010029308	GAA	-0.026816923	0.138909762	-0.193052835	0.84691759	0.864512005	negative
PRJNA729271	stage_3W_vs_E10	ordinary_muscle_development	ENSGALG00010029309	ALKBH4	2.501843431	0.162534562	15.39268571	1.83265E-53	2.32612E-52	positive
PRJNA729271	stage_3W_vs_E10	ordinary_muscle_development	ENSGALG00010029310	LRWD1	-1.129390391	0.092241897	-12.24378968	1.81362E-34	1.22901E-33	negative
PRJNA729271	stage_3W_vs_E10	ordinary_muscle_development	ENSGALG00010029311	ENSGALG00010029311	1.206689104	0.163942721	7.360431103	1.83317E-13	5.12871E-13	positive
PRJNA729271	stage_3W_vs_E10	ordinary_muscle_development	ENSGALG00010029312	SMURF2	0.562470434	0.0933737	6.023863639	1.70302E-09	3.82717E-09	positive
PRJNA729271	stage_3W_vs_E10	ordinary_muscle_development	ENSGALG00010029313	CCDC40	-1.169842588	0.238847058	-4.897873135	9.68795E-07	1.83781E-06	negative
PRJNA729271	stage_3W_vs_E10	ordinary_muscle_development	ENSGALG00010029314	MED13	0.104260386	0.126069278	0.827008671	0.408232147	0.447062932	positive
PRJNA729271	stage_3W_vs_E10	ordinary_muscle_development	ENSGALG00010029315	TBC1D16	-0.199392532	0.095714385	-2.083203407	0.037232687	0.047516094	negative
PRJNA729271	stage_3W_vs_E10	ordinary_muscle_development	ENSGALG00010029316	INTS2	-0.659024086	0.095120553	-6.928303749	4.25916E-12	1.10639E-11	negative
PRJNA729271	stage_3W_vs_E10	ordinary_muscle_development	ENSGALG00010029317	KPNA2	-3.832793765	0.20692914	-18.52225244	1.36601E-76	3.128E-75	negative
PRJNA729271	stage_3W_vs_E10	ordinary_muscle_development	ENSGALG00010029318	C17orf58	1.306554212	0.210182889	6.216272961	5.09102E-10	1.1826E-09	positive
PRJNA729271	stage_3W_vs_E10	ordinary_muscle_development	ENSGALG00010029319	BRIP1	-1.424308715	0.127165202	-11.20045967	4.05623E-29	2.25701E-28	negative
PRJNA729271	stage_3W_vs_E10	ordinary_muscle_development	ENSGALG00010029320	TBX4	-6.809992744	1.003623255	-6.785407479	1.15759E-11	2.95203E-11	negative
PRJNA729271	stage_3W_vs_E10	ordinary_muscle_development	ENSGALG00010029321	TBX2	-1.8474441	0.177350912	-10.41688527	2.07642E-25	9.87135E-25	negative
PRJNA729271	stage_3W_vs_E10	ordinary_muscle_development	ENSGALG00010029322	BPTF	-0.250658575	0.096907346	-2.586579726	0.009693374	0.013196718	negative
PRJNA729271	stage_3W_vs_E10	ordinary_muscle_development	ENSGALG00010029323	NOL11	0.381386975	0.090838211	4.198530246	2.68653E-05	4.58564E-05	positive
PRJNA729271	stage_3W_vs_E10	ordinary_muscle_development	ENSGALG00010029324	BCAS3	0.859621923	0.126373988	6.802206159	1.03029E-11	2.63294E-11	positive
PRJNA729271	stage_3W_vs_E10	ordinary_muscle_development	ENSGALG00010029325	LRRC75A	-1.516100138	0.172212373	-8.803665556	1.32419E-18	4.78297E-18	negative
PRJNA729271	stage_3W_vs_E10	ordinary_muscle_development	ENSGALG00010029326	NCBP3	-0.581194645	0.081394624	-7.140454948	9.30225E-13	2.5156E-12	negative
PRJNA729271	stage_3W_vs_E10	ordinary_muscle_development	ENSGALG00010029327	CRK	-0.613647355	0.054868969	-11.1838689	4.89102E-29	2.70903E-28	negative
PRJNA729271	stage_3W_vs_E10	ordinary_muscle_development	ENSGALG00010029328	ENSGALG00010029328	-0.240420443	0.08245193	-2.91588619	0.003546799	0.005050677	negative
PRJNA729271	stage_3W_vs_E10	ordinary_muscle_development	ENSGALG00010029329	ENSGALG00010029329	0.671961317	0.180283779	3.727242233	0.000193586	0.000308865	positive
PRJNA729271	stage_3W_vs_E10	ordinary_muscle_development	ENSGALG00010029330	AFMID	0.863760982	0.126018875	6.854219124	7.17031E-12	1.8428E-11	positive
PRJNA729271	stage_3W_vs_E10	ordinary_muscle_development	ENSGALG00010029331	TK1	-3.581320864	0.281247466	-12.73370003	3.84192E-37	2.80265E-36	negative
PRJNA729271	stage_3W_vs_E10	ordinary_muscle_development	ENSGALG00010029332	YWHAE	-1.269424429	0.148948691	-8.522561816	1.56062E-17	5.37449E-17	negative
PRJNA729271	stage_3W_vs_E10	ordinary_muscle_development	ENSGALG00010029333	ENSGALG00010029333	-1.340475518	0.176011389	-7.615845332	2.61971E-14	7.65888E-14	negative
PRJNA729271	stage_3W_vs_E10	ordinary_muscle_development	ENSGALG00010029335	RNF222	-0.913386993	1.507705825	-0.605812472	0.544639287	0.582064916	negative
PRJNA729271	stage_3W_vs_E10	ordinary_muscle_development	ENSGALG00010029336	SYNGR2	2.329878846	0.110093372	21.16275305	2.10562E-99	8.57332E-98	positive
PRJNA729271	stage_3W_vs_E10	ordinary_muscle_development	ENSGALG00010029337	MYO1C	1.156368348	0.133715607	8.647968433	5.24243E-18	1.84978E-17	positive
PRJNA729271	stage_3W_vs_E10	ordinary_muscle_development	ENSGALG00010029338	INPP5K	0.188519647	0.12002862	1.570622472	0.116270367	0.139284212	positive
PRJNA729271	stage_3W_vs_E10	ordinary_muscle_development	ENSGALG00010029339	PITPNA	0.796208146	0.126825255	6.277993661	3.4297E-10	8.04165E-10	positive
PRJNA729271	stage_3W_vs_E10	ordinary_muscle_development	ENSGALG00010029340	NDEL1	0.970502992	0.103471486	9.379424463	6.63338E-21	2.63211E-20	positive
PRJNA729271	stage_3W_vs_E10	ordinary_muscle_development	ENSGALG00010029341	SLC43A2	3.529955304	0.326069213	10.82578532	2.59842E-27	1.34371E-26	positive
PRJNA729271	stage_3W_vs_E10	ordinary_muscle_development	ENSGALG00010029342	TMC6	-0.442776931	0.129787672	-3.411548459	0.00064595	0.000984651	negative
PRJNA729271	stage_3W_vs_E10	ordinary_muscle_development	ENSGALG00010029343	SCARF1	1.788370407	0.130037344	13.75274483	4.90333E-43	4.37108E-42	positive
PRJNA729271	stage_3W_vs_E10	ordinary_muscle_development	ENSGALG00010029344	ENSGALG00010029344	1.925366228	0.163515991	11.7747886	5.26489E-32	3.26969E-31	positive
PRJNA729271	stage_3W_vs_E10	ordinary_muscle_development	ENSGALG00010029345	RILP	2.418970075	0.242849157	9.960792555	2.26252E-23	9.88403E-23	positive
PRJNA729271	stage_3W_vs_E10	ordinary_muscle_development	ENSGALG00010029346	ARL16	-1.105008367	0.131029553	-8.433275878	3.36121E-17	1.13674E-16	negative
PRJNA729271	stage_3W_vs_E10	ordinary_muscle_development	ENSGALG00010029347	PRPF8	-0.571066406	0.070033509	-8.154188096	3.51533E-16	1.12732E-15	negative
PRJNA729271	stage_3W_vs_E10	ordinary_muscle_development	ENSGALG00010029348	HGS	0.530405112	0.123373996	4.299164558	1.71443E-05	2.97577E-05	positive
PRJNA729271	stage_3W_vs_E10	ordinary_muscle_development	ENSGALG00010029349	MYH10	-2.799996582	0.107667685	-26.00591422	4.2453E-149	3.97E-147	negative
PRJNA729271	stage_3W_vs_E10	ordinary_muscle_development	ENSGALG00010029350	MRPL12	0.712773265	0.121476337	5.867589391	4.42177E-09	9.70819E-09	positive
PRJNA729271	stage_3W_vs_E10	ordinary_muscle_development	ENSGALG00010029351	ENSGALG00010029351	-1.093589251	0.201110545	-5.437751926	5.3957E-08	1.10969E-07	negative
PRJNA729271	stage_3W_vs_E10	ordinary_muscle_development	ENSGALG00010029352	AATF	-1.134164256	0.112807487	-10.05398029	8.82301E-24	3.9266E-23	negative
PRJNA729271	stage_3W_vs_E10	ordinary_muscle_development	ENSGALG00010029353	GCGR	3.606476821	0.889088477	4.056375621	4.98401E-05	8.34795E-05	positive
PRJNA729271	stage_3W_vs_E10	ordinary_muscle_development	ENSGALG00010029354	TLCD2	-0.936129943	0.288004148	-3.250404375	0.00115241	0.0017183	negative
PRJNA729271	stage_3W_vs_E10	ordinary_muscle_development	ENSGALG00010029355	MCRIP1	0.998039174	0.172432186	5.788009753	7.12253E-09	1.55086E-08	positive
PRJNA729271	stage_3W_vs_E10	ordinary_muscle_development	ENSGALG00010029357	ARHGDIA	-1.095999413	0.09770052	-11.21794863	3.32903E-29	1.86023E-28	negative
PRJNA729271	stage_3W_vs_E10	ordinary_muscle_development	ENSGALG00010029359	ENSGALG00010029359	-3.285644954	0.534033173	-6.15251097	7.62657E-10	1.75166E-09	negative
PRJNA729271	stage_3W_vs_E10	ordinary_muscle_development	ENSGALG00010029360	ACACA	-0.966596065	0.086314443	-11.19854377	4.14491E-29	2.30547E-28	negative
PRJNA729271	stage_3W_vs_E10	ordinary_muscle_development	ENSGALG00010029361	ALYREF	-2.139482421	0.136557977	-15.66720941	2.53466E-55	3.37373E-54	negative
PRJNA729271	stage_3W_vs_E10	ordinary_muscle_development	ENSGALG00010029362	TADA2A	-0.702675854	0.114578108	-6.13272351	8.63872E-10	1.97755E-09	negative
PRJNA729271	stage_3W_vs_E10	ordinary_muscle_development	ENSGALG00010029363	PIK3R6	2.090378758	0.190984492	10.94528011	7.0001E-28	3.71264E-27	positive
PRJNA729271	stage_3W_vs_E10	ordinary_muscle_development	ENSGALG00010029364	ANAPC11	-0.086822806	0.129416737	-0.670877723	0.502298429	0.540320277	negative
PRJNA729271	stage_3W_vs_E10	ordinary_muscle_development	ENSGALG00010029365	PIK3R5	2.131435162	0.183909073	11.589614	4.65224E-31	2.78079E-30	positive
PRJNA729271	stage_3W_vs_E10	ordinary_muscle_development	ENSGALG00010029366	MGAT5B	2.033707938	0.341107089	5.962080539	2.49046E-09	5.5401E-09	positive
PRJNA729271	stage_3W_vs_E10	ordinary_muscle_development	ENSGALG00010029367	DUSP14	-0.854915528	0.118522424	-7.213112051	5.46874E-13	1.49283E-12	negative
PRJNA729271	stage_3W_vs_E10	ordinary_muscle_development	ENSGALG00010029368	DDX52	-0.424095958	0.092168321	-4.601320188	4.19821E-06	7.62475E-06	negative
PRJNA729271	stage_3W_vs_E10	ordinary_muscle_development	ENSGALG00010029369	MFSD11	-0.042399937	0.085016588	-0.498725458	0.617972808	0.652594772	negative
PRJNA729271	stage_3W_vs_E10	ordinary_muscle_development	ENSGALG00010029370	SRSF2	-1.686691835	0.12791952	-13.18557037	1.06247E-39	8.5086E-39	negative
PRJNA729271	stage_3W_vs_E10	ordinary_muscle_development	ENSGALG00010029371	AUTS2	-2.818455061	0.183821409	-15.33257243	4.63271E-53	5.79888E-52	negative
PRJNA729271	stage_3W_vs_E10	ordinary_muscle_development	ENSGALG00010029372	METTL23	-0.160072027	0.142343484	-1.124547625	0.260780779	0.29665809	negative
PRJNA729271	stage_3W_vs_E10	ordinary_muscle_development	ENSGALG00010029373	GATSL2	1.990574486	0.168495628	11.81380494	3.31225E-32	2.07302E-31	positive
PRJNA729271	stage_3W_vs_E10	ordinary_muscle_development	ENSGALG00010029374	RCC1L	-0.564093283	0.118934294	-4.742898501	2.10682E-06	3.90666E-06	negative
PRJNA729271	stage_3W_vs_E10	ordinary_muscle_development	ENSGALG00010029375	JMJD6	1.011003772	0.160008828	6.318424953	2.64243E-10	6.23709E-10	positive
PRJNA729271	stage_3W_vs_E10	ordinary_muscle_development	ENSGALG00010029376	ENSGALG00010029376	-0.300875153	0.206946252	-1.453880659	0.145979396	0.172549241	negative
PRJNA729271	stage_3W_vs_E10	ordinary_muscle_development	ENSGALG00010029377	NCF1C	2.439818746	0.283731785	8.599032161	8.03912E-18	2.80923E-17	positive
PRJNA729271	stage_3W_vs_E10	ordinary_muscle_development	ENSGALG00010029378	ST6GALNAC1	-2.348254366	0.352644159	-6.658991251	2.75713E-11	6.89165E-11	negative
PRJNA729271	stage_3W_vs_E10	ordinary_muscle_development	ENSGALG00010029379	GTF2I	0.201393058	0.112459398	1.790806827	0.073324299	0.090154866	positive

PRJNA729271	stage_3W_vs_E10	ordinary_muscle_development	ENSGALG00010029380	RGS9	0.009159857	0.148812798	0.061552882	0.9509189	0.9572588	positive
PRJNA729271	stage_3W_vs_E10	ordinary_muscle_development	ENSGALG00010029382	GTF2IRD1	0.392772812	0.206230268	1.904535238	0.056840512	0.070861204	positive
PRJNA729271	stage_3W_vs_E10	ordinary_muscle_development	ENSGALG00010029383	GNA13	0.099781826	0.078849341	1.265474442	0.205701304	0.237713498	positive
PRJNA729271	stage_3W_vs_E10	ordinary_muscle_development	ENSGALG00010029384	WDR81	-0.362733338	0.075596532	-4.798280122	1.60034E-06	2.98632E-06	negative
PRJNA729271	stage_3W_vs_E10	ordinary_muscle_development	ENSGALG00010029385	CLIP2	-1.138026144	0.123239649	-9.234253355	2.60094E-20	1.0075E-19	negative
PRJNA729271	stage_3W_vs_E10	ordinary_muscle_development	ENSGALG00010029386	SLC16A6	-1.402942329	0.240499874	-5.833443091	5.42951E-09	1.18901E-08	negative
PRJNA729271	stage_3W_vs_E10	ordinary_muscle_development	ENSGALG00010029387	SERPINF1	2.094925175	0.17589699	11.90995464	1.05034E-32	6.68044E-32	positive
PRJNA729271	stage_3W_vs_E10	ordinary_muscle_development	ENSGALG00010029388	ST6GALNAC2	1.438974792	0.319560431	4.502981756	6.70066E-06	1.19835E-05	positive
PRJNA729271	stage_3W_vs_E10	ordinary_muscle_development	ENSGALG00010029389	SMYD4	0.254352714	0.095236938	2.670735952	0.007568516	0.010425319	positive
PRJNA729271	stage_3W_vs_E10	ordinary_muscle_development	ENSGALG00010029390	RPA1	-1.20663037	0.080383359	-15.01094727	6.22523E-51	7.27113E-50	negative
PRJNA729271	stage_3W_vs_E10	ordinary_muscle_development	ENSGALG00010029391	ARSG	-0.338317435	0.143865189	-2.351628196	0.018691448	0.024654885	negative
PRJNA729271	stage_3W_vs_E10	ordinary_muscle_development	ENSGALG00010029392	RTN4RL1	2.198802788	0.521747955	4.21430073	2.50553E-05	4.28882E-05	positive
PRJNA729271	stage_3W_vs_E10	ordinary_muscle_development	ENSGALG00010029394	CYGB	-1.091039902	0.143244741	-7.616613997	2.60417E-14	7.61497E-14	negative
PRJNA729271	stage_3W_vs_E10	ordinary_muscle_development	ENSGALG00010029395	DPH1	-0.522428775	0.120399336	-4.339133362	1.43046E-05	2.49723E-05	negative
PRJNA729271	stage_3W_vs_E10	ordinary_muscle_development	ENSGALG00010029396	WIPI1	2.336761065	0.154025065	15.17130384	5.47778E-52	6.61119E-51	positive
PRJNA729271	stage_3W_vs_E10	ordinary_muscle_development	ENSGALG00010029397	OVCA2	-0.818436036	0.157067057	-5.210742786	1.88086E-07	3.73467E-07	negative
PRJNA729271	stage_3W_vs_E10	ordinary_muscle_development	ENSGALG00010029398	RHBDF2	0.42730821	0.12382212	3.450984437	0.000558546	0.000855375	positive
PRJNA729271	stage_3W_vs_E10	ordinary_muscle_development	ENSGALG00010029399	PRKAR1A	-0.970316868	0.053880298	-18.00875088	1.6634E-72	3.4742E-71	negative
PRJNA729271	stage_3W_vs_E10	ordinary_muscle_development	ENSGALG00010029400	AANAT	1.789169898	0.630867761	2.836045855	0.004567589	0.006432255	positive
PRJNA729271	stage_3W_vs_E10	ordinary_muscle_development	ENSGALG00010029401	HIC1	-1.295989317	0.20249039	-6.400250974	1.55122E-10	3.70366E-10	negative
PRJNA729271	stage_3W_vs_E10	ordinary_muscle_development	ENSGALG00010029402	SMG6	-0.177005791	0.088956626	-1.989798841	0.046613099	0.058762991	negative
PRJNA729271	stage_3W_vs_E10	ordinary_muscle_development	ENSGALG00010029403	FAM20A	1.613426829	0.157897079	10.21821832	1.64333E-24	7.54518E-24	positive
PRJNA729271	stage_3W_vs_E10	ordinary_muscle_development	ENSGALG00010029404	SRR	-0.681589528	0.197458812	-3.451806088	0.000556848	0.000852954	negative
PRJNA729271	stage_3W_vs_E10	ordinary_muscle_development	ENSGALG00010029405	POLDIP2	0.515754881	0.079086633	6.521391272	6.96582E-11	1.69611E-10	positive
PRJNA729271	stage_3W_vs_E10	ordinary_muscle_development	ENSGALG00010029406	TMEM199	0.370095225	0.101465556	3.647496147	0.000264808	0.000417172	positive
PRJNA729271	stage_3W_vs_E10	ordinary_muscle_development	ENSGALG00010029407	UBE2O	0.323671959	0.188730118	1.714998978	0.086345414	0.105289542	positive
PRJNA729271	stage_3W_vs_E10	ordinary_muscle_development	ENSGALG00010029408	KIF12	2.168632658	0.461804601	4.695996215	2.6531E-06	4.89085E-06	positive
PRJNA729271	stage_3W_vs_E10	ordinary_muscle_development	ENSGALG00010029409	ENSGALG00010029409	3.42179171	0.188813706	18.12258119	2.11439E-73	4.50707E-72	positive
PRJNA729271	stage_3W_vs_E10	ordinary_muscle_development	ENSGALG00010029410	VTN	1.303332464	0.88815857	1.467454696	0.142252405	0.168501153	positive
PRJNA729271	stage_3W_vs_E10	ordinary_muscle_development	ENSGALG00010029411	SARM1	-3.506735432	0.259817567	-13.49691429	1.63064E-41	1.37659E-40	negative
PRJNA729271	stage_3W_vs_E10	ordinary_muscle_development	ENSGALG00010029412	SLC46A1	-0.309478384	0.113166086	-2.734727306	0.006243197	0.008663139	negative
PRJNA729271	stage_3W_vs_E10	ordinary_muscle_development	ENSGALG00010029413	B3GNTL1	0.220132788	0.218322639	1.008291163	0.313314704	0.351237172	positive
PRJNA729271	stage_3W_vs_E10	ordinary_muscle_development	ENSGALG00010029416	UNC119	-4.729684105	0.523225355	-9.039478031	1.57422E-19	5.91147E-19	negative
PRJNA729271	stage_3W_vs_E10	ordinary_muscle_development	ENSGALG00010029417	ENSGALG00010029417	3.895433232	0.209250171	18.61615316	2.37688E-77	5.54796E-76	positive
PRJNA729271	stage_3W_vs_E10	ordinary_muscle_development	ENSGALG00010029418	ABCA5	0.324238175	0.132377268	2.449349339	0.014311457	0.01912284	positive
PRJNA729271	stage_3W_vs_E10	ordinary_muscle_development	ENSGALG00010029419	RFC2	-0.631014855	0.132970727	-4.745517062	2.07974E-06	3.85843E-06	negative
PRJNA729271	stage_3W_vs_E10	ordinary_muscle_development	ENSGALG00010029420	LAT2	0.875733425	0.304177161	2.87902426	0.003989076	0.005648414	positive
PRJNA729271	stage_3W_vs_E10	ordinary_muscle_development	ENSGALG00010029421	TBCD	-0.324103833	0.09340357	-3.469929802	0.000520594	0.000800384	negative
PRJNA729271	stage_3W_vs_E10	ordinary_muscle_development	ENSGALG00010029422	EIF4H	-0.721450663	0.087405824	-8.254034254	1.53135E-16	5.01967E-16	negative
PRJNA729271	stage_3W_vs_E10	ordinary_muscle_development	ENSGALG00010029423	MYBBP1A	-1.197675625	0.121678684	-9.842936999	7.35323E-23	3.15434E-22	negative
PRJNA729271	stage_3W_vs_E10	ordinary_muscle_development	ENSGALG00010029424	MAP2K6	-0.291972804	0.218438533	-1.336635983	0.181341488	0.211195956	negative
PRJNA729271	stage_3W_vs_E10	ordinary_muscle_development	ENSGALG00010029425	SPNS2	2.486137642	0.162756234	15.27522225	1.11846E-52	1.37858E-51	positive
PRJNA729271	stage_3W_vs_E10	ordinary_muscle_development	ENSGALG00010029426	ZNF750	-5.349699332	1.037597646	-5.155851456	2.52481E-07	4.97243E-07	negative
PRJNA729271	stage_3W_vs_E10	ordinary_muscle_development	ENSGALG00010029427	SPNS3	0.193298938	0.284359293	0.679770075	0.496650057	0.534982386	positive
PRJNA729271	stage_3W_vs_E10	ordinary_muscle_development	ENSGALG00010029428	FN3K	0.944805141	0.254963219	3.705652698	0.000210847	0.000335003	positive
PRJNA729271	stage_3W_vs_E10	ordinary_muscle_development	ENSGALG00010029429	UBE2G1	1.987175117	0.089361035	22.23760188	1.4869E-109	7.5098E-108	positive
PRJNA729271	stage_3W_vs_E10	ordinary_muscle_development	ENSGALG00010029430	FN3KRP	-0.913073341	0.098022946	-9.314893943	1.22074E-20	4.79271E-20	negative
PRJNA729271	stage_3W_vs_E10	ordinary_muscle_development	ENSGALG00010029432	ENSGALG00010029432	-1.60521686	5.461767061	-0.293900645			negative
PRJNA729271	stage_3W_vs_E10	ordinary_muscle_development	ENSGALG00010029436	WDR45B	-0.390884923	0.080087571	-4.880718909	1.057E-06	1.99859E-06	negative
PRJNA729271	stage_3W_vs_E10	ordinary_muscle_development	ENSGALG00010029437	ENSGALG00010029437	-6.46540866	2.925413228	-2.210083895	0.02709934	0.035093803	negative
PRJNA729271	stage_3W_vs_E10	ordinary_muscle_development	ENSGALG00010029438	ENSGALG00010029438	-1.880671811	0.272574434	-6.899663274	5.21259E-12	1.34826E-11	negative
PRJNA729271	stage_3W_vs_E10	ordinary_muscle_development	ENSGALG00010029439	ENSGALG00010029439	-2.687822729	0.222778807	-12.06498393	1.61694E-33	1.05717E-32	negative
PRJNA729271	stage_3W_vs_E10	ordinary_muscle_development	ENSGALG00010029440	FOXK2	0.818891875	0.18142912	4.513563604	6.37473E-06	1.14344E-05	positive
PRJNA729271	stage_3W_vs_E10	ordinary_muscle_development	ENSGALG00010029442	ENSGALG00010029442	-1.179122544	0.285156394	-4.135002995	3.5495E-05	6.0021E-05	negative
PRJNA729271	stage_3W_vs_E10	ordinary_muscle_development	ENSGALG00010029443	ENSGALG00010029443	-1.229311255	0.318935573	-3.854418755	0.000116005	0.000188529	negative
PRJNA729271	stage_3W_vs_E10	ordinary_muscle_development	ENSGALG00010029445	SPHK1	0.131827303	0.136889655	0.963018745	0.335538116	0.373837432	positive
PRJNA729271	stage_3W_vs_E10	ordinary_muscle_development	ENSGALG00010029446	PRPSAP1	0.208171011	0.080195563	2.595792116	0.009437314	0.012863821	positive
PRJNA729271	stage_3W_vs_E10	ordinary_muscle_development	ENSGALG00010029449	UBALD2	1.809655432	0.181070429	9.994207487	1.61574E-23	7.09704E-23	positive
PRJNA729271	stage_3W_vs_E10	ordinary_muscle_development	ENSGALG00010029451	ENSGALG00010029451	-2.108771109	0.223582792	-9.431723664	4.03404E-21	1.61708E-20	negative
PRJNA729271	stage_3W_vs_E10	ordinary_muscle_development	ENSGALG00010029452	ENSGALG00010029452	-2.079881017	1.021489113	-2.036126465	0.041737658	0.052888133	negative
PRJNA729271	stage_3W_vs_E10	ordinary_muscle_development	ENSGALG00010029453	ENSGALG00010029453	-5.683854841	1.714448723	-3.315266747	0.000915557	0.001375518	negative
PRJNA729271	stage_3W_vs_E10	ordinary_muscle_development	ENSGALG00010029454	ENSGALG00010029454	-1.259239175	0.292338319	-4.307472171	1.65131E-05	2.86896E-05	negative
PRJNA729271	stage_3W_vs_E10	ordinary_muscle_development	ENSGALG00010029455	ENSGALG00010029455	-1.160557809	0.257809594	-4.501608298	6.74412E-06	1.20567E-05	negative
PRJNA729271	stage_3W_vs_E10	ordinary_muscle_development	ENSGALG00010029456	RNF157	-2.725075629	0.51654831	-5.275548437	1.3236E-07	2.65544E-07	negative
PRJNA729271	stage_3W_vs_E10	ordinary_muscle_development	ENSGALG00010029457	ENSGALG00010029457	-1.725824905	0.192157074	-8.981323806	2.67528E-19	9.95334E-19	negative
PRJNA729271	stage_3W_vs_E10	ordinary_muscle_development	ENSGALG00010029460	ENSGALG00010029460	-2.062379821	0.288182465	-7.156506975	8.27587E-13	2.2418E-12	negative
PRJNA729271	stage_3W_vs_E10	ordinary_muscle_development	ENSGALG00010029461	ENSGALG00010029461	-1.089432554	0.204349135	-5.331231531	9.7549E-08	1.97675E-07	negative
PRJNA729271	stage_3W_vs_E10	ordinary_muscle_development	ENSGALG00010029462	ENSGALG00010029462	0.485762694	1.80662789	0.268878111	0.788023482	0.811480561	positive
PRJNA729271	stage_3W_vs_E10	ordinary_muscle_development	ENSGALG00010029463	ENSGALG00010029463	-0.282652865	0.434999815	-0.649776978	0.515836292	0.553855337	negative
PRJNA729271	stage_3W_vs_E10	ordinary_muscle_development	ENSGALG00010029464	ENSGALG00010029464	0.27562756	0.528905789	0.521127895	0.60227768	0.637739424	positive
PRJNA729271	stage_3W_vs_E10	ordinary_muscle_development	ENSGALG00010029465	ENSGALG00010029465	1.082124421	2.363791663	0.457791792	0.647102034	0.680220238	positive
PRJNA729271	stage_3W_vs_E10	ordinary_muscle_development	ENSGALG00010029466	SLC39A11	-1.40660617	0.124316411	-11.31472632	1.10948E-29	6.30911E-29	negative
PRJNA729271	stage_3W_vs_E10	ordinary_muscle_development	ENSGALG00010029468	ENSGALG00010029468	-2.254212069	0.242601303	-9.291838278	1.51646E-20	5.91845E-20	negative
PRJNA729271	stage_3W_vs_E10	ordinary_muscle_development	ENSGALG00010029469	ENSGALG00010029469	-3.008435859	0.418621119	-7.186536287	6.64556E-13	1.80727E-12	negative
PRJNA729271	stage_3W_vs_E10	ordinary_muscle_development	ENSGALG00010029470	SSTR2	0.085468663	0.919919082	0.092908892	0.925975942	0.934437672	positive
PRJNA729271	stage_3W_vs_E10	ordinary_muscle_development	ENSGALG00010029472	ENSGALG00010029472	-0.598974235	0.80008379	-0.748639383	0.454074588	0.492687414	negative
PRJNA729271	stage_3W_vs_E10	ordinary_muscle_development	ENSGALG00010029473	COG1	1.240885669	0.099912233	12.41975709	2.0418E-35	1.42425E-34	positive
PRJNA729271	stage_3W_vs_E10	ordinary_muscle_development	ENSGALG00010029474	ENSGALG00010029474	-1.005931212	1.077828041	-0.933294713	0.350667824	0.389168525	negative
PRJNA729271	stage_3W_vs_E10	ordinary_muscle_development	ENSGALG00010029477	FAM104A	-0.075369413	0.057938777	-1.300845772	0.193311251	0.224181661	negative
PRJNA729271	stage_3W_vs_E10	ordinary_muscle_development	ENSGALG00010029479	ENSGALG00010029479	-1.987724842	0.55604077	-3.574782553	0.000350519	0.000547261	negative
PRJNA729271	stage_3W_vs_E10	ordinary_muscle_development	ENSGALG00010029480	ENSGALG00010029480	0.577296382	0.115386637	5.003147652	5.64017E-07	1.08456E-06	positive
PRJNA729271	stage_3W_vs_E10	ordinary_muscle_development	ENSGALG00010029481	ENSGALG00010029481	-1.500378532	0.279693344	-5.364369814	8.12323E-08	1.65537E-07	negative
PRJNA729271	stage_3W_vs_E10	ordinary_muscle_development	ENSGALG00010029482	DDX25	-0.954515737	0.191249802	-4.990937123	6.00871E-07	1.15405E-06	negative
PRJNA729271	stage_3W_vs_E10	ordinary_muscle_development	ENSGALG00010029484	ENSGALG00010029484	-2.31951416	0.622323714	-3.727182669	0.000193632	0.000308904	negative
PRJNA729271	stage_3W_vs_E10	ordinary_muscle_development	ENSGALG00010029487	CDC42EP4	-2.177493235	0.147896382	-14.72310006	4.58166E-49	5.01216E-48	negative
PRJNA729271	stage_3W_vs_E10	ordinary_muscle_development	ENSGALG00010029489	ENSGALG00010029489	-1.268856708	0.938071804	-1.352622158	0.17617637	0.20556037	negative
PRJNA729271	stage_3W_vs_E10	ordinary_muscle_development	ENSGALG00010029490	ENSGALG00010029490	0.179343291	0.534363276	0.33562054	0.737157033	0.764695569	positive
PRJNA729271	stage_3W_vs_E10	ordinary_muscle_development	ENSGALG00010029491	ENSGALG00010029491	-0.371048275	0.199437598	-1.860473044	0.062818629	0.077891876	negative
PRJNA729271	stage_3W_vs_E10	ordinary_muscle_development	ENSGALG00010029492	SDK2	-2.103535354	0.23275048	-9.037727258	1.59963E-19	6.00534E-19	negative
PRJNA729271	stage_3W_vs_E10	ordinary_muscle_development	ENSGALG00010029494	MYH7	-6.513467381	0.58924148	-11.05398652	2.09692E-28	1.1371E-27	negative
PRJNA729271	stage_3W_vs_E10	ordinary_muscle_development	ENSGALG00010029495	RPL38	0.296444846	0.188614519	1.571696854	0.116020872	0.139008311	positive
PRJNA729271	stage_3W_vs_E10	ordinary_muscle_development	ENSGALG00010029496	TTYH2	-1.628997877	0.14977887	-10.87601925	1.4997E-27	7.84488E-27	negative
PRJNA729271	stage_3W_vs_E10	ordinary_muscle_development	ENSGALG00010029497	ENSGALG00010029497	-0.701614543	0.591740043	-1.185680353	0.235748556	0.269876427	negative
PRJNA729271	stage_3W_vs_E10	ordinary_muscle_development	ENSGALG00010029498	ENSGALG00010029498	-0.046666781	0.148947022	-0.313311272	0.754044188	0.780036433	negative
PRJNA729271	stage_3W_vs_E10	ordinary_muscle_development	ENSGALG00010029501	ENSGALG00010029501	-1.315324226	0.395195127	-3.3282906	0.000873807	0.001315657	negative
PRJNA729271	stage_3W_vs_E10	ordinary_muscle_development	ENSGALG00010029502	BTBD17	-1.307645916	0.5857586	-2.232397297	0.025588716	0.03324744	negative
PRJNA729271	stage_3W_vs_E10	ordinary_muscle_development	ENSGALG00010029504	GPR142	-4.820816001	0.885882798	-5.44182144	5.27385E-08	1.08571E-07	negative
PRJNA729271	stage_3W_vs_E10	ordinary_muscle_development	ENSGALG00010029505	VEZF1	0.072256426	0.109998981	0.656882681	0.511256355	0.549263331	positive
PRJNA729271	stage_3W_vs_E10	ordinary_muscle_development	ENSGALG00010029506	OXLD1	-1.429134458	0.168998298	-8.45650209	2.75517E-17	9.35932E-17	negative
PRJNA729271	stage_3W_vs_E10	ordinary_muscle_development	ENSGALG00010029507	VPS37D	-0.155336602	0.172084433	-0.902676665	0.366697525	0.405375276	negative
PRJNA729271	stage_3W_vs_E10	ordinary_muscle_development	ENSGALG00010029508	ENSGALG00010029508	-1.392439399	0.150673785	-9.241417829	2.43252E-20	9.43017E-20	negative
PRJNA729271	stage_3W_vs_E10	ordinary_muscle_development	ENSGALG00010029509	DNAJC30	1.022283642	0.168610657	6.06298358	1.33619E-09	3.02202E-09	positive
PRJNA729271	stage_3W_vs_E10	ordinary_muscle_development	ENSGALG00010029510	PDE6G	-1.489435815	0.34203276	-4.354658354	1.33275E-05	2.3317E-05	negative
PRJNA729271	stage_3W_vs_E10	ordinary_muscle_development	ENSGALG00010029511	CUEDC1	-0.198051829	0.073912294	-2.679551928	0.007372077	0.010165359	negative
PRJNA729271	stage_3W_vs_E10	ordinary_muscle_development	ENSGALG00010029512	TSPAN10	0.015702182	0.638222675	0.024602984	0.980371639	0.983288604	positive
PRJNA729271	stage_3W_vs_E10	ordinary_muscle_development	ENSGALG00010029513	WBSCR22	-0.41429766	0.086192561	-4.806652148	1.53479E-06	2.86833E-06	negative
PRJNA729271	stage_3W_vs_E10	ordinary_muscle_development	ENSGALG00010029514	MRPS23	0.575968404	0.09697671	5.93924463	2.86338E-09	6.34629E-09	positive
PRJNA729271	stage_3W_vs_E10	ordinary_muscle_development	ENSGALG00010029515	NPLOC4	0.947600422	0.152320471	6.221096994	4.93691E-10	1.14827E-09	positive
PRJNA729271	stage_3W_vs_E10	ordinary_muscle_development	ENSGALG00010029516	ENSGALG00010029516	3.402157703	0.227014458	14.98652437	8.99381E-51	1.04627E-49	positive
PRJNA729271	stage_3W_vs_E10	ordinary_muscle_development	ENSGALG00010029517	MSI2	0.81081206	0.111197981	7.291607764	3.06278E-13	8.45618E-13	positive
PRJNA729271	stage_3W_vs_E10	ordinary_muscle_development	ENSGALG00010029518	FAAP100	-0.801558709	0.188461509	-4.253169317	2.10766E-05	3.63308E-05	negative
PRJNA729271	stage_3W_vs_E10	ordinary_muscle_development	ENSGALG00010029519	STX1A	-0.917808247	0.117921399	-7.783220485	7.07013E-15	2.12661E-14	negative
PRJNA729271	stage_3W_vs_E10	ordinary_muscle_development	ENSGALG00010029520	MKS1	-0.281788842	0.10956921	-2.571788578	0.010117466	0.013740529	negative
PRJNA729271	stage_3W_vs_E10	ordinary_muscle_development	ENSGALG00010029521	ACTG1	-0.931938544	0.098413098	-9.469659653	2.80757E-21	1.13264E-20	negative
PRJNA729271	stage_3W_vs_E10	ordinary_muscle_development	ENSGALG00010029522	AKAP1	1.136264895	0.111990493	10.14608348	3.44929E-24	1.56145E-23	positive
PRJNA729271	stage_3W_vs_E10	ordinary_muscle_development	ENSGALG00010029523	FSCN2	2.579990412	0.665749523	3.875316951	0.000106486	0.000173565	positive
PRJNA729271	stage_3W_vs_E10	ordinary_muscle_development	ENSGALG00010029524	ENSGALG00010029524	0.389716686	0.148342741	2.62713688	0.008610669	0.011791369	positive
PRJNA729271	stage_3W_vs_E10	ordinary_muscle_development	ENSGALG00010029525	SLC38A10	-1.372483324	0.138753197	-9.891543778	4.52988E-23	1.96177E-22	negative
PRJNA729271	stage_3W_vs_E10	ordinary_muscle_development	ENSGALG00010029526	BCL7B	0.903552765	0.111952583	8.070852342	6.98091E-16	2.20598E-15	positive
PRJNA729271	stage_3W_vs_E10	ordinary_muscle_development	ENSGALG00010029527	NDUFAF8	1.370383973	0.152504452	8.985862112	2.56714E-19	9.56326E-19	positive
PRJNA729271	stage_3W_vs_E10	ordinary_muscle_development	ENSGALG00010029528	TBL2	-0.321736801	0.096862779	-3.321573114	0.000895115	0.001345923	negative
PRJNA729271	stage_3W_vs_E10	ordinary_muscle_development	ENSGALG00010029529	TEPSIN	0.961866535	0.126689156	7.592335148	3.14191E-14	9.14865E-14	positive
PRJNA729271	stage_3W_vs_E10	ordinary_muscle_development	ENSGALG00010029530	ENSGALG00010029530	2.592873575	0.359884964	7.204728819	5.81595E-13	1.58522E-12	positive
PRJNA729271	stage_3W_vs_E10	ordinary_muscle_development	ENSGALG00010029531	CEP131	-1.036267905	0.172352013	-6.012508274	1.82675E-09	4.0957E-09	negative
PRJNA729271	stage_3W_vs_E10	ordinary_muscle_development	ENSGALG00010029532	EVI2A	4.396010212	0.44639253	9.847857919	7.00204E-23	3.00903E-22	positive
PRJNA729271	stage_3W_vs_E10	ordinary_muscle_development	ENSGALG00010029533	MLXIPL	0.276589741	0.715067269	0.386802408	0.698902495	0.728347952	positive
PRJNA729271	stage_3W_vs_E10	ordinary_muscle_development	ENSGALG00010029534	NF1	-0.467474949	0.091012889	-5.136359851	2.80111E-07	5.50089E-07	negative
PRJNA729271	stage_3W_vs_E10	ordinary_muscle_development	ENSGALG00010029536	BAZ1B	-0.825751497	0.077703355	-10.62697355	2.2324E-26	1.10969E-25	negative
PRJNA729271	stage_3W_vs_E10	ordinary_muscle_development	ENSGALG00010029537	MYH1B	4.3334866	0.206075437	21.02864207	3.58773E-98	1.41316E-96	positive
PRJNA729271	stage_3W_vs_E10	ordinary_muscle_development	ENSGALG00010029538	FZD9	0.233995426	0.173120053	1.351636752	0.176491547	0.205861831	positive
PRJNA729271	stage_3W_vs_E10	ordinary_muscle_development	ENSGALG00010029539	FKBP6	-1.418347155	0.3719035	-3.813750485	0.000136874	0.000221254	negative

PRJNA729271	stage_3W_vs_E10	ordinary_muscle_development	ENSGALG00010029541	ENSGALG00010029541	1.477714548	0.220228007	6.709930161	1.94718E-11	4.90261E-11	positive
PRJNA729271	stage_3W_vs_E10	ordinary_muscle_development	ENSGALG00010029542	AATK	1.547216749	0.141876986	10.90533985	1.08686E-27	5.72668E-27	positive
PRJNA729271	stage_3W_vs_E10	ordinary_muscle_development	ENSGALG00010029543	NSUN5	-0.278024751	0.13629358	-2.039896161	0.041360669	0.052451697	negative
PRJNA729271	stage_3W_vs_E10	ordinary_muscle_development	ENSGALG00010029544	ENSGALG00010029544	-1.13433395	0.179308952	-6.326142333	2.51366E-10	5.94477E-10	negative
PRJNA729271	stage_3W_vs_E10	ordinary_muscle_development	ENSGALG00010029545	OMG	3.800893849	0.291885971	13.02184493	9.19228E-39	7.11763E-38	positive
PRJNA729271	stage_3W_vs_E10	ordinary_muscle_development	ENSGALG00010029546	WSB1	0.357656533	0.165286817	2.163853955	0.03047556	0.039266066	positive
PRJNA729271	stage_3W_vs_E10	ordinary_muscle_development	ENSGALG00010029547	HIP1	-0.737474926	0.101135424	-7.291954642	3.0549E-13	8.43604E-13	negative
PRJNA729271	stage_3W_vs_E10	ordinary_muscle_development	ENSGALG00010029548	BAIAP2	0.98984164	0.220750224	4.483989293	7.32603E-06	1.30648E-05	positive
PRJNA729271	stage_3W_vs_E10	ordinary_muscle_development	ENSGALG00010029549	KSR1	1.458756944	0.12434197	11.7318146	8.75611E-32	5.38024E-31	positive
PRJNA729271	stage_3W_vs_E10	ordinary_muscle_development	ENSGALG00010029550	CHMP6	1.161965662	0.239336103	4.85495355	1.20415E-06	2.26884E-06	positive
PRJNA729271	stage_3W_vs_E10	ordinary_muscle_development	ENSGALG00010029552	NOS2	-0.264033276	0.129561871	-2.03789335	0.0415606	0.052677588	negative
PRJNA729271	stage_3W_vs_E10	ordinary_muscle_development	ENSGALG00010029553	MYH1C	5.703161741	0.194787981	29.27881748	1.9302E-188	3.1086E-186	positive
PRJNA729271	stage_3W_vs_E10	ordinary_muscle_development	ENSGALG00010029554	ENSGALG00010029554	8.828436897	0.365026274	24.18575739	3.1418E-129	2.1583E-127	positive
PRJNA729271	stage_3W_vs_E10	ordinary_muscle_development	ENSGALG00010029555	LYRM9	0.961613743	0.154523477	6.223091524	4.87453E-10	1.13436E-09	positive
PRJNA729271	stage_3W_vs_E10	ordinary_muscle_development	ENSGALG00010029556	NPTX1	2.417313467	0.558912343	4.325031461	1.5251E-05	2.65818E-05	positive
PRJNA729271	stage_3W_vs_E10	ordinary_muscle_development	ENSGALG00010029557	ENSGALG00010029557	3.417162732	0.554642797	6.161015247	7.228E-10	1.66381E-09	positive
PRJNA729271	stage_3W_vs_E10	ordinary_muscle_development	ENSGALG00010029558	ENSGALG00010029558	-1.629136717	0.793025275	-2.054331393	0.039943628	0.050756807	negative
PRJNA729271	stage_3W_vs_E10	ordinary_muscle_development	ENSGALG00010029560	ENSGALG00010029560	4.236580949	0.166958466	25.3750591	4.7549E-142	3.961E-140	positive
PRJNA729271	stage_3W_vs_E10	ordinary_muscle_development	ENSGALG00010029561	SCO1	0.006205611	0.104188435	0.059561423	0.952504944	0.958722253	positive
PRJNA729271	stage_3W_vs_E10	ordinary_muscle_development	ENSGALG00010029562	ENSGALG00010029562	4.036764509	0.681031804	5.927424361	3.07723E-09	6.80672E-09	positive
PRJNA729271	stage_3W_vs_E10	ordinary_muscle_development	ENSGALG00010029563	ADPRM	0.758654053	0.125267962	6.056249649	1.39332E-09	3.14813E-09	positive
PRJNA729271	stage_3W_vs_E10	ordinary_muscle_development	ENSGALG00010029564	NLK	0.597869387	0.08680942	6.887148734	5.69217E-12	1.4689E-11	positive
PRJNA729271	stage_3W_vs_E10	ordinary_muscle_development	ENSGALG00010029566	TMEM97	-0.899565471	0.147485659	-6.09934199	1.06506E-09	2.42241E-09	negative
PRJNA729271	stage_3W_vs_E10	ordinary_muscle_development	ENSGALG00010029567	IFT20	-0.827264953	0.120517966	-6.8642459	6.68434E-12	1.72003E-11	negative
PRJNA729271	stage_3W_vs_E10	ordinary_muscle_development	ENSGALG00010029568	ENSGALG00010029568	-2.711715684	1.149845414	-2.358330651	0.018357334	0.024240644	negative
PRJNA729271	stage_3W_vs_E10	ordinary_muscle_development	ENSGALG00010029569	TMEM220	1.264631455	0.20009432	6.320176669	2.61264E-10	6.1688E-10	positive
PRJNA729271	stage_3W_vs_E10	ordinary_muscle_development	ENSGALG00010029570	TNFAIP1	-0.734484374	0.079558775	-9.231971899	2.65695E-20	1.02864E-19	negative
PRJNA729271	stage_3W_vs_E10	ordinary_muscle_development	ENSGALG00010029571	EPX	1.324299268	0.713717288	1.855495572	0.063525491	0.07872121	positive
PRJNA729271	stage_3W_vs_E10	ordinary_muscle_development	ENSGALG00010029572	TSPOAP1	-0.014047574	0.118406712	-0.118638325	0.905561901	0.916628475	negative
PRJNA729271	stage_3W_vs_E10	ordinary_muscle_development	ENSGALG00010029573	MAP2K4	-0.170035949	0.060168875	-2.82597852	0.004713642	0.006628917	negative
PRJNA729271	stage_3W_vs_E10	ordinary_muscle_development	ENSGALG00010029574	SGSM2	-0.02027688	0.130917608	-0.154882757	0.876913747	0.891115029	negative
PRJNA729271	stage_3W_vs_E10	ordinary_muscle_development	ENSGALG00010029575	ANKFN1	-4.146335601	0.749039345	-5.535537794	3.10275E-08	6.47891E-08	negative
PRJNA729271	stage_3W_vs_E10	ordinary_muscle_development	ENSGALG00010029576	ATP2A3	1.650707942	0.130274566	12.67099173	8.5625E-37	6.19019E-36	positive
PRJNA729271	stage_3W_vs_E10	ordinary_muscle_development	ENSGALG00010029577	NOG	-6.462726959	0.588647007	-10.97895154	4.82491E-28	2.57405E-27	negative
PRJNA729271	stage_3W_vs_E10	ordinary_muscle_development	ENSGALG00010029578	C17orf67	-6.020991293	0.779000197	-7.729126791	1.08287E-14	3.22171E-14	negative
PRJNA729271	stage_3W_vs_E10	ordinary_muscle_development	ENSGALG00010029579	GALNT17	-0.615380578	0.184959393	-3.327111791	0.000877512	0.001320824	negative
PRJNA729271	stage_3W_vs_E10	ordinary_muscle_development	ENSGALG00010029580	P2RX1	1.634096068	0.334548464	4.884482347	1.03701E-06	1.9631E-06	positive
PRJNA729271	stage_3W_vs_E10	ordinary_muscle_development	ENSGALG00010029581	DGKE	-0.024130732	0.105226096	-0.229322696	0.818618115	0.839823736	negative
PRJNA729271	stage_3W_vs_E10	ordinary_muscle_development	ENSGALG00010029582	SCPEP1	0.240310539	0.150040278	1.601640183	0.1092352	0.131312352	positive
PRJNA729271	stage_3W_vs_E10	ordinary_muscle_development	ENSGALG00010029583	LIMK1	-1.314769537	0.20892278	-6.293088472	3.11211E-10	7.31711E-10	negative
PRJNA729271	stage_3W_vs_E10	ordinary_muscle_development	ENSGALG00010029584	TRIM25	1.23288414	0.232851763	5.294716802	1.19201E-07	2.40275E-07	positive
PRJNA729271	stage_3W_vs_E10	ordinary_muscle_development	ENSGALG00010029585	ENSGALG00010029585	0.511401719	0.357640224	1.429933451	0.15273612	0.179948798	positive
PRJNA729271	stage_3W_vs_E10	ordinary_muscle_development	ENSGALG00010029586	COIL	-0.226498658	0.188401836	-1.202210457	0.229281999	0.262960403	negative
PRJNA729271	stage_3W_vs_E10	ordinary_muscle_development	ENSGALG00010029588	RAB11FIP4	-3.048275229	0.150908984	-20.19942843	9.90369E-91	3.3077E-89	negative
PRJNA729271	stage_3W_vs_E10	ordinary_muscle_development	ENSGALG00010029590	UTP6	-0.097391545	0.098078396	-0.992996913	0.320711459	0.358807703	negative
PRJNA729271	stage_3W_vs_E10	ordinary_muscle_development	ENSGALG00010029591	SUZ12	-1.105145516	0.080640657	-13.70456988	9.53305E-43	8.41545E-42	negative
PRJNA729271	stage_3W_vs_E10	ordinary_muscle_development	ENSGALG00010029592	MNT	0.58865364	0.165983465	3.546459522	0.000390445	0.000607047	positive
PRJNA729271	stage_3W_vs_E10	ordinary_muscle_development	ENSGALG00010029593	PAFAH1B1	0.67560894	0.078850393	8.5682381	1.0508E-17	3.657E-17	positive
PRJNA729271	stage_3W_vs_E10	ordinary_muscle_development	ENSGALG00010029594	CRLF3	-0.987668947	0.068733981	-14.36944185	8.04772E-47	8.18034E-46	negative
PRJNA729271	stage_3W_vs_E10	ordinary_muscle_development	ENSGALG00010029595	CLUH	0.987279933	0.134958874	7.315413223	2.56591E-13	7.12506E-13	positive
PRJNA729271	stage_3W_vs_E10	ordinary_muscle_development	ENSGALG00010029596	ATAD5	-2.064915063	0.132920241	-15.53499338	2.01093E-54	2.60953E-53	negative
PRJNA729271	stage_3W_vs_E10	ordinary_muscle_development	ENSGALG00010029597	ADAP2	1.820082719	0.203162832	8.958738666	3.28413E-19	1.21686E-18	positive
PRJNA729271	stage_3W_vs_E10	ordinary_muscle_development	ENSGALG00010029598	SUPT4H1	0.140733296	0.149239663	0.943001964	0.345679898	0.384221311	positive
PRJNA729271	stage_3W_vs_E10	ordinary_muscle_development	ENSGALG00010029599	RNF43	-3.91659536	0.48869937	-8.014324552	1.10744E-15	3.4603E-15	negative
PRJNA729271	stage_3W_vs_E10	ordinary_muscle_development	ENSGALG00010029600	RHOT1	1.227617858	0.108507133	11.31370654	1.12245E-29	6.37788E-29	positive
PRJNA729271	stage_3W_vs_E10	ordinary_muscle_development	ENSGALG00010029601	HSF5	-2.703865526	0.535422174	-5.049969271	4.41881E-07	8.56865E-07	negative
PRJNA729271	stage_3W_vs_E10	ordinary_muscle_development	ENSGALG00010029602	MTMR4	-0.991516077	0.088164644	-11.24618704	2.41822E-29	1.35703E-28	negative
PRJNA729271	stage_3W_vs_E10	ordinary_muscle_development	ENSGALG00010029603	RHBDL3	-0.478125912	0.442500917	-1.080508297	0.279915894	0.316859108	negative
PRJNA729271	stage_3W_vs_E10	ordinary_muscle_development	ENSGALG00010029604	ENSGALG00010029604	0.159647193	0.119076145	1.340715169	0.180012948	0.209749813	positive
PRJNA729271	stage_3W_vs_E10	ordinary_muscle_development	ENSGALG00010029605	C17orf75	-0.957637623	0.0939512	-10.19292592	2.13248E-24	9.74165E-24	negative
PRJNA729271	stage_3W_vs_E10	ordinary_muscle_development	ENSGALG00010029606	ABHD11	0.921990975	0.207138385	4.451087002	8.54367E-06	1.51598E-05	positive
PRJNA729271	stage_3W_vs_E10	ordinary_muscle_development	ENSGALG00010029607	ZNF207	-0.763748907	0.065373133	-11.6829173	1.55853E-31	9.47605E-31	negative
PRJNA729271	stage_3W_vs_E10	ordinary_muscle_development	ENSGALG00010029608	ENSGALG00010029608	-2.817750679	0.344657975	-8.175498263	2.94646E-16	9.50777E-16	negative
PRJNA729271	stage_3W_vs_E10	ordinary_muscle_development	ENSGALG00010029609	METTL27	-1.213188237	0.156655745	-7.74429459	9.61139E-15	2.86838E-14	negative
PRJNA729271	stage_3W_vs_E10	ordinary_muscle_development	ENSGALG00010029610	ERN1	0.113461462	0.079469427	1.427737269	0.153367495	0.180648613	positive
PRJNA729271	stage_3W_vs_E10	ordinary_muscle_development	ENSGALG00010029611	YWHAG	0.978877275	0.130519982	7.499826921	6.39022E-14	1.83273E-13	positive
PRJNA729271	stage_3W_vs_E10	ordinary_muscle_development	ENSGALG00010029612	HSPB1	2.010409161	0.195762496	10.2696339	9.65704E-25	4.46931E-24	positive
PRJNA729271	stage_3W_vs_E10	ordinary_muscle_development	ENSGALG00010029614	MDH2	1.402556225	0.128503876	10.91450518	9.82629E-28	5.18878E-27	positive
PRJNA729271	stage_3W_vs_E10	ordinary_muscle_development	ENSGALG00010029615	ENSGALG00010029615	-1.108008371	0.304683902	-3.636583243	0.000276278	0.000434958	negative
PRJNA729271	stage_3W_vs_E10	ordinary_muscle_development	ENSGALG00010029616	TMEM120A	0.809027732	0.135526552	5.969514601	2.3796E-09	5.29918E-09	positive
PRJNA729271	stage_3W_vs_E10	ordinary_muscle_development	ENSGALG00010029617	POR	-0.568220821	0.095649118	-5.940680218	2.83842E-09	6.29385E-09	negative
PRJNA729271	stage_3W_vs_E10	ordinary_muscle_development	ENSGALG00010029618	TEX2	1.32947242	0.112430718	11.82481483	2.90543E-32	1.82312E-31	positive
PRJNA729271	stage_3W_vs_E10	ordinary_muscle_development	ENSGALG00010029619	ENSGALG00010029619	0.623949755	0.102198511	6.105272438	1.02625E-09	2.33672E-09	positive
PRJNA729271	stage_3W_vs_E10	ordinary_muscle_development	ENSGALG00010029620	ENSGALG00010029620	0.540830417	0.393808144	1.373334769	0.169648298	0.198566867	positive
PRJNA729271	stage_3W_vs_E10	ordinary_muscle_development	ENSGALG00010029621	TAF15	-2.0526888	0.122808729	-16.71451867	1.02746E-62	1.65112E-61	negative
PRJNA729271	stage_3W_vs_E10	ordinary_muscle_development	ENSGALG00010029622	MMP28	-0.332435001	0.158769185	-2.093825706	0.036275509	0.046343514	negative
PRJNA729271	stage_3W_vs_E10	ordinary_muscle_development	ENSGALG00010029623	ENSGALG00010029623	0.227329952	0.393667721	0.577466579	0.56362431	0.600318493	positive
PRJNA729271	stage_3W_vs_E10	ordinary_muscle_development	ENSGALG00010029624	RASL10B	-2.767560646	0.342803063	-8.073325313	6.8409E-16	2.16362E-15	negative
PRJNA729271	stage_3W_vs_E10	ordinary_muscle_development	ENSGALG00010029625	PECAM1	2.791873377	0.174266201	16.02073932	9.15552E-58	1.29346E-56	positive
PRJNA729271	stage_3W_vs_E10	ordinary_muscle_development	ENSGALG00010029626	AP2B1	-0.809090712	0.109454544	-7.392024842	1.44609E-13	4.06776E-13	negative
PRJNA729271	stage_3W_vs_E10	ordinary_muscle_development	ENSGALG00010029627	PEX12	-0.296725787	0.146750581	-2.021973515	0.043179088	0.054647768	negative
PRJNA729271	stage_3W_vs_E10	ordinary_muscle_development	ENSGALG00010029628	UNC45B	1.390257732	0.160540155	8.659875352	4.7228E-18	1.67131E-17	positive
PRJNA729271	stage_3W_vs_E10	ordinary_muscle_development	ENSGALG00010029629	ENSGALG00010029629	2.484650132	0.255056839	9.741554618	2.00464E-22	8.42727E-22	positive
PRJNA729271	stage_3W_vs_E10	ordinary_muscle_development	ENSGALG00010029630	RAD51D	0.265309362	0.119264811	2.224540155	0.026112124	0.033894084	positive
PRJNA729271	stage_3W_vs_E10	ordinary_muscle_development	ENSGALG00010029631	LIG3	-0.379016411	0.087090909	-4.351962959	1.34924E-05	2.35914E-05	negative
PRJNA729271	stage_3W_vs_E10	ordinary_muscle_development	ENSGALG00010029632	TMEM132E	1.984824442	0.225083155	8.818182956	1.16332E-18	4.21032E-18	positive
PRJNA729271	stage_3W_vs_E10	ordinary_muscle_development	ENSGALG00010029633	POLG2	-0.296393434	0.176925556	-1.675243763	0.093886414	0.113948219	negative
PRJNA729271	stage_3W_vs_E10	ordinary_muscle_development	ENSGALG00010029634	DDX5	-0.079250601	0.091131665	-0.869627488	0.384504011	0.423445193	negative
PRJNA729271	stage_3W_vs_E10	ordinary_muscle_development	ENSGALG00010029635	ENSGALG00010029635	3.075197927	0.574636869	5.351549982	8.7204E-08	1.77257E-07	positive
PRJNA729271	stage_3W_vs_E10	ordinary_muscle_development	ENSGALG00010029636	SPATA20	0.726203647	0.075372369	9.634878883	5.69591E-22	2.35892E-21	positive
PRJNA729271	stage_3W_vs_E10	ordinary_muscle_development	ENSGALG00010029638	EPN3	2.383113997	0.195059448	12.21737279	2.51068E-34	1.69109E-33	positive
PRJNA729271	stage_3W_vs_E10	ordinary_muscle_development	ENSGALG00010029639	ENSGALG00010029639	-0.442095091	0.759519868	-0.582071793	0.560518324	0.597405376	negative
PRJNA729271	stage_3W_vs_E10	ordinary_muscle_development	ENSGALG00010029641	ENSGALG00010029641	-0.040935863	0.721653771	-0.056725074	0.9547642	0.960729405	negative
PRJNA729271	stage_3W_vs_E10	ordinary_muscle_development	ENSGALG00010029642	NAT9	1.664284157	0.143791237	11.57430864	5.56178E-31	3.31625E-30	positive
PRJNA729271	stage_3W_vs_E10	ordinary_muscle_development	ENSGALG00010029644	MYCBPAP	0.190969073	0.166238629	1.148764722	0.25065301	0.28587736	positive
PRJNA729271	stage_3W_vs_E10	ordinary_muscle_development	ENSGALG00010029645	ENSGALG00010029645	-2.934804723	0.402234719	-7.296249148	2.95901E-13	8.18215E-13	negative
PRJNA729271	stage_3W_vs_E10	ordinary_muscle_development	ENSGALG00010029646	MRPS17	0.460848726	0.108274993	4.256280343	2.07856E-05	3.58505E-05	positive
PRJNA729271	stage_3W_vs_E10	ordinary_muscle_development	ENSGALG00010029647	RSAD1	0.614988726	0.154212938	3.987919129	6.66554E-05	0.000110584	positive
PRJNA729271	stage_3W_vs_E10	ordinary_muscle_development	ENSGALG00010029648	NIPSNAP2	0.964138664	0.12189834	7.909366673	2.58702E-15	7.94466E-15	positive
PRJNA729271	stage_3W_vs_E10	ordinary_muscle_development	ENSGALG00010029649	PSPH	4.414741626	0.257378409	17.15272714	5.99708E-66	1.04985E-64	positive
PRJNA729271	stage_3W_vs_E10	ordinary_muscle_development	ENSGALG00010029650	ACSF2	-0.188182172	0.134997014	-1.393972847	0.163325804	0.191661879	negative
PRJNA729271	stage_3W_vs_E10	ordinary_muscle_development	ENSGALG00010029651	ENSGALG00010029651	3.342006173	0.275073195	12.14951597	5.77062E-34	3.83166E-33	positive
PRJNA729271	stage_3W_vs_E10	ordinary_muscle_development	ENSGALG00010029652	LRRC59	-0.8418356	0.102097003	-8.2454487	1.64542E-16	5.38017E-16	negative
PRJNA729271	stage_3W_vs_E10	ordinary_muscle_development	ENSGALG00010029653	CCT6A	-0.411149029	0.09025478	-4.555426634	5.22794E-06	9.43228E-06	negative
PRJNA729271	stage_3W_vs_E10	ordinary_muscle_development	ENSGALG00010029654	EME1	-2.840527188	0.139107853	-20.41960344	1.11969E-92	3.91083E-91	negative
PRJNA729271	stage_3W_vs_E10	ordinary_muscle_development	ENSGALG00010029655	PHKG1	5.501445973	0.12822976	42.90303559	0	0	positive
PRJNA729271	stage_3W_vs_E10	ordinary_muscle_development	ENSGALG00010029656	MRPL27	0.517989642	0.112880817	4.588819029	4.45761E-06	8.08266E-06	positive
PRJNA729271	stage_3W_vs_E10	ordinary_muscle_development	ENSGALG00010029657	CHCHD2	2.274188771	0.146723873	15.49978695	3.48044E-54	4.49234E-53	positive
PRJNA729271	stage_3W_vs_E10	ordinary_muscle_development	ENSGALG00010029658	XYLT2	0.41973601	0.124926297	3.359869132	0.000779794	0.001179127	positive
PRJNA729271	stage_3W_vs_E10	ordinary_muscle_development	ENSGALG00010029659	VKORC1L1	-0.895112889	0.14655247	-6.107798046	1.01015E-09	2.3015E-09	negative
PRJNA729271	stage_3W_vs_E10	ordinary_muscle_development	ENSGALG00010029660	CD300LG	2.439953954	0.301389781	8.095675789	5.69473E-16	1.80941E-15	positive
PRJNA729271	stage_3W_vs_E10	ordinary_muscle_development	ENSGALG00010029661	KRABZFP	6.124502975	0.568857462	10.76632265	4.96435E-27	2.53911E-26	positive
PRJNA729271	stage_3W_vs_E10	ordinary_muscle_development	ENSGALG00010029663	GUSB	0.194668483	0.153747381	1.266158045	0.205456505	0.237449533	positive
PRJNA729271	stage_3W_vs_E10	ordinary_muscle_development	ENSGALG00010029664	RAB37	2.629964041	0.369177723	7.123842734	1.04959E-12	2.82837E-12	positive
PRJNA729271	stage_3W_vs_E10	ordinary_muscle_development	ENSGALG00010029665	ASL1	1.675322812	0.188740503	8.876329043	6.91032E-19	2.52687E-18	positive
PRJNA729271	stage_3W_vs_E10	ordinary_muscle_development	ENSGALG00010029666	SLC9A3R1	-0.254412493	0.199344457	-1.276245634	0.201868702	0.233563764	negative
PRJNA729271	stage_3W_vs_E10	ordinary_muscle_development	ENSGALG00010029667	TMEM104	0.134110429	0.092540004	1.449215723	0.147277348	0.17392742	positive
PRJNA729271	stage_3W_vs_E10	ordinary_muscle_development	ENSGALG00010029668	CRCP	-0.591127868	0.182350073	-3.241719936	0.001188107	0.001768496	negative
PRJNA729271	stage_3W_vs_E10	ordinary_muscle_development	ENSGALG00010029669	HID1	0.555672316	0.073868326	7.522470681	5.37507E-14	1.54863E-13	positive
PRJNA729271	stage_3W_vs_E10	ordinary_muscle_development	ENSGALG00010029670	TPST1	0.159104411	0.169836166	0.936811132	0.348855718	0.387365071	positive
PRJNA729271	stage_3W_vs_E10	ordinary_muscle_development	ENSGALG00010029671	GRIN2C	1.619602164	0.210187525	7.705510437	1.30321E-14	3.86537E-14	positive
PRJNA729271	stage_3W_vs_E10	ordinary_muscle_development	ENSGALG00010029672	KCTD7	-1.58704979	0.234477265	-6.76845915	1.30161E-11	3.30626E-11	negative
PRJNA729271	stage_3W_vs_E10	ordinary_muscle_development	ENSGALG00010029673	TMEM248	-0.450663643	0.090404992	-4.984941999	6.19804E-07	1.18931E-06	negative
PRJNA729271	stage_3W_vs_E10	ordinary_muscle_development	ENSGALG00010029674	FDXR	-1.133268327	0.116211499	-9.751774465	1.81272E-22	7.63152E-22	negative
PRJNA729271	stage_3W_vs_E10	ordinary_muscle_development	ENSGALG00010029675	DHRS13	0.178317169	0.223769268	0.796879616	0.425520951	0.463963155	positive
PRJNA729271	stage_3W_vs_E10	ordinary_muscle_development	ENSGALG00010029676	FADS6	-1.916472252	1.157593035	-1.655566502	0.097809639	0.11841232	negative
PRJNA729271	stage_3W_vs_E10	ordinary_muscle_development	ENSGALG00010029677	PHF12	-0.04215047	0.100559524	-0.419159399	0.675099642	0.706284324	negative
PRJNA729271	stage_3W_vs_E10	ordinary_muscle_development	ENSGALG00010029682	CDR2L	-2.46953896	0.14676376	-16.82662636	1.55726E-63	2.56506E-62	negative
PRJNA729271	stage_3W_vs_E10	ordinary_muscle_development	ENSGALG00010029683	MRPL58	0.703814699	0.108996355	6.457231497	1.06636E-10	2.56886E-10	positive
PRJNA729271	stage_3W_vs_E10	ordinary_muscle_development	ENSGALG00010029684	ENSGALG00010029684	-0.889381987	0.650850747	-1.36649146	0.171784759	0.200937709	negative
PRJNA729271	stage_3W_vs_E10	ordinary_muscle_development	ENSGALG00010029685	ATP5PD	0.66454002	0.115071346	5.775026029	7.69412E-09	1.67131E-08	positive

PRJNA729271	stage_3W_vs_E10	ordinary_muscle_development	ENSGALG00010029686	KCTD2	-0.181331713	0.059892176	-3.027636093	0.002464747	0.0035609	negative
PRJNA729271	stage_3W_vs_E10	ordinary_muscle_development	ENSGALG00010029687	SLC16A5	-3.63013292	0.708199899	-5.12585913	2.96184E-07	5.80788E-07	negative
PRJNA729271	stage_3W_vs_E10	ordinary_muscle_development	ENSGALG00010029689	ENSGALG00010029689	1.618336909	0.175756409	9.207840082	3.32753E-20	1.28278E-19	positive
PRJNA729271	stage_3W_vs_E10	ordinary_muscle_development	ENSGALG00010029690	ENSGALG00010029690	1.489521695	0.160697324	9.269113248	1.877E-20	7.30392E-20	positive
PRJNA729271	stage_3W_vs_E10	ordinary_muscle_development	ENSGALG00010029691	NUFIP2	0.800479792	0.140556332	5.695081686	1.23313E-08	2.64685E-08	positive
PRJNA729271	stage_3W_vs_E10	ordinary_muscle_development	ENSGALG00010029692	CRYBA1	-1.371554566	0.580039401	-2.364588619	0.018050108	0.023858866	negative
PRJNA729271	stage_3W_vs_E10	ordinary_muscle_development	ENSGALG00010029693	HN1	-1.046619487	0.126848565	-8.250936747	1.57158E-16	5.14686E-16	negative
PRJNA729271	stage_3W_vs_E10	ordinary_muscle_development	ENSGALG00010029694	ARMC7	0.308155042	0.090688229	3.397960752	0.000678902	0.001032708	positive
PRJNA729271	stage_3W_vs_E10	ordinary_muscle_development	ENSGALG00010029695	TAOK1	0.122226495	0.086468648	1.413535404	0.157498359	0.185288428	positive
PRJNA729271	stage_3W_vs_E10	ordinary_muscle_development	ENSGALG00010029696	SUMO2	-0.491963036	0.108150666	-4.548867356	5.39354E-06	9.71535E-06	negative
PRJNA729271	stage_3W_vs_E10	ordinary_muscle_development	ENSGALG00010029697	GIT1	1.853455303	0.164216513	11.2866561	1.52738E-29	8.63804E-29	positive
PRJNA729271	stage_3W_vs_E10	ordinary_muscle_development	ENSGALG00010029698	NUP85	-0.813293183	0.086246677	-9.429849535	4.10678E-21	1.64487E-20	negative
PRJNA729271	stage_3W_vs_E10	ordinary_muscle_development	ENSGALG00010029699	ANKRD13B	-0.928110262	0.175854648	-5.277712439	1.30807E-07	2.625E-07	negative
PRJNA729271	stage_3W_vs_E10	ordinary_muscle_development	ENSGALG00010029700	GGA3	0.192479689	0.079701927	2.414994174	0.015735461	0.020929117	positive
PRJNA729271	stage_3W_vs_E10	ordinary_muscle_development	ENSGALG00010029701	MRPS7	0.181696391	0.065999206	2.753008745	0.005905032	0.008215918	positive
PRJNA729271	stage_3W_vs_E10	ordinary_muscle_development	ENSGALG00010029702	CORO6	5.418496051	0.169544135	31.95920661	4.0245E-224	8.5786E-222	positive
PRJNA729271	stage_3W_vs_E10	ordinary_muscle_development	ENSGALG00010029703	MIF4GD	-0.111698876	0.115317379	-0.968621359	0.332734143	0.371141316	negative
PRJNA729271	stage_3W_vs_E10	ordinary_muscle_development	ENSGALG00010029704	SLC25A19	1.253118419	0.098665003	12.70073862	5.85743E-37	4.25155E-36	positive
PRJNA729271	stage_3W_vs_E10	ordinary_muscle_development	ENSGALG00010029705	GRB2	-0.77015618	0.068009572	-11.32423211	9.95474E-30	5.67193E-29	negative
PRJNA729271	stage_3W_vs_E10	ordinary_muscle_development	ENSGALG00010029706	SSH2	1.803677984	0.216637604	8.325784404	8.37721E-17	2.78058E-16	positive
PRJNA729271	stage_3W_vs_E10	ordinary_muscle_development	ENSGALG00010029707	NSRP1	0.17227604	0.22316389	0.77197095	0.440131633	0.478777338	positive
PRJNA729271	stage_3W_vs_E10	ordinary_muscle_development	ENSGALG00010029708	TMEM94	-0.147356705	0.08501719	-1.733257763	0.083049864	0.101433079	negative
PRJNA729271	stage_3W_vs_E10	ordinary_muscle_development	ENSGALG00010029709	SLC6A4	1.501866003	0.194701643	7.713679125	1.22242E-14	3.62869E-14	positive
PRJNA729271	stage_3W_vs_E10	ordinary_muscle_development	ENSGALG00010029710	BLMH	-0.099465106	0.082822442	-1.200943891	0.229772967	0.26348131	negative
PRJNA729271	stage_3W_vs_E10	ordinary_muscle_development	ENSGALG00010029711	CASKIN2	0.689298173	0.111247072	6.196101724	5.78787E-10	1.33932E-09	positive
PRJNA729271	stage_3W_vs_E10	ordinary_muscle_development	ENSGALG00010029713	CPD	0.063980832	0.116695311	0.548272521	0.583504793	0.619354274	positive
PRJNA729271	stage_3W_vs_E10	ordinary_muscle_development	ENSGALG00010029716	GOSR1	0.117417146	0.062916342	1.866242409	0.062007458	0.076938718	positive
PRJNA729271	stage_3W_vs_E10	ordinary_muscle_development	ENSGALG00010029717	TRARG1	-2.914264126	0.652290037	-4.467742816	7.90493E-06	1.40678E-05	negative
PRJNA729271	stage_3W_vs_E10	ordinary_muscle_development	ENSGALG00010029718	ABR	0.189225373	0.11105935	1.703822084	0.088414331	0.107667456	positive
PRJNA729271	stage_3W_vs_E10	ordinary_muscle_development	ENSGALG00010029720	TIMM22	0.629997392	0.105951175	5.946110498	2.74589E-09	6.09894E-09	positive
PRJNA729271	stage_3W_vs_E10	ordinary_muscle_development	ENSGALG00010029721	EXOC7	0.354403945	0.104965295	3.376391648	0.000734433	0.001113208	positive
PRJNA729271	stage_3W_vs_E10	ordinary_muscle_development	ENSGALG00010029722	SPECC1	0.683038224	0.142014216	4.809646829	1.51197E-06	2.82715E-06	positive
PRJNA729271	stage_3W_vs_E10	ordinary_muscle_development	ENSGALG00010029723	ADORA2B	1.158968297	0.179377288	6.461064901	1.03969E-10	2.50503E-10	positive
PRJNA729271	stage_3W_vs_E10	ordinary_muscle_development	ENSGALG00010029724	CACNA1G	-2.605776223	0.220534571	-11.81572671	3.23737E-32	2.02703E-31	negative
PRJNA729271	stage_3W_vs_E10	ordinary_muscle_development	ENSGALG00010029725	TSEN54	-0.33159246	0.068100572	-4.869158204	1.12075E-06	2.11499E-06	negative
PRJNA729271	stage_3W_vs_E10	ordinary_muscle_development	ENSGALG00010029726	SEPTIN9	-1.640727826	0.125762515	-13.04623899	6.67627E-39	5.19445E-38	negative
PRJNA729271	stage_3W_vs_E10	ordinary_muscle_development	ENSGALG00010029727	ZSWIM7	0.103521303	0.138441514	0.747761996	0.454603729	0.493187714	positive
PRJNA729271	stage_3W_vs_E10	ordinary_muscle_development	ENSGALG00010029728	TNRC6C	0.015471254	0.103778311	0.149079836	0.881490637	0.895452154	positive
PRJNA729271	stage_3W_vs_E10	ordinary_muscle_development	ENSGALG00010029729	RASL10A	0.246085689	1.044687967	0.235559035	0.813774864	0.835505501	positive
PRJNA729271	stage_3W_vs_E10	ordinary_muscle_development	ENSGALG00010029730	TTC19	1.516586364	0.131392954	11.54237211	8.06694E-31	4.77852E-30	positive
PRJNA729271	stage_3W_vs_E10	ordinary_muscle_development	ENSGALG00010029731	NCOR1	0.490569924	0.184627664	2.657077016	0.007882143	0.010839817	positive
PRJNA729271	stage_3W_vs_E10	ordinary_muscle_development	ENSGALG00010029732	PIGL	0.784979512	0.177631993	4.419133618	9.90974E-06	1.74959E-05	positive
PRJNA729271	stage_3W_vs_E10	ordinary_muscle_development	ENSGALG00010029733	ENSGALG00010029733	3.316414878	0.163978221	20.22472776	5.93169E-91	1.99028E-89	positive
PRJNA729271	stage_3W_vs_E10	ordinary_muscle_development	ENSGALG00010029734	SEC14L1	-0.417119079	0.084205245	-4.953599716	7.2853E-07	1.39113E-06	negative
PRJNA729271	stage_3W_vs_E10	ordinary_muscle_development	ENSGALG00010029735	SRP68	-0.604451125	0.114837241	-5.263546199	1.41303E-07	2.82821E-07	negative
PRJNA729271	stage_3W_vs_E10	ordinary_muscle_development	ENSGALG00010029736	ENSGALG00010029736	2.143665104	0.133052383	16.11143718	2.12038E-58	3.05516E-57	positive
PRJNA729271	stage_3W_vs_E10	ordinary_muscle_development	ENSGALG00010029737	ENSGALG00010029737	-6.268033181	0.581613749	-10.77696873	4.42219E-27	2.26501E-26	negative
PRJNA729271	stage_3W_vs_E10	ordinary_muscle_development	ENSGALG00010029738	TRPV2	1.187217798	0.16672709	7.120725239	1.07361E-12	2.89099E-12	positive
PRJNA729271	stage_3W_vs_E10	ordinary_muscle_development	ENSGALG00010029739	P2RX5	1.830367658	0.20315447	9.009733618	2.06558E-19	7.7226E-19	positive
PRJNA729271	stage_3W_vs_E10	ordinary_muscle_development	ENSGALG00010029740	HASPIN	-3.754214508	0.25459411	-14.74588124	3.27023E-49	3.58834E-48	negative
PRJNA729271	stage_3W_vs_E10	ordinary_muscle_development	ENSGALG00010029741	EMC6	1.358722802	0.161810077	8.397022162	4.57944E-17	1.53869E-16	positive
PRJNA729271	stage_3W_vs_E10	ordinary_muscle_development	ENSGALG00010029742	TAX1BP3	-1.152050187	0.119116908	-9.671592448	3.98134E-22	1.65927E-21	negative
PRJNA729271	stage_3W_vs_E10	ordinary_muscle_development	ENSGALG00010029743	MYOCD	3.313916531	0.254255948	13.03378173	7.86126E-39	6.09679E-38	positive
PRJNA729271	stage_3W_vs_E10	ordinary_muscle_development	ENSGALG00010029744	CTNS	1.225500549	0.087957013	13.93294863	3.99515E-44	3.69322E-43	positive
PRJNA729271	stage_3W_vs_E10	ordinary_muscle_development	ENSGALG00010029745	SHPK	0.993133463	0.16762796	5.924628928	3.13003E-09	6.91613E-09	positive
PRJNA729271	stage_3W_vs_E10	ordinary_muscle_development	ENSGALG00010029746	TRPV1	-0.665674656	0.339318256	-1.961800296	0.049785739	0.062501671	negative
PRJNA729271	stage_3W_vs_E10	ordinary_muscle_development	ENSGALG00010029747	TRPV3	-0.20071372	0.3437624	-0.583873398	0.559305492	0.596244253	negative
PRJNA729271	stage_3W_vs_E10	ordinary_muscle_development	ENSGALG00010029749	ASPA	3.585075225	0.282079833	12.70943471	5.24119E-37	3.8119E-36	positive
PRJNA729271	stage_3W_vs_E10	ordinary_muscle_development	ENSGALG00010029750	TEN1	-2.888179042	0.262006795	-11.02329825	2.95046E-28	1.59103E-27	negative
PRJNA729271	stage_3W_vs_E10	ordinary_muscle_development	ENSGALG00010029752	ENSGALG00010029752	-0.036474314	0.172332551	-0.211650754	0.83237951	0.851831474	negative
PRJNA729271	stage_3W_vs_E10	ordinary_muscle_development	ENSGALG00010029753	AKAP10	-0.051150046	0.07472569	-0.684504163	0.493656862	0.532051172	negative
PRJNA729271	stage_3W_vs_E10	ordinary_muscle_development	ENSGALG00010029754	NLE1	-0.902722704	0.179913459	-5.017538497	5.23377E-07	1.00842E-06	negative
PRJNA729271	stage_3W_vs_E10	ordinary_muscle_development	ENSGALG00010029755	ACOX1	0.401257778	0.119276644	3.364093464	0.000767955	0.001161953	positive
PRJNA729271	stage_3W_vs_E10	ordinary_muscle_development	ENSGALG00010029756	ELAC2	-0.146112161	0.083151429	-1.757181606	0.078886859	0.096617239	negative
PRJNA729271	stage_3W_vs_E10	ordinary_muscle_development	ENSGALG00010029757	ULK2	1.280350211	0.209047523	6.124684911	9.08632E-10	2.07509E-09	positive
PRJNA729271	stage_3W_vs_E10	ordinary_muscle_development	ENSGALG00010029758	DNAH9	0.814552275	0.304496004	2.675083628	0.007471061	0.010294993	positive
PRJNA729271	stage_3W_vs_E10	ordinary_muscle_development	ENSGALG00010029759	ALDH3A2	0.533131839	0.107600002	4.954756789	7.24208E-07	1.38306E-06	positive
PRJNA729271	stage_3W_vs_E10	ordinary_muscle_development	ENSGALG00010029760	SHISA6	-7.210194331	0.863937532	-8.345735735	7.07719E-17	2.35717E-16	negative
PRJNA729271	stage_3W_vs_E10	ordinary_muscle_development	ENSGALG00010029761	MRPL38	0.285004855	0.118350569	2.40814098	0.016033988	0.02131444	positive
PRJNA729271	stage_3W_vs_E10	ordinary_muscle_development	ENSGALG00010029762	SLC47A2	5.226058233	0.463146731	11.2838068	1.57768E-29	8.9121E-29	positive
PRJNA729271	stage_3W_vs_E10	ordinary_muscle_development	ENSGALG00010029763	GEMIN4	-0.558620973	0.098367114	-5.678940332	1.35532E-08	2.90096E-08	negative
PRJNA729271	stage_3W_vs_E10	ordinary_muscle_development	ENSGALG00010029764	NXN	-1.300534869	0.126380822	-10.29060303	7.76883E-25	3.60811E-24	negative
PRJNA729271	stage_3W_vs_E10	ordinary_muscle_development	ENSGALG00010029765	KCNJ16	-1.178099218	0.302149279	-3.899063481	9.65655E-05	0.000157768	negative
PRJNA729271	stage_3W_vs_E10	ordinary_muscle_development	ENSGALG00010029766	ENSGALG00010029766	0.519893976	0.145683475	3.568654414	0.000358819	0.000559798	positive
PRJNA729271	stage_3W_vs_E10	ordinary_muscle_development	ENSGALG00010029767	GLOD4	-0.183870787	0.086766461	-2.119145857	0.034078142	0.043655561	negative
PRJNA729271	stage_3W_vs_E10	ordinary_muscle_development	ENSGALG00010029768	KCNJ2	0.736638213	0.175470037	4.19808547	2.69181E-05	4.59411E-05	positive
PRJNA729271	stage_3W_vs_E10	ordinary_muscle_development	ENSGALG00010029770	TLCD3A	-1.758774216	0.236230049	-7.445175668	9.68155E-14	2.7468E-13	negative
PRJNA729271	stage_3W_vs_E10	ordinary_muscle_development	ENSGALG00010029771	SOX9	-4.519984332	0.268583622	-16.82896484	1.49696E-63	2.47135E-62	negative
PRJNA729271	stage_3W_vs_E10	ordinary_muscle_development	ENSGALG00010029772	VPS53	-0.107640988	0.069260173	-1.554154191	0.120147641	0.143644017	negative
PRJNA729271	stage_3W_vs_E10	ordinary_muscle_development	ENSGALG00010029773	TRIM65	1.239301032	0.131702449	9.409855667	4.96819E-21	1.97949E-20	positive
PRJNA729271	stage_3W_vs_E10	ordinary_muscle_development	ENSGALG00010029774	RFLNB	-0.799410841	0.125646227	-6.362394294	1.98633E-10	4.72152E-10	negative
PRJNA729271	stage_3W_vs_E10	ordinary_muscle_development	ENSGALG00010029775	ENSGALG00010029775	-0.144315804	0.142277117	-1.014328985	0.310425798	0.348402783	negative
PRJNA729271	stage_3W_vs_E10	ordinary_muscle_development	ENSGALG00010029776	WBP2	1.083999698	0.136053943	7.967425809	1.62014E-15	5.01792E-15	positive
PRJNA729271	stage_3W_vs_E10	ordinary_muscle_development	ENSGALG00010029777	ENSGALG00010029777	-0.763117874	0.141811483	-5.38121357	7.39854E-08	1.51151E-07	negative
PRJNA729271	stage_3W_vs_E10	ordinary_muscle_development	ENSGALG00010029778	DOC2B	0.296742137	0.346171557	0.857211204	0.391328172	0.430306619	positive
PRJNA729271	stage_3W_vs_E10	ordinary_muscle_development	ENSGALG00010029780	TEX14	0.217886691	0.25440793	0.856446145	0.39175105	0.43070627	positive
PRJNA729271	stage_3W_vs_E10	ordinary_muscle_development	ENSGALG00010029781	UNC13D	1.654157575	0.244062925	6.777586457	1.222E-11	3.10971E-11	positive
PRJNA729271	stage_3W_vs_E10	ordinary_muscle_development	ENSGALG00010029782	UNK	-0.112184913	0.12206463	-0.919061595	0.358063356	0.396768469	negative
PRJNA729271	stage_3W_vs_E10	ordinary_muscle_development	ENSGALG00010029783	RAD51C	-0.227988814	0.121375852	-1.878370447	0.060330505	0.074993993	negative
PRJNA729271	stage_3W_vs_E10	ordinary_muscle_development	ENSGALG00010029784	CLDN3	-4.894325949	0.761218076	-6.429597649	1.27942E-10	3.06888E-10	negative
PRJNA729271	stage_3W_vs_E10	ordinary_muscle_development	ENSGALG00010029785	ENSGALG00010029785	2.121896798	0.246193261	8.618825653	6.76443E-18	2.37525E-17	positive
PRJNA729271	stage_3W_vs_E10	ordinary_muscle_development	ENSGALG00010029787	H3-3B	-1.260719951	0.06428063	-19.61275025	1.20345E-85	3.56E-84	negative
PRJNA729271	stage_3W_vs_E10	ordinary_muscle_development	ENSGALG00010029791	GALK1	-0.576794049	0.114928897	-5.018703426	5.20214E-07	1.00286E-06	negative
PRJNA729271	stage_3W_vs_E10	ordinary_muscle_development	ENSGALG00010029804	ITGB4	0.735016468	0.226904672	3.239318349	0.001198158	0.001782175	positive
PRJNA729271	stage_3W_vs_E10	ordinary_muscle_development	ENSGALG00010029812	SAP30BP	-0.266974422	0.115475175	-2.311963774	0.02077968	0.027292629	negative
PRJNA729271	stage_3W_vs_E10	ordinary_muscle_development	ENSGALG00010029835	RECQL5	-0.546813276	0.110214867	-4.961338628	7.0009E-07	1.33929E-06	negative
PRJNA729271	stage_3W_vs_E10	ordinary_muscle_development	ENSGALG00010029841	SMIM5	-0.319092004	0.135685795	-2.351697942	0.018687944	0.024652507	negative
PRJNA729271	stage_3W_vs_E10	ordinary_muscle_development	ENSGALG00010029859	CSNK1D	-0.571612441	0.053097041	-10.76542935	5.01274E-27	2.56205E-26	negative
PRJNA729271	stage_3W_vs_E10	ordinary_muscle_development	ENSGALG00010029864	SLC16A3	1.006040763	0.338963684	2.967989816	0.002997543	0.004299365	positive
PRJNA729271	stage_3W_vs_E10	ordinary_muscle_development	ENSGALG00010029879	ENSGALG00010029879	-0.882426327	0.338646209	-2.60574695	0.009167416	0.012518306	negative
PRJNA729271	stage_3W_vs_E10	ordinary_muscle_development	ENSGALG00010029895	FASN	-2.123062941	0.168656551	-12.58808465	2.4556E-36	1.75685E-35	negative
PRJNA729271	stage_3W_vs_E10	ordinary_muscle_development	ENSGALG00010029907	NTN1	-0.424974269	0.271134766	-1.567391281	0.117023261	0.140128225	negative
PRJNA729271	stage_3W_vs_E10	ordinary_muscle_development	ENSGALG00010029921	STX8	0.087678443	0.104423642	0.839641689	0.401109317	0.440193788	positive
PRJNA729271	stage_3W_vs_E10	ordinary_muscle_development	ENSGALG00010029925	GGNBP2	0.68826234	0.124681817	5.520150069	3.3871E-08	7.05504E-08	positive
PRJNA729271	stage_3W_vs_E10	ordinary_muscle_development	ENSGALG00010029926	BLTP2	0.114432121	0.132554474	0.863283729	0.387981488	0.426982892	positive
PRJNA729271	stage_3W_vs_E10	ordinary_muscle_development	ENSGALG00010029927	ENSGALG00010029927	-0.680531004	0.716625927	-0.949632129	0.342299207	0.380697231	negative
PRJNA729271	stage_3W_vs_E10	ordinary_muscle_development	ENSGALG00010029928	USP43	-0.25178251	0.137812314	-1.826995742	0.06770041	0.083573279	negative
PRJNA729271	stage_3W_vs_E10	ordinary_muscle_development	ENSGALG00010029929	GDPD1	0.485134668	0.093751967	5.174661205	2.28325E-07	4.50834E-07	positive
PRJNA729271	stage_3W_vs_E10	ordinary_muscle_development	ENSGALG00010029930	ARHGAP44	-0.834818194	0.270447115	-3.08680754	0.002023185	0.002942907	negative
PRJNA729271	stage_3W_vs_E10	ordinary_muscle_development	ENSGALG00010029932	RASA4B	3.128261691	0.262664246	11.90973546	1.05311E-32	6.69509E-32	positive
PRJNA729271	stage_3W_vs_E10	ordinary_muscle_development	ENSGALG00010029933	TVP23B	0.124198834	0.094304133	1.317003093	0.187837588	0.218287969	positive
PRJNA729271	stage_3W_vs_E10	ordinary_muscle_development	ENSGALG00010029934	TEKT3	1.684852468	0.478358265	3.522156071	0.000428052	0.000663099	positive
PRJNA729271	stage_3W_vs_E10	ordinary_muscle_development	ENSGALG00010029935	SYNRG	-0.18586162	0.073683256	-2.522440391	0.01165437	0.015740784	negative
PRJNA729271	stage_3W_vs_E10	ordinary_muscle_development	ENSGALG00010029936	SERPINF2	3.329260214	0.499184859	6.669393427			positive
PRJNA729271	stage_3W_vs_E10	ordinary_muscle_development	ENSGALG00010029937	PMP22	1.494842417	0.124152928	12.04033155	2.18077E-33	1.42069E-32	positive
PRJNA729271	stage_3W_vs_E10	ordinary_muscle_development	ENSGALG00010029938	ENSGALG00010029938	-1.309195921	0.314077137	-4.168389761	3.06759E-05	5.21336E-05	negative
PRJNA729271	stage_3W_vs_E10	ordinary_muscle_development	ENSGALG00010029939	ENSGALG00010029939	0.488507296	1.554892637	0.314174294	0.753388665	0.779469572	positive
PRJNA729271	stage_3W_vs_E10	ordinary_muscle_development	ENSGALG00010029941	COX10	1.103253189	0.172474703	6.396608735	1.58866E-10	3.79242E-10	positive
PRJNA729271	stage_3W_vs_E10	ordinary_muscle_development	ENSGALG00010029942	ENSGALG00010029942	-1.684741092	1.515231623	-1.111870335	0.266193905	0.30257847	negative
PRJNA729271	stage_3W_vs_E10	ordinary_muscle_development	ENSGALG00010029943	HS3ST3A1	-1.506242386	0.197036144	-7.644497904	2.09762E-14	6.1611E-14	negative
PRJNA729271	stage_3W_vs_E10	ordinary_muscle_development	ENSGALG00010029945	CCL4	5.244150685	0.681491012	7.6951135	1.41369E-14	4.18533E-14	positive
PRJNA729271	stage_3W_vs_E10	ordinary_muscle_development	ENSGALG00010029946	METRNL	-0.776530376	0.137140219	-5.662309567	1.49349E-08	3.18918E-08	negative
PRJNA729271	stage_3W_vs_E10	ordinary_muscle_development	ENSGALG00010029947	CCL5	7.87400308	0.97826088	8.048980838	8.34863E-16	2.62787E-15	positive
PRJNA729271	stage_3W_vs_E10	ordinary_muscle_development	ENSGALG00010029948	MYADML2	1.280103001	0.21892129	5.847320755	4.99554E-09	1.0948E-08	positive
PRJNA729271	stage_3W_vs_E10	ordinary_muscle_development	ENSGALG00010029950	RFFL	0.395075518	0.10322167	3.827447474	0.000129479	0.000209651	positive
PRJNA729271	stage_3W_vs_E10	ordinary_muscle_development	ENSGALG00010029956	CCL15	6.373771933	0.181309748	35.15404991	1.0081E-270	3.6529E-268	positive
PRJNA729271	stage_3W_vs_E10	ordinary_muscle_development	ENSGALG00010029962	ENSGALG00010029962	-0.146855391	0.759294454	-0.19341033	0.846637626	0.86428709	negative
PRJNA729271	stage_3W_vs_E10	ordinary_muscle_development	ENSGALG00010029966	RABGEF1	0.265904039	0.080638261	3.297492237	0.000975524	0.001462882	positive
PRJNA729271	stage_3W_vs_E10	ordinary_muscle_development	ENSGALG00010030004	FLOT2	0.46431417	0.070585099	6.578076362	4.76573E-11	1.17282E-10	positive
PRJNA729271	stage_3W_vs_E10	ordinary_muscle_development	ENSGALG00010030014	ENSGALG00010030014	-0.171497356	0.306135502	-0.560200808	0.575342476	0.611541336	negative
PRJNA729271	stage_3W_vs_E10	ordinary_muscle_development	ENSGALG00010030102	GPS1	0.313010385	0.08298127	3.772060662	0.000161905	0.000259977	positive

PRJNA729271	stage_3W_vs_E10	ordinary_muscle_development	ENSGALG00010030103	C1QTNF1	2.313836305	0.208657442	11.0891626	1.41607E-28	7.70205E-28	positive
PRJNA729271	stage_3W_vs_E10	ordinary_muscle_development	ENSGALG00010030104	P4HB	-1.596145355	0.110213377	-14.48231961	1.56718E-47	1.63309E-46	negative
PRJNA729271	stage_3W_vs_E10	ordinary_muscle_development	ENSGALG00010030105	RAB40B	1.443814398	0.191346009	7.545568396	4.50319E-14	1.30313E-13	positive
PRJNA729271	stage_3W_vs_E10	ordinary_muscle_development	ENSGALG00010030106	GPRC5C	-2.151811892	0.213629838	-10.07261864	7.30079E-24	3.25515E-23	negative
PRJNA729271	stage_3W_vs_E10	ordinary_muscle_development	ENSGALG00010030107	RPTOR	0.726851565	0.138176864	5.26029859	1.43822E-07	2.87623E-07	positive
PRJNA729271	stage_3W_vs_E10	ordinary_muscle_development	ENSGALG00010030108	LLGL2	0.60957102	0.11862108	5.138808546	2.76486E-07	5.43493E-07	positive
PRJNA729271	stage_3W_vs_E10	ordinary_muscle_development	ENSGALG00010030109	FBF1	-0.081819351	0.089868506	-0.91043409	0.362593626	0.401389538	negative
PRJNA729271	stage_5W_vs_E10	ordinary_muscle_development	ENSGALG00010000007	ND1	0.518117359	0.243283862	2.129682398	0.033197843	0.042845939	positive
PRJNA729271	stage_5W_vs_E10	ordinary_muscle_development	ENSGALG00010000011	ND2	0.450958488	0.252908001	1.783093005	0.074571165	0.092078459	positive
PRJNA729271	stage_5W_vs_E10	ordinary_muscle_development	ENSGALG00010000017	COX1	1.86743048	0.272411322	6.85518674	7.12194E-12	1.89766E-11	positive
PRJNA729271	stage_5W_vs_E10	ordinary_muscle_development	ENSGALG00010000020	COX2	2.120067711	0.238999674	8.870588315	7.27605E-19	2.75801E-18	positive
PRJNA729271	stage_5W_vs_E10	ordinary_muscle_development	ENSGALG00010000022	ATP8	0.740810768	0.273219852	2.711409014	0.006699793	0.009341429	positive
PRJNA729271	stage_5W_vs_E10	ordinary_muscle_development	ENSGALG00010000023	ATP6	0.801298194	0.19879471	4.030782286	5.55915E-05	9.35453E-05	positive
PRJNA729271	stage_5W_vs_E10	ordinary_muscle_development	ENSGALG00010000024	COX3	1.577549914	0.221507887	7.121867928	1.06474E-12	2.96625E-12	positive
PRJNA729271	stage_5W_vs_E10	ordinary_muscle_development	ENSGALG00010000026	ND3	1.533759891	0.317881031	4.82494941	1.40039E-06	2.65967E-06	positive
PRJNA729271	stage_5W_vs_E10	ordinary_muscle_development	ENSGALG00010000028	ND4L	1.677442745	0.273708519	6.128573388	8.86705E-10	2.07504E-09	positive
PRJNA729271	stage_5W_vs_E10	ordinary_muscle_development	ENSGALG00010000029	ND4	1.541574695	0.254663474	6.053379661	1.41838E-09	3.28425E-09	positive
PRJNA729271	stage_5W_vs_E10	ordinary_muscle_development	ENSGALG00010000033	ND5	0.806415589	0.232338744	3.470861447	0.000518792	0.000804868	positive
PRJNA729271	stage_5W_vs_E10	ordinary_muscle_development	ENSGALG00010000034	CYTB	1.517582096	0.21899563	6.929736879	4.21624E-12	1.13638E-11	positive
PRJNA729271	stage_5W_vs_E10	ordinary_muscle_development	ENSGALG00010000037	ND6	-0.447870513	0.191632071	-2.337137579	0.019432032	0.025723041	negative
PRJNA729271	stage_5W_vs_E10	ordinary_muscle_development	ENSGALG00010000039	PDIA3	-0.913345709	0.065971336	-13.84458418	1.37172E-43	1.33983E-42	negative
PRJNA729271	stage_5W_vs_E10	ordinary_muscle_development	ENSGALG00010000042	CKMT1B	3.113792801	0.45745113	6.806831592	9.97716E-12	2.64144E-11	positive
PRJNA729271	stage_5W_vs_E10	ordinary_muscle_development	ENSGALG00010000043	PPIP5K1	0.559292888	0.123017343	4.546455609	5.45569E-06	9.89863E-06	positive
PRJNA729271	stage_5W_vs_E10	ordinary_muscle_development	ENSGALG00010000044	MAP1A	0.575492599	0.132499536	4.343355566	1.40323E-05	2.47533E-05	positive
PRJNA729271	stage_5W_vs_E10	ordinary_muscle_development	ENSGALG00010000045	TP53BP1	-1.354045705	0.07466746	-18.13434798	1.70712E-73	3.93397E-72	negative
PRJNA729271	stage_5W_vs_E10	ordinary_muscle_development	ENSGALG00010000046	TUBGCP4	-0.644730735	0.063037472	-10.22773777	1.48958E-24	7.26987E-24	negative
PRJNA729271	stage_5W_vs_E10	ordinary_muscle_development	ENSGALG00010000047	IQGAP1	-0.552670334	0.097234765	-5.683875853	1.31676E-08	2.88707E-08	negative
PRJNA729271	stage_5W_vs_E10	ordinary_muscle_development	ENSGALG00010000048	MAN2A2	2.508941108	0.147019118	17.0654071	2.68511E-65	5.0091E-64	positive
PRJNA729271	stage_5W_vs_E10	ordinary_muscle_development	ENSGALG00010000049	FES	2.034791438	0.18800324	10.82317216	2.6736E-27	1.45691E-26	positive
PRJNA729271	stage_5W_vs_E10	ordinary_muscle_development	ENSGALG00010000050	CIB1	0.652469267	0.130352732	5.005413044	5.57424E-07	1.0904E-06	positive
PRJNA729271	stage_5W_vs_E10	ordinary_muscle_development	ENSGALG00010000051	GDPGP1	0.04751671	0.193037879	0.246152257	0.805564372	0.828719345	positive
PRJNA729271	stage_5W_vs_E10	ordinary_muscle_development	ENSGALG00010000052	TTLL13	0.277600038	0.135242173	2.052614446	0.040109985	0.051224162	positive
PRJNA729271	stage_5W_vs_E10	ordinary_muscle_development	ENSGALG00010000053	HDDC3	0.56179848	0.118584813	4.737524692	2.16345E-06	4.04948E-06	positive
PRJNA729271	stage_5W_vs_E10	ordinary_muscle_development	ENSGALG00010000054	VPS33B	0.215473269	0.099647406	2.162357023	0.030590668	0.039654062	positive
PRJNA729271	stage_5W_vs_E10	ordinary_muscle_development	ENSGALG00010000055	PRC1	-1.666025147	0.214571125	-7.76444243	8.20052E-15	2.55133E-14	negative
PRJNA729271	stage_5W_vs_E10	ordinary_muscle_development	ENSGALG00010000056	UNC45A	0.279902754	0.081788024	3.4222951	0.000620949	0.000957924	positive
PRJNA729271	stage_5W_vs_E10	ordinary_muscle_development	ENSGALG00010000058	FURIN	1.662465105	0.171823135	9.675443911	3.83424E-22	1.68928E-21	positive
PRJNA729271	stage_5W_vs_E10	ordinary_muscle_development	ENSGALG00010000059	MFAP1	-0.305463544	0.111140411	-2.748447136	0.005987829	0.008387474	negative
PRJNA729271	stage_5W_vs_E10	ordinary_muscle_development	ENSGALG00010000060	HYPK	0.494086296	0.245516714	2.012434457	0.044174159	0.056088336	positive
PRJNA729271	stage_5W_vs_E10	ordinary_muscle_development	ENSGALG00010000061	SERINC4	3.3800448	0.331222823	10.20474608	1.88814E-24	9.15773E-24	positive
PRJNA729271	stage_5W_vs_E10	ordinary_muscle_development	ENSGALG00010000063	SERF2	0.38424489	0.127458557	3.014665296	0.00257263	0.003745883	positive
PRJNA729271	stage_5W_vs_E10	ordinary_muscle_development	ENSGALG00010000069	ENSGALG00010000069	1.672494688	0.234180509	7.141903891	9.2047E-13	2.57373E-12	positive
PRJNA729271	stage_5W_vs_E10	ordinary_muscle_development	ENSGALG00010000076	ENSGALG00010000076	1.012152838	0.194811829	5.195540972	2.04125E-07	4.11974E-07	positive
PRJNA729271	stage_5W_vs_E10	ordinary_muscle_development	ENSGALG00010000078	ENSGALG00010000078	1.64933436	0.466849574	3.532903211	0.000411023	0.000642417	positive
PRJNA729271	stage_5W_vs_E10	ordinary_muscle_development	ENSGALG00010000081	ENSGALG00010000081	8.595069047	0.298792088	28.76605307	5.7053E-182	7.9517E-180	positive
PRJNA729271	stage_5W_vs_E10	ordinary_muscle_development	ENSGALG00010000084	ENSGALG00010000084	1.79331209	0.262708413	6.826245365	8.71658E-12	2.31165E-11	positive
PRJNA729271	stage_5W_vs_E10	ordinary_muscle_development	ENSGALG00010000085	ENSGALG00010000085	-0.600999056	0.152618844	-3.93790859	8.21949E-05	0.000136677	negative
PRJNA729271	stage_5W_vs_E10	ordinary_muscle_development	ENSGALG00010000089	ENSGALG00010000089	2.583602747	0.280064015	9.225043589	2.83446E-20	1.14508E-19	positive
PRJNA729271	stage_5W_vs_E10	ordinary_muscle_development	ENSGALG00010000091	ENSGALG00010000091	2.477317928	0.908159059	2.727845859	0.006374939	0.008906481	positive
PRJNA729271	stage_5W_vs_E10	ordinary_muscle_development	ENSGALG00010000092	INPPL1	1.480251195	0.170632628	8.67507704	4.13271E-18	1.51616E-17	positive
PRJNA729271	stage_5W_vs_E10	ordinary_muscle_development	ENSGALG00010000093	ENSGALG00010000093	1.963985444	0.328217395	5.9837945	2.17998E-09	4.98956E-09	positive
PRJNA729271	stage_5W_vs_E10	ordinary_muscle_development	ENSGALG00010000098	ENSGALG00010000098	1.610761305	0.190352567	8.461988888	2.62856E-17	9.27481E-17	positive
PRJNA729271	stage_5W_vs_E10	ordinary_muscle_development	ENSGALG00010000105	CACNA1F	0.509711332	0.291675402	1.747529371	0.080545555	0.099000069	positive
PRJNA729271	stage_5W_vs_E10	ordinary_muscle_development	ENSGALG00010000107	ENSGALG00010000107	-1.394037641	0.573077485	-2.432546519	0.014993065	0.020117049	negative
PRJNA729271	stage_5W_vs_E10	ordinary_muscle_development	ENSGALG00010000121	ENSGALG00010000121	0.144444569	0.1373671	1.051522299	0.293018773	0.330967593	positive
PRJNA729271	stage_5W_vs_E10	ordinary_muscle_development	ENSGALG00010000123	ENSGALG00010000123	0.122900272	0.254813633	0.48231435	0.629582654	0.665258609	positive
PRJNA729271	stage_5W_vs_E10	ordinary_muscle_development	ENSGALG00010000124	ENSGALG00010000124	1.645521681	0.182100704	9.036327951	1.62023E-19	6.33709E-19	positive
PRJNA729271	stage_5W_vs_E10	ordinary_muscle_development	ENSGALG00010000126	ENSGALG00010000126	0.635601544	0.202701156	3.135658217	0.001714689	0.002537201	positive
PRJNA729271	stage_5W_vs_E10	ordinary_muscle_development	ENSGALG00010000127	ENSGALG00010000127	1.29655239	0.206065063	6.291956395	3.1349E-10	7.55199E-10	positive
PRJNA729271	stage_5W_vs_E10	ordinary_muscle_development	ENSGALG00010000133	USP39	-0.148119331	0.141966597	-1.043339306	0.296791173	0.334993619	negative
PRJNA729271	stage_5W_vs_E10	ordinary_muscle_development	ENSGALG00010000134	ENSGALG00010000134	0.748439035	0.127212155	5.883392446	4.01941E-09	9.06792E-09	positive
PRJNA729271	stage_5W_vs_E10	ordinary_muscle_development	ENSGALG00010000137	ENSGALG00010000137	1.230464631	0.54014099	2.278043425	0.02272399	0.029830125	positive
PRJNA729271	stage_5W_vs_E10	ordinary_muscle_development	ENSGALG00010000140	SEPHS3	-0.715512212	0.128404415	-5.57233343	2.5135E-08	5.41113E-08	negative
PRJNA729271	stage_5W_vs_E10	ordinary_muscle_development	ENSGALG00010000141	ENSGALG00010000141	-0.527026626	0.139949236	-3.765841403	0.000165989	0.000268259	negative
PRJNA729271	stage_5W_vs_E10	ordinary_muscle_development	ENSGALG00010000143	SCARF2	-1.234466061	0.159819338	-7.724134493	1.12616E-14	3.47534E-14	negative
PRJNA729271	stage_5W_vs_E10	ordinary_muscle_development	ENSGALG00010000145	ENSGALG00010000145	0.603549271	0.280515184	2.15157434	0.031430895	0.040685017	positive
PRJNA729271	stage_5W_vs_E10	ordinary_muscle_development	ENSGALG00010000146	ACADM	1.895376292	0.16228187	11.67953196	1.62186E-31	1.04252E-30	positive
PRJNA729271	stage_5W_vs_E10	ordinary_muscle_development	ENSGALG00010000147	ENSGALG00010000147	0.124757196	0.110476293	1.129266672	0.258785356	0.295106107	positive
PRJNA729271	stage_5W_vs_E10	ordinary_muscle_development	ENSGALG00010000148	ENSGALG00010000148	1.277159625	0.283410797	4.506390155	6.59398E-06	1.18925E-05	positive
PRJNA729271	stage_5W_vs_E10	ordinary_muscle_development	ENSGALG00010000149	ENSGALG00010000149	-0.68425577	0.140873268	-4.857243553	1.19031E-06	2.2708E-06	negative
PRJNA729271	stage_5W_vs_E10	ordinary_muscle_development	ENSGALG00010000150	B3GAT3	0.453684809	0.126055659	3.599083248	0.000319341	0.000504232	positive
PRJNA729271	stage_5W_vs_E10	ordinary_muscle_development	ENSGALG00010000151	TBX5	-4.245714508	0.252589825	-16.80873135	2.10631E-63	3.74613E-62	negative
PRJNA729271	stage_5W_vs_E10	ordinary_muscle_development	ENSGALG00010000153	NF2	0.878577881	0.093252799	9.421463935	4.44842E-21	1.86736E-20	positive
PRJNA729271	stage_5W_vs_E10	ordinary_muscle_development	ENSGALG00010000154	ENSGALG00010000154	7.232428007	3.934838601	1.83804947	0.066055116	0.082059385	positive
PRJNA729271	stage_5W_vs_E10	ordinary_muscle_development	ENSGALG00010000155	MAZ	-1.32259928	0.170554829	-7.754686783	8.85618E-15	2.75296E-14	negative
PRJNA729271	stage_5W_vs_E10	ordinary_muscle_development	ENSGALG00010000156	ENSGALG00010000156	2.849293403	0.478855048	5.950221078	2.67781E-09	6.10104E-09	positive
PRJNA729271	stage_5W_vs_E10	ordinary_muscle_development	ENSGALG00010000157	ENSGALG00010000157	10.05278435	5.306461835	1.894442034			positive
PRJNA729271	stage_5W_vs_E10	ordinary_muscle_development	ENSGALG00010000161	CDC42EP2	1.0617064	0.321179411	3.305649005	0.000947567	0.001440028	positive
PRJNA729271	stage_5W_vs_E10	ordinary_muscle_development	ENSGALG00010000166	PFDN6	0.237404475	0.129746233	1.829760056	0.067285826	0.083523855	positive
PRJNA729271	stage_5W_vs_E10	ordinary_muscle_development	ENSGALG00010000171	DPF2	-0.27693919	0.202675148	-1.366419085	0.171807462	0.202220801	negative
PRJNA729271	stage_5W_vs_E10	ordinary_muscle_development	ENSGALG00010000172	ENSGALG00010000172	-0.032664109	1.124422352	-0.02904968	0.976824968	0.980070459	negative
PRJNA729271	stage_5W_vs_E10	ordinary_muscle_development	ENSGALG00010000173	NIPSNAP1	-1.043288277	0.133025729	-7.842755592	4.40765E-15	1.38859E-14	negative
PRJNA729271	stage_5W_vs_E10	ordinary_muscle_development	ENSGALG00010000174	ENSGALG00010000174	0.54990675	0.098362476	5.590615159	2.26267E-08	4.8871E-08	positive
PRJNA729271	stage_5W_vs_E10	ordinary_muscle_development	ENSGALG00010000175	ENSGALG00010000175	-2.248885667	1.061251767	-2.119087796	0.034083048	0.043941455	negative
PRJNA729271	stage_5W_vs_E10	ordinary_muscle_development	ENSGALG00010000176	ENSGALG00010000176	0.061018127	0.505524055	0.120702717	0.903926507	0.916316856	positive
PRJNA729271	stage_5W_vs_E10	ordinary_muscle_development	ENSGALG00010000177	ENSGALG00010000177	0.36925048	0.210912893	1.750725023	0.079993287	0.09836297	positive
PRJNA729271	stage_5W_vs_E10	ordinary_muscle_development	ENSGALG00010000184	ENSGALG00010000184	1.158271395	3.492965407	0.331601164	0.740190441	0.768942911	positive
PRJNA729271	stage_5W_vs_E10	ordinary_muscle_development	ENSGALG00010000186	THOC5	-0.442880816	0.080215163	-5.521160856	3.36767E-08	7.1691E-08	negative
PRJNA729271	stage_5W_vs_E10	ordinary_muscle_development	ENSGALG00010000188	ENSGALG00010000188	1.469011036	1.328305093	1.105928934	0.26875728	0.305851988	positive
PRJNA729271	stage_5W_vs_E10	ordinary_muscle_development	ENSGALG00010000189	ENSGALG00010000189	1.409496628	0.580900483	2.426399476	0.015249474	0.020451622	positive
PRJNA729271	stage_5W_vs_E10	ordinary_muscle_development	ENSGALG00010000191	NEFH	2.678720038	0.40433719	6.624965749	3.47329E-11	8.92014E-11	positive
PRJNA729271	stage_5W_vs_E10	ordinary_muscle_development	ENSGALG00010000192	ENSGALG00010000192	1.607752372	0.681961448	2.357541435	0.018396402	0.02443022	positive
PRJNA729271	stage_5W_vs_E10	ordinary_muscle_development	ENSGALG00010000196	KSR2	-2.849843438	0.927773826	-3.071700622	0.002128431	0.00311979	negative
PRJNA729271	stage_5W_vs_E10	ordinary_muscle_development	ENSGALG00010000197	ENSGALG00010000197	0.217007377	0.501867969	0.432399338	0.665451199	0.699725436	positive
PRJNA729271	stage_5W_vs_E10	ordinary_muscle_development	ENSGALG00010000200	GAL3ST1	-4.078239708	0.357765954	-11.39918335	4.2206E-30	2.5608E-29	negative
PRJNA729271	stage_5W_vs_E10	ordinary_muscle_development	ENSGALG00010000202	ENSGALG00010000202	-0.458274626	0.302797348	-1.513469748	0.13016038	0.15578191	negative
PRJNA729271	stage_5W_vs_E10	ordinary_muscle_development	ENSGALG00010000205	RNF215	0.182914856	0.115141785	1.588605355	0.112149518	0.135320675	positive
PRJNA729271	stage_5W_vs_E10	ordinary_muscle_development	ENSGALG00010000210	CCDC157	-0.802392053	0.545186764	-1.47177464	0.141081744	0.167966375	negative
PRJNA729271	stage_5W_vs_E10	ordinary_muscle_development	ENSGALG00010000215	SF3A1	-0.445719039	0.090502666	-4.924927156	8.43918E-07	1.63101E-06	negative
PRJNA729271	stage_5W_vs_E10	ordinary_muscle_development	ENSGALG00010000221	TBC1D10A	0.041555994	0.088106803	0.471654774	0.637173224	0.672674864	positive
PRJNA729271	stage_5W_vs_E10	ordinary_muscle_development	ENSGALG00010000223	CASTOR1	1.151888614	0.153617631	7.498414109	6.45946E-14	1.91472E-13	positive
PRJNA729271	stage_5W_vs_E10	ordinary_muscle_development	ENSGALG00010000224	MYBPC2	11.07190195	0.251751584	43.97947289	0	0	positive
PRJNA729271	stage_5W_vs_E10	ordinary_muscle_development	ENSGALG00010000225	LIF	3.445477474	0.460427563	7.48321289	7.25272E-14	2.1411E-13	positive
PRJNA729271	stage_5W_vs_E10	ordinary_muscle_development	ENSGALG00010000226	ENSGALG00010000226	0.768254643	0.441024824	1.741975966	0.08151265	0.100112342	positive
PRJNA729271	stage_5W_vs_E10	ordinary_muscle_development	ENSGALG00010000227	ENSGALG00010000227	-0.088621831	0.291184939	-0.304348953	0.760862054	0.788721695	negative
PRJNA729271	stage_5W_vs_E10	ordinary_muscle_development	ENSGALG00010000231	ENSGALG00010000231	4.127446774	0.438104609	9.421144375	4.46198E-21	1.87251E-20	positive
PRJNA729271	stage_5W_vs_E10	ordinary_muscle_development	ENSGALG00010000232	ENSGALG00010000232	0.605013086	0.162096641	3.732422101	0.000189647	0.0003052	positive
PRJNA729271	stage_5W_vs_E10	ordinary_muscle_development	ENSGALG00010000233	HORMAD2	-1.379607744	0.52009879	-2.652587874	0.007987733	0.011046865	negative
PRJNA729271	stage_5W_vs_E10	ordinary_muscle_development	ENSGALG00010000234	ENSGALG00010000234	4.115961583	4.537147459	0.907169454			positive
PRJNA729271	stage_5W_vs_E10	ordinary_muscle_development	ENSGALG00010000235	ENSGALG00010000235	-2.392879088	0.250120894	-9.566890035	1.1017E-21	4.74424E-21	negative
PRJNA729271	stage_5W_vs_E10	ordinary_muscle_development	ENSGALG00010000239	ENSGALG00010000239	5.656323523	4.617344287	1.225016627	0.220568956	0.25457019	positive
PRJNA729271	stage_5W_vs_E10	ordinary_muscle_development	ENSGALG00010000242	ENSGALG00010000242	2.768435625	0.193135821	14.33413856	1.33898E-46	1.4366E-45	positive
PRJNA729271	stage_5W_vs_E10	ordinary_muscle_development	ENSGALG00010000246	ENSGALG00010000246	6.766916182	4.235001681	1.597854426	0.110075379	0.132961884	positive
PRJNA729271	stage_5W_vs_E10	ordinary_muscle_development	ENSGALG00010000247	ENSGALG00010000247	3.22668755	0.224839477	14.35107215	1.04903E-46	1.13222E-45	positive
PRJNA729271	stage_5W_vs_E10	ordinary_muscle_development	ENSGALG00010000249	ENSGALG00010000249	4.022243205	4.380243041	0.918269413			positive
PRJNA729271	stage_5W_vs_E10	ordinary_muscle_development	ENSGALG00010000254	ENSGALG00010000254	-2.892660191	0.691638922	-4.182327077	2.8854E-05	4.95603E-05	negative
PRJNA729271	stage_5W_vs_E10	ordinary_muscle_development	ENSGALG00010000255	ENSGALG00010000255	1.055630055	0.144462189	7.307310369	2.72543E-13	7.80886E-13	positive
PRJNA729271	stage_5W_vs_E10	ordinary_muscle_development	ENSGALG00010000256	ENSGALG00010000256	-1.075981035	0.426441194	-2.523163921	0.011630417	0.015816014	negative
PRJNA729271	stage_5W_vs_E10	ordinary_muscle_development	ENSGALG00010000262	HNRNPKL	1.673160778	3.470172962	0.482154866	0.629695937	0.665313988	positive
PRJNA729271	stage_5W_vs_E10	ordinary_muscle_development	ENSGALG00010000263	MTMR3	0.944059795	0.091555451	10.31134452	6.26191E-25	3.10418E-24	positive
PRJNA729271	stage_5W_vs_E10	ordinary_muscle_development	ENSGALG00010000267	ENSGALG00010000267	0.241627339	0.123055722	1.963560373	0.049581097	0.062553573	positive
PRJNA729271	stage_5W_vs_E10	ordinary_muscle_development	ENSGALG00010000269	ENSGALG00010000269	-1.371968178	0.506772529	-2.707266277	0.006783982	0.009453357	negative
PRJNA729271	stage_5W_vs_E10	ordinary_muscle_development	ENSGALG00010000271	RTN4R	-3.127698275	0.429350668	-7.284717383	3.22345E-13	9.19944E-13	negative
PRJNA729271	stage_5W_vs_E10	ordinary_muscle_development	ENSGALG00010000272	ENSGALG00010000272	0.030237632	0.382125681	0.07913007	0.936929166	0.945013448	positive
PRJNA729271	stage_5W_vs_E10	ordinary_muscle_development	ENSGALG00010000277	ENSGALG00010000277	3.809811007	0.570019208	6.683653737	2.33057E-11	6.03782E-11	positive
PRJNA729271	stage_5W_vs_E10	ordinary_muscle_development	ENSGALG00010000279	ENSGALG00010000279	-1.294205301	0.141512787	-9.145500737	5.93542E-20	2.36878E-19	negative
PRJNA729271	stage_5W_vs_E10	ordinary_muscle_development	ENSGALG00010000281	OSBP2	1.973485558	0.203831546	9.681943736	3.59809E-22	1.58765E-21	positive
PRJNA729271	stage_5W_vs_E10	ordinary_muscle_development	ENSGALG00010000282	ENSGALG00010000282	5.247562745	4.921586997	1.066233869	0.286317935	0.324055475	positive
PRJNA729271	stage_5W_vs_E10	ordinary_muscle_development	ENSGALG00010000284	ENSGALG00010000284	-1.125821889	0.157239191	-7.159931818	8.07172E-13	2.26261E-12	negative
PRJNA729271	stage_5W_vs_E10	ordinary_muscle_development	ENSGALG00010000285	MYLPF	5.771441957	0.145690974	39.61427263	0	0	positive
PRJNA729271	stage_5W_vs_E10	ordinary_muscle_development	ENSGALG00010000286	TCN2	1.826245517	0.138747482	13.16236875	1.44484E-39	1.24512E-38	positive

PRJNA729271	stage_5W_vs_E10	ordinary_muscle_development	ENSGALG00010000287	IQCD	-2.402444785	0.240974508	-9.96970513	2.06842E-23	9.61262E-23	negative
PRJNA729271	stage_5W_vs_E10	ordinary_muscle_development	ENSGALG00010000288	VARS2	0.371364761	0.155327603	2.390848459	0.016809489	0.022429674	positive
PRJNA729271	stage_5W_vs_E10	ordinary_muscle_development	ENSGALG00010000289	QPRT	-3.936565904	0.494066183	-7.967689428	1.61669E-15	5.23077E-15	negative
PRJNA729271	stage_5W_vs_E10	ordinary_muscle_development	ENSGALG00010000292	ABHD16A	0.695731701	0.134348824	5.178547015	2.23621E-07	4.4988E-07	positive
PRJNA729271	stage_5W_vs_E10	ordinary_muscle_development	ENSGALG00010000293	ENSGALG00010000293	2.773085352	0.350141393	7.919901529	2.37699E-15	7.61424E-15	positive
PRJNA729271	stage_5W_vs_E10	ordinary_muscle_development	ENSGALG00010000294	DUSP21	-1.624761375	0.313446791	-5.183531687	2.17723E-07	4.38624E-07	negative
PRJNA729271	stage_5W_vs_E10	ordinary_muscle_development	ENSGALG00010000298	SLC35E4	1.118199141	0.219295951	5.099041425	3.41378E-07	6.77289E-07	positive
PRJNA729271	stage_5W_vs_E10	ordinary_muscle_development	ENSGALG00010000301	UQCR10	2.257031505	0.154605909	14.59861091	2.86626E-48	3.25344E-47	positive
PRJNA729271	stage_5W_vs_E10	ordinary_muscle_development	ENSGALG00010000302	ENSGALG00010000302	0.905582476	0.231095388	3.918652315	8.90455E-05	0.000147544	positive
PRJNA729271	stage_5W_vs_E10	ordinary_muscle_development	ENSGALG00010000303	ENSGALG00010000303	-0.805095029	0.113920087	-7.067191114	1.58101E-12	4.36177E-12	negative
PRJNA729271	stage_5W_vs_E10	ordinary_muscle_development	ENSGALG00010000306	ENSGALG00010000306	1.784808114	0.207237427	8.612383088	7.15573E-18	2.60151E-17	positive
PRJNA729271	stage_5W_vs_E10	ordinary_muscle_development	ENSGALG00010000307	ZMAT5	0.931519534	0.112267024	8.297356617	1.06453E-16	3.64527E-16	positive
PRJNA729271	stage_5W_vs_E10	ordinary_muscle_development	ENSGALG00010000308	ENSGALG00010000308	-0.046869244	0.350282004	-0.133804317	0.893557313	0.906756739	negative
PRJNA729271	stage_5W_vs_E10	ordinary_muscle_development	ENSGALG00010000309	ENSGALG00010000309	5.30699029	4.277126822	1.240783945	0.214685574	0.248392991	positive
PRJNA729271	stage_5W_vs_E10	ordinary_muscle_development	ENSGALG00010000312	CABP7	-1.684082815	0.516109758	-3.263032308	0.00110227	0.001665006	negative
PRJNA729271	stage_5W_vs_E10	ordinary_muscle_development	ENSGALG00010000313	ENSGALG00010000313	6.322246968	3.781867717	1.6717261	0.094578345	0.115260897	positive
PRJNA729271	stage_5W_vs_E10	ordinary_muscle_development	ENSGALG00010000318	ENSGALG00010000318	5.520665589	4.723191525	1.168842203	0.242467213	0.277918888	positive
PRJNA729271	stage_5W_vs_E10	ordinary_muscle_development	ENSGALG00010000323	AP1B1	0.711588679	0.144814998	4.913777495	8.93381E-07	1.72316E-06	positive
PRJNA729271	stage_5W_vs_E10	ordinary_muscle_development	ENSGALG00010000324	ENSGALG00010000324	-0.172310313	0.175087387	-0.984138926	0.32504721	0.36464355	negative
PRJNA729271	stage_5W_vs_E10	ordinary_muscle_development	ENSGALG00010000326	ENSGALG00010000326	4.228233594	1.780794778	2.374351973	0.017579784	0.023392909	positive
PRJNA729271	stage_5W_vs_E10	ordinary_muscle_development	ENSGALG00010000331	ENSGALG00010000331	-0.130980886	0.171084156	-0.765593313	0.443918325	0.485813221	negative
PRJNA729271	stage_5W_vs_E10	ordinary_muscle_development	ENSGALG00010000338	ENSGALG00010000338	-1.221382561	0.197606813	-6.18087274	6.37482E-10	1.50788E-09	negative
PRJNA729271	stage_5W_vs_E10	ordinary_muscle_development	ENSGALG00010000340	NAA10	-0.189609354	0.133700288	-1.418167132	0.156141976	0.184863018	negative
PRJNA729271	stage_5W_vs_E10	ordinary_muscle_development	ENSGALG00010000341	EWSR1	-1.007212133	0.148263897	-6.793374202	1.09541E-11	2.89585E-11	negative
PRJNA729271	stage_5W_vs_E10	ordinary_muscle_development	ENSGALG00010000346	ENSGALG00010000346	-0.336371991	0.114471697	-2.938473006	0.003298334	0.004746758	negative
PRJNA729271	stage_5W_vs_E10	ordinary_muscle_development	ENSGALG00010000349	CHIR-IG1-5	3.087900933	0.434121396	7.1129895	1.13556E-12	3.15627E-12	positive
PRJNA729271	stage_5W_vs_E10	ordinary_muscle_development	ENSGALG00010000350	ENSGALG00010000350	0.238022529	0.165741231	1.436109333	0.150971233	0.179121553	positive
PRJNA729271	stage_5W_vs_E10	ordinary_muscle_development	ENSGALG00010000353	ENSGALG00010000353	0.725384132	0.11059999	6.558627472	5.43053E-11	1.37566E-10	positive
PRJNA729271	stage_5W_vs_E10	ordinary_muscle_development	ENSGALG00010000356	ENSGALG00010000356	0.451364595	0.12477665	3.617380302	0.0002976	0.000471032	positive
PRJNA729271	stage_5W_vs_E10	ordinary_muscle_development	ENSGALG00010000366	EMID1	-0.263037861	0.217178848	-1.211157825	0.225834923	0.260233501	negative
PRJNA729271	stage_5W_vs_E10	ordinary_muscle_development	ENSGALG00010000367	HSD17B10	0.122231968	0.139398299	0.876854082	0.3805659	0.421703441	positive
PRJNA729271	stage_5W_vs_E10	ordinary_muscle_development	ENSGALG00010000373	ENSGALG00010000373	3.351836073	0.45828528	7.313863703	2.59568E-13	7.44596E-13	positive
PRJNA729271	stage_5W_vs_E10	ordinary_muscle_development	ENSGALG00010000376	ASCC2	-0.669644517	0.086360453	-7.754064448	8.89971E-15	2.76471E-14	negative
PRJNA729271	stage_5W_vs_E10	ordinary_muscle_development	ENSGALG00010000377	ENSGALG00010000377	0.629174266	0.243381354	2.585137508	0.009734017	0.013352378	positive
PRJNA729271	stage_5W_vs_E10	ordinary_muscle_development	ENSGALG00010000381	ENSGALG00010000381	-0.935789698	0.310635694	-3.012498931	0.002591063	0.003771587	negative
PRJNA729271	stage_5W_vs_E10	ordinary_muscle_development	ENSGALG00010000382	ENSGALG00010000382	-0.034229602	0.511729474	-0.066890035	0.946669246	0.953724058	negative
PRJNA729271	stage_5W_vs_E10	ordinary_muscle_development	ENSGALG00010000384	ENSGALG00010000384	0.32108593	0.145018396	2.214104817	0.026821569	0.034962109	positive
PRJNA729271	stage_5W_vs_E10	ordinary_muscle_development	ENSGALG00010000386	ENSGALG00010000386	0.856512484	0.35509505	2.412065404	0.015862436	0.021226552	positive
PRJNA729271	stage_5W_vs_E10	ordinary_muscle_development	ENSGALG00010000387	DTX1	-1.158145829	0.459946437	-2.518001524	0.011802281	0.016034686	negative
PRJNA729271	stage_5W_vs_E10	ordinary_muscle_development	ENSGALG00010000388	ENSGALG00010000388	-0.107234177	0.184685732	-0.580630543	0.561489483	0.601047933	negative
PRJNA729271	stage_5W_vs_E10	ordinary_muscle_development	ENSGALG00010000391	ENSGALG00010000391	3.184939467	0.63795288	4.992436848	5.96222E-07	1.16433E-06	positive
PRJNA729271	stage_5W_vs_E10	ordinary_muscle_development	ENSGALG00010000402	PFKFB1	9.530840233	0.318338009	29.93937254	6.0507E-197	9.8545E-195	positive
PRJNA729271	stage_5W_vs_E10	ordinary_muscle_development	ENSGALG00010000405	ENSGALG00010000405	1.995117472	0.405127008	4.924671604	8.45021E-07	1.63293E-06	positive
PRJNA729271	stage_5W_vs_E10	ordinary_muscle_development	ENSGALG00010000406	NSMCE3	-0.480589939	0.099404804	-4.83467518	1.33363E-06	2.53588E-06	negative
PRJNA729271	stage_5W_vs_E10	ordinary_muscle_development	ENSGALG00010000407	WSB2	0.531840554	0.085334199	6.232443238	4.59216E-10	1.09515E-09	positive
PRJNA729271	stage_5W_vs_E10	ordinary_muscle_development	ENSGALG00010000408	ENSGALG00010000408	9.089304531	0.349988217	25.97031585	1.0722E-148	1.0501E-146	positive
PRJNA729271	stage_5W_vs_E10	ordinary_muscle_development	ENSGALG00010000411	ENSGALG00010000411	3.886254915	0.895314838	4.340657333	1.42057E-05	2.5041E-05	positive
PRJNA729271	stage_5W_vs_E10	ordinary_muscle_development	ENSGALG00010000412	RFC5	-1.326186619	0.109489526	-12.11245194	9.07404E-34	6.36017E-33	negative
PRJNA729271	stage_5W_vs_E10	ordinary_muscle_development	ENSGALG00010000422	ITIH6	-1.566194755	0.563193419	-2.780918069	0.005420542	0.007623787	negative
PRJNA729271	stage_5W_vs_E10	ordinary_muscle_development	ENSGALG00010000423	ENSGALG00010000423	2.754804348	0.200357825	13.74942233	5.13378E-43	4.90535E-42	positive
PRJNA729271	stage_5W_vs_E10	ordinary_muscle_development	ENSGALG00010000428	GNL3L	-0.731015252	0.149963043	-4.874636018	1.09009E-06	2.08648E-06	negative
PRJNA729271	stage_5W_vs_E10	ordinary_muscle_development	ENSGALG00010000431	AIFM3	-3.458229611	1.177696049	-2.936436455	0.003320068	0.00477614	negative
PRJNA729271	stage_5W_vs_E10	ordinary_muscle_development	ENSGALG00010000432	ENSGALG00010000432	1.162313821	0.182131158	6.381740686	1.75086E-10	4.28985E-10	positive
PRJNA729271	stage_5W_vs_E10	ordinary_muscle_development	ENSGALG00010000434	TP53	-0.01354469	0.221266589	-0.061214347	0.951188504	0.957663219	negative
PRJNA729271	stage_5W_vs_E10	ordinary_muscle_development	ENSGALG00010000435	P2RX6	-0.080866566	0.304603064	-0.265481788	0.790638338	0.815621856	negative
PRJNA729271	stage_5W_vs_E10	ordinary_muscle_development	ENSGALG00010000436	ENSGALG00010000436	2.280799803	0.362426344	6.293140224	3.11107E-10	7.49584E-10	positive
PRJNA729271	stage_5W_vs_E10	ordinary_muscle_development	ENSGALG00010000440	ENSGALG00010000440	-0.984667391	0.457183877	-2.153766658	0.031258476	0.040479908	negative
PRJNA729271	stage_5W_vs_E10	ordinary_muscle_development	ENSGALG00010000441	SLC7A4	-1.632835876	0.219469899	-7.439908095	1.00755E-13	2.94981E-13	negative
PRJNA729271	stage_5W_vs_E10	ordinary_muscle_development	ENSGALG00010000442	DGCR6	-0.773847931	0.131085084	-5.90340186	3.56081E-09	8.06595E-09	negative
PRJNA729271	stage_5W_vs_E10	ordinary_muscle_development	ENSGALG00010000443	TBX3	-1.425151388	0.158551367	-8.988578347	2.50449E-19	9.6962E-19	negative
PRJNA729271	stage_5W_vs_E10	ordinary_muscle_development	ENSGALG00010000444	RBM19	-0.447638616	0.11914246	-3.757171159	0.000171845	0.000277413	negative
PRJNA729271	stage_5W_vs_E10	ordinary_muscle_development	ENSGALG00010000449	PLBD2	1.363814223	0.10758223	12.67694696	7.93633E-37	6.26905E-36	positive
PRJNA729271	stage_5W_vs_E10	ordinary_muscle_development	ENSGALG00010000450	ENSGALG00010000450	0.752847586	0.188655462	3.990595227	6.59077E-05	0.000110405	positive
PRJNA729271	stage_5W_vs_E10	ordinary_muscle_development	ENSGALG00010000452	SLC8B1	2.308920516	0.223647025	10.32394918	5.49168E-25	2.73077E-24	positive
PRJNA729271	stage_5W_vs_E10	ordinary_muscle_development	ENSGALG00010000453	ACHE	-1.326550298	0.252658198	-5.250375044	1.5179E-07	3.09412E-07	negative
PRJNA729271	stage_5W_vs_E10	ordinary_muscle_development	ENSGALG00010000457	TPCN1	0.161872346	0.080600549	2.008328071	0.044608441	0.056614951	positive
PRJNA729271	stage_5W_vs_E10	ordinary_muscle_development	ENSGALG00010000458	ENSGALG00010000458	0.201460008	0.200488223	1.004847092	0.314970484	0.353969388	positive
PRJNA729271	stage_5W_vs_E10	ordinary_muscle_development	ENSGALG00010000462	ENSGALG00010000462	-0.924001214	0.249630299	-3.701478621	0.000214347	0.000343538	negative
PRJNA729271	stage_5W_vs_E10	ordinary_muscle_development	ENSGALG00010000465	ENSGALG00010000465	1.059867038	0.192262139	5.512614413	3.53542E-08	7.51627E-08	positive
PRJNA729271	stage_5W_vs_E10	ordinary_muscle_development	ENSGALG00010000466	FBXO21	-0.27741643	0.08213108	-3.37772778	0.000730874	0.001119757	negative
PRJNA729271	stage_5W_vs_E10	ordinary_muscle_development	ENSGALG00010000467	RNASEK	0.965402188	0.199019675	4.850787692	1.22972E-06	2.34414E-06	positive
PRJNA729271	stage_5W_vs_E10	ordinary_muscle_development	ENSGALG00010000468	TESC	-2.820043561	0.315813482	-8.92945906	4.28097E-19	1.6403E-18	negative
PRJNA729271	stage_5W_vs_E10	ordinary_muscle_development	ENSGALG00010000469	ENSGALG00010000469	6.80865083	0.385999062	17.63903466	1.23554E-69	2.58802E-68	positive
PRJNA729271	stage_5W_vs_E10	ordinary_muscle_development	ENSGALG00010000470	FBXW8	0.511955748	0.107635007	4.756405591	1.9707E-06	3.6973E-06	positive
PRJNA729271	stage_5W_vs_E10	ordinary_muscle_development	ENSGALG00010000471	ENSGALG00010000471	-0.254477366	0.206911814	-1.229883212	0.218740842	0.252681583	negative
PRJNA729271	stage_5W_vs_E10	ordinary_muscle_development	ENSGALG00010000475	RNFT2	-0.618983076	0.111088527	-5.571980224	2.5186E-08	5.42131E-08	negative
PRJNA729271	stage_5W_vs_E10	ordinary_muscle_development	ENSGALG00010000476	ENSGALG00010000476	8.549537743	0.214024429	39.94655083	0	0	positive
PRJNA729271	stage_5W_vs_E10	ordinary_muscle_development	ENSGALG00010000479	C12orf49	0.334571266	0.114358325	2.925639797	0.003437487	0.004937215	positive
PRJNA729271	stage_5W_vs_E10	ordinary_muscle_development	ENSGALG00010000483	ENSGALG00010000483	1.467463583	0.240323995	6.106188378	1.02039E-09	2.38019E-09	positive
PRJNA729271	stage_5W_vs_E10	ordinary_muscle_development	ENSGALG00010000485	MED13L	0.893773192	0.125998875	7.093501389	1.30761E-12	3.62197E-12	positive
PRJNA729271	stage_5W_vs_E10	ordinary_muscle_development	ENSGALG00010000491	ENSGALG00010000491	1.01556226	0.156325053	6.496477952	8.22222E-11	2.06089E-10	positive
PRJNA729271	stage_5W_vs_E10	ordinary_muscle_development	ENSGALG00010000495	ENSGALG00010000495	0.96560838	0.554664741	1.740886537	0.081703469	0.100338201	positive
PRJNA729271	stage_5W_vs_E10	ordinary_muscle_development	ENSGALG00010000501	ENSGALG00010000501	0.63678937	0.162541536	3.917702427	8.9397E-05	0.000148025	positive
PRJNA729271	stage_5W_vs_E10	ordinary_muscle_development	ENSGALG00010000507	TRPT1	1.636343184	0.290360887	5.635549608	1.74501E-08	3.7939E-08	positive
PRJNA729271	stage_5W_vs_E10	ordinary_muscle_development	ENSGALG00010000509	ENSGALG00010000509	-4.131266026	1.158226018	-3.566891058	0.000361242	0.000567917	negative
PRJNA729271	stage_5W_vs_E10	ordinary_muscle_development	ENSGALG00010000511	FERMT3	1.171171169	0.155811951	7.516568275	5.62327E-14	1.6737E-13	positive
PRJNA729271	stage_5W_vs_E10	ordinary_muscle_development	ENSGALG00010000525	ENSGALG00010000525	-4.093231398	0.618859276	-6.614155361	3.73679E-11	9.57821E-11	negative
PRJNA729271	stage_5W_vs_E10	ordinary_muscle_development	ENSGALG00010000526	POLD1	-1.704153125	0.166109923	-10.25918915	1.07606E-24	5.28015E-24	negative
PRJNA729271	stage_5W_vs_E10	ordinary_muscle_development	ENSGALG00010000529	STIP1	-0.110653973	0.192941783	-0.573509642	0.566299672	0.605436919	negative
PRJNA729271	stage_5W_vs_E10	ordinary_muscle_development	ENSGALG00010000532	ENSGALG00010000532	1.794937883	0.475286693	3.776537216	0.000159024	0.000257346	positive
PRJNA729271	stage_5W_vs_E10	ordinary_muscle_development	ENSGALG00010000546	FLRT1	-4.429105905	0.815292668	-5.43253494	5.55591E-08	1.16596E-07	negative
PRJNA729271	stage_5W_vs_E10	ordinary_muscle_development	ENSGALG00010000555	OTUB1	-0.858486892	0.103049204	-8.330844503	8.02677E-17	2.76558E-16	negative
PRJNA729271	stage_5W_vs_E10	ordinary_muscle_development	ENSGALG00010000561	ENSGALG00010000561	-1.10067074	0.27962769	-3.93620081	8.27817E-05	0.000137543	negative
PRJNA729271	stage_5W_vs_E10	ordinary_muscle_development	ENSGALG00010000564	NAA40	-1.36038601	0.09278921	-14.66103668	1.14511E-48	1.32048E-47	negative
PRJNA729271	stage_5W_vs_E10	ordinary_muscle_development	ENSGALG00010000569	SIPA1	1.303552932	0.224307481	5.811455449	6.1932E-09	1.38373E-08	positive
PRJNA729271	stage_5W_vs_E10	ordinary_muscle_development	ENSGALG00010000574	ENSGALG00010000574	3.882926252	0.17150447	22.64037936	1.7354E-113	9.3167E-112	positive
PRJNA729271	stage_5W_vs_E10	ordinary_muscle_development	ENSGALG00010000575	ENSGALG00010000575	0.974755322	0.160542402	6.071637846	1.26612E-09	2.93968E-09	positive
PRJNA729271	stage_5W_vs_E10	ordinary_muscle_development	ENSGALG00010000576	NOS1	4.779725104	0.420337995	11.37114693	5.82175E-30	3.5205E-29	positive
PRJNA729271	stage_5W_vs_E10	ordinary_muscle_development	ENSGALG00010000577	ENSGALG00010000577	0.136216711	0.270615777	0.503358348	0.61471235	0.651620266	positive
PRJNA729271	stage_5W_vs_E10	ordinary_muscle_development	ENSGALG00010000578	ENSGALG00010000578	1.570738077	0.371803589	4.224644744	2.39318E-05	4.14001E-05	positive
PRJNA729271	stage_5W_vs_E10	ordinary_muscle_development	ENSGALG00010000579	ENSGALG00010000579	-0.59564431	0.897196007	-0.663895409	0.506757255	0.547677185	negative
PRJNA729271	stage_5W_vs_E10	ordinary_muscle_development	ENSGALG00010000580	ENSGALG00010000580	0.60560989	0.493372018	1.227491362	0.219637967	0.253657264	positive
PRJNA729271	stage_5W_vs_E10	ordinary_muscle_development	ENSGALG00010000581	ENSGALG00010000581	-0.269818681	0.131315692	-2.054732962	0.039904804	0.050998073	negative
PRJNA729271	stage_5W_vs_E10	ordinary_muscle_development	ENSGALG00010000583	CCS	0.239805723	0.140169411	1.710827786	0.087112909	0.10662008	positive
PRJNA729271	stage_5W_vs_E10	ordinary_muscle_development	ENSGALG00010000584	RBM14	0.461910236	0.21197114	2.179118511	0.029322865	0.038078744	positive
PRJNA729271	stage_5W_vs_E10	ordinary_muscle_development	ENSGALG00010000585	SPTBN2	-2.911762246	0.5772835	-5.043903462	4.56129E-07	8.96974E-07	negative
PRJNA729271	stage_5W_vs_E10	ordinary_muscle_development	ENSGALG00010000586	ENSGALG00010000586	3.701681527	0.255442799	14.49123459	1.37646E-47	1.52304E-46	positive
PRJNA729271	stage_5W_vs_E10	ordinary_muscle_development	ENSGALG00010000589	ENSGALG00010000589	2.080345115	0.305556878	6.808372728	9.87089E-12	2.61474E-11	positive
PRJNA729271	stage_5W_vs_E10	ordinary_muscle_development	ENSGALG00010000590	DNAH5	1.341012497	0.430282427	3.116586722	0.001829579	0.00270031	positive
PRJNA729271	stage_5W_vs_E10	ordinary_muscle_development	ENSGALG00010000591	SBK2	4.912850758	0.303709315	16.17616096	7.42842E-59	1.15407E-57	positive
PRJNA729271	stage_5W_vs_E10	ordinary_muscle_development	ENSGALG00010000592	CCT5	-1.059399677	0.119332123	-8.877740954	6.82319E-19	2.59313E-18	negative
PRJNA729271	stage_5W_vs_E10	ordinary_muscle_development	ENSGALG00010000593	ATPSCKMT	-0.618957987	0.144597563	-4.280556143	1.86427E-05	3.25416E-05	negative
PRJNA729271	stage_5W_vs_E10	ordinary_muscle_development	ENSGALG00010000595	MYO10	-2.298023179	0.141059515	-16.29116038	1.14045E-59	1.80467E-58	negative
PRJNA729271	stage_5W_vs_E10	ordinary_muscle_development	ENSGALG00010000596	ENSGALG00010000596	-0.179023078	0.668690702	-0.267721799	0.788913469	0.814190156	negative
PRJNA729271	stage_5W_vs_E10	ordinary_muscle_development	ENSGALG00010000597	CDH9	-4.914665726	0.974709565	-5.042184773	4.60247E-07	9.04948E-07	negative
PRJNA729271	stage_5W_vs_E10	ordinary_muscle_development	ENSGALG00010000598	ENSGALG00010000598	-3.513447403	0.611378214	-5.746765782	9.09666E-09	2.01307E-08	negative
PRJNA729271	stage_5W_vs_E10	ordinary_muscle_development	ENSGALG00010000599	RETREG1	5.099575373	0.374624523	13.61249746	3.3751E-42	3.15017E-41	positive
PRJNA729271	stage_5W_vs_E10	ordinary_muscle_development	ENSGALG00010000601	ENSGALG00010000601	-2.813403041	0.377896585	-7.444902007	9.70164E-14	2.84436E-13	negative
PRJNA729271	stage_5W_vs_E10	ordinary_muscle_development	ENSGALG00010000603	ZNF622	-0.221536129	0.109367447	-2.025613065	0.042804452	0.054454145	negative
PRJNA729271	stage_5W_vs_E10	ordinary_muscle_development	ENSGALG00010000604	ENSGALG00010000604	-0.823194996	0.137097821	-6.004435309	1.91999E-09	4.40561E-09	negative
PRJNA729271	stage_5W_vs_E10	ordinary_muscle_development	ENSGALG00010000605	MARCHF11	-1.420339169	0.6720667	-2.113390186	0.034567385	0.044534197	negative
PRJNA729271	stage_5W_vs_E10	ordinary_muscle_development	ENSGALG00010000606	UBE2QL1	0.780919131	0.154063105	5.068826389	4.00276E-07	7.90033E-07	positive
PRJNA729271	stage_5W_vs_E10	ordinary_muscle_development	ENSGALG00010000607	ENSGALG00010000607	-1.764730714	0.290344587	-6.078056187	1.21648E-09	2.82941E-09	negative
PRJNA729271	stage_5W_vs_E10	ordinary_muscle_development	ENSGALG00010000608	ENSGALG00010000608	-0.389594886	0.936476772	-0.416021942	0.677393947	0.710890911	negative
PRJNA729271	stage_5W_vs_E10	ordinary_muscle_development	ENSGALG00010000610	ENSGALG00010000610	-0.438827434	0.080430985	-5.455950024	4.87117E-08	1.02702E-07	negative
PRJNA729271	stage_5W_vs_E10	ordinary_muscle_development	ENSGALG00010000611	C7orf57	1.410725382	0.323822706	4.356474569	1.32174E-05	2.33699E-05	positive
PRJNA729271	stage_5W_vs_E10	ordinary_muscle_development	ENSGALG00010000612	ENSGALG00010000612	-0.5612408	0.413873853	-1.356067304	0.175077755	0.205685853	negative
PRJNA729271	stage_5W_vs_E10	ordinary_muscle_development	ENSGALG00010000613	ENSGALG00010000613	0.021115694	0.177089802	0.119237212	0.905087426	0.917365376	positive
PRJNA729271	stage_5W_vs_E10	ordinary_muscle_development	ENSGALG00010000614	KAT8	1.75146544	0.182499741	9.597084535	8.22377E-22	3.5647E-21	positive
PRJNA729271	stage_5W_vs_E10	ordinary_muscle_development	ENSGALG00010000615	ENSGALG00010000615	-0.938941058	0.606334866	-1.548551981	0.121489457	0.145861814	negative
PRJNA729271	stage_5W_vs_E10	ordinary_muscle_development	ENSGALG00010000616	PSENEN	0.74721171	0.505947385	1.476856551	0.139714094	0.166446601	positive

PRJNA729271	stage_5W_vs_E10	ordinary_muscle_development	ENSGALG00010000619	ENSGALG00010000619	-0.078415537	0.315770736	-0.248330603	0.803878623	0.82727871	negative
PRJNA729271	stage_5W_vs_E10	ordinary_muscle_development	ENSGALG00010000621	UPP1	-0.134660477	0.237032148	-0.568110604	0.569959868	0.608900964	negative
PRJNA729271	stage_5W_vs_E10	ordinary_muscle_development	ENSGALG00010000622	ENSGALG00010000622	-0.697163798	0.470883773	-1.480543263	0.138728324	0.165367357	negative
PRJNA729271	stage_5W_vs_E10	ordinary_muscle_development	ENSGALG00010000623	ENSGALG00010000623	0.384142588	0.244424431	1.571621081	0.116038454	0.139768772	positive
PRJNA729271	stage_5W_vs_E10	ordinary_muscle_development	ENSGALG00010000625	ENSGALG00010000625	3.600217541	0.353661661	10.17983552	2.43973E-24	1.17723E-23	positive
PRJNA729271	stage_5W_vs_E10	ordinary_muscle_development	ENSGALG00010000626	ABCA13	-1.029387127	0.69515743	-1.480797131	0.138660641	0.165300271	negative
PRJNA729271	stage_5W_vs_E10	ordinary_muscle_development	ENSGALG00010000627	ENSGALG00010000627	1.090717508	0.226941755	4.806156142	1.5386E-06	2.91034E-06	positive
PRJNA729271	stage_5W_vs_E10	ordinary_muscle_development	ENSGALG00010000630	ENSGALG00010000630	0.794288198	0.365827829	2.171207699	0.029915476	0.038813536	positive
PRJNA729271	stage_5W_vs_E10	ordinary_muscle_development	ENSGALG00010000632	ENSGALG00010000632	-0.624037673	0.433797447	-1.438546211	0.150279136	0.178373409	negative
PRJNA729271	stage_5W_vs_E10	ordinary_muscle_development	ENSGALG00010000633	VWC2	-4.37581543	0.666160145	-6.568713943	5.07517E-11	1.28835E-10	negative
PRJNA729271	stage_5W_vs_E10	ordinary_muscle_development	ENSGALG00010000634	ENSGALG00010000634	1.456236496	0.18390468	7.918430869	2.40527E-15	7.70312E-15	positive
PRJNA729271	stage_5W_vs_E10	ordinary_muscle_development	ENSGALG00010000635	ENSGALG00010000635	-0.841787677	0.136075689	-6.186172427	6.16426E-10	1.45903E-09	negative
PRJNA729271	stage_5W_vs_E10	ordinary_muscle_development	ENSGALG00010000636	ENSGALG00010000636	0.565266021	0.143352544	3.943187933	8.04056E-05	0.000133748	positive
PRJNA729271	stage_5W_vs_E10	ordinary_muscle_development	ENSGALG00010000639	SCN1B	4.646681736	0.266294955	17.44937953	3.47992E-68	6.92878E-67	positive
PRJNA729271	stage_5W_vs_E10	ordinary_muscle_development	ENSGALG00010000641	ENSGALG00010000641	-0.779893172	0.448122994	-1.740355178	0.081796671	0.100435641	negative
PRJNA729271	stage_5W_vs_E10	ordinary_muscle_development	ENSGALG00010000643	VKORC1	0.305208915	0.210929841	1.446968879	0.14790564	0.175671276	positive
PRJNA729271	stage_5W_vs_E10	ordinary_muscle_development	ENSGALG00010000645	ENSGALG00010000645	-1.869336177	0.573552736	-3.259222842	0.001117179	0.001685419	negative
PRJNA729271	stage_5W_vs_E10	ordinary_muscle_development	ENSGALG00010000646	ENSGALG00010000646	-0.347419245	0.520239606	-0.667806221	0.504257295	0.54513794	negative
PRJNA729271	stage_5W_vs_E10	ordinary_muscle_development	ENSGALG00010000649	PAPD7	-0.305809631	0.084634476	-3.613298551	0.000302326	0.000478356	negative
PRJNA729271	stage_5W_vs_E10	ordinary_muscle_development	ENSGALG00010000650	BCKDK	1.175598438	0.169371517	6.940945331	3.89485E-12	1.05151E-11	positive
PRJNA729271	stage_5W_vs_E10	ordinary_muscle_development	ENSGALG00010000651	ENSGALG00010000651	-5.373771715	0.654935595	-8.205038414	2.30517E-16	7.76877E-16	negative
PRJNA729271	stage_5W_vs_E10	ordinary_muscle_development	ENSGALG00010000652	SRD5A1	0.015410538	0.230449164	0.066871748	0.946683805	0.953724058	positive
PRJNA729271	stage_5W_vs_E10	ordinary_muscle_development	ENSGALG00010000653	NSUN2	-0.741711435	0.093034959	-7.97239491	1.55629E-15	5.0376E-15	negative
PRJNA729271	stage_5W_vs_E10	ordinary_muscle_development	ENSGALG00010000654	ENSGALG00010000654	-1.471809068	0.179496397	-8.199658004	2.41072E-16	8.11693E-16	negative
PRJNA729271	stage_5W_vs_E10	ordinary_muscle_development	ENSGALG00010000655	ENSGALG00010000655	-2.713856576	0.974024926	-2.786229083	0.005332517	0.007504353	negative
PRJNA729271	stage_5W_vs_E10	ordinary_muscle_development	ENSGALG00010000656	ENSGALG00010000656	1.19445914	0.412211422	2.897685691	0.003759271	0.005377012	positive
PRJNA729271	stage_5W_vs_E10	ordinary_muscle_development	ENSGALG00010000659	ENSGALG00010000659	1.16172166	0.556488094	2.087594814	0.0368344	0.047307694	positive
PRJNA729271	stage_5W_vs_E10	ordinary_muscle_development	ENSGALG00010000660	ENSGALG00010000660	1.899848214	0.174392506	10.89409321	1.22986E-27	6.79891E-27	positive
PRJNA729271	stage_5W_vs_E10	ordinary_muscle_development	ENSGALG00010000661	ENSGALG00010000661	-2.013217372	0.728373795	-2.763989296	0.005709941	0.00801371	negative
PRJNA729271	stage_5W_vs_E10	ordinary_muscle_development	ENSGALG00010000670	ENSGALG00010000670	-3.435344676	0.711741269	-4.826676245	1.38831E-06	2.63707E-06	negative
PRJNA729271	stage_5W_vs_E10	ordinary_muscle_development	ENSGALG00010000674	GRB10	1.094917592	0.169343326	6.465667216	1.00853E-10	2.51222E-10	positive
PRJNA729271	stage_5W_vs_E10	ordinary_muscle_development	ENSGALG00010000684	COBL	1.29786865	0.135528611	9.576344341	1.00541E-21	4.34248E-21	positive
PRJNA729271	stage_5W_vs_E10	ordinary_muscle_development	ENSGALG00010000686	ENSGALG00010000686	-0.152048807	0.133692171	-1.137305242	0.255410704	0.291578968	negative
PRJNA729271	stage_5W_vs_E10	ordinary_muscle_development	ENSGALG00010000687	IKZF1	2.466493882	0.280434578	8.795255918	1.42722E-18	5.34149E-18	positive
PRJNA729271	stage_5W_vs_E10	ordinary_muscle_development	ENSGALG00010000688	ENSGALG00010000688	1.928832174	0.277172802	6.958951809	3.42814E-12	9.2967E-12	positive
PRJNA729271	stage_5W_vs_E10	ordinary_muscle_development	ENSGALG00010000695	FIGNL1	-2.129305342	0.153747705	-13.84934713	1.28374E-43	1.25474E-42	negative
PRJNA729271	stage_5W_vs_E10	ordinary_muscle_development	ENSGALG00010000697	ENSGALG00010000697	0.996261442	0.245507412	4.057968902	4.95014E-05	8.35786E-05	positive
PRJNA729271	stage_5W_vs_E10	ordinary_muscle_development	ENSGALG00010000702	DDC	-2.247473688	0.4846587	-4.637229637	3.5311E-06	6.50112E-06	negative
PRJNA729271	stage_5W_vs_E10	ordinary_muscle_development	ENSGALG00010000704	ENSGALG00010000704	-0.592088796	0.09745252	-6.075664287	1.23475E-09	2.86869E-09	negative
PRJNA729271	stage_5W_vs_E10	ordinary_muscle_development	ENSGALG00010000705	ENSGALG00010000705	2.708424401	1.092888177	2.478226462	0.013203731	0.017833403	positive
PRJNA729271	stage_5W_vs_E10	ordinary_muscle_development	ENSGALG00010000710	ENSGALG00010000710	0.899278755	0.505798081	1.777940226	0.075413673	0.093055349	positive
PRJNA729271	stage_5W_vs_E10	ordinary_muscle_development	ENSGALG00010000711	SPRY2	-0.146046056	0.111136717	-1.314111656	0.188808632	0.220672563	negative
PRJNA729271	stage_5W_vs_E10	ordinary_muscle_development	ENSGALG00010000714	FBXL7	-1.606019628	0.098716402	-16.26902519	1.63744E-59	2.58266E-58	negative
PRJNA729271	stage_5W_vs_E10	ordinary_muscle_development	ENSGALG00010000715	PCDH20	-2.469585194	0.279142496	-8.847041322	8.98701E-19	3.39325E-18	negative
PRJNA729271	stage_5W_vs_E10	ordinary_muscle_development	ENSGALG00010000717	NDFIP2	0.897811384	0.06684992	13.43025366	4.02032E-41	3.63534E-40	positive
PRJNA729271	stage_5W_vs_E10	ordinary_muscle_development	ENSGALG00010000718	EEF1G	-0.00711691	0.15097567	-0.047139451	0.962402085	0.967541838	negative
PRJNA729271	stage_5W_vs_E10	ordinary_muscle_development	ENSGALG00010000720	RBM26	-0.230486379	0.051663187	-4.461327146	8.14536E-06	1.4605E-05	negative
PRJNA729271	stage_5W_vs_E10	ordinary_muscle_development	ENSGALG00010000721	ENSGALG00010000721	-1.689378538	0.159134453	-10.61604518	2.5097E-26	1.31805E-25	negative
PRJNA729271	stage_5W_vs_E10	ordinary_muscle_development	ENSGALG00010000722	ANKH	0.840289587	0.169867184	4.946744674	7.54649E-07	1.46356E-06	positive
PRJNA729271	stage_5W_vs_E10	ordinary_muscle_development	ENSGALG00010000724	OBI1	-1.920926931	0.149260155	-12.86965651	6.66981E-38	5.43445E-37	negative
PRJNA729271	stage_5W_vs_E10	ordinary_muscle_development	ENSGALG00010000725	ENSGALG00010000725	-2.222219535	0.706821168	-3.14396291	0.001666766	0.002468809	negative
PRJNA729271	stage_5W_vs_E10	ordinary_muscle_development	ENSGALG00010000727	ENSGALG00010000727	-0.444543985	0.150483031	-2.954113716	0.003135684	0.004524362	negative
PRJNA729271	stage_5W_vs_E10	ordinary_muscle_development	ENSGALG00010000728	OTULIN	-0.770057562	0.296978849	-2.592971065	0.009515078	0.013061942	negative
PRJNA729271	stage_5W_vs_E10	ordinary_muscle_development	ENSGALG00010000729	EDNRB	0.234750352	0.15607318	1.504104367	0.132554496	0.158451049	positive
PRJNA729271	stage_5W_vs_E10	ordinary_muscle_development	ENSGALG00010000730	INTS5	1.746159262	0.221714426	7.875713346	3.38804E-15	1.07602E-14	positive
PRJNA729271	stage_5W_vs_E10	ordinary_muscle_development	ENSGALG00010000731	ENSGALG00010000731	-1.54007111	0.453600988	-3.395211099	0.000685757	0.001053641	negative
PRJNA729271	stage_5W_vs_E10	ordinary_muscle_development	ENSGALG00010000732	ENSGALG00010000732	-0.098510213	0.145582158	-0.676664054	0.498619128	0.539912045	negative
PRJNA729271	stage_5W_vs_E10	ordinary_muscle_development	ENSGALG00010000733	ENSGALG00010000733	-1.660245955	0.475448705	-3.49195599	0.000479497	0.000745502	negative
PRJNA729271	stage_5W_vs_E10	ordinary_muscle_development	ENSGALG00010000734	SLAIN1	-1.645339812	0.356381279	-4.61679642	3.89709E-06	7.15405E-06	negative
PRJNA729271	stage_5W_vs_E10	ordinary_muscle_development	ENSGALG00010000735	ENSGALG00010000735	2.84661763	0.526449576	5.407199019	6.4018E-08	1.33748E-07	positive
PRJNA729271	stage_5W_vs_E10	ordinary_muscle_development	ENSGALG00010000737	ENSGALG00010000737	-0.983335972	0.797957337	-1.232316474	0.217830888	0.251730744	negative
PRJNA729271	stage_5W_vs_E10	ordinary_muscle_development	ENSGALG00010000741	FAM105A	0.077348831	0.193483965	0.399768689	0.689326894	0.722402888	positive
PRJNA729271	stage_5W_vs_E10	ordinary_muscle_development	ENSGALG00010000753	TRIO	-0.058732129	0.068394704	-0.858723351	0.390493164	0.431807163	negative
PRJNA729271	stage_5W_vs_E10	ordinary_muscle_development	ENSGALG00010000757	SCEL	-3.362225323	0.48925904	-6.872076032	6.32742E-12	1.68999E-11	negative
PRJNA729271	stage_5W_vs_E10	ordinary_muscle_development	ENSGALG00010000760	CDH10	-2.821175016	0.384792171	-7.331685082	2.27277E-13	6.53646E-13	negative
PRJNA729271	stage_5W_vs_E10	ordinary_muscle_development	ENSGALG00010000762	ENSGALG00010000762	0.892472439	0.296939613	3.005568808	0.002650846	0.003852418	positive
PRJNA729271	stage_5W_vs_E10	ordinary_muscle_development	ENSGALG00010000768	ENSGALG00010000768	-1.820802677	0.382366679	-4.76192822	1.91752E-06	3.60125E-06	negative
PRJNA729271	stage_5W_vs_E10	ordinary_muscle_development	ENSGALG00010000770	ENSGALG00010000770	-1.058437739	0.565934238	-1.870248638	0.061449298	0.076626598	negative
PRJNA729271	stage_5W_vs_E10	ordinary_muscle_development	ENSGALG00010000777	MYCBP2	0.339541012	0.082506675	4.115315664	3.8665E-05	6.57583E-05	positive
PRJNA729271	stage_5W_vs_E10	ordinary_muscle_development	ENSGALG00010000783	DAP	-1.093408705	0.16316331	-6.701314796	2.06553E-11	5.36748E-11	negative
PRJNA729271	stage_5W_vs_E10	ordinary_muscle_development	ENSGALG00010000784	FBXL3	0.481823377	0.095748506	5.032176439	4.84943E-07	9.52472E-07	positive
PRJNA729271	stage_5W_vs_E10	ordinary_muscle_development	ENSGALG00010000785	CLN5	0.283066394	0.093307599	3.03369068	0.00241582	0.003525351	positive
PRJNA729271	stage_5W_vs_E10	ordinary_muscle_development	ENSGALG00010000786	ANKRD33B	0.955578527	0.326544055	2.926338764	0.003429772	0.004927111	positive
PRJNA729271	stage_5W_vs_E10	ordinary_muscle_development	ENSGALG00010000787	SNRNP48	-0.289801958	0.108924834	-2.660568292	0.007800891	0.010797738	negative
PRJNA729271	stage_5W_vs_E10	ordinary_muscle_development	ENSGALG00010000788	GATD3AL1	0.726209412	0.104580126	6.944048005	3.81021E-12	1.02962E-11	positive
PRJNA729271	stage_5W_vs_E10	ordinary_muscle_development	ENSGALG00010000789	ROPN1L	0.631094944	0.231490617	2.726222563	0.006406378	0.008946956	positive
PRJNA729271	stage_5W_vs_E10	ordinary_muscle_development	ENSGALG00010000790	MARCHF6	1.187636506	0.099038611	11.99165148	3.92991E-33	2.69716E-32	positive
PRJNA729271	stage_5W_vs_E10	ordinary_muscle_development	ENSGALG00010000791	ACOD1	2.202603336	0.495667066	4.44371532	8.84186E-06	1.58163E-05	positive
PRJNA729271	stage_5W_vs_E10	ordinary_muscle_development	ENSGALG00010000792	ENSGALG00010000792	-2.213528752	1.832728758	-1.207777606	0.227132817	0.26158352	negative
PRJNA729271	stage_5W_vs_E10	ordinary_muscle_development	ENSGALG00010000793	ENSGALG00010000793	2.017311526	1.290484001	1.563220872	0.118000652	0.141943523	positive
PRJNA729271	stage_5W_vs_E10	ordinary_muscle_development	ENSGALG00010000795	ENSGALG00010000795	-0.632513633	0.231673902	-2.730189409	0.006329795	0.008845967	negative
PRJNA729271	stage_5W_vs_E10	ordinary_muscle_development	ENSGALG00010000796	ENSGALG00010000796	-0.736320302	0.202164087	-3.642191409	0.000270327	0.000429318	negative
PRJNA729271	stage_5W_vs_E10	ordinary_muscle_development	ENSGALG00010000797	ENSGALG00010000797	3.109442065	0.413361987	7.52232223	5.38118E-14	1.6056E-13	positive
PRJNA729271	stage_5W_vs_E10	ordinary_muscle_development	ENSGALG00010000798	ENSGALG00010000798	-6.776874796	0.809555336	-8.371107561	5.70792E-17	1.98361E-16	negative
PRJNA729271	stage_5W_vs_E10	ordinary_muscle_development	ENSGALG00010000799	ENSGALG00010000799	-2.430522499	0.301714183	-8.055711788	7.90176E-16	2.59484E-15	negative
PRJNA729271	stage_5W_vs_E10	ordinary_muscle_development	ENSGALG00010000800	LMO7	0.083858569	0.151791476	0.55245901	0.580633911	0.618981285	positive
PRJNA729271	stage_5W_vs_E10	ordinary_muscle_development	ENSGALG00010000802	SEMA5A	-2.756275767	0.128365807	-21.47204014	2.8426E-102	1.25621E-100	negative
PRJNA729271	stage_5W_vs_E10	ordinary_muscle_development	ENSGALG00010000803	MTRR	0.047663555	0.098300202	0.484877492	0.627763249	0.663755803	positive
PRJNA729271	stage_5W_vs_E10	ordinary_muscle_development	ENSGALG00010000804	ENSGALG00010000804	-0.717249982	0.502406587	-1.42762854	0.153398804	0.18182318	negative
PRJNA729271	stage_5W_vs_E10	ordinary_muscle_development	ENSGALG00010000805	FASTKD3	0.164216376	0.131514983	1.248651464	0.21179258	0.245261121	positive
PRJNA729271	stage_5W_vs_E10	ordinary_muscle_development	ENSGALG00010000806	CFAP90	1.514013	0.42655651	3.549384345	0.000386133	0.00060495	positive
PRJNA729271	stage_5W_vs_E10	ordinary_muscle_development	ENSGALG00010000807	ENSGALG00010000807	-0.721943548	1.001892454	-0.720579883	0.471168034	0.513193617	negative
PRJNA729271	stage_5W_vs_E10	ordinary_muscle_development	ENSGALG00010000808	ADCY2	-2.327575639	0.188996321	-12.31545477	7.47924E-35	5.4781E-34	negative
PRJNA729271	stage_5W_vs_E10	ordinary_muscle_development	ENSGALG00010000810	UCHL3	-0.885545125	0.13818715	-6.408302974	1.47148E-10	3.62371E-10	negative
PRJNA729271	stage_5W_vs_E10	ordinary_muscle_development	ENSGALG00010000811	ENSGALG00010000811	6.751158325	1.045382445	6.458075085	1.06043E-10	2.63562E-10	positive
PRJNA729271	stage_5W_vs_E10	ordinary_muscle_development	ENSGALG00010000812	ENSGALG00010000812	-0.771851275	0.096449486	-8.002647849	1.21772E-15	3.96559E-15	negative
PRJNA729271	stage_5W_vs_E10	ordinary_muscle_development	ENSGALG00010000813	ENSGALG00010000813	-1.670426036	0.466688881	-3.57931398	0.000344497	0.000542417	negative
PRJNA729271	stage_5W_vs_E10	ordinary_muscle_development	ENSGALG00010000814	CDH18	-1.67106466	0.292406923	-5.714860108	1.09794E-08	2.41718E-08	negative
PRJNA729271	stage_5W_vs_E10	ordinary_muscle_development	ENSGALG00010000815	ENSGALG00010000815	-2.129604421	0.185385873	-11.48741483	1.52611E-30	9.43323E-30	negative
PRJNA729271	stage_5W_vs_E10	ordinary_muscle_development	ENSGALG00010000816	TBC1D4	1.457676606	0.10391769	14.02722294	1.06231E-44	1.06635E-43	positive
PRJNA729271	stage_5W_vs_E10	ordinary_muscle_development	ENSGALG00010000817	ENSGALG00010000817	0.682020677	0.115551314	5.90231867	3.58428E-09	8.11657E-09	positive
PRJNA729271	stage_5W_vs_E10	ordinary_muscle_development	ENSGALG00010000818	KLF12	-1.256716751	0.079978171	-15.71324687	1.22729E-55	1.72883E-54	negative
PRJNA729271	stage_5W_vs_E10	ordinary_muscle_development	ENSGALG00010000820	KLF5	-0.047020255	0.195001443	-0.24112773	0.809456121	0.831955363	negative
PRJNA729271	stage_5W_vs_E10	ordinary_muscle_development	ENSGALG00010000821	ENSGALG00010000821	3.788912024	0.14836167	25.53834853	7.3974E-144	6.5381E-142	positive
PRJNA729271	stage_5W_vs_E10	ordinary_muscle_development	ENSGALG00010000822	BASP1	-3.397684771	0.166092264	-20.45661056	5.24609E-93	1.91198E-91	negative
PRJNA729271	stage_5W_vs_E10	ordinary_muscle_development	ENSGALG00010000823	PIBF1	-0.567204095	0.163231683	-3.474840706	0.000511157	0.000793278	negative
PRJNA729271	stage_5W_vs_E10	ordinary_muscle_development	ENSGALG00010000843	DIS3	-1.179235855	0.08568265	-13.76283133	4.26491E-43	4.08862E-42	negative
PRJNA729271	stage_5W_vs_E10	ordinary_muscle_development	ENSGALG00010000853	ENSGALG00010000853	0.545876129	0.305312717	1.787924635	0.073788168	0.091181542	positive
PRJNA729271	stage_5W_vs_E10	ordinary_muscle_development	ENSGALG00010000857	ENSGALG00010000857	-0.325355263	0.782207286	-0.415945068			negative
PRJNA729271	stage_5W_vs_E10	ordinary_muscle_development	ENSGALG00010000859	BORA	-2.586552205	0.196088734	-13.19072318	9.92287E-40	8.60754E-39	negative
PRJNA729271	stage_5W_vs_E10	ordinary_muscle_development	ENSGALG00010000864	MZT1	-0.147235422	0.129094256	-1.140526519	0.254067011	0.290182123	negative
PRJNA729271	stage_5W_vs_E10	ordinary_muscle_development	ENSGALG00010000870	DACH1	0.282599492	0.143541154	1.968769828	0.048979529	0.061821514	positive
PRJNA729271	stage_5W_vs_E10	ordinary_muscle_development	ENSGALG00010000901	PCDH9	-1.50183919	0.243795686	-6.160236929	7.26362E-10	1.70808E-09	negative
PRJNA729271	stage_5W_vs_E10	ordinary_muscle_development	ENSGALG00010001066	SLC33A1	-0.656668139	0.089178182	-7.363551528	1.79081E-13	5.17396E-13	negative
PRJNA729271	stage_5W_vs_E10	ordinary_muscle_development	ENSGALG00010001070	C3orf33	-0.298769356	0.185272432	-1.612594774	0.106832575	0.129292658	negative
PRJNA729271	stage_5W_vs_E10	ordinary_muscle_development	ENSGALG00010001082	P2RY1	3.38813046	0.17993958	18.82926732	4.34734E-79	1.14781E-77	positive
PRJNA729271	stage_5W_vs_E10	ordinary_muscle_development	ENSGALG00010001104	MBNL1	2.739230077	0.122490095	22.36287006	9.0502E-111	4.6851E-109	positive
PRJNA729271	stage_5W_vs_E10	ordinary_muscle_development	ENSGALG00010001111	SUCNR1	4.573474358	0.418770378	10.92119833	9.12834E-28	5.06567E-27	positive
PRJNA729271	stage_5W_vs_E10	ordinary_muscle_development	ENSGALG00010001112	TSC22D2	-0.174316456	0.090578191	-1.924485955	0.0542937	0.068131519	negative
PRJNA729271	stage_5W_vs_E10	ordinary_muscle_development	ENSGALG00010001113	IGSF10	2.116543309	0.296550189	7.137217885	9.52388E-13	2.6599E-12	positive
PRJNA729271	stage_5W_vs_E10	ordinary_muscle_development	ENSGALG00010001114	MED12L	1.003201126	0.101498172	9.883932935	4.88761E-23	2.23066E-22	positive
PRJNA729271	stage_5W_vs_E10	ordinary_muscle_development	ENSGALG00010001115	PFN2	-1.170839005	0.095994196	-12.19697709	3.22578E-34	2.29994E-33	negative
PRJNA729271	stage_5W_vs_E10	ordinary_muscle_development	ENSGALG00010001116	RNF13	0.380488456	0.070327635	5.410226775	6.2945E-08	1.3162E-07	positive
PRJNA729271	stage_5W_vs_E10	ordinary_muscle_development	ENSGALG00010001117	P2RY13	0.838938024	0.316091822	2.654095945	0.007952121	0.011001167	positive
PRJNA729271	stage_5W_vs_E10	ordinary_muscle_development	ENSGALG00010001118	P2RY12	0.374703467	0.317425542	1.18044523	0.237823182	0.273049269	positive
PRJNA729271	stage_5W_vs_E10	ordinary_muscle_development	ENSGALG00010001119	COMMD2	-0.751579925	0.099649586	-7.542228235	4.62008E-14	1.38136E-13	negative
PRJNA729271	stage_5W_vs_E10	ordinary_muscle_development	ENSGALG00010001120	GPR87	-4.259480184	1.147030109	-3.713485943	0.000204424	0.000328288	negative
PRJNA729271	stage_5W_vs_E10	ordinary_muscle_development	ENSGALG00010001121	WWTR1	0.646556657	0.091684333	7.05198628	1.76382E-12	4.85686E-12	positive
PRJNA729271	stage_5W_vs_E10	ordinary_muscle_development	ENSGALG00010001122	P2RY14	3.749504755	0.422675594	8.870880658	7.25697E-19	2.75222E-18	positive
PRJNA729271	stage_5W_vs_E10	ordinary_muscle_development	ENSGALG00010001123	CP	-0.727048229	0.155962219	-4.661694572	3.13616E-06	5.80719E-06	negative
PRJNA729271	stage_5W_vs_E10	ordinary_muscle_development	ENSGALG00010001124	GPR171	3.649164052	0.563250885	6.478754225	9.2483E-11	2.3077E-10	positive

PRJNA729271	stage_5W_vs_E10	ordinary_muscle_development	ENSGALG00010001125	CLRN1	1.655218975	0.266901704	6.201605128	5.58902E-10	1.32677E-09	positive
PRJNA729271	stage_5W_vs_E10	ordinary_muscle_development	ENSGALG00010001126	MINDY4B	4.641714021	1.183684108	3.921412809	8.80313E-05	0.00014593	positive
PRJNA729271	stage_5W_vs_E10	ordinary_muscle_development	ENSGALG00010001127	SIAH2	0.65200636	0.100888454	6.462645951	1.02888E-10	2.55983E-10	positive
PRJNA729271	stage_5W_vs_E10	ordinary_muscle_development	ENSGALG00010001129	TM4SF4	-0.275134181	1.001776296	-0.274646328	0.78358797	0.809674053	negative
PRJNA729271	stage_5W_vs_E10	ordinary_muscle_development	ENSGALG00010001131	HPS3	0.234180165	0.100280231	2.335257534	0.019529971	0.025847967	positive
PRJNA729271	stage_5W_vs_E10	ordinary_muscle_development	ENSGALG00010001132	SELENOT	0.097163572	0.094923215	1.023601784	0.306023402	0.344823478	positive
PRJNA729271	stage_5W_vs_E10	ordinary_muscle_development	ENSGALG00010001133	EIF2A	-0.089085986	0.073908601	-1.205353427	0.228066893	0.262492426	negative
PRJNA729271	stage_5W_vs_E10	ordinary_muscle_development	ENSGALG00010001134	ENSGALG00010001134	0.188371264	0.645193021	0.2919611	0.770316366	0.797552552	positive
PRJNA729271	stage_5W_vs_E10	ordinary_muscle_development	ENSGALG00010001135	GYG1	2.330694608	0.159206049	14.63948524	1.57254E-48	1.8019E-47	positive
PRJNA729271	stage_5W_vs_E10	ordinary_muscle_development	ENSGALG00010001136	SERP1	-1.061578454	0.127192742	-8.34621883	7.04832E-17	2.43773E-16	negative
PRJNA729271	stage_5W_vs_E10	ordinary_muscle_development	ENSGALG00010001138	SPTSSB	-6.918440206	0.994755276	-6.954916827	3.5277E-12	9.55419E-12	negative
PRJNA729271	stage_5W_vs_E10	ordinary_muscle_development	ENSGALG00010001140	NMD3	0.344645438	0.121471105	2.837262717	0.004550215	0.006451034	positive
PRJNA729271	stage_5W_vs_E10	ordinary_muscle_development	ENSGALG00010001141	MTAP	-1.179478828	0.113480477	-10.39367171	2.64953E-25	1.32843E-24	negative
PRJNA729271	stage_5W_vs_E10	ordinary_muscle_development	ENSGALG00010001142	PPM1L	2.185742707	0.146084334	14.96219779	1.29672E-50	1.59153E-49	positive
PRJNA729271	stage_5W_vs_E10	ordinary_muscle_development	ENSGALG00010001143	CDKN2A	3.73583718	0.41093855	9.090987389	9.81437E-20	3.88156E-19	positive
PRJNA729271	stage_5W_vs_E10	ordinary_muscle_development	ENSGALG00010001144	CDKN2B	4.202262749	1.413102417	2.973784984	0.002941511	0.004254785	positive
PRJNA729271	stage_5W_vs_E10	ordinary_muscle_development	ENSGALG00010001145	TRIM36	-3.403637689	0.226471666	-15.02897799	4.74266E-51	5.90592E-50	negative
PRJNA729271	stage_5W_vs_E10	ordinary_muscle_development	ENSGALG00010001146	DIPK1C	1.772672183	0.241368802	7.344247357	2.0692E-13	5.96165E-13	positive
PRJNA729271	stage_5W_vs_E10	ordinary_muscle_development	ENSGALG00010001147	PGGT1B	-0.067947464	0.112957177	-0.601532958	0.547485067	0.587227767	negative
PRJNA729271	stage_5W_vs_E10	ordinary_muscle_development	ENSGALG00010001149	CIDEA	1.718926963	0.620820089	2.768800484	0.005626308	0.007902455	positive
PRJNA729271	stage_5W_vs_E10	ordinary_muscle_development	ENSGALG00010001150	ENSGALG00010001150	-0.455884137	0.6530273	-0.69810885	0.485109124	0.52667641	negative
PRJNA729271	stage_5W_vs_E10	ordinary_muscle_development	ENSGALG00010001151	CCDC112	1.70842788	0.215492807	7.9280042	2.22696E-15	7.14945E-15	positive
PRJNA729271	stage_5W_vs_E10	ordinary_muscle_development	ENSGALG00010001152	FEM1C	2.646591349	0.197409371	13.40661459	5.53064E-41	4.97929E-40	positive
PRJNA729271	stage_5W_vs_E10	ordinary_muscle_development	ENSGALG00010001153	TSHZ1	-2.257561043	0.118711053	-19.01727759	1.22691E-80	3.36182E-79	negative
PRJNA729271	stage_5W_vs_E10	ordinary_muscle_development	ENSGALG00010001154	PTGR3	-0.859685408	0.087033035	-9.877690772	5.20178E-23	2.37255E-22	negative
PRJNA729271	stage_5W_vs_E10	ordinary_muscle_development	ENSGALG00010001155	CYB5A	-0.078241481	0.094076646	-0.831678045	0.405590696	0.447109069	negative
PRJNA729271	stage_5W_vs_E10	ordinary_muscle_development	ENSGALG00010001156	ALDH7A1	-0.506183241	0.181344282	-2.791283163	0.005249952	0.007395341	negative
PRJNA729271	stage_5W_vs_E10	ordinary_muscle_development	ENSGALG00010001157	TIMM21	1.780749521	0.091127349	19.5413292	4.88923E-85	1.46727E-83	positive
PRJNA729271	stage_5W_vs_E10	ordinary_muscle_development	ENSGALG00010001158	FRMPD1	-0.140874079	0.364774902	-0.386194549	0.699352591	0.731604778	negative
PRJNA729271	stage_5W_vs_E10	ordinary_muscle_development	ENSGALG00010001159	RTTN	-0.910853872	0.114633911	-7.945762838	1.92999E-15	6.21811E-15	negative
PRJNA729271	stage_5W_vs_E10	ordinary_muscle_development	ENSGALG00010001160	TRMT10B	-0.613421117	0.12983213	-4.72472505	2.30427E-06	4.30085E-06	negative
PRJNA729271	stage_5W_vs_E10	ordinary_muscle_development	ENSGALG00010001161	B3GALNT1	0.738758287	0.097033748	7.613415956	2.66945E-14	8.08814E-14	positive
PRJNA729271	stage_5W_vs_E10	ordinary_muscle_development	ENSGALG00010001162	ENSGALG00010001162	1.74263733	0.396202651	4.398348488	1.09078E-05	1.93998E-05	positive
PRJNA729271	stage_5W_vs_E10	ordinary_muscle_development	ENSGALG00010001163	ARL14	0.051978868	0.465580378	0.111643168	0.911106343	0.922949643	positive
PRJNA729271	stage_5W_vs_E10	ordinary_muscle_development	ENSGALG00010001164	KPNA4	1.096874085	0.074548474	14.71356868	5.27506E-49	6.18124E-48	positive
PRJNA729271	stage_5W_vs_E10	ordinary_muscle_development	ENSGALG00010001165	ENSGALG00010001165	1.706345212	0.159078438	10.7264393	7.64869E-27	4.10014E-26	positive
PRJNA729271	stage_5W_vs_E10	ordinary_muscle_development	ENSGALG00010001166	TRIM59	-0.269945889	0.096649058	-2.793052474	0.005221322	0.007357871	negative
PRJNA729271	stage_5W_vs_E10	ordinary_muscle_development	ENSGALG00010001167	SMC4	-1.475165409	0.154227637	-9.564857766	1.12356E-21	4.83594E-21	negative
PRJNA729271	stage_5W_vs_E10	ordinary_muscle_development	ENSGALG00010001168	DOK6	-2.604533364	0.183556391	-14.18928181	1.06746E-45	1.11156E-44	negative
PRJNA729271	stage_5W_vs_E10	ordinary_muscle_development	ENSGALG00010001169	ENSGALG00010001169	-0.333730995	0.104095585	-3.206005296	0.001345915	0.00201394	negative
PRJNA729271	stage_5W_vs_E10	ordinary_muscle_development	ENSGALG00010001170	DCAF10	-0.168689567	0.070410489	-2.395801678	0.016584061	0.022143143	negative
PRJNA729271	stage_5W_vs_E10	ordinary_muscle_development	ENSGALG00010001171	POLR1E	-0.307237754	0.127687073	-2.406177432	0.016120433	0.021555874	negative
PRJNA729271	stage_5W_vs_E10	ordinary_muscle_development	ENSGALG00010001172	TMEM200C	-3.373366675	0.301608974	-11.18456999	4.85252E-29	2.83275E-28	negative
PRJNA729271	stage_5W_vs_E10	ordinary_muscle_development	ENSGALG00010001173	IFT80	0.143744524	0.11078308	1.297531398	0.194448409	0.226679242	positive
PRJNA729271	stage_5W_vs_E10	ordinary_muscle_development	ENSGALG00010001174	ENSGALG00010001174	-2.087648864	0.144972565	-14.40030304	5.15171E-47	5.61037E-46	negative
PRJNA729271	stage_5W_vs_E10	ordinary_muscle_development	ENSGALG00010001177	SEH1L	-0.780666986	0.072891743	-10.70995084	9.14103E-27	4.89108E-26	negative
PRJNA729271	stage_5W_vs_E10	ordinary_muscle_development	ENSGALG00010001178	ZBTB5	-0.406144953	0.065545391	-6.196392239	5.77721E-10	1.36988E-09	negative
PRJNA729271	stage_5W_vs_E10	ordinary_muscle_development	ENSGALG00010001179	GRHPR	1.626247208	0.184347596	8.821635015	1.128E-18	4.23915E-18	positive
PRJNA729271	stage_5W_vs_E10	ordinary_muscle_development	ENSGALG00010001180	SPIRE1	0.045028524	0.161012609	0.279658372	0.779739619	0.80615733	positive
PRJNA729271	stage_5W_vs_E10	ordinary_muscle_development	ENSGALG00010001181	MFSD1	0.60852115	0.104791452	5.80697319	6.36123E-09	1.41987E-08	positive
PRJNA729271	stage_5W_vs_E10	ordinary_muscle_development	ENSGALG00010001182	ZCCHC7	-0.276026575	0.08498645	-3.247889232	0.001162645	0.00175055	negative
PRJNA729271	stage_5W_vs_E10	ordinary_muscle_development	ENSGALG00010001183	RARRES1	1.266906365	0.188524951	6.72009917	1.81601E-11	4.73948E-11	positive
PRJNA729271	stage_5W_vs_E10	ordinary_muscle_development	ENSGALG00010001185	PTPN2	-0.833367989	0.068705942	-12.12948937	7.37076E-34	5.19899E-33	negative
PRJNA729271	stage_5W_vs_E10	ordinary_muscle_development	ENSGALG00010001186	ENSGALG00010001186	-0.244689336	0.374511087	-0.653356722	0.513526333	0.554001503	negative
PRJNA729271	stage_5W_vs_E10	ordinary_muscle_development	ENSGALG00010001187	GFM1	0.904995496	0.181755125	4.979202068	6.3847E-07	1.24373E-06	positive
PRJNA729271	stage_5W_vs_E10	ordinary_muscle_development	ENSGALG00010001188	PSMG2	0.053362008	0.112429561	0.474626132	0.635053477	0.670730118	positive
PRJNA729271	stage_5W_vs_E10	ordinary_muscle_development	ENSGALG00010001190	LXN	0.196396472	0.427518077	0.459387526	0.645955905	0.68095497	positive
PRJNA729271	stage_5W_vs_E10	ordinary_muscle_development	ENSGALG00010001191	TOMM5	0.105102516	0.151273644	0.694784054	0.487190643	0.528698687	positive
PRJNA729271	stage_5W_vs_E10	ordinary_muscle_development	ENSGALG00010001192	ENSGALG00010001192	3.700861264	0.220537235	16.78111756	3.35463E-63	5.89399E-62	positive
PRJNA729271	stage_5W_vs_E10	ordinary_muscle_development	ENSGALG00010001193	PRELID3A	0.83416961	0.228184728	3.655676775	0.000256504	0.00040844	positive
PRJNA729271	stage_5W_vs_E10	ordinary_muscle_development	ENSGALG00010001194	POT1	-1.085666408	0.111981157	-9.695081158	3.16386E-22	1.39988E-21	negative
PRJNA729271	stage_5W_vs_E10	ordinary_muscle_development	ENSGALG00010001195	ZNF608	-0.784450366	0.142724223	-5.496266486	3.87916E-08	8.22775E-08	negative
PRJNA729271	stage_5W_vs_E10	ordinary_muscle_development	ENSGALG00010001196	LSMEM1	5.265207376	0.20541666	25.63184203	6.7398E-145	6.1443E-143	positive
PRJNA729271	stage_5W_vs_E10	ordinary_muscle_development	ENSGALG00010001197	CEP76	-0.739349211	0.145723101	-5.073658246	3.9024E-07	7.71169E-07	negative
PRJNA729271	stage_5W_vs_E10	ordinary_muscle_development	ENSGALG00010001198	IFRD1	0.813395821	0.176233121	4.615453768	3.92237E-06	7.19772E-06	positive
PRJNA729271	stage_5W_vs_E10	ordinary_muscle_development	ENSGALG00010001199	AFG3L2	0.543543142	0.0901352	6.030309366	1.63646E-09	3.77294E-09	positive
PRJNA729271	stage_5W_vs_E10	ordinary_muscle_development	ENSGALG00010001200	CTTNBP2	1.68030964	0.127186368	13.21139732	7.54114E-40	6.57695E-39	positive
PRJNA729271	stage_5W_vs_E10	ordinary_muscle_development	ENSGALG00010001201	TUBB6	-1.072458548	0.165641572	-6.4745736	9.50801E-11	2.37046E-10	negative
PRJNA729271	stage_5W_vs_E10	ordinary_muscle_development	ENSGALG00010001202	CFTR	3.278946661	0.247026867	13.27364388	3.29176E-40	2.89352E-39	positive
PRJNA729271	stage_5W_vs_E10	ordinary_muscle_development	ENSGALG00010001203	TMX3	-1.054370095	0.100844691	-10.45538528	1.38435E-25	7.00145E-25	negative
PRJNA729271	stage_5W_vs_E10	ordinary_muscle_development	ENSGALG00010001204	SNX2	0.122491575	0.081657813	1.500059468	0.133598999	0.159594321	positive
PRJNA729271	stage_5W_vs_E10	ordinary_muscle_development	ENSGALG00010001205	CEP192	-0.340415164	0.105119725	-3.238356684	0.001202204	0.001806547	negative
PRJNA729271	stage_5W_vs_E10	ordinary_muscle_development	ENSGALG00010001206	RSRC1	0.106707933	0.077158491	1.382970705	0.166673848	0.196737576	positive
PRJNA729271	stage_5W_vs_E10	ordinary_muscle_development	ENSGALG00010001208	MOV10L1	-2.229882025	0.769568892	-2.897572977	0.003760622	0.005378414	negative
PRJNA729271	stage_5W_vs_E10	ordinary_muscle_development	ENSGALG00010001209	CDH7	-1.425175972	0.246933855	-5.771488781	7.85742E-09	1.74576E-08	negative
PRJNA729271	stage_5W_vs_E10	ordinary_muscle_development	ENSGALG00010001210	PANX2	-4.754064952	0.361800532	-13.14001648	1.94184E-39	1.66059E-38	negative
PRJNA729271	stage_5W_vs_E10	ordinary_muscle_development	ENSGALG00010001213	PP1C	-0.559173402	0.218816048	-2.555449689	0.010605069	0.014482803	negative
PRJNA729271	stage_5W_vs_E10	ordinary_muscle_development	ENSGALG00010001214	TES	-2.53625623	0.173817109	-14.59152232	3.18021E-48	3.58733E-47	negative
PRJNA729271	stage_5W_vs_E10	ordinary_muscle_development	ENSGALG00010001215	PRDM6	-2.946524576	0.724215547	-4.068574043	4.73017E-05	7.99579E-05	negative
PRJNA729271	stage_5W_vs_E10	ordinary_muscle_development	ENSGALG00010001216	SHOX2	-5.068324958	0.571220574	-8.872798337	7.13304E-19	2.70734E-18	negative
PRJNA729271	stage_5W_vs_E10	ordinary_muscle_development	ENSGALG00010001218	ENSGALG00010001218	-0.677193351	0.112426439	-6.023435058	1.70754E-09	3.93306E-09	negative
PRJNA729271	stage_5W_vs_E10	ordinary_muscle_development	ENSGALG00010001219	TFEC	2.290991966	0.388186772	5.901777258	3.59606E-09	8.14197E-09	positive
PRJNA729271	stage_5W_vs_E10	ordinary_muscle_development	ENSGALG00010001220	PLXNB2	-0.537346015	0.082774457	-6.491688787	8.48795E-11	2.12602E-10	negative
PRJNA729271	stage_5W_vs_E10	ordinary_muscle_development	ENSGALG00010001221	GRAMD2B	-0.946232771	0.157521787	-6.006996161	1.88992E-09	4.33867E-09	negative
PRJNA729271	stage_5W_vs_E10	ordinary_muscle_development	ENSGALG00010001222	ZNF800	-0.678950776	0.102904848	-6.597850235	4.17162E-11	1.06589E-10	negative
PRJNA729271	stage_5W_vs_E10	ordinary_muscle_development	ENSGALG00010001223	GRM8	-3.527863068	0.634598755	-5.559202633	2.7101E-08	5.81624E-08	negative
PRJNA729271	stage_5W_vs_E10	ordinary_muscle_development	ENSGALG00010001224	DNAJB9	1.753489578	0.188550656	9.299832832	1.40667E-20	5.75166E-20	positive
PRJNA729271	stage_5W_vs_E10	ordinary_muscle_development	ENSGALG00010001225	SOCS6	-0.050261739	0.097745407	-0.514210757	0.607104648	0.644309699	negative
PRJNA729271	stage_5W_vs_E10	ordinary_muscle_development	ENSGALG00010001226	VEPH1	-2.47061257	0.646039948	-3.824241175	0.000131175	0.000213687	negative
PRJNA729271	stage_5W_vs_E10	ordinary_muscle_development	ENSGALG00010001227	ENSGALG00010001227	-4.312154183	0.995401478	-4.332075327	1.4771E-05	2.60059E-05	negative
PRJNA729271	stage_5W_vs_E10	ordinary_muscle_development	ENSGALG00010001228	THAP5	-0.433737029	0.112377838	-3.859631365	0.000113558	0.000186013	negative
PRJNA729271	stage_5W_vs_E10	ordinary_muscle_development	ENSGALG00010001229	ZNF407	0.407545575	0.103210235	3.94869341	7.85789E-05	0.000130784	positive
PRJNA729271	stage_5W_vs_E10	ordinary_muscle_development	ENSGALG00010001230	PTX3	-3.831773087	0.587168893	-6.525844835	6.76193E-11	1.70459E-10	negative
PRJNA729271	stage_5W_vs_E10	ordinary_muscle_development	ENSGALG00010001231	CNDP1	1.52789618	0.52021404	2.937052946	0.003313475	0.004767129	positive
PRJNA729271	stage_5W_vs_E10	ordinary_muscle_development	ENSGALG00010001232	TSPAN12	-0.2597844	0.103972963	-2.498576473	0.012469324	0.016885542	negative
PRJNA729271	stage_5W_vs_E10	ordinary_muscle_development	ENSGALG00010001233	CNDP2	-0.672486868	0.096906683	-6.939530372	3.93406E-12	1.0617E-11	negative
PRJNA729271	stage_5W_vs_E10	ordinary_muscle_development	ENSGALG00010001234	AASS	1.897543925	0.09837034	19.28979738	6.54292E-83	1.88174E-81	positive
PRJNA729271	stage_5W_vs_E10	ordinary_muscle_development	ENSGALG00010001235	CCDC102B	-1.554640735	0.312822671	-4.969718876	6.705E-07	1.30438E-06	negative
PRJNA729271	stage_5W_vs_E10	ordinary_muscle_development	ENSGALG00010001237	MELK	-3.129692282	0.277913667	-11.26138312	2.03539E-29	1.20469E-28	negative
PRJNA729271	stage_5W_vs_E10	ordinary_muscle_development	ENSGALG00010001238	GOLIM4	-0.547891425	0.122357771	-4.477782012	7.54226E-06	1.35588E-05	negative
PRJNA729271	stage_5W_vs_E10	ordinary_muscle_development	ENSGALG00010001239	ENSGALG00010001239	0.176852703	0.252352175	0.700817035	0.483417206	0.525075489	positive
PRJNA729271	stage_5W_vs_E10	ordinary_muscle_development	ENSGALG00010001242	ENSGALG00010001242	-1.687933741	0.654835647	-2.577644862	0.009947618	0.013632478	negative
PRJNA729271	stage_5W_vs_E10	ordinary_muscle_development	ENSGALG00010001244	ENSGALG00010001244	0.558370832	0.154505515	3.613921682	0.0003016	0.000477311	positive
PRJNA729271	stage_5W_vs_E10	ordinary_muscle_development	ENSGALG00010001245	SERPINI1	-0.787041264	0.224808104	-3.500947022	0.000463608	0.000721494	negative
PRJNA729271	stage_5W_vs_E10	ordinary_muscle_development	ENSGALG00010001248	PTPRZ1	-2.187705369	0.260649039	-8.393299202	4.72687E-17	1.64821E-16	negative
PRJNA729271	stage_5W_vs_E10	ordinary_muscle_development	ENSGALG00010001249	PDCD10	0.424284645	0.1390511	3.051285798	0.002278636	0.003331198	positive
PRJNA729271	stage_5W_vs_E10	ordinary_muscle_development	ENSGALG00010001250	ENSGALG00010001250	-3.552646421	0.765697512	-4.639751817	3.48828E-06	6.42718E-06	negative
PRJNA729271	stage_5W_vs_E10	ordinary_muscle_development	ENSGALG00010001251	ZNF277	-0.412008858	0.124591075	-3.306889025	0.000943383	0.001433819	negative
PRJNA729271	stage_5W_vs_E10	ordinary_muscle_development	ENSGALG00010001252	SNCAIP	-2.405507391	0.124962861	-19.2497784	1.41768E-82	4.04513E-81	negative
PRJNA729271	stage_5W_vs_E10	ordinary_muscle_development	ENSGALG00010001253	ENSGALG00010001253	-1.016069758	0.839176879	-1.210793318	0.225974627	0.260373785	negative
PRJNA729271	stage_5W_vs_E10	ordinary_muscle_development	ENSGALG00010001254	NETO1	-1.649184471	0.411068436	-4.011946252	6.02202E-05	0.000101181	negative
PRJNA729271	stage_5W_vs_E10	ordinary_muscle_development	ENSGALG00010001258	ENSGALG00010001258	-1.21323888	0.131684094	-9.213253048	3.16388E-20	1.27638E-19	negative
PRJNA729271	stage_5W_vs_E10	ordinary_muscle_development	ENSGALG00010001259	ENSGALG00010001259	0.43763414	0.174057936	2.514301564	0.01192684	0.016191772	positive
PRJNA729271	stage_5W_vs_E10	ordinary_muscle_development	ENSGALG00010001260	ENSGALG00010001260	-4.009602296	1.01371587	-3.955351212	7.64223E-05	0.000127312	negative
PRJNA729271	stage_5W_vs_E10	ordinary_muscle_development	ENSGALG00010001262	ENSGALG00010001262	0.124348609	0.146096857	0.851138153	0.394692614	0.435924218	positive
PRJNA729271	stage_5W_vs_E10	ordinary_muscle_development	ENSGALG00010001264	FAM3C	0.671612085	0.113087754	5.93885777	2.87015E-09	6.5249E-09	positive
PRJNA729271	stage_5W_vs_E10	ordinary_muscle_development	ENSGALG00010001265	OPN1LW	1.49113167	0.601657769	2.478371835	0.013198352	0.017827799	positive
PRJNA729271	stage_5W_vs_E10	ordinary_muscle_development	ENSGALG00010001267	BCHE	-0.745791861	0.251421078	-2.966306035	0.003014004	0.004354429	negative
PRJNA729271	stage_5W_vs_E10	ordinary_muscle_development	ENSGALG00010001268	CBLN2	-3.435660447	0.431466662	-7.962748345	1.68259E-15	5.44038E-15	negative
PRJNA729271	stage_5W_vs_E10	ordinary_muscle_development	ENSGALG00010001270	DRAP1	-0.069439095	0.110618443	-0.627735238	0.530177397	0.570436563	negative
PRJNA729271	stage_5W_vs_E10	ordinary_muscle_development	ENSGALG00010001271	ENSGALG00010001271	0.142176383	0.186616877	0.761862405	0.44614213	0.487998639	positive
PRJNA729271	stage_5W_vs_E10	ordinary_muscle_development	ENSGALG00010001273	ENSGALG00010001273	-2.433067169	0.620826511	-3.919077434	8.88886E-05	0.000147301	negative
PRJNA729271	stage_5W_vs_E10	ordinary_muscle_development	ENSGALG00010001274	DLGAP1	-2.354425298	0.489284154	-4.81197946	1.49443E-06	2.82975E-06	negative
PRJNA729271	stage_5W_vs_E10	ordinary_muscle_development	ENSGALG00010001276	ENSGALG00010001276	-1.919640597	1.194090577	-1.607617239			negative
PRJNA729271	stage_5W_vs_E10	ordinary_muscle_development	ENSGALG00010001277	ENSGALG00010001277	0.858062319	0.162033862	5.295574084	1.18643E-07	2.43657E-07	positive
PRJNA729271	stage_5W_vs_E10	ordinary_muscle_development	ENSGALG00010001279	LPIN2	-0.752367528	0.138119871	-5.447206997	5.11669E-08	1.07644E-07	negative
PRJNA729271	stage_5W_vs_E10	ordinary_muscle_development	ENSGALG00010001281	LRRN3	-0.266680054	0.147251028	-1.811057329	0.070131978	0.086945178	negative
PRJNA729271	stage_5W_vs_E10	ordinary_muscle_development	ENSGALG00010001283	ENSGALG00010001283	0.888233013	0.180217885	4.928661845	8.27947E-07	1.60079E-06	positive
PRJNA729271	stage_5W_vs_E10	ordinary_muscle_development	ENSGALG00010001285	ENSGALG00010001285	1.225405565	0.219892846	5.572739575	2.50764E-08	5.40013E-08	positive
PRJNA729271	stage_5W_vs_E10	ordinary_muscle_development	ENSGALG00010001286	SRFBP1	-1.050299654	0.114430438	-9.178498925	4.37142E-20	1.75086E-19	negative
PRJNA729271	stage_5W_vs_E10	ordinary_muscle_development	ENSGALG00010001287	PLPP4	-5.038212406	0.439299555	-11.46874006	1.89397E-30	1.16475E-29	negative

PRJNA729271	stage_5W_vs_E10	ordinary_muscle_development	ENSGALG00010001288	TGIF1	-0.846147494	0.1568055	-5.396159537	6.80824E-08	1.41932E-07	negative
PRJNA729271	stage_5W_vs_E10	ordinary_muscle_development	ENSGALG00010001289	ENSGALG00010001289	1.518452364	0.350892589	4.327399363	1.5088E-05	2.65446E-05	positive
PRJNA729271	stage_5W_vs_E10	ordinary_muscle_development	ENSGALG00010001291	ENSGALG00010001291	1.112565033	0.14335285	7.761024859	8.42458E-15	2.62048E-14	positive
PRJNA729271	stage_5W_vs_E10	ordinary_muscle_development	ENSGALG00010001292	LOX	0.607955066	0.142245909	4.273972246	1.92021E-05	3.34697E-05	positive
PRJNA729271	stage_5W_vs_E10	ordinary_muscle_development	ENSGALG00010001295	ENSGALG00010001295	0.980844785	0.127380939	7.700090725	1.3597E-14	4.17559E-14	positive
PRJNA729271	stage_5W_vs_E10	ordinary_muscle_development	ENSGALG00010001297	MYL12A	-0.139124059	0.222883257	-0.624201483	0.532495273	0.572590429	negative
PRJNA729271	stage_5W_vs_E10	ordinary_muscle_development	ENSGALG00010001298	DOCK4	1.065734719	0.131219245	8.121786706	4.5937E-16	1.52637E-15	positive
PRJNA729271	stage_5W_vs_E10	ordinary_muscle_development	ENSGALG00010001300	MRPL49	0.138837118	0.181639314	0.764356103	0.44465506	0.48658275	positive
PRJNA729271	stage_5W_vs_E10	ordinary_muscle_development	ENSGALG00010001301	GMPS	-1.112781343	0.077642264	-14.33215989	1.37768E-46	1.47594E-45	negative
PRJNA729271	stage_5W_vs_E10	ordinary_muscle_development	ENSGALG00010001302	ENSGALG00010001302	-1.318229706	0.414048685	-3.183755329	0.001453778	0.002168178	negative
PRJNA729271	stage_5W_vs_E10	ordinary_muscle_development	ENSGALG00010001304	SYVN1	0.312022767	0.177315064	1.75970817	0.0784573	0.09658077	positive
PRJNA729271	stage_5W_vs_E10	ordinary_muscle_development	ENSGALG00010001305	WNT16	-1.770705033	0.396164531	-4.469620302	7.83586E-06	1.40657E-05	negative
PRJNA729271	stage_5W_vs_E10	ordinary_muscle_development	ENSGALG00010001306	CEP120	-0.137776932	0.085870304	-1.604477049	0.108608939	0.131289015	negative
PRJNA729271	stage_5W_vs_E10	ordinary_muscle_development	ENSGALG00010001307	CALU	-0.666797495	0.156158654	-4.270000268	1.95473E-05	3.40509E-05	negative
PRJNA729271	stage_5W_vs_E10	ordinary_muscle_development	ENSGALG00010001308	MGMT	-0.155717316	0.139862449	-1.113360424	0.265553665	0.302419891	negative
PRJNA729271	stage_5W_vs_E10	ordinary_muscle_development	ENSGALG00010001313	EMILIN2	1.017123847	0.176493341	5.762958768	8.2652E-09	1.83355E-08	positive
PRJNA729271	stage_5W_vs_E10	ordinary_muscle_development	ENSGALG00010001320	CPED1	-0.298074031	0.198116463	-1.504539431	0.132442528	0.158330264	negative
PRJNA729271	stage_5W_vs_E10	ordinary_muscle_development	ENSGALG00010001330	ENSGALG00010001330	0.773454784	0.144552352	5.350689717	8.76196E-08	1.81332E-07	positive
PRJNA729271	stage_5W_vs_E10	ordinary_muscle_development	ENSGALG00010001333	PLCH1	-1.65632341	0.310936688	-5.326883166	9.99124E-08	2.06201E-07	negative
PRJNA729271	stage_5W_vs_E10	ordinary_muscle_development	ENSGALG00010001334	EBF3	-0.845742767	0.159214213	-5.31198033	1.0844E-07	2.23273E-07	negative
PRJNA729271	stage_5W_vs_E10	ordinary_muscle_development	ENSGALG00010001343	ENSGALG00010001343	-0.818139237	0.32984455	-2.480378219	0.013124309	0.017732742	negative
PRJNA729271	stage_5W_vs_E10	ordinary_muscle_development	ENSGALG00010001347	MYOM1	3.052045712	0.164789703	18.52085212	1.402E-76	3.54043E-75	positive
PRJNA729271	stage_5W_vs_E10	ordinary_muscle_development	ENSGALG00010001349	ING3	0.659478579	0.104937289	6.284501771	3.28907E-10	7.91024E-10	positive
PRJNA729271	stage_5W_vs_E10	ordinary_muscle_development	ENSGALG00010001351	CAPN1	1.175540591	0.155654023	7.552266029	4.2775E-14	1.2813E-13	positive
PRJNA729271	stage_5W_vs_E10	ordinary_muscle_development	ENSGALG00010001353	GLRX3	0.116785384	0.078861901	1.480884711	0.138637298	0.165286037	positive
PRJNA729271	stage_5W_vs_E10	ordinary_muscle_development	ENSGALG00010001358	TCERG1L	-1.811351316	0.497177873	-3.643266155	0.0002692	0.00042767	negative
PRJNA729271	stage_5W_vs_E10	ordinary_muscle_development	ENSGALG00010001363	HIBADH	1.892858903	0.210196609	9.005182877	2.15307E-19	8.3605E-19	positive
PRJNA729271	stage_5W_vs_E10	ordinary_muscle_development	ENSGALG00010001364	SMCHD1	0.604478437	0.183977062	3.285618498	0.001017587	0.001541751	positive
PRJNA729271	stage_5W_vs_E10	ordinary_muscle_development	ENSGALG00010001365	ENSGALG00010001365	1.626015197	0.270438955	6.012503633	1.8268E-09	4.19908E-09	positive
PRJNA729271	stage_5W_vs_E10	ordinary_muscle_development	ENSGALG00010001367	ENSGALG00010001367	0.757561667	0.432942376	1.749797916	0.08015319	0.098542875	positive
PRJNA729271	stage_5W_vs_E10	ordinary_muscle_development	ENSGALG00010001368	WIPF3	1.508500273	0.140440379	10.74121475	6.5181E-27	3.50055E-26	positive
PRJNA729271	stage_5W_vs_E10	ordinary_muscle_development	ENSGALG00010001369	PPP2R2D	0.153037845	0.12412835	1.232900016	0.21761307	0.251559382	positive
PRJNA729271	stage_5W_vs_E10	ordinary_muscle_development	ENSGALG00010001372	ENSGALG00010001372	0.495107684	0.307463388	1.610298019	0.107332812	0.129843858	positive
PRJNA729271	stage_5W_vs_E10	ordinary_muscle_development	ENSGALG00010001373	IMPA2	1.142264455	0.139794324	8.171036012	3.05752E-16	1.02471E-15	positive
PRJNA729271	stage_5W_vs_E10	ordinary_muscle_development	ENSGALG00010001378	POLA2	-2.284577518	0.140038265	-16.31395186	7.85428E-60	1.24833E-58	negative
PRJNA729271	stage_5W_vs_E10	ordinary_muscle_development	ENSGALG00010001380	SCRN1	-4.294038317	0.189033611	-22.71573976	3.1315E-114	1.7129E-112	negative
PRJNA729271	stage_5W_vs_E10	ordinary_muscle_development	ENSGALG00010001381	MDFIC	-0.182454954	0.130605943	-1.396988137	0.162417139	0.191916233	negative
PRJNA729271	stage_5W_vs_E10	ordinary_muscle_development	ENSGALG00010001382	BNIP3	1.910499539	0.193041538	9.896831315	4.29673E-23	1.96731E-22	positive
PRJNA729271	stage_5W_vs_E10	ordinary_muscle_development	ENSGALG00010001385	MME	2.028421522	0.253674672	7.996153139	1.28367E-15	4.17566E-15	positive
PRJNA729271	stage_5W_vs_E10	ordinary_muscle_development	ENSGALG00010001386	FKBP14	-1.598797367	0.185568374	-8.615678041	6.95289E-18	2.52904E-17	negative
PRJNA729271	stage_5W_vs_E10	ordinary_muscle_development	ENSGALG00010001388	MPPE1	0.670421135	0.165428371	4.052636982	5.06436E-05	8.53977E-05	positive
PRJNA729271	stage_5W_vs_E10	ordinary_muscle_development	ENSGALG00010001395	GNAL	-0.173919772	0.107449917	-1.618612429	0.105530679	0.127813101	negative
PRJNA729271	stage_5W_vs_E10	ordinary_muscle_development	ENSGALG00010001397	KLHL7	-0.202839819	0.064453426	-3.147075841	0.001649122	0.002444174	negative
PRJNA729271	stage_5W_vs_E10	ordinary_muscle_development	ENSGALG00010001398	WDR11	-0.507862126	0.097314096	-5.218793022	1.80093E-07	3.64893E-07	negative
PRJNA729271	stage_5W_vs_E10	ordinary_muscle_development	ENSGALG00010001400	SNX24	1.418973386	0.159142463	8.916371884	4.81814E-19	1.84126E-18	positive
PRJNA729271	stage_5W_vs_E10	ordinary_muscle_development	ENSGALG00010001402	NUP42	0.019055345	0.132568879	0.143739204	0.885706412	0.89967165	positive
PRJNA729271	stage_5W_vs_E10	ordinary_muscle_development	ENSGALG00010001406	EML3	0.914758273	0.159804701	5.724226301	1.03906E-08	2.29207E-08	positive
PRJNA729271	stage_5W_vs_E10	ordinary_muscle_development	ENSGALG00010001407	FOXP2	-2.496648913	0.156115774	-15.99229116	1.44613E-57	2.161E-56	negative
PRJNA729271	stage_5W_vs_E10	ordinary_muscle_development	ENSGALG00010001409	GPNMB	-2.403954017	0.955922601	-2.514799854	0.011909998	0.016173451	negative
PRJNA729271	stage_5W_vs_E10	ordinary_muscle_development	ENSGALG00010001411	FGFR2	-5.376468162	0.37295171	-14.41599011	4.1051E-47	4.48743E-46	negative
PRJNA729271	stage_5W_vs_E10	ordinary_muscle_development	ENSGALG00010001412	ENSGALG00010001412	-2.205418598	0.242485769	-9.095043412	9.45487E-20	3.74244E-19	negative
PRJNA729271	stage_5W_vs_E10	ordinary_muscle_development	ENSGALG00010001414	CCDC126	1.444396977	0.171238031	8.435024429	3.31134E-17	1.16274E-16	positive
PRJNA729271	stage_5W_vs_E10	ordinary_muscle_development	ENSGALG00010001415	MC5R	-0.459108705	0.2678801	-1.713858947	0.086554634	0.106017359	negative
PRJNA729271	stage_5W_vs_E10	ordinary_muscle_development	ENSGALG00010001416	AP4M1	0.15177043	0.094106294	1.612755352	0.10679767	0.129261207	positive
PRJNA729271	stage_5W_vs_E10	ordinary_muscle_development	ENSGALG00010001418	RNMT	-1.222175185	0.068623616	-17.80983353	5.92859E-71	1.28645E-69	negative
PRJNA729271	stage_5W_vs_E10	ordinary_muscle_development	ENSGALG00010001419	KCND2	-3.737711207	0.32271997	-11.58190244	5.09037E-31	3.21924E-30	negative
PRJNA729271	stage_5W_vs_E10	ordinary_muscle_development	ENSGALG00010001420	FAM221A	-3.728709292	0.585346753	-6.370086233	1.88922E-10	4.62181E-10	negative
PRJNA729271	stage_5W_vs_E10	ordinary_muscle_development	ENSGALG00010001422	AP1S1	-1.660833225	0.142675336	-11.64064706	2.56066E-31	1.6351E-30	negative
PRJNA729271	stage_5W_vs_E10	ordinary_muscle_development	ENSGALG00010001423	FAM210A	1.575426807	0.136448226	11.54596764	7.73658E-31	4.84414E-30	positive
PRJNA729271	stage_5W_vs_E10	ordinary_muscle_development	ENSGALG00010001426	ATE1	0.58481899	0.09090536	6.433272945	1.24885E-10	3.08804E-10	positive
PRJNA729271	stage_5W_vs_E10	ordinary_muscle_development	ENSGALG00010001427	ASB11	8.604187173	0.46290302	18.58745092	4.06021E-77	1.0398E-75	positive
PRJNA729271	stage_5W_vs_E10	ordinary_muscle_development	ENSGALG00010001432	NSMCE4A	0.01696461	0.114751283	0.14783809	0.882470548	0.89676182	positive
PRJNA729271	stage_5W_vs_E10	ordinary_muscle_development	ENSGALG00010001436	ENSGALG00010001436	0.081238936	1.252755957	0.064848174	0.948294885	0.955147964	positive
PRJNA729271	stage_5W_vs_E10	ordinary_muscle_development	ENSGALG00010001440	CDCA7L	-1.428828091	0.150710879	-9.480590228	2.52849E-21	1.07322E-20	negative
PRJNA729271	stage_5W_vs_E10	ordinary_muscle_development	ENSGALG00010001441	HMGCLL1	-0.598974711	0.30115625	-1.988916752	0.046710392	0.059117012	negative
PRJNA729271	stage_5W_vs_E10	ordinary_muscle_development	ENSGALG00010001442	LDLRAD4	0.610243072	0.113870203	5.35911112	8.36324E-08	1.73402E-07	positive
PRJNA729271	stage_5W_vs_E10	ordinary_muscle_development	ENSGALG00010001443	ANKRD26	-0.198724834	0.145766559	-1.363308806	0.172785208	0.20327259	negative
PRJNA729271	stage_5W_vs_E10	ordinary_muscle_development	ENSGALG00010001448	GPR37	-4.248017928	0.341184237	-12.45080362	1.38444E-35	1.04409E-34	negative
PRJNA729271	stage_5W_vs_E10	ordinary_muscle_development	ENSGALG00010001450	GPR149	0.613052299	0.411398974	1.490164872	0.13618089	0.162544632	positive
PRJNA729271	stage_5W_vs_E10	ordinary_muscle_development	ENSGALG00010001453	TAX1BP1	0.964417271	0.073520981	13.11757891	2.61145E-39	2.21882E-38	positive
PRJNA729271	stage_5W_vs_E10	ordinary_muscle_development	ENSGALG00010001454	VEGFD	2.412468903	0.164645853	14.6524729	1.299E-48	1.49319E-47	positive
PRJNA729271	stage_5W_vs_E10	ordinary_muscle_development	ENSGALG00010001456	LSM8	-1.197196433	0.117921798	-10.15246084	3.23111E-24	1.5534E-23	negative
PRJNA729271	stage_5W_vs_E10	ordinary_muscle_development	ENSGALG00010001457	NDC80	-2.881725565	0.228226647	-12.62659556	1.50656E-36	1.17596E-35	negative
PRJNA729271	stage_5W_vs_E10	ordinary_muscle_development	ENSGALG00010001458	JAZF1	-0.763206816	0.158736298	-4.808016974	1.52435E-06	2.88565E-06	negative
PRJNA729271	stage_5W_vs_E10	ordinary_muscle_development	ENSGALG00010001460	MALSU1	0.018349233	0.106658923	0.172036548	0.863408796	0.88016812	positive
PRJNA729271	stage_5W_vs_E10	ordinary_muscle_development	ENSGALG00010001461	ASB9	-2.871160353	0.232216975	-12.3641278	4.08623E-35	3.02811E-34	negative
PRJNA729271	stage_5W_vs_E10	ordinary_muscle_development	ENSGALG00010001462	DHX36	-0.497456281	0.109216791	-4.554760099	5.24454E-06	9.52865E-06	negative
PRJNA729271	stage_5W_vs_E10	ordinary_muscle_development	ENSGALG00010001463	DSEL	0.042182787	0.158535921	0.266077156	0.790179788	0.81526486	positive
PRJNA729271	stage_5W_vs_E10	ordinary_muscle_development	ENSGALG00010001465	TACC2	1.342524928	0.157396116	8.529593748	1.46863E-17	5.25753E-17	positive
PRJNA729271	stage_5W_vs_E10	ordinary_muscle_development	ENSGALG00010001466	PRKX	0.124749641	0.130293591	0.957450325	0.338340008	0.378150853	positive
PRJNA729271	stage_5W_vs_E10	ordinary_muscle_development	ENSGALG00010001468	ANOS1	-0.666061123	0.15323143	-4.346765705	1.3816E-05	2.43866E-05	negative
PRJNA729271	stage_5W_vs_E10	ordinary_muscle_development	ENSGALG00010001469	ARHGEF26	-0.717757993	0.133251046	-5.386509261	7.18392E-08	1.49592E-07	negative
PRJNA729271	stage_5W_vs_E10	ordinary_muscle_development	ENSGALG00010001470	GPR85	-3.16048663	0.338261787	-9.343315595	9.33637E-21	3.84899E-20	negative
PRJNA729271	stage_5W_vs_E10	ordinary_muscle_development	ENSGALG00010001471	BMP5	-2.241985193	0.370721354	-6.047628943	1.46993E-09	3.39873E-09	negative
PRJNA729271	stage_5W_vs_E10	ordinary_muscle_development	ENSGALG00010001472	ENSGALG00010001472	0.647394848	0.190298945	3.401988638	0.000668974	0.001029055	positive
PRJNA729271	stage_5W_vs_E10	ordinary_muscle_development	ENSGALG00010001475	BMT2	-0.890999843	0.138179059	-6.448153932	1.13221E-10	2.8068E-10	negative
PRJNA729271	stage_5W_vs_E10	ordinary_muscle_development	ENSGALG00010001476	IGF2BP3	-2.681787371	0.195174312	-13.74047306	5.8095E-43	5.54734E-42	negative
PRJNA729271	stage_5W_vs_E10	ordinary_muscle_development	ENSGALG00010001477	NLGN4	-1.03606616	0.222472808	-4.657046264	3.20778E-06	5.93375E-06	negative
PRJNA729271	stage_5W_vs_E10	ordinary_muscle_development	ENSGALG00010001479	TMEM168	0.26340549	0.154803219	1.70155047	0.088839669	0.108577656	positive
PRJNA729271	stage_5W_vs_E10	ordinary_muscle_development	ENSGALG00010001480	JAKMIP3	-1.807239801	0.328096502	-5.508256847	3.62404E-08	7.69791E-08	negative
PRJNA729271	stage_5W_vs_E10	ordinary_muscle_development	ENSGALG00010001482	TRA2A	-0.965691238	0.066188394	-14.59003871	3.25013E-48	3.66335E-47	negative
PRJNA729271	stage_5W_vs_E10	ordinary_muscle_development	ENSGALG00010001483	ENSGALG00010001483	-2.351231923	0.340655056	-6.902090193	5.12429E-12	1.37498E-11	negative
PRJNA729271	stage_5W_vs_E10	ordinary_muscle_development	ENSGALG00010001486	WNT2	0.970032349	0.266786638	3.63598551	0.00027692	0.000439452	positive
PRJNA729271	stage_5W_vs_E10	ordinary_muscle_development	ENSGALG00010001487	CDH19	-0.80329263	0.24342477	-3.299962579	0.000966977	0.001468448	negative
PRJNA729271	stage_5W_vs_E10	ordinary_muscle_development	ENSGALG00010001488	GRPR	-2.687680217	0.403265302	-6.664794124	2.65037E-11	6.8443E-11	negative
PRJNA729271	stage_5W_vs_E10	ordinary_muscle_development	ENSGALG00010001491	PHF3	0.197782874	0.088398653	2.237396912	0.02526041	0.033013222	positive
PRJNA729271	stage_5W_vs_E10	ordinary_muscle_development	ENSGALG00010001492	ENSGALG00010001492	2.167704464	0.278269027	7.789959544	6.70307E-15	2.09218E-14	positive
PRJNA729271	stage_5W_vs_E10	ordinary_muscle_development	ENSGALG00010001495	CCNL1	0.778781273	0.10898648	7.145668649	8.95589E-13	2.50609E-12	positive
PRJNA729271	stage_5W_vs_E10	ordinary_muscle_development	ENSGALG00010001496	PTP4A1	1.115818919	0.190209996	5.866247542	4.45768E-09	1.00364E-08	positive
PRJNA729271	stage_5W_vs_E10	ordinary_muscle_development	ENSGALG00010001497	LEKR1	-1.001212284	0.294002215	-3.405458303	0.000660531	0.001016498	negative
PRJNA729271	stage_5W_vs_E10	ordinary_muscle_development	ENSGALG00010001499	TIPARP	1.682020841	0.18642947	9.022290511	1.84197E-19	7.18111E-19	positive
PRJNA729271	stage_5W_vs_E10	ordinary_muscle_development	ENSGALG00010001501	CAPZA2	1.779146943	0.106234905	16.74729175	5.92635E-63	1.03248E-61	positive
PRJNA729271	stage_5W_vs_E10	ordinary_muscle_development	ENSGALG00010001502	LGSN	1.767976205	0.932436075	1.896083016	0.057949068	0.072480088	positive
PRJNA729271	stage_5W_vs_E10	ordinary_muscle_development	ENSGALG00010001504	RBMS3	-0.338032479	0.099421659	-3.399988333	0.000673887	0.001036282	negative
PRJNA729271	stage_5W_vs_E10	ordinary_muscle_development	ENSGALG00010001505	SSR3	-0.503118858	0.097239547	-5.174014848	2.29117E-07	4.60617E-07	negative
PRJNA729271	stage_5W_vs_E10	ordinary_muscle_development	ENSGALG00010001506	DPYSL4	-3.041767123	0.308110597	-9.872322322	5.48791E-23	2.50149E-22	negative
PRJNA729271	stage_5W_vs_E10	ordinary_muscle_development	ENSGALG00010001507	METTL4	-0.032683887	0.097214397	-0.336204184	0.736716898	0.765883342	negative
PRJNA729271	stage_5W_vs_E10	ordinary_muscle_development	ENSGALG00010001508	CD109	-2.130798475	0.292550227	-7.283530405	3.25196E-13	9.27896E-13	negative
PRJNA729271	stage_5W_vs_E10	ordinary_muscle_development	ENSGALG00010001510	AP1S2	0.354010581	0.098011475	3.611929923	0.000303927	0.000480836	positive
PRJNA729271	stage_5W_vs_E10	ordinary_muscle_development	ENSGALG00010001512	PLCL2	2.27668521	0.172980754	13.16149428	1.46166E-39	1.25812E-38	positive
PRJNA729271	stage_5W_vs_E10	ordinary_muscle_development	ENSGALG00010001513	ZRSR2	-0.01307841	0.093525692	-0.139837622	0.888788287	0.902508124	negative
PRJNA729271	stage_5W_vs_E10	ordinary_muscle_development	ENSGALG00010001514	MET	0.981052778	0.151823246	6.461808743	1.03459E-10	2.5736E-10	positive
PRJNA729271	stage_5W_vs_E10	ordinary_muscle_development	ENSGALG00010001516	CLTRN	-0.980192333	0.545839329	-1.795752486	0.07253389	0.089731052	negative
PRJNA729271	stage_5W_vs_E10	ordinary_muscle_development	ENSGALG00010001517	CAV2	2.690836774	0.164028316	16.40470894	1.76973E-60	2.87581E-59	positive
PRJNA729271	stage_5W_vs_E10	ordinary_muscle_development	ENSGALG00010001518	SLC17A5	-0.974895697	0.16685709	-5.84269866	5.13619E-09	1.15353E-08	negative
PRJNA729271	stage_5W_vs_E10	ordinary_muscle_development	ENSGALG00010001520	KCNAB1	-2.45406365	0.216828044	-11.31801776	1.06861E-29	6.39269E-29	negative
PRJNA729271	stage_5W_vs_E10	ordinary_muscle_development	ENSGALG00010001521	TBC1D5	-0.026727801	0.074426233	-0.359118016	0.719506804	0.75003604	negative
PRJNA729271	stage_5W_vs_E10	ordinary_muscle_development	ENSGALG00010001524	ACE2	3.024115977	1.48347925	2.038529341	0.041497024	0.052886224	positive
PRJNA729271	stage_5W_vs_E10	ordinary_muscle_development	ENSGALG00010001525	EEF1A1	-1.386625574	0.176710086	-7.846895486	4.26462E-15	1.34616E-14	negative
PRJNA729271	stage_5W_vs_E10	ordinary_muscle_development	ENSGALG00010001526	PPP1R3A	6.812163003	0.149174139	45.66584435	0	0	positive
PRJNA729271	stage_5W_vs_E10	ordinary_muscle_development	ENSGALG00010001527	STK32C	-0.537872146	0.233527551	-2.303249206	0.021264826	0.02799579	negative
PRJNA729271	stage_5W_vs_E10	ordinary_muscle_development	ENSGALG00010001528	NR1D2	3.953644008	0.180087722	21.95398981	7.9325E-107	3.7211E-105	positive
PRJNA729271	stage_5W_vs_E10	ordinary_muscle_development	ENSGALG00010001531	ENSGALG00010001531	21.18980889	5.49263209	3.857860592			positive
PRJNA729271	stage_5W_vs_E10	ordinary_muscle_development	ENSGALG00010001532	ENSGALG00010001532	-1.455245462	0.27735324	-5.246902698	1.54678E-07	3.15205E-07	negative
PRJNA729271	stage_5W_vs_E10	ordinary_muscle_development	ENSGALG00010001533	ENSGALG00010001533	0.609326598	0.150351943	4.052668594	5.06367E-05	8.53961E-05	positive
PRJNA729271	stage_5W_vs_E10	ordinary_muscle_development	ENSGALG00010001534	SLITRK3	-3.357724625	0.392814625	-8.54786052	1.25391E-17	4.50556E-17	negative
PRJNA729271	stage_5W_vs_E10	ordinary_muscle_development	ENSGALG00010001535	RAP2B	-1.025956888	0.106831567	-9.60349939	7.72753E-22	3.35662E-21	negative
PRJNA729271	stage_5W_vs_E10	ordinary_muscle_development	ENSGALG00010001540	TRABD	-0.813744801	0.131165667	-6.203946656	5.50645E-10	1.30781E-09	negative
PRJNA729271	stage_5W_vs_E10	ordinary_muscle_development	ENSGALG00010001542	MTO1	0.939117814	0.127678121	7.355354299	1.90421E-13	5.49723E-13	positive
PRJNA729271	stage_5W_vs_E10	ordinary_muscle_development	ENSGALG00010001545	SELENOO	2.358857	0.128606617	18.34164561	3.84966E-75	9.40991E-74	positive
PRJNA729271	stage_5W_vs_E10	ordinary_muscle_development	ENSGALG00010001546	CGAS	-0.815897962	0.167487139	-4.871406632	1.10807E-06	2.11976E-06	negative

PRJNA729271	stage_5W_vs_E10	ordinary_muscle_development	ENSGALG00010001547	PWWP2B	1.035186925	0.109824057	9.425866734	4.26568E-21	1.79168E-20	positive
PRJNA729271	stage_5W_vs_E10	ordinary_muscle_development	ENSGALG00010001548	AKAP17A	-0.000550803	0.096083463	-0.005732546	0.995426115	0.996067919	negative
PRJNA729271	stage_5W_vs_E10	ordinary_muscle_development	ENSGALG00010001549	ENSGALG00010001549	-1.755356814	0.533305082	-3.291468381	0.000996658	0.001511305	negative
PRJNA729271	stage_5W_vs_E10	ordinary_muscle_development	ENSGALG00010001551	FHIP1B	2.404618489	0.159659579	15.06090966	2.92731E-51	3.65788E-50	positive
PRJNA729271	stage_5W_vs_E10	ordinary_muscle_development	ENSGALG00010001552	TUBGCP6	-0.010394942	0.109042246	-0.095329493	0.924053117	0.934392218	negative
PRJNA729271	stage_5W_vs_E10	ordinary_muscle_development	ENSGALG00010001553	CNGA4	0.312262192	0.408658414	0.76411541	0.44479847	0.486702938	positive
PRJNA729271	stage_5W_vs_E10	ordinary_muscle_development	ENSGALG00010001554	ENSGALG00010001554	0.599392599	0.183107126	3.27345315	0.00106242	0.001606989	positive
PRJNA729271	stage_5W_vs_E10	ordinary_muscle_development	ENSGALG00010001556	EPB41L3	-1.529348577	0.11787452	-12.97437797	1.70979E-38	1.41538E-37	negative
PRJNA729271	stage_5W_vs_E10	ordinary_muscle_development	ENSGALG00010001557	THRB	-2.948030292	0.360978727	-8.166770154	3.16756E-16	1.06085E-15	negative
PRJNA729271	stage_5W_vs_E10	ordinary_muscle_development	ENSGALG00010001558	CCKBR	-1.234659056	0.606002345	-2.0373833	0.041611646	0.053025126	negative
PRJNA729271	stage_5W_vs_E10	ordinary_muscle_development	ENSGALG00010001559	HDAC10	-1.955185211	0.135053783	-14.47708585	1.69117E-47	1.86272E-46	negative
PRJNA729271	stage_5W_vs_E10	ordinary_muscle_development	ENSGALG00010001567	INPP5A	-0.575907273	0.08366443	-6.883537876	5.83841E-12	1.56313E-11	negative
PRJNA729271	stage_5W_vs_E10	ordinary_muscle_development	ENSGALG00010001570	SMPD1	2.098253845	0.162221229	12.93452071	2.8741E-38	2.36302E-37	positive
PRJNA729271	stage_5W_vs_E10	ordinary_muscle_development	ENSGALG00010001585	ENSGALG00010001585	0.246924621	0.137368044	1.79754049	0.072249856	0.089394935	positive
PRJNA729271	stage_5W_vs_E10	ordinary_muscle_development	ENSGALG00010001586	ZBTB14	0.024584447	0.096156919	0.255670079	0.798205601	0.822257848	positive
PRJNA729271	stage_5W_vs_E10	ordinary_muscle_development	ENSGALG00010001588	ENSGALG00010001588	1.415300221	0.580451256	2.43827575	0.014757512	0.019821778	positive
PRJNA729271	stage_5W_vs_E10	ordinary_muscle_development	ENSGALG00010001595	AKAIN1	1.047365855	0.265731692	3.94144126	8.09935E-05	0.000134695	positive
PRJNA729271	stage_5W_vs_E10	ordinary_muscle_development	ENSGALG00010001599	TBLX1	-0.299552482	0.075199609	-3.983431368	6.79273E-05	0.00011367	negative
PRJNA729271	stage_5W_vs_E10	ordinary_muscle_development	ENSGALG00010001604	ENSGALG00010001604	5.659795309	0.421874134	13.41583865	4.88377E-41	4.40512E-40	positive
PRJNA729271	stage_5W_vs_E10	ordinary_muscle_development	ENSGALG00010001605	APBB1	-0.573464632	0.115077248	-4.983301574	6.25084E-07	1.21799E-06	negative
PRJNA729271	stage_5W_vs_E10	ordinary_muscle_development	ENSGALG00010001606	PIGA	-0.043486846	0.108957185	-0.399118666	0.689805769	0.722817511	negative
PRJNA729271	stage_5W_vs_E10	ordinary_muscle_development	ENSGALG00010001608	ENSGALG00010001608	-0.091293957	0.487980184	-0.187085377	0.851593693	0.869591446	negative
PRJNA729271	stage_5W_vs_E10	ordinary_muscle_development	ENSGALG00010001609	ENSGALG00010001609	2.42404358	0.452044196	5.36240394	8.21216E-08	1.70342E-07	positive
PRJNA729271	stage_5W_vs_E10	ordinary_muscle_development	ENSGALG00010001611	MAPK12	-1.270518505	0.163849061	-7.754200692	8.89016E-15	2.76233E-14	negative
PRJNA729271	stage_5W_vs_E10	ordinary_muscle_development	ENSGALG00010001612	HPX	5.347309495	0.926294964	5.772793446	7.7968E-09	1.73282E-08	positive
PRJNA729271	stage_5W_vs_E10	ordinary_muscle_development	ENSGALG00010001616	OPNVA	-1.720734872	0.690694679	-2.491310452	0.012727286	0.017215566	negative
PRJNA729271	stage_5W_vs_E10	ordinary_muscle_development	ENSGALG00010001617	TRIM3	-0.553997711	0.094294166	-5.875206621	4.22316E-09	9.5187E-09	negative
PRJNA729271	stage_5W_vs_E10	ordinary_muscle_development	ENSGALG00010001620	MOSPD2	0.033998965	0.144016072	0.236077575	0.813372475	0.835151521	positive
PRJNA729271	stage_5W_vs_E10	ordinary_muscle_development	ENSGALG00010001621	DLGAP2	-1.31155487	0.262931092	-4.988207585	6.09421E-07	1.18906E-06	negative
PRJNA729271	stage_5W_vs_E10	ordinary_muscle_development	ENSGALG00010001627	ARFIP2	-0.27819998	0.120869186	-2.301661731	0.021354256	0.028108422	negative
PRJNA729271	stage_5W_vs_E10	ordinary_muscle_development	ENSGALG00010001628	MAPK11	-2.225295889	0.214313245	-10.38338011	2.95137E-25	1.47619E-24	negative
PRJNA729271	stage_5W_vs_E10	ordinary_muscle_development	ENSGALG00010001632	FANCB	-1.268743245	0.278698543	-4.552385641	5.3041E-06	9.62962E-06	negative
PRJNA729271	stage_5W_vs_E10	ordinary_muscle_development	ENSGALG00010001633	B3GAT2	-2.728578903	0.424254723	-6.431463828	1.26381E-10	3.12396E-10	negative
PRJNA729271	stage_5W_vs_E10	ordinary_muscle_development	ENSGALG00010001635	TIMM10B	-0.259303015	0.152483011	-1.700537081	0.089029948	0.108782693	negative
PRJNA729271	stage_5W_vs_E10	ordinary_muscle_development	ENSGALG00010001639	ENSGALG00010001639	0.0074876	0.159129529	0.047053495	0.962470592	0.967543605	positive
PRJNA729271	stage_5W_vs_E10	ordinary_muscle_development	ENSGALG00010001642	ENSGALG00010001642	1.125541206	0.120892174	9.310290034	1.27484E-20	5.2333E-20	positive
PRJNA729271	stage_5W_vs_E10	ordinary_muscle_development	ENSGALG00010001647	ARL8BL	-0.827769737	0.156125217	-5.301960525	1.14566E-07	2.35483E-07	negative
PRJNA729271	stage_5W_vs_E10	ordinary_muscle_development	ENSGALG00010001649	UBE2E2	-0.85861438	0.234948777	-3.654474775	0.000257709	0.000410313	negative
PRJNA729271	stage_5W_vs_E10	ordinary_muscle_development	ENSGALG00010001654	RPS11	0.264497461	0.169421523	1.561179806	0.118481332	0.142474441	positive
PRJNA729271	stage_5W_vs_E10	ordinary_muscle_development	ENSGALG00010001657	SMAP1	-1.202689056	0.099094688	-12.13676612	6.74391E-34	4.77076E-33	negative
PRJNA729271	stage_5W_vs_E10	ordinary_muscle_development	ENSGALG00010001661	TCTN3	-0.488534463	0.105470889	-4.631936533	3.62261E-06	6.66647E-06	negative
PRJNA729271	stage_5W_vs_E10	ordinary_muscle_development	ENSGALG00010001664	SPAM1	0.595589306	2.0670095	0.288140575	0.773239135	0.800121449	positive
PRJNA729271	stage_5W_vs_E10	ordinary_muscle_development	ENSGALG00010001666	PIEZO2	-3.817584381	0.184971707	-20.63874765	1.23214E-94	4.651E-93	negative
PRJNA729271	stage_5W_vs_E10	ordinary_muscle_development	ENSGALG00010001668	UBE2E1	-0.2480607	0.086458466	-2.869131396	0.004116008	0.005866424	negative
PRJNA729271	stage_5W_vs_E10	ordinary_muscle_development	ENSGALG00010001670	SHOX	-1.36734869	0.164018673	-8.336542807	7.64941E-17	2.64122E-16	negative
PRJNA729271	stage_5W_vs_E10	ordinary_muscle_development	ENSGALG00010001672	ENSGALG00010001672	2.482870874	0.541343972	4.586493991	4.50751E-06	8.23084E-06	positive
PRJNA729271	stage_5W_vs_E10	ordinary_muscle_development	ENSGALG00010001674	ENSGALG00010001674	0.102626693	0.105771306	0.9702697	0.331912077	0.371596937	positive
PRJNA729271	stage_5W_vs_E10	ordinary_muscle_development	ENSGALG00010001677	SDHAF4	0.521139066	0.106882224	4.875825423	1.08354E-06	2.07422E-06	positive
PRJNA729271	stage_5W_vs_E10	ordinary_muscle_development	ENSGALG00010001678	SYAP1	0.738573567	0.058517817	12.62134527	1.61046E-36	1.25571E-35	positive
PRJNA729271	stage_5W_vs_E10	ordinary_muscle_development	ENSGALG00010001681	NAPG	0.146570261	0.09181323	1.596395874	0.110400437	0.133310091	positive
PRJNA729271	stage_5W_vs_E10	ordinary_muscle_development	ENSGALG00010001686	WASL	0.366197337	0.086087104	4.253800169	2.10173E-05	3.65064E-05	positive
PRJNA729271	stage_5W_vs_E10	ordinary_muscle_development	ENSGALG00010001688	ENSGALG00010001688	-0.722726714	0.166829429	-4.33212965	1.47674E-05	2.60026E-05	negative
PRJNA729271	stage_5W_vs_E10	ordinary_muscle_development	ENSGALG00010001689	ILK	0.262100041	0.067539979	3.880665104	0.000104171	0.000171353	positive
PRJNA729271	stage_5W_vs_E10	ordinary_muscle_development	ENSGALG00010001691	CTPS2	0.698814232	0.13522185	5.167909137	2.36727E-07	4.75126E-07	positive
PRJNA729271	stage_5W_vs_E10	ordinary_muscle_development	ENSGALG00010001692	ALDH18A1	-1.38827506	0.255737423	-5.428517446	5.68241E-08	1.19216E-07	negative
PRJNA729271	stage_5W_vs_E10	ordinary_muscle_development	ENSGALG00010001693	NKIRAS1	1.129755021	0.195725003	5.772154834	7.82641E-09	1.73914E-08	positive
PRJNA729271	stage_5W_vs_E10	ordinary_muscle_development	ENSGALG00010001694	ENSGALG00010001694	-1.051987878	0.223114112	-4.715021691	2.41685E-06	4.50575E-06	negative
PRJNA729271	stage_5W_vs_E10	ordinary_muscle_development	ENSGALG00010001695	LMOD2	0.132553867	0.472314127	0.280647686	0.778980637	0.805430083	positive
PRJNA729271	stage_5W_vs_E10	ordinary_muscle_development	ENSGALG00010001696	FAM135A	0.39490365	0.090681891	4.354823715	1.33174E-05	2.35352E-05	positive
PRJNA729271	stage_5W_vs_E10	ordinary_muscle_development	ENSGALG00010001697	TAF10	-0.419764752	0.119769595	-3.5047689	0.000457004	0.000711522	negative
PRJNA729271	stage_5W_vs_E10	ordinary_muscle_development	ENSGALG00010001698	RPL15	0.018679569	0.176461203	0.105856519	0.915696193	0.926951345	positive
PRJNA729271	stage_5W_vs_E10	ordinary_muscle_development	ENSGALG00010001699	ASB15	8.007636742	0.249462044	32.09961974	4.4631E-226	9.9526E-224	positive
PRJNA729271	stage_5W_vs_E10	ordinary_muscle_development	ENSGALG00010001700	ENSGALG00010001700	1.650860507	0.200492688	8.234018528	1.81036E-16	6.13254E-16	positive
PRJNA729271	stage_5W_vs_E10	ordinary_muscle_development	ENSGALG00010001701	NDUFA5	1.972747491	0.169055728	11.66921415	1.83107E-31	1.1744E-30	positive
PRJNA729271	stage_5W_vs_E10	ordinary_muscle_development	ENSGALG00010001702	PLEKHA1	0.36852614	0.083521267	4.412362881	1.02249E-05	1.8212E-05	positive
PRJNA729271	stage_5W_vs_E10	ordinary_muscle_development	ENSGALG00010001704	ENSGALG00010001704	-1.107460396	0.146518057	-7.558524983	4.07666E-14	1.2219E-13	negative
PRJNA729271	stage_5W_vs_E10	ordinary_muscle_development	ENSGALG00010001705	HTRA1	2.150796551	0.15465632	13.90694255	5.74847E-44	5.6683E-43	positive
PRJNA729271	stage_5W_vs_E10	ordinary_muscle_development	ENSGALG00010001706	IQUB	1.928246413	0.347433001	5.549980592	2.85701E-08	6.11614E-08	positive
PRJNA729271	stage_5W_vs_E10	ordinary_muscle_development	ENSGALG00010001707	ENSGALG00010001707	3.62810211	1.060454208	3.42127183	0.00062329	0.000961433	positive
PRJNA729271	stage_5W_vs_E10	ordinary_muscle_development	ENSGALG00010001708	AADAC	3.131627084	0.250900326	12.48155844	9.41261E-36	7.15447E-35	positive
PRJNA729271	stage_5W_vs_E10	ordinary_muscle_development	ENSGALG00010001710	LCMT2	-0.774390676	0.081839457	-9.462314448	3.01201E-21	1.2736E-20	negative
PRJNA729271	stage_5W_vs_E10	ordinary_muscle_development	ENSGALG00010001711	CREB5	-0.355143268	0.176072207	-2.017031954	0.043692179	0.055520131	negative
PRJNA729271	stage_5W_vs_E10	ordinary_muscle_development	ENSGALG00010001712	ATP6AP1	2.012837019	0.158788339	12.67622693	8.00955E-37	6.32E-36	positive
PRJNA729271	stage_5W_vs_E10	ordinary_muscle_development	ENSGALG00010001713	PNPLA4	0.788476089	0.221268864	3.563429919	0.00036604	0.000575212	positive
PRJNA729271	stage_5W_vs_E10	ordinary_muscle_development	ENSGALG00010001716	TRIL	-0.721214976	0.151363701	-4.764781606	1.89058E-06	3.55204E-06	negative
PRJNA729271	stage_5W_vs_E10	ordinary_muscle_development	ENSGALG00010001717	STARD10	0.064584331	0.523643017	0.123336566	0.901840586	0.91433023	positive
PRJNA729271	stage_5W_vs_E10	ordinary_muscle_development	ENSGALG00010001721	TM4SF1	-0.255611397	0.162411162	-1.57385363	0.115521292	0.139192114	negative
PRJNA729271	stage_5W_vs_E10	ordinary_muscle_development	ENSGALG00010001725	MTA2	-0.440621676	0.187104756	-2.354946421	0.018525377	0.024599243	negative
PRJNA729271	stage_5W_vs_E10	ordinary_muscle_development	ENSGALG00010001727	COL9A1	-11.86563844	0.846040029	-14.02491376	1.09745E-44	1.09935E-43	negative
PRJNA729271	stage_5W_vs_E10	ordinary_muscle_development	ENSGALG00010001728	STS	1.228063912	0.323904115	3.791442757	0.000149775	0.000242812	positive
PRJNA729271	stage_5W_vs_E10	ordinary_muscle_development	ENSGALG00010001729	ENSGALG00010001729	3.244636477	0.239784972	13.53144218	1.02003E-41	9.39345E-41	positive
PRJNA729271	stage_5W_vs_E10	ordinary_muscle_development	ENSGALG00010001731	ENSGALG00010001731	1.209831834	0.191463867	6.318851959	2.63514E-10	6.382E-10	positive
PRJNA729271	stage_5W_vs_E10	ordinary_muscle_development	ENSGALG00010001738	PUDP	1.004607962	0.119233711	8.425536311	3.59102E-17	1.25881E-16	positive
PRJNA729271	stage_5W_vs_E10	ordinary_muscle_development	ENSGALG00010001741	SATB1	0.617995597	0.196187937	3.150018322	0.001632602	0.002421423	positive
PRJNA729271	stage_5W_vs_E10	ordinary_muscle_development	ENSGALG00010001745	CADPS2	-1.218116022	0.114427443	-10.64531368	1.83366E-26	9.69323E-26	negative
PRJNA729271	stage_5W_vs_E10	ordinary_muscle_development	ENSGALG00010001749	VAPA	0.934952631	0.106062289	8.815127788	1.19549E-18	4.48694E-18	positive
PRJNA729271	stage_5W_vs_E10	ordinary_muscle_development	ENSGALG00010001751	AVPR2C	-0.717702142	0.439725989	-1.632157664	0.102646277	0.124475678	negative
PRJNA729271	stage_5W_vs_E10	ordinary_muscle_development	ENSGALG00010001752	ARRB1	2.228681251	0.104787529	21.26857336	2.21928E-100	9.43356E-99	positive
PRJNA729271	stage_5W_vs_E10	ordinary_muscle_development	ENSGALG00010001753	PNPLA8	0.581845661	0.204595609	2.84388147	0.004456762	0.006325347	positive
PRJNA729271	stage_5W_vs_E10	ordinary_muscle_development	ENSGALG00010001754	APCDD1	-4.213900136	0.204565992	-20.59922133	2.78891E-94	1.04458E-92	negative
PRJNA729271	stage_5W_vs_E10	ordinary_muscle_development	ENSGALG00010001755	CUZD1	0.372496723	1.486240727	0.250630141	0.802100076	0.825624244	positive
PRJNA729271	stage_5W_vs_E10	ordinary_muscle_development	ENSGALG00010001756	ENSGALG00010001756	0.264855408	0.21863025	1.211430749	0.225730361	0.260133692	positive
PRJNA729271	stage_5W_vs_E10	ordinary_muscle_development	ENSGALG00010001758	COL19A1	0.314617148	0.280409952	1.121989951	0.261866713	0.298479044	positive
PRJNA729271	stage_5W_vs_E10	ordinary_muscle_development	ENSGALG00010001759	C10orf88	-0.284572138	0.090861242	-3.13194198	0.001736542	0.002568225	negative
PRJNA729271	stage_5W_vs_E10	ordinary_muscle_development	ENSGALG00010001760	RAB5A	0.531448419	0.095852758	5.544424923	2.94922E-08	6.30701E-08	positive
PRJNA729271	stage_5W_vs_E10	ordinary_muscle_development	ENSGALG00010001761	ELOB	0.701555084	0.149096614	4.705372356	2.53403E-06	4.71754E-06	positive
PRJNA729271	stage_5W_vs_E10	ordinary_muscle_development	ENSGALG00010001763	ENSGALG00010001763	0.620939069	0.264215405	2.350124396	0.018767138	0.024897461	positive
PRJNA729271	stage_5W_vs_E10	ordinary_muscle_development	ENSGALG00010001764	ENSGALG00010001764	1.674837429	0.381685044	4.388009057	1.14393E-05	2.02978E-05	positive
PRJNA729271	stage_5W_vs_E10	ordinary_muscle_development	ENSGALG00010001767	ENSGALG00010001767	0.922747811	0.172280796	5.356068885	8.50521E-08	1.7627E-07	positive
PRJNA729271	stage_5W_vs_E10	ordinary_muscle_development	ENSGALG00010001771	ENSGALG00010001771	0.571586379	0.135305913	4.224400577	2.39578E-05	4.14401E-05	positive
PRJNA729271	stage_5W_vs_E10	ordinary_muscle_development	ENSGALG00010001773	NRCAM	-2.352173414	0.257415975	-9.13763573	6.38325E-20	2.5433E-19	negative
PRJNA729271	stage_5W_vs_E10	ordinary_muscle_development	ENSGALG00010001776	LMBRD1	0.982619472	0.131347172	7.481085844	7.3711E-14	2.17472E-13	positive
PRJNA729271	stage_5W_vs_E10	ordinary_muscle_development	ENSGALG00010001778	KAT2B	3.210698276	0.147095025	21.82737504	1.2754E-105	5.8502E-104	positive
PRJNA729271	stage_5W_vs_E10	ordinary_muscle_development	ENSGALG00010001779	ENSGALG00010001779	0.91179741	0.60551634	1.505818009	0.132113899	0.157950426	positive
PRJNA729271	stage_5W_vs_E10	ordinary_muscle_development	ENSGALG00010001780	ENSGALG00010001780	0.771649484	0.237391542	3.25053487	0.001151881	0.001734523	positive
PRJNA729271	stage_5W_vs_E10	ordinary_muscle_development	ENSGALG00010001783	SGO1	-3.115244965	0.253752536	-12.27670474	1.2082E-34	8.78277E-34	negative
PRJNA729271	stage_5W_vs_E10	ordinary_muscle_development	ENSGALG00010001785	CNTN1	0.389710948	0.213011579	1.82952941	0.067320338	0.083559539	positive
PRJNA729271	stage_5W_vs_E10	ordinary_muscle_development	ENSGALG00010001786	P2RY8	1.204227153	0.205282547	5.866193537	4.45913E-09	1.00381E-08	positive
PRJNA729271	stage_5W_vs_E10	ordinary_muscle_development	ENSGALG00010001788	HOXA1	-1.270433549	0.40393235	-3.145164159	0.001659937	0.002459448	negative
PRJNA729271	stage_5W_vs_E10	ordinary_muscle_development	ENSGALG00010001790	HOXA2	-1.736440435	0.258723505	-6.711568158	1.92544E-11	5.01514E-11	negative
PRJNA729271	stage_5W_vs_E10	ordinary_muscle_development	ENSGALG00010001793	IKZF5	0.883980637	0.103172837	8.567959037	1.05335E-17	3.79715E-17	positive
PRJNA729271	stage_5W_vs_E10	ordinary_muscle_development	ENSGALG00010001794	ENSGALG00010001794	-2.453575025	0.677374113	-3.622185997	0.000292124	0.000462718	negative
PRJNA729271	stage_5W_vs_E10	ordinary_muscle_development	ENSGALG00010001795	ENSGALG00010001795	1.558259751	0.478753268	3.25482844	0.001134609	0.001710113	positive
PRJNA729271	stage_5W_vs_E10	ordinary_muscle_development	ENSGALG00010001796	SLCO2B1	2.878291805	0.154881675	18.58381117	4.34522E-77	1.11083E-75	positive
PRJNA729271	stage_5W_vs_E10	ordinary_muscle_development	ENSGALG00010001798	PSTK	0.223704445	0.130329359	1.716454735	0.086078846	0.10546132	positive
PRJNA729271	stage_5W_vs_E10	ordinary_muscle_development	ENSGALG00010001800	ENSGALG00010001800	1.010887577	0.181986181	5.554749114	2.78011E-08	5.95767E-08	positive
PRJNA729271	stage_5W_vs_E10	ordinary_muscle_development	ENSGALG00010001801	HOXA3	-1.304893003	0.15028709	-8.682668608	3.8659E-18	1.42043E-17	negative
PRJNA729271	stage_5W_vs_E10	ordinary_muscle_development	ENSGALG00010001803	PPP4R1	-0.929764753	0.09757483	-9.528735551	1.59211E-21	6.81357E-21	negative
PRJNA729271	stage_5W_vs_E10	ordinary_muscle_development	ENSGALG00010001805	ENSGALG00010001805	1.827076258	0.256929483	7.111197351	1.1504E-12	3.19692E-12	positive
PRJNA729271	stage_5W_vs_E10	ordinary_muscle_development	ENSGALG00010001806	ENSGALG00010001806	4.083299388	0.904937998	4.512242163	6.41459E-06	1.1579E-05	positive
PRJNA729271	stage_5W_vs_E10	ordinary_muscle_development	ENSGALG00010001807	ASMTL	0.255956621	0.103884401	2.463859994	0.013744979	0.01852473	positive
PRJNA729271	stage_5W_vs_E10	ordinary_muscle_development	ENSGALG00010001808	HOXA4	-1.088052514	0.153645239	-7.081589512	1.4251E-12	3.94215E-12	negative
PRJNA729271	stage_5W_vs_E10	ordinary_muscle_development	ENSGALG00010001811	ACADSB	0.33283641	0.14910811	2.232181802	0.02560295	0.033427739	positive
PRJNA729271	stage_5W_vs_E10	ordinary_muscle_development	ENSGALG00010001813	HOXA5	-0.472785367	0.104190532	-4.537699922	5.68711E-06	1.03056E-05	negative
PRJNA729271	stage_5W_vs_E10	ordinary_muscle_development	ENSGALG00010001817	SLC25A6	-1.628289617	0.113099378	-14.39698123	5.40534E-47	5.88216E-46	negative
PRJNA729271	stage_5W_vs_E10	ordinary_muscle_development	ENSGALG00010001818	ADGRB3	-1.410103033	0.250093269	-5.638308618	1.71729E-08	3.73586E-08	negative
PRJNA729271	stage_5W_vs_E10	ordinary_muscle_development	ENSGALG00010001819	HOXA6	0.487371766	0.143658452	3.392572875	0.000692395	0.001063277	positive
PRJNA729271	stage_5W_vs_E10	ordinary_muscle_development	ENSGALG00010001820	PGM2L1	3.251989964	0.153202545	21.22673597	5.40914E-100	2.27262E-98	positive
PRJNA729271	stage_5W_vs_E10	ordinary_muscle_development	ENSGALG00010001822	KCNE3	3.298216065	0.301193426	10.95049156	6.6088E-28	3.68866E-27	positive
PRJNA729271	stage_5W_vs_E10	ordinary_muscle_development	ENSGALG00010001823	RALBP1	-0.356394512	0.076505866	-4.658394566	3.18685E-06	5.89653E-06	negative
PRJNA729271	stage_5W_vs_E10	ordinary_muscle_development	ENSGALG00010001825	LIPT2	2.232883659	0.142998735	15.61470918	5.78081E-55	7.98027E-54	positive

PRJNA729271	stage_5W_vs_E10	ordinary_muscle_development	ENSGALG00010001826	HOXA7	1.47388633	0.133034644	11.07896624	1.58694E-28	9.06333E-28	positive
PRJNA729271	stage_5W_vs_E10	ordinary_muscle_development	ENSGALG00010001827	PDZRN4	-2.601267081	0.195443118	-13.30958649	2.03605E-40	1.80065E-39	negative
PRJNA729271	stage_5W_vs_E10	ordinary_muscle_development	ENSGALG00010001828	ENSGALG00010001828	-3.332069848	1.120878834	-2.972729743	0.002951642	0.004267736	negative
PRJNA729271	stage_5W_vs_E10	ordinary_muscle_development	ENSGALG00010001829	POLD3	-0.513060161	0.121953324	-4.207020739	2.58759E-05	4.46248E-05	negative
PRJNA729271	stage_5W_vs_E10	ordinary_muscle_development	ENSGALG00010001831	CSF2RA	2.551921868	0.249608318	10.22370522	1.55289E-24	7.56865E-24	positive
PRJNA729271	stage_5W_vs_E10	ordinary_muscle_development	ENSGALG00010001832	BUB3	-1.151403476	0.087353394	-13.18098154	1.12912E-39	9.77111E-39	negative
PRJNA729271	stage_5W_vs_E10	ordinary_muscle_development	ENSGALG00010001834	ZCRB1	0.41704974	0.083953708	4.967615511	6.77812E-07	1.31789E-06	positive
PRJNA729271	stage_5W_vs_E10	ordinary_muscle_development	ENSGALG00010001835	MECP2	0.40324623	0.191288681	2.108050664	0.035026607	0.045101774	positive
PRJNA729271	stage_5W_vs_E10	ordinary_muscle_development	ENSGALG00010001836	TWSG1	-0.884063806	0.140254961	-6.303262287	2.91445E-10	7.03145E-10	negative
PRJNA729271	stage_5W_vs_E10	ordinary_muscle_development	ENSGALG00010001837	TAB3	1.214036234	0.137873888	8.805410896	1.30374E-18	4.88566E-18	positive
PRJNA729271	stage_5W_vs_E10	ordinary_muscle_development	ENSGALG00010001838	PPHLN1	-0.892270447	0.066578035	-13.4018742	5.89553E-41	5.30122E-40	negative
PRJNA729271	stage_5W_vs_E10	ordinary_muscle_development	ENSGALG00010001839	ENSGALG00010001839	8.392177132	0.597989124	14.03399627	9.65538E-45	9.73937E-44	positive
PRJNA729271	stage_5W_vs_E10	ordinary_muscle_development	ENSGALG00010001840	HOXA9	-0.181046175	0.145838628	-1.241414411	0.214452698	0.248143359	negative
PRJNA729271	stage_5W_vs_E10	ordinary_muscle_development	ENSGALG00010001842	GPR26	-0.399234899	0.523899998	-0.762044092	0.446033689	0.487944024	negative
PRJNA729271	stage_5W_vs_E10	ordinary_muscle_development	ENSGALG00010001843	PRICKLE1	-1.847421374	0.12242669	-15.09002135	1.88381E-51	2.37442E-50	negative
PRJNA729271	stage_5W_vs_E10	ordinary_muscle_development	ENSGALG00010001844	HOXA10	-1.007318488	0.167367554	-6.01860075	1.75931E-09	4.04974E-09	negative
PRJNA729271	stage_5W_vs_E10	ordinary_muscle_development	ENSGALG00010001845	ANKRD12	1.387492427	0.133634471	10.3827434	2.97112E-25	1.48556E-24	positive
PRJNA729271	stage_5W_vs_E10	ordinary_muscle_development	ENSGALG00010001848	HOXA11	-0.263597399	0.358902268	-0.734454537	0.462671758	0.50458409	negative
PRJNA729271	stage_5W_vs_E10	ordinary_muscle_development	ENSGALG00010001849	NDUFV2	0.593713238	0.09483755	6.260318168	3.84193E-10	9.2078E-10	positive
PRJNA729271	stage_5W_vs_E10	ordinary_muscle_development	ENSGALG00010001850	MYOM2	4.979155005	0.187788136	26.51474749	6.5526E-155	6.9328E-153	positive
PRJNA729271	stage_5W_vs_E10	ordinary_muscle_development	ENSGALG00010001852	CPXM2	-1.498847488	0.387223792	-3.87075257	0.0001085	0.000178009	negative
PRJNA729271	stage_5W_vs_E10	ordinary_muscle_development	ENSGALG00010001853	ENSGALG00010001853	1.177361581	0.186710749	6.305805028	2.867E-10	6.92388E-10	positive
PRJNA729271	stage_5W_vs_E10	ordinary_muscle_development	ENSGALG00010001854	CHRDL2	0.834275756	0.299351742	2.786941375	0.00532081	0.007488605	positive
PRJNA729271	stage_5W_vs_E10	ordinary_muscle_development	ENSGALG00010001856	CHST15	-0.160989661	0.116594876	-1.380761027	0.16735245	0.197458182	negative
PRJNA729271	stage_5W_vs_E10	ordinary_muscle_development	ENSGALG00010001857	HNRNPA2B1	-0.477920831	0.187717933	-2.545951913	0.01089802	0.014866064	negative
PRJNA729271	stage_5W_vs_E10	ordinary_muscle_development	ENSGALG00010001858	ADAMTS20	0.63808817	0.161075522	3.961422332	7.45046E-05	0.000124303	positive
PRJNA729271	stage_5W_vs_E10	ordinary_muscle_development	ENSGALG00010001860	RNF169	0.597122901	0.128649376	4.641475293	3.4593E-06	6.38028E-06	positive
PRJNA729271	stage_5W_vs_E10	ordinary_muscle_development	ENSGALG00010001861	CBX3	-1.047324928	0.074642054	-14.03129839	1.00298E-44	1.0089E-43	negative
PRJNA729271	stage_5W_vs_E10	ordinary_muscle_development	ENSGALG00010001864	ENSGALG00010001864	-0.233611758	0.198994837	-1.173958891	0.240411492	0.275715213	negative
PRJNA729271	stage_5W_vs_E10	ordinary_muscle_development	ENSGALG00010001865	CYBB	2.068175518	0.31542528	6.556784284	5.49805E-11	1.39204E-10	positive
PRJNA729271	stage_5W_vs_E10	ordinary_muscle_development	ENSGALG00010001866	PUS7L	-0.389543784	0.121689522	-3.201128384	0.001368905	0.002047073	negative
PRJNA729271	stage_5W_vs_E10	ordinary_muscle_development	ENSGALG00010001868	ENSGALG00010001868	-0.34996705	0.125230115	-2.794591782	0.005196529	0.007324357	negative
PRJNA729271	stage_5W_vs_E10	ordinary_muscle_development	ENSGALG00010001869	CRLF2	7.617509909	0.922482805	8.257617236	1.48609E-16	5.04944E-16	positive
PRJNA729271	stage_5W_vs_E10	ordinary_muscle_development	ENSGALG00010001870	PLP2	1.673161131	0.164316454	10.18255379	2.37252E-24	1.14556E-23	positive
PRJNA729271	stage_5W_vs_E10	ordinary_muscle_development	ENSGALG00010001871	ENSGALG00010001871	-1.144942354	0.124879467	-9.168379554	4.80183E-20	1.92007E-19	negative
PRJNA729271	stage_5W_vs_E10	ordinary_muscle_development	ENSGALG00010001873	ENSGALG00010001873	0.327510351	0.200615124	1.632530713	0.102567734	0.124390839	positive
PRJNA729271	stage_5W_vs_E10	ordinary_muscle_development	ENSGALG00010001877	OAT	0.292144714	0.136858057	2.134654847	0.032789214	0.042348717	positive
PRJNA729271	stage_5W_vs_E10	ordinary_muscle_development	ENSGALG00010001878	XK	-0.081015934	0.194399554	-0.416749591	0.676861577	0.710435088	negative
PRJNA729271	stage_5W_vs_E10	ordinary_muscle_development	ENSGALG00010001879	IRAK4	0.767619761	0.164588074	4.663884465	3.10296E-06	5.74864E-06	positive
PRJNA729271	stage_5W_vs_E10	ordinary_muscle_development	ENSGALG00010001880	MTCL1	2.874975773	0.143739072	20.00135198	5.35996E-89	1.77786E-87	positive
PRJNA729271	stage_5W_vs_E10	ordinary_muscle_development	ENSGALG00010001883	KBTBD11	-0.930798383	0.089014499	-10.45670535	1.3652E-25	6.91185E-25	negative
PRJNA729271	stage_5W_vs_E10	ordinary_muscle_development	ENSGALG00010001885	SMPX	3.560998999	0.37131937	9.590124536	8.79788E-22	3.80672E-21	positive
PRJNA729271	stage_5W_vs_E10	ordinary_muscle_development	ENSGALG00010001886	NEU3	-0.436798717	0.085699948	-5.096837605	3.45374E-07	6.8473E-07	negative
PRJNA729271	stage_5W_vs_E10	ordinary_muscle_development	ENSGALG00010001888	SNX10	1.563777674	0.200069376	7.816177096	5.44518E-15	1.70692E-14	positive
PRJNA729271	stage_5W_vs_E10	ordinary_muscle_development	ENSGALG00010001890	KLHL34	7.484842431	0.250667024	29.85970119	6.5691E-196	1.0464E-193	positive
PRJNA729271	stage_5W_vs_E10	ordinary_muscle_development	ENSGALG00010001892	RAB12	1.541627691	0.070478145	21.87384027	4.6109E-106	2.1285E-104	positive
PRJNA729271	stage_5W_vs_E10	ordinary_muscle_development	ENSGALG00010001895	P4HA3	-1.585695721	0.240670385	-6.588661577	4.43809E-11	1.13138E-10	negative
PRJNA729271	stage_5W_vs_E10	ordinary_muscle_development	ENSGALG00010001896	ARHGEF10	-0.20157496	0.134362609	-1.500231064	0.133554559	0.159554383	negative
PRJNA729271	stage_5W_vs_E10	ordinary_muscle_development	ENSGALG00010001897	TLR7	3.201105268	0.175352062	18.25530436	1.87762E-74	4.49852E-73	positive
PRJNA729271	stage_5W_vs_E10	ordinary_muscle_development	ENSGALG00010001898	LHPP	0.42385517	0.132410507	3.201069014	0.001369187	0.002047284	positive
PRJNA729271	stage_5W_vs_E10	ordinary_muscle_development	ENSGALG00010001899	TWF1	-0.609342292	0.113039976	-5.390502661	7.02609E-08	1.46432E-07	negative
PRJNA729271	stage_5W_vs_E10	ordinary_muscle_development	ENSGALG00010001901	PRPS2	1.049120816	0.18195753	5.765745533	8.12976E-09	1.80433E-08	positive
PRJNA729271	stage_5W_vs_E10	ordinary_muscle_development	ENSGALG00010001902	WDR45	0.25018878	0.142617784	1.754260748	0.079385838	0.097657449	positive
PRJNA729271	stage_5W_vs_E10	ordinary_muscle_development	ENSGALG00010001903	NARS2	-0.284834366	0.118480863	-2.404053784	0.016214388	0.02167351	negative
PRJNA729271	stage_5W_vs_E10	ordinary_muscle_development	ENSGALG00010001906	SKAP2	-0.093391687	0.128193745	-0.72851984	0.466295428	0.50815369	negative
PRJNA729271	stage_5W_vs_E10	ordinary_muscle_development	ENSGALG00010001908	FAM83B	-4.066072123	0.429345495	-9.47039662	2.78783E-21	1.18053E-20	negative
PRJNA729271	stage_5W_vs_E10	ordinary_muscle_development	ENSGALG00010001910	FAM53B	0.264172118	0.095861607	2.755765597	0.005855494	0.00821004	positive
PRJNA729271	stage_5W_vs_E10	ordinary_muscle_development	ENSGALG00010001911	USP35	0.585479259	0.340839465	1.717756656	0.08584101	0.105196605	positive
PRJNA729271	stage_5W_vs_E10	ordinary_muscle_development	ENSGALG00010001912	TMEM117	4.595726959	0.205474678	22.36639085	8.3636E-111	4.3452E-109	positive
PRJNA729271	stage_5W_vs_E10	ordinary_muscle_development	ENSGALG00010001914	ENSGALG00010001914	-0.8900132	0.157690673	-5.644044664	1.66101E-08	3.61941E-08	negative
PRJNA729271	stage_5W_vs_E10	ordinary_muscle_development	ENSGALG00010001915	EEF1AKMT2	-0.316877492	0.168180186	-1.884154727	0.059544049	0.074359524	negative
PRJNA729271	stage_5W_vs_E10	ordinary_muscle_development	ENSGALG00010001918	CNKSR2	-1.028409263	0.148142382	-6.942032704	3.86498E-12	1.04364E-11	negative
PRJNA729271	stage_5W_vs_E10	ordinary_muscle_development	ENSGALG00010001921	ENSGALG00010001921	0.036711805	0.286695916	0.128051371	0.89810832	0.911078389	positive
PRJNA729271	stage_5W_vs_E10	ordinary_muscle_development	ENSGALG00010001923	RAPGEF5	2.168293245	0.100879197	21.49395829	1.7733E-102	7.8605E-101	positive
PRJNA729271	stage_5W_vs_E10	ordinary_muscle_development	ENSGALG00010001925	FRMPD4	-3.102492397	0.224763941	-13.80333691	2.43323E-43	2.36076E-42	negative
PRJNA729271	stage_5W_vs_E10	ordinary_muscle_development	ENSGALG00010001928	KCTD21	0.210990724	0.1686366	1.25115618	0.210877502	0.244279501	positive
PRJNA729271	stage_5W_vs_E10	ordinary_muscle_development	ENSGALG00010001929	POLR2I	-0.571331276	0.127337843	-4.486735944	7.23226E-06	1.30129E-05	negative
PRJNA729271	stage_5W_vs_E10	ordinary_muscle_development	ENSGALG00010001935	ABRAXAS2	0.086041117	0.130367416	0.659989434	0.50926061	0.550013601	positive
PRJNA729271	stage_5W_vs_E10	ordinary_muscle_development	ENSGALG00010001938	ALG8	-1.068574871	0.107472269	-9.942796238	2.71108E-23	1.2527E-22	negative
PRJNA729271	stage_5W_vs_E10	ordinary_muscle_development	ENSGALG00010001940	PTPRM	0.682992326	0.110868938	6.160357783	7.25808E-10	1.70706E-09	positive
PRJNA729271	stage_5W_vs_E10	ordinary_muscle_development	ENSGALG00010001947	TINAG	6.388788242	0.301598709	21.18307557	1.36803E-99	5.68182E-98	positive
PRJNA729271	stage_5W_vs_E10	ordinary_muscle_development	ENSGALG00010001948	EMX2	-3.229512751	0.288024039	-11.21265004	3.5345E-29	2.07778E-28	negative
PRJNA729271	stage_5W_vs_E10	ordinary_muscle_development	ENSGALG00010001952	ENSGALG00010001952	0.228831507	0.07294861	3.13688644	0.001707522	0.002527112	positive
PRJNA729271	stage_5W_vs_E10	ordinary_muscle_development	ENSGALG00010001957	NDUFC2	1.174918073	0.11167535	10.5208363	6.92561E-26	3.55603E-25	positive
PRJNA729271	stage_5W_vs_E10	ordinary_muscle_development	ENSGALG00010001958	RAB11FIP2	-0.185968446	0.112113229	-1.658755603	0.09716505	0.11826412	negative
PRJNA729271	stage_5W_vs_E10	ordinary_muscle_development	ENSGALG00010001966	ARHGAP6	1.203063614	0.141188653	8.520965282	1.58229E-17	5.65466E-17	positive
PRJNA729271	stage_5W_vs_E10	ordinary_muscle_development	ENSGALG00010001967	LAMA1	-5.109425799	0.249914737	-20.86205943	1.18459E-96	4.69144E-95	negative
PRJNA729271	stage_5W_vs_E10	ordinary_muscle_development	ENSGALG00010001969	PLEKHA8	-2.17947428	0.129399914	-16.84293449	1.18225E-63	2.11304E-62	negative
PRJNA729271	stage_5W_vs_E10	ordinary_muscle_development	ENSGALG00010001971	MLIP	4.349339024	0.204742729	21.24294738	3.83083E-100	1.61418E-98	positive
PRJNA729271	stage_5W_vs_E10	ordinary_muscle_development	ENSGALG00010001972	FAM204A	-0.1547819	0.159077137	-0.97299903	0.330553784	0.370190613	negative
PRJNA729271	stage_5W_vs_E10	ordinary_muscle_development	ENSGALG00010001974	ENSGALG00010001974	0.68688788	0.40832079	1.682226074	0.09252501	0.112843922	positive
PRJNA729271	stage_5W_vs_E10	ordinary_muscle_development	ENSGALG00010001976	THRSP	9.028273203	1.423409772	6.342708461	2.2576E-10	5.48968E-10	positive
PRJNA729271	stage_5W_vs_E10	ordinary_muscle_development	ENSGALG00010001982	DMD	2.53180369	0.121826679	20.78201351	6.29619E-96	2.44674E-94	positive
PRJNA729271	stage_5W_vs_E10	ordinary_muscle_development	ENSGALG00010001987	KCTD14	-2.198950374	0.419692667	-5.23943006	1.61073E-07	3.27639E-07	negative
PRJNA729271	stage_5W_vs_E10	ordinary_muscle_development	ENSGALG00010001991	TOMM7	0.962895461	0.130207145	7.395104647	1.41297E-13	4.10276E-13	positive
PRJNA729271	stage_5W_vs_E10	ordinary_muscle_development	ENSGALG00010001995	LRRC1	-1.510407344	0.143438489	-10.53000038	6.28342E-26	3.23202E-25	negative
PRJNA729271	stage_5W_vs_E10	ordinary_muscle_development	ENSGALG00010001997	INTS4	-0.347086952	0.078271931	-4.434373169	9.23406E-06	1.64979E-05	negative
PRJNA729271	stage_5W_vs_E10	ordinary_muscle_development	ENSGALG00010002005	COLQ	-1.929604458	0.237923575	-8.110186051	5.05423E-16	1.67416E-15	negative
PRJNA729271	stage_5W_vs_E10	ordinary_muscle_development	ENSGALG00010002006	ENSGALG00010002006	3.205287646	0.403385809	7.94596036	1.92692E-15	6.20958E-15	positive
PRJNA729271	stage_5W_vs_E10	ordinary_muscle_development	ENSGALG00010002012	MID1	-1.830952308	0.140404423	-13.04056002	7.19268E-39	6.01951E-38	negative
PRJNA729271	stage_5W_vs_E10	ordinary_muscle_development	ENSGALG00010002017	ARHGAP28	-2.120148124	0.119671402	-17.71641418	3.13255E-70	6.65781E-69	negative
PRJNA729271	stage_5W_vs_E10	ordinary_muscle_development	ENSGALG00010002022	ERNI	-2.222893506	0.74873316	-2.96887279	0.002988943	0.004319084	negative
PRJNA729271	stage_5W_vs_E10	ordinary_muscle_development	ENSGALG00010002023	CLCN4	0.064119881	0.243294411	0.263548514	0.792127836	0.816809631	positive
PRJNA729271	stage_5W_vs_E10	ordinary_muscle_development	ENSGALG00010002024	KLHL31	4.49338448	0.165776422	27.10508791	8.5765E-162	9.9453E-160	positive
PRJNA729271	stage_5W_vs_E10	ordinary_muscle_development	ENSGALG00010002027	ENSGALG00010002027	7.209117786	0.535913871	13.45200819	2.99623E-41	2.71779E-40	positive
PRJNA729271	stage_5W_vs_E10	ordinary_muscle_development	ENSGALG00010002034	WWC3	-0.372058178	0.099901106	-3.724264872	0.000195885	0.000314959	negative
PRJNA729271	stage_5W_vs_E10	ordinary_muscle_development	ENSGALG00010002039	AAMDC	1.851864442	0.122699147	15.09272462	1.80819E-51	2.28307E-50	positive
PRJNA729271	stage_5W_vs_E10	ordinary_muscle_development	ENSGALG00010002041	GCLC	0.31530743	0.139278324	2.263865774	0.023582372	0.030909348	positive
PRJNA729271	stage_5W_vs_E10	ordinary_muscle_development	ENSGALG00010002046	FAM126A	-0.742930577	0.088681241	-8.377539202	5.40458E-17	1.87999E-16	negative
PRJNA729271	stage_5W_vs_E10	ordinary_muscle_development	ENSGALG00010002050	ENSGALG00010002050	3.421166595	1.101001574	3.107322165	0.001887905	0.002780167	positive
PRJNA729271	stage_5W_vs_E10	ordinary_muscle_development	ENSGALG00010002054	RSF1	-0.133885802	0.100096219	-1.337571026	0.181036315	0.212307555	negative
PRJNA729271	stage_5W_vs_E10	ordinary_muscle_development	ENSGALG00010002056	PRLHR	-1.801738161	0.46191226	-3.900606928	9.59518E-05	0.000158336	negative
PRJNA729271	stage_5W_vs_E10	ordinary_muscle_development	ENSGALG00010002062	GSTA4	-0.195096201	0.363523674	-0.536680869	0.591488073	0.629626175	negative
PRJNA729271	stage_5W_vs_E10	ordinary_muscle_development	ENSGALG00010002065	CLNS1A	-0.616688411	0.063567007	-9.701391352	2.97415E-22	1.31634E-21	negative
PRJNA729271	stage_5W_vs_E10	ordinary_muscle_development	ENSGALG00010002072	NEURL4	0.262137447	0.16107846	1.627389819	0.103654332	0.125635049	positive
PRJNA729271	stage_5W_vs_E10	ordinary_muscle_development	ENSGALG00010002079	ARX	2.031062588	0.190511005	10.66112998	1.54706E-26	8.20515E-26	positive
PRJNA729271	stage_5W_vs_E10	ordinary_muscle_development	ENSGALG00010002084	HACL1	-0.694785509	0.092327409	-7.525235614	5.26253E-14	1.57182E-13	negative
PRJNA729271	stage_5W_vs_E10	ordinary_muscle_development	ENSGALG00010002087	POLA1	-1.581418211	0.075389019	-20.97677125	1.0691E-97	4.29271E-96	negative
PRJNA729271	stage_5W_vs_E10	ordinary_muscle_development	ENSGALG00010002088	OZFL1	-0.034756374	0.13684284	-0.253987521	0.799505199	0.82342105	negative
PRJNA729271	stage_5W_vs_E10	ordinary_muscle_development	ENSGALG00010002090	AQP11	-0.769912697	0.313274975	-2.457625912	0.013985877	0.018833639	negative
PRJNA729271	stage_5W_vs_E10	ordinary_muscle_development	ENSGALG00010002097	PCYT1B	-1.590897895	0.453955203	-3.504526181	0.000457421	0.000712094	negative
PRJNA729271	stage_5W_vs_E10	ordinary_muscle_development	ENSGALG00010002098	ENSGALG00010002098	0.257972131	0.451007866	0.571990315	0.567328536	0.606402708	positive
PRJNA729271	stage_5W_vs_E10	ordinary_muscle_development	ENSGALG00010002100	BTD	0.681415445	0.116490184	5.849552503	4.92897E-09	1.1082E-08	positive
PRJNA729271	stage_5W_vs_E10	ordinary_muscle_development	ENSGALG00010002102	PAK1	-1.053571514	0.273020316	-3.858949141	0.000113876	0.00018649	negative
PRJNA729271	stage_5W_vs_E10	ordinary_muscle_development	ENSGALG00010002106	ENSGALG00010002106	2.834107556	0.85882152	3.299995969	0.000966862	0.001468427	positive
PRJNA729271	stage_5W_vs_E10	ordinary_muscle_development	ENSGALG00010002113	GDPD4	-8.24015764	0.518224132	-15.90076017	6.26005E-57	9.21213E-56	negative
PRJNA729271	stage_5W_vs_E10	ordinary_muscle_development	ENSGALG00010002118	ENSGALG00010002118	2.07768717	0.264162991	7.86517291	3.68593E-15	1.16858E-14	positive
PRJNA729271	stage_5W_vs_E10	ordinary_muscle_development	ENSGALG00010002122	ENSGALG00010002122	3.636229002	0.505818668	7.188799523	6.53635E-13	1.8401E-12	positive
PRJNA729271	stage_5W_vs_E10	ordinary_muscle_development	ENSGALG00010002126	ENSGALG00010002126	3.648400537	0.295753775	12.33593902	5.80087E-35	4.27254E-34	positive
PRJNA729271	stage_5W_vs_E10	ordinary_muscle_development	ENSGALG00010002134	ANKRD28	1.069327663	0.120523246	8.872376875	7.1601E-19	2.7169E-18	positive
PRJNA729271	stage_5W_vs_E10	ordinary_muscle_development	ENSGALG00010002135	ELOVL5	0.195520803	0.168139549	1.162848384	0.244891023	0.280453138	positive
PRJNA729271	stage_5W_vs_E10	ordinary_muscle_development	ENSGALG00010002136	ACAP1	0.740774582	0.234981988	3.152473892	0.001618933	0.002401887	positive
PRJNA729271	stage_5W_vs_E10	ordinary_muscle_development	ENSGALG00010002137	PDK3	1.263102163	0.131505778	9.604917648	7.62188E-22	3.31471E-21	positive
PRJNA729271	stage_5W_vs_E10	ordinary_muscle_development	ENSGALG00010002138	MYO7A	-0.922740826	0.172989996	-5.334070459	9.60352E-08	1.9838E-07	negative
PRJNA729271	stage_5W_vs_E10	ordinary_muscle_development	ENSGALG00010002140	ENSGALG00010002140	0.342269016	0.433111432	0.790256251	0.429378131	0.471181468	positive
PRJNA729271	stage_5W_vs_E10	ordinary_muscle_development	ENSGALG00010002142	CLDN34	0.226832871	0.258834555	0.876362394	0.380833055	0.421934964	positive
PRJNA729271	stage_5W_vs_E10	ordinary_muscle_development	ENSGALG00010002145	ENSGALG00010002145	0.474572182	0.465967247	1.018466825	0.308456159	0.347294704	positive
PRJNA729271	stage_5W_vs_E10	ordinary_muscle_development	ENSGALG00010002146	CACUL1	0.645313738	0.088089132	7.32569071	2.37671E-13	6.8327E-13	positive
PRJNA729271	stage_5W_vs_E10	ordinary_muscle_development	ENSGALG00010002148	POLR2A	1.342942305	0.176943043	7.589686968	3.20679E-14	9.67176E-14	positive
PRJNA729271	stage_5W_vs_E10	ordinary_muscle_development	ENSGALG00010002149	ENSGALG00010002149	-2.498078271	0.367759292	-6.79269926	1.10055E-11	2.90784E-11	negative
PRJNA729271	stage_5W_vs_E10	ordinary_muscle_development	ENSGALG00010002150	EIF3A	0.102761353	0.132470139	0.775732209	0.437907118	0.479814323	positive

PRJNA729271	stage_5W_vs_E10	ordinary_muscle_development	ENSGALG00010002159	GALNT15	1.59853335	0.189237599	8.447229062	2.98298E-17	1.05024E-16	positive
PRJNA729271	stage_5W_vs_E10	ordinary_muscle_development	ENSGALG00010002160	CAPN5	0.179339492	0.166181324	1.079179581	0.28050768	0.318050597	positive
PRJNA729271	stage_5W_vs_E10	ordinary_muscle_development	ENSGALG00010002161	CDKL5	-0.129352402	0.174614517	-0.740788362	0.458821773	0.500837533	negative
PRJNA729271	stage_5W_vs_E10	ordinary_muscle_development	ENSGALG00010002173	GCM1	-1.140351732	0.925282179	-1.232436718	0.217785992	0.251718998	negative
PRJNA729271	stage_5W_vs_E10	ordinary_muscle_development	ENSGALG00010002174	ENSGALG00010002174	-0.004490677	0.459760991	-0.009767417	0.992206853	0.993214019	negative
PRJNA729271	stage_5W_vs_E10	ordinary_muscle_development	ENSGALG00010002177	FBXO9	1.121647592	0.155029316	7.235067655	4.65297E-13	1.31986E-12	positive
PRJNA729271	stage_5W_vs_E10	ordinary_muscle_development	ENSGALG00010002181	FIS1	0.358501068	0.258779269	1.385354663	0.165944047	0.195924001	positive
PRJNA729271	stage_5W_vs_E10	ordinary_muscle_development	ENSGALG00010002185	ENSGALG00010002185	0.21916508	0.333149014	0.657859008	0.510628733	0.551450118	positive
PRJNA729271	stage_5W_vs_E10	ordinary_muscle_development	ENSGALG00010002186	CILK1	-0.15979966	0.07471525	-2.138782381	0.032453297	0.041937286	negative
PRJNA729271	stage_5W_vs_E10	ordinary_muscle_development	ENSGALG00010002192	ENSGALG00010002192	0.998253799	0.194805704	5.124356115	2.98556E-07	5.94365E-07	positive
PRJNA729271	stage_5W_vs_E10	ordinary_muscle_development	ENSGALG00010002194	SHROOM2	-2.131543275	0.133685487	-15.94446286	3.11305E-57	4.62807E-56	negative
PRJNA729271	stage_5W_vs_E10	ordinary_muscle_development	ENSGALG00010002196	ENSGALG00010002196	-0.965789678	0.101730872	-9.493575126	2.23243E-21	9.49227E-21	negative
PRJNA729271	stage_5W_vs_E10	ordinary_muscle_development	ENSGALG00010002197	GSTA2	-0.560573924	0.188894087	-2.96766263	0.003000735	0.004335691	negative
PRJNA729271	stage_5W_vs_E10	ordinary_muscle_development	ENSGALG00010002202	SFXN4	0.29180689	0.101457107	2.876160168	0.004025454	0.005743007	positive
PRJNA729271	stage_5W_vs_E10	ordinary_muscle_development	ENSGALG00010002206	DPH3P1	1.158106621	0.108869783	10.63753957	1.99323E-26	1.05138E-25	positive
PRJNA729271	stage_5W_vs_E10	ordinary_muscle_development	ENSGALG00010002210	GPR143	-5.591803144	1.231349375	-4.541199481	5.59351E-06	1.01436E-05	negative
PRJNA729271	stage_5W_vs_E10	ordinary_muscle_development	ENSGALG00010002211	ACER3	-0.58182044	0.174624685	-3.33183387	0.000862757	0.001314725	negative
PRJNA729271	stage_5W_vs_E10	ordinary_muscle_development	ENSGALG00010002215	YLEC18	0.80593554	0.352414802	2.28689469	0.022201971	0.029168636	positive
PRJNA729271	stage_5W_vs_E10	ordinary_muscle_development	ENSGALG00010002225	PRDX3	1.231934074	0.105340402	11.69479188	1.35519E-31	8.74598E-31	positive
PRJNA729271	stage_5W_vs_E10	ordinary_muscle_development	ENSGALG00010002226	OXNAD1	-0.202073483	0.134863979	-1.4983503	0.134042262	0.160110637	negative
PRJNA729271	stage_5W_vs_E10	ordinary_muscle_development	ENSGALG00010002227	ENSGALG00010002227	1.777135106	1.126812775	1.577134326	0.11476462	0.138406953	positive
PRJNA729271	stage_5W_vs_E10	ordinary_muscle_development	ENSGALG00010002231	TMEM14A	-0.881662941	0.137426033	-6.415545293	1.4032E-10	3.45907E-10	negative
PRJNA729271	stage_5W_vs_E10	ordinary_muscle_development	ENSGALG00010002235	MXRA5	0.14944067	0.201234462	0.742619675	0.457711973	0.499701367	positive
PRJNA729271	stage_5W_vs_E10	ordinary_muscle_development	ENSGALG00010002238	ENSGALG00010002238	0.067896022	0.422384003	0.160744775	0.872294422	0.888164348	positive
PRJNA729271	stage_5W_vs_E10	ordinary_muscle_development	ENSGALG00010002239	AGPAT5	1.548135854	0.127314548	12.1599289	5.08029E-34	3.60974E-33	positive
PRJNA729271	stage_5W_vs_E10	ordinary_muscle_development	ENSGALG00010002243	ARSH	0.040475016	0.176581752	0.229214036	0.818702564	0.840088749	positive
PRJNA729271	stage_5W_vs_E10	ordinary_muscle_development	ENSGALG00010002247	RFTN1	0.122689601	0.184798867	0.663908839	0.506748659	0.547677185	positive
PRJNA729271	stage_5W_vs_E10	ordinary_muscle_development	ENSGALG00010002251	GRK5	-0.474906574	0.15188585	-3.126733486	0.001767601	0.002611495	negative
PRJNA729271	stage_5W_vs_E10	ordinary_muscle_development	ENSGALG00010002253	ENSGALG00010002253	-0.716400856	0.323026926	-2.217774431	0.026570216	0.034659403	negative
PRJNA729271	stage_5W_vs_E10	ordinary_muscle_development	ENSGALG00010002254	ENSGALG00010002254	3.525090962	0.46622311	7.560952872	4.00127E-14	1.1998E-13	positive
PRJNA729271	stage_5W_vs_E10	ordinary_muscle_development	ENSGALG00010002259	TASL	3.011288017	0.481801242	6.250062795	4.10288E-10	9.81049E-10	positive
PRJNA729271	stage_5W_vs_E10	ordinary_muscle_development	ENSGALG00010002261	NROB1	-3.750111775	0.474019234	-7.911307198	2.547E-15	8.14625E-15	negative
PRJNA729271	stage_5W_vs_E10	ordinary_muscle_development	ENSGALG00010002269	MCPH1	-2.208954801	0.152501202	-14.48483533	1.51084E-47	1.66537E-46	negative
PRJNA729271	stage_5W_vs_E10	ordinary_muscle_development	ENSGALG00010002270	IMMP2L	0.072656443	0.247957644	0.293019574	0.76950719	0.796828598	positive
PRJNA729271	stage_5W_vs_E10	ordinary_muscle_development	ENSGALG00010002271	ENSGALG00010002271	3.350772231	0.681043829	4.920053732	8.65205E-07	1.67059E-06	positive
PRJNA729271	stage_5W_vs_E10	ordinary_muscle_development	ENSGALG00010002274	ENSGALG00010002274	4.227450814	0.778146267	5.432720033	5.55015E-08	1.16492E-07	positive
PRJNA729271	stage_5W_vs_E10	ordinary_muscle_development	ENSGALG00010002275	IL1RAPL1	-3.816740608	0.364213628	-10.47940087	1.07422E-25	5.47498E-25	negative
PRJNA729271	stage_5W_vs_E10	ordinary_muscle_development	ENSGALG00010002277	RGS10	-0.642919762	0.17790379	-3.613862096	0.00030167	0.000477369	negative
PRJNA729271	stage_5W_vs_E10	ordinary_muscle_development	ENSGALG00010002279	ENSGALG00010002279	-2.13271084	1.081643196	-1.971732312	0.048640173	0.061403876	negative
PRJNA729271	stage_5W_vs_E10	ordinary_muscle_development	ENSGALG00010002280	ST7	1.930937675	0.141120114	13.6829373	1.28399E-42	1.21564E-41	positive
PRJNA729271	stage_5W_vs_E10	ordinary_muscle_development	ENSGALG00010002281	TGFBR2	2.133678627	0.154844342	13.77950659	3.38578E-43	3.25876E-42	positive
PRJNA729271	stage_5W_vs_E10	ordinary_muscle_development	ENSGALG00010002282	TSKU	-1.308525601	0.1659145	-7.886746481	3.10167E-15	9.86801E-15	negative
PRJNA729271	stage_5W_vs_E10	ordinary_muscle_development	ENSGALG00010002283	ENSGALG00010002283	-1.364914015	0.948979585	-1.438296499	0.150349945	0.178428234	negative
PRJNA729271	stage_5W_vs_E10	ordinary_muscle_development	ENSGALG00010002285	CAV1	2.711120258	0.157427403	17.22139983	1.83496E-66	3.53224E-65	positive
PRJNA729271	stage_5W_vs_E10	ordinary_muscle_development	ENSGALG00010002288	ANGPT2	-0.671856317	0.178294321	-3.76824295	0.000164401	0.00026584	negative
PRJNA729271	stage_5W_vs_E10	ordinary_muscle_development	ENSGALG00010002289	ENSGALG00010002289	2.300591323	0.937411654	2.454195351	0.014120025	0.019005184	positive
PRJNA729271	stage_5W_vs_E10	ordinary_muscle_development	ENSGALG00010002291	RNASEH1	0.152974361	0.117249351	1.304692607	0.19199754	0.224110512	positive
PRJNA729271	stage_5W_vs_E10	ordinary_muscle_development	ENSGALG00010002293	GYG2	-2.126872234	0.372850348	-5.70435899	1.16782E-08	2.56711E-08	negative
PRJNA729271	stage_5W_vs_E10	ordinary_muscle_development	ENSGALG00010002294	DAZL	1.186962895	0.832417225	1.425923034	0.15389056	0.182331508	positive
PRJNA729271	stage_5W_vs_E10	ordinary_muscle_development	ENSGALG00010002295	YAF2	0.382941662	0.113913953	3.361674771	0.000774713	0.001185418	positive
PRJNA729271	stage_5W_vs_E10	ordinary_muscle_development	ENSGALG00010002298	ENSGALG00010002298	-1.761840685	0.474849503	-3.710313849	0.000207002	0.000332189	negative
PRJNA729271	stage_5W_vs_E10	ordinary_muscle_development	ENSGALG00010002303	ENSGALG00010002303	3.357554186	0.772041545	4.348929415	1.36804E-05	2.4159E-05	positive
PRJNA729271	stage_5W_vs_E10	ordinary_muscle_development	ENSGALG00010002308	GXYLT1	-1.155720682	0.104464309	-11.0633066	1.88999E-28	1.07729E-27	negative
PRJNA729271	stage_5W_vs_E10	ordinary_muscle_development	ENSGALG00010002309	GADL1	8.152032438	0.325428267	25.05016705	1.7385E-138	1.4E-136	positive
PRJNA729271	stage_5W_vs_E10	ordinary_muscle_development	ENSGALG00010002310	PRRG1	-0.675526152	0.106879277	-6.320459585	2.60787E-10	6.31913E-10	negative
PRJNA729271	stage_5W_vs_E10	ordinary_muscle_development	ENSGALG00010002312	ENSGALG00010002312	0.152138925	0.190995783	0.796556458	0.425708674	0.467473275	positive
PRJNA729271	stage_5W_vs_E10	ordinary_muscle_development	ENSGALG00010002315	ENSGALG00010002315	-0.391956239	0.171267571	-2.288560746	0.022104887	0.029043721	negative
PRJNA729271	stage_5W_vs_E10	ordinary_muscle_development	ENSGALG00010002324	SNTG2	-7.018417318	0.881145927	-7.965102154	1.65087E-15	5.34019E-15	negative
PRJNA729271	stage_5W_vs_E10	ordinary_muscle_development	ENSGALG00010002328	ENSGALG00010002328	-0.1395575	0.265302637	-0.526031335	0.598866435	0.636778828	negative
PRJNA729271	stage_5W_vs_E10	ordinary_muscle_development	ENSGALG00010002329	STT3B	-0.161878538	0.072649051	-2.228226451	0.025865418	0.03376434	negative
PRJNA729271	stage_5W_vs_E10	ordinary_muscle_development	ENSGALG00010002330	KHDRBS2	-3.994943264	0.325291529	-12.28111681	1.14408E-34	8.33338E-34	negative
PRJNA729271	stage_5W_vs_E10	ordinary_muscle_development	ENSGALG00010002331	ENSGALG00010002331	-3.222092881	0.355377141	-9.06668582	1.22692E-19	4.83135E-19	negative
PRJNA729271	stage_5W_vs_E10	ordinary_muscle_development	ENSGALG00010002332	ENSGALG00010002332	-1.881483064	0.628095986	-2.99553429	0.002739645	0.003976285	negative
PRJNA729271	stage_5W_vs_E10	ordinary_muscle_development	ENSGALG00010002333	ENSGALG00010002333	2.341059624	0.697859615	3.354628316	0.000794717	0.001214616	positive
PRJNA729271	stage_5W_vs_E10	ordinary_muscle_development	ENSGALG00010002334	OSBPL10	-1.424851521	0.170732176	-8.345536002	7.08916E-17	2.4501E-16	negative
PRJNA729271	stage_5W_vs_E10	ordinary_muscle_development	ENSGALG00010002335	TMEM47	0.515283693	0.134251104	3.838208249	0.000123935	0.000202279	positive
PRJNA729271	stage_5W_vs_E10	ordinary_muscle_development	ENSGALG00010002336	PRIM2	-2.077546635	0.123687197	-16.79678001	2.57657E-63	4.54348E-62	negative
PRJNA729271	stage_5W_vs_E10	ordinary_muscle_development	ENSGALG00010002338	CD99	0.431362135	0.148284368	2.909019619	0.003625641	0.005193563	positive
PRJNA729271	stage_5W_vs_E10	ordinary_muscle_development	ENSGALG00010002339	ZNFY4	-0.456339852	0.3153973	-1.446873048	0.147932483	0.175688762	negative
PRJNA729271	stage_5W_vs_E10	ordinary_muscle_development	ENSGALG00010002340	RAB23	-0.705695586	0.137409362	-5.135716937	2.8107E-07	5.6071E-07	negative
PRJNA729271	stage_5W_vs_E10	ordinary_muscle_development	ENSGALG00010002341	FAM49A	0.009373585	0.110474469	0.084848427	0.932381893	0.941343982	positive
PRJNA729271	stage_5W_vs_E10	ordinary_muscle_development	ENSGALG00010002342	BAG2	3.092505125	0.123414544	25.05786618	1.4331E-138	1.1605E-136	positive
PRJNA729271	stage_5W_vs_E10	ordinary_muscle_development	ENSGALG00010002343	GPD1L	-0.109900136	0.081988927	-1.340426574	0.180106701	0.211251548	negative
PRJNA729271	stage_5W_vs_E10	ordinary_muscle_development	ENSGALG00010002345	SCML2	-1.980572125	0.111005066	-17.84217778	3.32482E-71	7.27994E-70	negative
PRJNA729271	stage_5W_vs_E10	ordinary_muscle_development	ENSGALG00010002346	FBXO15	-0.87544513	0.376662555	-2.32421598	0.020113921	0.026567458	negative
PRJNA729271	stage_5W_vs_E10	ordinary_muscle_development	ENSGALG00010002347	ZNF451	-0.26183903	0.062266747	-4.205118179	2.60946E-05	4.49858E-05	negative
PRJNA729271	stage_5W_vs_E10	ordinary_muscle_development	ENSGALG00010002348	THAP12	-0.314494648	0.154496225	-2.035613794	0.041789152	0.053237278	negative
PRJNA729271	stage_5W_vs_E10	ordinary_muscle_development	ENSGALG00010002349	ZBED1	0.85323726	0.129436628	6.591930539	4.34144E-11	1.10771E-10	positive
PRJNA729271	stage_5W_vs_E10	ordinary_muscle_development	ENSGALG00010002350	BEND6	0.263998097	0.248684361	1.061579006	0.288426849	0.326111324	positive
PRJNA729271	stage_5W_vs_E10	ordinary_muscle_development	ENSGALG00010002352	ENSGALG00010002352	0.256815237	0.106945027	2.401376156	0.016333537	0.021826737	positive
PRJNA729271	stage_5W_vs_E10	ordinary_muscle_development	ENSGALG00010002353	CMTM8	0.45456849	0.138594348	3.279848689	0.001038628	0.001572314	positive
PRJNA729271	stage_5W_vs_E10	ordinary_muscle_development	ENSGALG00010002355	UFM1	-0.946502656	0.101324657	-9.341286537	9.51706E-21	3.91903E-20	negative
PRJNA729271	stage_5W_vs_E10	ordinary_muscle_development	ENSGALG00010002356	WNT11	-4.18874378	0.330235502	-12.68411103	7.2431E-37	5.73709E-36	negative
PRJNA729271	stage_5W_vs_E10	ordinary_muscle_development	ENSGALG00010002357	RAB31	-0.648698171	0.142477723	-4.552979627	5.28914E-06	9.60367E-06	negative
PRJNA729271	stage_5W_vs_E10	ordinary_muscle_development	ENSGALG00010002358	FREM2	-3.563900151	0.235122856	-15.15760827	6.74823E-52	8.687E-51	negative
PRJNA729271	stage_5W_vs_E10	ordinary_muscle_development	ENSGALG00010002359	CMTM6	0.471174074	0.115425146	4.08207477	4.46354E-05	7.55477E-05	positive
PRJNA729271	stage_5W_vs_E10	ordinary_muscle_development	ENSGALG00010002360	DHRSX	1.261586978	0.163086411	7.735696489	1.02839E-14	3.18446E-14	positive
PRJNA729271	stage_5W_vs_E10	ordinary_muscle_development	ENSGALG00010002361	ENSGALG00010002361	0.428049081	0.099113863	4.318760948	1.56908E-05	2.75548E-05	positive
PRJNA729271	stage_5W_vs_E10	ordinary_muscle_development	ENSGALG00010002362	TRPC4	-0.205940138	0.327216655	-0.629369362	0.529107271	0.569411975	negative
PRJNA729271	stage_5W_vs_E10	ordinary_muscle_development	ENSGALG00010002364	VSNL1	-0.712798233	0.396255432	-1.798835236	0.072044748	0.089148764	negative
PRJNA729271	stage_5W_vs_E10	ordinary_muscle_development	ENSGALG00010002365	RAI2	1.542436392	0.138033829	11.17433602	5.44561E-29	3.17259E-28	positive
PRJNA729271	stage_5W_vs_E10	ordinary_muscle_development	ENSGALG00010002366	ENSGALG00010002366	2.580650455	0.756704268	3.410381788	0.00064872	0.000999156	positive
PRJNA729271	stage_5W_vs_E10	ordinary_muscle_development	ENSGALG00010002367	STK31	-3.382707457	0.684331361	-4.943084083	7.68963E-07	1.49012E-06	negative
PRJNA729271	stage_5W_vs_E10	ordinary_muscle_development	ENSGALG00010002368	UVRAG	1.226737892	0.094978786	12.91591459	3.66068E-38	3.00122E-37	positive
PRJNA729271	stage_5W_vs_E10	ordinary_muscle_development	ENSGALG00010002369	GEMIN8	-0.502852559	0.120838266	-4.161368541	3.16346E-05	5.41438E-05	negative
PRJNA729271	stage_5W_vs_E10	ordinary_muscle_development	ENSGALG00010002370	ENSGALG00010002370	-0.091090313	0.157625045	-0.577892384	0.563336777	0.602669318	negative
PRJNA729271	stage_5W_vs_E10	ordinary_muscle_development	ENSGALG00010002371	BAG3	2.84656396	0.154656101	18.40576565	1.18104E-75	2.93135E-74	positive
PRJNA729271	stage_5W_vs_E10	ordinary_muscle_development	ENSGALG00010002372	SMC6	-0.191981323	0.127007449	-1.511575303	0.130641939	0.156280837	negative
PRJNA729271	stage_5W_vs_E10	ordinary_muscle_development	ENSGALG00010002373	POSTN	0.041283535	0.25561425	0.161507173	0.871693963	0.887802416	positive
PRJNA729271	stage_5W_vs_E10	ordinary_muscle_development	ENSGALG00010002374	DST	1.055862339	0.114232956	9.243062376	2.39541E-20	9.71231E-20	positive
PRJNA729271	stage_5W_vs_E10	ordinary_muscle_development	ENSGALG00010002375	NPY	-2.793053174	0.522981424	-5.340635528	9.26213E-08	1.91491E-07	negative
PRJNA729271	stage_5W_vs_E10	ordinary_muscle_development	ENSGALG00010002376	GEN1	-1.486818792	0.193210615	-7.695326649	1.41133E-14	4.32958E-14	negative
PRJNA729271	stage_5W_vs_E10	ordinary_muscle_development	ENSGALG00010002377	ART1A	5.528342472	0.242607464	22.78719035	6.1433E-115	3.4514E-113	positive
PRJNA729271	stage_5W_vs_E10	ordinary_muscle_development	ENSGALG00010002378	ENSGALG00010002378	1.25600518	0.44752549	2.806555626	0.005007427	0.007068132	positive
PRJNA729271	stage_5W_vs_E10	ordinary_muscle_development	ENSGALG00010002379	NHS	-1.670186978	0.136048222	-12.27643369	1.21225E-34	8.80781E-34	negative
PRJNA729271	stage_5W_vs_E10	ordinary_muscle_development	ENSGALG00010002380	CMPK2	5.486267222	0.74150476	7.398829409	1.3739E-13	3.99252E-13	positive
PRJNA729271	stage_5W_vs_E10	ordinary_muscle_development	ENSGALG00010002381	TDRD3	0.44203865	0.09660738	4.575619903	4.74812E-06	8.65384E-06	positive
PRJNA729271	stage_5W_vs_E10	ordinary_muscle_development	ENSGALG00010002382	ART1D	2.807356691	0.524274644	5.354744354	8.56775E-08	1.7749E-07	positive
PRJNA729271	stage_5W_vs_E10	ordinary_muscle_development	ENSGALG00010002383	INPP5F	0.394418946	0.124376828	3.171161	0.00151831	0.002258842	positive
PRJNA729271	stage_5W_vs_E10	ordinary_muscle_development	ENSGALG00010002384	ENSGALG00010002384	-1.455647522	0.312060149	-4.664637656	3.09161E-06	5.72836E-06	negative
PRJNA729271	stage_5W_vs_E10	ordinary_muscle_development	ENSGALG00010002385	PALS2	-2.464781015	0.128131452	-19.23634654	1.8371E-82	5.22132E-81	negative
PRJNA729271	stage_5W_vs_E10	ordinary_muscle_development	ENSGALG00010002386	MHCY2B2	-1.59280451	0.42035156	-3.789219935	0.000151121	0.000244913	negative
PRJNA729271	stage_5W_vs_E10	ordinary_muscle_development	ENSGALG00010002387	MCMBP	-0.164409965	0.109099018	-1.506979336	0.131815954	0.157607214	negative
PRJNA729271	stage_5W_vs_E10	ordinary_muscle_development	ENSGALG00010002388	ENSGALG00010002388	4.087543303	0.72757303	5.618052255	1.93122E-08	4.18807E-08	positive
PRJNA729271	stage_5W_vs_E10	ordinary_muscle_development	ENSGALG00010002389	COL21A1	-0.543116419	0.322430718	-1.684443782	0.092095935	0.112377354	negative
PRJNA729271	stage_5W_vs_E10	ordinary_muscle_development	ENSGALG00010002391	REPS2	-0.136162647	0.160720119	-0.847203502	0.396881702	0.438138639	negative
PRJNA729271	stage_5W_vs_E10	ordinary_muscle_development	ENSGALG00010002392	DFNA5	-1.539895001	0.207926996	-7.405940705	1.30224E-13	3.79112E-13	negative
PRJNA729271	stage_5W_vs_E10	ordinary_muscle_development	ENSGALG00010002393	GPM6B	-0.869862345	0.107298427	-8.10694407	5.19087E-16	1.71824E-15	negative
PRJNA729271	stage_5W_vs_E10	ordinary_muscle_development	ENSGALG00010002395	CLN8	-0.025887653	0.091298386	-0.283549952	0.776755296	0.803415728	negative
PRJNA729271	stage_5W_vs_E10	ordinary_muscle_development	ENSGALG00010002396	SEC23IP	0.15307655	0.088039668	1.738722471	0.082083591	0.100779405	positive
PRJNA729271	stage_5W_vs_E10	ordinary_muscle_development	ENSGALG00010002398	ENSGALG00010002398	-1.256297399	0.399217532	-3.146899373	0.001650117	0.002445399	negative
PRJNA729271	stage_5W_vs_E10	ordinary_muscle_development	ENSGALG00010002399	CSMD1	-2.693782654	0.404860234	-6.653611364	2.85987E-11	7.37351E-11	negative
PRJNA729271	stage_5W_vs_E10	ordinary_muscle_development	ENSGALG00010002400	RBBP7	-2.167003919	0.160961618	-13.46286122	2.58701E-41	2.35397E-40	negative
PRJNA729271	stage_5W_vs_E10	ordinary_muscle_development	ENSGALG00010002401	ZRANB1	0.013159146	0.1617844	0.081337548	0.935173514	0.943439594	positive
PRJNA729271	stage_5W_vs_E10	ordinary_muscle_development	ENSGALG00010002402	TXLNG	-0.109804983	0.127126107	-0.863748489	0.38772607	0.429047209	negative
PRJNA729271	stage_5W_vs_E10	ordinary_muscle_development	ENSGALG00010002403	ZNFY3	-0.167502684	0.265408794	-0.631112035	0.527967273	0.568480584	negative
PRJNA729271	stage_5W_vs_E10	ordinary_muscle_development	ENSGALG00010002404	OSBPL3	-0.117427904	0.252959117	-0.464216926	0.642492335	0.67755012	negative
PRJNA729271	stage_5W_vs_E10	ordinary_muscle_development	ENSGALG00010002405	KCNQ5	1.171639437	0.197492135	5.932587844	2.98197E-09	6.77274E-09	positive
PRJNA729271	stage_5W_vs_E10	ordinary_muscle_development	ENSGALG00010002406	DIAPH3	-1.956082365	0.249257467	-7.847637987	4.23946E-15	1.33851E-14	negative
PRJNA729271	stage_5W_vs_E10	ordinary_muscle_development	ENSGALG00010002408	OFD1	-0.361169552	0.116987191	-3.087257237	0.002020127	0.002967644	negative
PRJNA729271	stage_5W_vs_E10	ordinary_muscle_development	ENSGALG00010002409	CYCS	1.352427806	0.166748841	8.110567946	5.03837E-16	1.66967E-15	positive

PRJNA729271	stage_5W_vs_E10	ordinary_muscle_development	ENSGALG00010002410	RNF121	0.398227005	0.104662832	3.804856012	0.000141887	0.000230695	positive
PRJNA729271	stage_5W_vs_E10	ordinary_muscle_development	ENSGALG00010002411	ENSGALG00010002411	-1.767096478	0.200296917	-8.822384814	1.12047E-18	4.21304E-18	negative
PRJNA729271	stage_5W_vs_E10	ordinary_muscle_development	ENSGALG00010002413	TSSC1	-0.808763599	0.085930917	-9.411788324	4.87766E-21	2.04222E-20	negative
PRJNA729271	stage_5W_vs_E10	ordinary_muscle_development	ENSGALG00010002414	ENSGALG00010002414	3.076642811	0.355662274	8.650461497	5.12916E-18	1.87461E-17	positive
PRJNA729271	stage_5W_vs_E10	ordinary_muscle_development	ENSGALG00010002415	PCDH17	-5.887020293	0.536442233	-10.97419244	5.08583E-28	2.8529E-27	negative
PRJNA729271	stage_5W_vs_E10	ordinary_muscle_development	ENSGALG00010002416	SH3BGRL2	-1.753642227	0.287955859	-6.089968908	1.12933E-09	2.6305E-09	negative
PRJNA729271	stage_5W_vs_E10	ordinary_muscle_development	ENSGALG00010002418	TRAPPC2	-0.301533695	0.083662017	-3.604188701	0.00031313	0.000494909	negative
PRJNA729271	stage_5W_vs_E10	ordinary_muscle_development	ENSGALG00010002419	CTBP2	-1.603037408	0.116126817	-13.80419654	2.40438E-43	2.33433E-42	negative
PRJNA729271	stage_5W_vs_E10	ordinary_muscle_development	ENSGALG00010002420	TMEM272	-0.380636512	0.726657717	-0.523818165	0.600405018	0.638321163	negative
PRJNA729271	stage_5W_vs_E10	ordinary_muscle_development	ENSGALG00010002421	ENSGALG00010002421	-0.157549227	0.187841187	-0.838736325	0.401617289	0.443065881	negative
PRJNA729271	stage_5W_vs_E10	ordinary_muscle_development	ENSGALG00010002422	TRAPPC12	0.201529295	0.103679459	1.943772627	0.051922876	0.065350269	positive
PRJNA729271	stage_5W_vs_E10	ordinary_muscle_development	ENSGALG00010002423	ENSGALG00010002423	4.168052164	0.564557565	7.382864786	1.54919E-13	4.49111E-13	positive
PRJNA729271	stage_5W_vs_E10	ordinary_muscle_development	ENSGALG00010002424	CASK	-0.292362176	0.056969459	-5.131910681	2.86816E-07	5.7162E-07	negative
PRJNA729271	stage_5W_vs_E10	ordinary_muscle_development	ENSGALG00010002425	FDX2	0.339175823	0.134451185	2.522668907	0.0116468	0.015833094	positive
PRJNA729271	stage_5W_vs_E10	ordinary_muscle_development	ENSGALG00010002427	CYB5R4	-0.100328957	0.1135196	-0.883802948	0.376802594	0.417884744	negative
PRJNA729271	stage_5W_vs_E10	ordinary_muscle_development	ENSGALG00010002428	IBSP	-2.278114645	0.70713684	-3.22160368	0.001274753	0.001911009	negative
PRJNA729271	stage_5W_vs_E10	ordinary_muscle_development	ENSGALG00010002429	ENSGALG00010002429	0.891677617	0.543362314	1.641036917	0.100789752	0.122350209	positive
PRJNA729271	stage_5W_vs_E10	ordinary_muscle_development	ENSGALG00010002430	NUMA1	1.861083025	0.174161624	10.68595357	1.18426E-26	6.31328E-26	positive
PRJNA729271	stage_5W_vs_E10	ordinary_muscle_development	ENSGALG00010002431	BCKDHB	0.938444928	0.147639507	6.356326619	2.06635E-10	5.03985E-10	positive
PRJNA729271	stage_5W_vs_E10	ordinary_muscle_development	ENSGALG00010002432	RAVER1	-0.763074051	0.188117665	-4.056365746	4.98422E-05	8.41247E-05	negative
PRJNA729271	stage_5W_vs_E10	ordinary_muscle_development	ENSGALG00010002433	GRHL1	-1.841746545	0.331320766	-5.558802021	2.71633E-08	5.82788E-08	negative
PRJNA729271	stage_5W_vs_E10	ordinary_muscle_development	ENSGALG00010002434	GPR34	3.243596253	0.245955578	13.18773203	1.03245E-39	8.94521E-39	positive
PRJNA729271	stage_5W_vs_E10	ordinary_muscle_development	ENSGALG00010002435	ATP7B	-2.669508231	0.732830401	-3.642736747	0.000269755	0.000428457	negative
PRJNA729271	stage_5W_vs_E10	ordinary_muscle_development	ENSGALG00010002436	ENSGALG00010002436	4.879609938	0.873607847	5.585583916	2.32916E-08	5.02549E-08	positive
PRJNA729271	stage_5W_vs_E10	ordinary_muscle_development	ENSGALG00010002437	TTK	-2.893361507	0.198475835	-14.57790317	3.8826E-48	4.36266E-47	negative
PRJNA729271	stage_5W_vs_E10	ordinary_muscle_development	ENSGALG00010002438	RARB	-1.861949005	0.094837701	-19.63300435	8.07931E-86	2.48114E-84	negative
PRJNA729271	stage_5W_vs_E10	ordinary_muscle_development	ENSGALG00010002439	GPR82	4.633640965	0.850813983	5.446126953	5.14784E-08	1.08252E-07	positive
PRJNA729271	stage_5W_vs_E10	ordinary_muscle_development	ENSGALG00010002440	LRRC51	0.546409516	0.269817414	2.025108419	0.042856233	0.054515234	positive
PRJNA729271	stage_5W_vs_E10	ordinary_muscle_development	ENSGALG00010002441	USO1	-0.32409651	0.068212694	-4.751263872	2.02149E-06	3.78915E-06	negative
PRJNA729271	stage_5W_vs_E10	ordinary_muscle_development	ENSGALG00010002442	ENSGALG00010002442	-0.288464317	0.471431975	-0.611889588	0.540610811	0.58049883	negative
PRJNA729271	stage_5W_vs_E10	ordinary_muscle_development	ENSGALG00010002443	TYK2	0.569589743	0.137832348	4.13248234	3.58866E-05	6.11469E-05	positive
PRJNA729271	stage_5W_vs_E10	ordinary_muscle_development	ENSGALG00010002444	KLF11	0.810100117	0.182295348	4.443888046	8.83476E-06	1.58079E-05	positive
PRJNA729271	stage_5W_vs_E10	ordinary_muscle_development	ENSGALG00010002445	EDRF1	0.872708678	0.111137582	7.852507363	4.07801E-15	1.28894E-14	positive
PRJNA729271	stage_5W_vs_E10	ordinary_muscle_development	ENSGALG00010002446	RIMS1	-5.366005741	0.353119945	-15.19598602	3.75964E-52	4.85703E-51	negative
PRJNA729271	stage_5W_vs_E10	ordinary_muscle_development	ENSGALG00010002447	YLEC12	0.265437168	0.562452541	0.471928117	0.636978098	0.672517848	positive
PRJNA729271	stage_5W_vs_E10	ordinary_muscle_development	ENSGALG00010002448	RAB9A	-0.133087976	0.136328346	-0.976231134	0.328949938	0.368622292	negative
PRJNA729271	stage_5W_vs_E10	ordinary_muscle_development	ENSGALG00010002449	ALG11	-0.596139505	0.141370868	-4.216848316	2.4774E-05	4.28111E-05	negative
PRJNA729271	stage_5W_vs_E10	ordinary_muscle_development	ENSGALG00010002453	TOP2B	-0.872141387	0.063309928	-13.77574444	3.56687E-43	3.4285E-42	negative
PRJNA729271	stage_5W_vs_E10	ordinary_muscle_development	ENSGALG00010002454	NEK5	-3.731445696	0.767888323	-4.859359863	1.17766E-06	2.24755E-06	negative
PRJNA729271	stage_5W_vs_E10	ordinary_muscle_development	ENSGALG00010002455	NYX	1.903027652	0.35468592	5.365388214	8.07753E-08	1.6767E-07	positive
PRJNA729271	stage_5W_vs_E10	ordinary_muscle_development	ENSGALG00010002456	ELOVL4	-3.376503301	0.61984908	-5.44729904	5.11404E-08	1.07604E-07	negative
PRJNA729271	stage_5W_vs_E10	ordinary_muscle_development	ENSGALG00010002457	ENSGALG00010002457	2.345124405	0.44112779	5.316201925	1.05956E-07	2.1825E-07	positive
PRJNA729271	stage_5W_vs_E10	ordinary_muscle_development	ENSGALG00010002458	ENSGALG00010002458	-0.137541497	0.198164288	-0.694078124	0.487633217	0.529108488	negative
PRJNA729271	stage_5W_vs_E10	ordinary_muscle_development	ENSGALG00010002459	G3BP2	-0.766874216	0.074658682	-10.27173532	9.44895E-25	4.64752E-24	negative
PRJNA729271	stage_5W_vs_E10	ordinary_muscle_development	ENSGALG00010002460	LAMTOR1	1.197142332	0.180544806	6.630721526	3.3405E-11	8.58977E-11	positive
PRJNA729271	stage_5W_vs_E10	ordinary_muscle_development	ENSGALG00010002461	TOMT	-0.02766245	0.344283964	-0.080347774	0.935960663	0.944167987	negative
PRJNA729271	stage_5W_vs_E10	ordinary_muscle_development	ENSGALG00010002462	RRM2	-3.55423829	0.277022222	-12.83015586	1.1114E-37	8.98483E-37	negative
PRJNA729271	stage_5W_vs_E10	ordinary_muscle_development	ENSGALG00010002463	ASRGL1	0.381879946	0.101341462	3.768249806	0.000164396	0.00026584	positive
PRJNA729271	stage_5W_vs_E10	ordinary_muscle_development	ENSGALG00010002464	NGLY1	0.561230028	0.083225064	6.743521762	1.54593E-11	4.04992E-11	positive
PRJNA729271	stage_5W_vs_E10	ordinary_muscle_development	ENSGALG00010002466	UROS	1.131189641	0.175961575	6.428617379	1.2877E-10	3.18193E-10	positive
PRJNA729271	stage_5W_vs_E10	ordinary_muscle_development	ENSGALG00010002467	NEK3	-1.410601039	0.22383648	-6.301926457	2.93969E-10	7.08998E-10	negative
PRJNA729271	stage_5W_vs_E10	ordinary_muscle_development	ENSGALG00010002468	ENSGALG00010002468	1.020213938	0.543090084	1.87853538	0.060307962	0.075274598	positive
PRJNA729271	stage_5W_vs_E10	ordinary_muscle_development	ENSGALG00010002469	MEPE	-2.77065652	1.331936764	-2.080171218	0.037509831	0.048124004	negative
PRJNA729271	stage_5W_vs_E10	ordinary_muscle_development	ENSGALG00010002470	OXSM	0.374074054	0.162094571	2.307751894	0.02101294	0.027686807	positive
PRJNA729271	stage_5W_vs_E10	ordinary_muscle_development	ENSGALG00010002471	OGFRL1	-0.099917714	0.153112397	-0.652577556	0.514028659	0.554460888	negative
PRJNA729271	stage_5W_vs_E10	ordinary_muscle_development	ENSGALG00010002472	CDC37	-0.119342959	0.214784081	-0.555641548	0.578455923	0.617249603	negative
PRJNA729271	stage_5W_vs_E10	ordinary_muscle_development	ENSGALG00010002473	ENSGALG00010002473	3.000869864	0.627448966	4.782651698	1.72998E-06	3.25917E-06	positive
PRJNA729271	stage_5W_vs_E10	ordinary_muscle_development	ENSGALG00010002474	HPCAL1	0.104361352	0.141192835	0.739140562	0.459821649	0.5018156	positive
PRJNA729271	stage_5W_vs_E10	ordinary_muscle_development	ENSGALG00010002475	ENSGALG00010002475	0.053905921	0.188221496	0.286396201	0.774574684	0.801389011	positive
PRJNA729271	stage_5W_vs_E10	ordinary_muscle_development	ENSGALG00010002476	DDX3X	0.516447095	0.144971585	3.562402215	0.000367477	0.000577407	positive
PRJNA729271	stage_5W_vs_E10	ordinary_muscle_development	ENSGALG00010002478	PPME1	-1.1858875	0.057857564	-20.49667176	2.30531E-93	8.45963E-92	negative
PRJNA729271	stage_5W_vs_E10	ordinary_muscle_development	ENSGALG00010002481	CKAP2	-3.100578971	0.246955057	-12.55523579	3.72071E-36	2.87483E-35	negative
PRJNA729271	stage_5W_vs_E10	ordinary_muscle_development	ENSGALG00010002483	COQ2	1.312973306	0.138034662	9.511910156	1.87193E-21	7.98518E-21	positive
PRJNA729271	stage_5W_vs_E10	ordinary_muscle_development	ENSGALG00010002487	BCCIP	-0.547493542	0.118905871	-4.604428171	4.13601E-06	7.57728E-06	negative
PRJNA729271	stage_5W_vs_E10	ordinary_muscle_development	ENSGALG00010002492	RPL8	-0.017631282	0.129598002	-0.136045937	0.891784966	0.905211701	negative
PRJNA729271	stage_5W_vs_E10	ordinary_muscle_development	ENSGALG00010002500	OSR1	-2.553677537	0.201909731	-12.64761994	1.15315E-36	9.0498E-36	negative
PRJNA729271	stage_5W_vs_E10	ordinary_muscle_development	ENSGALG00010002501	TCEANC	0.503302711	0.093687812	5.372125777	7.78138E-08	1.61638E-07	positive
PRJNA729271	stage_5W_vs_E10	ordinary_muscle_development	ENSGALG00010002504	ENSGALG00010002504	0.936568461	0.065586959	14.27979691	2.92452E-46	3.08972E-45	positive
PRJNA729271	stage_5W_vs_E10	ordinary_muscle_development	ENSGALG00010002507	C2CD3	-0.716716184	0.112704978	-6.359223855	2.02776E-10	4.94987E-10	negative
PRJNA729271	stage_5W_vs_E10	ordinary_muscle_development	ENSGALG00010002511	VPS36	-0.199953156	0.077617732	-2.576127262	0.009991386	0.01368599	negative
PRJNA729271	stage_5W_vs_E10	ordinary_muscle_development	ENSGALG00010002512	DAXX	0.990556892	0.22949199	4.316302676	1.58664E-05	2.78498E-05	positive
PRJNA729271	stage_5W_vs_E10	ordinary_muscle_development	ENSGALG00010002518	MAST1	-0.07624	0.376836863	-0.202315663	0.839669956	0.859110327	negative
PRJNA729271	stage_5W_vs_E10	ordinary_muscle_development	ENSGALG00010002520	TTC32	0.82532674	0.164050721	5.030924178	4.88121E-07	9.58455E-07	positive
PRJNA729271	stage_5W_vs_E10	ordinary_muscle_development	ENSGALG00010002522	HPSE	2.168899272	0.2849152	7.612437927	2.68973E-14	8.1479E-14	positive
PRJNA729271	stage_5W_vs_E10	ordinary_muscle_development	ENSGALG00010002523	DNAJB13	4.247845653	0.415475109	10.22406773	1.54709E-24	7.54293E-24	positive
PRJNA729271	stage_5W_vs_E10	ordinary_muscle_development	ENSGALG00010002526	ZBTB22	-0.00678441	0.10725832	-0.063252998	0.949565043	0.956360846	negative
PRJNA729271	stage_5W_vs_E10	ordinary_muscle_development	ENSGALG00010002527	LRRC3B	-3.315648149	0.234661555	-14.12949021	2.49947E-45	2.58047E-44	negative
PRJNA729271	stage_5W_vs_E10	ordinary_muscle_development	ENSGALG00010002528	MED14	-0.052628885	0.116776099	-0.450681988	0.652218766	0.686857819	negative
PRJNA729271	stage_5W_vs_E10	ordinary_muscle_development	ENSGALG00010002530	STAG3	-0.475972576	0.123050007	-3.868123121	0.000109676	0.000179857	negative
PRJNA729271	stage_5W_vs_E10	ordinary_muscle_development	ENSGALG00010002533	LPAR2	-0.694978092	0.145174872	-4.787178965	1.69142E-06	3.18984E-06	negative
PRJNA729271	stage_5W_vs_E10	ordinary_muscle_development	ENSGALG00010002536	RGL2	1.76679774	0.141585184	12.47869085	9.75778E-36	7.40519E-35	positive
PRJNA729271	stage_5W_vs_E10	ordinary_muscle_development	ENSGALG00010002539	SNAP91	0.565693749	0.190260308	2.973262024	0.002946528	0.004261192	positive
PRJNA729271	stage_5W_vs_E10	ordinary_muscle_development	ENSGALG00010002543	UCP3	10.6137872	0.502909076	21.10478358	7.18831E-99	2.94335E-97	positive
PRJNA729271	stage_5W_vs_E10	ordinary_muscle_development	ENSGALG00010002544	THSD1	0.65809632	0.12570037	5.235436615	1.64595E-07	3.34475E-07	positive
PRJNA729271	stage_5W_vs_E10	ordinary_muscle_development	ENSGALG00010002546	ATP6AP2	0.765136455	0.062768511	12.18981367	3.52226E-34	2.51008E-33	positive
PRJNA729271	stage_5W_vs_E10	ordinary_muscle_development	ENSGALG00010002547	PBX4	-1.500987885	0.112414756	-13.35223182	1.14953E-40	1.02538E-39	negative
PRJNA729271	stage_5W_vs_E10	ordinary_muscle_development	ENSGALG00010002551	WDR35	-1.133893602	0.112771666	-10.05477389	8.75221E-24	4.12429E-23	negative
PRJNA729271	stage_5W_vs_E10	ordinary_muscle_development	ENSGALG00010002553	ENSGALG00010002553	0.289721526	0.226626843	1.278407806	0.201105673	0.233706946	positive
PRJNA729271	stage_5W_vs_E10	ordinary_muscle_development	ENSGALG00010002554	EGFL6	-3.68105749	0.293602221	-12.53756693	4.65056E-36	3.58372E-35	negative
PRJNA729271	stage_5W_vs_E10	ordinary_muscle_development	ENSGALG00010002556	ENSGALG00010002556	4.612715575	0.229841202	20.06914137	1.37357E-89	4.61947E-88	positive
PRJNA729271	stage_5W_vs_E10	ordinary_muscle_development	ENSGALG00010002557	ENSGALG00010002557	1.277250203	0.406672305	3.140735643	0.001685241	0.002495438	positive
PRJNA729271	stage_5W_vs_E10	ordinary_muscle_development	ENSGALG00010002559	NEK10	-1.6633408	0.588439373	-2.826698682	0.004703056	0.006660556	negative
PRJNA729271	stage_5W_vs_E10	ordinary_muscle_development	ENSGALG00010002561	PAAF1	-0.636708895	0.085400978	-7.455522292	8.95125E-14	2.62968E-13	negative
PRJNA729271	stage_5W_vs_E10	ordinary_muscle_development	ENSGALG00010002565	MRPS18C	0.731679864	0.114839593	6.37132059	1.87407E-10	4.58708E-10	positive
PRJNA729271	stage_5W_vs_E10	ordinary_muscle_development	ENSGALG00010002566	TMSB4X	-1.17400697	0.115744721	-10.14307138	3.55735E-24	1.70685E-23	negative
PRJNA729271	stage_5W_vs_E10	ordinary_muscle_development	ENSGALG00010002567	COA4	-0.20948224	0.239999944	-0.87284287	0.382748729	0.423927778	negative
PRJNA729271	stage_5W_vs_E10	ordinary_muscle_development	ENSGALG00010002569	GTPBP6	0.087393032	0.118210976	0.739297102	0.459726609	0.501749657	positive
PRJNA729271	stage_5W_vs_E10	ordinary_muscle_development	ENSGALG00010002570	ADAM12	-3.030495234	0.271250302	-11.1723202	5.57064E-29	3.24153E-28	negative
PRJNA729271	stage_5W_vs_E10	ordinary_muscle_development	ENSGALG00010002571	BCOR	0.573844169	0.141461007	4.056553696	4.98021E-05	8.40668E-05	positive
PRJNA729271	stage_5W_vs_E10	ordinary_muscle_development	ENSGALG00010002573	MVB12A	1.260649314	0.094522967	13.33696298	1.41091E-40	1.25544E-39	positive
PRJNA729271	stage_5W_vs_E10	ordinary_muscle_development	ENSGALG00010002575	BGV	0.050228162	0.403682551	0.124424901	0.900978858	0.913520464	positive
PRJNA729271	stage_5W_vs_E10	ordinary_muscle_development	ENSGALG00010002576	HELQ	-0.55064982	0.109489452	-5.029249949	4.92402E-07	9.66076E-07	negative
PRJNA729271	stage_5W_vs_E10	ordinary_muscle_development	ENSGALG00010002577	MRPL48	-0.052091103	0.103670524	-0.502467823	0.615338481	0.652127623	negative
PRJNA729271	stage_5W_vs_E10	ordinary_muscle_development	ENSGALG00010002579	MATN3	-1.08544178	0.575579252	-1.885825066	0.059318532	0.074097046	negative
PRJNA729271	stage_5W_vs_E10	ordinary_muscle_development	ENSGALG00010002581	ATP13A1	0.003465973	0.077502217	0.044720956	0.96432973	0.9690765	positive
PRJNA729271	stage_5W_vs_E10	ordinary_muscle_development	ENSGALG00010002582	ENSGALG00010002582	-3.691000721	0.548785305	-6.725764503	1.74673E-11	4.56195E-11	negative
PRJNA729271	stage_5W_vs_E10	ordinary_muscle_development	ENSGALG00010002584	ENSGALG00010002584	0.068270595	0.451262984	0.151287824	0.879748674	0.894497547	positive
PRJNA729271	stage_5W_vs_E10	ordinary_muscle_development	ENSGALG00010002586	GPR63	-0.374676684	0.147974546	-2.532034682	0.011340277	0.015440289	negative
PRJNA729271	stage_5W_vs_E10	ordinary_muscle_development	ENSGALG00010002587	DOCK1	-0.348850283	0.066781603	-5.223748288	1.75337E-07	3.55605E-07	negative
PRJNA729271	stage_5W_vs_E10	ordinary_muscle_development	ENSGALG00010002588	FAM175A	-0.930001958	0.089469617	-10.39461201	2.62352E-25	1.31585E-24	negative
PRJNA729271	stage_5W_vs_E10	ordinary_muscle_development	ENSGALG00010002589	SLC4A7	-0.213755846	0.145160598	-1.472547298	0.140873145	0.167772264	negative
PRJNA729271	stage_5W_vs_E10	ordinary_muscle_development	ENSGALG00010002590	ATP6V1C2	-1.520569819	0.261312095	-5.818979852	5.92079E-09	1.32421E-08	negative
PRJNA729271	stage_5W_vs_E10	ordinary_muscle_development	ENSGALG00010002591	ENSGALG00010002591	0.592003118	0.21380156	2.768937315	0.005623945	0.007899902	positive
PRJNA729271	stage_5W_vs_E10	ordinary_muscle_development	ENSGALG00010002592	RPS6KA3	1.306586902	0.101064603	12.92823468	3.11898E-38	2.56E-37	positive
PRJNA729271	stage_5W_vs_E10	ordinary_muscle_development	ENSGALG00010002597	ENSGALG00010002597	-0.605085805	0.118444253	-5.108612619	3.24533E-07	6.45105E-07	negative
PRJNA729271	stage_5W_vs_E10	ordinary_muscle_development	ENSGALG00010002598	NMT2	0.123781786	0.102610171	1.206330565	0.227690057	0.262183617	positive
PRJNA729271	stage_5W_vs_E10	ordinary_muscle_development	ENSGALG00010002599	KIFC1	-1.404471212	0.219919087	-6.386308865	1.69938E-10	4.16511E-10	negative
PRJNA729271	stage_5W_vs_E10	ordinary_muscle_development	ENSGALG00010002600	TRPA1	-2.406794122	0.607891927	-3.959246729	7.51865E-05	0.000125389	negative
PRJNA729271	stage_5W_vs_E10	ordinary_muscle_development	ENSGALG00010002601	PLCXD1	2.328414139	0.186790922	12.46534959	1.15365E-35	8.74131E-35	positive
PRJNA729271	stage_5W_vs_E10	ordinary_muscle_development	ENSGALG00010002603	INSYN2A	-0.238386864	0.260595912	-0.914775914	0.360309282	0.400604667	negative
PRJNA729271	stage_5W_vs_E10	ordinary_muscle_development	ENSGALG00010002604	BLEC3	-0.276670796	0.395643742	-0.69929274	0.484369103	0.52603058	negative
PRJNA729271	stage_5W_vs_E10	ordinary_muscle_development	ENSGALG00010002605	EOMES	4.141566095	0.677112619	6.116509983	9.56469E-10	2.23471E-09	positive
PRJNA729271	stage_5W_vs_E10	ordinary_muscle_development	ENSGALG00010002608	RPP38	-0.628707691	0.132313605	-4.751648114	2.01765E-06	3.78245E-06	negative
PRJNA729271	stage_5W_vs_E10	ordinary_muscle_development	ENSGALG00010002609	PDIA6	-1.159141915	0.125493657	-9.236657393	2.54318E-20	1.03028E-19	negative
PRJNA729271	stage_5W_vs_E10	ordinary_muscle_development	ENSGALG00010002611	NPS	-0.198890558	0.414132366	-0.480258424	0.631043648	0.666640746	negative
PRJNA729271	stage_5W_vs_E10	ordinary_muscle_development	ENSGALG00010002612	JPH1	4.71057402	0.214330491	21.97808623	4.6673E-107	2.2036E-105	positive
PRJNA729271	stage_5W_vs_E10	ordinary_muscle_development	ENSGALG00010002613	ENSGALG00010002613	-0.065999976	0.104972238	-0.628737439	0.529520963	0.569814875	negative
PRJNA729271	stage_5W_vs_E10	ordinary_muscle_development	ENSGALG00010002614	GPAT3	0.550979857	0.14475713	3.806236401	0.000141098	0.000229438	positive
PRJNA729271	stage_5W_vs_E10	ordinary_muscle_development	ENSGALG00010002616	ZNFB3	-0.779478446	0.171985268	-4.532239633	5.83616E-06	1.05638E-05	negative
PRJNA729271	stage_5W_vs_E10	ordinary_muscle_development	ENSGALG00010002619	RAB6A	-0.772852264	0.068742328	-11.24274205	2.51449E-29	1.48462E-28	negative
PRJNA729271	stage_5W_vs_E10	ordinary_muscle_development	ENSGALG00010002621	SLC25A15	-1.342572007	0.165356491	-8.119257967	4.69043E-16	1.55721E-15	negative

PRJNA729271	stage_5W_vs_E10	ordinary_muscle_development	ENSGALG00010002622	AZI2	0.557293557	0.079846145	6.97959251	2.96037E-12	8.04625E-12	positive
PRJNA729271	stage_5W_vs_E10	ordinary_muscle_development	ENSGALG00010002623	MID1IP1	2.266093923	0.135511109	16.72256944	8.97642E-63	1.55638E-61	positive
PRJNA729271	stage_5W_vs_E10	ordinary_muscle_development	ENSGALG00010002625	FHL5	1.402891408	0.218320252	6.425841831	1.31142E-10	3.23943E-10	positive
PRJNA729271	stage_5W_vs_E10	ordinary_muscle_development	ENSGALG00010002626	CHD7	1.025352524	0.135581619	7.562621931	3.95025E-14	1.18524E-13	positive
PRJNA729271	stage_5W_vs_E10	ordinary_muscle_development	ENSGALG00010002627	MRPS31	0.724467465	0.146318296	4.951311509	7.3715E-07	1.43039E-06	positive
PRJNA729271	stage_5W_vs_E10	ordinary_muscle_development	ENSGALG00010002628	TRIM7.2	-0.069844243	0.192192152	-0.363408404	0.716299825	0.747015322	negative
PRJNA729271	stage_5W_vs_E10	ordinary_muscle_development	ENSGALG00010002632	HPGD	2.176061178	0.272906003	7.973665484	1.54036E-15	4.98716E-15	positive
PRJNA729271	stage_5W_vs_E10	ordinary_muscle_development	ENSGALG00010002633	ENSGALG00010002633	-1.062777164	0.132217688	-8.038086151	9.12525E-16	2.98579E-15	negative
PRJNA729271	stage_5W_vs_E10	ordinary_muscle_development	ENSGALG00010002634	RGN	1.113573787	0.183327602	6.074228728	1.24585E-09	2.89401E-09	positive
PRJNA729271	stage_5W_vs_E10	ordinary_muscle_development	ENSGALG00010002638	FABP9	4.379669935	0.50413142	8.687555986	3.7032E-18	1.36273E-17	positive
PRJNA729271	stage_5W_vs_E10	ordinary_muscle_development	ENSGALG00010002644	KCNF1	-1.807425693	0.52654589	-3.432608111	0.000597805	0.000923302	negative
PRJNA729271	stage_5W_vs_E10	ordinary_muscle_development	ENSGALG00010002646	FOXO1	1.239182535	0.169403795	7.314963257	2.57452E-13	7.38671E-13	positive
PRJNA729271	stage_5W_vs_E10	ordinary_muscle_development	ENSGALG00010002647	FAM168A	-0.198627502	0.098262822	-2.021390164	0.043239392	0.054968864	negative
PRJNA729271	stage_5W_vs_E10	ordinary_muscle_development	ENSGALG00010002649	UFL1	0.539980066	0.079071098	6.829044747	8.54819E-12	2.26851E-11	positive
PRJNA729271	stage_5W_vs_E10	ordinary_muscle_development	ENSGALG00010002653	EIF1AX	-0.417800668	0.063790421	-6.549583196	5.76979E-11	1.45804E-10	negative
PRJNA729271	stage_5W_vs_E10	ordinary_muscle_development	ENSGALG00010002655	FABP4	7.718585392	0.946263979	8.156905008	3.43719E-16	1.1485E-15	positive
PRJNA729271	stage_5W_vs_E10	ordinary_muscle_development	ENSGALG00010002656	GLRA3	2.715521078	0.544679234	4.985541784	6.17884E-07	1.20493E-06	positive
PRJNA729271	stage_5W_vs_E10	ordinary_muscle_development	ENSGALG00010002658	FBXL12	-0.551430582	0.152012448	-3.627535705	0.000286139	0.000453635	negative
PRJNA729271	stage_5W_vs_E10	ordinary_muscle_development	ENSGALG00010002661	ACBD7	-2.723877608	0.220374323	-12.36023132	4.28921E-35	3.17366E-34	negative
PRJNA729271	stage_5W_vs_E10	ordinary_muscle_development	ENSGALG00010002664	ZNFB1	-0.130036664	0.14144137	-0.919367963	0.357903141	0.398051563	negative
PRJNA729271	stage_5W_vs_E10	ordinary_muscle_development	ENSGALG00010002667	ENSGALG00010002667	3.272879527	1.568721582	2.086335501			positive
PRJNA729271	stage_5W_vs_E10	ordinary_muscle_development	ENSGALG00010002669	IL4I1	5.393039942	1.102903743	4.889855508	1.0091E-06	1.93708E-06	positive
PRJNA729271	stage_5W_vs_E10	ordinary_muscle_development	ENSGALG00010002673	SLC66A3	-0.256964716	0.150383623	-1.708728062	0.087501334	0.107059326	negative
PRJNA729271	stage_5W_vs_E10	ordinary_muscle_development	ENSGALG00010002678	CCNI	1.42919088	0.172781593	8.27166168	1.32105E-16	4.50661E-16	positive
PRJNA729271	stage_5W_vs_E10	ordinary_muscle_development	ENSGALG00010002681	PLEKHB1	0.185636473	0.158048668	1.174552591	0.24017376	0.275464673	positive
PRJNA729271	stage_5W_vs_E10	ordinary_muscle_development	ENSGALG00010002682	UBL5	-0.150758873	0.166487405	-0.905527197	0.365186158	0.405840172	negative
PRJNA729271	stage_5W_vs_E10	ordinary_muscle_development	ENSGALG00010002685	CA8	1.84580265	0.177431458	10.4029053	2.40487E-25	1.20701E-24	positive
PRJNA729271	stage_5W_vs_E10	ordinary_muscle_development	ENSGALG00010002689	COG6	-0.633094008	0.062644691	-10.10610792	5.19055E-24	2.47165E-23	negative
PRJNA729271	stage_5W_vs_E10	ordinary_muscle_development	ENSGALG00010002693	MSC	-2.136209698	0.170050638	-12.56219748	3.40736E-36	2.63834E-35	negative
PRJNA729271	stage_5W_vs_E10	ordinary_muscle_development	ENSGALG00010002700	LHFPL6	-1.123736519	0.171005568	-6.571344635	4.98629E-11	1.26645E-10	negative
PRJNA729271	stage_5W_vs_E10	ordinary_muscle_development	ENSGALG00010002701	TMEM196	-2.66815302	0.630544603	-4.231505605	2.32132E-05	4.02194E-05	negative
PRJNA729271	stage_5W_vs_E10	ordinary_muscle_development	ENSGALG00010002703	PIN1	0.160282542	0.096601913	1.65920671	0.097074146	0.118163398	positive
PRJNA729271	stage_5W_vs_E10	ordinary_muscle_development	ENSGALG00010002704	KCNB2	-0.201286049	0.261340714	-0.770205476	0.441178016	0.48303311	negative
PRJNA729271	stage_5W_vs_E10	ordinary_muscle_development	ENSGALG00010002705	JADE3	-1.435149488	0.056610393	-25.35134305	8.6851E-142	7.4054E-140	negative
PRJNA729271	stage_5W_vs_E10	ordinary_muscle_development	ENSGALG00010002706	ROCK2	1.621952552	0.144080769	11.25724525	2.13323E-29	1.26106E-28	positive
PRJNA729271	stage_5W_vs_E10	ordinary_muscle_development	ENSGALG00010002707	RELT	-4.653627813	0.317137431	-14.67385226	9.48062E-49	1.09674E-47	negative
PRJNA729271	stage_5W_vs_E10	ordinary_muscle_development	ENSGALG00010002712	PTPRE	1.586475484	0.12338942	12.85746769	7.80933E-38	6.35175E-37	positive
PRJNA729271	stage_5W_vs_E10	ordinary_muscle_development	ENSGALG00010002713	OLFM2	0.382246336	0.177420098	2.154470318	0.031203307	0.040415686	positive
PRJNA729271	stage_5W_vs_E10	ordinary_muscle_development	ENSGALG00010002716	NHLRC3	-0.555976174	0.110545335	-5.029395188	4.92029E-07	9.65475E-07	negative
PRJNA729271	stage_5W_vs_E10	ordinary_muscle_development	ENSGALG00010002722	TRIM7.1	2.769328949	0.149053317	18.57945201	4.71293E-77	1.20271E-75	positive
PRJNA729271	stage_5W_vs_E10	ordinary_muscle_development	ENSGALG00010002725	TSPAN7	0.022650485	0.179093631	0.126472866	0.899357626	0.912068061	positive
PRJNA729271	stage_5W_vs_E10	ordinary_muscle_development	ENSGALG00010002728	RP2	-0.086655146	0.111666118	-0.776020043	0.437737152	0.479700613	negative
PRJNA729271	stage_5W_vs_E10	ordinary_muscle_development	ENSGALG00010002730	E2F6	0.52071207	0.10108655	5.151150867	2.58893E-07	5.18107E-07	positive
PRJNA729271	stage_5W_vs_E10	ordinary_muscle_development	ENSGALG00010002733	RDH8	0.549417356	0.3808692	1.442535538	0.149151354	0.177078292	positive
PRJNA729271	stage_5W_vs_E10	ordinary_muscle_development	ENSGALG00010002739	HEP21	-2.905279374	1.015077512	-2.862125639	0.004208101	0.005992378	negative
PRJNA729271	stage_5W_vs_E10	ordinary_muscle_development	ENSGALG00010002741	PDK4	7.136905292	0.57411795	12.43107848	1.77227E-35	1.33104E-34	positive
PRJNA729271	stage_5W_vs_E10	ordinary_muscle_development	ENSGALG00010002742	PROSER1	0.244946694	0.120766328	2.028269795	0.04253272	0.054146476	positive
PRJNA729271	stage_5W_vs_E10	ordinary_muscle_development	ENSGALG00010002747	FUT9	-1.585029131	0.564732869	-2.806688292	0.005005365	0.00706591	negative
PRJNA729271	stage_5W_vs_E10	ordinary_muscle_development	ENSGALG00010002750	ENSGALG00010002750	-2.339960992	0.235131663	-9.951705201	2.47899E-23	1.14728E-22	negative
PRJNA729271	stage_5W_vs_E10	ordinary_muscle_development	ENSGALG00010002752	NDUFA4	1.603836338	0.154174039	10.40276526	2.40841E-25	1.20837E-24	positive
PRJNA729271	stage_5W_vs_E10	ordinary_muscle_development	ENSGALG00010002753	SLC9A7	-0.029893491	0.154270378	-0.193773371	0.846353339	0.865093551	negative
PRJNA729271	stage_5W_vs_E10	ordinary_muscle_development	ENSGALG00010002758	GREB1	-5.796410664	0.510431149	-11.35591093	6.93137E-30	4.17582E-29	negative
PRJNA729271	stage_5W_vs_E10	ordinary_muscle_development	ENSGALG00010002760	RAB2A	0.675143956	0.092512332	7.297880593	2.92336E-13	8.35781E-13	positive
PRJNA729271	stage_5W_vs_E10	ordinary_muscle_development	ENSGALG00010002762	PPAN	-1.255008981	0.150757885	-8.324665634	8.45671E-17	2.91164E-16	negative
PRJNA729271	stage_5W_vs_E10	ordinary_muscle_development	ENSGALG00010002763	SEPTIN11	-1.895200833	0.116039006	-16.33244629	5.80109E-60	9.2403E-59	negative
PRJNA729271	stage_5W_vs_E10	ordinary_muscle_development	ENSGALG00010002765	MAP7D2	-4.817534161	0.460087464	-10.47090942	1.17509E-25	5.97645E-25	negative
PRJNA729271	stage_5W_vs_E10	ordinary_muscle_development	ENSGALG00010002766	NANOS1	-0.569661205	0.327985898	-1.736846643	0.082414244	0.101151097	negative
PRJNA729271	stage_5W_vs_E10	ordinary_muscle_development	ENSGALG00010002768	TRIM39.2	5.823858754	0.908866638	6.407825431	1.4761E-10	3.63446E-10	positive
PRJNA729271	stage_5W_vs_E10	ordinary_muscle_development	ENSGALG00010002774	TBX18	0.043984496	0.116919201	0.376195658	0.706771443	0.738536236	positive
PRJNA729271	stage_5W_vs_E10	ordinary_muscle_development	ENSGALG00010002779	FBXO8	0.742868569	0.085415431	8.697123677	3.40402E-18	1.25423E-17	positive
PRJNA729271	stage_5W_vs_E10	ordinary_muscle_development	ENSGALG00010002781	EIF3G	0.425102479	0.137783712	3.085288331	0.002033549	0.002986151	positive
PRJNA729271	stage_5W_vs_E10	ordinary_muscle_development	ENSGALG00010002782	GSTA3	-0.219700163	0.147603977	-1.488443385	0.136634002	0.163058609	negative
PRJNA729271	stage_5W_vs_E10	ordinary_muscle_development	ENSGALG00010002784	TRIM27.2	1.016806501	0.526214688	1.932303533	0.053322053	0.067022473	positive
PRJNA729271	stage_5W_vs_E10	ordinary_muscle_development	ENSGALG00010002787	CEP44	-0.548933483	0.132153159	-4.153767403	3.27046E-05	5.59288E-05	negative
PRJNA729271	stage_5W_vs_E10	ordinary_muscle_development	ENSGALG00010002788	ENSGALG00010002788	5.615164316	0.630009162	8.912829614	4.97464E-19	1.89906E-18	positive
PRJNA729271	stage_5W_vs_E10	ordinary_muscle_development	ENSGALG00010002790	BCLAF3	0.880642655	0.101554043	8.671665186	4.25846E-18	1.56072E-17	positive
PRJNA729271	stage_5W_vs_E10	ordinary_muscle_development	ENSGALG00010002792	ENSGALG00010002792	-0.111575343	0.230263027	-0.484556051	0.627991296	0.663900068	negative
PRJNA729271	stage_5W_vs_E10	ordinary_muscle_development	ENSGALG00010002796	PRDM8	3.654730579	0.699185047	5.227129204	1.72162E-07	3.49362E-07	positive
PRJNA729271	stage_5W_vs_E10	ordinary_muscle_development	ENSGALG00010002800	TUBGCP5	-0.149798023	0.075969029	-1.971830161	0.048628998	0.061395116	negative
PRJNA729271	stage_5W_vs_E10	ordinary_muscle_development	ENSGALG00010002801	FGF5	-3.408860631	0.562895479	-6.055938908	1.39601E-09	3.234E-09	negative
PRJNA729271	stage_5W_vs_E10	ordinary_muscle_development	ENSGALG00010002805	FAM171A1	-1.326032021	0.101006346	-13.128205	2.26973E-39	1.93187E-38	negative
PRJNA729271	stage_5W_vs_E10	ordinary_muscle_development	ENSGALG00010002807	P2RY6	3.198338503	0.213814496	14.95847362	1.37135E-50	1.68029E-49	positive
PRJNA729271	stage_5W_vs_E10	ordinary_muscle_development	ENSGALG00010002809	SH3KBP1	-0.201765111	0.110095636	-1.832634953	0.066856877	0.083019826	negative
PRJNA729271	stage_5W_vs_E10	ordinary_muscle_development	ENSGALG00010002810	TRIM27.1	-0.011624017	0.318137713	-0.03653769	0.970853626	0.975024133	negative
PRJNA729271	stage_5W_vs_E10	ordinary_muscle_development	ENSGALG00010002812	TRIM13	-0.576377138	0.103054923	-5.592912225	2.23292E-08	4.82358E-08	negative
PRJNA729271	stage_5W_vs_E10	ordinary_muscle_development	ENSGALG00010002814	TERF1	-2.410564407	0.155901824	-15.46206669	6.25587E-54	8.36521E-53	negative
PRJNA729271	stage_5W_vs_E10	ordinary_muscle_development	ENSGALG00010002817	SHFM1	0.607884608	0.091506254	6.643093583	3.07167E-11	7.90691E-11	positive
PRJNA729271	stage_5W_vs_E10	ordinary_muscle_development	ENSGALG00010002819	BMP3	-1.903678808	0.349085714	-5.453327747	4.94358E-08	1.04183E-07	negative
PRJNA729271	stage_5W_vs_E10	ordinary_muscle_development	ENSGALG00010002824	NXPH1	-3.091118552	0.579022796	-5.338509245	9.37139E-08	1.93612E-07	negative
PRJNA729271	stage_5W_vs_E10	ordinary_muscle_development	ENSGALG00010002825	TRIM41	1.177340839	0.119362902	9.863540691	5.98991E-23	2.72859E-22	positive
PRJNA729271	stage_5W_vs_E10	ordinary_muscle_development	ENSGALG00010002827	SBSPON	0.539834276	0.335169977	1.610628376	0.107260745	0.129778339	positive
PRJNA729271	stage_5W_vs_E10	ordinary_muscle_development	ENSGALG00010002832	LPIN1	2.421844143	0.201400845	12.02499496	2.62604E-33	1.81259E-32	positive
PRJNA729271	stage_5W_vs_E10	ordinary_muscle_development	ENSGALG00010002834	MRPL53	-0.001549538	0.11336494	-0.013668581	0.98909439	0.990666334	negative
PRJNA729271	stage_5W_vs_E10	ordinary_muscle_development	ENSGALG00010002835	CYFIP1	-0.756893647	0.084679443	-8.938339972	3.9506E-19	1.51693E-18	negative
PRJNA729271	stage_5W_vs_E10	ordinary_muscle_development	ENSGALG00010002837	DNMT1	-0.337720438	0.130285096	-2.592164791	0.009537407	0.013088877	negative
PRJNA729271	stage_5W_vs_E10	ordinary_muscle_development	ENSGALG00010002838	ENSGALG00010002838	1.152081867	0.259588041	4.438116116	9.07497E-06	1.62237E-05	positive
PRJNA729271	stage_5W_vs_E10	ordinary_muscle_development	ENSGALG00010002840	SRPX	0.748930103	0.169767132	4.411514129	1.0265E-05	1.82813E-05	positive
PRJNA729271	stage_5W_vs_E10	ordinary_muscle_development	ENSGALG00010002848	TRIB2	-1.885606982	0.108271806	-17.41549399	6.29433E-68	1.2464E-66	negative
PRJNA729271	stage_5W_vs_E10	ordinary_muscle_development	ENSGALG00010002853	CEP162	-0.494538083	0.169321524	-2.920704174	0.003492413	0.005012627	negative
PRJNA729271	stage_5W_vs_E10	ordinary_muscle_development	ENSGALG00010002855	RACK1	0.0986582	0.125311116	0.787306048	0.43110273	0.472930755	positive
PRJNA729271	stage_5W_vs_E10	ordinary_muscle_development	ENSGALG00010002857	DCAF15	-0.763358278	0.150724486	-5.06459367	4.09272E-07	8.07239E-07	negative
PRJNA729271	stage_5W_vs_E10	ordinary_muscle_development	ENSGALG00010002860	NIPA2	-0.037913314	0.108951142	-0.347984547	0.727851789	0.757645363	negative
PRJNA729271	stage_5W_vs_E10	ordinary_muscle_development	ENSGALG00010002864	RNASEH2B	-1.08050772	0.09520361	-11.34944059	7.46387E-30	4.49289E-29	negative
PRJNA729271	stage_5W_vs_E10	ordinary_muscle_development	ENSGALG00010002866	ENSGALG00010002866	2.89831152	0.252310854	11.48706635	1.53227E-30	9.46731E-30	positive
PRJNA729271	stage_5W_vs_E10	ordinary_muscle_development	ENSGALG00010002868	PPP1R9A	0.983582069	0.067028368	14.67411624	9.4438E-49	1.09335E-47	positive
PRJNA729271	stage_5W_vs_E10	ordinary_muscle_development	ENSGALG00010002869	ENSGALG00010002869	0.399940324	0.306563548	1.304591907	0.192031846	0.224132507	positive
PRJNA729271	stage_5W_vs_E10	ordinary_muscle_development	ENSGALG00010002870	FABP5	4.352761921	0.216112342	20.14120008	3.21452E-90	1.10414E-88	positive
PRJNA729271	stage_5W_vs_E10	ordinary_muscle_development	ENSGALG00010002878	P2RY2	6.181046224	0.24997229	24.72692562	5.4918E-135	4.2118E-133	positive
PRJNA729271	stage_5W_vs_E10	ordinary_muscle_development	ENSGALG00010002880	RASGEF1B	-0.124359961	0.207793031	-0.598479944	0.549519737	0.58932292	negative
PRJNA729271	stage_5W_vs_E10	ordinary_muscle_development	ENSGALG00010002881	ENSGALG00010002881	5.921759933	0.311939376	18.98368844	2.32674E-80	6.32759E-79	positive
PRJNA729271	stage_5W_vs_E10	ordinary_muscle_development	ENSGALG00010002883	MAP3K15	4.227130107	0.413585697	10.22068737	1.60201E-24	7.79755E-24	positive
PRJNA729271	stage_5W_vs_E10	ordinary_muscle_development	ENSGALG00010002884	ADHFE1	3.960582252	0.252108095	15.70985749	1.2947E-55	1.822E-54	positive
PRJNA729271	stage_5W_vs_E10	ordinary_muscle_development	ENSGALG00010002886	HERC2	0.311724217	0.105852142	2.944902297	0.003230567	0.004654778	positive
PRJNA729271	stage_5W_vs_E10	ordinary_muscle_development	ENSGALG00010002888	ENSGALG00010002888	-3.597745217	0.381595194	-9.428172243	4.17297E-21	1.75376E-20	negative
PRJNA729271	stage_5W_vs_E10	ordinary_muscle_development	ENSGALG00010002890	VXN	0.565709305	0.164712968	3.434515884	0.000593613	0.000916925	positive
PRJNA729271	stage_5W_vs_E10	ordinary_muscle_development	ENSGALG00010002894	FCHSD2	-0.405698675	0.072373197	-5.605648086	2.07477E-08	4.49131E-08	negative
PRJNA729271	stage_5W_vs_E10	ordinary_muscle_development	ENSGALG00010002896	OCA2	-2.90323112	0.321413984	-9.032684532	1.67511E-19	6.5482E-19	negative
PRJNA729271	stage_5W_vs_E10	ordinary_muscle_development	ENSGALG00010002904	PON1	3.252659832	0.178805252	18.19107549	6.07366E-74	1.42456E-72	positive
PRJNA729271	stage_5W_vs_E10	ordinary_muscle_development	ENSGALG00010002907	GABRG3	-1.728919609	0.369840962	-4.674765065	2.9429E-06	5.4584E-06	negative
PRJNA729271	stage_5W_vs_E10	ordinary_muscle_development	ENSGALG00010002908	S1PR2	0.089961149	0.181194748	0.49648872	0.619549644	0.656179386	positive
PRJNA729271	stage_5W_vs_E10	ordinary_muscle_development	ENSGALG00010002911	MYBL1	-0.934950244	0.236453066	-3.954062679	7.68353E-05	0.000127985	negative
PRJNA729271	stage_5W_vs_E10	ordinary_muscle_development	ENSGALG00010002912	RPS3	-0.58683786	0.192322707	-3.051318632	0.002278386	0.003331169	negative
PRJNA729271	stage_5W_vs_E10	ordinary_muscle_development	ENSGALG00010002913	NXNL1	3.057485198	0.563879975	5.422226948	5.88611E-08	1.23311E-07	positive
PRJNA729271	stage_5W_vs_E10	ordinary_muscle_development	ENSGALG00010002914	SLC27A1	2.570241799	0.304580214	8.438636787	3.2106E-17	1.12796E-16	positive
PRJNA729271	stage_5W_vs_E10	ordinary_muscle_development	ENSGALG00010002916	SDCBP	-0.795867941	0.122837104	-6.47905163	9.23009E-11	2.30355E-10	negative
PRJNA729271	stage_5W_vs_E10	ordinary_muscle_development	ENSGALG00010002917	PGLS	-0.334619714	0.097178653	-3.443345858	0.000574564	0.000888353	negative
PRJNA729271	stage_5W_vs_E10	ordinary_muscle_development	ENSGALG00010002918	LRATD1	-4.38167708	0.409914544	-10.68924522	1.14297E-26	6.09768E-26	negative
PRJNA729271	stage_5W_vs_E10	ordinary_muscle_development	ENSGALG00010002919	ENSGALG00010002919	1.541289131	0.202844026	7.598395489	2.99825E-14	9.06164E-14	positive
PRJNA729271	stage_5W_vs_E10	ordinary_muscle_development	ENSGALG00010002920	VCPIP1	1.56408567	0.101247573	15.44813008	7.7664E-54	1.03564E-52	positive
PRJNA729271	stage_5W_vs_E10	ordinary_muscle_development	ENSGALG00010002922	TMEM106B	-0.258484006	0.09200866	-2.8093443	0.004964253	0.007012654	negative
PRJNA729271	stage_5W_vs_E10	ordinary_muscle_development	ENSGALG00010002923	SERPINH1	-0.458153113	0.1585382	-2.889859426	0.003854141	0.00550673	negative
PRJNA729271	stage_5W_vs_E10	ordinary_muscle_development	ENSGALG00010002925	INTS6	0.12704429	0.122229899	1.039387992	0.298624326	0.336957777	positive
PRJNA729271	stage_5W_vs_E10	ordinary_muscle_development	ENSGALG00010002926	GALNTL6	-2.139236835	0.369067979	-5.796321976	6.77851E-09	1.51021E-08	negative
PRJNA729271	stage_5W_vs_E10	ordinary_muscle_development	ENSGALG00010002927	GABRA5	-4.125528762	0.607692416	-6.788843593	1.13036E-11	2.98444E-11	negative
PRJNA729271	stage_5W_vs_E10	ordinary_muscle_development	ENSGALG00010002928	ASB4	11.34016533	0.545588289	20.78520664	5.89101E-96	2.29543E-94	positive
PRJNA729271	stage_5W_vs_E10	ordinary_muscle_development	ENSGALG00010002929	PDHA2	0.369259986	0.113224842	3.261298325	0.001109033	0.00167435	positive
PRJNA729271	stage_5W_vs_E10	ordinary_muscle_development	ENSGALG00010002930	SERAF	-5.407737804	0.299992086	-18.02626824	1.21201E-72	2.7493E-71	negative
PRJNA729271	stage_5W_vs_E10	ordinary_muscle_development	ENSGALG00010002934	VWDE	-8.094942861	0.986733075	-8.203781819	2.32941E-16	7.84862E-16	negative
PRJNA729271	stage_5W_vs_E10	ordinary_muscle_development	ENSGALG00010002935	SYTL5	-3.360105244	0.684144147	-4.911399536	9.04286E-07	1.74234E-06	negative
PRJNA729271	stage_5W_vs_E10	ordinary_muscle_development	ENSGALG00010002937	GALNT7	-0.294198593	0.108811836	-2.70373704	0.006856452	0.009545167	negative
PRJNA729271	stage_5W_vs_E10	ordinary_muscle_development	ENSGALG00010002939	HMGB2	-3.021713686	0.173018532	-17.46468224	2.66179E-68	5.32175E-67	negative

PRJNA729271	stage_5W_vs_E10	ordinary_muscle_development	ENSGALG00010002940	BG1	1.226888268	0.4233913	2.897764478	0.003758327	0.005376192	positive
PRJNA729271	stage_5W_vs_E10	ordinary_muscle_development	ENSGALG00010002942	GABRB3	-4.289117277	0.624444337	-6.868694326	6.47922E-12	1.7299E-11	negative
PRJNA729271	stage_5W_vs_E10	ordinary_muscle_development	ENSGALG00010002943	SAP30	-0.45101592	0.150117242	-3.004424509	0.002660838	0.003865776	negative
PRJNA729271	stage_5W_vs_E10	ordinary_muscle_development	ENSGALG00010002946	GDPD5	-0.791536507	0.119424093	-6.627946557	3.40389E-11	8.74656E-11	negative
PRJNA729271	stage_5W_vs_E10	ordinary_muscle_development	ENSGALG00010002947	ENSGALG00010002947	-1.096124236	0.701483738	-1.562579681	0.118151491	0.142113174	negative
PRJNA729271	stage_5W_vs_E10	ordinary_muscle_development	ENSGALG00010002948	DLX6	-5.257510021	1.603059143	-3.279673145	0.001039274	0.001573129	negative
PRJNA729271	stage_5W_vs_E10	ordinary_muscle_development	ENSGALG00010002949	ENSGALG00010002949	0.647438755	1.183047277	0.547263637	0.584197622	0.622459904	positive
PRJNA729271	stage_5W_vs_E10	ordinary_muscle_development	ENSGALG00010002951	ENSGALG00010002951	-3.490413575	4.310423066	-0.809761251	0.418077407	0.459685353	negative
PRJNA729271	stage_5W_vs_E10	ordinary_muscle_development	ENSGALG00010002952	NBAS	0.030605715	0.064655338	0.473367179	0.635951241	0.671531525	positive
PRJNA729271	stage_5W_vs_E10	ordinary_muscle_development	ENSGALG00010002953	WDFY2	-0.414979371	0.133519965	-3.107994908	0.001883613	0.002774974	negative
PRJNA729271	stage_5W_vs_E10	ordinary_muscle_development	ENSGALG00010002954	CHST7	-0.015149253	0.126560102	-0.119700073	0.904720743	0.917057844	negative
PRJNA729271	stage_5W_vs_E10	ordinary_muscle_development	ENSGALG00010002955	BLEC1	0.476575761	0.467754559	1.018858614	0.308270094	0.347112174	positive
PRJNA729271	stage_5W_vs_E10	ordinary_muscle_development	ENSGALG00010002956	SCRG1	0.039348783	0.397909	0.098888898	0.921226484	0.93204251	positive
PRJNA729271	stage_5W_vs_E10	ordinary_muscle_development	ENSGALG00010002957	ADGRG2	-1.865594281	0.295027441	-6.32346021	2.5577E-10	6.20276E-10	negative
PRJNA729271	stage_5W_vs_E10	ordinary_muscle_development	ENSGALG00010002958	ENSGALG00010002958	0.623567621	0.181201773	3.441288749	0.00057895	0.000895039	positive
PRJNA729271	stage_5W_vs_E10	ordinary_muscle_development	ENSGALG00010002959	DLX5	-6.615550583	0.894372056	-7.396866367	1.39436E-13	4.04953E-13	negative
PRJNA729271	stage_5W_vs_E10	ordinary_muscle_development	ENSGALG00010002960	BLEC2	2.56181678	0.682162276	3.755436019	0.00017304	0.000279249	positive
PRJNA729271	stage_5W_vs_E10	ordinary_muscle_development	ENSGALG00010002965	PRSS35	0.49588109	0.211528917	2.344270927	0.019064317	0.025275522	positive
PRJNA729271	stage_5W_vs_E10	ordinary_muscle_development	ENSGALG00010002967	OLFM4	-2.305174657	0.876780124	-2.629136534	0.008560198	0.011800293	negative
PRJNA729271	stage_5W_vs_E10	ordinary_muscle_development	ENSGALG00010002972	ME1	3.082954172	0.150150793	20.53238687	1.10606E-93	4.07334E-92	positive
PRJNA729271	stage_5W_vs_E10	ordinary_muscle_development	ENSGALG00010002978	DYNLT3	1.554136719	0.101248036	15.34979628	3.553E-53	4.68189E-52	positive
PRJNA729271	stage_5W_vs_E10	ordinary_muscle_development	ENSGALG00010002981	KLHL35	-5.402701586	0.37825116	-14.28337082	2.77831E-46	2.93953E-45	negative
PRJNA729271	stage_5W_vs_E10	ordinary_muscle_development	ENSGALG00010002982	HAND2	-3.356932655	0.315062647	-10.65481006	1.65582E-26	8.76915E-26	negative
PRJNA729271	stage_5W_vs_E10	ordinary_muscle_development	ENSGALG00010002983	NSMAF	0.838132687	0.111559648	7.512866028	5.78467E-14	1.71998E-13	positive
PRJNA729271	stage_5W_vs_E10	ordinary_muscle_development	ENSGALG00010002985	ENSGALG00010002985	-0.33116661	0.223318964	-1.482930978	0.13809275	0.164663903	negative
PRJNA729271	stage_5W_vs_E10	ordinary_muscle_development	ENSGALG00010002987	PCDH8	-1.878559676	0.376794136	-4.985639365	6.17572E-07	1.20448E-06	negative
PRJNA729271	stage_5W_vs_E10	ordinary_muscle_development	ENSGALG00010002991	BLB1	6.332713641	0.306819934	20.6398377	1.20466E-94	4.55916E-93	positive
PRJNA729271	stage_5W_vs_E10	ordinary_muscle_development	ENSGALG00010002992	ENSGALG00010002992	0.291800615	0.241356656	1.209001732	0.22666218	0.261103735	positive
PRJNA729271	stage_5W_vs_E10	ordinary_muscle_development	ENSGALG00010002994	DUSP4	-3.978469911	0.192023907	-20.71861765	2.35346E-95	9.0727E-94	negative
PRJNA729271	stage_5W_vs_E10	ordinary_muscle_development	ENSGALG00010002995	RWDD2A	-0.895847726	0.129354804	-6.92550799	4.34414E-12	1.16976E-11	negative
PRJNA729271	stage_5W_vs_E10	ordinary_muscle_development	ENSGALG00010002998	LECT1	-7.563609488	0.302412555	-25.01089771	4.6532E-138	3.7059E-136	negative
PRJNA729271	stage_5W_vs_E10	ordinary_muscle_development	ENSGALG00010002999	CMTM7	0.064242861	0.204624638	0.31395467	0.753555467	0.781538816	positive
PRJNA729271	stage_5W_vs_E10	ordinary_muscle_development	ENSGALG00010003001	MAP6	-3.048750279	0.340324368	-8.958366103	3.29525E-19	1.27E-18	negative
PRJNA729271	stage_5W_vs_E10	ordinary_muscle_development	ENSGALG00010003002	PHKA2	-0.987384585	0.18865222	-5.233887971	1.65981E-07	3.37196E-07	negative
PRJNA729271	stage_5W_vs_E10	ordinary_muscle_development	ENSGALG00010003006	CYP7B1	-0.868035109	0.201568995	-4.306392005	1.65939E-05	2.90774E-05	negative
PRJNA729271	stage_5W_vs_E10	ordinary_muscle_development	ENSGALG00010003009	TNKS	-0.102205958	0.05813959	-1.757940793	0.078757584	0.096942186	negative
PRJNA729271	stage_5W_vs_E10	ordinary_muscle_development	ENSGALG00010003017	PGM3	-1.434318079	0.156341435	-9.174267059	4.54657E-20	1.82001E-19	negative
PRJNA729271	stage_5W_vs_E10	ordinary_muscle_development	ENSGALG00010003018	MOGAT2	-1.558965552	0.988401055	-1.577260106	0.114735688	0.138383574	negative
PRJNA729271	stage_5W_vs_E10	ordinary_muscle_development	ENSGALG00010003019	TAPBP	3.693921306	0.251438381	14.69115928	7.34464E-49	8.55792E-48	positive
PRJNA729271	stage_5W_vs_E10	ordinary_muscle_development	ENSGALG00010003022	BLB2	6.90804765	0.265611743	26.00806562	4.0139E-149	3.9579E-147	positive
PRJNA729271	stage_5W_vs_E10	ordinary_muscle_development	ENSGALG00010003024	SUGT1	0.800236449	0.071704136	11.16025506	6.38082E-29	3.70553E-28	positive
PRJNA729271	stage_5W_vs_E10	ordinary_muscle_development	ENSGALG00010003025	MFHAS1	1.589548975	0.122601773	12.96513855	1.92882E-38	1.59397E-37	positive
PRJNA729271	stage_5W_vs_E10	ordinary_muscle_development	ENSGALG00010003027	SCIN	-0.751422543	0.254756975	-2.949566127	0.003182205	0.004587375	negative
PRJNA729271	stage_5W_vs_E10	ordinary_muscle_development	ENSGALG00010003030	PPEF1	-0.771547912	0.673562054	-1.145474136	0.252012803	0.28794936	negative
PRJNA729271	stage_5W_vs_E10	ordinary_muscle_development	ENSGALG00010003034	ERI1	-1.008509216	0.065491985	-15.39897161	1.66293E-53	2.20129E-52	negative
PRJNA729271	stage_5W_vs_E10	ordinary_muscle_development	ENSGALG00010003035	DGAT2	3.764641909	0.455871526	8.258120322	1.47984E-16	5.03057E-16	positive
PRJNA729271	stage_5W_vs_E10	ordinary_muscle_development	ENSGALG00010003042	TOX	-2.320440513	0.294977018	-7.866512887	3.64663E-15	1.15638E-14	negative
PRJNA729271	stage_5W_vs_E10	ordinary_muscle_development	ENSGALG00010003052	ENSGALG00010003052	0.430142824	0.093621838	4.594471048	4.33848E-06	7.93118E-06	positive
PRJNA729271	stage_5W_vs_E10	ordinary_muscle_development	ENSGALG00010003057	DDX1	0.013845436	0.089655228	0.15442977	0.877270882	0.89222856	positive
PRJNA729271	stage_5W_vs_E10	ordinary_muscle_development	ENSGALG00010003061	ENSGALG00010003061	-0.691397567	0.324384194	-2.131415709	0.03305491	0.042676665	negative
PRJNA729271	stage_5W_vs_E10	ordinary_muscle_development	ENSGALG00010003063	GGH	0.018020419	0.088736169	0.203078628	0.839073576	0.858621354	positive
PRJNA729271	stage_5W_vs_E10	ordinary_muscle_development	ENSGALG00010003064	VAV1	2.392211139	0.249007819	9.606971994	7.47138E-22	3.25218E-21	positive
PRJNA729271	stage_5W_vs_E10	ordinary_muscle_development	ENSGALG00010003068	BRD2	0.563374356	0.126160951	4.465520825	7.98742E-06	1.43307E-05	positive
PRJNA729271	stage_5W_vs_E10	ordinary_muscle_development	ENSGALG00010003072	ELF1	1.193343017	0.073658822	16.20095173	4.96526E-59	7.7472E-58	positive
PRJNA729271	stage_5W_vs_E10	ordinary_muscle_development	ENSGALG00010003074	TTPA	1.861587056	0.351831689	5.291129577	1.21563E-07	2.49477E-07	positive
PRJNA729271	stage_5W_vs_E10	ordinary_muscle_development	ENSGALG00010003077	EIF2S3	-0.069347233	0.081859044	-0.847154209	0.396909173	0.438138639	negative
PRJNA729271	stage_5W_vs_E10	ordinary_muscle_development	ENSGALG00010003081	DOP1A	0.46438018	0.103734112	4.476639079	7.58273E-06	1.36265E-05	positive
PRJNA729271	stage_5W_vs_E10	ordinary_muscle_development	ENSGALG00010003087	TWISTNB	-0.122003638	0.180220782	-0.676967644	0.498426484	0.539800649	negative
PRJNA729271	stage_5W_vs_E10	ordinary_muscle_development	ENSGALG00010003090	DMA	3.040901974	0.184464043	16.48506626	4.69779E-61	7.77335E-60	positive
PRJNA729271	stage_5W_vs_E10	ordinary_muscle_development	ENSGALG00010003092	ART1F	5.765101359	1.346435077	4.281752205	1.85427E-05	3.2375E-05	positive
PRJNA729271	stage_5W_vs_E10	ordinary_muscle_development	ENSGALG00010003097	TFPI2	1.497341773	0.135663484	11.03717621	2.52855E-28	1.43337E-27	positive
PRJNA729271	stage_5W_vs_E10	ordinary_muscle_development	ENSGALG00010003101	KLHL15	-0.491151927	0.073918796	-6.644479523	3.0429E-11	7.83426E-11	negative
PRJNA729271	stage_5W_vs_E10	ordinary_muscle_development	ENSGALG00010003103	YTHDF3	0.454895381	0.086315563	5.270143217	1.36317E-07	2.78809E-07	positive
PRJNA729271	stage_5W_vs_E10	ordinary_muscle_development	ENSGALG00010003107	GNG11	0.094765948	0.181476044	0.522195362	0.601534315	0.639249624	positive
PRJNA729271	stage_5W_vs_E10	ordinary_muscle_development	ENSGALG00010003108	PPP1R3B	-0.258195034	0.182730087	-1.41298589	0.157659873	0.186599156	negative
PRJNA729271	stage_5W_vs_E10	ordinary_muscle_development	ENSGALG00010003109	ART5	5.753305034	0.557894991	10.31252319	6.18556E-25	3.06739E-24	positive
PRJNA729271	stage_5W_vs_E10	ordinary_muscle_development	ENSGALG00010003110	BF1	6.701907313	0.256211982	26.1576655	8.0625E-151	8.1157E-149	positive
PRJNA729271	stage_5W_vs_E10	ordinary_muscle_development	ENSGALG00010003113	WBP4	-0.353632799	0.118066432	-2.995201868	0.002742633	0.003979824	negative
PRJNA729271	stage_5W_vs_E10	ordinary_muscle_development	ENSGALG00010003115	UBE3D	-0.019629942	0.154154576	-0.127339338	0.898671827	0.9115639	negative
PRJNA729271	stage_5W_vs_E10	ordinary_muscle_development	ENSGALG00010003117	ID2	-1.939090654	0.126926077	-15.27732283	1.08299E-52	1.41678E-51	negative
PRJNA729271	stage_5W_vs_E10	ordinary_muscle_development	ENSGALG00010003121	ARMC1	0.145547192	0.082495375	1.76430729	0.077680257	0.095697376	positive
PRJNA729271	stage_5W_vs_E10	ordinary_muscle_development	ENSGALG00010003125	APOO	0.841127276	0.106322862	7.911066933	2.55192E-15	8.16019E-15	positive
PRJNA729271	stage_5W_vs_E10	ordinary_muscle_development	ENSGALG00010003126	ENSGALG00010003126	-2.718811844	0.528726244	-5.142191963	2.71551E-07	5.42317E-07	negative
PRJNA729271	stage_5W_vs_E10	ordinary_muscle_development	ENSGALG00010003131	TAP1	4.660040065	0.345010627	13.50694645	1.42302E-41	1.30302E-40	positive
PRJNA729271	stage_5W_vs_E10	ordinary_muscle_development	ENSGALG00010003132	TPBG	-3.972094396	0.166623247	-23.83877688	1.324E-125	8.9678E-124	negative
PRJNA729271	stage_5W_vs_E10	ordinary_muscle_development	ENSGALG00010003133	SAT1	2.230946604	0.167178496	13.34469834	1.27184E-40	1.13379E-39	positive
PRJNA729271	stage_5W_vs_E10	ordinary_muscle_development	ENSGALG00010003136	NUP98	0.075065539	0.104873541	0.71577195	0.474132175	0.515995636	positive
PRJNA729271	stage_5W_vs_E10	ordinary_muscle_development	ENSGALG00010003152	ENSGALG00010003152	1.343006318	0.127052791	10.570459	4.0849E-26	2.12149E-25	positive
PRJNA729271	stage_5W_vs_E10	ordinary_muscle_development	ENSGALG00010003154	MTFR1	-0.796049152	0.08220614	-9.683572929	3.54119E-22	1.5635E-21	negative
PRJNA729271	stage_5W_vs_E10	ordinary_muscle_development	ENSGALG00010003159	TAP2	5.073657406	0.263489451	19.25563772	1.26606E-82	3.61964E-81	positive
PRJNA729271	stage_5W_vs_E10	ordinary_muscle_development	ENSGALG00010003160	KIDINS220	0.167427924	0.151465074	1.105389649	0.268990787	0.30604564	positive
PRJNA729271	stage_5W_vs_E10	ordinary_muscle_development	ENSGALG00010003164	C3	4.464126252	0.458654531	9.733090912	2.1787E-22	9.70208E-22	positive
PRJNA729271	stage_5W_vs_E10	ordinary_muscle_development	ENSGALG00010003165	BMP2K	0.259009186	0.109859741	2.357635141	0.01839176	0.024428531	positive
PRJNA729271	stage_5W_vs_E10	ordinary_muscle_development	ENSGALG00010003166	PGAP2	0.662830105	0.110325649	6.007942018	1.87893E-09	4.31618E-09	positive
PRJNA729271	stage_5W_vs_E10	ordinary_muscle_development	ENSGALG00010003169	MTRF1	0.575410131	0.10837017	5.309672693	1.09822E-07	2.26054E-07	positive
PRJNA729271	stage_5W_vs_E10	ordinary_muscle_development	ENSGALG00010003170	C4	1.323706822	0.406245668	3.258390002	0.001120463	0.001689846	positive
PRJNA729271	stage_5W_vs_E10	ordinary_muscle_development	ENSGALG00010003171	PAQR3	-1.03504463	0.111165221	-9.310867343	1.26793E-20	5.2064E-20	negative
PRJNA729271	stage_5W_vs_E10	ordinary_muscle_development	ENSGALG00010003176	PRAG1	-1.528836874	0.263049812	-5.811967169	6.1743E-09	1.38005E-08	negative
PRJNA729271	stage_5W_vs_E10	ordinary_muscle_development	ENSGALG00010003178	CENPA	-2.384863979	0.263777951	-9.041180153	1.54989E-19	6.07181E-19	negative
PRJNA729271	stage_5W_vs_E10	ordinary_muscle_development	ENSGALG00010003180	IBTK	0.437818147	0.126818765	3.452313591	0.000555801	0.000860536	positive
PRJNA729271	stage_5W_vs_E10	ordinary_muscle_development	ENSGALG00010003183	NAA16	1.289990941	0.087504016	14.74207693	3.45979E-49	4.0772E-48	positive
PRJNA729271	stage_5W_vs_E10	ordinary_muscle_development	ENSGALG00010003184	BET1	-0.308836335	0.082005187	-3.766058548	0.000165845	0.000268056	negative
PRJNA729271	stage_5W_vs_E10	ordinary_muscle_development	ENSGALG00010003186	CYP21A1	-1.683609322	0.267829881	-6.286114589	3.2551E-10	7.83114E-10	negative
PRJNA729271	stage_5W_vs_E10	ordinary_muscle_development	ENSGALG00010003187	RHOG	1.473814265	0.239473942	6.154382611	7.53705E-10	1.77094E-09	positive
PRJNA729271	stage_5W_vs_E10	ordinary_muscle_development	ENSGALG00010003189	ANTXR2	0.560969745	0.126811458	4.423651883	9.70463E-06	1.73195E-05	positive
PRJNA729271	stage_5W_vs_E10	ordinary_muscle_development	ENSGALG00010003191	CC2D1A	1.488721655	0.160823799	9.256849209	2.10554E-20	8.55614E-20	positive
PRJNA729271	stage_5W_vs_E10	ordinary_muscle_development	ENSGALG00010003192	RGCC	2.899091135	0.307810994	9.418413224	4.57956E-21	1.9213E-20	positive
PRJNA729271	stage_5W_vs_E10	ordinary_muscle_development	ENSGALG00010003193	ACOT9	0.709726763	0.136631926	5.194443104	2.05334E-07	4.14239E-07	positive
PRJNA729271	stage_5W_vs_E10	ordinary_muscle_development	ENSGALG00010003198	PRKG2	-1.067686623	0.335792359	-3.179603685	0.001474766	0.002197444	negative
PRJNA729271	stage_5W_vs_E10	ordinary_muscle_development	ENSGALG00010003201	MBOAT2	-0.329091855	0.127931615	-2.57240444	0.010099483	0.013827525	negative
PRJNA729271	stage_5W_vs_E10	ordinary_muscle_development	ENSGALG00010003202	FAM46A	1.13931483	0.102695402	11.0941172	1.33976E-28	7.67583E-28	positive
PRJNA729271	stage_5W_vs_E10	ordinary_muscle_development	ENSGALG00010003204	PKD2	0.062213523	0.143390759	0.433874005	0.664379938	0.698751066	positive
PRJNA729271	stage_5W_vs_E10	ordinary_muscle_development	ENSGALG00010003205	ENSGALG00010003205	6.644783126	0.26394327	25.17504281	7.518E-140	6.2271E-138	positive
PRJNA729271	stage_5W_vs_E10	ordinary_muscle_development	ENSGALG00010003206	ENSGALG00010003206	-2.616119283	0.706723988	-3.70175532	0.000214113	0.000343239	negative
PRJNA729271	stage_5W_vs_E10	ordinary_muscle_development	ENSGALG00010003210	EYA1	-1.287312623	0.203759757	-6.31779624	2.6532E-10	6.42467E-10	negative
PRJNA729271	stage_5W_vs_E10	ordinary_muscle_development	ENSGALG00010003212	LTB4R	1.964604355	0.758837708	2.588965118	0.009626485	0.013208623	positive
PRJNA729271	stage_5W_vs_E10	ordinary_muscle_development	ENSGALG00010003214	PRDX4	-1.14544	0.095542839	-11.98875831	4.0696E-33	2.79171E-32	negative
PRJNA729271	stage_5W_vs_E10	ordinary_muscle_development	ENSGALG00010003218	MAP3K7	0.038030836	0.060775137	0.625763072	0.531470357	0.571627644	positive
PRJNA729271	stage_5W_vs_E10	ordinary_muscle_development	ENSGALG00010003220	SPP1	1.275687355	0.94061128	1.356232252	0.175025283	0.205640876	positive
PRJNA729271	stage_5W_vs_E10	ordinary_muscle_development	ENSGALG00010003222	ITGA8	-2.173101834	0.310143713	-7.006757642	2.43904E-12	6.65799E-12	negative
PRJNA729271	stage_5W_vs_E10	ordinary_muscle_development	ENSGALG00010003223	AREG	-3.70303808	1.030277431	-3.594214499	0.000325372	0.000513363	negative
PRJNA729271	stage_5W_vs_E10	ordinary_muscle_development	ENSGALG00010003229	STIM1	1.207014806	0.105342	11.4580586	2.14264E-30	1.31377E-29	positive
PRJNA729271	stage_5W_vs_E10	ordinary_muscle_development	ENSGALG00010003239	RRM1	-2.249365347	0.119875289	-18.76421213	1.48178E-78	3.89808E-77	negative
PRJNA729271	stage_5W_vs_E10	ordinary_muscle_development	ENSGALG00010003240	PLACL2	7.481199875	0.85560343	8.743770313	2.25458E-18	8.36235E-18	positive
PRJNA729271	stage_5W_vs_E10	ordinary_muscle_development	ENSGALG00010003242	ASAP2	-1.306065304	0.126853896	-10.29582337	7.35873E-25	3.63547E-24	negative
PRJNA729271	stage_5W_vs_E10	ordinary_muscle_development	ENSGALG00010003243	VPS50	-0.204198303	0.092729384	-2.20208842	0.027659063	0.03600522	negative
PRJNA729271	stage_5W_vs_E10	ordinary_muscle_development	ENSGALG00010003245	ENSGALG00010003245	-0.802044343	0.450565547	-1.780083607	0.075062279	0.092676979	negative
PRJNA729271	stage_5W_vs_E10	ordinary_muscle_development	ENSGALG00010003246	VWA8	1.277141051	0.108919897	11.72550728	9.43342E-32	6.11527E-31	positive
PRJNA729271	stage_5W_vs_E10	ordinary_muscle_development	ENSGALG00010003247	ENSGALG00010003247	0.615517964	0.211781789	2.906378155	0.003656393	0.005235544	positive
PRJNA729271	stage_5W_vs_E10	ordinary_muscle_development	ENSGALG00010003248	MRI1	0.033746862	0.096558641	0.34949603	0.726716951	0.756634657	positive
PRJNA729271	stage_5W_vs_E10	ordinary_muscle_development	ENSGALG00010003249	YJU2B	2.43186992	0.312359382	7.785487047	6.94452E-15	2.16661E-14	positive
PRJNA729271	stage_5W_vs_E10	ordinary_muscle_development	ENSGALG00010003250	CD1G	2.678658709	0.683623371	3.91832524	8.91663E-05	0.00014771	positive
PRJNA729271	stage_5W_vs_E10	ordinary_muscle_development	ENSGALG00010003251	ELOF1	-0.182656436	0.155236933	-1.176630023	0.239343209	0.274642164	negative
PRJNA729271	stage_5W_vs_E10	ordinary_muscle_development	ENSGALG00010003252	PHEX	-3.806707817	0.201290637	-18.91149972	9.17111E-80	2.44816E-78	negative
PRJNA729271	stage_5W_vs_E10	ordinary_muscle_development	ENSGALG00010003253	ITGB1BP1	-0.114240369	0.115058309	-0.992891084	0.320763035	0.360088305	negative
PRJNA729271	stage_5W_vs_E10	ordinary_muscle_development	ENSGALG00010003254	DGKH	0.186651977	0.169987851	1.098031273	0.272190834	0.309370825	positive
PRJNA729271	stage_5W_vs_E10	ordinary_muscle_development	ENSGALG00010003255	CALCR	-0.370631072	0.325489414	-1.138688561	0.254833075	0.290988296	negative
PRJNA729271	stage_5W_vs_E10	ordinary_muscle_development	ENSGALG00010003256	ENSGALG00010003256	-0.880533879	1.407174746	-0.625745936	0.531481598	0.571627644	negative
PRJNA729271	stage_5W_vs_E10	ordinary_muscle_development	ENSGALG00010003257	ENSGALG00010003257	1.08926001	0.794795334	1.370491198	0.170533621	0.200868262	positive
PRJNA729271	stage_5W_vs_E10	ordinary_muscle_development	ENSGALG00010003259	VEGFC	1.116095896	0.140765286	7.928772265	2.21323E-15	7.10852E-15	positive
PRJNA729271	stage_5W_vs_E10	ordinary_muscle_development	ENSGALG00010003260	ACP5	1.636269437	0.264783009	6.179661759	6.42391E-10	1.51875E-09	positive

PRJNA729271	stage_5W_vs_E10	ordinary_muscle_development	ENSGALG00010003261	PEX2	-0.916048437	0.173786233	-5.271122	1.35592E-07	2.77444E-07	negative
PRJNA729271	stage_5W_vs_E10	ordinary_muscle_development	ENSGALG00010003262	ENSGALG00010003262	-2.574026517	0.620986878	-4.145057821	3.39728E-05	5.79882E-05	negative
PRJNA729271	stage_5W_vs_E10	ordinary_muscle_development	ENSGALG00010003263	ENSGALG00010003263	1.721550537	0.759605715	2.266373863	0.023428503	0.030718783	positive
PRJNA729271	stage_5W_vs_E10	ordinary_muscle_development	ENSGALG00010003264	ENSGALG00010003264	-0.844537857	0.39467341	-2.139839764	0.032367718	0.041830428	negative
PRJNA729271	stage_5W_vs_E10	ordinary_muscle_development	ENSGALG00010003268	SMS	-0.685345428	0.064428388	-10.63732083	1.99792E-26	1.05347E-25	negative
PRJNA729271	stage_5W_vs_E10	ordinary_muscle_development	ENSGALG00010003269	ENSGALG00010003269	-0.450538515	0.460658632	-0.9780312	0.328058893	0.367709067	negative
PRJNA729271	stage_5W_vs_E10	ordinary_muscle_development	ENSGALG00010003271	AKAP11	0.333662974	0.08392211	3.975864924	7.01239E-05	0.000117197	positive
PRJNA729271	stage_5W_vs_E10	ordinary_muscle_development	ENSGALG00010003272	CPSF3	-0.279775304	0.066976301	-4.177228377	2.95083E-05	5.0666E-05	negative
PRJNA729271	stage_5W_vs_E10	ordinary_muscle_development	ENSGALG00010003273	NFIX	1.597575634	0.197395247	8.09328317	5.80776E-16	1.91718E-15	positive
PRJNA729271	stage_5W_vs_E10	ordinary_muscle_development	ENSGALG00010003274	BACH2	-0.258043731	0.148445768	-1.738303043	0.08215743	0.100852977	negative
PRJNA729271	stage_5W_vs_E10	ordinary_muscle_development	ENSGALG00010003275	MBTPS2	-0.186592609	0.130382039	-1.431122034	0.152395244	0.180707547	negative
PRJNA729271	stage_5W_vs_E10	ordinary_muscle_development	ENSGALG00010003276	TNFSF11	0.927210334	0.350716853	2.643757566	0.008199136	0.011323025	positive
PRJNA729271	stage_5W_vs_E10	ordinary_muscle_development	ENSGALG00010003278	ENSGALG00010003278	1.011194927	0.498702999	2.02764958	0.042596025	0.054218194	positive
PRJNA729271	stage_5W_vs_E10	ordinary_muscle_development	ENSGALG00010003279	ENSGALG00010003279	1.979561686	0.364767257	5.426917153	5.73357E-08	1.20255E-07	positive
PRJNA729271	stage_5W_vs_E10	ordinary_muscle_development	ENSGALG00010003280	ENSGALG00010003280	-0.363829635	0.166998141	-2.178644817	0.029358064	0.038121037	negative
PRJNA729271	stage_5W_vs_E10	ordinary_muscle_development	ENSGALG00010003287	ENSGALG00010003287	-2.499989436	1.929706967	-1.295528015	0.195138144	0.227337035	negative
PRJNA729271	stage_5W_vs_E10	ordinary_muscle_development	ENSGALG00010003292	GADD45GIP1	0.707125689	0.124442803	5.682334972	1.32868E-08	2.91277E-08	positive
PRJNA729271	stage_5W_vs_E10	ordinary_muscle_development	ENSGALG00010003294	ENSGALG00010003294	0.820654478	4.422040719	0.18558275	0.852771982	0.870610641	positive
PRJNA729271	stage_5W_vs_E10	ordinary_muscle_development	ENSGALG00010003295	IAH1	0.248764279	0.133663939	1.861117369	0.062727604	0.07814668	positive
PRJNA729271	stage_5W_vs_E10	ordinary_muscle_development	ENSGALG00010003296	ENSGALG00010003296	1.870321143	0.577101693	3.240886601	0.001191586	0.001791891	positive
PRJNA729271	stage_5W_vs_E10	ordinary_muscle_development	ENSGALG00010003298	IL7	8.044858178	0.969870546	8.294775226	1.08791E-16	3.72267E-16	positive
PRJNA729271	stage_5W_vs_E10	ordinary_muscle_development	ENSGALG00010003299	RAD23A	1.888964708	0.120309917	15.70082297	1.49293E-55	2.0969E-54	positive
PRJNA729271	stage_5W_vs_E10	ordinary_muscle_development	ENSGALG00010003300	CASP8AP2	-0.061746918	0.111461108	-0.553977246	0.579594418	0.618191397	negative
PRJNA729271	stage_5W_vs_E10	ordinary_muscle_development	ENSGALG00010003303	MTHFD2L	-0.052826706	0.195049752	-0.270837085	0.786516336	0.811889637	negative
PRJNA729271	stage_5W_vs_E10	ordinary_muscle_development	ENSGALG00010003304	ENSGALG00010003304	-1.365114558	0.77093453	-1.770726962	0.076606117	0.094454377	negative
PRJNA729271	stage_5W_vs_E10	ordinary_muscle_development	ENSGALG00010003305	CALR	-0.688469507	0.078280142	-8.794944497	1.43119E-18	5.35494E-18	negative
PRJNA729271	stage_5W_vs_E10	ordinary_muscle_development	ENSGALG00010003307	COL1A2	0.346665495	0.220386331	1.572989998	0.115721134	0.139409727	positive
PRJNA729271	stage_5W_vs_E10	ordinary_muscle_development	ENSGALG00010003308	MDN1	-1.239495309	0.099734172	-12.4279902	1.84208E-35	1.38203E-34	negative
PRJNA729271	stage_5W_vs_E10	ordinary_muscle_development	ENSGALG00010003311	ENSGALG00010003311	4.397696281	1.15866584	3.795482813	0.000147356	0.000239132	positive
PRJNA729271	stage_5W_vs_E10	ordinary_muscle_development	ENSGALG00010003315	EPGN	-0.933979781	0.369611182	-2.526925122	0.011506603	0.015653516	negative
PRJNA729271	stage_5W_vs_E10	ordinary_muscle_development	ENSGALG00010003317	LYRM2	0.328958634	0.178634204	1.84152098	0.065545249	0.081470921	positive
PRJNA729271	stage_5W_vs_E10	ordinary_muscle_development	ENSGALG00010003320	OR51E2	0.113794206	0.497390795	0.228782291	0.819038131	0.840373591	positive
PRJNA729271	stage_5W_vs_E10	ordinary_muscle_development	ENSGALG00010003323	STMN2	-2.413064566	0.49275657	-4.897072334	9.7275E-07	1.87102E-06	negative
PRJNA729271	stage_5W_vs_E10	ordinary_muscle_development	ENSGALG00010003324	ENSGALG00010003324	0.148073275	0.587068368	0.252224926	0.800867213	0.824650532	positive
PRJNA729271	stage_5W_vs_E10	ordinary_muscle_development	ENSGALG00010003330	HEY1	0.077877475	0.121734221	0.639733623	0.522345792	0.562845841	positive
PRJNA729271	stage_5W_vs_E10	ordinary_muscle_development	ENSGALG00010003331	EPSTI1	4.899719146	0.611182425	8.01678671	1.08547E-15	3.54368E-15	positive
PRJNA729271	stage_5W_vs_E10	ordinary_muscle_development	ENSGALG00010003333	ENSGALG00010003333	17.71925962	2.702628265	6.556306631	5.51568E-11	1.39626E-10	positive
PRJNA729271	stage_5W_vs_E10	ordinary_muscle_development	ENSGALG00010003334	TAC1	-5.984716865	1.086612006	-5.507685202	3.63583E-08	7.72181E-08	negative
PRJNA729271	stage_5W_vs_E10	ordinary_muscle_development	ENSGALG00010003339	ENSGALG00010003339	-1.230531713	0.498518118	-2.468379116	0.013572649	0.018300981	negative
PRJNA729271	stage_5W_vs_E10	ordinary_muscle_development	ENSGALG00010003344	ZFAND1	-0.052304829	0.19096235	-0.273901263	0.784160501	0.81015014	negative
PRJNA729271	stage_5W_vs_E10	ordinary_muscle_development	ENSGALG00010003346	ENSGALG00010003346	3.475785475	5.413575368	0.642049891	0.52084079	0.561432829	positive
PRJNA729271	stage_5W_vs_E10	ordinary_muscle_development	ENSGALG00010003347	GPM6A	-2.422212905	0.383541283	-6.315390315	2.6948E-10	6.51889E-10	negative
PRJNA729271	stage_5W_vs_E10	ordinary_muscle_development	ENSGALG00010003348	ADAM17	-0.191661496	0.083691946	-2.290082927	0.02201651	0.028943344	negative
PRJNA729271	stage_5W_vs_E10	ordinary_muscle_development	ENSGALG00010003351	ENSGALG00010003351	0.14846821	1.563699481	0.094946767	0.92435711	0.934596152	positive
PRJNA729271	stage_5W_vs_E10	ordinary_muscle_development	ENSGALG00010003352	FARSA	-0.040243714	0.102161223	-0.393923568	0.69363747	0.726307529	negative
PRJNA729271	stage_5W_vs_E10	ordinary_muscle_development	ENSGALG00010003356	ENSGALG00010003356	2.163935002	1.110220372	1.949104032	0.051283002	0.064588332	positive
PRJNA729271	stage_5W_vs_E10	ordinary_muscle_development	ENSGALG00010003358	DNAJC15	0.650104548	0.133658075	4.863937697	1.15073E-06	2.19761E-06	positive
PRJNA729271	stage_5W_vs_E10	ordinary_muscle_development	ENSGALG00010003363	HBE1	-9.323517301	0.589651141	-15.81192107	2.57494E-56	3.7138E-55	negative
PRJNA729271	stage_5W_vs_E10	ordinary_muscle_development	ENSGALG00010003364	ANKRD6	-2.659573674	0.167491316	-15.87887505	8.8756E-57	1.29689E-55	negative
PRJNA729271	stage_5W_vs_E10	ordinary_muscle_development	ENSGALG00010003366	CASD1	0.100578817	0.108830047	0.924182428	0.355391343	0.395652651	positive
PRJNA729271	stage_5W_vs_E10	ordinary_muscle_development	ENSGALG00010003371	ENSGALG00010003371	-3.493156278	0.390351866	-8.948737235	3.59572E-19	1.38359E-18	negative
PRJNA729271	stage_5W_vs_E10	ordinary_muscle_development	ENSGALG00010003372	ENOX1	-1.406560533	0.143630812	-9.792888536	1.20795E-22	5.43764E-22	negative
PRJNA729271	stage_5W_vs_E10	ordinary_muscle_development	ENSGALG00010003374	SYCE2	-0.909969981	0.507868031	-1.791744953	0.07317383	0.090453238	negative
PRJNA729271	stage_5W_vs_E10	ordinary_muscle_development	ENSGALG00010003375	YWHAQ	-1.182129919	0.086253312	-13.70532791	9.43401E-43	8.95521E-42	negative
PRJNA729271	stage_5W_vs_E10	ordinary_muscle_development	ENSGALG00010003379	ENSGALG00010003379	-3.221954278	0.531179186	-6.065663651	1.3141E-09	3.04891E-09	negative
PRJNA729271	stage_5W_vs_E10	ordinary_muscle_development	ENSGALG00010003382	ASNS	0.280727334	0.59950583	0.468264561	0.639595408	0.6748388	positive
PRJNA729271	stage_5W_vs_E10	ordinary_muscle_development	ENSGALG00010003383	UBE2J1	0.824286703	0.085871206	9.599104802	8.06418E-22	3.49657E-21	positive
PRJNA729271	stage_5W_vs_E10	ordinary_muscle_development	ENSGALG00010003388	PXDN	-0.224787811	0.186042578	-1.208260031	0.226947257	0.261390584	negative
PRJNA729271	stage_5W_vs_E10	ordinary_muscle_development	ENSGALG00010003389	ENSGALG00010003389	-4.586306382	0.477667756	-9.601456926	7.88223E-22	3.42074E-21	negative
PRJNA729271	stage_5W_vs_E10	ordinary_muscle_development	ENSGALG00010003395	ENSGALG00010003395	2.981445479	0.991830165	3.006004037	0.002647055	0.003847294	positive
PRJNA729271	stage_5W_vs_E10	ordinary_muscle_development	ENSGALG00010003397	SPATA4	1.282163872	0.426092165	3.009123328	0.002620027	0.003809921	positive
PRJNA729271	stage_5W_vs_E10	ordinary_muscle_development	ENSGALG00010003400	PKIA	1.103180787	0.114582214	9.62785369	6.099E-22	2.66521E-21	positive
PRJNA729271	stage_5W_vs_E10	ordinary_muscle_development	ENSGALG00010003401	ENSGALG00010003401	-0.001552071	1.724380545	-0.000900074	0.999281845	0.999419743	negative
PRJNA729271	stage_5W_vs_E10	ordinary_muscle_development	ENSGALG00010003403	GCDH	-0.194409694	0.150250424	-1.293904467	0.195698421	0.227971439	negative
PRJNA729271	stage_5W_vs_E10	ordinary_muscle_development	ENSGALG00010003405	GABRR2	-1.760019313	0.273948449	-6.424636889	1.32185E-10	3.26353E-10	negative
PRJNA729271	stage_5W_vs_E10	ordinary_muscle_development	ENSGALG00010003406	C1GALT1	-0.789587534	0.106230552	-7.432772602	1.06345E-13	3.11093E-13	negative
PRJNA729271	stage_5W_vs_E10	ordinary_muscle_development	ENSGALG00010003407	CHMP4C	0.822572007	0.209670229	3.923170254	8.73913E-05	0.000144902	positive
PRJNA729271	stage_5W_vs_E10	ordinary_muscle_development	ENSGALG00010003408	ENSGALG00010003408	10.99812691	3.66343258	3.002137114	0.002680914	0.003894163	positive
PRJNA729271	stage_5W_vs_E10	ordinary_muscle_development	ENSGALG00010003412	ASB5	1.52375224	0.146828284	10.37778419	3.12954E-25	1.56369E-24	positive
PRJNA729271	stage_5W_vs_E10	ordinary_muscle_development	ENSGALG00010003419	ENSGALG00010003419	-3.271322115	0.998716924	-3.275524864	0.001054658	0.001595915	negative
PRJNA729271	stage_5W_vs_E10	ordinary_muscle_development	ENSGALG00010003420	ENSGALG00010003420	1.313203842	0.631573938	2.079255907	0.037593835	0.048218976	positive
PRJNA729271	stage_5W_vs_E10	ordinary_muscle_development	ENSGALG00010003428	SNX16	0.740093034	0.154858978	4.779141923	1.76045E-06	3.31485E-06	positive
PRJNA729271	stage_5W_vs_E10	ordinary_muscle_development	ENSGALG00010003431	PM20D2	2.809810489	0.285457864	9.843170704	7.33616E-23	3.33347E-22	positive
PRJNA729271	stage_5W_vs_E10	ordinary_muscle_development	ENSGALG00010003432	LACC1	2.360267525	0.306745304	7.694551458	1.41991E-14	4.35498E-14	positive
PRJNA729271	stage_5W_vs_E10	ordinary_muscle_development	ENSGALG00010003437	PNRC1	1.500926582	0.178989101	8.385575304	5.04783E-17	1.75801E-16	positive
PRJNA729271	stage_5W_vs_E10	ordinary_muscle_development	ENSGALG00010003438	KCNS3	-1.256908977	0.178711877	-7.033158602	2.0191E-12	5.544E-12	negative
PRJNA729271	stage_5W_vs_E10	ordinary_muscle_development	ENSGALG00010003439	GMIP	1.050623679	0.228115353	4.605668434	4.11143E-06	7.53416E-06	positive
PRJNA729271	stage_5W_vs_E10	ordinary_muscle_development	ENSGALG00010003442	SPCS3	0.110737616	0.089833946	1.232692333	0.217690573	0.251628777	positive
PRJNA729271	stage_5W_vs_E10	ordinary_muscle_development	ENSGALG00010003447	ZC2HC1A	-0.59492064	0.126159564	-4.715620616	2.40975E-06	4.4931E-06	negative
PRJNA729271	stage_5W_vs_E10	ordinary_muscle_development	ENSGALG00010003449	BORCS6	1.307969833	0.109657932	11.92772662	8.48594E-33	5.73979E-32	positive
PRJNA729271	stage_5W_vs_E10	ordinary_muscle_development	ENSGALG00010003454	BPNT2	-1.108595876	0.120550809	-9.19608822	3.71218E-20	1.49301E-19	negative
PRJNA729271	stage_5W_vs_E10	ordinary_muscle_development	ENSGALG00010003456	CDS1	-3.205868386	0.38936124	-8.233660812	1.81577E-16	6.14801E-16	negative
PRJNA729271	stage_5W_vs_E10	ordinary_muscle_development	ENSGALG00010003466	TSC22D1	-0.060221445	0.100571879	-0.598790097	0.549312867	0.589144654	negative
PRJNA729271	stage_5W_vs_E10	ordinary_muscle_development	ENSGALG00010003468	ILVBL	1.068577951	0.135143703	7.906975488	2.63718E-15	8.42536E-15	positive
PRJNA729271	stage_5W_vs_E10	ordinary_muscle_development	ENSGALG00010003470	METTL3	0.802739325	0.186755135	4.298352085	1.72073E-05	3.01086E-05	positive
PRJNA729271	stage_5W_vs_E10	ordinary_muscle_development	ENSGALG00010003471	ADI1	0.954911496	0.133342035	7.161368851	7.98754E-13	2.23945E-12	positive
PRJNA729271	stage_5W_vs_E10	ordinary_muscle_development	ENSGALG00010003472	FAM110B	-2.791071113	0.132939185	-20.99509725	7.2714E-98	2.9359E-96	negative
PRJNA729271	stage_5W_vs_E10	ordinary_muscle_development	ENSGALG00010003473	ENSGALG00010003473	-0.62947098	0.25865225	-2.433657466	0.014947132	0.020060989	negative
PRJNA729271	stage_5W_vs_E10	ordinary_muscle_development	ENSGALG00010003474	RPS7	-0.201326888	0.166146412	-1.211743815	0.225610461	0.260016191	negative
PRJNA729271	stage_5W_vs_E10	ordinary_muscle_development	ENSGALG00010003475	ENSGALG00010003475	8.922784145	2.073506659	4.303233899	1.68323E-05	2.94809E-05	positive
PRJNA729271	stage_5W_vs_E10	ordinary_muscle_development	ENSGALG00010003478	COLEC11	1.409692612	0.177956886	7.921540111	2.34587E-15	7.51953E-15	positive
PRJNA729271	stage_5W_vs_E10	ordinary_muscle_development	ENSGALG00010003479	SYDE1	0.391970589	0.20184311	1.941956749	0.052142337	0.065619306	positive
PRJNA729271	stage_5W_vs_E10	ordinary_muscle_development	ENSGALG00010003485	RNGTT	-1.18296821	0.104353399	-11.33617329	8.68602E-30	5.20694E-29	negative
PRJNA729271	stage_5W_vs_E10	ordinary_muscle_development	ENSGALG00010003489	SGCE	-0.373801228	0.11951152	-3.127742218	0.001761546	0.00260308	negative
PRJNA729271	stage_5W_vs_E10	ordinary_muscle_development	ENSGALG00010003503	NUFIP1	-0.775624392	0.08852184	-8.761955124	1.91893E-18	7.15218E-18	negative
PRJNA729271	stage_5W_vs_E10	ordinary_muscle_development	ENSGALG00010003506	DYNC1I1	-3.171561073	0.199170559	-15.92384481	4.32936E-57	6.41001E-56	negative
PRJNA729271	stage_5W_vs_E10	ordinary_muscle_development	ENSGALG00010003507	WDFY3	0.642888998	0.097451492	6.59701548	4.19517E-11	1.07171E-10	positive
PRJNA729271	stage_5W_vs_E10	ordinary_muscle_development	ENSGALG00010003509	GPALPP1	-0.690093043	0.075518394	-9.138078883	6.35716E-20	2.5336E-19	negative
PRJNA729271	stage_5W_vs_E10	ordinary_muscle_development	ENSGALG00010003511	UBXN2B	-0.983492988	0.144880457	-6.788306759	1.13457E-11	2.99447E-11	negative
PRJNA729271	stage_5W_vs_E10	ordinary_muscle_development	ENSGALG00010003512	ENSGALG00010003512	1.207747031	0.418142564	2.888361853	0.003872541	0.005532474	positive
PRJNA729271	stage_5W_vs_E10	ordinary_muscle_development	ENSGALG00010003517	GTF2F2	-0.854421506	0.089080531	-9.591562783	8.67608E-22	3.75739E-21	negative
PRJNA729271	stage_5W_vs_E10	ordinary_muscle_development	ENSGALG00010003519	KCTD4	-3.205767983	0.765875515	-4.185755937	2.84219E-05	4.88411E-05	negative
PRJNA729271	stage_5W_vs_E10	ordinary_muscle_development	ENSGALG00010003520	ENSGALG00010003520	7.242863496	1.61393892	4.487693684	7.19983E-06	1.29577E-05	positive
PRJNA729271	stage_5W_vs_E10	ordinary_muscle_development	ENSGALG00010003522	ENSGALG00010003522	2.509079362	0.640785351	3.915631587	9.01678E-05	0.000149233	positive
PRJNA729271	stage_5W_vs_E10	ordinary_muscle_development	ENSGALG00010003525	NOL10	-0.993082752	0.068916836	-14.40987151	4.48544E-47	4.89582E-46	negative
PRJNA729271	stage_5W_vs_E10	ordinary_muscle_development	ENSGALG00010003528	GDAP1	-1.444598712	0.207671536	-6.956170968	3.49646E-12	9.47665E-12	negative
PRJNA729271	stage_5W_vs_E10	ordinary_muscle_development	ENSGALG00010003531	PPM1K	-0.421737065	0.150660268	-2.799258686	0.005122009	0.007224943	negative
PRJNA729271	stage_5W_vs_E10	ordinary_muscle_development	ENSGALG00010003539	ENSGALG00010003539	0.476902662	0.217900884	2.188621969	0.028624328	0.037214964	positive
PRJNA729271	stage_5W_vs_E10	ordinary_muscle_development	ENSGALG00010003541	CNR1	-0.760196143	0.219217117	-3.467777304	0.000524782	0.000813814	negative
PRJNA729271	stage_5W_vs_E10	ordinary_muscle_development	ENSGALG00010003543	SOX11	-3.640513419	0.083674853	-43.50785558	0	0	negative
PRJNA729271	stage_5W_vs_E10	ordinary_muscle_development	ENSGALG00010003552	PI15	-2.9991509	0.389448831	-7.701013992	1.34991E-14	4.14729E-14	negative
PRJNA729271	stage_5W_vs_E10	ordinary_muscle_development	ENSGALG00010003556	ENSGALG00010003556	9.066896434	1.828468264	4.958738751	7.09523E-07	1.37862E-06	positive
PRJNA729271	stage_5W_vs_E10	ordinary_muscle_development	ENSGALG00010003558	TPT1	1.838154369	0.184655462	9.954508534	2.41011E-23	1.11576E-22	positive
PRJNA729271	stage_5W_vs_E10	ordinary_muscle_development	ENSGALG00010003560	ADGRL3	-2.600559505	0.141038975	-18.43858765	6.44016E-76	1.60395E-74	negative
PRJNA729271	stage_5W_vs_E10	ordinary_muscle_development	ENSGALG00010003571	RPL7	-0.246549088	0.157476752	-1.565622131	0.117437106	0.141300809	negative
PRJNA729271	stage_5W_vs_E10	ordinary_muscle_development	ENSGALG00010003577	ENSGALG00010003577	8.383989156	1.549342692	5.41132004	6.25618E-08	1.30837E-07	positive
PRJNA729271	stage_5W_vs_E10	ordinary_muscle_development	ENSGALG00010003582	RDH10	-1.169875549	0.142317848	-8.220160487	2.03231E-16	6.86355E-16	negative
PRJNA729271	stage_5W_vs_E10	ordinary_muscle_development	ENSGALG00010003583	RSAD2	6.556662736	0.798870671	8.207414509	2.26002E-16	7.6237E-16	positive
PRJNA729271	stage_5W_vs_E10	ordinary_muscle_development	ENSGALG00010003585	PNPLA6	1.056674587	0.142681639	7.405820367	1.30342E-13	3.79379E-13	positive
PRJNA729271	stage_5W_vs_E10	ordinary_muscle_development	ENSGALG00010003589	SLC25A30	3.790298675	0.387386522	9.784281233	1.31526E-22	5.90789E-22	positive
PRJNA729271	stage_5W_vs_E10	ordinary_muscle_development	ENSGALG00010003595	SNX13	0.492054367	0.09424909	5.220786403	1.78165E-07	3.61138E-07	positive
PRJNA729271	stage_5W_vs_E10	ordinary_muscle_development	ENSGALG00010003596	CRISPLD1	-3.060279405	0.155488268	-19.68173839	3.09212E-86	9.55657E-85	negative
PRJNA729271	stage_5W_vs_E10	ordinary_muscle_development	ENSGALG00010003597	RNF144A	-1.053082152	0.147247986	-7.151759306	8.56727E-13	2.3992E-12	negative
PRJNA729271	stage_5W_vs_E10	ordinary_muscle_development	ENSGALG00010003604	ODC	-1.63993701	0.216658692	-7.56921863	3.75476E-14	1.12869E-13	negative
PRJNA729271	stage_5W_vs_E10	ordinary_muscle_development	ENSGALG00010003617	COG3	-0.045321069	0.08325033	-0.544395072	0.586169636	0.62427176	negative
PRJNA729271	stage_5W_vs_E10	ordinary_muscle_development	ENSGALG00010003619	HNF4G	-1.308469841	0.545928186	-2.396780153	0.016539844	0.022088174	negative
PRJNA729271	stage_5W_vs_E10	ordinary_muscle_development	ENSGALG00010003627	MCOLN1	0.481621934	0.120075378	4.010996623	6.0463E-05	0.000101578	positive
PRJNA729271	stage_5W_vs_E10	ordinary_muscle_development	ENSGALG00010003632	TECRL	-2.333638418	0.548799341	-4.252261695	2.11622E-05	3.67537E-05	negative
PRJNA729271	stage_5W_vs_E10	ordinary_muscle_development	ENSGALG00010003634	CYP4F11	1.547933509	0.302239638	5.121543684	3.03045E-07	6.03135E-07	positive
PRJNA729271	stage_5W_vs_E10	ordinary_muscle_development	ENSGALG00010003639	ENSGALG00010003639	0.497595263	0.765321922	0.650177721	0.515577431	0.556028393	positive
PRJNA729271	stage_5W_vs_E10	ordinary_muscle_development	ENSGALG00010003641	ENSGALG00010003641	9.04687018	2.44095121	3.706288821	0.000210318	0.000337305	positive

PRJNA729271	stage_5W_vs_E10	ordinary_muscle_development	ENSGALG00010003650	SPACA1	-2.983261971	0.952417788	-3.13230392	0.001734402	0.002565323	negative
PRJNA729271	stage_5W_vs_E10	ordinary_muscle_development	ENSGALG00010003652	MYCN	-0.924649512	0.142235035	-6.500856241	7.98641E-11	2.00386E-10	negative
PRJNA729271	stage_5W_vs_E10	ordinary_muscle_development	ENSGALG00010003660	IGFBP7	3.513383118	0.164767567	21.32326875	6.9062E-101	2.95297E-99	positive
PRJNA729271	stage_5W_vs_E10	ordinary_muscle_development	ENSGALG00010003665	RDH14	1.779871778	0.091524096	19.44702928	3.08812E-84	9.13515E-83	positive
PRJNA729271	stage_5W_vs_E10	ordinary_muscle_development	ENSGALG00010003666	SLC25A13	-0.569129015	0.078027664	-7.293938951	3.01022E-13	8.59766E-13	negative
PRJNA729271	stage_5W_vs_E10	ordinary_muscle_development	ENSGALG00010003669	ENSGALG00010003669	7.721836318	1.461583503	5.283198873	1.26947E-07	2.60232E-07	positive
PRJNA729271	stage_5W_vs_E10	ordinary_muscle_development	ENSGALG00010003671	ENSGALG00010003671	0.034867473	0.412863138	0.084452861	0.93269638	0.941530332	positive
PRJNA729271	stage_5W_vs_E10	ordinary_muscle_development	ENSGALG00010003674	AKIRIN2	0.183793157	0.132891632	1.383030328	0.166655567	0.196732017	positive
PRJNA729271	stage_5W_vs_E10	ordinary_muscle_development	ENSGALG00010003676	POLR2B	0.292095231	0.078163778	3.73696406	0.000186256	0.000299975	positive
PRJNA729271	stage_5W_vs_E10	ordinary_muscle_development	ENSGALG00010003678	ZNFY1	-0.192791837	0.445801282	-0.432461377	0.665406117	0.699725436	negative
PRJNA729271	stage_5W_vs_E10	ordinary_muscle_development	ENSGALG00010003680	TRAPPC5	0.398032781	0.107009473	3.719603243	0.000199536	0.000320616	positive
PRJNA729271	stage_5W_vs_E10	ordinary_muscle_development	ENSGALG00010003681	NT5C1B	0.071605646	0.12941474	0.553303637	0.580055511	0.618546651	positive
PRJNA729271	stage_5W_vs_E10	ordinary_muscle_development	ENSGALG00010003683	ENSGALG00010003683	7.61895774	1.550279954	4.914568959	8.8978E-07	1.71657E-06	positive
PRJNA729271	stage_5W_vs_E10	ordinary_muscle_development	ENSGALG00010003691	ERICH1	-0.23834793	0.155132596	-1.536414243	0.124436816	0.149239752	negative
PRJNA729271	stage_5W_vs_E10	ordinary_muscle_development	ENSGALG00010003692	ZNFY2	-0.481998642	0.261146833	-1.845699742	0.064935815	0.080745015	negative
PRJNA729271	stage_5W_vs_E10	ordinary_muscle_development	ENSGALG00010003695	NOA1	0.510412436	0.100900188	5.058587539	4.22373E-07	8.32513E-07	positive
PRJNA729271	stage_5W_vs_E10	ordinary_muscle_development	ENSGALG00010003698	MACC1	-4.643722717	1.191563419	-3.897167908	9.73241E-05	0.000160509	negative
PRJNA729271	stage_5W_vs_E10	ordinary_muscle_development	ENSGALG00010003700	REST	-0.607719316	0.06787154	-8.953963903	3.42941E-19	1.321E-18	negative
PRJNA729271	stage_5W_vs_E10	ordinary_muscle_development	ENSGALG00010003701	SARAF	1.450772683	0.081632814	17.77193037	1.16608E-70	2.50035E-69	positive
PRJNA729271	stage_5W_vs_E10	ordinary_muscle_development	ENSGALG00010003703	TDRP	-0.872745788	0.23010902	-3.792749145	0.000148989	0.000241565	negative
PRJNA729271	stage_5W_vs_E10	ordinary_muscle_development	ENSGALG00010003713	SPERT	0.485883815	0.264106166	1.839729159	0.06580801	0.081759416	positive
PRJNA729271	stage_5W_vs_E10	ordinary_muscle_development	ENSGALG00010003714	ORC3	-0.531632945	0.109748623	-4.844096735	1.27189E-06	2.42006E-06	negative
PRJNA729271	stage_5W_vs_E10	ordinary_muscle_development	ENSGALG00010003716	YLEC8	0.528376703	0.68390835	0.772584079	0.43976857	0.481562697	positive
PRJNA729271	stage_5W_vs_E10	ordinary_muscle_development	ENSGALG00010003717	LEPROTL1	-0.004885269	0.097524273	-0.050092853	0.960048395	0.965376448	negative
PRJNA729271	stage_5W_vs_E10	ordinary_muscle_development	ENSGALG00010003719	RPGR	-0.913349715	0.116040131	-7.870981411	3.5187E-15	1.11654E-14	negative
PRJNA729271	stage_5W_vs_E10	ordinary_muscle_development	ENSGALG00010003720	ENSGALG00010003720	9.146953666	2.603081497	3.513894466	0.000441588	0.000688408	positive
PRJNA729271	stage_5W_vs_E10	ordinary_muscle_development	ENSGALG00010003722	MHCY11	3.914425387	0.651766126	6.005874241	1.90304E-09	4.3674E-09	positive
PRJNA729271	stage_5W_vs_E10	ordinary_muscle_development	ENSGALG00010003724	ENSGALG00010003724	0.121047934	0.217564753	0.556376584	0.577953443	0.616849655	positive
PRJNA729271	stage_5W_vs_E10	ordinary_muscle_development	ENSGALG00010003726	ENPEP	-4.580076042	0.601249171	-7.617600589	2.58435E-14	7.83684E-14	negative
PRJNA729271	stage_5W_vs_E10	ordinary_muscle_development	ENSGALG00010003727	FBXO25	0.953541952	0.152423822	6.255859086	3.95334E-10	9.4701E-10	positive
PRJNA729271	stage_5W_vs_E10	ordinary_muscle_development	ENSGALG00010003729	RARS2	-0.960327332	0.106233676	-9.039763733	1.57011E-19	6.14768E-19	negative
PRJNA729271	stage_5W_vs_E10	ordinary_muscle_development	ENSGALG00010003731	ENSGALG00010003731	2.839107245	0.573260385	4.952561385	7.32429E-07	1.42161E-06	positive
PRJNA729271	stage_5W_vs_E10	ordinary_muscle_development	ENSGALG00010003733	STAU2	-0.07817344	0.071028574	-1.100591434	0.271074516	0.308247047	negative
PRJNA729271	stage_5W_vs_E10	ordinary_muscle_development	ENSGALG00010003734	ZC3H13	-0.421011532	0.142011627	-2.964627207	0.003030499	0.004376952	negative
PRJNA729271	stage_5W_vs_E10	ordinary_muscle_development	ENSGALG00010003736	ENSGALG00010003736	7.422541788	3.006257991	2.469030206	0.013547978	0.018269415	positive
PRJNA729271	stage_5W_vs_E10	ordinary_muscle_development	ENSGALG00010003737	SRP72	-0.637104821	0.057282769	-11.12210244	9.79365E-29	5.64673E-28	negative
PRJNA729271	stage_5W_vs_E10	ordinary_muscle_development	ENSGALG00010003740	PITX2	-4.217437308	0.367168068	-11.48639457	1.54423E-30	9.53307E-30	negative
PRJNA729271	stage_5W_vs_E10	ordinary_muscle_development	ENSGALG00010003742	ZFHX4	-0.993056713	0.16629306	-5.971726728	2.34756E-09	5.36716E-09	negative
PRJNA729271	stage_5W_vs_E10	ordinary_muscle_development	ENSGALG00010003743	ARL9	0.000417915	0.176988583	0.002361255	0.998115993	0.998391506	positive
PRJNA729271	stage_5W_vs_E10	ordinary_muscle_development	ENSGALG00010003746	HOPX	4.093781771	0.406521769	10.07026458	7.47767E-24	3.52943E-23	positive
PRJNA729271	stage_5W_vs_E10	ordinary_muscle_development	ENSGALG00010003749	MHCY15	2.98013256	0.94625476	3.149397693	0.001636074	0.002425828	positive
PRJNA729271	stage_5W_vs_E10	ordinary_muscle_development	ENSGALG00010003753	SLCO5A1	0.798492254	0.159840001	4.995572128	5.86616E-07	1.14611E-06	positive
PRJNA729271	stage_5W_vs_E10	ordinary_muscle_development	ENSGALG00010003754	FAM110C	0.963672447	0.245958341	3.918031176	8.92752E-05	0.000147857	positive
PRJNA729271	stage_5W_vs_E10	ordinary_muscle_development	ENSGALG00010003755	SLC35A1	-0.830618015	0.092871098	-8.943772956	3.76105E-19	1.44606E-18	negative
PRJNA729271	stage_5W_vs_E10	ordinary_muscle_development	ENSGALG00010003756	MHCY7	1.803186966	0.371527628	4.85343977	1.21338E-06	2.31329E-06	positive
PRJNA729271	stage_5W_vs_E10	ordinary_muscle_development	ENSGALG00010003761	UGT8	1.988205485	0.330401215	6.017548948	1.77078E-09	4.07484E-09	positive
PRJNA729271	stage_5W_vs_E10	ordinary_muscle_development	ENSGALG00010003762	EVI5L	-0.170629622	0.099771822	-1.710198513	0.087229171	0.106753363	negative
PRJNA729271	stage_5W_vs_E10	ordinary_muscle_development	ENSGALG00010003764	HDAC9	0.228341799	0.193598769	1.179458938	0.238215478	0.273434697	positive
PRJNA729271	stage_5W_vs_E10	ordinary_muscle_development	ENSGALG00010003765	ENSGALG00010003765	0.595366476	0.881307752	0.675548893	0.499327099	0.540452979	positive
PRJNA729271	stage_5W_vs_E10	ordinary_muscle_development	ENSGALG00010003767	ENSGALG00010003767	0.269482249	0.412375973	0.653486785	0.513442506	0.553952298	positive
PRJNA729271	stage_5W_vs_E10	ordinary_muscle_development	ENSGALG00010003768	ARSJ	-2.059271633	0.135719506	-15.17299677	5.33828E-52	6.87808E-51	negative
PRJNA729271	stage_5W_vs_E10	ordinary_muscle_development	ENSGALG00010003769	C6orf163	-0.330360487	0.534205596	-0.618414501	0.536302141	0.576369248	negative
PRJNA729271	stage_5W_vs_E10	ordinary_muscle_development	ENSGALG00010003772	ENSGALG00010003772	2.344994727	0.440711146	5.320933558	1.03236E-07	2.128E-07	positive
PRJNA729271	stage_5W_vs_E10	ordinary_muscle_development	ENSGALG00010003775	NCOA2	0.504315359	0.114385341	4.408916004	1.03889E-05	1.84929E-05	positive
PRJNA729271	stage_5W_vs_E10	ordinary_muscle_development	ENSGALG00010003777	ENSGALG00010003777	12.25545899	0.798809833	15.34214838	3.99743E-53	5.25796E-52	positive
PRJNA729271	stage_5W_vs_E10	ordinary_muscle_development	ENSGALG00010003778	LCA5	0.21370774	0.124421944	1.717604894	0.085868707	0.105221651	positive
PRJNA729271	stage_5W_vs_E10	ordinary_muscle_development	ENSGALG00010003785	ENSGALG00010003785	6.022652807	1.041749045	5.78128949	7.41302E-09	1.64904E-08	positive
PRJNA729271	stage_5W_vs_E10	ordinary_muscle_development	ENSGALG00010003786	ENSGALG00010003786	0.027139055	0.602313712	0.045058006	0.964061073	0.968873692	positive
PRJNA729271	stage_5W_vs_E10	ordinary_muscle_development	ENSGALG00010003787	SMIM8	1.357669835	0.18805141	7.219673778	5.21124E-13	1.47303E-12	positive
PRJNA729271	stage_5W_vs_E10	ordinary_muscle_development	ENSGALG00010003788	NUDT9	-0.022203148	0.127156915	-0.174612196	0.861384365	0.878475084	negative
PRJNA729271	stage_5W_vs_E10	ordinary_muscle_development	ENSGALG00010003790	ELOVL6	-0.625541473	0.182038915	-3.436306318	0.000589704	0.000911178	negative
PRJNA729271	stage_5W_vs_E10	ordinary_muscle_development	ENSGALG00010003791	ITGB8	-2.340437856	0.180031232	-13.00017683	1.22061E-38	1.01449E-37	negative
PRJNA729271	stage_5W_vs_E10	ordinary_muscle_development	ENSGALG00010003793	TRAM1L1	-0.534828784	0.085228877	-6.275206318	3.49171E-10	8.38925E-10	negative
PRJNA729271	stage_5W_vs_E10	ordinary_muscle_development	ENSGALG00010003795	LCP1	2.570438191	0.200348592	12.82982906	1.1161E-37	9.01778E-37	positive
PRJNA729271	stage_5W_vs_E10	ordinary_muscle_development	ENSGALG00010003796	ENSGALG00010003796	3.817126738	0.300218644	12.7144893	4.91309E-37	3.91723E-36	positive
PRJNA729271	stage_5W_vs_E10	ordinary_muscle_development	ENSGALG00010003797	ENSGALG00010003797	0.294349924	0.174860725	1.683339266	0.092309433	0.112609429	positive
PRJNA729271	stage_5W_vs_E10	ordinary_muscle_development	ENSGALG00010003803	LACTB2	0.910584143	0.132718004	6.861044608	6.83588E-12	1.82311E-11	positive
PRJNA729271	stage_5W_vs_E10	ordinary_muscle_development	ENSGALG00010003804	PSME1	3.684423905	0.25189671	14.62672502	1.89701E-48	2.16343E-47	positive
PRJNA729271	stage_5W_vs_E10	ordinary_muscle_development	ENSGALG00010003806	EGF	3.673322896	0.315870626	11.62920068	2.92827E-31	1.86737E-30	positive
PRJNA729271	stage_5W_vs_E10	ordinary_muscle_development	ENSGALG00010003808	TWIST1	-3.481391429	0.171276451	-20.32615348	7.54938E-92	2.70193E-90	negative
PRJNA729271	stage_5W_vs_E10	ordinary_muscle_development	ENSGALG00010003810	HMGN2P46	0.222394853	0.091236343	2.437568678	0.014786405	0.019858143	positive
PRJNA729271	stage_5W_vs_E10	ordinary_muscle_development	ENSGALG00010003812	BG8	-0.148996474	0.407241839	-0.365867305	0.714464094	0.745315369	negative
PRJNA729271	stage_5W_vs_E10	ordinary_muscle_development	ENSGALG00010003813	LRIT3	-3.781930025	0.87378908	-4.328195567	1.50336E-05	2.64553E-05	negative
PRJNA729271	stage_5W_vs_E10	ordinary_muscle_development	ENSGALG00010003814	TPP1	1.355312204	0.132305135	10.24383674	1.26133E-24	6.17251E-24	positive
PRJNA729271	stage_5W_vs_E10	ordinary_muscle_development	ENSGALG00010003815	RASAL3	5.871370921	0.561204395	10.46209006	1.28979E-25	6.54603E-25	positive
PRJNA729271	stage_5W_vs_E10	ordinary_muscle_development	ENSGALG00010003816	KLHL8	-0.944901314	0.182384965	-5.180807063	2.20928E-07	4.44709E-07	negative
PRJNA729271	stage_5W_vs_E10	ordinary_muscle_development	ENSGALG00010003817	BF2	6.329387627	0.281736803	22.46560763	9.0078E-112	4.7479E-110	positive
PRJNA729271	stage_5W_vs_E10	ordinary_muscle_development	ENSGALG00010003818	GAB2	0.436319567	0.160993367	2.710171074	0.006724851	0.009374251	positive
PRJNA729271	stage_5W_vs_E10	ordinary_muscle_development	ENSGALG00010003819	PHIP	-0.055704732	0.094233675	-0.591134027	0.554430622	0.594149924	negative
PRJNA729271	stage_5W_vs_E10	ordinary_muscle_development	ENSGALG00010003820	SH3YL1	-1.019753419	0.303644403	-3.358380424	0.000784006	0.001198499	negative
PRJNA729271	stage_5W_vs_E10	ordinary_muscle_development	ENSGALG00010003821	RUBCNL	1.285524435	0.155645604	8.259304484	1.46524E-16	4.98208E-16	positive
PRJNA729271	stage_5W_vs_E10	ordinary_muscle_development	ENSGALG00010003822	ENSGALG00010003822	-0.543341218	0.16469277	-3.299120031	0.000969884	0.001472708	negative
PRJNA729271	stage_5W_vs_E10	ordinary_muscle_development	ENSGALG00010003823	PTPN13	-0.01733497	0.149090083	-0.116271784	0.907437147	0.919554072	negative
PRJNA729271	stage_5W_vs_E10	ordinary_muscle_development	ENSGALG00010003824	SP4	0.199450218	0.158807623	1.255923452	0.209143725	0.242503588	positive
PRJNA729271	stage_5W_vs_E10	ordinary_muscle_development	ENSGALG00010003826	ENSGALG00010003826	-0.258491936	0.106867465	-2.418808536	0.015571434	0.020856398	negative
PRJNA729271	stage_5W_vs_E10	ordinary_muscle_development	ENSGALG00010003827	RRH	-0.426699892	0.743049987	-0.574254626	0.565795509	0.605076428	negative
PRJNA729271	stage_5W_vs_E10	ordinary_muscle_development	ENSGALG00010003829	ENSGALG00010003829	-0.271364081	0.134504547	-2.017508607	0.043642464	0.055461819	negative
PRJNA729271	stage_5W_vs_E10	ordinary_muscle_development	ENSGALG00010003830	DMB2	2.579377452	0.228343375	11.29604681	1.37259E-29	8.18078E-29	positive
PRJNA729271	stage_5W_vs_E10	ordinary_muscle_development	ENSGALG00010003831	IRAK1BP1	-0.423383734	0.171467417	-2.469178928	0.013542349	0.018263523	negative
PRJNA729271	stage_5W_vs_E10	ordinary_muscle_development	ENSGALG00010003832	ART1B	6.018195761	0.934224343	6.441917088	1.17974E-10	2.92113E-10	positive
PRJNA729271	stage_5W_vs_E10	ordinary_muscle_development	ENSGALG00010003833	ACP1	0.041649415	0.157772885	0.263983353	0.791792746	0.816638384	positive
PRJNA729271	stage_5W_vs_E10	ordinary_muscle_development	ENSGALG00010003834	MAPK10	-0.942363516	0.437308929	-2.154914874	0.031168496	0.040377813	negative
PRJNA729271	stage_5W_vs_E10	ordinary_muscle_development	ENSGALG00010003835	ENSGALG00010003835	2.06881039	0.16455586	12.57208582	3.00685E-36	2.33196E-35	positive
PRJNA729271	stage_5W_vs_E10	ordinary_muscle_development	ENSGALG00010003836	CD1F	4.151578983	0.328581371	12.63485806	1.35639E-36	1.06045E-35	positive
PRJNA729271	stage_5W_vs_E10	ordinary_muscle_development	ENSGALG00010003837	ALKAL2	0.777104538	0.247501661	3.139795249	0.00169066	0.002503178	positive
PRJNA729271	stage_5W_vs_E10	ordinary_muscle_development	ENSGALG00010003838	GAR1	-1.705712038	0.185513882	-9.194525046	3.76655E-20	1.51362E-19	negative
PRJNA729271	stage_5W_vs_E10	ordinary_muscle_development	ENSGALG00010003839	ENSGALG00010003839	4.766927931	0.716053749	6.657220831	2.79054E-11	7.19858E-11	positive
PRJNA729271	stage_5W_vs_E10	ordinary_muscle_development	ENSGALG00010003841	UBE2W	0.666464586	0.142273867	4.684378094	2.80811E-06	5.21373E-06	positive
PRJNA729271	stage_5W_vs_E10	ordinary_muscle_development	ENSGALG00010003842	ENSGALG00010003842	-0.176288451	0.144943216	-1.21625872	0.223886381	0.258131809	negative
PRJNA729271	stage_5W_vs_E10	ordinary_muscle_development	ENSGALG00010003843	LRCH1	0.085276866	0.109383242	0.779615454	0.435617257	0.477594141	positive
PRJNA729271	stage_5W_vs_E10	ordinary_muscle_development	ENSGALG00010003844	TMEM18	-1.429040476	0.120319251	-11.87707255	1.55726E-32	1.04454E-31	negative
PRJNA729271	stage_5W_vs_E10	ordinary_muscle_development	ENSGALG00010003845	CFI	1.087392702	0.866074851	1.255541251			positive
PRJNA729271	stage_5W_vs_E10	ordinary_muscle_development	ENSGALG00010003848	ESD	-0.121663627	0.124287725	-0.978886913	0.327635859	0.367291707	negative
PRJNA729271	stage_5W_vs_E10	ordinary_muscle_development	ENSGALG00010003850	PLA2G12A	-0.055190222	0.099352931	-0.555496668	0.578554989	0.617309868	negative
PRJNA729271	stage_5W_vs_E10	ordinary_muscle_development	ENSGALG00010003851	PHF14	-1.454351888	0.065484606	-22.20906524	2.8072E-109	1.3935E-107	negative
PRJNA729271	stage_5W_vs_E10	ordinary_muscle_development	ENSGALG00010003852	TCEB1	-0.112808763	0.107466899	-1.049707062	0.293852816	0.33188379	negative
PRJNA729271	stage_5W_vs_E10	ordinary_muscle_development	ENSGALG00010003853	ENSGALG00010003853	0.299629779	0.233012888	1.285893591	0.198480201	0.230859454	positive
PRJNA729271	stage_5W_vs_E10	ordinary_muscle_development	ENSGALG00010003858	SUCLA2	2.184855409	0.124194468	17.59221202	2.82627E-69	5.83572E-68	positive
PRJNA729271	stage_5W_vs_E10	ordinary_muscle_development	ENSGALG00010003860	TMEM70	0.407363486	0.114050369	3.571785789	0.000354555	0.000557708	positive
PRJNA729271	stage_5W_vs_E10	ordinary_muscle_development	ENSGALG00010003861	NUDT15	-0.682714521	0.105357374	-6.479987989	9.17299E-11	2.29048E-10	negative
PRJNA729271	stage_5W_vs_E10	ordinary_muscle_development	ENSGALG00010003862	PCK2	-0.726317818	0.146013497	-4.974319727	6.54772E-07	1.2748E-06	negative
PRJNA729271	stage_5W_vs_E10	ordinary_muscle_development	ENSGALG00010003863	HTR1B	-3.223254464	0.322162762	-10.00504976	1.44819E-23	6.7649E-23	negative
PRJNA729271	stage_5W_vs_E10	ordinary_muscle_development	ENSGALG00010003865	AKAP8L	-0.643391869	0.190407948	-3.379017929	0.000727453	0.001114868	negative
PRJNA729271	stage_5W_vs_E10	ordinary_muscle_development	ENSGALG00010003868	HBBA	-3.546654442	0.399069448	-8.887311357	6.26054E-19	2.3818E-18	negative
PRJNA729271	stage_5W_vs_E10	ordinary_muscle_development	ENSGALG00010003869	LY96	2.957976087	0.270781828	10.9238353	8.86707E-28	4.92445E-27	positive
PRJNA729271	stage_5W_vs_E10	ordinary_muscle_development	ENSGALG00010003870	MED4	-1.027365289	0.099207227	-10.35575051	3.94084E-25	1.9677E-24	negative
PRJNA729271	stage_5W_vs_E10	ordinary_muscle_development	ENSGALG00010003871	ENSGALG00010003871	-0.336634964	0.199887707	-1.684120393	0.092158404	0.112444113	negative
PRJNA729271	stage_5W_vs_E10	ordinary_muscle_development	ENSGALG00010003874	YES1	-0.720811743	0.098180938	-7.341666885	2.1095E-13	6.07413E-13	negative
PRJNA729271	stage_5W_vs_E10	ordinary_muscle_development	ENSGALG00010003875	AKAP8	-0.874097875	0.112320538	-7.782173161	7.12892E-15	2.22366E-14	negative
PRJNA729271	stage_5W_vs_E10	ordinary_muscle_development	ENSGALG00010003878	ARHGAP24	0.623803704	0.089010636	7.008192863	2.41416E-12	6.59379E-12	positive
PRJNA729271	stage_5W_vs_E10	ordinary_muscle_development	ENSGALG00010003879	CASP6	-1.379784482	0.120539427	-11.44674835	2.44132E-30	1.49438E-29	negative
PRJNA729271	stage_5W_vs_E10	ordinary_muscle_development	ENSGALG00010003882	ENSGALG00010003882	-0.343710406	0.122055321	-2.816021474	0.004862242	0.006873239	negative
PRJNA729271	stage_5W_vs_E10	ordinary_muscle_development	ENSGALG00010003884	TYMS	-3.401969628	0.19743877	-17.23050455	1.56778E-66	3.02193E-65	negative
PRJNA729271	stage_5W_vs_E10	ordinary_muscle_development	ENSGALG00010003887	GIPC1	-0.754206977	0.144716228	-5.211626835	1.87192E-07	3.78853E-07	negative
PRJNA729271	stage_5W_vs_E10	ordinary_muscle_development	ENSGALG00010003889	ITM2B	0.283982751	0.087757186	3.236005679	0.00121215	0.001820737	positive
PRJNA729271	stage_5W_vs_E10	ordinary_muscle_development	ENSGALG00010003892	OPRK1	-4.766981273	2.563370122	-1.859653911	0.062934507	0.078377496	negative
PRJNA729271	stage_5W_vs_E10	ordinary_muscle_development	ENSGALG00010003894	COPS4	0.155366152	0.124614118	1.246778089	0.212478877	0.24601656	positive
PRJNA729271	stage_5W_vs_E10	ordinary_muscle_development	ENSGALG00010003895	THSD7A	0.697242747	0.160255469	4.350820289	1.35629E-05	2.39603E-05	positive
PRJNA729271	stage_5W_vs_E10	ordinary_muscle_development	ENSGALG00010003896	MCUB	-1.152903805	0.135722656	-8.494556737	1.9869E-17	7.05538E-17	negative
PRJNA729271	stage_5W_vs_E10	ordinary_muscle_development	ENSGALG00010003898	NDST4	-2.459698591	0.512505688	-4.799358616	1.59175E-06	3.00656E-06	negative
PRJNA729271	stage_5W_vs_E10	ordinary_muscle_development	ENSGALG00010003899	LIN54	0.341763622	0.165576072	2.064088232	0.039009352	0.049915303	positive

PRJNA729271	stage_5W_vs_E10	ordinary_muscle_development	ENSGALG00010003900	RB1	-0.487998157	0.088036841	-5.543112998	2.97141E-08	6.35259E-08	negative
PRJNA729271	stage_5W_vs_E10	ordinary_muscle_development	ENSGALG00010003902	ENSGALG00010003902	1.077663472	0.124691269	8.642653833	5.49221E-18	2.00528E-17	positive
PRJNA729271	stage_5W_vs_E10	ordinary_muscle_development	ENSGALG00010003903	COL28A1	8.255121785	0.277046388	29.79689377	4.2859E-195	6.7527E-193	positive
PRJNA729271	stage_5W_vs_E10	ordinary_muscle_development	ENSGALG00010003909	DNAJB1	0.167166426	0.15668254	1.066911638	0.286011739	0.323759481	positive
PRJNA729271	stage_5W_vs_E10	ordinary_muscle_development	ENSGALG00010003910	THAP9	1.110985689	0.147763787	7.518660079	5.53405E-14	1.64884E-13	positive
PRJNA729271	stage_5W_vs_E10	ordinary_muscle_development	ENSGALG00010003913	PSMB5	0.550921549	0.116938772	4.711196619	2.46266E-06	4.58763E-06	positive
PRJNA729271	stage_5W_vs_E10	ordinary_muscle_development	ENSGALG00010003914	CAMK2D	0.879653311	0.105849056	8.310450215	9.53397E-17	3.27398E-16	positive
PRJNA729271	stage_5W_vs_E10	ordinary_muscle_development	ENSGALG00010003915	MIOS	-0.127804971	0.09302457	-1.373884025	0.16947769	0.199770604	negative
PRJNA729271	stage_5W_vs_E10	ordinary_muscle_development	ENSGALG00010003918	HTR1E	0.390914202	0.637972055	0.612745023	0.540044947	0.579934176	positive
PRJNA729271	stage_5W_vs_E10	ordinary_muscle_development	ENSGALG00010003920	LPAR6	1.822100446	0.151460086	12.03023508	2.46458E-33	1.70358E-32	positive
PRJNA729271	stage_5W_vs_E10	ordinary_muscle_development	ENSGALG00010003921	COLEC12	0.248890577	0.168291652	1.478924083	0.139160606	0.165828098	positive
PRJNA729271	stage_5W_vs_E10	ordinary_muscle_development	ENSGALG00010003923	ENSGALG00010003923	-0.933355103	0.121522947	-7.68048446	1.58488E-14	4.85171E-14	negative
PRJNA729271	stage_5W_vs_E10	ordinary_muscle_development	ENSGALG00010003925	SEC31A	-1.076484477	0.086202808	-12.48781225	8.70135E-36	6.61732E-35	negative
PRJNA729271	stage_5W_vs_E10	ordinary_muscle_development	ENSGALG00010003928	ENSGALG00010003928	0.648240822	0.155934841	4.157126259	3.22276E-05	5.51261E-05	positive
PRJNA729271	stage_5W_vs_E10	ordinary_muscle_development	ENSGALG00010003929	RCBTB2	0.786028276	0.096901843	8.111592647	4.99605E-16	1.65602E-15	positive
PRJNA729271	stage_5W_vs_E10	ordinary_muscle_development	ENSGALG00010003933	TECR	2.148370127	0.206505708	10.40344185	2.39136E-25	1.20065E-24	positive
PRJNA729271	stage_5W_vs_E10	ordinary_muscle_development	ENSGALG00010003937	ENSGALG00010003937	1.581717694	0.205868323	7.683152362	1.55221E-14	4.7547E-14	positive
PRJNA729271	stage_5W_vs_E10	ordinary_muscle_development	ENSGALG00010003948	SCD5	-2.645563415	0.143712562	-18.40871369	1.11848E-75	2.78084E-74	negative
PRJNA729271	stage_5W_vs_E10	ordinary_muscle_development	ENSGALG00010003958	CYSLTR2	4.342251232	0.360118948	12.05782496	1.7638E-33	1.22385E-32	positive
PRJNA729271	stage_5W_vs_E10	ordinary_muscle_development	ENSGALG00010003960	ASPH	1.289716331	0.195994704	6.58036317	4.69301E-11	1.19489E-10	positive
PRJNA729271	stage_5W_vs_E10	ordinary_muscle_development	ENSGALG00010003962	UMAD1	1.178088314	0.203656927	5.784670972	7.26544E-09	1.61746E-08	positive
PRJNA729271	stage_5W_vs_E10	ordinary_muscle_development	ENSGALG00010003965	SNTG1	-3.7826671	0.560730423	-6.745963734	1.52015E-11	3.98526E-11	negative
PRJNA729271	stage_5W_vs_E10	ordinary_muscle_development	ENSGALG00010003968	DSN1	0.224045739	0.172873863	1.296007012	0.194973069	0.22716298	positive
PRJNA729271	stage_5W_vs_E10	ordinary_muscle_development	ENSGALG00010003972	PENK	-2.403677341	0.241144827	-9.967774857	2.10901E-23	9.7981E-23	negative
PRJNA729271	stage_5W_vs_E10	ordinary_muscle_development	ENSGALG00010003977	MYO6	-1.586822878	0.121447627	-13.06590268	5.15693E-39	4.33583E-38	negative
PRJNA729271	stage_5W_vs_E10	ordinary_muscle_development	ENSGALG00010003978	ANK2	-0.437908278	0.120290189	-3.6404322	0.000272181	0.000432121	negative
PRJNA729271	stage_5W_vs_E10	ordinary_muscle_development	ENSGALG00010003980	CHCHD7	0.129235677	0.120467725	1.072782586	0.283368676	0.320993198	positive
PRJNA729271	stage_5W_vs_E10	ordinary_muscle_development	ENSGALG00010003981	TMEM150C	0.775636641	0.163390583	4.747131868	2.06321E-06	3.86686E-06	positive
PRJNA729271	stage_5W_vs_E10	ordinary_muscle_development	ENSGALG00010003982	GLCCI1	-0.344463634	0.121160703	-2.843031009	0.004468672	0.006339767	negative
PRJNA729271	stage_5W_vs_E10	ordinary_muscle_development	ENSGALG00010003985	PRMT5	-0.066002248	0.134638892	-0.490216806	0.62398049	0.660345238	negative
PRJNA729271	stage_5W_vs_E10	ordinary_muscle_development	ENSGALG00010003986	FNDC3A	0.726908834	0.063405213	11.46449643	1.98913E-30	1.2212E-29	positive
PRJNA729271	stage_5W_vs_E10	ordinary_muscle_development	ENSGALG00010003988	ENOPH1	-1.07326385	0.090502683	-11.85891751	1.9346E-32	1.29584E-31	negative
PRJNA729271	stage_5W_vs_E10	ordinary_muscle_development	ENSGALG00010003990	ICA1	-0.74881405	0.173778468	-4.309015135	1.63983E-05	2.87451E-05	negative
PRJNA729271	stage_5W_vs_E10	ordinary_muscle_development	ENSGALG00010003993	ENSGALG00010003993	0.49696658	0.134506706	3.694734603	0.000220117	0.000352473	positive
PRJNA729271	stage_5W_vs_E10	ordinary_muscle_development	ENSGALG00010003994	SDR16C5	1.663967168	0.084146689	19.77460064	4.92713E-87	1.54921E-85	positive
PRJNA729271	stage_5W_vs_E10	ordinary_muscle_development	ENSGALG00010003995	MLNR	-3.317474502	0.291154811	-11.39419435	4.46945E-30	2.70952E-29	negative
PRJNA729271	stage_5W_vs_E10	ordinary_muscle_development	ENSGALG00010004000	ENSGALG00010004000	1.55466747	0.175280039	8.869620724	7.33955E-19	2.78067E-18	positive
PRJNA729271	stage_5W_vs_E10	ordinary_muscle_development	ENSGALG00010004001	LARP7	-0.558293555	0.113662317	-4.911861481	9.02158E-07	1.73847E-06	negative
PRJNA729271	stage_5W_vs_E10	ordinary_muscle_development	ENSGALG00010004002	CDADC1	1.320463979	0.121791119	10.84203834	2.17569E-27	1.18916E-26	positive
PRJNA729271	stage_5W_vs_E10	ordinary_muscle_development	ENSGALG00010004003	HNRNPDL	-0.432814773	0.068362192	-6.331200945	2.4326E-10	5.90629E-10	negative
PRJNA729271	stage_5W_vs_E10	ordinary_muscle_development	ENSGALG00010004006	NKAIN3	-1.49782415	0.326310656	-4.590178471	4.42867E-06	8.09197E-06	negative
PRJNA729271	stage_5W_vs_E10	ordinary_muscle_development	ENSGALG00010004007	ENSGALG00010004007	-1.544702526	0.165231511	-9.348716334	8.87179E-21	3.66372E-20	negative
PRJNA729271	stage_5W_vs_E10	ordinary_muscle_development	ENSGALG00010004016	ENSGALG00010004016	-2.107341533	0.54978023	-3.833061679	0.000126558	0.000206467	negative
PRJNA729271	stage_5W_vs_E10	ordinary_muscle_development	ENSGALG00010004018	HNRNPD	-0.600496141	0.161926317	-3.70845304	0.000208529	0.00033451	negative
PRJNA729271	stage_5W_vs_E10	ordinary_muscle_development	ENSGALG00010004029	ARL4A	-0.49943193	0.194977685	-2.561482513	0.010422649	0.014251136	negative
PRJNA729271	stage_5W_vs_E10	ordinary_muscle_development	ENSGALG00010004034	NEDD8	0.208767171	0.112887538	1.849337633	0.064409075	0.080138158	positive
PRJNA729271	stage_5W_vs_E10	ordinary_muscle_development	ENSGALG00010004036	ADCYAP1	-3.299535528	0.877664747	-3.759448626	0.000170288	0.000275053	negative
PRJNA729271	stage_5W_vs_E10	ordinary_muscle_development	ENSGALG00010004038	SENP6	0.21996719	0.087698602	2.508217756	0.012134186	0.016451691	positive
PRJNA729271	stage_5W_vs_E10	ordinary_muscle_development	ENSGALG00010004039	ALPK1	4.863942915	0.218720498	22.23816682	1.4683E-109	7.3901E-108	positive
PRJNA729271	stage_5W_vs_E10	ordinary_muscle_development	ENSGALG00010004040	LGALS1B	-2.668336314	0.22627257	-11.79257527	4.26303E-32	2.81515E-31	negative
PRJNA729271	stage_5W_vs_E10	ordinary_muscle_development	ENSGALG00010004042	PLAG1	-1.553194808	0.126349356	-12.29285897	9.89437E-35	7.23242E-34	negative
PRJNA729271	stage_5W_vs_E10	ordinary_muscle_development	ENSGALG00010004043	CAB39L	0.645551016	0.201105803	3.210006904	0.001327318	0.001986933	positive
PRJNA729271	stage_5W_vs_E10	ordinary_muscle_development	ENSGALG00010004044	RNF138	-0.247291819	0.133822855	-1.847904225	0.064616199	0.080368269	negative
PRJNA729271	stage_5W_vs_E10	ordinary_muscle_development	ENSGALG00010004046	ENSGALG00010004046	-0.045465677	0.121917944	-0.372920304	0.70920776	0.740728778	negative
PRJNA729271	stage_5W_vs_E10	ordinary_muscle_development	ENSGALG00010004047	PDE7A	-0.960150826	0.119224356	-8.053311045	8.05838E-16	2.64567E-15	negative
PRJNA729271	stage_5W_vs_E10	ordinary_muscle_development	ENSGALG00010004049	SETDB2	0.159465738	0.138155851	1.154245276	0.248399625	0.28424667	positive
PRJNA729271	stage_5W_vs_E10	ordinary_muscle_development	ENSGALG00010004055	FILIP1	2.173361356	0.175269758	12.40009335	2.61019E-35	1.94723E-34	positive
PRJNA729271	stage_5W_vs_E10	ordinary_muscle_development	ENSGALG00010004056	SHROOM3	-2.443743947	0.186978298	-13.0696662	4.90804E-39	4.13136E-38	negative
PRJNA729271	stage_5W_vs_E10	ordinary_muscle_development	ENSGALG00010004057	ENSGALG00010004057	-0.559707545	0.419322754	-1.334789347	0.181945301	0.213270026	negative
PRJNA729271	stage_5W_vs_E10	ordinary_muscle_development	ENSGALG00010004058	TMEM30A	0.000723426	0.061963246	0.011675087	0.99068484	0.991985131	positive
PRJNA729271	stage_5W_vs_E10	ordinary_muscle_development	ENSGALG00010004060	ETV1	-1.548067971	0.09365331	-16.52977314	2.23985E-61	3.77081E-60	negative
PRJNA729271	stage_5W_vs_E10	ordinary_muscle_development	ENSGALG00010004063	ENSGALG00010004063	2.378454656	0.407358105	5.838731646	5.25997E-09	1.18042E-08	positive
PRJNA729271	stage_5W_vs_E10	ordinary_muscle_development	ENSGALG00010004065	TIFA	1.664808674	0.254707516	6.536158404	6.31191E-11	1.59337E-10	positive
PRJNA729271	stage_5W_vs_E10	ordinary_muscle_development	ENSGALG00010004070	ARL11	-0.141822229	0.404077654	-0.350977659	0.725605109	0.755716222	negative
PRJNA729271	stage_5W_vs_E10	ordinary_muscle_development	ENSGALG00010004072	SGK3	0.094247361	0.135055884	0.697839726	0.485277432	0.526819681	positive
PRJNA729271	stage_5W_vs_E10	ordinary_muscle_development	ENSGALG00010004076	COX7A2	-1.703115674	0.138173554	-12.32591636	6.56924E-35	4.82866E-34	negative
PRJNA729271	stage_5W_vs_E10	ordinary_muscle_development	ENSGALG00010004079	AP1AR	-0.851137615	0.151653575	-5.612380831	1.99562E-08	4.32448E-08	negative
PRJNA729271	stage_5W_vs_E10	ordinary_muscle_development	ENSGALG00010004081	RPS20	0.343164562	0.167960348	2.043128431	0.041039726	0.052323936	positive
PRJNA729271	stage_5W_vs_E10	ordinary_muscle_development	ENSGALG00010004082	FAM241A	0.804028076	0.122733623	6.551000938	5.71527E-11	1.44502E-10	positive
PRJNA729271	stage_5W_vs_E10	ordinary_muscle_development	ENSGALG00010004083	PHF11	0.1778017	0.183009749	0.971542231	0.331278334	0.370916071	positive
PRJNA729271	stage_5W_vs_E10	ordinary_muscle_development	ENSGALG00010004085	RB1CC1	1.181638802	0.140693844	8.398653202	4.51628E-17	1.5763E-16	positive
PRJNA729271	stage_5W_vs_E10	ordinary_muscle_development	ENSGALG00010004086	UTP3	-0.388025099	0.258160781	-1.503036585	0.13282961	0.158740638	negative
PRJNA729271	stage_5W_vs_E10	ordinary_muscle_development	ENSGALG00010004087	NCOA7	-0.040723909	0.109219224	-0.372863927	0.709249721	0.740728778	negative
PRJNA729271	stage_5W_vs_E10	ordinary_muscle_development	ENSGALG00010004094	DGKB	-2.196855846	0.206503479	-10.63834787	1.97602E-26	1.04306E-25	negative
PRJNA729271	stage_5W_vs_E10	ordinary_muscle_development	ENSGALG00010004095	ANKRD50	-0.862968996	0.113745754	-7.586823853	3.27842E-14	9.88368E-14	negative
PRJNA729271	stage_5W_vs_E10	ordinary_muscle_development	ENSGALG00010004096	COL12A1	-4.114345903	0.414436863	-9.927557779	3.15898E-23	1.45502E-22	negative
PRJNA729271	stage_5W_vs_E10	ordinary_muscle_development	ENSGALG00010004097	ENSGALG00010004097	-1.030338717	0.120661878	-8.539057504	1.35321E-17	4.85236E-17	negative
PRJNA729271	stage_5W_vs_E10	ordinary_muscle_development	ENSGALG00010004101	ARID1B	0.42693283	0.166398758	2.565721257	0.010296153	0.014079504	positive
PRJNA729271	stage_5W_vs_E10	ordinary_muscle_development	ENSGALG00010004102	PCMTD1	2.712035804	0.187630572	14.45412534	2.3611E-47	2.59078E-46	positive
PRJNA729271	stage_5W_vs_E10	ordinary_muscle_development	ENSGALG00010004104	SCARB2	1.357849299	0.094066194	14.43504029	3.11464E-47	3.40987E-46	positive
PRJNA729271	stage_5W_vs_E10	ordinary_muscle_development	ENSGALG00010004108	PERPB	0.989642781	0.241504497	4.097823405	4.17053E-05	7.07453E-05	positive
PRJNA729271	stage_5W_vs_E10	ordinary_muscle_development	ENSGALG00010004109	AGMO	-0.714692259	0.517823269	-1.380185677	0.167529483	0.197618803	negative
PRJNA729271	stage_5W_vs_E10	ordinary_muscle_development	ENSGALG00010004113	ECHDC1	0.000799113	0.195567039	0.004086135	0.996739745	0.997152502	positive
PRJNA729271	stage_5W_vs_E10	ordinary_muscle_development	ENSGALG00010004116	SPRY1	-0.389512817	0.095859484	-4.063372758	4.83687E-05	8.17043E-05	negative
PRJNA729271	stage_5W_vs_E10	ordinary_muscle_development	ENSGALG00010004117	STBD1	6.331809846	0.189190002	33.46799403	1.4088E-245	3.9271E-243	positive
PRJNA729271	stage_5W_vs_E10	ordinary_muscle_development	ENSGALG00010004122	TRIM55	1.320933551	0.194911355	6.777099005	1.22613E-11	3.23082E-11	positive
PRJNA729271	stage_5W_vs_E10	ordinary_muscle_development	ENSGALG00010004123	NDUFAF4	-0.322926088	0.11224015	-2.877099569	0.004013489	0.005727629	negative
PRJNA729271	stage_5W_vs_E10	ordinary_muscle_development	ENSGALG00010004125	MANEA	0.158454589	0.09326337	1.699001323	0.089318934	0.109126598	positive
PRJNA729271	stage_5W_vs_E10	ordinary_muscle_development	ENSGALG00010004127	EPHA7	-4.411277367	0.202248311	-21.81119513	1.8167E-105	8.2808E-104	negative
PRJNA729271	stage_5W_vs_E10	ordinary_muscle_development	ENSGALG00010004131	NUDT6	0.872130377	0.159668446	5.462133569	4.70446E-08	9.92882E-08	positive
PRJNA729271	stage_5W_vs_E10	ordinary_muscle_development	ENSGALG00010004133	CRH	2.365613248	0.995920611	2.375303033	0.017534548	0.023343431	positive
PRJNA729271	stage_5W_vs_E10	ordinary_muscle_development	ENSGALG00010004135	NUP54	-0.877227166	0.067026984	-13.0876718	3.87298E-39	3.2715E-38	negative
PRJNA729271	stage_5W_vs_E10	ordinary_muscle_development	ENSGALG00010004137	CROT	-0.871826682	0.084911681	-10.26745289	9.87782E-25	4.85188E-24	negative
PRJNA729271	stage_5W_vs_E10	ordinary_muscle_development	ENSGALG00010004139	PPEF2	2.37603173	0.592138283	4.012629819	6.0046E-05	0.0001009	positive
PRJNA729271	stage_5W_vs_E10	ordinary_muscle_development	ENSGALG00010004141	KPNA3	0.867628867	0.140852308	6.159848402	7.28146E-10	1.712E-09	positive
PRJNA729271	stage_5W_vs_E10	ordinary_muscle_development	ENSGALG00010004142	RBBP8	-1.460811326	0.116292782	-12.56149612	3.4377E-36	2.66041E-35	negative
PRJNA729271	stage_5W_vs_E10	ordinary_muscle_development	ENSGALG00010004143	TCF24	1.091553821	0.409845057	2.663332895	0.007737083	0.010712486	positive
PRJNA729271	stage_5W_vs_E10	ordinary_muscle_development	ENSGALG00010004146	NAAA	3.156235702	0.170120794	18.55290954	7.72548E-77	1.96803E-75	positive
PRJNA729271	stage_5W_vs_E10	ordinary_muscle_development	ENSGALG00010004147	SPRYD7	0.721613617	0.091346984	7.899698337	2.79579E-15	8.91051E-15	positive
PRJNA729271	stage_5W_vs_E10	ordinary_muscle_development	ENSGALG00010004148	ENSGALG00010004148	0.258361216	0.155140744	1.665334385	0.095846062	0.116737137	positive
PRJNA729271	stage_5W_vs_E10	ordinary_muscle_development	ENSGALG00010004149	SDAD1	-1.049730041	0.12364418	-8.489926821	2.06767E-17	7.33503E-17	negative
PRJNA729271	stage_5W_vs_E10	ordinary_muscle_development	ENSGALG00010004154	COPS5	1.554908056	0.128945433	12.05865168	1.74618E-33	1.21221E-32	positive
PRJNA729271	stage_5W_vs_E10	ordinary_muscle_development	ENSGALG00010004158	JCHAIN	7.726233516	0.468874084	16.4782695	5.25681E-61	8.68842E-60	positive
PRJNA729271	stage_5W_vs_E10	ordinary_muscle_development	ENSGALG00010004172	ST8SIA6	-1.147793081	0.129975873	-8.830816472	1.03914E-18	3.91638E-18	negative
PRJNA729271	stage_5W_vs_E10	ordinary_muscle_development	ENSGALG00010004177	CSPP1	0.101601487	0.128297764	0.791919391	0.428407679	0.470187727	positive
PRJNA729271	stage_5W_vs_E10	ordinary_muscle_development	ENSGALG00010004181	BBS12	-1.141300187	0.13372435	-8.53472225	1.40493E-17	5.03322E-17	negative
PRJNA729271	stage_5W_vs_E10	ordinary_muscle_development	ENSGALG00010004187	ARFGEF1	0.917634436	0.094087219	9.753019036	1.79062E-22	7.99848E-22	positive
PRJNA729271	stage_5W_vs_E10	ordinary_muscle_development	ENSGALG00010004188	BMI1	-0.37077163	0.116619762	-3.179320766	0.001476206	0.002199138	negative
PRJNA729271	stage_5W_vs_E10	ordinary_muscle_development	ENSGALG00010004191	CABLES1	0.526647481	0.126392764	4.166753423	3.08968E-05	5.29436E-05	positive
PRJNA729271	stage_5W_vs_E10	ordinary_muscle_development	ENSGALG00010004200	ABCB1	3.789167495	0.181005646	20.93397408	2.62659E-97	1.04883E-95	positive
PRJNA729271	stage_5W_vs_E10	ordinary_muscle_development	ENSGALG00010004201	CPA6	-9.901717676	0.947411066	-10.45134264	1.44465E-25	7.2988E-25	negative
PRJNA729271	stage_5W_vs_E10	ordinary_muscle_development	ENSGALG00010004210	SYNCRIP	-0.830637743	0.070769631	-11.73720607	8.21559E-32	5.34253E-31	negative
PRJNA729271	stage_5W_vs_E10	ordinary_muscle_development	ENSGALG00010004211	RNF146	0.200709045	0.075652559	2.653037098	0.00797711	0.011034279	positive
PRJNA729271	stage_5W_vs_E10	ordinary_muscle_development	ENSGALG00010004212	RSPO3	-2.15137554	0.387181632	-5.556502073	2.75234E-08	5.90079E-08	negative
PRJNA729271	stage_5W_vs_E10	ordinary_muscle_development	ENSGALG00010004214	REPS1	-0.045186154	0.126205003	-0.35803774	0.720315071	0.750770616	negative
PRJNA729271	stage_5W_vs_E10	ordinary_muscle_development	ENSGALG00010004216	SNX14	0.319893112	0.073812271	4.333874396	1.46508E-05	2.58067E-05	positive
PRJNA729271	stage_5W_vs_E10	ordinary_muscle_development	ENSGALG00010004217	CCDC28A	-0.069112392	0.11300533	-0.611585237	0.540812208	0.580672071	negative
PRJNA729271	stage_5W_vs_E10	ordinary_muscle_development	ENSGALG00010004219	FGF2	-0.219760376	0.182574738	-1.203673511	0.228715794	0.263155694	negative
PRJNA729271	stage_5W_vs_E10	ordinary_muscle_development	ENSGALG00010004222	SPAG6	-2.242636593	0.255831867	-8.766056467	1.85033E-18	6.90184E-18	negative
PRJNA729271	stage_5W_vs_E10	ordinary_muscle_development	ENSGALG00010004224	NT5E	0.816603412	0.258239178	3.162198002	0.001565831	0.002327152	positive
PRJNA729271	stage_5W_vs_E10	ordinary_muscle_development	ENSGALG00010004225	CETN4	-1.515694039	0.128025211	-11.83902781	2.4528E-32	1.63539E-31	negative
PRJNA729271	stage_5W_vs_E10	ordinary_muscle_development	ENSGALG00010004226	RUNDC3B	0.658683478	0.193740202	3.399828587	0.000674281	0.001036778	positive
PRJNA729271	stage_5W_vs_E10	ordinary_muscle_development	ENSGALG00010004230	TAF1B	-0.049751576	0.117380255	-0.423849619	0.671675489	0.705604886	negative
PRJNA729271	stage_5W_vs_E10	ordinary_muscle_development	ENSGALG00010004231	ARMC3	-1.065246603	0.430015625	-2.477227664	0.013240741	0.017878393	negative
PRJNA729271	stage_5W_vs_E10	ordinary_muscle_development	ENSGALG00010004233	NHSL1	-2.976136968	0.126216115	-23.57969073	6.2282E-123	3.8746E-121	negative
PRJNA729271	stage_5W_vs_E10	ordinary_muscle_development	ENSGALG00010004235	ADAD1	-1.399336608	0.437781426	-3.196427541	0.001391408	0.002078793	negative
PRJNA729271	stage_5W_vs_E10	ordinary_muscle_development	ENSGALG00010004236	DBF4	-1.474735235	0.126930476	-11.61844875	3.32112E-31	2.11139E-30	negative
PRJNA729271	stage_5W_vs_E10	ordinary_muscle_development	ENSGALG00010004237	RUFY3	0.775836715	0.090970932	8.528402354	1.48383E-17	5.30932E-17	positive
PRJNA729271	stage_5W_vs_E10	ordinary_muscle_development	ENSGALG00010004238	CCNC	-0.954785052	0.125369964	-7.61574	2.62185E-14	7.94724E-14	negative
PRJNA729271	stage_5W_vs_E10	ordinary_muscle_development	ENSGALG00010004240	ENSGALG00010004240	1.174358273	0.174397953	6.733784742	1.65306E-11	4.32666E-11	positive
PRJNA729271	stage_5W_vs_E10	ordinary_muscle_development	ENSGALG00010004241	PREX2	0.694310317	0.114431069	6.067498303	1.29918E-09	3.01499E-09	positive

PRJNA729271	stage_5W_vs_E10	ordinary_muscle_development	ENSGALG00010004242	ADAM22	-2.249797379	0.179016982	-12.56750817	3.18608E-36	2.46964E-35	negative
PRJNA729271	stage_5W_vs_E10	ordinary_muscle_development	ENSGALG00010004243	BLTP1	0.578481698	0.112743528	5.130952619	2.88279E-07	5.74458E-07	positive
PRJNA729271	stage_5W_vs_E10	ordinary_muscle_development	ENSGALG00010004244	TMEM241	-0.101875043	0.082110988	-1.240699271	0.214716864	0.248394973	negative
PRJNA729271	stage_5W_vs_E10	ordinary_muscle_development	ENSGALG00010004245	ARFGEF3	-1.156268132	0.228089324	-5.06936541	3.99144E-07	7.88121E-07	negative
PRJNA729271	stage_5W_vs_E10	ordinary_muscle_development	ENSGALG00010004246	C8orf34	-4.328885178	0.395861044	-10.93536543	7.80907E-28	4.35523E-27	negative
PRJNA729271	stage_5W_vs_E10	ordinary_muscle_development	ENSGALG00010004247	MEOX2	-0.564951786	0.10438044	-5.412429627	6.21753E-08	1.30048E-07	negative
PRJNA729271	stage_5W_vs_E10	ordinary_muscle_development	ENSGALG00010004248	GRSF1	-0.187202884	0.159721329	-1.172059397	0.24117321	0.276523151	negative
PRJNA729271	stage_5W_vs_E10	ordinary_muscle_development	ENSGALG00010004249	USP45	1.485881579	0.101124084	14.69364686	7.07986E-49	8.25604E-48	positive
PRJNA729271	stage_5W_vs_E10	ordinary_muscle_development	ENSGALG00010004250	MOB1B	-0.414023412	0.08826807	-4.690522989	2.72508E-06	5.06605E-06	negative
PRJNA729271	stage_5W_vs_E10	ordinary_muscle_development	ENSGALG00010004251	CCN2	-0.155344774	0.266158016	-0.583656192	0.559451646	0.599220543	negative
PRJNA729271	stage_5W_vs_E10	ordinary_muscle_development	ENSGALG00010004255	RPS12	-0.069565776	0.151105292	-0.460379483	0.645243859	0.680253817	negative
PRJNA729271	stage_5W_vs_E10	ordinary_muscle_development	ENSGALG00010004256	ENSGALG00010004256	-4.967608519	0.579900027	-8.566318834	1.06846E-17	3.85065E-17	negative
PRJNA729271	stage_5W_vs_E10	ordinary_muscle_development	ENSGALG00010004257	ENSGALG00010004257	-2.63106042	0.65846362	-3.9957567	6.44879E-05	0.000108151	negative
PRJNA729271	stage_5W_vs_E10	ordinary_muscle_development	ENSGALG00010004265	PIP4K2A	0.329215984	0.092951382	3.541808365	0.000397394	0.000622055	positive
PRJNA729271	stage_5W_vs_E10	ordinary_muscle_development	ENSGALG00010004275	ADARB2	-3.592384751	0.883653219	-4.065378446	4.79546E-05	8.10425E-05	negative
PRJNA729271	stage_5W_vs_E10	ordinary_muscle_development	ENSGALG00010004281	VIM	-0.550078219	0.171627268	-3.205074728	0.001350274	0.002020046	negative
PRJNA729271	stage_5W_vs_E10	ordinary_muscle_development	ENSGALG00010004283	DCK	-1.874429248	0.131930386	-14.20771442	8.20603E-46	8.59439E-45	negative
PRJNA729271	stage_5W_vs_E10	ordinary_muscle_development	ENSGALG00010004285	SLC18B1	-0.055425712	0.119248914	-0.46479008	0.642081792	0.677202056	negative
PRJNA729271	stage_5W_vs_E10	ordinary_muscle_development	ENSGALG00010004296	ENSGALG00010004296	-8.102684011	1.006555331	-8.04991416	8.28521E-16	2.71768E-15	negative
PRJNA729271	stage_5W_vs_E10	ordinary_muscle_development	ENSGALG00010004297	SULF1	-3.420268034	0.186795933	-18.31018466	6.86366E-75	1.65815E-73	negative
PRJNA729271	stage_5W_vs_E10	ordinary_muscle_development	ENSGALG00010004300	VNN2	2.888293202	0.245648114	11.75784807	6.43536E-32	4.20751E-31	positive
PRJNA729271	stage_5W_vs_E10	ordinary_muscle_development	ENSGALG00010004302	CRPPA	-0.048935422	0.128783929	-0.379980815	0.703959656	0.736105556	negative
PRJNA729271	stage_5W_vs_E10	ordinary_muscle_development	ENSGALG00010004306	PNISR	0.157850219	0.144232448	1.094415446	0.273772813	0.31092509	positive
PRJNA729271	stage_5W_vs_E10	ordinary_muscle_development	ENSGALG00010004307	NPC2	1.42895827	0.140542834	10.16742178	2.77147E-24	1.33464E-23	positive
PRJNA729271	stage_5W_vs_E10	ordinary_muscle_development	ENSGALG00010004308	DSG2	-2.585411539	0.474568932	-5.447915706	5.09635E-08	1.07247E-07	negative
PRJNA729271	stage_5W_vs_E10	ordinary_muscle_development	ENSGALG00010004309	MRPS28	-0.42762253	0.137396196	-3.112331658	0.001856158	0.002737867	negative
PRJNA729271	stage_5W_vs_E10	ordinary_muscle_development	ENSGALG00010004312	PDLIM5	6.400664129	0.181456617	35.27379841	1.4814E-272	4.8802E-270	positive
PRJNA729271	stage_5W_vs_E10	ordinary_muscle_development	ENSGALG00010004319	TAAR1	7.044086779	0.814614845	8.647137751	5.28072E-18	1.92903E-17	positive
PRJNA729271	stage_5W_vs_E10	ordinary_muscle_development	ENSGALG00010004323	TRDMT1	-0.345443585	0.160491123	-2.152415529	0.031364642	0.040602883	negative
PRJNA729271	stage_5W_vs_E10	ordinary_muscle_development	ENSGALG00010004325	LTBP2	0.826443533	0.113144915	7.304292298	2.7873E-13	7.97511E-13	positive
PRJNA729271	stage_5W_vs_E10	ordinary_muscle_development	ENSGALG00010004326	ENSGALG00010004326	-0.234450092	0.951094856	-0.246505478	0.805290965	0.828496879	negative
PRJNA729271	stage_5W_vs_E10	ordinary_muscle_development	ENSGALG00010004328	ENSGALG00010004328	-0.76335814	0.697085559	-1.095070942	0.273485558	0.310647533	negative
PRJNA729271	stage_5W_vs_E10	ordinary_muscle_development	ENSGALG00010004329	COQ3	1.137630336	0.120332364	9.454067874	3.25913E-21	1.37449E-20	positive
PRJNA729271	stage_5W_vs_E10	ordinary_muscle_development	ENSGALG00010004330	STX7	0.236787982	0.10331874	2.29182027	0.021916017	0.028819075	positive
PRJNA729271	stage_5W_vs_E10	ordinary_muscle_development	ENSGALG00010004332	ENSGALG00010004332	-1.147041929	0.902304064	-1.271236576	0.203644498	0.236543553	negative
PRJNA729271	stage_5W_vs_E10	ordinary_muscle_development	ENSGALG00010004333	DIO2	-0.606854412	0.317644721	-1.910481654	0.056071226	0.070252608	negative
PRJNA729271	stage_5W_vs_E10	ordinary_muscle_development	ENSGALG00010004334	ENSGALG00010004334	-0.588078287	0.237440346	-2.476741201	0.0132588	0.017899442	negative
PRJNA729271	stage_5W_vs_E10	ordinary_muscle_development	ENSGALG00010004336	ENSGALG00010004336	-4.648086273	0.663376577	-7.00670846	2.4399E-12	6.65907E-12	negative
PRJNA729271	stage_5W_vs_E10	ordinary_muscle_development	ENSGALG00010004337	BMPR1B	-2.22996425	0.245009577	-9.101539113	8.90606E-20	3.52811E-19	negative
PRJNA729271	stage_5W_vs_E10	ordinary_muscle_development	ENSGALG00010004338	ENSGALG00010004338	1.873745769	0.164106419	11.41787006	3.40476E-30	2.07361E-29	positive
PRJNA729271	stage_5W_vs_E10	ordinary_muscle_development	ENSGALG00010004339	B4GALT6	-3.471906567	0.135880817	-25.55111627	5.336E-144	4.7451E-142	negative
PRJNA729271	stage_5W_vs_E10	ordinary_muscle_development	ENSGALG00010004340	SLC4A4	1.105765893	0.169924845	6.507382084	7.64717E-11	1.92174E-10	positive
PRJNA729271	stage_5W_vs_E10	ordinary_muscle_development	ENSGALG00010004341	ENSGALG00010004341	-1.621974337	0.171919273	-9.434511363	3.92821E-21	1.65329E-20	negative
PRJNA729271	stage_5W_vs_E10	ordinary_muscle_development	ENSGALG00010004342	ZNF438	0.044918649	0.131203233	0.342359313	0.732080504	0.761664291	positive
PRJNA729271	stage_5W_vs_E10	ordinary_muscle_development	ENSGALG00010004344	SOSTDC1	-5.331360444	0.392669027	-13.577237	5.46494E-42	5.06161E-41	negative
PRJNA729271	stage_5W_vs_E10	ordinary_muscle_development	ENSGALG00010004345	FAXC	-2.524735335	0.194996728	-12.94757798	2.42484E-38	1.99705E-37	negative
PRJNA729271	stage_5W_vs_E10	ordinary_muscle_development	ENSGALG00010004346	CEP128	-0.392124863	0.106923756	-3.667331544	0.000245095	0.000391003	negative
PRJNA729271	stage_5W_vs_E10	ordinary_muscle_development	ENSGALG00010004350	MOXD1	2.164637426	0.238877849	9.061691702	1.28443E-19	5.05094E-19	positive
PRJNA729271	stage_5W_vs_E10	ordinary_muscle_development	ENSGALG00010004352	CHMP2A	0.713636662	0.056131169	12.71373229	4.9609E-37	3.95317E-36	positive
PRJNA729271	stage_5W_vs_E10	ordinary_muscle_development	ENSGALG00010004355	ANKMY2	0.142774727	0.123456096	1.156481797	0.247484141	0.283288527	positive
PRJNA729271	stage_5W_vs_E10	ordinary_muscle_development	ENSGALG00010004356	TRAPPC8	0.378141239	0.07741467	4.88461992	1.03629E-06	1.98795E-06	positive
PRJNA729271	stage_5W_vs_E10	ordinary_muscle_development	ENSGALG00010004358	GC	7.84373764	2.105183376	3.725916579	0.000194607	0.000312938	positive
PRJNA729271	stage_5W_vs_E10	ordinary_muscle_development	ENSGALG00010004362	FBXL4	1.995657482	0.120760309	16.52577326	2.3935E-61	4.02481E-60	positive
PRJNA729271	stage_5W_vs_E10	ordinary_muscle_development	ENSGALG00010004364	TSHR	0.915233859	0.455707193	2.008381417	0.044602776	0.056612718	positive
PRJNA729271	stage_5W_vs_E10	ordinary_muscle_development	ENSGALG00010004365	TPD52	-1.130619296	0.155956961	-7.249559682	4.18129E-13	1.18722E-12	negative
PRJNA729271	stage_5W_vs_E10	ordinary_muscle_development	ENSGALG00010004366	UNC5C	-5.56116569	0.398014288	-13.97227651	2.30151E-44	2.29438E-43	negative
PRJNA729271	stage_5W_vs_E10	ordinary_muscle_development	ENSGALG00010004367	TSPAN13	0.509385959	0.178234772	2.857949402	0.004263884	0.006067046	positive
PRJNA729271	stage_5W_vs_E10	ordinary_muscle_development	ENSGALG00010004368	GJD4	-7.190660122	0.440087386	-16.33916434	5.19602E-60	8.30389E-59	negative
PRJNA729271	stage_5W_vs_E10	ordinary_muscle_development	ENSGALG00010004369	UBE2M	0.89461042	0.233892768	3.824874231	0.000130839	0.000213187	positive
PRJNA729271	stage_5W_vs_E10	ordinary_muscle_development	ENSGALG00010004370	ATP6V1H	0.476408639	0.074401491	6.403213635	1.5214E-10	3.74155E-10	positive
PRJNA729271	stage_5W_vs_E10	ordinary_muscle_development	ENSGALG00010004372	SYNJ2	-0.318725119	0.188731139	-1.688778658	0.091261855	0.111425252	negative
PRJNA729271	stage_5W_vs_E10	ordinary_muscle_development	ENSGALG00010004373	MMS22L	-1.277017994	0.126197732	-10.11918337	4.54189E-24	2.16847E-23	negative
PRJNA729271	stage_5W_vs_E10	ordinary_muscle_development	ENSGALG00010004374	ILDR1	-2.018985658	0.603262592	-3.34677748	0.000817568	0.001248225	negative
PRJNA729271	stage_5W_vs_E10	ordinary_muscle_development	ENSGALG00010004375	ENSGALG00010004375	-0.035245392	0.21761523	-0.161961973	0.8713358	0.887499994	negative
PRJNA729271	stage_5W_vs_E10	ordinary_muscle_development	ENSGALG00010004377	AGR2	-3.340166587	0.752567564	-4.438361082	9.06465E-06	1.62072E-05	negative
PRJNA729271	stage_5W_vs_E10	ordinary_muscle_development	ENSGALG00010004378	TRIM28	-0.206433486	0.15592227	-1.323951256	0.185519234	0.21710813	negative
PRJNA729271	stage_5W_vs_E10	ordinary_muscle_development	ENSGALG00010004379	ZBTB10	-0.254704946	0.060448121	-4.213612316	2.51319E-05	4.34087E-05	negative
PRJNA729271	stage_5W_vs_E10	ordinary_muscle_development	ENSGALG00010004380	ISCA2	0.136570678	0.085142748	1.604020084	0.108709626	0.131388812	positive
PRJNA729271	stage_5W_vs_E10	ordinary_muscle_development	ENSGALG00010004381	ENSGALG00010004381	0.082373183	0.449986434	0.183057037	0.854753265	0.872326169	positive
PRJNA729271	stage_5W_vs_E10	ordinary_muscle_development	ENSGALG00010004383	ENSGALG00010004383	0.110300805	0.46082485	0.239355159	0.810830197	0.832954196	positive
PRJNA729271	stage_5W_vs_E10	ordinary_muscle_development	ENSGALG00010004384	ENSGALG00010004384	1.824870832	0.537068111	3.397838741	0.000679204	0.001043905	positive
PRJNA729271	stage_5W_vs_E10	ordinary_muscle_development	ENSGALG00010004385	ADAMTS3	-4.055422104	0.210230381	-19.29037127	6.4707E-83	1.86467E-81	negative
PRJNA729271	stage_5W_vs_E10	ordinary_muscle_development	ENSGALG00010004386	CFAP44	0.407791929	0.360881698	1.129987836	0.25848135	0.294782626	positive
PRJNA729271	stage_5W_vs_E10	ordinary_muscle_development	ENSGALG00010004389	ENSGALG00010004389	-0.290009334	0.137846655	-2.103854701	0.035391124	0.045550909	negative
PRJNA729271	stage_5W_vs_E10	ordinary_muscle_development	ENSGALG00010004390	PNLDC1	-0.188354311	0.530584205	-0.354994192	0.722593942	0.752875157	negative
PRJNA729271	stage_5W_vs_E10	ordinary_muscle_development	ENSGALG00010004393	ZBTB45	1.810303027	0.207726063	8.714857461	2.91125E-18	1.07512E-17	positive
PRJNA729271	stage_5W_vs_E10	ordinary_muscle_development	ENSGALG00010004394	CCNY	-0.655037573	0.087889178	-7.452994683	9.1245E-14	2.67949E-13	negative
PRJNA729271	stage_5W_vs_E10	ordinary_muscle_development	ENSGALG00010004395	BOC	-1.4818897	0.191663202	-7.731738216	1.06088E-14	3.28367E-14	negative
PRJNA729271	stage_5W_vs_E10	ordinary_muscle_development	ENSGALG00010004397	ENSGALG00010004397	0.347675316	0.170747729	2.036192912	0.041730988	0.053167853	positive
PRJNA729271	stage_5W_vs_E10	ordinary_muscle_development	ENSGALG00010004398	COX18	-0.954682529	0.128183242	-7.447795176	9.4913E-14	2.78382E-13	negative
PRJNA729271	stage_5W_vs_E10	ordinary_muscle_development	ENSGALG00010004399	POU3F2	-1.242977776	0.614164594	-2.023851243	0.04298546	0.054665226	negative
PRJNA729271	stage_5W_vs_E10	ordinary_muscle_development	ENSGALG00010004404	RGS20	-1.213935734	0.437231559	-2.776413798	0.005496221	0.007726479	negative
PRJNA729271	stage_5W_vs_E10	ordinary_muscle_development	ENSGALG00010004406	ENSGALG00010004406	-1.253485903	0.15179486	-8.257762522	1.48428E-16	5.04448E-16	negative
PRJNA729271	stage_5W_vs_E10	ordinary_muscle_development	ENSGALG00010004408	SERAC1	0.24265923	0.109343484	2.219238148	0.026470527	0.034548018	positive
PRJNA729271	stage_5W_vs_E10	ordinary_muscle_development	ENSGALG00010004416	STXBP6	0.15038958	0.21275825	0.70685663	0.479655578	0.521263128	positive
PRJNA729271	stage_5W_vs_E10	ordinary_muscle_development	ENSGALG00010004420	ENSGALG00010004420	2.691948086	0.276302698	9.742749898	1.98119E-22	8.83611E-22	positive
PRJNA729271	stage_5W_vs_E10	ordinary_muscle_development	ENSGALG00010004421	RAP1GDS1	-1.136127307	0.075815798	-14.9853637	9.15232E-51	1.13194E-49	negative
PRJNA729271	stage_5W_vs_E10	ordinary_muscle_development	ENSGALG00010004424	MRPL18	0.902495623	0.145071237	6.221051413	4.93834E-10	1.17636E-09	positive
PRJNA729271	stage_5W_vs_E10	ordinary_muscle_development	ENSGALG00010004425	CREM	3.631239172	0.240490533	15.09930193	1.63657E-51	2.06999E-50	positive
PRJNA729271	stage_5W_vs_E10	ordinary_muscle_development	ENSGALG00010004429	ENSGALG00010004429	0.678402078	0.349627241	1.940358181	0.052336175	0.065846095	positive
PRJNA729271	stage_5W_vs_E10	ordinary_muscle_development	ENSGALG00010004430	TCEA1	-1.139990115	0.095615983	-11.92258948	9.02589E-33	6.09363E-32	negative
PRJNA729271	stage_5W_vs_E10	ordinary_muscle_development	ENSGALG00010004433	AHR1A	-1.176124574	0.159591205	-7.369607699	1.71131E-13	4.94921E-13	negative
PRJNA729271	stage_5W_vs_E10	ordinary_muscle_development	ENSGALG00010004434	TCP1	-0.902114184	0.086813902	-10.39135631	2.71465E-25	1.36014E-24	negative
PRJNA729271	stage_5W_vs_E10	ordinary_muscle_development	ENSGALG00010004436	NOVA1	0.321253047	0.151485459	2.120685703	0.03394826	0.043779363	positive
PRJNA729271	stage_5W_vs_E10	ordinary_muscle_development	ENSGALG00010004440	GTF2H5	-0.258459958	0.104607554	-2.470758054	0.0134827	0.018186464	negative
PRJNA729271	stage_5W_vs_E10	ordinary_muscle_development	ENSGALG00010004443	ANKRD17	-0.412901707	0.094020697	-4.391604391	1.12517E-05	1.99821E-05	negative
PRJNA729271	stage_5W_vs_E10	ordinary_muscle_development	ENSGALG00010004445	EPHA6	-3.874462913	0.591362971	-6.551750965	5.68663E-11	1.43828E-10	negative
PRJNA729271	stage_5W_vs_E10	ordinary_muscle_development	ENSGALG00010004446	ACAT2	-1.916517143	0.129239268	-14.829217	9.48327E-50	1.13322E-48	negative
PRJNA729271	stage_5W_vs_E10	ordinary_muscle_development	ENSGALG00010004451	CLDND1	-0.284091505	0.08525174	-3.332383639	0.000861055	0.001312269	negative
PRJNA729271	stage_5W_vs_E10	ordinary_muscle_development	ENSGALG00010004454	SRI	-0.029073276	0.163838362	-0.177450966	0.859154177	0.87638563	negative
PRJNA729271	stage_5W_vs_E10	ordinary_muscle_development	ENSGALG00010004457	AGA	0.149269143	0.126829007	1.176932205	0.239222567	0.274525461	positive
PRJNA729271	stage_5W_vs_E10	ordinary_muscle_development	ENSGALG00010004460	ENSGALG00010004460	-3.059885007	0.896716975	-3.412319709	0.000644125	0.00099241	negative
PRJNA729271	stage_5W_vs_E10	ordinary_muscle_development	ENSGALG00010004468	LYPLA1	0.579636081	0.122387299	4.736080327	2.17891E-06	4.07737E-06	positive
PRJNA729271	stage_5W_vs_E10	ordinary_muscle_development	ENSGALG00010004471	C1H3ORF52	0.764442008	0.256373918	2.981746399	0.002866093	0.004149837	positive
PRJNA729271	stage_5W_vs_E10	ordinary_muscle_development	ENSGALG00010004472	NEIL3	-2.332623714	0.235896813	-9.888322275	4.67802E-23	2.13703E-22	negative
PRJNA729271	stage_5W_vs_E10	ordinary_muscle_development	ENSGALG00010004473	JDP2	-0.140897398	0.13953131	-1.009790549	0.312595649	0.351709535	negative
PRJNA729271	stage_5W_vs_E10	ordinary_muscle_development	ENSGALG00010004477	TULP4	-0.505979619	0.126577918	-3.997376698	6.40483E-05	0.000107451	negative
PRJNA729271	stage_5W_vs_E10	ordinary_muscle_development	ENSGALG00010004481	ZNF704	0.055393304	0.079816629	0.694007065	0.487677778	0.529108488	positive
PRJNA729271	stage_5W_vs_E10	ordinary_muscle_development	ENSGALG00010004483	STEAP4	1.562072684	0.600275139	2.602261166	0.00926113	0.012721766	positive
PRJNA729271	stage_5W_vs_E10	ordinary_muscle_development	ENSGALG00010004487	ABHD10	-0.322389702	0.100830687	-3.197337153	0.001387027	0.002072676	negative
PRJNA729271	stage_5W_vs_E10	ordinary_muscle_development	ENSGALG00010004489	LIG1	-2.22769561	0.26384294	-8.443264053	3.08597E-17	1.08518E-16	negative
PRJNA729271	stage_5W_vs_E10	ordinary_muscle_development	ENSGALG00010004494	WTAP	-0.440827064	0.057893197	-7.614488191	2.64738E-14	8.02296E-14	negative
PRJNA729271	stage_5W_vs_E10	ordinary_muscle_development	ENSGALG00010004495	MRPL15	0.192031214	0.114088903	1.68317171	0.092341856	0.112639502	positive
PRJNA729271	stage_5W_vs_E10	ordinary_muscle_development	ENSGALG00010004501	TAGLN3	-2.828545832	0.35890267	-7.881094436	3.24526E-15	1.03135E-14	negative
PRJNA729271	stage_5W_vs_E10	ordinary_muscle_development	ENSGALG00010004503	ENSGALG00010004503	-0.059228605	0.185178578	-0.319845879	0.749085166	0.777403128	negative
PRJNA729271	stage_5W_vs_E10	ordinary_muscle_development	ENSGALG00010004504	ZNF804B	-3.012910091	0.770693577	-3.909348905	9.25452E-05	0.000152923	negative
PRJNA729271	stage_5W_vs_E10	ordinary_muscle_development	ENSGALG00010004505	ENSGALG00010004505	1.181141453	0.131916838	8.953682299	3.43817E-19	1.32403E-18	positive
PRJNA729271	stage_5W_vs_E10	ordinary_muscle_development	ENSGALG00010004507	CUL2	0.74965932	0.089939431	8.335157464	7.73951E-17	2.67169E-16	positive
PRJNA729271	stage_5W_vs_E10	ordinary_muscle_development	ENSGALG00010004515	ENSGALG00010004515	-0.119445508	0.253909753	-0.47042505	0.638051371	0.673454789	negative
PRJNA729271	stage_5W_vs_E10	ordinary_muscle_development	ENSGALG00010004516	ENSGALG00010004516	-0.03688031	0.290749035	-0.126845857	0.8990624	0.911832458	negative
PRJNA729271	stage_5W_vs_E10	ordinary_muscle_development	ENSGALG00010004522	TSPAN5	-0.974251779	0.064094165	-15.20031943	3.51901E-52	4.55429E-51	negative
PRJNA729271	stage_5W_vs_E10	ordinary_muscle_development	ENSGALG00010004533	PAG1	0.036195038	0.113910084	0.317750955	0.750673856	0.778773085	positive
PRJNA729271	stage_5W_vs_E10	ordinary_muscle_development	ENSGALG00010004534	CUBN	2.039219185	0.212981582	9.574626889	1.02226E-21	4.41132E-21	positive
PRJNA729271	stage_5W_vs_E10	ordinary_muscle_development	ENSGALG00010004542	ERMARD	-0.445751533	0.132651136	-3.360329545	0.000778496	0.001190577	negative
PRJNA729271	stage_5W_vs_E10	ordinary_muscle_development	ENSGALG00010004545	TMPRSS7	-0.345848233	0.625487865	-0.552925568	0.580314379	0.618755612	negative
PRJNA729271	stage_5W_vs_E10	ordinary_muscle_development	ENSGALG00010004553	BATF	0.896858902	0.339078994	2.644985147	0.00816945	0.011285255	positive
PRJNA729271	stage_5W_vs_E10	ordinary_muscle_development	ENSGALG00010004565	ENSGALG00010004565	3.832903126	0.377856636	10.14380261	3.53082E-24	1.6958E-23	positive
PRJNA729271	stage_5W_vs_E10	ordinary_muscle_development	ENSGALG00010004568	RSU1	0.259567179	0.067580151	3.840878934	0.000122595	0.000200182	positive
PRJNA729271	stage_5W_vs_E10	ordinary_muscle_development	ENSGALG00010004570	ENSGALG00010004570	-0.277471846	0.605512556	-0.458242927	0.646777923	0.681672798	negative
PRJNA729271	stage_5W_vs_E10	ordinary_muscle_development	ENSGALG00010004576	STEAP2	0.629244125	0.210925559	2.983252132	0.002852029	0.004132364	positive
PRJNA729271	stage_5W_vs_E10	ordinary_muscle_development	ENSGALG00010004577	ZPLD1	-3.941840969	0.438775839	-8.983723842	2.61754E-19	1.01285E-18	negative

PRJNA729271	stage_5W_vs_E10	ordinary_muscle_development	ENSGALG00010004583	XKR4	0.600601447	0.110945535	5.413480106	6.18115E-08	1.29361E-07	positive
PRJNA729271	stage_5W_vs_E10	ordinary_muscle_development	ENSGALG00010004584	EIF4E	-0.104563189	0.066686613	-1.567978693	0.116886105	0.140719609	negative
PRJNA729271	stage_5W_vs_E10	ordinary_muscle_development	ENSGALG00010004586	ENSGALG00010004586	-4.744535312	0.428205923	-11.08003196	1.56817E-28	8.95962E-28	negative
PRJNA729271	stage_5W_vs_E10	ordinary_muscle_development	ENSGALG00010004588	MINDY3	-0.807387963	0.154302903	-5.232487187	1.67244E-07	3.39715E-07	negative
PRJNA729271	stage_5W_vs_E10	ordinary_muscle_development	ENSGALG00010004591	RRS1	-0.107465695	0.162414387	-0.661675957	0.508178916	0.549013801	negative
PRJNA729271	stage_5W_vs_E10	ordinary_muscle_development	ENSGALG00010004602	DYNLT2	-0.733860122	0.348853093	-2.103636566	0.035410162	0.045571366	negative
PRJNA729271	stage_5W_vs_E10	ordinary_muscle_development	ENSGALG00010004609	CFAP69	-3.238069493	0.697584388	-4.64183194	3.45334E-06	6.37089E-06	negative
PRJNA729271	stage_5W_vs_E10	ordinary_muscle_development	ENSGALG00010004610	TGS1	-0.628051438	0.12401518	-5.064310978	4.0988E-07	8.08328E-07	negative
PRJNA729271	stage_5W_vs_E10	ordinary_muscle_development	ENSGALG00010004622	ENSGALG00010004622	2.351001929	0.222864931	10.54899899	5.13411E-26	2.65592E-25	positive
PRJNA729271	stage_5W_vs_E10	ordinary_muscle_development	ENSGALG00010004623	MBIP	-0.939496672	0.129791514	-7.238506145	4.53654E-13	1.28734E-12	negative
PRJNA729271	stage_5W_vs_E10	ordinary_muscle_development	ENSGALG00010004624	PHF10	0.104726384	0.076271863	1.373067084	0.169731493	0.200020975	positive
PRJNA729271	stage_5W_vs_E10	ordinary_muscle_development	ENSGALG00010004630	ENSGALG00010004630	2.26933342	0.306251982	7.410020343	1.2628E-13	3.68073E-13	positive
PRJNA729271	stage_5W_vs_E10	ordinary_muscle_development	ENSGALG00010004639	HBS1L	1.409220217	0.079031752	17.83106382	4.05629E-71	8.86817E-70	positive
PRJNA729271	stage_5W_vs_E10	ordinary_muscle_development	ENSGALG00010004641	METAP1	-0.611841191	0.081663509	-7.492222667	6.77169E-14	2.00318E-13	negative
PRJNA729271	stage_5W_vs_E10	ordinary_muscle_development	ENSGALG00010004650	GTPBP10	-0.446043442	0.128669816	-3.466574033	0.000527137	0.000817377	negative
PRJNA729271	stage_5W_vs_E10	ordinary_muscle_development	ENSGALG00010004666	C6orf120	-0.029565396	0.11764065	-0.251319559	0.801567054	0.825134184	negative
PRJNA729271	stage_5W_vs_E10	ordinary_muscle_development	ENSGALG00010004669	ELMOD1	-2.799463714	0.371179609	-7.542073017	4.62559E-14	1.38272E-13	negative
PRJNA729271	stage_5W_vs_E10	ordinary_muscle_development	ENSGALG00010004673	RAB18	0.218254617	0.085009676	2.567409122	0.010246164	0.01401379	positive
PRJNA729271	stage_5W_vs_E10	ordinary_muscle_development	ENSGALG00010004689	BBX	0.449910518	0.083796975	5.369054436	7.91505E-08	1.64368E-07	positive
PRJNA729271	stage_5W_vs_E10	ordinary_muscle_development	ENSGALG00010004705	ENSGALG00010004705	5.191577512	0.226189468	22.95233969	1.3963E-116	8.0957E-115	positive
PRJNA729271	stage_5W_vs_E10	ordinary_muscle_development	ENSGALG00010004707	OTUD1	3.929801319	0.296367096	13.25991099	3.95365E-40	3.46482E-39	positive
PRJNA729271	stage_5W_vs_E10	ordinary_muscle_development	ENSGALG00010004715	WDR27	0.134640356	0.322196962	0.417882141	0.676033291	0.709668493	positive
PRJNA729271	stage_5W_vs_E10	ordinary_muscle_development	ENSGALG00010004716	ADH6	1.335746994	0.962846107	1.387290227	0.165353282	0.195258316	positive
PRJNA729271	stage_5W_vs_E10	ordinary_muscle_development	ENSGALG00010004717	ALKBH8	-0.085245194	0.176437992	-0.483145342	0.628992542	0.664813103	negative
PRJNA729271	stage_5W_vs_E10	ordinary_muscle_development	ENSGALG00010004721	LAMA2	2.934098174	0.158847097	18.47121053	3.52099E-76	8.86056E-75	positive
PRJNA729271	stage_5W_vs_E10	ordinary_muscle_development	ENSGALG00010004728	ENSGALG00010004728	-0.928180855	0.218132893	-4.255116425	2.0894E-05	3.6301E-05	negative
PRJNA729271	stage_5W_vs_E10	ordinary_muscle_development	ENSGALG00010004729	ENSGALG00010004729	9.801756456	1.082178469	9.05743067	1.33559E-19	5.24646E-19	positive
PRJNA729271	stage_5W_vs_E10	ordinary_muscle_development	ENSGALG00010004730	CLDN12	-0.244690529	0.063967905	-3.825207824	0.000130662	0.000212922	negative
PRJNA729271	stage_5W_vs_E10	ordinary_muscle_development	ENSGALG00010004735	ENSGALG00010004735	2.840116818	0.164708202	17.24332352	1.25604E-66	2.44052E-65	positive
PRJNA729271	stage_5W_vs_E10	ordinary_muscle_development	ENSGALG00010004736	CD47	0.293850909	0.101181995	2.904181807	0.003682143	0.005271894	positive
PRJNA729271	stage_5W_vs_E10	ordinary_muscle_development	ENSGALG00010004739	ENSGALG00010004739	0.207742188	0.699956689	0.296792918	0.766624612	0.794184088	positive
PRJNA729271	stage_5W_vs_E10	ordinary_muscle_development	ENSGALG00010004746	LYN	0.618834858	0.14143267	4.375473191	1.21169E-05	2.1466E-05	positive
PRJNA729271	stage_5W_vs_E10	ordinary_muscle_development	ENSGALG00010004751	PTER	0.58030734	0.125773969	4.613890666	3.952E-06	7.24934E-06	positive
PRJNA729271	stage_5W_vs_E10	ordinary_muscle_development	ENSGALG00010004755	PAX9	-3.65493331	0.741859564	-4.926718597	8.36221E-07	1.61635E-06	negative
PRJNA729271	stage_5W_vs_E10	ordinary_muscle_development	ENSGALG00010004757	TRMT10A	0.444417499	0.07548067	5.887831918	3.91295E-09	8.84287E-09	positive
PRJNA729271	stage_5W_vs_E10	ordinary_muscle_development	ENSGALG00010004758	FLT3	6.088949787	0.764891703	7.960538415	1.71292E-15	5.53597E-15	positive
PRJNA729271	stage_5W_vs_E10	ordinary_muscle_development	ENSGALG00010004760	THBS2	-3.906674501	0.24175862	-16.15940105	9.75044E-59	1.51158E-57	negative
PRJNA729271	stage_5W_vs_E10	ordinary_muscle_development	ENSGALG00010004762	IFT57	-1.155574346	0.113138423	-10.21380993	1.71976E-24	8.36229E-24	negative
PRJNA729271	stage_5W_vs_E10	ordinary_muscle_development	ENSGALG00010004765	ENSGALG00010004765	3.546420595	0.198974788	17.82346717	4.64653E-71	1.01128E-69	positive
PRJNA729271	stage_5W_vs_E10	ordinary_muscle_development	ENSGALG00010004769	HTR2A	-0.985028478	0.376225046	-2.61818953	0.008839769	0.012164858	negative
PRJNA729271	stage_5W_vs_E10	ordinary_muscle_development	ENSGALG00010004777	CDK14	-1.184160824	0.124768813	-9.490839896	2.2918E-21	9.73898E-21	negative
PRJNA729271	stage_5W_vs_E10	ordinary_muscle_development	ENSGALG00010004778	ENSGALG00010004778	-4.273156473	0.541583986	-7.890108613	3.01924E-15	9.61209E-15	negative
PRJNA729271	stage_5W_vs_E10	ordinary_muscle_development	ENSGALG00010004780	ENSGALG00010004780	0.109022886	0.260625769	0.418311997	0.675719019	0.70938996	positive
PRJNA729271	stage_5W_vs_E10	ordinary_muscle_development	ENSGALG00010004781	ENSGALG00010004781	-1.190936805	0.084422242	-14.10690804	3.44348E-45	3.53243E-44	negative
PRJNA729271	stage_5W_vs_E10	ordinary_muscle_development	ENSGALG00010004787	RIOK3	0.470981408	0.114697556	4.106289847	4.02065E-05	6.82987E-05	positive
PRJNA729271	stage_5W_vs_E10	ordinary_muscle_development	ENSGALG00010004791	FZD1	-0.947792388	0.118589477	-7.992213232	1.32538E-15	4.30554E-15	negative
PRJNA729271	stage_5W_vs_E10	ordinary_muscle_development	ENSGALG00010004798	PTPRK	-2.460173612	0.114751012	-21.43923247	5.7555E-102	2.50529E-100	negative
PRJNA729271	stage_5W_vs_E10	ordinary_muscle_development	ENSGALG00010004800	ZC3H12C	0.13684452	0.278060539	0.492139305	0.622620868	0.659087817	positive
PRJNA729271	stage_5W_vs_E10	ordinary_muscle_development	ENSGALG00010004803	RMC1	1.848056334	0.139360862	13.26094223	3.89965E-40	3.41957E-39	positive
PRJNA729271	stage_5W_vs_E10	ordinary_muscle_development	ENSGALG00010004808	THEMIS	4.204836028	0.670785926	6.268521544	3.64492E-10	8.74576E-10	positive
PRJNA729271	stage_5W_vs_E10	ordinary_muscle_development	ENSGALG00010004824	ATM	-0.273202913	0.111646564	-2.447033784	0.014403734	0.019372935	negative
PRJNA729271	stage_5W_vs_E10	ordinary_muscle_development	ENSGALG00010004832	C6orf58	1.237449176	2.003726893	0.617573773	0.536856338	0.576894701	positive
PRJNA729271	stage_5W_vs_E10	ordinary_muscle_development	ENSGALG00010004835	HHLA2	-0.471891119	0.410458078	-1.149669465	0.250280035	0.286172526	negative
PRJNA729271	stage_5W_vs_E10	ordinary_muscle_development	ENSGALG00010004837	CENPC	-2.011427917	0.14136076	-14.22904006	6.05066E-46	6.35539E-45	negative
PRJNA729271	stage_5W_vs_E10	ordinary_muscle_development	ENSGALG00010004841	AKAP9	-0.200379435	0.194701967	-1.029159788	0.303404595	0.34205877	negative
PRJNA729271	stage_5W_vs_E10	ordinary_muscle_development	ENSGALG00010004846	TTLL2	-0.206337362	0.36870148	-0.559632583	0.575730075	0.614567158	negative
PRJNA729271	stage_5W_vs_E10	ordinary_muscle_development	ENSGALG00010004851	MTCL3	-4.006560945	0.534340861	-7.498136937	6.47313E-14	1.91838E-13	negative
PRJNA729271	stage_5W_vs_E10	ordinary_muscle_development	ENSGALG00010004852	ENSGALG00010004852	5.236204018	0.22173363	23.6148392	2.7134E-123	1.71E-121	positive
PRJNA729271	stage_5W_vs_E10	ordinary_muscle_development	ENSGALG00010004855	ZEB1	0.207408704	0.09201964	2.253961267	0.024198605	0.031691252	positive
PRJNA729271	stage_5W_vs_E10	ordinary_muscle_development	ENSGALG00010004859	NPC1	1.344791162	0.19137488	7.026999371	2.11022E-12	5.78545E-12	positive
PRJNA729271	stage_5W_vs_E10	ordinary_muscle_development	ENSGALG00010004866	SLC25A21	-2.400677846	0.328135112	-7.316126068	2.55232E-13	7.32736E-13	negative
PRJNA729271	stage_5W_vs_E10	ordinary_muscle_development	ENSGALG00010004868	NPAT	0.058963699	0.113687108	0.51864895	0.604005568	0.641678445	positive
PRJNA729271	stage_5W_vs_E10	ordinary_muscle_development	ENSGALG00010004871	C11orf87	-0.034519478	0.313492288	-0.110112688	0.912320006	0.923985361	negative
PRJNA729271	stage_5W_vs_E10	ordinary_muscle_development	ENSGALG00010004872	KIAA0408	-0.882849382	0.255430589	-3.456318155	0.000547609	0.000848486	negative
PRJNA729271	stage_5W_vs_E10	ordinary_muscle_development	ENSGALG00010004876	ENSGALG00010004876	-0.06617334	0.653461279	-0.101265893	0.91933939	0.930379422	negative
PRJNA729271	stage_5W_vs_E10	ordinary_muscle_development	ENSGALG00010004879	ENSGALG00010004879	-1.840867482	0.632551721	-2.910224447	0.003611693	0.005174094	negative
PRJNA729271	stage_5W_vs_E10	ordinary_muscle_development	ENSGALG00010004880	STAP1	4.034292455	0.567454048	7.10946105	1.16497E-12	3.23615E-12	positive
PRJNA729271	stage_5W_vs_E10	ordinary_muscle_development	ENSGALG00010004881	MIPOL1	-1.631726977	0.158471271	-10.29667374	7.29399E-25	3.60595E-24	negative
PRJNA729271	stage_5W_vs_E10	ordinary_muscle_development	ENSGALG00010004883	KRIT1	0.084935049	0.073939998	1.148702344	0.250678739	0.286560593	positive
PRJNA729271	stage_5W_vs_E10	ordinary_muscle_development	ENSGALG00010004887	MYH15	-4.758820594	0.721803052	-6.592962696	4.31135E-11	1.10062E-10	negative
PRJNA729271	stage_5W_vs_E10	ordinary_muscle_development	ENSGALG00010004890	ACAT1	-0.521769166	0.098887529	-5.276389972	1.31754E-07	2.69817E-07	negative
PRJNA729271	stage_5W_vs_E10	ordinary_muscle_development	ENSGALG00010004891	ANKIB1	-0.393806853	0.077689892	-5.068958704	3.99998E-07	7.89592E-07	negative
PRJNA729271	stage_5W_vs_E10	ordinary_muscle_development	ENSGALG00010004892	HEY2	-2.184731219	0.314343035	-6.950149923	3.64898E-12	9.87344E-12	negative
PRJNA729271	stage_5W_vs_E10	ordinary_muscle_development	ENSGALG00010004896	UBA6	0.06378671	0.082280385	0.77523592	0.438200266	0.480026667	positive
PRJNA729271	stage_5W_vs_E10	ordinary_muscle_development	ENSGALG00010004899	GATAD1	-0.653315419	0.111154423	-5.877547687	4.16389E-09	9.38656E-09	negative
PRJNA729271	stage_5W_vs_E10	ordinary_muscle_development	ENSGALG00010004901	CUL5	1.224933096	0.092107946	13.29888626	2.34942E-40	2.07398E-39	positive
PRJNA729271	stage_5W_vs_E10	ordinary_muscle_development	ENSGALG00010004902	ANKRD29	3.36467033	0.298194232	11.28348563	1.58345E-29	9.41432E-29	positive
PRJNA729271	stage_5W_vs_E10	ordinary_muscle_development	ENSGALG00010004903	ENSGALG00010004903	3.453721792	0.461525623	7.483272039	7.24946E-14	2.14068E-13	positive
PRJNA729271	stage_5W_vs_E10	ordinary_muscle_development	ENSGALG00010004907	ENSGALG00010004907	-1.263843075	0.119904072	-10.54045164	5.62282E-26	2.90149E-25	negative
PRJNA729271	stage_5W_vs_E10	ordinary_muscle_development	ENSGALG00010004917	ENSGALG00010004917	-0.594618592	0.095039979	-6.256510118	3.93688E-10	9.43223E-10	negative
PRJNA729271	stage_5W_vs_E10	ordinary_muscle_development	ENSGALG00010004921	RRAGD	3.059499807	0.132597526	23.07358133	8.5302E-118	5.0468E-116	positive
PRJNA729271	stage_5W_vs_E10	ordinary_muscle_development	ENSGALG00010004924	ENSGALG00010004924	2.081549755	0.338167313	6.155384253	7.48957E-10	1.76007E-09	positive
PRJNA729271	stage_5W_vs_E10	ordinary_muscle_development	ENSGALG00010004927	KIF25	-3.423585394	0.292767701	-11.69386304	1.3701E-31	8.83433E-31	negative
PRJNA729271	stage_5W_vs_E10	ordinary_muscle_development	ENSGALG00010004928	HDDC2	-0.667672537	0.123459629	-5.408023174	6.37242E-08	1.33153E-07	negative
PRJNA729271	stage_5W_vs_E10	ordinary_muscle_development	ENSGALG00010004929	ENSGALG00010004929	2.386515689	0.158047384	15.10000119	1.6193E-51	2.04994E-50	positive
PRJNA729271	stage_5W_vs_E10	ordinary_muscle_development	ENSGALG00010004933	SPTLC2	-0.773770559	0.092029744	-8.407831255	4.17661E-17	1.45985E-16	negative
PRJNA729271	stage_5W_vs_E10	ordinary_muscle_development	ENSGALG00010004934	CIP2A	-2.63177582	0.166739188	-15.78378692	4.02309E-56	5.76233E-55	negative
PRJNA729271	stage_5W_vs_E10	ordinary_muscle_development	ENSGALG00010004935	SVIL	-0.265984841	0.069176325	-3.84502704	0.000120539	0.000196958	negative
PRJNA729271	stage_5W_vs_E10	ordinary_muscle_development	ENSGALG00010004939	DDX10	-0.559478729	0.111229403	-5.029953526	4.90599E-07	9.62929E-07	negative
PRJNA729271	stage_5W_vs_E10	ordinary_muscle_development	ENSGALG00010004942	GABRR1	-3.195455678	0.823942094	-3.878252734	0.000105209	0.000172845	negative
PRJNA729271	stage_5W_vs_E10	ordinary_muscle_development	ENSGALG00010004944	ALKBH1	0.00696919	0.098922146	0.070451265	0.943834489	0.951316384	positive
PRJNA729271	stage_5W_vs_E10	ordinary_muscle_development	ENSGALG00010004946	SH2D1B	4.197581896	0.415781736	10.095638	5.77543E-24	2.74205E-23	positive
PRJNA729271	stage_5W_vs_E10	ordinary_muscle_development	ENSGALG00010004949	RAB39A	-0.436176036	0.327897641	-1.330220108	0.183445761	0.214889793	negative
PRJNA729271	stage_5W_vs_E10	ordinary_muscle_development	ENSGALG00010004950	SLIRP	0.129273113	0.110759123	1.167155448	0.243147599	0.278654685	positive
PRJNA729271	stage_5W_vs_E10	ordinary_muscle_development	ENSGALG00010004951	AFDN	-0.739553959	0.062925227	-11.75290088	6.82354E-32	4.45528E-31	negative
PRJNA729271	stage_5W_vs_E10	ordinary_muscle_development	ENSGALG00010004952	PEX1	1.085935528	0.12120043	8.959832329	3.25172E-19	1.25356E-18	positive
PRJNA729271	stage_5W_vs_E10	ordinary_muscle_development	ENSGALG00010004957	ENSGALG00010004957	-0.291655074	0.147132506	-1.982261306	0.047450002	0.060011149	negative
PRJNA729271	stage_5W_vs_E10	ordinary_muscle_development	ENSGALG00010004958	TPD52L1	-3.237904565	0.45118404	-7.176460781	7.15392E-13	2.01117E-12	negative
PRJNA729271	stage_5W_vs_E10	ordinary_muscle_development	ENSGALG00010004962	ENSGALG00010004962	-0.406265961	0.095400235	-4.258542561	2.05764E-05	3.57663E-05	negative
PRJNA729271	stage_5W_vs_E10	ordinary_muscle_development	ENSGALG00010004963	ENSGALG00010004963	0.525962847	0.243476398	2.160221078	0.030755559	0.039860679	positive
PRJNA729271	stage_5W_vs_E10	ordinary_muscle_development	ENSGALG00010004965	JCAD	0.511635354	0.1007558	5.077974194	3.8148E-07	7.54271E-07	positive
PRJNA729271	stage_5W_vs_E10	ordinary_muscle_development	ENSGALG00010004966	LAMA3	1.764888806	0.210929122	8.367212592	5.89972E-17	2.04928E-16	positive
PRJNA729271	stage_5W_vs_E10	ordinary_muscle_development	ENSGALG00010004967	SLC35F2	-2.829329452	0.322121174	-8.783432086	1.58561E-18	5.92966E-18	negative
PRJNA729271	stage_5W_vs_E10	ordinary_muscle_development	ENSGALG00010004968	TRAT1	7.85070333	0.926810199	8.470669978	2.43986E-17	8.62158E-17	positive
PRJNA729271	stage_5W_vs_E10	ordinary_muscle_development	ENSGALG00010004969	YTHDC1	-0.08045799	0.077914584	-1.032643512	0.301770758	0.340322659	negative
PRJNA729271	stage_5W_vs_E10	ordinary_muscle_development	ENSGALG00010004970	RBM48	0.255802444	0.168190737	1.52090685	0.128283215	0.15367481	positive
PRJNA729271	stage_5W_vs_E10	ordinary_muscle_development	ENSGALG00010004973	SLN	10.00223991	0.1949032	51.31901339	0	0	positive
PRJNA729271	stage_5W_vs_E10	ordinary_muscle_development	ENSGALG00010004974	MTPAP	-0.396464252	0.062005939	-6.393972221	1.61631E-10	3.96822E-10	negative
PRJNA729271	stage_5W_vs_E10	ordinary_muscle_development	ENSGALG00010004975	SNW1	-0.10276393	0.069757987	-1.473149304	0.140710781	0.167592669	negative
PRJNA729271	stage_5W_vs_E10	ordinary_muscle_development	ENSGALG00010004976	ENSGALG00010004976	2.099571869	0.227748372	9.218822733	3.0038E-20	1.21315E-19	positive
PRJNA729271	stage_5W_vs_E10	ordinary_muscle_development	ENSGALG00010004977	RNF217	-0.165475593	0.137289559	-1.205303555	0.228086138	0.262493733	negative
PRJNA729271	stage_5W_vs_E10	ordinary_muscle_development	ENSGALG00010004980	FNDC1	0.574364429	0.37752105	1.52141034	0.128156896	0.153548868	positive
PRJNA729271	stage_5W_vs_E10	ordinary_muscle_development	ENSGALG00010004981	ENSGALG00010004981	0.043079702	0.207151376	0.207962425	0.835258307	0.855019008	positive
PRJNA729271	stage_5W_vs_E10	ordinary_muscle_development	ENSGALG00010004983	CLXN	-1.417957013	0.286186393	-4.954662582	7.24559E-07	1.40727E-06	negative
PRJNA729271	stage_5W_vs_E10	ordinary_muscle_development	ENSGALG00010004987	MAP3K8	0.841066883	0.274346597	3.065709183	0.002171544	0.003179448	positive
PRJNA729271	stage_5W_vs_E10	ordinary_muscle_development	ENSGALG00010004988	HJURP	-2.654392047	0.22248901	-11.93044117	8.2137E-33	5.56084E-32	negative
PRJNA729271	stage_5W_vs_E10	ordinary_muscle_development	ENSGALG00010004990	ENSGALG00010004990	6.043341339	2.376890749	2.542540645	0.01100498	0.015007732	positive
PRJNA729271	stage_5W_vs_E10	ordinary_muscle_development	ENSGALG00010004991	FAM133B	-0.474288822	0.063711987	-7.444263579	9.74867E-14	2.85699E-13	negative
PRJNA729271	stage_5W_vs_E10	ordinary_muscle_development	ENSGALG00010004993	SULT1B	2.738795556	0.245380094	11.16144149	6.29623E-29	3.65787E-28	positive
PRJNA729271	stage_5W_vs_E10	ordinary_muscle_development	ENSGALG00010004995	PIT54	11.32812394	2.19351983	5.164359029	2.41264E-07	4.83964E-07	positive
PRJNA729271	stage_5W_vs_E10	ordinary_muscle_development	ENSGALG00010004996	GPR158	0.186749207	0.216470928	0.862698785	0.388303101	0.429587349	positive
PRJNA729271	stage_5W_vs_E10	ordinary_muscle_development	ENSGALG00010004999	ADCK1	-0.68856098	0.195784853	-3.516926717	0.000436574	0.000681093	negative
PRJNA729271	stage_5W_vs_E10	ordinary_muscle_development	ENSGALG00010005003	EXPH5	0.535461161	0.420996068	1.27189112	0.203411807	0.236311144	positive
PRJNA729271	stage_5W_vs_E10	ordinary_muscle_development	ENSGALG00010005004	CDK6	-0.355220462	0.097223151	-3.653661296	0.000258527	0.000411571	negative
PRJNA729271	stage_5W_vs_E10	ordinary_muscle_development	ENSGALG00010005010	NKAIN2	-4.034384742	0.340747756	-11.83979841	2.43036E-32	1.62117E-31	negative
PRJNA729271	stage_5W_vs_E10	ordinary_muscle_development	ENSGALG00010005012	ENSGALG00010005012	11.38520733	1.059470947	10.74612509	6.18038E-27	3.32164E-26	positive
PRJNA729271	stage_5W_vs_E10	ordinary_muscle_development	ENSGALG00010005014	SULT1B1	1.05545919	0.152174467	6.935849439	4.03787E-12	1.08911E-11	positive
PRJNA729271	stage_5W_vs_E10	ordinary_muscle_development	ENSGALG00010005015	NRXN3	-2.292504632	0.414555242	-5.530034123	3.20169E-08	6.82577E-08	negative
PRJNA729271	stage_5W_vs_E10	ordinary_muscle_development	ENSGALG00010005016	STX11	0.172306088	0.172408435	0.999406372	0.317597874	0.356756137	positive
PRJNA729271	stage_5W_vs_E10	ordinary_muscle_development	ENSGALG00010005017	ENSGALG00010005017	0.624163619	0.171603616	3.637240481	0.000275575	0.000437413	positive
PRJNA729271	stage_5W_vs_E10	ordinary_muscle_development	ENSGALG00010005023	POGLUT3	-0.166618579	0.180849788	-0.92130923	0.356889004	0.397258955	negative

PRJNA729271	stage_5W_vs_E10	ordinary_muscle_development	ENSGALG00010005025	THOC1	-1.086606254	0.060190243	-18.05286368	7.49044E-73	1.70714E-71	negative
PRJNA729271	stage_5W_vs_E10	ordinary_muscle_development	ENSGALG00010005029	FAT4	-2.345567287	0.197915448	-11.85136034	2.11726E-32	1.41623E-31	negative
PRJNA729271	stage_5W_vs_E10	ordinary_muscle_development	ENSGALG00010005033	USP14	0.480286294	0.134536127	3.569942921	0.000357059	0.000561585	positive
PRJNA729271	stage_5W_vs_E10	ordinary_muscle_development	ENSGALG00010005035	NECTIN3	-0.693374963	0.099114365	-6.995706049	2.63926E-12	7.191E-12	negative
PRJNA729271	stage_5W_vs_E10	ordinary_muscle_development	ENSGALG00010005037	THNSL1	0.105584306	0.142644156	0.740193701	0.459182469	0.501193516	positive
PRJNA729271	stage_5W_vs_E10	ordinary_muscle_development	ENSGALG00010005042	SLC46A3	2.424815839	0.198101929	12.24024346	1.89463E-34	1.36699E-33	positive
PRJNA729271	stage_5W_vs_E10	ordinary_muscle_development	ENSGALG00010005045	UTRN	0.044999341	0.099402323	0.452699083	0.65076544	0.685476713	positive
PRJNA729271	stage_5W_vs_E10	ordinary_muscle_development	ENSGALG00010005048	ROCK1	0.023128536	0.114606192	0.20180878	0.840066217	0.859273151	positive
PRJNA729271	stage_5W_vs_E10	ordinary_muscle_development	ENSGALG00010005057	FLVCR2	-1.377783942	0.292856119	-4.704644536	2.54309E-06	4.73318E-06	negative
PRJNA729271	stage_5W_vs_E10	ordinary_muscle_development	ENSGALG00010005060	POMP	0.398146281	0.138286468	2.87914129	0.003987596	0.005692359	positive
PRJNA729271	stage_5W_vs_E10	ordinary_muscle_development	ENSGALG00010005065	ENKUR	-0.483094639	0.414033127	-1.166801899	0.24329038	0.278796273	negative
PRJNA729271	stage_5W_vs_E10	ordinary_muscle_development	ENSGALG00010005068	PLCXD2	2.503354828	0.258412589	9.687433729	3.40989E-22	1.50644E-21	positive
PRJNA729271	stage_5W_vs_E10	ordinary_muscle_development	ENSGALG00010005070	ALB	6.904158707	1.403636563	4.91876522	8.70919E-07	1.68118E-06	positive
PRJNA729271	stage_5W_vs_E10	ordinary_muscle_development	ENSGALG00010005075	TRDN	6.741195381	0.167016055	40.36255903	0	0	positive
PRJNA729271	stage_5W_vs_E10	ordinary_muscle_development	ENSGALG00010005077	ERG28	-0.63298314	0.105486088	-6.000631454	1.96552E-09	4.50794E-09	negative
PRJNA729271	stage_5W_vs_E10	ordinary_muscle_development	ENSGALG00010005081	GREB1L	0.61141917	0.17948318	3.406554139	0.000657885	0.001012749	positive
PRJNA729271	stage_5W_vs_E10	ordinary_muscle_development	ENSGALG00010005082	ENSGALG00010005082	-0.623446481	0.087646936	-7.113157753	1.13418E-12	3.15302E-12	negative
PRJNA729271	stage_5W_vs_E10	ordinary_muscle_development	ENSGALG00010005087	ENPP1	1.77953274	0.214265624	8.30526477	9.95971E-17	3.41614E-16	positive
PRJNA729271	stage_5W_vs_E10	ordinary_muscle_development	ENSGALG00010005092	ESCO1	2.088138021	0.139667362	14.95079445	1.53902E-50	1.88254E-49	positive
PRJNA729271	stage_5W_vs_E10	ordinary_muscle_development	ENSGALG00010005104	ENPP3	-0.077362496	0.200081365	-0.38665518	0.699011502	0.731353524	negative
PRJNA729271	stage_5W_vs_E10	ordinary_muscle_development	ENSGALG00010005107	TTLL5	-0.721972139	0.074661444	-9.669946111	4.04591E-22	1.78037E-21	negative
PRJNA729271	stage_5W_vs_E10	ordinary_muscle_development	ENSGALG00010005109	ABHD3	-0.12976911	0.375509716	-0.345581231	0.729657453	0.759306827	negative
PRJNA729271	stage_5W_vs_E10	ordinary_muscle_development	ENSGALG00010005111	RASSF6	-0.410516811	0.407515328	-1.007365324	0.313759249	0.352854396	negative
PRJNA729271	stage_5W_vs_E10	ordinary_muscle_development	ENSGALG00010005114	PHLDB2	-0.942921844	0.100420081	-9.389773747	6.01293E-21	2.50524E-20	negative
PRJNA729271	stage_5W_vs_E10	ordinary_muscle_development	ENSGALG00010005118	PRTFDC1	-1.058925367	0.138892752	-7.624050583	2.45837E-14	7.45639E-14	negative
PRJNA729271	stage_5W_vs_E10	ordinary_muscle_development	ENSGALG00010005119	MIB1	-1.565710284	0.057750648	-27.11156219	7.1942E-162	8.4097E-160	negative
PRJNA729271	stage_5W_vs_E10	ordinary_muscle_development	ENSGALG00010005122	IL8L1	3.157502586	0.896940399	3.520303677	0.000431053	0.000672636	positive
PRJNA729271	stage_5W_vs_E10	ordinary_muscle_development	ENSGALG00010005123	MTIF3	1.970946203	0.103416229	19.05838403	5.59772E-81	1.55141E-79	positive
PRJNA729271	stage_5W_vs_E10	ordinary_muscle_development	ENSGALG00010005126	MED23	-0.829495343	0.058902121	-14.08260564	4.85848E-45	4.95593E-44	negative
PRJNA729271	stage_5W_vs_E10	ordinary_muscle_development	ENSGALG00010005127	GATA6	-1.89112057	0.327943352	-5.766607435	8.08832E-09	1.79541E-08	negative
PRJNA729271	stage_5W_vs_E10	ordinary_muscle_development	ENSGALG00010005131	IL8L2	2.303020643	0.674186142	3.416001159	0.00063548	0.000979507	positive
PRJNA729271	stage_5W_vs_E10	ordinary_muscle_development	ENSGALG00010005132	LNX2	-0.39247548	0.096324954	-4.074494319	4.61144E-05	7.79964E-05	negative
PRJNA729271	stage_5W_vs_E10	ordinary_muscle_development	ENSGALG00010005138	ENSGALG00010005138	-2.437522117	0.971880389	-2.508047436	0.012140037	0.016458084	negative
PRJNA729271	stage_5W_vs_E10	ordinary_muscle_development	ENSGALG00010005140	DCT	-4.15001051	1.113435629	-3.727211885	0.00019361	0.000311439	negative
PRJNA729271	stage_5W_vs_E10	ordinary_muscle_development	ENSGALG00010005141	CCDC170	-0.483233802	0.31137706	-1.551924869	0.120680202	0.144950242	negative
PRJNA729271	stage_5W_vs_E10	ordinary_muscle_development	ENSGALG00010005143	GTF3A	-0.458015567	0.137082712	-3.341162144	0.000834285	0.001272809	negative
PRJNA729271	stage_5W_vs_E10	ordinary_muscle_development	ENSGALG00010005145	AKAP7	1.925292557	0.174740098	11.01803522	3.12812E-28	1.76429E-27	positive
PRJNA729271	stage_5W_vs_E10	ordinary_muscle_development	ENSGALG00010005146	TGFB3	0.006116361	0.204666851	0.029884471	0.976159191	0.979673715	positive
PRJNA729271	stage_5W_vs_E10	ordinary_muscle_development	ENSGALG00010005148	TTC39C	-1.034172378	0.088003521	-11.75148861	6.93856E-32	4.5263E-31	negative
PRJNA729271	stage_5W_vs_E10	ordinary_muscle_development	ENSGALG00010005150	ALCAM	-0.693980715	0.186206438	-3.726942646	0.000193817	0.000311737	negative
PRJNA729271	stage_5W_vs_E10	ordinary_muscle_development	ENSGALG00010005154	CWF19L2	-0.391628516	0.14995879	-2.611574266	0.009012641	0.012389817	negative
PRJNA729271	stage_5W_vs_E10	ordinary_muscle_development	ENSGALG00010005157	IMPA1	-0.167078293	0.109331634	-1.528178872	0.126468133	0.151600694	negative
PRJNA729271	stage_5W_vs_E10	ordinary_muscle_development	ENSGALG00010005158	IFT43	-1.439312185	0.203089587	-7.087080171	1.36971E-12	3.79109E-12	negative
PRJNA729271	stage_5W_vs_E10	ordinary_muscle_development	ENSGALG00010005159	ARHGAP21	0.500487846	0.09825635	5.093694671	3.51152E-07	6.95729E-07	positive
PRJNA729271	stage_5W_vs_E10	ordinary_muscle_development	ENSGALG00010005163	GPC6	-3.252875	0.390981853	-8.319759539	8.81419E-17	3.03328E-16	negative
PRJNA729271	stage_5W_vs_E10	ordinary_muscle_development	ENSGALG00010005165	METTL21C	4.394360449	0.560916581	7.834249506	4.71651E-15	1.4846E-14	positive
PRJNA729271	stage_5W_vs_E10	ordinary_muscle_development	ENSGALG00010005166	CBLB	0.25024429	0.12378081	2.021672745	0.043210171	0.054936535	positive
PRJNA729271	stage_5W_vs_E10	ordinary_muscle_development	ENSGALG00010005167	GUCY1A2	0.732935005	0.177621513	4.126386462	3.68508E-05	6.27234E-05	positive
PRJNA729271	stage_5W_vs_E10	ordinary_muscle_development	ENSGALG00010005170	GPATCH2L	0.308723109	0.060181478	5.12986916	2.89944E-07	5.77616E-07	positive
PRJNA729271	stage_5W_vs_E10	ordinary_muscle_development	ENSGALG00010005172	OSBPL1A	1.261934148	0.080454908	15.68498647	1.91604E-55	2.68598E-54	positive
PRJNA729271	stage_5W_vs_E10	ordinary_muscle_development	ENSGALG00010005174	TPP2	1.707599114	0.184984515	9.231038124	2.68022E-20	1.08428E-19	positive
PRJNA729271	stage_5W_vs_E10	ordinary_muscle_development	ENSGALG00010005176	BAMBI	-3.695190998	0.31846761	-11.60303554	3.97716E-31	2.52183E-30	negative
PRJNA729271	stage_5W_vs_E10	ordinary_muscle_development	ENSGALG00010005177	AASDHPPT	0.705042816	0.128041431	5.506364697	3.66319E-08	7.77878E-08	positive
PRJNA729271	stage_5W_vs_E10	ordinary_muscle_development	ENSGALG00010005178	EPHA5	-0.550261918	0.200676268	-2.742037834	0.006105931	0.008545473	negative
PRJNA729271	stage_5W_vs_E10	ordinary_muscle_development	ENSGALG00010005180	WAC	0.951530975	0.115426522	8.243607755	1.67094E-16	5.6669E-16	positive
PRJNA729271	stage_5W_vs_E10	ordinary_muscle_development	ENSGALG00010005181	IMPACT	0.97710709	0.131839744	7.411324244	1.25044E-13	3.64619E-13	positive
PRJNA729271	stage_5W_vs_E10	ordinary_muscle_development	ENSGALG00010005182	NEPRO	-0.980751883	0.093071912	-10.53757098	5.7977E-26	2.98854E-25	negative
PRJNA729271	stage_5W_vs_E10	ordinary_muscle_development	ENSGALG00010005184	FGF14	-2.392881009	0.31034409	-7.710412684	1.25412E-14	3.85871E-14	negative
PRJNA729271	stage_5W_vs_E10	ordinary_muscle_development	ENSGALG00010005186	ESR1	0.87039001	0.19055655	4.567620535	4.93292E-06	8.97658E-06	positive
PRJNA729271	stage_5W_vs_E10	ordinary_muscle_development	ENSGALG00010005187	KBTBD3	0.921376842	0.259586041	3.549408277	0.000386098	0.00060495	positive
PRJNA729271	stage_5W_vs_E10	ordinary_muscle_development	ENSGALG00010005190	ENSGALG00010005190	3.14695651	0.275029758	11.44224003	2.57159E-30	1.57279E-29	positive
PRJNA729271	stage_5W_vs_E10	ordinary_muscle_development	ENSGALG00010005191	MSANTD4	-0.582470094	0.106369265	-5.475924772	4.35233E-08	9.20037E-08	negative
PRJNA729271	stage_5W_vs_E10	ordinary_muscle_development	ENSGALG00010005193	EPB41L2	1.015690489	0.098280635	10.33459431	4.91487E-25	2.44647E-24	positive
PRJNA729271	stage_5W_vs_E10	ordinary_muscle_development	ENSGALG00010005194	ENSGALG00010005194	2.79971144	1.10816513	2.526438852	0.011522545	0.015673731	positive
PRJNA729271	stage_5W_vs_E10	ordinary_muscle_development	ENSGALG00010005198	TRPC3	-0.027706383	0.492855243	-0.056216066	0.955169683	0.961429649	negative
PRJNA729271	stage_5W_vs_E10	ordinary_muscle_development	ENSGALG00010005203	ENSGALG00010005203	0.402780399	0.105867211	3.804581182	0.000142044	0.0002309	positive
PRJNA729271	stage_5W_vs_E10	ordinary_muscle_development	ENSGALG00010005204	ITGBL1	1.496497862	0.31016895	4.824782957	1.40156E-06	2.66155E-06	positive
PRJNA729271	stage_5W_vs_E10	ordinary_muscle_development	ENSGALG00010005208	ENSGALG00010005208	-0.231642173	0.111515664	-2.077216456	0.037781586	0.048451215	negative
PRJNA729271	stage_5W_vs_E10	ordinary_muscle_development	ENSGALG00010005211	ESRRB	2.430927195	0.340223592	7.145087091	8.99388E-13	2.51624E-12	positive
PRJNA729271	stage_5W_vs_E10	ordinary_muscle_development	ENSGALG00010005212	TAF13	0.520456018	0.100843803	5.161011412	2.45619E-07	4.92291E-07	positive
PRJNA729271	stage_5W_vs_E10	ordinary_muscle_development	ENSGALG00010005213	ENSGALG00010005213	0.617068079	0.15876102	3.886773199	0.000101586	0.000167252	positive
PRJNA729271	stage_5W_vs_E10	ordinary_muscle_development	ENSGALG00010005214	BBS7	-0.599210201	0.121193188	-4.944256434	7.6435E-07	1.48198E-06	negative
PRJNA729271	stage_5W_vs_E10	ordinary_muscle_development	ENSGALG00010005215	VASH1	-1.18548133	0.142378231	-8.326282203	8.34208E-17	2.87285E-16	negative
PRJNA729271	stage_5W_vs_E10	ordinary_muscle_development	ENSGALG00010005216	ENSGALG00010005216	0.368451064	0.163974662	2.246999988	0.024640028	0.032246046	positive
PRJNA729271	stage_5W_vs_E10	ordinary_muscle_development	ENSGALG00010005217	CCNA2	-2.119082424	0.145259359	-14.58826772	3.3356E-48	3.75676E-47	negative
PRJNA729271	stage_5W_vs_E10	ordinary_muscle_development	ENSGALG00010005218	GTPBP8	0.280317733	0.118901437	2.35756387	0.018395291	0.02443022	positive
PRJNA729271	stage_5W_vs_E10	ordinary_muscle_development	ENSGALG00010005219	NALCN	-0.565511412	0.184179375	-3.070438334	0.002137448	0.003132374	negative
PRJNA729271	stage_5W_vs_E10	ordinary_muscle_development	ENSGALG00010005220	ANGEL1	0.09987649	0.085768611	1.164487665	0.244226439	0.279780466	positive
PRJNA729271	stage_5W_vs_E10	ordinary_muscle_development	ENSGALG00010005221	ENSGALG00010005221	1.309830365	0.302390835	4.331580902	1.48043E-05	2.60612E-05	positive
PRJNA729271	stage_5W_vs_E10	ordinary_muscle_development	ENSGALG00010005222	ABCB1	4.32199436	0.983685783	4.393673705	1.11451E-05	1.98024E-05	positive
PRJNA729271	stage_5W_vs_E10	ordinary_muscle_development	ENSGALG00010005223	GRIA4	-4.023427539	0.502121509	-8.012856392	1.12074E-15	3.65471E-15	negative
PRJNA729271	stage_5W_vs_E10	ordinary_muscle_development	ENSGALG00010005224	ENSGALG00010005224	2.707705127	0.37916888	7.141158654	9.25475E-13	2.58623E-12	positive
PRJNA729271	stage_5W_vs_E10	ordinary_muscle_development	ENSGALG00010005226	MYCT1	1.675949958	0.166035605	10.09391904	5.8775E-24	2.78778E-23	positive
PRJNA729271	stage_5W_vs_E10	ordinary_muscle_development	ENSGALG00010005227	EXOSC9	-0.789787217	0.106527032	-7.413960582	1.22582E-13	3.57583E-13	negative
PRJNA729271	stage_5W_vs_E10	ordinary_muscle_development	ENSGALG00010005228	ZNF521	-1.839152634	0.132550368	-13.87512283	8.96394E-44	8.79704E-43	negative
PRJNA729271	stage_5W_vs_E10	ordinary_muscle_development	ENSGALG00010005229	CIPC	0.079412262	0.072893969	1.08942157	0.275968024	0.313172826	positive
PRJNA729271	stage_5W_vs_E10	ordinary_muscle_development	ENSGALG00010005231	ENSGALG00010005231	2.219063687	0.421150839	5.269047284	1.37134E-07	2.80439E-07	positive
PRJNA729271	stage_5W_vs_E10	ordinary_muscle_development	ENSGALG00010005232	GATA3	-1.467098429	0.444947729	-3.297237704	0.000976408	0.001482304	negative
PRJNA729271	stage_5W_vs_E10	ordinary_muscle_development	ENSGALG00010005236	TMTC4	-1.728744422	0.092394031	-18.7105639	4.06056E-78	1.06242E-76	negative
PRJNA729271	stage_5W_vs_E10	ordinary_muscle_development	ENSGALG00010005237	RAP2A	-1.856934148	0.112131441	-16.56033425	1.34843E-61	2.28069E-60	negative
PRJNA729271	stage_5W_vs_E10	ordinary_muscle_development	ENSGALG00010005238	TMEM63C	-0.538618605	0.168163215	-3.202951399	0.001360269	0.002034789	negative
PRJNA729271	stage_5W_vs_E10	ordinary_muscle_development	ENSGALG00010005239	ENSGALG00010005239	-1.511653295	0.189278957	-7.986377971	1.38961E-15	4.51118E-15	negative
PRJNA729271	stage_5W_vs_E10	ordinary_muscle_development	ENSGALG00010005240	VIP	-5.351994811	0.434338591	-12.32217198	6.88155E-35	5.0531E-34	negative
PRJNA729271	stage_5W_vs_E10	ordinary_muscle_development	ENSGALG00010005241	C4orf54	4.021866873	0.187414379	21.4597562	3.7024E-102	1.62134E-100	positive
PRJNA729271	stage_5W_vs_E10	ordinary_muscle_development	ENSGALG00010005242	CCDC80	0.372394725	0.233919608	1.591977384	0.111389786	0.134459939	positive
PRJNA729271	stage_5W_vs_E10	ordinary_muscle_development	ENSGALG00010005243	NGB	-2.715514438	0.557621358	-4.869817842	1.11701E-06	2.13603E-06	negative
PRJNA729271	stage_5W_vs_E10	ordinary_muscle_development	ENSGALG00010005244	FBXO5	-1.597391927	0.100422763	-15.90667169	5.69623E-57	8.39094E-56	negative
PRJNA729271	stage_5W_vs_E10	ordinary_muscle_development	ENSGALG00010005245	KIAA1217	1.727260509	0.135105097	12.78456955	1.99967E-37	1.6085E-36	positive
PRJNA729271	stage_5W_vs_E10	ordinary_muscle_development	ENSGALG00010005246	ENSGALG00010005246	-0.237180732	0.196747639	-1.205507387	0.228007488	0.262465735	negative
PRJNA729271	stage_5W_vs_E10	ordinary_muscle_development	ENSGALG00010005247	THAP7	1.18386157	0.117118817	10.10820978	5.08041E-24	2.42239E-23	positive
PRJNA729271	stage_5W_vs_E10	ordinary_muscle_development	ENSGALG00010005248	TMEM200A	-0.962074064	0.24031298	-4.003421131	6.2433E-05	0.000104814	negative
PRJNA729271	stage_5W_vs_E10	ordinary_muscle_development	ENSGALG00010005249	PDGFD	-0.451409468	0.228923749	-1.971876969	0.048623653	0.061393715	negative
PRJNA729271	stage_5W_vs_E10	ordinary_muscle_development	ENSGALG00010005250	SLC35A5	-0.337656364	0.090565913	-3.728294146	0.00019278	0.000310155	negative
PRJNA729271	stage_5W_vs_E10	ordinary_muscle_development	ENSGALG00010005251	MTRF1L	-0.102618014	0.105246361	-0.975026722	0.329547005	0.369234277	negative
PRJNA729271	stage_5W_vs_E10	ordinary_muscle_development	ENSGALG00010005252	KLHL14	-3.292451937	0.245747584	-13.39769811	6.23677E-41	5.59764E-40	negative
PRJNA729271	stage_5W_vs_E10	ordinary_muscle_development	ENSGALG00010005253	POMT2	-0.660688336	0.097646805	-6.766102948	1.32297E-11	3.47905E-11	negative
PRJNA729271	stage_5W_vs_E10	ordinary_muscle_development	ENSGALG00010005254	ENSGALG00010005254	-0.221031563	0.739995413	-0.298693153	0.765174182	0.792794836	negative
PRJNA729271	stage_5W_vs_E10	ordinary_muscle_development	ENSGALG00010005255	BZW2	1.000544969	0.09287703	10.77279246	4.62749E-27	2.49723E-26	positive
PRJNA729271	stage_5W_vs_E10	ordinary_muscle_development	ENSGALG00010005256	OPRM1	-5.974376924	0.733619951	-8.143694719	3.83395E-16	1.27872E-15	negative
PRJNA729271	stage_5W_vs_E10	ordinary_muscle_development	ENSGALG00010005258	ITIH5	3.624105079	0.278764762	13.00058536	1.2141E-38	1.00966E-37	positive
PRJNA729271	stage_5W_vs_E10	ordinary_muscle_development	ENSGALG00010005259	SLC27A5	1.608106672	0.676349884	2.377625414	0.017424517	0.023205473	positive
PRJNA729271	stage_5W_vs_E10	ordinary_muscle_development	ENSGALG00010005260	ANXA5	-0.006835427	0.150002836	-0.045568651	0.963654056	0.968666126	negative
PRJNA729271	stage_5W_vs_E10	ordinary_muscle_development	ENSGALG00010005261	ATG3	-0.237838273	0.110379316	-2.154735887	0.031182508	0.040392355	negative
PRJNA729271	stage_5W_vs_E10	ordinary_muscle_development	ENSGALG00010005262	ENSGALG00010005262	-0.162037384	0.124948383	-1.296834581	0.19468811	0.226917089	negative
PRJNA729271	stage_5W_vs_E10	ordinary_muscle_development	ENSGALG00010005263	ENSGALG00010005263	2.569198851	0.317695805	8.086977585	6.11635E-16	2.01675E-15	positive
PRJNA729271	stage_5W_vs_E10	ordinary_muscle_development	ENSGALG00010005264	ITIH2	2.789745572	1.044702671	2.670372776			positive
PRJNA729271	stage_5W_vs_E10	ordinary_muscle_development	ENSGALG00010005265	DYNC2H1	-0.967031908	0.095683108	-10.10661051	5.164E-24	2.46062E-23	negative
PRJNA729271	stage_5W_vs_E10	ordinary_muscle_development	ENSGALG00010005266	SLC19A2	-0.744760879	0.277818927	-2.680742047	0.007345912	0.010195906	negative
PRJNA729271	stage_5W_vs_E10	ordinary_muscle_development	ENSGALG00010005267	CYP51A1	-0.764793289	0.181621131	-4.210926807	2.54325E-05	4.39124E-05	negative
PRJNA729271	stage_5W_vs_E10	ordinary_muscle_development	ENSGALG00010005268	MTTP	3.193412583	0.898022923	3.556047959	0.000376475	0.000590713	positive
PRJNA729271	stage_5W_vs_E10	ordinary_muscle_development	ENSGALG00010005269	KIN	0.17968072	0.106652123	1.684736466	0.092039428	0.112317857	positive
PRJNA729271	stage_5W_vs_E10	ordinary_muscle_development	ENSGALG00010005270	GSTZ1	-0.144345899	0.215798533	-0.668891938	0.503564413	0.544551341	negative
PRJNA729271	stage_5W_vs_E10	ordinary_muscle_development	ENSGALG00010005272	MBNL2	2.403081364	0.092542014	25.96746351	1.1548E-148	1.1234E-146	positive
PRJNA729271	stage_5W_vs_E10	ordinary_muscle_development	ENSGALG00010005273	ENSGALG00010005273	4.093963409	0.623881788	6.562081931	5.30616E-11	1.34557E-10	positive
PRJNA729271	stage_5W_vs_E10	ordinary_muscle_development	ENSGALG00010005275	L3MBTL3	-0.276071276	0.172575274	-1.599715119	0.109661796	0.132495435	negative
PRJNA729271	stage_5W_vs_E10	ordinary_muscle_development	ENSGALG00010005277	ENSGALG00010005277	1.810807214	0.200000815	9.053999175	1.37826E-19	5.40526E-19	positive
PRJNA729271	stage_5W_vs_E10	ordinary_muscle_development	ENSGALG00010005278	DAPP1	0.211532089	0.130211316	1.624529233	0.104262907	0.126330421	positive
PRJNA729271	stage_5W_vs_E10	ordinary_muscle_development	ENSGALG00010005279	CCDC181	0.497468901	0.316654767	1.571013462	0.116179522	0.139915434	positive
PRJNA729271	stage_5W_vs_E10	ordinary_muscle_development	ENSGALG00010005280	TMEM244	-2.611040979	0.672196948	-3.884339234	0.000102609	0.000168859	negative
PRJNA729271	stage_5W_vs_E10	ordinary_muscle_development	ENSGALG00010005281	TMED8	-1.049547875	0.069409699	-15.12105504	1.17643E-51	1.50108E-50	negative
PRJNA729271	stage_5W_vs_E10	ordinary_muscle_development	ENSGALG00010005282	FLT1	1.887569966	0.125311632	15.06300682	2.8359E-51	3.54977E-50	positive

PRJNA729271	stage_5W_vs_E10	ordinary_muscle_development	ENSGALG00010005283	ATP5C1	0.852600283	0.132120391	6.453207385	1.09507E-10	2.71799E-10	positive
PRJNA729271	stage_5W_vs_E10	ordinary_muscle_development	ENSGALG00010005284	ENSGALG00010005284	0.309175313	0.220306455	1.403387442	0.160501336	0.189776235	positive
PRJNA729271	stage_5W_vs_E10	ordinary_muscle_development	ENSGALG00010005285	MPP7	0.914504114	0.127646841	7.164330187	7.81678E-13	2.19199E-12	positive
PRJNA729271	stage_5W_vs_E10	ordinary_muscle_development	ENSGALG00010005286	ENSGALG00010005286	2.044047828	0.596594703	3.426191715	0.000612108	0.000944788	positive
PRJNA729271	stage_5W_vs_E10	ordinary_muscle_development	ENSGALG00010005287	ENSGALG00010005287	-0.722388443	0.422017517	-1.711749901	0.086942768	0.106429814	negative
PRJNA729271	stage_5W_vs_E10	ordinary_muscle_development	ENSGALG00010005288	ENSGALG00010005288	-0.470000498	0.095250284	-4.934373714	8.04084E-07	1.55631E-06	negative
PRJNA729271	stage_5W_vs_E10	ordinary_muscle_development	ENSGALG00010005291	IFNGR1	0.522808334	0.096036256	5.443864195	5.2137E-08	1.09589E-07	positive
PRJNA729271	stage_5W_vs_E10	ordinary_muscle_development	ENSGALG00010005292	TAF3	0.183468517	0.09869056	1.85902804	0.063023164	0.078481165	positive
PRJNA729271	stage_5W_vs_E10	ordinary_muscle_development	ENSGALG00010005293	BLZF1	0.114208884	0.096395718	1.184792089	0.236099661	0.27117786	positive
PRJNA729271	stage_5W_vs_E10	ordinary_muscle_development	ENSGALG00010005295	ENSGALG00010005295	2.514439722	0.54840323	4.585019901	4.53943E-06	8.28704E-06	positive
PRJNA729271	stage_5W_vs_E10	ordinary_muscle_development	ENSGALG00010005296	PAN3	0.82272414	0.101029248	8.143425322	3.8425E-16	1.28127E-15	positive
PRJNA729271	stage_5W_vs_E10	ordinary_muscle_development	ENSGALG00010005297	IL22RA2	-0.527548065	0.600888748	-0.877946321	0.37997285	0.421078476	negative
PRJNA729271	stage_5W_vs_E10	ordinary_muscle_development	ENSGALG00010005299	ODAD2	-3.983968909	0.669656392	-5.94927332	2.69336E-09	6.1355E-09	negative
PRJNA729271	stage_5W_vs_E10	ordinary_muscle_development	ENSGALG00010005300	QRFPR	-0.30699123	0.819287301	-0.374705221	0.707879709	0.739564392	negative
PRJNA729271	stage_5W_vs_E10	ordinary_muscle_development	ENSGALG00010005301	ENSGALG00010005301	1.820807532	0.353239854	5.154592578	2.54183E-07	5.09103E-07	positive
PRJNA729271	stage_5W_vs_E10	ordinary_muscle_development	ENSGALG00010005302	PSPC1	-1.458796677	0.081083829	-17.991216	2.28293E-72	5.14635E-71	negative
PRJNA729271	stage_5W_vs_E10	ordinary_muscle_development	ENSGALG00010005303	ENSGALG00010005303	2.753957326	0.278737354	9.880115753	5.07742E-23	2.31656E-22	positive
PRJNA729271	stage_5W_vs_E10	ordinary_muscle_development	ENSGALG00010005304	ENSGALG00010005304	1.338930745	0.207607685	6.449331302	1.12345E-10	2.78556E-10	positive
PRJNA729271	stage_5W_vs_E10	ordinary_muscle_development	ENSGALG00010005305	SCAF8	-0.016399938	0.086531043	-0.189526647	0.849680072	0.867881943	negative
PRJNA729271	stage_5W_vs_E10	ordinary_muscle_development	ENSGALG00010005307	TIAM2	-2.000562054	0.157910117	-12.66899228	8.78355E-37	6.91943E-36	negative
PRJNA729271	stage_5W_vs_E10	ordinary_muscle_development	ENSGALG00010005308	ENSGALG00010005308	2.66528023	0.54476526	4.892529732	9.95481E-07	1.91296E-06	positive
PRJNA729271	stage_5W_vs_E10	ordinary_muscle_development	ENSGALG00010005309	ASXL3	-2.524619277	0.145746275	-17.32201582	3.20916E-67	6.27757E-66	negative
PRJNA729271	stage_5W_vs_E10	ordinary_muscle_development	ENSGALG00010005310	LAMTOR3	0.726006144	0.076188951	9.52902142	1.58773E-21	6.79885E-21	positive
PRJNA729271	stage_5W_vs_E10	ordinary_muscle_development	ENSGALG00010005311	YME1L1	0.311064139	0.078251399	3.975189463	7.03232E-05	0.000117503	positive
PRJNA729271	stage_5W_vs_E10	ordinary_muscle_development	ENSGALG00010005313	MPHOSPH8	0.061069113	0.141427157	0.431806129	0.665882324	0.700077195	positive
PRJNA729271	stage_5W_vs_E10	ordinary_muscle_development	ENSGALG00010005314	IL20RA	-0.3363013	0.630745944	-0.533180282	0.59390879	0.632110133	negative
PRJNA729271	stage_5W_vs_E10	ordinary_muscle_development	ENSGALG00010005315	ENSGALG00010005315	2.943776975	0.494555765	5.952366115	2.64293E-09	6.02572E-09	positive
PRJNA729271	stage_5W_vs_E10	ordinary_muscle_development	ENSGALG00010005316	PARP4	0.661916744	0.107732039	6.144103005	8.04166E-10	1.88645E-09	positive
PRJNA729271	stage_5W_vs_E10	ordinary_muscle_development	ENSGALG00010005317	CHCHD3	0.639074441	0.109596138	5.831176674	5.50379E-09	1.23342E-08	positive
PRJNA729271	stage_5W_vs_E10	ordinary_muscle_development	ENSGALG00010005318	SLC35D3	-2.659679961	0.483916103	-5.49615924	3.88152E-08	8.23155E-08	negative
PRJNA729271	stage_5W_vs_E10	ordinary_muscle_development	ENSGALG00010005319	NOL4	-3.450040581	1.015353771	-3.397870456	0.000679126	0.001043895	negative
PRJNA729271	stage_5W_vs_E10	ordinary_muscle_development	ENSGALG00010005321	HS6ST3	-3.360399154	0.30245597	-11.11037469	1.1169E-28	6.42949E-28	negative
PRJNA729271	stage_5W_vs_E10	ordinary_muscle_development	ENSGALG00010005322	TFB1M	0.148916219	0.123345774	1.207307025	0.227313926	0.261771301	positive
PRJNA729271	stage_5W_vs_E10	ordinary_muscle_development	ENSGALG00010005323	DNAJB14	0.586303557	0.063639669	9.212863065	3.1754E-20	1.28032E-19	positive
PRJNA729271	stage_5W_vs_E10	ordinary_muscle_development	ENSGALG00010005324	CENPJ	-2.677270706	0.203701504	-13.1431072	1.86412E-39	1.59507E-38	negative
PRJNA729271	stage_5W_vs_E10	ordinary_muscle_development	ENSGALG00010005326	MASTL	-2.390165622	0.191174949	-12.5025043	7.23361E-36	5.52139E-35	negative
PRJNA729271	stage_5W_vs_E10	ordinary_muscle_development	ENSGALG00010005329	VIPAS39	0.054054709	0.118278506	0.457012103	0.647662346	0.682555308	positive
PRJNA729271	stage_5W_vs_E10	ordinary_muscle_development	ENSGALG00010005331	ENSGALG00010005331	1.87791969	0.798467322	2.351905504	0.018677519	0.024789942	positive
PRJNA729271	stage_5W_vs_E10	ordinary_muscle_development	ENSGALG00010005332	ENSGALG00010005332	2.78728926	0.679447192	4.102289759	4.09082E-05	6.94499E-05	positive
PRJNA729271	stage_5W_vs_E10	ordinary_muscle_development	ENSGALG00010005333	PLXNA4	-1.433877413	0.160221277	-8.949357021	3.57559E-19	1.37621E-18	negative
PRJNA729271	stage_5W_vs_E10	ordinary_muscle_development	ENSGALG00010005334	NME7	-0.177167079	0.133520701	-1.32688847	0.184545579	0.216090812	negative
PRJNA729271	stage_5W_vs_E10	ordinary_muscle_development	ENSGALG00010005335	AHSA1	0.523664252	0.133729331	3.91585187	9.00855E-05	0.000149114	positive
PRJNA729271	stage_5W_vs_E10	ordinary_muscle_development	ENSGALG00010005336	PEX7	0.594611266	0.125757871	4.728223046	2.26493E-06	4.23179E-06	positive
PRJNA729271	stage_5W_vs_E10	ordinary_muscle_development	ENSGALG00010005337	DCTN1	0.295493942	0.153976884	1.919079888	0.054974224	0.068937744	positive
PRJNA729271	stage_5W_vs_E10	ordinary_muscle_development	ENSGALG00010005338	PCGF1	0.810161838	0.117124825	6.917080475	4.61046E-12	1.23963E-11	positive
PRJNA729271	stage_5W_vs_E10	ordinary_muscle_development	ENSGALG00010005341	UGGT2	-0.300120441	0.119270615	-2.516298254	0.011859478	0.016107864	negative
PRJNA729271	stage_5W_vs_E10	ordinary_muscle_development	ENSGALG00010005342	MAP3K5	0.365343018	0.145583724	2.509504553	0.012090066	0.016394939	positive
PRJNA729271	stage_5W_vs_E10	ordinary_muscle_development	ENSGALG00010005343	RNF17	-4.939285036	1.784097237	-2.768506634	0.005631384	0.007908052	negative
PRJNA729271	stage_5W_vs_E10	ordinary_muscle_development	ENSGALG00010005344	ENSGALG00010005344	2.13953996	0.685825517	3.119656396	0.001810621	0.002673147	positive
PRJNA729271	stage_5W_vs_E10	ordinary_muscle_development	ENSGALG00010005345	NOX3	2.284924489	0.491598398	4.647949415	3.35251E-06	6.19119E-06	positive
PRJNA729271	stage_5W_vs_E10	ordinary_muscle_development	ENSGALG00010005346	FBXO30	1.549670216	0.220189746	7.037885487	1.95179E-12	5.36326E-12	positive
PRJNA729271	stage_5W_vs_E10	ordinary_muscle_development	ENSGALG00010005347	DOK1	0.461466198	0.14783177	3.121563093	0.001798937	0.002656167	positive
PRJNA729271	stage_5W_vs_E10	ordinary_muscle_development	ENSGALG00010005349	ACBD5	1.515954544	0.103273241	14.6790643	8.77936E-49	1.01887E-47	positive
PRJNA729271	stage_5W_vs_E10	ordinary_muscle_development	ENSGALG00010005350	ARHGAP20	-0.91488515	0.278298886	-3.287419375	0.001011101	0.001532244	negative
PRJNA729271	stage_5W_vs_E10	ordinary_muscle_development	ENSGALG00010005351	DTNA	1.999148651	0.163230555	12.24739232	1.73485E-34	1.25482E-33	positive
PRJNA729271	stage_5W_vs_E10	ordinary_muscle_development	ENSGALG00010005352	ENSGALG00010005352	3.578273072	0.591859013	6.045820025	1.48652E-09	3.43599E-09	positive
PRJNA729271	stage_5W_vs_E10	ordinary_muscle_development	ENSGALG00010005353	ENSGALG00010005353	0.350063235	0.103412669	3.385109766	0.000711498	0.001091455	positive
PRJNA729271	stage_5W_vs_E10	ordinary_muscle_development	ENSGALG00010005354	M1AP	-0.549583623	0.355559815	-1.545685423	0.122180559	0.146618694	negative
PRJNA729271	stage_5W_vs_E10	ordinary_muscle_development	ENSGALG00010005355	FDX1	0.016640716	0.104175889	0.159736727	0.873088468	0.888785543	positive
PRJNA729271	stage_5W_vs_E10	ordinary_muscle_development	ENSGALG00010005356	DNAJC3	0.692999183	0.084118866	8.2383324	1.74627E-16	5.91823E-16	positive
PRJNA729271	stage_5W_vs_E10	ordinary_muscle_development	ENSGALG00010005357	TNIP3	0.33123328	0.448199473	0.739030945	0.459888207	0.501850452	positive
PRJNA729271	stage_5W_vs_E10	ordinary_muscle_development	ENSGALG00010005358	SHPRH	0.196485538	0.089973784	2.183808778	0.028976302	0.037645559	positive
PRJNA729271	stage_5W_vs_E10	ordinary_muscle_development	ENSGALG00010005359	MEIS2	-2.450824786	0.134808781	-18.18000857	7.43222E-74	1.74039E-72	negative
PRJNA729271	stage_5W_vs_E10	ordinary_muscle_development	ENSGALG00010005360	CAMK1D	-0.202118096	0.151465561	-1.334416186	0.182067498	0.213396004	negative
PRJNA729271	stage_5W_vs_E10	ordinary_muscle_development	ENSGALG00010005361	ENSGALG00010005361	2.722457957	0.414640233	6.565831623	5.17433E-11	1.31283E-10	positive
PRJNA729271	stage_5W_vs_E10	ordinary_muscle_development	ENSGALG00010005362	ATP1B1	1.690685961	0.174190242	9.705974009	2.84347E-22	1.25928E-21	positive
PRJNA729271	stage_5W_vs_E10	ordinary_muscle_development	ENSGALG00010005363	RDX	0.976800819	0.085381775	11.44039016	2.62701E-30	1.60601E-29	positive
PRJNA729271	stage_5W_vs_E10	ordinary_muscle_development	ENSGALG00010005364	MAP7	-4.302975724	0.417908351	-10.29645786	7.31037E-25	3.61281E-24	negative
PRJNA729271	stage_5W_vs_E10	ordinary_muscle_development	ENSGALG00010005366	AUP1	0.898664911	0.11381646	7.895737695	2.88603E-15	9.19407E-15	positive
PRJNA729271	stage_5W_vs_E10	ordinary_muscle_development	ENSGALG00010005367	H2AZ1	-1.128542159	0.095647931	-11.7989187	3.95359E-32	2.612E-31	negative
PRJNA729271	stage_5W_vs_E10	ordinary_muscle_development	ENSGALG00010005368	CCDC3	-1.042962143	0.228897657	-4.55645617	5.20239E-06	9.45563E-06	negative
PRJNA729271	stage_5W_vs_E10	ordinary_muscle_development	ENSGALG00010005369	DPT	3.412868209	0.312893492	10.90744391	1.06201E-27	5.88222E-27	positive
PRJNA729271	stage_5W_vs_E10	ordinary_muscle_development	ENSGALG00010005371	GRM1	-2.937808586	1.324230832	-2.218501877	0.026520632	0.034604065	negative
PRJNA729271	stage_5W_vs_E10	ordinary_muscle_development	ENSGALG00010005372	CDIN1	-1.132181912	0.111491696	-10.15485415	3.15281E-24	1.51676E-23	negative
PRJNA729271	stage_5W_vs_E10	ordinary_muscle_development	ENSGALG00010005376	HTRA2	0.267728933	0.095308621	2.809073634	0.004968428	0.007017245	positive
PRJNA729271	stage_5W_vs_E10	ordinary_muscle_development	ENSGALG00010005377	EMCN	2.578177504	0.140495109	18.35065669	3.26147E-75	8.0127E-74	positive
PRJNA729271	stage_5W_vs_E10	ordinary_muscle_development	ENSGALG00010005378	ENSGALG00010005378	-5.264290851	0.818416089	-6.432291502	1.25694E-10	3.10752E-10	negative
PRJNA729271	stage_5W_vs_E10	ordinary_muscle_development	ENSGALG00010005379	DZIP1	-0.958895958	0.169458288	-5.658595821	1.52616E-08	3.33511E-08	negative
PRJNA729271	stage_5W_vs_E10	ordinary_muscle_development	ENSGALG00010005380	XCL1	9.037524926	1.012626748	8.924833301	4.46371E-19	1.70942E-18	positive
PRJNA729271	stage_5W_vs_E10	ordinary_muscle_development	ENSGALG00010005381	FGF9	-3.880312208	0.255717853	-15.17419359	5.2418E-52	6.75978E-51	negative
PRJNA729271	stage_5W_vs_E10	ordinary_muscle_development	ENSGALG00010005382	RAB32	-0.877542305	0.128833133	-6.81146444	9.66102E-12	2.56008E-11	negative
PRJNA729271	stage_5W_vs_E10	ordinary_muscle_development	ENSGALG00010005383	OPTN	3.282568	0.138255146	23.74282691	1.3032E-124	8.6253E-123	positive
PRJNA729271	stage_5W_vs_E10	ordinary_muscle_development	ENSGALG00010005384	MAPRE2	0.758016046	0.172683894	4.389616372	1.13551E-05	2.01533E-05	positive
PRJNA729271	stage_5W_vs_E10	ordinary_muscle_development	ENSGALG00010005385	PDSS1	-0.160986464	0.101129397	-1.591885934	0.111410337	0.134473547	negative
PRJNA729271	stage_5W_vs_E10	ordinary_muscle_development	ENSGALG00010005386	CPOX	-0.653101516	0.141469159	-4.616564627	3.90145E-06	7.16113E-06	negative
PRJNA729271	stage_5W_vs_E10	ordinary_muscle_development	ENSGALG00010005387	CLDN10	-3.136188272	0.58477258	-5.363090505	8.181E-08	1.6972E-07	negative
PRJNA729271	stage_5W_vs_E10	ordinary_muscle_development	ENSGALG00010005388	ENSGALG00010005388	3.790094904	0.457116906	8.291303278	1.12014E-16	3.83207E-16	positive
PRJNA729271	stage_5W_vs_E10	ordinary_muscle_development	ENSGALG00010005389	MCM10	-2.249310536	0.18846747	-11.93474151	7.80008E-33	5.29317E-32	negative
PRJNA729271	stage_5W_vs_E10	ordinary_muscle_development	ENSGALG00010005391	ADGB	0.208503971	0.387827393	0.537620537	0.590839049	0.629027691	positive
PRJNA729271	stage_5W_vs_E10	ordinary_muscle_development	ENSGALG00010005392	ENSGALG00010005392	0.29521344	0.109171166	2.70413381	0.006848271	0.009536524	positive
PRJNA729271	stage_5W_vs_E10	ordinary_muscle_development	ENSGALG00010005394	PHYH	2.902842427	0.135250165	21.46276445	3.4704E-102	1.52436E-100	positive
PRJNA729271	stage_5W_vs_E10	ordinary_muscle_development	ENSGALG00010005395	ENSGALG00010005395	-3.171282002	0.432972436	-7.32444318	2.39893E-13	6.89382E-13	negative
PRJNA729271	stage_5W_vs_E10	ordinary_muscle_development	ENSGALG00010005396	MICU2	0.543926559	0.122625818	4.435661012	9.17902E-06	1.64016E-05	positive
PRJNA729271	stage_5W_vs_E10	ordinary_muscle_development	ENSGALG00010005398	SEPHS1	-0.961941482	0.086794015	-11.08303934	1.51636E-28	8.67389E-28	negative
PRJNA729271	stage_5W_vs_E10	ordinary_muscle_development	ENSGALG00010005399	NDNF	-5.723779068	0.28168379	-20.31987382	8.57966E-92	3.06311E-90	negative
PRJNA729271	stage_5W_vs_E10	ordinary_muscle_development	ENSGALG00010005401	BCLAF1	-1.431621974	0.087524724	-16.3567722	3.89218E-60	6.26858E-59	negative
PRJNA729271	stage_5W_vs_E10	ordinary_muscle_development	ENSGALG00010005402	ABCC4	1.569367746	0.168812536	9.296511859	1.45129E-20	5.92909E-20	positive
PRJNA729271	stage_5W_vs_E10	ordinary_muscle_development	ENSGALG00010005403	ENSGALG00010005403	-0.967684342	0.148446916	-6.518723092	7.09084E-11	1.78565E-10	negative
PRJNA729271	stage_5W_vs_E10	ordinary_muscle_development	ENSGALG00010005404	BEND7	1.220491504	0.18704819	6.525011022	6.79965E-11	1.71351E-10	positive
PRJNA729271	stage_5W_vs_E10	ordinary_muscle_development	ENSGALG00010005405	CEBPD	5.383261204	0.474043072	11.35605924	6.91961E-30	4.17047E-29	positive
PRJNA729271	stage_5W_vs_E10	ordinary_muscle_development	ENSGALG00010005407	DPH6	-0.72481859	0.122361103	-5.923602939	3.14963E-09	7.15019E-09	negative
PRJNA729271	stage_5W_vs_E10	ordinary_muscle_development	ENSGALG00010005408	APBB1IP	1.114686517	0.202087899	5.515849887	3.47098E-08	7.38427E-08	positive
PRJNA729271	stage_5W_vs_E10	ordinary_muscle_development	ENSGALG00010005409	PRKCQ	2.608238212	0.217790608	11.9758985	4.75261E-33	3.24184E-32	positive
PRJNA729271	stage_5W_vs_E10	ordinary_muscle_development	ENSGALG00010005411	STXBP5	1.47018526	0.094887129	15.49404297	3.80586E-54	5.13649E-53	positive
PRJNA729271	stage_5W_vs_E10	ordinary_muscle_development	ENSGALG00010005412	MTFR2	-2.52631083	0.173943212	-14.52376784	8.56689E-48	9.51548E-47	negative
PRJNA729271	stage_5W_vs_E10	ordinary_muscle_development	ENSGALG00010005413	GAD2	-6.234502963	0.860943067	-7.241481118	4.43811E-13	1.2599E-12	negative
PRJNA729271	stage_5W_vs_E10	ordinary_muscle_development	ENSGALG00010005414	ENSGALG00010005414	-1.47760546	0.231299958	-6.388265147	1.67778E-10	4.11497E-10	negative
PRJNA729271	stage_5W_vs_E10	ordinary_muscle_development	ENSGALG00010005415	PRKDC	-0.88373742	0.088357141	-10.00187888	1.49533E-23	6.9806E-23	negative
PRJNA729271	stage_5W_vs_E10	ordinary_muscle_development	ENSGALG00010005416	PPP3CA	1.770201928	0.162025394	10.92545976	8.70982E-28	4.8464E-27	positive
PRJNA729271	stage_5W_vs_E10	ordinary_muscle_development	ENSGALG00010005417	LOXL3	1.258204432	0.163819658	7.680423994	1.58563E-14	4.85298E-14	positive
PRJNA729271	stage_5W_vs_E10	ordinary_muscle_development	ENSGALG00010005420	DHRS4	-0.405955472	0.120260483	-3.375634803	0.000736456	0.00112807	negative
PRJNA729271	stage_5W_vs_E10	ordinary_muscle_development	ENSGALG00010005421	ENSGALG00010005421	2.363217775	0.323488417	7.305416976	2.76408E-13	7.91337E-13	positive
PRJNA729271	stage_5W_vs_E10	ordinary_muscle_development	ENSGALG00010005423	ST3GAL6	1.488909538	0.149803751	9.93906714	2.8145E-23	1.29924E-22	positive
PRJNA729271	stage_5W_vs_E10	ordinary_muscle_development	ENSGALG00010005424	ENSGALG00010005424	1.052139441	0.134344733	7.831638959	4.81551E-15	1.51469E-14	positive
PRJNA729271	stage_5W_vs_E10	ordinary_muscle_development	ENSGALG00010005425	ELAPOR2	-0.721768484	0.252457707	-2.858967913	0.004250218	0.006048194	negative
PRJNA729271	stage_5W_vs_E10	ordinary_muscle_development	ENSGALG00010005426	ENSGALG00010005426	3.830246987	0.873064629	4.387128808	1.14857E-05	2.03751E-05	positive
PRJNA729271	stage_5W_vs_E10	ordinary_muscle_development	ENSGALG00010005427	ENSGALG00010005427	1.228426465	0.137654134	8.924007061	4.49715E-19	1.72177E-18	positive
PRJNA729271	stage_5W_vs_E10	ordinary_muscle_development	ENSGALG00010005428	ZNF770	0.048036564	0.10308811	0.465975796	0.641232825	0.67636951	positive
PRJNA729271	stage_5W_vs_E10	ordinary_muscle_development	ENSGALG00010005429	ZDHHC20	-1.676247145	0.118989807	-14.08731708	4.54503E-45	4.646E-44	negative
PRJNA729271	stage_5W_vs_E10	ordinary_muscle_development	ENSGALG00010005430	MCM4	-2.076246675	0.136603523	-15.19907123	3.5867E-52	4.63776E-51	negative
PRJNA729271	stage_5W_vs_E10	ordinary_muscle_development	ENSGALG00010005431	GRM3	-4.920934683	0.896825535	-5.487059064	4.08681E-08	8.65424E-08	negative
PRJNA729271	stage_5W_vs_E10	ordinary_muscle_development	ENSGALG00010005433	GPC5	-1.603543114	0.167038198	-9.599858796	8.0054E-22	3.47316E-21	negative
PRJNA729271	stage_5W_vs_E10	ordinary_muscle_development	ENSGALG00010005434	PRDM5	-1.437660794	0.222852241	-6.451183926	1.1098E-10	2.7536E-10	negative
PRJNA729271	stage_5W_vs_E10	ordinary_muscle_development	ENSGALG00010005435	MAD2L1	-2.889450347	0.176170256	-16.40146532	1.8668E-60	3.03016E-59	negative
PRJNA729271	stage_5W_vs_E10	ordinary_muscle_development	ENSGALG00010005436	AQR	-0.957212614	0.055591491	-17.2186892	1.92294E-66	3.69669E-65	negative
PRJNA729271	stage_5W_vs_E10	ordinary_muscle_development	ENSGALG00010005437	PDE7B	3.121702227	0.157787279	19.78424521	4.06945E-87	1.29074E-85	positive
PRJNA729271	stage_5W_vs_E10	ordinary_muscle_development	ENSGALG00010005438	GPR180	-0.625693708	0.140635144	-4.449056542	8.62483E-06	1.54399E-05	negative
PRJNA729271	stage_5W_vs_E10	ordinary_muscle_development	ENSGALG00010005439	TBX19	0.297895558	0.547248482	0.544351548	0.586199581	0.62427176	positive
PRJNA729271	stage_5W_vs_E10	ordinary_muscle_development	ENSGALG00010005440	ENSGALG00010005440	4.003666019	0.471971979	8.482846849	2.1975E-17	7.78415E-17	positive
PRJNA729271	stage_5W_vs_E10	ordinary_muscle_development	ENSGALG00010005441	UBE2V2	0.08406036	0.095491004	0.88029612	0.378698914	0.419891437	positive
PRJNA729271	stage_5W_vs_E10	ordinary_muscle_development	ENSGALG00010005442	SASH1	0.453257372	0.097623244	4.642924689	3.43512E-06	6.3397E-06	positive

PRJNA729271	stage_5W_vs_E10	ordinary_muscle_development	ENSGALG00010005443	BANK1	2.006733754	0.420514783	4.772088481	1.82325E-06	3.42865E-06	positive
PRJNA729271	stage_5W_vs_E10	ordinary_muscle_development	ENSGALG00010005444	ACTC1	-1.439696177	0.426603433	-3.374788072	0.000738726	0.001131427	negative
PRJNA729271	stage_5W_vs_E10	ordinary_muscle_development	ENSGALG00010005445	SFT2D2	-1.501258119	0.106946343	-14.03748909	9.19118E-45	9.27759E-44	negative
PRJNA729271	stage_5W_vs_E10	ordinary_muscle_development	ENSGALG00010005446	ENSGALG00010005446	3.084654716	0.371002105	8.31438603	9.22285E-17	3.17165E-16	positive
PRJNA729271	stage_5W_vs_E10	ordinary_muscle_development	ENSGALG00010005447	MYO3A	-3.481416131	0.468463882	-7.43155719	1.07327E-13	3.13839E-13	negative
PRJNA729271	stage_5W_vs_E10	ordinary_muscle_development	ENSGALG00010005448	ENSGALG00010005448	0.651302329	0.147491032	4.415877488	1.00601E-05	1.79384E-05	positive
PRJNA729271	stage_5W_vs_E10	ordinary_muscle_development	ENSGALG00010005449	TIPRL	-0.072283971	0.08637309	-0.836880693	0.402659634	0.444013038	negative
PRJNA729271	stage_5W_vs_E10	ordinary_muscle_development	ENSGALG00010005450	ENSGALG00010005450	3.875971999	0.412594772	9.39413744	5.76885E-21	2.40493E-20	positive
PRJNA729271	stage_5W_vs_E10	ordinary_muscle_development	ENSGALG00010005453	UST	0.283137203	0.114221194	2.478849964	0.013180674	0.017807239	positive
PRJNA729271	stage_5W_vs_E10	ordinary_muscle_development	ENSGALG00010005454	GPR161	-1.682163287	0.100124271	-16.80075435	2.4096E-63	4.26461E-62	negative
PRJNA729271	stage_5W_vs_E10	ordinary_muscle_development	ENSGALG00010005455	ENSGALG00010005455	2.6328006	0.678533895	3.880131296	0.0001044	0.00017168	positive
PRJNA729271	stage_5W_vs_E10	ordinary_muscle_development	ENSGALG00010005456	RPL7L1	-0.877076159	0.071019825	-12.34973691	4.8871E-35	3.60868E-34	negative
PRJNA729271	stage_5W_vs_E10	ordinary_muscle_development	ENSGALG00010005457	SEMA3D	-0.541556581	0.160315663	-3.378064067	0.000729981	0.001118506	negative
PRJNA729271	stage_5W_vs_E10	ordinary_muscle_development	ENSGALG00010005458	PRKD1	0.694409171	0.174344136	3.982979803	6.80565E-05	0.000113873	positive
PRJNA729271	stage_5W_vs_E10	ordinary_muscle_development	ENSGALG00010005459	ZC3H12D	5.472663443	0.501139777	10.92043316	9.20557E-28	5.10462E-27	positive
PRJNA729271	stage_5W_vs_E10	ordinary_muscle_development	ENSGALG00010005460	TGDS	-1.130467487	0.10347804	-10.92470907	8.78214E-28	4.87924E-27	negative
PRJNA729271	stage_5W_vs_E10	ordinary_muscle_development	ENSGALG00010005462	AHI1	0.457573033	0.151229901	3.025678314	0.002480761	0.003616113	positive
PRJNA729271	stage_5W_vs_E10	ordinary_muscle_development	ENSGALG00010005463	SKA3	-2.977596603	0.257886996	-11.54612932	7.72205E-31	4.83713E-30	negative
PRJNA729271	stage_5W_vs_E10	ordinary_muscle_development	ENSGALG00010005464	SLC39A8	-3.843638566	0.326614871	-11.76810644	5.69893E-32	3.73783E-31	negative
PRJNA729271	stage_5W_vs_E10	ordinary_muscle_development	ENSGALG00010005465	ENSGALG00010005465	-1.009344897	0.091876675	-10.98586667	4.46928E-28	2.51191E-27	negative
PRJNA729271	stage_5W_vs_E10	ordinary_muscle_development	ENSGALG00010005466	LATS2	0.592274193	0.089301944	6.632265399	3.30573E-11	8.50189E-11	positive
PRJNA729271	stage_5W_vs_E10	ordinary_muscle_development	ENSGALG00010005467	ENSGALG00010005467	3.754200297	0.250562545	14.98308653	9.47143E-51	1.1704E-49	positive
PRJNA729271	stage_5W_vs_E10	ordinary_muscle_development	ENSGALG00010005468	PPIL4	-0.60675174	0.105756652	-5.737244237	9.62294E-09	2.12597E-08	negative
PRJNA729271	stage_5W_vs_E10	ordinary_muscle_development	ENSGALG00010005470	SEMA3A	-1.841414287	0.141767094	-12.98901057	1.41241E-38	1.17189E-37	negative
PRJNA729271	stage_5W_vs_E10	ordinary_muscle_development	ENSGALG00010005472	PFKP	-0.95935297	0.114952588	-8.34564045	7.0829E-17	2.44852E-16	negative
PRJNA729271	stage_5W_vs_E10	ordinary_muscle_development	ENSGALG00010005473	ENSGALG00010005473	2.903258413	0.655766121	4.427277223	9.54301E-06	1.70394E-05	positive
PRJNA729271	stage_5W_vs_E10	ordinary_muscle_development	ENSGALG00010005474	COL8A1	1.944924961	0.343793156	5.657253294	1.53815E-08	3.36028E-08	positive
PRJNA729271	stage_5W_vs_E10	ordinary_muscle_development	ENSGALG00010005475	TMEM181	-0.330736315	0.092260797	-3.584797927	0.000337339	0.000531388	negative
PRJNA729271	stage_5W_vs_E10	ordinary_muscle_development	ENSGALG00010005476	NFKB1	1.481013238	0.114339991	12.9527143	2.26791E-38	1.87099E-37	positive
PRJNA729271	stage_5W_vs_E10	ordinary_muscle_development	ENSGALG00010005477	TMEM161B	0.336329668	0.161944987	2.076814316	0.0378187	0.048487907	positive
PRJNA729271	stage_5W_vs_E10	ordinary_muscle_development	ENSGALG00010005479	ENSGALG00010005479	0.098635103	0.626308936	0.157486341	0.874861572	0.890340412	positive
PRJNA729271	stage_5W_vs_E10	ordinary_muscle_development	ENSGALG00010005481	ENSGALG00010005481	-2.569091924	0.573779842	-4.477487246	7.55267E-06	1.35759E-05	negative
PRJNA729271	stage_5W_vs_E10	ordinary_muscle_development	ENSGALG00010005482	XPO4	-1.517462858	0.07655247	-19.82251992	1.90335E-87	6.13091E-86	negative
PRJNA729271	stage_5W_vs_E10	ordinary_muscle_development	ENSGALG00010005484	GINM1	1.23476421	0.122597747	10.07167132	7.37146E-24	3.4827E-23	positive
PRJNA729271	stage_5W_vs_E10	ordinary_muscle_development	ENSGALG00010005485	PITRM1	-0.602333514	0.0970913	-6.203784598	5.51212E-10	1.30895E-09	negative
PRJNA729271	stage_5W_vs_E10	ordinary_muscle_development	ENSGALG00010005486	IRF5	2.964484593	0.311402279	9.519790929	1.73528E-21	7.41316E-21	positive
PRJNA729271	stage_5W_vs_E10	ordinary_muscle_development	ENSGALG00010005487	ENSGALG00010005487	-1.73259084	0.212669322	-8.146877137	3.73443E-16	1.24581E-15	negative
PRJNA729271	stage_5W_vs_E10	ordinary_muscle_development	ENSGALG00010005489	G2E3	-1.479543443	0.086992728	-17.00766816	7.20489E-65	1.32364E-63	negative
PRJNA729271	stage_5W_vs_E10	ordinary_muscle_development	ENSGALG00010005490	DCBLD2	-0.981369618	0.11545876	-8.499741552	1.90013E-17	6.75722E-17	negative
PRJNA729271	stage_5W_vs_E10	ordinary_muscle_development	ENSGALG00010005491	GGACT	0.396760188	0.17289402	2.294817295	0.021743597	0.028605322	positive
PRJNA729271	stage_5W_vs_E10	ordinary_muscle_development	ENSGALG00010005492	TNPO3	0.42912311	0.096636558	4.440587689	8.97135E-06	1.60424E-05	positive
PRJNA729271	stage_5W_vs_E10	ordinary_muscle_development	ENSGALG00010005493	KLF6	0.9750396	0.181045875	5.385594111	7.22057E-08	1.50334E-07	positive
PRJNA729271	stage_5W_vs_E10	ordinary_muscle_development	ENSGALG00010005494	PPDPFL	0.085593538	0.445814123	0.19199378	0.847747078	0.866273803	positive
PRJNA729271	stage_5W_vs_E10	ordinary_muscle_development	ENSGALG00010005495	TXN	-1.172061135	0.146655897	-7.991912782	1.32861E-15	4.31508E-15	negative
PRJNA729271	stage_5W_vs_E10	ordinary_muscle_development	ENSGALG00010005497	MKX	-2.829868146	0.205527564	-13.76880111	3.92676E-43	3.76942E-42	negative
PRJNA729271	stage_5W_vs_E10	ordinary_muscle_development	ENSGALG00010005498	SMO	0.16866216	0.137199953	1.229316457	0.218953179	0.252886561	positive
PRJNA729271	stage_5W_vs_E10	ordinary_muscle_development	ENSGALG00010005499	PDE5A	-0.970450672	0.11314739	-8.57687193	9.74881E-18	3.51777E-17	negative
PRJNA729271	stage_5W_vs_E10	ordinary_muscle_development	ENSGALG00010005500	KATNA1	-0.634598289	0.132845569	-4.776962398	1.77963E-06	3.35053E-06	negative
PRJNA729271	stage_5W_vs_E10	ordinary_muscle_development	ENSGALG00010005502	SCFD1	-0.679276575	0.072782469	-9.332969635	1.02945E-20	4.23437E-20	negative
PRJNA729271	stage_5W_vs_E10	ordinary_muscle_development	ENSGALG00010005503	DYNLT1	-1.206946878	0.147437069	-8.186183358	2.69641E-16	9.05991E-16	negative
PRJNA729271	stage_5W_vs_E10	ordinary_muscle_development	ENSGALG00010005504	LATS1	-0.183691026	0.079742743	-2.303545376	0.021248177	0.027976413	negative
PRJNA729271	stage_5W_vs_E10	ordinary_muscle_development	ENSGALG00010005505	FABP2	0.517916744	0.978018022	0.529557464	0.596418792	0.63454862	positive
PRJNA729271	stage_5W_vs_E10	ordinary_muscle_development	ENSGALG00010005507	USP53	1.77364542	0.119170247	14.88329059	4.23164E-50	5.09449E-49	positive
PRJNA729271	stage_5W_vs_E10	ordinary_muscle_development	ENSGALG00010005508	SS18	-1.456543796	0.065793626	-22.1380685	1.3594E-108	6.6344E-107	negative
PRJNA729271	stage_5W_vs_E10	ordinary_muscle_development	ENSGALG00010005509	COCH	0.463157662	0.452244703	1.024130651	0.305773569	0.344568754	positive
PRJNA729271	stage_5W_vs_E10	ordinary_muscle_development	ENSGALG00010005510	MYOZ2	-1.728111507	0.319823851	-5.403322808	6.54176E-08	1.36554E-07	negative
PRJNA729271	stage_5W_vs_E10	ordinary_muscle_development	ENSGALG00010005511	AHCYL2	1.114487812	0.097739202	11.40266941	4.05492E-30	2.4644E-29	positive
PRJNA729271	stage_5W_vs_E10	ordinary_muscle_development	ENSGALG00010005512	SMARCAD1	-2.004135532	0.112976237	-17.73944311	2.07986E-70	4.43347E-69	negative
PRJNA729271	stage_5W_vs_E10	ordinary_muscle_development	ENSGALG00010005513	TMEM30CP	0.152581811	0.262573032	0.581102369	0.561171462	0.600796244	positive
PRJNA729271	stage_5W_vs_E10	ordinary_muscle_development	ENSGALG00010005514	EEF1AKMT1	0.422596478	0.143751357	2.939773838	0.003284519	0.004727815	positive
PRJNA729271	stage_5W_vs_E10	ordinary_muscle_development	ENSGALG00010005515	PCCA	-0.277910059	0.097711455	-2.844191181	0.004452432	0.006319998	negative
PRJNA729271	stage_5W_vs_E10	ordinary_muscle_development	ENSGALG00010005516	LARP4B	0.819719482	0.13178835	6.219969228	4.97252E-10	1.18411E-09	positive
PRJNA729271	stage_5W_vs_E10	ordinary_muscle_development	ENSGALG00010005518	HPGDS	-0.933276277	0.653798791	-1.427467119	0.153445296	0.181863415	negative
PRJNA729271	stage_5W_vs_E10	ordinary_muscle_development	ENSGALG00010005519	PTBP3	-0.435191875	0.251201888	-1.732438707	0.083195481	0.102049463	negative
PRJNA729271	stage_5W_vs_E10	ordinary_muscle_development	ENSGALG00010005521	SYTL3	3.203245366	0.381298735	8.400881181	4.43141E-17	1.54816E-16	positive
PRJNA729271	stage_5W_vs_E10	ordinary_muscle_development	ENSGALG00010005522	SYNPO2	2.249635794	0.178077436	12.63290756	1.39044E-36	1.08591E-35	positive
PRJNA729271	stage_5W_vs_E10	ordinary_muscle_development	ENSGALG00010005523	ENSGALG00010005523	4.476202435	0.595095361	7.52182377	5.40174E-14	1.61107E-13	positive
PRJNA729271	stage_5W_vs_E10	ordinary_muscle_development	ENSGALG00010005524	STRIP2	1.314456796	0.275317411	4.774332254	1.80304E-06	3.39285E-06	positive
PRJNA729271	stage_5W_vs_E10	ordinary_muscle_development	ENSGALG00010005526	STRN3	0.832815752	0.069629021	11.96075625	5.70401E-33	3.88167E-32	positive
PRJNA729271	stage_5W_vs_E10	ordinary_muscle_development	ENSGALG00010005527	NUP43	-1.088109084	0.097175129	-11.19740299	4.19862E-29	2.45696E-28	negative
PRJNA729271	stage_5W_vs_E10	ordinary_muscle_development	ENSGALG00010005529	ENSGALG00010005529	2.846845247	0.362444847	7.85456125	4.01174E-15	1.26827E-14	positive
PRJNA729271	stage_5W_vs_E10	ordinary_muscle_development	ENSGALG00010005530	IL17D	-0.48984782	0.211536783	-2.315662614	0.020576697	0.027149028	negative
PRJNA729271	stage_5W_vs_E10	ordinary_muscle_development	ENSGALG00010005531	SUSD1	0.729276217	0.253605897	2.875627997	0.004032246	0.005752132	positive
PRJNA729271	stage_5W_vs_E10	ordinary_muscle_development	ENSGALG00010005532	GTPBP4	-0.146075227	0.097760362	-1.494217324	0.135118826	0.161316809	negative
PRJNA729271	stage_5W_vs_E10	ordinary_muscle_development	ENSGALG00010005533	OPN4-1	-3.999750909	1.036284489	-3.859703539	0.000113525	0.000185979	negative
PRJNA729271	stage_5W_vs_E10	ordinary_muscle_development	ENSGALG00010005534	ENSGALG00010005534	-0.534561269	0.162970012	-3.280120444	0.001037628	0.001570965	negative
PRJNA729271	stage_5W_vs_E10	ordinary_muscle_development	ENSGALG00010005535	AP4S1	1.121602803	0.152407691	7.35922706	1.84978E-13	5.34222E-13	positive
PRJNA729271	stage_5W_vs_E10	ordinary_muscle_development	ENSGALG00010005536	TAF4B	-1.563016041	0.208208202	-7.506985932	6.05042E-14	1.79678E-13	negative
PRJNA729271	stage_5W_vs_E10	ordinary_muscle_development	ENSGALG00010005537	GRIN3A	-3.784989613	0.349251184	-10.83744248	2.28777E-27	1.24948E-26	negative
PRJNA729271	stage_5W_vs_E10	ordinary_muscle_development	ENSGALG00010005538	ENSGALG00010005538	2.776881868	0.315339472	8.806007852	1.29682E-18	4.86098E-18	positive
PRJNA729271	stage_5W_vs_E10	ordinary_muscle_development	ENSGALG00010005539	IFT88	-0.990692228	0.134077795	-7.388935856	1.48009E-13	4.29334E-13	negative
PRJNA729271	stage_5W_vs_E10	ordinary_muscle_development	ENSGALG00010005540	PCMT1	1.418966921	0.13711671	10.34860682	4.2462E-25	2.11653E-24	positive
PRJNA729271	stage_5W_vs_E10	ordinary_muscle_development	ENSGALG00010005542	KCTD1	-0.701427595	0.139390344	-5.032110367	4.8511E-07	9.52671E-07	negative
PRJNA729271	stage_5W_vs_E10	ordinary_muscle_development	ENSGALG00010005543	SEC24D	-0.845171759	0.202396954	-4.175812644	2.96924E-05	5.0958E-05	negative
PRJNA729271	stage_5W_vs_E10	ordinary_muscle_development	ENSGALG00010005544	AQP4	6.226049508	0.309037723	20.14656801	2.8843E-90	9.93063E-89	positive
PRJNA729271	stage_5W_vs_E10	ordinary_muscle_development	ENSGALG00010005545	RPL34	0.38894042	0.166919742	2.330104361	0.019800636	0.026178304	positive
PRJNA729271	stage_5W_vs_E10	ordinary_muscle_development	ENSGALG00010005546	NRF1	1.810977423	0.151464816	11.95642306	6.00952E-33	4.08766E-32	positive
PRJNA729271	stage_5W_vs_E10	ordinary_muscle_development	ENSGALG00010005547	LRP11	-1.487892975	0.224401211	-6.630503316	3.34544E-11	8.60095E-11	negative
PRJNA729271	stage_5W_vs_E10	ordinary_muscle_development	ENSGALG00010005548	EZR	0.140448164	0.177213849	0.792534926	0.428048836	0.469865041	positive
PRJNA729271	stage_5W_vs_E10	ordinary_muscle_development	ENSGALG00010005549	RNF20	-0.736318633	0.11409497	-6.453559088	1.09253E-10	2.71308E-10	negative
PRJNA729271	stage_5W_vs_E10	ordinary_muscle_development	ENSGALG00010005550	ENSGALG00010005550	2.978738965	0.538692418	5.529572839	3.21011E-08	6.84274E-08	positive
PRJNA729271	stage_5W_vs_E10	ordinary_muscle_development	ENSGALG00010005551	METTL14	-1.649346108	0.085474232	-19.29641324	5.75692E-83	1.66228E-81	negative
PRJNA729271	stage_5W_vs_E10	ordinary_muscle_development	ENSGALG00010005552	WDR37	0.455975601	0.085774146	5.316002804	1.06072E-07	2.18458E-07	positive
PRJNA729271	stage_5W_vs_E10	ordinary_muscle_development	ENSGALG00010005553	PPP1R14C	-0.195292779	0.166428575	-1.173432979	0.240622219	0.275935052	negative
PRJNA729271	stage_5W_vs_E10	ordinary_muscle_development	ENSGALG00010005554	CRYL1	2.512359915	0.161658111	15.54119304	1.82553E-54	2.48228E-53	positive
PRJNA729271	stage_5W_vs_E10	ordinary_muscle_development	ENSGALG00010005555	PRSS12	-4.65198187	0.192916712	-24.11393924	1.7853E-128	1.2685E-126	negative
PRJNA729271	stage_5W_vs_E10	ordinary_muscle_development	ENSGALG00010005556	IYD	-1.523108823	0.843110298	-1.806535667	0.070834708	0.087748833	negative
PRJNA729271	stage_5W_vs_E10	ordinary_muscle_development	ENSGALG00010005557	CMSS1	-0.44558741	0.168498176	-2.644464297	0.008182034	0.011300484	negative
PRJNA729271	stage_5W_vs_E10	ordinary_muscle_development	ENSGALG00010005558	TAGAP	3.127140415	0.20432231	15.30493861	7.08688E-53	9.28792E-52	positive
PRJNA729271	stage_5W_vs_E10	ordinary_muscle_development	ENSGALG00010005559	FILIP1L	0.383852762	0.131050087	2.929053846	0.003399955	0.004886214	positive
PRJNA729271	stage_5W_vs_E10	ordinary_muscle_development	ENSGALG00010005560	RSPH3	-1.101847106	0.379678299	-2.902054469	0.003707241	0.00530678	negative
PRJNA729271	stage_5W_vs_E10	ordinary_muscle_development	ENSGALG00010005561	CLYBL	0.947753817	0.197928198	4.78837187	1.6814E-06	3.17177E-06	positive
PRJNA729271	stage_5W_vs_E10	ordinary_muscle_development	ENSGALG00010005562	HECTD1	0.832920993	0.085379048	9.755566654	1.74623E-22	7.80498E-22	positive
PRJNA729271	stage_5W_vs_E10	ordinary_muscle_development	ENSGALG00010005564	TM9SF2	0.310323493	0.057930422	5.356831185	8.46942E-08	1.75553E-07	positive
PRJNA729271	stage_5W_vs_E10	ordinary_muscle_development	ENSGALG00010005565	PGAP4	-1.047049996	0.178351088	-5.870723918	4.33896E-09	9.77514E-09	negative
PRJNA729271	stage_5W_vs_E10	ordinary_muscle_development	ENSGALG00010005567	NDST3	-1.210237699	0.241765245	-5.005838197	5.56195E-07	1.08815E-06	negative
PRJNA729271	stage_5W_vs_E10	ordinary_muscle_development	ENSGALG00010005568	PLEKHG1	-0.270406324	0.133081997	-2.031877561	0.042166053	0.053689119	negative
PRJNA729271	stage_5W_vs_E10	ordinary_muscle_development	ENSGALG00010005572	ENSGALG00010005572	1.347373894	0.153588503	8.772622079	1.74553E-18	6.521E-18	positive
PRJNA729271	stage_5W_vs_E10	ordinary_muscle_development	ENSGALG00010005573	CHST9	0.201684721	0.283405663	0.711646758	0.476683545	0.518305302	positive
PRJNA729271	stage_5W_vs_E10	ordinary_muscle_development	ENSGALG00010005575	ENSGALG00010005575	3.807861489	0.435491896	8.743817101	2.25364E-18	8.36103E-18	positive
PRJNA729271	stage_5W_vs_E10	ordinary_muscle_development	ENSGALG00010005578	HEATR5A	0.286837001	0.120449371	2.381390617	0.017247412	0.022975943	positive
PRJNA729271	stage_5W_vs_E10	ordinary_muscle_development	ENSGALG00010005581	GR42L5	4.142960594	0.722085947	5.737489573	9.60902E-09	2.12321E-08	positive
PRJNA729271	stage_5W_vs_E10	ordinary_muscle_development	ENSGALG00010005583	ALDOB	-0.82950349	1.011553199	-0.820029526			negative
PRJNA729271	stage_5W_vs_E10	ordinary_muscle_development	ENSGALG00010005587	LEF1	-5.891434635	0.230293459	-25.58229253	2.4017E-144	2.1623E-142	negative
PRJNA729271	stage_5W_vs_E10	ordinary_muscle_development	ENSGALG00010005588	PLAGL1	-0.141718901	0.127552122	-1.111066588	0.266539688	0.303495112	negative
PRJNA729271	stage_5W_vs_E10	ordinary_muscle_development	ENSGALG00010005590	TBC1D23	-0.140681043	0.057196583	-2.459605709	0.013908973	0.018738783	negative
PRJNA729271	stage_5W_vs_E10	ordinary_muscle_development	ENSGALG00010005593	ENSGALG00010005593	4.037301217	1.051823904	3.838381314	0.000123848	0.00020216	positive
PRJNA729271	stage_5W_vs_E10	ordinary_muscle_development	ENSGALG00010005594	MRPL50	0.625684283	0.150354613	4.161390664	3.16315E-05	5.41438E-05	positive
PRJNA729271	stage_5W_vs_E10	ordinary_muscle_development	ENSGALG00010005596	PARD3	-0.202210855	0.054309024	-3.723338012	0.000196606	0.000316048	negative
PRJNA729271	stage_5W_vs_E10	ordinary_muscle_development	ENSGALG00010005597	ENSGALG00010005597	2.333525866	0.473009472	4.933359698	8.08271E-07	1.5642E-06	positive
PRJNA729271	stage_5W_vs_E10	ordinary_muscle_development	ENSGALG00010005601	TMEM215	-2.778288247	0.42745692	-6.499574846	8.05473E-11	2.0203E-10	negative
PRJNA729271	stage_5W_vs_E10	ordinary_muscle_development	ENSGALG00010005609	GJB6	-0.704228523	0.541107631	-1.301457386	0.193101942	0.225261797	negative
PRJNA729271	stage_5W_vs_E10	ordinary_muscle_development	ENSGALG00010005610	MTHFD1L	-0.854025913	0.190875901	-4.474246935	7.66811E-06	1.37748E-05	negative
PRJNA729271	stage_5W_vs_E10	ordinary_muscle_development	ENSGALG00010005617	DTD2	0.540743936	0.210098425	2.573764829	0.010059863	0.013775882	positive
PRJNA729271	stage_5W_vs_E10	ordinary_muscle_development	ENSGALG00010005618	PHACTR2	-0.671630292	0.068778734	-9.765086576	1.58976E-22	7.11881E-22	negative
PRJNA729271	stage_5W_vs_E10	ordinary_muscle_development	ENSGALG00010005619	CELF2	0.658488757	0.076814876	8.572411842	1.0134E-17	3.65585E-17	positive
PRJNA729271	stage_5W_vs_E10	ordinary_muscle_development	ENSGALG00010005621	CDH2	-2.441996757	0.143214701	-17.05129944	3.41848E-65	6.34455E-64	negative
PRJNA729271	stage_5W_vs_E10	ordinary_muscle_development	ENSGALG00010005629	NRP1	0.874912193	0.173204383	5.051328261	4.38748E-07	8.63966E-07	positive
PRJNA729271	stage_5W_vs_E10	ordinary_muscle_development	ENSGALG00010005630	NIT2	-0.447449666	0.166013335	-2.695263404	0.007033301	0.009779155	negative
PRJNA729271	stage_5W_vs_E10	ordinary_muscle_development	ENSGALG00010005632	ITGB1	0.330079872	0.093451738	3.532089167	0.00041229	0.000644329	positive
PRJNA729271	stage_5W_vs_E10	ordinary_muscle_development	ENSGALG00010005634	HADH	1.088111494	0.086688277	12.55200285	3.87581E-36	2.99307E-35	positive

PRJNA729271	stage_5W_vs_E10	ordinary_muscle_development	ENSGALG00010005636	ENSGALG00010005636	3.847557279	0.575216186	6.688889104	2.24871E-11	5.83305E-11	positive
PRJNA729271	stage_5W_vs_E10	ordinary_muscle_development	ENSGALG00010005637	TOPORS	-0.398036686	0.072881545	-5.461419466	4.72343E-08	9.9674E-08	negative
PRJNA729271	stage_5W_vs_E10	ordinary_muscle_development	ENSGALG00010005643	ENSGALG00010005643	-1.343890111	1.099622941	-1.222137208	0.221655744	0.255722701	negative
PRJNA729271	stage_5W_vs_E10	ordinary_muscle_development	ENSGALG00010005645	FUCA2	0.33175323	0.157270679	2.109441075	0.034906527	0.044955141	positive
PRJNA729271	stage_5W_vs_E10	ordinary_muscle_development	ENSGALG00010005649	TOMM70	-0.619397624	0.079399935	-7.800983984	6.14263E-15	1.92098E-14	negative
PRJNA729271	stage_5W_vs_E10	ordinary_muscle_development	ENSGALG00010005651	USP6NL	0.023359808	0.121479891	0.192293623	0.847512213	0.866094863	positive
PRJNA729271	stage_5W_vs_E10	ordinary_muscle_development	ENSGALG00010005656	CYP2U1	1.902919637	0.130022626	14.63529616	1.67246E-48	1.91487E-47	positive
PRJNA729271	stage_5W_vs_E10	ordinary_muscle_development	ENSGALG00010005660	UBAC2	1.291186958	0.100629755	12.83106532	1.09843E-37	8.88493E-37	positive
PRJNA729271	stage_5W_vs_E10	ordinary_muscle_development	ENSGALG00010005668	LNP1	-1.768742781	0.402870776	-4.390347697	1.1317E-05	2.00881E-05	negative
PRJNA729271	stage_5W_vs_E10	ordinary_muscle_development	ENSGALG00010005670	SGMS2	0.463024133	0.206050684	2.247137087	0.024631267	0.032237492	positive
PRJNA729271	stage_5W_vs_E10	ordinary_muscle_development	ENSGALG00010005675	GJA3	0.203382801	0.284674253	0.714440451	0.474954866	0.51661945	positive
PRJNA729271	stage_5W_vs_E10	ordinary_muscle_development	ENSGALG00010005679	TMEM45A	-2.33558549	0.208403181	-11.20705299	3.76522E-29	2.2078E-28	negative
PRJNA729271	stage_5W_vs_E10	ordinary_muscle_development	ENSGALG00010005680	ENSGALG00010005680	2.894291755	0.445246135	6.50043095	8.00902E-11	2.00919E-10	positive
PRJNA729271	stage_5W_vs_E10	ordinary_muscle_development	ENSGALG00010005681	EPC1	0.206936812	0.066583375	3.107935148	0.001883994	0.002775253	positive
PRJNA729271	stage_5W_vs_E10	ordinary_muscle_development	ENSGALG00010005688	ENSGALG00010005688	2.903937594	0.485702354	5.978841913	2.2473E-09	5.14118E-09	positive
PRJNA729271	stage_5W_vs_E10	ordinary_muscle_development	ENSGALG00010005692	SEC24B	0.680546375	0.143978967	4.726706903	2.2819E-06	4.2613E-06	positive
PRJNA729271	stage_5W_vs_E10	ordinary_muscle_development	ENSGALG00010005694	ENSGALG00010005694	-0.112053647	0.074276755	-1.508596415	0.131401951	0.157125167	negative
PRJNA729271	stage_5W_vs_E10	ordinary_muscle_development	ENSGALG00010005695	KIF5B	0.036016455	0.08363864	0.430619806	0.666744838	0.700933161	positive
PRJNA729271	stage_5W_vs_E10	ordinary_muscle_development	ENSGALG00010005696	NUBPL	-1.102363396	0.142305126	-7.746477095	9.4477E-15	2.93117E-14	negative
PRJNA729271	stage_5W_vs_E10	ordinary_muscle_development	ENSGALG00010005699	PRPF18	0.489948436	0.093074679	5.264035726	1.40927E-07	2.87953E-07	positive
PRJNA729271	stage_5W_vs_E10	ordinary_muscle_development	ENSGALG00010005704	AKAP12	-1.06842375	0.093738859	-11.39787448	4.28452E-30	2.5985E-29	negative
PRJNA729271	stage_5W_vs_E10	ordinary_muscle_development	ENSGALG00010005705	ECHDC3	1.58664679	0.122839238	12.91644927	3.63534E-38	2.98213E-37	positive
PRJNA729271	stage_5W_vs_E10	ordinary_muscle_development	ENSGALG00010005712	ENSGALG00010005712	0.630565188	0.108489596	5.812218066	6.16505E-09	1.3782E-08	positive
PRJNA729271	stage_5W_vs_E10	ordinary_muscle_development	ENSGALG00010005714	ARHGAP5	-0.793804469	0.068401894	-11.60500707	3.88655E-31	2.46761E-30	negative
PRJNA729271	stage_5W_vs_E10	ordinary_muscle_development	ENSGALG00010005716	ZBTB2	-0.473824251	0.112589266	-4.208431842	2.57149E-05	4.4363E-05	negative
PRJNA729271	stage_5W_vs_E10	ordinary_muscle_development	ENSGALG00010005720	COL25A1	-4.462901633	0.233501468	-19.1129489	1.97015E-81	5.49179E-80	negative
PRJNA729271	stage_5W_vs_E10	ordinary_muscle_development	ENSGALG00010005724	PROSER2	0.955330899	0.117608667	8.122963419	4.54936E-16	1.51246E-15	positive
PRJNA729271	stage_5W_vs_E10	ordinary_muscle_development	ENSGALG00010005738	ELAVL2	-4.288082761	0.531318193	-8.070649216	6.99253E-16	2.30095E-15	negative
PRJNA729271	stage_5W_vs_E10	ordinary_muscle_development	ENSGALG00010005740	ENSGALG00010005740	2.396117036	0.321617098	7.450216569	9.31871E-14	2.73431E-13	positive
PRJNA729271	stage_5W_vs_E10	ordinary_muscle_development	ENSGALG00010005742	GPR183	-0.950343274	0.211050687	-4.502914857	6.70277E-06	1.20812E-05	negative
PRJNA729271	stage_5W_vs_E10	ordinary_muscle_development	ENSGALG00010005743	ETNPPL	5.866656165	0.223270992	26.27594441	3.6126E-152	3.7403E-150	positive
PRJNA729271	stage_5W_vs_E10	ordinary_muscle_development	ENSGALG00010005748	AIG1	0.30705474	0.180114858	1.704771852	0.088236983	0.107891378	positive
PRJNA729271	stage_5W_vs_E10	ordinary_muscle_development	ENSGALG00010005752	RMND1	0.14553864	0.099097267	1.468644342	0.141929281	0.168905167	positive
PRJNA729271	stage_5W_vs_E10	ordinary_muscle_development	ENSGALG00010005755	ZMYM2	0.649444031	0.097191271	6.682123074	2.35505E-11	6.10015E-11	positive
PRJNA729271	stage_5W_vs_E10	ordinary_muscle_development	ENSGALG00010005756	TET2	0.482878297	0.164065163	2.943210412	0.003248276	0.004678898	positive
PRJNA729271	stage_5W_vs_E10	ordinary_muscle_development	ENSGALG00010005757	ARHGAP12	0.487138207	0.133522054	3.648372616	0.000263907	0.000419628	positive
PRJNA729271	stage_5W_vs_E10	ordinary_muscle_development	ENSGALG00010005758	HIVEP2	1.312214036	0.105270729	12.46513677	1.15673E-35	8.75916E-35	positive
PRJNA729271	stage_5W_vs_E10	ordinary_muscle_development	ENSGALG00010005760	GPR18	-1.400581292	0.340541591	-4.112805394	3.9088E-05	6.6461E-05	negative
PRJNA729271	stage_5W_vs_E10	ordinary_muscle_development	ENSGALG00010005763	AKAP6	2.228148549	0.142525033	15.63338384	4.31279E-55	5.97076E-54	positive
PRJNA729271	stage_5W_vs_E10	ordinary_muscle_development	ENSGALG00010005768	CXXC4	-5.52899519	0.952279097	-5.806065897	6.39578E-09	1.42714E-08	negative
PRJNA729271	stage_5W_vs_E10	ordinary_muscle_development	ENSGALG00010005769	TFG	-0.677495216	0.10643753	-6.365191089	1.95047E-10	4.76602E-10	negative
PRJNA729271	stage_5W_vs_E10	ordinary_muscle_development	ENSGALG00010005772	SVEP1	2.512817339	0.140705606	17.85868679	2.47392E-71	5.44149E-70	positive
PRJNA729271	stage_5W_vs_E10	ordinary_muscle_development	ENSGALG00010005777	ENSGALG00010005777	1.475687241	0.937463614	1.574127486	0.115457979	0.139127393	positive
PRJNA729271	stage_5W_vs_E10	ordinary_muscle_development	ENSGALG00010005779	ARMT1	0.39649309	0.111322377	3.561665686	0.000368509	0.000578841	positive
PRJNA729271	stage_5W_vs_E10	ordinary_muscle_development	ENSGALG00010005784	RASL11A	1.080201314	0.432777331	2.495974802	0.012561152	0.017001952	positive
PRJNA729271	stage_5W_vs_E10	ordinary_muscle_development	ENSGALG00010005785	RPL21	-0.024136462	0.139839412	-0.172601286	0.862964841	0.879918934	negative
PRJNA729271	stage_5W_vs_E10	ordinary_muscle_development	ENSGALG00010005789	RNASET2	0.72986572	0.154864871	4.712919827	2.44192E-06	4.55016E-06	positive
PRJNA729271	stage_5W_vs_E10	ordinary_muscle_development	ENSGALG00010005790	MUSK	-0.13925485	0.153871138	-0.905009554	0.365460326	0.406051466	negative
PRJNA729271	stage_5W_vs_E10	ordinary_muscle_development	ENSGALG00010005792	ADGRG6	-0.841858377	0.160207034	-5.25481532	1.48173E-07	3.02332E-07	negative
PRJNA729271	stage_5W_vs_E10	ordinary_muscle_development	ENSGALG00010005799	UFSP2	-0.413250583	0.098390082	-4.200124402	2.66768E-05	4.59568E-05	negative
PRJNA729271	stage_5W_vs_E10	ordinary_muscle_development	ENSGALG00010005800	FRMD4A	-0.750182264	0.109379128	-6.858550434	6.95628E-12	1.85488E-11	negative
PRJNA729271	stage_5W_vs_E10	ordinary_muscle_development	ENSGALG00010005814	UPF2	-0.035194583	0.124161454	-0.283458207	0.776825614	0.803431129	negative
PRJNA729271	stage_5W_vs_E10	ordinary_muscle_development	ENSGALG00010005817	VTA1	0.020091175	0.070048268	0.286819014	0.774250904	0.801111203	positive
PRJNA729271	stage_5W_vs_E10	ordinary_muscle_development	ENSGALG00010005821	DOCK9	1.272366184	0.153935315	8.265589897	1.39006E-16	4.73757E-16	positive
PRJNA729271	stage_5W_vs_E10	ordinary_muscle_development	ENSGALG00010005824	NMBR	-5.767573094	1.332630883	-4.327959952	1.50497E-05	2.64804E-05	negative
PRJNA729271	stage_5W_vs_E10	ordinary_muscle_development	ENSGALG00010005826	LRP2BP	-0.397021769	0.210084255	-1.88982163	0.05878182	0.07345827	negative
PRJNA729271	stage_5W_vs_E10	ordinary_muscle_development	ENSGALG00010005828	LPAR1	-1.92598182	0.156025922	-12.34398617	5.24913E-35	3.8701E-34	negative
PRJNA729271	stage_5W_vs_E10	ordinary_muscle_development	ENSGALG00010005829	NPAS3	-0.024045859	0.13515065	-0.177918933	0.858786642	0.876134035	negative
PRJNA729271	stage_5W_vs_E10	ordinary_muscle_development	ENSGALG00010005830	USP12	-0.121983528	0.185920679	-0.656105216	0.511756429	0.552421018	negative
PRJNA729271	stage_5W_vs_E10	ordinary_muscle_development	ENSGALG00010005831	SEC61A2	0.664561907	0.104971007	6.330909159	2.43721E-10	5.91648E-10	positive
PRJNA729271	stage_5W_vs_E10	ordinary_muscle_development	ENSGALG00010005836	NEBL	-0.087745615	0.245966487	-0.356738091	0.721287887	0.751676463	negative
PRJNA729271	stage_5W_vs_E10	ordinary_muscle_development	ENSGALG00010005839	RPS6KA2	-0.361061607	0.186540293	-1.935568991	0.052920511	0.066529299	negative
PRJNA729271	stage_5W_vs_E10	ordinary_muscle_development	ENSGALG00010005841	ENSGALG00010005841	2.096893752	0.307041782	6.829343351	8.53042E-12	2.26421E-11	positive
PRJNA729271	stage_5W_vs_E10	ordinary_muscle_development	ENSGALG00010005843	GPR12	-0.392895191	0.439079995	-0.894814602	0.370886203	0.411669769	negative
PRJNA729271	stage_5W_vs_E10	ordinary_muscle_development	ENSGALG00010005848	WASF3	1.265256547	0.187722779	6.740026739	1.58357E-11	4.1478E-11	positive
PRJNA729271	stage_5W_vs_E10	ordinary_muscle_development	ENSGALG00010005849	SNX25	0.097981815	0.098071045	0.999090148	0.317751023	0.35690051	positive
PRJNA729271	stage_5W_vs_E10	ordinary_muscle_development	ENSGALG00010005851	SLC15A1	0.248580348	0.909459713	0.273327498	0.784601479	0.810432441	positive
PRJNA729271	stage_5W_vs_E10	ordinary_muscle_development	ENSGALG00010005853	COMMD3	-0.906282488	0.143053358	-6.335275892	2.36917E-10	5.75517E-10	negative
PRJNA729271	stage_5W_vs_E10	ordinary_muscle_development	ENSGALG00010005858	CFAP97	-0.950521644	0.113930243	-8.343014262	7.24204E-17	2.50175E-16	negative
PRJNA729271	stage_5W_vs_E10	ordinary_muscle_development	ENSGALG00010005859	ENSGALG00010005859	1.972426338	0.517116025	3.814281981	0.000136579	0.00022234	positive
PRJNA729271	stage_5W_vs_E10	ordinary_muscle_development	ENSGALG00010005860	ABI3BP	1.302230717	0.212254546	6.135231225	8.50354E-10	1.99255E-09	positive
PRJNA729271	stage_5W_vs_E10	ordinary_muscle_development	ENSGALG00010005861	MPC1L	1.079454301	0.131893261	8.184302148	2.73886E-16	9.20042E-16	positive
PRJNA729271	stage_5W_vs_E10	ordinary_muscle_development	ENSGALG00010005866	DNAJC1	1.418001172	0.1476398	9.604464207	7.6555E-22	3.32733E-21	positive
PRJNA729271	stage_5W_vs_E10	ordinary_muscle_development	ENSGALG00010005871	EGLN3	1.536659558	0.195899793	7.84411016	4.36034E-15	1.37488E-14	positive
PRJNA729271	stage_5W_vs_E10	ordinary_muscle_development	ENSGALG00010005872	STK24	0.328071514	0.135451749	2.422054475	0.015433037	0.02068632	positive
PRJNA729271	stage_5W_vs_E10	ordinary_muscle_development	ENSGALG00010005875	CDK8	-1.002333912	0.155599037	-6.44177451	1.18085E-10	2.92338E-10	negative
PRJNA729271	stage_5W_vs_E10	ordinary_muscle_development	ENSGALG00010005879	FAM107B	-2.280028642	0.124874917	-18.25849981	1.7709E-74	4.24987E-73	negative
PRJNA729271	stage_5W_vs_E10	ordinary_muscle_development	ENSGALG00010005880	SLC25A4	5.610643399	0.179718963	31.21898384	5.8879E-214	1.1691E-211	positive
PRJNA729271	stage_5W_vs_E10	ordinary_muscle_development	ENSGALG00010005881	CORO2A	-0.185284443	0.279530425	-0.662841775	0.507431896	0.548324536	negative
PRJNA729271	stage_5W_vs_E10	ordinary_muscle_development	ENSGALG00010005884	MYB	-2.552915442	0.304049203	-8.396389203	4.60418E-17	1.60581E-16	negative
PRJNA729271	stage_5W_vs_E10	ordinary_muscle_development	ENSGALG00010005885	SLITRK5	0.713032492	0.381670076	1.868190713	0.061735493	0.076963616	positive
PRJNA729271	stage_5W_vs_E10	ordinary_muscle_development	ENSGALG00010005887	CCNH	-0.041921054	0.084920812	-0.493648764	0.621554255	0.658054848	negative
PRJNA729271	stage_5W_vs_E10	ordinary_muscle_development	ENSGALG00010005888	IMPG2	0.142470943	0.229232228	0.621513579	0.534261763	0.574404707	positive
PRJNA729271	stage_5W_vs_E10	ordinary_muscle_development	ENSGALG00010005890	SNX32	-0.659859938	0.089563699	-7.367493124	1.73866E-13	5.02732E-13	negative
PRJNA729271	stage_5W_vs_E10	ordinary_muscle_development	ENSGALG00010005891	RNF6	-0.159596564	0.118375632	-1.348221428	0.177587175	0.208414387	negative
PRJNA729271	stage_5W_vs_E10	ordinary_muscle_development	ENSGALG00010005892	ENSGALG00010005892	1.753912551	0.836261234	2.097326145	0.035964714	0.046231689	positive
PRJNA729271	stage_5W_vs_E10	ordinary_muscle_development	ENSGALG00010005893	SFT2D1	0.078876792	0.067154794	1.174551921	0.240174028	0.275464673	positive
PRJNA729271	stage_5W_vs_E10	ordinary_muscle_development	ENSGALG00010005895	MLLT10	0.652046481	0.099191783	6.573593717	4.91151E-11	1.24855E-10	positive
PRJNA729271	stage_5W_vs_E10	ordinary_muscle_development	ENSGALG00010005899	ENSGALG00010005899	-0.727875859	0.088839641	-8.193142765	2.54492E-16	8.55884E-16	negative
PRJNA729271	stage_5W_vs_E10	ordinary_muscle_development	ENSGALG00010005900	SPTSSA	-0.327549737	0.087728085	-3.733692979	0.000188692	0.000303765	negative
PRJNA729271	stage_5W_vs_E10	ordinary_muscle_development	ENSGALG00010005901	ENSGALG00010005901	0.482226049	0.24903372	1.936388573	0.052820128	0.066426146	positive
PRJNA729271	stage_5W_vs_E10	ordinary_muscle_development	ENSGALG00010005902	EAPP	-0.774747597	0.10370268	-7.470854115	7.96759E-14	2.34689E-13	negative
PRJNA729271	stage_5W_vs_E10	ordinary_muscle_development	ENSGALG00010005903	PRR18	-2.344127682	0.303452849	-7.724849814	1.11985E-14	3.45662E-14	negative
PRJNA729271	stage_5W_vs_E10	ordinary_muscle_development	ENSGALG00010005905	CITED2	0.545858431	0.20083265	2.717976534	0.00656825	0.009162427	positive
PRJNA729271	stage_5W_vs_E10	ordinary_muscle_development	ENSGALG00010005909	BRMS1L	-0.869984837	0.103276691	-8.423825604	3.64387E-17	1.27658E-16	negative
PRJNA729271	stage_5W_vs_E10	ordinary_muscle_development	ENSGALG00010005910	ENSGALG00010005910	2.665832888	0.424239202	6.283796671	3.30403E-10	7.9449E-10	positive
PRJNA729271	stage_5W_vs_E10	ordinary_muscle_development	ENSGALG00010005911	TBXT	-1.607522185	0.445272873	-3.610195641	0.000305966	0.000483901	negative
PRJNA729271	stage_5W_vs_E10	ordinary_muscle_development	ENSGALG00010005913	NFKBIA	2.271900852	0.142680739	15.92296807	4.39046E-57	6.49385E-56	positive
PRJNA729271	stage_5W_vs_E10	ordinary_muscle_development	ENSGALG00010005915	PAPSS1	-2.309613904	0.101392514	-22.7789391	7.4165E-115	4.1347E-113	negative
PRJNA729271	stage_5W_vs_E10	ordinary_muscle_development	ENSGALG00010005926	NUDT5	-0.20813212	0.235639629	-0.883264502	0.377093378	0.418175237	negative
PRJNA729271	stage_5W_vs_E10	ordinary_muscle_development	ENSGALG00010005934	SLITRK6	-6.625878969	0.364822779	-18.16191133	1.03363E-73	2.40488E-72	negative
PRJNA729271	stage_5W_vs_E10	ordinary_muscle_development	ENSGALG00010005935	ATP8A2	-3.205211887	0.2435603	-13.15982893	1.49424E-39	1.2854E-38	negative
PRJNA729271	stage_5W_vs_E10	ordinary_muscle_development	ENSGALG00010005941	TXLNB	4.413401057	0.181500766	24.31615669	1.3227E-130	9.6348E-129	positive
PRJNA729271	stage_5W_vs_E10	ordinary_muscle_development	ENSGALG00010005943	PROX2	-2.6451952	0.965077048	-2.740916079	0.006126815	0.008573047	negative
PRJNA729271	stage_5W_vs_E10	ordinary_muscle_development	ENSGALG00010005949	SLITRK1	0.386006436	0.362421073	1.06507724	0.286840978	0.324622109	positive
PRJNA729271	stage_5W_vs_E10	ordinary_muscle_development	ENSGALG00010005956	TXNL4B	-0.036285536	0.112217858	-0.323349037	0.746430921	0.774759484	negative
PRJNA729271	stage_5W_vs_E10	ordinary_muscle_development	ENSGALG00010005963	UGCG	1.26291229	0.103358348	12.21877394	2.46778E-34	1.76907E-33	positive
PRJNA729271	stage_5W_vs_E10	ordinary_muscle_development	ENSGALG00010005964	HSPA14	-0.557301104	0.114919586	-4.849487568	1.23781E-06	2.35893E-06	negative
PRJNA729271	stage_5W_vs_E10	ordinary_muscle_development	ENSGALG00010005966	PLXDC2	1.442317569	0.153173041	9.416262517	4.67431E-21	1.95934E-20	positive
PRJNA729271	stage_5W_vs_E10	ordinary_muscle_development	ENSGALG00010005967	GJD2	-2.315518446	0.280230604	-8.262903534	1.42171E-16	4.83863E-16	negative
PRJNA729271	stage_5W_vs_E10	ordinary_muscle_development	ENSGALG00010005969	HECA	0.709222695	0.065962371	10.75192848	5.80354E-27	3.12142E-26	positive
PRJNA729271	stage_5W_vs_E10	ordinary_muscle_development	ENSGALG00010005970	SHISA2	-3.961259	0.125245728	-31.62789705	1.5271E-219	3.2552E-217	negative
PRJNA729271	stage_5W_vs_E10	ordinary_muscle_development	ENSGALG00010005975	ABRACL	-0.678521228	0.10773607	-6.297994967	3.0152E-10	7.2709E-10	negative
PRJNA729271	stage_5W_vs_E10	ordinary_muscle_development	ENSGALG00010005978	VPS16	0.299136408	0.114569287	2.610965086	0.009028711	0.012410732	positive
PRJNA729271	stage_5W_vs_E10	ordinary_muscle_development	ENSGALG00010005979	SUV39H2	0.001857163	0.092737682	0.020025984	0.984022644	0.986268029	positive
PRJNA729271	stage_5W_vs_E10	ordinary_muscle_development	ENSGALG00010005981	AREL1	0.033772251	0.100211798	0.337008734	0.736110316	0.765417434	positive
PRJNA729271	stage_5W_vs_E10	ordinary_muscle_development	ENSGALG00010005984	IDH3B	1.677523683	0.11764229	14.25952931	3.91083E-46	4.12273E-45	positive
PRJNA729271	stage_5W_vs_E10	ordinary_muscle_development	ENSGALG00010005985	ENSGALG00010005985	-4.104181923	0.324820904	-12.63521489	1.35025E-36	1.05622E-35	negative
PRJNA729271	stage_5W_vs_E10	ordinary_muscle_development	ENSGALG00010005988	NUP58	-0.428360047	0.090077377	-4.755467614	1.97988E-06	3.71355E-06	negative
PRJNA729271	stage_5W_vs_E10	ordinary_muscle_development	ENSGALG00010005989	FCF1	-0.38425653	0.101202922	-3.796891651	0.000146522	0.000237885	negative
PRJNA729271	stage_5W_vs_E10	ordinary_muscle_development	ENSGALG00010005990	SLC2A12	2.467921586	0.150336437	16.4159909	1.46962E-60	2.39081E-59	positive
PRJNA729271	stage_5W_vs_E10	ordinary_muscle_development	ENSGALG00010005991	FARP1	-0.865109479	0.090121533	-9.599364914	8.04385E-22	3.4888E-21	negative
PRJNA729271	stage_5W_vs_E10	ordinary_muscle_development	ENSGALG00010005997	ENSGALG00010005997	1.095106025	0.23363091	4.687333638	2.76788E-06	5.14298E-06	positive
PRJNA729271	stage_5W_vs_E10	ordinary_muscle_development	ENSGALG00010005998	YLPM1	0.423144832	0.132998339	3.181579824	0.001464742	0.002183405	positive
PRJNA729271	stage_5W_vs_E10	ordinary_muscle_development	ENSGALG00010005999	PDE10A	0.644452801	0.293232312	2.197755069	0.027966565	0.03638917	positive
PRJNA729271	stage_5W_vs_E10	ordinary_muscle_development	ENSGALG00010006001	NOP56	-1.142015088	0.141009648	-8.098843593	5.54841E-16	1.83449E-15	negative
PRJNA729271	stage_5W_vs_E10	ordinary_muscle_development	ENSGALG00010006004	DCLRE1C	-1.17551972	0.155001341	-7.583932598	3.35235E-14	1.01003E-13	negative
PRJNA729271	stage_5W_vs_E10	ordinary_muscle_development	ENSGALG00010006009	MALRD1	1.784327585	0.60419885	2.953212482	0.003144854	0.00453669	positive
PRJNA729271	stage_5W_vs_E10	ordinary_muscle_development	ENSGALG00010006010	IPO5	-0.25737116	0.080853287	-3.183187325	0.001456633	0.002171989	negative
PRJNA729271	stage_5W_vs_E10	ordinary_muscle_development	ENSGALG00010006013	TRMT10C	-0.262181654	0.106600303	-2.45948319	0.013913721	0.018743438	negative
PRJNA729271	stage_5W_vs_E10	ordinary_muscle_development	ENSGALG00010006015	CDC123	-0.741150339	0.11277206	-6.572109599	4.96073E-11	1.26036E-10	negative

PRJNA729271	stage_5W_vs_E10	ordinary_muscle_development	ENSGALG00010006016	MTMR6	0.277119928	0.076759971	3.610214093	0.000305944	0.000483901	positive
PRJNA729271	stage_5W_vs_E10	ordinary_muscle_development	ENSGALG00010006019	SLC10A2	-4.697104875	1.18694319	-3.95731229	7.57978E-05	0.00012632	negative
PRJNA729271	stage_5W_vs_E10	ordinary_muscle_development	ENSGALG00010006021	ENSGALG00010006021	0.508856541	0.125552159	4.052949355	5.0576E-05	8.53035E-05	positive
PRJNA729271	stage_5W_vs_E10	ordinary_muscle_development	ENSGALG00010006022	AFG2A	-0.962752504	0.093074873	-10.34384975	4.46243E-25	2.22354E-24	negative
PRJNA729271	stage_5W_vs_E10	ordinary_muscle_development	ENSGALG00010006024	AMER2	-1.541851238	0.369706192	-4.170477187	3.03962E-05	5.21104E-05	negative
PRJNA729271	stage_5W_vs_E10	ordinary_muscle_development	ENSGALG00010006025	TNFAIP3	1.277539404	0.148648291	8.59437666	8.3718E-18	3.0307E-17	positive
PRJNA729271	stage_5W_vs_E10	ordinary_muscle_development	ENSGALG00010006028	ARL5B	-0.548944478	0.128622292	-4.267879773	1.9734E-05	3.43679E-05	negative
PRJNA729271	stage_5W_vs_E10	ordinary_muscle_development	ENSGALG00010006029	HSDL2	-0.551606699	0.135553005	-4.069306318	4.71533E-05	7.97163E-05	negative
PRJNA729271	stage_5W_vs_E10	ordinary_muscle_development	ENSGALG00010006031	ERCC5	0.473951854	0.097168408	4.877633212	1.07366E-06	2.05639E-06	positive
PRJNA729271	stage_5W_vs_E10	ordinary_muscle_development	ENSGALG00010006033	NSUN6	-0.497335792	0.177100379	-2.808214158	0.004981709	0.007033885	negative
PRJNA729271	stage_5W_vs_E10	ordinary_muscle_development	ENSGALG00010006035	ADH1C	1.533133437	0.693826884	2.209677186	0.027127574	0.035341919	positive
PRJNA729271	stage_5W_vs_E10	ordinary_muscle_development	ENSGALG00010006037	METTL21EP	8.006939076	0.674768623	11.86619947	1.77342E-32	1.18843E-31	positive
PRJNA729271	stage_5W_vs_E10	ordinary_muscle_development	ENSGALG00010006038	ENSGALG00010006038	0.522134505	0.195435293	2.671648994	0.007547956	0.010463621	positive
PRJNA729271	stage_5W_vs_E10	ordinary_muscle_development	ENSGALG00010006039	PCNP	-0.856322659	0.126211447	-6.784825625	1.16227E-11	3.06645E-11	negative
PRJNA729271	stage_5W_vs_E10	ordinary_muscle_development	ENSGALG00010006041	QKI	-0.011003123	0.08594608	-0.128023561	0.898130327	0.911078389	negative
PRJNA729271	stage_5W_vs_E10	ordinary_muscle_development	ENSGALG00010006047	ZBTB11	0.224815583	0.135071955	1.664413491	0.096029826	0.116941303	positive
PRJNA729271	stage_5W_vs_E10	ordinary_muscle_development	ENSGALG00010006049	AIMP1	-0.382926306	0.124617849	-3.072804655	0.002120573	0.003109529	negative
PRJNA729271	stage_5W_vs_E10	ordinary_muscle_development	ENSGALG00010006052	RALGAPA1	-0.348567137	0.081755233	-4.263545253	2.01209E-05	3.50165E-05	negative
PRJNA729271	stage_5W_vs_E10	ordinary_muscle_development	ENSGALG00010006053	TMEM243	-1.16635153	0.248063489	-4.701826669	2.57844E-06	4.79714E-06	negative
PRJNA729271	stage_5W_vs_E10	ordinary_muscle_development	ENSGALG00010006054	ENSGALG00010006054	1.733040106	2.255515043	0.768356705	0.442275295	0.484197915	positive
PRJNA729271	stage_5W_vs_E10	ordinary_muscle_development	ENSGALG00010006055	ENSGALG00010006055	2.449696752	0.567155475	4.319268456	1.56547E-05	2.74949E-05	positive
PRJNA729271	stage_5W_vs_E10	ordinary_muscle_development	ENSGALG00010006059	BIVM	-0.476243164	0.05648651	-8.431095617	3.42444E-17	1.20129E-16	negative
PRJNA729271	stage_5W_vs_E10	ordinary_muscle_development	ENSGALG00010006060	ENSGALG00010006060	1.175391202	0.351317801	3.345663661	0.000820859	0.001253118	positive
PRJNA729271	stage_5W_vs_E10	ordinary_muscle_development	ENSGALG00010006061	RPL24	-0.088480314	0.159075763	-0.556214928	0.578063936	0.616922158	negative
PRJNA729271	stage_5W_vs_E10	ordinary_muscle_development	ENSGALG00010006062	ENSGALG00010006062	1.857666469	0.645189891	2.879255387	0.003986154	0.00569086	positive
PRJNA729271	stage_5W_vs_E10	ordinary_muscle_development	ENSGALG00010006063	POGLUT2	-1.443155415	0.102897368	-14.0251927	1.09315E-44	1.09579E-43	negative
PRJNA729271	stage_5W_vs_E10	ordinary_muscle_development	ENSGALG00010006064	DMTF1	-0.043655288	0.082702442	-0.527859724	0.597596701	0.635615217	negative
PRJNA729271	stage_5W_vs_E10	ordinary_muscle_development	ENSGALG00010006065	WDR54	0.329227104	0.16061174	2.049832122	0.040380816	0.051538252	positive
PRJNA729271	stage_5W_vs_E10	ordinary_muscle_development	ENSGALG00010006066	ARHGAP18	0.221686218	0.120655138	1.837354148	0.066157631	0.082179695	positive
PRJNA729271	stage_5W_vs_E10	ordinary_muscle_development	ENSGALG00010006068	PACRG	0.266412674	0.274075126	0.97204251	0.331029401	0.370665984	positive
PRJNA729271	stage_5W_vs_E10	ordinary_muscle_development	ENSGALG00010006069	TTR	5.690681357	1.389530381	4.095399019	4.21442E-05	7.14647E-05	positive
PRJNA729271	stage_5W_vs_E10	ordinary_muscle_development	ENSGALG00010006070	TMEM242	0.551118563	0.114380497	4.818291395	1.44793E-06	2.74457E-06	positive
PRJNA729271	stage_5W_vs_E10	ordinary_muscle_development	ENSGALG00010006071	TEX30	-0.327405224	0.24411609	-1.341186581	0.179859882	0.21097912	negative
PRJNA729271	stage_5W_vs_E10	ordinary_muscle_development	ENSGALG00010006072	ENSGALG00010006072	0.303350282	0.233387452	1.299771173	0.193679408	0.225873604	positive
PRJNA729271	stage_5W_vs_E10	ordinary_muscle_development	ENSGALG00010006073	CEP97	-0.258000486	0.106852863	-2.414539769	0.015755103	0.021086816	negative
PRJNA729271	stage_5W_vs_E10	ordinary_muscle_development	ENSGALG00010006075	SOX21	-4.846086706	0.439854907	-11.01746651	3.14795E-28	1.77478E-27	negative
PRJNA729271	stage_5W_vs_E10	ordinary_muscle_development	ENSGALG00010006076	PODXL	3.286791832	0.163004199	20.16384765	2.03431E-90	7.03754E-89	positive
PRJNA729271	stage_5W_vs_E10	ordinary_muscle_development	ENSGALG00010006077	TBCK	-0.997984875	0.102350385	-9.750670494	1.83254E-22	8.18319E-22	negative
PRJNA729271	stage_5W_vs_E10	ordinary_muscle_development	ENSGALG00010006078	SPATA13	-1.953600262	0.168796046	-11.57373235	5.59928E-31	3.5303E-30	negative
PRJNA729271	stage_5W_vs_E10	ordinary_muscle_development	ENSGALG00010006079	ENSGALG00010006079	-0.000597753	0.801305003	-0.000745974	0.999404799	0.999473752	negative
PRJNA729271	stage_5W_vs_E10	ordinary_muscle_development	ENSGALG00010006080	ENSGALG00010006080	-0.415041166	0.206962297	-2.005395048	0.044920831	0.056975856	negative
PRJNA729271	stage_5W_vs_E10	ordinary_muscle_development	ENSGALG00010006081	ZDHHC14	0.285722079	0.078971836	3.618025018	0.00029686	0.000469963	positive
PRJNA729271	stage_5W_vs_E10	ordinary_muscle_development	ENSGALG00010006083	CACNB2	-0.673131226	0.173102182	-3.888635135	0.00010081	0.000165993	negative
PRJNA729271	stage_5W_vs_E10	ordinary_muscle_development	ENSGALG00010006084	TMEM68	-1.288504374	0.152914754	-8.426292053	3.56791E-17	1.25101E-16	negative
PRJNA729271	stage_5W_vs_E10	ordinary_muscle_development	ENSGALG00010006085	DLST	0.443235719	0.090221421	4.912754821	8.98056E-07	1.73103E-06	positive
PRJNA729271	stage_5W_vs_E10	ordinary_muscle_development	ENSGALG00010006086	ENSGALG00010006086	0.56399387	0.290523526	1.941301891	0.05222167	0.06571344	positive
PRJNA729271	stage_5W_vs_E10	ordinary_muscle_development	ENSGALG00010006087	ENSGALG00010006087	0.717409158	0.572939817	1.252154479	0.21051358	0.243916414	positive
PRJNA729271	stage_5W_vs_E10	ordinary_muscle_development	ENSGALG00010006091	RTKN	-1.564389478	0.104755616	-14.93370513	1.989E-50	2.42682E-49	negative
PRJNA729271	stage_5W_vs_E10	ordinary_muscle_development	ENSGALG00010006096	EDIL3	-0.789728827	0.237118541	-3.330523306	0.000866829	0.001320652	negative
PRJNA729271	stage_5W_vs_E10	ordinary_muscle_development	ENSGALG00010006100	RPS6KL1	-1.737682785	0.20963982	-8.288896556	1.14304E-16	3.90855E-16	negative
PRJNA729271	stage_5W_vs_E10	ordinary_muscle_development	ENSGALG00010006101	ENSGALG00010006101	0.919454303	0.114380659	8.038546978	9.091E-16	2.97526E-15	positive
PRJNA729271	stage_5W_vs_E10	ordinary_muscle_development	ENSGALG00010006103	NXPE3	-0.443789078	0.12041896	-3.685375433	0.000228366	0.000365239	negative
PRJNA729271	stage_5W_vs_E10	ordinary_muscle_development	ENSGALG00010006106	SHOC1	0.956581763	0.381596583	2.506788069	0.012183374	0.016514299	positive
PRJNA729271	stage_5W_vs_E10	ordinary_muscle_development	ENSGALG00010006107	PGF	3.060952556	0.190293523	16.08542692	3.22819E-58	4.94637E-57	positive
PRJNA729271	stage_5W_vs_E10	ordinary_muscle_development	ENSGALG00010006108	MKLN1	0.475461853	0.061351798	7.749762341	9.20647E-15	2.85817E-14	positive
PRJNA729271	stage_5W_vs_E10	ordinary_muscle_development	ENSGALG00010006111	WBP1	0.106908864	0.084685943	1.262415701	0.206799242	0.239977185	positive
PRJNA729271	stage_5W_vs_E10	ordinary_muscle_development	ENSGALG00010006113	EIF2B2	0.298393912	0.168285918	1.773136548	0.076206083	0.093993122	positive
PRJNA729271	stage_5W_vs_E10	ordinary_muscle_development	ENSGALG00010006118	NPNT	-3.969010706	0.212976454	-18.63591316	1.64326E-77	4.2534E-76	negative
PRJNA729271	stage_5W_vs_E10	ordinary_muscle_development	ENSGALG00010006120	MOGS	0.585816276	0.134873108	4.343462403	1.40254E-05	2.47443E-05	positive
PRJNA729271	stage_5W_vs_E10	ordinary_muscle_development	ENSGALG00010006121	ENSGALG00010006121	-0.756662433	0.327036657	-2.313693026	0.020684567	0.027273974	negative
PRJNA729271	stage_5W_vs_E10	ordinary_muscle_development	ENSGALG00010006123	MLH3	0.350639777	0.103113955	3.400507478	0.000672609	0.001034426	positive
PRJNA729271	stage_5W_vs_E10	ordinary_muscle_development	ENSGALG00010006126	ENSGALG00010006126	6.00077318	0.281224832	21.33799185	5.0414E-101	2.16198E-99	positive
PRJNA729271	stage_5W_vs_E10	ordinary_muscle_development	ENSGALG00010006128	ACYP1	1.712690951	0.118472094	14.45649259	2.2813E-47	2.50511E-46	positive
PRJNA729271	stage_5W_vs_E10	ordinary_muscle_development	ENSGALG00010006131	ENSGALG00010006131	0.694741839	0.127569521	5.445986108	5.15192E-08	1.08322E-07	positive
PRJNA729271	stage_5W_vs_E10	ordinary_muscle_development	ENSGALG00010006132	MIPEP	-1.030249025	0.135388849	-7.609555992	2.75039E-14	8.32295E-14	negative
PRJNA729271	stage_5W_vs_E10	ordinary_muscle_development	ENSGALG00010006134	K123	4.355884305	0.708208331	6.150569137	7.72054E-10	1.81259E-09	positive
PRJNA729271	stage_5W_vs_E10	ordinary_muscle_development	ENSGALG00010006135	ZC2HC1C	-0.414711345	0.150867789	-2.748839554	0.005980665	0.008378249	negative
PRJNA729271	stage_5W_vs_E10	ordinary_muscle_development	ENSGALG00010006139	GAREM1	0.333280532	0.129765111	2.568336983	0.010218776	0.013980289	positive
PRJNA729271	stage_5W_vs_E10	ordinary_muscle_development	ENSGALG00010006146	CCDC142	0.888443827	0.181518163	4.894517516	9.85472E-07	1.89448E-06	positive
PRJNA729271	stage_5W_vs_E10	ordinary_muscle_development	ENSGALG00010006147	ENSGALG00010006147	3.570727135	0.21539724	16.57740428	1.0152E-61	1.72109E-60	positive
PRJNA729271	stage_5W_vs_E10	ordinary_muscle_development	ENSGALG00010006151	ENSGALG00010006151	-1.07216353	0.168346064	-6.368806634	1.90505E-10	4.65974E-10	negative
PRJNA729271	stage_5W_vs_E10	ordinary_muscle_development	ENSGALG00010006155	TNFRSF19	-2.193824734	0.196151035	-11.1843648	4.86376E-29	2.83703E-28	negative
PRJNA729271	stage_5W_vs_E10	ordinary_muscle_development	ENSGALG00010006156	NFKBIZ	1.965314176	0.195621219	10.04652861	9.51607E-24	4.47697E-23	positive
PRJNA729271	stage_5W_vs_E10	ordinary_muscle_development	ENSGALG00010006157	SNX9	0.820190164	0.073409399	11.17282215	5.53925E-29	3.22585E-28	positive
PRJNA729271	stage_5W_vs_E10	ordinary_muscle_development	ENSGALG00010006163	ENSGALG00010006163	2.153932019	0.487518107	4.41815799	9.95456E-06	1.77568E-05	positive
PRJNA729271	stage_5W_vs_E10	ordinary_muscle_development	ENSGALG00010006172	ENSGALG00010006172	1.406745427	0.190512581	7.384002793	1.536E-13	4.45376E-13	positive
PRJNA729271	stage_5W_vs_E10	ordinary_muscle_development	ENSGALG00010006173	TBPL1	-0.011546082	0.082497138	-0.139957364	0.888693677	0.902508124	negative
PRJNA729271	stage_5W_vs_E10	ordinary_muscle_development	ENSGALG00010006175	IL2RA	7.391314169	0.491730157	15.03124034	4.58343E-51	5.71745E-50	positive
PRJNA729271	stage_5W_vs_E10	ordinary_muscle_development	ENSGALG00010006176	MMR1L3	3.576051385	0.213694822	16.73438485	7.36137E-63	1.28095E-61	positive
PRJNA729271	stage_5W_vs_E10	ordinary_muscle_development	ENSGALG00010006177	NEK9	-0.063815857	0.113797637	-0.560783672	0.574945019	0.613774344	negative
PRJNA729271	stage_5W_vs_E10	ordinary_muscle_development	ENSGALG00010006178	RPS23	-0.195147761	0.140902114	-1.384988172	0.166056084	0.196040311	negative
PRJNA729271	stage_5W_vs_E10	ordinary_muscle_development	ENSGALG00010006180	CD200L	3.232248855	1.100287835	2.937639365			positive
PRJNA729271	stage_5W_vs_E10	ordinary_muscle_development	ENSGALG00010006183	PRKN	1.921226536	0.138008762	13.92104752	4.71936E-44	4.67262E-43	positive
PRJNA729271	stage_5W_vs_E10	ordinary_muscle_development	ENSGALG00010006184	LBX2	-2.499231889	0.307821005	-8.119107683	4.69624E-16	1.55878E-15	negative
PRJNA729271	stage_5W_vs_E10	ordinary_muscle_development	ENSGALG00010006187	TCF21	-2.862805935	0.637835289	-4.488315373	7.17886E-06	1.29248E-05	negative
PRJNA729271	stage_5W_vs_E10	ordinary_muscle_development	ENSGALG00010006188	TMED10	-0.185317344	0.07499534	-2.471051463	0.013471643	0.018176623	negative
PRJNA729271	stage_5W_vs_E10	ordinary_muscle_development	ENSGALG00010006193	RBM17	-0.797023747	0.100263013	-7.949329683	1.87523E-15	6.0484E-15	negative
PRJNA729271	stage_5W_vs_E10	ordinary_muscle_development	ENSGALG00010006197	ENSGALG00010006197	-2.683993435	0.648673881	-4.137662258	3.50862E-05	5.98183E-05	negative
PRJNA729271	stage_5W_vs_E10	ordinary_muscle_development	ENSGALG00010006198	ENSGALG00010006198	2.680140326	0.670197006	3.99903357	6.36017E-05	0.000106739	positive
PRJNA729271	stage_5W_vs_E10	ordinary_muscle_development	ENSGALG00010006200	GSTCD	-1.556464908	0.105024734	-14.81998427	1.0881E-49	1.29704E-48	negative
PRJNA729271	stage_5W_vs_E10	ordinary_muscle_development	ENSGALG00010006201	SSTR1	-2.085836884	0.365375428	-5.708749747	1.13809E-08	2.5029E-08	negative
PRJNA729271	stage_5W_vs_E10	ordinary_muscle_development	ENSGALG00010006202	SACS	-1.111538849	0.200396323	-5.546702829	2.91107E-08	6.22635E-08	negative
PRJNA729271	stage_5W_vs_E10	ordinary_muscle_development	ENSGALG00010006203	MMR1L4	3.456438726	0.174101537	19.85300521	1.03803E-87	3.35106E-86	positive
PRJNA729271	stage_5W_vs_E10	ordinary_muscle_development	ENSGALG00010006205	CGGBP1	-0.662022214	0.074679786	-8.864811309	7.66337E-19	2.90179E-18	negative
PRJNA729271	stage_5W_vs_E10	ordinary_muscle_development	ENSGALG00010006207	ENSGALG00010006207	-0.613979329	0.212250918	-2.89270517	0.003819396	0.005459778	negative
PRJNA729271	stage_5W_vs_E10	ordinary_muscle_development	ENSGALG00010006212	FOS	3.782833544	0.366393996	10.32449655	5.46045E-25	2.71617E-24	positive
PRJNA729271	stage_5W_vs_E10	ordinary_muscle_development	ENSGALG00010006219	CLEC14A	2.021555032	0.182980642	11.04791749	2.24359E-28	1.27533E-27	positive
PRJNA729271	stage_5W_vs_E10	ordinary_muscle_development	ENSGALG00010006220	SGCG	1.26723788	0.171055685	7.408335379	1.27895E-13	3.72555E-13	positive
PRJNA729271	stage_5W_vs_E10	ordinary_muscle_development	ENSGALG00010006223	ATG10	-0.565825444	0.114956472	-4.922084266	8.56273E-07	1.65357E-06	negative
PRJNA729271	stage_5W_vs_E10	ordinary_muscle_development	ENSGALG00010006227	AGPAT4	-1.522408848	0.148329664	-10.26368432	1.02711E-24	5.04166E-24	negative
PRJNA729271	stage_5W_vs_E10	ordinary_muscle_development	ENSGALG00010006229	INTS12	-0.171993615	0.134101155	-1.282566253	0.19964408	0.232120072	negative
PRJNA729271	stage_5W_vs_E10	ordinary_muscle_development	ENSGALG00010006232	ZNF654	-0.154081478	0.104176315	-1.479045203	0.139128234	0.165803153	negative
PRJNA729271	stage_5W_vs_E10	ordinary_muscle_development	ENSGALG00010006236	C3orf38	0.286046516	0.085376635	3.350407462	0.000806928	0.001232239	positive
PRJNA729271	stage_5W_vs_E10	ordinary_muscle_development	ENSGALG00010006237	ENSGALG00010006237	2.5472422	0.295063372	8.6328648	5.98341E-18	2.18297E-17	positive
PRJNA729271	stage_5W_vs_E10	ordinary_muscle_development	ENSGALG00010006239	EYA4	1.621770179	0.16228013	9.993646025	1.62493E-23	7.57828E-23	positive
PRJNA729271	stage_5W_vs_E10	ordinary_muscle_development	ENSGALG00010006241	MRC1	2.213303664	0.244328234	9.058730639	1.31977E-19	5.18572E-19	positive
PRJNA729271	stage_5W_vs_E10	ordinary_muscle_development	ENSGALG00010006242	ENSGALG00010006242	1.59767609	0.532349097	3.001181177	0.002689345	0.003906018	positive
PRJNA729271	stage_5W_vs_E10	ordinary_muscle_development	ENSGALG00010006244	EPHA3	-2.28507863	0.162662939	-14.04793648	7.93115E-45	8.0393E-44	negative
PRJNA729271	stage_5W_vs_E10	ordinary_muscle_development	ENSGALG00010006245	PFKFB3	3.504667889	0.508482528	6.892405723	5.48566E-12	1.47004E-11	positive
PRJNA729271	stage_5W_vs_E10	ordinary_muscle_development	ENSGALG00010006246	SUPT20H	-0.197408752	0.100179811	-1.970544256	0.048776028	0.061570019	negative
PRJNA729271	stage_5W_vs_E10	ordinary_muscle_development	ENSGALG00010006249	C9orf72	0.488874818	0.104901343	4.660329432	3.15704E-06	5.8436E-06	positive
PRJNA729271	stage_5W_vs_E10	ordinary_muscle_development	ENSGALG00010006251	SEMA3E	-3.23578288	0.376919626	-8.584808679	9.09877E-18	3.28895E-17	negative
PRJNA729271	stage_5W_vs_E10	ordinary_muscle_development	ENSGALG00010006252	COX7C	1.104497114	0.136616664	8.08464413	6.2346E-16	2.05481E-15	positive
PRJNA729271	stage_5W_vs_E10	ordinary_muscle_development	ENSGALG00010006254	PROS1	1.341971973	0.137278773	9.775524226	1.43413E-22	6.43981E-22	positive
PRJNA729271	stage_5W_vs_E10	ordinary_muscle_development	ENSGALG00010006259	PPA2	0.500097065	0.087245124	5.732091863	9.91995E-09	2.19058E-08	positive
PRJNA729271	stage_5W_vs_E10	ordinary_muscle_development	ENSGALG00010006260	MAP3K4	1.202342533	0.158972759	7.563198511	3.93277E-14	1.18024E-13	positive
PRJNA729271	stage_5W_vs_E10	ordinary_muscle_development	ENSGALG00010006261	PCLO	-3.564431889	0.616876987	-5.778189112	7.55089E-09	1.6792E-08	negative
PRJNA729271	stage_5W_vs_E10	ordinary_muscle_development	ENSGALG00010006263	EXOSC8	-1.062912809	0.150944816	-7.041731127	1.89866E-12	5.22023E-12	negative
PRJNA729271	stage_5W_vs_E10	ordinary_muscle_development	ENSGALG00010006266	CENPW	-2.587262216	0.226384872	-11.42860028	3.0092E-30	1.83656E-29	negative
PRJNA729271	stage_5W_vs_E10	ordinary_muscle_development	ENSGALG00010006269	ENSGALG00010006269	2.394195007	1.251404174	1.913206825	0.05572158	0.069826602	positive
PRJNA729271	stage_5W_vs_E10	ordinary_muscle_development	ENSGALG00010006270	STAM	-0.261048634	0.093534676	-2.790928951	0.005255701	0.007402719	negative
PRJNA729271	stage_5W_vs_E10	ordinary_muscle_development	ENSGALG00010006273	ARL13B	-0.355124942	0.099195663	-3.580045029	0.000343535	0.000541019	negative
PRJNA729271	stage_5W_vs_E10	ordinary_muscle_development	ENSGALG00010006277	GNG10	-0.92894273	0.159959446	-5.80736401	6.34641E-09	1.41721E-08	negative
PRJNA729271	stage_5W_vs_E10	ordinary_muscle_development	ENSGALG00010006279	HACD1	3.562219342	0.141360918	25.19946388	4.0602E-140	3.3824E-138	positive
PRJNA729271	stage_5W_vs_E10	ordinary_muscle_development	ENSGALG00010006281	TRMT11	0.138792635	0.081456984	1.703876425	0.088404176	0.10808186	positive
PRJNA729271	stage_5W_vs_E10	ordinary_muscle_development	ENSGALG00010006283	DNAJC25	0.286229166	0.107364706	2.665952115	0.007677063	0.010634463	positive
PRJNA729271	stage_5W_vs_E10	ordinary_muscle_development	ENSGALG00010006285	FZD8	-1.156081443	0.155692004	-7.42543875	1.12407E-13	3.28495E-13	negative
PRJNA729271	stage_5W_vs_E10	ordinary_muscle_development	ENSGALG00010006287	ECPAS	1.652023937	0.122367923	13.50046563	1.55391E-41	1.42196E-40	positive
PRJNA729271	stage_5W_vs_E10	ordinary_muscle_development	ENSGALG00010006288	ARF5	-3.149052402	0.18710336	-16.83054971	1.45743E-63	2.60165E-62	negative
PRJNA729271	stage_5W_vs_E10	ordinary_muscle_development	ENSGALG00010006289	SKIDA1	-2.286390916	0.163013044	-14.02581576	1.08359E-44	1.08696E-43	negative
PRJNA729271	stage_5W_vs_E10	ordinary_muscle_development	ENSGALG00010006291	ENSGALG00010006291	0.14604116	0.210864561	0.692582763	0.488571431	0.530038387	positive

PRJNA729271	stage_5W_vs_E10	ordinary_muscle_development	ENSGALG00010006293	SLC22A3	0.692678201	0.355872371	1.946423094	0.051603938	0.064981242	positive
PRJNA729271	stage_5W_vs_E10	ordinary_muscle_development	ENSGALG00010006300	GCC1	-0.138984471	0.120710133	-1.151390253	0.249571722	0.285452703	negative
PRJNA729271	stage_5W_vs_E10	ordinary_muscle_development	ENSGALG00010006301	ENSGALG00010006301	0.751226491	0.146128278	5.140870081	2.73469E-07	5.46072E-07	positive
PRJNA729271	stage_5W_vs_E10	ordinary_muscle_development	ENSGALG00010006310	ALG5	-0.464395358	0.111512466	-4.16451519	3.12015E-05	5.34403E-05	negative
PRJNA729271	stage_5W_vs_E10	ordinary_muscle_development	ENSGALG00010006311	ABCD4	-0.359351132	0.117094441	-3.06890001	0.002148485	0.003147275	negative
PRJNA729271	stage_5W_vs_E10	ordinary_muscle_development	ENSGALG00010006323	ANAPC10	0.109319255	0.106364427	1.027780221	0.304053222	0.342710067	positive
PRJNA729271	stage_5W_vs_E10	ordinary_muscle_development	ENSGALG00010006327	DENND6B	-0.39276329	0.113808435	-3.451091218	0.000558325	0.000864166	negative
PRJNA729271	stage_5W_vs_E10	ordinary_muscle_development	ENSGALG00010006328	SYNDIG1L	-2.149431301	0.45316315	-4.743173189	2.10396E-06	3.94017E-06	negative
PRJNA729271	stage_5W_vs_E10	ordinary_muscle_development	ENSGALG00010006330	STX19	-0.702544018	0.426568043	-1.646968239	0.099564574	0.120961236	negative
PRJNA729271	stage_5W_vs_E10	ordinary_muscle_development	ENSGALG00010006331	SMAD9	-1.098331246	0.157486741	-6.974118816	3.07794E-12	8.35795E-12	negative
PRJNA729271	stage_5W_vs_E10	ordinary_muscle_development	ENSGALG00010006336	ALDH8A1	1.642042464	0.863510661	1.901589105	0.057224893	0.071629951	positive
PRJNA729271	stage_5W_vs_E10	ordinary_muscle_development	ENSGALG00010006339	ENSGALG00010006339	2.326889986	0.737823176	3.153723092	0.00161202	0.00239261	positive
PRJNA729271	stage_5W_vs_E10	ordinary_muscle_development	ENSGALG00010006341	HHIP	-0.173856847	0.218633353	-0.795198192	0.426498224	0.468269334	negative
PRJNA729271	stage_5W_vs_E10	ordinary_muscle_development	ENSGALG00010006342	RFXAP	0.080835115	0.084868023	0.952480236	0.340853485	0.380578591	positive
PRJNA729271	stage_5W_vs_E10	ordinary_muscle_development	ENSGALG00010006343	SEC23A	0.101817305	0.094789219	1.074144361	0.282757985	0.320376533	positive
PRJNA729271	stage_5W_vs_E10	ordinary_muscle_development	ENSGALG00010006344	HINT3	0.620578767	0.105211362	5.898400678	3.67042E-09	8.30514E-09	positive
PRJNA729271	stage_5W_vs_E10	ordinary_muscle_development	ENSGALG00010006345	SMC2	-3.096129079	0.250478366	-12.36086427	4.25557E-35	3.15038E-34	negative
PRJNA729271	stage_5W_vs_E10	ordinary_muscle_development	ENSGALG00010006346	NSUN3	-0.431817473	0.135751383	-3.180943453	0.001467963	0.002187982	negative
PRJNA729271	stage_5W_vs_E10	ordinary_muscle_development	ENSGALG00010006347	PPP6R2	1.733053708	0.135520531	12.78812664	1.91023E-37	1.53741E-36	positive
PRJNA729271	stage_5W_vs_E10	ordinary_muscle_development	ENSGALG00010006351	IGF2R	1.379477697	0.173961099	7.929805592	2.19489E-15	7.05119E-15	positive
PRJNA729271	stage_5W_vs_E10	ordinary_muscle_development	ENSGALG00010006352	GEMIN2	0.554896748	0.097396276	5.697309684	1.21713E-08	2.67307E-08	positive
PRJNA729271	stage_5W_vs_E10	ordinary_muscle_development	ENSGALG00010006353	ABCE1	-0.955058532	0.081174807	-11.76545493	5.88085E-32	3.85182E-31	negative
PRJNA729271	stage_5W_vs_E10	ordinary_muscle_development	ENSGALG00010006355	PTGR1	-1.006859095	0.152705828	-6.593455574	4.29705E-11	1.09716E-10	negative
PRJNA729271	stage_5W_vs_E10	ordinary_muscle_development	ENSGALG00010006359	SGK1	1.908688702	0.163755239	11.65574128	2.14506E-31	1.37395E-30	positive
PRJNA729271	stage_5W_vs_E10	ordinary_muscle_development	ENSGALG00010006361	SERTM1	-1.691115032	0.26265469	-6.438548759	1.20621E-10	2.98413E-10	negative
PRJNA729271	stage_5W_vs_E10	ordinary_muscle_development	ENSGALG00010006363	TRAPPC6A	-0.258657732	0.102896901	-2.513756298	0.011945295	0.016213788	negative
PRJNA729271	stage_5W_vs_E10	ordinary_muscle_development	ENSGALG00010006372	SBF1	0.477203512	0.113324312	4.210954412	2.54294E-05	4.39122E-05	positive
PRJNA729271	stage_5W_vs_E10	ordinary_muscle_development	ENSGALG00010006378	CAAP1	-1.168656668	0.104851979	-11.14577597	7.50869E-29	4.34311E-28	negative
PRJNA729271	stage_5W_vs_E10	ordinary_muscle_development	ENSGALG00010006381	SPART	1.569666362	0.156497191	10.02999703	1.1252E-23	5.27312E-23	positive
PRJNA729271	stage_5W_vs_E10	ordinary_muscle_development	ENSGALG00010006382	PNN	-0.47474459	0.196856202	-2.411631364	0.01588133	0.021249873	negative
PRJNA729271	stage_5W_vs_E10	ordinary_muscle_development	ENSGALG00010006387	OTUD4	-0.113409589	0.135894306	-0.834542609	0.403975287	0.445429925	negative
PRJNA729271	stage_5W_vs_E10	ordinary_muscle_development	ENSGALG00010006404	SMAD1	-0.283746256	0.109792702	-2.584381745	0.009755375	0.013379143	negative
PRJNA729271	stage_5W_vs_E10	ordinary_muscle_development	ENSGALG00010006408	ENSGALG00010006408	-0.475307367	0.671594793	-0.707729381	0.479113328	0.520751927	negative
PRJNA729271	stage_5W_vs_E10	ordinary_muscle_development	ENSGALG00010006411	RGS17	-1.366672011	0.391654728	-3.489481712	0.000483958	0.000752034	negative
PRJNA729271	stage_5W_vs_E10	ordinary_muscle_development	ENSGALG00010006421	LMF2	-0.907894125	0.113468455	-8.001290993	1.23121E-15	4.00864E-15	negative
PRJNA729271	stage_5W_vs_E10	ordinary_muscle_development	ENSGALG00010006434	MMAA	-0.004992913	0.117672096	-0.042430727	0.966155334	0.970843782	negative
PRJNA729271	stage_5W_vs_E10	ordinary_muscle_development	ENSGALG00010006446	LSAMP	-1.226776094	0.185529809	-6.612285642	3.78431E-11	9.69487E-11	negative
PRJNA729271	stage_5W_vs_E10	ordinary_muscle_development	ENSGALG00010006454	SOD2	1.487359946	0.105366044	14.1161222	3.02167E-45	3.10412E-44	positive
PRJNA729271	stage_5W_vs_E10	ordinary_muscle_development	ENSGALG00010006457	NCAPH2	-2.916898153	0.213223048	-13.68003217	1.33634E-42	1.26437E-41	negative
PRJNA729271	stage_5W_vs_E10	ordinary_muscle_development	ENSGALG00010006463	SCO2	-0.070267234	0.136698501	-0.514030758	0.607230486	0.644396068	negative
PRJNA729271	stage_5W_vs_E10	ordinary_muscle_development	ENSGALG00010006465	PLAA	0.26322772	0.116034351	2.26853271	0.023296758	0.030557099	positive
PRJNA729271	stage_5W_vs_E10	ordinary_muscle_development	ENSGALG00010006472	DCLK1	-2.039109586	0.324942344	-6.27529659	3.48968E-10	8.38577E-10	negative
PRJNA729271	stage_5W_vs_E10	ordinary_muscle_development	ENSGALG00010006478	ENSGALG00010006478	0.451890783	0.13225254	3.416877921	0.000633437	0.000976461	positive
PRJNA729271	stage_5W_vs_E10	ordinary_muscle_development	ENSGALG00010006480	IFT74	-0.521455268	0.119332897	-4.369752858	1.24387E-05	2.20226E-05	negative
PRJNA729271	stage_5W_vs_E10	ordinary_muscle_development	ENSGALG00010006486	CEP43	-0.10098395	0.113048316	-0.893281326	0.371706535	0.41251713	negative
PRJNA729271	stage_5W_vs_E10	ordinary_muscle_development	ENSGALG00010006492	GAP43	-4.91780357	0.514026738	-9.56721355	1.09826E-21	4.73083E-21	negative
PRJNA729271	stage_5W_vs_E10	ordinary_muscle_development	ENSGALG00010006496	UBE2H	0.800703712	0.113835719	7.033852958	2.00907E-12	5.51752E-12	positive
PRJNA729271	stage_5W_vs_E10	ordinary_muscle_development	ENSGALG00010006499	CCR6	2.201627432	0.679109186	3.241934402	0.001187213	0.001785501	positive
PRJNA729271	stage_5W_vs_E10	ordinary_muscle_development	ENSGALG00010006503	LRRC19	5.777181668	1.004764395	5.749787411	8.93557E-09	1.97893E-08	positive
PRJNA729271	stage_5W_vs_E10	ordinary_muscle_development	ENSGALG00010006506	ZNF827	-0.278793476	0.105826394	-2.634441804	0.008427575	0.011630743	negative
PRJNA729271	stage_5W_vs_E10	ordinary_muscle_development	ENSGALG00010006515	FBXO33	0.085230547	0.103468875	0.82373126	0.410092273	0.451659259	positive
PRJNA729271	stage_5W_vs_E10	ordinary_muscle_development	ENSGALG00010006516	NBEA	-0.819110504	0.127151606	-6.441998856	1.1791E-10	2.92005E-10	negative
PRJNA729271	stage_5W_vs_E10	ordinary_muscle_development	ENSGALG00010006517	ZBTB20	4.422670698	0.144376096	30.63298436	4.4537E-206	7.9699E-204	positive
PRJNA729271	stage_5W_vs_E10	ordinary_muscle_development	ENSGALG00010006521	ZNF410	-0.683324936	0.104670968	-6.528313903	6.65142E-11	1.67761E-10	negative
PRJNA729271	stage_5W_vs_E10	ordinary_muscle_development	ENSGALG00010006524	TAB2	-0.021062655	0.09182397	-0.22938079	0.818572966	0.840015231	negative
PRJNA729271	stage_5W_vs_E10	ordinary_muscle_development	ENSGALG00010006526	LSM6	-0.767456565	0.160940687	-4.768567728	1.8554E-06	3.48775E-06	negative
PRJNA729271	stage_5W_vs_E10	ordinary_muscle_development	ENSGALG00010006528	ZC3HC1	-0.486299858	0.083478466	-5.825452702	5.6958E-09	1.27546E-08	negative
PRJNA729271	stage_5W_vs_E10	ordinary_muscle_development	ENSGALG00010006531	FAM161B	-0.865106147	0.14372295	-6.019262374	1.75214E-09	4.03387E-09	negative
PRJNA729271	stage_5W_vs_E10	ordinary_muscle_development	ENSGALG00010006532	KLHDC10	0.642186241	0.102690309	6.253620692	4.01044E-10	9.60214E-10	positive
PRJNA729271	stage_5W_vs_E10	ordinary_muscle_development	ENSGALG00010006537	DRD3	-4.726938701	0.5871526	-8.050613597	8.238E-16	2.7028E-15	negative
PRJNA729271	stage_5W_vs_E10	ordinary_muscle_development	ENSGALG00010006538	TEK	1.862861732	0.104617067	17.80648019	6.29459E-71	1.36383E-69	positive
PRJNA729271	stage_5W_vs_E10	ordinary_muscle_development	ENSGALG00010006542	RFC3	-1.934450526	0.142528021	-13.57242259	5.83607E-42	5.39846E-41	negative
PRJNA729271	stage_5W_vs_E10	ordinary_muscle_development	ENSGALG00010006544	MOB3B	-2.915192731	0.162965106	-17.88844744	1.45095E-71	3.21583E-70	negative
PRJNA729271	stage_5W_vs_E10	ordinary_muscle_development	ENSGALG00010006546	SMOC2	-0.126471728	0.208353741	-0.607004836	0.543847703	0.58362867	negative
PRJNA729271	stage_5W_vs_E10	ordinary_muscle_development	ENSGALG00010006548	TMEM209	-0.956678375	0.082301466	-11.6240746	3.10945E-31	1.98029E-30	negative
PRJNA729271	stage_5W_vs_E10	ordinary_muscle_development	ENSGALG00010006549	ZDHHC23	-0.026971487	0.181379226	-0.148702186	0.881788636	0.896257365	negative
PRJNA729271	stage_5W_vs_E10	ordinary_muscle_development	ENSGALG00010006552	CPA2	5.797635224	1.660306383	3.491906845	0.000479585	0.000745559	positive
PRJNA729271	stage_5W_vs_E10	ordinary_muscle_development	ENSGALG00010006557	COQ6	0.10102825	0.10987391	0.91949263	0.357837959	0.398040152	positive
PRJNA729271	stage_5W_vs_E10	ordinary_muscle_development	ENSGALG00010006558	USP10	-0.820229429	0.089190281	-9.196399159	3.70146E-20	1.48911E-19	negative
PRJNA729271	stage_5W_vs_E10	ordinary_muscle_development	ENSGALG00010006564	SLC10A7	-1.108908664	0.14452148	-7.67296781	1.68062E-14	5.1307E-14	negative
PRJNA729271	stage_5W_vs_E10	ordinary_muscle_development	ENSGALG00010006566	CPA5	6.628358029	2.807407122	2.361024867	0.018224509	0.024215258	positive
PRJNA729271	stage_5W_vs_E10	ordinary_muscle_development	ENSGALG00010006568	ENTPD5	-0.584310323	0.199643512	-2.926768404	0.003425038	0.004921286	negative
PRJNA729271	stage_5W_vs_E10	ordinary_muscle_development	ENSGALG00010006570	ENSGALG00010006570	2.410621226	0.479870449	5.023483384	5.07426E-07	9.94745E-07	positive
PRJNA729271	stage_5W_vs_E10	ordinary_muscle_development	ENSGALG00010006574	CPA1	7.251333669	2.556840788	2.836052094	0.0045675	0.006473006	positive
PRJNA729271	stage_5W_vs_E10	ordinary_muscle_development	ENSGALG00010006575	SSBP2	-1.438071282	0.117858256	-12.20170174	3.04395E-34	2.1735E-33	negative
PRJNA729271	stage_5W_vs_E10	ordinary_muscle_development	ENSGALG00010006576	CCDC191	-0.129549053	0.114231144	-1.13409573	0.256754395	0.292997556	negative
PRJNA729271	stage_5W_vs_E10	ordinary_muscle_development	ENSGALG00010006580	ALDH6A1	0.830472384	0.138821165	5.982318213	2.19984E-09	5.03421E-09	positive
PRJNA729271	stage_5W_vs_E10	ordinary_muscle_development	ENSGALG00010006581	CEP41	-1.443072368	0.114082618	-12.64936226	1.12787E-36	8.85613E-36	negative
PRJNA729271	stage_5W_vs_E10	ordinary_muscle_development	ENSGALG00010006585	TTC29	-0.435572856	0.187478742	-2.323318643	0.020162041	0.026621314	negative
PRJNA729271	stage_5W_vs_E10	ordinary_muscle_development	ENSGALG00010006588	ACOT12	-2.056604744	0.774472607	-2.65549062	0.007919314	0.010958516	negative
PRJNA729271	stage_5W_vs_E10	ordinary_muscle_development	ENSGALG00010006592	STARD13	-0.206363094	0.114615562	-1.800480584	0.071784787	0.088849841	negative
PRJNA729271	stage_5W_vs_E10	ordinary_muscle_development	ENSGALG00010006598	ENSGALG00010006598	0.578836988	0.151871025	3.811372094	0.000138198	0.000224874	positive
PRJNA729271	stage_5W_vs_E10	ordinary_muscle_development	ENSGALG00010006602	EDNRA	-0.964238463	0.118715824	-8.122240384	4.57656E-16	1.52115E-15	negative
PRJNA729271	stage_5W_vs_E10	ordinary_muscle_development	ENSGALG00010006603	MEST	-0.647194517	0.225624061	-2.868464089	0.0041247	0.005878235	negative
PRJNA729271	stage_5W_vs_E10	ordinary_muscle_development	ENSGALG00010006606	SULT	-0.857991695	1.200180544	-0.714885522			negative
PRJNA729271	stage_5W_vs_E10	ordinary_muscle_development	ENSGALG00010006608	KL	3.03940553	0.410503472	7.404092143	1.32051E-13	3.84121E-13	positive
PRJNA729271	stage_5W_vs_E10	ordinary_muscle_development	ENSGALG00010006614	TMEM184C	-0.356035331	0.055568595	-6.407132175	1.48282E-10	3.65039E-10	negative
PRJNA729271	stage_5W_vs_E10	ordinary_muscle_development	ENSGALG00010006615	GRAMD1C	-0.248451364	0.102528065	-2.423252245	0.015382242	0.020622049	negative
PRJNA729271	stage_5W_vs_E10	ordinary_muscle_development	ENSGALG00010006616	COPG2	-0.818660131	0.069484941	-11.78183537	4.8427E-32	3.18923E-31	negative
PRJNA729271	stage_5W_vs_E10	ordinary_muscle_development	ENSGALG00010006619	LIN52	-0.182062215	0.102044943	-1.784137555	0.074401316	0.091892219	negative
PRJNA729271	stage_5W_vs_E10	ordinary_muscle_development	ENSGALG00010006621	DACT2	-2.332088337	0.211113253	-11.04662215	2.27619E-28	1.29284E-27	negative
PRJNA729271	stage_5W_vs_E10	ordinary_muscle_development	ENSGALG00010006623	ENSGALG00010006623	0.364620815	0.18811629	1.938273474	0.052589867	0.066159531	positive
PRJNA729271	stage_5W_vs_E10	ordinary_muscle_development	ENSGALG00010006627	SF3B5	-0.677909148	0.111483889	-6.080781311	1.19598E-09	2.78397E-09	negative
PRJNA729271	stage_5W_vs_E10	ordinary_muscle_development	ENSGALG00010006633	FBXO18	0.309213599	0.093667523	3.301182612	0.000962782	0.001462537	positive
PRJNA729271	stage_5W_vs_E10	ordinary_muscle_development	ENSGALG00010006634	PRMT9	0.557844267	0.095552195	5.838110444	5.27962E-09	1.18464E-08	positive
PRJNA729271	stage_5W_vs_E10	ordinary_muscle_development	ENSGALG00010006635	ENSGALG00010006635	-3.014174849	0.488295778	-6.172846425	6.70714E-10	1.58313E-09	negative
PRJNA729271	stage_5W_vs_E10	ordinary_muscle_development	ENSGALG00010006641	ENSGALG00010006641	-3.139034856	0.511048379	-6.142343828	8.13126E-10	1.90624E-09	negative
PRJNA729271	stage_5W_vs_E10	ordinary_muscle_development	ENSGALG00010006651	ENSGALG00010006651	0.579684854	0.112478628	5.153733323	2.55351E-07	5.11302E-07	positive
PRJNA729271	stage_5W_vs_E10	ordinary_muscle_development	ENSGALG00010006653	CFL2	0.583580755	0.104682277	5.574780881	2.47842E-08	5.33957E-08	positive
PRJNA729271	stage_5W_vs_E10	ordinary_muscle_development	ENSGALG00010006657	PDS5B	-1.165769589	0.08864829	-13.15050289	1.69046E-39	1.45076E-38	negative
PRJNA729271	stage_5W_vs_E10	ordinary_muscle_development	ENSGALG00010006664	ENSGALG00010006664	2.342593632	1.372692698	1.706568146	0.08790235	0.107522748	positive
PRJNA729271	stage_5W_vs_E10	ordinary_muscle_development	ENSGALG00010006670	ANKRD16	0.187408628	0.187178927	1.001227175	0.316716991	0.355821794	positive
PRJNA729271	stage_5W_vs_E10	ordinary_muscle_development	ENSGALG00010006673	BAZ1A	-0.993013428	0.08274409	-12.00101932	3.50947E-33	2.41547E-32	negative
PRJNA729271	stage_5W_vs_E10	ordinary_muscle_development	ENSGALG00010006678	ENSGALG00010006678	-1.898111833	1.241479175	-1.528911537	0.126286376	0.151407859	negative
PRJNA729271	stage_5W_vs_E10	ordinary_muscle_development	ENSGALG00010006681	CRISPLD2	-0.77902887	0.206540371	-3.771799515	0.000162074	0.000262166	negative
PRJNA729271	stage_5W_vs_E10	ordinary_muscle_development	ENSGALG00010006682	ARHGAP10	-1.149733337	0.122455097	-9.389019862	6.05612E-21	2.52251E-20	negative
PRJNA729271	stage_5W_vs_E10	ordinary_muscle_development	ENSGALG00010006685	ENSGALG00010006685	-0.398792154	0.119329591	-3.3419385	0.000831955	0.001269522	negative
PRJNA729271	stage_5W_vs_E10	ordinary_muscle_development	ENSGALG00010006694	ENSGALG00010006694	-4.930809972	0.975042403	-5.057021065	4.25856E-07	8.3915E-07	negative
PRJNA729271	stage_5W_vs_E10	ordinary_muscle_development	ENSGALG00010006701	LINGO2	-6.813277774	0.841956287	-8.092198941	5.85971E-16	1.93345E-15	negative
PRJNA729271	stage_5W_vs_E10	ordinary_muscle_development	ENSGALG00010006715	IRF8	2.21478295	0.160371812	13.81030071	2.20907E-43	2.14615E-42	positive
PRJNA729271	stage_5W_vs_E10	ordinary_muscle_development	ENSGALG00010006716	ENSGALG00010006716	1.195566682	0.096904018	12.33763785	5.67979E-35	4.18549E-34	positive
PRJNA729271	stage_5W_vs_E10	ordinary_muscle_development	ENSGALG00010006720	SRP54	0.054254142	0.091221831	0.594749531	0.552010908	0.591819386	positive
PRJNA729271	stage_5W_vs_E10	ordinary_muscle_development	ENSGALG00010006728	ENSGALG00010006728	0.318476237	0.136738771	2.329085121	0.019854557	0.026236831	positive
PRJNA729271	stage_5W_vs_E10	ordinary_muscle_development	ENSGALG00010006730	HAPLN1	-8.842932035	0.540399858	-16.36368312	3.47463E-60	5.60854E-59	negative
PRJNA729271	stage_5W_vs_E10	ordinary_muscle_development	ENSGALG00010006735	NR3C2	0.118674258	0.112444665	1.055401409	0.291241779	0.32908868	positive
PRJNA729271	stage_5W_vs_E10	ordinary_muscle_development	ENSGALG00010006739	VCAN	-3.35827793	0.253855625	-13.22908615	5.96077E-40	5.20804E-39	negative
PRJNA729271	stage_5W_vs_E10	ordinary_muscle_development	ENSGALG00010006740	BRCA2	-1.441152062	0.154099327	-9.352098335	8.59258E-21	3.55145E-20	negative
PRJNA729271	stage_5W_vs_E10	ordinary_muscle_development	ENSGALG00010006748	FAM177A1	1.15908839	0.076948033	15.06326204	2.82497E-51	3.53915E-50	positive
PRJNA729271	stage_5W_vs_E10	ordinary_muscle_development	ENSGALG00010006752	ZAR1L	1.031717992	0.402885264	2.560823351	0.010442444	0.014276856	positive
PRJNA729271	stage_5W_vs_E10	ordinary_muscle_development	ENSGALG00010006760	TASOR2	0.430308893	0.102523716	4.197164399	2.70278E-05	4.65116E-05	positive
PRJNA729271	stage_5W_vs_E10	ordinary_muscle_development	ENSGALG00010006761	PPP2R3C	-0.382424769	0.096092149	-3.979771226	6.89816E-05	0.000115354	negative
PRJNA729271	stage_5W_vs_E10	ordinary_muscle_development	ENSGALG00010006767	ENSGALG00010006767	7.362297754	0.330355223	22.28600377	5.0512E-110	2.56E-108	positive
PRJNA729271	stage_5W_vs_E10	ordinary_muscle_development	ENSGALG00010006794	PERPA	-4.674021419	0.576822822	-8.103045233	5.36003E-16	1.77302E-15	negative
PRJNA729271	stage_5W_vs_E10	ordinary_muscle_development	ENSGALG00010006803	COX4I1	1.265643977	0.103914248	12.17969621	3.98764E-34	2.84034E-33	positive
PRJNA729271	stage_5W_vs_E10	ordinary_muscle_development	ENSGALG00010006804	PSMA6	0.355461489	0.102180869	3.478747961	0.000503762	0.000782137	positive
PRJNA729271	stage_5W_vs_E10	ordinary_muscle_development	ENSGALG00010006814	ENSGALG00010006814	0.854443027	0.36476364	2.342456686	0.019157257	0.025389453	positive
PRJNA729271	stage_5W_vs_E10	ordinary_muscle_development	ENSGALG00010006816	ENSGALG00010006816	5.77300177	0.503522741	11.4652255	1.97245E-30	1.21147E-29	positive
PRJNA729271	stage_5W_vs_E10	ordinary_muscle_development	ENSGALG00010006819	EMC8	0.621375383	0.131767955	4.715679021	2.40906E-06	4.49239E-06	positive
PRJNA729271	stage_5W_vs_E10	ordinary_muscle_development	ENSGALG00010006821	XRCC4	-0.781263366	0.128429697	-6.083198692	1.17808E-09	2.74274E-09	negative
PRJNA729271	stage_5W_vs_E10	ordinary_muscle_development	ENSGALG00010006828	GTF2A1	0.137176102	0.106029995	1.293748069	0.195752456	0.228002886	positive
PRJNA729271	stage_5W_vs_E10	ordinary_muscle_development	ENSGALG00010006833	LTV1	-0.634089606	0.087796848	-7.222236585	5.11394E-13	1.44694E-12	negative

PRJNA729271	stage_5W_vs_E10	ordinary_muscle_development	ENSGALG00010006835	FRY	1.99821409	0.152164061	13.13197133	2.1596E-39	1.8403E-38	positive
PRJNA729271	stage_5W_vs_E10	ordinary_muscle_development	ENSGALG00010006838	ASB13	1.408429916	0.121864055	11.55738591	6.77396E-31	4.25796E-30	positive
PRJNA729271	stage_5W_vs_E10	ordinary_muscle_development	ENSGALG00010006840	ENSGALG00010006840	-3.425725298	0.296272093	-11.56276741	6.36247E-31	4.00452E-30	negative
PRJNA729271	stage_5W_vs_E10	ordinary_muscle_development	ENSGALG00010006843	STON2	1.999735552	0.191670837	10.43317585	1.74941E-25	8.82009E-25	positive
PRJNA729271	stage_5W_vs_E10	ordinary_muscle_development	ENSGALG00010006845	CALML3	-8.990623705	0.935779848	-9.607626974	7.42402E-22	3.23351E-21	negative
PRJNA729271	stage_5W_vs_E10	ordinary_muscle_development	ENSGALG00010006847	SEL1L	0.128332644	0.086806568	1.478374818	0.139307482	0.165989474	positive
PRJNA729271	stage_5W_vs_E10	ordinary_muscle_development	ENSGALG00010006850	IRF2BPL	-1.184300481	0.160608964	-7.373813103	1.65816E-13	4.79836E-13	negative
PRJNA729271	stage_5W_vs_E10	ordinary_muscle_development	ENSGALG00010006851	TMEM167A	-0.676761702	0.105278659	-6.42828953	1.29048E-10	3.18825E-10	negative
PRJNA729271	stage_5W_vs_E10	ordinary_muscle_development	ENSGALG00010006853	NET1	-0.64413547	0.084042967	-7.664358996	1.79727E-14	5.47758E-14	negative
PRJNA729271	stage_5W_vs_E10	ordinary_muscle_development	ENSGALG00010006855	DCLK2	0.170722914	0.123501185	1.382358506	0.16686165	0.196943214	positive
PRJNA729271	stage_5W_vs_E10	ordinary_muscle_development	ENSGALG00010006865	EFCAB2	-0.93254082	0.354425449	-2.631133914	0.00851005	0.011735626	negative
PRJNA729271	stage_5W_vs_E10	ordinary_muscle_development	ENSGALG00010006877	GSE1	0.723481734	0.241540432	2.995282109	0.002741911	0.003979175	positive
PRJNA729271	stage_5W_vs_E10	ordinary_muscle_development	ENSGALG00010006883	LRBA	-0.183369899	0.070605236	-2.597114739	0.009401051	0.012909079	negative
PRJNA729271	stage_5W_vs_E10	ordinary_muscle_development	ENSGALG00010006885	RHOU	-0.802205728	0.153414782	-5.228998905	1.7043E-07	3.45993E-07	negative
PRJNA729271	stage_5W_vs_E10	ordinary_muscle_development	ENSGALG00010006901	TUBAL3	-3.781214176	0.263937689	-14.3261623	1.50194E-46	1.60669E-45	negative
PRJNA729271	stage_5W_vs_E10	ordinary_muscle_development	ENSGALG00010006903	B3GLCT	-1.01443976	0.146539062	-6.922657681	4.43248E-12	1.19266E-11	negative
PRJNA729271	stage_5W_vs_E10	ordinary_muscle_development	ENSGALG00010006912	ENSGALG00010006912	-0.908736421	0.183841216	-4.943050525	7.69095E-07	1.49018E-06	negative
PRJNA729271	stage_5W_vs_E10	ordinary_muscle_development	ENSGALG00010006913	HSPH1	-1.028170498	0.185883613	-5.531259484	3.1794E-08	6.78125E-08	negative
PRJNA729271	stage_5W_vs_E10	ordinary_muscle_development	ENSGALG00010006922	SMYD3	-0.909043471	0.163843935	-5.548227786	2.8858E-08	6.17412E-08	negative
PRJNA729271	stage_5W_vs_E10	ordinary_muscle_development	ENSGALG00010006923	ENSGALG00010006923	0.250193068	0.227726338	1.098656705	0.271917834	0.309109011	positive
PRJNA729271	stage_5W_vs_E10	ordinary_muscle_development	ENSGALG00010006926	ID4	-3.192249012	0.232633989	-13.7221952	7.47669E-43	7.12054E-42	negative
PRJNA729271	stage_5W_vs_E10	ordinary_muscle_development	ENSGALG00010006927	UCN3	-5.031616045	1.046782566	-4.806744215	1.53408E-06	2.90294E-06	negative
PRJNA729271	stage_5W_vs_E10	ordinary_muscle_development	ENSGALG00010006939	FBXO31	1.945825951	0.126671141	15.36124117	2.97818E-53	3.93158E-52	positive
PRJNA729271	stage_5W_vs_E10	ordinary_muscle_development	ENSGALG00010006942	ALOX5AP	0.600466524	0.183639995	3.269802549	0.001076226	0.001627065	positive
PRJNA729271	stage_5W_vs_E10	ordinary_muscle_development	ENSGALG00010006944	MAB21L2	-5.47437791	0.53202061	-10.28978542	7.83508E-25	3.86817E-24	negative
PRJNA729271	stage_5W_vs_E10	ordinary_muscle_development	ENSGALG00010006946	PLPPR1	-6.694880596	0.5168906	-12.95221966	2.28257E-38	1.88095E-37	negative
PRJNA729271	stage_5W_vs_E10	ordinary_muscle_development	ENSGALG00010006948	ENSGALG00010006948	-10.84713713	0.759494232	-14.28205334	2.83134E-46	2.99346E-45	negative
PRJNA729271	stage_5W_vs_E10	ordinary_muscle_development	ENSGALG00010006953	MAP1LC3B	1.670544514	0.127778392	13.07376384	4.65063E-39	3.91696E-38	positive
PRJNA729271	stage_5W_vs_E10	ordinary_muscle_development	ENSGALG00010006957	PALM2AKAP2	0.832135505	0.091384072	9.105914001	8.55431E-20	3.39247E-19	positive
PRJNA729271	stage_5W_vs_E10	ordinary_muscle_development	ENSGALG00010006958	KIF26B	-4.663563998	0.245045334	-19.03143358	9.36552E-81	2.58086E-79	negative
PRJNA729271	stage_5W_vs_E10	ordinary_muscle_development	ENSGALG00010006961	ZCCHC14	0.468894398	0.117452023	3.992220709	6.54574E-05	0.000109701	positive
PRJNA729271	stage_5W_vs_E10	ordinary_muscle_development	ENSGALG00010006963	ENSGALG00010006963	2.191845625	0.415777744	5.271676167	1.35183E-07	2.76646E-07	positive
PRJNA729271	stage_5W_vs_E10	ordinary_muscle_development	ENSGALG00010006966	PGBD5	-2.750672452	0.258286582	-10.64969164	1.74944E-26	9.25815E-26	negative
PRJNA729271	stage_5W_vs_E10	ordinary_muscle_development	ENSGALG00010006983	GTPBP2	0.621704373	0.124529575	4.992423493	5.96263E-07	1.16433E-06	positive
PRJNA729271	stage_5W_vs_E10	ordinary_muscle_development	ENSGALG00010006988	USPL1	0.083287133	0.104368592	0.798009543	0.424864952	0.4666882	positive
PRJNA729271	stage_5W_vs_E10	ordinary_muscle_development	ENSGALG00010006992	SLC35B4	-1.649878352	0.105453524	-15.64554968	3.5628E-55	4.94189E-54	negative
PRJNA729271	stage_5W_vs_E10	ordinary_muscle_development	ENSGALG00010006998	POLH	0.697564494	0.121393983	5.746285575	9.12252E-09	2.01848E-08	positive
PRJNA729271	stage_5W_vs_E10	ordinary_muscle_development	ENSGALG00010007000	LRGUK	-1.613280509	0.529307686	-3.047906828	0.002304414	0.003366846	negative
PRJNA729271	stage_5W_vs_E10	ordinary_muscle_development	ENSGALG00010007001	SNX30	-0.927079829	0.093619438	-9.902642506	4.05419E-23	1.85849E-22	negative
PRJNA729271	stage_5W_vs_E10	ordinary_muscle_development	ENSGALG00010007002	EXOC4	0.121166592	0.119710428	1.012164049	0.311459623	0.350540199	positive
PRJNA729271	stage_5W_vs_E10	ordinary_muscle_development	ENSGALG00010007004	ENSGALG00010007004	-0.119815026	0.605867741	-0.197757726	0.843234623	0.862209781	negative
PRJNA729271	stage_5W_vs_E10	ordinary_muscle_development	ENSGALG00010007007	ACSL1	1.951749715	0.216879036	8.999254858	2.27255E-19	8.81236E-19	positive
PRJNA729271	stage_5W_vs_E10	ordinary_muscle_development	ENSGALG00010007010	ENSGALG00010007010	0.585007813	1.014645617	0.576563682	0.564234239	0.603513842	positive
PRJNA729271	stage_5W_vs_E10	ordinary_muscle_development	ENSGALG00010007011	HMGB1	-1.786755091	0.101982498	-17.5202131	1.00448E-68	2.04208E-67	negative
PRJNA729271	stage_5W_vs_E10	ordinary_muscle_development	ENSGALG00010007014	ENSGALG00010007014	-0.580224136	0.638663679	-0.908497157	0.363615615	0.404156763	negative
PRJNA729271	stage_5W_vs_E10	ordinary_muscle_development	ENSGALG00010007017	SRD5A2	2.343665745	0.33626014	6.969799461	3.17393E-12	8.61538E-12	positive
PRJNA729271	stage_5W_vs_E10	ordinary_muscle_development	ENSGALG00010007021	SULT6B1	0.017575929	0.248314384	0.070780956	0.943572089	0.951129919	positive
PRJNA729271	stage_5W_vs_E10	ordinary_muscle_development	ENSGALG00010007025	ENSGALG00010007025	1.39431567	0.753148956	1.851314615	0.064124305	0.079804396	positive
PRJNA729271	stage_5W_vs_E10	ordinary_muscle_development	ENSGALG00010007028	KATNAL1	-0.092061203	0.138626056	-0.664097399	0.506627975	0.547619127	negative
PRJNA729271	stage_5W_vs_E10	ordinary_muscle_development	ENSGALG00010007033	CEBPZOS	1.087099763	0.185953283	5.846090712	5.0326E-09	1.13114E-08	positive
PRJNA729271	stage_5W_vs_E10	ordinary_muscle_development	ENSGALG00010007038	CENPU	-2.161601369	0.208750303	-10.35496157	3.97346E-25	1.98263E-24	negative
PRJNA729271	stage_5W_vs_E10	ordinary_muscle_development	ENSGALG00010007041	NAE1	-0.459444359	0.080399507	-5.714517131	1.10016E-08	2.42169E-08	negative
PRJNA729271	stage_5W_vs_E10	ordinary_muscle_development	ENSGALG00010007049	ENSGALG00010007049	-0.559811786	0.528456128	-1.059334457	0.289447488	0.327163236	negative
PRJNA729271	stage_5W_vs_E10	ordinary_muscle_development	ENSGALG00010007051	E2F3	-2.00247113	0.158280555	-12.65140326	1.09894E-36	8.63838E-36	negative
PRJNA729271	stage_5W_vs_E10	ordinary_muscle_development	ENSGALG00010007066	INIP	-0.611154427	0.154118708	-3.965478528	7.32488E-05	0.000122278	negative
PRJNA729271	stage_5W_vs_E10	ordinary_muscle_development	ENSGALG00010007067	CASP3	-1.613828877	0.109157039	-14.78446914	1.84506E-49	2.18856E-48	negative
PRJNA729271	stage_5W_vs_E10	ordinary_muscle_development	ENSGALG00010007068	NCAM2	-5.064339077	0.327043372	-15.48522158	4.36556E-54	5.87002E-53	negative
PRJNA729271	stage_5W_vs_E10	ordinary_muscle_development	ENSGALG00010007070	UBL3	1.175416312	0.052199684	22.51769018	2.7851E-112	1.4788E-110	positive
PRJNA729271	stage_5W_vs_E10	ordinary_muscle_development	ENSGALG00010007085	PRIMPOL	-0.444842697	0.169384803	-2.626225553	0.008633759	0.01189717	negative
PRJNA729271	stage_5W_vs_E10	ordinary_muscle_development	ENSGALG00010007090	CEBPZ	0.024775059	0.093105806	0.266095751	0.790165468	0.81526486	positive
PRJNA729271	stage_5W_vs_E10	ordinary_muscle_development	ENSGALG00010007092	DYNC1LI2	0.464089401	0.06116675	7.587282297	3.26685E-14	9.85084E-14	positive
PRJNA729271	stage_5W_vs_E10	ordinary_muscle_development	ENSGALG00010007097	ZCCHC9	0.015968735	0.168301467	0.094881736	0.924408764	0.934596152	positive
PRJNA729271	stage_5W_vs_E10	ordinary_muscle_development	ENSGALG00010007098	NDUFAF7	0.591832025	0.128011024	4.623289524	3.77702E-06	6.93978E-06	positive
PRJNA729271	stage_5W_vs_E10	ordinary_muscle_development	ENSGALG00010007102	CMTM3	-1.163761346	0.165662484	-7.024893753	2.14229E-12	5.87004E-12	negative
PRJNA729271	stage_5W_vs_E10	ordinary_muscle_development	ENSGALG00010007106	SLC7A1	-2.187461257	0.242964363	-9.003218541	2.19195E-19	8.50666E-19	negative
PRJNA729271	stage_5W_vs_E10	ordinary_muscle_development	ENSGALG00010007108	MBOAT1	0.251381613	0.190569298	1.319108671	0.187132785	0.21889039	positive
PRJNA729271	stage_5W_vs_E10	ordinary_muscle_development	ENSGALG00010007110	ARL6	0.024570265	0.102771272	0.239077174	0.811045741	0.833116577	positive
PRJNA729271	stage_5W_vs_E10	ordinary_muscle_development	ENSGALG00010007114	CKLF	0.184867168	0.147253068	1.255438485	0.209319626	0.242668798	positive
PRJNA729271	stage_5W_vs_E10	ordinary_muscle_development	ENSGALG00010007115	ENSGALG00010007115	1.103897094	0.277995253	3.970920663	7.15954E-05	0.000119573	positive
PRJNA729271	stage_5W_vs_E10	ordinary_muscle_development	ENSGALG00010007118	ENSGALG00010007118	-0.932403924	0.131371047	-7.097484176	1.27048E-12	3.52385E-12	negative
PRJNA729271	stage_5W_vs_E10	ordinary_muscle_development	ENSGALG00010007123	ENSGALG00010007123	2.247395916	0.244752822	9.182308488	4.21947E-20	1.69187E-19	positive
PRJNA729271	stage_5W_vs_E10	ordinary_muscle_development	ENSGALG00010007126	ENSGALG00010007126	1.921012712	1.079464838	1.779597302	0.075141888	0.092754026	positive
PRJNA729271	stage_5W_vs_E10	ordinary_muscle_development	ENSGALG00010007130	IRF2	1.475803101	0.122220177	12.07495466	1.43243E-33	9.96788E-33	positive
PRJNA729271	stage_5W_vs_E10	ordinary_muscle_development	ENSGALG00010007133	BLVRA	0.616123697	0.189340953	3.254043497	0.001137749	0.001714667	positive
PRJNA729271	stage_5W_vs_E10	ordinary_muscle_development	ENSGALG00010007135	TK2	-0.755060708	0.118109817	-6.392870006	1.62801E-10	3.99491E-10	negative
PRJNA729271	stage_5W_vs_E10	ordinary_muscle_development	ENSGALG00010007137	ENSGALG00010007137	1.063098927	0.360134751	2.951947634	0.003157765	0.004554408	positive
PRJNA729271	stage_5W_vs_E10	ordinary_muscle_development	ENSGALG00010007141	PRKD3	0.136487446	0.08241484	1.656102796	0.097701003	0.118826652	positive
PRJNA729271	stage_5W_vs_E10	ordinary_muscle_development	ENSGALG00010007143	CKMT2	11.19654123	0.316775754	35.34532259	1.183E-273	4.0828E-271	positive
PRJNA729271	stage_5W_vs_E10	ordinary_muscle_development	ENSGALG00010007146	ENSGALG00010007146	1.697938297	0.141446921	12.00406687	3.38257E-33	2.33034E-32	positive
PRJNA729271	stage_5W_vs_E10	ordinary_muscle_development	ENSGALG00010007155	FOXL1	-2.091880145	0.740381726	-2.825407588	0.004722051	0.006685498	negative
PRJNA729271	stage_5W_vs_E10	ordinary_muscle_development	ENSGALG00010007160	ENSGALG00010007160	1.011160169	0.294274459	3.436112578	0.000590126	0.000911732	positive
PRJNA729271	stage_5W_vs_E10	ordinary_muscle_development	ENSGALG00010007162	ENSGALG00010007162	2.595727213	0.410403771	6.32481326	2.53539E-10	6.15071E-10	positive
PRJNA729271	stage_5W_vs_E10	ordinary_muscle_development	ENSGALG00010007165	ENSGALG00010007165	0.53076692	0.32711323	1.62257858	0.104679522	0.126803416	positive
PRJNA729271	stage_5W_vs_E10	ordinary_muscle_development	ENSGALG00010007166	RASGRF2	1.677480382	0.184385779	9.097666821	9.22931E-20	3.65516E-19	positive
PRJNA729271	stage_5W_vs_E10	ordinary_muscle_development	ENSGALG00010007168	MINA	-0.30883197	0.108296555	-2.85172479	0.004348273	0.006180449	negative
PRJNA729271	stage_5W_vs_E10	ordinary_muscle_development	ENSGALG00010007169	QPCT	0.297752895	0.218426711	1.36317071	0.172828716	0.203290776	positive
PRJNA729271	stage_5W_vs_E10	ordinary_muscle_development	ENSGALG00010007175	FOXC2	-2.985293134	0.419644653	-7.113859585	1.12842E-12	3.14064E-12	negative
PRJNA729271	stage_5W_vs_E10	ordinary_muscle_development	ENSGALG00010007181	GJB2	2.252142962	0.576986487	3.903285456	9.48957E-05	0.000156629	positive
PRJNA729271	stage_5W_vs_E10	ordinary_muscle_development	ENSGALG00010007182	ENSGALG00010007182	0.905849672	0.294972211	3.070966134	0.002133674	0.003127158	positive
PRJNA729271	stage_5W_vs_E10	ordinary_muscle_development	ENSGALG00010007183	VOPP1	1.293191218	0.316218742	4.089546397	4.32218E-05	7.32148E-05	positive
PRJNA729271	stage_5W_vs_E10	ordinary_muscle_development	ENSGALG00010007184	VGLL3	0.180560977	0.093885038	1.923213539	0.054453238	0.068308064	positive
PRJNA729271	stage_5W_vs_E10	ordinary_muscle_development	ENSGALG00010007187	ENPP6	-0.880651424	0.280537732	-3.139155002	0.001694358	0.002508397	negative
PRJNA729271	stage_5W_vs_E10	ordinary_muscle_development	ENSGALG00010007188	MAB21L1	-2.019347276	0.222956165	-9.057149329	1.33904E-19	5.25858E-19	negative
PRJNA729271	stage_5W_vs_E10	ordinary_muscle_development	ENSGALG00010007190	ENSGALG00010007190	0.31156661	0.148983624	2.09128092	0.036502889	0.046894388	positive
PRJNA729271	stage_5W_vs_E10	ordinary_muscle_development	ENSGALG00010007201	ENSGALG00010007201	3.476047465	0.252680827	13.75667284	4.64413E-43	4.44334E-42	positive
PRJNA729271	stage_5W_vs_E10	ordinary_muscle_development	ENSGALG00010007203	SLC30A6	0.055867746	0.105490204	0.529601267	0.596388416	0.63454862	positive
PRJNA729271	stage_5W_vs_E10	ordinary_muscle_development	ENSGALG00010007204	LANCL2	-1.210676293	0.088128809	-13.73757688	6.04658E-43	5.76993E-42	negative
PRJNA729271	stage_5W_vs_E10	ordinary_muscle_development	ENSGALG00010007207	EPM2A	1.752265743	0.102952245	17.02018005	5.81914E-65	1.07177E-63	positive
PRJNA729271	stage_5W_vs_E10	ordinary_muscle_development	ENSGALG00010007214	MTHFSD	1.027411718	0.139297921	7.37564286	1.63554E-13	4.73385E-13	positive
PRJNA729271	stage_5W_vs_E10	ordinary_muscle_development	ENSGALG00010007217	MSH3	0.013400293	0.143452802	0.093412554	0.925575818	0.935645546	positive
PRJNA729271	stage_5W_vs_E10	ordinary_muscle_development	ENSGALG00010007223	FOXF1	-1.377945438	0.540383719	-2.549938846	0.010774182	0.014705439	negative
PRJNA729271	stage_5W_vs_E10	ordinary_muscle_development	ENSGALG00010007224	DHFR	-2.948476636	0.21892782	-13.46780246	2.41961E-41	2.20441E-40	negative
PRJNA729271	stage_5W_vs_E10	ordinary_muscle_development	ENSGALG00010007226	ENSGALG00010007226	3.673511014	1.484495493	2.474585495	0.013339089	0.018004479	positive
PRJNA729271	stage_5W_vs_E10	ordinary_muscle_development	ENSGALG00010007230	ENSGALG00010007230	-1.500955703	0.354593926	-4.2328861	2.30711E-05	3.9978E-05	negative
PRJNA729271	stage_5W_vs_E10	ordinary_muscle_development	ENSGALG00010007243	BEAN1	-1.493189162	0.189946603	-7.861099599	3.80776E-15	1.20562E-14	negative
PRJNA729271	stage_5W_vs_E10	ordinary_muscle_development	ENSGALG00010007251	SPAST	-0.262135038	0.063665319	-4.117391422	3.83185E-05	6.51986E-05	negative
PRJNA729271	stage_5W_vs_E10	ordinary_muscle_development	ENSGALG00010007252	NFATC1	1.327766331	0.152925054	8.682464363	3.87285E-18	1.42263E-17	positive
PRJNA729271	stage_5W_vs_E10	ordinary_muscle_development	ENSGALG00010007259	ANKRD34B	-1.281287383	0.097447875	-13.14843846	1.73725E-39	1.48915E-38	negative
PRJNA729271	stage_5W_vs_E10	ordinary_muscle_development	ENSGALG00010007265	CD200R1A	0.128968072	0.286744174	0.449767018	0.652878442	0.68750258	positive
PRJNA729271	stage_5W_vs_E10	ordinary_muscle_development	ENSGALG00010007270	CDH5	0.419926918	0.10845622	3.871856478	0.00010801	0.000177264	positive
PRJNA729271	stage_5W_vs_E10	ordinary_muscle_development	ENSGALG00010007271	CADM2	-3.422970217	0.321534717	-10.64572513	1.82557E-26	9.65755E-26	negative
PRJNA729271	stage_5W_vs_E10	ordinary_muscle_development	ENSGALG00010007275	DPY30	0.25991244	0.098910527	2.627753063	0.008595088	0.011846135	positive
PRJNA729271	stage_5W_vs_E10	ordinary_muscle_development	ENSGALG00010007277	FAM151B	-0.880197806	0.192743735	-4.566674022	4.95524E-06	9.0132E-06	negative
PRJNA729271	stage_5W_vs_E10	ordinary_muscle_development	ENSGALG00010007283	STOX2	-0.983999351	0.123306151	-7.980131885	1.46177E-15	4.74012E-15	negative
PRJNA729271	stage_5W_vs_E10	ordinary_muscle_development	ENSGALG00010007284	ENSGALG00010007284	0.098687266	0.082223968	1.200225053	0.230051948	0.264567041	positive
PRJNA729271	stage_5W_vs_E10	ordinary_muscle_development	ENSGALG00010007287	ENSGALG00010007287	1.460868513	0.620374756	2.35481618	0.018531871	0.024603583	positive
PRJNA729271	stage_5W_vs_E10	ordinary_muscle_development	ENSGALG00010007294	TRAPPC11	0.584951977	0.064391624	9.084286745	1.04381E-19	4.12599E-19	positive
PRJNA729271	stage_5W_vs_E10	ordinary_muscle_development	ENSGALG00010007300	CCDC125	1.149781959	0.242516082	4.741054486	2.12609E-06	3.98109E-06	positive
PRJNA729271	stage_5W_vs_E10	ordinary_muscle_development	ENSGALG00010007302	CDH11	-3.18234637	0.175439953	-18.13923409	1.56193E-73	3.60512E-72	negative
PRJNA729271	stage_5W_vs_E10	ordinary_muscle_development	ENSGALG00010007304	F5	1.856822515	0.355131443	5.228550017	1.70845E-07	3.46785E-07	positive
PRJNA729271	stage_5W_vs_E10	ordinary_muscle_development	ENSGALG00010007318	RMDN2	1.371979563	0.123410155	11.11723389	1.03429E-28	5.95869E-28	positive
PRJNA729271	stage_5W_vs_E10	ordinary_muscle_development	ENSGALG00010007319	ATP9B	0.237561977	0.101884073	2.33168904	0.019717056	0.026078905	positive
PRJNA729271	stage_5W_vs_E10	ordinary_muscle_development	ENSGALG00010007320	ENSGALG00010007320	1.290956705	0.303011199	4.26042572	2.04038E-05	3.54875E-05	positive
PRJNA729271	stage_5W_vs_E10	ordinary_muscle_development	ENSGALG00010007321	CD200	1.690372838	0.151162775	11.18246762	4.96888E-29	2.89718E-28	positive
PRJNA729271	stage_5W_vs_E10	ordinary_muscle_development	ENSGALG00010007322	AK6	-0.371111046	0.109210263	-3.398133439	0.000678473	0.001043002	negative
PRJNA729271	stage_5W_vs_E10	ordinary_muscle_development	ENSGALG00010007324	ENSGALG00010007324	-0.121547404	0.141231446	-0.860625641	0.389444259	0.430784076	negative
PRJNA729271	stage_5W_vs_E10	ordinary_muscle_development	ENSGALG00010007326	RWDD4	0.975632154	0.115732305	8.430076252	3.4544E-17	1.21151E-16	positive
PRJNA729271	stage_5W_vs_E10	ordinary_muscle_development	ENSGALG00010007328	NOVA1	0.231838255	0.137296522	1.688595255	0.091297021	0.111458798	positive
PRJNA729271	stage_5W_vs_E10	ordinary_muscle_development	ENSGALG00010007330	MMP13	-1.775660335	0.714645079	-2.484674404	0.012966997	0.017526728	negative
PRJNA729271	stage_5W_vs_E10	ordinary_muscle_development	ENSGALG00010007333	QTRT2	-1.864522525	0.078868723	-23.64083578	1.4665E-123	9.2823E-122	negative
PRJNA729271	stage_5W_vs_E10	ordinary_muscle_development	ENSGALG00010007337	PRORP	-0.7920711	0.101956515	-7.768714935	7.92864E-15	2.46886E-14	negative

PRJNA729271	stage_5W_vs_E10	ordinary_muscle_development	ENSGALG00010007339	ENSGALG00010007339	0.829418873	0.223890056	3.704581105	0.00021174	0.000339511	positive
PRJNA729271	stage_5W_vs_E10	ordinary_muscle_development	ENSGALG00010007344	RAD17	0.140537795	0.10829605	1.297718573	0.194384059	0.226622451	positive
PRJNA729271	stage_5W_vs_E10	ordinary_muscle_development	ENSGALG00010007353	ING2	-0.735434213	0.126645997	-5.807007198	6.35994E-09	1.4198E-08	negative
PRJNA729271	stage_5W_vs_E10	ordinary_muscle_development	ENSGALG00010007357	NOX4	0.858380558	0.117645878	7.296307949	2.95772E-13	8.45436E-13	positive
PRJNA729271	stage_5W_vs_E10	ordinary_muscle_development	ENSGALG00010007360	ROBO1	-3.053059496	0.186911523	-16.33424975	5.63208E-60	8.98097E-59	negative
PRJNA729271	stage_5W_vs_E10	ordinary_muscle_development	ENSGALG00010007365	MEMO1	2.185047639	0.086396597	25.29089927	4.0225E-141	3.3703E-139	positive
PRJNA729271	stage_5W_vs_E10	ordinary_muscle_development	ENSGALG00010007369	ENSGALG00010007369	-0.203016484	0.212994692	-0.953152788	0.340512663	0.38025663	negative
PRJNA729271	stage_5W_vs_E10	ordinary_muscle_development	ENSGALG00010007374	CMIP	0.287243391	0.133656055	2.14912367	0.031624598	0.04091383	positive
PRJNA729271	stage_5W_vs_E10	ordinary_muscle_development	ENSGALG00010007375	CHORDC1	-0.375872859	0.075138102	-5.00242682	5.66131E-07	1.10683E-06	negative
PRJNA729271	stage_5W_vs_E10	ordinary_muscle_development	ENSGALG00010007376	CDKN2AIP	-0.733160505	0.061761697	-11.87079595	1.67863E-32	1.12543E-31	negative
PRJNA729271	stage_5W_vs_E10	ordinary_muscle_development	ENSGALG00010007381	EGFR	-0.330403313	0.098233453	-3.363450057	0.000769748	0.001178069	negative
PRJNA729271	stage_5W_vs_E10	ordinary_muscle_development	ENSGALG00010007382	EIF2AK2	1.456373453	0.256203236	5.684445963	1.31237E-08	2.87789E-08	positive
PRJNA729271	stage_5W_vs_E10	ordinary_muscle_development	ENSGALG00010007387	ENSGALG00010007387	-3.073256552	0.584903202	-5.254299411	1.48589E-07	3.03138E-07	negative
PRJNA729271	stage_5W_vs_E10	ordinary_muscle_development	ENSGALG00010007388	CYP1B1	-3.17912907	0.203028974	-15.65849938	2.90675E-55	4.04739E-54	negative
PRJNA729271	stage_5W_vs_E10	ordinary_muscle_development	ENSGALG00010007391	NANP	0.348134398	0.138889096	2.506563926	0.012191101	0.016522675	positive
PRJNA729271	stage_5W_vs_E10	ordinary_muscle_development	ENSGALG00010007392	NAALAD2	2.177338472	0.200159722	10.87800507	1.46739E-27	8.08432E-27	positive
PRJNA729271	stage_5W_vs_E10	ordinary_muscle_development	ENSGALG00010007393	PLCG2	1.559756659	0.125645694	12.41392852	2.19607E-35	1.64252E-34	positive
PRJNA729271	stage_5W_vs_E10	ordinary_muscle_development	ENSGALG00010007399	WWC2	-1.109815108	0.111450801	-9.957892653	2.32948E-23	1.07981E-22	negative
PRJNA729271	stage_5W_vs_E10	ordinary_muscle_development	ENSGALG00010007401	SEC61G	-0.241510295	0.22025316	-1.096512284	0.272854651	0.310028076	negative
PRJNA729271	stage_5W_vs_E10	ordinary_muscle_development	ENSGALG00010007403	SDR42E1	-2.161339054	0.267027371	-8.094073089	5.7702E-16	1.90522E-15	negative
PRJNA729271	stage_5W_vs_E10	ordinary_muscle_development	ENSGALG00010007404	FOLH1	1.40424635	0.171402498	8.19268309	2.55466E-16	8.58961E-16	positive
PRJNA729271	stage_5W_vs_E10	ordinary_muscle_development	ENSGALG00010007408	HCLS1	3.294010228	0.242089631	13.60657296	3.66008E-42	3.40957E-41	positive
PRJNA729271	stage_5W_vs_E10	ordinary_muscle_development	ENSGALG00010007411	ENSGALG00010007411	1.390867428	0.578231573	2.405381326	0.016155598	0.021596919	positive
PRJNA729271	stage_5W_vs_E10	ordinary_muscle_development	ENSGALG00010007413	GPATCH11	-0.276227425	0.098798485	-2.79586701	0.005176069	0.007297649	negative
PRJNA729271	stage_5W_vs_E10	ordinary_muscle_development	ENSGALG00010007414	ROBO2	-4.689637506	0.239890747	-19.54905543	4.20229E-85	1.26637E-83	negative
PRJNA729271	stage_5W_vs_E10	ordinary_muscle_development	ENSGALG00010007416	DCTD	-2.736510927	0.25374236	-10.78460423	4.06991E-27	2.20371E-26	negative
PRJNA729271	stage_5W_vs_E10	ordinary_muscle_development	ENSGALG00010007418	NME6	0.217262679	0.147695786	1.471014749	0.141287128	0.168196199	positive
PRJNA729271	stage_5W_vs_E10	ordinary_muscle_development	ENSGALG00010007419	RABL2A	-1.218124582	0.166713657	-7.306687424	2.73808E-13	7.84359E-13	negative
PRJNA729271	stage_5W_vs_E10	ordinary_muscle_development	ENSGALG00010007423	HEATR5B	-0.421567407	0.066329647	-6.355640696	2.0756E-10	5.06154E-10	negative
PRJNA729271	stage_5W_vs_E10	ordinary_muscle_development	ENSGALG00010007425	ARSA	0.093735241	0.129502423	0.723810711	0.469181953	0.511145615	positive
PRJNA729271	stage_5W_vs_E10	ordinary_muscle_development	ENSGALG00010007429	C21orf91	0.556199506	0.208895818	2.662568888	0.007754669	0.010734785	positive
PRJNA729271	stage_5W_vs_E10	ordinary_muscle_development	ENSGALG00010007430	MEF2C	0.461119632	0.147000639	3.136854613	0.001707708	0.002527129	positive
PRJNA729271	stage_5W_vs_E10	ordinary_muscle_development	ENSGALG00010007432	NAT	1.888138436	0.187765037	10.05585738	8.65646E-24	4.0805E-23	positive
PRJNA729271	stage_5W_vs_E10	ordinary_muscle_development	ENSGALG00010007433	RNF144B	3.209088264	0.267770032	11.98449373	4.28454E-33	2.93083E-32	positive
PRJNA729271	stage_5W_vs_E10	ordinary_muscle_development	ENSGALG00010007434	ENSGALG00010007434	0.565355064	0.250893819	2.253363861	0.024236216	0.031737642	positive
PRJNA729271	stage_5W_vs_E10	ordinary_muscle_development	ENSGALG00010007436	PNAT3	2.047002132	0.261306076	7.833733395	4.73592E-15	1.49039E-14	positive
PRJNA729271	stage_5W_vs_E10	ordinary_muscle_development	ENSGALG00010007437	TUSC1	-0.035980545	0.139822708	-0.257329767	0.796924215	0.821054553	negative
PRJNA729271	stage_5W_vs_E10	ordinary_muscle_development	ENSGALG00010007439	HSD17B2	-7.093091349	0.450683725	-15.73851229	8.23573E-56	1.16579E-54	negative
PRJNA729271	stage_5W_vs_E10	ordinary_muscle_development	ENSGALG00010007441	STRN	1.478051101	0.13534238	10.9208298	9.16546E-28	5.08432E-27	positive
PRJNA729271	stage_5W_vs_E10	ordinary_muscle_development	ENSGALG00010007455	TRPC6	0.973647689	0.209736787	4.642236126	3.44659E-06	6.36006E-06	positive
PRJNA729271	stage_5W_vs_E10	ordinary_muscle_development	ENSGALG00010007457	SHANK3	0.482245762	0.178622153	2.699809374	0.006937922	0.009652095	positive
PRJNA729271	stage_5W_vs_E10	ordinary_muscle_development	ENSGALG00010007465	PNAT10	1.831823087	0.194677741	9.409514829	4.98432E-21	2.08567E-20	positive
PRJNA729271	stage_5W_vs_E10	ordinary_muscle_development	ENSGALG00010007467	CHODL	-2.488002046	0.332502348	-7.482660079	7.28331E-14	2.1497E-13	negative
PRJNA729271	stage_5W_vs_E10	ordinary_muscle_development	ENSGALG00010007479	NFX1	-0.495448165	0.097686713	-5.071807117	3.94056E-07	7.78392E-07	negative
PRJNA729271	stage_5W_vs_E10	ordinary_muscle_development	ENSGALG00010007480	TENM3	-0.464597828	0.106128677	-4.377684178	1.19947E-05	2.12573E-05	negative
PRJNA729271	stage_5W_vs_E10	ordinary_muscle_development	ENSGALG00010007485	CTSC	1.224999279	0.15542843	7.881436361	3.23639E-15	1.02876E-14	positive
PRJNA729271	stage_5W_vs_E10	ordinary_muscle_development	ENSGALG00010007500	MPHOSPH6	0.026463794	0.116709449	0.226749371	0.820618634	0.841816497	positive
PRJNA729271	stage_5W_vs_E10	ordinary_muscle_development	ENSGALG00010007506	RAB38	-0.389301422	0.555208578	-0.701180489	0.483190384	0.524868452	negative
PRJNA729271	stage_5W_vs_E10	ordinary_muscle_development	ENSGALG00010007513	GALNT12	-0.156354604	0.129161078	-1.210539638	0.22607189	0.26046515	negative
PRJNA729271	stage_5W_vs_E10	ordinary_muscle_development	ENSGALG00010007514	VIT	-4.296023147	0.376789126	-11.40166435	4.10201E-30	2.49094E-29	negative
PRJNA729271	stage_5W_vs_E10	ordinary_muscle_development	ENSGALG00010007521	SLC12A7	-0.499031969	0.137211706	-3.63694894	0.000275887	0.00043786	negative
PRJNA729271	stage_5W_vs_E10	ordinary_muscle_development	ENSGALG00010007529	CDH13	2.56331823	0.134205562	19.09994034	2.52776E-81	7.0326E-80	positive
PRJNA729271	stage_5W_vs_E10	ordinary_muscle_development	ENSGALG00010007532	DCUN1D5	1.075004025	0.265945396	4.042198287	5.29524E-05	8.92287E-05	positive
PRJNA729271	stage_5W_vs_E10	ordinary_muscle_development	ENSGALG00010007536	GABBR2	-1.714025779	0.222863189	-7.690932668	1.46066E-14	4.47712E-14	negative
PRJNA729271	stage_5W_vs_E10	ordinary_muscle_development	ENSGALG00010007539	RPS3A	-0.307764703	0.175372368	-1.754921295	0.079272771	0.097537287	negative
PRJNA729271	stage_5W_vs_E10	ordinary_muscle_development	ENSGALG00010007542	MRPL36	0.940739063	0.109928258	8.55775463	1.15087E-17	4.14045E-17	positive
PRJNA729271	stage_5W_vs_E10	ordinary_muscle_development	ENSGALG00010007544	MLYCD	1.016701216	0.101237939	10.04268979	9.89388E-24	4.64717E-23	positive
PRJNA729271	stage_5W_vs_E10	ordinary_muscle_development	ENSGALG00010007547	ENSGALG00010007547	1.71879642	0.555855272	3.092165366	0.001987021	0.002921379	positive
PRJNA729271	stage_5W_vs_E10	ordinary_muscle_development	ENSGALG00010007548	HSBP1	-0.60397932	0.087773327	-6.881126004	5.93813E-12	1.58924E-11	negative
PRJNA729271	stage_5W_vs_E10	ordinary_muscle_development	ENSGALG00010007549	SH3D19	0.612550493	0.157891984	3.8795541	0.000104648	0.00017202	positive
PRJNA729271	stage_5W_vs_E10	ordinary_muscle_development	ENSGALG00010007551	FEZ2	-0.069821056	0.111647513	-0.625370454	0.531727949	0.571850172	negative
PRJNA729271	stage_5W_vs_E10	ordinary_muscle_development	ENSGALG00010007555	NDUFS6	2.049857504	0.129353082	15.8469939	1.47468E-56	2.14184E-55	positive
PRJNA729271	stage_5W_vs_E10	ordinary_muscle_development	ENSGALG00010007564	IRX4	-8.862901422	0.947294196	-9.35601787	8.27986E-21	3.42611E-20	negative
PRJNA729271	stage_5W_vs_E10	ordinary_muscle_development	ENSGALG00010007566	FHIP1A	0.601100185	0.173292727	3.468698282	0.000522986	0.000811116	positive
PRJNA729271	stage_5W_vs_E10	ordinary_muscle_development	ENSGALG00010007567	TRIP13	-2.918390468	0.148839824	-19.6075915	1.33192E-85	4.05591E-84	negative
PRJNA729271	stage_5W_vs_E10	ordinary_muscle_development	ENSGALG00010007569	GPR89A	-0.183234542	0.082811821	-2.212661662	0.02692098	0.035085381	negative
PRJNA729271	stage_5W_vs_E10	ordinary_muscle_development	ENSGALG00010007574	ENSGALG00010007574	0.257835344	0.190378079	1.354333153	0.175630114	0.206290032	positive
PRJNA729271	stage_5W_vs_E10	ordinary_muscle_development	ENSGALG00010007575	MMP27	-4.068302441	0.364588493	-11.15861448	6.49966E-29	3.77303E-28	negative
PRJNA729271	stage_5W_vs_E10	ordinary_muscle_development	ENSGALG00010007577	WFDC1	0.28203268	0.119481603	2.360469509	0.018251819	0.024249323	positive
PRJNA729271	stage_5W_vs_E10	ordinary_muscle_development	ENSGALG00010007579	GATB	0.281542584	0.17926777	1.570514228	0.116295527	0.140043505	positive
PRJNA729271	stage_5W_vs_E10	ordinary_muscle_development	ENSGALG00010007580	BRD9	-0.84994297	0.10303945	-8.248714185	1.60108E-16	5.43377E-16	negative
PRJNA729271	stage_5W_vs_E10	ordinary_muscle_development	ENSGALG00010007581	LPCAT1	-0.530586696	0.116102673	-4.569978296	4.87775E-06	8.8834E-06	negative
PRJNA729271	stage_5W_vs_E10	ordinary_muscle_development	ENSGALG00010007584	ELP2	-0.492693091	0.116379892	-4.233489844	2.30093E-05	3.98756E-05	negative
PRJNA729271	stage_5W_vs_E10	ordinary_muscle_development	ENSGALG00010007585	KCNG4	-1.553113125	0.671896284	-2.311537006	0.020803212	0.027425425	negative
PRJNA729271	stage_5W_vs_E10	ordinary_muscle_development	ENSGALG00010007586	MMP7	4.654016909	1.486050055	3.131803598	0.001737361	0.002569174	positive
PRJNA729271	stage_5W_vs_E10	ordinary_muscle_development	ENSGALG00010007588	TTC27	-1.214500745	0.104655325	-11.60476779	3.89744E-31	2.47344E-30	negative
PRJNA729271	stage_5W_vs_E10	ordinary_muscle_development	ENSGALG00010007590	FBXW7	-1.039036559	0.141203836	-7.358415966	1.86105E-13	5.3737E-13	negative
PRJNA729271	stage_5W_vs_E10	ordinary_muscle_development	ENSGALG00010007593	SEC61B	-1.509317667	0.119662207	-12.61315255	1.78699E-36	1.39111E-35	negative
PRJNA729271	stage_5W_vs_E10	ordinary_muscle_development	ENSGALG00010007595	ATP2C2	0.576998669	0.162755786	3.545180691	0.000392344	0.000614548	positive
PRJNA729271	stage_5W_vs_E10	ordinary_muscle_development	ENSGALG00010007596	ENSGALG00010007596	-4.034342269	0.536463012	-7.520261745	5.46667E-14	1.6301E-13	negative
PRJNA729271	stage_5W_vs_E10	ordinary_muscle_development	ENSGALG00010007597	SMKR1	-0.68788985	0.672786674	-1.022448684	0.306568589	0.345384089	negative
PRJNA729271	stage_5W_vs_E10	ordinary_muscle_development	ENSGALG00010007601	TMEM154	0.931671463	0.30125989	3.092583822	0.001984222	0.002917559	positive
PRJNA729271	stage_5W_vs_E10	ordinary_muscle_development	ENSGALG00010007603	ALG2	-0.391653329	0.106257208	-3.68589892	0.000227897	0.000364529	negative
PRJNA729271	stage_5W_vs_E10	ordinary_muscle_development	ENSGALG00010007604	ENSGALG00010007604	-4.571359036	0.627699857	-7.282714792	3.27168E-13	9.33158E-13	negative
PRJNA729271	stage_5W_vs_E10	ordinary_muscle_development	ENSGALG00010007610	TLDC1	0.044481605	0.158221612	0.281134825	0.778606991	0.80510118	positive
PRJNA729271	stage_5W_vs_E10	ordinary_muscle_development	ENSGALG00010007614	BIRC6	0.177494234	0.088032459	2.016236244	0.043775278	0.05562085	positive
PRJNA729271	stage_5W_vs_E10	ordinary_muscle_development	ENSGALG00010007624	TPPP	1.234268139	0.247621729	4.984490432	6.21253E-07	1.21117E-06	positive
PRJNA729271	stage_5W_vs_E10	ordinary_muscle_development	ENSGALG00010007626	DLK2	-3.588243269	0.392718234	-9.136940832	6.42439E-20	2.55899E-19	negative
PRJNA729271	stage_5W_vs_E10	ordinary_muscle_development	ENSGALG00010007633	ENSGALG00010007633	0.501709901	0.10303031	4.869536927	1.1186E-06	2.13878E-06	positive
PRJNA729271	stage_5W_vs_E10	ordinary_muscle_development	ENSGALG00010007640	TMEM123	-0.645440076	0.218207233	-2.95792247	0.0030972	0.004470615	negative
PRJNA729271	stage_5W_vs_E10	ordinary_muscle_development	ENSGALG00010007642	ZNF318	0.661807577	0.106818305	6.195638268	5.80493E-10	1.376E-09	positive
PRJNA729271	stage_5W_vs_E10	ordinary_muscle_development	ENSGALG00010007643	COTL1	-0.412605815	0.164045043	-2.515198307	0.011896545	0.016156696	negative
PRJNA729271	stage_5W_vs_E10	ordinary_muscle_development	ENSGALG00010007650	KLHL36	-0.163067798	0.088562502	-1.841273617	0.065581472	0.081501692	negative
PRJNA729271	stage_5W_vs_E10	ordinary_muscle_development	ENSGALG00010007651	CTNNAL1	-0.362880386	0.136404613	-2.660323417	0.007806565	0.01080456	negative
PRJNA729271	stage_5W_vs_E10	ordinary_muscle_development	ENSGALG00010007658	BIRC2	1.741408776	0.118638365	14.67829377	8.87968E-49	1.02969E-47	positive
PRJNA729271	stage_5W_vs_E10	ordinary_muscle_development	ENSGALG00010007660	FAM98A	1.269188221	0.09652547	13.14873904	1.73036E-39	1.48412E-38	positive
PRJNA729271	stage_5W_vs_E10	ordinary_muscle_development	ENSGALG00010007663	ARFIP1	-0.470896931	0.110674456	-4.254793269	2.09242E-05	3.63491E-05	negative
PRJNA729271	stage_5W_vs_E10	ordinary_muscle_development	ENSGALG00010007671	FAM206A	0.183085573	0.102056948	1.793955012	0.07282035	0.090054686	positive
PRJNA729271	stage_5W_vs_E10	ordinary_muscle_development	ENSGALG00010007673	FHDC1	-2.328518594	0.344565058	-6.757848886	1.40055E-11	3.67973E-11	negative
PRJNA729271	stage_5W_vs_E10	ordinary_muscle_development	ENSGALG00010007678	CDH8	0.359440528	0.285390414	1.259469521	0.207860792	0.241151127	positive
PRJNA729271	stage_5W_vs_E10	ordinary_muscle_development	ENSGALG00010007679	TMEM135	1.048687174	0.141890535	7.390818354	1.45928E-13	4.23553E-13	positive
PRJNA729271	stage_5W_vs_E10	ordinary_muscle_development	ENSGALG00010007680	CHMP2B	-0.063697427	0.125965512	-0.505673539	0.613085847	0.650086273	negative
PRJNA729271	stage_5W_vs_E10	ordinary_muscle_development	ENSGALG00010007695	RASGRP3	1.26126049	0.142590903	8.845308232	9.1276E-19	3.44453E-18	positive
PRJNA729271	stage_5W_vs_E10	ordinary_muscle_development	ENSGALG00010007698	ADAMTS18	-1.419095744	0.400424251	-3.543980512	0.000394134	0.000617219	negative
PRJNA729271	stage_5W_vs_E10	ordinary_muscle_development	ENSGALG00010007699	SLC39A6	-1.691438659	0.107992084	-15.66261714	2.72451E-55	3.80094E-54	negative
PRJNA729271	stage_5W_vs_E10	ordinary_muscle_development	ENSGALG00010007707	ENSGALG00010007707	5.582016927	0.706309387	7.90307623	2.72103E-15	8.67605E-15	positive
PRJNA729271	stage_5W_vs_E10	ordinary_muscle_development	ENSGALG00010007713	SCLT1	-0.596969586	0.214070499	-2.788658824	0.005292679	0.007450459	negative
PRJNA729271	stage_5W_vs_E10	ordinary_muscle_development	ENSGALG00010007719	LTBP1	-2.548829856	0.191306345	-13.32328969	1.69472E-40	1.50429E-39	negative
PRJNA729271	stage_5W_vs_E10	ordinary_muscle_development	ENSGALG00010007724	RBM11	1.81365657	0.331899858	5.464469259	4.64294E-08	9.80469E-08	positive
PRJNA729271	stage_5W_vs_E10	ordinary_muscle_development	ENSGALG00010007730	COG2	-0.336501939	0.064950703	-5.180882169	2.20839E-07	4.44592E-07	negative
PRJNA729271	stage_5W_vs_E10	ordinary_muscle_development	ENSGALG00010007731	ENSGALG00010007731	0.390665425	0.268166364	1.456802485	0.14517091	0.172550417	positive
PRJNA729271	stage_5W_vs_E10	ordinary_muscle_development	ENSGALG00010007741	ENSGALG00010007741	-0.005919153	0.206043342	-0.028727707	0.977081759	0.98026025	negative
PRJNA729271	stage_5W_vs_E10	ordinary_muscle_development	ENSGALG00010007748	CSGALNACT1	-2.863009043	0.271798838	-10.53355881	6.05029E-26	3.11431E-25	negative
PRJNA729271	stage_5W_vs_E10	ordinary_muscle_development	ENSGALG00010007751	HSPA13	-0.620419429	0.098607413	-6.291813242	3.13779E-10	7.5577E-10	negative
PRJNA729271	stage_5W_vs_E10	ordinary_muscle_development	ENSGALG00010007753	PRSS23	2.008179871	0.227609932	8.822900885	1.11532E-18	4.19584E-18	positive
PRJNA729271	stage_5W_vs_E10	ordinary_muscle_development	ENSGALG00010007758	DEK	-0.135077293	0.07672628	-1.760508817	0.078321574	0.09642188	negative
PRJNA729271	stage_5W_vs_E10	ordinary_muscle_development	ENSGALG00010007759	AGT	-0.838102217	1.283047123	-0.653212343			negative
PRJNA729271	stage_5W_vs_E10	ordinary_muscle_development	ENSGALG00010007761	ENSGALG00010007761	1.955441563	0.114567288	17.06806191	2.56577E-65	4.79882E-64	positive
PRJNA729271	stage_5W_vs_E10	ordinary_muscle_development	ENSGALG00010007762	SH2D4A	-0.659146132	0.334013921	-1.973409164	0.048448964	0.061183807	negative
PRJNA729271	stage_5W_vs_E10	ordinary_muscle_development	ENSGALG00010007763	FZD4	0.568402313	0.099842271	5.693002633	1.24824E-08	2.73975E-08	positive
PRJNA729271	stage_5W_vs_E10	ordinary_muscle_development	ENSGALG00010007765	NUDT7	0.435110297	0.09630612	4.517992196	6.24288E-06	1.12733E-05	positive
PRJNA729271	stage_5W_vs_E10	ordinary_muscle_development	ENSGALG00010007766	ENSGALG00010007766	1.923654637	0.127259001	15.11605952	1.26913E-51	1.61653E-50	positive
PRJNA729271	stage_5W_vs_E10	ordinary_muscle_development	ENSGALG00010007767	VAT1L	-2.034755382	0.325419222	-6.252720326	4.03364E-10	9.6545E-10	negative
PRJNA729271	stage_5W_vs_E10	ordinary_muscle_development	ENSGALG00010007770	ENSGALG00010007770	-0.19937332	0.404079215	-0.493401573	0.621728871	0.658191644	negative
PRJNA729271	stage_5W_vs_E10	ordinary_muscle_development	ENSGALG00010007771	CAPN9	-2.223870946	0.527442476	-4.216328886	2.48312E-05	4.28996E-05	negative
PRJNA729271	stage_5W_vs_E10	ordinary_muscle_development	ENSGALG00010007772	CLEC3A	-3.747991627	0.672872216	-5.570138788	2.54536E-08	5.47728E-08	negative
PRJNA729271	stage_5W_vs_E10	ordinary_muscle_development	ENSGALG00010007774	ENSGALG00010007774	0.483688885	0.284332199	1.701140026	0.088916697	0.108662635	positive
PRJNA729271	stage_5W_vs_E10	ordinary_muscle_development	ENSGALG00010007777	KIF13A	0.453703987	0.124839847	3.634288228	0.000278749	0.000442161	positive
PRJNA729271	stage_5W_vs_E10	ordinary_muscle_development	ENSGALG00010007780	SIRT5	0.773253808	0.142465465	5.427657907	5.70983E-08	1.19774E-07	positive
PRJNA729271	stage_5W_vs_E10	ordinary_muscle_development	ENSGALG00010007781	MBP	4.134920137	0.234955799	17.59871499	2.51977E-69	5.21772E-68	positive
PRJNA729271	stage_5W_vs_E10	ordinary_muscle_development	ENSGALG00010007783	NOL7	-0.425280211	0.110537234	-3.847393295	0.000119381	0.000195132	negative

PRJNA729271	stage_5W_vs_E10	ordinary_muscle_development	ENSGALG00010007784	INTS10	-0.683259789	0.09517719	-7.178818698	7.03163E-13	1.97756E-12	negative
PRJNA729271	stage_5W_vs_E10	ordinary_muscle_development	ENSGALG00010007786	SAMSN1	2.341548786	0.223838707	10.46087526	1.30643E-25	6.62355E-25	positive
PRJNA729271	stage_5W_vs_E10	ordinary_muscle_development	ENSGALG00010007787	C1orf198	-1.983232896	0.120149575	-16.50636626	3.30175E-61	5.52005E-60	negative
PRJNA729271	stage_5W_vs_E10	ordinary_muscle_development	ENSGALG00010007788	PALM2AKAP2	-0.152011197	0.169795334	-0.895261336	0.370647404	0.411436217	negative
PRJNA729271	stage_5W_vs_E10	ordinary_muscle_development	ENSGALG00010007789	WWOX	-1.030724048	0.115326895	-8.937412642	3.98388E-19	1.5293E-18	negative
PRJNA729271	stage_5W_vs_E10	ordinary_muscle_development	ENSGALG00010007792	ENSGALG00010007792	1.052587055	0.226946771	4.638034944	3.51737E-06	6.47831E-06	positive
PRJNA729271	stage_5W_vs_E10	ordinary_muscle_development	ENSGALG00010007795	ZNF236	0.131031895	0.116219345	1.127453398	0.259550833	0.295932457	positive
PRJNA729271	stage_5W_vs_E10	ordinary_muscle_development	ENSGALG00010007796	MCUR1	-0.620055003	0.103304776	-6.002191033	1.94672E-09	4.46554E-09	negative
PRJNA729271	stage_5W_vs_E10	ordinary_muscle_development	ENSGALG00010007798	HGSNAT	0.72735921	0.097372891	7.469832737	8.02968E-14	2.36421E-13	positive
PRJNA729271	stage_5W_vs_E10	ordinary_muscle_development	ENSGALG00010007800	MAF	0.438122149	0.114380518	3.830391363	0.00012794	0.000208674	positive
PRJNA729271	stage_5W_vs_E10	ordinary_muscle_development	ENSGALG00010007801	TTC13	0.041403604	0.161201976	0.256843031	0.79729995	0.821383282	positive
PRJNA729271	stage_5W_vs_E10	ordinary_muscle_development	ENSGALG00010007802	ENSGALG00010007802	4.679679552	0.718832015	6.510115654	7.5093E-11	1.88807E-10	positive
PRJNA729271	stage_5W_vs_E10	ordinary_muscle_development	ENSGALG00010007803	ME3	0.563159837	0.177621487	3.170561428	0.001521447	0.002263044	positive
PRJNA729271	stage_5W_vs_E10	ordinary_muscle_development	ENSGALG00010007805	ENSGALG00010007805	9.125832468	1.518051879	6.011541894	1.83767E-09	4.22274E-09	positive
PRJNA729271	stage_5W_vs_E10	ordinary_muscle_development	ENSGALG00010007806	DYNLRB2	-1.761330975	0.439518656	-4.0074089	6.13885E-05	0.000103109	negative
PRJNA729271	stage_5W_vs_E10	ordinary_muscle_development	ENSGALG00010007809	ARV1	-0.43181504	0.121417274	-3.556454755	0.000375893	0.000589928	negative
PRJNA729271	stage_5W_vs_E10	ordinary_muscle_development	ENSGALG00010007811	PRCP	0.405693019	0.182875478	2.218411264	0.026526804	0.034609003	positive
PRJNA729271	stage_5W_vs_E10	ordinary_muscle_development	ENSGALG00010007812	POMK	-0.195229716	0.122292432	-1.596416983	0.110395727	0.133310091	negative
PRJNA729271	stage_5W_vs_E10	ordinary_muscle_development	ENSGALG00010007817	CDYL2	-1.484038555	0.140733046	-10.54506102	5.35381E-26	2.76661E-25	negative
PRJNA729271	stage_5W_vs_E10	ordinary_muscle_development	ENSGALG00010007818	ENSGALG00010007818	0.479000132	0.224795932	2.130822062	0.033103804	0.042732179	positive
PRJNA729271	stage_5W_vs_E10	ordinary_muscle_development	ENSGALG00010007819	NRIP1	1.926919813	0.166441964	11.57712736	5.38194E-31	3.40067E-30	positive
PRJNA729271	stage_5W_vs_E10	ordinary_muscle_development	ENSGALG00010007821	FAM89A	-2.079409972	0.126845421	-16.39325999	2.13672E-60	3.4644E-59	negative
PRJNA729271	stage_5W_vs_E10	ordinary_muscle_development	ENSGALG00010007826	ENSGALG00010007826	8.60205667	1.375783426	6.252478776	4.03989E-10	9.66785E-10	positive
PRJNA729271	stage_5W_vs_E10	ordinary_muscle_development	ENSGALG00010007830	SMIM19	0.776593453	0.130578738	5.947319324	2.72569E-09	6.20624E-09	positive
PRJNA729271	stage_5W_vs_E10	ordinary_muscle_development	ENSGALG00010007835	GUCY1A1	1.515014636	0.18622519	8.135390481	4.10613E-16	1.36792E-15	positive
PRJNA729271	stage_5W_vs_E10	ordinary_muscle_development	ENSGALG00010007845	TFAP2B	-7.576600231	1.094265546	-6.923913721	4.39334E-12	1.18257E-11	negative
PRJNA729271	stage_5W_vs_E10	ordinary_muscle_development	ENSGALG00010007846	ENSGALG00010007846	0.423943776	0.148753547	2.849974226	0.004372277	0.006212131	positive
PRJNA729271	stage_5W_vs_E10	ordinary_muscle_development	ENSGALG00010007847	CMC2	0.133521394	0.116849717	1.142676231	0.253173042	0.289206655	positive
PRJNA729271	stage_5W_vs_E10	ordinary_muscle_development	ENSGALG00010007849	CXADR	-3.580057218	0.317445568	-11.27770421	1.69102E-29	1.00333E-28	negative
PRJNA729271	stage_5W_vs_E10	ordinary_muscle_development	ENSGALG00010007850	ENSGALG00010007850	-1.782889077	0.281350215	-6.336903194	2.34429E-10	5.69569E-10	negative
PRJNA729271	stage_5W_vs_E10	ordinary_muscle_development	ENSGALG00010007851	AvBD7	-0.521266155	1.012771117	-0.514692951	0.6067676	0.644093472	negative
PRJNA729271	stage_5W_vs_E10	ordinary_muscle_development	ENSGALG00010007852	SLC20A2	0.594563554	0.20795825	2.859052497	0.004249085	0.006047769	positive
PRJNA729271	stage_5W_vs_E10	ordinary_muscle_development	ENSGALG00010007853	C1orf131	0.584225995	0.110415036	5.291181513	1.21529E-07	2.49442E-07	positive
PRJNA729271	stage_5W_vs_E10	ordinary_muscle_development	ENSGALG00010007854	FAM181B	0.740058449	0.117696515	6.287853523	3.21885E-10	7.74781E-10	positive
PRJNA729271	stage_5W_vs_E10	ordinary_muscle_development	ENSGALG00010007855	RNF182	-1.781112797	0.228877839	-7.781936432	7.14228E-15	2.22735E-14	negative
PRJNA729271	stage_5W_vs_E10	ordinary_muscle_development	ENSGALG00010007856	PDCD2	0.935650998	0.129327744	7.234727576	4.66465E-13	1.32291E-12	positive
PRJNA729271	stage_5W_vs_E10	ordinary_muscle_development	ENSGALG00010007857	ENSGALG00010007857	0.430972566	0.331052906	1.301823843	0.192976611	0.225144557	positive
PRJNA729271	stage_5W_vs_E10	ordinary_muscle_development	ENSGALG00010007859	CENPN	-1.992265212	0.163587171	-12.17861522	4.04084E-34	2.87682E-33	negative
PRJNA729271	stage_5W_vs_E10	ordinary_muscle_development	ENSGALG00010007860	DEFB4A	-0.528448414	0.981516045	-0.538400179	0.590300803	0.628500818	negative
PRJNA729271	stage_5W_vs_E10	ordinary_muscle_development	ENSGALG00010007861	INTU	-2.345538416	0.182076366	-12.88216848	5.67192E-38	4.62659E-37	negative
PRJNA729271	stage_5W_vs_E10	ordinary_muscle_development	ENSGALG00010007862	ATMIN	0.442130229	0.086139159	5.132743742	2.85549E-07	5.6933E-07	positive
PRJNA729271	stage_5W_vs_E10	ordinary_muscle_development	ENSGALG00010007863	AvBD1	-0.910193999	0.928718264	-0.980053945	0.327059491	0.366702299	negative
PRJNA729271	stage_5W_vs_E10	ordinary_muscle_development	ENSGALG00010007864	TYR	-4.247909911	1.160928279	-3.659063173	0.000253139	0.000403258	negative
PRJNA729271	stage_5W_vs_E10	ordinary_muscle_development	ENSGALG00010007865	GABRR3	0.846745517	0.425918493	1.988045906	0.046806613	0.059233618	positive
PRJNA729271	stage_5W_vs_E10	ordinary_muscle_development	ENSGALG00010007866	TBP	-0.412607054	0.086639234	-4.762358046	1.91344E-06	3.59405E-06	negative
PRJNA729271	stage_5W_vs_E10	ordinary_muscle_development	ENSGALG00010007867	ZNF516	-1.53960386	0.107783308	-14.28425136	2.74342E-46	2.90474E-45	negative
PRJNA729271	stage_5W_vs_E10	ordinary_muscle_development	ENSGALG00010007868	INHBA	-2.091182571	0.302059411	-6.923083643	4.41917E-12	1.1893E-11	negative
PRJNA729271	stage_5W_vs_E10	ordinary_muscle_development	ENSGALG00010007869	GCSH	-1.420740133	0.125615565	-11.31022365	1.16791E-29	6.97237E-29	negative
PRJNA729271	stage_5W_vs_E10	ordinary_muscle_development	ENSGALG00010007870	AvBD3	-3.122472279	0.924487051	-3.377518676	0.00073143	0.00112049	negative
PRJNA729271	stage_5W_vs_E10	ordinary_muscle_development	ENSGALG00010007871	HSPA4L	-0.895878568	0.088839713	-10.08421278	6.48832E-24	3.06946E-23	negative
PRJNA729271	stage_5W_vs_E10	ordinary_muscle_development	ENSGALG00010007872	KLHL2	-0.659900576	0.077970933	-8.463417683	2.59654E-17	9.16629E-17	negative
PRJNA729271	stage_5W_vs_E10	ordinary_muscle_development	ENSGALG00010007873	ENSGALG00010007873	0.654902168	0.767224745	0.853598861	0.39332729	0.434581833	positive
PRJNA729271	stage_5W_vs_E10	ordinary_muscle_development	ENSGALG00010007874	ENSGALG00010007874	-0.889757902	0.607553685	-1.464492644	0.143059391	0.170180212	negative
PRJNA729271	stage_5W_vs_E10	ordinary_muscle_development	ENSGALG00010007875	AvBD4	0.188683641	1.600132245	0.117917529	0.906133004	0.918296713	positive
PRJNA729271	stage_5W_vs_E10	ordinary_muscle_development	ENSGALG00010007877	MSMO1	-0.225389487	0.187364439	-1.202946984	0.228996837	0.263437234	negative
PRJNA729271	stage_5W_vs_E10	ordinary_muscle_development	ENSGALG00010007878	PSMB1	0.350819735	0.122151851	2.871996867	0.00407887	0.005814636	positive
PRJNA729271	stage_5W_vs_E10	ordinary_muscle_development	ENSGALG00010007879	PLK4	-2.44291951	0.196960757	-12.40307738	2.51477E-35	1.87701E-34	negative
PRJNA729271	stage_5W_vs_E10	ordinary_muscle_development	ENSGALG00010007880	BCO1	2.449757033	0.280629771	8.729498021	2.55805E-18	9.47101E-18	positive
PRJNA729271	stage_5W_vs_E10	ordinary_muscle_development	ENSGALG00010007881	AvBD14	-2.804308887	0.87913511	-3.189849723	0.001423468	0.002125159	negative
PRJNA729271	stage_5W_vs_E10	ordinary_muscle_development	ENSGALG00010007882	CPE	-0.930076249	0.227585064	-4.086719187	4.37516E-05	7.40864E-05	negative
PRJNA729271	stage_5W_vs_E10	ordinary_muscle_development	ENSGALG00010007883	DHTKD1	0.211531303	0.121001876	1.748165486	0.080435376	0.098873031	positive
PRJNA729271	stage_5W_vs_E10	ordinary_muscle_development	ENSGALG00010007884	GLI3	-1.534207589	0.113503896	-13.51678345	1.24501E-41	1.14146E-40	negative
PRJNA729271	stage_5W_vs_E10	ordinary_muscle_development	ENSGALG00010007885	USP38	0.905162011	0.103789824	8.721105582	2.75498E-18	1.01845E-17	positive
PRJNA729271	stage_5W_vs_E10	ordinary_muscle_development	ENSGALG00010007886	GRM5	-3.385353775	0.97574701	-3.469499513	0.000521429	0.000808873	negative
PRJNA729271	stage_5W_vs_E10	ordinary_muscle_development	ENSGALG00010007887	CDT1	-1.400269976	0.127512834	-10.98140428	4.69567E-28	2.63711E-27	negative
PRJNA729271	stage_5W_vs_E10	ordinary_muscle_development	ENSGALG00010007889	DCAF6	2.422112912	0.097000652	24.97006839	1.293E-137	1.0186E-135	positive
PRJNA729271	stage_5W_vs_E10	ordinary_muscle_development	ENSGALG00010007890	ENSGALG00010007890	-1.398430996	0.126240447	-11.07751935	1.61279E-28	9.20733E-28	negative
PRJNA729271	stage_5W_vs_E10	ordinary_muscle_development	ENSGALG00010007891	GDI1	-0.80202339	0.056036435	-14.31253427	1.82731E-46	1.94756E-45	negative
PRJNA729271	stage_5W_vs_E10	ordinary_muscle_development	ENSGALG00010007892	ENSGALG00010007892	-1.026709897	0.495732916	-2.071094863	0.038349932	0.049110545	negative
PRJNA729271	stage_5W_vs_E10	ordinary_muscle_development	ENSGALG00010007893	MFSD8	0.608325101	0.148688776	4.091264444	4.29028E-05	7.2683E-05	positive
PRJNA729271	stage_5W_vs_E10	ordinary_muscle_development	ENSGALG00010007894	CD88	2.151866225	0.259355526	8.296974647	1.06796E-16	3.65614E-16	positive
PRJNA729271	stage_5W_vs_E10	ordinary_muscle_development	ENSGALG00010007895	GOLGB1	-0.600940447	0.112109611	-5.360293731	8.30867E-08	1.72295E-07	negative
PRJNA729271	stage_5W_vs_E10	ordinary_muscle_development	ENSGALG00010007896	TIMMDC1	0.780433279	0.104977017	7.434325199	1.05103E-13	3.07647E-13	positive
PRJNA729271	stage_5W_vs_E10	ordinary_muscle_development	ENSGALG00010007897	EFHC1	-0.324842845	0.363332539	-0.894064832	0.371287204	0.41208331	negative
PRJNA729271	stage_5W_vs_E10	ordinary_muscle_development	ENSGALG00010007898	GAB1	0.415092832	0.089392313	4.643495837	3.42563E-06	6.323E-06	positive
PRJNA729271	stage_5W_vs_E10	ordinary_muscle_development	ENSGALG00010007899	APRT	-0.618148839	0.15314149	-4.036455702	5.42648E-05	9.13658E-05	negative
PRJNA729271	stage_5W_vs_E10	ordinary_muscle_development	ENSGALG00010007900	FHOD3	0.822914118	0.199938198	4.115842419	3.85768E-05	6.56303E-05	positive
PRJNA729271	stage_5W_vs_E10	ordinary_muscle_development	ENSGALG00010007901	FSTL5	-1.706055543	0.322649367	-5.287645718	1.23901E-07	2.5413E-07	negative
PRJNA729271	stage_5W_vs_E10	ordinary_muscle_development	ENSGALG00010007902	C7orf25	2.475178131	0.141688017	17.46921288	2.45863E-68	4.93599E-67	positive
PRJNA729271	stage_5W_vs_E10	ordinary_muscle_development	ENSGALG00010007903	PSMA2	0.003070086	0.103657088	0.029617716	0.976371936	0.979751555	positive
PRJNA729271	stage_5W_vs_E10	ordinary_muscle_development	ENSGALG00010007904	ENSGALG00010007904	0.00515388	0.123991312	0.041566462	0.96684431	0.971468756	positive
PRJNA729271	stage_5W_vs_E10	ordinary_muscle_development	ENSGALG00010007905	MRPL32	-0.341242128	0.159341454	-2.141577851	0.032227468	0.041656603	negative
PRJNA729271	stage_5W_vs_E10	ordinary_muscle_development	ENSGALG00010007906	NAF1	-1.074876462	0.115063753	-9.341573154	9.49132E-21	3.90954E-20	negative
PRJNA729271	stage_5W_vs_E10	ordinary_muscle_development	ENSGALG00010007907	IRX2	-8.518415702	1.029954656	-8.270670608	1.33208E-16	4.54316E-16	negative
PRJNA729271	stage_5W_vs_E10	ordinary_muscle_development	ENSGALG00010007908	IRX1	-3.758439274	0.614819388	-6.113078647	9.77272E-10	2.28182E-09	negative
PRJNA729271	stage_5W_vs_E10	ordinary_muscle_development	ENSGALG00010007909	GALNS	-0.070511926	0.107426212	-0.656375428	0.511582596	0.552274502	negative
PRJNA729271	stage_5W_vs_E10	ordinary_muscle_development	ENSGALG00010007910	ANKS6	0.004860411	0.148514306	0.032726888	0.973892382	0.977669511	positive
PRJNA729271	stage_5W_vs_E10	ordinary_muscle_development	ENSGALG00010007911	IF16	7.543381535	0.800260242	9.426160568	4.25375E-21	1.78719E-20	positive
PRJNA729271	stage_5W_vs_E10	ordinary_muscle_development	ENSGALG00010007912	TRAPPC2L	-0.222131398	0.115255307	-1.927298646	0.053942422	0.06771416	negative
PRJNA729271	stage_5W_vs_E10	ordinary_muscle_development	ENSGALG00010007914	PAQR8	0.252006222	0.125128148	2.013985073	0.044011101	0.05589109	positive
PRJNA729271	stage_5W_vs_E10	ordinary_muscle_development	ENSGALG00010007915	HECW1	-0.327568026	0.139868094	-2.341978191	0.019181836	0.025419703	negative
PRJNA729271	stage_5W_vs_E10	ordinary_muscle_development	ENSGALG00010007916	MCM3	-3.016934129	0.205684867	-14.66774959	1.03729E-48	1.199E-47	negative
PRJNA729271	stage_5W_vs_E10	ordinary_muscle_development	ENSGALG00010007917	POGLUT1	-2.753669841	0.084166628	-32.71688426	8.9856E-235	2.1708E-232	negative
PRJNA729271	stage_5W_vs_E10	ordinary_muscle_development	ENSGALG00010007918	NPY1R	-1.911470998	0.729793929	-2.61919279	0.008813812	0.012131441	negative
PRJNA729271	stage_5W_vs_E10	ordinary_muscle_development	ENSGALG00010007920	FAT3	-3.845181713	0.185592592	-20.71840079	2.36408E-95	9.08948E-94	negative
PRJNA729271	stage_5W_vs_E10	ordinary_muscle_development	ENSGALG00010007921	FAM120B	-0.454324673	0.101902569	-4.458422161	8.25652E-06	1.4797E-05	negative
PRJNA729271	stage_5W_vs_E10	ordinary_muscle_development	ENSGALG00010007922	IL17A	-0.665286883	0.510398087	-1.303466649	0.192415495	0.224544127	negative
PRJNA729271	stage_5W_vs_E10	ordinary_muscle_development	ENSGALG00010007923	YAP1	-1.947308579	0.092062925	-21.15193032	2.64878E-99	1.09698E-97	negative
PRJNA729271	stage_5W_vs_E10	ordinary_muscle_development	ENSGALG00010007924	ABHD18	-0.132471257	0.116321137	-1.138840804	0.25476956	0.290938688	negative
PRJNA729271	stage_5W_vs_E10	ordinary_muscle_development	ENSGALG00010007925	STK17A	-1.065718426	0.24148781	-4.41313549	1.01884E-05	1.81516E-05	negative
PRJNA729271	stage_5W_vs_E10	ordinary_muscle_development	ENSGALG00010007926	EIF3E	0.191006942	0.13142606	1.453341456	0.146128972	0.173646455	positive
PRJNA729271	stage_5W_vs_E10	ordinary_muscle_development	ENSGALG00010007927	DLL1	0.366349367	0.163185712	2.244984336	0.024769138	0.032397461	positive
PRJNA729271	stage_5W_vs_E10	ordinary_muscle_development	ENSGALG00010007928	C11orf70	-2.633659223	0.709674068	-3.711082796	0.000206375	0.000331253	negative
PRJNA729271	stage_5W_vs_E10	ordinary_muscle_development	ENSGALG00010007929	LRRC14B	7.583258227	0.329802284	22.99334662	5.4335E-117	3.1886E-115	positive
PRJNA729271	stage_5W_vs_E10	ordinary_muscle_development	ENSGALG00010007930	CBFA2T3	0.545135808	0.197055797	2.766403304	0.005667839	0.007957703	positive
PRJNA729271	stage_5W_vs_E10	ordinary_muscle_development	ENSGALG00010007931	GBE1	1.554436119	0.189087982	8.220702888	2.02314E-16	6.83735E-16	positive
PRJNA729271	stage_5W_vs_E10	ordinary_muscle_development	ENSGALG00010007932	NPY5R	0.51843356	0.181736983	2.852658558	0.004335518	0.006163528	positive
PRJNA729271	stage_5W_vs_E10	ordinary_muscle_development	ENSGALG00010007933	CEP126	0.1502529	0.459215124	0.327195016	0.74352039	0.772070209	positive
PRJNA729271	stage_5W_vs_E10	ordinary_muscle_development	ENSGALG00010007934	PKHD1	-1.151846412	0.35966677	-3.202537756	0.001362224	0.002037503	negative
PRJNA729271	stage_5W_vs_E10	ordinary_muscle_development	ENSGALG00010007935	LARP1B	2.137191745	0.122156647	17.49550099	1.55043E-68	3.13876E-67	positive
PRJNA729271	stage_5W_vs_E10	ordinary_muscle_development	ENSGALG00010007936	ENSGALG00010007936	2.397523749	0.139066162	17.24016621	1.32655E-66	2.57407E-65	positive
PRJNA729271	stage_5W_vs_E10	ordinary_muscle_development	ENSGALG00010007937	ANGPTL5	2.554330038	0.403910101	6.324006342	2.54867E-10	6.1819E-10	positive
PRJNA729271	stage_5W_vs_E10	ordinary_muscle_development	ENSGALG00010007938	BCL9	-0.990573933	0.106884916	-9.267668203	1.9026E-20	7.74015E-20	negative
PRJNA729271	stage_5W_vs_E10	ordinary_muscle_development	ENSGALG00010007940	GLIPR2	-1.3969244	0.271902946	-5.13758464	2.78292E-07	5.55396E-07	negative
PRJNA729271	stage_5W_vs_E10	ordinary_muscle_development	ENSGALG00010007941	MRPL19	-0.797539939	0.075246823	-10.59898484	3.01232E-26	1.5746E-25	negative
PRJNA729271	stage_5W_vs_E10	ordinary_muscle_development	ENSGALG00010007942	PGRMC2	-0.481639752	0.086612346	-5.560867163	2.68437E-08	5.76274E-08	negative
PRJNA729271	stage_5W_vs_E10	ordinary_muscle_development	ENSGALG00010007943	BLOC1S4	0.284976333	0.100566794	2.833702082	0.004601221	0.006520158	positive
PRJNA729271	stage_5W_vs_E10	ordinary_muscle_development	ENSGALG00010007944	ACSF3	-0.700354677	0.145247103	-4.821815114	1.42258E-06	2.69934E-06	negative
PRJNA729271	stage_5W_vs_E10	ordinary_muscle_development	ENSGALG00010007945	ENSGALG00010007945	-1.479655178	0.124180943	-11.91531597	9.84921E-33	6.64329E-32	negative
PRJNA729271	stage_5W_vs_E10	ordinary_muscle_development	ENSGALG00010007946	ENSGALG00010007946	-0.453616256	0.160350028	-2.828912863	0.004670641	0.006615943	negative
PRJNA729271	stage_5W_vs_E10	ordinary_muscle_development	ENSGALG00010007948	ENSGALG00010007948	-2.090618879	0.474039358	-4.410222154	1.03265E-05	1.83839E-05	negative
PRJNA729271	stage_5W_vs_E10	ordinary_muscle_development	ENSGALG00010007950	ARC	0.120227795	0.548864105	0.219048383	0.826612359	0.847464087	positive
PRJNA729271	stage_5W_vs_E10	ordinary_muscle_development	ENSGALG00010007951	TGFBR1	-0.851960967	0.114455223	-7.443618061	9.79645E-14	2.87042E-13	negative
PRJNA729271	stage_5W_vs_E10	ordinary_muscle_development	ENSGALG00010007952	EMC2	-0.570760783	0.110246146	-5.177149529	2.25302E-07	4.53198E-07	negative
PRJNA729271	stage_5W_vs_E10	ordinary_muscle_development	ENSGALG00010007953	SCGN	-4.155429608	0.815489868	-5.095623832	3.47595E-07	6.88963E-07	negative
PRJNA729271	stage_5W_vs_E10	ordinary_muscle_development	ENSGALG00010007954	PSCA	0.7847568	0.590646852	1.328639605	0.183966899	0.215465433	positive
PRJNA729271	stage_5W_vs_E10	ordinary_muscle_development	ENSGALG00010007955	TMA16	-0.018239767	0.12211469	-0.149365871	0.881264941	0.89578789	negative
PRJNA729271	stage_5W_vs_E10	ordinary_muscle_development	ENSGALG00010007956	HS1BP3	0.354782216	0.131836817	2.691070867	0.007122306	0.00990006	positive
PRJNA729271	stage_5W_vs_E10	ordinary_muscle_development	ENSGALG00010007957	CDH15	0.153926853	0.163352945	0.942296162	0.346041033	0.38589512	positive
PRJNA729271	stage_5W_vs_E10	ordinary_muscle_development	ENSGALG00010007958	ENSGALG00010007958	3.4870209	0.477056568	7.309449524	2.68239E-13	7.6886E-13	positive
PRJNA729271	stage_5W_vs_E10	ordinary_muscle_development	ENSGALG00010007960	TSNAX	-0.710208822	0.063472924	-11.18916189	4.60768E-29	2.69308E-28	negative

PRJNA729271	stage_5W_vs_E10	ordinary_muscle_development	ENSGALG00010007962	ADCY8	-2.534352684	0.507815531	-4.9906955	6.01623E-07	1.17464E-06	negative
PRJNA729271	stage_5W_vs_E10	ordinary_muscle_development	ENSGALG00010007963	LDAH	0.081183735	0.084669558	0.958830271	0.337644257	0.377462501	positive
PRJNA729271	stage_5W_vs_E10	ordinary_muscle_development	ENSGALG00010007964	RECQL4	-2.78882586	0.171978192	-16.21615987	3.87689E-59	6.06208E-58	negative
PRJNA729271	stage_5W_vs_E10	ordinary_muscle_development	ENSGALG00010007965	EGLN1	1.609330833	0.15746708	10.22010974	1.61158E-24	7.84152E-24	positive
PRJNA729271	stage_5W_vs_E10	ordinary_muscle_development	ENSGALG00010007966	TMEM74	-1.671426909	0.230319046	-7.257006924	3.95749E-13	1.1239E-12	negative
PRJNA729271	stage_5W_vs_E10	ordinary_muscle_development	ENSGALG00010007968	SLC22A31	-0.558909283	0.223643773	-2.499105051	0.01245074	0.016861952	negative
PRJNA729271	stage_5W_vs_E10	ordinary_muscle_development	ENSGALG00010007970	SPRTN	0.995013841	0.106041161	9.383279353	6.39521E-21	2.65917E-20	positive
PRJNA729271	stage_5W_vs_E10	ordinary_muscle_development	ENSGALG00010007972	EXOC8	-0.181867467	0.112621241	-1.614859377	0.106341151	0.128740916	negative
PRJNA729271	stage_5W_vs_E10	ordinary_muscle_development	ENSGALG00010007973	CARMIL1	-0.059443626	0.134337767	-0.442493775	0.658131934	0.692632134	negative
PRJNA729271	stage_5W_vs_E10	ordinary_muscle_development	ENSGALG00010007975	ANKRD11	0.648118204	0.1760596	3.681243192	0.000232099	0.000371006	positive
PRJNA729271	stage_5W_vs_E10	ordinary_muscle_development	ENSGALG00010007976	ENSGALG00010007976	0.014775758	0.378457281	0.039042076	0.968856843	0.973288512	positive
PRJNA729271	stage_5W_vs_E10	ordinary_muscle_development	ENSGALG00010007977	APOB	3.178582298	1.377471062	2.307549237	0.021024221	0.027699153	positive
PRJNA729271	stage_5W_vs_E10	ordinary_muscle_development	ENSGALG00010007978	JADE1	1.36211666	0.115940681	11.74839281	7.19747E-32	4.69098E-31	positive
PRJNA729271	stage_5W_vs_E10	ordinary_muscle_development	ENSGALG00010007979	RAB30	-0.3659608	0.089890948	-4.071164118	4.67788E-05	7.90923E-05	negative
PRJNA729271	stage_5W_vs_E10	ordinary_muscle_development	ENSGALG00010007980	MARCHF1	1.149377658	0.221758016	5.18302642	2.18314E-07	4.39692E-07	positive
PRJNA729271	stage_5W_vs_E10	ordinary_muscle_development	ENSGALG00010007981	TDRD15	-5.273575009	0.960770913	-5.488899526	4.04446E-08	8.56706E-08	negative
PRJNA729271	stage_5W_vs_E10	ordinary_muscle_development	ENSGALG00010007982	RECK	-0.588837652	0.146705666	-4.013734895	5.97655E-05	0.000100452	negative
PRJNA729271	stage_5W_vs_E10	ordinary_muscle_development	ENSGALG00010007983	RCHY1	0.272489298	0.087285199	3.121827087	0.001797325	0.002654057	positive
PRJNA729271	stage_5W_vs_E10	ordinary_muscle_development	ENSGALG00010007984	EIF3H	0.084974684	0.122141454	0.695707157	0.486612242	0.528110546	positive
PRJNA729271	stage_5W_vs_E10	ordinary_muscle_development	ENSGALG00010007985	APELA	-5.811178864	0.509427677	-11.40726961	3.84613E-30	2.33849E-29	negative
PRJNA729271	stage_5W_vs_E10	ordinary_muscle_development	ENSGALG00010007986	MYC	-0.628403234	0.191620018	-3.279423732	0.001040193	0.001574355	negative
PRJNA729271	stage_5W_vs_E10	ordinary_muscle_development	ENSGALG00010007987	ENSGALG00010007987	0.88672673	0.295039616	3.00544972	0.002651884	0.00385354	positive
PRJNA729271	stage_5W_vs_E10	ordinary_muscle_development	ENSGALG00010007988	TARBP1	-0.205564035	0.124154209	-1.655715396	0.097779468	0.118912106	negative
PRJNA729271	stage_5W_vs_E10	ordinary_muscle_development	ENSGALG00010007989	SPG7	0.300575599	0.097413765	3.085555719	0.002031722	0.002983769	positive
PRJNA729271	stage_5W_vs_E10	ordinary_muscle_development	ENSGALG00010007990	TRHR	-4.569890442	1.154799176	-3.957303171	7.58007E-05	0.00012632	negative
PRJNA729271	stage_5W_vs_E10	ordinary_muscle_development	ENSGALG00010007991	PCF11	0.671883933	0.10984763	6.116508236	9.56479E-10	2.23471E-09	positive
PRJNA729271	stage_5W_vs_E10	ordinary_muscle_development	ENSGALG00010007992	LAPTM4A	0.421718344	0.099014247	4.259168308	2.05189E-05	3.56749E-05	positive
PRJNA729271	stage_5W_vs_E10	ordinary_muscle_development	ENSGALG00010007993	LRRC14	0.623956908	0.174046043	3.585010603	0.000337065	0.000531118	positive
PRJNA729271	stage_5W_vs_E10	ordinary_muscle_development	ENSGALG00010007994	FAM65B	0.755960797	0.166593194	4.5377652	5.68535E-06	1.03037E-05	positive
PRJNA729271	stage_5W_vs_E10	ordinary_muscle_development	ENSGALG00010007995	KIAA0319	-6.031398743	1.024591071	-5.886639963	3.94126E-09	8.90408E-09	negative
PRJNA729271	stage_5W_vs_E10	ordinary_muscle_development	ENSGALG00010007996	NUDCD1	-1.226857576	0.076947512	-15.94408371	3.132E-57	4.65147E-56	negative
PRJNA729271	stage_5W_vs_E10	ordinary_muscle_development	ENSGALG00010007997	TMEM192	-0.314654734	0.138456753	-2.272584951	0.023051203	0.030245967	negative
PRJNA729271	stage_5W_vs_E10	ordinary_muscle_development	ENSGALG00010007998	RPL13	-0.314152136	0.144851682	-2.168784862	0.030099021	0.039041191	negative
PRJNA729271	stage_5W_vs_E10	ordinary_muscle_development	ENSGALG00010007999	SDC1	-1.28380951	0.208228066	-6.165400937	7.03048E-10	1.65541E-09	negative
PRJNA729271	stage_5W_vs_E10	ordinary_muscle_development	ENSGALG00010008000	C8orf82	-0.81058755	0.177295163	-4.571966524	4.83168E-06	8.80171E-06	negative
PRJNA729271	stage_5W_vs_E10	ordinary_muscle_development	ENSGALG00010008001	ALDH5A1	0.40392388	0.136412048	2.961057213	0.00306585	0.004425803	positive
PRJNA729271	stage_5W_vs_E10	ordinary_muscle_development	ENSGALG00010008002	GJA8	-1.156026137	0.175201325	-6.598272804	4.15975E-11	1.06304E-10	negative
PRJNA729271	stage_5W_vs_E10	ordinary_muscle_development	ENSGALG00010008004	ENY2	-1.207383264	0.171873606	-7.02483234	2.14323E-12	5.87151E-12	negative
PRJNA729271	stage_5W_vs_E10	ordinary_muscle_development	ENSGALG00010008005	ANKRD42	-0.342447962	0.348750021	-0.981929581	0.326134542	0.365778411	negative
PRJNA729271	stage_5W_vs_E10	ordinary_muscle_development	ENSGALG00010008006	CPNE7	-2.259284886	0.835125737	-2.705323027	0.0068238	0.009504274	negative
PRJNA729271	stage_5W_vs_E10	ordinary_muscle_development	ENSGALG00010008007	CBR4	0.296842114	0.122894975	2.415412943	0.015717379	0.021040212	positive
PRJNA729271	stage_5W_vs_E10	ordinary_muscle_development	ENSGALG00010008008	BTC	3.531186104	0.199948124	17.66051131	8.4471E-70	1.77708E-68	positive
PRJNA729271	stage_5W_vs_E10	ordinary_muscle_development	ENSGALG00010008009	GPLD1	-0.842588021	1.307645294	-0.644355181	0.519345141	0.559903891	negative
PRJNA729271	stage_5W_vs_E10	ordinary_muscle_development	ENSGALG00010008010	CERK	-0.487695219	0.092887739	-5.250372362	1.51792E-07	3.09412E-07	negative
PRJNA729271	stage_5W_vs_E10	ordinary_muscle_development	ENSGALG00010008011	ADNP2	-0.18623603	0.10071558	-1.84912831	0.064439287	0.080168867	negative
PRJNA729271	stage_5W_vs_E10	ordinary_muscle_development	ENSGALG00010008012	CCDC90B	-0.451610958	0.081088365	-5.569368142	2.55665E-08	5.49913E-08	negative
PRJNA729271	stage_5W_vs_E10	ordinary_muscle_development	ENSGALG00010008013	ENSGALG00010008013	-0.284267017	0.240249537	-1.183215667	0.236723685	0.271873054	negative
PRJNA729271	stage_5W_vs_E10	ordinary_muscle_development	ENSGALG00010008015	ARHGAP39	-0.917706135	0.160488836	-5.718192982	1.07663E-08	2.37101E-08	negative
PRJNA729271	stage_5W_vs_E10	ordinary_muscle_development	ENSGALG00010008016	GSR	-0.761439446	0.144130798	-5.282975311	1.27103E-07	2.60481E-07	negative
PRJNA729271	stage_5W_vs_E10	ordinary_muscle_development	ENSGALG00010008017	GMNN	-1.939449892	0.137683921	-14.0862483	4.61432E-45	4.7135E-44	negative
PRJNA729271	stage_5W_vs_E10	ordinary_muscle_development	ENSGALG00010008018	ENSGALG00010008018	0.428826225	0.208671436	2.055030786	0.039876031	0.050965794	positive
PRJNA729271	stage_5W_vs_E10	ordinary_muscle_development	ENSGALG00010008019	PPP2CB	-0.959645361	0.101081865	-9.493744118	2.22882E-21	9.47967E-21	negative
PRJNA729271	stage_5W_vs_E10	ordinary_muscle_development	ENSGALG00010008020	COA6	0.864926726	0.131265528	6.589138386	4.42386E-11	1.12795E-10	positive
PRJNA729271	stage_5W_vs_E10	ordinary_muscle_development	ENSGALG00010008022	SLC35F3	-0.144993314	0.384805318	-0.376796545	0.706324809	0.738259165	negative
PRJNA729271	stage_5W_vs_E10	ordinary_muscle_development	ENSGALG00010008023	ATAD2	-0.509465117	0.077642522	-6.561676584	5.32061E-11	1.349E-10	negative
PRJNA729271	stage_5W_vs_E10	ordinary_muscle_development	ENSGALG00010008024	MRS2	0.602323887	0.095466614	6.309262085	2.80369E-10	6.77325E-10	positive
PRJNA729271	stage_5W_vs_E10	ordinary_muscle_development	ENSGALG00010008025	TBC1D22A	-0.100101614	0.097299257	-1.028801422	0.303572998	0.34222201	negative
PRJNA729271	stage_5W_vs_E10	ordinary_muscle_development	ENSGALG00010008026	PURG	0.777760155	0.122139057	6.3678251	1.91727E-10	4.68728E-10	positive
PRJNA729271	stage_5W_vs_E10	ordinary_muscle_development	ENSGALG00010008027	C6orf62	0.58261273	0.107219882	5.433812468	5.51626E-08	1.15831E-07	positive
PRJNA729271	stage_5W_vs_E10	ordinary_muscle_development	ENSGALG00010008028	KCNK1	-2.939467695	0.170011062	-17.28986142	5.60869E-67	1.09419E-65	negative
PRJNA729271	stage_5W_vs_E10	ordinary_muscle_development	ENSGALG00010008029	LY6E	0.946200591	0.314383628	3.009700594	0.002615053	0.003803833	positive
PRJNA729271	stage_5W_vs_E10	ordinary_muscle_development	ENSGALG00010008030	GJA5	-2.215064436	0.150589614	-14.7092776	5.62042E-49	6.58061E-48	negative
PRJNA729271	stage_5W_vs_E10	ordinary_muscle_development	ENSGALG00010008031	MAP3K21	-1.8894012	0.183292824	-10.3081024	6.47675E-25	3.20849E-24	negative
PRJNA729271	stage_5W_vs_E10	ordinary_muscle_development	ENSGALG00010008032	PUM2	-0.596105639	0.054646634	-10.9083688	1.05126E-27	5.82491E-27	negative
PRJNA729271	stage_5W_vs_E10	ordinary_muscle_development	ENSGALG00010008033	ACOT13	-0.021908122	0.121088546	-0.180926458	0.856425301	0.873848004	negative
PRJNA729271	stage_5W_vs_E10	ordinary_muscle_development	ENSGALG00010008034	DPEP1	-3.020888085	0.544224631	-5.550811031	2.84347E-08	6.08986E-08	negative
PRJNA729271	stage_5W_vs_E10	ordinary_muscle_development	ENSGALG00010008035	TDP2	-0.688978423	0.106569197	-6.465080381	1.01245E-10	2.52156E-10	negative
PRJNA729271	stage_5W_vs_E10	ordinary_muscle_development	ENSGALG00010008036	SPOCK3	-4.793605875	0.199581829	-24.01824805	1.7929E-127	1.2616E-125	negative
PRJNA729271	stage_5W_vs_E10	ordinary_muscle_development	ENSGALG00010008037	ENSGALG00010008037	4.979667827	0.878423947	5.668866205	1.43746E-08	3.14599E-08	positive
PRJNA729271	stage_5W_vs_E10	ordinary_muscle_development	ENSGALG00010008038	WRN	0.043590641	0.075916802	0.574189635	0.565839482	0.605078812	positive
PRJNA729271	stage_5W_vs_E10	ordinary_muscle_development	ENSGALG00010008039	CHMP1A	-0.201188963	0.090942204	-2.212272788	0.026947822	0.035114049	negative
PRJNA729271	stage_5W_vs_E10	ordinary_muscle_development	ENSGALG00010008040	ENSGALG00010008040	0.888695134	0.265102181	3.352273947	0.000801507	0.001224477	positive
PRJNA729271	stage_5W_vs_E10	ordinary_muscle_development	ENSGALG00010008041	ENSGALG00010008041	-2.705948679	0.24689357	-10.95998038	5.95123E-28	3.32805E-27	negative
PRJNA729271	stage_5W_vs_E10	ordinary_muscle_development	ENSGALG00010008042	ENSGALG00010008042	1.123740436	0.806444333	1.39345072	0.163483537	0.193129085	positive
PRJNA729271	stage_5W_vs_E10	ordinary_muscle_development	ENSGALG00010008043	IL17REL	-3.933160292	0.400436932	-9.822171686	9.03753E-23	4.09116E-22	negative
PRJNA729271	stage_5W_vs_E10	ordinary_muscle_development	ENSGALG00010008045	DCDC2	-2.702324934	0.868704079	-3.110754283	0.001866101	0.002751133	negative
PRJNA729271	stage_5W_vs_E10	ordinary_muscle_development	ENSGALG00010008046	RHOB	-0.777357143	0.169069378	-4.597858898	4.26855E-06	7.81023E-06	negative
PRJNA729271	stage_5W_vs_E10	ordinary_muscle_development	ENSGALG00010008047	ASAP1	-1.614371335	0.131467358	-12.27963623	1.16521E-34	8.47877E-34	negative
PRJNA729271	stage_5W_vs_E10	ordinary_muscle_development	ENSGALG00010008048	ENSGALG00010008048	2.403820001	0.749218183	3.208437885	0.001334581	0.001997187	positive
PRJNA729271	stage_5W_vs_E10	ordinary_muscle_development	ENSGALG00010008050	CDK10	-0.752011919	0.158853902	-4.733984566	2.20154E-06	4.11813E-06	negative
PRJNA729271	stage_5W_vs_E10	ordinary_muscle_development	ENSGALG00010008051	ENSGALG00010008051	-0.166351883	0.079022658	-2.105116279	0.035281188	0.045413446	negative
PRJNA729271	stage_5W_vs_E10	ordinary_muscle_development	ENSGALG00010008052	RBFA	0.202479912	0.12331804	1.641932616	0.10060397	0.122142101	positive
PRJNA729271	stage_5W_vs_E10	ordinary_muscle_development	ENSGALG00010008054	CRISP3	-4.812983743	0.890691816	-5.403646533	6.52996E-08	1.36346E-07	negative
PRJNA729271	stage_5W_vs_E10	ordinary_muscle_development	ENSGALG00010008056	GTF2E2	-0.467243661	0.071210649	-6.561429641	5.32943E-11	1.351E-10	negative
PRJNA729271	stage_5W_vs_E10	ordinary_muscle_development	ENSGALG00010008057	BAG1	-1.075425745	0.135218353	-7.953252773	1.81677E-15	5.86114E-15	negative
PRJNA729271	stage_5W_vs_E10	ordinary_muscle_development	ENSGALG00010008058	USP25	3.304930379	0.137999934	23.94878228	9.5141E-127	6.5984E-125	positive
PRJNA729271	stage_5W_vs_E10	ordinary_muscle_development	ENSGALG00010008059	ENSGALG00010008059	0.48024137	0.093265853	5.149166099	2.61647E-07	5.23475E-07	positive
PRJNA729271	stage_5W_vs_E10	ordinary_muscle_development	ENSGALG00010008062	VPS9D1	0.281981989	0.138752953	2.03225938	0.042127404	0.053644621	positive
PRJNA729271	stage_5W_vs_E10	ordinary_muscle_development	ENSGALG00010008063	KCNV1	-0.714546639	0.293049703	-2.43831211	0.014756027	0.019821778	negative
PRJNA729271	stage_5W_vs_E10	ordinary_muscle_development	ENSGALG00010008064	DDX60	3.070697448	0.627355541	4.894668566	9.84715E-07	1.89328E-06	positive
PRJNA729271	stage_5W_vs_E10	ordinary_muscle_development	ENSGALG00010008065	CHMP5	0.076024071	0.086442845	0.879472109	0.37914535	0.420322129	positive
PRJNA729271	stage_5W_vs_E10	ordinary_muscle_development	ENSGALG00010008066	PCNX2	-3.685396271	0.187978328	-19.60543171	1.38968E-85	4.22294E-84	negative
PRJNA729271	stage_5W_vs_E10	ordinary_muscle_development	ENSGALG00010008067	MPC2	1.656707274	0.145200419	11.40979676	3.73601E-30	2.27249E-29	positive
PRJNA729271	stage_5W_vs_E10	ordinary_muscle_development	ENSGALG00010008069	DLG2	-2.997702367	0.227040088	-13.20340562	8.38565E-40	7.3003E-39	negative
PRJNA729271	stage_5W_vs_E10	ordinary_muscle_development	ENSGALG00010008071	SMIM18	0.348230571	0.314194561	1.108327815	0.267720267	0.304767554	positive
PRJNA729271	stage_5W_vs_E10	ordinary_muscle_development	ENSGALG00010008072	NRSN1	1.117324736	0.198648993	5.624618175	1.85919E-08	4.03669E-08	positive
PRJNA729271	stage_5W_vs_E10	ordinary_muscle_development	ENSGALG00010008073	ZNF276	0.084668661	0.084082167	1.006975246	0.31394667	0.352983011	positive
PRJNA729271	stage_5W_vs_E10	ordinary_muscle_development	ENSGALG00010008074	RHAG	-3.430966438	0.449373792	-7.634994513	2.2583E-14	6.85817E-14	negative
PRJNA729271	stage_5W_vs_E10	ordinary_muscle_development	ENSGALG00010008075	SDHA	1.207954043	0.136498967	8.849547117	8.78752E-19	3.32052E-18	positive
PRJNA729271	stage_5W_vs_E10	ordinary_muscle_development	ENSGALG00010008076	ENSGALG00010008076	2.919816855	1.760503708	1.658512187	0.09721413	0.118313922	positive
PRJNA729271	stage_5W_vs_E10	ordinary_muscle_development	ENSGALG00010008077	ENSGALG00010008077	1.839757899	1.476874397	1.24571047	0.212870709	0.24643087	positive
PRJNA729271	stage_5W_vs_E10	ordinary_muscle_development	ENSGALG00010008078	GUCY1B1	0.408015447	0.141447906	2.884563364	0.003919569	0.005598556	positive
PRJNA729271	stage_5W_vs_E10	ordinary_muscle_development	ENSGALG00010008079	CCDC127	0.67039818	0.108182145	6.196939271	5.75717E-10	1.36535E-09	positive
PRJNA729271	stage_5W_vs_E10	ordinary_muscle_development	ENSGALG00010008081	TOP1MT	-0.843012093	0.142408955	-5.919656487	3.22615E-09	7.32159E-09	negative
PRJNA729271	stage_5W_vs_E10	ordinary_muscle_development	ENSGALG00010008083	FANCA	-2.086021575	0.258347995	-8.074463975	6.77737E-16	2.23116E-15	negative
PRJNA729271	stage_5W_vs_E10	ordinary_muscle_development	ENSGALG00010008084	CYP2AC1	4.517700729	1.326247483	3.406378362			positive
PRJNA729271	stage_5W_vs_E10	ordinary_muscle_development	ENSGALG00010008086	RHPN1	1.077607895	0.208836271	5.160060993	2.46869E-07	4.94729E-07	positive
PRJNA729271	stage_5W_vs_E10	ordinary_muscle_development	ENSGALG00010008087	CPNE8	-0.442877976	0.183702985	-2.410837121	0.015915955	0.021294237	negative
PRJNA729271	stage_5W_vs_E10	ordinary_muscle_development	ENSGALG00010008088	SLC35A4	0.276823361	0.071794011	3.85580017	0.000115352	0.000188844	positive
PRJNA729271	stage_5W_vs_E10	ordinary_muscle_development	ENSGALG00010008091	ENSGALG00010008091	1.482715421	0.271902499	5.453114363	4.94952E-08	1.04278E-07	positive
PRJNA729271	stage_5W_vs_E10	ordinary_muscle_development	ENSGALG00010008092	SPIRE2	0.441480749	0.652359388	0.676744687	0.498567959	0.539912045	positive
PRJNA729271	stage_5W_vs_E10	ordinary_muscle_development	ENSGALG00010008093	EFR3A	0.247834647	0.092410291	2.681894459	0.007320655	0.010165044	positive
PRJNA729271	stage_5W_vs_E10	ordinary_muscle_development	ENSGALG00010008094	PIM3	0.530946358	0.14224146	3.73271168	0.000189429	0.000304883	positive
PRJNA729271	stage_5W_vs_E10	ordinary_muscle_development	ENSGALG00010008096	CPSF1	-1.151356871	0.153043418	-7.523073426	5.35034E-14	1.59706E-13	negative
PRJNA729271	stage_5W_vs_E10	ordinary_muscle_development	ENSGALG00010008098	ENSGALG00010008098	0.475539464	0.166766297	2.851532195	0.004350908	0.006183588	positive
PRJNA729271	stage_5W_vs_E10	ordinary_muscle_development	ENSGALG00010008099	PANK3	-0.175930327	0.076940825	-2.286566695	0.022221127	0.029188513	negative
PRJNA729271	stage_5W_vs_E10	ordinary_muscle_development	ENSGALG00010008100	ENSGALG00010008100	1.515435178	0.23564359	6.431047732	1.26727E-10	3.13199E-10	positive
PRJNA729271	stage_5W_vs_E10	ordinary_muscle_development	ENSGALG00010008101	CENPQ	-3.061841334	0.213657128	-14.33063041	1.40836E-46	1.5077E-45	negative
PRJNA729271	stage_5W_vs_E10	ordinary_muscle_development	ENSGALG00010008103	RBPMS	0.099705948	0.108870498	0.915821554	0.359760497	0.400025192	positive
PRJNA729271	stage_5W_vs_E10	ordinary_muscle_development	ENSGALG00010008104	TCF25	0.373323779	0.09455478	3.948227452	7.8732E-05	0.000131024	positive
PRJNA729271	stage_5W_vs_E10	ordinary_muscle_development	ENSGALG00010008105	MMUT	0.188433664	0.096505987	1.95255931	0.050871837	0.064092766	positive
PRJNA729271	stage_5W_vs_E10	ordinary_muscle_development	ENSGALG00010008106	ENSGALG00010008106	0.614667393	0.37230795	1.650964994	0.098745723	0.120016708	positive
PRJNA729271	stage_5W_vs_E10	ordinary_muscle_development	ENSGALG00010008107	TUBB3	-1.955219154	0.209274702	-9.342835692	9.37879E-21	3.86539E-20	negative
PRJNA729271	stage_5W_vs_E10	ordinary_muscle_development	ENSGALG00010008108	ENSGALG00010008108	1.150999047	0.332788198	3.458653441	0.000542883	0.000841254	positive
PRJNA729271	stage_5W_vs_E10	ordinary_muscle_development	ENSGALG00010008110	DEF8	0.625741342	0.152335803	4.107644637	3.99714E-05	6.79233E-05	positive
PRJNA729271	stage_5W_vs_E10	ordinary_muscle_development	ENSGALG00010008111	SYTL2	-0.013787681	0.258406733	-0.053356509	0.957447857	0.963188758	negative
PRJNA729271	stage_5W_vs_E10	ordinary_muscle_development	ENSGALG00010008113	DBNDD1	-1.703411805	0.188031907	-9.059163583	1.31455E-19	5.16658E-19	negative
PRJNA729271	stage_5W_vs_E10	ordinary_muscle_development	ENSGALG00010008115	ENSGALG00010008115	-0.142898983	0.201950439	-0.707594319	0.479197222	0.520804059	negative
PRJNA729271	stage_5W_vs_E10	ordinary_muscle_development	ENSGALG00010008116	URAH	-0.896560858	0.484434937	-1.85073534	0.064207637	0.079901245	negative
PRJNA729271	stage_5W_vs_E10	ordinary_muscle_development	ENSGALG00010008117	ENSGALG00010008117	2.006276844	0.179622422	11.16941207	5.75606E-29	3.34808E-28	positive
PRJNA729271	stage_5W_vs_E10	ordinary_muscle_development	ENSGALG00010008118	ENSGALG00010008118	0.300641937	0.618861268	0.485798599	0.627109968	0.663161814	positive

PRJNA729271	stage_5W_vs_E10	ordinary_muscle_development	ENSGALG00010008119	CCDC83	-0.89191891	0.538736083	-1.655576707	0.09780757	0.118936303	negative
PRJNA729271	stage_5W_vs_E10	ordinary_muscle_development	ENSGALG00010008120	CDC5L	-0.412823034	0.064860556	-6.3647779	1.95573E-10	4.77726E-10	negative
PRJNA729271	stage_5W_vs_E10	ordinary_muscle_development	ENSGALG00010008121	MPZL1	-0.823993725	0.135750192	-6.069926792	1.27969E-09	2.97022E-09	negative
PRJNA729271	stage_5W_vs_E10	ordinary_muscle_development	ENSGALG00010008123	ENSGALG00010008123	-1.0479157	0.166048105	-6.310916342	2.77388E-10	6.70347E-10	negative
PRJNA729271	stage_5W_vs_E10	ordinary_muscle_development	ENSGALG00010008124	ENSGALG00010008124	-0.073561123	0.215061969	-0.34204617	0.732316147	0.761800097	negative
PRJNA729271	stage_5W_vs_E10	ordinary_muscle_development	ENSGALG00010008126	CDH3	-4.361673505	0.332502447	-13.11771852	2.60665E-39	2.21603E-38	negative
PRJNA729271	stage_5W_vs_E10	ordinary_muscle_development	ENSGALG00010008127	TBC1D2	-0.325365825	0.13461005	-2.417099055	0.015644759	0.020948802	negative
PRJNA729271	stage_5W_vs_E10	ordinary_muscle_development	ENSGALG00010008128	RARS	-0.765312939	0.07088168	-10.79704847	3.55446E-27	1.93111E-26	negative
PRJNA729271	stage_5W_vs_E10	ordinary_muscle_development	ENSGALG00010008129	TANGO6	0.466230717	0.117261195	3.976001742	7.00836E-05	0.000117143	positive
PRJNA729271	stage_5W_vs_E10	ordinary_muscle_development	ENSGALG00010008130	RCSD1	0.972570298	0.120180315	8.092592357	5.84081E-16	1.92765E-15	positive
PRJNA729271	stage_5W_vs_E10	ordinary_muscle_development	ENSGALG00010008131	OC90	-3.524598004	0.94281813	-3.738364689	0.000185221	0.000298342	negative
PRJNA729271	stage_5W_vs_E10	ordinary_muscle_development	ENSGALG00010008132	TDO2	1.712132292	0.639336977	2.677981024	0.007406741	0.010277687	positive
PRJNA729271	stage_5W_vs_E10	ordinary_muscle_development	ENSGALG00010008133	CREG1	2.905048927	0.167214002	17.37323963	1.31583E-67	2.59143E-66	positive
PRJNA729271	stage_5W_vs_E10	ordinary_muscle_development	ENSGALG00010008134	SLC2A13	-0.402409335	0.078154788	-5.148876302	2.62052E-07	5.24212E-07	negative
PRJNA729271	stage_5W_vs_E10	ordinary_muscle_development	ENSGALG00010008135	WWC1	-0.116769238	0.131747304	-0.886312167	0.375449335	0.416447666	negative
PRJNA729271	stage_5W_vs_E10	ordinary_muscle_development	ENSGALG00010008136	ELP1	0.245553516	0.113238772	2.16845795	0.030123861	0.039069914	positive
PRJNA729271	stage_5W_vs_E10	ordinary_muscle_development	ENSGALG00010008137	HHLA1	-2.146165152	0.899347234	-2.386358763	0.017016142	0.022680366	negative
PRJNA729271	stage_5W_vs_E10	ordinary_muscle_development	ENSGALG00010008139	ENSGALG00010008139	1.113439635	0.405486457	2.745935445	0.006033864	0.008450324	positive
PRJNA729271	stage_5W_vs_E10	ordinary_muscle_development	ENSGALG00010008141	APTX	0.139516935	0.154582572	0.902539873	0.36677015	0.407381864	positive
PRJNA729271	stage_5W_vs_E10	ordinary_muscle_development	ENSGALG00010008142	CD247	3.470202206	0.320095432	10.84114881	2.19695E-27	1.20033E-26	positive
PRJNA729271	stage_5W_vs_E10	ordinary_muscle_development	ENSGALG00010008143	DCTN6	0.732486524	0.115130757	6.362214084	1.98866E-10	4.85607E-10	positive
PRJNA729271	stage_5W_vs_E10	ordinary_muscle_development	ENSGALG00010008144	NTPCR	-0.03882682	0.133751222	-0.290291329	0.771593372	0.798589498	negative
PRJNA729271	stage_5W_vs_E10	ordinary_muscle_development	ENSGALG00010008145	ENSGALG00010008145	1.270853461	0.209307522	6.071704673	1.2656E-09	2.93893E-09	positive
PRJNA729271	stage_5W_vs_E10	ordinary_muscle_development	ENSGALG00010008146	SOX4	-2.193447967	0.120213288	-18.24630202	2.21398E-74	5.29566E-73	negative
PRJNA729271	stage_5W_vs_E10	ordinary_muscle_development	ENSGALG00010008147	CDKAL1	0.424382019	0.1125068	3.772056613	0.000161908	0.000261925	positive
PRJNA729271	stage_5W_vs_E10	ordinary_muscle_development	ENSGALG00010008148	ABCD2	2.058863758	0.138105388	14.9079177	2.9274E-50	3.53606E-49	positive
PRJNA729271	stage_5W_vs_E10	ordinary_muscle_development	ENSGALG00010008149	DNAJA1	1.832347487	0.281450086	6.510381697	7.49601E-11	1.88506E-10	positive
PRJNA729271	stage_5W_vs_E10	ordinary_muscle_development	ENSGALG00010008150	TERT	-2.255048254	0.59596732	-3.783845486	0.000154424	0.000250097	negative
PRJNA729271	stage_5W_vs_E10	ordinary_muscle_development	ENSGALG00010008151	SIPA1L2	-0.680575405	0.121973569	-5.57969577	2.4094E-08	5.19551E-08	negative
PRJNA729271	stage_5W_vs_E10	ordinary_muscle_development	ENSGALG00010008152	ENSGALG00010008152	-3.059335504	0.168596874	-18.14586134	1.38448E-73	3.21088E-72	negative
PRJNA729271	stage_5W_vs_E10	ordinary_muscle_development	ENSGALG00010008153	SMU1	-0.396382529	0.061929753	-6.400518496	1.5485E-10	3.80562E-10	negative
PRJNA729271	stage_5W_vs_E10	ordinary_muscle_development	ENSGALG00010008154	KCNQ3	0.402934757	0.223768382	1.800677796	0.071753679	0.088818922	positive
PRJNA729271	stage_5W_vs_E10	ordinary_muscle_development	ENSGALG00010008155	MAP10	0.273295717	0.225306864	1.212993303	0.225132381	0.25950647	positive
PRJNA729271	stage_5W_vs_E10	ordinary_muscle_development	ENSGALG00010008156	HAS3	-4.956499205	1.154238576	-4.294172202	1.75346E-05	3.06629E-05	negative
PRJNA729271	stage_5W_vs_E10	ordinary_muscle_development	ENSGALG00010008157	CTSO	-0.727597443	0.156414964	-4.651712512	3.2919E-06	6.08313E-06	negative
PRJNA729271	stage_5W_vs_E10	ordinary_muscle_development	ENSGALG00010008158	CLPTM1L	-0.395874815	0.075564342	-5.238910404	1.61527E-07	3.28471E-07	negative
PRJNA729271	stage_5W_vs_E10	ordinary_muscle_development	ENSGALG00010008159	SUPT3H	-1.680929593	0.134180533	-12.52737302	5.28855E-36	4.06887E-35	negative
PRJNA729271	stage_5W_vs_E10	ordinary_muscle_development	ENSGALG00010008160	TXNL4A	-0.138880287	0.092369206	-1.503534498	0.132701268	0.158600336	negative
PRJNA729271	stage_5W_vs_E10	ordinary_muscle_development	ENSGALG00010008161	TMEFF1	-2.899315912	0.367541455	-7.888405168	3.06073E-15	9.74204E-15	negative
PRJNA729271	stage_5W_vs_E10	ordinary_muscle_development	ENSGALG00010008162	HSBP1L1	1.69575566	0.194513897	8.717915218	2.83371E-18	1.04702E-17	positive
PRJNA729271	stage_5W_vs_E10	ordinary_muscle_development	ENSGALG00010008163	CHTF8	-3.232053818	0.249533581	-12.9523802	2.2778E-38	1.87809E-37	negative
PRJNA729271	stage_5W_vs_E10	ordinary_muscle_development	ENSGALG00010008164	TG	0.863599792	0.290563817	2.972151873	0.002957204	0.004275351	positive
PRJNA729271	stage_5W_vs_E10	ordinary_muscle_development	ENSGALG00010008165	MRPL1	0.199902734	0.085489302	2.338336258	0.019369812	0.025652391	positive
PRJNA729271	stage_5W_vs_E10	ordinary_muscle_development	ENSGALG00010008166	RUNX2	-2.684519611	0.399317	-6.722778171	1.78292E-11	4.65563E-11	negative
PRJNA729271	stage_5W_vs_E10	ordinary_muscle_development	ENSGALG00010008167	UTP4	0.250175167	0.174445621	1.434115484	0.15153931	0.179736707	positive
PRJNA729271	stage_5W_vs_E10	ordinary_muscle_development	ENSGALG00010008168	CAVIN4	5.294920522	0.124866244	42.4047393	0	0	positive
PRJNA729271	stage_5W_vs_E10	ordinary_muscle_development	ENSGALG00010008169	HTR4	-2.237920071	0.417185885	-5.36432356	8.12531E-08	1.68613E-07	negative
PRJNA729271	stage_5W_vs_E10	ordinary_muscle_development	ENSGALG00010008170	PICALM	-0.152158402	0.070847114	-2.147700773	0.031737534	0.041056274	negative
PRJNA729271	stage_5W_vs_E10	ordinary_muscle_development	ENSGALG00010008171	KIF21A	0.194278025	0.179930964	1.07973648	0.280259544	0.317794109	positive
PRJNA729271	stage_5W_vs_E10	ordinary_muscle_development	ENSGALG00010008172	SNTB2	-0.427315382	0.133794339	-3.193822587	0.001404024	0.002096778	negative
PRJNA729271	stage_5W_vs_E10	ordinary_muscle_development	ENSGALG00010008174	SLC26A2	-0.464844763	0.12337831	-3.767637608	0.0001648	0.000266455	negative
PRJNA729271	stage_5W_vs_E10	ordinary_muscle_development	ENSGALG00010008175	ENSGALG00010008175	-1.222437902	0.451809587	-2.705648435	0.006817117	0.009496792	negative
PRJNA729271	stage_5W_vs_E10	ordinary_muscle_development	ENSGALG00010008176	SLA	3.123650558	0.292142084	10.69223068	1.10677E-26	5.91104E-26	positive
PRJNA729271	stage_5W_vs_E10	ordinary_muscle_development	ENSGALG00010008177	NREP	-1.297669271	0.18464397	-7.027953702	2.09584E-12	5.74929E-12	negative
PRJNA729271	stage_5W_vs_E10	ordinary_muscle_development	ENSGALG00010008178	PGR	0.86588188	0.281121454	3.080098896	0.002069319	0.003037138	positive
PRJNA729271	stage_5W_vs_E10	ordinary_muscle_development	ENSGALG00010008179	SEMA6A	-0.90331966	0.097073846	-9.305489595	1.33376E-20	5.469E-20	negative
PRJNA729271	stage_5W_vs_E10	ordinary_muscle_development	ENSGALG00010008180	POU2F1	0.798330536	0.157670024	5.063299382	4.12062E-07	8.12521E-07	positive
PRJNA729271	stage_5W_vs_E10	ordinary_muscle_development	ENSGALG00010008181	DISC1	0.05604653	0.130448339	0.42964541	0.667453599	0.701576499	positive
PRJNA729271	stage_5W_vs_E10	ordinary_muscle_development	ENSGALG00010008182	CLIC5	6.788862675	0.284515816	23.86110818	7.7655E-126	5.3095E-124	positive
PRJNA729271	stage_5W_vs_E10	ordinary_muscle_development	ENSGALG00010008183	ALG10	-0.885344246	0.089134869	-9.932636432	3.00211E-23	1.38452E-22	negative
PRJNA729271	stage_5W_vs_E10	ordinary_muscle_development	ENSGALG00010008184	SLC66A2	-0.110608124	0.085996942	-1.286186708	0.198377909	0.230765237	negative
PRJNA729271	stage_5W_vs_E10	ordinary_muscle_development	ENSGALG00010008185	PDGFC	-1.955967242	0.092481121	-21.14990839	2.76478E-99	1.14175E-97	negative
PRJNA729271	stage_5W_vs_E10	ordinary_muscle_development	ENSGALG00010008186	NOP53	0.445379484	0.121539647	3.664479004	0.000247843	0.000395082	positive
PRJNA729271	stage_5W_vs_E10	ordinary_muscle_development	ENSGALG00010008187	ENSGALG00010008187	4.354411852	0.374044121	11.64143909	2.53698E-31	1.6207E-30	positive
PRJNA729271	stage_5W_vs_E10	ordinary_muscle_development	ENSGALG00010008188	VPS4A	0.843403579	0.112862886	7.472815951	7.84966E-14	2.31262E-13	positive
PRJNA729271	stage_5W_vs_E10	ordinary_muscle_development	ENSGALG00010008189	EXOC3	0.366831492	0.114253422	3.210682749	0.0013242	0.001982471	positive
PRJNA729271	stage_5W_vs_E10	ordinary_muscle_development	ENSGALG00010008190	TSNARE1	-0.202942304	0.16982129	-1.195034518	0.232073544	0.266849597	negative
PRJNA729271	stage_5W_vs_E10	ordinary_muscle_development	ENSGALG00010008192	HNRNPU	-0.654896647	0.082987941	-7.891467595	2.98653E-15	9.51006E-15	negative
PRJNA729271	stage_5W_vs_E10	ordinary_muscle_development	ENSGALG00010008193	CNOT6L	1.663919486	0.12964772	12.83415921	1.05543E-37	8.54182E-37	positive
PRJNA729271	stage_5W_vs_E10	ordinary_muscle_development	ENSGALG00010008194	PDF	0.790071957	0.164550391	4.801398235	1.57562E-06	2.97726E-06	positive
PRJNA729271	stage_5W_vs_E10	ordinary_muscle_development	ENSGALG00010008195	ENSGALG00010008195	2.783238586	0.423370678	6.573999403	4.89814E-11	1.24537E-10	positive
PRJNA729271	stage_5W_vs_E10	ordinary_muscle_development	ENSGALG00010008196	PDCD6	0.275197828	0.146933803	1.872937496	0.061077017	0.076188585	positive
PRJNA729271	stage_5W_vs_E10	ordinary_muscle_development	ENSGALG00010008197	STARD4	-0.052652148	0.157779387	-0.333707393	0.73860037	0.767511102	negative
PRJNA729271	stage_5W_vs_E10	ordinary_muscle_development	ENSGALG00010008198	GLRB	-3.623491981	0.414280436	-8.746471399	2.20128E-18	8.17933E-18	negative
PRJNA729271	stage_5W_vs_E10	ordinary_muscle_development	ENSGALG00010008199	TPRKB	0.496889528	0.112531123	4.415574252	1.00742E-05	1.79591E-05	positive
PRJNA729271	stage_5W_vs_E10	ordinary_muscle_development	ENSGALG00010008200	COG8	-0.05887357	0.128276757	-0.458957421	0.646264743	0.681230997	negative
PRJNA729271	stage_5W_vs_E10	ordinary_muscle_development	ENSGALG00010008201	ENPP4	1.8578083	0.240840965	7.713838473	1.22089E-14	3.76048E-14	positive
PRJNA729271	stage_5W_vs_E10	ordinary_muscle_development	ENSGALG00010008202	KCNG2	1.114327642	0.19483353	5.719383316	1.06911E-08	2.35585E-08	positive
PRJNA729271	stage_5W_vs_E10	ordinary_muscle_development	ENSGALG00010008203	CXCL13	5.165334468	1.261472351	4.094687023	4.22739E-05	7.16595E-05	positive
PRJNA729271	stage_5W_vs_E10	ordinary_muscle_development	ENSGALG00010008204	ENSGALG00010008204	0.482205797	0.153878012	3.133688758	0.001726239	0.002553508	positive
PRJNA729271	stage_5W_vs_E10	ordinary_muscle_development	ENSGALG00010008205	ENSGALG00010008205	-0.647381865	0.182901441	-3.539512111	0.000400867	0.000627424	negative
PRJNA729271	stage_5W_vs_E10	ordinary_muscle_development	ENSGALG00010008206	CXCL13L2	-0.50494396	0.729471576	-0.692205121	0.488808523	0.530216235	negative
PRJNA729271	stage_5W_vs_E10	ordinary_muscle_development	ENSGALG00010008207	CAMK4	-0.828090356	0.160361584	-5.163894833	2.41864E-07	4.85099E-07	negative
PRJNA729271	stage_5W_vs_E10	ordinary_muscle_development	ENSGALG00010008208	ILDR2	1.571352258	0.152461582	10.30654566	6.58249E-25	3.25976E-24	positive
PRJNA729271	stage_5W_vs_E10	ordinary_muscle_development	ENSGALG00010008209	PPARGC1B	2.106327122	0.236891163	8.891539449	6.0268E-19	2.29468E-18	positive
PRJNA729271	stage_5W_vs_E10	ordinary_muscle_development	ENSGALG00010008210	CTDP1	0.269609197	0.085259998	3.162200363	0.001565818	0.002327152	positive
PRJNA729271	stage_5W_vs_E10	ordinary_muscle_development	ENSGALG00010008211	ADGRB1	-0.449845129	0.2370957	-1.89731458	0.057786432	0.072282907	negative
PRJNA729271	stage_5W_vs_E10	ordinary_muscle_development	ENSGALG00010008212	GTSE1	-2.960587511	0.231792409	-12.77258181	2.33286E-37	1.87443E-36	negative
PRJNA729271	stage_5W_vs_E10	ordinary_muscle_development	ENSGALG00010008213	ISCA1	0.845445038	0.11705569	7.222588153	5.10073E-13	1.44348E-12	positive
PRJNA729271	stage_5W_vs_E10	ordinary_muscle_development	ENSGALG00010008214	CCNG2	-0.381496168	0.136006047	-2.80499415	0.005031749	0.00710039	negative
PRJNA729271	stage_5W_vs_E10	ordinary_muscle_development	ENSGALG00010008215	SELENOW	2.517393714	0.127812036	19.69606137	2.33061E-86	7.23387E-85	positive
PRJNA729271	stage_5W_vs_E10	ordinary_muscle_development	ENSGALG00010008217	ARHGEF37	3.623758194	0.162577084	22.28947708	4.6742E-110	2.3773E-108	positive
PRJNA729271	stage_5W_vs_E10	ordinary_muscle_development	ENSGALG00010008218	ARHGAP42	-0.528537826	0.103145067	-5.124218178	2.98775E-07	5.94719E-07	negative
PRJNA729271	stage_5W_vs_E10	ordinary_muscle_development	ENSGALG00010008219	NIP7	-0.755453443	0.120873017	-6.249975899	4.10516E-10	9.81433E-10	negative
PRJNA729271	stage_5W_vs_E10	ordinary_muscle_development	ENSGALG00010008221	ENSGALG00010008221	3.387518433	0.518949195	6.527649459	6.68098E-11	1.68477E-10	positive
PRJNA729271	stage_5W_vs_E10	ordinary_muscle_development	ENSGALG00010008222	STYXL2	3.24535911	0.274801081	11.80984839	3.47187E-32	2.30108E-31	positive
PRJNA729271	stage_5W_vs_E10	ordinary_muscle_development	ENSGALG00010008223	ENSGALG00010008223	-1.246400374	0.153170744	-8.137326611	4.04102E-16	1.34654E-15	negative
PRJNA729271	stage_5W_vs_E10	ordinary_muscle_development	ENSGALG00010008224	ENSGALG00010008224	-0.20334456	0.212353186	-0.957577156	0.338276023	0.378108494	negative
PRJNA729271	stage_5W_vs_E10	ordinary_muscle_development	ENSGALG00010008226	COX20	-0.566245749	0.165908104	-3.413008384	0.0006425	0.000990011	negative
PRJNA729271	stage_5W_vs_E10	ordinary_muscle_development	ENSGALG00010008227	DUSP1	3.292093297	0.176892046	18.61074806	2.62921E-77	6.75716E-76	positive
PRJNA729271	stage_5W_vs_E10	ordinary_muscle_development	ENSGALG00010008228	TMED6	2.692392373	0.679714268	3.961064963	7.46162E-05	0.000124461	positive
PRJNA729271	stage_5W_vs_E10	ordinary_muscle_development	ENSGALG00010008229	PIGY	1.146939544	0.122080199	9.394967881	5.72353E-21	2.38672E-20	positive
PRJNA729271	stage_5W_vs_E10	ordinary_muscle_development	ENSGALG00010008230	TRMU	-0.127892234	0.116009426	-1.102429672	0.270274918	0.3074584	negative
PRJNA729271	stage_5W_vs_E10	ordinary_muscle_development	ENSGALG00010008231	ZCCHC6	-0.077508772	0.110472507	-0.701611419	0.482921528	0.52461572	negative
PRJNA729271	stage_5W_vs_E10	ordinary_muscle_development	ENSGALG00010008232	PYURF	1.289158663	0.127222663	10.13308975	3.93999E-24	1.88669E-23	positive
PRJNA729271	stage_5W_vs_E10	ordinary_muscle_development	ENSGALG00010008233	CYR1B	-0.51797544	0.071699551	-7.224249453	5.03877E-13	1.42622E-12	negative
PRJNA729271	stage_5W_vs_E10	ordinary_muscle_development	ENSGALG00010008234	COMMD10	-1.542404012	0.121979264	-12.64480507	1.19521E-36	9.3646E-36	negative
PRJNA729271	stage_5W_vs_E10	ordinary_muscle_development	ENSGALG00010008236	ENSGALG00010008236	0.908569951	0.705558824	1.287730973	0.197839628	0.230206744	positive
PRJNA729271	stage_5W_vs_E10	ordinary_muscle_development	ENSGALG00010008237	GRIA2	-4.208122892	0.486511027	-8.649594061	5.16829E-18	1.88844E-17	negative
PRJNA729271	stage_5W_vs_E10	ordinary_muscle_development	ENSGALG00010008238	NTAQ1	-0.443025276	0.1657272	-2.673220067	0.007512695	0.010419722	negative
PRJNA729271	stage_5W_vs_E10	ordinary_muscle_development	ENSGALG00010008240	RCAN2	-0.078886807	0.137951619	-0.571844008	0.56742766	0.606419223	negative
PRJNA729271	stage_5W_vs_E10	ordinary_muscle_development	ENSGALG00010008241	HERC3	0.40008141	0.083296077	4.803124302	1.56209E-06	2.95247E-06	positive
PRJNA729271	stage_5W_vs_E10	ordinary_muscle_development	ENSGALG00010008242	CNTN5	-6.276306889	1.395675282	-4.496967863			negative
PRJNA729271	stage_5W_vs_E10	ordinary_muscle_development	ENSGALG00010008243	YTHDC2	0.005818768	0.103720003	0.056100734	0.955261559	0.9614301	positive
PRJNA729271	stage_5W_vs_E10	ordinary_muscle_development	ENSGALG00010008245	FBXO32	4.035919557	0.491864811	8.205343148	2.29933E-16	7.75269E-16	positive
PRJNA729271	stage_5W_vs_E10	ordinary_muscle_development	ENSGALG00010008246	TERF2	-0.660142192	0.0594619	-11.1019358	1.22756E-28	7.04692E-28	negative
PRJNA729271	stage_5W_vs_E10	ordinary_muscle_development	ENSGALG00010008247	GASK1B	0.632434535	0.161069037	3.926481145	8.61976E-05	0.000142956	positive
PRJNA729271	stage_5W_vs_E10	ordinary_muscle_development	ENSGALG00010008248	TPK1	-0.745889707	0.164116617	-4.544876209	5.49675E-06	9.9719E-06	negative
PRJNA729271	stage_5W_vs_E10	ordinary_muscle_development	ENSGALG00010008250	KLHL38	5.232692478	0.59313446	8.822101611	1.12331E-18	4.22262E-18	positive
PRJNA729271	stage_5W_vs_E10	ordinary_muscle_development	ENSGALG00010008251	AHRR	-3.822346687	0.354348236	-10.78697817	3.96617E-27	2.15075E-26	negative
PRJNA729271	stage_5W_vs_E10	ordinary_muscle_development	ENSGALG00010008252	ZFP92	-2.165985079	0.13177498	-16.43699797	1.03942E-60	1.69666E-59	negative
PRJNA729271	stage_5W_vs_E10	ordinary_muscle_development	ENSGALG00010008253	ENSGALG00010008253	-0.275781066	0.722136896	-0.381895826	0.702538636	0.734725652	negative
PRJNA729271	stage_5W_vs_E10	ordinary_muscle_development	ENSGALG00010008255	MSANTD3	-1.908386029	0.131196167	-14.54605023	6.18739E-48	6.92023E-47	negative
PRJNA729271	stage_5W_vs_E10	ordinary_muscle_development	ENSGALG00010008257	DESI2	-0.605861626	0.060030761	-10.09251945	5.96193E-24	2.82597E-23	negative
PRJNA729271	stage_5W_vs_E10	ordinary_muscle_development	ENSGALG00010008258	CCDC82	0.787732943	0.131956183	5.969655431	2.37755E-09	5.43317E-09	positive
PRJNA729271	stage_5W_vs_E10	ordinary_muscle_development	ENSGALG00010008259	MCC	-0.652472949	0.109431651	-5.962378711	2.48592E-09	5.67634E-09	negative
PRJNA729271	stage_5W_vs_E10	ordinary_muscle_development	ENSGALG00010008261	CRX	-0.717323253	0.405824402	-1.767570526	0.077132729	0.095063252	negative
PRJNA729271	stage_5W_vs_E10	ordinary_muscle_development	ENSGALG00010008262	CYB5B	-1.241698056	0.073286495	-16.94306794	2.16532E-64	3.92819E-63	negative
PRJNA729271	stage_5W_vs_E10	ordinary_muscle_development	ENSGALG00010008263	C1orf101	-1.588634401	0.559233473	-2.840735536	0.004500962	0.006383703	negative
PRJNA729271	stage_5W_vs_E10	ordinary_muscle_development	ENSGALG00010008264	CNTNAP2	-2.87446285	0.3375829	-8.514835468	1.66827E-17	5.95165E-17	negative
PRJNA729271	stage_5W_vs_E10	ordinary_muscle_development	ENSGALG00010008265	ENSGALG00010008265	0.951679524	0.644231385	1.477232476	0.139613331	0.16634023	positive

PRJNA729271	stage_5W_vs_E10	ordinary_muscle_development	ENSGALG00010008266	ECSCR	0.987994047	0.187145342	5.279287403	1.29687E-07	2.65698E-07	positive
PRJNA729271	stage_5W_vs_E10	ordinary_muscle_development	ENSGALG00010008267	MAML2	-0.364494651	0.118802884	-3.068062311	0.002154517	0.003155155	negative
PRJNA729271	stage_5W_vs_E10	ordinary_muscle_development	ENSGALG00010008268	ENSGALG00010008268	2.321370621	0.231096081	10.04504539	9.66032E-24	4.54189E-23	positive
PRJNA729271	stage_5W_vs_E10	ordinary_muscle_development	ENSGALG00010008269	ENSGALG00010008269	0.548501867	0.320923101	1.709137999	0.08742539	0.106975437	positive
PRJNA729271	stage_5W_vs_E10	ordinary_muscle_development	ENSGALG00010008270	ENSGALG00010008270	0.631953948	0.1651788	3.825878067	0.000130307	0.000212391	positive
PRJNA729271	stage_5W_vs_E10	ordinary_muscle_development	ENSGALG00010008272	DCP2	-1.020411161	0.085748605	-11.9000322	1.18302E-32	7.96831E-32	negative
PRJNA729271	stage_5W_vs_E10	ordinary_muscle_development	ENSGALG00010008273	TMEM144	0.450087639	0.119909074	3.75357448	0.000174331	0.000281207	positive
PRJNA729271	stage_5W_vs_E10	ordinary_muscle_development	ENSGALG00010008274	NFAT5	-0.312003971	0.1098154	-2.841167731	0.004494866	0.006375681	negative
PRJNA729271	stage_5W_vs_E10	ordinary_muscle_development	ENSGALG00010008275	CELSR1	-1.88923971	0.174869099	-10.80373676	3.30474E-27	1.79746E-26	negative
PRJNA729271	stage_5W_vs_E10	ordinary_muscle_development	ENSGALG00010008276	DNAJC18	0.815548457	0.093479239	8.724380533	2.67642E-18	9.90165E-18	positive
PRJNA729271	stage_5W_vs_E10	ordinary_muscle_development	ENSGALG00010008277	MZB1	7.899502299	1.021799932	7.730967731	1.06732E-14	3.3022E-14	positive
PRJNA729271	stage_5W_vs_E10	ordinary_muscle_development	ENSGALG00010008278	CYP39A1	1.010352778	0.160071565	6.311881662	2.75663E-10	6.664E-10	positive
PRJNA729271	stage_5W_vs_E10	ordinary_muscle_development	ENSGALG00010008279	MAPK15	-0.667108707	0.175642237	-3.798110956	0.000145803	0.000236771	negative
PRJNA729271	stage_5W_vs_E10	ordinary_muscle_development	ENSGALG00010008282	FAM83H	-2.706789278	0.294858002	-9.179975656	4.31189E-20	1.72845E-19	negative
PRJNA729271	stage_5W_vs_E10	ordinary_muscle_development	ENSGALG00010008283	NQO1	-0.391372225	0.195654308	-2.000325105	0.04546517	0.057621547	negative
PRJNA729271	stage_5W_vs_E10	ordinary_muscle_development	ENSGALG00010008284	BCKDHA	1.609848147	0.12883476	12.49544877	7.9049E-36	6.02426E-35	positive
PRJNA729271	stage_5W_vs_E10	ordinary_muscle_development	ENSGALG00010008285	ADSS2	-1.25861099	0.089721131	-14.02803298	1.05024E-44	1.05497E-43	negative
PRJNA729271	stage_5W_vs_E10	ordinary_muscle_development	ENSGALG00010008289	MTMR2	-0.030962586	0.069177882	-0.447579271	0.654456866	0.68911465	negative
PRJNA729271	stage_5W_vs_E10	ordinary_muscle_development	ENSGALG00010008290	FAM13A	1.008024703	0.163375979	6.169968858	6.83034E-10	1.61011E-09	positive
PRJNA729271	stage_5W_vs_E10	ordinary_muscle_development	ENSGALG00010008291	PTGFRN	-0.098876647	0.161018548	-0.614069921	0.539169124	0.579133005	negative
PRJNA729271	stage_5W_vs_E10	ordinary_muscle_development	ENSGALG00010008292	REEP5	0.705986245	0.113484554	6.220989727	4.94029E-10	1.17663E-09	positive
PRJNA729271	stage_5W_vs_E10	ordinary_muscle_development	ENSGALG00010008293	PLA2G7	1.403692517	0.112477176	12.47979874	9.62296E-36	7.30669E-35	positive
PRJNA729271	stage_5W_vs_E10	ordinary_muscle_development	ENSGALG00010008294	NOB1	0.389207732	0.154470584	2.519623625	0.011748038	0.015963983	positive
PRJNA729271	stage_5W_vs_E10	ordinary_muscle_development	ENSGALG00010008295	DMAC2	0.155195613	0.133349224	1.16382839	0.244493564	0.280064345	positive
PRJNA729271	stage_5W_vs_E10	ordinary_muscle_development	ENSGALG00010008296	PALLD	-0.206340923	0.153107914	-1.347682929	0.177760385	0.208600775	negative
PRJNA729271	stage_5W_vs_E10	ordinary_muscle_development	ENSGALG00010008297	ENSGALG00010008297	-0.198622555	0.297006848	-0.668747393	0.50365663	0.544569808	negative
PRJNA729271	stage_5W_vs_E10	ordinary_muscle_development	ENSGALG00010008298	ENSGALG00010008298	-1.190069625	0.13198088	-9.016985092	1.93337E-19	7.52731E-19	negative
PRJNA729271	stage_5W_vs_E10	ordinary_muscle_development	ENSGALG00010008299	ANKRD66	2.396312157	0.246226822	9.732132897	2.19932E-22	9.7909E-22	positive
PRJNA729271	stage_5W_vs_E10	ordinary_muscle_development	ENSGALG00010008300	CUL1	0.004035623	0.085254561	0.047336149	0.962245317	0.967451334	positive
PRJNA729271	stage_5W_vs_E10	ordinary_muscle_development	ENSGALG00010008301	FAM91A1	-0.726517309	0.105998954	-6.854004508	7.18108E-12	1.91306E-11	negative
PRJNA729271	stage_5W_vs_E10	ordinary_muscle_development	ENSGALG00010008302	ENSGALG00010008302	0.566352434	0.144693666	3.91414807	9.0724E-05	0.000150068	positive
PRJNA729271	stage_5W_vs_E10	ordinary_muscle_development	ENSGALG00010008303	TEX10	-1.423705679	0.072951442	-19.51579907	8.05991E-85	2.41381E-83	negative
PRJNA729271	stage_5W_vs_E10	ordinary_muscle_development	ENSGALG00010008306	ENSGALG00010008306	0.081753703	0.314643494	0.259829631	0.794995193	0.819358314	positive
PRJNA729271	stage_5W_vs_E10	ordinary_muscle_development	ENSGALG00010008307	ENSGALG00010008307	0.141622557	0.971254063	0.14581412	0.884068129	0.898070469	positive
PRJNA729271	stage_5W_vs_E10	ordinary_muscle_development	ENSGALG00010008308	WWP2	-1.964477278	0.126310157	-15.55280527	1.52287E-54	2.07267E-53	negative
PRJNA729271	stage_5W_vs_E10	ordinary_muscle_development	ENSGALG00010008309	ENSGALG00010008309	0.939923729	0.278020854	3.380766995	0.000722838	0.001108382	positive
PRJNA729271	stage_5W_vs_E10	ordinary_muscle_development	ENSGALG00010008310	EXOSC5	0.443064518	0.1806854	2.452132366	0.014201241	0.01910945	positive
PRJNA729271	stage_5W_vs_E10	ordinary_muscle_development	ENSGALG00010008311	SRP19	-0.634028788	0.115509198	-5.488989597	4.04239E-08	8.56394E-08	negative
PRJNA729271	stage_5W_vs_E10	ordinary_muscle_development	ENSGALG00010008312	ZBTB18	2.017778494	0.216857241	9.30463972	1.34447E-20	5.51135E-20	positive
PRJNA729271	stage_5W_vs_E10	ordinary_muscle_development	ENSGALG00010008313	PSMD7	1.065050893	0.177931801	5.985725362	2.15428E-09	4.9315E-09	positive
PRJNA729271	stage_5W_vs_E10	ordinary_muscle_development	ENSGALG00010008314	INVS	-0.055472783	0.106676353	-0.520010114	0.603056525	0.640811109	negative
PRJNA729271	stage_5W_vs_E10	ordinary_muscle_development	ENSGALG00010008316	ADGRF5	3.54036565	0.133506406	26.51832038	5.9595E-155	6.3517E-153	positive
PRJNA729271	stage_5W_vs_E10	ordinary_muscle_development	ENSGALG00010008317	CEP57	0.010027867	0.110942753	0.09038776	0.927979083	0.937813345	positive
PRJNA729271	stage_5W_vs_E10	ordinary_muscle_development	ENSGALG00010008318	ADPRH	2.245848569	0.133035747	16.88154211	6.15167E-64	1.10494E-62	positive
PRJNA729271	stage_5W_vs_E10	ordinary_muscle_development	ENSGALG00010008319	ERP44	-0.164088162	0.065257281	-2.514480535	0.011920788	0.016185072	negative
PRJNA729271	stage_5W_vs_E10	ordinary_muscle_development	ENSGALG00010008320	GPRIN3	0.883015034	0.114465663	7.714235107	1.2171E-14	3.7496E-14	positive
PRJNA729271	stage_5W_vs_E10	ordinary_muscle_development	ENSGALG00010008321	ZFHX3	0.65680359	0.101226149	6.488477522	8.67081E-11	2.16957E-10	positive
PRJNA729271	stage_5W_vs_E10	ordinary_muscle_development	ENSGALG00010008323	AKT3	0.439003159	0.104577839	4.197860315	2.69449E-05	4.63855E-05	positive
PRJNA729271	stage_5W_vs_E10	ordinary_muscle_development	ENSGALG00010008324	SCRIB	0.40524631	0.103814012	3.903580078	9.47802E-05	0.000156456	positive
PRJNA729271	stage_5W_vs_E10	ordinary_muscle_development	ENSGALG00010008325	AADAT	-0.098520052	0.134607288	-0.731907266	0.464225171	0.506125901	negative
PRJNA729271	stage_5W_vs_E10	ordinary_muscle_development	ENSGALG00010008327	TGFB1	0.995742391	0.127158104	7.830742663	4.84996E-15	1.52495E-14	positive
PRJNA729271	stage_5W_vs_E10	ordinary_muscle_development	ENSGALG00010008328	ENSGALG00010008328	-0.613257116	0.494288513	-1.240686564	0.21472156	0.248394973	negative
PRJNA729271	stage_5W_vs_E10	ordinary_muscle_development	ENSGALG00010008330	EZH2	-2.092411185	0.080921487	-25.85730038	2.014E-147	1.9081E-145	negative
PRJNA729271	stage_5W_vs_E10	ordinary_muscle_development	ENSGALG00010008331	CCDC97	0.477068166	0.264746445	1.801981385	0.071548334	0.088572304	positive
PRJNA729271	stage_5W_vs_E10	ordinary_muscle_development	ENSGALG00010008332	DHX38	-0.382842797	0.093909479	-4.076721545	4.56751E-05	7.72624E-05	negative
PRJNA729271	stage_5W_vs_E10	ordinary_muscle_development	ENSGALG00010008333	APC	-0.461776574	0.092307006	-5.002616767	5.65573E-07	1.10589E-06	negative
PRJNA729271	stage_5W_vs_E10	ordinary_muscle_development	ENSGALG00010008334	STX17	1.034984781	0.132867497	7.789600941	6.72212E-15	2.09768E-14	positive
PRJNA729271	stage_5W_vs_E10	ordinary_muscle_development	ENSGALG00010008335	HNRNPUL1	-0.675407813	0.1912435	-3.531664142	0.000412953	0.000645295	negative
PRJNA729271	stage_5W_vs_E10	ordinary_muscle_development	ENSGALG00010008336	PAIP2B	0.509185621	0.109118677	4.666347085	3.06602E-06	5.68238E-06	positive
PRJNA729271	stage_5W_vs_E10	ordinary_muscle_development	ENSGALG00010008339	FAM76B	-1.35551682	0.116026287	-11.68284231	1.55991E-31	1.00403E-30	negative
PRJNA729271	stage_5W_vs_E10	ordinary_muscle_development	ENSGALG00010008340	PDIA4	-0.781083938	0.105808029	-7.382085697	1.55829E-13	4.51567E-13	negative
PRJNA729271	stage_5W_vs_E10	ordinary_muscle_development	ENSGALG00010008341	DHODH	-1.657365121	0.112339601	-14.75316908	2.9355E-49	3.47064E-48	negative
PRJNA729271	stage_5W_vs_E10	ordinary_muscle_development	ENSGALG00010008342	PLD3	0.876733035	0.148872636	5.889148315	3.88191E-09	8.7741E-09	positive
PRJNA729271	stage_5W_vs_E10	ordinary_muscle_development	ENSGALG00010008343	TNFRSF21	1.994694682	0.223098997	8.940850084	3.86188E-19	1.48365E-18	positive
PRJNA729271	stage_5W_vs_E10	ordinary_muscle_development	ENSGALG00010008344	MFAP3L	0.297889737	0.309149135	0.963579398	0.33525684	0.374936185	positive
PRJNA729271	stage_5W_vs_E10	ordinary_muscle_development	ENSGALG00010008346	NR4A3	3.281111136	0.523728059	6.264913784	3.73032E-10	8.94622E-10	positive
PRJNA729271	stage_5W_vs_E10	ordinary_muscle_development	ENSGALG00010008347	PUF60	-0.741633476	0.055638584	-13.32948149	1.55978E-40	1.38706E-39	negative
PRJNA729271	stage_5W_vs_E10	ordinary_muscle_development	ENSGALG00010008348	ENSGALG00010008348	-1.548486629	0.105987966	-14.61002311	2.42436E-48	2.75832E-47	negative
PRJNA729271	stage_5W_vs_E10	ordinary_muscle_development	ENSGALG00010008350	LRRC6	-0.651380124	0.182717725	-3.564953109	0.000363921	0.000572043	negative
PRJNA729271	stage_5W_vs_E10	ordinary_muscle_development	ENSGALG00010008351	IST1	-0.187597382	0.068356927	-2.744379976	0.006062532	0.008488013	negative
PRJNA729271	stage_5W_vs_E10	ordinary_muscle_development	ENSGALG00010008352	ENSGALG00010008352	0.64370901	0.152597082	4.218357277	2.46089E-05	4.25409E-05	positive
PRJNA729271	stage_5W_vs_E10	ordinary_muscle_development	ENSGALG00010008354	TMEM71	2.474435956	0.220148495	11.2398495	2.59825E-29	1.53345E-28	positive
PRJNA729271	stage_5W_vs_E10	ordinary_muscle_development	ENSGALG00010008355	MAFA	4.082067301	0.177632957	22.98034874	7.3296E-117	4.284E-115	positive
PRJNA729271	stage_5W_vs_E10	ordinary_muscle_development	ENSGALG00010008356	ENSGALG00010008356	-0.763858538	0.132403137	-5.769187602	7.96546E-09	1.76841E-08	negative
PRJNA729271	stage_5W_vs_E10	ordinary_muscle_development	ENSGALG00010008358	HPF1	-1.776364652	0.117409782	-15.12961374	1.033E-51	1.31924E-50	negative
PRJNA729271	stage_5W_vs_E10	ordinary_muscle_development	ENSGALG00010008360	ZC3H3	-0.047688469	0.062727799	-0.76024457	0.447108408	0.48886146	negative
PRJNA729271	stage_5W_vs_E10	ordinary_muscle_development	ENSGALG00010008361	MATR3	-1.26596441	0.057208684	-22.12888541	1.6665E-108	8.0787E-107	negative
PRJNA729271	stage_5W_vs_E10	ordinary_muscle_development	ENSGALG00010008362	RAMP3	0.564588986	0.262202805	2.153253036	0.031298798	0.040521264	positive
PRJNA729271	stage_5W_vs_E10	ordinary_muscle_development	ENSGALG00010008364	ENSGALG00010008364	-2.723015124	0.627687738	-4.338168418	1.43675E-05	2.532E-05	negative
PRJNA729271	stage_5W_vs_E10	ordinary_muscle_development	ENSGALG00010008366	RPRD1A	-0.41265113	0.108743347	-3.794725308	0.000147807	0.00023981	negative
PRJNA729271	stage_5W_vs_E10	ordinary_muscle_development	ENSGALG00010008367	ENSGALG00010008367	-2.922266324	0.667674262	-4.376784447	1.20443E-05	2.13426E-05	negative
PRJNA729271	stage_5W_vs_E10	ordinary_muscle_development	ENSGALG00010008368	SNCA	0.392853756	0.325997227	1.205083122	0.228171215	0.262570797	positive
PRJNA729271	stage_5W_vs_E10	ordinary_muscle_development	ENSGALG00010008372	CLCN3	-0.017215112	0.06936571	-0.248178989	0.803995922	0.827340685	negative
PRJNA729271	stage_5W_vs_E10	ordinary_muscle_development	ENSGALG00010008373	MACIR	-1.06568651	0.16800973	-6.343004717	2.25327E-10	5.48005E-10	negative
PRJNA729271	stage_5W_vs_E10	ordinary_muscle_development	ENSGALG00010008374	ZNF821	-0.786614085	0.088089072	-8.92975789	4.26942E-19	1.63674E-18	negative
PRJNA729271	stage_5W_vs_E10	ordinary_muscle_development	ENSGALG00010008375	ENSGALG00010008375	-0.554992768	0.098611811	-5.628055741	1.82252E-08	3.95886E-08	negative
PRJNA729271	stage_5W_vs_E10	ordinary_muscle_development	ENSGALG00010008376	MMRN1	1.375230195	0.258221488	5.325777525	1.00522E-07	2.07412E-07	positive
PRJNA729271	stage_5W_vs_E10	ordinary_muscle_development	ENSGALG00010008377	AKT2	0.301618343	0.116756281	2.583315785	0.009785571	0.013418016	positive
PRJNA729271	stage_5W_vs_E10	ordinary_muscle_development	ENSGALG00010008380	SIL1	0.883287261	0.171611096	5.147028856	2.64645E-07	5.29034E-07	positive
PRJNA729271	stage_5W_vs_E10	ordinary_muscle_development	ENSGALG00010008381	C18orf21	0.526314947	0.113748801	4.626993348	3.71012E-06	6.82292E-06	positive
PRJNA729271	stage_5W_vs_E10	ordinary_muscle_development	ENSGALG00010008382	ENSGALG00010008382	0.055842342	0.131479696	0.424722171	0.67103922	0.704987569	positive
PRJNA729271	stage_5W_vs_E10	ordinary_muscle_development	ENSGALG00010008383	PLD5	-3.666156826	0.441280014	-8.308005598	9.73239E-17	3.34054E-16	negative
PRJNA729271	stage_5W_vs_E10	ordinary_muscle_development	ENSGALG00010008385	CTNNA1	-0.716509802	0.091506245	-7.830173767	4.87196E-15	1.53154E-14	negative
PRJNA729271	stage_5W_vs_E10	ordinary_muscle_development	ENSGALG00010008386	SESN3	-0.947265073	0.228179375	-4.151405337	3.3044E-05	5.64627E-05	negative
PRJNA729271	stage_5W_vs_E10	ordinary_muscle_development	ENSGALG00010008387	PHF20L1	0.241730941	0.112412067	2.150400279	0.031523566	0.04080133	positive
PRJNA729271	stage_5W_vs_E10	ordinary_muscle_development	ENSGALG00010008388	ADCY1	-0.914542957	0.41709861	-2.192630075	0.028334042	0.036847474	negative
PRJNA729271	stage_5W_vs_E10	ordinary_muscle_development	ENSGALG00010008389	GALNT1	-0.843680576	0.088800215	-9.500884393	2.08115E-21	8.85941E-21	negative
PRJNA729271	stage_5W_vs_E10	ordinary_muscle_development	ENSGALG00010008391	ENSGALG00010008391	-0.160338086	0.072272306	-2.218527348	0.026518897	0.034604065	negative
PRJNA729271	stage_5W_vs_E10	ordinary_muscle_development	ENSGALG00010008392	CD36	2.737678553	0.369459487	7.40995602	1.26341E-13	3.68178E-13	positive
PRJNA729271	stage_5W_vs_E10	ordinary_muscle_development	ENSGALG00010008394	CD2AP	1.082885355	0.110624031	9.788879952	1.2568E-22	5.6523E-22	positive
PRJNA729271	stage_5W_vs_E10	ordinary_muscle_development	ENSGALG00010008395	ENDOD1	0.440805083	0.146746059	3.003863186	0.002665752	0.003872527	positive
PRJNA729271	stage_5W_vs_E10	ordinary_muscle_development	ENSGALG00010008396	LRRTM2	-2.951292408	0.465134106	-6.345035489	2.22374E-10	5.40915E-10	negative
PRJNA729271	stage_5W_vs_E10	ordinary_muscle_development	ENSGALG00010008397	ATXN1L	-0.332770995	0.07266861	-4.579294877	4.66546E-06	8.50747E-06	negative
PRJNA729271	stage_5W_vs_E10	ordinary_muscle_development	ENSGALG00010008399	INO80C	-0.270892477	0.111955491	-2.419644403	0.015535691	0.020816218	negative
PRJNA729271	stage_5W_vs_E10	ordinary_muscle_development	ENSGALG00010008400	MAP1LC3C	-1.979453625	0.265261156	-7.462282275	8.50365E-14	2.50122E-13	negative
PRJNA729271	stage_5W_vs_E10	ordinary_muscle_development	ENSGALG00010008401	PSMD8	0.771018692	0.155958913	4.943729585	7.6642E-07	1.48559E-06	positive
PRJNA729271	stage_5W_vs_E10	ordinary_muscle_development	ENSGALG00010008403	GNAT3	-1.524993318	0.46779241	-3.259978757	0.001114206	0.001681809	negative
PRJNA729271	stage_5W_vs_E10	ordinary_muscle_development	ENSGALG00010008405	LRATD2	0.557000767	0.144327347	3.859287783	0.000113718	0.000186253	positive
PRJNA729271	stage_5W_vs_E10	ordinary_muscle_development	ENSGALG00010008407	GNAI1	-1.00291986	0.194667458	-5.151964629	2.57772E-07	5.16006E-07	negative
PRJNA729271	stage_5W_vs_E10	ordinary_muscle_development	ENSGALG00010008408	SMARCA4	-0.687818296	0.154128923	-4.462616645	8.09648E-06	1.45228E-05	negative
PRJNA729271	stage_5W_vs_E10	ordinary_muscle_development	ENSGALG00010008409	CWC15	0.285113525	0.084858417	3.359873238	0.000779782	0.001192294	positive
PRJNA729271	stage_5W_vs_E10	ordinary_muscle_development	ENSGALG00010008410	NAPRT	-0.857514334	0.134067835	-6.396122789	1.59372E-10	3.91475E-10	negative
PRJNA729271	stage_5W_vs_E10	ordinary_muscle_development	ENSGALG00010008411	ENSGALG00010008411	0.506838388	0.394067556	1.286171319	0.198383278	0.230765237	positive
PRJNA729271	stage_5W_vs_E10	ordinary_muscle_development	ENSGALG00010008412	NEK1	-1.11935142	0.105798438	-10.58003732	3.68811E-26	1.91816E-25	negative
PRJNA729271	stage_5W_vs_E10	ordinary_muscle_development	ENSGALG00010008415	ENSGALG00010008415	0.629511285	0.364003839	1.729408368	0.083736033	0.102695135	positive
PRJNA729271	stage_5W_vs_E10	ordinary_muscle_development	ENSGALG00010008416	MSX2	-5.829151974	0.553255287	-10.53609809	5.88919E-26	3.03462E-25	negative
PRJNA729271	stage_5W_vs_E10	ordinary_muscle_development	ENSGALG00010008417	IGFBP1	-0.134044907	0.883682452	-0.151689	0.879432234	0.894238529	negative
PRJNA729271	stage_5W_vs_E10	ordinary_muscle_development	ENSGALG00010008418	TMEM245	1.678913333	0.088609326	18.94736611	4.64246E-80	1.25079E-78	positive
PRJNA729271	stage_5W_vs_E10	ordinary_muscle_development	ENSGALG00010008422	EXO1	-3.803856164	0.239540169	-15.87982585	8.74208E-57	1.27996E-55	negative
PRJNA729271	stage_5W_vs_E10	ordinary_muscle_development	ENSGALG00010008424	PPIP5K2	-0.758806194	0.121399159	-6.250506167	4.09125E-10	9.78429E-10	negative
PRJNA729271	stage_5W_vs_E10	ordinary_muscle_development	ENSGALG00010008425	MAGI2	-0.364470905	0.107783222	-3.381518011	0.000720865	0.001105474	negative
PRJNA729271	stage_5W_vs_E10	ordinary_muscle_development	ENSGALG00010008426	AP1G1	-0.580871961	0.056302458	-10.31699108	5.90446E-25	2.931E-24	negative
PRJNA729271	stage_5W_vs_E10	ordinary_muscle_development	ENSGALG00010008429	IGFBP3	1.34915786	0.118687178	11.36734296	6.08106E-30	3.67424E-29	positive
PRJNA729271	stage_5W_vs_E10	ordinary_muscle_development	ENSGALG00010008430	ENSGALG00010008430	-0.355300426	0.421107026	-0.843729514	0.398820571	0.440081012	negative
PRJNA729271	stage_5W_vs_E10	ordinary_muscle_development	ENSGALG00010008431	SH3RF1	-0.598950657	0.12729791	-4.705109907	2.53729E-06	4.72301E-06	negative
PRJNA729271	stage_5W_vs_E10	ordinary_muscle_development	ENSGALG00010008433	EBAG9	0.864469745	0.09532004	9.069129113	1.19972E-19	4.72553E-19	positive
PRJNA729271	stage_5W_vs_E10	ordinary_muscle_development	ENSGALG00010008434	PTCHD4	-1.442504525	0.482608495	-2.988974585	0.002799154	0.004058998	negative
PRJNA729271	stage_5W_vs_E10	ordinary_muscle_development	ENSGALG00010008437	SHKBP1	0.48769307	0.159286321	3.061738542	0.002200556	0.003220624	positive
PRJNA729271	stage_5W_vs_E10	ordinary_muscle_development	ENSGALG00010008438	SYBU	0.560286476	0.262549741	2.134020296	0.032841119	0.042411977	positive
PRJNA729271	stage_5W_vs_E10	ordinary_muscle_development	ENSGALG00010008440	ENSGALG00010008440	0.385227738	0.316696168	1.216395325	0.223834364	0.258092364	positive
PRJNA729271	stage_5W_vs_E10	ordinary_muscle_development	ENSGALG00010008441	PHLPP2	0.152859096	0.086719802	1.762678113	0.077954794	0.096002952	positive

PRJNA729271	stage_5W_vs_E10	ordinary_muscle_development	ENSGALG00010008443	RSFR	5.263503428	0.439059326	11.98813719	4.10022E-33	2.81006E-32	positive
PRJNA729271	stage_5W_vs_E10	ordinary_muscle_development	ENSGALG00010008444	SLC7A11	-3.169526669	0.469888102	-6.745279688	1.52733E-11	4.00264E-11	negative
PRJNA729271	stage_5W_vs_E10	ordinary_muscle_development	ENSGALG00010008447	FRRS1L	-0.254787753	0.156117025	-1.632030546	0.102673052	0.124497732	negative
PRJNA729271	stage_5W_vs_E10	ordinary_muscle_development	ENSGALG00010008448	TNS3	-0.921739474	0.081247498	-11.34483521	7.86743E-30	4.72992E-29	negative
PRJNA729271	stage_5W_vs_E10	ordinary_muscle_development	ENSGALG00010008449	SRA1	0.016846565	0.109944131	0.153228419	0.878218147	0.893129309	positive
PRJNA729271	stage_5W_vs_E10	ordinary_muscle_development	ENSGALG00010008450	GIN1	-0.5706679	0.179584682	-3.177709222	0.001484435	0.002210261	negative
PRJNA729271	stage_5W_vs_E10	ordinary_muscle_development	ENSGALG00010008451	OPN3	1.107597801	0.307416425	3.602923308	0.000314658	0.000497055	positive
PRJNA729271	stage_5W_vs_E10	ordinary_muscle_development	ENSGALG00010008452	ENSGALG00010008452	0.407893915	0.42607577	0.957327178	0.338402142	0.378191137	positive
PRJNA729271	stage_5W_vs_E10	ordinary_muscle_development	ENSGALG00010008453	AMOTL1	1.229665371	0.120090076	10.2395253	1.31882E-24	6.44731E-24	positive
PRJNA729271	stage_5W_vs_E10	ordinary_muscle_development	ENSGALG00010008455	FKBP1B	1.634294224	0.150667686	10.84701215	2.06051E-27	1.12706E-26	positive
PRJNA729271	stage_5W_vs_E10	ordinary_muscle_development	ENSGALG00010008456	NHLRC1	0.344580494	0.128677612	2.677858948	0.007409441	0.010280449	positive
PRJNA729271	stage_5W_vs_E10	ordinary_muscle_development	ENSGALG00010008457	MARVELD3	0.39134384	0.120831497	3.238756849	0.001200519	0.001804575	positive
PRJNA729271	stage_5W_vs_E10	ordinary_muscle_development	ENSGALG00010008459	EPB41L4B	-2.559713246	0.24143941	-10.60188658	2.92029E-26	1.52814E-25	negative
PRJNA729271	stage_5W_vs_E10	ordinary_muscle_development	ENSGALG00010008464	SF3B6	-0.481928143	0.111228064	-4.332792706	1.4723E-05	2.59275E-05	negative
PRJNA729271	stage_5W_vs_E10	ordinary_muscle_development	ENSGALG00010008466	C11orf97	-1.211601549	0.361514251	-3.35146276	0.000803858	0.001227682	negative
PRJNA729271	stage_5W_vs_E10	ordinary_muscle_development	ENSGALG00010008468	EPPK1	-2.022732608	0.299847282	-6.745876095	1.52106E-11	3.98695E-11	negative
PRJNA729271	stage_5W_vs_E10	ordinary_muscle_development	ENSGALG00010008469	NOCT	2.254017004	0.276125587	8.163013888	3.26767E-16	1.09337E-15	positive
PRJNA729271	stage_5W_vs_E10	ordinary_muscle_development	ENSGALG00010008473	CSMD3	-4.708686584	0.504659371	-9.33042534	1.05447E-20	4.33481E-20	negative
PRJNA729271	stage_5W_vs_E10	ordinary_muscle_development	ENSGALG00010008474	ENSGALG00010008474	1.392307681	0.139514759	9.979644395	1.87136E-23	8.71079E-23	positive
PRJNA729271	stage_5W_vs_E10	ordinary_muscle_development	ENSGALG00010008476	RNF166	0.331214628	0.134477492	2.462974462	0.013778973	0.018568819	positive
PRJNA729271	stage_5W_vs_E10	ordinary_muscle_development	ENSGALG00010008486	CTU2	-0.916764243	0.140598128	-6.520458365	7.00929E-11	1.76572E-10	negative
PRJNA729271	stage_5W_vs_E10	ordinary_muscle_development	ENSGALG00010008488	CCSER1	-1.158271081	0.157539474	-7.352259425	1.94884E-13	5.6227E-13	negative
PRJNA729271	stage_5W_vs_E10	ordinary_muscle_development	ENSGALG00010008492	ANKRD49	0.448215549	0.123632206	3.625394742	0.00028852	0.00045721	positive
PRJNA729271	stage_5W_vs_E10	ordinary_muscle_development	ENSGALG00010008497	PAM	-0.785009368	0.130096297	-6.0340639	1.59887E-09	3.68979E-09	negative
PRJNA729271	stage_5W_vs_E10	ordinary_muscle_development	ENSGALG00010008498	ENSGALG00010008498	2.508010356	0.147673299	16.98350598	1.08789E-64	1.98601E-63	positive
PRJNA729271	stage_5W_vs_E10	ordinary_muscle_development	ENSGALG00010008500	ENSGALG00010008500	0.718212024	0.152510073	4.709275982	2.48598E-06	4.63047E-06	positive
PRJNA729271	stage_5W_vs_E10	ordinary_muscle_development	ENSGALG00010008501	ENSGALG00010008501	-1.027682721	0.417713628	-2.460256624	0.01388377	0.018708306	negative
PRJNA729271	stage_5W_vs_E10	ordinary_muscle_development	ENSGALG00010008504	RNF139	1.397028435	0.087117803	16.03608434	7.15242E-58	1.07882E-56	positive
PRJNA729271	stage_5W_vs_E10	ordinary_muscle_development	ENSGALG00010008505	PTPN3	-0.022030587	0.270350434	-0.081489002	0.935053071	0.943419772	negative
PRJNA729271	stage_5W_vs_E10	ordinary_muscle_development	ENSGALG00010008509	MGARP	1.083984064	0.358958337	3.019804673	0.002529378	0.003685127	positive
PRJNA729271	stage_5W_vs_E10	ordinary_muscle_development	ENSGALG00010008514	PIEZO1	2.566656342	0.165847087	15.47604112	5.0352E-54	6.75164E-53	positive
PRJNA729271	stage_5W_vs_E10	ordinary_muscle_development	ENSGALG00010008515	TAFA5	-1.563469727	0.223758702	-6.987302438	2.80222E-12	7.62782E-12	negative
PRJNA729271	stage_5W_vs_E10	ordinary_muscle_development	ENSGALG00010008516	MRE11	-1.16550651	0.079508	-14.65898416	1.18026E-48	1.35884E-47	negative
PRJNA729271	stage_5W_vs_E10	ordinary_muscle_development	ENSGALG00010008522	NUDT12	0.842348675	0.149636483	5.629300139	1.80942E-08	3.93099E-08	positive
PRJNA729271	stage_5W_vs_E10	ordinary_muscle_development	ENSGALG00010008527	CHUK	0.202665885	0.055186512	3.672380782	0.000240301	0.000383566	positive
PRJNA729271	stage_5W_vs_E10	ordinary_muscle_development	ENSGALG00010008529	GPR83	-0.383261334	0.522232601	-0.733890096	0.463015723	0.504921224	negative
PRJNA729271	stage_5W_vs_E10	ordinary_muscle_development	ENSGALG00010008533	ENSGALG00010008533	-1.205182567	0.66467314	-1.813195833	0.069801622	0.086557833	negative
PRJNA729271	stage_5W_vs_E10	ordinary_muscle_development	ENSGALG00010008534	RAB33B	1.031692887	0.071872975	14.35439231	9.99982E-47	1.08089E-45	positive
PRJNA729271	stage_5W_vs_E10	ordinary_muscle_development	ENSGALG00010008542	MOCOS	2.867034295	0.153803543	18.6408859	1.4974E-77	3.8828E-76	positive
PRJNA729271	stage_5W_vs_E10	ordinary_muscle_development	ENSGALG00010008561	EEF1D	0.973162376	0.114725681	8.482515604	2.20377E-17	7.80444E-17	positive
PRJNA729271	stage_5W_vs_E10	ordinary_muscle_development	ENSGALG00010008580	PANX1	-1.193183453	0.249857735	-4.775451335	1.79305E-06	3.37522E-06	negative
PRJNA729271	stage_5W_vs_E10	ordinary_muscle_development	ENSGALG00010008588	PYCR3	-2.327516167	0.187449529	-12.4167619	2.11968E-35	1.58621E-34	negative
PRJNA729271	stage_5W_vs_E10	ordinary_muscle_development	ENSGALG00010008592	KIAA0513	1.402358453	0.139338412	10.06440675	7.93644E-24	3.74353E-23	positive
PRJNA729271	stage_5W_vs_E10	ordinary_muscle_development	ENSGALG00010008596	ENSGALG00010008596	0.638866173	0.154282962	4.140873146	3.45986E-05	5.90147E-05	positive
PRJNA729271	stage_5W_vs_E10	ordinary_muscle_development	ENSGALG00010008618	EIF4EBP3	0.204172215	0.116749038	1.748812829	0.080323379	0.098743734	positive
PRJNA729271	stage_5W_vs_E10	ordinary_muscle_development	ENSGALG00010008623	TRPS1	-1.972632783	0.133045517	-14.82675118	9.83804E-50	1.17465E-48	negative
PRJNA729271	stage_5W_vs_E10	ordinary_muscle_development	ENSGALG00010008636	AGTPBP1	4.786667222	0.238501493	20.06975791	1.35664E-89	4.57313E-88	positive
PRJNA729271	stage_5W_vs_E10	ordinary_muscle_development	ENSGALG00010008637	RGS7	-5.886286621	1.002586189	-5.871102837	4.32906E-09	9.75434E-09	negative
PRJNA729271	stage_5W_vs_E10	ordinary_muscle_development	ENSGALG00010008653	ENSGALG00010008653	-1.052041972	0.159678007	-6.588521429	4.44228E-11	1.13205E-10	negative
PRJNA729271	stage_5W_vs_E10	ordinary_muscle_development	ENSGALG00010008656	CCDC6	0.396715625	0.071421863	5.554540397	2.78343E-08	5.96303E-08	positive
PRJNA729271	stage_5W_vs_E10	ordinary_muscle_development	ENSGALG00010008658	TSTA3	-1.959248083	0.125928266	-15.55844564	1.39444E-54	1.89967E-53	negative
PRJNA729271	stage_5W_vs_E10	ordinary_muscle_development	ENSGALG00010008666	SLC16A9	1.24428938	0.188666321	6.595185476	4.24725E-11	1.08483E-10	positive
PRJNA729271	stage_5W_vs_E10	ordinary_muscle_development	ENSGALG00010008668	ENSGALG00010008668	-0.04438457	0.321913636	-0.137877261	0.890337422	0.903932264	negative
PRJNA729271	stage_5W_vs_E10	ordinary_muscle_development	ENSGALG00010008674	RXFP1	2.250941533	0.268615968	8.379775609	5.30287E-17	1.8455E-16	positive
PRJNA729271	stage_5W_vs_E10	ordinary_muscle_development	ENSGALG00010008686	ENSGALG00010008686	-1.403097116	0.411446908	-3.410153509	0.000649263	0.000999689	negative
PRJNA729271	stage_5W_vs_E10	ordinary_muscle_development	ENSGALG00010008688	HBEGF	2.535294564	0.228254934	11.1072936	1.1561E-28	6.64989E-28	positive
PRJNA729271	stage_5W_vs_E10	ordinary_muscle_development	ENSGALG00010008690	YIF1B	-0.493697244	0.124697318	-3.959164901	7.52123E-05	0.000125412	negative
PRJNA729271	stage_5W_vs_E10	ordinary_muscle_development	ENSGALG00010008693	C4orf46	-0.6271981	0.106051737	-5.914076654	3.33742E-09	7.56584E-09	negative
PRJNA729271	stage_5W_vs_E10	ordinary_muscle_development	ENSGALG00010008696	PFN4	-0.051104256	0.214016573	-0.238786442	0.811271184	0.833289102	negative
PRJNA729271	stage_5W_vs_E10	ordinary_muscle_development	ENSGALG00010008699	NAA35	0.520341573	0.100026368	5.202044047	1.97108E-07	3.98367E-07	positive
PRJNA729271	stage_5W_vs_E10	ordinary_muscle_development	ENSGALG00010008700	ENSGALG00010008700	0.174778827	0.25862284	0.675805845	0.499163923	0.540337421	positive
PRJNA729271	stage_5W_vs_E10	ordinary_muscle_development	ENSGALG00010008708	ZC3H4	1.169129066	0.244927454	4.773368792	1.8117E-06	3.40825E-06	positive
PRJNA729271	stage_5W_vs_E10	ordinary_muscle_development	ENSGALG00010008716	TATDN1	-0.020081218	0.111360131	-0.180326821	0.856896001	0.874266737	negative
PRJNA729271	stage_5W_vs_E10	ordinary_muscle_development	ENSGALG00010008729	TMEM254	-1.097087926	0.12082175	-9.080218817	1.08357E-19	4.27732E-19	negative
PRJNA729271	stage_5W_vs_E10	ordinary_muscle_development	ENSGALG00010008731	NDUFB9	0.969964225	0.087535468	11.08081389	1.55453E-28	8.88522E-28	positive
PRJNA729271	stage_5W_vs_E10	ordinary_muscle_development	ENSGALG00010008739	VSTM5	-0.445699836	0.249291173	-1.787868503	0.073797226	0.091184962	negative
PRJNA729271	stage_5W_vs_E10	ordinary_muscle_development	ENSGALG00010008745	GRAMD4	1.77747843	0.151941506	11.69843893	1.2982E-31	8.38563E-31	positive
PRJNA729271	stage_5W_vs_E10	ordinary_muscle_development	ENSGALG00010008750	PFDN1	0.690107441	0.165932104	4.15897481	3.19679E-05	5.47013E-05	positive
PRJNA729271	stage_5W_vs_E10	ordinary_muscle_development	ENSGALG00010008752	GOLM1	-1.204446623	0.17344843	-6.944119483	3.80828E-12	1.02949E-11	negative
PRJNA729271	stage_5W_vs_E10	ordinary_muscle_development	ENSGALG00010008756	ITSN2	1.54294469	0.07257907	21.25881048	2.73255E-100	1.15476E-98	positive
PRJNA729271	stage_5W_vs_E10	ordinary_muscle_development	ENSGALG00010008762	SAE1	-0.528180989	0.102446533	-5.155674588	2.52719E-07	5.06242E-07	negative
PRJNA729271	stage_5W_vs_E10	ordinary_muscle_development	ENSGALG00010008764	ETFDH	-0.103065327	0.124263551	-0.829409157	0.406872922	0.44835206	negative
PRJNA729271	stage_5W_vs_E10	ordinary_muscle_development	ENSGALG00010008768	PRXL2C	2.598533346	0.145627933	17.84364639	3.23856E-71	7.1018E-70	positive
PRJNA729271	stage_5W_vs_E10	ordinary_muscle_development	ENSGALG00010008775	MED17	-0.161168634	0.090344447	-1.78393513	0.074434207	0.091925009	negative
PRJNA729271	stage_5W_vs_E10	ordinary_muscle_development	ENSGALG00010008780	KARS1	0.275518689	0.057336187	4.805319317	1.54505E-06	2.92178E-06	positive
PRJNA729271	stage_5W_vs_E10	ordinary_muscle_development	ENSGALG00010008782	CCDC9	-0.116908371	0.296642887	-0.39410475	0.693503704	0.726219925	negative
PRJNA729271	stage_5W_vs_E10	ordinary_muscle_development	ENSGALG00010008790	GPN1	0.150782416	0.095607874	1.577092033	0.11477435	0.138407172	positive
PRJNA729271	stage_5W_vs_E10	ordinary_muscle_development	ENSGALG00010008793	C11orf54	0.694189145	0.115040821	6.034285396	1.59668E-09	3.68532E-09	positive
PRJNA729271	stage_5W_vs_E10	ordinary_muscle_development	ENSGALG00010008799	ENSGALG00010008799	0.378910734	0.174748512	2.168320225	0.030134331	0.039079997	positive
PRJNA729271	stage_5W_vs_E10	ordinary_muscle_development	ENSGALG00010008807	BRD1	-0.153201824	0.06248132	-2.451962032	0.014207965	0.019116723	negative
PRJNA729271	stage_5W_vs_E10	ordinary_muscle_development	ENSGALG00010008811	TERF2IP	0.824311657	0.094359215	8.735889322	2.41747E-18	8.95735E-18	positive
PRJNA729271	stage_5W_vs_E10	ordinary_muscle_development	ENSGALG00010008814	ENSGALG00010008814	0.727125388	0.235175441	3.091842346	0.001989185	0.002924263	positive
PRJNA729271	stage_5W_vs_E10	ordinary_muscle_development	ENSGALG00010008821	ENSGALG00010008821	-0.217647819	0.138851487	-1.567486409	0.117001041	0.140834586	negative
PRJNA729271	stage_5W_vs_E10	ordinary_muscle_development	ENSGALG00010008824	GREM2	-1.173661096	0.309365562	-3.793767765	0.000148378	0.000240683	negative
PRJNA729271	stage_5W_vs_E10	ordinary_muscle_development	ENSGALG00010008825	PPID	0.18490921	0.108886312	1.698185998	0.089472662	0.109296981	positive
PRJNA729271	stage_5W_vs_E10	ordinary_muscle_development	ENSGALG00010008830	DHX34	0.270551707	0.197875146	1.367284937	0.171536011	0.201934093	positive
PRJNA729271	stage_5W_vs_E10	ordinary_muscle_development	ENSGALG00010008832	MTSS1	0.506915943	0.124204774	4.081291952	4.47861E-05	7.57938E-05	positive
PRJNA729271	stage_5W_vs_E10	ordinary_muscle_development	ENSGALG00010008837	ENSGALG00010008837	-0.949863531	0.099855641	-9.512367299	1.86372E-21	7.9525E-21	negative
PRJNA729271	stage_5W_vs_E10	ordinary_muscle_development	ENSGALG00010008839	ZNF512	-0.361812001	0.07461272	-4.849199998	1.2396E-06	2.36204E-06	negative
PRJNA729271	stage_5W_vs_E10	ordinary_muscle_development	ENSGALG00010008846	SLC35D2	0.742408497	0.283381369	2.619821127	0.00879759	0.012110263	positive
PRJNA729271	stage_5W_vs_E10	ordinary_muscle_development	ENSGALG00010008850	CYSTM1	2.634349453	0.123372299	21.35284391	3.6692E-101	1.57818E-99	positive
PRJNA729271	stage_5W_vs_E10	ordinary_muscle_development	ENSGALG00010008852	KHDRBS3	0.654388462	0.083351003	7.850996828	4.12743E-15	1.30399E-14	positive
PRJNA729271	stage_5W_vs_E10	ordinary_muscle_development	ENSGALG00010008859	ENSGALG00010008859	-1.080540976	0.221356296	-4.881455814	1.05306E-06	2.01852E-06	negative
PRJNA729271	stage_5W_vs_E10	ordinary_muscle_development	ENSGALG00010008860	SQLE	-1.409566058	0.245457246	-5.742613347	9.32264E-09	2.06182E-08	negative
PRJNA729271	stage_5W_vs_E10	ordinary_muscle_development	ENSGALG00010008862	ZNF367	-0.674081263	0.146021042	-4.616329622	3.90586E-06	7.16833E-06	negative
PRJNA729271	stage_5W_vs_E10	ordinary_muscle_development	ENSGALG00010008864	ADAT1	0.411713867	0.138277695	2.977442358	0.002906643	0.004204769	positive
PRJNA729271	stage_5W_vs_E10	ordinary_muscle_development	ENSGALG00010008866	WASHC5	-0.262806148	0.073651829	-3.56822295	0.000359411	0.000565161	negative
PRJNA729271	stage_5W_vs_E10	ordinary_muscle_development	ENSGALG00010008871	PRR7	-1.445623682	0.196608449	-7.352805474	1.94089E-13	5.60088E-13	negative
PRJNA729271	stage_5W_vs_E10	ordinary_muscle_development	ENSGALG00010008877	FNDC4	-0.057044394	0.26240831	-0.217387911	0.827906051	0.848571504	negative
PRJNA729271	stage_5W_vs_E10	ordinary_muscle_development	ENSGALG00010008879	HABP4	-1.659229357	0.179459582	-9.245699422	2.33707E-20	9.47841E-20	negative
PRJNA729271	stage_5W_vs_E10	ordinary_muscle_development	ENSGALG00010008880	FMN2	-1.334255749	0.296142275	-4.505455187	6.62308E-06	1.19435E-05	negative
PRJNA729271	stage_5W_vs_E10	ordinary_muscle_development	ENSGALG00010008884	FAM135B	-0.297193242	0.529541476	-0.561227506	0.574642454	0.613496529	negative
PRJNA729271	stage_5W_vs_E10	ordinary_muscle_development	ENSGALG00010008888	FBXO46	1.404234521	0.141887375	9.896824998	4.297E-23	1.96731E-22	positive
PRJNA729271	stage_5W_vs_E10	ordinary_muscle_development	ENSGALG00010008892	ENSGALG00010008892	-0.638393181	0.131502734	-4.854600079	1.2063E-06	2.3004E-06	negative
PRJNA729271	stage_5W_vs_E10	ordinary_muscle_development	ENSGALG00010008893	ENSGALG00010008893	5.122670444	0.138087923	37.09716497	3.1212E-301	1.2567E-298	positive
PRJNA729271	stage_5W_vs_E10	ordinary_muscle_development	ENSGALG00010008897	NSMCE2	-0.126026894	0.164465648	-0.76628096	0.443509145	0.485438726	negative
PRJNA729271	stage_5W_vs_E10	ordinary_muscle_development	ENSGALG00010008903	ENSGALG00010008903	-0.242114477	0.596296403	-0.406030417	0.684720252	0.718023796	negative
PRJNA729271	stage_5W_vs_E10	ordinary_muscle_development	ENSGALG00010008905	GABARAPL2	0.807967449	0.09364076	8.62837348	6.22306E-18	2.26869E-17	positive
PRJNA729271	stage_5W_vs_E10	ordinary_muscle_development	ENSGALG00010008906	DBN1	-3.425777331	0.214097382	-16.00102391	1.25691E-57	1.88406E-56	negative
PRJNA729271	stage_5W_vs_E10	ordinary_muscle_development	ENSGALG00010008910	TRIB1	2.074596492	0.261042325	7.947356769	1.90533E-15	6.14138E-15	positive
PRJNA729271	stage_5W_vs_E10	ordinary_muscle_development	ENSGALG00010008918	PLAC9	-0.599705902	0.246394009	-2.433930534	0.014935861	0.020047718	negative
PRJNA729271	stage_5W_vs_E10	ordinary_muscle_development	ENSGALG00010008921	OPA3	0.649211597	0.127551159	5.08981339	3.58416E-07	7.09635E-07	positive
PRJNA729271	stage_5W_vs_E10	ordinary_muscle_development	ENSGALG00010008922	ENSGALG00010008922	2.122899815	1.380168577	1.538145303	0.124013091	0.148743877	positive
PRJNA729271	stage_5W_vs_E10	ordinary_muscle_development	ENSGALG00010008928	CFC1	-6.612298555	0.915705804	-7.220985742	5.16121E-13	1.45974E-12	negative
PRJNA729271	stage_5W_vs_E10	ordinary_muscle_development	ENSGALG00010008931	FNIP2	0.026759616	0.132331082	0.202217167	0.839746953	0.859128465	positive
PRJNA729271	stage_5W_vs_E10	ordinary_muscle_development	ENSGALG00010008932	VASP	1.352118373	0.120311075	11.23851956	2.63769E-29	1.55546E-28	positive
PRJNA729271	stage_5W_vs_E10	ordinary_muscle_development	ENSGALG00010008936	IFT172	-0.684493658	0.095954382	-7.13353205	9.78255E-13	2.73004E-12	negative
PRJNA729271	stage_5W_vs_E10	ordinary_muscle_development	ENSGALG00010008938	ZBED4	-0.205853498	0.090762024	-2.268057592	0.023325697	0.030592289	negative
PRJNA729271	stage_5W_vs_E10	ordinary_muscle_development	ENSGALG00010008939	RANBP9	1.152001323	0.102852267	11.20054386	4.05238E-29	2.37522E-28	positive
PRJNA729271	stage_5W_vs_E10	ordinary_muscle_development	ENSGALG00010008943	ENSGALG00010008943	3.772949861	0.451198689	8.362058559	6.16332E-17	2.13879E-16	positive
PRJNA729271	stage_5W_vs_E10	ordinary_muscle_development	ENSGALG00010008945	FER1L6	0.905983864	0.562999562	1.609208827	0.107570687	0.130109905	positive
PRJNA729271	stage_5W_vs_E10	ordinary_muscle_development	ENSGALG00010008946	COL22A1	-2.682711928	0.622182091	-4.311779411	1.61946E-05	2.83983E-05	negative
PRJNA729271	stage_5W_vs_E10	ordinary_muscle_development	ENSGALG00010008955	KRTCAP3	-2.556712444	0.438276907	-5.833555006	5.42587E-09	1.21633E-08	negative
PRJNA729271	stage_5W_vs_E10	ordinary_muscle_development	ENSGALG00010008956	LVRN	0.405137822	0.483506907	0.837915274	0.40207829	0.443439459	positive
PRJNA729271	stage_5W_vs_E10	ordinary_muscle_development	ENSGALG00010008960	LRRC2	9.634859378	0.397449607	24.24171318	8.0863E-130	5.8314E-128	positive
PRJNA729271	stage_5W_vs_E10	ordinary_muscle_development	ENSGALG00010008966	NRBP1	0.145277038	0.116344262	1.248682452	0.211781242	0.245261121	positive
PRJNA729271	stage_5W_vs_E10	ordinary_muscle_development	ENSGALG00010008969	PPP1R37	0.431886487	0.187475658	2.303693677	0.021239845	0.027967983	positive
PRJNA729271	stage_5W_vs_E10	ordinary_muscle_development	ENSGALG00010008971	ANXA11	0.830253912	0.10366659	8.008886084	1.15752E-15	3.77294E-15	positive
PRJNA729271	stage_5W_vs_E10	ordinary_muscle_development	ENSGALG00010008973	KCNK9	-3.19660179	0.906062361	-3.528015207	0.000418688	0.000653904	negative
PRJNA729271	stage_5W_vs_E10	ordinary_muscle_development	ENSGALG00010008974	SMCO4	-0.318728255	0.102299733	-3.115631347	0.001835516	0.002708797	negative
PRJNA729271	stage_5W_vs_E10	ordinary_muscle_development	ENSGALG00010008980	ENSGALG00010008980	-0.688647187	0.276330093	-2.492117954	0.012698386	0.017179681	negative

PRJNA729271	stage_5W_vs_E10	ordinary_muscle_development	ENSGALG00010008983	ENSGALG00010008983	-0.702655118	0.362738809	-1.937082827	0.05273522	0.06632512	negative
PRJNA729271	stage_5W_vs_E10	ordinary_muscle_development	ENSGALG00010008988	PPM1G	-0.68791342	0.080744876	-8.51959221	1.60116E-17	5.71928E-17	negative
PRJNA729271	stage_5W_vs_E10	ordinary_muscle_development	ENSGALG00010008991	ENSGALG00010008991	0.548484414	0.128280477	4.275665521	1.90567E-05	3.32323E-05	positive
PRJNA729271	stage_5W_vs_E10	ordinary_muscle_development	ENSGALG00010008995	ECD	-1.022379196	0.102039407	-10.01945451	1.25192E-23	5.8613E-23	negative
PRJNA729271	stage_5W_vs_E10	ordinary_muscle_development	ENSGALG00010008996	CHRM3	-1.131176652	0.241299473	-4.687853805	2.76085E-06	5.13058E-06	negative
PRJNA729271	stage_5W_vs_E10	ordinary_muscle_development	ENSGALG00010008997	CHST6	-1.970759481	0.135817279	-14.51037371	1.04152E-47	1.15507E-46	negative
PRJNA729271	stage_5W_vs_E10	ordinary_muscle_development	ENSGALG00010008999	TRAPPC9	-0.710313386	0.065964656	-10.76809054	4.86996E-27	2.62514E-26	negative
PRJNA729271	stage_5W_vs_E10	ordinary_muscle_development	ENSGALG00010009000	FAM149B1	-0.577938989	0.103850786	-5.565090189	2.62017E-08	5.63158E-08	negative
PRJNA729271	stage_5W_vs_E10	ordinary_muscle_development	ENSGALG00010009002	ENSGALG00010009002	0.972348751	0.097467456	9.976137615	1.93867E-23	9.02119E-23	positive
PRJNA729271	stage_5W_vs_E10	ordinary_muscle_development	ENSGALG00010009003	ALG12	-1.421132344	0.13676727	-10.39088038	2.72823E-25	1.36647E-24	negative
PRJNA729271	stage_5W_vs_E10	ordinary_muscle_development	ENSGALG00010009007	TMEM65	-0.814990465	0.10063186	-8.098731997	5.5535E-16	1.83576E-15	negative
PRJNA729271	stage_5W_vs_E10	ordinary_muscle_development	ENSGALG00010009011	ACP6	-0.498172071	0.123524138	-4.032993717	5.50708E-05	9.2712E-05	negative
PRJNA729271	stage_5W_vs_E10	ordinary_muscle_development	ENSGALG00010009012	DEUP1	-2.374499763	0.878344595	-2.703380626	0.00686381	0.009554492	negative
PRJNA729271	stage_5W_vs_E10	ordinary_muscle_development	ENSGALG00010009013	LIPG	0.19525669	0.222784379	0.876437978	0.38079198	0.421921705	positive
PRJNA729271	stage_5W_vs_E10	ordinary_muscle_development	ENSGALG00010009015	TMEM231	-0.252095365	0.092736114	-2.718416317	0.006559525	0.009151137	negative
PRJNA729271	stage_5W_vs_E10	ordinary_muscle_development	ENSGALG00010009016	DNAJC9	-1.858528843	0.114075576	-16.29208374	1.12336E-59	1.77957E-58	negative
PRJNA729271	stage_5W_vs_E10	ordinary_muscle_development	ENSGALG00010009019	ZNF513	0.703263741	0.13424782	5.238548687	1.61844E-07	3.29069E-07	positive
PRJNA729271	stage_5W_vs_E10	ordinary_muscle_development	ENSGALG00010009020	ARHGAP35	0.389151575	0.182753999	2.129373784	0.033223348	0.042871221	positive
PRJNA729271	stage_5W_vs_E10	ordinary_muscle_development	ENSGALG00010009021	HNF4beta	-0.620346878	0.455137968	-1.362986438	0.172886785	0.203342579	negative
PRJNA729271	stage_5W_vs_E10	ordinary_muscle_development	ENSGALG00010009024	PMAIP1	1.583431029	0.252105378	6.280830019	3.3677E-10	8.09533E-10	positive
PRJNA729271	stage_5W_vs_E10	ordinary_muscle_development	ENSGALG00010009025	UTP23	-0.128833717	0.097824886	-1.316983056	0.187844304	0.219651758	negative
PRJNA729271	stage_5W_vs_E10	ordinary_muscle_development	ENSGALG00010009026	RAPGEF2	-0.118486308	0.13708331	-0.864337958	0.387402264	0.428721623	negative
PRJNA729271	stage_5W_vs_E10	ordinary_muscle_development	ENSGALG00010009028	PDLIM7	-1.264595125	0.115234404	-10.97411086	5.09042E-28	2.85438E-27	negative
PRJNA729271	stage_5W_vs_E10	ordinary_muscle_development	ENSGALG00010009029	IGSF3	0.163602011	0.162734201	1.005332685	0.314736681	0.353761492	positive
PRJNA729271	stage_5W_vs_E10	ordinary_muscle_development	ENSGALG00010009030	ZDHHC7	-0.622326601	0.092459315	-6.730815622	1.68715E-11	4.4119E-11	negative
PRJNA729271	stage_5W_vs_E10	ordinary_muscle_development	ENSGALG00010009031	SPIN1	-0.819599707	0.079741688	-10.27818351	8.83775E-25	4.35873E-24	negative
PRJNA729271	stage_5W_vs_E10	ordinary_muscle_development	ENSGALG00010009034	SNX17	1.608710026	0.118890292	13.53104617	1.02554E-41	9.43221E-41	positive
PRJNA729271	stage_5W_vs_E10	ordinary_muscle_development	ENSGALG00010009035	ENSGALG00010009035	-1.783732025	0.084407107	-21.13248613	3.99926E-99	1.64685E-97	negative
PRJNA729271	stage_5W_vs_E10	ordinary_muscle_development	ENSGALG00010009037	ENSGALG00010009037	2.69570826	0.179804181	14.99246706	8.22407E-51	1.018E-49	positive
PRJNA729271	stage_5W_vs_E10	ordinary_muscle_development	ENSGALG00010009038	RAD21	-0.807508814	0.124393058	-6.491590683	8.49348E-11	2.12704E-10	negative
PRJNA729271	stage_5W_vs_E10	ordinary_muscle_development	ENSGALG00010009039	SLC36A4	-0.369657064	0.171997134	-2.149204784	0.03161817	0.040909165	negative
PRJNA729271	stage_5W_vs_E10	ordinary_muscle_development	ENSGALG00010009040	CRELD2	-1.600768532	0.144988949	-11.04062444	2.43336E-28	1.38157E-27	negative
PRJNA729271	stage_5W_vs_E10	ordinary_muscle_development	ENSGALG00010009043	TFAM	-0.78054839	0.084374964	-9.250947833	2.22511E-20	9.03443E-20	negative
PRJNA729271	stage_5W_vs_E10	ordinary_muscle_development	ENSGALG00010009047	SUPT5H	0.244377245	0.110170555	2.218172039	0.026543104	0.034627153	positive
PRJNA729271	stage_5W_vs_E10	ordinary_muscle_development	ENSGALG00010009050	PIK3CG	2.221826974	0.091257881	24.346686	6.2853E-131	4.6247E-129	positive
PRJNA729271	stage_5W_vs_E10	ordinary_muscle_development	ENSGALG00010009051	TROJANZ	3.729856332	0.236934067	15.74216988	7.77318E-56	1.10247E-54	positive
PRJNA729271	stage_5W_vs_E10	ordinary_muscle_development	ENSGALG00010009054	RYR2	-1.640896767	0.501267134	-3.273497613	0.001062253	0.001606904	negative
PRJNA729271	stage_5W_vs_E10	ordinary_muscle_development	ENSGALG00010009057	NXNL2	1.7364146	0.360257592	4.819925079	1.43612E-06	2.7243E-06	positive
PRJNA729271	stage_5W_vs_E10	ordinary_muscle_development	ENSGALG00010009059	MED29	1.037659769	0.12590493	8.241613518	1.69904E-16	5.75948E-16	positive
PRJNA729271	stage_5W_vs_E10	ordinary_muscle_development	ENSGALG00010009065	EIF2B4	0.53113387	0.094693205	5.608996625	2.03503E-08	4.4066E-08	positive
PRJNA729271	stage_5W_vs_E10	ordinary_muscle_development	ENSGALG00010009067	ENSGALG00010009067	-1.096691148	1.621515853	-0.676336988	0.498826714	0.540072693	negative
PRJNA729271	stage_5W_vs_E10	ordinary_muscle_development	ENSGALG00010009068	AGO2	-0.175731294	0.081967357	-2.143918026	0.032039456	0.041432055	negative
PRJNA729271	stage_5W_vs_E10	ordinary_muscle_development	ENSGALG00010009069	GRID2	-3.495123986	0.346512244	-10.0865815	6.33369E-24	2.99729E-23	negative
PRJNA729271	stage_5W_vs_E10	ordinary_muscle_development	ENSGALG00010009070	ENSGALG00010009070	1.215087187	1.252522351	0.970112178	0.331990579	0.37165612	positive
PRJNA729271	stage_5W_vs_E10	ordinary_muscle_development	ENSGALG00010009073	PRKAR2B	1.275026009	0.403669693	3.158587407	0.001585358	0.002354725	positive
PRJNA729271	stage_5W_vs_E10	ordinary_muscle_development	ENSGALG00010009076	TMEM214	0.20438307	0.115964703	1.762459309	0.077991726	0.096032115	positive
PRJNA729271	stage_5W_vs_E10	ordinary_muscle_development	ENSGALG00010009078	PTTG2	-2.162416964	0.173491217	-12.46412929	1.17144E-35	8.86225E-35	negative
PRJNA729271	stage_5W_vs_E10	ordinary_muscle_development	ENSGALG00010009079	KCTD15	-1.218335989	0.099211991	-12.2801284	1.15814E-34	8.43158E-34	negative
PRJNA729271	stage_5W_vs_E10	ordinary_muscle_development	ENSGALG00010009080	TLL1	-0.4136179	0.16955639	-2.439412045	0.014711183	0.01976811	negative
PRJNA729271	stage_5W_vs_E10	ordinary_muscle_development	ENSGALG00010009081	HBP1	1.216308837	0.12198916	9.970630501	2.04924E-23	9.52652E-23	positive
PRJNA729271	stage_5W_vs_E10	ordinary_muscle_development	ENSGALG00010009083	MED30	0.296916976	0.127769473	2.323849101	0.020133583	0.026586161	positive
PRJNA729271	stage_5W_vs_E10	ordinary_muscle_development	ENSGALG00010009085	PAF1	-0.244888776	0.089154905	-2.746778495	0.006018377	0.008429449	negative
PRJNA729271	stage_5W_vs_E10	ordinary_muscle_development	ENSGALG00010009086	UBE2D1	1.787982298	0.170637032	10.47827822	1.08705E-25	5.53645E-25	positive
PRJNA729271	stage_5W_vs_E10	ordinary_muscle_development	ENSGALG00010009088	ENSGALG00010009088	-0.460184802	0.144783165	-3.178441371	0.001480692	0.00220514	negative
PRJNA729271	stage_5W_vs_E10	ordinary_muscle_development	ENSGALG00010009089	AGBL5	-0.587688967	0.159800647	-3.67763821	0.000235404	0.00037608	negative
PRJNA729271	stage_5W_vs_E10	ordinary_muscle_development	ENSGALG00010009091	EXT1	-0.375497483	0.11233472	-3.34266629	0.000829776	0.00126633	negative
PRJNA729271	stage_5W_vs_E10	ordinary_muscle_development	ENSGALG00010009092	EED	-1.000071572	0.117714543	-8.495735094	1.96684E-17	6.9893E-17	negative
PRJNA729271	stage_5W_vs_E10	ordinary_muscle_development	ENSGALG00010009093	SLU7	0.410014949	0.131870042	3.109234991	0.001875725	0.002764027	positive
PRJNA729271	stage_5W_vs_E10	ordinary_muscle_development	ENSGALG00010009094	DARS	0.023711263	0.095338044	0.24870725	0.80358724	0.827037564	positive
PRJNA729271	stage_5W_vs_E10	ordinary_muscle_development	ENSGALG00010009096	ENSGALG00010009096	1.920481168	1.30458147	1.472105201	0.140992471	0.167886788	positive
PRJNA729271	stage_5W_vs_E10	ordinary_muscle_development	ENSGALG00010009097	C1QTNF2	0.431756283	0.181625651	2.377176791	0.017445724	0.023231582	positive
PRJNA729271	stage_5W_vs_E10	ordinary_muscle_development	ENSGALG00010009098	CAD	-1.210187649	0.197981074	-6.112643107	9.79944E-10	2.28732E-09	negative
PRJNA729271	stage_5W_vs_E10	ordinary_muscle_development	ENSGALG00010009099	CXCR4	0.003876874	0.190780363	0.020321139	0.983787193	0.986100225	positive
PRJNA729271	stage_5W_vs_E10	ordinary_muscle_development	ENSGALG00010009100	ENSGALG00010009100	-0.457208072	0.155305031	-2.943936	0.00324067	0.004668871	negative
PRJNA729271	stage_5W_vs_E10	ordinary_muscle_development	ENSGALG00010009101	HIKESHI	0.031403614	0.098510286	0.318785128	0.749889456	0.778070699	positive
PRJNA729271	stage_5W_vs_E10	ordinary_muscle_development	ENSGALG00010009102	HTRA3	1.241319255	0.21685125	5.724289129	1.03868E-08	2.29157E-08	positive
PRJNA729271	stage_5W_vs_E10	ordinary_muscle_development	ENSGALG00010009103	AP3S1	-1.57698154	0.124572654	-12.65913095	9.95958E-37	7.8331E-36	negative
PRJNA729271	stage_5W_vs_E10	ordinary_muscle_development	ENSGALG00010009104	MTR	0.432992722	0.122995687	3.520389492	0.000430914	0.00067249	positive
PRJNA729271	stage_5W_vs_E10	ordinary_muscle_development	ENSGALG00010009106	THSD7B	-1.105314289	0.23829533	-4.638421958	3.51079E-06	6.46702E-06	negative
PRJNA729271	stage_5W_vs_E10	ordinary_muscle_development	ENSGALG00010009109	ENSGALG00010009109	-0.264091054	0.218995984	-1.205917339	0.227849364	0.26232538	negative
PRJNA729271	stage_5W_vs_E10	ordinary_muscle_development	ENSGALG00010009111	LRFN1	0.857310926	0.243374616	3.52259796	0.000427339	0.000667128	positive
PRJNA729271	stage_5W_vs_E10	ordinary_muscle_development	ENSGALG00010009112	ENSGALG00010009112	7.905158759	0.26469418	29.86525336	5.5644E-196	8.9618E-194	positive
PRJNA729271	stage_5W_vs_E10	ordinary_muscle_development	ENSGALG00010009113	RPLP1	0.249796998	0.176376448	1.416271849	0.156695927	0.185488563	positive
PRJNA729271	stage_5W_vs_E10	ordinary_muscle_development	ENSGALG00010009114	SETD7	1.625938378	0.149900733	10.84676737	2.06604E-27	1.12966E-26	positive
PRJNA729271	stage_5W_vs_E10	ordinary_muscle_development	ENSGALG00010009115	COG5	-0.01728203	0.114965739	-0.150323304	0.880509548	0.895082818	negative
PRJNA729271	stage_5W_vs_E10	ordinary_muscle_development	ENSGALG00010009116	S1PR3	-1.193041859	0.185170378	-6.44294122	1.1718E-10	2.90247E-10	negative
PRJNA729271	stage_5W_vs_E10	ordinary_muscle_development	ENSGALG00010009117	ERGIC1	-0.014820473	0.067074355	-0.220955885	0.825126782	0.846021979	negative
PRJNA729271	stage_5W_vs_E10	ordinary_muscle_development	ENSGALG00010009118	ACTN2	4.072526835	0.143907922	28.29953203	3.502E-176	4.7441E-174	positive
PRJNA729271	stage_5W_vs_E10	ordinary_muscle_development	ENSGALG00010009119	CISD1	1.249197406	0.153012312	8.16403198	3.24023E-16	1.08444E-15	positive
PRJNA729271	stage_5W_vs_E10	ordinary_muscle_development	ENSGALG00010009120	ATG12	0.073384512	0.089665557	0.818424763	0.413114687	0.454677098	positive
PRJNA729271	stage_5W_vs_E10	ordinary_muscle_development	ENSGALG00010009121	SYCN	0.427859038	0.773278652	0.553305121	0.580054495	0.618546651	positive
PRJNA729271	stage_5W_vs_E10	ordinary_muscle_development	ENSGALG00010009123	ENSGALG00010009123	-1.814988664	0.136186246	-13.32725377	1.60705E-40	1.42734E-39	negative
PRJNA729271	stage_5W_vs_E10	ordinary_muscle_development	ENSGALG00010009125	CD72L1	7.197999383	0.931354946	7.728524356	1.088E-14	3.3626E-14	positive
PRJNA729271	stage_5W_vs_E10	ordinary_muscle_development	ENSGALG00010009126	JPH3	2.076721693	0.13532342	15.34635834	3.74635E-53	4.93218E-52	positive
PRJNA729271	stage_5W_vs_E10	ordinary_muscle_development	ENSGALG00010009127	SCOC	0.258031097	0.074372189	3.469456801	0.000521512	0.000808916	positive
PRJNA729271	stage_5W_vs_E10	ordinary_muscle_development	ENSGALG00010009129	ENSGALG00010009129	-0.581276177	0.451026049	-1.288786265	0.197472402	0.229816336	negative
PRJNA729271	stage_5W_vs_E10	ordinary_muscle_development	ENSGALG00010009130	HEATR1	-1.148151468	0.074105763	-15.49341683	3.84311E-54	5.18195E-53	negative
PRJNA729271	stage_5W_vs_E10	ordinary_muscle_development	ENSGALG00010009131	CDKL2	-1.917170607	0.30795443	-6.225500991	4.8002E-10	1.14382E-09	negative
PRJNA729271	stage_5W_vs_E10	ordinary_muscle_development	ENSGALG00010009133	BOD1L1	0.131518671	0.12930778	1.017097904	0.309106857	0.348000303	positive
PRJNA729271	stage_5W_vs_E10	ordinary_muscle_development	ENSGALG00010009135	TENM4	-1.843756046	0.185795587	-9.923572867	3.28772E-23	1.51239E-22	negative
PRJNA729271	stage_5W_vs_E10	ordinary_muscle_development	ENSGALG00010009136	KHK	-1.997146602	0.500793557	-3.987963855	6.66428E-05	0.000111597	negative
PRJNA729271	stage_5W_vs_E10	ordinary_muscle_development	ENSGALG00010009137	IPMK	0.119157546	0.092180333	1.292656929	0.196129741	0.228382117	positive
PRJNA729271	stage_5W_vs_E10	ordinary_muscle_development	ENSGALG00010009138	ENSGALG00010009138	-0.190681029	0.11123976	-1.714144553	0.086502181	0.105962065	negative
PRJNA729271	stage_5W_vs_E10	ordinary_muscle_development	ENSGALG00010009139	CKS2	-3.6813169	0.31672016	-11.6232478	3.13969E-31	1.99868E-30	negative
PRJNA729271	stage_5W_vs_E10	ordinary_muscle_development	ENSGALG00010009141	MAML3	0.113914916	0.117641192	0.968325075	0.332882045	0.372510248	positive
PRJNA729271	stage_5W_vs_E10	ordinary_muscle_development	ENSGALG00010009142	LGALS8	-0.109133369	0.134281842	-0.812718735	0.416379337	0.457957242	negative
PRJNA729271	stage_5W_vs_E10	ordinary_muscle_development	ENSGALG00010009143	MCCC2	0.276952048	0.135732168	2.040430444	0.041307472	0.052660669	positive
PRJNA729271	stage_5W_vs_E10	ordinary_muscle_development	ENSGALG00010009144	CREBZF	0.171091134	0.159440521	1.073071846	0.283238882	0.320871246	positive
PRJNA729271	stage_5W_vs_E10	ordinary_muscle_development	ENSGALG00010009147	HNMT	2.912657846	0.226871842	12.83834	9.99956E-38	8.10647E-37	positive
PRJNA729271	stage_5W_vs_E10	ordinary_muscle_development	ENSGALG00010009149	FUT4	-1.319807659	0.213633643	-6.177901763	6.49591E-10	1.53552E-09	negative
PRJNA729271	stage_5W_vs_E10	ordinary_muscle_development	ENSGALG00010009150	ENSGALG00010009150	-0.764091125	0.42043719	-1.817372827	0.069160046	0.085806288	negative
PRJNA729271	stage_5W_vs_E10	ordinary_muscle_development	ENSGALG00010009151	CCNJL	0.972269402	0.358751106	2.710150258	0.006725273	0.009374251	positive
PRJNA729271	stage_5W_vs_E10	ordinary_muscle_development	ENSGALG00010009152	RPP25L	0.517894094	0.123022497	4.209751113	2.55652E-05	4.4131E-05	positive
PRJNA729271	stage_5W_vs_E10	ordinary_muscle_development	ENSGALG00010009153	SECISBP2	0.039596236	0.12458762	0.317818381	0.750622707	0.778773085	positive
PRJNA729271	stage_5W_vs_E10	ordinary_muscle_development	ENSGALG00010009155	PAK4	-0.313292863	0.222019271	-1.411106618	0.158213179	0.187192885	negative
PRJNA729271	stage_5W_vs_E10	ordinary_muscle_development	ENSGALG00010009157	ABHD1	1.117217337	0.137496492	8.125424314	4.458E-16	1.48276E-15	positive
PRJNA729271	stage_5W_vs_E10	ordinary_muscle_development	ENSGALG00010009159	PTK2	0.357936625	0.098876198	3.620048429	0.000294548	0.000466354	positive
PRJNA729271	stage_5W_vs_E10	ordinary_muscle_development	ENSGALG00010009160	ENSGALG00010009160	-3.093908476	0.394210149	-7.848373482	4.21468E-15	1.33126E-14	negative
PRJNA729271	stage_5W_vs_E10	ordinary_muscle_development	ENSGALG00010009163	SAMD12	-0.453596193	0.178161712	-2.545980215	0.010897136	0.014866064	negative
PRJNA729271	stage_5W_vs_E10	ordinary_muscle_development	ENSGALG00010009164	TMEM128	0.428039766	0.100923474	4.241231004	2.22297E-05	3.85569E-05	positive
PRJNA729271	stage_5W_vs_E10	ordinary_muscle_development	ENSGALG00010009166	CLGN	-0.650488372	0.232901261	-2.792979179	0.005222505	0.007358823	negative
PRJNA729271	stage_5W_vs_E10	ordinary_muscle_development	ENSGALG00010009167	DCTN3	-0.035863014	0.110804715	-0.323659643	0.746195728	0.77457083	negative
PRJNA729271	stage_5W_vs_E10	ordinary_muscle_development	ENSGALG00010009168	MRPS12	0.571682985	0.175218657	3.262683298	0.001103628	0.001666884	positive
PRJNA729271	stage_5W_vs_E10	ordinary_muscle_development	ENSGALG00010009170	RPL26L1	0.093135354	0.191304865	0.486842582	0.626369892	0.662572545	positive
PRJNA729271	stage_5W_vs_E10	ordinary_muscle_development	ENSGALG00010009172	SPOPL	0.62534981	0.132100201	4.733905068	2.20241E-06	4.11921E-06	positive
PRJNA729271	stage_5W_vs_E10	ordinary_muscle_development	ENSGALG00010009173	LYAR	-0.869387982	0.143867458	-6.042978697	1.51295E-09	3.49652E-09	negative
PRJNA729271	stage_5W_vs_E10	ordinary_muscle_development	ENSGALG00010009178	ENSGALG00010009178	0.786606202	0.25312007	3.10764058	0.001885873	0.002777738	positive
PRJNA729271	stage_5W_vs_E10	ordinary_muscle_development	ENSGALG00010009180	NFKBIB	0.577962057	0.146792133	3.937282225	8.24097E-05	0.000137003	positive
PRJNA729271	stage_5W_vs_E10	ordinary_muscle_development	ENSGALG00010009181	ARID3C	-6.244930659	0.933530869	-6.689581312	2.2381E-11	5.8076E-11	negative
PRJNA729271	stage_5W_vs_E10	ordinary_muscle_development	ENSGALG00010009186	PREB	-0.466785378	0.099794196	-4.677480244	2.90422E-06	5.38802E-06	negative
PRJNA729271	stage_5W_vs_E10	ordinary_muscle_development	ENSGALG00010009188	ENSGALG00010009188	-1.195756798	0.494229074	-2.419438397	0.015544493	0.020824162	negative
PRJNA729271	stage_5W_vs_E10	ordinary_muscle_development	ENSGALG00010009189	ENSGALG00010009189	-1.404304356	0.247545617	-5.672911403	1.40391E-08	3.07536E-08	negative
PRJNA729271	stage_5W_vs_E10	ordinary_muscle_development	ENSGALG00010009190	ZBTB49	0.087845189	0.139326709	0.63049781	0.528368916	0.568746706	positive
PRJNA729271	stage_5W_vs_E10	ordinary_muscle_development	ENSGALG00010009192	LSM14A	-0.216557344	0.059576834	-3.634925333	0.000278061	0.000441118	negative
PRJNA729271	stage_5W_vs_E10	ordinary_muscle_development	ENSGALG00010009193	EDARADD	-0.861132556	0.217288046	-3.963092189	7.39852E-05	0.000123493	negative
PRJNA729271	stage_5W_vs_E10	ordinary_muscle_development	ENSGALG00010009195	ENSGALG00010009195	3.967676073	0.610245504	6.501770264	7.93803E-11	1.99207E-10	positive
PRJNA729271	stage_5W_vs_E10	ordinary_muscle_development	ENSGALG00010009197	FH	0.652685827	0.102669643	6.357145175	2.05538E-10	5.01392E-10	positive
PRJNA729271	stage_5W_vs_E10	ordinary_muscle_development	ENSGALG00010009198	KCNN2	0.052194056	0.177131716	0.294662396	0.768251792	0.795695304	positive
PRJNA729271	stage_5W_vs_E10	ordinary_muscle_development	ENSGALG00010009199	SEMA4D	-1.823797679	0.150281557	-12.13587159	6.81801E-34	4.81848E-33	negative
PRJNA729271	stage_5W_vs_E10	ordinary_muscle_development	ENSGALG00010009200	ADGRA3	-2.177758292	0.095478339	-22.80892509	3.7393E-115	2.109E-113	negative
PRJNA729271	stage_5W_vs_E10	ordinary_muscle_development	ENSGALG00010009202	FABP6	1.644226747	0.284773925	5.773796695	7.75049E-09	1.72279E-08	positive

PRJNA729271	stage_5W_vs_E10	ordinary_muscle_development	ENSGALG00010009205	SIRT2	0.484683749	0.097840215	4.953829578	7.27669E-07	1.41294E-06	positive
PRJNA729271	stage_5W_vs_E10	ordinary_muscle_development	ENSGALG00010009208	SLC5A6	-0.323122905	0.193978482	-1.665766748	0.095759881	0.116651776	negative
PRJNA729271	stage_5W_vs_E10	ordinary_muscle_development	ENSGALG00010009209	SLC6A19	5.287476749	1.707609423	3.096420457	0.001958724	0.002880944	positive
PRJNA729271	stage_5W_vs_E10	ordinary_muscle_development	ENSGALG00010009210	PCDH15	-2.984856696	0.326849416	-9.132207514	6.71165E-20	2.66974E-19	negative
PRJNA729271	stage_5W_vs_E10	ordinary_muscle_development	ENSGALG00010009212	NXPH2	-2.044850424	0.295123129	-6.928804356	4.24412E-12	1.14347E-11	negative
PRJNA729271	stage_5W_vs_E10	ordinary_muscle_development	ENSGALG00010009213	DUS4L	-0.664090633	0.093822515	-7.078158525	1.46083E-12	4.03943E-12	negative
PRJNA729271	stage_5W_vs_E10	ordinary_muscle_development	ENSGALG00010009214	PCDH10	-5.437365642	0.305855567	-17.77755983	1.05472E-70	2.26491E-69	negative
PRJNA729271	stage_5W_vs_E10	ordinary_muscle_development	ENSGALG00010009216	NSG1	-4.226832403	0.24820293	-17.0297442	4.94198E-65	9.13699E-64	negative
PRJNA729271	stage_5W_vs_E10	ordinary_muscle_development	ENSGALG00010009219	TNFRSF11B	1.213286272	0.180576448	6.718961871	1.83024E-11	4.77317E-11	positive
PRJNA729271	stage_5W_vs_E10	ordinary_muscle_development	ENSGALG00010009220	GADD45G	0.075892196	0.191568308	0.396162586	0.69198509	0.72483913	positive
PRJNA729271	stage_5W_vs_E10	ordinary_muscle_development	ENSGALG00010009223	ENSGALG00010009223	0.194191781	0.108843408	1.784139103	0.074401065	0.091892219	positive
PRJNA729271	stage_5W_vs_E10	ordinary_muscle_development	ENSGALG00010009224	ENSGALG00010009224	5.386634242	0.446075886	12.0756006	1.42123E-33	9.89942E-33	positive
PRJNA729271	stage_5W_vs_E10	ordinary_muscle_development	ENSGALG00010009225	HNRNPL	-0.527727207	0.125204336	-4.214927566	2.49858E-05	4.31617E-05	negative
PRJNA729271	stage_5W_vs_E10	ordinary_muscle_development	ENSGALG00010009227	ENSGALG00010009227	-0.391632872	0.174253381	-2.247490807	0.024608677	0.032210834	negative
PRJNA729271	stage_5W_vs_E10	ordinary_muscle_development	ENSGALG00010009228	ATP6V0E1	1.102044989	0.099138509	11.11621508	1.04617E-28	6.02471E-28	positive
PRJNA729271	stage_5W_vs_E10	ordinary_muscle_development	ENSGALG00010009232	ECH1	0.087215262	0.165499614	0.526981662	0.598206323	0.636170261	positive
PRJNA729271	stage_5W_vs_E10	ordinary_muscle_development	ENSGALG00010009234	PCDH18	-1.161099191	0.128844065	-9.011662195	2.02957E-19	7.89125E-19	negative
PRJNA729271	stage_5W_vs_E10	ordinary_muscle_development	ENSGALG00010009235	KLHDC4	0.718714833	0.130335068	5.514362649	3.50046E-08	7.44522E-08	positive
PRJNA729271	stage_5W_vs_E10	ordinary_muscle_development	ENSGALG00010009236	DTWD2	-0.562026748	0.118703786	-4.734699431	2.1938E-06	4.1047E-06	negative
PRJNA729271	stage_5W_vs_E10	ordinary_muscle_development	ENSGALG00010009237	DENND3	-0.875435147	0.178962	-4.891737603	9.99496E-07	1.91992E-06	negative
PRJNA729271	stage_5W_vs_E10	ordinary_muscle_development	ENSGALG00010009239	STX18	0.270785954	0.082133185	3.296912878	0.000977538	0.001483708	positive
PRJNA729271	stage_5W_vs_E10	ordinary_muscle_development	ENSGALG00010009243	BCAP29	0.42018009	0.099392377	4.227488092	2.36315E-05	4.09098E-05	positive
PRJNA729271	stage_5W_vs_E10	ordinary_muscle_development	ENSGALG00010009246	RUSC2	2.811444031	0.141416029	19.88066034	5.98411E-88	1.94048E-86	positive
PRJNA729271	stage_5W_vs_E10	ordinary_muscle_development	ENSGALG00010009250	ERO1B	-0.206558841	0.10876183	-1.899185053	0.057540149	0.072012128	negative
PRJNA729271	stage_5W_vs_E10	ordinary_muscle_development	ENSGALG00010009252	SIRT1	-0.056211286	0.096581275	-0.582010191	0.560559816	0.600274419	negative
PRJNA729271	stage_5W_vs_E10	ordinary_muscle_development	ENSGALG00010009253	SLC45A4	-0.274549288	0.141843129	-1.935583972	0.052918675	0.066529299	negative
PRJNA729271	stage_5W_vs_E10	ordinary_muscle_development	ENSGALG00010009254	SLC26A4	-0.517903729	0.774393358	-0.668786378	0.503631757	0.544569808	negative
PRJNA729271	stage_5W_vs_E10	ordinary_muscle_development	ENSGALG00010009256	CREBRF	1.983165719	0.172629619	11.48798061	1.51615E-30	9.37566E-30	positive
PRJNA729271	stage_5W_vs_E10	ordinary_muscle_development	ENSGALG00010009257	ENSGALG00010009257	2.452315908	0.186725887	13.13324011	2.12372E-39	1.81185E-38	positive
PRJNA729271	stage_5W_vs_E10	ordinary_muscle_development	ENSGALG00010009258	DNAJC12	-0.562736633	0.169124093	-3.327359346	0.000876732	0.001335039	negative
PRJNA729271	stage_5W_vs_E10	ordinary_muscle_development	ENSGALG00010009259	MGAT4D	2.897098569	0.774806607	3.739124761	0.000184662	0.000297475	positive
PRJNA729271	stage_5W_vs_E10	ordinary_muscle_development	ENSGALG00010009263	COLEC10	0.188362874	0.220529619	0.854138665	0.393028163	0.434284435	positive
PRJNA729271	stage_5W_vs_E10	ordinary_muscle_development	ENSGALG00010009266	PCDH7	-1.926618379	0.242941684	-7.930373856	2.18487E-15	7.02367E-15	negative
PRJNA729271	stage_5W_vs_E10	ordinary_muscle_development	ENSGALG00010009268	PWWP2A	0.105109719	0.133363256	0.788146016	0.430611302	0.472463161	positive
PRJNA729271	stage_5W_vs_E10	ordinary_muscle_development	ENSGALG00010009269	ENSGALG00010009269	-4.206106467	0.506295446	-8.307612684	9.76466E-17	3.35082E-16	negative
PRJNA729271	stage_5W_vs_E10	ordinary_muscle_development	ENSGALG00010009273	MAL2	-0.118327501	0.484756219	-0.244096921	0.807155757	0.829944151	negative
PRJNA729271	stage_5W_vs_E10	ordinary_muscle_development	ENSGALG00010009275	CBLL1	0.138129254	0.143229838	0.964388818	0.334851026	0.374540136	positive
PRJNA729271	stage_5W_vs_E10	ordinary_muscle_development	ENSGALG00010009276	ENSGALG00010009276	-0.484300563	0.330579122	-1.465006502	0.142919144	0.170055245	negative
PRJNA729271	stage_5W_vs_E10	ordinary_muscle_development	ENSGALG00010009277	ACTN4	-0.061887041	0.128318275	-0.482293271	0.629597627	0.665258609	negative
PRJNA729271	stage_5W_vs_E10	ordinary_muscle_development	ENSGALG00010009279	ELMOD2	-2.25515189	0.114550443	-19.68697665	2.78844E-86	8.63641E-85	negative
PRJNA729271	stage_5W_vs_E10	ordinary_muscle_development	ENSGALG00010009280	HSD17B4	0.115055524	0.083346495	1.380448263	0.167448669	0.197555629	positive
PRJNA729271	stage_5W_vs_E10	ordinary_muscle_development	ENSGALG00010009284	CCN3	-3.466614038	0.681095657	-5.089760893	3.58515E-07	7.09735E-07	negative
PRJNA729271	stage_5W_vs_E10	ordinary_muscle_development	ENSGALG00010009286	ENSGALG00010009286	-0.153780126	0.188061139	-0.817713465	0.41352082	0.455089537	negative
PRJNA729271	stage_5W_vs_E10	ordinary_muscle_development	ENSGALG00010009289	GPR137B	0.624774067	0.144331693	4.328737864	1.49966E-05	2.63966E-05	positive
PRJNA729271	stage_5W_vs_E10	ordinary_muscle_development	ENSGALG00010009294	ACOX3	0.79105956	0.139789612	5.658929504	1.5232E-08	3.32913E-08	positive
PRJNA729271	stage_5W_vs_E10	ordinary_muscle_development	ENSGALG00010009297	CTNNA3	4.769409833	0.204191566	23.3575261	1.1557E-120	7.0383E-119	positive
PRJNA729271	stage_5W_vs_E10	ordinary_muscle_development	ENSGALG00010009299	MAP4K1	3.867003007	0.290134724	13.32830125	1.58465E-40	1.40831E-39	positive
PRJNA729271	stage_5W_vs_E10	ordinary_muscle_development	ENSGALG00010009303	TTC1	0.321971958	0.096458437	3.337934653	0.000844036	0.001286738	positive
PRJNA729271	stage_5W_vs_E10	ordinary_muscle_development	ENSGALG00010009306	TBC1D9	-1.422038873	0.109049825	-13.04026741	7.22034E-39	6.03917E-38	negative
PRJNA729271	stage_5W_vs_E10	ordinary_muscle_development	ENSGALG00010009307	DIRAS2	3.80467906	0.310543805	12.25166626	1.64579E-34	1.191E-33	positive
PRJNA729271	stage_5W_vs_E10	ordinary_muscle_development	ENSGALG00010009309	TRIM54	2.672952618	0.148964868	17.94351011	5.39378E-72	1.20839E-70	positive
PRJNA729271	stage_5W_vs_E10	ordinary_muscle_development	ENSGALG00010009311	BNIP1	-0.127844442	0.112774875	-1.13362521	0.256951794	0.293199736	negative
PRJNA729271	stage_5W_vs_E10	ordinary_muscle_development	ENSGALG00010009313	UNC13B	-0.615475503	0.189561144	-3.246844215	0.001166922	0.00175626	negative
PRJNA729271	stage_5W_vs_E10	ordinary_muscle_development	ENSGALG00010009317	LRRTM3	-4.797449499	0.678493278	-7.070739914	1.5411E-12	4.25328E-12	negative
PRJNA729271	stage_5W_vs_E10	ordinary_muscle_development	ENSGALG00010009320	GPR20	-2.874970932	0.25578992	-11.23957868	2.60623E-29	1.53754E-28	negative
PRJNA729271	stage_5W_vs_E10	ordinary_muscle_development	ENSGALG00010009322	ENSGALG00010009322	2.218261694	0.166726969	13.3047563	2.172E-40	1.91971E-39	positive
PRJNA729271	stage_5W_vs_E10	ordinary_muscle_development	ENSGALG00010009323	RNF150	0.833706058	0.106946761	7.795524152	6.41417E-15	2.0046E-14	positive
PRJNA729271	stage_5W_vs_E10	ordinary_muscle_development	ENSGALG00010009326	DLD	0.705378406	0.131799544	5.351903235	8.70339E-08	1.80197E-07	positive
PRJNA729271	stage_5W_vs_E10	ordinary_muscle_development	ENSGALG00010009327	NID1	2.380298589	0.163059233	14.59775412	2.9025E-48	3.292E-47	positive
PRJNA729271	stage_5W_vs_E10	ordinary_muscle_development	ENSGALG00010009328	ENSGALG00010009328	-1.562368367	0.140673745	-11.10632524	1.16871E-28	6.71971E-28	negative
PRJNA729271	stage_5W_vs_E10	ordinary_muscle_development	ENSGALG00010009330	EIF3K	0.154544368	0.141596785	1.091439809	0.275079403	0.312262194	positive
PRJNA729271	stage_5W_vs_E10	ordinary_muscle_development	ENSGALG00010009332	ENSGALG00010009332	0.398774669	0.16354855	2.438264774	0.01475796	0.019821778	positive
PRJNA729271	stage_5W_vs_E10	ordinary_muscle_development	ENSGALG00010009333	ADRA1B	2.070975271	0.316267432	6.548177472	5.82435E-11	1.47131E-10	positive
PRJNA729271	stage_5W_vs_E10	ordinary_muscle_development	ENSGALG00010009334	SS18L2	0.798729088	0.163798124	4.87630182	1.08093E-06	2.06949E-06	positive
PRJNA729271	stage_5W_vs_E10	ordinary_muscle_development	ENSGALG00010009335	PTP4A3	3.223144501	0.195048427	16.52484233	2.43075E-61	4.0827E-60	positive
PRJNA729271	stage_5W_vs_E10	ordinary_muscle_development	ENSGALG00010009336	LAMB1	0.937565589	0.109941258	8.527877601	1.49057E-17	5.33214E-17	positive
PRJNA729271	stage_5W_vs_E10	ordinary_muscle_development	ENSGALG00010009337	ZNF330	0.004971723	0.101391149	0.049035075	0.960891344	0.96615705	positive
PRJNA729271	stage_5W_vs_E10	ordinary_muscle_development	ENSGALG00010009338	KYNU	-0.197108555	0.578832343	-0.340527887	0.733459026	0.76287949	negative
PRJNA729271	stage_5W_vs_E10	ordinary_muscle_development	ENSGALG00010009340	SYK	1.825369538	0.137467588	13.27854488	3.08328E-40	2.71684E-39	positive
PRJNA729271	stage_5W_vs_E10	ordinary_muscle_development	ENSGALG00010009344	ENPP2	-0.659800236	0.258003893	-2.557326668	0.010548011	0.014410313	negative
PRJNA729271	stage_5W_vs_E10	ordinary_muscle_development	ENSGALG00010009345	ZFAT	0.338837285	0.125306942	2.704058356	0.006849826	0.009537774	positive
PRJNA729271	stage_5W_vs_E10	ordinary_muscle_development	ENSGALG00010009346	STC2	-1.563747226	0.307993722	-5.077204868	3.83028E-07	7.57227E-07	negative
PRJNA729271	stage_5W_vs_E10	ordinary_muscle_development	ENSGALG00010009347	TESK1	2.900242321	0.141133103	20.54969575	7.7447E-94	2.86376E-92	positive
PRJNA729271	stage_5W_vs_E10	ordinary_muscle_development	ENSGALG00010009348	ERGIC2	0.038588516	0.082387454	0.468378546	0.639513908	0.674801929	positive
PRJNA729271	stage_5W_vs_E10	ordinary_muscle_development	ENSGALG00010009351	GTF3C2	0.505488716	0.121844649	4.148632889	3.34467E-05	5.71238E-05	positive
PRJNA729271	stage_5W_vs_E10	ordinary_muscle_development	ENSGALG00010009352	HSF1	1.48712728	0.178241909	8.343308778	7.22402E-17	2.49612E-16	positive
PRJNA729271	stage_5W_vs_E10	ordinary_muscle_development	ENSGALG00010009355	GARRE1	0.194166288	0.072436823	2.680491506	0.007351413	0.010201889	positive
PRJNA729271	stage_5W_vs_E10	ordinary_muscle_development	ENSGALG00010009357	LYST	0.270745063	0.116622723	2.3215464	0.020257373	0.026742315	positive
PRJNA729271	stage_5W_vs_E10	ordinary_muscle_development	ENSGALG00010009359	REEP3	-1.052912889	0.111341971	-9.456567702	3.18217E-21	1.34256E-20	negative
PRJNA729271	stage_5W_vs_E10	ordinary_muscle_development	ENSGALG00010009360	TNFAIP8	0.329725191	0.12811461	2.573673612	0.010062515	0.013778212	positive
PRJNA729271	stage_5W_vs_E10	ordinary_muscle_development	ENSGALG00010009361	LRRK2	1.025454778	0.087855649	11.67204145	1.77121E-31	1.13651E-30	positive
PRJNA729271	stage_5W_vs_E10	ordinary_muscle_development	ENSGALG00010009362	IL15	5.42478663	0.308540002	17.5821177	3.37723E-69	6.94369E-68	positive
PRJNA729271	stage_5W_vs_E10	ordinary_muscle_development	ENSGALG00010009363	TAF2	-0.358799315	0.077626802	-4.622106113	3.79864E-06	6.97684E-06	negative
PRJNA729271	stage_5W_vs_E10	ordinary_muscle_development	ENSGALG00010009365	CA5B	1.03505536	0.160372372	6.454075262	1.08882E-10	2.70432E-10	positive
PRJNA729271	stage_5W_vs_E10	ordinary_muscle_development	ENSGALG00010009367	DMXL1	0.52420328	0.119278131	4.394797909	1.10876E-05	1.97027E-05	positive
PRJNA729271	stage_5W_vs_E10	ordinary_muscle_development	ENSGALG00010009368	DSCC1	-2.895681891	0.201934294	-14.33972324	1.23547E-46	1.3285E-45	negative
PRJNA729271	stage_5W_vs_E10	ordinary_muscle_development	ENSGALG00010009370	ENSGALG00010009370	1.747972948	0.451482891	3.871626111	0.000108112	0.000177412	positive
PRJNA729271	stage_5W_vs_E10	ordinary_muscle_development	ENSGALG00010009371	PHTF2	2.394543607	0.154508111	15.49785047	3.58693E-54	4.85005E-53	positive
PRJNA729271	stage_5W_vs_E10	ordinary_muscle_development	ENSGALG00010009372	RAB28	-0.397964075	0.099084212	-4.016422644	5.90882E-05	9.93371E-05	negative
PRJNA729271	stage_5W_vs_E10	ordinary_muscle_development	ENSGALG00010009373	ARHGAP15	1.220300673	0.215257608	5.669024592	1.43613E-08	3.14356E-08	positive
PRJNA729271	stage_5W_vs_E10	ordinary_muscle_development	ENSGALG00010009376	CDO1	-0.515018373	0.250793457	-2.053555863	0.040018698	0.051130103	negative
PRJNA729271	stage_5W_vs_E10	ordinary_muscle_development	ENSGALG00010009378	ENSGALG00010009378	-1.157035766	0.085081271	-13.59918283	4.04932E-42	3.76491E-41	negative
PRJNA729271	stage_5W_vs_E10	ordinary_muscle_development	ENSGALG00010009379	RYR1	4.36010289	0.219475292	19.86603067	8.00912E-88	2.59134E-86	positive
PRJNA729271	stage_5W_vs_E10	ordinary_muscle_development	ENSGALG00010009381	INPP4B	-1.037593698	0.116176849	-8.931157193	4.21576E-19	1.6166E-18	negative
PRJNA729271	stage_5W_vs_E10	ordinary_muscle_development	ENSGALG00010009383	ASXL2	0.978633493	0.11119716	8.800885717	1.35741E-18	5.08151E-18	positive
PRJNA729271	stage_5W_vs_E10	ordinary_muscle_development	ENSGALG00010009385	GPI	3.594181249	0.177942093	20.19860051	1.00711E-90	3.50074E-89	positive
PRJNA729271	stage_5W_vs_E10	ordinary_muscle_development	ENSGALG00010009386	TMEM60	0.311665553	0.111985367	2.78309178	0.005384357	0.007574365	positive
PRJNA729271	stage_5W_vs_E10	ordinary_muscle_development	ENSGALG00010009387	DEPTOR	3.13148377	0.138210034	22.65742712	1.1787E-113	6.375E-112	positive
PRJNA729271	stage_5W_vs_E10	ordinary_muscle_development	ENSGALG00010009388	TMED7	-0.325775685	0.073794026	-4.414662043	1.01168E-05	1.80284E-05	negative
PRJNA729271	stage_5W_vs_E10	ordinary_muscle_development	ENSGALG00010009389	CPEB4	2.021446029	0.125432999	16.11574342	1.97771E-58	3.03997E-57	positive
PRJNA729271	stage_5W_vs_E10	ordinary_muscle_development	ENSGALG00010009391	RSBN1L	0.16927516	0.107518474	1.574382096	0.11539914	0.139068053	positive
PRJNA729271	stage_5W_vs_E10	ordinary_muscle_development	ENSGALG00010009392	MAPRE3	1.248902396	0.105792015	11.80526143	3.6665E-32	2.42564E-31	positive
PRJNA729271	stage_5W_vs_E10	ordinary_muscle_development	ENSGALG00010009393	MARVELD2	-0.459279017	0.133859043	-3.431064543	0.000601218	0.000928374	negative
PRJNA729271	stage_5W_vs_E10	ordinary_muscle_development	ENSGALG00010009394	BANP	-0.4006155	0.097875042	-4.093132343	4.25585E-05	7.21334E-05	negative
PRJNA729271	stage_5W_vs_E10	ordinary_muscle_development	ENSGALG00010009396	AUH	0.434101809	0.11196483	3.877126502	0.000105697	0.000173607	positive
PRJNA729271	stage_5W_vs_E10	ordinary_muscle_development	ENSGALG00010009398	WTIP	0.188707773	0.138559746	1.361923485	0.173222034	0.203687302	positive
PRJNA729271	stage_5W_vs_E10	ordinary_muscle_development	ENSGALG00010009400	CNTFR	-0.263228749	0.283628192	-0.928076816	0.353367725	0.393550916	negative
PRJNA729271	stage_5W_vs_E10	ordinary_muscle_development	ENSGALG00010009404	GNG4	-1.264042008	0.407084873	-3.105106801	0.001902103	0.002800507	negative
PRJNA729271	stage_5W_vs_E10	ordinary_muscle_development	ENSGALG00010009407	PTPN12	0.018980116	0.087488297	0.216944624	0.828251501	0.848805536	positive
PRJNA729271	stage_5W_vs_E10	ordinary_muscle_development	ENSGALG00010009408	ENSGALG00010009408	-0.410640054	0.093520539	-4.390907681	1.12878E-05	2.00413E-05	negative
PRJNA729271	stage_5W_vs_E10	ordinary_muscle_development	ENSGALG00010009410	JMJD1C	0.115846628	0.1082454	1.070222184	0.284519311	0.322170722	positive
PRJNA729271	stage_5W_vs_E10	ordinary_muscle_development	ENSGALG00010009411	SMARCA5	-1.649115818	0.098000179	-16.82768172	1.52975E-63	2.7274E-62	negative
PRJNA729271	stage_5W_vs_E10	ordinary_muscle_development	ENSGALG00010009415	MMP19	-5.075901795	0.470245023	-10.79416378	3.66786E-27	1.99197E-26	negative
PRJNA729271	stage_5W_vs_E10	ordinary_muscle_development	ENSGALG00010009416	COL14A1	-4.14539113	0.47399447	-8.745652937	2.2173E-18	8.23462E-18	negative
PRJNA729271	stage_5W_vs_E10	ordinary_muscle_development	ENSGALG00010009424	ENSGALG00010009424	-0.924960871	1.281971549	-0.721514351	0.470593114	0.512605936	negative
PRJNA729271	stage_5W_vs_E10	ordinary_muscle_development	ENSGALG00010009431	SMN	0.093504969	0.149860995	0.623944667	0.532663925	0.572729292	positive
PRJNA729271	stage_5W_vs_E10	ordinary_muscle_development	ENSGALG00010009432	BOP1	0.237352506	0.179178176	1.324673082	0.185279605	0.216845206	positive
PRJNA729271	stage_5W_vs_E10	ordinary_muscle_development	ENSGALG00010009435	GTDC1	-0.535291657	0.090832581	-5.893167957	3.78861E-09	8.57123E-09	negative
PRJNA729271	stage_5W_vs_E10	ordinary_muscle_development	ENSGALG00010009437	DPYSL5	-5.313115783	0.301605661	-17.61610098	1.85348E-69	3.85454E-68	negative
PRJNA729271	stage_5W_vs_E10	ordinary_muscle_development	ENSGALG00010009439	B3GALNT2	1.038208294	0.097920159	10.60260018	2.89809E-26	1.51707E-25	positive
PRJNA729271	stage_5W_vs_E10	ordinary_muscle_development	ENSGALG00010009440	DOK3	0.054042374	0.214265299	0.252221776	0.800869648	0.824650532	positive
PRJNA729271	stage_5W_vs_E10	ordinary_muscle_development	ENSGALG00010009441	ZNF518B	-1.091461447	0.099158133	-11.0072811	3.52481E-28	1.98493E-27	negative
PRJNA729271	stage_5W_vs_E10	ordinary_muscle_development	ENSGALG00010009444	NFIL3	1.095443264	0.221608994	4.943135413	7.6876E-07	1.48993E-06	positive
PRJNA729271	stage_5W_vs_E10	ordinary_muscle_development	ENSGALG00010009446	ENSGALG00010009446	1.525206882	0.249637888	6.109677073	9.98329E-10	2.32949E-09	positive
PRJNA729271	stage_5W_vs_E10	ordinary_muscle_development	ENSGALG00010009447	DDX41	-0.317381132	0.106320359	-2.985139777	0.002834488	0.00410859	negative
PRJNA729271	stage_5W_vs_E10	ordinary_muscle_development	ENSGALG00010009451	ROR2	-1.73134334	0.14137594	-12.24637897	1.75666E-34	1.26996E-33	negative
PRJNA729271	stage_5W_vs_E10	ordinary_muscle_development	ENSGALG00010009452	TBCE	-0.142583771	0.086646332	-1.645583473	0.099849544	0.121276952	negative
PRJNA729271	stage_5W_vs_E10	ordinary_muscle_development	ENSGALG00010009453	GSAP	-0.173843104	0.115069421	-1.510767172	0.130847781	0.156489577	negative
PRJNA729271	stage_5W_vs_E10	ordinary_muscle_development	ENSGALG00010009457	BDP1	-0.070824036	0.12985977	-0.545388587	0.585486288	0.623603773	negative
PRJNA729271	stage_5W_vs_E10	ordinary_muscle_development	ENSGALG00010009458	SCX	-5.398246262	0.51488406	-10.48439299	1.01898E-25	5.20257E-25	negative
PRJNA729271	stage_5W_vs_E10	ordinary_muscle_development	ENSGALG00010009461	ZEB2	0.546614742	0.125478548	4.356240562	1.32315E-05	2.3392E-05	positive

PRJNA729271	stage_5W_vs_E10	ordinary_muscle_development	ENSGALG00010009462	NRBF2	2.006406931	0.119569491	16.78025826	3.40353E-63	5.97265E-62	positive
PRJNA729271	stage_5W_vs_E10	ordinary_muscle_development	ENSGALG00010009463	MRPL13	-0.355310087	0.109690698	-3.239199791	0.001198656	0.001801962	negative
PRJNA729271	stage_5W_vs_E10	ordinary_muscle_development	ENSGALG00010009464	SPTLC1	0.296291388	0.147871099	2.003713984	0.045100707	0.057184636	positive
PRJNA729271	stage_5W_vs_E10	ordinary_muscle_development	ENSGALG00010009465	GGPS1	0.611175256	0.09901481	6.172564047	6.71913E-10	1.5857E-09	positive
PRJNA729271	stage_5W_vs_E10	ordinary_muscle_development	ENSGALG00010009466	FAM193B	-0.679077701	0.11285185	-6.017426379	1.77212E-09	4.07728E-09	negative
PRJNA729271	stage_5W_vs_E10	ordinary_muscle_development	ENSGALG00010009467	EGR2	1.690338642	0.302186302	5.5936971	2.22285E-08	4.80253E-08	positive
PRJNA729271	stage_5W_vs_E10	ordinary_muscle_development	ENSGALG00010009469	DNMT3A	0.502018899	0.210743998	2.382126679	0.017212975	0.022932176	positive
PRJNA729271	stage_5W_vs_E10	ordinary_muscle_development	ENSGALG00010009470	ZNF469	-1.559116177	0.212754623	-7.328236424	2.33201E-13	6.70551E-13	negative
PRJNA729271	stage_5W_vs_E10	ordinary_muscle_development	ENSGALG00010009472	ADO	0.163109926	0.084695083	1.925848815	0.054123254	0.067935276	positive
PRJNA729271	stage_5W_vs_E10	ordinary_muscle_development	ENSGALG00010009474	ACVR2A	-1.00876708	0.096326073	-10.47241989	1.15648E-25	5.88389E-25	negative
PRJNA729271	stage_5W_vs_E10	ordinary_muscle_development	ENSGALG00010009477	ZNF365	0.873751726	0.243628438	3.586411075	0.00033526	0.00052839	positive
PRJNA729271	stage_5W_vs_E10	ordinary_muscle_development	ENSGALG00010009479	MTBP	-2.970736358	0.148643169	-19.98569044	7.33653E-89	2.42792E-87	negative
PRJNA729271	stage_5W_vs_E10	ordinary_muscle_development	ENSGALG00010009482	ARID4B	0.110547276	0.108170548	1.021972047	0.306794131	0.345584467	positive
PRJNA729271	stage_5W_vs_E10	ordinary_muscle_development	ENSGALG00010009484	RTKN2	-1.762232856	0.136582159	-12.90236491	4.36493E-38	3.57052E-37	negative
PRJNA729271	stage_5W_vs_E10	ordinary_muscle_development	ENSGALG00010009486	B4GALT1	0.287218587	0.128103458	2.242083011	0.024956008	0.032630104	positive
PRJNA729271	stage_5W_vs_E10	ordinary_muscle_development	ENSGALG00010009489	MROH1	-0.023667216	0.138410032	-0.170993499	0.864228878	0.880880218	negative
PRJNA729271	stage_5W_vs_E10	ordinary_muscle_development	ENSGALG00010009490	SNTB1	0.417220557	0.159823567	2.610507104	0.009040809	0.012426184	positive
PRJNA729271	stage_5W_vs_E10	ordinary_muscle_development	ENSGALG00010009491	RBM34	-0.25724891	0.103106914	-2.49497245	0.012596691	0.017048463	negative
PRJNA729271	stage_5W_vs_E10	ordinary_muscle_development	ENSGALG00010009492	CDC42SE2	-0.86170001	0.125350531	-6.874322788	6.2285E-12	1.6648E-11	negative
PRJNA729271	stage_5W_vs_E10	ordinary_muscle_development	ENSGALG00010009493	ENSGALG00010009493	1.186852041	0.385902069	3.075526503	0.002101313	0.003081911	positive
PRJNA729271	stage_5W_vs_E10	ordinary_muscle_development	ENSGALG00010009494	UBA2	-0.780475453	0.075191869	-10.37978531	3.06464E-25	1.53179E-24	negative
PRJNA729271	stage_5W_vs_E10	ordinary_muscle_development	ENSGALG00010009497	HS3ST1	3.145660971	0.186203219	16.89369811	5.00643E-64	9.01468E-63	positive
PRJNA729271	stage_5W_vs_E10	ordinary_muscle_development	ENSGALG00010009498	ORC4	-1.731944288	0.118594289	-14.60394348	2.65059E-48	3.011E-47	negative
PRJNA729271	stage_5W_vs_E10	ordinary_muscle_development	ENSGALG00010009499	MXD3	-1.403332675	0.248287752	-5.652041492	1.58553E-08	3.46119E-08	negative
PRJNA729271	stage_5W_vs_E10	ordinary_muscle_development	ENSGALG00010009500	CCDC146	1.488722934	0.562348243	2.647332775	0.008112947	0.011212545	positive
PRJNA729271	stage_5W_vs_E10	ordinary_muscle_development	ENSGALG00010009501	HGH1	-0.396202746	0.113295123	-3.497085617	0.000470371	0.000731705	negative
PRJNA729271	stage_5W_vs_E10	ordinary_muscle_development	ENSGALG00010009502	ARID5B	-1.195460136	0.09771931	-12.23361213	2.05589E-34	1.47965E-33	negative
PRJNA729271	stage_5W_vs_E10	ordinary_muscle_development	ENSGALG00010009503	IL11RA	-2.169473211	0.136824508	-15.85588166	1.28018E-56	1.86308E-55	negative
PRJNA729271	stage_5W_vs_E10	ordinary_muscle_development	ENSGALG00010009506	HAS2	-2.065572963	0.259850978	-7.949067496	1.87921E-15	6.05987E-15	negative
PRJNA729271	stage_5W_vs_E10	ordinary_muscle_development	ENSGALG00010009508	TOMM20	0.868471525	0.090074983	9.641650745	5.33235E-22	2.33724E-21	positive
PRJNA729271	stage_5W_vs_E10	ordinary_muscle_development	ENSGALG00010009511	ZFPM1	-0.95660001	0.259940486	-3.680073178	0.000233167	0.000372671	negative
PRJNA729271	stage_5W_vs_E10	ordinary_muscle_development	ENSGALG00010009514	PRELID1	-1.444975275	0.092680253	-15.59097257	8.38472E-55	1.15201E-53	negative
PRJNA729271	stage_5W_vs_E10	ordinary_muscle_development	ENSGALG00010009515	MBD5	0.820055824	0.095732273	8.566137601	1.07014E-17	3.85479E-17	positive
PRJNA729271	stage_5W_vs_E10	ordinary_muscle_development	ENSGALG00010009518	FGL2	7.418581873	0.288205913	25.74056098	4.1116E-146	3.7961E-144	positive
PRJNA729271	stage_5W_vs_E10	ordinary_muscle_development	ENSGALG00010009520	SPINK4	0.123310385	0.619856988	0.198933605	0.842314682	0.861390667	positive
PRJNA729271	stage_5W_vs_E10	ordinary_muscle_development	ENSGALG00010009522	TAF5L	0.683767972	0.080732721	8.469527145	2.46391E-17	8.70446E-17	positive
PRJNA729271	stage_5W_vs_E10	ordinary_muscle_development	ENSGALG00010009524	FAM185A	1.337740709	0.160626459	8.32827119	8.20314E-17	2.82568E-16	positive
PRJNA729271	stage_5W_vs_E10	ordinary_muscle_development	ENSGALG00010009525	ENSGALG00010009525	0.415866197	0.156953038	2.64962184	0.008058191	0.011137931	positive
PRJNA729271	stage_5W_vs_E10	ordinary_muscle_development	ENSGALG00010009526	ENSGALG00010009526	-0.662306563	0.243903522	-2.71544485	0.00661868	0.009230998	negative
PRJNA729271	stage_5W_vs_E10	ordinary_muscle_development	ENSGALG00010009527	ENSGALG00010009527	-1.846471272	0.536719338	-3.440292052	0.000581087	0.000898246	negative
PRJNA729271	stage_5W_vs_E10	ordinary_muscle_development	ENSGALG00010009530	KIF3C	-1.327944989	0.157972511	-8.406177631	4.23589E-17	1.48021E-16	negative
PRJNA729271	stage_5W_vs_E10	ordinary_muscle_development	ENSGALG00010009531	FBXL13	-0.691086354	0.447235676	-1.545239774	0.122288277	0.146735809	negative
PRJNA729271	stage_5W_vs_E10	ordinary_muscle_development	ENSGALG00010009534	XPA	0.170782659	0.131253412	1.301167386	0.193201166	0.225352128	positive
PRJNA729271	stage_5W_vs_E10	ordinary_muscle_development	ENSGALG00010009538	CRIM1	1.844772661	0.073751858	25.01323634	4.3884E-138	3.5143E-136	positive
PRJNA729271	stage_5W_vs_E10	ordinary_muscle_development	ENSGALG00010009540	RAB10	0.77295514	0.089932144	8.594870554	8.33587E-18	3.01845E-17	positive
PRJNA729271	stage_5W_vs_E10	ordinary_muscle_development	ENSGALG00010009541	PMPCB	0.008189796	0.09262121	0.088422464	0.929540908	0.938998917	positive
PRJNA729271	stage_5W_vs_E10	ordinary_muscle_development	ENSGALG00010009542	WDR1	-0.038976284	0.14315657	-0.272263329	0.78541955	0.810988487	negative
PRJNA729271	stage_5W_vs_E10	ordinary_muscle_development	ENSGALG00010009543	MAF1	2.174017434	0.129532864	16.7835201	3.2216E-63	5.67402E-62	positive
PRJNA729271	stage_5W_vs_E10	ordinary_muscle_development	ENSGALG00010009546	GAREM2	-3.536144489	0.840061461	-4.20938783	2.56064E-05	4.41888E-05	negative
PRJNA729271	stage_5W_vs_E10	ordinary_muscle_development	ENSGALG00010009548	PIK3C3	-0.208161765	0.082307035	-2.529088383	0.011435923	0.015566128	negative
PRJNA729271	stage_5W_vs_E10	ordinary_muscle_development	ENSGALG00010009551	HADHA	0.394165059	0.130226332	3.026769265	0.002471826	0.003603813	positive
PRJNA729271	stage_5W_vs_E10	ordinary_muscle_development	ENSGALG00010009554	ABCB10	0.638860067	0.101990298	6.263929778	3.75395E-10	9.0014E-10	positive
PRJNA729271	stage_5W_vs_E10	ordinary_muscle_development	ENSGALG00010009555	EPC2	-0.200736046	0.077908827	-2.576550726	0.009979156	0.013670529	negative
PRJNA729271	stage_5W_vs_E10	ordinary_muscle_development	ENSGALG00010009557	CIDEC	3.801826565	1.055069535	3.603389576	0.000314094	0.000496272	positive
PRJNA729271	stage_5W_vs_E10	ordinary_muscle_development	ENSGALG00010009558	TMEM26	-3.941649949	0.204841318	-19.24245555	1.63287E-82	4.64998E-81	negative
PRJNA729271	stage_5W_vs_E10	ordinary_muscle_development	ENSGALG00010009560	RAB24	1.085582477	0.142670027	7.609043735	2.76131E-14	8.35425E-14	positive
PRJNA729271	stage_5W_vs_E10	ordinary_muscle_development	ENSGALG00010009562	HADHB	0.992869233	0.18953606	5.23841866	1.61958E-07	3.29255E-07	positive
PRJNA729271	stage_5W_vs_E10	ordinary_muscle_development	ENSGALG00010009565	TMEM8B	0.457405014	0.227777595	2.008121186	0.044630416	0.056637881	positive
PRJNA729271	stage_5W_vs_E10	ordinary_muscle_development	ENSGALG00010009567	NSD1	-0.033583644	0.106677412	-0.314814951	0.75290216	0.780917124	negative
PRJNA729271	stage_5W_vs_E10	ordinary_muscle_development	ENSGALG00010009569	ZC3H18	-0.16774771	0.143889508	-1.165809189	0.243691601	0.279189832	negative
PRJNA729271	stage_5W_vs_E10	ordinary_muscle_development	ENSGALG00010009570	SHARPIN	2.322409907	0.134767849	17.23267031	1.51016E-66	2.91475E-65	positive
PRJNA729271	stage_5W_vs_E10	ordinary_muscle_development	ENSGALG00010009571	DNAJC2	0.40775487	0.185140101	2.202412489	0.027636185	0.035978668	positive
PRJNA729271	stage_5W_vs_E10	ordinary_muscle_development	ENSGALG00010009574	TAF1C	-0.656929752	0.152892807	-4.296668801	1.73384E-05	3.0327E-05	negative
PRJNA729271	stage_5W_vs_E10	ordinary_muscle_development	ENSGALG00010009575	RHOBTB1	-3.711122901	0.259602191	-14.29542209	2.33685E-46	2.47607E-45	negative
PRJNA729271	stage_5W_vs_E10	ordinary_muscle_development	ENSGALG00010009578	KIF5C	-1.389085265	0.157684881	-8.809248273	1.25988E-18	4.72373E-18	negative
PRJNA729271	stage_5W_vs_E10	ordinary_muscle_development	ENSGALG00010009582	TIGD5	-0.885775542	0.140648814	-6.297781809	3.01935E-10	7.27969E-10	negative
PRJNA729271	stage_5W_vs_E10	ordinary_muscle_development	ENSGALG00010009583	LYPD6B	-2.969661969	0.319376233	-9.298318616	1.42684E-20	5.8325E-20	negative
PRJNA729271	stage_5W_vs_E10	ordinary_muscle_development	ENSGALG00010009591	LYPD6	-2.412567633	0.493530402	-4.888387068	1.01665E-06	1.95107E-06	negative
PRJNA729271	stage_5W_vs_E10	ordinary_muscle_development	ENSGALG00010009593	NCBP1	-0.886441622	0.063226096	-14.0201859	1.17307E-44	1.17428E-43	negative
PRJNA729271	stage_5W_vs_E10	ordinary_muscle_development	ENSGALG00010009594	HINT2	0.785322665	0.163597209	4.800342684	1.58394E-06	2.9926E-06	positive
PRJNA729271	stage_5W_vs_E10	ordinary_muscle_development	ENSGALG00010009595	ENSGALG00010009595	1.04314322	0.186096462	5.605389852	2.07787E-08	4.49734E-08	positive
PRJNA729271	stage_5W_vs_E10	ordinary_muscle_development	ENSGALG00010009596	URB2	-0.806434762	0.098564965	-8.181758688	2.79731E-16	9.39457E-16	negative
PRJNA729271	stage_5W_vs_E10	ordinary_muscle_development	ENSGALG00010009597	CDK1	-3.35676629	0.261037784	-12.85931193	7.62524E-38	6.20594E-37	negative
PRJNA729271	stage_5W_vs_E10	ordinary_muscle_development	ENSGALG00010009600	LRRC17	-2.001629196	0.217740075	-9.192745955	3.82939E-20	1.53801E-19	negative
PRJNA729271	stage_5W_vs_E10	ordinary_muscle_development	ENSGALG00010009601	ZHX2	0.112321918	0.136656726	0.821927475	0.411118175	0.452685958	positive
PRJNA729271	stage_5W_vs_E10	ordinary_muscle_development	ENSGALG00010009602	TSTD2	0.109320061	0.081063851	1.348567335	0.177475979	0.208300754	positive
PRJNA729271	stage_5W_vs_E10	ordinary_muscle_development	ENSGALG00010009603	HINT1	1.32329526	0.17708071	7.472836874	7.84841E-14	2.31262E-13	positive
PRJNA729271	stage_5W_vs_E10	ordinary_muscle_development	ENSGALG00010009605	ENSGALG00010009605	-0.892566909	0.189056203	-4.721172308	2.34489E-06	4.37442E-06	negative
PRJNA729271	stage_5W_vs_E10	ordinary_muscle_development	ENSGALG00010009606	CHSY3	-0.273507775	0.214337364	-1.276062042	0.201933588	0.234650262	negative
PRJNA729271	stage_5W_vs_E10	ordinary_muscle_development	ENSGALG00010009608	AFAP1	-1.796995636	0.10760295	-16.70024503	1.30528E-62	2.25506E-61	negative
PRJNA729271	stage_5W_vs_E10	ordinary_muscle_development	ENSGALG00010009609	MMADHC	-0.506716506	0.072647489	-6.975003743	3.05863E-12	8.30706E-12	negative
PRJNA729271	stage_5W_vs_E10	ordinary_muscle_development	ENSGALG00010009612	DERL1	-0.319838693	0.063814762	-5.011985949	5.38711E-07	1.05465E-06	negative
PRJNA729271	stage_5W_vs_E10	ordinary_muscle_development	ENSGALG00010009615	ENSGALG00010009615	3.567927087	0.394855631	9.036029399	1.62466E-19	6.3527E-19	positive
PRJNA729271	stage_5W_vs_E10	ordinary_muscle_development	ENSGALG00010009616	ARMC10	-0.339781305	0.179603294	-1.891843396	0.058511849	0.073133505	negative
PRJNA729271	stage_5W_vs_E10	ordinary_muscle_development	ENSGALG00010009617	SLC25A46	0.991570132	0.097372731	10.18324247	2.35578E-24	1.13786E-23	positive
PRJNA729271	stage_5W_vs_E10	ordinary_muscle_development	ENSGALG00010009619	ENSGALG00010009619	-0.440067762	0.192560945	-2.285342763	0.022292737	0.02927727	negative
PRJNA729271	stage_5W_vs_E10	ordinary_muscle_development	ENSGALG00010009620	TMOD1	-0.976972521	0.232958195	-4.193767555	2.74359E-05	4.71972E-05	negative
PRJNA729271	stage_5W_vs_E10	ordinary_muscle_development	ENSGALG00010009623	XPO5	-0.950980635	0.111826869	-8.50404415	1.83098E-17	6.51769E-17	negative
PRJNA729271	stage_5W_vs_E10	ordinary_muscle_development	ENSGALG00010009629	ENSGALG00010009629	-0.486834767	0.644574341	-0.755281022	0.450080383	0.492036741	negative
PRJNA729271	stage_5W_vs_E10	ordinary_muscle_development	ENSGALG00010009634	TBC1D31	-0.940069369	0.107362854	-8.756002069	2.02298E-18	7.53033E-18	negative
PRJNA729271	stage_5W_vs_E10	ordinary_muscle_development	ENSGALG00010009635	TDRD7	0.887529072	0.139096491	6.380671917	1.76313E-10	4.31917E-10	positive
PRJNA729271	stage_5W_vs_E10	ordinary_muscle_development	ENSGALG00010009637	CYBA	0.782808513	0.135649529	5.77081629	7.88884E-09	1.75247E-08	positive
PRJNA729271	stage_5W_vs_E10	ordinary_muscle_development	ENSGALG00010009638	RND3	-1.33918434	0.150950348	-8.871687639	7.20456E-19	2.73306E-18	negative
PRJNA729271	stage_5W_vs_E10	ordinary_muscle_development	ENSGALG00010009640	NPR2	-0.322034489	0.18871401	-1.706468371	0.08792091	0.107536376	negative
PRJNA729271	stage_5W_vs_E10	ordinary_muscle_development	ENSGALG00010009643	PSMC2	0.561986494	0.108613819	5.174171201	2.28925E-07	4.60295E-07	positive
PRJNA729271	stage_5W_vs_E10	ordinary_muscle_development	ENSGALG00010009644	SLC27A6	0.717353136	0.21352595	3.359559515	0.000780668	0.001193523	positive
PRJNA729271	stage_5W_vs_E10	ordinary_muscle_development	ENSGALG00010009646	MVD	-0.027277562	0.169403889	-0.161020873	0.872076961	0.888005307	negative
PRJNA729271	stage_5W_vs_E10	ordinary_muscle_development	ENSGALG00010009647	GALNT2	-1.64456759	0.078945841	-20.83159255	2.23892E-96	8.8428E-95	negative
PRJNA729271	stage_5W_vs_E10	ordinary_muscle_development	ENSGALG00010009649	TENM2	-1.38957122	0.314187835	-4.422740368	9.74568E-06	1.73906E-05	negative
PRJNA729271	stage_5W_vs_E10	ordinary_muscle_development	ENSGALG00010009650	KLHL29	0.321497977	0.19126421	1.680910282	0.092780342	0.113145807	positive
PRJNA729271	stage_5W_vs_E10	ordinary_muscle_development	ENSGALG00010009652	CIAO2B	0.179167911	0.081668005	2.19385683	0.028245704	0.036742482	positive
PRJNA729271	stage_5W_vs_E10	ordinary_muscle_development	ENSGALG00010009653	ABLIM2	3.123936292	0.183321978	17.04070801	4.09741E-65	7.59488E-64	positive
PRJNA729271	stage_5W_vs_E10	ordinary_muscle_development	ENSGALG00010009654	NDUFA12	1.626325038	0.139600542	11.64984763	2.29869E-31	1.47171E-30	positive
PRJNA729271	stage_5W_vs_E10	ordinary_muscle_development	ENSGALG00010009657	MSMP	1.804566744	0.702638067	2.568273524	0.010220647	0.013980593	positive
PRJNA729271	stage_5W_vs_E10	ordinary_muscle_development	ENSGALG00010009658	NAPEPLD	1.84671564	0.122268109	15.10382109	1.52815E-51	1.93624E-50	positive
PRJNA729271	stage_5W_vs_E10	ordinary_muscle_development	ENSGALG00010009659	APOLD1	-0.132626734	0.256795741	-0.516467809	0.605527716	0.643012765	negative
PRJNA729271	stage_5W_vs_E10	ordinary_muscle_development	ENSGALG00010009663	FER	-0.219096029	0.122668685	-1.786079543	0.07408638	0.09153154	negative
PRJNA729271	stage_5W_vs_E10	ordinary_muscle_development	ENSGALG00010009665	DGKQ	0.373376698	0.170755312	2.186618346	0.028770397	0.037398162	positive
PRJNA729271	stage_5W_vs_E10	ordinary_muscle_development	ENSGALG00010009666	RRAD	1.4923398	0.364407776	4.095246858	4.21719E-05	7.14949E-05	positive
PRJNA729271	stage_5W_vs_E10	ordinary_muscle_development	ENSGALG00010009668	TFAP2A	-5.258246775	0.767354163	-6.852437932	7.26019E-12	1.93343E-11	negative
PRJNA729271	stage_5W_vs_E10	ordinary_muscle_development	ENSGALG00010009671	MINAR2	1.352852232	0.90435992	1.495922367	0.134673887	0.160812092	positive
PRJNA729271	stage_5W_vs_E10	ordinary_muscle_development	ENSGALG00010009672	DDX47	-0.156571551	0.11980568	-1.306879197	0.191253742	0.223350225	negative
PRJNA729271	stage_5W_vs_E10	ordinary_muscle_development	ENSGALG00010009676	ACO1	-0.413222979	0.09611463	-4.299272409	1.7136E-05	2.99983E-05	negative
PRJNA729271	stage_5W_vs_E10	ordinary_muscle_development	ENSGALG00010009677	SLC26A5	2.757271206	0.160719559	17.15579123	5.68899E-66	1.07935E-64	positive
PRJNA729271	stage_5W_vs_E10	ordinary_muscle_development	ENSGALG00010009678	JARID2	-0.636639639	0.101560606	-6.26856872	3.64382E-10	8.74456E-10	negative
PRJNA729271	stage_5W_vs_E10	ordinary_muscle_development	ENSGALG00010009679	ANK3	2.053787393	0.102836542	19.97137743	9.7721E-89	3.21194E-87	positive
PRJNA729271	stage_5W_vs_E10	ordinary_muscle_development	ENSGALG00010009683	ADAMTS19	2.214664123	0.238471438	9.286915636	1.58824E-20	6.48129E-20	positive
PRJNA729271	stage_5W_vs_E10	ordinary_muscle_development	ENSGALG00010009686	MAT2B	1.219757739	0.086773697	14.05676819	7.00111E-45	7.11649E-44	positive
PRJNA729271	stage_5W_vs_E10	ordinary_muscle_development	ENSGALG00010009689	ENSGALG00010009689	-2.115293485	0.54044614	-3.913976491	9.07885E-05	0.00015014	negative
PRJNA729271	stage_5W_vs_E10	ordinary_muscle_development	ENSGALG00010009690	RBM43	0.372641796	0.185706781	2.006613832	0.044790797	0.056821513	positive
PRJNA729271	stage_5W_vs_E10	ordinary_muscle_development	ENSGALG00010009695	ATAD2B	0.347692313	0.076423436	4.549550915	5.37605E-06	9.75781E-06	positive
PRJNA729271	stage_5W_vs_E10	ordinary_muscle_development	ENSGALG00010009697	ENSGALG00010009697	-1.403947122	0.299651205	-4.685271065	2.7959E-06	5.19304E-06	negative
PRJNA729271	stage_5W_vs_E10	ordinary_muscle_development	ENSGALG00010009699	POLR1C	-1.100443801	0.112581558	-9.774636429	1.44676E-22	6.4925E-22	negative
PRJNA729271	stage_5W_vs_E10	ordinary_muscle_development	ENSGALG00010009700	SH3TC1	0.752468855	0.207254567	3.630650291	0.000282708	0.000448392	positive
PRJNA729271	stage_5W_vs_E10	ordinary_muscle_development	ENSGALG00010009702	ENSGALG00010009702	-0.837136327	0.125164133	-6.688308427	2.25765E-11	5.85414E-11	negative
PRJNA729271	stage_5W_vs_E10	ordinary_muscle_development	ENSGALG00010009704	RGP1	0.141296145	0.130976095	1.078793388	0.280679843	0.318220909	positive
PRJNA729271	stage_5W_vs_E10	ordinary_muscle_development	ENSGALG00010009705	DTNBP1	-0.324650615	0.072497362	-4.478102475	7.53095E-06	1.35402E-05	negative
PRJNA729271	stage_5W_vs_E10	ordinary_muscle_development	ENSGALG00010009710	ENSGALG00010009710	-0.977581119	0.113560185	-8.608484728	7.40326E-18	2.68813E-17	negative
PRJNA729271	stage_5W_vs_E10	ordinary_muscle_development	ENSGALG00010009712	UBXN2A	3.661431373	0.177829179	20.5895984	3.40177E-94	1.27084E-92	positive
PRJNA729271	stage_5W_vs_E10	ordinary_muscle_development	ENSGALG00010009715	NUDCD2	-0.631408033	0.094350885	-6.692126265	2.19951E-11	5.71155E-11	negative
PRJNA729271	stage_5W_vs_E10	ordinary_muscle_development	ENSGALG00010009717	EDN1	2.738881861	0.250556744	10.93118394	8.17745E-28	4.55542E-27	positive

PRJNA729271	stage_5W_vs_E10	ordinary_muscle_development	ENSGALG00010009718	ENSGALG00010009718	-0.845744736	0.184409838	-4.586223508	4.51335E-06	8.24047E-06	negative
PRJNA729271	stage_5W_vs_E10	ordinary_muscle_development	ENSGALG00010009719	YIPF3	0.077336253	0.082384562	0.938722627	0.347873173	0.387848753	positive
PRJNA729271	stage_5W_vs_E10	ordinary_muscle_development	ENSGALG00010009720	RELN	-0.80134256	0.169025305	-4.740962079	2.12706E-06	3.98239E-06	negative
PRJNA729271	stage_5W_vs_E10	ordinary_muscle_development	ENSGALG00010009721	ZHX1	1.219104065	0.155939644	7.817794309	5.37569E-15	1.68586E-14	positive
PRJNA729271	stage_5W_vs_E10	ordinary_muscle_development	ENSGALG00010009722	GBA2	0.318319581	0.096701762	3.291766082	0.000995604	0.001509864	positive
PRJNA729271	stage_5W_vs_E10	ordinary_muscle_development	ENSGALG00010009724	ISOC1	0.598315501	0.143061895	4.182214293	2.88684E-05	4.9579E-05	positive
PRJNA729271	stage_5W_vs_E10	ordinary_muscle_development	ENSGALG00010009726	PRR16	0.091289527	0.145676156	0.626660736	0.530881647	0.571120957	positive
PRJNA729271	stage_5W_vs_E10	ordinary_muscle_development	ENSGALG00010009727	CDH20	3.470641524	0.212790853	16.31010676	8.36464E-60	1.32799E-58	positive
PRJNA729271	stage_5W_vs_E10	ordinary_muscle_development	ENSGALG00010009728	CCDC149	-1.134797128	0.104235879	-10.8868188	1.33212E-27	7.35027E-27	negative
PRJNA729271	stage_5W_vs_E10	ordinary_muscle_development	ENSGALG00010009730	C8orf76	-0.377800084	0.118325043	-3.192900451	0.001408515	0.002103052	negative
PRJNA729271	stage_5W_vs_E10	ordinary_muscle_development	ENSGALG00010009731	NMI	1.263208021	0.241683916	5.226694602	1.72567E-07	3.50135E-07	positive
PRJNA729271	stage_5W_vs_E10	ordinary_muscle_development	ENSGALG00010009732	HMMR	-2.968694708	0.24731979	-12.00346608	3.40723E-33	2.34621E-32	negative
PRJNA729271	stage_5W_vs_E10	ordinary_muscle_development	ENSGALG00010009736	WISP1	-1.792346666	0.33338416	-5.376220233	7.60657E-08	1.58143E-07	negative
PRJNA729271	stage_5W_vs_E10	ordinary_muscle_development	ENSGALG00010009738	ENSGALG00010009738	-1.135831747	0.262042632	-4.334530377	1.46072E-05	2.57361E-05	negative
PRJNA729271	stage_5W_vs_E10	ordinary_muscle_development	ENSGALG00010009740	LRRC73	-2.486715394	0.376295617	-6.608409135	3.88472E-11	9.94507E-11	negative
PRJNA729271	stage_5W_vs_E10	ordinary_muscle_development	ENSGALG00010009741	C2H6orf52	0.011331023	0.070901699	0.159813134	0.873028278	0.888785543	positive
PRJNA729271	stage_5W_vs_E10	ordinary_muscle_development	ENSGALG00010009743	CCNG1	1.87554599	0.172673869	10.86178239	1.75294E-27	9.60637E-27	positive
PRJNA729271	stage_5W_vs_E10	ordinary_muscle_development	ENSGALG00010009744	DAPK1	0.001022562	0.102905665	0.009936884	0.992071644	0.993167992	positive
PRJNA729271	stage_5W_vs_E10	ordinary_muscle_development	ENSGALG00010009749	NDRG1	1.378951161	0.167027675	8.255824458	1.50857E-16	5.12342E-16	positive
PRJNA729271	stage_5W_vs_E10	ordinary_muscle_development	ENSGALG00010009751	ENSGALG00010009751	-0.25724297	0.218095797	-1.179495314	0.238201002	0.273434697	negative
PRJNA729271	stage_5W_vs_E10	ordinary_muscle_development	ENSGALG00010009761	PDP2	0.73301575	0.116053373	6.316195133	2.68082E-10	6.48721E-10	positive
PRJNA729271	stage_5W_vs_E10	ordinary_muscle_development	ENSGALG00010009762	MAN2A1	0.310037669	0.102654775	3.020197252	0.002526101	0.003680723	positive
PRJNA729271	stage_5W_vs_E10	ordinary_muscle_development	ENSGALG00010009765	STX10	-0.703218589	0.099421316	-7.073116883	1.51492E-12	4.18421E-12	negative
PRJNA729271	stage_5W_vs_E10	ordinary_muscle_development	ENSGALG00010009769	TMCC3	2.13796105	0.224901827	9.506196909	1.97761E-21	8.431E-21	positive
PRJNA729271	stage_5W_vs_E10	ordinary_muscle_development	ENSGALG00010009772	ENSGALG00010009772	-0.765311005	0.590152654	-1.296801768	0.194699402	0.226917089	negative
PRJNA729271	stage_5W_vs_E10	ordinary_muscle_development	ENSGALG00010009774	PIAS2	-0.843285832	0.062571362	-13.47718526	2.13082E-41	1.94498E-40	negative
PRJNA729271	stage_5W_vs_E10	ordinary_muscle_development	ENSGALG00010009775	KIAA1614	-0.600654698	0.145125922	-4.138851886	3.49048E-05	5.9523E-05	negative
PRJNA729271	stage_5W_vs_E10	ordinary_muscle_development	ENSGALG00010009777	ST3GAL1	-1.927668093	0.191836094	-10.04851616	9.32611E-24	4.39045E-23	negative
PRJNA729271	stage_5W_vs_E10	ordinary_muscle_development	ENSGALG00010009778	ENSGALG00010009778	0.268896352	0.111256469	2.416905324	0.015653088	0.020958019	positive
PRJNA729271	stage_5W_vs_E10	ordinary_muscle_development	ENSGALG00010009779	PPP1R3E	2.998986474	0.343138126	8.739881256	2.33356E-18	8.65308E-18	positive
PRJNA729271	stage_5W_vs_E10	ordinary_muscle_development	ENSGALG00010009780	MFSD2B	-1.500215351	0.267975036	-5.598339959	2.16414E-08	4.67918E-08	negative
PRJNA729271	stage_5W_vs_E10	ordinary_muscle_development	ENSGALG00010009784	ENSGALG00010009784	-0.950657182	0.180470155	-5.26766979	1.38166E-07	2.82471E-07	negative
PRJNA729271	stage_5W_vs_E10	ordinary_muscle_development	ENSGALG00010009786	DIPK2B	0.635790458	0.160222994	3.968159894	7.24297E-05	0.000120939	positive
PRJNA729271	stage_5W_vs_E10	ordinary_muscle_development	ENSGALG00010009788	PAK1IP1	0.216445638	0.092255465	2.346155194	0.018968208	0.025152701	positive
PRJNA729271	stage_5W_vs_E10	ordinary_muscle_development	ENSGALG00010009791	GABRG2	-2.095537023	0.372685802	-5.622798108	1.87889E-08	4.07702E-08	negative
PRJNA729271	stage_5W_vs_E10	ordinary_muscle_development	ENSGALG00010009793	TNFAIP6	-1.507891259	0.292381296	-5.157276748	2.50567E-07	5.0207E-07	negative
PRJNA729271	stage_5W_vs_E10	ordinary_muscle_development	ENSGALG00010009795	NDUFA6	0.898083496	0.11484677	7.819841128	5.289E-15	1.65976E-14	positive
PRJNA729271	stage_5W_vs_E10	ordinary_muscle_development	ENSGALG00010009796	ENSGALG00010009796	-0.937540693	0.198183919	-4.73065977	2.23791E-06	4.18454E-06	negative
PRJNA729271	stage_5W_vs_E10	ordinary_muscle_development	ENSGALG00010009798	YIPF4	0.698773421	0.077497472	9.016725373	1.93795E-19	7.54314E-19	positive
PRJNA729271	stage_5W_vs_E10	ordinary_muscle_development	ENSGALG00010009801	ENSGALG00010009801	-0.239541728	0.602437686	-0.397620755	0.690909759	0.723922	negative
PRJNA729271	stage_5W_vs_E10	ordinary_muscle_development	ENSGALG00010009804	XPR1	0.867001757	0.117091541	7.404478128	1.31667E-13	3.83159E-13	positive
PRJNA729271	stage_5W_vs_E10	ordinary_muscle_development	ENSGALG00010009806	WDCP	-0.383691361	0.173803925	-2.207610449	0.02727144	0.03551977	negative
PRJNA729271	stage_5W_vs_E10	ordinary_muscle_development	ENSGALG00010009809	SMDT1	0.979044055	0.090132509	10.86227447	1.74352E-27	9.56197E-27	positive
PRJNA729271	stage_5W_vs_E10	ordinary_muscle_development	ENSGALG00010009811	ENSGALG00010009811	0.101712279	0.087504398	1.162367621	0.24508617	0.28065445	positive
PRJNA729271	stage_5W_vs_E10	ordinary_muscle_development	ENSGALG00010009822	ENSGALG00010009822	-0.658216322	0.204120159	-3.224651224	0.001261262	0.001891695	negative
PRJNA729271	stage_5W_vs_E10	ordinary_muscle_development	ENSGALG00010009830	RNASEL	-0.94436638	0.197994685	-4.769655207	1.84542E-06	3.46988E-06	negative
PRJNA729271	stage_5W_vs_E10	ordinary_muscle_development	ENSGALG00010009831	N6AMT1	-0.873455446	0.12296313	-7.10339307	1.21731E-12	3.37894E-12	negative
PRJNA729271	stage_5W_vs_E10	ordinary_muscle_development	ENSGALG00010009833	SOD3	3.582449825	0.311399379	11.50435763	1.2542E-30	7.80239E-30	positive
PRJNA729271	stage_5W_vs_E10	ordinary_muscle_development	ENSGALG00010009840	ENSGALG00010009840	-0.040228788	0.178608805	-0.225234068	0.821797188	0.842965837	negative
PRJNA729271	stage_5W_vs_E10	ordinary_muscle_development	ENSGALG00010009841	GABRA1	-1.305475485	0.251846474	-5.183616295	2.17624E-07	4.38486E-07	negative
PRJNA729271	stage_5W_vs_E10	ordinary_muscle_development	ENSGALG00010009842	PHETA2	-0.074745564	0.090215755	-0.828520076	0.40737603	0.448838215	negative
PRJNA729271	stage_5W_vs_E10	ordinary_muscle_development	ENSGALG00010009847	ENSGALG00010009847	-0.718987133	0.119007796	-6.041512906	1.52676E-09	3.52787E-09	negative
PRJNA729271	stage_5W_vs_E10	ordinary_muscle_development	ENSGALG00010009850	RIF1	-1.79834872	0.106164523	-16.93926254	2.31002E-64	4.18547E-63	negative
PRJNA729271	stage_5W_vs_E10	ordinary_muscle_development	ENSGALG00010009852	ENSGALG00010009852	-0.47935769	0.253144205	-1.893615104	0.058276117	0.072863997	negative
PRJNA729271	stage_5W_vs_E10	ordinary_muscle_development	ENSGALG00010009857	ORC5	-1.353299193	0.141079723	-9.592442916	8.60237E-22	3.72658E-21	negative
PRJNA729271	stage_5W_vs_E10	ordinary_muscle_development	ENSGALG00010009858	ST8SIA5	-1.447050795	0.25210557	-5.73986046	9.47546E-09	2.09498E-08	negative
PRJNA729271	stage_5W_vs_E10	ordinary_muscle_development	ENSGALG00010009866	NAGA	0.954080958	0.114320697	8.345653796	7.0821E-17	2.44852E-16	positive
PRJNA729271	stage_5W_vs_E10	ordinary_muscle_development	ENSGALG00010009868	LTN1	0.596333334	0.095072439	6.272410198	3.55502E-10	8.53427E-10	positive
PRJNA729271	stage_5W_vs_E10	ordinary_muscle_development	ENSGALG00010009876	SELENOI	-0.686893534	0.111206054	-6.176763832	6.54288E-10	1.54587E-09	negative
PRJNA729271	stage_5W_vs_E10	ordinary_muscle_development	ENSGALG00010009883	ENSGALG00010009883	-0.200377121	0.202232888	-0.990823614	0.321771717	0.361136743	negative
PRJNA729271	stage_5W_vs_E10	ordinary_muscle_development	ENSGALG00010009884	GFOD1	1.440794687	0.11215782	12.8461367	9.0414E-38	7.33791E-37	positive
PRJNA729271	stage_5W_vs_E10	ordinary_muscle_development	ENSGALG00010009910	ENSGALG00010009910	0.365411742	0.176240684	2.073367705	0.038138072	0.048860823	positive
PRJNA729271	stage_5W_vs_E10	ordinary_muscle_development	ENSGALG00010009914	RWDD2B	-0.291390976	0.124661869	-2.337450731	0.01941576	0.025703849	negative
PRJNA729271	stage_5W_vs_E10	ordinary_muscle_development	ENSGALG00010009941	CACNA1E	-2.553997337	0.44722976	-5.710705243	1.12509E-08	2.47544E-08	negative
PRJNA729271	stage_5W_vs_E10	ordinary_muscle_development	ENSGALG00010009943	DHX15	-0.66778273	0.069172527	-9.653872078	4.73339E-22	2.07911E-21	negative
PRJNA729271	stage_5W_vs_E10	ordinary_muscle_development	ENSGALG00010009948	WBP2NL	1.108340721	0.064411881	17.20708526	2.34964E-66	4.49908E-65	positive
PRJNA729271	stage_5W_vs_E10	ordinary_muscle_development	ENSGALG00010009972	PTK2B	2.70600768	0.257729572	10.49940704	8.69244E-26	4.45062E-25	positive
PRJNA729271	stage_5W_vs_E10	ordinary_muscle_development	ENSGALG00010009985	USP16	-0.718076242	0.116462534	-6.16572745	7.01598E-10	1.65226E-09	negative
PRJNA729271	stage_5W_vs_E10	ordinary_muscle_development	ENSGALG00010009989	TBC1D7	-0.726865597	0.136031176	-5.343375072	9.12317E-08	1.88672E-07	negative
PRJNA729271	stage_5W_vs_E10	ordinary_muscle_development	ENSGALG00010009991	LHFPL3	-4.399079608	0.584748066	-7.523034044	5.35195E-14	1.59721E-13	negative
PRJNA729271	stage_5W_vs_E10	ordinary_muscle_development	ENSGALG00010009993	ENSGALG00010009993	-0.707168459	0.369046205	-1.916205749	0.055338912	0.06935906	negative
PRJNA729271	stage_5W_vs_E10	ordinary_muscle_development	ENSGALG00010009995	SEPTIN3	0.42747954	0.37192468	1.149371264	0.250402924	0.286290455	positive
PRJNA729271	stage_5W_vs_E10	ordinary_muscle_development	ENSGALG00010009999	GABRB2	-3.029015453	0.248274524	-12.20026686	3.09807E-34	2.20997E-33	negative
PRJNA729271	stage_5W_vs_E10	ordinary_muscle_development	ENSGALG00010010006	PPARGC1A	1.356386232	0.267996502	5.06120872	4.16607E-07	8.21371E-07	positive
PRJNA729271	stage_5W_vs_E10	ordinary_muscle_development	ENSGALG00010010007	CTIF	0.761535212	0.094511441	8.057598185	7.78081E-16	2.55685E-15	positive
PRJNA729271	stage_5W_vs_E10	ordinary_muscle_development	ENSGALG00010010009	ELF2	-0.206242737	0.088532283	-2.329576634	0.019828538	0.026208498	negative
PRJNA729271	stage_5W_vs_E10	ordinary_muscle_development	ENSGALG00010010011	EPB41L4A	1.097519146	0.121842493	9.007687849	2.10446E-19	8.17807E-19	positive
PRJNA729271	stage_5W_vs_E10	ordinary_muscle_development	ENSGALG00010010014	GLUL	2.812519555	0.392409963	7.167299045	7.64918E-13	2.14666E-12	positive
PRJNA729271	stage_5W_vs_E10	ordinary_muscle_development	ENSGALG00010010017	CCDC59	-0.763556812	0.107555574	-7.099183996	1.25496E-12	3.48145E-12	negative
PRJNA729271	stage_5W_vs_E10	ordinary_muscle_development	ENSGALG00010010022	KMT2E	0.678204317	0.101170673	6.703566303	2.03393E-11	5.28918E-11	positive
PRJNA729271	stage_5W_vs_E10	ordinary_muscle_development	ENSGALG00010010035	ATP10B	-1.180494281	0.321921328	-3.667027249	0.000245387	0.000391425	negative
PRJNA729271	stage_5W_vs_E10	ordinary_muscle_development	ENSGALG00010010037	NAA15	0.306585848	0.138248994	2.217635285	0.02657971	0.034668667	positive
PRJNA729271	stage_5W_vs_E10	ordinary_muscle_development	ENSGALG00010010042	METTL25	-0.882600917	0.118185972	-7.467899121	8.14853E-14	2.39872E-13	negative
PRJNA729271	stage_5W_vs_E10	ordinary_muscle_development	ENSGALG00010010049	ZBTB7C	0.097742291	0.279848655	0.349268398	0.726887821	0.756750626	positive
PRJNA729271	stage_5W_vs_E10	ordinary_muscle_development	ENSGALG00010010052	ZNF346	-0.492349144	0.114944933	-4.283347939	1.84102E-05	3.2159E-05	negative
PRJNA729271	stage_5W_vs_E10	ordinary_muscle_development	ENSGALG00010010053	CHRNA2	-5.576426885	0.90175211	-6.183990948	6.2501E-10	1.4791E-09	negative
PRJNA729271	stage_5W_vs_E10	ordinary_muscle_development	ENSGALG00010010059	ENSGALG00010010059	-1.295528748	0.133938045	-9.672597122	3.94244E-22	1.73589E-21	negative
PRJNA729271	stage_5W_vs_E10	ordinary_muscle_development	ENSGALG00010010061	CYP2D6	1.023479074	0.203468231	5.030166477	4.90054E-07	9.6212E-07	positive
PRJNA729271	stage_5W_vs_E10	ordinary_muscle_development	ENSGALG00010010062	RGS7BP	-0.895428934	0.177137843	-5.054983854	4.30427E-07	8.48042E-07	negative
PRJNA729271	stage_5W_vs_E10	ordinary_muscle_development	ENSGALG00010010064	ENSGALG00010010064	-0.045205454	0.088693174	-0.509683572	0.610273164	0.647340615	negative
PRJNA729271	stage_5W_vs_E10	ordinary_muscle_development	ENSGALG00010010071	GLT8D2	-1.22964879	0.269210753	-4.567606518	4.93325E-06	8.97658E-06	negative
PRJNA729271	stage_5W_vs_E10	ordinary_muscle_development	ENSGALG00010010077	CCT8	-0.729538366	0.115414202	-6.32104501	2.598E-10	6.29628E-10	negative
PRJNA729271	stage_5W_vs_E10	ordinary_muscle_development	ENSGALG00010010080	CDC42EP3	2.681200891	0.117907931	22.73978413	1.8112E-114	9.9821E-113	positive
PRJNA729271	stage_5W_vs_E10	ordinary_muscle_development	ENSGALG00010010084	HK3	0.485068408	0.226489543	2.141681255	0.03221914	0.041650328	positive
PRJNA729271	stage_5W_vs_E10	ordinary_muscle_development	ENSGALG00010010085	TDG	-1.523208342	0.104837663	-14.52920931	7.91285E-48	8.80928E-47	negative
PRJNA729271	stage_5W_vs_E10	ordinary_muscle_development	ENSGALG00010010094	SLC2A9	2.084055657	0.242247961	8.602985336	7.76689E-18	2.81805E-17	positive
PRJNA729271	stage_5W_vs_E10	ordinary_muscle_development	ENSGALG00010010095	C12orf73	0.533499837	0.098835614	5.397850168	6.74442E-08	1.40642E-07	positive
PRJNA729271	stage_5W_vs_E10	ordinary_muscle_development	ENSGALG00010010100	IRF2BP2	0.702318343	0.162488296	4.322270336	1.54432E-05	2.71398E-05	positive
PRJNA729271	stage_5W_vs_E10	ordinary_muscle_development	ENSGALG00010010106	C7orf73	-1.08158421	0.207415424	-5.214579464	1.84235E-07	3.72972E-07	negative
PRJNA729271	stage_5W_vs_E10	ordinary_muscle_development	ENSGALG00010010107	MGST2	0.835396919	0.304336039	2.744981902	0.006051423	0.008473279	positive
PRJNA729271	stage_5W_vs_E10	ordinary_muscle_development	ENSGALG00010010111	CNOT4	1.364332123	0.132689702	10.28212517	8.4836E-25	4.18692E-24	positive
PRJNA729271	stage_5W_vs_E10	ordinary_muscle_development	ENSGALG00010010115	NEB	4.729003791	0.171691262	27.54364858	5.2727E-167	6.6459E-165	positive
PRJNA729271	stage_5W_vs_E10	ordinary_muscle_development	ENSGALG00010010116	DRD5	-4.768136917	0.767884941	-6.209441888	5.31731E-10	1.26372E-09	negative
PRJNA729271	stage_5W_vs_E10	ordinary_muscle_development	ENSGALG00010010119	SRPK2	-1.098747094	0.125887916	-8.727979025	2.59264E-18	9.59415E-18	negative
PRJNA729271	stage_5W_vs_E10	ordinary_muscle_development	ENSGALG00010010122	THBS4	-5.031031903	0.251904822	-19.97195551	9.65965E-89	3.1822E-87	negative
PRJNA729271	stage_5W_vs_E10	ordinary_muscle_development	ENSGALG00010010124	ENSGALG00010010124	0.827416415	0.146346105	5.653832864	1.56909E-08	3.42632E-08	positive
PRJNA729271	stage_5W_vs_E10	ordinary_muscle_development	ENSGALG00010010125	AHCTF1	-0.132076897	0.066875967	-1.974953083	0.048273473	0.060978125	negative
PRJNA729271	stage_5W_vs_E10	ordinary_muscle_development	ENSGALG00010010127	FAM13C	1.86345122	0.333815626	5.582276794	2.3739E-08	5.11973E-08	positive
PRJNA729271	stage_5W_vs_E10	ordinary_muscle_development	ENSGALG00010010136	EPHX2	-0.041509216	0.119135987	-0.348418784	0.727525698	0.757360312	negative
PRJNA729271	stage_5W_vs_E10	ordinary_muscle_development	ENSGALG00010010142	ENSGALG00010010142	0.939073876	0.294407779	3.189704693	0.001424182	0.002126007	positive
PRJNA729271	stage_5W_vs_E10	ordinary_muscle_development	ENSGALG00010010146	PHACTR1	-4.530374249	0.367742498	-12.31941991	7.12046E-35	5.22588E-34	negative
PRJNA729271	stage_5W_vs_E10	ordinary_muscle_development	ENSGALG00010010150	NTRK2	0.468969039	0.297693554	1.575341595	0.115177618	0.13882934	positive
PRJNA729271	stage_5W_vs_E10	ordinary_muscle_development	ENSGALG00010010153	ENSGALG00010010153	3.669648288	0.433411942	8.466883192	2.52047E-17	8.89992E-17	positive
PRJNA729271	stage_5W_vs_E10	ordinary_muscle_development	ENSGALG00010010154	IQSEC3	-7.33261172	0.864925051	-8.477742329	2.29606E-17	8.11938E-17	negative
PRJNA729271	stage_5W_vs_E10	ordinary_muscle_development	ENSGALG00010010155	BRINP3	-4.797036165	0.633128028	-7.576723752	3.5439E-14	1.06663E-13	negative
PRJNA729271	stage_5W_vs_E10	ordinary_muscle_development	ENSGALG00010010156	ENSGALG00010010156	-0.268224758	0.098107672	-2.73398351	0.006257318	0.008749742	negative
PRJNA729271	stage_5W_vs_E10	ordinary_muscle_development	ENSGALG00010010161	SEPSECS	-0.474513194	0.065722669	-7.219931891	5.20136E-13	1.47052E-12	negative
PRJNA729271	stage_5W_vs_E10	ordinary_muscle_development	ENSGALG00010010164	ENSGALG00010010164	1.062794275	0.409550021	2.59502923	0.009458287	0.012985213	positive
PRJNA729271	stage_5W_vs_E10	ordinary_muscle_development	ENSGALG00010010167	PUS7	-0.595504065	0.070403872	-8.458399411	2.71072E-17	9.56008E-17	negative
PRJNA729271	stage_5W_vs_E10	ordinary_muscle_development	ENSGALG00010010168	SNCB	-5.148678874	0.528651743	-9.739263972	2.05034E-22	9.13889E-22	negative
PRJNA729271	stage_5W_vs_E10	ordinary_muscle_development	ENSGALG00010010171	ENSGALG00010010171	2.827261412	0.379061821	7.458576037	8.74625E-14	2.57102E-13	positive
PRJNA729271	stage_5W_vs_E10	ordinary_muscle_development	ENSGALG00010010178	LGI2	-4.132161101	0.217729511	-18.97841539	2.57238E-80	6.96946E-79	negative
PRJNA729271	stage_5W_vs_E10	ordinary_muscle_development	ENSGALG00010010179	CDHR2	0.17211079	0.872956284	0.197158544	0.843703471	0.862567485	positive
PRJNA729271	stage_5W_vs_E10	ordinary_muscle_development	ENSGALG00010010183	NKX3-2	-4.249090188	0.428840757	-9.90831706	3.83044E-23	1.7587E-22	negative
PRJNA729271	stage_5W_vs_E10	ordinary_muscle_development	ENSGALG00010010185	PHYHIPL	-1.84379234	0.352854128	-5.225367068	1.7381E-07	3.52557E-07	negative
PRJNA729271	stage_5W_vs_E10	ordinary_muscle_development	ENSGALG00010010186	TNPO1	0.627453945	0.072142168	8.697464554	3.39382E-18	1.2511E-17	positive
PRJNA729271	stage_5W_vs_E10	ordinary_muscle_development	ENSGALG00010010188	DSP	-5.10106385	0.355339077	-14.35548235	9.84383E-47	1.06482E-45	negative
PRJNA729271	stage_5W_vs_E10	ordinary_muscle_development	ENSGALG00010010190	RNF44	0.333184583	0.180356601	1.84736561	0.06469417	0.080458343	positive
PRJNA729271	stage_5W_vs_E10	ordinary_muscle_development	ENSGALG00010010191	RINT1	-0.585736897	0.095273041	-6.147981522	7.84752E-10	1.8418E-09	negative
PRJNA729271	stage_5W_vs_E10	ordinary_muscle_development	ENSGALG00010010195	ENSGALG00010010195	3.243221019	0.281590633	11.51750322	1.07686E-30	6.7136E-30	positive
PRJNA729271	stage_5W_vs_E10	ordinary_muscle_development	ENSGALG00010010196	EFCAB10	-0.947591694	0.371453308	-2.551038515	0.010740246	0.014663263	negative

PRJNA729271	stage_5W_vs_E10	ordinary_muscle_development	ENSGALG00010010197	SCCPDH	-0.305358146	0.121535388	-2.512503988	0.011987775	0.016265287	negative
PRJNA729271	stage_5W_vs_E10	ordinary_muscle_development	ENSGALG00010010201	RIOK1	-0.415116058	0.099228635	-4.183430097	2.87143E-05	4.93262E-05	negative
PRJNA729271	stage_5W_vs_E10	ordinary_muscle_development	ENSGALG00010010205	ARL5A	0.79594478	0.082558366	9.64099487	5.36653E-22	2.35151E-21	positive
PRJNA729271	stage_5W_vs_E10	ordinary_muscle_development	ENSGALG00010010206	SCARA5	1.707808373	0.158165314	10.7976163	3.53255E-27	1.91992E-26	positive
PRJNA729271	stage_5W_vs_E10	ordinary_muscle_development	ENSGALG00010010214	FAF2	-1.17098131	0.080394884	-14.56537092	4.66446E-48	5.22903E-47	negative
PRJNA729271	stage_5W_vs_E10	ordinary_muscle_development	ENSGALG00010010215	ENSGALG00010010215	0.775296128	0.12369653	6.267727402	3.66355E-10	8.78756E-10	positive
PRJNA729271	stage_5W_vs_E10	ordinary_muscle_development	ENSGALG00010010216	ATXN7L1	0.602413503	0.128554158	4.686067819	2.78504E-06	5.17354E-06	positive
PRJNA729271	stage_5W_vs_E10	ordinary_muscle_development	ENSGALG00010010218	PBK	-2.965673764	0.226393459	-13.09964418	3.30805E-39	2.8041E-38	negative
PRJNA729271	stage_5W_vs_E10	ordinary_muscle_development	ENSGALG00010010220	LHX9	-8.691769051	0.902676048	-9.628890753	6.03777E-22	2.63925E-21	negative
PRJNA729271	stage_5W_vs_E10	ordinary_muscle_development	ENSGALG00010010222	PAH	0.934030808	1.06385617	0.877967186	0.379961527	0.421078476	positive
PRJNA729271	stage_5W_vs_E10	ordinary_muscle_development	ENSGALG00010010223	ENSGALG00010010223	2.563812358	0.19466023	13.17070443	1.29385E-39	1.11699E-38	positive
PRJNA729271	stage_5W_vs_E10	ordinary_muscle_development	ENSGALG00010010226	BICC1	-0.256856145	0.164180718	-1.564472049	0.117706752	0.14160174	negative
PRJNA729271	stage_5W_vs_E10	ordinary_muscle_development	ENSGALG00010010231	C1orf53	-0.75612756	0.246698008	-3.06499256	0.002176754	0.003186754	negative
PRJNA729271	stage_5W_vs_E10	ordinary_muscle_development	ENSGALG00010010234	ENSGALG00010010234	1.89168592	0.268278982	7.051189418	1.77395E-12	4.88383E-12	positive
PRJNA729271	stage_5W_vs_E10	ordinary_muscle_development	ENSGALG00010010243	ESCO2	-0.679417587	0.112091849	-6.061257738	1.35061E-09	3.13134E-09	negative
PRJNA729271	stage_5W_vs_E10	ordinary_muscle_development	ENSGALG00010010246	CLTB	0.865886261	0.066446697	13.03129128	8.12213E-39	6.7778E-38	positive
PRJNA729271	stage_5W_vs_E10	ordinary_muscle_development	ENSGALG00010010252	WDR7	1.145817487	0.09557633	11.98850689	4.08197E-33	2.79887E-32	positive
PRJNA729271	stage_5W_vs_E10	ordinary_muscle_development	ENSGALG00010010253	IL10RB	0.976931327	0.122810718	7.954772528	1.79461E-15	5.7948E-15	positive
PRJNA729271	stage_5W_vs_E10	ordinary_muscle_development	ENSGALG00010010254	WDR41	1.445212696	0.111702021	12.93810695	2.74305E-38	2.25656E-37	positive
PRJNA729271	stage_5W_vs_E10	ordinary_muscle_development	ENSGALG00010010255	CACNB4	0.618232356	0.174909691	3.53458035	0.000408423	0.000638698	positive
PRJNA729271	stage_5W_vs_E10	ordinary_muscle_development	ENSGALG00010010257	ENSGALG00010010257	0.881344871	0.136503562	6.456570511	1.07102E-10	2.66058E-10	positive
PRJNA729271	stage_5W_vs_E10	ordinary_muscle_development	ENSGALG00010010259	IGF1	0.580177839	0.396104823	1.464707838	0.143000646	0.170138255	positive
PRJNA729271	stage_5W_vs_E10	ordinary_muscle_development	ENSGALG00010010262	HSPA9	0.303498765	0.145671311	2.083449125	0.037210304	0.047756629	positive
PRJNA729271	stage_5W_vs_E10	ordinary_muscle_development	ENSGALG00010010263	RAB4A	-0.529182247	0.137517273	-3.848114739	0.00011903	0.000194602	negative
PRJNA729271	stage_5W_vs_E10	ordinary_muscle_development	ENSGALG00010010266	EFNA5	-1.848216423	0.227887153	-8.110226478	5.05255E-16	1.67398E-15	negative
PRJNA729271	stage_5W_vs_E10	ordinary_muscle_development	ENSGALG00010010267	CCSER2	1.206723483	0.106860546	11.29250723	1.42901E-29	8.51358E-29	positive
PRJNA729271	stage_5W_vs_E10	ordinary_muscle_development	ENSGALG00010010270	SSR1	-0.781293246	0.077862933	-10.03421292	1.07816E-23	5.05921E-23	negative
PRJNA729271	stage_5W_vs_E10	ordinary_muscle_development	ENSGALG00010010271	HUNK	-2.360366869	0.11666533	-20.23194781	5.12384E-91	1.79396E-89	negative
PRJNA729271	stage_5W_vs_E10	ordinary_muscle_development	ENSGALG00010010274	MIS18A	-2.020552593	0.100878683	-20.02952988	3.04519E-89	1.0194E-87	negative
PRJNA729271	stage_5W_vs_E10	ordinary_muscle_development	ENSGALG00010010275	PDE8B	-4.962009749	1.095795667	-4.528225377	5.94811E-06	1.07611E-05	negative
PRJNA729271	stage_5W_vs_E10	ordinary_muscle_development	ENSGALG00010010279	CCSAP	1.245202873	0.240097909	5.186229552	2.14594E-07	4.32501E-07	positive
PRJNA729271	stage_5W_vs_E10	ordinary_muscle_development	ENSGALG00010010280	HSP90B1	-0.869992456	0.135532348	-6.419076087	1.37104E-10	3.38152E-10	negative
PRJNA729271	stage_5W_vs_E10	ordinary_muscle_development	ENSGALG00010010284	CCDC25	0.764972074	0.094301303	8.111999027	4.97937E-16	1.65087E-15	positive
PRJNA729271	stage_5W_vs_E10	ordinary_muscle_development	ENSGALG00010010285	ENSGALG00010010285	3.802910848	0.572256198	6.645469039	3.02253E-11	7.78319E-11	positive
PRJNA729271	stage_5W_vs_E10	ordinary_muscle_development	ENSGALG00010010287	STAM2	0.137136984	0.097167405	1.411347598	0.158142146	0.187124115	positive
PRJNA729271	stage_5W_vs_E10	ordinary_muscle_development	ENSGALG00010010288	ENSGALG00010010288	0.950174399	0.163349923	5.81680348	5.99836E-09	1.34135E-08	positive
PRJNA729271	stage_5W_vs_E10	ordinary_muscle_development	ENSGALG00010010290	ENSGALG00010010290	0.278459656	0.169686932	1.641020037	0.100793256	0.122350209	positive
PRJNA729271	stage_5W_vs_E10	ordinary_muscle_development	ENSGALG00010010291	TCF20	-0.701037405	0.117796659	-5.951250313	2.66102E-09	6.06565E-09	negative
PRJNA729271	stage_5W_vs_E10	ordinary_muscle_development	ENSGALG00010010293	ENSGALG00010010293	3.215288293	0.475730899	6.758628247	1.39304E-11	3.66132E-11	positive
PRJNA729271	stage_5W_vs_E10	ordinary_muscle_development	ENSGALG00010010295	ENSGALG00010010295	8.104786493	0.181413002	44.67588534	0	0	positive
PRJNA729271	stage_5W_vs_E10	ordinary_muscle_development	ENSGALG00010010298	KCNIP4	-3.408425056	0.475709515	-7.164929329	7.78267E-13	2.18327E-12	negative
PRJNA729271	stage_5W_vs_E10	ordinary_muscle_development	ENSGALG00010010300	DENND1B	2.201031351	0.090482661	24.32544887	1.0548E-130	7.7217E-129	positive
PRJNA729271	stage_5W_vs_E10	ordinary_muscle_development	ENSGALG00010010301	ENSGALG00010010301	-0.575056104	0.530471411	-1.084047307	0.278343824	0.31569591	negative
PRJNA729271	stage_5W_vs_E10	ordinary_muscle_development	ENSGALG00010010302	WDR36	-0.731465499	0.104757296	-6.982477828	2.90019E-12	7.89006E-12	negative
PRJNA729271	stage_5W_vs_E10	ordinary_muscle_development	ENSGALG00010010303	ETF1	1.05852609	0.184505411	5.737100514	9.63111E-09	2.12745E-08	positive
PRJNA729271	stage_5W_vs_E10	ordinary_muscle_development	ENSGALG00010010305	URB1	-1.150854189	0.103452013	-11.12452195	9.53154E-29	5.4978E-28	negative
PRJNA729271	stage_5W_vs_E10	ordinary_muscle_development	ENSGALG00010010308	PACRGL	-0.979459302	0.228306502	-4.290106906	1.78587E-05	3.12145E-05	negative
PRJNA729271	stage_5W_vs_E10	ordinary_muscle_development	ENSGALG00010010310	NT5DC3	1.450413917	0.118440076	12.2459725	1.76548E-34	1.2757E-33	positive
PRJNA729271	stage_5W_vs_E10	ordinary_muscle_development	ENSGALG00010010311	CTSV	0.856101506	0.189473496	4.518317993	6.23328E-06	1.12573E-05	positive
PRJNA729271	stage_5W_vs_E10	ordinary_muscle_development	ENSGALG00010010313	GRID1	-3.247875187	0.224141286	-14.49030317	1.39526E-47	1.54232E-46	negative
PRJNA729271	stage_5W_vs_E10	ordinary_muscle_development	ENSGALG00010010314	CRB1	-1.430226732	0.29080896	-4.918097207	8.73895E-07	1.68647E-06	negative
PRJNA729271	stage_5W_vs_E10	ordinary_muscle_development	ENSGALG00010010317	ENSGALG00010010317	0.028386013	0.107652376	0.263682174	0.792024833	0.816806465	positive
PRJNA729271	stage_5W_vs_E10	ordinary_muscle_development	ENSGALG00010010320	SYPL1	2.08339605	0.571041709	3.648413096	0.000263865	0.000419608	positive
PRJNA729271	stage_5W_vs_E10	ordinary_muscle_development	ENSGALG00010010321	NUP133	-1.140554732	0.073590666	-15.49863309	3.54351E-54	4.79581E-53	negative
PRJNA729271	stage_5W_vs_E10	ordinary_muscle_development	ENSGALG00010010322	ENSGALG00010010322	0.45845548	0.066981363	6.844522982	7.67311E-12	2.04002E-11	positive
PRJNA729271	stage_5W_vs_E10	ordinary_muscle_development	ENSGALG00010010323	FBP2	10.71595039	0.241134544	44.43971484	0	0	positive
PRJNA729271	stage_5W_vs_E10	ordinary_muscle_development	ENSGALG00010010327	FBP1	0.228259249	0.577544477	0.395223672	0.692677824	0.725407489	positive
PRJNA729271	stage_5W_vs_E10	ordinary_muscle_development	ENSGALG00010010330	ALPK2	1.53057114	0.15370387	9.957921915	2.32879E-23	1.07981E-22	positive
PRJNA729271	stage_5W_vs_E10	ordinary_muscle_development	ENSGALG00010010333	WAPL	0.391156848	0.063180882	6.191063475	5.97596E-10	1.41515E-09	positive
PRJNA729271	stage_5W_vs_E10	ordinary_muscle_development	ENSGALG00010010334	ZBTB41	0.406422073	0.086826199	4.680869096	2.85661E-06	5.30174E-06	positive
PRJNA729271	stage_5W_vs_E10	ordinary_muscle_development	ENSGALG00010010336	FMNL2	-0.962756103	0.089207672	-10.79230163	3.74296E-27	2.03199E-26	negative
PRJNA729271	stage_5W_vs_E10	ordinary_muscle_development	ENSGALG00010010337	SCARA3	-0.565840681	0.155416132	-3.640810486	0.000271781	0.000431533	negative
PRJNA729271	stage_5W_vs_E10	ordinary_muscle_development	ENSGALG00010010338	CNST	1.856449007	0.155001952	11.97693953	4.69332E-33	3.20441E-32	positive
PRJNA729271	stage_5W_vs_E10	ordinary_muscle_development	ENSGALG00010010345	ENSGALG00010010345	-0.522981019	0.140953831	-3.710300144	0.000207014	0.000332189	negative
PRJNA729271	stage_5W_vs_E10	ordinary_muscle_development	ENSGALG00010010346	TFB2M	0.472378779	0.139769409	3.379700775	0.000725648	0.001112455	positive
PRJNA729271	stage_5W_vs_E10	ordinary_muscle_development	ENSGALG00010010347	FBXW11	0.221608156	0.083883153	2.641867275	0.008245036	0.011385329	positive
PRJNA729271	stage_5W_vs_E10	ordinary_muscle_development	ENSGALG00010010349	RREB1	0.771612744	0.082216139	9.38517372	6.28128E-21	2.61404E-20	positive
PRJNA729271	stage_5W_vs_E10	ordinary_muscle_development	ENSGALG00010010351	NAMPTP1	4.209302729	0.212505429	19.80797733	2.54088E-87	8.13026E-86	positive
PRJNA729271	stage_5W_vs_E10	ordinary_muscle_development	ENSGALG00010010352	MALT1	0.081905267	0.115379726	0.709875728	0.477781203	0.519381921	positive
PRJNA729271	stage_5W_vs_E10	ordinary_muscle_development	ENSGALG00010010356	STAB2	1.347944792	0.278909	4.83291967	1.34545E-06	2.55802E-06	positive
PRJNA729271	stage_5W_vs_E10	ordinary_muscle_development	ENSGALG00010010358	ASPM	-2.491194723	0.182160563	-13.67581812	1.41606E-42	1.33893E-41	negative
PRJNA729271	stage_5W_vs_E10	ordinary_muscle_development	ENSGALG00010010361	PIGM	-1.216669068	0.174247332	-6.982425786	2.90126E-12	7.8915E-12	negative
PRJNA729271	stage_5W_vs_E10	ordinary_muscle_development	ENSGALG00010010366	CLU	3.603660406	0.235081683	15.32939683	4.86483E-53	6.39308E-52	positive
PRJNA729271	stage_5W_vs_E10	ordinary_muscle_development	ENSGALG00010010368	ENSGALG00010010368	-0.235050307	0.080275425	-2.928048112	0.003410973	0.004901561	negative
PRJNA729271	stage_5W_vs_E10	ordinary_muscle_development	ENSGALG00010010369	ENSGALG00010010369	-0.079835617	0.249921459	-0.319442823	0.749390742	0.777608906	negative
PRJNA729271	stage_5W_vs_E10	ordinary_muscle_development	ENSGALG00010010371	SOCS1L	-2.150977281	0.212333738	-10.13017198	4.05935E-24	1.94256E-23	negative
PRJNA729271	stage_5W_vs_E10	ordinary_muscle_development	ENSGALG00010010374	CMTM4	-0.041455725	0.112045999	-0.369988449	0.711391097	0.74253719	negative
PRJNA729271	stage_5W_vs_E10	ordinary_muscle_development	ENSGALG00010010379	SLC35F6	0.895433998	0.187548306	4.774417957	1.80228E-06	3.39185E-06	positive
PRJNA729271	stage_5W_vs_E10	ordinary_muscle_development	ENSGALG00010010381	MAST4	1.499553449	0.131525811	11.40121045	4.12346E-30	2.50291E-29	positive
PRJNA729271	stage_5W_vs_E10	ordinary_muscle_development	ENSGALG00010010382	SEC11C	0.722850068	0.151813075	4.761448021	1.92209E-06	3.60936E-06	positive
PRJNA729271	stage_5W_vs_E10	ordinary_muscle_development	ENSGALG00010010383	SLIT2	-0.663491919	0.160261423	-4.140060088	3.47215E-05	5.92173E-05	negative
PRJNA729271	stage_5W_vs_E10	ordinary_muscle_development	ENSGALG00010010386	PRPF40A	0.54063036	0.146370491	3.693574817	0.000221123	0.000354047	positive
PRJNA729271	stage_5W_vs_E10	ordinary_muscle_development	ENSGALG00010010390	KCNK3	0.00645073	0.451690966	0.01428129	0.988605566	0.990266518	positive
PRJNA729271	stage_5W_vs_E10	ordinary_muscle_development	ENSGALG00010010393	FANCC	-2.680344135	0.15225278	-17.60456618	2.27242E-69	4.71902E-68	negative
PRJNA729271	stage_5W_vs_E10	ordinary_muscle_development	ENSGALG00010010394	MRPL42	0.299818959	0.124009542	2.417708782	0.015618571	0.020916086	positive
PRJNA729271	stage_5W_vs_E10	ordinary_muscle_development	ENSGALG00010010395	GRP	0.083667145	0.847139757	0.098764276	0.921325433	0.93204251	positive
PRJNA729271	stage_5W_vs_E10	ordinary_muscle_development	ENSGALG00010010397	PICK1	-0.538894775	0.093230593	-5.780235382	7.45962E-09	1.65916E-08	negative
PRJNA729271	stage_5W_vs_E10	ordinary_muscle_development	ENSGALG00010010399	FGF18	0.679744389	0.180484678	3.7662166	0.00016574	0.000267916	positive
PRJNA729271	stage_5W_vs_E10	ordinary_muscle_development	ENSGALG00010010403	ENSGALG00010010403	-1.3944934	0.467646026	-2.981942161	0.002864261	0.004147599	negative
PRJNA729271	stage_5W_vs_E10	ordinary_muscle_development	ENSGALG00010010404	COP1	0.049940018	0.116765861	0.427693658	0.668874168	0.702865815	positive
PRJNA729271	stage_5W_vs_E10	ordinary_muscle_development	ENSGALG00010010405	EVA1C	2.370679428	0.231617728	10.23531078	1.37752E-24	6.73202E-24	positive
PRJNA729271	stage_5W_vs_E10	ordinary_muscle_development	ENSGALG00010010406	PTCH1	-1.263574801	0.095495718	-13.23174308	5.75373E-40	5.03321E-39	negative
PRJNA729271	stage_5W_vs_E10	ordinary_muscle_development	ENSGALG00010010407	SOX10	-2.120229779	0.309648865	-6.847206682	7.53059E-12	2.00323E-11	negative
PRJNA729271	stage_5W_vs_E10	ordinary_muscle_development	ENSGALG00010010408	GAS8	-0.35863753	0.118393967	-3.029187542	0.002452124	0.003576169	negative
PRJNA729271	stage_5W_vs_E10	ordinary_muscle_development	ENSGALG00010010410	CACNA2D1	2.469051754	0.158084368	15.61856993	5.44124E-55	7.51866E-54	positive
PRJNA729271	stage_5W_vs_E10	ordinary_muscle_development	ENSGALG00010010411	CD180	2.3006254	0.421792092	5.454406196	4.91368E-08	1.03583E-07	positive
PRJNA729271	stage_5W_vs_E10	ordinary_muscle_development	ENSGALG00010010416	SLC35B3	-0.627433312	0.128373899	-4.887545822	1.02101E-06	1.95916E-06	negative
PRJNA729271	stage_5W_vs_E10	ordinary_muscle_development	ENSGALG00010010417	OTOF	0.023870729	0.675469119	0.035339483	0.97180904	0.975780829	positive
PRJNA729271	stage_5W_vs_E10	ordinary_muscle_development	ENSGALG00010010418	BRINP2	-5.543663715	0.590800747	-9.383305188	6.39364E-21	2.65917E-20	negative
PRJNA729271	stage_5W_vs_E10	ordinary_muscle_development	ENSGALG00010010420	NPM1	-1.275288624	0.112397133	-11.34627361	7.73911E-30	4.65664E-29	negative
PRJNA729271	stage_5W_vs_E10	ordinary_muscle_development	ENSGALG00010010421	POLR2F	0.576454472	0.090653505	6.358876801	2.03234E-10	4.96023E-10	positive
PRJNA729271	stage_5W_vs_E10	ordinary_muscle_development	ENSGALG00010010422	CDH1	-3.449060337	0.907169136	-3.802003619	0.000143531	0.000233211	negative
PRJNA729271	stage_5W_vs_E10	ordinary_muscle_development	ENSGALG00010010423	C21orf59	0.213448136	0.098268438	2.172092499	0.029848687	0.038737283	positive
PRJNA729271	stage_5W_vs_E10	ordinary_muscle_development	ENSGALG00010010425	BLOC1S5	0.600621338	0.078941848	7.608402259	2.77505E-14	8.39406E-14	positive
PRJNA729271	stage_5W_vs_E10	ordinary_muscle_development	ENSGALG00010010430	ASTN1	-5.603671467	0.698494332	-8.022500983	1.03614E-15	3.3849E-15	negative
PRJNA729271	stage_5W_vs_E10	ordinary_muscle_development	ENSGALG00010010431	SOCS2	-0.380795669	0.245666643	-1.550050365	0.121129428	0.145453654	negative
PRJNA729271	stage_5W_vs_E10	ordinary_muscle_development	ENSGALG00010010433	ENSGALG00010010433	-2.046478999	1.631102119	-1.254660254	0.20960212	0.242940051	negative
PRJNA729271	stage_5W_vs_E10	ordinary_muscle_development	ENSGALG00010010434	PROM1	-2.696397368	0.155505255	-17.33959005	2.36417E-67	4.6309E-66	negative
PRJNA729271	stage_5W_vs_E10	ordinary_muscle_development	ENSGALG00010010435	GAS1	-0.910374222	0.16586982	-5.488486231	4.05393E-08	8.58587E-08	negative
PRJNA729271	stage_5W_vs_E10	ordinary_muscle_development	ENSGALG00010010438	LDB3	4.394766393	0.185172542	23.73335885	1.6322E-124	1.0754E-122	positive
PRJNA729271	stage_5W_vs_E10	ordinary_muscle_development	ENSGALG00010010439	HGF	1.302481346	0.239392548	5.440776484	5.30488E-08	1.11457E-07	positive
PRJNA729271	stage_5W_vs_E10	ordinary_muscle_development	ENSGALG00010010444	TXNDC5	-1.736313372	0.14013164	-12.39058765	2.93882E-35	2.19014E-34	negative
PRJNA729271	stage_5W_vs_E10	ordinary_muscle_development	ENSGALG00010010445	LMAN1L	-0.878282217	0.083428263	-10.52739421	6.4598E-26	3.32156E-25	negative
PRJNA729271	stage_5W_vs_E10	ordinary_muscle_development	ENSGALG00010010446	C22orf23	-0.502165705	0.615191026	-0.816276056	0.414342266	0.455836789	negative
PRJNA729271	stage_5W_vs_E10	ordinary_muscle_development	ENSGALG00010010447	BTF3	-0.316573586	0.097177526	-3.257683125	0.001123258	0.001693708	negative
PRJNA729271	stage_5W_vs_E10	ordinary_muscle_development	ENSGALG00010010448	GALNT13	-1.002187041	0.37571926	-2.667382668	0.007644458	0.01059235	negative
PRJNA729271	stage_5W_vs_E10	ordinary_muscle_development	ENSGALG00010010449	PAPPA2	-2.524745211	0.752772315	-3.353929418	0.000796727	0.00121756	negative
PRJNA729271	stage_5W_vs_E10	ordinary_muscle_development	ENSGALG00010010451	RANBP17	-1.656165199	0.501347677	-3.303426497	0.00095511	0.001451187	negative
PRJNA729271	stage_5W_vs_E10	ordinary_muscle_development	ENSGALG00010010453	DRC1	-1.443976436	0.472911134	-3.053377964	0.002262807	0.003309059	negative
PRJNA729271	stage_5W_vs_E10	ordinary_muscle_development	ENSGALG00010010456	BMP6	0.870773603	0.174129941	5.000711531	5.71191E-07	1.11643E-06	positive
PRJNA729271	stage_5W_vs_E10	ordinary_muscle_development	ENSGALG00010010457	AGGF1	-0.523147185	0.091810015	-5.698149458	1.21115E-08	2.66034E-08	negative
PRJNA729271	stage_5W_vs_E10	ordinary_muscle_development	ENSGALG00010010460	SEMA3C	0.682472156	0.142105064	4.802588568	1.56628E-06	2.95999E-06	positive
PRJNA729271	stage_5W_vs_E10	ordinary_muscle_development	ENSGALG00010010461	SLC7A5	2.138695571	0.261255794	8.186212964	2.69575E-16	9.05978E-16	positive
PRJNA729271	stage_5W_vs_E10	ordinary_muscle_development	ENSGALG00010010463	ACAA2	-0.313944389	0.128969205	-2.434258546	0.014922332	0.020031413	negative
PRJNA729271	stage_5W_vs_E10	ordinary_muscle_development	ENSGALG00010010465	BMPR1A	-0.939457961	0.098219848	-9.564848463	1.12366E-21	4.83594E-21	negative
PRJNA729271	stage_5W_vs_E10	ordinary_muscle_development	ENSGALG00010010466	CRADD	0.991675228	0.124078247	7.992337497	1.32404E-15	4.30216E-15	positive
PRJNA729271	stage_5W_vs_E10	ordinary_muscle_development	ENSGALG00010010467	FGFBP2	-9.106451127	0.588878172	-15.46406636	6.06462E-54	8.11696E-53	negative
PRJNA729271	stage_5W_vs_E10	ordinary_muscle_development	ENSGALG00010010468	ENSGALG00010010468	4.70100679	1.31758743	3.567889829	0.000359868	0.000565819	positive
PRJNA729271	stage_5W_vs_E10	ordinary_muscle_development	ENSGALG00010010471	KCNJ3	-1.368697257	0.169369844	-8.081115399	6.4177E-16	2.11372E-15	negative
PRJNA729271	stage_5W_vs_E10	ordinary_muscle_development	ENSGALG00010010472	MMRN2	1.897463447	0.136306699	13.92054433	4.75271E-44	4.70242E-43	positive
PRJNA729271	stage_5W_vs_E10	ordinary_muscle_development	ENSGALG00010010473	TRIM35	0.604554194	0.179150326	3.37456375	0.000739328	0.00113223	positive

PRJNA729271	stage_5W_vs_E10	ordinary_muscle_development	ENSGALG00010010475	CCDC71L	0.866885259	0.107401716	8.071428406	6.94805E-16	2.28683E-15	positive
PRJNA729271	stage_5W_vs_E10	ordinary_muscle_development	ENSGALG00010010478	ENSGALG00010010478	0.512122189	0.218692483	2.341745731	0.019193786	0.025433214	positive
PRJNA729271	stage_5W_vs_E10	ordinary_muscle_development	ENSGALG00010010483	FGFBP1	-5.262760085	1.091126541	-4.823235333	1.41248E-06	2.68124E-06	negative
PRJNA729271	stage_5W_vs_E10	ordinary_muscle_development	ENSGALG00010010486	ENSGALG00010010486	-1.659704921	0.47299092	-3.508957258	0.000449867	0.000700712	negative
PRJNA729271	stage_5W_vs_E10	ordinary_muscle_development	ENSGALG00010010494	ENSGALG00010010494	0.727309856	0.264015639	2.754798389	0.005872831	0.008232755	positive
PRJNA729271	stage_5W_vs_E10	ordinary_muscle_development	ENSGALG00010010496	MRPL17	0.264300775	0.142740438	1.851618076	0.064080686	0.079756958	positive
PRJNA729271	stage_5W_vs_E10	ordinary_muscle_development	ENSGALG00010010499	NR4A2	2.697844094	0.458520049	5.883808352	4.00932E-09	9.04656E-09	positive
PRJNA729271	stage_5W_vs_E10	ordinary_muscle_development	ENSGALG00010010505	CD38	3.182374581	0.192858918	16.50104963	3.6057E-61	6.00743E-60	positive
PRJNA729271	stage_5W_vs_E10	ordinary_muscle_development	ENSGALG00010010511	GABRP	-2.278758274	0.465922551	-4.890852069	1.004E-06	1.92781E-06	negative
PRJNA729271	stage_5W_vs_E10	ordinary_muscle_development	ENSGALG00010010512	PLXNC1	0.374081339	0.198890004	1.880845345	0.05999296	0.074900771	positive
PRJNA729271	stage_5W_vs_E10	ordinary_muscle_development	ENSGALG00010010515	RXRG	-1.143026004	0.385711938	-2.963418785	0.003042423	0.004393298	negative
PRJNA729271	stage_5W_vs_E10	ordinary_muscle_development	ENSGALG00010010517	STMN4	-7.643524631	0.620263093	-12.32303634	6.80817E-35	5.00175E-34	negative
PRJNA729271	stage_5W_vs_E10	ordinary_muscle_development	ENSGALG00010010521	SNCG	0.965071629	0.382984204	2.519873193	0.011739712	0.015954165	positive
PRJNA729271	stage_5W_vs_E10	ordinary_muscle_development	ENSGALG00010010526	ENSGALG00010010526	0.738952746	0.250621241	2.948484111	0.003193366	0.004603007	positive
PRJNA729271	stage_5W_vs_E10	ordinary_muscle_development	ENSGALG00010010528	GPD2	2.527634346	0.124477887	20.3058905	1.14058E-91	4.06209E-90	positive
PRJNA729271	stage_5W_vs_E10	ordinary_muscle_development	ENSGALG00010010534	SYNJ1	0.965131225	0.120048864	8.039486515	9.02157E-16	2.95387E-15	positive
PRJNA729271	stage_5W_vs_E10	ordinary_muscle_development	ENSGALG00010010535	LMX1A	-2.698480892	0.461985157	-5.841055386	5.18712E-09	1.16461E-08	negative
PRJNA729271	stage_5W_vs_E10	ordinary_muscle_development	ENSGALG00010010537	ENSGALG00010010537	-0.16092101	0.227792849	-0.706435741	0.479917201	0.521508346	negative
PRJNA729271	stage_5W_vs_E10	ordinary_muscle_development	ENSGALG00010010544	ENSGALG00010010544	-0.748233292	0.169273642	-4.420258729	9.85828E-06	1.75893E-05	negative
PRJNA729271	stage_5W_vs_E10	ordinary_muscle_development	ENSGALG00010010546	MICALL1	-0.193109943	0.168362163	-1.146991344	0.251385199	0.287277551	negative
PRJNA729271	stage_5W_vs_E10	ordinary_muscle_development	ENSGALG00010010547	BST1	1.221328489	0.164585951	7.420612052	1.1658E-13	3.40555E-13	positive
PRJNA729271	stage_5W_vs_E10	ordinary_muscle_development	ENSGALG00010010567	ERMN	0.624347194	0.462047388	1.35126225	0.17661144	0.207353217	positive
PRJNA729271	stage_5W_vs_E10	ordinary_muscle_development	ENSGALG00010010572	AQP3	3.763897593	0.146093357	25.76364633	2.2669E-146	2.1064E-144	positive
PRJNA729271	stage_5W_vs_E10	ordinary_muscle_development	ENSGALG00010010573	CEP83	-0.841709428	0.158998669	-5.293814299	1.19791E-07	2.4598E-07	negative
PRJNA729271	stage_5W_vs_E10	ordinary_muscle_development	ENSGALG00010010575	PAXBP1	0.317652167	0.123011544	2.582295581	0.009814549	0.013456478	positive
PRJNA729271	stage_5W_vs_E10	ordinary_muscle_development	ENSGALG00010010577	PBX1	-0.335163417	0.145791464	-2.298923464	0.021509286	0.028307345	negative
PRJNA729271	stage_5W_vs_E10	ordinary_muscle_development	ENSGALG00010010578	GLUD2	0.794389677	0.111374513	7.132598437	9.84916E-13	2.74757E-12	positive
PRJNA729271	stage_5W_vs_E10	ordinary_muscle_development	ENSGALG00010010585	FBXL5	0.760795888	0.163486197	4.653578727	3.26223E-06	6.02984E-06	positive
PRJNA729271	stage_5W_vs_E10	ordinary_muscle_development	ENSGALG00010010586	CYTIP	1.822232259	0.241595867	7.542481087	4.61113E-14	1.37896E-13	positive
PRJNA729271	stage_5W_vs_E10	ordinary_muscle_development	ENSGALG00010010588	SLC25A3	0.424363492	0.101040736	4.199924776	2.67004E-05	4.59835E-05	positive
PRJNA729271	stage_5W_vs_E10	ordinary_muscle_development	ENSGALG00010010593	EIF3L	0.035283736	0.103905213	0.339576187	0.734175714	0.763515352	positive
PRJNA729271	stage_5W_vs_E10	ordinary_muscle_development	ENSGALG00010010596	SHLD2	0.564583169	0.133475794	4.229854369	2.33843E-05	4.04916E-05	positive
PRJNA729271	stage_5W_vs_E10	ordinary_muscle_development	ENSGALG00010010599	F2RL1	-1.346268202	0.201850638	-6.669625705	2.56457E-11	6.62864E-11	negative
PRJNA729271	stage_5W_vs_E10	ordinary_muscle_development	ENSGALG00010010604	HIVEP1	0.146773693	0.100211342	1.464641526	0.143018746	0.170145825	positive
PRJNA729271	stage_5W_vs_E10	ordinary_muscle_development	ENSGALG00010010605	NUF2	-2.857316194	0.269986222	-10.58319261	3.56596E-26	1.8573E-25	negative
PRJNA729271	stage_5W_vs_E10	ordinary_muscle_development	ENSGALG00010010614	IKBIP	-0.058678097	0.142060792	-0.413049205	0.679570564	0.712968683	negative
PRJNA729271	stage_5W_vs_E10	ordinary_muscle_development	ENSGALG00010010618	APAF1	0.003882566	0.136039422	0.028540004	0.977231462	0.980342587	positive
PRJNA729271	stage_5W_vs_E10	ordinary_muscle_development	ENSGALG00010010619	LRRC52	-0.855417731	0.275269703	-3.107562221	0.001886373	0.002778193	negative
PRJNA729271	stage_5W_vs_E10	ordinary_muscle_development	ENSGALG00010010620	ANKRD54	0.42705868	0.110804609	3.854159885	0.000116128	0.000190028	positive
PRJNA729271	stage_5W_vs_E10	ordinary_muscle_development	ENSGALG00010010623	SMAD2	-0.287770444	0.077367385	-3.719531729	0.000199592	0.000320671	negative
PRJNA729271	stage_5W_vs_E10	ordinary_muscle_development	ENSGALG00010010625	FAM169A	-7.711724686	0.539056172	-14.30597604	2.00801E-46	2.13231E-45	negative
PRJNA729271	stage_5W_vs_E10	ordinary_muscle_development	ENSGALG00010010626	ADTRP	1.152217719	0.610815339	1.886360157	0.059246438	0.074019748	positive
PRJNA729271	stage_5W_vs_E10	ordinary_muscle_development	ENSGALG00010010629	ENSGALG00010010629	-1.231271793	0.360158713	-3.418692233	0.000629229	0.000970077	negative
PRJNA729271	stage_5W_vs_E10	ordinary_muscle_development	ENSGALG00010010633	EPCIP	0.820236183	0.336896222	2.434685012	0.014904758	0.020011529	positive
PRJNA729271	stage_5W_vs_E10	ordinary_muscle_development	ENSGALG00010010635	ALDH9A1	-0.567400016	0.077165163	-7.353059261	1.93721E-13	5.59137E-13	negative
PRJNA729271	stage_5W_vs_E10	ordinary_muscle_development	ENSGALG00010010638	ACVR1C	1.550357017	0.433497801	3.576389577	0.000348372	0.000548459	positive
PRJNA729271	stage_5W_vs_E10	ordinary_muscle_development	ENSGALG00010010642	CC2D2A	-1.092148304	0.121395823	-8.996588857	2.3284E-19	9.02168E-19	negative
PRJNA729271	stage_5W_vs_E10	ordinary_muscle_development	ENSGALG00010010645	FAM219A	0.416515397	0.085376378	4.878578927	1.06853E-06	2.04737E-06	positive
PRJNA729271	stage_5W_vs_E10	ordinary_muscle_development	ENSGALG00010010646	MCM3AP	-0.541016284	0.058449128	-9.256190887	2.11855E-20	8.60421E-20	negative
PRJNA729271	stage_5W_vs_E10	ordinary_muscle_development	ENSGALG00010010648	CENPM	-2.617394449	0.272467729	-9.606254876	7.52358E-22	3.27392E-21	negative
PRJNA729271	stage_5W_vs_E10	ordinary_muscle_development	ENSGALG00010010651	NUAK1	0.803169713	0.133704245	6.00706216	1.88915E-09	4.3376E-09	positive
PRJNA729271	stage_5W_vs_E10	ordinary_muscle_development	ENSGALG00010010655	C1QTNF7	0.757845561	0.249670674	3.035380761	0.002402322	0.00350636	positive
PRJNA729271	stage_5W_vs_E10	ordinary_muscle_development	ENSGALG00010010657	TMEM170B	-1.727522117	0.182417419	-9.470159839	2.79416E-21	1.18287E-20	negative
PRJNA729271	stage_5W_vs_E10	ordinary_muscle_development	ENSGALG00010010659	ADAMTS1	0.012540319	0.152200618	0.082393351	0.934333923	0.942920714	positive
PRJNA729271	stage_5W_vs_E10	ordinary_muscle_development	ENSGALG00010010661	CPEB2	0.217675467	0.182358043	1.193670778	0.232606776	0.267399097	positive
PRJNA729271	stage_5W_vs_E10	ordinary_muscle_development	ENSGALG00010010662	MGST3	-1.061893826	0.153514743	-6.917210733	4.60623E-12	1.23872E-11	negative
PRJNA729271	stage_5W_vs_E10	ordinary_muscle_development	ENSGALG00010010663	EFR3B	-0.922336482	0.170713264	-5.40284017	6.55939E-08	1.36902E-07	negative
PRJNA729271	stage_5W_vs_E10	ordinary_muscle_development	ENSGALG00010010666	C12orf75	-0.286682553	0.380186911	-0.754056874	0.450815071	0.492769286	negative
PRJNA729271	stage_5W_vs_E10	ordinary_muscle_development	ENSGALG00010010671	SIGMAR1	-0.916317762	0.143742384	-6.374722192	1.83295E-10	4.4887E-10	negative
PRJNA729271	stage_5W_vs_E10	ordinary_muscle_development	ENSGALG00010010678	KCNIP1	-1.539989161	0.370790107	-4.153263887	3.27766E-05	5.60322E-05	negative
PRJNA729271	stage_5W_vs_E10	ordinary_muscle_development	ENSGALG00010010680	ENSGALG00010010680	-1.23968006	0.65300337	-1.898428274	0.057639688	0.072111797	negative
PRJNA729271	stage_5W_vs_E10	ordinary_muscle_development	ENSGALG00010010682	SMIM13	-0.863975063	0.106155835	-8.138743007	3.99403E-16	1.33119E-15	negative
PRJNA729271	stage_5W_vs_E10	ordinary_muscle_development	ENSGALG00010010685	STIM2	0.328120018	0.108095708	3.035458354	0.002401704	0.00350581	positive
PRJNA729271	stage_5W_vs_E10	ordinary_muscle_development	ENSGALG00010010688	IQGAP2	-0.429137018	0.169417254	-2.533018375	0.011308502	0.015401366	negative
PRJNA729271	stage_5W_vs_E10	ordinary_muscle_development	ENSGALG00010010689	POFUT2	-0.531320652	0.150118286	-3.539346636	0.000401119	0.00062775	negative
PRJNA729271	stage_5W_vs_E10	ordinary_muscle_development	ENSGALG00010010690	MTPN	-0.267375158	0.135246142	-1.976952205	0.048047033	0.060702671	negative
PRJNA729271	stage_5W_vs_E10	ordinary_muscle_development	ENSGALG00010010693	KCNMB1	1.384719123	0.329709234	4.19981906	2.67128E-05	4.5997E-05	positive
PRJNA729271	stage_5W_vs_E10	ordinary_muscle_development	ENSGALG00010010697	NEDD9	-0.13414777	0.138496426	-0.968600952	0.332744328	0.372406108	negative
PRJNA729271	stage_5W_vs_E10	ordinary_muscle_development	ENSGALG00010010698	GPRIN2	-1.905434064	0.192694884	-9.888347985	4.67682E-23	2.13703E-22	negative
PRJNA729271	stage_5W_vs_E10	ordinary_muscle_development	ENSGALG00010010699	DNAJC27	-0.713885087	0.153924925	-4.637878408	3.52004E-06	6.4824E-06	negative
PRJNA729271	stage_5W_vs_E10	ordinary_muscle_development	ENSGALG00010010706	SREBF2	1.121520951	0.13829368	8.109705002	5.07428E-16	1.68041E-15	positive
PRJNA729271	stage_5W_vs_E10	ordinary_muscle_development	ENSGALG00010010710	UCK2	-0.26266631	0.100566489	-2.611867161	0.009004924	0.012381556	negative
PRJNA729271	stage_5W_vs_E10	ordinary_muscle_development	ENSGALG00010010711	ENSGALG00010010711	-1.325237784	0.431909941	-3.068319705	0.002152662	0.003152757	negative
PRJNA729271	stage_5W_vs_E10	ordinary_muscle_development	ENSGALG00010010712	ADAMTS5	0.464753853	0.145373458	3.196964981	0.001388818	0.002075138	positive
PRJNA729271	stage_5W_vs_E10	ordinary_muscle_development	ENSGALG00010010713	NSA2	-0.043988432	0.090956393	-0.483621113	0.628654788	0.664504569	negative
PRJNA729271	stage_5W_vs_E10	ordinary_muscle_development	ENSGALG00010010719	SH3BP4	-0.342166135	0.165646343	-2.065642555	0.038862243	0.049731457	negative
PRJNA729271	stage_5W_vs_E10	ordinary_muscle_development	ENSGALG00010010722	TBC1D19	-1.326658367	0.170705596	-7.771616143	7.7491E-15	2.41399E-14	negative
PRJNA729271	stage_5W_vs_E10	ordinary_muscle_development	ENSGALG00010010724	ADCY3	0.024263273	0.148069844	0.163863703	0.869838441	0.886161668	positive
PRJNA729271	stage_5W_vs_E10	ordinary_muscle_development	ENSGALG00010010728	ELOVL2	-0.693825709	0.969244846	-0.715841526	0.474089208	0.515987616	negative
PRJNA729271	stage_5W_vs_E10	ordinary_muscle_development	ENSGALG00010010732	CCKAR	-5.706199007	1.165005816	-4.898000446	9.68168E-07	1.8627E-06	negative
PRJNA729271	stage_5W_vs_E10	ordinary_muscle_development	ENSGALG00010010733	GFM2	-0.651170365	0.128194712	-5.079541562	3.78347E-07	7.48279E-07	negative
PRJNA729271	stage_5W_vs_E10	ordinary_muscle_development	ENSGALG00010010734	AMN1	1.960036648	0.141060605	13.89499674	6.79266E-44	6.68883E-43	positive
PRJNA729271	stage_5W_vs_E10	ordinary_muscle_development	ENSGALG00010010740	CCDC134	0.547518187	0.097000454	5.644490952	1.65671E-08	3.61112E-08	positive
PRJNA729271	stage_5W_vs_E10	ordinary_muscle_development	ENSGALG00010010742	LCP2	3.180444856	0.258173035	12.31904355	7.15377E-35	5.24767E-34	positive
PRJNA729271	stage_5W_vs_E10	ordinary_muscle_development	ENSGALG00010010743	KLHL20	-0.218946001	0.084913	-2.578474459	0.009923764	0.01360236	negative
PRJNA729271	stage_5W_vs_E10	ordinary_muscle_development	ENSGALG00010010745	ATVR1	-0.105533027	0.063647112	-1.65809609	0.097298073	0.118406143	negative
PRJNA729271	stage_5W_vs_E10	ordinary_muscle_development	ENSGALG00010010748	SF3B1	-0.160802668	0.07185023	-2.238025812	0.025219372	0.032962561	negative
PRJNA729271	stage_5W_vs_E10	ordinary_muscle_development	ENSGALG00010010749	NCOA4	-0.068562679	0.098340753	-0.697194984	0.485680778	0.527148003	negative
PRJNA729271	stage_5W_vs_E10	ordinary_muscle_development	ENSGALG00010010751	SLC30A5	-0.288942917	0.079024423	-3.656374918	0.000255807	0.000407375	negative
PRJNA729271	stage_5W_vs_E10	ordinary_muscle_development	ENSGALG00010010752	ENSGALG00010010752	-1.072721041	0.263489031	-4.071217065	4.67681E-05	7.90836E-05	negative
PRJNA729271	stage_5W_vs_E10	ordinary_muscle_development	ENSGALG00010010753	ENSGALG00010010753	-1.26970224	1.162708505	-1.092021116			negative
PRJNA729271	stage_5W_vs_E10	ordinary_muscle_development	ENSGALG00010010754	DNAI1	-2.30668904	0.311070604	-7.415323108	1.21329E-13	3.54048E-13	negative
PRJNA729271	stage_5W_vs_E10	ordinary_muscle_development	ENSGALG00010010756	CENPL	-3.136653508	0.211332567	-14.84226287	7.80771E-50	9.36086E-49	negative
PRJNA729271	stage_5W_vs_E10	ordinary_muscle_development	ENSGALG00010010759	ENSGALG00010010759	-1.157660731	0.330948138	-3.498012521	0.000468739	0.000729245	negative
PRJNA729271	stage_5W_vs_E10	ordinary_muscle_development	ENSGALG00010010760	FOXI1	3.830240651	0.636761488	6.015188929	1.79677E-09	4.13334E-09	positive
PRJNA729271	stage_5W_vs_E10	ordinary_muscle_development	ENSGALG00010010761	MEI1	0.365619461	0.273534186	1.336649969	0.18133692	0.212591286	positive
PRJNA729271	stage_5W_vs_E10	ordinary_muscle_development	ENSGALG00010010762	MRPL39	0.062535873	0.111264468	0.562047114	0.574083919	0.612945375	positive
PRJNA729271	stage_5W_vs_E10	ordinary_muscle_development	ENSGALG00010010765	ENSGALG00010010765	3.196681846	0.800757455	3.99207254	6.54983E-05	0.000109757	positive
PRJNA729271	stage_5W_vs_E10	ordinary_muscle_development	ENSGALG00010010766	CENPO	-2.857302361	0.254614609	-11.22206763	3.17753E-29	1.87153E-28	negative
PRJNA729271	stage_5W_vs_E10	ordinary_muscle_development	ENSGALG00010010768	ENSGALG00010010768	0.644233513	0.091668138	7.027889157	2.09681E-12	5.75086E-12	positive
PRJNA729271	stage_5W_vs_E10	ordinary_muscle_development	ENSGALG00010010769	HEXB	0.210543307	0.12025459	1.750813063	0.079978116	0.098352659	positive
PRJNA729271	stage_5W_vs_E10	ordinary_muscle_development	ENSGALG00010010771	ENSGALG00010010771	-0.633218574	0.072398599	-8.746282129	2.20497E-18	8.19095E-18	negative
PRJNA729271	stage_5W_vs_E10	ordinary_muscle_development	ENSGALG00010010774	KPTN	-0.325495794	0.137159572	-2.373117594	0.017638647	0.02346638	negative
PRJNA729271	stage_5W_vs_E10	ordinary_muscle_development	ENSGALG00010010776	GCAT	0.127802396	0.194699928	0.656406999	0.511562288	0.552274502	positive
PRJNA729271	stage_5W_vs_E10	ordinary_muscle_development	ENSGALG00010010778	UPP2	2.954181136	0.367097637	8.047398948	8.45721E-16	2.77284E-15	positive
PRJNA729271	stage_5W_vs_E10	ordinary_muscle_development	ENSGALG00010010779	DOCK2	2.122056878	0.185289692	11.45264401	2.28081E-30	1.3979E-29	positive
PRJNA729271	stage_5W_vs_E10	ordinary_muscle_development	ENSGALG00010010780	PARG	-0.899418724	0.050094106	-17.95458187	4.41897E-72	9.93069E-71	negative
PRJNA729271	stage_5W_vs_E10	ordinary_muscle_development	ENSGALG00010010781	NAPA	0.44306098	0.097680408	4.535822381	5.73795E-06	1.03899E-05	positive
PRJNA729271	stage_5W_vs_E10	ordinary_muscle_development	ENSGALG00010010783	F2RL2	-0.53237514	0.148132716	-3.593906566	0.000325757	0.000513858	negative
PRJNA729271	stage_5W_vs_E10	ordinary_muscle_development	ENSGALG00010010784	CCDC148	0.937989503	0.2997042	3.129717579	0.001749744	0.002586168	positive
PRJNA729271	stage_5W_vs_E10	ordinary_muscle_development	ENSGALG00010010786	NABP1	0.252876213	0.154487476	1.636871923	0.101657234	0.123327888	positive
PRJNA729271	stage_5W_vs_E10	ordinary_muscle_development	ENSGALG00010010789	ENSGALG00010010789	-1.095286146	0.297892233	-3.676786521	0.000236191	0.000377296	negative
PRJNA729271	stage_5W_vs_E10	ordinary_muscle_development	ENSGALG00010010791	H-10	-0.720711338	0.296531658	-2.43047013	0.015079249	0.020228942	negative
PRJNA729271	stage_5W_vs_E10	ordinary_muscle_development	ENSGALG00010010792	XRCC6	-1.073887742	0.149503245	-7.183039671	6.81783E-13	1.91818E-12	negative
PRJNA729271	stage_5W_vs_E10	ordinary_muscle_development	ENSGALG00010010796	CAVIN2	2.567682918	0.145964955	17.59109177	2.8827E-69	5.94377E-68	positive
PRJNA729271	stage_5W_vs_E10	ordinary_muscle_development	ENSGALG00010010799	DARS2	-0.460986684	0.108203328	-4.260374355	2.04085E-05	3.54903E-05	negative
PRJNA729271	stage_5W_vs_E10	ordinary_muscle_development	ENSGALG00010010800	BICRA	0.756793964	0.189914147	3.984926741	6.75009E-05	0.000112969	positive
PRJNA729271	stage_5W_vs_E10	ordinary_muscle_development	ENSGALG00010010805	PTRHD1	-0.59320804	0.167111865	-3.549766153	0.000385573	0.000604204	negative
PRJNA729271	stage_5W_vs_E10	ordinary_muscle_development	ENSGALG00010010806	INSYN2B	1.943975094	0.754258907	2.577331308	0.009956647	0.013643562	positive
PRJNA729271	stage_5W_vs_E10	ordinary_muscle_development	ENSGALG00010010809	CENPH	-2.493101915	0.161087302	-15.47671282	4.98292E-54	6.68772E-53	negative
PRJNA729271	stage_5W_vs_E10	ordinary_muscle_development	ENSGALG00010010810	GALT	0.44492321	0.113187079	3.930865713	8.46405E-05	0.000140502	positive
PRJNA729271	stage_5W_vs_E10	ordinary_muscle_development	ENSGALG00010010813	EHD2	2.51472218	0.148081951	16.98196274	1.11688E-64	2.03637E-63	positive
PRJNA729271	stage_5W_vs_E10	ordinary_muscle_development	ENSGALG00010010816	AP2S1	-1.056117781	0.113517419	-9.303574664	1.35801E-20	5.56529E-20	negative
PRJNA729271	stage_5W_vs_E10	ordinary_muscle_development	ENSGALG00010010821	RGS8	-0.987770805	0.408263943	-2.41944169	0.015544353	0.020824162	negative
PRJNA729271	stage_5W_vs_E10	ordinary_muscle_development	ENSGALG00010010822	OGDHL	-0.984784334	0.364491127	-2.701806053	0.006896398	0.009596168	negative
PRJNA729271	stage_5W_vs_E10	ordinary_muscle_development	ENSGALG00010010823	TMEFF2	-2.068805845	0.179303733	-11.53799648	8.48792E-31	5.30541E-30	negative
PRJNA729271	stage_5W_vs_E10	ordinary_muscle_development	ENSGALG00010010824	PKP4	0.094825782	0.109593422	0.865250679	0.386901215	0.428199825	positive
PRJNA729271	stage_5W_vs_E10	ordinary_muscle_development	ENSGALG00010010825	MAK	4.187106763	0.365056576	11.46974754	1.87205E-30	1.15175E-29	positive
PRJNA729271	stage_5W_vs_E10	ordinary_muscle_development	ENSGALG00010010826	NCOA1	0.414820227	0.162082812	2.559310414	0.010488004	0.01433509	positive
PRJNA729271	stage_5W_vs_E10	ordinary_muscle_development	ENSGALG00010010828	SPDL1	-1.045363349	0.125846869	-8.306629774	9.84585E-17	3.37789E-16	negative
PRJNA729271	stage_5W_vs_E10	ordinary_muscle_development	ENSGALG00010010829	LAMB4	-3.022301731	0.261341831	-11.56455404	6.23142E-31	3.92544E-30	negative

PRJNA729271	stage_5W_vs_E10	ordinary_muscle_development	ENSGALG00010010831	DESI1	-0.08970096	0.092949139	-0.96505424	0.334517645	0.374224996	negative
PRJNA729271	stage_5W_vs_E10	ordinary_muscle_development	ENSGALG00010010834	KGD4	1.122087521	0.125080177	8.970946031	2.93978E-19	1.13542E-18	positive
PRJNA729271	stage_5W_vs_E10	ordinary_muscle_development	ENSGALG00010010836	DIP2A	-0.277743384	0.106104207	-2.617647236	0.008853828	0.012181893	negative
PRJNA729271	stage_5W_vs_E10	ordinary_muscle_development	ENSGALG00010010837	RBPJ	-0.339011099	0.12317416	-2.752290727	0.005917996	0.008294464	negative
PRJNA729271	stage_5W_vs_E10	ordinary_muscle_development	ENSGALG00010010838	JAM2	1.63870427	0.173598859	9.439602741	3.74195E-21	1.57536E-20	positive
PRJNA729271	stage_5W_vs_E10	ordinary_muscle_development	ENSGALG00010010840	RGS16	3.666235052	0.537332179	6.823032742	8.91383E-12	2.36338E-11	positive
PRJNA729271	stage_5W_vs_E10	ordinary_muscle_development	ENSGALG00010010841	PMM1	1.132094883	0.130227127	8.69323395	3.52266E-18	1.29728E-17	positive
PRJNA729271	stage_5W_vs_E10	ordinary_muscle_development	ENSGALG00010010843	ZNF395	0.557219899	0.179695769	3.100907171	0.001929288	0.002839377	positive
PRJNA729271	stage_5W_vs_E10	ordinary_muscle_development	ENSGALG00010010844	CDC14B	0.429359261	0.174795139	2.456356989	0.014035365	0.01889677	positive
PRJNA729271	stage_5W_vs_E10	ordinary_muscle_development	ENSGALG00010010845	FKRP	0.925030197	0.253412978	3.650287383	0.000261947	0.000416741	positive
PRJNA729271	stage_5W_vs_E10	ordinary_muscle_development	ENSGALG00010010848	RGR	1.286253564	0.568944802	2.260770394	0.023773479	0.031154197	positive
PRJNA729271	stage_5W_vs_E10	ordinary_muscle_development	ENSGALG00010010849	ACBD6	-0.596097611	0.110834085	-5.378287824	7.51975E-08	1.5636E-07	negative
PRJNA729271	stage_5W_vs_E10	ordinary_muscle_development	ENSGALG00010010850	ENSGALG00010010850	3.71688389	0.105714731	35.15956454	8.3027E-271	2.6744E-268	positive
PRJNA729271	stage_5W_vs_E10	ordinary_muscle_development	ENSGALG00010010851	PNOC	5.234102597	0.752961082	6.951358741	3.61785E-12	9.79284E-12	positive
PRJNA729271	stage_5W_vs_E10	ordinary_muscle_development	ENSGALG00010010853	SMIM20	0.499692709	0.146080771	3.420660407	0.000624693	0.000963495	positive
PRJNA729271	stage_5W_vs_E10	ordinary_muscle_development	ENSGALG00010010854	LRIT1	2.214295011	0.551723056	4.013417577	5.98459E-05	0.000100576	positive
PRJNA729271	stage_5W_vs_E10	ordinary_muscle_development	ENSGALG00010010855	ATP5PF	0.85642202	0.148557314	5.764926667	8.16934E-09	1.81284E-08	positive
PRJNA729271	stage_5W_vs_E10	ordinary_muscle_development	ENSGALG00010010856	CSDC2	-6.644028243	0.912451064	-7.281517335	3.30086E-13	9.41109E-13	negative
PRJNA729271	stage_5W_vs_E10	ordinary_muscle_development	ENSGALG00010010857	SEL1L3	0.561412446	0.06054031	9.273365955	1.80362E-20	7.33956E-20	positive
PRJNA729271	stage_5W_vs_E10	ordinary_muscle_development	ENSGALG00010010859	ENSGALG00010010859	-1.516543093	0.274690289	-5.520919934	3.37229E-08	7.17789E-08	negative
PRJNA729271	stage_5W_vs_E10	ordinary_muscle_development	ENSGALG00010010861	ENSGALG00010010861	0.258832585	0.240965483	1.074147974	0.282756366	0.320376533	positive
PRJNA729271	stage_5W_vs_E10	ordinary_muscle_development	ENSGALG00010010862	STRN4	0.329532997	0.152898494	2.155240306	0.031143034	0.040348434	positive
PRJNA729271	stage_5W_vs_E10	ordinary_muscle_development	ENSGALG00010010865	ENSGALG00010010865	1.372522054	0.156573799	8.765975316	1.85167E-18	6.90504E-18	positive
PRJNA729271	stage_5W_vs_E10	ordinary_muscle_development	ENSGALG00010010867	QSOX1	-0.139555117	0.162523199	-0.858678135	0.390518117	0.431807163	negative
PRJNA729271	stage_5W_vs_E10	ordinary_muscle_development	ENSGALG00010010868	SLC39A10	-2.198183828	0.111158392	-19.7752396	4.86511E-87	1.53304E-85	negative
PRJNA729271	stage_5W_vs_E10	ordinary_muscle_development	ENSGALG00010010869	ENSGALG00010010869	5.218437192	0.17349809	30.07777887	9.4627E-199	1.5766E-196	positive
PRJNA729271	stage_5W_vs_E10	ordinary_muscle_development	ENSGALG00010010870	NOL6	-1.329396432	0.12487947	-10.64543619	1.83125E-26	9.68402E-26	negative
PRJNA729271	stage_5W_vs_E10	ordinary_muscle_development	ENSGALG00010010871	GCNT2	-3.847549832	1.181065885	-3.257692803	0.001123219	0.001693708	negative
PRJNA729271	stage_5W_vs_E10	ordinary_muscle_development	ENSGALG00010010874	ENSGALG00010010874	3.475383722	0.259302672	13.4028072	5.82187E-41	5.23824E-40	positive
PRJNA729271	stage_5W_vs_E10	ordinary_muscle_development	ENSGALG00010010876	ENSGALG00010010876	0.374218363	0.159860342	2.340908069	0.019236903	0.025485688	positive
PRJNA729271	stage_5W_vs_E10	ordinary_muscle_development	ENSGALG00010010880	ELP3	0.244413793	0.106064212	2.304394549	0.021200506	0.027918718	positive
PRJNA729271	stage_5W_vs_E10	ordinary_muscle_development	ENSGALG00010010882	CEP350	0.46233335	0.09839732	4.69863763	2.61903E-06	4.8714E-06	positive
PRJNA729271	stage_5W_vs_E10	ordinary_muscle_development	ENSGALG00010010884	DNAH7	-2.52142419	0.311244068	-8.101115658	5.44574E-16	1.80096E-15	negative
PRJNA729271	stage_5W_vs_E10	ordinary_muscle_development	ENSGALG00010010885	SNRPD2	-0.987696677	0.275900803	-3.579897799	0.000343729	0.000541265	negative
PRJNA729271	stage_5W_vs_E10	ordinary_muscle_development	ENSGALG00010010887	GABPA	-0.03484003	0.066335738	-0.525207549	0.599438918	0.637340799	negative
PRJNA729271	stage_5W_vs_E10	ordinary_muscle_development	ENSGALG00010010888	GNPAT	1.105510316	0.118614192	9.320219587	1.161E-20	4.77003E-20	positive
PRJNA729271	stage_5W_vs_E10	ordinary_muscle_development	ENSGALG00010010889	CCL19	3.229581171	0.638903366	5.054882074	4.30657E-07	8.48379E-07	positive
PRJNA729271	stage_5W_vs_E10	ordinary_muscle_development	ENSGALG00010010890	NOL12	-0.473235677	0.13574203	-3.486287017	0.000489775	0.000760747	negative
PRJNA729271	stage_5W_vs_E10	ordinary_muscle_development	ENSGALG00010010891	POLR3H	-0.684666228	0.109059794	-6.277897662	3.43182E-10	8.24808E-10	negative
PRJNA729271	stage_5W_vs_E10	ordinary_muscle_development	ENSGALG00010010893	CCL21	1.456699172	0.484480412	3.006724598	0.002640789	0.003838956	positive
PRJNA729271	stage_5W_vs_E10	ordinary_muscle_development	ENSGALG00010010894	SPAG1	0.237309322	0.142383472	1.666691496	0.095575765	0.116447063	positive
PRJNA729271	stage_5W_vs_E10	ordinary_muscle_development	ENSGALG00010010896	LY86	3.757963319	0.292414792	12.8514816	8.43785E-38	6.85191E-37	positive
PRJNA729271	stage_5W_vs_E10	ordinary_muscle_development	ENSGALG00010010897	CEP170	-0.13475	0.090738251	-1.485040746	0.137533031	0.164072193	negative
PRJNA729271	stage_5W_vs_E10	ordinary_muscle_development	ENSGALG00010010898	PHF24	-4.617979212	3.569493153	-1.293735277	0.195756876	0.228002886	negative
PRJNA729271	stage_5W_vs_E10	ordinary_muscle_development	ENSGALG00010010900	SLC34A2	-1.532617369	1.074017926	-1.426994217	0.153581561	0.181995155	negative
PRJNA729271	stage_5W_vs_E10	ordinary_muscle_development	ENSGALG00010010904	TOR1AIP1	-0.793848667	0.082921651	-9.573478772	1.03368E-21	4.45927E-21	negative
PRJNA729271	stage_5W_vs_E10	ordinary_muscle_development	ENSGALG00010010905	ENC1	0.069666166	0.148073769	0.470482834	0.638010096	0.673454789	positive
PRJNA729271	stage_5W_vs_E10	ordinary_muscle_development	ENSGALG00010010906	ATP6V1C1	0.882744389	0.138959071	6.352549606	2.11775E-10	5.16086E-10	positive
PRJNA729271	stage_5W_vs_E10	ordinary_muscle_development	ENSGALG00010010908	STK17B	1.000971125	0.122746797	8.154763728	3.49863E-16	1.16823E-15	positive
PRJNA729271	stage_5W_vs_E10	ordinary_muscle_development	ENSGALG00010010909	SLIT3	-2.438117545	0.329529488	-7.398784135	1.37437E-13	3.99309E-13	negative
PRJNA729271	stage_5W_vs_E10	ordinary_muscle_development	ENSGALG00010010911	ENSGALG00010010911	-1.330835646	0.358427058	-3.712988777	0.000204826	0.000328897	negative
PRJNA729271	stage_5W_vs_E10	ordinary_muscle_development	ENSGALG00010010912	BAALC	-0.324106201	0.340939525	-0.950626657	0.341793931	0.381599248	negative
PRJNA729271	stage_5W_vs_E10	ordinary_muscle_development	ENSGALG00010010913	IFT56	-1.932265652	0.14979587	-12.89932526	4.54055E-38	3.71E-37	negative
PRJNA729271	stage_5W_vs_E10	ordinary_muscle_development	ENSGALG00010010915	APP	-0.892449611	0.110229375	-8.09629568	5.6658E-16	1.87203E-15	negative
PRJNA729271	stage_5W_vs_E10	ordinary_muscle_development	ENSGALG00010010916	ENSGALG00010010916	-2.092364823	0.625539709	-3.344895283	0.000823136	0.001256462	negative
PRJNA729271	stage_5W_vs_E10	ordinary_muscle_development	ENSGALG00010010917	TANC1	0.237099542	0.068757188	3.44836006	0.000564002	0.00087258	positive
PRJNA729271	stage_5W_vs_E10	ordinary_muscle_development	ENSGALG00010010918	TOR1AIP2	1.144537406	0.118828467	9.631845262	5.86664E-22	2.56599E-21	positive
PRJNA729271	stage_5W_vs_E10	ordinary_muscle_development	ENSGALG00010010919	CDHR1	-2.829496186	0.636744098	-4.443694407	8.84272E-06	1.58163E-05	negative
PRJNA729271	stage_5W_vs_E10	ordinary_muscle_development	ENSGALG00010010920	UBN2	0.918401302	0.113434337	8.096325388	5.66442E-16	1.872E-15	positive
PRJNA729271	stage_5W_vs_E10	ordinary_muscle_development	ENSGALG00010010921	ENSGALG00010010921	-0.431904798	0.131989956	-3.272255039	0.001066933	0.001613479	negative
PRJNA729271	stage_5W_vs_E10	ordinary_muscle_development	ENSGALG00010010922	DYM	1.176086143	0.101372709	11.60160518	4.04421E-31	2.56322E-30	positive
PRJNA729271	stage_5W_vs_E10	ordinary_muscle_development	ENSGALG00010010923	F13A1	2.80865309	0.23083933	12.16713412	4.65128E-34	3.30978E-33	positive
PRJNA729271	stage_5W_vs_E10	ordinary_muscle_development	ENSGALG00010010924	ENSGALG00010010924	-1.33766005	0.211747209	-6.317249963	2.66259E-10	6.44526E-10	negative
PRJNA729271	stage_5W_vs_E10	ordinary_muscle_development	ENSGALG00010010928	HECW2	-0.222351816	0.116650693	-1.90613369	0.056632863	0.070913385	negative
PRJNA729271	stage_5W_vs_E10	ordinary_muscle_development	ENSGALG00010010929	FMC1	0.068025473	0.14959203	0.454739959	0.64929633	0.684174226	positive
PRJNA729271	stage_5W_vs_E10	ordinary_muscle_development	ENSGALG00010010930	FAM163A	-5.464003088	0.817061905	-6.687379568	2.27202E-11	5.88929E-11	negative
PRJNA729271	stage_5W_vs_E10	ordinary_muscle_development	ENSGALG00010010931	CYYR1	-2.376383273	0.196565975	-12.08949451	1.20023E-33	8.39234E-33	negative
PRJNA729271	stage_5W_vs_E10	ordinary_muscle_development	ENSGALG00010010932	GHITM	0.896236285	0.088206118	10.16070429	2.96924E-24	1.42892E-23	positive
PRJNA729271	stage_5W_vs_E10	ordinary_muscle_development	ENSGALG00010010933	ANAPC4	-0.69528449	0.11150828	-6.235272282	4.50993E-10	1.07607E-09	negative
PRJNA729271	stage_5W_vs_E10	ordinary_muscle_development	ENSGALG00010010934	ENSGALG00010010934	-0.054185223	0.120050878	-0.451352159	0.651735758	0.686399027	negative
PRJNA729271	stage_5W_vs_E10	ordinary_muscle_development	ENSGALG00010010935	LGALS1A	0.718482732	0.227828134	3.153617241	0.001612604	0.002393232	positive
PRJNA729271	stage_5W_vs_E10	ordinary_muscle_development	ENSGALG00010010937	CDK7	0.028072371	0.116263472	0.241454776	0.809202663	0.831753836	positive
PRJNA729271	stage_5W_vs_E10	ordinary_muscle_development	ENSGALG00010010938	FZD6	0.173275568	0.12074676	1.435032859	0.151277734	0.179470516	positive
PRJNA729271	stage_5W_vs_E10	ordinary_muscle_development	ENSGALG00010010939	ZCCHC4	-0.403120788	0.142089237	-2.837095872	0.004552594	0.006453623	negative
PRJNA729271	stage_5W_vs_E10	ordinary_muscle_development	ENSGALG00010010940	NRG3	-3.08140802	0.382955189	-8.046393179	8.52697E-16	2.79445E-15	negative
PRJNA729271	stage_5W_vs_E10	ordinary_muscle_development	ENSGALG00010010941	RIPK4	-5.890899894	0.821861514	-7.167752466	7.6239E-13	2.13998E-12	negative
PRJNA729271	stage_5W_vs_E10	ordinary_muscle_development	ENSGALG00010010942	NRN1	2.533310642	0.337973997	7.495578541	6.60066E-14	1.95418E-13	positive
PRJNA729271	stage_5W_vs_E10	ordinary_muscle_development	ENSGALG00010010943	WDSUB1	0.015391595	0.104083079	0.147877975	0.882439071	0.89676182	positive
PRJNA729271	stage_5W_vs_E10	ordinary_muscle_development	ENSGALG00010010945	PI4K2B	-0.860221214	0.1011486	-8.504529115	1.82334E-17	6.4921E-17	negative
PRJNA729271	stage_5W_vs_E10	ordinary_muscle_development	ENSGALG00010010946	SH2D4B	0.403251244	0.325499162	1.238870298	0.215393535	0.249092811	positive
PRJNA729271	stage_5W_vs_E10	ordinary_muscle_development	ENSGALG00010010947	LUC7L2	-0.528249648	0.070763959	-7.464953297	8.33292E-14	2.4515E-13	negative
PRJNA729271	stage_5W_vs_E10	ordinary_muscle_development	ENSGALG00010010948	PRDM15	-0.243960574	0.084856022	-2.874994218	0.004040349	0.005762556	negative
PRJNA729271	stage_5W_vs_E10	ordinary_muscle_development	ENSGALG00010010949	FZD3	-2.851466005	0.236602541	-12.05171334	1.8996E-33	1.31682E-32	negative
PRJNA729271	stage_5W_vs_E10	ordinary_muscle_development	ENSGALG00010010951	ENSGALG00010010951	-1.132839106	0.136917417	-8.273886044	1.29662E-16	4.425E-16	negative
PRJNA729271	stage_5W_vs_E10	ordinary_muscle_development	ENSGALG00010010952	ENSGALG00010010952	3.276030066	0.467074985	7.013927473	2.3172E-12	6.33373E-12	positive
PRJNA729271	stage_5W_vs_E10	ordinary_muscle_development	ENSGALG00010010953	EXTL3	0.140489235	0.110338583	1.273255756	0.202927301	0.235773307	positive
PRJNA729271	stage_5W_vs_E10	ordinary_muscle_development	ENSGALG00010010954	MTERF2	0.957656503	0.226068351	4.236136989	2.27398E-05	3.94275E-05	positive
PRJNA729271	stage_5W_vs_E10	ordinary_muscle_development	ENSGALG00010010955	GTF3C3	0.031321316	0.083171916	0.376585238	0.70648186	0.738370074	positive
PRJNA729271	stage_5W_vs_E10	ordinary_muscle_development	ENSGALG00010010956	AXDND1	3.106361068	0.570358607	5.446329785	5.14198E-08	1.08144E-07	positive
PRJNA729271	stage_5W_vs_E10	ordinary_muscle_development	ENSGALG00010010957	ZMAT2	0.046748857	0.075483277	0.619327341	0.535700736	0.575780971	positive
PRJNA729271	stage_5W_vs_E10	ordinary_muscle_development	ENSGALG00010010958	LCORL	0.242606158	0.160437873	1.512150174	0.130495663	0.156138594	positive
PRJNA729271	stage_5W_vs_E10	ordinary_muscle_development	ENSGALG00010010959	TMEM263	-1.471739903	0.09066943	-16.23193076	2.99877E-59	4.70933E-58	negative
PRJNA729271	stage_5W_vs_E10	ordinary_muscle_development	ENSGALG00010010960	POLDIP3	-0.322579705	0.070414217	-4.581172911	4.62375E-06	8.43566E-06	negative
PRJNA729271	stage_5W_vs_E10	ordinary_muscle_development	ENSGALG00010010961	C2orf66	0.916178722	0.367412358	2.493598004	0.012645567	0.017109819	positive
PRJNA729271	stage_5W_vs_E10	ordinary_muscle_development	ENSGALG00010010962	ANKDD1B	0.592263959	0.272995437	2.169501315	0.030044645	0.038974147	positive
PRJNA729271	stage_5W_vs_E10	ordinary_muscle_development	ENSGALG00010010963	TSPAN14	-0.32750381	0.074319197	-4.406718925	1.04948E-05	1.86745E-05	negative
PRJNA729271	stage_5W_vs_E10	ordinary_muscle_development	ENSGALG00010010964	INTS9	-0.761692868	0.093652148	-8.133213031	4.18059E-16	1.39209E-15	negative
PRJNA729271	stage_5W_vs_E10	ordinary_muscle_development	ENSGALG00010010965	BAZ2B	0.032491476	0.132315125	0.245561312	0.806021841	0.829013453	positive
PRJNA729271	stage_5W_vs_E10	ordinary_muscle_development	ENSGALG00010010966	ENSGALG00010010966	3.270944087	0.509636252	6.418193508	1.37901E-10	3.4006E-10	positive
PRJNA729271	stage_5W_vs_E10	ordinary_muscle_development	ENSGALG00010010967	PRXL2A	-0.008667008	0.390490128	-0.022195203	0.982292244	0.985010452	negative
PRJNA729271	stage_5W_vs_E10	ordinary_muscle_development	ENSGALG00010010968	RRP7A	-0.120150993	0.246871754	-0.486693967	0.626475221	0.662635605	negative
PRJNA729271	stage_5W_vs_E10	ordinary_muscle_development	ENSGALG00010010970	TPM2	-1.489725911	0.352528965	-4.225825556	2.38066E-05	4.11885E-05	negative
PRJNA729271	stage_5W_vs_E10	ordinary_muscle_development	ENSGALG00010010971	LCORL	0.149043811	0.125080805	1.191580204	0.233425891	0.268276902	positive
PRJNA729271	stage_5W_vs_E10	ordinary_muscle_development	ENSGALG00010010972	EXOSC3	-0.596838084	0.167647088	-3.560086187	0.000370733	0.000582145	negative
PRJNA729271	stage_5W_vs_E10	ordinary_muscle_development	ENSGALG00010010973	PGAP1	-0.235138049	0.112999763	-2.080872054	0.037445619	0.048050128	negative
PRJNA729271	stage_5W_vs_E10	ordinary_muscle_development	ENSGALG00010010974	NCAPG	-2.467107229	0.193688469	-12.73750181	3.65925E-37	2.92236E-36	negative
PRJNA729271	stage_5W_vs_E10	ordinary_muscle_development	ENSGALG00010010975	ATP8B1	-3.139020314	0.910055031	-3.449264283	0.000562116	0.000869942	negative
PRJNA729271	stage_5W_vs_E10	ordinary_muscle_development	ENSGALG00010010976	DYDC2	-0.122347946	0.868597604	-0.140856877	0.88798301	0.901857744	negative
PRJNA729271	stage_5W_vs_E10	ordinary_muscle_development	ENSGALG00010010977	FARS2	-0.203200529	0.082662306	-2.458200574	0.013963516	0.018807022	negative
PRJNA729271	stage_5W_vs_E10	ordinary_muscle_development	ENSGALG00010010978	SERHL	-0.004625032	0.135380027	-0.034163323	0.972746914	0.976654881	negative
PRJNA729271	stage_5W_vs_E10	ordinary_muscle_development	ENSGALG00010010979	ENSGALG00010010979	-2.113596629	0.411252752	-5.1394103	2.75602E-07	5.50255E-07	negative
PRJNA729271	stage_5W_vs_E10	ordinary_muscle_development	ENSGALG00010010980	POLK	0.50806916	0.12572827	4.041009703	5.32216E-05	8.96718E-05	positive
PRJNA729271	stage_5W_vs_E10	ordinary_muscle_development	ENSGALG00010010981	RIC8B	-0.916054159	0.065866302	-13.90778178	5.68143E-44	5.60601E-43	negative
PRJNA729271	stage_5W_vs_E10	ordinary_muscle_development	ENSGALG00010010982	ZBTB21	0.771053125	0.119585145	6.447733344	1.13535E-10	2.81411E-10	positive
PRJNA729271	stage_5W_vs_E10	ordinary_muscle_development	ENSGALG00010010983	MED28	-0.017267377	0.089530251	-0.192866396	0.847063603	0.865697449	negative
PRJNA729271	stage_5W_vs_E10	ordinary_muscle_development	ENSGALG00010010984	MAT1A	-2.940172273	0.391304398	-7.513772625	5.74473E-14	1.70845E-13	negative
PRJNA729271	stage_5W_vs_E10	ordinary_muscle_development	ENSGALG00010010985	CERT1	1.040560259	0.10806237	9.629256278	6.01633E-22	2.63067E-21	positive
PRJNA729271	stage_5W_vs_E10	ordinary_muscle_development	ENSGALG00010010986	NARS	-0.149497975	0.1301035	-1.14906959	0.250527287	0.28641005	negative
PRJNA729271	stage_5W_vs_E10	ordinary_muscle_development	ENSGALG00010010987	MARCHF7	-0.303575982	0.081243295	-3.736628136	0.000186504	0.000300342	negative
PRJNA729271	stage_5W_vs_E10	ordinary_muscle_development	ENSGALG00010010988	ANKRD44	-0.403363698	0.086028199	-4.688738162	2.74895E-06	5.10912E-06	negative
PRJNA729271	stage_5W_vs_E10	ordinary_muscle_development	ENSGALG00010010989	TRIQK	-1.548989268	0.181504629	-8.534158476	1.41179E-17	5.05657E-17	negative
PRJNA729271	stage_5W_vs_E10	ordinary_muscle_development	ENSGALG00010010990	MED18	0.464769269	0.119459966	3.890585984	0.000100002	0.000164757	positive
PRJNA729271	stage_5W_vs_E10	ordinary_muscle_development	ENSGALG00010010991	C2CD2	-0.657167254	0.163367066	-4.022642198	5.75489E-05	9.67828E-05	negative
PRJNA729271	stage_5W_vs_E10	ordinary_muscle_development	ENSGALG00010010992	PCDHA6	1.055393741	0.292952921	3.602605283	0.000315044	0.000497609	positive
PRJNA729271	stage_5W_vs_E10	ordinary_muscle_development	ENSGALG00010010993	ENSGALG00010010993	-0.588576679	0.395779437	-1.487133044	0.136979676	0.163457677	negative
PRJNA729271	stage_5W_vs_E10	ordinary_muscle_development	ENSGALG00010010994	NAA20	-0.012540848	0.14993116	-0.083644042	0.933339449	0.94211388	negative
PRJNA729271	stage_5W_vs_E10	ordinary_muscle_development	ENSGALG00010010995	HEBP1	-0.645492647	0.183889351	-3.510223095	0.000447731	0.00069766	negative
PRJNA729271	stage_5W_vs_E10	ordinary_muscle_development	ENSGALG00010010996	RFX4	-1.896543976	0.936586337	-2.024953707	0.042872118	0.054530655	negative
PRJNA729271	stage_5W_vs_E10	ordinary_muscle_development	ENSGALG00010010997	LAP3	-1.597388086	0.15095444	-10.58192183	3.61467E-26	1.88131E-25	negative
PRJNA729271	stage_5W_vs_E10	ordinary_muscle_development	ENSGALG00010010998	SOAT1	0.024357291	0.166326068	0.146443017	0.88357167	0.897629055	positive
PRJNA729271	stage_5W_vs_E10	ordinary_muscle_development	ENSGALG00010011001	ANGPT1	-1.138302156	0.250267015	-4.548350713	5.4068E-06	9.81116E-06	negative

PRJNA729271	stage_5W_vs_E10	ordinary_muscle_development	ENSGALG00010011002	RPRM	-1.736136591	0.682387489	-2.544209292	0.010952544	0.014939035	negative
PRJNA729271	stage_5W_vs_E10	ordinary_muscle_development	ENSGALG00010011003	FECH	-0.892445597	0.112871122	-7.906766399	2.64161E-15	8.43394E-15	negative
PRJNA729271	stage_5W_vs_E10	ordinary_muscle_development	ENSGALG00010011004	UMODL1	-2.334100185	0.460039033	-5.073700312	3.90153E-07	7.71103E-07	negative
PRJNA729271	stage_5W_vs_E10	ordinary_muscle_development	ENSGALG00010011006	TMEM64	-0.410014659	0.101141889	-4.053856056	5.03802E-05	8.49833E-05	negative
PRJNA729271	stage_5W_vs_E10	ordinary_muscle_development	ENSGALG00010011007	ENSGALG00010011007	-1.020284937	0.184973827	-5.515834073	3.4713E-08	7.38427E-08	negative
PRJNA729271	stage_5W_vs_E10	ordinary_muscle_development	ENSGALG00010011009	SFTPA1	4.022416667	1.139608064	3.52964918	0.000416111	0.000649949	positive
PRJNA729271	stage_5W_vs_E10	ordinary_muscle_development	ENSGALG00010011013	ENSGALG00010011013	4.113637346	0.333941931	12.31842115	7.20919E-35	5.28564E-34	positive
PRJNA729271	stage_5W_vs_E10	ordinary_muscle_development	ENSGALG00010011015	LYRM4	-0.290964687	0.185153042	-1.571482078	0.116070714	0.139796012	negative
PRJNA729271	stage_5W_vs_E10	ordinary_muscle_development	ENSGALG00010011018	QDPR	-0.925498755	0.105713224	-8.754805856	2.04455E-18	7.60867E-18	negative
PRJNA729271	stage_5W_vs_E10	ordinary_muscle_development	ENSGALG00010011019	NECAB1	-2.938493192	0.239217281	-12.28378309	1.10698E-34	8.07128E-34	negative
PRJNA729271	stage_5W_vs_E10	ordinary_muscle_development	ENSGALG00010011020	SFTPA2	2.307529416	0.85997446	2.683253425	0.007290972	0.010127708	positive
PRJNA729271	stage_5W_vs_E10	ordinary_muscle_development	ENSGALG00010011021	PPP1R3G	2.153634701	0.183445843	11.73989374	7.95864E-32	5.18241E-31	positive
PRJNA729271	stage_5W_vs_E10	ordinary_muscle_development	ENSGALG00010011022	C5orf22	0.151811703	0.194362236	0.781076127	0.434757722	0.476723896	positive
PRJNA729271	stage_5W_vs_E10	ordinary_muscle_development	ENSGALG00010011025	POLR3B	-0.483440382	0.092042567	-5.252356599	1.50165E-07	3.06311E-07	negative
PRJNA729271	stage_5W_vs_E10	ordinary_muscle_development	ENSGALG00010011027	FAM234B	-0.295397892	0.101322632	-2.915418659	0.003552117	0.005093277	negative
PRJNA729271	stage_5W_vs_E10	ordinary_muscle_development	ENSGALG00010011028	HMBOX1	-1.005958977	0.087079289	-11.55221858	7.1939E-31	4.51801E-30	negative
PRJNA729271	stage_5W_vs_E10	ordinary_muscle_development	ENSGALG00010011029	HMGCR	-0.576857301	0.125673128	-4.5901404	4.42948E-06	8.09243E-06	negative
PRJNA729271	stage_5W_vs_E10	ordinary_muscle_development	ENSGALG00010011033	DROSHA	-0.982616682	0.10232583	-9.602821544	7.77853E-22	3.37777E-21	negative
PRJNA729271	stage_5W_vs_E10	ordinary_muscle_development	ENSGALG00010011034	FAM180A	1.175917387	0.39962952	2.94251883	0.00325554	0.00468843	positive
PRJNA729271	stage_5W_vs_E10	ordinary_muscle_development	ENSGALG00010011038	GSG1	-2.95669166	0.547333496	-5.401992899	6.59046E-08	1.3753E-07	negative
PRJNA729271	stage_5W_vs_E10	ordinary_muscle_development	ENSGALG00010011040	CDH6	-2.568707117	0.22340181	-11.49814818	1.34776E-30	8.37008E-30	negative
PRJNA729271	stage_5W_vs_E10	ordinary_muscle_development	ENSGALG00010011042	SLC13A4	-0.037566115	0.549999947	-0.068302035	0.945545204	0.952709422	negative
PRJNA729271	stage_5W_vs_E10	ordinary_muscle_development	ENSGALG00010011044	MBL2	0.413401074	0.628906143	0.657333497	0.510966503	0.55177378	positive
PRJNA729271	stage_5W_vs_E10	ordinary_muscle_development	ENSGALG00010011045	ENSGALG00010011045	-2.494680272	0.432340669	-5.770172579	7.91904E-09	1.75864E-08	negative
PRJNA729271	stage_5W_vs_E10	ordinary_muscle_development	ENSGALG00010011049	ABL2	-0.636353986	0.130595312	-4.872716897	1.10074E-06	2.10602E-06	negative
PRJNA729271	stage_5W_vs_E10	ordinary_muscle_development	ENSGALG00010011050	SYT10	2.843291489	0.405370666	7.01405337	2.31511E-12	6.32922E-12	positive
PRJNA729271	stage_5W_vs_E10	ordinary_muscle_development	ENSGALG00010011055	TOR3A	-0.907192674	0.139344107	-6.510448801	7.49266E-11	1.88454E-10	negative
PRJNA729271	stage_5W_vs_E10	ordinary_muscle_development	ENSGALG00010011056	ENSGALG00010011056	-1.339958712	0.541678904	-2.473714042	0.013371668	0.018045091	negative
PRJNA729271	stage_5W_vs_E10	ordinary_muscle_development	ENSGALG00010011059	ENSGALG00010011059	-1.733856409	0.208885313	-8.300518517	1.03658E-16	3.55122E-16	negative
PRJNA729271	stage_5W_vs_E10	ordinary_muscle_development	ENSGALG00010011061	KIF13B	0.869244594	0.154616631	5.621934644	1.88831E-08	4.09684E-08	positive
PRJNA729271	stage_5W_vs_E10	ordinary_muscle_development	ENSGALG00010011062	TMEM55A	-0.372565755	0.122274581	-3.046959986	0.002311685	0.003377129	negative
PRJNA729271	stage_5W_vs_E10	ordinary_muscle_development	ENSGALG00010011064	MYLIP	1.043889309	0.09657515	10.80908809	3.11754E-27	1.69627E-26	positive
PRJNA729271	stage_5W_vs_E10	ordinary_muscle_development	ENSGALG00010011067	TMTC2	-0.435500593	0.103036756	-4.226652816	2.37193E-05	4.10521E-05	negative
PRJNA729271	stage_5W_vs_E10	ordinary_muscle_development	ENSGALG00010011069	ENSGALG00010011069	-0.801891999	0.527885336	-1.519064739	0.128746201	0.154178469	negative
PRJNA729271	stage_5W_vs_E10	ordinary_muscle_development	ENSGALG00010011076	PKP2	-3.267804784	0.319806733	-10.21806123	1.646E-24	8.0063E-24	negative
PRJNA729271	stage_5W_vs_E10	ordinary_muscle_development	ENSGALG00010011077	RASGEF1A	0.668131264	0.145864748	4.580484808	4.63899E-06	8.4624E-06	positive
PRJNA729271	stage_5W_vs_E10	ordinary_muscle_development	ENSGALG00010011080	EMP1	0.293890036	0.255442406	1.150513889	0.249932277	0.28584254	positive
PRJNA729271	stage_5W_vs_E10	ordinary_muscle_development	ENSGALG00010011085	UGT1A1	3.688311068	1.320897661	2.792276175	0.005233866	0.007373398	positive
PRJNA729271	stage_5W_vs_E10	ordinary_muscle_development	ENSGALG00010011086	OTUD6A	0.410973128	0.113250458	3.628887127	0.000284646	0.000451366	positive
PRJNA729271	stage_5W_vs_E10	ordinary_muscle_development	ENSGALG00010011090	GRIN2B	-2.683050609	0.431634567	-6.216023494	5.09912E-10	1.21326E-09	negative
PRJNA729271	stage_5W_vs_E10	ordinary_muscle_development	ENSGALG00010011091	YARS2	-0.035405938	0.101308505	-0.349486334	0.726724229	0.756634657	negative
PRJNA729271	stage_5W_vs_E10	ordinary_muscle_development	ENSGALG00010011094	CSGALNACT2	-0.394588979	0.091065425	-4.33302736	1.47073E-05	2.5903E-05	negative
PRJNA729271	stage_5W_vs_E10	ordinary_muscle_development	ENSGALG00010011095	TNN	-3.696912117	0.503431834	-7.343421424	2.08202E-13	5.99738E-13	negative
PRJNA729271	stage_5W_vs_E10	ordinary_muscle_development	ENSGALG00010011096	LDB2	-0.731435826	0.115275179	-6.345128539	2.2224E-10	5.4077E-10	negative
PRJNA729271	stage_5W_vs_E10	ordinary_muscle_development	ENSGALG00010011099	CCBE1	0.147818813	0.197623715	0.747981145	0.45447153	0.496425383	positive
PRJNA729271	stage_5W_vs_E10	ordinary_muscle_development	ENSGALG00010011101	ENSGALG00010011101	-0.751140712	0.831075653	-0.903817491	0.366092185	0.406722329	negative
PRJNA729271	stage_5W_vs_E10	ordinary_muscle_development	ENSGALG00010011104	ABCG1	-0.793403369	0.26053095	-3.045332504	0.002324232	0.003395117	negative
PRJNA729271	stage_5W_vs_E10	ordinary_muscle_development	ENSGALG00010011106	USP40	0.428213567	0.115800307	3.697862105	0.000217423	0.000348332	positive
PRJNA729271	stage_5W_vs_E10	ordinary_muscle_development	ENSGALG00010011107	ENSGALG00010011107	0.986225458	0.190328462	5.181702445	2.1987E-07	4.42723E-07	positive
PRJNA729271	stage_5W_vs_E10	ordinary_muscle_development	ENSGALG00010011109	ENSGALG00010011109	-0.961973617	1.253078741	-0.767688083	0.442672518	0.484559594	negative
PRJNA729271	stage_5W_vs_E10	ordinary_muscle_development	ENSGALG00010011111	MRPS14	0.848796748	0.116821426	7.265762626	3.70939E-13	1.0551E-12	positive
PRJNA729271	stage_5W_vs_E10	ordinary_muscle_development	ENSGALG00010011116	TAPT1	1.178754327	0.127257332	9.262761625	1.99212E-20	8.09979E-20	positive
PRJNA729271	stage_5W_vs_E10	ordinary_muscle_development	ENSGALG00010011117	SLC6A15	-8.906197518	0.879509393	-10.1263245	4.22223E-24	2.01918E-23	negative
PRJNA729271	stage_5W_vs_E10	ordinary_muscle_development	ENSGALG00010011118	GMPR2	2.487701677	0.135799054	18.31899121	5.83851E-75	1.41757E-73	positive
PRJNA729271	stage_5W_vs_E10	ordinary_muscle_development	ENSGALG00010011119	ENSGALG00010011119	-0.332593777	0.291726746	-1.140086679	0.254250191	0.290368462	negative
PRJNA729271	stage_5W_vs_E10	ordinary_muscle_development	ENSGALG00010011120	ENSGALG00010011120	2.475289565	3.002383782	0.824441425	0.409688785	0.45124916	positive
PRJNA729271	stage_5W_vs_E10	ordinary_muscle_development	ENSGALG00010011121	NUP35	-0.334428386	0.104740494	-3.192923522	0.001408402	0.002103052	negative
PRJNA729271	stage_5W_vs_E10	ordinary_muscle_development	ENSGALG00010011124	CACYBP	0.558105404	0.067552251	8.261832839	1.43453E-16	4.87994E-16	positive
PRJNA729271	stage_5W_vs_E10	ordinary_muscle_development	ENSGALG00010011126	ATXN1	1.248326568	0.138303357	9.02600337	1.78056E-19	6.94543E-19	positive
PRJNA729271	stage_5W_vs_E10	ordinary_muscle_development	ENSGALG00010011128	GCNT4	1.764080597	0.236132454	7.470724883	7.97542E-14	2.34872E-13	positive
PRJNA729271	stage_5W_vs_E10	ordinary_muscle_development	ENSGALG00010011129	ENSGALG00010011129	0.730963311	0.115944626	6.30441732	2.8928E-10	6.98387E-10	positive
PRJNA729271	stage_5W_vs_E10	ordinary_muscle_development	ENSGALG00010011130	DNM1L	-0.027693294	0.11532274	-0.240137321	0.810223802	0.832668971	negative
PRJNA729271	stage_5W_vs_E10	ordinary_muscle_development	ENSGALG00010011131	RET	0.118805312	0.495398335	0.239817746	0.810471549	0.832821857	positive
PRJNA729271	stage_5W_vs_E10	ordinary_muscle_development	ENSGALG00010011133	CDC37L1	0.735482073	0.119065761	6.177108049	6.52864E-10	1.54276E-09	positive
PRJNA729271	stage_5W_vs_E10	ordinary_muscle_development	ENSGALG00010011136	ENSGALG00010011136	-1.244880395	0.408308442	-3.048872537	0.002297019	0.00335638	negative
PRJNA729271	stage_5W_vs_E10	ordinary_muscle_development	ENSGALG00010011139	UBASH3A	6.658222098	0.563669217	11.81228617	3.37264E-32	2.23737E-31	positive
PRJNA729271	stage_5W_vs_E10	ordinary_muscle_development	ENSGALG00010011141	MSX1	-4.643204638	0.841601465	-5.517106175	3.44627E-08	7.33319E-08	negative
PRJNA729271	stage_5W_vs_E10	ordinary_muscle_development	ENSGALG00010011142	DUSP19	-0.236877409	0.151671967	-1.56177449	0.118341123	0.142329454	negative
PRJNA729271	stage_5W_vs_E10	ordinary_muscle_development	ENSGALG00010011143	AK3	2.562086709	0.163015885	15.71679172	1.16053E-55	1.63637E-54	positive
PRJNA729271	stage_5W_vs_E10	ordinary_muscle_development	ENSGALG00010011144	ATF7IP	0.080931425	0.062106752	1.303101874	0.192539984	0.224671314	positive
PRJNA729271	stage_5W_vs_E10	ordinary_muscle_development	ENSGALG00010011146	BMS1	-0.771750506	0.129226993	-5.972053401	2.34286E-09	5.35727E-09	negative
PRJNA729271	stage_5W_vs_E10	ordinary_muscle_development	ENSGALG00010011148	ENSGALG00010011148	-0.206424636	0.184116868	-1.121160914	0.262219374	0.298834014	negative
PRJNA729271	stage_5W_vs_E10	ordinary_muscle_development	ENSGALG00010011150	FGD4	2.545836687	0.162397711	15.67655527	2.18803E-55	3.06134E-54	positive
PRJNA729271	stage_5W_vs_E10	ordinary_muscle_development	ENSGALG00010011152	SLC18A3	-0.87470696	0.665098035	-1.315154929	0.188457839	0.220315862	negative
PRJNA729271	stage_5W_vs_E10	ordinary_muscle_development	ENSGALG00010011154	SLC26A7	-4.626251807	0.611929227	-7.560109249	4.02731E-14	1.20736E-13	negative
PRJNA729271	stage_5W_vs_E10	ordinary_muscle_development	ENSGALG00010011157	ENSGALG00010011157	-4.933658259	1.574356484	-3.133761831	0.001725809	0.002553133	negative
PRJNA729271	stage_5W_vs_E10	ordinary_muscle_development	ENSGALG00010011161	ENSGALG00010011161	0.054276586	0.094568203	0.573941176	0.566007608	0.605213948	positive
PRJNA729271	stage_5W_vs_E10	ordinary_muscle_development	ENSGALG00010011166	SOX7	0.802594521	0.142205359	5.643911903	1.66229E-08	3.62166E-08	positive
PRJNA729271	stage_5W_vs_E10	ordinary_muscle_development	ENSGALG00010011170	RBM24	0.707639208	0.14286304	4.953270001	7.29766E-07	1.41682E-06	positive
PRJNA729271	stage_5W_vs_E10	ordinary_muscle_development	ENSGALG00010011175	GPR52	0.130119505	0.188655223	0.689721189	0.490369538	0.531790099	positive
PRJNA729271	stage_5W_vs_E10	ordinary_muscle_development	ENSGALG00010011178	RSPO2	-5.249697294	0.336253841	-15.61230431	6.00291E-55	8.279E-54	negative
PRJNA729271	stage_5W_vs_E10	ordinary_muscle_development	ENSGALG00010011181	ENSGALG00010011181	-0.142720737	0.280479153	-0.508846151	0.610860067	0.647915753	negative
PRJNA729271	stage_5W_vs_E10	ordinary_muscle_development	ENSGALG00010011184	FAM8A1	1.026182946	0.135528169	7.571731777	3.68281E-14	1.10752E-13	positive
PRJNA729271	stage_5W_vs_E10	ordinary_muscle_development	ENSGALG00010011187	STK32B	-6.772955037	0.776522867	-8.722157873	2.72949E-18	1.00929E-17	negative
PRJNA729271	stage_5W_vs_E10	ordinary_muscle_development	ENSGALG00010011190	ENSGALG00010011190	-0.313012005	0.21396815	-1.462890642	0.143497305	0.17067313	negative
PRJNA729271	stage_5W_vs_E10	ordinary_muscle_development	ENSGALG00010011192	RPL17	-0.14759553	0.123547065	-1.194650235	0.232223713	0.267001089	negative
PRJNA729271	stage_5W_vs_E10	ordinary_muscle_development	ENSGALG00010011193	FAM20B	-0.829845929	0.079028319	-10.50061477	8.58194E-26	4.39559E-25	negative
PRJNA729271	stage_5W_vs_E10	ordinary_muscle_development	ENSGALG00010011198	PINX1	-0.913251615	0.068598024	-13.31308925	1.94279E-40	1.72132E-39	negative
PRJNA729271	stage_5W_vs_E10	ordinary_muscle_development	ENSGALG00010011199	ENSGALG00010011199	-3.435778104	1.672345495	-2.054466685	0.039930545	0.05102647	negative
PRJNA729271	stage_5W_vs_E10	ordinary_muscle_development	ENSGALG00010011200	PCDHB4	-0.195992599	0.58436502	-0.335394132	0.737327794	0.766353533	negative
PRJNA729271	stage_5W_vs_E10	ordinary_muscle_development	ENSGALG00010011203	ENSGALG00010011203	-0.62574719	0.105039792	-5.957239418	2.56534E-09	5.85401E-09	negative
PRJNA729271	stage_5W_vs_E10	ordinary_muscle_development	ENSGALG00010011205	CYTL1	-9.203076295	0.954886939	-9.637870114	5.53239E-22	2.42345E-21	negative
PRJNA729271	stage_5W_vs_E10	ordinary_muscle_development	ENSGALG00010011210	NUP153	-0.21564746	0.098063048	-2.199069524	0.027872979	0.036273912	negative
PRJNA729271	stage_5W_vs_E10	ordinary_muscle_development	ENSGALG00010011211	SLC37A1	0.486169697	0.150898966	3.221822599	0.00127378	0.001909747	positive
PRJNA729271	stage_5W_vs_E10	ordinary_muscle_development	ENSGALG00010011212	NCKAP1	-0.493896524	0.063274178	-7.805656909	5.91924E-15	1.85312E-14	negative
PRJNA729271	stage_5W_vs_E10	ordinary_muscle_development	ENSGALG00010011213	XKR6	0.283019455	0.182071165	1.554444133	0.120078513	0.144275369	positive
PRJNA729271	stage_5W_vs_E10	ordinary_muscle_development	ENSGALG00010011218	ENSGALG00010011218	-0.772189261	0.592630483	-1.302986064	0.192579519	0.224699359	negative
PRJNA729271	stage_5W_vs_E10	ordinary_muscle_development	ENSGALG00010011222	RCL1	-0.441559023	0.090419642	-4.883441392	1.0425E-06	1.99935E-06	negative
PRJNA729271	stage_5W_vs_E10	ordinary_muscle_development	ENSGALG00010011231	PLBD1	1.736052062	0.204722684	8.48001808	2.25159E-17	7.96797E-17	positive
PRJNA729271	stage_5W_vs_E10	ordinary_muscle_development	ENSGALG00010011235	ENSGALG00010011235	-0.674877155	0.093712188	-7.201594266	5.95126E-13	1.67926E-12	negative
PRJNA729271	stage_5W_vs_E10	ordinary_muscle_development	ENSGALG00010011237	ENSGALG00010011237	-0.229536182	0.259682289	-0.883911579	0.376743946	0.417851672	negative
PRJNA729271	stage_5W_vs_E10	ordinary_muscle_development	ENSGALG00010011239	RALGPS2	-2.476822177	0.079732831	-31.06401896	7.3782E-212	1.4452E-209	negative
PRJNA729271	stage_5W_vs_E10	ordinary_muscle_development	ENSGALG00010011243	ALX1	-8.240828794	0.895372378	-9.203800562	3.45508E-20	1.39115E-19	negative
PRJNA729271	stage_5W_vs_E10	ordinary_muscle_development	ENSGALG00010011244	PDE9A	-0.981982064	0.17245376	-5.694176009	1.23969E-08	2.72139E-08	negative
PRJNA729271	stage_5W_vs_E10	ordinary_muscle_development	ENSGALG00010011246	DNAJB5	2.027248644	0.233088	8.697353117	3.39715E-18	1.25201E-17	positive
PRJNA729271	stage_5W_vs_E10	ordinary_muscle_development	ENSGALG00010011247	ENSGALG00010011247	-0.510709553	0.097454377	-5.240498861	1.60143E-07	3.25793E-07	negative
PRJNA729271	stage_5W_vs_E10	ordinary_muscle_development	ENSGALG00010011250	ENSGALG00010011250	0.023090861	0.099427364	0.23223849	0.816352784	0.837796206	positive
PRJNA729271	stage_5W_vs_E10	ordinary_muscle_development	ENSGALG00010011251	FRZB	1.08392438	0.175099787	6.190323798	6.00408E-10	1.42158E-09	positive
PRJNA729271	stage_5W_vs_E10	ordinary_muscle_development	ENSGALG00010011253	KDM4C	0.921206366	0.108682551	8.476120167	2.32828E-17	8.23133E-17	positive
PRJNA729271	stage_5W_vs_E10	ordinary_muscle_development	ENSGALG00010011262	ENSGALG00010011262	-1.379535311	0.208985246	-6.601113415	4.08081E-11	1.04323E-10	negative
PRJNA729271	stage_5W_vs_E10	ordinary_muscle_development	ENSGALG00010011267	EVC2	-1.118480835	0.166202974	-6.729607831	1.70121E-11	4.44627E-11	negative
PRJNA729271	stage_5W_vs_E10	ordinary_muscle_development	ENSGALG00010011271	ANGPTL1	-2.911350074	0.162745746	-17.88894732	1.43799E-71	3.19199E-70	negative
PRJNA729271	stage_5W_vs_E10	ordinary_muscle_development	ENSGALG00010011273	RUNX1T1	-1.046542486	0.099251177	-10.54438362	5.39253E-26	2.78463E-25	negative
PRJNA729271	stage_5W_vs_E10	ordinary_muscle_development	ENSGALG00010011274	ENSGALG00010011274	-0.502401744	0.532763492	-0.943010832	0.345675362	0.385516995	negative
PRJNA729271	stage_5W_vs_E10	ordinary_muscle_development	ENSGALG00010011275	WDR4	-1.324870627	0.105898215	-12.51079278	6.51705E-36	4.99021E-35	negative
PRJNA729271	stage_5W_vs_E10	ordinary_muscle_development	ENSGALG00010011278	ENSGALG00010011278	1.134723435	0.524059408	2.165257254	0.03036799	0.039375974	positive
PRJNA729271	stage_5W_vs_E10	ordinary_muscle_development	ENSGALG00010011280	FAM199X	-0.275632616	0.08189861	-3.365534716	0.000763955	0.001169697	negative
PRJNA729271	stage_5W_vs_E10	ordinary_muscle_development	ENSGALG00010011281	ENSGALG00010011281	-1.947071937	0.679998077	-2.863349179	0.004191883	0.005969871	negative
PRJNA729271	stage_5W_vs_E10	ordinary_muscle_development	ENSGALG00010011282	CRY1	0.500214314	0.127287209	3.929808173	8.50137E-05	0.000141057	positive
PRJNA729271	stage_5W_vs_E10	ordinary_muscle_development	ENSGALG00010011283	LONRF3	1.707390044	0.140853916	12.12170801	8.10522E-34	5.70316E-33	positive
PRJNA729271	stage_5W_vs_E10	ordinary_muscle_development	ENSGALG00010011285	ENSGALG00010011285	2.089733087	0.260228253	8.030385109	9.71672E-16	3.17573E-15	positive
PRJNA729271	stage_5W_vs_E10	ordinary_muscle_development	ENSGALG00010011287	ENSGALG00010011287	0.22307193	0.117427431	1.899657755	0.057478048	0.071940618	positive
PRJNA729271	stage_5W_vs_E10	ordinary_muscle_development	ENSGALG00010011288	ENSGALG00010011288	-0.390695748	0.299121983	-1.306141874	0.191504315	0.223606819	negative
PRJNA729271	stage_5W_vs_E10	ordinary_muscle_development	ENSGALG00010011293	NDUFV3	1.313937217	0.167488783	7.844926629	4.33207E-15	1.36626E-14	positive
PRJNA729271	stage_5W_vs_E10	ordinary_muscle_development	ENSGALG00010011295	FAM167A	-0.584612106	0.373192681	-1.566515464	0.117227991	0.141094499	negative
PRJNA729271	stage_5W_vs_E10	ordinary_muscle_development	ENSGALG00010011297	OSR2	-0.963891021	0.186397045	-5.171171147	2.32631E-07	4.67164E-07	negative
PRJNA729271	stage_5W_vs_E10	ordinary_muscle_development	ENSGALG00010011300	EVC	-1.107749662	0.162937721	-6.798607798	1.05635E-11	2.79463E-11	negative
PRJNA729271	stage_5W_vs_E10	ordinary_muscle_development	ENSGALG00010011304	COMMD5	-0.851142829	0.093822683	-9.071823551	1.17042E-19	4.61261E-19	negative
PRJNA729271	stage_5W_vs_E10	ordinary_muscle_development	ENSGALG00010011306	EC12	0.688426693	0.138134342	4.983747574	6.23644E-07	1.21538E-06	positive
PRJNA729271	stage_5W_vs_E10	ordinary_muscle_development	ENSGALG00010011307	ENSGALG00010011307	-2.632101803	0.483062442	-5.448781721	5.0716E-08	1.06788E-07	negative
PRJNA729271	stage_5W_vs_E10	ordinary_muscle_development	ENSGALG00010011308	SPIC	6.902599217	1.368151319	5.045201593	4.53043E-07	8.91268E-07	positive

PRJNA729271	stage_5W_vs_E10	ordinary_muscle_development	ENSGALG00010011311	DOCK8	1.548646813	0.189321787	8.179971458	2.83911E-16	9.53276E-16	positive
PRJNA729271	stage_5W_vs_E10	ordinary_muscle_development	ENSGALG00010011312	VPS13B	-0.293588805	0.053165647	-5.52215242	3.34872E-08	7.13085E-08	negative
PRJNA729271	stage_5W_vs_E10	ordinary_muscle_development	ENSGALG00010011313	RASSF9	-0.648845344	0.252318119	-2.571536862	0.010124824	0.013859601	negative
PRJNA729271	stage_5W_vs_E10	ordinary_muscle_development	ENSGALG00010011316	PARPBP	-2.598899618	0.218736775	-11.88140231	1.47866E-32	9.93265E-32	negative
PRJNA729271	stage_5W_vs_E10	ordinary_muscle_development	ENSGALG00010011317	BLK	3.272706488	0.35166948	9.306199911	1.32488E-20	5.4341E-20	positive
PRJNA729271	stage_5W_vs_E10	ordinary_muscle_development	ENSGALG00010011318	PRP4K	-0.552040585	0.098174884	-5.623032734	1.87634E-08	4.0721E-08	negative
PRJNA729271	stage_5W_vs_E10	ordinary_muscle_development	ENSGALG00010011321	MAP2	-2.753963176	0.155874784	-17.66779144	7.42473E-70	1.56654E-68	negative
PRJNA729271	stage_5W_vs_E10	ordinary_muscle_development	ENSGALG00010011322	GATA4	-3.391455776	1.308155659	-2.592547572	0.0095268	0.013076797	negative
PRJNA729271	stage_5W_vs_E10	ordinary_muscle_development	ENSGALG00010011323	GUCY2C	-1.018859277	0.873721309	-1.166114717	0.243568067	0.279070361	negative
PRJNA729271	stage_5W_vs_E10	ordinary_muscle_development	ENSGALG00010011326	COX6C	0.679389827	0.135628016	5.009214532	5.46526E-07	1.06952E-06	positive
PRJNA729271	stage_5W_vs_E10	ordinary_muscle_development	ENSGALG00010011329	PCDHGC5	-0.545164948	0.117071928	-4.656666692	3.2137E-06	5.94393E-06	negative
PRJNA729271	stage_5W_vs_E10	ordinary_muscle_development	ENSGALG00010011333	ENSGALG00010011333	-1.239963281	0.132157823	-9.382443271	6.44613E-21	2.67957E-20	negative
PRJNA729271	stage_5W_vs_E10	ordinary_muscle_development	ENSGALG00010011335	VCP	0.912550235	0.173635773	5.255542801	1.47589E-07	3.01182E-07	positive
PRJNA729271	stage_5W_vs_E10	ordinary_muscle_development	ENSGALG00010011339	ENSGALG00010011339	-0.189748152	0.301008803	-0.630374097	0.528449854	0.568746706	negative
PRJNA729271	stage_5W_vs_E10	ordinary_muscle_development	ENSGALG00010011340	PKNOX1	-0.223148953	0.108976903	-2.047672011	0.040592148	0.051776064	negative
PRJNA729271	stage_5W_vs_E10	ordinary_muscle_development	ENSGALG00010011342	RASAL2	0.588127206	0.066185794	8.886003613	6.33463E-19	2.40935E-18	positive
PRJNA729271	stage_5W_vs_E10	ordinary_muscle_development	ENSGALG00010011343	PXDC1	-0.512264371	0.141364388	-3.623715828	0.000290401	0.00046014	negative
PRJNA729271	stage_5W_vs_E10	ordinary_muscle_development	ENSGALG00010011345	CRMP1	-2.731157956	0.233982174	-11.67250442	1.7616E-31	1.13084E-30	negative
PRJNA729271	stage_5W_vs_E10	ordinary_muscle_development	ENSGALG00010011347	RGS22	-1.558523481	0.414641802	-3.758722524	0.000170783	0.000275822	negative
PRJNA729271	stage_5W_vs_E10	ordinary_muscle_development	ENSGALG00010011348	SLC22A23	0.157259199	0.163173934	0.963751962	0.335170296	0.374868321	positive
PRJNA729271	stage_5W_vs_E10	ordinary_muscle_development	ENSGALG00010011349	ENSGALG00010011349	-0.545121429	0.091475999	-5.959174382	2.53515E-09	5.78603E-09	negative
PRJNA729271	stage_5W_vs_E10	ordinary_muscle_development	ENSGALG00010011351	KANK1	-2.05279654	0.111781404	-18.36438321	2.53311E-75	6.23386E-74	negative
PRJNA729271	stage_5W_vs_E10	ordinary_muscle_development	ENSGALG00010011352	NTS	-0.779869554	0.546188002	-1.427840873	0.153337665	0.181765576	negative
PRJNA729271	stage_5W_vs_E10	ordinary_muscle_development	ENSGALG00010011353	ENSGALG00010011353	-3.015436091	0.21489118	-14.03238648	9.8771E-45	9.94226E-44	negative
PRJNA729271	stage_5W_vs_E10	ordinary_muscle_development	ENSGALG00010011357	UNC80	0.79280042	0.403892272	1.962900689	0.049657715	0.062628433	positive
PRJNA729271	stage_5W_vs_E10	ordinary_muscle_development	ENSGALG00010011359	RNF19A	0.173546262	0.086620598	2.003521857	0.045121304	0.057205747	positive
PRJNA729271	stage_5W_vs_E10	ordinary_muscle_development	ENSGALG00010011360	TCF4	1.069091863	0.170012353	6.288318718	3.20923E-10	7.7272E-10	positive
PRJNA729271	stage_5W_vs_E10	ordinary_muscle_development	ENSGALG00010011361	NUP37	-1.358639799	0.092157591	-14.74257073	3.43458E-49	4.05079E-48	negative
PRJNA729271	stage_5W_vs_E10	ordinary_muscle_development	ENSGALG00010011363	FDFT1	1.089587255	0.146158004	7.454858623	8.99642E-14	2.64241E-13	positive
PRJNA729271	stage_5W_vs_E10	ordinary_muscle_development	ENSGALG00010011365	PSMG4	-0.647151012	0.181012571	-3.575171645	0.000349998	0.000550958	negative
PRJNA729271	stage_5W_vs_E10	ordinary_muscle_development	ENSGALG00010011367	IER3IP1	0.315910921	0.113229442	2.79000686	0.005270692	0.007423114	positive
PRJNA729271	stage_5W_vs_E10	ordinary_muscle_development	ENSGALG00010011368	ENSGALG00010011368	-2.810984118	0.3094426	-9.084024373	1.04633E-19	4.13482E-19	negative
PRJNA729271	stage_5W_vs_E10	ordinary_muscle_development	ENSGALG00010011370	MGAT4C	-5.386868024	1.574826278	-3.420610959	0.000624806	0.000963567	negative
PRJNA729271	stage_5W_vs_E10	ordinary_muscle_development	ENSGALG00010011371	ENSGALG00010011371	1.020362528	0.19566989	5.214714075	1.84101E-07	3.72754E-07	positive
PRJNA729271	stage_5W_vs_E10	ordinary_muscle_development	ENSGALG00010011372	VMA21	-0.260813841	0.092747466	-2.812085909	0.004922136	0.006956548	negative
PRJNA729271	stage_5W_vs_E10	ordinary_muscle_development	ENSGALG00010011374	ENSGALG00010011374	-0.361257	0.093137326	-3.878756414	0.000104992	0.000172546	negative
PRJNA729271	stage_5W_vs_E10	ordinary_muscle_development	ENSGALG00010011375	C12orf50	0.396189678	0.137946117	2.87206111	0.004078041	0.005814026	positive
PRJNA729271	stage_5W_vs_E10	ordinary_muscle_development	ENSGALG00010011377	SEC16B	0.782838697	0.325419931	2.405626153	0.016144777	0.021584444	positive
PRJNA729271	stage_5W_vs_E10	ordinary_muscle_development	ENSGALG00010011378	TUBB2B	-3.137991566	0.194565364	-16.12821264	1.61631E-58	2.49238E-57	negative
PRJNA729271	stage_5W_vs_E10	ordinary_muscle_development	ENSGALG00010011379	CELF4	-3.488715674	0.798449667	-4.369362049	1.2461E-05	2.20594E-05	negative
PRJNA729271	stage_5W_vs_E10	ordinary_muscle_development	ENSGALG00010011381	RLIG1	-0.043241875	0.118985176	-0.363422373	0.716289391	0.747015322	negative
PRJNA729271	stage_5W_vs_E10	ordinary_muscle_development	ENSGALG00010011382	ENSGALG00010011382	0.161218758	0.196186201	0.821764006	0.411211223	0.452754021	positive
PRJNA729271	stage_5W_vs_E10	ordinary_muscle_development	ENSGALG00010011384	PUM3	-1.23475069	0.082698079	-14.93082667	2.07675E-50	2.52538E-49	negative
PRJNA729271	stage_5W_vs_E10	ordinary_muscle_development	ENSGALG00010011386	CTSB	2.035438437	0.12602969	16.15046766	1.12704E-58	1.74535E-57	positive
PRJNA729271	stage_5W_vs_E10	ordinary_muscle_development	ENSGALG00010011388	GPR50	-0.347850381	0.167493626	-2.076797717	0.037820233	0.048487907	negative
PRJNA729271	stage_5W_vs_E10	ordinary_muscle_development	ENSGALG00010011389	RC3H1	0.94318667	0.085542526	11.02593893	2.86513E-28	1.61974E-27	positive
PRJNA729271	stage_5W_vs_E10	ordinary_muscle_development	ENSGALG00010011394	SERPINC1	3.493335453	0.906171671	3.855048181	0.000115707	0.000189384	positive
PRJNA729271	stage_5W_vs_E10	ordinary_muscle_development	ENSGALG00010011398	ENSGALG00010011398	0.545375013	0.186172268	2.929410582	0.003396055	0.004881093	positive
PRJNA729271	stage_5W_vs_E10	ordinary_muscle_development	ENSGALG00010011399	JAKMIP1	-2.312276935	0.262073737	-8.823001336	1.11432E-18	4.19316E-18	negative
PRJNA729271	stage_5W_vs_E10	ordinary_muscle_development	ENSGALG00010011400	BPHL	0.927223637	0.199025169	4.658826025	3.18018E-06	5.88493E-06	positive
PRJNA729271	stage_5W_vs_E10	ordinary_muscle_development	ENSGALG00010011401	ANKRD46	-0.469986308	0.068724842	-6.838666931	7.99335E-12	2.12399E-11	negative
PRJNA729271	stage_5W_vs_E10	ordinary_muscle_development	ENSGALG00010011403	SV2C	-5.392576665	0.647072014	-8.333812233	7.828E-17	2.70048E-16	negative
PRJNA729271	stage_5W_vs_E10	ordinary_muscle_development	ENSGALG00010011406	UBE2R2	-0.075466446	0.115893971	-0.651168003	0.514938043	0.555400471	negative
PRJNA729271	stage_5W_vs_E10	ordinary_muscle_development	ENSGALG00010011407	CEP290	-0.754924562	0.181089292	-4.168797369	3.06211E-05	5.24897E-05	negative
PRJNA729271	stage_5W_vs_E10	ordinary_muscle_development	ENSGALG00010011411	ENSGALG00010011411	-0.42042169	0.223279622	-1.882938026	0.059708767	0.074558803	negative
PRJNA729271	stage_5W_vs_E10	ordinary_muscle_development	ENSGALG00010011412	KANSL1L	0.47446886	0.12054267	3.936107114	8.2814E-05	0.00013758	positive
PRJNA729271	stage_5W_vs_E10	ordinary_muscle_development	ENSGALG00010011413	ZBTB37	1.254473165	0.103378376	12.13477341	6.9101E-34	4.88119E-33	positive
PRJNA729271	stage_5W_vs_E10	ordinary_muscle_development	ENSGALG00010011414	PABPC1	0.257643534	0.114162883	2.256806471	0.02402017	0.031468941	positive
PRJNA729271	stage_5W_vs_E10	ordinary_muscle_development	ENSGALG00010011416	ENSGALG00010011416	0.328407894	0.438688827	0.748612396	0.454090858	0.496046951	positive
PRJNA729271	stage_5W_vs_E10	ordinary_muscle_development	ENSGALG00010011417	WFS1	-0.104383953	0.145394982	-0.717933675	0.472798183	0.514698435	negative
PRJNA729271	stage_5W_vs_E10	ordinary_muscle_development	ENSGALG00010011418	RIPK1	-0.423117255	0.086494644	-4.891831889	9.99018E-07	1.91925E-06	negative
PRJNA729271	stage_5W_vs_E10	ordinary_muscle_development	ENSGALG00010011419	RNF180	-0.118599026	0.151027231	-0.785282394	0.432288016	0.474159273	negative
PRJNA729271	stage_5W_vs_E10	ordinary_muscle_development	ENSGALG00010011420	HMGB3	-2.103606943	0.100223919	-20.98907093	8.25434E-98	3.32352E-96	negative
PRJNA729271	stage_5W_vs_E10	ordinary_muscle_development	ENSGALG00010011421	ENSGALG00010011421	0.861931988	0.116942719	7.370548548	1.69927E-13	4.91538E-13	positive
PRJNA729271	stage_5W_vs_E10	ordinary_muscle_development	ENSGALG00010011422	KATNAL2	-2.351275584	0.264894304	-8.876278382	6.91347E-19	2.62676E-18	negative
PRJNA729271	stage_5W_vs_E10	ordinary_muscle_development	ENSGALG00010011423	GDF6	-0.556964496	0.360093203	-1.546723157	0.121930017	0.146354388	negative
PRJNA729271	stage_5W_vs_E10	ordinary_muscle_development	ENSGALG00010011424	ACADL	0.812874431	0.097342075	8.350699666	6.78598E-17	2.3498E-16	positive
PRJNA729271	stage_5W_vs_E10	ordinary_muscle_development	ENSGALG00010011425	WASHC3	-0.115709473	0.080106733	-1.444441297	0.148614881	0.176484737	negative
PRJNA729271	stage_5W_vs_E10	ordinary_muscle_development	ENSGALG00010011426	NQO2	0.758286351	0.127000446	5.97073771	2.36183E-09	5.3981E-09	positive
PRJNA729271	stage_5W_vs_E10	ordinary_muscle_development	ENSGALG00010011427	TMTC3	-0.797195661	0.088387409	-9.019335145	1.89234E-19	7.37551E-19	negative
PRJNA729271	stage_5W_vs_E10	ordinary_muscle_development	ENSGALG00010011428	UQCRB	1.329146627	0.162960144	8.15626814	3.45535E-16	1.1543E-15	positive
PRJNA729271	stage_5W_vs_E10	ordinary_muscle_development	ENSGALG00010011429	UBAP2	0.252275419	0.107264286	2.351905076	0.018677541	0.024789942	positive
PRJNA729271	stage_5W_vs_E10	ordinary_muscle_development	ENSGALG00010011430	ENSGALG00010011430	-0.361067122	0.339915462	-1.062226238	0.288132991	0.325855325	negative
PRJNA729271	stage_5W_vs_E10	ordinary_muscle_development	ENSGALG00010011431	DRAM1	4.192714829	0.399904117	10.48430025	1.01998E-25	5.20584E-25	positive
PRJNA729271	stage_5W_vs_E10	ordinary_muscle_development	ENSGALG00010011433	RNASE6	5.682016689	0.330286027	17.20332144	2.50738E-66	4.79478E-65	positive
PRJNA729271	stage_5W_vs_E10	ordinary_muscle_development	ENSGALG00010011434	NPL	0.540033359	0.180315799	2.994930905	0.00274507	0.003982962	positive
PRJNA729271	stage_5W_vs_E10	ordinary_muscle_development	ENSGALG00010011436	UBAP2L2	2.431280086	2.237728888	1.08649448			positive
PRJNA729271	stage_5W_vs_E10	ordinary_muscle_development	ENSGALG00010011437	DMRT1	-2.08854978	0.575807043	-3.627169566	0.000286545	0.00045418	negative
PRJNA729271	stage_5W_vs_E10	ordinary_muscle_development	ENSGALG00010011438	ENSGALG00010011438	3.762851747	0.441597639	8.52099607	1.58187E-17	5.65455E-17	positive
PRJNA729271	stage_5W_vs_E10	ordinary_muscle_development	ENSGALG00010011439	ENSGALG00010011439	-0.533005501	0.333705732	-1.597232082	0.110213984	0.133107124	negative
PRJNA729271	stage_5W_vs_E10	ordinary_muscle_development	ENSGALG00010011440	FUNDC1	0.233015174	0.111361192	2.092427082	0.036400328	0.046775067	positive
PRJNA729271	stage_5W_vs_E10	ordinary_muscle_development	ENSGALG00010011441	CD99L2	1.177917205	0.07966915	14.78511069	1.82757E-49	2.16958E-48	positive
PRJNA729271	stage_5W_vs_E10	ordinary_muscle_development	ENSGALG00010011442	ENSGALG00010011442	1.497229153	2.050946814	0.730018518	0.465378866	0.507269263	positive
PRJNA729271	stage_5W_vs_E10	ordinary_muscle_development	ENSGALG00010011443	ENSGALG00010011443	-1.877137571	0.178015798	-10.5447808	5.36979E-26	2.77388E-25	negative
PRJNA729271	stage_5W_vs_E10	ordinary_muscle_development	ENSGALG00010011445	MTERF3	-0.159552871	0.081764334	-1.951374916	0.051012463	0.064258769	negative
PRJNA729271	stage_5W_vs_E10	ordinary_muscle_development	ENSGALG00010011446	ENSGALG00010011446	0.116425575	0.225021067	0.517398556	0.604877968	0.642397716	positive
PRJNA729271	stage_5W_vs_E10	ordinary_muscle_development	ENSGALG00010011447	LAMC1	2.144103467	0.130303007	16.45475045	7.75408E-61	1.27288E-59	positive
PRJNA729271	stage_5W_vs_E10	ordinary_muscle_development	ENSGALG00010011448	AvBD10	5.077505841	2.616016593	1.940930289	0.052266734	0.065764436	positive
PRJNA729271	stage_5W_vs_E10	ordinary_muscle_development	ENSGALG00010011449	DUSP22	-1.130983923	0.145449394	-7.775789856	7.49781E-15	2.33722E-14	negative
PRJNA729271	stage_5W_vs_E10	ordinary_muscle_development	ENSGALG00010011450	PTDSS1	-0.892032796	0.069869687	-12.76709302	2.5033E-37	2.00916E-36	negative
PRJNA729271	stage_5W_vs_E10	ordinary_muscle_development	ENSGALG00010011451	CRHBP	-9.181336191	0.818375895	-11.21897193	3.29074E-29	1.93742E-28	negative
PRJNA729271	stage_5W_vs_E10	ordinary_muscle_development	ENSGALG00010011452	KITLG	1.336845834	0.188566348	7.089524976	1.34573E-12	3.72615E-12	positive
PRJNA729271	stage_5W_vs_E10	ordinary_muscle_development	ENSGALG00010011453	GNPTAB	0.290838726	0.124397029	2.337987716	0.019387885	0.025671636	positive
PRJNA729271	stage_5W_vs_E10	ordinary_muscle_development	ENSGALG00010011456	KIAA1328	-0.694821654	0.09755536	-7.122331918	1.06116E-12	2.95742E-12	negative
PRJNA729271	stage_5W_vs_E10	ordinary_muscle_development	ENSGALG00010011457	ENSGALG00010011457	1.276488744	0.262060809	4.870963914	1.11055E-06	2.12423E-06	positive
PRJNA729271	stage_5W_vs_E10	ordinary_muscle_development	ENSGALG00010011458	TPGS2	-0.695634347	0.129286223	-5.38057597	7.42479E-08	1.5443E-07	negative
PRJNA729271	stage_5W_vs_E10	ordinary_muscle_development	ENSGALG00010011459	ENSGALG00010011459	0.39282327	0.276520341	1.420594479	0.155434684	0.184055694	positive
PRJNA729271	stage_5W_vs_E10	ordinary_muscle_development	ENSGALG00010011460	LAMC2	-1.143752806	0.618649491	-1.848789698	0.064488185	0.080215931	negative
PRJNA729271	stage_5W_vs_E10	ordinary_muscle_development	ENSGALG00010011461	IRF4	2.42501587	0.342705752	7.076087451	1.48281E-12	4.0971E-12	positive
PRJNA729271	stage_5W_vs_E10	ordinary_muscle_development	ENSGALG00010011463	IMP3	-0.55026582	0.131051517	-4.198851207	2.68273E-05	4.61885E-05	negative
PRJNA729271	stage_5W_vs_E10	ordinary_muscle_development	ENSGALG00010011464	FANCG	-1.769793004	0.164186995	-10.77913024	4.31951E-27	2.33624E-26	negative
PRJNA729271	stage_5W_vs_E10	ordinary_muscle_development	ENSGALG00010011465	PPP2R2C	-1.675820216	0.344380155	-4.866192759	1.13769E-06	2.17355E-06	negative
PRJNA729271	stage_5W_vs_E10	ordinary_muscle_development	ENSGALG00010011466	MTMR1	-0.188616134	0.066970238	-2.816417259	0.004856255	0.006866116	negative
PRJNA729271	stage_5W_vs_E10	ordinary_muscle_development	ENSGALG00010011467	ENSGALG00010011467	4.307862245	3.967595105	1.085761559			positive
PRJNA729271	stage_5W_vs_E10	ordinary_muscle_development	ENSGALG00010011468	EFHC2	-1.392483822	0.299120322	-4.655263182	3.23567E-06	5.98304E-06	negative
PRJNA729271	stage_5W_vs_E10	ordinary_muscle_development	ENSGALG00010011469	CPS1	1.405578085	0.331833636	4.235791468	2.27748E-05	3.94834E-05	positive
PRJNA729271	stage_5W_vs_E10	ordinary_muscle_development	ENSGALG00010011471	NMNAT2	-2.74719295	0.423898817	-6.480775219	9.12525E-11	2.27896E-10	negative
PRJNA729271	stage_5W_vs_E10	ordinary_muscle_development	ENSGALG00010011475	NDP	-3.269168427	0.343053104	-9.52962788	1.57848E-21	6.76125E-21	negative
PRJNA729271	stage_5W_vs_E10	ordinary_muscle_development	ENSGALG00010011477	SDC2	0.062775188	0.167326662	0.375165483	0.707537399	0.739260045	positive
PRJNA729271	stage_5W_vs_E10	ordinary_muscle_development	ENSGALG00010011478	HDAC3	-0.875548031	0.078111214	-11.20899268	3.68362E-29	2.16289E-28	negative
PRJNA729271	stage_5W_vs_E10	ordinary_muscle_development	ENSGALG00010011480	BLTP3B	-0.08208537	0.064951179	-1.263801089	0.20630143	0.239437844	negative
PRJNA729271	stage_5W_vs_E10	ordinary_muscle_development	ENSGALG00010011482	DMRT2	-0.711039077	0.160887474	-4.419480651	9.89384E-06	1.76506E-05	negative
PRJNA729271	stage_5W_vs_E10	ordinary_muscle_development	ENSGALG00010011484	CHPT1	0.920582402	0.078289576	11.75868423	6.37195E-32	4.16794E-31	positive
PRJNA729271	stage_5W_vs_E10	ordinary_muscle_development	ENSGALG00010011485	MTM1	1.176004392	0.076151909	15.44287484	8.42593E-54	1.12152E-52	positive
PRJNA729271	stage_5W_vs_E10	ordinary_muscle_development	ENSGALG00010011487	MAN2B2	-0.332631153	0.158569234	-2.09770296	0.035931393	0.046192953	negative
PRJNA729271	stage_5W_vs_E10	ordinary_muscle_development	ENSGALG00010011493	ENSGALG00010011493	1.200692515	0.261052231	4.599434031	4.2364E-06	7.75533E-06	positive
PRJNA729271	stage_5W_vs_E10	ordinary_muscle_development	ENSGALG00010011496	ACTR6	-1.183681539	0.088105649	-13.43479741	3.78102E-41	3.42109E-40	negative
PRJNA729271	stage_5W_vs_E10	ordinary_muscle_development	ENSGALG00010011497	CPQ	0.453254223	0.249580269	1.816065926	0.06936026	0.086039963	positive
PRJNA729271	stage_5W_vs_E10	ordinary_muscle_development	ENSGALG00010011499	SMG7	0.317413415	0.146688571	2.163859214	0.030475156	0.039511394	positive
PRJNA729271	stage_5W_vs_E10	ordinary_muscle_development	ENSGALG00010011503	NCF2	2.210124682	0.363260509	6.084131435	1.17124E-09	2.72726E-09	positive
PRJNA729271	stage_5W_vs_E10	ordinary_muscle_development	ENSGALG00010011505	MYL1	5.813722431	0.152231681	38.1899641	4.1327E-319	1.872E-316	positive
PRJNA729271	stage_5W_vs_E10	ordinary_muscle_development	ENSGALG00010011509	EXOC2	0.362247386	0.067280158	5.384163761	7.27822E-08	1.51469E-07	positive
PRJNA729271	stage_5W_vs_E10	ordinary_muscle_development	ENSGALG00010011510	ENSGALG00010011510	1.158187998	0.259903793	4.456218148	8.34181E-06	1.49462E-05	positive
PRJNA729271	stage_5W_vs_E10	ordinary_muscle_development	ENSGALG00010011515	SERINC5	-0.368388111	0.086324683	-4.267471344	1.97701E-05	3.44267E-05	negative
PRJNA729271	stage_5W_vs_E10	ordinary_muscle_development	ENSGALG00010011523	PIGO	0.123137438	0.120478785	1.022067395	0.306749004	0.345560489	positive
PRJNA729271	stage_5W_vs_E10	ordinary_muscle_development	ENSGALG00010011525	ENSGALG00010011525	-1.984074132	0.246379613	-8.052915221	8.08449E-16	2.65304E-15	negative
PRJNA729271	stage_5W_vs_E10	ordinary_muscle_development	ENSGALG00010011529	SCYL2	0.249487883	0.082296695	3.03156625	0.002432885	0.003549539	positive
PRJNA729271	stage_5W_vs_E10	ordinary_muscle_development	ENSGALG00010011530	ARPC5	-0.997427158	0.120007433	-8.311378139	9.4597E-17	3.25002E-16	negative
PRJNA729271	stage_5W_vs_E10	ordinary_muscle_development	ENSGALG00010011531	MTDH	0.303935333	0.071922778	4.225856438	2.38034E-05	4.11878E-05	positive
PRJNA729271	stage_5W_vs_E10	ordinary_muscle_development	ENSGALG00010011533	ENSGALG00010011533	-0.369759283	0.273542649	-1.351742715	0.176457635	0.207189423	negative
PRJNA729271	stage_5W_vs_E10	ordinary_muscle_development	ENSGALG00010011536	FCHSD1	-0.670784391	0.177280652	-3.783742809	0.000154487	0.000250173	negative

PRJNA729271	stage_5W_vs_E10	ordinary_muscle_development	ENSGALG00010011538	ENSGALG00010011538	2.888903711	0.253689784	11.38754451	4.82393E-30	2.92197E-29	positive
PRJNA729271	stage_5W_vs_E10	ordinary_muscle_development	ENSGALG00010011539	LANCL1	-0.061020334	0.106625773	-0.572285035	0.567128886	0.606234013	negative
PRJNA729271	stage_5W_vs_E10	ordinary_muscle_development	ENSGALG00010011544	STOML2	-0.358426434	0.092544474	-3.873018222	0.000107496	0.000176501	negative
PRJNA729271	stage_5W_vs_E10	ordinary_muscle_development	ENSGALG00010011545	USP12-like	-0.540540143	0.077011118	-7.019888382	2.2348E-12	6.11428E-12	negative
PRJNA729271	stage_5W_vs_E10	ordinary_muscle_development	ENSGALG00010011546	MAOA	2.624859409	0.125609029	20.89705993	5.69461E-97	2.26146E-95	positive
PRJNA729271	stage_5W_vs_E10	ordinary_muscle_development	ENSGALG00010011549	KIAA0232	0.743639675	0.143894887	5.167936754	2.36692E-07	4.75122E-07	positive
PRJNA729271	stage_5W_vs_E10	ordinary_muscle_development	ENSGALG00010011551	GMDS	-1.310512227	0.155723282	-8.415647336	3.90731E-17	1.36737E-16	negative
PRJNA729271	stage_5W_vs_E10	ordinary_muscle_development	ENSGALG00010011555	ENSGALG00010011555	2.228290413	4.209975359	0.529288232	0.596605518	0.634700696	positive
PRJNA729271	stage_5W_vs_E10	ordinary_muscle_development	ENSGALG00010011556	LAPTM4B	-0.679292119	0.144945859	-4.686523125	2.77885E-06	5.16271E-06	negative
PRJNA729271	stage_5W_vs_E10	ordinary_muscle_development	ENSGALG00010011558	MYBPC1	-0.67633818	0.560043548	-1.207652839			negative
PRJNA729271	stage_5W_vs_E10	ordinary_muscle_development	ENSGALG00010011559	MTX3	0.110154104	0.127112504	0.866587477	0.386168077	0.427453706	positive
PRJNA729271	stage_5W_vs_E10	ordinary_muscle_development	ENSGALG00010011562	ENSGALG00010011562	-8.871453926	0.892338086	-9.941808007	2.73812E-23	1.26479E-22	negative
PRJNA729271	stage_5W_vs_E10	ordinary_muscle_development	ENSGALG00010011564	ARAP3	1.505863074	0.121461159	12.39789809	2.68268E-35	2.00028E-34	positive
PRJNA729271	stage_5W_vs_E10	ordinary_muscle_development	ENSGALG00010011570	RGL1	-0.889588184	0.136985226	-6.4940447	8.35619E-11	2.09374E-10	negative
PRJNA729271	stage_5W_vs_E10	ordinary_muscle_development	ENSGALG00010011571	ATOSB	1.132880743	0.114498463	9.894287783	4.40736E-23	2.01656E-22	positive
PRJNA729271	stage_5W_vs_E10	ordinary_muscle_development	ENSGALG00010011572	PTCD2	0.729284576	0.102420196	7.120515305	1.07524E-12	2.99494E-12	positive
PRJNA729271	stage_5W_vs_E10	ordinary_muscle_development	ENSGALG00010011573	CMAS	0.237042573	0.177982061	1.331834076	0.182914718	0.214337011	positive
PRJNA729271	stage_5W_vs_E10	ordinary_muscle_development	ENSGALG00010011574	GAS2L3	-1.311866573	0.154343086	-8.499678267	1.90117E-17	6.75925E-17	negative
PRJNA729271	stage_5W_vs_E10	ordinary_muscle_development	ENSGALG00010011575	ENSGALG00010011575	-1.999355107	0.470737713	-4.247280492	2.16381E-05	3.75577E-05	negative
PRJNA729271	stage_5W_vs_E10	ordinary_muscle_development	ENSGALG00010011577	ENSGALG00010011577	0.295581583	0.160532374	1.841258401	0.065583701	0.081501692	positive
PRJNA729271	stage_5W_vs_E10	ordinary_muscle_development	ENSGALG00010011578	MYLK4	3.52237438	0.328472777	10.72348951	7.89667E-27	4.22994E-26	positive
PRJNA729271	stage_5W_vs_E10	ordinary_muscle_development	ENSGALG00010011584	ENSGALG00010011584	-3.43039065	1.062551203	-3.228447384	0.001244642	0.001867413	negative
PRJNA729271	stage_5W_vs_E10	ordinary_muscle_development	ENSGALG00010011586	ENSGALG00010011586	0.003095541	0.253352009	0.01221834	0.990251418	0.991619641	positive
PRJNA729271	stage_5W_vs_E10	ordinary_muscle_development	ENSGALG00010011588	COLGALT2	-0.170002502	0.198994291	-0.854308434	0.392934115	0.434213616	negative
PRJNA729271	stage_5W_vs_E10	ordinary_muscle_development	ENSGALG00010011589	RERG	-2.47611141	0.226638599	-10.92537381	8.71807E-28	4.84913E-27	negative
PRJNA729271	stage_5W_vs_E10	ordinary_muscle_development	ENSGALG00010011593	WRNIP1	-0.960848865	0.104634699	-9.182889365	4.19676E-20	1.68323E-19	negative
PRJNA729271	stage_5W_vs_E10	ordinary_muscle_development	ENSGALG00010011595	ENSGALG00010011595	0.290307119	0.164661726	1.763051599	0.077891787	0.095933508	positive
PRJNA729271	stage_5W_vs_E10	ordinary_muscle_development	ENSGALG00010011597	TBC1D14	-0.342604606	0.130561658	-2.624082841	0.008688265	0.011966591	negative
PRJNA729271	stage_5W_vs_E10	ordinary_muscle_development	ENSGALG00010011601	ANO4	0.812950772	0.454374536	1.789164462	0.073588333	0.09095011	positive
PRJNA729271	stage_5W_vs_E10	ordinary_muscle_development	ENSGALG00010011603	ENSGALG00010011603	0.956130546	0.273089817	3.501157807	0.000463241	0.000721001	positive
PRJNA729271	stage_5W_vs_E10	ordinary_muscle_development	ENSGALG00010011605	APPL2	-0.788969601	0.142471653	-5.537730393	3.06416E-08	6.54317E-08	negative
PRJNA729271	stage_5W_vs_E10	ordinary_muscle_development	ENSGALG00010011607	TSEN15	0.042600069	0.162456723	0.262224108	0.793148668	0.817745924	positive
PRJNA729271	stage_5W_vs_E10	ordinary_muscle_development	ENSGALG00010011609	ENSGALG00010011609	-0.756141767	0.923759841	-0.818547997	0.413044347	0.454634203	negative
PRJNA729271	stage_5W_vs_E10	ordinary_muscle_development	ENSGALG00010011612	ENSGALG00010011612	-0.988844278	0.487865474	-2.026878986	0.04267479	0.054298726	negative
PRJNA729271	stage_5W_vs_E10	ordinary_muscle_development	ENSGALG00010011617	MAMLD1	1.385038324	0.096156636	14.4039807	4.88471E-47	5.3236E-46	positive
PRJNA729271	stage_5W_vs_E10	ordinary_muscle_development	ENSGALG00010011619	PCDH1	0.002533728	0.160316726	0.015804516	0.987390345	0.989233001	positive
PRJNA729271	stage_5W_vs_E10	ordinary_muscle_development	ENSGALG00010011621	WASHC4	0.447790408	0.088066641	5.084676844	3.68253E-07	7.28613E-07	positive
PRJNA729271	stage_5W_vs_E10	ordinary_muscle_development	ENSGALG00010011622	BACH1	0.403951542	0.160107086	2.52300852	0.011635558	0.015821521	positive
PRJNA729271	stage_5W_vs_E10	ordinary_muscle_development	ENSGALG00010011625	ERBB4	2.283787298	0.216163978	10.56506877	4.32651E-26	2.24616E-25	positive
PRJNA729271	stage_5W_vs_E10	ordinary_muscle_development	ENSGALG00010011626	SERPINB1	3.395332477	0.553460471	6.134733473	8.53021E-10	1.99782E-09	positive
PRJNA729271	stage_5W_vs_E10	ordinary_muscle_development	ENSGALG00010011628	MATN2	1.219570042	0.192044729	6.350447864	2.14689E-10	5.23011E-10	positive
PRJNA729271	stage_5W_vs_E10	ordinary_muscle_development	ENSGALG00010011630	DELE1	0.43486939	0.101300915	4.292847619	1.76396E-05	3.08427E-05	positive
PRJNA729271	stage_5W_vs_E10	ordinary_muscle_development	ENSGALG00010011633	TADA2B	-0.256846856	0.087823312	-2.924586323	0.003449144	0.004952976	negative
PRJNA729271	stage_5W_vs_E10	ordinary_muscle_development	ENSGALG00010011634	AVD	8.49130578	0.882733073	9.619335723	6.62577E-22	2.89104E-21	positive
PRJNA729271	stage_5W_vs_E10	ordinary_muscle_development	ENSGALG00010011635	PCDH12	-0.380647264	0.197281677	-1.929460807	0.053673681	0.067417678	negative
PRJNA729271	stage_5W_vs_E10	ordinary_muscle_development	ENSGALG00010011636	GRIK1	0.553229936	0.396162959	1.396470629	0.162572819	0.19208453	positive
PRJNA729271	stage_5W_vs_E10	ordinary_muscle_development	ENSGALG00010011637	CECR2	-0.085358068	0.155609715	-0.548539454	0.583321546	0.621709251	negative
PRJNA729271	stage_5W_vs_E10	ordinary_muscle_development	ENSGALG00010011638	GRPEL1	0.264874933	0.081768641	3.239321708	0.001198143	0.001801378	positive
PRJNA729271	stage_5W_vs_E10	ordinary_muscle_development	ENSGALG00010011639	ENSGALG00010011639	-0.332565229	0.146379996	-2.271930857	0.023090687	0.030295031	negative
PRJNA729271	stage_5W_vs_E10	ordinary_muscle_development	ENSGALG00010011641	ENSGALG00010011641	-1.748255599	0.583803089	-2.994598063	0.002748067	0.003986512	negative
PRJNA729271	stage_5W_vs_E10	ordinary_muscle_development	ENSGALG00010011643	C1orf21	0.873225275	0.080305675	10.87376797	1.53718E-27	8.45276E-27	positive
PRJNA729271	stage_5W_vs_E10	ordinary_muscle_development	ENSGALG00010011647	FBXO38	0.210488815	0.083417	2.523332346	0.011624848	0.015809924	positive
PRJNA729271	stage_5W_vs_E10	ordinary_muscle_development	ENSGALG00010011648	ENSGALG00010011648	0.781243005	0.219781456	3.554635679	0.000378503	0.000593729	positive
PRJNA729271	stage_5W_vs_E10	ordinary_muscle_development	ENSGALG00010011649	MRPS27	0.060825505	0.137377303	0.442762407	0.657937598	0.692477888	positive
PRJNA729271	stage_5W_vs_E10	ordinary_muscle_development	ENSGALG00010011650	RPL30	0.052242503	0.155382805	0.336218046	0.736706446	0.765883342	positive
PRJNA729271	stage_5W_vs_E10	ordinary_muscle_development	ENSGALG00010011651	SERPINB6	0.14549022	0.128111846	1.135650015	0.256103068	0.292300312	positive
PRJNA729271	stage_5W_vs_E10	ordinary_muscle_development	ENSGALG00010011653	NR2C1	-0.099532291	0.089131656	-1.116688445	0.26412756	0.300866718	negative
PRJNA729271	stage_5W_vs_E10	ordinary_muscle_development	ENSGALG00010011654	CLDN8	-2.652136894	1.364434345	-1.943762926	0.051924046	0.065350269	negative
PRJNA729271	stage_5W_vs_E10	ordinary_muscle_development	ENSGALG00010011655	SORCS2	-0.570871133	0.184943784	-3.086727869	0.002023728	0.002972632	negative
PRJNA729271	stage_5W_vs_E10	ordinary_muscle_development	ENSGALG00010011657	ALDH1L2	0.171744934	0.142418288	1.205919096	0.227848687	0.26232538	positive
PRJNA729271	stage_5W_vs_E10	ordinary_muscle_development	ENSGALG00010011658	TENM1	-1.05402318	0.295308373	-3.569228903	0.000358033	0.000563057	negative
PRJNA729271	stage_5W_vs_E10	ordinary_muscle_development	ENSGALG00010011659	CD14	1.463381029	0.152111225	9.620467041	6.55329E-22	2.86028E-21	positive
PRJNA729271	stage_5W_vs_E10	ordinary_muscle_development	ENSGALG00010011660	ENSGALG00010011660	-0.786746274	0.088226684	-8.917327979	4.77673E-19	1.8264E-18	negative
PRJNA729271	stage_5W_vs_E10	ordinary_muscle_development	ENSGALG00010011662	RIDA	-1.051921104	0.171506537	-6.133416949	8.60113E-10	2.01411E-09	negative
PRJNA729271	stage_5W_vs_E10	ordinary_muscle_development	ENSGALG00010011663	UTP20	-0.542936279	0.137343032	-3.953140338	7.71322E-05	0.000128435	negative
PRJNA729271	stage_5W_vs_E10	ordinary_muscle_development	ENSGALG00010011664	SERPINB10B	2.599689569	0.549901391	4.727555909	2.27239E-06	4.24407E-06	positive
PRJNA729271	stage_5W_vs_E10	ordinary_muscle_development	ENSGALG00010011667	ENSGALG00010011667	-0.481265685	0.411522051	-1.169477271	0.242211394	0.27764762	negative
PRJNA729271	stage_5W_vs_E10	ordinary_muscle_development	ENSGALG00010011669	ENSGALG00010011669	-5.888115511	0.777643938	-7.571737174	3.68266E-14	1.10752E-13	negative
PRJNA729271	stage_5W_vs_E10	ordinary_muscle_development	ENSGALG00010011670	TMCO6	-0.00549188	0.171891902	-0.031949612	0.974512234	0.97808855	negative
PRJNA729271	stage_5W_vs_E10	ordinary_muscle_development	ENSGALG00010011671	SLC41A2	-1.30552097	0.194726621	-6.704378507	2.02265E-11	5.26173E-11	negative
PRJNA729271	stage_5W_vs_E10	ordinary_muscle_development	ENSGALG00010011673	POP1	-1.360408771	0.126446427	-10.75877592	5.38811E-27	2.89906E-26	negative
PRJNA729271	stage_5W_vs_E10	ordinary_muscle_development	ENSGALG00010011674	ENSGALG00010011674	-1.518337173	0.509018134	-2.982874424	0.002855551	0.004137053	negative
PRJNA729271	stage_5W_vs_E10	ordinary_muscle_development	ENSGALG00010011677	EDEM3	0.019937554	0.079670335	0.250250663	0.802393508	0.825867634	positive
PRJNA729271	stage_5W_vs_E10	ordinary_muscle_development	ENSGALG00010011679	OVAL	-7.871410674	1.214507677	-6.481153497	9.1024E-11	2.27364E-10	negative
PRJNA729271	stage_5W_vs_E10	ordinary_muscle_development	ENSGALG00010011681	MAP1B	-1.651363207	0.358827252	-4.60211202	4.18228E-06	7.65915E-06	negative
PRJNA729271	stage_5W_vs_E10	ordinary_muscle_development	ENSGALG00010011682	USP15	1.606974682	0.122394111	13.12950982	2.23096E-39	1.89999E-38	positive
PRJNA729271	stage_5W_vs_E10	ordinary_muscle_development	ENSGALG00010011683	NIPAL2	-1.638216827	0.290476855	-5.63974995	1.70297E-08	3.7064E-08	negative
PRJNA729271	stage_5W_vs_E10	ordinary_muscle_development	ENSGALG00010011685	NDUFA2	1.168115837	0.111664824	10.4609115	1.30593E-25	6.62334E-25	positive
PRJNA729271	stage_5W_vs_E10	ordinary_muscle_development	ENSGALG00010011688	PDE3A	0.386254507	0.147383075	2.620752127	0.008773603	0.01207839	positive
PRJNA729271	stage_5W_vs_E10	ordinary_muscle_development	ENSGALG00010011692	ARL1	-0.624363299	0.089762032	-6.955761625	3.50662E-12	9.50243E-12	negative
PRJNA729271	stage_5W_vs_E10	ordinary_muscle_development	ENSGALG00010011696	ZFC3H1	0.410153244	0.109164363	3.757208246	0.00017182	0.000277403	positive
PRJNA729271	stage_5W_vs_E10	ordinary_muscle_development	ENSGALG00010011699	TIAM1	-1.115450386	0.144728366	-7.70719949	1.28609E-14	3.95373E-14	negative
PRJNA729271	stage_5W_vs_E10	ordinary_muscle_development	ENSGALG00010011702	NUP205	-0.660869862	0.083982434	-7.869143964	3.57076E-15	1.13256E-14	negative
PRJNA729271	stage_5W_vs_E10	ordinary_muscle_development	ENSGALG00010011703	YWHAZ	-1.173516536	0.094479353	-12.42087815	2.01339E-35	1.509E-34	negative
PRJNA729271	stage_5W_vs_E10	ordinary_muscle_development	ENSGALG00010011708	IK	0.450828924	0.091473684	4.928509541	8.28593E-07	1.60182E-06	positive
PRJNA729271	stage_5W_vs_E10	ordinary_muscle_development	ENSGALG00010011712	DCAF12L2	0.05606562	0.076192868	0.735838165	0.461829192	0.503740999	positive
PRJNA729271	stage_5W_vs_E10	ordinary_muscle_development	ENSGALG00010011713	AVPR1A	-3.273764265	0.804799349	-4.06780183	4.74587E-05	8.02139E-05	negative
PRJNA729271	stage_5W_vs_E10	ordinary_muscle_development	ENSGALG00010011715	SLCO1B3	2.747514667	1.744477772	1.574978317	0.11526145	0.13891367	positive
PRJNA729271	stage_5W_vs_E10	ordinary_muscle_development	ENSGALG00010011720	IKZF2	0.836894137	0.172234834	4.859029489	1.17963E-06	2.25101E-06	positive
PRJNA729271	stage_5W_vs_E10	ordinary_muscle_development	ENSGALG00010011723	SPRY3	2.394619802	0.155580589	15.39150751	1.86633E-53	2.46829E-52	positive
PRJNA729271	stage_5W_vs_E10	ordinary_muscle_development	ENSGALG00010011724	ARHGEF28	-0.045025735	0.15042902	-0.299315483	0.764699342	0.79241614	negative
PRJNA729271	stage_5W_vs_E10	ordinary_muscle_development	ENSGALG00010011726	WDR55	-0.671143806	0.088719791	-7.564758629	3.88585E-14	1.16688E-13	negative
PRJNA729271	stage_5W_vs_E10	ordinary_muscle_development	ENSGALG00010011729	FAM129A	-0.482891062	0.232536297	-2.076626612	0.037836035	0.048503876	negative
PRJNA729271	stage_5W_vs_E10	ordinary_muscle_development	ENSGALG00010011734	ENSGALG00010011734	-1.693876974	0.299658516	-5.65269092	1.57955E-08	3.44865E-08	negative
PRJNA729271	stage_5W_vs_E10	ordinary_muscle_development	ENSGALG00010011738	FGD6	0.717533833	0.11728321	6.117958699	9.47816E-10	2.21555E-09	positive
PRJNA729271	stage_5W_vs_E10	ordinary_muscle_development	ENSGALG00010011745	AFAP1L1	-0.686592414	0.192940953	-3.558562367	0.00037289	0.000585405	negative
PRJNA729271	stage_5W_vs_E10	ordinary_muscle_development	ENSGALG00010011746	SOD1	0.68840379	0.142342041	4.836264701	1.32302E-06	2.51635E-06	positive
PRJNA729271	stage_5W_vs_E10	ordinary_muscle_development	ENSGALG00010011747	UTP15	-0.961817028	0.117841426	-8.161960189	3.29631E-16	1.10245E-15	negative
PRJNA729271	stage_5W_vs_E10	ordinary_muscle_development	ENSGALG00010011751	ZNF706	1.238270697	0.109421136	11.31655863	1.08654E-29	6.49459E-29	positive
PRJNA729271	stage_5W_vs_E10	ordinary_muscle_development	ENSGALG00010011752	RNF2	-2.538020029	0.13137662	-19.31865835	3.74252E-83	1.0915E-81	negative
PRJNA729271	stage_5W_vs_E10	ordinary_muscle_development	ENSGALG00010011758	SERPINB11	0.102875861	0.512214806	0.200845152	0.840819656	0.859922452	positive
PRJNA729271	stage_5W_vs_E10	ordinary_muscle_development	ENSGALG00010011759	CREB3	0.423236994	0.130436583	3.244772176	0.001175446	0.001768538	positive
PRJNA729271	stage_5W_vs_E10	ordinary_muscle_development	ENSGALG00010011764	PCYOX1L	-0.905042042	0.135907252	-6.659262334	2.75205E-11	7.10184E-11	negative
PRJNA729271	stage_5W_vs_E10	ordinary_muscle_development	ENSGALG00010011770	CHST11	-3.718175135	0.308554628	-12.05029772	1.93251E-33	1.33835E-32	negative
PRJNA729271	stage_5W_vs_E10	ordinary_muscle_development	ENSGALG00010011772	GRHL2	-2.492995724	0.460830678	-5.409786809	6.30998E-08	1.31924E-07	negative
PRJNA729271	stage_5W_vs_E10	ordinary_muscle_development	ENSGALG00010011774	ANKRA2	-0.531856932	0.094264815	-5.642157487	1.67932E-08	3.65767E-08	negative
PRJNA729271	stage_5W_vs_E10	ordinary_muscle_development	ENSGALG00010011775	VEZT	0.339857666	0.080126963	4.241489402	2.22041E-05	3.85217E-05	positive
PRJNA729271	stage_5W_vs_E10	ordinary_muscle_development	ENSGALG00010011776	IL17RA	-0.149207643	0.137029798	-1.088870054	0.276211194	0.313399707	negative
PRJNA729271	stage_5W_vs_E10	ordinary_muscle_development	ENSGALG00010011780	SERPINB5	-4.672819788	0.757635133	-6.167638724	6.93173E-10	1.63268E-09	negative
PRJNA729271	stage_5W_vs_E10	ordinary_muscle_development	ENSGALG00010011784	GRPEL2	0.089596147	0.1341071	0.66809399	0.504073598	0.544979996	positive
PRJNA729271	stage_5W_vs_E10	ordinary_muscle_development	ENSGALG00010011789	TXNRD1	-0.644054655	0.135385297	-4.757197935	1.96299E-06	3.6833E-06	negative
PRJNA729271	stage_5W_vs_E10	ordinary_muscle_development	ENSGALG00010011790	AMY2A	1.749159432	1.110349684	1.575323033	0.1151819	0.13882934	positive
PRJNA729271	stage_5W_vs_E10	ordinary_muscle_development	ENSGALG00010011792	HDHD5	1.365609042	0.141104909	9.677969781	3.7407E-22	1.64907E-21	positive
PRJNA729271	stage_5W_vs_E10	ordinary_muscle_development	ENSGALG00010011793	TLN1	0.678707757	0.113985557	5.954331199	2.61137E-09	5.9553E-09	positive
PRJNA729271	stage_5W_vs_E10	ordinary_muscle_development	ENSGALG00010011796	METAP2	0.413399316	0.089324537	4.628059982	3.69107E-06	6.7896E-06	positive
PRJNA729271	stage_5W_vs_E10	ordinary_muscle_development	ENSGALG00010011797	SCAF4	0.109249201	0.156456393	0.698272528	0.485006776	0.526604736	positive
PRJNA729271	stage_5W_vs_E10	ordinary_muscle_development	ENSGALG00010011802	VPS4B	0.276238816	0.092625085	2.982332655	0.00286061	0.004143554	positive
PRJNA729271	stage_5W_vs_E10	ordinary_muscle_development	ENSGALG00010011808	SMARCA2	0.542563095	0.122647904	4.423745363	9.70043E-06	1.73141E-05	positive
PRJNA729271	stage_5W_vs_E10	ordinary_muscle_development	ENSGALG00010011810	FGFR4	-0.546071493	0.126309412	-4.323284281	1.53723E-05	2.70186E-05	negative
PRJNA729271	stage_5W_vs_E10	ordinary_muscle_development	ENSGALG00010011814	ENSGALG00010011814	2.207157362	0.528508377	4.176201286	2.96418E-05	5.08831E-05	positive
PRJNA729271	stage_5W_vs_E10	ordinary_muscle_development	ENSGALG00010011817	KDSR	0.63849758	0.08976164	7.113256655	1.13336E-12	3.15137E-12	positive
PRJNA729271	stage_5W_vs_E10	ordinary_muscle_development	ENSGALG00010011819	USP44	0.795944867	0.139009514	5.725830154	1.02929E-08	2.27121E-08	positive
PRJNA729271	stage_5W_vs_E10	ordinary_muscle_development	ENSGALG00010011823	ENSGALG00010011823	-3.295276126	0.655912695	-5.023955401	5.0618E-07	9.9257E-07	negative
PRJNA729271	stage_5W_vs_E10	ordinary_muscle_development	ENSGALG00010011825	TAFA2	-5.058441881	0.433071298	-11.68039051	1.60556E-31	1.0325E-30	negative
PRJNA729271	stage_5W_vs_E10	ordinary_muscle_development	ENSGALG00010011837	VLDLR	1.85322823	0.134462071	13.78253525	3.24667E-43	3.12902E-42	positive
PRJNA729271	stage_5W_vs_E10	ordinary_muscle_development	ENSGALG00010011839	CBS	-3.249042323	0.462358227	-7.027110436	2.10854E-12	5.78194E-12	negative
PRJNA729271	stage_5W_vs_E10	ordinary_muscle_development	ENSGALG00010011841	KCNMB4	-3.329883243	0.26773792	-12.43709986	1.64364E-35	1.23635E-34	negative
PRJNA729271	stage_5W_vs_E10	ordinary_muscle_development	ENSGALG00010011843	BCL2	0.723489306	0.11192857	6.463848357	1.02073E-10	2.54087E-10	positive
PRJNA729271	stage_5W_vs_E10	ordinary_muscle_development	ENSGALG00010011846	UBAP1	0.555880065	0.105087258	5.289699944	1.22517E-07	2.514E-07	positive
PRJNA729271	stage_5W_vs_E10	ordinary_muscle_development	ENSGALG00010011850	SPAG16	-2.810949118	0.399076869	-7.04362828	1.87297E-12	5.15157E-12	negative
PRJNA729271	stage_5W_vs_E10	ordinary_muscle_development	ENSGALG00010011856	NFYB	-0.170928375	0.129236122	-1.32260527	0.185966681	0.217579065	negative

PRJNA729271	stage_5W_vs_E10	ordinary_muscle_development	ENSGALG00010011857	ENSGALG00010011857	0.529994503	0.244951526	2.163670957	0.030489611	0.0395266	positive
PRJNA729271	stage_5W_vs_E10	ordinary_muscle_development	ENSGALG00010011859	NTN4	2.783655068	0.198714128	14.00834001	1.38607E-44	1.38559E-43	positive
PRJNA729271	stage_5W_vs_E10	ordinary_muscle_development	ENSGALG00010011860	CECR1	0.819783069	0.155837414	5.260502272	1.43662E-07	2.93294E-07	positive
PRJNA729271	stage_5W_vs_E10	ordinary_muscle_development	ENSGALG00010011861	ENSGALG00010011861	-3.886309607	0.738217379	-5.264451525	1.40608E-07	2.87342E-07	negative
PRJNA729271	stage_5W_vs_E10	ordinary_muscle_development	ENSGALG00010011866	KLF8	0.496941409	0.187732003	2.647078821	0.008119043	0.011219899	positive
PRJNA729271	stage_5W_vs_E10	ordinary_muscle_development	ENSGALG00010011867	ENSGALG00010011867	0.264743333	0.275630151	0.960502079	0.336802585	0.376606763	positive
PRJNA729271	stage_5W_vs_E10	ordinary_muscle_development	ENSGALG00010011868	SNRPF	-1.780209775	0.12627967	-14.09735851	3.94253E-45	4.0358E-44	negative
PRJNA729271	stage_5W_vs_E10	ordinary_muscle_development	ENSGALG00010011870	AvBD6	0.12961811	1.564309255	0.082859645	0.933963142	0.94267779	positive
PRJNA729271	stage_5W_vs_E10	ordinary_muscle_development	ENSGALG00010011871	CCT2	-1.289241407	0.098345879	-13.10925708	2.91439E-39	2.47476E-38	negative
PRJNA729271	stage_5W_vs_E10	ordinary_muscle_development	ENSGALG00010011873	RFX3	-0.319772143	0.194549594	-1.643653613	0.100247773	0.121719841	negative
PRJNA729271	stage_5W_vs_E10	ordinary_muscle_development	ENSGALG00010011874	KCNS2	-7.296395837	0.842480475	-8.660611203	4.69241E-18	1.71672E-17	negative
PRJNA729271	stage_5W_vs_E10	ordinary_muscle_development	ENSGALG00010011875	PSD2	-1.834427698	0.295138982	-6.215470713	5.1171E-10	1.21714E-09	negative
PRJNA729271	stage_5W_vs_E10	ordinary_muscle_development	ENSGALG00010011876	AMDHD1	0.049699585	0.45124527	0.11013874	0.912299345	0.923985361	positive
PRJNA729271	stage_5W_vs_E10	ordinary_muscle_development	ENSGALG00010011880	U2AF1	-0.368384069	0.156641201	-2.351769954	0.018684326	0.024794407	negative
PRJNA729271	stage_5W_vs_E10	ordinary_muscle_development	ENSGALG00010011881	RIOK2	-0.951767759	0.097881506	-9.723673056	2.39001E-22	1.06202E-21	negative
PRJNA729271	stage_5W_vs_E10	ordinary_muscle_development	ENSGALG00010011882	ENSGALG00010011882	0.075914265	0.292254181	0.259754247	0.795053345	0.819359988	positive
PRJNA729271	stage_5W_vs_E10	ordinary_muscle_development	ENSGALG00010011883	HCFC2	0.912879362	0.106906581	8.53903801	1.35344E-17	4.85236E-17	positive
PRJNA729271	stage_5W_vs_E10	ordinary_muscle_development	ENSGALG00010011885	NCALD	-0.493459992	0.212204934	-2.325393586	0.020050924	0.026489076	negative
PRJNA729271	stage_5W_vs_E10	ordinary_muscle_development	ENSGALG00010011886	NTNG1	-4.454204869	0.211725955	-21.03759493	2.97068E-98	1.20616E-96	negative
PRJNA729271	stage_5W_vs_E10	ordinary_muscle_development	ENSGALG00010011887	PGM5	2.004771015	0.132342597	15.14834274	7.77013E-52	9.96708E-51	positive
PRJNA729271	stage_5W_vs_E10	ordinary_muscle_development	ENSGALG00010011888	CAND1	-1.242291067	0.070591657	-17.59827041	2.53962E-69	5.25133E-68	negative
PRJNA729271	stage_5W_vs_E10	ordinary_muscle_development	ENSGALG00010011891	HAL	0.40014225	0.559727272	0.714887894	0.474678318	0.516413939	positive
PRJNA729271	stage_5W_vs_E10	ordinary_muscle_development	ENSGALG00010011893	LIX1	-2.450635194	0.45269418	-5.413445326	6.18235E-08	1.29368E-07	negative
PRJNA729271	stage_5W_vs_E10	ordinary_muscle_development	ENSGALG00010011894	VWC2L	-2.584218664	0.391585884	-6.599366245	4.12919E-11	1.05542E-10	negative
PRJNA729271	stage_5W_vs_E10	ordinary_muscle_development	ENSGALG00010011895	PHF21B	-3.11464176	0.903044641	-3.449045172	0.000562573	0.000870555	negative
PRJNA729271	stage_5W_vs_E10	ordinary_muscle_development	ENSGALG00010011896	SIK1	4.629323328	0.298123799	15.5281911	2.23599E-54	3.03755E-53	positive
PRJNA729271	stage_5W_vs_E10	ordinary_muscle_development	ENSGALG00010011898	ENSGALG00010011898	1.688423174	0.109608287	15.4041562	1.53479E-53	2.03353E-52	positive
PRJNA729271	stage_5W_vs_E10	ordinary_muscle_development	ENSGALG00010011899	PHLPP1	0.012860903	0.085263428	0.150837273	0.880104083	0.894796148	positive
PRJNA729271	stage_5W_vs_E10	ordinary_muscle_development	ENSGALG00010011902	TP53I3	-1.679170485	0.26209592	-6.406702115	1.48701E-10	3.65946E-10	negative
PRJNA729271	stage_5W_vs_E10	ordinary_muscle_development	ENSGALG00010011903	LTA4H	-0.063728912	0.115829255	-0.55019703	0.582184241	0.620588364	negative
PRJNA729271	stage_5W_vs_E10	ordinary_muscle_development	ENSGALG00010011904	KIF24	-3.176998492	0.338120638	-9.396050216	5.66498E-21	2.36367E-20	negative
PRJNA729271	stage_5W_vs_E10	ordinary_muscle_development	ENSGALG00010011906	ENSGALG00010011906	-3.766129141	0.964703095	-3.903925632	9.46449E-05	0.000156268	negative
PRJNA729271	stage_5W_vs_E10	ordinary_muscle_development	ENSGALG00010011907	BIK	-1.515208043	0.270342702	-5.604767693	2.08534E-08	4.51216E-08	negative
PRJNA729271	stage_5W_vs_E10	ordinary_muscle_development	ENSGALG00010011908	ENSGALG00010011908	-0.974671268	0.205886358	-4.734025503	2.2011E-06	4.11783E-06	negative
PRJNA729271	stage_5W_vs_E10	ordinary_muscle_development	ENSGALG00010011909	C8orf48	-1.13370909	0.210956346	-5.374140731	7.69487E-08	1.59887E-07	negative
PRJNA729271	stage_5W_vs_E10	ordinary_muscle_development	ENSGALG00010011911	NUDT2	-0.604753043	0.140966248	-4.290055611	1.78628E-05	3.1218E-05	negative
PRJNA729271	stage_5W_vs_E10	ordinary_muscle_development	ENSGALG00010011912	TCP11X2	1.370841553	0.125561459	10.91769369	9.48744E-28	5.25891E-27	positive
PRJNA729271	stage_5W_vs_E10	ordinary_muscle_development	ENSGALG00010011913	CXXC5	0.082140399	0.112656996	0.729119378	0.465928642	0.507792155	positive
PRJNA729271	stage_5W_vs_E10	ordinary_muscle_development	ENSGALG00010011915	RRM2B	0.953434777	0.120928424	7.88429008	3.16329E-15	1.00596E-14	positive
PRJNA729271	stage_5W_vs_E10	ordinary_muscle_development	ENSGALG00010011916	MCAT	0.106752565	0.098114539	1.088040227	0.276577349	0.313766039	positive
PRJNA729271	stage_5W_vs_E10	ordinary_muscle_development	ENSGALG00010011917	HSF2BP	-0.171510752	0.177589947	-0.965768358	0.334160105	0.373853869	negative
PRJNA729271	stage_5W_vs_E10	ordinary_muscle_development	ENSGALG00010011921	MEGF10	0.539430682	0.167311262	3.224114603	0.001263628	0.001894919	positive
PRJNA729271	stage_5W_vs_E10	ordinary_muscle_development	ENSGALG00010011922	PIK3R1	0.321720986	0.173111357	1.858462616	0.063103347	0.078574265	positive
PRJNA729271	stage_5W_vs_E10	ordinary_muscle_development	ENSGALG00010011923	BARD1	-2.62754035	0.168831898	-15.56305637	1.29752E-54	1.76929E-53	negative
PRJNA729271	stage_5W_vs_E10	ordinary_muscle_development	ENSGALG00010011925	RRP1B	-0.653346344	0.44689031	-1.461983688	0.14374568	0.170954515	negative
PRJNA729271	stage_5W_vs_E10	ordinary_muscle_development	ENSGALG00010011926	DYRK2	0.352918348	0.195037473	1.809489956	0.070374919	0.0872389	positive
PRJNA729271	stage_5W_vs_E10	ordinary_muscle_development	ENSGALG00010011927	ENSGALG00010011927	-1.566724462	0.531096333	-2.949981695	0.003177928	0.004582121	negative
PRJNA729271	stage_5W_vs_E10	ordinary_muscle_development	ENSGALG00010011929	ELK3	-0.992828777	0.135713529	-7.315621238	2.56193E-13	7.35205E-13	negative
PRJNA729271	stage_5W_vs_E10	ordinary_muscle_development	ENSGALG00010011930	TSPO	0.269580785	0.151031295	1.784933282	0.074272139	0.091748309	positive
PRJNA729271	stage_5W_vs_E10	ordinary_muscle_development	ENSGALG00010011931	ENSGALG00010011931	1.332977573	1.297679174	1.027201175			positive
PRJNA729271	stage_5W_vs_E10	ordinary_muscle_development	ENSGALG00010011932	VAV3	1.040497829	0.16502944	6.304922496	2.88338E-10	6.96229E-10	positive
PRJNA729271	stage_5W_vs_E10	ordinary_muscle_development	ENSGALG00010011935	ST8SIA4	0.54147255	0.124546884	4.347539915	1.37673E-05	2.43095E-05	positive
PRJNA729271	stage_5W_vs_E10	ordinary_muscle_development	ENSGALG00010011936	CKAP4	-1.699729455	0.175674057	-9.675472195	3.83318E-22	1.68928E-21	negative
PRJNA729271	stage_5W_vs_E10	ordinary_muscle_development	ENSGALG00010011937	SLC1A1	-1.348300292	0.325383833	-4.14372244	3.41713E-05	5.83133E-05	negative
PRJNA729271	stage_5W_vs_E10	ordinary_muscle_development	ENSGALG00010011941	ENSGALG00010011941	-2.765505197	0.243617214	-11.3518464	7.26129E-30	4.37276E-29	negative
PRJNA729271	stage_5W_vs_E10	ordinary_muscle_development	ENSGALG00010011943	TTLL12	-0.797151691	0.056062342	-14.21902219	6.98217E-46	7.3285E-45	negative
PRJNA729271	stage_5W_vs_E10	ordinary_muscle_development	ENSGALG00010011945	ENSGALG00010011945	0.506880983	0.471473834	1.075098864	0.282330468	0.319942157	positive
PRJNA729271	stage_5W_vs_E10	ordinary_muscle_development	ENSGALG00010011947	THAP1	0.904389647	0.113268058	7.984507399	1.41085E-15	4.57909E-15	positive
PRJNA729271	stage_5W_vs_E10	ordinary_muscle_development	ENSGALG00010011949	ABCA12	-2.66904251	0.453205864	-5.889249729	3.87953E-09	8.77008E-09	negative
PRJNA729271	stage_5W_vs_E10	ordinary_muscle_development	ENSGALG00010011950	PDXK	-0.109357192	0.165274774	-0.661668987	0.508183385	0.549013801	negative
PRJNA729271	stage_5W_vs_E10	ordinary_muscle_development	ENSGALG00010011952	UBE2D2	0.783295871	0.173728049	4.508747299	6.52115E-06	1.1767E-05	positive
PRJNA729271	stage_5W_vs_E10	ordinary_muscle_development	ENSGALG00010011953	CDK17	0.536752029	0.058072983	9.242714975	2.40321E-20	9.74118E-20	positive
PRJNA729271	stage_5W_vs_E10	ordinary_muscle_development	ENSGALG00010011954	ENSGALG00010011954	1.847791718	0.441359319	4.186592735	2.83173E-05	4.8673E-05	positive
PRJNA729271	stage_5W_vs_E10	ordinary_muscle_development	ENSGALG00010011957	ENSGALG00010011957	3.504322872	0.209991567	16.68792195	1.60459E-62	2.75576E-61	positive
PRJNA729271	stage_5W_vs_E10	ordinary_muscle_development	ENSGALG00010011960	CFAP54	-0.477525124	0.292945202	-1.630083447	0.103083862	0.12498541	negative
PRJNA729271	stage_5W_vs_E10	ordinary_muscle_development	ENSGALG00010011961	AGPAT3	0.063531809	0.103591349	0.613292617	0.539682874	0.579631243	positive
PRJNA729271	stage_5W_vs_E10	ordinary_muscle_development	ENSGALG00010011962	CPSF6	-1.110658514	0.060525155	-18.3503622	3.27919E-75	8.04262E-74	negative
PRJNA729271	stage_5W_vs_E10	ordinary_muscle_development	ENSGALG00010011963	ZCCHC2	0.235424538	0.100696964	2.337950695	0.019389806	0.025671834	positive
PRJNA729271	stage_5W_vs_E10	ordinary_muscle_development	ENSGALG00010011964	ENSGALG00010011964	3.289610492	4.307764278	0.76364682	0.44507774	0.486971757	positive
PRJNA729271	stage_5W_vs_E10	ordinary_muscle_development	ENSGALG00010011965	ATIC	-0.656829039	0.147211213	-4.46181393	8.12688E-06	1.45755E-05	negative
PRJNA729271	stage_5W_vs_E10	ordinary_muscle_development	ENSGALG00010011966	SLC25A24	-0.009148872	0.138101593	-0.066247403	0.947180857	0.954158491	negative
PRJNA729271	stage_5W_vs_E10	ordinary_muscle_development	ENSGALG00010011967	MTMR9	0.155661144	0.137435524	1.132612145	0.257377166	0.293592667	positive
PRJNA729271	stage_5W_vs_E10	ordinary_muscle_development	ENSGALG00010011968	ENSGALG00010011968	6.268303775	0.223385561	28.06046978	2.9767E-173	3.9225E-171	positive
PRJNA729271	stage_5W_vs_E10	ordinary_muscle_development	ENSGALG00010011969	TMEM173	2.443136231	0.29030656	8.415711425	3.90518E-17	1.36695E-16	positive
PRJNA729271	stage_5W_vs_E10	ordinary_muscle_development	ENSGALG00010011971	RNF170	-0.232337619	0.0902349	-2.574808848	0.01002955	0.013735669	negative
PRJNA729271	stage_5W_vs_E10	ordinary_muscle_development	ENSGALG00010011972	IPO11	-0.146304208	0.08157667	-1.793456497	0.072899961	0.090137757	negative
PRJNA729271	stage_5W_vs_E10	ordinary_muscle_development	ENSGALG00010011973	TRAPPC10	0.284270885	0.049914223	5.695188008	1.23236E-08	2.70571E-08	positive
PRJNA729271	stage_5W_vs_E10	ordinary_muscle_development	ENSGALG00010011976	TNFRSF11A	0.495186976	0.089434039	5.536896062	3.07879E-08	6.57344E-08	positive
PRJNA729271	stage_5W_vs_E10	ordinary_muscle_development	ENSGALG00010011977	GATD3A	1.42174857	0.118220033	12.02629147	2.58514E-33	1.78521E-32	positive
PRJNA729271	stage_5W_vs_E10	ordinary_muscle_development	ENSGALG00010011978	ENSGALG00010011978	3.124678358	5.419719684	0.57653874	0.564251092	0.603513842	positive
PRJNA729271	stage_5W_vs_E10	ordinary_muscle_development	ENSGALG00010011979	NEDD1	0.054150594	0.077302979	0.700498149	0.48361626	0.525252337	positive
PRJNA729271	stage_5W_vs_E10	ordinary_muscle_development	ENSGALG00010011980	AvBD9	22.38897425	2.273949172	9.845855186	7.14291E-23	3.24668E-22	positive
PRJNA729271	stage_5W_vs_E10	ordinary_muscle_development	ENSGALG00010011981	SCUBE1	-4.406506525	0.357284939	-12.33331171	5.9932E-35	4.41195E-34	negative
PRJNA729271	stage_5W_vs_E10	ordinary_muscle_development	ENSGALG00010011982	STK3	-1.027522705	0.106008689	-9.692815863	3.23485E-22	1.43085E-21	negative
PRJNA729271	stage_5W_vs_E10	ordinary_muscle_development	ENSGALG00010011983	LYZ	2.345810007	0.526866033	4.452384212	8.4922E-06	1.521E-05	positive
PRJNA729271	stage_5W_vs_E10	ordinary_muscle_development	ENSGALG00010011984	FN1	0.291646409	0.240234415	1.214007612	0.224744818	0.259080335	positive
PRJNA729271	stage_5W_vs_E10	ordinary_muscle_development	ENSGALG00010011987	H2BC32	-2.367802623	0.375625035	-6.303633686	2.90747E-10	7.01812E-10	negative
PRJNA729271	stage_5W_vs_E10	ordinary_muscle_development	ENSGALG00010011988	HINTW	3.054697042	3.134343119	0.974589228	0.329764059	0.369391811	positive
PRJNA729271	stage_5W_vs_E10	ordinary_muscle_development	ENSGALG00010011989	H1-010	-1.348337063	0.137224241	-9.825793541	8.71847E-23	3.94796E-22	negative
PRJNA729271	stage_5W_vs_E10	ordinary_muscle_development	ENSGALG00010011991	CSNK1A1	-0.157560356	0.056477009	-2.789814093	0.005273831	0.007426092	negative
PRJNA729271	stage_5W_vs_E10	ordinary_muscle_development	ENSGALG00010011992	YEATS4	-1.463750413	0.08835476	-16.56674088	1.2122E-61	2.05267E-60	negative
PRJNA729271	stage_5W_vs_E10	ordinary_muscle_development	ENSGALG00010011993	RELCH	-0.120661958	0.052147771	-2.31384691	0.020676121	0.02727028	negative
PRJNA729271	stage_5W_vs_E10	ordinary_muscle_development	ENSGALG00010011996	LRRC70	1.516097092	0.218378398	6.942523177	3.85158E-12	1.04022E-11	positive
PRJNA729271	stage_5W_vs_E10	ordinary_muscle_development	ENSGALG00010011997	DENND5B	-0.637510079	0.134830168	-4.728245067	2.26469E-06	4.23179E-06	negative
PRJNA729271	stage_5W_vs_E10	ordinary_muscle_development	ENSGALG00010011998	MPPED1	-0.748872448	0.076153601	-9.833710205	8.05937E-23	3.6575E-22	negative
PRJNA729271	stage_5W_vs_E10	ordinary_muscle_development	ENSGALG00010012001	EEIG2	0.618673077	0.252631422	2.448915786	0.014328695	0.019276214	positive
PRJNA729271	stage_5W_vs_E10	ordinary_muscle_development	ENSGALG00010012004	LGR5	-3.521424215	0.206919793	-17.01830537	6.00846E-65	1.10524E-63	negative
PRJNA729271	stage_5W_vs_E10	ordinary_muscle_development	ENSGALG00010012007	MREG	-0.794228221	0.214573902	-3.701420417	0.000214396	0.000343579	negative
PRJNA729271	stage_5W_vs_E10	ordinary_muscle_development	ENSGALG00010012008	ZNF366	4.803797703	0.17527374	27.40740108	2.2385E-165	2.7971E-163	positive
PRJNA729271	stage_5W_vs_E10	ordinary_muscle_development	ENSGALG00010012010	HENMT1	-1.405173132	0.18132296	-7.749559874	9.22116E-15	2.86211E-14	negative
PRJNA729271	stage_5W_vs_E10	ordinary_muscle_development	ENSGALG00010012011	HOOK3	0.879812099	0.068230147	12.89477073	4.81694E-38	3.9336E-37	positive
PRJNA729271	stage_5W_vs_E10	ordinary_muscle_development	ENSGALG00010012013	FOXD1	-4.178594154	0.80893679	-5.165538525	2.39748E-07	4.80989E-07	negative
PRJNA729271	stage_5W_vs_E10	ordinary_muscle_development	ENSGALG00010012015	ENSGALG00010012015	1.065867617	0.215376468	4.948858274	7.46501E-07	1.44814E-06	positive
PRJNA729271	stage_5W_vs_E10	ordinary_muscle_development	ENSGALG00010012016	WDR91	-0.431321531	0.064533379	-6.683696701	2.32989E-11	6.03713E-11	negative
PRJNA729271	stage_5W_vs_E10	ordinary_muscle_development	ENSGALG00010012017	N4BP3	-0.702711503	0.132164051	-5.316964009	1.05513E-07	2.17369E-07	negative
PRJNA729271	stage_5W_vs_E10	ordinary_muscle_development	ENSGALG00010012019	ENSGALG00010012019	7.435259474	0.30067092	24.72889455	5.2304E-135	4.0327E-133	positive
PRJNA729271	stage_5W_vs_E10	ordinary_muscle_development	ENSGALG00010012021	ENSGALG00010012021	-0.04313989	0.130662178	-0.330163563	0.741276377	0.769960665	negative
PRJNA729271	stage_5W_vs_E10	ordinary_muscle_development	ENSGALG00010012024	UBR5	0.252027841	0.0758083	3.324541499	0.000885641	0.001347754	positive
PRJNA729271	stage_5W_vs_E10	ordinary_muscle_development	ENSGALG00010012026	SYT1	-4.175092528	0.302511458	-13.80143602	2.49826E-43	2.42223E-42	negative
PRJNA729271	stage_5W_vs_E10	ordinary_muscle_development	ENSGALG00010012028	TMEM140	5.336353293	0.169822006	31.42321429	9.7515E-217	1.9908E-214	positive
PRJNA729271	stage_5W_vs_E10	ordinary_muscle_development	ENSGALG00010012031	GRK6	-0.074637107	0.10823126	-0.689607669	0.490440944	0.531827746	negative
PRJNA729271	stage_5W_vs_E10	ordinary_muscle_development	ENSGALG00010012034	ARHGEF9	1.24292915	0.150636363	8.251189308	1.56826E-16	5.32363E-16	positive
PRJNA729271	stage_5W_vs_E10	ordinary_muscle_development	ENSGALG00010012035	ENSGALG00010012035	-0.784671841	0.550401279	-1.425635934	0.153973458	0.182414816	negative
PRJNA729271	stage_5W_vs_E10	ordinary_muscle_development	ENSGALG00010012039	ODF1	-3.785492182	0.978777218	-3.867572837	0.000109924	0.000180243	negative
PRJNA729271	stage_5W_vs_E10	ordinary_muscle_development	ENSGALG00010012040	B4GALT7	-0.033117127	0.151636479	-0.218398154	0.827118904	0.847884619	negative
PRJNA729271	stage_5W_vs_E10	ordinary_muscle_development	ENSGALG00010012041	ICOSLG	0.476155184	0.150651135	3.160647835	0.001574187	0.002339092	positive
PRJNA729271	stage_5W_vs_E10	ordinary_muscle_development	ENSGALG00010012042	FNDC7	-4.265545755	0.471818523	-9.040649214	1.55744E-19	6.09973E-19	negative
PRJNA729271	stage_5W_vs_E10	ordinary_muscle_development	ENSGALG00010012043	NUP50	-0.327511083	0.09588956	-3.41550303	0.000636643	0.000981195	negative
PRJNA729271	stage_5W_vs_E10	ordinary_muscle_development	ENSGALG00010012044	ENSGALG00010012044	3.187756203	0.603328896	5.283612678	1.26661E-07	2.59718E-07	positive
PRJNA729271	stage_5W_vs_E10	ordinary_muscle_development	ENSGALG00010012045	ENSGALG00010012045	0.695055528	0.300786532	2.310793383	0.02084427	0.027477055	positive
PRJNA729271	stage_5W_vs_E10	ordinary_muscle_development	ENSGALG00010012046	LOXHD1	-0.464592509	0.277233432	-1.675817037	0.093774036	0.114319146	negative
PRJNA729271	stage_5W_vs_E10	ordinary_muscle_development	ENSGALG00010012047	LMAN2	0.04881495	0.090881121	0.537129705	0.591178023	0.62934235	positive
PRJNA729271	stage_5W_vs_E10	ordinary_muscle_development	ENSGALG00010012048	PDE6H	-1.613678055	0.535805198	-3.011687943	0.002597995	0.003780538	negative
PRJNA729271	stage_5W_vs_E10	ordinary_muscle_development	ENSGALG00010012049	IFNAR1	0.807494399	0.144886608	5.573285271	2.4998E-08	5.38404E-08	positive
PRJNA729271	stage_5W_vs_E10	ordinary_muscle_development	ENSGALG00010012051	ARK2C	-2.147947689	0.208484062	-10.30269494	6.85145E-25	3.39064E-24	negative
PRJNA729271	stage_5W_vs_E10	ordinary_muscle_development	ENSGALG00010012052	KLF10	2.052820823	0.12299936	16.68968701	1.55785E-62	2.67866E-61	positive
PRJNA729271	stage_5W_vs_E10	ordinary_muscle_development	ENSGALG00010012054	FAM60A	-2.166619925	0.093228231	-23.23995526	1.7974E-119	1.0811E-117	negative
PRJNA729271	stage_5W_vs_E10	ordinary_muscle_development	ENSGALG00010012055	ENSGALG00010012055	1.190446727	0.156391121	7.611984119	2.6992E-14	8.17486E-14	positive
PRJNA729271	stage_5W_vs_E10	ordinary_muscle_development	ENSGALG00010012058	ENSGALG00010012058	-2.481921047	0.334466758	-7.42053131	1.16651E-13	3.40694E-13	negative
PRJNA729271	stage_5W_vs_E10	ordinary_muscle_development	ENSGALG00010012059	ARHGDIB	-0.814919442	0.14634859	-5.568345006	2.5717E-08	5.52987E-08	negative
PRJNA729271	stage_5W_vs_E10	ordinary_muscle_development	ENSGALG00010012060	ENSGALG00010012060	1.391362518	0.124142329	11.20780099	3.73354E-29	2.19011E-28	positive

PRJNA729271	stage_5W_vs_E10	ordinary_muscle_development	ENSGALG00010012061	AZIN1	1.080448758	0.120443673	8.970572948	2.94976E-19	1.13897E-18	positive
PRJNA729271	stage_5W_vs_E10	ordinary_muscle_development	ENSGALG00010012062	HMGA2	-6.694815462	0.193793758	-34.54608406	1.632E-261	5.0333E-259	negative
PRJNA729271	stage_5W_vs_E10	ordinary_muscle_development	ENSGALG00010012063	PTPRD	-0.664545493	0.196662904	-3.379109534	0.00072721	0.001114615	negative
PRJNA729271	stage_5W_vs_E10	ordinary_muscle_development	ENSGALG00010012064	RGS14	-0.13030396	0.171347078	-0.760467946	0.446974923	0.488752377	negative
PRJNA729271	stage_5W_vs_E10	ordinary_muscle_development	ENSGALG00010012066	STXBP3	-0.781885454	0.123795031	-6.315968001	2.68476E-10	6.49567E-10	negative
PRJNA729271	stage_5W_vs_E10	ordinary_muscle_development	ENSGALG00010012067	ENSGALG00010012067	0.095198763	0.084015888	1.133104288	0.257170461	0.293426151	positive
PRJNA729271	stage_5W_vs_E10	ordinary_muscle_development	ENSGALG00010012069	ERP27	-1.165896527	0.65515074	-1.779585147	0.075143879	0.092754026	negative
PRJNA729271	stage_5W_vs_E10	ordinary_muscle_development	ENSGALG00010012070	DNAJC28	0.85215551	0.161021582	5.292181945	1.20866E-07	2.48116E-07	positive
PRJNA729271	stage_5W_vs_E10	ordinary_muscle_development	ENSGALG00010012071	ENSGALG00010012071	-0.910813361	0.154000555	-5.914351173	3.33186E-09	7.55441E-09	negative
PRJNA729271	stage_5W_vs_E10	ordinary_muscle_development	ENSGALG00010012072	CAPRIN2	-0.224710059	0.151488429	-1.483348002	0.137981975	0.16454535	negative
PRJNA729271	stage_5W_vs_E10	ordinary_muscle_development	ENSGALG00010012074	KIAA0930	-0.266071006	0.088327324	-3.01232954	0.00259251	0.003773314	negative
PRJNA729271	stage_5W_vs_E10	ordinary_muscle_development	ENSGALG00010012075	PIGN	-0.48148916	0.098494196	-4.888502895	1.01606E-06	1.95018E-06	negative
PRJNA729271	stage_5W_vs_E10	ordinary_muscle_development	ENSGALG00010012077	IFNGR2	1.111944678	0.067927038	16.36969179	3.14804E-60	5.08705E-59	positive
PRJNA729271	stage_5W_vs_E10	ordinary_muscle_development	ENSGALG00010012078	TMBIM4	-0.197036466	0.083228321	-2.3674209	0.017912549	0.023816695	negative
PRJNA729271	stage_5W_vs_E10	ordinary_muscle_development	ENSGALG00010012079	ENSGALG00010012079	-0.078670907	0.367582016	-0.214022732	0.83052933	0.850778985	negative
PRJNA729271	stage_5W_vs_E10	ordinary_muscle_development	ENSGALG00010012081	CHIC1	0.079390603	0.125153584	0.634345427	0.525855416	0.566501245	positive
PRJNA729271	stage_5W_vs_E10	ordinary_muscle_development	ENSGALG00010012082	HAUS1	-2.703693615	0.215657434	-12.53698317	4.68493E-36	3.60829E-35	negative
PRJNA729271	stage_5W_vs_E10	ordinary_muscle_development	ENSGALG00010012083	MGP	3.825254444	0.277673878	13.77606879	3.55089E-43	3.4154E-42	positive
PRJNA729271	stage_5W_vs_E10	ordinary_muscle_development	ENSGALG00010012084	TMEM50B	0.774741139	0.105520202	7.342111986	2.10249E-13	6.05516E-13	positive
PRJNA729271	stage_5W_vs_E10	ordinary_muscle_development	ENSGALG00010012085	IPO8	-0.318511028	0.096859235	-3.288390893	0.001007618	0.001527125	negative
PRJNA729271	stage_5W_vs_E10	ordinary_muscle_development	ENSGALG00010012086	CIBAR1	-1.829902921	0.135582626	-13.49658862	1.63786E-41	1.49784E-40	negative
PRJNA729271	stage_5W_vs_E10	ordinary_muscle_development	ENSGALG00010012087	TMEM171	0.463584692	0.171614918	2.701307654	0.006906742	0.009609639	positive
PRJNA729271	stage_5W_vs_E10	ordinary_muscle_development	ENSGALG00010012088	ATP5F1AZ	0.457515186	0.169912435	2.692652752	0.007088606	0.009854161	positive
PRJNA729271	stage_5W_vs_E10	ordinary_muscle_development	ENSGALG00010012089	ENSGALG00010012089	-1.991432955	0.297006797	-6.705008027	2.01395E-11	5.24004E-11	negative
PRJNA729271	stage_5W_vs_E10	ordinary_muscle_development	ENSGALG00010012090	HELB	0.288246618	0.223757916	1.288207465	0.197673754	0.230032198	positive
PRJNA729271	stage_5W_vs_E10	ordinary_muscle_development	ENSGALG00010012091	ENSGALG00010012091	-0.98315525	0.446365827	-2.202577325	0.027624554	0.035966757	negative
PRJNA729271	stage_5W_vs_E10	ordinary_muscle_development	ENSGALG00010012092	OC3	9.511516893	0.697618603	13.63426499	2.50502E-42	2.34866E-41	positive
PRJNA729271	stage_5W_vs_E10	ordinary_muscle_development	ENSGALG00010012093	ENSGALG00010012093	-1.507511948	0.069240748	-21.77203454	4.2724E-105	1.9413E-103	negative
PRJNA729271	stage_5W_vs_E10	ordinary_muscle_development	ENSGALG00010012094	ENSGALG00010012094	0.88705265	0.330740452	2.682020435	0.007317899	0.010162723	positive
PRJNA729271	stage_5W_vs_E10	ordinary_muscle_development	ENSGALG00010012095	RNF38	0.358875304	0.086323889	4.157311598	3.22015E-05	5.5088E-05	positive
PRJNA729271	stage_5W_vs_E10	ordinary_muscle_development	ENSGALG00010012096	ENSGALG00010012096	-2.476791952	0.169278887	-14.63142862	1.7703E-48	2.02211E-47	negative
PRJNA729271	stage_5W_vs_E10	ordinary_muscle_development	ENSGALG00010012097	GART	-0.148840822	0.192296738	-0.774016366	0.438921113	0.480726417	negative
PRJNA729271	stage_5W_vs_E10	ordinary_muscle_development	ENSGALG00010012098	ART4	1.49747889	0.546910595	2.738068898	0.006180113	0.008646789	positive
PRJNA729271	stage_5W_vs_E10	ordinary_muscle_development	ENSGALG00010012099	UPK3A	-3.28778887	0.493213073	-6.666061895	2.62758E-11	6.78668E-11	negative
PRJNA729271	stage_5W_vs_E10	ordinary_muscle_development	ENSGALG00010012100	UBE2F	0.890137487	0.111330237	7.995469259	1.29081E-15	4.19608E-15	positive
PRJNA729271	stage_5W_vs_E10	ordinary_muscle_development	ENSGALG00010012101	ENSGALG00010012101	-2.095967897	0.13893365	-15.08610685	1.99894E-51	2.51735E-50	negative
PRJNA729271	stage_5W_vs_E10	ordinary_muscle_development	ENSGALG00010012102	SON	0.485019036	0.104852129	4.625743328	3.73257E-06	6.86246E-06	positive
PRJNA729271	stage_5W_vs_E10	ordinary_muscle_development	ENSGALG00010012103	ENSGALG00010012103	1.064074486	0.207998026	5.115791269	3.12428E-07	6.21555E-07	positive
PRJNA729271	stage_5W_vs_E10	ordinary_muscle_development	ENSGALG00010012105	DONSON	-2.234112027	0.213750395	-10.45196679	1.43518E-25	7.25344E-25	negative
PRJNA729271	stage_5W_vs_E10	ordinary_muscle_development	ENSGALG00010012106	TMTC1	-0.597051903	0.139571106	-4.277761485	1.88782E-05	3.29369E-05	negative
PRJNA729271	stage_5W_vs_E10	ordinary_muscle_development	ENSGALG00010012107	CLCC1	0.11011695	0.085229978	1.291997875	0.19635788	0.228592681	positive
PRJNA729271	stage_5W_vs_E10	ordinary_muscle_development	ENSGALG00010012108	PSTPIP2	0.938424017	0.33396966	2.809907995	0.004955566	0.007001066	positive
PRJNA729271	stage_5W_vs_E10	ordinary_muscle_development	ENSGALG00010012110	ENSGALG00010012110	0.732154111	0.113013195	6.47848342	9.26491E-11	2.31144E-10	positive
PRJNA729271	stage_5W_vs_E10	ordinary_muscle_development	ENSGALG00010012111	DRD1	0.435083804	0.313808159	1.386464282	0.16560518	0.19553984	positive
PRJNA729271	stage_5W_vs_E10	ordinary_muscle_development	ENSGALG00010012112	MBD2	1.862754113	0.189810435	9.813760292	9.8238E-23	4.44155E-22	positive
PRJNA729271	stage_5W_vs_E10	ordinary_muscle_development	ENSGALG00010012113	WDR47	-0.683651497	0.096336001	-7.096531811	1.27927E-12	3.54685E-12	negative
PRJNA729271	stage_5W_vs_E10	ordinary_muscle_development	ENSGALG00010012114	TAL2	1.418197589	0.305602149	4.640666278	3.47288E-06	6.40287E-06	positive
PRJNA729271	stage_5W_vs_E10	ordinary_muscle_development	ENSGALG00010012116	RBM12B	-0.637708438	0.078476921	-8.126063413	4.43457E-16	1.47531E-15	negative
PRJNA729271	stage_5W_vs_E10	ordinary_muscle_development	ENSGALG00010012118	ENSGALG00010012118	-0.705128564	0.763489067	-0.923560788	0.355715038	0.395982603	negative
PRJNA729271	stage_5W_vs_E10	ordinary_muscle_development	ENSGALG00010012119	EPG5	0.613162354	0.122578649	5.00219538	5.66811E-07	1.10801E-06	positive
PRJNA729271	stage_5W_vs_E10	ordinary_muscle_development	ENSGALG00010012120	ENSGALG00010012120	0.231095346	0.187864513	1.230117077	0.218653266	0.252600549	positive
PRJNA729271	stage_5W_vs_E10	ordinary_muscle_development	ENSGALG00010012121	SREK1	-0.484380138	0.100632497	-4.813357022	1.48416E-06	2.81177E-06	negative
PRJNA729271	stage_5W_vs_E10	ordinary_muscle_development	ENSGALG00010012122	GRIP1	-4.1418672	0.287897084	-14.38662436	6.27865E-47	6.82226E-46	negative
PRJNA729271	stage_5W_vs_E10	ordinary_muscle_development	ENSGALG00010012123	ENSGALG00010012123	-2.148454411	0.213176677	-10.07828081	6.89214E-24	3.25943E-23	negative
PRJNA729271	stage_5W_vs_E10	ordinary_muscle_development	ENSGALG00010012124	ERAP1	3.274421599	0.146206837	22.3958172	4.3231E-111	2.2622E-109	positive
PRJNA729271	stage_5W_vs_E10	ordinary_muscle_development	ENSGALG00010012125	RNF152	0.46599932	0.147841838	3.152012496	0.001621493	0.002405439	positive
PRJNA729271	stage_5W_vs_E10	ordinary_muscle_development	ENSGALG00010012127	ENSGALG00010012127	-0.620687788	0.117138558	-5.298748759	1.16599E-07	2.39527E-07	negative
PRJNA729271	stage_5W_vs_E10	ordinary_muscle_development	ENSGALG00010012128	ENSGALG00010012128	2.579677464	0.766779479	3.364301647	0.000767376	0.001174688	positive
PRJNA729271	stage_5W_vs_E10	ordinary_muscle_development	ENSGALG00010012129	CRYZL1	-0.824976462	0.099341051	-8.30448699	1.00252E-16	3.43696E-16	negative
PRJNA729271	stage_5W_vs_E10	ordinary_muscle_development	ENSGALG00010012130	TMEM67	-1.621802091	0.171240539	-9.470900426	2.77442E-21	1.17519E-20	negative
PRJNA729271	stage_5W_vs_E10	ordinary_muscle_development	ENSGALG00010012131	SLC14A1	1.692920678	0.240717149	7.032821243	2.02399E-12	5.55638E-12	positive
PRJNA729271	stage_5W_vs_E10	ordinary_muscle_development	ENSGALG00010012132	MC4R	-4.995820888	1.155122634	-4.324926842	1.52582E-05	2.68343E-05	negative
PRJNA729271	stage_5W_vs_E10	ordinary_muscle_development	ENSGALG00010012133	RAB3IP	-2.14759604	0.237172283	-9.055004279	1.36562E-19	5.36007E-19	negative
PRJNA729271	stage_5W_vs_E10	ordinary_muscle_development	ENSGALG00010012135	CAMSAP2	-0.33182422	0.09245592	-3.588999174	0.00033195	0.000523457	negative
PRJNA729271	stage_5W_vs_E10	ordinary_muscle_development	ENSGALG00010012136	ENSGALG00010012136	1.164342776	0.171759295	6.778921473	1.21076E-11	3.19091E-11	positive
PRJNA729271	stage_5W_vs_E10	ordinary_muscle_development	ENSGALG00010012137	ENSGALG00010012137	-0.469213868	0.99068803	-0.473624242	0.635767885	0.67138682	negative
PRJNA729271	stage_5W_vs_E10	ordinary_muscle_development	ENSGALG00010012138	DDX11	-2.194506179	0.176090768	-12.46235794	1.19776E-35	9.05662E-35	negative
PRJNA729271	stage_5W_vs_E10	ordinary_muscle_development	ENSGALG00010012140	GLOD5	-1.264622817	0.834451808	-1.515513305	0.129642463	0.155213307	negative
PRJNA729271	stage_5W_vs_E10	ordinary_muscle_development	ENSGALG00010012141	ENSGALG00010012141	0.162163524	0.094506653	1.715895329	0.086181202	0.105577799	positive
PRJNA729271	stage_5W_vs_E10	ordinary_muscle_development	ENSGALG00010012144	H1-01	-0.879800686	0.157752968	-5.577078508	2.44592E-08	5.27112E-08	negative
PRJNA729271	stage_5W_vs_E10	ordinary_muscle_development	ENSGALG00010012145	ENSGALG00010012145	8.090259433	0.865510319	9.347386454	8.98402E-21	3.70901E-20	positive
PRJNA729271	stage_5W_vs_E10	ordinary_muscle_development	ENSGALG00010012147	ARFGAP3	-0.557230697	0.123151924	-4.524742103	6.04692E-06	1.0933E-05	negative
PRJNA729271	stage_5W_vs_E10	ordinary_muscle_development	ENSGALG00010012148	SETBP1	-0.716209826	0.109974057	-6.512534347	7.38933E-11	1.85952E-10	negative
PRJNA729271	stage_5W_vs_E10	ordinary_muscle_development	ENSGALG00010012149	SFXN1	-0.579306475	0.174177539	-3.325953963	0.000881165	0.001341507	negative
PRJNA729271	stage_5W_vs_E10	ordinary_muscle_development	ENSGALG00010012151	MYRFL	-0.687610993	0.524032154	-1.312154204	0.18946811	0.221371938	negative
PRJNA729271	stage_5W_vs_E10	ordinary_muscle_development	ENSGALG00010012154	CWC27	-0.255856665	0.114394832	-2.236610352	0.025311818	0.033068481	negative
PRJNA729271	stage_5W_vs_E10	ordinary_muscle_development	ENSGALG00010012156	ENSGALG00010012156	-3.101620438	0.64338411	-4.820791173	1.4299E-06	2.71288E-06	negative
PRJNA729271	stage_5W_vs_E10	ordinary_muscle_development	ENSGALG00010012157	H2BC32	-2.465076631	0.199095835	-12.38135713	3.2972E-35	2.45217E-34	negative
PRJNA729271	stage_5W_vs_E10	ordinary_muscle_development	ENSGALG00010012159	WASHC1	-0.245555798	0.103360114	-2.375730717	0.017514239	0.023318536	negative
PRJNA729271	stage_5W_vs_E10	ordinary_muscle_development	ENSGALG00010012160	SYT4	-4.979485995	0.487910088	-10.2057451	1.86881E-24	9.06877E-24	negative
PRJNA729271	stage_5W_vs_E10	ordinary_muscle_development	ENSGALG00010012163	PDP1	2.74416707	0.220539942	12.44294818	1.5276E-35	1.15086E-34	positive
PRJNA729271	stage_5W_vs_E10	ordinary_muscle_development	ENSGALG00010012168	ENSGALG00010012168	-3.814620547	0.61568883	-6.195695555	5.80282E-10	1.37573E-09	negative
PRJNA729271	stage_5W_vs_E10	ordinary_muscle_development	ENSGALG00010012169	H3C33	-3.00758957	0.228951327	-13.136371	2.03767E-39	1.74151E-38	negative
PRJNA729271	stage_5W_vs_E10	ordinary_muscle_development	ENSGALG00010012171	CDH17	-0.881767049	0.403830645	-2.183507023	0.028998492	0.037667636	negative
PRJNA729271	stage_5W_vs_E10	ordinary_muscle_development	ENSGALG00010012172	CNOT2	-0.706126033	0.090122702	-7.83516272	4.68236E-15	1.47417E-14	negative
PRJNA729271	stage_5W_vs_E10	ordinary_muscle_development	ENSGALG00010012175	PACSIN2	0.357958354	0.154586564	2.315585165	0.02058093	0.027152141	positive
PRJNA729271	stage_5W_vs_E10	ordinary_muscle_development	ENSGALG00010012179	ENSGALG00010012179	-1.834685264	0.180422701	-10.16881609	2.73209E-24	1.31654E-23	negative
PRJNA729271	stage_5W_vs_E10	ordinary_muscle_development	ENSGALG00010012180	DDX59	-0.386899395	0.107943582	-3.584274184	0.000338017	0.000532387	negative
PRJNA729271	stage_5W_vs_E10	ordinary_muscle_development	ENSGALG00010012186	KIF20A	-2.981155837	0.248976861	-11.97362607	4.88462E-33	3.32719E-32	negative
PRJNA729271	stage_5W_vs_E10	ordinary_muscle_development	ENSGALG00010012187	ENSGALG00010012187	-3.371388323	0.880351745	-3.829592369	0.000128356	0.000209282	negative
PRJNA729271	stage_5W_vs_E10	ordinary_muscle_development	ENSGALG00010012188	ADAMTS6	-1.172669547	0.186248967	-6.296247246	3.04938E-10	7.34964E-10	negative
PRJNA729271	stage_5W_vs_E10	ordinary_muscle_development	ENSGALG00010012190	CAST	0.676160427	0.13260288	5.099138334	3.41203E-07	6.77035E-07	positive
PRJNA729271	stage_5W_vs_E10	ordinary_muscle_development	ENSGALG00010012191	BEST3	5.803941899	0.372925606	15.56327	1.2932E-54	1.76505E-53	positive
PRJNA729271	stage_5W_vs_E10	ordinary_muscle_development	ENSGALG00010012193	TTLL1	-0.545720916	0.215828495	-2.528493354	0.011455326	0.015591074	negative
PRJNA729271	stage_5W_vs_E10	ordinary_muscle_development	ENSGALG00010012194	LRRFIP1	2.030481393	0.15977299	12.70853973	5.30152E-37	4.21302E-36	positive
PRJNA729271	stage_5W_vs_E10	ordinary_muscle_development	ENSGALG00010012195	ITSN1	0.01029502	0.072128858	0.142730943	0.886502678	0.900417372	positive
PRJNA729271	stage_5W_vs_E10	ordinary_muscle_development	ENSGALG00010012196	CENPK	-3.217037384	0.266162702	-12.0867325	1.24126E-33	8.67086E-33	negative
PRJNA729271	stage_5W_vs_E10	ordinary_muscle_development	ENSGALG00010012198	TMEM5	0.765568786	0.100203553	7.640136159	2.16992E-14	6.59669E-14	positive
PRJNA729271	stage_5W_vs_E10	ordinary_muscle_development	ENSGALG00010012199	ENSGALG00010012199	1.118005006	0.286345681	3.904389269	9.44636E-05	0.000155987	positive
PRJNA729271	stage_5W_vs_E10	ordinary_muscle_development	ENSGALG00010012200	GEM	1.490686898	0.238764365	6.243339101	4.28327E-10	1.02266E-09	positive
PRJNA729271	stage_5W_vs_E10	ordinary_muscle_development	ENSGALG00010012205	SLC6A13	-4.719039812	0.423535238	-11.14202406	7.83189E-29	4.52464E-28	negative
PRJNA729271	stage_5W_vs_E10	ordinary_muscle_development	ENSGALG00010012206	RIBC2	-2.989559509	0.739241077	-4.04409279	5.25261E-05	8.85206E-05	negative
PRJNA729271	stage_5W_vs_E10	ordinary_muscle_development	ENSGALG00010012207	GRIA3	-2.228038246	0.266921984	-8.347151523	6.9929E-17	2.41972E-16	negative
PRJNA729271	stage_5W_vs_E10	ordinary_muscle_development	ENSGALG00010012209	KIF14	-1.360157161	0.229142391	-5.935860034	2.92309E-09	6.64214E-09	negative
PRJNA729271	stage_5W_vs_E10	ordinary_muscle_development	ENSGALG00010012210	PPWD1	-1.223087379	0.077284626	-15.82575277	2.06712E-56	2.98435E-55	negative
PRJNA729271	stage_5W_vs_E10	ordinary_muscle_development	ENSGALG00010012211	ENSGALG00010012211	2.262620226	3.991197156	0.566902645	0.570780327	0.609687608	positive
PRJNA729271	stage_5W_vs_E10	ordinary_muscle_development	ENSGALG00010012212	RAD54B	-3.198313249	0.202767925	-15.77327007	4.75242E-56	6.80022E-55	negative
PRJNA729271	stage_5W_vs_E10	ordinary_muscle_development	ENSGALG00010012215	RAB17	4.978202214	0.767713861	6.484450091	8.9056E-11	2.22602E-10	positive
PRJNA729271	stage_5W_vs_E10	ordinary_muscle_development	ENSGALG00010012216	BRD8	-0.105514899	0.058122771	-1.815379722	0.069465575	0.086161261	negative
PRJNA729271	stage_5W_vs_E10	ordinary_muscle_development	ENSGALG00010012219	KDM5A	-0.632629749	0.077843705	-8.126922389	4.40327E-16	1.46523E-15	negative
PRJNA729271	stage_5W_vs_E10	ordinary_muscle_development	ENSGALG00010012226	SLCO4C1	1.975632538	0.492117121	4.014557616	5.95574E-05	0.000100114	positive
PRJNA729271	stage_5W_vs_E10	ordinary_muscle_development	ENSGALG00010012228	NHP2	-1.253855334	0.117794	-10.64447539	1.85024E-26	9.77731E-26	negative
PRJNA729271	stage_5W_vs_E10	ordinary_muscle_development	ENSGALG00010012229	FSBP	0.264726623	0.202560438	1.306901909	0.191246028	0.223350225	positive
PRJNA729271	stage_5W_vs_E10	ordinary_muscle_development	ENSGALG00010012230	PTPRQ	0.105110685	0.396567657	0.265051078	0.790970114	0.815906049	positive
PRJNA729271	stage_5W_vs_E10	ordinary_muscle_development	ENSGALG00010012232	PCSK1	0.563839681	0.43134652	1.307161773	0.191157775	0.22327413	positive
PRJNA729271	stage_5W_vs_E10	ordinary_muscle_development	ENSGALG00010012234	C1GALT1C1	-0.301977322	0.065194728	-4.631928542	3.62275E-06	6.66647E-06	negative
PRJNA729271	stage_5W_vs_E10	ordinary_muscle_development	ENSGALG00010012236	PRLH	-1.608096449	0.598013762	-2.68906261	0.007165298	0.009956955	negative
PRJNA729271	stage_5W_vs_E10	ordinary_muscle_development	ENSGALG00010012240	TRIM14	0.080713164	0.227863437	0.354217269	0.723176063	0.753427516	positive
PRJNA729271	stage_5W_vs_E10	ordinary_muscle_development	ENSGALG00010012242	CCDC77	-2.422903587	0.149712833	-16.18367338	6.57518E-59	1.02371E-57	negative
PRJNA729271	stage_5W_vs_E10	ordinary_muscle_development	ENSGALG00010012243	ENSGALG00010012243	-2.574422599	1.654494988	-1.556017164	0.119704011	0.143849249	negative
PRJNA729271	stage_5W_vs_E10	ordinary_muscle_development	ENSGALG00010012244	ENSGALG00010012244	-1.153953085	0.084861883	-13.59801414	4.11454E-42	3.8231E-41	negative
PRJNA729271	stage_5W_vs_E10	ordinary_muscle_development	ENSGALG00010012245	ENSGALG00010012245	-0.485066987	0.079278487	-6.118519706	9.44486E-10	2.20883E-09	negative
PRJNA729271	stage_5W_vs_E10	ordinary_muscle_development	ENSGALG00010012247	TRIM23	1.027276306	0.125703709	8.172203653	3.02807E-16	1.01508E-15	positive
PRJNA729271	stage_5W_vs_E10	ordinary_muscle_development	ENSGALG00010012248	H2AC39	-2.433748315	0.21002169	-11.58808082	4.73626E-31	2.99791E-30	negative
PRJNA729271	stage_5W_vs_E10	ordinary_muscle_development	ENSGALG00010012250	NANS	-0.739772273	0.111471406	-6.636430801	3.2137E-11	8.26958E-11	negative
PRJNA729271	stage_5W_vs_E10	ordinary_muscle_development	ENSGALG00010012253	MYF6	2.175424864	0.289412165	7.51670153	5.61755E-14	1.67234E-13	positive
PRJNA729271	stage_5W_vs_E10	ordinary_muscle_development	ENSGALG00010012259	IL1RAPL2	-1.840976514	0.233645238	-7.879366734	3.29044E-15	1.04548E-14	negative
PRJNA729271	stage_5W_vs_E10	ordinary_muscle_development	ENSGALG00010012260	ENSGALG00010012260	2.116658165	0.302468929	6.99793586	2.59761E-12	7.07884E-12	positive
PRJNA729271	stage_5W_vs_E10	ordinary_muscle_development	ENSGALG00010012263	SHLD3	0.5461337	0.127714363	4.276212068	1.901E-05	3.31589E-05	positive
PRJNA729271	stage_5W_vs_E10	ordinary_muscle_development	ENSGALG00010012264	MYF5	1.304607294	0.155762642	8.375610964	5.49381E-17	1.91058E-16	positive
PRJNA729271	stage_5W_vs_E10	ordinary_muscle_development	ENSGALG00010012266	H3C33	-2.603658069	0.20331584	-12.80597747	1.51802E-37	1.22311E-36	negative

PRJNA729271	stage_5W_vs_E10	ordinary_muscle_development	ENSGALG00010012268	ENSGALG00010012268	-0.855887184	0.169525559	-5.048720599	4.44779E-07	8.75365E-07	negative
PRJNA729271	stage_5W_vs_E10	ordinary_muscle_development	ENSGALG00010012271	ENSGALG00010012271	-0.361071137	0.307301093	-1.174975113	0.240004672	0.275335792	negative
PRJNA729271	stage_5W_vs_E10	ordinary_muscle_development	ENSGALG00010012274	B4GALNT3	-0.335530523	0.278361738	-1.205375875	0.22805823	0.262492426	negative
PRJNA729271	stage_5W_vs_E10	ordinary_muscle_development	ENSGALG00010012275	VIRMA	-0.081042789	0.073811814	-1.097965021	0.272219764	0.309379447	negative
PRJNA729271	stage_5W_vs_E10	ordinary_muscle_development	ENSGALG00010012277	H3C33	-2.45662883	0.210388175	-11.67664878	1.67781E-31	1.07753E-30	negative
PRJNA729271	stage_5W_vs_E10	ordinary_muscle_development	ENSGALG00010012282	ENSGALG00010012282	1.043444898	1.476148579	0.706869832			positive
PRJNA729271	stage_5W_vs_E10	ordinary_muscle_development	ENSGALG00010012283	CUPIN1A	-0.264966213	0.790558204	-0.335163448	0.737501793	0.766479422	negative
PRJNA729271	stage_5W_vs_E10	ordinary_muscle_development	ENSGALG00010012285	FBLN1	-2.736400916	0.199668045	-13.7047514	9.50924E-43	9.02071E-42	negative
PRJNA729271	stage_5W_vs_E10	ordinary_muscle_development	ENSGALG00010012286	ENSGALG00010012286	2.55149559	2.66900548	0.955972406	0.339086176	0.378838764	positive
PRJNA729271	stage_5W_vs_E10	ordinary_muscle_development	ENSGALG00010012288	MCTS1	0.575537524	0.11357005	5.067687524	4.02678E-07	7.94665E-07	positive
PRJNA729271	stage_5W_vs_E10	ordinary_muscle_development	ENSGALG00010012290	ENSGALG00010012290	0.026474583	0.097554684	0.27138198	0.78609726	0.811514834	positive
PRJNA729271	stage_5W_vs_E10	ordinary_muscle_development	ENSGALG00010012291	NR5A2	-1.60917141	0.20354733	-7.905637528	2.66566E-15	8.50886E-15	negative
PRJNA729271	stage_5W_vs_E10	ordinary_muscle_development	ENSGALG00010012293	SRGAP1	-1.594890015	0.107293341	-14.86476239	5.58126E-50	6.70815E-49	negative
PRJNA729271	stage_5W_vs_E10	ordinary_muscle_development	ENSGALG00010012294	ENSGALG00010012294	-0.197072483	0.529449424	-0.372221545	0.709727906	0.741174784	negative
PRJNA729271	stage_5W_vs_E10	ordinary_muscle_development	ENSGALG00010012295	CUL4B	-0.259971257	0.065706832	-3.956533107	7.60454E-05	0.000126699	negative
PRJNA729271	stage_5W_vs_E10	ordinary_muscle_development	ENSGALG00010012297	ABLIM3	-0.313481591	0.195803612	-1.601000039	0.109376911	0.132173265	negative
PRJNA729271	stage_5W_vs_E10	ordinary_muscle_development	ENSGALG00010012300	NINJ2	-2.859322881	0.232444057	-12.30112277	8.93238E-35	6.53583E-34	negative
PRJNA729271	stage_5W_vs_E10	ordinary_muscle_development	ENSGALG00010012303	CA9	1.412699327	0.211545747	6.677985008	2.4225E-11	6.27038E-11	positive
PRJNA729271	stage_5W_vs_E10	ordinary_muscle_development	ENSGALG00010012304	ESRP1	-6.145582271	0.854851243	-7.18906631	6.52359E-13	1.83718E-12	negative
PRJNA729271	stage_5W_vs_E10	ordinary_muscle_development	ENSGALG00010012307	ENSGALG00010012307	-2.03856407	0.173267445	-11.76541885	5.88336E-32	3.85182E-31	negative
PRJNA729271	stage_5W_vs_E10	ordinary_muscle_development	ENSGALG00010012308	TRABD2A	1.750902973	0.344716203	5.079259281	3.78909E-07	7.49289E-07	positive
PRJNA729271	stage_5W_vs_E10	ordinary_muscle_development	ENSGALG00010012309	TRAPPC13	-0.397857869	0.126185316	-3.152964873	0.001616213	0.002398342	negative
PRJNA729271	stage_5W_vs_E10	ordinary_muscle_development	ENSGALG00010012311	ENSGALG00010012311	-4.077982508	0.550460904	-7.408305434	1.27923E-13	3.72564E-13	negative
PRJNA729271	stage_5W_vs_E10	ordinary_muscle_development	ENSGALG00010012312	SH3TC2	1.56439655	0.176635516	8.856636446	8.24649E-19	3.11934E-18	positive
PRJNA729271	stage_5W_vs_E10	ordinary_muscle_development	ENSGALG00010012314	ADRB2	5.175183492	0.261628979	19.78062027	4.37279E-87	1.38392E-85	positive
PRJNA729271	stage_5W_vs_E10	ordinary_muscle_development	ENSGALG00010012315	ATP6V1E1	0.71792376	0.100572836	7.138346564	9.44602E-13	2.63866E-12	positive
PRJNA729271	stage_5W_vs_E10	ordinary_muscle_development	ENSGALG00010012316	ENSGALG00010012316	-3.071930515	1.034132441	-2.970538775	0.002972779	0.004296583	negative
PRJNA729271	stage_5W_vs_E10	ordinary_muscle_development	ENSGALG00010012317	ENSGALG00010012317	-5.162882069	1.154550103	-4.471769614	7.7575E-06	1.39302E-05	negative
PRJNA729271	stage_5W_vs_E10	ordinary_muscle_development	ENSGALG00010012318	POLR1D	-0.690977888	0.14339161	-4.818816716	1.44412E-06	2.73807E-06	negative
PRJNA729271	stage_5W_vs_E10	ordinary_muscle_development	ENSGALG00010012320	DPY19L4	-0.614672541	0.061906981	-9.9289697	3.11457E-23	1.43502E-22	negative
PRJNA729271	stage_5W_vs_E10	ordinary_muscle_development	ENSGALG00010012322	ENSGALG00010012322	-2.326533165	0.279011205	-8.338493668	7.52429E-17	2.59863E-16	negative
PRJNA729271	stage_5W_vs_E10	ordinary_muscle_development	ENSGALG00010012323	SMAD7B	0.307684035	3.546010974	0.086769059	0.930855082	0.940260935	positive
PRJNA729271	stage_5W_vs_E10	ordinary_muscle_development	ENSGALG00010012324	ENSGALG00010012324	-2.025746992	0.164327501	-12.32749832	6.44157E-35	4.73721E-34	negative
PRJNA729271	stage_5W_vs_E10	ordinary_muscle_development	ENSGALG00010012325	MLPH	-2.943713345	0.600516028	-4.90197298	9.48789E-07	1.82566E-06	negative
PRJNA729271	stage_5W_vs_E10	ordinary_muscle_development	ENSGALG00010012326	SGTB	-0.047680768	0.116834099	-0.408106613	0.683195405	0.716616065	negative
PRJNA729271	stage_5W_vs_E10	ordinary_muscle_development	ENSGALG00010012327	UBTD2	-1.090572751	0.090145743	-12.0978841	1.08368E-33	7.58841E-33	negative
PRJNA729271	stage_5W_vs_E10	ordinary_muscle_development	ENSGALG00010012329	ATXN10	-0.251525186	0.107717575	-2.335043153	0.019541167	0.025860423	negative
PRJNA729271	stage_5W_vs_E10	ordinary_muscle_development	ENSGALG00010012331	ENSGALG00010012331	-0.843930989	0.26548494	-3.178828108	0.001478718	0.002202652	negative
PRJNA729271	stage_5W_vs_E10	ordinary_muscle_development	ENSGALG00010012334	ARHGEF39	-2.507053278	0.245967762	-10.19260924	2.13944E-24	1.03439E-23	negative
PRJNA729271	stage_5W_vs_E10	ordinary_muscle_development	ENSGALG00010012336	ENSGALG00010012336	4.3419759	4.094048548	1.06055799	0.288890823	0.326559501	positive
PRJNA729271	stage_5W_vs_E10	ordinary_muscle_development	ENSGALG00010012337	ACSS3	0.672513675	0.154419084	4.355120209	1.32994E-05	2.35091E-05	positive
PRJNA729271	stage_5W_vs_E10	ordinary_muscle_development	ENSGALG00010012338	H3C33	-2.796249733	0.226119227	-12.36626255	3.9791E-35	2.95174E-34	negative
PRJNA729271	stage_5W_vs_E10	ordinary_muscle_development	ENSGALG00010012339	WNK1	1.22752345	0.154714399	7.934125465	2.11984E-15	6.81763E-15	positive
PRJNA729271	stage_5W_vs_E10	ordinary_muscle_development	ENSGALG00010012340	ENHO	-1.192984319	0.164912339	-7.234051281	4.68795E-13	1.329E-12	negative
PRJNA729271	stage_5W_vs_E10	ordinary_muscle_development	ENSGALG00010012341	GOPC	-0.411006205	0.081026451	-5.072494239	3.92635E-07	7.75797E-07	negative
PRJNA729271	stage_5W_vs_E10	ordinary_muscle_development	ENSGALG00010012342	ENSGALG00010012342	-0.998353415	1.67613925	-0.595626774	0.551424586	0.591234513	negative
PRJNA729271	stage_5W_vs_E10	ordinary_muscle_development	ENSGALG00010012343	BCL2L13	1.66293685	0.170218752	9.769410438	1.52336E-22	6.8278E-22	positive
PRJNA729271	stage_5W_vs_E10	ordinary_muscle_development	ENSGALG00010012345	H2BC32	-2.17293347	0.165424523	-13.13549789	2.06131E-39	1.76068E-38	negative
PRJNA729271	stage_5W_vs_E10	ordinary_muscle_development	ENSGALG00010012346	NLN	-0.515436619	0.140335658	-3.672884193	0.000239828	0.000382853	negative
PRJNA729271	stage_5W_vs_E10	ordinary_muscle_development	ENSGALG00010012347	ENSGALG00010012347	-2.066271871	0.502658905	-4.110683907	3.94489E-05	6.70511E-05	negative
PRJNA729271	stage_5W_vs_E10	ordinary_muscle_development	ENSGALG00010012350	H2AC32	-2.324962794	0.19242323	-12.0825474	1.3061E-33	9.11062E-33	negative
PRJNA729271	stage_5W_vs_E10	ordinary_muscle_development	ENSGALG00010012352	PTPRC	3.662782487	0.19270742	19.00696132	1.49357E-80	4.06943E-79	positive
PRJNA729271	stage_5W_vs_E10	ordinary_muscle_development	ENSGALG00010012353	PIGG	0.174491755	0.152460005	1.144508392	0.25241286	0.288361002	positive
PRJNA729271	stage_5W_vs_E10	ordinary_muscle_development	ENSGALG00010012354	DCBLD1	0.279698842	0.150318559	1.860707316	0.06278552	0.07821211	positive
PRJNA729271	stage_5W_vs_E10	ordinary_muscle_development	ENSGALG00010012358	STK10	0.868244176	0.148792452	5.835270303	5.37034E-09	1.20444E-08	positive
PRJNA729271	stage_5W_vs_E10	ordinary_muscle_development	ENSGALG00010012360	ROS1	1.134516725	0.395716584	2.866993121	0.00414392	0.005905046	positive
PRJNA729271	stage_5W_vs_E10	ordinary_muscle_development	ENSGALG00010012361	WIF1	-7.811471638	0.535147646	-14.59685322	2.94111E-48	3.33318E-47	negative
PRJNA729271	stage_5W_vs_E10	ordinary_muscle_development	ENSGALG00010012362	COL6A3	3.687506185	0.292452347	12.60891295	1.88576E-36	1.46642E-35	positive
PRJNA729271	stage_5W_vs_E10	ordinary_muscle_development	ENSGALG00010012366	INTS8	-0.764474401	0.068966763	-11.08467854	1.48884E-28	8.51985E-28	negative
PRJNA729271	stage_5W_vs_E10	ordinary_muscle_development	ENSGALG00010012367	RAD52	-2.541597171	0.159681638	-15.91665269	4.85674E-57	7.17619E-56	negative
PRJNA729271	stage_5W_vs_E10	ordinary_muscle_development	ENSGALG00010012371	ATP6V1G3	1.59424538	0.428480985	3.720691079	0.000198678	0.000319308	positive
PRJNA729271	stage_5W_vs_E10	ordinary_muscle_development	ENSGALG00010012372	LEMD3	0.067783072	0.073649826	0.920342599	0.357393754	0.397698639	positive
PRJNA729271	stage_5W_vs_E10	ordinary_muscle_development	ENSGALG00010012373	WNT7B	-9.081343193	0.964819024	-9.412483555	4.8455E-21	2.02934E-20	negative
PRJNA729271	stage_5W_vs_E10	ordinary_muscle_development	ENSGALG00010012376	VGLL2	1.430870992	0.186267542	7.681805296	1.56862E-14	4.80396E-14	positive
PRJNA729271	stage_5W_vs_E10	ordinary_muscle_development	ENSGALG00010012378	RPS19BP1	-0.255094923	0.092434453	-2.75973855	0.005784764	0.008115578	negative
PRJNA729271	stage_5W_vs_E10	ordinary_muscle_development	ENSGALG00010012382	SH3PXD2B	-1.710754144	0.134950594	-12.67689229	7.94187E-37	6.27001E-36	negative
PRJNA729271	stage_5W_vs_E10	ordinary_muscle_development	ENSGALG00010012384	NRK	0.759042091	0.121759566	6.233942142	4.54841E-10	1.08508E-09	positive
PRJNA729271	stage_5W_vs_E10	ordinary_muscle_development	ENSGALG00010012387	ERBIN	-0.071797327	0.089073783	-0.806043303	0.420217881	0.461863678	negative
PRJNA729271	stage_5W_vs_E10	ordinary_muscle_development	ENSGALG00010012390	MMP16	-1.429526668	0.174876087	-8.174511983	2.97066E-16	9.96291E-16	negative
PRJNA729271	stage_5W_vs_E10	ordinary_muscle_development	ENSGALG00010012391	NEURL1B	-0.918304812	0.158557893	-5.791605798	6.97166E-09	1.55277E-08	negative
PRJNA729271	stage_5W_vs_E10	ordinary_muscle_development	ENSGALG00010012393	ENSGALG00010012393	1.940457051	1.603540546	1.210107881	0.2262375	0.260635237	positive
PRJNA729271	stage_5W_vs_E10	ordinary_muscle_development	ENSGALG00010012398	ENSGALG00010012398	-5.380249981	0.927856302	-5.798581059	6.68784E-09	1.49093E-08	negative
PRJNA729271	stage_5W_vs_E10	ordinary_muscle_development	ENSGALG00010012399	ERC1	-0.784052777	0.086073999	-9.109054825	8.31028E-20	3.29749E-19	negative
PRJNA729271	stage_5W_vs_E10	ordinary_muscle_development	ENSGALG00010012401	RIPK2	0.587464761	0.131418956	4.470167597	7.81583E-06	1.40315E-05	positive
PRJNA729271	stage_5W_vs_E10	ordinary_muscle_development	ENSGALG00010012404	VAMP7	0.27824315	0.052429454	5.307000717	1.11444E-07	2.29197E-07	positive
PRJNA729271	stage_5W_vs_E10	ordinary_muscle_development	ENSGALG00010012405	MSRB3	2.86403333	0.125453622	22.82941925	2.3404E-115	1.3304E-113	positive
PRJNA729271	stage_5W_vs_E10	ordinary_muscle_development	ENSGALG00010012406	ENSGALG00010012406	4.898698085	0.238105667	20.57363076	4.72897E-94	1.7576E-92	positive
PRJNA729271	stage_5W_vs_E10	ordinary_muscle_development	ENSGALG00010012407	ATF4	0.993318214	0.128831183	7.710231231	1.2559E-14	3.86338E-14	positive
PRJNA729271	stage_5W_vs_E10	ordinary_muscle_development	ENSGALG00010012410	CACNA1I	-2.027246292	0.409073332	-4.955703858	7.20689E-07	1.39994E-06	negative
PRJNA729271	stage_5W_vs_E10	ordinary_muscle_development	ENSGALG00010012412	PURA	1.435530753	0.131009544	10.95745171	6.11984E-28	3.41838E-27	positive
PRJNA729271	stage_5W_vs_E10	ordinary_muscle_development	ENSGALG00010012413	NEK7	0.284415301	0.073042375	3.893839729	9.86699E-05	0.000162654	positive
PRJNA729271	stage_5W_vs_E10	ordinary_muscle_development	ENSGALG00010012414	ENSGALG00010012414	7.463103014	0.203319926	36.70620557	5.8145E-295	2.2779E-292	positive
PRJNA729271	stage_5W_vs_E10	ordinary_muscle_development	ENSGALG00010012415	BID	-2.161753055	0.116673163	-18.52828013	1.22128E-76	3.09485E-75	negative
PRJNA729271	stage_5W_vs_E10	ordinary_muscle_development	ENSGALG00010012418	ENSGALG00010012418	0.656661062	0.783141455	0.838496108	0.401752133	0.443128965	positive
PRJNA729271	stage_5W_vs_E10	ordinary_muscle_development	ENSGALG00010012419	HACE1	0.429604946	0.096844818	4.436013748	9.164E-06	1.63768E-05	positive
PRJNA729271	stage_5W_vs_E10	ordinary_muscle_development	ENSGALG00010012426	MIEF1	-0.254287649	0.081204431	-3.131450413	0.001739452	0.002571741	negative
PRJNA729271	stage_5W_vs_E10	ordinary_muscle_development	ENSGALG00010012427	C7	5.657440245	0.447339113	12.64687142	1.16419E-36	9.13147E-36	positive
PRJNA729271	stage_5W_vs_E10	ordinary_muscle_development	ENSGALG00010012428	CCNE2	-3.257683937	0.237504661	-13.71629477	8.11055E-43	7.71913E-42	negative
PRJNA729271	stage_5W_vs_E10	ordinary_muscle_development	ENSGALG00010012429	CXCR7	0.780818548	0.101458151	7.695966638	1.40428E-14	4.30978E-14	positive
PRJNA729271	stage_5W_vs_E10	ordinary_muscle_development	ENSGALG00010012431	LAMP2	0.681598112	0.079449767	8.578981863	9.57166E-18	3.45557E-17	positive
PRJNA729271	stage_5W_vs_E10	ordinary_muscle_development	ENSGALG00010012433	ENSGALG00010012433	0.175981207	0.742942461	0.236870573	0.8127572	0.83457889	positive
PRJNA729271	stage_5W_vs_E10	ordinary_muscle_development	ENSGALG00010012436	ENSGALG00010012436	5.275352575	0.199630661	26.42556281	6.9683E-154	7.2666E-152	positive
PRJNA729271	stage_5W_vs_E10	ordinary_muscle_development	ENSGALG00010012437	ENSGALG00010012437	3.479542104	3.7846234	0.919389259	0.357892005	0.398051563	positive
PRJNA729271	stage_5W_vs_E10	ordinary_muscle_development	ENSGALG00010012439	TENT2	0.539272671	0.10170017	5.302573947	1.14181E-07	2.34726E-07	positive
PRJNA729271	stage_5W_vs_E10	ordinary_muscle_development	ENSGALG00010012441	FST	0.323900015	0.239168733	1.354274077	0.175648953	0.206290032	positive
PRJNA729271	stage_5W_vs_E10	ordinary_muscle_development	ENSGALG00010012442	CPLX1	-4.63608732	0.36663759	-12.64487725	1.19411E-36	9.36106E-36	negative
PRJNA729271	stage_5W_vs_E10	ordinary_muscle_development	ENSGALG00010012445	SOBP	-0.224226578	0.164067812	-1.366670127	0.171728725	0.202144541	negative
PRJNA729271	stage_5W_vs_E10	ordinary_muscle_development	ENSGALG00010012447	COPS8	0.466036456	0.116623042	3.996092419	6.43966E-05	0.000108011	positive
PRJNA729271	stage_5W_vs_E10	ordinary_muscle_development	ENSGALG00010012449	SUB1	-1.464691327	0.109818728	-13.33735467	1.40352E-40	1.24963E-39	negative
PRJNA729271	stage_5W_vs_E10	ordinary_muscle_development	ENSGALG00010012450	PPFIA2	-2.850601592	0.292963382	-9.730231707	2.24082E-22	9.97258E-22	negative
PRJNA729271	stage_5W_vs_E10	ordinary_muscle_development	ENSGALG00010012454	IQCA1	1.950431832	0.421496212	4.627400623	3.70284E-06	6.81038E-06	positive
PRJNA729271	stage_5W_vs_E10	ordinary_muscle_development	ENSGALG00010012456	ENSGALG00010012456	-1.598309052	0.093633763	-17.06979413	2.49077E-65	4.66455E-64	negative
PRJNA729271	stage_5W_vs_E10	ordinary_muscle_development	ENSGALG00010012458	MRPS30	-0.053490599	0.10890089	-0.491186058	0.623294859	0.659753102	negative
PRJNA729271	stage_5W_vs_E10	ordinary_muscle_development	ENSGALG00010012464	ENSGALG00010012464	0.987099865	0.23737143	4.158461133	3.20399E-05	5.4818E-05	positive
PRJNA729271	stage_5W_vs_E10	ordinary_muscle_development	ENSGALG00010012466	PDSS2	0.398422362	0.121205376	3.2871674	0.001012007	0.001533455	positive
PRJNA729271	stage_5W_vs_E10	ordinary_muscle_development	ENSGALG00010012469	CETN2	-0.774153339	0.131237196	-5.898886603	3.65963E-09	8.28201E-09	negative
PRJNA729271	stage_5W_vs_E10	ordinary_muscle_development	ENSGALG00010012470	CHD1	0.099921725	0.105691753	0.945407011	0.344451124	0.384269918	positive
PRJNA729271	stage_5W_vs_E10	ordinary_muscle_development	ENSGALG00010012474	ASB18	-1.129478768	1.02669654	-1.100109646	0.271284351	0.308437264	negative
PRJNA729271	stage_5W_vs_E10	ordinary_muscle_development	ENSGALG00010012477	ENSGALG00010012477	-0.055340793	0.257479938	-0.214932449	0.829819986	0.850232607	negative
PRJNA729271	stage_5W_vs_E10	ordinary_muscle_development	ENSGALG00010012483	ENSGALG00010012483	1.255887667	0.252772527	4.968450015	6.74902E-07	1.31276E-06	positive
PRJNA729271	stage_5W_vs_E10	ordinary_muscle_development	ENSGALG00010012485	C6	6.215179379	1.216081784	5.110823514	3.20758E-07	6.37863E-07	positive
PRJNA729271	stage_5W_vs_E10	ordinary_muscle_development	ENSGALG00010012486	RADX	1.218085369	0.107108546	11.37243862	5.73622E-30	3.47022E-29	positive
PRJNA729271	stage_5W_vs_E10	ordinary_muscle_development	ENSGALG00010012489	GBX2	-3.337705171	1.032754531	-3.23184752	0.001229927	0.001845909	negative
PRJNA729271	stage_5W_vs_E10	ordinary_muscle_development	ENSGALG00010012497	PLCXD3	0.49005215	0.398634889	1.229325791	0.218949681	0.252886561	positive
PRJNA729271	stage_5W_vs_E10	ordinary_muscle_development	ENSGALG00010012498	KCNT2	0.939279464	0.214201249	4.385032611	1.15968E-05	2.05698E-05	positive
PRJNA729271	stage_5W_vs_E10	ordinary_muscle_development	ENSGALG00010012506	TP53INP1	-0.49079239	0.126520502	-3.879153056	0.000104821	0.000172285	negative
PRJNA729271	stage_5W_vs_E10	ordinary_muscle_development	ENSGALG00010012508	MDM1	-0.769369555	0.069877596	-11.01024643	3.4107E-28	1.92216E-27	negative
PRJNA729271	stage_5W_vs_E10	ordinary_muscle_development	ENSGALG00010012509	MICAL3	0.688320265	0.093471913	7.363926177	1.78578E-13	5.16048E-13	positive
PRJNA729271	stage_5W_vs_E10	ordinary_muscle_development	ENSGALG00010012511	OXCT1	-0.716172977	0.122251324	-5.858202224	4.67905E-09	1.05282E-08	negative
PRJNA729271	stage_5W_vs_E10	ordinary_muscle_development	ENSGALG00010012517	MGAT3	1.057435998	0.175270494	6.033166079	1.60778E-09	3.70977E-09	positive
PRJNA729271	stage_5W_vs_E10	ordinary_muscle_development	ENSGALG00010012519	ENSGALG00010012519	1.637190628	1.671184584	0.979658767	0.327254586	0.366892662	positive
PRJNA729271	stage_5W_vs_E10	ordinary_muscle_development	ENSGALG00010012521	HOMER1	0.696318938	0.134174236	5.189662018	2.10676E-07	4.24722E-07	positive
PRJNA729271	stage_5W_vs_E10	ordinary_muscle_development	ENSGALG00010012529	BEND3	-0.944924978	0.126090529	-7.494020233	6.67954E-14	1.97632E-13	negative
PRJNA729271	stage_5W_vs_E10	ordinary_muscle_development	ENSGALG00010012532	FAM174A	0.85448911	0.151317253	5.647003841	1.63268E-08	3.56036E-08	positive
PRJNA729271	stage_5W_vs_E10	ordinary_muscle_development	ENSGALG00010012535	JMY	-0.545218608	0.104875828	-5.198706121	2.00681E-07	4.05417E-07	negative
PRJNA729271	stage_5W_vs_E10	ordinary_muscle_development	ENSGALG00010012536	FBXO4	0.257475658	0.13461823	1.912635892	0.055794681	0.069912163	positive
PRJNA729271	stage_5W_vs_E10	ordinary_muscle_development	ENSGALG00010012538	RAP1B	-0.413046557	0.088056834	-4.690681444	2.72297E-06	5.06278E-06	negative
PRJNA729271	stage_5W_vs_E10	ordinary_muscle_development	ENSGALG00010012547	ENSGALG00010012547	-2.2844597	0.490745908	-4.655076412	3.23861E-06	5.9877E-06	negative
PRJNA729271	stage_5W_vs_E10	ordinary_muscle_development	ENSGALG00010012550	TAB1	-0.036303249	0.096508658	-0.376165719	0.706793699	0.738536236	negative
PRJNA729271	stage_5W_vs_E10	ordinary_muscle_development	ENSGALG00010012551	PCGF3	-0.562222944	0.156509673	-3.592256847	0.000327826	0.000517067	negative
PRJNA729271	stage_5W_vs_E10	ordinary_muscle_development	ENSGALG00010012553	ENSGALG00010012553	0.597248101	0.223902498	2.667447247	0.007642989	0.010592324	positive

PRJNA729271	stage_5W_vs_E10	ordinary_muscle_development	ENSGALG00010012555	ENSGALG00010012555	-1.74034827	0.213221364	-8.162166461	3.29068E-16	1.10082E-15	negative
PRJNA729271	stage_5W_vs_E10	ordinary_muscle_development	ENSGALG00010012561	OSGIN2	-0.872124844	0.128375009	-6.793571838	1.09391E-11	2.89241E-11	negative
PRJNA729271	stage_5W_vs_E10	ordinary_muscle_development	ENSGALG00010012565	SYNGR1	1.259439801	0.121982853	10.32472815	5.44729E-25	2.71055E-24	positive
PRJNA729271	stage_5W_vs_E10	ordinary_muscle_development	ENSGALG00010012569	C6orf203	1.223001823	0.120998328	10.10759271	5.1125E-24	2.43689E-23	positive
PRJNA729271	stage_5W_vs_E10	ordinary_muscle_development	ENSGALG00010012571	AGAP1	0.09384868	0.082439966	1.138388154	0.254958438	0.29110529	positive
PRJNA729271	stage_5W_vs_E10	ordinary_muscle_development	ENSGALG00010012574	PHAX	-0.616456724	0.105695841	-5.832365	5.46472E-09	1.22485E-08	negative
PRJNA729271	stage_5W_vs_E10	ordinary_muscle_development	ENSGALG00010012578	NDUFS4	0.558821215	0.153020619	3.651934095	0.000260273	0.000414213	positive
PRJNA729271	stage_5W_vs_E10	ordinary_muscle_development	ENSGALG00010012582	CD24	-2.487610432	0.225024566	-11.05483938	2.07708E-28	1.18207E-27	negative
PRJNA729271	stage_5W_vs_E10	ordinary_muscle_development	ENSGALG00010012583	NDUFAF6	-0.094981205	0.104937357	-0.905122904	0.36540028	0.406015872	negative
PRJNA729271	stage_5W_vs_E10	ordinary_muscle_development	ENSGALG00010012586	CDC73	0.604763599	0.133725196	4.522435695	6.11321E-06	1.10515E-05	positive
PRJNA729271	stage_5W_vs_E10	ordinary_muscle_development	ENSGALG00010012587	MBLAC2	0.979110677	0.146132745	6.700145677	2.08212E-11	5.40963E-11	positive
PRJNA729271	stage_5W_vs_E10	ordinary_muscle_development	ENSGALG00010012588	PEX26	0.411384267	0.105496521	3.899505548	9.63893E-05	0.000159003	positive
PRJNA729271	stage_5W_vs_E10	ordinary_muscle_development	ENSGALG00010012593	TUBA8B	5.04928248	0.955765523	5.28297198	1.27105E-07	2.60481E-07	positive
PRJNA729271	stage_5W_vs_E10	ordinary_muscle_development	ENSGALG00010012599	DMGDH	-3.762757012	0.201701004	-18.65512283	1.14738E-77	2.98052E-76	negative
PRJNA729271	stage_5W_vs_E10	ordinary_muscle_development	ENSGALG00010012600	NUP107	-1.20996163	0.100840907	-11.99871827	3.60841E-33	2.48239E-32	negative
PRJNA729271	stage_5W_vs_E10	ordinary_muscle_development	ENSGALG00010012601	TUBA8A	4.474261372	0.237026675	18.87661532	1.7761E-79	4.73244E-78	positive
PRJNA729271	stage_5W_vs_E10	ordinary_muscle_development	ENSGALG00010012604	ENSGALG00010012604	1.889741554	2.125983559	0.888878724			positive
PRJNA729271	stage_5W_vs_E10	ordinary_muscle_development	ENSGALG00010012606	ATP5PO	0.244369729	0.114357942	2.136884644	0.032607375	0.042121371	positive
PRJNA729271	stage_5W_vs_E10	ordinary_muscle_development	ENSGALG00010012607	LMNB1	-3.53097857	0.166032767	-21.26675742	2.30686E-100	9.77718E-99	negative
PRJNA729271	stage_5W_vs_E10	ordinary_muscle_development	ENSGALG00010012610	RPL3	-1.326474472	0.155075147	-8.553752777	1.1915E-17	4.28343E-17	negative
PRJNA729271	stage_5W_vs_E10	ordinary_muscle_development	ENSGALG00010012614	LHFPL2	-1.064442224	0.145441461	-7.31869863	2.50387E-13	7.19113E-13	negative
PRJNA729271	stage_5W_vs_E10	ordinary_muscle_development	ENSGALG00010012618	B3GALT2	-0.934044581	0.334458245	-2.792709094	0.005226867	0.007364254	negative
PRJNA729271	stage_5W_vs_E10	ordinary_muscle_development	ENSGALG00010012624	PDGFB	0.963552417	0.182930585	5.267311728	1.38436E-07	2.82983E-07	positive
PRJNA729271	stage_5W_vs_E10	ordinary_muscle_development	ENSGALG00010012629	NUS1	0.194499109	0.110367507	1.762285964	0.078020994	0.096059994	positive
PRJNA729271	stage_5W_vs_E10	ordinary_muscle_development	ENSGALG00010012631	GLRX2	0.252085003	0.15538743	1.622299844	0.104739162	0.126865059	positive
PRJNA729271	stage_5W_vs_E10	ordinary_muscle_development	ENSGALG00010012633	USP18	2.464280253	0.541460366	4.551173838	5.33474E-06	9.68405E-06	positive
PRJNA729271	stage_5W_vs_E10	ordinary_muscle_development	ENSGALG00010012640	PRRC1	-0.542453005	0.0742469	-7.306069436	2.7507E-13	7.87817E-13	negative
PRJNA729271	stage_5W_vs_E10	ordinary_muscle_development	ENSGALG00010012648	MRPS6	-0.385521199	0.133087308	-2.896754057	0.003770452	0.00539194	negative
PRJNA729271	stage_5W_vs_E10	ordinary_muscle_development	ENSGALG00010012650	DSE	-0.886840488	0.105848563	-8.378389516	5.36568E-17	1.86691E-16	negative
PRJNA729271	stage_5W_vs_E10	ordinary_muscle_development	ENSGALG00010012653	CBX7	2.352346572	0.249804902	9.416735045	4.65333E-21	1.95111E-20	positive
PRJNA729271	stage_5W_vs_E10	ordinary_muscle_development	ENSGALG00010012654	TENT5B	3.129515375	0.308550791	10.14262632	3.5736E-24	1.71408E-23	positive
PRJNA729271	stage_5W_vs_E10	ordinary_muscle_development	ENSGALG00010012662	SLC35E3	-1.712286475	0.109443446	-15.64539985	3.57119E-55	4.9488E-54	negative
PRJNA729271	stage_5W_vs_E10	ordinary_muscle_development	ENSGALG00010012667	SCAMP1	-0.655429874	0.089726216	-7.304775616	2.7773E-13	7.94963E-13	negative
PRJNA729271	stage_5W_vs_E10	ordinary_muscle_development	ENSGALG00010012669	ERCC8	-0.218040763	0.149610377	-1.457390643	0.145008578	0.172399881	negative
PRJNA729271	stage_5W_vs_E10	ordinary_muscle_development	ENSGALG00010012670	ENSGALG00010012670	-0.62044071	0.225594219	-2.750250929	0.005954964	0.008344664	negative
PRJNA729271	stage_5W_vs_E10	ordinary_muscle_development	ENSGALG00010012671	NBN	0.353429146	0.183790973	1.922995136	0.054480661	0.06833655	positive
PRJNA729271	stage_5W_vs_E10	ordinary_muscle_development	ENSGALG00010012672	RO60	-0.828210577	0.091650022	-9.036665326	1.61524E-19	6.31927E-19	negative
PRJNA729271	stage_5W_vs_E10	ordinary_muscle_development	ENSGALG00010012673	ENSGALG00010012673	-1.611805942	0.334137754	-4.823776783	1.40865E-06	2.67431E-06	negative
PRJNA729271	stage_5W_vs_E10	ordinary_muscle_development	ENSGALG00010012674	ENSGALG00010012674	0.026296171	0.138746093	0.189527293	0.849679566	0.867881943	positive
PRJNA729271	stage_5W_vs_E10	ordinary_muscle_development	ENSGALG00010012676	ENSGALG00010012676	0.646966307	0.209252462	3.091797818	0.001989483	0.002924405	positive
PRJNA729271	stage_5W_vs_E10	ordinary_muscle_development	ENSGALG00010012680	TRPM8	-1.142257452	0.337738992	-3.382071596	0.000719414	0.001103365	negative
PRJNA729271	stage_5W_vs_E10	ordinary_muscle_development	ENSGALG00010012682	ENSGALG00010012682	-0.963440362	0.112063523	-8.597270013	8.16348E-18	2.9575E-17	negative
PRJNA729271	stage_5W_vs_E10	ordinary_muscle_development	ENSGALG00010012683	MDM2	0.951879073	0.085981756	11.0707099	1.74017E-28	9.92668E-28	positive
PRJNA729271	stage_5W_vs_E10	ordinary_muscle_development	ENSGALG00010012685	NPTXR	0.029791756	0.265276167	0.112304685	0.910581827	0.922482778	positive
PRJNA729271	stage_5W_vs_E10	ordinary_muscle_development	ENSGALG00010012688	NDUFAF2	0.510969662	0.113016676	4.521188184	6.14935E-06	1.11141E-05	positive
PRJNA729271	stage_5W_vs_E10	ordinary_muscle_development	ENSGALG00010012690	NT5DC1	1.328125287	0.335280877	3.961231849	7.45641E-05	0.000124388	positive
PRJNA729271	stage_5W_vs_E10	ordinary_muscle_development	ENSGALG00010012693	UCHL5	-0.538613673	0.078215476	-6.886280019	5.72702E-12	1.53416E-11	negative
PRJNA729271	stage_5W_vs_E10	ordinary_muscle_development	ENSGALG00010012696	DNAL4	-1.129257545	0.095566499	-11.81645824	3.20932E-32	2.13097E-31	negative
PRJNA729271	stage_5W_vs_E10	ordinary_muscle_development	ENSGALG00010012699	RNF128	-0.014124888	0.144688793	-0.097622542	0.922232024	0.932720708	negative
PRJNA729271	stage_5W_vs_E10	ordinary_muscle_development	ENSGALG00010012702	PTPRO	-2.205199184	0.340625487	-6.473970001	9.54609E-11	2.37954E-10	negative
PRJNA729271	stage_5W_vs_E10	ordinary_muscle_development	ENSGALG00010012704	SMIM15	-0.035276969	0.067941098	-0.519228713	0.603601258	0.64129592	negative
PRJNA729271	stage_5W_vs_E10	ordinary_muscle_development	ENSGALG00010012705	CPM	1.265669287	0.18559417	6.819553026	9.13242E-12	2.42089E-11	positive
PRJNA729271	stage_5W_vs_E10	ordinary_muscle_development	ENSGALG00010012706	TBC1D8B	1.267050183	0.103524218	12.23916689	1.91993E-34	1.38455E-33	positive
PRJNA729271	stage_5W_vs_E10	ordinary_muscle_development	ENSGALG00010012708	SPP2	1.158217871	1.106572386	1.046671583	0.295251074	0.333385084	positive
PRJNA729271	stage_5W_vs_E10	ordinary_muscle_development	ENSGALG00010012709	PPP1R8	-1.210748285	0.058609379	-20.65792737	8.28447E-95	3.14354E-93	negative
PRJNA729271	stage_5W_vs_E10	ordinary_muscle_development	ENSGALG00010012711	ELL2	-0.296657779	0.127902669	-2.319402571	0.020373218	0.0268879	negative
PRJNA729271	stage_5W_vs_E10	ordinary_muscle_development	ENSGALG00010012712	EEF1E1	0.423517981	0.133209107	3.17934704	0.001476073	0.002199138	positive
PRJNA729271	stage_5W_vs_E10	ordinary_muscle_development	ENSGALG00010012714	RGS2	0.649496188	0.172967049	3.755028428	0.000173322	0.000279673	positive
PRJNA729271	stage_5W_vs_E10	ordinary_muscle_development	ENSGALG00010012719	COL10A1	-6.348843721	0.846002529	-7.504520975	6.16536E-14	1.83016E-13	negative
PRJNA729271	stage_5W_vs_E10	ordinary_muscle_development	ENSGALG00010012720	ACO2	0.949370202	0.119927204	7.916220577	2.44839E-15	7.83604E-15	positive
PRJNA729271	stage_5W_vs_E10	ordinary_muscle_development	ENSGALG00010012722	ENSGALG00010012722	0.37800332	0.098054173	3.855045716	0.000115708	0.000189384	positive
PRJNA729271	stage_5W_vs_E10	ordinary_muscle_development	ENSGALG00010012725	ENSGALG00010012725	1.721341319	0.256806159	6.702881758	2.04349E-11	5.31307E-11	positive
PRJNA729271	stage_5W_vs_E10	ordinary_muscle_development	ENSGALG00010012726	IL7R	6.709445012	0.462865809	14.49544314	1.29464E-47	1.4336E-46	positive
PRJNA729271	stage_5W_vs_E10	ordinary_muscle_development	ENSGALG00010012730	CAP2	2.359048192	0.167717378	14.06561572	6.17828E-45	6.29333E-44	positive
PRJNA729271	stage_5W_vs_E10	ordinary_muscle_development	ENSGALG00010012732	MGST1	-0.917355942	0.175548604	-5.225652167	1.73542E-07	3.52064E-07	negative
PRJNA729271	stage_5W_vs_E10	ordinary_muscle_development	ENSGALG00010012733	ENSGALG00010012733	-3.101232857	1.217478952	-2.547257881	0.010857317	0.014813329	negative
PRJNA729271	stage_5W_vs_E10	ordinary_muscle_development	ENSGALG00010012734	TMEM19	-0.538016423	0.145762584	-3.691046156	0.000223334	0.000357427	negative
PRJNA729271	stage_5W_vs_E10	ordinary_muscle_development	ENSGALG00010012738	RAB21	0.823697037	0.068506749	12.02358964	2.6711E-33	1.84282E-32	positive
PRJNA729271	stage_5W_vs_E10	ordinary_muscle_development	ENSGALG00010012741	ENSGALG00010012741	0.187073521	0.151799634	1.232371359	0.217810395	0.251727131	positive
PRJNA729271	stage_5W_vs_E10	ordinary_muscle_development	ENSGALG00010012742	ENSGALG00010012742	-2.118809762	0.174507114	-12.14168126	6.35075E-34	4.49483E-33	negative
PRJNA729271	stage_5W_vs_E10	ordinary_muscle_development	ENSGALG00010012743	ENSGALG00010012743	2.41309442	0.495502177	4.869997609	1.116E-06	2.13437E-06	positive
PRJNA729271	stage_5W_vs_E10	ordinary_muscle_development	ENSGALG00010012744	ARL4C	-2.343512051	0.233821342	-10.02266104	1.21195E-23	5.676E-23	negative
PRJNA729271	stage_5W_vs_E10	ordinary_muscle_development	ENSGALG00010012745	MARCHF3	-0.13686179	0.309486058	-0.442222796	0.658327992	0.692788169	negative
PRJNA729271	stage_5W_vs_E10	ordinary_muscle_development	ENSGALG00010012747	TOB2	1.777027361	0.145152025	12.24252548	1.8421E-34	1.32974E-33	positive
PRJNA729271	stage_5W_vs_E10	ordinary_muscle_development	ENSGALG00010012748	ENSGALG00010012748	0.428105654	0.214828859	1.992775347	0.046286052	0.058605549	positive
PRJNA729271	stage_5W_vs_E10	ordinary_muscle_development	ENSGALG00010012749	RGS4	2.408003198	0.176920893	13.61062084	3.4629E-42	3.23003E-41	positive
PRJNA729271	stage_5W_vs_E10	ordinary_muscle_development	ENSGALG00010012751	AP3B1	-0.108961478	0.074941883	-1.453946371	0.145961175	0.17346128	negative
PRJNA729271	stage_5W_vs_E10	ordinary_muscle_development	ENSGALG00010012753	GTPBP1	-0.506682943	0.053305618	-9.505244661	1.99578E-21	8.50599E-21	negative
PRJNA729271	stage_5W_vs_E10	ordinary_muscle_development	ENSGALG00010012755	CLVS2	-3.35372833	0.41954585	-7.993711123	1.30936E-15	4.25543E-15	negative
PRJNA729271	stage_5W_vs_E10	ordinary_muscle_development	ENSGALG00010012756	RPA2	-1.68535942	0.106035719	-15.89426129	6.94422E-57	1.01982E-55	negative
PRJNA729271	stage_5W_vs_E10	ordinary_muscle_development	ENSGALG00010012758	NAB1	-0.261831325	0.128870854	-2.031734229	0.042180569	0.053702885	negative
PRJNA729271	stage_5W_vs_E10	ordinary_muscle_development	ENSGALG00010012759	ATP1B4	7.009219053	0.197624764	35.46731152	1.5693E-275	5.5479E-273	positive
PRJNA729271	stage_5W_vs_E10	ordinary_muscle_development	ENSGALG00010012760	PLEKHF2	0.8419176	0.10552046	7.978714282	1.47865E-15	4.79273E-15	positive
PRJNA729271	stage_5W_vs_E10	ordinary_muscle_development	ENSGALG00010012761	SMPDL3A	2.285377292	0.22469711	10.17092427	2.67359E-24	1.28921E-23	positive
PRJNA729271	stage_5W_vs_E10	ordinary_muscle_development	ENSGALG00010012763	SMPDL3B	-0.210905143	0.134697923	-1.56576389	0.117403903	0.141272586	negative
PRJNA729271	stage_5W_vs_E10	ordinary_muscle_development	ENSGALG00010012765	PHF5A	0.202447748	0.106902418	1.893762111	0.058256593	0.072852154	positive
PRJNA729271	stage_5W_vs_E10	ordinary_muscle_development	ENSGALG00010012767	JOSD1	-0.723867186	0.071726452	-10.09205344	5.99031E-24	2.83849E-23	negative
PRJNA729271	stage_5W_vs_E10	ordinary_muscle_development	ENSGALG00010012768	EPS8	-0.658129175	0.116604748	-5.644102724	1.66045E-08	3.61874E-08	negative
PRJNA729271	stage_5W_vs_E10	ordinary_muscle_development	ENSGALG00010012769	NEMP2	-0.709537078	0.082927478	-8.556115528	1.16734E-17	4.19763E-17	negative
PRJNA729271	stage_5W_vs_E10	ordinary_muscle_development	ENSGALG00010012771	ITGA2	-2.438772017	0.201034607	-12.13110546	7.22672E-34	5.09987E-33	negative
PRJNA729271	stage_5W_vs_E10	ordinary_muscle_development	ENSGALG00010012772	TOMM22	-0.611986025	0.074823874	-8.179020796	2.8616E-16	9.60603E-16	negative
PRJNA729271	stage_5W_vs_E10	ordinary_muscle_development	ENSGALG00010012773	XKR8	-0.489971667	0.128784618	-3.8045822	0.000142044	0.0002309	negative
PRJNA729271	stage_5W_vs_E10	ordinary_muscle_development	ENSGALG00010012775	KCNE2	3.482940282	0.145365828	23.95982825	7.2989E-127	5.111E-125	positive
PRJNA729271	stage_5W_vs_E10	ordinary_muscle_development	ENSGALG00010012778	TBC1D15	0.199435046	0.151678454	1.314854157	0.188558922	0.220398482	positive
PRJNA729271	stage_5W_vs_E10	ordinary_muscle_development	ENSGALG00010012780	FABP7	-3.170979873	0.258044915	-12.28848037	1.04451E-34	7.62346E-34	negative
PRJNA729271	stage_5W_vs_E10	ordinary_muscle_development	ENSGALG00010012781	SPIN1L	1.272649695	3.183825741	0.399723414	0.689360245	0.722402888	positive
PRJNA729271	stage_5W_vs_E10	ordinary_muscle_development	ENSGALG00010012786	TEF	1.221794997	0.087741103	13.92500149	4.46532E-44	4.42714E-43	positive
PRJNA729271	stage_5W_vs_E10	ordinary_muscle_development	ENSGALG00010012788	RGS1	3.764594696	0.631087031	5.965254413	2.44253E-09	5.57903E-09	positive
PRJNA729271	stage_5W_vs_E10	ordinary_muscle_development	ENSGALG00010012790	PITX1	-6.189093697	0.642334201	-9.635317075	5.67166E-22	2.48296E-21	negative
PRJNA729271	stage_5W_vs_E10	ordinary_muscle_development	ENSGALG00010012791	CFAP418	0.202877391	0.190625872	1.064269972	0.287206417	0.324920738	positive
PRJNA729271	stage_5W_vs_E10	ordinary_muscle_development	ENSGALG00010012792	CBY1	-0.284781772	0.144730589	-1.967668163	0.049106231	0.06196525	negative
PRJNA729271	stage_5W_vs_E10	ordinary_muscle_development	ENSGALG00010012795	TBCA	-0.686970479	0.137053688	-5.012418764	5.37501E-07	1.05242E-06	negative
PRJNA729271	stage_5W_vs_E10	ordinary_muscle_development	ENSGALG00010012796	MOCS2	0.959296826	0.120759172	7.943883738	1.95947E-15	6.31168E-15	positive
PRJNA729271	stage_5W_vs_E10	ordinary_muscle_development	ENSGALG00010012797	ZC3H7B	0.288427309	0.070290787	4.103344453	4.0722E-05	6.91502E-05	positive
PRJNA729271	stage_5W_vs_E10	ordinary_muscle_development	ENSGALG00010012798	MFSD6	0.746809073	0.095186688	7.845730203	4.30441E-15	1.35783E-14	positive
PRJNA729271	stage_5W_vs_E10	ordinary_muscle_development	ENSGALG00010012801	DMC1	2.392469469	0.43238795	5.533154815	3.14522E-08	6.70933E-08	positive
PRJNA729271	stage_5W_vs_E10	ordinary_muscle_development	ENSGALG00010012803	NUP155	-1.099983737	0.090747448	-12.12137379	8.13834E-34	5.72369E-33	negative
PRJNA729271	stage_5W_vs_E10	ordinary_muscle_development	ENSGALG00010012804	ENSGALG00010012804	0.539723508	0.160612894	3.360399622	0.000778298	0.001190401	positive
PRJNA729271	stage_5W_vs_E10	ordinary_muscle_development	ENSGALG00010012808	EYA3	-0.42160015	0.093137854	-4.526625127	5.99331E-06	1.08415E-05	negative
PRJNA729271	stage_5W_vs_E10	ordinary_muscle_development	ENSGALG00010012809	RHOBTB3	5.370558723	0.238033821	22.56216659	1.02E-112	5.4358E-111	positive
PRJNA729271	stage_5W_vs_E10	ordinary_muscle_development	ENSGALG00010012812	DECR1	0.503429309	0.239840793	2.099014527	0.03581562	0.046064544	positive
PRJNA729271	stage_5W_vs_E10	ordinary_muscle_development	ENSGALG00010012818	RGS18	1.929560962	0.192233557	10.03758653	1.04192E-23	4.89076E-23	positive
PRJNA729271	stage_5W_vs_E10	ordinary_muscle_development	ENSGALG00010012819	RANGAP1	-0.267238932	0.067902695	-3.935615978	8.29836E-05	0.000137846	negative
PRJNA729271	stage_5W_vs_E10	ordinary_muscle_development	ENSGALG00010012821	ZBTB33	0.04416903	0.051962672	0.850014607	0.395316965	0.436580527	positive
PRJNA729271	stage_5W_vs_E10	ordinary_muscle_development	ENSGALG00010012824	HIBCH	1.002408286	0.117320789	8.544165917	1.29468E-17	4.64629E-17	positive
PRJNA729271	stage_5W_vs_E10	ordinary_muscle_development	ENSGALG00010012827	ENSGALG00010012827	-0.386582698	0.191505125	-2.018654588	0.043523133	0.055315021	negative
PRJNA729271	stage_5W_vs_E10	ordinary_muscle_development	ENSGALG00010012829	RGS5	5.072251529	0.180466254	28.10637119	8.1883E-174	1.0889E-171	positive
PRJNA729271	stage_5W_vs_E10	ordinary_muscle_development	ENSGALG00010012830	ARRDC3	1.351443619	0.149068379	9.065930882	1.23545E-19	4.86361E-19	positive
PRJNA729271	stage_5W_vs_E10	ordinary_muscle_development	ENSGALG00010012831	ENSGALG00010012831	-1.668523305	0.385887722	-4.323856943	1.53325E-05	2.6955E-05	negative
PRJNA729271	stage_5W_vs_E10	ordinary_muscle_development	ENSGALG00010012832	CHADL	-3.05914148	0.362429489	-8.440652797	3.1557E-17	1.10916E-16	negative
PRJNA729271	stage_5W_vs_E10	ordinary_muscle_development	ENSGALG00010012833	JAK2	0.845939593	0.105347713	8.029975839	9.74919E-16	3.18563E-15	positive
PRJNA729271	stage_5W_vs_E10	ordinary_muscle_development	ENSGALG00010012834	TPH2	-3.655074232	0.717077725	-5.097179993	3.44751E-07	6.83699E-07	negative
PRJNA729271	stage_5W_vs_E10	ordinary_muscle_development	ENSGALG00010012836	ENSGALG00010012836	-0.58226487	1.152498424	-0.505219667			negative
PRJNA729271	stage_5W_vs_E10	ordinary_muscle_development	ENSGALG00010012837	PKIB	1.899398676	0.262637254	7.232023052	4.75851E-13	1.34795E-12	positive
PRJNA729271	stage_5W_vs_E10	ordinary_muscle_development	ENSGALG00010012838	C2orf88	0.468717437	0.228743034	2.049100377	0.040452301	0.05161131	positive
PRJNA729271	stage_5W_vs_E10	ordinary_muscle_development	ENSGALG00010012839	TGFBI	-1.00611956	0.180051912	-5.587941534	2.29777E-08	4.95997E-08	negative
PRJNA729271	stage_5W_vs_E10	ordinary_muscle_development	ENSGALG00010012841	L3MBTL2	-0.811112661	0.094950955	-8.54243816	1.31419E-17	4.71515E-17	negative
PRJNA729271	stage_5W_vs_E10	ordinary_muscle_development	ENSGALG00010012842	DDX17	-0.004389302	0.049096271	-0.089401944	0.928762479	0.938408764	negative
PRJNA729271	stage_5W_vs_E10	ordinary_muscle_development	ENSGALG00010012843	ENSGALG00010012843	0.246312502	0.158324931	1.555740474	0.119769818	0.143916398	positive
PRJNA729271	stage_5W_vs_E10	ordinary_muscle_development	ENSGALG00010012845	HDHD2	-0.121649457	0.102933148	-1.181829751	0.23727326	0.272439473	negative
PRJNA729271	stage_5W_vs_E10	ordinary_muscle_development	ENSGALG00010012847	RLN3	1.53026165	0.669212683	2.286659666	0.022215696	0.029184023	positive

PRJNA729271	stage_5W_vs_E10	ordinary_muscle_development	ENSGALG00010012848	SERINC1	0.90064949	0.092478718	9.738991967	2.05583E-22	9.16057E-22	positive
PRJNA729271	stage_5W_vs_E10	ordinary_muscle_development	ENSGALG00010012849	TRHDE	-3.639704761	0.360784396	-10.08830981	6.22318E-24	2.94691E-23	negative
PRJNA729271	stage_5W_vs_E10	ordinary_muscle_development	ENSGALG00010012850	CALB1	1.317369912	1.118383514	1.177923222	0.238827216	0.274115172	positive
PRJNA729271	stage_5W_vs_E10	ordinary_muscle_development	ENSGALG00010012851	ENSGALG00010012851	-1.223303109	0.270616086	-4.52043752	6.1712E-06	1.11508E-05	negative
PRJNA729271	stage_5W_vs_E10	ordinary_muscle_development	ENSGALG00010012852	MSTN	1.045006989	0.419013139	2.493971885	0.012632255	0.017094999	positive
PRJNA729271	stage_5W_vs_E10	ordinary_muscle_development	ENSGALG00010012854	LECT2	-0.384755227	0.776093365	-0.495758944	0.620064495	0.656573515	negative
PRJNA729271	stage_5W_vs_E10	ordinary_muscle_development	ENSGALG00010012856	AVR2	1.455278548	1.074397252	1.354506954	0.175574696	0.206252956	positive
PRJNA729271	stage_5W_vs_E10	ordinary_muscle_development	ENSGALG00010012857	NSDHL	-1.767138598	0.223432979	-7.909032059	2.59398E-15	8.28918E-15	negative
PRJNA729271	stage_5W_vs_E10	ordinary_muscle_development	ENSGALG00010012859	FBXL21	0.976094967	0.111255333	8.773466796	1.73248E-18	6.47391E-18	positive
PRJNA729271	stage_5W_vs_E10	ordinary_muscle_development	ENSGALG00010012860	ENSGALG00010012860	1.041659995	0.260904809	3.992490593	6.53829E-05	0.000109596	positive
PRJNA729271	stage_5W_vs_E10	ordinary_muscle_development	ENSGALG00010012861	WDR70	-0.465289312	0.083479586	-5.573689718	2.494E-08	5.37234E-08	negative
PRJNA729271	stage_5W_vs_E10	ordinary_muscle_development	ENSGALG00010012863	KCNE1	1.071746887	0.422026498	2.539525108	0.011100308	0.015132038	positive
PRJNA729271	stage_5W_vs_E10	ordinary_muscle_development	ENSGALG00010012865	EP300	0.604554385	0.12304904	4.913117453	8.96395E-07	1.72829E-06	positive
PRJNA729271	stage_5W_vs_E10	ordinary_muscle_development	ENSGALG00010012867	A4GALT	0.908039017	0.102067173	8.896484407	5.76436E-19	2.19533E-18	positive
PRJNA729271	stage_5W_vs_E10	ordinary_muscle_development	ENSGALG00010012868	CTHRC1	-4.315598493	0.599200094	-7.202266044	5.922E-13	1.67133E-12	negative
PRJNA729271	stage_5W_vs_E10	ordinary_muscle_development	ENSGALG00010012869	ENSGALG00010012869	0.894847202	0.215910385	4.144530615	3.4051E-05	5.81149E-05	positive
PRJNA729271	stage_5W_vs_E10	ordinary_muscle_development	ENSGALG00010012870	KCNC2	-2.695289404	0.220248375	-12.2374996	1.95977E-34	1.41187E-33	negative
PRJNA729271	stage_5W_vs_E10	ordinary_muscle_development	ENSGALG00010012871	ENSGALG00010012871	2.158483423	0.347772673	6.206592967	5.41456E-10	1.28641E-09	positive
PRJNA729271	stage_5W_vs_E10	ordinary_muscle_development	ENSGALG00010012873	PTAFR	2.949535418	0.220609618	13.36993123	9.06246E-41	8.10366E-40	positive
PRJNA729271	stage_5W_vs_E10	ordinary_muscle_development	ENSGALG00010012875	HSF2	-0.676778148	0.134026326	-5.049591144	4.42757E-07	8.71504E-07	negative
PRJNA729271	stage_5W_vs_E10	ordinary_muscle_development	ENSGALG00010012876	KDELR3	-1.241295284	0.163737833	-7.580992486	3.42921E-14	1.03275E-13	negative
PRJNA729271	stage_5W_vs_E10	ordinary_muscle_development	ENSGALG00010012877	SLC25A32	-0.500545455	0.095883577	-5.220346075	1.78589E-07	3.61948E-07	negative
PRJNA729271	stage_5W_vs_E10	ordinary_muscle_development	ENSGALG00010012878	GDNF	0.260204488	0.318573659	0.816779668	0.414054354	0.455642109	positive
PRJNA729271	stage_5W_vs_E10	ordinary_muscle_development	ENSGALG00010012879	RCAN1	0.061385816	0.17362569	0.353552609	0.723674196	0.7538923	positive
PRJNA729271	stage_5W_vs_E10	ordinary_muscle_development	ENSGALG00010012880	CLK4	1.460050708	0.139100314	10.49638683	8.975E-26	4.59204E-25	positive
PRJNA729271	stage_5W_vs_E10	ordinary_muscle_development	ENSGALG00010012882	ENSGALG00010012882	1.294676779	0.574581962	2.253249953	0.024243393	0.031744172	positive
PRJNA729271	stage_5W_vs_E10	ordinary_muscle_development	ENSGALG00010012883	DCAF13	-0.649783204	0.067250629	-9.662113404	4.36759E-22	1.91959E-21	negative
PRJNA729271	stage_5W_vs_E10	ordinary_muscle_development	ENSGALG00010012884	ENSGALG00010012884	-0.028744631	0.212834878	-0.135056017	0.892567585	0.905942661	negative
PRJNA729271	stage_5W_vs_E10	ordinary_muscle_development	ENSGALG00010012885	PMS1	0.365199057	0.129422929	2.821749291	0.00477625	0.006758272	positive
PRJNA729271	stage_5W_vs_E10	ordinary_muscle_development	ENSGALG00010012887	KCNJ4	0.090210362	0.432256977	0.208696137	0.834685456	0.854553304	positive
PRJNA729271	stage_5W_vs_E10	ordinary_muscle_development	ENSGALG00010012888	ENSGALG00010012888	3.313006611	3.604549308	0.919118128	0.358033789	0.39813577	positive
PRJNA729271	stage_5W_vs_E10	ordinary_muscle_development	ENSGALG00010012889	RIC1	0.728443581	0.104850883	6.947424382	3.72015E-12	1.00604E-11	positive
PRJNA729271	stage_5W_vs_E10	ordinary_muscle_development	ENSGALG00010012890	MFAP3	-0.307838196	0.070144924	-4.388602635	1.14081E-05	2.0245E-05	negative
PRJNA729271	stage_5W_vs_E10	ordinary_muscle_development	ENSGALG00010012891	CAPS2	-3.16391404	0.342840499	-9.228530604	2.7437E-20	1.10903E-19	negative
PRJNA729271	stage_5W_vs_E10	ordinary_muscle_development	ENSGALG00010012892	ORMDL1	0.301788676	0.161898206	1.864064358	0.062312667	0.077663122	positive
PRJNA729271	stage_5W_vs_E10	ordinary_muscle_development	ENSGALG00010012893	ENSGALG00010012893	1.607659524	0.187681277	8.565902542	1.07233E-17	3.8617E-17	positive
PRJNA729271	stage_5W_vs_E10	ordinary_muscle_development	ENSGALG00010012894	CLIC6	-0.312224374	0.671821673	-0.464742931	0.642115561	0.677202056	negative
PRJNA729271	stage_5W_vs_E10	ordinary_muscle_development	ENSGALG00010012896	STK38L	0.536639514	0.099099083	5.415181432	6.12266E-08	1.28161E-07	positive
PRJNA729271	stage_5W_vs_E10	ordinary_muscle_development	ENSGALG00010012897	ENSGALG00010012897	0.790238179	0.300444128	2.630233395	0.008532627	0.011765641	positive
PRJNA729271	stage_5W_vs_E10	ordinary_muscle_development	ENSGALG00010012898	FAM114A2	1.076234181	0.114520052	9.397779347	5.57267E-21	2.3265E-20	positive
PRJNA729271	stage_5W_vs_E10	ordinary_muscle_development	ENSGALG00010012899	ENSGALG00010012899	0.99897788	0.130541828	7.652550081	1.97032E-14	5.99745E-14	positive
PRJNA729271	stage_5W_vs_E10	ordinary_muscle_development	ENSGALG00010012900	RBX1	0.450864746	0.172223476	2.617905272	0.008847136	0.012173841	positive
PRJNA729271	stage_5W_vs_E10	ordinary_muscle_development	ENSGALG00010012903	RGMB	0.056203346	0.092645624	0.606648693	0.544084079	0.583839112	positive
PRJNA729271	stage_5W_vs_E10	ordinary_muscle_development	ENSGALG00010012904	ERMP1	-0.176872965	0.135905675	-1.301439147	0.193108181	0.225261797	negative
PRJNA729271	stage_5W_vs_E10	ordinary_muscle_development	ENSGALG00010012905	NKAIN1	-3.205006664	0.284377592	-11.27025036	1.84046E-29	1.09021E-28	negative
PRJNA729271	stage_5W_vs_E10	ordinary_muscle_development	ENSGALG00010012908	STRAP	-0.432460163	0.083244587	-5.195054442	2.0466E-07	4.12938E-07	negative
PRJNA729271	stage_5W_vs_E10	ordinary_muscle_development	ENSGALG00010012910	XPNPEP3	0.097728667	0.099908738	0.978179375	0.327985616	0.367655363	positive
PRJNA729271	stage_5W_vs_E10	ordinary_muscle_development	ENSGALG00010012911	CSNK1E	-0.910901868	0.085789501	-10.61787119	2.4611E-26	1.29346E-25	negative
PRJNA729271	stage_5W_vs_E10	ordinary_muscle_development	ENSGALG00010012912	NKAPL	-0.067551971	0.100626182	-0.671316054	0.50201921	0.543001899	negative
PRJNA729271	stage_5W_vs_E10	ordinary_muscle_development	ENSGALG00010012913	EGFLAM	-1.554391128	0.155976835	-9.965525514	2.1573E-23	1.0016E-22	negative
PRJNA729271	stage_5W_vs_E10	ordinary_muscle_development	ENSGALG00010012915	ENSGALG00010012915	5.903832277	0.278178627	21.22316997	5.83541E-100	2.43759E-98	positive
PRJNA729271	stage_5W_vs_E10	ordinary_muscle_development	ENSGALG00010012917	RUNX1	1.318145007	0.247402326	5.327941047	9.93324E-08	2.05103E-07	positive
PRJNA729271	stage_5W_vs_E10	ordinary_muscle_development	ENSGALG00010012918	GLRX	1.543241353	0.277462295	5.561985837	2.66722E-08	5.7293E-08	positive
PRJNA729271	stage_5W_vs_E10	ordinary_muscle_development	ENSGALG00010012921	EBF1	-0.007371574	0.140318987	-0.0525344	0.958102886	0.963687553	negative
PRJNA729271	stage_5W_vs_E10	ordinary_muscle_development	ENSGALG00010012922	SNRNP40	-1.321246692	0.130524657	-10.12258312	4.38682E-24	2.09651E-23	negative
PRJNA729271	stage_5W_vs_E10	ordinary_muscle_development	ENSGALG00010012923	MLANA	-4.636092128	0.941452558	-4.924403347	8.46181E-07	1.63495E-06	negative
PRJNA729271	stage_5W_vs_E10	ordinary_muscle_development	ENSGALG00010012924	GLIPR1	0.402978505	0.201058685	2.004283005	0.045039753	0.057112346	positive
PRJNA729271	stage_5W_vs_E10	ordinary_muscle_development	ENSGALG00010012925	SETD4	-1.110255727	0.191176621	-5.807486917	6.34175E-09	1.41639E-08	negative
PRJNA729271	stage_5W_vs_E10	ordinary_muscle_development	ENSGALG00010012926	OSGEPL1	0.514407063	0.164724941	3.122824388	0.001791246	0.00264535	positive
PRJNA729271	stage_5W_vs_E10	ordinary_muscle_development	ENSGALG00010012927	ENSGALG00010012927	3.680546953	3.683291515	0.999254862			positive
PRJNA729271	stage_5W_vs_E10	ordinary_muscle_development	ENSGALG00010012930	IL6ST	0.787832696	0.09086658	8.670213983	4.31309E-18	1.58034E-17	positive
PRJNA729271	stage_5W_vs_E10	ordinary_muscle_development	ENSGALG00010012932	ATF6	0.49189332	0.118266205	4.159204386	3.19358E-05	5.46528E-05	positive
PRJNA729271	stage_5W_vs_E10	ordinary_muscle_development	ENSGALG00010012933	ZCCHC17	-0.310828694	0.146617295	-2.120000191	0.034006029	0.043849959	negative
PRJNA729271	stage_5W_vs_E10	ordinary_muscle_development	ENSGALG00010012934	NDUFA1	1.437788572	0.151555217	9.486895935	2.38016E-21	1.01115E-20	positive
PRJNA729271	stage_5W_vs_E10	ordinary_muscle_development	ENSGALG00010012938	UPF3B	0.527348846	0.150212552	3.510684296	0.000446955	0.00069655	positive
PRJNA729271	stage_5W_vs_E10	ordinary_muscle_development	ENSGALG00010012939	GJA1	-2.903066648	0.207333794	-14.00189804	1.51763E-44	1.51606E-43	negative
PRJNA729271	stage_5W_vs_E10	ordinary_muscle_development	ENSGALG00010012940	FBN2	-3.99413865	0.22518596	-17.73706784	2.16965E-70	4.61808E-69	negative
PRJNA729271	stage_5W_vs_E10	ordinary_muscle_development	ENSGALG00010012943	ASNSD1	-0.346272816	0.123360242	-2.807005005	0.005000446	0.007059654	negative
PRJNA729271	stage_5W_vs_E10	ordinary_muscle_development	ENSGALG00010012944	NCK1	-0.314549121	0.05275646	-5.962286359	2.48733E-09	5.67866E-09	negative
PRJNA729271	stage_5W_vs_E10	ordinary_muscle_development	ENSGALG00010012945	CLINT1	0.024660519	0.062967666	0.391637817	0.695325841	0.727865102	positive
PRJNA729271	stage_5W_vs_E10	ordinary_muscle_development	ENSGALG00010012948	ST13P5	-0.673811611	0.095364009	-7.065680439	1.59831E-12	4.40865E-12	negative
PRJNA729271	stage_5W_vs_E10	ordinary_muscle_development	ENSGALG00010012949	ENSGALG00010012949	-0.500070932	0.174903509	-2.859124642	0.004248118	0.006046988	negative
PRJNA729271	stage_5W_vs_E10	ordinary_muscle_development	ENSGALG00010012952	BRD10	1.476824018	0.186297414	7.92723843	2.24073E-15	7.19047E-15	positive
PRJNA729271	stage_5W_vs_E10	ordinary_muscle_development	ENSGALG00010012954	RIMS2	0.203201839	0.327465636	0.620528741	0.534909738	0.574973426	positive
PRJNA729271	stage_5W_vs_E10	ordinary_muscle_development	ENSGALG00010012955	RPL39L	-0.058063004	0.155219242	-0.374070919	0.708351554	0.73995069	negative
PRJNA729271	stage_5W_vs_E10	ordinary_muscle_development	ENSGALG00010012956	FABP3	2.9870104	0.168311621	17.7469053	1.82117E-70	3.89349E-69	positive
PRJNA729271	stage_5W_vs_E10	ordinary_muscle_development	ENSGALG00010012957	DERA	-0.358674176	0.228680138	-1.568453559	0.11677532	0.140609592	negative
PRJNA729271	stage_5W_vs_E10	ordinary_muscle_development	ENSGALG00010012958	OLFML2B	1.182782176	0.204853362	5.773799196	7.75038E-09	1.72279E-08	positive
PRJNA729271	stage_5W_vs_E10	ordinary_muscle_development	ENSGALG00010012959	TBC1D32	0.040799626	0.146990522	0.27756637	0.781345251	0.807644542	positive
PRJNA729271	stage_5W_vs_E10	ordinary_muscle_development	ENSGALG00010012962	SLC25A17	-0.596536611	0.20950987	-2.847295981	0.004409235	0.006262185	negative
PRJNA729271	stage_5W_vs_E10	ordinary_muscle_development	ENSGALG00010012963	UIMC1	-0.427149555	0.09350541	-4.568180132	4.91977E-06	8.95655E-06	negative
PRJNA729271	stage_5W_vs_E10	ordinary_muscle_development	ENSGALG00010012964	CBR3	-0.387118006	0.072822343	-5.315923542	1.06118E-07	2.18522E-07	negative
PRJNA729271	stage_5W_vs_E10	ordinary_muscle_development	ENSGALG00010012965	ENSGALG00010012965	-0.676114825	0.086021772	-7.859810482	3.84715E-15	1.21783E-14	negative
PRJNA729271	stage_5W_vs_E10	ordinary_muscle_development	ENSGALG00010012966	GLIPR1L	1.606275899	0.265173294	6.057457268	1.3829E-09	3.20415E-09	positive
PRJNA729271	stage_5W_vs_E10	ordinary_muscle_development	ENSGALG00010012967	ECRG4	-1.836090357	0.210086308	-8.739695471	2.3374E-18	8.6651E-18	negative
PRJNA729271	stage_5W_vs_E10	ordinary_muscle_development	ENSGALG00010012968	LSM11	-1.109117468	0.078954608	-14.04753303	7.97645E-45	8.07957E-44	negative
PRJNA729271	stage_5W_vs_E10	ordinary_muscle_development	ENSGALG00010012969	SLC12A2	0.118570379	0.142375875	0.832798246	0.404958524	0.446446137	positive
PRJNA729271	stage_5W_vs_E10	ordinary_muscle_development	ENSGALG00010012970	UNC5A	-1.787902564	0.201943874	-8.853462732	8.4845E-19	3.20853E-18	negative
PRJNA729271	stage_5W_vs_E10	ordinary_muscle_development	ENSGALG00010012971	ENSGALG00010012971	-2.558620577	0.893563543	-2.863389623	0.004191348	0.005969696	negative
PRJNA729271	stage_5W_vs_E10	ordinary_muscle_development	ENSGALG00010012973	GLDC	-5.22301905	0.358501273	-14.56903908	4.42065E-48	4.95955E-47	negative
PRJNA729271	stage_5W_vs_E10	ordinary_muscle_development	ENSGALG00010012974	SLC49A3	-0.783290149	0.217835056	-3.595794733	0.000323403	0.000510423	negative
PRJNA729271	stage_5W_vs_E10	ordinary_muscle_development	ENSGALG00010012975	KRR1	-0.529920299	0.093945079	-5.640745694	1.69315E-08	3.68613E-08	negative
PRJNA729271	stage_5W_vs_E10	ordinary_muscle_development	ENSGALG00010012976	SLC9A2	3.293647249	0.22754369	14.47479054	1.74858E-47	1.9245E-46	positive
PRJNA729271	stage_5W_vs_E10	ordinary_muscle_development	ENSGALG00010012977	NKRF	-0.56959975	0.100378024	-5.674546376	1.39057E-08	3.04705E-08	negative
PRJNA729271	stage_5W_vs_E10	ordinary_muscle_development	ENSGALG00010012978	HSD17B7	-0.017768281	0.209958208	-0.084627705	0.932557373	0.941455573	negative
PRJNA729271	stage_5W_vs_E10	ordinary_muscle_development	ENSGALG00010012979	HTR1A	1.545814767	0.530303266	2.914963696	0.003557299	0.005100203	positive
PRJNA729271	stage_5W_vs_E10	ordinary_muscle_development	ENSGALG00010012980	KICS2	-0.374597197	0.078257685	-4.786714539	1.69534E-06	3.19598E-06	negative
PRJNA729271	stage_5W_vs_E10	ordinary_muscle_development	ENSGALG00010012982	DDR2	-1.455966922	0.245123874	-5.939718957	2.85511E-09	6.49174E-09	negative
PRJNA729271	stage_5W_vs_E10	ordinary_muscle_development	ENSGALG00010012985	ARNTL2	-0.419190629	0.171292025	-2.447227929	0.014395977	0.019364299	negative
PRJNA729271	stage_5W_vs_E10	ordinary_muscle_development	ENSGALG00010012986	TMEM184B	0.429375842	0.140420095	3.057794839	0.002229722	0.003261992	positive
PRJNA729271	stage_5W_vs_E10	ordinary_muscle_development	ENSGALG00010012988	RPL18A	-0.257639469	0.134981187	-1.908706501	0.056299964	0.070527005	negative
PRJNA729271	stage_5W_vs_E10	ordinary_muscle_development	ENSGALG00010012991	MFSD9	0.056664255	0.1021397	0.554772089	0.57905056	0.617758673	positive
PRJNA729271	stage_5W_vs_E10	ordinary_muscle_development	ENSGALG00010012992	HARS1	-0.554470228	0.127696161	-4.342105711	1.41124E-05	2.48855E-05	negative
PRJNA729271	stage_5W_vs_E10	ordinary_muscle_development	ENSGALG00010012993	DOP1B	0.181871872	0.098762667	1.841504253	0.065547698	0.081470921	positive
PRJNA729271	stage_5W_vs_E10	ordinary_muscle_development	ENSGALG00010012995	SERINC2	0.477449448	0.151590711	3.149595674	0.001634966	0.002424432	positive
PRJNA729271	stage_5W_vs_E10	ordinary_muscle_development	ENSGALG00010012997	UBE2A	0.733129845	0.057531265	12.7431552	3.40347E-37	2.72259E-36	positive
PRJNA729271	stage_5W_vs_E10	ordinary_muscle_development	ENSGALG00010012998	MAFF	-0.123551558	0.200643923	-0.615775231	0.538042877	0.577998333	negative
PRJNA729271	stage_5W_vs_E10	ordinary_muscle_development	ENSGALG00010013001	ADGRV1	-2.044098108	0.350743249	-5.827904347	5.61277E-09	1.25726E-08	negative
PRJNA729271	stage_5W_vs_E10	ordinary_muscle_development	ENSGALG00010013004	UAP1	-0.450034386	0.154523464	-2.912401625	0.003586612	0.005139179	negative
PRJNA729271	stage_5W_vs_E10	ordinary_muscle_development	ENSGALG00010013005	SLC40A1	0.93582329	0.090685551	10.31943108	5.75632E-25	2.86138E-24	positive
PRJNA729271	stage_5W_vs_E10	ordinary_muscle_development	ENSGALG00010013006	GRIK2	-6.044670873	1.174255433	-5.147662682	2.63752E-07	5.27468E-07	negative
PRJNA729271	stage_5W_vs_E10	ordinary_muscle_development	ENSGALG00010013008	STEEP1	-0.261198804	0.110011287	-2.374290966	0.017582689	0.023394628	negative
PRJNA729271	stage_5W_vs_E10	ordinary_muscle_development	ENSGALG00010013012	TMEM182	2.05924924	0.151494509	13.5928969	4.41263E-42	4.09744E-41	positive
PRJNA729271	stage_5W_vs_E10	ordinary_muscle_development	ENSGALG00010013013	DCSTAMP	5.71561615	1.062107261	5.381392593	7.39118E-08	1.53775E-07	positive
PRJNA729271	stage_5W_vs_E10	ordinary_muscle_development	ENSGALG00010013015	PLA2G6	0.81568043	0.14940877	5.459387895	4.77779E-08	1.00792E-07	positive
PRJNA729271	stage_5W_vs_E10	ordinary_muscle_development	ENSGALG00010013016	QRSL1	0.860918715	0.122208085	7.044695246	1.85868E-12	5.11322E-12	positive
PRJNA729271	stage_5W_vs_E10	ordinary_muscle_development	ENSGALG00010013017	SCMH1	0.759271082	0.158453162	4.791769837	1.65316E-06	3.11892E-06	positive
PRJNA729271	stage_5W_vs_E10	ordinary_muscle_development	ENSGALG00010013018	ID1	-0.06308267	0.192762367	-0.32725615	0.743474155	0.772070209	negative
PRJNA729271	stage_5W_vs_E10	ordinary_muscle_development	ENSGALG00010013019	DPYS	0.189393805	0.942388943	0.200972016	0.840720456	0.859881669	positive
PRJNA729271	stage_5W_vs_E10	ordinary_muscle_development	ENSGALG00010013021	SLC25A5	-1.425841769	0.164604992	-8.662202496	4.62735E-18	1.69335E-17	negative
PRJNA729271	stage_5W_vs_E10	ordinary_muscle_development	ENSGALG00010013027	UHMK1	1.36174991	0.125189399	10.87751771	1.47526E-27	8.12456E-27	positive
PRJNA729271	stage_5W_vs_E10	ordinary_muscle_development	ENSGALG00010013029	MYLK2	9.624272492	0.253700941	37.9355017	6.6912E-315	2.8526E-312	positive
PRJNA729271	stage_5W_vs_E10	ordinary_muscle_development	ENSGALG00010013032	BAIAP2L2	2.099436153	0.38028167	5.520739799	3.37575E-08	7.18419E-08	positive
PRJNA729271	stage_5W_vs_E10	ordinary_muscle_development	ENSGALG00010013033	MAP3K1	0.118437914	0.125347271	0.944878282	0.34472102	0.38448224	positive
PRJNA729271	stage_5W_vs_E10	ordinary_muscle_development	ENSGALG00010013036	ENSGALG00010013036	1.720918293	0.401713203	4.283947551	1.83606E-05	3.20802E-05	positive
PRJNA729271	stage_5W_vs_E10	ordinary_muscle_development	ENSGALG00010013037	MORC4	0.709834713	0.089693228	7.914027965	2.49192E-15	7.97184E-15	positive
PRJNA729271	stage_5W_vs_E10	ordinary_muscle_development	ENSGALG00010013040	SLC25A43	2.030126171	0.203922844	9.955364174	2.38946E-23	1.10656E-22	positive
PRJNA729271	stage_5W_vs_E10	ordinary_muscle_development	ENSGALG00010013041	LRP12	0.283872227	0.075030654	3.783416642	0.00015469	0.000250473	positive
PRJNA729271	stage_5W_vs_E10	ordinary_muscle_development	ENSGALG00010013042	SLC25A48	-1.150979491	0.313594186	-3.670283258	0.000242282	0.000386643	negative
PRJNA729271	stage_5W_vs_E10	ordinary_muscle_development	ENSGALG00010013044	ENSGALG00010013044	0.408394805	0.126492472	3.228609556	0.001243936	0.001866548	positive
PRJNA729271	stage_5W_vs_E10	ordinary_muscle_development	ENSGALG00010013045	FSD1L	1.586534605	0.143894101	11.02570986	2.87243E-28	1.62323E-27	positive

PRJNA729271	stage_5W_vs_E10	ordinary_muscle_development	ENSGALG00010013046	SLC12A5	-0.770889781	0.382721101	-2.014233807	0.043984992	0.055862828	negative
PRJNA729271	stage_5W_vs_E10	ordinary_muscle_development	ENSGALG00010013047	CTPS1	-0.192575058	0.100863736	-1.909259622	0.056228608	0.070443706	negative
PRJNA729271	stage_5W_vs_E10	ordinary_muscle_development	ENSGALG00010013048	WDR75	-1.653887527	0.090320032	-18.31141441	6.71039E-75	1.62654E-73	negative
PRJNA729271	stage_5W_vs_E10	ordinary_muscle_development	ENSGALG00010013051	XPOT	0.299412008	0.078661973	3.806311959	0.000141054	0.000229393	positive
PRJNA729271	stage_5W_vs_E10	ordinary_muscle_development	ENSGALG00010013053	MRTFA	1.142143733	0.17407657	6.561157144	5.33918E-11	1.35323E-10	positive
PRJNA729271	stage_5W_vs_E10	ordinary_muscle_development	ENSGALG00010013056	ENSGALG00010013056	2.533696253	0.562420545	4.504985238	6.63776E-06	1.19684E-05	positive
PRJNA729271	stage_5W_vs_E10	ordinary_muscle_development	ENSGALG00010013058	ENSGALG00010013058	0.853005554	0.274075968	3.112296061	0.001856382	0.002737918	positive
PRJNA729271	stage_5W_vs_E10	ordinary_muscle_development	ENSGALG00010013062	ENSGALG00010013062	0.096495314	0.106584418	0.905341664	0.36528441	0.405918241	positive
PRJNA729271	stage_5W_vs_E10	ordinary_muscle_development	ENSGALG00010013063	PARP12	1.828600155	0.216864688	8.431986636	3.39846E-17	1.19246E-16	positive
PRJNA729271	stage_5W_vs_E10	ordinary_muscle_development	ENSGALG00010013065	FKTN	-0.239561332	0.104397945	-2.294693939	0.02175067	0.028612031	negative
PRJNA729271	stage_5W_vs_E10	ordinary_muscle_development	ENSGALG00010013071	ENSGALG00010013071	-0.898681816	0.225947319	-3.977395352	6.96743E-05	0.000116485	negative
PRJNA729271	stage_5W_vs_E10	ordinary_muscle_development	ENSGALG00010013076	RTN4IP1	-0.523924186	0.085667102	-6.115815425	9.60645E-10	2.24408E-09	negative
PRJNA729271	stage_5W_vs_E10	ordinary_muscle_development	ENSGALG00010013078	COL5A2	-0.961008149	0.242028714	-3.970636932	7.16807E-05	0.000119702	negative
PRJNA729271	stage_5W_vs_E10	ordinary_muscle_development	ENSGALG00010013079	ENSGALG00010013079	-1.282199898	0.207400479	-6.182241746	6.31976E-10	1.4951E-09	negative
PRJNA729271	stage_5W_vs_E10	ordinary_muscle_development	ENSGALG00010013080	ARHGEF7	-0.263392962	0.100057329	-2.63242047	0.008477887	0.011697341	negative
PRJNA729271	stage_5W_vs_E10	ordinary_muscle_development	ENSGALG00010013081	NCOA5	-0.966959551	0.102705777	-9.41485063	4.73756E-21	1.98471E-20	negative
PRJNA729271	stage_5W_vs_E10	ordinary_muscle_development	ENSGALG00010013082	CHAF1B	-2.124122735	0.116660761	-18.207688	4.4849E-74	1.0605E-72	negative
PRJNA729271	stage_5W_vs_E10	ordinary_muscle_development	ENSGALG00010013084	SGSM3	0.69160991	0.11627076	5.948270297	2.70991E-09	6.17127E-09	positive
PRJNA729271	stage_5W_vs_E10	ordinary_muscle_development	ENSGALG00010013085	LYSMD3	-0.189086543	0.090409858	-2.091437218	0.036488889	0.046884713	negative
PRJNA729271	stage_5W_vs_E10	ordinary_muscle_development	ENSGALG00010013086	CXCL14	-1.565819817	0.516824537	-3.029693262	0.002448022	0.003570546	negative
PRJNA729271	stage_5W_vs_E10	ordinary_muscle_development	ENSGALG00010013087	GPN2	-0.625568971	0.094678296	-6.607311258	3.91363E-11	1.00155E-10	negative
PRJNA729271	stage_5W_vs_E10	ordinary_muscle_development	ENSGALG00010013089	TBK1	0.588531611	0.076163038	7.727260181	1.09886E-14	3.39471E-14	positive
PRJNA729271	stage_5W_vs_E10	ordinary_muscle_development	ENSGALG00010013093	GPATCH3	0.094939105	0.161269367	0.588698939	0.55606325	0.595811414	positive
PRJNA729271	stage_5W_vs_E10	ordinary_muscle_development	ENSGALG00010013095	ADSL	2.65518663	0.12761919	20.80554374	3.85564E-96	1.51456E-94	positive
PRJNA729271	stage_5W_vs_E10	ordinary_muscle_development	ENSGALG00010013096	CLDN14	-1.549976095	0.457044721	-3.391300725	0.000695617	0.001067885	negative
PRJNA729271	stage_5W_vs_E10	ordinary_muscle_development	ENSGALG00010013097	ENSGALG00010013097	-0.601696046	0.445761215	-1.349816956	0.177074705	0.207863447	negative
PRJNA729271	stage_5W_vs_E10	ordinary_muscle_development	ENSGALG00010013099	SIM2	0.636476764	0.202073074	3.149735644	0.001634183	0.002423519	positive
PRJNA729271	stage_5W_vs_E10	ordinary_muscle_development	ENSGALG00010013100	ZFPM2	-1.642006651	0.181902165	-9.026867017	1.76657E-19	6.89271E-19	negative
PRJNA729271	stage_5W_vs_E10	ordinary_muscle_development	ENSGALG00010013103	AIRIM	0.158657337	0.147179326	1.07798657	0.28103975	0.318604033	positive
PRJNA729271	stage_5W_vs_E10	ordinary_muscle_development	ENSGALG00010013104	HLCS	-0.288771041	0.10971543	-2.63200026	0.00848838	0.011709097	negative
PRJNA729271	stage_5W_vs_E10	ordinary_muscle_development	ENSGALG00010013106	SETD9	0.035236667	0.180922447	0.194761167	0.845579925	0.864363964	positive
PRJNA729271	stage_5W_vs_E10	ordinary_muscle_development	ENSGALG00010013109	ENSGALG00010013109	2.588726817	1.466372487	1.765395109	0.077497385	0.095496437	positive
PRJNA729271	stage_5W_vs_E10	ordinary_muscle_development	ENSGALG00010013110	ANKRD10	1.512150025	0.154432597	9.791650588	1.22283E-22	5.50293E-22	positive
PRJNA729271	stage_5W_vs_E10	ordinary_muscle_development	ENSGALG00010013111	RFESD	2.04208104	0.277438846	7.360472655	1.8326E-13	5.29366E-13	positive
PRJNA729271	stage_5W_vs_E10	ordinary_muscle_development	ENSGALG00010013112	CD40	0.741017425	0.160883211	4.605933842	4.10619E-06	7.5255E-06	positive
PRJNA729271	stage_5W_vs_E10	ordinary_muscle_development	ENSGALG00010013114	PIGP	-0.254678332	0.125046161	-2.036674538	0.041682668	0.053110959	negative
PRJNA729271	stage_5W_vs_E10	ordinary_muscle_development	ENSGALG00010013115	ENSGALG00010013115	-2.361841796	0.385179098	-6.131801562	8.68894E-10	2.03434E-09	negative
PRJNA729271	stage_5W_vs_E10	ordinary_muscle_development	ENSGALG00010013116	TARS	-0.872700673	0.168419962	-5.181693802	2.1988E-07	4.42723E-07	negative
PRJNA729271	stage_5W_vs_E10	ordinary_muscle_development	ENSGALG00010013117	ARSK	0.080401411	0.131953098	0.609318104	0.542313609	0.58206855	positive
PRJNA729271	stage_5W_vs_E10	ordinary_muscle_development	ENSGALG00010013119	RNF114	0.599306195	0.078891935	7.596545746	3.0414E-14	9.1863E-14	positive
PRJNA729271	stage_5W_vs_E10	ordinary_muscle_development	ENSGALG00010013121	PCBD2	0.044790588	0.164685726	0.271976143	0.785640363	0.811158705	positive
PRJNA729271	stage_5W_vs_E10	ordinary_muscle_development	ENSGALG00010013123	PPFIBP1	0.514741452	0.10815456	4.759313437	1.94253E-06	3.64632E-06	positive
PRJNA729271	stage_5W_vs_E10	ordinary_muscle_development	ENSGALG00010013125	POU3F3	-6.072699164	0.704713613	-8.617258207	6.85764E-18	2.49564E-17	negative
PRJNA729271	stage_5W_vs_E10	ordinary_muscle_development	ENSGALG00010013129	RASSF3	0.094578635	0.111102696	0.851272187	0.394618171	0.435875211	positive
PRJNA729271	stage_5W_vs_E10	ordinary_muscle_development	ENSGALG00010013131	SPATA2	0.002041156	0.11802622	0.01729409	0.986202	0.98831568	positive
PRJNA729271	stage_5W_vs_E10	ordinary_muscle_development	ENSGALG00010013132	SKIC3	-0.406416962	0.089721308	-4.529770797	5.90477E-06	1.06867E-05	negative
PRJNA729271	stage_5W_vs_E10	ordinary_muscle_development	ENSGALG00010013133	TXNDC15	-0.090741691	0.120516485	-0.752940068	0.451485929	0.493424455	negative
PRJNA729271	stage_5W_vs_E10	ordinary_muscle_development	ENSGALG00010013134	ENSGALG00010013134	2.256624974	5.373894896	0.419923541	0.67454131	0.708204859	positive
PRJNA729271	stage_5W_vs_E10	ordinary_muscle_development	ENSGALG00010013135	TTC3	-0.412564262	0.085406794	-4.830578988	1.36137E-06	2.58726E-06	negative
PRJNA729271	stage_5W_vs_E10	ordinary_muscle_development	ENSGALG00010013137	TMEM121	-6.481877773	0.396682233	-16.34022709	5.10624E-60	8.17192E-59	negative
PRJNA729271	stage_5W_vs_E10	ordinary_muscle_development	ENSGALG00010013139	TMEM185A	0.576046412	0.082874029	6.950867694	3.63046E-12	9.82516E-12	positive
PRJNA729271	stage_5W_vs_E10	ordinary_muscle_development	ENSGALG00010013143	ENSGALG00010013143	0.519739059	0.421558262	1.232899712	0.217613183	0.251559382	positive
PRJNA729271	stage_5W_vs_E10	ordinary_muscle_development	ENSGALG00010013144	TEDC1	-1.512792495	0.173145291	-8.73712755	2.39112E-18	8.86202E-18	negative
PRJNA729271	stage_5W_vs_E10	ordinary_muscle_development	ENSGALG00010013145	ENSGALG00010013145	0.291147117	0.279260086	1.042566166	0.297149266	0.33537169	positive
PRJNA729271	stage_5W_vs_E10	ordinary_muscle_development	ENSGALG00010013146	TNRC6B	1.045970408	0.119254174	8.770933336	1.77191E-18	6.61444E-18	positive
PRJNA729271	stage_5W_vs_E10	ordinary_muscle_development	ENSGALG00010013149	MCTP1	0.481563204	0.198932211	2.42074022	0.015488942	0.020757416	positive
PRJNA729271	stage_5W_vs_E10	ordinary_muscle_development	ENSGALG00010013150	GNS	1.084606427	0.103054129	10.52462854	6.65233E-26	3.41813E-25	positive
PRJNA729271	stage_5W_vs_E10	ordinary_muscle_development	ENSGALG00010013152	TBXAS1	0.720358285	0.221145874	3.257389674	0.00112442	0.001695284	positive
PRJNA729271	stage_5W_vs_E10	ordinary_muscle_development	ENSGALG00010013154	ENSGALG00010013154	-1.331456587	0.427982354	-3.111008142	0.001864498	0.002749049	negative
PRJNA729271	stage_5W_vs_E10	ordinary_muscle_development	ENSGALG00010013156	ABCC9	2.201689944	0.129633011	16.98402223	1.07836E-64	1.9711E-63	positive
PRJNA729271	stage_5W_vs_E10	ordinary_muscle_development	ENSGALG00010013158	CRIP1	3.438291854	0.184571199	18.62853939	1.88601E-77	4.87304E-76	positive
PRJNA729271	stage_5W_vs_E10	ordinary_muscle_development	ENSGALG00010013159	ENSGALG00010013159	0.241365436	1.400520068	0.172339863	0.863170347	0.880001155	positive
PRJNA729271	stage_5W_vs_E10	ordinary_muscle_development	ENSGALG00010013160	CRYBG1	-4.798627659	0.606699962	-7.909391722	2.5865E-15	8.26709E-15	negative
PRJNA729271	stage_5W_vs_E10	ordinary_muscle_development	ENSGALG00010013162	IDS	-0.016650824	0.112211928	-0.148387287	0.882037132	0.896447078	negative
PRJNA729271	stage_5W_vs_E10	ordinary_muscle_development	ENSGALG00010013163	ENSGALG00010013163	-0.658950794	0.482701976	-1.36512968	0.172212295	0.202647931	negative
PRJNA729271	stage_5W_vs_E10	ordinary_muscle_development	ENSGALG00010013164	FAM83F	-2.894004077	0.519554539	-5.570164165	2.54499E-08	5.47728E-08	negative
PRJNA729271	stage_5W_vs_E10	ordinary_muscle_development	ENSGALG00010013165	NUDC	-0.346896942	0.081296399	-4.267064036	1.98062E-05	3.44854E-05	negative
PRJNA729271	stage_5W_vs_E10	ordinary_muscle_development	ENSGALG00010013168	VPS26C	-0.146778562	0.138376322	-1.060720215	0.28881707	0.326501593	negative
PRJNA729271	stage_5W_vs_E10	ordinary_muscle_development	ENSGALG00010013170	COL3A1	2.633791106	0.234906093	11.21210212	3.55645E-29	2.08961E-28	positive
PRJNA729271	stage_5W_vs_E10	ordinary_muscle_development	ENSGALG00010013171	SLC9A8	0.322613197	0.136578797	2.362103077	0.018171589	0.024147156	positive
PRJNA729271	stage_5W_vs_E10	ordinary_muscle_development	ENSGALG00010013172	TNFSF13B	4.377692946	0.407651969	10.73879995	6.69084E-27	3.59199E-26	positive
PRJNA729271	stage_5W_vs_E10	ordinary_muscle_development	ENSGALG00010013173	KCNJ8	0.282923327	0.155850451	1.815351356	0.069469931	0.086161261	positive
PRJNA729271	stage_5W_vs_E10	ordinary_muscle_development	ENSGALG00010013174	ADAMTS12	0.621796851	0.165282181	3.762031988	0.000168538	0.000272257	positive
PRJNA729271	stage_5W_vs_E10	ordinary_muscle_development	ENSGALG00010013175	HIPK2	2.784475979	0.162811426	17.10246046	1.42269E-65	2.67123E-64	positive
PRJNA729271	stage_5W_vs_E10	ordinary_muscle_development	ENSGALG00010013177	OXR1	0.796777209	0.06949139	11.4658407	1.95848E-30	1.2034E-29	positive
PRJNA729271	stage_5W_vs_E10	ordinary_muscle_development	ENSGALG00010013179	TBC1D30	0.749807472	0.183054426	4.096090367	4.20186E-05	7.126E-05	positive
PRJNA729271	stage_5W_vs_E10	ordinary_muscle_development	ENSGALG00010013180	C5orf24	-0.19308427	0.059699935	-3.234245919	0.001219644	0.001831045	negative
PRJNA729271	stage_5W_vs_E10	ordinary_muscle_development	ENSGALG00010013181	DYRK1A	0.621761303	0.079189325	7.851579767	4.10829E-15	1.29823E-14	positive
PRJNA729271	stage_5W_vs_E10	ordinary_muscle_development	ENSGALG00010013183	CLTA	-1.42041314	0.138257424	-10.27368436	9.25992E-25	4.56384E-24	negative
PRJNA729271	stage_5W_vs_E10	ordinary_muscle_development	ENSGALG00010013184	LDHB	-1.68436254	0.11497002	-14.65045007	1.33826E-48	1.53709E-47	negative
PRJNA729271	stage_5W_vs_E10	ordinary_muscle_development	ENSGALG00010013185	ENSGALG00010013185	-0.972951361	0.326245501	-2.982267523	0.002861219	0.004144021	negative
PRJNA729271	stage_5W_vs_E10	ordinary_muscle_development	ENSGALG00010013186	DDX46	-0.717918496	0.080638782	-8.902893559	5.44094E-19	2.07379E-18	negative
PRJNA729271	stage_5W_vs_E10	ordinary_muscle_development	ENSGALG00010013187	KDF1	-3.621309449	0.283777487	-12.76108788	2.70398E-37	2.16752E-36	negative
PRJNA729271	stage_5W_vs_E10	ordinary_muscle_development	ENSGALG00010013188	ENSGALG00010013188	0.866110293	0.362985289	2.386075467	0.017029256	0.022693672	positive
PRJNA729271	stage_5W_vs_E10	ordinary_muscle_development	ENSGALG00010013190	SLC45A2	-4.483941927	0.782391286	-5.731073451	9.9797E-09	2.20277E-08	negative
PRJNA729271	stage_5W_vs_E10	ordinary_muscle_development	ENSGALG00010013191	ABRA	6.310513445	0.418813364	15.06760287	2.64538E-51	3.32566E-50	positive
PRJNA729271	stage_5W_vs_E10	ordinary_muscle_development	ENSGALG00010013192	B4GALT5	0.408302008	0.082414746	4.954234896	7.26154E-07	1.41018E-06	positive
PRJNA729271	stage_5W_vs_E10	ordinary_muscle_development	ENSGALG00010013194	GRAP2	0.185157824	0.335002182	0.552706324	0.580464522	0.618846223	positive
PRJNA729271	stage_5W_vs_E10	ordinary_muscle_development	ENSGALG00010013196	MRPS9	-0.217091234	0.11467081	-1.893169108	0.058335384	0.072931809	negative
PRJNA729271	stage_5W_vs_E10	ordinary_muscle_development	ENSGALG00010013198	CAMLG	-0.806794581	0.087795637	-9.189460948	3.94815E-20	1.58484E-19	negative
PRJNA729271	stage_5W_vs_E10	ordinary_muscle_development	ENSGALG00010013199	RALYL	-3.595109859	0.649357097	-5.536414206	3.08727E-08	6.59057E-08	negative
PRJNA729271	stage_5W_vs_E10	ordinary_muscle_development	ENSGALG00010013202	ENSGALG00010013202	0.336372758	0.652700101	0.515355762	0.606304439	0.643654906	positive
PRJNA729271	stage_5W_vs_E10	ordinary_muscle_development	ENSGALG00010013203	AMACR	0.484386847	0.120566625	4.017586506	5.87973E-05	9.88593E-05	positive
PRJNA729271	stage_5W_vs_E10	ordinary_muscle_development	ENSGALG00010013204	ENSGALG00010013204	0.29836454	0.365379356	0.816588391	0.414163691	0.45572783	positive
PRJNA729271	stage_5W_vs_E10	ordinary_muscle_development	ENSGALG00010013205	KCNJ6	-1.539434127	0.433447141	-3.551607523	0.000382886	0.000600122	negative
PRJNA729271	stage_5W_vs_E10	ordinary_muscle_development	ENSGALG00010013206	AFF2	-0.39392205	0.112237756	-3.509710677	0.000448595	0.00069888	negative
PRJNA729271	stage_5W_vs_E10	ordinary_muscle_development	ENSGALG00010013207	C1QTNF3	-2.35495243	0.199398951	-11.81025487	3.45512E-32	2.29103E-31	negative
PRJNA729271	stage_5W_vs_E10	ordinary_muscle_development	ENSGALG00010013208	ENSGALG00010013208	0.782424671	0.297314844	2.631636751	0.008497466	0.011720504	positive
PRJNA729271	stage_5W_vs_E10	ordinary_muscle_development	ENSGALG00010013209	KIAA1549	-1.038504489	0.104404035	-9.946976544	2.59961E-23	1.20196E-22	negative
PRJNA729271	stage_5W_vs_E10	ordinary_muscle_development	ENSGALG00010013212	ENSGALG00010013212	0.973349103	0.152373805	6.387903107	1.68176E-10	4.12332E-10	positive
PRJNA729271	stage_5W_vs_E10	ordinary_muscle_development	ENSGALG00010013213	SH2D1A	3.881813738	0.351664548	11.03839941	2.49437E-28	1.4151E-27	positive
PRJNA729271	stage_5W_vs_E10	ordinary_muscle_development	ENSGALG00010013214	RAI14	0.350693052	0.169211077	2.072518294	0.038217132	0.048953458	positive
PRJNA729271	stage_5W_vs_E10	ordinary_muscle_development	ENSGALG00010013215	SF3A3	-1.012663239	0.082986491	-12.20274808	3.00508E-34	2.14786E-33	negative
PRJNA729271	stage_5W_vs_E10	ordinary_muscle_development	ENSGALG00010013217	TGFBRAP1	0.248596095	0.083668099	2.971217219	0.002966219	0.004287529	positive
PRJNA729271	stage_5W_vs_E10	ordinary_muscle_development	ENSGALG00010013220	CSTF1	-0.10623472	0.087175597	-1.218629106	0.222984997	0.257194838	negative
PRJNA729271	stage_5W_vs_E10	ordinary_muscle_development	ENSGALG00010013221	C2orf49	0.294140198	0.11819918	2.488513021	0.012827854	0.017345125	positive
PRJNA729271	stage_5W_vs_E10	ordinary_muscle_development	ENSGALG00010013224	ATP6V0A4	-0.336119031	0.783316037	-0.429097599	0.667852199	0.70189368	negative
PRJNA729271	stage_5W_vs_E10	ordinary_muscle_development	ENSGALG00010013225	GNE	-1.244368188	0.097907543	-12.70962534	5.22843E-37	4.1595E-36	negative
PRJNA729271	stage_5W_vs_E10	ordinary_muscle_development	ENSGALG00010013226	DUSP6	-0.989610959	0.114669169	-8.630139802	6.1277E-18	2.23449E-17	negative
PRJNA729271	stage_5W_vs_E10	ordinary_muscle_development	ENSGALG00010013227	CITED4	1.802452741	0.161650759	11.15028939	7.13739E-29	4.1333E-28	positive
PRJNA729271	stage_5W_vs_E10	ordinary_muscle_development	ENSGALG00010013228	RTFDC1	0.53728905	0.084753791	6.339410261	2.30646E-10	5.6066E-10	positive
PRJNA729271	stage_5W_vs_E10	ordinary_muscle_development	ENSGALG00010013230	ENSGALG00010013230	5.79546851	0.340187378	17.0361068	4.43275E-65	8.20597E-64	positive
PRJNA729271	stage_5W_vs_E10	ordinary_muscle_development	ENSGALG00010013232	FHL2	0.375551891	0.421456452	0.891081129	0.372885648	0.41373067	positive
PRJNA729271	stage_5W_vs_E10	ordinary_muscle_development	ENSGALG00010013233	LRRCC1	-1.105374507	0.133595337	-8.274050039	1.29484E-16	4.42032E-16	negative
PRJNA729271	stage_5W_vs_E10	ordinary_muscle_development	ENSGALG00010013235	GCNT7	0.637887615	0.251260212	2.538752994	0.011124834	0.015161194	positive
PRJNA729271	stage_5W_vs_E10	ordinary_muscle_development	ENSGALG00010013236	MON2	0.165453078	0.082789243	1.998485208	0.045664082	0.057858468	positive
PRJNA729271	stage_5W_vs_E10	ordinary_muscle_development	ENSGALG00010013238	RAD1	-0.267354413	0.135082151	-1.979198666	0.047793643	0.060398331	negative
PRJNA729271	stage_5W_vs_E10	ordinary_muscle_development	ENSGALG00010013241	SEC24A	0.292964334	0.135678753	2.159249893	0.030830785	0.039954603	positive
PRJNA729271	stage_5W_vs_E10	ordinary_muscle_development	ENSGALG00010013242	SULT4A1	-0.640744981	0.16039077	-3.994899346	6.47217E-05	0.000108531	negative
PRJNA729271	stage_5W_vs_E10	ordinary_muscle_development	ENSGALG00010013244	TRIM24	0.098382383	0.14812216	0.6641976	0.506563849	0.547590647	positive
PRJNA729271	stage_5W_vs_E10	ordinary_muscle_development	ENSGALG00010013246	LNPEP	2.46898165	0.174769061	14.12710945	2.5854E-45	2.66539E-44	positive
PRJNA729271	stage_5W_vs_E10	ordinary_muscle_development	ENSGALG00010013247	ATG5	0.027002505	0.092790162	0.29100612	0.771046639	0.798137615	positive
PRJNA729271	stage_5W_vs_E10	ordinary_muscle_development	ENSGALG00010013248	ENSGALG00010013248	0.30926914	0.682550689	0.453107945	0.650471014	0.685216377	positive
PRJNA729271	stage_5W_vs_E10	ordinary_muscle_development	ENSGALG00010013249	AURKA	-3.716129995	0.234082919	-15.87527191	9.40026E-57	1.37079E-55	negative
PRJNA729271	stage_5W_vs_E10	ordinary_muscle_development	ENSGALG00010013250	E2F5	-0.10773897	0.096141073	-1.120634155	0.26244362	0.29904255	negative
PRJNA729271	stage_5W_vs_E10	ordinary_muscle_development	ENSGALG00010013251	MTA1	-0.746731127	0.082996167	-8.997176084	2.31598E-19	8.97837E-19	negative
PRJNA729271	stage_5W_vs_E10	ordinary_muscle_development	ENSGALG00010013252	PPM1H	-1.418406165	0.185298856	-7.654694667	1.93772E-14	5.90069E-14	negative
PRJNA729271	stage_5W_vs_E10	ordinary_muscle_development	ENSGALG00010013253	SHB	0.610176207	0.099270988	6.146571291	7.91757E-10	1.85794E-09	positive
PRJNA729271	stage_5W_vs_E10	ordinary_muscle_development	ENSGALG00010013254	SAR1B	2.056438989	0.141476643	14.53553702	7.21455E-48	8.05662E-47	positive
PRJNA729271	stage_5W_vs_E10	ordinary_muscle_development	ENSGALG00010013256	BRIX1	-0.913030762	0.118603647	-7.698167671	1.38031E-14	4.238E-14	negative
PRJNA729271	stage_5W_vs_E10	ordinary_muscle_development	ENSGALG00010013257	STX12	-0.382846303	0.090123463	-4.248020331	2.15668E-05	3.74474E-05	negative

PRJNA729271	stage_5W_vs_E10	ordinary_muscle_development	ENSGALG00010013258	RBIS	0.710148802	0.145190127	4.891164558	1.00241E-06	1.92501E-06	positive
PRJNA729271	stage_5W_vs_E10	ordinary_muscle_development	ENSGALG00010013259	PTPRB	3.402101641	0.138928791	24.48809644	1.9781E-132	1.4934E-130	positive
PRJNA729271	stage_5W_vs_E10	ordinary_muscle_development	ENSGALG00010013260	FAM210B	-0.520367826	0.084205072	-6.179768216	6.41958E-10	1.51822E-09	negative
PRJNA729271	stage_5W_vs_E10	ordinary_muscle_development	ENSGALG00010013261	JADE2	2.329959113	0.197116155	11.82023421	3.06828E-32	2.03919E-31	positive
PRJNA729271	stage_5W_vs_E10	ordinary_muscle_development	ENSGALG00010013262	CA13	-2.074305256	0.265038437	-7.82643181	5.01911E-15	1.57677E-14	negative
PRJNA729271	stage_5W_vs_E10	ordinary_muscle_development	ENSGALG00010013263	ATP11A	-0.599510044	0.11358769	-5.277949065	1.30638E-07	2.67608E-07	negative
PRJNA729271	stage_5W_vs_E10	ordinary_muscle_development	ENSGALG00010013264	DNAJC21	0.459810843	0.12170298	3.778139553	0.000158004	0.000255754	positive
PRJNA729271	stage_5W_vs_E10	ordinary_muscle_development	ENSGALG00010013265	PACS2	0.71482048	0.122339837	5.842908573	5.12972E-09	1.15226E-08	positive
PRJNA729271	stage_5W_vs_E10	ordinary_muscle_development	ENSGALG00010013266	KIAA1755	2.076641934	0.188102952	11.0399221	2.45246E-28	1.39187E-27	positive
PRJNA729271	stage_5W_vs_E10	ordinary_muscle_development	ENSGALG00010013267	PNPLA3	-0.132557396	0.179532226	-0.738348759	0.460302544	0.502226976	negative
PRJNA729271	stage_5W_vs_E10	ordinary_muscle_development	ENSGALG00010013268	PTPRR	2.447896341	0.255656009	9.574961083	1.01896E-21	4.39839E-21	positive
PRJNA729271	stage_5W_vs_E10	ordinary_muscle_development	ENSGALG00010013269	INPP5B	1.036711767	0.100839893	10.28077024	8.60372E-25	4.24476E-24	positive
PRJNA729271	stage_5W_vs_E10	ordinary_muscle_development	ENSGALG00010013271	GULP1	0.365727406	0.139355691	2.624416713	0.008679752	0.011957126	positive
PRJNA729271	stage_5W_vs_E10	ordinary_muscle_development	ENSGALG00010013273	STAG2	0.931939165	0.116255337	8.016312948	1.08967E-15	3.55657E-15	positive
PRJNA729271	stage_5W_vs_E10	ordinary_muscle_development	ENSGALG00010013274	MTF1	1.471403755	0.119033318	12.36127644	4.2338E-35	3.13587E-34	positive
PRJNA729271	stage_5W_vs_E10	ordinary_muscle_development	ENSGALG00010013275	MAPRE1	-1.209884175	0.076388423	-15.83858031	1.68584E-56	2.44607E-55	negative
PRJNA729271	stage_5W_vs_E10	ordinary_muscle_development	ENSGALG00010013276	POC1B	-1.627522639	0.111223552	-14.6328957	1.73254E-48	1.98209E-47	negative
PRJNA729271	stage_5W_vs_E10	ordinary_muscle_development	ENSGALG00010013277	YRDC	-1.141163991	0.096982962	-11.76664401	5.79857E-32	3.79974E-31	negative
PRJNA729271	stage_5W_vs_E10	ordinary_muscle_development	ENSGALG00010013278	SAMM50	0.558157324	0.103017055	5.418106026	6.02337E-08	1.26132E-07	positive
PRJNA729271	stage_5W_vs_E10	ordinary_muscle_development	ENSGALG00010013280	FRS2	-0.225981393	0.112190828	-2.014259068	0.043982341	0.055862828	negative
PRJNA729271	stage_5W_vs_E10	ordinary_muscle_development	ENSGALG00010013281	BIRC8	1.841678688	0.138739647	13.27435038	3.26087E-40	2.8681E-39	positive
PRJNA729271	stage_5W_vs_E10	ordinary_muscle_development	ENSGALG00010013282	SEC22B	-0.983801182	0.090312368	-10.89331617	1.2404E-27	6.85458E-27	negative
PRJNA729271	stage_5W_vs_E10	ordinary_muscle_development	ENSGALG00010013283	PAWR	-0.508463259	0.279796196	-1.817262941	0.069176861	0.085819805	negative
PRJNA729271	stage_5W_vs_E10	ordinary_muscle_development	ENSGALG00010013284	MANEAL	-1.226506645	0.323924496	-3.786396706	0.000152848	0.0002476	negative
PRJNA729271	stage_5W_vs_E10	ordinary_muscle_development	ENSGALG00010013285	ATP2B1	-0.679089819	0.108346453	-6.267762349	3.66273E-10	8.78704E-10	negative
PRJNA729271	stage_5W_vs_E10	ordinary_muscle_development	ENSGALG00010013286	RNF14	1.497799533	0.118841377	12.60335052	2.02362E-36	1.57278E-35	positive
PRJNA729271	stage_5W_vs_E10	ordinary_muscle_development	ENSGALG00010013288	CA3A	6.063816526	0.775640609	7.817817238	5.37471E-15	1.68586E-14	positive
PRJNA729271	stage_5W_vs_E10	ordinary_muscle_development	ENSGALG00010013289	TM9SF4	-1.007119026	0.075164873	-13.39879908	6.14494E-41	5.52207E-40	negative
PRJNA729271	stage_5W_vs_E10	ordinary_muscle_development	ENSGALG00010013290	AKR1D1	-4.269843694	0.340222576	-12.55014802	3.96768E-36	3.06238E-35	negative
PRJNA729271	stage_5W_vs_E10	ordinary_muscle_development	ENSGALG00010013292	PRDM1	-1.003420919	0.285345964	-3.516506442	0.000437266	0.000682037	negative
PRJNA729271	stage_5W_vs_E10	ordinary_muscle_development	ENSGALG00010013293	TUBGCP3	-1.014424847	0.059480607	-17.05471586	3.22437E-65	5.99195E-64	negative
PRJNA729271	stage_5W_vs_E10	ordinary_muscle_development	ENSGALG00010013295	CA3B	0.356024661	0.398709428	0.892942669	0.371887876	0.412686783	positive
PRJNA729271	stage_5W_vs_E10	ordinary_muscle_development	ENSGALG00010013296	AGXT2	0.402615908	0.399587243	1.007579482	0.313656383	0.352766083	positive
PRJNA729271	stage_5W_vs_E10	ordinary_muscle_development	ENSGALG00010013297	FCHO2	0.612494761	0.125439811	4.882778084	1.04602E-06	2.00556E-06	positive
PRJNA729271	stage_5W_vs_E10	ordinary_muscle_development	ENSGALG00010013298	EPYC	-6.950391556	0.40728433	-17.06520738	2.69431E-65	5.01979E-64	negative
PRJNA729271	stage_5W_vs_E10	ordinary_muscle_development	ENSGALG00010013299	PLAGL2	-0.905966923	0.111547983	-8.121768783	4.59438E-16	1.52637E-15	negative
PRJNA729271	stage_5W_vs_E10	ordinary_muscle_development	ENSGALG00010013300	GNPDA1	-1.202890701	0.099500342	-12.08931218	1.2029E-33	8.40693E-33	negative
PRJNA729271	stage_5W_vs_E10	ordinary_muscle_development	ENSGALG00010013301	PARVB	-2.245095971	0.141662156	-15.84824089	1.44571E-56	2.10187E-55	negative
PRJNA729271	stage_5W_vs_E10	ordinary_muscle_development	ENSGALG00010013302	ENSGALG00010013302	2.842217096	0.570297905	4.983741081	6.23665E-07	1.21538E-06	positive
PRJNA729271	stage_5W_vs_E10	ordinary_muscle_development	ENSGALG00010013303	TFPI	-0.035181778	0.233668691	-0.150562652	0.880320725	0.894953633	negative
PRJNA729271	stage_5W_vs_E10	ordinary_muscle_development	ENSGALG00010013304	KCNJ15	0.048091156	0.237485809	0.202501175	0.83952494	0.859022589	positive
PRJNA729271	stage_5W_vs_E10	ordinary_muscle_development	ENSGALG00010013305	THOC2	-0.525770236	0.091330744	-5.756771614	8.57378E-09	1.89939E-08	negative
PRJNA729271	stage_5W_vs_E10	ordinary_muscle_development	ENSGALG00010013306	ARSB	-0.020994055	0.150161412	-0.139809922	0.888810173	0.902508124	negative
PRJNA729271	stage_5W_vs_E10	ordinary_muscle_development	ENSGALG00010013307	POFUT1	-0.950430397	0.089546945	-10.61376681	2.57168E-26	1.35011E-25	negative
PRJNA729271	stage_5W_vs_E10	ordinary_muscle_development	ENSGALG00010013309	ENSGALG00010013309	-1.405991949	0.294030122	-4.78179562	1.73736E-06	3.27265E-06	negative
PRJNA729271	stage_5W_vs_E10	ordinary_muscle_development	ENSGALG00010013310	MYO16	-2.801899483	0.304124525	-9.213000757	3.17133E-20	1.27903E-19	negative
PRJNA729271	stage_5W_vs_E10	ordinary_muscle_development	ENSGALG00010013311	RSPO1	-4.012372617	0.271133402	-14.7985183	1.49745E-49	1.7806E-48	negative
PRJNA729271	stage_5W_vs_E10	ordinary_muscle_development	ENSGALG00010013312	ENSGALG00010013312	2.418452512	0.560164872	4.317394096	1.57882E-05	2.77159E-05	positive
PRJNA729271	stage_5W_vs_E10	ordinary_muscle_development	ENSGALG00010013313	GAK	0.03356954	0.094143772	0.356577385	0.72140821	0.751747808	positive
PRJNA729271	stage_5W_vs_E10	ordinary_muscle_development	ENSGALG00010013314	KIT	0.33187878	0.205466326	1.615246583	0.106257306	0.128660902	positive
PRJNA729271	stage_5W_vs_E10	ordinary_muscle_development	ENSGALG00010013316	KDM7A	1.947799428	0.12953961	15.03632309	4.24482E-51	5.29962E-50	positive
PRJNA729271	stage_5W_vs_E10	ordinary_muscle_development	ENSGALG00010013317	PREP	-0.497525714	0.109677678	-4.536253153	5.72624E-06	1.03726E-05	negative
PRJNA729271	stage_5W_vs_E10	ordinary_muscle_development	ENSGALG00010013318	KERA	-6.352606689	0.493432067	-12.87432884	6.27824E-38	5.11829E-37	negative
PRJNA729271	stage_5W_vs_E10	ordinary_muscle_development	ENSGALG00010013319	ENSGALG00010013319	2.299032536	1.559050902	1.474635968			positive
PRJNA729271	stage_5W_vs_E10	ordinary_muscle_development	ENSGALG00010013320	CALCRL	0.259973561	0.123853839	2.099035148	0.035813802	0.046064544	positive
PRJNA729271	stage_5W_vs_E10	ordinary_muscle_development	ENSGALG00010013322	IRS2	0.743530659	0.513239262	1.448701832	0.147420869	0.175109849	positive
PRJNA729271	stage_5W_vs_E10	ordinary_muscle_development	ENSGALG00010013323	NOTCH2	-0.796253191	0.1088066	-7.318059669	2.51582E-13	7.22401E-13	negative
PRJNA729271	stage_5W_vs_E10	ordinary_muscle_development	ENSGALG00010013324	PARVG	1.277098305	0.147627302	8.650827383	5.11274E-18	1.86908E-17	positive
PRJNA729271	stage_5W_vs_E10	ordinary_muscle_development	ENSGALG00010013325	CA2	3.087506173	0.316883024	9.743362497	1.96928E-22	8.78569E-22	positive
PRJNA729271	stage_5W_vs_E10	ordinary_muscle_development	ENSGALG00010013326	FGF20	-6.045623517	1.01681912	-5.945623362	2.75407E-09	6.26888E-09	negative
PRJNA729271	stage_5W_vs_E10	ordinary_muscle_development	ENSGALG00010013327	SLF1	-1.360450549	0.100090736	-13.59217254	4.45653E-42	4.13555E-41	negative
PRJNA729271	stage_5W_vs_E10	ordinary_muscle_development	ENSGALG00010013328	SLC37A3	-0.502045852	0.079421431	-6.321289459	2.5939E-10	6.28738E-10	negative
PRJNA729271	stage_5W_vs_E10	ordinary_muscle_development	ENSGALG00010013329	LIFR	-0.344419736	0.1227651	-2.805518322	0.005023573	0.007090232	negative
PRJNA729271	stage_5W_vs_E10	ordinary_muscle_development	ENSGALG00010013330	ERG	-0.113390048	0.128853644	-0.879991009	0.37886418	0.42004255	negative
PRJNA729271	stage_5W_vs_E10	ordinary_muscle_development	ENSGALG00010013331	KIF3B	-0.667639008	0.150825257	-4.426572979	9.5742E-06	1.7093E-05	negative
PRJNA729271	stage_5W_vs_E10	ordinary_muscle_development	ENSGALG00010013332	LUM	-0.629710676	0.32072358	-1.963406234	0.04959899	0.062570701	negative
PRJNA729271	stage_5W_vs_E10	ordinary_muscle_development	ENSGALG00010013333	PPP1R12A	-0.013381823	0.098250525	-0.136201037	0.891662355	0.90515063	negative
PRJNA729271	stage_5W_vs_E10	ordinary_muscle_development	ENSGALG00010013334	SH3BGRL3	0.664735325	0.121014085	5.493040947	3.95071E-08	8.37583E-08	positive
PRJNA729271	stage_5W_vs_E10	ordinary_muscle_development	ENSGALG00010013335	POPDC3	3.758041881	0.17573634	21.38454623	1.8609E-101	8.02783E-100	positive
PRJNA729271	stage_5W_vs_E10	ordinary_muscle_development	ENSGALG00010013336	MTNR1A	0.57361835	0.705224801	0.813383689	0.415998108	0.457572665	positive
PRJNA729271	stage_5W_vs_E10	ordinary_muscle_development	ENSGALG00010013337	RAB19	1.484148305	0.252174715	5.885396979	3.971E-09	8.96567E-09	positive
PRJNA729271	stage_5W_vs_E10	ordinary_muscle_development	ENSGALG00010013339	ATP6V0D2	3.333544224	0.537891282	6.197431223	5.73921E-10	1.36131E-09	positive
PRJNA729271	stage_5W_vs_E10	ordinary_muscle_development	ENSGALG00010013340	LIN28B	-6.73759078	0.666658123	-10.10651569	5.169E-24	2.4622E-23	negative
PRJNA729271	stage_5W_vs_E10	ordinary_muscle_development	ENSGALG00010013343	SHISAL1	-7.575842684	0.672880439	-11.25882437	2.09535E-29	1.23917E-28	negative
PRJNA729271	stage_5W_vs_E10	ordinary_muscle_development	ENSGALG00010013344	OSMR	1.431399537	0.123157266	11.62253425	3.16603E-31	2.01456E-30	positive
PRJNA729271	stage_5W_vs_E10	ordinary_muscle_development	ENSGALG00010013345	DCN	0.266141282	0.213783944	1.244907718	0.213165674	0.24675263	positive
PRJNA729271	stage_5W_vs_E10	ordinary_muscle_development	ENSGALG00010013346	HMGCS2	1.296341539	0.692342308	1.872399713	0.061151325	0.076268151	positive
PRJNA729271	stage_5W_vs_E10	ordinary_muscle_development	ENSGALG00010013347	ENSGALG00010013347	0.238229868	0.315135041	0.755961215	0.44967245	0.49162786	positive
PRJNA729271	stage_5W_vs_E10	ordinary_muscle_development	ENSGALG00010013348	MKRN4P	0.443869179	0.070084819	6.333314236	2.3995E-10	5.8269E-10	positive
PRJNA729271	stage_5W_vs_E10	ordinary_muscle_development	ENSGALG00010013349	ETS2	-0.511582503	0.102215381	-5.004946377	5.58776E-07	1.09275E-06	negative
PRJNA729271	stage_5W_vs_E10	ordinary_muscle_development	ENSGALG00010013351	EDAR	-3.69076559	0.413712613	-8.921085502	4.6174E-19	1.76641E-18	negative
PRJNA729271	stage_5W_vs_E10	ordinary_muscle_development	ENSGALG00010013352	ASXL1	-0.56483182	0.089542885	-6.307947498	2.8276E-10	6.82987E-10	negative
PRJNA729271	stage_5W_vs_E10	ordinary_muscle_development	ENSGALG00010013353	NDFIP1	-0.247889347	0.084912277	-2.919358141	0.00350753	0.005031834	negative
PRJNA729271	stage_5W_vs_E10	ordinary_muscle_development	ENSGALG00010013354	WWP1	3.696329576	0.14539952	25.42188289	1.445E-142	1.2467E-140	positive
PRJNA729271	stage_5W_vs_E10	ordinary_muscle_development	ENSGALG00010013355	FAM171B	-2.010089986	0.151117544	-13.30149984	2.26871E-40	2.00396E-39	negative
PRJNA729271	stage_5W_vs_E10	ordinary_muscle_development	ENSGALG00010013356	AMER1	-0.891096565	0.066232512	-13.45406556	2.914E-41	2.64818E-40	negative
PRJNA729271	stage_5W_vs_E10	ordinary_muscle_development	ENSGALG00010013357	RICTOR	0.22103958	0.109917972	2.010950312	0.044330705	0.056272316	positive
PRJNA729271	stage_5W_vs_E10	ordinary_muscle_development	ENSGALG00010013358	RPS16	-0.076015561	0.174187686	-0.436400314	0.662546311	0.696923714	negative
PRJNA729271	stage_5W_vs_E10	ordinary_muscle_development	ENSGALG00010013359	BTG1	0.449081942	0.13255541	3.387880908	0.000704348	0.001080831	positive
PRJNA729271	stage_5W_vs_E10	ordinary_muscle_development	ENSGALG00010013360	PRR5	-0.789844428	0.115252796	-6.85314765	7.22425E-12	1.92421E-11	negative
PRJNA729271	stage_5W_vs_E10	ordinary_muscle_development	ENSGALG00010013361	ENSGALG00010013361	0.251572262	0.230803086	1.089986562	0.275719064	0.3129148	positive
PRJNA729271	stage_5W_vs_E10	ordinary_muscle_development	ENSGALG00010013362	ENSGALG00010013362	2.461889806	0.366730123	6.713083135	1.90554E-11	4.9651E-11	positive
PRJNA729271	stage_5W_vs_E10	ordinary_muscle_development	ENSGALG00010013363	HS3ST5	3.160007686	0.225554944	14.00992425	1.35549E-44	1.35596E-43	positive
PRJNA729271	stage_5W_vs_E10	ordinary_muscle_development	ENSGALG00010013364	OTOGL	3.045830945	0.35747726	8.520348809	1.59074E-17	5.68344E-17	positive
PRJNA729271	stage_5W_vs_E10	ordinary_muscle_development	ENSGALG00010013365	CEP85	1.946867542	0.227271308	8.566270677	1.0689E-17	3.8513E-17	positive
PRJNA729271	stage_5W_vs_E10	ordinary_muscle_development	ENSGALG00010013367	NOL4L	-0.344265649	0.181332011	-1.89853765	0.057625293	0.072106235	negative
PRJNA729271	stage_5W_vs_E10	ordinary_muscle_development	ENSGALG00010013369	MTMR8	-0.359274772	0.062824145	-5.718737156	1.07319E-08	2.36447E-08	negative
PRJNA729271	stage_5W_vs_E10	ordinary_muscle_development	ENSGALG00010013370	CNKSR1	3.192140509	0.234674432	13.6024214	3.87392E-42	3.60414E-41	positive
PRJNA729271	stage_5W_vs_E10	ordinary_muscle_development	ENSGALG00010013371	FAT1	-1.345267667	0.15671226	-8.58431668	9.13779E-18	3.30223E-17	negative
PRJNA729271	stage_5W_vs_E10	ordinary_muscle_development	ENSGALG00010013372	SPRY4	-1.900418899	0.302325769	-6.285997065	3.25756E-10	7.83577E-10	negative
PRJNA729271	stage_5W_vs_E10	ordinary_muscle_development	ENSGALG00010013373	ENSGALG00010013373	-0.062795232	0.574159545	-0.10936896	0.912909852	0.924518152	negative
PRJNA729271	stage_5W_vs_E10	ordinary_muscle_development	ENSGALG00010013374	RMDN1	-0.013938862	0.168619019	-0.082664828	0.934118052	0.942768498	negative
PRJNA729271	stage_5W_vs_E10	ordinary_muscle_development	ENSGALG00010013375	PSMG1	-0.273424512	0.088815937	-3.078552341	0.00208009	0.003052329	negative
PRJNA729271	stage_5W_vs_E10	ordinary_muscle_development	ENSGALG00010013376	ARHGAP8	3.184339045	0.372469816	8.549253943	1.23887E-17	4.45261E-17	positive
PRJNA729271	stage_5W_vs_E10	ordinary_muscle_development	ENSGALG00010013378	SH3RF3	-0.379774525	0.144685978	-2.624819155	0.0086695	0.011945285	negative
PRJNA729271	stage_5W_vs_E10	ordinary_muscle_development	ENSGALG00010013379	ZNF593	0.317744038	0.131963283	2.407821566	0.016048022	0.021464989	positive
PRJNA729271	stage_5W_vs_E10	ordinary_muscle_development	ENSGALG00010013380	PHGDH	-2.056181439	0.611882061	-3.360421183	0.000778237	0.001190401	negative
PRJNA729271	stage_5W_vs_E10	ordinary_muscle_development	ENSGALG00010013381	DENND2A	-1.302135909	0.089664901	-14.52224777	8.75902E-48	9.72144E-47	negative
PRJNA729271	stage_5W_vs_E10	ordinary_muscle_development	ENSGALG00010013382	ASB12	2.075007925	0.220642909	9.404371696	5.2342E-21	2.18806E-20	positive
PRJNA729271	stage_5W_vs_E10	ordinary_muscle_development	ENSGALG00010013383	SEPTIN10	0.838460674	0.138879999	6.037303274	1.56711E-09	3.61939E-09	positive
PRJNA729271	stage_5W_vs_E10	ordinary_muscle_development	ENSGALG00010013385	PGRMC1	-0.3405099	0.155793634	-2.185647073	0.028841436	0.037483781	negative
PRJNA729271	stage_5W_vs_E10	ordinary_muscle_development	ENSGALG00010013386	ITGAV	0.236930617	0.121856464	1.944341804	0.051854247	0.065273757	positive
PRJNA729271	stage_5W_vs_E10	ordinary_muscle_development	ENSGALG00010013387	NDST1	-0.577147525	0.093678775	-6.160920902	7.23231E-10	1.70155E-09	negative
PRJNA729271	stage_5W_vs_E10	ordinary_muscle_development	ENSGALG00010013388	SOWAHC	0.44406952	0.213044619	2.084396794	0.037124089	0.047658637	positive
PRJNA729271	stage_5W_vs_E10	ordinary_muscle_development	ENSGALG00010013389	NAP1L1	-1.444114967	0.062506845	-23.10330918	4.2886E-118	2.5477E-116	negative
PRJNA729271	stage_5W_vs_E10	ordinary_muscle_development	ENSGALG00010013390	SLC16A2	-2.763388556	0.136196313	-20.28974563	1.58413E-91	5.58684E-90	negative
PRJNA729271	stage_5W_vs_E10	ordinary_muscle_development	ENSGALG00010013391	HMGN1	0.049494582	0.087697071	0.564381252	0.572494695	0.611338633	positive
PRJNA729271	stage_5W_vs_E10	ordinary_muscle_development	ENSGALG00010013392	HDAC2	-1.509697469	0.069908769	-21.59525173	1.9906E-103	8.9053E-102	negative
PRJNA729271	stage_5W_vs_E10	ordinary_muscle_development	ENSGALG00010013393	ADCK2	-1.221071302	0.167591759	-7.285986568	3.19325E-13	9.11683E-13	negative
PRJNA729271	stage_5W_vs_E10	ordinary_muscle_development	ENSGALG00010013394	DNMT3B	-7.197320597	0.334182546	-21.53709308	6.9962E-103	3.1203E-101	negative
PRJNA729271	stage_5W_vs_E10	ordinary_muscle_development	ENSGALG00010013396	FYB1	1.986263523	0.18577255	10.69191074	1.11059E-26	5.92929E-26	positive
PRJNA729271	stage_5W_vs_E10	ordinary_muscle_development	ENSGALG00010013397	MATN1	-7.765279908	0.458529903	-16.93516575	2.4766E-64	4.48168E-63	negative
PRJNA729271	stage_5W_vs_E10	ordinary_muscle_development	ENSGALG00010013398	CHAMP1	1.214978935	0.155290842	7.823893005	5.12143E-15	1.60821E-14	positive
PRJNA729271	stage_5W_vs_E10	ordinary_muscle_development	ENSGALG00010013399	FGF1	4.384167857	0.321468048	13.63795836	2.38134E-42	2.2356E-41	positive
PRJNA729271	stage_5W_vs_E10	ordinary_muscle_development	ENSGALG00010013400	BBS10	0.060981894	0.168205486	0.362544023	0.716945532	0.74752737	positive
PRJNA729271	stage_5W_vs_E10	ordinary_muscle_development	ENSGALG00010013402	MARCKS	-1.551791603	0.155117583	-10.00396974	1.46408E-23	6.83692E-23	negative
PRJNA729271	stage_5W_vs_E10	ordinary_muscle_development	ENSGALG00010013403	CPNE3	0.818267354	0.103184693	7.930123481	2.18928E-15	7.03472E-15	positive
PRJNA729271	stage_5W_vs_E10	ordinary_muscle_development	ENSGALG00010013404	ENSGALG00010013404	-0.101543131	0.232180527	-0.437345597	0.661860732	0.696253089	negative
PRJNA729271	stage_5W_vs_E10	ordinary_muscle_development	ENSGALG00010013405	RPS14	-0.220346493	0.175393944	-1.256294757	0.209009122	0.242366978	negative
PRJNA729271	stage_5W_vs_E10	ordinary_muscle_development	ENSGALG00010013406	ENSGALG00010013406	0.833905051	0.277521931	3.004825772	0.00265733	0.003861067	positive
PRJNA729271	stage_5W_vs_E10	ordinary_muscle_development	ENSGALG00010013407	ZC3H15	1.398789399	0.112498728	12.4338241	1.71242E-35	1.28676E-34	positive

PRJNA729271	stage_5W_vs_E10	ordinary_muscle_development	ENSGALG00010013408	RLIM	-0.212438394	0.065423567	-3.247123385	0.001165778	0.001755085	negative
PRJNA729271	stage_5W_vs_E10	ordinary_muscle_development	ENSGALG00010013409	DAB2	-0.169650006	0.147826925	-1.147625889	0.251123038	0.287000586	negative
PRJNA729271	stage_5W_vs_E10	ordinary_muscle_development	ENSGALG00010013410	KIAA0825	-0.600450999	0.103854046	-5.781681333	7.39577E-09	1.64546E-08	negative
PRJNA729271	stage_5W_vs_E10	ordinary_muscle_development	ENSGALG00010013411	ENSGALG00010013411	1.148117671	0.446864262	2.569276108	0.010191122	0.013946405	positive
PRJNA729271	stage_5W_vs_E10	ordinary_muscle_development	ENSGALG00010013412	COMMD7	-0.968290128	0.102961889	-9.404354698	5.23504E-21	2.18806E-20	negative
PRJNA729271	stage_5W_vs_E10	ordinary_muscle_development	ENSGALG00010013413	WRB	-0.526562145	0.085235678	-6.177719893	6.5034E-10	1.53704E-09	negative
PRJNA729271	stage_5W_vs_E10	ordinary_muscle_development	ENSGALG00010013414	LAPTM5	2.449965347	0.172915279	14.16858799	1.43354E-45	1.49168E-44	positive
PRJNA729271	stage_5W_vs_E10	ordinary_muscle_development	ENSGALG00010013416	NDUFB2	0.576201952	0.110683696	5.205843075	1.93118E-07	3.90519E-07	positive
PRJNA729271	stage_5W_vs_E10	ordinary_muscle_development	ENSGALG00010013417	AKR1B1	-0.223029512	0.088410718	-2.522652423	0.011647346	0.015833094	negative
PRJNA729271	stage_5W_vs_E10	ordinary_muscle_development	ENSGALG00010013418	HCN1	-2.177600153	0.86992421	-2.503206749	0.012307363	0.016677126	negative
PRJNA729271	stage_5W_vs_E10	ordinary_muscle_development	ENSGALG00010013419	ENSGALG00010013419	2.705249846	0.334163923	8.095577236	5.69934E-16	1.88225E-15	positive
PRJNA729271	stage_5W_vs_E10	ordinary_muscle_development	ENSGALG00010013420	ENSGALG00010013420	3.110844652	4.195476205	0.741475937			positive
PRJNA729271	stage_5W_vs_E10	ordinary_muscle_development	ENSGALG00010013423	ZNF804A	-1.469338768	0.425464077	-3.453496654	0.000553369	0.000857137	negative
PRJNA729271	stage_5W_vs_E10	ordinary_muscle_development	ENSGALG00010013425	ENSGALG00010013425	0.758310922	0.221253399	3.42734135	0.000609522	0.000940998	positive
PRJNA729271	stage_5W_vs_E10	ordinary_muscle_development	ENSGALG00010013427	NEXMIF	-2.784405008	0.197511336	-14.09744407	3.93775E-45	4.03376E-44	negative
PRJNA729271	stage_5W_vs_E10	ordinary_muscle_development	ENSGALG00010013428	BPGM	4.421312119	0.15277625	28.93978692	3.7728E-184	5.4146E-182	positive
PRJNA729271	stage_5W_vs_E10	ordinary_muscle_development	ENSGALG00010013429	PDE4DIP	2.228489066	0.187526054	11.88362372	1.43987E-32	9.68489E-32	positive
PRJNA729271	stage_5W_vs_E10	ordinary_muscle_development	ENSGALG00010013431	TMEM175	-0.53294398	0.08809151	-6.049890424	1.44944E-09	3.35404E-09	negative
PRJNA729271	stage_5W_vs_E10	ordinary_muscle_development	ENSGALG00010013434	CORIN	-5.876400709	0.479943372	-12.24394596	1.81013E-34	1.30732E-33	negative
PRJNA729271	stage_5W_vs_E10	ordinary_muscle_development	ENSGALG00010013435	SDC3	-1.767102715	0.180265392	-9.802784083	1.09524E-22	4.93951E-22	negative
PRJNA729271	stage_5W_vs_E10	ordinary_muscle_development	ENSGALG00010013436	NPAS2	0.401618369	0.209320379	1.918677824	0.05502512	0.068983663	positive
PRJNA729271	stage_5W_vs_E10	ordinary_muscle_development	ENSGALG00010013437	ENSGALG00010013437	0.488143442	0.124759212	3.912684569	9.12757E-05	0.000150894	positive
PRJNA729271	stage_5W_vs_E10	ordinary_muscle_development	ENSGALG00010013438	RPL37	0.336829442	0.187506723	1.796359282	0.072437395	0.089619328	positive
PRJNA729271	stage_5W_vs_E10	ordinary_muscle_development	ENSGALG00010013440	EEA1	0.260487881	0.098300557	2.649912555	0.008051261	0.011129413	positive
PRJNA729271	stage_5W_vs_E10	ordinary_muscle_development	ENSGALG00010013442	ENSGALG00010013442	1.252778288	0.104145638	12.02909991	2.4987E-33	1.72634E-32	positive
PRJNA729271	stage_5W_vs_E10	ordinary_muscle_development	ENSGALG00010013444	OSBPL8	-1.060617788	0.103411608	-10.25627401	1.10904E-24	5.4401E-24	negative
PRJNA729271	stage_5W_vs_E10	ordinary_muscle_development	ENSGALG00010013446	ATP5I	1.080102694	0.145389998	7.429002752	1.09419E-13	3.1983E-13	positive
PRJNA729271	stage_5W_vs_E10	ordinary_muscle_development	ENSGALG00010013449	LCA5L	0.285541278	0.212288787	1.345060575	0.178605674	0.209541824	positive
PRJNA729271	stage_5W_vs_E10	ordinary_muscle_development	ENSGALG00010013450	CD74	6.254919668	0.189993889	32.92168874	1.0758E-237	2.7356E-235	positive
PRJNA729271	stage_5W_vs_E10	ordinary_muscle_development	ENSGALG00010013451	PRKAB2	4.322002838	0.166899134	25.89589726	7.4074E-148	7.0639E-146	positive
PRJNA729271	stage_5W_vs_E10	ordinary_muscle_development	ENSGALG00010013452	LSS	-0.539130165	0.225067327	-2.395417288	0.016601459	0.022162291	negative
PRJNA729271	stage_5W_vs_E10	ordinary_muscle_development	ENSGALG00010013453	PUM1	-0.149640064	0.089397297	-1.673876831	0.094154808	0.11477369	negative
PRJNA729271	stage_5W_vs_E10	ordinary_muscle_development	ENSGALG00010013457	SH3BGR	3.517534633	0.147500502	23.84761132	1.0721E-125	7.2959E-124	positive
PRJNA729271	stage_5W_vs_E10	ordinary_muscle_development	ENSGALG00010013458	LAMA4	0.998411403	0.143678191	6.948941909	3.68036E-12	9.95648E-12	positive
PRJNA729271	stage_5W_vs_E10	ordinary_muscle_development	ENSGALG00010013459	ATP10D	-0.593289895	0.14472071	-4.099550743	4.13953E-05	7.02358E-05	negative
PRJNA729271	stage_5W_vs_E10	ordinary_muscle_development	ENSGALG00010013461	S100B	3.26831579	0.573822827	5.695687996	1.22875E-08	2.6982E-08	positive
PRJNA729271	stage_5W_vs_E10	ordinary_muscle_development	ENSGALG00010013463	ENSGALG00010013463	2.023975785	0.209064555	9.681104428	3.62776E-22	1.60025E-21	positive
PRJNA729271	stage_5W_vs_E10	ordinary_muscle_development	ENSGALG00010013464	ABCB7	-0.26472836	0.075129794	-3.523613534	0.000425705	0.000664648	negative
PRJNA729271	stage_5W_vs_E10	ordinary_muscle_development	ENSGALG00010013465	CALD1	-0.232834751	0.157088863	-1.482184964	0.138291086	0.164886839	negative
PRJNA729271	stage_5W_vs_E10	ordinary_muscle_development	ENSGALG00010013466	BRAF	0.227847659	0.061759452	3.689275919	0.000224893	0.000359844	positive
PRJNA729271	stage_5W_vs_E10	ordinary_muscle_development	ENSGALG00010013468	DEPDC1B	-3.480926212	0.283633212	-12.27263262	1.27054E-34	9.21745E-34	negative
PRJNA729271	stage_5W_vs_E10	ordinary_muscle_development	ENSGALG00010013470	RPL31	-0.321050107	0.14641143	-2.192794006	0.028322224	0.036835409	negative
PRJNA729271	stage_5W_vs_E10	ordinary_muscle_development	ENSGALG00010013472	ENSGALG00010013472	0.683244201	0.239540469	2.852312195	0.004340245	0.006169643	positive
PRJNA729271	stage_5W_vs_E10	ordinary_muscle_development	ENSGALG00010013476	ZDHHC17	-0.504560466	0.130477472	-3.867031282	0.000110168	0.000180603	negative
PRJNA729271	stage_5W_vs_E10	ordinary_muscle_development	ENSGALG00010013477	RFC1	-0.107795749	0.089648133	-1.202431608	0.229196349	0.263645828	negative
PRJNA729271	stage_5W_vs_E10	ordinary_muscle_development	ENSGALG00010013480	TBC1D8	1.066450411	0.152771649	6.980682727	2.93749E-12	7.98554E-12	positive
PRJNA729271	stage_5W_vs_E10	ordinary_muscle_development	ENSGALG00010013482	ENSGALG00010013482	-0.358297749	0.235095774	-1.524050148	0.127496185	0.152769876	negative
PRJNA729271	stage_5W_vs_E10	ordinary_muscle_development	ENSGALG00010013483	ENSGALG00010013483	-1.023662748	0.210345385	-4.866580493	1.13546E-06	2.16958E-06	negative
PRJNA729271	stage_5W_vs_E10	ordinary_muscle_development	ENSGALG00010013484	TCOF1	-0.479901365	0.102453613	-4.684084353	2.81214E-06	5.22054E-06	negative
PRJNA729271	stage_5W_vs_E10	ordinary_muscle_development	ENSGALG00010013486	PLPP6	1.471183441	0.161266252	9.122698798	7.32764E-20	2.91237E-19	positive
PRJNA729271	stage_5W_vs_E10	ordinary_muscle_development	ENSGALG00010013487	ENSGALG00010013487	-0.260883728	0.093533821	-2.789191396	0.005283983	0.007439663	negative
PRJNA729271	stage_5W_vs_E10	ordinary_muscle_development	ENSGALG00010013488	UPRT	-1.040348257	0.123149909	-8.447819963	2.96793E-17	1.04519E-16	negative
PRJNA729271	stage_5W_vs_E10	ordinary_muscle_development	ENSGALG00010013491	CNOT11	0.932644933	0.110562218	8.435475935	3.29858E-17	1.15854E-16	positive
PRJNA729271	stage_5W_vs_E10	ordinary_muscle_development	ENSGALG00010013492	B3GALT5	-1.443990956	0.617587748	-2.33811464	0.019381302	0.025665263	negative
PRJNA729271	stage_5W_vs_E10	ordinary_muscle_development	ENSGALG00010013493	ENSGALG00010013493	0.288161863	0.127278215	2.264031299	0.02357219	0.030898796	positive
PRJNA729271	stage_5W_vs_E10	ordinary_muscle_development	ENSGALG00010013494	ASB1	0.364537089	0.144738528	2.518590553	0.011782558	0.016009391	positive
PRJNA729271	stage_5W_vs_E10	ordinary_muscle_development	ENSGALG00010013497	FAM172A	-0.528777516	0.108297144	-4.882654315	1.04667E-06	2.00655E-06	negative
PRJNA729271	stage_5W_vs_E10	ordinary_muscle_development	ENSGALG00010013498	KDR	0.80269064	0.125281823	6.407079809	1.48333E-10	3.65103E-10	positive
PRJNA729271	stage_5W_vs_E10	ordinary_muscle_development	ENSGALG00010013501	AKR1B10	-1.637019243	0.138211373	-11.84431646	2.30288E-32	1.53826E-31	negative
PRJNA729271	stage_5W_vs_E10	ordinary_muscle_development	ENSGALG00010013502	PAFAH2	1.026930818	0.209699115	4.897163347	9.723E-07	1.8704E-06	positive
PRJNA729271	stage_5W_vs_E10	ordinary_muscle_development	ENSGALG00010013504	IGSF5	-2.201178589	0.863992311	-2.547683075	0.010844094	0.014796681	negative
PRJNA729271	stage_5W_vs_E10	ordinary_muscle_development	ENSGALG00010013506	RNF149	-0.349480484	0.104955718	-3.32978985	0.000869116	0.001323719	negative
PRJNA729271	stage_5W_vs_E10	ordinary_muscle_development	ENSGALG00010013508	TUBE1	-0.086541588	0.193390389	-0.447496842	0.654516368	0.689127243	negative
PRJNA729271	stage_5W_vs_E10	ordinary_muscle_development	ENSGALG00010013509	ENSGALG00010013509	0.652955788	0.19235904	3.394463754	0.000687631	0.001056319	positive
PRJNA729271	stage_5W_vs_E10	ordinary_muscle_development	ENSGALG00010013510	MRPS33	-0.32485264	0.088641597	-3.66478776	0.000247544	0.000394649	negative
PRJNA729271	stage_5W_vs_E10	ordinary_muscle_development	ENSGALG00010013511	FAM78B	0.730478333	0.227365044	3.212799654	0.001314479	0.001968731	positive
PRJNA729271	stage_5W_vs_E10	ordinary_muscle_development	ENSGALG00010013512	ENSGALG00010013512	3.458255513	3.427256841	1.009044747			positive
PRJNA729271	stage_5W_vs_E10	ordinary_muscle_development	ENSGALG00010013514	LMBRD2	0.709422226	0.136014314	5.215790934	1.83035E-07	3.7075E-07	positive
PRJNA729271	stage_5W_vs_E10	ordinary_muscle_development	ENSGALG00010013516	CSRP2	-1.091944324	0.134359666	-8.127024713	4.39956E-16	1.46433E-15	negative
PRJNA729271	stage_5W_vs_E10	ordinary_muscle_development	ENSGALG00010013517	STMN1	-3.528936295	0.218148642	-16.17675117	7.35757E-59	1.14429E-57	negative
PRJNA729271	stage_5W_vs_E10	ordinary_muscle_development	ENSGALG00010013520	CREG2	-0.563095665	0.191558889	-2.939543386	0.003286962	0.004730863	negative
PRJNA729271	stage_5W_vs_E10	ordinary_muscle_development	ENSGALG00010013523	TMEM178B	-1.823511754	0.194019745	-9.398588544	5.52999E-21	2.30934E-20	negative
PRJNA729271	stage_5W_vs_E10	ordinary_muscle_development	ENSGALG00010013524	ZDHHC15	0.661252539	0.121376326	5.447953184	5.09528E-08	1.0724E-07	positive
PRJNA729271	stage_5W_vs_E10	ordinary_muscle_development	ENSGALG00010013526	SIRPA	0.727313546	0.231167967	3.146255753	0.001653753	0.002450537	positive
PRJNA729271	stage_5W_vs_E10	ordinary_muscle_development	ENSGALG00010013527	ADARB1	0.16190419	0.100513017	1.610778324	0.107228047	0.129749607	positive
PRJNA729271	stage_5W_vs_E10	ordinary_muscle_development	ENSGALG00010013528	PRDX6	0.689168556	0.131460278	5.242409095	1.58493E-07	3.22573E-07	positive
PRJNA729271	stage_5W_vs_E10	ordinary_muscle_development	ENSGALG00010013531	ARSI	-3.487459348	0.248345639	-14.04276458	8.53183E-45	8.61804E-44	negative
PRJNA729271	stage_5W_vs_E10	ordinary_muscle_development	ENSGALG00010013532	WDR19	-0.563754946	0.076534235	-7.366049319	1.75759E-13	5.08001E-13	negative
PRJNA729271	stage_5W_vs_E10	ordinary_muscle_development	ENSGALG00010013533	SKP2	-1.138637097	0.082559116	-13.79177926	2.85627E-43	2.76195E-42	negative
PRJNA729271	stage_5W_vs_E10	ordinary_muscle_development	ENSGALG00010013537	LMO3	-2.70842851	0.445577354	-6.07846985	1.21335E-09	2.82258E-09	negative
PRJNA729271	stage_5W_vs_E10	ordinary_muscle_development	ENSGALG00010013538	E2F7	-2.131530206	0.270441074	-7.881680735	3.23006E-15	1.02697E-14	negative
PRJNA729271	stage_5W_vs_E10	ordinary_muscle_development	ENSGALG00010013539	NSFL1C	0.348731559	0.091869823	3.795931531	0.00014709	0.000238727	positive
PRJNA729271	stage_5W_vs_E10	ordinary_muscle_development	ENSGALG00010013544	ENSGALG00010013544	-1.056286212	0.291992687	-3.617509139	0.000297452	0.000470849	negative
PRJNA729271	stage_5W_vs_E10	ordinary_muscle_development	ENSGALG00010013546	IDUA	-0.083457356	0.132263966	-0.630990873	0.528046492	0.568488955	negative
PRJNA729271	stage_5W_vs_E10	ordinary_muscle_development	ENSGALG00010013549	PCP4	-4.01620856	0.369565306	-10.86738526	1.64858E-27	9.05844E-27	negative
PRJNA729271	stage_5W_vs_E10	ordinary_muscle_development	ENSGALG00010013550	KLHL5	-0.692158049	0.125543355	-5.513298986	3.52169E-08	7.48818E-08	negative
PRJNA729271	stage_5W_vs_E10	ordinary_muscle_development	ENSGALG00010013551	UBE2N	-0.409141066	0.111711873	-3.66246715	0.000249798	0.000398111	negative
PRJNA729271	stage_5W_vs_E10	ordinary_muscle_development	ENSGALG00010013552	FGF16	0.071708277	0.405032121	0.177043432	0.859474274	0.876650454	positive
PRJNA729271	stage_5W_vs_E10	ordinary_muscle_development	ENSGALG00010013553	TADA1	0.160688504	0.112169234	1.432554171	0.151985287	0.180236172	positive
PRJNA729271	stage_5W_vs_E10	ordinary_muscle_development	ENSGALG00010013554	ENSGALG00010013554	3.458005468	5.403335067	0.63997613	0.522188118	0.562717773	positive
PRJNA729271	stage_5W_vs_E10	ordinary_muscle_development	ENSGALG00010013556	RASL11B	-3.282935109	0.178650496	-18.37629998	2.03376E-75	5.02205E-74	negative
PRJNA729271	stage_5W_vs_E10	ordinary_muscle_development	ENSGALG00010013558	SNAPC3	-0.375028443	0.08691162	-4.315055263	1.59563E-05	2.79906E-05	negative
PRJNA729271	stage_5W_vs_E10	ordinary_muscle_development	ENSGALG00010013561	AGK	0.020298158	0.108845872	0.186485327	0.852064184	0.870010591	positive
PRJNA729271	stage_5W_vs_E10	ordinary_muscle_development	ENSGALG00010013562	RERGL	1.08995276	0.157722182	6.910586367	4.82654E-12	1.29653E-11	positive
PRJNA729271	stage_5W_vs_E10	ordinary_muscle_development	ENSGALG00010013563	FKBP1A	0.382104708	0.099158997	3.853454768	0.000116463	0.000190555	positive
PRJNA729271	stage_5W_vs_E10	ordinary_muscle_development	ENSGALG00010013565	ENSGALG00010013565	-0.531360535	5.324803546	-0.099789698	0.920511287	0.931500357	negative
PRJNA729271	stage_5W_vs_E10	ordinary_muscle_development	ENSGALG00010013569	ENSGALG00010013569	-3.04393042	0.553322806	-5.501183733	3.7725E-08	8.00386E-08	negative
PRJNA729271	stage_5W_vs_E10	ordinary_muscle_development	ENSGALG00010013572	DENND11	0.296243451	0.104385861	2.837965296	0.004540212	0.006438111	positive
PRJNA729271	stage_5W_vs_E10	ordinary_muscle_development	ENSGALG00010013573	ENSGALG00010013573	1.164339806	0.461830057	2.521143413	0.011697417	0.015899668	positive
PRJNA729271	stage_5W_vs_E10	ordinary_muscle_development	ENSGALG00010013574	NR2F1	-1.901287355	0.161010734	-11.80845094	3.53005E-32	2.33858E-31	negative
PRJNA729271	stage_5W_vs_E10	ordinary_muscle_development	ENSGALG00010013576	CAMK2A	0.523298508	0.186149995	2.81116585	0.004936233	0.006975792	positive
PRJNA729271	stage_5W_vs_E10	ordinary_muscle_development	ENSGALG00010013579	H2AC32	-2.446391093	0.177454118	-13.78604858	3.0924E-43	2.98431E-42	negative
PRJNA729271	stage_5W_vs_E10	ordinary_muscle_development	ENSGALG00010013580	RANBP3L	-0.918861736	0.316877227	-2.899740526	0.003734717	0.005344	negative
PRJNA729271	stage_5W_vs_E10	ordinary_muscle_development	ENSGALG00010013582	MAP4K4	-1.300564615	0.107618115	-12.08499711	1.26775E-33	8.84737E-33	negative
PRJNA729271	stage_5W_vs_E10	ordinary_muscle_development	ENSGALG00010013583	FASLG	2.321388814	0.307234698	7.555750809	4.1645E-14	1.24797E-13	positive
PRJNA729271	stage_5W_vs_E10	ordinary_muscle_development	ENSGALG00010013587	SLC26A1	-0.362613468	0.495822587	-0.73133713	0.464573256	0.506429221	negative
PRJNA729271	stage_5W_vs_E10	ordinary_muscle_development	ENSGALG00010013589	DSCAM	-6.213123529	0.928705654	-6.69008905	2.23035E-11	5.78852E-11	negative
PRJNA729271	stage_5W_vs_E10	ordinary_muscle_development	ENSGALG00010013592	H3C33	-2.638700624	0.184532004	-14.29941998	2.20641E-46	2.33957E-45	negative
PRJNA729271	stage_5W_vs_E10	ordinary_muscle_development	ENSGALG00010013596	SDCBP2	-0.224884008	0.123270978	-1.824306185	0.068105796	0.084519992	negative
PRJNA729271	stage_5W_vs_E10	ordinary_muscle_development	ENSGALG00010013598	PAQR7	2.28199612	0.22492397	10.1456333	3.46523E-24	1.66485E-23	positive
PRJNA729271	stage_5W_vs_E10	ordinary_muscle_development	ENSGALG00010013604	FYN	-0.687578487	0.111913323	-6.143848359	8.05457E-10	1.88918E-09	negative
PRJNA729271	stage_5W_vs_E10	ordinary_muscle_development	ENSGALG00010013609	PIGC	1.578032045	0.16190484	9.746663802	1.9063E-22	8.50734E-22	positive
PRJNA729271	stage_5W_vs_E10	ordinary_muscle_development	ENSGALG00010013612	TWIST2	-5.317250746	0.296688052	-17.92202521	7.93848E-72	1.77028E-70	negative
PRJNA729271	stage_5W_vs_E10	ordinary_muscle_development	ENSGALG00010013614	ATRX	-0.862924521	0.086889896	-9.931241254	3.04442E-23	1.40314E-22	negative
PRJNA729271	stage_5W_vs_E10	ordinary_muscle_development	ENSGALG00010013617	PIK3C2G	0.738832851	0.160407685	4.605969164	4.10549E-06	7.52518E-06	positive
PRJNA729271	stage_5W_vs_E10	ordinary_muscle_development	ENSGALG00010013619	IL1R2	0.717392497	0.198086704	3.62160853	0.000292777	0.000463651	positive
PRJNA729271	stage_5W_vs_E10	ordinary_muscle_development	ENSGALG00010013621	PDGFRA	0.085376688	0.164670315	0.518470425	0.604130089	0.641763697	positive
PRJNA729271	stage_5W_vs_E10	ordinary_muscle_development	ENSGALG00010013624	SST1	-7.416490816	1.740043611	-4.262244215	2.02384E-05	3.52168E-05	negative
PRJNA729271	stage_5W_vs_E10	ordinary_muscle_development	ENSGALG00010013625	SSBP1	-0.614679431	0.11333219	-5.423696742	5.83789E-08	1.22372E-07	negative
PRJNA729271	stage_5W_vs_E10	ordinary_muscle_development	ENSGALG00010013627	MYCBP	-0.060256883	0.14825377	-0.406444186	0.684416259	0.717844694	negative
PRJNA729271	stage_5W_vs_E10	ordinary_muscle_development	ENSGALG00010013632	SLC1A3	-7.584786665	0.717360017	-10.57319406	3.96747E-26	2.06124E-25	negative
PRJNA729271	stage_5W_vs_E10	ordinary_muscle_development	ENSGALG00010013635	BCL6	0.996073027	0.159257221	6.254492093	3.98812E-10	9.55184E-10	positive
PRJNA729271	stage_5W_vs_E10	ordinary_muscle_development	ENSGALG00010013638	COMMD8	-0.361119714	0.117398036	-3.076028568	0.002097777	0.003077037	negative
PRJNA729271	stage_5W_vs_E10	ordinary_muscle_development	ENSGALG00010013639	ENSGALG00010013639	0.972916925	0.454195037	2.142068597	0.032187962	0.041616673	positive
PRJNA729271	stage_5W_vs_E10	ordinary_muscle_development	ENSGALG00010013640	SNPH	-3.110254324	0.753527311	-4.127593357	3.6658E-05	6.24171E-05	negative
PRJNA729271	stage_5W_vs_E10	ordinary_muscle_development	ENSGALG00010013645	RRAGC	-0.258791062	0.121913819	-2.122737711	0.033775837	0.043568636	negative
PRJNA729271	stage_5W_vs_E10	ordinary_muscle_development	ENSGALG00010013646	CLDN1	-0.057526583	0.336399325	-0.171006833	0.864218393	0.880880218	negative
PRJNA729271	stage_5W_vs_E10	ordinary_muscle_development	ENSGALG00010013648	NAV3	-0.501598071	0.118553658	-4.23097928	2.32676E-05	4.03088E-05	negative
PRJNA729271	stage_5W_vs_E10	ordinary_muscle_development	ENSGALG00010013650	BACE2	-0.923227737	0.161752595	-5.707653343	1.14544E-08	2.51869E-08	negative
PRJNA729271	stage_5W_vs_E10	ordinary_muscle_development	ENSGALG00010013651	ENSGALG00010013651	0.165932988	0.159488809	1.040405207	0.298151684	0.336476849	positive
PRJNA729271	stage_5W_vs_E10	ordinary_muscle_development	ENSGALG00010013654	SLC6A7	-6.086802561	0.915053263	-6.651856029	2.8942E-11	7.45935E-11	negative

PRJNA729271	stage_5W_vs_E10	ordinary_muscle_development	ENSGALG00010013657	MAGT1	-0.913875096	0.105144939	-8.691574746	3.5745E-18	1.31604E-17	negative
PRJNA729271	stage_5W_vs_E10	ordinary_muscle_development	ENSGALG00010013661	GABRA4	-0.617989308	0.357676813	-1.727786889	0.084026439	0.103042575	negative
PRJNA729271	stage_5W_vs_E10	ordinary_muscle_development	ENSGALG00010013663	OPA1	0.060745829	0.106610926	0.569789898	0.568820208	0.607817834	positive
PRJNA729271	stage_5W_vs_E10	ordinary_muscle_development	ENSGALG00010013664	REV3L	0.269747148	0.096321921	2.800475178	0.005102743	0.007198468	positive
PRJNA729271	stage_5W_vs_E10	ordinary_muscle_development	ENSGALG00010013666	COX7B	0.805152807	0.143505018	5.610624756	2.01597E-08	4.36664E-08	positive
PRJNA729271	stage_5W_vs_E10	ordinary_muscle_development	ENSGALG00010013667	SOWAHA	-0.624218936	0.455675171	-1.369876999	0.170725301	0.201077699	negative
PRJNA729271	stage_5W_vs_E10	ordinary_muscle_development	ENSGALG00010013669	NPR3	0.281967325	0.254749188	1.106842879	0.268361866	0.305425977	positive
PRJNA729271	stage_5W_vs_E10	ordinary_muscle_development	ENSGALG00010013672	ENSGALG00010013672	-4.571149757	0.509237018	-8.97646792	2.79598E-19	1.08103E-18	negative
PRJNA729271	stage_5W_vs_E10	ordinary_muscle_development	ENSGALG00010013675	CHRNA6	1.436267212	0.370843858	3.872970201	0.000107517	0.000176516	positive
PRJNA729271	stage_5W_vs_E10	ordinary_muscle_development	ENSGALG00010013677	GABRG1	-3.529655667	0.469449346	-7.518714634	5.53174E-14	1.64849E-13	negative
PRJNA729271	stage_5W_vs_E10	ordinary_muscle_development	ENSGALG00010013678	ATP7A	0.578496759	0.101652701	5.690913806	1.26361E-08	2.77264E-08	positive
PRJNA729271	stage_5W_vs_E10	ordinary_muscle_development	ENSGALG00010013679	MFSD4B	2.228687393	0.213149863	10.45596446	1.37592E-25	6.96366E-25	positive
PRJNA729271	stage_5W_vs_E10	ordinary_muscle_development	ENSGALG00010013686	UQCRQ	2.255740344	0.137995634	16.34646165	4.60983E-60	7.39972E-59	positive
PRJNA729271	stage_5W_vs_E10	ordinary_muscle_development	ENSGALG00010013690	CREB3L2	-0.718422313	0.101861699	-7.052918992	1.75203E-12	4.82532E-12	negative
PRJNA729271	stage_5W_vs_E10	ordinary_muscle_development	ENSGALG00010013696	CETN3	-0.468884867	0.111567162	-4.202713941	2.63734E-05	4.54545E-05	negative
PRJNA729271	stage_5W_vs_E10	ordinary_muscle_development	ENSGALG00010013697	HDAC4	0.092120319	0.067333945	1.368111128	0.171277294	0.201645904	positive
PRJNA729271	stage_5W_vs_E10	ordinary_muscle_development	ENSGALG00010013698	TFAP2E	-4.575325756	0.75871243	-6.03038197	1.63573E-09	3.77185E-09	negative
PRJNA729271	stage_5W_vs_E10	ordinary_muscle_development	ENSGALG00010013709	PSMF1	0.521787799	0.100663117	5.183505297	2.17754E-07	4.38625E-07	positive
PRJNA729271	stage_5W_vs_E10	ordinary_muscle_development	ENSGALG00010013715	IL1R1	0.291915861	0.233952518	1.24775687	0.212120108	0.245620783	positive
PRJNA729271	stage_5W_vs_E10	ordinary_muscle_development	ENSGALG00010013719	NCDN	-0.334304229	0.140790034	-2.374487875	0.017573313	0.023388594	negative
PRJNA729271	stage_5W_vs_E10	ordinary_muscle_development	ENSGALG00010013720	SLC16A10	0.806137923	0.189786168	4.247611563	2.16062E-05	3.75112E-05	positive
PRJNA729271	stage_5W_vs_E10	ordinary_muscle_development	ENSGALG00010013732	FNTA	0.34897734	0.075738376	4.607668656	4.07209E-06	7.46678E-06	positive
PRJNA729271	stage_5W_vs_E10	ordinary_muscle_development	ENSGALG00010013737	AFF4	0.651632344	0.084665945	7.696510578	1.39832E-14	4.29239E-14	positive
PRJNA729271	stage_5W_vs_E10	ordinary_muscle_development	ENSGALG00010013740	GNPDA2	-0.972853514	0.120379372	-8.081563306	6.39417E-16	2.10644E-15	negative
PRJNA729271	stage_5W_vs_E10	ordinary_muscle_development	ENSGALG00010013742	RSPO4	-2.570930191	1.246310708	-2.062832466	0.039128548	0.050054568	negative
PRJNA729271	stage_5W_vs_E10	ordinary_muscle_development	ENSGALG00010013744	ENSGALG00010013744	-0.803030053	0.32078694	-2.503312801	0.012303675	0.016673688	negative
PRJNA729271	stage_5W_vs_E10	ordinary_muscle_development	ENSGALG00010013746	ENSGALG00010013746	-0.000987929	0.086879263	-0.011371292	0.990927217	0.992159288	negative
PRJNA729271	stage_5W_vs_E10	ordinary_muscle_development	ENSGALG00010013749	NELL2	-2.517884801	0.464285411	-5.423140035	5.85611E-08	1.22718E-07	negative
PRJNA729271	stage_5W_vs_E10	ordinary_muscle_development	ENSGALG00010013751	FUT10	-0.103651934	0.171402221	-0.604729236	0.545358919	0.585077162	negative
PRJNA729271	stage_5W_vs_E10	ordinary_muscle_development	ENSGALG00010013753	PLEKHA5	0.117578477	0.124399316	0.945169808	0.344572191	0.384375397	positive
PRJNA729271	stage_5W_vs_E10	ordinary_muscle_development	ENSGALG00010013755	ANGPT4	1.701481635	0.153813776	11.0619587	1.91861E-28	1.09317E-27	positive
PRJNA729271	stage_5W_vs_E10	ordinary_muscle_development	ENSGALG00010013757	DGKI	-2.016006976	0.297825901	-6.769078741	1.29605E-11	3.4101E-11	negative
PRJNA729271	stage_5W_vs_E10	ordinary_muscle_development	ENSGALG00010013758	FAM3B	-2.047792475	0.561458585	-3.647272532	0.000265039	0.000421382	negative
PRJNA729271	stage_5W_vs_E10	ordinary_muscle_development	ENSGALG00010013760	RPF2	-1.092215617	0.116915593	-9.341915753	9.46066E-21	3.89802E-20	negative
PRJNA729271	stage_5W_vs_E10	ordinary_muscle_development	ENSGALG00010013766	DNM3	-0.209450079	0.109256998	-1.91704039	0.055232801	0.069232052	negative
PRJNA729271	stage_5W_vs_E10	ordinary_muscle_development	ENSGALG00010013767	PGK2	5.432288719	0.171875682	31.60591807	3.0617E-219	6.4318E-217	positive
PRJNA729271	stage_5W_vs_E10	ordinary_muscle_development	ENSGALG00010013768	FAM110A	-1.498724467	0.245654471	-6.1009452	1.05443E-09	2.45842E-09	negative
PRJNA729271	stage_5W_vs_E10	ordinary_muscle_development	ENSGALG00010013771	PELO	0.314112112	0.102961844	3.050762288	0.002282612	0.003336674	positive
PRJNA729271	stage_5W_vs_E10	ordinary_muscle_development	ENSGALG00010013772	KIAA0319L	1.622216392	0.143011634	11.34324769	8.0115E-30	4.81055E-29	positive
PRJNA729271	stage_5W_vs_E10	ordinary_muscle_development	ENSGALG00010013775	DBX2	-1.080358876	0.363367684	-2.973183702	0.00294728	0.004261854	negative
PRJNA729271	stage_5W_vs_E10	ordinary_muscle_development	ENSGALG00010013777	CPLANE1	-1.185705859	0.096180392	-12.32793744	6.40656E-35	4.71387E-34	negative
PRJNA729271	stage_5W_vs_E10	ordinary_muscle_development	ENSGALG00010013778	ENSGALG00010013778	-4.095821603	0.70462637	-5.812756626	6.14524E-09	1.37398E-08	negative
PRJNA729271	stage_5W_vs_E10	ordinary_muscle_development	ENSGALG00010013779	SLC52A3	-1.395374111	0.278163492	-5.016381189	5.26538E-07	1.03151E-06	negative
PRJNA729271	stage_5W_vs_E10	ordinary_muscle_development	ENSGALG00010013783	NDUFA10	0.589838102	0.09576924	6.158951463	7.32282E-10	1.72144E-09	positive
PRJNA729271	stage_5W_vs_E10	ordinary_muscle_development	ENSGALG00010013784	ANO6	1.882093357	0.094305339	19.95744236	1.29155E-88	4.23554E-87	positive
PRJNA729271	stage_5W_vs_E10	ordinary_muscle_development	ENSGALG00010013787	ZCCHC10	-0.506504701	0.105232019	-4.813218519	1.48519E-06	2.81335E-06	negative
PRJNA729271	stage_5W_vs_E10	ordinary_muscle_development	ENSGALG00010013791	AEBP2	-0.191008572	0.06553029	-2.914813478	0.003559012	0.005102154	negative
PRJNA729271	stage_5W_vs_E10	ordinary_muscle_development	ENSGALG00010013792	FGF10	-0.295079908	0.234627519	-1.257652599	0.208517422	0.241835496	negative
PRJNA729271	stage_5W_vs_E10	ordinary_muscle_development	ENSGALG00010013793	METTL13	-1.093363464	0.132906948	-8.226533556	1.92709E-16	6.51731E-16	negative
PRJNA729271	stage_5W_vs_E10	ordinary_muscle_development	ENSGALG00010013794	GTF3C6	-0.764859214	0.179474783	-4.261652813	2.02921E-05	3.53059E-05	negative
PRJNA729271	stage_5W_vs_E10	ordinary_muscle_development	ENSGALG00010013797	IL1RL1	0.573335479	0.317157166	1.807733013	0.070648064	0.087532583	positive
PRJNA729271	stage_5W_vs_E10	ordinary_muscle_development	ENSGALG00010013802	PYROXD1	0.729994573	0.14036417	5.200718765	1.98519E-07	4.01106E-07	positive
PRJNA729271	stage_5W_vs_E10	ordinary_muscle_development	ENSGALG00010013805	HSPA4	0.448977585	0.145267896	3.090686912	0.001996941	0.002934755	positive
PRJNA729271	stage_5W_vs_E10	ordinary_muscle_development	ENSGALG00010013808	PLAAT1	1.496551718	0.136481906	10.96520234	5.61748E-28	3.14383E-27	positive
PRJNA729271	stage_5W_vs_E10	ordinary_muscle_development	ENSGALG00010013809	MX1	8.122167451	1.211965439	6.701649395	2.0608E-11	5.35616E-11	positive
PRJNA729271	stage_5W_vs_E10	ordinary_muscle_development	ENSGALG00010013814	AMD1	-0.154683913	0.110521878	-1.399577319	0.16163993	0.191075745	negative
PRJNA729271	stage_5W_vs_E10	ordinary_muscle_development	ENSGALG00010013815	SRXN1	-0.097571898	0.252288086	-0.386747944	0.698942819	0.731334451	negative
PRJNA729271	stage_5W_vs_E10	ordinary_muscle_development	ENSGALG00010013820	MASP1	-3.317954894	0.496712152	-6.679834353	2.39212E-11	6.19397E-11	negative
PRJNA729271	stage_5W_vs_E10	ordinary_muscle_development	ENSGALG00010013823	GUF1	-0.036904714	0.113636327	-0.32476159	0.745361517	0.773760308	negative
PRJNA729271	stage_5W_vs_E10	ordinary_muscle_development	ENSGALG00010013828	PTN	-8.198987479	0.27776954	-29.5172303	1.7305E-191	2.5859E-189	negative
PRJNA729271	stage_5W_vs_E10	ordinary_muscle_development	ENSGALG00010013831	TCERG1	-0.12390004	0.113178686	-1.094729449	0.273635183	0.310793134	negative
PRJNA729271	stage_5W_vs_E10	ordinary_muscle_development	ENSGALG00010013833	TMPRSS2	-2.909785682	1.28939575	-2.256704881	0.024026522	0.031474418	negative
PRJNA729271	stage_5W_vs_E10	ordinary_muscle_development	ENSGALG00010013836	IL18R1	1.568269986	0.251126437	6.244941802	4.23958E-10	1.0129E-09	positive
PRJNA729271	stage_5W_vs_E10	ordinary_muscle_development	ENSGALG00010013837	IFI27L2	2.810779829	0.334165528	8.411339876	4.05353E-17	1.41717E-16	positive
PRJNA729271	stage_5W_vs_E10	ordinary_muscle_development	ENSGALG00010013842	CDK19	0.519515101	0.111335573	4.666209443	3.06807E-06	5.68546E-06	positive
PRJNA729271	stage_5W_vs_E10	ordinary_muscle_development	ENSGALG00010013846	YIPF7	8.549853195	0.318964041	26.80506924	2.8202E-158	3.1445E-156	positive
PRJNA729271	stage_5W_vs_E10	ordinary_muscle_development	ENSGALG00010013848	ITPA	-1.411740273	0.105832136	-13.33942907	1.36501E-40	1.21609E-39	negative
PRJNA729271	stage_5W_vs_E10	ordinary_muscle_development	ENSGALG00010013857	IL18RAP	2.529492855	0.574241742	4.404926828	1.0582E-05	1.88249E-05	positive
PRJNA729271	stage_5W_vs_E10	ordinary_muscle_development	ENSGALG00010013859	MYOC	1.761266707	0.245592395	7.17150346	7.41786E-13	2.08255E-12	positive
PRJNA729271	stage_5W_vs_E10	ordinary_muscle_development	ENSGALG00010013861	TCF15	2.27940465	0.155855329	14.62513128	1.94196E-48	2.21295E-47	positive
PRJNA729271	stage_5W_vs_E10	ordinary_muscle_development	ENSGALG00010013866	KCTD8	-0.496496322	0.37195144	-1.334841778	0.181928137	0.213267152	negative
PRJNA729271	stage_5W_vs_E10	ordinary_muscle_development	ENSGALG00010013867	RECQL	-0.972057679	0.128211552	-7.58167002	3.41135E-14	1.02759E-13	negative
PRJNA729271	stage_5W_vs_E10	ordinary_muscle_development	ENSGALG00010013871	ENSGALG00010013871	0.564878892	0.233347475	2.420762822	0.015487979	0.020757416	positive
PRJNA729271	stage_5W_vs_E10	ordinary_muscle_development	ENSGALG00010013872	GLS2	3.086556096	0.196752916	15.68747323	1.84245E-55	2.58531E-54	positive
PRJNA729271	stage_5W_vs_E10	ordinary_muscle_development	ENSGALG00010013878	ARID2	-0.403799271	0.082233999	-4.91036892	9.09052E-07	1.75083E-06	negative
PRJNA729271	stage_5W_vs_E10	ordinary_muscle_development	ENSGALG00010013880	ZMYM4	-0.674446089	0.090537781	-7.449333128	9.38132E-14	2.75212E-13	negative
PRJNA729271	stage_5W_vs_E10	ordinary_muscle_development	ENSGALG00010013889	ENSGALG00010013889	-0.21791279	0.422337096	-0.515968859	0.605876159	0.643288524	negative
PRJNA729271	stage_5W_vs_E10	ordinary_muscle_development	ENSGALG00010013890	SFPQ	-1.495488036	0.07274049	-20.55922404	6.36427E-94	2.35934E-92	negative
PRJNA729271	stage_5W_vs_E10	ordinary_muscle_development	ENSGALG00010013893	CSNK2A1	-0.4243052	0.079276792	-5.352199431	8.68916E-08	1.79953E-07	negative
PRJNA729271	stage_5W_vs_E10	ordinary_muscle_development	ENSGALG00010013897	AMOT	-0.993973036	0.075671227	-13.13541591	2.06355E-39	1.76155E-38	negative
PRJNA729271	stage_5W_vs_E10	ordinary_muscle_development	ENSGALG00010013898	EMB	-1.286144724	0.295350928	-4.354632406	1.3329E-05	2.35529E-05	negative
PRJNA729271	stage_5W_vs_E10	ordinary_muscle_development	ENSGALG00010013899	WNT5B	0.458209119	0.141243483	3.244108042	0.00117819	0.001772483	positive
PRJNA729271	stage_5W_vs_E10	ordinary_muscle_development	ENSGALG00010013901	NFXL1	-0.392091957	0.122544184	-3.199596635	0.0013762	0.002057134	negative
PRJNA729271	stage_5W_vs_E10	ordinary_muscle_development	ENSGALG00010013908	TBC1D20	1.066347672	0.115526756	9.230309169	2.69852E-20	1.09107E-19	positive
PRJNA729271	stage_5W_vs_E10	ordinary_muscle_development	ENSGALG00010013910	ADIPOR2	1.685557067	0.207869776	8.108716438	5.11573E-16	1.69375E-15	positive
PRJNA729271	stage_5W_vs_E10	ordinary_muscle_development	ENSGALG00010013911	TNIK	0.272619749	0.148040096	1.841526423	0.065544452	0.081470921	positive
PRJNA729271	stage_5W_vs_E10	ordinary_muscle_development	ENSGALG00010013914	NRG1	-0.901928792	0.233482196	-3.862944614	0.000112028	0.000183611	negative
PRJNA729271	stage_5W_vs_E10	ordinary_muscle_development	ENSGALG00010013915	ENSGALG00010013915	1.036743043	0.191548303	5.412436576	6.21729E-08	1.30048E-07	positive
PRJNA729271	stage_5W_vs_E10	ordinary_muscle_development	ENSGALG00010013917	TRPC5	-2.377108143	0.623959041	-3.809718247	0.000139125	0.000226307	negative
PRJNA729271	stage_5W_vs_E10	ordinary_muscle_development	ENSGALG00010013921	PPP2R2B	0.988387574	0.105709257	9.350056989	8.76005E-21	3.61964E-20	positive
PRJNA729271	stage_5W_vs_E10	ordinary_muscle_development	ENSGALG00010013922	ENSGALG00010013922	-3.043316931	0.954488288	-3.188427737	0.001430488	0.002134864	negative
PRJNA729271	stage_5W_vs_E10	ordinary_muscle_development	ENSGALG00010013923	FMR1NB	1.88861942	0.503295935	3.752502829	0.000175078	0.000282318	positive
PRJNA729271	stage_5W_vs_E10	ordinary_muscle_development	ENSGALG00010013928	CCNO	1.146198343	0.493961172	2.320421945	0.020318063	0.026819991	positive
PRJNA729271	stage_5W_vs_E10	ordinary_muscle_development	ENSGALG00010013930	SLC22A16	1.874632507	0.304267908	6.16112465	7.22301E-10	1.69964E-09	positive
PRJNA729271	stage_5W_vs_E10	ordinary_muscle_development	ENSGALG00010013934	STAT1	2.396713341	0.282484542	8.484405297	2.16825E-17	7.68241E-17	positive
PRJNA729271	stage_5W_vs_E10	ordinary_muscle_development	ENSGALG00010013936	DLGAP3	-2.978250928	0.45011514	-6.616642411	3.67449E-11	9.42685E-11	negative
PRJNA729271	stage_5W_vs_E10	ordinary_muscle_development	ENSGALG00010013938	ENSGALG00010013938	0.844658184	0.411658478	2.051842071	0.040185013	0.051315458	positive
PRJNA729271	stage_5W_vs_E10	ordinary_muscle_development	ENSGALG00010013940	UPF3A	-1.884495499	0.267557574	-7.043327055	1.87703E-12	5.16174E-12	negative
PRJNA729271	stage_5W_vs_E10	ordinary_muscle_development	ENSGALG00010013942	CACNA2D4	1.524212892	0.496116287	3.072289568	0.002124235	0.003114271	positive
PRJNA729271	stage_5W_vs_E10	ordinary_muscle_development	ENSGALG00010013944	METTL24	-3.47326408	0.254856942	-13.62828908	2.7188E-42	2.5458E-41	negative
PRJNA729271	stage_5W_vs_E10	ordinary_muscle_development	ENSGALG00010013945	SCAF11	-0.507593791	0.077307707	-6.565888552	5.17235E-11	1.31256E-10	negative
PRJNA729271	stage_5W_vs_E10	ordinary_muscle_development	ENSGALG00010013947	ENSGALG00010013947	-0.159066441	0.182215441	-0.872958078	0.382685928	0.42389061	negative
PRJNA729271	stage_5W_vs_E10	ordinary_muscle_development	ENSGALG00010013949	PRRC2C	0.022265743	0.126143629	0.176511037	0.859892479	0.877015302	positive
PRJNA729271	stage_5W_vs_E10	ordinary_muscle_development	ENSGALG00010013953	DHX29	-0.376179282	0.081729578	-4.602731251	4.16986E-06	7.63737E-06	negative
PRJNA729271	stage_5W_vs_E10	ordinary_muscle_development	ENSGALG00010013954	LPL	1.883342273	0.334003651	5.638687675	1.71351E-08	3.72821E-08	positive
PRJNA729271	stage_5W_vs_E10	ordinary_muscle_development	ENSGALG00010013958	TUSC3	0.638790064	0.175710876	3.635461158	0.000277484	0.000440253	positive
PRJNA729271	stage_5W_vs_E10	ordinary_muscle_development	ENSGALG00010013959	STK32A	-0.442939943	0.429087214	-1.032284179	0.30193901	0.340485915	negative
PRJNA729271	stage_5W_vs_E10	ordinary_muscle_development	ENSGALG00010013964	SLC38A1	-1.36380438	0.161374067	-8.451199184	2.88326E-17	1.01612E-16	negative
PRJNA729271	stage_5W_vs_E10	ordinary_muscle_development	ENSGALG00010013965	SLC2A2	1.119151716	1.04187227	1.074173628			positive
PRJNA729271	stage_5W_vs_E10	ordinary_muscle_development	ENSGALG00010013967	MLLT3	-0.373488718	0.154731035	-2.413793178	0.015787421	0.02112812	negative
PRJNA729271	stage_5W_vs_E10	ordinary_muscle_development	ENSGALG00010013971	ENSGALG00010013971	-1.120603967	0.391345229	-2.863466534	0.004190331	0.005968833	negative
PRJNA729271	stage_5W_vs_E10	ordinary_muscle_development	ENSGALG00010013973	ENSGALG00010013973	1.138721131	0.237263013	4.799404317	1.59138E-06	3.00627E-06	positive
PRJNA729271	stage_5W_vs_E10	ordinary_muscle_development	ENSGALG00010013976	CDC40	-0.30863603	0.075988638	-4.061607585	4.87359E-05	8.2315E-05	negative
PRJNA729271	stage_5W_vs_E10	ordinary_muscle_development	ENSGALG00010013977	SLC38A2	0.049964947	0.192763633	0.259203185	0.795478474	0.819739832	positive
PRJNA729271	stage_5W_vs_E10	ordinary_muscle_development	ENSGALG00010013980	FMO4	1.017987519	0.379630295	2.681523398	0.007328779	0.01017535	positive
PRJNA729271	stage_5W_vs_E10	ordinary_muscle_development	ENSGALG00010013981	RLF	0.416970191	0.107185743	3.890164686	0.000100176	0.000165006	positive
PRJNA729271	stage_5W_vs_E10	ordinary_muscle_development	ENSGALG00010013989	ENSGALG00010013989	1.565451522	0.204036679	7.672402494	1.68804E-14	5.15228E-14	positive
PRJNA729271	stage_5W_vs_E10	ordinary_muscle_development	ENSGALG00010013994	CHRM2	-4.214465688	0.495259838	-8.509605186	1.74527E-17	6.22022E-17	negative
PRJNA729271	stage_5W_vs_E10	ordinary_muscle_development	ENSGALG00010013995	REM1	0.685208974	0.210205066	3.259716742	0.001115235	0.001682837	positive
PRJNA729271	stage_5W_vs_E10	ordinary_muscle_development	ENSGALG00010014000	STAT4	8.721431167	0.921945602	9.459811017	3.085E-21	1.30333E-20	positive
PRJNA729271	stage_5W_vs_E10	ordinary_muscle_development	ENSGALG00010014001	CDC16	-0.997603566	0.087409152	-11.41303334	3.59954E-30	2.1904E-29	negative
PRJNA729271	stage_5W_vs_E10	ordinary_muscle_development	ENSGALG00010014002	ZDHHC18	0.220181032	0.272111852	0.809156348	0.41842522	0.460032885	positive
PRJNA729271	stage_5W_vs_E10	ordinary_muscle_development	ENSGALG00010014010	EIF5A2	-0.160552806	0.10255556	-1.565520244	0.117460975	0.141317798	negative
PRJNA729271	stage_5W_vs_E10	ordinary_muscle_development	ENSGALG00010014011	SFN	-6.577870475	0.781882843	-8.412859468	4.00134E-17	1.39994E-16	negative
PRJNA729271	stage_5W_vs_E10	ordinary_muscle_development	ENSGALG00010014014	PARP8	1.272934979	0.195904922	6.497718215	8.15474E-11	2.04433E-10	positive
PRJNA729271	stage_5W_vs_E10	ordinary_muscle_development	ENSGALG00010014018	DPYSL3	-1.570974987	0.239010527	-6.5728276	4.93686E-11	1.25455E-10	negative
PRJNA729271	stage_5W_vs_E10	ordinary_muscle_development	ENSGALG00010014019	IRAG2	1.86414878	0.219848562	8.479240285	2.26669E-17	8.01945E-17	positive
PRJNA729271	stage_5W_vs_E10	ordinary_muscle_development	ENSGALG00010014020	PDXP	-1.046942391	0.109163497	-9.590590472	8.75823E-22	3.7907E-21	negative
PRJNA729271	stage_5W_vs_E10	ordinary_muscle_development	ENSGALG00010014026	SH3BP1	4.074348472	0.314037287	12.97409144	1.71619E-38	1.41988E-37	positive
PRJNA729271	stage_5W_vs_E10	ordinary_muscle_development	ENSGALG00010014030	WASF1	-2.869510816	0.381627801	-7.51913464	5.514E-14	1.64388E-13	negative
PRJNA729271	stage_5W_vs_E10	ordinary_muscle_development	ENSGALG00010014031	RPL22L1	-0.614585551	0.188737026	-3.256306211	0.00112872	0.00170159	negative

PRJNA729271	stage_5W_vs_E10	ordinary_muscle_development	ENSGALG00010014032	PSD3	-0.70011888	0.078256508	-8.946462024	3.67058E-19	1.41165E-18	negative
PRJNA729271	stage_5W_vs_E10	ordinary_muscle_development	ENSGALG00010014033	SLC38A4	2.271237903	0.474867859	4.782884042	1.72798E-06	3.25592E-06	positive
PRJNA729271	stage_5W_vs_E10	ordinary_muscle_development	ENSGALG00010014035	PSIP1	-1.088351888	0.11866187	-9.171875452	4.6486E-20	1.86034E-19	negative
PRJNA729271	stage_5W_vs_E10	ordinary_muscle_development	ENSGALG00010014041	SKIV2L2	-0.04240533	0.098647113	-0.42986894	0.66729098	0.701456433	negative
PRJNA729271	stage_5W_vs_E10	ordinary_muscle_development	ENSGALG00010014042	SGCZ	-2.588215024	0.383586803	-6.747403734	1.50514E-11	3.94735E-11	negative
PRJNA729271	stage_5W_vs_E10	ordinary_muscle_development	ENSGALG00010014045	GOLT1B	-1.420355129	0.111615843	-12.72538996	4.27336E-37	3.41092E-36	negative
PRJNA729271	stage_5W_vs_E10	ordinary_muscle_development	ENSGALG00010014047	HM13	0.061029776	0.167346509	0.36469106	0.715342033	0.746123823	positive
PRJNA729271	stage_5W_vs_E10	ordinary_muscle_development	ENSGALG00010014056	FMO3	4.699904802	0.253900042	18.51084688	1.68825E-76	4.25587E-75	positive
PRJNA729271	stage_5W_vs_E10	ordinary_muscle_development	ENSGALG00010014062	GGA1	0.905292003	0.103670713	8.732379477	2.4937E-18	9.23746E-18	positive
PRJNA729271	stage_5W_vs_E10	ordinary_muscle_development	ENSGALG00010014065	CHUNK-1	-4.166882574	0.990700796	-4.205994978	2.59936E-05	4.48224E-05	negative
PRJNA729271	stage_5W_vs_E10	ordinary_muscle_development	ENSGALG00010014066	BCL2L1	0.458865226	0.116284002	3.946073569	7.94432E-05	0.000132193	positive
PRJNA729271	stage_5W_vs_E10	ordinary_muscle_development	ENSGALG00010014076	PRRX1	-3.101181045	0.249648073	-12.42221105	1.98012E-35	1.48484E-34	negative
PRJNA729271	stage_5W_vs_E10	ordinary_muscle_development	ENSGALG00010014077	SLITRK2	0.309674884	0.099980539	3.097351619	0.001952581	0.002872201	positive
PRJNA729271	stage_5W_vs_E10	ordinary_muscle_development	ENSGALG00010014080	MYO1B	0.055007502	0.160725866	0.342244239	0.732167095	0.761699709	positive
PRJNA729271	stage_5W_vs_E10	ordinary_muscle_development	ENSGALG00010014082	HNRNPR	-0.987705802	0.04334827	-22.78535687	6.4059E-115	3.5851E-113	negative
PRJNA729271	stage_5W_vs_E10	ordinary_muscle_development	ENSGALG00010014083	ENSGALG00010014083	-0.655584238	0.146139487	-4.486017092	7.25669E-06	1.30536E-05	negative
PRJNA729271	stage_5W_vs_E10	ordinary_muscle_development	ENSGALG00010014089	FIG4	0.075498252	0.114262632	0.660743154	0.508777044	0.549614194	positive
PRJNA729271	stage_5W_vs_E10	ordinary_muscle_development	ENSGALG00010014091	CHIC2	0.426771106	0.098836088	4.317968418	1.57472E-05	2.76506E-05	positive
PRJNA729271	stage_5W_vs_E10	ordinary_muscle_development	ENSGALG00010014092	TYRP1	-1.942795635	1.027729628	-1.890376207	0.058707663	0.073371924	negative
PRJNA729271	stage_5W_vs_E10	ordinary_muscle_development	ENSGALG00010014093	PLD1	0.690157972	0.09314334	7.40963307	1.26649E-13	3.69001E-13	positive
PRJNA729271	stage_5W_vs_E10	ordinary_muscle_development	ENSGALG00010014096	JAKMIP2	-2.915213644	0.161588392	-18.04098426	9.28749E-73	2.11338E-71	negative
PRJNA729271	stage_5W_vs_E10	ordinary_muscle_development	ENSGALG00010014099	TPX2	-2.269474854	0.144395112	-15.71711691	1.15459E-55	1.62958E-54	negative
PRJNA729271	stage_5W_vs_E10	ordinary_muscle_development	ENSGALG00010014102	TRIM2	0.864571856	0.1373202	6.296028213	3.05369E-10	7.3588E-10	positive
PRJNA729271	stage_5W_vs_E10	ordinary_muscle_development	ENSGALG00010014104	ALDH1A1	4.779220794	0.516834362	9.247103411	2.30659E-20	9.36001E-20	positive
PRJNA729271	stage_5W_vs_E10	ordinary_muscle_development	ENSGALG00010014105	AHR1B	-3.667081172	0.813625228	-4.507088826	6.57231E-06	1.18563E-05	negative
PRJNA729271	stage_5W_vs_E10	ordinary_muscle_development	ENSGALG00010014109	AMIGO2	-0.913435886	0.164791673	-5.542973568	2.97378E-08	6.35672E-08	negative
PRJNA729271	stage_5W_vs_E10	ordinary_muscle_development	ENSGALG00010014111	GORAB	-0.315514313	0.082787	-3.811157708	0.000138317	0.000225043	negative
PRJNA729271	stage_5W_vs_E10	ordinary_muscle_development	ENSGALG00010014114	ANXA1	2.488041541	0.332132741	7.491105923	6.82957E-14	2.01947E-13	positive
PRJNA729271	stage_5W_vs_E10	ordinary_muscle_development	ENSGALG00010014120	SOX5	-2.171802917	0.135942136	-15.97593634	1.8801E-57	2.80659E-56	negative
PRJNA729271	stage_5W_vs_E10	ordinary_muscle_development	ENSGALG00010014122	ABCA1	0.271537838	0.140829418	1.928132929	0.053838594	0.067607244	positive
PRJNA729271	stage_5W_vs_E10	ordinary_muscle_development	ENSGALG00010014125	PLPP1	3.517892088	0.138643172	25.3737132	4.9203E-142	4.2201E-140	positive
PRJNA729271	stage_5W_vs_E10	ordinary_muscle_development	ENSGALG00010014128	COL4A1	3.424010054	0.144537754	23.68938184	4.6393E-124	3.0021E-122	positive
PRJNA729271	stage_5W_vs_E10	ordinary_muscle_development	ENSGALG00010014131	CDC23	-0.73079749	0.118010928	-6.192625555	5.91702E-10	1.40188E-09	negative
PRJNA729271	stage_5W_vs_E10	ordinary_muscle_development	ENSGALG00010014134	RPAP3	-0.24311886	0.114230109	-2.128325553	0.033310101	0.042979341	negative
PRJNA729271	stage_5W_vs_E10	ordinary_muscle_development	ENSGALG00010014135	ZBTB24	0.566642777	0.153488331	3.691764529	0.000222704	0.000356459	positive
PRJNA729271	stage_5W_vs_E10	ordinary_muscle_development	ENSGALG00010014142	PPIL6	0.51367381	0.5183285	0.991019808	0.321675908	0.36105717	positive
PRJNA729271	stage_5W_vs_E10	ordinary_muscle_development	ENSGALG00010014143	NFATC2	0.432040813	0.151544775	2.850911967	0.004359404	0.006194447	positive
PRJNA729271	stage_5W_vs_E10	ordinary_muscle_development	ENSGALG00010014145	NTMT2	7.2322403	0.251297585	28.77958537	3.8634E-182	5.437E-180	positive
PRJNA729271	stage_5W_vs_E10	ordinary_muscle_development	ENSGALG00010014147	ENSGALG00010014147	-2.141543178	0.377044406	-5.679816871	1.34839E-08	2.95553E-08	negative
PRJNA729271	stage_5W_vs_E10	ordinary_muscle_development	ENSGALG00010014148	KCNG1	-1.630501533	0.387150943	-4.211539612	2.53636E-05	4.38038E-05	negative
PRJNA729271	stage_5W_vs_E10	ordinary_muscle_development	ENSGALG00010014149	LGALS2	6.158378607	0.501627092	12.27680622	1.20668E-34	8.77616E-34	positive
PRJNA729271	stage_5W_vs_E10	ordinary_muscle_development	ENSGALG00010014151	ATP23	0.185098175	0.0904672	2.046025236	0.040753888	0.051973219	positive
PRJNA729271	stage_5W_vs_E10	ordinary_muscle_development	ENSGALG00010014153	FOCAD	0.379375295	0.132567112	2.861760277	0.004212954	0.005998701	positive
PRJNA729271	stage_5W_vs_E10	ordinary_muscle_development	ENSGALG00010014154	DNAI7	-1.820102517	0.495443533	-3.673683065	0.000239079	0.0003817	negative
PRJNA729271	stage_5W_vs_E10	ordinary_muscle_development	ENSGALG00010014155	ENSGALG00010014155	-5.078149477	1.40138278	-3.623670527	0.000290452	0.00046017	negative
PRJNA729271	stage_5W_vs_E10	ordinary_muscle_development	ENSGALG00010014156	CD164	0.467474336	0.092220753	5.069079588	3.99744E-07	7.89198E-07	positive
PRJNA729271	stage_5W_vs_E10	ordinary_muscle_development	ENSGALG00010014159	CDC42EP1	-0.459795049	0.150051311	-3.064252117	0.002182149	0.00319433	negative
PRJNA729271	stage_5W_vs_E10	ordinary_muscle_development	ENSGALG00010014161	GFRA3	0.677169288	0.249859732	2.710197768	0.00672431	0.009374251	positive
PRJNA729271	stage_5W_vs_E10	ordinary_muscle_development	ENSGALG00010014162	COL18A1	2.478220079	0.188105936	13.17459798	1.2288E-39	1.0621E-38	positive
PRJNA729271	stage_5W_vs_E10	ordinary_muscle_development	ENSGALG00010014165	LUZP1	-1.284954931	0.119828767	-10.72325918	7.91637E-27	4.23893E-26	negative
PRJNA729271	stage_5W_vs_E10	ordinary_muscle_development	ENSGALG00010014166	FGF12	-1.321795299	0.204505657	-6.463367903	1.02398E-10	2.54852E-10	negative
PRJNA729271	stage_5W_vs_E10	ordinary_muscle_development	ENSGALG00010014167	SLC38A9	0.444981876	0.108119843	4.115635603	3.86114E-05	6.56815E-05	positive
PRJNA729271	stage_5W_vs_E10	ordinary_muscle_development	ENSGALG00010014170	CEMIP2	-0.98139622	0.107493723	-9.129800266	6.86258E-20	2.72903E-19	negative
PRJNA729271	stage_5W_vs_E10	ordinary_muscle_development	ENSGALG00010014171	DLC1	0.225900274	0.132810411	1.70092293	0.08895746	0.108703287	positive
PRJNA729271	stage_5W_vs_E10	ordinary_muscle_development	ENSGALG00010014173	MND1	-2.670682402	0.205304036	-13.00842623	1.09576E-38	9.13339E-38	negative
PRJNA729271	stage_5W_vs_E10	ordinary_muscle_development	ENSGALG00010014176	ABHD17B	-0.328309666	0.090940247	-3.610169079	0.000305997	0.000483901	negative
PRJNA729271	stage_5W_vs_E10	ordinary_muscle_development	ENSGALG00010014178	LRTM2	-2.01287342	0.485056774	-4.149768705	3.32811E-05	5.68545E-05	negative
PRJNA729271	stage_5W_vs_E10	ordinary_muscle_development	ENSGALG00010014179	CTBPL	-0.73373341	0.042827891	-17.13213951	8.54536E-66	1.61493E-64	negative
PRJNA729271	stage_5W_vs_E10	ordinary_muscle_development	ENSGALG00010014180	SLC19A1	-0.991425612	0.14646267	-6.769135167	1.29554E-11	3.40939E-11	negative
PRJNA729271	stage_5W_vs_E10	ordinary_muscle_development	ENSGALG00010014184	SLC44A1	1.009870761	0.132260613	7.635461044	2.25014E-14	6.83482E-14	positive
PRJNA729271	stage_5W_vs_E10	ordinary_muscle_development	ENSGALG00010014186	MOCS3	-0.872734229	0.112546204	-7.754452808	8.87252E-15	2.75744E-14	negative
PRJNA729271	stage_5W_vs_E10	ordinary_muscle_development	ENSGALG00010014189	DDX4	-1.474966088	0.536070907	-2.751438419	0.005933417	0.008315275	negative
PRJNA729271	stage_5W_vs_E10	ordinary_muscle_development	ENSGALG00010014190	HACD4	2.038109797	0.220721907	9.233835569	2.61111E-20	1.05691E-19	positive
PRJNA729271	stage_5W_vs_E10	ordinary_muscle_development	ENSGALG00010014192	KDM1A	-1.944948269	0.062973401	-30.88523461	1.8857E-209	3.5043E-207	negative
PRJNA729271	stage_5W_vs_E10	ordinary_muscle_development	ENSGALG00010014193	CARD10	-1.549820909	0.362379251	-4.276792631	1.89605E-05	3.30765E-05	negative
PRJNA729271	stage_5W_vs_E10	ordinary_muscle_development	ENSGALG00010014194	LRIG3	-1.951554558	0.094717138	-20.60402785	2.52539E-94	9.50795E-93	negative
PRJNA729271	stage_5W_vs_E10	ordinary_muscle_development	ENSGALG00010014197	SH3GL2	-2.826904574	0.36537948	-7.736900215	1.0187E-14	3.15581E-14	negative
PRJNA729271	stage_5W_vs_E10	ordinary_muscle_development	ENSGALG00010014198	KDM3B	-0.540024783	0.045949092	-11.75267576	6.84175E-32	4.46516E-31	negative
PRJNA729271	stage_5W_vs_E10	ordinary_muscle_development	ENSGALG00010014199	LNX1	-1.121475499	0.250254383	-4.481342079	7.41751E-06	1.33379E-05	negative
PRJNA729271	stage_5W_vs_E10	ordinary_muscle_development	ENSGALG00010014200	DCP1B	-0.070526308	0.090420532	-0.779981123	0.435401986	0.477394235	negative
PRJNA729271	stage_5W_vs_E10	ordinary_muscle_development	ENSGALG00010014203	TMEM38B	1.436097731	0.169107056	8.492240133	2.02692E-17	7.19573E-17	positive
PRJNA729271	stage_5W_vs_E10	ordinary_muscle_development	ENSGALG00010014204	KIFAP3	-0.699626815	0.08475307	-8.254884615	1.52049E-16	5.16269E-16	negative
PRJNA729271	stage_5W_vs_E10	ordinary_muscle_development	ENSGALG00010014205	DPM1	-0.368821572	0.08628657	-4.274379775	1.9167E-05	3.34126E-05	negative
PRJNA729271	stage_5W_vs_E10	ordinary_muscle_development	ENSGALG00010014209	CCDC50	-0.52396889	0.107627212	-4.868368125	1.12524E-06	2.15062E-06	negative
PRJNA729271	stage_5W_vs_E10	ordinary_muscle_development	ENSGALG00010014210	ENSGALG00010014210	0.134327358	0.254158297	0.528518483	0.597139522	0.635175561	positive
PRJNA729271	stage_5W_vs_E10	ordinary_muscle_development	ENSGALG00010014213	ETFRF1	0.764346158	0.152151567	5.023583887	5.07161E-07	9.94359E-07	positive
PRJNA729271	stage_5W_vs_E10	ordinary_muscle_development	ENSGALG00010014214	REEP2	0.177265778	0.133967434	1.323200515	0.185768705	0.21736498	positive
PRJNA729271	stage_5W_vs_E10	ordinary_muscle_development	ENSGALG00010014217	RASA3	-0.138404613	0.10994421	-1.258862228	0.208080099	0.241386237	negative
PRJNA729271	stage_5W_vs_E10	ordinary_muscle_development	ENSGALG00010014218	IL31RA	1.351244658	0.167100295	8.086428902	6.14396E-16	2.0254E-15	positive
PRJNA729271	stage_5W_vs_E10	ordinary_muscle_development	ENSGALG00010014220	C9orf85	-0.497152644	0.133069912	-3.736025945	0.000186951	0.000301029	negative
PRJNA729271	stage_5W_vs_E10	ordinary_muscle_development	ENSGALG00010014221	SCYL3	1.458972803	0.130961306	11.14048756	7.96819E-29	4.60155E-28	positive
PRJNA729271	stage_5W_vs_E10	ordinary_muscle_development	ENSGALG00010014222	TBX22	-2.568741489	0.448752614	-5.724181668	1.03933E-08	2.29232E-08	negative
PRJNA729271	stage_5W_vs_E10	ordinary_muscle_development	ENSGALG00010014224	NMUR2	-6.371440554	0.967166025	-6.587742323	4.46565E-11	1.1378E-10	negative
PRJNA729271	stage_5W_vs_E10	ordinary_muscle_development	ENSGALG00010014225	ZMPSTE24	-0.422507244	0.076647222	-5.512362169	3.54049E-08	7.52485E-08	negative
PRJNA729271	stage_5W_vs_E10	ordinary_muscle_development	ENSGALG00010014226	SLC16A7	1.200009096	0.258922586	4.634625022	3.57585E-06	6.58184E-06	positive
PRJNA729271	stage_5W_vs_E10	ordinary_muscle_development	ENSGALG00010014234	GLRA1	-0.675744166	0.730568683	-0.924956382	0.354988597	0.395264995	negative
PRJNA729271	stage_5W_vs_E10	ordinary_muscle_development	ENSGALG00010014236	WDR44	-0.208594357	0.082618085	-2.52480261	0.011576329	0.015745415	negative
PRJNA729271	stage_5W_vs_E10	ordinary_muscle_development	ENSGALG00010014238	PHLDA1	-2.722683034	0.123435684	-22.05750357	8.0928E-108	3.8972E-106	negative
PRJNA729271	stage_5W_vs_E10	ordinary_muscle_development	ENSGALG00010014240	PIGV	0.252774045	0.131564461	1.921294271	0.054694619	0.068598988	positive
PRJNA729271	stage_5W_vs_E10	ordinary_muscle_development	ENSGALG00010014241	ENSGALG00010014241	-8.863969723	1.197259445	-7.403549635	1.32592E-13	3.85617E-13	negative
PRJNA729271	stage_5W_vs_E10	ordinary_muscle_development	ENSGALG00010014243	ENSGALG00010014243	0.385027175	0.14437802	2.666799112	0.007657743	0.010608715	positive
PRJNA729271	stage_5W_vs_E10	ordinary_muscle_development	ENSGALG00010014244	SESN1	4.01737805	0.369820956	10.86303516	1.72905E-27	9.48622E-27	positive
PRJNA729271	stage_5W_vs_E10	ordinary_muscle_development	ENSGALG00010014246	KRAS	-1.381733905	0.115338274	-11.97983858	4.53205E-33	3.09576E-32	negative
PRJNA729271	stage_5W_vs_E10	ordinary_muscle_development	ENSGALG00010014249	ENSGALG00010014249	0.320372709	0.101282934	3.163146015	0.00156074	0.002320063	positive
PRJNA729271	stage_5W_vs_E10	ordinary_muscle_development	ENSGALG00010014250	ENSGALG00010014250	4.890120235	0.253146006	19.31739045	3.83556E-83	1.1164E-81	positive
PRJNA729271	stage_5W_vs_E10	ordinary_muscle_development	ENSGALG00010014257	NOP16	-0.698209497	0.126326732	-5.527013041	3.25729E-08	6.94023E-08	negative
PRJNA729271	stage_5W_vs_E10	ordinary_muscle_development	ENSGALG00010014258	ENSGALG00010014258	-1.233667475	0.537650192	-2.294554144	0.021758689	0.028619981	negative
PRJNA729271	stage_5W_vs_E10	ordinary_muscle_development	ENSGALG00010014260	ADNP	-0.658018125	0.075791515	-8.681949784	3.89041E-18	1.42836E-17	negative
PRJNA729271	stage_5W_vs_E10	ordinary_muscle_development	ENSGALG00010014263	ZNF131	-0.247244213	0.120141003	-2.057950296	0.039594905	0.05062434	negative
PRJNA729271	stage_5W_vs_E10	ordinary_muscle_development	ENSGALG00010014265	ZNF462	-0.701317492	0.139836178	-5.015279316	5.29565E-07	1.0373E-06	negative
PRJNA729271	stage_5W_vs_E10	ordinary_muscle_development	ENSGALG00010014267	SCFD2	-0.847873664	0.076410167	-11.09634613	1.30678E-28	7.49277E-28	negative
PRJNA729271	stage_5W_vs_E10	ordinary_muscle_development	ENSGALG00010014272	PARD6B	-1.242401294	0.256406614	-4.845433879	1.26335E-06	2.40508E-06	negative
PRJNA729271	stage_5W_vs_E10	ordinary_muscle_development	ENSGALG00010014274	FIRRM	-0.686963671	0.150325216	-4.569849885	4.88074E-06	8.88772E-06	negative
PRJNA729271	stage_5W_vs_E10	ordinary_muscle_development	ENSGALG00010014278	G3BP1	-0.216769586	0.139458988	-1.554360813	0.120098375	0.144287273	negative
PRJNA729271	stage_5W_vs_E10	ordinary_muscle_development	ENSGALG00010014284	TMEM131L	-0.873206994	0.085544936	-10.20758254	1.83377E-24	8.90766E-24	negative
PRJNA729271	stage_5W_vs_E10	ordinary_muscle_development	ENSGALG00010014288	ARMC2	-1.689988499	0.251491014	-6.719876281	1.81879E-11	4.74588E-11	negative
PRJNA729271	stage_5W_vs_E10	ordinary_muscle_development	ENSGALG00010014291	OSTN	-4.697495502	0.556160838	-8.446289594	3.00707E-17	1.05821E-16	negative
PRJNA729271	stage_5W_vs_E10	ordinary_muscle_development	ENSGALG00010014292	NIM1K	4.293804648	0.442342978	9.706957867	2.81617E-22	1.24757E-21	positive
PRJNA729271	stage_5W_vs_E10	ordinary_muscle_development	ENSGALG00010014294	TMPO	-0.409419752	0.102196121	-4.006216169	6.16992E-05	0.000103618	negative
PRJNA729271	stage_5W_vs_E10	ordinary_muscle_development	ENSGALG00010014295	CACNA1C	-1.567924864	0.179973625	-8.71197024	2.98638E-18	1.10259E-17	negative
PRJNA729271	stage_5W_vs_E10	ordinary_muscle_development	ENSGALG00010014298	METTL18	-0.623300922	0.225915626	-2.75899872	0.005797876	0.008133186	negative
PRJNA729271	stage_5W_vs_E10	ordinary_muscle_development	ENSGALG00010014300	ELFN2	-0.287155403	0.448420575	-0.640370713	0.521931618	0.562546158	negative
PRJNA729271	stage_5W_vs_E10	ordinary_muscle_development	ENSGALG00010014301	ARID1A	0.23384902	0.119258831	1.960852868	0.049896189	0.062918248	positive
PRJNA729271	stage_5W_vs_E10	ordinary_muscle_development	ENSGALG00010014302	FOXO3	1.415945306	0.233518906	6.063514651	1.33179E-09	3.08868E-09	positive
PRJNA729271	stage_5W_vs_E10	ordinary_muscle_development	ENSGALG00010014303	GABRA2	0.826791386	0.358654545	2.305258356	0.021152108	0.027860046	positive
PRJNA729271	stage_5W_vs_E10	ordinary_muscle_development	ENSGALG00010014305	ARL10	-1.077429978	0.294381551	-3.659977919	0.000252237	0.00040191	negative
PRJNA729271	stage_5W_vs_E10	ordinary_muscle_development	ENSGALG00010014306	ENSGALG00010014306	0.245669198	0.311080679	0.789728242	0.429686494	0.471484158	positive
PRJNA729271	stage_5W_vs_E10	ordinary_muscle_development	ENSGALG00010014308	ENSGALG00010014308	2.238405021	0.19885574	11.25642648	2.15314E-29	1.27231E-28	positive
PRJNA729271	stage_5W_vs_E10	ordinary_muscle_development	ENSGALG00010014310	COL6A1	2.26437461	0.237722953	9.525267049	1.64618E-21	7.03668E-21	positive
PRJNA729271	stage_5W_vs_E10	ordinary_muscle_development	ENSGALG00010014312	KIAA1191	0.030644255	0.082940266	0.369473801	0.711774598	0.742777019	positive
PRJNA729271	stage_5W_vs_E10	ordinary_muscle_development	ENSGALG00010014314	ENSGALG00010014314	0.056400598	0.168730175	0.334265034	0.738179573	0.767128829	positive
PRJNA729271	stage_5W_vs_E10	ordinary_muscle_development	ENSGALG00010014317	RAD23B	0.91341562	0.146261177	6.245099606	4.2353E-10	1.01205E-09	positive
PRJNA729271	stage_5W_vs_E10	ordinary_muscle_development	ENSGALG00010014318	ENSGALG00010014318	1.463797063	0.466343897	3.138878999	0.001695955	0.002510505	positive
PRJNA729271	stage_5W_vs_E10	ordinary_muscle_development	ENSGALG00010014319	ENSGALG00010014319	0.015089238	0.11605148	0.130021931	0.896549075	0.909601655	positive
PRJNA729271	stage_5W_vs_E10	ordinary_muscle_development	ENSGALG00010014320	UTS2B	-2.726966935	1.014720398	-2.687407232	0.00720091	0.010004523	negative
PRJNA729271	stage_5W_vs_E10	ordinary_muscle_development	ENSGALG00010014323	KLF4	-0.046005386	0.178076272	-0.258346525	0.796139483	0.820304365	negative
PRJNA729271	stage_5W_vs_E10	ordinary_muscle_development	ENSGALG00010014324	COL4A2	3.338202535	0.1422083	23.47403449	7.5138E-122	4.5955E-120	positive
PRJNA729271	stage_5W_vs_E10	ordinary_muscle_development	ENSGALG00010014328	RASSF8	-0.941598344	0.092113263	-10.22217989	1.57753E-24	7.68356E-24	negative
PRJNA729271	stage_5W_vs_E10	ordinary_muscle_development	ENSGALG00010014329	IER5	0.826418658	0.15536653	5.319155031	1.0425E-07	2.14799E-07	positive
PRJNA729271	stage_5W_vs_E10	ordinary_muscle_development	ENSGALG00010014334	HMGCS1	0.008494446	0.158244777	0.053679154	0.957190792	0.963037449	positive
PRJNA729271	stage_5W_vs_E10	ordinary_muscle_development	ENSGALG00010014338	BHLHE41	1.766714763	0.220403894	8.015805581	1.09417E-15	3.57048E-15	positive

PRJNA729271	stage_5W_vs_E10	ordinary_muscle_development	ENSGALG00010014340	EFNB1	-2.217412671	0.138374743	-16.02469227	8.59152E-58	1.29319E-56	negative
PRJNA729271	stage_5W_vs_E10	ordinary_muscle_development	ENSGALG00010014344	LSG1	-0.979748195	0.089680121	-10.92492057	8.7617E-28	4.87153E-27	negative
PRJNA729271	stage_5W_vs_E10	ordinary_muscle_development	ENSGALG00010014346	AFG1L	0.464331874	0.160445386	2.894018238	0.003803461	0.005437535	positive
PRJNA729271	stage_5W_vs_E10	ordinary_muscle_development	ENSGALG00010014347	KIAA0040	2.408083357	0.332865754	7.234398029	4.67599E-13	1.32587E-12	positive
PRJNA729271	stage_5W_vs_E10	ordinary_muscle_development	ENSGALG00010014350	PTGER4	-0.028991839	0.170768083	-0.169773174	0.865188525	0.881796349	negative
PRJNA729271	stage_5W_vs_E10	ordinary_muscle_development	ENSGALG00010014355	RAB20	0.689227695	0.166553065	4.138186795	3.50061E-05	5.96887E-05	positive
PRJNA729271	stage_5W_vs_E10	ordinary_muscle_development	ENSGALG00010014359	TMEM271	1.266295606	0.356089102	3.556120074	0.000376372	0.000590615	positive
PRJNA729271	stage_5W_vs_E10	ordinary_muscle_development	ENSGALG00010014361	SSPN	2.514436817	0.149059826	16.86864189	7.65359E-64	1.373E-62	positive
PRJNA729271	stage_5W_vs_E10	ordinary_muscle_development	ENSGALG00010014362	NAXD	1.544848829	0.106777971	14.46786084	1.93394E-47	2.12689E-46	positive
PRJNA729271	stage_5W_vs_E10	ordinary_muscle_development	ENSGALG00010014367	OPHN1	-0.060079691	0.1095634	-0.548355484	0.583447837	0.621798132	negative
PRJNA729271	stage_5W_vs_E10	ordinary_muscle_development	ENSGALG00010014371	TTC33	-0.33775654	0.102327403	-3.300743783	0.000964289	0.001464672	negative
PRJNA729271	stage_5W_vs_E10	ordinary_muscle_development	ENSGALG00010014372	CCDC171	-0.767733209	0.127219767	-6.034700656	1.59258E-09	3.67703E-09	negative
PRJNA729271	stage_5W_vs_E10	ordinary_muscle_development	ENSGALG00010014375	H2BC35	-2.214319327	0.247100615	-8.961205245	3.21149E-19	1.23837E-18	negative
PRJNA729271	stage_5W_vs_E10	ordinary_muscle_development	ENSGALG00010014379	CARS2	-0.42725407	0.091061805	-4.691913019	2.70662E-06	5.03303E-06	negative
PRJNA729271	stage_5W_vs_E10	ordinary_muscle_development	ENSGALG00010014380	SNX3	0.771956326	0.117366295	6.577325538	4.78985E-11	1.21827E-10	positive
PRJNA729271	stage_5W_vs_E10	ordinary_muscle_development	ENSGALG00010014388	MFNG	3.393138611	0.217842067	15.57614038	1.0575E-54	1.45019E-53	positive
PRJNA729271	stage_5W_vs_E10	ordinary_muscle_development	ENSGALG00010014389	COL6A2	2.32003664	0.26446524	8.77255792	1.74653E-18	6.52303E-18	positive
PRJNA729271	stage_5W_vs_E10	ordinary_muscle_development	ENSGALG00010014390	SPARC	1.595204489	0.159038746	10.03028843	1.12188E-23	5.26099E-23	positive
PRJNA729271	stage_5W_vs_E10	ordinary_muscle_development	ENSGALG00010014393	ATP8A1	1.465935609	0.144023717	10.17843197	2.47518E-24	1.19393E-23	positive
PRJNA729271	stage_5W_vs_E10	ordinary_muscle_development	ENSGALG00010014396	ING1	-0.373636698	0.119603319	-3.123965961	0.001784311	0.002635377	negative
PRJNA729271	stage_5W_vs_E10	ordinary_muscle_development	ENSGALG00010014397	PRKAA1	-0.404150066	0.076964396	-5.25113023	1.51169E-07	3.08303E-07	negative
PRJNA729271	stage_5W_vs_E10	ordinary_muscle_development	ENSGALG00010014399	GAS6	2.947181396	0.189828335	15.52550833	2.33149E-54	3.16432E-53	positive
PRJNA729271	stage_5W_vs_E10	ordinary_muscle_development	ENSGALG00010014400	RIPOR3	-0.585529796	0.1016681	-5.759228296	8.44994E-09	1.87338E-08	negative
PRJNA729271	stage_5W_vs_E10	ordinary_muscle_development	ENSGALG00010014403	H2BC37	-1.177746869	0.188701613	-6.241318487	4.33898E-10	1.03562E-09	negative
PRJNA729271	stage_5W_vs_E10	ordinary_muscle_development	ENSGALG00010014407	YIPF6	-0.408521419	0.091160503	-4.481342326	7.4175E-06	1.33379E-05	negative
PRJNA729271	stage_5W_vs_E10	ordinary_muscle_development	ENSGALG00010014408	ENSGALG00010014408	-0.622912408	0.33436713	-1.86295946	0.06246797	0.077843297	negative
PRJNA729271	stage_5W_vs_E10	ordinary_muscle_development	ENSGALG00010014411	ATP13A3	-0.624360793	0.081428327	-7.667611681	1.75228E-14	5.34386E-14	negative
PRJNA729271	stage_5W_vs_E10	ordinary_muscle_development	ENSGALG00010014414	TMEM255B	1.192565224	0.502536378	2.373092329	0.017639854	0.02346638	positive
PRJNA729271	stage_5W_vs_E10	ordinary_muscle_development	ENSGALG00010014416	H2BC32	-2.645450735	0.270042451	-9.796425429	1.1664E-22	5.25226E-22	negative
PRJNA729271	stage_5W_vs_E10	ordinary_muscle_development	ENSGALG00010014418	RAC2	3.326284517	0.290783999	11.43902186	2.66877E-30	1.63085E-29	positive
PRJNA729271	stage_5W_vs_E10	ordinary_muscle_development	ENSGALG00010014427	TMEM267	-1.06012358	0.13869962	-7.643305597	2.11715E-14	6.43895E-14	negative
PRJNA729271	stage_5W_vs_E10	ordinary_muscle_development	ENSGALG00010014431	ENSGALG00010014431	-1.491510236	0.158545551	-9.407455631	5.08292E-21	2.12632E-20	negative
PRJNA729271	stage_5W_vs_E10	ordinary_muscle_development	ENSGALG00010014439	ENSGALG00010014439	-0.788533863	0.841117094	-0.937484054			negative
PRJNA729271	stage_5W_vs_E10	ordinary_muscle_development	ENSGALG00010014442	SHISA3	-5.597771864	0.997838986	-5.609894923	2.02449E-08	4.38444E-08	negative
PRJNA729271	stage_5W_vs_E10	ordinary_muscle_development	ENSGALG00010014449	ENSGALG00010014449	-1.318671344	0.155069627	-8.503737101	1.83583E-17	6.53175E-17	negative
PRJNA729271	stage_5W_vs_E10	ordinary_muscle_development	ENSGALG00010014453	NR2E1	1.010887736	0.344333776	2.935778618	0.003327117	0.004785805	positive
PRJNA729271	stage_5W_vs_E10	ordinary_muscle_development	ENSGALG00010014455	C4orf19	-2.234891043	0.267107121	-8.367021572	5.90929E-17	2.05211E-16	negative
PRJNA729271	stage_5W_vs_E10	ordinary_muscle_development	ENSGALG00010014465	SIMC1	2.461404647	0.146525807	16.79843773	2.50557E-63	4.42366E-62	positive
PRJNA729271	stage_5W_vs_E10	ordinary_muscle_development	ENSGALG00010014469	TMCO3	-0.689036709	0.12860216	-5.35789373	8.41977E-08	1.74549E-07	negative
PRJNA729271	stage_5W_vs_E10	ordinary_muscle_development	ENSGALG00010014470	RPS6KA1	-0.719333462	0.12935799	-5.560796538	2.68546E-08	5.76422E-08	negative
PRJNA729271	stage_5W_vs_E10	ordinary_muscle_development	ENSGALG00010014474	PTPN1	-0.178736393	0.077805022	-2.297234647	0.021605388	0.028428658	negative
PRJNA729271	stage_5W_vs_E10	ordinary_muscle_development	ENSGALG00010014476	C5orf34	-0.099162441	0.181727656	-0.545665106	0.585296162	0.62344708	negative
PRJNA729271	stage_5W_vs_E10	ordinary_muscle_development	ENSGALG00010014477	ITPR2	0.68815406	0.097932848	7.02679516	2.11331E-12	5.79283E-12	positive
PRJNA729271	stage_5W_vs_E10	ordinary_muscle_development	ENSGALG00010014482	THOC3	-1.769781635	0.090701495	-19.51215504	8.65552E-85	2.58684E-83	negative
PRJNA729271	stage_5W_vs_E10	ordinary_muscle_development	ENSGALG00010014487	OSTM1	0.463237626	0.083362511	5.556905846	2.74599E-08	5.88803E-08	positive
PRJNA729271	stage_5W_vs_E10	ordinary_muscle_development	ENSGALG00010014488	ENSGALG00010014488	-1.370533144	0.410238446	-3.340820827	0.000835311	0.001274241	negative
PRJNA729271	stage_5W_vs_E10	ordinary_muscle_development	ENSGALG00010014492	CPLX2	-1.292065777	0.319821313	-4.039961456	5.346E-05	9.00526E-05	negative
PRJNA729271	stage_5W_vs_E10	ordinary_muscle_development	ENSGALG00010014494	KLHL9	-1.099421509	0.068497546	-16.05052404	5.66834E-58	8.60342E-57	negative
PRJNA729271	stage_5W_vs_E10	ordinary_muscle_development	ENSGALG00010014504	SEC63	-0.698430112	0.046359978	-15.06536774	2.73639E-51	3.43411E-50	negative
PRJNA729271	stage_5W_vs_E10	ordinary_muscle_development	ENSGALG00010014505	CYTH4	3.109265642	0.209070942	14.87182109	5.02282E-50	6.04198E-49	positive
PRJNA729271	stage_5W_vs_E10	ordinary_muscle_development	ENSGALG00010014512	HMGN4	-1.316297325	0.111265577	-11.83022959	2.72396E-32	1.81201E-31	negative
PRJNA729271	stage_5W_vs_E10	ordinary_muscle_development	ENSGALG00010014515	ETNK1	-1.806247648	0.113718382	-15.88351518	8.24267E-57	1.20806E-55	negative
PRJNA729271	stage_5W_vs_E10	ordinary_muscle_development	ENSGALG00010014517	DCUN1D2	1.804733697	0.127041586	14.2058499	8.4274E-46	8.8135E-45	positive
PRJNA729271	stage_5W_vs_E10	ordinary_muscle_development	ENSGALG00010014522	FAT2	-6.501644082	0.583172094	-11.14875721	7.26134E-29	4.20172E-28	negative
PRJNA729271	stage_5W_vs_E10	ordinary_muscle_development	ENSGALG00010014537	GKAP1	2.451400422	0.120115517	20.40869066	1.39985E-92	5.06004E-91	positive
PRJNA729271	stage_5W_vs_E10	ordinary_muscle_development	ENSGALG00010014539	PAIP1	-0.34987301	0.085488029	-4.092654993	4.26462E-05	7.22737E-05	negative
PRJNA729271	stage_5W_vs_E10	ordinary_muscle_development	ENSGALG00010014547	DHDDS	0.091339832	0.107168713	0.85229942	0.394047928	0.435344871	positive
PRJNA729271	stage_5W_vs_E10	ordinary_muscle_development	ENSGALG00010014550	SCML4	-6.116005695	0.984979851	-6.209269857	5.32313E-10	1.2649E-09	negative
PRJNA729271	stage_5W_vs_E10	ordinary_muscle_development	ENSGALG00010014555	VGLL1	-6.752192012	0.899648511	-7.505366741	6.12569E-14	1.81876E-13	negative
PRJNA729271	stage_5W_vs_E10	ordinary_muscle_development	ENSGALG00010014567	UTP11	-0.484951443	0.117440709	-4.129329993	3.63822E-05	6.19683E-05	negative
PRJNA729271	stage_5W_vs_E10	ordinary_muscle_development	ENSGALG00010014570	KIF27	-0.852375819	0.164584257	-5.178963247	2.23122E-07	4.49002E-07	negative
PRJNA729271	stage_5W_vs_E10	ordinary_muscle_development	ENSGALG00010014572	IL1RAP	0.364721373	0.124618333	2.92670721	0.003425712	0.004921766	positive
PRJNA729271	stage_5W_vs_E10	ordinary_muscle_development	ENSGALG00010014573	CEBPB	4.103386241	0.347578132	11.80565136	3.64954E-32	2.41553E-31	positive
PRJNA729271	stage_5W_vs_E10	ordinary_muscle_development	ENSGALG00010014576	HRH2	1.136714557	0.262996972	4.32215835	1.5451E-05	2.71503E-05	positive
PRJNA729271	stage_5W_vs_E10	ordinary_muscle_development	ENSGALG00010014578	ENSGALG00010014578	5.494914817	0.302260442	18.17940442	7.51454E-74	1.754E-72	positive
PRJNA729271	stage_5W_vs_E10	ordinary_muscle_development	ENSGALG00010014579	NNT	0.070601622	0.14473501	0.487799199	0.625692079	0.66195217	positive
PRJNA729271	stage_5W_vs_E10	ordinary_muscle_development	ENSGALG00010014592	QNG1	0.044657767	0.092956445	0.480416035	0.630931595	0.666570952	positive
PRJNA729271	stage_5W_vs_E10	ordinary_muscle_development	ENSGALG00010014599	SSTR3	-0.374534676	0.376291627	-0.99533088	0.319575363	0.358866199	negative
PRJNA729271	stage_5W_vs_E10	ordinary_muscle_development	ENSGALG00010014602	FRK	-1.310032975	1.163246731	-1.126186681	0.26008651	0.296519896	negative
PRJNA729271	stage_5W_vs_E10	ordinary_muscle_development	ENSGALG00010014603	ENSGALG00010014603	4.269234084	0.221663539	19.2599744	1.16436E-82	3.33546E-81	positive
PRJNA729271	stage_5W_vs_E10	ordinary_muscle_development	ENSGALG00010014611	TMEM189	0.370861472	0.104253858	3.557292534	0.000374697	0.000588177	positive
PRJNA729271	stage_5W_vs_E10	ordinary_muscle_development	ENSGALG00010014614	GHR	0.757400369	0.090429817	8.375560125	5.49618E-17	1.91094E-16	positive
PRJNA729271	stage_5W_vs_E10	ordinary_muscle_development	ENSGALG00010014617	GRTP1	1.406758233	0.203236583	6.921776647	4.46014E-12	1.19988E-11	positive
PRJNA729271	stage_5W_vs_E10	ordinary_muscle_development	ENSGALG00010014624	GPRC6A	0.645884881	0.417269858	1.547882909	0.12165049	0.146030961	positive
PRJNA729271	stage_5W_vs_E10	ordinary_muscle_development	ENSGALG00010014626	C2CD5	-0.422866526	0.091993371	-4.5967065	4.29222E-06	7.85254E-06	negative
PRJNA729271	stage_5W_vs_E10	ordinary_muscle_development	ENSGALG00010014632	NWD2	-1.332384961	0.173558007	-7.676885596	1.63003E-14	4.9815E-14	negative
PRJNA729271	stage_5W_vs_E10	ordinary_muscle_development	ENSGALG00010014634	CCDC152	3.042564279	0.448161832	6.788985717	1.12925E-11	2.98204E-11	positive
PRJNA729271	stage_5W_vs_E10	ordinary_muscle_development	ENSGALG00010014641	DOCK11	0.192367328	0.096481549	1.993825038	0.046171178	0.058465207	positive
PRJNA729271	stage_5W_vs_E10	ordinary_muscle_development	ENSGALG00010014645	SLC36A1	-0.023151139	0.120725551	-0.191766691	0.847924964	0.866333428	negative
PRJNA729271	stage_5W_vs_E10	ordinary_muscle_development	ENSGALG00010014648	C1QTNF6	0.639760194	0.274564233	2.330092992	0.019801237	0.026178304	positive
PRJNA729271	stage_5W_vs_E10	ordinary_muscle_development	ENSGALG00010014653	FAM162B	1.754246387	0.490690769	3.5750548	0.000350155	0.000551145	positive
PRJNA729271	stage_5W_vs_E10	ordinary_muscle_development	ENSGALG00010014654	LAMP1	1.568673407	0.104370213	15.02989555	4.67743E-51	5.82969E-50	positive
PRJNA729271	stage_5W_vs_E10	ordinary_muscle_development	ENSGALG00010014655	FHL3	-1.438924109	0.134878548	-10.66829481	1.43231E-26	7.60208E-26	negative
PRJNA729271	stage_5W_vs_E10	ordinary_muscle_development	ENSGALG00010014658	SELENOP1	2.911014385	0.17346251	16.78180708	3.3159E-63	5.83301E-62	positive
PRJNA729271	stage_5W_vs_E10	ordinary_muscle_development	ENSGALG00010014668	GP5	-0.705560876	0.162523497	-4.341285348	1.41652E-05	2.49725E-05	negative
PRJNA729271	stage_5W_vs_E10	ordinary_muscle_development	ENSGALG00010014672	ISL1	-4.971027032	1.231354105	-4.03704102	5.41296E-05	9.11488E-05	negative
PRJNA729271	stage_5W_vs_E10	ordinary_muscle_development	ENSGALG00010014676	UBE2V1	-0.582416959	0.058732884	-9.916369105	3.53378E-23	1.62404E-22	negative
PRJNA729271	stage_5W_vs_E10	ordinary_muscle_development	ENSGALG00010014677	ENSGALG00010014677	-1.351707806	0.090219605	-14.98241772	9.56724E-51	1.18124E-49	negative
PRJNA729271	stage_5W_vs_E10	ordinary_muscle_development	ENSGALG00010014682	IL13RA1	0.567840956	0.126208959	4.499212742	6.82056E-06	1.22873E-05	positive
PRJNA729271	stage_5W_vs_E10	ordinary_muscle_development	ENSGALG00010014687	CUL4A	2.167329281	0.11436804	18.95048025	4.37572E-80	1.18112E-78	positive
PRJNA729271	stage_5W_vs_E10	ordinary_muscle_development	ENSGALG00010014690	CSF3R	4.390423984	0.365442928	12.01397987	3.0005E-33	2.06909E-32	positive
PRJNA729271	stage_5W_vs_E10	ordinary_muscle_development	ENSGALG00010014691	PABIR2	0.113636689	0.054208114	2.096304043	0.036055228	0.046343933	positive
PRJNA729271	stage_5W_vs_E10	ordinary_muscle_development	ENSGALG00010014696	SPINK5	0.724502337	0.63221122	1.14598146	0.251802823	0.287732119	positive
PRJNA729271	stage_5W_vs_E10	ordinary_muscle_development	ENSGALG00010014697	ENSGALG00010014697	1.468720818	0.663860665	2.212393195	0.026939509	0.035106372	positive
PRJNA729271	stage_5W_vs_E10	ordinary_muscle_development	ENSGALG00010014702	ENSGALG00010014702	0.517340799	0.269604477	1.918888009	0.054998508	0.068956931	positive
PRJNA729271	stage_5W_vs_E10	ordinary_muscle_development	ENSGALG00010014703	PCID2	-0.98966456	0.081039082	-12.21218867	2.67592E-34	1.91449E-33	negative
PRJNA729271	stage_5W_vs_E10	ordinary_muscle_development	ENSGALG00010014705	ARAP2	0.598273931	0.216180428	2.767475005	0.005649238	0.007932355	positive
PRJNA729271	stage_5W_vs_E10	ordinary_muscle_development	ENSGALG00010014707	KPNA5	1.498016835	0.129354849	11.58067783	5.16361E-31	3.26413E-30	positive
PRJNA729271	stage_5W_vs_E10	ordinary_muscle_development	ENSGALG00010014710	NPEPL1	1.454554499	0.099424933	14.62967546	1.81652E-48	2.07326E-47	positive
PRJNA729271	stage_5W_vs_E10	ordinary_muscle_development	ENSGALG00010014713	ST8SIA1	-2.715203156	0.138375582	-19.62198179	1.00363E-85	3.07561E-84	negative
PRJNA729271	stage_5W_vs_E10	ordinary_muscle_development	ENSGALG00010014716	TMEM35A	-2.188613495	0.136252615	-16.06290999	4.6425E-58	7.06118E-57	negative
PRJNA729271	stage_5W_vs_E10	ordinary_muscle_development	ENSGALG00010014717	ELOVL7	1.934659657	0.18232266	10.61120927	2.64306E-26	1.38708E-25	positive
PRJNA729271	stage_5W_vs_E10	ordinary_muscle_development	ENSGALG00010014719	ENSGALG00010014719	-0.080539836	1.169689448	-0.068855743	0.945104447	0.952463948	negative
PRJNA729271	stage_5W_vs_E10	ordinary_muscle_development	ENSGALG00010014722	KCTD17	-2.927974811	0.422348937	-6.932596621	4.13185E-12	1.11384E-11	negative
PRJNA729271	stage_5W_vs_E10	ordinary_muscle_development	ENSGALG00010014726	PWP2	-1.788164079	0.064841103	-27.5776321	2.0641E-167	2.6245E-165	negative
PRJNA729271	stage_5W_vs_E10	ordinary_muscle_development	ENSGALG00010014728	INTS13	0.419333246	0.111984428	3.744567463	0.000180705	0.000291197	positive
PRJNA729271	stage_5W_vs_E10	ordinary_muscle_development	ENSGALG00010014729	MRPS15	0.551599709	0.095371435	5.783699345	7.30755E-09	1.62658E-08	positive
PRJNA729271	stage_5W_vs_E10	ordinary_muscle_development	ENSGALG00010014734	ZUP1	-0.61332078	0.099709538	-6.151074321	7.69598E-10	1.80712E-09	negative
PRJNA729271	stage_5W_vs_E10	ordinary_muscle_development	ENSGALG00010014735	STX16	0.500066162	0.10486025	4.768882013	1.85251E-06	3.48277E-06	positive
PRJNA729271	stage_5W_vs_E10	ordinary_muscle_development	ENSGALG00010014736	KIF2A	-1.231266144	0.090080593	-13.66849511	1.566E-42	1.47589E-41	negative
PRJNA729271	stage_5W_vs_E10	ordinary_muscle_development	ENSGALG00010014737	RMI1	-0.325611455	0.114937178	-2.832951529	0.004612038	0.006534847	negative
PRJNA729271	stage_5W_vs_E10	ordinary_muscle_development	ENSGALG00010014738	BRWD1	0.427380952	0.086643607	4.932631107	8.11293E-07	1.56984E-06	positive
PRJNA729271	stage_5W_vs_E10	ordinary_muscle_development	ENSGALG00010014739	TRMT2B	0.909328818	0.175792214	5.172747976	2.30676E-07	4.6343E-07	positive
PRJNA729271	stage_5W_vs_E10	ordinary_muscle_development	ENSGALG00010014740	TRMT9B	-3.697774059	0.595989374	-6.204429513	5.48957E-10	1.30402E-09	negative
PRJNA729271	stage_5W_vs_E10	ordinary_muscle_development	ENSGALG00010014742	PROZ	20.49415553	2.845982257	7.201083379	5.9736E-13	1.68523E-12	positive
PRJNA729271	stage_5W_vs_E10	ordinary_muscle_development	ENSGALG00010014744	IL2RB	6.081553165	0.423387358	14.36404053	8.70033E-47	9.4324E-46	positive
PRJNA729271	stage_5W_vs_E10	ordinary_muscle_development	ENSGALG00010014745	LSM10	0.625680865	0.188783209	3.314282383	0.000918787	0.001397169	positive
PRJNA729271	stage_5W_vs_E10	ordinary_muscle_development	ENSGALG00010014748	FGFR1OP2	0.04776487	0.108571829	0.439937971	0.659982034	0.694327158	positive
PRJNA729271	stage_5W_vs_E10	ordinary_muscle_development	ENSGALG00010014750	LONRF1	0.927172331	0.181120201	5.119099494	3.06998E-07	6.10919E-07	positive
PRJNA729271	stage_5W_vs_E10	ordinary_muscle_development	ENSGALG00010014753	ENSGALG00010014753	-2.145614132	0.441474856	-4.860104948	1.17324E-06	2.23941E-06	negative
PRJNA729271	stage_5W_vs_E10	ordinary_muscle_development	ENSGALG00010014754	DIMT1	-0.485272885	0.143640959	-3.37837403	0.000729158	0.001117364	negative
PRJNA729271	stage_5W_vs_E10	ordinary_muscle_development	ENSGALG00010014758	FXN	-0.941347731	0.103482944	-9.096646204	9.31642E-20	3.68865E-19	negative
PRJNA729271	stage_5W_vs_E10	ordinary_muscle_development	ENSGALG00010014762	STK40	0.146532827	0.104476771	1.402539779	0.16075412	0.190059623	positive
PRJNA729271	stage_5W_vs_E10	ordinary_muscle_development	ENSGALG00010014764	ENSGALG00010014764	7.236095974	2.287578407	3.163212222	0.001560385	0.002319773	positive
PRJNA729271	stage_5W_vs_E10	ordinary_muscle_development	ENSGALG00010014766	F10	-0.21740403	0.22098381	-0.983800715	0.325213508	0.364773643	negative
PRJNA729271	stage_5W_vs_E10	ordinary_muscle_development	ENSGALG00010014767	LRRC15	1.056966282	0.248080853	4.260571783	2.03905E-05	3.54686E-05	positive
PRJNA729271	stage_5W_vs_E10	ordinary_muscle_development	ENSGALG00010014769	CCDC69	0.546445501	0.324329299	1.684847783	0.092017943	0.112301094	positive
PRJNA729271	stage_5W_vs_E10	ordinary_muscle_development	ENSGALG00010014777	APCDD1L	-2.086962478	0.22699956	-9.193685121	3.79609E-20	1.52506E-19	negative
PRJNA729271	stage_5W_vs_E10	ordinary_muscle_development	ENSGALG00010014780	EVA1B	0.323891866	0.125684646	2.577020163	0.009965614	0.013654558	positive
PRJNA729271	stage_5W_vs_E10	ordinary_muscle_development	ENSGALG00010014783	ENSGALG00010014783	-0.165022062	0.119792744	-1.377563079	0.168338227	0.198524335	negative
PRJNA729271	stage_5W_vs_E10	ordinary_muscle_development	ENSGALG00010014789	F7	0.425903757	0.913112232	0.466430896	0.640907101	0.676124339	positive

PRJNA729271	stage_5W_vs_E10	ordinary_muscle_development	ENSGALG00010014792	ENSGALG00010014792	1.300680334	0.27053425	4.807821322	1.52584E-06	2.8881E-06	positive
PRJNA729271	stage_5W_vs_E10	ordinary_muscle_development	ENSGALG00010014793	TLR2A	1.188467617	0.175254969	6.781363307	1.19047E-11	3.13914E-11	positive
PRJNA729271	stage_5W_vs_E10	ordinary_muscle_development	ENSGALG00010014795	THRAP3	-0.663859313	0.095604112	-6.943836421	3.81592E-12	1.03098E-11	negative
PRJNA729271	stage_5W_vs_E10	ordinary_muscle_development	ENSGALG00010014796	PRLR	-1.159478386	0.250854098	-4.622122559	3.79833E-06	6.97684E-06	negative
PRJNA729271	stage_5W_vs_E10	ordinary_muscle_development	ENSGALG00010014797	ZDHHC9	0.091035862	0.092332646	0.985955298	0.324155049	0.363670855	positive
PRJNA729271	stage_5W_vs_E10	ordinary_muscle_development	ENSGALG00010014801	MPST	-0.83028763	0.088642664	-9.366681793	7.48486E-21	3.10157E-20	negative
PRJNA729271	stage_5W_vs_E10	ordinary_muscle_development	ENSGALG00010014802	ENSGALG00010014802	-1.695192501	0.444283286	-3.815566677	0.000135871	0.000221236	negative
PRJNA729271	stage_5W_vs_E10	ordinary_muscle_development	ENSGALG00010014805	SPEF2	-1.470949854	0.376212298	-3.909893058	9.2337E-05	0.000152614	negative
PRJNA729271	stage_5W_vs_E10	ordinary_muscle_development	ENSGALG00010014806	MAP7D1	1.624545456	0.226386275	7.175989161	7.17862E-13	2.01773E-12	positive
PRJNA729271	stage_5W_vs_E10	ordinary_muscle_development	ENSGALG00010014807	HES1	0.317706873	0.22445468	1.415461121	0.15693334	0.185754431	positive
PRJNA729271	stage_5W_vs_E10	ordinary_muscle_development	ENSGALG00010014810	ENTREP1	3.615596681	0.215845799	16.75083183	5.584E-63	9.75182E-62	positive
PRJNA729271	stage_5W_vs_E10	ordinary_muscle_development	ENSGALG00010014812	RWDD1	-0.592982442	0.084390392	-7.026658244	2.11539E-12	5.79741E-12	negative
PRJNA729271	stage_5W_vs_E10	ordinary_muscle_development	ENSGALG00010014813	SASH3	3.233231133	0.249982537	12.93382798	2.90012E-38	2.38306E-37	positive
PRJNA729271	stage_5W_vs_E10	ordinary_muscle_development	ENSGALG00010014816	TST	0.404318529	0.108841321	3.714752137	0.000203403	0.000326684	positive
PRJNA729271	stage_5W_vs_E10	ordinary_muscle_development	ENSGALG00010014818	ENSGALG00010014818	0.263792441	0.453043546	0.582267296	0.560386651	0.600133321	positive
PRJNA729271	stage_5W_vs_E10	ordinary_muscle_development	ENSGALG00010014821	C3AR1L	2.505073051	0.228352683	10.97019319	5.31587E-28	2.97849E-27	positive
PRJNA729271	stage_5W_vs_E10	ordinary_muscle_development	ENSGALG00010014824	ANXA6	0.243330221	0.090727053	2.682002913	0.007318282	0.010162723	positive
PRJNA729271	stage_5W_vs_E10	ordinary_muscle_development	ENSGALG00010014829	ENSGALG00010014829	4.743114368	0.385920462	12.29039357	1.02008E-34	7.44891E-34	positive
PRJNA729271	stage_5W_vs_E10	ordinary_muscle_development	ENSGALG00010014830	TRAPPC3L	-2.899188092	0.601672787	-4.818546148	1.44608E-06	2.74143E-06	negative
PRJNA729271	stage_5W_vs_E10	ordinary_muscle_development	ENSGALG00010014831	VAPB	0.253577991	0.060745561	4.174428327	2.98735E-05	5.12506E-05	positive
PRJNA729271	stage_5W_vs_E10	ordinary_muscle_development	ENSGALG00010014837	APBA1	-0.734247204	0.114294597	-6.424163735	1.32596E-10	3.27314E-10	negative
PRJNA729271	stage_5W_vs_E10	ordinary_muscle_development	ENSGALG00010014839	TM7SF3	0.47286325	0.108652215	4.352081089	1.34851E-05	2.38258E-05	positive
PRJNA729271	stage_5W_vs_E10	ordinary_muscle_development	ENSGALG00010014840	NCF4	2.205455457	0.208952016	10.55484173	4.82452E-26	2.50023E-25	positive
PRJNA729271	stage_5W_vs_E10	ordinary_muscle_development	ENSGALG00010014847	PAICS	-1.69362256	0.092749629	-18.26015449	1.71804E-74	4.12986E-73	negative
PRJNA729271	stage_5W_vs_E10	ordinary_muscle_development	ENSGALG00010014851	CALHM4	0.157673719	0.465151346	0.33897294	0.734630115	0.763933103	positive
PRJNA729271	stage_5W_vs_E10	ordinary_muscle_development	ENSGALG00010014856	MMGT1	0.676512506	0.071355814	9.480832366	2.52263E-21	1.07105E-20	positive
PRJNA729271	stage_5W_vs_E10	ordinary_muscle_development	ENSGALG00010014859	TRAPPC3	0.287512227	0.128418155	2.238875236	0.025164035	0.0328932	positive
PRJNA729271	stage_5W_vs_E10	ordinary_muscle_development	ENSGALG00010014861	MED21	-0.659775142	0.099908704	-6.603780439	4.00803E-11	1.02481E-10	negative
PRJNA729271	stage_5W_vs_E10	ordinary_muscle_development	ENSGALG00010014862	ENSGALG00010014862	0.078965896	0.319563241	0.247105693	0.804826428	0.828077731	positive
PRJNA729271	stage_5W_vs_E10	ordinary_muscle_development	ENSGALG00010014865	TLR2	4.14500629	0.37198363	11.14298039	7.74822E-29	4.47809E-28	positive
PRJNA729271	stage_5W_vs_E10	ordinary_muscle_development	ENSGALG00010014866	FAM26E	0.05512501	0.149893227	0.367761851	0.713050817	0.743948146	positive
PRJNA729271	stage_5W_vs_E10	ordinary_muscle_development	ENSGALG00010014867	TP63	0.183787331	0.271287527	0.677463255	0.498112077	0.539500452	positive
PRJNA729271	stage_5W_vs_E10	ordinary_muscle_development	ENSGALG00010014870	ENSGALG00010014870	3.325065333	1.932075708	1.72098087	0.085254295	0.104504102	positive
PRJNA729271	stage_5W_vs_E10	ordinary_muscle_development	ENSGALG00010014873	CALHM6	2.988990837	0.673688819	4.436752927	9.1326E-06	1.63227E-05	positive
PRJNA729271	stage_5W_vs_E10	ordinary_muscle_development	ENSGALG00010014876	PCNT	-0.139664413	0.103432438	-1.350296055	0.176921037	0.207699881	negative
PRJNA729271	stage_5W_vs_E10	ordinary_muscle_development	ENSGALG00010014877	ENSGALG00010014877	-0.388105539	0.091361089	-4.248039765	2.15649E-05	3.74474E-05	negative
PRJNA729271	stage_5W_vs_E10	ordinary_muscle_development	ENSGALG00010014878	TMCO1	0.727813975	0.129849092	5.605075576	2.08164E-08	4.50484E-08	positive
PRJNA729271	stage_5W_vs_E10	ordinary_muscle_development	ENSGALG00010014880	SFRP2	-2.226032722	0.293226779	-7.591505564	3.1621E-14	9.54092E-14	negative
PRJNA729271	stage_5W_vs_E10	ordinary_muscle_development	ENSGALG00010014881	ENSGALG00010014881	-0.03269323	0.453650148	-0.072067055	0.942548544	0.950284562	negative
PRJNA729271	stage_5W_vs_E10	ordinary_muscle_development	ENSGALG00010014882	XPNPEP2	0.182432621	0.618697797	0.294865478	0.768096645	0.795595317	positive
PRJNA729271	stage_5W_vs_E10	ordinary_muscle_development	ENSGALG00010014883	PPAT	-1.142759698	0.122953577	-9.294237139	1.48266E-20	6.05554E-20	negative
PRJNA729271	stage_5W_vs_E10	ordinary_muscle_development	ENSGALG00010014886	RABGAP1L	0.539866181	0.069825933	7.731599999	1.06203E-14	3.28654E-14	positive
PRJNA729271	stage_5W_vs_E10	ordinary_muscle_development	ENSGALG00010014887	MTMR12	-0.260990159	0.13232886	-1.972284499	0.048577138	0.061340327	negative
PRJNA729271	stage_5W_vs_E10	ordinary_muscle_development	ENSGALG00010014889	PVALB	1.247538652	0.418725448	2.979371464	0.002888404	0.004179219	positive
PRJNA729271	stage_5W_vs_E10	ordinary_muscle_development	ENSGALG00010014893	SLC9A6	0.023468086	0.088567159	0.264975034	0.791028695	0.815908413	positive
PRJNA729271	stage_5W_vs_E10	ordinary_muscle_development	ENSGALG00010014894	RAB22A	-0.066698583	0.078479737	-0.849882857	0.395390217	0.436628158	negative
PRJNA729271	stage_5W_vs_E10	ordinary_muscle_development	ENSGALG00010014897	COL8A2	-3.404775553	0.252415146	-13.48879259	1.82058E-41	1.66389E-40	negative
PRJNA729271	stage_5W_vs_E10	ordinary_muscle_development	ENSGALG00010014898	ENSGALG00010014898	1.628388616	0.711343079	2.289174751	0.022069201	0.029004721	positive
PRJNA729271	stage_5W_vs_E10	ordinary_muscle_development	ENSGALG00010014900	UBLCP1	-0.458196937	0.084594462	-5.41639399	6.0813E-08	1.27327E-07	negative
PRJNA729271	stage_5W_vs_E10	ordinary_muscle_development	ENSGALG00010014902	AASDH	-0.015948153	0.127388747	-0.125192793	0.900370923	0.912967928	negative
PRJNA729271	stage_5W_vs_E10	ordinary_muscle_development	ENSGALG00010014903	MCF2L	1.82024201	0.153694786	11.84322547	2.33304E-32	1.55769E-31	positive
PRJNA729271	stage_5W_vs_E10	ordinary_muscle_development	ENSGALG00010014906	IFT27	-0.054827359	0.108803138	-0.503913404	0.614322231	0.65125435	negative
PRJNA729271	stage_5W_vs_E10	ordinary_muscle_development	ENSGALG00010014907	ENSGALG00010014907	-0.47211869	0.070885314	-6.660317435	2.73237E-11	7.05229E-11	negative
PRJNA729271	stage_5W_vs_E10	ordinary_muscle_development	ENSGALG00010014908	ENSGALG00010014908	-0.531356292	0.102341292	-5.192002988	2.08044E-07	4.1959E-07	negative
PRJNA729271	stage_5W_vs_E10	ordinary_muscle_development	ENSGALG00010014909	FBXL14	-0.129759499	0.097881315	-1.325682014	0.184945048	0.216523582	negative
PRJNA729271	stage_5W_vs_E10	ordinary_muscle_development	ENSGALG00010014912	ENSGALG00010014912	3.101419767	0.312801834	9.914966696	3.58376E-23	1.64648E-22	positive
PRJNA729271	stage_5W_vs_E10	ordinary_muscle_development	ENSGALG00010014914	PTAR1	-0.1645583	0.182366721	-0.902348296	0.366871878	0.407463632	negative
PRJNA729271	stage_5W_vs_E10	ordinary_muscle_development	ENSGALG00010014916	DYNLT2B	-0.165063159	0.155075477	-1.0644053	0.287145134	0.324890228	negative
PRJNA729271	stage_5W_vs_E10	ordinary_muscle_development	ENSGALG00010014917	EIF3D	0.083075165	0.105116531	0.790314944	0.429343861	0.471179533	positive
PRJNA729271	stage_5W_vs_E10	ordinary_muscle_development	ENSGALG00010014918	PLRG1	-0.469576266	0.084599159	-5.550602044	2.84687E-08	6.09624E-08	negative
PRJNA729271	stage_5W_vs_E10	ordinary_muscle_development	ENSGALG00010014922	MAN1A1	0.644887555	0.164597985	3.917955353	8.93032E-05	0.000147886	positive
PRJNA729271	stage_5W_vs_E10	ordinary_muscle_development	ENSGALG00010014923	CIMIP1	-3.272022868	0.478923212	-6.832040687	8.37151E-12	2.22244E-11	negative
PRJNA729271	stage_5W_vs_E10	ordinary_muscle_development	ENSGALG00010014928	SUCO	-1.027055913	0.081890278	-12.54185403	4.40566E-36	3.39681E-35	negative
PRJNA729271	stage_5W_vs_E10	ordinary_muscle_development	ENSGALG00010014929	FHL1	1.322298851	0.284929376	4.640795091	3.47071E-06	6.39969E-06	positive
PRJNA729271	stage_5W_vs_E10	ordinary_muscle_development	ENSGALG00010014931	RNF145	-0.354320026	0.108948414	-3.252181578	0.001145228	0.001725043	negative
PRJNA729271	stage_5W_vs_E10	ordinary_muscle_development	ENSGALG00010014934	MAMDC2	1.383025365	0.204119796	6.775557253	1.23928E-11	3.26487E-11	positive
PRJNA729271	stage_5W_vs_E10	ordinary_muscle_development	ENSGALG00010014935	PCYT1A	0.431932819	0.131664507	3.280556229	0.001036026	0.001568703	positive
PRJNA729271	stage_5W_vs_E10	ordinary_muscle_development	ENSGALG00010014936	FOXRED2	-0.167937602	0.170307318	-0.986085643	0.324091088	0.363627241	negative
PRJNA729271	stage_5W_vs_E10	ordinary_muscle_development	ENSGALG00010014940	VAMP4	-0.112087473	0.085651867	-1.308640161	0.19065627	0.222706313	negative
PRJNA729271	stage_5W_vs_E10	ordinary_muscle_development	ENSGALG00010014942	PMEPA1	-1.558765784	0.14692035	-10.60959755	2.68905E-26	1.40917E-25	negative
PRJNA729271	stage_5W_vs_E10	ordinary_muscle_development	ENSGALG00010014943	APLN	-0.010127663	0.304703029	-0.033237815	0.973484943	0.977328179	negative
PRJNA729271	stage_5W_vs_E10	ordinary_muscle_development	ENSGALG00010014944	FGB	1.486844798	0.916498481	1.622310161			positive
PRJNA729271	stage_5W_vs_E10	ordinary_muscle_development	ENSGALG00010014945	TXN2	0.472304511	0.104729736	4.50974603	6.49053E-06	1.17132E-05	positive
PRJNA729271	stage_5W_vs_E10	ordinary_muscle_development	ENSGALG00010014946	PCK1	4.117102533	1.041605661	3.952649921	7.72905E-05	0.000128684	positive
PRJNA729271	stage_5W_vs_E10	ordinary_muscle_development	ENSGALG00010014948	RBM38	3.707255837	0.258986462	14.31447732	1.77695E-46	1.89668E-45	positive
PRJNA729271	stage_5W_vs_E10	ordinary_muscle_development	ENSGALG00010014949	AGO3	0.089575261	0.109328015	0.819325777	0.41260057	0.454180229	positive
PRJNA729271	stage_5W_vs_E10	ordinary_muscle_development	ENSGALG00010014952	C21orf58	-1.703632364	0.711449283	-2.394594251	0.016638766	0.022208004	negative
PRJNA729271	stage_5W_vs_E10	ordinary_muscle_development	ENSGALG00010014953	ENSGALG00010014953	2.106104528	0.328414454	6.412947124	1.42733E-10	3.51617E-10	positive
PRJNA729271	stage_5W_vs_E10	ordinary_muscle_development	ENSGALG00010014956	FGG	4.771985776	1.524515191	3.130166104	0.001747075	0.002582749	positive
PRJNA729271	stage_5W_vs_E10	ordinary_muscle_development	ENSGALG00010014965	ABHD13	-0.079648216	0.09951962	-0.800326768	0.423521498	0.465353556	negative
PRJNA729271	stage_5W_vs_E10	ordinary_muscle_development	ENSGALG00010014967	HNRNPH1	-0.733402644	0.107742657	-6.806984941	9.96654E-12	2.63911E-11	negative
PRJNA729271	stage_5W_vs_E10	ordinary_muscle_development	ENSGALG00010014970	SLC51A	0.716558786	0.51929283	1.379874214	0.167625377	0.197715831	positive
PRJNA729271	stage_5W_vs_E10	ordinary_muscle_development	ENSGALG00010014971	SMC5	-0.670673864	0.125846738	-5.32929081	9.8597E-08	2.03614E-07	negative
PRJNA729271	stage_5W_vs_E10	ordinary_muscle_development	ENSGALG00010014972	FGA	4.489516987	1.657245859	2.709022902	0.006748168	0.009405259	positive
PRJNA729271	stage_5W_vs_E10	ordinary_muscle_development	ENSGALG00010014974	LIG4	0.267108671	0.112589014	2.372422153	0.017671887	0.023506836	positive
PRJNA729271	stage_5W_vs_E10	ordinary_muscle_development	ENSGALG00010014976	ITGB2	3.841507764	0.209090563	18.37245883	2.18295E-75	5.38126E-74	positive
PRJNA729271	stage_5W_vs_E10	ordinary_muscle_development	ENSGALG00010014980	ENSGALG00010014980	1.612177159	0.727325661	2.216582262	0.026651651	0.034759374	positive
PRJNA729271	stage_5W_vs_E10	ordinary_muscle_development	ENSGALG00010014982	ENSGALG00010014982	-0.222623539	0.37915814	-0.587152207	0.557101487	0.596835629	negative
PRJNA729271	stage_5W_vs_E10	ordinary_muscle_development	ENSGALG00010014984	LPP	1.100021904	0.091767332	11.9870751	4.15312E-33	2.84497E-32	positive
PRJNA729271	stage_5W_vs_E10	ordinary_muscle_development	ENSGALG00010014985	ENSGALG00010014985	0.260724221	0.10778426	2.41894522	0.015565584	0.020850489	positive
PRJNA729271	stage_5W_vs_E10	ordinary_muscle_development	ENSGALG00010014987	KLF9	6.902841746	0.290307389	23.77769915	5.6823E-125	3.7956E-123	positive
PRJNA729271	stage_5W_vs_E10	ordinary_muscle_development	ENSGALG00010014990	AGO1	-1.036770248	0.048377955	-21.43063407	6.9231E-102	3.0045E-100	negative
PRJNA729271	stage_5W_vs_E10	ordinary_muscle_development	ENSGALG00010014992	ARL15	-1.03555823	0.098267991	-10.53810322	5.76499E-26	2.97273E-25	negative
PRJNA729271	stage_5W_vs_E10	ordinary_muscle_development	ENSGALG00010014994	ENSGALG00010014994	-0.078711508	0.120078933	-0.655498065	0.512147132	0.552760438	negative
PRJNA729271	stage_5W_vs_E10	ordinary_muscle_development	ENSGALG00010014995	LRAT	-1.137624141	0.258758752	-4.396466324	1.10027E-05	1.95639E-05	negative
PRJNA729271	stage_5W_vs_E10	ordinary_muscle_development	ENSGALG00010014997	RAE1	-0.584076311	0.09009874	-6.482624616	9.01406E-11	2.25274E-10	negative
PRJNA729271	stage_5W_vs_E10	ordinary_muscle_development	ENSGALG00010014998	NALF1	-1.263819484	0.562777887	-2.24568078	0.024724462	0.032350702	negative
PRJNA729271	stage_5W_vs_E10	ordinary_muscle_development	ENSGALG00010015002	ADAM19	-1.272702728	0.201535857	-6.315018814	2.70129E-10	6.53348E-10	negative
PRJNA729271	stage_5W_vs_E10	ordinary_muscle_development	ENSGALG00010015006	HSPB3	3.907559566	0.329985368	11.84161464	2.3783E-32	1.58717E-31	positive
PRJNA729271	stage_5W_vs_E10	ordinary_muscle_development	ENSGALG00010015009	AGO4	0.554729869	0.104282999	5.319466005	1.04072E-07	2.14462E-07	positive
PRJNA729271	stage_5W_vs_E10	ordinary_muscle_development	ENSGALG00010015013	SNX18	-1.029596525	0.111232508	-9.256255568	2.11727E-20	8.60142E-20	negative
PRJNA729271	stage_5W_vs_E10	ordinary_muscle_development	ENSGALG00010015016	ARGLU1	-0.024611113	0.080133846	-0.307125076	0.758748171	0.786586665	negative
PRJNA729271	stage_5W_vs_E10	ordinary_muscle_development	ENSGALG00010015017	FAM184A	-0.100375785	0.191903435	-0.523053612	0.600936946	0.638699298	negative
PRJNA729271	stage_5W_vs_E10	ordinary_muscle_development	ENSGALG00010015018	MAP7D3	-0.274028449	0.069245801	-3.957329488	7.57924E-05	0.00012632	negative
PRJNA729271	stage_5W_vs_E10	ordinary_muscle_development	ENSGALG00010015020	NIPAL4	-1.378953746	0.834557417	-1.652317405	0.098469863	0.11969146	negative
PRJNA729271	stage_5W_vs_E10	ordinary_muscle_development	ENSGALG00010015021	RBM46	-1.750185615	0.881571386	-1.985302204	0.047110859	0.059603029	negative
PRJNA729271	stage_5W_vs_E10	ordinary_muscle_development	ENSGALG00010015024	CRACD	-1.884023628	0.388652396	-4.847580117	1.24977E-06	2.38078E-06	negative
PRJNA729271	stage_5W_vs_E10	ordinary_muscle_development	ENSGALG00010015025	TRAF3IP1	-0.385140197	0.18668276	-2.06307319	0.039105675	0.050029723	negative
PRJNA729271	stage_5W_vs_E10	ordinary_muscle_development	ENSGALG00010015027	CLSPN	-2.491793878	0.184938939	-13.47360322	2.23678E-41	2.04041E-40	negative
PRJNA729271	stage_5W_vs_E10	ordinary_muscle_development	ENSGALG00010015028	MCM9	-2.379958394	0.273963054	-8.687150906	3.71642E-18	1.36725E-17	negative
PRJNA729271	stage_5W_vs_E10	ordinary_muscle_development	ENSGALG00010015029	TFRC	-1.311006707	0.18272988	-7.174561208	7.25395E-13	2.03771E-12	negative
PRJNA729271	stage_5W_vs_E10	ordinary_muscle_development	ENSGALG00010015031	MYH9	0.676809872	0.109521037	6.179724807	6.42134E-10	1.51839E-09	positive
PRJNA729271	stage_5W_vs_E10	ordinary_muscle_development	ENSGALG00010015032	C1orf216	-1.027533884	0.249908734	-4.111636548	3.92864E-05	6.67906E-05	negative
PRJNA729271	stage_5W_vs_E10	ordinary_muscle_development	ENSGALG00010015033	BMP7	-1.504984122	0.325168985	-4.62831386	3.68655E-06	6.78215E-06	negative
PRJNA729271	stage_5W_vs_E10	ordinary_muscle_development	ENSGALG00010015035	SLC35F1	-3.881082208	0.139590435	-27.80335353	3.9511E-170	5.1135E-168	negative
PRJNA729271	stage_5W_vs_E10	ordinary_muscle_development	ENSGALG00010015037	CYFIP2	-0.155898295	0.192314573	-0.810642128	0.417571217	0.459163616	negative
PRJNA729271	stage_5W_vs_E10	ordinary_muscle_development	ENSGALG00010015038	PCSK5	-0.368088898	0.206858924	-1.77941996	0.075170937	0.092779524	negative
PRJNA729271	stage_5W_vs_E10	ordinary_muscle_development	ENSGALG00010015039	FAM114A1	-0.247471866	0.127193976	-1.945625685	0.051699719	0.065096198	negative
PRJNA729271	stage_5W_vs_E10	ordinary_muscle_development	ENSGALG00010015040	ZC4H2	-1.16163871	0.088807314	-13.08043963	4.25969E-39	3.59377E-38	negative
PRJNA729271	stage_5W_vs_E10	ordinary_muscle_development	ENSGALG00010015041	TFAP2C	-6.50048436	0.668443241	-9.72481127	2.36343E-22	1.05086E-21	negative
PRJNA729271	stage_5W_vs_E10	ordinary_muscle_development	ENSGALG00010015042	EFNB2	-0.21470874	0.133320281	-1.6104732	0.107294592	0.129808455	negative
PRJNA729271	stage_5W_vs_E10	ordinary_muscle_development	ENSGALG00010015043	COQ10B	3.179141284	0.221731709	14.33778371	1.27048E-46	1.36513E-45	positive
PRJNA729271	stage_5W_vs_E10	ordinary_muscle_development	ENSGALG00010015044	ZC3H12B	-0.331420214	0.129983901	-2.549702012	0.010781503	0.014714046	negative
PRJNA729271	stage_5W_vs_E10	ordinary_muscle_development	ENSGALG00010015046	PSMB2	0.022706057	0.093922584	0.241752903	0.808971634	0.831575338	positive
PRJNA729271	stage_5W_vs_E10	ordinary_muscle_development	ENSGALG00010015047	NPY2R	-2.364823227	0.208451818	-11.34469946	7.87965E-30	4.7353E-29	negative
PRJNA729271	stage_5W_vs_E10	ordinary_muscle_development	ENSGALG00010015049	DCK2	-2.761283884	0.251736269	-10.96895531	5.38914E-28	3.01721E-27	negative
PRJNA729271	stage_5W_vs_E10	ordinary_muscle_development	ENSGALG00010015050	HIVEP3	0.529491705	0.174484554	3.034605026	0.002408509	0.003515036	positive
PRJNA729271	stage_5W_vs_E10	ordinary_muscle_development	ENSGALG00010015052	ENSGALG00010015052	-0.550281999	0.10908552	-5.044500845	4.54707E-07	8.94298E-07	negative
PRJNA729271	stage_5W_vs_E10	ordinary_muscle_development	ENSGALG00010015054	ENSGALG00010015054	-2.225257541	0.177916232	-12.50733287	6.80716E-36	5.20685E-35	negative
PRJNA729271	stage_5W_vs_E10	ordinary_muscle_development	ENSGALG00010015055	ZNF335	0.859660516	0.182019234	4.722910302	2.32493E-06	4.33886E-06	positive
PRJNA729271	stage_5W_vs_E10	ordinary_muscle_development	ENSGALG00010015056	ENSGALG00010015056	-0.58198144	0.454264817	-1.281150153	0.200140931	0.232660421	negative

PRJNA729271	stage_5W_vs_E10	ordinary_muscle_development	ENSGALG00010015057	LAS1L	-0.833459475	0.113645339	-7.333864106	2.2361E-13	6.43355E-13	negative
PRJNA729271	stage_5W_vs_E10	ordinary_muscle_development	ENSGALG00010015059	ENSGALG00010015059	0.493323369	0.164551207	2.997993008	0.002717639	0.003945926	positive
PRJNA729271	stage_5W_vs_E10	ordinary_muscle_development	ENSGALG00010015061	MMP9	-0.503565147	0.474284268	-1.061736982	0.288355106	0.326081078	negative
PRJNA729271	stage_5W_vs_E10	ordinary_muscle_development	ENSGALG00010015062	ENSGALG00010015062	0.177555941	0.237002006	0.749174844	0.453751829	0.495713956	positive
PRJNA729271	stage_5W_vs_E10	ordinary_muscle_development	ENSGALG00010015063	EEF1AKMT4	-2.782107929	0.271818753	-10.23515817	1.3797E-24	6.74037E-24	negative
PRJNA729271	stage_5W_vs_E10	ordinary_muscle_development	ENSGALG00010015064	RFK	-0.152544288	0.148326139	-1.028438342	0.30374368	0.342387794	negative
PRJNA729271	stage_5W_vs_E10	ordinary_muscle_development	ENSGALG00010015065	F11	4.083679043	1.241169659	3.290186007	0.001001212	0.001517892	positive
PRJNA729271	stage_5W_vs_E10	ordinary_muscle_development	ENSGALG00010015066	RUFY1	-0.556246301	0.071988601	-7.726866397	1.10226E-14	3.40377E-14	negative
PRJNA729271	stage_5W_vs_E10	ordinary_muscle_development	ENSGALG00010015068	ESM1	-1.871483125	0.330930659	-5.655212284	1.55654E-08	3.39943E-08	negative
PRJNA729271	stage_5W_vs_E10	ordinary_muscle_development	ENSGALG00010015069	HSPD1	-1.420830401	0.102847957	-13.8148627	2.07349E-43	2.01849E-42	negative
PRJNA729271	stage_5W_vs_E10	ordinary_muscle_development	ENSGALG00010015070	EDN2	0.552111265	0.439819592	1.25531303	0.209365147	0.24270216	positive
PRJNA729271	stage_5W_vs_E10	ordinary_muscle_development	ENSGALG00010015071	SNAI1	-1.840214201	0.128238853	-14.34989598	1.06697E-46	1.15073E-45	negative
PRJNA729271	stage_5W_vs_E10	ordinary_muscle_development	ENSGALG00010015072	MAP9	-0.219381192	0.138736368	-1.581281069	0.113813786	0.137283085	negative
PRJNA729271	stage_5W_vs_E10	ordinary_muscle_development	ENSGALG00010015075	ENSGALG00010015075	3.268441224	0.16701491	19.56975709	2.80013E-85	8.45581E-84	positive
PRJNA729271	stage_5W_vs_E10	ordinary_muscle_development	ENSGALG00010015076	ITK	4.625587535	0.561244348	8.241664351	1.69831E-16	5.75838E-16	positive
PRJNA729271	stage_5W_vs_E10	ordinary_muscle_development	ENSGALG00010015080	ASF1A	-1.384235957	0.080649036	-17.16370117	4.96465E-66	9.43153E-65	negative
PRJNA729271	stage_5W_vs_E10	ordinary_muscle_development	ENSGALG00010015083	CEP135	-2.348592119	0.196823941	-11.93245146	8.0177E-33	5.43068E-32	negative
PRJNA729271	stage_5W_vs_E10	ordinary_muscle_development	ENSGALG00010015084	FOXO6	1.166323775	0.202416279	5.7620058	8.31201E-09	1.84337E-08	positive
PRJNA729271	stage_5W_vs_E10	ordinary_muscle_development	ENSGALG00010015085	PSMD2	0.501676335	0.163453161	3.069236056	0.002146069	0.003144372	positive
PRJNA729271	stage_5W_vs_E10	ordinary_muscle_development	ENSGALG00010015086	FOXN3	0.261888284	0.096303319	2.719410796	0.006539833	0.009124543	positive
PRJNA729271	stage_5W_vs_E10	ordinary_muscle_development	ENSGALG00010015087	GABRA3	-5.698087819	0.891436213	-6.392030904	1.63697E-10	4.01622E-10	negative
PRJNA729271	stage_5W_vs_E10	ordinary_muscle_development	ENSGALG00010015088	ENSGALG00010015088	0.837769002	1.080485603	0.775363411			positive
PRJNA729271	stage_5W_vs_E10	ordinary_muscle_development	ENSGALG00010015090	GZMA	3.48778613	0.497611282	7.009057586	2.39929E-12	6.5544E-12	positive
PRJNA729271	stage_5W_vs_E10	ordinary_muscle_development	ENSGALG00010015091	MSN	-0.429260961	0.121301915	-3.538781399	0.000401979	0.000629027	negative
PRJNA729271	stage_5W_vs_E10	ordinary_muscle_development	ENSGALG00010015092	HSPE1	-0.722648682	0.122894471	-5.880237533	4.09678E-09	9.23959E-09	negative
PRJNA729271	stage_5W_vs_E10	ordinary_muscle_development	ENSGALG00010015094	CDK5RAP1	1.884438302	0.213109048	8.842601105	9.35156E-19	3.52813E-18	positive
PRJNA729271	stage_5W_vs_E10	ordinary_muscle_development	ENSGALG00010015095	RBFOX2	-0.455537206	0.133207593	-3.419754053	0.000626778	0.000966505	negative
PRJNA729271	stage_5W_vs_E10	ordinary_muscle_development	ENSGALG00010015096	GABRE	-2.773648826	1.065156825	-2.603981648	0.009214769	0.012660481	negative
PRJNA729271	stage_5W_vs_E10	ordinary_muscle_development	ENSGALG00010015097	MFSD2A	-1.253128424	0.168075139	-7.455763118	8.93492E-14	2.62541E-13	negative
PRJNA729271	stage_5W_vs_E10	ordinary_muscle_development	ENSGALG00010015098	HAVCR1	2.018790202	0.214082925	9.429944988	4.10304E-21	1.72544E-20	positive
PRJNA729271	stage_5W_vs_E10	ordinary_muscle_development	ENSGALG00010015100	MOB4	0.498869921	0.088958873	5.607871399	2.0483E-08	4.43467E-08	positive
PRJNA729271	stage_5W_vs_E10	ordinary_muscle_development	ENSGALG00010015101	CAP1	-0.769142247	0.099501364	-7.729966857	1.07575E-14	3.32614E-14	negative
PRJNA729271	stage_5W_vs_E10	ordinary_muscle_development	ENSGALG00010015103	ITPK1	-1.005824403	0.093463162	-10.76172019	5.21868E-27	2.80894E-26	negative
PRJNA729271	stage_5W_vs_E10	ordinary_muscle_development	ENSGALG00010015104	CNGA2	1.230667388	0.286317815	4.298256423	1.72147E-05	3.01179E-05	positive
PRJNA729271	stage_5W_vs_E10	ordinary_muscle_development	ENSGALG00010015105	ENSGALG00010015105	8.735286726	0.938283745	9.309856184	1.28006E-20	5.25323E-20	positive
PRJNA729271	stage_5W_vs_E10	ordinary_muscle_development	ENSGALG00010015107	MB	0.568025668	0.563941653	1.007241911	0.313818537	0.352893693	positive
PRJNA729271	stage_5W_vs_E10	ordinary_muscle_development	ENSGALG00010015108	ENSGALG00010015108	0.444603484	0.089024211	4.994186203	5.90844E-07	1.15422E-06	positive
PRJNA729271	stage_5W_vs_E10	ordinary_muscle_development	ENSGALG00010015109	PPT1	0.921866257	0.107856151	8.547182981	1.26129E-17	4.53015E-17	positive
PRJNA729271	stage_5W_vs_E10	ordinary_muscle_development	ENSGALG00010015110	ENSGALG00010015110	4.065977521	0.683087	5.952356757	2.64308E-09	6.02572E-09	positive
PRJNA729271	stage_5W_vs_E10	ordinary_muscle_development	ENSGALG00010015111	ADAMTS2	1.348773111	0.142263081	9.480837183	2.52251E-21	1.07105E-20	positive
PRJNA729271	stage_5W_vs_E10	ordinary_muscle_development	ENSGALG00010015112	CBLN4	-2.001404585	0.367730873	-5.442579704	5.25145E-08	1.10366E-07	negative
PRJNA729271	stage_5W_vs_E10	ordinary_muscle_development	ENSGALG00010015115	CLDN2	-0.546895017	0.446733582	-1.22420843	0.22087361	0.254901511	negative
PRJNA729271	stage_5W_vs_E10	ordinary_muscle_development	ENSGALG00010015117	RASD2	-0.26681094	0.206767679	-1.290389973	0.196915292	0.229204782	negative
PRJNA729271	stage_5W_vs_E10	ordinary_muscle_development	ENSGALG00010015118	GPX8	-1.116872636	0.187406023	-5.959641079	2.52793E-09	5.77044E-09	negative
PRJNA729271	stage_5W_vs_E10	ordinary_muscle_development	ENSGALG00010015119	TLR1A	1.107861264	0.212521712	5.212932154	1.85879E-07	3.76248E-07	positive
PRJNA729271	stage_5W_vs_E10	ordinary_muscle_development	ENSGALG00010015121	UNC79	-4.81557251	0.315886231	-15.244642	1.7871E-52	2.32531E-51	negative
PRJNA729271	stage_5W_vs_E10	ordinary_muscle_development	ENSGALG00010015122	ENSGALG00010015122	-3.996067971	0.220429038	-18.12859144	1.89553E-73	4.36121E-72	negative
PRJNA729271	stage_5W_vs_E10	ordinary_muscle_development	ENSGALG00010015123	CEP85L	1.389021004	0.250822021	5.53787502	3.06164E-08	6.53874E-08	positive
PRJNA729271	stage_5W_vs_E10	ordinary_muscle_development	ENSGALG00010015124	UXS1	-0.091634098	0.096713783	-0.947477131	0.343395704	0.38326948	negative
PRJNA729271	stage_5W_vs_E10	ordinary_muscle_development	ENSGALG00010015126	MCM5	-2.945630945	0.182664993	-16.12586464	1.67893E-58	2.58345E-57	negative
PRJNA729271	stage_5W_vs_E10	ordinary_muscle_development	ENSGALG00010015127	RPL11	0.130777101	0.151601496	0.862637268	0.388336933	0.42959199	positive
PRJNA729271	stage_5W_vs_E10	ordinary_muscle_development	ENSGALG00010015128	OCRL	0.568970473	0.106559506	5.339462361	9.32226E-08	1.9268E-07	positive
PRJNA729271	stage_5W_vs_E10	ordinary_muscle_development	ENSGALG00010015129	PRRG3	-1.374709562	0.140771696	-9.765525334	1.5829E-22	7.09245E-22	negative
PRJNA729271	stage_5W_vs_E10	ordinary_muscle_development	ENSGALG00010015131	DOK5	0.429718806	0.245985595	1.746926708	0.080650052	0.099103298	positive
PRJNA729271	stage_5W_vs_E10	ordinary_muscle_development	ENSGALG00010015132	GCNT1	-1.43172774	0.293567286	-4.877000298	1.07711E-06	2.06272E-06	negative
PRJNA729271	stage_5W_vs_E10	ordinary_muscle_development	ENSGALG00010015133	RFTN2	-1.636156075	0.128770869	-12.7059489	5.48007E-37	4.35014E-36	negative
PRJNA729271	stage_5W_vs_E10	ordinary_muscle_development	ENSGALG00010015134	PLN	2.178266479	0.525200347	4.147496266	3.36131E-05	5.74013E-05	positive
PRJNA729271	stage_5W_vs_E10	ordinary_muscle_development	ENSGALG00010015136	TCEB3	0.751118095	0.090478507	8.301619006	1.02702E-16	3.51931E-16	positive
PRJNA729271	stage_5W_vs_E10	ordinary_muscle_development	ENSGALG00010015137	AMN	1.344409885	0.439535485	3.058706136	0.002222951	0.003252415	positive
PRJNA729271	stage_5W_vs_E10	ordinary_muscle_development	ENSGALG00010015138	ENSGALG00010015138	0.941613533	0.453739251	2.0752305	0.037965177	0.048652218	positive
PRJNA729271	stage_5W_vs_E10	ordinary_muscle_development	ENSGALG00010015140	ASCC3	0.370757127	0.072965705	5.081251875	3.74956E-07	7.41774E-07	positive
PRJNA729271	stage_5W_vs_E10	ordinary_muscle_development	ENSGALG00010015141	PFDN4	0.17873708	0.136130936	1.312979146	0.189189975	0.22108261	positive
PRJNA729271	stage_5W_vs_E10	ordinary_muscle_development	ENSGALG00010015142	HNRNPAB	-1.026859086	0.128767531	-7.974518687	1.52976E-15	4.95505E-15	negative
PRJNA729271	stage_5W_vs_E10	ordinary_muscle_development	ENSGALG00010015143	EXOC1	0.20366009	0.07968218	2.555905116	0.010591199	0.014466588	positive
PRJNA729271	stage_5W_vs_E10	ordinary_muscle_development	ENSGALG00010015145	CLIC2	2.373274745	0.123890611	19.15621144	8.5903E-82	2.40844E-80	positive
PRJNA729271	stage_5W_vs_E10	ordinary_muscle_development	ENSGALG00010015146	ENSGALG00010015146	1.304954955	0.127395806	10.24331172	1.2682E-24	6.20401E-24	positive
PRJNA729271	stage_5W_vs_E10	ordinary_muscle_development	ENSGALG00010015148	ENSGALG00010015148	-0.366942254	0.076866755	-4.773744537	1.80832E-06	3.40233E-06	negative
PRJNA729271	stage_5W_vs_E10	ordinary_muscle_development	ENSGALG00010015149	ENSGALG00010015149	1.412823771	0.138475224	10.20271883	1.92798E-24	9.3434E-24	positive
PRJNA729271	stage_5W_vs_E10	ordinary_muscle_development	ENSGALG00010015151	ENSGALG00010015151	-4.576999546	0.488355981	-9.372260664	7.09953E-21	2.94611E-20	negative
PRJNA729271	stage_5W_vs_E10	ordinary_muscle_development	ENSGALG00010015152	BOLL	3.096230857	0.276088064	11.21464946	3.45553E-29	2.03279E-28	positive
PRJNA729271	stage_5W_vs_E10	ordinary_muscle_development	ENSGALG00010015153	ERCC6L	-3.223403368	0.251135151	-12.83533329	1.03955E-37	8.418E-37	negative
PRJNA729271	stage_5W_vs_E10	ordinary_muscle_development	ENSGALG00010015154	CYP24A1	-1.69980931	0.694741351	-2.446679339	0.014417905	0.019390196	negative
PRJNA729271	stage_5W_vs_E10	ordinary_muscle_development	ENSGALG00010015155	HMOX1	-0.345150343	0.178754751	-1.930859691	0.053500406	0.067223335	negative
PRJNA729271	stage_5W_vs_E10	ordinary_muscle_development	ENSGALG00010015156	CNOT6	-0.710740677	0.074449847	-9.546569974	1.34061E-21	5.7594E-21	negative
PRJNA729271	stage_5W_vs_E10	ordinary_muscle_development	ENSGALG00010015157	CDC42BPB	-0.355018876	0.070070005	-5.066631249	4.04918E-07	7.98868E-07	negative
PRJNA729271	stage_5W_vs_E10	ordinary_muscle_development	ENSGALG00010015158	PIN4	0.180464026	0.165026287	1.09354715	0.274153639	0.311308813	positive
PRJNA729271	stage_5W_vs_E10	ordinary_muscle_development	ENSGALG00010015160	SIM1	-6.931298397	0.592491479	-11.69856215	1.29631E-31	8.3772E-31	negative
PRJNA729271	stage_5W_vs_E10	ordinary_muscle_development	ENSGALG00010015161	GFPT2	-0.819211354	0.201063875	-4.0743836	4.61364E-05	7.80244E-05	negative
PRJNA729271	stage_5W_vs_E10	ordinary_muscle_development	ENSGALG00010015163	PLK2	-1.003212953	0.2098604	-4.78038235	1.74962E-06	3.29532E-06	negative
PRJNA729271	stage_5W_vs_E10	ordinary_muscle_development	ENSGALG00010015165	KLF3	0.923997335	0.116552852	7.927711038	2.23222E-15	7.16475E-15	positive
PRJNA729271	stage_5W_vs_E10	ordinary_muscle_development	ENSGALG00010015166	YTHDF2	0.309530084	0.181084929	1.709308916	0.087393743	0.106945741	positive
PRJNA729271	stage_5W_vs_E10	ordinary_muscle_development	ENSGALG00010015167	RAB3C	-3.843602818	0.499730051	-7.691358191	1.45581E-14	4.4632E-14	negative
PRJNA729271	stage_5W_vs_E10	ordinary_muscle_development	ENSGALG00010015169	ENSGALG00010015169	-0.462675491	0.616257202	-0.750783098	0.452783203	0.494693037	negative
PRJNA729271	stage_5W_vs_E10	ordinary_muscle_development	ENSGALG00010015170	EXOC1L	-1.878504974	0.886912994	-2.118026217	0.034172847	0.044049393	negative
PRJNA729271	stage_5W_vs_E10	ordinary_muscle_development	ENSGALG00010015171	NHSL2	2.517900071	0.19808892	12.71095865	5.14004E-37	4.09143E-36	positive
PRJNA729271	stage_5W_vs_E10	ordinary_muscle_development	ENSGALG00010015172	PPP4R3A	0.174067288	0.076783547	2.26698679	0.023391033	0.030672428	positive
PRJNA729271	stage_5W_vs_E10	ordinary_muscle_development	ENSGALG00010015173	ENS-1	0.411657263	0.668043858	0.616212929	0.537753996	0.577730817	positive
PRJNA729271	stage_5W_vs_E10	ordinary_muscle_development	ENSGALG00010015174	ST6GAL2	-5.059460573	0.225194272	-22.4670926	8.711E-112	4.6083E-110	negative
PRJNA729271	stage_5W_vs_E10	ordinary_muscle_development	ENSGALG00010015175	MAPK9	0.025670683	0.069247508	0.370709121	0.710854196	0.742137105	positive
PRJNA729271	stage_5W_vs_E10	ordinary_muscle_development	ENSGALG00010015177	TC2N	-0.377402657	0.162930033	-2.316347999	0.020539276	0.027104588	negative
PRJNA729271	stage_5W_vs_E10	ordinary_muscle_development	ENSGALG00010015180	PITHD1	0.948472659	0.125288918	7.570283747	3.7241E-14	1.1197E-13	positive
PRJNA729271	stage_5W_vs_E10	ordinary_muscle_development	ENSGALG00010015181	VPS13A	1.359900771	0.120021744	11.3304533	9.27236E-30	5.55154E-29	positive
PRJNA729271	stage_5W_vs_E10	ordinary_muscle_development	ENSGALG00010015182	PHYKPL	-1.258976233	0.330237903	-3.812331116	0.000137662	0.000224053	negative
PRJNA729271	stage_5W_vs_E10	ordinary_muscle_development	ENSGALG00010015183	SLC5A7	-6.24581131	0.948258874	-6.586609922	4.49982E-11	1.14591E-10	negative
PRJNA729271	stage_5W_vs_E10	ordinary_muscle_development	ENSGALG00010015186	ENSGALG00010015186	0.580177234	0.371934851	1.55988941	0.118786018	0.142793442	positive
PRJNA729271	stage_5W_vs_E10	ordinary_muscle_development	ENSGALG00010015188	SULT1C3	-1.141186316	1.19400268	-0.955765288	0.339190829	0.378897277	negative
PRJNA729271	stage_5W_vs_E10	ordinary_muscle_development	ENSGALG00010015190	FBLN5	0.668917556	0.192182418	3.480638679	0.00050022	0.000776804	positive
PRJNA729271	stage_5W_vs_E10	ordinary_muscle_development	ENSGALG00010015191	TOM1	5.788019486	0.186625916	31.01401777	3.4887E-211	6.6538E-209	positive
PRJNA729271	stage_5W_vs_E10	ordinary_muscle_development	ENSGALG00010015192	ENSGALG00010015192	2.96893112	0.212591221	13.96544557	2.53316E-44	2.52185E-43	positive
PRJNA729271	stage_5W_vs_E10	ordinary_muscle_development	ENSGALG00010015193	LYPLA2	-0.940026129	0.107680081	-8.729805144	2.55112E-18	9.44774E-18	negative
PRJNA729271	stage_5W_vs_E10	ordinary_muscle_development	ENSGALG00010015194	TMLHE	-0.047934149	0.113724921	-0.421492044	0.673395819	0.7072071	negative
PRJNA729271	stage_5W_vs_E10	ordinary_muscle_development	ENSGALG00010015196	PDE4D	1.661822262	0.217523032	7.639753107	2.17639E-14	6.61496E-14	positive
PRJNA729271	stage_5W_vs_E10	ordinary_muscle_development	ENSGALG00010015198	NME5	0.564127473	0.220569039	2.557600447	0.010539711	0.014401689	positive
PRJNA729271	stage_5W_vs_E10	ordinary_muscle_development	ENSGALG00010015199	ENSGALG00010015199	-1.288289183	0.180536391	-7.135897514	9.61576E-13	2.68504E-12	negative
PRJNA729271	stage_5W_vs_E10	ordinary_muscle_development	ENSGALG00010015200	TBC1D1	-0.613834469	0.08633084	-7.110257132	1.15827E-12	3.21816E-12	negative
PRJNA729271	stage_5W_vs_E10	ordinary_muscle_development	ENSGALG00010015201	GCC2	0.337305898	0.108714624	3.102672704	0.001917816	0.002823067	positive
PRJNA729271	stage_5W_vs_E10	ordinary_muscle_development	ENSGALG00010015203	HMGXB4	-0.56216312	0.066525535	-8.450335963	2.90466E-17	1.02341E-16	negative
PRJNA729271	stage_5W_vs_E10	ordinary_muscle_development	ENSGALG00010015204	ENSGALG00010015204	-0.055686921	0.142272055	-0.39141152	0.695493078	0.727987592	negative
PRJNA729271	stage_5W_vs_E10	ordinary_muscle_development	ENSGALG00010015207	AKR1E2	-0.174263188	0.17866401	-0.975368169	0.329377667	0.369073074	negative
PRJNA729271	stage_5W_vs_E10	ordinary_muscle_development	ENSGALG00010015208	GNA14	2.213238566	0.580932698	3.809802018	0.000139078	0.000226256	positive
PRJNA729271	stage_5W_vs_E10	ordinary_muscle_development	ENSGALG00010015212	GNAQ	0.510467923	0.09732907	5.244763192	1.56483E-07	3.1866E-07	positive
PRJNA729271	stage_5W_vs_E10	ordinary_muscle_development	ENSGALG00010015213	RUBCN	1.193842856	0.116520821	10.24574705	1.23666E-24	6.05586E-24	positive
PRJNA729271	stage_5W_vs_E10	ordinary_muscle_development	ENSGALG00010015218	PLCL1	-1.751802571	0.161292264	-10.86104523	1.76715E-27	9.68058E-27	negative
PRJNA729271	stage_5W_vs_E10	ordinary_muscle_development	ENSGALG00010015227	FYTTD1	-1.279910892	0.076652198	-16.69764118	1.3635E-62	2.35284E-61	negative
PRJNA729271	stage_5W_vs_E10	ordinary_muscle_development	ENSGALG00010015233	ENSGALG00010015233	0.474333515	0.718261713	0.660390922	0.509002996	0.549817306	positive
PRJNA729271	stage_5W_vs_E10	ordinary_muscle_development	ENSGALG00010015234	TRIP11	0.188725301	0.14376137	1.312767829	0.189261193	0.221148005	positive
PRJNA729271	stage_5W_vs_E10	ordinary_muscle_development	ENSGALG00010015241	ENSGALG00010015241	0.235221723	0.163625069	1.437565306	0.150557431	0.178659841	positive
PRJNA729271	stage_5W_vs_E10	ordinary_muscle_development	ENSGALG00010015248	ENSGALG00010015248	0.640442807	0.13516907	4.738086935	2.15745E-06	4.03878E-06	positive
PRJNA729271	stage_5W_vs_E10	ordinary_muscle_development	ENSGALG00010015250	RORB	-2.742795889	0.570881641	-4.804491316	1.55145E-06	2.93344E-06	negative
PRJNA729271	stage_5W_vs_E10	ordinary_muscle_development	ENSGALG00010015253	ENSGALG00010015253	-0.479756814	0.073680292	-6.511331605	7.44875E-11	1.87382E-10	negative
PRJNA729271	stage_5W_vs_E10	ordinary_muscle_development	ENSGALG00010015260	RASGEF1C	0.218633689	0.144610714	1.511877526	0.130565022	0.156201717	positive
PRJNA729271	stage_5W_vs_E10	ordinary_muscle_development	ENSGALG00010015261	IQCG	-0.299246613	0.428266746	-0.698738849	0.484715251	0.526327632	negative
PRJNA729271	stage_5W_vs_E10	ordinary_muscle_development	ENSGALG00010015283	ENSGALG00010015283	-3.476949827	0.943481219	-3.685234805	0.000228492	0.0003654	negative
PRJNA729271	stage_5W_vs_E10	ordinary_muscle_development	ENSGALG00010015285	ALG13	-0.971595568	0.43433233	-2.236986522	0.025287221	0.033042303	negative
PRJNA729271	stage_5W_vs_E10	ordinary_muscle_development	ENSGALG00010015291	ZFP64	0.172808247	0.092462583	1.868953267	0.061629316	0.076837858	positive
PRJNA729271	stage_5W_vs_E10	ordinary_muscle_development	ENSGALG00010015296	RPL35A	-0.387216894	0.195436895	-1.981288607	0.047558917	0.060138402	negative
PRJNA729271	stage_5W_vs_E10	ordinary_muscle_development	ENSGALG00010015298	ENSGALG00010015298	3.052759272	0.233242664	13.08833994	3.83907E-39	3.24663E-38	positive
PRJNA729271	stage_5W_vs_E10	ordinary_muscle_development	ENSGALG00010015299	GALE	-0.946741077	0.096948199	-9.765432323	1.58435E-22	7.09676E-22	negative
PRJNA729271	stage_5W_vs_E10	ordinary_muscle_development	ENSGALG00010015300	ATXN3	1.208949426	0.126812692	9.533347249	1.52292E-21	6.52519E-21	positive
PRJNA729271	stage_5W_vs_E10	ordinary_muscle_development	ENSGALG00010015306	BCAT1	1.175051439	0.090714984	12.95322324	2.25292E-38	1.86075E-37	positive
PRJNA729271	stage_5W_vs_E10	ordinary_muscle_development	ENSGALG00010015310	PGM2	-1.59560505	0.09544372	-16.71775834	9.73112E-63	1.68522E-61	negative

PRJNA729271	stage_5W_vs_E10	ordinary_muscle_development	ENSGALG00010015314	GMEB1	-0.025700533	0.068794336	-0.373585009	0.708713089	0.740275003	negative
PRJNA729271	stage_5W_vs_E10	ordinary_muscle_development	ENSGALG00010015318	SALL4	-0.902523076	0.192433949	-4.69004083	2.73151E-06	5.07735E-06	negative
PRJNA729271	stage_5W_vs_E10	ordinary_muscle_development	ENSGALG00010015320	TRPM6	-2.624992964	0.423555263	-6.197521775	5.73591E-10	1.36075E-09	negative
PRJNA729271	stage_5W_vs_E10	ordinary_muscle_development	ENSGALG00010015327	LARGE1	0.183719143	0.12610583	1.456864781	0.14515371	0.172544122	positive
PRJNA729271	stage_5W_vs_E10	ordinary_muscle_development	ENSGALG00010015328	ENSGALG00010015328	0.462353267	0.412409573	1.12110217	0.262244375	0.298839011	positive
PRJNA729271	stage_5W_vs_E10	ordinary_muscle_development	ENSGALG00010015333	MED7	-0.015717091	0.068826587	-0.228357846	0.819368057	0.840652604	negative
PRJNA729271	stage_5W_vs_E10	ordinary_muscle_development	ENSGALG00010015339	ENSGALG00010015339	3.258963073	0.745483361	4.371610749	1.23333E-05	2.18414E-05	positive
PRJNA729271	stage_5W_vs_E10	ordinary_muscle_development	ENSGALG00010015348	CPSF2	-0.749465091	0.061753888	-12.13632234	6.78057E-34	4.79436E-33	negative
PRJNA729271	stage_5W_vs_E10	ordinary_muscle_development	ENSGALG00010015353	RELL1	0.580482728	0.091347898	6.354636973	2.08919E-10	5.09298E-10	positive
PRJNA729271	stage_5W_vs_E10	ordinary_muscle_development	ENSGALG00010015356	ENSGALG00010015356	-0.874126992	0.088684974	-9.856540012	6.42245E-23	2.92471E-22	negative
PRJNA729271	stage_5W_vs_E10	ordinary_muscle_development	ENSGALG00010015362	ATP9A	-0.932342383	0.135666523	-6.872309861	6.31706E-12	1.68754E-11	negative
PRJNA729271	stage_5W_vs_E10	ordinary_muscle_development	ENSGALG00010015364	CLOCK	2.786791092	0.202226652	13.78053319	3.33798E-43	3.21488E-42	positive
PRJNA729271	stage_5W_vs_E10	ordinary_muscle_development	ENSGALG00010015370	ENSGALG00010015370	2.069755121	0.142494056	14.52520319	8.38932E-48	9.32539E-47	positive
PRJNA729271	stage_5W_vs_E10	ordinary_muscle_development	ENSGALG00010015374	LIMS1	-0.953640056	0.150907688	-6.319360329	2.62648E-10	6.36211E-10	negative
PRJNA729271	stage_5W_vs_E10	ordinary_muscle_development	ENSGALG00010015384	TAF12	-0.074166987	0.117241338	-0.632600996	0.526994237	0.567601535	negative
PRJNA729271	stage_5W_vs_E10	ordinary_muscle_development	ENSGALG00010015386	SLC24A4	1.497606601	0.242779002	6.168600214	6.88972E-10	1.62358E-09	positive
PRJNA729271	stage_5W_vs_E10	ordinary_muscle_development	ENSGALG00010015388	PSMD1	0.61121381	0.12223879	5.000162448	5.7282E-07	1.11946E-06	positive
PRJNA729271	stage_5W_vs_E10	ordinary_muscle_development	ENSGALG00010015390	CCNB3	-3.331826899	0.212680515	-15.66587755	2.58832E-55	3.61791E-54	negative
PRJNA729271	stage_5W_vs_E10	ordinary_muscle_development	ENSGALG00010015391	SYN3	-2.930175822	0.596349519	-4.913520892	8.94552E-07	1.72519E-06	negative
PRJNA729271	stage_5W_vs_E10	ordinary_muscle_development	ENSGALG00010015392	TMEM165	-0.722530599	0.08256242	-8.751325324	2.10862E-18	7.84104E-18	negative
PRJNA729271	stage_5W_vs_E10	ordinary_muscle_development	ENSGALG00010015393	DCX	-2.399810625	0.378215093	-6.345094807	2.22289E-10	5.40798E-10	negative
PRJNA729271	stage_5W_vs_E10	ordinary_muscle_development	ENSGALG00010015397	SRD5A3	-0.201842986	0.084414912	-2.391082107	0.016798796	0.022417468	negative
PRJNA729271	stage_5W_vs_E10	ordinary_muscle_development	ENSGALG00010015404	BEND4	-4.156976139	0.424328966	-9.796588184	1.16453E-22	5.24544E-22	negative
PRJNA729271	stage_5W_vs_E10	ordinary_muscle_development	ENSGALG00010015407	TIMP3	0.407948256	0.17553734	2.323997024	0.020125654	0.026578111	positive
PRJNA729271	stage_5W_vs_E10	ordinary_muscle_development	ENSGALG00010015409	FAM13B	0.24633656	0.132234343	1.862878839	0.062479315	0.077850741	positive
PRJNA729271	stage_5W_vs_E10	ordinary_muscle_development	ENSGALG00010015410	HTR2B	-1.791771094	0.221059258	-8.105388152	5.25774E-16	1.73998E-15	negative
PRJNA729271	stage_5W_vs_E10	ordinary_muscle_development	ENSGALG00010015413	CARNMT1	0.797550604	0.10602704	7.522143448	5.38854E-14	1.60747E-13	positive
PRJNA729271	stage_5W_vs_E10	ordinary_muscle_development	ENSGALG00010015416	CAPN6	2.126077138	0.162384442	13.09286232	3.61715E-39	3.06254E-38	positive
PRJNA729271	stage_5W_vs_E10	ordinary_muscle_development	ENSGALG00010015422	ENSGALG00010015422	-1.167206766	0.529808821	-2.203071599	0.027589703	0.035924608	negative
PRJNA729271	stage_5W_vs_E10	ordinary_muscle_development	ENSGALG00010015430	HMGCL	0.816359033	0.130656137	6.248149172	4.15345E-10	9.92814E-10	positive
PRJNA729271	stage_5W_vs_E10	ordinary_muscle_development	ENSGALG00010015431	RIN3	-0.168933049	0.157982831	-1.069312715	0.284928783	0.322609179	negative
PRJNA729271	stage_5W_vs_E10	ordinary_muscle_development	ENSGALG00010015436	SLC30A9	-0.210325549	0.099117731	-2.121977029	0.033839667	0.043643204	negative
PRJNA729271	stage_5W_vs_E10	ordinary_muscle_development	ENSGALG00010015440	ENSGALG00010015440	0.565149688	0.384984523	1.46798028	0.14210958	0.16910585	positive
PRJNA729271	stage_5W_vs_E10	ordinary_muscle_development	ENSGALG00010015443	PAK3	-0.141509533	0.101893307	-1.388801067	0.164893251	0.194762685	negative
PRJNA729271	stage_5W_vs_E10	ordinary_muscle_development	ENSGALG00010015449	HDX	-0.975120207	0.094750258	-10.29147813	7.69854E-25	3.80205E-24	negative
PRJNA729271	stage_5W_vs_E10	ordinary_muscle_development	ENSGALG00010015466	NMRK1	0.489936396	0.125147482	3.914872184	9.04521E-05	0.000149669	positive
PRJNA729271	stage_5W_vs_E10	ordinary_muscle_development	ENSGALG00010015469	TMEM33	-0.204915684	0.073579518	-2.784955522	0.005353507	0.007532428	negative
PRJNA729271	stage_5W_vs_E10	ordinary_muscle_development	ENSGALG00010015472	LGMN	0.638151432	0.134137283	4.757450098	1.96054E-06	3.67918E-06	positive
PRJNA729271	stage_5W_vs_E10	ordinary_muscle_development	ENSGALG00010015477	ARMC9	-1.385202862	0.107857292	-12.84292265	9.4248E-38	7.6448E-37	negative
PRJNA729271	stage_5W_vs_E10	ordinary_muscle_development	ENSGALG00010015478	FBXO7	-0.02341321	0.063021223	-0.371513104	0.710255398	0.741604538	negative
PRJNA729271	stage_5W_vs_E10	ordinary_muscle_development	ENSGALG00010015481	GOLGA5	0.042591032	0.071896305	0.59239529	0.553585919	0.593332439	positive
PRJNA729271	stage_5W_vs_E10	ordinary_muscle_development	ENSGALG00010015483	SGCD	1.70847499	0.1621582	10.53585319	5.90454E-26	3.04145E-25	positive
PRJNA729271	stage_5W_vs_E10	ordinary_muscle_development	ENSGALG00010015485	BCAS1	0.879752857	0.189588152	4.640336693	3.47842E-06	6.41064E-06	positive
PRJNA729271	stage_5W_vs_E10	ordinary_muscle_development	ENSGALG00010015486	MTFR1L	1.912527142	0.118812787	16.09698073	2.6786E-58	4.10861E-57	positive
PRJNA729271	stage_5W_vs_E10	ordinary_muscle_development	ENSGALG00010015487	RPS6KA6	-0.136989189	0.118436546	-1.156646265	0.247416912	0.283233939	negative
PRJNA729271	stage_5W_vs_E10	ordinary_muscle_development	ENSGALG00010015490	RANBP2	0.298255435	0.082907094	3.597465782	0.000321333	0.000507321	positive
PRJNA729271	stage_5W_vs_E10	ordinary_muscle_development	ENSGALG00010015493	OSTF1	-0.027148862	0.141503662	-0.191859783	0.84785204	0.866319986	negative
PRJNA729271	stage_5W_vs_E10	ordinary_muscle_development	ENSGALG00010015494	CHRDL1	-0.592310826	0.24016539	-2.466262214	0.013653135	0.018406081	negative
PRJNA729271	stage_5W_vs_E10	ordinary_muscle_development	ENSGALG00010015498	MID2	0.88385891	0.134778983	6.557839304	5.4593E-11	1.38271E-10	positive
PRJNA729271	stage_5W_vs_E10	ordinary_muscle_development	ENSGALG00010015502	BPIFB6	0.520250541	1.451883018	0.35832814	0.720097762	0.750598091	positive
PRJNA729271	stage_5W_vs_E10	ordinary_muscle_development	ENSGALG00010015505	CHGA	-1.549387681	0.513665912	-3.016333468	0.002558517	0.003726832	negative
PRJNA729271	stage_5W_vs_E10	ordinary_muscle_development	ENSGALG00010015507	AMMECR1	-1.179241345	0.072261168	-16.31915693	7.21248E-60	1.14758E-58	negative
PRJNA729271	stage_5W_vs_E10	ordinary_muscle_development	ENSGALG00010015508	FUCA1	0.710866316	0.10465997	6.79215093	1.10474E-11	2.91786E-11	positive
PRJNA729271	stage_5W_vs_E10	ordinary_muscle_development	ENSGALG00010015511	BPIFB4	-3.544732697	0.644354608	-5.501214165	3.77185E-08	8.00365E-08	negative
PRJNA729271	stage_5W_vs_E10	ordinary_muscle_development	ENSGALG00010015516	SELENON	-0.758157054	0.113214029	-6.696670541	2.13222E-11	5.53879E-11	negative
PRJNA729271	stage_5W_vs_E10	ordinary_muscle_development	ENSGALG00010015520	UBE3A	0.949370348	0.078252832	12.13208933	7.14041E-34	5.04141E-33	positive
PRJNA729271	stage_5W_vs_E10	ordinary_muscle_development	ENSGALG00010015521	DKC1	-1.362108066	0.082794264	-16.45172026	8.15196E-61	1.33517E-59	negative
PRJNA729271	stage_5W_vs_E10	ordinary_muscle_development	ENSGALG00010015523	MRPL22	-0.239950225	0.145652311	-1.6474179	0.099472179	0.120859114	negative
PRJNA729271	stage_5W_vs_E10	ordinary_muscle_development	ENSGALG00010015532	NXT2	-1.063155585	0.112219632	-9.473882271	2.69632E-21	1.14278E-20	negative
PRJNA729271	stage_5W_vs_E10	ordinary_muscle_development	ENSGALG00010015533	PSAT1	-1.725622384	0.279074883	-6.18336686	6.27487E-10	1.48472E-09	negative
PRJNA729271	stage_5W_vs_E10	ordinary_muscle_development	ENSGALG00010015540	ACSL4	0.830576925	0.151967772	5.465480689	4.61654E-08	9.75036E-08	positive
PRJNA729271	stage_5W_vs_E10	ordinary_muscle_development	ENSGALG00010015541	RASEF	-1.16347629	0.332957003	-3.494373985	0.000475175	0.00073894	negative
PRJNA729271	stage_5W_vs_E10	ordinary_muscle_development	ENSGALG00010015542	CNGA3	-2.413317761	0.341026523	-7.076627772	1.47705E-12	4.08272E-12	negative
PRJNA729271	stage_5W_vs_E10	ordinary_muscle_development	ENSGALG00010015544	RTCB	-0.005127915	0.073352473	-0.069907861	0.944266996	0.951686143	negative
PRJNA729271	stage_5W_vs_E10	ordinary_muscle_development	ENSGALG00010015551	VWA3B	-1.884688952	0.498984603	-3.77704831	0.000158698	0.000256848	negative
PRJNA729271	stage_5W_vs_E10	ordinary_muscle_development	ENSGALG00010015553	ASCL4	-4.55441405	0.862016595	-5.283441269	1.2678E-07	2.59925E-07	negative
PRJNA729271	stage_5W_vs_E10	ordinary_muscle_development	ENSGALG00010015555	PNRC2	-0.178471807	0.102383903	-1.74316276	0.081305188	0.099874467	negative
PRJNA729271	stage_5W_vs_E10	ordinary_muscle_development	ENSGALG00010015557	IRS4	0.585948727	0.192178401	3.048983264	0.002296173	0.003355482	positive
PRJNA729271	stage_5W_vs_E10	ordinary_muscle_development	ENSGALG00010015558	MPP1	0.924269899	0.259479085	3.56202081	0.000368011	0.000578121	positive
PRJNA729271	stage_5W_vs_E10	ordinary_muscle_development	ENSGALG00010015562	IRF9	2.406330044	0.268151463	8.973771829	2.8653E-19	1.10724E-18	positive
PRJNA729271	stage_5W_vs_E10	ordinary_muscle_development	ENSGALG00010015563	B3GNT7	-2.132640828	0.321732855	-6.628607531	3.38868E-11	8.70903E-11	negative
PRJNA729271	stage_5W_vs_E10	ordinary_muscle_development	ENSGALG00010015564	PKD2L2	-1.315354082	0.324281178	-4.056214706	4.98744E-05	8.41692E-05	negative
PRJNA729271	stage_5W_vs_E10	ordinary_muscle_development	ENSGALG00010015565	ENSGALG00010015565	-0.188818164	0.452426966	-0.41734507	0.676426027	0.710029348	negative
PRJNA729271	stage_5W_vs_E10	ordinary_muscle_development	ENSGALG00010015567	ENSGALG00010015567	5.842685009	0.422193284	13.83888668	1.48489E-43	1.44841E-42	positive
PRJNA729271	stage_5W_vs_E10	ordinary_muscle_development	ENSGALG00010015570	LIMCH1	1.303675273	0.127837065	10.19794437	2.02513E-24	9.80438E-24	positive
PRJNA729271	stage_5W_vs_E10	ordinary_muscle_development	ENSGALG00010015571	DUSP15	0.425996471	0.304582287	1.39862523	0.161925396	0.191381981	positive
PRJNA729271	stage_5W_vs_E10	ordinary_muscle_development	ENSGALG00010015573	MAN1C1	1.537341938	0.122408078	12.5591543	3.54097E-36	2.73887E-35	positive
PRJNA729271	stage_5W_vs_E10	ordinary_muscle_development	ENSGALG00010015574	C14orf132	0.593252785	0.254539866	2.330687108	0.019769864	0.026141597	positive
PRJNA729271	stage_5W_vs_E10	ordinary_muscle_development	ENSGALG00010015580	GEMIN5	-0.354337495	0.071770544	-4.937088029	7.92977E-07	1.53563E-06	negative
PRJNA729271	stage_5W_vs_E10	ordinary_muscle_development	ENSGALG00010015583	PRDM4	1.233521469	0.134296196	9.185081229	4.11217E-20	1.64976E-19	positive
PRJNA729271	stage_5W_vs_E10	ordinary_muscle_development	ENSGALG00010015584	TTLL9	1.888335282	0.234665924	8.04690877	8.49114E-16	2.78334E-15	positive
PRJNA729271	stage_5W_vs_E10	ordinary_muscle_development	ENSGALG00010015585	BDKRB1	2.542891031	0.294926522	8.622117166	6.57274E-18	2.39316E-17	positive
PRJNA729271	stage_5W_vs_E10	ordinary_muscle_development	ENSGALG00010015588	BDKRB2	0.520415766	0.431623273	1.205717577	0.227926406	0.262393237	positive
PRJNA729271	stage_5W_vs_E10	ordinary_muscle_development	ENSGALG00010015589	FRMD3	0.648814678	0.179728624	3.609968545	0.000306234	0.000484222	positive
PRJNA729271	stage_5W_vs_E10	ordinary_muscle_development	ENSGALG00010015596	UCHL1	-2.430398204	0.504242351	-4.819900983	1.43629E-06	2.7243E-06	negative
PRJNA729271	stage_5W_vs_E10	ordinary_muscle_development	ENSGALG00010015605	LDLRAP1	-0.186458606	0.058584383	-3.182735674	0.001458907	0.002175155	negative
PRJNA729271	stage_5W_vs_E10	ordinary_muscle_development	ENSGALG00010015606	TMEM131	0.374791405	0.10853016	3.453338716	0.000553693	0.000857547	positive
PRJNA729271	stage_5W_vs_E10	ordinary_muscle_development	ENSGALG00010015610	ENSGALG00010015610	1.185235093	0.286593556	4.135595756	3.54035E-05	6.03521E-05	positive
PRJNA729271	stage_5W_vs_E10	ordinary_muscle_development	ENSGALG00010015613	PWP1	-0.789300681	0.081793267	-9.649946848	4.91812E-22	2.15894E-21	negative
PRJNA729271	stage_5W_vs_E10	ordinary_muscle_development	ENSGALG00010015614	ATG2B	1.004377278	0.106945463	9.391490324	5.91572E-21	2.46545E-20	positive
PRJNA729271	stage_5W_vs_E10	ordinary_muscle_development	ENSGALG00010015616	NADK2	-0.423731434	0.118597681	-3.572847555	0.00035312	0.000555571	negative
PRJNA729271	stage_5W_vs_E10	ordinary_muscle_development	ENSGALG00010015617	SRSF10	-0.250740255	0.056282085	-4.455063353	8.38684E-06	1.50231E-05	negative
PRJNA729271	stage_5W_vs_E10	ordinary_muscle_development	ENSGALG00010015618	NCL	-1.405765352	0.101189695	-13.8923766	7.04581E-44	6.9334E-43	negative
PRJNA729271	stage_5W_vs_E10	ordinary_muscle_development	ENSGALG00010015620	PDRG1	1.291850148	0.143475244	9.003993381	2.17653E-19	8.44907E-19	positive
PRJNA729271	stage_5W_vs_E10	ordinary_muscle_development	ENSGALG00010015621	IDNK	0.739228885	0.152805829	4.837700835	1.3135E-06	2.49857E-06	positive
PRJNA729271	stage_5W_vs_E10	ordinary_muscle_development	ENSGALG00010015624	ITGA1	1.6859426	0.129022901	13.0670027	5.08291E-39	4.27608E-38	positive
PRJNA729271	stage_5W_vs_E10	ordinary_muscle_development	ENSGALG00010015626	XKR7	-0.677485257	0.577407201	-1.173323187	0.240666227	0.275963687	negative
PRJNA729271	stage_5W_vs_E10	ordinary_muscle_development	ENSGALG00010015628	UBQLN1	0.551314705	0.118480548	4.653208584	3.26809E-06	6.03991E-06	positive
PRJNA729271	stage_5W_vs_E10	ordinary_muscle_development	ENSGALG00010015631	GSKIP	-0.304773893	0.106657107	-2.857511331	0.004269774	0.00607351	negative
PRJNA729271	stage_5W_vs_E10	ordinary_muscle_development	ENSGALG00010015632	COL4A5	0.086013406	0.161878856	0.531344288	0.595180221	0.633416835	positive
PRJNA729271	stage_5W_vs_E10	ordinary_muscle_development	ENSGALG00010015633	CCM2L	1.132116875	0.18445049	6.13778187	8.36817E-10	1.96114E-09	positive
PRJNA729271	stage_5W_vs_E10	ordinary_muscle_development	ENSGALG00010015634	ENSGALG00010015634	-1.697966035	1.15368831	-1.471771899	0.141082484	0.167966375	negative
PRJNA729271	stage_5W_vs_E10	ordinary_muscle_development	ENSGALG00010015635	ABTB3	-2.568658923	0.133080021	-19.3016119	5.20597E-83	1.50921E-81	negative
PRJNA729271	stage_5W_vs_E10	ordinary_muscle_development	ENSGALG00010015636	APBB2	0.676625559	0.124160957	5.449583965	5.04878E-08	1.06323E-07	positive
PRJNA729271	stage_5W_vs_E10	ordinary_muscle_development	ENSGALG00010015642	ENSGALG00010015642	-3.278669067	1.026010919	-3.195549878	0.001395647	0.002084912	negative
PRJNA729271	stage_5W_vs_E10	ordinary_muscle_development	ENSGALG00010015643	ENSGALG00010015643	-1.366883778	0.120771429	-11.31793989	1.06956E-29	6.39573E-29	negative
PRJNA729271	stage_5W_vs_E10	ordinary_muscle_development	ENSGALG00010015645	MACO1	0.382198802	0.094255971	4.054902794	5.01552E-05	8.46135E-05	positive
PRJNA729271	stage_5W_vs_E10	ordinary_muscle_development	ENSGALG00010015647	PDZD2	0.226243432	0.136713698	1.654870248	0.09795082	0.119080529	positive
PRJNA729271	stage_5W_vs_E10	ordinary_muscle_development	ENSGALG00010015650	ENSGALG00010015650	-0.994999057	1.295762964	-0.767886631	0.442554541	0.484467039	negative
PRJNA729271	stage_5W_vs_E10	ordinary_muscle_development	ENSGALG00010015653	HCK	2.285928177	0.518292632	4.410497149	1.03134E-05	1.83629E-05	positive
PRJNA729271	stage_5W_vs_E10	ordinary_muscle_development	ENSGALG00010015655	ENSGALG00010015655	6.651176137	0.360089908	18.47087626	3.54287E-76	8.90014E-75	positive
PRJNA729271	stage_5W_vs_E10	ordinary_muscle_development	ENSGALG00010015657	NMUR1	-4.175002032	1.196859156	-3.488298528	0.000486105	0.000755127	negative
PRJNA729271	stage_5W_vs_E10	ordinary_muscle_development	ENSGALG00010015660	KLHL3	-0.041429856	0.126384052	-0.327809205	0.743055927	0.771698478	negative
PRJNA729271	stage_5W_vs_E10	ordinary_muscle_development	ENSGALG00010015668	ZNF217	0.649850908	0.134887436	4.81772748	1.45203E-06	2.75197E-06	positive
PRJNA729271	stage_5W_vs_E10	ordinary_muscle_development	ENSGALG00010015669	INPP4A	0.733973237	0.13018496	5.637926521	1.7211E-08	3.7436E-08	positive
PRJNA729271	stage_5W_vs_E10	ordinary_muscle_development	ENSGALG00010015670	ENSGALG00010015670	-1.856094323	0.132260075	-14.03367057	9.69983E-45	9.77741E-44	negative
PRJNA729271	stage_5W_vs_E10	ordinary_muscle_development	ENSGALG00010015674	RFKL	1.527436473	0.193179939	7.906806874	2.64075E-15	8.43306E-15	positive
PRJNA729271	stage_5W_vs_E10	ordinary_muscle_development	ENSGALG00010015676	TGM2	2.697240085	0.192200038	14.03350447	9.72259E-45	9.79353E-44	positive
PRJNA729271	stage_5W_vs_E10	ordinary_muscle_development	ENSGALG00010015677	GRHL3	-3.21485264	0.276982674	-11.60669221	3.81074E-31	2.4216E-30	negative
PRJNA729271	stage_5W_vs_E10	ordinary_muscle_development	ENSGALG00010015680	ENSGALG00010015680	-0.611451082	0.105016605	-5.822422873	5.80006E-09	1.29801E-08	negative
PRJNA729271	stage_5W_vs_E10	ordinary_muscle_development	ENSGALG00010015684	PDE6D	-0.867679449	0.101356442	-8.560673944	1.1221E-17	4.03893E-17	negative
PRJNA729271	stage_5W_vs_E10	ordinary_muscle_development	ENSGALG00010015686	CNOT8	-1.003283788	0.05513264	-18.19763744	5.3882E-74	1.26995E-72	negative
PRJNA729271	stage_5W_vs_E10	ordinary_muscle_development	ENSGALG00010015691	EXOC3L4	-1.231244745	0.826324076	-1.490026469	0.136217276	0.162574675	negative
PRJNA729271	stage_5W_vs_E10	ordinary_muscle_development	ENSGALG00010015693	COA5	0.050615247	0.108020773	0.468569565	0.639377336	0.67475606	positive
PRJNA729271	stage_5W_vs_E10	ordinary_muscle_development	ENSGALG00010015695	ASAH1	1.096577565	0.093818435	11.68829525	1.46294E-31	9.42456E-31	positive
PRJNA729271	stage_5W_vs_E10	ordinary_muscle_development	ENSGALG00010015697	FAXDC2	0.643856834	0.346222089	1.859664229	0.062933046	0.078377496	positive
PRJNA729271	stage_5W_vs_E10	ordinary_muscle_development	ENSGALG00010015698	TNFAIP2	0.506245588	0.200337366	2.526965372	0.011505285	0.015653191	positive
PRJNA729271	stage_5W_vs_E10	ordinary_muscle_development	ENSGALG00010015701	UNC50	-0.2713993	0.088199646	-3.077101908	0.002090238	0.0030666	negative
PRJNA729271	stage_5W_vs_E10	ordinary_muscle_development	ENSGALG00010015703	GK5	-0.158899928	0.189710739	-0.837590583	0.402260683	0.443606863	negative
PRJNA729271	stage_5W_vs_E10	ordinary_muscle_development	ENSGALG00010015705	NPY6R	-5.634164471	1.176881841	-4.787366307	1.68984E-06	3.18728E-06	negative
PRJNA729271	stage_5W_vs_E10	ordinary_muscle_development	ENSGALG00010015706	ENSGALG00010015706	1.52928931	0.161717351	9.456556787	3.1825E-21	1.34256E-20	positive

PRJNA729271	stage_5W_vs_E10	ordinary_muscle_development	ENSGALG00010015709	HNRNPA0	-1.980382737	0.114876312	-17.23926112	1.34748E-66	2.61119E-65	negative
PRJNA729271	stage_5W_vs_E10	ordinary_muscle_development	ENSGALG00010015711	LBFABP	1.007497173	0.680816469	1.479836665	0.138916841	0.165578457	positive
PRJNA729271	stage_5W_vs_E10	ordinary_muscle_development	ENSGALG00010015716	MGAT4A	0.388813776	0.131776414	2.950556666	0.003172019	0.004574056	positive
PRJNA729271	stage_5W_vs_E10	ordinary_muscle_development	ENSGALG00010015719	XRN1	0.443918898	0.099396206	4.466155353	7.96378E-06	1.429E-05	positive
PRJNA729271	stage_5W_vs_E10	ordinary_muscle_development	ENSGALG00010015729	RHCE	0.047273543	0.198423071	0.238246201	0.811690146	0.83366036	positive
PRJNA729271	stage_5W_vs_E10	ordinary_muscle_development	ENSGALG00010015731	PHF6	-1.61101676	0.071113592	-22.65413291	1.2702E-113	6.8444E-112	negative
PRJNA729271	stage_5W_vs_E10	ordinary_muscle_development	ENSGALG00010015733	COL4A6	4.72192E-05	0.152902417	0.000308819	0.999753598	0.999753598	positive
PRJNA729271	stage_5W_vs_E10	ordinary_muscle_development	ENSGALG00010015734	TSHZ2	-1.630707642	0.168278832	-9.690509648	3.30873E-22	1.46309E-21	negative
PRJNA729271	stage_5W_vs_E10	ordinary_muscle_development	ENSGALG00010015738	CRACDL	-2.983366522	0.438207842	-6.808108477	9.88903E-12	2.61907E-11	negative
PRJNA729271	stage_5W_vs_E10	ordinary_muscle_development	ENSGALG00010015742	HPRT1	1.251188195	0.085824124	14.57851409	3.84801E-48	4.32715E-47	positive
PRJNA729271	stage_5W_vs_E10	ordinary_muscle_development	ENSGALG00010015746	PAPOLB	-0.111947806	0.072198552	-1.550554728	0.121008429	0.145320395	negative
PRJNA729271	stage_5W_vs_E10	ordinary_muscle_development	ENSGALG00010015749	ATR	-1.148015493	0.134925652	-8.508504342	1.76191E-17	6.27646E-17	negative
PRJNA729271	stage_5W_vs_E10	ordinary_muscle_development	ENSGALG00010015751	MYOM3	-3.266930116	0.541588206	-6.032129355	1.61813E-09	3.73306E-09	negative
PRJNA729271	stage_5W_vs_E10	ordinary_muscle_development	ENSGALG00010015753	RPRD1B	-0.541170622	0.078922132	-6.857019795	7.03119E-12	1.87451E-11	negative
PRJNA729271	stage_5W_vs_E10	ordinary_muscle_development	ENSGALG00010015756	PCM1	-0.297457464	0.103360242	-2.877871213	0.004003686	0.005714763	negative
PRJNA729271	stage_5W_vs_E10	ordinary_muscle_development	ENSGALG00010015758	PCDH19	0.599277005	0.169757966	3.530184873	0.000415269	0.000648774	positive
PRJNA729271	stage_5W_vs_E10	ordinary_muscle_development	ENSGALG00010015759	EIF5	0.725897559	0.091571338	7.927126296	2.24275E-15	7.19537E-15	positive
PRJNA729271	stage_5W_vs_E10	ordinary_muscle_development	ENSGALG00010015764	TTI1	0.211913072	0.097404089	2.175607562	0.029584617	0.038401453	positive
PRJNA729271	stage_5W_vs_E10	ordinary_muscle_development	ENSGALG00010015765	ENSGALG00010015765	0.79378485	0.371687132	2.135626393	0.032709878	0.042250017	positive
PRJNA729271	stage_5W_vs_E10	ordinary_muscle_development	ENSGALG00010015766	TLE1	-0.47447131	0.100664728	-4.713381927	2.43639E-06	4.5416E-06	negative
PRJNA729271	stage_5W_vs_E10	ordinary_muscle_development	ENSGALG00010015769	TMEM50A	-0.138506242	0.100352775	-1.380193449	0.167527091	0.197618803	negative
PRJNA729271	stage_5W_vs_E10	ordinary_muscle_development	ENSGALG00010015774	PLS1	-2.777527022	0.412495536	-6.73347171	1.65662E-11	4.3352E-11	negative
PRJNA729271	stage_5W_vs_E10	ordinary_muscle_development	ENSGALG00010015776	SPOCK1	-6.179142986	0.273371801	-22.60343954	4.009E-113	2.1443E-111	negative
PRJNA729271	stage_5W_vs_E10	ordinary_muscle_development	ENSGALG00010015778	PABIR3	-1.618682538	0.088281948	-18.33537395	4.32035E-75	1.05073E-73	negative
PRJNA729271	stage_5W_vs_E10	ordinary_muscle_development	ENSGALG00010015782	ATG4A	0.312529316	0.082459558	3.790092057	0.000150591	0.000244109	positive
PRJNA729271	stage_5W_vs_E10	ordinary_muscle_development	ENSGALG00010015785	FGL1	1.966210267	0.190704876	10.31022547	6.33525E-25	3.13947E-24	positive
PRJNA729271	stage_5W_vs_E10	ordinary_muscle_development	ENSGALG00010015788	TRPC1	-0.034472682	0.128368624	-0.268544457	0.78828026	0.813652522	negative
PRJNA729271	stage_5W_vs_E10	ordinary_muscle_development	ENSGALG00010015792	MARK3	0.258009417	0.081562926	3.163317331	0.001559822	0.002319173	positive
PRJNA729271	stage_5W_vs_E10	ordinary_muscle_development	ENSGALG00010015793	RSRP1	2.221550503	0.232784314	9.543385757	1.38244E-21	5.93379E-21	positive
PRJNA729271	stage_5W_vs_E10	ordinary_muscle_development	ENSGALG00010015795	PSMD10	-0.383112859	0.135038108	-2.837072168	0.004552932	0.006453623	negative
PRJNA729271	stage_5W_vs_E10	ordinary_muscle_development	ENSGALG00010015796	LARP1	0.29798853	0.141036934	2.112840387	0.034614432	0.044590845	positive
PRJNA729271	stage_5W_vs_E10	ordinary_muscle_development	ENSGALG00010015797	PIP5K1B	-1.068240684	0.166860169	-6.402011283	1.53343E-10	3.76986E-10	negative
PRJNA729271	stage_5W_vs_E10	ordinary_muscle_development	ENSGALG00010015798	IL22RA1	-1.446231994	0.281892445	-5.130438999	2.89067E-07	5.75949E-07	negative
PRJNA729271	stage_5W_vs_E10	ordinary_muscle_development	ENSGALG00010015800	BVES	2.859998444	0.181449826	15.76192439	5.68744E-56	8.11412E-55	positive
PRJNA729271	stage_5W_vs_E10	ordinary_muscle_development	ENSGALG00010015801	VSTM2L	-3.064151218	0.313860855	-9.762769625	1.62651E-22	7.27889E-22	negative
PRJNA729271	stage_5W_vs_E10	ordinary_muscle_development	ENSGALG00010015802	TRPC7	-3.101287079	0.936180487	-3.312702113	0.000923993	0.001404939	negative
PRJNA729271	stage_5W_vs_E10	ordinary_muscle_development	ENSGALG00010015803	SYF2	0.516907943	0.159840144	3.233905639	0.001221098	0.001833038	positive
PRJNA729271	stage_5W_vs_E10	ordinary_muscle_development	ENSGALG00010015804	CKB	-2.450127808	0.160833547	-15.23393502	2.1053E-52	2.73443E-51	negative
PRJNA729271	stage_5W_vs_E10	ordinary_muscle_development	ENSGALG00010015807	ENSGALG00010015807	-0.030650805	0.942012309	-0.032537584	0.974043344	0.977753343	negative
PRJNA729271	stage_5W_vs_E10	ordinary_muscle_development	ENSGALG00010015809	PCOLCE2	4.166827326	0.23193199	17.96572922	3.61496E-72	8.13647E-71	positive
PRJNA729271	stage_5W_vs_E10	ordinary_muscle_development	ENSGALG00010015810	LIPT1	-0.412766122	0.106901889	-3.861167725	0.000112846	0.000184909	negative
PRJNA729271	stage_5W_vs_E10	ordinary_muscle_development	ENSGALG00010015811	CTNNBL1	-0.59726204	0.056437374	-10.5827398	3.58324E-26	1.86563E-25	negative
PRJNA729271	stage_5W_vs_E10	ordinary_muscle_development	ENSGALG00010015813	ENSGALG00010015813	1.086140779	0.138757918	7.82759493	4.97291E-15	1.56259E-14	positive
PRJNA729271	stage_5W_vs_E10	ordinary_muscle_development	ENSGALG00010015814	PAQR9	1.467435642	0.417499999	3.514815918	0.000440059	0.000686172	positive
PRJNA729271	stage_5W_vs_E10	ordinary_muscle_development	ENSGALG00010015816	MITD1	-0.003228326	0.21397083	-0.015087692	0.98796222	0.989737534	negative
PRJNA729271	stage_5W_vs_E10	ordinary_muscle_development	ENSGALG00010015820	ENSGALG00010015820	3.187806126	0.51144653	6.232921604	4.57815E-10	1.09199E-09	positive
PRJNA729271	stage_5W_vs_E10	ordinary_muscle_development	ENSGALG00010015822	WFDC2	-1.735979006	0.609253452	-2.849354404	0.004380805	0.006223637	negative
PRJNA729271	stage_5W_vs_E10	ordinary_muscle_development	ENSGALG00010015825	RUNX3	-1.718060494	0.482306103	-3.562178629	0.00036779	0.000577836	negative
PRJNA729271	stage_5W_vs_E10	ordinary_muscle_development	ENSGALG00010015827	ENSGALG00010015827	1.933842435	0.148129109	13.05511418	5.94209E-39	4.97865E-38	positive
PRJNA729271	stage_5W_vs_E10	ordinary_muscle_development	ENSGALG00010015829	SMAD5	-0.378029927	0.048077264	-7.86296664	3.75141E-15	1.18856E-14	negative
PRJNA729271	stage_5W_vs_E10	ordinary_muscle_development	ENSGALG00010015831	LYG2	3.88058695	0.623942889	6.219458573	4.98873E-10	1.18777E-09	positive
PRJNA729271	stage_5W_vs_E10	ordinary_muscle_development	ENSGALG00010015832	RAB39B	-1.541144278	0.087250552	-17.66343294	8.02095E-70	1.68988E-68	negative
PRJNA729271	stage_5W_vs_E10	ordinary_muscle_development	ENSGALG00010015834	STARD8	0.143983502	0.133679793	1.077077537	0.281445628	0.31903921	positive
PRJNA729271	stage_5W_vs_E10	ordinary_muscle_development	ENSGALG00010015837	GDA	3.511454844	0.157935738	22.23344058	1.6314E-109	8.1824E-108	positive
PRJNA729271	stage_5W_vs_E10	ordinary_muscle_development	ENSGALG00010015838	IFNLR1	-0.633417515	1.269915776	-0.498787027	0.617929428	0.654647497	negative
PRJNA729271	stage_5W_vs_E10	ordinary_muscle_development	ENSGALG00010015839	U2SURP	-0.487217859	0.071543172	-6.810123766	9.75148E-12	2.58358E-11	negative
PRJNA729271	stage_5W_vs_E10	ordinary_muscle_development	ENSGALG00010015840	MTUS1	1.768685276	0.145949667	12.11846048	8.43283E-34	5.92218E-33	positive
PRJNA729271	stage_5W_vs_E10	ordinary_muscle_development	ENSGALG00010015843	ENSGALG00010015843	-2.945647381	0.569389206	-5.173346017	2.29939E-07	4.62141E-07	negative
PRJNA729271	stage_5W_vs_E10	ordinary_muscle_development	ENSGALG00010015845	SAP30L	1.671717552	0.125167591	13.35583392	1.09525E-40	9.77562E-40	positive
PRJNA729271	stage_5W_vs_E10	ordinary_muscle_development	ENSGALG00010015848	UBE2B	2.157813384	0.161270416	13.38009436	7.9047E-41	7.07712E-40	positive
PRJNA729271	stage_5W_vs_E10	ordinary_muscle_development	ENSGALG00010015852	ZFAND5	1.582085318	0.285804564	5.535549523	3.10254E-08	6.62219E-08	positive
PRJNA729271	stage_5W_vs_E10	ordinary_muscle_development	ENSGALG00010015855	NIPAL3	-1.055223577	0.082055152	-12.85993087	7.56443E-38	6.15991E-37	negative
PRJNA729271	stage_5W_vs_E10	ordinary_muscle_development	ENSGALG00010015856	TXNDC9	0.648189134	0.133149444	4.8681325	1.12658E-06	2.1529E-06	positive
PRJNA729271	stage_5W_vs_E10	ordinary_muscle_development	ENSGALG00010015858	CHST2	-0.634188602	0.091889496	-6.901644143	5.14041E-12	1.3788E-11	negative
PRJNA729271	stage_5W_vs_E10	ordinary_muscle_development	ENSGALG00010015861	PPP2CA	-1.104254195	0.068058735	-16.22501803	3.35619E-59	5.25924E-58	negative
PRJNA729271	stage_5W_vs_E10	ordinary_muscle_development	ENSGALG00010015862	PDGFRL	0.691796389	0.209119737	3.308135325	0.000939194	0.001427752	positive
PRJNA729271	stage_5W_vs_E10	ordinary_muscle_development	ENSGALG00010015863	ENSGALG00010015863	-3.284848742	0.595730759	-5.513982097	3.50804E-08	7.46025E-08	negative
PRJNA729271	stage_5W_vs_E10	ordinary_muscle_development	ENSGALG00010015865	VRK1	-1.960093258	0.143951482	-13.61634647	3.2019E-42	2.99043E-41	negative
PRJNA729271	stage_5W_vs_E10	ordinary_muscle_development	ENSGALG00010015866	ARHGEF6	0.746083109	0.070514578	10.58055126	3.66794E-26	1.90835E-25	positive
PRJNA729271	stage_5W_vs_E10	ordinary_muscle_development	ENSGALG00010015867	PICALML	0.098528974	0.056842066	1.733381302	0.083027919	0.101878412	positive
PRJNA729271	stage_5W_vs_E10	ordinary_muscle_development	ENSGALG00010015869	SLC7A2	2.817701218	0.193800656	14.53917276	6.84144E-48	7.64585E-47	positive
PRJNA729271	stage_5W_vs_E10	ordinary_muscle_development	ENSGALG00010015870	EIF5B	0.358988064	0.156099112	2.299744429	0.021462703	0.028248605	positive
PRJNA729271	stage_5W_vs_E10	ordinary_muscle_development	ENSGALG00010015872	TMC1	-2.442964446	0.428224377	-5.704870108	1.16432E-08	2.55981E-08	negative
PRJNA729271	stage_5W_vs_E10	ordinary_muscle_development	ENSGALG00010015874	CLIC4	-0.699579427	0.114663348	-6.101159964	1.05301E-09	2.45551E-09	negative
PRJNA729271	stage_5W_vs_E10	ordinary_muscle_development	ENSGALG00010015875	DNTTIP1	0.995194075	0.139358645	7.141243931	9.249E-13	2.58512E-12	positive
PRJNA729271	stage_5W_vs_E10	ordinary_muscle_development	ENSGALG00010015876	SKP1	-0.450224559	0.077532524	-5.806912167	6.36355E-09	1.42016E-08	negative
PRJNA729271	stage_5W_vs_E10	ordinary_muscle_development	ENSGALG00010015877	REV1	0.308288285	0.104276688	2.956444938	0.003112078	0.004491196	positive
PRJNA729271	stage_5W_vs_E10	ordinary_muscle_development	ENSGALG00010015879	TRMT61A	-1.476021722	0.124780917	-11.82890586	2.76725E-32	1.83997E-31	negative
PRJNA729271	stage_5W_vs_E10	ordinary_muscle_development	ENSGALG00010015881	GAB3	2.366125113	0.174474404	13.56144548	6.77869E-42	6.26241E-41	positive
PRJNA729271	stage_5W_vs_E10	ordinary_muscle_development	ENSGALG00010015886	AFF3	-2.433132209	0.217453684	-11.18919745	4.60583E-29	2.69308E-28	negative
PRJNA729271	stage_5W_vs_E10	ordinary_muscle_development	ENSGALG00010015887	SLC9A9	0.671473592	0.180558776	3.718864329	0.00020012	0.000321448	positive
PRJNA729271	stage_5W_vs_E10	ordinary_muscle_development	ENSGALG00010015889	SH3BP5	1.287904899	0.165049989	7.803120161	6.0395E-15	1.88954E-14	positive
PRJNA729271	stage_5W_vs_E10	ordinary_muscle_development	ENSGALG00010015892	UBE2C	-2.685849455	0.227497746	-11.80604864	3.63234E-32	2.40524E-31	negative
PRJNA729271	stage_5W_vs_E10	ordinary_muscle_development	ENSGALG00010015893	TCF7	-0.368031633	0.418775778	-0.878827412	0.379494861	0.420613091	negative
PRJNA729271	stage_5W_vs_E10	ordinary_muscle_development	ENSGALG00010015894	CEP78	-2.099812885	0.121701517	-17.25379391	1.04787E-66	2.03877E-65	negative
PRJNA729271	stage_5W_vs_E10	ordinary_muscle_development	ENSGALG00010015895	RBMXL1	-1.654444884	0.085737336	-19.29666775	5.72864E-83	1.65742E-81	negative
PRJNA729271	stage_5W_vs_E10	ordinary_muscle_development	ENSGALG00010015896	LONRF2	-0.672902748	0.07936814	-8.47824766	2.28611E-17	8.08618E-17	negative
PRJNA729271	stage_5W_vs_E10	ordinary_muscle_development	ENSGALG00010015898	ENSGALG00010015898	1.46628699	0.218624188	6.706883647	1.98825E-11	5.17594E-11	positive
PRJNA729271	stage_5W_vs_E10	ordinary_muscle_development	ENSGALG00010015899	SMARCA1	-0.717525292	0.116258666	-6.17180049	6.75167E-10	1.59286E-09	negative
PRJNA729271	stage_5W_vs_E10	ordinary_muscle_development	ENSGALG00010015900	ENSGALG00010015900	1.258700243	0.16048896	7.84290861	4.40228E-15	1.3878E-14	positive
PRJNA729271	stage_5W_vs_E10	ordinary_muscle_development	ENSGALG00010015902	ENSGALG00010015902	1.291568742	0.484128077	2.667824494	0.007634413	0.010581451	positive
PRJNA729271	stage_5W_vs_E10	ordinary_muscle_development	ENSGALG00010015904	MTMR7	1.299396997	0.260231975	4.993225743	5.93791E-07	1.15982E-06	positive
PRJNA729271	stage_5W_vs_E10	ordinary_muscle_development	ENSGALG00010015905	ANPEP	0.881803968	0.217437922	4.055428606	5.00425E-05	8.44332E-05	positive
PRJNA729271	stage_5W_vs_E10	ordinary_muscle_development	ENSGALG00010015906	BAG5	0.994462519	0.100701783	9.875321851	5.32617E-23	2.42853E-22	positive
PRJNA729271	stage_5W_vs_E10	ordinary_muscle_development	ENSGALG00010015908	CHRD	1.136678928	0.137011446	8.296233348	1.07464E-16	3.67815E-16	positive
PRJNA729271	stage_5W_vs_E10	ordinary_muscle_development	ENSGALG00010015909	TNNC2	4.221093186	0.106580861	39.60460769	0	0	positive
PRJNA729271	stage_5W_vs_E10	ordinary_muscle_development	ENSGALG00010015910	C5orf15	0.248038355	0.093097503	2.664285798	0.007715198	0.010683206	positive
PRJNA729271	stage_5W_vs_E10	ordinary_muscle_development	ENSGALG00010015911	TM9SF2L	0.436401653	0.078239764	5.577747588	2.43653E-08	5.25245E-08	positive
PRJNA729271	stage_5W_vs_E10	ordinary_muscle_development	ENSGALG00010015912	ENSGALG00010015912	0.135790936	0.270060828	0.502816115	0.615093563	0.651929014	positive
PRJNA729271	stage_5W_vs_E10	ordinary_muscle_development	ENSGALG00010015913	CHST10	-0.818868921	0.105417426	-7.767870595	7.98165E-15	2.4843E-14	negative
PRJNA729271	stage_5W_vs_E10	ordinary_muscle_development	ENSGALG00010015914	SRRM1	-0.584366455	0.11804834	-4.950230159	7.41258E-07	1.43816E-06	negative
PRJNA729271	stage_5W_vs_E10	ordinary_muscle_development	ENSGALG00010015916	COA8	1.656206519	0.144281892	11.47896313	1.68287E-30	1.03801E-29	positive
PRJNA729271	stage_5W_vs_E10	ordinary_muscle_development	ENSGALG00010015917	TMEM63A	1.248130138	0.106373528	11.73346569	8.58693E-32	5.57401E-31	positive
PRJNA729271	stage_5W_vs_E10	ordinary_muscle_development	ENSGALG00010015918	BICD1	-0.831275526	0.190568458	-4.362083511	1.2883E-05	2.27897E-05	negative
PRJNA729271	stage_5W_vs_E10	ordinary_muscle_development	ENSGALG00010015919	ENSGALG00010015919	0.812477689	0.229891372	3.534180873	0.000409041	0.000639595	positive
PRJNA729271	stage_5W_vs_E10	ordinary_muscle_development	ENSGALG00010015921	SNX21	1.909200363	0.109676883	17.40750003	7.23759E-68	1.43123E-66	positive
PRJNA729271	stage_5W_vs_E10	ordinary_muscle_development	ENSGALG00010015923	RCAN3	-0.615402785	0.121492977	-5.065336265	4.0768E-07	8.04208E-07	negative
PRJNA729271	stage_5W_vs_E10	ordinary_muscle_development	ENSGALG00010015927	GLA	2.518004049	0.166207342	15.14977629	7.60249E-52	9.76069E-51	positive
PRJNA729271	stage_5W_vs_E10	ordinary_muscle_development	ENSGALG00010015928	CAPN7	0.478381978	0.122222545	3.914024015	9.07706E-05	0.000150128	positive
PRJNA729271	stage_5W_vs_E10	ordinary_muscle_development	ENSGALG00010015929	SLC6A12	-5.464865929	1.127999654	-4.844740786	1.26777E-06	2.41286E-06	negative
PRJNA729271	stage_5W_vs_E10	ordinary_muscle_development	ENSGALG00010015931	RBM47	2.272063227	0.27271961	8.33113256	8.00726E-17	2.76017E-16	positive
PRJNA729271	stage_5W_vs_E10	ordinary_muscle_development	ENSGALG00010015932	ACOT8	1.768792682	0.142420152	12.41953937	2.04737E-35	1.53368E-34	positive
PRJNA729271	stage_5W_vs_E10	ordinary_muscle_development	ENSGALG00010015933	ENSGALG00010015933	-1.484545907	1.054496012	-1.407825056	0.159182879	0.18827873	negative
PRJNA729271	stage_5W_vs_E10	ordinary_muscle_development	ENSGALG00010015934	TMC2	-1.848933332	1.288779615	-1.434638872	0.151390032	0.179589042	negative
PRJNA729271	stage_5W_vs_E10	ordinary_muscle_development	ENSGALG00010015935	EPHX1	0.196934526	0.112219159	1.754910017	0.0792747	0.097537287	positive
PRJNA729271	stage_5W_vs_E10	ordinary_muscle_development	ENSGALG00010015936	RPL36AL	-0.211318282	0.139600406	-1.513736874	0.130092589	0.15572649	negative
PRJNA729271	stage_5W_vs_E10	ordinary_muscle_development	ENSGALG00010015937	ENSGALG00010015937	0.52111621	0.194068228	2.685221671	0.007248172	0.010069221	positive
PRJNA729271	stage_5W_vs_E10	ordinary_muscle_development	ENSGALG00010015939	SUN2	0.902172155	0.079598392	11.33405004	8.89924E-30	5.33035E-29	positive
PRJNA729271	stage_5W_vs_E10	ordinary_muscle_development	ENSGALG00010015942	AP3S2	-0.677176927	0.132862715	-5.096816884	3.45412E-07	6.8473E-07	negative
PRJNA729271	stage_5W_vs_E10	ordinary_muscle_development	ENSGALG00010015946	TLE4	0.527520572	0.098706733	5.344322052	9.07561E-08	1.87742E-07	positive
PRJNA729271	stage_5W_vs_E10	ordinary_muscle_development	ENSGALG00010015954	CTSA	1.344936588	0.096316892	13.96366261	2.59735E-44	2.58398E-43	positive
PRJNA729271	stage_5W_vs_E10	ordinary_muscle_development	ENSGALG00010015957	ENSGALG00010015957	3.889020172	0.213469224	18.21817729	3.70285E-74	8.81327E-73	positive
PRJNA729271	stage_5W_vs_E10	ordinary_muscle_development	ENSGALG00010015959	TRNAU1AP	1.215678504	0.129638542	9.377446569	6.75897E-21	2.8072E-20	positive
PRJNA729271	stage_5W_vs_E10	ordinary_muscle_development	ENSGALG00010015960	CHRNA9	2.757659731	0.847079524	3.255490957	0.001131965	0.001706305	positive
PRJNA729271	stage_5W_vs_E10	ordinary_muscle_development	ENSGALG00010015963	DIPK2A	-0.478934824	0.109359074	-4.379470357	1.18968E-05	2.10889E-05	negative
PRJNA729271	stage_5W_vs_E10	ordinary_muscle_development	ENSGALG00010015964	KLC1	0.13648692	0.087413625	1.561391839	0.118431326	0.142426124	positive
PRJNA729271	stage_5W_vs_E10	ordinary_muscle_development	ENSGALG00010015967	ZSWIM1	-0.895444072	0.227806819	-3.930716725	8.4693E-05	0.000140557	negative
PRJNA729271	stage_5W_vs_E10	ordinary_muscle_development	ENSGALG00010015970	THPO	0.515331491	0.388161763	1.327620442	0.184303528	0.215842259	positive
PRJNA729271	stage_5W_vs_E10	ordinary_muscle_development	ENSGALG00010015971	ENSGALG00010015971	-1.11643664	0.159450565	-7.001772888	2.52744E-12	6.89409E-12	negative
PRJNA729271	stage_5W_vs_E10	ordinary_muscle_development	ENSGALG00010015972	FSTL4	-2.365247563	0.306679152	-7.712449788	1.23425E-14	3.7999E-14	negative
PRJNA729271	stage_5W_vs_E10	ordinary_muscle_development	ENSGALG00010015974	VPS37A	2.662829285	0.162993015	16.33707604	5.37704E-60	8.58372E-59	positive

PRJNA729271	stage_5W_vs_E10	ordinary_muscle_development	ENSGALG00010015975	BTK	2.642922295	0.17036424	15.51336298	2.81728E-54	3.8165E-53	positive
PRJNA729271	stage_5W_vs_E10	ordinary_muscle_development	ENSGALG00010015979	ENSGALG00010015979	4.165097521	0.202195965	20.59931073	2.78377E-94	1.04458E-92	positive
PRJNA729271	stage_5W_vs_E10	ordinary_muscle_development	ENSGALG00010015980	EXOSC7	-1.304830725	0.094593917	-13.79402364	2.76876E-43	2.67912E-42	negative
PRJNA729271	stage_5W_vs_E10	ordinary_muscle_development	ENSGALG00010015983	CNOT7	-0.561136455	0.06525699	-8.598871215	8.0504E-18	2.91726E-17	negative
PRJNA729271	stage_5W_vs_E10	ordinary_muscle_development	ENSGALG00010015984	PDCL3	0.22970791	0.107991412	2.127094242	0.033412254	0.043107309	positive
PRJNA729271	stage_5W_vs_E10	ordinary_muscle_development	ENSGALG00010015985	TIMM8A	-0.985090538	0.112932576	-8.722820055	2.71357E-18	1.00366E-17	negative
PRJNA729271	stage_5W_vs_E10	ordinary_muscle_development	ENSGALG00010015986	NEURL2	3.20511635	0.230898269	13.88107572	8.24965E-44	8.10154E-43	positive
PRJNA729271	stage_5W_vs_E10	ordinary_muscle_development	ENSGALG00010015987	POLR2H	-0.695323452	0.098261006	-7.076290799	1.48064E-12	4.09187E-12	negative
PRJNA729271	stage_5W_vs_E10	ordinary_muscle_development	ENSGALG00010015989	ENAH	-1.075980276	0.163295108	-6.589176413	4.42273E-11	1.12786E-10	negative
PRJNA729271	stage_5W_vs_E10	ordinary_muscle_development	ENSGALG00010015992	ATP10A	-0.686735285	0.157688296	-4.355017488	1.33056E-05	2.35173E-05	negative
PRJNA729271	stage_5W_vs_E10	ordinary_muscle_development	ENSGALG00010015994	ENSGALG00010015994	-0.108635321	0.109290036	-0.994009377	0.320218302	0.359504631	negative
PRJNA729271	stage_5W_vs_E10	ordinary_muscle_development	ENSGALG00010016004	XRCC3	-0.853953476	0.171724148	-4.972821155	6.59855E-07	1.28418E-06	negative
PRJNA729271	stage_5W_vs_E10	ordinary_muscle_development	ENSGALG00010016006	PLTP	0.184332781	0.317038375	0.581421039	0.560956721	0.600610701	positive
PRJNA729271	stage_5W_vs_E10	ordinary_muscle_development	ENSGALG00010016008	TAF7	-0.860570782	0.097229398	-8.85093189	8.67915E-19	3.28128E-18	negative
PRJNA729271	stage_5W_vs_E10	ordinary_muscle_development	ENSGALG00010016011	ADD1	-0.544326902	0.080886251	-6.729535516	1.70206E-11	4.44768E-11	negative
PRJNA729271	stage_5W_vs_E10	ordinary_muscle_development	ENSGALG00010016015	ZDHHC3	0.779465546	0.082221303	9.480092351	2.54058E-21	1.07804E-20	positive
PRJNA729271	stage_5W_vs_E10	ordinary_muscle_development	ENSGALG00010016016	ZDHHC2	-0.102247077	0.278341319	-0.367344228	0.713362267	0.744219524	negative
PRJNA729271	stage_5W_vs_E10	ordinary_muscle_development	ENSGALG00010016017	RAPGEF6	0.64695761	0.071453462	9.05425144	1.37508E-19	5.39571E-19	positive
PRJNA729271	stage_5W_vs_E10	ordinary_muscle_development	ENSGALG00010016022	EPHB3	-1.929885242	0.169254523	-11.4022669	4.07371E-30	2.47479E-29	negative
PRJNA729271	stage_5W_vs_E10	ordinary_muscle_development	ENSGALG00010016025	PCIF1	0.199496207	0.108125973	1.845035023	0.065032444	0.080858233	positive
PRJNA729271	stage_5W_vs_E10	ordinary_muscle_development	ENSGALG00010016027	COPS2	1.184217041	0.149813052	7.904631989	2.68726E-15	8.57404E-15	positive
PRJNA729271	stage_5W_vs_E10	ordinary_muscle_development	ENSGALG00010016030	TGM4	2.276602582	0.70378071	3.234818102	0.001217202	0.001827758	positive
PRJNA729271	stage_5W_vs_E10	ordinary_muscle_development	ENSGALG00010016040	RPSA2	-0.15854445	0.105951866	-1.496381858	0.134554174	0.160695622	negative
PRJNA729271	stage_5W_vs_E10	ordinary_muscle_development	ENSGALG00010016041	ENSGALG00010016041	0.635735263	0.091050631	6.982217029	2.90558E-12	7.90149E-12	positive
PRJNA729271	stage_5W_vs_E10	ordinary_muscle_development	ENSGALG00010016043	ADAMTSL1	0.183998913	0.192379153	0.956438941	0.338850519	0.378604661	positive
PRJNA729271	stage_5W_vs_E10	ordinary_muscle_development	ENSGALG00010016047	SRP9	0.091526053	0.089056907	1.027725484	0.304078977	0.342712446	positive
PRJNA729271	stage_5W_vs_E10	ordinary_muscle_development	ENSGALG00010016049	ZFYVE21	0.237245511	0.123015397	1.928583876	0.053782542	0.067542709	positive
PRJNA729271	stage_5W_vs_E10	ordinary_muscle_development	ENSGALG00010016053	DRP2	2.099200131	0.464471358	4.519546993	6.19721E-06	1.11964E-05	positive
PRJNA729271	stage_5W_vs_E10	ordinary_muscle_development	ENSGALG00010016057	SOHO1	-3.23250931	0.702022862	-4.604564161	4.13331E-06	7.57329E-06	negative
PRJNA729271	stage_5W_vs_E10	ordinary_muscle_development	ENSGALG00010016059	EPDR1	0.989119413	0.200623405	4.930229416	8.21331E-07	1.58863E-06	positive
PRJNA729271	stage_5W_vs_E10	ordinary_muscle_development	ENSGALG00010016060	ENSGALG00010016060	0.110055996	1.380148761	0.079742126	0.936442355	0.944588165	positive
PRJNA729271	stage_5W_vs_E10	ordinary_muscle_development	ENSGALG00010016066	C14orf2	1.068930542	0.149530523	7.148577567	8.76817E-13	2.45499E-12	positive
PRJNA729271	stage_5W_vs_E10	ordinary_muscle_development	ENSGALG00010016067	AHSG	3.173584283	0.854814967	3.712597935	0.000205143	0.000329369	positive
PRJNA729271	stage_5W_vs_E10	ordinary_muscle_development	ENSGALG00010016069	GALK2	0.576990699	0.144937946	3.980949879	6.86404E-05	0.00011481	positive
PRJNA729271	stage_5W_vs_E10	ordinary_muscle_development	ENSGALG00010016073	SAXO1	4.567465107	0.984357581	4.640046659	3.48331E-06	6.41883E-06	positive
PRJNA729271	stage_5W_vs_E10	ordinary_muscle_development	ENSGALG00010016076	RAB3GAP2	0.149494079	0.090742306	1.647457356	0.099464074	0.120859114	positive
PRJNA729271	stage_5W_vs_E10	ordinary_muscle_development	ENSGALG00010016083	CDH22	-1.58325756	0.428156325	-3.697849281	0.000217434	0.000348332	negative
PRJNA729271	stage_5W_vs_E10	ordinary_muscle_development	ENSGALG00010016086	TDRD9	-2.018879236	0.362676919	-5.56660523	2.5975E-08	5.58368E-08	negative
PRJNA729271	stage_5W_vs_E10	ordinary_muscle_development	ENSGALG00010016087	IARS2	0.232843315	0.090329581	2.577708354	0.00994579	0.013631262	positive
PRJNA729271	stage_5W_vs_E10	ordinary_muscle_development	ENSGALG00010016091	HMX1	-2.640665513	0.842605819	-3.133927458	0.001724835	0.002551953	negative
PRJNA729271	stage_5W_vs_E10	ordinary_muscle_development	ENSGALG00010016094	FGF7	0.242000112	0.185428378	1.305086713	0.191863323	0.223989921	positive
PRJNA729271	stage_5W_vs_E10	ordinary_muscle_development	ENSGALG00010016097	FNIP1	1.310920232	0.110145948	11.90166556	1.16008E-32	7.81749E-32	positive
PRJNA729271	stage_5W_vs_E10	ordinary_muscle_development	ENSGALG00010016100	SFRP4	-1.173506146	0.412846547	-2.842475378	0.004476469	0.006350207	negative
PRJNA729271	stage_5W_vs_E10	ordinary_muscle_development	ENSGALG00010016103	BPNT1	-1.204705034	0.093005553	-12.9530441	2.25819E-38	1.86403E-37	negative
PRJNA729271	stage_5W_vs_E10	ordinary_muscle_development	ENSGALG00010016107	UVSSA	0.079570522	0.0921453	0.863533159	0.387844396	0.429145384	positive
PRJNA729271	stage_5W_vs_E10	ordinary_muscle_development	ENSGALG00010016109	MLH1	-0.140229207	0.10185291	-1.376781543	0.168579799	0.19877688	negative
PRJNA729271	stage_5W_vs_E10	ordinary_muscle_development	ENSGALG00010016111	EPB41	-0.086529697	0.102547062	-0.843804737	0.398778528	0.44006812	negative
PRJNA729271	stage_5W_vs_E10	ordinary_muscle_development	ENSGALG00010016113	DTWD1	-0.229240672	0.149346346	-1.534960029	0.124793647	0.149642944	negative
PRJNA729271	stage_5W_vs_E10	ordinary_muscle_development	ENSGALG00010016114	HAUS6	-1.596265271	0.152206883	-10.48747103	9.86331E-26	5.04121E-25	negative
PRJNA729271	stage_5W_vs_E10	ordinary_muscle_development	ENSGALG00010016115	RD3L	-1.345274468	0.483962054	-2.779710637	0.005440736	0.007650704	negative
PRJNA729271	stage_5W_vs_E10	ordinary_muscle_development	ENSGALG00010016117	ENSGALG00010016117	0.172647807	0.241598363	0.714606693	0.474852108	0.516546439	positive
PRJNA729271	stage_5W_vs_E10	ordinary_muscle_development	ENSGALG00010016118	EPRS	-0.021348978	0.087590201	-0.243737062	0.807434467	0.83017184	negative
PRJNA729271	stage_5W_vs_E10	ordinary_muscle_development	ENSGALG00010016119	FETUB	2.126316134	1.419189814	1.498260566			positive
PRJNA729271	stage_5W_vs_E10	ordinary_muscle_development	ENSGALG00010016121	SLC35C2	-0.461732932	0.100957178	-4.573552301	4.79524E-06	8.73752E-06	negative
PRJNA729271	stage_5W_vs_E10	ordinary_muscle_development	ENSGALG00010016122	AFG2B	0.001294499	0.133351541	0.009707418	0.992254723	0.993214019	positive
PRJNA729271	stage_5W_vs_E10	ordinary_muscle_development	ENSGALG00010016127	ENSGALG00010016127	0.307572161	0.16977952	1.81159754	0.070048405	0.086848998	positive
PRJNA729271	stage_5W_vs_E10	ordinary_muscle_development	ENSGALG00010016129	CENPI	-2.549070136	0.239188589	-10.65715614	1.61459E-26	8.56017E-26	negative
PRJNA729271	stage_5W_vs_E10	ordinary_muscle_development	ENSGALG00010016131	SMYD1	0.742196407	0.217527642	3.411963645	0.000644967	0.000993602	positive
PRJNA729271	stage_5W_vs_E10	ordinary_muscle_development	ENSGALG00010016137	STARD3NL	0.130677014	0.140478937	0.930224961	0.352254621	0.392371531	positive
PRJNA729271	stage_5W_vs_E10	ordinary_muscle_development	ENSGALG00010016139	LURAP1L	-0.06987093	0.19264128	-0.362699679	0.716829239	0.747459882	negative
PRJNA729271	stage_5W_vs_E10	ordinary_muscle_development	ENSGALG00010016141	RRP15	-1.029222299	0.178585183	-5.76320095	8.25334E-09	1.8312E-08	negative
PRJNA729271	stage_5W_vs_E10	ordinary_muscle_development	ENSGALG00010016147	FABP1	3.017187964	0.799032945	3.776049514	0.000159335	0.000257822	positive
PRJNA729271	stage_5W_vs_E10	ordinary_muscle_development	ENSGALG00010016150	POLG	0.053357045	0.096180925	0.554757041	0.579060855	0.617758673	positive
PRJNA729271	stage_5W_vs_E10	ordinary_muscle_development	ENSGALG00010016159	ASPG	-1.949683641	0.19402343	-10.04870204	9.30854E-24	4.3836E-23	negative
PRJNA729271	stage_5W_vs_E10	ordinary_muscle_development	ENSGALG00010016165	KK34	4.090990799	0.91155398	4.48793038	7.19184E-06	1.29466E-05	positive
PRJNA729271	stage_5W_vs_E10	ordinary_muscle_development	ENSGALG00010016168	PLIN2	1.466401904	0.229336165	6.394115411	1.61479E-10	3.96518E-10	positive
PRJNA729271	stage_5W_vs_E10	ordinary_muscle_development	ENSGALG00010016169	RHOH	0.91543123	0.118675791	7.713715	1.22207E-14	3.76332E-14	positive
PRJNA729271	stage_5W_vs_E10	ordinary_muscle_development	ENSGALG00010016173	ENSGALG00010016173	-3.836826878	0.550246221	-6.972927261	3.10413E-12	8.42749E-12	negative
PRJNA729271	stage_5W_vs_E10	ordinary_muscle_development	ENSGALG00010016177	ENSGALG00010016177	3.264598713	1.545843325	2.111856137	0.034698791	0.044695545	positive
PRJNA729271	stage_5W_vs_E10	ordinary_muscle_development	ENSGALG00010016181	FANCI	-2.562217013	0.180352053	-14.20675267	8.31948E-46	8.70692E-45	negative
PRJNA729271	stage_5W_vs_E10	ordinary_muscle_development	ENSGALG00010016182	ELMO2	-0.404908107	0.062665583	-6.461411347	1.03731E-10	2.57992E-10	negative
PRJNA729271	stage_5W_vs_E10	ordinary_muscle_development	ENSGALG00010016190	THNSL2	0.400320576	0.153085741	2.61500891	0.008922513	0.0122729	positive
PRJNA729271	stage_5W_vs_E10	ordinary_muscle_development	ENSGALG00010016193	TCAIM	-0.322266133	0.136125998	-2.367410617	0.017913046	0.023816695	negative
PRJNA729271	stage_5W_vs_E10	ordinary_muscle_development	ENSGALG00010016197	SNRPN	-1.676968794	0.117154522	-14.31416184	1.78503E-46	1.9039E-45	negative
PRJNA729271	stage_5W_vs_E10	ordinary_muscle_development	ENSGALG00010016199	RLBP1	-5.782941083	0.606114132	-9.541010149	1.41448E-21	6.06886E-21	negative
PRJNA729271	stage_5W_vs_E10	ordinary_muscle_development	ENSGALG00010016200	MICU3	0.816223253	0.117552226	6.943494664	3.82517E-12	1.03328E-11	positive
PRJNA729271	stage_5W_vs_E10	ordinary_muscle_development	ENSGALG00010016205	CD40LG	7.971308785	1.004274219	7.937382673	2.06492E-15	6.64396E-15	positive
PRJNA729271	stage_5W_vs_E10	ordinary_muscle_development	ENSGALG00010016206	KNG1	3.33370527	1.151352308	2.895469308	0.00378592	0.005413526	positive
PRJNA729271	stage_5W_vs_E10	ordinary_muscle_development	ENSGALG00010016209	ADAMTS7	-1.167149395	0.166777412	-6.998246215	2.59186E-12	7.06451E-12	negative
PRJNA729271	stage_5W_vs_E10	ordinary_muscle_development	ENSGALG00010016211	TACC3	-1.775619426	0.161973023	-10.96243923	5.79169E-28	3.24008E-27	negative
PRJNA729271	stage_5W_vs_E10	ordinary_muscle_development	ENSGALG00010016215	SPATA17	-3.279884451	0.396818863	-8.265444903	1.39175E-16	4.7411E-16	negative
PRJNA729271	stage_5W_vs_E10	ordinary_muscle_development	ENSGALG00010016218	N4BP2	0.179877099	0.075692426	2.376421367	0.017481487	0.023277067	positive
PRJNA729271	stage_5W_vs_E10	ordinary_muscle_development	ENSGALG00010016221	TMEM200B	-0.606431325	0.203059873	-2.986465595	0.002822226	0.004091635	negative
PRJNA729271	stage_5W_vs_E10	ordinary_muscle_development	ENSGALG00010016224	MOSPD1	1.840971743	0.115267403	15.97131278	2.02479E-57	3.01638E-56	positive
PRJNA729271	stage_5W_vs_E10	ordinary_muscle_development	ENSGALG00010016225	P4HA2	1.496050357	0.15624261	9.575175139	1.01685E-21	4.39059E-21	positive
PRJNA729271	stage_5W_vs_E10	ordinary_muscle_development	ENSGALG00010016229	ENSGALG00010016229	0.57081937	0.22551528	2.531178249	0.011368006	0.015475136	positive
PRJNA729271	stage_5W_vs_E10	ordinary_muscle_development	ENSGALG00010016235	DDX27	-0.286301544	0.123737858	-2.313774859	0.020680075	0.027270532	negative
PRJNA729271	stage_5W_vs_E10	ordinary_muscle_development	ENSGALG00010016236	GPATCH2	-0.756698572	0.067047117	-11.2860718	1.53756E-29	9.14898E-29	negative
PRJNA729271	stage_5W_vs_E10	ordinary_muscle_development	ENSGALG00010016240	EIF4A2	0.948110682	0.10032404	9.450483448	3.37269E-21	1.42073E-20	positive
PRJNA729271	stage_5W_vs_E10	ordinary_muscle_development	ENSGALG00010016241	TOPAZ1	1.031627178	0.802742677	1.285128108	0.198747521	0.231133289	positive
PRJNA729271	stage_5W_vs_E10	ordinary_muscle_development	ENSGALG00010016242	PDS5A	0.495902641	0.092191622	5.379042397	7.48831E-08	1.55729E-07	positive
PRJNA729271	stage_5W_vs_E10	ordinary_muscle_development	ENSGALG00010016245	FAM96A	-0.466258207	0.106629671	-4.372687282	1.22726E-05	2.17366E-05	negative
PRJNA729271	stage_5W_vs_E10	ordinary_muscle_development	ENSGALG00010016247	KIF26A	-2.539413143	0.167702598	-15.14236021	8.51038E-52	1.0907E-50	negative
PRJNA729271	stage_5W_vs_E10	ordinary_muscle_development	ENSGALG00010016251	MPDZ	-0.385553325	0.073245768	-5.263830773	1.41084E-07	2.88193E-07	negative
PRJNA729271	stage_5W_vs_E10	ordinary_muscle_development	ENSGALG00010016253	EDN3	-1.326133363	0.215113764	-6.164800139	7.05722E-10	1.66143E-09	negative
PRJNA729271	stage_5W_vs_E10	ordinary_muscle_development	ENSGALG00010016257	ENSGALG00010016257	-0.662443968	0.11202715	-5.913244829	3.35433E-09	7.60297E-09	negative
PRJNA729271	stage_5W_vs_E10	ordinary_muscle_development	ENSGALG00010016258	CTNNB1	-2.103464128	0.077381608	-27.18299857	1.0318E-162	1.2259E-160	negative
PRJNA729271	stage_5W_vs_E10	ordinary_muscle_development	ENSGALG00010016260	INTS6L	1.157784063	0.144127131	8.033075066	9.50595E-16	3.10755E-15	positive
PRJNA729271	stage_5W_vs_E10	ordinary_muscle_development	ENSGALG00010016262	SPG11	0.612667148	0.060691826	10.09472265	5.82956E-24	2.76685E-23	positive
PRJNA729271	stage_5W_vs_E10	ordinary_muscle_development	ENSGALG00010016263	HGFAC	0.499958759	1.359169369	0.367841397			positive
PRJNA729271	stage_5W_vs_E10	ordinary_muscle_development	ENSGALG00010016266	PDLIM4	-0.746400824	0.121280989	-6.154310181	7.5405E-10	1.77147E-09	negative
PRJNA729271	stage_5W_vs_E10	ordinary_muscle_development	ENSGALG00010016270	RFC4	-1.874825236	0.113694403	-16.49003981	4.32664E-61	7.17559E-60	negative
PRJNA729271	stage_5W_vs_E10	ordinary_muscle_development	ENSGALG00010016274	ZNF831	4.011682513	0.466254415	8.604063332	7.69425E-18	2.79239E-17	positive
PRJNA729271	stage_5W_vs_E10	ordinary_muscle_development	ENSGALG00010016276	ACTR2	-0.503236156	0.107569561	-4.678239371	2.89349E-06	5.3688E-06	negative
PRJNA729271	stage_5W_vs_E10	ordinary_muscle_development	ENSGALG00010016279	PRELID3B	-0.802828239	0.106834684	-7.514677887	5.70512E-14	1.69702E-13	negative
PRJNA729271	stage_5W_vs_E10	ordinary_muscle_development	ENSGALG00010016281	SLC22A4	-1.64244375	0.225764915	-7.275017678	3.46375E-13	9.86386E-13	negative
PRJNA729271	stage_5W_vs_E10	ordinary_muscle_development	ENSGALG00010016286	ULK4	-1.584937565	0.145014577	-10.92950515	8.33014E-28	4.6387E-27	negative
PRJNA729271	stage_5W_vs_E10	ordinary_muscle_development	ENSGALG00010016287	BCL11B	-3.150234342	0.342131026	-9.207683902	3.33237E-20	1.34249E-19	negative
PRJNA729271	stage_5W_vs_E10	ordinary_muscle_development	ENSGALG00010016289	IRF1	5.09850073	0.255634993	19.94445543	1.67464E-88	5.47943E-87	positive
PRJNA729271	stage_5W_vs_E10	ordinary_muscle_development	ENSGALG00010016292	PHKA1	6.148609788	0.147874829	41.57982692	0	0	positive
PRJNA729271	stage_5W_vs_E10	ordinary_muscle_development	ENSGALG00010016295	UBE2K	0.139599973	0.09360052	1.491444418	0.135844854	0.162170248	positive
PRJNA729271	stage_5W_vs_E10	ordinary_muscle_development	ENSGALG00010016299	SRSF4	-1.158732622	0.110946233	-10.44409161	1.55941E-25	7.8731E-25	negative
PRJNA729271	stage_5W_vs_E10	ordinary_muscle_development	ENSGALG00010016303	ENSGALG00010016303	0.795717918	0.203403132	3.912023928	9.15258E-05	0.000151291	positive
PRJNA729271	stage_5W_vs_E10	ordinary_muscle_development	ENSGALG00010016304	EIF3J	1.463822931	0.099579089	14.7001036	6.43612E-49	7.51745E-48	positive
PRJNA729271	stage_5W_vs_E10	ordinary_muscle_development	ENSGALG00010016305	DENND4C	0.944069588	0.096177662	9.81589252	9.6183E-23	4.35135E-22	positive
PRJNA729271	stage_5W_vs_E10	ordinary_muscle_development	ENSGALG00010016307	RAD50	-0.327606711	0.128428181	-2.550894268	0.010744692	0.014667951	negative
PRJNA729271	stage_5W_vs_E10	ordinary_muscle_development	ENSGALG00010016308	RPS6	-0.618961134	0.140741905	-4.397845349	1.09331E-05	1.94424E-05	negative
PRJNA729271	stage_5W_vs_E10	ordinary_muscle_development	ENSGALG00010016310	HDAC8	-0.478262797	0.093152266	-5.134204668	2.8334E-07	5.65081E-07	negative
PRJNA729271	stage_5W_vs_E10	ordinary_muscle_development	ENSGALG00010016312	MECR	0.146975296	0.146256904	1.004911853	0.314939296	0.353961782	positive
PRJNA729271	stage_5W_vs_E10	ordinary_muscle_development	ENSGALG00010016314	ACER2	-0.266442464	0.161483398	-1.649968151	0.09894945	0.120254237	negative
PRJNA729271	stage_5W_vs_E10	ordinary_muscle_development	ENSGALG00010016315	RPS4Y1	-0.198767515	0.136466438	-1.4565304	0.145246052	0.172625576	negative
PRJNA729271	stage_5W_vs_E10	ordinary_muscle_development	ENSGALG00010016317	CTDSPL2	-0.665170049	0.057464217	-11.57537835	5.49284E-31	3.4647E-30	negative
PRJNA729271	stage_5W_vs_E10	ordinary_muscle_development	ENSGALG00010016319	RBM41	0.787425599	0.185579255	4.243069072	2.20484E-05	3.8256E-05	positive
PRJNA729271	stage_5W_vs_E10	ordinary_muscle_development	ENSGALG00010016322	EBF4	-1.045540851	0.13550293	-7.716001788	1.20036E-14	3.70038E-14	negative
PRJNA729271	stage_5W_vs_E10	ordinary_muscle_development	ENSGALG00010016324	TUBB1	-1.319640565	0.213728769	-6.174370322	6.64277E-10	1.56845E-09	negative
PRJNA729271	stage_5W_vs_E10	ordinary_muscle_development	ENSGALG00010016328	USH2A	5.077782994	0.609449752	8.331750034	7.9656E-17	2.74646E-16	positive
PRJNA729271	stage_5W_vs_E10	ordinary_muscle_development	ENSGALG00010016332	ENSGALG00010016332	-1.066650698	0.245185122	-4.35038916	1.35896E-05	2.40046E-05	negative
PRJNA729271	stage_5W_vs_E10	ordinary_muscle_development	ENSGALG00010016335	ABHD4	2.803832919	0.183125767	15.31096889	6.45945E-53	8.48095E-52	positive
PRJNA729271	stage_5W_vs_E10	ordinary_muscle_development	ENSGALG00010016336	SLC24A2	0.119315011	0.355101104	0.336002928	0.736868659	0.765986174	positive
PRJNA729271	stage_5W_vs_E10	ordinary_muscle_development	ENSGALG00010016338	PTPRU	-1.098296908	0.151322669	-7.257980028	3.92914E-13	1.11628E-12	negative
PRJNA729271	stage_5W_vs_E10	ordinary_muscle_development	ENSGALG00010016339	SETD3	0.806508723	0.121867832	6.617896682	3.64346E-11	9.34889E-11	positive

PRJNA729271	stage_5W_vs_E10	ordinary_muscle_development	ENSGALG00010016340	RGS12	-2.046121822	0.103301976	-19.80718952	2.58094E-87	8.24026E-86	negative
PRJNA729271	stage_5W_vs_E10	ordinary_muscle_development	ENSGALG00010016341	SLX4	-1.044951929	0.066012913	-15.82950781	1.94739E-56	2.81991E-55	negative
PRJNA729271	stage_5W_vs_E10	ordinary_muscle_development	ENSGALG00010016344	SMIM14	0.186389221	0.112054719	1.663376809	0.096237032	0.117173942	positive
PRJNA729271	stage_5W_vs_E10	ordinary_muscle_development	ENSGALG00010016345	CTSZ	1.064468276	0.11036128	9.645305622	5.14577E-22	2.25682E-21	positive
PRJNA729271	stage_5W_vs_E10	ordinary_muscle_development	ENSGALG00010016346	TRAP1	-0.02125986	0.104098962	-0.2042274	0.838175801	0.857944935	negative
PRJNA729271	stage_5W_vs_E10	ordinary_muscle_development	ENSGALG00010016347	ANO10	0.301937713	0.090082208	3.351801854	0.000802875	0.001226438	positive
PRJNA729271	stage_5W_vs_E10	ordinary_muscle_development	ENSGALG00010016350	CASC4	0.093895806	0.062975693	1.490984888	0.135965463	0.162300863	positive
PRJNA729271	stage_5W_vs_E10	ordinary_muscle_development	ENSGALG00010016353	C1orf232	-0.172083649	0.150348627	-1.144564155	0.252389748	0.288357326	negative
PRJNA729271	stage_5W_vs_E10	ordinary_muscle_development	ENSGALG00010016355	MCF2L2	1.107427903	0.228462583	4.847305357	1.2515E-06	2.38377E-06	positive
PRJNA729271	stage_5W_vs_E10	ordinary_muscle_development	ENSGALG00010016361	NELFCD	-0.965388884	0.083486516	-11.56341089	6.31496E-31	3.97634E-30	negative
PRJNA729271	stage_5W_vs_E10	ordinary_muscle_development	ENSGALG00010016362	NUP62	-0.377353161	0.170302341	-2.215783759	0.026706315	0.034824401	negative
PRJNA729271	stage_5W_vs_E10	ordinary_muscle_development	ENSGALG00010016366	KIF3A	-0.576175002	0.099007592	-5.819503209	5.90228E-09	1.32027E-08	negative
PRJNA729271	stage_5W_vs_E10	ordinary_muscle_development	ENSGALG00010016368	CCNK	-0.035669046	0.148904668	-0.23954283	0.810684689	0.832922779	negative
PRJNA729271	stage_5W_vs_E10	ordinary_muscle_development	ENSGALG00010016369	MSANTD1	-4.882274995	0.417406893	-11.69668033	1.32538E-31	8.55738E-31	negative
PRJNA729271	stage_5W_vs_E10	ordinary_muscle_development	ENSGALG00010016370	TMEM255A	4.531551309	0.185216185	24.46628143	3.3771E-132	2.5233E-130	positive
PRJNA729271	stage_5W_vs_E10	ordinary_muscle_development	ENSGALG00010016372	DNAAF6	-0.27586085	0.199667415	-1.381601749	0.167094017	0.197191428	negative
PRJNA729271	stage_5W_vs_E10	ordinary_muscle_development	ENSGALG00010016373	SRL	3.289246198	0.13165594	24.98365208	9.2046E-138	7.2907E-136	positive
PRJNA729271	stage_5W_vs_E10	ordinary_muscle_development	ENSGALG00010016374	FRMD5	7.660158319	0.335335598	22.8432602	1.7052E-115	9.7309E-114	positive
PRJNA729271	stage_5W_vs_E10	ordinary_muscle_development	ENSGALG00010016375	CCNI2	-1.569023591	0.140286688	-11.18440823	4.86138E-29	2.83678E-28	negative
PRJNA729271	stage_5W_vs_E10	ordinary_muscle_development	ENSGALG00010016377	FRMPD3	4.002095462	0.277306577	14.43202505	3.25385E-47	3.55959E-46	positive
PRJNA729271	stage_5W_vs_E10	ordinary_muscle_development	ENSGALG00010016378	WDR76	-1.32135803	0.176181349	-7.499988164	6.38236E-14	1.89225E-13	negative
PRJNA729271	stage_5W_vs_E10	ordinary_muscle_development	ENSGALG00010016379	TFAP4	-1.32039669	0.15741942	-8.387762386	4.95482E-17	1.72603E-16	negative
PRJNA729271	stage_5W_vs_E10	ordinary_muscle_development	ENSGALG00010016382	UGDH	-1.304108868	0.186290829	-7.000392208	2.55247E-12	6.95975E-12	negative
PRJNA729271	stage_5W_vs_E10	ordinary_muscle_development	ENSGALG00010016383	FAM110D	1.06453425	0.175273992	6.073543691	1.25118E-09	2.90592E-09	positive
PRJNA729271	stage_5W_vs_E10	ordinary_muscle_development	ENSGALG00010016384	GNAS	-0.15955421	0.074839998	-2.131937658	0.033011972	0.042628823	negative
PRJNA729271	stage_5W_vs_E10	ordinary_muscle_development	ENSGALG00010016385	HTT	0.345320511	0.106632321	3.238422536	0.001201927	0.001806317	positive
PRJNA729271	stage_5W_vs_E10	ordinary_muscle_development	ENSGALG00010016387	CCDC85C	-0.570825971	0.159059417	-3.588759357	0.000332255	0.000523881	negative
PRJNA729271	stage_5W_vs_E10	ordinary_muscle_development	ENSGALG00010016390	ENSGALG00010016390	3.426046563	0.308809796	11.09435841	1.33615E-28	7.65818E-28	positive
PRJNA729271	stage_5W_vs_E10	ordinary_muscle_development	ENSGALG00010016392	STMN3	0.055112842	0.32556093	0.169285798	0.865571846	0.882125003	positive
PRJNA729271	stage_5W_vs_E10	ordinary_muscle_development	ENSGALG00010016393	BLM	-2.079521725	0.127529444	-16.30620862	8.91578E-60	1.41394E-58	negative
PRJNA729271	stage_5W_vs_E10	ordinary_muscle_development	ENSGALG00010016394	PDIK1L	0.208240736	0.096860309	2.149907818	0.031562507	0.040840791	positive
PRJNA729271	stage_5W_vs_E10	ordinary_muscle_development	ENSGALG00010016395	ENSGALG00010016395	-0.582235214	0.161970146	-3.594706976	0.000324757	0.000512448	negative
PRJNA729271	stage_5W_vs_E10	ordinary_muscle_development	ENSGALG00010016397	LIAS	0.230829789	0.104410263	2.210795967	0.027049969	0.035243982	positive
PRJNA729271	stage_5W_vs_E10	ordinary_muscle_development	ENSGALG00010016398	SEPTIN6	-0.419226337	0.068706972	-6.101656464	1.04975E-09	2.44829E-09	negative
PRJNA729271	stage_5W_vs_E10	ordinary_muscle_development	ENSGALG00010016399	ACSM5	6.585776295	0.32786668	20.08675077	9.63657E-90	3.27124E-88	positive
PRJNA729271	stage_5W_vs_E10	ordinary_muscle_development	ENSGALG00010016400	ENSGALG00010016400	0.912891892	0.086600616	10.54140186	5.56629E-26	2.87334E-25	positive
PRJNA729271	stage_5W_vs_E10	ordinary_muscle_development	ENSGALG00010016402	SEPTIN8	-0.898672933	0.07815072	-11.49922784	1.33101E-30	8.26959E-30	negative
PRJNA729271	stage_5W_vs_E10	ordinary_muscle_development	ENSGALG00010016403	GRK4	-0.600423577	0.13461777	-4.460210379	8.18792E-06	1.46795E-05	negative
PRJNA729271	stage_5W_vs_E10	ordinary_muscle_development	ENSGALG00010016404	ESRRG	0.44328517	0.150685815	2.941784326	0.003263271	0.004698164	positive
PRJNA729271	stage_5W_vs_E10	ordinary_muscle_development	ENSGALG00010016407	RPL9	-0.236559781	0.180752927	-1.308746608	0.190620198	0.222682122	negative
PRJNA729271	stage_5W_vs_E10	ordinary_muscle_development	ENSGALG00010016409	GMEB2	1.606037399	0.206087374	7.792992672	6.54404E-15	2.04387E-14	positive
PRJNA729271	stage_5W_vs_E10	ordinary_muscle_development	ENSGALG00010016410	TRAK1	1.265887725	0.096723225	13.08773277	3.86987E-39	3.27078E-38	positive
PRJNA729271	stage_5W_vs_E10	ordinary_muscle_development	ENSGALG00010016411	CTSH	1.303533081	0.122857852	10.61009177	2.67486E-26	1.40225E-25	positive
PRJNA729271	stage_5W_vs_E10	ordinary_muscle_development	ENSGALG00010016412	TMSB15B	-2.848180625	0.215591823	-13.21098631	7.58243E-40	6.60898E-39	negative
PRJNA729271	stage_5W_vs_E10	ordinary_muscle_development	ENSGALG00010016415	RASGRF1	3.816134017	0.508659171	7.502339942	6.26885E-14	1.86012E-13	positive
PRJNA729271	stage_5W_vs_E10	ordinary_muscle_development	ENSGALG00010016416	CYP46A1	1.16753145	0.111600001	10.46175123	1.29441E-25	6.56719E-25	positive
PRJNA729271	stage_5W_vs_E10	ordinary_muscle_development	ENSGALG00010016417	NOP14	-0.772380745	0.10756785	-7.180405157	6.95051E-13	1.95513E-12	negative
PRJNA729271	stage_5W_vs_E10	ordinary_muscle_development	ENSGALG00010016418	NFIB	0.066178471	0.098127219	0.674415023	0.500047494	0.541076393	positive
PRJNA729271	stage_5W_vs_E10	ordinary_muscle_development	ENSGALG00010016421	ENSGALG00010016421	-3.256705693	0.666793276	-4.884130975	1.03886E-06	1.99263E-06	negative
PRJNA729271	stage_5W_vs_E10	ordinary_muscle_development	ENSGALG00010016422	KCTD3	0.14171857	0.090499116	1.565966344	0.117356496	0.141227266	positive
PRJNA729271	stage_5W_vs_E10	ordinary_muscle_development	ENSGALG00010016423	TRMT44	-0.807110004	0.112831549	-7.153229819	8.47595E-13	2.37409E-12	negative
PRJNA729271	stage_5W_vs_E10	ordinary_muscle_development	ENSGALG00010016425	TRIM63	4.206322757	0.413930462	10.16190676	2.93284E-24	1.41187E-23	positive
PRJNA729271	stage_5W_vs_E10	ordinary_muscle_development	ENSGALG00010016426	HELZ2	3.573569008	0.366869353	9.740712813	2.02131E-22	9.01229E-22	positive
PRJNA729271	stage_5W_vs_E10	ordinary_muscle_development	ENSGALG00010016428	ADRA1D	0.853635622	0.202985043	4.205411441	2.60608E-05	4.49329E-05	positive
PRJNA729271	stage_5W_vs_E10	ordinary_muscle_development	ENSGALG00010016429	ENSGALG00010016429	-0.592586793	0.39884646	-1.485751666	0.137344819	0.163866421	negative
PRJNA729271	stage_5W_vs_E10	ordinary_muscle_development	ENSGALG00010016431	B3GNT5	-3.845759826	0.161887234	-23.75579434	9.5723E-125	6.3647E-123	negative
PRJNA729271	stage_5W_vs_E10	ordinary_muscle_development	ENSGALG00010016433	TMED3	-0.640752239	0.146621923	-4.370098472	1.24191E-05	2.19905E-05	negative
PRJNA729271	stage_5W_vs_E10	ordinary_muscle_development	ENSGALG00010016434	GPR139	-3.255497532	0.758696021	-4.290911568	1.77941E-05	3.11054E-05	negative
PRJNA729271	stage_5W_vs_E10	ordinary_muscle_development	ENSGALG00010016436	SLC30A2	1.604347929	0.56502151	2.839445757	0.004519198	0.00640894	positive
PRJNA729271	stage_5W_vs_E10	ordinary_muscle_development	ENSGALG00010016441	ENSGALG00010016441	-2.385861248	0.742784085	-3.212052191	0.001317904	0.001973453	negative
PRJNA729271	stage_5W_vs_E10	ordinary_muscle_development	ENSGALG00010016445	ZDHHC21	0.039525339	0.138638602	0.285096201	0.775570441	0.802247452	positive
PRJNA729271	stage_5W_vs_E10	ordinary_muscle_development	ENSGALG00010016447	ID3	-1.155656053	0.113176929	-10.21105683	1.76927E-24	8.59725E-24	negative
PRJNA729271	stage_5W_vs_E10	ordinary_muscle_development	ENSGALG00010016449	FMR1	0.259527949	0.106786917	2.430334693	0.015084886	0.02023463	positive
PRJNA729271	stage_5W_vs_E10	ordinary_muscle_development	ENSGALG00010016450	ENSGALG00010016450	0.37555448	0.253165702	1.483433486	0.137959276	0.164531817	positive
PRJNA729271	stage_5W_vs_E10	ordinary_muscle_development	ENSGALG00010016451	USP46	-0.131383057	0.147957157	-0.887980414	0.374551287	0.41554695	negative
PRJNA729271	stage_5W_vs_E10	ordinary_muscle_development	ENSGALG00010016452	GLCE	-0.26751304	0.11457646	-2.334799311	0.019553907	0.025874921	negative
PRJNA729271	stage_5W_vs_E10	ordinary_muscle_development	ENSGALG00010016453	MYCL	-1.88141994	0.184556973	-10.19425011	2.10362E-24	1.01809E-23	negative
PRJNA729271	stage_5W_vs_E10	ordinary_muscle_development	ENSGALG00010016454	ANKS3	-0.199843425	0.095931151	-2.083196359	0.037233329	0.047781948	negative
PRJNA729271	stage_5W_vs_E10	ordinary_muscle_development	ENSGALG00010016455	RBM27	-0.700529615	0.092245435	-7.594192762	3.09717E-14	9.35086E-14	negative
PRJNA729271	stage_5W_vs_E10	ordinary_muscle_development	ENSGALG00010016456	LAMP3	1.067996317	0.347880552	3.070008685	0.002140525	0.003136567	positive
PRJNA729271	stage_5W_vs_E10	ordinary_muscle_development	ENSGALG00010016457	SMOX	-0.073011322	0.180914961	-0.403567075	0.686531101	0.719646247	negative
PRJNA729271	stage_5W_vs_E10	ordinary_muscle_development	ENSGALG00010016458	ZC3H12A	1.956934471	0.087945197	22.25174918	1.0848E-109	5.4787E-108	positive
PRJNA729271	stage_5W_vs_E10	ordinary_muscle_development	ENSGALG00010016459	PAQR5	3.124746229	0.271964062	11.48955566	1.48876E-30	9.21808E-30	positive
PRJNA729271	stage_5W_vs_E10	ordinary_muscle_development	ENSGALG00010016460	LBR	-1.379518919	0.069757231	-19.77599888	4.79242E-87	1.51342E-85	negative
PRJNA729271	stage_5W_vs_E10	ordinary_muscle_development	ENSGALG00010016461	HPCAL4	-3.330849537	0.370230809	-8.996683835	2.32638E-19	9.01629E-19	negative
PRJNA729271	stage_5W_vs_E10	ordinary_muscle_development	ENSGALG00010016462	RNF24	-1.160383325	0.159601159	-7.270519412	3.58108E-13	1.0192E-12	negative
PRJNA729271	stage_5W_vs_E10	ordinary_muscle_development	ENSGALG00010016463	FREM1	-2.132559296	0.144749932	-14.73271362	3.97421E-49	4.67204E-48	negative
PRJNA729271	stage_5W_vs_E10	ordinary_muscle_development	ENSGALG00010016464	PANK2	-0.593015619	0.091826685	-6.457987856	1.06104E-10	2.63669E-10	negative
PRJNA729271	stage_5W_vs_E10	ordinary_muscle_development	ENSGALG00010016465	STK35	-1.688977586	0.221363607	-7.629879214	2.34974E-14	7.13287E-14	negative
PRJNA729271	stage_5W_vs_E10	ordinary_muscle_development	ENSGALG00010016466	TMEM164	0.391396316	0.233403508	1.67690846	0.093560384	0.114068278	positive
PRJNA729271	stage_5W_vs_E10	ordinary_muscle_development	ENSGALG00010016468	TTC39B	0.312234915	0.161294545	1.935805794	0.052891491	0.066510121	positive
PRJNA729271	stage_5W_vs_E10	ordinary_muscle_development	ENSGALG00010016469	MAVS	0.835065661	0.102100515	8.178858432	2.86546E-16	9.61676E-16	positive
PRJNA729271	stage_5W_vs_E10	ordinary_muscle_development	ENSGALG00010016470	TNIP1	1.675173576	0.158090492	10.59629557	3.10018E-26	1.61936E-25	positive
PRJNA729271	stage_5W_vs_E10	ordinary_muscle_development	ENSGALG00010016471	POMGNT2	0.931662005	0.135648865	6.868188707	6.50222E-12	1.73572E-11	positive
PRJNA729271	stage_5W_vs_E10	ordinary_muscle_development	ENSGALG00010016472	TFDP1	-1.442713468	0.122596435	-11.76798874	5.70689E-32	3.74135E-31	negative
PRJNA729271	stage_5W_vs_E10	ordinary_muscle_development	ENSGALG00010016473	RAB9B	-0.456004437	0.160295657	-2.844771004	0.004444336	0.006310182	negative
PRJNA729271	stage_5W_vs_E10	ordinary_muscle_development	ENSGALG00010016474	MAPK8IP3	-0.714161214	0.111809189	-6.387321277	1.68817E-10	4.13834E-10	negative
PRJNA729271	stage_5W_vs_E10	ordinary_muscle_development	ENSGALG00010016475	MCCC1	0.356960673	0.084442462	4.227265115	2.36549E-05	4.09455E-05	positive
PRJNA729271	stage_5W_vs_E10	ordinary_muscle_development	ENSGALG00010016477	CNIH3	0.016937824	0.206837981	0.081889333	0.934734717	0.943193854	positive
PRJNA729271	stage_5W_vs_E10	ordinary_muscle_development	ENSGALG00010016479	ENSGALG00010016479	-4.068900213	0.54125413	-7.517541184	5.5816E-14	1.66198E-13	negative
PRJNA729271	stage_5W_vs_E10	ordinary_muscle_development	ENSGALG00010016480	GPX3	4.325099986	0.13323904	32.46120643	3.7637E-231	8.6596E-229	positive
PRJNA729271	stage_5W_vs_E10	ordinary_muscle_development	ENSGALG00010016482	EML1	1.745165706	0.140042002	12.46173064	1.20722E-35	9.12339E-35	positive
PRJNA729271	stage_5W_vs_E10	ordinary_muscle_development	ENSGALG00010016483	WDR26	0.133999954	0.103225072	1.298133792	0.194241364	0.226492525	positive
PRJNA729271	stage_5W_vs_E10	ordinary_muscle_development	ENSGALG00010016489	CCK	-5.838466231	0.665884643	-8.767984505	1.81893E-18	6.78645E-18	negative
PRJNA729271	stage_5W_vs_E10	ordinary_muscle_development	ENSGALG00010016491	DNAJC8	-0.35140105	0.054325317	-6.468458329	9.90078E-11	2.46753E-10	negative
PRJNA729271	stage_5W_vs_E10	ordinary_muscle_development	ENSGALG00010016496	PLP1	2.251226847	0.335183939	6.716392363	1.86279E-11	4.85631E-11	positive
PRJNA729271	stage_5W_vs_E10	ordinary_muscle_development	ENSGALG00010016498	KIF23	-2.722053188	0.208860317	-13.03288836	7.95387E-39	6.64503E-38	negative
PRJNA729271	stage_5W_vs_E10	ordinary_muscle_development	ENSGALG00010016500	ENSGALG00010016500	0.576673113	0.106864503	5.396301815	6.80285E-08	1.4184E-07	positive
PRJNA729271	stage_5W_vs_E10	ordinary_muscle_development	ENSGALG00010016502	YIPF5	-1.574708827	0.133752495	-11.77330433	5.35838E-32	3.52243E-31	negative
PRJNA729271	stage_5W_vs_E10	ordinary_muscle_development	ENSGALG00010016504	SNRK	0.896991123	0.084739044	10.5853344	3.48534E-26	1.81727E-25	positive
PRJNA729271	stage_5W_vs_E10	ordinary_muscle_development	ENSGALG00010016505	KCTD16	-2.925323693	0.383570969	-7.626551359	2.41117E-14	7.31629E-14	negative
PRJNA729271	stage_5W_vs_E10	ordinary_muscle_development	ENSGALG00010016507	SPATA18	-0.56159424	0.144284399	-3.892272778	9.93095E-05	0.000163653	negative
PRJNA729271	stage_5W_vs_E10	ordinary_muscle_development	ENSGALG00010016508	CREBBP	0.576030745	0.119623351	4.81537042	1.46927E-06	2.78393E-06	positive
PRJNA729271	stage_5W_vs_E10	ordinary_muscle_development	ENSGALG00010016510	THG1L	-1.011329211	0.115533962	-8.753523119	2.06794E-18	7.69372E-18	negative
PRJNA729271	stage_5W_vs_E10	ordinary_muscle_development	ENSGALG00010016514	GASK1A	-2.151257423	0.251962147	-8.538018306	1.36543E-17	4.89294E-17	negative
PRJNA729271	stage_5W_vs_E10	ordinary_muscle_development	ENSGALG00010016518	TMEM42	0.223720045	0.142305317	1.57211304	0.115924338	0.139642922	positive
PRJNA729271	stage_5W_vs_E10	ordinary_muscle_development	ENSGALG00010016519	MEX3B	-3.330451425	0.181890574	-18.3101925	6.86267E-75	1.65815E-73	negative
PRJNA729271	stage_5W_vs_E10	ordinary_muscle_development	ENSGALG00010016520	CHMP1B	0.319345721	0.082923547	3.851086143	0.000117595	0.000192343	positive
PRJNA729271	stage_5W_vs_E10	ordinary_muscle_development	ENSGALG00010016526	EIF1B	1.692230264	0.113357211	14.92829839	2.157E-50	2.61817E-49	positive
PRJNA729271	stage_5W_vs_E10	ordinary_muscle_development	ENSGALG00010016527	MRPS34	-0.406336632	0.09929753	-4.09211218	4.27462E-05	7.24346E-05	negative
PRJNA729271	stage_5W_vs_E10	ordinary_muscle_development	ENSGALG00010016529	DCUN1D1	-0.116207084	0.097878425	-1.187259443	0.2351253	0.27010154	negative
PRJNA729271	stage_5W_vs_E10	ordinary_muscle_development	ENSGALG00010016533	CNIH4	0.063755465	0.110426145	0.577358424	0.563697354	0.603010565	positive
PRJNA729271	stage_5W_vs_E10	ordinary_muscle_development	ENSGALG00010016534	TRIT1	-0.213919232	0.145644637	-1.468775206	0.141893771	0.168876773	negative
PRJNA729271	stage_5W_vs_E10	ordinary_muscle_development	ENSGALG00010016535	BNC2	-1.35861598	0.132248694	-10.27319013	9.3075E-25	4.58573E-24	negative
PRJNA729271	stage_5W_vs_E10	ordinary_muscle_development	ENSGALG00010016541	UBE2J2	-0.273390896	0.079548387	-3.436787432	0.000588657	0.000909658	negative
PRJNA729271	stage_5W_vs_E10	ordinary_muscle_development	ENSGALG00010016542	ENOX2	-0.152585379	0.077297146	-1.97401052	0.048380547	0.061102729	negative
PRJNA729271	stage_5W_vs_E10	ordinary_muscle_development	ENSGALG00010016543	TCEA3	5.149801261	0.174017065	29.59365662	1.8033E-192	2.7807E-190	positive
PRJNA729271	stage_5W_vs_E10	ordinary_muscle_development	ENSGALG00010016544	ADCY9	1.603063156	0.13548907	11.83167879	2.67733E-32	1.78263E-31	positive
PRJNA729271	stage_5W_vs_E10	ordinary_muscle_development	ENSGALG00010016546	C1QTNF12	-0.514102109	0.407380814	-1.261969368	0.206959809	0.240144287	negative
PRJNA729271	stage_5W_vs_E10	ordinary_muscle_development	ENSGALG00010016547	SGCB	2.185951557	0.143240691	15.26068842	1.39766E-52	1.8235E-51	positive
PRJNA729271	stage_5W_vs_E10	ordinary_muscle_development	ENSGALG00010016548	EVL	0.368994175	0.108628323	3.396850508	0.000681662	0.001047571	positive
PRJNA729271	stage_5W_vs_E10	ordinary_muscle_development	ENSGALG00010016549	PTPRA	-0.772100232	0.103620786	-7.45120995	9.2488E-14	2.71544E-13	negative
PRJNA729271	stage_5W_vs_E10	ordinary_muscle_development	ENSGALG00010016550	FBLIM1	-1.936659627	0.147031062	-13.17177205	1.27568E-39	1.10196E-38	negative
PRJNA729271	stage_5W_vs_E10	ordinary_muscle_development	ENSGALG00010016552	ENSGALG00010016552	2.739993176	0.448428935	6.110206014	9.95026E-10	2.32215E-09	positive
PRJNA729271	stage_5W_vs_E10	ordinary_muscle_development	ENSGALG00010016553	SLC25A14	-0.311600462	0.096661753	-3.223616922	0.001265826	0.001898019	negative
PRJNA729271	stage_5W_vs_E10	ordinary_muscle_development	ENSGALG00010016556	ASAP3	-0.686856829	0.19675206	-3.490976552	0.000481258	0.000747999	negative
PRJNA729271	stage_5W_vs_E10	ordinary_muscle_development	ENSGALG00010016558	NR2F2	-0.907876622	0.109947442	-8.257369169	1.48918E-16	5.05875E-16	negative
PRJNA729271	stage_5W_vs_E10	ordinary_muscle_development	ENSGALG00010016559	ROGDI	-0.598698226	0.117078227	-5.113659838	3.15976E-07	6.28527E-07	negative
PRJNA729271	stage_5W_vs_E10	ordinary_muscle_development	ENSGALG00010016560	DCUN1D4	1.044170528	0.169401717	6.163872158	7.09873E-10	1.67093E-09	positive
PRJNA729271	stage_5W_vs_E10	ordinary_muscle_development	ENSGALG00010016561	DEGS2	2.391209001	0.564544154	4.235645671	2.27896E-05	3.95043E-05	positive
PRJNA729271	stage_5W_vs_E10	ordinary_muscle_development	ENSGALG00010016562	GLIS2	0.333818094	0.222417452	1.500862864	0.133391035	0.15937216	positive

PRJNA729271	stage_5W_vs_E10	ordinary_muscle_development	ENSGALG00010016564	SPEN	0.977724161	0.165913344	5.892980861	3.7929E-09	8.57961E-09	positive
PRJNA729271	stage_5W_vs_E10	ordinary_muscle_development	ENSGALG00010016565	NVL	-0.615297933	0.098537167	-6.244323376	4.25639E-10	1.01675E-09	negative
PRJNA729271	stage_5W_vs_E10	ordinary_muscle_development	ENSGALG00010016566	CNTLN	-2.320620521	0.216832978	-10.70234121	9.92386E-27	5.30603E-26	negative
PRJNA729271	stage_5W_vs_E10	ordinary_muscle_development	ENSGALG00010016567	E2F2	-1.844107843	0.278780573	-6.614908007	3.71783E-11	9.53298E-11	negative
PRJNA729271	stage_5W_vs_E10	ordinary_muscle_development	ENSGALG00010016568	PAM16	1.54313619	0.134530444	11.47053521	1.85508E-30	1.1418E-29	positive
PRJNA729271	stage_5W_vs_E10	ordinary_muscle_development	ENSGALG00010016569	YY1	0.43293737	0.086803307	4.987567717	6.11442E-07	1.19285E-06	positive
PRJNA729271	stage_5W_vs_E10	ordinary_muscle_development	ENSGALG00010016572	RAB33A	-0.264723278	0.115638389	-2.289233529	0.022065787	0.029002864	negative
PRJNA729271	stage_5W_vs_E10	ordinary_muscle_development	ENSGALG00010016573	DEGS1	-0.467112836	0.128772874	-3.627416402	0.000286271	0.000453795	negative
PRJNA729271	stage_5W_vs_E10	ordinary_muscle_development	ENSGALG00010016575	CWH43	-0.354449441	0.399892778	-0.886361196	0.375422922	0.416447666	negative
PRJNA729271	stage_5W_vs_E10	ordinary_muscle_development	ENSGALG00010016576	MRPS26	0.296300668	0.106637115	2.778588547	0.005459563	0.007676435	positive
PRJNA729271	stage_5W_vs_E10	ordinary_muscle_development	ENSGALG00010016577	ZBTB17	1.187798133	0.185463653	6.404479327	1.50883E-10	3.71128E-10	positive
PRJNA729271	stage_5W_vs_E10	ordinary_muscle_development	ENSGALG00010016580	MARS2	-0.601681161	0.112933112	-5.327765685	9.94283E-08	2.05272E-07	negative
PRJNA729271	stage_5W_vs_E10	ordinary_muscle_development	ENSGALG00010016581	H2AFYMACROH2A1	0.179331405	0.07660593	2.340959815	0.019234237	0.025484485	positive
PRJNA729271	stage_5W_vs_E10	ordinary_muscle_development	ENSGALG00010016583	VPS41	0.104407196	0.083134857	1.255877493	0.20916039	0.242503588	positive
PRJNA729271	stage_5W_vs_E10	ordinary_muscle_development	ENSGALG00010016584	EHBP1	0.420269729	0.160466739	2.619045745	0.008817612	0.012135519	positive
PRJNA729271	stage_5W_vs_E10	ordinary_muscle_development	ENSGALG00010016588	ATP11B	-0.676486124	0.100654651	-6.720863073	1.80651E-11	4.71554E-11	negative
PRJNA729271	stage_5W_vs_E10	ordinary_muscle_development	ENSGALG00010016589	FBXO28	0.005554612	0.078492497	0.07076615	0.943583873	0.951129919	positive
PRJNA729271	stage_5W_vs_E10	ordinary_muscle_development	ENSGALG00010016591	HOMER2	-0.956481708	0.205633739	-4.651385089	3.29713E-06	6.09124E-06	negative
PRJNA729271	stage_5W_vs_E10	ordinary_muscle_development	ENSGALG00010016592	TMEM129	0.481064793	0.103278362	4.657943676	3.19384E-06	5.9087E-06	positive
PRJNA729271	stage_5W_vs_E10	ordinary_muscle_development	ENSGALG00010016597	FRYL	0.577793772	0.125424093	4.606720739	4.09069E-06	7.49994E-06	positive
PRJNA729271	stage_5W_vs_E10	ordinary_muscle_development	ENSGALG00010016598	AIFM1	-0.115400102	0.088866382	-1.298579956	0.19408812	0.226332044	negative
PRJNA729271	stage_5W_vs_E10	ordinary_muscle_development	ENSGALG00010016599	ENSGALG00010016599	-0.157679632	0.283663376	-0.555868841	0.57830052	0.617129209	negative
PRJNA729271	stage_5W_vs_E10	ordinary_muscle_development	ENSGALG00010016600	GNL2	-0.537180499	0.110402706	-4.86564614	1.14083E-06	2.17928E-06	negative
PRJNA729271	stage_5W_vs_E10	ordinary_muscle_development	ENSGALG00010016602	HSPB7	4.291016103	0.513781285	8.351834192	6.7211E-17	2.32901E-16	positive
PRJNA729271	stage_5W_vs_E10	ordinary_muscle_development	ENSGALG00010016603	ENSGALG00010016603	0.380831221	0.125894085	3.025012823	0.002486226	0.00362335	positive
PRJNA729271	stage_5W_vs_E10	ordinary_muscle_development	ENSGALG00010016604	TP53BP2	-1.175294346	0.063683174	-18.4553356	4.72421E-76	1.18065E-74	negative
PRJNA729271	stage_5W_vs_E10	ordinary_muscle_development	ENSGALG00010016605	WDPCP	-0.616963195	0.169196714	-3.646425403	0.000265914	0.00042268	negative
PRJNA729271	stage_5W_vs_E10	ordinary_muscle_development	ENSGALG00010016606	VASN	-1.441351862	0.182290778	-7.906883064	2.63913E-15	8.42976E-15	negative
PRJNA729271	stage_5W_vs_E10	ordinary_muscle_development	ENSGALG00010016607	FAM103A1	-0.442028802	0.115053324	-3.841947265	0.000122062	0.000199402	negative
PRJNA729271	stage_5W_vs_E10	ordinary_muscle_development	ENSGALG00010016609	SOX2	-2.533012187	0.675650361	-3.748998496	0.000177542	0.000286228	negative
PRJNA729271	stage_5W_vs_E10	ordinary_muscle_development	ENSGALG00010016611	DNALI1	-0.198889337	0.210189177	-0.94623967	0.344026357	0.383855134	negative
PRJNA729271	stage_5W_vs_E10	ordinary_muscle_development	ENSGALG00010016612	GRIA1	-1.681896044	0.344671301	-4.879710142	1.06242E-06	2.0362E-06	negative
PRJNA729271	stage_5W_vs_E10	ordinary_muscle_development	ENSGALG00010016613	SNIP1	-0.642817458	0.078778799	-8.159777287	3.35643E-16	1.12229E-15	negative
PRJNA729271	stage_5W_vs_E10	ordinary_muscle_development	ENSGALG00010016614	DNAJC19	0.997950561	0.139071917	7.175787744	7.1892E-13	2.02031E-12	positive
PRJNA729271	stage_5W_vs_E10	ordinary_muscle_development	ENSGALG00010016615	BCORL1	1.178159144	0.124804234	9.440057503	3.72575E-21	1.56899E-20	positive
PRJNA729271	stage_5W_vs_E10	ordinary_muscle_development	ENSGALG00010016616	SLBP	-2.347426408	0.239383703	-9.806124549	1.0596E-22	4.78173E-22	negative
PRJNA729271	stage_5W_vs_E10	ordinary_muscle_development	ENSGALG00010016618	TRPM3	-2.075787833	0.494227595	-4.200064612	2.66839E-05	4.59635E-05	negative
PRJNA729271	stage_5W_vs_E10	ordinary_muscle_development	ENSGALG00010016619	SLC25A47	0.981184797	0.329242632	2.980126816	0.002881291	0.004171009	positive
PRJNA729271	stage_5W_vs_E10	ordinary_muscle_development	ENSGALG00010016620	ENSGALG00010016620	0.063299667	0.122233525	0.517858478	0.604557014	0.642170154	positive
PRJNA729271	stage_5W_vs_E10	ordinary_muscle_development	ENSGALG00010016622	KIAA1143	-0.576734856	0.1346409	-4.283504155	1.83973E-05	3.21403E-05	negative
PRJNA729271	stage_5W_vs_E10	ordinary_muscle_development	ENSGALG00010016623	DNAJA3	1.424787415	0.141122922	10.09607365	5.74984E-24	2.7308E-23	positive
PRJNA729271	stage_5W_vs_E10	ordinary_muscle_development	ENSGALG00010016624	MDH1	0.659361089	0.154747757	4.26087656	2.03627E-05	3.54245E-05	positive
PRJNA729271	stage_5W_vs_E10	ordinary_muscle_development	ENSGALG00010016626	CAPN2	0.558500756	0.229426674	2.434332266	0.014919293	0.020029189	positive
PRJNA729271	stage_5W_vs_E10	ordinary_muscle_development	ENSGALG00010016629	LARS1	-0.698171986	0.071843923	-9.71789894	2.52947E-22	1.12227E-21	negative
PRJNA729271	stage_5W_vs_E10	ordinary_muscle_development	ENSGALG00010016630	ENSGALG00010016630	1.990736026	0.334965935	5.943099937	2.79682E-09	6.36419E-09	positive
PRJNA729271	stage_5W_vs_E10	ordinary_muscle_development	ENSGALG00010016631	FAM53A	1.089689668	0.091639538	11.89104275	1.31751E-32	8.87011E-32	positive
PRJNA729271	stage_5W_vs_E10	ordinary_muscle_development	ENSGALG00010016637	POU6F2	-2.837050258	0.802903384	-3.533488977	0.000410113	0.000641134	negative
PRJNA729271	stage_5W_vs_E10	ordinary_muscle_development	ENSGALG00010016640	OCIAD1	0.951319156	0.10372785	9.171299276	4.67352E-20	1.86979E-19	positive
PRJNA729271	stage_5W_vs_E10	ordinary_muscle_development	ENSGALG00010016643	BTBD1	1.059768955	0.17331247	6.114787659	9.66856E-10	2.25787E-09	positive
PRJNA729271	stage_5W_vs_E10	ordinary_muscle_development	ENSGALG00010016644	NMRAL1	-2.644119725	0.213178423	-12.40331777	2.50723E-35	1.87235E-34	negative
PRJNA729271	stage_5W_vs_E10	ordinary_muscle_development	ENSGALG00010016647	EGR1	2.213626826	0.204277427	10.8363751	2.31461E-27	1.26367E-26	positive
PRJNA729271	stage_5W_vs_E10	ordinary_muscle_development	ENSGALG00010016648	ADRA2C	-1.482918568	0.282423676	-5.250687866	1.51532E-07	3.08969E-07	negative
PRJNA729271	stage_5W_vs_E10	ordinary_muscle_development	ENSGALG00010016651	ENSGALG00010016651	2.682353266	0.180115757	14.89238538	3.69353E-50	4.45035E-49	positive
PRJNA729271	stage_5W_vs_E10	ordinary_muscle_development	ENSGALG00010016652	FXR1	2.344775128	0.098855736	23.71916102	2.2874E-124	1.4935E-122	positive
PRJNA729271	stage_5W_vs_E10	ordinary_muscle_development	ENSGALG00010016653	EPHA2	-0.149657194	0.208917179	-0.716346998	0.473777114	0.515686661	negative
PRJNA729271	stage_5W_vs_E10	ordinary_muscle_development	ENSGALG00010016654	SMIM3	0.791703157	0.183798576	4.307449904	1.65147E-05	2.89422E-05	positive
PRJNA729271	stage_5W_vs_E10	ordinary_muscle_development	ENSGALG00010016655	TRANK1	1.623311428	0.323554978	5.017111589	5.24541E-07	1.02774E-06	positive
PRJNA729271	stage_5W_vs_E10	ordinary_muscle_development	ENSGALG00010016656	CAPN8	-5.059168681	1.188356961	-4.257280303	2.06929E-05	3.59601E-05	negative
PRJNA729271	stage_5W_vs_E10	ordinary_muscle_development	ENSGALG00010016657	SLC25A29	2.10722536	0.279243353	7.546197022	4.48152E-14	1.34131E-13	positive
PRJNA729271	stage_5W_vs_E10	ordinary_muscle_development	ENSGALG00010016658	ZAR1	-1.580587472	0.584125628	-2.705903313	0.006811888	0.00949133	negative
PRJNA729271	stage_5W_vs_E10	ordinary_muscle_development	ENSGALG00010016661	UBE4B	-0.648985462	0.128853108	-5.036630249	4.73799E-07	9.30963E-07	negative
PRJNA729271	stage_5W_vs_E10	ordinary_muscle_development	ENSGALG00010016664	TM6SF1	2.36381492	0.248891485	9.497371609	2.15255E-21	9.15796E-21	positive
PRJNA729271	stage_5W_vs_E10	ordinary_muscle_development	ENSGALG00010016665	ENSGALG00010016665	6.923491147	0.991592293	6.982195403	2.90603E-12	7.90149E-12	positive
PRJNA729271	stage_5W_vs_E10	ordinary_muscle_development	ENSGALG00010016667	WARS1	-0.336348809	0.1437587	-2.339676191	0.019300465	0.025565224	negative
PRJNA729271	stage_5W_vs_E10	ordinary_muscle_development	ENSGALG00010016671	CASS4	-0.082204479	0.246514448	-0.333467186	0.738781655	0.767644451	negative
PRJNA729271	stage_5W_vs_E10	ordinary_muscle_development	ENSGALG00010016672	DCTN4	0.23579189	0.080622482	2.92464192	0.003448528	0.004952582	positive
PRJNA729271	stage_5W_vs_E10	ordinary_muscle_development	ENSGALG00010016673	LRPAP1	0.187212168	0.079645217	2.350576402	0.018744359	0.024869518	positive
PRJNA729271	stage_5W_vs_E10	ordinary_muscle_development	ENSGALG00010016674	SLC10A4	-1.253498396	0.394996121	-3.173444826	0.001506415	0.002241607	negative
PRJNA729271	stage_5W_vs_E10	ordinary_muscle_development	ENSGALG00010016675	SKIL	-0.394102874	0.140220992	-2.810583988	0.004945168	0.006987222	negative
PRJNA729271	stage_5W_vs_E10	ordinary_muscle_development	ENSGALG00010016676	NLRC3	4.718008838	0.405118198	11.6460057	2.40468E-31	1.53753E-30	positive
PRJNA729271	stage_5W_vs_E10	ordinary_muscle_development	ENSGALG00010016677	MEAF6	0.370642344	0.088245377	4.200133278	2.66758E-05	4.59568E-05	positive
PRJNA729271	stage_5W_vs_E10	ordinary_muscle_development	ENSGALG00010016678	NOX1	3.080233694	0.313823363	9.815182873	9.68622E-23	4.38071E-22	positive
PRJNA729271	stage_5W_vs_E10	ordinary_muscle_development	ENSGALG00010016680	BPIFB3	0.339363375	0.865444319	0.39212618	0.694964985	0.727580448	positive
PRJNA729271	stage_5W_vs_E10	ordinary_muscle_development	ENSGALG00010016681	SUSD4	2.629573225	0.278026299	9.458001749	3.13884E-21	1.3253E-20	positive
PRJNA729271	stage_5W_vs_E10	ordinary_muscle_development	ENSGALG00010016682	GPR141	0.999764036	0.441560192	2.264162518	0.023564121	0.030891013	positive
PRJNA729271	stage_5W_vs_E10	ordinary_muscle_development	ENSGALG00010016683	RBM22	-0.643038148	0.074476027	-8.634162863	5.91587E-18	2.15887E-17	negative
PRJNA729271	stage_5W_vs_E10	ordinary_muscle_development	ENSGALG00010016691	ENSGALG00010016691	1.133369919	0.29876587	3.793505327	0.000148535	0.0002409	positive
PRJNA729271	stage_5W_vs_E10	ordinary_muscle_development	ENSGALG00010016692	PRKCI	-1.790319513	0.075711221	-23.64668653	1.2767E-123	8.1523E-122	negative
PRJNA729271	stage_5W_vs_E10	ordinary_muscle_development	ENSGALG00010016696	DOK7	1.097294485	0.129291708	8.486967212	2.121E-17	7.52235E-17	positive
PRJNA729271	stage_5W_vs_E10	ordinary_muscle_development	ENSGALG00010016699	SYNPO	2.70409426	0.144733924	18.68320977	6.7816E-78	1.76797E-76	positive
PRJNA729271	stage_5W_vs_E10	ordinary_muscle_development	ENSGALG00010016700	CLUAP1	-0.873415704	0.127776741	-6.835482727	8.17293E-12	2.17091E-11	negative
PRJNA729271	stage_5W_vs_E10	ordinary_muscle_development	ENSGALG00010016702	WDR25	0.088136743	0.099397335	0.886711336	0.375234334	0.416272897	positive
PRJNA729271	stage_5W_vs_E10	ordinary_muscle_development	ENSGALG00010016704	CSTF2	-0.846821413	0.074778111	-11.32445574	9.92937E-30	5.94245E-29	negative
PRJNA729271	stage_5W_vs_E10	ordinary_muscle_development	ENSGALG00010016706	RTN4	1.991858791	0.125466209	15.87565929	9.34241E-57	1.36373E-55	positive
PRJNA729271	stage_5W_vs_E10	ordinary_muscle_development	ENSGALG00010016707	KIF1B	1.765057048	0.130417757	13.53387062	9.86875E-42	9.09393E-41	positive
PRJNA729271	stage_5W_vs_E10	ordinary_muscle_development	ENSGALG00010016712	ENSGALG00010016712	0.607524526	0.157013083	3.869260545	0.000109166	0.000179061	positive
PRJNA729271	stage_5W_vs_E10	ordinary_muscle_development	ENSGALG00010016720	INTS15	-0.235235718	0.100492433	-2.34083016	0.019240918	0.025488677	negative
PRJNA729271	stage_5W_vs_E10	ordinary_muscle_development	ENSGALG00010016724	PGD	-1.510204378	0.110998711	-13.60560284	3.70897E-42	3.4529E-41	negative
PRJNA729271	stage_5W_vs_E10	ordinary_muscle_development	ENSGALG00010016729	ELMO1	0.98060781	0.09413466	10.41707499	2.07229E-25	1.04225E-24	positive
PRJNA729271	stage_5W_vs_E10	ordinary_muscle_development	ENSGALG00010016731	C4orf48	-1.949783451	0.278767761	-6.994293193	2.666E-12	7.26247E-12	negative
PRJNA729271	stage_5W_vs_E10	ordinary_muscle_development	ENSGALG00010016732	ARHGAP26	0.144585936	0.076639495	1.88657213	0.059217898	0.073990469	positive
PRJNA729271	stage_5W_vs_E10	ordinary_muscle_development	ENSGALG00010016733	PHC3	1.202166698	0.152094628	7.904070717	2.6994E-15	8.60897E-15	positive
PRJNA729271	stage_5W_vs_E10	ordinary_muscle_development	ENSGALG00010016735	ENSGALG00010016735	-1.321906284	0.34822732	-3.796101593	0.000146989	0.00023859	negative
PRJNA729271	stage_5W_vs_E10	ordinary_muscle_development	ENSGALG00010016736	CENPS	-3.289319942	0.274687794	-11.97475831	4.8184E-33	3.28517E-32	negative
PRJNA729271	stage_5W_vs_E10	ordinary_muscle_development	ENSGALG00010016738	NAA60	1.036376377	0.150029434	6.907820365	4.92157E-12	1.32132E-11	positive
PRJNA729271	stage_5W_vs_E10	ordinary_muscle_development	ENSGALG00010016741	NELFA	-0.288608445	0.089936879	-3.209011126	0.001331923	0.001993416	negative
PRJNA729271	stage_5W_vs_E10	ordinary_muscle_development	ENSGALG00010016742	TRMT12	-0.372834812	0.143438713	-2.599262111	0.009342441	0.012831029	negative
PRJNA729271	stage_5W_vs_E10	ordinary_muscle_development	ENSGALG00010016744	CRYBG2	-7.047867439	1.035850956	-6.803939699	1.01796E-11	2.69406E-11	negative
PRJNA729271	stage_5W_vs_E10	ordinary_muscle_development	ENSGALG00010016745	BEGAIN	0.884262957	0.36476334	2.424210058	0.015341729	0.020571541	positive
PRJNA729271	stage_5W_vs_E10	ordinary_muscle_development	ENSGALG00010016746	HDGFL3	-1.798269521	0.124746292	-14.41541458	4.13946E-47	4.52159E-46	negative
PRJNA729271	stage_5W_vs_E10	ordinary_muscle_development	ENSGALG00010016750	SYTL4	0.88679878	0.124961981	7.096548686	1.27911E-12	3.54685E-12	positive
PRJNA729271	stage_5W_vs_E10	ordinary_muscle_development	ENSGALG00010016751	DFFA	-1.110012683	0.091562543	-12.12299965	7.97846E-34	5.61669E-33	negative
PRJNA729271	stage_5W_vs_E10	ordinary_muscle_development	ENSGALG00010016752	ENSGALG00010016752	1.620175198	0.172038537	9.417513209	4.61898E-21	1.93727E-20	positive
PRJNA729271	stage_5W_vs_E10	ordinary_muscle_development	ENSGALG00010016754	ENSGALG00010016754	0.310141994	0.184325199	1.682580545	0.09245632	0.112769637	positive
PRJNA729271	stage_5W_vs_E10	ordinary_muscle_development	ENSGALG00010016756	NUDT16L1	-0.199357133	0.10704516	-1.862364756	0.062551694	0.077934227	negative
PRJNA729271	stage_5W_vs_E10	ordinary_muscle_development	ENSGALG00010016757	SRPX2	-0.537362205	0.279096695	-1.92536212	0.054184071	0.067999836	negative
PRJNA729271	stage_5W_vs_E10	ordinary_muscle_development	ENSGALG00010016758	NR3C1	0.427843948	0.068442558	6.251139047	4.0747E-10	9.74633E-10	positive
PRJNA729271	stage_5W_vs_E10	ordinary_muscle_development	ENSGALG00010016760	DLK1	0.31312814	0.262685534	1.192026582	0.233250822	0.268096953	positive
PRJNA729271	stage_5W_vs_E10	ordinary_muscle_development	ENSGALG00010016761	GPR160	3.810332217	0.341634396	11.15324529	6.90415E-29	4.00142E-28	positive
PRJNA729271	stage_5W_vs_E10	ordinary_muscle_development	ENSGALG00010016762	EML6	-0.028583978	0.12549985	-0.227761053	0.819832006	0.841069073	negative
PRJNA729271	stage_5W_vs_E10	ordinary_muscle_development	ENSGALG00010016763	UBALD1	1.22651273	0.172400826	7.114308895	1.12475E-12	3.13103E-12	positive
PRJNA729271	stage_5W_vs_E10	ordinary_muscle_development	ENSGALG00010016766	AOAH	2.349356958	0.323218157	7.268641647	3.6312E-13	1.03326E-12	positive
PRJNA729271	stage_5W_vs_E10	ordinary_muscle_development	ENSGALG00010016767	SLAIN2	1.145069076	0.091861978	12.46510361	1.15721E-35	8.75916E-35	positive
PRJNA729271	stage_5W_vs_E10	ordinary_muscle_development	ENSGALG00010016768	GALNT10	0.27527507	0.099948351	2.754173207	0.005884062	0.008247701	positive
PRJNA729271	stage_5W_vs_E10	ordinary_muscle_development	ENSGALG00010016769	BNC1	-8.902423536	0.532651776	-16.71340253	1.04688E-62	1.8108E-61	negative
PRJNA729271	stage_5W_vs_E10	ordinary_muscle_development	ENSGALG00010016772	TSPAN6	-1.096880233	0.126906741	-8.643199155	5.46605E-18	1.99623E-17	negative
PRJNA729271	stage_5W_vs_E10	ordinary_muscle_development	ENSGALG00010016773	RPS27A	-0.263942016	0.161331692	-1.636020873	0.101835219	0.123523138	negative
PRJNA729271	stage_5W_vs_E10	ordinary_muscle_development	ENSGALG00010016774	PEX14	-0.021853203	0.119559499	-0.182780985	0.854969869	0.872485796	negative
PRJNA729271	stage_5W_vs_E10	ordinary_muscle_development	ENSGALG00010016777	TNMD	-2.68979912	0.489795694	-5.491675715	3.98138E-08	8.43591E-08	negative
PRJNA729271	stage_5W_vs_E10	ordinary_muscle_development	ENSGALG00010016778	MTIF2	0.118470926	0.108366109	1.093247023	0.274285356	0.311433984	positive
PRJNA729271	stage_5W_vs_E10	ordinary_muscle_development	ENSGALG00010016779	CTNNA2	-2.658599369	0.155209241	-17.12913063	8.99894E-66	1.69843E-64	negative
PRJNA729271	stage_5W_vs_E10	ordinary_muscle_development	ENSGALG00010016780	PRORSD1P	-1.960668786	0.144083546	-13.60786047	3.59618E-42	3.3522E-41	negative
PRJNA729271	stage_5W_vs_E10	ordinary_muscle_development	ENSGALG00010016781	CDIP1	1.875833459	0.143302647	13.09001266	3.75545E-39	3.17777E-38	positive
PRJNA729271	stage_5W_vs_E10	ordinary_muscle_development	ENSGALG00010016783	CLBA1	-0.109293019	0.304300462	-0.359161527	0.719474256	0.75003604	negative
PRJNA729271	stage_5W_vs_E10	ordinary_muscle_development	ENSGALG00010016785	PDGFRB	1.232193848	0.194134661	6.347108966	2.19399E-10	5.34215E-10	positive
PRJNA729271	stage_5W_vs_E10	ordinary_muscle_development	ENSGALG00010016786	DISP1	-0.69278055	0.112182907	-6.175455518	6.5973E-10	1.55847E-09	negative
PRJNA729271	stage_5W_vs_E10	ordinary_muscle_development	ENSGALG00010016787	PPP2R5C	-0.49364085	0.07586499	-6.506833373	7.67515E-11	1.9281E-10	negative
PRJNA729271	stage_5W_vs_E10	ordinary_muscle_development	ENSGALG00010016788	ANLN	-2.758985281	0.289539605	-9.528870096	1.59004E-21	6.80676E-21	negative
PRJNA729271	stage_5W_vs_E10	ordinary_muscle_development	ENSGALG00010016789	DISP3	1.581014062	0.111341303	14.19970864	9.19941E-46	9.61395E-45	positive
PRJNA729271	stage_5W_vs_E10	ordinary_muscle_development	ENSGALG00010016791	LRRTM1	-6.039259592	0.830441623	-7.272346935	3.53295E-13	1.00589E-12	negative
PRJNA729271	stage_5W_vs_E10	ordinary_muscle_development	ENSGALG00010016792	HMOX2	-1.118839673	0.124222234	-9.006758587	2.12237E-19	8.24543E-19	negative

PRJNA729271	stage_5W_vs_E10	ordinary_muscle_development	ENSGALG00010016793	SLC9A1	1.112454357	0.154356591	7.207041514	5.71806E-13	1.6144E-12	positive
PRJNA729271	stage_5W_vs_E10	ordinary_muscle_development	ENSGALG00010016795	GUCA2B	1.988348634	1.235772211	1.608992835	0.107617908	0.13015616	positive
PRJNA729271	stage_5W_vs_E10	ordinary_muscle_development	ENSGALG00010016796	BROX	0.472595323	0.085013955	5.559032293	2.71275E-08	5.82106E-08	positive
PRJNA729271	stage_5W_vs_E10	ordinary_muscle_development	ENSGALG00010016797	SEC62	-0.205632816	0.10939362	-1.879751457	0.060141959	0.075080329	negative
PRJNA729271	stage_5W_vs_E10	ordinary_muscle_development	ENSGALG00010016799	WDTC1	1.734608129	0.131890102	13.15192048	1.65906E-39	1.42465E-38	positive
PRJNA729271	stage_5W_vs_E10	ordinary_muscle_development	ENSGALG00010016800	RPL14	-0.54435445	0.169120318	-3.218740691	0.001287549	0.001929592	negative
PRJNA729271	stage_5W_vs_E10	ordinary_muscle_development	ENSGALG00010016802	TMEM222	-0.365194075	0.085747397	-4.258952337	2.05387E-05	3.57051E-05	negative
PRJNA729271	stage_5W_vs_E10	ordinary_muscle_development	ENSGALG00010016804	CSF1R	3.013696476	0.135754692	22.19957506	3.4671E-109	1.7094E-107	positive
PRJNA729271	stage_5W_vs_E10	ordinary_muscle_development	ENSGALG00010016805	ENSGALG00010016805	-7.261667245	1.069024506	-6.792797736	1.0998E-11	2.90675E-11	negative
PRJNA729271	stage_5W_vs_E10	ordinary_muscle_development	ENSGALG00010016807	VSIG4	5.678408951	0.229520155	24.74034989	3.938E-135	3.0525E-133	positive
PRJNA729271	stage_5W_vs_E10	ordinary_muscle_development	ENSGALG00010016808	ENTPD3	-2.423416982	0.606335245	-3.996826844	6.41972E-05	0.000107689	negative
PRJNA729271	stage_5W_vs_E10	ordinary_muscle_development	ENSGALG00010016809	SYTL1	1.800930234	0.485520169	3.709279965	0.000207849	0.000333493	positive
PRJNA729271	stage_5W_vs_E10	ordinary_muscle_development	ENSGALG00010016810	SH3GL3	-2.337931735	0.205551761	-11.37393191	5.63889E-30	3.41277E-29	negative
PRJNA729271	stage_5W_vs_E10	ordinary_muscle_development	ENSGALG00010016812	LRRC31	-0.962538081	0.133894203	-7.188795765	6.53653E-13	1.8401E-12	negative
PRJNA729271	stage_5W_vs_E10	ordinary_muscle_development	ENSGALG00010016813	ENSGALG00010016813	-2.795318734	0.611451634	-4.571610535	4.8399E-06	8.81557E-06	negative
PRJNA729271	stage_5W_vs_E10	ordinary_muscle_development	ENSGALG00010016814	HMGXB3	0.52435275	0.099939966	5.24667732	1.54867E-07	3.15546E-07	positive
PRJNA729271	stage_5W_vs_E10	ordinary_muscle_development	ENSGALG00010016816	MAP3K6	3.605098963	0.259184188	13.90940932	5.55362E-44	5.48363E-43	positive
PRJNA729271	stage_5W_vs_E10	ordinary_muscle_development	ENSGALG00010016817	GGCT	-0.87631098	0.104061844	-8.421059497	3.73095E-17	1.30628E-16	negative
PRJNA729271	stage_5W_vs_E10	ordinary_muscle_development	ENSGALG00010016820	EIF2AK3	-0.715517527	0.109733855	-6.520481096	7.00822E-11	1.76572E-10	negative
PRJNA729271	stage_5W_vs_E10	ordinary_muscle_development	ENSGALG00010016823	HSF3	1.080833878	0.079097539	13.66457028	1.65277E-42	1.55362E-41	positive
PRJNA729271	stage_5W_vs_E10	ordinary_muscle_development	ENSGALG00010016825	ENSGALG00010016825	3.535374228	0.290893696	12.15349208	5.49666E-34	3.90177E-33	positive
PRJNA729271	stage_5W_vs_E10	ordinary_muscle_development	ENSGALG00010016828	LACTBL1	4.688010852	0.377662869	12.41321623	2.2157E-35	1.65635E-34	positive
PRJNA729271	stage_5W_vs_E10	ordinary_muscle_development	ENSGALG00010016831	AIDA	-0.649467816	0.067638311	-9.602070242	7.83546E-22	3.40147E-21	negative
PRJNA729271	stage_5W_vs_E10	ordinary_muscle_development	ENSGALG00010016832	DYNC1H1	0.031261849	0.094183835	0.331923724	0.739946856	0.76874496	positive
PRJNA729271	stage_5W_vs_E10	ordinary_muscle_development	ENSGALG00010016834	ENSGALG00010016834	1.431716334	0.07429313	19.27118061	9.37723E-83	2.69154E-81	positive
PRJNA729271	stage_5W_vs_E10	ordinary_muscle_development	ENSGALG00010016835	CCDC88A	-1.624218478	0.152229716	-10.66952315	1.4135E-26	7.50501E-26	negative
PRJNA729271	stage_5W_vs_E10	ordinary_muscle_development	ENSGALG00010016836	HEPH	1.829117745	0.1491969	12.25975702	1.48946E-34	1.07895E-33	positive
PRJNA729271	stage_5W_vs_E10	ordinary_muscle_development	ENSGALG00010016840	LRRC34	-0.450553865	0.211402932	-2.131256465	0.03306802	0.042689788	negative
PRJNA729271	stage_5W_vs_E10	ordinary_muscle_development	ENSGALG00010016842	NPY7R	0.266366786	0.436091861	0.610804304	0.541329144	0.581184056	positive
PRJNA729271	stage_5W_vs_E10	ordinary_muscle_development	ENSGALG00010016845	MIA3	1.369434301	0.137466129	9.961976162	2.23574E-23	1.03736E-22	positive
PRJNA729271	stage_5W_vs_E10	ordinary_muscle_development	ENSGALG00010016847	EPHB2	-1.601331628	0.107254452	-14.93021127	2.09601E-50	2.54666E-49	negative
PRJNA729271	stage_5W_vs_E10	ordinary_muscle_development	ENSGALG00010016848	ENSGALG00010016848	0.212039628	0.171493991	1.236425991	0.21630026	0.25008154	positive
PRJNA729271	stage_5W_vs_E10	ordinary_muscle_development	ENSGALG00010016849	WASF2	0.986857158	0.152699435	6.462742689	1.02822E-10	2.55864E-10	positive
PRJNA729271	stage_5W_vs_E10	ordinary_muscle_development	ENSGALG00010016851	TEC	1.977077536	0.138016681	14.32491723	1.5291E-46	1.63454E-45	positive
PRJNA729271	stage_5W_vs_E10	ordinary_muscle_development	ENSGALG00010016852	AHDC1	-0.181187618	0.170391767	-1.063358992	0.287619182	0.325325013	negative
PRJNA729271	stage_5W_vs_E10	ordinary_muscle_development	ENSGALG00010016854	TAF1A	-0.250649676	0.106527681	-2.352906534	0.018627316	0.024727808	negative
PRJNA729271	stage_5W_vs_E10	ordinary_muscle_development	ENSGALG00010016856	NOD1	-0.152591234	0.138689982	-1.100232559	0.271230808	0.308400577	negative
PRJNA729271	stage_5W_vs_E10	ordinary_muscle_development	ENSGALG00010016858	ENSGALG00010016858	-2.893940232	0.20486845	-14.12584626	2.63219E-45	2.7117E-44	negative
PRJNA729271	stage_5W_vs_E10	ordinary_muscle_development	ENSGALG00010016859	RPIA	0.498476718	0.13051853	3.819202676	0.000133884	0.00021805	positive
PRJNA729271	stage_5W_vs_E10	ordinary_muscle_development	ENSGALG00010016860	VSX1	-1.569808941	0.518276147	-3.028904474	0.002454423	0.003579161	negative
PRJNA729271	stage_5W_vs_E10	ordinary_muscle_development	ENSGALG00010016861	FGR	1.243893902	0.121419888	10.24456475	1.25187E-24	6.12829E-24	positive
PRJNA729271	stage_5W_vs_E10	ordinary_muscle_development	ENSGALG00010016862	TXK	6.337087664	0.506472276	12.51221038	6.40177E-36	4.90972E-35	positive
PRJNA729271	stage_5W_vs_E10	ordinary_muscle_development	ENSGALG00010016863	MYNN	-0.312040757	0.08313946	-3.753220884	0.000174577	0.000281573	negative
PRJNA729271	stage_5W_vs_E10	ordinary_muscle_development	ENSGALG00010016864	ENSGALG00010016864	0.34933324	0.267660762	1.305134293	0.191847123	0.22398905	positive
PRJNA729271	stage_5W_vs_E10	ordinary_muscle_development	ENSGALG00010016865	CFAP36	-1.65423607	0.14106285	-11.72694351	9.27476E-32	6.01511E-31	negative
PRJNA729271	stage_5W_vs_E10	ordinary_muscle_development	ENSGALG00010016866	ZNRF2	0.445285447	0.103546766	4.300331777	1.70543E-05	2.98589E-05	positive
PRJNA729271	stage_5W_vs_E10	ordinary_muscle_development	ENSGALG00010016867	FLT4	0.224441939	0.119546777	1.877440323	0.060457769	0.075442094	positive
PRJNA729271	stage_5W_vs_E10	ordinary_muscle_development	ENSGALG00010016868	NIPAL1	2.686342546	0.469788948	5.718190169	1.07665E-08	2.37101E-08	positive
PRJNA729271	stage_5W_vs_E10	ordinary_muscle_development	ENSGALG00010016869	HSP90AA1	0.482062811	0.156346817	3.083291504	0.002047245	0.003005045	positive
PRJNA729271	stage_5W_vs_E10	ordinary_muscle_development	ENSGALG00010016872	EDA2R	2.14505356	0.280242909	7.654265264	1.94421E-14	5.91919E-14	positive
PRJNA729271	stage_5W_vs_E10	ordinary_muscle_development	ENSGALG00010016873	ENTPD6	0.923289731	0.138924642	6.645975259	3.01216E-11	7.75786E-11	positive
PRJNA729271	stage_5W_vs_E10	ordinary_muscle_development	ENSGALG00010016874	ADAMTSL3	-0.256969362	0.145314675	-1.768364835	0.076999931	0.094907653	negative
PRJNA729271	stage_5W_vs_E10	ordinary_muscle_development	ENSGALG00010016875	SESN2	1.963486414	0.21890006	8.969784725	2.97095E-19	1.14684E-18	positive
PRJNA729271	stage_5W_vs_E10	ordinary_muscle_development	ENSGALG00010016876	MTURN	-0.992945013	0.193086847	-5.142478776	2.71137E-07	5.41564E-07	negative
PRJNA729271	stage_5W_vs_E10	ordinary_muscle_development	ENSGALG00010016877	UBE2D3	0.475902443	0.135627765	3.50888657	0.000449987	0.000700823	positive
PRJNA729271	stage_5W_vs_E10	ordinary_muscle_development	ENSGALG00010016878	SUCLG1	1.316659115	0.125405639	10.49920186	8.71136E-26	4.45873E-25	positive
PRJNA729271	stage_5W_vs_E10	ordinary_muscle_development	ENSGALG00010016879	RNF130	-0.178379353	0.101074784	-1.764825469	0.077593103	0.095598132	negative
PRJNA729271	stage_5W_vs_E10	ordinary_muscle_development	ENSGALG00010016880	AR	1.40674069	0.201581872	6.978507922	2.98331E-12	8.10556E-12	positive
PRJNA729271	stage_5W_vs_E10	ordinary_muscle_development	ENSGALG00010016881	C1QB	3.003460447	0.29019938	10.34964461	4.20042E-25	2.09443E-24	positive
PRJNA729271	stage_5W_vs_E10	ordinary_muscle_development	ENSGALG00010016882	ENSGALG00010016882	-0.746299938	0.050040949	-14.91378468	2.68115E-50	3.24401E-49	negative
PRJNA729271	stage_5W_vs_E10	ordinary_muscle_development	ENSGALG00010016883	TMEM251	0.103968019	0.077948967	1.333795977	0.18227073	0.213616933	positive
PRJNA729271	stage_5W_vs_E10	ordinary_muscle_development	ENSGALG00010016884	SBK1	-1.253283076	0.285343443	-4.392191613	1.12214E-05	1.99306E-05	negative
PRJNA729271	stage_5W_vs_E10	ordinary_muscle_development	ENSGALG00010016885	CDCA4	-0.646013696	0.153721207	-4.202502096	2.63981E-05	4.54874E-05	negative
PRJNA729271	stage_5W_vs_E10	ordinary_muscle_development	ENSGALG00010016887	TJP2	0.32262373	0.139096028	2.319431655	0.020371643	0.0268879	positive
PRJNA729271	stage_5W_vs_E10	ordinary_muscle_development	ENSGALG00010016888	ENSGALG00010016888	-0.605212147	0.0895509	-6.75830333	1.39617E-11	3.66887E-11	negative
PRJNA729271	stage_5W_vs_E10	ordinary_muscle_development	ENSGALG00010016889	FUNDC2	2.132923009	0.086815619	24.56842484	2.7488E-133	2.097E-131	positive
PRJNA729271	stage_5W_vs_E10	ordinary_muscle_development	ENSGALG00010016890	UBR7	-0.775670422	0.075847264	-10.2267423	1.50497E-24	7.34249E-24	negative
PRJNA729271	stage_5W_vs_E10	ordinary_muscle_development	ENSGALG00010016891	ENSGALG00010016891	-1.305478209	1.165892047	-1.119724774			negative
PRJNA729271	stage_5W_vs_E10	ordinary_muscle_development	ENSGALG00010016892	MAL	0.844074674	0.43885357	1.923362897	0.054434491	0.068290457	positive
PRJNA729271	stage_5W_vs_E10	ordinary_muscle_development	ENSGALG00010016893	TBC1D9B	0.129092871	0.050874058	2.537498972	0.01116477	0.015212759	positive
PRJNA729271	stage_5W_vs_E10	ordinary_muscle_development	ENSGALG00010016894	BTBD7	0.36145697	0.09186696	3.934569849	8.33458E-05	0.000138416	positive
PRJNA729271	stage_5W_vs_E10	ordinary_muscle_development	ENSGALG00010016895	ENSGALG00010016895	0.600350892	0.160764984	3.734338651	0.000188209	0.00030302	positive
PRJNA729271	stage_5W_vs_E10	ordinary_muscle_development	ENSGALG00010016896	SPTBN1	0.123593367	0.098752471	1.251547075	0.21073495	0.24413388	positive
PRJNA729271	stage_5W_vs_E10	ordinary_muscle_development	ENSGALG00010016898	ENSGALG00010016898	1.026378255	0.626326034	1.638728393	0.101269838	0.122878478	positive
PRJNA729271	stage_5W_vs_E10	ordinary_muscle_development	ENSGALG00010016899	SAXO2	-1.249278578	0.574696308	-2.173806517	0.029719669	0.038573299	negative
PRJNA729271	stage_5W_vs_E10	ordinary_muscle_development	ENSGALG00010016900	CISD2	-0.680681277	0.092118343	-7.389204559	1.4771E-13	4.28553E-13	negative
PRJNA729271	stage_5W_vs_E10	ordinary_muscle_development	ENSGALG00010016901	MECOM	-0.483843031	0.192576451	-2.51247247	0.011988846	0.016265287	negative
PRJNA729271	stage_5W_vs_E10	ordinary_muscle_development	ENSGALG00010016902	MRNIP	-1.526473649	0.147575652	-10.34366865	4.47087E-25	2.22699E-24	negative
PRJNA729271	stage_5W_vs_E10	ordinary_muscle_development	ENSGALG00010016903	PPM1A	1.69796814	0.147096199	11.54324962	7.98505E-31	4.99539E-30	positive
PRJNA729271	stage_5W_vs_E10	ordinary_muscle_development	ENSGALG00010016904	EAF1	0.823925954	0.069933176	11.78161781	4.85521E-32	3.19602E-31	positive
PRJNA729271	stage_5W_vs_E10	ordinary_muscle_development	ENSGALG00010016905	C1QC	4.155915759	0.370786698	11.20837339	3.70948E-29	2.17688E-28	positive
PRJNA729271	stage_5W_vs_E10	ordinary_muscle_development	ENSGALG00010016907	SQSTM1	3.636307548	0.159406445	22.81154663	3.5218E-115	1.9941E-113	positive
PRJNA729271	stage_5W_vs_E10	ordinary_muscle_development	ENSGALG00010016908	CMC4	1.547896904	0.159174006	9.724558296	2.36931E-22	1.05315E-21	positive
PRJNA729271	stage_5W_vs_E10	ordinary_muscle_development	ENSGALG00010016909	MRPS5	-0.371614791	0.1347895	-2.75700103	0.005833417	0.008180667	negative
PRJNA729271	stage_5W_vs_E10	ordinary_muscle_development	ENSGALG00010016910	NSD2	-1.081093851	0.111345117	-9.709396116	2.74961E-22	1.21883E-21	negative
PRJNA729271	stage_5W_vs_E10	ordinary_muscle_development	ENSGALG00010016911	AXIN1	-0.213294988	0.099781152	-2.137628037	0.032546944	0.042050803	negative
PRJNA729271	stage_5W_vs_E10	ordinary_muscle_development	ENSGALG00010016912	CCDC88C	1.37417845	0.159601038	8.610084678	7.30066E-18	2.65221E-17	positive
PRJNA729271	stage_5W_vs_E10	ordinary_muscle_development	ENSGALG00010016913	BRCC3	-0.453990479	0.112735381	-4.027045238	5.64822E-05	9.50109E-05	negative
PRJNA729271	stage_5W_vs_E10	ordinary_muscle_development	ENSGALG00010016915	PLOD2	-1.912528038	0.168334592	-11.36146777	6.50425E-30	3.92339E-29	negative
PRJNA729271	stage_5W_vs_E10	ordinary_muscle_development	ENSGALG00010016916	ACYP2	4.019766473	0.157307074	25.55362821	5.0038E-144	4.4772E-142	positive
PRJNA729271	stage_5W_vs_E10	ordinary_muscle_development	ENSGALG00010016917	METTL6	1.474892389	0.169830907	8.684475748	3.80493E-18	1.39839E-17	positive
PRJNA729271	stage_5W_vs_E10	ordinary_muscle_development	ENSGALG00010016918	RCOR1	-0.312668539	0.074678709	-4.186849842	2.82853E-05	4.86237E-05	negative
PRJNA729271	stage_5W_vs_E10	ordinary_muscle_development	ENSGALG00010016919	ARHGDIG	0.298387074	0.199466865	1.495923012	0.134673719	0.160812092	positive
PRJNA729271	stage_5W_vs_E10	ordinary_muscle_development	ENSGALG00010016920	C1QA	4.102689509	0.317834002	12.90827755	4.04242E-38	3.31045E-37	positive
PRJNA729271	stage_5W_vs_E10	ordinary_muscle_development	ENSGALG00010016921	EFL1	-0.094987221	0.074835715	-1.269276581	0.204342436	0.237316209	negative
PRJNA729271	stage_5W_vs_E10	ordinary_muscle_development	ENSGALG00010016922	ENSGALG00010016922	-0.611979123	0.198762719	-3.078943207	0.002077363	0.003048636	negative
PRJNA729271	stage_5W_vs_E10	ordinary_muscle_development	ENSGALG00010016923	ENSGALG00010016923	-3.412139329	0.1840886	-18.53531034	1.0717E-76	2.72053E-75	negative
PRJNA729271	stage_5W_vs_E10	ordinary_muscle_development	ENSGALG00010016924	LETM1	0.234434416	0.117205622	2.000197712	0.045478919	0.057633933	positive
PRJNA729271	stage_5W_vs_E10	ordinary_muscle_development	ENSGALG00010016925	PHACTR4	-0.02546943	0.134672128	-0.189121764	0.849997384	0.868083709	negative
PRJNA729271	stage_5W_vs_E10	ordinary_muscle_development	ENSGALG00010016926	ENSGALG00010016926	-0.009721783	0.184641738	-0.052652144	0.95800907	0.96366006	negative
PRJNA729271	stage_5W_vs_E10	ordinary_muscle_development	ENSGALG00010016927	MGAT4B	1.598661707	0.114901784	13.91328882	5.26039E-44	5.20118E-43	positive
PRJNA729271	stage_5W_vs_E10	ordinary_muscle_development	ENSGALG00010016928	RGS11	3.780231468	0.376887267	10.03013846	1.12359E-23	5.26728E-23	positive
PRJNA729271	stage_5W_vs_E10	ordinary_muscle_development	ENSGALG00010016930	C10H15ORF48	5.9034298	0.382693474	15.42600071	1.09445E-53	1.45409E-52	positive
PRJNA729271	stage_5W_vs_E10	ordinary_muscle_development	ENSGALG00010016931	SLC30A4	0.487957804	0.082143278	5.940325417	2.84457E-09	6.4698E-09	positive
PRJNA729271	stage_5W_vs_E10	ordinary_muscle_development	ENSGALG00010016932	OGT	0.689307045	0.092211232	7.475304617	7.70252E-14	2.27112E-13	positive
PRJNA729271	stage_5W_vs_E10	ordinary_muscle_development	ENSGALG00010016934	SLC25A38	-0.297727196	0.140834282	-2.114025029	0.03451313	0.04446825	negative
PRJNA729271	stage_5W_vs_E10	ordinary_muscle_development	ENSGALG00010016935	PRIMA1	-3.344841282	0.272125758	-12.29152767	1.00587E-34	7.34883E-34	negative
PRJNA729271	stage_5W_vs_E10	ordinary_muscle_development	ENSGALG00010016936	RCC1	-1.206460194	0.165799302	-7.276630117	3.42262E-13	9.75248E-13	negative
PRJNA729271	stage_5W_vs_E10	ordinary_muscle_development	ENSGALG00010016937	DHRS7	-0.260379602	0.172157754	-1.512447719	0.130420003	0.156079751	negative
PRJNA729271	stage_5W_vs_E10	ordinary_muscle_development	ENSGALG00010016938	PSME4	1.624253826	0.166231922	9.771010321	1.49949E-22	6.72498E-22	positive
PRJNA729271	stage_5W_vs_E10	ordinary_muscle_development	ENSGALG00010016939	FAM234A	0.414737156	0.079631391	5.208211863	1.90669E-07	3.85622E-07	positive
PRJNA729271	stage_5W_vs_E10	ordinary_muscle_development	ENSGALG00010016940	FGFR3	-4.623594049	0.221090644	-20.91266264	4.10664E-97	1.63532E-95	negative
PRJNA729271	stage_5W_vs_E10	ordinary_muscle_development	ENSGALG00010016941	SLC8A1	1.844285253	0.169792971	10.86196465	1.74944E-27	9.59084E-27	positive
PRJNA729271	stage_5W_vs_E10	ordinary_muscle_development	ENSGALG00010016942	ZBTB40	1.100735736	0.15046291	7.315661605	2.56116E-13	7.3513E-13	positive
PRJNA729271	stage_5W_vs_E10	ordinary_muscle_development	ENSGALG00010016943	PCNX4	-0.718876748	0.111212419	-6.463997063	1.01973E-10	2.53881E-10	negative
PRJNA729271	stage_5W_vs_E10	ordinary_muscle_development	ENSGALG00010016944	OPRD1	-6.390446637	0.903571754	-7.072428514	1.52245E-12	4.20342E-12	negative
PRJNA729271	stage_5W_vs_E10	ordinary_muscle_development	ENSGALG00010016945	BDH2	0.024658613	0.146120275	0.168755588	0.865988893	0.882487978	positive
PRJNA729271	stage_5W_vs_E10	ordinary_muscle_development	ENSGALG00010016946	TAF1	0.707688737	0.106342394	6.654812946	2.83661E-11	7.31482E-11	positive
PRJNA729271	stage_5W_vs_E10	ordinary_muscle_development	ENSGALG00010016947	CANX	-0.437539703	0.071393101	-6.128599217	8.86561E-10	2.07504E-09	negative
PRJNA729271	stage_5W_vs_E10	ordinary_muscle_development	ENSGALG00010016948	CCR8	3.53429149	0.47498282	7.440882795	1.00015E-13	2.9293E-13	positive
PRJNA729271	stage_5W_vs_E10	ordinary_muscle_development	ENSGALG00010016949	LUC7L	-0.12312349	0.095684431	-1.286766188	0.198175795	0.230560892	negative
PRJNA729271	stage_5W_vs_E10	ordinary_muscle_development	ENSGALG00010016950	ENSGALG00010016950	3.903180381	0.981636792	3.976196098	7.00264E-05	0.000117061	positive
PRJNA729271	stage_5W_vs_E10	ordinary_muscle_development	ENSGALG00010016952	GINS1	-3.155342756	0.233186939	-13.53138715	1.02079E-41	9.39451E-41	negative
PRJNA729271	stage_5W_vs_E10	ordinary_muscle_development	ENSGALG00010016953	GJA4	1.049521543	0.162457943	6.460266089	1.04519E-10	2.59908E-10	positive
PRJNA729271	stage_5W_vs_E10	ordinary_muscle_development	ENSGALG00010016955	ENSGALG00010016955	-6.250289744	0.273061535	-22.88967483	5.8878E-116	3.3733E-114	negative
PRJNA729271	stage_5W_vs_E10	ordinary_muscle_development	ENSGALG00010016956	CENPE	-2.889838733	0.257264804	-11.23293466	2.80988E-29	1.65633E-28	negative
PRJNA729271	stage_5W_vs_E10	ordinary_muscle_development	ENSGALG00010016957	WNT4	-1.922971336	0.241929005	-7.948494371	1.88792E-15	6.08661E-15	negative
PRJNA729271	stage_5W_vs_E10	ordinary_muscle_development	ENSGALG00010016958	TLR15	2.671207059	0.492591349	5.422764859	5.86842E-08	1.22959E-07	positive
PRJNA729271	stage_5W_vs_E10	ordinary_muscle_development	ENSGALG00010016959	PERCC1	-0.491953877	1.328769389	-0.370232668	0.711209138	0.742454192	negative
PRJNA729271	stage_5W_vs_E10	ordinary_muscle_development	ENSGALG00010016960	GJB3	-5.430614515	1.067158386	-5.088855213	3.60232E-07	7.13035E-07	negative
PRJNA729271	stage_5W_vs_E10	ordinary_muscle_development	ENSGALG00010016961	EIF4A3	-0.897305313	0.075145723	-11.94086999	7.24613E-33	4.92187E-32	negative

PRJNA729271	stage_5W_vs_E10	ordinary_muscle_development	ENSGALG00010016962	SQOR	2.402185395	0.136100547	17.65007888	1.01614E-69	2.13154E-68	positive
PRJNA729271	stage_5W_vs_E10	ordinary_muscle_development	ENSGALG00010016963	CD8A	5.742284985	0.650608764	8.826018491	1.08468E-18	4.08481E-18	positive
PRJNA729271	stage_5W_vs_E10	ordinary_muscle_development	ENSGALG00010016964	MAML1	0.421956118	0.085064044	4.960452111	7.03293E-07	1.36689E-06	positive
PRJNA729271	stage_5W_vs_E10	ordinary_muscle_development	ENSGALG00010016965	SLC25A33	-0.61487876	0.340401616	-1.806333259	0.0708663	0.087780466	negative
PRJNA729271	stage_5W_vs_E10	ordinary_muscle_development	ENSGALG00010016966	KDM3A	0.112760605	0.158429498	0.711739963	0.476625816	0.518281411	positive
PRJNA729271	stage_5W_vs_E10	ordinary_muscle_development	ENSGALG00010016967	ERLEC1	0.15891417	0.075113165	2.115663339	0.034373451	0.044296156	positive
PRJNA729271	stage_5W_vs_E10	ordinary_muscle_development	ENSGALG00010016968	C15orf61	0.805843803	0.147320469	5.470005677	4.50021E-08	9.50883E-08	positive
PRJNA729271	stage_5W_vs_E10	ordinary_muscle_development	ENSGALG00010016969	CHMP3	1.406842421	0.082755425	17.00000239	8.21166E-65	1.50478E-63	positive
PRJNA729271	stage_5W_vs_E10	ordinary_muscle_development	ENSGALG00010016970	SYNE3	1.476161599	0.098856208	14.93241174	2.02797E-50	2.47228E-49	positive
PRJNA729271	stage_5W_vs_E10	ordinary_muscle_development	ENSGALG00010016972	ASB3	-1.644840095	0.118037597	-13.93488298	3.88837E-44	3.85776E-43	negative
PRJNA729271	stage_5W_vs_E10	ordinary_muscle_development	ENSGALG00010016973	PLSCR1	3.120414904	0.120417777	25.9132413	4.7234E-148	4.5644E-146	positive
PRJNA729271	stage_5W_vs_E10	ordinary_muscle_development	ENSGALG00010016974	CFAP96	-1.880770496	0.749432676	-2.50959233	0.012087061	0.016392398	negative
PRJNA729271	stage_5W_vs_E10	ordinary_muscle_development	ENSGALG00010016975	MALL	1.841398776	0.212012286	8.685339938	3.77611E-18	1.3885E-17	positive
PRJNA729271	stage_5W_vs_E10	ordinary_muscle_development	ENSGALG00010016976	ENSGALG00010016976	6.266131859	0.907938857	6.901491011	5.14595E-12	1.38003E-11	positive
PRJNA729271	stage_5W_vs_E10	ordinary_muscle_development	ENSGALG00010016977	CLCN7	0.338158108	0.119608023	2.82721927	0.004695416	0.006650387	positive
PRJNA729271	stage_5W_vs_E10	ordinary_muscle_development	ENSGALG00010016978	FAM181A	1.764750271	0.364302136	4.84419414	1.27127E-06	2.41919E-06	positive
PRJNA729271	stage_5W_vs_E10	ordinary_muscle_development	ENSGALG00010016979	PLSCR5	-2.649052082	0.666331072	-3.975579399	7.02081E-05	0.000117324	negative
PRJNA729271	stage_5W_vs_E10	ordinary_muscle_development	ENSGALG00010016980	NPHP1	-0.570473412	0.102567949	-5.561907205	2.66842E-08	5.73019E-08	negative
PRJNA729271	stage_5W_vs_E10	ordinary_muscle_development	ENSGALG00010016981	ENSGALG00010016981	1.309333959	0.458319809	2.856812936	0.004279179	0.006085226	positive
PRJNA729271	stage_5W_vs_E10	ordinary_muscle_development	ENSGALG00010016982	GLRX5	-0.52236243	0.141597344	-3.689069396	0.000225076	0.000360096	negative
PRJNA729271	stage_5W_vs_E10	ordinary_muscle_development	ENSGALG00010016983	ZFAND2A	0.133073112	0.285471057	0.466152726	0.641106185	0.676285143	positive
PRJNA729271	stage_5W_vs_E10	ordinary_muscle_development	ENSGALG00010016984	RNF103	1.476453246	0.114409993	12.9049326	4.22185E-38	3.45543E-37	positive
PRJNA729271	stage_5W_vs_E10	ordinary_muscle_development	ENSGALG00010016985	ENSGALG00010016985	2.113785052	0.183022416	11.54932331	7.44039E-31	4.66473E-30	positive
PRJNA729271	stage_5W_vs_E10	ordinary_muscle_development	ENSGALG00010016986	GFER	0.354486123	0.08641336	4.102214302	4.09215E-05	6.94645E-05	positive
PRJNA729271	stage_5W_vs_E10	ordinary_muscle_development	ENSGALG00010016987	ASB2	2.952958994	0.318317098	9.276784094	1.74671E-20	7.11595E-20	positive
PRJNA729271	stage_5W_vs_E10	ordinary_muscle_development	ENSGALG00010016988	ECI1	0.722405661	0.110356726	6.546095427	5.90608E-11	1.4917E-10	positive
PRJNA729271	stage_5W_vs_E10	ordinary_muscle_development	ENSGALG00010016989	RHOG2	0.156326703	0.093008699	1.680775082	0.09280661	0.11316832	positive
PRJNA729271	stage_5W_vs_E10	ordinary_muscle_development	ENSGALG00010016991	PDLIM3	5.428483271	0.199135832	27.26020332	1.2579E-163	1.5069E-161	positive
PRJNA729271	stage_5W_vs_E10	ordinary_muscle_development	ENSGALG00010016993	ENSGALG00010016993	1.195582005	0.179862219	6.647210363	2.987E-11	7.69443E-11	positive
PRJNA729271	stage_5W_vs_E10	ordinary_muscle_development	ENSGALG00010016994	ATL2	1.225802681	0.115527445	10.61048901	2.66351E-26	1.39731E-25	positive
PRJNA729271	stage_5W_vs_E10	ordinary_muscle_development	ENSGALG00010016998	DNASE1L2	0.230744131	0.364607074	0.632856979	0.526827044	0.567505797	positive
PRJNA729271	stage_5W_vs_E10	ordinary_muscle_development	ENSGALG00010016999	ITGB1BP2	3.524612819	0.172297304	20.45657558	5.24985E-93	1.91198E-91	positive
PRJNA729271	stage_5W_vs_E10	ordinary_muscle_development	ENSGALG00010017001	TPCN3	-1.096465384	0.121628728	-9.014855326	1.9713E-19	7.67089E-19	negative
PRJNA729271	stage_5W_vs_E10	ordinary_muscle_development	ENSGALG00010017003	CPZ	-0.297459162	0.398915953	-0.745668755	0.455867538	0.497875223	negative
PRJNA729271	stage_5W_vs_E10	ordinary_muscle_development	ENSGALG00010017004	CX3CR1	1.005337836	0.403156486	2.493666533	0.012643126	0.017108114	positive
PRJNA729271	stage_5W_vs_E10	ordinary_muscle_development	ENSGALG00010017006	DNAH3	0.210125514	0.245839696	0.854725732	0.392703002	0.433991309	positive
PRJNA729271	stage_5W_vs_E10	ordinary_muscle_development	ENSGALG00010017008	RTN1	-0.375069653	0.167656162	-2.237136103	0.025277446	0.033032508	negative
PRJNA729271	stage_5W_vs_E10	ordinary_muscle_development	ENSGALG00010017010	NONO	-1.608212067	0.055606029	-28.92154116	6.4001E-184	9.0951E-182	negative
PRJNA729271	stage_5W_vs_E10	ordinary_muscle_development	ENSGALG00010017012	SYNGR3	2.444104358	0.177680772	13.75559288	4.714E-43	4.50722E-42	positive
PRJNA729271	stage_5W_vs_E10	ordinary_muscle_development	ENSGALG00010017016	ZIC1	-6.353858344	0.937282436	-6.779022097	1.20992E-11	3.18927E-11	negative
PRJNA729271	stage_5W_vs_E10	ordinary_muscle_development	ENSGALG00010017017	ENSGALG00010017017	0.864592849	0.269404452	3.209274539	0.001330704	0.001991796	positive
PRJNA729271	stage_5W_vs_E10	ordinary_muscle_development	ENSGALG00010017018	HP1BP3	-1.262802428	0.084092676	-15.0167945	5.69982E-51	7.07353E-50	negative
PRJNA729271	stage_5W_vs_E10	ordinary_muscle_development	ENSGALG00010017020	GJB5	-3.409506226	0.583886623	-5.839329233	5.24114E-09	1.17656E-08	negative
PRJNA729271	stage_5W_vs_E10	ordinary_muscle_development	ENSGALG00010017022	CCR4	2.566599738	0.529714444	4.845251566	1.26451E-06	2.40698E-06	positive
PRJNA729271	stage_5W_vs_E10	ordinary_muscle_development	ENSGALG00010017024	BUB1	-3.339313641	0.220583666	-15.13853539	9.02002E-52	1.15499E-50	negative
PRJNA729271	stage_5W_vs_E10	ordinary_muscle_development	ENSGALG00010017033	LDAF1	0.495745106	0.111265852	4.455500922	8.36975E-06	1.49944E-05	positive
PRJNA729271	stage_5W_vs_E10	ordinary_muscle_development	ENSGALG00010017034	ZMYM3	0.23165572	0.090591286	2.557152361	0.010553298	0.014416177	positive
PRJNA729271	stage_5W_vs_E10	ordinary_muscle_development	ENSGALG00010017035	PIK3CD	1.850880084	0.200307286	9.240203499	2.46029E-20	9.96977E-20	positive
PRJNA729271	stage_5W_vs_E10	ordinary_muscle_development	ENSGALG00010017038	ZNF598	0.020562935	0.131474989	0.156401874	0.87571626	0.890960005	positive
PRJNA729271	stage_5W_vs_E10	ordinary_muscle_development	ENSGALG00010017039	MAP2K5	-0.754843084	0.078984433	-9.556858904	1.21385E-21	5.21789E-21	negative
PRJNA729271	stage_5W_vs_E10	ordinary_muscle_development	ENSGALG00010017041	JKAMP	0.152997163	0.090527076	1.690070747	0.091014421	0.111141873	positive
PRJNA729271	stage_5W_vs_E10	ordinary_muscle_development	ENSGALG00010017042	HNRNPLL	-1.118755567	0.071002397	-15.75658877	6.18844E-56	8.81153E-55	negative
PRJNA729271	stage_5W_vs_E10	ordinary_muscle_development	ENSGALG00010017045	GLB1	-0.107169348	0.099505041	-1.077024304	0.281469408	0.319041216	negative
PRJNA729271	stage_5W_vs_E10	ordinary_muscle_development	ENSGALG00010017049	TRAF3	0.186362678	0.090139025	2.067502715	0.038686809	0.049524445	positive
PRJNA729271	stage_5W_vs_E10	ordinary_muscle_development	ENSGALG00010017050	L3HYPDH	-1.287460991	0.158314079	-8.1323215	4.21146E-16	1.40205E-15	negative
PRJNA729271	stage_5W_vs_E10	ordinary_muscle_development	ENSGALG00010017054	GALM	-0.707266472	0.152509131	-4.637535263	3.52588E-06	6.49234E-06	negative
PRJNA729271	stage_5W_vs_E10	ordinary_muscle_development	ENSGALG00010017055	GPR135	-1.553820551	0.343341266	-4.525586364	6.02283E-06	1.08908E-05	negative
PRJNA729271	stage_5W_vs_E10	ordinary_muscle_development	ENSGALG00010017056	AGTR1	3.393876256	0.202428886	16.76577052	4.34347E-63	7.59452E-62	positive
PRJNA729271	stage_5W_vs_E10	ordinary_muscle_development	ENSGALG00010017057	NDUFAB1	0.638902101	0.121914928	5.240556757	1.60093E-07	3.25736E-07	positive
PRJNA729271	stage_5W_vs_E10	ordinary_muscle_development	ENSGALG00010017060	CLSTN1	-1.621759861	0.102677266	-15.79473167	3.38229E-56	4.86372E-55	negative
PRJNA729271	stage_5W_vs_E10	ordinary_muscle_development	ENSGALG00010017063	TMPPE	0.402364272	0.10870241	3.701521158	0.000214311	0.000343518	positive
PRJNA729271	stage_5W_vs_E10	ordinary_muscle_development	ENSGALG00010017065	VRK2	-0.412009697	0.135351523	-3.043997495	0.002334571	0.003409875	negative
PRJNA729271	stage_5W_vs_E10	ordinary_muscle_development	ENSGALG00010017068	ZP2	-3.517880292	1.13552493	-3.098021188	0.001948175	0.00286601	negative
PRJNA729271	stage_5W_vs_E10	ordinary_muscle_development	ENSGALG00010017070	BLOC1S6	0.384592382	0.104919711	3.665587496	0.000246771	0.000393504	positive
PRJNA729271	stage_5W_vs_E10	ordinary_muscle_development	ENSGALG00010017077	CTNNBIP1	-0.580735201	0.105356617	-5.512090425	3.54597E-08	7.53537E-08	negative
PRJNA729271	stage_5W_vs_E10	ordinary_muscle_development	ENSGALG00010017080	PALB2	-0.996796286	0.10450178	-9.538558008	1.44832E-21	6.21107E-21	negative
PRJNA729271	stage_5W_vs_E10	ordinary_muscle_development	ENSGALG00010017083	ENSGALG00010017083	-0.32315682	0.434821975	-0.743193395	0.457364602	0.499434978	negative
PRJNA729271	stage_5W_vs_E10	ordinary_muscle_development	ENSGALG00010017085	ENSGALG00010017085	-1.073871037	0.121808628	-8.816050683	1.18568E-18	4.45128E-18	negative
PRJNA729271	stage_5W_vs_E10	ordinary_muscle_development	ENSGALG00010017089	CRTAP	-0.942080077	0.158165953	-5.956276049	2.5805E-09	5.88767E-09	negative
PRJNA729271	stage_5W_vs_E10	ordinary_muscle_development	ENSGALG00010017094	SORBS2	0.253307501	0.148285698	1.708239589	0.087591896	0.107161084	positive
PRJNA729271	stage_5W_vs_E10	ordinary_muscle_development	ENSGALG00010017096	DCTN5	-0.696913413	0.092171426	-7.561057105	3.99807E-14	1.19909E-13	negative
PRJNA729271	stage_5W_vs_E10	ordinary_muscle_development	ENSGALG00010017098	DAAM1	-1.228916783	0.125036402	-9.828472083	8.4897E-23	3.84677E-22	negative
PRJNA729271	stage_5W_vs_E10	ordinary_muscle_development	ENSGALG00010017100	SUSD5	-7.778422271	0.620870333	-12.52825568	5.23003E-36	4.02599E-35	negative
PRJNA729271	stage_5W_vs_E10	ordinary_muscle_development	ENSGALG00010017101	CSMD2	-0.517055947	0.236722533	-2.184227839	0.02894551	0.037608925	negative
PRJNA729271	stage_5W_vs_E10	ordinary_muscle_development	ENSGALG00010017104	FANCL	-2.83588771	0.183125623	-15.48602356	4.31146E-54	5.80265E-53	negative
PRJNA729271	stage_5W_vs_E10	ordinary_muscle_development	ENSGALG00010017105	PLK1	-3.705031776	0.237912148	-15.57310886	1.10884E-54	1.51772E-53	negative
PRJNA729271	stage_5W_vs_E10	ordinary_muscle_development	ENSGALG00010017106	CRYM	-1.238589188	0.12137678	-10.20449869	1.89296E-24	9.17675E-24	negative
PRJNA729271	stage_5W_vs_E10	ordinary_muscle_development	ENSGALG00010017109	ITGB1BP3	0.330436433	0.127589446	2.58984144	0.009602015	0.013176295	positive
PRJNA729271	stage_5W_vs_E10	ordinary_muscle_development	ENSGALG00010017110	BRF1	-0.202999008	0.072530288	-2.798817078	0.005129018	0.007234127	negative
PRJNA729271	stage_5W_vs_E10	ordinary_muscle_development	ENSGALG00010017111	GJB1	-2.138254155	0.499610289	-4.279844117	1.87024E-05	3.26381E-05	negative
PRJNA729271	stage_5W_vs_E10	ordinary_muscle_development	ENSGALG00010017112	LZIC	-0.423514926	0.063439295	-6.675908429	2.45705E-11	6.35755E-11	negative
PRJNA729271	stage_5W_vs_E10	ordinary_muscle_development	ENSGALG00010017115	LGALSL	2.457934211	0.191847406	12.81192306	1.40606E-37	1.13416E-36	positive
PRJNA729271	stage_5W_vs_E10	ordinary_muscle_development	ENSGALG00010017116	ENSGALG00010017116	-2.119476697	0.294943206	-7.186050255	6.66925E-13	1.8771E-12	negative
PRJNA729271	stage_5W_vs_E10	ordinary_muscle_development	ENSGALG00010017119	BTBD6	-1.668018499	0.152023244	-10.97212804	5.20331E-28	2.91655E-27	negative
PRJNA729271	stage_5W_vs_E10	ordinary_muscle_development	ENSGALG00010017120	ERN2	1.481398081	0.141047208	10.50285293	8.38082E-26	4.2941E-25	positive
PRJNA729271	stage_5W_vs_E10	ordinary_muscle_development	ENSGALG00010017122	NCBP2	-1.172316322	0.193271368	-6.065649215	1.31422E-09	3.04891E-09	negative
PRJNA729271	stage_5W_vs_E10	ordinary_muscle_development	ENSGALG00010017127	SOX8	-1.307245639	0.135964067	-9.614640591	6.93511E-22	3.02329E-21	negative
PRJNA729271	stage_5W_vs_E10	ordinary_muscle_development	ENSGALG00010017131	TLR3	4.339572615	0.374281881	11.59439673	4.39955E-31	2.78721E-30	positive
PRJNA729271	stage_5W_vs_E10	ordinary_muscle_development	ENSGALG00010017132	TMEM108	-2.056247048	0.290950226	-7.067349897	1.5792E-12	4.35762E-12	negative
PRJNA729271	stage_5W_vs_E10	ordinary_muscle_development	ENSGALG00010017133	WDR20	0.366959027	0.112797969	3.253241443	0.001140965	0.001719052	positive
PRJNA729271	stage_5W_vs_E10	ordinary_muscle_development	ENSGALG00010017135	ENSGALG00010017135	0.54347878	0.133686376	4.065326587	4.79652E-05	8.10511E-05	positive
PRJNA729271	stage_5W_vs_E10	ordinary_muscle_development	ENSGALG00010017136	ENSGALG00010017136	0.064080673	0.409269661	0.15657323	0.875581202	0.890885127	positive
PRJNA729271	stage_5W_vs_E10	ordinary_muscle_development	ENSGALG00010017139	COL9A2	-9.454402681	0.611546871	-15.45981694	6.47824E-54	8.6466E-53	negative
PRJNA729271	stage_5W_vs_E10	ordinary_muscle_development	ENSGALG00010017140	UQCRC2	0.568407871	0.115156054	4.935979041	7.97497E-07	1.54397E-06	positive
PRJNA729271	stage_5W_vs_E10	ordinary_muscle_development	ENSGALG00010017141	AFTPH	0.638414328	0.075774093	8.425232182	3.60036E-17	1.26178E-16	positive
PRJNA729271	stage_5W_vs_E10	ordinary_muscle_development	ENSGALG00010017142	RBP7	2.201098787	0.694476487	3.169436013	0.001527351	0.002271594	positive
PRJNA729271	stage_5W_vs_E10	ordinary_muscle_development	ENSGALG00010017143	FAM149A	0.566737734	0.269583686	2.102270143	0.035529618	0.045716984	positive
PRJNA729271	stage_5W_vs_E10	ordinary_muscle_development	ENSGALG00010017144	CPB1	0.717977033	0.74940894	0.958057757	0.338033645	0.377866715	positive
PRJNA729271	stage_5W_vs_E10	ordinary_muscle_development	ENSGALG00010017147	SRSF7L	-1.232822048	0.071795642	-17.17126568	4.35812E-66	8.29016E-65	negative
PRJNA729271	stage_5W_vs_E10	ordinary_muscle_development	ENSGALG00010017148	MOK	-0.757035904	0.498523714	-1.518555452	0.128874432	0.15431928	negative
PRJNA729271	stage_5W_vs_E10	ordinary_muscle_development	ENSGALG00010017152	CYP4V2	3.569035431	0.196161477	18.1943748	5.71879E-74	1.3435E-72	positive
PRJNA729271	stage_5W_vs_E10	ordinary_muscle_development	ENSGALG00010017153	SLC35F4	-1.183945322	0.614151034	-1.927775508	0.053883055	0.067651355	negative
PRJNA729271	stage_5W_vs_E10	ordinary_muscle_development	ENSGALG00010017154	PIGZ	0.532262422	0.188756385	2.819837976	0.00480479	0.006797329	positive
PRJNA729271	stage_5W_vs_E10	ordinary_muscle_development	ENSGALG00010017162	BCL11A	-2.597128616	0.215684066	-12.04135597	2.15386E-33	1.48951E-32	negative
PRJNA729271	stage_5W_vs_E10	ordinary_muscle_development	ENSGALG00010017163	CDV3	-0.397488907	0.124785961	-3.185365599	0.001445712	0.002156814	negative
PRJNA729271	stage_5W_vs_E10	ordinary_muscle_development	ENSGALG00010017167	SMAP2	-0.256151851	0.097226105	-2.634599527	0.00842366	0.011626448	negative
PRJNA729271	stage_5W_vs_E10	ordinary_muscle_development	ENSGALG00010017169	CCDC198	0.171422968	0.251104465	0.682675904	0.494811659	0.536406776	positive
PRJNA729271	stage_5W_vs_E10	ordinary_muscle_development	ENSGALG00010017174	DHX57	-0.455250754	0.097491597	-4.669640946	3.01727E-06	5.59418E-06	negative
PRJNA729271	stage_5W_vs_E10	ordinary_muscle_development	ENSGALG00010017180	ENSGALG00010017180	-2.890475127	0.592450758	-4.878844509	1.06709E-06	2.04488E-06	negative
PRJNA729271	stage_5W_vs_E10	ordinary_muscle_development	ENSGALG00010017183	NFYC	-0.133741267	0.093952539	-1.423498164	0.154591794	0.183087512	negative
PRJNA729271	stage_5W_vs_E10	ordinary_muscle_development	ENSGALG00010017184	CARD11	2.417281928	0.209994821	11.51115022	1.15922E-30	7.22394E-30	positive
PRJNA729271	stage_5W_vs_E10	ordinary_muscle_development	ENSGALG00010017185	NAA30	0.34166959	0.064221836	5.320146725	1.03684E-07	2.13692E-07	positive
PRJNA729271	stage_5W_vs_E10	ordinary_muscle_development	ENSGALG00010017187	ZNF839	0.789026657	0.138665183	5.690156971	1.26923E-08	2.78454E-08	positive
PRJNA729271	stage_5W_vs_E10	ordinary_muscle_development	ENSGALG00010017188	ENSGALG00010017188	2.752108106	0.154210737	17.84641042	3.08222E-71	6.76921E-70	positive
PRJNA729271	stage_5W_vs_E10	ordinary_muscle_development	ENSGALG00010017190	STK26	-0.381159719	0.068120406	-5.595382378	2.20136E-08	4.75682E-08	negative
PRJNA729271	stage_5W_vs_E10	ordinary_muscle_development	ENSGALG00010017198	TOPBP1	-0.977048791	0.069824588	-13.9929045	1.72232E-44	1.71935E-43	negative
PRJNA729271	stage_5W_vs_E10	ordinary_muscle_development	ENSGALG00010017200	KCNQ4	1.270897474	0.306817758	4.142190087	3.44005E-05	5.86906E-05	positive
PRJNA729271	stage_5W_vs_E10	ordinary_muscle_development	ENSGALG00010017201	AP5M1	-1.419593151	0.155003757	-9.158443477	5.26512E-20	2.103E-19	negative
PRJNA729271	stage_5W_vs_E10	ordinary_muscle_development	ENSGALG00010017207	ENSGALG00010017207	8.776805921	0.28047013	31.29319303	5.7755E-215	1.1627E-212	positive
PRJNA729271	stage_5W_vs_E10	ordinary_muscle_development	ENSGALG00010017208	CINP	-0.574685436	0.173391458	-3.314381475	0.000918461	0.00139682	negative
PRJNA729271	stage_5W_vs_E10	ordinary_muscle_development	ENSGALG00010017211	UBFD1	1.557606943	0.127520328	12.21457764	2.59847E-34	1.86068E-33	positive
PRJNA729271	stage_5W_vs_E10	ordinary_muscle_development	ENSGALG00010017212	TINAGL1	3.481157877	0.134774716	25.82945808	4.1402E-147	3.8969E-145	positive
PRJNA729271	stage_5W_vs_E10	ordinary_muscle_development	ENSGALG00010017215	FRMD7	4.102370991	0.582595349	7.041544354	1.90121E-12	5.22625E-12	positive
PRJNA729271	stage_5W_vs_E10	ordinary_muscle_development	ENSGALG00010017219	EARS2	-1.61514649	0.154874565	-10.42873948	1.83304E-25	9.23527E-25	negative
PRJNA729271	stage_5W_vs_E10	ordinary_muscle_development	ENSGALG00010017221	SPPL2A	0.180851146	0.075673	2.389903213	0.016852813	0.022478328	positive
PRJNA729271	stage_5W_vs_E10	ordinary_muscle_development	ENSGALG00010017223	NOC2L	-1.06538799	0.077067332	-13.82411923	1.8233E-43	1.77732E-42	negative
PRJNA729271	stage_5W_vs_E10	ordinary_muscle_development	ENSGALG00010017226	PEF1	-0.170731773	0.075886944	-2.249817484	0.024460534	0.032022709	negative
PRJNA729271	stage_5W_vs_E10	ordinary_muscle_development	ENSGALG00010017228	RAP2C	-0.580401284	0.07219732	-8.039097304	9.05027E-16	2.96259E-15	negative
PRJNA729271	stage_5W_vs_E10	ordinary_muscle_development	ENSGALG00010017240	MELTF	-0.533136314	0.245458262	-2.172003948	0.029855365	0.038742481	negative
PRJNA729271	stage_5W_vs_E10	ordinary_muscle_development	ENSGALG00010017246	GGA2	0.804338652	0.129664545	6.203227358	5.53168E-10	1.31338E-09	positive
PRJNA729271	stage_5W_vs_E10	ordinary_muscle_development	ENSGALG00010017248	TECPR2	0.752544481	0.083909757	8.968497939	3.00585E-19	1.1597E-18	positive

PRJNA729271	stage_5W_vs_E10	ordinary_muscle_development	ENSGALG00010017250	COL16A1	-0.689952781	0.17027904	-4.051894942	5.08045E-05	8.56591E-05	negative
PRJNA729271	stage_5W_vs_E10	ordinary_muscle_development	ENSGALG00010017252	EXOC5	-0.162799087	0.087971204	-1.85059519	0.064227812	0.07991949	negative
PRJNA729271	stage_5W_vs_E10	ordinary_muscle_development	ENSGALG00010017256	CARHSP1	0.294475939	0.128795114	2.286390616	0.022231417	0.029199383	positive
PRJNA729271	stage_5W_vs_E10	ordinary_muscle_development	ENSGALG00010017263	COG7	-0.443430459	0.072969066	-6.076965001	1.22478E-09	2.84735E-09	negative
PRJNA729271	stage_5W_vs_E10	ordinary_muscle_development	ENSGALG00010017264	PIAS1	0.610478867	0.086056567	7.093925374	1.3036E-12	3.61157E-12	positive
PRJNA729271	stage_5W_vs_E10	ordinary_muscle_development	ENSGALG00010017266	PMM2	-1.275698136	0.110445807	-11.55044422	7.34399E-31	4.60628E-30	negative
PRJNA729271	stage_5W_vs_E10	ordinary_muscle_development	ENSGALG00010017272	NRXN1	2.000931265	0.134081998	14.92319098	2.32866E-50	2.82223E-49	positive
PRJNA729271	stage_5W_vs_E10	ordinary_muscle_development	ENSGALG00010017278	CCR5	1.953389027	0.227105533	8.601239276	7.88599E-18	2.85911E-17	positive
PRJNA729271	stage_5W_vs_E10	ordinary_muscle_development	ENSGALG00010017280	SOS1	1.253655209	0.15484706	8.096086631	5.67554E-16	1.87482E-15	positive
PRJNA729271	stage_5W_vs_E10	ordinary_muscle_development	ENSGALG00010017283	INF2	1.28230484	0.136456303	9.397182886	5.60435E-21	2.33904E-20	positive
PRJNA729271	stage_5W_vs_E10	ordinary_muscle_development	ENSGALG00010017284	ENSGALG00010017284	1.092637542	0.196401617	5.563281816	2.64748E-08	5.68774E-08	positive
PRJNA729271	stage_5W_vs_E10	ordinary_muscle_development	ENSGALG00010017285	IL21R	6.349581546	0.807895559	7.8594089	3.8595E-15	1.22147E-14	positive
PRJNA729271	stage_5W_vs_E10	ordinary_muscle_development	ENSGALG00010017289	ADGRB2	-1.331751004	0.314797006	-4.230507205	2.33165E-05	4.03839E-05	negative
PRJNA729271	stage_5W_vs_E10	ordinary_muscle_development	ENSGALG00010017290	DACT1	-1.418633464	0.157092823	-9.030542829	1.70822E-19	6.67224E-19	negative
PRJNA729271	stage_5W_vs_E10	ordinary_muscle_development	ENSGALG00010017296	SCNN1G	1.912067838	0.225170117	8.491658948	2.03708E-17	7.23004E-17	positive
PRJNA729271	stage_5W_vs_E10	ordinary_muscle_development	ENSGALG00010017299	USP31	1.241576717	0.110540544	11.23186721	2.84404E-29	1.67579E-28	positive
PRJNA729271	stage_5W_vs_E10	ordinary_muscle_development	ENSGALG00010017300	TRPM7	-0.529650218	0.104869576	-5.050561272	4.40514E-07	8.67325E-07	negative
PRJNA729271	stage_5W_vs_E10	ordinary_muscle_development	ENSGALG00010017301	LHCGR	-2.289762918	0.563506461	-4.063419101	4.83591E-05	8.16975E-05	negative
PRJNA729271	stage_5W_vs_E10	ordinary_muscle_development	ENSGALG00010017304	ANKRD9	6.067499594	0.297446091	20.39865299	1.71885E-92	6.19771E-91	positive
PRJNA729271	stage_5W_vs_E10	ordinary_muscle_development	ENSGALG00010017305	HS3ST2	3.381263967	0.476513756	7.095837056	1.28571E-12	3.56404E-12	positive
PRJNA729271	stage_5W_vs_E10	ordinary_muscle_development	ENSGALG00010017307	TMEM186	0.415189488	0.156926682	2.645754581	0.008150893	0.011261766	positive
PRJNA729271	stage_5W_vs_E10	ordinary_muscle_development	ENSGALG00010017310	SAMD11	-0.939753854	0.177342876	-5.299078695	1.16388E-07	2.39128E-07	negative
PRJNA729271	stage_5W_vs_E10	ordinary_muscle_development	ENSGALG00010017312	PTP4A2	1.548695901	0.154639154	10.01490151	1.31092E-23	6.12963E-23	positive
PRJNA729271	stage_5W_vs_E10	ordinary_muscle_development	ENSGALG00010017313	MAP4K3	0.631517665	0.082528142	7.652149313	1.97648E-14	6.01491E-14	positive
PRJNA729271	stage_5W_vs_E10	ordinary_muscle_development	ENSGALG00010017314	CALML4	0.80895277	0.220162863	3.674337984	0.000238467	0.000380848	positive
PRJNA729271	stage_5W_vs_E10	ordinary_muscle_development	ENSGALG00010017315	HS3ST4	-2.27986098	0.947193157	-2.406965215	0.016085702	0.021513402	negative
PRJNA729271	stage_5W_vs_E10	ordinary_muscle_development	ENSGALG00010017318	ADSS1	4.232509824	0.123661821	34.22648801	9.7619E-257	2.8877E-254	positive
PRJNA729271	stage_5W_vs_E10	ordinary_muscle_development	ENSGALG00010017322	KHDRBS1	-1.39488144	0.071206463	-19.58925331	1.9097E-85	5.79104E-84	negative
PRJNA729271	stage_5W_vs_E10	ordinary_muscle_development	ENSGALG00010017323	ENSGALG00010017323	1.232887659	0.104654092	11.78059679	4.91439E-32	3.23204E-31	positive
PRJNA729271	stage_5W_vs_E10	ordinary_muscle_development	ENSGALG00010017326	STON1	-0.16833823	0.123783708	-1.359938503	0.173849385	0.204375251	negative
PRJNA729271	stage_5W_vs_E10	ordinary_muscle_development	ENSGALG00010017327	CLN6	1.07157718	0.131482402	8.149966583	3.64025E-16	1.21495E-15	positive
PRJNA729271	stage_5W_vs_E10	ordinary_muscle_development	ENSGALG00010017332	FGFR1	-0.420151416	0.159041781	-2.641767544	0.008247464	0.011387597	negative
PRJNA729271	stage_5W_vs_E10	ordinary_muscle_development	ENSGALG00010017334	KDM8	-0.421787569	0.158375502	-2.663212201	0.007739859	0.010715306	negative
PRJNA729271	stage_5W_vs_E10	ordinary_muscle_development	ENSGALG00010017335	THUMPD2	-0.01819264	0.137719521	-0.132099211	0.89490582	0.908061593	negative
PRJNA729271	stage_5W_vs_E10	ordinary_muscle_development	ENSGALG00010017337	ABAT	1.006172366	0.201790267	4.986228425	6.15693E-07	1.20098E-06	positive
PRJNA729271	stage_5W_vs_E10	ordinary_muscle_development	ENSGALG00010017338	MBNL3	0.499129256	0.074597687	6.690948141	2.21729E-11	5.75567E-11	positive
PRJNA729271	stage_5W_vs_E10	ordinary_muscle_development	ENSGALG00010017340	SIVA1	-0.831641471	0.107596562	-7.729256926	1.08176E-14	3.34402E-14	negative
PRJNA729271	stage_5W_vs_E10	ordinary_muscle_development	ENSGALG00010017341	TA3	-0.421373484	0.081245168	-5.186443618	2.14348E-07	4.32064E-07	negative
PRJNA729271	stage_5W_vs_E10	ordinary_muscle_development	ENSGALG00010017343	DLG1	-0.028393633	0.099593039	-0.285096563	0.775570164	0.802247452	negative
PRJNA729271	stage_5W_vs_E10	ordinary_muscle_development	ENSGALG00010017344	RERE	1.483615023	0.153220312	9.682887341	3.56503E-22	1.57354E-21	positive
PRJNA729271	stage_5W_vs_E10	ordinary_muscle_development	ENSGALG00010017346	GM2A	-0.554058665	0.158049431	-3.505603661	0.000455573	0.000709371	negative
PRJNA729271	stage_5W_vs_E10	ordinary_muscle_development	ENSGALG00010017347	TMEM39B	-0.964697575	0.12402907	-7.77799571	7.36826E-15	2.29733E-14	negative
PRJNA729271	stage_5W_vs_E10	ordinary_muscle_development	ENSGALG00010017349	ENSGALG00010017349	1.161343396	0.113785565	10.20642111	1.85584E-24	9.00885E-24	positive
PRJNA729271	stage_5W_vs_E10	ordinary_muscle_development	ENSGALG00010017351	CCR2	3.110178292	0.205219224	15.15539446	6.97951E-52	8.9688E-51	positive
PRJNA729271	stage_5W_vs_E10	ordinary_muscle_development	ENSGALG00010017352	GPCPD1	1.617569872	0.238359871	6.786250825	1.15085E-11	3.03688E-11	positive
PRJNA729271	stage_5W_vs_E10	ordinary_muscle_development	ENSGALG00010017354	FEM1B	-0.071456114	0.105273737	-0.678764869	0.497286857	0.538727428	negative
PRJNA729271	stage_5W_vs_E10	ordinary_muscle_development	ENSGALG00010017356	NSMCE1	-0.858631431	0.121539754	-7.064613893	1.61063E-12	4.4418E-12	negative
PRJNA729271	stage_5W_vs_E10	ordinary_muscle_development	ENSGALG00010017359	EHD3	0.864228242	0.095329954	9.065652539	1.23861E-19	4.8734E-19	positive
PRJNA729271	stage_5W_vs_E10	ordinary_muscle_development	ENSGALG00010017361	METTL22	0.11148721	0.151531014	0.735738557	0.46188982	0.50376922	positive
PRJNA729271	stage_5W_vs_E10	ordinary_muscle_development	ENSGALG00010017362	ENO1	-0.224770061	0.1301948	-1.726413503	0.084273048	0.103318771	negative
PRJNA729271	stage_5W_vs_E10	ordinary_muscle_development	ENSGALG00010017363	TIMM9	-0.237668878	0.14334877	-1.657976399	0.09732223	0.118425598	negative
PRJNA729271	stage_5W_vs_E10	ordinary_muscle_development	ENSGALG00010017364	AKT1	-0.424920369	0.073934748	-5.747234936	9.07146E-09	2.00811E-08	negative
PRJNA729271	stage_5W_vs_E10	ordinary_muscle_development	ENSGALG00010017367	TIMD4	0.524644405	0.308944798	1.698181707	0.089473472	0.109296981	positive
PRJNA729271	stage_5W_vs_E10	ordinary_muscle_development	ENSGALG00010017368	ERLIN2	-0.300892191	0.050642022	-5.941551689	2.82337E-09	6.42359E-09	negative
PRJNA729271	stage_5W_vs_E10	ordinary_muscle_development	ENSGALG00010017369	CAPN14	-0.2236281	0.531159397	-0.421018815	0.673741343	0.707518711	negative
PRJNA729271	stage_5W_vs_E10	ordinary_muscle_development	ENSGALG00010017371	TMEM114	-0.673770139	1.190793145	-0.565816273	0.571518683	0.610386333	negative
PRJNA729271	stage_5W_vs_E10	ordinary_muscle_development	ENSGALG00010017372	ENSGALG00010017372	0.84571413	0.110441896	7.657548085	1.89516E-14	5.77352E-14	positive
PRJNA729271	stage_5W_vs_E10	ordinary_muscle_development	ENSGALG00010017373	RP1-2705.3	-0.361486477	0.107924033	-3.349453006	0.000809713	0.001236362	negative
PRJNA729271	stage_5W_vs_E10	ordinary_muscle_development	ENSGALG00010017375	BDH1A	-0.605670505	0.161567726	-3.748709721	0.000177747	0.000286526	negative
PRJNA729271	stage_5W_vs_E10	ordinary_muscle_development	ENSGALG00010017376	GALNT14	-0.326406622	0.279630615	-1.167277848	0.243098181	0.278620079	negative
PRJNA729271	stage_5W_vs_E10	ordinary_muscle_development	ENSGALG00010017377	USP8	0.278788072	0.075613424	3.687018199	0.000226897	0.00036297	positive
PRJNA729271	stage_5W_vs_E10	ordinary_muscle_development	ENSGALG00010017378	ENSGALG00010017378	-0.932139974	0.563220561	-1.655017659	0.097920916	0.119064145	negative
PRJNA729271	stage_5W_vs_E10	ordinary_muscle_development	ENSGALG00010017380	MARCKSL1	-1.722811793	0.135581683	-12.70681814	5.4195E-37	4.30442E-36	negative
PRJNA729271	stage_5W_vs_E10	ordinary_muscle_development	ENSGALG00010017381	CA6	-0.01484956	0.676097255	-0.021963645	0.982476956	0.985116577	negative
PRJNA729271	stage_5W_vs_E10	ordinary_muscle_development	ENSGALG00010017383	ENSGALG00010017383	0.120702699	0.151470129	0.796874604	0.425523861	0.467305733	positive
PRJNA729271	stage_5W_vs_E10	ordinary_muscle_development	ENSGALG00010017384	ARID4A	0.073514527	0.092766568	0.792467898	0.428087903	0.469872342	positive
PRJNA729271	stage_5W_vs_E10	ordinary_muscle_development	ENSGALG00010017386	ENSGALG00010017386	-0.826777571	0.6463429	-1.279162455	0.200839854	0.233435465	negative
PRJNA729271	stage_5W_vs_E10	ordinary_muscle_development	ENSGALG00010017387	HS6ST2	-0.863334328	0.115105083	-7.500401429	6.36227E-14	1.88668E-13	negative
PRJNA729271	stage_5W_vs_E10	ordinary_muscle_development	ENSGALG00010017388	PLPBP	0.347082258	0.082768108	4.193429908	2.74768E-05	4.72507E-05	positive
PRJNA729271	stage_5W_vs_E10	ordinary_muscle_development	ENSGALG00010017390	DDX24	-1.796020684	0.105825373	-16.97155065	1.33363E-64	2.42852E-63	negative
PRJNA729271	stage_5W_vs_E10	ordinary_muscle_development	ENSGALG00010017391	CRAMP1	0.424730709	0.116648709	3.641109387	0.000271466	0.00043108	positive
PRJNA729271	stage_5W_vs_E10	ordinary_muscle_development	ENSGALG00010017393	VDAC1	3.292060029	0.163615866	20.12066505	4.86508E-90	1.66712E-88	positive
PRJNA729271	stage_5W_vs_E10	ordinary_muscle_development	ENSGALG00010017395	BRF2	-0.346631197	0.127347658	-2.721928322	0.006490222	0.009058812	negative
PRJNA729271	stage_5W_vs_E10	ordinary_muscle_development	ENSGALG00010017396	CCR8L	3.688624353	0.271594449	13.58136872	5.16521E-42	4.78707E-41	positive
PRJNA729271	stage_5W_vs_E10	ordinary_muscle_development	ENSGALG00010017398	SLC22A5	-0.047368691	0.183348529	-0.258353267	0.79613428	0.820304365	negative
PRJNA729271	stage_5W_vs_E10	ordinary_muscle_development	ENSGALG00010017399	CPXM1	-0.10727433	0.290856108	-0.368822684	0.712259896	0.743229947	negative
PRJNA729271	stage_5W_vs_E10	ordinary_muscle_development	ENSGALG00010017400	GPC4	-1.31676789	0.119172129	-11.04929396	2.20946E-28	1.25642E-27	negative
PRJNA729271	stage_5W_vs_E10	ordinary_muscle_development	ENSGALG00010017402	SLC2A5	2.33134768	0.226947286	10.2726396	9.36078E-25	4.60885E-24	positive
PRJNA729271	stage_5W_vs_E10	ordinary_muscle_development	ENSGALG00010017403	ITGA11	-0.178498007	0.292549146	-0.610147079	0.541764382	0.58160826	negative
PRJNA729271	stage_5W_vs_E10	ordinary_muscle_development	ENSGALG00010017405	XCR1	6.518137171	0.901208764	7.232660655	4.73622E-13	1.34216E-12	positive
PRJNA729271	stage_5W_vs_E10	ordinary_muscle_development	ENSGALG00010017406	IFT140	-1.134176531	0.125864898	-9.011063074	2.04069E-19	7.93235E-19	negative
PRJNA729271	stage_5W_vs_E10	ordinary_muscle_development	ENSGALG00010017407	EPN2	0.46696128	0.085025498	5.492014669	3.97374E-08	8.4222E-08	positive
PRJNA729271	stage_5W_vs_E10	ordinary_muscle_development	ENSGALG00010017409	GPC3	-1.699628104	0.344006718	-4.940682884	7.78494E-07	1.50799E-06	negative
PRJNA729271	stage_5W_vs_E10	ordinary_muscle_development	ENSGALG00010017411	RAB11FIP1	-0.683183521	0.140302287	-4.869368384	1.11956E-06	2.14033E-06	negative
PRJNA729271	stage_5W_vs_E10	ordinary_muscle_development	ENSGALG00010017413	ZIC3	-6.478339303	0.995874432	-6.505176852	7.7602E-11	1.94811E-10	negative
PRJNA729271	stage_5W_vs_E10	ordinary_muscle_development	ENSGALG00010017414	ENSGALG00010017414	0.206460277	0.197841466	1.043564227	0.296687051	0.334902173	positive
PRJNA729271	stage_5W_vs_E10	ordinary_muscle_development	ENSGALG00010017415	HDAC1	-1.75702091	0.06549233	-26.82788832	1.5281E-158	1.717E-156	negative
PRJNA729271	stage_5W_vs_E10	ordinary_muscle_development	ENSGALG00010017418	CHGB	-0.823357498	0.287302663	-2.865819233	0.004159317	0.005926403	negative
PRJNA729271	stage_5W_vs_E10	ordinary_muscle_development	ENSGALG00010017419	B9D1	-1.115961264	0.103451191	-10.78732161	3.95138E-27	2.14353E-26	negative
PRJNA729271	stage_5W_vs_E10	ordinary_muscle_development	ENSGALG00010017421	GPR157	2.445070233	0.261818119	9.338812172	9.74209E-21	4.01055E-20	positive
PRJNA729271	stage_5W_vs_E10	ordinary_muscle_development	ENSGALG00010017422	ENSGALG00010017422	-0.115548742	0.232736397	-0.496479038	0.619556473	0.656179386	negative
PRJNA729271	stage_5W_vs_E10	ordinary_muscle_development	ENSGALG00010017423	TMEM204	0.450575593	0.132750995	3.394140986	0.000688442	0.001057431	positive
PRJNA729271	stage_5W_vs_E10	ordinary_muscle_development	ENSGALG00010017424	APOD	3.983814679	0.479428274	8.309511332	9.6097E-17	3.29921E-16	positive
PRJNA729271	stage_5W_vs_E10	ordinary_muscle_development	ENSGALG00010017425	H6PD	0.751234252	0.12580462	5.971436111	2.35174E-09	5.37589E-09	positive
PRJNA729271	stage_5W_vs_E10	ordinary_muscle_development	ENSGALG00010017428	PSMA3	0.257977492	0.107945623	2.389883762	0.016853706	0.022478328	positive
PRJNA729271	stage_5W_vs_E10	ordinary_muscle_development	ENSGALG00010017429	TELO2	-0.262486161	0.104432778	-2.513446112	0.011955804	0.016226534	negative
PRJNA729271	stage_5W_vs_E10	ordinary_muscle_development	ENSGALG00010017430	CACNA1H	-2.892332221	0.219734348	-13.16285889	1.4355E-39	1.23854E-38	negative
PRJNA729271	stage_5W_vs_E10	ordinary_muscle_development	ENSGALG00010017431	FYCO1	3.751812908	0.117208885	32.00962885	8.0099E-225	1.7329E-222	positive
PRJNA729271	stage_5W_vs_E10	ordinary_muscle_development	ENSGALG00010017433	RNPS1	-0.894231115	0.079918123	-11.18934078	4.5984E-29	2.68982E-28	negative
PRJNA729271	stage_5W_vs_E10	ordinary_muscle_development	ENSGALG00010017434	FGF13	2.626070366	0.148382357	17.69799605	4.34495E-70	9.20761E-69	positive
PRJNA729271	stage_5W_vs_E10	ordinary_muscle_development	ENSGALG00010017435	ACTR10	-0.824120402	0.091007167	-9.055554953	1.35875E-19	5.33453E-19	negative
PRJNA729271	stage_5W_vs_E10	ordinary_muscle_development	ENSGALG00010017437	DRG2	0.113154622	0.13989024	0.808881463	0.418583332	0.460171817	positive
PRJNA729271	stage_5W_vs_E10	ordinary_muscle_development	ENSGALG00010017438	ADRB3	1.556911989	0.863188498	1.803675551	0.071282185	0.088280525	positive
PRJNA729271	stage_5W_vs_E10	ordinary_muscle_development	ENSGALG00010017439	PPP4R4	-1.744504483	0.275143321	-6.340348282	2.29246E-10	5.57351E-10	negative
PRJNA729271	stage_5W_vs_E10	ordinary_muscle_development	ENSGALG00010017441	SPSB1	0.231091454	0.256969743	0.899294412	0.368495863	0.409173245	positive
PRJNA729271	stage_5W_vs_E10	ordinary_muscle_development	ENSGALG00010017442	LCK	2.575684566	0.238215977	10.81239219	3.00724E-27	1.63687E-26	positive
PRJNA729271	stage_5W_vs_E10	ordinary_muscle_development	ENSGALG00010017445	F9	4.775520248	1.16610669	4.095268715	4.21679E-05	7.14949E-05	positive
PRJNA729271	stage_5W_vs_E10	ordinary_muscle_development	ENSGALG00010017447	CAPN13	-0.994994716	0.573961279	-1.733557212	0.082996678	0.1018487	negative
PRJNA729271	stage_5W_vs_E10	ordinary_muscle_development	ENSGALG00010017449	TRMT6	-1.135645609	0.088340646	-12.85530109	8.03125E-38	6.52539E-37	negative
PRJNA729271	stage_5W_vs_E10	ordinary_muscle_development	ENSGALG00010017450	ENSGALG00010017450	2.327421797	0.209559649	11.10624973	1.16969E-28	6.72273E-28	positive
PRJNA729271	stage_5W_vs_E10	ordinary_muscle_development	ENSGALG00010017451	GABPB1	-0.875371097	0.078502315	-11.15089531	7.08895E-29	4.10689E-28	negative
PRJNA729271	stage_5W_vs_E10	ordinary_muscle_development	ENSGALG00010017452	ARMH4	-3.088937922	0.445349698	-6.935982971	4.03406E-12	1.08829E-11	negative
PRJNA729271	stage_5W_vs_E10	ordinary_muscle_development	ENSGALG00010017453	PPP1R2	1.356291167	0.138002423	9.828024328	8.52752E-23	3.8627E-22	positive
PRJNA729271	stage_5W_vs_E10	ordinary_muscle_development	ENSGALG00010017454	ENSGALG00010017454	1.182218083	0.158739662	7.447528018	9.51054E-14	2.7889E-13	positive
PRJNA729271	stage_5W_vs_E10	ordinary_muscle_development	ENSGALG00010017455	CCR9	3.225787437	0.694510001	4.644695444	3.40578E-06	6.28877E-06	positive
PRJNA729271	stage_5W_vs_E10	ordinary_muscle_development	ENSGALG00010017457	ZMAT4	-2.06264052	0.268588743	-7.679549415	1.59649E-14	4.88313E-14	negative
PRJNA729271	stage_5W_vs_E10	ordinary_muscle_development	ENSGALG00010017458	ENSGALG00010017458	0.735266557	0.172187822	4.270142617	1.95348E-05	3.40333E-05	positive
PRJNA729271	stage_5W_vs_E10	ordinary_muscle_development	ENSGALG00010017459	FAM167B	1.745310376	0.289923312	6.019903549	1.74521E-09	4.0192E-09	positive
PRJNA729271	stage_5W_vs_E10	ordinary_muscle_development	ENSGALG00010017462	MCM8	-0.568848097	0.120727337	-4.71184165	2.45488E-06	4.57371E-06	negative
PRJNA729271	stage_5W_vs_E10	ordinary_muscle_development	ENSGALG00010017463	PPP1R36	0.380369539	0.349221215	1.089193678	0.276068486	0.313262307	positive
PRJNA729271	stage_5W_vs_E10	ordinary_muscle_development	ENSGALG00010017464	SH3BP2	0.915624729	0.128713601	7.113659503	1.13006E-12	3.14396E-12	positive
PRJNA729271	stage_5W_vs_E10	ordinary_muscle_development	ENSGALG00010017465	TMEM234	-0.060206417	0.239041654	-0.251865799	0.801144796	0.824875253	negative
PRJNA729271	stage_5W_vs_E10	ordinary_muscle_development	ENSGALG00010017466	SPIA4	6.697178913	2.727136398	2.455755025			positive
PRJNA729271	stage_5W_vs_E10	ordinary_muscle_development	ENSGALG00010017467	SFRP1	-4.726952373	0.118629465	-39.84636005	0	0	negative
PRJNA729271	stage_5W_vs_E10	ordinary_muscle_development	ENSGALG00010017468	AMDHD2	-0.138224702	0.129092033	-1.070745408	0.284283919	0.321954482	negative
PRJNA729271	stage_5W_vs_E10	ordinary_muscle_development	ENSGALG00010017470	HSPA2	3.134538213	0.169884291	18.45101858	5.11715E-76	1.27664E-74	positive
PRJNA729271	stage_5W_vs_E10	ordinary_muscle_development	ENSGALG00010017471	CRLS1	0.876481313	0.089056632	9.841842129	7.43371E-23	3.37568E-22	positive
PRJNA729271	stage_5W_vs_E10	ordinary_muscle_development	ENSGALG00010017472	LZTFL1	-1.381075928	0.172323098	-8.014456251	1.10625E-15	3.60827E-15	negative
PRJNA729271	stage_5W_vs_E10	ordinary_muscle_development	ENSGALG00010017473	EIF3I	0.091252913	0.10464365	0.87203488	0.383189349	0.424383376	positive
PRJNA729271	stage_5W_vs_E10	ordinary_muscle_development	ENSGALG00010017475	STUM	1.89808651	0.24790789	7.656418312	1.9119E-14	5.82329E-14	positive
PRJNA729271	stage_5W_vs_E10	ordinary_muscle_development	ENSGALG00010017476	ATP6V0C	1.530871296	0.111999841	13.66851316	1.56562E-42	1.47589E-41	positive

PRJNA729271	stage_5W_vs_E10	ordinary_muscle_development	ENSGALG00010017477	MCF2	1.728083911	0.182055832	9.492054684	2.26524E-21	9.62895E-21	positive
PRJNA729271	stage_5W_vs_E10	ordinary_muscle_development	ENSGALG00010017479	SSTR5	1.448072175	0.486000627	2.979568532	0.002886547	0.004177365	positive
PRJNA729271	stage_5W_vs_E10	ordinary_muscle_development	ENSGALG00010017480	DCDC2B	0.570897865	0.201266246	2.8365306	0.004560661	0.006463946	positive
PRJNA729271	stage_5W_vs_E10	ordinary_muscle_development	ENSGALG00010017481	SPIA1	6.983744274	2.485780918	2.809476983			positive
PRJNA729271	stage_5W_vs_E10	ordinary_muscle_development	ENSGALG00010017482	CORO2B	-0.854062158	0.14530744	-5.87762168	4.16203E-09	9.38382E-09	negative
PRJNA729271	stage_5W_vs_E10	ordinary_muscle_development	ENSGALG00010017484	IQCC	0.035956488	0.135255577	0.265841077	0.790361606	0.815394411	positive
PRJNA729271	stage_5W_vs_E10	ordinary_muscle_development	ENSGALG00010017485	LBH	-0.681094849	0.108051131	-6.303449483	2.91093E-10	7.0253E-10	negative
PRJNA729271	stage_5W_vs_E10	ordinary_muscle_development	ENSGALG00010017487	C1QTNF8	0.034991363	0.509637046	0.068659378	0.945260753	0.952521002	positive
PRJNA729271	stage_5W_vs_E10	ordinary_muscle_development	ENSGALG00010017489	TNFRSF1B	1.294976653	0.177507505	7.295334644	2.97918E-13	8.51235E-13	positive
PRJNA729271	stage_5W_vs_E10	ordinary_muscle_development	ENSGALG00010017491	CCDC28B	1.186826002	0.197869765	5.998015934	1.99743E-09	4.57896E-09	positive
PRJNA729271	stage_5W_vs_E10	ordinary_muscle_development	ENSGALG00010017493	YPEL5	0.056850667	0.134629298	0.422275598	0.672823867	0.70665763	positive
PRJNA729271	stage_5W_vs_E10	ordinary_muscle_development	ENSGALG00010017494	TBC1D24	-1.541675836	0.097413726	-15.8260638	2.05693E-56	2.9726E-55	negative
PRJNA729271	stage_5W_vs_E10	ordinary_muscle_development	ENSGALG00010017495	NT5M	0.286154744	0.134299197	2.130725644	0.033111751	0.042738632	positive
PRJNA729271	stage_5W_vs_E10	ordinary_muscle_development	ENSGALG00010017496	PARP1	-1.514462511	0.103925376	-14.57259594	4.19637E-48	4.71157E-47	negative
PRJNA729271	stage_5W_vs_E10	ordinary_muscle_development	ENSGALG00010017499	SNRPD1	-1.598877495	0.099602176	-16.05263611	5.47868E-58	8.32426E-57	negative
PRJNA729271	stage_5W_vs_E10	ordinary_muscle_development	ENSGALG00010017500	ACAP2	0.197143493	0.100730946	1.957129382	0.050332259	0.063435014	positive
PRJNA729271	stage_5W_vs_E10	ordinary_muscle_development	ENSGALG00010017502	NAT8B	0.614756167	0.118804552	5.174516944	2.28501E-07	4.59507E-07	positive
PRJNA729271	stage_5W_vs_E10	ordinary_muscle_development	ENSGALG00010017503	MED9	0.9623635	0.125447469	7.671446104	1.70068E-14	5.18867E-14	positive
PRJNA729271	stage_5W_vs_E10	ordinary_muscle_development	ENSGALG00010017505	TNFRSF8	1.179195288	0.302346214	3.900149007	9.61335E-05	0.000158618	positive
PRJNA729271	stage_5W_vs_E10	ordinary_muscle_development	ENSGALG00010017509	RASD1	0.566092228	0.302739925	1.86989618	0.061498236	0.076681027	positive
PRJNA729271	stage_5W_vs_E10	ordinary_muscle_development	ENSGALG00010017510	HDC	4.549930128	0.379254132	11.99704826	3.68195E-33	2.53057E-32	positive
PRJNA729271	stage_5W_vs_E10	ordinary_muscle_development	ENSGALG00010017513	TNIP2	0.674995483	0.144277781	4.678443753	2.8906E-06	5.36414E-06	positive
PRJNA729271	stage_5W_vs_E10	ordinary_muscle_development	ENSGALG00010017514	TXLNA	-0.290063296	0.097003806	-2.990225931	0.002787712	0.00404281	negative
PRJNA729271	stage_5W_vs_E10	ordinary_muscle_development	ENSGALG00010017516	AAK1	-0.034192798	0.080361535	-0.425487127	0.670481604	0.704452802	negative
PRJNA729271	stage_5W_vs_E10	ordinary_muscle_development	ENSGALG00010017517	LIN9	-0.724621519	0.104141477	-6.958049162	3.45017E-12	9.35294E-12	negative
PRJNA729271	stage_5W_vs_E10	ordinary_muscle_development	ENSGALG00010017518	PEMT	1.241394639	0.147108519	8.438631905	3.21073E-17	1.12796E-16	positive
PRJNA729271	stage_5W_vs_E10	ordinary_muscle_development	ENSGALG00010017519	NTN3	-2.174307704	0.206469985	-10.53086579	6.22592E-26	3.20357E-25	negative
PRJNA729271	stage_5W_vs_E10	ordinary_muscle_development	ENSGALG00010017520	ZBTB1	-0.817254253	0.081516731	-10.02560143	1.17641E-23	5.51134E-23	negative
PRJNA729271	stage_5W_vs_E10	ordinary_muscle_development	ENSGALG00010017521	MIIP	0.523111545	0.116298912	4.497991722	6.85984E-06	1.23566E-05	positive
PRJNA729271	stage_5W_vs_E10	ordinary_muscle_development	ENSGALG00010017522	XXYLT1	-0.596835281	0.107904823	-5.531126996	3.1818E-08	6.78537E-08	negative
PRJNA729271	stage_5W_vs_E10	ordinary_muscle_development	ENSGALG00010017524	ACBD3	-0.261223231	0.060153226	-4.342630442	1.40787E-05	2.48321E-05	negative
PRJNA729271	stage_5W_vs_E10	ordinary_muscle_development	ENSGALG00010017525	ENSGALG00010017525	-4.9956607	0.830637173	-6.014251303	1.8072E-09	4.15601E-09	negative
PRJNA729271	stage_5W_vs_E10	ordinary_muscle_development	ENSGALG00010017526	PLEKHA2	1.949390918	0.170063819	11.46270226	2.03078E-30	1.24624E-29	positive
PRJNA729271	stage_5W_vs_E10	ordinary_muscle_development	ENSGALG00010017528	RAI1	0.450286684	0.177272575	2.540080918	0.011082682	0.015109432	positive
PRJNA729271	stage_5W_vs_E10	ordinary_muscle_development	ENSGALG00010017529	MFN2	1.256408649	0.118288038	10.62160355	2.36464E-26	1.24367E-25	positive
PRJNA729271	stage_5W_vs_E10	ordinary_muscle_development	ENSGALG00010017530	TLR1B	1.087230173	0.212032584	5.127656096	2.93372E-07	5.84204E-07	positive
PRJNA729271	stage_5W_vs_E10	ordinary_muscle_development	ENSGALG00010017531	SACM1L	-0.579221304	0.112732119	-5.138032616	2.7763E-07	5.54151E-07	negative
PRJNA729271	stage_5W_vs_E10	ordinary_muscle_development	ENSGALG00010017532	MIXL1	0.664779524	0.320675892	2.073057377	0.03816694	0.048893486	positive
PRJNA729271	stage_5W_vs_E10	ordinary_muscle_development	ENSGALG00010017533	FAM193A	-0.01681776	0.078466752	-0.214329764	0.830289909	0.850593839	negative
PRJNA729271	stage_5W_vs_E10	ordinary_muscle_development	ENSGALG00010017534	ATP11C	-0.846129744	0.084568129	-10.00530288	1.44449E-23	6.7498E-23	negative
PRJNA729271	stage_5W_vs_E10	ordinary_muscle_development	ENSGALG00010017535	LIMD1	0.045254916	0.089970038	0.502999852	0.614964376	0.651839754	positive
PRJNA729271	stage_5W_vs_E10	ordinary_muscle_development	ENSGALG00010017536	GSC	-2.98486764	0.474062197	-6.296362922	3.0471E-10	7.34538E-10	negative
PRJNA729271	stage_5W_vs_E10	ordinary_muscle_development	ENSGALG00010017537	MANBA	1.006113757	0.187528952	5.365111599	8.08992E-08	1.67903E-07	positive
PRJNA729271	stage_5W_vs_E10	ordinary_muscle_development	ENSGALG00010017538	H3-3A	-0.481536481	0.076162975	-6.322448439	2.57451E-10	6.24247E-10	negative
PRJNA729271	stage_5W_vs_E10	ordinary_muscle_development	ENSGALG00010017539	KPNA6	1.150882255	0.138358781	8.318100577	8.93841E-17	3.0753E-16	positive
PRJNA729271	stage_5W_vs_E10	ordinary_muscle_development	ENSGALG00010017540	EIF4G1	0.882556357	0.16454559	5.363597748	8.15805E-08	1.69268E-07	positive
PRJNA729271	stage_5W_vs_E10	ordinary_muscle_development	ENSGALG00010017541	SREBF1	1.868254608	0.227529567	8.211041021	2.19279E-16	7.39862E-16	positive
PRJNA729271	stage_5W_vs_E10	ordinary_muscle_development	ENSGALG00010017542	PLOD1	-0.32486265	0.125319179	-2.592281982	0.009534159	0.013085658	negative
PRJNA729271	stage_5W_vs_E10	ordinary_muscle_development	ENSGALG00010017543	ZBTB25	0.451381343	0.176268834	2.560755252	0.010444491	0.014278307	positive
PRJNA729271	stage_5W_vs_E10	ordinary_muscle_development	ENSGALG00010017544	SDE2	0.326790837	0.068657486	4.759726216	1.93856E-06	3.63935E-06	positive
PRJNA729271	stage_5W_vs_E10	ordinary_muscle_development	ENSGALG00010017545	DICER1	-0.053341474	0.120734816	-0.441806896	0.658628948	0.693004254	negative
PRJNA729271	stage_5W_vs_E10	ordinary_muscle_development	ENSGALG00010017546	NFU1	0.792902935	0.108680808	7.295703384	2.97103E-13	8.49074E-13	positive
PRJNA729271	stage_5W_vs_E10	ordinary_muscle_development	ENSGALG00010017547	RNF4	0.01406875	0.065813953	0.213765465	0.830729957	0.850924368	positive
PRJNA729271	stage_5W_vs_E10	ordinary_muscle_development	ENSGALG00010017550	TM2D2	0.528478535	0.076478519	6.91015646	4.84119E-12	1.30022E-11	positive
PRJNA729271	stage_5W_vs_E10	ordinary_muscle_development	ENSGALG00010017551	NALF2	-0.841711012	0.286923932	-2.933568516	0.003350897	0.004819533	negative
PRJNA729271	stage_5W_vs_E10	ordinary_muscle_development	ENSGALG00010017552	LARS2	-0.10141033	0.103432467	-0.980449689	0.326864193	0.366511679	negative
PRJNA729271	stage_5W_vs_E10	ordinary_muscle_development	ENSGALG00010017553	EDA	0.378010189	0.109455345	3.453556236	0.000553247	0.000857039	positive
PRJNA729271	stage_5W_vs_E10	ordinary_muscle_development	ENSGALG00010017554	FAM131A	-3.28136233	0.394837223	-8.310671183	9.51623E-17	3.26867E-16	negative
PRJNA729271	stage_5W_vs_E10	ordinary_muscle_development	ENSGALG00010017555	CELA2A	1.65407548	1.223068774	1.352397768	0.176248103	0.206960163	positive
PRJNA729271	stage_5W_vs_E10	ordinary_muscle_development	ENSGALG00010017556	TOM1L2	-0.11497945	0.115589462	-0.994722599	0.3198712	0.359142761	negative
PRJNA729271	stage_5W_vs_E10	ordinary_muscle_development	ENSGALG00010017558	ENSGALG00010017558	-1.752256082	0.075528186	-23.20002877	4.5502E-119	2.7142E-117	negative
PRJNA729271	stage_5W_vs_E10	ordinary_muscle_development	ENSGALG00010017559	DUSP10	2.118905132	0.149760153	14.14865765	1.90356E-45	1.97228E-44	positive
PRJNA729271	stage_5W_vs_E10	ordinary_muscle_development	ENSGALG00010017561	CFAP99	0.090522411	0.576028322	0.157149236	0.875127235	0.89054825	positive
PRJNA729271	stage_5W_vs_E10	ordinary_muscle_development	ENSGALG00010017562	SLC30A10	-3.681176323	0.620730889	-5.930390109	3.02216E-09	6.86295E-09	negative
PRJNA729271	stage_5W_vs_E10	ordinary_muscle_development	ENSGALG00010017563	ALK	1.198315795	0.154253596	7.768478807	7.94343E-15	2.47294E-14	positive
PRJNA729271	stage_5W_vs_E10	ordinary_muscle_development	ENSGALG00010017565	ENSGALG00010017565	-0.600769539	0.188927592	-3.179893062	0.001473294	0.002195476	negative
PRJNA729271	stage_5W_vs_E10	ordinary_muscle_development	ENSGALG00010017568	CDCP1	-2.347466188	0.247906274	-9.469168126	2.82081E-21	1.1938E-20	negative
PRJNA729271	stage_5W_vs_E10	ordinary_muscle_development	ENSGALG00010017569	CLMN	-1.475025428	0.24724314	-5.965890207	2.43304E-09	5.55822E-09	negative
PRJNA729271	stage_5W_vs_E10	ordinary_muscle_development	ENSGALG00010017570	DRC3	-0.589516971	0.381180407	-1.546556327	0.121970268	0.146378459	negative
PRJNA729271	stage_5W_vs_E10	ordinary_muscle_development	ENSGALG00010017571	ADAM9	1.140461243	0.149868538	7.609744242	2.74639E-14	8.31431E-14	positive
PRJNA729271	stage_5W_vs_E10	ordinary_muscle_development	ENSGALG00010017573	ENSGALG00010017573	0.603312608	0.227314851	2.654083557	0.007952413	0.011001167	positive
PRJNA729271	stage_5W_vs_E10	ordinary_muscle_development	ENSGALG00010017574	DYNC1LI1	-0.228118975	0.056308197	-4.051256987	5.09432E-05	8.5883E-05	negative
PRJNA729271	stage_5W_vs_E10	ordinary_muscle_development	ENSGALG00010017575	CLCN2	1.646947375	0.765928812	2.150261682	0.031534522	0.040811865	positive
PRJNA729271	stage_5W_vs_E10	ordinary_muscle_development	ENSGALG00010017576	ENSGALG00010017576	-2.273367192	0.290755816	-7.818819328	5.33211E-15	1.67292E-14	negative
PRJNA729271	stage_5W_vs_E10	ordinary_muscle_development	ENSGALG00010017577	CLPX	1.245334416	0.137621505	9.048981203	1.44308E-19	5.65491E-19	positive
PRJNA729271	stage_5W_vs_E10	ordinary_muscle_development	ENSGALG00010017578	BSDC1	0.243375628	0.100576839	2.419797939	0.015529133	0.020809355	positive
PRJNA729271	stage_5W_vs_E10	ordinary_muscle_development	ENSGALG00010017579	P3H2	-0.471449384	0.145909616	-3.23110564	0.001233124	0.001850515	negative
PRJNA729271	stage_5W_vs_E10	ordinary_muscle_development	ENSGALG00010017580	LYPLAL1	-0.008866972	0.155741631	-0.056933859	0.954597883	0.96096231	negative
PRJNA729271	stage_5W_vs_E10	ordinary_muscle_development	ENSGALG00010017581	EFCAB11	-2.668581961	0.513622398	-5.195610571	2.04049E-07	4.11877E-07	negative
PRJNA729271	stage_5W_vs_E10	ordinary_muscle_development	ENSGALG00010017582	CLCN6	0.109940763	0.102865093	1.068785914	0.285166148	0.3228275	positive
PRJNA729271	stage_5W_vs_E10	ordinary_muscle_development	ENSGALG00010017583	MTHFD1	-1.510983345	0.107428484	-14.065016	6.23088E-45	6.34246E-44	negative
PRJNA729271	stage_5W_vs_E10	ordinary_muscle_development	ENSGALG00010017584	WNT11B	0.163613527	0.628771609	0.260211379	0.794700727	0.819113066	positive
PRJNA729271	stage_5W_vs_E10	ordinary_muscle_development	ENSGALG00010017585	ENSGALG00010017585	-0.647618017	0.123988019	-5.223230627	1.75828E-07	3.56502E-07	negative
PRJNA729271	stage_5W_vs_E10	ordinary_muscle_development	ENSGALG00010017586	ZFYVE28	0.339969541	0.160754199	2.114840818	0.034443517	0.044382503	positive
PRJNA729271	stage_5W_vs_E10	ordinary_muscle_development	ENSGALG00010017587	MB21D2	-0.458189744	0.147329845	-3.109958772	0.001871135	0.002757712	negative
PRJNA729271	stage_5W_vs_E10	ordinary_muscle_development	ENSGALG00010017588	NOX5	-1.075902142	0.491204546	-2.190334255	0.028500004	0.037059977	negative
PRJNA729271	stage_5W_vs_E10	ordinary_muscle_development	ENSGALG00010017589	ATPAF2	0.384216201	0.146600189	2.620843825	0.008771243	0.012076289	positive
PRJNA729271	stage_5W_vs_E10	ordinary_muscle_development	ENSGALG00010017590	ENSGALG00010017590	-0.863076692	0.194209668	-4.444045967	8.82827E-06	1.57982E-05	negative
PRJNA729271	stage_5W_vs_E10	ordinary_muscle_development	ENSGALG00010017591	ESR2	-0.787776117	0.247838777	-3.178582968	0.001479969	0.002204289	negative
PRJNA729271	stage_5W_vs_E10	ordinary_muscle_development	ENSGALG00010017592	GID4	1.296044186	0.108492122	11.94597504	6.81464E-33	4.63095E-32	positive
PRJNA729271	stage_5W_vs_E10	ordinary_muscle_development	ENSGALG00010017593	MTHFR	0.743095043	0.132432587	5.611119306	2.01022E-08	4.35483E-08	positive
PRJNA729271	stage_5W_vs_E10	ordinary_muscle_development	ENSGALG00010017594	CEP68	0.116629158	0.102967348	1.132680994	0.257348242	0.293582777	positive
PRJNA729271	stage_5W_vs_E10	ordinary_muscle_development	ENSGALG00010017595	CCNF	-2.623043269	0.130471278	-20.10437325	6.75686E-90	2.29908E-88	negative
PRJNA729271	stage_5W_vs_E10	ordinary_muscle_development	ENSGALG00010017596	TDP1	-2.243928086	0.103079467	-21.76891436	4.5733E-105	2.0716E-103	negative
PRJNA729271	stage_5W_vs_E10	ordinary_muscle_development	ENSGALG00010017597	CNOT10	-0.113344027	0.136040994	-0.833160823	0.404754034	0.446254638	negative
PRJNA729271	stage_5W_vs_E10	ordinary_muscle_development	ENSGALG00010017598	ENSGALG00010017598	0.535250755	0.118004108	4.535865441	5.73677E-06	1.03891E-05	positive
PRJNA729271	stage_5W_vs_E10	ordinary_muscle_development	ENSGALG00010017599	KCNK13	0.810415608	0.221453128	3.659535606	0.000252673	0.00040256	positive
PRJNA729271	stage_5W_vs_E10	ordinary_muscle_development	ENSGALG00010017600	MXD4	0.597771361	0.103390115	5.781707105	7.39464E-09	1.64546E-08	positive
PRJNA729271	stage_5W_vs_E10	ordinary_muscle_development	ENSGALG00010017601	ENSGALG00010017601	0.87467263	0.116463086	7.510299279	5.89923E-14	1.7526E-13	positive
PRJNA729271	stage_5W_vs_E10	ordinary_muscle_development	ENSGALG00010017603	CYP3A5	2.296211298	1.349470889	1.701564158	0.088837101	0.108577656	positive
PRJNA729271	stage_5W_vs_E10	ordinary_muscle_development	ENSGALG00010017604	GATM	2.008562309	0.241328392	8.322942425	8.58061E-17	2.9536E-16	positive
PRJNA729271	stage_5W_vs_E10	ordinary_muscle_development	ENSGALG00010017605	GFPT1	-1.287474677	0.07245759	-17.76866555	1.23596E-70	2.64627E-69	negative
PRJNA729271	stage_5W_vs_E10	ordinary_muscle_development	ENSGALG00010017606	ENSGALG00010017606	0.7477516	0.254098928	2.942757788	0.003253028	0.004685279	positive
PRJNA729271	stage_5W_vs_E10	ordinary_muscle_development	ENSGALG00010017608	RBBP4	-1.318981056	0.081308858	-16.22186175	3.5332E-59	5.53065E-58	negative
PRJNA729271	stage_5W_vs_E10	ordinary_muscle_development	ENSGALG00010017609	TRIM71	-2.345246123	0.223479868	-10.49421653	9.18364E-26	4.69714E-25	negative
PRJNA729271	stage_5W_vs_E10	ordinary_muscle_development	ENSGALG00010017610	PSMC1	0.210272105	0.119199639	1.764033078	0.07772641	0.09573796	positive
PRJNA729271	stage_5W_vs_E10	ordinary_muscle_development	ENSGALG00010017612	CLDN11	-0.255607751	0.417000654	-0.612967267	0.539897984	0.579819313	negative
PRJNA729271	stage_5W_vs_E10	ordinary_muscle_development	ENSGALG00010017613	HAUS3	-1.841897056	0.122997651	-14.97505886	1.06873E-50	1.31617E-49	negative
PRJNA729271	stage_5W_vs_E10	ordinary_muscle_development	ENSGALG00010017615	AGTRAP	0.615152117	0.134797155	4.563539322	5.02984E-06	9.14659E-06	positive
PRJNA729271	stage_5W_vs_E10	ordinary_muscle_development	ENSGALG00010017616	PDCD7	0.961457806	0.155714886	6.174475863	6.63833E-10	1.56765E-09	positive
PRJNA729271	stage_5W_vs_E10	ordinary_muscle_development	ENSGALG00010017619	POLN	0.682318286	0.32556871	2.09577353	0.036102286	0.046400304	positive
PRJNA729271	stage_5W_vs_E10	ordinary_muscle_development	ENSGALG00010017620	UBAP1L	0.42077666	0.330871188	1.271723484	0.203471384	0.236361413	positive
PRJNA729271	stage_5W_vs_E10	ordinary_muscle_development	ENSGALG00010017621	SYNE2	1.344477921	0.155926986	8.622483891	6.55172E-18	2.38671E-17	positive
PRJNA729271	stage_5W_vs_E10	ordinary_muscle_development	ENSGALG00010017622	RAB1B	0.01543708	0.112570878	0.137132092	0.890926386	0.90446687	positive
PRJNA729271	stage_5W_vs_E10	ordinary_muscle_development	ENSGALG00010017625	YBX1	-0.595741694	0.091296636	-6.525341108	6.78469E-11	1.71004E-10	negative
PRJNA729271	stage_5W_vs_E10	ordinary_muscle_development	ENSGALG00010017626	COPS7B	-0.796415899	0.141061706	-5.645868898	1.64349E-08	3.58285E-08	negative
PRJNA729271	stage_5W_vs_E10	ordinary_muscle_development	ENSGALG00010017628	NRDE2	-0.102693248	0.104690241	-0.980924749	0.326629852	0.366305586	negative
PRJNA729271	stage_5W_vs_E10	ordinary_muscle_development	ENSGALG00010017629	ENSGALG00010017629	-0.560270021	0.144678389	-3.872520452	0.000107716	0.000176822	negative
PRJNA729271	stage_5W_vs_E10	ordinary_muscle_development	ENSGALG00010017631	NAT8L	-3.86304289	0.274987961	-14.04804368	7.91915E-45	8.03276E-44	negative
PRJNA729271	stage_5W_vs_E10	ordinary_muscle_development	ENSGALG00010017632	AWAT1	0.697619678	0.605375848	1.15237448	0.249167223	0.285035033	positive
PRJNA729271	stage_5W_vs_E10	ordinary_muscle_development	ENSGALG00010017633	HLX	0.798766125	0.150683284	5.300960416	1.15195E-07	2.36744E-07	positive
PRJNA729271	stage_5W_vs_E10	ordinary_muscle_development	ENSGALG00010017634	PPIH	-0.721560178	0.098765144	-7.305818077	2.75585E-13	7.89135E-13	negative
PRJNA729271	stage_5W_vs_E10	ordinary_muscle_development	ENSGALG00010017636	ZBTB80S	-0.382717293	0.159560481	-2.398571949	0.016459143	0.021988504	negative
PRJNA729271	stage_5W_vs_E10	ordinary_muscle_development	ENSGALG00010017638	GFRA2	0.494854711	0.25796083	1.918332764	0.055068831	0.069032491	positive
PRJNA729271	stage_5W_vs_E10	ordinary_muscle_development	ENSGALG00010017639	ENSGALG00010017639	-1.029089569	0.140095119	-7.345648979	2.04763E-13	5.9042E-13	negative
PRJNA729271	stage_5W_vs_E10	ordinary_muscle_development	ENSGALG00010017641	GPRC5B	1.478987213	0.159192058	9.290584153	1.53444E-20	6.2635E-20	positive
PRJNA729271	stage_5W_vs_E10	ordinary_muscle_development	ENSGALG00010017642	MTARC1	3.223065194	0.121596764	26.50617571	8.227E-155	8.6414E-153	positive
PRJNA729271	stage_5W_vs_E10	ordinary_muscle_development	ENSGALG00010017643	CALM1	0.96187314	0.060905292	15.79293215	3.4802E-56	4.9946E-55	positive
PRJNA729271	stage_5W_vs_E10	ordinary_muscle_development	ENSGALG00010017644	RBBP6	0.419379413	0.097165573	4.31613175	1.58787E-05	2.7868E-05	positive
PRJNA729271	stage_5W_vs_E10	ordinary_muscle_development	ENSGALG00010017645	SYNC	1.30915913	0.270099605	4.846949444	1.25374E-06	2.38742E-06	positive
PRJNA729271	stage_5W_vs_E10	ordinary_muscle_development	ENSGALG00010017646	MAEA	-0.448776765	0.087071384	-5.154124624	2.54818E-07	5.10306E-07	negative

PRJNA729271	stage_5W_vs_E10	ordinary_muscle_development	ENSGALG00010017647	ITGA9	-0.452047187	0.16744528	-2.699671122	0.006940805	0.009655179	negative
PRJNA729271	stage_5W_vs_E10	ordinary_muscle_development	ENSGALG00010017648	ENSGALG00010017648	-1.070054529	0.16177377	-6.614511929	3.7278E-11	9.55685E-11	negative
PRJNA729271	stage_5W_vs_E10	ordinary_muscle_development	ENSGALG00010017649	ENSGALG00010017649	-0.596825448	0.114717583	-5.202562963	1.96559E-07	3.97311E-07	negative
PRJNA729271	stage_5W_vs_E10	ordinary_muscle_development	ENSGALG00010017651	PPCS	-0.240283894	0.124156016	-1.935338318	0.052948793	0.06655908	negative
PRJNA729271	stage_5W_vs_E10	ordinary_muscle_development	ENSGALG00010017652	C1orf115	2.563541312	0.169709804	15.10544033	1.49107E-51	1.89091E-50	positive
PRJNA729271	stage_5W_vs_E10	ordinary_muscle_development	ENSGALG00010017653	ENSGALG00010017653	1.454161663	0.172625171	8.423810102	3.64435E-17	1.27658E-16	positive
PRJNA729271	stage_5W_vs_E10	ordinary_muscle_development	ENSGALG00010017654	KBTBD13	5.636493605	0.314773219	17.90652212	1.04888E-71	2.33183E-70	positive
PRJNA729271	stage_5W_vs_E10	ordinary_muscle_development	ENSGALG00010017655	ENSGALG00010017655	-1.230772389	0.113156502	-10.87672706	1.48811E-27	8.19221E-27	negative
PRJNA729271	stage_5W_vs_E10	ordinary_muscle_development	ENSGALG00010017656	FSD2	7.997271248	0.207835023	38.47893939	0	0	positive
PRJNA729271	stage_5W_vs_E10	ordinary_muscle_development	ENSGALG00010017657	DIS3L2	0.129427772	0.094940668	1.363249008	0.172804047	0.203278255	positive
PRJNA729271	stage_5W_vs_E10	ordinary_muscle_development	ENSGALG00010017658	WHAMM	2.671372646	0.1615824	16.53257192	2.13822E-61	3.6039E-60	positive
PRJNA729271	stage_5W_vs_E10	ordinary_muscle_development	ENSGALG00010017659	TNRC6A	-0.621595548	0.079256324	-7.84285111	4.4043E-15	1.38814E-14	negative
PRJNA729271	stage_5W_vs_E10	ordinary_muscle_development	ENSGALG00010017660	CLIP4	4.040835341	0.246801386	16.37282274	2.99018E-60	4.83735E-59	positive
PRJNA729271	stage_5W_vs_E10	ordinary_muscle_development	ENSGALG00010017661	NHSL3	0.267141303	0.1716437	1.556371145	0.119619863	0.143760045	positive
PRJNA729271	stage_5W_vs_E10	ordinary_muscle_development	ENSGALG00010017662	GOLGA4	0.813695963	0.165427932	4.918733831	8.71058E-07	1.68122E-06	positive
PRJNA729271	stage_5W_vs_E10	ordinary_muscle_development	ENSGALG00010017663	ZMYND12	-0.406137462	0.224634469	-1.807992625	0.070607648	0.087489987	negative
PRJNA729271	stage_5W_vs_E10	ordinary_muscle_development	ENSGALG00010017664	ENSGALG00010017664	0.460302851	0.074543051	6.174993474	6.61662E-10	1.56278E-09	positive
PRJNA729271	stage_5W_vs_E10	ordinary_muscle_development	ENSGALG00010017665	CTBP1	-0.188110349	0.063130059	-2.97972712	0.002885053	0.00417562	negative
PRJNA729271	stage_5W_vs_E10	ordinary_muscle_development	ENSGALG00010017667	SLC2A1	-0.512206232	0.111524913	-4.59275167	4.37439E-06	7.99481E-06	negative
PRJNA729271	stage_5W_vs_E10	ordinary_muscle_development	ENSGALG00010017668	ENSGALG00010017668	5.621148438	0.586102028	9.590733646	8.74609E-22	3.78658E-21	positive
PRJNA729271	stage_5W_vs_E10	ordinary_muscle_development	ENSGALG00010017669	ENSGALG00010017669	0.639217443	0.177702989	3.59711137	0.000321771	0.000507958	positive
PRJNA729271	stage_5W_vs_E10	ordinary_muscle_development	ENSGALG00010017670	FAM179A	2.937443263	0.329269478	8.921091873	4.61713E-19	1.76641E-18	positive
PRJNA729271	stage_5W_vs_E10	ordinary_muscle_development	ENSGALG00010017671	ANTXR1	-0.303184716	0.184757857	-1.640984159	0.100800703	0.122350209	negative
PRJNA729271	stage_5W_vs_E10	ordinary_muscle_development	ENSGALG00010017672	SPRED2	-0.695780954	0.078697637	-8.841192445	9.47024E-19	3.57198E-18	negative
PRJNA729271	stage_5W_vs_E10	ordinary_muscle_development	ENSGALG00010017673	WDR89	-0.133214344	0.091046993	-1.463138306	0.143429538	0.170606528	negative
PRJNA729271	stage_5W_vs_E10	ordinary_muscle_development	ENSGALG00010017675	ENSGALG00010017675	0.484371129	0.499612465	0.969493683	0.332298931	0.371972585	positive
PRJNA729271	stage_5W_vs_E10	ordinary_muscle_development	ENSGALG00010017676	SPON2	2.254892214	0.262772011	8.581173489	9.39101E-18	3.39204E-17	positive
PRJNA729271	stage_5W_vs_E10	ordinary_muscle_development	ENSGALG00010017677	WDR43	-0.925703243	0.110697205	-8.362480695	6.1413E-17	2.13166E-16	negative
PRJNA729271	stage_5W_vs_E10	ordinary_muscle_development	ENSGALG00010017678	YARS	-0.583664478	0.138379126	-4.217865055	2.46626E-05	4.26288E-05	negative
PRJNA729271	stage_5W_vs_E10	ordinary_muscle_development	ENSGALG00010017679	RASL12	2.906124259	0.181457135	16.01548626	9.96248E-58	1.49644E-56	positive
PRJNA729271	stage_5W_vs_E10	ordinary_muscle_development	ENSGALG00010017681	ENSGALG00010017681	4.358645766	1.248417339	3.491337095	0.000480609	0.000747071	positive
PRJNA729271	stage_5W_vs_E10	ordinary_muscle_development	ENSGALG00010017684	TRMT61B	-0.059091804	0.09974077	-0.592453855	0.553546712	0.593332439	negative
PRJNA729271	stage_5W_vs_E10	ordinary_muscle_development	ENSGALG00010017685	PHC2	0.65645701	0.183738946	3.572770086	0.000353225	0.000555676	positive
PRJNA729271	stage_5W_vs_E10	ordinary_muscle_development	ENSGALG00010017687	TTC7B	0.458581311	0.098379325	4.661358583	3.14129E-06	5.81594E-06	positive
PRJNA729271	stage_5W_vs_E10	ordinary_muscle_development	ENSGALG00010017688	SPDYA	-1.200410237	0.605233221	-1.983384579	0.047324488	0.059857632	negative
PRJNA729271	stage_5W_vs_E10	ordinary_muscle_development	ENSGALG00010017691	LZTS1	-2.506901883	0.274282483	-9.13985411	6.25366E-20	2.49372E-19	negative
PRJNA729271	stage_5W_vs_E10	ordinary_muscle_development	ENSGALG00010017692	EPCAM	-4.742094379	1.00360185	-4.725075364	2.3003E-06	4.29399E-06	negative
PRJNA729271	stage_5W_vs_E10	ordinary_muscle_development	ENSGALG00010017693	MTFMT	-0.143238597	0.118490989	-1.208856456	0.226717997	0.26114728	negative
PRJNA729271	stage_5W_vs_E10	ordinary_muscle_development	ENSGALG00010017694	ATP6V1B2	0.056664168	0.070820868	0.800105523	0.423649662	0.465423817	positive
PRJNA729271	stage_5W_vs_E10	ordinary_muscle_development	ENSGALG00010017695	FNDC5	-2.206665381	0.210781673	-10.46896227	1.19951E-25	6.09851E-25	negative
PRJNA729271	stage_5W_vs_E10	ordinary_muscle_development	ENSGALG00010017698	ENSGALG00010017698	0.681828735	0.797311298	0.855160007	0.392462573	0.43382483	positive
PRJNA729271	stage_5W_vs_E10	ordinary_muscle_development	ENSGALG00010017699	RNF19B	0.903073819	0.240281508	3.758399164	0.000171004	0.000276117	positive
PRJNA729271	stage_5W_vs_E10	ordinary_muscle_development	ENSGALG00010017700	VPS35L	-0.42991655	0.079489609	-5.408462254	6.35682E-08	1.32865E-07	negative
PRJNA729271	stage_5W_vs_E10	ordinary_muscle_development	ENSGALG00010017701	PPP2R5E	0.337113689	0.099095794	3.40189706	0.000669198	0.00102929	positive
PRJNA729271	stage_5W_vs_E10	ordinary_muscle_development	ENSGALG00010017703	RPS6KA5	0.890119153	0.140018191	6.357167936	2.05507E-10	5.01392E-10	positive
PRJNA729271	stage_5W_vs_E10	ordinary_muscle_development	ENSGALG00010017704	ZNF362	-0.214115439	0.199887555	-1.071179441	0.284088754	0.321783737	negative
PRJNA729271	stage_5W_vs_E10	ordinary_muscle_development	ENSGALG00010017705	MAK16	0.462642919	0.103690063	4.461786461	8.12792E-06	1.45756E-05	positive
PRJNA729271	stage_5W_vs_E10	ordinary_muscle_development	ENSGALG00010017706	FGFRL1	-1.33479934	0.145109099	-9.198591629	3.62672E-20	1.45945E-19	negative
PRJNA729271	stage_5W_vs_E10	ordinary_muscle_development	ENSGALG00010017707	AK2	-0.813138373	0.096179183	-8.454411346	2.80499E-17	9.89012E-17	negative
PRJNA729271	stage_5W_vs_E10	ordinary_muscle_development	ENSGALG00010017708	MEIS1	-1.839759323	0.105225435	-17.48397931	1.89781E-68	3.8313E-67	negative
PRJNA729271	stage_5W_vs_E10	ordinary_muscle_development	ENSGALG00010017710	RAB41	-0.473083939	0.118100469	-4.005775266	6.18144E-05	0.0001038	negative
PRJNA729271	stage_5W_vs_E10	ordinary_muscle_development	ENSGALG00010017711	LRRFIP2	1.240585469	0.134122035	9.249676769	2.25173E-20	9.13995E-20	positive
PRJNA729271	stage_5W_vs_E10	ordinary_muscle_development	ENSGALG00010017712	MSH2	-1.885306218	0.07494364	-25.15632048	1.2052E-139	9.9256E-138	negative
PRJNA729271	stage_5W_vs_E10	ordinary_muscle_development	ENSGALG00010017713	ALKBH5	3.298529148	0.149027295	22.13372489	1.4969E-108	7.2809E-107	positive
PRJNA729271	stage_5W_vs_E10	ordinary_muscle_development	ENSGALG00010017714	ATAD3A	-1.321316584	0.156880274	-8.422452061	3.68685E-17	1.29116E-16	negative
PRJNA729271	stage_5W_vs_E10	ordinary_muscle_development	ENSGALG00010017715	CILP	2.999328413	0.168590596	17.79060328	8.35753E-71	1.79736E-69	positive
PRJNA729271	stage_5W_vs_E10	ordinary_muscle_development	ENSGALG00010017717	LLGL1	-0.188452204	0.149996888	-1.256374096	0.208980369	0.242353024	negative
PRJNA729271	stage_5W_vs_E10	ordinary_muscle_development	ENSGALG00010017718	ATOH8	1.270609956	0.29845982	4.257222821	2.06982E-05	3.59651E-05	positive
PRJNA729271	stage_5W_vs_E10	ordinary_muscle_development	ENSGALG00010017719	ENSGALG00010017719	3.767095945	1.773387008	2.124237928	0.033650253	0.043410504	positive
PRJNA729271	stage_5W_vs_E10	ordinary_muscle_development	ENSGALG00010017720	ENSGALG00010017720	-2.162854943	0.394788828	-5.478511014	4.2892E-08	9.06825E-08	negative
PRJNA729271	stage_5W_vs_E10	ordinary_muscle_development	ENSGALG00010017721	VWA1	1.208476793	0.12030639	10.04499258	9.66549E-24	4.54285E-23	positive
PRJNA729271	stage_5W_vs_E10	ordinary_muscle_development	ENSGALG00010017723	KCNK12	-2.74375899	0.431868178	-6.353232613	2.10837E-10	5.13885E-10	negative
PRJNA729271	stage_5W_vs_E10	ordinary_muscle_development	ENSGALG00010017724	FLII	1.951693266	0.127671799	15.28680007	9.36394E-53	1.22611E-51	positive
PRJNA729271	stage_5W_vs_E10	ordinary_muscle_development	ENSGALG00010017725	ENSGALG00010017725	1.325893941	0.395467133	3.35272853	0.000800192	0.001222597	positive
PRJNA729271	stage_5W_vs_E10	ordinary_muscle_development	ENSGALG00010017726	TTI2	0.103736048	0.125184732	0.828663738	0.407294711	0.448782731	positive
PRJNA729271	stage_5W_vs_E10	ordinary_muscle_development	ENSGALG00010017727	AZIN2	0.530579924	0.153650428	3.453162675	0.000554055	0.000858015	positive
PRJNA729271	stage_5W_vs_E10	ordinary_muscle_development	ENSGALG00010017729	MIEF2	2.276676207	0.139238335	16.35092959	4.28394E-60	6.89187E-59	positive
PRJNA729271	stage_5W_vs_E10	ordinary_muscle_development	ENSGALG00010017730	TARP	8.120685934	0.537238812	15.11559804	1.27805E-51	1.62646E-50	positive
PRJNA729271	stage_5W_vs_E10	ordinary_muscle_development	ENSGALG00010017732	RHOJ	-0.3556535	0.079439984	-4.477008721	7.56962E-06	1.36047E-05	negative
PRJNA729271	stage_5W_vs_E10	ordinary_muscle_development	ENSGALG00010017733	TRIM62	-0.42956313	0.133671689	-3.213568497	0.001310965	0.001963671	negative
PRJNA729271	stage_5W_vs_E10	ordinary_muscle_development	ENSGALG00010017734	TOP3A	-0.372547349	0.119225271	-3.124734756	0.001779655	0.002629035	negative
PRJNA729271	stage_5W_vs_E10	ordinary_muscle_development	ENSGALG00010017736	ENSGALG00010017736	5.782664612	4.325493693	1.336879677	0.181261916	0.212520542	positive
PRJNA729271	stage_5W_vs_E10	ordinary_muscle_development	ENSGALG00010017737	ENSGALG00010017737	0.010188315	0.191065799	0.053323595	0.957474081	0.963188758	positive
PRJNA729271	stage_5W_vs_E10	ordinary_muscle_development	ENSGALG00010017738	NDUFS5	0.45937505	0.112110736	4.097511682	4.17615E-05	7.08323E-05	positive
PRJNA729271	stage_5W_vs_E10	ordinary_muscle_development	ENSGALG00010017739	MRPL20	-0.326616554	0.099784687	-3.273213183	0.001063322	0.001608186	negative
PRJNA729271	stage_5W_vs_E10	ordinary_muscle_development	ENSGALG00010017741	SMCR8	1.952673125	0.13827163	14.12200844	2.77955E-45	2.86148E-44	positive
PRJNA729271	stage_5W_vs_E10	ordinary_muscle_development	ENSGALG00010017742	DGLUCY	-0.419136223	0.244777502	-1.712315145	0.086838607	0.106311285	negative
PRJNA729271	stage_5W_vs_E10	ordinary_muscle_development	ENSGALG00010017743	KCNH5	-4.633945033	0.57993743	-7.990422399	1.34477E-15	4.36659E-15	negative
PRJNA729271	stage_5W_vs_E10	ordinary_muscle_development	ENSGALG00010017744	ETAA1	-0.964161332	0.099975736	-9.643953292	5.21404E-22	2.28607E-21	negative
PRJNA729271	stage_5W_vs_E10	ordinary_muscle_development	ENSGALG00010017745	SHMT1	-0.893234197	0.189866829	-4.704530024	2.54451E-06	4.73523E-06	negative
PRJNA729271	stage_5W_vs_E10	ordinary_muscle_development	ENSGALG00010017746	MSH6	-0.702706221	0.08935951	-7.863810109	3.72622E-15	1.18084E-14	negative
PRJNA729271	stage_5W_vs_E10	ordinary_muscle_development	ENSGALG00010017747	DUSP26	0.940766968	0.124696445	7.544457002	4.54176E-14	1.35878E-13	positive
PRJNA729271	stage_5W_vs_E10	ordinary_muscle_development	ENSGALG00010017748	ENSGALG00010017748	-4.673492452	0.905216969	-5.162842294	2.43228E-07	4.87634E-07	negative
PRJNA729271	stage_5W_vs_E10	ordinary_muscle_development	ENSGALG00010017751	ST3GAL5	0.095294675	0.126381008	0.754026869	0.450833088	0.492769286	positive
PRJNA729271	stage_5W_vs_E10	ordinary_muscle_development	ENSGALG00010017753	GNRHR	1.05101116	0.340373823	3.087814308	0.002016344	0.002962387	positive
PRJNA729271	stage_5W_vs_E10	ordinary_muscle_development	ENSGALG00010017754	PDZD11	-1.117883015	0.162367458	-6.884895707	5.78299E-12	1.54858E-11	negative
PRJNA729271	stage_5W_vs_E10	ordinary_muscle_development	ENSGALG00010017755	C1D	-0.953286863	0.141109177	-6.75566879	1.42178E-11	3.73346E-11	negative
PRJNA729271	stage_5W_vs_E10	ordinary_muscle_development	ENSGALG00010017756	EIF2B5	-0.35412076	0.119232605	-2.969999368	0.002978004	0.004303705	negative
PRJNA729271	stage_5W_vs_E10	ordinary_muscle_development	ENSGALG00010017757	ENSGALG00010017757	1.419672541	0.138514222	10.24929078	1.19215E-24	5.84186E-24	positive
PRJNA729271	stage_5W_vs_E10	ordinary_muscle_development	ENSGALG00010017759	ENSGALG00010017759	3.005733686	0.877643102	3.424779022	0.0006153	0.000949512	positive
PRJNA729271	stage_5W_vs_E10	ordinary_muscle_development	ENSGALG00010017761	PARP16	0.434813723	0.107853656	4.031515864	5.54183E-05	9.32646E-05	positive
PRJNA729271	stage_5W_vs_E10	ordinary_muscle_development	ENSGALG00010017762	FBXO11	-0.431336596	0.056175274	-7.678406604	1.6108E-14	4.9248E-14	negative
PRJNA729271	stage_5W_vs_E10	ordinary_muscle_development	ENSGALG00010017763	POLR1A	-0.452960106	0.087051855	-5.203336657	1.95742E-07	3.9577E-07	negative
PRJNA729271	stage_5W_vs_E10	ordinary_muscle_development	ENSGALG00010017764	IGDCC3	-1.699573659	0.24351529	-6.979330357	2.9659E-12	8.05976E-12	negative
PRJNA729271	stage_5W_vs_E10	ordinary_muscle_development	ENSGALG00010017765	SYT16	-3.27382522	0.309998628	-10.56077324	4.52915E-26	2.34968E-25	negative
PRJNA729271	stage_5W_vs_E10	ordinary_muscle_development	ENSGALG00010017766	DNAAF10	0.773086417	0.15379942	5.026588627	4.99282E-07	9.79176E-07	positive
PRJNA729271	stage_5W_vs_E10	ordinary_muscle_development	ENSGALG00010017767	KIF4B	-3.242809999	0.223169248	-14.53072066	7.74018E-48	8.6303E-47	negative
PRJNA729271	stage_5W_vs_E10	ordinary_muscle_development	ENSGALG00010017768	FOXN2	0.036998031	0.083735489	0.441844093	0.658602029	0.693004254	positive
PRJNA729271	stage_5W_vs_E10	ordinary_muscle_development	ENSGALG00010017770	PTCD3	-0.227227943	0.070729743	-3.212622191	0.001315292	0.001969744	negative
PRJNA729271	stage_5W_vs_E10	ordinary_muscle_development	ENSGALG00010017771	PNO1	0.344894325	0.157849353	2.184958743	0.028891872	0.037545964	positive
PRJNA729271	stage_5W_vs_E10	ordinary_muscle_development	ENSGALG00010017772	ENSGALG00010017772	3.350566239	0.331919311	10.09452035	5.84159E-24	2.77165E-23	positive
PRJNA729271	stage_5W_vs_E10	ordinary_muscle_development	ENSGALG00010017773	DVL3	-1.587033387	0.084940551	-18.68404873	6.67583E-78	1.74353E-76	negative
PRJNA729271	stage_5W_vs_E10	ordinary_muscle_development	ENSGALG00010017774	ASH2L	0.525507841	0.163338902	3.217285251	0.001294099	0.001939208	positive
PRJNA729271	stage_5W_vs_E10	ordinary_muscle_development	ENSGALG00010017775	PRPSAP2	-1.291459793	0.087200535	-14.81022788	1.25813E-49	1.49849E-48	negative
PRJNA729271	stage_5W_vs_E10	ordinary_muscle_development	ENSGALG00010017776	GPR68	1.401853976	0.360828773	3.885094762	0.00010229	0.000168354	positive
PRJNA729271	stage_5W_vs_E10	ordinary_muscle_development	ENSGALG00010017778	PPP3R1	1.037857648	0.078627437	13.19968817	8.8099E-40	7.65126E-39	positive
PRJNA729271	stage_5W_vs_E10	ordinary_muscle_development	ENSGALG00010017779	SNAPC1	0.06746581	0.124701416	0.541018798	0.588494626	0.626669821	positive
PRJNA729271	stage_5W_vs_E10	ordinary_muscle_development	ENSGALG00010017780	TAF9	0.136397242	0.137881048	0.989238503	0.322546464	0.361950221	positive
PRJNA729271	stage_5W_vs_E10	ordinary_muscle_development	ENSGALG00010017781	CYP26C1	1.434462178	0.301846941	4.752283308	2.01132E-06	3.77107E-06	positive
PRJNA729271	stage_5W_vs_E10	ordinary_muscle_development	ENSGALG00010017782	IGDCC4	-0.484770089	0.116403305	-4.164573239	3.11935E-05	5.34331E-05	negative
PRJNA729271	stage_5W_vs_E10	ordinary_muscle_development	ENSGALG00010017783	STAR	-0.598985565	0.439713543	-1.362217686	0.173129196	0.203594653	negative
PRJNA729271	stage_5W_vs_E10	ordinary_muscle_development	ENSGALG00010017786	KAZALD1	-2.755140891	0.170715239	-16.13881047	1.3614E-58	2.10603E-57	negative
PRJNA729271	stage_5W_vs_E10	ordinary_muscle_development	ENSGALG00010017787	CNRIP1	2.427481689	0.234413563	10.35555133	3.94905E-25	1.97112E-24	positive
PRJNA729271	stage_5W_vs_E10	ordinary_muscle_development	ENSGALG00010017789	IMMT	1.240036329	0.130151016	9.527673031	1.60848E-21	6.87959E-21	positive
PRJNA729271	stage_5W_vs_E10	ordinary_muscle_development	ENSGALG00010017790	R3HCC1L	0.243510725	0.176996303	1.375795547	0.168884941	0.199120483	positive
PRJNA729271	stage_5W_vs_E10	ordinary_muscle_development	ENSGALG00010017791	CCNL2	-0.002938166	0.112126116	-0.026204116	0.979094533	0.982143616	negative
PRJNA729271	stage_5W_vs_E10	ordinary_muscle_development	ENSGALG00010017792	LSM1	-0.029178716	0.123825021	-0.235644747	0.813708348	0.835318874	negative
PRJNA729271	stage_5W_vs_E10	ordinary_muscle_development	ENSGALG00010017793	FAM83G	-6.434826873	0.857758091	-7.501913353	6.28929E-14	1.86543E-13	negative
PRJNA729271	stage_5W_vs_E10	ordinary_muscle_development	ENSGALG00010017795	MACF1	-0.211754484	0.114222055	-1.853884382	0.063755703	0.07937292	negative
PRJNA729271	stage_5W_vs_E10	ordinary_muscle_development	ENSGALG00010017796	MXI1	1.505178709	0.132460863	11.36319568	6.37685E-30	3.84814E-29	positive
PRJNA729271	stage_5W_vs_E10	ordinary_muscle_development	ENSGALG00010017797	PPP1R21	-0.23433241	0.092472746	-2.534069979	0.011274621	0.015359552	negative
PRJNA729271	stage_5W_vs_E10	ordinary_muscle_development	ENSGALG00010017798	BAG4	0.432395353	0.157971475	2.737173613	0.006196958	0.008669521	positive
PRJNA729271	stage_5W_vs_E10	ordinary_muscle_development	ENSGALG00010017799	GRAPL	2.713705845	0.300880954	9.019201147	1.89466E-19	7.38255E-19	positive
PRJNA729271	stage_5W_vs_E10	ordinary_muscle_development	ENSGALG00010017801	SMNDC1	-0.404948666	0.07215392	-5.612289225	1.99667E-08	4.32613E-08	negative
PRJNA729271	stage_5W_vs_E10	ordinary_muscle_development	ENSGALG00010017803	ENSGALG00010017803	1.032964595	0.118127094	8.744518785	2.23968E-18	8.31136E-18	positive
PRJNA729271	stage_5W_vs_E10	ordinary_muscle_development	ENSGALG00010017804	DUSP5	-0.32911357	0.248161992	-1.326204578	0.184771943	0.216338394	negative
PRJNA729271	stage_5W_vs_E10	ordinary_muscle_development	ENSGALG00010017805	DPP8	1.382305075	0.106068116	13.03223937	8.02182E-39	6.69795E-38	positive
PRJNA729271	stage_5W_vs_E10	ordinary_muscle_development	ENSGALG00010017806	ENSGALG00010017806	1.917946582	0.761112257	2.519926022	0.01173795	0.015953266	positive
PRJNA729271	stage_5W_vs_E10	ordinary_muscle_development	ENSGALG00010017807	MRPL35	0.45757194	0.146888334	3.115100618	0.001838822	0.002713399	positive
PRJNA729271	stage_5W_vs_E10	ordinary_muscle_development	ENSGALG00010017808	SFRP5	1.056272858	0.361776293	2.919685116	0.003503852	0.005027553	positive
PRJNA729271	stage_5W_vs_E10	ordinary_muscle_development	ENSGALG00010017809	MOSMO	-0.193520625	0.105138377	-1.840627849	0.065676114	0.081602543	negative

PRJNA729271	stage_5W_vs_E10	ordinary_muscle_development	ENSGALG00010017810	HIF1A	-0.804681782	0.078869798	-10.20266068	1.92914E-24	9.34588E-24	negative
PRJNA729271	stage_5W_vs_E10	ordinary_muscle_development	ENSGALG00010017811	HACD3	-0.887382193	0.066811445	-13.2818889	2.94861E-40	2.59976E-39	negative
PRJNA729271	stage_5W_vs_E10	ordinary_muscle_development	ENSGALG00010017812	ENSGALG00010017812	3.304546501	1.76052609	1.877022169	0.060515055	0.075507078	positive
PRJNA729271	stage_5W_vs_E10	ordinary_muscle_development	ENSGALG00010017813	DDHD2	-0.069673818	0.162318732	-0.429240775	0.667748013	0.701835069	negative
PRJNA729271	stage_5W_vs_E10	ordinary_muscle_development	ENSGALG00010017814	ENSGALG00010017814	-3.399481376	0.278314145	-12.21454762	2.59943E-34	1.86068E-33	negative
PRJNA729271	stage_5W_vs_E10	ordinary_muscle_development	ENSGALG00010017815	VWA9	-0.758318981	0.086709664	-8.745495599	2.22039E-18	8.24188E-18	negative
PRJNA729271	stage_5W_vs_E10	ordinary_muscle_development	ENSGALG00010017816	PRKCH	1.558263495	0.281513429	5.53530785	3.10682E-08	6.6284E-08	positive
PRJNA729271	stage_5W_vs_E10	ordinary_muscle_development	ENSGALG00010017817	REEP1	2.56900192	0.116526815	22.04644423	1.0333E-107	4.9595E-106	positive
PRJNA729271	stage_5W_vs_E10	ordinary_muscle_development	ENSGALG00010017818	SLC38A6	-0.579792813	0.138696512	-4.18029844	2.91127E-05	4.99927E-05	negative
PRJNA729271	stage_5W_vs_E10	ordinary_muscle_development	ENSGALG00010017820	FLRT2	-0.355299477	0.134992207	-2.631999914	0.008488388	0.011709097	negative
PRJNA729271	stage_5W_vs_E10	ordinary_muscle_development	ENSGALG00010017821	ENSGALG00010017821	-3.81254547	0.403334726	-9.452559442	3.30645E-21	1.39363E-20	negative
PRJNA729271	stage_5W_vs_E10	ordinary_muscle_development	ENSGALG00010017822	VWA3A	-2.111756252	0.273425029	-7.723346531	1.13315E-14	3.49616E-14	negative
PRJNA729271	stage_5W_vs_E10	ordinary_muscle_development	ENSGALG00010017823	PPIE	-0.329774882	0.090948508	-3.625951544	0.000287899	0.000456276	negative
PRJNA729271	stage_5W_vs_E10	ordinary_muscle_development	ENSGALG00010017824	YAE1	-0.039062098	0.124759702	-0.313098681	0.754205691	0.782101266	negative
PRJNA729271	stage_5W_vs_E10	ordinary_muscle_development	ENSGALG00010017825	PLPP5	-1.7749644	0.157240352	-11.28822451	1.50037E-29	8.93135E-29	negative
PRJNA729271	stage_5W_vs_E10	ordinary_muscle_development	ENSGALG00010017826	PABPC4	3.330178487	0.140001721	23.78669682	4.5859E-125	3.0917E-123	positive
PRJNA729271	stage_5W_vs_E10	ordinary_muscle_development	ENSGALG00010017828	ENSGALG00010017828	0.633576669	0.236604636	2.677786366	0.007411047	0.010281693	positive
PRJNA729271	stage_5W_vs_E10	ordinary_muscle_development	ENSGALG00010017830	DENND4A	1.268942876	0.12767105	9.939159091	2.81191E-23	1.29846E-22	positive
PRJNA729271	stage_5W_vs_E10	ordinary_muscle_development	ENSGALG00010017831	PLEK	2.037640953	0.180963902	11.25993047	2.06922E-29	1.22422E-28	positive
PRJNA729271	stage_5W_vs_E10	ordinary_muscle_development	ENSGALG00010017832	POU3F1	-3.836296042	0.614429097	-6.24367573	4.27406E-10	1.0208E-09	negative
PRJNA729271	stage_5W_vs_E10	ordinary_muscle_development	ENSGALG00010017834	ENSGALG00010017834	-1.82468351	0.419743025	-4.347144332	1.37921E-05	2.43475E-05	negative
PRJNA729271	stage_5W_vs_E10	ordinary_muscle_development	ENSGALG00010017838	B3GNT2	-0.8475663	0.126703328	-6.689376757	2.24123E-11	5.81468E-11	negative
PRJNA729271	stage_5W_vs_E10	ordinary_muscle_development	ENSGALG00010017841	GALC	-1.177264813	0.268803457	-4.379649075	1.18871E-05	2.10742E-05	negative
PRJNA729271	stage_5W_vs_E10	ordinary_muscle_development	ENSGALG00010017843	NSD3	-0.082381683	0.071503518	-1.15213468	0.249265734	0.285125222	negative
PRJNA729271	stage_5W_vs_E10	ordinary_muscle_development	ENSGALG00010017848	CYP3A4	0.88936566	0.197959374	4.492667564	7.03365E-06	1.26665E-05	positive
PRJNA729271	stage_5W_vs_E10	ordinary_muscle_development	ENSGALG00010017849	FBXO48	2.645366418	0.175253647	15.09450136	1.76013E-51	2.22434E-50	positive
PRJNA729271	stage_5W_vs_E10	ordinary_muscle_development	ENSGALG00010017850	TRMT5	-0.887886218	0.096191666	-9.230386128	2.69658E-20	1.0906E-19	negative
PRJNA729271	stage_5W_vs_E10	ordinary_muscle_development	ENSGALG00010017852	LETM2	0.348479566	0.115579687	3.015058924	0.002569293	0.003741777	positive
PRJNA729271	stage_5W_vs_E10	ordinary_muscle_development	ENSGALG00010017855	SDR42E2	-1.067244423	0.327391091	-3.259845647	0.001114729	0.001682248	negative
PRJNA729271	stage_5W_vs_E10	ordinary_muscle_development	ENSGALG00010017856	FASTKD5	0.518940747	0.115637492	4.487651355	7.20126E-06	1.29587E-05	positive
PRJNA729271	stage_5W_vs_E10	ordinary_muscle_development	ENSGALG00010017858	GPR65	3.694839961	0.281033697	13.14732005	1.76313E-39	1.51044E-38	positive
PRJNA729271	stage_5W_vs_E10	ordinary_muscle_development	ENSGALG00010017859	EXOC6	1.880787356	0.133916077	14.04452252	8.32274E-45	8.4127E-44	positive
PRJNA729271	stage_5W_vs_E10	ordinary_muscle_development	ENSGALG00010017860	CYSLTR1	0.632536055	0.202859744	3.118095506	0.001820238	0.002687071	positive
PRJNA729271	stage_5W_vs_E10	ordinary_muscle_development	ENSGALG00010017861	PRSS56	-0.019635993	0.548154397	-0.035822012	0.971424281	0.975462069	negative
PRJNA729271	stage_5W_vs_E10	ordinary_muscle_development	ENSGALG00010017865	UBOX5	0.206507053	0.136067591	1.51767994	0.129095104	0.154570752	positive
PRJNA729271	stage_5W_vs_E10	ordinary_muscle_development	ENSGALG00010017866	RALA	-0.628937576	0.095971089	-6.553406679	5.62391E-11	1.42291E-10	negative
PRJNA729271	stage_5W_vs_E10	ordinary_muscle_development	ENSGALG00010017867	APLF	1.074583409	0.109048694	9.854161161	6.57635E-23	2.99385E-22	positive
PRJNA729271	stage_5W_vs_E10	ordinary_muscle_development	ENSGALG00010017868	CHRND	0.788987836	0.241985322	3.260478069	0.001112246	0.001679025	positive
PRJNA729271	stage_5W_vs_E10	ordinary_muscle_development	ENSGALG00010017870	RAB11A	0.404236682	0.073758879	5.480515551	4.24088E-08	8.97263E-08	positive
PRJNA729271	stage_5W_vs_E10	ordinary_muscle_development	ENSGALG00010017873	HSPG2	2.266600984	0.109602694	20.68015751	5.22699E-95	1.98859E-93	positive
PRJNA729271	stage_5W_vs_E10	ordinary_muscle_development	ENSGALG00010017876	COMMD1	-0.679214357	0.138547032	-4.902410022	9.4668E-07	1.82184E-06	negative
PRJNA729271	stage_5W_vs_E10	ordinary_muscle_development	ENSGALG00010017878	CHRNG	-0.070178854	0.251604602	-0.27892516	0.78030226	0.806681496	negative
PRJNA729271	stage_5W_vs_E10	ordinary_muscle_development	ENSGALG00010017880	CDK13	0.264271944	0.084480367	3.128205456	0.001758772	0.002599245	positive
PRJNA729271	stage_5W_vs_E10	ordinary_muscle_development	ENSGALG00010017881	KCNK10	-2.487314912	0.554593546	-4.484933025	7.29368E-06	1.31185E-05	negative
PRJNA729271	stage_5W_vs_E10	ordinary_muscle_development	ENSGALG00010017882	LZTS3	2.298663588	0.199504406	11.52186877	1.02366E-30	6.38469E-30	positive
PRJNA729271	stage_5W_vs_E10	ordinary_muscle_development	ENSGALG00010017886	DDRGK1	0.668947978	0.101438107	6.594641762	4.26284E-11	1.08862E-10	positive
PRJNA729271	stage_5W_vs_E10	ordinary_muscle_development	ENSGALG00010017887	PROKR1	6.647850057	0.387506874	17.15543775	5.72371E-66	1.08451E-64	positive
PRJNA729271	stage_5W_vs_E10	ordinary_muscle_development	ENSGALG00010017888	MPLKIP	-0.623948321	0.114481759	-5.450198567	5.03136E-08	1.05971E-07	negative
PRJNA729271	stage_5W_vs_E10	ordinary_muscle_development	ENSGALG00010017890	ANAPC1	-0.31879953	0.065369728	-4.876867929	1.07784E-06	2.06383E-06	negative
PRJNA729271	stage_5W_vs_E10	ordinary_muscle_development	ENSGALG00010017891	GOLGA7B	4.629700968	0.322699519	14.34678608	1.1159E-46	1.20171E-45	positive
PRJNA729271	stage_5W_vs_E10	ordinary_muscle_development	ENSGALG00010017894	KAT6A	0.145712127	0.081208352	1.794299764	0.072765336	0.08999741	positive
PRJNA729271	stage_5W_vs_E10	ordinary_muscle_development	ENSGALG00010017895	MNAT1	0.280855547	0.081462413	3.447670364	0.000565444	0.000874438	positive
PRJNA729271	stage_5W_vs_E10	ordinary_muscle_development	ENSGALG00010017898	NHERF2	1.216087117	0.114447977	10.62567595	2.26366E-26	1.19186E-25	positive
PRJNA729271	stage_5W_vs_E10	ordinary_muscle_development	ENSGALG00010017901	HTR7L	-2.801702799	0.781490587	-3.585075552	0.000336981	0.000531043	negative
PRJNA729271	stage_5W_vs_E10	ordinary_muscle_development	ENSGALG00010017903	ENSGALG00010017903	-4.932722878	0.493926293	-9.98675906	1.74184E-23	8.1157E-23	negative
PRJNA729271	stage_5W_vs_E10	ordinary_muscle_development	ENSGALG00010017904	MEGF11	-3.894235166	0.368834534	-10.55821732	4.65416E-26	2.41367E-25	negative
PRJNA729271	stage_5W_vs_E10	ordinary_muscle_development	ENSGALG00010017905	EIF4E2	-0.377012455	0.088199006	-4.274565806	1.9151E-05	3.33887E-05	negative
PRJNA729271	stage_5W_vs_E10	ordinary_muscle_development	ENSGALG00010017909	NTHL1	-0.838499035	0.142769039	-5.873115375	4.27681E-09	9.63811E-09	negative
PRJNA729271	stage_5W_vs_E10	ordinary_muscle_development	ENSGALG00010017915	BFSP1	4.54946353	0.418758933	10.86415877	1.7079E-27	9.37727E-27	positive
PRJNA729271	stage_5W_vs_E10	ordinary_muscle_development	ENSGALG00010017918	SIX4	0.284130787	0.201790927	1.408045403	0.159117626	0.188216908	positive
PRJNA729271	stage_5W_vs_E10	ordinary_muscle_development	ENSGALG00010017920	AP3M2	-0.69376766	0.081667296	-8.495048718	1.9785E-17	7.02728E-17	negative
PRJNA729271	stage_5W_vs_E10	ordinary_muscle_development	ENSGALG00010017921	SPATA7	0.490547188	0.205382906	2.388451881	0.016919524	0.022561959	positive
PRJNA729271	stage_5W_vs_E10	ordinary_muscle_development	ENSGALG00010017922	MERTK	0.870666165	0.13065743	6.663732532	2.66959E-11	6.89272E-11	positive
PRJNA729271	stage_5W_vs_E10	ordinary_muscle_development	ENSGALG00010017927	PCSK2	0.7154907	0.124577413	5.743342072	9.28259E-09	2.05328E-08	positive
PRJNA729271	stage_5W_vs_E10	ordinary_muscle_development	ENSGALG00010017930	TSC2	-0.390556814	0.078616766	-4.967856538	6.7697E-07	1.31643E-06	negative
PRJNA729271	stage_5W_vs_E10	ordinary_muscle_development	ENSGALG00010017933	SIX1	0.992017345	0.19577352	5.067168147	4.03778E-07	7.96727E-07	positive
PRJNA729271	stage_5W_vs_E10	ordinary_muscle_development	ENSGALG00010017937	PTPN21	1.644494503	0.119280611	13.78677132	3.06159E-43	2.95654E-42	positive
PRJNA729271	stage_5W_vs_E10	ordinary_muscle_development	ENSGALG00010017941	DIS3L	0.669190282	0.136258403	4.911185433	9.05274E-07	1.74378E-06	positive
PRJNA729271	stage_5W_vs_E10	ordinary_muscle_development	ENSGALG00010017946	PTGDR	1.048977181	0.251377699	4.172912659	3.0073E-05	5.15746E-05	positive
PRJNA729271	stage_5W_vs_E10	ordinary_muscle_development	ENSGALG00010017948	PLAT	2.078136039	0.156098704	13.31296151	1.94612E-40	1.72321E-39	positive
PRJNA729271	stage_5W_vs_E10	ordinary_muscle_development	ENSGALG00010017949	SFXN3	0.934036874	0.138643278	6.736979139	1.61713E-11	4.23417E-11	positive
PRJNA729271	stage_5W_vs_E10	ordinary_muscle_development	ENSGALG00010017950	FBLN7	0.025278563	0.310379301	0.081444099	0.93508878	0.943419772	positive
PRJNA729271	stage_5W_vs_E10	ordinary_muscle_development	ENSGALG00010017954	PTGER2	1.427615407	0.25683683	5.558452848	2.72176E-08	5.83868E-08	positive
PRJNA729271	stage_5W_vs_E10	ordinary_muscle_development	ENSGALG00010017955	IKBKB	0.675284858	0.063258397	10.67502324	1.33224E-26	7.08395E-26	positive
PRJNA729271	stage_5W_vs_E10	ordinary_muscle_development	ENSGALG00010017956	TIPIN	-0.93295617	0.082233041	-11.34527138	7.8283E-30	4.70835E-29	negative
PRJNA729271	stage_5W_vs_E10	ordinary_muscle_development	ENSGALG00010017957	THUMPD1	-0.010483227	0.098570002	-0.106353118	0.915302188	0.926681932	negative
PRJNA729271	stage_5W_vs_E10	ordinary_muscle_development	ENSGALG00010017958	CAPN10	-0.993227185	0.09305046	-10.67407069	1.34598E-26	7.15173E-26	negative
PRJNA729271	stage_5W_vs_E10	ordinary_muscle_development	ENSGALG00010017959	PDZD7	1.720758977	0.279173227	6.163767913	7.1034E-10	1.67176E-09	positive
PRJNA729271	stage_5W_vs_E10	ordinary_muscle_development	ENSGALG00010017962	TXNDC16	-0.653802194	0.151222693	-4.323439685	1.53615E-05	2.70028E-05	negative
PRJNA729271	stage_5W_vs_E10	ordinary_muscle_development	ENSGALG00010017963	LPAR4	-1.86587806	0.170558305	-10.93982529	7.4343E-28	4.14781E-27	negative
PRJNA729271	stage_5W_vs_E10	ordinary_muscle_development	ENSGALG00010017970	AMPH	4.730790082	0.660092294	7.166861545	7.67366E-13	2.15311E-12	positive
PRJNA729271	stage_5W_vs_E10	ordinary_muscle_development	ENSGALG00010017972	RNPEPL1	1.621151746	0.171359473	9.460531841	3.06381E-21	1.29513E-20	positive
PRJNA729271	stage_5W_vs_E10	ordinary_muscle_development	ENSGALG00010017973	PKD1	1.831513311	0.129010243	14.19665031	9.60972E-46	1.00355E-44	positive
PRJNA729271	stage_5W_vs_E10	ordinary_muscle_development	ENSGALG00010017974	MXRA8	0.465866047	0.183955034	2.532499584	0.01132525	0.015421322	positive
PRJNA729271	stage_5W_vs_E10	ordinary_muscle_development	ENSGALG00010017976	GPR137C	-0.899034028	0.108489976	-8.286793515	1.16342E-16	3.97638E-16	negative
PRJNA729271	stage_5W_vs_E10	ordinary_muscle_development	ENSGALG00010017979	ENSGALG00010017979	0.10881757	0.161352889	0.674407324	0.500052388	0.541076393	positive
PRJNA729271	stage_5W_vs_E10	ordinary_muscle_development	ENSGALG00010017983	SLC4A11	2.587253924	0.202025944	12.80654292	1.507E-37	1.21491E-36	positive
PRJNA729271	stage_5W_vs_E10	ordinary_muscle_development	ENSGALG00010017985	ABCC5	0.381622198	0.267943997	1.424261048	0.154370919	0.182840862	positive
PRJNA729271	stage_5W_vs_E10	ordinary_muscle_development	ENSGALG00010017987	ERI2	-2.728964754	0.25565481	-10.67441193	1.34104E-26	7.12811E-26	negative
PRJNA729271	stage_5W_vs_E10	ordinary_muscle_development	ENSGALG00010017991	RAB26	-0.518146352	0.244534849	-2.118905976	0.034098414	0.043957356	negative
PRJNA729271	stage_5W_vs_E10	ordinary_muscle_development	ENSGALG00010017992	ENSGALG00010017992	6.362279171	0.559520902	11.370941	5.8355E-30	3.52734E-29	positive
PRJNA729271	stage_5W_vs_E10	ordinary_muscle_development	ENSGALG00010017993	REXO5	-1.294495969	0.098481961	-13.14449824	1.83015E-39	1.56693E-38	negative
PRJNA729271	stage_5W_vs_E10	ordinary_muscle_development	ENSGALG00010017996	ZC3H14	-0.062296305	0.092302955	-0.67491127	0.49973214	0.540850995	negative
PRJNA729271	stage_5W_vs_E10	ordinary_muscle_development	ENSGALG00010017997	ENSGALG00010017997	-1.620068039	0.451915886	-3.58488845	0.000337222	0.000531308	negative
PRJNA729271	stage_5W_vs_E10	ordinary_muscle_development	ENSGALG00010017999	GPR174	5.027301301	0.542058456	9.274463388	1.78515E-20	7.26848E-20	positive
PRJNA729271	stage_5W_vs_E10	ordinary_muscle_development	ENSGALG00010018000	MAP2K1	0.320907578	0.073204265	4.383727912	1.16665E-05	2.06884E-05	positive
PRJNA729271	stage_5W_vs_E10	ordinary_muscle_development	ENSGALG00010018001	TRAF7	-0.197867743	0.086385778	-2.290512936	0.0219916	0.028913219	negative
PRJNA729271	stage_5W_vs_E10	ordinary_muscle_development	ENSGALG00010018002	ZC3H8	0.308587594	0.079697742	3.871974128	0.000107957	0.000177199	positive
PRJNA729271	stage_5W_vs_E10	ordinary_muscle_development	ENSGALG00010018004	CDC42BPA	0.000839828	0.062243767	0.013492558	0.989234823	0.990670082	positive
PRJNA729271	stage_5W_vs_E10	ordinary_muscle_development	ENSGALG00010018006	DCUN1D3	-0.008579466	0.104374226	-0.082199089	0.934488398	0.943010953	negative
PRJNA729271	stage_5W_vs_E10	ordinary_muscle_development	ENSGALG00010018007	DNAAF9	-0.292352792	0.179630707	-1.627521251	0.103626439	0.125611744	negative
PRJNA729271	stage_5W_vs_E10	ordinary_muscle_development	ENSGALG00010018008	ABCF3	2.144155035	0.131753864	16.2739442	1.51103E-59	2.38588E-58	positive
PRJNA729271	stage_5W_vs_E10	ordinary_muscle_development	ENSGALG00010018010	ENSGALG00010018010	-0.766732284	0.119920336	-6.39368027	1.6194E-10	3.97514E-10	negative
PRJNA729271	stage_5W_vs_E10	ordinary_muscle_development	ENSGALG00010018011	LIPA	1.206026529	0.185287191	6.50895792	7.56739E-11	1.90235E-10	positive
PRJNA729271	stage_5W_vs_E10	ordinary_muscle_development	ENSGALG00010018012	DVL1	1.554433974	0.137941455	11.26879493	1.87113E-29	1.10793E-28	positive
PRJNA729271	stage_5W_vs_E10	ordinary_muscle_development	ENSGALG00010018014	LYRM1	0.308129166	0.096769108	3.184168708	0.001451704	0.002165306	positive
PRJNA729271	stage_5W_vs_E10	ordinary_muscle_development	ENSGALG00010018016	IFIT5	9.229454462	0.856881947	10.77097551	4.71974E-27	2.54511E-26	positive
PRJNA729271	stage_5W_vs_E10	ordinary_muscle_development	ENSGALG00010018019	NAPB	0.406999811	0.353150149	1.152483756	0.249122341	0.285006182	positive
PRJNA729271	stage_5W_vs_E10	ordinary_muscle_development	ENSGALG00010018020	SNAPC5	-0.578977576	0.104061303	-5.563812471	2.63944E-08	5.6713E-08	negative
PRJNA729271	stage_5W_vs_E10	ordinary_muscle_development	ENSGALG00010018021	ATRN	1.435413763	0.070729249	20.29448601	1.43851E-91	5.08566E-90	positive
PRJNA729271	stage_5W_vs_E10	ordinary_muscle_development	ENSGALG00010018022	XPO6	-0.288520707	0.11663034	-2.473804895	0.013368268	0.018042183	negative
PRJNA729271	stage_5W_vs_E10	ordinary_muscle_development	ENSGALG00010018025	CPTP	-0.779883692	0.137682193	-5.664375851	1.47561E-08	3.22803E-08	negative
PRJNA729271	stage_5W_vs_E10	ordinary_muscle_development	ENSGALG00010018026	CCDC39	-1.728158009	0.72922438	-2.369857695	0.017794933	0.023666167	negative
PRJNA729271	stage_5W_vs_E10	ordinary_muscle_development	ENSGALG00010018027	EML5	-1.282862551	0.332814584	-3.854586342	0.000115925	0.000189719	negative
PRJNA729271	stage_5W_vs_E10	ordinary_muscle_development	ENSGALG00010018030	POLB	0.798938677	0.10065402	7.937474112	2.0634E-15	6.64054E-15	positive
PRJNA729271	stage_5W_vs_E10	ordinary_muscle_development	ENSGALG00010018032	ERO1A	-0.002549883	0.12442318	-0.020493629	0.983649594	0.986030489	negative
PRJNA729271	stage_5W_vs_E10	ordinary_muscle_development	ENSGALG00010018033	COQ8A	1.753201778	0.213274576	8.220397457	2.0283E-16	6.85319E-16	positive
PRJNA729271	stage_5W_vs_E10	ordinary_muscle_development	ENSGALG00010018035	ITM2A	1.289587859	0.086353464	14.93382891	1.98531E-50	2.42436E-49	positive
PRJNA729271	stage_5W_vs_E10	ordinary_muscle_development	ENSGALG00010018036	RPL4	-0.370405443	0.107084123	-3.459013666	0.000542157	0.000840309	negative
PRJNA729271	stage_5W_vs_E10	ordinary_muscle_development	ENSGALG00010018037	GZF1	-0.122017516	0.084082394	-1.451166062	0.146733625	0.174336384	negative
PRJNA729271	stage_5W_vs_E10	ordinary_muscle_development	ENSGALG00010018039	TTC14	0.013114786	0.132278737	0.099145079	0.92102308	0.931953197	positive
PRJNA729271	stage_5W_vs_E10	ordinary_muscle_development	ENSGALG00010018042	ZWILCH	-1.670476759	0.140968303	-11.85001683	2.15148E-32	1.43845E-31	negative
PRJNA729271	stage_5W_vs_E10	ordinary_muscle_development	ENSGALG00010018043	PSMC6	0.055570944	0.091127553	0.609814945	0.541984401	0.581758285	positive
PRJNA729271	stage_5W_vs_E10	ordinary_muscle_development	ENSGALG00010018044	INTS11	-0.927963748	0.061312992	-15.13486331	9.53787E-52	1.21915E-50	negative
PRJNA729271	stage_5W_vs_E10	ordinary_muscle_development	ENSGALG00010018045	PSEN2	1.044145052	0.073343309	14.23640495	5.44574E-46	5.72414E-45	positive
PRJNA729271	stage_5W_vs_E10	ordinary_muscle_development	ENSGALG00010018046	CAPN15	-0.333431105	0.103701885	-3.215284895	0.001303151	0.001952168	negative
PRJNA729271	stage_5W_vs_E10	ordinary_muscle_development	ENSGALG00010018048	GFRA4	-4.087690895	0.170608501	-23.95947958	7.3602E-127	5.1291E-125	negative
PRJNA729271	stage_5W_vs_E10	ordinary_muscle_development	ENSGALG00010018049	SUGCT	-1.921541113	0.196335924	-9.787007243	1.28029E-22	5.75437E-22	negative
PRJNA729271	stage_5W_vs_E10	ordinary_muscle_development	ENSGALG00010018051	GNPNAT1	-1.184609888	0.137724799	-8.601282398	7.88302E-18	2.85876E-17	negative
PRJNA729271	stage_5W_vs_E10	ordinary_muscle_development	ENSGALG00010018052	PPP1RL	-0.235947905	0.131606859	-1.792823774	0.073001107	0.090255121	negative
PRJNA729271	stage_5W_vs_E10	ordinary_muscle_development	ENSGALG00010018053	DIAPH2	2.288192284	0.091184186	25.09417894	5.757E-139	4.688E-137	positive

PRJNA729271	stage_5W_vs_E10	ordinary_muscle_development	ENSGALG00010018054	SMAD6	-0.770765885	0.159922311	-4.819626976	1.43827E-06	2.72769E-06	negative
PRJNA729271	stage_5W_vs_E10	ordinary_muscle_development	ENSGALG00010018055	PEX5L	0.334184128	0.479119807	0.697495956	0.485492469	0.527013655	positive
PRJNA729271	stage_5W_vs_E10	ordinary_muscle_development	ENSGALG00010018057	SSTR4	-0.603040504	0.181072744	-3.330377006	0.000867285	0.001321208	negative
PRJNA729271	stage_5W_vs_E10	ordinary_muscle_development	ENSGALG00010018058	ITPKB	0.231653459	0.091121634	2.54224436	0.011014314	0.015019048	positive
PRJNA729271	stage_5W_vs_E10	ordinary_muscle_development	ENSGALG00010018060	CASKIN1	-2.242978179	0.347878663	-6.447587669	1.13644E-10	2.81634E-10	negative
PRJNA729271	stage_5W_vs_E10	ordinary_muscle_development	ENSGALG00010018061	NDUFAF5	0.970762904	0.134965051	7.192698374	6.35232E-13	1.78999E-12	positive
PRJNA729271	stage_5W_vs_E10	ordinary_muscle_development	ENSGALG00010018062	GOLGA7	-0.990708826	0.095022856	-10.42600559	1.88653E-25	9.49818E-25	negative
PRJNA729271	stage_5W_vs_E10	ordinary_muscle_development	ENSGALG00010018063	DECR2	-0.082417259	0.099139908	-0.831322723	0.405791341	0.447296235	negative
PRJNA729271	stage_5W_vs_E10	ordinary_muscle_development	ENSGALG00010018064	TTC8	-1.436661549	0.162116759	-8.861894079	7.86663E-19	2.97798E-18	negative
PRJNA729271	stage_5W_vs_E10	ordinary_muscle_development	ENSGALG00010018067	ENSGALG00010018067	-0.081885963	0.178672606	-0.458301722	0.646735687	0.681672798	negative
PRJNA729271	stage_5W_vs_E10	ordinary_muscle_development	ENSGALG00010018068	ADAM33	-0.267947274	0.139006479	-1.927588374	0.053906346	0.067674735	negative
PRJNA729271	stage_5W_vs_E10	ordinary_muscle_development	ENSGALG00010018069	ENSGALG00010018069	-0.977508903	0.384973929	-2.539156108	0.011112023	0.015146584	negative
PRJNA729271	stage_5W_vs_E10	ordinary_muscle_development	ENSGALG00010018071	GPAT4	-0.062399819	0.07980515	-0.781902161	0.434272075	0.476263428	negative
PRJNA729271	stage_5W_vs_E10	ordinary_muscle_development	ENSGALG00010018075	MLST8	1.354147175	0.102581405	13.20070798	8.69143E-40	7.55743E-39	positive
PRJNA729271	stage_5W_vs_E10	ordinary_muscle_development	ENSGALG00010018076	FOXA2	-4.284519646	1.450676962	-2.953462251	0.00314231	0.004533472	negative
PRJNA729271	stage_5W_vs_E10	ordinary_muscle_development	ENSGALG00010018078	NKX6-3	5.765460374	0.694467687	8.301985081	1.02386E-16	3.50931E-16	positive
PRJNA729271	stage_5W_vs_E10	ordinary_muscle_development	ENSGALG00010018080	BRICD5	2.026158269	0.320959248	6.312820966	2.73994E-10	6.62541E-10	positive
PRJNA729271	stage_5W_vs_E10	ordinary_muscle_development	ENSGALG00010018081	DIO3	-4.683215425	0.26726729	-17.52259103	9.63363E-69	1.96123E-67	negative
PRJNA729271	stage_5W_vs_E10	ordinary_muscle_development	ENSGALG00010018082	RAB11FIP3	-0.465934514	0.177538671	-2.624411415	0.008679887	0.011957126	negative
PRJNA729271	stage_5W_vs_E10	ordinary_muscle_development	ENSGALG00010018083	PAX1	-5.46975658	1.015301599	-5.387321942	7.15153E-08	1.49003E-07	negative
PRJNA729271	stage_5W_vs_E10	ordinary_muscle_development	ENSGALG00010018085	PGP	2.565003257	0.159297524	16.10196567	2.47127E-58	3.79461E-57	positive
PRJNA729271	stage_5W_vs_E10	ordinary_muscle_development	ENSGALG00010018087	ENSGALG00010018087	-0.83320823	0.056485667	-14.75079032	3.04083E-49	3.59224E-48	negative
PRJNA729271	stage_5W_vs_E10	ordinary_muscle_development	ENSGALG00010018089	TASP1	0.536863976	0.092203138	5.822621524	5.79316E-09	1.29686E-08	positive
PRJNA729271	stage_5W_vs_E10	ordinary_muscle_development	ENSGALG00010018092	ENSGALG00010018092	-4.265790534	0.219280574	-19.45357243	2.71817E-84	8.05725E-83	negative
PRJNA729271	stage_5W_vs_E10	ordinary_muscle_development	ENSGALG00010018095	E4F1	-0.25011386	0.070572403	-3.544074605	0.000393994	0.000617065	negative
PRJNA729271	stage_5W_vs_E10	ordinary_muscle_development	ENSGALG00010018099	SMAD3	-1.753961327	0.131236072	-13.36493313	9.69221E-41	8.66144E-40	negative
PRJNA729271	stage_5W_vs_E10	ordinary_muscle_development	ENSGALG00010018102	ZDHHC4	1.015638603	0.165553397	6.134809797	8.52611E-10	1.99719E-09	positive
PRJNA729271	stage_5W_vs_E10	ordinary_muscle_development	ENSGALG00010018103	BTRC	-0.394878189	0.108121017	-3.652187166	0.000260016	0.000413896	negative
PRJNA729271	stage_5W_vs_E10	ordinary_muscle_development	ENSGALG00010018111	NKX2-2	-1.709216282	0.960210373	-1.78004355	0.075068834	0.092677178	negative
PRJNA729271	stage_5W_vs_E10	ordinary_muscle_development	ENSGALG00010018115	KLHL4	-1.715873013	0.199822602	-8.586981618	8.92839E-18	3.22816E-17	negative
PRJNA729271	stage_5W_vs_E10	ordinary_muscle_development	ENSGALG00010018119	GRID2IP	-3.085113562	0.844778102	-3.651980982	0.000260225	0.000414183	negative
PRJNA729271	stage_5W_vs_E10	ordinary_muscle_development	ENSGALG00010018121	FERMT2	-0.731859426	0.160210178	-4.568120678	4.92117E-06	8.95797E-06	negative
PRJNA729271	stage_5W_vs_E10	ordinary_muscle_development	ENSGALG00010018123	USP13	4.171470383	0.140685964	29.65093507	3.2987E-193	5.1414E-191	positive
PRJNA729271	stage_5W_vs_E10	ordinary_muscle_development	ENSGALG00010018128	SEPTIN12	0.176222268	0.192872733	0.913671234	0.360889623	0.401188365	positive
PRJNA729271	stage_5W_vs_E10	ordinary_muscle_development	ENSGALG00010018137	NDUFB5	1.28125686	0.127543565	10.04564095	9.60214E-24	4.516E-23	positive
PRJNA729271	stage_5W_vs_E10	ordinary_muscle_development	ENSGALG00010018140	CHPF	-1.078081018	0.106192219	-10.15216585	3.2409E-24	1.55759E-23	negative
PRJNA729271	stage_5W_vs_E10	ordinary_muscle_development	ENSGALG00010018142	DDHD1	-1.404314684	0.145944996	-9.622218816	6.44262E-22	2.81282E-21	negative
PRJNA729271	stage_5W_vs_E10	ordinary_muscle_development	ENSGALG00010018143	MRPL47	0.261034202	0.123919619	2.106480024	0.035162677	0.04526492	positive
PRJNA729271	stage_5W_vs_E10	ordinary_muscle_development	ENSGALG00010018145	ENSGALG00010018145	-2.15147187	0.376783004	-5.7101086	1.12904E-08	2.48375E-08	negative
PRJNA729271	stage_5W_vs_E10	ordinary_muscle_development	ENSGALG00010018147	ISM1	-0.781658361	0.289091774	-2.703841589	0.006854296	0.009543081	negative
PRJNA729271	stage_5W_vs_E10	ordinary_muscle_development	ENSGALG00010018148	XRN2	-0.512242991	0.055489729	-9.231311791	2.67338E-20	1.08181E-19	negative
PRJNA729271	stage_5W_vs_E10	ordinary_muscle_development	ENSGALG00010018149	ENSGALG00010018149	1.194444889	0.292458298	4.084154548	4.42376E-05	7.48918E-05	positive
PRJNA729271	stage_5W_vs_E10	ordinary_muscle_development	ENSGALG00010018150	AAGAB	-1.646309657	0.08166235	-20.15995945	2.20062E-90	7.59477E-89	negative
PRJNA729271	stage_5W_vs_E10	ordinary_muscle_development	ENSGALG00010018154	SLC16A12	-2.723036585	0.30001564	-9.076315423	1.12313E-19	4.42985E-19	negative
PRJNA729271	stage_5W_vs_E10	ordinary_muscle_development	ENSGALG00010018157	FBXL2	-0.808788375	0.154162183	-5.246347435	1.55144E-07	3.16067E-07	negative
PRJNA729271	stage_5W_vs_E10	ordinary_muscle_development	ENSGALG00010018160	ENSGALG00010018160	0.936765708	0.164731136	5.686634193	1.29568E-08	2.8417E-08	positive
PRJNA729271	stage_5W_vs_E10	ordinary_muscle_development	ENSGALG00010018162	ENSGALG00010018162	-0.449970172	0.362870327	-1.240030222	0.214964216	0.248635997	negative
PRJNA729271	stage_5W_vs_E10	ordinary_muscle_development	ENSGALG00010018163	CCT7	-0.852368455	0.081293172	-10.48511746	1.0112E-25	5.16467E-25	negative
PRJNA729271	stage_5W_vs_E10	ordinary_muscle_development	ENSGALG00010018165	DACH2	1.323971985	0.180515568	7.334392245	2.2273E-13	6.40951E-13	positive
PRJNA729271	stage_5W_vs_E10	ordinary_muscle_development	ENSGALG00010018166	MSS51	6.656186968	0.23590654	28.21535582	3.7899E-175	5.0865E-173	positive
PRJNA729271	stage_5W_vs_E10	ordinary_muscle_development	ENSGALG00010018168	ENSGALG00010018168	-6.810894841	0.674257782	-10.10132181	5.45025E-24	2.59191E-23	negative
PRJNA729271	stage_5W_vs_E10	ordinary_muscle_development	ENSGALG00010018172	ACTL6A	-1.793845775	0.094791573	-18.92410593	7.22042E-80	1.93457E-78	negative
PRJNA729271	stage_5W_vs_E10	ordinary_muscle_development	ENSGALG00010018174	BMP4	0.806411502	0.203971832	3.95354345	7.70023E-05	0.000128249	positive
PRJNA729271	stage_5W_vs_E10	ordinary_muscle_development	ENSGALG00010018177	KIF20B	-2.69021031	0.189558963	-14.19194462	1.02769E-45	1.07245E-44	negative
PRJNA729271	stage_5W_vs_E10	ordinary_muscle_development	ENSGALG00010018179	PRADC1	0.648697785	0.117294722	5.530494233	3.1933E-08	6.80889E-08	positive
PRJNA729271	stage_5W_vs_E10	ordinary_muscle_development	ENSGALG00010018183	CYP17A1	-0.611059329	0.378534188	-1.61427778	0.106467188	0.128882736	negative
PRJNA729271	stage_5W_vs_E10	ordinary_muscle_development	ENSGALG00010018185	GNB4	-0.519874455	0.069638846	-7.465293956	8.31139E-14	2.44567E-13	negative
PRJNA729271	stage_5W_vs_E10	ordinary_muscle_development	ENSGALG00010018186	SPTLC3	-3.587503736	0.633482638	-5.663144527	1.48624E-08	3.2508E-08	negative
PRJNA729271	stage_5W_vs_E10	ordinary_muscle_development	ENSGALG00010018188	UBP1	-0.254838298	0.052478374	-4.856063133	1.19743E-06	2.28407E-06	negative
PRJNA729271	stage_5W_vs_E10	ordinary_muscle_development	ENSGALG00010018189	MIS18BP1	-1.468179014	0.122241732	-12.01045653	3.13112E-33	2.15813E-32	negative
PRJNA729271	stage_5W_vs_E10	ordinary_muscle_development	ENSGALG00010018192	IQCH	-1.874759172	0.228966192	-8.187930099	2.65757E-16	8.93356E-16	negative
PRJNA729271	stage_5W_vs_E10	ordinary_muscle_development	ENSGALG00010018193	ANK1	4.321331211	0.24659602	17.52392928	9.40963E-69	1.91832E-67	positive
PRJNA729271	stage_5W_vs_E10	ordinary_muscle_development	ENSGALG00010018198	BTBD3	-0.58744558	0.103035641	-5.701382289	1.1884E-08	2.61156E-08	negative
PRJNA729271	stage_5W_vs_E10	ordinary_muscle_development	ENSGALG00010018199	BORCS7	-0.75803096	0.121240168	-6.252308696	4.04429E-10	9.67679E-10	negative
PRJNA729271	stage_5W_vs_E10	ordinary_muscle_development	ENSGALG00010018206	KIZ	-0.269977192	0.128577653	-2.099720957	0.035753395	0.04600084	negative
PRJNA729271	stage_5W_vs_E10	ordinary_muscle_development	ENSGALG00010018209	GMPPA	-0.007406603	0.131826305	-0.056184558	0.955194783	0.961429649	negative
PRJNA729271	stage_5W_vs_E10	ordinary_muscle_development	ENSGALG00010018215	CFAP70	3.478006301	0.264249287	13.161838	1.45503E-39	1.25316E-38	positive
PRJNA729271	stage_5W_vs_E10	ordinary_muscle_development	ENSGALG00010018216	FANCM	-0.476614635	0.087063236	-5.474350117	4.3912E-08	9.2812E-08	negative
PRJNA729271	stage_5W_vs_E10	ordinary_muscle_development	ENSGALG00010018217	ENSGALG00010018217	-2.424558954	0.57690609	-4.202692595	2.63759E-05	4.54545E-05	negative
PRJNA729271	stage_5W_vs_E10	ordinary_muscle_development	ENSGALG00010018219	SLC24A5	-4.145859674	0.741797764	-5.588935252	2.28466E-08	4.93314E-08	negative
PRJNA729271	stage_5W_vs_E10	ordinary_muscle_development	ENSGALG00010018226	JAG1	0.118645239	0.101988459	1.163320246	0.244699594	0.2802782	positive
PRJNA729271	stage_5W_vs_E10	ordinary_muscle_development	ENSGALG00010018229	AS3MT	0.403192027	0.190250619	2.119267881	0.034067834	0.043925748	positive
PRJNA729271	stage_5W_vs_E10	ordinary_muscle_development	ENSGALG00010018231	SMYD5	-1.037002391	0.082225058	-12.61175621	1.81894E-36	1.41522E-35	negative
PRJNA729271	stage_5W_vs_E10	ordinary_muscle_development	ENSGALG00010018233	RASSF2	0.865284607	0.183446619	4.716819591	2.3956E-06	4.46786E-06	positive
PRJNA729271	stage_5W_vs_E10	ordinary_muscle_development	ENSGALG00010018240	SOUL	-0.16443417	0.668479196	-0.245982479	0.805695795	0.828795724	negative
PRJNA729271	stage_5W_vs_E10	ordinary_muscle_development	ENSGALG00010018241	RALGAPA2	0.674029974	0.090983855	7.408237139	1.27989E-13	3.72681E-13	positive
PRJNA729271	stage_5W_vs_E10	ordinary_muscle_development	ENSGALG00010018244	CHM	0.267449722	0.081892427	3.265866369	0.001091297	0.001648776	positive
PRJNA729271	stage_5W_vs_E10	ordinary_muscle_development	ENSGALG00010018251	USP48	0.757990099	0.165965665	4.567150078	4.944E-06	8.99389E-06	positive
PRJNA729271	stage_5W_vs_E10	ordinary_muscle_development	ENSGALG00010018255	MFN1	0.725167545	0.11290527	6.422796237	1.33794E-10	3.30212E-10	positive
PRJNA729271	stage_5W_vs_E10	ordinary_muscle_development	ENSGALG00010018258	OSCP1	-0.637885958	0.282894407	-2.254855317	0.024142412	0.031620517	negative
PRJNA729271	stage_5W_vs_E10	ordinary_muscle_development	ENSGALG00010018259	WDR90	-0.461474007	0.130613139	-3.533136178	0.000410661	0.000641921	negative
PRJNA729271	stage_5W_vs_E10	ordinary_muscle_development	ENSGALG00010018261	PRNP	0.648362637	0.195044722	3.324174216	0.000886808	0.001349247	positive
PRJNA729271	stage_5W_vs_E10	ordinary_muscle_development	ENSGALG00010018263	SLX4IP	-1.654186549	0.119344715	-13.86057649	1.09789E-43	1.07454E-42	negative
PRJNA729271	stage_5W_vs_E10	ordinary_muscle_development	ENSGALG00010018264	CFAP61	-0.442358294	0.322749609	-1.370592811	0.170501925	0.200857167	negative
PRJNA729271	stage_5W_vs_E10	ordinary_muscle_development	ENSGALG00010018265	PDCD4	0.115779683	0.065380996	1.770846114	0.076586295	0.094437971	positive
PRJNA729271	stage_5W_vs_E10	ordinary_muscle_development	ENSGALG00010018267	ADPRHL2	-0.522679173	0.077423116	-6.750944676	1.46886E-11	3.85568E-11	negative
PRJNA729271	stage_5W_vs_E10	ordinary_muscle_development	ENSGALG00010018273	PROM2	-4.538613867	1.044614446	-4.344774172	1.39419E-05	2.45999E-05	negative
PRJNA729271	stage_5W_vs_E10	ordinary_muscle_development	ENSGALG00010018274	ZNF711	-0.128339548	0.097599215	-1.314964965	0.188521678	0.220372719	negative
PRJNA729271	stage_5W_vs_E10	ordinary_muscle_development	ENSGALG00010018275	ZNF639	-0.317086481	0.084426648	-3.755762996	0.000172814	0.000278916	negative
PRJNA729271	stage_5W_vs_E10	ordinary_muscle_development	ENSGALG00010018276	ARHGAP17	0.279769605	0.07179395	3.896840954	9.74556E-05	0.000160689	positive
PRJNA729271	stage_5W_vs_E10	ordinary_muscle_development	ENSGALG00010018278	PUSL1	0.693231501	0.150833731	4.595997849	4.30683E-06	7.8763E-06	positive
PRJNA729271	stage_5W_vs_E10	ordinary_muscle_development	ENSGALG00010018283	POF1B	0.446515394	0.178507935	2.501375611	0.012371189	0.016757349	positive
PRJNA729271	stage_5W_vs_E10	ordinary_muscle_development	ENSGALG00010018284	ENSGALG00010018284	1.618547343	0.167033558	9.689953124	3.32681E-22	1.47064E-21	positive
PRJNA729271	stage_5W_vs_E10	ordinary_muscle_development	ENSGALG00010018285	BBIP1	-0.130034496	0.192179775	-0.676629454	0.498641086	0.539912045	negative
PRJNA729271	stage_5W_vs_E10	ordinary_muscle_development	ENSGALG00010018287	MKKS	0.142893999	0.102951169	1.387978404	0.165143621	0.195026625	positive
PRJNA729271	stage_5W_vs_E10	ordinary_muscle_development	ENSGALG00010018288	CRNKL1	-0.319293769	0.062184074	-5.134655037	2.82662E-07	5.63807E-07	negative
PRJNA729271	stage_5W_vs_E10	ordinary_muscle_development	ENSGALG00010018289	HEYL	-0.807921423	0.20980297	-3.850857903	0.000117705	0.0001925	negative
PRJNA729271	stage_5W_vs_E10	ordinary_muscle_development	ENSGALG00010018290	RHOT2	-0.082675736	0.134638987	-0.614054944	0.53917902	0.579133005	negative
PRJNA729271	stage_5W_vs_E10	ordinary_muscle_development	ENSGALG00010018294	FKBP3	0.373399595	0.177905129	2.0988692	0.035828433	0.046076935	positive
PRJNA729271	stage_5W_vs_E10	ordinary_muscle_development	ENSGALG00010018296	PIK3CA	0.64612138	0.096699394	6.681752125	2.36102E-11	6.11453E-11	positive
PRJNA729271	stage_5W_vs_E10	ordinary_muscle_development	ENSGALG00010018298	ENSGALG00010018298	-0.189286663	0.200075225	-0.946077474	0.344109072	0.383917873	negative
PRJNA729271	stage_5W_vs_E10	ordinary_muscle_development	ENSGALG00010018299	SHOC2	0.509741908	0.082609832	6.170475044	6.80851E-10	1.60575E-09	positive
PRJNA729271	stage_5W_vs_E10	ordinary_muscle_development	ENSGALG00010018301	ENSGALG00010018301	-0.109813167	0.312985157	-0.350857428	0.725695311	0.75572624	negative
PRJNA729271	stage_5W_vs_E10	ordinary_muscle_development	ENSGALG00010018302	RAB11FIP5	1.55410626	0.09980657	15.57118199	1.14276E-54	1.56267E-53	positive
PRJNA729271	stage_5W_vs_E10	ordinary_muscle_development	ENSGALG00010018306	DES	4.524000548	0.150906178	29.97889546	1.8493E-197	3.046E-195	positive
PRJNA729271	stage_5W_vs_E10	ordinary_muscle_development	ENSGALG00010018308	ACAP3	-0.682225899	0.177289995	-3.848078969	0.000119048	0.000194609	negative
PRJNA729271	stage_5W_vs_E10	ordinary_muscle_development	ENSGALG00010018309	DPYSL2	-1.162745528	0.124292129	-9.35494095	8.36464E-21	3.4602E-20	negative
PRJNA729271	stage_5W_vs_E10	ordinary_muscle_development	ENSGALG00010018310	APOOL	-0.357514478	0.112738985	-3.171169927	0.001518263	0.002258842	negative
PRJNA729271	stage_5W_vs_E10	ordinary_muscle_development	ENSGALG00010018311	NKX2-3	2.432746824	0.737572296	3.298316433	0.000972665	0.001476775	positive
PRJNA729271	stage_5W_vs_E10	ordinary_muscle_development	ENSGALG00010018312	PRPF39	-0.575594005	0.079584194	-7.232516629	4.74125E-13	1.34332E-12	negative
PRJNA729271	stage_5W_vs_E10	ordinary_muscle_development	ENSGALG00010018313	MYEF2	-1.570952059	0.082481704	-19.04606704	7.08279E-81	1.95926E-79	negative
PRJNA729271	stage_5W_vs_E10	ordinary_muscle_development	ENSGALG00010018314	SLC24A3	-1.524015111	0.132578449	-11.49519491	1.39466E-30	8.65393E-30	negative
PRJNA729271	stage_5W_vs_E10	ordinary_muscle_development	ENSGALG00010018315	CLASP2	0.747353368	0.117285053	6.372110908	1.86444E-10	4.56427E-10	positive
PRJNA729271	stage_5W_vs_E10	ordinary_muscle_development	ENSGALG00010018317	ZMAT3	-0.677367196	0.140741727	-4.812838469	1.48802E-06	2.81834E-06	negative
PRJNA729271	stage_5W_vs_E10	ordinary_muscle_development	ENSGALG00010018319	STUB1	0.752330957	0.201710153	3.729762472	0.00019166	0.000308406	positive
PRJNA729271	stage_5W_vs_E10	ordinary_muscle_development	ENSGALG00010018322	NT5C1A	4.803031954	0.240397277	19.97956057	8.29508E-89	2.73889E-87	positive
PRJNA729271	stage_5W_vs_E10	ordinary_muscle_development	ENSGALG00010018323	GOT1	2.000523494	0.142434904	14.04517741	8.24616E-45	8.34111E-44	positive
PRJNA729271	stage_5W_vs_E10	ordinary_muscle_development	ENSGALG00010018325	ENSGALG00010018325	1.492758395	0.189951457	7.858630916	3.88355E-15	1.22881E-14	positive
PRJNA729271	stage_5W_vs_E10	ordinary_muscle_development	ENSGALG00010018326	LCMT1	-0.990388823	0.11271291	-8.786826875	1.53844E-18	5.75474E-18	negative
PRJNA729271	stage_5W_vs_E10	ordinary_muscle_development	ENSGALG00010018327	SNAP25	-0.700101084	0.520038147	-1.346249479	0.178222073	0.209125633	negative
PRJNA729271	stage_5W_vs_E10	ordinary_muscle_development	ENSGALG00010018328	ENSGALG00010018328	2.625669019	0.553335876	4.745163172	2.08338E-06	3.90264E-06	positive
PRJNA729271	stage_5W_vs_E10	ordinary_muscle_development	ENSGALG00010018329	ENSGALG00010018329	2.955393598	0.219745629	13.44915758	3.114E-41	2.82285E-40	positive
PRJNA729271	stage_5W_vs_E10	ordinary_muscle_development	ENSGALG00010018333	ENSGALG00010018333	1.038774484	0.216637687	4.794985103	1.62687E-06	3.0709E-06	positive
PRJNA729271	stage_5W_vs_E10	ordinary_muscle_development	ENSGALG00010018336	JMJD8	-0.390677161	0.151097873	-2.585590086	0.009721247	0.013336122	negative
PRJNA729271	stage_5W_vs_E10	ordinary_muscle_development	ENSGALG00010018337	TMEM151B	-1.794741435	0.330814512	-5.425219779	5.78833E-08	1.21351E-07	negative
PRJNA729271	stage_5W_vs_E10	ordinary_muscle_development	ENSGALG00010018338	PDCD6IP	0.324455475	0.084739565	3.828854631	0.000128741	0.000209863	positive
PRJNA729271	stage_5W_vs_E10	ordinary_muscle_development	ENSGALG00010018339	FAM76A	0.194045741	0.115939672	1.673678548	0.094193792	0.114801901	positive
PRJNA729271	stage_5W_vs_E10	ordinary_muscle_development	ENSGALG00010018340	DNPEP	1.201298533	0.097050255	12.37810793	3.43342E-35	2.55087E-34	positive
PRJNA729271	stage_5W_vs_E10	ordinary_muscle_development	ENSGALG00010018342	BNIP3L	-0.876524392	0.106467262	-8.232806753	1.82877E-16	6.18913E-16	negative
PRJNA729271	stage_5W_vs_E10	ordinary_muscle_development	ENSGALG00010018344	SFXN5	-1.262352785	0.199742074	-6.319914278	2.61708E-10	6.3404E-10	negative
PRJNA729271	stage_5W_vs_E10	ordinary_muscle_development	ENSGALG00010018345	WDR24	0.672775804	0.170998761	3.934389932	8.34083E-05	0.000138504	positive

PRJNA729271	stage_5W_vs_E10	ordinary_muscle_development	ENSGALG00010018347	CTXN2	-5.355956567	0.96375169	-5.557403035	2.73818E-08	5.87303E-08	negative
PRJNA729271	stage_5W_vs_E10	ordinary_muscle_development	ENSGALG00010018348	TOGARAM1	0.612594666	0.107404336	5.703630676	1.17282E-08	2.57772E-08	positive
PRJNA729271	stage_5W_vs_E10	ordinary_muscle_development	ENSGALG00010018349	KCNMB2	1.075275096	0.191531148	5.614100396	1.97587E-08	4.28298E-08	positive
PRJNA729271	stage_5W_vs_E10	ordinary_muscle_development	ENSGALG00010018350	CNNM1	-1.091369697	0.261464506	-4.174064437	2.99213E-05	5.13265E-05	negative
PRJNA729271	stage_5W_vs_E10	ordinary_muscle_development	ENSGALG00010018351	RIN2	0.714707801	0.086801601	8.233808945	1.81353E-16	6.14184E-16	positive
PRJNA729271	stage_5W_vs_E10	ordinary_muscle_development	ENSGALG00010018354	AARS2	-0.018812189	0.119156871	-0.157877501	0.874553328	0.890139786	negative
PRJNA729271	stage_5W_vs_E10	ordinary_muscle_development	ENSGALG00010018355	PRKCB	0.745834584	0.151808054	4.913010651	8.96884E-07	1.729E-06	positive
PRJNA729271	stage_5W_vs_E10	ordinary_muscle_development	ENSGALG00010018356	ADRA1A	-2.467904748	0.478547947	-5.157068927	2.50845E-07	5.02558E-07	negative
PRJNA729271	stage_5W_vs_E10	ordinary_muscle_development	ENSGALG00010018359	CACNG3	-4.651674048	0.864459722	-5.381018835	7.40654E-08	1.54073E-07	negative
PRJNA729271	stage_5W_vs_E10	ordinary_muscle_development	ENSGALG00010018361	ASIC4	-1.461564351	0.259285506	-5.636891836	1.73147E-08	3.76559E-08	negative
PRJNA729271	stage_5W_vs_E10	ordinary_muscle_development	ENSGALG00010018363	TCTE1	0.247202952	0.392145741	0.630385406	0.528442457	0.568746706	positive
PRJNA729271	stage_5W_vs_E10	ordinary_muscle_development	ENSGALG00010018365	HPSE2	-1.763524765	0.261269489	-6.749830493	1.48018E-11	3.8847E-11	negative
PRJNA729271	stage_5W_vs_E10	ordinary_muscle_development	ENSGALG00010018367	PAK5	-0.912695397	0.219314125	-4.161589667	3.1604E-05	5.41041E-05	negative
PRJNA729271	stage_5W_vs_E10	ordinary_muscle_development	ENSGALG00010018370	ZBTB8A	-0.674782035	0.115299948	-5.852405373	4.84514E-09	1.08986E-08	negative
PRJNA729271	stage_5W_vs_E10	ordinary_muscle_development	ENSGALG00010018371	ENSGALG00010018371	0.509754794	0.136556842	3.732912886	0.000189278	0.000304674	positive
PRJNA729271	stage_5W_vs_E10	ordinary_muscle_development	ENSGALG00010018372	NLGN3	-1.012702795	0.472025331	-2.145441628	0.031917554	0.041285467	negative
PRJNA729271	stage_5W_vs_E10	ordinary_muscle_development	ENSGALG00010018374	HPS1	-0.407052734	0.135991492	-2.993222048	0.002760488	0.004004131	negative
PRJNA729271	stage_5W_vs_E10	ordinary_muscle_development	ENSGALG00010018376	PYGB	-0.413734643	0.143080727	-2.89161686	0.00383265	0.005477104	negative
PRJNA729271	stage_5W_vs_E10	ordinary_muscle_development	ENSGALG00010018377	RAP1GAP	0.852465493	0.307541436	2.771871989	0.005573495	0.007832071	positive
PRJNA729271	stage_5W_vs_E10	ordinary_muscle_development	ENSGALG00010018378	ARPP21	0.127964308	0.145534434	0.879271695	0.379253979	0.420410403	positive
PRJNA729271	stage_5W_vs_E10	ordinary_muscle_development	ENSGALG00010018379	SRBD1	0.834017752	0.126022042	6.618030762	3.64015E-11	9.34207E-11	positive
PRJNA729271	stage_5W_vs_E10	ordinary_muscle_development	ENSGALG00010018380	SLC20A1	-1.401334039	0.144063303	-9.727210236	2.30837E-22	1.027E-21	negative
PRJNA729271	stage_5W_vs_E10	ordinary_muscle_development	ENSGALG00010018381	HAPSTR1	0.543265805	0.067427696	8.057012671	7.81815E-16	2.56796E-15	positive
PRJNA729271	stage_5W_vs_E10	ordinary_muscle_development	ENSGALG00010018383	METRN	-1.842786135	0.166525033	-11.06612081	1.83159E-28	1.04441E-27	negative
PRJNA729271	stage_5W_vs_E10	ordinary_muscle_development	ENSGALG00010018384	LAMP5	-0.703362434	0.244854807	-2.872569429	0.004071486	0.005805252	negative
PRJNA729271	stage_5W_vs_E10	ordinary_muscle_development	ENSGALG00010018385	SPR	-1.199936052	0.143674638	-8.351759677	6.72534E-17	2.32992E-16	negative
PRJNA729271	stage_5W_vs_E10	ordinary_muscle_development	ENSGALG00010018387	NT5DC4	-2.894664531	0.372277273	-7.77556069	7.5114E-15	2.34095E-14	negative
PRJNA729271	stage_5W_vs_E10	ordinary_muscle_development	ENSGALG00010018388	SMC3	-1.165224122	0.066526558	-17.51517219	1.09753E-68	2.225E-67	negative
PRJNA729271	stage_5W_vs_E10	ordinary_muscle_development	ENSGALG00010018391	STAC	-1.493883374	0.221881625	-6.732794458	1.66435E-11	4.35465E-11	negative
PRJNA729271	stage_5W_vs_E10	ordinary_muscle_development	ENSGALG00010018393	PRKCE	-0.052019461	0.077108508	-0.67462674	0.49991294	0.541006276	negative
PRJNA729271	stage_5W_vs_E10	ordinary_muscle_development	ENSGALG00010018394	KLHL28	0.189706451	0.097432895	1.947047262	0.051529069	0.064892602	positive
PRJNA729271	stage_5W_vs_E10	ordinary_muscle_development	ENSGALG00010018395	MED12	0.128289306	0.078954295	1.624855308	0.104193393	0.126256749	positive
PRJNA729271	stage_5W_vs_E10	ordinary_muscle_development	ENSGALG00010018396	TBL1XR1	0.020392572	0.0747771	0.27271146	0.785075025	0.810748253	positive
PRJNA729271	stage_5W_vs_E10	ordinary_muscle_development	ENSGALG00010018397	FAM173A	2.425102006	0.159147629	15.23806553	1.97636E-52	2.56926E-51	positive
PRJNA729271	stage_5W_vs_E10	ordinary_muscle_development	ENSGALG00010018398	LRFN5	-0.118511339	0.188653601	-0.62819548	0.529875891	0.570154483	negative
PRJNA729271	stage_5W_vs_E10	ordinary_muscle_development	ENSGALG00010018399	ABHD12	0.368473332	0.162806626	2.263257592	0.023619815	0.030955625	positive
PRJNA729271	stage_5W_vs_E10	ordinary_muscle_development	ENSGALG00010018401	ENSGALG00010018401	-0.740567405	0.103492813	-7.155737536	8.32242E-13	2.33153E-12	negative
PRJNA729271	stage_5W_vs_E10	ordinary_muscle_development	ENSGALG00010018404	IL2RG	3.099442524	0.253710799	12.21643912	2.53967E-34	1.8197E-33	positive
PRJNA729271	stage_5W_vs_E10	ordinary_muscle_development	ENSGALG00010018405	DCLK3	-1.400889207	0.192390585	-7.281485258	3.30165E-13	9.41148E-13	negative
PRJNA729271	stage_5W_vs_E10	ordinary_muscle_development	ENSGALG00010018406	TMEM260	-0.835262739	0.132224033	-6.317026633	2.66644E-10	6.4535E-10	negative
PRJNA729271	stage_5W_vs_E10	ordinary_muscle_development	ENSGALG00010018410	EXOC6B	0.409975064	0.098984124	4.141826454	3.44551E-05	5.87768E-05	positive
PRJNA729271	stage_5W_vs_E10	ordinary_muscle_development	ENSGALG00010018413	SLC15A2	-1.681266684	0.360756378	-4.660393506	3.15605E-06	5.84253E-06	negative
PRJNA729271	stage_5W_vs_E10	ordinary_muscle_development	ENSGALG00010018414	EPAS1	2.037023631	0.137653652	14.79818084	1.50498E-49	1.78809E-48	positive
PRJNA729271	stage_5W_vs_E10	ordinary_muscle_development	ENSGALG00010018415	PELI2	-2.119192985	0.133368688	-15.88973403	7.46436E-57	1.0951E-55	negative
PRJNA729271	stage_5W_vs_E10	ordinary_muscle_development	ENSGALG00010018416	DUT	-2.126929893	0.135324695	-15.71723399	1.15246E-55	1.62816E-54	negative
PRJNA729271	stage_5W_vs_E10	ordinary_muscle_development	ENSGALG00010018418	PLCB4	-0.49159759	0.119782479	-4.104085953	4.05917E-05	6.89449E-05	negative
PRJNA729271	stage_5W_vs_E10	ordinary_muscle_development	ENSGALG00010018419	USP7	0.723877488	0.133589134	5.418685375	6.00389E-08	1.25742E-07	positive
PRJNA729271	stage_5W_vs_E10	ordinary_muscle_development	ENSGALG00010018420	RHOQ	3.089002399	0.131452016	23.49908727	4.1673E-122	2.5596E-120	positive
PRJNA729271	stage_5W_vs_E10	ordinary_muscle_development	ENSGALG00010018422	ENSGALG00010018422	0.199037683	0.216205411	0.920595288	0.357261763	0.397612811	positive
PRJNA729271	stage_5W_vs_E10	ordinary_muscle_development	ENSGALG00010018425	CYP26B1	-2.541433768	0.234606327	-10.83275888	2.40789E-27	1.3141E-26	negative
PRJNA729271	stage_5W_vs_E10	ordinary_muscle_development	ENSGALG00010018426	ENSGALG00010018426	3.255697452	0.347665832	9.364444689	7.64512E-21	3.16617E-20	positive
PRJNA729271	stage_5W_vs_E10	ordinary_muscle_development	ENSGALG00010018427	PIGF	-0.228852913	0.144643019	-1.582191207	0.113605927	0.137043768	negative
PRJNA729271	stage_5W_vs_E10	ordinary_muscle_development	ENSGALG00010018428	PPP2R5D	-0.292755543	0.06445454	-4.542046893	5.57106E-06	1.01042E-05	negative
PRJNA729271	stage_5W_vs_E10	ordinary_muscle_development	ENSGALG00010018430	LOXL4	-1.570629408	0.26051339	-6.028977668	1.65E-09	3.80356E-09	negative
PRJNA729271	stage_5W_vs_E10	ordinary_muscle_development	ENSGALG00010018434	HSPBAP1	1.555416078	0.159966336	9.72339632	2.39652E-22	1.06459E-21	positive
PRJNA729271	stage_5W_vs_E10	ordinary_muscle_development	ENSGALG00010018436	FOXO4	0.978494503	0.145363733	6.731352336	1.68093E-11	4.39645E-11	positive
PRJNA729271	stage_5W_vs_E10	ordinary_muscle_development	ENSGALG00010018438	PPP2R2A	0.42450059	0.098439786	4.312286799	1.61575E-05	2.83367E-05	positive
PRJNA729271	stage_5W_vs_E10	ordinary_muscle_development	ENSGALG00010018440	MEA1	0.605185753	0.101400165	5.968291617	2.39751E-09	5.47791E-09	positive
PRJNA729271	stage_5W_vs_E10	ordinary_muscle_development	ENSGALG00010018441	CRIPT	0.711135173	0.093527723	7.60346935	2.88296E-14	8.71682E-14	positive
PRJNA729271	stage_5W_vs_E10	ordinary_muscle_development	ENSGALG00010018443	PLCB1	1.527956348	0.146735982	10.41296299	2.1638E-25	1.08715E-24	positive
PRJNA729271	stage_5W_vs_E10	ordinary_muscle_development	ENSGALG00010018444	NUBP2	-0.384579092	0.09909766	-3.880809008	0.00010411	0.000171271	negative
PRJNA729271	stage_5W_vs_E10	ordinary_muscle_development	ENSGALG00010018446	FBN1	1.782292868	0.232077185	7.679741844	1.5941E-14	4.87786E-14	positive
PRJNA729271	stage_5W_vs_E10	ordinary_muscle_development	ENSGALG00010018448	KLHDC3	2.245321987	0.164693738	13.63331732	2.53777E-42	2.37783E-41	positive
PRJNA729271	stage_5W_vs_E10	ordinary_muscle_development	ENSGALG00010018451	PIK3R4	-0.199290966	0.112027892	-1.7789406	0.075249501	0.092860677	negative
PRJNA729271	stage_5W_vs_E10	ordinary_muscle_development	ENSGALG00010018452	SPSB3	0.662513034	0.119244324	5.555929295	2.76139E-08	5.9193E-08	positive
PRJNA729271	stage_5W_vs_E10	ordinary_muscle_development	ENSGALG00010018453	TMX4	0.068133673	0.092466164	0.736849784	0.461213704	0.503145379	positive
PRJNA729271	stage_5W_vs_E10	ordinary_muscle_development	ENSGALG00010018455	RBM20	4.205080805	0.246376777	17.06768331	2.58246E-65	4.82381E-64	positive
PRJNA729271	stage_5W_vs_E10	ordinary_muscle_development	ENSGALG00010018458	SEMA5B	-1.75054693	0.108524237	-16.13046984	1.5583E-58	2.4055E-57	negative
PRJNA729271	stage_5W_vs_E10	ordinary_muscle_development	ENSGALG00010018459	SNX12	0.522830212	0.102401039	5.10571198	3.29551E-07	6.54542E-07	positive
PRJNA729271	stage_5W_vs_E10	ordinary_muscle_development	ENSGALG00010018462	KTN1	0.561636036	0.116425902	4.823978409	1.40723E-06	2.67196E-06	positive
PRJNA729271	stage_5W_vs_E10	ordinary_muscle_development	ENSGALG00010018464	ENSGALG00010018464	-0.77172597	0.929208119	-0.830520046	0.406244819	0.447728	negative
PRJNA729271	stage_5W_vs_E10	ordinary_muscle_development	ENSGALG00010018465	NAALADL2	-3.421693648	0.21474438	-15.93379837	3.6923E-57	5.47239E-56	negative
PRJNA729271	stage_5W_vs_E10	ordinary_muscle_development	ENSGALG00010018466	DYSF	2.419815738	0.182460857	13.26210879	3.83945E-40	3.36882E-39	positive
PRJNA729271	stage_5W_vs_E10	ordinary_muscle_development	ENSGALG00010018467	TLX1	-3.073889539	0.936912708	-3.280870795	0.001034871	0.001567119	negative
PRJNA729271	stage_5W_vs_E10	ordinary_muscle_development	ENSGALG00010018469	KLHDC7A	0.017423058	0.202567361	0.086011183	0.931457527	0.940775175	positive
PRJNA729271	stage_5W_vs_E10	ordinary_muscle_development	ENSGALG00010018470	IQCB1	1.08657938	0.158054806	6.87470002	6.21204E-12	1.66071E-11	positive
PRJNA729271	stage_5W_vs_E10	ordinary_muscle_development	ENSGALG00010018471	HHEX	1.68447937	0.179628872	9.377553584	6.75212E-21	2.80516E-20	positive
PRJNA729271	stage_5W_vs_E10	ordinary_muscle_development	ENSGALG00010018472	LMF1	0.181649311	0.14141705	1.284493711	0.198969263	0.2313726	positive
PRJNA729271	stage_5W_vs_E10	ordinary_muscle_development	ENSGALG00010018476	ENSGALG00010018476	0.036885373	0.376241537	0.098036418	0.921903375	0.932453383	positive
PRJNA729271	stage_5W_vs_E10	ordinary_muscle_development	ENSGALG00010018480	EME2	0.871079938	0.241601591	3.605439575	0.000311625	0.000492585	positive
PRJNA729271	stage_5W_vs_E10	ordinary_muscle_development	ENSGALG00010018482	TWNK	-0.551692861	0.128836479	-4.282116876	1.85124E-05	3.23258E-05	negative
PRJNA729271	stage_5W_vs_E10	ordinary_muscle_development	ENSGALG00010018484	NLGN1	-3.648371578	0.289025022	-12.62303018	1.57637E-36	1.22979E-35	negative
PRJNA729271	stage_5W_vs_E10	ordinary_muscle_development	ENSGALG00010018485	ATP2C1	-0.05997749	0.066918524	-0.896276349	0.370105188	0.410865796	negative
PRJNA729271	stage_5W_vs_E10	ordinary_muscle_development	ENSGALG00010018486	ENSGALG00010018486	0.598514829	0.100687565	5.94427753	2.77679E-09	6.3196E-09	positive
PRJNA729271	stage_5W_vs_E10	ordinary_muscle_development	ENSGALG00010018487	ENSGALG00010018487	-1.70676477	0.130647643	-13.06387721	5.29602E-39	4.4502E-38	negative
PRJNA729271	stage_5W_vs_E10	ordinary_muscle_development	ENSGALG00010018488	ENSGALG00010018488	1.018939208	0.154685723	6.587157405	4.48327E-11	1.14189E-10	positive
PRJNA729271	stage_5W_vs_E10	ordinary_muscle_development	ENSGALG00010018489	SLC7A3	-1.626356995	0.20933059	-7.769323142	7.89066E-15	2.45757E-14	negative
PRJNA729271	stage_5W_vs_E10	ordinary_muscle_development	ENSGALG00010018490	NME3	-0.286652731	0.121035402	-2.368337916	0.017868208	0.023761438	negative
PRJNA729271	stage_5W_vs_E10	ordinary_muscle_development	ENSGALG00010018491	TIA1	0.11411765	0.120559629	0.94656604	0.343859956	0.383699004	positive
PRJNA729271	stage_5W_vs_E10	ordinary_muscle_development	ENSGALG00010018492	ENSGALG00010018492	-0.089095719	0.176498757	-0.504795168	0.61370271	0.650692764	negative
PRJNA729271	stage_5W_vs_E10	ordinary_muscle_development	ENSGALG00010018494	HAO1	0.667809707	0.28843698	2.315270765	0.020598119	0.027172345	positive
PRJNA729271	stage_5W_vs_E10	ordinary_muscle_development	ENSGALG00010018495	ENSGALG00010018495	0.24182818	0.668530377	0.361731028	0.717553038	0.748106976	positive
PRJNA729271	stage_5W_vs_E10	ordinary_muscle_development	ENSGALG00010018496	GNG13	-2.29289537	0.585136298	-3.918566288	8.90772E-05	0.00014758	negative
PRJNA729271	stage_5W_vs_E10	ordinary_muscle_development	ENSGALG00010018497	ASTE1	-1.019881309	0.114383825	-8.916307091	4.82095E-19	1.84185E-18	negative
PRJNA729271	stage_5W_vs_E10	ordinary_muscle_development	ENSGALG00010018498	ENSGALG00010018498	2.535707599	0.383418844	6.613414126	3.75556E-11	9.62291E-11	positive
PRJNA729271	stage_5W_vs_E10	ordinary_muscle_development	ENSGALG00010018501	ATG14	0.51237312	0.135090013	3.792827525	0.000148942	0.000241516	positive
PRJNA729271	stage_5W_vs_E10	ordinary_muscle_development	ENSGALG00010018502	ARHGEF10L	1.058224975	0.122102856	8.666668482	4.44948E-18	1.62949E-17	positive
PRJNA729271	stage_5W_vs_E10	ordinary_muscle_development	ENSGALG00010018503	BMP2	-3.272602035	0.198294643	-16.50373397	3.44891E-61	5.75943E-60	negative
PRJNA729271	stage_5W_vs_E10	ordinary_muscle_development	ENSGALG00010018504	SLCO3A1	0.045564612	0.181152654	0.25152605	0.801407425	0.825028453	positive
PRJNA729271	stage_5W_vs_E10	ordinary_muscle_development	ENSGALG00010018505	CHTF18	-1.523937109	0.146036355	-10.43532692	1.71024E-25	8.62559E-25	negative
PRJNA729271	stage_5W_vs_E10	ordinary_muscle_development	ENSGALG00010018506	ENSGALG00010018506	0.578636408	0.144698335	3.998915459	6.36334E-05	0.000106768	positive
PRJNA729271	stage_5W_vs_E10	ordinary_muscle_development	ENSGALG00010018508	ENSGALG00010018508	-0.286979025	0.101769419	-2.819894499	0.004803944	0.006796795	negative
PRJNA729271	stage_5W_vs_E10	ordinary_muscle_development	ENSGALG00010018509	ENSGALG00010018509	-1.318365843	0.113178065	-11.64859859	2.33263E-31	1.49278E-30	negative
PRJNA729271	stage_5W_vs_E10	ordinary_muscle_development	ENSGALG00010018510	ENSGALG00010018510	0.264223164	0.127013786	2.080271544	0.037500633	0.048116463	positive
PRJNA729271	stage_5W_vs_E10	ordinary_muscle_development	ENSGALG00010018511	SOCS5	-0.002207238	0.100823333	-0.021892131	0.982534002	0.985116577	negative
PRJNA729271	stage_5W_vs_E10	ordinary_muscle_development	ENSGALG00010018512	FERMT1	0.180075083	0.170922553	1.053547818	0.29208999	0.329944234	positive
PRJNA729271	stage_5W_vs_E10	ordinary_muscle_development	ENSGALG00010018513	RPUSD1	0.111287856	0.155557126	0.715414709	0.474352826	0.516158263	positive
PRJNA729271	stage_5W_vs_E10	ordinary_muscle_development	ENSGALG00010018514	EAF2	-0.410080239	0.243541931	-1.683817806	0.092216885	0.112505997	negative
PRJNA729271	stage_5W_vs_E10	ordinary_muscle_development	ENSGALG00010018515	SEMA4G	-0.728482785	0.186124733	-3.913949384	9.07987E-05	0.00015014	negative
PRJNA729271	stage_5W_vs_E10	ordinary_muscle_development	ENSGALG00010018516	LRRN4	1.000376849	0.154142548	6.489946214	8.5867E-11	2.15001E-10	positive
PRJNA729271	stage_5W_vs_E10	ordinary_muscle_development	ENSGALG00010018517	ECT2	-2.741895207	0.202265401	-13.55592796	7.3082E-42	6.74729E-41	negative
PRJNA729271	stage_5W_vs_E10	ordinary_muscle_development	ENSGALG00010018518	ENSGALG00010018518	0.899478945	0.775620349	1.15968972	0.246175157	0.281834682	positive
PRJNA729271	stage_5W_vs_E10	ordinary_muscle_development	ENSGALG00010018519	OGDH	2.283606995	0.13616775	16.77054218	4.00835E-63	7.01703E-62	positive
PRJNA729271	stage_5W_vs_E10	ordinary_muscle_development	ENSGALG00010018520	ENSGALG00010018520	0.423937697	0.124733978	3.39873467	0.000676984	0.001040823	positive
PRJNA729271	stage_5W_vs_E10	ordinary_muscle_development	ENSGALG00010018521	ENSGALG00010018521	0.67047155	0.15898585	4.217177508	2.47379E-05	4.27538E-05	positive
PRJNA729271	stage_5W_vs_E10	ordinary_muscle_development	ENSGALG00010018522	NEK11	-0.702223459	0.273660738	-2.566036563	0.010286798	0.014068039	negative
PRJNA729271	stage_5W_vs_E10	ordinary_muscle_development	ENSGALG00010018523	TEX11	0.221579256	0.168228194	1.317135078	0.187793351	0.219609893	positive
PRJNA729271	stage_5W_vs_E10	ordinary_muscle_development	ENSGALG00010018524	SEC22A	-0.21724081	0.117010086	-1.856599017	0.063368224	0.078897303	negative
PRJNA729271	stage_5W_vs_E10	ordinary_muscle_development	ENSGALG00010018525	ST8SIA2	-1.397300389	0.290885497	-4.803609674	1.5583E-06	2.9457E-06	negative
PRJNA729271	stage_5W_vs_E10	ordinary_muscle_development	ENSGALG00010018526	ENSGALG00010018526	0.437337582	0.101072937	4.326950381	1.51188E-05	2.65923E-05	positive
PRJNA729271	stage_5W_vs_E10	ordinary_muscle_development	ENSGALG00010018527	CCT4	-0.945237387	0.081656646	-11.57575567	5.46873E-31	3.45099E-30	negative
PRJNA729271	stage_5W_vs_E10	ordinary_muscle_development	ENSGALG00010018528	ENSGALG00010018528	-0.81113609	0.79405413	-1.021512337	0.307011767	0.345775879	negative
PRJNA729271	stage_5W_vs_E10	ordinary_muscle_development	ENSGALG00010018529	UBR2	0.822701725	0.092653195	8.879367041	6.72419E-19	2.55618E-18	positive
PRJNA729271	stage_5W_vs_E10	ordinary_muscle_development	ENSGALG00010018530	FAM161A	-0.591948748	0.226218397	-2.61671357	0.008878081	0.012212943	negative
PRJNA729271	stage_5W_vs_E10	ordinary_muscle_development	ENSGALG00010018531	ADCY5	0.473495409	0.135337831	3.498618274	0.000467676	0.000727668	positive
PRJNA729271	stage_5W_vs_E10	ordinary_muscle_development	ENSGALG00010018532	MFSD13A	-0.755899452	0.132631798	-5.699232487	1.20348E-08	2.6439E-08	negative
PRJNA729271	stage_5W_vs_E10	ordinary_muscle_development	ENSGALG00010018533	PCYOX1	1.008736826	0.17324856	5.822483169	5.79796E-09	1.29774E-08	positive
PRJNA729271	stage_5W_vs_E10	ordinary_muscle_development	ENSGALG00010018534	NCEH1	2.783969427	0.194680995	14.30016027	2.18306E-46	2.31651E-45	positive
PRJNA729271	stage_5W_vs_E10	ordinary_muscle_development	ENSGALG00010018535	FAM174B	0.127865738	0.102985192	1.241593424	0.214386609	0.248086692	positive
PRJNA729271	stage_5W_vs_E10	ordinary_muscle_development	ENSGALG00010018536	ENSGALG00010018536	1.431848623	0.238098082	6.013692397	1.81344E-09	4.16905E-09	positive
PRJNA729271	stage_5W_vs_E10	ordinary_muscle_development	ENSGALG00010018537	ENSGALG00010018537	0.312349713	0.664606819	0.46997669	0.638371671	0.673743802	positive

PRJNA729271	stage_5W_vs_E10	ordinary_muscle_development	ENSGALG00010018539	CHD2	-0.072092757	0.088534246	-0.814292322	0.415477507	0.457034717	negative
PRJNA729271	stage_5W_vs_E10	ordinary_muscle_development	ENSGALG00010018540	TNFSF10	0.975343122	0.108925878	8.954191034	3.42236E-19	1.31864E-18	positive
PRJNA729271	stage_5W_vs_E10	ordinary_muscle_development	ENSGALG00010018542	ZMIZ2	2.100382683	0.262521179	8.000812331	1.23601E-15	4.02335E-15	positive
PRJNA729271	stage_5W_vs_E10	ordinary_muscle_development	ENSGALG00010018543	MRPL3	-0.714192516	0.077995272	-9.156869347	5.34247E-20	2.13272E-19	negative
PRJNA729271	stage_5W_vs_E10	ordinary_muscle_development	ENSGALG00010018544	HACD2	-0.926115198	0.102744109	-9.013803417	1.99031E-19	7.7407E-19	negative
PRJNA729271	stage_5W_vs_E10	ordinary_muscle_development	ENSGALG00010018546	SNRPGP15	-1.114275425	0.13658059	-8.158373182	3.39567E-16	1.13515E-15	negative
PRJNA729271	stage_5W_vs_E10	ordinary_muscle_development	ENSGALG00010018548	RGMA	-1.342904066	0.124903596	-10.75152446	5.82902E-27	3.13396E-26	negative
PRJNA729271	stage_5W_vs_E10	ordinary_muscle_development	ENSGALG00010018549	GHSR	-2.569936282	0.558838376	-4.598711172	4.25113E-06	7.78031E-06	negative
PRJNA729271	stage_5W_vs_E10	ordinary_muscle_development	ENSGALG00010018551	FBXO34	1.296826521	0.111389498	11.64226924	2.51241E-31	1.6057E-30	positive
PRJNA729271	stage_5W_vs_E10	ordinary_muscle_development	ENSGALG00010018552	DTD1	0.367368287	0.103423167	3.552088916	0.000382186	0.00059909	positive
PRJNA729271	stage_5W_vs_E10	ordinary_muscle_development	ENSGALG00010018553	ENSGALG00010018553	-1.003259551	0.489484776	-2.049623603	0.040401175	0.051555158	negative
PRJNA729271	stage_5W_vs_E10	ordinary_muscle_development	ENSGALG00010018554	PPIA	-1.619857558	0.123852967	-13.07887568	4.34824E-39	3.6644E-38	negative
PRJNA729271	stage_5W_vs_E10	ordinary_muscle_development	ENSGALG00010018555	UBN1	-0.258267418	0.074400893	-3.471294612	0.000517955	0.000803657	negative
PRJNA729271	stage_5W_vs_E10	ordinary_muscle_development	ENSGALG00010018556	MRPL2	1.866414861	0.125844102	14.8311667	9.21179E-50	1.10169E-48	positive
PRJNA729271	stage_5W_vs_E10	ordinary_muscle_development	ENSGALG00010018557	TBCC	0.263743614	0.102646256	2.569442132	0.01018624	0.01394104	positive
PRJNA729271	stage_5W_vs_E10	ordinary_muscle_development	ENSGALG00010018558	ENSGALG00010018558	0.728989958	0.234870772	3.103791725	0.001910578	0.002812698	positive
PRJNA729271	stage_5W_vs_E10	ordinary_muscle_development	ENSGALG00010018559	DLG3	-2.384013454	0.140589155	-16.95730692	1.69958E-64	3.08714E-63	negative
PRJNA729271	stage_5W_vs_E10	ordinary_muscle_development	ENSGALG00010018560	ENSGALG00010018560	1.203123268	0.080077577	15.02447148	5.07646E-51	6.30534E-50	positive
PRJNA729271	stage_5W_vs_E10	ordinary_muscle_development	ENSGALG00010018561	ACTR1A	0.07898727	0.059240351	1.333335609	0.182421693	0.213776574	positive
PRJNA729271	stage_5W_vs_E10	ordinary_muscle_development	ENSGALG00010018562	ENSGALG00010018562	-4.393290348	0.798071416	-5.504883722	3.69412E-08	7.84331E-08	negative
PRJNA729271	stage_5W_vs_E10	ordinary_muscle_development	ENSGALG00010018564	DLGAP5	-2.567685492	0.155109283	-16.5540414	1.49707E-61	2.5262E-60	negative
PRJNA729271	stage_5W_vs_E10	ordinary_muscle_development	ENSGALG00010018566	ENSGALG00010018566	1.351989788	0.144330021	9.367349734	7.43766E-21	3.08378E-20	positive
PRJNA729271	stage_5W_vs_E10	ordinary_muscle_development	ENSGALG00010018568	FAM136A	-1.41371681	0.197813258	-7.146724265	8.88731E-13	2.48786E-12	negative
PRJNA729271	stage_5W_vs_E10	ordinary_muscle_development	ENSGALG00010018573	SEC23B	-0.70961577	0.079955482	-8.875135928	6.98481E-19	2.65247E-18	negative
PRJNA729271	stage_5W_vs_E10	ordinary_muscle_development	ENSGALG00010018574	ENSGALG00010018574	-1.701838848	0.261609073	-6.505274555	7.75516E-11	1.94719E-10	negative
PRJNA729271	stage_5W_vs_E10	ordinary_muscle_development	ENSGALG00010018576	PTK7	-2.048546321	0.115060902	-17.80401765	6.57762E-71	1.4209E-69	negative
PRJNA729271	stage_5W_vs_E10	ordinary_muscle_development	ENSGALG00010018577	NARFL	0.510383706	0.082021076	6.222592184	4.89007E-10	1.16505E-09	positive
PRJNA729271	stage_5W_vs_E10	ordinary_muscle_development	ENSGALG00010018579	FNDC3B	0.57438751	0.101436333	5.662542166	1.49147E-08	3.26174E-08	positive
PRJNA729271	stage_5W_vs_E10	ordinary_muscle_development	ENSGALG00010018581	SUFU	-1.148483191	0.092950972	-12.35579545	4.53251E-35	3.35026E-34	negative
PRJNA729271	stage_5W_vs_E10	ordinary_muscle_development	ENSGALG00010018582	RCC2	-2.242166715	0.112602245	-19.91227363	3.18526E-88	1.03754E-86	negative
PRJNA729271	stage_5W_vs_E10	ordinary_muscle_development	ENSGALG00010018583	PPL	3.250929033	0.21289313	15.27023926	1.20729E-52	1.57655E-51	positive
PRJNA729271	stage_5W_vs_E10	ordinary_muscle_development	ENSGALG00010018585	GDPD2	-2.124339398	0.61401113	-3.459773437	0.00054063	0.000838032	negative
PRJNA729271	stage_5W_vs_E10	ordinary_muscle_development	ENSGALG00010018587	SRF	0.58635039	0.173510766	3.379331467	0.000726623	0.001113833	positive
PRJNA729271	stage_5W_vs_E10	ordinary_muscle_development	ENSGALG00010018588	POLR3F	0.469424465	0.102648891	4.573108005	4.80542E-06	8.75498E-06	positive
PRJNA729271	stage_5W_vs_E10	ordinary_muscle_development	ENSGALG00010018590	TGFA	-0.905842581	0.55425109	-1.634354171	0.102184505	0.123936441	negative
PRJNA729271	stage_5W_vs_E10	ordinary_muscle_development	ENSGALG00010018593	LGALS3	0.341178251	0.329883267	1.034239334	0.30102429	0.339533661	positive
PRJNA729271	stage_5W_vs_E10	ordinary_muscle_development	ENSGALG00010018594	BICRAL	0.166373556	0.104613181	1.590368951	0.111751665	0.134874303	positive
PRJNA729271	stage_5W_vs_E10	ordinary_muscle_development	ENSGALG00010018598	ENSGALG00010018598	0.457786461	0.078222938	5.852330207	4.84733E-09	1.09018E-08	positive
PRJNA729271	stage_5W_vs_E10	ordinary_muscle_development	ENSGALG00010018601	CPNE4	-4.565258768	0.430486022	-10.60489432	2.82784E-26	1.48137E-25	negative
PRJNA729271	stage_5W_vs_E10	ordinary_muscle_development	ENSGALG00010018602	MCTP2	-0.859549215	0.223971347	-3.837764188	0.00012416	0.000202623	negative
PRJNA729271	stage_5W_vs_E10	ordinary_muscle_development	ENSGALG00010018604	FAM43A	0.406680845	0.124390707	3.269382859	0.001077824	0.001629098	positive
PRJNA729271	stage_5W_vs_E10	ordinary_muscle_development	ENSGALG00010018606	DZANK1	-0.373935743	0.242762732	-1.540334217	0.123478907	0.14813994	negative
PRJNA729271	stage_5W_vs_E10	ordinary_muscle_development	ENSGALG00010018607	HAGHL	0.532782359	0.116423306	4.576251757	4.73381E-06	8.62884E-06	positive
PRJNA729271	stage_5W_vs_E10	ordinary_muscle_development	ENSGALG00010018608	TRIM8	0.409702188	0.126356267	3.242436613	0.001185123	0.001782542	positive
PRJNA729271	stage_5W_vs_E10	ordinary_muscle_development	ENSGALG00010018609	ENSGALG00010018609	0.389434048	0.12490875	3.117748344	0.001822383	0.002689964	positive
PRJNA729271	stage_5W_vs_E10	ordinary_muscle_development	ENSGALG00010018611	NUDCD3	0.667239634	0.112673762	5.921872314	3.18297E-09	7.22473E-09	positive
PRJNA729271	stage_5W_vs_E10	ordinary_muscle_development	ENSGALG00010018613	PADI3	1.376702724	0.387299104	3.554624092	0.00037852	0.000593729	positive
PRJNA729271	stage_5W_vs_E10	ordinary_muscle_development	ENSGALG00010018615	HTR2C	-0.618724428	0.281640051	-2.196862364	0.028030277	0.036468797	negative
PRJNA729271	stage_5W_vs_E10	ordinary_muscle_development	ENSGALG00010018616	ENSGALG00010018616	-0.162718615	0.849669957	-0.191508024	0.848127594	0.866479383	negative
PRJNA729271	stage_5W_vs_E10	ordinary_muscle_development	ENSGALG00010018618	FAHD1	0.341752073	0.171325074	1.994758071	0.046069272	0.058346361	positive
PRJNA729271	stage_5W_vs_E10	ordinary_muscle_development	ENSGALG00010018619	MAPK1IP1L	-0.214366006	0.076653749	-2.796549533	0.005165149	0.00728367	negative
PRJNA729271	stage_5W_vs_E10	ordinary_muscle_development	ENSGALG00010018623	ARRDC4	1.536384858	0.131376362	11.69453038	1.35937E-31	8.76906E-31	positive
PRJNA729271	stage_5W_vs_E10	ordinary_muscle_development	ENSGALG00010018624	ENSGALG00010018624	2.50727323	0.381157307	6.578053685	4.76646E-11	1.21253E-10	positive
PRJNA729271	stage_5W_vs_E10	ordinary_muscle_development	ENSGALG00010018627	MYLK	-0.679895859	0.180112672	-3.774836339	0.000160113	0.000259051	negative
PRJNA729271	stage_5W_vs_E10	ordinary_muscle_development	ENSGALG00010018628	XDH	4.863487749	0.564489939	8.615720873	6.95029E-18	2.52873E-17	positive
PRJNA729271	stage_5W_vs_E10	ordinary_muscle_development	ENSGALG00010018631	KAT14	-0.264616683	0.052734836	-5.017872527	5.22468E-07	1.02382E-06	negative
PRJNA729271	stage_5W_vs_E10	ordinary_muscle_development	ENSGALG00010018633	ENSGALG00010018633	0.906695696	0.183652338	4.937022337	7.93244E-07	1.53594E-06	positive
PRJNA729271	stage_5W_vs_E10	ordinary_muscle_development	ENSGALG00010018638	SEC14L5	1.527651665	0.159824948	9.558280393	1.19729E-21	5.14826E-21	positive
PRJNA729271	stage_5W_vs_E10	ordinary_muscle_development	ENSGALG00010018641	ENSGALG00010018641	-2.192390269	0.687564713	-3.188631161	0.001429481	0.002133697	negative
PRJNA729271	stage_5W_vs_E10	ordinary_muscle_development	ENSGALG00010018644	ESF1	-0.649604379	0.098318127	-6.607167966	3.91741E-11	1.00235E-10	negative
PRJNA729271	stage_5W_vs_E10	ordinary_muscle_development	ENSGALG00010018649	HAGH	3.230908737	0.104858281	30.81214683	1.8017E-208	3.2645E-206	positive
PRJNA729271	stage_5W_vs_E10	ordinary_muscle_development	ENSGALG00010018654	ENSGALG00010018654	1.630801456	0.217926443	7.483265611	7.24981E-14	2.14068E-13	positive
PRJNA729271	stage_5W_vs_E10	ordinary_muscle_development	ENSGALG00010018655	BIRC5	-2.716415245	0.213689698	-12.71196164	5.07453E-37	4.0415E-36	negative
PRJNA729271	stage_5W_vs_E10	ordinary_muscle_development	ENSGALG00010018662	PADI1	1.204610179	0.874400989	1.377640458	0.168314323	0.198512296	positive
PRJNA729271	stage_5W_vs_E10	ordinary_muscle_development	ENSGALG00010018665	IL13RA2	-1.18309923	0.334538877	-3.536507452	0.000405455	0.000634125	negative
PRJNA729271	stage_5W_vs_E10	ordinary_muscle_development	ENSGALG00010018668	OVOL2	-3.076086011	0.784113677	-3.923010274	8.74494E-05	0.000144982	negative
PRJNA729271	stage_5W_vs_E10	ordinary_muscle_development	ENSGALG00010018670	MRAS	1.3914466	0.213069513	6.53048192	6.55584E-11	1.65379E-10	positive
PRJNA729271	stage_5W_vs_E10	ordinary_muscle_development	ENSGALG00010018672	PKDCC	-1.634362113	0.099332122	-16.45351057	7.91449E-61	1.29774E-59	negative
PRJNA729271	stage_5W_vs_E10	ordinary_muscle_development	ENSGALG00010018673	NAGPA	-0.041451643	0.091784204	-0.451620657	0.651542286	0.686245127	negative
PRJNA729271	stage_5W_vs_E10	ordinary_muscle_development	ENSGALG00010018684	PADI2	3.473552	1.03570903	3.353791362	0.000797124	0.001218039	positive
PRJNA729271	stage_5W_vs_E10	ordinary_muscle_development	ENSGALG00010018685	ARL3	-1.948712903	0.105550316	-18.46240717	4.14452E-76	1.03756E-74	negative
PRJNA729271	stage_5W_vs_E10	ordinary_muscle_development	ENSGALG00010018693	ENSGALG00010018693	0.775521405	0.164811075	4.705517557	2.53223E-06	4.71479E-06	positive
PRJNA729271	stage_5W_vs_E10	ordinary_muscle_development	ENSGALG00010018694	MGME1	-1.720291708	0.106971987	-16.0817028	3.42825E-58	5.24736E-57	negative
PRJNA729271	stage_5W_vs_E10	ordinary_muscle_development	ENSGALG00010018698	MRPS16	0.939140973	0.114584014	8.196090701	2.48331E-16	8.35746E-16	positive
PRJNA729271	stage_5W_vs_E10	ordinary_muscle_development	ENSGALG00010018701	SFXN2	-2.693220684	0.141642458	-19.01421881	1.3006E-80	3.55031E-79	negative
PRJNA729271	stage_5W_vs_E10	ordinary_muscle_development	ENSGALG00010018704	PGPEP1L	4.829572996	0.417796061	11.55964225	6.59831E-31	4.14935E-30	positive
PRJNA729271	stage_5W_vs_E10	ordinary_muscle_development	ENSGALG00010018707	SLC45A1	0.258208466	0.100039429	2.581066967	0.009849547	0.013501909	positive
PRJNA729271	stage_5W_vs_E10	ordinary_muscle_development	ENSGALG00010018709	SNX5	-0.337436156	0.114579197	-2.945003664	0.003229508	0.004653715	negative
PRJNA729271	stage_5W_vs_E10	ordinary_muscle_development	ENSGALG00010018713	ENSGALG00010018713	-0.394437654	0.13803428	-2.857534051	0.004269468	0.00607351	negative
PRJNA729271	stage_5W_vs_E10	ordinary_muscle_development	ENSGALG00010018715	DNAJC13	-0.139902129	0.103328249	-1.353958199	0.175749714	0.206391648	negative
PRJNA729271	stage_5W_vs_E10	ordinary_muscle_development	ENSGALG00010018717	MEIOB	-5.511002922	0.986228426	-5.587957898	2.29755E-08	4.95997E-08	negative
PRJNA729271	stage_5W_vs_E10	ordinary_muscle_development	ENSGALG00010018719	ERRFI1	-0.051437747	0.085431403	-0.602094139	0.547111476	0.586913917	negative
PRJNA729271	stage_5W_vs_E10	ordinary_muscle_development	ENSGALG00010018721	LRCH2	0.242003853	0.188923432	1.280962613	0.200206798	0.232718327	positive
PRJNA729271	stage_5W_vs_E10	ordinary_muscle_development	ENSGALG00010018724	RRBP1	-0.701864124	0.141703543	-4.953045693	7.30608E-07	1.41826E-06	negative
PRJNA729271	stage_5W_vs_E10	ordinary_muscle_development	ENSGALG00010018726	EBF2	-0.654599152	0.156068071	-4.194318213	2.73694E-05	4.70939E-05	negative
PRJNA729271	stage_5W_vs_E10	ordinary_muscle_development	ENSGALG00010018727	PARK7	2.693578086	0.131637917	20.46202296	4.69494E-93	1.71851E-91	positive
PRJNA729271	stage_5W_vs_E10	ordinary_muscle_development	ENSGALG00010018729	HS3ST6	-7.268837256	0.788996378	-9.212763781	3.17834E-20	1.28115E-19	negative
PRJNA729271	stage_5W_vs_E10	ordinary_muscle_development	ENSGALG00010018730	IGF1R	-0.340266395	0.087732022	-3.878474337	0.000105114	0.000172727	negative
PRJNA729271	stage_5W_vs_E10	ordinary_muscle_development	ENSGALG00010018732	ENSGALG00010018732	4.187325801	0.218388089	19.17378289	6.12859E-82	1.72829E-80	positive
PRJNA729271	stage_5W_vs_E10	ordinary_muscle_development	ENSGALG00010018736	EEF2KMT	-0.149853309	0.134561698	-1.113640152	0.265433594	0.302306902	negative
PRJNA729271	stage_5W_vs_E10	ordinary_muscle_development	ENSGALG00010018747	FLRT3	-3.016432405	0.127569238	-23.64545293	1.3146E-123	8.3573E-122	negative
PRJNA729271	stage_5W_vs_E10	ordinary_muscle_development	ENSGALG00010018750	SOCS4	0.16666272	0.12647867	1.31771404	0.187599393	0.219400774	positive
PRJNA729271	stage_5W_vs_E10	ordinary_muscle_development	ENSGALG00010018752	PLS3	-0.859213625	0.141040211	-6.091976305	1.11525E-09	2.59897E-09	negative
PRJNA729271	stage_5W_vs_E10	ordinary_muscle_development	ENSGALG00010018753	SYNM	3.267409879	0.153497733	21.28637222	1.51837E-100	6.47315E-99	positive
PRJNA729271	stage_5W_vs_E10	ordinary_muscle_development	ENSGALG00010018756	EML4	-2.522185993	0.063936566	-39.44825572	0	0	negative
PRJNA729271	stage_5W_vs_E10	ordinary_muscle_development	ENSGALG00010018758	ENSGALG00010018758	-0.549274918	0.131509526	-4.176693021	2.95778E-05	5.07793E-05	negative
PRJNA729271	stage_5W_vs_E10	ordinary_muscle_development	ENSGALG00010018764	TMEM177	-0.532540074	0.094290322	-5.647876275	1.62442E-08	3.54288E-08	negative
PRJNA729271	stage_5W_vs_E10	ordinary_muscle_development	ENSGALG00010018767	NDUFB6	1.488625221	0.16910414	8.803008709	1.33196E-18	4.98884E-18	positive
PRJNA729271	stage_5W_vs_E10	ordinary_muscle_development	ENSGALG00010018774	MSRB1	0.954737753	0.123166043	7.751631305	9.07195E-15	2.81761E-14	positive
PRJNA729271	stage_5W_vs_E10	ordinary_muscle_development	ENSGALG00010018780	WDHD1	-3.044687252	0.210569262	-14.45931483	2.18967E-47	2.40631E-46	negative
PRJNA729271	stage_5W_vs_E10	ordinary_muscle_development	ENSGALG00010018783	ENSGALG00010018783	0.087387633	0.178621725	0.489232947	0.624676787	0.661022779	positive
PRJNA729271	stage_5W_vs_E10	ordinary_muscle_development	ENSGALG00010018786	DSTN	-1.248798352	0.114937051	-10.86506341	1.69106E-27	9.28832E-27	negative
PRJNA729271	stage_5W_vs_E10	ordinary_muscle_development	ENSGALG00010018796	PER3	2.051291838	0.308531254	6.648570634	2.95953E-11	7.62502E-11	positive
PRJNA729271	stage_5W_vs_E10	ordinary_muscle_development	ENSGALG00010018799	ENSGALG00010018799	-1.600747142	0.342236882	-4.677307521	2.90666E-06	5.39187E-06	negative
PRJNA729271	stage_5W_vs_E10	ordinary_muscle_development	ENSGALG00010018806	GCH1	1.624104506	0.308833596	5.258833652	1.44972E-07	2.95884E-07	positive
PRJNA729271	stage_5W_vs_E10	ordinary_muscle_development	ENSGALG00010018807	RD3	-2.865891499	0.260477458	-11.00245496	3.71868E-28	2.09248E-27	negative
PRJNA729271	stage_5W_vs_E10	ordinary_muscle_development	ENSGALG00010018808	MATCAP2	-1.324266508	0.092378437	-14.33523394	1.31802E-46	1.41516E-45	negative
PRJNA729271	stage_5W_vs_E10	ordinary_muscle_development	ENSGALG00010018811	VAMP3	1.228415866	0.123141005	9.975684898	1.94753E-23	9.05952E-23	positive
PRJNA729271	stage_5W_vs_E10	ordinary_muscle_development	ENSGALG00010018815	ENSGALG00010018815	2.055605592	0.170232845	12.07525838	1.42715E-33	9.93592E-33	positive
PRJNA729271	stage_5W_vs_E10	ordinary_muscle_development	ENSGALG00010018819	LRRC28	0.079929971	0.079097462	1.010525114	0.312243774	0.351368178	positive
PRJNA729271	stage_5W_vs_E10	ordinary_muscle_development	ENSGALG00010018822	TRAF5	2.094212956	0.190269132	11.00658282	3.55223E-28	1.99959E-27	positive
PRJNA729271	stage_5W_vs_E10	ordinary_muscle_development	ENSGALG00010018823	RAB40C	-0.550222598	0.077427047	-7.106335825	1.19164E-12	3.30898E-12	negative
PRJNA729271	stage_5W_vs_E10	ordinary_muscle_development	ENSGALG00010018829	PTPN4	2.062981328	0.138159373	14.93189554	2.04373E-50	2.4894E-49	positive
PRJNA729271	stage_5W_vs_E10	ordinary_muscle_development	ENSGALG00010018831	WBP1L	1.587243863	0.240135363	6.609788083	3.84871E-11	9.85462E-11	positive
PRJNA729271	stage_5W_vs_E10	ordinary_muscle_development	ENSGALG00010018832	EEPD1	0.852771222	0.184572957	4.620239269	3.83298E-06	7.03813E-06	positive
PRJNA729271	stage_5W_vs_E10	ordinary_muscle_development	ENSGALG00010018834	SAMD4A	1.476571853	0.171469466	8.611281557	7.22483E-18	2.62598E-17	positive
PRJNA729271	stage_5W_vs_E10	ordinary_muscle_development	ENSGALG00010018835	CAMK2B	0.769589604	0.178889739	4.302033251	1.69238E-05	2.96376E-05	positive
PRJNA729271	stage_5W_vs_E10	ordinary_muscle_development	ENSGALG00010018837	RCOR3	-1.041785292	0.095374126	-10.92314384	8.93485E-28	4.96019E-27	negative
PRJNA729271	stage_5W_vs_E10	ordinary_muscle_development	ENSGALG00010018838	WFIKKN1	-6.60989174	0.450900525	-14.65931259	1.17456E-48	1.35336E-47	negative
PRJNA729271	stage_5W_vs_E10	ordinary_muscle_development	ENSGALG00010018839	RPL3L	10.98477282	0.249405854	44.04376507	0	0	positive
PRJNA729271	stage_5W_vs_E10	ordinary_muscle_development	ENSGALG00010018843	MCRIP2	1.205600976	0.236529071	5.097051997	3.44984E-07	6.84068E-07	positive
PRJNA729271	stage_5W_vs_E10	ordinary_muscle_development	ENSGALG00010018847	CGRRF1	0.140721491	0.156676087	0.898168275	0.369095844	0.409808063	positive
PRJNA729271	stage_5W_vs_E10	ordinary_muscle_development	ENSGALG00010018848	MEF2A	1.388394599	0.114609893	12.11409039	8.89454E-34	6.23737E-33	positive
PRJNA729271	stage_5W_vs_E10	ordinary_muscle_development	ENSGALG00010018849	KCNH1	-2.86217451	0.448302666	-6.384469087	1.71993E-10	4.21478E-10	negative
PRJNA729271	stage_5W_vs_E10	ordinary_muscle_development	ENSGALG00010018852	NDUFB10	1.955125331	0.115782917	16.88612945	5.69166E-64	1.02358E-62	positive
PRJNA729271	stage_5W_vs_E10	ordinary_muscle_development	ENSGALG00010018854	LYSMD4	0.89997525	0.170950617	5.26453349	1.40546E-07	2.87255E-07	positive
PRJNA729271	stage_5W_vs_E10	ordinary_muscle_development	ENSGALG00010018855	SYNDIG1	-2.993510016	0.779860115	-3.838521756	0.000123777	0.000202067	negative
PRJNA729271	stage_5W_vs_E10	ordinary_muscle_development	ENSGALG00010018857	ENSGALG00010018857	0.43164977	0.082345156	5.241957154	1.58882E-07	3.23319E-07	positive
PRJNA729271	stage_5W_vs_E10	ordinary_muscle_development	ENSGALG00010018858	EPB41L5	-1.252792431	0.118720625	-10.55244135	4.94941E-26	2.5622E-25	negative
PRJNA729271	stage_5W_vs_E10	ordinary_muscle_development	ENSGALG00010018860	AGTR2	1.411448906	0.214976328	6.565601522	5.18232E-11	1.31463E-10	positive
PRJNA729271	stage_5W_vs_E10	ordinary_muscle_development	ENSGALG00010018864	ABCA3	1.442517023	0.118793033	12.1431113	6.24071E-34	4.42126E-33	positive

PRJNA729271	stage_5W_vs_E10	ordinary_muscle_development	ENSGALG00010018865	ENSGALG00010018865	-2.591244678	0.234865424	-11.03289124	2.652E-28	1.50218E-27	negative
PRJNA729271	stage_5W_vs_E10	ordinary_muscle_development	ENSGALG00010018867	RPS2	-0.407307027	0.155959755	-2.611616225	0.009011535	0.012389472	negative
PRJNA729271	stage_5W_vs_E10	ordinary_muscle_development	ENSGALG00010018872	KCTD9	0.038291028	0.101165011	0.378500708	0.705058668	0.737148398	positive
PRJNA729271	stage_5W_vs_E10	ordinary_muscle_development	ENSGALG00010018874	ENSGALG00010018874	-0.594105872	0.117933604	-5.037630097	4.71331E-07	9.2624E-07	negative
PRJNA729271	stage_5W_vs_E10	ordinary_muscle_development	ENSGALG00010018876	GMFB	-0.867964786	0.098104448	-8.847354063	8.96187E-19	3.38464E-18	negative
PRJNA729271	stage_5W_vs_E10	ordinary_muscle_development	ENSGALG00010018877	CST7	5.533126262	0.789598128	7.007522016	2.42576E-12	6.62297E-12	positive
PRJNA729271	stage_5W_vs_E10	ordinary_muscle_development	ENSGALG00010018880	TMEM17	-0.286311272	0.258456656	-1.107772871	0.267959919	0.305016415	negative
PRJNA729271	stage_5W_vs_E10	ordinary_muscle_development	ENSGALG00010018881	ENSGALG00010018881	0.698692125	0.639887132	1.091899008	0.274877494	0.31205743	positive
PRJNA729271	stage_5W_vs_E10	ordinary_muscle_development	ENSGALG00010018882	HHAT	-1.211783946	0.168291488	-7.20050647	5.99893E-13	1.69205E-12	negative
PRJNA729271	stage_5W_vs_E10	ordinary_muscle_development	ENSGALG00010018883	TBL3	-1.707948788	0.078295469	-21.81414602	1.7032E-105	7.788E-104	negative
PRJNA729271	stage_5W_vs_E10	ordinary_muscle_development	ENSGALG00010018885	EFEMP1	0.716228032	0.207487422	3.451910601	0.000556632	0.00086173	positive
PRJNA729271	stage_5W_vs_E10	ordinary_muscle_development	ENSGALG00010018887	ADAMTS17	-2.938518864	0.204638652	-14.35954954	9.28285E-47	1.00489E-45	negative
PRJNA729271	stage_5W_vs_E10	ordinary_muscle_development	ENSGALG00010018891	GNRH1	-4.06035845	0.456137592	-8.901608902	5.50429E-19	2.09739E-18	negative
PRJNA729271	stage_5W_vs_E10	ordinary_muscle_development	ENSGALG00010018892	APMAP	-0.53901462	0.124124675	-4.342525936	1.40854E-05	2.48409E-05	negative
PRJNA729271	stage_5W_vs_E10	ordinary_muscle_development	ENSGALG00010018894	SERTAD4	-1.032343426	0.093349538	-11.05890237	1.98512E-28	1.13062E-27	negative
PRJNA729271	stage_5W_vs_E10	ordinary_muscle_development	ENSGALG00010018895	SEPTIN7	-1.059022567	0.089961784	-11.77191608	5.4473E-32	3.57765E-31	negative
PRJNA729271	stage_5W_vs_E10	ordinary_muscle_development	ENSGALG00010018897	CCDC85A	-0.590577621	0.16775748	-3.520424956	0.000430856	0.000672473	negative
PRJNA729271	stage_5W_vs_E10	ordinary_muscle_development	ENSGALG00010018899	ACSS1	0.257371398	0.099022393	2.599123176	0.009346223	0.012835007	positive
PRJNA729271	stage_5W_vs_E10	ordinary_muscle_development	ENSGALG00010018901	TCF7L2	-0.300948074	0.147858595	-2.035377608	0.041812893	0.053262842	negative
PRJNA729271	stage_5W_vs_E10	ordinary_muscle_development	ENSGALG00010018902	NOXO1	2.30464681	0.477240156	4.829113351	1.37142E-06	2.60603E-06	positive
PRJNA729271	stage_5W_vs_E10	ordinary_muscle_development	ENSGALG00010018903	CAMTA1	-0.106660561	0.096831998	-1.101501191	0.270678587	0.307845124	negative
PRJNA729271	stage_5W_vs_E10	ordinary_muscle_development	ENSGALG00010018904	UGP2	2.372090938	0.271203009	8.746550954	2.19973E-18	8.17566E-18	positive
PRJNA729271	stage_5W_vs_E10	ordinary_muscle_development	ENSGALG00010018908	ENSGALG00010018908	0.088308752	0.237500382	0.371825725	0.710022609	0.741429128	positive
PRJNA729271	stage_5W_vs_E10	ordinary_muscle_development	ENSGALG00010018909	TTBK1	0.169627486	0.13181339	1.286875984	0.198137517	0.230534862	positive
PRJNA729271	stage_5W_vs_E10	ordinary_muscle_development	ENSGALG00010018911	CNIH1	0.323446403	0.093799158	3.448286861	0.000564155	0.000872724	positive
PRJNA729271	stage_5W_vs_E10	ordinary_muscle_development	ENSGALG00010018913	ENSGALG00010018913	1.692666128	0.217037196	7.798967922	6.24155E-15	1.95149E-14	positive
PRJNA729271	stage_5W_vs_E10	ordinary_muscle_development	ENSGALG00010018915	DOCK5	-0.081667665	0.187991739	-0.434421563	0.663982342	0.698383575	negative
PRJNA729271	stage_5W_vs_E10	ordinary_muscle_development	ENSGALG00010018916	CERS3	-1.321582932	0.337062454	-3.920884438	8.82246E-05	0.000146234	negative
PRJNA729271	stage_5W_vs_E10	ordinary_muscle_development	ENSGALG00010018917	DNAJC11	-0.161437654	0.105833639	-1.525390752	0.127161664	0.152394239	negative
PRJNA729271	stage_5W_vs_E10	ordinary_muscle_development	ENSGALG00010018919	RALB	0.041166265	0.107669484	0.382339204	0.702209779	0.734487714	positive
PRJNA729271	stage_5W_vs_E10	ordinary_muscle_development	ENSGALG00010018920	ACAD11	-0.504953227	0.117306959	-4.304546237	1.67328E-05	2.93138E-05	negative
PRJNA729271	stage_5W_vs_E10	ordinary_muscle_development	ENSGALG00010018922	EEF2K	2.036790008	0.114006624	17.86554087	2.18801E-71	4.82728E-70	positive
PRJNA729271	stage_5W_vs_E10	ordinary_muscle_development	ENSGALG00010018923	VPS54	-0.252160814	0.092922188	-2.713677114	0.006654099	0.009279504	negative
PRJNA729271	stage_5W_vs_E10	ordinary_muscle_development	ENSGALG00010018925	LINS1	-0.070354718	0.12877662	-0.546331452	0.58483812	0.623096549	negative
PRJNA729271	stage_5W_vs_E10	ordinary_muscle_development	ENSGALG00010018926	SLC22A7	-0.188640109	1.092326924	-0.172695651	0.862890663	0.879918934	negative
PRJNA729271	stage_5W_vs_E10	ordinary_muscle_development	ENSGALG00010018932	CDKN3	-2.436523626	0.24033468	-10.13804429	3.74522E-24	1.79402E-23	negative
PRJNA729271	stage_5W_vs_E10	ordinary_muscle_development	ENSGALG00010018933	PHF13	-0.060312041	0.088961237	-0.677958659	0.497797906	0.539200466	negative
PRJNA729271	stage_5W_vs_E10	ordinary_muscle_development	ENSGALG00010018936	SYT14	1.326304407	0.219270738	6.048706816	1.46013E-09	3.37661E-09	positive
PRJNA729271	stage_5W_vs_E10	ordinary_muscle_development	ENSGALG00010018937	INHBB	-4.160967545	0.452499436	-9.195519842	3.73186E-20	1.50051E-19	negative
PRJNA729271	stage_5W_vs_E10	ordinary_muscle_development	ENSGALG00010018941	KLHL21	1.805746675	0.143732207	12.56327102	3.36143E-36	2.60417E-35	positive
PRJNA729271	stage_5W_vs_E10	ordinary_muscle_development	ENSGALG00010018943	ACKR4	4.152520682	0.342998146	12.10653973	9.75221E-34	6.8322E-33	positive
PRJNA729271	stage_5W_vs_E10	ordinary_muscle_development	ENSGALG00010018944	NEFL	-5.831896064	0.33356937	-17.48330806	1.92028E-68	3.87128E-67	negative
PRJNA729271	stage_5W_vs_E10	ordinary_muscle_development	ENSGALG00010018945	HABP2	-1.405953142	0.701096578	-2.005363007	0.044924253	0.056975856	negative
PRJNA729271	stage_5W_vs_E10	ordinary_muscle_development	ENSGALG00010018947	ENSGALG00010018947	1.590973283	0.207517185	7.666706177	1.76469E-14	5.38057E-14	positive
PRJNA729271	stage_5W_vs_E10	ordinary_muscle_development	ENSGALG00010018948	POLR3E	1.042143361	0.107710736	9.675389828	3.83627E-22	1.68966E-21	positive
PRJNA729271	stage_5W_vs_E10	ordinary_muscle_development	ENSGALG00010018950	TTL	0.544944568	0.096950946	5.620827784	1.90045E-08	4.12236E-08	positive
PRJNA729271	stage_5W_vs_E10	ordinary_muscle_development	ENSGALG00010018951	YKT6	-0.926910948	0.092517037	-10.01881361	1.26006E-23	5.89752E-23	negative
PRJNA729271	stage_5W_vs_E10	ordinary_muscle_development	ENSGALG00010018955	ZBTB48	1.043722915	0.141461562	7.378137935	1.60519E-13	4.64786E-13	positive
PRJNA729271	stage_5W_vs_E10	ordinary_muscle_development	ENSGALG00010018958	PELI3	-0.506401854	0.118486844	-4.273907841	1.92076E-05	3.34754E-05	negative
PRJNA729271	stage_5W_vs_E10	ordinary_muscle_development	ENSGALG00010018959	UBA5	-0.439554174	0.117507347	-3.740652699	0.000183543	0.000295705	negative
PRJNA729271	stage_5W_vs_E10	ordinary_muscle_development	ENSGALG00010018960	RBFOX1	-1.075558963	0.222774455	-4.828017481	1.37899E-06	2.62006E-06	negative
PRJNA729271	stage_5W_vs_E10	ordinary_muscle_development	ENSGALG00010018961	ASB7	-0.357391223	0.100602453	-3.552510031	0.000381574	0.000598261	negative
PRJNA729271	stage_5W_vs_E10	ordinary_muscle_development	ENSGALG00010018968	GLI2	-1.440618166	0.19732399	-7.300775584	2.86114E-13	8.18153E-13	negative
PRJNA729271	stage_5W_vs_E10	ordinary_muscle_development	ENSGALG00010018969	DIEXF	-0.754594146	0.091322686	-8.262942993	1.42124E-16	4.83816E-16	negative
PRJNA729271	stage_5W_vs_E10	ordinary_muscle_development	ENSGALG00010018971	POLR1B	0.245167683	0.099033737	2.475597611	0.01330134	0.017955198	positive
PRJNA729271	stage_5W_vs_E10	ordinary_muscle_development	ENSGALG00010018973	NEFM	-6.531651736	0.787065038	-8.298744606	1.05217E-16	3.60379E-16	negative
PRJNA729271	stage_5W_vs_E10	ordinary_muscle_development	ENSGALG00010018974	TAS1R1	0.847375247	0.29238321	2.898166577	0.003753512	0.005370364	positive
PRJNA729271	stage_5W_vs_E10	ordinary_muscle_development	ENSGALG00010018977	NPHP3	-0.366868538	0.112589797	-3.258452779	0.001120215	0.001689648	negative
PRJNA729271	stage_5W_vs_E10	ordinary_muscle_development	ENSGALG00010018983	MDGA2	-3.780044933	0.242140404	-15.6109632	6.13044E-55	8.44684E-54	negative
PRJNA729271	stage_5W_vs_E10	ordinary_muscle_development	ENSGALG00010018985	NOL9	-0.482749669	0.08984861	-5.372923057	7.74704E-08	1.60948E-07	negative
PRJNA729271	stage_5W_vs_E10	ordinary_muscle_development	ENSGALG00010018986	CTDSP1	1.839401112	0.191354755	9.61251842	7.07958E-22	3.08534E-21	positive
PRJNA729271	stage_5W_vs_E10	ordinary_muscle_development	ENSGALG00010018987	MRPS10	-0.062942071	0.108704379	-0.579020564	0.562575298	0.602032406	negative
PRJNA729271	stage_5W_vs_E10	ordinary_muscle_development	ENSGALG00010018990	GCK	-1.977583617	0.434312884	-4.553361625	5.27954E-06	9.58744E-06	negative
PRJNA729271	stage_5W_vs_E10	ordinary_muscle_development	ENSGALG00010018991	RYBP	-2.751771376	0.136824773	-20.11164578	5.83552E-90	1.99025E-88	negative
PRJNA729271	stage_5W_vs_E10	ordinary_muscle_development	ENSGALG00010018993	ENSGALG00010018993	0.286557205	0.484493028	0.591457851	0.554213688	0.593961362	positive
PRJNA729271	stage_5W_vs_E10	ordinary_muscle_development	ENSGALG00010018998	RPS29	2.051098337	0.439936036	4.662264895	3.12748E-06	5.7926E-06	positive
PRJNA729271	stage_5W_vs_E10	ordinary_muscle_development	ENSGALG00010018999	THUMPD3	-0.971722081	0.073221856	-13.27092932	3.41321E-40	2.99846E-39	negative
PRJNA729271	stage_5W_vs_E10	ordinary_muscle_development	ENSGALG00010019000	TRERF1	0.387267839	0.119045874	3.253097524	0.001141543	0.00171967	positive
PRJNA729271	stage_5W_vs_E10	ordinary_muscle_development	ENSGALG00010019001	ALDH1A3	0.505297489	0.159903347	3.160018219	0.001577593	0.002343912	positive
PRJNA729271	stage_5W_vs_E10	ordinary_muscle_development	ENSGALG00010019004	ECE2	0.265657806	0.198709204	1.33691747	0.181249578	0.212520542	positive
PRJNA729271	stage_5W_vs_E10	ordinary_muscle_development	ENSGALG00010019005	ENSGALG00010019005	-1.436218881	0.220329901	-6.518492844	7.10173E-11	1.78808E-10	negative
PRJNA729271	stage_5W_vs_E10	ordinary_muscle_development	ENSGALG00010019006	ENSGALG00010019006	-0.758408024	0.07961868	-9.525503565	1.64244E-21	7.02275E-21	negative
PRJNA729271	stage_5W_vs_E10	ordinary_muscle_development	ENSGALG00010019008	HERPUD2	0.153335237	0.045198616	3.392476372	0.000692639	0.001063538	positive
PRJNA729271	stage_5W_vs_E10	ordinary_muscle_development	ENSGALG00010019009	NRAP	4.301602549	0.218355897	19.69996052	2.15788E-86	6.71213E-85	positive
PRJNA729271	stage_5W_vs_E10	ordinary_muscle_development	ENSGALG00010019011	VIL1	1.774686167	0.400489305	4.431294787	9.36689E-06	1.67332E-05	positive
PRJNA729271	stage_5W_vs_E10	ordinary_muscle_development	ENSGALG00010019012	ADAM28	0.385829972	0.358427562	1.076451737	0.281725275	0.319281302	positive
PRJNA729271	stage_5W_vs_E10	ordinary_muscle_development	ENSGALG00010019013	LRRK1	0.465773848	0.099906047	4.662118671	3.12971E-06	5.79597E-06	positive
PRJNA729271	stage_5W_vs_E10	ordinary_muscle_development	ENSGALG00010019015	GSG1L	-4.941476528	1.073981452	-4.601081816	4.20302E-06	7.69616E-06	negative
PRJNA729271	stage_5W_vs_E10	ordinary_muscle_development	ENSGALG00010019016	PLEKHG5	0.686630791	0.144389927	4.75539262	1.98061E-06	3.71445E-06	positive
PRJNA729271	stage_5W_vs_E10	ordinary_muscle_development	ENSGALG00010019017	IRAK2	0.839509119	0.082103908	10.22495934	1.53292E-24	7.47635E-24	positive
PRJNA729271	stage_5W_vs_E10	ordinary_muscle_development	ENSGALG00010019018	SH3BGRL	-0.872202186	0.110012209	-7.928230787	2.2229E-15	7.138E-15	negative
PRJNA729271	stage_5W_vs_E10	ordinary_muscle_development	ENSGALG00010019019	TBX20	-0.242930782	0.319326599	-0.760759621	0.446800657	0.488635545	negative
PRJNA729271	stage_5W_vs_E10	ordinary_muscle_development	ENSGALG00010019020	KCNK2	1.705045471	0.440707397	3.86888326	0.000109335	0.000179318	positive
PRJNA729271	stage_5W_vs_E10	ordinary_muscle_development	ENSGALG00010019021	LRR1	-1.395733787	0.155610121	-8.969428076	2.98058E-19	1.15025E-18	negative
PRJNA729271	stage_5W_vs_E10	ordinary_muscle_development	ENSGALG00010019023	AP2M1	-0.121111482	0.071614046	-1.691169391	0.090804455	0.110894817	negative
PRJNA729271	stage_5W_vs_E10	ordinary_muscle_development	ENSGALG00010019024	CNPY3	-0.340190534	0.103182533	-3.296977935	0.000977312	0.00148352	negative
PRJNA729271	stage_5W_vs_E10	ordinary_muscle_development	ENSGALG00010019025	DNAAF2	-0.681193774	0.248280862	-2.743641894	0.006076178	0.008506171	negative
PRJNA729271	stage_5W_vs_E10	ordinary_muscle_development	ENSGALG00010019026	CHSY1	-1.880353151	0.126211354	-14.89844683	3.3733E-50	4.06789E-49	negative
PRJNA729271	stage_5W_vs_E10	ordinary_muscle_development	ENSGALG00010019027	GNLY	3.814833484	0.310815217	12.27363807	1.25486E-34	9.10823E-34	positive
PRJNA729271	stage_5W_vs_E10	ordinary_muscle_development	ENSGALG00010019028	TNFRSF25	7.344596047	0.701108228	10.47569512	1.11714E-25	5.68572E-25	positive
PRJNA729271	stage_5W_vs_E10	ordinary_muscle_development	ENSGALG00010019029	CENPF	-2.476964099	0.260701957	-9.501133517	2.07618E-21	8.84084E-21	negative
PRJNA729271	stage_5W_vs_E10	ordinary_muscle_development	ENSGALG00010019030	CDR2	-2.503021242	0.155709968	-16.07489411	3.82647E-58	5.83838E-57	negative
PRJNA729271	stage_5W_vs_E10	ordinary_muscle_development	ENSGALG00010019032	GNMT	-1.819972259	0.192428474	-9.457915557	3.14143E-21	1.326E-20	negative
PRJNA729271	stage_5W_vs_E10	ordinary_muscle_development	ENSGALG00010019033	CYP2AB4	-4.098360366	1.168804554	-3.506454824	0.000454119	0.000707182	negative
PRJNA729271	stage_5W_vs_E10	ordinary_muscle_development	ENSGALG00010019034	CASP7	0.991827347	0.167565476	5.919043515	3.23819E-09	7.34778E-09	positive
PRJNA729271	stage_5W_vs_E10	ordinary_muscle_development	ENSGALG00010019035	ENSGALG00010019035	-1.4542483	0.504688959	-2.881474369	0.003958194	0.005652057	negative
PRJNA729271	stage_5W_vs_E10	ordinary_muscle_development	ENSGALG00010019036	REL	0.847758664	0.109248	7.759946742	8.49651E-15	2.64228E-14	positive
PRJNA729271	stage_5W_vs_E10	ordinary_muscle_development	ENSGALG00010019038	PLEKHS1	-2.381082285	0.486857353	-4.89071854	1.00469E-06	1.92886E-06	negative
PRJNA729271	stage_5W_vs_E10	ordinary_muscle_development	ENSGALG00010019040	HMGN5	-2.769800718	0.083804834	-33.05060807	1.5246E-239	4.0181E-237	negative
PRJNA729271	stage_5W_vs_E10	ordinary_muscle_development	ENSGALG00010019041	KATNIP	-0.346468745	0.090181883	-3.841888565	0.000122091	0.000199404	negative
PRJNA729271	stage_5W_vs_E10	ordinary_muscle_development	ENSGALG00010019042	PEX6	0.562308154	0.105954277	5.307083098	1.11393E-07	2.29125E-07	positive
PRJNA729271	stage_5W_vs_E10	ordinary_muscle_development	ENSGALG00010019043	METTL9	-1.058113447	0.104330993	-10.14188996	3.60065E-24	1.72648E-23	negative
PRJNA729271	stage_5W_vs_E10	ordinary_muscle_development	ENSGALG00010019045	LBX1	0.460739684	0.266754549	1.727204602	0.084130925	0.103153253	positive
PRJNA729271	stage_5W_vs_E10	ordinary_muscle_development	ENSGALG00010019046	SELENOS	0.70853955	0.080917615	8.756307884	2.0175E-18	7.51186E-18	positive
PRJNA729271	stage_5W_vs_E10	ordinary_muscle_development	ENSGALG00010019047	GHRL	-0.475505805	0.448144597	-1.061054419	0.288665171	0.326355327	negative
PRJNA729271	stage_5W_vs_E10	ordinary_muscle_development	ENSGALG00010019049	POLD2	-0.60380457	0.163324937	-3.696952728	0.000218203	0.000349486	negative
PRJNA729271	stage_5W_vs_E10	ordinary_muscle_development	ENSGALG00010019050	TNS1	1.572909335	0.133412008	11.78986327	4.40255E-32	2.90596E-31	positive
PRJNA729271	stage_5W_vs_E10	ordinary_muscle_development	ENSGALG00010019051	IGSF6	3.081250423	0.24959334	12.34508271	5.1781E-35	3.81967E-34	positive
PRJNA729271	stage_5W_vs_E10	ordinary_muscle_development	ENSGALG00010019059	BRWD3	0.534461129	0.077760553	6.873165259	6.27927E-12	1.67775E-11	positive
PRJNA729271	stage_5W_vs_E10	ordinary_muscle_development	ENSGALG00010019062	PUS10	-1.178004904	0.102045955	-11.54386671	7.92795E-31	4.96182E-30	negative
PRJNA729271	stage_5W_vs_E10	ordinary_muscle_development	ENSGALG00010019064	ENSGALG00010019064	-1.014382831	0.172314019	-5.886827051	3.9368E-09	8.8954E-09	negative
PRJNA729271	stage_5W_vs_E10	ordinary_muscle_development	ENSGALG00010019065	CAND2	3.960264354	0.120820262	32.77814737	1.2063E-235	2.9636E-233	positive
PRJNA729271	stage_5W_vs_E10	ordinary_muscle_development	ENSGALG00010019067	GTF3C1	-0.606268193	0.058762193	-10.3173173	5.88444E-25	2.92206E-24	negative
PRJNA729271	stage_5W_vs_E10	ordinary_muscle_development	ENSGALG00010019068	SNRPA1	0.289433877	0.163329414	1.77208666	0.076380173	0.09419983	positive
PRJNA729271	stage_5W_vs_E10	ordinary_muscle_development	ENSGALG00010019072	AEBP1	1.745304785	0.211188713	8.264195366	1.4064E-16	4.78877E-16	positive
PRJNA729271	stage_5W_vs_E10	ordinary_muscle_development	ENSGALG00010019076	ESPN	-0.753564742	0.421915743	-1.786055045	0.074090346	0.09153154	negative
PRJNA729271	stage_5W_vs_E10	ordinary_muscle_development	ENSGALG00010019079	CAPG	1.768181188	0.240752084	7.344406571	2.06674E-13	5.95574E-13	positive
PRJNA729271	stage_5W_vs_E10	ordinary_muscle_development	ENSGALG00010019081	PCSK6	1.128497277	0.136367115	8.275435616	1.27987E-16	4.37025E-16	positive
PRJNA729271	stage_5W_vs_E10	ordinary_muscle_development	ENSGALG00010019082	POLR3K	1.528645492	0.175929866	8.688948196	3.6581E-18	1.34648E-17	positive
PRJNA729271	stage_5W_vs_E10	ordinary_muscle_development	ENSGALG00010019083	OTOA	-0.90173168	0.236510949	-3.812642427	0.000137489	0.000223796	negative
PRJNA729271	stage_5W_vs_E10	ordinary_muscle_development	ENSGALG00010019087	PEX13	0.653806984	0.086798599	7.532460108	4.97931E-14	1.48784E-13	positive
PRJNA729271	stage_5W_vs_E10	ordinary_muscle_development	ENSGALG00010019093	RPL32	0.288229575	0.151780525	1.898989183	0.057565898	0.072038133	positive
PRJNA729271	stage_5W_vs_E10	ordinary_muscle_development	ENSGALG00010019094	SNRNP25	0.100745833	0.168594953	0.597561384	0.550132637	0.589936567	positive
PRJNA729271	stage_5W_vs_E10	ordinary_muscle_development	ENSGALG00010019097	STC1	-3.031216878	0.283911274	-10.67663443	1.30933E-26	6.96466E-26	negative
PRJNA729271	stage_5W_vs_E10	ordinary_muscle_development	ENSGALG00010019101	POLE2	-2.094885787	0.123896804	-16.90831177	3.90742E-64	7.05331E-63	negative
PRJNA729271	stage_5W_vs_E10	ordinary_muscle_development	ENSGALG00010019104	POLL	0.120816706	0.120461039	1.002952548	0.315883757	0.354940702	positive
PRJNA729271	stage_5W_vs_E10	ordinary_muscle_development	ENSGALG00010019108	KDELR2	-0.653428827	0.099779011	-6.548760308	5.80167E-11	1.46584E-10	negative
PRJNA729271	stage_5W_vs_E10	ordinary_muscle_development	ENSGALG00010019110	MBD4	0.919883448	0.108013027	8.516412091	1.64572E-17	5.87267E-17	positive
PRJNA729271	stage_5W_vs_E10	ordinary_muscle_development	ENSGALG00010019113	KLHDC1	0.992737412	0.134131365	7.401232446	1.34926E-13	3.92171E-13	positive
PRJNA729271	stage_5W_vs_E10	ordinary_muscle_development	ENSGALG00010019114	TENT5D	0.301963852	0.325788245	0.92687154	0.353993235	0.394186982	positive
PRJNA729271	stage_5W_vs_E10	ordinary_muscle_development	ENSGALG00010019115	DBNL	-0.92883908	0.103195036	-9.000811603	2.24055E-19	8.69293E-19	negative

PRJNA729271	stage_5W_vs_E10	ordinary_muscle_development	ENSGALG00010019120	DAGLB	1.463015795	0.078585004	18.61698449	2.34027E-77	6.02526E-76	positive
PRJNA729271	stage_5W_vs_E10	ordinary_muscle_development	ENSGALG00010019123	PLEKHO2	1.850246212	0.117621385	15.73052568	9.34322E-56	1.32127E-54	positive
PRJNA729271	stage_5W_vs_E10	ordinary_muscle_development	ENSGALG00010019124	BMPER	-1.250186799	0.250564308	-4.989484773	6.05405E-07	1.18171E-06	negative
PRJNA729271	stage_5W_vs_E10	ordinary_muscle_development	ENSGALG00010019125	ENSGALG00010019125	1.975037839	0.198342823	9.957697535	2.33406E-23	1.08159E-22	positive
PRJNA729271	stage_5W_vs_E10	ordinary_muscle_development	ENSGALG00010019127	TMBIM1	1.303465832	0.122024652	10.68198766	1.23597E-26	6.58651E-26	positive
PRJNA729271	stage_5W_vs_E10	ordinary_muscle_development	ENSGALG00010019130	RPS6KC1	-0.932671265	0.084638974	-11.01940655	3.08083E-28	1.73829E-27	negative
PRJNA729271	stage_5W_vs_E10	ordinary_muscle_development	ENSGALG00010019132	ENSGALG00010019132	-0.961112944	0.153988049	-6.241477516	4.33457E-10	1.03474E-09	negative
PRJNA729271	stage_5W_vs_E10	ordinary_muscle_development	ENSGALG00010019135	KIAA1841	-1.129688778	0.196609219	-5.745858621	9.14557E-09	2.02328E-08	negative
PRJNA729271	stage_5W_vs_E10	ordinary_muscle_development	ENSGALG00010019139	DPCD	-1.733548945	0.139948209	-12.38707492	3.0704E-35	2.28584E-34	negative
PRJNA729271	stage_5W_vs_E10	ordinary_muscle_development	ENSGALG00010019141	ACOT7	-0.253208187	0.152751306	-1.657649898	0.097388152	0.118486404	negative
PRJNA729271	stage_5W_vs_E10	ordinary_muscle_development	ENSGALG00010019144	IFT122	-1.527177589	0.076144817	-20.05622501	1.78103E-89	5.97593E-88	negative
PRJNA729271	stage_5W_vs_E10	ordinary_muscle_development	ENSGALG00010019145	RAC1	-0.145546922	0.076906135	-1.892526805	0.058420826	0.07303233	negative
PRJNA729271	stage_5W_vs_E10	ordinary_muscle_development	ENSGALG00010019146	KLHDC2	1.517371185	0.085166183	17.81659254	5.2541E-71	1.1418E-69	positive
PRJNA729271	stage_5W_vs_E10	ordinary_muscle_development	ENSGALG00010019148	ENSGALG00010019148	0.530961781	0.21738672	2.442475701	0.014586912	0.019606573	positive
PRJNA729271	stage_5W_vs_E10	ordinary_muscle_development	ENSGALG00010019149	COX5B	1.355014072	0.18023654	7.517976484	5.56305E-14	1.65714E-13	positive
PRJNA729271	stage_5W_vs_E10	ordinary_muscle_development	ENSGALG00010019158	ENSGALG00010019158	2.744381799	0.251151775	10.92718456	8.54589E-28	4.75701E-27	positive
PRJNA729271	stage_5W_vs_E10	ordinary_muscle_development	ENSGALG00010019166	PNPT1	-0.54492512	0.110193397	-4.945170371	7.60773E-07	1.47524E-06	negative
PRJNA729271	stage_5W_vs_E10	ordinary_muscle_development	ENSGALG00010019169	GPR153	-3.048269678	0.262915083	-11.59412249	4.41366E-31	2.79493E-30	negative
PRJNA729271	stage_5W_vs_E10	ordinary_muscle_development	ENSGALG00010019172	NEMF	-0.008086776	0.08736495	-0.092563157	0.926250615	0.936262389	negative
PRJNA729271	stage_5W_vs_E10	ordinary_muscle_development	ENSGALG00010019173	RHO	-3.776782649	1.042841637	-3.621626252	0.000292757	0.000463651	negative
PRJNA729271	stage_5W_vs_E10	ordinary_muscle_development	ENSGALG00010019174	ENSGALG00010019174	0.338315515	0.436283884	0.775448115	0.438074913	0.479961895	positive
PRJNA729271	stage_5W_vs_E10	ordinary_muscle_development	ENSGALG00010019183	ANGEL2	0.226863278	0.079473913	2.854562828	0.004309611	0.006127899	positive
PRJNA729271	stage_5W_vs_E10	ordinary_muscle_development	ENSGALG00010019184	PNKD	-0.813421994	0.193240575	-4.209374727	2.56078E-05	4.41888E-05	negative
PRJNA729271	stage_5W_vs_E10	ordinary_muscle_development	ENSGALG00010019185	ANKDD1A	0.165049123	0.213178859	0.774228382	0.438795747	0.480642663	positive
PRJNA729271	stage_5W_vs_E10	ordinary_muscle_development	ENSGALG00010019188	ENSGALG00010019188	1.90377187	0.372570085	5.109835556	3.22439E-07	6.41119E-07	positive
PRJNA729271	stage_5W_vs_E10	ordinary_muscle_development	ENSGALG00010019189	ICMT	-0.166163297	0.094891545	-1.751086435	0.079931022	0.098303086	negative
PRJNA729271	stage_5W_vs_E10	ordinary_muscle_development	ENSGALG00010019193	ENSGALG00010019193	4.883633272	1.010109096	4.834758238	1.33308E-06	2.53515E-06	positive
PRJNA729271	stage_5W_vs_E10	ordinary_muscle_development	ENSGALG00010019194	ENSGALG00010019194	0.142358471	0.189362378	0.751778009	0.452184569	0.494113481	positive
PRJNA729271	stage_5W_vs_E10	ordinary_muscle_development	ENSGALG00010019204	ARF6	-0.075462606	0.230023163	-0.328065248	0.742862329	0.771552699	negative
PRJNA729271	stage_5W_vs_E10	ordinary_muscle_development	ENSGALG00010019205	ENSGALG00010019205	-0.477030502	0.264713832	-1.802061113	0.071535791	0.088571905	negative
PRJNA729271	stage_5W_vs_E10	ordinary_muscle_development	ENSGALG00010019206	RNF207	0.813899569	0.283167604	2.874267949	0.004049652	0.005775257	positive
PRJNA729271	stage_5W_vs_E10	ordinary_muscle_development	ENSGALG00010019208	PLXND1	0.78255761	0.158019891	4.952272826	7.33517E-07	1.42353E-06	positive
PRJNA729271	stage_5W_vs_E10	ordinary_muscle_development	ENSGALG00010019209	VCPKMT	0.06994211	0.086273202	0.810704932	0.41753514	0.459158778	positive
PRJNA729271	stage_5W_vs_E10	ordinary_muscle_development	ENSGALG00010019212	RPL22	-0.274751553	0.114000203	-2.410097048	0.015948278	0.021335513	negative
PRJNA729271	stage_5W_vs_E10	ordinary_muscle_development	ENSGALG00010019213	RHBDF1	-0.262510129	0.110888466	-2.367334838	0.017916715	0.023819388	negative
PRJNA729271	stage_5W_vs_E10	ordinary_muscle_development	ENSGALG00010019215	ENSGALG00010019215	-0.187405993	0.102814518	-1.82275808	0.068340039	0.08480343	negative
PRJNA729271	stage_5W_vs_E10	ordinary_muscle_development	ENSGALG00010019218	RMND5A	1.148147658	0.137526571	8.348551503	6.91052E-17	2.39236E-16	positive
PRJNA729271	stage_5W_vs_E10	ordinary_muscle_development	ENSGALG00010019221	VASH2	-3.131233243	0.233447966	-13.41298147	5.07565E-41	4.57535E-40	negative
PRJNA729271	stage_5W_vs_E10	ordinary_muscle_development	ENSGALG00010019223	AAMP	-0.149947612	0.126157383	-1.1885758	0.234606635	0.269548445	negative
PRJNA729271	stage_5W_vs_E10	ordinary_muscle_development	ENSGALG00010019225	PROK2	-7.016685919	0.908203034	-7.725900108	1.11066E-14	3.42896E-14	negative
PRJNA729271	stage_5W_vs_E10	ordinary_muscle_development	ENSGALG00010019227	CLCN5	0.566467479	0.072096117	7.857114934	3.93082E-15	1.2435E-14	positive
PRJNA729271	stage_5W_vs_E10	ordinary_muscle_development	ENSGALG00010019230	SPG21	-0.303353686	0.098079636	-3.092932418	0.001981893	0.00291443	negative
PRJNA729271	stage_5W_vs_E10	ordinary_muscle_development	ENSGALG00010019231	ENSGALG00010019231	3.60046158	0.3111671	11.57082989	5.79197E-31	3.6502E-30	positive
PRJNA729271	stage_5W_vs_E10	ordinary_muscle_development	ENSGALG00010019232	SOS2	1.294778569	0.092863869	13.94275926	3.48213E-44	3.45946E-43	positive
PRJNA729271	stage_5W_vs_E10	ordinary_muscle_development	ENSGALG00010019233	ARPC2	-0.525282501	0.053752234	-9.772291521	1.48065E-22	6.64251E-22	negative
PRJNA729271	stage_5W_vs_E10	ordinary_muscle_development	ENSGALG00010019236	MPG	-0.06704205	0.20919139	-0.320481882	0.748603062	0.776958427	negative
PRJNA729271	stage_5W_vs_E10	ordinary_muscle_development	ENSGALG00010019237	SORCS3	-1.775115822	0.337428342	-5.260719392	1.43493E-07	2.92989E-07	negative
PRJNA729271	stage_5W_vs_E10	ordinary_muscle_development	ENSGALG00010019239	CYTH3	0.352625001	0.081114863	4.347230453	1.37867E-05	2.43409E-05	positive
PRJNA729271	stage_5W_vs_E10	ordinary_muscle_development	ENSGALG00010019240	ENSGALG00010019240	0.385629979	0.202728301	1.902201012	0.057144881	0.071535974	positive
PRJNA729271	stage_5W_vs_E10	ordinary_muscle_development	ENSGALG00010019243	SERTAD2	0.77708877	0.154315936	5.035700061	4.76106E-07	9.35369E-07	positive
PRJNA729271	stage_5W_vs_E10	ordinary_muscle_development	ENSGALG00010019246	IL17RC	0.888123459	0.135010895	6.578161387	4.76301E-11	1.21186E-10	positive
PRJNA729271	stage_5W_vs_E10	ordinary_muscle_development	ENSGALG00010019247	HBZ	-6.315453718	0.581639249	-10.85802537	1.82656E-27	9.99849E-27	negative
PRJNA729271	stage_5W_vs_E10	ordinary_muscle_development	ENSGALG00010019248	RMI2	-0.58324093	0.171024016	-3.410286718	0.000648946	0.000999307	negative
PRJNA729271	stage_5W_vs_E10	ordinary_muscle_development	ENSGALG00010019250	CXCR1	0.154142849	0.25462658	0.605368258	0.544934337	0.584664931	positive
PRJNA729271	stage_5W_vs_E10	ordinary_muscle_development	ENSGALG00010019252	TATDN3	-0.186749305	0.118499085	-1.575955661	0.115036022	0.138688109	negative
PRJNA729271	stage_5W_vs_E10	ordinary_muscle_development	ENSGALG00010019253	HBAD	-4.54084542	0.427478991	-10.62238265	2.34498E-26	1.23377E-25	negative
PRJNA729271	stage_5W_vs_E10	ordinary_muscle_development	ENSGALG00010019255	ENSGALG00010019255	3.365429228	0.19800949	16.99630273	8.74654E-65	1.60077E-63	positive
PRJNA729271	stage_5W_vs_E10	ordinary_muscle_development	ENSGALG00010019256	BBS9	-1.134427298	0.107029845	-10.59916795	3.00643E-26	1.57209E-25	negative
PRJNA729271	stage_5W_vs_E10	ordinary_muscle_development	ENSGALG00010019257	SLC1A4	-0.555582701	0.158204694	-3.51179657	0.000445089	0.000693716	negative
PRJNA729271	stage_5W_vs_E10	ordinary_muscle_development	ENSGALG00010019259	L2HGDH	0.029964098	0.109501958	0.273639835	0.784361418	0.810242215	positive
PRJNA729271	stage_5W_vs_E10	ordinary_muscle_development	ENSGALG00010019260	TRAIL-LIKE	-0.468803625	0.176758398	-2.652228305	0.007996245	0.011057582	negative
PRJNA729271	stage_5W_vs_E10	ordinary_muscle_development	ENSGALG00010019262	TMC3	-4.139714018	0.536037242	-7.722810451	1.13792E-14	3.51015E-14	negative
PRJNA729271	stage_5W_vs_E10	ordinary_muscle_development	ENSGALG00010019263	SNRPB2	-0.914698648	0.102607939	-8.91450168	4.90015E-19	1.87112E-18	negative
PRJNA729271	stage_5W_vs_E10	ordinary_muscle_development	ENSGALG00010019264	HK2	0.839111531	0.214622148	3.909715471	9.24049E-05	0.000152709	positive
PRJNA729271	stage_5W_vs_E10	ordinary_muscle_development	ENSGALG00010019265	RUFY4	5.787415872	0.654253902	8.845825536	9.08541E-19	3.42951E-18	positive
PRJNA729271	stage_5W_vs_E10	ordinary_muscle_development	ENSGALG00010019266	ATP5S	-0.278540865	0.160224303	-1.73844329	0.082132734	0.100831201	negative
PRJNA729271	stage_5W_vs_E10	ordinary_muscle_development	ENSGALG00010019267	STARD5	-0.156018462	0.186065471	-0.838513784	0.40174221	0.443128965	negative
PRJNA729271	stage_5W_vs_E10	ordinary_muscle_development	ENSGALG00010019268	NSL1	-1.264002321	0.095533219	-13.23102405	5.80905E-40	5.07854E-39	negative
PRJNA729271	stage_5W_vs_E10	ordinary_muscle_development	ENSGALG00010019269	SOCS1	0.742956053	0.252546776	2.941855221	0.003262524	0.004697555	positive
PRJNA729271	stage_5W_vs_E10	ordinary_muscle_development	ENSGALG00010019270	ENSGALG00010019270	-0.044160781	0.19803862	-0.222990754	0.823542702	0.844457518	negative
PRJNA729271	stage_5W_vs_E10	ordinary_muscle_development	ENSGALG00010019271	CDKL1	-0.69328064	0.200217792	-3.462632535	0.000534918	0.000829267	negative
PRJNA729271	stage_5W_vs_E10	ordinary_muscle_development	ENSGALG00010019274	ENSGALG00010019274	-2.656057024	0.893724898	-2.971895525	0.002959674	0.004278495	negative
PRJNA729271	stage_5W_vs_E10	ordinary_muscle_development	ENSGALG00010019275	CTAGE1	1.048697486	0.592946051	1.768622093	0.076956961	0.094862756	positive
PRJNA729271	stage_5W_vs_E10	ordinary_muscle_development	ENSGALG00010019277	IGFBP5	0.272166522	0.132830803	2.048971434	0.040464908	0.051622852	positive
PRJNA729271	stage_5W_vs_E10	ordinary_muscle_development	ENSGALG00010019278	JPT2	-1.934077431	0.073859362	-26.18594816	3.8418E-151	3.9216E-149	negative
PRJNA729271	stage_5W_vs_E10	ordinary_muscle_development	ENSGALG00010019280	TLNRD1	0.159554585	0.116545293	1.369034996	0.170988336	0.201354775	positive
PRJNA729271	stage_5W_vs_E10	ordinary_muscle_development	ENSGALG00010019281	ENSGALG00010019281	-0.368858701	1.162792182	-0.317218078	0.751078134	0.779136731	negative
PRJNA729271	stage_5W_vs_E10	ordinary_muscle_development	ENSGALG00010019282	IGFBP2	-2.720999346	0.15784361	-17.23857778	1.3635E-66	2.63871E-65	negative
PRJNA729271	stage_5W_vs_E10	ordinary_muscle_development	ENSGALG00010019283	NT5C3A	2.043101962	0.255519408	7.995877799	1.28654E-15	4.18407E-15	positive
PRJNA729271	stage_5W_vs_E10	ordinary_muscle_development	ENSGALG00010019284	CHD5	1.163079295	0.131963737	8.813628033	1.2116E-18	4.54623E-18	positive
PRJNA729271	stage_5W_vs_E10	ordinary_muscle_development	ENSGALG00010019285	BATF3	-0.271969027	0.272755453	-0.997116734	0.318707851	0.35794749	negative
PRJNA729271	stage_5W_vs_E10	ordinary_muscle_development	ENSGALG00010019287	AHSA2	-0.687662245	0.098782718	-6.96136184	3.36999E-12	9.14414E-12	negative
PRJNA729271	stage_5W_vs_E10	ordinary_muscle_development	ENSGALG00010019288	RPL37A	0.27528068	0.156727531	1.756428354	0.079015295	0.097251142	positive
PRJNA729271	stage_5W_vs_E10	ordinary_muscle_development	ENSGALG00010019289	FKBP9	-1.738822582	0.166669561	-10.43275432	1.7572E-25	8.85624E-25	negative
PRJNA729271	stage_5W_vs_E10	ordinary_muscle_development	ENSGALG00010019291	ADD2	-0.78726745	0.27610049	-2.851380126	0.00435299	0.00618594	negative
PRJNA729271	stage_5W_vs_E10	ordinary_muscle_development	ENSGALG00010019292	ENSGALG00010019292	-0.638834599	0.097781632	-6.53327811	6.43455E-11	1.62399E-10	negative
PRJNA729271	stage_5W_vs_E10	ordinary_muscle_development	ENSGALG00010019294	SMARCAL1	-0.412509429	0.09812798	-4.203790081	2.62482E-05	4.52454E-05	negative
PRJNA729271	stage_5W_vs_E10	ordinary_muscle_development	ENSGALG00010019295	MAP4K5	0.193083646	0.13002511	1.48497199	0.137551244	0.164072193	positive
PRJNA729271	stage_5W_vs_E10	ordinary_muscle_development	ENSGALG00010019296	ENSGALG00010019296	0.279629181	0.102243608	2.734930681	0.006239341	0.008726288	positive
PRJNA729271	stage_5W_vs_E10	ordinary_muscle_development	ENSGALG00010019297	SLC25A26	1.413869883	0.146525081	9.649336964	4.94746E-22	2.17116E-21	positive
PRJNA729271	stage_5W_vs_E10	ordinary_muscle_development	ENSGALG00010019298	PGAP6	0.678922107	0.092737915	7.320868763	2.46371E-13	7.07858E-13	positive
PRJNA729271	stage_5W_vs_E10	ordinary_muscle_development	ENSGALG00010019300	MESDC2	-0.443663495	0.066478837	-6.673755391	2.49339E-11	6.44812E-11	negative
PRJNA729271	stage_5W_vs_E10	ordinary_muscle_development	ENSGALG00010019302	USP34	-0.083566002	0.094732632	-0.882124776	0.377709335	0.418826256	negative
PRJNA729271	stage_5W_vs_E10	ordinary_muscle_development	ENSGALG00010019305	SNRNP27	0.289299152	0.118904701	2.433033757	0.014972905	0.020093719	positive
PRJNA729271	stage_5W_vs_E10	ordinary_muscle_development	ENSGALG00010019307	ATF3	3.431945822	0.486174314	7.059085029	1.67602E-12	4.62038E-12	positive
PRJNA729271	stage_5W_vs_E10	ordinary_muscle_development	ENSGALG00010019310	CRELD1	1.045228089	0.096897656	10.78692855	3.96831E-27	2.15111E-26	positive
PRJNA729271	stage_5W_vs_E10	ordinary_muscle_development	ENSGALG00010019312	KBTBD2	0.242011601	0.072604617	3.333281153	0.000858282	0.00130818	positive
PRJNA729271	stage_5W_vs_E10	ordinary_muscle_development	ENSGALG00010019313	MRPL28	-0.20769396	0.100002597	-2.076885667	0.037812113	0.048486074	negative
PRJNA729271	stage_5W_vs_E10	ordinary_muscle_development	ENSGALG00010019314	GKN2	-3.791181154	1.283774111	-2.953152835	0.003145462	0.004537115	negative
PRJNA729271	stage_5W_vs_E10	ordinary_muscle_development	ENSGALG00010019316	CLEC16A	0.211252388	0.088127653	2.397118061	0.016524598	0.022070852	positive
PRJNA729271	stage_5W_vs_E10	ordinary_muscle_development	ENSGALG00010019318	NOG2	0.348973738	0.278554703	1.252801459	0.210277971	0.243682378	positive
PRJNA729271	stage_5W_vs_E10	ordinary_muscle_development	ENSGALG00010019319	ACTR3	-0.913125807	0.066958259	-13.63723942	2.40493E-42	2.25628E-41	negative
PRJNA729271	stage_5W_vs_E10	ordinary_muscle_development	ENSGALG00010019322	EGR3	2.358795853	0.300642411	7.845851977	4.30024E-15	1.35681E-14	positive
PRJNA729271	stage_5W_vs_E10	ordinary_muscle_development	ENSGALG00010019323	LRIG1	-0.645156577	0.120039153	-5.374551225	7.67737E-08	1.59546E-07	negative
PRJNA729271	stage_5W_vs_E10	ordinary_muscle_development	ENSGALG00010019325	SLITRK4	-2.478442418	0.211160053	-11.73726935	8.20944E-32	5.34093E-31	negative
PRJNA729271	stage_5W_vs_E10	ordinary_muscle_development	ENSGALG00010019331	CEMIP	-1.110508676	0.325628287	-3.410356904	0.000648779	0.000999156	negative
PRJNA729271	stage_5W_vs_E10	ordinary_muscle_development	ENSGALG00010019334	ATL1	-1.21939569	0.13138148	-9.28133625	1.67366E-20	6.82412E-20	negative
PRJNA729271	stage_5W_vs_E10	ordinary_muscle_development	ENSGALG00010019335	HIPK3	1.529057164	0.115153506	13.27842472	3.08823E-40	2.71955E-39	positive
PRJNA729271	stage_5W_vs_E10	ordinary_muscle_development	ENSGALG00010019336	DEXI	1.244000892	0.23853434	5.215185752	1.83633E-07	3.71858E-07	positive
PRJNA729271	stage_5W_vs_E10	ordinary_muscle_development	ENSGALG00010019337	XPO1	-0.535681395	0.08413755	-6.366733945	1.93096E-10	4.71914E-10	negative
PRJNA729271	stage_5W_vs_E10	ordinary_muscle_development	ENSGALG00010019344	DNAJC24	-0.451949296	0.08736573	-5.173072949	2.30275E-07	4.62753E-07	negative
PRJNA729271	stage_5W_vs_E10	ordinary_muscle_development	ENSGALG00010019345	ENSGALG00010019345	0.093906364	0.186907467	0.502421683	0.61537093	0.652127623	positive
PRJNA729271	stage_5W_vs_E10	ordinary_muscle_development	ENSGALG00010019346	POU3F4	-1.363884923	0.584600869	-2.333018979	0.019647149	0.025989896	negative
PRJNA729271	stage_5W_vs_E10	ordinary_muscle_development	ENSGALG00010019350	SAV1	-0.101283182	0.088709174	-1.141737211	0.253563269	0.289629596	negative
PRJNA729271	stage_5W_vs_E10	ordinary_muscle_development	ENSGALG00010019355	ENTPD4	0.605490255	0.097780223	6.19235913	5.92703E-10	1.40403E-09	positive
PRJNA729271	stage_5W_vs_E10	ordinary_muscle_development	ENSGALG00010019356	AVL9	-0.236842721	0.096341585	-2.458364394	0.013957148	0.018800191	negative
PRJNA729271	stage_5W_vs_E10	ordinary_muscle_development	ENSGALG00010019365	ENSGALG00010019365	0.242920539	0.178278582	1.362589585	0.173011893	0.203473216	positive
PRJNA729271	stage_5W_vs_E10	ordinary_muscle_development	ENSGALG00010019371	TMEM87A	1.051706189	0.076612088	13.72767957	6.93182E-43	6.60597E-42	positive
PRJNA729271	stage_5W_vs_E10	ordinary_muscle_development	ENSGALG00010019373	DENND5A	0.164330488	0.061616834	2.666973892	0.007653762	0.010604213	positive
PRJNA729271	stage_5W_vs_E10	ordinary_muscle_development	ENSGALG00010019376	ENSGALG00010019376	5.688138502	0.313773312	18.12817817	1.90982E-73	4.38715E-72	positive
PRJNA729271	stage_5W_vs_E10	ordinary_muscle_development	ENSGALG00010019388	GPR75	-0.126661738	0.135046195	-0.937914157	0.348288527	0.388252111	negative
PRJNA729271	stage_5W_vs_E10	ordinary_muscle_development	ENSGALG00010019390	TVP23A	-0.293691363	0.214980198	-1.36613216	0.171897486	0.202310332	negative
PRJNA729271	stage_5W_vs_E10	ordinary_muscle_development	ENSGALG00010019391	GANC	3.574919024	0.130803744	27.33040299	1.8466E-164	2.2306E-162	positive
PRJNA729271	stage_5W_vs_E10	ordinary_muscle_development	ENSGALG00010019392	NIN	-1.599582666	0.108760742	-14.70735334	5.78251E-49	6.75948E-48	negative
PRJNA729271	stage_5W_vs_E10	ordinary_muscle_development	ENSGALG00010019398	SLC35F5	1.090867457	0.120164081	9.078149179	1.10437E-19	4.35824E-19	positive
PRJNA729271	stage_5W_vs_E10	ordinary_muscle_development	ENSGALG00010019399	CD93	0.69513321	0.151768313	4.580226234	4.64473E-06	8.4718E-06	positive
PRJNA729271	stage_5W_vs_E10	ordinary_muscle_development	ENSGALG00010019403	PACC1	-0.465720173	0.107764636	-4.321641959	1.54872E-05	2.72106E-05	negative
PRJNA729271	stage_5W_vs_E10	ordinary_muscle_development	ENSGALG00010019411	LSM5	-0.963379564	0.158560245	-6.075795147	1.23375E-09	2.86681E-09	negative
PRJNA729271	stage_5W_vs_E10	ordinary_muscle_development	ENSGALG00010019416	GPR39	0.239865977	0.497065416	0.482564203	0.629405201	0.665200758	positive
PRJNA729271	stage_5W_vs_E10	ordinary_muscle_development	ENSGALG00010019418	ZNF703	0.667206459	0.233494148	2.857486862	0.004270103	0.00607351	positive
PRJNA729271	stage_5W_vs_E10	ordinary_muscle_development	ENSGALG00010019422	IMMP1L	0.861812302	0.139681546	6.169836502	6.83606E-10	1.6112E-09	positive

PRJNA729271	stage_5W_vs_E10	ordinary_muscle_development	ENSGALG00010019423	LYPD1	-0.817512762	0.180910852	-4.518870767	6.21703E-06	1.12294E-05	negative
PRJNA729271	stage_5W_vs_E10	ordinary_muscle_development	ENSGALG00010019426	GRM7	-2.570764676	0.265799083	-9.67183424	3.97194E-22	1.74835E-21	negative
PRJNA729271	stage_5W_vs_E10	ordinary_muscle_development	ENSGALG00010019430	ENSGALG00010019430	0.609244141	0.403160719	1.511169399	0.130745297	0.156391573	positive
PRJNA729271	stage_5W_vs_E10	ordinary_muscle_development	ENSGALG00010019441	LMCD1	2.479999016	0.137575885	18.02640789	1.20895E-72	2.74667E-71	positive
PRJNA729271	stage_5W_vs_E10	ordinary_muscle_development	ENSGALG00010019443	NCKAP5	-0.964492845	0.135986155	-7.092581203	1.31633E-12	3.64544E-12	negative
PRJNA729271	stage_5W_vs_E10	ordinary_muscle_development	ENSGALG00010019451	CAV3	3.619662996	0.143068827	25.30015152	3.182E-141	2.6816E-139	positive
PRJNA729271	stage_5W_vs_E10	ordinary_muscle_development	ENSGALG00010019458	ELP4	-0.175218015	0.102654418	-1.706872607	0.087845733	0.107462563	negative
PRJNA729271	stage_5W_vs_E10	ordinary_muscle_development	ENSGALG00010019461	MGAT5	0.54033315	0.070038431	7.714809451	1.21163E-14	3.73354E-14	positive
PRJNA729271	stage_5W_vs_E10	ordinary_muscle_development	ENSGALG00010019470	TGFB2	-1.204576101	0.20052515	-6.007107343	1.88862E-09	4.33707E-09	negative
PRJNA729271	stage_5W_vs_E10	ordinary_muscle_development	ENSGALG00010019471	NUBP1	0.04273586	0.093995152	0.45466025	0.649353682	0.684174226	positive
PRJNA729271	stage_5W_vs_E10	ordinary_muscle_development	ENSGALG00010019472	CAPN3	7.930979565	0.255616326	31.02688975	2.3392E-211	4.521E-209	positive
PRJNA729271	stage_5W_vs_E10	ordinary_muscle_development	ENSGALG00010019473	OXTR	0.785450209	0.442114058	1.776578227	0.075637661	0.093323791	positive
PRJNA729271	stage_5W_vs_E10	ordinary_muscle_development	ENSGALG00010019474	TMEM163	-0.743546017	0.547591162	-1.357848828	0.174511658	0.205120538	negative
PRJNA729271	stage_5W_vs_E10	ordinary_muscle_development	ENSGALG00010019477	NCAPH	-2.62886713	0.180960423	-14.52730425	8.13597E-48	9.05072E-47	negative
PRJNA729271	stage_5W_vs_E10	ordinary_muscle_development	ENSGALG00010019481	PYGL	0.225099294	0.098563523	2.283799184	0.022383336	0.02939359	positive
PRJNA729271	stage_5W_vs_E10	ordinary_muscle_development	ENSGALG00010019483	SCUBE2	-1.937035941	0.261609738	-7.404296011	1.31848E-13	3.83608E-13	negative
PRJNA729271	stage_5W_vs_E10	ordinary_muscle_development	ENSGALG00010019488	TEKT5	-3.509122742	1.312435179	-2.673749377	0.007500848	0.010404287	negative
PRJNA729271	stage_5W_vs_E10	ordinary_muscle_development	ENSGALG00010019495	EMP2	0.424031059	0.196888193	2.15366423	0.031266513	0.040486699	positive
PRJNA729271	stage_5W_vs_E10	ordinary_muscle_development	ENSGALG00010019498	ENSGALG00010019498	-0.744582395	0.291046735	-2.558291527	0.010518787	0.014375808	negative
PRJNA729271	stage_5W_vs_E10	ordinary_muscle_development	ENSGALG00010019509	ZNF106	1.733153619	0.222169838	7.801030215	6.14038E-15	1.92069E-14	positive
PRJNA729271	stage_5W_vs_E10	ordinary_muscle_development	ENSGALG00010019515	ENSGALG00010019515	-0.284985211	0.109379033	-2.605483002	0.009174483	0.01260752	negative
PRJNA729271	stage_5W_vs_E10	ordinary_muscle_development	ENSGALG00010019520	CD6	5.684604279	0.966882209	5.87931418	4.1197E-09	9.28983E-09	positive
PRJNA729271	stage_5W_vs_E10	ordinary_muscle_development	ENSGALG00010019524	HHIPL2	1.764706171	0.354681938	4.975461052	6.50925E-07	1.26766E-06	positive
PRJNA729271	stage_5W_vs_E10	ordinary_muscle_development	ENSGALG00010019529	SNAP23	0.033115835	0.067498654	0.490614744	0.623698956	0.66013263	positive
PRJNA729271	stage_5W_vs_E10	ordinary_muscle_development	ENSGALG00010019534	PPP2R5A	0.996795168	0.104475017	9.540990746	1.41474E-21	6.06886E-21	positive
PRJNA729271	stage_5W_vs_E10	ordinary_muscle_development	ENSGALG00010019540	UNC5D	-0.258538309	0.361292052	-0.715593679	0.474242278	0.516076713	negative
PRJNA729271	stage_5W_vs_E10	ordinary_muscle_development	ENSGALG00010019546	HSD17B12	-1.036864423	0.078470858	-13.21336935	7.34611E-40	6.41071E-39	negative
PRJNA729271	stage_5W_vs_E10	ordinary_muscle_development	ENSGALG00010019557	TRIM9	-1.015401362	0.343632463	-2.954905233	0.003127651	0.004513221	negative
PRJNA729271	stage_5W_vs_E10	ordinary_muscle_development	ENSGALG00010019572	NFKBIE	0.57031753	0.183171724	3.113567524	0.001848402	0.002726981	positive
PRJNA729271	stage_5W_vs_E10	ordinary_muscle_development	ENSGALG00010019577	MXD1	0.208416268	0.188216205	1.107323719	0.268153993	0.305213359	positive
PRJNA729271	stage_5W_vs_E10	ordinary_muscle_development	ENSGALG00010019580	CD5	4.180412328	0.435993324	9.588248487	8.9593E-22	3.8731E-21	positive
PRJNA729271	stage_5W_vs_E10	ordinary_muscle_development	ENSGALG00010019581	SLC35B2	-0.203703192	0.133898176	-1.521329097	0.128177272	0.153560588	negative
PRJNA729271	stage_5W_vs_E10	ordinary_muscle_development	ENSGALG00010019584	RAD18	-1.492646227	0.119304502	-12.51123132	6.48117E-36	4.96536E-35	negative
PRJNA729271	stage_5W_vs_E10	ordinary_muscle_development	ENSGALG00010019585	PDE1C	-0.618408112	0.211510222	-2.923774122	0.003458156	0.004964934	negative
PRJNA729271	stage_5W_vs_E10	ordinary_muscle_development	ENSGALG00010019586	GLYR1	-0.700355089	0.090808656	-7.71242653	1.23448E-14	3.7999E-14	negative
PRJNA729271	stage_5W_vs_E10	ordinary_muscle_development	ENSGALG00010019587	CCNT2	-0.224357358	0.128447415	-1.74668643	0.080691745	0.099146126	negative
PRJNA729271	stage_5W_vs_E10	ordinary_muscle_development	ENSGALG00010019588	CHMP7	1.072872632	0.092592135	11.58708167	4.79182E-31	3.03175E-30	positive
PRJNA729271	stage_5W_vs_E10	ordinary_muscle_development	ENSGALG00010019591	ENSGALG00010019591	-2.078711945	0.227380013	-9.142017006	6.12982E-20	2.44568E-19	negative
PRJNA729271	stage_5W_vs_E10	ordinary_muscle_development	ENSGALG00010019592	ALKBH3	0.293653013	0.132560387	2.215239559	0.026743626	0.034863644	positive
PRJNA729271	stage_5W_vs_E10	ordinary_muscle_development	ENSGALG00010019594	TMX1	-0.679030295	0.342567378	-1.982180264	0.047459069	0.060017379	negative
PRJNA729271	stage_5W_vs_E10	ordinary_muscle_development	ENSGALG00010019595	GRIN2A	-2.436083193	0.234456263	-10.3903524	2.74338E-25	1.37358E-24	negative
PRJNA729271	stage_5W_vs_E10	ordinary_muscle_development	ENSGALG00010019598	KCNAB2	-0.766813508	0.135443117	-5.661517007	1.50041E-08	3.28079E-08	negative
PRJNA729271	stage_5W_vs_E10	ordinary_muscle_development	ENSGALG00010019600	MAP3K19	-1.418982133	0.247903182	-5.723936756	1.04084E-08	2.29494E-08	negative
PRJNA729271	stage_5W_vs_E10	ordinary_muscle_development	ENSGALG00010019602	SLC29A1	1.83142068	0.193734479	9.453251101	3.28467E-21	1.38485E-20	positive
PRJNA729271	stage_5W_vs_E10	ordinary_muscle_development	ENSGALG00010019610	CORO7	0.398439907	0.100977084	3.945844851	7.95191E-05	0.000132304	positive
PRJNA729271	stage_5W_vs_E10	ordinary_muscle_development	ENSGALG00010019613	FRMD6	-0.575920137	0.124953588	-4.609072442	4.04469E-06	7.41843E-06	negative
PRJNA729271	stage_5W_vs_E10	ordinary_muscle_development	ENSGALG00010019614	MICALL2	0.726747017	0.156479562	4.644357421	3.41137E-06	6.29827E-06	positive
PRJNA729271	stage_5W_vs_E10	ordinary_muscle_development	ENSGALG00010019617	ANO3	-1.226939744	0.145248726	-8.447163562	2.98465E-17	1.05057E-16	negative
PRJNA729271	stage_5W_vs_E10	ordinary_muscle_development	ENSGALG00010019618	NPHP4	-0.392820277	0.106772759	-3.679030883	0.000234122	0.000374074	negative
PRJNA729271	stage_5W_vs_E10	ordinary_muscle_development	ENSGALG00010019621	ENSGALG00010019621	0.399311903	0.15120078	2.64093811	0.008267682	0.011414425	positive
PRJNA729271	stage_5W_vs_E10	ordinary_muscle_development	ENSGALG00010019622	C2orf42	0.97567066	0.142038781	6.869044167	6.46335E-12	1.72598E-11	positive
PRJNA729271	stage_5W_vs_E10	ordinary_muscle_development	ENSGALG00010019624	SRGAP3	0.217385497	0.130118635	1.670671516	0.094786578	0.115504956	positive
PRJNA729271	stage_5W_vs_E10	ordinary_muscle_development	ENSGALG00010019625	INTS1	-0.29366944	0.076688882	-3.829361353	0.000128476	0.000209455	negative
PRJNA729271	stage_5W_vs_E10	ordinary_muscle_development	ENSGALG00010019630	AJAP1	-1.407014426	0.389665324	-3.610827908	0.000305221	0.000482831	negative
PRJNA729271	stage_5W_vs_E10	ordinary_muscle_development	ENSGALG00010019634	GNG2	-1.900806173	0.2846214	-6.678367036	2.41619E-11	6.25517E-11	negative
PRJNA729271	stage_5W_vs_E10	ordinary_muscle_development	ENSGALG00010019635	LRRC57	0.86463483	0.07720123	11.19975465	4.08864E-29	2.39454E-28	positive
PRJNA729271	stage_5W_vs_E10	ordinary_muscle_development	ENSGALG00010019640	MGRN1	-0.778995566	0.118188605	-6.591122451	4.36514E-11	1.11337E-10	negative
PRJNA729271	stage_5W_vs_E10	ordinary_muscle_development	ENSGALG00010019641	CAPN11	1.309305106	0.081623631	16.04076044	6.63371E-58	1.00162E-56	positive
PRJNA729271	stage_5W_vs_E10	ordinary_muscle_development	ENSGALG00010019643	RAB3GAP1	-0.367755552	0.071105562	-5.171966048	2.31644E-07	4.6531E-07	negative
PRJNA729271	stage_5W_vs_E10	ordinary_muscle_development	ENSGALG00010019645	ENSGALG00010019645	3.275767616	0.161399589	20.29600964	1.3946E-91	4.94249E-90	positive
PRJNA729271	stage_5W_vs_E10	ordinary_muscle_development	ENSGALG00010019646	VPS37C	1.562499658	0.125234077	12.47663328	1.00132E-35	7.59503E-35	positive
PRJNA729271	stage_5W_vs_E10	ordinary_muscle_development	ENSGALG00010019648	PRDM16	-1.28436896	0.192435733	-6.674274771	2.48458E-11	6.42762E-11	negative
PRJNA729271	stage_5W_vs_E10	ordinary_muscle_development	ENSGALG00010019649	TMEM63B	-0.022318929	0.076628244	-0.291262439	0.770850612	0.797991689	negative
PRJNA729271	stage_5W_vs_E10	ordinary_muscle_development	ENSGALG00010019650	KBTBD8	0.693838028	0.087003701	7.97481047	1.52615E-15	4.94446E-15	positive
PRJNA729271	stage_5W_vs_E10	ordinary_muscle_development	ENSGALG00010019652	TNFRSF10B	1.07243648	0.318892637	3.363001701	0.000770999	0.001179859	positive
PRJNA729271	stage_5W_vs_E10	ordinary_muscle_development	ENSGALG00010019655	RTRAF	0.556848269	0.098425378	5.657568025	1.53533E-08	3.35463E-08	positive
PRJNA729271	stage_5W_vs_E10	ordinary_muscle_development	ENSGALG00010019657	PPP1R17	-2.151213397	0.274912776	-7.825076126	5.0735E-15	1.59351E-14	negative
PRJNA729271	stage_5W_vs_E10	ordinary_muscle_development	ENSGALG00010019659	MRPL14	0.933334647	0.125137323	7.45848342	8.7524E-14	2.57231E-13	positive
PRJNA729271	stage_5W_vs_E10	ordinary_muscle_development	ENSGALG00010019660	ENSGALG00010019660	0.914643942	0.505499927	1.809384912	0.070391226	0.087251652	positive
PRJNA729271	stage_5W_vs_E10	ordinary_muscle_development	ENSGALG00010019667	ITPRID1	0.530235596	0.617411031	0.858804863	0.390448184	0.431795714	positive
PRJNA729271	stage_5W_vs_E10	ordinary_muscle_development	ENSGALG00010019670	ENSGALG00010019670	-1.169283051	0.126454944	-9.246637697	2.31665E-20	9.39824E-20	negative
PRJNA729271	stage_5W_vs_E10	ordinary_muscle_development	ENSGALG00010019673	NID2	0.517964261	0.132639658	3.905048217	9.42066E-05	0.00015558	positive
PRJNA729271	stage_5W_vs_E10	ordinary_muscle_development	ENSGALG00010019675	MAFK	1.840590869	0.137062003	13.42889227	4.09491E-41	3.70048E-40	positive
PRJNA729271	stage_5W_vs_E10	ordinary_muscle_development	ENSGALG00010019679	RHOBTB2	-1.577286564	0.172239188	-9.157535999	5.30958E-20	2.12017E-19	negative
PRJNA729271	stage_5W_vs_E10	ordinary_muscle_development	ENSGALG00010019680	CCDC34	-0.54851079	0.204037243	-2.688287591	0.007181951	0.009979139	negative
PRJNA729271	stage_5W_vs_E10	ordinary_muscle_development	ENSGALG00010019682	TMEM158	-1.325896342	0.367945883	-3.603509113	0.00031395	0.000496097	negative
PRJNA729271	stage_5W_vs_E10	ordinary_muscle_development	ENSGALG00010019683	TMEM184A	0.499906154	0.294956189	1.694848838	0.0901041	0.110058054	positive
PRJNA729271	stage_5W_vs_E10	ordinary_muscle_development	ENSGALG00010019684	PIGQ	-0.406726678	0.131800922	-3.085916793	0.002029256	0.00298045	negative
PRJNA729271	stage_5W_vs_E10	ordinary_muscle_development	ENSGALG00010019690	ENSGALG00010019690	-0.4864266	0.091982966	-5.28822479	1.23509E-07	2.53371E-07	negative
PRJNA729271	stage_5W_vs_E10	ordinary_muscle_development	ENSGALG00010019692	MMEL1	-0.17217231	0.300355251	-0.5732289	0.566489719	0.605595433	negative
PRJNA729271	stage_5W_vs_E10	ordinary_muscle_development	ENSGALG00010019693	HAUS2	-0.466118174	0.119746148	-3.892552544	9.9195E-05	0.000163483	negative
PRJNA729271	stage_5W_vs_E10	ordinary_muscle_development	ENSGALG00010019695	PGA5	11.00510203	2.385061208	4.614180128	3.9465E-06	7.24016E-06	positive
PRJNA729271	stage_5W_vs_E10	ordinary_muscle_development	ENSGALG00010019697	EIF4EBP1	2.234114466	0.145219236	15.38442512	2.08218E-53	2.75124E-52	positive
PRJNA729271	stage_5W_vs_E10	ordinary_muscle_development	ENSGALG00010019700	HES5B	-2.695172714	0.267436713	-10.0777963	6.92621E-24	3.27447E-23	negative
PRJNA729271	stage_5W_vs_E10	ordinary_muscle_development	ENSGALG00010019706	NPRL3	0.570228297	0.098688276	5.77807537	7.556E-09	1.68008E-08	positive
PRJNA729271	stage_5W_vs_E10	ordinary_muscle_development	ENSGALG00010019713	NUDT14	-0.098938449	0.115429273	-0.857134814	0.391370383	0.432684041	negative
PRJNA729271	stage_5W_vs_E10	ordinary_muscle_development	ENSGALG00010019716	VEGFA	0.259782407	0.131145131	1.980877254	0.04760504	0.060186224	positive
PRJNA729271	stage_5W_vs_E10	ordinary_muscle_development	ENSGALG00010019717	ZRANB3	-0.886999941	0.143299916	-6.189814778	6.0235E-10	1.42594E-09	negative
PRJNA729271	stage_5W_vs_E10	ordinary_muscle_development	ENSGALG00010019724	HES5A	-4.211520488	0.311320166	-13.52793987	1.0698E-41	9.83305E-41	negative
PRJNA729271	stage_5W_vs_E10	ordinary_muscle_development	ENSGALG00010019726	PEBP4	5.250764042	0.223250822	23.51957315	2.5723E-122	1.5866E-120	positive
PRJNA729271	stage_5W_vs_E10	ordinary_muscle_development	ENSGALG00010019731	PANK4	1.504212068	0.133302477	11.28420189	1.57061E-29	9.34179E-29	positive
PRJNA729271	stage_5W_vs_E10	ordinary_muscle_development	ENSGALG00010019732	HBA1	-3.20208133	0.403784843	-7.930167226	2.18851E-15	7.0338E-15	negative
PRJNA729271	stage_5W_vs_E10	ordinary_muscle_development	ENSGALG00010019735	ENSGALG00010019735	-0.154445273	0.171498482	-0.900563496	0.367820451	0.408454565	negative
PRJNA729271	stage_5W_vs_E10	ordinary_muscle_development	ENSGALG00010019738	ADRA2A	-1.774673635	0.357570419	-4.963144427	6.9361E-07	1.34825E-06	negative
PRJNA729271	stage_5W_vs_E10	ordinary_muscle_development	ENSGALG00010019743	KCNU1	3.845742535	0.432568553	8.890481076	6.08449E-19	2.31604E-18	positive
PRJNA729271	stage_5W_vs_E10	ordinary_muscle_development	ENSGALG00010019744	KBP	0.179529668	0.437312384	0.410529576	0.681417512	0.714802927	positive
PRJNA729271	stage_5W_vs_E10	ordinary_muscle_development	ENSGALG00010019752	GPAM	-0.329554313	0.168247385	-1.958748496	0.050142248	0.063212027	negative
PRJNA729271	stage_5W_vs_E10	ordinary_muscle_development	ENSGALG00010019761	MRPS18A	0.715409714	0.087172713	8.206807981	2.27146E-16	7.66051E-16	positive
PRJNA729271	stage_5W_vs_E10	ordinary_muscle_development	ENSGALG00010019767	SUCLG2	-1.176544951	0.079228954	-14.84993671	6.96344E-50	8.35555E-49	negative
PRJNA729271	stage_5W_vs_E10	ordinary_muscle_development	ENSGALG00010019771	VWCE	-2.9248223	0.420508293	-6.955444993	3.51451E-12	9.52024E-12	negative
PRJNA729271	stage_5W_vs_E10	ordinary_muscle_development	ENSGALG00010019772	ENSGALG00010019772	0.231220439	0.165295633	1.398829692	0.16186406	0.191325088	positive
PRJNA729271	stage_5W_vs_E10	ordinary_muscle_development	ENSGALG00010019778	ABHD17C	0.129126641	0.132831609	0.972107783	0.33099693	0.370658259	positive
PRJNA729271	stage_5W_vs_E10	ordinary_muscle_development	ENSGALG00010019785	ACSL5	2.038725526	0.212593597	9.589778573	8.82743E-22	3.81837E-21	positive
PRJNA729271	stage_5W_vs_E10	ordinary_muscle_development	ENSGALG00010019786	R3HDM1	0.764727415	0.087199662	8.769843773	1.78914E-18	6.67704E-18	positive
PRJNA729271	stage_5W_vs_E10	ordinary_muscle_development	ENSGALG00010019789	ARNT2	-2.199756128	0.176018169	-12.49732421	7.72064E-36	5.88694E-35	negative
PRJNA729271	stage_5W_vs_E10	ordinary_muscle_development	ENSGALG00010019794	RSPH9	-0.30804607	0.484262401	-0.636113951	0.524702153	0.565342876	negative
PRJNA729271	stage_5W_vs_E10	ordinary_muscle_development	ENSGALG00010019799	DOK2	1.459769895	0.263522012	5.539460952	3.03404E-08	6.48076E-08	positive
PRJNA729271	stage_5W_vs_E10	ordinary_muscle_development	ENSGALG00010019800	EXT2	-0.460980835	0.060671674	-7.597958093	3.0084E-14	9.09041E-14	negative
PRJNA729271	stage_5W_vs_E10	ordinary_muscle_development	ENSGALG00010019801	FIBIN	-4.557435118	0.188579957	-24.16712348	4.9336E-129	3.5402E-127	negative
PRJNA729271	stage_5W_vs_E10	ordinary_muscle_development	ENSGALG00010019803	FAH	-1.792951346	0.506809575	-3.537721928	0.000403595	0.000631284	negative
PRJNA729271	stage_5W_vs_E10	ordinary_muscle_development	ENSGALG00010019804	ELFN1	-2.060862686	0.297239701	-6.933335877	4.11031E-12	1.10824E-11	negative
PRJNA729271	stage_5W_vs_E10	ordinary_muscle_development	ENSGALG00010019807	ZDHHC6	0.698848726	0.078460042	8.907065355	5.24012E-19	1.99883E-18	positive
PRJNA729271	stage_5W_vs_E10	ordinary_muscle_development	ENSGALG00010019810	ZFAND6	1.054502581	0.124539139	8.467238413	2.51279E-17	8.87499E-17	positive
PRJNA729271	stage_5W_vs_E10	ordinary_muscle_development	ENSGALG00010019811	UBXN4	0.653671574	0.110554144	5.91268268	3.3658E-09	7.62778E-09	positive
PRJNA729271	stage_5W_vs_E10	ordinary_muscle_development	ENSGALG00010019813	ENSGALG00010019813	3.88247736	0.217276675	17.86881798	2.0632E-71	4.55885E-70	positive
PRJNA729271	stage_5W_vs_E10	ordinary_muscle_development	ENSGALG00010019816	ENSGALG00010019816	5.127051756	0.685520151	7.479067896	7.48516E-14	2.20747E-13	positive
PRJNA729271	stage_5W_vs_E10	ordinary_muscle_development	ENSGALG00010019820	BBOX1	0.255422626	0.154194288	1.656498619	0.097620885	0.118749137	positive
PRJNA729271	stage_5W_vs_E10	ordinary_muscle_development	ENSGALG00010019823	LCT	-1.638829528	0.226949345	-7.221124734	5.15593E-13	1.45853E-12	negative
PRJNA729271	stage_5W_vs_E10	ordinary_muscle_development	ENSGALG00010019825	BCL2A1	3.375901253	0.275399892	12.25817929	1.51874E-34	1.09961E-33	positive
PRJNA729271	stage_5W_vs_E10	ordinary_muscle_development	ENSGALG00010019830	JAG2	-0.386215891	0.098682895	-3.913706546	9.08901E-05	0.000150274	negative
PRJNA729271	stage_5W_vs_E10	ordinary_muscle_development	ENSGALG00010019832	MAD2L1BP	-2.570601556	0.146751679	-17.51667565	1.06892E-68	2.17002E-67	negative
PRJNA729271	stage_5W_vs_E10	ordinary_muscle_development	ENSGALG00010019833	MTHFS	-0.655280153	0.131448171	-4.985083821	6.1935E-07	1.20762E-06	negative
PRJNA729271	stage_5W_vs_E10	ordinary_muscle_development	ENSGALG00010019834	PLCH2	-1.604668332	0.244459353	-6.564151933	5.23298E-11	1.32725E-10	negative
PRJNA729271	stage_5W_vs_E10	ordinary_muscle_development	ENSGALG00010019836	MINAR1	2.555931172	0.480108811	5.323649788	1.01706E-07	2.09824E-07	positive
PRJNA729271	stage_5W_vs_E10	ordinary_muscle_development	ENSGALG00010019838	ALX4	-3.973337868	0.225278678	-17.63743422	1.27103E-69	2.65852E-68	negative
PRJNA729271	stage_5W_vs_E10	ordinary_muscle_development	ENSGALG00010019843	MCM6	-2.18348089	0.11169386	-19.54879963	4.22342E-85	1.27009E-83	negative
PRJNA729271	stage_5W_vs_E10	ordinary_muscle_development	ENSGALG00010019846	ENSGALG00010019846	0.354842395	0.071993267	4.928827509	8.27246E-07	1.59964E-06	positive
PRJNA729271	stage_5W_vs_E10	ordinary_muscle_development	ENSGALG00010019848	MAD1L1	-0.636785579	0.126889872	-5.018411384	5.21005E-07	1.02109E-06	negative
PRJNA729271	stage_5W_vs_E10	ordinary_muscle_development	ENSGALG00010019854	CCDC14	-0.644046342	0.149229978	-4.315797347	1.59028E-05	2.79035E-05	negative
PRJNA729271	stage_5W_vs_E10	ordinary_muscle_development	ENSGALG00010019855	TTC7A	1.045983193	0.124332184	8.412811247	4.00299E-17	1.40018E-16	positive
PRJNA729271	stage_5W_vs_E10	ordinary_muscle_development	ENSGALG00010019859	BIN3	-0.622983284	0.137802155	-4.520852986	6.1591E-06	1.11303E-05	negative

PRJNA729271	stage_5W_vs_E10	ordinary_muscle_development	ENSGALG00010019865	ENSGALG00010019865	0.33435469	0.135691396	2.464081728	0.013736479	0.018516717	positive
PRJNA729271	stage_5W_vs_E10	ordinary_muscle_development	ENSGALG00010019867	PEX11A	-0.663878045	0.378148505	-1.7556014	0.079156493	0.097416655	negative
PRJNA729271	stage_5W_vs_E10	ordinary_muscle_development	ENSGALG00010019871	TAFA1	-3.292951944	0.634439835	-5.190329739	2.09922E-07	4.2326E-07	negative
PRJNA729271	stage_5W_vs_E10	ordinary_muscle_development	ENSGALG00010019872	VTI1A	0.13669759	0.10900935	1.253998768	0.209842453	0.243197118	positive
PRJNA729271	stage_5W_vs_E10	ordinary_muscle_development	ENSGALG00010019873	LGR4	-1.848457165	0.09933633	-18.60806788	2.76407E-77	7.09118E-76	negative
PRJNA729271	stage_5W_vs_E10	ordinary_muscle_development	ENSGALG00010019875	PLEKHH1	-2.930953304	0.306576894	-9.560255063	1.17467E-21	5.05247E-21	negative
PRJNA729271	stage_5W_vs_E10	ordinary_muscle_development	ENSGALG00010019876	MRM2	0.86910952	0.223400022	3.890373476	0.00010009	0.000164883	positive
PRJNA729271	stage_5W_vs_E10	ordinary_muscle_development	ENSGALG00010019878	MCFD2	0.072065092	0.15011414	0.480068649	0.63117858	0.666734697	positive
PRJNA729271	stage_5W_vs_E10	ordinary_muscle_development	ENSGALG00010019891	PIGH	-1.77945547	0.110767165	-16.0648281	4.5011E-58	6.86052E-57	negative
PRJNA729271	stage_5W_vs_E10	ordinary_muscle_development	ENSGALG00010019896	DDB1	0.046494799	0.090960862	0.511151691	0.609244843	0.646391743	positive
PRJNA729271	stage_5W_vs_E10	ordinary_muscle_development	ENSGALG00010019898	NUDT1	-1.786452661	0.190527725	-9.376339633	6.83028E-21	2.836E-20	negative
PRJNA729271	stage_5W_vs_E10	ordinary_muscle_development	ENSGALG00010019899	ENSGALG00010019899	-0.286570759	0.454167611	-0.630980176	0.528053487	0.568488955	negative
PRJNA729271	stage_5W_vs_E10	ordinary_muscle_development	ENSGALG00010019905	ENSGALG00010019905	-0.364849681	0.124790068	-2.923707683	0.003458894	0.004965501	negative
PRJNA729271	stage_5W_vs_E10	ordinary_muscle_development	ENSGALG00010019906	ENSGALG00010019906	1.547402121	0.232734164	6.648796625	2.95499E-11	7.61468E-11	positive
PRJNA729271	stage_5W_vs_E10	ordinary_muscle_development	ENSGALG00010019910	ENSGALG00010019910	-0.485715754	0.259574436	-1.871200266	0.061317328	0.076468611	negative
PRJNA729271	stage_5W_vs_E10	ordinary_muscle_development	ENSGALG00010019912	ARG2	-5.915684318	0.264320442	-22.38072948	6.0644E-111	3.162E-109	negative
PRJNA729271	stage_5W_vs_E10	ordinary_muscle_development	ENSGALG00010019916	NOC3L	-0.399047537	0.114319807	-3.490624668	0.000481893	0.000748905	negative
PRJNA729271	stage_5W_vs_E10	ordinary_muscle_development	ENSGALG00010019919	LIN7C	0.133368838	0.107202663	1.244081389	0.21346961	0.247064995	positive
PRJNA729271	stage_5W_vs_E10	ordinary_muscle_development	ENSGALG00010019921	CALM2	0.74764724	0.112628882	6.638148461	3.17648E-11	8.17526E-11	positive
PRJNA729271	stage_5W_vs_E10	ordinary_muscle_development	ENSGALG00010019925	RDH11	-1.90840267	0.147918778	-12.90169305	4.40316E-38	3.59976E-37	negative
PRJNA729271	stage_5W_vs_E10	ordinary_muscle_development	ENSGALG00010019926	KIF7	-0.86762544	0.101525466	-8.545889741	1.2755E-17	4.57972E-17	negative
PRJNA729271	stage_5W_vs_E10	ordinary_muscle_development	ENSGALG00010019931	ZFYVE26	1.070730364	0.134162455	7.980849511	1.4533E-15	4.7137E-15	positive
PRJNA729271	stage_5W_vs_E10	ordinary_muscle_development	ENSGALG00010019932	KALRN	-0.225605968	0.117501446	-1.920027162	0.054854469	0.068793522	negative
PRJNA729271	stage_5W_vs_E10	ordinary_muscle_development	ENSGALG00010019934	TKFC	-0.066131392	0.152761993	-0.432904751	0.665083969	0.699440769	negative
PRJNA729271	stage_5W_vs_E10	ordinary_muscle_development	ENSGALG00010019935	ENSGALG00010019935	1.645015254	1.429929183	1.150417289	0.249972043	0.285865464	positive
PRJNA729271	stage_5W_vs_E10	ordinary_muscle_development	ENSGALG00010019938	TAFA4	-4.804449945	0.606117725	-7.926595359	2.25236E-15	7.22459E-15	negative
PRJNA729271	stage_5W_vs_E10	ordinary_muscle_development	ENSGALG00010019947	ENSGALG00010019947	-0.313568701	0.287674657	-1.090011558	0.275708053	0.3129148	negative
PRJNA729271	stage_5W_vs_E10	ordinary_muscle_development	ENSGALG00010019948	TICRR	-1.820440581	0.131696981	-13.82294847	1.85321E-43	1.80526E-42	negative
PRJNA729271	stage_5W_vs_E10	ordinary_muscle_development	ENSGALG00010019954	CYB561A3	1.559994798	0.124733033	12.50666931	6.86425E-36	5.24775E-35	positive
PRJNA729271	stage_5W_vs_E10	ordinary_muscle_development	ENSGALG00010019956	SLC29A3	-0.502273178	0.174677025	-2.875439276	0.004034657	0.005755005	negative
PRJNA729271	stage_5W_vs_E10	ordinary_muscle_development	ENSGALG00010019961	HTATSF1	-0.388194096	0.069909588	-5.552801923	2.81127E-08	6.02177E-08	negative
PRJNA729271	stage_5W_vs_E10	ordinary_muscle_development	ENSGALG00010019962	SNX8	0.759438315	0.142155645	5.342301478	9.17739E-08	1.89766E-07	positive
PRJNA729271	stage_5W_vs_E10	ordinary_muscle_development	ENSGALG00010019967	CEP170B	-0.339086373	0.1076998	-3.148440123	0.001641443	0.002433291	negative
PRJNA729271	stage_5W_vs_E10	ordinary_muscle_development	ENSGALG00010019968	RHCG	1.047766924	0.934230818	1.121528966	0.262062769	0.298679024	positive
PRJNA729271	stage_5W_vs_E10	ordinary_muscle_development	ENSGALG00010019972	PPP1CB	-0.049397685	0.091527078	-0.53970569	0.589400015	0.627587837	negative
PRJNA729271	stage_5W_vs_E10	ordinary_muscle_development	ENSGALG00010019975	ENSGALG00010019975	0.401978754	0.195457626	2.056603073	0.039724422	0.050780977	positive
PRJNA729271	stage_5W_vs_E10	ordinary_muscle_development	ENSGALG00010019977	ZMIZ1	-0.256609326	0.088721667	-2.892296026	0.003824374	0.005466355	negative
PRJNA729271	stage_5W_vs_E10	ordinary_muscle_development	ENSGALG00010019979	ENSGALG00010019979	-3.342652875	0.537757942	-6.215906103	5.10293E-10	1.21397E-09	negative
PRJNA729271	stage_5W_vs_E10	ordinary_muscle_development	ENSGALG00010019982	PLCE1	-0.130262522	0.112819997	-1.154604899	0.24825226	0.284100466	negative
PRJNA729271	stage_5W_vs_E10	ordinary_muscle_development	ENSGALG00010019985	UMPS	-1.890081605	0.089634745	-21.08648391	1.05842E-98	4.30949E-97	negative
PRJNA729271	stage_5W_vs_E10	ordinary_muscle_development	ENSGALG00010019989	ENSGALG00010019989	1.059134443	0.278939316	3.797006664	0.000146454	0.000237801	positive
PRJNA729271	stage_5W_vs_E10	ordinary_muscle_development	ENSGALG00010019990	MARCHF8	0.647272408	0.106918783	6.053869938	1.41407E-09	3.27479E-09	positive
PRJNA729271	stage_5W_vs_E10	ordinary_muscle_development	ENSGALG00010019991	EIF3B	0.086895994	0.075243835	1.154858655	0.248148313	0.284003931	positive
PRJNA729271	stage_5W_vs_E10	ordinary_muscle_development	ENSGALG00010019992	CHAC2	-0.517430933	0.146237256	-3.538297598	0.000402716	0.000630045	negative
PRJNA729271	stage_5W_vs_E10	ordinary_muscle_development	ENSGALG00010019994	PLD4	5.140354468	0.567318645	9.060788874	1.2951E-19	5.09155E-19	positive
PRJNA729271	stage_5W_vs_E10	ordinary_muscle_development	ENSGALG00010019996	ITGB5	0.421515523	0.124446486	3.387122732	0.000706298	0.001083708	positive
PRJNA729271	stage_5W_vs_E10	ordinary_muscle_development	ENSGALG00010019997	FSHR	-0.596549619	0.447982619	-1.331635635	0.182979949	0.214396117	negative
PRJNA729271	stage_5W_vs_E10	ordinary_muscle_development	ENSGALG00010019998	PLEK2	-5.403430832	0.772067012	-6.998655231	2.58431E-12	7.04524E-12	negative
PRJNA729271	stage_5W_vs_E10	ordinary_muscle_development	ENSGALG00010019999	ERLIN1	-0.804596463	0.064535384	-12.46752428	1.1226E-35	8.51049E-35	negative
PRJNA729271	stage_5W_vs_E10	ordinary_muscle_development	ENSGALG00010020003	ENSGALG00010020003	1.560405524	0.186939823	8.347100681	6.99591E-17	2.42018E-16	positive
PRJNA729271	stage_5W_vs_E10	ordinary_muscle_development	ENSGALG00010020005	EOGT	0.819236134	0.163370059	5.014603878	5.31428E-07	1.04067E-06	positive
PRJNA729271	stage_5W_vs_E10	ordinary_muscle_development	ENSGALG00010020006	PLB1	-1.465086704	0.390279598	-3.753941308	0.000174076	0.000280858	negative
PRJNA729271	stage_5W_vs_E10	ordinary_muscle_development	ENSGALG00010020008	EIF2S1	-0.578574948	0.079445984	-7.282620442	3.27397E-13	9.33627E-13	negative
PRJNA729271	stage_5W_vs_E10	ordinary_muscle_development	ENSGALG00010020009	MACROD2	-0.688872163	0.118076201	-5.834132191	5.40712E-09	1.2125E-08	negative
PRJNA729271	stage_5W_vs_E10	ordinary_muscle_development	ENSGALG00010020010	SORBS3	2.739131205	0.205051745	13.3582438	1.06036E-40	9.4701E-40	positive
PRJNA729271	stage_5W_vs_E10	ordinary_muscle_development	ENSGALG00010020011	TLE3	-0.535834537	0.203789655	-2.629351015	0.008554801	0.011793974	negative
PRJNA729271	stage_5W_vs_E10	ordinary_muscle_development	ENSGALG00010020013	ADAM8	0.807950635	0.525063061	1.538768758	0.123860759	0.148573461	positive
PRJNA729271	stage_5W_vs_E10	ordinary_muscle_development	ENSGALG00010020015	ATP6V1D	0.779607078	0.078400435	9.943912688	2.68086E-23	1.23913E-22	positive
PRJNA729271	stage_5W_vs_E10	ordinary_muscle_development	ENSGALG00010020017	ENSGALG00010020017	4.163451319	0.285291067	14.59369675	3.08043E-48	3.48291E-47	positive
PRJNA729271	stage_5W_vs_E10	ordinary_muscle_development	ENSGALG00010020018	ENSGALG00010020018	1.631515686	0.20452479	7.977104815	1.49806E-15	4.85453E-15	positive
PRJNA729271	stage_5W_vs_E10	ordinary_muscle_development	ENSGALG00010020019	PEX10	1.219140341	0.097609572	12.48996702	8.46887E-36	6.45067E-35	positive
PRJNA729271	stage_5W_vs_E10	ordinary_muscle_development	ENSGALG00010020023	FOSL2	0.832761033	0.208582582	3.992476394	6.53868E-05	0.000109596	positive
PRJNA729271	stage_5W_vs_E10	ordinary_muscle_development	ENSGALG00010020025	LOXL2	0.143792505	0.139505365	1.030731002	0.302666982	0.341280273	positive
PRJNA729271	stage_5W_vs_E10	ordinary_muscle_development	ENSGALG00010020027	RER1	-0.22884003	0.070883477	-3.228397362	0.001244859	0.001867547	negative
PRJNA729271	stage_5W_vs_E10	ordinary_muscle_development	ENSGALG00010020030	MRPL33	0.705485256	0.13486129	5.231191665	1.68421E-07	3.42009E-07	positive
PRJNA729271	stage_5W_vs_E10	ordinary_muscle_development	ENSGALG00010020032	CHST12	-0.20270637	0.124370082	-1.629864404	0.103130158	0.125020628	negative
PRJNA729271	stage_5W_vs_E10	ordinary_muscle_development	ENSGALG00010020036	TMF1	0.185184996	0.083879156	2.207759418	0.027261048	0.035509426	positive
PRJNA729271	stage_5W_vs_E10	ordinary_muscle_development	ENSGALG00010020037	MPP5	-0.215916369	0.089941503	-2.400631082	0.016366828	0.021869208	negative
PRJNA729271	stage_5W_vs_E10	ordinary_muscle_development	ENSGALG00010020038	SKI	-0.250835812	0.091009421	-2.756152156	0.005848578	0.008201136	negative
PRJNA729271	stage_5W_vs_E10	ordinary_muscle_development	ENSGALG00010020041	CDH23	0.52597638	0.152513472	3.448720776	0.000563249	0.000871508	positive
PRJNA729271	stage_5W_vs_E10	ordinary_muscle_development	ENSGALG00010020046	BDNF	-2.093383746	0.256140962	-8.17278006	3.01363E-16	1.01047E-15	negative
PRJNA729271	stage_5W_vs_E10	ordinary_muscle_development	ENSGALG00010020048	ENSGALG00010020048	-0.903176435	0.2687426	-3.360749042	0.000777314	0.001189147	negative
PRJNA729271	stage_5W_vs_E10	ordinary_muscle_development	ENSGALG00010020053	LFNG	0.461323682	0.134938566	3.418768238	0.000629053	0.00096991	positive
PRJNA729271	stage_5W_vs_E10	ordinary_muscle_development	ENSGALG00010020055	R3HCC1	-0.77485092	0.084822075	-9.135014954	6.53977E-20	2.6028E-19	negative
PRJNA729271	stage_5W_vs_E10	ordinary_muscle_development	ENSGALG00010020056	UACA	-1.483594903	0.165911667	-8.942077024	3.81923E-19	1.46765E-18	negative
PRJNA729271	stage_5W_vs_E10	ordinary_muscle_development	ENSGALG00010020057	RBKS	0.374894983	0.091811686	4.083303555	4.44E-05	7.51579E-05	positive
PRJNA729271	stage_5W_vs_E10	ordinary_muscle_development	ENSGALG00010020059	PRKCZ	-0.079481066	0.140185439	-0.566970912	0.570733944	0.609682992	negative
PRJNA729271	stage_5W_vs_E10	ordinary_muscle_development	ENSGALG00010020061	UBA3	0.204050581	0.0724193	2.817627106	0.004837996	0.006840967	positive
PRJNA729271	stage_5W_vs_E10	ordinary_muscle_development	ENSGALG00010020063	ENSGALG00010020063	1.848040558	0.128448157	14.38744322	6.20476E-47	6.74704E-46	positive
PRJNA729271	stage_5W_vs_E10	ordinary_muscle_development	ENSGALG00010020064	TMEM138	-0.221753149	0.134487156	-1.648879761	0.09917227	0.120514928	negative
PRJNA729271	stage_5W_vs_E10	ordinary_muscle_development	ENSGALG00010020065	TTYH3	-1.955397363	0.112746733	-17.34327295	2.21743E-67	4.34935E-66	negative
PRJNA729271	stage_5W_vs_E10	ordinary_muscle_development	ENSGALG00010020066	ENSGALG00010020066	2.05940115	0.386933131	5.322369641	1.02424E-07	2.11246E-07	positive
PRJNA729271	stage_5W_vs_E10	ordinary_muscle_development	ENSGALG00010020069	TMEM216	-0.626549271	0.201434568	-3.110435703	0.001868116	0.002753822	negative
PRJNA729271	stage_5W_vs_E10	ordinary_muscle_development	ENSGALG00010020070	ARL6IP5	0.946116443	0.114397472	8.270431385	1.33475E-16	4.55122E-16	positive
PRJNA729271	stage_5W_vs_E10	ordinary_muscle_development	ENSGALG00010020071	ENSGALG00010020071	0.527574708	0.143568388	3.674727523	0.000238103	0.000380309	positive
PRJNA729271	stage_5W_vs_E10	ordinary_muscle_development	ENSGALG00010020074	ENSGALG00010020074	-0.08434765	0.207747977	-0.406009489	0.684735628	0.718023796	negative
PRJNA729271	stage_5W_vs_E10	ordinary_muscle_development	ENSGALG00010020075	CPSF7	-0.804635057	0.148970715	-5.40129687	6.61608E-08	1.38045E-07	negative
PRJNA729271	stage_5W_vs_E10	ordinary_muscle_development	ENSGALG00010020080	SLC4A1AP	-0.167651888	0.103797562	-1.615181364	0.106271425	0.128667249	negative
PRJNA729271	stage_5W_vs_E10	ordinary_muscle_development	ENSGALG00010020083	CFAP74	-2.120729522	0.47209787	-4.492139567	7.05112E-06	1.26964E-05	negative
PRJNA729271	stage_5W_vs_E10	ordinary_muscle_development	ENSGALG00010020084	ENSGALG00010020084	-0.498823337	0.08799944	-5.668483116	1.44067E-08	3.15256E-08	negative
PRJNA729271	stage_5W_vs_E10	ordinary_muscle_development	ENSGALG00010020087	ENSGALG00010020087	3.272894624	0.475138375	6.8882978	5.64639E-12	1.51284E-11	positive
PRJNA729271	stage_5W_vs_E10	ordinary_muscle_development	ENSGALG00010020088	LMOD3	4.206491785	0.194009236	21.68191506	3.0397E-104	1.3683E-102	positive
PRJNA729271	stage_5W_vs_E10	ordinary_muscle_development	ENSGALG00010020090	MORF4L2	-0.613247508	0.053842999	-11.38954959	4.7142E-30	2.8567E-29	negative
PRJNA729271	stage_5W_vs_E10	ordinary_muscle_development	ENSGALG00010020091	SUPT7L	-0.078928644	0.08382809	-0.941553656	0.346421206	0.386289359	negative
PRJNA729271	stage_5W_vs_E10	ordinary_muscle_development	ENSGALG00010020092	SDHAF2	1.329199851	0.136905064	9.708916626	2.76258E-22	1.2242E-21	positive
PRJNA729271	stage_5W_vs_E10	ordinary_muscle_development	ENSGALG00010020094	CLEC3B	4.086874589	0.227632258	17.95384638	4.47789E-72	1.00475E-70	positive
PRJNA729271	stage_5W_vs_E10	ordinary_muscle_development	ENSGALG00010020095	LRFN2	0.166125013	0.315674457	0.526254214	0.598711591	0.636660884	positive
PRJNA729271	stage_5W_vs_E10	ordinary_muscle_development	ENSGALG00010020096	GPHN	1.842039546	0.115592266	15.93566436	3.58372E-57	5.31688E-56	positive
PRJNA729271	stage_5W_vs_E10	ordinary_muscle_development	ENSGALG00010020097	RETSAT	1.164176794	0.22858081	5.093064429	3.52322E-07	6.97855E-07	positive
PRJNA729271	stage_5W_vs_E10	ordinary_muscle_development	ENSGALG00010020098	GABRD	1.916727304	0.330179005	5.805115626	6.43216E-09	1.43503E-08	positive
PRJNA729271	stage_5W_vs_E10	ordinary_muscle_development	ENSGALG00010020100	MOCS1	2.480586897	0.157082731	15.79159513	3.55476E-56	5.09656E-55	positive
PRJNA729271	stage_5W_vs_E10	ordinary_muscle_development	ENSGALG00010020101	KIF15	-3.114552236	0.208591764	-14.9313289	2.06117E-50	2.50854E-49	negative
PRJNA729271	stage_5W_vs_E10	ordinary_muscle_development	ENSGALG00010020102	TMEM127	0.793405352	0.200251698	3.962040569	7.43119E-05	0.000124024	positive
PRJNA729271	stage_5W_vs_E10	ordinary_muscle_development	ENSGALG00010020103	PSAP	1.525820925	0.104992831	14.53262015	7.52848E-48	8.40072E-47	positive
PRJNA729271	stage_5W_vs_E10	ordinary_muscle_development	ENSGALG00010020104	BRAT1	0.229177366	0.113382429	2.021277609	0.043251036	0.054978844	positive
PRJNA729271	stage_5W_vs_E10	ordinary_muscle_development	ENSGALG00010020106	ENSGALG00010020106	2.290647871	0.162033817	14.13685066	2.25137E-45	2.32764E-44	positive
PRJNA729271	stage_5W_vs_E10	ordinary_muscle_development	ENSGALG00010020108	RPAP1	-0.624026073	0.117513058	-5.310270033	1.09463E-07	2.25347E-07	negative
PRJNA729271	stage_5W_vs_E10	ordinary_muscle_development	ENSGALG00010020109	WDFY4	2.538537494	0.358896755	7.073169255	1.51435E-12	4.18343E-12	positive
PRJNA729271	stage_5W_vs_E10	ordinary_muscle_development	ENSGALG00010020111	ADRA2B	1.433033826	0.121730428	11.77219081	5.42959E-32	3.56763E-31	positive
PRJNA729271	stage_5W_vs_E10	ordinary_muscle_development	ENSGALG00010020113	CIAO1	0.071044439	0.10444942	0.680180315	0.496390295	0.537997407	positive
PRJNA729271	stage_5W_vs_E10	ordinary_muscle_development	ENSGALG00010020116	GNA12	0.364025408	0.071245597	5.109444266	3.23108E-07	6.4236E-07	positive
PRJNA729271	stage_5W_vs_E10	ordinary_muscle_development	ENSGALG00010020117	AKAP13	-0.249239174	0.102448722	-2.432818762	0.014981798	0.020103792	negative
PRJNA729271	stage_5W_vs_E10	ordinary_muscle_development	ENSGALG00010020118	GNB1	-0.554866266	0.093390758	-5.941340219	2.82701E-09	6.43088E-09	negative
PRJNA729271	stage_5W_vs_E10	ordinary_muscle_development	ENSGALG00010020120	DAAM2	-0.652355503	0.102396047	-6.370905141	1.87916E-10	4.59875E-10	negative
PRJNA729271	stage_5W_vs_E10	ordinary_muscle_development	ENSGALG00010020121	ENSGALG00010020121	-2.718072584	0.292347302	-9.297409505	1.43909E-20	5.88092E-20	negative
PRJNA729271	stage_5W_vs_E10	ordinary_muscle_development	ENSGALG00010020123	ENSGALG00010020123	-1.077883675	0.167748402	-6.425597289	1.31353E-10	3.24409E-10	negative
PRJNA729271	stage_5W_vs_E10	ordinary_muscle_development	ENSGALG00010020125	NAV2	0.085272073	0.130382783	0.654013291	0.513103243	0.553664153	positive
PRJNA729271	stage_5W_vs_E10	ordinary_muscle_development	ENSGALG00010020126	NADK	0.30457753	0.104510011	2.914338333	0.003564434	0.005109421	positive
PRJNA729271	stage_5W_vs_E10	ordinary_muscle_development	ENSGALG00010020127	FRMD4B	-1.151146579	0.14143317	-8.139155613	3.98044E-16	1.32697E-15	negative
PRJNA729271	stage_5W_vs_E10	ordinary_muscle_development	ENSGALG00010020128	KIF6	-0.328572781	0.532301825	-0.617267809	0.537058096	0.577025951	negative
PRJNA729271	stage_5W_vs_E10	ordinary_muscle_development	ENSGALG00010020129	MGAT2	-0.360947279	0.117267663	-3.077977939	0.002084103	0.003057909	negative
PRJNA729271	stage_5W_vs_E10	ordinary_muscle_development	ENSGALG00010020130	PDXDC1	-0.11797801	0.073978288	-1.594765354	0.110764717	0.133738824	negative
PRJNA729271	stage_5W_vs_E10	ordinary_muscle_development	ENSGALG00010020131	SNRNP200	-0.461561127	0.124781546	-3.698953431	0.00021649	0.000346897	negative
PRJNA729271	stage_5W_vs_E10	ordinary_muscle_development	ENSGALG00010020132	SHC4	-0.934430618	0.234458564	-3.985482987	6.7343E-05	0.000112744	negative
PRJNA729271	stage_5W_vs_E10	ordinary_muscle_development	ENSGALG00010020133	ENSGALG00010020133	1.969681561	0.370092077	5.322139224	1.02554E-07	2.11454E-07	positive
PRJNA729271	stage_5W_vs_E10	ordinary_muscle_development	ENSGALG00010020136	KCNK17	2.18148354	0.273790057	7.967723747	1.61624E-15	5.23049E-15	positive
PRJNA729271	stage_5W_vs_E10	ordinary_muscle_development	ENSGALG00010020137	NTAN1	-0.493886671	0.085184889	-5.797820204	6.71824E-09	1.49725E-08	negative
PRJNA729271	stage_5W_vs_E10	ordinary_muscle_development	ENSGALG00010020138	ENSGALG00010020138	0.462380346	0.085215372	5.426020404	5.76244E-08	1.20843E-07	positive
PRJNA729271	stage_5W_vs_E10	ordinary_muscle_development	ENSGALG00010020140	SECISBP2L	0.708373103	0.094612797	7.487074985	7.04255E-14	2.0816E-13	positive
PRJNA729271	stage_5W_vs_E10	ordinary_muscle_development	ENSGALG00010020141	THOC7	-0.106177489	0.127816604	-0.830701847	0.406142083	0.447648809	negative
PRJNA729271	stage_5W_vs_E10	ordinary_muscle_development	ENSGALG00010020142	SLC35E2B	-1.596278597	0.071528029	-22.3168264	2.5367E-110	1.2993E-108	negative

PRJNA729271	stage_5W_vs_E10	ordinary_muscle_development	ENSGALG00010020143	KCNK5	-1.134190325	0.183124327	-6.19355355	5.88227E-10	1.39388E-09	negative
PRJNA729271	stage_5W_vs_E10	ordinary_muscle_development	ENSGALG00010020147	ABHD2	1.847461435	0.145764348	12.67430243	8.20857E-37	6.47351E-36	positive
PRJNA729271	stage_5W_vs_E10	ordinary_muscle_development	ENSGALG00010020149	FAHD2A	1.114899215	0.172454547	6.464887325	1.01374E-10	2.52434E-10	positive
PRJNA729271	stage_5W_vs_E10	ordinary_muscle_development	ENSGALG00010020152	ATXN7	0.968517619	0.107227024	9.032402301	1.67944E-19	6.56158E-19	positive
PRJNA729271	stage_5W_vs_E10	ordinary_muscle_development	ENSGALG00010020153	AXL	-0.855677221	0.122225829	-7.000788878	2.54525E-12	6.94138E-12	negative
PRJNA729271	stage_5W_vs_E10	ordinary_muscle_development	ENSGALG00010020155	MFGE8	2.353674668	0.13413083	17.5476039	6.20428E-69	1.27201E-67	positive
PRJNA729271	stage_5W_vs_E10	ordinary_muscle_development	ENSGALG00010020160	SYT7	-3.567941772	0.335954642	-10.62030798	2.39769E-26	1.26059E-25	negative
PRJNA729271	stage_5W_vs_E10	ordinary_muscle_development	ENSGALG00010020163	CDC2L1	-0.299073184	0.096105841	-3.11191476	0.001858782	0.002741178	negative
PRJNA729271	stage_5W_vs_E10	ordinary_muscle_development	ENSGALG00010020164	C10orf71	4.872609045	0.14779313	32.9691174	2.2516E-238	5.8279E-236	positive
PRJNA729271	stage_5W_vs_E10	ordinary_muscle_development	ENSGALG00010020166	HAPLN3	0.519618099	0.1714711	3.030353791	0.002442674	0.003563104	positive
PRJNA729271	stage_5W_vs_E10	ordinary_muscle_development	ENSGALG00010020168	COX7A2L	1.172108674	0.11478681	10.2111791	1.76704E-24	8.5893E-24	positive
PRJNA729271	stage_5W_vs_E10	ordinary_muscle_development	ENSGALG00010020170	SORCS1	-1.374291185	0.308241973	-4.458481661	8.25423E-06	1.47947E-05	negative
PRJNA729271	stage_5W_vs_E10	ordinary_muscle_development	ENSGALG00010020171	RRN3	-0.132165349	0.076296959	-1.732249239	0.083229196	0.102082179	negative
PRJNA729271	stage_5W_vs_E10	ordinary_muscle_development	ENSGALG00010020175	ACAN	-10.39706323	0.573025082	-18.14416777	1.42781E-73	3.3061E-72	negative
PRJNA729271	stage_5W_vs_E10	ordinary_muscle_development	ENSGALG00010020176	ENSGALG00010020176	0.211265103	0.251245697	0.840870531	0.400420469	0.441812796	positive
PRJNA729271	stage_5W_vs_E10	ordinary_muscle_development	ENSGALG00010020177	KCNG3	-3.565436543	0.291533271	-12.22994731	2.15078E-34	1.54641E-33	negative
PRJNA729271	stage_5W_vs_E10	ordinary_muscle_development	ENSGALG00010020179	PSMD6	0.610537947	0.134815976	4.528676533	5.93543E-06	1.07395E-05	positive
PRJNA729271	stage_5W_vs_E10	ordinary_muscle_development	ENSGALG00010020180	TCF7L1	-0.761659505	0.213688834	-3.564339287	0.000364774	0.000573284	negative
PRJNA729271	stage_5W_vs_E10	ordinary_muscle_development	ENSGALG00010020181	LEUTX	3.008123084	0.212917092	14.12814283	2.54775E-45	2.62844E-44	positive
PRJNA729271	stage_5W_vs_E10	ordinary_muscle_development	ENSGALG00010020183	XPNPEP1	-0.822485309	0.069682592	-11.80331107	3.7525E-32	2.48141E-31	negative
PRJNA729271	stage_5W_vs_E10	ordinary_muscle_development	ENSGALG00010020184	RSL1D1	-1.350988434	0.072406672	-18.65834179	1.08032E-77	2.81135E-76	negative
PRJNA729271	stage_5W_vs_E10	ordinary_muscle_development	ENSGALG00010020186	MGA	0.262857003	0.094838709	2.771621488	0.005577785	0.00783734	positive
PRJNA729271	stage_5W_vs_E10	ordinary_muscle_development	ENSGALG00010020187	MMP23B	-0.467477069	0.208171941	-2.245629585	0.024727744	0.032351511	negative
PRJNA729271	stage_5W_vs_E10	ordinary_muscle_development	ENSGALG00010020193	VSTM4	-2.016630716	0.181526084	-11.10931645	1.13021E-28	6.50355E-28	negative
PRJNA729271	stage_5W_vs_E10	ordinary_muscle_development	ENSGALG00010020194	PRICKLE2	-1.540249947	0.157897723	-9.754731874	1.76065E-22	7.86704E-22	negative
PRJNA729271	stage_5W_vs_E10	ordinary_muscle_development	ENSGALG00010020199	DET1	-0.165383725	0.084640488	-1.953955239	0.050706511	0.063901137	negative
PRJNA729271	stage_5W_vs_E10	ordinary_muscle_development	ENSGALG00010020202	MAPKBP1	0.189163335	0.091196029	2.074249696	0.038056127	0.048760149	positive
PRJNA729271	stage_5W_vs_E10	ordinary_muscle_development	ENSGALG00010020204	GSPT2	0.117257823	0.086581212	1.354310244	0.175637419	0.206290032	positive
PRJNA729271	stage_5W_vs_E10	ordinary_muscle_development	ENSGALG00010020206	MRPS11	0.317900199	0.081698478	3.891139792	9.97744E-05	0.0001644	positive
PRJNA729271	stage_5W_vs_E10	ordinary_muscle_development	ENSGALG00010020211	ADD3	-0.612435598	0.083508625	-7.333800533	2.23716E-13	6.43533E-13	negative
PRJNA729271	stage_5W_vs_E10	ordinary_muscle_development	ENSGALG00010020212	JMJD7	-0.100630303	0.141580975	-0.710761479	0.477232055	0.518862784	negative
PRJNA729271	stage_5W_vs_E10	ordinary_muscle_development	ENSGALG00010020215	PRMT3	-0.375606017	0.080941749	-4.6404485	3.47654E-06	6.40799E-06	negative
PRJNA729271	stage_5W_vs_E10	ordinary_muscle_development	ENSGALG00010020224	MRPL46	-0.062729307	0.0880457	-0.712463042	0.476178089	0.517872254	negative
PRJNA729271	stage_5W_vs_E10	ordinary_muscle_development	ENSGALG00010020226	TCIM	2.293454312	0.197617122	11.60554453	3.86221E-31	2.45323E-30	positive
PRJNA729271	stage_5W_vs_E10	ordinary_muscle_development	ENSGALG00010020228	TM9SF3	-0.65731874	0.08323792	-7.89686645	2.86003E-15	9.11323E-15	negative
PRJNA729271	stage_5W_vs_E10	ordinary_muscle_development	ENSGALG00010020230	ENSGALG00010020230	0.170788156	0.219218621	0.779076866	0.435934437	0.477869596	positive
PRJNA729271	stage_5W_vs_E10	ordinary_muscle_development	ENSGALG00010020236	SLC35G1	2.161515798	0.287242739	7.525049396	5.27004E-14	1.57374E-13	positive
PRJNA729271	stage_5W_vs_E10	ordinary_muscle_development	ENSGALG00010020237	MIB2	0.367234776	0.07534447	4.874077382	1.09318E-06	2.09211E-06	positive
PRJNA729271	stage_5W_vs_E10	ordinary_muscle_development	ENSGALG00010020240	SV2B	-1.10673827	0.373413789	-2.963838787	0.003038274	0.004387744	negative
PRJNA729271	stage_5W_vs_E10	ordinary_muscle_development	ENSGALG00010020244	SLC25A37	-2.202117062	0.218957085	-10.05729986	8.53059E-24	4.02248E-23	negative
PRJNA729271	stage_5W_vs_E10	ordinary_muscle_development	ENSGALG00010020246	ADAMTS9	-0.917565064	0.130085898	-7.053532128	1.74432E-12	4.80501E-12	negative
PRJNA729271	stage_5W_vs_E10	ordinary_muscle_development	ENSGALG00010020248	NOLC1	-0.714442692	0.081002692	-8.819987064	1.14473E-18	4.29978E-18	negative
PRJNA729271	stage_5W_vs_E10	ordinary_muscle_development	ENSGALG00010020250	ENSGALG00010020250	-0.197896963	0.246993107	-0.801224638	0.42300161	0.464852793	negative
PRJNA729271	stage_5W_vs_E10	ordinary_muscle_development	ENSGALG00010020252	DMTN	0.930692695	0.189080163	4.922212239	8.55713E-07	1.65271E-06	positive
PRJNA729271	stage_5W_vs_E10	ordinary_muscle_development	ENSGALG00010020254	ENSGALG00010020254	-1.430807307	0.13558521	-10.55282733	4.92911E-26	2.55261E-25	negative
PRJNA729271	stage_5W_vs_E10	ordinary_muscle_development	ENSGALG00010020257	ZNF710	1.066199333	0.16086784	6.62779666	3.40734E-11	8.75389E-11	positive
PRJNA729271	stage_5W_vs_E10	ordinary_muscle_development	ENSGALG00010020259	ENSGALG00010020259	-1.543767491	0.218118302	-7.077661428	1.46607E-12	4.05316E-12	negative
PRJNA729271	stage_5W_vs_E10	ordinary_muscle_development	ENSGALG00010020261	SSU72	0.38610953	0.091402467	4.224279097	2.39707E-05	4.14575E-05	positive
PRJNA729271	stage_5W_vs_E10	ordinary_muscle_development	ENSGALG00010020265	ENSGALG00010020265	-1.387103079	0.22979506	-6.036261514	1.57726E-09	3.64224E-09	negative
PRJNA729271	stage_5W_vs_E10	ordinary_muscle_development	ENSGALG00010020268	CPLANE2	0.007111822	0.099361141	0.071575483	0.942939752	0.95061286	positive
PRJNA729271	stage_5W_vs_E10	ordinary_muscle_development	ENSGALG00010020269	ENSGALG00010020269	-2.221859606	0.757966357	-2.931343305	0.003374996	0.004853231	negative
PRJNA729271	stage_5W_vs_E10	ordinary_muscle_development	ENSGALG00010020272	ENSGALG00010020272	-1.428472517	0.094845977	-15.06097116	2.92459E-51	3.65763E-50	negative
PRJNA729271	stage_5W_vs_E10	ordinary_muscle_development	ENSGALG00010020275	ELOVL3	-1.694985008	0.518659937	-3.268008354	0.001083072	0.00163686	negative
PRJNA729271	stage_5W_vs_E10	ordinary_muscle_development	ENSGALG00010020277	IDH2	0.616831883	0.124849379	4.940608334	7.78792E-07	1.50836E-06	positive
PRJNA729271	stage_5W_vs_E10	ordinary_muscle_development	ENSGALG00010020282	MAGI1	-0.716214239	0.137862305	-5.195141899	2.04564E-07	4.12801E-07	negative
PRJNA729271	stage_5W_vs_E10	ordinary_muscle_development	ENSGALG00010020283	FBXO42	-0.023692159	0.223210049	-0.106142889	0.915468983	0.926786067	negative
PRJNA729271	stage_5W_vs_E10	ordinary_muscle_development	ENSGALG00010020284	HEG1	2.033854624	0.116268335	17.49276472	1.62671E-68	3.28858E-67	positive
PRJNA729271	stage_5W_vs_E10	ordinary_muscle_development	ENSGALG00010020285	ADAMTS14	-3.815809113	0.275371516	-13.85694921	1.15479E-43	1.12947E-42	negative
PRJNA729271	stage_5W_vs_E10	ordinary_muscle_development	ENSGALG00010020287	GALNT16	-0.015932049	0.161714119	-0.098519842	0.921519517	0.932130175	negative
PRJNA729271	stage_5W_vs_E10	ordinary_muscle_development	ENSGALG00010020290	NELL1	-5.472978814	0.640684605	-8.542391646	1.31472E-17	4.71588E-17	negative
PRJNA729271	stage_5W_vs_E10	ordinary_muscle_development	ENSGALG00010020291	SNX29	0.354111069	0.145339439	2.436441702	0.01483256	0.019918284	positive
PRJNA729271	stage_5W_vs_E10	ordinary_muscle_development	ENSGALG00010020292	NPM2	-0.635521857	0.243090009	-2.614347913	0.008939795	0.012293172	negative
PRJNA729271	stage_5W_vs_E10	ordinary_muscle_development	ENSGALG00010020293	SEMA4B	-0.357369741	0.113685969	-3.143481495	0.00166951	0.002472621	negative
PRJNA729271	stage_5W_vs_E10	ordinary_muscle_development	ENSGALG00010020295	LGI1	-0.150697952	0.149490666	-1.008075992	0.313417982	0.352525308	negative
PRJNA729271	stage_5W_vs_E10	ordinary_muscle_development	ENSGALG00010020298	ENSGALG00010020298	-0.230935586	0.174795296	-1.321177348	0.186442237	0.218117855	negative
PRJNA729271	stage_5W_vs_E10	ordinary_muscle_development	ENSGALG00010020300	EHD4	1.255552192	0.128628873	9.761044837	1.65442E-22	7.39691E-22	positive
PRJNA729271	stage_5W_vs_E10	ordinary_muscle_development	ENSGALG00010020302	HAAO	3.183588685	1.434921763	2.218649663	0.026510568	0.034597162	positive
PRJNA729271	stage_5W_vs_E10	ordinary_muscle_development	ENSGALG00010020303	EXD2	-0.567622273	0.075564365	-7.511771859	5.83324E-14	1.7337E-13	negative
PRJNA729271	stage_5W_vs_E10	ordinary_muscle_development	ENSGALG00010020304	ZFP36L2	1.202244218	0.15521417	7.745711715	9.50479E-15	2.94826E-14	positive
PRJNA729271	stage_5W_vs_E10	ordinary_muscle_development	ENSGALG00010020305	XPO7	-0.004116405	0.138306708	-0.029762876	0.976256166	0.979703208	negative
PRJNA729271	stage_5W_vs_E10	ordinary_muscle_development	ENSGALG00010020307	SHQ1	-1.373675197	0.229781001	-5.978193123	2.25626E-09	5.16088E-09	negative
PRJNA729271	stage_5W_vs_E10	ordinary_muscle_development	ENSGALG00010020309	GXYLT2	-1.188583264	0.159922648	-7.432238503	1.06775E-13	3.12289E-13	negative
PRJNA729271	stage_5W_vs_E10	ordinary_muscle_development	ENSGALG00010020311	SGPL1	-0.539378476	0.134823712	-4.000620262	6.31767E-05	0.00010605	negative
PRJNA729271	stage_5W_vs_E10	ordinary_muscle_development	ENSGALG00010020313	FRA10AC1	-0.059776516	0.115541218	-0.517360966	0.604904203	0.642397716	negative
PRJNA729271	stage_5W_vs_E10	ordinary_muscle_development	ENSGALG00010020314	THADA	-0.352971665	0.079955476	-4.414602765	1.01196E-05	1.80311E-05	negative
PRJNA729271	stage_5W_vs_E10	ordinary_muscle_development	ENSGALG00010020315	ANO5	0.400106867	0.108427909	3.690072694	0.00022419	0.000358758	positive
PRJNA729271	stage_5W_vs_E10	ordinary_muscle_development	ENSGALG00010020316	PDPK1	0.479357691	0.112391696	4.265063254	1.99846E-05	3.47876E-05	positive
PRJNA729271	stage_5W_vs_E10	ordinary_muscle_development	ENSGALG00010020319	NTRK3	-1.005993231	0.163503714	-6.152724018	7.61633E-10	1.78899E-09	negative
PRJNA729271	stage_5W_vs_E10	ordinary_muscle_development	ENSGALG00010020321	SZRD1	0.856693946	0.169574259	5.052028256	4.37143E-07	8.61039E-07	positive
PRJNA729271	stage_5W_vs_E10	ordinary_muscle_development	ENSGALG00010020323	ARHGAP25	1.890347217	0.151159695	12.50563002	6.95462E-36	5.31124E-35	positive
PRJNA729271	stage_5W_vs_E10	ordinary_muscle_development	ENSGALG00010020324	PLEKHH2	0.727366837	0.184761462	3.936788706	8.25793E-05	0.000137269	positive
PRJNA729271	stage_5W_vs_E10	ordinary_muscle_development	ENSGALG00010020329	PPP4R2	-0.137078655	0.106123899	-1.291685069	0.19646623	0.22870045	negative
PRJNA729271	stage_5W_vs_E10	ordinary_muscle_development	ENSGALG00010020334	CABP4	0.147081596	0.50335209	0.292204202	0.770130499	0.797417071	positive
PRJNA729271	stage_5W_vs_E10	ordinary_muscle_development	ENSGALG00010020336	OSBP	0.300905416	0.134576914	2.235936357	0.02535594	0.033123139	positive
PRJNA729271	stage_5W_vs_E10	ordinary_muscle_development	ENSGALG00010020338	NECAP2	0.045407335	0.166375688	0.272920493	0.784914333	0.810640061	positive
PRJNA729271	stage_5W_vs_E10	ordinary_muscle_development	ENSGALG00010020340	DCAF5	0.711471425	0.0969511	7.338456485	2.16071E-13	6.22036E-13	positive
PRJNA729271	stage_5W_vs_E10	ordinary_muscle_development	ENSGALG00010020341	DYNC2LI1	-1.303998187	0.131885125	-9.887378813	4.7223E-23	2.1559E-22	negative
PRJNA729271	stage_5W_vs_E10	ordinary_muscle_development	ENSGALG00010020342	CPPED1	-1.173216486	0.108330325	-10.82999135	2.48179E-27	1.35391E-26	negative
PRJNA729271	stage_5W_vs_E10	ordinary_muscle_development	ENSGALG00010020343	PATL1	1.307807196	0.174030542	7.514814246	5.69918E-14	1.6956E-13	positive
PRJNA729271	stage_5W_vs_E10	ordinary_muscle_development	ENSGALG00010020345	AGBL1	-0.326403256	0.454584508	-0.718025471	0.472741582	0.514675471	negative
PRJNA729271	stage_5W_vs_E10	ordinary_muscle_development	ENSGALG00010020346	PCBD1	-1.638062843	0.277376841	-5.905550151	3.51472E-09	7.96278E-09	negative
PRJNA729271	stage_5W_vs_E10	ordinary_muscle_development	ENSGALG00010020350	PDZRN3	0.266402568	0.10812042	2.463943159	0.01374179	0.018522154	positive
PRJNA729271	stage_5W_vs_E10	ordinary_muscle_development	ENSGALG00010020351	CROCC	-0.217091483	0.149665544	-1.450510765	0.146916139	0.174538926	negative
PRJNA729271	stage_5W_vs_E10	ordinary_muscle_development	ENSGALG00010020352	ABCG5	-2.001666792	0.770747093	-2.597047476	0.009402893	0.012910385	negative
PRJNA729271	stage_5W_vs_E10	ordinary_muscle_development	ENSGALG00010020356	STX3	-0.318580631	0.398675586	-0.799097415	0.424233928	0.466030374	negative
PRJNA729271	stage_5W_vs_E10	ordinary_muscle_development	ENSGALG00010020357	RBP4	0.97340211	0.731581704	1.330544632	0.183338891	0.214781963	positive
PRJNA729271	stage_5W_vs_E10	ordinary_muscle_development	ENSGALG00010020362	CDS2	-0.102613364	0.12707479	-0.807503712	0.419376337	0.461008646	negative
PRJNA729271	stage_5W_vs_E10	ordinary_muscle_development	ENSGALG00010020364	MRPL16	1.201613897	0.11039557	10.88462063	1.36466E-27	7.5269E-27	positive
PRJNA729271	stage_5W_vs_E10	ordinary_muscle_development	ENSGALG00010020365	UNC5B	-0.811247395	0.142043571	-5.711257385	1.12144E-08	2.46817E-08	negative
PRJNA729271	stage_5W_vs_E10	ordinary_muscle_development	ENSGALG00010020369	CNTN3	-3.850054367	0.328552558	-11.71822978	1.02798E-31	6.65796E-31	negative
PRJNA729271	stage_5W_vs_E10	ordinary_muscle_development	ENSGALG00010020370	CEP55	-1.919647943	0.228792075	-8.3903603	4.84655E-17	1.68912E-16	negative
PRJNA729271	stage_5W_vs_E10	ordinary_muscle_development	ENSGALG00010020373	MS4A4A	-1.001459255	0.217302441	-4.608596428	4.05396E-06	7.43449E-06	negative
PRJNA729271	stage_5W_vs_E10	ordinary_muscle_development	ENSGALG00010020374	PCNA	-2.121407764	0.096923768	-21.88738444	3.4262E-106	1.5969E-104	negative
PRJNA729271	stage_5W_vs_E10	ordinary_muscle_development	ENSGALG00010020375	LRPPRC	-0.145122135	0.10188208	-1.424412767	0.154327021	0.182803805	negative
PRJNA729271	stage_5W_vs_E10	ordinary_muscle_development	ENSGALG00010020377	TMEM230	-0.323938476	0.099822524	-3.245144132	0.001173912	0.001766413	negative
PRJNA729271	stage_5W_vs_E10	ordinary_muscle_development	ENSGALG00010020379	COL13A1	-2.282917647	0.20139559	-11.33548977	8.7541E-30	5.24558E-29	negative
PRJNA729271	stage_5W_vs_E10	ordinary_muscle_development	ENSGALG00010020382	MFAP2	-4.856693305	0.168981803	-28.74092486	1.1761E-181	1.6236E-179	negative
PRJNA729271	stage_5W_vs_E10	ordinary_muscle_development	ENSGALG00010020383	BAAT	-0.571072594	0.106688737	-5.352698066	8.66524E-08	1.79484E-07	negative
PRJNA729271	stage_5W_vs_E10	ordinary_muscle_development	ENSGALG00010020385	SLC12A8	-3.09180587	0.483534635	-6.394176646	1.61415E-10	3.96426E-10	negative
PRJNA729271	stage_5W_vs_E10	ordinary_muscle_development	ENSGALG00010020386	PPM1B	0.896057833	0.071942985	12.45511049	1.3117E-35	9.89747E-35	positive
PRJNA729271	stage_5W_vs_E10	ordinary_muscle_development	ENSGALG00010020388	MYOF	1.808517033	0.166022735	10.89318904	1.24213E-27	6.86154E-27	positive
PRJNA729271	stage_5W_vs_E10	ordinary_muscle_development	ENSGALG00010020389	ACTN1	-0.876899881	0.157295416	-5.574859732	2.47729E-08	5.33795E-08	negative
PRJNA729271	stage_5W_vs_E10	ordinary_muscle_development	ENSGALG00010020390	KLHL25	-0.07525976	0.223563114	-0.336637646	0.736390074	0.765633405	negative
PRJNA729271	stage_5W_vs_E10	ordinary_muscle_development	ENSGALG00010020392	SLC23A2	-0.275078453	0.082399065	-3.33836864	0.000842719	0.001284865	negative
PRJNA729271	stage_5W_vs_E10	ordinary_muscle_development	ENSGALG00010020393	SHISA9	-1.833172613	0.313774362	-5.842327587	5.14765E-09	1.15593E-08	negative
PRJNA729271	stage_5W_vs_E10	ordinary_muscle_development	ENSGALG00010020396	CHL1	-0.897670908	0.197073887	-4.554996707	5.23864E-06	9.51913E-06	negative
PRJNA729271	stage_5W_vs_E10	ordinary_muscle_development	ENSGALG00010020399	ATP13A2	0.357988338	0.131074361	2.731185071	0.006310702	0.008820136	positive
PRJNA729271	stage_5W_vs_E10	ordinary_muscle_development	ENSGALG00010020402	SLC3A1	2.736733844	0.316073964	8.658523485	4.77914E-18	1.74801E-17	positive
PRJNA729271	stage_5W_vs_E10	ordinary_muscle_development	ENSGALG00010020405	UBE2I	-0.84856886	0.082239354	-10.31828211	5.82561E-25	2.89483E-24	negative
PRJNA729271	stage_5W_vs_E10	ordinary_muscle_development	ENSGALG00010020413	CYP26A1	-0.276634255	0.345304575	-0.801131162	0.423055717	0.464877009	negative
PRJNA729271	stage_5W_vs_E10	ordinary_muscle_development	ENSGALG00010020415	TSR3	-0.147024863	0.094613646	-1.55394987	0.120196375	0.144381077	negative
PRJNA729271	stage_5W_vs_E10	ordinary_muscle_development	ENSGALG00010020416	ENSGALG00010020416	-6.094424469	1.103478365	-5.522921575	3.33409E-08	7.10073E-08	negative
PRJNA729271	stage_5W_vs_E10	ordinary_muscle_development	ENSGALG00010020417	ENSGALG00010020417	5.633140652	0.744670221	7.564611146	3.89027E-14	1.16797E-13	positive
PRJNA729271	stage_5W_vs_E10	ordinary_muscle_development	ENSGALG00010020422	ERCC4	-0.220917609	0.089518743	-2.467836347	0.013593245	0.018327047	negative
PRJNA729271	stage_5W_vs_E10	ordinary_muscle_development	ENSGALG00010020428	SEMA4C	-0.055800406	0.151007713	-0.369520238	0.711739991	0.742777019	negative
PRJNA729271	stage_5W_vs_E10	ordinary_muscle_development	ENSGALG00010020429	ZNF148	0.016531829	0.102417938	0.161415369	0.871766262	0.887813671	positive
PRJNA729271	stage_5W_vs_E10	ordinary_muscle_development	ENSGALG00010020433	GNPTG	-0.450098926	0.073193842	-6.149409786	7.77718E-10	1.82559E-09	negative
PRJNA729271	stage_5W_vs_E10	ordinary_muscle_development	ENSGALG00010020436	CNTN4	-3.199062514	0.270394809	-11.83107963	2.69651E-32	1.79458E-31	negative
PRJNA729271	stage_5W_vs_E10	ordinary_muscle_development	ENSGALG00010020437	SNX4	-1.069943962	0.085201152	-12.55785789	3.59946E-36	2.78262E-35	negative
PRJNA729271	stage_5W_vs_E10	ordinary_muscle_development	ENSGALG00010020438	HTR7	0.385464669	0.281244916	1.37056582	0.170510344	0.200857167	positive
PRJNA729271	stage_5W_vs_E10	ordinary_muscle_development	ENSGALG00010020440	PAX7	-0.297539458	0.187146716	-1.589872715	0.111863499	0.134986798	negative

PRJNA729271	stage_5W_vs_E10	ordinary_muscle_development	ENSGALG00010020443	PREPL	0.341318797	0.135868648	2.512123302	0.012000715	0.016279866	positive
PRJNA729271	stage_5W_vs_E10	ordinary_muscle_development	ENSGALG00010020444	ENSGALG00010020444	-3.428253958	1.199661159	-2.857685216	0.004267435	0.006071503	negative
PRJNA729271	stage_5W_vs_E10	ordinary_muscle_development	ENSGALG00010020449	PRPF19	-0.406817082	0.097657543	-4.165751765	3.10328E-05	5.31703E-05	negative
PRJNA729271	stage_5W_vs_E10	ordinary_muscle_development	ENSGALG00010020450	ANKRD39	0.483488478	0.181389425	2.665472248	0.007688028	0.010648635	positive
PRJNA729271	stage_5W_vs_E10	ordinary_muscle_development	ENSGALG00010020453	ENSGALG00010020453	-0.717915328	0.143696476	-4.996053825	5.85153E-07	1.14341E-06	negative
PRJNA729271	stage_5W_vs_E10	ordinary_muscle_development	ENSGALG00010020455	ENSGALG00010020455	0.015774166	0.688572046	0.022908519	0.981723245	0.984507987	positive
PRJNA729271	stage_5W_vs_E10	ordinary_muscle_development	ENSGALG00010020457	IL5RA	2.38416654	0.946947441	2.517739039	0.011811079	0.016045136	positive
PRJNA729271	stage_5W_vs_E10	ordinary_muscle_development	ENSGALG00010020460	MAPK8	0.193652526	0.141096085	1.372486878	0.169911921	0.200217323	positive
PRJNA729271	stage_5W_vs_E10	ordinary_muscle_development	ENSGALG00010020462	CNNM4	0.557039918	0.181659845	3.066389932	0.002166606	0.003172537	positive
PRJNA729271	stage_5W_vs_E10	ordinary_muscle_development	ENSGALG00010020467	ALDH4A1	0.989714929	0.13078865	7.567284548	3.81107E-14	1.14514E-13	positive
PRJNA729271	stage_5W_vs_E10	ordinary_muscle_development	ENSGALG00010020468	ENSGALG00010020468	0.308751227	0.44487728	0.694014374	0.487673195	0.529108488	positive
PRJNA729271	stage_5W_vs_E10	ordinary_muscle_development	ENSGALG00010020473	TMEM109	3.300754113	0.158782604	20.7878825	5.57157E-96	2.17682E-94	positive
PRJNA729271	stage_5W_vs_E10	ordinary_muscle_development	ENSGALG00010020476	TMEM132A	-1.132067048	0.113801927	-9.947696634	2.58088E-23	1.19406E-22	negative
PRJNA729271	stage_5W_vs_E10	ordinary_muscle_development	ENSGALG00010020478	ENSGALG00010020478	3.111504455	0.661995755	4.700187926	2.59922E-06	4.83518E-06	positive
PRJNA729271	stage_5W_vs_E10	ordinary_muscle_development	ENSGALG00010020481	RPP30	-0.788743324	0.125003592	-6.309765291	2.79459E-10	6.75239E-10	negative
PRJNA729271	stage_5W_vs_E10	ordinary_muscle_development	ENSGALG00010020484	TRNT1	0.189993847	0.12457246	1.525167339	0.127217365	0.152448388	positive
PRJNA729271	stage_5W_vs_E10	ordinary_muscle_development	ENSGALG00010020485	LMAN2L	-0.347458418	0.131935635	-2.633544893	0.008449866	0.011660397	negative
PRJNA729271	stage_5W_vs_E10	ordinary_muscle_development	ENSGALG00010020486	MRTFB	1.598844356	0.111737312	14.30895668	1.92378E-46	2.04587E-45	positive
PRJNA729271	stage_5W_vs_E10	ordinary_muscle_development	ENSGALG00010020489	IFFO2	-1.330057143	0.188716778	-7.047900858	1.81637E-12	4.99872E-12	negative
PRJNA729271	stage_5W_vs_E10	ordinary_muscle_development	ENSGALG00010020493	DEPDC7	-1.404983085	0.162684902	-8.636222929	5.81021E-18	2.12085E-17	negative
PRJNA729271	stage_5W_vs_E10	ordinary_muscle_development	ENSGALG00010020497	OSBPL11	-0.283954186	0.069925321	-4.060820649	4.89005E-05	8.25834E-05	negative
PRJNA729271	stage_5W_vs_E10	ordinary_muscle_development	ENSGALG00010020498	FRMPD2	-0.029352474	0.527495439	-0.055644981	0.95562463	0.96166197	negative
PRJNA729271	stage_5W_vs_E10	ordinary_muscle_development	ENSGALG00010020507	ANKRD1	8.148613512	0.585285375	13.9224622	4.62686E-44	4.58416E-43	positive
PRJNA729271	stage_5W_vs_E10	ordinary_muscle_development	ENSGALG00010020509	CRBN	0.805266928	0.102820309	7.831788645	4.80978E-15	1.5133E-14	positive
PRJNA729271	stage_5W_vs_E10	ordinary_muscle_development	ENSGALG00010020514	CAMKMT	-1.169095114	0.141299711	-8.273867725	1.29682E-16	4.425E-16	negative
PRJNA729271	stage_5W_vs_E10	ordinary_muscle_development	ENSGALG00010020517	UBR4	0.758578174	0.094096208	8.061729474	7.52225E-16	2.47245E-15	positive
PRJNA729271	stage_5W_vs_E10	ordinary_muscle_development	ENSGALG00010020520	ARHGAP22	-0.081426493	0.200453389	-0.406211608	0.684587125	0.717971955	negative
PRJNA729271	stage_5W_vs_E10	ordinary_muscle_development	ENSGALG00010020521	ENSGALG00010020521	0.004219165	0.838421866	0.005032269	0.995984847	0.996466065	positive
PRJNA729271	stage_5W_vs_E10	ordinary_muscle_development	ENSGALG00010020524	ENSGALG00010020524	0.337150428	0.406271536	0.829864753	0.406615255	0.448102191	positive
PRJNA729271	stage_5W_vs_E10	ordinary_muscle_development	ENSGALG00010020525	ENSGALG00010020525	1.376802308	0.722648059	1.905218301	0.0567517	0.07105605	positive
PRJNA729271	stage_5W_vs_E10	ordinary_muscle_development	ENSGALG00010020530	SRSF5A	-0.734575094	0.069644679	-10.54746901	5.21839E-26	2.69856E-25	negative
PRJNA729271	stage_5W_vs_E10	ordinary_muscle_development	ENSGALG00010020533	ENSGALG00010020533	0.015664899	0.097964374	0.159904042	0.872956665	0.888776207	positive
PRJNA729271	stage_5W_vs_E10	ordinary_muscle_development	ENSGALG00010020535	LMLN	-0.72089743	0.15471846	-4.659414448	3.1711E-06	5.86889E-06	negative
PRJNA729271	stage_5W_vs_E10	ordinary_muscle_development	ENSGALG00010020537	CCDC167	-0.977709568	0.10051933	-9.726582668	2.32265E-22	1.03304E-21	negative
PRJNA729271	stage_5W_vs_E10	ordinary_muscle_development	ENSGALG00010020540	PCGF5	1.861544439	0.161532871	11.52424536	9.95809E-31	6.21898E-30	positive
PRJNA729271	stage_5W_vs_E10	ordinary_muscle_development	ENSGALG00010020544	BMERB1	0.357457825	0.163243298	2.189724347	0.028544234	0.037114162	positive
PRJNA729271	stage_5W_vs_E10	ordinary_muscle_development	ENSGALG00010020545	STYX	1.626778047	0.141384939	11.50602077	1.23025E-30	7.65671E-30	positive
PRJNA729271	stage_5W_vs_E10	ordinary_muscle_development	ENSGALG00010020546	LRRC20	3.007770824	0.236666582	12.70889534	5.27747E-37	4.19621E-36	positive
PRJNA729271	stage_5W_vs_E10	ordinary_muscle_development	ENSGALG00010020548	CMTR1	0.409715729	0.18492535	2.215573633	0.026720716	0.034840045	positive
PRJNA729271	stage_5W_vs_E10	ordinary_muscle_development	ENSGALG00010020550	HECTD2	1.417722864	0.212641877	6.667185619	2.60755E-11	6.73854E-11	positive
PRJNA729271	stage_5W_vs_E10	ordinary_muscle_development	ENSGALG00010020554	ZFP36L1	0.904247728	0.210691849	4.29180213	1.77229E-05	3.09846E-05	positive
PRJNA729271	stage_5W_vs_E10	ordinary_muscle_development	ENSGALG00010020556	EMC1	-0.146520741	0.086031078	-1.703114098	0.088546718	0.108247	negative
PRJNA729271	stage_5W_vs_E10	ordinary_muscle_development	ENSGALG00010020557	ENSGALG00010020557	3.878953161	0.255927446	15.15645634	6.86761E-52	8.83283E-51	positive
PRJNA729271	stage_5W_vs_E10	ordinary_muscle_development	ENSGALG00010020558	EIF4EBP2	0.228875519	0.100555964	2.276100891	0.022839972	0.029976946	positive
PRJNA729271	stage_5W_vs_E10	ordinary_muscle_development	ENSGALG00010020562	LRRN1	-4.936346355	0.237808247	-20.75767519	1.04501E-95	4.05012E-94	negative
PRJNA729271	stage_5W_vs_E10	ordinary_muscle_development	ENSGALG00010020568	PLA2G4F	2.605265322	0.379993376	6.856080893	7.07753E-12	1.88617E-11	positive
PRJNA729271	stage_5W_vs_E10	ordinary_muscle_development	ENSGALG00010020569	DTX3L	3.802108759	0.250118197	15.20124807	3.46947E-52	4.49419E-51	positive
PRJNA729271	stage_5W_vs_E10	ordinary_muscle_development	ENSGALG00010020571	MRTO4	-0.758446206	0.083184647	-9.117622458	7.67904E-20	3.04953E-19	negative
PRJNA729271	stage_5W_vs_E10	ordinary_muscle_development	ENSGALG00010020572	PPP1R3C	3.006185436	0.271880692	11.0570023	2.02761E-28	1.15437E-27	positive
PRJNA729271	stage_5W_vs_E10	ordinary_muscle_development	ENSGALG00010020574	DTL	-3.192628963	0.19184573	-16.64164726	3.47914E-62	5.93995E-61	negative
PRJNA729271	stage_5W_vs_E10	ordinary_muscle_development	ENSGALG00010020576	AKR7L	0.599514914	0.104476482	5.738276248	9.5645E-09	2.1137E-08	positive
PRJNA729271	stage_5W_vs_E10	ordinary_muscle_development	ENSGALG00010020578	TNKS2	0.241126354	0.063175049	3.816797284	0.000135195	0.000220161	positive
PRJNA729271	stage_5W_vs_E10	ordinary_muscle_development	ENSGALG00010020580	ENSGALG00010020580	-0.431810961	0.116539596	-3.70527249	0.000211164	0.000338623	negative
PRJNA729271	stage_5W_vs_E10	ordinary_muscle_development	ENSGALG00010020582	KANSL3	0.748519915	0.14820745	5.050487784	4.40683E-07	8.67541E-07	positive
PRJNA729271	stage_5W_vs_E10	ordinary_muscle_development	ENSGALG00010020584	PARP9	3.598956683	0.287492642	12.5184306	5.9194E-36	4.54458E-35	positive
PRJNA729271	stage_5W_vs_E10	ordinary_muscle_development	ENSGALG00010020586	C11orf16	0.696712723	0.097548426	7.142224112	9.18327E-13	2.56823E-12	positive
PRJNA729271	stage_5W_vs_E10	ordinary_muscle_development	ENSGALG00010020588	PALD1	-0.633925739	0.109852504	-5.770699014	7.89434E-09	1.75342E-08	negative
PRJNA729271	stage_5W_vs_E10	ordinary_muscle_development	ENSGALG00010020590	INTS7	-0.983061488	0.080323062	-12.23884485	1.92756E-34	1.38936E-33	negative
PRJNA729271	stage_5W_vs_E10	ordinary_muscle_development	ENSGALG00010020594	PQLC2	0.030588384	0.150402202	0.203377234	0.838840193	0.858503749	positive
PRJNA729271	stage_5W_vs_E10	ordinary_muscle_development	ENSGALG00010020595	ZFYVE27	0.462151292	0.134031321	3.448084287	0.000564578	0.000873239	positive
PRJNA729271	stage_5W_vs_E10	ordinary_muscle_development	ENSGALG00010020596	SUMF1	0.5908213	0.120747863	4.89301661	9.9302E-07	1.90849E-06	positive
PRJNA729271	stage_5W_vs_E10	ordinary_muscle_development	ENSGALG00010020597	ARID5A	1.85768386	0.238422097	7.791575886	6.61785E-15	2.06603E-14	positive
PRJNA729271	stage_5W_vs_E10	ordinary_muscle_development	ENSGALG00010020599	PARN	-1.404383077	0.068973299	-20.36125729	3.68993E-92	1.3239E-90	negative
PRJNA729271	stage_5W_vs_E10	ordinary_muscle_development	ENSGALG00010020601	AKIP1	-1.390739387	0.181239572	-7.67348639	1.67383E-14	5.11141E-14	negative
PRJNA729271	stage_5W_vs_E10	ordinary_muscle_development	ENSGALG00010020602	DENND2B	-0.91360522	0.097854969	-9.336319181	9.97413E-21	4.10491E-20	negative
PRJNA729271	stage_5W_vs_E10	ordinary_muscle_development	ENSGALG00010020603	VPS39	0.551875817	0.092811483	5.946201879	2.74436E-09	6.24775E-09	positive
PRJNA729271	stage_5W_vs_E10	ordinary_muscle_development	ENSGALG00010020605	STAMBP	-0.198368497	0.089884044	-2.206937821	0.027318404	0.035577741	negative
PRJNA729271	stage_5W_vs_E10	ordinary_muscle_development	ENSGALG00010020607	ENSGALG00010020607	3.977905551	0.238482018	16.68010686	1.82891E-62	3.13729E-61	positive
PRJNA729271	stage_5W_vs_E10	ordinary_muscle_development	ENSGALG00010020608	CAPZB	1.032223911	0.099406692	10.38384732	2.93696E-25	1.46949E-24	positive
PRJNA729271	stage_5W_vs_E10	ordinary_muscle_development	ENSGALG00010020609	AVPI1	-0.427433166	0.235927002	-1.811717871	0.070029801	0.086833359	negative
PRJNA729271	stage_5W_vs_E10	ordinary_muscle_development	ENSGALG00010020611	LPGAT1	-0.166271713	0.099796061	-1.666114982	0.095690515	0.116577073	negative
PRJNA729271	stage_5W_vs_E10	ordinary_muscle_development	ENSGALG00010020612	RPL27A	-0.036222654	0.192809977	-0.187867115	0.850980822	0.869026844	negative
PRJNA729271	stage_5W_vs_E10	ordinary_muscle_development	ENSGALG00010020613	ACTG2	0.254127747	0.251901348	1.008838377	0.313052153	0.35217968	positive
PRJNA729271	stage_5W_vs_E10	ordinary_muscle_development	ENSGALG00010020614	FAIM	-0.277222009	0.110485669	-2.509121875	0.012103172	0.016411176	negative
PRJNA729271	stage_5W_vs_E10	ordinary_muscle_development	ENSGALG00010020616	PI4K2A	0.999715727	0.130650459	7.651834799	1.98132E-14	6.02838E-14	positive
PRJNA729271	stage_5W_vs_E10	ordinary_muscle_development	ENSGALG00010020617	DGUOK	-0.762331866	0.117599784	-6.482425729	9.02595E-11	2.25532E-10	negative
PRJNA729271	stage_5W_vs_E10	ordinary_muscle_development	ENSGALG00010020618	ENSGALG00010020618	-0.359131499	0.329885695	-1.088654356	0.276306337	0.313483121	negative
PRJNA729271	stage_5W_vs_E10	ordinary_muscle_development	ENSGALG00010020619	QARS	-0.233164107	0.100003265	-2.331564952	0.019723589	0.026085167	negative
PRJNA729271	stage_5W_vs_E10	ordinary_muscle_development	ENSGALG00010020620	MARF1	0.704444977	0.103074337	6.834339167	8.23839E-12	2.1879E-11	positive
PRJNA729271	stage_5W_vs_E10	ordinary_muscle_development	ENSGALG00010020622	ABCG2	1.748798345	0.116512526	15.00953077	6.3596E-51	7.88558E-50	positive
PRJNA729271	stage_5W_vs_E10	ordinary_muscle_development	ENSGALG00010020623	NEK2	-2.27179672	0.228583792	-9.938573053	2.8285E-23	1.30529E-22	negative
PRJNA729271	stage_5W_vs_E10	ordinary_muscle_development	ENSGALG00010020624	MINOS1	0.766145413	0.135922545	5.636632335	1.73408E-08	3.7707E-08	positive
PRJNA729271	stage_5W_vs_E10	ordinary_muscle_development	ENSGALG00010020625	SLC30A1	-0.336621031	0.076804888	-4.38280738	1.1716E-05	2.07735E-05	negative
PRJNA729271	stage_5W_vs_E10	ordinary_muscle_development	ENSGALG00010020626	MORN4	-0.572132458	0.119521165	-4.786871503	1.69401E-06	3.1939E-06	negative
PRJNA729271	stage_5W_vs_E10	ordinary_muscle_development	ENSGALG00010020629	TRIM66	-2.861961382	0.247233664	-11.57593728	5.45716E-31	3.44519E-30	negative
PRJNA729271	stage_5W_vs_E10	ordinary_muscle_development	ENSGALG00010020630	QRICH1	0.096079029	0.126124303	0.761780454	0.446191048	0.488005677	positive
PRJNA729271	stage_5W_vs_E10	ordinary_muscle_development	ENSGALG00010020632	BLOC1S2	-0.315218913	0.114143198	-2.761609261	0.005751727	0.008070011	negative
PRJNA729271	stage_5W_vs_E10	ordinary_muscle_development	ENSGALG00010020633	HOGA1	-0.45200248	0.368397269	-1.226943081	0.219843985	0.253874965	negative
PRJNA729271	stage_5W_vs_E10	ordinary_muscle_development	ENSGALG00010020635	NDE1	-0.426284076	0.13417165	-3.177154613	0.001487277	0.002214038	negative
PRJNA729271	stage_5W_vs_E10	ordinary_muscle_development	ENSGALG00010020636	NBL1	-0.694552585	0.178734141	-3.885953638	0.000101929	0.000167798	negative
PRJNA729271	stage_5W_vs_E10	ordinary_muscle_development	ENSGALG00010020638	IMPDH2	1.02594663	0.111453611	9.205144787	3.41211E-20	1.37423E-19	positive
PRJNA729271	stage_5W_vs_E10	ordinary_muscle_development	ENSGALG00010020640	PTPN14	-0.388358567	0.092611191	-4.193430227	2.74768E-05	4.72507E-05	negative
PRJNA729271	stage_5W_vs_E10	ordinary_muscle_development	ENSGALG00010020642	ANKRD2	4.191808283	0.628758941	6.666797102	2.61446E-11	6.75519E-11	positive
PRJNA729271	stage_5W_vs_E10	ordinary_muscle_development	ENSGALG00010020646	CEP70	-0.839414809	0.117711899	-7.13109564	9.95731E-13	2.7772E-12	negative
PRJNA729271	stage_5W_vs_E10	ordinary_muscle_development	ENSGALG00010020648	TET3	-0.687131763	0.150868657	-4.554503091	5.25096E-06	9.53911E-06	negative
PRJNA729271	stage_5W_vs_E10	ordinary_muscle_development	ENSGALG00010020649	MYH11	3.799816449	0.21198116	17.92525546	7.49057E-72	1.67555E-70	positive
PRJNA729271	stage_5W_vs_E10	ordinary_muscle_development	ENSGALG00010020651	LMO1	-1.025995409	0.194471865	-5.275803811	1.32175E-07	2.70605E-07	negative
PRJNA729271	stage_5W_vs_E10	ordinary_muscle_development	ENSGALG00010020653	NDUFAF3	0.910337292	0.118568867	7.677709262	1.61959E-14	4.95063E-14	positive
PRJNA729271	stage_5W_vs_E10	ordinary_muscle_development	ENSGALG00010020654	SAYSD1	0.117552153	0.149196009	0.787904141	0.430752779	0.472582617	positive
PRJNA729271	stage_5W_vs_E10	ordinary_muscle_development	ENSGALG00010020655	RIC3	-1.847510921	0.142864606	-12.93190082	2.97375E-38	2.44218E-37	negative
PRJNA729271	stage_5W_vs_E10	ordinary_muscle_development	ENSGALG00010020657	DALRD3	0.640965687	0.093140193	6.881730318	5.91299E-12	1.5828E-11	positive
PRJNA729271	stage_5W_vs_E10	ordinary_muscle_development	ENSGALG00010020659	RAD51B	-0.833987028	0.136569434	-6.106688768	1.01719E-09	2.37312E-09	negative
PRJNA729271	stage_5W_vs_E10	ordinary_muscle_development	ENSGALG00010020662	BFAR	0.01872066	0.087173669	0.21475132	0.829961208	0.850317198	positive
PRJNA729271	stage_5W_vs_E10	ordinary_muscle_development	ENSGALG00010020664	ESYT3	1.696089871	0.4342069	3.906178996	9.37671E-05	0.000154889	positive
PRJNA729271	stage_5W_vs_E10	ordinary_muscle_development	ENSGALG00010020666	TMCO4	1.100266541	0.205455461	5.355255763	8.54355E-08	1.77039E-07	positive
PRJNA729271	stage_5W_vs_E10	ordinary_muscle_development	ENSGALG00010020667	UBTD1	-0.111652206	0.157692487	-0.708037578	0.478921922	0.520582921	negative
PRJNA729271	stage_5W_vs_E10	ordinary_muscle_development	ENSGALG00010020670	ENSGALG00010020670	-0.224860666	0.135129792	-1.664034725	0.09610549	0.117023612	negative
PRJNA729271	stage_5W_vs_E10	ordinary_muscle_development	ENSGALG00010020673	MMS19	-0.102051614	0.128364219	-0.795016044	0.42660417	0.468350181	negative
PRJNA729271	stage_5W_vs_E10	ordinary_muscle_development	ENSGALG00010020674	TRPV4	-8.159798901	0.94523584	-8.632553439	5.99973E-18	2.18838E-17	negative
PRJNA729271	stage_5W_vs_E10	ordinary_muscle_development	ENSGALG00010020675	SMYD2	-0.587884417	0.179110484	-3.282244592	0.001029842	0.001559829	negative
PRJNA729271	stage_5W_vs_E10	ordinary_muscle_development	ENSGALG00010020676	CYP2C23a	7.66270945	1.372273209	5.583953256	2.35112E-08	5.0721E-08	positive
PRJNA729271	stage_5W_vs_E10	ordinary_muscle_development	ENSGALG00010020677	CYR61	1.400709826	0.232754092	6.01798152	1.76605E-09	4.06462E-09	positive
PRJNA729271	stage_5W_vs_E10	ordinary_muscle_development	ENSGALG00010020679	OTUD3	0.408060919	0.108370468	3.765425466	0.000166266	0.000268676	positive
PRJNA729271	stage_5W_vs_E10	ordinary_muscle_development	ENSGALG00010020680	CFAP221	0.354114124	0.521419045	0.679135385	0.497052083	0.538513338	positive
PRJNA729271	stage_5W_vs_E10	ordinary_muscle_development	ENSGALG00010020681	BCL10	-0.236336572	0.088421085	-2.672853111	0.007520917	0.010430128	negative
PRJNA729271	stage_5W_vs_E10	ordinary_muscle_development	ENSGALG00010020682	ZDHHC16	0.954598566	0.167199887	5.709325427	1.13425E-08	2.49483E-08	positive
PRJNA729271	stage_5W_vs_E10	ordinary_muscle_development	ENSGALG00010020684	P4HTM	1.348661184	0.120894457	11.15569081	6.71691E-29	3.89602E-28	positive
PRJNA729271	stage_5W_vs_E10	ordinary_muscle_development	ENSGALG00010020685	RPL6	-0.27720803	0.116091115	-2.387848802	0.016947312	0.022596936	negative
PRJNA729271	stage_5W_vs_E10	ordinary_muscle_development	ENSGALG00010020686	C1orf52	-0.242448869	0.12516116	-1.937093493	0.052733916	0.06632512	negative
PRJNA729271	stage_5W_vs_E10	ordinary_muscle_development	ENSGALG00010020691	ENSGALG00010020691	1.320934071	0.163015638	8.103112613	5.35706E-16	1.77244E-15	positive
PRJNA729271	stage_5W_vs_E10	ordinary_muscle_development	ENSGALG00010020692	TUB	-0.68321434	0.154726308	-4.415631379	1.00716E-05	1.79566E-05	negative
PRJNA729271	stage_5W_vs_E10	ordinary_muscle_development	ENSGALG00010020693	PTGFR	-0.454621674	0.190487789	-2.386618468	0.017004128	0.022666437	negative
PRJNA729271	stage_5W_vs_E10	ordinary_muscle_development	ENSGALG00010020696	MOB1A	-0.824523272	0.101946156	-8.087830882	6.07367E-16	2.00314E-15	negative
PRJNA729271	stage_5W_vs_E10	ordinary_muscle_development	ENSGALG00010020699	PLA2G10	0.01718204	0.416340766	0.041269176	0.967081306	0.971639532	positive
PRJNA729271	stage_5W_vs_E10	ordinary_muscle_development	ENSGALG00010020700	MTHFD2	1.226848463	0.132165038	9.282700506	1.65236E-20	6.73917E-20	positive
PRJNA729271	stage_5W_vs_E10	ordinary_muscle_development	ENSGALG00010020701	PTPN11	-0.644931684	0.085401193	-7.551787801	4.29324E-14	1.28575E-13	negative
PRJNA729271	stage_5W_vs_E10	ordinary_muscle_development	ENSGALG00010020704	PROX1	0.468617131	0.236597239	1.980653423	0.047630153	0.060212722	positive
PRJNA729271	stage_5W_vs_E10	ordinary_muscle_development	ENSGALG00010020705	EXOSC1	1.763290265	0.172981794	10.19350199	2.11987E-24	1.02528E-23	positive
PRJNA729271	stage_5W_vs_E10	ordinary_muscle_development	ENSGALG00010020707	SLC4A5	-2.745864895	0.580645227	-4.728989008	2.25641E-06	4.21803E-06	negative
PRJNA729271	stage_5W_vs_E10	ordinary_muscle_development	ENSGALG00010020708	FAM214A	0.027101928	0.134232645	0.201902658	0.839992824	0.859258714	positive
PRJNA729271	stage_5W_vs_E10	ordinary_muscle_development	ENSGALG00010020712	ZC3H7A	-0.94734275	0.071781847	-13.19752548	9.06646E-40	7.86937E-39	negative

PRJNA729271	stage_5W_vs_E10	ordinary_muscle_development	ENSGALG00010020713	SCTR	-4.624369727	1.330124724	-3.476643689	0.000507732	0.000788132	negative
PRJNA729271	stage_5W_vs_E10	ordinary_muscle_development	ENSGALG00010020714	PGAM1	4.70152189	0.144443299	32.54925586	2.1452E-232	5.0152E-230	positive
PRJNA729271	stage_5W_vs_E10	ordinary_muscle_development	ENSGALG00010020715	ENSGALG00010020715	-0.619350818	0.860461745	-0.719788906	0.471654976	0.51360821	negative
PRJNA729271	stage_5W_vs_E10	ordinary_muscle_development	ENSGALG00010020716	RRAS	0.346847903	0.179532583	1.931949604	0.053365726	0.067071552	positive
PRJNA729271	stage_5W_vs_E10	ordinary_muscle_development	ENSGALG00010020719	RPH3A	-1.953231052	0.669363585	-2.918041997	0.003522369	0.005052121	negative
PRJNA729271	stage_5W_vs_E10	ordinary_muscle_development	ENSGALG00010020720	ENSGALG00010020720	-5.340223629	0.501759689	-10.64299054	1.87997E-26	9.9308E-26	negative
PRJNA729271	stage_5W_vs_E10	ordinary_muscle_development	ENSGALG00010020721	PTDSS2	0.15651615	0.170151078	0.919865752	0.357642918	0.397884265	positive
PRJNA729271	stage_5W_vs_E10	ordinary_muscle_development	ENSGALG00010020723	RPS24	-0.244461034	0.190133356	-1.285734597	0.198535702	0.230905481	negative
PRJNA729271	stage_5W_vs_E10	ordinary_muscle_development	ENSGALG00010020724	TACR1	-3.228207506	0.284237009	-11.35744961	6.81039E-30	4.10635E-29	negative
PRJNA729271	stage_5W_vs_E10	ordinary_muscle_development	ENSGALG00010020725	FOPNL	-0.785299251	0.113567783	-6.914806576	4.68502E-12	1.25875E-11	negative
PRJNA729271	stage_5W_vs_E10	ordinary_muscle_development	ENSGALG00010020728	TMEM37	1.598155196	0.208389213	7.669087935	1.73224E-14	5.28383E-14	positive
PRJNA729271	stage_5W_vs_E10	ordinary_muscle_development	ENSGALG00010020730	RRP12	0.38035781	0.461851636	0.823549773	0.410195425	0.451738542	positive
PRJNA729271	stage_5W_vs_E10	ordinary_muscle_development	ENSGALG00010020732	ARIH2	1.850741632	0.142699162	12.96953398	1.82134E-38	1.50601E-37	positive
PRJNA729271	stage_5W_vs_E10	ordinary_muscle_development	ENSGALG00010020733	TMEM229B	-1.198253435	0.109372107	-10.95574976	6.23598E-28	3.48192E-27	negative
PRJNA729271	stage_5W_vs_E10	ordinary_muscle_development	ENSGALG00010020736	LRRTM4	-0.494236581	0.229513084	-2.153413535	0.031286193	0.040508563	negative
PRJNA729271	stage_5W_vs_E10	ordinary_muscle_development	ENSGALG00010020737	POLR3A	-0.458313119	0.116519192	-3.933370218	8.37631E-05	0.000139077	negative
PRJNA729271	stage_5W_vs_E10	ordinary_muscle_development	ENSGALG00010020739	PKP3	-4.50863718	0.762267989	-5.914766521	3.32347E-09	7.53655E-09	negative
PRJNA729271	stage_5W_vs_E10	ordinary_muscle_development	ENSGALG00010020742	THAP10	-1.42470927	0.209602177	-6.797206443	1.06667E-11	2.82143E-11	negative
PRJNA729271	stage_5W_vs_E10	ordinary_muscle_development	ENSGALG00010020743	PURB	0.941499049	0.167399008	5.624280922	1.86282E-08	4.04397E-08	positive
PRJNA729271	stage_5W_vs_E10	ordinary_muscle_development	ENSGALG00010020744	CHST3	-1.803104981	0.120599387	-14.95119522	1.52979E-50	1.87283E-49	negative
PRJNA729271	stage_5W_vs_E10	ordinary_muscle_development	ENSGALG00010020745	ABCC1	1.202865273	0.166305304	7.232873762	4.72879E-13	1.34032E-12	positive
PRJNA729271	stage_5W_vs_E10	ordinary_muscle_development	ENSGALG00010020747	DBI	-0.477129947	0.121392938	-3.930458839	8.47839E-05	0.000140692	negative
PRJNA729271	stage_5W_vs_E10	ordinary_muscle_development	ENSGALG00010020750	AIFM2	0.8525993	0.286126763	2.979795707	0.002884407	0.004175103	positive
PRJNA729271	stage_5W_vs_E10	ordinary_muscle_development	ENSGALG00010020753	SYDE2	1.653794091	0.095628077	17.29402228	5.21808E-67	1.01935E-65	positive
PRJNA729271	stage_5W_vs_E10	ordinary_muscle_development	ENSGALG00010020754	SLC25A20	-0.675494336	0.115253616	-5.86093835	4.60259E-09	1.03578E-08	negative
PRJNA729271	stage_5W_vs_E10	ordinary_muscle_development	ENSGALG00010020756	MACROH2A2	-4.794053663	0.175236298	-27.35765201	8.7571E-165	1.0757E-162	negative
PRJNA729271	stage_5W_vs_E10	ordinary_muscle_development	ENSGALG00010020759	ARHGAP19	-2.958641511	0.175101394	-16.89673304	4.75534E-64	8.57321E-63	negative
PRJNA729271	stage_5W_vs_E10	ordinary_muscle_development	ENSGALG00010020760	TXNDC11	-0.397690971	0.096201683	-4.133929448	3.56613E-05	6.07773E-05	negative
PRJNA729271	stage_5W_vs_E10	ordinary_muscle_development	ENSGALG00010020762	TTBK2	0.065294458	0.141761428	0.460593966	0.645089942	0.680141017	positive
PRJNA729271	stage_5W_vs_E10	ordinary_muscle_development	ENSGALG00010020764	PRKAR2A	0.096439843	0.098930063	0.974828471	0.329645352	0.369289615	positive
PRJNA729271	stage_5W_vs_E10	ordinary_muscle_development	ENSGALG00010020765	GLT1D1	-2.850841944	0.219490367	-12.98846044	1.4226E-38	1.17967E-37	negative
PRJNA729271	stage_5W_vs_E10	ordinary_muscle_development	ENSGALG00010020766	C2orf76	0.250792391	0.121379481	2.066184403	0.038811071	0.049670358	positive
PRJNA729271	stage_5W_vs_E10	ordinary_muscle_development	ENSGALG00010020767	UBXN10	-0.488855253	0.178084599	-2.745073169	0.006049741	0.008471741	negative
PRJNA729271	stage_5W_vs_E10	ordinary_muscle_development	ENSGALG00010020768	DNAH8	-0.250028771	0.653152532	-0.382803034	0.701865811	0.734180915	negative
PRJNA729271	stage_5W_vs_E10	ordinary_muscle_development	ENSGALG00010020770	B4GALNT4	-4.515396815	0.204886369	-22.03854186	1.2304E-107	5.8665E-106	negative
PRJNA729271	stage_5W_vs_E10	ordinary_muscle_development	ENSGALG00010020771	LINGO1	3.801261641	0.199830545	19.02242546	1.11218E-80	3.05322E-79	positive
PRJNA729271	stage_5W_vs_E10	ordinary_muscle_development	ENSGALG00010020773	ZCCHC24	1.139353503	0.08944566	12.73794066	3.63873E-37	2.90824E-36	positive
PRJNA729271	stage_5W_vs_E10	ordinary_muscle_development	ENSGALG00010020775	UBR1	1.245727431	0.080864639	15.40509477	1.51267E-53	2.00605E-52	positive
PRJNA729271	stage_5W_vs_E10	ordinary_muscle_development	ENSGALG00010020776	MCOLN3	0.083378911	0.440203402	0.189409965	0.849771515	0.867914184	positive
PRJNA729271	stage_5W_vs_E10	ordinary_muscle_development	ENSGALG00010020778	SLIT1	-1.363807202	0.226763749	-6.014220561	1.80754E-09	4.15614E-09	negative
PRJNA729271	stage_5W_vs_E10	ordinary_muscle_development	ENSGALG00010020779	SLC15A4	0.118972601	0.06577598	1.808754527	0.070489148	0.087365558	positive
PRJNA729271	stage_5W_vs_E10	ordinary_muscle_development	ENSGALG00010020781	SNN	-0.409786236	0.139487703	-2.937794712	0.003305558	0.004756683	negative
PRJNA729271	stage_5W_vs_E10	ordinary_muscle_development	ENSGALG00010020782	PPIF	-0.265519711	0.158641442	-1.673709638	0.094187679	0.114801901	negative
PRJNA729271	stage_5W_vs_E10	ordinary_muscle_development	ENSGALG00010020786	DDX19	-0.284726575	0.131418869	-2.166557796	0.030268588	0.039250598	negative
PRJNA729271	stage_5W_vs_E10	ordinary_muscle_development	ENSGALG00010020787	TMEM132C	-4.018403811	0.307070983	-13.08623748	3.9468E-39	3.3319E-38	negative
PRJNA729271	stage_5W_vs_E10	ordinary_muscle_development	ENSGALG00010020790	PRPH2L	2.714704308	0.304071314	8.927854036	4.34352E-19	1.66383E-18	positive
PRJNA729271	stage_5W_vs_E10	ordinary_muscle_development	ENSGALG00010020794	STEAP3	0.182445706	0.141486893	1.289488388	0.197228351	0.229550742	positive
PRJNA729271	stage_5W_vs_E10	ordinary_muscle_development	ENSGALG00010020797	DGCR8	-0.990667206	0.067085869	-14.76715165	2.38585E-49	2.8254E-48	negative
PRJNA729271	stage_5W_vs_E10	ordinary_muscle_development	ENSGALG00010020798	C1QL2	-1.159546415	0.568820672	-2.038509624	0.041498993	0.052886224	negative
PRJNA729271	stage_5W_vs_E10	ordinary_muscle_development	ENSGALG00010020803	ABCC6	2.214957011	0.15690907	14.11618214	3.0191E-45	3.10368E-44	positive
PRJNA729271	stage_5W_vs_E10	ordinary_muscle_development	ENSGALG00010020806	HTD2	0.429426392	0.173289796	2.478082394	0.013209064	0.017838943	positive
PRJNA729271	stage_5W_vs_E10	ordinary_muscle_development	ENSGALG00010020809	ENSGALG00010020809	0.436170658	0.221559619	1.968637875	0.04899469	0.061835266	positive
PRJNA729271	stage_5W_vs_E10	ordinary_muscle_development	ENSGALG00010020810	IFITM5	1.393981674	0.241924558	5.762051139	8.30978E-09	1.84316E-08	positive
PRJNA729271	stage_5W_vs_E10	ordinary_muscle_development	ENSGALG00010020811	MCOLN2	0.426300243	0.657306161	0.64855659	0.516625022	0.557095647	positive
PRJNA729271	stage_5W_vs_E10	ordinary_muscle_development	ENSGALG00010020813	NPY4R	1.217367401	0.542724151	2.243068415	0.024892403	0.03255575	positive
PRJNA729271	stage_5W_vs_E10	ordinary_muscle_development	ENSGALG00010020816	TRMT2A	0.117471601	0.090865316	1.292810136	0.196076734	0.228338737	positive
PRJNA729271	stage_5W_vs_E10	ordinary_muscle_development	ENSGALG00010020818	TMEM62	0.719916038	0.195454128	3.683299221	0.000230235	0.000368066	positive
PRJNA729271	stage_5W_vs_E10	ordinary_muscle_development	ENSGALG00010020820	VWA5B1	-0.583578885	0.476747788	-1.224083047	0.2209209	0.254915495	negative
PRJNA729271	stage_5W_vs_E10	ordinary_muscle_development	ENSGALG00010020827	ENSGALG00010020827	-0.507901661	0.092182122	-5.509763187	3.59317E-08	7.63456E-08	negative
PRJNA729271	stage_5W_vs_E10	ordinary_muscle_development	ENSGALG00010020828	CAMK2N1	-0.476136283	0.228072695	-2.087651408	0.036829291	0.047305323	negative
PRJNA729271	stage_5W_vs_E10	ordinary_muscle_development	ENSGALG00010020831	MUL1	0.728602882	0.144569304	5.03981732	4.65976E-07	9.15965E-07	positive
PRJNA729271	stage_5W_vs_E10	ordinary_muscle_development	ENSGALG00010020833	RANBP1	-2.228720474	0.08397153	-26.54138232	3.2294E-155	3.4933E-153	negative
PRJNA729271	stage_5W_vs_E10	ordinary_muscle_development	ENSGALG00010020835	PTGR2	-0.231173099	0.11540046	-2.003225109	0.045153132	0.057241092	negative
PRJNA729271	stage_5W_vs_E10	ordinary_muscle_development	ENSGALG00010020839	PGGHG	-1.321561094	0.149749276	-8.825158481	1.09305E-18	4.11419E-18	negative
PRJNA729271	stage_5W_vs_E10	ordinary_muscle_development	ENSGALG00010020840	LPAR3	-3.131754328	0.314211525	-9.967025646	2.12497E-23	9.8691E-23	negative
PRJNA729271	stage_5W_vs_E10	ordinary_muscle_development	ENSGALG00010020843	HMG20A	-1.47765882	0.084791598	-17.42694868	5.15229E-68	1.02165E-66	negative
PRJNA729271	stage_5W_vs_E10	ordinary_muscle_development	ENSGALG00010020845	RAB35	0.188257296	0.092552594	2.034057473	0.041945801	0.053422756	positive
PRJNA729271	stage_5W_vs_E10	ordinary_muscle_development	ENSGALG00010020847	MIDEAS	0.23437829	0.090074559	2.602047599	0.009266899	0.012728485	positive
PRJNA729271	stage_5W_vs_E10	ordinary_muscle_development	ENSGALG00010020854	ENSGALG00010020854	12.2047758	0.380978903	32.03530619	3.517E-225	7.7242E-223	positive
PRJNA729271	stage_5W_vs_E10	ordinary_muscle_development	ENSGALG00010020855	DNAL1	0.417404388	0.099194355	4.207944962	2.57704E-05	4.44533E-05	positive
PRJNA729271	stage_5W_vs_E10	ordinary_muscle_development	ENSGALG00010020856	VTG2	-4.693302993	0.986898223	-4.755609936	1.97848E-06	3.71141E-06	negative
PRJNA729271	stage_5W_vs_E10	ordinary_muscle_development	ENSGALG00010020861	CDA	1.331405143	0.176968714	7.52339275	5.33728E-14	1.59349E-13	positive
PRJNA729271	stage_5W_vs_E10	ordinary_muscle_development	ENSGALG00010020865	BICDL1	-1.908722048	0.832428856	-2.292955168	0.021850586	0.028738249	negative
PRJNA729271	stage_5W_vs_E10	ordinary_muscle_development	ENSGALG00010020866	LCOR	-0.228318618	0.09758972	-2.339576516	0.019305617	0.025569711	negative
PRJNA729271	stage_5W_vs_E10	ordinary_muscle_development	ENSGALG00010020870	RIOX1	-0.310517965	0.148693416	-2.088310111	0.03676987	0.047233185	negative
PRJNA729271	stage_5W_vs_E10	ordinary_muscle_development	ENSGALG00010020873	PINK1	2.821596982	0.136270696	20.70582357	3.06944E-95	1.17392E-93	positive
PRJNA729271	stage_5W_vs_E10	ordinary_muscle_development	ENSGALG00010020875	ENSGALG00010020875	0.438520379	0.173241128	2.531271778	0.011364975	0.015472463	positive
PRJNA729271	stage_5W_vs_E10	ordinary_muscle_development	ENSGALG00010020878	XYLT1	-0.179615963	0.12442966	-1.443514058	0.148875715	0.176765522	negative
PRJNA729271	stage_5W_vs_E10	ordinary_muscle_development	ENSGALG00010020880	PXK	2.574156663	0.16343973	15.74988325	6.88082E-56	9.76861E-55	positive
PRJNA729271	stage_5W_vs_E10	ordinary_muscle_development	ENSGALG00010020886	GBF1	-0.220157214	0.086367017	-2.54908901	0.010800473	0.014738548	negative
PRJNA729271	stage_5W_vs_E10	ordinary_muscle_development	ENSGALG00010020887	ARPP19	-0.989432072	0.071347788	-13.86773289	9.93681E-44	9.7452E-43	negative
PRJNA729271	stage_5W_vs_E10	ordinary_muscle_development	ENSGALG00010020889	PPRC1	-0.189995643	0.282652981	-0.672186945	0.501464691	0.542483073	negative
PRJNA729271	stage_5W_vs_E10	ordinary_muscle_development	ENSGALG00010020890	RPS15A	-0.326632225	0.188392516	-1.733785564	0.082956138	0.101807571	negative
PRJNA729271	stage_5W_vs_E10	ordinary_muscle_development	ENSGALG00010020894	PSMD13	0.434421946	0.120845138	3.594864914	0.00032456	0.000512193	positive
PRJNA729271	stage_5W_vs_E10	ordinary_muscle_development	ENSGALG00010020896	MARCO	3.922938966	0.391043237	10.03198264	1.1028E-23	5.17315E-23	positive
PRJNA729271	stage_5W_vs_E10	ordinary_muscle_development	ENSGALG00010020898	ENSGALG00010020898	0.027341147	0.131426426	0.208033866	0.835202525	0.855019008	positive
PRJNA729271	stage_5W_vs_E10	ordinary_muscle_development	ENSGALG00010020900	PDHB	0.314079225	0.116229663	2.702229498	0.00688762	0.009586716	positive
PRJNA729271	stage_5W_vs_E10	ordinary_muscle_development	ENSGALG00010020903	AGMAT	1.803458294	0.379258695	4.755219371	1.98231E-06	3.71715E-06	positive
PRJNA729271	stage_5W_vs_E10	ordinary_muscle_development	ENSGALG00010020905	KCTD6	-0.643696564	0.090422493	-7.11876591	1.08898E-12	3.03261E-12	negative
PRJNA729271	stage_5W_vs_E10	ordinary_muscle_development	ENSGALG00010020906	SIRT3	1.553760952	0.109095389	14.24222389	5.01067E-46	5.27066E-45	positive
PRJNA729271	stage_5W_vs_E10	ordinary_muscle_development	ENSGALG00010020912	PDE8A	1.349418037	0.143624775	9.395440577	5.69789E-21	2.37672E-20	positive
PRJNA729271	stage_5W_vs_E10	ordinary_muscle_development	ENSGALG00010020913	TMEM132B	-3.260516796	0.277790562	-11.73732029	8.2045E-32	5.34011E-31	negative
PRJNA729271	stage_5W_vs_E10	ordinary_muscle_development	ENSGALG00010020915	EN1	-4.617469178	0.408660919	-11.29902313	1.32686E-29	7.91148E-29	negative
PRJNA729271	stage_5W_vs_E10	ordinary_muscle_development	ENSGALG00010020916	ARL6IP1	-0.47788112	0.098239907	-4.864429711	1.14787E-06	2.19244E-06	negative
PRJNA729271	stage_5W_vs_E10	ordinary_muscle_development	ENSGALG00010020918	ENSGALG00010020918	-3.388753984	0.368237989	-9.202619189	3.49329E-20	1.40614E-19	negative
PRJNA729271	stage_5W_vs_E10	ordinary_muscle_development	ENSGALG00010020920	ACOX2	2.326517164	0.205222772	11.33654486	8.64923E-30	5.18703E-29	positive
PRJNA729271	stage_5W_vs_E10	ordinary_muscle_development	ENSGALG00010020921	LDB1	-0.273508261	0.15202547	-1.799094985	0.072003657	0.089105524	negative
PRJNA729271	stage_5W_vs_E10	ordinary_muscle_development	ENSGALG00010020922	INSIG2	0.239465776	0.080386794	2.978919336	0.002892669	0.004184973	positive
PRJNA729271	stage_5W_vs_E10	ordinary_muscle_development	ENSGALG00010020924	DNAJC16	1.139025045	0.119348839	9.543662563	1.37875E-21	5.91972E-21	positive
PRJNA729271	stage_5W_vs_E10	ordinary_muscle_development	ENSGALG00010020925	RIC8A	0.070664187	0.103990698	0.679524116	0.496805833	0.53828678	positive
PRJNA729271	stage_5W_vs_E10	ordinary_muscle_development	ENSGALG00010020927	ENSGALG00010020927	-0.206510041	0.116981113	-1.765328053	0.077508647	0.095502197	negative
PRJNA729271	stage_5W_vs_E10	ordinary_muscle_development	ENSGALG00010020928	AACS	-0.895985676	0.151776166	-5.903335828	3.56224E-09	8.06792E-09	negative
PRJNA729271	stage_5W_vs_E10	ordinary_muscle_development	ENSGALG00010020932	CASP9	0.004491006	0.122298516	0.036721672	0.970706928	0.974944355	positive
PRJNA729271	stage_5W_vs_E10	ordinary_muscle_development	ENSGALG00010020934	ABCD3	-0.093239197	0.153885773	-0.605898746	0.544581992	0.584330148	negative
PRJNA729271	stage_5W_vs_E10	ordinary_muscle_development	ENSGALG00010020935	BRI3BP	0.025362738	0.065190552	0.389055426	0.697235146	0.729600306	positive
PRJNA729271	stage_5W_vs_E10	ordinary_muscle_development	ENSGALG00010020936	BET1L	-0.152162836	0.126485352	-1.203007571	0.228973391	0.263431169	negative
PRJNA729271	stage_5W_vs_E10	ordinary_muscle_development	ENSGALG00010020938	SMG1	0.528572082	0.079859242	6.618796599	3.62135E-11	9.2971E-11	positive
PRJNA729271	stage_5W_vs_E10	ordinary_muscle_development	ENSGALG00010020939	CTRC	6.552776529	2.120187789	3.090658556	0.001997131	0.002934755	positive
PRJNA729271	stage_5W_vs_E10	ordinary_muscle_development	ENSGALG00010020940	ENSGALG00010020940	-1.276076543	0.135709159	-9.403024453	5.30168E-21	2.21463E-20	negative
PRJNA729271	stage_5W_vs_E10	ordinary_muscle_development	ENSGALG00010020941	ENSGALG00010020941	2.893892703	0.18316667	15.79923197	3.1493E-56	4.53318E-55	positive
PRJNA729271	stage_5W_vs_E10	ordinary_muscle_development	ENSGALG00010020943	FAM107A	0.844061597	0.373933677	2.257249475	0.023992491	0.031435519	positive
PRJNA729271	stage_5W_vs_E10	ordinary_muscle_development	ENSGALG00010020944	MYO5A	-1.473811764	0.157316696	-9.368438345	7.36136E-21	3.05302E-20	negative
PRJNA729271	stage_5W_vs_E10	ordinary_muscle_development	ENSGALG00010020945	NUMB	0.296498697	0.086919178	3.411199988	0.000646776	0.000996283	positive
PRJNA729271	stage_5W_vs_E10	ordinary_muscle_development	ENSGALG00010020946	CTBS	0.868272952	0.21548699	4.029352085	5.59308E-05	9.41053E-05	positive
PRJNA729271	stage_5W_vs_E10	ordinary_muscle_development	ENSGALG00010020949	HPS6	0.743513695	0.109766299	6.773606319	1.25611E-11	3.30802E-11	positive
PRJNA729271	stage_5W_vs_E10	ordinary_muscle_development	ENSGALG00010020950	EFHD2	-1.636299795	0.269850073	-6.063736715	1.32995E-09	3.08491E-09	negative
PRJNA729271	stage_5W_vs_E10	ordinary_muscle_development	ENSGALG00010020951	ENSGALG00010020951	-1.282584817	0.266774726	-4.807744847	1.52642E-06	2.88883E-06	negative
PRJNA729271	stage_5W_vs_E10	ordinary_muscle_development	ENSGALG00010020952	GNG5	-1.144385324	0.099946842	-11.44993983	2.35309E-30	1.44159E-29	negative
PRJNA729271	stage_5W_vs_E10	ordinary_muscle_development	ENSGALG00010020954	PLIN1	10.53771029	1.405789762	7.495936149	6.58268E-14	1.94932E-13	positive
PRJNA729271	stage_5W_vs_E10	ordinary_muscle_development	ENSGALG00010020955	FHAD1	-0.482606895	0.932059483	-0.517785511	0.604607929	0.642177177	negative
PRJNA729271	stage_5W_vs_E10	ordinary_muscle_development	ENSGALG00010020957	RPF1	-0.511909443	0.103294297	-4.955834497	7.20205E-07	1.39919E-06	negative
PRJNA729271	stage_5W_vs_E10	ordinary_muscle_development	ENSGALG00010020959	ENSGALG00010020959	-1.460489512	0.365912393	-3.991363889	6.56944E-05	0.000110073	negative
PRJNA729271	stage_5W_vs_E10	ordinary_muscle_development	ENSGALG00010020960	CLEC19A	0.730472254	0.38753368	1.884925857	0.059439849	0.074242189	positive
PRJNA729271	stage_5W_vs_E10	ordinary_muscle_development	ENSGALG00010020962	MYO5C	-3.388499429	0.371169883	-9.129241315	6.8981E-20	2.7424E-19	negative
PRJNA729271	stage_5W_vs_E10	ordinary_muscle_development	ENSGALG00010020963	CCDC93	1.235287777	0.099132686	12.46095342	1.21904E-35	9.20795E-35	positive
PRJNA729271	stage_5W_vs_E10	ordinary_muscle_development	ENSGALG00010020965	DHX37	-0.894122583	0.095243911	-9.387713884	6.13166E-21	2.55251E-20	negative
PRJNA729271	stage_5W_vs_E10	ordinary_muscle_development	ENSGALG00010020966	PAPLN	0.486284092	0.248878473	1.953901784	0.050712834	0.063903549	positive
PRJNA729271	stage_5W_vs_E10	ordinary_muscle_development	ENSGALG00010020967	VPS51	0.084581644	0.076194929	1.110069196	0.26696921	0.303936436	positive
PRJNA729271	stage_5W_vs_E10	ordinary_muscle_development	ENSGALG00010020969	RBP3	-0.913966326	0.334389084	-2.733242108	0.006271421	0.008766926	negative
PRJNA729271	stage_5W_vs_E10	ordinary_muscle_development	ENSGALG00010020970	ARMH3	0.36884848	0.078921984	4.673583452	2.95989E-06	5.48921E-06	positive
PRJNA729271	stage_5W_vs_E10	ordinary_muscle_development	ENSGALG00010020971	TMEM51	-0.850186188	0.288850716	-2.943341114	0.003246904	0.004677388	negative

PRJNA729271	stage_5W_vs_E10	ordinary_muscle_development	ENSGALG00010020973	GNB5	-1.05306338	0.165367642	-6.36801353	1.91492E-10	4.68232E-10	negative
PRJNA729271	stage_5W_vs_E10	ordinary_muscle_development	ENSGALG00010020975	HCCS	0.756688916	0.130302482	5.807171927	6.35369E-09	1.41862E-08	positive
PRJNA729271	stage_5W_vs_E10	ordinary_muscle_development	ENSGALG00010020976	GDF10	-1.098155653	0.310017007	-3.54224326	0.000396739	0.000621097	negative
PRJNA729271	stage_5W_vs_E10	ordinary_muscle_development	ENSGALG00010020978	BCL2L10	1.134193568	0.115657221	9.806508924	1.05557E-22	4.76504E-22	positive
PRJNA729271	stage_5W_vs_E10	ordinary_muscle_development	ENSGALG00010020979	ENSGALG00010020979	0.889725273	0.244064454	3.645452091	0.000266922	0.000424143	positive
PRJNA729271	stage_5W_vs_E10	ordinary_muscle_development	ENSGALG00010020981	CIT	-2.131092176	0.237270846	-8.981685743	2.6665E-19	1.03124E-18	negative
PRJNA729271	stage_5W_vs_E10	ordinary_muscle_development	ENSGALG00010020982	TLCD4	0.07411758	0.152537823	0.485896408	0.627040615	0.663136854	positive
PRJNA729271	stage_5W_vs_E10	ordinary_muscle_development	ENSGALG00010020984	PSEN1	-1.091545481	0.060557427	-18.02496479	1.24091E-72	2.81047E-71	negative
PRJNA729271	stage_5W_vs_E10	ordinary_muscle_development	ENSGALG00010020986	CFAP20DC	-2.757884379	0.356485352	-7.736318941	1.02337E-14	3.16959E-14	negative
PRJNA729271	stage_5W_vs_E10	ordinary_muscle_development	ENSGALG00010020987	NADSYN1	-0.080005968	0.093593958	-0.854819792	0.392650919	0.433966838	negative
PRJNA729271	stage_5W_vs_E10	ordinary_muscle_development	ENSGALG00010020989	MAPK4	1.420669033	0.152745011	9.300919359	1.39236E-20	5.6992E-20	positive
PRJNA729271	stage_5W_vs_E10	ordinary_muscle_development	ENSGALG00010020990	UBB	-1.137653154	0.106764158	-10.65575917	1.63902E-26	8.6865E-26	negative
PRJNA729271	stage_5W_vs_E10	ordinary_muscle_development	ENSGALG00010020991	ALG14	0.518467801	0.140310611	3.695143198	0.000219763	0.000351946	positive
PRJNA729271	stage_5W_vs_E10	ordinary_muscle_development	ENSGALG00010020992	KCNH7	3.173044557	0.430692985	7.367300292	1.74118E-13	5.03359E-13	positive
PRJNA729271	stage_5W_vs_E10	ordinary_muscle_development	ENSGALG00010020994	DHCR7	-1.415699404	0.145795104	-9.710198552	2.72805E-22	1.20964E-21	negative
PRJNA729271	stage_5W_vs_E10	ordinary_muscle_development	ENSGALG00010020998	SYT17	-0.197616532	0.290770781	-0.679629951	0.4967388	0.538254385	negative
PRJNA729271	stage_5W_vs_E10	ordinary_muscle_development	ENSGALG00010020999	IFIH1	3.607968208	0.334525406	10.78533391	4.03774E-27	2.18711E-26	positive
PRJNA729271	stage_5W_vs_E10	ordinary_muscle_development	ENSGALG00010021000	NFKB2	1.453847682	0.114014464	12.75143196	3.06071E-37	2.4511E-36	positive
PRJNA729271	stage_5W_vs_E10	ordinary_muscle_development	ENSGALG00010021002	SLC44A3	0.260939675	0.158506738	1.646237111	0.099714951	0.121133777	positive
PRJNA729271	stage_5W_vs_E10	ordinary_muscle_development	ENSGALG00010021003	KAZN	-0.697568923	0.121675145	-5.733043718	9.86442E-09	2.17865E-08	negative
PRJNA729271	stage_5W_vs_E10	ordinary_muscle_development	ENSGALG00010021004	PSD	-0.065952823	0.174716861	-0.377484023	0.705813936	0.737778394	negative
PRJNA729271	stage_5W_vs_E10	ordinary_muscle_development	ENSGALG00010021010	LEO1	-0.537846309	0.112892742	-4.764223968	1.89582E-06	3.56142E-06	negative
PRJNA729271	stage_5W_vs_E10	ordinary_muscle_development	ENSGALG00010021011	SCARB1	0.683078727	0.106506382	6.413500445	1.42216E-10	3.50402E-10	positive
PRJNA729271	stage_5W_vs_E10	ordinary_muscle_development	ENSGALG00010021012	KCNIP2	-1.364296489	0.727697534	-1.874812576	0.060818513	0.075872652	negative
PRJNA729271	stage_5W_vs_E10	ordinary_muscle_development	ENSGALG00010021013	FAP	0.091863816	0.296705417	0.309612871	0.756855367	0.7847928	positive
PRJNA729271	stage_5W_vs_E10	ordinary_muscle_development	ENSGALG00010021014	FBXL15	0.924411542	0.089626721	10.31401719	6.09012E-25	3.02109E-24	positive
PRJNA729271	stage_5W_vs_E10	ordinary_muscle_development	ENSGALG00010021015	RBM25	-0.312147314	0.135454534	-2.304443448	0.021197763	0.027917643	negative
PRJNA729271	stage_5W_vs_E10	ordinary_muscle_development	ENSGALG00010021016	FHIT	-0.521816829	0.220055466	-2.371296832	0.017725789	0.023576373	negative
PRJNA729271	stage_5W_vs_E10	ordinary_muscle_development	ENSGALG00010021017	COQ7	0.461831227	0.157469513	2.932829469	0.003358883	0.00483054	positive
PRJNA729271	stage_5W_vs_E10	ordinary_muscle_development	ENSGALG00010021018	F3	0.665211239	0.220852897	3.012010474	0.002595236	0.003776903	positive
PRJNA729271	stage_5W_vs_E10	ordinary_muscle_development	ENSGALG00010021019	VSIG10	-1.107526762	0.098163029	-11.28252434	1.60085E-29	9.50998E-29	negative
PRJNA729271	stage_5W_vs_E10	ordinary_muscle_development	ENSGALG00010021020	ZDHHC13	-0.160276226	0.120411215	-1.331073898	0.183164697	0.214595237	negative
PRJNA729271	stage_5W_vs_E10	ordinary_muscle_development	ENSGALG00010021021	GCG	1.804861316	0.34691709	5.202572506	1.96549E-07	3.97311E-07	positive
PRJNA729271	stage_5W_vs_E10	ordinary_muscle_development	ENSGALG00010021022	ZFYVE1	1.531915602	0.077753064	19.70231809	2.0597E-86	6.42051E-85	positive
PRJNA729271	stage_5W_vs_E10	ordinary_muscle_development	ENSGALG00010021023	MGEA5	0.419437278	0.097335216	4.309203766	1.63843E-05	2.87241E-05	positive
PRJNA729271	stage_5W_vs_E10	ordinary_muscle_development	ENSGALG00010021024	ENSGALG00010021024	0.127900893	0.18817312	0.679697999	0.496695703	0.538247923	positive
PRJNA729271	stage_5W_vs_E10	ordinary_muscle_development	ENSGALG00010021025	CUEDC2	0.816210372	0.115034633	7.095344704	1.29029E-12	3.57607E-12	positive
PRJNA729271	stage_5W_vs_E10	ordinary_muscle_development	ENSGALG00010021026	ADGRA2	-0.6438727	0.135072143	-4.766880009	1.87101E-06	3.51572E-06	negative
PRJNA729271	stage_5W_vs_E10	ordinary_muscle_development	ENSGALG00010021027	PLA2G4A	0.40083208	0.166739604	2.403940459	0.016219415	0.021678231	positive
PRJNA729271	stage_5W_vs_E10	ordinary_muscle_development	ENSGALG00010021028	ENSGALG00010021028	0.309464137	0.33040684	0.936615407	0.348956424	0.388966731	positive
PRJNA729271	stage_5W_vs_E10	ordinary_muscle_development	ENSGALG00010021029	PRDM2	-0.536534436	0.075625803	-7.094594865	1.29731E-12	3.59482E-12	negative
PRJNA729271	stage_5W_vs_E10	ordinary_muscle_development	ENSGALG00010021030	CSRP3	0.753476298	0.551929544	1.36516754	0.172200398	0.202647931	positive
PRJNA729271	stage_5W_vs_E10	ordinary_muscle_development	ENSGALG00010021031	DDX54	-0.266124735	0.110488635	-2.40861638	0.01601312	0.021420282	negative
PRJNA729271	stage_5W_vs_E10	ordinary_muscle_development	ENSGALG00010021032	TMC7	0.061539137	0.119264996	0.515986576	0.605863785	0.643288524	positive
PRJNA729271	stage_5W_vs_E10	ordinary_muscle_development	ENSGALG00010021033	RNF122	-2.542123715	0.23589617	-10.77645183	4.4471E-27	2.40346E-26	negative
PRJNA729271	stage_5W_vs_E10	ordinary_muscle_development	ENSGALG00010021034	NPM3	-0.875816568	0.278857435	-3.14073235	0.00168526	0.002495438	negative
PRJNA729271	stage_5W_vs_E10	ordinary_muscle_development	ENSGALG00010021036	E2F8	-2.142610405	0.202565531	-10.5773692	3.79463E-26	1.97215E-25	negative
PRJNA729271	stage_5W_vs_E10	ordinary_muscle_development	ENSGALG00010021038	PTGS2	0.146705499	0.300592696	0.488054104	0.625511519	0.66180945	positive
PRJNA729271	stage_5W_vs_E10	ordinary_muscle_development	ENSGALG00010021040	FGF8	-6.340399689	0.944924773	-6.709951807	1.94689E-11	5.07009E-11	negative
PRJNA729271	stage_5W_vs_E10	ordinary_muscle_development	ENSGALG00010021041	GLTP	-0.532759706	0.189077938	-2.817672501	0.004837312	0.006840667	negative
PRJNA729271	stage_5W_vs_E10	ordinary_muscle_development	ENSGALG00010021042	LRRC38	0.243021883	0.09880411	2.459633325	0.013907903	0.018738783	positive
PRJNA729271	stage_5W_vs_E10	ordinary_muscle_development	ENSGALG00010021043	PTPRG	-0.251584405	0.090493771	-2.780129532	0.005433722	0.007641583	negative
PRJNA729271	stage_5W_vs_E10	ordinary_muscle_development	ENSGALG00010021044	EIF4G2	0.346042179	0.068018371	5.087481123	3.6285E-07	7.18121E-07	positive
PRJNA729271	stage_5W_vs_E10	ordinary_muscle_development	ENSGALG00010021045	DPP4	2.659802436	0.259071435	10.26667581	9.95768E-25	4.88945E-24	positive
PRJNA729271	stage_5W_vs_E10	ordinary_muscle_development	ENSGALG00010021046	ENSGALG00010021046	-2.369857285	0.254739291	-9.303069318	1.36449E-20	5.59023E-20	negative
PRJNA729271	stage_5W_vs_E10	ordinary_muscle_development	ENSGALG00010021047	TMC5	-1.910419945	0.569975964	-3.351755274	0.00080301	0.001226515	negative
PRJNA729271	stage_5W_vs_E10	ordinary_muscle_development	ENSGALG00010021048	API5	-0.409681578	0.075868526	-5.399888434	6.66824E-08	1.39114E-07	negative
PRJNA729271	stage_5W_vs_E10	ordinary_muscle_development	ENSGALG00010021050	CTR9	-0.037126123	0.094691681	-0.392073757	0.695003717	0.727580448	negative
PRJNA729271	stage_5W_vs_E10	ordinary_muscle_development	ENSGALG00010021051	GDE1	0.274003124	0.135926279	2.015821565	0.043818638	0.055671063	positive
PRJNA729271	stage_5W_vs_E10	ordinary_muscle_development	ENSGALG00010021052	CEP15	-1.529737056	0.151641338	-10.08786311	6.25156E-24	2.95938E-23	negative
PRJNA729271	stage_5W_vs_E10	ordinary_muscle_development	ENSGALG00010021053	ENSGALG00010021053	-5.494061369	0.783799941	-7.00952001	2.39137E-12	6.53401E-12	negative
PRJNA729271	stage_5W_vs_E10	ordinary_muscle_development	ENSGALG00010021055	IRAG1	0.069632311	0.222047989	0.313591272	0.75383149	0.781769152	positive
PRJNA729271	stage_5W_vs_E10	ordinary_muscle_development	ENSGALG00010021057	ZDHHC8	-0.658500663	0.081930398	-8.037317989	9.18261E-16	3.00388E-15	negative
PRJNA729271	stage_5W_vs_E10	ordinary_muscle_development	ENSGALG00010021058	CCP110	-0.398152233	0.095584283	-4.16545713	3.10729E-05	5.32327E-05	negative
PRJNA729271	stage_5W_vs_E10	ordinary_muscle_development	ENSGALG00010021059	ENSGALG00010021059	-2.485230368	1.385081825	-1.794284151	0.072767826	0.08999741	negative
PRJNA729271	stage_5W_vs_E10	ordinary_muscle_development	ENSGALG00010021062	MYO9A	-1.348355241	0.102006222	-13.2183627	6.87441E-40	6.00268E-39	negative
PRJNA729271	stage_5W_vs_E10	ordinary_muscle_development	ENSGALG00010021064	LYVE1	2.521092552	0.285786905	8.821581762	1.12854E-18	4.24007E-18	positive
PRJNA729271	stage_5W_vs_E10	ordinary_muscle_development	ENSGALG00010021065	CNNM2	-0.471141842	0.112841782	-4.175242824	2.97668E-05	5.10737E-05	negative
PRJNA729271	stage_5W_vs_E10	ordinary_muscle_development	ENSGALG00010021067	SLC4A10	-3.221487466	0.32998081	-9.762650946	1.62842E-22	7.28517E-22	negative
PRJNA729271	stage_5W_vs_E10	ordinary_muscle_development	ENSGALG00010021069	PGAM5	-0.018844202	0.071620714	-0.263111064	0.792464976	0.817099148	negative
PRJNA729271	stage_5W_vs_E10	ordinary_muscle_development	ENSGALG00010021070	RNF141	0.364550038	0.08582578	4.247558708	2.16113E-05	3.75156E-05	positive
PRJNA729271	stage_5W_vs_E10	ordinary_muscle_development	ENSGALG00010021071	HIF1AN	1.616896649	0.094960516	17.02704151	5.17555E-65	9.54448E-64	positive
PRJNA729271	stage_5W_vs_E10	ordinary_muscle_development	ENSGALG00010021072	ENSGALG00010021072	0.592995381	0.089953009	6.592279509	4.33124E-11	1.10531E-10	positive
PRJNA729271	stage_5W_vs_E10	ordinary_muscle_development	ENSGALG00010021073	NDUFB8	0.523501455	0.107696246	4.860907186	1.16849E-06	2.23064E-06	positive
PRJNA729271	stage_5W_vs_E10	ordinary_muscle_development	ENSGALG00010021074	TTC17	0.525037684	0.06937704	7.567888279	3.7934E-14	1.14006E-13	positive
PRJNA729271	stage_5W_vs_E10	ordinary_muscle_development	ENSGALG00010021075	DHRS3	-1.288282385	0.133516734	-9.648845847	4.97121E-22	2.18092E-21	negative
PRJNA729271	stage_5W_vs_E10	ordinary_muscle_development	ENSGALG00010021077	SEC31B	0.718154239	0.078567732	9.140574931	6.21212E-20	2.47784E-19	positive
PRJNA729271	stage_5W_vs_E10	ordinary_muscle_development	ENSGALG00010021078	ENSGALG00010021078	-3.133968119	0.293914694	-10.66284939	1.51872E-26	8.05777E-26	negative
PRJNA729271	stage_5W_vs_E10	ordinary_muscle_development	ENSGALG00010021080	TTLL7	2.219259321	0.173771089	12.7711654	2.37571E-37	1.9078E-36	positive
PRJNA729271	stage_5W_vs_E10	ordinary_muscle_development	ENSGALG00010021081	KCNH6	-1.873776575	0.242472357	-7.727794607	1.09426E-14	3.38121E-14	negative
PRJNA729271	stage_5W_vs_E10	ordinary_muscle_development	ENSGALG00010021082	SENP8	0.180331597	0.163807972	1.100871925	0.270952402	0.308132361	positive
PRJNA729271	stage_5W_vs_E10	ordinary_muscle_development	ENSGALG00010021083	SCD	0.12799807	0.561220695	0.228070831			positive
PRJNA729271	stage_5W_vs_E10	ordinary_muscle_development	ENSGALG00010021084	ENSGALG00010021084	-0.84919024	0.587946878	-1.444331573	0.148645728	0.176506909	negative
PRJNA729271	stage_5W_vs_E10	ordinary_muscle_development	ENSGALG00010021085	AMPD3	1.977059643	0.184051013	10.74191123	6.46911E-27	3.47553E-26	positive
PRJNA729271	stage_5W_vs_E10	ordinary_muscle_development	ENSGALG00010021086	PSMD14	0.057108579	0.109365073	0.522182977	0.601542937	0.639249624	positive
PRJNA729271	stage_5W_vs_E10	ordinary_muscle_development	ENSGALG00010021087	THBS1	-1.355001453	0.356286343	-3.803124869	0.000142882	0.00023221	negative
PRJNA729271	stage_5W_vs_E10	ordinary_muscle_development	ENSGALG00010021088	ANKLE2	-0.298084796	0.087569161	-3.403992835	0.000664085	0.00102175	negative
PRJNA729271	stage_5W_vs_E10	ordinary_muscle_development	ENSGALG00010021090	ADM	3.091271772	0.25650888	12.05132459	1.90858E-33	1.32241E-32	positive
PRJNA729271	stage_5W_vs_E10	ordinary_muscle_development	ENSGALG00010021091	TANK	0.227277755	0.082208568	2.764648009	0.005698425	0.007999096	positive
PRJNA729271	stage_5W_vs_E10	ordinary_muscle_development	ENSGALG00010021092	VPS13D	1.073783147	0.094702678	11.33846659	8.46141E-30	5.07649E-29	positive
PRJNA729271	stage_5W_vs_E10	ordinary_muscle_development	ENSGALG00010021093	GRAMD2	2.454814078	0.219890372	11.16380882	6.13075E-29	3.56316E-28	positive
PRJNA729271	stage_5W_vs_E10	ordinary_muscle_development	ENSGALG00010021094	ENSGALG00010021094	3.305350429	0.31400909	10.52628899	6.53607E-26	3.35958E-25	positive
PRJNA729271	stage_5W_vs_E10	ordinary_muscle_development	ENSGALG00010021095	NT5C2	0.054507342	0.105959442	0.514417034	0.606960452	0.644203834	positive
PRJNA729271	stage_5W_vs_E10	ordinary_muscle_development	ENSGALG00010021096	ZFAND4	-1.192983639	0.108229933	-11.02267745	2.97088E-28	1.67691E-27	negative
PRJNA729271	stage_5W_vs_E10	ordinary_muscle_development	ENSGALG00010021097	KATNBL1	-0.25061381	0.129808064	-1.930649011	0.053526473	0.067250257	negative
PRJNA729271	stage_5W_vs_E10	ordinary_muscle_development	ENSGALG00010021098	SDK1	-0.523047676	0.142354115	-3.674271552	0.000238529	0.000380905	negative
PRJNA729271	stage_5W_vs_E10	ordinary_muscle_development	ENSGALG00010021099	HMCN1	-1.910096947	0.264666888	-7.216984945	5.31529E-13	1.50185E-12	negative
PRJNA729271	stage_5W_vs_E10	ordinary_muscle_development	ENSGALG00010021100	EMC7	0.902461042	0.08552339	10.55221315	4.96144E-26	2.56752E-25	positive
PRJNA729271	stage_5W_vs_E10	ordinary_muscle_development	ENSGALG00010021101	TMEM240	-4.372442171	0.683309597	-6.398918133	1.56482E-10	3.84506E-10	negative
PRJNA729271	stage_5W_vs_E10	ordinary_muscle_development	ENSGALG00010021102	GOLGA3	1.392993109	0.111993439	12.43816707	1.62183E-35	1.22058E-34	positive
PRJNA729271	stage_5W_vs_E10	ordinary_muscle_development	ENSGALG00010021103	RBMS1	0.020233872	0.074259489	0.272475237	0.785256629	0.810878025	positive
PRJNA729271	stage_5W_vs_E10	ordinary_muscle_development	ENSGALG00010021104	INA	-2.48861647	0.439758593	-5.659051376	1.52212E-08	3.32727E-08	negative
PRJNA729271	stage_5W_vs_E10	ordinary_muscle_development	ENSGALG00010021105	ENSGALG00010021105	2.754043637	0.410712282	6.705530267	2.00676E-11	5.22227E-11	positive
PRJNA729271	stage_5W_vs_E10	ordinary_muscle_development	ENSGALG00010021106	ITGB6	-2.63248332	0.216772089	-12.14401415	6.17221E-34	4.37487E-33	negative
PRJNA729271	stage_5W_vs_E10	ordinary_muscle_development	ENSGALG00010021108	PCGF6	0.066719712	0.106471392	0.626644499	0.530892292	0.571120957	positive
PRJNA729271	stage_5W_vs_E10	ordinary_muscle_development	ENSGALG00010021110	PKLR	5.135832854	0.168775043	30.43004911	2.2002E-203	3.8423E-201	positive
PRJNA729271	stage_5W_vs_E10	ordinary_muscle_development	ENSGALG00010021111	AVEN	0.680924952	0.078517467	8.672273551	4.23577E-18	1.55279E-17	positive
PRJNA729271	stage_5W_vs_E10	ordinary_muscle_development	ENSGALG00010021112	TAF5	-1.014942267	0.072765654	-13.94809509	3.23121E-44	3.21237E-43	negative
PRJNA729271	stage_5W_vs_E10	ordinary_muscle_development	ENSGALG00010021113	SYP	-1.822031363	0.314892027	-5.786209904	7.19922E-09	1.60296E-08	negative
PRJNA729271	stage_5W_vs_E10	ordinary_muscle_development	ENSGALG00010021114	SBF2	-0.289228805	0.098671516	-2.931228958	0.003376238	0.004854536	negative
PRJNA729271	stage_5W_vs_E10	ordinary_muscle_development	ENSGALG00010021115	ALPL	0.165408638	0.242681352	0.681587755	0.49549966	0.537112442	positive
PRJNA729271	stage_5W_vs_E10	ordinary_muscle_development	ENSGALG00010021116	FAM21C	0.745931997	0.063854313	11.68177936	1.57954E-31	1.01622E-30	positive
PRJNA729271	stage_5W_vs_E10	ordinary_muscle_development	ENSGALG00010021118	USMG5	0.871900385	0.179876443	4.847218289	1.25205E-06	2.3845E-06	positive
PRJNA729271	stage_5W_vs_E10	ordinary_muscle_development	ENSGALG00010021120	PDCD11	-1.516704169	0.088663491	-17.10629878	1.33199E-65	2.50418E-64	negative
PRJNA729271	stage_5W_vs_E10	ordinary_muscle_development	ENSGALG00010021121	PLA2R1	1.109755331	0.091570114	12.1191869	8.35842E-34	5.87278E-33	positive
PRJNA729271	stage_5W_vs_E10	ordinary_muscle_development	ENSGALG00010021122	CYP1A1	3.288319945	0.925908147	3.551453734	0.000383109	0.000600408	positive
PRJNA729271	stage_5W_vs_E10	ordinary_muscle_development	ENSGALG00010021123	RYR3	7.269268481	0.216123217	33.63483373	5.1958E-248	1.4767E-245	positive
PRJNA729271	stage_5W_vs_E10	ordinary_muscle_development	ENSGALG00010021124	CALHM2	0.504875502	0.131279803	3.845797231	0.000120161	0.000196385	positive
PRJNA729271	stage_5W_vs_E10	ordinary_muscle_development	ENSGALG00010021125	LY75	2.937825833	0.123882164	23.71467958	2.5444E-124	1.6538E-122	positive
PRJNA729271	stage_5W_vs_E10	ordinary_muscle_development	ENSGALG00010021126	ECE1	1.000564217	0.103876124	9.632282954	5.84169E-22	2.55585E-21	positive
PRJNA729271	stage_5W_vs_E10	ordinary_muscle_development	ENSGALG00010021127	SWAP70	-0.174339691	0.077762375	-2.241954305	0.024964326	0.032638036	negative
PRJNA729271	stage_5W_vs_E10	ordinary_muscle_development	ENSGALG00010021129	TXNRD3	-0.509919483	0.086530568	-5.892940433	3.79383E-09	8.58037E-09	negative
PRJNA729271	stage_5W_vs_E10	ordinary_muscle_development	ENSGALG00010021130	ENSGALG00010021130	4.526319895	1.101957001	4.107528598	3.99915E-05	6.79495E-05	positive
PRJNA729271	stage_5W_vs_E10	ordinary_muscle_development	ENSGALG00010021131	EDC3	-0.558123547	0.116689436	-4.782982656	1.72713E-06	3.25507E-06	negative
PRJNA729271	stage_5W_vs_E10	ordinary_muscle_development	ENSGALG00010021133	ENSGALG00010021133	-0.303823852	0.102442621	-2.965795387	0.003019013	0.004361231	negative
PRJNA729271	stage_5W_vs_E10	ordinary_muscle_development	ENSGALG00010021134	CHFR	-0.881366061	0.094238588	-9.352496492	8.56029E-21	3.53912E-20	negative
PRJNA729271	stage_5W_vs_E10	ordinary_muscle_development	ENSGALG00010021135	CLK3	0.540072315	0.107045251	5.045271128	4.52879E-07	8.91065E-07	positive
PRJNA729271	stage_5W_vs_E10	ordinary_muscle_development	ENSGALG00010021136	WEE1	-2.22302437	0.151026574	-14.7194253	4.83754E-49	5.67315E-48	negative
PRJNA729271	stage_5W_vs_E10	ordinary_muscle_development	ENSGALG00010021137	PLAU	0.798547139	0.190255204	4.197242039	2.70185E-05	4.65015E-05	positive
PRJNA729271	stage_5W_vs_E10	ordinary_muscle_development	ENSGALG00010021138	DDX18	-0.790180456	0.100506099	-7.862014967	3.78003E-15	1.19711E-14	negative
PRJNA729271	stage_5W_vs_E10	ordinary_muscle_development	ENSGALG00010021139	ARHGAP29	0.966498818	0.095666866	10.10275402	5.37122E-24	2.556E-23	positive

PRJNA729271	stage_5W_vs_E10	ordinary_muscle_development	ENSGALG00010021140	SLC11A1	0.580426987	0.43413322	1.336978975	0.1812295	0.212516916	positive
PRJNA729271	stage_5W_vs_E10	ordinary_muscle_development	ENSGALG00010021141	SGSM1	-0.547384372	0.156886006	-3.489057989	0.000484726	0.000753146	negative
PRJNA729271	stage_5W_vs_E10	ordinary_muscle_development	ENSGALG00010021142	WNT6	-7.594162488	0.928850167	-8.175874605	2.93727E-16	9.85322E-16	negative
PRJNA729271	stage_5W_vs_E10	ordinary_muscle_development	ENSGALG00010021143	ABCA4	-0.867355426	0.356646427	-2.431975646	0.015016717	0.020146919	negative
PRJNA729271	stage_5W_vs_E10	ordinary_muscle_development	ENSGALG00010021144	FOXK1	0.397016669	0.108781655	3.649665657	0.000262582	0.000417613	positive
PRJNA729271	stage_5W_vs_E10	ordinary_muscle_development	ENSGALG00010021145	EIF4G3	0.399029115	0.157362169	2.535737256	0.011221089	0.015288061	positive
PRJNA729271	stage_5W_vs_E10	ordinary_muscle_development	ENSGALG00010021146	AP5Z1	0.202303455	0.08549686	2.366209169	0.017971288	0.023885367	positive
PRJNA729271	stage_5W_vs_E10	ordinary_muscle_development	ENSGALG00010021147	ARID3B	-0.43698564	0.086179436	-5.070648628	3.96462E-07	7.83038E-07	negative
PRJNA729271	stage_5W_vs_E10	ordinary_muscle_development	ENSGALG00010021148	GPATCH8	-0.025952387	0.105655152	-0.245632949	0.805966381	0.829013453	negative
PRJNA729271	stage_5W_vs_E10	ordinary_muscle_development	ENSGALG00010021149	ENSGALG00010021149	-3.163770355	1.345538149	-2.351304834	0.018707701	0.024823153	negative
PRJNA729271	stage_5W_vs_E10	ordinary_muscle_development	ENSGALG00010021150	ENSGALG00010021150	0.502704399	0.257367697	1.953253669	0.050789544	0.063994649	positive
PRJNA729271	stage_5W_vs_E10	ordinary_muscle_development	ENSGALG00010021151	WNT10A	-4.978289179	0.812899234	-6.124115971	9.11885E-10	2.13327E-09	negative
PRJNA729271	stage_5W_vs_E10	ordinary_muscle_development	ENSGALG00010021152	STK11IP	0.991286726	0.126960167	7.807856207	5.81689E-15	1.82186E-14	positive
PRJNA729271	stage_5W_vs_E10	ordinary_muscle_development	ENSGALG00010021153	PTPRF	-2.96778567	0.161295003	-18.39973723	1.32003E-75	3.27075E-74	negative
PRJNA729271	stage_5W_vs_E10	ordinary_muscle_development	ENSGALG00010021154	CHST13	-2.718306332	0.215050076	-12.64034118	1.26504E-36	9.90639E-36	negative
PRJNA729271	stage_5W_vs_E10	ordinary_muscle_development	ENSGALG00010021155	RADIL	-1.647938541	0.21920612	-7.517757891	5.57236E-14	1.65957E-13	negative
PRJNA729271	stage_5W_vs_E10	ordinary_muscle_development	ENSGALG00010021156	DDOST	-1.080056867	0.081142779	-13.31057274	2.00935E-40	1.77812E-39	negative
PRJNA729271	stage_5W_vs_E10	ordinary_muscle_development	ENSGALG00010021157	ENSGALG00010021157	9.400200253	0.803338914	11.7014128	1.25349E-31	8.10408E-31	positive
PRJNA729271	stage_5W_vs_E10	ordinary_muscle_development	ENSGALG00010021158	ACTG1L	-2.245461731	0.129254411	-17.37241853	1.33479E-67	2.62522E-66	negative
PRJNA729271	stage_5W_vs_E10	ordinary_muscle_development	ENSGALG00010021159	MMD2	1.681019926	0.238563331	7.04643049	1.83566E-12	5.05085E-12	positive
PRJNA729271	stage_5W_vs_E10	ordinary_muscle_development	ENSGALG00010021160	ZNF143	0.435764704	0.164350663	2.651432582	0.008015111	0.011081556	positive
PRJNA729271	stage_5W_vs_E10	ordinary_muscle_development	ENSGALG00010021161	ENSGALG00010021161	-1.909630119	0.112291342	-17.00603165	7.40894E-65	1.3594E-63	negative
PRJNA729271	stage_5W_vs_E10	ordinary_muscle_development	ENSGALG00010021162	WIPI2	-0.213465868	0.09505955	-2.2456015	0.024729544	0.032351511	negative
PRJNA729271	stage_5W_vs_E10	ordinary_muscle_development	ENSGALG00010021163	INHA	-1.751285833	0.383306592	-4.568890462	4.90313E-06	8.92738E-06	negative
PRJNA729271	stage_5W_vs_E10	ordinary_muscle_development	ENSGALG00010021164	SLC41A3	0.353609328	0.213676657	1.65488048	0.097948745	0.119080529	positive
PRJNA729271	stage_5W_vs_E10	ordinary_muscle_development	ENSGALG00010021165	PTRF	3.523812099	0.105580812	33.37549737	3.1091E-244	8.5032E-242	positive
PRJNA729271	stage_5W_vs_E10	ordinary_muscle_development	ENSGALG00010021166	NEURL1	1.455758528	0.292324099	4.979947019	6.36017E-07	1.23912E-06	positive
PRJNA729271	stage_5W_vs_E10	ordinary_muscle_development	ENSGALG00010021167	KIAA1671	0.45675642	0.126099032	3.622204012	0.000292104	0.000462718	positive
PRJNA729271	stage_5W_vs_E10	ordinary_muscle_development	ENSGALG00010021168	IVNS1ABP	0.54456151	0.105789591	5.147590647	2.63854E-07	5.27598E-07	positive
PRJNA729271	stage_5W_vs_E10	ordinary_muscle_development	ENSGALG00010021169	CAMK2G	2.327196915	0.140038584	16.61825516	5.14071E-62	8.76643E-61	positive
PRJNA729271	stage_5W_vs_E10	ordinary_muscle_development	ENSGALG00010021170	UBL7	-0.317277839	0.139058453	-2.281614908	0.022512086	0.029554631	negative
PRJNA729271	stage_5W_vs_E10	ordinary_muscle_development	ENSGALG00010021171	ENSGALG00010021171	-0.886626922	0.21485676	-4.126595427	3.68173E-05	6.26738E-05	negative
PRJNA729271	stage_5W_vs_E10	ordinary_muscle_development	ENSGALG00010021172	SLC29A4	-2.018593784	0.305221485	-6.613537652	3.75242E-11	9.61658E-11	negative
PRJNA729271	stage_5W_vs_E10	ordinary_muscle_development	ENSGALG00010021173	KLF15	2.467740804	0.287246442	8.591023035	8.61983E-18	3.11816E-17	positive
PRJNA729271	stage_5W_vs_E10	ordinary_muscle_development	ENSGALG00010021175	IPO7	0.294274425	0.097610484	3.014782965	0.002571632	0.003744807	positive
PRJNA729271	stage_5W_vs_E10	ordinary_muscle_development	ENSGALG00010021176	ENSGALG00010021176	-0.223372257	0.118265739	-1.888731755	0.058927781	0.073634327	negative
PRJNA729271	stage_5W_vs_E10	ordinary_muscle_development	ENSGALG00010021177	OBSL1	0.879468879	0.187727858	4.684807511	2.80223E-06	5.20414E-06	positive
PRJNA729271	stage_5W_vs_E10	ordinary_muscle_development	ENSGALG00010021178	NDST2	-0.429913532	0.097787724	-4.396395736	1.10063E-05	1.95678E-05	negative
PRJNA729271	stage_5W_vs_E10	ordinary_muscle_development	ENSGALG00010021180	TMEM198	-0.474201995	0.144789196	-3.275120022	0.001056171	0.001598037	negative
PRJNA729271	stage_5W_vs_E10	ordinary_muscle_development	ENSGALG00010021181	DAGLA	0.48218379	0.127611503	3.778529195	0.000157757	0.000255382	positive
PRJNA729271	stage_5W_vs_E10	ordinary_muscle_development	ENSGALG00010021182	ZSWIM8	0.411070045	0.069017945	5.955987857	2.58506E-09	5.8962E-09	positive
PRJNA729271	stage_5W_vs_E10	ordinary_muscle_development	ENSGALG00010021183	CFAP100	2.7357134	0.457386308	5.981187787	2.21516E-09	5.06848E-09	positive
PRJNA729271	stage_5W_vs_E10	ordinary_muscle_development	ENSGALG00010021184	DRAXIN	-0.885918397	0.441401602	-2.007057504	0.04474354	0.0567715	negative
PRJNA729271	stage_5W_vs_E10	ordinary_muscle_development	ENSGALG00010021186	SWT1	0.572737087	0.090123049	6.355056705	2.0835E-10	5.07995E-10	positive
PRJNA729271	stage_5W_vs_E10	ordinary_muscle_development	ENSGALG00010021187	SEMA7A	1.923502869	0.278139908	6.915594677	4.65905E-12	1.252E-11	positive
PRJNA729271	stage_5W_vs_E10	ordinary_muscle_development	ENSGALG00010021188	TNRC18	-0.600620421	0.133661959	-4.493577864	7.00364E-06	1.2614E-05	negative
PRJNA729271	stage_5W_vs_E10	ordinary_muscle_development	ENSGALG00010021189	ENSGALG00010021189	4.155110401	0.359732929	11.55054226	7.33561E-31	4.60302E-30	positive
PRJNA729271	stage_5W_vs_E10	ordinary_muscle_development	ENSGALG00010021190	ENSGALG00010021190	0.424974878	0.102937933	4.128457454	3.65205E-05	6.21904E-05	positive
PRJNA729271	stage_5W_vs_E10	ordinary_muscle_development	ENSGALG00010021191	MYRF	-1.337745448	0.215264225	-6.214434593	5.15098E-10	1.22499E-09	negative
PRJNA729271	stage_5W_vs_E10	ordinary_muscle_development	ENSGALG00010021192	ENSGALG00010021192	-3.049061117	0.183524641	-16.61390595	5.5274E-62	9.41477E-61	negative
PRJNA729271	stage_5W_vs_E10	ordinary_muscle_development	ENSGALG00010021193	USP37	-0.118106887	0.15729141	-0.750879443	0.452725213	0.494666965	negative
PRJNA729271	stage_5W_vs_E10	ordinary_muscle_development	ENSGALG00010021194	CYP11A1	0.087144289	0.366333627	0.237882308	0.811972378	0.833891145	positive
PRJNA729271	stage_5W_vs_E10	ordinary_muscle_development	ENSGALG00010021195	GCLM	-0.546735177	0.09089336	-6.0151278	1.79745E-09	4.13425E-09	negative
PRJNA729271	stage_5W_vs_E10	ordinary_muscle_development	ENSGALG00010021196	ATP6V0A1	2.11400777	0.120083941	17.60441692	2.27842E-69	4.7247E-68	positive
PRJNA729271	stage_5W_vs_E10	ordinary_muscle_development	ENSGALG00010021197	CHCHD1	0.45857638	0.105499136	4.346731141	1.38181E-05	2.43874E-05	positive
PRJNA729271	stage_5W_vs_E10	ordinary_muscle_development	ENSGALG00010021199	TMEM258	-0.921806804	0.138484269	-6.656400831	2.80614E-11	7.23755E-11	negative
PRJNA729271	stage_5W_vs_E10	ordinary_muscle_development	ENSGALG00010021200	FTO	-0.453721101	0.165417369	-2.742886698	0.00609017	0.008524238	negative
PRJNA729271	stage_5W_vs_E10	ordinary_muscle_development	ENSGALG00010021201	TRMT1L	-0.292353813	0.089466669	-3.267740005	0.001084099	0.001638242	negative
PRJNA729271	stage_5W_vs_E10	ordinary_muscle_development	ENSGALG00010021202	UROC1	-2.218673153	0.61861368	-3.586524557	0.000335115	0.000528257	negative
PRJNA729271	stage_5W_vs_E10	ordinary_muscle_development	ENSGALG00010021203	FUT11	-0.11406165	0.082966645	-1.374789235	0.169196796	0.199471945	negative
PRJNA729271	stage_5W_vs_E10	ordinary_muscle_development	ENSGALG00010021204	NAGLU	0.54547498	0.09473052	5.758175749	8.50278E-09	1.88452E-08	positive
PRJNA729271	stage_5W_vs_E10	ordinary_muscle_development	ENSGALG00010021206	HSD17B1	0.502383187	0.420620275	1.194386523	0.232326806	0.267098434	positive
PRJNA729271	stage_5W_vs_E10	ordinary_muscle_development	ENSGALG00010021207	MAD2L2	-0.173179837	0.122593144	-1.412638845	0.157761941	0.186704714	negative
PRJNA729271	stage_5W_vs_E10	ordinary_muscle_development	ENSGALG00010021210	ENSGALG00010021210	-0.501250146	0.064028728	-7.8285195	4.93648E-15	1.55148E-14	negative
PRJNA729271	stage_5W_vs_E10	ordinary_muscle_development	ENSGALG00010021211	FEN1	-2.569205376	0.158332275	-16.22666871	3.26716E-59	5.12527E-58	negative
PRJNA729271	stage_5W_vs_E10	ordinary_muscle_development	ENSGALG00010021212	MYO18B	3.882796534	0.161057536	24.10813317	2.054E-128	1.4523E-126	positive
PRJNA729271	stage_5W_vs_E10	ordinary_muscle_development	ENSGALG00010021213	FBXO44	0.928100483	0.193556202	4.794992212	1.62681E-06	3.0709E-06	positive
PRJNA729271	stage_5W_vs_E10	ordinary_muscle_development	ENSGALG00010021214	FBXL18	-0.106518285	0.115759683	-0.92016739	0.357485293	0.397769964	negative
PRJNA729271	stage_5W_vs_E10	ordinary_muscle_development	ENSGALG00010021215	SH3PXD2A	-0.326704804	0.111714225	-2.924469153	0.003450443	0.00495435	negative
PRJNA729271	stage_5W_vs_E10	ordinary_muscle_development	ENSGALG00010021217	FBXO2	-1.398662637	0.212776899	-6.573376357	4.91869E-11	1.25016E-10	negative
PRJNA729271	stage_5W_vs_E10	ordinary_muscle_development	ENSGALG00010021218	DPF3	0.357658469	0.174490519	2.049730096	0.040390776	0.051546426	positive
PRJNA729271	stage_5W_vs_E10	ordinary_muscle_development	ENSGALG00010021219	STRA6	-6.537299858	0.482716938	-13.54271901	8.74896E-42	8.06718E-41	negative
PRJNA729271	stage_5W_vs_E10	ordinary_muscle_development	ENSGALG00010021220	SYNPO2L	3.466932002	0.205742543	16.85082704	1.03458E-63	1.85138E-62	positive
PRJNA729271	stage_5W_vs_E10	ordinary_muscle_development	ENSGALG00010021222	ENSGALG00010021222	5.281770049	0.302362665	17.4683275	2.49708E-68	5.00625E-67	positive
PRJNA729271	stage_5W_vs_E10	ordinary_muscle_development	ENSGALG00010021223	DFFB	0.230674906	0.112040562	2.058851749	0.039508443	0.050518249	positive
PRJNA729271	stage_5W_vs_E10	ordinary_muscle_development	ENSGALG00010021224	MYOZ1	7.908008419	0.140963083	56.09985436	0	0	positive
PRJNA729271	stage_5W_vs_E10	ordinary_muscle_development	ENSGALG00010021227	CHCHD4	-1.193768246	0.100023816	-11.93484007	7.79085E-33	5.28938E-32	negative
PRJNA729271	stage_5W_vs_E10	ordinary_muscle_development	ENSGALG00010021228	ESRP2	-6.097693701	0.956395538	-6.375702792	1.82126E-10	4.46082E-10	negative
PRJNA729271	stage_5W_vs_E10	ordinary_muscle_development	ENSGALG00010021229	GLO1	1.997884554	0.17823964	11.20897994	3.68415E-29	2.16289E-28	positive
PRJNA729271	stage_5W_vs_E10	ordinary_muscle_development	ENSGALG00010021230	C1orf174	0.171640366	0.058919398	2.91313847	0.003578159	0.005128081	positive
PRJNA729271	stage_5W_vs_E10	ordinary_muscle_development	ENSGALG00010021231	STN1	-1.386892495	0.120620242	-11.49800788	1.34995E-30	8.38011E-30	negative
PRJNA729271	stage_5W_vs_E10	ordinary_muscle_development	ENSGALG00010021232	ACTB	-0.71943692	0.109336391	-6.580031709	4.70348E-11	1.19735E-10	negative
PRJNA729271	stage_5W_vs_E10	ordinary_muscle_development	ENSGALG00010021233	COASY	0.335683824	0.062014575	5.412982735	6.19835E-08	1.29684E-07	positive
PRJNA729271	stage_5W_vs_E10	ordinary_muscle_development	ENSGALG00010021234	ISLR2	-1.3817724	0.342131572	-4.038716429	5.37445E-05	9.05213E-05	negative
PRJNA729271	stage_5W_vs_E10	ordinary_muscle_development	ENSGALG00010021235	XRCC5	-1.858096828	0.140731027	-13.20317823	8.411E-40	7.31798E-39	negative
PRJNA729271	stage_5W_vs_E10	ordinary_muscle_development	ENSGALG00010021236	BTBD9	0.402777829	0.073773962	5.459620378	4.77154E-08	1.00675E-07	positive
PRJNA729271	stage_5W_vs_E10	ordinary_muscle_development	ENSGALG00010021237	FSCN1	-3.789148853	0.223998142	-16.91598338	3.43042E-64	6.2E-63	negative
PRJNA729271	stage_5W_vs_E10	ordinary_muscle_development	ENSGALG00010021238	NDUFS8	1.505200541	0.14707906	10.23395539	1.39695E-24	6.82235E-24	positive
PRJNA729271	stage_5W_vs_E10	ordinary_muscle_development	ENSGALG00010021239	USP54	0.177465559	0.070342909	2.522863506	0.011640357	0.015826562	positive
PRJNA729271	stage_5W_vs_E10	ordinary_muscle_development	ENSGALG00010021240	SLK	0.215644078	0.078598518	2.743615056	0.006076675	0.008506171	positive
PRJNA729271	stage_5W_vs_E10	ordinary_muscle_development	ENSGALG00010021241	TMEM43	-0.469910398	0.171983471	-2.732299768	0.006289389	0.008791195	negative
PRJNA729271	stage_5W_vs_E10	ordinary_muscle_development	ENSGALG00010021242	BCAR3	3.927248313	0.223977222	17.53414156	7.86281E-69	1.60523E-67	positive
PRJNA729271	stage_5W_vs_E10	ordinary_muscle_development	ENSGALG00010021243	DYL1	-1.300887144	0.095327211	-13.64654574	2.11677E-42	1.9885E-41	negative
PRJNA729271	stage_5W_vs_E10	ordinary_muscle_development	ENSGALG00010021244	PPP3CB	0.638302878	0.087395828	7.303585209	2.80199E-13	8.01557E-13	positive
PRJNA729271	stage_5W_vs_E10	ordinary_muscle_development	ENSGALG00010021245	ZFAND3	0.797856928	0.070514958	11.31471888	1.10957E-29	6.62954E-29	positive
PRJNA729271	stage_5W_vs_E10	ordinary_muscle_development	ENSGALG00010021246	PECR	0.297367532	0.115769563	2.568615835	0.010210558	0.013970364	positive
PRJNA729271	stage_5W_vs_E10	ordinary_muscle_development	ENSGALG00010021247	GNAO1	-2.002351432	0.228003493	-8.782108569	1.60438E-18	5.99832E-18	negative
PRJNA729271	stage_5W_vs_E10	ordinary_muscle_development	ENSGALG00010021248	ANXA7	0.488656788	0.168484762	2.900302576	0.003728026	0.005334953	positive
PRJNA729271	stage_5W_vs_E10	ordinary_muscle_development	ENSGALG00010021249	ENSGALG00010021249	-1.198087722	0.426506761	-2.809070881	0.004968471	0.007017245	negative
PRJNA729271	stage_5W_vs_E10	ordinary_muscle_development	ENSGALG00010021250	ISLR	0.439187572	0.145848239	3.011264144	0.002601624	0.003785439	positive
PRJNA729271	stage_5W_vs_E10	ordinary_muscle_development	ENSGALG00010021251	RNF216	0.247778238	0.084057701	2.947716105	0.003201309	0.004613998	positive
PRJNA729271	stage_5W_vs_E10	ordinary_muscle_development	ENSGALG00010021252	XPC	0.667019381	0.131501632	5.072327788	3.92979E-07	7.7637E-07	positive
PRJNA729271	stage_5W_vs_E10	ordinary_muscle_development	ENSGALG00010021253	TMEM169	-1.808763868	0.116748541	-15.49281776	3.8791E-54	5.22561E-53	negative
PRJNA729271	stage_5W_vs_E10	ordinary_muscle_development	ENSGALG00010021254	FNBP1L	-0.469176577	0.108489893	-4.32461092	1.52801E-05	2.68663E-05	negative
PRJNA729271	stage_5W_vs_E10	ordinary_muscle_development	ENSGALG00010021255	EIF3F	0.343154498	0.092086996	3.72641647	0.000194221	0.000312353	positive
PRJNA729271	stage_5W_vs_E10	ordinary_muscle_development	ENSGALG00010021256	ENSGALG00010021256	1.085116152	0.289123801	3.753119424	0.000174648	0.000281655	positive
PRJNA729271	stage_5W_vs_E10	ordinary_muscle_development	ENSGALG00010021257	RGS6	-1.362493045	0.326610165	-4.171618619	3.02444E-05	5.18562E-05	negative
PRJNA729271	stage_5W_vs_E10	ordinary_muscle_development	ENSGALG00010021258	LSM3	-1.314401285	0.115632118	-11.36709515	6.09835E-30	3.68315E-29	negative
PRJNA729271	stage_5W_vs_E10	ordinary_muscle_development	ENSGALG00010021259	CEP104	-0.534152267	0.134127427	-3.98242386	6.8216E-05	0.000114113	negative
PRJNA729271	stage_5W_vs_E10	ordinary_muscle_development	ENSGALG00010021260	KDM4A	0.257270242	0.067644752	3.803255008	0.000142807	0.000232114	positive
PRJNA729271	stage_5W_vs_E10	ordinary_muscle_development	ENSGALG00010021261	JUP	0.52134612	0.128296478	4.063604316	4.83207E-05	8.16422E-05	positive
PRJNA729271	stage_5W_vs_E10	ordinary_muscle_development	ENSGALG00010021262	MDGA1	-2.126952201	0.361367824	-5.885837248	3.96044E-09	8.94323E-09	negative
PRJNA729271	stage_5W_vs_E10	ordinary_muscle_development	ENSGALG00010021263	STOML1	0.595350829	0.242551759	2.454531075	0.014106847	0.018991246	positive
PRJNA729271	stage_5W_vs_E10	ordinary_muscle_development	ENSGALG00010021264	CBFB	-1.593678339	0.170495719	-9.347321746	8.98952E-21	3.71022E-20	negative
PRJNA729271	stage_5W_vs_E10	ordinary_muscle_development	ENSGALG00010021265	SIPA1L1	-0.9196075	0.090569151	-10.15365039	3.19196E-24	1.53508E-23	negative
PRJNA729271	stage_5W_vs_E10	ordinary_muscle_development	ENSGALG00010021266	COQ5	-0.176264736	0.100410936	-1.755433657	0.079185159	0.097443661	negative
PRJNA729271	stage_5W_vs_E10	ordinary_muscle_development	ENSGALG00010021267	LRRC47	-0.02899257	0.079418417	-0.365061039	0.715065843	0.745889421	negative
PRJNA729271	stage_5W_vs_E10	ordinary_muscle_development	ENSGALG00010021269	NUDT13	-0.130513861	0.179732152	-0.72615756	0.467742186	0.509653686	negative
PRJNA729271	stage_5W_vs_E10	ordinary_muscle_development	ENSGALG00010021271	ENSGALG00010021271	-0.963294278	0.475741087	-2.024828849	0.042884942	0.05454218	negative
PRJNA729271	stage_5W_vs_E10	ordinary_muscle_development	ENSGALG00010021272	ENSGALG00010021272	1.643464328	0.355308288	4.625460155	3.73768E-06	6.87097E-06	positive
PRJNA729271	stage_5W_vs_E10	ordinary_muscle_development	ENSGALG00010021275	MAP3K2	0.2447825	0.091870322	2.664434993	0.007711777	0.010679489	positive
PRJNA729271	stage_5W_vs_E10	ordinary_muscle_development	ENSGALG00010021277	ENSGALG00010021277	-0.41301048	0.249378319	-1.656160333	0.097689354	0.118822454	negative
PRJNA729271	stage_5W_vs_E10	ordinary_muscle_development	ENSGALG00010021287	COL17A1	-6.829628978	0.482454406	-14.15600912	1.71459E-45	1.77902E-44	negative
PRJNA729271	stage_5W_vs_E10	ordinary_muscle_development	ENSGALG00010021290	ERCC3	0.086544993	0.068202251	1.268946288	0.204460222	0.237414957	positive
PRJNA729271	stage_5W_vs_E10	ordinary_muscle_development	ENSGALG00010021293	PCNX1	0.16386299	0.068995525	2.37497996	0.017549903	0.023361727	positive
PRJNA729271	stage_5W_vs_E10	ordinary_muscle_development	ENSGALG00010021296	GATC	0.075670637	0.122552161	0.617456569	0.53693362	0.576934975	positive
PRJNA729271	stage_5W_vs_E10	ordinary_muscle_development	ENSGALG00010021298	RPGRIP1L	-1.51836377	0.150134474	-10.11335855	4.82029E-24	2.29987E-23	negative
PRJNA729271	stage_5W_vs_E10	ordinary_muscle_development	ENSGALG00010021299	CYP27C1	-5.178703075	0.202354578	-25.59222098	1.8622E-144	1.687E-142	negative
PRJNA729271	stage_5W_vs_E10	ordinary_muscle_development	ENSGALG00010021301	SMURF1	0.16473178	0.080561266	2.044801297	0.040874452	0.052122388	positive
PRJNA729271	stage_5W_vs_E10	ordinary_muscle_development	ENSGALG00010021302	P4HA1	-0.597722573	0.060264254	-9.918360084	3.46401E-23	1.59248E-22	negative
PRJNA729271	stage_5W_vs_E10	ordinary_muscle_development	ENSGALG00010021307	MAP3K9	-0.326483283	0.136189568	-2.397270872	0.016517708	0.022064711	negative

PRJNA729271	stage_5W_vs_E10	ordinary_muscle_development	ENSGALG00010021309	ST3GAL3	0.261430916	0.126809569	2.061602437	0.039245602	0.050195447	positive
PRJNA729271	stage_5W_vs_E10	ordinary_muscle_development	ENSGALG00010021311	TRIAP1	-0.018929968	0.121148894	-0.156253738	0.87583302	0.891016257	negative
PRJNA729271	stage_5W_vs_E10	ordinary_muscle_development	ENSGALG00010021313	KPNA7	4.114492906	1.232293898	3.338889297	0.000841141	0.001282864	positive
PRJNA729271	stage_5W_vs_E10	ordinary_muscle_development	ENSGALG00010021315	COX6A1	-0.396114243	0.135929089	-2.914124172	0.00356688	0.005112422	negative
PRJNA729271	stage_5W_vs_E10	ordinary_muscle_development	ENSGALG00010021318	TP73	-1.882357367	0.269779685	-6.977387376	3.0072E-12	8.16891E-12	negative
PRJNA729271	stage_5W_vs_E10	ordinary_muscle_development	ENSGALG00010021320	SLC6A6	1.159646496	0.195280286	5.938369515	2.87871E-09	6.54334E-09	positive
PRJNA729271	stage_5W_vs_E10	ordinary_muscle_development	ENSGALG00010021325	ARPC1A	0.081468682	0.06416063	1.269761252	0.204169687	0.237134584	positive
PRJNA729271	stage_5W_vs_E10	ordinary_muscle_development	ENSGALG00010021327	IGSF1	3.368804983	0.420051349	8.019983735	1.05759E-15	3.45343E-15	positive
PRJNA729271	stage_5W_vs_E10	ordinary_muscle_development	ENSGALG00010021329	PSMC3IP	-2.179901073	0.170347881	-12.79676073	1.70934E-37	1.37649E-36	negative
PRJNA729271	stage_5W_vs_E10	ordinary_muscle_development	ENSGALG00010021330	COMMD4	-0.698874193	0.132213509	-5.285951461	1.25053E-07	2.56458E-07	negative
PRJNA729271	stage_5W_vs_E10	ordinary_muscle_development	ENSGALG00010021332	OIT3	0.556797573	0.130016441	4.282516645	1.84791E-05	3.22717E-05	positive
PRJNA729271	stage_5W_vs_E10	ordinary_muscle_development	ENSGALG00010021335	NFATC3	0.687964156	0.101661993	6.767171626	1.31324E-11	3.45408E-11	positive
PRJNA729271	stage_5W_vs_E10	ordinary_muscle_development	ENSGALG00010021338	PDIA5	-1.114303251	0.158831991	-7.015609682	2.28948E-12	6.26152E-12	negative
PRJNA729271	stage_5W_vs_E10	ordinary_muscle_development	ENSGALG00010021341	L3MBTL1	-0.510752059	0.157740139	-3.237933359	0.001203989	0.001809042	negative
PRJNA729271	stage_5W_vs_E10	ordinary_muscle_development	ENSGALG00010021342	SFR1	-2.856343358	0.189486368	-15.07413641	2.39629E-51	3.01512E-50	negative
PRJNA729271	stage_5W_vs_E10	ordinary_muscle_development	ENSGALG00010021345	NEIL1	0.750057469	0.152102598	4.931260074	8.17009E-07	1.58069E-06	positive
PRJNA729271	stage_5W_vs_E10	ordinary_muscle_development	ENSGALG00010021346	RPS21	0.175794861	0.278516614	0.631182675	0.527921089	0.568473084	positive
PRJNA729271	stage_5W_vs_E10	ordinary_muscle_development	ENSGALG00010021348	SLC4A3	0.172307151	0.116456087	1.479589042	0.138982953	0.165643636	positive
PRJNA729271	stage_5W_vs_E10	ordinary_muscle_development	ENSGALG00010021350	CABLES2	-0.271146477	0.135797315	-1.996699845	0.045857799	0.058088683	negative
PRJNA729271	stage_5W_vs_E10	ordinary_muscle_development	ENSGALG00010021352	CFAP43	-0.761315774	0.230048718	-3.309367607	0.00093507	0.001421632	negative
PRJNA729271	stage_5W_vs_E10	ordinary_muscle_development	ENSGALG00010021353	IPO13	0.997083572	0.178725715	5.578847864	2.42117E-08	5.22012E-08	positive
PRJNA729271	stage_5W_vs_E10	ordinary_muscle_development	ENSGALG00010021354	ENSGALG00010021354	0.082467566	0.112616941	0.732283836	0.463995343	0.505913382	positive
PRJNA729271	stage_5W_vs_E10	ordinary_muscle_development	ENSGALG00010021358	MMP15	0.620013753	0.129229268	4.797781217	1.60433E-06	3.02993E-06	positive
PRJNA729271	stage_5W_vs_E10	ordinary_muscle_development	ENSGALG00010021360	PTPRN	-0.239781469	0.265997209	-0.901443551	0.367352535	0.407966211	negative
PRJNA729271	stage_5W_vs_E10	ordinary_muscle_development	ENSGALG00010021361	WRAP73	-0.995598869	0.095201168	-10.45784297	1.34891E-25	6.83177E-25	negative
PRJNA729271	stage_5W_vs_E10	ordinary_muscle_development	ENSGALG00010021362	ARPC1B	1.331482045	0.119901077	11.10483806	1.18833E-28	6.8271E-28	positive
PRJNA729271	stage_5W_vs_E10	ordinary_muscle_development	ENSGALG00010021363	ENSGALG00010021363	-0.043049581	0.07437857	-0.578790108	0.562730807	0.602154366	negative
PRJNA729271	stage_5W_vs_E10	ordinary_muscle_development	ENSGALG00010021367	GRIP2	-0.578228038	0.18037545	-3.205691445	0.001347384	0.00201593	negative
PRJNA729271	stage_5W_vs_E10	ordinary_muscle_development	ENSGALG00010021372	CD86	5.253607875	0.55434429	9.477157008	2.61305E-21	1.10814E-20	positive
PRJNA729271	stage_5W_vs_E10	ordinary_muscle_development	ENSGALG00010021373	CEP250	-0.573895507	0.154263705	-3.720223808	0.000199046	0.000319864	negative
PRJNA729271	stage_5W_vs_E10	ordinary_muscle_development	ENSGALG00010021375	TUBG1	-0.710301508	0.117710062	-6.03433125	1.59622E-09	3.68486E-09	negative
PRJNA729271	stage_5W_vs_E10	ordinary_muscle_development	ENSGALG00010021378	KIF11	-2.798786871	0.187482547	-14.92825289	2.15848E-50	2.61817E-49	negative
PRJNA729271	stage_5W_vs_E10	ordinary_muscle_development	ENSGALG00010021381	DNAJB2	2.287974818	0.146966787	15.5679719	1.20157E-54	1.64154E-53	positive
PRJNA729271	stage_5W_vs_E10	ordinary_muscle_development	ENSGALG00010021382	TPRG1L	0.368455949	0.086870295	4.241449259	2.22081E-05	3.8524E-05	positive
PRJNA729271	stage_5W_vs_E10	ordinary_muscle_development	ENSGALG00010021383	MSI1	-0.882898112	0.119664031	-7.378141159	1.60515E-13	4.64786E-13	negative
PRJNA729271	stage_5W_vs_E10	ordinary_muscle_development	ENSGALG00010021384	ARTN	0.293665803	0.421218343	0.697181896	0.485688967	0.527148003	positive
PRJNA729271	stage_5W_vs_E10	ordinary_muscle_development	ENSGALG00010021385	GDF5	-7.462051319	0.614539334	-12.14251214	6.28658E-34	4.45159E-33	negative
PRJNA729271	stage_5W_vs_E10	ordinary_muscle_development	ENSGALG00010021387	SLC2A14	1.385520964	0.093270825	14.85481614	6.47453E-50	7.77534E-49	positive
PRJNA729271	stage_5W_vs_E10	ordinary_muscle_development	ENSGALG00010021388	DPH2	-1.324078403	0.159295828	-8.312072053	9.40453E-17	3.23183E-16	negative
PRJNA729271	stage_5W_vs_E10	ordinary_muscle_development	ENSGALG00010021390	GSTO2	-1.47711699	0.217219019	-6.800127349	1.04527E-11	2.76582E-11	negative
PRJNA729271	stage_5W_vs_E10	ordinary_muscle_development	ENSGALG00010021391	ENSGALG00010021391	3.265584854	0.359150879	9.092515267	9.67739E-20	3.82843E-19	positive
PRJNA729271	stage_5W_vs_E10	ordinary_muscle_development	ENSGALG00010021393	CCDC174	-0.115190697	0.114569449	-1.005422461	0.314693469	0.353740351	negative
PRJNA729271	stage_5W_vs_E10	ordinary_muscle_development	ENSGALG00010021394	MAN2C1	-0.469648737	0.098515916	-4.767237196	1.86769E-06	3.50995E-06	negative
PRJNA729271	stage_5W_vs_E10	ordinary_muscle_development	ENSGALG00010021396	MED6	-0.048233924	0.079296265	-0.608274856	0.543005195	0.582767681	negative
PRJNA729271	stage_5W_vs_E10	ordinary_muscle_development	ENSGALG00010021397	ENSGALG00010021397	3.837392894	1.743557681	2.200898162	0.027743234	0.036108304	positive
PRJNA729271	stage_5W_vs_E10	ordinary_muscle_development	ENSGALG00010021400	B4GALT2	-0.019453518	0.092322705	-0.210712175	0.833111873	0.853183313	negative
PRJNA729271	stage_5W_vs_E10	ordinary_muscle_development	ENSGALG00010021401	PDAP1	-0.765630783	0.095224831	-8.040243026	8.96605E-16	2.93635E-15	negative
PRJNA729271	stage_5W_vs_E10	ordinary_muscle_development	ENSGALG00010021402	ENSGALG00010021402	0.091634826	0.088922106	1.030506702	0.302772208	0.341372367	positive
PRJNA729271	stage_5W_vs_E10	ordinary_muscle_development	ENSGALG00010021404	UQCC1	0.116821527	0.094358505	1.238060378	0.215693674	0.249400112	positive
PRJNA729271	stage_5W_vs_E10	ordinary_muscle_development	ENSGALG00010021405	IDE	1.592288122	0.1138324	13.98800455	1.84517E-44	1.84072E-43	positive
PRJNA729271	stage_5W_vs_E10	ordinary_muscle_development	ENSGALG00010021407	SIN3A	-0.136061379	0.111698795	-1.218109641	0.223182311	0.257401941	negative
PRJNA729271	stage_5W_vs_E10	ordinary_muscle_development	ENSGALG00010021408	TTC9	0.978010075	0.205503138	4.759100448	1.94458E-06	3.6497E-06	positive
PRJNA729271	stage_5W_vs_E10	ordinary_muscle_development	ENSGALG00010021409	MEGF6	-1.856361725	0.261936009	-7.087081051	1.3697E-12	3.79109E-12	negative
PRJNA729271	stage_5W_vs_E10	ordinary_muscle_development	ENSGALG00010021411	MCU	0.961166571	0.129009105	7.450377764	9.30733E-14	2.73152E-13	positive
PRJNA729271	stage_5W_vs_E10	ordinary_muscle_development	ENSGALG00010021412	ZNF319	0.513821928	0.123754179	4.15195617	3.29645E-05	5.63402E-05	positive
PRJNA729271	stage_5W_vs_E10	ordinary_muscle_development	ENSGALG00010021413	SYNJ2BP	1.463152996	0.149299166	9.800141795	1.12428E-22	5.06729E-22	positive
PRJNA729271	stage_5W_vs_E10	ordinary_muscle_development	ENSGALG00010021414	BUD31	0.562740552	0.111247974	5.058434155	4.22713E-07	8.3307E-07	positive
PRJNA729271	stage_5W_vs_E10	ordinary_muscle_development	ENSGALG00010021415	SIRT4	0.737484528	0.150134817	4.912148563	9.00838E-07	1.73616E-06	positive
PRJNA729271	stage_5W_vs_E10	ordinary_muscle_development	ENSGALG00010021416	PLEKHH3	1.396432918	0.121509902	11.49233842	1.44156E-30	8.93349E-30	positive
PRJNA729271	stage_5W_vs_E10	ordinary_muscle_development	ENSGALG00010021417	STK16	-1.327373353	0.165924294	-7.999873424	1.24547E-15	4.05233E-15	negative
PRJNA729271	stage_5W_vs_E10	ordinary_muscle_development	ENSGALG00010021419	MANSC1	-1.257241604	0.201287486	-6.245999846	4.21098E-10	1.0064E-09	negative
PRJNA729271	stage_5W_vs_E10	ordinary_muscle_development	ENSGALG00010021420	PLXNB3	-1.532283148	0.191372327	-8.006816724	1.17716E-15	3.83522E-15	negative
PRJNA729271	stage_5W_vs_E10	ordinary_muscle_development	ENSGALG00010021421	GLB1L	-0.355170568	0.170239735	-2.086296533	0.036951771	0.047441622	negative
PRJNA729271	stage_5W_vs_E10	ordinary_muscle_development	ENSGALG00010021424	PTCD1	-0.080834839	0.108821913	-0.742817658	0.457592084	0.499608109	negative
PRJNA729271	stage_5W_vs_E10	ordinary_muscle_development	ENSGALG00010021425	PTPN9	-1.140148725	0.125765192	-9.065693811	1.23814E-19	4.87288E-19	negative
PRJNA729271	stage_5W_vs_E10	ordinary_muscle_development	ENSGALG00010021426	SLC38A7	-0.141061078	0.145572972	-0.969005966	0.332542212	0.372216167	negative
PRJNA729271	stage_5W_vs_E10	ordinary_muscle_development	ENSGALG00010021427	RPLP0	0.254487801	0.135409076	1.879399877	0.060189913	0.075133723	positive
PRJNA729271	stage_5W_vs_E10	ordinary_muscle_development	ENSGALG00010021429	MED8	0.81567864	0.114353633	7.132949087	9.82409E-13	2.7411E-12	positive
PRJNA729271	stage_5W_vs_E10	ordinary_muscle_development	ENSGALG00010021431	CPSF4	-0.847441228	0.107861435	-7.856758352	3.94202E-15	1.24677E-14	negative
PRJNA729271	stage_5W_vs_E10	ordinary_muscle_development	ENSGALG00010021432	SYT9	0.427538843	0.29102431	1.46908292	0.1418103	0.168791287	positive
PRJNA729271	stage_5W_vs_E10	ordinary_muscle_development	ENSGALG00010021433	ENSGALG00010021433	4.909030957	0.270587382	18.14212813	1.48181E-73	3.42564E-72	positive
PRJNA729271	stage_5W_vs_E10	ordinary_muscle_development	ENSGALG00010021434	ANKZF1	-0.033905077	0.178843372	-0.189579726	0.849638476	0.867881943	negative
PRJNA729271	stage_5W_vs_E10	ordinary_muscle_development	ENSGALG00010021435	GCN1	0.518144491	0.089009472	5.821228658	5.84166E-09	1.30711E-08	positive
PRJNA729271	stage_5W_vs_E10	ordinary_muscle_development	ENSGALG00010021438	GOT2	1.402612716	0.110061985	12.74384358	3.37356E-37	2.70015E-36	positive
PRJNA729271	stage_5W_vs_E10	ordinary_muscle_development	ENSGALG00010021439	ITPRIP	0.108008407	0.186446073	0.579301056	0.562386053	0.601874324	positive
PRJNA729271	stage_5W_vs_E10	ordinary_muscle_development	ENSGALG00010021441	ENSGALG00010021441	0.632394235	0.318582331	1.98502608	0.04714157	0.059636678	positive
PRJNA729271	stage_5W_vs_E10	ordinary_muscle_development	ENSGALG00010021443	SNUPN	-0.003271541	0.135459041	-0.024151516	0.980731751	0.98360538	negative
PRJNA729271	stage_5W_vs_E10	ordinary_muscle_development	ENSGALG00010021444	ATG9A	2.178806313	0.245870299	8.861608439	7.88682E-19	2.98484E-18	positive
PRJNA729271	stage_5W_vs_E10	ordinary_muscle_development	ENSGALG00010021446	HOXB8	-0.630698733	0.596922846	-1.056583338	0.290701788	0.328529738	negative
PRJNA729271	stage_5W_vs_E10	ordinary_muscle_development	ENSGALG00010021447	OCSTAMP	1.985780496	0.623142085	3.186721845	0.001438951	0.002147168	positive
PRJNA729271	stage_5W_vs_E10	ordinary_muscle_development	ENSGALG00010021448	SZT2	0.648489497	0.100101738	6.478304067	9.27593E-11	2.31379E-10	positive
PRJNA729271	stage_5W_vs_E10	ordinary_muscle_development	ENSGALG00010021450	OLFML1	4.202599954	0.230927247	18.19880504	5.27459E-74	1.2452E-72	positive
PRJNA729271	stage_5W_vs_E10	ordinary_muscle_development	ENSGALG00010021452	SLC25A1	-0.826801178	0.119167886	-6.93812069	3.9735E-12	1.07215E-11	negative
PRJNA729271	stage_5W_vs_E10	ordinary_muscle_development	ENSGALG00010021454	ATP5J2	0.895278944	0.174281349	5.136975074	2.79196E-07	5.57124E-07	positive
PRJNA729271	stage_5W_vs_E10	ordinary_muscle_development	ENSGALG00010021455	CFAP58	-0.317234628	0.520119549	-0.60992637	0.541910584	0.581722129	negative
PRJNA729271	stage_5W_vs_E10	ordinary_muscle_development	ENSGALG00010021457	USB1	0.626102675	0.113650347	5.509025654	3.60825E-08	7.66549E-08	positive
PRJNA729271	stage_5W_vs_E10	ordinary_muscle_development	ENSGALG00010021458	USP30	0.111224509	0.081246085	1.368982992	0.171004591	0.201357559	positive
PRJNA729271	stage_5W_vs_E10	ordinary_muscle_development	ENSGALG00010021459	ARHGEF16	-0.053369004	0.128230651	-0.416195373	0.677267045	0.710809197	negative
PRJNA729271	stage_5W_vs_E10	ordinary_muscle_development	ENSGALG00010021461	SNX33	0.622515012	0.109834238	5.667768271	1.44669E-08	3.16526E-08	positive
PRJNA729271	stage_5W_vs_E10	ordinary_muscle_development	ENSGALG00010021462	SETD5	-0.575243236	0.095383411	-6.030851989	1.63098E-09	3.76149E-09	negative
PRJNA729271	stage_5W_vs_E10	ordinary_muscle_development	ENSGALG00010021464	ENSGALG00010021464	1.701119019	0.410200712	4.147040634	3.368E-05	5.75088E-05	positive
PRJNA729271	stage_5W_vs_E10	ordinary_muscle_development	ENSGALG00010021465	ABCB6	1.894233723	0.211592025	8.95229261	3.48175E-19	1.34045E-18	positive
PRJNA729271	stage_5W_vs_E10	ordinary_muscle_development	ENSGALG00010021466	HES4	-0.394527178	0.180469902	-2.186110667	0.02880751	0.037443046	negative
PRJNA729271	stage_5W_vs_E10	ordinary_muscle_development	ENSGALG00010021467	LHFPL4	0.606994434	0.252236539	2.406449266	0.016108441	0.021541826	positive
PRJNA729271	stage_5W_vs_E10	ordinary_muscle_development	ENSGALG00010021469	CSPG4	-0.04327274	0.161880962	-0.267312103	0.789228869	0.814457671	negative
PRJNA729271	stage_5W_vs_E10	ordinary_muscle_development	ENSGALG00010021470	ENSGALG00010021470	0.115002726	0.541692162	0.212302732	0.831870864	0.851972598	positive
PRJNA729271	stage_5W_vs_E10	ordinary_muscle_development	ENSGALG00010021473	SLC8A3	1.845461622	0.131392674	14.04539212	8.22121E-45	8.32168E-44	positive
PRJNA729271	stage_5W_vs_E10	ordinary_muscle_development	ENSGALG00010021474	PPFIBP2	-0.113592733	0.181521087	-0.625782575	0.531457563	0.571627644	negative
PRJNA729271	stage_5W_vs_E10	ordinary_muscle_development	ENSGALG00010021476	ALKBH2	0.031784471	0.122021897	0.260481697	0.794492232	0.8189564	positive
PRJNA729271	stage_5W_vs_E10	ordinary_muscle_development	ENSGALG00010021477	ZFAND2B	0.467703524	0.119084845	3.927481501	8.584E-05	0.000142395	positive
PRJNA729271	stage_5W_vs_E10	ordinary_muscle_development	ENSGALG00010021478	SLC13A3	-0.461753741	0.196682264	-2.347714182	0.01888901	0.025049972	negative
PRJNA729271	stage_5W_vs_E10	ordinary_muscle_development	ENSGALG00010021479	SKAP1	1.866351584	0.406039098	4.596482444	4.29683E-06	7.859E-06	positive
PRJNA729271	stage_5W_vs_E10	ordinary_muscle_development	ENSGALG00010021480	ENSGALG00010021480	0.141121128	0.075139278	1.878127287	0.060363754	0.075337749	positive
PRJNA729271	stage_5W_vs_E10	ordinary_muscle_development	ENSGALG00010021481	MICU1	0.497351268	0.174921294	2.843286004	0.004465098	0.006335317	positive
PRJNA729271	stage_5W_vs_E10	ordinary_muscle_development	ENSGALG00010021483	UNG	-2.194626415	0.159760966	-13.73693755	6.1002E-43	5.81726E-42	negative
PRJNA729271	stage_5W_vs_E10	ordinary_muscle_development	ENSGALG00010021484	MARCHF5	0.816643738	0.070945073	11.51092949	1.16219E-30	7.23934E-30	positive
PRJNA729271	stage_5W_vs_E10	ordinary_muscle_development	ENSGALG00010021485	SMOC1	-3.105845015	0.225314634	-13.78447976	3.16037E-43	3.04787E-42	negative
PRJNA729271	stage_5W_vs_E10	ordinary_muscle_development	ENSGALG00010021487	KLHL17	-1.914091184	0.159534572	-11.99797106	3.64113E-33	2.50371E-32	negative
PRJNA729271	stage_5W_vs_E10	ordinary_muscle_development	ENSGALG00010021488	HYI	-0.402470206	0.134732142	-2.987187768	0.002815567	0.00408239	negative
PRJNA729271	stage_5W_vs_E10	ordinary_muscle_development	ENSGALG00010021489	CNPPD1	1.034326744	0.085309191	12.12444673	7.83878E-34	5.52373E-33	positive
PRJNA729271	stage_5W_vs_E10	ordinary_muscle_development	ENSGALG00010021490	ACACB	5.432377978	0.329241638	16.49966878	3.68911E-61	6.13933E-60	positive
PRJNA729271	stage_5W_vs_E10	ordinary_muscle_development	ENSGALG00010021492	PARP6	0.511287229	0.157135993	3.253788128	0.001138772	0.00171603	positive
PRJNA729271	stage_5W_vs_E10	ordinary_muscle_development	ENSGALG00010021493	UNC93B1	0.84425158	0.130130798	6.487715397	8.71477E-11	2.1802E-10	positive
PRJNA729271	stage_5W_vs_E10	ordinary_muscle_development	ENSGALG00010021494	SLC2A10	-1.449364582	0.247674493	-5.851892788	4.8601E-09	1.09288E-08	negative
PRJNA729271	stage_5W_vs_E10	ordinary_muscle_development	ENSGALG00010021496	MTMR14	0.831815796	0.11538856	7.208823804	5.64372E-13	1.59372E-12	positive
PRJNA729271	stage_5W_vs_E10	ordinary_muscle_development	ENSGALG00010021498	B3GALT6	-1.628134756	0.11606189	-14.02815989	1.04837E-44	1.05382E-43	negative
PRJNA729271	stage_5W_vs_E10	ordinary_muscle_development	ENSGALG00010021501	SLC23A3	3.920345856	0.558203465	7.023148555	2.16923E-12	5.93825E-12	positive
PRJNA729271	stage_5W_vs_E10	ordinary_muscle_development	ENSGALG00010021502	DNAJB12	0.88996172	0.146026131	6.094537405	1.09754E-09	2.55852E-09	positive
PRJNA729271	stage_5W_vs_E10	ordinary_muscle_development	ENSGALG00010021503	KCNS1	-3.149055846	0.750856084	-4.193953958	2.74134E-05	4.7164E-05	negative
PRJNA729271	stage_5W_vs_E10	ordinary_muscle_development	ENSGALG00010021504	ODR4	-0.746851928	0.079418168	-9.404043771	5.25054E-21	2.19391E-20	negative
PRJNA729271	stage_5W_vs_E10	ordinary_muscle_development	ENSGALG00010021505	IHH	-4.822559571	0.582558393	-8.278242372	1.25007E-16	4.26949E-16	negative
PRJNA729271	stage_5W_vs_E10	ordinary_muscle_development	ENSGALG00010021506	MAX	0.679249795	0.251404584	2.701819453	0.00689612	0.009596168	positive
PRJNA729271	stage_5W_vs_E10	ordinary_muscle_development	ENSGALG00010021509	DDIT4	1.882436165	0.277862792	6.7746968	1.24667E-11	3.28376E-11	positive
PRJNA729271	stage_5W_vs_E10	ordinary_muscle_development	ENSGALG00010021510	NTSR1	2.23872871	0.388005027	5.769844597	7.93447E-09	1.7618E-08	positive
PRJNA729271	stage_5W_vs_E10	ordinary_muscle_development	ENSGALG00010021512	CPNE9	0.834686217	0.432949954	1.927904621	0.053866991	0.067637044	positive
PRJNA729271	stage_5W_vs_E10	ordinary_muscle_development	ENSGALG00010021513	SDF4	0.100825228	0.097250607	1.036756806	0.299849211	0.338234577	positive
PRJNA729271	stage_5W_vs_E10	ordinary_muscle_development	ENSGALG00010021514	TPR	-0.636289965	0.079450614	-8.008622321	1.16001E-15	3.78019E-15	negative
PRJNA729271	stage_5W_vs_E10	ordinary_muscle_development	ENSGALG00010021516	SRSF5	-1.169953553	0.153428017	-7.625423161	2.43235E-14	7.37902E-14	negative
PRJNA729271	stage_5W_vs_E10	ordinary_muscle_development	ENSGALG00010021518	CPEB3	2.451225821	0.145875619	16.80353329	2.2993E-63	4.07935E-62	positive
PRJNA729271	stage_5W_vs_E10	ordinary_muscle_development	ENSGALG00010021519	FNTB	0.53800413	0.099708568	5.395766308	6.82317E-08	1.42223E-07	positive
PRJNA729271	stage_5W_vs_E10	ordinary_muscle_development	ENSGALG00010021521	ENSGALG00010021521	1.363145764	0.261376751	5.215252532	1.83567E-07	3.71776E-07	positive

PRJNA729271	stage_5W_vs_E10	ordinary_muscle_development	ENSGALG00010021523	CELF6	-1.158445103	0.161689783	-7.164615367	7.80053E-13	2.18786E-12	negative
PRJNA729271	stage_5W_vs_E10	ordinary_muscle_development	ENSGALG00010021525	ENSGALG00010021525	3.849470221	0.726699357	5.29719778	1.17593E-07	2.41535E-07	positive
PRJNA729271	stage_5W_vs_E10	ordinary_muscle_development	ENSGALG00010021526	KCNJ12	3.730777938	0.213594978	17.46659948	2.57385E-68	5.15304E-67	positive
PRJNA729271	stage_5W_vs_E10	ordinary_muscle_development	ENSGALG00010021528	BRPF1	0.203908215	0.167165833	1.219796004	0.222542217	0.256707415	positive
PRJNA729271	stage_5W_vs_E10	ordinary_muscle_development	ENSGALG00010021529	BTAF1	0.523267941	0.075513968	6.9294192	4.22572E-12	1.13872E-11	positive
PRJNA729271	stage_5W_vs_E10	ordinary_muscle_development	ENSGALG00010021530	MLF2	-0.507651602	0.061069385	-8.312702037	9.35472E-17	3.21548E-16	negative
PRJNA729271	stage_5W_vs_E10	ordinary_muscle_development	ENSGALG00010021531	RAB15	-0.871033644	0.26704301	-3.261772871	0.001107178	0.001671724	negative
PRJNA729271	stage_5W_vs_E10	ordinary_muscle_development	ENSGALG00010021532	DPP10	-2.077582222	0.186192425	-11.1582532	6.52612E-29	3.78688E-28	negative
PRJNA729271	stage_5W_vs_E10	ordinary_muscle_development	ENSGALG00010021533	SSX2IP	-5.05091868	0.363731947	-13.88637626	7.66145E-44	7.529E-43	negative
PRJNA729271	stage_5W_vs_E10	ordinary_muscle_development	ENSGALG00010021534	KIFC3	0.426302122	0.170416336	2.501533192	0.012365685	0.016751458	positive
PRJNA729271	stage_5W_vs_E10	ordinary_muscle_development	ENSGALG00010021535	SUSD6	0.771114036	0.085544095	9.014228661	1.98261E-19	7.7128E-19	positive
PRJNA729271	stage_5W_vs_E10	ordinary_muscle_development	ENSGALG00010021538	TNFRSF18	3.994743556	0.395403491	10.10295469	5.36024E-24	2.55161E-23	positive
PRJNA729271	stage_5W_vs_E10	ordinary_muscle_development	ENSGALG00010021539	HEXA	-0.16735828	0.119692865	-1.398231043	0.162043698	0.191506188	negative
PRJNA729271	stage_5W_vs_E10	ordinary_muscle_development	ENSGALG00010021540	STK4	-0.735323552	0.077687185	-9.465184642	2.93043E-21	1.23947E-20	negative
PRJNA729271	stage_5W_vs_E10	ordinary_muscle_development	ENSGALG00010021541	CYP27A1	2.781013726	0.276182383	10.06948268	7.53735E-24	3.55644E-23	positive
PRJNA729271	stage_5W_vs_E10	ordinary_muscle_development	ENSGALG00010021543	PLEKHD1	0.426099898	0.265644137	1.604025233	0.108708491	0.131388812	positive
PRJNA729271	stage_5W_vs_E10	ordinary_muscle_development	ENSGALG00010021545	CYB5R2	-1.335117338	0.142476574	-9.37078499	7.19951E-21	2.98674E-20	negative
PRJNA729271	stage_5W_vs_E10	ordinary_muscle_development	ENSGALG00010021546	OGG1	-1.323900543	0.188747124	-7.0141495	2.31352E-12	6.32606E-12	negative
PRJNA729271	stage_5W_vs_E10	ordinary_muscle_development	ENSGALG00010021549	ANAPC16	0.374709949	0.090921851	4.121230989	3.76853E-05	6.41363E-05	positive
PRJNA729271	stage_5W_vs_E10	ordinary_muscle_development	ENSGALG00010021550	MAP2K3	1.475386701	0.171564943	8.599581464	8.00073E-18	2.89999E-17	positive
PRJNA729271	stage_5W_vs_E10	ordinary_muscle_development	ENSGALG00010021552	ENSGALG00010021552	1.121834481	0.172447013	6.505386581	7.74938E-11	1.94607E-10	positive
PRJNA729271	stage_5W_vs_E10	ordinary_muscle_development	ENSGALG00010021553	SLC39A9	-0.725501536	0.087238833	-8.316268242	9.07762E-17	3.12245E-16	negative
PRJNA729271	stage_5W_vs_E10	ordinary_muscle_development	ENSGALG00010021554	SNX11	0.294012141	0.148409662	1.981084903	0.047581753	0.06016203	positive
PRJNA729271	stage_5W_vs_E10	ordinary_muscle_development	ENSGALG00010021556	CAMK1	-0.890150282	0.14624929	-6.086527218	1.15386E-09	2.68721E-09	negative
PRJNA729271	stage_5W_vs_E10	ordinary_muscle_development	ENSGALG00010021558	CHURC1	1.76674485	0.179503723	9.842385548	7.39365E-23	3.35854E-22	positive
PRJNA729271	stage_5W_vs_E10	ordinary_muscle_development	ENSGALG00010021559	MYO1H	-0.276353217	0.607886048	-0.454613521	0.649387306	0.684174226	negative
PRJNA729271	stage_5W_vs_E10	ordinary_muscle_development	ENSGALG00010021561	TGIF2	-2.002790324	0.10112813	-19.80448295	2.72344E-87	8.67612E-86	negative
PRJNA729271	stage_5W_vs_E10	ordinary_muscle_development	ENSGALG00010021562	LRP6	0.094551228	0.082326494	1.148490888	0.250765972	0.286637708	positive
PRJNA729271	stage_5W_vs_E10	ordinary_muscle_development	ENSGALG00010021564	PRKAG3	11.10389356	0.629734814	17.63264999	1.38329E-69	2.88916E-68	positive
PRJNA729271	stage_5W_vs_E10	ordinary_muscle_development	ENSGALG00010021565	CBX1	-1.789345355	0.106092595	-16.86588354	8.01939E-64	1.43685E-62	negative
PRJNA729271	stage_5W_vs_E10	ordinary_muscle_development	ENSGALG00010021566	ENSGALG00010021566	-0.24712765	0.098358499	-2.512519536	0.011987247	0.016265287	negative
PRJNA729271	stage_5W_vs_E10	ordinary_muscle_development	ENSGALG00010021568	TADA3	0.468813732	0.142282998	3.294938527	0.000984432	0.001493245	positive
PRJNA729271	stage_5W_vs_E10	ordinary_muscle_development	ENSGALG00010021570	KATNB1	-0.908672979	0.108181747	-8.399503616	4.4837E-17	1.56568E-16	negative
PRJNA729271	stage_5W_vs_E10	ordinary_muscle_development	ENSGALG00010021572	MYL9	-0.392936355	0.113601335	-3.458906134	0.000542374	0.000840555	negative
PRJNA729271	stage_5W_vs_E10	ordinary_muscle_development	ENSGALG00010021573	NATD1	1.186205462	0.195732272	6.060346884	1.35828E-09	3.14862E-09	positive
PRJNA729271	stage_5W_vs_E10	ordinary_muscle_development	ENSGALG00010021574	TTLL4	1.673732469	0.225618033	7.418433923	1.18513E-13	3.45992E-13	positive
PRJNA729271	stage_5W_vs_E10	ordinary_muscle_development	ENSGALG00010021575	ADGRL2	-0.31428798	0.083046062	-3.784501904	0.000154017	0.000249466	negative
PRJNA729271	stage_5W_vs_E10	ordinary_muscle_development	ENSGALG00010021576	ERH	-1.492264623	0.122876749	-12.14440194	6.14301E-34	4.35631E-33	negative
PRJNA729271	stage_5W_vs_E10	ordinary_muscle_development	ENSGALG00010021577	ARIH1	0.718720034	0.076408284	9.406310336	5.13859E-21	2.14899E-20	positive
PRJNA729271	stage_5W_vs_E10	ordinary_muscle_development	ENSGALG00010021578	ENSGALG00010021578	6.797444126	0.930601207	7.304357735	2.78594E-13	7.9728E-13	positive
PRJNA729271	stage_5W_vs_E10	ordinary_muscle_development	ENSGALG00010021579	C20H20ORF24	0.438260329	0.11193195	3.915417628	9.02479E-05	0.000149348	positive
PRJNA729271	stage_5W_vs_E10	ordinary_muscle_development	ENSGALG00010021580	KCTD10	-1.235546339	0.110653412	-11.16591269	5.98731E-29	3.48119E-28	negative
PRJNA729271	stage_5W_vs_E10	ordinary_muscle_development	ENSGALG00010021582	STK36	-0.043964612	0.146520018	-0.300058742	0.764132349	0.791941827	negative
PRJNA729271	stage_5W_vs_E10	ordinary_muscle_development	ENSGALG00010021583	NFE2L1	1.746146337	0.220881503	7.905353364	2.67175E-15	8.52641E-15	positive
PRJNA729271	stage_5W_vs_E10	ordinary_muscle_development	ENSGALG00010021584	OVCH2	2.035117427	0.353987104	5.749128717	8.97045E-09	1.98635E-08	positive
PRJNA729271	stage_5W_vs_E10	ordinary_muscle_development	ENSGALG00010021586	BBS4	-0.310350365	0.123987354	-2.5030808	0.012311743	0.016681503	negative
PRJNA729271	stage_5W_vs_E10	ordinary_muscle_development	ENSGALG00010021588	RNF25	0.509546068	0.138684623	3.674135286	0.000238656	0.000381067	positive
PRJNA729271	stage_5W_vs_E10	ordinary_muscle_development	ENSGALG00010021593	DCAF7	-1.099595653	0.057795609	-19.02559155	1.04699E-80	2.87972E-79	negative
PRJNA729271	stage_5W_vs_E10	ordinary_muscle_development	ENSGALG00010021594	ADPGK	-1.693289843	0.092279666	-18.34954465	3.3289E-75	8.15075E-74	negative
PRJNA729271	stage_5W_vs_E10	ordinary_muscle_development	ENSGALG00010021595	UBE3B	0.535082498	0.084023855	6.368221221	1.91233E-10	4.67677E-10	positive
PRJNA729271	stage_5W_vs_E10	ordinary_muscle_development	ENSGALG00010021597	GGT7	-0.975527519	0.189418479	-5.150117994	2.60323E-07	5.20897E-07	negative
PRJNA729271	stage_5W_vs_E10	ordinary_muscle_development	ENSGALG00010021599	ARPC4	-0.135622726	0.103521156	-1.31009671	0.190163121	0.222166071	negative
PRJNA729271	stage_5W_vs_E10	ordinary_muscle_development	ENSGALG00010021600	FMN1	0.142799264	0.130534856	1.093955043	0.273974696	0.311129992	positive
PRJNA729271	stage_5W_vs_E10	ordinary_muscle_development	ENSGALG00010021601	LPAR5	1.965496568	0.348989564	5.631963728	1.78169E-08	3.87191E-08	positive
PRJNA729271	stage_5W_vs_E10	ordinary_muscle_development	ENSGALG00010021602	PLEKHG4	0.508629909	0.097789323	5.201282646	1.97918E-07	3.99947E-07	positive
PRJNA729271	stage_5W_vs_E10	ordinary_muscle_development	ENSGALG00010021604	SPTB	1.648289174	0.189397546	8.702801124	3.23791E-18	1.19454E-17	positive
PRJNA729271	stage_5W_vs_E10	ordinary_muscle_development	ENSGALG00010021605	MAP3K3	-0.086998406	0.11285114	-0.770912955	0.440758528	0.482610278	negative
PRJNA729271	stage_5W_vs_E10	ordinary_muscle_development	ENSGALG00010021606	RNF8	-0.273302844	0.094984031	-2.87735571	0.004010233	0.005723545	negative
PRJNA729271	stage_5W_vs_E10	ordinary_muscle_development	ENSGALG00010021607	SLC9A5	2.001190036	0.184880687	10.82422435	2.64307E-27	1.44082E-26	positive
PRJNA729271	stage_5W_vs_E10	ordinary_muscle_development	ENSGALG00010021609	ENSGALG00010021609	1.288981386	0.204219499	6.311744934	2.75907E-10	6.66878E-10	positive
PRJNA729271	stage_5W_vs_E10	ordinary_muscle_development	ENSGALG00010021610	TMEM11	-0.012232772	0.090957375	-0.134489058	0.893015863	0.906334192	negative
PRJNA729271	stage_5W_vs_E10	ordinary_muscle_development	ENSGALG00010021611	GREM1	0.662219281	0.599035449	1.105475947	0.268953411	0.306027137	positive
PRJNA729271	stage_5W_vs_E10	ordinary_muscle_development	ENSGALG00010021612	BCS1L	-0.218508312	0.134317608	-1.626803183	0.103778905	0.125765006	negative
PRJNA729271	stage_5W_vs_E10	ordinary_muscle_development	ENSGALG00010021613	ADGRL4	2.709339054	0.149491652	18.12368126	2.07253E-73	4.75336E-72	positive
PRJNA729271	stage_5W_vs_E10	ordinary_muscle_development	ENSGALG00010021615	FLVCR1	-1.525725941	0.17927655	-8.510460195	1.73244E-17	6.17604E-17	negative
PRJNA729271	stage_5W_vs_E10	ordinary_muscle_development	ENSGALG00010021617	SORBS1	1.402632377	0.111024751	12.63351067	1.37982E-36	1.07819E-35	positive
PRJNA729271	stage_5W_vs_E10	ordinary_muscle_development	ENSGALG00010021618	NEO1	-2.266912956	0.108267251	-20.93812251	2.40761E-97	9.64043E-96	negative
PRJNA729271	stage_5W_vs_E10	ordinary_muscle_development	ENSGALG00010021619	FHOD1	4.277878033	0.178657299	23.94460267	1.0517E-126	7.2596E-125	positive
PRJNA729271	stage_5W_vs_E10	ordinary_muscle_development	ENSGALG00010021621	ZNF518A	0.068460579	0.137465873	0.498018726	0.618470843	0.65512532	positive
PRJNA729271	stage_5W_vs_E10	ordinary_muscle_development	ENSGALG00010021622	PTHLH	-1.718765124	0.260868173	-6.58863481	4.43889E-11	1.13138E-10	negative
PRJNA729271	stage_5W_vs_E10	ordinary_muscle_development	ENSGALG00010021623	FAR1	1.720047274	0.12938859	13.29365496	2.51963E-40	2.22289E-39	positive
PRJNA729271	stage_5W_vs_E10	ordinary_muscle_development	ENSGALG00010021624	HSD11B2	1.666519531	0.36089666	4.617719462	3.8798E-06	7.12321E-06	positive
PRJNA729271	stage_5W_vs_E10	ordinary_muscle_development	ENSGALG00010021625	HCN4	-2.221760749	0.912174736	-2.435674506	0.014864053	0.019958726	negative
PRJNA729271	stage_5W_vs_E10	ordinary_muscle_development	ENSGALG00010021626	ZNF142	0.977533258	0.212789574	4.593896398	4.35045E-06	7.95206E-06	positive
PRJNA729271	stage_5W_vs_E10	ordinary_muscle_development	ENSGALG00010021627	NCOA6	0.603938788	0.103639278	5.827315678	5.6326E-09	1.2615E-08	positive
PRJNA729271	stage_5W_vs_E10	ordinary_muscle_development	ENSGALG00010021628	REC114	-0.169757546	0.550504407	-0.308367279	0.757802877	0.785662878	negative
PRJNA729271	stage_5W_vs_E10	ordinary_muscle_development	ENSGALG00010021629	DHRS7B	1.362522457	0.101279015	13.45315663	2.95005E-41	2.67926E-40	positive
PRJNA729271	stage_5W_vs_E10	ordinary_muscle_development	ENSGALG00010021630	VTG1	-1.98477093	0.325901624	-6.090092173	1.12846E-09	2.62889E-09	negative
PRJNA729271	stage_5W_vs_E10	ordinary_muscle_development	ENSGALG00010021632	LIMD2	-3.729520029	0.168710422	-22.10604411	2.7647E-108	1.3358E-106	negative
PRJNA729271	stage_5W_vs_E10	ordinary_muscle_development	ENSGALG00010021633	CCNJ	-1.047657471	0.108934663	-9.61730128	6.7581E-22	2.94701E-21	negative
PRJNA729271	stage_5W_vs_E10	ordinary_muscle_development	ENSGALG00010021634	PLCD4	5.291469713	0.241599109	21.90185936	2.4939E-106	1.1661E-104	positive
PRJNA729271	stage_5W_vs_E10	ordinary_muscle_development	ENSGALG00010021635	FOXJ2	0.728743479	0.113786164	6.404499925	1.50863E-10	3.71128E-10	positive
PRJNA729271	stage_5W_vs_E10	ordinary_muscle_development	ENSGALG00010021636	C1orf159	-0.210301018	0.19769165	-1.063783009	0.28742701	0.325133019	negative
PRJNA729271	stage_5W_vs_E10	ordinary_muscle_development	ENSGALG00010021637	ATP6V0D1	0.429762576	0.102909075	4.176138765	2.96499E-05	5.08911E-05	positive
PRJNA729271	stage_5W_vs_E10	ordinary_muscle_development	ENSGALG00010021638	SRC	-0.725656281	0.174767567	-4.152122116	3.29406E-05	5.6306E-05	negative
PRJNA729271	stage_5W_vs_E10	ordinary_muscle_development	ENSGALG00010021639	SCG5	0.960939563	0.343872685	2.794463145	0.005198596	0.007326558	positive
PRJNA729271	stage_5W_vs_E10	ordinary_muscle_development	ENSGALG00010021640	PLEKHG3	0.164947607	0.107927553	1.528317867	0.126433636	0.151571876	positive
PRJNA729271	stage_5W_vs_E10	ordinary_muscle_development	ENSGALG00010021642	NPTN	-0.007710688	0.090308484	-0.085381655	0.931957977	0.941143436	negative
PRJNA729271	stage_5W_vs_E10	ordinary_muscle_development	ENSGALG00010021643	ENSGALG00010021643	0.303235449	0.129296027	2.345280485	0.019012771	0.025209487	positive
PRJNA729271	stage_5W_vs_E10	ordinary_muscle_development	ENSGALG00010021644	TARDBP	-0.280905433	0.15392775	-1.824917417	0.068013492	0.08441267	negative
PRJNA729271	stage_5W_vs_E10	ordinary_muscle_development	ENSGALG00010021645	ASCC1	-0.22786562	0.139264689	-1.63620529	0.10179663	0.123486664	negative
PRJNA729271	stage_5W_vs_E10	ordinary_muscle_development	ENSGALG00010021646	RPUSD3	-0.172596035	0.168449077	-1.02461847	0.305543246	0.344335978	negative
PRJNA729271	stage_5W_vs_E10	ordinary_muscle_development	ENSGALG00010021647	ARHGAP11B	-1.668918931	0.137735186	-12.11686704	8.59836E-34	6.03258E-33	negative
PRJNA729271	stage_5W_vs_E10	ordinary_muscle_development	ENSGALG00010021648	BRE	0.055133539	0.067254613	0.819773333	0.412345336	0.453933746	positive
PRJNA729271	stage_5W_vs_E10	ordinary_muscle_development	ENSGALG00010021649	PXN	0.641124652	0.096280922	6.658896029	2.75892E-11	7.11829E-11	positive
PRJNA729271	stage_5W_vs_E10	ordinary_muscle_development	ENSGALG00010021650	CD276	-1.684398224	0.112449568	-14.97914356	1.00504E-50	1.23983E-49	negative
PRJNA729271	stage_5W_vs_E10	ordinary_muscle_development	ENSGALG00010021651	RNF113B	-0.076355101	0.156557867	-0.487711682	0.625754076	0.661969445	negative
PRJNA729271	stage_5W_vs_E10	ordinary_muscle_development	ENSGALG00010021652	BORCS5	-0.290842772	0.097071628	-2.996166636	0.00273397	0.003968843	negative
PRJNA729271	stage_5W_vs_E10	ordinary_muscle_development	ENSGALG00010021655	USP22	-0.673005686	0.094036687	-7.156841758	8.25569E-13	2.31328E-12	negative
PRJNA729271	stage_5W_vs_E10	ordinary_muscle_development	ENSGALG00010021656	SETD6	-0.149351834	0.079339725	-1.882434472	0.059777049	0.074637637	negative
PRJNA729271	stage_5W_vs_E10	ordinary_muscle_development	ENSGALG00010021657	JAGN1	0.513289938	0.142616363	3.599095692	0.000319326	0.000504232	positive
PRJNA729271	stage_5W_vs_E10	ordinary_muscle_development	ENSGALG00010021658	STRADA	-0.509741837	0.090973813	-5.603171065	2.10465E-08	4.55192E-08	negative
PRJNA729271	stage_5W_vs_E10	ordinary_muscle_development	ENSGALG00010021659	SPOCK2	-1.445567642	0.150818745	-9.584800865	9.26362E-22	4.00346E-21	negative
PRJNA729271	stage_5W_vs_E10	ordinary_muscle_development	ENSGALG00010021660	RNF223	0.981429773	0.479030872	2.048781883	0.040483448	0.051641959	positive
PRJNA729271	stage_5W_vs_E10	ordinary_muscle_development	ENSGALG00010021661	DUSP16	0.326141969	0.120922028	2.697126188	0.006994076	0.009726483	positive
PRJNA729271	stage_5W_vs_E10	ordinary_muscle_development	ENSGALG00010021662	RASGRP1	1.670193803	0.159690743	10.45892688	1.33357E-25	6.75643E-25	positive
PRJNA729271	stage_5W_vs_E10	ordinary_muscle_development	ENSGALG00010021663	CNOT9	0.518331339	0.139799618	3.707673508	0.000209172	0.000335504	positive
PRJNA729271	stage_5W_vs_E10	ordinary_muscle_development	ENSGALG00010021664	TYSND1	-0.127908796	0.164778125	-0.776248641	0.437602191	0.479588974	negative
PRJNA729271	stage_5W_vs_E10	ordinary_muscle_development	ENSGALG00010021665	CNOT1	0.186950019	0.09145315	2.044216292	0.040932184	0.052191416	positive
PRJNA729271	stage_5W_vs_E10	ordinary_muscle_development	ENSGALG00010021666	CCDC47	0.883746151	0.092163117	9.588935087	8.89988E-22	3.84856E-21	positive
PRJNA729271	stage_5W_vs_E10	ordinary_muscle_development	ENSGALG00010021667	TBX15	-0.002604111	0.191785494	-0.013578247	0.98916646	0.990670064	negative
PRJNA729271	stage_5W_vs_E10	ordinary_muscle_development	ENSGALG00010021668	PYGO1	-0.828217582	0.131748605	-6.286348013	3.25021E-10	7.82068E-10	negative
PRJNA729271	stage_5W_vs_E10	ordinary_muscle_development	ENSGALG00010021669	ENSGALG00010021669	-0.792932204	0.074609241	-10.62780146	2.21267E-26	1.16543E-25	negative
PRJNA729271	stage_5W_vs_E10	ordinary_muscle_development	ENSGALG00010021670	BLCAP	1.895451559	0.108557825	17.46029423	2.87449E-68	5.73908E-67	positive
PRJNA729271	stage_5W_vs_E10	ordinary_muscle_development	ENSGALG00010021671	TNFRSF13B	3.589674081	0.721299118	4.976678868	6.46845E-07	1.25988E-06	positive
PRJNA729271	stage_5W_vs_E10	ordinary_muscle_development	ENSGALG00010021674	ING4	-1.411474195	0.128023886	-11.02508477	2.89246E-28	1.63391E-27	negative
PRJNA729271	stage_5W_vs_E10	ordinary_muscle_development	ENSGALG00010021675	DDX42	-0.584467838	0.059730093	-9.785148602	1.30404E-22	5.85927E-22	negative
PRJNA729271	stage_5W_vs_E10	ordinary_muscle_development	ENSGALG00010021676	TP53INP2	2.795258423	0.275573325	10.14342889	3.54436E-24	1.70117E-23	positive
PRJNA729271	stage_5W_vs_E10	ordinary_muscle_development	ENSGALG00010021677	ENSGALG00010021677	-0.320083216	0.15360803	-2.083766164	0.037181443	0.047728038	negative
PRJNA729271	stage_5W_vs_E10	ordinary_muscle_development	ENSGALG00010021678	DNAAF4	0.369889637	0.163142265	2.267282706	0.023372961	0.030651504	positive
PRJNA729271	stage_5W_vs_E10	ordinary_muscle_development	ENSGALG00010021679	MASP2	-4.096124845	1.260079252	-3.25068827	0.00115126	0.001733768	negative
PRJNA729271	stage_5W_vs_E10	ordinary_muscle_development	ENSGALG00010021680	PIGT	0.514379839	0.118521683	4.339964014	1.42506E-05	2.5117E-05	positive
PRJNA729271	stage_5W_vs_E10	ordinary_muscle_development	ENSGALG00010021681	BTBD10	-0.128855568	0.080277414	-1.605128546	0.108465518	0.131126579	negative
PRJNA729271	stage_5W_vs_E10	ordinary_muscle_development	ENSGALG00010021682	MYL4	-6.721564989	0.304932358	-22.04280661	1.1198E-107	5.3568E-106	negative
PRJNA729271	stage_5W_vs_E10	ordinary_muscle_development	ENSGALG00010021683	HFM1	-1.550055766	0.443399922	-3.495841316	0.00047257	0.000735046	negative
PRJNA729271	stage_5W_vs_E10	ordinary_muscle_development	ENSGALG00010021684	BIN1	2.494906107	0.12119168	20.58644703	3.63035E-94	1.35275E-92	positive
PRJNA729271	stage_5W_vs_E10	ordinary_muscle_development	ENSGALG00010021685	DBNDD2	1.621899411	0.139241862	11.64807325	2.34705E-31	1.50135E-30	positive
PRJNA729271	stage_5W_vs_E10	ordinary_muscle_development	ENSGALG00010021686	CCPG1	2.293308319	0.164970459	13.90132713	6.21777E-44	6.12689E-43	positive
PRJNA729271	stage_5W_vs_E10	ordinary_muscle_development	ENSGALG00010021687	ZNF384	-0.213799694	0.107113839	-1.996004408	0.045933443	0.058179418	negative
PRJNA729271	stage_5W_vs_E10	ordinary_muscle_development	ENSGALG00010021688	ITPR1	0.20086985	0.116897251	1.718345376	0.085733637	0.105073905	positive
PRJNA729271	stage_5W_vs_E10	ordinary_muscle_development	ENSGALG00010021689	MPRIP	0.434964013	0.109286421	3.980037125	6.89045E-05	0.000115238	positive

PRJNA729271	stage_5W_vs_E10	ordinary_muscle_development	ENSGALG00010021691	PIGB	-1.125061805	0.153920966	-7.309347373	2.68443E-13	7.69292E-13	negative
PRJNA729271	stage_5W_vs_E10	ordinary_muscle_development	ENSGALG00010021693	PPA1	-0.239209585	0.091837757	-2.604697602	0.009195538	0.012635257	negative
PRJNA729271	stage_5W_vs_E10	ordinary_muscle_development	ENSGALG00010021694	ENSGALG00010021694	3.728429831	1.100472072	3.388027672	0.000703972	0.001080367	positive
PRJNA729271	stage_5W_vs_E10	ordinary_muscle_development	ENSGALG00010021695	FNBP4	0.051802293	0.071483793	0.72467186	0.468653362	0.510608124	positive
PRJNA729271	stage_5W_vs_E10	ordinary_muscle_development	ENSGALG00010021697	PIANP	-1.484889482	0.239590183	-6.197622378	5.73225E-10	1.36011E-09	negative
PRJNA729271	stage_5W_vs_E10	ordinary_muscle_development	ENSGALG00010021698	GYPC	-0.348371176	0.135288452	-2.575025226	0.010023278	0.013728377	negative
PRJNA729271	stage_5W_vs_E10	ordinary_muscle_development	ENSGALG00010021699	SYS1	-0.459475482	0.086705715	-5.299252537	1.16278E-07	2.38935E-07	negative
PRJNA729271	stage_5W_vs_E10	ordinary_muscle_development	ENSGALG00010021700	FLCN	0.725840641	0.077993163	9.306465011	1.32158E-20	5.42209E-20	positive
PRJNA729271	stage_5W_vs_E10	ordinary_muscle_development	ENSGALG00010021701	DR1	-0.500457269	0.077209647	-6.481797156	9.06364E-11	2.26435E-10	negative
PRJNA729271	stage_5W_vs_E10	ordinary_muscle_development	ENSGALG00010021702	PLD6	-0.085890046	0.314474644	-0.273122325	0.784759186	0.810537581	negative
PRJNA729271	stage_5W_vs_E10	ordinary_muscle_development	ENSGALG00010021703	CD82	0.276213762	0.17346728	1.592310448	0.111314967	0.134380815	positive
PRJNA729271	stage_5W_vs_E10	ordinary_muscle_development	ENSGALG00010021704	COPS7A	1.670546926	0.10195178	16.38565726	2.42137E-60	3.92154E-59	positive
PRJNA729271	stage_5W_vs_E10	ordinary_muscle_development	ENSGALG00010021705	CCDC18	-0.609748018	0.192660204	-3.164888266	0.001551425	0.002307162	negative
PRJNA729271	stage_5W_vs_E10	ordinary_muscle_development	ENSGALG00010021706	ENSGALG00010021706	2.17839567	0.158980117	13.70231515	9.8338E-43	9.3225E-42	positive
PRJNA729271	stage_5W_vs_E10	ordinary_muscle_development	ENSGALG00010021707	DLX2	-3.624995851	1.10718533	-3.2740642	0.001060125	0.001603853	negative
PRJNA729271	stage_5W_vs_E10	ordinary_muscle_development	ENSGALG00010021708	CNTNAP5	-3.031980439	0.330083122	-9.185505813	4.09598E-20	1.64372E-19	negative
PRJNA729271	stage_5W_vs_E10	ordinary_muscle_development	ENSGALG00010021710	ENSGALG00010021710	-1.390443479	0.328637407	-4.230934917	2.32722E-05	4.0312E-05	negative
PRJNA729271	stage_5W_vs_E10	ordinary_muscle_development	ENSGALG00010021711	ENSGALG00010021711	0.934251362	0.220482702	4.237300042	2.26224E-05	3.92285E-05	positive
PRJNA729271	stage_5W_vs_E10	ordinary_muscle_development	ENSGALG00010021713	RAB27A	0.303133138	0.080482244	3.766459827	0.000165579	0.000267685	positive
PRJNA729271	stage_5W_vs_E10	ordinary_muscle_development	ENSGALG00010021714	TSPAN18	0.238020137	0.118318652	2.011687353	0.044252905	0.056178477	positive
PRJNA729271	stage_5W_vs_E10	ordinary_muscle_development	ENSGALG00010021715	MMAB	-0.383713865	0.133993613	-2.863672793	0.004187603	0.005965534	negative
PRJNA729271	stage_5W_vs_E10	ordinary_muscle_development	ENSGALG00010021716	TFCP2L1	0.000370661	0.388416931	0.000954287	0.999238589	0.999419743	positive
PRJNA729271	stage_5W_vs_E10	ordinary_muscle_development	ENSGALG00010021717	SDC4	0.769445448	0.198393678	3.878376854	0.000105156	0.000172776	positive
PRJNA729271	stage_5W_vs_E10	ordinary_muscle_development	ENSGALG00010021718	SST7	-3.667030038	0.50148549	-7.312335279	2.62539E-13	7.5282E-13	negative
PRJNA729271	stage_5W_vs_E10	ordinary_muscle_development	ENSGALG00010021719	BHLHE40	4.204626048	0.213749648	19.67079754	3.83698E-86	1.18334E-84	positive
PRJNA729271	stage_5W_vs_E10	ordinary_muscle_development	ENSGALG00010021720	CACNB1	1.411460151	0.260978808	5.40833243	6.36143E-08	1.32942E-07	positive
PRJNA729271	stage_5W_vs_E10	ordinary_muscle_development	ENSGALG00010021721	TP53I11	-0.684739227	0.143478707	-4.77241008	1.82034E-06	3.42363E-06	negative
PRJNA729271	stage_5W_vs_E10	ordinary_muscle_development	ENSGALG00010021722	AARS1	0.376141123	0.158933911	2.366651148	0.017949843	0.023859053	positive
PRJNA729271	stage_5W_vs_E10	ordinary_muscle_development	ENSGALG00010021723	COPS3	1.309580982	0.105984075	12.35639389	4.4989E-35	3.32712E-34	positive
PRJNA729271	stage_5W_vs_E10	ordinary_muscle_development	ENSGALG00010021725	SH2D6	1.708764463	0.20957092	8.153633432	3.5315E-16	1.17893E-15	positive
PRJNA729271	stage_5W_vs_E10	ordinary_muscle_development	ENSGALG00010021726	ARNTL	-0.751351115	0.148904494	-5.04585922	4.51488E-07	8.88449E-07	negative
PRJNA729271	stage_5W_vs_E10	ordinary_muscle_development	ENSGALG00010021727	MVK	-1.106683135	0.105479757	-10.49190069	9.41158E-26	4.81202E-25	negative
PRJNA729271	stage_5W_vs_E10	ordinary_muscle_development	ENSGALG00010021728	TMED5	-0.06846185	0.064940466	-1.0542248	0.291780008	0.329645457	negative
PRJNA729271	stage_5W_vs_E10	ordinary_muscle_development	ENSGALG00010021729	RBPJL	-4.019782008	0.700386173	-5.739379448	9.50241E-09	2.1003E-08	negative
PRJNA729271	stage_5W_vs_E10	ordinary_muscle_development	ENSGALG00010021730	ARL8B	0.275813435	0.072139783	3.823319452	0.000131667	0.000214464	positive
PRJNA729271	stage_5W_vs_E10	ordinary_muscle_development	ENSGALG00010021731	SRM	-0.51028675	0.119560345	-4.268026763	1.9721E-05	3.43494E-05	negative
PRJNA729271	stage_5W_vs_E10	ordinary_muscle_development	ENSGALG00010021732	CASR	-1.675239651	0.302324482	-5.541197456	3.0041E-08	6.41965E-08	negative
PRJNA729271	stage_5W_vs_E10	ordinary_muscle_development	ENSGALG00010021733	ENSGALG00010021733	1.402444744	0.611917367	2.29188583	0.021912232	0.028816712	positive
PRJNA729271	stage_5W_vs_E10	ordinary_muscle_development	ENSGALG00010021734	PARD3B	0.293144496	0.064015728	4.579257411	4.6663E-06	8.50792E-06	positive
PRJNA729271	stage_5W_vs_E10	ordinary_muscle_development	ENSGALG00010021735	PIGBOS1	1.008915128	0.145052956	6.955495137	3.51326E-12	9.51863E-12	positive
PRJNA729271	stage_5W_vs_E10	ordinary_muscle_development	ENSGALG00010021736	EDEM1	0.701059407	0.082521654	8.495459941	1.97151E-17	7.00416E-17	positive
PRJNA729271	stage_5W_vs_E10	ordinary_muscle_development	ENSGALG00010021738	PRDM11	-0.2556459	0.073363641	-3.484640324	0.000492799	0.000765362	negative
PRJNA729271	stage_5W_vs_E10	ordinary_muscle_development	ENSGALG00010021739	EXOSC10	0.338740455	0.1103769	3.068943366	0.002148173	0.003147137	positive
PRJNA729271	stage_5W_vs_E10	ordinary_muscle_development	ENSGALG00010021740	PDLIM1	0.72588419	0.158910583	4.567878207	4.92686E-06	8.96721E-06	positive
PRJNA729271	stage_5W_vs_E10	ordinary_muscle_development	ENSGALG00010021741	RPL19	0.005648898	0.146046108	0.038678865	0.969146424	0.973503214	positive
PRJNA729271	stage_5W_vs_E10	ordinary_muscle_development	ENSGALG00010021742	MATN4	-8.816621946	0.536077673	-16.44653822	8.88008E-61	1.45279E-59	negative
PRJNA729271	stage_5W_vs_E10	ordinary_muscle_development	ENSGALG00010021743	SYT13	-5.455199701	0.520370815	-10.48329296	1.0309E-25	5.2579E-25	negative
PRJNA729271	stage_5W_vs_E10	ordinary_muscle_development	ENSGALG00010021744	LITAF	4.03508488	0.338408525	11.9237093	8.90535E-33	6.02066E-32	positive
PRJNA729271	stage_5W_vs_E10	ordinary_muscle_development	ENSGALG00010021745	RSL24D1	0.179357859	0.125706745	1.426795822	0.153638756	0.182048048	positive
PRJNA729271	stage_5W_vs_E10	ordinary_muscle_development	ENSGALG00010021746	MTOR	0.517788484	0.11061733	4.680898392	2.8562E-06	5.30166E-06	positive
PRJNA729271	stage_5W_vs_E10	ordinary_muscle_development	ENSGALG00010021747	AKTIP	1.298005043	0.114831362	11.30357616	1.25981E-29	7.5148E-29	positive
PRJNA729271	stage_5W_vs_E10	ordinary_muscle_development	ENSGALG00010021748	CDCA7	-2.394309848	0.17884905	-13.38732217	7.17202E-41	6.4291E-40	negative
PRJNA729271	stage_5W_vs_E10	ordinary_muscle_development	ENSGALG00010021749	CLASP1	0.057661434	0.09113591	0.632697183	0.52693141	0.567576041	positive
PRJNA729271	stage_5W_vs_E10	ordinary_muscle_development	ENSGALG00010021750	HELLS	-3.02648429	0.1530666	-19.77233627	5.15334E-87	1.61683E-85	negative
PRJNA729271	stage_5W_vs_E10	ordinary_muscle_development	ENSGALG00010021751	MTCH2	-0.920349259	0.094202505	-9.769902156	1.51598E-22	6.79684E-22	negative
PRJNA729271	stage_5W_vs_E10	ordinary_muscle_development	ENSGALG00010021752	CSTB	0.248666947	0.109370949	2.273610578	0.022989411	0.030167618	positive
PRJNA729271	stage_5W_vs_E10	ordinary_muscle_development	ENSGALG00010021753	MTF2	-1.485376537	0.098298676	-15.11084991	1.37357E-51	1.74648E-50	negative
PRJNA729271	stage_5W_vs_E10	ordinary_muscle_development	ENSGALG00010021754	SLC17A9	-1.379204919	0.476394653	-2.895089	0.00379051	0.005419555	negative
PRJNA729271	stage_5W_vs_E10	ordinary_muscle_development	ENSGALG00010021755	ENSGALG00010021755	2.74395822	0.153598534	17.86448185	2.22993E-71	4.91229E-70	positive
PRJNA729271	stage_5W_vs_E10	ordinary_muscle_development	ENSGALG00010021756	USP42	-1.415631716	0.128372689	-11.02751474	2.81538E-28	1.59223E-27	negative
PRJNA729271	stage_5W_vs_E10	ordinary_muscle_development	ENSGALG00010021757	ZP4	-0.38948512	0.393212362	-0.990521047	0.321919507	0.36127464	negative
PRJNA729271	stage_5W_vs_E10	ordinary_muscle_development	ENSGALG00010021758	RASSF10	-4.371602938	0.431175255	-10.13880758	3.71608E-24	1.78065E-23	negative
PRJNA729271	stage_5W_vs_E10	ordinary_muscle_development	ENSGALG00010021759	NIFK	-0.646597336	0.094237674	-6.861346517	6.82145E-12	1.8196E-11	negative
PRJNA729271	stage_5W_vs_E10	ordinary_muscle_development	ENSGALG00010021760	CSTA	2.211741791	0.392190058	5.639464193	1.7058E-08	3.71199E-08	positive
PRJNA729271	stage_5W_vs_E10	ordinary_muscle_development	ENSGALG00010021761	MAFB	0.565078164	0.169519943	3.333402275	0.000857908	0.001307748	positive
PRJNA729271	stage_5W_vs_E10	ordinary_muscle_development	ENSGALG00010021762	LAMB2	3.268599498	0.198586663	16.45931022	7.19153E-61	1.18187E-59	positive
PRJNA729271	stage_5W_vs_E10	ordinary_muscle_development	ENSGALG00010021764	FAM222A	-2.222723922	0.931506732	-2.386159806	0.017025351	0.022690554	negative
PRJNA729271	stage_5W_vs_E10	ordinary_muscle_development	ENSGALG00010021765	EIF2AK1	-0.216213099	0.105968652	-2.040349619	0.041315516	0.052666292	negative
PRJNA729271	stage_5W_vs_E10	ordinary_muscle_development	ENSGALG00010021766	AGRN	-0.755675161	0.149586288	-5.051767604	4.3774E-07	8.62098E-07	negative
PRJNA729271	stage_5W_vs_E10	ordinary_muscle_development	ENSGALG00010021767	CCDC58	-0.10936098	0.214098812	-0.510796764	0.609493375	0.6466081	negative
PRJNA729271	stage_5W_vs_E10	ordinary_muscle_development	ENSGALG00010021768	DIPK1A	1.113913021	0.149631958	7.444352355	9.74212E-14	2.85565E-13	positive
PRJNA729271	stage_5W_vs_E10	ordinary_muscle_development	ENSGALG00010021769	ENSGALG00010021769	-0.6829612	0.179039865	-3.814576153	0.000136417	0.000222101	negative
PRJNA729271	stage_5W_vs_E10	ordinary_muscle_development	ENSGALG00010021770	RALGAPB	0.136322579	0.097982018	1.39130202	0.164133862	0.193881536	positive
PRJNA729271	stage_5W_vs_E10	ordinary_muscle_development	ENSGALG00010021771	ANKRD61	0.804566568	0.143763779	5.596448393	2.18787E-08	4.72908E-08	positive
PRJNA729271	stage_5W_vs_E10	ordinary_muscle_development	ENSGALG00010021772	TSN	-0.845944461	0.083812499	-10.09329716	5.91487E-24	2.80458E-23	negative
PRJNA729271	stage_5W_vs_E10	ordinary_muscle_development	ENSGALG00010021773	ENSGALG00010021773	-4.134127038	1.033711646	-3.999303921	6.35291E-05	0.00010663	negative
PRJNA729271	stage_5W_vs_E10	ordinary_muscle_development	ENSGALG00010021774	FBXL20	0.806428773	0.072422109	11.13511857	8.46323E-29	4.88548E-28	positive
PRJNA729271	stage_5W_vs_E10	ordinary_muscle_development	ENSGALG00010021775	CCDC36	0.51265837	0.375785782	1.364230351	0.172495079	0.202964215	positive
PRJNA729271	stage_5W_vs_E10	ordinary_muscle_development	ENSGALG00010021776	PMS2	-0.840592423	0.095717474	-8.782016357	1.6057E-18	6.0017E-18	negative
PRJNA729271	stage_5W_vs_E10	ordinary_muscle_development	ENSGALG00010021778	RBL2	0.670377058	0.103812213	6.457593383	1.06381E-10	2.64312E-10	positive
PRJNA729271	stage_5W_vs_E10	ordinary_muscle_development	ENSGALG00010021779	UNC13C	0.236203949	0.246886095	0.956732492	0.338702294	0.37846822	positive
PRJNA729271	stage_5W_vs_E10	ordinary_muscle_development	ENSGALG00010021781	RAPGEF4	-0.467781287	0.26878298	-1.740367964	0.081794427	0.100435641	negative
PRJNA729271	stage_5W_vs_E10	ordinary_muscle_development	ENSGALG00010021782	ADAM11	-0.174286229	0.300446286	-0.580091142	0.561853157	0.601392816	negative
PRJNA729271	stage_5W_vs_E10	ordinary_muscle_development	ENSGALG00010021783	GIT2	-0.772125879	0.048624102	-15.87948888	8.78917E-57	1.28556E-55	negative
PRJNA729271	stage_5W_vs_E10	ordinary_muscle_development	ENSGALG00010021784	ANGPTL7	1.546539818	0.876592857	1.764262401	0.077687811	0.095698549	positive
PRJNA729271	stage_5W_vs_E10	ordinary_muscle_development	ENSGALG00010021785	CCDC71	-0.023518679	0.090076702	-0.261096137	0.79401837	0.818584373	negative
PRJNA729271	stage_5W_vs_E10	ordinary_muscle_development	ENSGALG00010021786	FAM162A	0.204369768	0.173215481	1.17985856	0.238056474	0.273295469	positive
PRJNA729271	stage_5W_vs_E10	ordinary_muscle_development	ENSGALG00010021788	RPL5	-0.090021271	0.123933308	-0.726368662	0.467612797	0.509551007	negative
PRJNA729271	stage_5W_vs_E10	ordinary_muscle_development	ENSGALG00010021789	CHD9	0.181851611	0.090909965	2.000348488	0.045462647	0.057621547	positive
PRJNA729271	stage_5W_vs_E10	ordinary_muscle_development	ENSGALG00010021790	PHF21A	0.406836461	0.128149842	3.174693425	0.001499949	0.002232213	positive
PRJNA729271	stage_5W_vs_E10	ordinary_muscle_development	ENSGALG00010021791	PLEKHM2	1.809922576	0.184064142	9.833107938	8.10772E-23	3.67829E-22	positive
PRJNA729271	stage_5W_vs_E10	ordinary_muscle_development	ENSGALG00010021792	KLHDC8B	2.850470763	0.262716974	10.84996802	1.99495E-27	1.09161E-26	positive
PRJNA729271	stage_5W_vs_E10	ordinary_muscle_development	ENSGALG00010021793	ENSGALG00010021793	-0.576366476	0.162549489	-3.545790756	0.000391437	0.000613193	negative
PRJNA729271	stage_5W_vs_E10	ordinary_muscle_development	ENSGALG00010021794	ENSGALG00010021794	-1.407653358	0.205855453	-6.838066902	8.02689E-12	2.13251E-11	negative
PRJNA729271	stage_5W_vs_E10	ordinary_muscle_development	ENSGALG00010021795	NDRG3	-0.195411237	0.106704105	-1.831337577	0.067050172	0.083245589	negative
PRJNA729271	stage_5W_vs_E10	ordinary_muscle_development	ENSGALG00010021797	SLC25A34	-0.76593308	0.193907305	-3.949996001	7.81525E-05	0.00013009	negative
PRJNA729271	stage_5W_vs_E10	ordinary_muscle_development	ENSGALG00010021798	RANBP10	0.457115406	0.182018705	2.511365002	0.012026527	0.016313355	positive
PRJNA729271	stage_5W_vs_E10	ordinary_muscle_development	ENSGALG00010021799	ENSGALG00010021799	-0.609846904	0.223826198	-2.724644883	0.006437068	0.008987218	negative
PRJNA729271	stage_5W_vs_E10	ordinary_muscle_development	ENSGALG00010021800	TMEM82	-0.871587034	0.380689569	-2.289495447	0.022050582	0.028985507	negative
PRJNA729271	stage_5W_vs_E10	ordinary_muscle_development	ENSGALG00010021801	VAMP1	2.952703298	0.2419864	12.20193901	3.03509E-34	2.16824E-33	positive
PRJNA729271	stage_5W_vs_E10	ordinary_muscle_development	ENSGALG00010021803	TLL2	-3.472005851	0.175244711	-19.81232887	2.33051E-87	7.49019E-86	negative
PRJNA729271	stage_5W_vs_E10	ordinary_muscle_development	ENSGALG00010021805	FUT8	-0.313125674	0.108643347	-2.882143106	0.003949803	0.00564063	negative
PRJNA729271	stage_5W_vs_E10	ordinary_muscle_development	ENSGALG00010021807	CDC42	-0.114666069	0.082996029	-1.381585011	0.167099159	0.197191428	negative
PRJNA729271	stage_5W_vs_E10	ordinary_muscle_development	ENSGALG00010021810	ANKRD13A	-0.964310282	0.097151874	-9.925802214	3.21507E-23	1.47991E-22	negative
PRJNA729271	stage_5W_vs_E10	ordinary_muscle_development	ENSGALG00010021812	TEAD1	-0.384255199	0.083596771	-4.596531589	4.29582E-06	7.85814E-06	negative
PRJNA729271	stage_5W_vs_E10	ordinary_muscle_development	ENSGALG00010021817	EVI5	0.237526909	0.100685962	2.359086655	0.018319977	0.024337647	positive
PRJNA729271	stage_5W_vs_E10	ordinary_muscle_development	ENSGALG00010021821	PPP1R19L	-1.275158888	0.120402457	-10.59080458	3.28754E-26	1.71598E-25	negative
PRJNA729271	stage_5W_vs_E10	ordinary_muscle_development	ENSGALG00010021823	ENSGALG00010021823	-0.002255884	0.259545035	-0.008691685	0.993065126	0.993956567	negative
PRJNA729271	stage_5W_vs_E10	ordinary_muscle_development	ENSGALG00010021826	FBXO40	6.426588849	0.207609564	30.95516756	2.1647E-210	4.075E-208	positive
PRJNA729271	stage_5W_vs_E10	ordinary_muscle_development	ENSGALG00010021827	CCZ1	-0.584376114	0.096734813	-6.041011444	1.53151E-09	3.53829E-09	negative
PRJNA729271	stage_5W_vs_E10	ordinary_muscle_development	ENSGALG00010021829	ERCC6	-0.172432701	0.097411009	-1.770156187	0.076701126	0.094563479	negative
PRJNA729271	stage_5W_vs_E10	ordinary_muscle_development	ENSGALG00010021831	ENSGALG00010021831	0.596411897	0.392328348	1.520185579	0.128464341	0.153879069	positive
PRJNA729271	stage_5W_vs_E10	ordinary_muscle_development	ENSGALG00010021835	NUDT8	-0.189683521	0.176150357	-1.076827346	0.281557406	0.319116006	negative
PRJNA729271	stage_5W_vs_E10	ordinary_muscle_development	ENSGALG00010021837	ENSGALG00010021837	0.554150559	0.324940223	1.705392316	0.088121281	0.107763263	positive
PRJNA729271	stage_5W_vs_E10	ordinary_muscle_development	ENSGALG00010021839	C1orf50	0.057095511	0.121878042	0.468464293	0.6394526	0.67478636	positive
PRJNA729271	stage_5W_vs_E10	ordinary_muscle_development	ENSGALG00010021840	ENSGALG00010021840	0.522475895	0.218898235	2.386843796	0.01699371	0.022654633	positive
PRJNA729271	stage_5W_vs_E10	ordinary_muscle_development	ENSGALG00010021841	CENPT	-1.952230119	0.096677144	-20.19329539	1.1213E-90	3.88834E-89	negative
PRJNA729271	stage_5W_vs_E10	ordinary_muscle_development	ENSGALG00010021842	ENSGALG00010021842	-2.894941154	0.194283924	-14.90056972	3.26781E-50	3.94396E-49	negative
PRJNA729271	stage_5W_vs_E10	ordinary_muscle_development	ENSGALG00010021843	ENSGALG00010021843	2.6191337	0.376413671	6.958125865	3.44829E-12	9.3496E-12	positive
PRJNA729271	stage_5W_vs_E10	ordinary_muscle_development	ENSGALG00010021847	P3H1	-1.137618518	0.151764797	-7.495931481	6.58292E-14	1.94932E-13	negative
PRJNA729271	stage_5W_vs_E10	ordinary_muscle_development	ENSGALG00010021848	LARGE2	1.390015855	0.137068504	10.14103031	3.63248E-24	1.74116E-23	positive
PRJNA729271	stage_5W_vs_E10	ordinary_muscle_development	ENSGALG00010021851	ENSGALG00010021851	0.943527354	0.22220433	4.246214967	2.17412E-05	3.77321E-05	positive
PRJNA729271	stage_5W_vs_E10	ordinary_muscle_development	ENSGALG00010021853	ENSGALG00010021853	0.387387131	0.590111229	0.656464599	0.511525238	0.552274502	positive
PRJNA729271	stage_5W_vs_E10	ordinary_muscle_development	ENSGALG00010021854	TAPBPL	1.924025554	0.152705574	12.59957648	2.12281E-36	1.64899E-35	positive
PRJNA729271	stage_5W_vs_E10	ordinary_muscle_development	ENSGALG00010021860	ENSGALG00010021860	2.359211275	0.251378064	9.385111958	6.28496E-21	2.61483E-20	positive
PRJNA729271	stage_5W_vs_E10	ordinary_muscle_development	ENSGALG00010021862	PDK1	-0.769969955	0.107155548	-7.185535116	6.69445E-13	1.88383E-12	negative
PRJNA729271	stage_5W_vs_E10	ordinary_muscle_development	ENSGALG00010021863	CLDN19	1.193267958	0.299889856	3.979020742	6.91997E-05	0.000115705	positive
PRJNA729271	stage_5W_vs_E10	ordinary_muscle_development	ENSGALG00010021864	GFI1	4.095447758	0.445379542	9.195410597	3.73565E-20	1.50162E-19	positive
PRJNA729271	stage_5W_vs_E10	ordinary_muscle_development	ENSGALG00010021866	SCNN1A	1.801292289	0.358813162	5.020139956	5.16338E-07	1.01208E-06	positive
PRJNA729271	stage_5W_vs_E10	ordinary_muscle_development	ENSGALG00010021867	CXCL12	0.01643418	0.182328805	0.090134853	0.928180053	0.937951051	positive
PRJNA729271	stage_5W_vs_E10	ordinary_muscle_development	ENSGALG00010021869	GJC3	0.262057401	0.12165367	2.15412656	0.031230248	0.040446966	positive

PRJNA729271	stage_5W_vs_E10	ordinary_muscle_development	ENSGALG00010021870	ENSGALG00010021870	-0.365738822	0.169519499	-2.157502966	0.030966496	0.040126887	negative
PRJNA729271	stage_5W_vs_E10	ordinary_muscle_development	ENSGALG00010021871	ENSGALG00010021871	4.742846437	0.250623594	18.92418175	7.21004E-80	1.93457E-78	positive
PRJNA729271	stage_5W_vs_E10	ordinary_muscle_development	ENSGALG00010021874	TMCC1	0.636585977	0.078769468	8.081633559	6.39049E-16	2.10571E-15	positive
PRJNA729271	stage_5W_vs_E10	ordinary_muscle_development	ENSGALG00010021877	PLEKHN1	0.956571204	0.147632801	6.479394813	9.20912E-11	2.29871E-10	positive
PRJNA729271	stage_5W_vs_E10	ordinary_muscle_development	ENSGALG00010021879	CREBL2	1.206080291	0.154563602	7.803132671	6.0389E-15	1.88954E-14	positive
PRJNA729271	stage_5W_vs_E10	ordinary_muscle_development	ENSGALG00010021881	PHF20	-0.619068021	0.049570549	-12.48862556	8.61287E-36	6.55691E-35	negative
PRJNA729271	stage_5W_vs_E10	ordinary_muscle_development	ENSGALG00010021887	NUTF2	-0.977707512	0.111051275	-8.804108861	1.31896E-18	4.94142E-18	negative
PRJNA729271	stage_5W_vs_E10	ordinary_muscle_development	ENSGALG00010021892	NDUFV1	0.937232458	0.121933051	7.686451271	1.51272E-14	4.63571E-14	positive
PRJNA729271	stage_5W_vs_E10	ordinary_muscle_development	ENSGALG00010021897	ENSGALG00010021897	0.380162864	0.209532239	1.814340672	0.069625294	0.086346563	positive
PRJNA729271	stage_5W_vs_E10	ordinary_muscle_development	ENSGALG00010021902	LMTK2	0.920269036	0.119096785	7.727068671	1.10051E-14	3.39909E-14	positive
PRJNA729271	stage_5W_vs_E10	ordinary_muscle_development	ENSGALG00010021903	MORC2	-0.383497437	0.079007901	-4.853912494	1.21049E-06	2.30809E-06	negative
PRJNA729271	stage_5W_vs_E10	ordinary_muscle_development	ENSGALG00010021905	RBSN	-0.323351319	0.073751545	-4.384332808	1.16342E-05	2.06335E-05	negative
PRJNA729271	stage_5W_vs_E10	ordinary_muscle_development	ENSGALG00010021908	EDC4	0.11038717	0.108539272	1.017025154	0.309141463	0.348012233	positive
PRJNA729271	stage_5W_vs_E10	ordinary_muscle_development	ENSGALG00010021913	ENSGALG00010021913	1.75365285	0.175487924	9.993011517	1.63536E-23	7.62451E-23	positive
PRJNA729271	stage_5W_vs_E10	ordinary_muscle_development	ENSGALG00010021916	MRPS25	0.38989157	0.148729441	2.621482118	0.008754836	0.012054844	positive
PRJNA729271	stage_5W_vs_E10	ordinary_muscle_development	ENSGALG00010021919	PARVA	0.344377312	0.124619871	2.763422147	0.005719874	0.008026873	positive
PRJNA729271	stage_5W_vs_E10	ordinary_muscle_development	ENSGALG00010021922	MRPL51	-0.222947847	0.138636744	-1.608143996	0.107803645	0.130348168	negative
PRJNA729271	stage_5W_vs_E10	ordinary_muscle_development	ENSGALG00010021924	ENSGALG00010021924	0.317385889	0.472357278	0.671919125	0.501635184	0.542627015	positive
PRJNA729271	stage_5W_vs_E10	ordinary_muscle_development	ENSGALG00010021928	ENSGALG00010021928	4.367040903	0.887022244	4.923259742	8.51144E-07	1.64432E-06	positive
PRJNA729271	stage_5W_vs_E10	ordinary_muscle_development	ENSGALG00010021931	ITGA6	0.934475389	0.121682722	7.679606232	1.59578E-14	4.88199E-14	positive
PRJNA729271	stage_5W_vs_E10	ordinary_muscle_development	ENSGALG00010021932	RPAP2	0.875091247	0.162117388	5.397886423	6.74305E-08	1.40634E-07	positive
PRJNA729271	stage_5W_vs_E10	ordinary_muscle_development	ENSGALG00010021933	PRR14L	-0.327916324	0.089820093	-3.650812577	0.000261412	0.00041598	negative
PRJNA729271	stage_5W_vs_E10	ordinary_muscle_development	ENSGALG00010021934	EFTUD2	-1.098897286	0.067566577	-16.26391839	1.77982E-59	2.80419E-58	negative
PRJNA729271	stage_5W_vs_E10	ordinary_muscle_development	ENSGALG00010021935	CETP	6.416979707	0.29598717	21.6799252	3.1739E-104	1.4243E-102	positive
PRJNA729271	stage_5W_vs_E10	ordinary_muscle_development	ENSGALG00010021938	CABP2	0.016014283	0.16655465	0.096150321	0.923401186	0.933838009	positive
PRJNA729271	stage_5W_vs_E10	ordinary_muscle_development	ENSGALG00010021943	OCM3	-5.335183566	0.571325377	-9.338257645			negative
PRJNA729271	stage_5W_vs_E10	ordinary_muscle_development	ENSGALG00010021945	C1QTNF4	-1.997774642	0.218097202	-9.160019599	5.18879E-20	2.07308E-19	negative
PRJNA729271	stage_5W_vs_E10	ordinary_muscle_development	ENSGALG00010021947	CDK2AP2	0.92442637	0.248227058	3.724116042	0.000196001	0.00031511	positive
PRJNA729271	stage_5W_vs_E10	ordinary_muscle_development	ENSGALG00010021949	ENSGALG00010021949	-1.079953083	0.247529349	-4.362929434	1.28332E-05	2.27072E-05	negative
PRJNA729271	stage_5W_vs_E10	ordinary_muscle_development	ENSGALG00010021951	PSKH1	0.10914628	0.143492868	0.760639059	0.446872684	0.488677446	positive
PRJNA729271	stage_5W_vs_E10	ordinary_muscle_development	ENSGALG00010021953	PITPNM1	0.468292607	0.177473818	2.638657416	0.008323505	0.0114904	positive
PRJNA729271	stage_5W_vs_E10	ordinary_muscle_development	ENSGALG00010021958	BHLHA15	1.478399194	0.373628898	3.956865221	7.59397E-05	0.000126537	positive
PRJNA729271	stage_5W_vs_E10	ordinary_muscle_development	ENSGALG00010021960	CDKN1B	0.186226254	0.14501688	1.284169494	0.199082657	0.23148589	positive
PRJNA729271	stage_5W_vs_E10	ordinary_muscle_development	ENSGALG00010021965	PEX16	0.317591367	0.10241293	3.101086609	0.001928119	0.002837945	positive
PRJNA729271	stage_5W_vs_E10	ordinary_muscle_development	ENSGALG00010021966	ADGRA1	-0.453034042	0.159304827	-2.843818673	0.004457641	0.006325975	negative
PRJNA729271	stage_5W_vs_E10	ordinary_muscle_development	ENSGALG00010021969	CCDC103	-0.851896689	0.287521453	-2.962897828	0.003047577	0.004400302	negative
PRJNA729271	stage_5W_vs_E10	ordinary_muscle_development	ENSGALG00010021970	ERGIC3	-0.506269623	0.091460908	-5.535366248	3.10579E-08	6.62801E-08	negative
PRJNA729271	stage_5W_vs_E10	ordinary_muscle_development	ENSGALG00010021971	ICOS	2.341807221	0.363088034	6.449695405	1.12075E-10	2.77935E-10	positive
PRJNA729271	stage_5W_vs_E10	ordinary_muscle_development	ENSGALG00010021972	PISD	-0.171499048	0.184266141	-0.930713841	0.352001608	0.392149974	negative
PRJNA729271	stage_5W_vs_E10	ordinary_muscle_development	ENSGALG00010021974	GFAP	4.135746561	0.803398502	5.147814629	2.63539E-07	5.27114E-07	positive
PRJNA729271	stage_5W_vs_E10	ordinary_muscle_development	ENSGALG00010021978	NR2C2	0.416990333	0.073822673	5.648540125	1.61816E-08	3.52976E-08	positive
PRJNA729271	stage_5W_vs_E10	ordinary_muscle_development	ENSGALG00010021980	GLMN	-0.052039942	0.133329823	-0.390309842	0.696307446	0.728787381	negative
PRJNA729271	stage_5W_vs_E10	ordinary_muscle_development	ENSGALG00010021983	CTLA4	-0.635052379	0.511466322	-1.241630879	0.214372783	0.248086692	negative
PRJNA729271	stage_5W_vs_E10	ordinary_muscle_development	ENSGALG00010021988	FAM180B	4.702126424	0.262348717	17.92319197	7.7737E-72	1.73621E-70	positive
PRJNA729271	stage_5W_vs_E10	ordinary_muscle_development	ENSGALG00010021989	NCAPD2	-1.777865533	0.152229463	-11.67885312	1.63486E-31	1.05041E-30	negative
PRJNA729271	stage_5W_vs_E10	ordinary_muscle_development	ENSGALG00010021994	ENSGALG00010021994	-4.887911018	0.416221048	-11.74354598	7.62222E-32	4.96558E-31	negative
PRJNA729271	stage_5W_vs_E10	ordinary_muscle_development	ENSGALG00010021995	CD28	4.012519967	0.470990913	8.519315033	1.605E-17	5.73157E-17	positive
PRJNA729271	stage_5W_vs_E10	ordinary_muscle_development	ENSGALG00010021999	ANKRD22	-4.338599973	0.645669647	-6.719535284	1.82305E-11	4.75614E-11	negative
PRJNA729271	stage_5W_vs_E10	ordinary_muscle_development	ENSGALG00010022002	KIF18B	-2.111387024	0.224235855	-9.415920685	4.68954E-21	1.96516E-20	negative
PRJNA729271	stage_5W_vs_E10	ordinary_muscle_development	ENSGALG00010022005	NLRC5	4.826048561	0.180898645	26.67819082	8.4308E-157	9.2579E-155	positive
PRJNA729271	stage_5W_vs_E10	ordinary_muscle_development	ENSGALG00010022006	SFI1	-0.211865774	0.10979055	-1.929726867	0.053640689	0.067382077	negative
PRJNA729271	stage_5W_vs_E10	ordinary_muscle_development	ENSGALG00010022012	ENSGALG00010022012	-0.669456458	2.998349023	-0.223275027	0.823321463	0.844290386	negative
PRJNA729271	stage_5W_vs_E10	ordinary_muscle_development	ENSGALG00010022017	CNP1	2.606367543	0.586941712	4.440590078	8.97125E-06	1.60424E-05	positive
PRJNA729271	stage_5W_vs_E10	ordinary_muscle_development	ENSGALG00010022019	BTBD8	0.062740373	0.154722543	0.405502466	0.685108206	0.718362518	positive
PRJNA729271	stage_5W_vs_E10	ordinary_muscle_development	ENSGALG00010022021	TECPR1	1.432509443	0.07786494	18.39736134	1.37919E-75	3.4115E-74	positive
PRJNA729271	stage_5W_vs_E10	ordinary_muscle_development	ENSGALG00010022022	STAMBPL1	-1.392342817	0.257870906	-5.399379238	6.68719E-08	1.39489E-07	negative
PRJNA729271	stage_5W_vs_E10	ordinary_muscle_development	ENSGALG00010022024	MICAL2	2.4177916	0.183157476	13.20061651	8.70199E-40	7.56207E-39	positive
PRJNA729271	stage_5W_vs_E10	ordinary_muscle_development	ENSGALG00010022027	EIF4ENIF1	-0.397896392	0.067639608	-5.882594614	4.03884E-09	9.11034E-09	negative
PRJNA729271	stage_5W_vs_E10	ordinary_muscle_development	ENSGALG00010022028	FGD5	0.69648665	0.115126204	6.049766473	1.45056E-09	3.35608E-09	positive
PRJNA729271	stage_5W_vs_E10	ordinary_muscle_development	ENSGALG00010022029	ENSGALG00010022029	1.515680397	0.627456482	2.415594452	0.015709548	0.02103167	positive
PRJNA729271	stage_5W_vs_E10	ordinary_muscle_development	ENSGALG00010022030	ZNF341	0.023304185	0.15844923	0.147076667	0.883071506	0.897246704	positive
PRJNA729271	stage_5W_vs_E10	ordinary_muscle_development	ENSGALG00010022032	KBTBD4	0.324919625	0.082066246	3.959235866	7.519E-05	0.000125389	positive
PRJNA729271	stage_5W_vs_E10	ordinary_muscle_development	ENSGALG00010022033	RAPH1	-0.406903068	0.117009502	-3.477521577	0.000506072	0.00078564	negative
PRJNA729271	stage_5W_vs_E10	ordinary_muscle_development	ENSGALG00010022034	ENSGALG00010022034	1.910321796	0.105079341	18.17980377	7.46002E-74	1.74408E-72	positive
PRJNA729271	stage_5W_vs_E10	ordinary_muscle_development	ENSGALG00010022037	DRG1	0.63287036	0.093660308	6.757081786	1.40799E-11	3.69791E-11	positive
PRJNA729271	stage_5W_vs_E10	ordinary_muscle_development	ENSGALG00010022038	GAPDH	5.348098297	0.13718078	38.98576984	0	0	positive
PRJNA729271	stage_5W_vs_E10	ordinary_muscle_development	ENSGALG00010022041	MAPK8IP1	-0.086233098	0.152178137	-0.566658918	0.570945937	0.609819568	negative
PRJNA729271	stage_5W_vs_E10	ordinary_muscle_development	ENSGALG00010022042	AIP	0.121457085	0.086090752	1.410802927	0.158302731	0.187283553	positive
PRJNA729271	stage_5W_vs_E10	ordinary_muscle_development	ENSGALG00010022044	PXMP4	0.392598639	0.059680876	6.578298897	4.75861E-11	1.21096E-10	positive
PRJNA729271	stage_5W_vs_E10	ordinary_muscle_development	ENSGALG00010022046	CRY2	1.216226556	0.103325068	11.7708759	5.51489E-32	3.61878E-31	positive
PRJNA729271	stage_5W_vs_E10	ordinary_muscle_development	ENSGALG00010022049	EPHX4	-0.598737057	0.593804196	-1.008307217	0.313306999	0.352427825	negative
PRJNA729271	stage_5W_vs_E10	ordinary_muscle_development	ENSGALG00010022050	ENSGALG00010022050	0.184414195	0.20324974	0.907328073	0.364233333	0.404812311	positive
PRJNA729271	stage_5W_vs_E10	ordinary_muscle_development	ENSGALG00010022051	BAIAP2L1	-0.193559561	0.114610882	-1.688841035	0.091249898	0.111420037	negative
PRJNA729271	stage_5W_vs_E10	ordinary_muscle_development	ENSGALG00010022052	IFFO1	0.350073548	0.105261503	3.325750989	0.000881807	0.001342202	positive
PRJNA729271	stage_5W_vs_E10	ordinary_muscle_development	ENSGALG00010022054	ENSGALG00010022054	2.330180646	0.421492573	5.528402622	3.2316E-08	6.88752E-08	positive
PRJNA729271	stage_5W_vs_E10	ordinary_muscle_development	ENSGALG00010022055	E2F1	-1.342634223	0.164330235	-8.170342005	3.07516E-16	1.03039E-15	negative
PRJNA729271	stage_5W_vs_E10	ordinary_muscle_development	ENSGALG00010022058	NLRP3	-0.715786105	0.130282887	-5.49409152	3.92727E-08	8.32735E-08	negative
PRJNA729271	stage_5W_vs_E10	ordinary_muscle_development	ENSGALG00010022059	NPTX2	-1.649840284	0.251811809	-6.551878126	5.68179E-11	1.4373E-10	negative
PRJNA729271	stage_5W_vs_E10	ordinary_muscle_development	ENSGALG00010022062	MITF	1.719090842	0.175047992	9.82068301	9.172E-23	4.15074E-22	positive
PRJNA729271	stage_5W_vs_E10	ordinary_muscle_development	ENSGALG00010022065	PABPC1L	-0.175829574	0.258641094	-0.679820718	0.496617987	0.538203942	negative
PRJNA729271	stage_5W_vs_E10	ordinary_muscle_development	ENSGALG00010022066	DAD1	-0.630494473	0.140110037	-4.499995037	6.7955E-06	1.22437E-05	negative
PRJNA729271	stage_5W_vs_E10	ordinary_muscle_development	ENSGALG00010022067	TMEM130	-1.527320349	0.254859057	-5.992803893	2.06254E-09	4.72671E-09	negative
PRJNA729271	stage_5W_vs_E10	ordinary_muscle_development	ENSGALG00010022069	NOP2	-0.882116181	0.135841942	-6.493695315	8.37561E-11	2.09824E-10	negative
PRJNA729271	stage_5W_vs_E10	ordinary_muscle_development	ENSGALG00010022071	ACTA2	-0.0414333	0.198078269	-0.209176405	0.834310531	0.85422979	negative
PRJNA729271	stage_5W_vs_E10	ordinary_muscle_development	ENSGALG00010022072	SMTN	0.649161813	0.312697379	2.07600657	0.037893343	0.048568752	positive
PRJNA729271	stage_5W_vs_E10	ordinary_muscle_development	ENSGALG00010022073	PTPMT1	0.778049487	0.13348088	5.82892089	5.57869E-09	1.24982E-08	positive
PRJNA729271	stage_5W_vs_E10	ordinary_muscle_development	ENSGALG00010022076	YWHAB	-1.35861088	0.07023888	-19.34271858	2.34766E-83	6.88853E-82	negative
PRJNA729271	stage_5W_vs_E10	ordinary_muscle_development	ENSGALG00010022079	ABI2	-0.395358627	0.076417647	-5.173656146	2.29557E-07	4.61438E-07	negative
PRJNA729271	stage_5W_vs_E10	ordinary_muscle_development	ENSGALG00010022081	FAS	1.545439733	0.173981378	8.88278822	6.5205E-19	2.4794E-18	positive
PRJNA729271	stage_5W_vs_E10	ordinary_muscle_development	ENSGALG00010022082	TGFBR3	1.441730237	0.144395786	9.98457277	1.78067E-23	8.29394E-23	positive
PRJNA729271	stage_5W_vs_E10	ordinary_muscle_development	ENSGALG00010022084	SELENOM	-1.308392131	0.202973745	-6.446115128	1.14754E-10	2.84285E-10	negative
PRJNA729271	stage_5W_vs_E10	ordinary_muscle_development	ENSGALG00010022085	MLLT6	0.630834236	0.17747293	3.554537793	0.000378644	0.000593859	positive
PRJNA729271	stage_5W_vs_E10	ordinary_muscle_development	ENSGALG00010022087	SLC35C1	-0.139219243	0.074841196	-1.86019531	0.062857898	0.078295543	negative
PRJNA729271	stage_5W_vs_E10	ordinary_muscle_development	ENSGALG00010022088	DKK3	-1.918279153	0.270944911	-7.079960075	1.44196E-12	3.98802E-12	negative
PRJNA729271	stage_5W_vs_E10	ordinary_muscle_development	ENSGALG00010022090	TRRAP	-0.93134449	0.091102533	-10.2230362	1.56365E-24	7.61852E-24	negative
PRJNA729271	stage_5W_vs_E10	ordinary_muscle_development	ENSGALG00010022092	CTRL	5.182428939	1.79297197	2.890412692	0.003847364	0.005497589	positive
PRJNA729271	stage_5W_vs_E10	ordinary_muscle_development	ENSGALG00010022093	CHD4	-0.796867263	0.13650943	-5.83745212	5.30051E-09	1.18897E-08	negative
PRJNA729271	stage_5W_vs_E10	ordinary_muscle_development	ENSGALG00010022097	INPP5J	-1.650896721	0.319211843	-5.171790332	2.31862E-07	4.65683E-07	negative
PRJNA729271	stage_5W_vs_E10	ordinary_muscle_development	ENSGALG00010022098	CDC7	-3.118430216	0.178724216	-17.44828026	3.54754E-68	7.05371E-67	negative
PRJNA729271	stage_5W_vs_E10	ordinary_muscle_development	ENSGALG00010022100	ENTPD1	0.386711533	0.165691812	2.333920603	0.01959988	0.02593102	positive
PRJNA729271	stage_5W_vs_E10	ordinary_muscle_development	ENSGALG00010022101	ENSGALG00010022101	1.113496647	0.18315174	6.079640018	1.20453E-09	2.80341E-09	positive
PRJNA729271	stage_5W_vs_E10	ordinary_muscle_development	ENSGALG00010022104	EXOC3L1	0.310678492	0.142065348	2.186870314	0.028751993	0.03737759	positive
PRJNA729271	stage_5W_vs_E10	ordinary_muscle_development	ENSGALG00010022105	DLG5	-1.413903443	0.085651388	-16.50765366	3.23207E-61	5.41606E-60	negative
PRJNA729271	stage_5W_vs_E10	ordinary_muscle_development	ENSGALG00010022108	USP47	0.78037036	0.088021635	8.865665312	7.60486E-19	2.88039E-18	positive
PRJNA729271	stage_5W_vs_E10	ordinary_muscle_development	ENSGALG00010022111	PCGF2	-2.130287033	0.204575887	-10.41318731	2.15871E-25	1.08497E-24	negative
PRJNA729271	stage_5W_vs_E10	ordinary_muscle_development	ENSGALG00010022115	E2F4	-0.260146966	0.186073723	-1.398085459	0.162087406	0.191542226	negative
PRJNA729271	stage_5W_vs_E10	ordinary_muscle_development	ENSGALG00010022116	CYP20A1	-0.929275005	0.104904352	-8.858307441	8.12384E-19	3.07374E-18	negative
PRJNA729271	stage_5W_vs_E10	ordinary_muscle_development	ENSGALG00010022117	FAM20C	0.09867046	0.121565513	0.811664902	0.416983939	0.45858742	positive
PRJNA729271	stage_5W_vs_E10	ordinary_muscle_development	ENSGALG00010022120	FOXL3	-6.142838165	0.934810889	-6.571209465	4.99082E-11	1.26738E-10	negative
PRJNA729271	stage_5W_vs_E10	ordinary_muscle_development	ENSGALG00010022122	PSMB3	-0.160485926	0.158087338	-1.01517255	0.310023583	0.348978165	negative
PRJNA729271	stage_5W_vs_E10	ordinary_muscle_development	ENSGALG00010022124	ENSGALG00010022124	-0.101882726	0.157643118	-0.646287181	0.518093388	0.558595929	negative
PRJNA729271	stage_5W_vs_E10	ordinary_muscle_development	ENSGALG00010022125	CBFA2T2	0.246234481	0.187220194	1.315213258	0.18843824	0.220310719	positive
PRJNA729271	stage_5W_vs_E10	ordinary_muscle_development	ENSGALG00010022129	ELMO3	0.487388401	0.241928373	2.014597933	0.043946794	0.055824098	positive
PRJNA729271	stage_5W_vs_E10	ordinary_muscle_development	ENSGALG00010022134	CH25H	0.00130668	0.237527562	0.005501172	0.995610722	0.996160517	positive
PRJNA729271	stage_5W_vs_E10	ordinary_muscle_development	ENSGALG00010022135	CHST1	-4.742095206	0.285466861	-16.61171873	5.73269E-62	9.75297E-61	negative
PRJNA729271	stage_5W_vs_E10	ordinary_muscle_development	ENSGALG00010022137	PDDC1	-0.677741173	0.129221571	-5.244799045	1.56453E-07	3.18643E-07	negative
PRJNA729271	stage_5W_vs_E10	ordinary_muscle_development	ENSGALG00010022138	SPAG17	0.41675543	0.213466631	1.952321205	0.050900081	0.064122778	positive
PRJNA729271	stage_5W_vs_E10	ordinary_muscle_development	ENSGALG00010022142	TMEM208	1.090656796	0.104949163	10.39223906	2.68964E-25	1.34807E-24	positive
PRJNA729271	stage_5W_vs_E10	ordinary_muscle_development	ENSGALG00010022143	MATCAP1	-0.933865591	0.203099755	-4.59806361	4.26436E-06	7.80354E-06	negative
PRJNA729271	stage_5W_vs_E10	ordinary_muscle_development	ENSGALG00010022144	SNTA1	3.263485005	0.186664714	17.48313824	1.92601E-68	3.87744E-67	positive
PRJNA729271	stage_5W_vs_E10	ordinary_muscle_development	ENSGALG00010022148	TALDO1	-0.357822958	0.086324725	-4.145080759	3.39694E-05	5.79882E-05	negative
PRJNA729271	stage_5W_vs_E10	ordinary_muscle_development	ENSGALG00010022149	NBEAL1	0.119584236	0.106406792	1.123840256	0.2610808	0.297630059	positive
PRJNA729271	stage_5W_vs_E10	ordinary_muscle_development	ENSGALG00010022150	CPNE2	-0.851008767	0.131927006	-6.450603187	1.11406E-10	2.7637E-10	negative
PRJNA729271	stage_5W_vs_E10	ordinary_muscle_development	ENSGALG00010022151	PIP4K2B	-0.322377578	0.155018214	-2.079610966	0.03756123	0.048181419	negative
PRJNA729271	stage_5W_vs_E10	ordinary_muscle_development	ENSGALG00010022152	ZNF644	-0.451148803	0.100845185	-4.473677167	7.68858E-06	1.38099E-05	negative
PRJNA729271	stage_5W_vs_E10	ordinary_muscle_development	ENSGALG00010022154	CPN1	1.368773713	0.194529394	7.036333599	1.97364E-12	5.42228E-12	positive
PRJNA729271	stage_5W_vs_E10	ordinary_muscle_development	ENSGALG00010022157	EPS8L2	1.169744134	0.136152807	8.591406653	8.5911E-18	3.10854E-17	positive
PRJNA729271	stage_5W_vs_E10	ordinary_muscle_development	ENSGALG00010022158	CWC25	-0.37147001	0.080776048	-4.59876435	4.25004E-06	7.77931E-06	negative
PRJNA729271	stage_5W_vs_E10	ordinary_muscle_development	ENSGALG00010022159	KIAA2013	0.394443624	0.089541713	4.405138246	1.05716E-05	1.88089E-05	positive
PRJNA729271	stage_5W_vs_E10	ordinary_muscle_development	ENSGALG00010022161	CELF1	0.087996839	0.094370742	0.932458903	0.351099416	0.391205015	positive
PRJNA729271	stage_5W_vs_E10	ordinary_muscle_development	ENSGALG00010022162	POLE	-2.367964382	0.16119095	-14.69043011	7.42411E-49	8.64357E-48	negative

PRJNA729271	stage_5W_vs_E10	ordinary_muscle_development	ENSGALG00010022164	PDGFA	-0.67407364	0.109194003	-6.173174536	6.69323E-10	1.5801E-09	negative
PRJNA729271	stage_5W_vs_E10	ordinary_muscle_development	ENSGALG00010022165	FAM192A	-0.087357884	0.089343568	-0.977774743	0.328185746	0.367822808	negative
PRJNA729271	stage_5W_vs_E10	ordinary_muscle_development	ENSGALG00010022166	SDHB	1.19830324	0.10795469	11.10005724	1.25364E-28	7.19377E-28	positive
PRJNA729271	stage_5W_vs_E10	ordinary_muscle_development	ENSGALG00010022167	WDR3	-0.779765039	0.072824993	-10.70738229	9.39817E-27	5.02681E-26	negative
PRJNA729271	stage_5W_vs_E10	ordinary_muscle_development	ENSGALG00010022169	TOX2	-0.352713112	0.235834403	-1.495596519	0.134758831	0.160900268	negative
PRJNA729271	stage_5W_vs_E10	ordinary_muscle_development	ENSGALG00010022170	RSPRY1	-0.59482634	0.141844825	-4.193500471	2.74683E-05	4.72472E-05	negative
PRJNA729271	stage_5W_vs_E10	ordinary_muscle_development	ENSGALG00010022171	FOXP1	-0.391068563	0.094757863	-4.127030223	3.67478E-05	6.25628E-05	negative
PRJNA729271	stage_5W_vs_E10	ordinary_muscle_development	ENSGALG00010022172	GIPC2	1.272313721	0.18201713	6.99007681	2.74736E-12	7.4813E-12	positive
PRJNA729271	stage_5W_vs_E10	ordinary_muscle_development	ENSGALG00010022173	RPL23	0.031595026	0.104084757	0.303550944	0.76147003	0.789295486	positive
PRJNA729271	stage_5W_vs_E10	ordinary_muscle_development	ENSGALG00010022174	KCNMA1	1.175543691	0.125307626	9.38126213	6.51877E-21	2.70899E-20	positive
PRJNA729271	stage_5W_vs_E10	ordinary_muscle_development	ENSGALG00010022176	ARL2BPL	-1.346565965	0.146954463	-9.163151229	5.04035E-20	2.01433E-19	negative
PRJNA729271	stage_5W_vs_E10	ordinary_muscle_development	ENSGALG00010022178	ENSGALG00010022178	0.802380463	0.326585927	2.456873973	0.014015184	0.018871351	positive
PRJNA729271	stage_5W_vs_E10	ordinary_muscle_development	ENSGALG00010022180	PLLP	1.057149402	0.392242306	2.695143756	0.007035827	0.009781729	positive
PRJNA729271	stage_5W_vs_E10	ordinary_muscle_development	ENSGALG00010022181	ENSGALG00010022181	-2.459778964	0.241442679	-10.18783826	2.24705E-24	1.08606E-23	negative
PRJNA729271	stage_5W_vs_E10	ordinary_muscle_development	ENSGALG00010022182	CNTNAP1	0.015184347	0.302196903	0.050246534	0.959925929	0.965358264	positive
PRJNA729271	stage_5W_vs_E10	ordinary_muscle_development	ENSGALG00010022184	PRKAR1B	-1.098170435	0.094361054	-11.63796275	2.64252E-31	1.68663E-30	negative
PRJNA729271	stage_5W_vs_E10	ordinary_muscle_development	ENSGALG00010022186	DNAJB4	1.661231698	0.112409078	14.77844788	2.01762E-49	2.39129E-48	positive
PRJNA729271	stage_5W_vs_E10	ordinary_muscle_development	ENSGALG00010022190	ACVR1B	1.368102362	0.163229207	8.381480182	5.22662E-17	1.8194E-16	positive
PRJNA729271	stage_5W_vs_E10	ordinary_muscle_development	ENSGALG00010022193	GDAP2	-0.525671341	0.082944495	-6.337627841	2.3333E-10	5.66993E-10	negative
PRJNA729271	stage_5W_vs_E10	ordinary_muscle_development	ENSGALG00010022194	ENSGALG00010022194	-1.720082791	0.093788807	-18.33995807	3.97103E-75	9.69026E-74	negative
PRJNA729271	stage_5W_vs_E10	ordinary_muscle_development	ENSGALG00010022196	PLXNA1	-0.017642742	0.108535386	-0.162552906	0.870870469	0.887088366	negative
PRJNA729271	stage_5W_vs_E10	ordinary_muscle_development	ENSGALG00010022199	RBM39	-0.373735028	0.061894988	-6.038211468	1.55832E-09	3.59965E-09	negative
PRJNA729271	stage_5W_vs_E10	ordinary_muscle_development	ENSGALG00010022200	DNMBP	0.287509945	0.10962915	2.62256841	0.008726975	0.012017623	positive
PRJNA729271	stage_5W_vs_E10	ordinary_muscle_development	ENSGALG00010022202	CX3CL1	3.124533154	0.158177761	19.75330248	7.51398E-87	2.34731E-85	positive
PRJNA729271	stage_5W_vs_E10	ordinary_muscle_development	ENSGALG00010022203	DNAAF5	-0.002762697	0.132316978	-0.02087938	0.983341875	0.985790199	negative
PRJNA729271	stage_5W_vs_E10	ordinary_muscle_development	ENSGALG00010022204	ENSGALG00010022204	0.821928895	0.331299331	2.480925308	0.013104183	0.0177072	positive
PRJNA729271	stage_5W_vs_E10	ordinary_muscle_development	ENSGALG00010022205	GALNT18	-0.899020038	0.109360122	-8.220729994	2.02268E-16	6.83735E-16	negative
PRJNA729271	stage_5W_vs_E10	ordinary_muscle_development	ENSGALG00010022207	ENSGALG00010022207	4.613461936	0.305373742	15.10759212	1.44318E-51	1.83177E-50	positive
PRJNA729271	stage_5W_vs_E10	ordinary_muscle_development	ENSGALG00010022208	CCL17	3.659763016	0.677291931	5.403523723	6.53443E-08	1.3642E-07	positive
PRJNA729271	stage_5W_vs_E10	ordinary_muscle_development	ENSGALG00010022209	ZFYVE19	-0.628573763	0.121988188	-5.152742849	2.56704E-07	5.1394E-07	negative
PRJNA729271	stage_5W_vs_E10	ordinary_muscle_development	ENSGALG00010022210	EZH1	1.000137431	0.083058314	12.04138857	2.15301E-33	1.48951E-32	positive
PRJNA729271	stage_5W_vs_E10	ordinary_muscle_development	ENSGALG00010022211	ENSGALG00010022211	0.151981668	0.503604385	0.301787817	0.762813818	0.790631841	positive
PRJNA729271	stage_5W_vs_E10	ordinary_muscle_development	ENSGALG00010022213	IDH1	-0.870987832	0.080840242	-10.77418635	4.55794E-27	2.46244E-26	negative
PRJNA729271	stage_5W_vs_E10	ordinary_muscle_development	ENSGALG00010022214	ENSGALG00010022214	-1.425444156	0.26515441	-5.375902132	7.62002E-08	1.584E-07	negative
PRJNA729271	stage_5W_vs_E10	ordinary_muscle_development	ENSGALG00010022215	ENSGALG00010022215	1.688160446	0.128645314	13.12259576	2.44417E-39	2.07912E-38	positive
PRJNA729271	stage_5W_vs_E10	ordinary_muscle_development	ENSGALG00010022216	ROMO1	0.021368923	0.145083742	0.147286817	0.882905637	0.897141059	positive
PRJNA729271	stage_5W_vs_E10	ordinary_muscle_development	ENSGALG00010022218	ENSGALG00010022218	4.903597914	0.246651253	19.8806933	5.98018E-88	1.94048E-86	positive
PRJNA729271	stage_5W_vs_E10	ordinary_muscle_development	ENSGALG00010022219	EIF4E3	0.757994112	0.136937129	5.535343956	3.10618E-08	6.62801E-08	positive
PRJNA729271	stage_5W_vs_E10	ordinary_muscle_development	ENSGALG00010022221	CUTC	0.407522401	0.083311684	4.891539581	1.0005E-06	1.9216E-06	positive
PRJNA729271	stage_5W_vs_E10	ordinary_muscle_development	ENSGALG00010022222	ENSGALG00010022222	-0.254264782	0.110681087	-2.297273991	0.021603145	0.028428287	negative
PRJNA729271	stage_5W_vs_E10	ordinary_muscle_development	ENSGALG00010022223	DOK4	-1.907063809	0.181856275	-10.48665388	9.94895E-26	5.08319E-25	negative
PRJNA729271	stage_5W_vs_E10	ordinary_muscle_development	ENSGALG00010022224	NHEJ1	1.080662741	0.144181276	7.495167004	6.6214E-14	1.95992E-13	positive
PRJNA729271	stage_5W_vs_E10	ordinary_muscle_development	ENSGALG00010022225	ENSGALG00010022225	0.034128088	0.540941672	0.063090145	0.949694722	0.956424998	positive
PRJNA729271	stage_5W_vs_E10	ordinary_muscle_development	ENSGALG00010022226	FBRSL1	0.332298719	0.132172407	2.514130794	0.011932617	0.016198097	positive
PRJNA729271	stage_5W_vs_E10	ordinary_muscle_development	ENSGALG00010022227	TMEM80	-0.075063303	0.161798637	-0.463930382	0.642697624	0.677717304	negative
PRJNA729271	stage_5W_vs_E10	ordinary_muscle_development	ENSGALG00010022228	FUBP1	-1.857241031	0.055956208	-33.19097402	1.453E-241	3.9003E-239	negative
PRJNA729271	stage_5W_vs_E10	ordinary_muscle_development	ENSGALG00010022229	RAPSN	0.233736698	0.179137006	1.304792928	0.191963368	0.22408867	positive
PRJNA729271	stage_5W_vs_E10	ordinary_muscle_development	ENSGALG00010022230	NFS1	-0.429874122	0.087592522	-4.907657778	9.21705E-07	1.77496E-06	negative
PRJNA729271	stage_5W_vs_E10	ordinary_muscle_development	ENSGALG00010022231	ZNF503	-2.587217446	0.211866697	-12.21153436	2.69753E-34	1.929E-33	negative
PRJNA729271	stage_5W_vs_E10	ordinary_muscle_development	ENSGALG00010022232	GPR27	-3.490616013	0.208136486	-16.77080304	3.99079E-63	6.99473E-62	negative
PRJNA729271	stage_5W_vs_E10	ordinary_muscle_development	ENSGALG00010022233	FAM46C	3.721183535	0.16393296	22.6994226	4.5392E-114	2.4642E-112	positive
PRJNA729271	stage_5W_vs_E10	ordinary_muscle_development	ENSGALG00010022234	COX15	0.197560749	0.09541223	2.07060194	0.038396011	0.04916521	positive
PRJNA729271	stage_5W_vs_E10	ordinary_muscle_development	ENSGALG00010022235	RAMP2	1.185340896	0.113088216	10.48155982	1.04998E-25	5.35329E-25	positive
PRJNA729271	stage_5W_vs_E10	ordinary_muscle_development	ENSGALG00010022236	SHF	1.089263464	0.181878562	5.988960159	2.11187E-09	4.839E-09	positive
PRJNA729271	stage_5W_vs_E10	ordinary_muscle_development	ENSGALG00010022237	SUN1	0.760882406	0.091070029	8.354915645	6.54795E-17	2.26955E-16	positive
PRJNA729271	stage_5W_vs_E10	ordinary_muscle_development	ENSGALG00010022238	COMTD1	0.295738675	0.165144652	1.790785663	0.073327697	0.090635709	positive
PRJNA729271	stage_5W_vs_E10	ordinary_muscle_development	ENSGALG00010022239	DEAF1	0.358180817	0.115889474	3.090710527	0.001996782	0.002934755	positive
PRJNA729271	stage_5W_vs_E10	ordinary_muscle_development	ENSGALG00010022240	ENSGALG00010022240	-0.438696556	0.131980056	-3.323960973	0.000887486	0.001350138	negative
PRJNA729271	stage_5W_vs_E10	ordinary_muscle_development	ENSGALG00010022241	POLR2C	0.167307993	0.094926339	1.762503389	0.077984284	0.09603111	positive
PRJNA729271	stage_5W_vs_E10	ordinary_muscle_development	ENSGALG00010022242	USP19	0.679159625	0.126077317	5.38685024	7.17031E-08	1.49373E-07	positive
PRJNA729271	stage_5W_vs_E10	ordinary_muscle_development	ENSGALG00010022243	ENSGALG00010022243	1.370309898	0.598677029	2.288896737	0.022085353	0.029020686	positive
PRJNA729271	stage_5W_vs_E10	ordinary_muscle_development	ENSGALG00010022244	ENTPD7	-1.117162185	0.176033916	-6.346289457	2.2057E-10	5.36887E-10	negative
PRJNA729271	stage_5W_vs_E10	ordinary_muscle_development	ENSGALG00010022245	PIKFYVE	1.019519482	0.099906536	10.20473257	1.88841E-24	9.15773E-24	positive
PRJNA729271	stage_5W_vs_E10	ordinary_muscle_development	ENSGALG00010022246	RHOV	-4.130839514	0.471740833	-8.756586729	2.01252E-18	7.49524E-18	negative
PRJNA729271	stage_5W_vs_E10	ordinary_muscle_development	ENSGALG00010022247	GALNT9	-2.099459425	0.220119181	-9.537830424	1.45852E-21	6.25294E-21	negative
PRJNA729271	stage_5W_vs_E10	ordinary_muscle_development	ENSGALG00010022248	FIGNL2	-3.093238109	0.241333963	-12.81725155	1.31272E-37	1.05946E-36	negative
PRJNA729271	stage_5W_vs_E10	ordinary_muscle_development	ENSGALG00010022250	SLC25A28	1.713115939	0.127471036	13.43925644	3.55996E-41	3.2251E-40	positive
PRJNA729271	stage_5W_vs_E10	ordinary_muscle_development	ENSGALG00010022251	WNK4	-0.185633681	0.151363277	-1.226411612	0.220043818	0.25406525	negative
PRJNA729271	stage_5W_vs_E10	ordinary_muscle_development	ENSGALG00010022252	COQ9	3.871124428	0.131521893	29.43330829	2.0591E-190	3.0456E-188	positive
PRJNA729271	stage_5W_vs_E10	ordinary_muscle_development	ENSGALG00010022253	ALPK3	3.922510066	0.21725496	18.05487003	7.22318E-73	1.64882E-71	positive
PRJNA729271	stage_5W_vs_E10	ordinary_muscle_development	ENSGALG00010022255	NOC4L	-2.51946526	0.094982527	-26.52556575	4.9163E-155	5.2786E-153	negative
PRJNA729271	stage_5W_vs_E10	ordinary_muscle_development	ENSGALG00010022256	PSMC3	0.244317527	0.092097924	2.652801668	0.007982676	0.011040925	positive
PRJNA729271	stage_5W_vs_E10	ordinary_muscle_development	ENSGALG00010022257	KCNK18	-0.985447134	1.156296039	-0.852244668	0.39407831	0.435345256	negative
PRJNA729271	stage_5W_vs_E10	ordinary_muscle_development	ENSGALG00010022258	ENSGALG00010022258	3.085087279	0.717651887	4.298863188	1.71676E-05	3.0046E-05	positive
PRJNA729271	stage_5W_vs_E10	ordinary_muscle_development	ENSGALG00010022259	GET4	0.106448735	0.085539695	1.244436686	0.213338888	0.246933417	positive
PRJNA729271	stage_5W_vs_E10	ordinary_muscle_development	ENSGALG00010022264	SCN8A	-0.907602199	0.256548	-3.537748098	0.000403555	0.000631284	negative
PRJNA729271	stage_5W_vs_E10	ordinary_muscle_development	ENSGALG00010022266	COA3	-0.241160006	0.147956746	-1.629935867	0.103115052	0.12501277	negative
PRJNA729271	stage_5W_vs_E10	ordinary_muscle_development	ENSGALG00010022269	CNTD1	-0.502545086	0.401454338	-1.251811325	0.210638623	0.24404179	negative
PRJNA729271	stage_5W_vs_E10	ordinary_muscle_development	ENSGALG00010022271	SLC18A2	-0.560039694	0.225609393	-2.482342102	0.01305219	0.017638588	negative
PRJNA729271	stage_5W_vs_E10	ordinary_muscle_development	ENSGALG00010022274	CCDC91	0.857575342	0.174307321	4.919904318	8.65865E-07	1.67165E-06	positive
PRJNA729271	stage_5W_vs_E10	ordinary_muscle_development	ENSGALG00010022275	VDAC2	1.601699501	0.124067067	12.90994899	3.95563E-38	3.2412E-37	positive
PRJNA729271	stage_5W_vs_E10	ordinary_muscle_development	ENSGALG00010022276	BECN1	0.614239545	0.088439679	6.94529367	3.77674E-12	1.02115E-11	positive
PRJNA729271	stage_5W_vs_E10	ordinary_muscle_development	ENSGALG00010022279	EP400	0.306313978	0.149128327	2.054029471	0.039972839	0.051076014	positive
PRJNA729271	stage_5W_vs_E10	ordinary_muscle_development	ENSGALG00010022280	PDZD8	0.1911268	0.067650516	2.825208323	0.004724988	0.006689004	positive
PRJNA729271	stage_5W_vs_E10	ordinary_muscle_development	ENSGALG00010022282	TMT1A	2.53026487	0.223117359	11.34051101	8.26604E-30	4.96133E-29	positive
PRJNA729271	stage_5W_vs_E10	ordinary_muscle_development	ENSGALG00010022290	NEXN	1.672080401	0.220094726	7.597094355	3.02854E-14	9.14936E-14	positive
PRJNA729271	stage_5W_vs_E10	ordinary_muscle_development	ENSGALG00010022293	DNAJC10	-0.753656025	0.064299771	-11.72097528	9.95196E-32	6.44853E-31	negative
PRJNA729271	stage_5W_vs_E10	ordinary_muscle_development	ENSGALG00010022294	ENSGALG00010022294	1.587666677	0.637131056	2.491899682	0.012706192	0.017188638	positive
PRJNA729271	stage_5W_vs_E10	ordinary_muscle_development	ENSGALG00010022296	CIAPIN1	-0.220804828	0.097705563	-2.259900282	0.02382744	0.031222089	negative
PRJNA729271	stage_5W_vs_E10	ordinary_muscle_development	ENSGALG00010022297	VPS18	-0.392955461	0.058309456	-6.739137823	1.59329E-11	4.1725E-11	negative
PRJNA729271	stage_5W_vs_E10	ordinary_muscle_development	ENSGALG00010022299	MRGBP	-0.373981365	0.080883118	-4.623725868	3.76908E-06	6.92607E-06	negative
PRJNA729271	stage_5W_vs_E10	ordinary_muscle_development	ENSGALG00010022302	ENSGALG00010022302	0.083394621	0.150833265	0.552892763	0.580336843	0.618755612	positive
PRJNA729271	stage_5W_vs_E10	ordinary_muscle_development	ENSGALG00010022303	DUSP13A	5.78788076	0.203204149	28.48308356	1.898E-178	2.5954E-176	positive
PRJNA729271	stage_5W_vs_E10	ordinary_muscle_development	ENSGALG00010022304	ADAP1	-1.466487981	0.192158956	-7.631640025	2.31786E-14	7.03757E-14	negative
PRJNA729271	stage_5W_vs_E10	ordinary_muscle_development	ENSGALG00010022307	ENSGALG00010022307	8.840411032	0.634275386	13.93781191	3.73207E-44	3.70523E-43	positive
PRJNA729271	stage_5W_vs_E10	ordinary_muscle_development	ENSGALG00010022308	OGFR	0.348776973	0.181145264	1.925399341	0.054179418	0.067999836	positive
PRJNA729271	stage_5W_vs_E10	ordinary_muscle_development	ENSGALG00010022309	WSCD2	-1.28594362	0.366344437	-3.510203761	0.000447763	0.00069766	negative
PRJNA729271	stage_5W_vs_E10	ordinary_muscle_development	ENSGALG00010022310	DCLRE1A	-2.032943423	0.104444833	-19.4642796	2.20574E-84	6.55168E-83	negative
PRJNA729271	stage_5W_vs_E10	ordinary_muscle_development	ENSGALG00010022311	DLL4	0.945587557	0.141679507	6.674130774	2.48702E-11	6.43278E-11	positive
PRJNA729271	stage_5W_vs_E10	ordinary_muscle_development	ENSGALG00010022312	ZNF592	-0.097625867	0.055992725	-1.743545554	0.081238364	0.099800838	negative
PRJNA729271	stage_5W_vs_E10	ordinary_muscle_development	ENSGALG00010022313	ENSGALG00010022313	0.641480284	0.372825587	1.720590827	0.085325099	0.104582049	positive
PRJNA729271	stage_5W_vs_E10	ordinary_muscle_development	ENSGALG00010022314	DUSP13B	6.791173072	0.551839329	12.30643181	8.36399E-35	6.12303E-34	positive
PRJNA729271	stage_5W_vs_E10	ordinary_muscle_development	ENSGALG00010022316	INO80	0.315868934	0.069323136	4.55647209	5.20199E-06	9.45563E-06	positive
PRJNA729271	stage_5W_vs_E10	ordinary_muscle_development	ENSGALG00010022317	PSME3	-1.508733657	0.074532698	-20.24257414	4.13024E-91	1.44958E-89	negative
PRJNA729271	stage_5W_vs_E10	ordinary_muscle_development	ENSGALG00010022320	COL9A3	-9.170090791	0.572731977	-16.01113813	1.06837E-57	1.60312E-56	negative
PRJNA729271	stage_5W_vs_E10	ordinary_muscle_development	ENSGALG00010022322	ENSGALG00010022322	2.577871958	0.373111439	6.90912067	4.87667E-12	1.30951E-11	positive
PRJNA729271	stage_5W_vs_E10	ordinary_muscle_development	ENSGALG00010022323	DUSP29	6.014216951	0.381677867	15.75731128	6.1181E-56	8.71995E-55	positive
PRJNA729271	stage_5W_vs_E10	ordinary_muscle_development	ENSGALG00010022325	MIGA1	0.092331597	0.107994239	0.8549678	0.392568974	0.433909355	positive
PRJNA729271	stage_5W_vs_E10	ordinary_muscle_development	ENSGALG00010022326	COX19	0.755877835	0.095423222	7.921319527	2.35003E-15	7.53122E-15	positive
PRJNA729271	stage_5W_vs_E10	ordinary_muscle_development	ENSGALG00010022328	ENSGALG00010022328	-0.786581313	0.244964626	-3.210999585	0.001322741	0.001980491	negative
PRJNA729271	stage_5W_vs_E10	ordinary_muscle_development	ENSGALG00010022332	KAT6B	-1.264702459	0.062586216	-20.20736433	8.43327E-91	2.94555E-89	negative
PRJNA729271	stage_5W_vs_E10	ordinary_muscle_development	ENSGALG00010022333	ENSGALG00010022333	1.451407929	0.131612587	11.02788084	2.80394E-28	1.587E-27	positive
PRJNA729271	stage_5W_vs_E10	ordinary_muscle_development	ENSGALG00010022334	PDE1A	-1.527695063	0.122907678	-12.42961459	1.80502E-35	1.35494E-34	negative
PRJNA729271	stage_5W_vs_E10	ordinary_muscle_development	ENSGALG00010022340	GFOD2	-0.657855521	0.0953713	-6.897835342	5.28009E-12	1.41574E-11	negative
PRJNA729271	stage_5W_vs_E10	ordinary_muscle_development	ENSGALG00010022343	GATA5	-2.894867447	0.614216873	-4.713103101	2.43973E-06	4.54665E-06	negative
PRJNA729271	stage_5W_vs_E10	ordinary_muscle_development	ENSGALG00010022345	USP33	-0.116569023	0.1229745	-0.94791215	0.343174179	0.383051727	negative
PRJNA729271	stage_5W_vs_E10	ordinary_muscle_development	ENSGALG00010022347	SEC11A	-1.448135224	0.105945524	-13.66867773	1.56208E-42	1.47411E-41	negative
PRJNA729271	stage_5W_vs_E10	ordinary_muscle_development	ENSGALG00010022348	G6PC1	2.597249998	0.620744623	4.184087791	2.86313E-05	4.91894E-05	positive
PRJNA729271	stage_5W_vs_E10	ordinary_muscle_development	ENSGALG00010022353	SLC39A13	0.628833362	0.095011028	6.618530214	3.62788E-11	9.31222E-11	positive
PRJNA729271	stage_5W_vs_E10	ordinary_muscle_development	ENSGALG00010022354	FAR2	0.176359834	0.136459418	1.292397671	0.196219463	0.228449889	positive
PRJNA729271	stage_5W_vs_E10	ordinary_muscle_development	ENSGALG00010022356	TCFL5	-1.119310147	0.237852467	-4.705900945	2.52747E-06	4.70654E-06	negative
PRJNA729271	stage_5W_vs_E10	ordinary_muscle_development	ENSGALG00010022357	ENSGALG00010022357	-5.178401581	0.799737185	-6.475129178	9.47309E-11	2.36216E-10	negative
PRJNA729271	stage_5W_vs_E10	ordinary_muscle_development	ENSGALG00010022359	ZZZ3	-0.464882795	0.079754054	-5.828955011	5.57755E-09	1.24975E-08	negative
PRJNA729271	stage_5W_vs_E10	ordinary_muscle_development	ENSGALG00010022363	KIF18A	-2.422162085	0.206065149	-11.75435097	6.70741E-32	4.38143E-31	negative
PRJNA729271	stage_5W_vs_E10	ordinary_muscle_development	ENSGALG00010022365	PLCG1	-1.589604683	0.111095046	-14.30851099	1.93615E-46	2.05751E-45	negative
PRJNA729271	stage_5W_vs_E10	ordinary_muscle_development	ENSGALG00010022366	AARSD1	0.714024396	0.113025333	6.317383686	2.66029E-10	6.44076E-10	positive
PRJNA729271	stage_5W_vs_E10	ordinary_muscle_development	ENSGALG00010022373	AK5	-3.816995434	0.233341035	-16.35801191	3.81376E-60	6.14911E-59	negative
PRJNA729271	stage_5W_vs_E10	ordinary_muscle_development	ENSGALG00010022375	NMB	-1.791243643	0.433485184	-4.132191159	3.59321E-05	6.12172E-05	negative
PRJNA729271	stage_5W_vs_E10	ordinary_muscle_development	ENSGALG00010022377	ZHX3	1.627360226	0.089354731	18.21235655	4.11833E-74	9.75411E-73	positive
PRJNA729271	stage_5W_vs_E10	ordinary_muscle_development	ENSGALG00010022379	METTL15	-0.901666589	0.123851781	-7.28020688	3.33309E-13	9.49923E-13	negative
PRJNA729271	stage_5W_vs_E10	ordinary_muscle_development	ENSGALG00010022382	CALB2	-3.977295601	0.87881308	-4.52575831	6.01793E-06	1.08833E-05	negative

PRJNA729271	stage_5W_vs_E10	ordinary_muscle_development	ENSGALG00010022385	ENSGALG00010022385	3.153867432	0.619615174	5.090042277	3.57984E-07	7.08876E-07	positive
PRJNA729271	stage_5W_vs_E10	ordinary_muscle_development	ENSGALG00010022386	SPI1	2.918624415	0.243521061	11.98510058	4.25328E-33	2.91082E-32	positive
PRJNA729271	stage_5W_vs_E10	ordinary_muscle_development	ENSGALG00010022388	CYP2W1	-0.771555783	0.445215175	-1.732995248	0.083096513	0.101945321	negative
PRJNA729271	stage_5W_vs_E10	ordinary_muscle_development	ENSGALG00010022391	ENSGALG00010022391	0.658812608	0.18049337	3.650065412	0.000262174	0.000417055	positive
PRJNA729271	stage_5W_vs_E10	ordinary_muscle_development	ENSGALG00010022392	EMILIN3	-3.799459034	0.191940166	-19.79501792	3.28635E-87	1.04464E-85	negative
PRJNA729271	stage_5W_vs_E10	ordinary_muscle_development	ENSGALG00010022395	PTGES3L	1.254052376	0.134058357	9.354525889	8.39755E-21	3.47282E-20	positive
PRJNA729271	stage_5W_vs_E10	ordinary_muscle_development	ENSGALG00010022396	PPP1R1C	-1.973314369	0.34474256	-5.724023082	1.04031E-08	2.29412E-08	negative
PRJNA729271	stage_5W_vs_E10	ordinary_muscle_development	ENSGALG00010022397	ATF1	-0.274908539	0.047743042	-5.758085996	8.5073E-09	1.88524E-08	negative
PRJNA729271	stage_5W_vs_E10	ordinary_muscle_development	ENSGALG00010022400	NHLRC2	0.100570383	0.072125239	1.394385449	0.16320124	0.192811311	positive
PRJNA729271	stage_5W_vs_E10	ordinary_muscle_development	ENSGALG00010022401	LCAT	1.823172056	0.437929352	4.163164786	3.13867E-05	5.37512E-05	positive
PRJNA729271	stage_5W_vs_E10	ordinary_muscle_development	ENSGALG00010022406	ITPRID2	0.889782173	0.104371264	8.525164322	1.52593E-17	5.45728E-17	positive
PRJNA729271	stage_5W_vs_E10	ordinary_muscle_development	ENSGALG00010022407	RUNDC1	0.786366595	0.095172535	8.262536993	1.42609E-16	4.85237E-16	positive
PRJNA729271	stage_5W_vs_E10	ordinary_muscle_development	ENSGALG00010022408	RAD51AP1	-0.389506525	0.137987868	-2.822759216	0.004761232	0.006737679	negative
PRJNA729271	stage_5W_vs_E10	ordinary_muscle_development	ENSGALG00010022409	CARNS1	9.046090809	0.338373072	26.73407421	1.8915E-157	2.0929E-155	positive
PRJNA729271	stage_5W_vs_E10	ordinary_muscle_development	ENSGALG00010022411	ADRB1	1.033114235	0.290070339	3.561599024	0.000368603	0.000578925	positive
PRJNA729271	stage_5W_vs_E10	ordinary_muscle_development	ENSGALG00010022412	CHAC1	6.799133898	0.650397214	10.45381769	1.40743E-25	7.1157E-25	positive
PRJNA729271	stage_5W_vs_E10	ordinary_muscle_development	ENSGALG00010022415	DIP2B	-0.161203174	0.109379346	-1.47379903	0.14053571	0.167397905	negative
PRJNA729271	stage_5W_vs_E10	ordinary_muscle_development	ENSGALG00010022416	CCDC186	-0.144830323	0.087113064	-1.662555712	0.096401403	0.117364214	negative
PRJNA729271	stage_5W_vs_E10	ordinary_muscle_development	ENSGALG00010022419	DUOX2	2.540664724	0.697418918	3.642953553	0.000269527	0.000428143	positive
PRJNA729271	stage_5W_vs_E10	ordinary_muscle_development	ENSGALG00010022421	CMKLR1	2.229987324	0.174749442	12.7610555	2.70511E-37	2.16752E-36	positive
PRJNA729271	stage_5W_vs_E10	ordinary_muscle_development	ENSGALG00010022422	RPS6KB2	1.632539669	0.154198921	10.58723145	3.41544E-26	1.7821E-25	positive
PRJNA729271	stage_5W_vs_E10	ordinary_muscle_development	ENSGALG00010022425	VWA2	-4.755772007	0.647499263	-7.344829991	2.06021E-13	5.9381E-13	negative
PRJNA729271	stage_5W_vs_E10	ordinary_muscle_development	ENSGALG00010022426	EXD1	-0.689528983	0.235259491	-2.930929507	0.003379495	0.004858736	negative
PRJNA729271	stage_5W_vs_E10	ordinary_muscle_development	ENSGALG00010022427	RPL27	0.003468848	0.138699737	0.025009767	0.980047173	0.983031194	positive
PRJNA729271	stage_5W_vs_E10	ordinary_muscle_development	ENSGALG00010022429	ADK	-1.527554925	0.131953042	-11.57650403	5.42121E-31	3.42398E-30	negative
PRJNA729271	stage_5W_vs_E10	ordinary_muscle_development	ENSGALG00010022431	LARP4	-0.366885529	0.13303715	-2.757767493	0.005819758	0.008163091	negative
PRJNA729271	stage_5W_vs_E10	ordinary_muscle_development	ENSGALG00010022433	LNPK	1.148287664	0.107498108	10.68193372	1.23668E-26	6.58792E-26	positive
PRJNA729271	stage_5W_vs_E10	ordinary_muscle_development	ENSGALG00010022435	PIGK	0.200918241	0.16399998	1.225111369	0.220533262	0.254549262	positive
PRJNA729271	stage_5W_vs_E10	ordinary_muscle_development	ENSGALG00010022436	AFAP1L2	0.06543452	0.155595401	0.420542766	0.674088995	0.707781237	positive
PRJNA729271	stage_5W_vs_E10	ordinary_muscle_development	ENSGALG00010022438	RPN2	-0.842590211	0.101454504	-8.305104044	9.9732E-17	3.41996E-16	negative
PRJNA729271	stage_5W_vs_E10	ordinary_muscle_development	ENSGALG00010022439	IFI35	2.230099644	0.203208315	10.97445075	5.07131E-28	2.84586E-27	positive
PRJNA729271	stage_5W_vs_E10	ordinary_muscle_development	ENSGALG00010022440	SORD	0.907389553	0.123872552	7.325186544	2.38567E-13	6.85708E-13	positive
PRJNA729271	stage_5W_vs_E10	ordinary_muscle_development	ENSGALG00010022441	FICD	0.21793551	0.118904644	1.832859523	0.066823465	0.082985445	positive
PRJNA729271	stage_5W_vs_E10	ordinary_muscle_development	ENSGALG00010022448	FERRY3	-0.665937526	0.122308019	-5.444757664	5.1876E-08	1.09056E-07	negative
PRJNA729271	stage_5W_vs_E10	ordinary_muscle_development	ENSGALG00010022449	ENSGALG00010022449	-0.54894496	0.103040734	-5.327455841	9.9598E-08	2.05593E-07	negative
PRJNA729271	stage_5W_vs_E10	ordinary_muscle_development	ENSGALG00010022451	CHP1	0.114466444	0.121139653	0.944913094	0.344703246	0.38448224	positive
PRJNA729271	stage_5W_vs_E10	ordinary_muscle_development	ENSGALG00010022452	AP3M1	-0.529035931	0.081819104	-6.465921803	1.00683E-10	2.50843E-10	negative
PRJNA729271	stage_5W_vs_E10	ordinary_muscle_development	ENSGALG00010022453	ENSGALG00010022453	3.142538167	0.233016911	13.48630942	1.88293E-41	1.71979E-40	positive
PRJNA729271	stage_5W_vs_E10	ordinary_muscle_development	ENSGALG00010022454	ABLIM1	2.083428356	0.140447277	14.83423819	8.79972E-50	1.05415E-48	positive
PRJNA729271	stage_5W_vs_E10	ordinary_muscle_development	ENSGALG00010022455	C7orf50	-1.343976	0.156044212	-8.612789804	7.13038E-18	2.59295E-17	negative
PRJNA729271	stage_5W_vs_E10	ordinary_muscle_development	ENSGALG00010022456	ATP5G3	-0.034059226	0.158321455	-0.215127041	0.829668272	0.850137255	negative
PRJNA729271	stage_5W_vs_E10	ordinary_muscle_development	ENSGALG00010022459	FGF6	2.895213257	0.742232726	3.90068122	9.59224E-05	0.000158305	positive
PRJNA729271	stage_5W_vs_E10	ordinary_muscle_development	ENSGALG00010022460	VAT1	-0.774429214	0.118161568	-6.553985613	5.60214E-11	1.4179E-10	negative
PRJNA729271	stage_5W_vs_E10	ordinary_muscle_development	ENSGALG00010022462	ITGA5	0.229244327	0.129772735	1.766506083	0.077310982	0.095274841	positive
PRJNA729271	stage_5W_vs_E10	ordinary_muscle_development	ENSGALG00010022463	DUSP12	0.010083234	0.118494426	0.085094587	0.932186194	0.941211959	positive
PRJNA729271	stage_5W_vs_E10	ordinary_muscle_development	ENSGALG00010022466	NEDD4	0.263834768	0.137493873	1.918883814	0.05499904	0.068956931	positive
PRJNA729271	stage_5W_vs_E10	ordinary_muscle_development	ENSGALG00010022467	SART3	-0.985190653	0.11732165	-8.397347376	4.56678E-17	1.59315E-16	negative
PRJNA729271	stage_5W_vs_E10	ordinary_muscle_development	ENSGALG00010022468	MYBPC3	-1.041236958	0.429556374	-2.423982091	0.015351363	0.020582555	negative
PRJNA729271	stage_5W_vs_E10	ordinary_muscle_development	ENSGALG00010022470	ENSGALG00010022470	0.662736814	0.168301356	3.937798428	8.22326E-05	0.000136724	positive
PRJNA729271	stage_5W_vs_E10	ordinary_muscle_development	ENSGALG00010022471	ST6GALNAC5	-3.156966518	0.18781993	-16.80847457	2.11545E-63	3.75778E-62	negative
PRJNA729271	stage_5W_vs_E10	ordinary_muscle_development	ENSGALG00010022472	QSER1	0.470952766	0.087468252	5.384270933	7.27389E-08	1.514E-07	positive
PRJNA729271	stage_5W_vs_E10	ordinary_muscle_development	ENSGALG00010022473	ZNF385A	0.674964101	0.463258079	1.456993698	0.14511812	0.172515964	positive
PRJNA729271	stage_5W_vs_E10	ordinary_muscle_development	ENSGALG00010022474	VCL	0.187497316	0.119693448	1.566479362	0.117236436	0.141094499	positive
PRJNA729271	stage_5W_vs_E10	ordinary_muscle_development	ENSGALG00010022476	OIP5	-1.359650967	0.116176694	-11.70330231	1.22588E-31	7.92912E-31	negative
PRJNA729271	stage_5W_vs_E10	ordinary_muscle_development	ENSGALG00010022478	ATP6V1A	-0.163991608	0.096872184	-1.692865809	0.090481013	0.110509124	negative
PRJNA729271	stage_5W_vs_E10	ordinary_muscle_development	ENSGALG00010022479	ENSGALG00010022479	2.029869535	0.638688246	3.178185205	0.001482001	0.002206862	positive
PRJNA729271	stage_5W_vs_E10	ordinary_muscle_development	ENSGALG00010022480	PIF1	-3.77744206	0.28997605	-13.02673813	8.62152E-39	7.19039E-38	negative
PRJNA729271	stage_5W_vs_E10	ordinary_muscle_development	ENSGALG00010022481	CORO1B	-0.629139013	0.150302281	-4.185824778	2.84132E-05	4.88321E-05	negative
PRJNA729271	stage_5W_vs_E10	ordinary_muscle_development	ENSGALG00010022482	PRTG	0.403924357	0.294118704	1.373337881	0.169647331	0.199954307	positive
PRJNA729271	stage_5W_vs_E10	ordinary_muscle_development	ENSGALG00010022483	TOP2A	-3.124304043	0.239337876	-13.05394742	6.03382E-39	5.05258E-38	negative
PRJNA729271	stage_5W_vs_E10	ordinary_muscle_development	ENSGALG00010022485	ATF2	-0.207287141	0.057793741	-3.586671101	0.000334926	0.000528035	negative
PRJNA729271	stage_5W_vs_E10	ordinary_muscle_development	ENSGALG00010022486	FAM183A	-1.682686483	0.678909913	-2.478512173	0.013193161	0.017822448	negative
PRJNA729271	stage_5W_vs_E10	ordinary_muscle_development	ENSGALG00010022489	NUSAP1	-2.245125455	0.240481229	-9.335969661	1.00071E-20	4.11731E-20	negative
PRJNA729271	stage_5W_vs_E10	ordinary_muscle_development	ENSGALG00010022493	ENSGALG00010022493	-0.69015078	0.170018988	-4.059257066	4.92291E-05	8.31286E-05	negative
PRJNA729271	stage_5W_vs_E10	ordinary_muscle_development	ENSGALG00010022494	FHIP2A	0.115337917	0.065730811	1.754700948	0.079310474	0.09757302	positive
PRJNA729271	stage_5W_vs_E10	ordinary_muscle_development	ENSGALG00010022496	TUBGCP2	0.025167835	0.072429609	0.347479926	0.728230797	0.757954485	positive
PRJNA729271	stage_5W_vs_E10	ordinary_muscle_development	ENSGALG00010022497	CORO1C	0.370814367	0.149006846	2.488572683	0.012825702	0.017343833	positive
PRJNA729271	stage_5W_vs_E10	ordinary_muscle_development	ENSGALG00010022498	NAA50	0.44477521	0.11384827	3.906736656	9.3551E-05	0.00015455	positive
PRJNA729271	stage_5W_vs_E10	ordinary_muscle_development	ENSGALG00010022500	ENSGALG00010022500	-0.373571053	0.441239584	-0.846639936	0.397195847	0.438421703	negative
PRJNA729271	stage_5W_vs_E10	ordinary_muscle_development	ENSGALG00010022502	ENSGALG00010022502	-2.64273463	0.630213863	-4.193393363	2.74812E-05	4.72527E-05	negative
PRJNA729271	stage_5W_vs_E10	ordinary_muscle_development	ENSGALG00010022504	RAD9A	1.278021668	0.143519186	8.904883775	5.3442E-19	2.038E-18	positive
PRJNA729271	stage_5W_vs_E10	ordinary_muscle_development	ENSGALG00010022505	ZNF511	1.131355925	0.119959649	9.431137375	4.05666E-21	1.70686E-20	positive
PRJNA729271	stage_5W_vs_E10	ordinary_muscle_development	ENSGALG00010022506	EBNA1BP2	-1.130817104	0.087038694	-12.9921194	1.35619E-38	1.12588E-37	negative
PRJNA729271	stage_5W_vs_E10	ordinary_muscle_development	ENSGALG00010022507	ENSGALG00010022507	-0.516784336	0.221414038	-2.33401794	0.019594783	0.025926643	negative
PRJNA729271	stage_5W_vs_E10	ordinary_muscle_development	ENSGALG00010022508	USF3	0.880448703	0.121189975	7.265029168	3.72958E-13	1.06063E-12	positive
PRJNA729271	stage_5W_vs_E10	ordinary_muscle_development	ENSGALG00010022511	IGFBP4	-0.059172536	0.271491498	-0.217953553	0.827465301	0.848179728	negative
PRJNA729271	stage_5W_vs_E10	ordinary_muscle_development	ENSGALG00010022512	PRAP1	1.372179956	0.870409614	1.576476103	0.114916122	0.138566607	positive
PRJNA729271	stage_5W_vs_E10	ordinary_muscle_development	ENSGALG00010022513	NDUFAF1	-0.003329639	0.085257758	-0.03905379	0.968847503	0.973288512	negative
PRJNA729271	stage_5W_vs_E10	ordinary_muscle_development	ENSGALG00010022515	CHRNA1	-2.089267764	0.172718395	-12.0963825	1.10368E-33	7.72473E-33	negative
PRJNA729271	stage_5W_vs_E10	ordinary_muscle_development	ENSGALG00010022516	TRUB1	-0.804405999	0.153387212	-5.24428332	1.56891E-07	3.19401E-07	negative
PRJNA729271	stage_5W_vs_E10	ordinary_muscle_development	ENSGALG00010022517	HNRNPA1	-0.811097594	0.181493693	-4.46901255	7.85815E-06	1.4104E-05	negative
PRJNA729271	stage_5W_vs_E10	ordinary_muscle_development	ENSGALG00010022518	ECHS1	-0.12676719	0.113232281	-1.119532245	0.262913143	0.299554001	negative
PRJNA729271	stage_5W_vs_E10	ordinary_muscle_development	ENSGALG00010022520	TMOD2	-3.955505967	0.226768401	-17.44293272	3.89561E-68	7.73518E-67	negative
PRJNA729271	stage_5W_vs_E10	ordinary_muscle_development	ENSGALG00010022521	RTF1	-0.159437607	0.091250556	-1.74725079	0.080593845	0.099042627	negative
PRJNA729271	stage_5W_vs_E10	ordinary_muscle_development	ENSGALG00010022522	TNS4	-1.826782853	0.37597543	-4.858782535	1.1811E-06	2.25352E-06	negative
PRJNA729271	stage_5W_vs_E10	ordinary_muscle_development	ENSGALG00010022524	SOX18	1.038150267	0.228725125	4.538855396	5.65604E-06	1.02519E-05	positive
PRJNA729271	stage_5W_vs_E10	ordinary_muscle_development	ENSGALG00010022525	ISCU	0.995685919	0.194112012	5.129439992	2.90605E-07	5.78855E-07	positive
PRJNA729271	stage_5W_vs_E10	ordinary_muscle_development	ENSGALG00010022526	PPP1CA	1.585646377	0.137409299	11.5395868	8.33256E-31	5.21055E-30	positive
PRJNA729271	stage_5W_vs_E10	ordinary_muscle_development	ENSGALG00010022528	SIDT1	-3.882862703	0.533711025	-7.275215461	3.45868E-13	9.85136E-13	negative
PRJNA729271	stage_5W_vs_E10	ordinary_muscle_development	ENSGALG00010022529	CCR7	0.547272461	0.336356628	1.627060136	0.103724326	0.125709374	positive
PRJNA729271	stage_5W_vs_E10	ordinary_muscle_development	ENSGALG00010022531	ENSGALG00010022531	1.64726326	0.37414353	4.402757576	1.06884E-05	1.90119E-05	positive
PRJNA729271	stage_5W_vs_E10	ordinary_muscle_development	ENSGALG00010022532	CBX5	-3.264552483	0.182686398	-17.86970747	2.03056E-71	4.49359E-70	negative
PRJNA729271	stage_5W_vs_E10	ordinary_muscle_development	ENSGALG00010022533	TMEM119	-0.768698055	0.133461886	-5.759682238	8.42724E-09	1.86864E-08	negative
PRJNA729271	stage_5W_vs_E10	ordinary_muscle_development	ENSGALG00010022534	CFAP57	0.304230964	0.490032985	0.620837726	0.534706398	0.574840113	positive
PRJNA729271	stage_5W_vs_E10	ordinary_muscle_development	ENSGALG00010022536	SMUG1	-0.162911003	0.101885108	-1.59896776	0.109827766	0.132684904	negative
PRJNA729271	stage_5W_vs_E10	ordinary_muscle_development	ENSGALG00010022538	WIPF1	0.579309835	0.157424455	3.67992276	0.000233305	0.00037285	positive
PRJNA729271	stage_5W_vs_E10	ordinary_muscle_development	ENSGALG00010022539	LYSMD2	0.683861634	0.101922532	6.709621698	1.9513E-11	5.08066E-11	positive
PRJNA729271	stage_5W_vs_E10	ordinary_muscle_development	ENSGALG00010022540	GPR146	1.343727459	0.238531249	5.633339289	1.76753E-08	3.84229E-08	positive
PRJNA729271	stage_5W_vs_E10	ordinary_muscle_development	ENSGALG00010022541	ITPKA	-1.620356135	0.127207249	-12.73792292	3.63956E-37	2.90824E-36	negative
PRJNA729271	stage_5W_vs_E10	ordinary_muscle_development	ENSGALG00010022542	ENSGALG00010022542	0.718620049	0.730366448	0.983917115	0.325156268	0.364737664	positive
PRJNA729271	stage_5W_vs_E10	ordinary_muscle_development	ENSGALG00010022544	SPICE1	-0.386786177	0.071206602	-5.431886447	5.57614E-08	1.17004E-07	negative
PRJNA729271	stage_5W_vs_E10	ordinary_muscle_development	ENSGALG00010022545	CSTF3	-0.863155846	0.068287084	-12.64010406	1.26886E-36	9.93095E-36	negative
PRJNA729271	stage_5W_vs_E10	ordinary_muscle_development	ENSGALG00010022546	MTG1	1.184056213	0.157315014	7.526657367	5.20557E-14	1.55513E-13	positive
PRJNA729271	stage_5W_vs_E10	ordinary_muscle_development	ENSGALG00010022547	SMARCE1	-1.194947041	0.062330585	-19.17111856	6.45068E-82	1.81558E-80	negative
PRJNA729271	stage_5W_vs_E10	ordinary_muscle_development	ENSGALG00010022548	ENSGALG00010022548	0.087809204	0.115258222	0.761847635	0.446150946	0.487998639	positive
PRJNA729271	stage_5W_vs_E10	ordinary_muscle_development	ENSGALG00010022550	TRIM45	-1.365676012	0.171056704	-7.983761991	1.4194E-15	4.60581E-15	negative
PRJNA729271	stage_5W_vs_E10	ordinary_muscle_development	ENSGALG00010022551	GPER1	-0.953218729	0.60581754	-1.573441946	0.115616521	0.139295276	negative
PRJNA729271	stage_5W_vs_E10	ordinary_muscle_development	ENSGALG00010022552	GPR155	1.118243843	0.105176216	10.63209803	2.11305E-26	1.11377E-25	positive
PRJNA729271	stage_5W_vs_E10	ordinary_muscle_development	ENSGALG00010022553	PRPF6	0.345408581	0.169379488	2.03925862	0.041424223	0.052800221	positive
PRJNA729271	stage_5W_vs_E10	ordinary_muscle_development	ENSGALG00010022554	SCG3	-3.521038371	0.359185104	-9.802851875	1.09451E-22	4.93773E-22	negative
PRJNA729271	stage_5W_vs_E10	ordinary_muscle_development	ENSGALG00010022557	WNT1	-0.659049594	0.427877981	-1.540274617	0.123493428	0.1481451	negative
PRJNA729271	stage_5W_vs_E10	ordinary_muscle_development	ENSGALG00010022558	NYAP2	-1.869288737	0.601710678	-3.10662384	0.00189237	0.002786459	negative
PRJNA729271	stage_5W_vs_E10	ordinary_muscle_development	ENSGALG00010022559	SSH1	0.174937353	0.087190773	2.006374605	0.044816296	0.056848885	positive
PRJNA729271	stage_5W_vs_E10	ordinary_muscle_development	ENSGALG00010022560	SAMD10	-4.406458946	1.448819092	-3.041414191	0.002354697	0.003438578	negative
PRJNA729271	stage_5W_vs_E10	ordinary_muscle_development	ENSGALG00010022562	GHRH	-3.855977624	1.135962642	-3.394458129	0.000687646	0.001056319	negative
PRJNA729271	stage_5W_vs_E10	ordinary_muscle_development	ENSGALG00010022565	ARF3	-1.571155989	0.176111391	-8.921376303	4.60529E-19	1.76271E-18	negative
PRJNA729271	stage_5W_vs_E10	ordinary_muscle_development	ENSGALG00010022567	PAOX	0.308018442	0.126160233	2.441485995	0.014626956	0.019658574	positive
PRJNA729271	stage_5W_vs_E10	ordinary_muscle_development	ENSGALG00010022568	SCRN3	2.968885402	0.166773092	17.801945	6.82566E-71	1.4701E-69	positive
PRJNA729271	stage_5W_vs_E10	ordinary_muscle_development	ENSGALG00010022569	KIAA1549L	-3.5983476	0.272495134	-13.20518114	8.19025E-40	7.13447E-39	negative
PRJNA729271	stage_5W_vs_E10	ordinary_muscle_development	ENSGALG00010022571	RIPOR1	0.930930896	0.107385465	8.669058679	4.35707E-18	1.59605E-17	positive
PRJNA729271	stage_5W_vs_E10	ordinary_muscle_development	ENSGALG00010022572	ENSGALG00010022572	-5.639818979	0.464375352	-12.14495764	6.10142E-34	4.32893E-33	negative
PRJNA729271	stage_5W_vs_E10	ordinary_muscle_development	ENSGALG00010022573	AP4E1	-0.433342714	0.083031064	-5.219043264	1.7985E-07	3.64452E-07	negative
PRJNA729271	stage_5W_vs_E10	ordinary_muscle_development	ENSGALG00010022574	ITPRIPL2	0.115987638	0.108956339	1.064533175	0.287087234	0.324850075	positive
PRJNA729271	stage_5W_vs_E10	ordinary_muscle_development	ENSGALG00010022575	TMEM41B	-0.041432734	0.110095133	-0.376335743	0.70666731	0.738510646	negative
PRJNA729271	stage_5W_vs_E10	ordinary_muscle_development	ENSGALG00010022578	ZNF512B	-0.365747033	0.135529123	-2.698660077	0.006961925	0.009683629	negative
PRJNA729271	stage_5W_vs_E10	ordinary_muscle_development	ENSGALG00010022579	TNFAIP8L3	-0.308605334	0.147323084	-2.094752069	0.036193038	0.046512819	negative
PRJNA729271	stage_5W_vs_E10	ordinary_muscle_development	ENSGALG00010022581	TMEM125	-2.269520942	0.690127908	-3.288551175	0.001007045	0.001526416	negative
PRJNA729271	stage_5W_vs_E10	ordinary_muscle_development	ENSGALG00010022582	ENSGALG00010022582	0.459665004	0.216604911	2.122135648	0.033826349	0.04362991	positive
PRJNA729271	stage_5W_vs_E10	ordinary_muscle_development	ENSGALG00010022583	FKBP11	-0.427446563	0.12384006	-3.451601706	0.00055727	0.000862625	negative
PRJNA729271	stage_5W_vs_E10	ordinary_muscle_development	ENSGALG00010022585	CIR1	-0.187153159	0.094715035	-1.975960397	0.048159263	0.060839159	negative
PRJNA729271	stage_5W_vs_E10	ordinary_muscle_development	ENSGALG00010022587	ENSGALG00010022587	-1.752921447	0.886544587	-1.977251311	0.04801323	0.060665252	negative

PRJNA729271	stage_5W_vs_E10	ordinary_muscle_development	ENSGALG00010022588	TCP11L1	0.160864013	0.0774925	2.075865584	0.037906385	0.048581171	positive
PRJNA729271	stage_5W_vs_E10	ordinary_muscle_development	ENSGALG00010022590	ENSGALG00010022590	-1.393526464	0.166049526	-8.39223393	4.76991E-17	1.66281E-16	negative
PRJNA729271	stage_5W_vs_E10	ordinary_muscle_development	ENSGALG00010022592	CCDC65	-0.385411011	0.438537131	-0.878856049	0.379479332	0.420613091	negative
PRJNA729271	stage_5W_vs_E10	ordinary_muscle_development	ENSGALG00010022593	MANBAL	-2.227085321	0.148395311	-15.00778765	6.52893E-51	8.08861E-50	negative
PRJNA729271	stage_5W_vs_E10	ordinary_muscle_development	ENSGALG00010022597	SLC04A1	2.023085906	0.222461768	9.094083545	9.53875E-20	3.77462E-19	positive
PRJNA729271	stage_5W_vs_E10	ordinary_muscle_development	ENSGALG00010022599	RNLS	-1.273476154	0.279504278	-4.556195578	5.20884E-06	9.46617E-06	negative
PRJNA729271	stage_5W_vs_E10	ordinary_muscle_development	ENSGALG00010022603	ATRNL1	-0.420786432	0.169809796	-2.477986795	0.013212604	0.017842061	negative
PRJNA729271	stage_5W_vs_E10	ordinary_muscle_development	ENSGALG00010022608	P3H4	-1.885561002	0.208323735	-9.051109824	1.41523E-19	5.54874E-19	negative
PRJNA729271	stage_5W_vs_E10	ordinary_muscle_development	ENSGALG00010022609	RBBP8NL	-7.411071303	1.061065745	-6.984554291	2.85762E-12	7.7757E-12	negative
PRJNA729271	stage_5W_vs_E10	ordinary_muscle_development	ENSGALG00010022614	DAO	-0.418597111	0.203632601	-2.055648793	0.039816381	0.050894043	negative
PRJNA729271	stage_5W_vs_E10	ordinary_muscle_development	ENSGALG00010022616	GLDN	2.013390131	0.336258103	5.987633045	2.12917E-09	4.87556E-09	positive
PRJNA729271	stage_5W_vs_E10	ordinary_muscle_development	ENSGALG00010022619	MADD	-0.217554047	0.132622209	-1.640404339	0.100921124	0.122475862	negative
PRJNA729271	stage_5W_vs_E10	ordinary_muscle_development	ENSGALG00010022622	MPPED2	-1.549035959	0.138493761	-11.18487898	4.83565E-29	2.82404E-28	negative
PRJNA729271	stage_5W_vs_E10	ordinary_muscle_development	ENSGALG00010022623	TOP1	-1.141278436	0.126735783	-9.005179174	2.15314E-19	8.3605E-19	negative
PRJNA729271	stage_5W_vs_E10	ordinary_muscle_development	ENSGALG00010022625	ENSGALG00010022625	0.41057222	0.123446379	3.325915469	0.000881287	0.001341551	positive
PRJNA729271	stage_5W_vs_E10	ordinary_muscle_development	ENSGALG00010022627	CTCF	-0.424034363	0.098499369	-4.304944973	1.67027E-05	2.92646E-05	negative
PRJNA729271	stage_5W_vs_E10	ordinary_muscle_development	ENSGALG00010022628	SVOP	1.680647899	0.547049969	3.072201802	0.00212486	0.003114871	positive
PRJNA729271	stage_5W_vs_E10	ordinary_muscle_development	ENSGALG00010022630	ENSGALG00010022630	-2.808049114	0.310694293	-9.037981005	1.59592E-19	6.24706E-19	negative
PRJNA729271	stage_5W_vs_E10	ordinary_muscle_development	ENSGALG00010022637	DMXL2	-0.596855081	0.110409716	-5.405820285	6.45125E-08	1.34742E-07	negative
PRJNA729271	stage_5W_vs_E10	ordinary_muscle_development	ENSGALG00010022642	SEC13	-0.511532587	0.068810134	-7.433971715	1.05385E-13	3.08409E-13	negative
PRJNA729271	stage_5W_vs_E10	ordinary_muscle_development	ENSGALG00010022643	SP3	-0.219505304	0.07719131	-2.843653045	0.004459958	0.006328644	negative
PRJNA729271	stage_5W_vs_E10	ordinary_muscle_development	ENSGALG00010022644	ARMC8	0.666185095	0.085305556	7.809398627	5.74615E-15	1.80048E-14	positive
PRJNA729271	stage_5W_vs_E10	ordinary_muscle_development	ENSGALG00010022645	ENSGALG00010022645	-1.979429294	0.778809592	-2.541608776	0.011034361	0.015044968	negative
PRJNA729271	stage_5W_vs_E10	ordinary_muscle_development	ENSGALG00010022647	SVIP	-0.687287913	0.134648757	-5.104301962	3.32018E-07	6.5935E-07	negative
PRJNA729271	stage_5W_vs_E10	ordinary_muscle_development	ENSGALG00010022649	PATZ1	-3.186351166	0.103227444	-30.86728718	3.2839E-209	6.0254E-207	negative
PRJNA729271	stage_5W_vs_E10	ordinary_muscle_development	ENSGALG00010022650	NR1H3	1.501747133	0.098500219	15.24612991	1.74685E-52	2.27498E-51	positive
PRJNA729271	stage_5W_vs_E10	ordinary_muscle_development	ENSGALG00010022652	CARMIL2	0.500541123	0.249999208	2.002170832	0.045266362	0.057379616	positive
PRJNA729271	stage_5W_vs_E10	ordinary_muscle_development	ENSGALG00010022655	NRIP3	-6.750987006	0.539555347	-12.51213068	6.40819E-36	4.91205E-35	negative
PRJNA729271	stage_5W_vs_E10	ordinary_muscle_development	ENSGALG00010022656	FKBP10	-1.708229966	0.211706388	-8.068863582	7.09555E-16	2.33432E-15	negative
PRJNA729271	stage_5W_vs_E10	ordinary_muscle_development	ENSGALG00010022657	MRPS35	0.002352528	0.11240362	0.02092929	0.983302062	0.985790199	positive
PRJNA729271	stage_5W_vs_E10	ordinary_muscle_development	ENSGALG00010022659	TIE1	1.335530109	0.150946977	8.847677079	8.93598E-19	3.37574E-18	positive
PRJNA729271	stage_5W_vs_E10	ordinary_muscle_development	ENSGALG00010022660	METAP1D	0.366153555	0.189164122	1.935639543	0.052911864	0.066529299	positive
PRJNA729271	stage_5W_vs_E10	ordinary_muscle_development	ENSGALG00010022661	ENSGALG00010022661	-0.910222361	0.385849888	-2.359006415	0.018323939	0.02434068	negative
PRJNA729271	stage_5W_vs_E10	ordinary_muscle_development	ENSGALG00010022662	GFRA1	-4.599084837	0.17294347	-26.59299499	8.1804E-156	8.9154E-154	negative
PRJNA729271	stage_5W_vs_E10	ordinary_muscle_development	ENSGALG00010022664	TRPC4AP	0.767072319	0.097428499	7.873182137	3.45733E-15	1.09755E-14	positive
PRJNA729271	stage_5W_vs_E10	ordinary_muscle_development	ENSGALG00010022666	ENSGALG00010022666	-0.862933872	0.106271707	-8.12007159	4.65909E-16	1.54751E-15	negative
PRJNA729271	stage_5W_vs_E10	ordinary_muscle_development	ENSGALG00010022667	PTEN	0.812382263	0.041661299	19.49968612	1.10458E-84	3.29442E-83	positive
PRJNA729271	stage_5W_vs_E10	ordinary_muscle_development	ENSGALG00010022669	MANSC4	-6.00251086	1.130854089	-5.307944604	1.10868E-07	2.28078E-07	negative
PRJNA729271	stage_5W_vs_E10	ordinary_muscle_development	ENSGALG00010022671	DBR1	-0.367709117	0.056606492	-6.495882488	8.25481E-11	2.0687E-10	negative
PRJNA729271	stage_5W_vs_E10	ordinary_muscle_development	ENSGALG00010022673	KLHL42	0.075233283	0.1510112	0.498196712	0.6183454	0.655040311	positive
PRJNA729271	stage_5W_vs_E10	ordinary_muscle_development	ENSGALG00010022674	ENSGALG00010022674	2.281284677	0.336504016	6.779368355	1.20702E-11	3.18221E-11	positive
PRJNA729271	stage_5W_vs_E10	ordinary_muscle_development	ENSGALG00010022681	PPP2R3A	1.002821667	0.093102637	10.77114141	4.71124E-27	2.54147E-26	positive
PRJNA729271	stage_5W_vs_E10	ordinary_muscle_development	ENSGALG00010022682	EIF1	1.155644571	0.155670788	7.423644386	1.13941E-13	3.32911E-13	positive
PRJNA729271	stage_5W_vs_E10	ordinary_muscle_development	ENSGALG00010022684	ENSGALG00010022684	-3.153571483	0.539630262	-5.843948544	5.09778E-09	1.14526E-08	negative
PRJNA729271	stage_5W_vs_E10	ordinary_muscle_development	ENSGALG00010022685	ATAD1	0.433992922	0.108222385	4.010195692	6.06685E-05	0.000101911	positive
PRJNA729271	stage_5W_vs_E10	ordinary_muscle_development	ENSGALG00010022689	MPL	0.326956539	0.426987993	0.765727713	0.443838334	0.485762356	positive
PRJNA729271	stage_5W_vs_E10	ordinary_muscle_development	ENSGALG00010022691	ASIC2	-4.374124099	0.654077789	-6.687467721	2.27065E-11	5.8868E-11	negative
PRJNA729271	stage_5W_vs_E10	ordinary_muscle_development	ENSGALG00010022695	PIK3IP1	2.45448831	0.202549385	12.11797463	8.48296E-34	5.95451E-33	positive
PRJNA729271	stage_5W_vs_E10	ordinary_muscle_development	ENSGALG00010022698	SEMA6D	0.50853229	0.117126511	4.341735156	1.41362E-05	2.49245E-05	positive
PRJNA729271	stage_5W_vs_E10	ordinary_muscle_development	ENSGALG00010022702	CDC20	-3.182672513	0.237335658	-13.41000565	5.28348E-41	4.75973E-40	negative
PRJNA729271	stage_5W_vs_E10	ordinary_muscle_development	ENSGALG00010022707	SALL1	-3.994039275	0.526912843	-7.580075773	3.45353E-14	1.03986E-13	negative
PRJNA729271	stage_5W_vs_E10	ordinary_muscle_development	ENSGALG00010022708	SPRED1	-0.211253304	0.130687134	-1.616481267	0.1059903	0.128348321	negative
PRJNA729271	stage_5W_vs_E10	ordinary_muscle_development	ENSGALG00010022709	RNF5	-0.468427966	0.085434361	-5.482898922	4.18412E-08	8.85514E-08	negative
PRJNA729271	stage_5W_vs_E10	ordinary_muscle_development	ENSGALG00010022711	LRRC49	-0.509903627	0.194294546	-2.624384666	0.008680569	0.011957126	negative
PRJNA729271	stage_5W_vs_E10	ordinary_muscle_development	ENSGALG00010022712	DDX23	0.089912046	0.201689692	0.445793959	0.655746086	0.69027157	positive
PRJNA729271	stage_5W_vs_E10	ordinary_muscle_development	ENSGALG00010022713	PNLIPRP1	6.835570462	2.794351574	2.446209892	0.014436693	0.019411861	positive
PRJNA729271	stage_5W_vs_E10	ordinary_muscle_development	ENSGALG00010022717	ELOVL1	1.087581818	0.170865214	6.365144755	1.95106E-10	4.76665E-10	positive
PRJNA729271	stage_5W_vs_E10	ordinary_muscle_development	ENSGALG00010022718	C12orf43	-1.038111019	0.145715087	-7.124252131	1.04647E-12	2.91761E-12	negative
PRJNA729271	stage_5W_vs_E10	ordinary_muscle_development	ENSGALG00010022719	MSL2	0.155202208	0.097457059	1.592518892	0.111268162	0.134335499	positive
PRJNA729271	stage_5W_vs_E10	ordinary_muscle_development	ENSGALG00010022724	LUZP2	-2.742011164	0.420123367	-6.526680918	6.72431E-11	1.69541E-10	negative
PRJNA729271	stage_5W_vs_E10	ordinary_muscle_development	ENSGALG00010022725	KRT222	-4.603331562	0.703510439	-6.5433735	6.01464E-11	1.51859E-10	negative
PRJNA729271	stage_5W_vs_E10	ordinary_muscle_development	ENSGALG00010022727	ACP2	0.462893708	0.083116998	5.569182257	2.55938E-08	5.50418E-08	positive
PRJNA729271	stage_5W_vs_E10	ordinary_muscle_development	ENSGALG00010022729	ENSGALG00010022729	-0.573819804	0.133940028	-4.284154722	1.83435E-05	3.20542E-05	negative
PRJNA729271	stage_5W_vs_E10	ordinary_muscle_development	ENSGALG00010022731	LRRC8B	-0.937129884	0.178588784	-5.247417361	1.54246E-07	3.1437E-07	negative
PRJNA729271	stage_5W_vs_E10	ordinary_muscle_development	ENSGALG00010022733	PCCB	0.28497792	0.115330821	2.470960658	0.013475064	0.018179547	positive
PRJNA729271	stage_5W_vs_E10	ordinary_muscle_development	ENSGALG00010022734	ENSGALG00010022734	0.55363484	0.119274501	4.641686481	3.45577E-06	6.37457E-06	positive
PRJNA729271	stage_5W_vs_E10	ordinary_muscle_development	ENSGALG00010022737	CYLD	0.641889459	0.079092648	8.115665353	4.8313E-16	1.60251E-15	positive
PRJNA729271	stage_5W_vs_E10	ordinary_muscle_development	ENSGALG00010022742	IFT46	-0.530178509	0.124922831	-4.244048141	2.19523E-05	3.8094E-05	negative
PRJNA729271	stage_5W_vs_E10	ordinary_muscle_development	ENSGALG00010022743	ZNF326	-1.502357572	0.153172863	-9.808248969	1.03753E-22	4.68798E-22	negative
PRJNA729271	stage_5W_vs_E10	ordinary_muscle_development	ENSGALG00010022745	PAPSS2	-0.808237923	0.238411389	-3.390097785	0.000698677	0.001072469	negative
PRJNA729271	stage_5W_vs_E10	ordinary_muscle_development	ENSGALG00010022747	NT5C3B	-0.225868689	0.137828995	-1.638760332	0.101263184	0.122878478	negative
PRJNA729271	stage_5W_vs_E10	ordinary_muscle_development	ENSGALG00010022748	ENSGALG00010022748	0.058125235	0.315807072	0.184052988	0.853971887	0.871590093	positive
PRJNA729271	stage_5W_vs_E10	ordinary_muscle_development	ENSGALG00010022749	HSPA12A	-1.03778207	0.130710046	-7.939573882	2.02877E-15	6.53054E-15	negative
PRJNA729271	stage_5W_vs_E10	ordinary_muscle_development	ENSGALG00010022751	SLC25A12	3.400219333	0.190966642	17.80530518	6.42809E-71	1.39067E-69	positive
PRJNA729271	stage_5W_vs_E10	ordinary_muscle_development	ENSGALG00010022754	SNX20	0.18561969	0.184387047	1.006685083	0.314086133	0.353112425	positive
PRJNA729271	stage_5W_vs_E10	ordinary_muscle_development	ENSGALG00010022755	MINPP1	-0.515370854	0.115900076	-4.446682625	8.72065E-06	1.56076E-05	negative
PRJNA729271	stage_5W_vs_E10	ordinary_muscle_development	ENSGALG00010022759	THSD4	-0.815908191	0.121432774	-6.719011371	1.82962E-11	4.77241E-11	negative
PRJNA729271	stage_5W_vs_E10	ordinary_muscle_development	ENSGALG00010022760	ELAVL4	-5.087620619	0.229067171	-22.21016914	2.739E-109	1.3643E-107	negative
PRJNA729271	stage_5W_vs_E10	ordinary_muscle_development	ENSGALG00010022764	MFAP5	2.772146465	0.222869948	12.43840407	1.61702E-35	1.2176E-34	positive
PRJNA729271	stage_5W_vs_E10	ordinary_muscle_development	ENSGALG00010022767	CACNB3	-0.179139887	0.468788646	-0.382133587	0.70236228	0.734594216	negative
PRJNA729271	stage_5W_vs_E10	ordinary_muscle_development	ENSGALG00010022768	ENSGALG00010022768	1.85260624	0.530226382	3.493991066	0.000475857	0.000739921	positive
PRJNA729271	stage_5W_vs_E10	ordinary_muscle_development	ENSGALG00010022769	FXYD6	0.857659966	0.097840996	8.765854806	1.85365E-18	6.91065E-18	positive
PRJNA729271	stage_5W_vs_E10	ordinary_muscle_development	ENSGALG00010022770	HAT1	-1.458186742	0.093629364	-15.57403228	1.09295E-54	1.49738E-53	negative
PRJNA729271	stage_5W_vs_E10	ordinary_muscle_development	ENSGALG00010022771	SGMS1	1.150041509	0.219641852	5.235985295	1.64107E-07	3.33529E-07	positive
PRJNA729271	stage_5W_vs_E10	ordinary_muscle_development	ENSGALG00010022776	CDH4	-0.522884281	0.178899008	-2.922790273	0.003469101	0.004979661	negative
PRJNA729271	stage_5W_vs_E10	ordinary_muscle_development	ENSGALG00010022780	DYNC1I2	-0.583792772	0.057964119	-10.07162339	7.37506E-24	3.48327E-23	negative
PRJNA729271	stage_5W_vs_E10	ordinary_muscle_development	ENSGALG00010022782	TMPRSS13	0.845326505	0.227286419	3.719212563	0.000199845	0.000321041	positive
PRJNA729271	stage_5W_vs_E10	ordinary_muscle_development	ENSGALG00010022783	STAG1	-0.224959506	0.055792165	-4.032098513	5.5281E-05	9.30444E-05	negative
PRJNA729271	stage_5W_vs_E10	ordinary_muscle_development	ENSGALG00010022784	DMRTA2	-2.85992307	0.90520385	-3.159424331	0.001580811	0.002348213	negative
PRJNA729271	stage_5W_vs_E10	ordinary_muscle_development	ENSGALG00010022786	KRT40	0.100327859	0.582211166	0.172322114	0.8631843	0.880001155	positive
PRJNA729271	stage_5W_vs_E10	ordinary_muscle_development	ENSGALG00010022787	ENO4	-1.506556709	0.335810272	-4.486333006	7.24595E-06	1.30359E-05	negative
PRJNA729271	stage_5W_vs_E10	ordinary_muscle_development	ENSGALG00010022788	CYBRD1	2.253642895	0.173314707	13.00318326	1.17355E-38	9.76497E-38	positive
PRJNA729271	stage_5W_vs_E10	ordinary_muscle_development	ENSGALG00010022789	ENSGALG00010022789	1.149285818	0.331816756	3.463615982	0.000532967	0.000826329	positive
PRJNA729271	stage_5W_vs_E10	ordinary_muscle_development	ENSGALG00010022792	DDB2	-0.965775433	0.127639544	-7.566428082	3.83626E-14	1.15223E-13	negative
PRJNA729271	stage_5W_vs_E10	ordinary_muscle_development	ENSGALG00010022795	TAF4	0.310789761	0.111065322	2.798261012	0.005137858	0.007245889	positive
PRJNA729271	stage_5W_vs_E10	ordinary_muscle_development	ENSGALG00010022796	RIMKLB	-1.852025032	0.138362463	-13.3853141	7.36852E-41	6.60116E-40	negative
PRJNA729271	stage_5W_vs_E10	ordinary_muscle_development	ENSGALG00010022797	EFHD1	1.760843391	0.1315232	13.388082	7.09904E-41	6.36761E-40	positive
PRJNA729271	stage_5W_vs_E10	ordinary_muscle_development	ENSGALG00010022799	DCAF17	-0.320179392	0.155324155	-2.061362525	0.039268467	0.050220261	negative
PRJNA729271	stage_5W_vs_E10	ordinary_muscle_development	ENSGALG00010022800	KLHL10	0.307757187	0.431324377	0.713516795	0.475526024	0.5172019	positive
PRJNA729271	stage_5W_vs_E10	ordinary_muscle_development	ENSGALG00010022801	LSM14B	-0.378463729	0.103236287	-3.665995181	0.000246379	0.000392921	negative
PRJNA729271	stage_5W_vs_E10	ordinary_muscle_development	ENSGALG00010022803	ENSGALG00010022803	1.110825999	0.153835901	7.220850225	5.16635E-13	1.46091E-12	positive
PRJNA729271	stage_5W_vs_E10	ordinary_muscle_development	ENSGALG00010022804	SNX19	-0.316377695	0.116337141	-2.719490032	0.006538267	0.009124114	negative
PRJNA729271	stage_5W_vs_E10	ordinary_muscle_development	ENSGALG00010022805	ENSGALG00010022805	-0.400949209	0.274802096	-1.459047128	0.144552137	0.171871317	negative
PRJNA729271	stage_5W_vs_E10	ordinary_muscle_development	ENSGALG00010022806	SPPL3	-0.512062396	0.064314184	-7.961889044	1.69432E-15	5.47708E-15	negative
PRJNA729271	stage_5W_vs_E10	ordinary_muscle_development	ENSGALG00010022807	ADCY6	-0.384771734	0.14892585	-2.583646381	0.009776197	0.013406431	negative
PRJNA729271	stage_5W_vs_E10	ordinary_muscle_development	ENSGALG00010022808	PSMA7	0.352294566	0.136723143	2.576700327	0.009974839	0.013665906	positive
PRJNA729271	stage_5W_vs_E10	ordinary_muscle_development	ENSGALG00010022809	MYO1E	-0.120748927	0.155625136	-0.775896042	0.43781037	0.47974458	negative
PRJNA729271	stage_5W_vs_E10	ordinary_muscle_development	ENSGALG00010022811	GIGYF2	0.210001525	0.09794341	2.144110821	0.032024008	0.041415774	positive
PRJNA729271	stage_5W_vs_E10	ordinary_muscle_development	ENSGALG00010022813	USP24	1.955696322	0.121848618	16.05021343	5.69678E-58	8.63754E-57	positive
PRJNA729271	stage_5W_vs_E10	ordinary_muscle_development	ENSGALG00010022815	LRRC8D	0.578499932	0.236629165	2.444753297	0.014495126	0.019485008	positive
PRJNA729271	stage_5W_vs_E10	ordinary_muscle_development	ENSGALG00010022816	METTL8	0.05886985	0.14276842	0.412345041	0.68008654	0.713458377	positive
PRJNA729271	stage_5W_vs_E10	ordinary_muscle_development	ENSGALG00010022817	KRT23	-4.532537839	1.039171515	-4.361684067	1.29065E-05	2.28285E-05	negative
PRJNA729271	stage_5W_vs_E10	ordinary_muscle_development	ENSGALG00010022819	PACSIN3	1.976364849	0.108445061	18.22457217	3.29442E-74	7.86699E-73	positive
PRJNA729271	stage_5W_vs_E10	ordinary_muscle_development	ENSGALG00010022820	SHTN1	-1.136806335	0.173549965	-6.550311508	5.74172E-11	1.4512E-10	negative
PRJNA729271	stage_5W_vs_E10	ordinary_muscle_development	ENSGALG00010022821	ENSGALG00010022821	4.273928169	1.372482118	3.114013736			positive
PRJNA729271	stage_5W_vs_E10	ordinary_muscle_development	ENSGALG00010022823	IGHMBP2	0.360529288	0.114481823	3.149227342	0.001637028	0.002426994	positive
PRJNA729271	stage_5W_vs_E10	ordinary_muscle_development	ENSGALG00010022824	PUS1	-1.551791149	0.087167743	-17.80235551	6.7758E-71	1.46154E-69	negative
PRJNA729271	stage_5W_vs_E10	ordinary_muscle_development	ENSGALG00010022825	SS18L1	1.443174966	0.26377807	5.471171142	4.47071E-08	9.44788E-08	positive
PRJNA729271	stage_5W_vs_E10	ordinary_muscle_development	ENSGALG00010022826	SNORC	-6.745492227	0.759964014	-8.876067943	6.92655E-19	2.63104E-18	negative
PRJNA729271	stage_5W_vs_E10	ordinary_muscle_development	ENSGALG00010022827	CMTR2	-0.104961492	0.094387707	-1.112025023	0.266127392	0.303049457	negative
PRJNA729271	stage_5W_vs_E10	ordinary_muscle_development	ENSGALG00010022828	PRKG1	-1.768890966	0.172252996	-10.26914488	9.70612E-25	4.77239E-24	negative
PRJNA729271	stage_5W_vs_E10	ordinary_muscle_development	ENSGALG00010022830	MTG2	-0.644812836	0.131745536	-4.894380923	9.86157E-07	1.89555E-06	negative
PRJNA729271	stage_5W_vs_E10	ordinary_muscle_development	ENSGALG00010022831	LRRC8C	0.947777179	0.088728105	10.68181472	1.23827E-26	6.59395E-26	positive
PRJNA729271	stage_5W_vs_E10	ordinary_muscle_development	ENSGALG00010022833	HRH3	-0.973447763	0.561712896	-1.73299878	0.083095886	0.101945321	negative
PRJNA729271	stage_5W_vs_E10	ordinary_muscle_development	ENSGALG00010022834	CHRNA7	-3.646908415	0.276480547	-13.19047019	9.95623E-40	8.63131E-39	negative
PRJNA729271	stage_5W_vs_E10	ordinary_muscle_development	ENSGALG00010022837	ITGB3	-1.076602488	0.151532061	-7.104783501	1.20511E-12	3.34574E-12	negative
PRJNA729271	stage_5W_vs_E10	ordinary_muscle_development	ENSGALG00010022838	ARFGAP2	0.482344542	0.079343398	6.079202011	1.20782E-09	2.81062E-09	positive
PRJNA729271	stage_5W_vs_E10	ordinary_muscle_development	ENSGALG00010022839	SYT12	-1.261928349	0.302452054	-4.172325278	3.01507E-05	5.17017E-05	negative
PRJNA729271	stage_5W_vs_E10	ordinary_muscle_development	ENSGALG00010022840	MARVELD1	0.134318842	0.192176791	0.698933731	0.484593446	0.526234792	positive
PRJNA729271	stage_5W_vs_E10	ordinary_muscle_development	ENSGALG00010022841	ENSGALG00010022841	-8.572341361	0.781424986	-10.97013982	5.31901E-28	2.9791E-27	negative
PRJNA729271	stage_5W_vs_E10	ordinary_muscle_development	ENSGALG00010022842	PHC1	-0.997059281	0.143239648	-6.960777234	3.38401E-12	9.18045E-12	negative

PRJNA729271	stage_5W_vs_E10	ordinary_muscle_development	ENSGALG00010022845	HYDIN	2.921290233	0.317032718	9.214475568	3.12804E-20	1.26227E-19	positive
PRJNA729271	stage_5W_vs_E10	ordinary_muscle_development	ENSGALG00010022848	SGIP1	-3.197379893	1.005491594	-3.17991708	0.001473172	0.002195476	negative
PRJNA729271	stage_5W_vs_E10	ordinary_muscle_development	ENSGALG00010022850	ULK1	2.233971393	0.249794661	8.943231148	3.77954E-19	1.45278E-18	positive
PRJNA729271	stage_5W_vs_E10	ordinary_muscle_development	ENSGALG00010022858	HERC4	0.452357741	0.127285158	3.553892281	0.000379575	0.000595254	positive
PRJNA729271	stage_5W_vs_E10	ordinary_muscle_development	ENSGALG00010022860	MOB3C	-1.395909706	0.133897947	-10.42517628	1.90306E-25	9.57476E-25	negative
PRJNA729271	stage_5W_vs_E10	ordinary_muscle_development	ENSGALG00010022862	ACADS	0.161174068	0.123386772	1.306250791	0.191467286	0.223581592	positive
PRJNA729271	stage_5W_vs_E10	ordinary_muscle_development	ENSGALG00010022863	FAM81A	-0.612870874	0.331486047	-1.848858736	0.064478213	0.08021041	negative
PRJNA729271	stage_5W_vs_E10	ordinary_muscle_development	ENSGALG00010022864	CSTPP1	-1.211366884	0.208713784	-5.803962063	6.47659E-09	1.44472E-08	negative
PRJNA729271	stage_5W_vs_E10	ordinary_muscle_development	ENSGALG00010022866	KLHL11	-0.501941912	0.099613705	-5.038884072	4.68254E-07	9.20317E-07	negative
PRJNA729271	stage_5W_vs_E10	ordinary_muscle_development	ENSGALG00010022867	ATPAF1	-0.258473848	0.13401831	-1.92864578	0.053774852	0.067538903	negative
PRJNA729271	stage_5W_vs_E10	ordinary_muscle_development	ENSGALG00010022868	VAC14	-0.60592706	0.102836051	-5.89216576	3.81167E-09	8.61854E-09	negative
PRJNA729271	stage_5W_vs_E10	ordinary_muscle_development	ENSGALG00010022872	KRT13	-3.839269469	0.5058029	-7.590445741	3.18807E-14	9.61729E-14	negative
PRJNA729271	stage_5W_vs_E10	ordinary_muscle_development	ENSGALG00010022873	NGEF	-0.129313138	0.374958663	-0.344873052	0.730189811	0.759806268	negative
PRJNA729271	stage_5W_vs_E10	ordinary_muscle_development	ENSGALG00010022874	EFCAB14	1.078729374	0.076159445	14.16409177	1.5283E-45	1.58801E-44	positive
PRJNA729271	stage_5W_vs_E10	ordinary_muscle_development	ENSGALG00010022875	SPON1	-0.394059755	0.244310824	-1.612944315	0.106756606	0.129222297	negative
PRJNA729271	stage_5W_vs_E10	ordinary_muscle_development	ENSGALG00010022877	KYAT3	0.048335696	0.101877901	0.474447313	0.635180961	0.670815886	positive
PRJNA729271	stage_5W_vs_E10	ordinary_muscle_development	ENSGALG00010022879	ENSGALG00010022879	1.950274334	0.277832688	7.019599993	2.22504E-12	6.08873E-12	positive
PRJNA729271	stage_5W_vs_E10	ordinary_muscle_development	ENSGALG00010022881	CCNB1	-2.788961597	0.212838898	-13.10362731	3.1389E-39	2.66228E-38	negative
PRJNA729271	stage_5W_vs_E10	ordinary_muscle_development	ENSGALG00010022885	M6PR	0.407017064	0.099270644	4.100074801	4.13017E-05	7.00934E-05	positive
PRJNA729271	stage_5W_vs_E10	ordinary_muscle_development	ENSGALG00010022886	PLA2G15	1.939565318	0.281230449	6.896711658	5.322E-12	1.42671E-11	positive
PRJNA729271	stage_5W_vs_E10	ordinary_muscle_development	ENSGALG00010022888	OSBPL2	1.210361529	0.090498006	13.37445517	8.52763E-41	7.63012E-40	positive
PRJNA729271	stage_5W_vs_E10	ordinary_muscle_development	ENSGALG00010022890	MYPN	5.683401556	0.166050377	34.22697168	9.6015E-257	2.8877E-254	positive
PRJNA729271	stage_5W_vs_E10	ordinary_muscle_development	ENSGALG00010022891	NTM	-0.854499597	0.220337127	-3.878146217	0.000105255	0.000172901	negative
PRJNA729271	stage_5W_vs_E10	ordinary_muscle_development	ENSGALG00010022900	MMP17	0.223959239	0.199601027	1.122034504	0.26184777	0.298479044	positive
PRJNA729271	stage_5W_vs_E10	ordinary_muscle_development	ENSGALG00010022911	SMPD3	-3.65163518	0.347464525	-10.50937555	7.82097E-26	4.01292E-25	negative
PRJNA729271	stage_5W_vs_E10	ordinary_muscle_development	ENSGALG00010022917	CYP4B7	-0.603383657	0.479741846	-1.257725715	0.208490969	0.241824166	negative
PRJNA729271	stage_5W_vs_E10	ordinary_muscle_development	ENSGALG00010022919	ACLY	-1.917013122	0.125654107	-15.25627106	1.49554E-52	1.94945E-51	negative
PRJNA729271	stage_5W_vs_E10	ordinary_muscle_development	ENSGALG00010022921	ENSGALG00010022921	0.769748604	0.198382752	3.880118588	0.000104406	0.00017168	positive
PRJNA729271	stage_5W_vs_E10	ordinary_muscle_development	ENSGALG00010022922	ATOH7	0.225566041	0.437696769	0.515347741	0.606310044	0.643654906	positive
PRJNA729271	stage_5W_vs_E10	ordinary_muscle_development	ENSGALG00010022923	CD63	1.721788679	0.126426719	13.61886706	3.09329E-42	2.89086E-41	positive
PRJNA729271	stage_5W_vs_E10	ordinary_muscle_development	ENSGALG00010022928	RNF111	0.358180698	0.08313396	4.308476336	1.64383E-05	2.88118E-05	positive
PRJNA729271	stage_5W_vs_E10	ordinary_muscle_development	ENSGALG00010022929	LRP4	-0.530651376	0.117235873	-4.526356681	6.00093E-06	1.08539E-05	negative
PRJNA729271	stage_5W_vs_E10	ordinary_muscle_development	ENSGALG00010022930	GTF2B	-0.374519357	0.118598046	-3.157888091	0.001589166	0.002359897	negative
PRJNA729271	stage_5W_vs_E10	ordinary_muscle_development	ENSGALG00010022931	RDH5	1.133063036	0.121422984	9.331536748	1.04347E-20	4.2908E-20	positive
PRJNA729271	stage_5W_vs_E10	ordinary_muscle_development	ENSGALG00010022932	OPCML	-1.044269822	0.204950116	-5.095238995	3.48302E-07	6.9027E-07	negative
PRJNA729271	stage_5W_vs_E10	ordinary_muscle_development	ENSGALG00010022933	UNC119B	-0.732938483	0.076778971	-9.546083633	1.34692E-21	5.78477E-21	negative
PRJNA729271	stage_5W_vs_E10	ordinary_muscle_development	ENSGALG00010022937	BLOC1S1	0.811529482	0.161338018	5.029995364	4.90492E-07	9.62849E-07	positive
PRJNA729271	stage_5W_vs_E10	ordinary_muscle_development	ENSGALG00010022942	NEU2	-1.560083776	0.503249535	-3.100020305	0.001935074	0.002847603	negative
PRJNA729271	stage_5W_vs_E10	ordinary_muscle_development	ENSGALG00010022945	KCNA4	-5.749563701	0.55722924	-10.31812993	5.83485E-25	2.89843E-24	negative
PRJNA729271	stage_5W_vs_E10	ordinary_muscle_development	ENSGALG00010022946	KRT17	-6.471106649	0.563191764	-11.49005909	1.48011E-30	9.16844E-30	negative
PRJNA729271	stage_5W_vs_E10	ordinary_muscle_development	ENSGALG00010022949	INSC	-2.686003601	0.269703953	-9.959081333	2.3018E-23	1.06767E-22	negative
PRJNA729271	stage_5W_vs_E10	ordinary_muscle_development	ENSGALG00010022950	ITGA7	4.168741383	0.154108326	27.05072134	3.7456E-161	4.2749E-159	positive
PRJNA729271	stage_5W_vs_E10	ordinary_muscle_development	ENSGALG00010022953	ENSGALG00010022953	-1.19334761	1.166613399	-1.022916085	0.306347524	0.345161862	negative
PRJNA729271	stage_5W_vs_E10	ordinary_muscle_development	ENSGALG00010022954	ADRM1	0.543614394	0.143473479	3.788953867	0.000151283	0.000245148	positive
PRJNA729271	stage_5W_vs_E10	ordinary_muscle_development	ENSGALG00010022956	PBLD	0.745170371	0.122641239	6.076017968	1.23204E-09	2.86329E-09	positive
PRJNA729271	stage_5W_vs_E10	ordinary_muscle_development	ENSGALG00010022957	SLTM	-0.056212601	0.143031085	-0.393009682	0.694312331	0.726961661	negative
PRJNA729271	stage_5W_vs_E10	ordinary_muscle_development	ENSGALG00010022958	SFSWAP	-0.151456262	0.098114697	-1.543665381	0.122669417	0.147180962	negative
PRJNA729271	stage_5W_vs_E10	ordinary_muscle_development	ENSGALG00010022959	ADAMTS8	0.372679114	0.264390136	1.409580252	0.158663661	0.18769524	positive
PRJNA729271	stage_5W_vs_E10	ordinary_muscle_development	ENSGALG00010022967	ADAMTS15	-0.523516073	0.162513681	-3.221366162	0.00127581	0.001912396	negative
PRJNA729271	stage_5W_vs_E10	ordinary_muscle_development	ENSGALG00010022969	TTN	4.531656571	0.575497504	7.874328801	3.42577E-15	1.08777E-14	positive
PRJNA729271	stage_5W_vs_E10	ordinary_muscle_development	ENSGALG00010022971	CKAP5	-1.17612775	0.058591823	-20.07324039	1.26483E-89	4.27359E-88	negative
PRJNA729271	stage_5W_vs_E10	ordinary_muscle_development	ENSGALG00010022972	ENSGALG00010022972	-0.03348617	1.091876723	-0.030668453			negative
PRJNA729271	stage_5W_vs_E10	ordinary_muscle_development	ENSGALG00010022974	INPP5D	2.259827678	0.154136167	14.66124225	1.14165E-48	1.31753E-47	positive
PRJNA729271	stage_5W_vs_E10	ordinary_muscle_development	ENSGALG00010022976	MINDY2	-0.14156386	0.066207572	-2.138182327	0.032501948	0.041996411	negative
PRJNA729271	stage_5W_vs_E10	ordinary_muscle_development	ENSGALG00010022978	ARL14EP	-0.19467633	0.087083165	-2.235521984	0.0253831	0.033155631	negative
PRJNA729271	stage_5W_vs_E10	ordinary_muscle_development	ENSGALG00010022979	LAMA5	-0.063051824	0.149215018	-0.422556823	0.672618635	0.706493269	negative
PRJNA729271	stage_5W_vs_E10	ordinary_muscle_development	ENSGALG00010022981	HNRNPH3	-1.482989682	0.129849803	-11.42080808	3.29159E-30	2.00638E-29	negative
PRJNA729271	stage_5W_vs_E10	ordinary_muscle_development	ENSGALG00010022983	PKN2	0.335633947	0.06110841	5.492434614	3.9643E-08	8.40342E-08	positive
PRJNA729271	stage_5W_vs_E10	ordinary_muscle_development	ENSGALG00010022986	ALG9	-0.569412598	0.10792814	-5.275849271	1.32143E-07	2.70576E-07	negative
PRJNA729271	stage_5W_vs_E10	ordinary_muscle_development	ENSGALG00010022987	F2	3.986338627	1.380448507	2.887712658			positive
PRJNA729271	stage_5W_vs_E10	ordinary_muscle_development	ENSGALG00010022990	SLC25A22	-1.291616082	0.101841273	-12.68263884	7.38046E-37	5.83951E-36	negative
PRJNA729271	stage_5W_vs_E10	ordinary_muscle_development	ENSGALG00010022991	TAL1	1.471657732	0.175224192	8.398713188	4.51398E-17	1.57587E-16	positive
PRJNA729271	stage_5W_vs_E10	ordinary_muscle_development	ENSGALG00010022992	SLC7A6	0.007977994	0.106941427	0.074601527	0.940531759	0.948383155	positive
PRJNA729271	stage_5W_vs_E10	ordinary_muscle_development	ENSGALG00010022994	LIPC	-2.714265228	0.889199491	-3.052481762	0.002269575	0.003318621	negative
PRJNA729271	stage_5W_vs_E10	ordinary_muscle_development	ENSGALG00010022997	ARHGAP1	-0.911677479	0.095431468	-9.553216504	1.25731E-21	5.40311E-21	negative
PRJNA729271	stage_5W_vs_E10	ordinary_muscle_development	ENSGALG00010022999	ENSGALG00010022999	1.294145832	0.511901895	2.528112994	0.011467744	0.015605046	positive
PRJNA729271	stage_5W_vs_E10	ordinary_muscle_development	ENSGALG00010023002	ATG16L1	1.426375194	0.133542339	10.68107094	1.24823E-26	6.64456E-26	positive
PRJNA729271	stage_5W_vs_E10	ordinary_muscle_development	ENSGALG00010023003	ENSGALG00010023003	-6.108614339	0.731454024	-8.351330553	6.74982E-17	2.33784E-16	negative
PRJNA729271	stage_5W_vs_E10	ordinary_muscle_development	ENSGALG00010023005	IRF7	3.287375717	0.346091312	9.498579164	2.12774E-21	9.05506E-21	positive
PRJNA729271	stage_5W_vs_E10	ordinary_muscle_development	ENSGALG00010023007	LMO4	-0.188194198	0.142508293	-1.320584187	0.186640048	0.218331652	negative
PRJNA729271	stage_5W_vs_E10	ordinary_muscle_development	ENSGALG00010023008	CDHR5	-1.022746025	0.961004597	-1.064246756	0.287216931	0.324920738	negative
PRJNA729271	stage_5W_vs_E10	ordinary_muscle_development	ENSGALG00010023012	ENSGALG00010023012	0.986490821	0.512888061	1.923403753	0.054429363	0.068289935	positive
PRJNA729271	stage_5W_vs_E10	ordinary_muscle_development	ENSGALG00010023013	ENSGALG00010023013	1.164345805	0.401379458	2.900860476	0.003721395	0.00532599	positive
PRJNA729271	stage_5W_vs_E10	ordinary_muscle_development	ENSGALG00010023015	METTL2B	-0.560689635	0.104379207	-5.371660209	7.8015E-08	1.62033E-07	negative
PRJNA729271	stage_5W_vs_E10	ordinary_muscle_development	ENSGALG00010023017	BUB1B	-3.050444584	0.203751221	-14.97141744	1.12889E-50	1.38908E-49	negative
PRJNA729271	stage_5W_vs_E10	ordinary_muscle_development	ENSGALG00010023019	CABP1	0.389143739	0.31618766	1.230736641	0.218421379	0.252352773	positive
PRJNA729271	stage_5W_vs_E10	ordinary_muscle_development	ENSGALG00010023024	PRMT7	-0.264202291	0.113683172	-2.324022869	0.020124268	0.026578111	negative
PRJNA729271	stage_5W_vs_E10	ordinary_muscle_development	ENSGALG00010023026	PHRF1	0.21690044	0.091350639	2.374372431	0.017578809	0.023392909	positive
PRJNA729271	stage_5W_vs_E10	ordinary_muscle_development	ENSGALG00010023028	TMEM61	-1.713439185	0.310047066	-5.526384134	3.26898E-08	6.96412E-08	negative
PRJNA729271	stage_5W_vs_E10	ordinary_muscle_development	ENSGALG00010023030	HS2ST1	-0.677781447	0.085517823	-7.925616211	2.27018E-15	7.28014E-15	negative
PRJNA729271	stage_5W_vs_E10	ordinary_muscle_development	ENSGALG00010023041	CD151	1.372663543	0.119344455	11.50169518	1.2935E-30	8.04343E-30	positive
PRJNA729271	stage_5W_vs_E10	ordinary_muscle_development	ENSGALG00010023042	ENSGALG00010023042	-1.29238087	0.669079934	-1.93157918	0.053411468	0.067118281	negative
PRJNA729271	stage_5W_vs_E10	ordinary_muscle_development	ENSGALG00010023043	CNP	1.478239308	0.145010111	10.19404302	2.1081E-24	1.01993E-23	positive
PRJNA729271	stage_5W_vs_E10	ordinary_muscle_development	ENSGALG00010023046	SLC7A6OS	-0.606546999	0.075406653	-8.043680136	8.71799E-16	2.85576E-15	negative
PRJNA729271	stage_5W_vs_E10	ordinary_muscle_development	ENSGALG00010023048	ARFGAP1	-0.3775767	0.083816202	-4.504817567	6.643E-06	1.19764E-05	negative
PRJNA729271	stage_5W_vs_E10	ordinary_muscle_development	ENSGALG00010023050	RUFY2	-0.675738425	0.096228516	-7.022226373	2.1836E-12	5.97646E-12	negative
PRJNA729271	stage_5W_vs_E10	ordinary_muscle_development	ENSGALG00010023052	RAD54L	-2.257639387	0.140736564	-16.04159802	6.54484E-58	9.89233E-57	negative
PRJNA729271	stage_5W_vs_E10	ordinary_muscle_development	ENSGALG00010023053	MAN1A2	-0.611920071	0.047734267	-12.81930372	1.27843E-37	1.03236E-36	negative
PRJNA729271	stage_5W_vs_E10	ordinary_muscle_development	ENSGALG00010023054	AQP9	1.838658796	0.231172729	7.953614625	1.81147E-15	5.84794E-15	positive
PRJNA729271	stage_5W_vs_E10	ordinary_muscle_development	ENSGALG00010023056	SAG	1.39273175	0.440244947	3.163538298	0.001558638	0.002317651	positive
PRJNA729271	stage_5W_vs_E10	ordinary_muscle_development	ENSGALG00010023057	PAFAH1B2	-1.440032484	0.075608716	-19.04585304	7.1118E-81	1.96353E-79	negative
PRJNA729271	stage_5W_vs_E10	ordinary_muscle_development	ENSGALG00010023058	PPARA	0.274337843	0.207083189	1.324771193	0.185247052	0.216824614	positive
PRJNA729271	stage_5W_vs_E10	ordinary_muscle_development	ENSGALG00010023059	SELENOF	-0.514968298	0.093553486	-5.504533532	3.70147E-08	7.85776E-08	negative
PRJNA729271	stage_5W_vs_E10	ordinary_muscle_development	ENSGALG00010023060	ALDH1A2	-1.598369081	0.167973154	-9.515622254	1.80629E-21	7.70969E-21	negative
PRJNA729271	stage_5W_vs_E10	ordinary_muscle_development	ENSGALG00010023062	STX2	0.121665226	0.103479346	1.175744052	0.23969717	0.275004786	positive
PRJNA729271	stage_5W_vs_E10	ordinary_muscle_development	ENSGALG00010023063	ENSGALG00010023063	-0.811894848	0.104919066	-7.738296534	1.00758E-14	3.12336E-14	negative
PRJNA729271	stage_5W_vs_E10	ordinary_muscle_development	ENSGALG00010023064	ATG13	0.466483867	0.068869228	6.773473135	1.25727E-11	3.31047E-11	positive
PRJNA729271	stage_5W_vs_E10	ordinary_muscle_development	ENSGALG00010023065	DNA2	-2.908343034	0.232230128	-12.52353888	5.55041E-36	4.26581E-35	negative
PRJNA729271	stage_5W_vs_E10	ordinary_muscle_development	ENSGALG00010023066	TLR21	1.875553869	0.185243896	10.12478096	4.28938E-24	2.05061E-23	positive
PRJNA729271	stage_5W_vs_E10	ordinary_muscle_development	ENSGALG00010023068	CDPF1	-0.639816344	0.127715606	-5.009695866	5.45161E-07	1.06699E-06	negative
PRJNA729271	stage_5W_vs_E10	ordinary_muscle_development	ENSGALG00010023069	ENSGALG00010023069	-0.059865611	0.285676678	-0.209557223	0.83401327	0.853985755	negative
PRJNA729271	stage_5W_vs_E10	ordinary_muscle_development	ENSGALG00010023070	SIDT2	2.088436997	0.200123637	10.43573378	1.70293E-25	8.5917E-25	positive
PRJNA729271	stage_5W_vs_E10	ordinary_muscle_development	ENSGALG00010023071	POLR2M	0.556194544	0.081984283	6.784160641	1.16763E-11	3.07948E-11	positive
PRJNA729271	stage_5W_vs_E10	ordinary_muscle_development	ENSGALG00010023073	POMGNT1	-0.025181692	0.086355075	-0.291606394	0.770587586	0.797776378	negative
PRJNA729271	stage_5W_vs_E10	ordinary_muscle_development	ENSGALG00010023074	CCNT1	0.002864082	0.168298936	0.017017824	0.986422397	0.988468209	positive
PRJNA729271	stage_5W_vs_E10	ordinary_muscle_development	ENSGALG00010023075	PKDREJ	-2.386142199	0.322357026	-7.402172139	1.33975E-13	3.89483E-13	negative
PRJNA729271	stage_5W_vs_E10	ordinary_muscle_development	ENSGALG00010023076	DNAJC7	0.896098983	0.080089752	11.18868475	4.63254E-29	2.70652E-28	positive
PRJNA729271	stage_5W_vs_E10	ordinary_muscle_development	ENSGALG00010023077	KANSL2	-0.014554919	0.15273057	-0.095298006	0.924078127	0.934392218	negative
PRJNA729271	stage_5W_vs_E10	ordinary_muscle_development	ENSGALG00010023078	TAGLN	2.338262953	0.195992283	11.93038277	8.21947E-33	5.56215E-32	positive
PRJNA729271	stage_5W_vs_E10	ordinary_muscle_development	ENSGALG00010023079	SLC25A16	0.820301202	0.095274454	8.609875621	7.31399E-18	2.65638E-17	positive
PRJNA729271	stage_5W_vs_E10	ordinary_muscle_development	ENSGALG00010023080	MCRS1	0.347788474	0.111858022	3.109195629	0.001875975	0.002764027	positive
PRJNA729271	stage_5W_vs_E10	ordinary_muscle_development	ENSGALG00010023081	PRLHR2	-4.28911253	0.651090663	-6.587581078	4.4705E-11	1.13884E-10	negative
PRJNA729271	stage_5W_vs_E10	ordinary_muscle_development	ENSGALG00010023083	HARBI1	0.213249837	0.159106837	1.340293362	0.180149989	0.211285224	positive
PRJNA729271	stage_5W_vs_E10	ordinary_muscle_development	ENSGALG00010023084	ENSGALG00010023084	0.800345967	0.241418569	3.315179811	0.000915842	0.001392983	positive
PRJNA729271	stage_5W_vs_E10	ordinary_muscle_development	ENSGALG00010023086	PCSK7	0.216629402	0.092194077	2.34971062	0.018788011	0.024920592	positive
PRJNA729271	stage_5W_vs_E10	ordinary_muscle_development	ENSGALG00010023087	SH3GLB1	1.258967928	0.117771663	10.68990532	1.13487E-26	6.05667E-26	positive
PRJNA729271	stage_5W_vs_E10	ordinary_muscle_development	ENSGALG00010023089	ENSGALG00010023089	1.113037809	0.756065944	1.472143824	0.140982043	0.167886788	positive
PRJNA729271	stage_5W_vs_E10	ordinary_muscle_development	ENSGALG00010023090	LRRC41	-0.163525746	0.093582886	-1.747389437	0.080569808	0.099021483	negative
PRJNA729271	stage_5W_vs_E10	ordinary_muscle_development	ENSGALG00010023091	ENSGALG00010023091	0.547733203	0.131570457	4.163040972	3.14037E-05	5.37703E-05	positive
PRJNA729271	stage_5W_vs_E10	ordinary_muscle_development	ENSGALG00010023093	POP5	-0.700135778	0.132896043	-5.26829666	1.37695E-07	2.81548E-07	negative
PRJNA729271	stage_5W_vs_E10	ordinary_muscle_development	ENSGALG00010023094	CCDC32	-0.323406945	0.102535444	-3.15409903	0.001609944	0.002389774	negative
PRJNA729271	stage_5W_vs_E10	ordinary_muscle_development	ENSGALG00010023096	SPATS2	0.451266881	0.170516879	2.646464579	0.008133802	0.011239224	positive
PRJNA729271	stage_5W_vs_E10	ordinary_muscle_development	ENSGALG00010023097	LURAP1	1.14965608	0.211428187	5.43757244	5.40114E-08	1.13447E-07	positive
PRJNA729271	stage_5W_vs_E10	ordinary_muscle_development	ENSGALG00010023101	CLCA2	0.986178238	1.207824213	0.816491529	0.414219067	0.455754165	positive
PRJNA729271	stage_5W_vs_E10	ordinary_muscle_development	ENSGALG00010023102	RNF10	0.642517452	0.101516456	6.32919508	2.46443E-10	5.98158E-10	positive
PRJNA729271	stage_5W_vs_E10	ordinary_muscle_development	ENSGALG00010023103	DGKD	0.677267907	0.125316253	5.404469812	6.50004E-08	1.35741E-07	positive
PRJNA729271	stage_5W_vs_E10	ordinary_muscle_development	ENSGALG00010023104	ENSGALG00010023104	1.254025348	0.157597556	7.957137051	1.76066E-15	5.6877E-15	positive
PRJNA729271	stage_5W_vs_E10	ordinary_muscle_development	ENSGALG00010023107	ZC3HAV1	1.624427426	0.291256593	5.577306961	2.44271E-08	5.26499E-08	positive
PRJNA729271	stage_5W_vs_E10	ordinary_muscle_development	ENSGALG00010023108	TET1	-0.17286565	0.061722631	-2.800685071	0.005099426	0.007194489	negative
PRJNA729271	stage_5W_vs_E10	ordinary_muscle_development	ENSGALG00010023110	LIMA1	-1.330518236	0.155016267	-8.58308782	9.23598E-18	3.33688E-17	negative
PRJNA729271	stage_5W_vs_E10	ordinary_muscle_development	ENSGALG00010023112	PCMT1L	2.951407779	0.129921671	22.71682434	3.0551E-114	1.6774E-112	positive

PRJNA729271	stage_5W_vs_E10	ordinary_muscle_development	ENSGALG00010023113	MYZAP	0.221711456	0.132137821	1.677880374	0.093370455	0.113846294	positive
PRJNA729271	stage_5W_vs_E10	ordinary_muscle_development	ENSGALG00010023114	UQCRHL	1.126697913	0.167450313	6.728550641	1.71361E-11	4.47708E-11	positive
PRJNA729271	stage_5W_vs_E10	ordinary_muscle_development	ENSGALG00010023115	RPLP2	0.149507484	0.136390151	1.09617507	0.273002167	0.310171376	positive
PRJNA729271	stage_5W_vs_E10	ordinary_muscle_development	ENSGALG00010023116	PROC	4.04623247	1.014099345	3.989976416	6.60799E-05	0.00011068	positive
PRJNA729271	stage_5W_vs_E10	ordinary_muscle_development	ENSGALG00010023117	RPUSD2	-0.394373567	0.104924652	-3.758635928	0.000170842	0.000275887	negative
PRJNA729271	stage_5W_vs_E10	ordinary_muscle_development	ENSGALG00010023118	PIDD1	0.521927364	0.139446587	3.742847901	0.000181946	0.000293165	positive
PRJNA729271	stage_5W_vs_E10	ordinary_muscle_development	ENSGALG00010023119	NDRG4	-0.616126512	0.173095912	-3.559451557	0.00037163	0.00058349	negative
PRJNA729271	stage_5W_vs_E10	ordinary_muscle_development	ENSGALG00010023120	CHRM4	-1.21126273	0.253644581	-4.775433101	1.79321E-06	3.37522E-06	negative
PRJNA729271	stage_5W_vs_E10	ordinary_muscle_development	ENSGALG00010023122	ENSGALG00010023122	1.43475211	0.683389361	2.099465097	0.035775921	0.046021653	positive
PRJNA729271	stage_5W_vs_E10	ordinary_muscle_development	ENSGALG00010023123	RNF214	0.512986563	0.143551655	3.5735329	0.000352197	0.000554299	positive
PRJNA729271	stage_5W_vs_E10	ordinary_muscle_development	ENSGALG00010023124	NSUN4	0.920199607	0.135415966	6.795355334	1.08046E-11	2.85737E-11	positive
PRJNA729271	stage_5W_vs_E10	ordinary_muscle_development	ENSGALG00010023125	COL20A1	0.77718744	0.282628011	2.749859924	0.005962075	0.008353013	positive
PRJNA729271	stage_5W_vs_E10	ordinary_muscle_development	ENSGALG00010023126	CGNL1	0.153991729	0.137729549	1.118073281	0.263535697	0.300216121	positive
PRJNA729271	stage_5W_vs_E10	ordinary_muscle_development	ENSGALG00010023127	CERS5	-1.02887095	0.098297253	-10.46693492	1.22547E-25	6.22832E-25	negative
PRJNA729271	stage_5W_vs_E10	ordinary_muscle_development	ENSGALG00010023128	IWS1	0.284370434	0.137223297	2.072318923	0.038235709	0.048972926	positive
PRJNA729271	stage_5W_vs_E10	ordinary_muscle_development	ENSGALG00010023130	DYNLT5	-4.240505195	0.947736224	-4.474351711	7.66435E-06	1.37698E-05	negative
PRJNA729271	stage_5W_vs_E10	ordinary_muscle_development	ENSGALG00010023131	SOX6	0.880399395	0.137095723	6.421785989	1.34685E-10	3.32355E-10	positive
PRJNA729271	stage_5W_vs_E10	ordinary_muscle_development	ENSGALG00010023134	KNL1	-2.858727333	0.217685087	-13.13239858	2.14745E-39	1.83102E-38	negative
PRJNA729271	stage_5W_vs_E10	ordinary_muscle_development	ENSGALG00010023135	GINS3	-1.910490073	0.074021111	-25.81007011	6.8353E-147	6.3921E-145	negative
PRJNA729271	stage_5W_vs_E10	ordinary_muscle_development	ENSGALG00010023136	MDK	-6.679185239	0.426474703	-15.66138668	2.77774E-55	3.87148E-54	negative
PRJNA729271	stage_5W_vs_E10	ordinary_muscle_development	ENSGALG00010023137	ETV6	0.139770721	0.113456352	1.231933854	0.217973794	0.251875808	positive
PRJNA729271	stage_5W_vs_E10	ordinary_muscle_development	ENSGALG00010023140	CCAR1	-0.38270321	0.083876544	-4.562696454	5.05008E-06	9.18225E-06	negative
PRJNA729271	stage_5W_vs_E10	ordinary_muscle_development	ENSGALG00010023142	TLK2	1.259145812	0.167998122	7.494999325	6.62987E-14	1.96202E-13	positive
PRJNA729271	stage_5W_vs_E10	ordinary_muscle_development	ENSGALG00010023144	C11orf58	-0.260408182	0.106663998	-2.441387794	0.014630934	0.019662098	negative
PRJNA729271	stage_5W_vs_E10	ordinary_muscle_development	ENSGALG00010023147	RAD51	-3.214261529	0.196708723	-16.3402084	5.1078E-60	8.17192E-59	negative
PRJNA729271	stage_5W_vs_E10	ordinary_muscle_development	ENSGALG00010023149	ADGRD1	-0.338658802	0.184847416	-1.832099192	0.066936644	0.083111757	negative
PRJNA729271	stage_5W_vs_E10	ordinary_muscle_development	ENSGALG00010023151	ENSGALG00010023151	0.770986618	0.485459152	1.588159612	0.112250251	0.135430947	positive
PRJNA729271	stage_5W_vs_E10	ordinary_muscle_development	ENSGALG00010023155	CFAP263	-1.609891373	0.385748811	-4.173418884	3.00063E-05	5.14662E-05	negative
PRJNA729271	stage_5W_vs_E10	ordinary_muscle_development	ENSGALG00010023157	STOX1	-3.935089284	0.452196942	-8.702158112	3.25631E-18	1.20102E-17	negative
PRJNA729271	stage_5W_vs_E10	ordinary_muscle_development	ENSGALG00010023158	ENSGALG00010023158	4.058489371	0.444371339	9.133103371	6.65633E-20	2.64846E-19	positive
PRJNA729271	stage_5W_vs_E10	ordinary_muscle_development	ENSGALG00010023159	DAW1	0.056652722	0.501564671	0.112951978	0.910068627	0.922027312	positive
PRJNA729271	stage_5W_vs_E10	ordinary_muscle_development	ENSGALG00010023160	ENSGALG00010023160	-1.276059113	0.111266211	-11.46852312	1.89872E-30	1.16717E-29	negative
PRJNA729271	stage_5W_vs_E10	ordinary_muscle_development	ENSGALG00010023161	ODF2L	-0.997477576	0.432596104	-2.305794176	0.021122136	0.027823097	negative
PRJNA729271	stage_5W_vs_E10	ordinary_muscle_development	ENSGALG00010023162	CHRNA4	-1.348416677	0.270559074	-4.983816134	6.23423E-07	1.21524E-06	negative
PRJNA729271	stage_5W_vs_E10	ordinary_muscle_development	ENSGALG00010023163	CRTAC1	-1.899599661	0.291617451	-6.514012288	7.31695E-11	1.84163E-10	negative
PRJNA729271	stage_5W_vs_E10	ordinary_muscle_development	ENSGALG00010023164	NKIRAS2	0.519352655	0.133174053	3.89980362	9.62707E-05	0.000158826	positive
PRJNA729271	stage_5W_vs_E10	ordinary_muscle_development	ENSGALG00010023165	CSNK2A2	0.036728327	0.130134578	0.282233421	0.777764533	0.80428743	positive
PRJNA729271	stage_5W_vs_E10	ordinary_muscle_development	ENSGALG00010023166	KDM2A	0.130726634	0.113376661	1.153029495	0.248898278	0.284794801	positive
PRJNA729271	stage_5W_vs_E10	ordinary_muscle_development	ENSGALG00010023167	GPD1	11.8786765	0.209872653	56.59944893	0	0	positive
PRJNA729271	stage_5W_vs_E10	ordinary_muscle_development	ENSGALG00010023168	SPHKAP	-3.333722418	0.389231296	-8.564887899	1.08181E-17	3.89489E-17	negative
PRJNA729271	stage_5W_vs_E10	ordinary_muscle_development	ENSGALG00010023169	RAN	-1.005797296	0.097060769	-10.36255231	3.67035E-25	1.83328E-24	negative
PRJNA729271	stage_5W_vs_E10	ordinary_muscle_development	ENSGALG00010023170	TCF12	-1.351232123	0.064987333	-20.7922383	5.08813E-96	1.99331E-94	negative
PRJNA729271	stage_5W_vs_E10	ordinary_muscle_development	ENSGALG00010023171	ENSGALG00010023171	-1.113042571	0.115506916	-9.636155181	5.62556E-22	2.46352E-21	negative
PRJNA729271	stage_5W_vs_E10	ordinary_muscle_development	ENSGALG00010023172	DHX58	3.883644464	0.322037632	12.05959825	1.72623E-33	1.19893E-32	positive
PRJNA729271	stage_5W_vs_E10	ordinary_muscle_development	ENSGALG00010023174	KCNQ2	-2.102190368	0.373577705	-5.627183685	1.83176E-08	3.97832E-08	negative
PRJNA729271	stage_5W_vs_E10	ordinary_muscle_development	ENSGALG00010023175	GRK2	0.631987775	0.136948015	4.614800551	3.93473E-06	7.21948E-06	positive
PRJNA729271	stage_5W_vs_E10	ordinary_muscle_development	ENSGALG00010023177	LZTS2	0.401520292	0.171322902	2.343646346	0.019096269	0.025313253	positive
PRJNA729271	stage_5W_vs_E10	ordinary_muscle_development	ENSGALG00010023178	CFAP20	-0.737204885	0.099756793	-7.390021905	1.46805E-13	4.26013E-13	negative
PRJNA729271	stage_5W_vs_E10	ordinary_muscle_development	ENSGALG00010023179	KAT2A	-0.750401257	0.132244162	-5.674362059	1.39206E-08	3.04987E-08	negative
PRJNA729271	stage_5W_vs_E10	ordinary_muscle_development	ENSGALG00010023180	EEF1A2	6.554937645	0.121649899	53.88362591	0	0	positive
PRJNA729271	stage_5W_vs_E10	ordinary_muscle_development	ENSGALG00010023181	PLEKHA7	-0.105160055	0.166389819	-0.632010152	0.527380244	0.567932885	negative
PRJNA729271	stage_5W_vs_E10	ordinary_muscle_development	ENSGALG00010023182	EPHB6	-2.194992427	0.145436548	-15.09244034	1.816E-51	2.29094E-50	negative
PRJNA729271	stage_5W_vs_E10	ordinary_muscle_development	ENSGALG00010023183	ENSGALG00010023183	-2.229573227	0.651243611	-3.423562533	0.00061806	0.000953671	negative
PRJNA729271	stage_5W_vs_E10	ordinary_muscle_development	ENSGALG00010023184	SMARCD1	-1.246242226	0.056658479	-21.99568781	3.167E-107	1.5051E-105	negative
PRJNA729271	stage_5W_vs_E10	ordinary_muscle_development	ENSGALG00010023185	FBXW4	2.407671867	0.116406584	20.68329626	4.89772E-95	1.86822E-93	positive
PRJNA729271	stage_5W_vs_E10	ordinary_muscle_development	ENSGALG00010023186	MIER1	-0.342379702	0.099683331	-3.434673569	0.000593268	0.000916489	negative
PRJNA729271	stage_5W_vs_E10	ordinary_muscle_development	ENSGALG00010023187	ENSGALG00010023187	5.267953076	0.229879122	22.91618757	3.2044E-116	1.8431E-114	positive
PRJNA729271	stage_5W_vs_E10	ordinary_muscle_development	ENSGALG00010023188	KIF1BP	0.297332447	0.058414465	5.090048244	3.57972E-07	7.08876E-07	positive
PRJNA729271	stage_5W_vs_E10	ordinary_muscle_development	ENSGALG00010023189	RMDN3	-0.457495553	0.085682386	-5.339435236	9.32366E-08	1.92681E-07	negative
PRJNA729271	stage_5W_vs_E10	ordinary_muscle_development	ENSGALG00010023190	SF3B3	-1.058041689	0.056496083	-18.72770007	2.94361E-78	7.71566E-77	negative
PRJNA729271	stage_5W_vs_E10	ordinary_muscle_development	ENSGALG00010023193	PPDPF	4.529216137	0.312636852	14.48714732	1.46085E-47	1.61272E-46	positive
PRJNA729271	stage_5W_vs_E10	ordinary_muscle_development	ENSGALG00010023194	MRC2	0.407242623	0.193240964	2.107434233	0.035079957	0.045162446	positive
PRJNA729271	stage_5W_vs_E10	ordinary_muscle_development	ENSGALG00010023195	ENSGALG00010023195	-5.418282694	0.212513741	-25.49615225	2.1748E-143	1.899E-141	negative
PRJNA729271	stage_5W_vs_E10	ordinary_muscle_development	ENSGALG00010023196	DGKZ	1.220204291	0.127022672	9.606192881	7.52811E-22	3.27491E-21	positive
PRJNA729271	stage_5W_vs_E10	ordinary_muscle_development	ENSGALG00010023198	PTK6	-1.675216861	0.554084813	-3.023394291	0.002499563	0.003642422	negative
PRJNA729271	stage_5W_vs_E10	ordinary_muscle_development	ENSGALG00010023199	RPS13	0.207885352	0.196774531	1.056464729	0.290755947	0.328565327	positive
PRJNA729271	stage_5W_vs_E10	ordinary_muscle_development	ENSGALG00010023200	VPS26A	0.246864267	0.0827693	2.98255834	0.002858502	0.004140914	positive
PRJNA729271	stage_5W_vs_E10	ordinary_muscle_development	ENSGALG00010023201	ZNF280D	-0.120461672	0.118216154	-1.018995015	0.308205333	0.347066214	negative
PRJNA729271	stage_5W_vs_E10	ordinary_muscle_development	ENSGALG00010023202	PID1	-0.421280723	0.157632458	-2.672550626	0.007527701	0.010438538	negative
PRJNA729271	stage_5W_vs_E10	ordinary_muscle_development	ENSGALG00010023203	COG4	-0.382821816	0.064886892	-5.899832825	3.6387E-09	8.23594E-09	negative
PRJNA729271	stage_5W_vs_E10	ordinary_muscle_development	ENSGALG00010023204	SRGN	5.115821259	0.306735774	16.67826739	1.8861E-62	3.23157E-61	positive
PRJNA729271	stage_5W_vs_E10	ordinary_muscle_development	ENSGALG00010023205	DNAJC17	-1.079753088	0.118632964	-9.101627827	8.89879E-20	3.52619E-19	negative
PRJNA729271	stage_5W_vs_E10	ordinary_muscle_development	ENSGALG00010023206	HSPB9	4.379295838	0.313489348	13.96952039	2.39232E-44	2.38328E-43	positive
PRJNA729271	stage_5W_vs_E10	ordinary_muscle_development	ENSGALG00010023207	MNS1	-1.097519697	0.168544662	-6.511744035	7.42832E-11	1.86901E-10	negative
PRJNA729271	stage_5W_vs_E10	ordinary_muscle_development	ENSGALG00010023208	BACE1	-0.522764418	0.079076112	-6.610901909	3.81986E-11	9.78247E-11	negative
PRJNA729271	stage_5W_vs_E10	ordinary_muscle_development	ENSGALG00010023209	PIK3C2A	-0.228508448	0.106776162	-2.140069878	0.03234912	0.04181012	negative
PRJNA729271	stage_5W_vs_E10	ordinary_muscle_development	ENSGALG00010023210	ZNHIT6	-0.430215682	0.143653006	-2.994825483	0.002746019	0.00398394	negative
PRJNA729271	stage_5W_vs_E10	ordinary_muscle_development	ENSGALG00010023211	TLK1	-0.284782565	0.076903554	-3.703113193	0.00021297	0.000341444	negative
PRJNA729271	stage_5W_vs_E10	ordinary_muscle_development	ENSGALG00010023212	RIMBP2	1.342221705	0.214703949	6.251499863	4.06529E-10	9.72544E-10	positive
PRJNA729271	stage_5W_vs_E10	ordinary_muscle_development	ENSGALG00010023213	FCSK	0.529564963	0.114969588	4.606130832	4.1023E-06	7.52028E-06	positive
PRJNA729271	stage_5W_vs_E10	ordinary_muscle_development	ENSGALG00010023214	SUPV3L1	-0.413447044	0.090520213	-4.567455486	4.9368E-06	8.98192E-06	negative
PRJNA729271	stage_5W_vs_E10	ordinary_muscle_development	ENSGALG00010023215	TRIP12	0.384274072	0.056230766	6.83387584	8.26506E-12	2.19458E-11	positive
PRJNA729271	stage_5W_vs_E10	ordinary_muscle_development	ENSGALG00010023216	SLC35D1	-0.133752235	0.123358924	-1.084252609	0.278252812	0.31561738	negative
PRJNA729271	stage_5W_vs_E10	ordinary_muscle_development	ENSGALG00010023217	CREB3L1	-0.462592936	0.206802886	-2.236878535	0.02529428	0.033048547	negative
PRJNA729271	stage_5W_vs_E10	ordinary_muscle_development	ENSGALG00010023218	SSH3	0.204415195	0.131282665	1.557061584	0.119455865	0.143574856	positive
PRJNA729271	stage_5W_vs_E10	ordinary_muscle_development	ENSGALG00010023219	TEX9	0.16364312	0.14994242	1.091373075	0.275108755	0.312271057	positive
PRJNA729271	stage_5W_vs_E10	ordinary_muscle_development	ENSGALG00010023220	ST3GAL2	-0.804307037	0.087664978	-9.174781723	4.5249E-20	1.81184E-19	negative
PRJNA729271	stage_5W_vs_E10	ordinary_muscle_development	ENSGALG00010023221	AMY1A	2.97130607	0.135791679	21.88135605	3.9104E-106	1.8109E-104	positive
PRJNA729271	stage_5W_vs_E10	ordinary_muscle_development	ENSGALG00010023222	ENSGALG00010023222	-1.076756818	0.605189905	-1.779204856	0.075206183	0.092815123	negative
PRJNA729271	stage_5W_vs_E10	ordinary_muscle_development	ENSGALG00010023223	CEP164	-1.481728526	0.102563824	-14.44689234	2.62254E-47	2.87547E-46	negative
PRJNA729271	stage_5W_vs_E10	ordinary_muscle_development	ENSGALG00010023225	PDPR	-0.151519674	0.122207476	-1.239856007	0.215028658	0.248690689	negative
PRJNA729271	stage_5W_vs_E10	ordinary_muscle_development	ENSGALG00010023226	ENSGALG00010023226	0.094227842	0.21064114	0.447338266	0.654630842	0.689197709	positive
PRJNA729271	stage_5W_vs_E10	ordinary_muscle_development	ENSGALG00010023227	RNPC3	-0.258233573	0.125796396	-2.052789914	0.040092957	0.051206927	negative
PRJNA729271	stage_5W_vs_E10	ordinary_muscle_development	ENSGALG00010023228	GORASP2	1.03720729	0.101619108	10.20681359	1.84835E-24	8.9755E-24	positive
PRJNA729271	stage_5W_vs_E10	ordinary_muscle_development	ENSGALG00010023229	HKDC1	-0.10552569	0.404966603	-0.260578746	0.794417382	0.818937483	negative
PRJNA729271	stage_5W_vs_E10	ordinary_muscle_development	ENSGALG00010023230	ENSGALG00010023230	1.424208399	0.083042067	17.15044487	6.23731E-66	1.18029E-64	positive
PRJNA729271	stage_5W_vs_E10	ordinary_muscle_development	ENSGALG00010023231	ASIC1	-3.031783759	0.308460109	-9.828770951	8.46455E-23	3.83657E-22	negative
PRJNA729271	stage_5W_vs_E10	ordinary_muscle_development	ENSGALG00010023233	SEZ6L	-2.289109213	1.102476591	-2.076333621	0.037863106	0.048534287	negative
PRJNA729271	stage_5W_vs_E10	ordinary_muscle_development	ENSGALG00010023234	ENSGALG00010023234	-0.101019632	0.09432765	-1.070944007	0.284194607	0.321878483	negative
PRJNA729271	stage_5W_vs_E10	ordinary_muscle_development	ENSGALG00010023235	ENSGALG00010023235	-0.456987763	0.360126191	-1.268965642	0.204453318	0.237414957	negative
PRJNA729271	stage_5W_vs_E10	ordinary_muscle_development	ENSGALG00010023237	GAD1	-4.774517106	0.788013098	-6.058931146	1.37029E-09	3.17544E-09	negative
PRJNA729271	stage_5W_vs_E10	ordinary_muscle_development	ENSGALG00010023238	NUCB2	-0.210872287	0.07823588	-2.695339866	0.007031687	0.009777849	negative
PRJNA729271	stage_5W_vs_E10	ordinary_muscle_development	ENSGALG00010023239	RBPMS2	0.735818043	0.203187409	3.621376188	0.00029304	0.000464017	positive
PRJNA729271	stage_5W_vs_E10	ordinary_muscle_development	ENSGALG00010023240	PIWIL1	-2.550467269	0.456439877	-5.587739804	2.30044E-08	4.96499E-08	negative
PRJNA729271	stage_5W_vs_E10	ordinary_muscle_development	ENSGALG00010023243	HK1	1.724797881	0.114162767	15.10823471	1.42917E-51	1.81559E-50	positive
PRJNA729271	stage_5W_vs_E10	ordinary_muscle_development	ENSGALG00010023244	PPIB	-1.365401122	0.118865193	-11.48697186	1.53395E-30	9.47363E-30	negative
PRJNA729271	stage_5W_vs_E10	ordinary_muscle_development	ENSGALG00010023245	TANC2	-1.667636346	0.15109877	-11.03673009	2.54113E-28	1.43994E-27	negative
PRJNA729271	stage_5W_vs_E10	ordinary_muscle_development	ENSGALG00010023246	SP5	-6.28686159	0.974196043	-6.453384443	1.09379E-10	2.71528E-10	negative
PRJNA729271	stage_5W_vs_E10	ordinary_muscle_development	ENSGALG00010023248	PRDM10	-0.148868946	0.121434083	-1.225923912	0.220227309	0.254256858	negative
PRJNA729271	stage_5W_vs_E10	ordinary_muscle_development	ENSGALG00010023249	IL12RB2	2.663583123	0.416089254	6.401470586	1.53887E-10	3.7826E-10	positive
PRJNA729271	stage_5W_vs_E10	ordinary_muscle_development	ENSGALG00010023250	FZD10	-5.474385938	0.476279268	-11.49406725	1.41299E-30	8.76018E-30	negative
PRJNA729271	stage_5W_vs_E10	ordinary_muscle_development	ENSGALG00010023251	ENSGALG00010023251	10.81444612	0.916504673	11.79966282	3.91878E-32	2.59019E-31	positive
PRJNA729271	stage_5W_vs_E10	ordinary_muscle_development	ENSGALG00010023252	GLG1	0.093764468	0.079812599	1.174807855	0.240071597	0.275390772	positive
PRJNA729271	stage_5W_vs_E10	ordinary_muscle_development	ENSGALG00010023254	MYO3B	-0.753640413	0.309065182	-2.438451356	0.014750344	0.019817057	negative
PRJNA729271	stage_5W_vs_E10	ordinary_muscle_development	ENSGALG00010023255	SNX22	3.330021569	0.301238886	11.05442134	2.08678E-28	1.18712E-27	positive
PRJNA729271	stage_5W_vs_E10	ordinary_muscle_development	ENSGALG00010023257	PAK6	2.850659605	0.288196343	9.891380217	4.53729E-23	2.07404E-22	positive
PRJNA729271	stage_5W_vs_E10	ordinary_muscle_development	ENSGALG00010023258	ENSGALG00010023258	-0.497829447	0.365622083	-1.361595674	0.173325521	0.203792459	negative
PRJNA729271	stage_5W_vs_E10	ordinary_muscle_development	ENSGALG00010023259	TSPAN15	1.476916242	0.289617424	5.099542083	3.40476E-07	6.75685E-07	positive
PRJNA729271	stage_5W_vs_E10	ordinary_muscle_development	ENSGALG00010023260	ENSGALG00010023260	-4.218560684	0.309702852	-13.62131689	2.99125E-42	2.7973E-41	negative
PRJNA729271	stage_5W_vs_E10	ordinary_muscle_development	ENSGALG00010023261	SNX1	-0.420104287	0.096253412	-4.36456516	1.27376E-05	2.25435E-05	negative
PRJNA729271	stage_5W_vs_E10	ordinary_muscle_development	ENSGALG00010023262	POLD4	2.45675137	0.206265356	11.910635	1.04181E-32	7.02373E-32	positive
PRJNA729271	stage_5W_vs_E10	ordinary_muscle_development	ENSGALG00010023263	SRP14	-1.03713789	0.073454647	-14.11943196	2.88306E-45	2.96593E-44	negative
PRJNA729271	stage_5W_vs_E10	ordinary_muscle_development	ENSGALG00010023264	RACGAP1	-2.779631631	0.232916244	-11.9340394	7.86617E-33	5.33054E-32	negative
PRJNA729271	stage_5W_vs_E10	ordinary_muscle_development	ENSGALG00010023265	RAB5C	-0.404032594	0.071437333	-5.655762596	1.55156E-08	3.38906E-08	negative
PRJNA729271	stage_5W_vs_E10	ordinary_muscle_development	ENSGALG00010023266	RFWD3	-1.29278873	0.143782766	-8.991263468	2.44404E-19	9.46471E-19	negative
PRJNA729271	stage_5W_vs_E10	ordinary_muscle_development	ENSGALG00010023267	DSCAML1	-4.357820609	0.908336218	-4.797585432	1.6059E-06	3.0325E-06	negative
PRJNA729271	stage_5W_vs_E10	ordinary_muscle_development	ENSGALG00010023268	KCNJ11	2.771772127	0.180552906	15.35157858	3.45672E-53	4.55915E-52	positive
PRJNA729271	stage_5W_vs_E10	ordinary_muscle_development	ENSGALG00010023269	OPRL1	-3.592599678	0.695429206	-5.166017829	2.39134E-07	4.79824E-07	negative
PRJNA729271	stage_5W_vs_E10	ordinary_muscle_development	ENSGALG00010023271	PATL2	0.844654116	0.411390075	2.053170862	0.040056009	0.051168754	positive
PRJNA729271	stage_5W_vs_E10	ordinary_muscle_development	ENSGALG00010023273	HDAC7	1.118766187	0.113062316	9.895128907	4.37047E-23	2.00031E-22	positive
PRJNA729271	stage_5W_vs_E10	ordinary_muscle_development	ENSGALG00010023274	ZBTB38	0.932567756	0.114854617	8.119549589	4.67917E-16	1.55383E-15	positive

PRJNA729271	stage_5W_vs_E10	ordinary_muscle_development	ENSGALG00010023275	TWIST3	-9.404615185	0.89481424	-10.51013134	7.75854E-26	3.9823E-25	negative
PRJNA729271	stage_5W_vs_E10	ordinary_muscle_development	ENSGALG00010023276	ABCC8	1.12505495	0.162403178	6.927542705	4.28213E-12	1.15349E-11	positive
PRJNA729271	stage_5W_vs_E10	ordinary_muscle_development	ENSGALG00010023278	ENSGALG00010023278	-1.442407532	0.34728617	-4.15336877	3.27616E-05	5.60132E-05	negative
PRJNA729271	stage_5W_vs_E10	ordinary_muscle_development	ENSGALG00010023279	FA2H	-0.61043162	0.445186592	-1.3711815	0.170318384	0.200663657	negative
PRJNA729271	stage_5W_vs_E10	ordinary_muscle_development	ENSGALG00010023281	B2M	5.36243446	0.199007404	26.94590434	6.3709E-160	7.2145E-158	positive
PRJNA729271	stage_5W_vs_E10	ordinary_muscle_development	ENSGALG00010023282	SERBP1	-0.78917941	0.087406901	-9.028799811	1.73565E-19	6.77389E-19	negative
PRJNA729271	stage_5W_vs_E10	ordinary_muscle_development	ENSGALG00010023285	SLC48A1	2.152680695	0.111078418	19.37982856	1.14227E-83	3.36528E-82	positive
PRJNA729271	stage_5W_vs_E10	ordinary_muscle_development	ENSGALG00010023286	HCRT	-1.080448629	0.610040455	-1.771109801	0.076542444	0.094391929	negative
PRJNA729271	stage_5W_vs_E10	ordinary_muscle_development	ENSGALG00010023287	RGS19	0.054818602	0.200232085	0.273775312	0.784257297	0.810192397	positive
PRJNA729271	stage_5W_vs_E10	ordinary_muscle_development	ENSGALG00010023289	NFRKB	-0.333602673	0.087523445	-3.811580707	0.000138081	0.000224709	negative
PRJNA729271	stage_5W_vs_E10	ordinary_muscle_development	ENSGALG00010023290	SLC16A14	-4.48129456	0.290102025	-15.44730535	7.8664E-54	1.04801E-52	negative
PRJNA729271	stage_5W_vs_E10	ordinary_muscle_development	ENSGALG00010023293	COL11A1	-7.353208777	0.491569123	-14.95864658	1.36779E-50	1.67734E-49	negative
PRJNA729271	stage_5W_vs_E10	ordinary_muscle_development	ENSGALG00010023295	ENSGALG00010023295	0.907659496	0.512776179	1.770089042	0.076712309	0.094569222	positive
PRJNA729271	stage_5W_vs_E10	ordinary_muscle_development	ENSGALG00010023299	PASK	-1.834663944	0.14622533	-12.54682714	4.13759E-36	3.19183E-35	negative
PRJNA729271	stage_5W_vs_E10	ordinary_muscle_development	ENSGALG00010023304	ENSGALG00010023304	-0.362181334	0.175213377	-2.067087222	0.038725936	0.049570156	negative
PRJNA729271	stage_5W_vs_E10	ordinary_muscle_development	ENSGALG00010023305	PLCB2	2.166078372	0.15699862	13.7967988	2.66423E-43	2.58142E-42	positive
PRJNA729271	stage_5W_vs_E10	ordinary_muscle_development	ENSGALG00010023309	PIK3R3	-1.228334496	0.201593461	-6.093126688	1.10726E-09	2.58077E-09	negative
PRJNA729271	stage_5W_vs_E10	ordinary_muscle_development	ENSGALG00010023311	ETS1	-0.115377935	0.133712381	-0.862881463	0.388202644	0.429508993	negative
PRJNA729271	stage_5W_vs_E10	ordinary_muscle_development	ENSGALG00010023312	UBR3	2.408182014	0.114095808	21.10666512	6.90783E-99	2.83652E-97	positive
PRJNA729271	stage_5W_vs_E10	ordinary_muscle_development	ENSGALG00010023317	RAPGEF3	1.172547316	0.148860969	7.876794883	3.35886E-15	1.06699E-14	positive
PRJNA729271	stage_5W_vs_E10	ordinary_muscle_development	ENSGALG00010023319	EIF2AK4	0.075843073	0.061904836	1.225155863	0.220516501	0.254549262	positive
PRJNA729271	stage_5W_vs_E10	ordinary_muscle_development	ENSGALG00010023320	ENSGALG00010023320	-0.846561664	0.140538528	-6.023698099	1.70476E-09	3.92729E-09	negative
PRJNA729271	stage_5W_vs_E10	ordinary_muscle_development	ENSGALG00010023322	PRKAB1	-0.487355952	0.092810061	-5.251111235	1.51184E-07	3.08303E-07	negative
PRJNA729271	stage_5W_vs_E10	ordinary_muscle_development	ENSGALG00010023324	LAG3	6.138680856	0.943188251	6.508436518	7.5937E-11	1.90863E-10	positive
PRJNA729271	stage_5W_vs_E10	ordinary_muscle_development	ENSGALG00010023328	TCEA2	-1.754397952	0.113208129	-15.49710231	3.62894E-54	4.90228E-53	negative
PRJNA729271	stage_5W_vs_E10	ordinary_muscle_development	ENSGALG00010023330	TSPAN1	2.140890009	1.358253171	1.576208364	0.114977792	0.138629437	positive
PRJNA729271	stage_5W_vs_E10	ordinary_muscle_development	ENSGALG00010023331	CD4	-0.957611722	0.320910716	-2.984044079	0.002844658	0.004122919	negative
PRJNA729271	stage_5W_vs_E10	ordinary_muscle_development	ENSGALG00010023332	PPP1R7	0.348953221	0.07501659	4.651680637	3.29241E-06	6.08329E-06	positive
PRJNA729271	stage_5W_vs_E10	ordinary_muscle_development	ENSGALG00010023333	ENDOU	-1.33993379	0.576957674	-2.322412632	0.020210728	0.026683168	negative
PRJNA729271	stage_5W_vs_E10	ordinary_muscle_development	ENSGALG00010023334	RPS17	-0.130471367	0.151665114	-0.860259576	0.389645971	0.430974311	negative
PRJNA729271	stage_5W_vs_E10	ordinary_muscle_development	ENSGALG00010023335	WDR59	0.108430673	0.069870713	1.551875864	0.120691929	0.144952317	positive
PRJNA729271	stage_5W_vs_E10	ordinary_muscle_development	ENSGALG00010023336	GPR162	-1.332682816	0.153453299	-8.684614976	3.80027E-18	1.39703E-17	negative
PRJNA729271	stage_5W_vs_E10	ordinary_muscle_development	ENSGALG00010023337	FLI1	0.563195029	0.11657489	4.831186439	1.35722E-06	2.57971E-06	positive
PRJNA729271	stage_5W_vs_E10	ordinary_muscle_development	ENSGALG00010023338	HDLBP	0.095960387	0.124082297	0.773360822	0.439308869	0.481095653	positive
PRJNA729271	stage_5W_vs_E10	ordinary_muscle_development	ENSGALG00010023340	P3H3	-1.287639815	0.160714391	-8.011975817	1.1288E-15	3.68015E-15	negative
PRJNA729271	stage_5W_vs_E10	ordinary_muscle_development	ENSGALG00010023342	FITM2	-0.344111257	0.136957831	-2.512534357	0.011986743	0.016265287	negative
PRJNA729271	stage_5W_vs_E10	ordinary_muscle_development	ENSGALG00010023343	TMEM233	4.524962331	0.33597032	13.46833951	2.40207E-41	2.18981E-40	positive
PRJNA729271	stage_5W_vs_E10	ordinary_muscle_development	ENSGALG00010023347	STAT5A	2.570831133	0.169183086	15.19555648	3.78436E-52	4.88462E-51	positive
PRJNA729271	stage_5W_vs_E10	ordinary_muscle_development	ENSGALG00010023349	ENSGALG00010023349	3.554968812	0.563976213	6.303402044	2.91182E-10	7.02628E-10	positive
PRJNA729271	stage_5W_vs_E10	ordinary_muscle_development	ENSGALG00010023350	ZNRF1	1.384338828	0.230729146	5.999843775	1.97507E-09	4.52914E-09	positive
PRJNA729271	stage_5W_vs_E10	ordinary_muscle_development	ENSGALG00010023351	GNB3	-0.200575427	0.397688181	-0.504353501	0.614012987	0.650974126	negative
PRJNA729271	stage_5W_vs_E10	ordinary_muscle_development	ENSGALG00010023354	SEPTIN2	-2.741745871	0.172446333	-15.89912541	6.42555E-57	9.44609E-56	negative
PRJNA729271	stage_5W_vs_E10	ordinary_muscle_development	ENSGALG00010023356	ENSGALG00010023356	0.347978116	0.188700961	1.844071772	0.065172681	0.081025647	positive
PRJNA729271	stage_5W_vs_E10	ordinary_muscle_development	ENSGALG00010023359	CDCA3	-2.798845519	0.277051754	-10.10224799	5.39901E-24	2.56839E-23	negative
PRJNA729271	stage_5W_vs_E10	ordinary_muscle_development	ENSGALG00010023362	GADD45A	0.508338232	0.149320181	3.404350489	0.000663216	0.001020522	positive
PRJNA729271	stage_5W_vs_E10	ordinary_muscle_development	ENSGALG00010023363	CPEB1	-0.797940777	0.217652009	-3.666130999	0.000246248	0.000392756	negative
PRJNA729271	stage_5W_vs_E10	ordinary_muscle_development	ENSGALG00010023364	CTRB1	7.793751467	2.216892866	3.515619355	0.00043873	0.000684173	positive
PRJNA729271	stage_5W_vs_E10	ordinary_muscle_development	ENSGALG00010023366	GDAP1L1	-3.882295289	0.488125099	-7.953484253	1.81338E-15	5.8528E-15	negative
PRJNA729271	stage_5W_vs_E10	ordinary_muscle_development	ENSGALG00010023368	ENSGALG00010023368	-1.940074749	0.235795343	-8.227790784	1.90698E-16	6.45079E-16	negative
PRJNA729271	stage_5W_vs_E10	ordinary_muscle_development	ENSGALG00010023369	ACE	2.017583965	0.476559259	4.233647605	2.29931E-05	3.98523E-05	positive
PRJNA729271	stage_5W_vs_E10	ordinary_muscle_development	ENSGALG00010023374	ENSGALG00010023374	-0.939359121	0.207865204	-4.51907825	6.21094E-06	1.12198E-05	negative
PRJNA729271	stage_5W_vs_E10	ordinary_muscle_development	ENSGALG00010023375	ST14	-0.164145924	0.148628636	-1.104403088	0.269418322	0.306508011	negative
PRJNA729271	stage_5W_vs_E10	ordinary_muscle_development	ENSGALG00010023377	OSER1	1.067903457	0.150053105	7.116836767	1.10432E-12	3.07475E-12	positive
PRJNA729271	stage_5W_vs_E10	ordinary_muscle_development	ENSGALG00010023379	CCDC60	-0.793700666	0.63675895	-1.246469587	0.212592048	0.246127934	negative
PRJNA729271	stage_5W_vs_E10	ordinary_muscle_development	ENSGALG00010023380	USP5	-0.673786678	0.09717421	-6.933801438	4.0968E-12	1.1048E-11	negative
PRJNA729271	stage_5W_vs_E10	ordinary_muscle_development	ENSGALG00010023381	HOXB7	-0.581619424	0.166401009	-3.495287851	0.000473551	0.000736493	negative
PRJNA729271	stage_5W_vs_E10	ordinary_muscle_development	ENSGALG00010023383	HOXB6	0.302099325	0.17026921	1.774245178	0.076022604	0.093774798	positive
PRJNA729271	stage_5W_vs_E10	ordinary_muscle_development	ENSGALG00010023384	AP3B2	-1.732596565	0.229943696	-7.534873096	4.8881E-14	1.46089E-13	negative
PRJNA729271	stage_5W_vs_E10	ordinary_muscle_development	ENSGALG00010023387	ENSGALG00010023387	1.665817349	0.182642899	9.120624753	7.46925E-20	2.96703E-19	positive
PRJNA729271	stage_5W_vs_E10	ordinary_muscle_development	ENSGALG00010023388	FARP2	-0.841439408	0.085044049	-9.894159806	4.413E-23	2.01851E-22	negative
PRJNA729271	stage_5W_vs_E10	ordinary_muscle_development	ENSGALG00010023389	GNG12	-0.244415465	0.110301976	-2.215875669	0.026700018	0.034819322	negative
PRJNA729271	stage_5W_vs_E10	ordinary_muscle_development	ENSGALG00010023390	HSPB8	2.525903532	0.250798695	10.07143811	7.38897E-24	3.4887E-23	positive
PRJNA729271	stage_5W_vs_E10	ordinary_muscle_development	ENSGALG00010023392	TPI1	4.562023368	0.149509055	30.51335824	1.7329E-204	3.0633E-202	positive
PRJNA729271	stage_5W_vs_E10	ordinary_muscle_development	ENSGALG00010023394	HOXB5	-0.983195003	0.176966635	-5.555821308	2.76309E-08	5.92208E-08	negative
PRJNA729271	stage_5W_vs_E10	ordinary_muscle_development	ENSGALG00010023396	ENSGALG00010023396	-0.099724955	0.147591788	-0.675680917	0.499243255	0.540402582	negative
PRJNA729271	stage_5W_vs_E10	ordinary_muscle_development	ENSGALG00010023397	ENSGALG00010023397	0.459539645	0.242675041	1.893641979	0.058272548	0.072863997	positive
PRJNA729271	stage_5W_vs_E10	ordinary_muscle_development	ENSGALG00010023401	LRRC23	1.321313313	0.374844287	3.524965857	0.000423537	0.000661406	positive
PRJNA729271	stage_5W_vs_E10	ordinary_muscle_development	ENSGALG00010023405	DNAJA2	-0.248588479	0.077090497	-3.224631932	0.001261347	0.001891695	negative
PRJNA729271	stage_5W_vs_E10	ordinary_muscle_development	ENSGALG00010023408	ZBTB44	0.35443452	0.127076077	2.789152203	0.005284622	0.007439841	positive
PRJNA729271	stage_5W_vs_E10	ordinary_muscle_development	ENSGALG00010023411	ENSGALG00010023411	3.830403024	0.180467043	21.22494479	5.61922E-100	2.35406E-98	positive
PRJNA729271	stage_5W_vs_E10	ordinary_muscle_development	ENSGALG00010023412	GSS	-0.752267064	0.108632353	-6.92488973	4.36315E-12	1.17466E-11	negative
PRJNA729271	stage_5W_vs_E10	ordinary_muscle_development	ENSGALG00010023414	SUDS3	-0.729220807	0.076965733	-9.474616541	2.67742E-21	1.13511E-20	negative
PRJNA729271	stage_5W_vs_E10	ordinary_muscle_development	ENSGALG00010023415	HOXB4	0.22524012	0.165989914	1.356950639	0.174796895	0.205402335	positive
PRJNA729271	stage_5W_vs_E10	ordinary_muscle_development	ENSGALG00010023421	OLFM3	-0.602918751	0.253331029	-2.379964088	0.017314325	0.023062961	negative
PRJNA729271	stage_5W_vs_E10	ordinary_muscle_development	ENSGALG00010023422	WLS	-2.510050257	0.156399319	-16.04898454	5.8107E-58	8.80105E-57	negative
PRJNA729271	stage_5W_vs_E10	ordinary_muscle_development	ENSGALG00010023426	KCNJ1	-0.397081448	0.510622222	-0.777642318	0.436779906	0.478760189	negative
PRJNA729271	stage_5W_vs_E10	ordinary_muscle_development	ENSGALG00010023428	HOXB3	-1.792273072	0.195253904	-9.179192005	4.34338E-20	1.74011E-19	negative
PRJNA729271	stage_5W_vs_E10	ordinary_muscle_development	ENSGALG00010023429	BMF	0.644491991	0.147305699	4.375200664	1.21321E-05	2.14902E-05	positive
PRJNA729271	stage_5W_vs_E10	ordinary_muscle_development	ENSGALG00010023431	BCAR1	-0.363173983	0.174629285	-2.079685444	0.037554394	0.048176913	negative
PRJNA729271	stage_5W_vs_E10	ordinary_muscle_development	ENSGALG00010023432	TMOD3	-1.246234407	0.175186528	-7.113757116	1.12926E-12	3.14237E-12	negative
PRJNA729271	stage_5W_vs_E10	ordinary_muscle_development	ENSGALG00010023434	ATN1	-0.217819793	0.187487319	-1.161784141	0.245323158	0.280881451	negative
PRJNA729271	stage_5W_vs_E10	ordinary_muscle_development	ENSGALG00010023437	KCNJ5	-1.396815012	0.259157582	-5.389828847	7.05248E-08	1.4696E-07	negative
PRJNA729271	stage_5W_vs_E10	ordinary_muscle_development	ENSGALG00010023438	HOXB2	-0.038802369	0.236130698	-0.164325815	0.869474661	0.885853322	negative
PRJNA729271	stage_5W_vs_E10	ordinary_muscle_development	ENSGALG00010023439	ACSS2	1.038018806	0.132702553	7.822146464	5.19301E-15	1.62999E-14	positive
PRJNA729271	stage_5W_vs_E10	ordinary_muscle_development	ENSGALG00010023441	ENSGALG00010023441	2.461364486	0.145102643	16.96291969	1.54474E-64	2.80941E-63	positive
PRJNA729271	stage_5W_vs_E10	ordinary_muscle_development	ENSGALG00010023442	STK25	0.136596334	0.112516545	1.214011097	0.224743487	0.259080335	positive
PRJNA729271	stage_5W_vs_E10	ordinary_muscle_development	ENSGALG00010023443	ENSGALG00010023443	0.583962301	0.182552872	3.198866681	0.00137969	0.002062137	positive
PRJNA729271	stage_5W_vs_E10	ordinary_muscle_development	ENSGALG00010023447	C12orf57	0.031092138	0.1145652	0.271392516	0.786089157	0.811514834	positive
PRJNA729271	stage_5W_vs_E10	ordinary_muscle_development	ENSGALG00010023448	FAM219B	-0.045181959	0.122073682	-0.370120391	0.71129279	0.742488045	negative
PRJNA729271	stage_5W_vs_E10	ordinary_muscle_development	ENSGALG00010023453	ARHGAP32	-0.917716984	0.178350305	-5.145586858	2.66686E-07	5.3282E-07	negative
PRJNA729271	stage_5W_vs_E10	ordinary_muscle_development	ENSGALG00010023454	HOXB1	1.34238751	0.395271378	3.396116146	0.000683494	0.001050274	positive
PRJNA729271	stage_5W_vs_E10	ordinary_muscle_development	ENSGALG00010023455	CFDP1	-0.706833446	0.097048848	-7.283274983	3.25812E-13	9.29472E-13	negative
PRJNA729271	stage_5W_vs_E10	ordinary_muscle_development	ENSGALG00010023456	MPI	0.757730979	0.144477782	5.244619401	1.56605E-07	3.18864E-07	positive
PRJNA729271	stage_5W_vs_E10	ordinary_muscle_development	ENSGALG00010023469	ENSGALG00010023469	0.840724152	0.21397894	3.929004188	8.52984E-05	0.000141513	positive
PRJNA729271	stage_5W_vs_E10	ordinary_muscle_development	ENSGALG00010023472	PTPN6	2.091713207	0.194355398	10.76231085	5.18533E-27	2.79203E-26	positive
PRJNA729271	stage_5W_vs_E10	ordinary_muscle_development	ENSGALG00010023473	BOK	-0.722865673	0.201647824	-3.584792822	0.000337346	0.000531388	negative
PRJNA729271	stage_5W_vs_E10	ordinary_muscle_development	ENSGALG00010023475	ENSGALG00010023475	-4.132580786	0.683004177	-6.050593722	1.44313E-09	3.34049E-09	negative
PRJNA729271	stage_5W_vs_E10	ordinary_muscle_development	ENSGALG00010023476	SCAMP2	0.228413241	0.15103061	1.512363893	0.130441315	0.156092369	positive
PRJNA729271	stage_5W_vs_E10	ordinary_muscle_development	ENSGALG00010023478	TAOK3	0.879764402	0.123185854	7.141764842	9.21401E-13	2.57584E-12	positive
PRJNA729271	stage_5W_vs_E10	ordinary_muscle_development	ENSGALG00010023479	MCCC2L	0.933485871	0.156306633	5.972144969	2.34154E-09	5.35511E-09	positive
PRJNA729271	stage_5W_vs_E10	ordinary_muscle_development	ENSGALG00010023482	MTCL2	-0.23938716	0.099118366	-2.415164513	0.015728104	0.021052624	negative
PRJNA729271	stage_5W_vs_E10	ordinary_muscle_development	ENSGALG00010023485	BARX2	-4.557544593	0.313241091	-14.54963837	5.87126E-48	6.57173E-47	negative
PRJNA729271	stage_5W_vs_E10	ordinary_muscle_development	ENSGALG00010023487	GRN	0.452348524	0.146135541	3.095403908	0.00196545	0.002890544	positive
PRJNA729271	stage_5W_vs_E10	ordinary_muscle_development	ENSGALG00010023489	ETV5	-0.797370904	0.218411312	-3.650776594	0.000261449	0.000415993	negative
PRJNA729271	stage_5W_vs_E10	ordinary_muscle_development	ENSGALG00010023490	PHB2	0.07581875	0.103001147	0.736096178	0.461672168	0.503607622	positive
PRJNA729271	stage_5W_vs_E10	ordinary_muscle_development	ENSGALG00010023496	PLEKHA3	0.531217116	0.103468794	5.134080475	2.83527E-07	5.65376E-07	positive
PRJNA729271	stage_5W_vs_E10	ordinary_muscle_development	ENSGALG00010023499	PEBP1	0.014936323	0.139833403	0.106815128	0.914935646	0.926375537	positive
PRJNA729271	stage_5W_vs_E10	ordinary_muscle_development	ENSGALG00010023501	ENSGALG00010023501	6.016771409	2.974639215	2.022689467	0.043105173	0.05480785	positive
PRJNA729271	stage_5W_vs_E10	ordinary_muscle_development	ENSGALG00010023506	RPE65	-1.13592024	0.600381423	-1.891997646	0.058491294	0.073114118	negative
PRJNA729271	stage_5W_vs_E10	ordinary_muscle_development	ENSGALG00010023510	ASPHD2	-0.92035653	0.257589191	-3.572962534	0.000352965	0.000555388	negative
PRJNA729271	stage_5W_vs_E10	ordinary_muscle_development	ENSGALG00010023513	CDCA9	-2.040244575	0.22195363	-9.192210905	3.84849E-20	1.54526E-19	negative
PRJNA729271	stage_5W_vs_E10	ordinary_muscle_development	ENSGALG00010023514	ULK3	0.379272877	0.107323065	3.533936321	0.00040942	0.000640119	positive
PRJNA729271	stage_5W_vs_E10	ordinary_muscle_development	ENSGALG00010023518	METTL5	0.387057926	0.119262541	3.245427463	0.001172744	0.001764839	positive
PRJNA729271	stage_5W_vs_E10	ordinary_muscle_development	ENSGALG00010023519	DGKG	-0.147774685	0.252068676	-0.586247712	0.557709063	0.597442382	negative
PRJNA729271	stage_5W_vs_E10	ordinary_muscle_development	ENSGALG00010023522	EMG1	-1.487816047	0.164390751	-9.050485116	1.42335E-19	5.57907E-19	negative
PRJNA729271	stage_5W_vs_E10	ordinary_muscle_development	ENSGALG00010023526	KNSTRN	-3.072903625	0.194945617	-15.7628762	5.60242E-56	8.00069E-55	negative
PRJNA729271	stage_5W_vs_E10	ordinary_muscle_development	ENSGALG00010023527	TMEM98	-1.943410395	0.238474772	-8.149333271	3.65936E-16	1.22105E-15	negative
PRJNA729271	stage_5W_vs_E10	ordinary_muscle_development	ENSGALG00010023533	FKBP7	-1.16656104	0.163514197	-7.134310425	9.72736E-13	2.71516E-12	negative
PRJNA729271	stage_5W_vs_E10	ordinary_muscle_development	ENSGALG00010023536	ENSGALG00010023536	-0.313479591	0.20857585	-1.502952481	0.132851298	0.158753468	negative
PRJNA729271	stage_5W_vs_E10	ordinary_muscle_development	ENSGALG00010023541	PRSS2	6.765153403	2.487711808	2.719428103	0.006539491	0.009124543	positive
PRJNA729271	stage_5W_vs_E10	ordinary_muscle_development	ENSGALG00010023543	ENSGALG00010023543	-2.414013636	0.857153827	-2.816313199	0.004857828	0.00686767	negative
PRJNA729271	stage_5W_vs_E10	ordinary_muscle_development	ENSGALG00010023544	SSB	-0.930605824	0.102977128	-9.037014759	1.61009E-19	6.30081E-19	negative
PRJNA729271	stage_5W_vs_E10	ordinary_muscle_development	ENSGALG00010023545	PTPRT	-2.693562905	0.148043369	-18.19441774	5.71431E-74	1.3435E-72	negative
PRJNA729271	stage_5W_vs_E10	ordinary_muscle_development	ENSGALG00010023546	SLC11A2	0.63446937	0.155476885	4.080795479	4.48818E-05	7.5947E-05	positive
PRJNA729271	stage_5W_vs_E10	ordinary_muscle_development	ENSGALG00010023551	ENSGALG00010023551	0.448696238	0.175445848	2.557462853	0.010543882	0.01440603	positive
PRJNA729271	stage_5W_vs_E10	ordinary_muscle_development	ENSGALG00010023552	SGPP2	-0.50068182	0.408228891	-1.226473264	0.220020631	0.254058714	negative
PRJNA729271	stage_5W_vs_E10	ordinary_muscle_development	ENSGALG00010023555	HPS4	-0.999188262	0.171115427	-5.839264638	5.24317E-09	1.17683E-08	negative
PRJNA729271	stage_5W_vs_E10	ordinary_muscle_development	ENSGALG00010023556	IVD	-0.058999816	0.121974571	-0.483705869	0.628594627	0.664489434	negative
PRJNA729271	stage_5W_vs_E10	ordinary_muscle_development	ENSGALG00010023559	PJVK	1.631847789	0.694427177	2.349919247	0.018777484	0.024908908	positive
PRJNA729271	stage_5W_vs_E10	ordinary_muscle_development	ENSGALG00010023569	LPCAT3	0.316162594	0.103040892	3.06832159	0.002152648	0.003152757	positive
PRJNA729271	stage_5W_vs_E10	ordinary_muscle_development	ENSGALG00010023571	APLP2	1.044734851	0.098606283	10.59501301	3.14297E-26	1.64112E-25	positive

PRJNA729271	stage_5W_vs_E10	ordinary_muscle_development	ENSGALG00010023572	MYBL2	-1.538322104	0.129502504	-11.87870548	1.52714E-32	1.02506E-31	negative
PRJNA729271	stage_5W_vs_E10	ordinary_muscle_development	ENSGALG00010023574	DEPDC1	-2.840119662	0.215441502	-13.18278807	1.1024E-39	9.54558E-39	negative
PRJNA729271	stage_5W_vs_E10	ordinary_muscle_development	ENSGALG00010023578	SRRD	-0.214640849	0.102831249	-2.087311505	0.036859985	0.047336359	negative
PRJNA729271	stage_5W_vs_E10	ordinary_muscle_development	ENSGALG00010023580	RDM1	-1.475761262	0.167206805	-8.825964098	1.08521E-18	4.08574E-18	negative
PRJNA729271	stage_5W_vs_E10	ordinary_muscle_development	ENSGALG00010023585	TFIP11	-0.090462776	0.096596329	-0.936503245	0.349014143	0.389001153	negative
PRJNA729271	stage_5W_vs_E10	ordinary_muscle_development	ENSGALG00010023588	FARSB	-0.136420309	0.134625833	-1.013329356	0.31090287	0.349940759	negative
PRJNA729271	stage_5W_vs_E10	ordinary_muscle_development	ENSGALG00010023590	S1PR1	1.404762707	0.114898488	12.22612004	2.25453E-34	1.61859E-33	positive
PRJNA729271	stage_5W_vs_E10	ordinary_muscle_development	ENSGALG00010023592	BAHD1	-0.330960019	0.104904674	-3.154864361	0.001605727	0.002383759	negative
PRJNA729271	stage_5W_vs_E10	ordinary_muscle_development	ENSGALG00010023593	CSK	-0.197044566	0.081896159	-2.4060294	0.016126967	0.021562622	negative
PRJNA729271	stage_5W_vs_E10	ordinary_muscle_development	ENSGALG00010023597	MOGAT1	0.455397305	0.339364826	1.341910741	0.179624939	0.21072058	positive
PRJNA729271	stage_5W_vs_E10	ordinary_muscle_development	ENSGALG00010023598	IL10RA	4.33717558	0.348733887	12.43692038	1.64734E-35	1.23849E-34	positive
PRJNA729271	stage_5W_vs_E10	ordinary_muscle_development	ENSGALG00010023600	CHST14	-2.335466843	0.085927494	-27.17950609	1.1347E-162	1.3372E-160	negative
PRJNA729271	stage_5W_vs_E10	ordinary_muscle_development	ENSGALG00010023601	TPST2	1.074568418	0.10534768	10.20020963	1.97845E-24	9.58157E-24	positive
PRJNA729271	stage_5W_vs_E10	ordinary_muscle_development	ENSGALG00010023603	C1S	3.927681495	0.39285306	9.997838619	1.55759E-23	7.2666E-23	positive
PRJNA729271	stage_5W_vs_E10	ordinary_muscle_development	ENSGALG00010023604	ENSGALG00010023604	0.218045903	0.416418477	0.523622064	0.600541432	0.638419366	positive
PRJNA729271	stage_5W_vs_E10	ordinary_muscle_development	ENSGALG00010023605	INSYN1	3.405767207	0.30483142	11.1726252	5.55154E-29	3.23171E-28	positive
PRJNA729271	stage_5W_vs_E10	ordinary_muscle_development	ENSGALG00010023606	LRRC7	-3.516048268	0.370023481	-9.502230119	2.05443E-21	8.75335E-21	negative
PRJNA729271	stage_5W_vs_E10	ordinary_muscle_development	ENSGALG00010023612	GPT2	-0.109700434	0.188593338	-0.581677142	0.560784171	0.600470308	negative
PRJNA729271	stage_5W_vs_E10	ordinary_muscle_development	ENSGALG00010023613	COPB1	-0.814692989	0.052012757	-15.66333013	2.69413E-55	3.76218E-54	negative
PRJNA729271	stage_5W_vs_E10	ordinary_muscle_development	ENSGALG00010023615	PRSS3	4.090708535	5.112064492	0.800206754	0.423591017	0.465394663	positive
PRJNA729271	stage_5W_vs_E10	ordinary_muscle_development	ENSGALG00010023617	ENSGALG00010023617	0.360818621	0.310490312	1.162093008	0.245197687	0.280759972	positive
PRJNA729271	stage_5W_vs_E10	ordinary_muscle_development	ENSGALG00010023620	IRS1	1.149316873	0.14127815	8.135135356	4.11478E-16	1.37049E-15	positive
PRJNA729271	stage_5W_vs_E10	ordinary_muscle_development	ENSGALG00010023621	CD79B	6.213888451	0.86601419	7.175273248	7.21629E-13	2.02753E-12	positive
PRJNA729271	stage_5W_vs_E10	ordinary_muscle_development	ENSGALG00010023623	OSBPL6	0.634216507	0.217715831	2.913047273	0.003579204	0.005129072	positive
PRJNA729271	stage_5W_vs_E10	ordinary_muscle_development	ENSGALG00010023624	PSMA1	0.213557966	0.07096215	3.009462998	0.0026171	0.003806046	positive
PRJNA729271	stage_5W_vs_E10	ordinary_muscle_development	ENSGALG00010023626	LOXL1	0.885954891	0.19517752	4.539226095	5.64611E-06	1.02364E-05	positive
PRJNA729271	stage_5W_vs_E10	ordinary_muscle_development	ENSGALG00010023627	IFT52	0.094178793	0.101490285	0.927958703	0.353428992	0.393588909	positive
PRJNA729271	stage_5W_vs_E10	ordinary_muscle_development	ENSGALG00010023631	ENSGALG00010023631	2.958341904	0.453955878	6.516804937	7.18207E-11	1.80799E-10	positive
PRJNA729271	stage_5W_vs_E10	ordinary_muscle_development	ENSGALG00010023635	SCN4A	9.187977542	0.317426421	28.94521988	3.2233E-184	4.6721E-182	positive
PRJNA729271	stage_5W_vs_E10	ordinary_muscle_development	ENSGALG00010023636	PDE3B	0.337471114	0.08118469	4.156831978	3.22691E-05	5.51907E-05	positive
PRJNA729271	stage_5W_vs_E10	ordinary_muscle_development	ENSGALG00010023637	RHBDD1	0.320675732	0.106334834	3.015716686	0.002563727	0.003734045	positive
PRJNA729271	stage_5W_vs_E10	ordinary_muscle_development	ENSGALG00010023638	DCTN2	0.313114526	0.131170664	2.387077394	0.016982916	0.022642325	positive
PRJNA729271	stage_5W_vs_E10	ordinary_muscle_development	ENSGALG00010023639	KLHL23	0.898133774	0.217502293	4.12930715	3.63858E-05	6.19683E-05	positive
PRJNA729271	stage_5W_vs_E10	ordinary_muscle_development	ENSGALG00010023641	LRRC40	-1.80731647	0.114431183	-15.79391576	3.42634E-56	4.92218E-55	negative
PRJNA729271	stage_5W_vs_E10	ordinary_muscle_development	ENSGALG00010023642	PAX6	-0.869046906	0.943980008	-0.920620033	0.357248839	0.397612811	negative
PRJNA729271	stage_5W_vs_E10	ordinary_muscle_development	ENSGALG00010023643	SCN4B	3.048957323	0.151824769	20.08208104	1.05866E-89	3.58533E-88	positive
PRJNA729271	stage_5W_vs_E10	ordinary_muscle_development	ENSGALG00010023646	DPH5	1.12262121	0.141791236	7.917423096	2.42484E-15	7.76236E-15	positive
PRJNA729271	stage_5W_vs_E10	ordinary_muscle_development	ENSGALG00010023648	SCN2B	2.593422754	0.359246643	7.219059128	5.23485E-13	1.47941E-12	positive
PRJNA729271	stage_5W_vs_E10	ordinary_muscle_development	ENSGALG00010023652	SLC30A7	-1.153437667	0.097264402	-11.85878536	1.93766E-32	1.29729E-31	negative
PRJNA729271	stage_5W_vs_E10	ordinary_muscle_development	ENSGALG00010023655	C1R	1.582392287	0.185136294	8.547174898	1.26138E-17	4.53015E-17	positive
PRJNA729271	stage_5W_vs_E10	ordinary_muscle_development	ENSGALG00010023658	ENSGALG00010023658	-0.922044229	0.330831503	-2.787050871	0.005319013	0.007486802	negative
PRJNA729271	stage_5W_vs_E10	ordinary_muscle_development	ENSGALG00010023660	CRYBB1	-0.631777375	0.584779344	-1.08036883	0.27997797	0.317524504	negative
PRJNA729271	stage_5W_vs_E10	ordinary_muscle_development	ENSGALG00010023663	PHOSPHO2	0.644801427	0.090992701	7.086298333	1.37747E-12	3.81183E-12	positive
PRJNA729271	stage_5W_vs_E10	ordinary_muscle_development	ENSGALG00010023664	SLC6A9	-1.713132613	0.329620496	-5.197287895	2.02217E-07	4.08293E-07	negative
PRJNA729271	stage_5W_vs_E10	ordinary_muscle_development	ENSGALG00010023665	C16orf87	-0.536758638	0.13981208	-3.839143508	0.000123464	0.000201579	negative
PRJNA729271	stage_5W_vs_E10	ordinary_muscle_development	ENSGALG00010023666	ENSGALG00010023666	2.332444767	0.247565039	9.42154343	4.44505E-21	1.86648E-20	positive
PRJNA729271	stage_5W_vs_E10	ordinary_muscle_development	ENSGALG00010023668	RCN1	-1.068612784	0.161424329	-6.619899201	3.59444E-11	9.22965E-11	negative
PRJNA729271	stage_5W_vs_E10	ordinary_muscle_development	ENSGALG00010023670	EXTL2	0.727907431	0.126551704	5.751858012	8.82679E-09	1.95514E-08	positive
PRJNA729271	stage_5W_vs_E10	ordinary_muscle_development	ENSGALG00010023671	ENSGALG00010023671	5.987544796	0.632522859	9.466131877	2.90399E-21	1.22864E-20	positive
PRJNA729271	stage_5W_vs_E10	ordinary_muscle_development	ENSGALG00010023676	ENSGALG00010023676	0.691752426	0.258047263	2.680719869	0.007346399	0.010195906	positive
PRJNA729271	stage_5W_vs_E10	ordinary_muscle_development	ENSGALG00010023677	MN1	0.74583388	0.161036541	4.631457416	3.63101E-06	6.68081E-06	positive
PRJNA729271	stage_5W_vs_E10	ordinary_muscle_development	ENSGALG00010023680	MPZL3	-4.143110335	0.408452187	-10.1434402	3.54394E-24	1.70117E-23	negative
PRJNA729271	stage_5W_vs_E10	ordinary_muscle_development	ENSGALG00010023686	MYLK3	-0.564876608	0.227088114	-2.487477651	0.012865254	0.01739245	negative
PRJNA729271	stage_5W_vs_E10	ordinary_muscle_development	ENSGALG00010023689	WT1	-2.272680912	0.252509806	-9.000366958	2.24964E-19	8.72588E-19	negative
PRJNA729271	stage_5W_vs_E10	ordinary_muscle_development	ENSGALG00010023693	SRSF11	-0.662840558	0.06925872	-9.570499741	1.0639E-21	4.58556E-21	negative
PRJNA729271	stage_5W_vs_E10	ordinary_muscle_development	ENSGALG00010023694	PPCDC	1.610400528	0.151774995	10.61044689	2.66471E-26	1.39743E-25	positive
PRJNA729271	stage_5W_vs_E10	ordinary_muscle_development	ENSGALG00010023695	CLSTN3	-0.636151216	0.131012198	-4.855664031	1.19984E-06	2.28838E-06	negative
PRJNA729271	stage_5W_vs_E10	ordinary_muscle_development	ENSGALG00010023696	LRRC4C	-0.245051028	0.530139212	-0.462239017	0.643909926	0.678946271	negative
PRJNA729271	stage_5W_vs_E10	ordinary_muscle_development	ENSGALG00010023698	ENSGALG00010023698	-1.853536066	0.150027358	-12.3546538	4.59731E-35	3.39643E-34	negative
PRJNA729271	stage_5W_vs_E10	ordinary_muscle_development	ENSGALG00010023699	PPIG	-0.217645737	0.203324877	-1.070433389	0.284424277	0.322088273	negative
PRJNA729271	stage_5W_vs_E10	ordinary_muscle_development	ENSGALG00010023702	COL4A4	-0.260407772	0.274407529	-0.948981875	0.342629831	0.382503034	negative
PRJNA729271	stage_5W_vs_E10	ordinary_muscle_development	ENSGALG00010023703	PITPNB	-1.017166151	0.081376232	-12.49954841	7.50766E-36	5.72755E-35	negative
PRJNA729271	stage_5W_vs_E10	ordinary_muscle_development	ENSGALG00010023704	ANKRD13C	-0.171013027	0.068770672	-2.486714484	0.012892883	0.017428176	negative
PRJNA729271	stage_5W_vs_E10	ordinary_muscle_development	ENSGALG00010023706	CDK5RAP3	-0.481041677	0.129245391	-3.721925189	0.00019771	0.000317787	negative
PRJNA729271	stage_5W_vs_E10	ordinary_muscle_development	ENSGALG00010023707	MPZL2	-0.464562515	0.271282591	-1.712467111	0.086810621	0.106289858	negative
PRJNA729271	stage_5W_vs_E10	ordinary_muscle_development	ENSGALG00010023710	CDC14A	0.518009089	0.120616452	4.294680197	1.74945E-05	3.05964E-05	positive
PRJNA729271	stage_5W_vs_E10	ordinary_muscle_development	ENSGALG00010023711	ENSGALG00010023711	-0.443464338	0.35345509	-1.254655401	0.209603882	0.242940051	negative
PRJNA729271	stage_5W_vs_E10	ordinary_muscle_development	ENSGALG00010023712	ARHGAP40	-2.109154688	0.16605374	-12.7016392	5.7904E-37	4.59397E-36	negative
PRJNA729271	stage_5W_vs_E10	ordinary_muscle_development	ENSGALG00010023714	EIF3M	-0.564538401	0.110637714	-5.102585549	3.35044E-07	6.65269E-07	negative
PRJNA729271	stage_5W_vs_E10	ordinary_muscle_development	ENSGALG00010023715	CD3E	3.606165864	0.339680926	10.61633313	2.50197E-26	1.31446E-25	positive
PRJNA729271	stage_5W_vs_E10	ordinary_muscle_development	ENSGALG00010023716	FASTKD1	-0.54922257	0.107547476	-5.106791816	3.27674E-07	6.50903E-07	negative
PRJNA729271	stage_5W_vs_E10	ordinary_muscle_development	ENSGALG00010023720	SCAMP5	-1.098714474	0.098953352	-11.10335781	1.20818E-28	6.93841E-28	negative
PRJNA729271	stage_5W_vs_E10	ordinary_muscle_development	ENSGALG00010023724	CYP2R1	-0.709377422	0.129337025	-5.484720436	4.14124E-08	8.76566E-08	negative
PRJNA729271	stage_5W_vs_E10	ordinary_muscle_development	ENSGALG00010023726	PNPO	-0.17751352	0.192049792	-0.924309878	0.355325001	0.395609178	negative
PRJNA729271	stage_5W_vs_E10	ordinary_muscle_development	ENSGALG00010023730	SP2	1.367475589	0.180719128	7.56685584	3.82366E-14	1.14868E-13	positive
PRJNA729271	stage_5W_vs_E10	ordinary_muscle_development	ENSGALG00010023731	ACTR5	-0.041658413	0.098974011	-0.420902542	0.673826249	0.707556612	negative
PRJNA729271	stage_5W_vs_E10	ordinary_muscle_development	ENSGALG00010023732	TM2D1	0.759470729	0.077236846	9.83301068	8.11556E-23	3.6807E-22	positive
PRJNA729271	stage_5W_vs_E10	ordinary_muscle_development	ENSGALG00010023733	ORC6	-1.995166095	0.19423432	-10.27195449	9.4275E-25	4.63855E-24	negative
PRJNA729271	stage_5W_vs_E10	ordinary_muscle_development	ENSGALG00010023736	KIF5A	-2.477229288	0.640129702	-3.869886494	0.000108886	0.000178622	negative
PRJNA729271	stage_5W_vs_E10	ordinary_muscle_development	ENSGALG00010023738	ENSGALG00010023738	0.926076053	0.387353397	2.390778187	0.016812707	0.022431902	positive
PRJNA729271	stage_5W_vs_E10	ordinary_muscle_development	ENSGALG00010023740	PPP1R16B	0.993225837	0.099700577	9.962087165	2.23324E-23	1.03653E-22	positive
PRJNA729271	stage_5W_vs_E10	ordinary_muscle_development	ENSGALG00010023743	CD3D	3.175759138	0.217611132	14.59373476	3.07871E-48	3.48291E-47	positive
PRJNA729271	stage_5W_vs_E10	ordinary_muscle_development	ENSGALG00010023744	MT4	1.61552455	0.316310904	5.107394425	3.26631E-07	6.49009E-07	positive
PRJNA729271	stage_5W_vs_E10	ordinary_muscle_development	ENSGALG00010023746	TTC28	-1.436600526	0.089791572	-15.99928021	1.29261E-57	1.93557E-56	negative
PRJNA729271	stage_5W_vs_E10	ordinary_muscle_development	ENSGALG00010023748	ENSGALG00010023748	-1.208866667	0.176481796	-6.849809401	7.39484E-12	1.9682E-11	negative
PRJNA729271	stage_5W_vs_E10	ordinary_muscle_development	ENSGALG00010023749	RPP25	-0.847173622	0.309635507	-2.736035118	0.006218439	0.008698733	negative
PRJNA729271	stage_5W_vs_E10	ordinary_muscle_development	ENSGALG00010023750	ENSGALG00010023750	-2.394715219	0.169274446	-14.14693876	1.95066E-45	2.01818E-44	negative
PRJNA729271	stage_5W_vs_E10	ordinary_muscle_development	ENSGALG00010023751	PRRG4	-0.769736804	0.281558967	-2.733838717	0.00626007	0.008752746	negative
PRJNA729271	stage_5W_vs_E10	ordinary_muscle_development	ENSGALG00010023752	C1QL1	-1.852527081	0.322265255	-5.748454276	9.0063E-09	1.99399E-08	negative
PRJNA729271	stage_5W_vs_E10	ordinary_muscle_development	ENSGALG00010023759	CALCA	-1.193078905	0.276781888	-4.310538212	1.62858E-05	2.85548E-05	negative
PRJNA729271	stage_5W_vs_E10	ordinary_muscle_development	ENSGALG00010023760	ZW10	-0.729727539	0.093590621	-7.797015709	6.33884E-15	1.98148E-14	negative
PRJNA729271	stage_5W_vs_E10	ordinary_muscle_development	ENSGALG00010023762	BBS5	3.004577367	0.152081093	19.75641623	7.0646E-87	2.21169E-85	positive
PRJNA729271	stage_5W_vs_E10	ordinary_muscle_development	ENSGALG00010023764	COX5A	1.096555393	0.177661578	6.172158344	6.7364E-10	1.58952E-09	positive
PRJNA729271	stage_5W_vs_E10	ordinary_muscle_development	ENSGALG00010023766	ENSGALG00010023766	-3.220909464	0.335189387	-9.609222693	7.30987E-22	3.18475E-21	negative
PRJNA729271	stage_5W_vs_E10	ordinary_muscle_development	ENSGALG00010023767	ENSGALG00010023767	3.440089747	0.208815401	16.47431045	5.61248E-61	9.24465E-60	positive
PRJNA729271	stage_5W_vs_E10	ordinary_muscle_development	ENSGALG00010023770	SCRN2	0.369894176	0.13785535	2.683205089	0.007292026	0.010128202	positive
PRJNA729271	stage_5W_vs_E10	ordinary_muscle_development	ENSGALG00010023773	OVOA	1.130630941	0.527919301	2.141673811	0.03221974	0.041650328	positive
PRJNA729271	stage_5W_vs_E10	ordinary_muscle_development	ENSGALG00010023775	ENSGALG00010023775	1.614163826	0.135887417	11.878685	1.52751E-32	1.02506E-31	positive
PRJNA729271	stage_5W_vs_E10	ordinary_muscle_development	ENSGALG00010023777	OAZ2	0.388910568	0.132401708	2.937353102	0.00331027	0.00476299	positive
PRJNA729271	stage_5W_vs_E10	ordinary_muscle_development	ENSGALG00010023778	FANCF	-3.560824089	0.243365444	-14.63159285	1.76604E-48	2.01882E-47	negative
PRJNA729271	stage_5W_vs_E10	ordinary_muscle_development	ENSGALG00010023781	COL4A3	0.620217705	0.264585272	2.344112734	0.019072406	0.025283933	positive
PRJNA729271	stage_5W_vs_E10	ordinary_muscle_development	ENSGALG00010023783	VPS35	-0.348162177	0.0632763	-5.502252429	3.7497E-08	7.95781E-08	negative
PRJNA729271	stage_5W_vs_E10	ordinary_muscle_development	ENSGALG00010023786	ENSGALG00010023786	-2.920711421	0.449142415	-6.502862619	7.88058E-11	1.97799E-10	negative
PRJNA729271	stage_5W_vs_E10	ordinary_muscle_development	ENSGALG00010023788	RTCA	-0.437844435	0.106017274	-4.129934847	3.62866E-05	6.18139E-05	negative
PRJNA729271	stage_5W_vs_E10	ordinary_muscle_development	ENSGALG00010023791	VTI1B	0.278857588	0.081395663	3.425951421	0.00061265	0.000945524	positive
PRJNA729271	stage_5W_vs_E10	ordinary_muscle_development	ENSGALG00010023792	ZNF609	-0.127057187	0.176912693	-0.718191471	0.472639236	0.514602698	negative
PRJNA729271	stage_5W_vs_E10	ordinary_muscle_development	ENSGALG00010023793	RBM45	-0.503869722	0.066781533	-7.545045708	4.52129E-14	1.35293E-13	negative
PRJNA729271	stage_5W_vs_E10	ordinary_muscle_development	ENSGALG00010023794	MT3	1.900220145	0.462501682	4.108569154	3.98118E-05	6.766E-05	positive
PRJNA729271	stage_5W_vs_E10	ordinary_muscle_development	ENSGALG00010023798	ARCN1	-0.848927688	0.057259049	-14.8260877	9.93574E-50	1.18534E-48	negative
PRJNA729271	stage_5W_vs_E10	ordinary_muscle_development	ENSGALG00010023799	NMT1	0.443308285	0.1266537	3.500160565	0.000464978	0.000723549	positive
PRJNA729271	stage_5W_vs_E10	ordinary_muscle_development	ENSGALG00010023800	PEX5	0.399093738	0.072557971	5.50034314	3.79053E-08	8.04094E-08	positive
PRJNA729271	stage_5W_vs_E10	ordinary_muscle_development	ENSGALG00010023802	ENSGALG00010023802	2.389839907	0.274438646	8.708102687	3.09003E-18	1.14057E-17	positive
PRJNA729271	stage_5W_vs_E10	ordinary_muscle_development	ENSGALG00010023805	GALNT6	0.257053664	0.310810864	0.827042081	0.408213211	0.449726418	positive
PRJNA729271	stage_5W_vs_E10	ordinary_muscle_development	ENSGALG00010023806	PATJ	-1.376729691	0.174403395	-7.893938601	2.92796E-15	9.3256E-15	negative
PRJNA729271	stage_5W_vs_E10	ordinary_muscle_development	ENSGALG00010023807	MRPL10	0.823880851	0.125276371	6.576506366	4.8163E-11	1.22478E-10	positive
PRJNA729271	stage_5W_vs_E10	ordinary_muscle_development	ENSGALG00010023808	DHX35	-0.455634678	0.117975673	-3.862107038	0.000112413	0.000184221	negative
PRJNA729271	stage_5W_vs_E10	ordinary_muscle_development	ENSGALG00010023809	CDON	-1.603921854	0.100587699	-15.94550702	3.06145E-57	4.55603E-56	negative
PRJNA729271	stage_5W_vs_E10	ordinary_muscle_development	ENSGALG00010023811	MRPL44	0.634883852	0.084529409	7.510804348	5.87652E-14	1.74621E-13	positive
PRJNA729271	stage_5W_vs_E10	ordinary_muscle_development	ENSGALG00010023813	CELA1	5.505093678	5.26634867	1.045334068	0.295868597	0.334004308	positive
PRJNA729271	stage_5W_vs_E10	ordinary_muscle_development	ENSGALG00010023815	PLCD3	0.595510181	0.138451288	4.301225295	1.69856E-05	2.97423E-05	positive
PRJNA729271	stage_5W_vs_E10	ordinary_muscle_development	ENSGALG00010023817	BBS2	0.013057727	0.117670277	0.110968782	0.911641103	0.923426819	positive
PRJNA729271	stage_5W_vs_E10	ordinary_muscle_development	ENSGALG00010023818	ALOX5	2.878615227	0.182877951	15.74063584	7.96394E-56	1.12842E-54	positive
PRJNA729271	stage_5W_vs_E10	ordinary_muscle_development	ENSGALG00010023819	BIN2	2.460953932	0.225938097	10.89216011	1.25625E-27	6.93423E-27	positive
PRJNA729271	stage_5W_vs_E10	ordinary_muscle_development	ENSGALG00010023820	CYP1C1	2.541025034	0.781077752	3.253229308	0.001141014	0.001719052	positive
PRJNA729271	stage_5W_vs_E10	ordinary_muscle_development	ENSGALG00010023821	KANK4	-0.254570297	0.124101596	-2.051305578	0.040237198	0.051373045	negative
PRJNA729271	stage_5W_vs_E10	ordinary_muscle_development	ENSGALG00010023822	CHEK2	-1.410707622	0.132772258	-10.62501792	2.27968E-26	1.19986E-25	negative
PRJNA729271	stage_5W_vs_E10	ordinary_muscle_development	ENSGALG00010023823	MUC2	3.306109557	0.518754758	6.373164793	1.85167E-10	4.53377E-10	positive
PRJNA729271	stage_5W_vs_E10	ordinary_muscle_development	ENSGALG00010023826	DBT	0.835274483	0.127451643	6.553658013	5.61445E-11	1.42076E-10	positive
PRJNA729271	stage_5W_vs_E10	ordinary_muscle_development	ENSGALG00010023828	CHEK1	-2.918018234	0.197931138	-14.74259316	3.43344E-49	4.05079E-48	negative
PRJNA729271	stage_5W_vs_E10	ordinary_muscle_development	ENSGALG00010023829	USP1	-1.391419975	0.101804859	-13.6675203	1.58712E-42	1.49288E-41	negative
PRJNA729271	stage_5W_vs_E10	ordinary_muscle_development	ENSGALG00010023830	AGFG1	1.314602547	0.112268423	11.70945947	1.14004E-31	7.38048E-31	positive

PRJNA729271	stage_5W_vs_E10	ordinary_muscle_development	ENSGALG00010023831	SATB2	-4.242584136	0.550811518	-7.702424514	1.33508E-14	4.10262E-14	negative
PRJNA729271	stage_5W_vs_E10	ordinary_muscle_development	ENSGALG00010023832	ENSGALG00010023832	-1.675784582	0.64257611	-2.607916098	0.009109528	0.012519447	negative
PRJNA729271	stage_5W_vs_E10	ordinary_muscle_development	ENSGALG00010023833	PIP4K2C	0.008425749	0.10845372	0.077689814	0.938074797	0.946037305	positive
PRJNA729271	stage_5W_vs_E10	ordinary_muscle_development	ENSGALG00010023835	INAFM2	0.368981684	0.121913736	3.02658008	0.002473373	0.003605707	positive
PRJNA729271	stage_5W_vs_E10	ordinary_muscle_development	ENSGALG00010023836	NDUFS3	1.282279087	0.147771197	8.677462952	4.04695E-18	1.48545E-17	positive
PRJNA729271	stage_5W_vs_E10	ordinary_muscle_development	ENSGALG00010023837	STT3A	-0.732603149	0.093087905	-7.870014323	3.54601E-15	1.12496E-14	negative
PRJNA729271	stage_5W_vs_E10	ordinary_muscle_development	ENSGALG00010023840	SMAGP	-3.351055989	0.679557334	-4.931233648	8.17119E-07	1.58069E-06	negative
PRJNA729271	stage_5W_vs_E10	ordinary_muscle_development	ENSGALG00010023841	OSBPL7	0.535206433	0.147533133	3.627703293	0.000285954	0.000453391	positive
PRJNA729271	stage_5W_vs_E10	ordinary_muscle_development	ENSGALG00010023847	PIK3AP1	1.456783928	0.182086768	8.000493075	1.23922E-15	4.03289E-15	positive
PRJNA729271	stage_5W_vs_E10	ordinary_muscle_development	ENSGALG00010023850	DAZAP2	1.596112004	0.200612945	7.956176528	1.77437E-15	5.73073E-15	positive
PRJNA729271	stage_5W_vs_E10	ordinary_muscle_development	ENSGALG00010023851	HSCB	0.100517054	0.150006985	0.670082488	0.502805208	0.543811482	positive
PRJNA729271	stage_5W_vs_E10	ordinary_muscle_development	ENSGALG00010023852	LRRC39	3.194626666	0.190140581	16.80139323	2.38378E-63	4.22408E-62	positive
PRJNA729271	stage_5W_vs_E10	ordinary_muscle_development	ENSGALG00010023854	SHCBP1	-2.748906109	0.224184732	-12.26179004	1.45255E-34	1.05274E-33	negative
PRJNA729271	stage_5W_vs_E10	ordinary_muscle_development	ENSGALG00010023857	ACBD4	0.486937607	0.154014767	3.161629338	0.001568891	0.002331462	positive
PRJNA729271	stage_5W_vs_E10	ordinary_muscle_development	ENSGALG00010023859	ENSGALG00010023859	3.361396718	0.753160363	4.463055789	8.0799E-06	1.44948E-05	positive
PRJNA729271	stage_5W_vs_E10	ordinary_muscle_development	ENSGALG00010023860	TRIP4	0.022720888	0.115041534	0.197501605	0.843435025	0.862353861	positive
PRJNA729271	stage_5W_vs_E10	ordinary_muscle_development	ENSGALG00010023861	ENSGALG00010023861	-0.620925898	0.156739027	-3.961527072	7.44719E-05	0.000124277	negative
PRJNA729271	stage_5W_vs_E10	ordinary_muscle_development	ENSGALG00010023862	TRMT13	-0.736370385	0.135378925	-5.439328065	5.34819E-08	1.12351E-07	negative
PRJNA729271	stage_5W_vs_E10	ordinary_muscle_development	ENSGALG00010023863	EYA2	-4.503442023	0.247234393	-18.21527327	3.90463E-74	9.2783E-73	negative
PRJNA729271	stage_5W_vs_E10	ordinary_muscle_development	ENSGALG00010023864	EPHA1	-5.090976172	0.657627596	-7.741427214	9.8307E-15	3.04804E-14	negative
PRJNA729271	stage_5W_vs_E10	ordinary_muscle_development	ENSGALG00010023866	AMBRA1	0.068748021	0.074742244	0.919801397	0.357676553	0.397891147	positive
PRJNA729271	stage_5W_vs_E10	ordinary_muscle_development	ENSGALG00010023867	ENSGALG00010023867	0.332558832	0.532454527	0.624576964	0.532248743	0.572367796	positive
PRJNA729271	stage_5W_vs_E10	ordinary_muscle_development	ENSGALG00010023869	DOCK7	-1.132481106	0.047889466	-23.64781223	1.2431E-123	8.0084E-122	negative
PRJNA729271	stage_5W_vs_E10	ordinary_muscle_development	ENSGALG00010023871	UQCRFS1	1.278133754	0.14031074	9.109308057	8.2909E-20	3.29071E-19	positive
PRJNA729271	stage_5W_vs_E10	ordinary_muscle_development	ENSGALG00010023874	HEXIM1	2.305615332	0.223831554	10.30067162	6.99711E-25	3.46154E-24	positive
PRJNA729271	stage_5W_vs_E10	ordinary_muscle_development	ENSGALG00010023878	ENSGALG00010023878	2.686224912	1.496973062	1.794437709	0.072743333	0.089982472	positive
PRJNA729271	stage_5W_vs_E10	ordinary_muscle_development	ENSGALG00010023879	EI24	0.700678062	0.116856345	5.996063462	2.02158E-09	4.63359E-09	positive
PRJNA729271	stage_5W_vs_E10	ordinary_muscle_development	ENSGALG00010023880	ENSGALG00010023880	-2.669552618	0.204480861	-13.05526887	5.93003E-39	4.97142E-38	negative
PRJNA729271	stage_5W_vs_E10	ordinary_muscle_development	ENSGALG00010023881	VSTM2B	-1.562523188	0.232642141	-6.716423696	1.86239E-11	4.85614E-11	negative
PRJNA729271	stage_5W_vs_E10	ordinary_muscle_development	ENSGALG00010023883	TBX21	6.450115386	0.341835735	18.86904946	2.04952E-79	5.45097E-78	positive
PRJNA729271	stage_5W_vs_E10	ordinary_muscle_development	ENSGALG00010023884	C2orf69	-0.840919261	0.126789561	-6.632401412	3.30269E-11	8.49556E-11	negative
PRJNA729271	stage_5W_vs_E10	ordinary_muscle_development	ENSGALG00010023887	RASA2	-0.344120634	0.128803362	-2.671674325	0.007547386	0.010463621	negative
PRJNA729271	stage_5W_vs_E10	ordinary_muscle_development	ENSGALG00010023888	CTH	-0.984572489	0.259121922	-3.799649538	0.000144901	0.000235385	negative
PRJNA729271	stage_5W_vs_E10	ordinary_muscle_development	ENSGALG00010023889	SAMD8	2.377850722	0.190040726	12.51232182	6.39279E-36	4.90543E-35	positive
PRJNA729271	stage_5W_vs_E10	ordinary_muscle_development	ENSGALG00010023890	SASS6	-0.954932768	0.120643362	-7.915336148	2.46586E-15	7.89021E-15	negative
PRJNA729271	stage_5W_vs_E10	ordinary_muscle_development	ENSGALG00010023891	ENSGALG00010023891	4.411477927	0.264223416	16.69601425	1.40118E-62	2.41499E-61	positive
PRJNA729271	stage_5W_vs_E10	ordinary_muscle_development	ENSGALG00010023896	POP4	-0.732354987	0.130667018	-5.604742469	2.08565E-08	4.51216E-08	negative
PRJNA729271	stage_5W_vs_E10	ordinary_muscle_development	ENSGALG00010023902	PTGER3	0.130187501	0.235127428	0.553689128	0.579791617	0.618356228	positive
PRJNA729271	stage_5W_vs_E10	ordinary_muscle_development	ENSGALG00010023903	TBKBP1	0.734793866	0.357860475	2.05329707	0.040043775	0.051157634	positive
PRJNA729271	stage_5W_vs_E10	ordinary_muscle_development	ENSGALG00010023909	FOXRED1	0.068427657	0.155048403	0.441330937	0.658973432	0.69331639	positive
PRJNA729271	stage_5W_vs_E10	ordinary_muscle_development	ENSGALG00010023910	RNF7	0.862820014	0.100442362	8.590200342	8.68178E-18	3.13978E-17	positive
PRJNA729271	stage_5W_vs_E10	ordinary_muscle_development	ENSGALG00010023911	TFCP2	-1.042216234	0.123406188	-8.445413113	3.02972E-17	1.06592E-16	negative
PRJNA729271	stage_5W_vs_E10	ordinary_muscle_development	ENSGALG00010023913	ZMYND8	-0.846542868	0.083814175	-10.10023504	5.51099E-24	2.61908E-23	negative
PRJNA729271	stage_5W_vs_E10	ordinary_muscle_development	ENSGALG00010023914	CCDC117	-0.439258025	0.066435194	-6.611827183	3.79605E-11	9.72323E-11	negative
PRJNA729271	stage_5W_vs_E10	ordinary_muscle_development	ENSGALG00010023917	ZRANB2	-0.423072798	0.080272721	-5.270442285	1.36095E-07	2.78394E-07	negative
PRJNA729271	stage_5W_vs_E10	ordinary_muscle_development	ENSGALG00010023918	MFSD14A	-0.849264153	0.081226896	-10.45545497	1.38333E-25	6.99874E-25	negative
PRJNA729271	stage_5W_vs_E10	ordinary_muscle_development	ENSGALG00010023920	ENSGALG00010023920	0.37104316	0.383978824	0.966311517	0.333888325	0.373578639	positive
PRJNA729271	stage_5W_vs_E10	ordinary_muscle_development	ENSGALG00010023921	GRK7	0.942758753	0.179781588	5.243911578	1.57208E-07	3.20001E-07	positive
PRJNA729271	stage_5W_vs_E10	ordinary_muscle_development	ENSGALG00010023923	CSNK1G1	-0.974830916	0.098483518	-9.898416886	4.22916E-23	1.93809E-22	negative
PRJNA729271	stage_5W_vs_E10	ordinary_muscle_development	ENSGALG00010023925	NCOA3	-0.173113295	0.077081154	-2.245857598	0.024713131	0.032338795	negative
PRJNA729271	stage_5W_vs_E10	ordinary_muscle_development	ENSGALG00010023926	ZYX	0.527953316	0.137396104	3.842563965	0.000121756	0.000198923	positive
PRJNA729271	stage_5W_vs_E10	ordinary_muscle_development	ENSGALG00010023927	KPNB1	-0.222184956	0.130283114	-1.705401023	0.088119658	0.107763263	negative
PRJNA729271	stage_5W_vs_E10	ordinary_muscle_development	ENSGALG00010023930	SRPRA	0.448147763	0.131215648	3.415353059	0.000636994	0.000981632	positive
PRJNA729271	stage_5W_vs_E10	ordinary_muscle_development	ENSGALG00010023931	CSRNP2	-1.373947559	0.151601792	-9.062871469	1.27061E-19	4.99795E-19	negative
PRJNA729271	stage_5W_vs_E10	ordinary_muscle_development	ENSGALG00010023937	XBP1	-0.613776439	0.110739035	-5.542548197	2.98101E-08	6.37125E-08	negative
PRJNA729271	stage_5W_vs_E10	ordinary_muscle_development	ENSGALG00010023940	ATP1B3	-0.0320568	0.071748613	-0.446793313	0.6550243	0.689561858	negative
PRJNA729271	stage_5W_vs_E10	ordinary_muscle_development	ENSGALG00010023942	NEGR1	2.692793203	0.220212417	12.22816244	2.19856E-34	1.57919E-33	positive
PRJNA729271	stage_5W_vs_E10	ordinary_muscle_development	ENSGALG00010023945	LETMD1	0.965699033	0.207411416	4.65595892	3.22476E-06	5.96363E-06	positive
PRJNA729271	stage_5W_vs_E10	ordinary_muscle_development	ENSGALG00010023946	ZNRF3	-0.906363443	0.129250519	-7.012454983	2.34172E-12	6.39956E-12	negative
PRJNA729271	stage_5W_vs_E10	ordinary_muscle_development	ENSGALG00010023947	ENSGALG00010023947	-9.439818189	0.943980893	-10.0000098	1.52382E-23	7.11132E-23	negative
PRJNA729271	stage_5W_vs_E10	ordinary_muscle_development	ENSGALG00010023948	SULF2	-1.828370305	0.221204165	-8.265532909	1.39072E-16	4.73872E-16	negative
PRJNA729271	stage_5W_vs_E10	ordinary_muscle_development	ENSGALG00010023953	SLC35A3	0.658196611	0.154493391	4.260354471	2.04103E-05	3.54903E-05	positive
PRJNA729271	stage_5W_vs_E10	ordinary_muscle_development	ENSGALG00010023954	PLEKHF1	0.813354382	0.09531564	8.533273061	1.42264E-17	5.09417E-17	positive
PRJNA729271	stage_5W_vs_E10	ordinary_muscle_development	ENSGALG00010023956	KREMEN1	-1.918232056	0.132380735	-14.49026598	1.39601E-47	1.54232E-46	negative
PRJNA729271	stage_5W_vs_E10	ordinary_muscle_development	ENSGALG00010023957	YBX3	2.322325346	0.138825116	16.72842362	8.13633E-63	1.4141E-61	positive
PRJNA729271	stage_5W_vs_E10	ordinary_muscle_development	ENSGALG00010023962	FPGT	-1.307718515	0.118586436	-11.02755554	2.8141E-28	1.59213E-27	negative
PRJNA729271	stage_5W_vs_E10	ordinary_muscle_development	ENSGALG00010023966	PEAK1	0.927659843	0.097929092	9.472770777	2.72517E-21	1.15467E-20	positive
PRJNA729271	stage_5W_vs_E10	ordinary_muscle_development	ENSGALG00010023967	PRPH	-2.826355155	0.365278124	-7.737542906	1.01356E-14	3.14125E-14	negative
PRJNA729271	stage_5W_vs_E10	ordinary_muscle_development	ENSGALG00010023968	TYW5	0.505896184	0.115090126	4.395652372	1.10441E-05	1.96277E-05	positive
PRJNA729271	stage_5W_vs_E10	ordinary_muscle_development	ENSGALG00010023972	ENSGALG00010023972	3.417761578	0.504847848	6.769884412	1.28885E-11	3.3924E-11	positive
PRJNA729271	stage_5W_vs_E10	ordinary_muscle_development	ENSGALG00010023974	PREX1	0.173885519	0.103024596	1.687805884	0.091448498	0.111634325	positive
PRJNA729271	stage_5W_vs_E10	ordinary_muscle_development	ENSGALG00010023978	TFDP2	0.136203607	0.139819458	0.974139138	0.329987459	0.369613493	positive
PRJNA729271	stage_5W_vs_E10	ordinary_muscle_development	ENSGALG00010023979	FAM118B	-0.733340393	0.117456336	-6.243514979	4.27845E-10	1.02168E-09	negative
PRJNA729271	stage_5W_vs_E10	ordinary_muscle_development	ENSGALG00010023981	ENSGALG00010023981	2.43235899	0.361142998	6.735168632	1.6374E-11	4.28646E-11	positive
PRJNA729271	stage_5W_vs_E10	ordinary_muscle_development	ENSGALG00010023984	FMNL1	3.908271768	0.195963249	19.94390163	1.69328E-88	5.52796E-87	positive
PRJNA729271	stage_5W_vs_E10	ordinary_muscle_development	ENSGALG00010023986	SYCE3	-0.215586113	0.614310912	-0.350939742	0.725633555	0.755716222	negative
PRJNA729271	stage_5W_vs_E10	ordinary_muscle_development	ENSGALG00010023987	TNNI3K	-2.019249438	0.478665963	-4.218493884	2.4594E-05	4.25202E-05	negative
PRJNA729271	stage_5W_vs_E10	ordinary_muscle_development	ENSGALG00010023989	ENSGALG00010023989	-0.156569287	0.101230222	-1.546665452	0.121943938	0.146358978	negative
PRJNA729271	stage_5W_vs_E10	ordinary_muscle_development	ENSGALG00010023991	TSPAN3	0.034523996	0.106110264	0.325359632	0.744908904	0.773401229	positive
PRJNA729271	stage_5W_vs_E10	ordinary_muscle_development	ENSGALG00010023992	FAM131B	-1.384175113	0.140706849	-9.837297355	7.7772E-23	3.53055E-22	negative
PRJNA729271	stage_5W_vs_E10	ordinary_muscle_development	ENSGALG00010023993	C19orf12	-0.327735903	0.175698425	-1.865332053	0.062134875	0.077448191	negative
PRJNA729271	stage_5W_vs_E10	ordinary_muscle_development	ENSGALG00010023996	MYO7B	5.498179664	0.460711776	11.93409839	7.8606E-33	5.32969E-32	positive
PRJNA729271	stage_5W_vs_E10	ordinary_muscle_development	ENSGALG00010023997	ARFGEF2	-0.090330807	0.063009432	-1.43360771	0.151684242	0.179893887	negative
PRJNA729271	stage_5W_vs_E10	ordinary_muscle_development	ENSGALG00010023999	DGCR14	-0.155758036	0.093540719	-1.665136182	0.09588559	0.116775468	negative
PRJNA729271	stage_5W_vs_E10	ordinary_muscle_development	ENSGALG00010024001	ENSGALG00010024001	4.50185847	0.450701849	9.988551148	1.71064E-23	7.97289E-23	positive
PRJNA729271	stage_5W_vs_E10	ordinary_muscle_development	ENSGALG00010024002	DGCR2	-0.297079431	0.06046415	-4.9133153	8.95491E-07	1.72677E-06	negative
PRJNA729271	stage_5W_vs_E10	ordinary_muscle_development	ENSGALG00010024003	ERICH3	-0.772421795	0.300699131	-2.568752997	0.010206518	0.013966155	negative
PRJNA729271	stage_5W_vs_E10	ordinary_muscle_development	ENSGALG00010024004	EIF4B	0.038742295	0.12524748	0.309325942	0.757073599	0.784962935	positive
PRJNA729271	stage_5W_vs_E10	ordinary_muscle_development	ENSGALG00010024005	STYK1	4.540665081	0.249222658	18.21931087	3.62694E-74	8.64679E-73	positive
PRJNA729271	stage_5W_vs_E10	ordinary_muscle_development	ENSGALG00010024008	CCNE1	0.126622319	0.103438626	1.224129932	0.220903216	0.254915382	positive
PRJNA729271	stage_5W_vs_E10	ordinary_muscle_development	ENSGALG00010024009	CSE1L	-0.971809033	0.061649009	-15.7635792	5.54043E-56	7.91998E-55	negative
PRJNA729271	stage_5W_vs_E10	ordinary_muscle_development	ENSGALG00010024010	ENSGALG00010024010	1.61864684	0.088411874	18.30802551	7.14127E-75	1.72234E-73	positive
PRJNA729271	stage_5W_vs_E10	ordinary_muscle_development	ENSGALG00010024011	AGPS	0.523189109	0.070074282	7.466207213	8.25393E-14	2.42925E-13	positive
PRJNA729271	stage_5W_vs_E10	ordinary_muscle_development	ENSGALG00010024013	PTPRJ	0.747888042	0.119607233	6.252866355	4.02987E-10	9.64706E-10	positive
PRJNA729271	stage_5W_vs_E10	ordinary_muscle_development	ENSGALG00010024016	CRYZ	0.07369021	0.103769937	0.710130622	0.477623139	0.519249036	positive
PRJNA729271	stage_5W_vs_E10	ordinary_muscle_development	ENSGALG00010024017	TYW3	-1.867563422	0.140732697	-13.27028803	3.44255E-40	3.0224E-39	negative
PRJNA729271	stage_5W_vs_E10	ordinary_muscle_development	ENSGALG00010024018	KRT18	-5.76932135	1.054900915	-5.469064692	4.52417E-08	9.55805E-08	negative
PRJNA729271	stage_5W_vs_E10	ordinary_muscle_development	ENSGALG00010024019	MAP3K14	1.292472728	0.105754599	12.22143284	2.38838E-34	1.71384E-33	positive
PRJNA729271	stage_5W_vs_E10	ordinary_muscle_development	ENSGALG00010024020	MAIP1	0.233034823	0.122122207	1.908210043	0.056364074	0.070601215	positive
PRJNA729271	stage_5W_vs_E10	ordinary_muscle_development	ENSGALG00010024021	IGLL1	15.27035805	1.20710504	12.65039706	1.11311E-36	8.74499E-36	positive
PRJNA729271	stage_5W_vs_E10	ordinary_muscle_development	ENSGALG00010024023	LHX8	-5.316702283	0.613731035	-8.662919067	4.59835E-18	1.68358E-17	negative
PRJNA729271	stage_5W_vs_E10	ordinary_muscle_development	ENSGALG00010024024	AGL	5.034785616	0.122586797	41.07118996	0	0	positive
PRJNA729271	stage_5W_vs_E10	ordinary_muscle_development	ENSGALG00010024025	CLCN1	7.590316447	0.33341522	22.76535681	1.0111E-114	5.6151E-113	positive
PRJNA729271	stage_5W_vs_E10	ordinary_muscle_development	ENSGALG00010024026	TIRAP	0.436243017	0.104789795	4.163029582	3.14053E-05	5.37703E-05	positive
PRJNA729271	stage_5W_vs_E10	ordinary_muscle_development	ENSGALG00010024029	SCAPER	-0.001522727	0.147378616	-0.010332073	0.991756345	0.992920861	negative
PRJNA729271	stage_5W_vs_E10	ordinary_muscle_development	ENSGALG00010024030	STAU1	0.116263797	0.089689022	1.296299072	0.194872468	0.227100533	positive
PRJNA729271	stage_5W_vs_E10	ordinary_muscle_development	ENSGALG00010024031	LIMS2	0.794865628	0.115170136	6.901664378	5.13968E-12	1.3788E-11	positive
PRJNA729271	stage_5W_vs_E10	ordinary_muscle_development	ENSGALG00010024034	LRP2	-1.224793787	0.249819793	-4.902709159	9.45239E-07	1.81931E-06	negative
PRJNA729271	stage_5W_vs_E10	ordinary_muscle_development	ENSGALG00010024035	OSBPL5	-0.728699817	0.137349486	-5.30544261	1.124E-07	2.3113E-07	negative
PRJNA729271	stage_5W_vs_E10	ordinary_muscle_development	ENSGALG00010024038	NKD1	-2.756225779	0.168578723	-16.34978446	4.36521E-60	7.01483E-59	negative
PRJNA729271	stage_5W_vs_E10	ordinary_muscle_development	ENSGALG00010024039	MRPL45	-0.110647245	0.107032179	-1.033775502	0.301241127	0.3397518	negative
PRJNA729271	stage_5W_vs_E10	ordinary_muscle_development	ENSGALG00010024040	ENSGALG00010024040	-5.168644385	0.981915686	-5.26383727	1.41079E-07	2.88193E-07	negative
PRJNA729271	stage_5W_vs_E10	ordinary_muscle_development	ENSGALG00010024042	DCPS	-0.625757306	0.131822903	-4.746954382	2.06503E-06	3.86975E-06	negative
PRJNA729271	stage_5W_vs_E10	ordinary_muscle_development	ENSGALG00010024045	PSTPIP1	2.941233379	0.2432414	12.09182883	1.16661E-33	8.16117E-33	positive
PRJNA729271	stage_5W_vs_E10	ordinary_muscle_development	ENSGALG00010024046	ENSGALG00010024046	-3.597536385	0.573372016	-6.274349431	3.51099E-10	8.43278E-10	negative
PRJNA729271	stage_5W_vs_E10	ordinary_muscle_development	ENSGALG00010024049	RCN2	-1.065720729	0.144380158	-7.381351753	1.5669E-13	4.53973E-13	negative
PRJNA729271	stage_5W_vs_E10	ordinary_muscle_development	ENSGALG00010024051	ENSGALG00010024051	-6.643160194	0.774532796	-8.576990187	9.73879E-18	3.51503E-17	negative
PRJNA729271	stage_5W_vs_E10	ordinary_muscle_development	ENSGALG00010024052	ENSGALG00010024052	0.794271314	0.166410964	4.772950623	1.81546E-06	3.41489E-06	positive
PRJNA729271	stage_5W_vs_E10	ordinary_muscle_development	ENSGALG00010024053	ISL2	-1.024587157	0.567054377	-1.806858739	0.070784307	0.087693892	negative
PRJNA729271	stage_5W_vs_E10	ordinary_muscle_development	ENSGALG00010024054	ENSGALG00010024054	-6.577998537	1.032516126	-6.37084339	1.87992E-10	4.59983E-10	negative
PRJNA729271	stage_5W_vs_E10	ordinary_muscle_development	ENSGALG00010024057	HSD3B1	-2.70614586	0.821304397	-3.29493653	0.000984439	0.001493245	negative
PRJNA729271	stage_5W_vs_E10	ordinary_muscle_development	ENSGALG00010024058	SLC44A5	0.025920251	0.105936969	0.244676161	0.806707188	0.829541762	positive
PRJNA729271	stage_5W_vs_E10	ordinary_muscle_development	ENSGALG00010024060	FRRS1	0.235410416	0.162097443	1.45227717	0.146424553	0.173983433	positive
PRJNA729271	stage_5W_vs_E10	ordinary_muscle_development	ENSGALG00010024061	ENSGALG00010024061	0.320430507	1.358599654	0.235853517	0.813546338	0.835270872	positive
PRJNA729271	stage_5W_vs_E10	ordinary_muscle_development	ENSGALG00010024062	ST3GAL4	-0.310497533	0.134466009	-2.309115399	0.020937178	0.027594508	negative
PRJNA729271	stage_5W_vs_E10	ordinary_muscle_development	ENSGALG00010024063	BRD7	0.118160123	0.104037168	1.135749126	0.256061574	0.292275968	positive
PRJNA729271	stage_5W_vs_E10	ordinary_muscle_development	ENSGALG00010024064	GPR17	-1.091860488	0.221486309	-4.929697438	8.23571E-07	1.59275E-06	negative
PRJNA729271	stage_5W_vs_E10	ordinary_muscle_development	ENSGALG00010024066	ENSGALG00010024066	8.246281372	2.111216218	3.905938814	9.38603E-05	0.000155026	positive
PRJNA729271	stage_5W_vs_E10	ordinary_muscle_development	ENSGALG00010024068	CASP2	-1.503905693	0.087572001	-17.17336226	4.2035E-66	8.00653E-65	negative
PRJNA729271	stage_5W_vs_E10	ordinary_muscle_development	ENSGALG00010024069	ENSGALG00010024069	9.662818723	2.214488834	4.363453351	1.28025E-05	2.26557E-05	positive
PRJNA729271	stage_5W_vs_E10	ordinary_muscle_development	ENSGALG00010024073	RABGGTB	0.257697484	0.125115853	2.059670915	0.039430012	0.05042241	positive

PRJNA729271	stage_5W_vs_E10	ordinary_muscle_development	ENSGALG00010024074	ADCY7	0.816599665	0.221090022	3.693516588	0.000221174	0.000354089	positive
PRJNA729271	stage_5W_vs_E10	ordinary_muscle_development	ENSGALG00010024075	SPATS2L	-0.499814585	0.137343013	-3.639170093	0.000273518	0.000434196	negative
PRJNA729271	stage_5W_vs_E10	ordinary_muscle_development	ENSGALG00010024077	SFT2D3	0.161103286	0.117189295	1.374726984	0.169216102	0.199478481	positive
PRJNA729271	stage_5W_vs_E10	ordinary_muscle_development	ENSGALG00010024078	KIRREL3	-1.977543952	0.250180814	-7.904458856	2.691E-15	8.58408E-15	negative
PRJNA729271	stage_5W_vs_E10	ordinary_muscle_development	ENSGALG00010024080	TENT4B	-0.073869561	0.088100309	-0.838471077	0.401766185	0.443128965	negative
PRJNA729271	stage_5W_vs_E10	ordinary_muscle_development	ENSGALG00010024081	CARS	-0.281382486	0.099166817	-2.837466162	0.004547317	0.006447555	negative
PRJNA729271	stage_5W_vs_E10	ordinary_muscle_development	ENSGALG00010024083	PALMD	-1.233377445	0.191577021	-6.438023933	1.21039E-10	2.99396E-10	negative
PRJNA729271	stage_5W_vs_E10	ordinary_muscle_development	ENSGALG00010024086	GABARAPL1	-0.20507607	0.215204805	-0.952934436	0.340623291	0.380350867	negative
PRJNA729271	stage_5W_vs_E10	ordinary_muscle_development	ENSGALG00010024089	ENSGALG00010024089	9.739629966	1.750274077	5.56463133	2.62707E-08	5.64558E-08	positive
PRJNA729271	stage_5W_vs_E10	ordinary_muscle_development	ENSGALG00010024091	ETFA	-0.807586053	0.117413653	-6.878127297	6.06445E-12	1.62245E-11	negative
PRJNA729271	stage_5W_vs_E10	ordinary_muscle_development	ENSGALG00010024095	RBM12	-1.218155087	0.053663333	-22.69995214	4.4848E-114	2.4439E-112	negative
PRJNA729271	stage_5W_vs_E10	ordinary_muscle_development	ENSGALG00010024096	MYO1D	-0.061059778	0.220714566	-0.276645891	0.782052023	0.808317461	negative
PRJNA729271	stage_5W_vs_E10	ordinary_muscle_development	ENSGALG00010024099	ENSGALG00010024099	9.321971377	1.908956163	4.883282057	1.04334E-06	2.0007E-06	positive
PRJNA729271	stage_5W_vs_E10	ordinary_muscle_development	ENSGALG00010024101	CNEP1R1	-0.017759126	0.090656823	-0.195893976	0.844693152	0.863518389	negative
PRJNA729271	stage_5W_vs_E10	ordinary_muscle_development	ENSGALG00010024102	SGF29	0.092510088	0.090542637	1.02172955	0.306908921	0.345686908	positive
PRJNA729271	stage_5W_vs_E10	ordinary_muscle_development	ENSGALG00010024104	PLPPR5	-1.320859569	0.170865581	-7.73040166	1.07208E-14	3.3155E-14	negative
PRJNA729271	stage_5W_vs_E10	ordinary_muscle_development	ENSGALG00010024106	WDR33	-0.165034845	0.06392602	-2.581653666	0.009832821	0.013480255	negative
PRJNA729271	stage_5W_vs_E10	ordinary_muscle_development	ENSGALG00010024107	NFE2L2	1.528042959	0.062576752	24.41870045	1.0826E-131	8.0474E-130	positive
PRJNA729271	stage_5W_vs_E10	ordinary_muscle_development	ENSGALG00010024110	CPNE1	-0.611330141	0.126315926	-4.839691719	1.30041E-06	2.474E-06	negative
PRJNA729271	stage_5W_vs_E10	ordinary_muscle_development	ENSGALG00010024112	NAP1L4	0.716095333	0.090410989	7.920445739	2.36661E-15	7.58266E-15	positive
PRJNA729271	stage_5W_vs_E10	ordinary_muscle_development	ENSGALG00010024113	ENSGALG00010024113	1.285351341	0.163485757	7.862160981	3.77562E-15	1.19597E-14	positive
PRJNA729271	stage_5W_vs_E10	ordinary_muscle_development	ENSGALG00010024115	POLR2D	-0.869567425	0.075967244	-11.4466102	2.44522E-30	1.49613E-29	negative
PRJNA729271	stage_5W_vs_E10	ordinary_muscle_development	ENSGALG00010024116	MCAM	0.687291486	0.1829303	3.757122168	0.000171879	0.000277437	positive
PRJNA729271	stage_5W_vs_E10	ordinary_muscle_development	ENSGALG00010024118	TMEM266	-1.199407087	0.139103711	-8.622394574	6.55683E-18	2.38797E-17	negative
PRJNA729271	stage_5W_vs_E10	ordinary_muscle_development	ENSGALG00010024119	AMMECR1L	-0.927508822	0.076497384	-12.12471299	7.81334E-34	5.50848E-33	negative
PRJNA729271	stage_5W_vs_E10	ordinary_muscle_development	ENSGALG00010024121	NRG4	1.108023611	0.167733791	6.605846107	3.95253E-11	1.01115E-10	positive
PRJNA729271	stage_5W_vs_E10	ordinary_muscle_development	ENSGALG00010024122	SOCS7	0.619094503	0.106119634	5.833929855	5.41369E-09	1.21379E-08	positive
PRJNA729271	stage_5W_vs_E10	ordinary_muscle_development	ENSGALG00010024123	PLPPR4	0.0014225	0.388403246	0.003662431	0.99707781	0.997421867	positive
PRJNA729271	stage_5W_vs_E10	ordinary_muscle_development	ENSGALG00010024124	DIDO1	-0.167694334	0.067146978	-2.497421909	0.012510002	0.01693588	negative
PRJNA729271	stage_5W_vs_E10	ordinary_muscle_development	ENSGALG00010024125	ENSGALG00010024125	-2.940377028	1.676827863	-1.753535406	0.079510148	0.09780207	negative
PRJNA729271	stage_5W_vs_E10	ordinary_muscle_development	ENSGALG00010024126	CDK5R1	-1.193405384	0.295048306	-4.044779654	5.23724E-05	8.82718E-05	negative
PRJNA729271	stage_5W_vs_E10	ordinary_muscle_development	ENSGALG00010024128	PHLDA2	-2.527835	0.236722824	-10.67845912	1.28385E-26	6.83164E-26	negative
PRJNA729271	stage_5W_vs_E10	ordinary_muscle_development	ENSGALG00010024129	GID8	-0.059419785	0.101422398	-0.585864528	0.557966556	0.597674049	negative
PRJNA729271	stage_5W_vs_E10	ordinary_muscle_development	ENSGALG00010024131	ENSGALG00010024131	0.224705839	1.302045091	0.172579153	0.862982239	0.879918934	positive
PRJNA729271	stage_5W_vs_E10	ordinary_muscle_development	ENSGALG00010024132	ENSGALG00010024132	-1.366729037	0.151436212	-9.025113727	1.79509E-19	7.00022E-19	negative
PRJNA729271	stage_5W_vs_E10	ordinary_muscle_development	ENSGALG00010024133	PSMD11	0.678686218	0.140828891	4.81922576	1.44116E-06	2.73282E-06	positive
PRJNA729271	stage_5W_vs_E10	ordinary_muscle_development	ENSGALG00010024135	FBXO22	1.260725157	0.087029619	14.48616189	1.48195E-47	1.63477E-46	positive
PRJNA729271	stage_5W_vs_E10	ordinary_muscle_development	ENSGALG00010024136	SAP130	-0.087742286	0.175486655	-0.499994066	0.617079256	0.653794592	negative
PRJNA729271	stage_5W_vs_E10	ordinary_muscle_development	ENSGALG00010024137	ENSGALG00010024137	2.902624481	0.71444423	4.062772657			positive
PRJNA729271	stage_5W_vs_E10	ordinary_muscle_development	ENSGALG00010024138	CBL	0.859074624	0.148535715	5.783623333	7.31085E-09	1.62707E-08	positive
PRJNA729271	stage_5W_vs_E10	ordinary_muscle_development	ENSGALG00010024139	SLC22A18	-2.311469964	0.827101984	-2.794661369	0.00519541	0.007323493	negative
PRJNA729271	stage_5W_vs_E10	ordinary_muscle_development	ENSGALG00010024140	KRT6A	-4.423359971	1.027646742	-4.304358484	1.6747E-05	2.93351E-05	negative
PRJNA729271	stage_5W_vs_E10	ordinary_muscle_development	ENSGALG00010024141	YTHDF1	-0.383804959	0.096307841	-3.985189105	6.74264E-05	0.000112857	negative
PRJNA729271	stage_5W_vs_E10	ordinary_muscle_development	ENSGALG00010024142	DNAJC6	-0.525836389	0.251943657	-2.087118982	0.036877381	0.047354503	negative
PRJNA729271	stage_5W_vs_E10	ordinary_muscle_development	ENSGALG00010024143	UBE2Q2	2.077373779	0.116546584	17.82440731	4.56907E-71	9.95921E-70	positive
PRJNA729271	stage_5W_vs_E10	ordinary_muscle_development	ENSGALG00010024144	HNRNPA3	-1.302901711	0.126839387	-10.27205938	9.41726E-25	4.63508E-24	negative
PRJNA729271	stage_5W_vs_E10	ordinary_muscle_development	ENSGALG00010024145	RAP1GAP3	-0.455689031	0.186297646	-2.446026784	0.014444027	0.019419921	negative
PRJNA729271	stage_5W_vs_E10	ordinary_muscle_development	ENSGALG00010024146	CDKN1C	-2.331816954	0.245400255	-9.502096698	2.05706E-21	8.762E-21	negative
PRJNA729271	stage_5W_vs_E10	ordinary_muscle_development	ENSGALG00010024147	ENSGALG00010024147	-0.830123622	0.290352512	-2.859019944	0.004249521	0.006047796	negative
PRJNA729271	stage_5W_vs_E10	ordinary_muscle_development	ENSGALG00010024148	UGGT1	-0.315870239	0.058653879	-5.385325631	7.23136E-08	1.50537E-07	negative
PRJNA729271	stage_5W_vs_E10	ordinary_muscle_development	ENSGALG00010024149	TCIRG1	1.072941726	0.152003417	7.058668455	1.68106E-12	4.63337E-12	positive
PRJNA729271	stage_5W_vs_E10	ordinary_muscle_development	ENSGALG00010024150	NHERF4	1.390066866	0.350899315	3.961440805	7.44989E-05	0.000124303	positive
PRJNA729271	stage_5W_vs_E10	ordinary_muscle_development	ENSGALG00010024151	LEPROT	-0.439233693	0.148307023	-2.96165133	0.003059941	0.004417713	negative
PRJNA729271	stage_5W_vs_E10	ordinary_muscle_development	ENSGALG00010024152	ZNF423	-1.269046118	0.108154909	-11.73359707	8.57361E-32	5.56785E-31	negative
PRJNA729271	stage_5W_vs_E10	ordinary_muscle_development	ENSGALG00010024153	KCNQ1	1.687500414	0.202471093	8.334525118	7.78099E-17	2.68537E-16	positive
PRJNA729271	stage_5W_vs_E10	ordinary_muscle_development	ENSGALG00010024154	VPS29L	1.286883591	0.464214131	2.772176687	0.00556828	0.007825502	positive
PRJNA729271	stage_5W_vs_E10	ordinary_muscle_development	ENSGALG00010024155	SNX7	-1.132866913	0.16677841	-6.792647296	1.10094E-11	2.90836E-11	negative
PRJNA729271	stage_5W_vs_E10	ordinary_muscle_development	ENSGALG00010024157	CHRNB4	-2.935291902	0.401037356	-7.3192481	2.49364E-13	7.16317E-13	negative
PRJNA729271	stage_5W_vs_E10	ordinary_muscle_development	ENSGALG00010024158	ENSGALG00010024158	0.185333607	0.347589995	0.533196034	0.593897886	0.632110133	positive
PRJNA729271	stage_5W_vs_E10	ordinary_muscle_development	ENSGALG00010024159	NLRX1	0.484797506	0.162566225	2.982153936	0.00286228	0.004145145	positive
PRJNA729271	stage_5W_vs_E10	ordinary_muscle_development	ENSGALG00010024162	SRCIN1	-2.799609949	0.467529783	-5.988088997	2.12321E-09	4.86345E-09	negative
PRJNA729271	stage_5W_vs_E10	ordinary_muscle_development	ENSGALG00010024163	HS6ST1	-0.951397001	0.097203739	-9.787658522	1.27208E-22	5.71921E-22	negative
PRJNA729271	stage_5W_vs_E10	ordinary_muscle_development	ENSGALG00010024164	SNAP29	1.793982184	0.112008994	16.01641195	9.81531E-58	1.47586E-56	positive
PRJNA729271	stage_5W_vs_E10	ordinary_muscle_development	ENSGALG00010024165	LEPR	-1.067809564	0.27860744	-3.832667085	0.000126761	0.000206776	negative
PRJNA729271	stage_5W_vs_E10	ordinary_muscle_development	ENSGALG00010024166	ENSGALG00010024166	6.000350885	0.886523121	6.768408793	1.30206E-11	3.4253E-11	positive
PRJNA729271	stage_5W_vs_E10	ordinary_muscle_development	ENSGALG00010024167	CHRNA3	-0.295950314	0.524296783	-0.56447097	0.572433652	0.611318484	negative
PRJNA729271	stage_5W_vs_E10	ordinary_muscle_development	ENSGALG00010024168	BIRC7	-2.901877945	0.500948195	-5.792770541	6.92347E-09	1.54227E-08	negative
PRJNA729271	stage_5W_vs_E10	ordinary_muscle_development	ENSGALG00010024169	FAM168B	-0.538523253	0.072445167	-7.433529092	1.05738E-13	3.09381E-13	negative
PRJNA729271	stage_5W_vs_E10	ordinary_muscle_development	ENSGALG00010024170	NUP160	-0.832895738	0.086821734	-9.593170999	8.54186E-22	3.70147E-21	negative
PRJNA729271	stage_5W_vs_E10	ordinary_muscle_development	ENSGALG00010024171	ENSGALG00010024171	-2.592127731	0.682169303	-3.799830514	0.000144795	0.000235239	negative
PRJNA729271	stage_5W_vs_E10	ordinary_muscle_development	ENSGALG00010024172	ABCG4	-4.516504671	0.513171252	-8.801164629	1.35404E-18	5.0702E-18	negative
PRJNA729271	stage_5W_vs_E10	ordinary_muscle_development	ENSGALG00010024173	CBLN1	-2.551654769	0.455998278	-5.595755273	2.19663E-08	4.74731E-08	negative
PRJNA729271	stage_5W_vs_E10	ordinary_muscle_development	ENSGALG00010024174	PLEKHB2	1.008799573	0.101311469	9.957407447	2.34087E-23	1.0844E-22	positive
PRJNA729271	stage_5W_vs_E10	ordinary_muscle_development	ENSGALG00010024175	DHRS9	5.566415089	0.395768967	14.06480941	6.2491E-45	6.35654E-44	positive
PRJNA729271	stage_5W_vs_E10	ordinary_muscle_development	ENSGALG00010024177	ENSGALG00010024177	0.943402498	0.415632034	2.269802181	0.023219588	0.030458636	positive
PRJNA729271	stage_5W_vs_E10	ordinary_muscle_development	ENSGALG00010024178	N4BP1	0.991441263	0.120670603	8.216096071	2.10235E-16	7.09678E-16	positive
PRJNA729271	stage_5W_vs_E10	ordinary_muscle_development	ENSGALG00010024179	ATG4C	-0.620248219	0.118258997	-5.244829033	1.56427E-07	3.18636E-07	negative
PRJNA729271	stage_5W_vs_E10	ordinary_muscle_development	ENSGALG00010024180	HINFP	0.298368104	0.140960114	2.116684601	0.034286625	0.044188194	positive
PRJNA729271	stage_5W_vs_E10	ordinary_muscle_development	ENSGALG00010024181	FOXD3	0.268854033	0.316614009	0.849153939	0.395795639	0.437042568	positive
PRJNA729271	stage_5W_vs_E10	ordinary_muscle_development	ENSGALG00010024183	MTX2	0.482767653	0.106426268	4.536170084	5.7285E-06	1.03754E-05	positive
PRJNA729271	stage_5W_vs_E10	ordinary_muscle_development	ENSGALG00010024184	ENSGALG00010024184	-3.193740825	0.225730755	-14.14845233	1.90913E-45	1.97663E-44	negative
PRJNA729271	stage_5W_vs_E10	ordinary_muscle_development	ENSGALG00010024185	CHRNA5	0.862989564	0.153631067	5.617285495	1.93981E-08	4.20606E-08	positive
PRJNA729271	stage_5W_vs_E10	ordinary_muscle_development	ENSGALG00010024186	HAO2	-1.33200074	0.744925012	-1.788100438	0.073759805	0.091154265	negative
PRJNA729271	stage_5W_vs_E10	ordinary_muscle_development	ENSGALG00010024188	TSSC4	-1.341887449	0.085438517	-15.70588417	1.37842E-55	1.93795E-54	negative
PRJNA729271	stage_5W_vs_E10	ordinary_muscle_development	ENSGALG00010024189	KAT7	-0.186923987	0.140607752	-1.329400288	0.183715943	0.215188896	negative
PRJNA729271	stage_5W_vs_E10	ordinary_muscle_development	ENSGALG00010024190	WARS2	-1.245836649	0.113968328	-10.93142872	8.15541E-28	4.5449E-27	negative
PRJNA729271	stage_5W_vs_E10	ordinary_muscle_development	ENSGALG00010024191	DPYD	-0.670479961	0.111971916	-5.987929701	2.12529E-09	4.86745E-09	negative
PRJNA729271	stage_5W_vs_E10	ordinary_muscle_development	ENSGALG00010024192	ALG6	-1.0452122	0.0977935	-10.6879516	1.15902E-26	6.18104E-26	negative
PRJNA729271	stage_5W_vs_E10	ordinary_muscle_development	ENSGALG00010024193	GSTK1	0.145600253	0.126811461	1.148163202	0.250901197	0.286769662	positive
PRJNA729271	stage_5W_vs_E10	ordinary_muscle_development	ENSGALG00010024194	NKAIN4	-0.40012877	0.086289955	-4.637026051	3.53458E-06	6.5067E-06	negative
PRJNA729271	stage_5W_vs_E10	ordinary_muscle_development	ENSGALG00010024195	PSMA4	0.172642497	0.120957181	1.42730258	0.153492697	0.181904721	positive
PRJNA729271	stage_5W_vs_E10	ordinary_muscle_development	ENSGALG00010024196	MRPL21	0.051865133	0.109996533	0.47151607	0.637272247	0.672730407	positive
PRJNA729271	stage_5W_vs_E10	ordinary_muscle_development	ENSGALG00010024197	KRT7	-4.467636868	0.311356189	-14.34895796	1.0815E-46	1.16553E-45	negative
PRJNA729271	stage_5W_vs_E10	ordinary_muscle_development	ENSGALG00010024198	PI4KA	0.138373375	0.064535784	2.144134081	0.032022145	0.041415774	positive
PRJNA729271	stage_5W_vs_E10	ordinary_muscle_development	ENSGALG00010024199	SLC35B1	-0.330181079	0.10237872	-3.225094812	0.00125931	0.001889029	negative
PRJNA729271	stage_5W_vs_E10	ordinary_muscle_development	ENSGALG00010024200	GCHFR	-3.790724964	0.802805274	-4.721848605	2.33711E-06	4.36045E-06	negative
PRJNA729271	stage_5W_vs_E10	ordinary_muscle_development	ENSGALG00010024201	IREB2	-0.295015868	0.069889351	-4.221184848	2.43022E-05	4.20207E-05	negative
PRJNA729271	stage_5W_vs_E10	ordinary_muscle_development	ENSGALG00010024203	SPOP	0.08019073	0.072747667	1.102313437	0.270325429	0.3074676	positive
PRJNA729271	stage_5W_vs_E10	ordinary_muscle_development	ENSGALG00010024204	PTBP2	0.089646342	0.108411897	0.826905014	0.408290901	0.449777825	positive
PRJNA729271	stage_5W_vs_E10	ordinary_muscle_development	ENSGALG00010024205	ANKRD13D	0.238287487	0.169313373	1.407375461	0.159316084	0.188420907	positive
PRJNA729271	stage_5W_vs_E10	ordinary_muscle_development	ENSGALG00010024206	KRT80	-2.426657489	0.62931736	-3.856015492	0.00011525	0.000188699	negative
PRJNA729271	stage_5W_vs_E10	ordinary_muscle_development	ENSGALG00010024207	KCTD18	-0.736038468	0.125459655	-5.866734334	4.44462E-09	1.00085E-08	negative
PRJNA729271	stage_5W_vs_E10	ordinary_muscle_development	ENSGALG00010024208	SERPIND1	2.259907111	0.582318757	3.880876381	0.000104081	0.000171243	positive
PRJNA729271	stage_5W_vs_E10	ordinary_muscle_development	ENSGALG00010024209	RWDD3	-0.485925526	0.144452877	-3.363903408	0.000768484	0.00117626	negative
PRJNA729271	stage_5W_vs_E10	ordinary_muscle_development	ENSGALG00010024210	NXPH3	-4.001330849	1.235522128	-3.238574815	0.001201285	0.00180554	negative
PRJNA729271	stage_5W_vs_E10	ordinary_muscle_development	ENSGALG00010024211	TRPM5	-1.948309019	0.992376535	-1.963275985	0.049614114	0.062584334	negative
PRJNA729271	stage_5W_vs_E10	ordinary_muscle_development	ENSGALG00010024212	RIMS4	0.24877248	0.199182276	1.248968962	0.211676426	0.245165785	positive
PRJNA729271	stage_5W_vs_E10	ordinary_muscle_development	ENSGALG00010024213	ENSGALG00010024213	-0.647834708	0.192559818	-3.364329667	0.000767298	0.001174688	negative
PRJNA729271	stage_5W_vs_E10	ordinary_muscle_development	ENSGALG00010024214	CRABP1	-10.33328126	0.534727618	-19.32438294	3.34965E-83	9.78894E-82	negative
PRJNA729271	stage_5W_vs_E10	ordinary_muscle_development	ENSGALG00010024215	ENSGALG00010024215	0.832943999	0.158277643	5.262549945	1.42071E-07	2.90127E-07	positive
PRJNA729271	stage_5W_vs_E10	ordinary_muscle_development	ENSGALG00010024218	NGFR	-2.565737235	0.233284554	-10.99831595	3.89335E-28	2.18906E-27	negative
PRJNA729271	stage_5W_vs_E10	ordinary_muscle_development	ENSGALG00010024220	SIAH1	2.190517368	0.200510391	10.9247075	8.78229E-28	4.87924E-27	positive
PRJNA729271	stage_5W_vs_E10	ordinary_muscle_development	ENSGALG00010024222	ENSGALG00010024222	-0.568885271	0.315696554	-1.802000255	0.071545365	0.088572304	negative
PRJNA729271	stage_5W_vs_E10	ordinary_muscle_development	ENSGALG00010024223	ENSGALG00010024223	-2.306282105	0.332122496	-6.944070738	3.8096E-12	1.02962E-11	negative
PRJNA729271	stage_5W_vs_E10	ordinary_muscle_development	ENSGALG00010024225	BCDIN3D	-0.133351189	0.182745431	-0.729710112	0.465567399	0.507436608	negative
PRJNA729271	stage_5W_vs_E10	ordinary_muscle_development	ENSGALG00010024226	POU2AF1	4.574081303	1.245590114	3.672220302	0.000240452	0.000383765	positive
PRJNA729271	stage_5W_vs_E10	ordinary_muscle_development	ENSGALG00010024227	MUC6	1.954858604	0.428173458	4.565576324	4.98124E-06	9.05936E-06	positive
PRJNA729271	stage_5W_vs_E10	ordinary_muscle_development	ENSGALG00010024230	LONP2	0.448552733	0.150544751	2.979530874	0.002886901	0.004177462	positive
PRJNA729271	stage_5W_vs_E10	ordinary_muscle_development	ENSGALG00010024233	FAIM2	-4.880986018	0.327295727	-14.9130759	2.70976E-50	3.2759E-49	negative
PRJNA729271	stage_5W_vs_E10	ordinary_muscle_development	ENSGALG00010024234	ENSGALG00010024234	0.953226375	0.270431327	3.524837098	0.000423743	0.000661657	positive
PRJNA729271	stage_5W_vs_E10	ordinary_muscle_development	ENSGALG00010024238	ATF7	1.069092255	0.206542827	5.176128715	2.26537E-07	4.5562E-07	positive
PRJNA729271	stage_5W_vs_E10	ordinary_muscle_development	ENSGALG00010024240	WDR61	-0.884604584	0.12725401	-6.951486901	3.61456E-12	9.78578E-12	negative
PRJNA729271	stage_5W_vs_E10	ordinary_muscle_development	ENSGALG00010024242	KMT2A	0.378865156	0.124940173	3.032372601	0.002426395	0.003540426	positive
PRJNA729271	stage_5W_vs_E10	ordinary_muscle_development	ENSGALG00010024243	MPP3	-0.031711254	0.20784678	-0.152570342	0.878737114	0.893594393	negative
PRJNA729271	stage_5W_vs_E10	ordinary_muscle_development	ENSGALG00010024245	TARBP2	0.326740018	0.165063867	1.979476332	0.047762402	0.060364113	positive
PRJNA729271	stage_5W_vs_E10	ordinary_muscle_development	ENSGALG00010024250	AP2A2	-0.227091912	0.053704178	-4.228570695	2.35181E-05	4.07184E-05	negative
PRJNA729271	stage_5W_vs_E10	ordinary_muscle_development	ENSGALG00010024251	ENSGALG00010024251	3.013425701	0.20613857	14.6184467	2.14232E-48	2.43935E-47	positive
PRJNA729271	stage_5W_vs_E10	ordinary_muscle_development	ENSGALG00010024254	ENSGALG00010024254	2.224428482	0.31006086	7.174167299	7.27486E-13	2.04319E-12	positive
PRJNA729271	stage_5W_vs_E10	ordinary_muscle_development	ENSGALG00010024255	CFAP97D1	0.879723766	0.677865933	1.2977843	0.194361466	0.226614338	positive
PRJNA729271	stage_5W_vs_E10	ordinary_muscle_development	ENSGALG00010024258	DNAJA4	0.427983101	0.151798835	2.819409648	0.004811207	0.006805743	positive
PRJNA729271	stage_5W_vs_E10	ordinary_muscle_development	ENSGALG00010024259	ENSGALG00010024259	1.139183307	0.134819056	8.449720268	2.92002E-17	1.02857E-16	positive

PRJNA729271	stage_5W_vs_E10	ordinary_muscle_development	ENSGALG00010024263	PHKB	4.686693125	0.131312932	35.69102502	5.4474E-279	2.0246E-276	positive
PRJNA729271	stage_5W_vs_E10	ordinary_muscle_development	ENSGALG00010024264	AMER3	-0.542051458	0.305158774	-1.77629321	0.075684602	0.09337376	negative
PRJNA729271	stage_5W_vs_E10	ordinary_muscle_development	ENSGALG00010024266	ENSGALG00010024266	0.142639976	0.24654877	0.578546694	0.562895082	0.602285688	positive
PRJNA729271	stage_5W_vs_E10	ordinary_muscle_development	ENSGALG00010024267	MAP3K12	-2.049464299	0.218308219	-9.387939252	6.11856E-21	2.54779E-20	negative
PRJNA729271	stage_5W_vs_E10	ordinary_muscle_development	ENSGALG00010024270	RAB18L	0.748743375	0.774394965	0.966875314	0.333606369	0.373291981	positive
PRJNA729271	stage_5W_vs_E10	ordinary_muscle_development	ENSGALG00010024274	ACSBG1	0.159132407	0.316840719	0.502247337	0.615493549	0.652209883	positive
PRJNA729271	stage_5W_vs_E10	ordinary_muscle_development	ENSGALG00010024277	ITGB3BP	-1.82906906	0.191963288	-9.528223235	1.59998E-21	6.84525E-21	negative
PRJNA729271	stage_5W_vs_E10	ordinary_muscle_development	ENSGALG00010024280	ENSGALG00010024280	3.389325812	0.123953787	27.34346323	1.2916E-164	1.5732E-162	positive
PRJNA729271	stage_5W_vs_E10	ordinary_muscle_development	ENSGALG00010024282	HIC2	-1.834605347	0.152031873	-12.06724161	1.5732E-33	1.09317E-32	negative
PRJNA729271	stage_5W_vs_E10	ordinary_muscle_development	ENSGALG00010024283	UBE4A	0.317568366	0.11041047	2.87625228	0.004024279	0.005741897	positive
PRJNA729271	stage_5W_vs_E10	ordinary_muscle_development	ENSGALG00010024285	CCND2	-0.702802992	0.097521653	-7.206635334	5.73513E-13	1.6189E-12	negative
PRJNA729271	stage_5W_vs_E10	ordinary_muscle_development	ENSGALG00010024286	ADA	1.58296904	0.179068378	8.840025586	9.56967E-19	3.60854E-18	positive
PRJNA729271	stage_5W_vs_E10	ordinary_muscle_development	ENSGALG00010024288	CHID1	0.338240543	0.134752051	2.510095698	0.012069845	0.016370581	positive
PRJNA729271	stage_5W_vs_E10	ordinary_muscle_development	ENSGALG00010024290	SOST	-0.713200351	0.488269519	-1.460669413	0.144106187	0.1713692	negative
PRJNA729271	stage_5W_vs_E10	ordinary_muscle_development	ENSGALG00010024291	ENSGALG00010024291	1.594943835	0.575270377	2.772511672	0.005562552	0.00781821	positive
PRJNA729271	stage_5W_vs_E10	ordinary_muscle_development	ENSGALG00010024300	MEOX1	-4.098652272	0.296101604	-13.84204684	1.42102E-43	1.38705E-42	negative
PRJNA729271	stage_5W_vs_E10	ordinary_muscle_development	ENSGALG00010024304	PKIG	-0.049182668	0.132402702	-0.371462724	0.710292915	0.741604538	negative
PRJNA729271	stage_5W_vs_E10	ordinary_muscle_development	ENSGALG00010024305	ENSGALG00010024305	5.002271862	0.725359446	6.896266243	5.33871E-12	1.43093E-11	positive
PRJNA729271	stage_5W_vs_E10	ordinary_muscle_development	ENSGALG00010024309	ABCB11	2.724561084	0.389834466	6.989020526	2.76812E-12	7.53642E-12	positive
PRJNA729271	stage_5W_vs_E10	ordinary_muscle_development	ENSGALG00010024311	KLHL6	2.099534488	0.123297705	17.02817175	5.07656E-65	9.37386E-64	positive
PRJNA729271	stage_5W_vs_E10	ordinary_muscle_development	ENSGALG00010024313	SERINC3	2.491752809	0.106756889	23.34044042	1.7234E-120	1.0452E-118	positive
PRJNA729271	stage_5W_vs_E10	ordinary_muscle_development	ENSGALG00010024314	ITFG1	1.7084133	0.145577654	11.73540892	8.39198E-32	5.45478E-31	positive
PRJNA729271	stage_5W_vs_E10	ordinary_muscle_development	ENSGALG00010024321	UBE2L3	0.106547081	0.08220204	1.29616103	0.194920012	0.227137678	positive
PRJNA729271	stage_5W_vs_E10	ordinary_muscle_development	ENSGALG00010024322	IDH3A	1.378410669	0.158897285	8.674853477	4.14083E-18	1.51876E-17	positive
PRJNA729271	stage_5W_vs_E10	ordinary_muscle_development	ENSGALG00010024323	TTPAL	-1.193416685	0.103685857	-11.50992742	1.17577E-30	7.3208E-30	negative
PRJNA729271	stage_5W_vs_E10	ordinary_muscle_development	ENSGALG00010024325	PCBP2	0.750988633	0.169218828	4.437973261	9.08099E-06	1.62325E-05	positive
PRJNA729271	stage_5W_vs_E10	ordinary_muscle_development	ENSGALG00010024326	KLHL24	1.261197954	0.178795214	7.053868629	1.74011E-12	4.79431E-12	positive
PRJNA729271	stage_5W_vs_E10	ordinary_muscle_development	ENSGALG00010024327	PPP1R1B	4.011544265	0.415686311	9.650412241	4.89585E-22	2.14981E-21	positive
PRJNA729271	stage_5W_vs_E10	ordinary_muscle_development	ENSGALG00010024328	YDJC	-2.385325717	0.155830937	-15.30713836	6.85127E-53	8.98725E-52	negative
PRJNA729271	stage_5W_vs_E10	ordinary_muscle_development	ENSGALG00010024330	SDF2L1	-1.409994349	0.103161712	-13.66780684	1.58088E-42	1.48895E-41	negative
PRJNA729271	stage_5W_vs_E10	ordinary_muscle_development	ENSGALG00010024332	EFCAB7	-0.228299022	0.165276681	-1.381314173	0.167182382	0.197273578	negative
PRJNA729271	stage_5W_vs_E10	ordinary_muscle_development	ENSGALG00010024333	ETV4	-4.174823372	0.24277427	-17.19631724	2.82954E-66	5.40371E-65	negative
PRJNA729271	stage_5W_vs_E10	ordinary_muscle_development	ENSGALG00010024334	USP28	1.936181774	0.122511591	15.80407015	2.91662E-56	4.20243E-55	positive
PRJNA729271	stage_5W_vs_E10	ordinary_muscle_development	ENSGALG00010024336	TSPAN4	-0.283757061	0.117099733	-2.423208441	0.015384097	0.020622629	negative
PRJNA729271	stage_5W_vs_E10	ordinary_muscle_development	ENSGALG00010024338	NDUFA9	0.530345352	0.114204481	4.643822609	3.42021E-06	6.3138E-06	positive
PRJNA729271	stage_5W_vs_E10	ordinary_muscle_development	ENSGALG00010024339	TOP3B	-0.235666702	0.087592444	-2.690491244	0.007134691	0.009915373	negative
PRJNA729271	stage_5W_vs_E10	ordinary_muscle_development	ENSGALG00010024340	NETO2	0.140534415	0.370981769	0.378817577	0.704823334	0.736955509	positive
PRJNA729271	stage_5W_vs_E10	ordinary_muscle_development	ENSGALG00010024342	HNF4A	1.018330516	1.391520996	0.731811104	0.464283871	0.506151828	positive
PRJNA729271	stage_5W_vs_E10	ordinary_muscle_development	ENSGALG00010024343	POLR2L	0.540087965	0.126470703	4.270459098	1.95071E-05	3.39891E-05	positive
PRJNA729271	stage_5W_vs_E10	ordinary_muscle_development	ENSGALG00010024344	YEATS2	-0.320350798	0.133698282	-2.396072654	0.016571805	0.022128818	negative
PRJNA729271	stage_5W_vs_E10	ordinary_muscle_development	ENSGALG00010024345	STARD3	0.222821678	0.093008723	2.395707305	0.016588331	0.022146804	positive
PRJNA729271	stage_5W_vs_E10	ordinary_muscle_development	ENSGALG00010024346	PPM1F	0.274479001	0.091603315	2.996387212	0.002731993	0.00396637	positive
PRJNA729271	stage_5W_vs_E10	ordinary_muscle_development	ENSGALG00010024347	OTOG	-1.901369053	0.297637667	-6.388200374	1.67849E-10	4.11601E-10	negative
PRJNA729271	stage_5W_vs_E10	ordinary_muscle_development	ENSGALG00010024349	R3HDML	-5.277186799	0.813727071	-6.485204915	8.86113E-11	2.21566E-10	negative
PRJNA729271	stage_5W_vs_E10	ordinary_muscle_development	ENSGALG00010024350	PGM1	7.538925229	0.135811982	55.51001548	0	0	positive
PRJNA729271	stage_5W_vs_E10	ordinary_muscle_development	ENSGALG00010024351	SP1	1.375048997	0.153184343	8.976433059	2.79687E-19	1.08108E-18	positive
PRJNA729271	stage_5W_vs_E10	ordinary_muscle_development	ENSGALG00010024354	C2CD2L	0.056319225	0.07818864	0.720299329	0.471340718	0.513343129	positive
PRJNA729271	stage_5W_vs_E10	ordinary_muscle_development	ENSGALG00010024356	PARL	0.131026105	0.118012581	1.110272341	0.266881688	0.30386066	positive
PRJNA729271	stage_5W_vs_E10	ordinary_muscle_development	ENSGALG00010024358	MAPK3	0.53596753	0.06180218	8.672307807	4.23449E-18	1.55272E-17	positive
PRJNA729271	stage_5W_vs_E10	ordinary_muscle_development	ENSGALG00010024360	FTL	-0.76445316	0.242236155	-3.155817754	0.001600488	0.002376468	negative
PRJNA729271	stage_5W_vs_E10	ordinary_muscle_development	ENSGALG00010024361	H2AX	-2.467714071	0.218899879	-11.27325464	1.77871E-29	1.0545E-28	negative
PRJNA729271	stage_5W_vs_E10	ordinary_muscle_development	ENSGALG00010024362	DHX8	-0.093503862	0.058117667	-1.608871574	0.107644426	0.13017737	negative
PRJNA729271	stage_5W_vs_E10	ordinary_muscle_development	ENSGALG00010024363	EDEM2	-0.225000703	0.113642334	-1.979902159	0.047714524	0.060308861	negative
PRJNA729271	stage_5W_vs_E10	ordinary_muscle_development	ENSGALG00010024365	ENSGALG00010024365	-0.058117607	0.109743138	-0.529578505	0.596404201	0.63454862	negative
PRJNA729271	stage_5W_vs_E10	ordinary_muscle_development	ENSGALG00010024366	PARD6A	-0.770825318	0.543925415	-1.417152605	0.156438315	0.18519874	negative
PRJNA729271	stage_5W_vs_E10	ordinary_muscle_development	ENSGALG00010024367	ENSGALG00010024367	-1.811970825	0.236102658	-7.674504143	1.6606E-14	5.07278E-14	negative
PRJNA729271	stage_5W_vs_E10	ordinary_muscle_development	ENSGALG00010024368	DPAGT1	0.17391983	0.079258041	2.194349359	0.028210304	0.036699726	positive
PRJNA729271	stage_5W_vs_E10	ordinary_muscle_development	ENSGALG00010024369	MYOD1	0.856823237	0.275577377	3.109192946	0.001875992	0.002764027	positive
PRJNA729271	stage_5W_vs_E10	ordinary_muscle_development	ENSGALG00010024370	HERPUD1	0.269111163	0.09275415	2.901338258	0.003715725	0.0053184	positive
PRJNA729271	stage_5W_vs_E10	ordinary_muscle_development	ENSGALG00010024371	YPEL1	0.48174562	0.105794369	4.553603614	5.27347E-06	9.57761E-06	positive
PRJNA729271	stage_5W_vs_E10	ordinary_muscle_development	ENSGALG00010024373	TCAP	13.31093467	0.478222994	27.83415861	1.6752E-170	2.1876E-168	positive
PRJNA729271	stage_5W_vs_E10	ordinary_muscle_development	ENSGALG00010024374	ENSGALG00010024374	1.670166198	0.581952837	2.869933938	0.004105576	0.005852131	positive
PRJNA729271	stage_5W_vs_E10	ordinary_muscle_development	ENSGALG00010024377	CIB2	-0.428976794	0.171759075	-2.497549518	0.0125055	0.016931367	negative
PRJNA729271	stage_5W_vs_E10	ordinary_muscle_development	ENSGALG00010024378	ZDHHC1	-0.595501671	0.18130515	-3.284527062	0.001021537	0.001547411	negative
PRJNA729271	stage_5W_vs_E10	ordinary_muscle_development	ENSGALG00010024382	ENSGALG00010024382	0.586006189	0.449562916	1.303502064	0.192403412	0.224544127	positive
PRJNA729271	stage_5W_vs_E10	ordinary_muscle_development	ENSGALG00010024383	DUSP28	1.017798633	0.19227391	5.293482783	1.20009E-07	2.46391E-07	positive
PRJNA729271	stage_5W_vs_E10	ordinary_muscle_development	ENSGALG00010024386	TPPP3	1.466610174	0.268417588	5.463912341	4.65754E-08	9.83408E-08	positive
PRJNA729271	stage_5W_vs_E10	ordinary_muscle_development	ENSGALG00010024387	PPIL2	-0.320096799	0.066785266	-4.792925443	1.64367E-06	3.10221E-06	negative
PRJNA729271	stage_5W_vs_E10	ordinary_muscle_development	ENSGALG00010024389	AAAS	-0.058746928	0.107627057	-0.545837912	0.58517736	0.623412159	negative
PRJNA729271	stage_5W_vs_E10	ordinary_muscle_development	ENSGALG00010024391	TBC1D2B	-0.420317938	0.111005875	-3.786447666	0.000152816	0.000247577	negative
PRJNA729271	stage_5W_vs_E10	ordinary_muscle_development	ENSGALG00010024392	CHMP4B	0.313580194	0.084801372	3.697819808	0.000217459	0.000348334	positive
PRJNA729271	stage_5W_vs_E10	ordinary_muscle_development	ENSGALG00010024393	OGFOD1	-1.359252374	0.108551953	-12.52167594	5.68225E-36	4.36482E-35	negative
PRJNA729271	stage_5W_vs_E10	ordinary_muscle_development	ENSGALG00010024396	ENSGALG00010024396	0.323754607	0.332115807	0.974824444	0.32964735	0.369289615	positive
PRJNA729271	stage_5W_vs_E10	ordinary_muscle_development	ENSGALG00010024398	PHB	-0.231535922	0.088556315	-2.61456138	0.008934211	0.012286659	negative
PRJNA729271	stage_5W_vs_E10	ordinary_muscle_development	ENSGALG00010024399	ROR1	-0.794514099	0.168797272	-4.706913149	2.51496E-06	4.68384E-06	negative
PRJNA729271	stage_5W_vs_E10	ordinary_muscle_development	ENSGALG00010024400	KCNA1	-3.031401016	0.399582705	-7.58641697	3.28873E-14	9.91269E-14	negative
PRJNA729271	stage_5W_vs_E10	ordinary_muscle_development	ENSGALG00010024401	KCNC1	2.222068273	0.215385885	10.31668473	5.92332E-25	2.93935E-24	positive
PRJNA729271	stage_5W_vs_E10	ordinary_muscle_development	ENSGALG00010024402	GPC1	-1.63183011	0.120109381	-13.58620028	4.8354E-42	4.48426E-41	negative
PRJNA729271	stage_5W_vs_E10	ordinary_muscle_development	ENSGALG00010024403	HMBS	-0.7109737	0.077450565	-9.179709617	4.32255E-20	1.73225E-19	negative
PRJNA729271	stage_5W_vs_E10	ordinary_muscle_development	ENSGALG00010024406	NUDT21	-1.555847229	0.067712313	-22.97731616	7.8597E-117	4.5753E-115	negative
PRJNA729271	stage_5W_vs_E10	ordinary_muscle_development	ENSGALG00010024407	HIRA	-0.868684691	0.082695159	-10.50466195	8.22168E-26	4.21703E-25	negative
PRJNA729271	stage_5W_vs_E10	ordinary_muscle_development	ENSGALG00010024408	ENSGALG00010024408	-2.521735791	1.394701643	-1.808082613	0.070593644	0.087480112	negative
PRJNA729271	stage_5W_vs_E10	ordinary_muscle_development	ENSGALG00010024410	MYG1	-0.295956599	0.109697352	-2.697937495	0.006977054	0.009703742	negative
PRJNA729271	stage_5W_vs_E10	ordinary_muscle_development	ENSGALG00010024412	PIGU	-0.401380397	0.101743901	-3.945006939	7.97977E-05	0.000132752	negative
PRJNA729271	stage_5W_vs_E10	ordinary_muscle_development	ENSGALG00010024413	AMFR	1.661870764	0.09192813	18.07793505	4.7556E-73	1.08898E-71	positive
PRJNA729271	stage_5W_vs_E10	ordinary_muscle_development	ENSGALG00010024415	SIK2	1.080414525	0.059490794	18.16103722	1.05022E-73	2.43957E-72	positive
PRJNA729271	stage_5W_vs_E10	ordinary_muscle_development	ENSGALG00010024416	VDR	0.594955451	0.287535698	2.069153339	0.0385317	0.049330241	positive
PRJNA729271	stage_5W_vs_E10	ordinary_muscle_development	ENSGALG00010024419	PGAP3	0.476682698	0.126984332	3.75387018	0.000174125	0.000280906	positive
PRJNA729271	stage_5W_vs_E10	ordinary_muscle_development	ENSGALG00010024420	TMEM106C	0.631771043	0.158355247	3.989580729	6.61902E-05	0.000110852	positive
PRJNA729271	stage_5W_vs_E10	ordinary_muscle_development	ENSGALG00010024421	DAPK2	-0.409895825	0.261353055	-1.568360563	0.116797009	0.140624026	negative
PRJNA729271	stage_5W_vs_E10	ordinary_muscle_development	ENSGALG00010024423	SERGEF	-0.055987906	0.119880966	-0.467029155	0.640479019	0.675721915	negative
PRJNA729271	stage_5W_vs_E10	ordinary_muscle_development	ENSGALG00010024424	SAMHD1	1.340519903	0.179211041	7.480118942	7.42554E-14	2.19034E-13	positive
PRJNA729271	stage_5W_vs_E10	ordinary_muscle_development	ENSGALG00010024425	MRPL40	0.462930372	0.089132815	5.193714268	2.06139E-07	4.15807E-07	positive
PRJNA729271	stage_5W_vs_E10	ordinary_muscle_development	ENSGALG00010024426	PHAF1	0.791924152	0.105324159	7.518922155	5.52297E-14	1.64621E-13	positive
PRJNA729271	stage_5W_vs_E10	ordinary_muscle_development	ENSGALG00010024427	C22orf39	0.06627173	0.136711133	0.484757377	0.627848461	0.663797479	positive
PRJNA729271	stage_5W_vs_E10	ordinary_muscle_development	ENSGALG00010024428	SAAL1	-1.652913385	0.065804011	-25.11873309	3.1049E-139	2.5427E-137	negative
PRJNA729271	stage_5W_vs_E10	ordinary_muscle_development	ENSGALG00010024430	ZNF652	-0.016499969	0.064569122	-0.255539622	0.798306345	0.822303189	negative
PRJNA729271	stage_5W_vs_E10	ordinary_muscle_development	ENSGALG00010024431	FEZ1	-3.25157663	0.160929	-20.20503837	8.84009E-91	3.08022E-89	negative
PRJNA729271	stage_5W_vs_E10	ordinary_muscle_development	ENSGALG00010024433	ENSGALG00010024433	-1.813838329	0.169620985	-10.69347837	1.09197E-26	5.83418E-26	negative
PRJNA729271	stage_5W_vs_E10	ordinary_muscle_development	ENSGALG00010024434	B3GNT9	-0.709502349	0.158769571	-4.468755217	7.86761E-06	1.41192E-05	negative
PRJNA729271	stage_5W_vs_E10	ordinary_muscle_development	ENSGALG00010024437	HERC1	0.15455392	0.081579144	1.894527359	0.058155046	0.072731441	positive
PRJNA729271	stage_5W_vs_E10	ordinary_muscle_development	ENSGALG00010024438	RBL1	-2.101019285	0.123122803	-17.06442051	2.73086E-65	5.08136E-64	negative
PRJNA729271	stage_5W_vs_E10	ordinary_muscle_development	ENSGALG00010024439	UFD1	0.568988554	0.112433882	5.060650268	4.17829E-07	8.23668E-07	positive
PRJNA729271	stage_5W_vs_E10	ordinary_muscle_development	ENSGALG00010024440	PKNOX2	1.999126656	0.205861541	9.711025415	2.70601E-22	1.20023E-21	positive
PRJNA729271	stage_5W_vs_E10	ordinary_muscle_development	ENSGALG00010024441	SAA	4.77802891	0.78992156	6.048738444	1.45984E-09	3.37649E-09	positive
PRJNA729271	stage_5W_vs_E10	ordinary_muscle_development	ENSGALG00010024446	GRM4	-5.08666103	0.598208462	-8.503157936	1.84502E-17	6.56282E-17	negative
PRJNA729271	stage_5W_vs_E10	ordinary_muscle_development	ENSGALG00010024449	CACHD1	-0.698163747	0.084926066	-8.220841711	2.0208E-16	6.83263E-16	negative
PRJNA729271	stage_5W_vs_E10	ordinary_muscle_development	ENSGALG00010024451	PHOSPHO1	2.836574036	0.267505469	10.60379829	2.86118E-26	1.4983E-25	positive
PRJNA729271	stage_5W_vs_E10	ordinary_muscle_development	ENSGALG00010024452	SLC16A1	0.601410444	0.251561113	2.390713085	0.016815688	0.022433815	positive
PRJNA729271	stage_5W_vs_E10	ordinary_muscle_development	ENSGALG00010024453	HTR3A	2.184436058	0.61472094	3.553540992	0.000380082	0.000595985	positive
PRJNA729271	stage_5W_vs_E10	ordinary_muscle_development	ENSGALG00010024454	HPS5	0.328608204	0.120586457	2.725083823	0.006428516	0.008977008	positive
PRJNA729271	stage_5W_vs_E10	ordinary_muscle_development	ENSGALG00010024457	CHD6	-0.406530686	0.101112926	-4.020560994	5.80597E-05	9.76306E-05	negative
PRJNA729271	stage_5W_vs_E10	ordinary_muscle_development	ENSGALG00010024459	AHCYL1	-0.340834988	0.093202461	-3.656931186	0.000255253	0.000406537	negative
PRJNA729271	stage_5W_vs_E10	ordinary_muscle_development	ENSGALG00010024460	IMP4	-0.249977364	0.156194625	-1.600422321	0.109504927	0.132316932	negative
PRJNA729271	stage_5W_vs_E10	ordinary_muscle_development	ENSGALG00010024462	ZBTB32	6.684635899	0.428650496	15.59460669	7.92097E-55	1.08932E-53	positive
PRJNA729271	stage_5W_vs_E10	ordinary_muscle_development	ENSGALG00010024463	FBXL22	-1.368539678	0.22976831	-5.956172459	2.58214E-09	5.89048E-09	negative
PRJNA729271	stage_5W_vs_E10	ordinary_muscle_development	ENSGALG00010024465	SENP1	-0.84313051	0.161862468	-5.208931513	1.89931E-07	3.84236E-07	negative
PRJNA729271	stage_5W_vs_E10	ordinary_muscle_development	ENSGALG00010024466	ADGRG3	4.678603449	0.680524199	6.874999389	6.19901E-12	1.65753E-11	positive
PRJNA729271	stage_5W_vs_E10	ordinary_muscle_development	ENSGALG00010024467	ENSGALG00010024467	1.827518501	0.277237492	6.59188801	4.34268E-11	1.10783E-10	positive
PRJNA729271	stage_5W_vs_E10	ordinary_muscle_development	ENSGALG00010024469	GTF2H1	0.175839222	0.128046375	1.37324639	0.169675762	0.199971557	positive
PRJNA729271	stage_5W_vs_E10	ordinary_muscle_development	ENSGALG00010024471	CCDC61	-0.516947589	0.117684905	-4.392641431	1.11982E-05	1.98943E-05	negative
PRJNA729271	stage_5W_vs_E10	ordinary_muscle_development	ENSGALG00010024472	LDHA	7.683831513	0.201250862	38.1803657	5.9637E-319	2.6195E-316	positive
PRJNA729271	stage_5W_vs_E10	ordinary_muscle_development	ENSGALG00010024473	RAVER2	-1.181121692	0.061108221	-19.32835996	3.1012E-83	9.08119E-82	negative
PRJNA729271	stage_5W_vs_E10	ordinary_muscle_development	ENSGALG00010024474	STRIP1	0.312066589	0.103662968	3.010396028	0.002609073	0.003795515	positive
PRJNA729271	stage_5W_vs_E10	ordinary_muscle_development	ENSGALG00010024475	ADGRG1	1.310044897	0.15694438	8.347192171	6.99049E-17	2.41946E-16	positive
PRJNA729271	stage_5W_vs_E10	ordinary_muscle_development	ENSGALG00010024476	KCNB1	-0.999367698	0.182350065	-5.480489956	4.2415E-08	8.97263E-08	negative
PRJNA729271	stage_5W_vs_E10	ordinary_muscle_development	ENSGALG00010024479	ERBB2	-0.123669472	0.129619835	-0.954093726	0.340036202	0.379783075	negative
PRJNA729271	stage_5W_vs_E10	ordinary_muscle_development	ENSGALG00010024480	CDC45	-2.740622478	0.219580085	-12.4811978	9.45534E-36	7.18319E-35	negative
PRJNA729271	stage_5W_vs_E10	ordinary_muscle_development	ENSGALG00010024482	USP3	-1.420769418	0.060261804	-23.57661604	6.6974E-123	4.1487E-121	negative
PRJNA729271	stage_5W_vs_E10	ordinary_muscle_development	ENSGALG00010024486	GPR37L1	-1.805580587	0.355430244	-5.079985784	3.77463E-07	7.46633E-07	negative
PRJNA729271	stage_5W_vs_E10	ordinary_muscle_development	ENSGALG00010024488	ADGRG5	3.196254516	0.412363766	7.751055685	9.11317E-15	2.82981E-14	positive

PRJNA729271	stage_5W_vs_E10	ordinary_muscle_development	ENSGALG00010024490	TGM3	0.10482423	0.974719658	0.107542953	0.914358251	0.925855596	positive
PRJNA729271	stage_5W_vs_E10	ordinary_muscle_development	ENSGALG00010024492	NTF3	-2.192014673	0.252637741	-8.676513103	4.08088E-18	1.49753E-17	negative
PRJNA729271	stage_5W_vs_E10	ordinary_muscle_development	ENSGALG00010024493	JAK1	0.512892117	0.060132312	8.529392895	1.47118E-17	5.26536E-17	positive
PRJNA729271	stage_5W_vs_E10	ordinary_muscle_development	ENSGALG00010024494	DDAH1	-0.5870123	0.178345679	-3.291429895	0.000996795	0.001511353	negative
PRJNA729271	stage_5W_vs_E10	ordinary_muscle_development	ENSGALG00010024495	CLDN5	0.217386249	0.171370699	1.26851469	0.204614208	0.237555703	positive
PRJNA729271	stage_5W_vs_E10	ordinary_muscle_development	ENSGALG00010024496	UEVLD	1.393616218	0.207942122	6.701942838	2.05666E-11	5.34637E-11	positive
PRJNA729271	stage_5W_vs_E10	ordinary_muscle_development	ENSGALG00010024497	ABI3	1.159786063	0.184017531	6.302584624	2.92723E-10	7.0611E-10	positive
PRJNA729271	stage_5W_vs_E10	ordinary_muscle_development	ENSGALG00010024498	CCDC102A	-1.079941884	0.121362803	-8.898458651	5.66276E-19	2.15721E-18	negative
PRJNA729271	stage_5W_vs_E10	ordinary_muscle_development	ENSGALG00010024499	CA12	-1.211658749	0.587214265	-2.063401421	0.039074505	0.049994258	negative
PRJNA729271	stage_5W_vs_E10	ordinary_muscle_development	ENSGALG00010024501	ARL8A	0.097998897	0.090343052	1.084741932	0.27803597	0.315396102	positive
PRJNA729271	stage_5W_vs_E10	ordinary_muscle_development	ENSGALG00010024502	VPS8	0.567127638	0.073031016	7.765572295	8.12774E-15	2.52923E-14	positive
PRJNA729271	stage_5W_vs_E10	ordinary_muscle_development	ENSGALG00010024504	CASC3	0.603678641	0.186634635	3.23454776	0.001218355	0.0018293	positive
PRJNA729271	stage_5W_vs_E10	ordinary_muscle_development	ENSGALG00010024505	DNASE2B	2.648430708	0.209875712	12.61904335	1.65823E-36	1.29226E-35	positive
PRJNA729271	stage_5W_vs_E10	ordinary_muscle_development	ENSGALG00010024506	RAB8B	0.297895642	0.081274605	3.665297928	0.000247051	0.000393906	positive
PRJNA729271	stage_5W_vs_E10	ordinary_muscle_development	ENSGALG00010024508	RPS27L	3.397315737	0.188798994	17.99435297	2.15727E-72	4.87066E-71	positive
PRJNA729271	stage_5W_vs_E10	ordinary_muscle_development	ENSGALG00010024509	SEPTIN5	0.306046648	0.107419413	2.84908135	0.004384567	0.006228371	positive
PRJNA729271	stage_5W_vs_E10	ordinary_muscle_development	ENSGALG00010024510	CNN3	-0.846074466	0.101195795	-8.360767042	6.23118E-17	2.1613E-16	negative
PRJNA729271	stage_5W_vs_E10	ordinary_muscle_development	ENSGALG00010024511	ENSGALG00010024511	-0.584154732	0.170463088	-3.426869347	0.000610583	0.000942534	negative
PRJNA729271	stage_5W_vs_E10	ordinary_muscle_development	ENSGALG00010024512	CCNDBP1	0.327655434	0.11518476	2.844607506	0.004446618	0.006312804	positive
PRJNA729271	stage_5W_vs_E10	ordinary_muscle_development	ENSGALG00010024513	SPTY2D1	0.212429026	0.139239533	1.525637306	0.127100216	0.152333193	positive
PRJNA729271	stage_5W_vs_E10	ordinary_muscle_development	ENSGALG00010024514	FAF1	0.60790661	0.068182037	8.915934955	4.83717E-19	1.84756E-18	positive
PRJNA729271	stage_5W_vs_E10	ordinary_muscle_development	ENSGALG00010024515	C3orf70	-0.851042495	0.156616853	-5.433913909	5.51312E-08	1.15782E-07	negative
PRJNA729271	stage_5W_vs_E10	ordinary_muscle_development	ENSGALG00010024516	EHHADH	0.452830798	0.240315946	1.8843144	0.059522461	0.074338968	positive
PRJNA729271	stage_5W_vs_E10	ordinary_muscle_development	ENSGALG00010024517	LACTB	0.179593303	0.097551003	1.841019549	0.065618694	0.081538189	positive
PRJNA729271	stage_5W_vs_E10	ordinary_muscle_development	ENSGALG00010024518	ENSGALG00010024518	0.290674837	1.33961853	0.216983291	0.828221367	0.848805536	positive
PRJNA729271	stage_5W_vs_E10	ordinary_muscle_development	ENSGALG00010024519	MIEN1	0.925540012	0.138923205	6.662242022	2.69681E-11	6.96177E-11	positive
PRJNA729271	stage_5W_vs_E10	ordinary_muscle_development	ENSGALG00010024520	MAP3K13	-0.366993007	0.137158923	-2.675677231	0.007457843	0.010345625	negative
PRJNA729271	stage_5W_vs_E10	ordinary_muscle_development	ENSGALG00010024521	HEPACAM	-2.344010007	0.586963726	-3.993449512	6.51189E-05	0.000109172	negative
PRJNA729271	stage_5W_vs_E10	ordinary_muscle_development	ENSGALG00010024522	DNTTIP2	-0.146311084	0.075747096	-1.93157351	0.053412168	0.067118281	negative
PRJNA729271	stage_5W_vs_E10	ordinary_muscle_development	ENSGALG00010024523	TOX3	-2.3053164	0.304765818	-7.564222303	3.90192E-14	1.17122E-13	negative
PRJNA729271	stage_5W_vs_E10	ordinary_muscle_development	ENSGALG00010024524	TMEM41A	0.991757336	0.132487341	7.485676219	7.11797E-14	2.10346E-13	positive
PRJNA729271	stage_5W_vs_E10	ordinary_muscle_development	ENSGALG00010024525	GP1BB	0.133633621	0.193237438	0.691551402	0.48921909	0.530621873	positive
PRJNA729271	stage_5W_vs_E10	ordinary_muscle_development	ENSGALG00010024526	TGM6	0.677342561	1.583567651	0.427732002	0.668846248	0.702865815	positive
PRJNA729271	stage_5W_vs_E10	ordinary_muscle_development	ENSGALG00010024527	ATP6V0B	0.670945637	0.099658582	6.732442112	1.66839E-11	4.36443E-11	positive
PRJNA729271	stage_5W_vs_E10	ordinary_muscle_development	ENSGALG00010024528	TBX1	-1.517851957	0.223722408	-6.784532552	1.16463E-11	3.07212E-11	negative
PRJNA729271	stage_5W_vs_E10	ordinary_muscle_development	ENSGALG00010024529	GRB7	-6.380948798	1.212304553	-5.263486625	1.41349E-07	2.88693E-07	negative
PRJNA729271	stage_5W_vs_E10	ordinary_muscle_development	ENSGALG00010024530	ENSGALG00010024530	1.832027249	0.514486918	3.560882087	0.000369611	0.000580446	positive
PRJNA729271	stage_5W_vs_E10	ordinary_muscle_development	ENSGALG00010024531	CEP63	-0.852520924	0.130514284	-6.532012437	6.48918E-11	1.63726E-10	negative
PRJNA729271	stage_5W_vs_E10	ordinary_muscle_development	ENSGALG00010024532	TMEM86A	0.355642368	0.215208446	1.652548371	0.098422813	0.119644303	positive
PRJNA729271	stage_5W_vs_E10	ordinary_muscle_development	ENSGALG00010024533	CDKN2C	3.069038881	0.221389619	13.86261423	1.06716E-43	1.04516E-42	positive
PRJNA729271	stage_5W_vs_E10	ordinary_muscle_development	ENSGALG00010024534	VWF	4.657818093	0.125403811	37.14255602	5.7816E-302	2.3944E-299	positive
PRJNA729271	stage_5W_vs_E10	ordinary_muscle_development	ENSGALG00010024535	ENSGALG00010024535	2.047451856	0.151471907	13.51704024	1.24068E-41	1.1382E-40	positive
PRJNA729271	stage_5W_vs_E10	ordinary_muscle_development	ENSGALG00010024536	URI1	-0.233169371	0.062243203	-3.746101711	0.000179604	0.000289455	negative
PRJNA729271	stage_5W_vs_E10	ordinary_muscle_development	ENSGALG00010024537	EPB42	-1.216869045	0.184587591	-6.592366482	4.3287E-11	1.10485E-10	negative
PRJNA729271	stage_5W_vs_E10	ordinary_muscle_development	ENSGALG00010024540	PTPN5	-2.262352995	0.305092403	-7.415304257	1.21346E-13	3.54048E-13	negative
PRJNA729271	stage_5W_vs_E10	ordinary_muscle_development	ENSGALG00010024541	WIPF2	0.30383502	0.204659584	1.484587309	0.137653179	0.164180271	positive
PRJNA729271	stage_5W_vs_E10	ordinary_muscle_development	ENSGALG00010024542	ZNFX1	4.044281867	0.424789363	9.520675949	1.72056E-21	7.35246E-21	positive
PRJNA729271	stage_5W_vs_E10	ordinary_muscle_development	ENSGALG00010024544	ZNF536	-2.001007733	0.251225441	-7.964988428	1.65239E-15	5.34392E-15	negative
PRJNA729271	stage_5W_vs_E10	ordinary_muscle_development	ENSGALG00010024545	PHACTR3	-0.874569705	0.286072927	-3.057156486	0.002234476	0.003268617	negative
PRJNA729271	stage_5W_vs_E10	ordinary_muscle_development	ENSGALG00010024546	KY	6.50709425	0.40153472	16.20555815	4.60685E-59	7.19573E-58	positive
PRJNA729271	stage_5W_vs_E10	ordinary_muscle_development	ENSGALG00010024547	PFKM	9.707401447	0.197740103	49.09171837	0	0	positive
PRJNA729271	stage_5W_vs_E10	ordinary_muscle_development	ENSGALG00010024548	CDC6	-2.620345317	0.307597657	-8.518742763	1.61295E-17	5.75854E-17	negative
PRJNA729271	stage_5W_vs_E10	ordinary_muscle_development	ENSGALG00010024549	GNB1L	-0.453625329	0.134959274	-3.361201609	0.000776042	0.001187326	negative
PRJNA729271	stage_5W_vs_E10	ordinary_muscle_development	ENSGALG00010024551	RNF11	1.742061082	0.126581771	13.76233772	4.29414E-43	4.11391E-42	positive
PRJNA729271	stage_5W_vs_E10	ordinary_muscle_development	ENSGALG00010024552	CD81	1.043264826	0.066215852	15.75551476	6.29447E-56	8.94494E-55	positive
PRJNA729271	stage_5W_vs_E10	ordinary_muscle_development	ENSGALG00010024553	ASB8	-0.139012338	0.114161623	-1.217680119	0.223345555	0.25754923	negative
PRJNA729271	stage_5W_vs_E10	ordinary_muscle_development	ENSGALG00010024554	ST6GALNAC3	-2.553415183	0.213043427	-11.98542107	4.23686E-33	2.90096E-32	negative
PRJNA729271	stage_5W_vs_E10	ordinary_muscle_development	ENSGALG00010024555	EPHB1	-0.389757019	0.162743927	-2.39490976	0.016624456	0.022190947	negative
PRJNA729271	stage_5W_vs_E10	ordinary_muscle_development	ENSGALG00010024556	ENSGALG00010024556	-0.717239841	0.213585754	-3.358088389	0.000784835	0.00119964	negative
PRJNA729271	stage_5W_vs_E10	ordinary_muscle_development	ENSGALG00010024557	TSPAN32	1.017100707	0.288121217	3.530113884	0.000415381	0.000648879	positive
PRJNA729271	stage_5W_vs_E10	ordinary_muscle_development	ENSGALG00010024558	PFDN5	0.164612754	0.108304876	1.519901597	0.128535709	0.153951835	positive
PRJNA729271	stage_5W_vs_E10	ordinary_muscle_development	ENSGALG00010024559	ENSGALG00010024559	-2.19244963	0.338012268	-6.486301945	8.79688E-11	2.19998E-10	negative
PRJNA729271	stage_5W_vs_E10	ordinary_muscle_development	ENSGALG00010024560	A4GNT	-1.398610207	0.617192582	-2.266083954	0.023446243	0.030739264	negative
PRJNA729271	stage_5W_vs_E10	ordinary_muscle_development	ENSGALG00010024561	ROBO3	-0.240651876	0.528983682	-0.454932514	0.649157791	0.684081583	negative
PRJNA729271	stage_5W_vs_E10	ordinary_muscle_development	ENSGALG00010024563	ESPL1	-1.514944812	0.166328165	-9.108167648	8.3785E-20	3.32366E-19	negative
PRJNA729271	stage_5W_vs_E10	ordinary_muscle_development	ENSGALG00010024564	PPP1R3D	2.803451649	0.19757134	14.1895664	1.06314E-45	1.10865E-44	positive
PRJNA729271	stage_5W_vs_E10	ordinary_muscle_development	ENSGALG00010024565	FAM217B	0.429832267	0.139152192	3.088936369	0.002008745	0.002951521	positive
PRJNA729271	stage_5W_vs_E10	ordinary_muscle_development	ENSGALG00010024566	MTSS2	0.263515977	0.221473206	1.189832315	0.234112306	0.269001813	positive
PRJNA729271	stage_5W_vs_E10	ordinary_muscle_development	ENSGALG00010024567	IKZF3	4.188426932	0.379470672	11.03755109	2.51802E-28	1.42796E-27	positive
PRJNA729271	stage_5W_vs_E10	ordinary_muscle_development	ENSGALG00010024568	DZIP1L	0.347787635	0.268348159	1.296031379	0.194964674	0.22716298	positive
PRJNA729271	stage_5W_vs_E10	ordinary_muscle_development	ENSGALG00010024569	ASCL2	-2.072642202	0.319416373	-6.488841454	8.64989E-11	2.16509E-10	negative
PRJNA729271	stage_5W_vs_E10	ordinary_muscle_development	ENSGALG00010024570	TPM1	5.385721941	0.164139833	32.8117913	3.9978E-236	9.9911E-234	positive
PRJNA729271	stage_5W_vs_E10	ordinary_muscle_development	ENSGALG00010024571	KCNA3	-1.428626763	0.361487391	-3.952079104	7.74751E-05	0.000128977	negative
PRJNA729271	stage_5W_vs_E10	ordinary_muscle_development	ENSGALG00010024573	ENSGALG00010024573	2.006110973	0.265281176	7.562206273	3.96289E-14	1.18879E-13	positive
PRJNA729271	stage_5W_vs_E10	ordinary_muscle_development	ENSGALG00010024575	TXNRD2	0.564778743	0.125171128	4.512052835	6.42032E-06	1.15879E-05	positive
PRJNA729271	stage_5W_vs_E10	ordinary_muscle_development	ENSGALG00010024576	CSAD	-0.491318046	0.163865138	-2.998307331	0.002714838	0.003942253	negative
PRJNA729271	stage_5W_vs_E10	ordinary_muscle_development	ENSGALG00010024578	ENSGALG00010024578	1.869146211	0.593877438	3.147360198	0.001647519	0.002442048	positive
PRJNA729271	stage_5W_vs_E10	ordinary_muscle_development	ENSGALG00010024579	ZNF740	-0.477241338	0.106609604	-4.476532305	7.58652E-06	1.36317E-05	negative
PRJNA729271	stage_5W_vs_E10	ordinary_muscle_development	ENSGALG00010024580	IGF2	-0.251145429	0.194301011	-1.29255853	0.19616379	0.228403417	negative
PRJNA729271	stage_5W_vs_E10	ordinary_muscle_development	ENSGALG00010024581	RARA	0.490701328	0.172449443	2.845479336	0.004434464	0.006296782	positive
PRJNA729271	stage_5W_vs_E10	ordinary_muscle_development	ENSGALG00010024582	TLN2	1.167986014	0.107399003	10.87520351	1.51318E-27	8.32391E-27	positive
PRJNA729271	stage_5W_vs_E10	ordinary_muscle_development	ENSGALG00010024583	ITGB7	3.837745663	0.47590448	8.064109135	7.37718E-16	2.42587E-15	positive
PRJNA729271	stage_5W_vs_E10	ordinary_muscle_development	ENSGALG00010024585	TBCCD1	-1.488335313	0.133743524	-11.12827945	9.13823E-29	5.27304E-28	negative
PRJNA729271	stage_5W_vs_E10	ordinary_muscle_development	ENSGALG00010024586	DLGAP4	0.117671039	0.096042121	1.225202422	0.220498962	0.254549262	positive
PRJNA729271	stage_5W_vs_E10	ordinary_muscle_development	ENSGALG00010024587	ENSGALG00010024587	3.610364954	0.178212776	20.25873253	2.97523E-91	1.04675E-89	positive
PRJNA729271	stage_5W_vs_E10	ordinary_muscle_development	ENSGALG00010024588	TTC39A	-4.413633335	0.409212958	-10.78566366	4.02329E-27	2.1801E-26	negative
PRJNA729271	stage_5W_vs_E10	ordinary_muscle_development	ENSGALG00010024589	ADORA1	1.837006814	0.460942139	3.985330608	6.73862E-05	0.000112803	positive
PRJNA729271	stage_5W_vs_E10	ordinary_muscle_development	ENSGALG00010024590	MRPL23	-0.068424331	0.104630674	-0.653960529	0.513137236	0.553664153	negative
PRJNA729271	stage_5W_vs_E10	ordinary_muscle_development	ENSGALG00010024592	COMT	-0.321016747	0.198438052	-1.617717688	0.105723452	0.12803588	negative
PRJNA729271	stage_5W_vs_E10	ordinary_muscle_development	ENSGALG00010024593	SRSF6	-0.771481971	0.075126696	-10.26907893	9.71275E-25	4.77404E-24	negative
PRJNA729271	stage_5W_vs_E10	ordinary_muscle_development	ENSGALG00010024594	TNNI2	6.341878989	0.183198532	34.61752075	1.377E-262	4.3392E-260	positive
PRJNA729271	stage_5W_vs_E10	ordinary_muscle_development	ENSGALG00010024595	MYBPHL	4.880292142	0.316412444	15.42383124	1.13186E-53	1.50241E-52	positive
PRJNA729271	stage_5W_vs_E10	ordinary_muscle_development	ENSGALG00010024596	EPS15	1.12073104	0.066627063	16.82095814	1.71365E-63	3.05151E-62	positive
PRJNA729271	stage_5W_vs_E10	ordinary_muscle_development	ENSGALG00010024597	DNAJB11	-1.199065564	0.101723317	-11.78751937	4.52678E-32	2.98661E-31	negative
PRJNA729271	stage_5W_vs_E10	ordinary_muscle_development	ENSGALG00010024598	LRRC3C	1.289428205	0.236715152	5.447172232	5.11769E-08	1.07649E-07	positive
PRJNA729271	stage_5W_vs_E10	ordinary_muscle_development	ENSGALG00010024599	ENSGALG00010024599	0.025910618	0.219228565	0.118189972	0.905917135	0.918142139	positive
PRJNA729271	stage_5W_vs_E10	ordinary_muscle_development	ENSGALG00010024600	CD9	0.211722572	0.334810995	0.632364453	0.527148756	0.567725776	positive
PRJNA729271	stage_5W_vs_E10	ordinary_muscle_development	ENSGALG00010024601	ENSGALG00010024601	-0.975690227	0.514154985	-1.897657818	0.057741173	0.072232528	negative
PRJNA729271	stage_5W_vs_E10	ordinary_muscle_development	ENSGALG00010024602	PROCR	0.797080933	0.244309548	3.262586093	0.001104006	0.001667282	positive
PRJNA729271	stage_5W_vs_E10	ordinary_muscle_development	ENSGALG00010024603	ENSGALG00010024603	0.096336152	1.130129148	0.085243489	0.932067817	0.941157994	positive
PRJNA729271	stage_5W_vs_E10	ordinary_muscle_development	ENSGALG00010024604	MFSD5	1.17152078	0.168787531	6.940801665	3.89881E-12	1.05239E-11	positive
PRJNA729271	stage_5W_vs_E10	ordinary_muscle_development	ENSGALG00010024605	LSP1P1	-0.479762911	0.195909234	-2.448904021	0.014329163	0.019276214	negative
PRJNA729271	stage_5W_vs_E10	ordinary_muscle_development	ENSGALG00010024607	RARG	-0.606508992	0.142208377	-4.264931527	1.99964E-05	3.48039E-05	negative
PRJNA729271	stage_5W_vs_E10	ordinary_muscle_development	ENSGALG00010024608	DENND2D	0.907177388	0.335720352	2.702181689	0.006888611	0.009587174	positive
PRJNA729271	stage_5W_vs_E10	ordinary_muscle_development	ENSGALG00010024609	PRR33	14.39956423	0.836411041	17.21589448	2.01806E-66	3.86927E-65	positive
PRJNA729271	stage_5W_vs_E10	ordinary_muscle_development	ENSGALG00010024610	PFKL	-0.945770263	0.138810413	-6.813395618	9.53215E-12	2.5264E-11	negative
PRJNA729271	stage_5W_vs_E10	ordinary_muscle_development	ENSGALG00010024611	ENSGALG00010024611	-1.626967429	0.263680213	-6.170229493	6.81909E-10	1.60798E-09	negative
PRJNA729271	stage_5W_vs_E10	ordinary_muscle_development	ENSGALG00010024612	ENSGALG00010024612	-2.122453277	0.668285637	-3.175967223	0.001493378	0.002222663	negative
PRJNA729271	stage_5W_vs_E10	ordinary_muscle_development	ENSGALG00010024613	ENSGALG00010024613	-0.29394994	0.096220252	-3.054969561	0.002250834	0.003291881	negative
PRJNA729271	stage_5W_vs_E10	ordinary_muscle_development	ENSGALG00010024614	ENSGALG00010024614	-6.347154745	0.878344323	-7.226271728	4.96434E-13	1.40586E-12	negative
PRJNA729271	stage_5W_vs_E10	ordinary_muscle_development	ENSGALG00010024615	SYT8	-3.827682323	0.809584601	-4.727958414	2.26789E-06	4.23676E-06	negative
PRJNA729271	stage_5W_vs_E10	ordinary_muscle_development	ENSGALG00010024616	ARVCF	-0.835520054	0.098952695	-8.443631065	3.07629E-17	1.08204E-16	negative
PRJNA729271	stage_5W_vs_E10	ordinary_muscle_development	ENSGALG00010024618	VPS13C	-0.030381779	0.122869282	-0.247269115	0.80469996	0.828006383	negative
PRJNA729271	stage_5W_vs_E10	ordinary_muscle_development	ENSGALG00010024619	ENSGALG00010024619	0.377833315	0.209592182	1.802707102	0.071434226	0.088453708	positive
PRJNA729271	stage_5W_vs_E10	ordinary_muscle_development	ENSGALG00010024620	MSANTD2	-0.618228669	0.131039714	-4.717872542	2.38324E-06	4.44538E-06	negative
PRJNA729271	stage_5W_vs_E10	ordinary_muscle_development	ENSGALG00010024621	THRA	0.962959369	0.185335122	5.195773787	2.0387E-07	4.11573E-07	positive
PRJNA729271	stage_5W_vs_E10	ordinary_muscle_development	ENSGALG00010024622	PRMT8	-1.368631651	0.215163279	-6.360897913	2.00578E-10	4.89704E-10	negative
PRJNA729271	stage_5W_vs_E10	ordinary_muscle_development	ENSGALG00010024623	NRDC	1.050911757	0.109389724	9.607042786	7.46625E-22	3.25092E-21	positive
PRJNA729271	stage_5W_vs_E10	ordinary_muscle_development	ENSGALG00010024624	CTSD	0.645035627	0.128725177	5.010951582	5.41615E-07	1.06019E-06	positive
PRJNA729271	stage_5W_vs_E10	ordinary_muscle_development	ENSGALG00010024625	SPRYD3	0.573569835	0.079238018	7.238568667	4.53445E-13	1.28699E-12	positive
PRJNA729271	stage_5W_vs_E10	ordinary_muscle_development	ENSGALG00010024626	TSHZ3	-1.991745547	0.102719925	-19.39006039	9.36269E-84	2.764E-82	negative
PRJNA729271	stage_5W_vs_E10	ordinary_muscle_development	ENSGALG00010024627	CEPT1	0.334400112	0.079049128	4.230282109	2.33398E-05	4.04195E-05	positive
PRJNA729271	stage_5W_vs_E10	ordinary_muscle_development	ENSGALG00010024628	TANGO2	0.150891258	0.111888826	1.348582009	0.177471262	0.208300754	positive
PRJNA729271	stage_5W_vs_E10	ordinary_muscle_development	ENSGALG00010024629	ENSGALG00010024629	0.963965134	0.172475807	5.588987527	2.28397E-08	4.93239E-08	positive
PRJNA729271	stage_5W_vs_E10	ordinary_muscle_development	ENSGALG00010024630	CFAP410	-1.271042169	0.10096488	-12.58895345	2.42872E-36	1.88561E-35	negative
PRJNA729271	stage_5W_vs_E10	ordinary_muscle_development	ENSGALG00010024631	EPB41L1	-0.578944207	0.150327927	-3.851208624	0.000117536	0.000192268	negative
PRJNA729271	stage_5W_vs_E10	ordinary_muscle_development	ENSGALG00010024632	IFITM10	-1.506451231	0.466030142	-3.232518874	0.00122704	0.001841768	negative
PRJNA729271	stage_5W_vs_E10	ordinary_muscle_development	ENSGALG00010024633	NRGN	4.101002821	0.278284335	14.73673617	3.74449E-49	4.40913E-48	positive
PRJNA729271	stage_5W_vs_E10	ordinary_muscle_development	ENSGALG00010024634	ENSGALG00010024634	2.146218573	0.163120528	13.15725614	1.54599E-39	1.32858E-38	positive
PRJNA729271	stage_5W_vs_E10	ordinary_muscle_development	ENSGALG00010024635	ENSGALG00010024635	-1.312870343	0.203869625	-6.439754528	1.19667E-10	2.96103E-10	negative
PRJNA729271	stage_5W_vs_E10	ordinary_muscle_development	ENSGALG00010024636	PANX3	-1.809360845	0.312043142	-5.798431695	6.6938E-09	1.49203E-08	negative

PRJNA729271	stage_5W_vs_E10	ordinary_muscle_development	ENSGALG00010024637	DUSP8	1.986543379	0.276939983	7.17319094	7.32695E-13	2.05742E-12	positive
PRJNA729271	stage_5W_vs_E10	ordinary_muscle_development	ENSGALG00010024638	ZNF507	0.327741118	0.129624841	2.528382016	0.011458959	0.015594556	positive
PRJNA729271	stage_5W_vs_E10	ordinary_muscle_development	ENSGALG00010024639	AAR2	-0.370788968	0.076729575	-4.832412667	1.34888E-06	2.5642E-06	negative
PRJNA729271	stage_5W_vs_E10	ordinary_muscle_development	ENSGALG00010024640	GSDMA	-0.645999429	0.295960525	-2.182721595	0.029056319	0.037739367	negative
PRJNA729271	stage_5W_vs_E10	ordinary_muscle_development	ENSGALG00010024641	TRA2B	-1.681058607	0.089235833	-18.83838084	3.65992E-79	9.69844E-78	negative
PRJNA729271	stage_5W_vs_E10	ordinary_muscle_development	ENSGALG00010024642	RAB3B	0.677271822	0.224963331	3.010587636	0.002607427	0.003793501	positive
PRJNA729271	stage_5W_vs_E10	ordinary_muscle_development	ENSGALG00010024643	ETV7	2.933377435	0.374022284	7.84278788	4.40652E-15	1.38853E-14	positive
PRJNA729271	stage_5W_vs_E10	ordinary_muscle_development	ENSGALG00010024644	EIF6	-0.693579541	0.075924196	-9.135158167	6.53112E-20	2.60007E-19	negative
PRJNA729271	stage_5W_vs_E10	ordinary_muscle_development	ENSGALG00010024645	PARP11	0.343990113	0.118480556	2.903346531	0.003691979	0.005285455	positive
PRJNA729271	stage_5W_vs_E10	ordinary_muscle_development	ENSGALG00010024646	RORA	1.506573763	0.192743722	7.816460883	5.43292E-15	1.70344E-14	positive
PRJNA729271	stage_5W_vs_E10	ordinary_muscle_development	ENSGALG00010024647	RPUSD4	-0.388142524	0.136265432	-2.848429851	0.004393554	0.006240525	negative
PRJNA729271	stage_5W_vs_E10	ordinary_muscle_development	ENSGALG00010024648	MMP24	-1.738070084	0.127109834	-13.67376569	1.45658E-42	1.37634E-41	negative
PRJNA729271	stage_5W_vs_E10	ordinary_muscle_development	ENSGALG00010024649	NCOR2	0.335412708	0.129601604	2.588028987	0.009652686	0.01324332	positive
PRJNA729271	stage_5W_vs_E10	ordinary_muscle_development	ENSGALG00010024650	PRKAG1	1.113649264	0.120602833	9.234022406	2.60655E-20	1.05536E-19	positive
PRJNA729271	stage_5W_vs_E10	ordinary_muscle_development	ENSGALG00010024651	PSMD3	0.230544411	0.127717999	1.805105104	0.071058237	0.088010693	positive
PRJNA729271	stage_5W_vs_E10	ordinary_muscle_development	ENSGALG00010024652	PUS3	-0.713789847	0.128450768	-5.556913819	2.74586E-08	5.88803E-08	negative
PRJNA729271	stage_5W_vs_E10	ordinary_muscle_development	ENSGALG00010024653	RFLNA	-3.103376243	0.256869987	-12.08150582	1.32275E-33	9.22234E-33	negative
PRJNA729271	stage_5W_vs_E10	ordinary_muscle_development	ENSGALG00010024654	CCDC92	-0.622093633	0.274002961	-2.270390188	0.023183919	0.0304146	negative
PRJNA729271	stage_5W_vs_E10	ordinary_muscle_development	ENSGALG00010024655	IGF2BP2	-2.387271898	0.168916997	-14.13281046	2.38435E-45	2.46338E-44	negative
PRJNA729271	stage_5W_vs_E10	ordinary_muscle_development	ENSGALG00010024656	HYLS1	-0.710815185	0.185016897	-3.841893345	0.000122089	0.000199404	negative
PRJNA729271	stage_5W_vs_E10	ordinary_muscle_development	ENSGALG00010024657	DPY19L3	-0.880816479	0.129033989	-6.826236127	8.71714E-12	2.31165E-11	negative
PRJNA729271	stage_5W_vs_E10	ordinary_muscle_development	ENSGALG00010024659	BEND5	0.385051818	0.108260276	3.556723027	0.00037551	0.00058939	positive
PRJNA729271	stage_5W_vs_E10	ordinary_muscle_development	ENSGALG00010024660	KMT2D	1.189550911	0.157088445	7.572491476	3.66133E-14	1.10152E-13	positive
PRJNA729271	stage_5W_vs_E10	ordinary_muscle_development	ENSGALG00010024662	MOB2	0.42206129	0.071312179	5.918502199	3.24887E-09	7.37084E-09	positive
PRJNA729271	stage_5W_vs_E10	ordinary_muscle_development	ENSGALG00010024663	ICE2	-1.025770364	0.125051118	-8.202808448	2.34835E-16	7.91061E-16	negative
PRJNA729271	stage_5W_vs_E10	ordinary_muscle_development	ENSGALG00010024664	DNAH10	-3.539795146	0.250092403	-14.1539491	1.76557E-45	1.83061E-44	negative
PRJNA729271	stage_5W_vs_E10	ordinary_muscle_development	ENSGALG00010024665	MAP1LC3A	3.128008254	0.147618331	21.18983616	1.18509E-99	4.93617E-98	positive
PRJNA729271	stage_5W_vs_E10	ordinary_muscle_development	ENSGALG00010024666	MED24	-1.224867444	0.116613062	-10.50368996	8.30681E-26	4.25768E-25	negative
PRJNA729271	stage_5W_vs_E10	ordinary_muscle_development	ENSGALG00010024667	RBM7	0.806111164	0.135503772	5.948994278	2.69795E-09	6.145E-09	positive
PRJNA729271	stage_5W_vs_E10	ordinary_muscle_development	ENSGALG00010024668	ENSGALG00010024668	0.731799497	0.185106555	3.953395919	7.70498E-05	0.000128313	positive
PRJNA729271	stage_5W_vs_E10	ordinary_muscle_development	ENSGALG00010024669	STIL	-1.319716569	0.183317298	-7.199083678	6.06185E-13	1.70947E-12	negative
PRJNA729271	stage_5W_vs_E10	ordinary_muscle_development	ENSGALG00010024670	SENP2	0.40564064	0.095983622	4.226144323	2.3773E-05	4.114E-05	positive
PRJNA729271	stage_5W_vs_E10	ordinary_muscle_development	ENSGALG00010024671	KCTD20	1.527999754	0.280175472	5.45372421	4.93257E-08	1.03966E-07	positive
PRJNA729271	stage_5W_vs_E10	ordinary_muscle_development	ENSGALG00010024672	DYNLRB1	-0.468177268	0.105708113	-4.428962475	9.46875E-06	1.6911E-05	negative
PRJNA729271	stage_5W_vs_E10	ordinary_muscle_development	ENSGALG00010024673	REXO2	1.284675487	0.097599138	13.16277494	1.43709E-39	1.23918E-38	positive
PRJNA729271	stage_5W_vs_E10	ordinary_muscle_development	ENSGALG00010024674	DHH	2.566520879	0.323505864	7.933460146	2.13123E-15	6.85275E-15	positive
PRJNA729271	stage_5W_vs_E10	ordinary_muscle_development	ENSGALG00010024675	NR1D1	5.05049439	0.408429867	12.36563433	4.01033E-35	2.97339E-34	positive
PRJNA729271	stage_5W_vs_E10	ordinary_muscle_development	ENSGALG00010024676	ENSGALG00010024676	0.772618986	0.166219059	4.648197329	3.34849E-06	6.18454E-06	positive
PRJNA729271	stage_5W_vs_E10	ordinary_muscle_development	ENSGALG00010024677	LMBR1L	-2.89959076	0.414789195	-6.990516616	2.73876E-12	7.45928E-12	negative
PRJNA729271	stage_5W_vs_E10	ordinary_muscle_development	ENSGALG00010024678	AMOTL2	0.502604707	0.091631945	5.485038053	4.1338E-08	8.7512E-08	positive
PRJNA729271	stage_5W_vs_E10	ordinary_muscle_development	ENSGALG00010024679	MSL1	0.479700888	0.11082208	4.328567792	1.50082E-05	2.64138E-05	positive
PRJNA729271	stage_5W_vs_E10	ordinary_muscle_development	ENSGALG00010024680	BRSK1	-1.03946587	0.267212558	-3.890033756	0.00010023	0.000165076	negative
PRJNA729271	stage_5W_vs_E10	ordinary_muscle_development	ENSGALG00010024681	ATP6V0A2	0.930730132	0.085401788	10.89825112	1.17494E-27	6.50276E-27	positive
PRJNA729271	stage_5W_vs_E10	ordinary_muscle_development	ENSGALG00010024682	ENSGALG00010024682	-2.182345642	0.17940484	-12.16436325	4.81183E-34	3.42067E-33	negative
PRJNA729271	stage_5W_vs_E10	ordinary_muscle_development	ENSGALG00010024683	SHISA4	3.053195135	0.174726981	17.47409082	2.25715E-68	4.53778E-67	positive
PRJNA729271	stage_5W_vs_E10	ordinary_muscle_development	ENSGALG00010024684	ENSGALG00010024684	-0.653168537	0.329879556	-1.98002127	0.047701139	0.060297201	negative
PRJNA729271	stage_5W_vs_E10	ordinary_muscle_development	ENSGALG00010024685	ENSGALG00010024685	-1.441370292	0.223556073	-6.447466513	1.13735E-10	2.81811E-10	negative
PRJNA729271	stage_5W_vs_E10	ordinary_muscle_development	ENSGALG00010024686	CMPK1	-0.982534349	0.133738766	-7.346668257	2.03208E-13	5.86054E-13	negative
PRJNA729271	stage_5W_vs_E10	ordinary_muscle_development	ENSGALG00010024687	RYK	-0.678902671	0.107988999	-6.28677624	3.24126E-10	7.80044E-10	negative
PRJNA729271	stage_5W_vs_E10	ordinary_muscle_development	ENSGALG00010024688	CADM1	-2.210141898	0.132527665	-16.67683426	1.93189E-62	3.30611E-61	negative
PRJNA729271	stage_5W_vs_E10	ordinary_muscle_development	ENSGALG00010024689	FOXD2	-0.160408541	0.312729262	-0.512931024	0.607999571	0.645144593	negative
PRJNA729271	stage_5W_vs_E10	ordinary_muscle_development	ENSGALG00010024690	TUBA1C	-4.534984308	0.651664889	-6.959074187	3.42516E-12	9.29037E-12	negative
PRJNA729271	stage_5W_vs_E10	ordinary_muscle_development	ENSGALG00010024691	TCTN2	-0.858379413	0.152424611	-5.631501417	1.78648E-08	3.88172E-08	negative
PRJNA729271	stage_5W_vs_E10	ordinary_muscle_development	ENSGALG00010024692	DRAM2	1.80822594	0.104909368	17.2360769	1.42377E-66	2.75167E-65	positive
PRJNA729271	stage_5W_vs_E10	ordinary_muscle_development	ENSGALG00010024693	ANXA2	0.835349057	0.201463651	4.146400862	3.37742E-05	5.76628E-05	positive
PRJNA729271	stage_5W_vs_E10	ordinary_muscle_development	ENSGALG00010024694	GTF2H3	-0.89021571	0.099281111	-8.966617129	3.05761E-19	1.17935E-18	negative
PRJNA729271	stage_5W_vs_E10	ordinary_muscle_development	ENSGALG00010024695	TRABD2B	-0.437451536	0.195834358	-2.233783391	0.025497329	0.033298836	negative
PRJNA729271	stage_5W_vs_E10	ordinary_muscle_development	ENSGALG00010024696	TRADD	0.260834515	0.114274953	2.282516921	0.02245884	0.029487399	positive
PRJNA729271	stage_5W_vs_E10	ordinary_muscle_development	ENSGALG00010024697	IGF2BP1	-2.949110879	0.128543788	-22.94246136	1.7523E-116	1.0119E-114	negative
PRJNA729271	stage_5W_vs_E10	ordinary_muscle_development	ENSGALG00010024698	ENSGALG00010024698	-1.798696973	0.372964505	-4.822702835	1.41626E-06	2.6877E-06	negative
PRJNA729271	stage_5W_vs_E10	ordinary_muscle_development	ENSGALG00010024699	FBXL8	-0.743136851	0.135613431	-5.479817478	4.25765E-08	9.00417E-08	negative
PRJNA729271	stage_5W_vs_E10	ordinary_muscle_development	ENSGALG00010024700	TOLLIP	0.786376975	0.084146246	9.345360207	9.15772E-21	3.77857E-20	positive
PRJNA729271	stage_5W_vs_E10	ordinary_muscle_development	ENSGALG00010024701	EIF2B1	-0.325807901	0.102340718	-3.18356082	0.001454755	0.002169412	negative
PRJNA729271	stage_5W_vs_E10	ordinary_muscle_development	ENSGALG00010024702	INAVA	-2.920277133	0.886461934	-3.294306299	0.000986649	0.00149644	negative
PRJNA729271	stage_5W_vs_E10	ordinary_muscle_development	ENSGALG00010024703	HSF4	-0.114467415	0.57864715	-0.197819026	0.84318666	0.862209781	negative
PRJNA729271	stage_5W_vs_E10	ordinary_muscle_development	ENSGALG00010024705	SLCO2A1	2.754828411	0.234160026	11.76472543	5.9319E-32	3.88185E-31	positive
PRJNA729271	stage_5W_vs_E10	ordinary_muscle_development	ENSGALG00010024706	ENSGALG00010024706	2.533584904	0.929095755	2.726936259	0.006392539	0.008929348	positive
PRJNA729271	stage_5W_vs_E10	ordinary_muscle_development	ENSGALG00010024707	ENSGALG00010024707	-1.376643107	0.376255158	-3.658801956	0.000253397	0.000403625	negative
PRJNA729271	stage_5W_vs_E10	ordinary_muscle_development	ENSGALG00010024708	BNIP2	0.793641111	0.062574407	12.6831583	7.3317E-37	5.80409E-36	positive
PRJNA729271	stage_5W_vs_E10	ordinary_muscle_development	ENSGALG00010024709	TEAD4	0.961344718	0.096775276	9.933784313	2.96774E-23	1.36911E-22	positive
PRJNA729271	stage_5W_vs_E10	ordinary_muscle_development	ENSGALG00010024710	HSDL1	-0.753238861	0.129837256	-5.801407732	6.57605E-09	1.46646E-08	negative
PRJNA729271	stage_5W_vs_E10	ordinary_muscle_development	ENSGALG00010024711	DDX55	-0.408339553	0.092620908	-4.408718954	1.03984E-05	1.85074E-05	negative
PRJNA729271	stage_5W_vs_E10	ordinary_muscle_development	ENSGALG00010024712	LRIF1	0.490221445	0.098540175	4.974838375	6.53021E-07	1.27157E-06	positive
PRJNA729271	stage_5W_vs_E10	ordinary_muscle_development	ENSGALG00010024713	RND2	-2.263021553	0.493973015	-4.581265546	4.62171E-06	8.43298E-06	negative
PRJNA729271	stage_5W_vs_E10	ordinary_muscle_development	ENSGALG00010024714	SRPRB	-0.850408144	0.175335307	-4.850181974	1.23348E-06	2.351E-06	negative
PRJNA729271	stage_5W_vs_E10	ordinary_muscle_development	ENSGALG00010024715	DNAAF1	-0.244065477	0.17944707	-1.360097307	0.173799133	0.204332747	negative
PRJNA729271	stage_5W_vs_E10	ordinary_muscle_development	ENSGALG00010024716	GTF2A2	-0.642694744	0.078671191	-8.169378613	3.09982E-16	1.03841E-15	negative
PRJNA729271	stage_5W_vs_E10	ordinary_muscle_development	ENSGALG00010024717	TF	2.998033393	0.652400294	4.595389394	4.31942E-06	7.89732E-06	positive
PRJNA729271	stage_5W_vs_E10	ordinary_muscle_development	ENSGALG00010024718	ENSGALG00010024718	0.920879371	0.192536661	4.782878052	1.72803E-06	3.25592E-06	positive
PRJNA729271	stage_5W_vs_E10	ordinary_muscle_development	ENSGALG00010024719	PYM1	-1.152485038	0.13991978	-8.236755634	1.76943E-16	5.99531E-16	negative
PRJNA729271	stage_5W_vs_E10	ordinary_muscle_development	ENSGALG00010024720	BUD13	-0.348258723	0.108820111	-3.20031581	0.001372771	0.00205243	negative
PRJNA729271	stage_5W_vs_E10	ordinary_muscle_development	ENSGALG00010024721	ENSGALG00010024721	2.36122117	0.737483824	3.201726048	0.001366068	0.002043042	positive
PRJNA729271	stage_5W_vs_E10	ordinary_muscle_development	ENSGALG00010024722	BRCA1	-3.177541598	0.263770728	-12.04660435	2.02106E-33	1.39901E-32	negative
PRJNA729271	stage_5W_vs_E10	ordinary_muscle_development	ENSGALG00010024723	ITCH	0.452943769	0.10049396	4.507174066	6.56967E-06	1.18531E-05	positive
PRJNA729271	stage_5W_vs_E10	ordinary_muscle_development	ENSGALG00010024724	HOXD3	-1.87138459	0.262621617	-7.125782762	1.03491E-12	2.88592E-12	negative
PRJNA729271	stage_5W_vs_E10	ordinary_muscle_development	ENSGALG00010024726	TMED2	-0.381590044	0.058802254	-6.489377865	8.61915E-11	2.15777E-10	negative
PRJNA729271	stage_5W_vs_E10	ordinary_muscle_development	ENSGALG00010024728	SLC5A9	-2.08210501	0.832190993	-2.501955715	0.012350937	0.016733044	negative
PRJNA729271	stage_5W_vs_E10	ordinary_muscle_development	ENSGALG00010024729	FOXM1	-2.425526695	0.171198283	-14.16793824	1.44686E-45	1.50447E-44	negative
PRJNA729271	stage_5W_vs_E10	ordinary_muscle_development	ENSGALG00010024731	AOX1	4.669070186	0.197449433	23.64691617	1.2698E-123	8.144E-122	positive
PRJNA729271	stage_5W_vs_E10	ordinary_muscle_development	ENSGALG00010024732	AHCY	-1.444771277	0.224995729	-6.421327581	1.35091E-10	3.33301E-10	negative
PRJNA729271	stage_5W_vs_E10	ordinary_muscle_development	ENSGALG00010024733	MBTPS1	-0.187766753	0.088176756	-2.129435927	0.033218211	0.042868409	negative
PRJNA729271	stage_5W_vs_E10	ordinary_muscle_development	ENSGALG00010024734	ZPR1	-0.010189223	0.115086218	-0.088535565	0.929451018	0.938973551	negative
PRJNA729271	stage_5W_vs_E10	ordinary_muscle_development	ENSGALG00010024735	OTUD7A	1.339813777	0.180563575	7.420177492	1.16963E-13	3.41536E-13	positive
PRJNA729271	stage_5W_vs_E10	ordinary_muscle_development	ENSGALG00010024736	DGKA	0.042539858	0.147415969	0.288570217	0.77291029	0.79983827	positive
PRJNA729271	stage_5W_vs_E10	ordinary_muscle_development	ENSGALG00010024737	STK38	-0.710925648	0.062669783	-11.34399408	7.94344E-30	4.77166E-29	negative
PRJNA729271	stage_5W_vs_E10	ordinary_muscle_development	ENSGALG00010024738	NBR1	0.160891341	0.078476397	2.050187664	0.040346121	0.051498505	positive
PRJNA729271	stage_5W_vs_E10	ordinary_muscle_development	ENSGALG00010024739	ASIP	-7.362922469	0.613794019	-11.99575466	3.73993E-33	2.56921E-32	negative
PRJNA729271	stage_5W_vs_E10	ordinary_muscle_development	ENSGALG00010024741	RILPL2	-1.015466979	0.158413649	-6.410224044	1.45306E-10	3.57895E-10	negative
PRJNA729271	stage_5W_vs_E10	ordinary_muscle_development	ENSGALG00010024742	PAK2	0.110625127	0.069854499	1.583650709	0.113273227	0.136653802	positive
PRJNA729271	stage_5W_vs_E10	ordinary_muscle_development	ENSGALG00010024744	KLF13	3.06487261	0.129826513	23.60744763	3.2318E-123	2.0192E-121	positive
PRJNA729271	stage_5W_vs_E10	ordinary_muscle_development	ENSGALG00010024745	PMEL	-7.300264332	0.723567017	-10.08927185	6.16249E-24	2.91913E-23	negative
PRJNA729271	stage_5W_vs_E10	ordinary_muscle_development	ENSGALG00010024746	HOXD4	-1.32435733	0.331832349	-3.991043471	6.57832E-05	0.000110209	negative
PRJNA729271	stage_5W_vs_E10	ordinary_muscle_development	ENSGALG00010024747	TULP3	-0.27691037	0.087336614	-3.170610317	0.001521191	0.002262896	negative
PRJNA729271	stage_5W_vs_E10	ordinary_muscle_development	ENSGALG00010024748	TRPM1	0.092904862	0.39413654	0.235717456	0.813651924	0.835318874	positive
PRJNA729271	stage_5W_vs_E10	ordinary_muscle_development	ENSGALG00010024749	APOA5	2.271474701	0.674106075	3.369610192	0.000752746	0.001152657	positive
PRJNA729271	stage_5W_vs_E10	ordinary_muscle_development	ENSGALG00010024750	APOA4	6.199940524	1.548299685	4.004354315	6.21871E-05	0.000104414	positive
PRJNA729271	stage_5W_vs_E10	ordinary_muscle_development	ENSGALG00010024751	MTMR10	1.383305855	0.055905167	24.74379237	3.616E-135	2.8179E-133	positive
PRJNA729271	stage_5W_vs_E10	ordinary_muscle_development	ENSGALG00010024752	MPP2	-0.115398088	0.218884377	-0.527210255	0.598047588	0.636048117	negative
PRJNA729271	stage_5W_vs_E10	ordinary_muscle_development	ENSGALG00010024753	HOXD8	-1.541617594	0.18176432	-8.481409283	2.22482E-17	7.8771E-17	negative
PRJNA729271	stage_5W_vs_E10	ordinary_muscle_development	ENSGALG00010024754	EIF2S2	1.18841807	0.144591527	8.219140457	2.04967E-16	6.92056E-16	positive
PRJNA729271	stage_5W_vs_E10	ordinary_muscle_development	ENSGALG00010024755	PIGX	0.387665421	0.15795839	2.454224947	0.014118863	0.019005184	positive
PRJNA729271	stage_5W_vs_E10	ordinary_muscle_development	ENSGALG00010024756	CDK2	-2.756805685	0.172020423	-16.02603709	8.40764E-58	1.26683E-56	negative
PRJNA729271	stage_5W_vs_E10	ordinary_muscle_development	ENSGALG00010024757	PPFIA1	-0.414562643	0.075878198	-5.463527808	4.66764E-08	9.85398E-08	negative
PRJNA729271	stage_5W_vs_E10	ordinary_muscle_development	ENSGALG00010024758	SPATA6	0.175697084	0.105840625	1.660015552	0.096911324	0.117975111	positive
PRJNA729271	stage_5W_vs_E10	ordinary_muscle_development	ENSGALG00010024759	APOC3	2.418632598	0.969930584	2.493614119			positive
PRJNA729271	stage_5W_vs_E10	ordinary_muscle_development	ENSGALG00010024760	ENSGALG00010024760	2.656790709	0.185364164	14.33281736	1.3647E-46	1.46312E-45	positive
PRJNA729271	stage_5W_vs_E10	ordinary_muscle_development	ENSGALG00010024761	NECAB2	1.40178664	0.23261379	6.026240484	1.67817E-09	3.86727E-09	positive
PRJNA729271	stage_5W_vs_E10	ordinary_muscle_development	ENSGALG00010024762	FAN1	-0.505154948	0.091174018	-5.540558129	3.01509E-08	6.44218E-08	negative
PRJNA729271	stage_5W_vs_E10	ordinary_muscle_development	ENSGALG00010024763	HOXD9	-1.444678897	0.320706355	-4.504678109	6.64736E-06	1.19828E-05	negative
PRJNA729271	stage_5W_vs_E10	ordinary_muscle_development	ENSGALG00010024764	ENSGALG00010024764	0.407645103	0.261292546	1.56010996	0.118733898	0.142742627	positive
PRJNA729271	stage_5W_vs_E10	ordinary_muscle_development	ENSGALG00010024765	SNRNP35	-0.205276853	0.135129564	-1.519111342	0.128734472	0.154177161	negative
PRJNA729271	stage_5W_vs_E10	ordinary_muscle_development	ENSGALG00010024767	CTTN	-1.148793604	0.069685769	-16.48534007	4.67656E-61	7.74706E-60	negative
PRJNA729271	stage_5W_vs_E10	ordinary_muscle_development	ENSGALG00010024769	RALY	3.188203872	0.204017344	15.62712174	4.75816E-55	6.58106E-54	positive
PRJNA729271	stage_5W_vs_E10	ordinary_muscle_development	ENSGALG00010024770	CDC27	0.242957337	0.072307477	3.36005827	0.00077926	0.001191621	positive
PRJNA729271	stage_5W_vs_E10	ordinary_muscle_development	ENSGALG00010024771	AOX2	4.972818605	0.263715256	18.85677258	2.58527E-79	6.86328E-78	positive
PRJNA729271	stage_5W_vs_E10	ordinary_muscle_development	ENSGALG00010024772	ELAPOR1	-0.604218033	0.238585733	-2.532498592	0.011325282	0.015421322	negative
PRJNA729271	stage_5W_vs_E10	ordinary_muscle_development	ENSGALG00010024773	APOA1	6.717641288	0.264052636	25.44053863	8.9844E-143	7.7981E-141	positive
PRJNA729271	stage_5W_vs_E10	ordinary_muscle_development	ENSGALG00010024775	ITFG2	0.719140101	0.094094688	7.642727913	2.12667E-14	6.46656E-14	positive
PRJNA729271	stage_5W_vs_E10	ordinary_muscle_development	ENSGALG00010024776	MPHOSPH10	-0.69473191	0.108133608	-6.424754726	1.32082E-10	3.26156E-10	negative
PRJNA729271	stage_5W_vs_E10	ordinary_muscle_development	ENSGALG00010024778	RAB5B	0.570213006	0.139755867	4.0800649	4.50231E-05	7.61772E-05	positive
PRJNA729271	stage_5W_vs_E10	ordinary_muscle_development	ENSGALG00010024779	ENSGALG00010024779	-5.151285786	1.204783999	-4.275692398	1.90544E-05	3.32323E-05	negative

PRJNA729271	stage_5W_vs_E10	ordinary_muscle_development	ENSGALG00010024780	HOXD11	-6.771275515	1.116974786	-6.062156101	1.34309E-09	3.11439E-09	negative
PRJNA729271	stage_5W_vs_E10	ordinary_muscle_development	ENSGALG00010024781	CEP19	-0.851310625	0.164860539	-5.163822896	2.41957E-07	4.85219E-07	negative
PRJNA729271	stage_5W_vs_E10	ordinary_muscle_development	ENSGALG00010024782	HOXD12	-4.854811271	2.178365665	-2.228648454	0.025837304	0.033730679	negative
PRJNA729271	stage_5W_vs_E10	ordinary_muscle_development	ENSGALG00010024783	UBE2T	-2.777637329	0.245689596	-11.30547397	1.23287E-29	7.3571E-29	negative
PRJNA729271	stage_5W_vs_E10	ordinary_muscle_development	ENSGALG00010024784	ENSGALG00010024784	0.585438769	0.139035991	4.210699432	2.54581E-05	4.39514E-05	positive
PRJNA729271	stage_5W_vs_E10	ordinary_muscle_development	ENSGALG00010024785	MCEE	0.789399273	0.200824302	3.930795551	8.46652E-05	0.000140527	positive
PRJNA729271	stage_5W_vs_E10	ordinary_muscle_development	ENSGALG00010024786	PDCD5	0.841052487	0.097142243	8.657947936	4.80333E-18	1.75641E-17	positive
PRJNA729271	stage_5W_vs_E10	ordinary_muscle_development	ENSGALG00010024787	SARS1	0.389337353	0.099654133	3.906886176	9.34932E-05	0.000154472	positive
PRJNA729271	stage_5W_vs_E10	ordinary_muscle_development	ENSGALG00010024788	SIK3	0.681158274	0.166064859	4.101760459	4.10019E-05	6.95927E-05	positive
PRJNA729271	stage_5W_vs_E10	ordinary_muscle_development	ENSGALG00010024789	SUOX	1.280817813	0.17138116	7.473504168	7.8087E-14	2.30195E-13	positive
PRJNA729271	stage_5W_vs_E10	ordinary_muscle_development	ENSGALG00010024791	SHANK2	-2.411322365	0.425323644	-5.669382361	1.43313E-08	3.13748E-08	negative
PRJNA729271	stage_5W_vs_E10	ordinary_muscle_development	ENSGALG00010024792	BZW1	-0.905171162	0.069319121	-13.05802999	5.71886E-39	4.79993E-38	negative
PRJNA729271	stage_5W_vs_E10	ordinary_muscle_development	ENSGALG00010024793	FKBP4	-1.098067668	0.117226062	-9.367095087	7.45562E-21	3.09034E-20	negative
PRJNA729271	stage_5W_vs_E10	ordinary_muscle_development	ENSGALG00010024794	DDX6	-0.796935345	0.064875574	-12.28445843	1.10322E-34	8.0479E-34	negative
PRJNA729271	stage_5W_vs_E10	ordinary_muscle_development	ENSGALG00010024795	NRROS	1.807650554	0.205166152	8.810666569	1.24404E-18	4.66555E-18	positive
PRJNA729271	stage_5W_vs_E10	ordinary_muscle_development	ENSGALG00010024797	IKZF4	1.291577313	0.23133776	5.583080397	2.36295E-08	5.09688E-08	positive
PRJNA729271	stage_5W_vs_E10	ordinary_muscle_development	ENSGALG00010024798	FBXO45	-0.301593888	0.085112865	-3.543458313	0.000394916	0.000618376	negative
PRJNA729271	stage_5W_vs_E10	ordinary_muscle_development	ENSGALG00010024799	ENSGALG00010024799	1.141083419	0.185481821	6.151995995	7.65138E-10	1.79693E-09	positive
PRJNA729271	stage_5W_vs_E10	ordinary_muscle_development	ENSGALG00010024800	ENSGALG00010024800	0.120476699	0.130944682	0.920057976	0.357542463	0.39780304	positive
PRJNA729271	stage_5W_vs_E10	ordinary_muscle_development	ENSGALG00010024801	CXCR5	4.382306782	1.013287152	4.324841949	1.52641E-05	2.68414E-05	positive
PRJNA729271	stage_5W_vs_E10	ordinary_muscle_development	ENSGALG00010024802	CD59	1.006452474	0.163826473	6.143405606	8.07707E-10	1.89384E-09	positive
PRJNA729271	stage_5W_vs_E10	ordinary_muscle_development	ENSGALG00010024803	CLK1	-0.125470524	0.139007931	-0.902614136	0.366730721	0.407369285	negative
PRJNA729271	stage_5W_vs_E10	ordinary_muscle_development	ENSGALG00010024804	WDR53	-0.038551905	0.106128724	-0.363256085	0.716413595	0.74708022	negative
PRJNA729271	stage_5W_vs_E10	ordinary_muscle_development	ENSGALG00010024805	ENSGALG00010024805	1.215641171	0.368048246	3.302939718	0.00095677	0.001453556	positive
PRJNA729271	stage_5W_vs_E10	ordinary_muscle_development	ENSGALG00010024806	ANKRD27	0.465523946	0.074737467	6.22878948	4.70053E-10	1.12026E-09	positive
PRJNA729271	stage_5W_vs_E10	ordinary_muscle_development	ENSGALG00010024807	PPIL3	-0.878131121	0.197446968	-4.447427731	8.69047E-06	1.55555E-05	negative
PRJNA729271	stage_5W_vs_E10	ordinary_muscle_development	ENSGALG00010024808	CELSR2	-1.14557038	0.259294184	-4.418033453	9.9603E-06	1.77648E-05	negative
PRJNA729271	stage_5W_vs_E10	ordinary_muscle_development	ENSGALG00010024809	KANSL1	0.59588759	0.170804503	3.488711239	0.000485355	0.000754043	positive
PRJNA729271	stage_5W_vs_E10	ordinary_muscle_development	ENSGALG00010024810	ADIPOR1	1.153840978	0.112762743	10.23246643	1.4186E-24	6.92575E-24	positive
PRJNA729271	stage_5W_vs_E10	ordinary_muscle_development	ENSGALG00010024811	NIF3L1	-0.749933577	0.140148677	-5.350985765	8.74764E-08	1.81061E-07	negative
PRJNA729271	stage_5W_vs_E10	ordinary_muscle_development	ENSGALG00010024812	SBNO1	0.659601918	0.111773	5.901263434	3.60728E-09	8.16611E-09	positive
PRJNA729271	stage_5W_vs_E10	ordinary_muscle_development	ENSGALG00010024813	RNF168	-0.448444018	0.096629457	-4.640862427	3.46958E-06	6.39842E-06	negative
PRJNA729271	stage_5W_vs_E10	ordinary_muscle_development	ENSGALG00010024814	APBA2	-3.717278389	0.178537141	-20.82075678	2.80717E-96	1.1057E-94	negative
PRJNA729271	stage_5W_vs_E10	ordinary_muscle_development	ENSGALG00010024816	NFIA	-0.272335696	0.122626731	-2.220850984	0.026361056	0.03440824	negative
PRJNA729271	stage_5W_vs_E10	ordinary_muscle_development	ENSGALG00010024817	UCKL1	3.061838426	0.220088749	13.91183528	5.3684E-44	5.30436E-43	positive
PRJNA729271	stage_5W_vs_E10	ordinary_muscle_development	ENSGALG00010024818	FBXO3	-0.171781386	0.07695446	-2.23224731	0.025598622	0.033425099	negative
PRJNA729271	stage_5W_vs_E10	ordinary_muscle_development	ENSGALG00010024820	NUDT19	0.829974054	0.139483107	5.950355356	2.67561E-09	6.09795E-09	positive
PRJNA729271	stage_5W_vs_E10	ordinary_muscle_development	ENSGALG00010024821	NECAP1	-0.369470591	0.106322565	-3.47499696	0.000510859	0.000792901	negative
PRJNA729271	stage_5W_vs_E10	ordinary_muscle_development	ENSGALG00010024822	RPS26	0.247354193	0.137169695	1.803271443	0.071345595	0.088351508	positive
PRJNA729271	stage_5W_vs_E10	ordinary_muscle_development	ENSGALG00010024823	ASB10	12.95842035	0.865253527	14.97644326	1.04671E-50	1.29014E-49	positive
PRJNA729271	stage_5W_vs_E10	ordinary_muscle_development	ENSGALG00010024824	DNAJC5	-0.669418877	0.107308838	-6.23824549	4.42506E-10	1.056E-09	negative
PRJNA729271	stage_5W_vs_E10	ordinary_muscle_development	ENSGALG00010024825	UBXN7	-0.564494545	0.076314208	-7.396978362	1.39318E-13	4.04693E-13	negative
PRJNA729271	stage_5W_vs_E10	ordinary_muscle_development	ENSGALG00010024826	RBM33	0.433771883	0.111796995	3.879995904	0.000104458	0.000171747	positive
PRJNA729271	stage_5W_vs_E10	ordinary_muscle_development	ENSGALG00010024827	ENSGALG00010024827	0.053732972	0.366113869	0.146765739	0.883316928	0.897433159	positive
PRJNA729271	stage_5W_vs_E10	ordinary_muscle_development	ENSGALG00010024828	TIMM17B	0.780985549	0.13332792	5.857629432	4.69521E-09	1.05629E-08	positive
PRJNA729271	stage_5W_vs_E10	ordinary_muscle_development	ENSGALG00010024829	ERBB3	-3.3262967	0.230870411	-14.40763538	4.63303E-47	5.0531E-46	negative
PRJNA729271	stage_5W_vs_E10	ordinary_muscle_development	ENSGALG00010024830	ORC2	-1.860114818	0.120130493	-15.48411875	4.44106E-54	5.96601E-53	negative
PRJNA729271	stage_5W_vs_E10	ordinary_muscle_development	ENSGALG00010024833	MAPT	2.721639964	0.200887836	13.54805755	8.13546E-42	7.50627E-41	positive
PRJNA729271	stage_5W_vs_E10	ordinary_muscle_development	ENSGALG00010024834	INSIG1	1.990351027	0.258473694	7.700400746	1.3564E-14	4.16636E-14	positive
PRJNA729271	stage_5W_vs_E10	ordinary_muscle_development	ENSGALG00010024835	ENTREP2	-2.229591384	0.161419452	-13.81240834	2.14537E-43	2.08706E-42	negative
PRJNA729271	stage_5W_vs_E10	ordinary_muscle_development	ENSGALG00010024836	TXNDC12	-0.899926668	0.106976531	-8.412374707	4.01792E-17	1.40506E-16	negative
PRJNA729271	stage_5W_vs_E10	ordinary_muscle_development	ENSGALG00010024837	TNFRSF1A	1.355698889	0.155936057	8.693941042	3.50079E-18	1.28956E-17	positive
PRJNA729271	stage_5W_vs_E10	ordinary_muscle_development	ENSGALG00010024838	EN2	-4.980535265	0.938097647	-5.309186396	1.10116E-07	2.26626E-07	negative
PRJNA729271	stage_5W_vs_E10	ordinary_muscle_development	ENSGALG00010024839	PA2G4	-1.151014341	0.099546436	-11.56258709	6.37585E-31	4.01119E-30	negative
PRJNA729271	stage_5W_vs_E10	ordinary_muscle_development	ENSGALG00010024840	BTF3L4	-1.295250778	0.102072729	-12.68948912	6.76255E-37	5.35938E-36	negative
PRJNA729271	stage_5W_vs_E10	ordinary_muscle_development	ENSGALG00010024841	ENSGALG00010024841	-2.277676133	0.222211614	-10.2500319	1.18304E-24	5.7992E-24	negative
PRJNA729271	stage_5W_vs_E10	ordinary_muscle_development	ENSGALG00010024842	ZFYVE9	0.083298346	0.075938495	1.09691858	0.272676986	0.309874787	positive
PRJNA729271	stage_5W_vs_E10	ordinary_muscle_development	ENSGALG00010024843	BCL9L	0.740426754	0.13340065	5.550398416	2.85019E-08	6.10244E-08	positive
PRJNA729271	stage_5W_vs_E10	ordinary_muscle_development	ENSGALG00010024844	TM4SF19	2.694778696	0.372950944	7.225558042	4.99048E-13	1.41283E-12	positive
PRJNA729271	stage_5W_vs_E10	ordinary_muscle_development	ENSGALG00010024845	HYCC2	0.563774063	0.110533209	5.100494856	3.38767E-07	6.72493E-07	positive
PRJNA729271	stage_5W_vs_E10	ordinary_muscle_development	ENSGALG00010024846	LMO2	0.766737145	0.14281603	5.368705072	7.9304E-08	1.64663E-07	positive
PRJNA729271	stage_5W_vs_E10	ordinary_muscle_development	ENSGALG00010024847	PPIL1	-0.083192955	0.150169657	-0.55399311	0.579583561	0.618191397	negative
PRJNA729271	stage_5W_vs_E10	ordinary_muscle_development	ENSGALG00010024848	ESYT1	0.174091867	0.085884687	2.027041985	0.042658119	0.054291395	positive
PRJNA729271	stage_5W_vs_E10	ordinary_muscle_development	ENSGALG00010024849	CENATAC	0.304202279	0.194065947	1.567520135	0.116993164	0.140834586	positive
PRJNA729271	stage_5W_vs_E10	ordinary_muscle_development	ENSGALG00010024850	CDK2AP1	-0.645049376	0.117679552	-5.481405769	4.21959E-08	8.92891E-08	negative
PRJNA729271	stage_5W_vs_E10	ordinary_muscle_development	ENSGALG00010024851	CPNE5	-3.710273774	0.9687982	-3.829769475	0.000128263	0.000209161	negative
PRJNA729271	stage_5W_vs_E10	ordinary_muscle_development	ENSGALG00010024852	CEP89	-0.987770857	0.097495139	-10.13148828	4.00506E-24	1.91722E-23	negative
PRJNA729271	stage_5W_vs_E10	ordinary_muscle_development	ENSGALG00010024853	TJP1	0.157481328	0.067607457	2.329348501	0.019840611	0.026220793	positive
PRJNA729271	stage_5W_vs_E10	ordinary_muscle_development	ENSGALG00010024854	C12orf65	0.946145981	0.150439799	6.289199968	3.19106E-10	7.68474E-10	positive
PRJNA729271	stage_5W_vs_E10	ordinary_muscle_development	ENSGALG00010024855	CAPRIN1	-0.231077686	0.071847151	-3.216240057	0.001298821	0.001946084	negative
PRJNA729271	stage_5W_vs_E10	ordinary_muscle_development	ENSGALG00010024856	SORT1	3.180693831	0.131876918	24.11865465	1.5931E-128	1.1375E-126	positive
PRJNA729271	stage_5W_vs_E10	ordinary_muscle_development	ENSGALG00010024857	SLC7A4	0.774064269	0.141619941	5.465785831	4.60861E-08	9.73502E-08	positive
PRJNA729271	stage_5W_vs_E10	ordinary_muscle_development	ENSGALG00010024858	SLC25A39	0.182913371	0.122199915	1.496837142	0.134435639	0.160567287	positive
PRJNA729271	stage_5W_vs_E10	ordinary_muscle_development	ENSGALG00010024859	TARSL2	0.225707406	0.103331898	2.184295553	0.028940537	0.037605835	positive
PRJNA729271	stage_5W_vs_E10	ordinary_muscle_development	ENSGALG00010024860	NEU4	-3.408627706	0.663645903	-5.136214493	2.80328E-07	5.59305E-07	negative
PRJNA729271	stage_5W_vs_E10	ordinary_muscle_development	ENSGALG00010024861	ORC1	0.223821566	0.063255651	3.538364746	0.000402613	0.000629953	positive
PRJNA729271	stage_5W_vs_E10	ordinary_muscle_development	ENSGALG00010024862	METTL16	0.610712683	0.119550144	5.108422821	3.24859E-07	6.45665E-07	positive
PRJNA729271	stage_5W_vs_E10	ordinary_muscle_development	ENSGALG00010024863	LMOD1	1.517861163	0.289964693	5.234641323	1.65306E-07	3.35871E-07	positive
PRJNA729271	stage_5W_vs_E10	ordinary_muscle_development	ENSGALG00010024864	MPHOSPH9	-0.923949866	0.131879074	-7.006038476	2.45161E-12	6.68976E-12	negative
PRJNA729271	stage_5W_vs_E10	ordinary_muscle_development	ENSGALG00010024865	SNAP47	1.082731741	0.142285097	7.609593434	2.74959E-14	8.32228E-14	positive
PRJNA729271	stage_5W_vs_E10	ordinary_muscle_development	ENSGALG00010024866	TRAPPC4	0.141755787	0.086543874	1.637964429	0.101429114	0.123061439	positive
PRJNA729271	stage_5W_vs_E10	ordinary_muscle_development	ENSGALG00010024867	TM2D3	0.267172927	0.106580285	2.506776241	0.012183781	0.016514299	positive
PRJNA729271	stage_5W_vs_E10	ordinary_muscle_development	ENSGALG00010024868	FAAP24	-2.017793928	0.217277085	-9.286731399	1.59099E-20	6.49069E-20	negative
PRJNA729271	stage_5W_vs_E10	ordinary_muscle_development	ENSGALG00010024869	NDUFB3	1.587783075	0.121786624	13.03741764	7.49529E-39	6.26553E-38	positive
PRJNA729271	stage_5W_vs_E10	ordinary_muscle_development	ENSGALG00010024870	TPD52L2	0.143576098	0.082868971	1.732567644	0.083172544	0.102029962	positive
PRJNA729271	stage_5W_vs_E10	ordinary_muscle_development	ENSGALG00010024871	POLQ	-1.545164232	0.140153816	-11.0247746	2.90245E-28	1.63892E-27	negative
PRJNA729271	stage_5W_vs_E10	ordinary_muscle_development	ENSGALG00010024872	RUNDC3A	-1.222562034	0.209431353	-5.837531078	5.298E-09	1.18859E-08	negative
PRJNA729271	stage_5W_vs_E10	ordinary_muscle_development	ENSGALG00010024873	HYOU1	-1.019376031	0.108950132	-9.356354257	8.25355E-21	3.4162E-20	negative
PRJNA729271	stage_5W_vs_E10	ordinary_muscle_development	ENSGALG00010024874	JMJD4	0.07664977	0.081864473	0.936300778	0.349118348	0.389087379	positive
PRJNA729271	stage_5W_vs_E10	ordinary_muscle_development	ENSGALG00010024876	CFLAR	0.343784981	0.095394928	3.603807744	0.000313589	0.000495581	positive
PRJNA729271	stage_5W_vs_E10	ordinary_muscle_development	ENSGALG00010024877	PRPF38A	-1.328130196	0.083484051	-15.90878956	5.50679E-57	8.12013E-56	negative
PRJNA729271	stage_5W_vs_E10	ordinary_muscle_development	ENSGALG00010024878	RHPN2	-2.290956857	0.293442591	-7.807172273	5.84853E-15	1.83138E-14	negative
PRJNA729271	stage_5W_vs_E10	ordinary_muscle_development	ENSGALG00010024879	D2HGDH	0.308430542	0.150414409	2.050538537	0.040311907	0.051459366	positive
PRJNA729271	stage_5W_vs_E10	ordinary_muscle_development	ENSGALG00010024880	NAT10	-1.368417864	0.139986312	-9.775369092	1.43633E-22	6.44769E-22	negative
PRJNA729271	stage_5W_vs_E10	ordinary_muscle_development	ENSGALG00010024881	CASP10	1.302721606	0.285633231	4.56081949	5.09544E-06	9.26356E-06	positive
PRJNA729271	stage_5W_vs_E10	ordinary_muscle_development	ENSGALG00010024882	MYL6	-1.532144418	0.164868309	-9.293140829	1.49801E-20	6.11653E-20	negative
PRJNA729271	stage_5W_vs_E10	ordinary_muscle_development	ENSGALG00010024883	ING5	1.487165589	0.12305546	12.08532789	1.26265E-33	8.81608E-33	positive
PRJNA729271	stage_5W_vs_E10	ordinary_muscle_development	ENSGALG00010024884	ZBTB46	1.631415559	0.132932843	12.27247925	1.27295E-34	9.23031E-34	positive
PRJNA729271	stage_5W_vs_E10	ordinary_muscle_development	ENSGALG00010024885	SLC4A1	-0.699213409	0.236909528	-2.951394207	0.003163429	0.004562123	negative
PRJNA729271	stage_5W_vs_E10	ordinary_muscle_development	ENSGALG00010024886	ABTB2	-1.800602102	0.143972158	-12.50659936	6.8703E-36	5.2496E-35	negative
PRJNA729271	stage_5W_vs_E10	ordinary_muscle_development	ENSGALG00010024887	CASP18	2.602391127	0.249621408	10.42535231	1.89954E-25	9.56036E-25	positive
PRJNA729271	stage_5W_vs_E10	ordinary_muscle_development	ENSGALG00010024888	PITPNM2	0.735586367	0.126661125	5.80751486	6.34069E-09	1.41637E-08	positive
PRJNA729271	stage_5W_vs_E10	ordinary_muscle_development	ENSGALG00010024889	SLC2A4RG	-0.177035567	0.504943472	-0.350604725	0.725884911	0.755869381	negative
PRJNA729271	stage_5W_vs_E10	ordinary_muscle_development	ENSGALG00010024890	SMARCC2	0.299261514	0.145907184	2.051040303	0.040263022	0.051401489	positive
PRJNA729271	stage_5W_vs_E10	ordinary_muscle_development	ENSGALG00010024891	PKP1	-1.453925811	0.471299929	-3.084926865	0.002036022	0.002989177	negative
PRJNA729271	stage_5W_vs_E10	ordinary_muscle_development	ENSGALG00010024893	ARL6IP4	-0.405166571	0.141659045	-2.860153203	0.004234364	0.006028001	negative
PRJNA729271	stage_5W_vs_E10	ordinary_muscle_development	ENSGALG00010024894	TUT4	1.038018492	0.121909107	8.51469198	1.67034E-17	5.95756E-17	positive
PRJNA729271	stage_5W_vs_E10	ordinary_muscle_development	ENSGALG00010024895	ENSGALG00010024895	1.349729725	0.413756497	3.262135418	0.001105763	0.001669761	positive
PRJNA729271	stage_5W_vs_E10	ordinary_muscle_development	ENSGALG00010024896	RNF41	0.693640901	0.100429617	6.90673652	4.9593E-12	1.3312E-11	positive
PRJNA729271	stage_5W_vs_E10	ordinary_muscle_development	ENSGALG00010024897	ADAL	0.373907058	0.192186419	1.945543604	0.051709587	0.065102967	positive
PRJNA729271	stage_5W_vs_E10	ordinary_muscle_development	ENSGALG00010024898	NABP2	-0.657105783	0.31718012	-2.071711754	0.03829233	0.049041114	negative
PRJNA729271	stage_5W_vs_E10	ordinary_muscle_development	ENSGALG00010024899	OGFOD2	-0.775961574	0.133256959	-5.82304728	5.77842E-09	1.29376E-08	negative
PRJNA729271	stage_5W_vs_E10	ordinary_muscle_development	ENSGALG00010024900	DTYMK	-1.592849534	0.105992476	-15.02794911	4.81689E-51	5.99321E-50	negative
PRJNA729271	stage_5W_vs_E10	ordinary_muscle_development	ENSGALG00010024901	ZGPAT	-0.504314527	0.131860766	-3.824598799	0.000130985	0.000213401	negative
PRJNA729271	stage_5W_vs_E10	ordinary_muscle_development	ENSGALG00010024902	LARP6	0.68527424	0.117918055	5.811444544	6.19361E-09	1.38373E-08	positive
PRJNA729271	stage_5W_vs_E10	ordinary_muscle_development	ENSGALG00010024903	CASP8	-0.556498561	0.11118868	-5.004992955	5.58641E-07	1.09263E-06	negative
PRJNA729271	stage_5W_vs_E10	ordinary_muscle_development	ENSGALG00010024904	ALS2CL	1.43192595	0.160017769	8.948543396	3.60204E-19	1.38566E-18	positive
PRJNA729271	stage_5W_vs_E10	ordinary_muscle_development	ENSGALG00010024905	GPX7	-2.030034226	0.210072019	-9.663515552	4.3082E-22	1.89407E-21	negative
PRJNA729271	stage_5W_vs_E10	ordinary_muscle_development	ENSGALG00010024907	PHLDB1	1.004931356	0.133155695	7.54704016	4.45262E-14	1.33293E-13	positive
PRJNA729271	stage_5W_vs_E10	ordinary_muscle_development	ENSGALG00010024908	ENSGALG00010024908	0.941260052	0.126504819	7.440507458	1.00299E-13	2.93705E-13	positive
PRJNA729271	stage_5W_vs_E10	ordinary_muscle_development	ENSGALG00010024910	UBTF	-0.925569501	0.123857288	-7.472870746	7.84639E-14	2.3126E-13	negative
PRJNA729271	stage_5W_vs_E10	ordinary_muscle_development	ENSGALG00010024911	CAT	0.983891277	0.153256391	6.419903724	1.36361E-10	3.36376E-10	positive
PRJNA729271	stage_5W_vs_E10	ordinary_muscle_development	ENSGALG00010024915	STXBP5L	0.490162215	0.171346835	2.86064353	0.004227821	0.006019278	positive
PRJNA729271	stage_5W_vs_E10	ordinary_muscle_development	ENSGALG00010024917	TRAK2	1.091596636	0.12236479	8.920839399	4.62767E-19	1.76987E-18	positive
PRJNA729271	stage_5W_vs_E10	ordinary_muscle_development	ENSGALG00010024918	ATG4B	-0.511023288	0.075734373	-6.747574035	1.50338E-11	3.94415E-11	negative
PRJNA729271	stage_5W_vs_E10	ordinary_muscle_development	ENSGALG00010024919	ANKRD52	-0.22139831	0.123438981	-1.793585044	0.072879425	0.090120054	negative
PRJNA729271	stage_5W_vs_E10	ordinary_muscle_development	ENSGALG00010024920	VIPR2	0.328921104	0.300111749	1.095995424	0.273080777	0.310212058	positive
PRJNA729271	stage_5W_vs_E10	ordinary_muscle_development	ENSGALG00010024921	STRADB	-0.459780621	0.109754758	-4.189163446	2.79985E-05	4.81364E-05	negative
PRJNA729271	stage_5W_vs_E10	ordinary_muscle_development	ENSGALG00010024924	MNX1	-5.877789214	0.952865969	-6.168537238	6.89246E-10	1.62396E-09	negative
PRJNA729271	stage_5W_vs_E10	ordinary_muscle_development	ENSGALG00010024925	GTF2E1	-0.679504597	0.084707549	-8.021771407	1.04231E-15	3.4043E-15	negative
PRJNA729271	stage_5W_vs_E10	ordinary_muscle_development	ENSGALG00010024926	ELF5	-5.54836248	0.97836518	-5.671054731	1.41921E-08	3.10841E-08	negative

PRJNA729271	stage_5W_vs_E10	ordinary_muscle_development	ENSGALG00010024927	NOM1	-0.503927792	0.115068472	-4.379373297	1.19021E-05	2.10958E-05	negative
PRJNA729271	stage_5W_vs_E10	ordinary_muscle_development	ENSGALG00010024928	THAP4	1.285435202	0.084551918	15.20290994	3.38255E-52	4.38552E-51	positive
PRJNA729271	stage_5W_vs_E10	ordinary_muscle_development	ENSGALG00010024930	TMEM237	-0.05739433	0.100060162	-0.573598214	0.566239721	0.605417478	negative
PRJNA729271	stage_5W_vs_E10	ordinary_muscle_development	ENSGALG00010024931	UBE3C	0.759587759	0.08839873	8.592745108	8.49158E-18	3.07329E-17	positive
PRJNA729271	stage_5W_vs_E10	ordinary_muscle_development	ENSGALG00010024932	ANKK1	-0.325214955	1.755275203	-0.185278613	0.853010512	0.870792829	negative
PRJNA729271	stage_5W_vs_E10	ordinary_muscle_development	ENSGALG00010024933	CS	0.579062944	0.157174865	3.684195585	0.000229426	0.000366854	positive
PRJNA729271	stage_5W_vs_E10	ordinary_muscle_development	ENSGALG00010024934	G6PC3	0.766722356	0.222083839	3.452400503	0.000555622	0.000860351	positive
PRJNA729271	stage_5W_vs_E10	ordinary_muscle_development	ENSGALG00010024935	KLHL30	2.019906305	0.388623699	5.197589108	2.0189E-07	4.07803E-07	positive
PRJNA729271	stage_5W_vs_E10	ordinary_muscle_development	ENSGALG00010024936	EHF	-0.516466933	0.561831176	-0.919256452	0.35796145	0.398085869	negative
PRJNA729271	stage_5W_vs_E10	ordinary_muscle_development	ENSGALG00010024937	ILKAP	-1.009230014	0.127736323	-7.900885149	2.7693E-15	8.82801E-15	negative
PRJNA729271	stage_5W_vs_E10	ordinary_muscle_development	ENSGALG00010024938	ENSGALG00010024938	0.464702282	0.176895773	2.626983535	0.00861455	0.01187183	positive
PRJNA729271	stage_5W_vs_E10	ordinary_muscle_development	ENSGALG00010024939	COQ10A	2.288692439	0.202966704	11.27619653	1.72024E-29	1.02025E-28	positive
PRJNA729271	stage_5W_vs_E10	ordinary_muscle_development	ENSGALG00010024940	RABL3	-0.18410278	0.091835756	-2.004696081	0.044995548	0.057061283	negative
PRJNA729271	stage_5W_vs_E10	ordinary_muscle_development	ENSGALG00010024941	PDHX	0.722574679	0.118150307	6.115724082	9.61195E-10	2.24501E-09	positive
PRJNA729271	stage_5W_vs_E10	ordinary_muscle_development	ENSGALG00010024942	DNAJB6	0.418543716	0.066066994	6.335140904	2.37124E-10	5.75925E-10	positive
PRJNA729271	stage_5W_vs_E10	ordinary_muscle_development	ENSGALG00010024943	ABCB9	0.593280478	0.175180419	3.386682607	0.000707432	0.001085333	positive
PRJNA729271	stage_5W_vs_E10	ordinary_muscle_development	ENSGALG00010024944	ENSGALG00010024944	-0.423259725	0.112984704	-3.746168388	0.000179556	0.00028941	negative
PRJNA729271	stage_5W_vs_E10	ordinary_muscle_development	ENSGALG00010024945	APIP	0.73595069	0.08067632	9.122264007	7.3571E-20	2.92328E-19	positive
PRJNA729271	stage_5W_vs_E10	ordinary_muscle_development	ENSGALG00010024946	KIF1A	-3.878896875	0.264316637	-14.67519001	9.29548E-49	1.07704E-47	negative
PRJNA729271	stage_5W_vs_E10	ordinary_muscle_development	ENSGALG00010024947	MPP4	0.628328754	0.319551207	1.96628503	0.049265695	0.062161059	positive
PRJNA729271	stage_5W_vs_E10	ordinary_muscle_development	ENSGALG00010024949	PAN2	-0.290277693	0.11543068	-2.514736051	0.011912153	0.016174862	negative
PRJNA729271	stage_5W_vs_E10	ordinary_muscle_development	ENSGALG00010024950	NCAM1	-0.930182732	0.155070487	-5.998451106	1.99208E-09	4.56743E-09	negative
PRJNA729271	stage_5W_vs_E10	ordinary_muscle_development	ENSGALG00010024953	NCAPG2	-2.124189217	0.223219603	-9.516140998	1.7973E-21	7.67358E-21	negative
PRJNA729271	stage_5W_vs_E10	ordinary_muscle_development	ENSGALG00010024956	VPS37B	-0.292591747	0.138647063	-2.110334978	0.034829513	0.044859942	negative
PRJNA729271	stage_5W_vs_E10	ordinary_muscle_development	ENSGALG00010024960	ARFRP1	-0.739446811	0.103035017	-7.176655373	7.14375E-13	2.0087E-12	negative
PRJNA729271	stage_5W_vs_E10	ordinary_muscle_development	ENSGALG00010024961	LSM12	0.515611794	0.203542998	2.533183642	0.011303172	0.015395553	positive
PRJNA729271	stage_5W_vs_E10	ordinary_muscle_development	ENSGALG00010024963	TNFRSF6B	0.2448068	0.588591	0.415920053	0.677468505	0.710917685	positive
PRJNA729271	stage_5W_vs_E10	ordinary_muscle_development	ENSGALG00010024964	ITM2C	-1.420355092	0.173309816	-8.195468214	2.4962E-16	8.39888E-16	negative
PRJNA729271	stage_5W_vs_E10	ordinary_muscle_development	ENSGALG00010024965	STAT2	1.525311538	0.217378526	7.016845535	2.26933E-12	6.20758E-12	positive
PRJNA729271	stage_5W_vs_E10	ordinary_muscle_development	ENSGALG00010024966	AGAP3	-0.328256978	0.116144836	-2.826272694	0.004709315	0.00666877	negative
PRJNA729271	stage_5W_vs_E10	ordinary_muscle_development	ENSGALG00010024968	TMEM101	0.658239369	0.13318733	4.942207098	7.72431E-07	1.49644E-06	positive
PRJNA729271	stage_5W_vs_E10	ordinary_muscle_development	ENSGALG00010024970	CAB39	0.521960253	0.077737209	6.714419765	1.88816E-11	4.92069E-11	positive
PRJNA729271	stage_5W_vs_E10	ordinary_muscle_development	ENSGALG00010024973	RTEL1	-1.905122325	0.091797772	-20.75347019	1.14055E-95	4.40859E-94	negative
PRJNA729271	stage_5W_vs_E10	ordinary_muscle_development	ENSGALG00010024974	CD44	0.334106448	0.166012574	2.012537001	0.04416336	0.056079536	positive
PRJNA729271	stage_5W_vs_E10	ordinary_muscle_development	ENSGALG00010024977	LRP3	1.486614755	0.10432455	14.2499033	4.48902E-46	4.72881E-45	positive
PRJNA729271	stage_5W_vs_E10	ordinary_muscle_development	ENSGALG00010024983	APOF	1.964122794	0.506695424	3.876338137	0.00010604	0.000174151	positive
PRJNA729271	stage_5W_vs_E10	ordinary_muscle_development	ENSGALG00010024984	SHISAL2A	-0.197320248	0.510562182	-0.386476426	0.699143858	0.731439208	negative
PRJNA729271	stage_5W_vs_E10	ordinary_muscle_development	ENSGALG00010024986	PTS	-1.129566305	0.171873262	-6.572088599	4.96143E-11	1.26036E-10	negative
PRJNA729271	stage_5W_vs_E10	ordinary_muscle_development	ENSGALG00010024989	TIMELESS	-1.548336112	0.166466724	-9.301174869	1.38902E-20	5.68914E-20	negative
PRJNA729271	stage_5W_vs_E10	ordinary_muscle_development	ENSGALG00010024994	NDUFB4	1.367839561	0.10396096	13.15724252	1.54627E-39	1.32858E-38	positive
PRJNA729271	stage_5W_vs_E10	ordinary_muscle_development	ENSGALG00010024996	COA7	0.059127031	0.125159643	0.472412913	0.636632089	0.672201496	positive
PRJNA729271	stage_5W_vs_E10	ordinary_muscle_development	ENSGALG00010024997	HES6	0.058218598	0.260145953	0.223792055	0.822919112	0.843937493	positive
PRJNA729271	stage_5W_vs_E10	ordinary_muscle_development	ENSGALG00010025003	ENSGALG00010025003	10.81761032	0.243120378	44.4948729	0	0	positive
PRJNA729271	stage_5W_vs_E10	ordinary_muscle_development	ENSGALG00010025005	HIP1R	-0.780262931	0.125482551	-6.21809907	5.03214E-10	1.19771E-09	negative
PRJNA729271	stage_5W_vs_E10	ordinary_muscle_development	ENSGALG00010025006	ENSGALG00010025006	0.155153229	0.306763802	0.505774241	0.613015144	0.650058856	positive
PRJNA729271	stage_5W_vs_E10	ordinary_muscle_development	ENSGALG00010025007	ZYG11B	0.553270502	0.101367169	5.458083782	4.81301E-08	1.0149E-07	positive
PRJNA729271	stage_5W_vs_E10	ordinary_muscle_development	ENSGALG00010025008	ENSGALG00010025008	-2.609847313	0.620001757	-4.20941922	2.56028E-05	4.41888E-05	negative
PRJNA729271	stage_5W_vs_E10	ordinary_muscle_development	ENSGALG00010025012	ENSGALG00010025012	4.143499138	0.74694996	5.547224528	2.9024E-08	6.20872E-08	positive
PRJNA729271	stage_5W_vs_E10	ordinary_muscle_development	ENSGALG00010025026	DENR	-0.131498828	0.116345433	-1.130244861	0.258373062	0.294705503	negative
PRJNA729271	stage_5W_vs_E10	ordinary_muscle_development	ENSGALG00010025028	HES6	-1.48305747	0.137538864	-10.7828248	4.14944E-27	2.24593E-26	negative
PRJNA729271	stage_5W_vs_E10	ordinary_muscle_development	ENSGALG00010025031	ECHDC2	0.354329665	0.122721607	2.887263905	0.003886081	0.005551271	positive
PRJNA729271	stage_5W_vs_E10	ordinary_muscle_development	ENSGALG00010025032	FSTL1	-0.989588411	0.18128381	-5.458779872	4.79418E-08	1.01108E-07	negative
PRJNA729271	stage_5W_vs_E10	ordinary_muscle_development	ENSGALG00010025039	CEBPA	0.222399962	0.30890066	0.71997244	0.471541964	0.513523724	positive
PRJNA729271	stage_5W_vs_E10	ordinary_muscle_development	ENSGALG00010025042	BCO2	0.498952188	0.480909774	1.037517256	0.299494858	0.337913752	positive
PRJNA729271	stage_5W_vs_E10	ordinary_muscle_development	ENSGALG00010025044	TMEM9	0.234490287	0.217066027	1.080271703	0.280021207	0.317548693	positive
PRJNA729271	stage_5W_vs_E10	ordinary_muscle_development	ENSGALG00010025047	PER2	1.454956191	0.292868904	4.967943573	6.76666E-07	1.31602E-06	positive
PRJNA729271	stage_5W_vs_E10	ordinary_muscle_development	ENSGALG00010025048	ESYT2	1.225930818	0.082177645	14.91805735	2.51489E-50	3.04539E-49	positive
PRJNA729271	stage_5W_vs_E10	ordinary_muscle_development	ENSGALG00010025055	ALS2	1.056652553	0.154117851	6.856133433	7.07493E-12	1.88582E-11	positive
PRJNA729271	stage_5W_vs_E10	ordinary_muscle_development	ENSGALG00010025056	RBMS2	0.26493179	0.172594076	1.534999325	0.124783994	0.149642944	positive
PRJNA729271	stage_5W_vs_E10	ordinary_muscle_development	ENSGALG00010025057	SCP2	0.161924557	0.066756908	2.425585044	0.015283734	0.020495673	positive
PRJNA729271	stage_5W_vs_E10	ordinary_muscle_development	ENSGALG00010025060	GPR156	-0.352034332	0.177286658	-1.985678656	0.047069016	0.059555289	negative
PRJNA729271	stage_5W_vs_E10	ordinary_muscle_development	ENSGALG00010025064	GPN3	-0.040222055	0.083389377	-0.482340268	0.629564246	0.665258609	negative
PRJNA729271	stage_5W_vs_E10	ordinary_muscle_development	ENSGALG00010025067	SLC1A2	-2.36183896	0.343031688	-6.885191787	5.77097E-12	1.54564E-11	negative
PRJNA729271	stage_5W_vs_E10	ordinary_muscle_development	ENSGALG00010025069	PODN	4.683279222	0.196940435	23.78018121	5.356E-125	3.5942E-123	positive
PRJNA729271	stage_5W_vs_E10	ordinary_muscle_development	ENSGALG00010025071	CDK15	-0.029958028	0.334220463	-0.08963553	0.928576849	0.938286611	negative
PRJNA729271	stage_5W_vs_E10	ordinary_muscle_development	ENSGALG00010025072	ENSGALG00010025072	0.585183994	0.642438846	0.910878908	0.36235918	0.402791128	positive
PRJNA729271	stage_5W_vs_E10	ordinary_muscle_development	ENSGALG00010025073	IL18	1.339267485	0.200299896	6.686311442	2.28866E-11	5.93136E-11	positive
PRJNA729271	stage_5W_vs_E10	ordinary_muscle_development	ENSGALG00010025080	BAZ2A	1.660182326	0.20247125	8.199595366	2.41198E-16	8.11927E-16	positive
PRJNA729271	stage_5W_vs_E10	ordinary_muscle_development	ENSGALG00010025092	COL1A1	2.354529464	0.220974002	10.65523295	1.64831E-26	8.73257E-26	positive
PRJNA729271	stage_5W_vs_E10	ordinary_muscle_development	ENSGALG00010025097	LMBR1	-0.235491129	0.121531255	-1.937700129	0.052659818	0.066241783	negative
PRJNA729271	stage_5W_vs_E10	ordinary_muscle_development	ENSGALG00010025098	ATP5B	0.837683095	0.138237449	6.059740693	1.36341E-09	3.16E-09	positive
PRJNA729271	stage_5W_vs_E10	ordinary_muscle_development	ENSGALG00010025102	ARPC3	-0.396107071	0.085711502	-4.621399245	3.8116E-06	6.99977E-06	negative
PRJNA729271	stage_5W_vs_E10	ordinary_muscle_development	ENSGALG00010025103	GSK3B	0.175670822	0.100190026	1.75337635	0.079537428	0.097827324	positive
PRJNA729271	stage_5W_vs_E10	ordinary_muscle_development	ENSGALG00010025113	SDHD	0.845051416	0.120214421	7.02953448	2.07224E-12	5.68561E-12	positive
PRJNA729271	stage_5W_vs_E10	ordinary_muscle_development	ENSGALG00010025114	CEBPG	0.452882148	0.097303496	4.654325558	3.25043E-06	6.0088E-06	positive
PRJNA729271	stage_5W_vs_E10	ordinary_muscle_development	ENSGALG00010025115	MGAT4F	0.226528999	0.253957466	0.891995826	0.37239517	0.413218096	positive
PRJNA729271	stage_5W_vs_E10	ordinary_muscle_development	ENSGALG00010025116	ANAPC7	-0.662147018	0.071351426	-9.280081082	1.6935E-20	6.90305E-20	negative
PRJNA729271	stage_5W_vs_E10	ordinary_muscle_development	ENSGALG00010025121	DYNC2I1	-0.425090135	0.155416818	-2.735161743	0.006234963	0.008721006	negative
PRJNA729271	stage_5W_vs_E10	ordinary_muscle_development	ENSGALG00010025125	PIK3CB	1.109069141	0.131074894	8.461339196	2.64324E-17	9.32436E-17	positive
PRJNA729271	stage_5W_vs_E10	ordinary_muscle_development	ENSGALG00010025126	ENSGALG00010025126	-0.327369635	0.134226295	-2.438938184	0.014730488	0.019792216	negative
PRJNA729271	stage_5W_vs_E10	ordinary_muscle_development	ENSGALG00010025127	PAMR1	-0.6236426	0.159326252	-3.914248851	9.06861E-05	0.000150022	negative
PRJNA729271	stage_5W_vs_E10	ordinary_muscle_development	ENSGALG00010025130	ATP2A2	-1.902899719	0.125854081	-15.1198889	1.19744E-51	1.52656E-50	negative
PRJNA729271	stage_5W_vs_E10	ordinary_muscle_development	ENSGALG00010025131	SUMO1	-0.287241606	0.081080653	-3.542665185	0.000396105	0.000620171	negative
PRJNA729271	stage_5W_vs_E10	ordinary_muscle_development	ENSGALG00010025138	CFAP91	-0.571029148	0.274064572	-2.083556963	0.037200485	0.047748254	negative
PRJNA729271	stage_5W_vs_E10	ordinary_muscle_development	ENSGALG00010025139	COPB2	-0.775470901	0.047077708	-16.47214637	5.81693E-61	9.57054E-60	negative
PRJNA729271	stage_5W_vs_E10	ordinary_muscle_development	ENSGALG00010025144	PIH1D2	-0.205308951	0.163132029	-1.258544701	0.208194831	0.241500007	negative
PRJNA729271	stage_5W_vs_E10	ordinary_muscle_development	ENSGALG00010025145	CPT2	-0.715984712	0.096090094	-7.451181288	9.25081E-14	2.71548E-13	negative
PRJNA729271	stage_5W_vs_E10	ordinary_muscle_development	ENSGALG00010025147	COX17	2.173571435	0.113460309	19.15710831	8.44358E-82	2.37189E-80	positive
PRJNA729271	stage_5W_vs_E10	ordinary_muscle_development	ENSGALG00010025149	IFT81	-1.412891157	0.143694079	-9.832633062	8.14606E-23	3.69337E-22	negative
PRJNA729271	stage_5W_vs_E10	ordinary_muscle_development	ENSGALG00010025154	TMEM183A	1.605307212	0.120923811	13.27536071	3.21718E-40	2.83139E-39	positive
PRJNA729271	stage_5W_vs_E10	ordinary_muscle_development	ENSGALG00010025155	CZIB	0.369983734	0.091838164	4.02864907	5.60983E-05	9.43761E-05	positive
PRJNA729271	stage_5W_vs_E10	ordinary_muscle_development	ENSGALG00010025156	DLAT	0.565364037	0.11545863	4.896680643	9.7469E-07	1.8745E-06	positive
PRJNA729271	stage_5W_vs_E10	ordinary_muscle_development	ENSGALG00010025157	NOP58	-1.424123031	0.140991298	-10.10078671	5.48008E-24	2.60524E-23	negative
PRJNA729271	stage_5W_vs_E10	ordinary_muscle_development	ENSGALG00010025160	MRPS22	0.435593951	0.130805332	3.33009323	0.000868169	0.001322416	positive
PRJNA729271	stage_5W_vs_E10	ordinary_muscle_development	ENSGALG00010025161	ENSGALG00010025161	-1.256661788	0.105976081	-11.85797562	1.95648E-32	1.30929E-31	negative
PRJNA729271	stage_5W_vs_E10	ordinary_muscle_development	ENSGALG00010025163	LDLRAD3	-1.508401697	0.137649179	-10.95830506	6.06241E-28	3.38761E-27	negative
PRJNA729271	stage_5W_vs_E10	ordinary_muscle_development	ENSGALG00010025164	C11orf52	1.322436408	0.125256608	10.55781751	4.67403E-26	2.4231E-25	positive
PRJNA729271	stage_5W_vs_E10	ordinary_muscle_development	ENSGALG00010025167	PTGES3	-0.51185968	0.188768749	-2.711570014	0.00669654	0.009337792	negative
PRJNA729271	stage_5W_vs_E10	ordinary_muscle_development	ENSGALG00010025168	POPDC2	0.585613816	0.212563185	2.755010546	0.005869024	0.008228214	positive
PRJNA729271	stage_5W_vs_E10	ordinary_muscle_development	ENSGALG00010025169	RBP2	2.238203423	2.222283798	1.007163633	0.313856146	0.352908606	positive
PRJNA729271	stage_5W_vs_E10	ordinary_muscle_development	ENSGALG00010025170	COMMD9	-0.113934184	0.097254991	-1.171499614	0.241398013	0.276737124	negative
PRJNA729271	stage_5W_vs_E10	ordinary_muscle_development	ENSGALG00010025172	RBP1	6.191213131	0.902181703	6.862490243	6.76704E-12	1.80541E-11	positive
PRJNA729271	stage_5W_vs_E10	ordinary_muscle_development	ENSGALG00010025182	PLA1A	3.692477014	0.191987495	19.23290379	1.96322E-82	5.56885E-81	positive
PRJNA729271	stage_5W_vs_E10	ordinary_muscle_development	ENSGALG00010025186	TMEM120B	-1.180485508	0.21129567	-5.586889256	2.31173E-08	4.98862E-08	negative
PRJNA729271	stage_5W_vs_E10	ordinary_muscle_development	ENSGALG00010025188	PPFIA4	-3.309240161	0.417605042	-7.924329996	2.2938E-15	7.35426E-15	negative
PRJNA729271	stage_5W_vs_E10	ordinary_muscle_development	ENSGALG00010025190	PRR5L	0.72713392	0.23991159	3.030841148	0.002438735	0.003557716	positive
PRJNA729271	stage_5W_vs_E10	ordinary_muscle_development	ENSGALG00010025192	CRYAB	2.208573471	0.181709784	12.15440039	5.43592E-34	3.86054E-33	positive
PRJNA729271	stage_5W_vs_E10	ordinary_muscle_development	ENSGALG00010025195	P2RX7	5.614785631	0.397909165	14.11072204	3.26221E-45	3.34884E-44	positive
PRJNA729271	stage_5W_vs_E10	ordinary_muscle_development	ENSGALG00010025200	PEPD	-0.177257203	0.080528412	-2.201175931	0.027723572	0.036085953	negative
PRJNA729271	stage_5W_vs_E10	ordinary_muscle_development	ENSGALG00010025204	TRAF6	1.383034428	0.116378133	11.88397157	1.43389E-32	9.64914E-32	positive
PRJNA729271	stage_5W_vs_E10	ordinary_muscle_development	ENSGALG00010025207	BMPR2	0.225760816	0.097571821	2.31379115	0.020679181	0.027270532	positive
PRJNA729271	stage_5W_vs_E10	ordinary_muscle_development	ENSGALG00010025210	HSPB2	1.985460814	0.295335714	6.722725087	1.78357E-11	4.65649E-11	positive
PRJNA729271	stage_5W_vs_E10	ordinary_muscle_development	ENSGALG00010025212	P2RX4	1.152954049	0.17898782	6.441522379	1.18281E-10	2.92774E-10	positive
PRJNA729271	stage_5W_vs_E10	ordinary_muscle_development	ENSGALG00010025214	LRP8	-2.048872949	0.212890829	-9.624054537	6.32862E-22	2.76389E-21	negative
PRJNA729271	stage_5W_vs_E10	ordinary_muscle_development	ENSGALG00010025215	MYOG	-1.465959029	0.214746182	-6.826473065	8.70276E-12	2.30868E-11	negative
PRJNA729271	stage_5W_vs_E10	ordinary_muscle_development	ENSGALG00010025216	DPP6	-2.567455679	0.266745162	-9.625125549	6.26304E-22	2.73607E-21	negative
PRJNA729271	stage_5W_vs_E10	ordinary_muscle_development	ENSGALG00010025219	NACA	-1.21237637	0.125854141	-9.633186131	5.79056E-22	2.53424E-21	negative
PRJNA729271	stage_5W_vs_E10	ordinary_muscle_development	ENSGALG00010025222	FAM117B	-1.104359908	0.144780479	-7.627823262	2.38751E-14	7.246E-14	negative
PRJNA729271	stage_5W_vs_E10	ordinary_muscle_development	ENSGALG00010025225	DMRTB1	2.360740704	0.668687051	3.530411875	0.000414913	0.000648288	positive
PRJNA729271	stage_5W_vs_E10	ordinary_muscle_development	ENSGALG00010025228	FDXACB1	-0.301463192	0.112885312	-2.670526282	0.007573244	0.010497674	negative
PRJNA729271	stage_5W_vs_E10	ordinary_muscle_development	ENSGALG00010025229	ENSGALG00010025229	-0.854235058	0.166077128	-5.143604483	2.69517E-07	5.38402E-07	negative
PRJNA729271	stage_5W_vs_E10	ordinary_muscle_development	ENSGALG00010025230	CHST8	-3.442374812	0.399073904	-8.625908076	6.35861E-18	2.31694E-17	negative
PRJNA729271	stage_5W_vs_E10	ordinary_muscle_development	ENSGALG00010025232	PRIM1	-1.585131786	0.154299081	-10.27311228	9.31501E-25	4.58787E-24	negative
PRJNA729271	stage_5W_vs_E10	ordinary_muscle_development	ENSGALG00010025233	WDR12	-1.295455088	0.07823338	-16.55885358	1.38202E-61	2.33478E-60	negative
PRJNA729271	stage_5W_vs_E10	ordinary_muscle_development	ENSGALG00010025236	ENSGALG00010025236	1.270545653	0.249102657	5.100490168	3.38775E-07	6.72493E-07	positive
PRJNA729271	stage_5W_vs_E10	ordinary_muscle_development	ENSGALG00010025238	IGSF9B	0.218371249	0.302117859	0.722801525	0.46980183	0.511782469	positive
PRJNA729271	stage_5W_vs_E10	ordinary_muscle_development	ENSGALG00010025239	RAG2	-2.297341634	0.637587878	-3.603176464	0.000314352	0.000496625	negative
PRJNA729271	stage_5W_vs_E10	ordinary_muscle_development	ENSGALG00010025242	CLSTN2	-4.184581048	0.31142536	-13.43686669	3.67678E-41	3.32885E-40	negative
PRJNA729271	stage_5W_vs_E10	ordinary_muscle_development	ENSGALG00010025243	PAXIP1	-0.547108025	0.090131909	-6.070081441	1.27845E-09	2.96784E-09	negative
PRJNA729271	stage_5W_vs_E10	ordinary_muscle_development	ENSGALG00010025245	ENSGALG00010025245	1.389134085	0.067981792	20.43391402	8.35303E-93	3.03452E-91	positive
PRJNA729271	stage_5W_vs_E10	ordinary_muscle_development	ENSGALG00010025247	SGCA	3.128517427	0.140165542	22.32016074	2.3544E-110	1.2102E-108	positive

PRJNA729271	stage_5W_vs_E10	ordinary_muscle_development	ENSGALG00010025248	C11orf74	-0.057007834	0.213710628	-0.266752451	0.789659767	0.814844331	negative
PRJNA729271	stage_5W_vs_E10	ordinary_muscle_development	ENSGALG00010025250	GLIS1	-4.816144665	0.509108875	-9.45995032	3.0809E-21	1.30197E-20	negative
PRJNA729271	stage_5W_vs_E10	ordinary_muscle_development	ENSGALG00010025252	PPP1R9B	-0.012478214	0.145154045	-0.085965321	0.931493985	0.940775175	negative
PRJNA729271	stage_5W_vs_E10	ordinary_muscle_development	ENSGALG00010025256	ANAPC5	-1.152865626	0.124441145	-9.264344371	1.96279E-20	7.9828E-20	negative
PRJNA729271	stage_5W_vs_E10	ordinary_muscle_development	ENSGALG00010025257	LPCAT2	0.578298334	0.165142603	3.501811903	0.000462106	0.000719311	positive
PRJNA729271	stage_5W_vs_E10	ordinary_muscle_development	ENSGALG00010025259	SHH	-6.708106372	1.053579774	-6.366965784	1.92804E-10	4.71281E-10	negative
PRJNA729271	stage_5W_vs_E10	ordinary_muscle_development	ENSGALG00010025262	CARF	-0.413395943	0.112680379	-3.66874826	0.000243741	0.000388886	negative
PRJNA729271	stage_5W_vs_E10	ordinary_muscle_development	ENSGALG00010025263	SAMD14	-1.597732486	0.815875479	-1.958304333	0.050194313	0.063266658	negative
PRJNA729271	stage_5W_vs_E10	ordinary_muscle_development	ENSGALG00010025268	ZBTB39	-1.184940624	0.113246903	-10.46333803	1.27291E-25	6.46262E-25	negative
PRJNA729271	stage_5W_vs_E10	ordinary_muscle_development	ENSGALG00010025270	NDC1	-2.164222019	0.124350012	-17.40427668	7.6567E-68	1.51204E-66	negative
PRJNA729271	stage_5W_vs_E10	ordinary_muscle_development	ENSGALG00010025271	RNF34	0.664707621	0.117833405	5.64107961	1.68987E-08	3.68009E-08	positive
PRJNA729271	stage_5W_vs_E10	ordinary_muscle_development	ENSGALG00010025272	NCAPD3	-2.148561188	0.186405347	-11.52628518	9.72501E-31	6.07604E-30	negative
PRJNA729271	stage_5W_vs_E10	ordinary_muscle_development	ENSGALG00010025274	PDK2	2.726746841	0.270025886	10.09809423	5.63261E-24	2.676E-23	positive
PRJNA729271	stage_5W_vs_E10	ordinary_muscle_development	ENSGALG00010025280	HSPB11	-0.614404422	0.15836805	-3.879598325	0.000104629	0.000172008	negative
PRJNA729271	stage_5W_vs_E10	ordinary_muscle_development	ENSGALG00010025286	ITGA3	-0.472351331	0.177084235	-2.667382168	0.007644469	0.01059235	negative
PRJNA729271	stage_5W_vs_E10	ordinary_muscle_development	ENSGALG00010025289	KDM2B	-0.993778581	0.075098642	-13.23297676	5.66005E-40	4.95425E-39	negative
PRJNA729271	stage_5W_vs_E10	ordinary_muscle_development	ENSGALG00010025291	DUS2	-0.186499746	0.093370426	-1.997417745	0.045779822	0.057994976	negative
PRJNA729271	stage_5W_vs_E10	ordinary_muscle_development	ENSGALG00010025292	ATP1A1	0.786567468	0.127514907	6.168435431	6.8969E-10	1.62475E-09	positive
PRJNA729271	stage_5W_vs_E10	ordinary_muscle_development	ENSGALG00010025297	SPSB4	-0.907650097	0.146381419	-6.200582713	5.62545E-10	1.3352E-09	negative
PRJNA729271	stage_5W_vs_E10	ordinary_muscle_development	ENSGALG00010025298	ENSGALG00010025298	-4.66289038	0.356980778	-13.06202088	5.42678E-39	4.55743E-38	negative
PRJNA729271	stage_5W_vs_E10	ordinary_muscle_development	ENSGALG00010025301	MORN3	-1.626988405	0.324439994	-5.01475907	5.31E-07	1.03997E-06	negative
PRJNA729271	stage_5W_vs_E10	ordinary_muscle_development	ENSGALG00010025303	TMCC2	2.571690165	0.188106814	13.67143545	1.50399E-42	1.42021E-41	positive
PRJNA729271	stage_5W_vs_E10	ordinary_muscle_development	ENSGALG00010025304	DDX28	-0.126575005	0.093281426	-1.356915422	0.174808086	0.205402335	negative
PRJNA729271	stage_5W_vs_E10	ordinary_muscle_development	ENSGALG00010025305	GPR182	2.099478213	0.411801235	5.09828052	3.42753E-07	6.7983E-07	positive
PRJNA729271	stage_5W_vs_E10	ordinary_muscle_development	ENSGALG00010025311	ENSGALG00010025311	0.349278257	0.690388713	0.505915364	0.612916066	0.650001344	positive
PRJNA729271	stage_5W_vs_E10	ordinary_muscle_development	ENSGALG00010025312	PLEKHM3	0.303382467	0.114233803	2.655802919	0.007911984	0.010949418	positive
PRJNA729271	stage_5W_vs_E10	ordinary_muscle_development	ENSGALG00010025313	DIO1	-0.852778456	0.898997453	-0.948588289	0.342830049	0.382697079	negative
PRJNA729271	stage_5W_vs_E10	ordinary_muscle_development	ENSGALG00010025315	LRRC42	0.013565776	0.119762189	0.113272614	0.909814428	0.921839879	positive
PRJNA729271	stage_5W_vs_E10	ordinary_muscle_development	ENSGALG00010025316	SLC25A36	-0.095744442	0.110047717	-0.870026605	0.384285865	0.425500238	negative
PRJNA729271	stage_5W_vs_E10	ordinary_muscle_development	ENSGALG00010025318	NUAK2	-2.217363087	0.45731553	-4.848650313	1.24304E-06	2.36828E-06	negative
PRJNA729271	stage_5W_vs_E10	ordinary_muscle_development	ENSGALG00010025319	LDLRAD1	1.127908508	0.34330918	3.285401539	0.001018371	0.001542777	positive
PRJNA729271	stage_5W_vs_E10	ordinary_muscle_development	ENSGALG00010025320	JAM3	-1.48345714	0.14482665	-10.24298454	1.27249E-24	6.22293E-24	negative
PRJNA729271	stage_5W_vs_E10	ordinary_muscle_development	ENSGALG00010025323	KLHDC8A	-2.280275025	0.192508323	-11.84507238	2.28221E-32	1.52516E-31	negative
PRJNA729271	stage_5W_vs_E10	ordinary_muscle_development	ENSGALG00010025325	DPEP2	1.339731376	0.242363594	5.52774842	3.24318E-08	6.91119E-08	positive
PRJNA729271	stage_5W_vs_E10	ordinary_muscle_development	ENSGALG00010025327	TMEM59	1.525081414	0.070824646	21.53320194	7.6091E-103	3.3832E-101	positive
PRJNA729271	stage_5W_vs_E10	ordinary_muscle_development	ENSGALG00010025329	MYO1A	-0.903022649	0.209157208	-4.317435004	1.57853E-05	2.77141E-05	negative
PRJNA729271	stage_5W_vs_E10	ordinary_muscle_development	ENSGALG00010025330	USH1C	-3.065305365	0.424189947	-7.2262565	4.9649E-13	1.40586E-12	negative
PRJNA729271	stage_5W_vs_E10	ordinary_muscle_development	ENSGALG00010025331	ENSGALG00010025331	2.455874577	0.336777252	7.292281647	3.04749E-13	8.7024E-13	positive
PRJNA729271	stage_5W_vs_E10	ordinary_muscle_development	ENSGALG00010025333	FZD5	0.90170991	0.196999972	4.577208325	4.71222E-06	8.59057E-06	positive
PRJNA729271	stage_5W_vs_E10	ordinary_muscle_development	ENSGALG00010025334	RHOF	2.12901613	0.217012025	9.810590578	1.01374E-22	4.58188E-22	positive
PRJNA729271	stage_5W_vs_E10	ordinary_muscle_development	ENSGALG00010025336	ADAM10	-0.036742387	0.063349283	-0.579996877	0.561916724	0.601416445	negative
PRJNA729271	stage_5W_vs_E10	ordinary_muscle_development	ENSGALG00010025337	NEMP1	-0.086345121	0.14633707	-0.59004271	0.555162016	0.594889734	negative
PRJNA729271	stage_5W_vs_E10	ordinary_muscle_development	ENSGALG00010025338	FADD	-1.084616649	0.098517614	-11.0093678	3.44412E-28	1.94025E-27	negative
PRJNA729271	stage_5W_vs_E10	ordinary_muscle_development	ENSGALG00010025340	METTL21A	1.003248168	0.116508576	8.610938352	7.2465E-18	2.63319E-17	positive
PRJNA729271	stage_5W_vs_E10	ordinary_muscle_development	ENSGALG00010025341	VPS26B	-1.183470805	0.132466487	-8.934214829	4.10451E-19	1.57435E-18	negative
PRJNA729271	stage_5W_vs_E10	ordinary_muscle_development	ENSGALG00010025342	TCEANC2	-1.136447185	0.123312872	-9.215965607	3.08489E-20	1.24521E-19	negative
PRJNA729271	stage_5W_vs_E10	ordinary_muscle_development	ENSGALG00010025343	NAB2	1.507039899	0.288787509	5.218507903	1.8037E-07	3.65404E-07	positive
PRJNA729271	stage_5W_vs_E10	ordinary_muscle_development	ENSGALG00010025344	ENSGALG00010025344	0.606167969	0.536350866	1.130170581	0.258404354	0.294718003	positive
PRJNA729271	stage_5W_vs_E10	ordinary_muscle_development	ENSGALG00010025345	CCM2	-0.070389888	0.089612249	-0.785494045	0.43216396	0.474059074	negative
PRJNA729271	stage_5W_vs_E10	ordinary_muscle_development	ENSGALG00010025346	PXYLP1	-2.476774362	0.129707289	-19.09510547	2.77297E-81	7.70004E-80	negative
PRJNA729271	stage_5W_vs_E10	ordinary_muscle_development	ENSGALG00010025348	HIPK1	0.718039245	0.092710381	7.744971387	9.56034E-15	2.96485E-14	positive
PRJNA729271	stage_5W_vs_E10	ordinary_muscle_development	ENSGALG00010025349	SETD1A	0.1813196	0.126851206	1.429388069	0.152892726	0.18125297	positive
PRJNA729271	stage_5W_vs_E10	ordinary_muscle_development	ENSGALG00010025351	CREB1	-0.462548732	0.067262294	-6.876790959	6.12158E-12	1.63743E-11	negative
PRJNA729271	stage_5W_vs_E10	ordinary_muscle_development	ENSGALG00010025353	SLC12A4	-0.437694789	0.077961518	-5.614241471	1.97426E-08	4.28013E-08	negative
PRJNA729271	stage_5W_vs_E10	ordinary_muscle_development	ENSGALG00010025354	ENSGALG00010025354	-4.236283925	1.705941377	-2.483252931	0.013018861	0.017595188	negative
PRJNA729271	stage_5W_vs_E10	ordinary_muscle_development	ENSGALG00010025355	STAT6	1.522108226	0.139943443	10.87659555	1.49025E-27	8.20092E-27	positive
PRJNA729271	stage_5W_vs_E10	ordinary_muscle_development	ENSGALG00010025357	OLFML3	-1.426958746	0.121604521	-11.73442182	8.49045E-32	5.51632E-31	negative
PRJNA729271	stage_5W_vs_E10	ordinary_muscle_development	ENSGALG00010025358	INCENP	-2.342586065	0.205939099	-11.37513989	5.56136E-30	3.36725E-29	negative
PRJNA729271	stage_5W_vs_E10	ordinary_muscle_development	ENSGALG00010025359	CDCP2	1.556424704	0.37769872	4.120810116	3.77543E-05	6.4246E-05	positive
PRJNA729271	stage_5W_vs_E10	ordinary_muscle_development	ENSGALG00010025360	THYN1	0.777666949	0.111021323	7.004662949	2.47581E-12	6.75454E-12	positive
PRJNA729271	stage_5W_vs_E10	ordinary_muscle_development	ENSGALG00010025362	G6PC2	-0.417713117	0.242631973	-1.7215914	0.085143562	0.104377193	negative
PRJNA729271	stage_5W_vs_E10	ordinary_muscle_development	ENSGALG00010025364	EPHA4	-3.537046889	0.188117262	-18.80235154	7.22414E-79	1.90389E-77	negative
PRJNA729271	stage_5W_vs_E10	ordinary_muscle_development	ENSGALG00010025365	SIKE1	0.485265182	0.091177436	5.32220691	1.02516E-07	2.11405E-07	positive
PRJNA729271	stage_5W_vs_E10	ordinary_muscle_development	ENSGALG00010025368	LRP1	1.516630041	0.138708379	10.9339468	7.93217E-28	4.42218E-27	positive
PRJNA729271	stage_5W_vs_E10	ordinary_muscle_development	ENSGALG00010025370	HPD	1.623769256	0.494726042	3.282158442	0.001030157	0.001560143	positive
PRJNA729271	stage_5W_vs_E10	ordinary_muscle_development	ENSGALG00010025373	CYB5RL	-0.881428566	0.092003547	-9.580375932	9.66923E-22	4.17751E-21	negative
PRJNA729271	stage_5W_vs_E10	ordinary_muscle_development	ENSGALG00010025377	MYO1G	3.782689736	0.278906457	13.56257498	6.6751E-42	6.17063E-41	positive
PRJNA729271	stage_5W_vs_E10	ordinary_muscle_development	ENSGALG00010025378	PAX3	-2.761833719	0.282845491	-9.764460835	1.59961E-22	7.16068E-22	negative
PRJNA729271	stage_5W_vs_E10	ordinary_muscle_development	ENSGALG00010025379	ACAD8	1.142408133	0.186122953	6.137921825	8.3608E-10	1.95973E-09	positive
PRJNA729271	stage_5W_vs_E10	ordinary_muscle_development	ENSGALG00010025380	SPC25	-2.253922423	0.226578536	-9.947643168	2.58226E-23	1.19432E-22	negative
PRJNA729271	stage_5W_vs_E10	ordinary_muscle_development	ENSGALG00010025381	MRPL37	0.634573061	0.122535414	5.178691144	2.23448E-07	4.49595E-07	positive
PRJNA729271	stage_5W_vs_E10	ordinary_muscle_development	ENSGALG00010025382	MAB21L3	-0.349387505	0.309681077	-1.128217158	0.259228219	0.295587873	negative
PRJNA729271	stage_5W_vs_E10	ordinary_muscle_development	ENSGALG00010025386	TPCN2	-0.622840328	0.099260365	-6.274814002	3.50053E-10	8.40903E-10	negative
PRJNA729271	stage_5W_vs_E10	ordinary_muscle_development	ENSGALG00010025387	PSMD9	-0.118656161	0.069280998	-1.712679734	0.086771476	0.106265	negative
PRJNA729271	stage_5W_vs_E10	ordinary_muscle_development	ENSGALG00010025389	SNED1	-1.888466423	0.152949021	-12.34703177	5.0542E-35	3.73017E-34	negative
PRJNA729271	stage_5W_vs_E10	ordinary_muscle_development	ENSGALG00010025390	BCL7A	-1.736112436	0.090368962	-19.21137965	2.9726E-82	8.4156E-81	negative
PRJNA729271	stage_5W_vs_E10	ordinary_muscle_development	ENSGALG00010025392	SLC6A2	0.048823334	0.239252036	0.204066537	0.838301504	0.858013013	positive
PRJNA729271	stage_5W_vs_E10	ordinary_muscle_development	ENSGALG00010025398	TSPAN2	0.357894271	0.143857728	2.487834857	0.01285234	0.017376613	positive
PRJNA729271	stage_5W_vs_E10	ordinary_muscle_development	ENSGALG00010025400	SLC22A15	-0.617343872	0.157637608	-3.916222018	8.99474E-05	0.000148902	negative
PRJNA729271	stage_5W_vs_E10	ordinary_muscle_development	ENSGALG00010025403	ENSGALG00010025403	0.378047929	0.130070739	2.906479436	0.003655209	0.005234367	positive
PRJNA729271	stage_5W_vs_E10	ordinary_muscle_development	ENSGALG00010025404	CERS6	-0.892931218	0.280353906	-3.185014371	0.001447468	0.002159211	negative
PRJNA729271	stage_5W_vs_E10	ordinary_muscle_development	ENSGALG00010025405	TAMALIN	2.326616722	0.163095448	14.26536886	3.59685E-46	3.79726E-45	positive
PRJNA729271	stage_5W_vs_E10	ordinary_muscle_development	ENSGALG00010025406	ENSGALG00010025406	-0.084757443	0.149021497	-0.568759844	0.569519129	0.608519813	negative
PRJNA729271	stage_5W_vs_E10	ordinary_muscle_development	ENSGALG00010025407	MLXIP	1.251548725	0.128207615	9.761890722	1.64067E-22	7.33773E-22	positive
PRJNA729271	stage_5W_vs_E10	ordinary_muscle_development	ENSGALG00010025413	NECAB3	0.812943721	0.121758529	6.676688082	2.44402E-11	6.32496E-11	positive
PRJNA729271	stage_5W_vs_E10	ordinary_muscle_development	ENSGALG00010025414	MMP2	0.26288447	0.221387395	1.187441	0.235053716	0.270040708	positive
PRJNA729271	stage_5W_vs_E10	ordinary_muscle_development	ENSGALG00010025415	ENSGALG00010025415	4.368642634	0.275932644	15.83227912	1.86347E-56	2.7011E-55	positive
PRJNA729271	stage_5W_vs_E10	ordinary_muscle_development	ENSGALG00010025416	ENSGALG00010025416	2.499657958	0.599828925	4.167284791	3.08249E-05	5.28266E-05	positive
PRJNA729271	stage_5W_vs_E10	ordinary_muscle_development	ENSGALG00010025417	B3GAT1	-2.165162984	0.204524328	-10.58633469	3.44831E-26	1.7986E-25	negative
PRJNA729271	stage_5W_vs_E10	ordinary_muscle_development	ENSGALG00010025418	CASQ2	4.493356388	0.209413513	21.45685976	3.9403E-102	1.72034E-100	positive
PRJNA729271	stage_5W_vs_E10	ordinary_muscle_development	ENSGALG00010025419	CCND1	-1.335255376	0.134780027	-9.906923203	3.88424E-23	1.78284E-22	negative
PRJNA729271	stage_5W_vs_E10	ordinary_muscle_development	ENSGALG00010025420	STK39	-1.491553045	0.209398452	-7.123037599	1.05574E-12	2.94288E-12	negative
PRJNA729271	stage_5W_vs_E10	ordinary_muscle_development	ENSGALG00010025421	NR4A1	7.532624669	0.397031625	18.97235431	2.88683E-80	7.80684E-79	positive
PRJNA729271	stage_5W_vs_E10	ordinary_muscle_development	ENSGALG00010025422	SSBP3	-1.31597507	0.158287106	-8.313848834	9.26472E-17	3.1853E-16	negative
PRJNA729271	stage_5W_vs_E10	ordinary_muscle_development	ENSGALG00010025423	NGF	-0.328082197	0.34630118	-0.947389775	0.343440199	0.383289628	negative
PRJNA729271	stage_5W_vs_E10	ordinary_muscle_development	ENSGALG00010025424	IRX6	-4.055929861	0.426516452	-9.50943356	1.91704E-21	8.17518E-21	negative
PRJNA729271	stage_5W_vs_E10	ordinary_muscle_development	ENSGALG00010025425	ENSGALG00010025425	0.284012387	0.139574185	2.034848972	0.041866072	0.053325897	positive
PRJNA729271	stage_5W_vs_E10	ordinary_muscle_development	ENSGALG00010025426	ENSGALG00010025426	0.852968118	0.153148115	5.569563261	2.55379E-08	5.49379E-08	positive
PRJNA729271	stage_5W_vs_E10	ordinary_muscle_development	ENSGALG00010025427	SLC35G2	-0.534611468	0.132765262	-4.026742082	5.6555E-05	9.51224E-05	negative
PRJNA729271	stage_5W_vs_E10	ordinary_muscle_development	ENSGALG00010025428	VANGL1	-0.201313312	0.110005424	-1.830030784	0.067245336	0.083480742	negative
PRJNA729271	stage_5W_vs_E10	ordinary_muscle_development	ENSGALG00010025429	SCN3B	-4.279175514	0.281352674	-15.20929393	3.06835E-52	3.98171E-51	negative
PRJNA729271	stage_5W_vs_E10	ordinary_muscle_development	ENSGALG00010025430	IRX5	-4.262814315	0.873857128	-4.878159343	1.0708E-06	2.05118E-06	negative
PRJNA729271	stage_5W_vs_E10	ordinary_muscle_development	ENSGALG00010025432	IRX3	-4.224651182	0.733646957	-5.758425279	8.49022E-09	1.88203E-08	negative
PRJNA729271	stage_5W_vs_E10	ordinary_muscle_development	ENSGALG00010025433	CHPF2	0.916605856	0.121502887	7.543901841	4.56115E-14	1.3643E-13	positive
PRJNA729271	stage_5W_vs_E10	ordinary_muscle_development	ENSGALG00010025434	SYT6	-1.082439168	0.245391685	-4.411067009	1.02862E-05	1.83169E-05	negative
PRJNA729271	stage_5W_vs_E10	ordinary_muscle_development	ENSGALG00010025435	SCLY	-0.306412764	0.101847327	-3.008549884	0.002624977	0.003816353	negative
PRJNA729271	stage_5W_vs_E10	ordinary_muscle_development	ENSGALG00010025438	EXOSC6	-0.117426689	0.122860668	-0.955771206	0.339187838	0.378897277	negative
PRJNA729271	stage_5W_vs_E10	ordinary_muscle_development	ENSGALG00010025442	ABCF2	2.509069424	0.146861485	17.08459794	1.93271E-65	3.62414E-64	positive
PRJNA729271	stage_5W_vs_E10	ordinary_muscle_development	ENSGALG00010025445	B3GNT4	0.960906231	0.302418066	3.177410141	0.001485967	0.002212315	positive
PRJNA729271	stage_5W_vs_E10	ordinary_muscle_development	ENSGALG00010025448	CHD1L	-0.353014822	0.109095	-3.235847865	0.00121282	0.001821555	negative
PRJNA729271	stage_5W_vs_E10	ordinary_muscle_development	ENSGALG00010025449	ENSGALG00010025449	0.363652548	0.11677159	3.114221099	0.001844312	0.002721224	positive
PRJNA729271	stage_5W_vs_E10	ordinary_muscle_development	ENSGALG00010025451	LRRC43	1.030225758	0.352875106	2.919519512	0.003505714	0.005029727	positive
PRJNA729271	stage_5W_vs_E10	ordinary_muscle_development	ENSGALG00010025452	TRIM33	-0.079767911	0.126538676	-0.630383639	0.528443613	0.568746706	negative
PRJNA729271	stage_5W_vs_E10	ordinary_muscle_development	ENSGALG00010025458	BCAS2	-0.207298191	0.116754781	-1.775500665	0.075815256	0.09352699	negative
PRJNA729271	stage_5W_vs_E10	ordinary_muscle_development	ENSGALG00010025459	DIABLO	-0.535077214	0.079781151	-6.706812402	1.98922E-11	5.17754E-11	negative
PRJNA729271	stage_5W_vs_E10	ordinary_muscle_development	ENSGALG00010025464	VPS33A	-0.223448135	0.090917672	-2.457697486	0.013983091	0.018831636	negative
PRJNA729271	stage_5W_vs_E10	ordinary_muscle_development	ENSGALG00010025465	ENSGALG00010025465	-0.541879625	0.084807537	-6.389522043	1.66405E-10	4.08198E-10	negative
PRJNA729271	stage_5W_vs_E10	ordinary_muscle_development	ENSGALG00010025470	ORAOV1	0.157379023	0.124282915	1.266296526	0.20540694	0.238437863	positive
PRJNA729271	stage_5W_vs_E10	ordinary_muscle_development	ENSGALG00010025472	ZCCHC8	0.134061474	0.09794596	1.368728973	0.17108401	0.201434711	positive
PRJNA729271	stage_5W_vs_E10	ordinary_muscle_development	ENSGALG00010025473	PARS2	0.381525051	0.079382293	4.806173229	1.53847E-06	2.91034E-06	positive
PRJNA729271	stage_5W_vs_E10	ordinary_muscle_development	ENSGALG00010025477	ENSGALG00010025477	0.827287437	0.070364856	11.75711128	6.49175E-32	4.24247E-31	positive
PRJNA729271	stage_5W_vs_E10	ordinary_muscle_development	ENSGALG00010025480	FGF19	-5.486727328	0.894341112	-6.134938061	8.51924E-10	1.9959E-09	negative
PRJNA729271	stage_5W_vs_E10	ordinary_muscle_development	ENSGALG00010025481	TMEM39A	-0.217664398	0.09112483	-2.388639812	0.016910873	0.022552498	negative
PRJNA729271	stage_5W_vs_E10	ordinary_muscle_development	ENSGALG00010025483	FGF4	-2.337800935	0.369512243	-6.326721191	2.50425E-10	6.0772E-10	negative
PRJNA729271	stage_5W_vs_E10	ordinary_muscle_development	ENSGALG00010025485	DENND2C	1.216664361	0.159880863	7.609818564	2.74481E-14	8.31126E-14	positive
PRJNA729271	stage_5W_vs_E10	ordinary_muscle_development	ENSGALG00010025486	B3GALT1	-2.621392548	0.211973416	-12.36660993	3.96193E-35	2.94051E-34	negative
PRJNA729271	stage_5W_vs_E10	ordinary_muscle_development	ENSGALG00010025487	FGF3	-6.369203688	0.932916964	-6.827192491	8.65925E-12	2.29756E-11	negative
PRJNA729271	stage_5W_vs_E10	ordinary_muscle_development	ENSGALG00010025488	SLC4A8	-2.558510276	0.202820258	-12.61466831	1.75294E-36	1.36533E-35	negative
PRJNA729271	stage_5W_vs_E10	ordinary_muscle_development	ENSGALG00010025491	ZMYND11	-0.411928671	0.064402786	-6.396131257	1.59363E-10	3.91475E-10	negative
PRJNA729271	stage_5W_vs_E10	ordinary_muscle_development	ENSGALG00010025496	ATG101	0.636506874	0.111262998	5.720741703	1.0606E-08	2.33745E-08	positive
PRJNA729271	stage_5W_vs_E10	ordinary_muscle_development	ENSGALG00010025499	STAC3	4.691221183	0.158618315	29.57553289	3.0845E-192	4.7063E-190	positive

PRJNA729271	stage_5W_vs_E10	ordinary_muscle_development	ENSGALG00010025504	AMPD1	5.965642369	0.197718931	30.17233771	5.4642E-200	9.2098E-198	positive
PRJNA729271	stage_5W_vs_E10	ordinary_muscle_development	ENSGALG00010025505	ENSGALG00010025505	-1.198966665	0.265570685	-4.514680016	6.34124E-06	1.1448E-05	negative
PRJNA729271	stage_5W_vs_E10	ordinary_muscle_development	ENSGALG00010025506	R3HDM2	0.761483914	0.17712919	4.2990312	1.71546E-05	3.00273E-05	positive
PRJNA729271	stage_5W_vs_E10	ordinary_muscle_development	ENSGALG00010025508	ENSGALG00010025508	-0.447777859	0.134578539	-3.327260522	0.000877043	0.001335372	negative
PRJNA729271	stage_5W_vs_E10	ordinary_muscle_development	ENSGALG00010025512	NRAS	-0.260075781	0.078066296	-3.331473292	0.000863876	0.001316292	negative
PRJNA729271	stage_5W_vs_E10	ordinary_muscle_development	ENSGALG00010025513	RSRC2	-0.111494824	0.08793725	-1.267890727	0.204836976	0.237795288	negative
PRJNA729271	stage_5W_vs_E10	ordinary_muscle_development	ENSGALG00010025520	ACOT11	0.80704212	0.296200217	2.724650669	0.006436955	0.008987218	positive
PRJNA729271	stage_5W_vs_E10	ordinary_muscle_development	ENSGALG00010025522	SUMO3	-0.580667789	0.10636903	-5.458992989	4.78843E-08	1.01002E-07	negative
PRJNA729271	stage_5W_vs_E10	ordinary_muscle_development	ENSGALG00010025523	B4GALT4	-1.26027355	0.134858726	-9.345139066	9.17688E-21	3.7854E-20	negative
PRJNA729271	stage_5W_vs_E10	ordinary_muscle_development	ENSGALG00010025527	KNTC1	-2.8428932	0.241313049	-11.78093442	4.89474E-32	3.22058E-31	negative
PRJNA729271	stage_5W_vs_E10	ordinary_muscle_development	ENSGALG00010025531	ENSGALG00010025531	-0.055092405	0.096913515	-0.568469788	0.569716015	0.608685313	negative
PRJNA729271	stage_5W_vs_E10	ordinary_muscle_development	ENSGALG00010025533	INHBC	-1.872096903	0.64598201	-2.898063526	0.003754745	0.005371598	negative
PRJNA729271	stage_5W_vs_E10	ordinary_muscle_development	ENSGALG00010025538	ANO1	2.942463878	0.209402065	14.05174239	7.51617E-45	7.63468E-44	positive
PRJNA729271	stage_5W_vs_E10	ordinary_muscle_development	ENSGALG00010025541	TTC21B	-0.592497835	0.128923164	-4.595743845	4.31208E-06	7.8849E-06	negative
PRJNA729271	stage_5W_vs_E10	ordinary_muscle_development	ENSGALG00010025544	CSDE1	0.554720208	0.08597966	6.451760892	1.10558E-10	2.7436E-10	positive
PRJNA729271	stage_5W_vs_E10	ordinary_muscle_development	ENSGALG00010025548	ENSGALG00010025548	-1.91592526	0.205666285	-9.315699251	1.21152E-20	4.97619E-20	negative
PRJNA729271	stage_5W_vs_E10	ordinary_muscle_development	ENSGALG00010025549	DHCR24	-1.283042487	0.246340039	-5.208420422	1.90455E-07	3.85242E-07	negative
PRJNA729271	stage_5W_vs_E10	ordinary_muscle_development	ENSGALG00010025553	UBE2G2	-0.285430236	0.096764066	-2.949754473	0.003180266	0.004585036	negative
PRJNA729271	stage_5W_vs_E10	ordinary_muscle_development	ENSGALG00010025554	DIP2C	0.035178606	0.071171358	0.494280382	0.621108178	0.657630609	positive
PRJNA729271	stage_5W_vs_E10	ordinary_muscle_development	ENSGALG00010025557	VPS29	-0.552211034	0.078076235	-7.072715943	1.5193E-12	4.19552E-12	negative
PRJNA729271	stage_5W_vs_E10	ordinary_muscle_development	ENSGALG00010025558	GALNT3	-2.702270571	0.235823593	-11.45886437	2.1228E-30	1.30216E-29	negative
PRJNA729271	stage_5W_vs_E10	ordinary_muscle_development	ENSGALG00010025559	SLC6A17	-4.625958779	1.19618811	-3.867250259	0.000110069	0.000180461	negative
PRJNA729271	stage_5W_vs_E10	ordinary_muscle_development	ENSGALG00010025562	GRAMD1B	-0.267203976	0.185938781	-1.437053496	0.150702794	0.178817698	negative
PRJNA729271	stage_5W_vs_E10	ordinary_muscle_development	ENSGALG00010025563	TSPEAR	0.093708587	0.236705987	0.395886001	0.692189129	0.724963695	positive
PRJNA729271	stage_5W_vs_E10	ordinary_muscle_development	ENSGALG00010025565	ENSGALG00010025565	1.952450401	0.243006687	8.034554226	9.39197E-16	3.07168E-15	positive
PRJNA729271	stage_5W_vs_E10	ordinary_muscle_development	ENSGALG00010025568	MYL3	-7.809367952	1.012939125	-7.709612318	1.26201E-14	3.88052E-14	negative
PRJNA729271	stage_5W_vs_E10	ordinary_muscle_development	ENSGALG00010025569	PPTC7	1.401835923	0.192890492	7.267522139	3.66141E-13	1.04165E-12	positive
PRJNA729271	stage_5W_vs_E10	ordinary_muscle_development	ENSGALG00010025571	SRSF3	-1.507956113	0.229650265	-6.566315573	5.15755E-11	1.30903E-10	negative
PRJNA729271	stage_5W_vs_E10	ordinary_muscle_development	ENSGALG00010025572	ENSGALG00010025572	-0.277819088	0.112458454	-2.470415323	0.013495627	0.018202206	negative
PRJNA729271	stage_5W_vs_E10	ordinary_muscle_development	ENSGALG00010025573	CLMP	-1.932223694	0.169084849	-11.42753891	3.0462E-30	1.85836E-29	negative
PRJNA729271	stage_5W_vs_E10	ordinary_muscle_development	ENSGALG00010025574	ARHGAP31	-0.60784693	0.099787643	-6.091404849	1.11924E-09	2.60784E-09	negative
PRJNA729271	stage_5W_vs_E10	ordinary_muscle_development	ENSGALG00010025575	TRPM2	-1.443728364	0.513148813	-2.813469169	0.004901009	0.006927364	negative
PRJNA729271	stage_5W_vs_E10	ordinary_muscle_development	ENSGALG00010025577	PTH2R	-3.417356769	0.205067882	-16.66451485	2.37407E-62	4.05804E-61	negative
PRJNA729271	stage_5W_vs_E10	ordinary_muscle_development	ENSGALG00010025578	ORMDL2	0.080925456	0.147810288	0.547495425	0.584038413	0.622336013	positive
PRJNA729271	stage_5W_vs_E10	ordinary_muscle_development	ENSGALG00010025579	CSRNP3	-3.849476437	0.349992014	-10.99875508	3.87444E-28	2.17928E-27	negative
PRJNA729271	stage_5W_vs_E10	ordinary_muscle_development	ENSGALG00010025580	RASSF7	-0.935537802	0.169975489	-5.503957109	3.7136E-08	7.88236E-08	negative
PRJNA729271	stage_5W_vs_E10	ordinary_muscle_development	ENSGALG00010025582	DMAP1	-0.445790186	0.115094806	-3.873243284	0.000107397	0.000176358	negative
PRJNA729271	stage_5W_vs_E10	ordinary_muscle_development	ENSGALG00010025585	SARNP	-0.936727823	0.096956369	-9.661333546	4.40097E-22	1.93368E-21	negative
PRJNA729271	stage_5W_vs_E10	ordinary_muscle_development	ENSGALG00010025586	TCTN1	0.545959482	0.125645418	4.345239879	1.39124E-05	2.45507E-05	positive
PRJNA729271	stage_5W_vs_E10	ordinary_muscle_development	ENSGALG00010025587	LRRC56	-3.423584806	0.323084354	-10.59656638	3.09122E-26	1.61526E-25	negative
PRJNA729271	stage_5W_vs_E10	ordinary_muscle_development	ENSGALG00010025588	ENSGALG00010025588	0.220075032	0.30781157	0.714966731	0.474629601	0.516413939	positive
PRJNA729271	stage_5W_vs_E10	ordinary_muscle_development	ENSGALG00010025590	HRAS	-0.261045548	0.114366817	-2.282528761	0.022458141	0.029487399	negative
PRJNA729271	stage_5W_vs_E10	ordinary_muscle_development	ENSGALG00010025591	OTOS	1.486329246	0.342928587	4.334223802	1.46275E-05	2.57688E-05	positive
PRJNA729271	stage_5W_vs_E10	ordinary_muscle_development	ENSGALG00010025592	HSPA8	-0.260778953	0.208198212	-1.252551361	0.210369026	0.243768409	negative
PRJNA729271	stage_5W_vs_E10	ordinary_muscle_development	ENSGALG00010025594	UPK1B	-4.4472138	0.524704602	-8.475652367	2.33766E-17	8.26246E-17	negative
PRJNA729271	stage_5W_vs_E10	ordinary_muscle_development	ENSGALG00010025595	ERI3	-0.302993841	0.094729312	-3.198522557	0.001381337	0.002064386	negative
PRJNA729271	stage_5W_vs_E10	ordinary_muscle_development	ENSGALG00010025597	SCN2A	-5.290553775	0.807512534	-6.551667689	5.6898E-11	1.43883E-10	negative
PRJNA729271	stage_5W_vs_E10	ordinary_muscle_development	ENSGALG00010025598	ENSGALG00010025598	2.661137082	0.394322357	6.74863354	1.49244E-11	3.91617E-11	positive
PRJNA729271	stage_5W_vs_E10	ordinary_muscle_development	ENSGALG00010025599	COPS9	0.90421008	0.142459053	6.347157722	2.19329E-10	5.34136E-10	positive
PRJNA729271	stage_5W_vs_E10	ordinary_muscle_development	ENSGALG00010025600	ENSGALG00010025600	-1.155079045	0.112493672	-10.26794688	9.82738E-25	4.82874E-24	negative
PRJNA729271	stage_5W_vs_E10	ordinary_muscle_development	ENSGALG00010025604	HVCN1	0.087738943	0.134331114	0.653154288	0.513656816	0.554101031	positive
PRJNA729271	stage_5W_vs_E10	ordinary_muscle_development	ENSGALG00010025606	CCDC12	-0.768187245	0.13244049	-5.800244642	6.62182E-09	1.47644E-08	negative
PRJNA729271	stage_5W_vs_E10	ordinary_muscle_development	ENSGALG00010025608	ENSGALG00010025608	-0.817894817	0.485103737	-1.686020443	0.091791862	0.112044045	negative
PRJNA729271	stage_5W_vs_E10	ordinary_muscle_development	ENSGALG00010025610	RNH1	-0.546910453	0.13381628	-4.087024799	4.3694E-05	7.39976E-05	negative
PRJNA729271	stage_5W_vs_E10	ordinary_muscle_development	ENSGALG00010025612	TMEM53	0.204376071	0.150411247	1.358781846	0.174215727	0.204789309	positive
PRJNA729271	stage_5W_vs_E10	ordinary_muscle_development	ENSGALG00010025613	CDKN1A	4.520551372	0.272479447	16.59043064	8.17322E-62	1.38887E-60	positive
PRJNA729271	stage_5W_vs_E10	ordinary_muscle_development	ENSGALG00010025618	ADIPOQ	7.823163271	0.221569942	35.30787256	4.4464E-273	1.4989E-270	positive
PRJNA729271	stage_5W_vs_E10	ordinary_muscle_development	ENSGALG00010025621	PPP1CC	-0.477292881	0.117683484	-4.055733788	4.99772E-05	8.43328E-05	negative
PRJNA729271	stage_5W_vs_E10	ordinary_muscle_development	ENSGALG00010025623	ENSGALG00010025623	2.937589532	0.386186299	7.606664298	2.81261E-14	8.50589E-14	positive
PRJNA729271	stage_5W_vs_E10	ordinary_muscle_development	ENSGALG00010025624	ARMH1	-0.070151839	0.394520757	-0.17781533	0.858868007	0.876155378	negative
PRJNA729271	stage_5W_vs_E10	ordinary_muscle_development	ENSGALG00010025628	ENSGALG00010025628	-5.855029208	0.422237519	-13.86667206	1.00848E-43	9.88368E-43	negative
PRJNA729271	stage_5W_vs_E10	ordinary_muscle_development	ENSGALG00010025629	ST6GAL1	3.197592666	0.14337506	22.30229354	3.5104E-110	1.7917E-108	positive
PRJNA729271	stage_5W_vs_E10	ordinary_muscle_development	ENSGALG00010025635	CHKA	-0.773185654	0.163608819	-4.725818932	2.2919E-06	4.27886E-06	negative
PRJNA729271	stage_5W_vs_E10	ordinary_muscle_development	ENSGALG00010025637	C6orf89	0.316120364	0.127813671	2.473290708	0.013387519	0.018064801	positive
PRJNA729271	stage_5W_vs_E10	ordinary_muscle_development	ENSGALG00010025639	MYL2	-7.247547482	0.499774663	-14.50163048	1.18306E-47	1.31104E-46	negative
PRJNA729271	stage_5W_vs_E10	ordinary_muscle_development	ENSGALG00010025640	SLC38A11	-6.872897442	0.993809459	-6.915709426	4.65528E-12	1.25122E-11	negative
PRJNA729271	stage_5W_vs_E10	ordinary_muscle_development	ENSGALG00010025642	RPS8	-0.228710284	0.117583985	-1.945080228	0.051765323	0.06516748	negative
PRJNA729271	stage_5W_vs_E10	ordinary_muscle_development	ENSGALG00010025644	ENSGALG00010025644	4.154190639	0.282217948	14.71979605	4.81109E-49	5.6467E-48	positive
PRJNA729271	stage_5W_vs_E10	ordinary_muscle_development	ENSGALG00010025645	NBEAL2	1.985673071	0.185130623	10.7257948	7.7022E-27	4.1273E-26	positive
PRJNA729271	stage_5W_vs_E10	ordinary_muscle_development	ENSGALG00010025647	JHY	-1.189002722	0.313286669	-3.795254763	0.000147492	0.000239326	negative
PRJNA729271	stage_5W_vs_E10	ordinary_muscle_development	ENSGALG00010025648	ENSGALG00010025648	3.328607241	0.486725533	6.838776718	7.98723E-12	2.12275E-11	positive
PRJNA729271	stage_5W_vs_E10	ordinary_muscle_development	ENSGALG00010025650	PPP1R1A	5.682055525	0.456088247	12.45823711	1.26128E-35	9.522E-35	positive
PRJNA729271	stage_5W_vs_E10	ordinary_muscle_development	ENSGALG00010025652	KMT5B	-0.316579851	0.077214048	-4.100029203	4.13098E-05	7.0099E-05	negative
PRJNA729271	stage_5W_vs_E10	ordinary_muscle_development	ENSGALG00010025653	IGSF11	-2.616280075	0.221967088	-11.78679283	4.566E-32	3.01111E-31	negative
PRJNA729271	stage_5W_vs_E10	ordinary_muscle_development	ENSGALG00010025654	CRTAM	5.907869366	0.679026972	8.700492924	3.30446E-18	1.21847E-17	positive
PRJNA729271	stage_5W_vs_E10	ordinary_muscle_development	ENSGALG00010025658	PDE1B	0.561627725	0.359986329	1.560136261	0.118727684	0.142742627	positive
PRJNA729271	stage_5W_vs_E10	ordinary_muscle_development	ENSGALG00010025659	COBLL1	1.504181546	0.130188004	11.55391814	7.05301E-31	4.43144E-30	positive
PRJNA729271	stage_5W_vs_E10	ordinary_muscle_development	ENSGALG00010025660	KIF2C	-3.265770103	0.207270833	-15.75605241	6.24117E-56	8.87789E-55	negative
PRJNA729271	stage_5W_vs_E10	ordinary_muscle_development	ENSGALG00010025661	SNRPE	-0.811053728	0.130428945	-6.218356871	5.02388E-10	1.19595E-09	negative
PRJNA729271	stage_5W_vs_E10	ordinary_muscle_development	ENSGALG00010025664	GPR55	3.701989345	0.595469756	6.216922536	5.07E-10	1.20653E-09	positive
PRJNA729271	stage_5W_vs_E10	ordinary_muscle_development	ENSGALG00010025666	CUX2	-4.075604128	0.477297968	-8.538909449	1.35495E-17	4.85656E-17	negative
PRJNA729271	stage_5W_vs_E10	ordinary_muscle_development	ENSGALG00010025674	GRB14	1.965724288	0.445231835	4.415057805	1.00983E-05	1.79976E-05	positive
PRJNA729271	stage_5W_vs_E10	ordinary_muscle_development	ENSGALG00010025679	ENSGALG00010025679	0.668147168	1.276500036	0.52342119	0.600681181	0.638494395	positive
PRJNA729271	stage_5W_vs_E10	ordinary_muscle_development	ENSGALG00010025685	NCKAP1L	2.702661499	0.190469591	14.18946449	1.06469E-45	1.10946E-44	positive
PRJNA729271	stage_5W_vs_E10	ordinary_muscle_development	ENSGALG00010025688	BEST4	2.359776696	0.489261347	4.823141472	1.41315E-06	2.68215E-06	positive
PRJNA729271	stage_5W_vs_E10	ordinary_muscle_development	ENSGALG00010025691	ENSGALG00010025691	-4.119605633	0.187528374	-21.96790564	5.84E-107	2.7484E-105	negative
PRJNA729271	stage_5W_vs_E10	ordinary_muscle_development	ENSGALG00010025692	ENSGALG00010025692	5.140167917	0.954217076	5.38679096	7.17268E-08	1.49401E-07	positive
PRJNA729271	stage_5W_vs_E10	ordinary_muscle_development	ENSGALG00010025696	MTCH1	0.259659019	0.104270852	2.490235693	0.012765833	0.017264485	positive
PRJNA729271	stage_5W_vs_E10	ordinary_muscle_development	ENSGALG00010025701	ENSGALG00010025701	1.158318548	3.255211182	0.355835146	0.721964026	0.752272918	positive
PRJNA729271	stage_5W_vs_E10	ordinary_muscle_development	ENSGALG00010025702	GTSF2	-0.340127665	0.187138711	-1.817516343	0.069138088	0.085786388	negative
PRJNA729271	stage_5W_vs_E10	ordinary_muscle_development	ENSGALG00010025703	UBASH3B	-1.238590825	0.142656255	-8.68234499	3.87691E-18	1.42376E-17	negative
PRJNA729271	stage_5W_vs_E10	ordinary_muscle_development	ENSGALG00010025705	ACSL3	-1.41854095	0.086092164	-16.47700416	5.36797E-61	8.86205E-60	negative
PRJNA729271	stage_5W_vs_E10	ordinary_muscle_development	ENSGALG00010025706	ENSGALG00010025706	1.156518785	3.056422803	0.37838966	0.705141148	0.73718146	positive
PRJNA729271	stage_5W_vs_E10	ordinary_muscle_development	ENSGALG00010025708	HOXC8	-2.413597602	0.246120013	-9.806588161	1.05475E-22	4.76278E-22	negative
PRJNA729271	stage_5W_vs_E10	ordinary_muscle_development	ENSGALG00010025710	ENSGALG00010025710	0.193345323	2.061446255	0.093791105	0.925275097	0.935406789	positive
PRJNA729271	stage_5W_vs_E10	ordinary_muscle_development	ENSGALG00010025719	HOXC11	-6.817597976	2.679982774	-2.543896193	0.010962366	0.014951025	negative
PRJNA729271	stage_5W_vs_E10	ordinary_muscle_development	ENSGALG00010025720	PHETA1	-2.067070675	0.272267829	-7.592048908	3.14886E-14	9.50297E-14	negative
PRJNA729271	stage_5W_vs_E10	ordinary_muscle_development	ENSGALG00010025723	SETD2	0.301766279	0.122130113	2.470858991	0.013478899	0.018183028	positive
PRJNA729271	stage_5W_vs_E10	ordinary_muscle_development	ENSGALG00010025727	GLI1	-2.933068582	0.242005032	-12.11986609	8.28945E-34	5.82714E-33	negative
PRJNA729271	stage_5W_vs_E10	ordinary_muscle_development	ENSGALG00010025734	PLK3	-0.162621527	0.186776144	-0.870676111	0.383931026	0.425172299	negative
PRJNA729271	stage_5W_vs_E10	ordinary_muscle_development	ENSGALG00010025736	KIF9	1.701947776	0.320614178	5.308398352	1.10593E-07	2.27575E-07	positive
PRJNA729271	stage_5W_vs_E10	ordinary_muscle_development	ENSGALG00010025737	ENSGALG00010025737	-0.708010262	0.152625524	-4.638871937	3.50316E-06	6.45378E-06	negative
PRJNA729271	stage_5W_vs_E10	ordinary_muscle_development	ENSGALG00010025738	SOX13	0.075547955	0.155363356	0.486266239	0.626778413	0.662907924	positive
PRJNA729271	stage_5W_vs_E10	ordinary_muscle_development	ENSGALG00010025743	SH2B3	0.879843515	0.107854067	8.15772221	3.41402E-16	1.14102E-15	positive
PRJNA729271	stage_5W_vs_E10	ordinary_muscle_development	ENSGALG00010025745	COX14	1.330521072	0.226693726	5.869245245	4.37783E-09	9.85965E-09	positive
PRJNA729271	stage_5W_vs_E10	ordinary_muscle_development	ENSGALG00010025748	ETNK2	-1.789587774	0.239114852	-7.4842184	7.19742E-14	2.12608E-13	negative
PRJNA729271	stage_5W_vs_E10	ordinary_muscle_development	ENSGALG00010025753	SORL1	-0.526208147	0.172033317	-3.058757201	0.002222572	0.003252189	negative
PRJNA729271	stage_5W_vs_E10	ordinary_muscle_development	ENSGALG00010025755	ENSGALG00010025755	-0.237857535	0.272386253	-0.873236193	0.382534351	0.423755095	negative
PRJNA729271	stage_5W_vs_E10	ordinary_muscle_development	ENSGALG00010025782	ENSGALG00010025782	1.158242993	4.564438882	0.253753643	0.799685889	0.82354863	positive
PRJNA729271	stage_5W_vs_E10	ordinary_muscle_development	ENSGALG00010025784	ENSGALG00010025784	0.675242661	2.459378008	0.274558307	0.783655602	0.809686218	positive
PRJNA729271	stage_5W_vs_E10	ordinary_muscle_development	ENSGALG00010025787	KCNE4	2.590839701	0.290140592	8.929600927	4.27548E-19	1.63863E-18	positive
PRJNA729271	stage_5W_vs_E10	ordinary_muscle_development	ENSGALG00010025790	LRP5	0.125315933	0.078142271	1.603689406	0.108782532	0.131465967	positive
PRJNA729271	stage_5W_vs_E10	ordinary_muscle_development	ENSGALG00010025791	FIGN	0.043947638	0.238009715	0.184646402	0.853506388	0.871176332	positive
PRJNA729271	stage_5W_vs_E10	ordinary_muscle_development	ENSGALG00010025794	ENSGALG00010025794	2.822360977	4.075615271	0.692499363	0.488623786	0.530055514	positive
PRJNA729271	stage_5W_vs_E10	ordinary_muscle_development	ENSGALG00010025799	SC5D	1.205021111	0.265488941	4.538874978	5.65552E-06	1.02519E-05	positive
PRJNA729271	stage_5W_vs_E10	ordinary_muscle_development	ENSGALG00010025800	ARL2BP	-0.846188415	0.139256571	-6.076470278	1.22857E-09	2.85569E-09	negative
PRJNA729271	stage_5W_vs_E10	ordinary_muscle_development	ENSGALG00010025801	PTPN7	3.145862503	0.359399443	8.753109022	2.07554E-18	7.72003E-18	positive
PRJNA729271	stage_5W_vs_E10	ordinary_muscle_development	ENSGALG00010025804	ENSGALG00010025804	0.674633045	2.257954404	0.298780632	0.765107429	0.792782343	positive
PRJNA729271	stage_5W_vs_E10	ordinary_muscle_development	ENSGALG00010025805	ATXN2	0.008408221	0.09414392	0.089312421	0.928833623	0.938415234	positive
PRJNA729271	stage_5W_vs_E10	ordinary_muscle_development	ENSGALG00010025809	ENSGALG00010025809	1.58695745	0.191642207	8.280834777	1.22315E-16	4.17952E-16	positive
PRJNA729271	stage_5W_vs_E10	ordinary_muscle_development	ENSGALG00010025822	KLF7	-0.00140387	0.09848566	-0.014254561	0.988626891	0.990266518	negative
PRJNA729271	stage_5W_vs_E10	ordinary_muscle_development	ENSGALG00010025829	ENSGALG00010025829	-0.313810803	0.258848395	-1.212334358	0.225384417	0.25977633	negative
PRJNA729271	stage_5W_vs_E10	ordinary_muscle_development	ENSGALG00010025830	TBCEL	0.21892517	0.192703365	1.136073415	0.25592584	0.292144043	positive
PRJNA729271	stage_5W_vs_E10	ordinary_muscle_development	ENSGALG00010025831	BRAP	0.192688831	0.069875207	2.757613748	0.005822495	0.008166141	positive
PRJNA729271	stage_5W_vs_E10	ordinary_muscle_development	ENSGALG00010025832	ENKD1	1.241058104	0.145698379	8.517995297	1.62339E-17	5.79439E-17	positive
PRJNA729271	stage_5W_vs_E10	ordinary_muscle_development	ENSGALG00010025835	PPP6R3	-0.021715785	0.119414271	-0.181852514	0.855698471	0.873167852	negative
PRJNA729271	stage_5W_vs_E10	ordinary_muscle_development	ENSGALG00010025836	ENSGALG00010025836	0.261598747	0.11428062	2.289091072	0.022074061	0.029008479	positive
PRJNA729271	stage_5W_vs_E10	ordinary_muscle_development	ENSGALG00010025837	ENSGALG00010025837	2.535799757	0.177113564	14.31736624	1.70462E-46	1.82081E-45	positive
PRJNA729271	stage_5W_vs_E10	ordinary_muscle_development	ENSGALG00010025846	GRIK4	0.085310966	0.219094778	0.389379274	0.696995601	0.729454963	positive
PRJNA729271	stage_5W_vs_E10	ordinary_muscle_development	ENSGALG00010025847	DEF6	0.233002552	0.125778774	1.852479121	0.063957053	0.079616754	positive
PRJNA729271	stage_5W_vs_E10	ordinary_muscle_development	ENSGALG00010025849	OBSCN	4.586019093	0.229074155	20.01980143	3.70194E-89	1.23355E-87	positive

PRJNA729271	stage_5W_vs_E10	ordinary_muscle_development	ENSGALG00010025852	BTBD19	-0.067837558	0.24543536	-0.27639684	0.782243283	0.808457497	negative
PRJNA729271	stage_5W_vs_E10	ordinary_muscle_development	ENSGALG00010025858	ENSGALG00010025858	1.158338881	2.976788379	0.389123691	0.697184649	0.729600136	positive
PRJNA729271	stage_5W_vs_E10	ordinary_muscle_development	ENSGALG00010025862	PTCH2	-3.826725611	0.324739102	-11.78400011	4.71988E-32	3.10976E-31	negative
PRJNA729271	stage_5W_vs_E10	ordinary_muscle_development	ENSGALG00010025865	LGR6	-3.638218091	0.676857177	-5.375163648	7.65132E-08	1.59028E-07	negative
PRJNA729271	stage_5W_vs_E10	ordinary_muscle_development	ENSGALG00010025869	FASTKD2	-0.066446948	0.093283108	-0.712314902	0.476269798	0.517933133	negative
PRJNA729271	stage_5W_vs_E10	ordinary_muscle_development	ENSGALG00010025870	ENSGALG00010025870	1.874606686	0.164191346	11.41720759	3.43081E-30	2.0886E-29	positive
PRJNA729271	stage_5W_vs_E10	ordinary_muscle_development	ENSGALG00010025871	ENSGALG00010025871	0.15702631	1.590204933	0.098745959	0.921339978	0.93204251	positive
PRJNA729271	stage_5W_vs_E10	ordinary_muscle_development	ENSGALG00010025874	MDH1B	-2.252831807	0.327634068	-6.876060905	6.15302E-12	1.64554E-11	negative
PRJNA729271	stage_5W_vs_E10	ordinary_muscle_development	ENSGALG00010025876	ENSGALG00010025876	1.642856362	1.784677425	0.920534064	0.357293741	0.397617871	positive
PRJNA729271	stage_5W_vs_E10	ordinary_muscle_development	ENSGALG00010025877	ENSGALG00010025877	1.158328834	3.503381349	0.330631672	0.740922719	0.769648475	positive
PRJNA729271	stage_5W_vs_E10	ordinary_muscle_development	ENSGALG00010025881	GAL	1.263816783	0.612740708	2.062563766	0.039154093	0.050082826	positive
PRJNA729271	stage_5W_vs_E10	ordinary_muscle_development	ENSGALG00010025882	LDHD	2.542897053	0.173229056	14.67939102	8.73716E-49	1.01478E-47	positive
PRJNA729271	stage_5W_vs_E10	ordinary_muscle_development	ENSGALG00010025886	ALDH2	1.388434598	0.109649517	12.66247798	9.54385E-37	7.51021E-36	positive
PRJNA729271	stage_5W_vs_E10	ordinary_muscle_development	ENSGALG00010025887	ZNF76	-0.500991765	0.117672744	-4.257500489	2.06725E-05	3.5929E-05	negative
PRJNA729271	stage_5W_vs_E10	ordinary_muscle_development	ENSGALG00010025889	TESMIN	1.359526167	0.583228009	2.331037168	0.0197514	0.026119564	positive
PRJNA729271	stage_5W_vs_E10	ordinary_muscle_development	ENSGALG00010025890	SCG2	1.180485905	0.511084108	2.309768368	0.020900981	0.027549307	positive
PRJNA729271	stage_5W_vs_E10	ordinary_muscle_development	ENSGALG00010025891	NUP93	-1.963684494	0.111206638	-17.65797908	8.83466E-70	1.85592E-68	negative
PRJNA729271	stage_5W_vs_E10	ordinary_muscle_development	ENSGALG00010025892	ZC3H11A	0.292616937	0.086240206	3.393045439	0.000691202	0.001061556	positive
PRJNA729271	stage_5W_vs_E10	ordinary_muscle_development	ENSGALG00010025894	DYTN	-1.783950984	0.616838385	-2.89208815	0.003826906	0.005469434	negative
PRJNA729271	stage_5W_vs_E10	ordinary_muscle_development	ENSGALG00010025897	MAPKAPK5	1.328106601	0.119722522	11.09320597	1.35348E-28	7.75138E-28	positive
PRJNA729271	stage_5W_vs_E10	ordinary_muscle_development	ENSGALG00010025898	ENSGALG00010025898	1.158397019	2.00257413	0.578454002	0.562957644	0.602308167	positive
PRJNA729271	stage_5W_vs_E10	ordinary_muscle_development	ENSGALG00010025899	ENSGALG00010025899	1.166397725	0.163966504	7.113634163	1.13027E-12	3.14396E-12	positive
PRJNA729271	stage_5W_vs_E10	ordinary_muscle_development	ENSGALG00010025900	FAM237A	1.057365932	0.570909366	1.852073191	0.064015314	0.079682436	positive
PRJNA729271	stage_5W_vs_E10	ordinary_muscle_development	ENSGALG00010025902	ENSGALG00010025902	1.158407531	1.873306446	0.618375885	0.53632759	0.576369248	positive
PRJNA729271	stage_5W_vs_E10	ordinary_muscle_development	ENSGALG00010025904	RPL10A	-0.189355759	0.138872035	-1.363526923	0.172716505	0.203208259	negative
PRJNA729271	stage_5W_vs_E10	ordinary_muscle_development	ENSGALG00010025905	CPT1A	0.666103403	0.174955456	3.80727425	0.000140507	0.000228529	positive
PRJNA729271	stage_5W_vs_E10	ordinary_muscle_development	ENSGALG00010025907	ARHGEF12	0.438244115	0.071493677	6.129830369	8.79728E-10	2.05938E-09	positive
PRJNA729271	stage_5W_vs_E10	ordinary_muscle_development	ENSGALG00010025910	HEATR3	-0.146367678	0.113176781	-1.293265952	0.195919092	0.228173489	negative
PRJNA729271	stage_5W_vs_E10	ordinary_muscle_development	ENSGALG00010025913	EIF2B3	-0.355670585	0.0782974	-4.542559359	5.55753E-06	1.00809E-05	negative
PRJNA729271	stage_5W_vs_E10	ordinary_muscle_development	ENSGALG00010025914	ENSGALG00010025914	-5.639694071	1.762371208	-3.200060263	0.001373989	0.002054039	negative
PRJNA729271	stage_5W_vs_E10	ordinary_muscle_development	ENSGALG00010025917	SCUBE3	-4.897012358	0.421171501	-11.62712183	3.00044E-31	1.91255E-30	negative
PRJNA729271	stage_5W_vs_E10	ordinary_muscle_development	ENSGALG00010025919	RGS9BP	-0.673825635	0.319505944	-2.10896119	0.034947932	0.045004466	negative
PRJNA729271	stage_5W_vs_E10	ordinary_muscle_development	ENSGALG00010025920	GTSF1	0.423978792	0.261066637	1.624025178	0.104370435	0.126450138	positive
PRJNA729271	stage_5W_vs_E10	ordinary_muscle_development	ENSGALG00010025924	TMEM116	1.911744524	0.17146953	11.14917926	7.22699E-29	4.18351E-28	positive
PRJNA729271	stage_5W_vs_E10	ordinary_muscle_development	ENSGALG00010025925	KLHL18	0.810290592	0.090925998	8.911539133	5.03289E-19	1.9208E-18	positive
PRJNA729271	stage_5W_vs_E10	ordinary_muscle_development	ENSGALG00010025926	ENSGALG00010025926	0.450523045	0.425899895	1.057814406	0.290140064	0.327920485	positive
PRJNA729271	stage_5W_vs_E10	ordinary_muscle_development	ENSGALG00010025927	ENSGALG00010025927	-2.524788867	1.16002526	-2.176494731	0.029518287	0.038322219	negative
PRJNA729271	stage_5W_vs_E10	ordinary_muscle_development	ENSGALG00010025929	NELFE	0.091187617	0.091015201	1.001894359	0.316394617	0.35548717	positive
PRJNA729271	stage_5W_vs_E10	ordinary_muscle_development	ENSGALG00010025931	TMEM136	-2.29049923	0.215466301	-10.63042909	2.1512E-26	1.13347E-25	negative
PRJNA729271	stage_5W_vs_E10	ordinary_muscle_development	ENSGALG00010025932	AP1S3	-0.633508823	0.316873618	-1.999247609	0.04558157	0.05775897	negative
PRJNA729271	stage_5W_vs_E10	ordinary_muscle_development	ENSGALG00010025933	SKIC2	0.437434283	0.102166731	4.281572687	1.85577E-05	3.23972E-05	positive
PRJNA729271	stage_5W_vs_E10	ordinary_muscle_development	ENSGALG00010025935	CATH3	-0.884231556	0.380014458	-2.326836619	0.019973963	0.026392214	negative
PRJNA729271	stage_5W_vs_E10	ordinary_muscle_development	ENSGALG00010025936	GOSR2	-0.668149331	0.069036167	-9.678250677	3.73044E-22	1.64505E-21	negative
PRJNA729271	stage_5W_vs_E10	ordinary_muscle_development	ENSGALG00010025938	ERP29	-0.866219529	0.090195125	-9.603839758	7.70205E-22	3.34656E-21	negative
PRJNA729271	stage_5W_vs_E10	ordinary_muscle_development	ENSGALG00010025939	TMEM136-1	-3.981735909	0.890458078	-4.471559088	7.76514E-06	1.39422E-05	negative
PRJNA729271	stage_5W_vs_E10	ordinary_muscle_development	ENSGALG00010025940	HECTD3	0.886794737	0.087705589	10.11104024	4.93574E-24	2.35418E-23	positive
PRJNA729271	stage_5W_vs_E10	ordinary_muscle_development	ENSGALG00010025941	ENSGALG00010025941	-4.061789976	0.878370957	-4.624230734	3.75991E-06	6.91009E-06	negative
PRJNA729271	stage_5W_vs_E10	ordinary_muscle_development	ENSGALG00010025942	ENSGALG00010025942	-0.316418598	1.317950848	-0.240083762	0.810265322	0.832668971	negative
PRJNA729271	stage_5W_vs_E10	ordinary_muscle_development	ENSGALG00010025943	CATH1	-1.360287918	0.826812249	-1.645219842	0.099924483	0.121357803	negative
PRJNA729271	stage_5W_vs_E10	ordinary_muscle_development	ENSGALG00010025946	KRT5	-9.850051452	1.273035171	-7.737454293	1.01427E-14	3.14277E-14	negative
PRJNA729271	stage_5W_vs_E10	ordinary_muscle_development	ENSGALG00010025948	WNT9B	-8.220123429	0.923232858	-8.903629633	5.40496E-19	2.06062E-18	negative
PRJNA729271	stage_5W_vs_E10	ordinary_muscle_development	ENSGALG00010025949	GPATCH1	-0.135578839	0.122989036	-1.102365247	0.270302914	0.307466118	negative
PRJNA729271	stage_5W_vs_E10	ordinary_muscle_development	ENSGALG00010025951	TBRG4	0.137632075	0.114317149	1.203949505	0.228609096	0.26305381	positive
PRJNA729271	stage_5W_vs_E10	ordinary_muscle_development	ENSGALG00010025953	POU2F3	-3.256732932	1.082342719	-3.008966453	0.002621381	0.003811507	negative
PRJNA729271	stage_5W_vs_E10	ordinary_muscle_development	ENSGALG00010025954	TUBA1A	-2.56193583	0.158870256	-16.1259627	1.67626E-58	2.58209E-57	negative
PRJNA729271	stage_5W_vs_E10	ordinary_muscle_development	ENSGALG00010025956	ENSGALG00010025956	1.583560223	0.49765341	3.1820544	0.001462343	0.002180054	positive
PRJNA729271	stage_5W_vs_E10	ordinary_muscle_development	ENSGALG00010025957	ENSGALG00010025957	-0.250676761	0.115440237	-2.171485146	0.029894519	0.038789817	negative
PRJNA729271	stage_5W_vs_E10	ordinary_muscle_development	ENSGALG00010025958	HOXC9	-4.110198397	0.425068477	-9.669497083	4.0637E-22	1.78766E-21	negative
PRJNA729271	stage_5W_vs_E10	ordinary_muscle_development	ENSGALG00010025959	UROD	-0.637777954	0.106250612	-6.002581444	1.94205E-09	4.45552E-09	negative
PRJNA729271	stage_5W_vs_E10	ordinary_muscle_development	ENSGALG00010025960	CMKLR2	1.942663197	0.158256658	12.27539629	1.22789E-34	8.91697E-34	positive
PRJNA729271	stage_5W_vs_E10	ordinary_muscle_development	ENSGALG00010025962	ENSGALG00010025962	1.055298318	0.184785396	5.710940047	1.12354E-08	2.4724E-08	positive
PRJNA729271	stage_5W_vs_E10	ordinary_muscle_development	ENSGALG00010025963	OAF	-0.16983685	0.245901421	-0.690670469	0.489772652	0.531182536	negative
PRJNA729271	stage_5W_vs_E10	ordinary_muscle_development	ENSGALG00010025964	HOXC10	-9.230248788	1.094367161	-8.434325439	3.33119E-17	1.16942E-16	negative
PRJNA729271	stage_5W_vs_E10	ordinary_muscle_development	ENSGALG00010025968	FADS2	0.652592231	0.155963419	4.184264715	2.86091E-05	4.9157E-05	positive
PRJNA729271	stage_5W_vs_E10	ordinary_muscle_development	ENSGALG00010025970	KLHL12	-0.106581245	0.110986758	-0.960305954	0.336901254	0.376688034	negative
PRJNA729271	stage_5W_vs_E10	ordinary_muscle_development	ENSGALG00010025971	TRAFD1	0.772170143	0.198078187	3.898309825	9.68664E-05	0.000159772	positive
PRJNA729271	stage_5W_vs_E10	ordinary_muscle_development	ENSGALG00010025972	WNT3	-6.993422653	1.318245216	-5.305099966	1.12611E-07	2.31532E-07	negative
PRJNA729271	stage_5W_vs_E10	ordinary_muscle_development	ENSGALG00010025974	EEF1B2	0.560943639	0.095552052	5.870555625	4.34337E-09	9.78355E-09	positive
PRJNA729271	stage_5W_vs_E10	ordinary_muscle_development	ENSGALG00010025976	NAA25	-0.390498463	0.099197271	-3.936584726	8.26494E-05	0.000137354	negative
PRJNA729271	stage_5W_vs_E10	ordinary_muscle_development	ENSGALG00010025978	TRIM29	-8.703809721	0.93587456	-9.300188392	1.40197E-20	5.73569E-20	negative
PRJNA729271	stage_5W_vs_E10	ordinary_muscle_development	ENSGALG00010025980	HECTD4	-0.221482159	0.064539616	-3.43172412	0.000599757	0.000926218	negative
PRJNA729271	stage_5W_vs_E10	ordinary_muscle_development	ENSGALG00010025982	SERPINE2	-1.029949744	0.199106698	-5.172853319	2.30546E-07	4.63233E-07	negative
PRJNA729271	stage_5W_vs_E10	ordinary_muscle_development	ENSGALG00010025983	RNF26	-1.350363353	0.086125668	-15.6789884	2.10581E-55	2.94915E-54	negative
PRJNA729271	stage_5W_vs_E10	ordinary_muscle_development	ENSGALG00010025984	NDUFS1	1.20487644	0.116975108	10.30028064	7.0256E-25	3.47445E-24	positive
PRJNA729271	stage_5W_vs_E10	ordinary_muscle_development	ENSGALG00010025985	CHCHD10	3.929848865	0.317763861	12.36719889	3.93299E-35	2.92053E-34	positive
PRJNA729271	stage_5W_vs_E10	ordinary_muscle_development	ENSGALG00010025986	RAB3IL1	-0.118624079	0.108221385	-1.096124192	0.273024429	0.310172356	negative
PRJNA729271	stage_5W_vs_E10	ordinary_muscle_development	ENSGALG00010025988	NSF	-0.432783124	0.060150043	-7.195059292	6.24336E-13	1.75997E-12	negative
PRJNA729271	stage_5W_vs_E10	ordinary_muscle_development	ENSGALG00010025990	FAM124B	1.176655482	0.239422595	4.914554876	8.89844E-07	1.71657E-06	positive
PRJNA729271	stage_5W_vs_E10	ordinary_muscle_development	ENSGALG00010025992	MMP11	-1.956105518	0.344948024	-5.670725393	1.42194E-08	3.11345E-08	negative
PRJNA729271	stage_5W_vs_E10	ordinary_muscle_development	ENSGALG00010025994	INO80D	0.855463686	0.107713817	7.942005118	1.98939E-15	6.40661E-15	positive
PRJNA729271	stage_5W_vs_E10	ordinary_muscle_development	ENSGALG00010025996	CUL3	1.66752586	0.096502004	17.27970184	6.68929E-67	1.30324E-65	positive
PRJNA729271	stage_5W_vs_E10	ordinary_muscle_development	ENSGALG00010025997	CCDC43	-0.003609249	0.149625095	-0.024121951	0.980755334	0.98360538	negative
PRJNA729271	stage_5W_vs_E10	ordinary_muscle_development	ENSGALG00010025998	SMARCB1	-1.296984347	0.145131682	-8.936603828	4.01314E-19	1.54012E-18	negative
PRJNA729271	stage_5W_vs_E10	ordinary_muscle_development	ENSGALG00010025999	DBF4B	-1.769356703	0.170365063	-10.38567809	2.88115E-25	1.44207E-24	negative
PRJNA729271	stage_5W_vs_E10	ordinary_muscle_development	ENSGALG00010026001	ANKS1A	1.049529376	0.091910848	11.41899346	3.36104E-30	2.04785E-29	positive
PRJNA729271	stage_5W_vs_E10	ordinary_muscle_development	ENSGALG00010026002	FTH1	1.842946672	0.141825367	12.99447841	1.31501E-38	1.09233E-37	positive
PRJNA729271	stage_5W_vs_E10	ordinary_muscle_development	ENSGALG00010026003	NECTIN1	-0.393643942	0.155751862	-2.527378717	0.011491751	0.015636246	negative
PRJNA729271	stage_5W_vs_E10	ordinary_muscle_development	ENSGALG00010026005	DERL3	0.521039074	0.113729344	4.581395232	4.61884E-06	8.42882E-06	positive
PRJNA729271	stage_5W_vs_E10	ordinary_muscle_development	ENSGALG00010026006	ITGA2B	0.08867804	0.301016361	0.294595415	0.768302965	0.795695304	positive
PRJNA729271	stage_5W_vs_E10	ordinary_muscle_development	ENSGALG00010026008	NRP2	-1.003638449	0.10132889	-9.904761159	3.96917E-23	1.82009E-22	negative
PRJNA729271	stage_5W_vs_E10	ordinary_muscle_development	ENSGALG00010026009	ENSGALG00010026009	-1.720083756	0.310470763	-5.540243922	3.02051E-08	6.4528E-08	negative
PRJNA729271	stage_5W_vs_E10	ordinary_muscle_development	ENSGALG00010026010	ZSWIM5	-0.829295217	0.130341831	-6.362464056	1.98543E-10	4.84899E-10	negative
PRJNA729271	stage_5W_vs_E10	ordinary_muscle_development	ENSGALG00010026011	GJC2	-0.931989531	0.165044647	-5.646893426	1.63373E-08	3.56211E-08	negative
PRJNA729271	stage_5W_vs_E10	ordinary_muscle_development	ENSGALG00010026013	RABIF	-0.769200707	0.114354372	-6.726465253	1.73834E-11	4.54086E-11	negative
PRJNA729271	stage_5W_vs_E10	ordinary_muscle_development	ENSGALG00010026016	SLC2A11	-0.812490609	0.391105486	-2.077420639	0.037762753	0.048431349	negative
PRJNA729271	stage_5W_vs_E10	ordinary_muscle_development	ENSGALG00010026017	PLEKHM1	0.908239979	0.145115824	6.258724614	3.88139E-10	9.30082E-10	positive
PRJNA729271	stage_5W_vs_E10	ordinary_muscle_development	ENSGALG00010026023	THY1	0.663003616	0.299284098	2.215298512	0.026739582	0.034861507	positive
PRJNA729271	stage_5W_vs_E10	ordinary_muscle_development	ENSGALG00010026030	IBA57	0.330549615	0.084009852	3.934653006	8.3317E-05	0.000138384	positive
PRJNA729271	stage_5W_vs_E10	ordinary_muscle_development	ENSGALG00010026036	TUB5A	-1.491729664	0.207282533	-7.196600893	6.17321E-13	1.74053E-12	negative
PRJNA729271	stage_5W_vs_E10	ordinary_muscle_development	ENSGALG00010026040	GUK1	-1.20967926	0.129170715	-9.364965237	7.60753E-21	3.15151E-20	negative
PRJNA729271	stage_5W_vs_E10	ordinary_muscle_development	ENSGALG00010026051	TAF11	-0.042618569	0.128376689	-0.331980588	0.739903917	0.76874496	negative
PRJNA729271	stage_5W_vs_E10	ordinary_muscle_development	ENSGALG00010026061	MPEG1	4.933775409	0.341681519	14.43969056	2.91146E-47	3.18985E-46	positive
PRJNA729271	stage_5W_vs_E10	ordinary_muscle_development	ENSGALG00010026062	ITGA4	-1.718368577	0.161022207	-10.67162478	1.38189E-26	7.33984E-26	negative
PRJNA729271	stage_5W_vs_E10	ordinary_muscle_development	ENSGALG00010026064	ENSGALG00010026064	0.245854753	0.149869945	1.640454016	0.100910802	0.122473589	positive
PRJNA729271	stage_5W_vs_E10	ordinary_muscle_development	ENSGALG00010026066	SELENOP2	0.247838302	0.580860524	0.426674377	0.669616516	0.703594882	positive
PRJNA729271	stage_5W_vs_E10	ordinary_muscle_development	ENSGALG00010026068	BLTP3A	0.806415899	0.111077842	7.259916876	3.87328E-13	1.10063E-12	positive
PRJNA729271	stage_5W_vs_E10	ordinary_muscle_development	ENSGALG00010026073	HPDL	-1.783778606	0.281392636	-6.339109057	2.31098E-10	5.61663E-10	negative
PRJNA729271	stage_5W_vs_E10	ordinary_muscle_development	ENSGALG00010026076	C1orf35	0.05767923	0.136469286	0.422653565	0.672548039	0.706470311	positive
PRJNA729271	stage_5W_vs_E10	ordinary_muscle_development	ENSGALG00010026078	UBE2E3	0.029760262	0.107178532	0.277669993	0.781265697	0.807619903	positive
PRJNA729271	stage_5W_vs_E10	ordinary_muscle_development	ENSGALG00010026081	KPNA1	0.603990813	0.107816063	5.602048475	2.11833E-08	4.58082E-08	positive
PRJNA729271	stage_5W_vs_E10	ordinary_muscle_development	ENSGALG00010026083	MUTYH	-1.956601725	0.136144773	-14.37147887	7.81446E-47	8.47834E-46	negative
PRJNA729271	stage_5W_vs_E10	ordinary_muscle_development	ENSGALG00010026088	SNRPC	-0.394646381	0.082299607	-4.795240143	1.6248E-06	3.0678E-06	negative
PRJNA729271	stage_5W_vs_E10	ordinary_muscle_development	ENSGALG00010026090	USP2	4.1350319	0.233993694	17.67155274	6.94585E-70	1.46764E-68	positive
PRJNA729271	stage_5W_vs_E10	ordinary_muscle_development	ENSGALG00010026091	DTX4	0.111902444	0.163063662	0.686250038	0.492555449	0.53400084	positive
PRJNA729271	stage_5W_vs_E10	ordinary_muscle_development	ENSGALG00010026093	TOE1	-1.259784753	0.13361478	-9.428483554	4.1606E-21	1.74907E-20	negative
PRJNA729271	stage_5W_vs_E10	ordinary_muscle_development	ENSGALG00010026094	C6orf106	1.594371352	0.077651725	20.53233653	1.10721E-93	4.07334E-92	positive
PRJNA729271	stage_5W_vs_E10	ordinary_muscle_development	ENSGALG00010026095	MIF	-1.909206496	0.202016991	-9.450722394	3.365E-21	1.4179E-20	negative
PRJNA729271	stage_5W_vs_E10	ordinary_muscle_development	ENSGALG00010026097	ARF1	-0.606639034	0.05608136	-10.81712423	2.85598E-27	1.55571E-26	negative
PRJNA729271	stage_5W_vs_E10	ordinary_muscle_development	ENSGALG00010026098	STAT3	1.245047345	0.10803902	11.52405258	9.9804E-31	6.22755E-30	positive
PRJNA729271	stage_5W_vs_E10	ordinary_muscle_development	ENSGALG00010026102	IPO9	-1.294593512	0.06029968	-21.46932653	3.0135E-102	1.3277E-100	negative
PRJNA729271	stage_5W_vs_E10	ordinary_muscle_development	ENSGALG00010026104	DDX51	-0.535469581	0.104843683	-5.107313705	3.26771E-07	6.49198E-07	negative
PRJNA729271	stage_5W_vs_E10	ordinary_muscle_development	ENSGALG00010026105	CNTF	2.161616154	0.187572946	11.52413609	9.97073E-31	6.22419E-30	positive
PRJNA729271	stage_5W_vs_E10	ordinary_muscle_development	ENSGALG00010026106	TESK2	0.840275097	0.118638106	7.082674551	1.41398E-12	3.91214E-12	positive
PRJNA729271	stage_5W_vs_E10	ordinary_muscle_development	ENSGALG00010026111	ENSGALG00010026111	-3.118621389	0.164838833	-18.91921536	7.92251E-80	2.11876E-78	negative
PRJNA729271	stage_5W_vs_E10	ordinary_muscle_development	ENSGALG00010026112	ZFP91	1.023527978	0.120543528	8.490940926	2.04971E-17	7.27307E-17	positive
PRJNA729271	stage_5W_vs_E10	ordinary_muscle_development	ENSGALG00010026113	MFRP	1.23309984	0.517210572	2.384135025	0.01711932	0.022811596	positive
PRJNA729271	stage_5W_vs_E10	ordinary_muscle_development	ENSGALG00010026114	CWC22	-0.489735162	0.08379619	-5.844360749	5.08517E-09	1.14261E-08	negative
PRJNA729271	stage_5W_vs_E10	ordinary_muscle_development	ENSGALG00010026115	MMACHC	0.805960402	0.177639269	4.537062147	5.70433E-06	1.03355E-05	positive
PRJNA729271	stage_5W_vs_E10	ordinary_muscle_development	ENSGALG00010026116	DLX3	-4.83808648	0.790788442	-6.118054117	9.47249E-10	2.21458E-09	negative
PRJNA729271	stage_5W_vs_E10	ordinary_muscle_development	ENSGALG00010026117	LZTR1	-0.035016901	0.072139398	-0.485406069	0.627388328	0.663407777	negative
PRJNA729271	stage_5W_vs_E10	ordinary_muscle_development	ENSGALG00010026120	C1QTNF5	1.175306416	0.193403113	6.076977766	1.22469E-09	2.84735E-09	positive

PRJNA729271	stage_5W_vs_E10	ordinary_muscle_development	ENSGALG00010026121	ENSGALG00010026121	2.014710116	0.257520245	7.823501872	5.13738E-15	1.61287E-14	positive
PRJNA729271	stage_5W_vs_E10	ordinary_muscle_development	ENSGALG00010026124	PACSIN1	-4.085505198	0.603405263	-6.770748365	1.28118E-11	3.37281E-11	negative
PRJNA729271	stage_5W_vs_E10	ordinary_muscle_development	ENSGALG00010026127	ENSGALG00010026127	-0.149799245	0.713346455	-0.209995079	0.833671514	0.853696121	negative
PRJNA729271	stage_5W_vs_E10	ordinary_muscle_development	ENSGALG00010026128	PRDX1	-1.589808753	0.142222908	-11.17828892	5.20845E-29	3.03565E-28	negative
PRJNA729271	stage_5W_vs_E10	ordinary_muscle_development	ENSGALG00010026132	DDTL	-0.380833297	0.185842038	-2.04923117	0.040439515	0.05159954	negative
PRJNA729271	stage_5W_vs_E10	ordinary_muscle_development	ENSGALG00010026137	LPXN	2.76208833	0.321330916	8.595775222	8.27046E-18	2.99551E-17	positive
PRJNA729271	stage_5W_vs_E10	ordinary_muscle_development	ENSGALG00010026141	WNT3A	-8.050663728	0.966025017	-8.333804598	7.82851E-17	2.70048E-16	negative
PRJNA729271	stage_5W_vs_E10	ordinary_muscle_development	ENSGALG00010026143	RNPEP	-0.135709288	0.141536811	-0.958826809	0.337646001	0.377462501	negative
PRJNA729271	stage_5W_vs_E10	ordinary_muscle_development	ENSGALG00010026148	RPS10	0.255441424	0.134108571	1.90473601	0.056814396	0.071128404	positive
PRJNA729271	stage_5W_vs_E10	ordinary_muscle_development	ENSGALG00010026149	LASP1	-0.673069049	0.129163202	-5.210996927	1.87829E-07	3.80036E-07	negative
PRJNA729271	stage_5W_vs_E10	ordinary_muscle_development	ENSGALG00010026154	ZNF385B	-1.498257965	0.179252098	-8.358384528	6.35829E-17	2.20487E-16	negative
PRJNA729271	stage_5W_vs_E10	ordinary_muscle_development	ENSGALG00010026162	MED1	-0.13185288	0.151915455	-0.867935919	0.385429414	0.426701241	negative
PRJNA729271	stage_5W_vs_E10	ordinary_muscle_development	ENSGALG00010026163	CTNND1	-0.265370549	0.081899386	-3.240201947	0.001194451	0.001796013	negative
PRJNA729271	stage_5W_vs_E10	ordinary_muscle_development	ENSGALG00010026166	WNT9A	-1.674046665	0.244536639	-6.845790775	7.60546E-12	2.02277E-11	negative
PRJNA729271	stage_5W_vs_E10	ordinary_muscle_development	ENSGALG00010026167	CABIN1	-0.673756548	0.060162938	-11.19886381	4.12996E-29	2.41776E-28	negative
PRJNA729271	stage_5W_vs_E10	ordinary_muscle_development	ENSGALG00010026173	CDK12	0.109460329	0.162494092	0.673626516	0.500548791	0.541573093	positive
PRJNA729271	stage_5W_vs_E10	ordinary_muscle_development	ENSGALG00010026183	SESTD1	-0.063063455	0.168535113	-0.37418585	0.70826605	0.739914696	negative
PRJNA729271	stage_5W_vs_E10	ordinary_muscle_development	ENSGALG00010026184	NUDT3	0.539822994	0.104225453	5.179377758	2.22627E-07	4.48067E-07	positive
PRJNA729271	stage_5W_vs_E10	ordinary_muscle_development	ENSGALG00010026192	SMIM29	2.579870767	0.120515231	21.40701016	1.1496E-101	4.97396E-100	positive
PRJNA729271	stage_5W_vs_E10	ordinary_muscle_development	ENSGALG00010026199	TBX6	-1.020150149	0.41182812	-2.477126014	0.013244513	0.01788182	negative
PRJNA729271	stage_5W_vs_E10	ordinary_muscle_development	ENSGALG00010026203	TMX2	0.381099426	0.104039466	3.663027507	0.000249252	0.000397284	positive
PRJNA729271	stage_5W_vs_E10	ordinary_muscle_development	ENSGALG00010026205	ENSGALG00010026205	4.468825071	1.305437544	3.423239274	0.000618796	0.000954704	positive
PRJNA729271	stage_5W_vs_E10	ordinary_muscle_development	ENSGALG00010026209	MED19	1.341234751	0.171090181	7.839343822	4.52907E-15	1.42653E-14	positive
PRJNA729271	stage_5W_vs_E10	ordinary_muscle_development	ENSGALG00010026213	AKR1A1	-1.325502658	0.124429296	-10.65265741	1.69457E-26	8.97109E-26	negative
PRJNA729271	stage_5W_vs_E10	ordinary_muscle_development	ENSGALG00010026218	FTSJ3	-0.913305963	0.168394775	-5.42360035	5.84104E-08	1.2242E-07	negative
PRJNA729271	stage_5W_vs_E10	ordinary_muscle_development	ENSGALG00010026220	ENSGALG00010026220	0.005971831	0.097034549	0.061543351	0.95092649	0.957465927	positive
PRJNA729271	stage_5W_vs_E10	ordinary_muscle_development	ENSGALG00010026221	CRKL	0.70107299	0.145873561	4.806031926	1.53955E-06	2.91177E-06	positive
PRJNA729271	stage_5W_vs_E10	ordinary_muscle_development	ENSGALG00010026229	CRHR2	1.910603945	0.210370967	9.082070459	1.06529E-19	4.20745E-19	positive
PRJNA729271	stage_5W_vs_E10	ordinary_muscle_development	ENSGALG00010026232	PSMC5	0.671692427	0.130879373	5.132148897	2.86453E-07	5.71054E-07	positive
PRJNA729271	stage_5W_vs_E10	ordinary_muscle_development	ENSGALG00010026234	ZDHHC5	0.732981122	0.159984149	4.581585905	4.61463E-06	8.42219E-06	positive
PRJNA729271	stage_5W_vs_E10	ordinary_muscle_development	ENSGALG00010026237	KLHL22	2.667041742	0.136928553	19.47761577	1.70015E-84	5.06029E-83	positive
PRJNA729271	stage_5W_vs_E10	ordinary_muscle_development	ENSGALG00010026239	GIP	-4.126679086	1.157574424	-3.564936301	0.000363945	0.000572043	negative
PRJNA729271	stage_5W_vs_E10	ordinary_muscle_development	ENSGALG00010026242	CCDC141	0.29531217	0.253928708	1.162972756	0.244840556	0.280417498	positive
PRJNA729271	stage_5W_vs_E10	ordinary_muscle_development	ENSGALG00010026244	CLP1	-0.853894228	0.131866049	-6.475466848	9.45192E-11	2.35729E-10	negative
PRJNA729271	stage_5W_vs_E10	ordinary_muscle_development	ENSGALG00010026249	YPEL4	0.011257834	0.571027091	0.01971506	0.984270677	0.986448417	positive
PRJNA729271	stage_5W_vs_E10	ordinary_muscle_development	ENSGALG00010026252	ENO2	0.426112704	0.335824184	1.268856515	0.204492244	0.237433121	positive
PRJNA729271	stage_5W_vs_E10	ordinary_muscle_development	ENSGALG00010026254	ENSGALG00010026254	-2.028150507	0.657398461	-3.085115995	0.002034728	0.002987579	negative
PRJNA729271	stage_5W_vs_E10	ordinary_muscle_development	ENSGALG00010026259	MED15	-0.11059734	0.089328757	-1.238093353	0.215681448	0.249400112	negative
PRJNA729271	stage_5W_vs_E10	ordinary_muscle_development	ENSGALG00010026260	ENSGALG00010026260	19.17570954	3.171550716	6.046162038	1.48337E-09	3.42926E-09	positive
PRJNA729271	stage_5W_vs_E10	ordinary_muscle_development	ENSGALG00010026261	IFT70A	-0.636677788	0.108575659	-5.86390903	4.52095E-09	1.01757E-08	negative
PRJNA729271	stage_5W_vs_E10	ordinary_muscle_development	ENSGALG00010026262	NAV1	0.162083013	0.160666316	1.008817633	0.313062103	0.35217968	positive
PRJNA729271	stage_5W_vs_E10	ordinary_muscle_development	ENSGALG00010026264	FZD7	-2.087987602	0.1434779	-14.55267744	5.61612E-48	6.29101E-47	negative
PRJNA729271	stage_5W_vs_E10	ordinary_muscle_development	ENSGALG00010026266	ENSGALG00010026266	-1.652749805	0.355406783	-4.650304624	3.31445E-06	6.12246E-06	negative
PRJNA729271	stage_5W_vs_E10	ordinary_muscle_development	ENSGALG00010026268	GARS1	-0.287962182	0.150963209	-1.907499075	0.056455991	0.07069802	negative
PRJNA729271	stage_5W_vs_E10	ordinary_muscle_development	ENSGALG00010026272	LRRC58	0.465286653	0.096810273	4.806170254	1.53849E-06	2.91034E-06	positive
PRJNA729271	stage_5W_vs_E10	ordinary_muscle_development	ENSGALG00010026277	SMARCD2	0.515889382	0.124205047	4.153529953	3.27385E-05	5.59803E-05	positive
PRJNA729271	stage_5W_vs_E10	ordinary_muscle_development	ENSGALG00010026287	NASP	-1.874294216	0.092316365	-20.30294652	1.21101E-91	4.30236E-90	negative
PRJNA729271	stage_5W_vs_E10	ordinary_muscle_development	ENSGALG00010026291	SERPING1	2.278350385	0.16843278	13.52676356	1.08705E-41	9.9853E-41	positive
PRJNA729271	stage_5W_vs_E10	ordinary_muscle_development	ENSGALG00010026295	SMPD4	-0.413383078	0.132065738	-3.130131126	0.001747283	0.002582793	negative
PRJNA729271	stage_5W_vs_E10	ordinary_muscle_development	ENSGALG00010026298	SNF8	0.141537319	0.115646355	1.223880505	0.220997309	0.254983363	positive
PRJNA729271	stage_5W_vs_E10	ordinary_muscle_development	ENSGALG00010026300	HMGA1	-1.452001804	0.11121687	-13.05558955	5.90511E-39	4.95339E-38	negative
PRJNA729271	stage_5W_vs_E10	ordinary_muscle_development	ENSGALG00010026305	ATP5MC1	0.983236761	0.167105232	5.883937614	4.00619E-09	9.0409E-09	positive
PRJNA729271	stage_5W_vs_E10	ordinary_muscle_development	ENSGALG00010026315	SYT2	-3.178560593	0.413157194	-7.693344426	1.43338E-14	4.39535E-14	negative
PRJNA729271	stage_5W_vs_E10	ordinary_muscle_development	ENSGALG00010026316	ENSGALG00010026316	1.351981253	0.346936372	3.896914137	9.74261E-05	0.000160659	positive
PRJNA729271	stage_5W_vs_E10	ordinary_muscle_development	ENSGALG00010026320	ENSGALG00010026320	1.020025107	0.174507883	5.845152016	5.06106E-09	1.13737E-08	positive
PRJNA729271	stage_5W_vs_E10	ordinary_muscle_development	ENSGALG00010026321	GPBP1L1	0.470649666	0.047444942	9.919912404	3.41055E-23	1.5684E-22	positive
PRJNA729271	stage_5W_vs_E10	ordinary_muscle_development	ENSGALG00010026325	TMEM69	0.269227944	0.138047313	1.950258481	0.051145319	0.064420524	positive
PRJNA729271	stage_5W_vs_E10	ordinary_muscle_development	ENSGALG00010026329	ENSGALG00010026329	1.179433139	0.27643819	4.266534726	1.98533E-05	3.45632E-05	positive
PRJNA729271	stage_5W_vs_E10	ordinary_muscle_development	ENSGALG00010026334	ENSGALG00010026334	1.742156576	0.126467809	13.77549421	3.57925E-43	3.43812E-42	positive
PRJNA729271	stage_5W_vs_E10	ordinary_muscle_development	ENSGALG00010026335	IPP	-1.12616338	0.106697155	-10.55476485	4.82847E-26	2.50138E-25	negative
PRJNA729271	stage_5W_vs_E10	ordinary_muscle_development	ENSGALG00010026344	TIMM10	0.342156637	0.17202728	1.988967319	0.04670481	0.059117012	positive
PRJNA729271	stage_5W_vs_E10	ordinary_muscle_development	ENSGALG00010026346	FZD2	0.01122303	0.163554509	0.068619511	0.945292488	0.952521002	positive
PRJNA729271	stage_5W_vs_E10	ordinary_muscle_development	ENSGALG00010026354	ENSGALG00010026354	1.656930489	0.245615299	6.746039408	1.51935E-11	3.98391E-11	positive
PRJNA729271	stage_5W_vs_E10	ordinary_muscle_development	ENSGALG00010026355	PPP1R12B	1.679578655	0.137258663	12.23659486	1.98174E-34	1.42699E-33	positive
PRJNA729271	stage_5W_vs_E10	ordinary_muscle_development	ENSGALG00010026360	CTDSPL	1.174435112	0.126585365	9.277811162	1.72996E-20	7.04969E-20	positive
PRJNA729271	stage_5W_vs_E10	ordinary_muscle_development	ENSGALG00010026362	ENSGALG00010026362	-0.019202759	0.382531779	-0.05019912	0.959963713	0.965358264	negative
PRJNA729271	stage_5W_vs_E10	ordinary_muscle_development	ENSGALG00010026367	RBP	2.483501539	0.540844408	4.591896494	4.39236E-06	8.02664E-06	positive
PRJNA729271	stage_5W_vs_E10	ordinary_muscle_development	ENSGALG00010026377	ENSGALG00010026377	-4.417493033	0.247802232	-17.82668782	4.3865E-71	9.57565E-70	negative
PRJNA729271	stage_5W_vs_E10	ordinary_muscle_development	ENSGALG00010026388	SNRPD3	-0.514759255	0.080245118	-6.414835791	1.40975E-10	3.47404E-10	negative
PRJNA729271	stage_5W_vs_E10	ordinary_muscle_development	ENSGALG00010026393	SLC43A3	-0.147479255	0.125422886	-1.175856019	0.239652417	0.274975206	negative
PRJNA729271	stage_5W_vs_E10	ordinary_muscle_development	ENSGALG00010026402	TFEB	2.407956804	0.164416078	14.64550688	1.43924E-48	1.65176E-47	positive
PRJNA729271	stage_5W_vs_E10	ordinary_muscle_development	ENSGALG00010026403	GUCD1	1.922654285	0.115960039	16.58031762	9.67163E-62	1.64157E-60	positive
PRJNA729271	stage_5W_vs_E10	ordinary_muscle_development	ENSGALG00010026409	VILL	-3.329005241	0.272161559	-12.23172463	2.10423E-34	1.51369E-33	negative
PRJNA729271	stage_5W_vs_E10	ordinary_muscle_development	ENSGALG00010026410	ENSGALG00010026410	1.048281814	0.318029006	3.296183035	0.000980081	0.001487413	positive
PRJNA729271	stage_5W_vs_E10	ordinary_muscle_development	ENSGALG00010026416	ENSGALG00010026416	1.066833484	1.427791532	0.747191351	0.454948064	0.496908461	positive
PRJNA729271	stage_5W_vs_E10	ordinary_muscle_development	ENSGALG00010026422	P2RX3	-3.194530917	0.388618132	-8.220231253	2.03111E-16	6.8611E-16	negative
PRJNA729271	stage_5W_vs_E10	ordinary_muscle_development	ENSGALG00010026427	PGC	3.972797207	1.055486755	3.763947949	0.000167252	0.000270239	positive
PRJNA729271	stage_5W_vs_E10	ordinary_muscle_development	ENSGALG00010026433	VPS11	0.016125124	0.071945167	0.224130748	0.822655568	0.843726912	positive
PRJNA729271	stage_5W_vs_E10	ordinary_muscle_development	ENSGALG00010026434	ENSGALG00010026434	0.547475715	0.142145071	3.851527954	0.000117383	0.000192039	positive
PRJNA729271	stage_5W_vs_E10	ordinary_muscle_development	ENSGALG00010026436	PLCD1	-0.708978953	0.076970857	-9.21100501	3.23086E-20	1.30195E-19	negative
PRJNA729271	stage_5W_vs_E10	ordinary_muscle_development	ENSGALG00010026438	TOMM6	0.448215446	0.121389692	3.692368262	0.000222175	0.000355652	positive
PRJNA729271	stage_5W_vs_E10	ordinary_muscle_development	ENSGALG00010026443	UPB1	0.428495039	0.365726364	1.171627426	0.241346672	0.276700151	positive
PRJNA729271	stage_5W_vs_E10	ordinary_muscle_development	ENSGALG00010026448	ADORA2A	-0.327442992	0.113829874	-2.87659979	0.004019851	0.005736143	negative
PRJNA729271	stage_5W_vs_E10	ordinary_muscle_development	ENSGALG00010026457	SSRP1	-1.134764264	0.099207151	-11.4383313	2.6901E-30	1.64319E-29	negative
PRJNA729271	stage_5W_vs_E10	ordinary_muscle_development	ENSGALG00010026458	DLEC1	0.801498291	0.187243428	4.280514947	1.86461E-05	3.25437E-05	positive
PRJNA729271	stage_5W_vs_E10	ordinary_muscle_development	ENSGALG00010026480	USP49	-0.183947752	0.214025782	-0.85946539	0.390083807	0.431425666	negative
PRJNA729271	stage_5W_vs_E10	ordinary_muscle_development	ENSGALG00010026481	DIXDC1	-1.597455667	0.138970187	-11.49495224	1.39858E-30	8.67457E-30	negative
PRJNA729271	stage_5W_vs_E10	ordinary_muscle_development	ENSGALG00010026484	TNKS1BP1	-0.03831112	0.11184925	-0.342524605	0.731956131	0.761589557	negative
PRJNA729271	stage_5W_vs_E10	ordinary_muscle_development	ENSGALG00010026485	ACAA1	0.43184633	0.105375832	4.098153452	4.16459E-05	7.06528E-05	positive
PRJNA729271	stage_5W_vs_E10	ordinary_muscle_development	ENSGALG00010026492	SPECC1L	-0.932485861	0.075825889	-12.29772402	9.31623E-35	6.81326E-34	negative
PRJNA729271	stage_5W_vs_E10	ordinary_muscle_development	ENSGALG00010026498	ENSGALG00010026498	-0.745111678	0.088249779	-8.443212966	3.08732E-17	1.08539E-16	negative
PRJNA729271	stage_5W_vs_E10	ordinary_muscle_development	ENSGALG00010026507	APLNR	-1.921405948	0.297047052	-6.468355556	9.90752E-11	2.46879E-10	negative
PRJNA729271	stage_5W_vs_E10	ordinary_muscle_development	ENSGALG00010026508	CSRP1	2.299846957	0.14814245	15.52456412	2.36606E-54	3.20824E-53	positive
PRJNA729271	stage_5W_vs_E10	ordinary_muscle_development	ENSGALG00010026516	ENSGALG00010026516	0.417310431	0.167550526	2.490654252	0.012750813	0.017245781	positive
PRJNA729271	stage_5W_vs_E10	ordinary_muscle_development	ENSGALG00010026522	MED20	0.248985093	0.128992632	1.93022724	0.053578688	0.067310026	positive
PRJNA729271	stage_5W_vs_E10	ordinary_muscle_development	ENSGALG00010026536	BCR	-0.273321131	0.097328336	-2.808237987	0.00498134	0.007033885	negative
PRJNA729271	stage_5W_vs_E10	ordinary_muscle_development	ENSGALG00010026537	FJX1	-3.318402721	0.186998118	-17.74564768	1.8624E-70	3.97578E-69	negative
PRJNA729271	stage_5W_vs_E10	ordinary_muscle_development	ENSGALG00010026539	ENSGALG00010026539	0.244047043	0.364744129	0.669091079	0.50343738	0.544454587	positive
PRJNA729271	stage_5W_vs_E10	ordinary_muscle_development	ENSGALG00010026550	MYD88	0.845296208	0.105034432	8.047801019	8.42948E-16	2.76438E-15	positive
PRJNA729271	stage_5W_vs_E10	ordinary_muscle_development	ENSGALG00010026553	PLPP3	-0.0531866	0.127928154	-0.415753675	0.67759026	0.710993979	negative
PRJNA729271	stage_5W_vs_E10	ordinary_muscle_development	ENSGALG00010026559	RAB36	-3.570569638	0.670294425	-5.326867572	9.9921E-08	2.06201E-07	negative
PRJNA729271	stage_5W_vs_E10	ordinary_muscle_development	ENSGALG00010026560	TNNI1	-6.060405845	0.592895607	-10.22170813	1.58523E-24	7.71846E-24	negative
PRJNA729271	stage_5W_vs_E10	ordinary_muscle_development	ENSGALG00010026565	RSPH14	-3.306741607	0.616117716	-5.367061397	8.00298E-08	1.66146E-07	negative
PRJNA729271	stage_5W_vs_E10	ordinary_muscle_development	ENSGALG00010026566	ENSGALG00010026566	0.163443388	0.123316564	1.325396874	0.185039553	0.216599235	positive
PRJNA729271	stage_5W_vs_E10	ordinary_muscle_development	ENSGALG00010026568	BYSL	0.101635617	0.111210223	0.913905344	0.360766585	0.401082348	positive
PRJNA729271	stage_5W_vs_E10	ordinary_muscle_development	ENSGALG00010026569	GNAZ	-1.872471947	0.214106686	-8.74550897	2.22013E-18	8.24188E-18	negative
PRJNA729271	stage_5W_vs_E10	ordinary_muscle_development	ENSGALG00010026581	DNER	-5.969371335	0.328365476	-18.17904676	7.56371E-74	1.76264E-72	negative
PRJNA729271	stage_5W_vs_E10	ordinary_muscle_development	ENSGALG00010026584	ENSGALG00010026584	-0.347681322	0.111025149	-3.131554661	0.001738834	0.002571091	negative
PRJNA729271	stage_5W_vs_E10	ordinary_muscle_development	ENSGALG00010026588	CCND3	1.514484096	0.142366569	10.63791945	1.98512E-26	1.04748E-25	positive
PRJNA729271	stage_5W_vs_E10	ordinary_muscle_development	ENSGALG00010026595	PRKAA2	2.996408576	0.26112929	11.4748084	1.76568E-30	1.0877E-29	positive
PRJNA729271	stage_5W_vs_E10	ordinary_muscle_development	ENSGALG00010026601	ENSGALG00010026601	1.420648822	0.450601155	3.152785576	0.001617206	0.002399569	positive
PRJNA729271	stage_5W_vs_E10	ordinary_muscle_development	ENSGALG00010026612	MFF	0.090629972	0.081978969	1.105527103	0.268931257	0.306025952	positive
PRJNA729271	stage_5W_vs_E10	ordinary_muscle_development	ENSGALG00010026625	ENSGALG00010026625	4.386657217	0.629740574	6.9658164	3.26505E-12	8.86105E-12	positive
PRJNA729271	stage_5W_vs_E10	ordinary_muscle_development	ENSGALG00010026628	FYB2	-1.432714593	0.78150434	-1.833277847	0.066761262	0.082922407	negative
PRJNA729271	stage_5W_vs_E10	ordinary_muscle_development	ENSGALG00010026630	XIRP1	1.528205081	0.243780233	6.268781769	3.63884E-10	8.73405E-10	positive
PRJNA729271	stage_5W_vs_E10	ordinary_muscle_development	ENSGALG00010026633	SLC5A1	-1.062837529	0.533969073	-1.990447731	0.046541636	0.058924012	negative
PRJNA729271	stage_5W_vs_E10	ordinary_muscle_development	ENSGALG00010026635	C8A	4.966290495	2.531722479	1.961625153	0.049806142	0.062810164	positive
PRJNA729271	stage_5W_vs_E10	ordinary_muscle_development	ENSGALG00010026638	LAD1	-4.761404978	0.634160309	-7.508204012	5.9944E-14	1.78051E-13	negative
PRJNA729271	stage_5W_vs_E10	ordinary_muscle_development	ENSGALG00010026643	YWHAH	-0.563289213	0.111997559	-5.029477603	4.91818E-07	9.65191E-07	negative
PRJNA729271	stage_5W_vs_E10	ordinary_muscle_development	ENSGALG00010026659	LIPH	-0.084986421	0.253189015	-0.335663935	0.737124305	0.766196974	negative
PRJNA729271	stage_5W_vs_E10	ordinary_muscle_development	ENSGALG00010026666	ENSGALG00010026666	-1.139665857	0.489124387	-2.330012337	0.019805499	0.026181551	negative
PRJNA729271	stage_5W_vs_E10	ordinary_muscle_development	ENSGALG00010026671	TAF8	0.224020987	0.155736015	1.438466156	0.150301833	0.178385743	positive
PRJNA729271	stage_5W_vs_E10	ordinary_muscle_development	ENSGALG00010026673	DEPDC5	0.373069763	0.121869941	3.061212295	0.002204427	0.003225964	positive
PRJNA729271	stage_5W_vs_E10	ordinary_muscle_development	ENSGALG00010026681	PLXDC1	3.192619019	0.25739615	12.40352281	2.50082E-35	1.86853E-34	positive
PRJNA729271	stage_5W_vs_E10	ordinary_muscle_development	ENSGALG00010026686	ENSGALG00010026686	0.410354584	0.415429623	0.987783635	0.323258624	0.3627213	positive
PRJNA729271	stage_5W_vs_E10	ordinary_muscle_development	ENSGALG00010026690	KRT14	-6.469178639	0.482821641	-13.39869236	6.15378E-41	5.52659E-40	negative
PRJNA729271	stage_5W_vs_E10	ordinary_muscle_development	ENSGALG00010026692	NMNAT3	4.54325704	0.49910856	9.102743182	8.80785E-20	3.49111E-19	positive
PRJNA729271	stage_5W_vs_E10	ordinary_muscle_development	ENSGALG00010026696	CSRNP1	-1.243754358	0.151580063	-8.205263514	2.30086E-16	7.75603E-16	negative
PRJNA729271	stage_5W_vs_E10	ordinary_muscle_development	ENSGALG00010026697	CHN1	-2.200284103	0.120288582	-18.29171201	9.63413E-75	2.31971E-73	negative
PRJNA729271	stage_5W_vs_E10	ordinary_muscle_development	ENSGALG00010026700	C8B	0.032995289	1.024167656	0.032216687	0.974299249	0.977942498	positive
PRJNA729271	stage_5W_vs_E10	ordinary_muscle_development	ENSGALG00010026704	WDFY1	-0.368394849	0.070409154	-5.232201005	1.67503E-07	3.40194E-07	negative

PRJNA729271	stage_5W_vs_E10	ordinary_muscle_development	ENSGALG00010026706	KRT24	-7.343466185	0.592822301	-12.38729746	3.06189E-35	2.28068E-34	negative
PRJNA729271	stage_5W_vs_E10	ordinary_muscle_development	ENSGALG00010026707	GORASP1	0.000491126	0.081698848	0.006011419	0.995203611	0.995959427	positive
PRJNA729271	stage_5W_vs_E10	ordinary_muscle_development	ENSGALG00010026715	WDR48	-0.410449397	0.068271031	-6.012057987	1.83183E-09	4.20998E-09	negative
PRJNA729271	stage_5W_vs_E10	ordinary_muscle_development	ENSGALG00010026719	ENSGALG00010026719	-1.065452699	0.742850362	-1.434276341	0.151493419	0.179696981	negative
PRJNA729271	stage_5W_vs_E10	ordinary_muscle_development	ENSGALG00010026724	OLA1	1.156590133	0.09770574	11.83748398	2.49836E-32	1.665E-31	positive
PRJNA729271	stage_5W_vs_E10	ordinary_muscle_development	ENSGALG00010026731	ENSGALG00010026731	-2.327919653	0.520528916	-4.472219662	7.74118E-06	1.39027E-05	negative
PRJNA729271	stage_5W_vs_E10	ordinary_muscle_development	ENSGALG00010026737	SCN11A	-7.389946451	1.151887948	-6.415508089	1.40354E-10	3.45933E-10	negative
PRJNA729271	stage_5W_vs_E10	ordinary_muscle_development	ENSGALG00010026740	DAB1	-4.782915119	0.173260014	-27.60541811	9.5795E-168	1.2288E-165	negative
PRJNA729271	stage_5W_vs_E10	ordinary_muscle_development	ENSGALG00010026743	TNNT2	-4.338997073	0.587735982	-7.382561573	1.55273E-13	4.50045E-13	negative
PRJNA729271	stage_5W_vs_E10	ordinary_muscle_development	ENSGALG00010026746	HOXD10	-4.815071902	0.54055087	-8.907712794	5.20961E-19	1.98772E-18	negative
PRJNA729271	stage_5W_vs_E10	ordinary_muscle_development	ENSGALG00010026755	ENSGALG00010026755	-0.182984365	0.188923563	-0.968562959	0.332763292	0.372406108	negative
PRJNA729271	stage_5W_vs_E10	ordinary_muscle_development	ENSGALG00010026759	OMA1	-0.744118183	0.11309664	-6.579489713	4.72066E-11	1.20151E-10	negative
PRJNA729271	stage_5W_vs_E10	ordinary_muscle_development	ENSGALG00010026767	ENSGALG00010026767	19.58695588	2.512681946	7.795238832	6.42868E-15	2.0087E-14	positive
PRJNA729271	stage_5W_vs_E10	ordinary_muscle_development	ENSGALG00010026770	ENSGALG00010026770	-0.211953111	0.573325103	-0.369690965	0.711612765	0.742715079	negative
PRJNA729271	stage_5W_vs_E10	ordinary_muscle_development	ENSGALG00010026775	BTG2	3.005161293	0.36597389	8.211408989	2.18608E-16	7.3777E-16	positive
PRJNA729271	stage_5W_vs_E10	ordinary_muscle_development	ENSGALG00010026780	MYSM1	0.324687171	0.079057624	4.106968503	4.00886E-05	6.81064E-05	positive
PRJNA729271	stage_5W_vs_E10	ordinary_muscle_development	ENSGALG00010026782	HOXB9	-4.561339707	0.62853381	-7.257111132	3.95445E-13	1.12326E-12	negative
PRJNA729271	stage_5W_vs_E10	ordinary_muscle_development	ENSGALG00010026786	ENSGALG00010026786	2.597848588	0.577146454	4.501194752	6.75726E-06	1.21778E-05	positive
PRJNA729271	stage_5W_vs_E10	ordinary_muscle_development	ENSGALG00010026796	DCAKD	-0.252232673	0.229474265	-1.0991763	0.271691174	0.308875574	negative
PRJNA729271	stage_5W_vs_E10	ordinary_muscle_development	ENSGALG00010026801	FMOD	1.171177104	0.491450462	2.383103066	0.017167387	0.022873543	positive
PRJNA729271	stage_5W_vs_E10	ordinary_muscle_development	ENSGALG00010026822	PRELP	0.533042318	0.199988857	2.665360088	0.007690593	0.010651169	positive
PRJNA729271	stage_5W_vs_E10	ordinary_muscle_development	ENSGALG00010026825	ADAM23	-0.753768901	0.136740771	-5.512393239	3.53987E-08	7.52462E-08	negative
PRJNA729271	stage_5W_vs_E10	ordinary_muscle_development	ENSGALG00010026827	FAM117A	2.046509	0.127923678	15.99789056	1.32178E-57	1.97722E-56	positive
PRJNA729271	stage_5W_vs_E10	ordinary_muscle_development	ENSGALG00010026834	ENSGALG00010026834	1.543498605	2.083404379	0.740854066	0.458781929	0.500831757	positive
PRJNA729271	stage_5W_vs_E10	ordinary_muscle_development	ENSGALG00010026836	ENSGALG00010026836	2.737420567	0.597703974	4.579893536	4.65213E-06	8.48422E-06	positive
PRJNA729271	stage_5W_vs_E10	ordinary_muscle_development	ENSGALG00010026839	FGGY	-0.285644984	0.120129928	-2.377800339	0.017416253	0.0231966	negative
PRJNA729271	stage_5W_vs_E10	ordinary_muscle_development	ENSGALG00010026846	MICAL1	1.100090061	0.223484553	4.922443402	8.54703E-07	1.65098E-06	positive
PRJNA729271	stage_5W_vs_E10	ordinary_muscle_development	ENSGALG00010026851	ENSGALG00010026851	1.642452529	1.37856128	1.191425113	0.233486739	0.26832556	positive
PRJNA729271	stage_5W_vs_E10	ordinary_muscle_development	ENSGALG00010026854	SMPD2	-0.435962464	0.155554322	-2.80263807	0.005068652	0.007151767	negative
PRJNA729271	stage_5W_vs_E10	ordinary_muscle_development	ENSGALG00010026856	UBE2Z	-0.098544621	0.077964854	-1.263962111	0.206243627	0.239389924	negative
PRJNA729271	stage_5W_vs_E10	ordinary_muscle_development	ENSGALG00010026874	TEAD3	0.714990394	0.160329913	4.45949469	8.21531E-06	1.47268E-05	positive
PRJNA729271	stage_5W_vs_E10	ordinary_muscle_development	ENSGALG00010026885	TULP1	3.401870898	0.637173305	5.339004114	9.34585E-08	1.93112E-07	positive
PRJNA729271	stage_5W_vs_E10	ordinary_muscle_development	ENSGALG00010026886	JUN	1.401014717	0.192879787	7.263667914	3.76732E-13	1.07115E-12	positive
PRJNA729271	stage_5W_vs_E10	ordinary_muscle_development	ENSGALG00010026889	ENSGALG00010026889	-0.066475342	0.274731007	-0.241965198	0.80880713	0.831524175	negative
PRJNA729271	stage_5W_vs_E10	ordinary_muscle_development	ENSGALG00010026893	FKBP5	3.02981206	0.386166158	7.845876703	4.29939E-15	1.35681E-14	positive
PRJNA729271	stage_5W_vs_E10	ordinary_muscle_development	ENSGALG00010026894	IP6K1	-0.156871736	0.066702505	-2.351811759	0.018682227	0.024793891	negative
PRJNA729271	stage_5W_vs_E10	ordinary_muscle_development	ENSGALG00010026897	ENSGALG00010026897	-0.449711067	0.227240682	-1.979007732	0.047815136	0.060420224	negative
PRJNA729271	stage_5W_vs_E10	ordinary_muscle_development	ENSGALG00010026898	PNPLA2	4.251343291	0.271530513	15.65696336	2.9778E-55	4.14234E-54	positive
PRJNA729271	stage_5W_vs_E10	ordinary_muscle_development	ENSGALG00010026899	HOOK1	-0.166834716	0.167712067	-0.994768703	0.319848771	0.359142761	negative
PRJNA729271	stage_5W_vs_E10	ordinary_muscle_development	ENSGALG00010026901	FAM212A	-0.863379107	0.177557851	-4.862522835	1.15899E-06	2.21309E-06	negative
PRJNA729271	stage_5W_vs_E10	ordinary_muscle_development	ENSGALG00010026907	ENSGALG00010026907	-0.770780861	0.135437891	-5.691028239	1.26277E-08	2.7712E-08	negative
PRJNA729271	stage_5W_vs_E10	ordinary_muscle_development	ENSGALG00010026909	ENSGALG00010026909	0.593981486	0.1548079	3.836893892	0.0001246	0.000203319	positive
PRJNA729271	stage_5W_vs_E10	ordinary_muscle_development	ENSGALG00010026916	ENSGALG00010026916	1.107457032	0.212038909	5.22289534	1.76147E-07	3.57098E-07	positive
PRJNA729271	stage_5W_vs_E10	ordinary_muscle_development	ENSGALG00010026917	AMT	-1.616708108	0.148593212	-10.88009394	1.43415E-27	7.90419E-27	negative
PRJNA729271	stage_5W_vs_E10	ordinary_muscle_development	ENSGALG00010026919	NICN1	-0.64436115	0.174912988	-3.683895396	0.000229697	0.000367246	negative
PRJNA729271	stage_5W_vs_E10	ordinary_muscle_development	ENSGALG00010026920	GATA2	2.333581016	0.198627389	11.7485359	7.1853E-32	4.68515E-31	positive
PRJNA729271	stage_5W_vs_E10	ordinary_muscle_development	ENSGALG00010026924	TPH1	0.460991563	0.350117645	1.31667618	0.187947191	0.219754339	positive
PRJNA729271	stage_5W_vs_E10	ordinary_muscle_development	ENSGALG00010026942	CDK18	-1.130894518	0.18489025	-6.116571957	9.56097E-10	2.23454E-09	negative
PRJNA729271	stage_5W_vs_E10	ordinary_muscle_development	ENSGALG00010026943	EEFSEC	-0.279319843	0.07169481	-3.895956229	9.78121E-05	0.000161259	negative
PRJNA729271	stage_5W_vs_E10	ordinary_muscle_development	ENSGALG00010026950	LHFPL5	-0.208728419	0.280944511	-0.742952471	0.457510457	0.499556615	negative
PRJNA729271	stage_5W_vs_E10	ordinary_muscle_development	ENSGALG00010026951	RUVBL1	-1.03809367	0.106821252	-9.718044403	2.52586E-22	1.12101E-21	negative
PRJNA729271	stage_5W_vs_E10	ordinary_muscle_development	ENSGALG00010026954	MFSD4A	0.976435425	0.177802148	5.491696467	3.98091E-08	8.43591E-08	positive
PRJNA729271	stage_5W_vs_E10	ordinary_muscle_development	ENSGALG00010026958	CLTCL1	0.130868484	0.084213746	1.554003826	0.120183504	0.144377581	positive
PRJNA729271	stage_5W_vs_E10	ordinary_muscle_development	ENSGALG00010026960	SEC61A1	-1.535401748	0.1025854	-14.96705912	1.20535E-50	1.48064E-49	negative
PRJNA729271	stage_5W_vs_E10	ordinary_muscle_development	ENSGALG00010026964	SRPK1	-0.560838609	0.060299411	-9.300896995	1.39266E-20	5.6992E-20	negative
PRJNA729271	stage_5W_vs_E10	ordinary_muscle_development	ENSGALG00010026972	TNNT3	6.158044035	0.158348857	38.88909677	0	0	positive
PRJNA729271	stage_5W_vs_E10	ordinary_muscle_development	ENSGALG00010026973	MGLL	2.044973545	0.123965342	16.49633285	3.89861E-61	6.48055E-60	positive
PRJNA729271	stage_5W_vs_E10	ordinary_muscle_development	ENSGALG00010026977	ELK4	0.533100903	0.097168832	5.486336405	4.10355E-08	8.68843E-08	positive
PRJNA729271	stage_5W_vs_E10	ordinary_muscle_development	ENSGALG00010026980	LIMK2	-1.042922801	0.119082514	-8.757984423	1.98773E-18	7.4048E-18	negative
PRJNA729271	stage_5W_vs_E10	ordinary_muscle_development	ENSGALG00010026982	ABTB1	1.889844606	0.084626633	22.33155848	1.8245E-110	9.4116E-109	positive
PRJNA729271	stage_5W_vs_E10	ordinary_muscle_development	ENSGALG00010026984	PGR2/3	-1.319246369	1.20114717	-1.098322006	0.272063906	0.309250809	negative
PRJNA729271	stage_5W_vs_E10	ordinary_muscle_development	ENSGALG00010026999	MLEC	-0.711007408	0.082962708	-8.570204908	1.03301E-17	3.72568E-17	negative
PRJNA729271	stage_5W_vs_E10	ordinary_muscle_development	ENSGALG00010027001	SLC26A8	0.151803863	0.255474226	0.594204219	0.552375531	0.592166506	positive
PRJNA729271	stage_5W_vs_E10	ordinary_muscle_development	ENSGALG00010027003	SEPTIN2L	-0.890686989	0.08272635	-10.76666613	4.94587E-27	2.66507E-26	negative
PRJNA729271	stage_5W_vs_E10	ordinary_muscle_development	ENSGALG00010027012	RILPL1	1.891417962	0.192031813	9.849503251	6.88836E-23	3.13294E-22	positive
PRJNA729271	stage_5W_vs_E10	ordinary_muscle_development	ENSGALG00010027015	ENSGALG00010027015	1.331221979	0.52541316	2.53366699	0.011287594	0.015375781	positive
PRJNA729271	stage_5W_vs_E10	ordinary_muscle_development	ENSGALG00010027025	ORAI1	2.217637567	0.127048511	17.45504567	3.15126E-68	6.28301E-67	positive
PRJNA729271	stage_5W_vs_E10	ordinary_muscle_development	ENSGALG00010027026	SLC45A3	1.357610327	0.201202438	6.747484479	1.5043E-11	3.94587E-11	positive
PRJNA729271	stage_5W_vs_E10	ordinary_muscle_development	ENSGALG00010027027	ARHGEF3	0.489482889	0.102270606	4.786154165	1.70007E-06	3.2045E-06	positive
PRJNA729271	stage_5W_vs_E10	ordinary_muscle_development	ENSGALG00010027036	NUCKS1	-1.536785786	0.066114469	-23.24431875	1.6238E-119	9.8069E-118	negative
PRJNA729271	stage_5W_vs_E10	ordinary_muscle_development	ENSGALG00010027040	CLIP1	1.584298653	0.1078528	14.68945322	7.53191E-49	8.76204E-48	positive
PRJNA729271	stage_5W_vs_E10	ordinary_muscle_development	ENSGALG00010027041	MAPK14	0.72787631	0.073767179	9.867210871	5.7748E-23	2.63143E-22	positive
PRJNA729271	stage_5W_vs_E10	ordinary_muscle_development	ENSGALG00010027043	IL17RD	-3.55131865	0.179253225	-19.81174203	2.35784E-87	7.56124E-86	negative
PRJNA729271	stage_5W_vs_E10	ordinary_muscle_development	ENSGALG00010027045	MAPK13	0.626280227	0.19143543	3.271495918	0.001069801	0.001617648	positive
PRJNA729271	stage_5W_vs_E10	ordinary_muscle_development	ENSGALG00010027046	HESX1	-0.92126782	0.330943018	-2.783765691	0.005373183	0.00755938	negative
PRJNA729271	stage_5W_vs_E10	ordinary_muscle_development	ENSGALG00010027050	GSTT1	2.375421036	0.151805512	15.64779172	3.43949E-55	4.77542E-54	positive
PRJNA729271	stage_5W_vs_E10	ordinary_muscle_development	ENSGALG00010027052	APPL1	0.197905594	0.084954122	2.32955847	0.019829499	0.026208498	positive
PRJNA729271	stage_5W_vs_E10	ordinary_muscle_development	ENSGALG00010027059	ASB14	7.524276944	0.210847908	35.68580317	6.5643E-279	2.3787E-276	positive
PRJNA729271	stage_5W_vs_E10	ordinary_muscle_development	ENSGALG00010027063	RAB7L1	0.757962502	0.087499811	8.662447271	4.61743E-18	1.69014E-17	positive
PRJNA729271	stage_5W_vs_E10	ordinary_muscle_development	ENSGALG00010027073	DNAH12	-1.106954149	0.311410742	-3.55464344	0.000378492	0.000593729	negative
PRJNA729271	stage_5W_vs_E10	ordinary_muscle_development	ENSGALG00010027074	BRPF3	-0.06464164	0.08101415	-0.797905545	0.424925305	0.466719126	negative
PRJNA729271	stage_5W_vs_E10	ordinary_muscle_development	ENSGALG00010027075	SLC41A1	1.741726309	0.094313163	18.46747849	3.77301E-76	9.4619E-75	positive
PRJNA729271	stage_5W_vs_E10	ordinary_muscle_development	ENSGALG00010027076	VDHAP	1.155698016	0.172615505	6.69521555	2.15354E-11	5.59318E-11	positive
PRJNA729271	stage_5W_vs_E10	ordinary_muscle_development	ENSGALG00010027077	ARF4	-0.703854175	0.104807303	-6.715697776	1.87168E-11	4.87863E-11	negative
PRJNA729271	stage_5W_vs_E10	ordinary_muscle_development	ENSGALG00010027078	PDE12	-0.987196748	0.124390985	-7.936240319	2.08402E-15	6.70392E-15	negative
PRJNA729271	stage_5W_vs_E10	ordinary_muscle_development	ENSGALG00010027079	PM20D1	0.910749792	0.094196991	9.668565625	4.10085E-22	1.80345E-21	positive
PRJNA729271	stage_5W_vs_E10	ordinary_muscle_development	ENSGALG00010027080	TBC1D22B	0.785967083	0.155795121	5.0448761	4.53815E-07	8.92666E-07	positive
PRJNA729271	stage_5W_vs_E10	ordinary_muscle_development	ENSGALG00010027081	PDE4B	1.663622457	0.290765017	5.721535805	1.05565E-08	2.3269E-08	positive
PRJNA729271	stage_5W_vs_E10	ordinary_muscle_development	ENSGALG00010027082	SLC26A9	-3.197408169	0.656180706	-4.872755537	1.10052E-06	2.10589E-06	negative
PRJNA729271	stage_5W_vs_E10	ordinary_muscle_development	ENSGALG00010027083	DENND6A	-0.621452833	0.088071829	-7.056204495	1.71112E-12	4.71534E-12	negative
PRJNA729271	stage_5W_vs_E10	ordinary_muscle_development	ENSGALG00010027084	OSBPL9	-0.033304656	0.094768201	-0.351432813	0.725263669	0.755439557	negative
PRJNA729271	stage_5W_vs_E10	ordinary_muscle_development	ENSGALG00010027085	PIM1	0.377653826	0.151240844	2.497035963	0.012523626	0.016952741	positive
PRJNA729271	stage_5W_vs_E10	ordinary_muscle_development	ENSGALG00010027086	CACNA1S	5.175211367	0.201121533	25.73176168	5.1584E-146	4.7323E-144	positive
PRJNA729271	stage_5W_vs_E10	ordinary_muscle_development	ENSGALG00010027087	RAB7B	0.53284925	0.331216125	1.608766026	0.107667512	0.130194426	positive
PRJNA729271	stage_5W_vs_E10	ordinary_muscle_development	ENSGALG00010027088	AGBL4	0.61399662	0.256141281	2.39710139	0.01652535	0.022070852	positive
PRJNA729271	stage_5W_vs_E10	ordinary_muscle_development	ENSGALG00010027092	ENSGALG00010027092	-1.957436229	0.203532195	-9.617329733	6.75623E-22	2.94701E-21	negative
PRJNA729271	stage_5W_vs_E10	ordinary_muscle_development	ENSGALG00010027093	SLMAP	0.462380411	0.076419561	6.050550495	1.44352E-09	3.34086E-09	positive
PRJNA729271	stage_5W_vs_E10	ordinary_muscle_development	ENSGALG00010027095	SCN5A	-1.78104318	0.403601838	-4.412871826	1.02008E-05	1.81715E-05	negative
PRJNA729271	stage_5W_vs_E10	ordinary_muscle_development	ENSGALG00010027096	YIPF1	1.182734416	0.10977616	10.77405531	4.56443E-27	2.46503E-26	positive
PRJNA729271	stage_5W_vs_E10	ordinary_muscle_development	ENSGALG00010027097	ENSGALG00010027097	-0.218431437	0.148612834	-1.469801975	0.141615394	0.168573141	negative
PRJNA729271	stage_5W_vs_E10	ordinary_muscle_development	ENSGALG00010027098	EXOG	0.147722188	0.146219704	1.010275524	0.312363304	0.3514754	positive
PRJNA729271	stage_5W_vs_E10	ordinary_muscle_development	ENSGALG00010027099	GNA11	-0.64459854	0.086152016	-7.48210629	7.31408E-14	2.15834E-13	negative
PRJNA729271	stage_5W_vs_E10	ordinary_muscle_development	ENSGALG00010027100	MIER2	-0.789359701	0.0943588	-8.365512272	5.98543E-17	2.07806E-16	negative
PRJNA729271	stage_5W_vs_E10	ordinary_muscle_development	ENSGALG00010027101	RNF220	-0.182147835	0.0800027	-2.276771088	0.022799899	0.029927061	negative
PRJNA729271	stage_5W_vs_E10	ordinary_muscle_development	ENSGALG00010027102	DUS3L	-1.031138484	0.146798425	-7.024179491	2.15328E-12	5.89715E-12	negative
PRJNA729271	stage_5W_vs_E10	ordinary_muscle_development	ENSGALG00010027103	ACVR2B	-1.929359355	0.192092822	-10.04389093	9.77409E-24	4.5924E-23	negative
PRJNA729271	stage_5W_vs_E10	ordinary_muscle_development	ENSGALG00010027105	CHERP	-0.538429259	0.12918664	-4.167840099	3.075E-05	5.27043E-05	negative
PRJNA729271	stage_5W_vs_E10	ordinary_muscle_development	ENSGALG00010027106	CYP2J23	0.601774135	0.145598884	4.133095796	3.57909E-05	6.0991E-05	positive
PRJNA729271	stage_5W_vs_E10	ordinary_muscle_development	ENSGALG00010027107	KIF21B	-0.428669037	0.199387043	-2.149934273	0.031560414	0.040840791	negative
PRJNA729271	stage_5W_vs_E10	ordinary_muscle_development	ENSGALG00010027108	ENSGALG00010027108	0.199623404	0.142001573	1.405783049	0.159788557	0.188948861	positive
PRJNA729271	stage_5W_vs_E10	ordinary_muscle_development	ENSGALG00010027109	C19orf44	0.189155044	0.125204968	1.510763088	0.130848822	0.156489577	positive
PRJNA729271	stage_5W_vs_E10	ordinary_muscle_development	ENSGALG00010027110	IKBKE	1.198978115	0.201498258	5.950315039	2.67627E-09	6.09849E-09	positive
PRJNA729271	stage_5W_vs_E10	ordinary_muscle_development	ENSGALG00010027111	CALR3	-2.778315492	0.892564824	-3.112732451	0.00185364	0.00273443	negative
PRJNA729271	stage_5W_vs_E10	ordinary_muscle_development	ENSGALG00010027112	XYLB	-1.800996923	0.194171187	-9.275304683	1.77112E-20	7.21337E-20	negative
PRJNA729271	stage_5W_vs_E10	ordinary_muscle_development	ENSGALG00010027113	PIAS4	-0.619901637	0.137075431	-4.522339506	6.11599E-06	1.10551E-05	negative
PRJNA729271	stage_5W_vs_E10	ordinary_muscle_development	ENSGALG00010027114	FLNB	-0.497381354	0.113650954	-4.376394011	1.20659E-05	2.13782E-05	negative
PRJNA729271	stage_5W_vs_E10	ordinary_muscle_development	ENSGALG00010027115	C9ORF152	-0.312589769	0.612510356	-0.510342015	0.609811872	0.646898645	negative
PRJNA729271	stage_5W_vs_E10	ordinary_muscle_development	ENSGALG00010027116	CCDC94	0.08447418	0.103490548	0.816250189	0.414357057	0.455836789	positive
PRJNA729271	stage_5W_vs_E10	ordinary_muscle_development	ENSGALG00010027117	RASSF5	3.427583394	0.228109156	15.02606673	4.95571E-51	6.16063E-50	positive
PRJNA729271	stage_5W_vs_E10	ordinary_muscle_development	ENSGALG00010027118	ABHD6	1.285731094	0.159578847	8.057027085	7.81723E-16	2.56796E-15	positive
PRJNA729271	stage_5W_vs_E10	ordinary_muscle_development	ENSGALG00010027119	NUB1	1.910116477	0.097394027	19.61225476	1.21523E-85	3.70838E-84	positive
PRJNA729271	stage_5W_vs_E10	ordinary_muscle_development	ENSGALG00010027121	ELF3	-0.257201112	0.890301321	-0.288892205	0.772663869	0.799640353	negative
PRJNA729271	stage_5W_vs_E10	ordinary_muscle_development	ENSGALG00010027122	DNASE1L3	5.489935361	0.247474587	22.18383484	4.9201E-109	2.4094E-107	positive
PRJNA729271	stage_5W_vs_E10	ordinary_muscle_development	ENSGALG00010027123	WDR86	-2.472480404	0.227687786	-10.8590823	1.80554E-27	9.88718E-27	negative
PRJNA729271	stage_5W_vs_E10	ordinary_muscle_development	ENSGALG00010027124	IP6K2	0.568312933	0.17380689	3.269795188	0.001076254	0.001627065	positive
PRJNA729271	stage_5W_vs_E10	ordinary_muscle_development	ENSGALG00010027125	CRYGN	1.453979349	0.404327663	3.596042228	0.000323095	0.000509993	positive
PRJNA729271	stage_5W_vs_E10	ordinary_muscle_development	ENSGALG00010027126	ENSGALG00010027126	7.546577369	0.535288162	14.09815853	3.89809E-45	3.99596E-44	positive
PRJNA729271	stage_5W_vs_E10	ordinary_muscle_development	ENSGALG00010027127	S1PR4	3.251251887	0.595195075	5.462497966	4.69481E-08	9.9099E-08	positive
PRJNA729271	stage_5W_vs_E10	ordinary_muscle_development	ENSGALG00010027128	EIF2D	0.29368831	0.090310026	3.252001187	0.001145955	0.001725958	positive
PRJNA729271	stage_5W_vs_E10	ordinary_muscle_development	ENSGALG00010027129	NCLN	0.608674116	0.128780347	4.726451887	2.28477E-06	4.2661E-06	positive
PRJNA729271	stage_5W_vs_E10	ordinary_muscle_development	ENSGALG00010027130	NCKIPSD	0.150518188	0.1448047	1.039456514	0.298592472	0.336948064	positive

PRJNA729271	stage_5W_vs_E10	ordinary_muscle_development	ENSGALG00010027131	UBA52	0.437422237	0.152251411	2.873025838	0.004065609	0.005797442	positive
PRJNA729271	stage_5W_vs_E10	ordinary_muscle_development	ENSGALG00010027133	CELF5	-3.464464529	0.345077036	-10.03968438	1.02E-23	4.78941E-23	negative
PRJNA729271	stage_5W_vs_E10	ordinary_muscle_development	ENSGALG00010027134	DYRK3	-0.035631167	0.148845009	-0.239384363	0.810807554	0.832954196	negative
PRJNA729271	stage_5W_vs_E10	ordinary_muscle_development	ENSGALG00010027135	PRKAG2	0.234618945	0.139179663	1.685727214	0.091848353	0.112103559	positive
PRJNA729271	stage_5W_vs_E10	ordinary_muscle_development	ENSGALG00010027136	MAPKAPK2	1.721781327	0.143914777	11.96389529	5.49237E-33	3.7394E-32	positive
PRJNA729271	stage_5W_vs_E10	ordinary_muscle_development	ENSGALG00010027137	FKBP8	0.815182313	0.089814207	9.076318076	1.1231E-19	4.42985E-19	positive
PRJNA729271	stage_5W_vs_E10	ordinary_muscle_development	ENSGALG00010027139	HSD11B1L	-0.043371733	0.144779592	-0.299570765	0.764504587	0.792270965	negative
PRJNA729271	stage_5W_vs_E10	ordinary_muscle_development	ENSGALG00010027141	PIGR	8.991340885	1.38570809	6.488625524	8.6623E-11	2.16782E-10	positive
PRJNA729271	stage_5W_vs_E10	ordinary_muscle_development	ENSGALG00010027143	GALNT11	-1.360001303	0.123319806	-11.02824718	2.79255E-28	1.58117E-27	negative
PRJNA729271	stage_5W_vs_E10	ordinary_muscle_development	ENSGALG00010027144	ENSGALG00010027144	-5.02138573	0.979462427	-5.126675196	2.94904E-07	5.87174E-07	negative
PRJNA729271	stage_5W_vs_E10	ordinary_muscle_development	ENSGALG00010027145	CELSR3	-1.103220998	0.170950865	-6.453439097	1.0934E-10	2.71477E-10	negative
PRJNA729271	stage_5W_vs_E10	ordinary_muscle_development	ENSGALG00010027146	KXD1	1.331177165	0.122204429	10.89303537	1.24423E-27	6.87051E-27	positive
PRJNA729271	stage_5W_vs_E10	ordinary_muscle_development	ENSGALG00010027147	KMT2C	1.257899339	0.103388249	12.16675353	4.67301E-34	3.32362E-33	positive
PRJNA729271	stage_5W_vs_E10	ordinary_muscle_development	ENSGALG00010027148	ELL	1.150194631	0.165537122	6.948257977	3.69824E-12	1.0003E-11	positive
PRJNA729271	stage_5W_vs_E10	ordinary_muscle_development	ENSGALG00010027149	C1orf116	2.092114634	0.224318002	9.326557014	1.09366E-20	4.49464E-20	positive
PRJNA729271	stage_5W_vs_E10	ordinary_muscle_development	ENSGALG00010027150	YOD1	-0.962263897	0.091995557	-10.45989529	1.32001E-25	6.69007E-25	negative
PRJNA729271	stage_5W_vs_E10	ordinary_muscle_development	ENSGALG00010027151	MICOS13	0.806406469	0.144069491	5.597343789	2.17661E-08	4.70543E-08	positive
PRJNA729271	stage_5W_vs_E10	ordinary_muscle_development	ENSGALG00010027152	C4BPS	5.751648124	0.428406768	13.42567054	4.27695E-41	3.86258E-40	positive
PRJNA729271	stage_5W_vs_E10	ordinary_muscle_development	ENSGALG00010027153	ENSGALG00010027153	3.312368891	0.227961607	14.53038052	7.77871E-48	8.6666E-47	positive
PRJNA729271	stage_5W_vs_E10	ordinary_muscle_development	ENSGALG00010027154	PLPP2	-0.049496244	0.138467337	-0.357457906	0.720749034	0.751168913	negative
PRJNA729271	stage_5W_vs_E10	ordinary_muscle_development	ENSGALG00010027155	ENSGALG00010027155	2.178460153	0.289830213	7.516332155	5.63343E-14	1.67638E-13	positive
PRJNA729271	stage_5W_vs_E10	ordinary_muscle_development	ENSGALG00010027156	ISYNA1	-1.608568733	0.150186844	-10.7104503	9.09185E-27	4.86656E-26	negative
PRJNA729271	stage_5W_vs_E10	ordinary_muscle_development	ENSGALG00010027157	ENSGALG00010027157	1.367001745	0.250886748	5.448680563	5.07449E-08	1.06833E-07	positive
PRJNA729271	stage_5W_vs_E10	ordinary_muscle_development	ENSGALG00010027158	XRCC2	-0.091732929	0.134720291	-0.680913978	0.495925923	0.537534305	negative
PRJNA729271	stage_5W_vs_E10	ordinary_muscle_development	ENSGALG00010027159	TMUB1	0.721935743	0.164400921	4.391312036	1.12669E-05	2.00065E-05	positive
PRJNA729271	stage_5W_vs_E10	ordinary_muscle_development	ENSGALG00010027160	FASTK	1.754960463	0.165903471	10.57820221	3.76105E-26	1.9554E-25	positive
PRJNA729271	stage_5W_vs_E10	ordinary_muscle_development	ENSGALG00010027161	C4BPG	1.098572163	0.203114227	5.408642128	6.35044E-08	1.32751E-07	positive
PRJNA729271	stage_5W_vs_E10	ordinary_muscle_development	ENSGALG00010027162	SLC4A2	0.863495657	0.129414999	6.672299683	2.51826E-11	6.5101E-11	positive
PRJNA729271	stage_5W_vs_E10	ordinary_muscle_development	ENSGALG00010027163	ENSGALG00010027163	-0.896264283	0.079933384	-11.21264036	3.53489E-29	2.07778E-28	negative
PRJNA729271	stage_5W_vs_E10	ordinary_muscle_development	ENSGALG00010027164	ENSGALG00010027164	2.5841707	0.461188203	5.603288814	2.10322E-08	4.5495E-08	positive
PRJNA729271	stage_5W_vs_E10	ordinary_muscle_development	ENSGALG00010027165	ENSGALG00010027165	-3.56460306	0.233409969	-15.27185439	1.17776E-52	1.53937E-51	negative
PRJNA729271	stage_5W_vs_E10	ordinary_muscle_development	ENSGALG00010027166	CDK5	0.000573136	0.100492117	0.005703291	0.995449457	0.996067919	positive
PRJNA729271	stage_5W_vs_E10	ordinary_muscle_development	ENSGALG00010027167	ENSGALG00010027167	-2.005768815	0.472997755	-4.240546161	2.22977E-05	3.86701E-05	negative
PRJNA729271	stage_5W_vs_E10	ordinary_muscle_development	ENSGALG00010027168	SSBP4	-1.240672725	0.184835931	-6.712291914	1.91591E-11	4.99121E-11	negative
PRJNA729271	stage_5W_vs_E10	ordinary_muscle_development	ENSGALG00010027169	ABCB8	-0.044570189	0.112589139	-0.395865791	0.692204038	0.724963695	negative
PRJNA729271	stage_5W_vs_E10	ordinary_muscle_development	ENSGALG00010027170	TLE2	-0.846992302	0.179702575	-4.713300867	2.43736E-06	4.54282E-06	negative
PRJNA729271	stage_5W_vs_E10	ordinary_muscle_development	ENSGALG00010027171	ATG9B	3.994319174	0.273741028	14.59159852	3.17666E-48	3.58611E-47	positive
PRJNA729271	stage_5W_vs_E10	ordinary_muscle_development	ENSGALG00010027172	ENSGALG00010027172	-1.821356551	0.547248253	-3.328208985	0.000874063	0.001331114	negative
PRJNA729271	stage_5W_vs_E10	ordinary_muscle_development	ENSGALG00010027173	ENSGALG00010027173	-1.06840995	0.098050098	-10.89657195	1.19682E-27	6.6188E-27	negative
PRJNA729271	stage_5W_vs_E10	ordinary_muscle_development	ENSGALG00010027174	SHISA5	-0.139025437	0.132792849	-1.046934669	0.295129711	0.333274007	negative
PRJNA729271	stage_5W_vs_E10	ordinary_muscle_development	ENSGALG00010027175	NOS3	1.272971456	0.163321261	7.79427891	6.47773E-15	2.02359E-14	positive
PRJNA729271	stage_5W_vs_E10	ordinary_muscle_development	ENSGALG00010027176	ENSGALG00010027176	0.51881943	0.170644528	3.040351985	0.002363018	0.003450383	positive
PRJNA729271	stage_5W_vs_E10	ordinary_muscle_development	ENSGALG00010027178	C4BPM	0.858340701	0.080976992	10.5998097	2.98587E-26	1.5619E-25	positive
PRJNA729271	stage_5W_vs_E10	ordinary_muscle_development	ENSGALG00010027183	CD34	0.576204009	0.15700745	3.669915081	0.000242631	0.000387157	positive
PRJNA729271	stage_5W_vs_E10	ordinary_muscle_development	ENSGALG00010027184	MPND	-0.23816132	0.346738749	-0.686860988	0.492170333	0.533623231	negative
PRJNA729271	stage_5W_vs_E10	ordinary_muscle_development	ENSGALG00010027185	ATRIP	-1.160633715	0.084680541	-13.70602629	9.34367E-43	8.87526E-42	negative
PRJNA729271	stage_5W_vs_E10	ordinary_muscle_development	ENSGALG00010027186	PLXNA2	-0.006849874	0.124442453	-0.055044512	0.956103002	0.962009789	negative
PRJNA729271	stage_5W_vs_E10	ordinary_muscle_development	ENSGALG00010027187	ENSGALG00010027187	-0.766368531	0.141061066	-5.432884855	5.54502E-08	1.16401E-07	negative
PRJNA729271	stage_5W_vs_E10	ordinary_muscle_development	ENSGALG00010027188	KCNH2	0.617033002	0.261085663	2.36333545	0.018111268	0.024069206	positive
PRJNA729271	stage_5W_vs_E10	ordinary_muscle_development	ENSGALG00010027189	ENSGALG00010027189	-1.910950736	0.329337951	-5.802400628	6.53721E-09	1.45802E-08	negative
PRJNA729271	stage_5W_vs_E10	ordinary_muscle_development	ENSGALG00010027190	IPPK	-0.026957875	0.11520361	-0.234002002	0.814983424	0.836450098	negative
PRJNA729271	stage_5W_vs_E10	ordinary_muscle_development	ENSGALG00010027192	SH3GL1	-1.366257622	0.117436618	-11.6340001	2.76815E-31	1.76604E-30	negative
PRJNA729271	stage_5W_vs_E10	ordinary_muscle_development	ENSGALG00010027193	CAMK1G	-1.866216282	0.511811716	-3.646294574	0.000266049	0.000422849	negative
PRJNA729271	stage_5W_vs_E10	ordinary_muscle_development	ENSGALG00010027194	ENSGALG00010027194	-1.888395996	0.265469949	-7.11340775	1.13212E-12	3.14852E-12	negative
PRJNA729271	stage_5W_vs_E10	ordinary_muscle_development	ENSGALG00010027195	CENPP	-3.05277031	0.2257428	-13.52322339	1.14066E-41	1.04711E-40	negative
PRJNA729271	stage_5W_vs_E10	ordinary_muscle_development	ENSGALG00010027196	ENSGALG00010027196	1.284315777	0.326681154	3.931404561	8.4451E-05	0.000140204	positive
PRJNA729271	stage_5W_vs_E10	ordinary_muscle_development	ENSGALG00010027197	ENSGALG00010027197	-2.441721514	0.598760972	-4.077957026	4.54332E-05	7.6862E-05	negative
PRJNA729271	stage_5W_vs_E10	ordinary_muscle_development	ENSGALG00010027198	ECM2	1.045173614	0.203102092	5.146050454	2.66028E-07	5.31579E-07	positive
PRJNA729271	stage_5W_vs_E10	ordinary_muscle_development	ENSGALG00010027199	LAMB3	-1.370953223	0.339323104	-4.040258997	5.33922E-05	8.99489E-05	negative
PRJNA729271	stage_5W_vs_E10	ordinary_muscle_development	ENSGALG00010027200	G0S2	3.77797229	0.317970294	11.88152591	1.47647E-32	9.92647E-32	positive
PRJNA729271	stage_5W_vs_E10	ordinary_muscle_development	ENSGALG00010027201	ASPN	0.646807316	0.31611	2.04614633	0.040741976	0.051962599	positive
PRJNA729271	stage_5W_vs_E10	ordinary_muscle_development	ENSGALG00010027202	CHAF1A	-1.760176574	0.087980576	-20.00642237	4.84176E-89	1.60966E-87	negative
PRJNA729271	stage_5W_vs_E10	ordinary_muscle_development	ENSGALG00010027203	ENSGALG00010027203	-1.152535102	0.310482257	-3.712080407	0.000205563	0.000330007	negative
PRJNA729271	stage_5W_vs_E10	ordinary_muscle_development	ENSGALG00010027204	OMD	-4.241509428	0.887394047	-4.779736174	1.75525E-06	3.3055E-06	negative
PRJNA729271	stage_5W_vs_E10	ordinary_muscle_development	ENSGALG00010027205	OGN	1.094844418	0.221869137	4.934640463	8.02985E-07	1.55439E-06	positive
PRJNA729271	stage_5W_vs_E10	ordinary_muscle_development	ENSGALG00010027206	ENSGALG00010027206	6.228553299	0.974245439	6.393207554	1.62442E-10	3.98678E-10	positive
PRJNA729271	stage_5W_vs_E10	ordinary_muscle_development	ENSGALG00010027207	NOL8	-0.437322772	0.124481751	-3.513147647	0.000442831	0.000690272	negative
PRJNA729271	stage_5W_vs_E10	ordinary_muscle_development	ENSGALG00010027208	UBXN6	0.726773525	0.094735726	7.671588685	1.69879E-14	5.18399E-14	positive
PRJNA729271	stage_5W_vs_E10	ordinary_muscle_development	ENSGALG00010027209	TRAF3IP3	2.632680164	0.233353974	11.28191699	1.61195E-29	9.57196E-29	positive
PRJNA729271	stage_5W_vs_E10	ordinary_muscle_development	ENSGALG00010027210	ENSGALG00010027210	0.239867292	0.257846004	0.930273449	0.352229521	0.392371531	positive
PRJNA729271	stage_5W_vs_E10	ordinary_muscle_development	ENSGALG00010027211	HDGFRP2	-0.775997793	0.102182483	-7.59423505	3.09616E-14	9.34976E-14	negative
PRJNA729271	stage_5W_vs_E10	ordinary_muscle_development	ENSGALG00010027212	IRF6	-3.748079535	0.567534378	-6.604145374	3.99817E-11	1.02247E-10	negative
PRJNA729271	stage_5W_vs_E10	ordinary_muscle_development	ENSGALG00010027213	KCNC4	0.793290545	0.339673491	2.335450267	0.019519911	0.025837011	positive
PRJNA729271	stage_5W_vs_E10	ordinary_muscle_development	ENSGALG00010027214	PLIN4	0.798496605	0.266306452	2.998412542	0.0027139	0.003941287	positive
PRJNA729271	stage_5W_vs_E10	ordinary_muscle_development	ENSGALG00010027215	ENSGALG00010027215	-1.615623494	0.19292245	-8.37447115	5.54724E-17	1.92823E-16	negative
PRJNA729271	stage_5W_vs_E10	ordinary_muscle_development	ENSGALG00010027216	ENSGALG00010027216	1.773662933	0.572345004	3.098940186	0.001942142	0.002857425	positive
PRJNA729271	stage_5W_vs_E10	ordinary_muscle_development	ENSGALG00010027217	SEMA6B	0.58164911	0.220532376	2.637477189	0.008352524	0.011529363	positive
PRJNA729271	stage_5W_vs_E10	ordinary_muscle_development	ENSGALG00010027218	IARS1	-0.140032943	0.16447514	-0.851392758	0.394551213	0.435834464	negative
PRJNA729271	stage_5W_vs_E10	ordinary_muscle_development	ENSGALG00010027219	ENSGALG00010027219	0.181173096	0.137935354	1.313463809	0.189026708	0.22090963	positive
PRJNA729271	stage_5W_vs_E10	ordinary_muscle_development	ENSGALG00010027220	ANKRD24	-1.232192402	0.612345424	-2.012250528	0.044193535	0.056108022	negative
PRJNA729271	stage_5W_vs_E10	ordinary_muscle_development	ENSGALG00010027221	LRRC61	-0.765342624	0.276265295	-2.770317653	0.005600165	0.007868022	negative
PRJNA729271	stage_5W_vs_E10	ordinary_muscle_development	ENSGALG00010027222	GUCA1B	0.679251352	0.947595956	0.716815376	0.473488023	0.515410701	positive
PRJNA729271	stage_5W_vs_E10	ordinary_muscle_development	ENSGALG00010027223	SIRT6	-0.553080371	0.084656154	-6.533256558	6.43548E-11	1.62399E-10	negative
PRJNA729271	stage_5W_vs_E10	ordinary_muscle_development	ENSGALG00010027224	RARRES2	1.018462262	0.120755511	8.434085157	3.33804E-17	1.17155E-16	positive
PRJNA729271	stage_5W_vs_E10	ordinary_muscle_development	ENSGALG00010027225	CREB3L3	-0.484666154	0.734150951	-0.660172344	0.509143238	0.549927812	negative
PRJNA729271	stage_5W_vs_E10	ordinary_muscle_development	ENSGALG00010027227	ENSGALG00010027227	1.330388337	0.183173452	7.262997589	3.78605E-13	1.07626E-12	positive
PRJNA729271	stage_5W_vs_E10	ordinary_muscle_development	ENSGALG00010027229	MDM4	0.550184592	0.080308739	6.850868242	7.34031E-12	1.95405E-11	positive
PRJNA729271	stage_5W_vs_E10	ordinary_muscle_development	ENSGALG00010027230	MAP2K2	-0.463494804	0.082371064	-5.626912939	1.83463E-08	3.98397E-08	negative
PRJNA729271	stage_5W_vs_E10	ordinary_muscle_development	ENSGALG00010027231	ENSGALG00010027231	-0.983074332	0.158593014	-6.198724076	5.69227E-10	1.35084E-09	negative
PRJNA729271	stage_5W_vs_E10	ordinary_muscle_development	ENSGALG00010027232	ARRDC2	2.979750512	0.326951373	9.113742169	7.95883E-20	3.15977E-19	positive
PRJNA729271	stage_5W_vs_E10	ordinary_muscle_development	ENSGALG00010027233	ENSGALG00010027233	2.329215931	0.254130975	9.165415318	4.93565E-20	1.97304E-19	positive
PRJNA729271	stage_5W_vs_E10	ordinary_muscle_development	ENSGALG00010027234	SLC6A11	-4.973242601	0.669409204	-7.429301197	1.09173E-13	3.19173E-13	negative
PRJNA729271	stage_5W_vs_E10	ordinary_muscle_development	ENSGALG00010027236	PEX11G	0.035334417	0.281886569	0.125349772	0.900246651	0.912905779	positive
PRJNA729271	stage_5W_vs_E10	ordinary_muscle_development	ENSGALG00010027237	CTC-575C13.4	0.036099507	0.164838085	0.218999797	0.826650206	0.847464087	positive
PRJNA729271	stage_5W_vs_E10	ordinary_muscle_development	ENSGALG00010027239	ENSGALG00010027239	-0.303334019	0.084466388	-3.591180189	0.000329184	0.000519151	negative
PRJNA729271	stage_5W_vs_E10	ordinary_muscle_development	ENSGALG00010027240	SLC6A1	0.271438609	0.320164613	0.847809528	0.396544056	0.437802277	positive
PRJNA729271	stage_5W_vs_E10	ordinary_muscle_development	ENSGALG00010027241	LRRN2	-1.759099471	0.271255128	-6.485036748	8.87102E-11	2.21775E-10	negative
PRJNA729271	stage_5W_vs_E10	ordinary_muscle_development	ENSGALG00010027242	ENSGALG00010027242	0.180588134	0.192434766	0.938438189	0.348019268	0.387981794	positive
PRJNA729271	stage_5W_vs_E10	ordinary_muscle_development	ENSGALG00010027243	HRH1	0.33990365	0.199689857	1.702157812	0.088725789	0.108456764	positive
PRJNA729271	stage_5W_vs_E10	ordinary_muscle_development	ENSGALG00010027244	ARHGEF18	0.465440371	0.090070813	5.167493845	2.37254E-07	4.76117E-07	positive
PRJNA729271	stage_5W_vs_E10	ordinary_muscle_development	ENSGALG00010027245	LRIG2	-0.100675411	0.066646878	-1.510579562	0.130895604	0.156532612	negative
PRJNA729271	stage_5W_vs_E10	ordinary_muscle_development	ENSGALG00010027246	ZNF767	-0.940586558	0.101423753	-9.273829159	1.7958E-20	7.3098E-20	negative
PRJNA729271	stage_5W_vs_E10	ordinary_muscle_development	ENSGALG00010027247	ATG7	-0.022599666	0.079943696	-0.28269479	0.77741081	0.803979002	negative
PRJNA729271	stage_5W_vs_E10	ordinary_muscle_development	ENSGALG00010027248	ENSGALG00010027248	-2.790786888	0.93861022	-2.97331824	0.002945988	0.004260836	negative
PRJNA729271	stage_5W_vs_E10	ordinary_muscle_development	ENSGALG00010027249	C2CD4C	3.007484388	1.175668067	2.558106725	0.010524379	0.014382094	positive
PRJNA729271	stage_5W_vs_E10	ordinary_muscle_development	ENSGALG00010027250	ENSGALG00010027250	-0.11681965	0.17214093	-0.678627968	0.497373617	0.538781151	negative
PRJNA729271	stage_5W_vs_E10	ordinary_muscle_development	ENSGALG00010027251	ENSGALG00010027251	0.172423845	0.201238184	0.856814753	0.391547271	0.432846071	positive
PRJNA729271	stage_5W_vs_E10	ordinary_muscle_development	ENSGALG00010027252	MAGI3	-0.625905077	0.072761335	-8.602165886	7.82256E-18	2.83754E-17	negative
PRJNA729271	stage_5W_vs_E10	ordinary_muscle_development	ENSGALG00010027253	VGLL4	-0.829045198	0.088100335	-9.410238853	4.95011E-21	2.07196E-20	negative
PRJNA729271	stage_5W_vs_E10	ordinary_muscle_development	ENSGALG00010027254	INSR	0.806513328	0.133319877	6.049460462	1.45332E-09	3.36193E-09	positive
PRJNA729271	stage_5W_vs_E10	ordinary_muscle_development	ENSGALG00010027255	ENSGALG00010027255	-0.577805972	0.184791321	-3.126802546	0.001767186	0.002611147	negative
PRJNA729271	stage_5W_vs_E10	ordinary_muscle_development	ENSGALG00010027256	PHTF1	-1.64893758	0.092029949	-17.91740191	8.62639E-72	1.92073E-70	negative
PRJNA729271	stage_5W_vs_E10	ordinary_muscle_development	ENSGALG00010027257	TAFA3	2.065183979	0.265719192	7.77205427	7.72233E-15	2.40617E-14	positive
PRJNA729271	stage_5W_vs_E10	ordinary_muscle_development	ENSGALG00010027258	ENSGALG00010027258	-1.460490738	0.17805786	-8.20233792	2.35756E-16	7.9398E-16	negative
PRJNA729271	stage_5W_vs_E10	ordinary_muscle_development	ENSGALG00010027259	ENSGALG00010027259	-0.509355373	0.086528772	-5.886543415	3.94356E-09	8.90789E-09	negative
PRJNA729271	stage_5W_vs_E10	ordinary_muscle_development	ENSGALG00010027260	LEMD2	0.292823737	0.100731621	2.906969365	0.003649489	0.005226692	positive
PRJNA729271	stage_5W_vs_E10	ordinary_muscle_development	ENSGALG00010027261	ENSGALG00010027261	-0.383991556	0.113439375	-3.384993569	0.000711799	0.001091802	negative
PRJNA729271	stage_5W_vs_E10	ordinary_muscle_development	ENSGALG00010027262	USE1	-0.452760866	0.135492166	-3.341601806	0.000832965	0.001270929	negative
PRJNA729271	stage_5W_vs_E10	ordinary_muscle_development	ENSGALG00010027263	TAMM41	-1.557149263	0.129942104	-11.98340811	4.34103E-33	2.96808E-32	negative
PRJNA729271	stage_5W_vs_E10	ordinary_muscle_development	ENSGALG00010027264	ENSGALG00010027264	1.015372668	0.093983871	10.80369068	3.3064E-27	1.79769E-26	positive
PRJNA729271	stage_5W_vs_E10	ordinary_muscle_development	ENSGALG00010027265	IP6K3	4.512938296	0.207749461	21.72298434	1.2443E-104	5.6186E-103	positive
PRJNA729271	stage_5W_vs_E10	ordinary_muscle_development	ENSGALG00010027267	MYO9B	-0.171189555	0.09550959	-1.792380803	0.073071988	0.09033505	negative
PRJNA729271	stage_5W_vs_E10	ordinary_muscle_development	ENSGALG00010027268	UQCRC1	0.749890275	0.126721318	5.917633182	3.26607E-09	7.40756E-09	positive
PRJNA729271	stage_5W_vs_E10	ordinary_muscle_development	ENSGALG00010027269	CDC25A	-1.195750805	0.101033651	-11.83517365	2.56811E-32	1.7107E-31	negative
PRJNA729271	stage_5W_vs_E10	ordinary_muscle_development	ENSGALG00010027270	ENSGALG00010027270	-3.17560323	0.430052919	-7.384215033	1.53355E-13	4.44755E-13	negative
PRJNA729271	stage_5W_vs_E10	ordinary_muscle_development	ENSGALG00010027271	INKA2	-1.184666912	0.149183702	-7.940994208	2.00567E-15	6.45762E-15	negative
PRJNA729271	stage_5W_vs_E10	ordinary_muscle_development	ENSGALG00010027272	DDX20	-0.630893299	0.104241296	-6.052239578	1.42846E-09	3.30706E-09	negative
PRJNA729271	stage_5W_vs_E10	ordinary_muscle_development	ENSGALG00010027273	HAUS8	-1.713619297	0.135327529	-12.66275467	9.51027E-37	7.48785E-36	negative
PRJNA729271	stage_5W_vs_E10	ordinary_muscle_development	ENSGALG00010027274	COPG1	-0.763019741	0.06109894	-12.48826481	8.65201E-36	6.58325E-35	negative
PRJNA729271	stage_5W_vs_E10	ordinary_muscle_development	ENSGALG00010027275	CPAMD8	-0.524080011	0.227654859	-2.302081377	0.021330583	0.028079812	negative
PRJNA729271	stage_5W_vs_E10	ordinary_muscle_development	ENSGALG00010027276	ENSGALG00010027276	0.779520124	0.138193617	5.640782404	1.69279E-08	3.6859E-08	positive

PRJNA729271	stage_5W_vs_E10	ordinary_muscle_development	ENSGALG00010027277	KCND3	1.819361739	0.389742019	4.668118011	3.03971E-06	5.63507E-06	positive
PRJNA729271	stage_5W_vs_E10	ordinary_muscle_development	ENSGALG00010027278	H1-10	-1.051042882	0.129476891	-8.117609795	4.75455E-16	1.57777E-15	negative
PRJNA729271	stage_5W_vs_E10	ordinary_muscle_development	ENSGALG00010027279	F2RL3	0.927568112	0.352365162	2.632405842	0.008478252	0.011697341	positive
PRJNA729271	stage_5W_vs_E10	ordinary_muscle_development	ENSGALG00010027280	HMCES	-0.687866328	0.126599765	-5.433393389	5.52923E-08	1.16087E-07	negative
PRJNA729271	stage_5W_vs_E10	ordinary_muscle_development	ENSGALG00010027281	ENSGALG00010027281	-0.050669407	0.2494991	-0.20308453	0.839068963	0.858621354	negative
PRJNA729271	stage_5W_vs_E10	ordinary_muscle_development	ENSGALG00010027282	ENSGALG00010027282	2.228432058	0.128442802	17.34960639	1.986E-67	3.90069E-66	positive
PRJNA729271	stage_5W_vs_E10	ordinary_muscle_development	ENSGALG00010027283	RAB7A	0.634821313	0.062667411	10.13000696	4.06621E-24	1.9452E-23	positive
PRJNA729271	stage_5W_vs_E10	ordinary_muscle_development	ENSGALG00010027284	CTTNBP2NL	1.500267456	0.089576057	16.74853192	5.8041E-63	1.0124E-61	positive
PRJNA729271	stage_5W_vs_E10	ordinary_muscle_development	ENSGALG00010027285	PTPRS	-0.643915647	0.112042184	-5.747082232	9.07966E-09	2.00961E-08	negative
PRJNA729271	stage_5W_vs_E10	ordinary_muscle_development	ENSGALG00010027286	RPN1	-0.193724939	0.062969415	-3.076492602	0.002094515	0.003072563	negative
PRJNA729271	stage_5W_vs_E10	ordinary_muscle_development	ENSGALG00010027287	DHX30	-0.506963558	0.089847929	-5.642462393	1.67635E-08	3.65175E-08	negative
PRJNA729271	stage_5W_vs_E10	ordinary_muscle_development	ENSGALG00010027288	DAG1	1.4231109	0.167797613	8.481115305	2.23045E-17	7.8951E-17	positive
PRJNA729271	stage_5W_vs_E10	ordinary_muscle_development	ENSGALG00010027289	SMARCC1	-1.07508783	0.13970524	-7.69540091	1.41051E-14	4.32798E-14	negative
PRJNA729271	stage_5W_vs_E10	ordinary_muscle_development	ENSGALG00010027290	CSPG5	-4.784790634	0.414300105	-11.54909346	7.46032E-31	4.6752E-30	negative
PRJNA729271	stage_5W_vs_E10	ordinary_muscle_development	ENSGALG00010027291	MST1	1.851346044	0.273755824	6.762764037	1.35384E-11	3.55892E-11	positive
PRJNA729271	stage_5W_vs_E10	ordinary_muscle_development	ENSGALG00010027292	KDM4B	-0.09786971	0.061259855	-1.597615772	0.110128514	0.133014983	negative
PRJNA729271	stage_5W_vs_E10	ordinary_muscle_development	ENSGALG00010027293	ELP6	-0.446963995	0.153155686	-2.918363711	0.003518737	0.005047411	negative
PRJNA729271	stage_5W_vs_E10	ordinary_muscle_development	ENSGALG00010027295	ENSGALG00010027295	2.505757488	0.236742191	10.58433007	3.52292E-26	1.83554E-25	positive
PRJNA729271	stage_5W_vs_E10	ordinary_muscle_development	ENSGALG00010027297	ENSGALG00010027297	3.569403872	2.054386197	1.737455147	0.082306865	0.10102786	positive
PRJNA729271	stage_5W_vs_E10	ordinary_muscle_development	ENSGALG00010027299	PGPEP1	0.104457513	0.16312807	0.640340519	0.521951244	0.562546158	positive
PRJNA729271	stage_5W_vs_E10	ordinary_muscle_development	ENSGALG00010027301	SCAP	-0.361098327	0.06506382	-5.549909684	2.85817E-08	6.11772E-08	negative
PRJNA729271	stage_5W_vs_E10	ordinary_muscle_development	ENSGALG00010027303	APEH	0.165634761	0.140001247	1.183094892	0.236771542	0.271885159	positive
PRJNA729271	stage_5W_vs_E10	ordinary_muscle_development	ENSGALG00010027304	PTPN23	1.110679296	0.136827105	8.117392325	4.76307E-16	1.58024E-15	positive
PRJNA729271	stage_5W_vs_E10	ordinary_muscle_development	ENSGALG00010027305	LSM4	-1.011541394	0.130844714	-7.730854091	1.06827E-14	3.30445E-14	negative
PRJNA729271	stage_5W_vs_E10	ordinary_muscle_development	ENSGALG00010027306	ENSGALG00010027306	4.465306172	0.3133725	14.24919599	4.53472E-46	4.77348E-45	positive
PRJNA729271	stage_5W_vs_E10	ordinary_muscle_development	ENSGALG00010027307	JUND	0.703776162	0.176199801	3.994193848	6.49147E-05	0.000108842	positive
PRJNA729271	stage_5W_vs_E10	ordinary_muscle_development	ENSGALG00010027309	ENSGALG00010027309	4.048759075	0.657211004	6.160516259	7.25082E-10	1.70562E-09	positive
PRJNA729271	stage_5W_vs_E10	ordinary_muscle_development	ENSGALG00010027310	ARHGEF2	-0.077351977	0.133219536	-0.580635383	0.56148622	0.601047933	negative
PRJNA729271	stage_5W_vs_E10	ordinary_muscle_development	ENSGALG00010027311	NFASC	2.518927198	0.239400041	10.52183277	6.85274E-26	3.51986E-25	positive
PRJNA729271	stage_5W_vs_E10	ordinary_muscle_development	ENSGALG00010027312	ENSGALG00010027312	4.533078387	0.607632417	7.460231783	8.63707E-14	2.53995E-13	positive
PRJNA729271	stage_5W_vs_E10	ordinary_muscle_development	ENSGALG00010027313	BSN	-0.468126188	0.234740967	-1.994224501	0.046127526	0.058415035	negative
PRJNA729271	stage_5W_vs_E10	ordinary_muscle_development	ENSGALG00010027314	ENSGALG00010027314	2.540194811	0.352297008	7.21037861	5.57965E-13	1.57594E-12	positive
PRJNA729271	stage_5W_vs_E10	ordinary_muscle_development	ENSGALG00010027315	RAB3A	0.508175375	0.226621615	2.242395878	0.024935798	0.032609563	positive
PRJNA729271	stage_5W_vs_E10	ordinary_muscle_development	ENSGALG00010027316	TRAIP	-3.673754797	0.227726154	-16.13233584	1.51192E-58	2.33639E-57	negative
PRJNA729271	stage_5W_vs_E10	ordinary_muscle_development	ENSGALG00010027317	MPV17L2	0.569635331	0.146799255	3.88036936	0.000104298	0.000171542	positive
PRJNA729271	stage_5W_vs_E10	ordinary_muscle_development	ENSGALG00010027319	MST1R	2.027307879	0.323801305	6.260962666	3.82608E-10	9.17133E-10	positive
PRJNA729271	stage_5W_vs_E10	ordinary_muscle_development	ENSGALG00010027320	MON1A	1.010245287	0.160662057	6.288014136	3.21553E-10	7.74108E-10	positive
PRJNA729271	stage_5W_vs_E10	ordinary_muscle_development	ENSGALG00010027321	IFI30	3.343472011	0.202937588	16.47537079	5.51494E-61	9.09432E-60	positive
PRJNA729271	stage_5W_vs_E10	ordinary_muscle_development	ENSGALG00010027322	CRABP2	-6.869441755	0.226971698	-30.2656314	3.2495E-201	5.5413E-199	negative
PRJNA729271	stage_5W_vs_E10	ordinary_muscle_development	ENSGALG00010027323	ISG20L2	-0.738667568	0.133130352	-5.548453505	2.88208E-08	6.16797E-08	negative
PRJNA729271	stage_5W_vs_E10	ordinary_muscle_development	ENSGALG00010027324	PIK3R2	-0.751519643	0.097801002	-7.684171219	1.53991E-14	4.71802E-14	negative
PRJNA729271	stage_5W_vs_E10	ordinary_muscle_development	ENSGALG00010027325	METTL25B	0.239872362	0.292573471	0.819870514	0.412289928	0.453907223	positive
PRJNA729271	stage_5W_vs_E10	ordinary_muscle_development	ENSGALG00010027326	MRPL24	0.35227761	0.118078565	2.983417096	0.002850492	0.00413055	positive
PRJNA729271	stage_5W_vs_E10	ordinary_muscle_development	ENSGALG00010027327	RBM6	-0.313524578	0.089147927	-3.516902614	0.000436614	0.000681093	negative
PRJNA729271	stage_5W_vs_E10	ordinary_muscle_development	ENSGALG00010027328	RBM15	-0.122348748	0.131402941	-0.931095958	0.351803929	0.391959873	negative
PRJNA729271	stage_5W_vs_E10	ordinary_muscle_development	ENSGALG00010027329	MINDY4	-0.998989679	0.120492309	-8.290899975	1.12395E-16	3.84418E-16	negative
PRJNA729271	stage_5W_vs_E10	ordinary_muscle_development	ENSGALG00010027330	ENSGALG00010027330	0.531673557	0.194804361	2.729269283	0.006347485	0.008868979	positive
PRJNA729271	stage_5W_vs_E10	ordinary_muscle_development	ENSGALG00010027331	SLC16A4	0.955360908	0.162361482	5.884159804	4.00081E-09	9.03017E-09	positive
PRJNA729271	stage_5W_vs_E10	ordinary_muscle_development	ENSGALG00010027332	HDGF	0.962854861	0.176621065	5.451529018	4.99386E-08	1.05197E-07	positive
PRJNA729271	stage_5W_vs_E10	ordinary_muscle_development	ENSGALG00010027333	PROK1	0.301882164	0.283479406	1.064917442	0.286913291	0.324678597	positive
PRJNA729271	stage_5W_vs_E10	ordinary_muscle_development	ENSGALG00010027334	PRCC	-0.237475067	0.114912932	-2.066565205	0.038775142	0.049628758	negative
PRJNA729271	stage_5W_vs_E10	ordinary_muscle_development	ENSGALG00010027335	MAST3	0.896963434	0.172780198	5.191355516	2.08769E-07	4.20993E-07	positive
PRJNA729271	stage_5W_vs_E10	ordinary_muscle_development	ENSGALG00010027336	RAD54L2	0.38618155	0.13749913	2.808610849	0.004975575	0.007026594	positive
PRJNA729271	stage_5W_vs_E10	ordinary_muscle_development	ENSGALG00010027337	AQP1	-1.24201462	0.266133856	-4.666879444	3.05809E-06	5.66841E-06	negative
PRJNA729271	stage_5W_vs_E10	ordinary_muscle_development	ENSGALG00010027339	LAMTOR5	0.693515422	0.107682419	6.440377432	1.19177E-10	2.94941E-10	positive
PRJNA729271	stage_5W_vs_E10	ordinary_muscle_development	ENSGALG00010027340	ENSGALG00010027340	3.342759148	0.737077304	4.535154084	5.75614E-06	1.04216E-05	positive
PRJNA729271	stage_5W_vs_E10	ordinary_muscle_development	ENSGALG00010027341	ENSGALG00010027341	3.99939186	0.543994125	7.351902668	1.95405E-13	5.63661E-13	positive
PRJNA729271	stage_5W_vs_E10	ordinary_muscle_development	ENSGALG00010027346	SIN3B	0.331247852	0.088847332	3.728281346	0.00019279	0.000310155	positive
PRJNA729271	stage_5W_vs_E10	ordinary_muscle_development	ENSGALG00010027348	ENSGALG00010027348	0.925883703	0.205429921	4.507053778	6.5734E-06	1.18568E-05	positive
PRJNA729271	stage_5W_vs_E10	ordinary_muscle_development	ENSGALG00010027350	INSRR	2.576124062	0.328938914	7.831618429	4.81629E-15	1.51469E-14	positive
PRJNA729271	stage_5W_vs_E10	ordinary_muscle_development	ENSGALG00010027351	TMEM38A	3.869421558	0.14786292	26.16897838	5.9943E-151	6.0761E-149	positive
PRJNA729271	stage_5W_vs_E10	ordinary_muscle_development	ENSGALG00010027354	ENSGALG00010027354	4.154770149	0.815726028	5.093340171	3.5181E-07	6.96936E-07	positive
PRJNA729271	stage_5W_vs_E10	ordinary_muscle_development	ENSGALG00010027355	ADCYAP1R1	-1.987834048	0.274635413	-7.238083481	4.55069E-13	1.2911E-12	negative
PRJNA729271	stage_5W_vs_E10	ordinary_muscle_development	ENSGALG00010027356	ENSGALG00010027356	0.949231655	0.219695701	4.320665583	1.55559E-05	2.7328E-05	positive
PRJNA729271	stage_5W_vs_E10	ordinary_muscle_development	ENSGALG00010027358	RNF126	-0.060268337	0.10106676	-0.596322048	0.550960105	0.590780198	negative
PRJNA729271	stage_5W_vs_E10	ordinary_muscle_development	ENSGALG00010027359	ENSGALG00010027359	0.808707025	0.116749039	6.926883814	4.30212E-12	1.15866E-11	positive
PRJNA729271	stage_5W_vs_E10	ordinary_muscle_development	ENSGALG00010027360	SMIM7	0.690528994	0.113596769	6.078773168	1.21106E-09	2.8177E-09	positive
PRJNA729271	stage_5W_vs_E10	ordinary_muscle_development	ENSGALG00010027361	FANCD2	-0.083412165	0.192947122	-0.432305826	0.665519154	0.699746129	negative
PRJNA729271	stage_5W_vs_E10	ordinary_muscle_development	ENSGALG00010027362	ENSGALG00010027362	-0.112219766	0.144529269	-0.776450104	0.437483271	0.479494897	negative
PRJNA729271	stage_5W_vs_E10	ordinary_muscle_development	ENSGALG00010027363	VIPR1	0.763282679	0.25604649	2.9810316	0.002872791	0.00415912	positive
PRJNA729271	stage_5W_vs_E10	ordinary_muscle_development	ENSGALG00010027364	FSTL3	1.928366054	0.233410444	8.261695682	1.43617E-16	4.88441E-16	positive
PRJNA729271	stage_5W_vs_E10	ordinary_muscle_development	ENSGALG00010027365	PLEKHA6	1.972725688	0.16696148	11.81545397	3.2479E-32	2.1556E-31	positive
PRJNA729271	stage_5W_vs_E10	ordinary_muscle_development	ENSGALG00010027366	USP4	0.815699283	0.082193267	9.924161836	3.26837E-23	1.50397E-22	positive
PRJNA729271	stage_5W_vs_E10	ordinary_muscle_development	ENSGALG00010027367	SEC22C	-0.391685562	0.095180583	-4.115183458	3.86872E-05	6.57872E-05	negative
PRJNA729271	stage_5W_vs_E10	ordinary_muscle_development	ENSGALG00010027368	EMC3	1.071376079	0.088369386	12.12383759	7.89728E-34	5.56225E-33	positive
PRJNA729271	stage_5W_vs_E10	ordinary_muscle_development	ENSGALG00010027369	ENSGALG00010027369	0.77082056	0.101714443	7.578280335	3.50165E-14	1.05413E-13	positive
PRJNA729271	stage_5W_vs_E10	ordinary_muscle_development	ENSGALG00010027370	NUP210L	-1.104485351	0.356304991	-3.099831265	0.001936309	0.002849132	negative
PRJNA729271	stage_5W_vs_E10	ordinary_muscle_development	ENSGALG00010027372	PIK3C2B	-0.150715775	0.173133097	-0.87051972	0.384016447	0.425234408	negative
PRJNA729271	stage_5W_vs_E10	ordinary_muscle_development	ENSGALG00010027373	MLN	0.936253283	1.332533243	0.702611577	0.482297846	0.52397746	positive
PRJNA729271	stage_5W_vs_E10	ordinary_muscle_development	ENSGALG00010027374	NKTR	-0.019886928	0.118693416	-0.167548702	0.86693833	0.883335586	negative
PRJNA729271	stage_5W_vs_E10	ordinary_muscle_development	ENSGALG00010027375	FUT7	2.107890179	0.410735562	5.131988494	2.86697E-07	5.71462E-07	positive
PRJNA729271	stage_5W_vs_E10	ordinary_muscle_development	ENSGALG00010027376	DSTYK	0.412689112	0.079811916	5.17077061	2.33131E-07	4.68102E-07	positive
PRJNA729271	stage_5W_vs_E10	ordinary_muscle_development	ENSGALG00010027378	ZBTB47	0.543737011	0.164386732	3.30766969	0.000940757	0.001429978	positive
PRJNA729271	stage_5W_vs_E10	ordinary_muscle_development	ENSGALG00010027379	OPTC	-0.758824739	0.644480906	-1.177420048	0.239027892	0.274323776	negative
PRJNA729271	stage_5W_vs_E10	ordinary_muscle_development	ENSGALG00010027380	GPX1	-0.050552101	0.132769637	-0.380750466	0.703388418	0.735561295	negative
PRJNA729271	stage_5W_vs_E10	ordinary_muscle_development	ENSGALG00010027381	KLHL40	1.511553574	0.196984162	7.673477715	1.67395E-14	5.11141E-14	positive
PRJNA729271	stage_5W_vs_E10	ordinary_muscle_development	ENSGALG00010027382	PALM	0.018512646	0.201358246	0.091938851	0.926746624	0.936698439	positive
PRJNA729271	stage_5W_vs_E10	ordinary_muscle_development	ENSGALG00010027383	ASS1	3.576881059	0.282138373	12.67775462	7.85499E-37	6.20818E-36	positive
PRJNA729271	stage_5W_vs_E10	ordinary_muscle_development	ENSGALG00010027384	HHATL	0.536134365	0.219216786	2.445681166	0.014457879	0.019436743	positive
PRJNA729271	stage_5W_vs_E10	ordinary_muscle_development	ENSGALG00010027385	CNTN2	1.292319498	0.629939909	2.051496469	0.040218623	0.051353853	positive
PRJNA729271	stage_5W_vs_E10	ordinary_muscle_development	ENSGALG00010027386	RHOA	-0.300878969	0.066071983	-4.553805626	5.2684E-06	9.56961E-06	negative
PRJNA729271	stage_5W_vs_E10	ordinary_muscle_development	ENSGALG00010027387	ENSGALG00010027387	-0.803056594	0.272478949	-2.947224347	0.003206405	0.004620883	negative
PRJNA729271	stage_5W_vs_E10	ordinary_muscle_development	ENSGALG00010027388	URM1	0.770681569	0.144071018	5.349317167	8.82867E-08	1.82686E-07	positive
PRJNA729271	stage_5W_vs_E10	ordinary_muscle_development	ENSGALG00010027391	TPM3	-1.235150019	0.114764201	-10.76250264	5.17455E-27	2.78726E-26	negative
PRJNA729271	stage_5W_vs_E10	ordinary_muscle_development	ENSGALG00010027392	RBBP5	-0.035012262	0.126985518	-0.275718543	0.782764252	0.808880576	negative
PRJNA729271	stage_5W_vs_E10	ordinary_muscle_development	ENSGALG00010027393	TEX264	1.025827113	0.18921426	5.42151059	5.90975E-08	1.23789E-07	positive
PRJNA729271	stage_5W_vs_E10	ordinary_muscle_development	ENSGALG00010027394	SLC27A4	-0.690780905	0.087829609	-7.865011763	3.69063E-15	1.16982E-14	negative
PRJNA729271	stage_5W_vs_E10	ordinary_muscle_development	ENSGALG00010027395	BCL2L15	0.967527859	0.252633921	3.829762268	0.000128267	0.000209161	positive
PRJNA729271	stage_5W_vs_E10	ordinary_muscle_development	ENSGALG00010027397	RFX5	0.812932972	0.125079311	6.49934002	8.06731E-11	2.02311E-10	positive
PRJNA729271	stage_5W_vs_E10	ordinary_muscle_development	ENSGALG00010027399	MAPKAP1	0.269291911	0.11138344	2.417701502	0.015618884	0.020916086	positive
PRJNA729271	stage_5W_vs_E10	ordinary_muscle_development	ENSGALG00010027401	ENSGALG00010027401	1.393181095	0.312617776	4.456499926	8.33086E-06	1.49284E-05	positive
PRJNA729271	stage_5W_vs_E10	ordinary_muscle_development	ENSGALG00010027402	AP4B1	0.109091894	0.087280737	1.249896571	0.211337332	0.244792603	positive
PRJNA729271	stage_5W_vs_E10	ordinary_muscle_development	ENSGALG00010027403	ACKR2	1.122489175	0.369677534	3.036400839	0.002394208	0.003495221	positive
PRJNA729271	stage_5W_vs_E10	ordinary_muscle_development	ENSGALG00010027405	DCLRE1B	-1.589625881	0.181428498	-8.76172098	1.92292E-18	7.16522E-18	negative
PRJNA729271	stage_5W_vs_E10	ordinary_muscle_development	ENSGALG00010027407	ENSGALG00010027407	-1.384976893	1.157147701	-1.196888601	0.231349978	0.266038709	negative
PRJNA729271	stage_5W_vs_E10	ordinary_muscle_development	ENSGALG00010027410	ALAS1	0.248200132	0.088043399	2.819065775	0.004816365	0.006812374	positive
PRJNA729271	stage_5W_vs_E10	ordinary_muscle_development	ENSGALG00010027412	ENSGALG00010027412	0.848631596	0.286163821	2.965544674	0.003021475	0.004364352	positive
PRJNA729271	stage_5W_vs_E10	ordinary_muscle_development	ENSGALG00010027414	PBX3	-1.557618625	0.075188399	-20.71620945	2.47414E-95	9.48747E-94	negative
PRJNA729271	stage_5W_vs_E10	ordinary_muscle_development	ENSGALG00010027417	WNT2B	-2.088752782	0.368329549	-5.670880296	1.42066E-08	3.11111E-08	negative
PRJNA729271	stage_5W_vs_E10	ordinary_muscle_development	ENSGALG00010027418	ENSGALG00010027418	2.975436028	0.86120532	3.454967078	0.00055036	0.000852658	positive
PRJNA729271	stage_5W_vs_E10	ordinary_muscle_development	ENSGALG00010027419	PTBP1	-1.488063072	0.084855343	-17.53646882	7.54739E-69	1.54301E-67	negative
PRJNA729271	stage_5W_vs_E10	ordinary_muscle_development	ENSGALG00010027421	PLPPR3	-3.180370327	0.277208409	-11.47284939	1.80612E-30	1.11214E-29	negative
PRJNA729271	stage_5W_vs_E10	ordinary_muscle_development	ENSGALG00010027423	PEX11B	1.194623917	0.210013403	5.688322257	1.28294E-08	2.81419E-08	positive
PRJNA729271	stage_5W_vs_E10	ordinary_muscle_development	ENSGALG00010027425	CFD	4.100432024	0.238164037	17.21683959	1.98538E-66	3.81166E-65	positive
PRJNA729271	stage_5W_vs_E10	ordinary_muscle_development	ENSGALG00010027427	UBAC1	2.950979801	0.115559587	25.5364343	7.7686E-144	6.8246E-142	positive
PRJNA729271	stage_5W_vs_E10	ordinary_muscle_development	ENSGALG00010027438	ST7L	0.366451558	0.083429274	4.392361829	1.12126E-05	1.99175E-05	positive
PRJNA729271	stage_5W_vs_E10	ordinary_muscle_development	ENSGALG00010027440	TWF2	0.541561787	0.19041037	2.844182215	0.004452558	0.006319998	positive
PRJNA729271	stage_5W_vs_E10	ordinary_muscle_development	ENSGALG00010027443	MED16	-0.392683963	0.157023052	-2.500804551	0.012391154	0.016782824	negative
PRJNA729271	stage_5W_vs_E10	ordinary_muscle_development	ENSGALG00010027444	SELENBP1	0.592208282	0.121800717	4.862108338	1.16142E-06	2.21744E-06	positive
PRJNA729271	stage_5W_vs_E10	ordinary_muscle_development	ENSGALG00010027445	PPM1M	-0.163000372	0.16441245	-0.991411369	0.321484751	0.360870554	negative
PRJNA729271	stage_5W_vs_E10	ordinary_muscle_development	ENSGALG00010027448	ADAMTSL5	2.173843128	0.316276767	6.873230527	6.2764E-12	1.67729E-11	positive
PRJNA729271	stage_5W_vs_E10	ordinary_muscle_development	ENSGALG00010027450	CAMSAP1	0.807088596	0.142838984	5.650338403	1.60132E-08	3.49513E-08	positive
PRJNA729271	stage_5W_vs_E10	ordinary_muscle_development	ENSGALG00010027455	R3HDM4	0.967976329	0.147579586	6.559012366	5.41653E-11	1.37236E-10	positive
PRJNA729271	stage_5W_vs_E10	ordinary_muscle_development	ENSGALG00010027457	CAPZA1	-0.137546548	0.128089508	-1.073831492	0.282898213	0.320510364	negative
PRJNA729271	stage_5W_vs_E10	ordinary_muscle_development	ENSGALG00010027460	WDR82	-0.468897843	0.073741088	-6.358705268	2.03461E-10	4.96494E-10	negative
PRJNA729271	stage_5W_vs_E10	ordinary_muscle_development	ENSGALG00010027467	GLYCTK	0.010645686	0.135843386	0.07836735	0.937535845	0.945559565	positive
PRJNA729271	stage_5W_vs_E10	ordinary_muscle_development	ENSGALG00010027468	BSG	0.775366883	0.115205313	6.730304896	1.69308E-11	4.42582E-11	positive
PRJNA729271	stage_5W_vs_E10	ordinary_muscle_development	ENSGALG00010027471	ENSGALG00010027471	1.268411117	0.129335291	9.807153992	1.04885E-22	4.73764E-22	positive
PRJNA729271	stage_5W_vs_E10	ordinary_muscle_development	ENSGALG00010027473	DNAH1	-1.930264691	0.373876122	-5.162845598	2.43224E-07	4.87634E-07	negative
PRJNA729271	stage_5W_vs_E10	ordinary_muscle_development	ENSGALG00010027475	ARID3A	-0.410761478	0.167899177	-2.446477027	0.014425999	0.019399282	negative
PRJNA729271	stage_5W_vs_E10	ordinary_muscle_development	ENSGALG00010027479	HCN2	-0.0096783	0.26916631	-0.03595658	0.971316981	0.975421895	negative

PRJNA729271	stage_5W_vs_E10	ordinary_muscle_development	ENSGALG00010027487	MPZ	3.641701326	0.538415907	6.763732798	1.34481E-11	3.53583E-11	positive
PRJNA729271	stage_5W_vs_E10	ordinary_muscle_development	ENSGALG00010027490	ATP2B4	0.657886732	0.067669377	9.722074656	2.42784E-22	1.07817E-21	positive
PRJNA729271	stage_5W_vs_E10	ordinary_muscle_development	ENSGALG00010027492	PCP4L1	2.139824846	0.495824271	4.315692012	1.59104E-05	2.79134E-05	positive
PRJNA729271	stage_5W_vs_E10	ordinary_muscle_development	ENSGALG00010027497	KCNT1	3.686885082	0.338760955	10.88344164	1.38243E-27	7.62202E-27	positive
PRJNA729271	stage_5W_vs_E10	ordinary_muscle_development	ENSGALG00010027499	MOV10	3.438141139	0.398935193	8.618294894	6.79585E-18	2.47378E-17	positive
PRJNA729271	stage_5W_vs_E10	ordinary_muscle_development	ENSGALG00010027501	BAP1	-0.551063176	0.067564509	-8.156104203	3.46004E-16	1.15561E-15	negative
PRJNA729271	stage_5W_vs_E10	ordinary_muscle_development	ENSGALG00010027502	THOP1	0.055220051	0.139101942	0.396975413	0.691385592	0.724315854	positive
PRJNA729271	stage_5W_vs_E10	ordinary_muscle_development	ENSGALG00010027510	RHOC	2.504654616	0.182720708	13.7075575	9.1486E-43	8.69567E-42	positive
PRJNA729271	stage_5W_vs_E10	ordinary_muscle_development	ENSGALG00010027511	POLRMT	0.004607028	0.082777327	0.055655679	0.955616108	0.96166197	positive
PRJNA729271	stage_5W_vs_E10	ordinary_muscle_development	ENSGALG00010027513	SEMA3G	1.08179288	0.182111424	5.940280157	2.84535E-09	6.47057E-09	positive
PRJNA729271	stage_5W_vs_E10	ordinary_muscle_development	ENSGALG00010027516	PPM1J	3.741304404	0.213085669	17.55774763	5.18939E-69	1.06544E-67	positive
PRJNA729271	stage_5W_vs_E10	ordinary_muscle_development	ENSGALG00010027520	FANCE	-1.381281745	0.178070598	-7.756933281	8.70077E-15	2.70523E-14	negative
PRJNA729271	stage_5W_vs_E10	ordinary_muscle_development	ENSGALG00010027522	PSMD5	0.141108089	0.104863222	1.345639454	0.178418821	0.209339551	positive
PRJNA729271	stage_5W_vs_E10	ordinary_muscle_development	ENSGALG00010027524	ENSGALG00010027524	1.397832867	0.111135599	12.57772385	2.79981E-36	2.17255E-35	positive
PRJNA729271	stage_5W_vs_E10	ordinary_muscle_development	ENSGALG00010027525	ENSGALG00010027525	6.06842889	0.263793258	23.00448816	4.2032E-117	2.4767E-115	positive
PRJNA729271	stage_5W_vs_E10	ordinary_muscle_development	ENSGALG00010027527	ENSGALG00010027527	-2.866322546	0.664320312	-4.314669434	1.59842E-05	2.80362E-05	negative
PRJNA729271	stage_5W_vs_E10	ordinary_muscle_development	ENSGALG00010027528	ENSGALG00010027528	5.634424203	2.335075465	2.412951654			positive
PRJNA729271	stage_5W_vs_E10	ordinary_muscle_development	ENSGALG00010027529	ACY1	2.31050507	0.140087859	16.49325706	4.10227E-61	6.81127E-60	positive
PRJNA729271	stage_5W_vs_E10	ordinary_muscle_development	ENSGALG00010027530	PHF19	0.35572753	0.140111611	2.53888687	0.011120578	0.015156819	positive
PRJNA729271	stage_5W_vs_E10	ordinary_muscle_development	ENSGALG00010027531	UFC1	0.203722145	0.169688837	1.200563039	0.229920746	0.264437135	positive
PRJNA729271	stage_5W_vs_E10	ordinary_muscle_development	ENSGALG00010027533	WDR18	-1.539712222	0.099590568	-15.46042216	6.41766E-54	8.57363E-53	negative
PRJNA729271	stage_5W_vs_E10	ordinary_muscle_development	ENSGALG00010027534	KCNN3	0.544456099	0.192433066	2.82932715	0.004664599	0.00660803	positive
PRJNA729271	stage_5W_vs_E10	ordinary_muscle_development	ENSGALG00010027536	USP21	0.840511415	0.305638698	2.750016345	0.005959229	0.008349833	positive
PRJNA729271	stage_5W_vs_E10	ordinary_muscle_development	ENSGALG00010027537	SGTA	-0.369435711	0.068222315	-5.415147077	6.12291E-08	1.28161E-07	negative
PRJNA729271	stage_5W_vs_E10	ordinary_muscle_development	ENSGALG00010027538	ABHD14A	0.894246064	0.135391203	6.604905245	3.97772E-11	1.01742E-10	positive
PRJNA729271	stage_5W_vs_E10	ordinary_muscle_development	ENSGALG00010027539	DDR2L	-0.082480142	0.108201052	-0.762285958	0.445889353	0.487822942	negative
PRJNA729271	stage_5W_vs_E10	ordinary_muscle_development	ENSGALG00010027540	PPOX	-0.772434142	0.119568126	-6.460201129	1.04564E-10	2.59975E-10	negative
PRJNA729271	stage_5W_vs_E10	ordinary_muscle_development	ENSGALG00010027543	TUBB4B	-0.284067159	0.152103135	-1.86759569	0.061818448	0.077060406	negative
PRJNA729271	stage_5W_vs_E10	ordinary_muscle_development	ENSGALG00010027545	PPARD	0.011232548	0.20282546	0.055380367	0.955835437	0.961807335	positive
PRJNA729271	stage_5W_vs_E10	ordinary_muscle_development	ENSGALG00010027547	B4GALT3	0.924848531	0.223231806	4.142996239	3.42798E-05	5.84915E-05	positive
PRJNA729271	stage_5W_vs_E10	ordinary_muscle_development	ENSGALG00010027548	PCBP4	-0.204207037	0.148917284	-1.37127828	0.170288224	0.200644432	negative
PRJNA729271	stage_5W_vs_E10	ordinary_muscle_development	ENSGALG00010027553	FGF22	-0.653809876	0.318491532	-2.052832837	0.040088793	0.05120612	negative
PRJNA729271	stage_5W_vs_E10	ordinary_muscle_development	ENSGALG00010027554	ENSGALG00010027554	-0.042345568	0.153270216	-0.276280476	0.782332651	0.808492213	negative
PRJNA729271	stage_5W_vs_E10	ordinary_muscle_development	ENSGALG00010027555	ENSGALG00010027555	1.179069466	0.343708513	3.430434282	0.000602616	0.000930434	positive
PRJNA729271	stage_5W_vs_E10	ordinary_muscle_development	ENSGALG00010027557	C6orf132	-2.204830003	0.838918798	-2.628180472	0.008584296	0.011832386	negative
PRJNA729271	stage_5W_vs_E10	ordinary_muscle_development	ENSGALG00010027559	ENSGALG00010027559	2.377461132	0.268045462	8.869619039	7.33966E-19	2.78067E-18	positive
PRJNA729271	stage_5W_vs_E10	ordinary_muscle_development	ENSGALG00010027561	ADAMTS4	1.785006493	0.3353205	5.323284716	1.0191E-07	2.10216E-07	positive
PRJNA729271	stage_5W_vs_E10	ordinary_muscle_development	ENSGALG00010027562	SLC39A3	0.154001243	0.148539572	1.036769133	0.299843464	0.338234577	positive
PRJNA729271	stage_5W_vs_E10	ordinary_muscle_development	ENSGALG00010027563	WDR77	-0.993019496	0.123146087	-8.063751921	7.39878E-16	2.43242E-15	negative
PRJNA729271	stage_5W_vs_E10	ordinary_muscle_development	ENSGALG00010027567	PARP3	1.63734696	0.128685019	12.72367971	4.36796E-37	3.48451E-36	positive
PRJNA729271	stage_5W_vs_E10	ordinary_muscle_development	ENSGALG00010027568	NELFB	-0.29433774	0.076322904	-3.856479839	0.000115032	0.000188362	negative
PRJNA729271	stage_5W_vs_E10	ordinary_muscle_development	ENSGALG00010027569	ATP5PB	0.939904773	0.125353256	7.49804832	6.4775E-14	1.91928E-13	positive
PRJNA729271	stage_5W_vs_E10	ordinary_muscle_development	ENSGALG00010027570	NDUFS2	0.53268906	0.137771593	3.866465127	0.000110424	0.000181002	positive
PRJNA729271	stage_5W_vs_E10	ordinary_muscle_development	ENSGALG00010027571	RBM10	0.029971205	0.101801214	0.294409114	0.768445303	0.795785859	positive
PRJNA729271	stage_5W_vs_E10	ordinary_muscle_development	ENSGALG00010027574	GNG7	-0.788113318	0.096388128	-8.176456337	2.92313E-16	9.80806E-16	negative
PRJNA729271	stage_5W_vs_E10	ordinary_muscle_development	ENSGALG00010027575	FCER1G	2.479109614	0.330383122	7.503741717	6.20215E-14	1.84071E-13	positive
PRJNA729271	stage_5W_vs_E10	ordinary_muscle_development	ENSGALG00010027576	GADD45B	1.783070851	0.417127812	4.274639091	1.91447E-05	3.33818E-05	positive
PRJNA729271	stage_5W_vs_E10	ordinary_muscle_development	ENSGALG00010027578	ENSGALG00010027578	2.200292871	0.23823792	9.23569542	2.56614E-20	1.03929E-19	positive
PRJNA729271	stage_5W_vs_E10	ordinary_muscle_development	ENSGALG00010027579	C26H6ORF125	0.1012799	0.158233308	0.640066884	0.522129117	0.562696026	positive
PRJNA729271	stage_5W_vs_E10	ordinary_muscle_development	ENSGALG00010027583	GLE1	-1.20044139	0.074726204	-16.06453064	4.52275E-58	6.88626E-57	negative
PRJNA729271	stage_5W_vs_E10	ordinary_muscle_development	ENSGALG00010027586	HJV	3.944176213	0.289433854	13.62721103	2.75925E-42	2.58201E-41	positive
PRJNA729271	stage_5W_vs_E10	ordinary_muscle_development	ENSGALG00010027590	SEMA3F	-2.248376519	0.241734125	-9.301030699	1.39091E-20	5.69525E-20	negative
PRJNA729271	stage_5W_vs_E10	ordinary_muscle_development	ENSGALG00010027592	MAP1S	-0.31063826	0.169451471	-1.83319896	0.066772988	0.082929866	negative
PRJNA729271	stage_5W_vs_E10	ordinary_muscle_development	ENSGALG00010027593	ENSGALG00010027593	3.901273576	0.429928921	9.074229206	1.14485E-19	4.51431E-19	positive
PRJNA729271	stage_5W_vs_E10	ordinary_muscle_development	ENSGALG00010027594	ITPR3	0.004060452	0.139075506	0.029196026	0.976708251	0.980021189	positive
PRJNA729271	stage_5W_vs_E10	ordinary_muscle_development	ENSGALG00010027595	GNAT1	-0.120490354	0.449496145	-0.26805648	0.788655844	0.813982232	negative
PRJNA729271	stage_5W_vs_E10	ordinary_muscle_development	ENSGALG00010027597	EXOSC2	-1.600831263	0.118893811	-13.46437844	2.53442E-41	2.30756E-40	negative
PRJNA729271	stage_5W_vs_E10	ordinary_muscle_development	ENSGALG00010027600	KIRREL1	-0.297204645	0.141309514	-2.103217512	0.03544676	0.045614417	negative
PRJNA729271	stage_5W_vs_E10	ordinary_muscle_development	ENSGALG00010027605	FCHO1	1.911428771	0.304231094	6.282818576	3.32489E-10	7.99374E-10	positive
PRJNA729271	stage_5W_vs_E10	ordinary_muscle_development	ENSGALG00010027607	ABL1	-0.971895077	0.079656803	-12.20103046	3.06915E-34	2.19042E-33	negative
PRJNA729271	stage_5W_vs_E10	ordinary_muscle_development	ENSGALG00010027608	GNAI2	0.054455905	0.144076759	0.377964534	0.705456941	0.737458413	positive
PRJNA729271	stage_5W_vs_E10	ordinary_muscle_development	ENSGALG00010027609	RPL36	-0.043902887	0.186843033	-0.23497203	0.814230443	0.835795642	negative
PRJNA729271	stage_5W_vs_E10	ordinary_muscle_development	ENSGALG00010027612	BAK1	-0.769839922	0.163958139	-4.695344342	2.66158E-06	4.94991E-06	negative
PRJNA729271	stage_5W_vs_E10	ordinary_muscle_development	ENSGALG00010027614	LONP1	-0.260153801	0.079804136	-3.259903712	0.0011145	0.001682079	negative
PRJNA729271	stage_5W_vs_E10	ordinary_muscle_development	ENSGALG00010027616	SLC38A3	3.914377437	0.267970596	14.60748862	2.51624E-48	2.86061E-47	positive
PRJNA729271	stage_5W_vs_E10	ordinary_muscle_development	ENSGALG00010027618	PPP1R26	0.372000775	0.07866532	4.728904346	2.25735E-06	4.21924E-06	positive
PRJNA729271	stage_5W_vs_E10	ordinary_muscle_development	ENSGALG00010027619	JAK3	3.749352168	0.334316613	11.21497415	3.44287E-29	2.02616E-28	positive
PRJNA729271	stage_5W_vs_E10	ordinary_muscle_development	ENSGALG00010027621	TSPO2	-6.468454989	0.499720422	-12.94414778	2.53561E-38	2.08709E-37	negative
PRJNA729271	stage_5W_vs_E10	ordinary_muscle_development	ENSGALG00010027622	FIBCD1	-2.799651711	1.16917435	-2.394554508	0.01664057	0.022208366	negative
PRJNA729271	stage_5W_vs_E10	ordinary_muscle_development	ENSGALG00010027623	APOBEC2	2.37186695	0.182709864	12.98160315	1.55589E-38	1.28872E-37	positive
PRJNA729271	stage_5W_vs_E10	ordinary_muscle_development	ENSGALG00010027624	ENSGALG00010027624	5.110139545	0.392917322	13.00563567	1.1365E-38	9.46756E-38	positive
PRJNA729271	stage_5W_vs_E10	ordinary_muscle_development	ENSGALG00010027626	SEMA3B	0.07937057	0.215733579	0.367910136	0.712940242	0.743886324	positive
PRJNA729271	stage_5W_vs_E10	ordinary_muscle_development	ENSGALG00010027627	OARD1	1.722952377	0.092950004	18.53633457	1.05149E-76	2.67391E-75	positive
PRJNA729271	stage_5W_vs_E10	ordinary_muscle_development	ENSGALG00010027628	PIERCE1	-0.584924303	0.251605672	-2.324765963	0.020084478	0.026530986	negative
PRJNA729271	stage_5W_vs_E10	ordinary_muscle_development	ENSGALG00010027632	LAMC3	-1.137428053	0.391135331	-2.908016641	0.00363729	0.005209735	negative
PRJNA729271	stage_5W_vs_E10	ordinary_muscle_development	ENSGALG00010027634	OASL	6.442610635	0.711563628	9.054159577	1.37623E-19	5.39879E-19	positive
PRJNA729271	stage_5W_vs_E10	ordinary_muscle_development	ENSGALG00010027635	CD48	3.28581609	0.225292969	14.58463662	3.5179E-48	3.959E-47	positive
PRJNA729271	stage_5W_vs_E10	ordinary_muscle_development	ENSGALG00010027636	SLC1A6	1.573104225	0.594776284	2.644867101	0.008172301	0.011288117	positive
PRJNA729271	stage_5W_vs_E10	ordinary_muscle_development	ENSGALG00010027638	MRPS2	0.745010546	0.141173826	5.277256901	1.31132E-07	2.68582E-07	positive
PRJNA729271	stage_5W_vs_E10	ordinary_muscle_development	ENSGALG00010027641	HYAL3	1.00873432	0.16419789	6.143406104	8.07704E-10	1.89384E-09	positive
PRJNA729271	stage_5W_vs_E10	ordinary_muscle_development	ENSGALG00010027642	DIPK1B	-1.12986678	0.172295571	-6.55772388	5.46353E-11	1.38354E-10	negative
PRJNA729271	stage_5W_vs_E10	ordinary_muscle_development	ENSGALG00010027644	NAA80	1.864101182	0.142511028	13.08039947	4.26194E-39	3.59377E-38	positive
PRJNA729271	stage_5W_vs_E10	ordinary_muscle_development	ENSGALG00010027645	SLAMF1	5.905216407	0.779542481	7.575233618	3.58482E-14	1.07872E-13	positive
PRJNA729271	stage_5W_vs_E10	ordinary_muscle_development	ENSGALG00010027648	NFYA	-0.268094098	0.072855092	-3.679826481	0.000233393	0.00037295	negative
PRJNA729271	stage_5W_vs_E10	ordinary_muscle_development	ENSGALG00010027649	FBLN2	-1.004263137	0.191682158	-5.239210312	1.61265E-07	3.27984E-07	negative
PRJNA729271	stage_5W_vs_E10	ordinary_muscle_development	ENSGALG00010027650	CCDC124	1.077652995	0.190757149	5.649345247	1.6106E-08	3.51485E-08	positive
PRJNA729271	stage_5W_vs_E10	ordinary_muscle_development	ENSGALG00010027653	WNT7A	-5.451753332	0.523362833	-10.41677587	2.07881E-25	1.04517E-24	negative
PRJNA729271	stage_5W_vs_E10	ordinary_muscle_development	ENSGALG00010027654	AIF1L	1.236539479	0.09109125	13.57473396	5.65487E-42	5.23418E-41	positive
PRJNA729271	stage_5W_vs_E10	ordinary_muscle_development	ENSGALG00010027663	COPA	-0.594505184	0.070786826	-8.398528657	4.52108E-17	1.57759E-16	negative
PRJNA729271	stage_5W_vs_E10	ordinary_muscle_development	ENSGALG00010027664	TREMB1	1.575938952	0.315854534	4.989445406	6.05529E-07	1.18179E-06	positive
PRJNA729271	stage_5W_vs_E10	ordinary_muscle_development	ENSGALG00010027665	TRAF1	0.467791214	0.128266029	3.647039031	0.00026528	0.000421718	positive
PRJNA729271	stage_5W_vs_E10	ordinary_muscle_development	ENSGALG00010027667	PTPDC1	-0.153162944	0.153095601	-1.000439875	0.317097681	0.356221877	negative
PRJNA729271	stage_5W_vs_E10	ordinary_muscle_development	ENSGALG00010027668	RANBP3	-0.446673078	0.054251243	-8.233416455	1.81948E-16	6.15913E-16	negative
PRJNA729271	stage_5W_vs_E10	ordinary_muscle_development	ENSGALG00010027671	BARX1	-5.871137444	0.935927925	-6.273065785	3.54007E-10	8.49981E-10	negative
PRJNA729271	stage_5W_vs_E10	ordinary_muscle_development	ENSGALG00010027672	CNTRL	-0.594217354	0.174435489	-3.406516402	0.000657976	0.001012781	negative
PRJNA729271	stage_5W_vs_E10	ordinary_muscle_development	ENSGALG00010027674	TREM2	1.237353873	0.282002832	4.387735627	1.14537E-05	2.03208E-05	positive
PRJNA729271	stage_5W_vs_E10	ordinary_muscle_development	ENSGALG00010027685	ENSGALG00010027685	3.91714898	0.294127174	13.31787513	1.8222E-40	1.61645E-39	positive
PRJNA729271	stage_5W_vs_E10	ordinary_muscle_development	ENSGALG00010027692	PHF2	0.606011007	0.112558806	5.383950199	7.28687E-08	1.51627E-07	positive
PRJNA729271	stage_5W_vs_E10	ordinary_muscle_development	ENSGALG00010027693	RAB14	-0.498814045	0.060179688	-8.288744268	1.1445E-16	3.91264E-16	negative
PRJNA729271	stage_5W_vs_E10	ordinary_muscle_development	ENSGALG00010027695	TREMB2	1.629427286	0.299067827	5.448353643	5.08382E-08	1.07014E-07	positive
PRJNA729271	stage_5W_vs_E10	ordinary_muscle_development	ENSGALG00010027697	CATH2	-1.140961322	1.163664875	-0.980489612	0.326844495	0.366511679	negative
PRJNA729271	stage_5W_vs_E10	ordinary_muscle_development	ENSGALG00010027698	FAM120A	0.15347623	0.066500855	2.307883559	0.021005614	0.02767967	positive
PRJNA729271	stage_5W_vs_E10	ordinary_muscle_development	ENSGALG00010027699	VANGL2	-2.188951889	0.130302435	-16.79900982	2.48152E-63	4.38654E-62	negative
PRJNA729271	stage_5W_vs_E10	ordinary_muscle_development	ENSGALG00010027700	TCF3	-1.426627718	0.096396611	-14.79956302	1.47437E-49	1.7546E-48	negative
PRJNA729271	stage_5W_vs_E10	ordinary_muscle_development	ENSGALG00010027702	FOXP4	-2.909674437	0.253272965	-11.48829459	1.51065E-30	9.34564E-30	negative
PRJNA729271	stage_5W_vs_E10	ordinary_muscle_development	ENSGALG00010027703	NCSTN	-0.068200613	0.101295698	-0.673282419	0.500767636	0.541769434	negative
PRJNA729271	stage_5W_vs_E10	ordinary_muscle_development	ENSGALG00010027704	MDFI	-4.016268584	0.196769068	-20.41107691	1.33315E-92	4.831E-91	negative
PRJNA729271	stage_5W_vs_E10	ordinary_muscle_development	ENSGALG00010027705	S100A11	0.251881881	0.229691319	1.096610365	0.272811755	0.310003636	positive
PRJNA729271	stage_5W_vs_E10	ordinary_muscle_development	ENSGALG00010027706	GSN	4.058293104	0.171890944	23.60969698	3.0644E-123	1.9228E-121	positive
PRJNA729271	stage_5W_vs_E10	ordinary_muscle_development	ENSGALG00010027707	HIGD1C	0.844158411	0.211784299	3.985934826	6.7215E-05	0.000112543	positive
PRJNA729271	stage_5W_vs_E10	ordinary_muscle_development	ENSGALG00010027709	PSMA5	0.944556423	0.106998479	8.827755622	1.06797E-18	4.02398E-18	positive
PRJNA729271	stage_5W_vs_E10	ordinary_muscle_development	ENSGALG00010027710	ENSGALG00010027710	3.723071517	0.284085623	13.10545561	3.06417E-39	2.60041E-38	positive
PRJNA729271	stage_5W_vs_E10	ordinary_muscle_development	ENSGALG00010027711	WNK2	1.286441942	0.186015281	6.91578637	4.65275E-12	1.25077E-11	positive
PRJNA729271	stage_5W_vs_E10	ordinary_muscle_development	ENSGALG00010027712	STOM	1.469556495	0.157188764	9.348991994	8.8487E-21	3.65523E-20	positive
PRJNA729271	stage_5W_vs_E10	ordinary_muscle_development	ENSGALG00010027713	ENSGALG00010027713	0.778165879	0.139648039	5.57233662	2.51345E-08	5.41113E-08	positive
PRJNA729271	stage_5W_vs_E10	ordinary_muscle_development	ENSGALG00010027714	SYPL2	7.345656474	0.162062473	45.32607916	0	0	positive
PRJNA729271	stage_5W_vs_E10	ordinary_muscle_development	ENSGALG00010027715	NINJ1	-0.095532354	0.123428903	-0.773986897	0.43893854	0.480726417	negative
PRJNA729271	stage_5W_vs_E10	ordinary_muscle_development	ENSGALG00010027717	ATXN7L2	1.193237589	0.228432192	5.223596458	1.75481E-07	3.55847E-07	positive
PRJNA729271	stage_5W_vs_E10	ordinary_muscle_development	ENSGALG00010027718	ENSGALG00010027718	2.907490572	0.458166293	6.34592858	2.21088E-10	5.38057E-10	positive
PRJNA729271	stage_5W_vs_E10	ordinary_muscle_development	ENSGALG00010027719	GPR61	-3.507934962	0.997302072	-3.517424721	0.000435756	0.000679901	negative
PRJNA729271	stage_5W_vs_E10	ordinary_muscle_development	ENSGALG00010027720	DAB2IP	0.61473557	0.087437667	7.030557757	2.0571E-12	5.64513E-12	positive
PRJNA729271	stage_5W_vs_E10	ordinary_muscle_development	ENSGALG00010027721	SUSD3	-1.657631574	0.194811285	-8.50890938	1.75577E-17	6.25612E-17	negative
PRJNA729271	stage_5W_vs_E10	ordinary_muscle_development	ENSGALG00010027722	GNAI3	-0.646495789	0.085223561	-7.585880998	3.30235E-14	9.95169E-14	negative
PRJNA729271	stage_5W_vs_E10	ordinary_muscle_development	ENSGALG00010027723	ENSGALG00010027723	2.162712472	1.030668753	2.098358437	0.035873494	0.046126701	positive
PRJNA729271	stage_5W_vs_E10	ordinary_muscle_development	ENSGALG00010027724	TTLL11	-0.371230181	0.112833653	-3.290066137	0.001001638	0.00151838	negative
PRJNA729271	stage_5W_vs_E10	ordinary_muscle_development	ENSGALG00010027725	BTN1A1	3.213626543	0.249943151	12.85742989	7.81315E-38	6.35175E-37	positive
PRJNA729271	stage_5W_vs_E10	ordinary_muscle_development	ENSGALG00010027727	GSTM2	1.095987227	0.171169615	6.402930946	1.52422E-10	3.74785E-10	positive
PRJNA729271	stage_5W_vs_E10	ordinary_muscle_development	ENSGALG00010027728	CRNN	0.190199775	1.679237626	0.113265551	0.909820028	0.921839879	positive
PRJNA729271	stage_5W_vs_E10	ordinary_muscle_development	ENSGALG00010027729	EPS8L3	1.208433095	0.246055948	4.911212696	9.05148E-07	1.74377E-06	positive
PRJNA729271	stage_5W_vs_E10	ordinary_muscle_development	ENSGALG00010027730	ENSGALG00010027730	-2.531812186	0.155940031	-16.23580659	2.81523E-59	4.4259E-58	negative
PRJNA729271	stage_5W_vs_E10	ordinary_muscle_development	ENSGALG00010027731	OLFML2A	1.122511082	0.102955216	10.90290635	1.11633E-27	6.18078E-27	positive

PRJNA729271	stage_5W_vs_E10	ordinary_muscle_development	ENSGALG00010027732	EEF2	0.449576104	0.117050341	3.840878198	0.000122595	0.000200182	positive
PRJNA729271	stage_5W_vs_E10	ordinary_muscle_development	ENSGALG00010027733	RPL35	-0.227390558	0.154100502	-1.475599074	0.140051554	0.166834917	negative
PRJNA729271	stage_5W_vs_E10	ordinary_muscle_development	ENSGALG00010027734	CSF1	1.291517378	0.13887178	9.30007075	1.40352E-20	5.74042E-20	positive
PRJNA729271	stage_5W_vs_E10	ordinary_muscle_development	ENSGALG00010027736	ENSGALG00010027736	2.768654205	1.616709389	1.712524356	0.08680008	0.106289858	positive
PRJNA729271	stage_5W_vs_E10	ordinary_muscle_development	ENSGALG00010027738	ENSGALG00010027738	1.182421173	0.247003183	4.787068557	1.69235E-06	3.19118E-06	positive
PRJNA729271	stage_5W_vs_E10	ordinary_muscle_development	ENSGALG00010027739	FGD3	-1.834588093	0.075175213	-24.40416238	1.5447E-131	1.1424E-129	negative
PRJNA729271	stage_5W_vs_E10	ordinary_muscle_development	ENSGALG00010027740	MARCHF2	0.032691658	0.14535147	0.224914534	0.822045762	0.843161147	positive
PRJNA729271	stage_5W_vs_E10	ordinary_muscle_development	ENSGALG00010027741	RAB11B	-0.931561464	0.062231182	-14.9693679	1.16423E-50	1.43134E-49	negative
PRJNA729271	stage_5W_vs_E10	ordinary_muscle_development	ENSGALG00010027743	C1orf74	1.17450632	0.188478492	6.231513774	4.61949E-10	1.10131E-09	positive
PRJNA729271	stage_5W_vs_E10	ordinary_muscle_development	ENSGALG00010027744	ENSGALG00010027744	1.68088543	1.332846629	1.261124418	0.207264024	0.24047803	positive
PRJNA729271	stage_5W_vs_E10	ordinary_muscle_development	ENSGALG00010027745	BICD2	0.449728555	0.138510911	3.246881786	0.001166768	0.001756211	positive
PRJNA729271	stage_5W_vs_E10	ordinary_muscle_development	ENSGALG00010027746	UQCR11	2.282432278	0.184390446	12.37825672	3.42706E-35	2.54745E-34	positive
PRJNA729271	stage_5W_vs_E10	ordinary_muscle_development	ENSGALG00010027748	ENSGALG00010027748	1.15835374	2.889617951	0.400867436	0.688517726	0.721624327	positive
PRJNA729271	stage_5W_vs_E10	ordinary_muscle_development	ENSGALG00010027751	ANGPTL4	-1.380422052	0.212135338	-6.507270622	7.65285E-11	1.92283E-10	negative
PRJNA729271	stage_5W_vs_E10	ordinary_muscle_development	ENSGALG00010027752	SCAI	-1.358135611	0.087085535	-15.59542126	7.82058E-55	1.07654E-53	negative
PRJNA729271	stage_5W_vs_E10	ordinary_muscle_development	ENSGALG00010027759	ENSGALG00010027759	-2.083154045	1.029368189	-2.023721023	0.042998864	0.054677475	negative
PRJNA729271	stage_5W_vs_E10	ordinary_muscle_development	ENSGALG00010027766	ENSGALG00010027766	-2.327294736	0.829641343	-2.805181727	0.005028822	0.00709695	negative
PRJNA729271	stage_5W_vs_E10	ordinary_muscle_development	ENSGALG00010027767	ENSGALG00010027767	-0.679865974	1.1564138	-0.587908908	0.556593437	0.596335418	negative
PRJNA729271	stage_5W_vs_E10	ordinary_muscle_development	ENSGALG00010027772	ENSGALG00010027772	-2.455967609	1.170293228	-2.098591661	0.035852912	0.046104326	negative
PRJNA729271	stage_5W_vs_E10	ordinary_muscle_development	ENSGALG00010027775	MVB12B	0.709104189	0.088133037	8.045838541	8.56569E-16	2.8065E-15	positive
PRJNA729271	stage_5W_vs_E10	ordinary_muscle_development	ENSGALG00010027776	TKT	-1.773053327	0.129568586	-13.68428394	1.26043E-42	1.19411E-41	negative
PRJNA729271	stage_5W_vs_E10	ordinary_muscle_development	ENSGALG00010027784	PEAR1	1.012267464	0.192008177	5.272001851	1.34944E-07	2.76194E-07	positive
PRJNA729271	stage_5W_vs_E10	ordinary_muscle_development	ENSGALG00010027787	DCP1A	-0.01426143	0.13812037	-0.103253629	0.917761677	0.928912472	negative
PRJNA729271	stage_5W_vs_E10	ordinary_muscle_development	ENSGALG00010027789	UAP1L1	-1.444332175	0.135010909	-10.69789236	1.0412E-26	5.56494E-26	negative
PRJNA729271	stage_5W_vs_E10	ordinary_muscle_development	ENSGALG00010027801	MBD3	-3.708836755	0.135511905	-27.36908432	6.402E-165	7.9314E-163	negative
PRJNA729271	stage_5W_vs_E10	ordinary_muscle_development	ENSGALG00010027803	SAPCD2	-1.833077809	0.17004147	-10.78018093	4.27045E-27	2.31057E-26	negative
PRJNA729271	stage_5W_vs_E10	ordinary_muscle_development	ENSGALG00010027811	MEX3D	-1.128817597	0.102789683	-10.98181809	4.67421E-28	2.62607E-27	negative
PRJNA729271	stage_5W_vs_E10	ordinary_muscle_development	ENSGALG00010027813	LRRC71	-2.460301029	0.338126099	-7.276282539	3.43145E-13	9.77571E-13	negative
PRJNA729271	stage_5W_vs_E10	ordinary_muscle_development	ENSGALG00010027814	ENSGALG00010027814	-0.313780009	0.133923979	-2.342971072	0.019130866	0.025356795	negative
PRJNA729271	stage_5W_vs_E10	ordinary_muscle_development	ENSGALG00010027816	ZMYND19	-0.961418379	0.122142065	-7.871312622	3.50939E-15	1.11383E-14	negative
PRJNA729271	stage_5W_vs_E10	ordinary_muscle_development	ENSGALG00010027821	NMRK2	4.195397403	0.226495315	18.52310894	1.34444E-76	3.401E-75	positive
PRJNA729271	stage_5W_vs_E10	ordinary_muscle_development	ENSGALG00010027822	DPH7	0.161192405	0.090367074	1.783751522	0.07446405	0.09195403	positive
PRJNA729271	stage_5W_vs_E10	ordinary_muscle_development	ENSGALG00010027826	ARHGEF11	0.82868388	0.164384253	5.04113908	4.62769E-07	9.09784E-07	positive
PRJNA729271	stage_5W_vs_E10	ordinary_muscle_development	ENSGALG00010027830	CACNA1D	-3.500557437	0.431413378	-8.114160614	4.89154E-16	1.62212E-15	negative
PRJNA729271	stage_5W_vs_E10	ordinary_muscle_development	ENSGALG00010027831	ATCAY	-3.488926659	0.24382678	-14.30903802	1.92153E-46	2.04498E-45	negative
PRJNA729271	stage_5W_vs_E10	ordinary_muscle_development	ENSGALG00010027838	ENSGALG00010027838	0.203192999	0.584812335	0.347449919	0.728253336	0.757954485	positive
PRJNA729271	stage_5W_vs_E10	ordinary_muscle_development	ENSGALG00010027842	SLAMF8	-2.155212465	0.257948205	-8.355214032	6.53142E-17	2.26436E-16	negative
PRJNA729271	stage_5W_vs_E10	ordinary_muscle_development	ENSGALG00010027847	NRTN	-1.089214083	0.360971041	-3.017455582	0.002549064	0.003713435	negative
PRJNA729271	stage_5W_vs_E10	ordinary_muscle_development	ENSGALG00010027860	ENSGALG00010027860	2.063354954	0.259254713	7.958794385	1.73724E-15	5.6133E-15	positive
PRJNA729271	stage_5W_vs_E10	ordinary_muscle_development	ENSGALG00010027861	PNPLA7	-0.213565644	0.07965587	-2.681103649	0.007337978	0.010187146	negative
PRJNA729271	stage_5W_vs_E10	ordinary_muscle_development	ENSGALG00010027863	CHDH	-2.258822106	0.154797431	-14.59211624	3.15264E-48	3.56177E-47	negative
PRJNA729271	stage_5W_vs_E10	ordinary_muscle_development	ENSGALG00010027864	ACTR8	-0.313159821	0.082538523	-3.794104974	0.000148177	0.000240383	negative
PRJNA729271	stage_5W_vs_E10	ordinary_muscle_development	ENSGALG00010027865	SELENOK	-0.128729828	0.123103268	-1.045706015	0.295696785	0.333862354	negative
PRJNA729271	stage_5W_vs_E10	ordinary_muscle_development	ENSGALG00010027866	KCNA2	-0.703698312	0.138788924	-5.070277174	3.97237E-07	7.84461E-07	negative
PRJNA729271	stage_5W_vs_E10	ordinary_muscle_development	ENSGALG00010027867	UBAP2L	-0.330237312	0.168611594	-1.958568237	0.050163372	0.063233158	negative
PRJNA729271	stage_5W_vs_E10	ordinary_muscle_development	ENSGALG00010027868	CACNA2D3	-3.528704978	0.313027107	-11.27284155	1.78708E-29	1.05902E-28	negative
PRJNA729271	stage_5W_vs_E10	ordinary_muscle_development	ENSGALG00010027869	NSMF	-0.85421751	0.165967469	-5.146897242	2.6483E-07	5.29332E-07	negative
PRJNA729271	stage_5W_vs_E10	ordinary_muscle_development	ENSGALG00010027870	HAX1	1.969995383	0.120528204	16.34468379	4.74627E-60	7.6103E-59	positive
PRJNA729271	stage_5W_vs_E10	ordinary_muscle_development	ENSGALG00010027872	KDM5B	-0.273989638	0.091823814	-2.983862521	0.002846346	0.004124954	negative
PRJNA729271	stage_5W_vs_E10	ordinary_muscle_development	ENSGALG00010027873	RFX2	0.232087338	0.198952076	1.166548964	0.243392564	0.278891321	positive
PRJNA729271	stage_5W_vs_E10	ordinary_muscle_development	ENSGALG00010027874	FRS3	-0.190434975	0.134812927	-1.41258691	0.15777722	0.186707552	negative
PRJNA729271	stage_5W_vs_E10	ordinary_muscle_development	ENSGALG00010027875	WNT5A	-1.654092753	0.16966126	-9.749383896	1.85591E-22	8.28501E-22	negative
PRJNA729271	stage_5W_vs_E10	ordinary_muscle_development	ENSGALG00010027876	ANAPC2	-0.363350114	0.110239861	-3.295995758	0.000980735	0.001488249	negative
PRJNA729271	stage_5W_vs_E10	ordinary_muscle_development	ENSGALG00010027877	OAZ1	0.856490162	0.114340152	7.490720839	6.84964E-14	2.02499E-13	positive
PRJNA729271	stage_5W_vs_E10	ordinary_muscle_development	ENSGALG00010027879	ENSGALG00010027879	4.269861195	0.416688794	10.2471227	1.21919E-24	5.97233E-24	positive
PRJNA729271	stage_5W_vs_E10	ordinary_muscle_development	ENSGALG00010027880	AMH	1.384915485	0.352807217	3.925417103	8.65796E-05	0.000143573	positive
PRJNA729271	stage_5W_vs_E10	ordinary_muscle_development	ENSGALG00010027881	ENSGALG00010027881	-1.629351022	0.316290871	-5.151432349	2.58504E-07	5.17402E-07	negative
PRJNA729271	stage_5W_vs_E10	ordinary_muscle_development	ENSGALG00010027882	CH1A	6.794164179	1.387798406	4.895642012	9.79853E-07	1.88418E-06	positive
PRJNA729271	stage_5W_vs_E10	ordinary_muscle_development	ENSGALG00010027883	ATP8B2	0.09676489	0.117521474	0.823380507	0.410291644	0.451810179	positive
PRJNA729271	stage_5W_vs_E10	ordinary_muscle_development	ENSGALG00010027884	SF3A2	-0.725428484	0.191505635	-3.788026842	0.000151848	0.000246037	negative
PRJNA729271	stage_5W_vs_E10	ordinary_muscle_development	ENSGALG00010027885	PLEKHJ1	-1.119503716	0.0764029	-14.65263382	1.29593E-48	1.49084E-47	negative
PRJNA729271	stage_5W_vs_E10	ordinary_muscle_development	ENSGALG00010027886	IL6R	2.147996832	0.192553946	11.15529896	6.74657E-29	3.91166E-28	positive
PRJNA729271	stage_5W_vs_E10	ordinary_muscle_development	ENSGALG00010027887	ERC2	-2.489282361	0.286004355	-8.703651955	3.21372E-18	1.18592E-17	negative
PRJNA729271	stage_5W_vs_E10	ordinary_muscle_development	ENSGALG00010027888	PFKFB2	-0.477681889	0.146551885	-3.259472841	0.001116195	0.00168411	negative
PRJNA729271	stage_5W_vs_E10	ordinary_muscle_development	ENSGALG00010027889	RBM8A	-0.706461713	0.164337764	-4.298839762	1.71695E-05	3.0046E-05	negative
PRJNA729271	stage_5W_vs_E10	ordinary_muscle_development	ENSGALG00010027890	ENSGALG00010027890	5.337915607	0.822933676	6.486446915	8.78842E-11	2.19824E-10	positive
PRJNA729271	stage_5W_vs_E10	ordinary_muscle_development	ENSGALG00010027891	CCDC66	-0.987222293	0.143499947	-6.879600375	6.00207E-12	1.60605E-11	negative
PRJNA729271	stage_5W_vs_E10	ordinary_muscle_development	ENSGALG00010027892	SHE	0.375912322	0.167654578	2.242183458	0.024949518	0.032624561	positive
PRJNA729271	stage_5W_vs_E10	ordinary_muscle_development	ENSGALG00010027893	DOT1L	0.70257064	0.157858335	4.450640127	8.56147E-06	1.53284E-05	positive
PRJNA729271	stage_5W_vs_E10	ordinary_muscle_development	ENSGALG00010027894	RSBN1	-0.000420493	0.068354138	-0.006151686	0.995091696	0.995916187	negative
PRJNA729271	stage_5W_vs_E10	ordinary_muscle_development	ENSGALG00010027895	LIX1L	-0.243119249	0.179106607	-1.357399674	0.174654252	0.205254855	negative
PRJNA729271	stage_5W_vs_E10	ordinary_muscle_development	ENSGALG00010027896	TOR4A	1.266638744	0.157664949	8.033737073	9.45477E-16	3.09152E-15	positive
PRJNA729271	stage_5W_vs_E10	ordinary_muscle_development	ENSGALG00010027897	AP3D1	0.039665755	0.09817126	0.404046508	0.68617852	0.719432741	positive
PRJNA729271	stage_5W_vs_E10	ordinary_muscle_development	ENSGALG00010027898	TASOR	-0.524434632	0.092839126	-5.64885362	1.61521E-08	3.52386E-08	negative
PRJNA729271	stage_5W_vs_E10	ordinary_muscle_development	ENSGALG00010027899	PMVK	-1.055887488	0.183961935	-5.739706353	9.48409E-09	2.09657E-08	negative
PRJNA729271	stage_5W_vs_E10	ordinary_muscle_development	ENSGALG00010027901	MOB3A	-0.617494142	0.099668427	-6.195483969	5.81062E-10	1.37712E-09	negative
PRJNA729271	stage_5W_vs_E10	ordinary_muscle_development	ENSGALG00010027902	UBE2Q1	0.231542779	0.07026793	3.295141604	0.000983721	0.001492468	positive
PRJNA729271	stage_5W_vs_E10	ordinary_muscle_development	ENSGALG00010027903	NDUFA8	0.606515947	0.125632277	4.827707981	1.38113E-06	2.62379E-06	positive
PRJNA729271	stage_5W_vs_E10	ordinary_muscle_development	ENSGALG00010027904	MORN5	-2.856702339	0.708376539	-4.032745558	5.5129E-05	9.27992E-05	negative
PRJNA729271	stage_5W_vs_E10	ordinary_muscle_development	ENSGALG00010027905	SYN2	0.281741363	0.271612457	1.037291755	0.299599907	0.338005966	positive
PRJNA729271	stage_5W_vs_E10	ordinary_muscle_development	ENSGALG00010027906	LHX6	2.162910327	0.172687307	12.52501048	5.44842E-36	4.18964E-35	positive
PRJNA729271	stage_5W_vs_E10	ordinary_muscle_development	ENSGALG00010027907	MKNK2	2.125484106	0.188434584	11.27969218	1.65323E-29	9.81312E-29	positive
PRJNA729271	stage_5W_vs_E10	ordinary_muscle_development	ENSGALG00010027908	PBXIP1	1.517936223	0.106412027	14.26470542	3.63122E-46	3.83075E-45	positive
PRJNA729271	stage_5W_vs_E10	ordinary_muscle_development	ENSGALG00010027909	TIMP4	0.284704163	0.250103187	1.138346799	0.254975699	0.29110529	positive
PRJNA729271	stage_5W_vs_E10	ordinary_muscle_development	ENSGALG00010027910	CHRNB2	-0.570241002	0.20190085	-2.824361566	0.004737491	0.006705393	negative
PRJNA729271	stage_5W_vs_E10	ordinary_muscle_development	ENSGALG00010027911	PTPN22	3.197974307	0.329520207	9.704941419	2.87241E-22	1.2717E-21	positive
PRJNA729271	stage_5W_vs_E10	ordinary_muscle_development	ENSGALG00010027912	RGS3	-1.077081457	0.131900259	-8.165878281	3.19105E-16	1.06847E-15	negative
PRJNA729271	stage_5W_vs_E10	ordinary_muscle_development	ENSGALG00010027913	PYGO2	-0.459000603	0.226442361	-2.027008557	0.042661538	0.054291395	negative
PRJNA729271	stage_5W_vs_E10	ordinary_muscle_development	ENSGALG00010027914	BTBD2	1.408414367	0.12523018	11.24660502	2.40679E-29	1.42161E-28	positive
PRJNA729271	stage_5W_vs_E10	ordinary_muscle_development	ENSGALG00010027915	GSTM3	0.164425773	0.08376201	1.963011307	0.04964486	0.062617669	positive
PRJNA729271	stage_5W_vs_E10	ordinary_muscle_development	ENSGALG00010027916	ADAR	0.451962832	0.128344743	3.521475229	0.000429153	0.000669887	positive
PRJNA729271	stage_5W_vs_E10	ordinary_muscle_development	ENSGALG00010027917	PPARG	0.642312304	0.452254884	1.420244042	0.155536646	0.184161386	positive
PRJNA729271	stage_5W_vs_E10	ordinary_muscle_development	ENSGALG00010027918	CSNK1G2	0.50437306	0.100756982	5.005837295	5.56197E-07	1.08815E-06	positive
PRJNA729271	stage_5W_vs_E10	ordinary_muscle_development	ENSGALG00010027919	TSEN2	-0.124463845	0.119038716	-1.045574489	0.295757532	0.333904933	negative
PRJNA729271	stage_5W_vs_E10	ordinary_muscle_development	ENSGALG00010027920	SCAMP4	-0.347157512	0.113811117	-3.050295265	0.002286165	0.003341531	negative
PRJNA729271	stage_5W_vs_E10	ordinary_muscle_development	ENSGALG00010027921	MKRN2OS	0.5003321	0.233413737	2.143541793	0.032069619	0.041467362	positive
PRJNA729271	stage_5W_vs_E10	ordinary_muscle_development	ENSGALG00010027922	SHC1	0.161563211	0.113330705	1.425590811	0.15398649	0.182415346	positive
PRJNA729271	stage_5W_vs_E10	ordinary_muscle_development	ENSGALG00010027923	AMPD2	0.180116589	0.151051723	1.192416647	0.233097916	0.26794245	positive
PRJNA729271	stage_5W_vs_E10	ordinary_muscle_development	ENSGALG00010027925	MKRN2	-0.375161326	0.070140681	-5.348698062	8.85892E-08	1.83286E-07	negative
PRJNA729271	stage_5W_vs_E10	ordinary_muscle_development	ENSGALG00010027926	CKS1B	-2.515298435	0.253253159	-9.931952861	3.02277E-23	1.39361E-22	negative
PRJNA729271	stage_5W_vs_E10	ordinary_muscle_development	ENSGALG00010027927	ENSGALG00010027927	1.426750046	0.195588678	7.294645365	2.99447E-13	8.55436E-13	positive
PRJNA729271	stage_5W_vs_E10	ordinary_muscle_development	ENSGALG00010027928	FLAD1	0.380608365	0.161629989	2.354812783	0.01853204	0.024603583	positive
PRJNA729271	stage_5W_vs_E10	ordinary_muscle_development	ENSGALG00010027929	CDK5RAP2	-0.943033763	0.137650599	-6.850923804	7.33746E-12	1.95365E-11	negative
PRJNA729271	stage_5W_vs_E10	ordinary_muscle_development	ENSGALG00010027930	RAF1	1.19754119	0.089249626	13.41788468	4.75081E-41	4.28786E-40	positive
PRJNA729271	stage_5W_vs_E10	ordinary_muscle_development	ENSGALG00010027931	ABHD17A	0.722227936	0.172072079	4.197240721	2.70187E-05	4.65015E-05	positive
PRJNA729271	stage_5W_vs_E10	ordinary_muscle_development	ENSGALG00010027932	POLE3	-2.026650914	0.078239699	-25.90310199	6.1448E-148	5.8986E-146	negative
PRJNA729271	stage_5W_vs_E10	ordinary_muscle_development	ENSGALG00010027933	ENSGALG00010027933	3.348512398	0.21640393	15.47343621	5.24319E-54	7.02404E-53	positive
PRJNA729271	stage_5W_vs_E10	ordinary_muscle_development	ENSGALG00010027934	CNBP	0.0466753	0.085156573	0.548111536	0.58361532	0.621930897	positive
PRJNA729271	stage_5W_vs_E10	ordinary_muscle_development	ENSGALG00010027935	ISY1	-0.593313711	0.089438187	-6.633785063	3.27186E-11	8.41776E-11	negative
PRJNA729271	stage_5W_vs_E10	ordinary_muscle_development	ENSGALG00010027936	ENSGALG00010027936	-0.274487115	0.086088932	-3.188413514	0.001430558	0.002134864	negative
PRJNA729271	stage_5W_vs_E10	ordinary_muscle_development	ENSGALG00010027937	HDHD3	-0.265850021	0.094329513	-2.818312239	0.004827684	0.006827717	negative
PRJNA729271	stage_5W_vs_E10	ordinary_muscle_development	ENSGALG00010027938	ENSGALG00010027938	-0.258693539	0.112685635	-2.295710006	0.021692468	0.028540648	negative
PRJNA729271	stage_5W_vs_E10	ordinary_muscle_development	ENSGALG00010027939	BSPRY	-2.929476563	0.548595934	-5.339953112	9.29706E-08	1.92186E-07	negative
PRJNA729271	stage_5W_vs_E10	ordinary_muscle_development	ENSGALG00010027940	ZBTB7B	1.932463375	0.394996127	4.892360309	9.96338E-07	1.91436E-06	positive
PRJNA729271	stage_5W_vs_E10	ordinary_muscle_development	ENSGALG00010027941	KLF2	0.86402924	0.151097076	5.718371677	1.0755E-08	2.3692E-08	positive
PRJNA729271	stage_5W_vs_E10	ordinary_muscle_development	ENSGALG00010027942	KHDC4	0.521077044	0.123583996	4.216379643	2.48256E-05	4.28951E-05	positive
PRJNA729271	stage_5W_vs_E10	ordinary_muscle_development	ENSGALG00010027943	RAB43	-0.248251216	0.094410502	-2.629487303	0.008551373	0.011790369	negative
PRJNA729271	stage_5W_vs_E10	ordinary_muscle_development	ENSGALG00010027944	WDR31	-3.040008992	0.348773867	-8.716275155	2.87504E-18	1.06202E-17	negative
PRJNA729271	stage_5W_vs_E10	ordinary_muscle_development	ENSGALG00010027946	AP1M1	-0.243851911	0.0926785	-2.63115943	0.008509411	0.011735626	negative
PRJNA729271	stage_5W_vs_E10	ordinary_muscle_development	ENSGALG00010027947	GP9	1.304951424	0.343535961	3.798587552	0.000145523	0.000236342	positive
PRJNA729271	stage_5W_vs_E10	ordinary_muscle_development	ENSGALG00010027948	PRPF4	-0.453531689	0.114599472	-3.957537333	7.57265E-05	0.00012624	negative
PRJNA729271	stage_5W_vs_E10	ordinary_muscle_development	ENSGALG00010027950	CDC26	0.012681387	0.128471178	0.098709974	0.92136855	0.93204251	positive
PRJNA729271	stage_5W_vs_E10	ordinary_muscle_development	ENSGALG00010027951	FAM32A	0.297744201	0.104635414	2.845539465	0.004433627	0.00629621	positive
PRJNA729271	stage_5W_vs_E10	ordinary_muscle_development	ENSGALG00010027952	CIB3	-0.422598588	0.539757231	-0.782941968	0.433661191	0.475629462	negative
PRJNA729271	stage_5W_vs_E10	ordinary_muscle_development	ENSGALG00010027953	SLC31A1	-1.988378292	0.122760346	-16.19723601	5.27451E-59	8.22086E-58	negative
PRJNA729271	stage_5W_vs_E10	ordinary_muscle_development	ENSGALG00010027954	CFAP92	-0.4016846	0.229490336	-1.750333401	0.080060801	0.098437637	negative
PRJNA729271	stage_5W_vs_E10	ordinary_muscle_development	ENSGALG00010027955	ENSGALG00010027955	0.341496072	0.116953495	2.919930443	0.003501095	0.005024592	positive
PRJNA729271	stage_5W_vs_E10	ordinary_muscle_development	ENSGALG00010027956	RAB8A	-0.842000775	0.066405198	-12.67974196	7.65836E-37	6.05608E-36	negative
PRJNA729271	stage_5W_vs_E10	ordinary_muscle_development	ENSGALG00010027957	ENTREP3	-0.762728595	0.488840795	-1.560280164	0.118693689	0.142717961	negative
PRJNA729271	stage_5W_vs_E10	ordinary_muscle_development	ENSGALG00010027958	ENSGALG00010027958	1.251599253	0.13860395	9.030040317	1.71609E-19	6.70115E-19	positive
PRJNA729271	stage_5W_vs_E10	ordinary_muscle_development	ENSGALG00010027959	TPM4	-2.243079832	0.172487108	-13.00433323	1.15603E-38	9.62471E-38	negative

PRJNA729271	stage_5W_vs_E10	ordinary_muscle_development	ENSGALG00010027960	PPP6C	0.143665319	0.120599458	1.191260079	0.233551501	0.268378706	positive
PRJNA729271	stage_5W_vs_E10	ordinary_muscle_development	ENSGALG00010027961	TINCR	-5.232768913	1.055257061	-4.958762283	7.09437E-07	1.37862E-06	negative
PRJNA729271	stage_5W_vs_E10	ordinary_muscle_development	ENSGALG00010027962	SCAMP3	0.990739232	0.126101899	7.856655922	3.94525E-15	1.24752E-14	positive
PRJNA729271	stage_5W_vs_E10	ordinary_muscle_development	ENSGALG00010027964	RABEPK	-1.185816582	0.139754541	-8.484994979	2.15728E-17	7.64542E-17	negative
PRJNA729271	stage_5W_vs_E10	ordinary_muscle_development	ENSGALG00010027965	ACAD9	0.933219482	0.109470136	8.524877374	1.52972E-17	5.46948E-17	positive
PRJNA729271	stage_5W_vs_E10	ordinary_muscle_development	ENSGALG00010027966	CLK2	0.106967163	0.100604467	1.063244674	0.287671008	0.325358244	positive
PRJNA729271	stage_5W_vs_E10	ordinary_muscle_development	ENSGALG00010027968	FKBP15	0.039266744	0.097270867	0.40368453	0.686444717	0.719607737	positive
PRJNA729271	stage_5W_vs_E10	ordinary_muscle_development	ENSGALG00010027969	HSPA5	-0.818835057	0.129521349	-6.322008423	2.58185E-10	6.25923E-10	negative
PRJNA729271	stage_5W_vs_E10	ordinary_muscle_development	ENSGALG00010027970	GRIN3B	-0.271727815	0.180948332	-1.501687317	0.133177881	0.159130606	negative
PRJNA729271	stage_5W_vs_E10	ordinary_muscle_development	ENSGALG00010027971	ENSGALG00010027971	-0.152811506	0.467450674	-0.326904023	0.743740478	0.772243426	negative
PRJNA729271	stage_5W_vs_E10	ordinary_muscle_development	ENSGALG00010027972	TMEM259	0.597144796	0.105702868	5.649277131	1.61124E-08	3.51572E-08	positive
PRJNA729271	stage_5W_vs_E10	ordinary_muscle_development	ENSGALG00010027973	GAPVD1	0.688274256	0.064687755	10.63994657	1.94241E-26	1.02569E-25	positive
PRJNA729271	stage_5W_vs_E10	ordinary_muscle_development	ENSGALG00010027974	ENSGALG00010027974	0.956304903	0.287591198	3.325223127	0.000883478	0.001344605	positive
PRJNA729271	stage_5W_vs_E10	ordinary_muscle_development	ENSGALG00010027975	IQSEC1	-0.429829788	0.11841464	-3.629870324	0.000283564	0.0004497	negative
PRJNA729271	stage_5W_vs_E10	ordinary_muscle_development	ENSGALG00010027976	LRSAM1	0.130062711	0.098251895	1.32376797	0.185580117	0.217161847	positive
PRJNA729271	stage_5W_vs_E10	ordinary_muscle_development	ENSGALG00010027977	BOLA1	1.071257717	0.156102792	6.862514797	6.76587E-12	1.80541E-11	positive
PRJNA729271	stage_5W_vs_E10	ordinary_muscle_development	ENSGALG00010027979	RPL12	-0.087716821	0.119614431	-0.73332975	0.463357334	0.50525574	negative
PRJNA729271	stage_5W_vs_E10	ordinary_muscle_development	ENSGALG00010027980	NUP210	1.753870267	0.14124657	12.41708221	2.11122E-35	1.58069E-34	positive
PRJNA729271	stage_5W_vs_E10	ordinary_muscle_development	ENSGALG00010027981	CNN2	-0.878828145	0.158393257	-5.548393666	2.88306E-08	6.16917E-08	negative
PRJNA729271	stage_5W_vs_E10	ordinary_muscle_development	ENSGALG00010027982	ENTPD2	1.847125974	0.24923547	7.411168152	1.25192E-13	3.64975E-13	positive
PRJNA729271	stage_5W_vs_E10	ordinary_muscle_development	ENSGALG00010027983	POGZ	-0.375953139	0.146849106	-2.560132294	0.010463232	0.01430258	negative
PRJNA729271	stage_5W_vs_E10	ordinary_muscle_development	ENSGALG00010027984	ARHGAP45	3.11814685	0.164715808	18.93046507	6.39949E-80	1.72098E-78	positive
PRJNA729271	stage_5W_vs_E10	ordinary_muscle_development	ENSGALG00010027985	PSMB4	0.487772836	0.094758116	5.147557325	2.639E-07	5.27619E-07	positive
PRJNA729271	stage_5W_vs_E10	ordinary_muscle_development	ENSGALG00010027986	CGN	-1.757084567	0.312469726	-5.623215374	1.87435E-08	4.0684E-08	negative
PRJNA729271	stage_5W_vs_E10	ordinary_muscle_development	ENSGALG00010027987	TMEM141	0.426486895	0.138813914	3.072364178	0.002123705	0.003113807	positive
PRJNA729271	stage_5W_vs_E10	ordinary_muscle_development	ENSGALG00010027988	HDAC11	1.023305437	0.128184137	7.983089506	1.42715E-15	4.62994E-15	positive
PRJNA729271	stage_5W_vs_E10	ordinary_muscle_development	ENSGALG00010027989	ENSGALG00010027989	0.394833054	0.141256981	2.795140119	0.005187722	0.007313367	positive
PRJNA729271	stage_5W_vs_E10	ordinary_muscle_development	ENSGALG00010027990	ENSGALG00010027990	0.718025567	0.193482072	3.711070288	0.000206385	0.000331253	positive
PRJNA729271	stage_5W_vs_E10	ordinary_muscle_development	ENSGALG00010027991	ENSGALG00010027991	0.395483555	0.146168461	2.705669565	0.006816684	0.009496792	positive
PRJNA729271	stage_5W_vs_E10	ordinary_muscle_development	ENSGALG00010027993	CLIC3	0.198392929	0.172552692	1.14975273	0.25024573	0.286155873	positive
PRJNA729271	stage_5W_vs_E10	ordinary_muscle_development	ENSGALG00010027994	TUFT1	-1.565730137	0.176505784	-8.870701567	7.26865E-19	2.75593E-18	negative
PRJNA729271	stage_5W_vs_E10	ordinary_muscle_development	ENSGALG00010027995	POLR2E	-0.113833023	0.082637989	-1.377490231	0.168360733	0.198534724	negative
PRJNA729271	stage_5W_vs_E10	ordinary_muscle_development	ENSGALG00010027996	GPX4	0.618755338	0.121922349	5.074995201	3.87506E-07	7.65975E-07	positive
PRJNA729271	stage_5W_vs_E10	ordinary_muscle_development	ENSGALG00010027998	SNX27	0.899499263	0.091806963	9.797723801	1.15151E-22	5.18843E-22	positive
PRJNA729271	stage_5W_vs_E10	ordinary_muscle_development	ENSGALG00010027999	ENSGALG00010027999	-1.558457023	0.520996644	-2.991299541	0.002777929	0.004029025	negative
PRJNA729271	stage_5W_vs_E10	ordinary_muscle_development	ENSGALG00010028000	MRPL9	1.301794272	0.165881924	7.847716253	4.23681E-15	1.33797E-14	positive
PRJNA729271	stage_5W_vs_E10	ordinary_muscle_development	ENSGALG00010028001	SBNO2	0.986902067	0.116052188	8.503950554	1.83246E-17	6.52135E-17	positive
PRJNA729271	stage_5W_vs_E10	ordinary_muscle_development	ENSGALG00010028002	ABCA2	0.08006388	0.120726516	0.663183884	0.507212792	0.548128638	positive
PRJNA729271	stage_5W_vs_E10	ordinary_muscle_development	ENSGALG00010028004	ENSGALG00010028004	-0.231411267	0.280165512	-0.825980562	0.408815118	0.450321084	negative
PRJNA729271	stage_5W_vs_E10	ordinary_muscle_development	ENSGALG00010028005	IER5L	-0.099022959	0.143727559	-0.688962922	0.490846601	0.532227819	negative
PRJNA729271	stage_5W_vs_E10	ordinary_muscle_development	ENSGALG00010028006	ENSGALG00010028006	3.154176173	0.810088414	3.893619659	9.87595E-05	0.000162784	positive
PRJNA729271	stage_5W_vs_E10	ordinary_muscle_development	ENSGALG00010028007	STK11	1.252714848	0.119254371	10.50456132	8.23046E-26	4.22004E-25	positive
PRJNA729271	stage_5W_vs_E10	ordinary_muscle_development	ENSGALG00010028008	NTMT1	0.518644996	0.113165438	4.583068861	4.58201E-06	8.36371E-06	positive
PRJNA729271	stage_5W_vs_E10	ordinary_muscle_development	ENSGALG00010028009	RRP9	-0.691163765	0.179574728	-3.848892175	0.000118653	0.000194008	negative
PRJNA729271	stage_5W_vs_E10	ordinary_muscle_development	ENSGALG00010028010	ASB6	-0.445590258	0.073871224	-6.031986951	1.61956E-09	3.73576E-09	negative
PRJNA729271	stage_5W_vs_E10	ordinary_muscle_development	ENSGALG00010028011	ENSGALG00010028011	-1.046721812	0.099315077	-10.53940498	5.68575E-26	2.93292E-25	negative
PRJNA729271	stage_5W_vs_E10	ordinary_muscle_development	ENSGALG00010028012	PRRX2	-3.606079241	0.255870765	-14.09336172	4.17218E-45	4.26787E-44	negative
PRJNA729271	stage_5W_vs_E10	ordinary_muscle_development	ENSGALG00010028013	CBARP	0.25610519	0.215868783	1.18639289	0.235467178	0.270472838	positive
PRJNA729271	stage_5W_vs_E10	ordinary_muscle_development	ENSGALG00010028014	ENSGALG00010028014	0.651024191	0.294059853	2.213917286	0.026834469	0.034975779	positive
PRJNA729271	stage_5W_vs_E10	ordinary_muscle_development	ENSGALG00010028015	DUSP7	-0.603730234	0.101749	-5.933525001	2.96499E-09	6.73523E-09	negative
PRJNA729271	stage_5W_vs_E10	ordinary_muscle_development	ENSGALG00010028016	PRF1	5.805400182	1.227909174	4.727874266	2.26883E-06	4.23797E-06	positive
PRJNA729271	stage_5W_vs_E10	ordinary_muscle_development	ENSGALG00010028017	ENSGALG00010028017	1.15056546	1.329461918	0.865436944	0.386799011	0.4281194	positive
PRJNA729271	stage_5W_vs_E10	ordinary_muscle_development	ENSGALG00010028019	RPL29	0.481042453	0.227131549	2.117902405	0.034183334	0.044058992	positive
PRJNA729271	stage_5W_vs_E10	ordinary_muscle_development	ENSGALG00010028020	ENSGALG00010028020	0.293111411	0.156520836	1.872667042	0.061114377	0.076228629	positive
PRJNA729271	stage_5W_vs_E10	ordinary_muscle_development	ENSGALG00010028021	ATP5F1D	1.294816099	0.145202801	8.917294204	4.77819E-19	1.82647E-18	positive
PRJNA729271	stage_5W_vs_E10	ordinary_muscle_development	ENSGALG00010028022	ENSGALG00010028022	-0.488189564	0.143497338	-3.402080967	0.000668748	0.001028816	negative
PRJNA729271	stage_5W_vs_E10	ordinary_muscle_development	ENSGALG00010028023	ENSGALG00010028023	1.022179161	0.902426596	1.132700615	0.257339999	0.293582777	positive
PRJNA729271	stage_5W_vs_E10	ordinary_muscle_development	ENSGALG00010028024	RBM3	-2.387671901	0.113181001	-21.09604855	8.64672E-99	3.53054E-97	negative
PRJNA729271	stage_5W_vs_E10	ordinary_muscle_development	ENSGALG00010028025	TNNC1	4.747244861	0.237085543	20.02334181	3.448E-89	1.15159E-87	positive
PRJNA729271	stage_5W_vs_E10	ordinary_muscle_development	ENSGALG00010028026	ANKMY1	0.789864671	0.16727654	4.721909429	2.33641E-06	4.35971E-06	positive
PRJNA729271	stage_5W_vs_E10	ordinary_muscle_development	ENSGALG00010028027	ENSGALG00010028027	1.329525088	0.230905245	5.757881712	8.5176E-09	1.88723E-08	positive
PRJNA729271	stage_5W_vs_E10	ordinary_muscle_development	ENSGALG00010028028	FAM174C	-0.378133622	0.166144677	-2.275929808	0.022850211	0.029987669	negative
PRJNA729271	stage_5W_vs_E10	ordinary_muscle_development	ENSGALG00010028029	EFNA2	-7.193881808	0.399241568	-18.01886972	1.38545E-72	3.13294E-71	negative
PRJNA729271	stage_5W_vs_E10	ordinary_muscle_development	ENSGALG00010028030	PFDN2	0.138296875	0.135427779	1.021185432	0.307166593	0.345923376	positive
PRJNA729271	stage_5W_vs_E10	ordinary_muscle_development	ENSGALG00010028031	NISCH	0.963092294	0.080700931	11.93409146	7.86125E-33	5.32969E-32	positive
PRJNA729271	stage_5W_vs_E10	ordinary_muscle_development	ENSGALG00010028032	ENSGALG00010028032	-4.352347114	0.335161123	-12.9858352	1.47223E-38	1.22013E-37	negative
PRJNA729271	stage_5W_vs_E10	ordinary_muscle_development	ENSGALG00010028033	ENSGALG00010028033	3.370105888	0.290425937	11.60401143	3.93205E-31	2.49432E-30	positive
PRJNA729271	stage_5W_vs_E10	ordinary_muscle_development	ENSGALG00010028034	MUM1L1	-0.537133031	0.077816415	-6.902567204	5.1071E-12	1.37063E-11	negative
PRJNA729271	stage_5W_vs_E10	ordinary_muscle_development	ENSGALG00010028035	ENSGALG00010028035	1.514355897	1.037548473	1.45955195	0.144413253	0.171720271	positive
PRJNA729271	stage_5W_vs_E10	ordinary_muscle_development	ENSGALG00010028036	NDUFS7	1.687025649	0.122140641	13.81215653	2.15289E-43	2.09296E-42	positive
PRJNA729271	stage_5W_vs_E10	ordinary_muscle_development	ENSGALG00010028037	ENSGALG00010028037	-0.088469534	0.28056487	-0.315326483	0.75251378	0.780570147	negative
PRJNA729271	stage_5W_vs_E10	ordinary_muscle_development	ENSGALG00010028039	STAB1	2.668243431	0.162251548	16.44510309	9.09295E-61	1.48593E-59	positive
PRJNA729271	stage_5W_vs_E10	ordinary_muscle_development	ENSGALG00010028042	DPP7	0.639449125	0.191434415	3.34030391	0.000836868	0.001276481	positive
PRJNA729271	stage_5W_vs_E10	ordinary_muscle_development	ENSGALG00010028043	NT5DC2	1.88161638	0.115609742	16.2755867	1.47103E-59	2.32525E-58	positive
PRJNA729271	stage_5W_vs_E10	ordinary_muscle_development	ENSGALG00010028044	GAMT	2.940831132	0.153641685	19.14084149	1.15391E-81	3.22273E-80	positive
PRJNA729271	stage_5W_vs_E10	ordinary_muscle_development	ENSGALG00010028045	ENSGALG00010028045	-0.562501856	0.590110789	-0.953213984	0.340481662	0.380251306	negative
PRJNA729271	stage_5W_vs_E10	ordinary_muscle_development	ENSGALG00010028046	SMIM4	2.200466391	0.203279319	10.82484142	2.62533E-27	1.43169E-26	positive
PRJNA729271	stage_5W_vs_E10	ordinary_muscle_development	ENSGALG00010028047	ENSGALG00010028047	0.70695558	0.29115005	2.428148577	0.015176125	0.020355133	positive
PRJNA729271	stage_5W_vs_E10	ordinary_muscle_development	ENSGALG00010028048	S100A10	1.761638895	0.218441999	8.064561325	7.34993E-16	2.41745E-15	positive
PRJNA729271	stage_5W_vs_E10	ordinary_muscle_development	ENSGALG00010028049	MAN1B1	0.093597132	0.060451104	1.548311373	0.121547348	0.145919232	positive
PRJNA729271	stage_5W_vs_E10	ordinary_muscle_development	ENSGALG00010028050	PBRM1	-0.794029082	0.087908475	-9.03245199	1.67867E-19	6.56036E-19	negative
PRJNA729271	stage_5W_vs_E10	ordinary_muscle_development	ENSGALG00010028051	GNL3	-0.662034106	0.093156705	-7.10667154	1.18875E-12	3.30157E-12	negative
PRJNA729271	stage_5W_vs_E10	ordinary_muscle_development	ENSGALG00010028052	ENSGALG00010028052	-4.230720924	0.224622768	-18.83478223	3.91735E-79	1.03617E-77	negative
PRJNA729271	stage_5W_vs_E10	ordinary_muscle_development	ENSGALG00010028053	ORM1	5.94322368	2.135731471	2.782757927	0.0053899	0.007581427	positive
PRJNA729271	stage_5W_vs_E10	ordinary_muscle_development	ENSGALG00010028054	RPS15	-0.455748271	0.140832973	-3.236090676	0.001211789	0.001820383	negative
PRJNA729271	stage_5W_vs_E10	ordinary_muscle_development	ENSGALG00010028055	ENSGALG00010028055	-0.318204152	0.118262725	-2.690654652	0.007131197	0.009911469	negative
PRJNA729271	stage_5W_vs_E10	ordinary_muscle_development	ENSGALG00010028056	SLC25A25	2.403727364	0.236030391	10.18397401	2.33813E-24	1.12971E-23	positive
PRJNA729271	stage_5W_vs_E10	ordinary_muscle_development	ENSGALG00010028057	APC2	-0.907365967	0.379646583	-2.39002801	0.016847088	0.022473637	negative
PRJNA729271	stage_5W_vs_E10	ordinary_muscle_development	ENSGALG00010028058	GLT8D1	-1.073964036	0.116010642	-9.257461394	2.0935E-20	8.50963E-20	negative
PRJNA729271	stage_5W_vs_E10	ordinary_muscle_development	ENSGALG00010028059	GZMM	4.053326687	0.549496343	7.376439791	1.62578E-13	4.70656E-13	positive
PRJNA729271	stage_5W_vs_E10	ordinary_muscle_development	ENSGALG00010028060	ENSGALG00010028060	0.461690094	0.142358218	3.243157304	0.001182129	0.001778224	positive
PRJNA729271	stage_5W_vs_E10	ordinary_muscle_development	ENSGALG00010028061	NAIF1	-0.730130827	0.09586391	-7.616326363	2.60997E-14	7.9129E-14	negative
PRJNA729271	stage_5W_vs_E10	ordinary_muscle_development	ENSGALG00010028062	ENSGALG00010028062	0.196957499	0.496862016	0.39640281	0.691807894	0.724705891	positive
PRJNA729271	stage_5W_vs_E10	ordinary_muscle_development	ENSGALG00010028064	SPCS1	-0.253531825	0.087937344	-2.883096244	0.003937871	0.005624144	negative
PRJNA729271	stage_5W_vs_E10	ordinary_muscle_development	ENSGALG00010028065	REEP6	2.589437318	0.778948198	3.324274099	0.00088649	0.001348906	positive
PRJNA729271	stage_5W_vs_E10	ordinary_muscle_development	ENSGALG00010028066	ENSGALG00010028066	1.970542516	0.18357879	10.73404239	7.04455E-27	3.77908E-26	positive
PRJNA729271	stage_5W_vs_E10	ordinary_muscle_development	ENSGALG00010028067	FAM102A	-0.389069655	0.112836949	-3.44806962	0.000564608	0.000873239	negative
PRJNA729271	stage_5W_vs_E10	ordinary_muscle_development	ENSGALG00010028068	POLE4	-0.841365011	0.113777688	-7.394815486	1.41605E-13	4.11088E-13	negative
PRJNA729271	stage_5W_vs_E10	ordinary_muscle_development	ENSGALG00010028069	NEK4	-1.148893607	0.11612722	-9.893404931	4.44642E-23	2.03315E-22	negative
PRJNA729271	stage_5W_vs_E10	ordinary_muscle_development	ENSGALG00010028070	MEX3A	-4.060263797	0.169879758	-23.90080983	3.0039E-126	2.0636E-124	negative
PRJNA729271	stage_5W_vs_E10	ordinary_muscle_development	ENSGALG00010028071	DPM2	-0.983133302	0.117038978	-8.400050281	4.46288E-17	1.55878E-16	negative
PRJNA729271	stage_5W_vs_E10	ordinary_muscle_development	ENSGALG00010028072	ENSGALG00010028072	-7.446131098	0.991157905	-7.512557849	5.79831E-14	1.72368E-13	negative
PRJNA729271	stage_5W_vs_E10	ordinary_muscle_development	ENSGALG00010028073	ST6GALNAC4	3.056263737	0.120725055	25.31590258	2.1346E-141	1.8094E-139	positive
PRJNA729271	stage_5W_vs_E10	ordinary_muscle_development	ENSGALG00010028074	ENSGALG00010028074	1.627901534	0.446782304	3.643612382	0.000268838	0.000427141	positive
PRJNA729271	stage_5W_vs_E10	ordinary_muscle_development	ENSGALG00010028075	RAB25	-0.84017617	0.521328551	-1.611605901	0.107047726	0.129542226	negative
PRJNA729271	stage_5W_vs_E10	ordinary_muscle_development	ENSGALG00010028076	ST6GALNAC6	1.460858295	0.152620385	9.571842537	1.05017E-21	4.52908E-21	positive
PRJNA729271	stage_5W_vs_E10	ordinary_muscle_development	ENSGALG00010028078	LAMTOR2	0.63974651	0.130960356	4.885039473	1.03408E-06	1.98399E-06	positive
PRJNA729271	stage_5W_vs_E10	ordinary_muscle_development	ENSGALG00010028079	ITIH3	7.687499712	1.527219133	5.033658591	4.81206E-07	9.45262E-07	positive
PRJNA729271	stage_5W_vs_E10	ordinary_muscle_development	ENSGALG00010028080	MUSTN1	4.229014454	0.404856097	10.44572254	1.53284E-25	7.74163E-25	positive
PRJNA729271	stage_5W_vs_E10	ordinary_muscle_development	ENSGALG00010028081	UBQLN4	-0.181624703	0.162402216	-1.118363455	0.263411796	0.30009856	negative
PRJNA729271	stage_5W_vs_E10	ordinary_muscle_development	ENSGALG00010028082	AK1	6.715020778	0.137111125	48.97502489	0	0	positive
PRJNA729271	stage_5W_vs_E10	ordinary_muscle_development	ENSGALG00010028083	ELAVL1	-0.353820913	0.049515232	-7.145698367	8.95395E-13	2.50603E-12	negative
PRJNA729271	stage_5W_vs_E10	ordinary_muscle_development	ENSGALG00010028084	ENG	1.866726498	0.19204553	9.720228823	2.47225E-22	1.09756E-21	positive
PRJNA729271	stage_5W_vs_E10	ordinary_muscle_development	ENSGALG00010028085	ENSGALG00010028085	-0.752497339	0.289039662	-2.603439727	0.009229349	0.012679312	negative
PRJNA729271	stage_5W_vs_E10	ordinary_muscle_development	ENSGALG00010028086	MATK	1.922390958	0.249447303	7.706601494	1.29212E-14	3.97145E-14	positive
PRJNA729271	stage_5W_vs_E10	ordinary_muscle_development	ENSGALG00010028087	STIMATE	-0.577250254	0.089497244	-6.449922144	1.11908E-10	2.77567E-10	negative
PRJNA729271	stage_5W_vs_E10	ordinary_muscle_development	ENSGALG00010028088	NAXE	0.834389576	0.114889123	7.262563735	3.79821E-13	1.07951E-12	positive
PRJNA729271	stage_5W_vs_E10	ordinary_muscle_development	ENSGALG00010028091	FPGS	-1.024945283	0.102749639	-9.975171595	1.95763E-23	9.10357E-23	negative
PRJNA729271	stage_5W_vs_E10	ordinary_muscle_development	ENSGALG00010028093	SFMBT1	-0.067570385	0.138288128	-0.488620288	0.625110547	0.66143349	negative
PRJNA729271	stage_5W_vs_E10	ordinary_muscle_development	ENSGALG00010028095	CDK9	-0.389133291	0.094819138	-4.103953038	4.0615E-05	6.89765E-05	negative
PRJNA729271	stage_5W_vs_E10	ordinary_muscle_development	ENSGALG00010028096	TOR2A	-0.997984438	0.155018973	-6.437821231	1.21201E-10	2.99745E-10	negative
PRJNA729271	stage_5W_vs_E10	ordinary_muscle_development	ENSGALG00010028097	TTC24	0.685586836	0.227547547	3.012938814	0.002587311	0.003766503	positive
PRJNA729271	stage_5W_vs_E10	ordinary_muscle_development	ENSGALG00010028098	RFT1	-0.582856401	0.121020442	-4.816181401	1.46332E-06	2.77301E-06	negative
PRJNA729271	stage_5W_vs_E10	ordinary_muscle_development	ENSGALG00010028099	PCASP2	3.878828075	0.241254653	16.07773371	3.65507E-58	5.58275E-57	positive
PRJNA729271	stage_5W_vs_E10	ordinary_muscle_development	ENSGALG00010028100	ENSGALG00010028100	1.608425683	0.457483513	3.51581125	0.000438413	0.000683752	positive
PRJNA729271	stage_5W_vs_E10	ordinary_muscle_development	ENSGALG00010028101	MRPL54	0.988511609	0.126849382	7.792797977	6.55414E-15	2.04658E-14	positive
PRJNA729271	stage_5W_vs_E10	ordinary_muscle_development	ENSGALG00010028102	CERCAM	-0.488323409	0.180487727	-2.705576812	0.006818588	0.009497927	negative
PRJNA729271	stage_5W_vs_E10	ordinary_muscle_development	ENSGALG00010028103	APBA3	0.771370085	0.129322107	5.964719425	2.45055E-09	5.59645E-09	positive
PRJNA729271	stage_5W_vs_E10	ordinary_muscle_development	ENSGALG00010028104	GOLGA1	0.36230094	0.115215065	3.144562205	0.001663355	0.002464262	positive
PRJNA729271	stage_5W_vs_E10	ordinary_muscle_development	ENSGALG00010028105	PRKCD	-0.25465814	0.140841672	-1.808116424	0.070588383	0.087480112	negative
PRJNA729271	stage_5W_vs_E10	ordinary_muscle_development	ENSGALG00010028106	ARPC5L	0.152157593	0.105691287	1.439641791	0.14996877	0.178019598	positive

PRJNA729271	stage_5W_vs_E10	ordinary_muscle_development	ENSGALG00010028107	NOTCH1	-0.115421264	0.124310634	-0.928490675	0.353153103	0.39334211	negative
PRJNA729271	stage_5W_vs_E10	ordinary_muscle_development	ENSGALG00010028108	IQGAP3	-2.178637393	0.213994128	-10.18082792	2.41498E-24	1.16567E-23	negative
PRJNA729271	stage_5W_vs_E10	ordinary_muscle_development	ENSGALG00010028109	TJP3	-0.734359102	0.224833839	-3.266230326	0.001089896	0.00164683	negative
PRJNA729271	stage_5W_vs_E10	ordinary_muscle_development	ENSGALG00010028110	COQ4	0.308970012	0.102055183	3.02747986	0.002466021	0.003595713	positive
PRJNA729271	stage_5W_vs_E10	ordinary_muscle_development	ENSGALG00010028111	TRUB2	-0.67397666	0.091332729	-7.379355343	1.59058E-13	4.6074E-13	negative
PRJNA729271	stage_5W_vs_E10	ordinary_muscle_development	ENSGALG00010028112	SWI5	-0.007010082	0.082139801	-0.085343298	0.93198847	0.941143436	negative
PRJNA729271	stage_5W_vs_E10	ordinary_muscle_development	ENSGALG00010028113	PIP5K1C	-0.713613677	0.097717207	-7.302845638	2.81744E-13	8.05818E-13	negative
PRJNA729271	stage_5W_vs_E10	ordinary_muscle_development	ENSGALG00010028114	PFKFB4	-0.460166015	0.091501026	-5.029080402	4.92838E-07	9.668E-07	negative
PRJNA729271	stage_5W_vs_E10	ordinary_muscle_development	ENSGALG00010028115	CACTIN	0.680148232	0.175107491	3.884175525	0.000102678	0.000168954	positive
PRJNA729271	stage_5W_vs_E10	ordinary_muscle_development	ENSGALG00010028116	HYAL1	1.14686096	0.148640912	7.715648029	1.20369E-14	3.70987E-14	positive
PRJNA729271	stage_5W_vs_E10	ordinary_muscle_development	ENSGALG00010028117	ENSGALG00010028117	0.69606141	0.231513314	3.006571837	0.002642116	0.003840501	positive
PRJNA729271	stage_5W_vs_E10	ordinary_muscle_development	ENSGALG00010028118	ENSGALG00010028118	0.357684271	0.077339082	4.624883837	3.74808E-06	6.88923E-06	positive
PRJNA729271	stage_5W_vs_E10	ordinary_muscle_development	ENSGALG00010028119	HYAL2	0.623507932	0.127152409	4.903626558	9.40833E-07	1.81131E-06	positive
PRJNA729271	stage_5W_vs_E10	ordinary_muscle_development	ENSGALG00010028120	TBXA2R	-0.306677509	0.255140108	-1.201996468	0.229364896	0.263818773	negative
PRJNA729271	stage_5W_vs_E10	ordinary_muscle_development	ENSGALG00010028121	TUSC2	0.566705099	0.095762125	5.917841712	3.26194E-09	7.39934E-09	positive
PRJNA729271	stage_5W_vs_E10	ordinary_muscle_development	ENSGALG00010028122	SEC16A	0.173777032	0.088627645	1.96075425	0.049907697	0.062927285	positive
PRJNA729271	stage_5W_vs_E10	ordinary_muscle_development	ENSGALG00010028123	RASSF1	0.290909755	0.1064044	2.734001166	0.006256982	0.008749742	positive
PRJNA729271	stage_5W_vs_E10	ordinary_muscle_development	ENSGALG00010028124	SUGP2	-1.061190452	0.090448606	-11.73252415	8.68301E-32	5.63385E-31	negative
PRJNA729271	stage_5W_vs_E10	ordinary_muscle_development	ENSGALG00010028125	HMG20B	-1.122107452	0.12325081	-9.10426023	8.68563E-20	3.44361E-19	negative
PRJNA729271	stage_5W_vs_E10	ordinary_muscle_development	ENSGALG00010028126	ZMYND10	0.843896005	0.081534598	10.35015845	4.17794E-25	2.08394E-24	positive
PRJNA729271	stage_5W_vs_E10	ordinary_muscle_development	ENSGALG00010028127	MFSD12	0.46599247	0.168574313	2.764314807	0.005704248	0.008006495	positive
PRJNA729271	stage_5W_vs_E10	ordinary_muscle_development	ENSGALG00010028128	DNM1	-2.067295116	0.144025588	-14.35366553	1.01052E-46	1.09147E-45	negative
PRJNA729271	stage_5W_vs_E10	ordinary_muscle_development	ENSGALG00010028129	CYB561D2	0.635097501	0.10604831	5.988756467	2.11452E-09	4.8443E-09	positive
PRJNA729271	stage_5W_vs_E10	ordinary_muscle_development	ENSGALG00010028130	TMEM115	0.604864555	0.126855682	4.768131335	1.85943E-06	3.49486E-06	positive
PRJNA729271	stage_5W_vs_E10	ordinary_muscle_development	ENSGALG00010028131	TMEM161A	0.903225286	0.203951301	4.428632139	9.48326E-06	1.69348E-05	positive
PRJNA729271	stage_5W_vs_E10	ordinary_muscle_development	ENSGALG00010028132	CACNA2D2	-0.60662083	0.135171747	-4.487778264	7.19698E-06	1.29542E-05	negative
PRJNA729271	stage_5W_vs_E10	ordinary_muscle_development	ENSGALG00010028133	ENSGALG00010028133	0.31262044	0.319929187	0.977155109	0.328492368	0.368137999	positive
PRJNA729271	stage_5W_vs_E10	ordinary_muscle_development	ENSGALG00010028134	INPP5E	-1.007701647	0.073051852	-13.79433394	2.75687E-43	2.6694E-42	negative
PRJNA729271	stage_5W_vs_E10	ordinary_muscle_development	ENSGALG00010028136	C3orf18	-1.178726031	0.163824981	-7.195032308	6.2446E-13	1.75997E-12	negative
PRJNA729271	stage_5W_vs_E10	ordinary_muscle_development	ENSGALG00010028137	NUP214	-0.351599829	0.066560295	-5.282425975	1.27484E-07	2.61222E-07	negative
PRJNA729271	stage_5W_vs_E10	ordinary_muscle_development	ENSGALG00010028138	ENSGALG00010028138	-0.299516932	0.504799492	-0.593338417	0.552954693	0.59274355	negative
PRJNA729271	stage_5W_vs_E10	ordinary_muscle_development	ENSGALG00010028139	FZR1	-0.797107408	0.088650528	-8.991569798	2.43724E-19	9.44089E-19	negative
PRJNA729271	stage_5W_vs_E10	ordinary_muscle_development	ENSGALG00010028140	HEMK1	-0.628136503	0.112940403	-5.56166336	2.67215E-08	5.73735E-08	negative
PRJNA729271	stage_5W_vs_E10	ordinary_muscle_development	ENSGALG00010028141	CISH	2.64699339	0.186300404	14.20819996	8.14934E-46	8.54119E-45	positive
PRJNA729271	stage_5W_vs_E10	ordinary_muscle_development	ENSGALG00010028142	PMPCA	-0.029295949	0.122286952	-0.239567255	0.810665753	0.832922779	negative
PRJNA729271	stage_5W_vs_E10	ordinary_muscle_development	ENSGALG00010028144	FAM78A	1.605432119	0.184819915	8.686467146	3.73885E-18	1.37515E-17	positive
PRJNA729271	stage_5W_vs_E10	ordinary_muscle_development	ENSGALG00010028145	ENSGALG00010028145	-0.219045348	0.144578784	-1.515058725	0.129757533	0.155338243	negative
PRJNA729271	stage_5W_vs_E10	ordinary_muscle_development	ENSGALG00010028146	MAPKAPK3	0.293808391	0.106348623	2.762691063	0.0057327	0.008044093	positive
PRJNA729271	stage_5W_vs_E10	ordinary_muscle_development	ENSGALG00010028147	SLC25A42	0.464778381	0.201471986	2.306913179	0.021059661	0.027743323	positive
PRJNA729271	stage_5W_vs_E10	ordinary_muscle_development	ENSGALG00010028148	ENSGALG00010028148	6.272410524	0.25553458	24.54622981	4.7451E-133	3.6011E-131	positive
PRJNA729271	stage_5W_vs_E10	ordinary_muscle_development	ENSGALG00010028149	SNAPC4	-0.208416381	0.090971975	-2.290995449	0.021963677	0.028879127	negative
PRJNA729271	stage_5W_vs_E10	ordinary_muscle_development	ENSGALG00010028150	ARMC6	-0.328780017	0.082552979	-3.982654806	6.81497E-05	0.000114015	negative
PRJNA729271	stage_5W_vs_E10	ordinary_muscle_development	ENSGALG00010028151	DOCK3	-1.397321567	0.261083206	-5.352016268	8.69796E-08	1.8011E-07	negative
PRJNA729271	stage_5W_vs_E10	ordinary_muscle_development	ENSGALG00010028152	ENSGALG00010028152	-0.279668528	0.360713477	-0.775320429	0.43815034	0.480008252	negative
PRJNA729271	stage_5W_vs_E10	ordinary_muscle_development	ENSGALG00010028153	MANF	-0.706094761	0.148343437	-4.759865173	1.93722E-06	3.63731E-06	negative
PRJNA729271	stage_5W_vs_E10	ordinary_muscle_development	ENSGALG00010028154	ENSGALG00010028154	-1.316058519	0.138248858	-9.51948926	1.74032E-21	7.43252E-21	negative
PRJNA729271	stage_5W_vs_E10	ordinary_muscle_development	ENSGALG00010028155	CIZ1	-0.358500006	0.078809851	-4.548923812	5.3921E-06	9.78571E-06	negative
PRJNA729271	stage_5W_vs_E10	ordinary_muscle_development	ENSGALG00010028156	DOHH	0.475135513	0.096635443	4.916783096	8.79779E-07	1.6976E-06	positive
PRJNA729271	stage_5W_vs_E10	ordinary_muscle_development	ENSGALG00010028157	PRRC2B	-0.270389032	0.145130341	-1.863077215	0.062451403	0.077829343	negative
PRJNA729271	stage_5W_vs_E10	ordinary_muscle_development	ENSGALG00010028158	DDX49	-0.478454265	0.099418729	-4.812516385	1.49042E-06	2.82252E-06	negative
PRJNA729271	stage_5W_vs_E10	ordinary_muscle_development	ENSGALG00010028159	DCAF1	0.182985894	0.090278291	2.026909158	0.042671704	0.054298726	positive
PRJNA729271	stage_5W_vs_E10	ordinary_muscle_development	ENSGALG00010028160	ENSGALG00010028160	6.609003831	0.547675465	12.06737248	1.5707E-33	1.09195E-32	positive
PRJNA729271	stage_5W_vs_E10	ordinary_muscle_development	ENSGALG00010028161	ENSGALG00010028161	2.570658992	0.589540309	4.360446522	1.29797E-05	2.29552E-05	positive
PRJNA729271	stage_5W_vs_E10	ordinary_muscle_development	ENSGALG00010028162	CARD9	1.051724033	0.175097237	6.006514148	1.89554E-09	4.3509E-09	positive
PRJNA729271	stage_5W_vs_E10	ordinary_muscle_development	ENSGALG00010028163	GBP	3.704550088	0.343744282	10.77705225	4.41818E-27	2.38872E-26	positive
PRJNA729271	stage_5W_vs_E10	ordinary_muscle_development	ENSGALG00010028164	BBLN	-0.309044368	0.142345168	-2.171091389	0.029924265	0.038821464	negative
PRJNA729271	stage_5W_vs_E10	ordinary_muscle_development	ENSGALG00010028165	NFIC	2.174649316	0.154594479	14.066798	6.07588E-45	6.19338E-44	positive
PRJNA729271	stage_5W_vs_E10	ordinary_muscle_development	ENSGALG00010028168	PTGES2	0.637222656	0.137844969	4.622748752	3.78688E-06	6.95702E-06	positive
PRJNA729271	stage_5W_vs_E10	ordinary_muscle_development	ENSGALG00010028169	DNLZ	1.410266379	0.142480429	9.897965536	4.24829E-23	1.94624E-22	positive
PRJNA729271	stage_5W_vs_E10	ordinary_muscle_development	ENSGALG00010028170	COPE	-1.147118864	0.095011955	-12.07341611	1.45947E-33	1.01512E-32	negative
PRJNA729271	stage_5W_vs_E10	ordinary_muscle_development	ENSGALG00010028171	RNF123	2.124225772	0.157868036	13.45570529	2.85007E-41	2.5917E-40	positive
PRJNA729271	stage_5W_vs_E10	ordinary_muscle_development	ENSGALG00010028172	CERS1	0.54949803	0.210148918	2.614803036	0.008927892	0.012279135	positive
PRJNA729271	stage_5W_vs_E10	ordinary_muscle_development	ENSGALG00010028173	GDF3	0.571839194	0.227229282	2.516573522	0.011850217	0.016096795	positive
PRJNA729271	stage_5W_vs_E10	ordinary_muscle_development	ENSGALG00010028174	GMPPB	-0.939710326	0.156318761	-6.011500582	1.83814E-09	4.22314E-09	negative
PRJNA729271	stage_5W_vs_E10	ordinary_muscle_development	ENSGALG00010028175	POMT1	0.022174259	0.094483737	0.234688634	0.81445041	0.835962236	positive
PRJNA729271	stage_5W_vs_E10	ordinary_muscle_development	ENSGALG00010028176	UPF1	0.204791332	0.094715412	2.162175385	0.03060466	0.039668653	positive
PRJNA729271	stage_5W_vs_E10	ordinary_muscle_development	ENSGALG00010028177	ENSGALG00010028177	6.200051222	0.388194281	15.97151615	2.0182E-57	3.00965E-56	positive
PRJNA729271	stage_5W_vs_E10	ordinary_muscle_development	ENSGALG00010028178	ENSGALG00010028178	-2.216913622	0.167213393	-13.25799077	4.05618E-40	3.55253E-39	negative
PRJNA729271	stage_5W_vs_E10	ordinary_muscle_development	ENSGALG00010028179	MCM2	-2.564735311	0.145023287	-17.68498953	5.47312E-70	1.15814E-68	negative
PRJNA729271	stage_5W_vs_E10	ordinary_muscle_development	ENSGALG00010028180	ODF2	-1.274739304	0.106437794	-11.97637843	4.72518E-33	3.22465E-32	negative
PRJNA729271	stage_5W_vs_E10	ordinary_muscle_development	ENSGALG00010028181	BGLAP	-0.73861474	0.282176086	-2.617566751	0.008855917	0.01218361	negative
PRJNA729271	stage_5W_vs_E10	ordinary_muscle_development	ENSGALG00010028182	SMG5	0.233445819	0.146806112	1.590164171	0.111797804	0.134918756	positive
PRJNA729271	stage_5W_vs_E10	ordinary_muscle_development	ENSGALG00010028183	CRTC1	0.325065091	0.097859248	3.321761588	0.000894511	0.001360682	positive
PRJNA729271	stage_5W_vs_E10	ordinary_muscle_development	ENSGALG00010028184	TMEM79	0.763546396	0.249773925	3.056949984	0.002236016	0.00327054	positive
PRJNA729271	stage_5W_vs_E10	ordinary_muscle_development	ENSGALG00010028185	GLMP	0.455894143	0.087068586	5.236034764	1.64063E-07	3.33487E-07	positive
PRJNA729271	stage_5W_vs_E10	ordinary_muscle_development	ENSGALG00010028186	CCT3	-0.093279271	0.132313349	-0.704987604	0.48081795	0.52244799	negative
PRJNA729271	stage_5W_vs_E10	ordinary_muscle_development	ENSGALG00010028187	TPRA1	0.707522351	0.069587001	10.16745003	2.77067E-24	1.33464E-23	positive
PRJNA729271	stage_5W_vs_E10	ordinary_muscle_development	ENSGALG00010028188	ENSGALG00010028188	-0.028360794	0.111459846	-0.254448528	0.799149064	0.823112747	negative
PRJNA729271	stage_5W_vs_E10	ordinary_muscle_development	ENSGALG00010028189	GPSM1	-0.214312955	0.107883129	-1.986528907	0.046974626	0.059441048	negative
PRJNA729271	stage_5W_vs_E10	ordinary_muscle_development	ENSGALG00010028190	ILF2	-1.199775545	0.08148297	-14.72424915	4.50445E-49	5.29108E-48	negative
PRJNA729271	stage_5W_vs_E10	ordinary_muscle_development	ENSGALG00010028191	KLHL26	0.580025764	0.099644767	5.820935508	5.85191E-09	1.30921E-08	positive
PRJNA729271	stage_5W_vs_E10	ordinary_muscle_development	ENSGALG00010028192	ENSGALG00010028192	2.540421912	0.310043119	8.193769688	2.53169E-16	8.51634E-16	positive
PRJNA729271	stage_5W_vs_E10	ordinary_muscle_development	ENSGALG00010028193	BRINP1	-5.537210561	0.282285577	-19.61563399	1.1371E-85	3.47728E-84	negative
PRJNA729271	stage_5W_vs_E10	ordinary_muscle_development	ENSGALG00010028194	ENSGALG00010028194	1.698900287	0.181533737	9.358592581	8.08059E-21	3.34556E-20	positive
PRJNA729271	stage_5W_vs_E10	ordinary_muscle_development	ENSGALG00010028195	ENSGALG00010028195	1.973102347	0.297615097	6.629711898	3.36343E-11	8.64566E-11	positive
PRJNA729271	stage_5W_vs_E10	ordinary_muscle_development	ENSGALG00010028196	ENSGALG00010028196	-1.639277729	0.099305001	-16.50750425	3.24008E-61	5.42321E-60	negative
PRJNA729271	stage_5W_vs_E10	ordinary_muscle_development	ENSGALG00010028197	INTS3	-0.080129408	0.154155758	-0.519795099	0.603206395	0.640923376	negative
PRJNA729271	stage_5W_vs_E10	ordinary_muscle_development	ENSGALG00010028198	UCK1	-0.259133275	0.092679201	-2.796024056	0.005173555	0.007294813	negative
PRJNA729271	stage_5W_vs_E10	ordinary_muscle_development	ENSGALG00010028199	DPP9	0.10296398	0.106701463	0.964972528	0.334558572	0.374241897	positive
PRJNA729271	stage_5W_vs_E10	ordinary_muscle_development	ENSGALG00010028201	ENSGALG00010028201	3.55130606	0.159876771	22.21277078	2.585E-109	1.292E-107	positive
PRJNA729271	stage_5W_vs_E10	ordinary_muscle_development	ENSGALG00010028202	QSOX2	-0.408410416	0.067448695	-6.05512702	1.40307E-09	3.24984E-09	negative
PRJNA729271	stage_5W_vs_E10	ordinary_muscle_development	ENSGALG00010028205	PRRT1B	-1.442712138	0.505545518	-2.853772977	0.004320339	0.006142552	negative
PRJNA729271	stage_5W_vs_E10	ordinary_muscle_development	ENSGALG00010028206	RBM15B	-0.502509145	0.096061311	-5.231129377	1.68478E-07	3.42076E-07	negative
PRJNA729271	stage_5W_vs_E10	ordinary_muscle_development	ENSGALG00010028207	ENSGALG00010028207	2.212232745	0.24215931	9.135443719	6.51391E-20	2.59393E-19	positive
PRJNA729271	stage_5W_vs_E10	ordinary_muscle_development	ENSGALG00010028211	GATAD2B	0.528460775	0.1447914	3.649807766	0.000262437	0.000417428	positive
PRJNA729271	stage_5W_vs_E10	ordinary_muscle_development	ENSGALG00010028216	NKX2-3	0.071948097	1.582958047	0.045451676	0.963747292	0.96869267	positive
PRJNA729271	stage_5W_vs_E10	ordinary_muscle_development	ENSGALG00010028221	TNFAIP8L1	-1.511210083	0.191612129	-7.886818516	3.09988E-15	9.86448E-15	negative
PRJNA729271	stage_5W_vs_E10	ordinary_muscle_development	ENSGALG00010028232	C9orf69	0.859545808	0.108991841	7.886331684	3.11199E-15	9.89868E-15	positive
PRJNA729271	stage_5W_vs_E10	ordinary_muscle_development	ENSGALG00010028239	EDMPN2	1.158409812	1.64549537	0.703988497	0.481439937	0.523084618	positive
PRJNA729271	stage_5W_vs_E10	ordinary_muscle_development	ENSGALG00010028242	ENSGALG00010028242	0.717689453	0.27914433	2.571033605	0.010139549	0.013878448	positive
PRJNA729271	stage_5W_vs_E10	ordinary_muscle_development	ENSGALG00010028245	JAC1	-0.248753294	1.48470997	-0.167543358	0.866942535	0.883335586	negative
PRJNA729271	stage_5W_vs_E10	ordinary_muscle_development	ENSGALG00010028247	MVP	0.257818899	0.093247008	2.764902654	0.005693979	0.007993629	positive
PRJNA729271	stage_5W_vs_E10	ordinary_muscle_development	ENSGALG00010028262	C1orf43	1.736243693	0.141984637	12.22839126	2.19238E-34	1.57553E-33	positive
PRJNA729271	stage_5W_vs_E10	ordinary_muscle_development	ENSGALG00010028270	RAPGEF1	1.14925569	0.181040785	6.348048522	2.18063E-10	5.31142E-10	positive
PRJNA729271	stage_5W_vs_E10	ordinary_muscle_development	ENSGALG00010028279	EDBETA	0.980315096	1.629315883	0.601672829	0.54739194	0.587171329	positive
PRJNA729271	stage_5W_vs_E10	ordinary_muscle_development	ENSGALG00010028283	ENSGALG00010028283	0.932084689	0.15141605	6.155785245	7.47064E-10	1.75591E-09	positive
PRJNA729271	stage_5W_vs_E10	ordinary_muscle_development	ENSGALG00010028286	KCTD12A	0.201918231	1.447828087	0.13946285	0.88908441	0.902723349	positive
PRJNA729271	stage_5W_vs_E10	ordinary_muscle_development	ENSGALG00010028292	ENSGALG00010028292	-1.071122505	0.513391623	-2.086365372	0.036945539	0.047437824	negative
PRJNA729271	stage_5W_vs_E10	ordinary_muscle_development	ENSGALG00010028301	FBN3	-4.532971499	0.186429737	-24.31463757	1.3726E-130	9.9479E-129	negative
PRJNA729271	stage_5W_vs_E10	ordinary_muscle_development	ENSGALG00010028311	ENSGALG00010028311	-3.143593992	0.272146392	-11.55111397	7.28698E-31	4.57448E-30	negative
PRJNA729271	stage_5W_vs_E10	ordinary_muscle_development	ENSGALG00010028314	MEF2D	2.376940206	0.213718589	11.12182249	9.82443E-29	5.66223E-28	positive
PRJNA729271	stage_5W_vs_E10	ordinary_muscle_development	ENSGALG00010028316	TIMM44	-0.385417827	0.08135496	-4.737484084	2.16388E-06	4.04976E-06	negative
PRJNA729271	stage_5W_vs_E10	ordinary_muscle_development	ENSGALG00010028318	NACC2	1.698611015	0.188335459	9.019071715	1.8969E-19	7.38929E-19	positive
PRJNA729271	stage_5W_vs_E10	ordinary_muscle_development	ENSGALG00010028321	RHBG	1.001779396	0.187081284	5.354781489	8.56599E-08	1.77479E-07	positive
PRJNA729271	stage_5W_vs_E10	ordinary_muscle_development	ENSGALG00010028322	STRBP	-0.020328436	0.109398193	-0.185820587	0.85258546	0.870481527	negative
PRJNA729271	stage_5W_vs_E10	ordinary_muscle_development	ENSGALG00010028326	CRB2	-1.887496162	0.266921948	-7.071341187	1.53443E-12	4.23569E-12	negative
PRJNA729271	stage_5W_vs_E10	ordinary_muscle_development	ENSGALG00010028328	HNRNPM	-1.366535746	0.166085338	-8.227913204	1.90503E-16	6.44571E-16	negative
PRJNA729271	stage_5W_vs_E10	ordinary_muscle_development	ENSGALG00010028329	HAPLN2	0.891458652	0.599467547	1.487084091	0.136992603	0.163459646	positive
PRJNA729271	stage_5W_vs_E10	ordinary_muscle_development	ENSGALG00010028337	ENSGALG00010028337	3.965831101	0.197118349	20.11903571	5.02762E-90	1.71876E-88	positive
PRJNA729271	stage_5W_vs_E10	ordinary_muscle_development	ENSGALG00010028339	BCAN	-2.215445233	0.428658894	-5.168317425	2.36211E-07	4.74221E-07	negative
PRJNA729271	stage_5W_vs_E10	ordinary_muscle_development	ENSGALG00010028344	LSM7	-1.573778027	0.095784065	-16.43047858	1.15742E-60	1.88503E-59	negative
PRJNA729271	stage_5W_vs_E10	ordinary_muscle_development	ENSGALG00010028346	DENND1A	-0.212951264	0.074534186	-2.857095171	0.004275376	0.006080414	negative
PRJNA729271	stage_5W_vs_E10	ordinary_muscle_development	ENSGALG00010028348	NES	-4.912408282	0.278741809	-17.62350719	1.62604E-69	3.38642E-68	negative
PRJNA729271	stage_5W_vs_E10	ordinary_muscle_development	ENSGALG00010028353	ENSGALG00010028353	1.158414341	1.620473986	0.714861424	0.474694676	0.516413939	positive
PRJNA729271	stage_5W_vs_E10	ordinary_muscle_development	ENSGALG00010028355	RBM18	-0.232248892	0.074644493	-3.111400233	0.001862024	0.00274568	negative
PRJNA729271	stage_5W_vs_E10	ordinary_muscle_development	ENSGALG00010028357	SPPL2C	-0.615224521	0.093633851	-6.570535284	5.01347E-11	1.27291E-10	negative
PRJNA729271	stage_5W_vs_E10	ordinary_muscle_development	ENSGALG00010028360	ENSGALG00010028360	-6.451294939	0.989478289	-6.519895397	7.03564E-11	1.77205E-10	negative
PRJNA729271	stage_5W_vs_E10	ordinary_muscle_development	ENSGALG00010028362	TMPRSS9	-2.88016746	0.439686264	-6.550505889	5.73425E-11	1.44956E-10	negative
PRJNA729271	stage_5W_vs_E10	ordinary_muscle_development	ENSGALG00010028364	MHM2	9.251424717	0.671030995	13.78688137	3.05692E-43	2.954E-42	positive
PRJNA729271	stage_5W_vs_E10	ordinary_muscle_development	ENSGALG00010028366	MRRF	0.025155981	0.087867156	0.286295605	0.774651724	0.801411515	positive
PRJNA729271	stage_5W_vs_E10	ordinary_muscle_development	ENSGALG00010028368	TIMM13	0.279655808	0.121149963	2.308344154	0.020980002	0.027648435	positive

PRJNA729271	stage_5W_vs_E10	ordinary_muscle_development	ENSGALG00010028370	S100A12	1.342109375	0.671804945	1.997766444	0.045741987	0.057952111	positive
PRJNA729271	stage_5W_vs_E10	ordinary_muscle_development	ENSGALG00010028372	PTGS1	-0.23639678	0.16527898	-1.430289445	0.152633963	0.180975811	negative
PRJNA729271	stage_5W_vs_E10	ordinary_muscle_development	ENSGALG00010028374	FDPS	0.594174401	0.13084951	4.540898942	5.60149E-06	1.01568E-05	positive
PRJNA729271	stage_5W_vs_E10	ordinary_muscle_development	ENSGALG00010028375	LMNB2	-2.001528839	0.15283736	-13.09580878	3.47948E-39	2.9477E-38	negative
PRJNA729271	stage_5W_vs_E10	ordinary_muscle_development	ENSGALG00010028377	PDCL	-0.130498076	0.059902258	-2.178516801	0.029367583	0.038129981	negative
PRJNA729271	stage_5W_vs_E10	ordinary_muscle_development	ENSGALG00010028380	RUSC1	0.896038604	0.14193983	6.312805963	2.74021E-10	6.62541E-10	positive
PRJNA729271	stage_5W_vs_E10	ordinary_muscle_development	ENSGALG00010028384	ENSGALG00010028384	-1.419178301	1.16546189	-1.217696016	0.223339512	0.25754923	negative
PRJNA729271	stage_5W_vs_E10	ordinary_muscle_development	ENSGALG00010028386	NCAN	-7.838697698	0.883233916	-8.874996253	6.99358E-19	2.6551E-18	negative
PRJNA729271	stage_5W_vs_E10	ordinary_muscle_development	ENSGALG00010028391	MED26	0.528793472	0.113402901	4.662962496	3.1169E-06	5.77373E-06	positive
PRJNA729271	stage_5W_vs_E10	ordinary_muscle_development	ENSGALG00010028392	ASH1L	1.02775725	0.120763701	8.510481591	1.73212E-17	6.17604E-17	positive
PRJNA729271	stage_5W_vs_E10	ordinary_muscle_development	ENSGALG00010028394	ZBTB26	-0.22724566	0.104126943	-2.182390585	0.029080719	0.037767675	negative
PRJNA729271	stage_5W_vs_E10	ordinary_muscle_development	ENSGALG00010028399	SLC35E1	-0.075042608	0.079811553	-0.940247438	0.347090654	0.387006079	negative
PRJNA729271	stage_5W_vs_E10	ordinary_muscle_development	ENSGALG00010028405	S100A6	0.617809149	0.279972161	2.206680646	0.027336378	0.035597952	positive
PRJNA729271	stage_5W_vs_E10	ordinary_muscle_development	ENSGALG00010028411	LHX2	-5.330070422	0.98243113	-5.425388365	5.78287E-08	1.21254E-07	negative
PRJNA729271	stage_5W_vs_E10	ordinary_muscle_development	ENSGALG00010028412	ENSGALG00010028412	2.196343064	0.314376762	6.986340377	2.82149E-12	7.67884E-12	positive
PRJNA729271	stage_5W_vs_E10	ordinary_muscle_development	ENSGALG00010028414	KBTBD12	7.777624108	0.350268869	22.20472558	3.0918E-109	1.5295E-107	positive
PRJNA729271	stage_5W_vs_E10	ordinary_muscle_development	ENSGALG00010028415	FSD1	0.448967787	0.168022387	2.672071235	0.007538464	0.010452462	positive
PRJNA729271	stage_5W_vs_E10	ordinary_muscle_development	ENSGALG00010028418	ENSGALG00010028418	1.840929686	0.124106185	14.83350473	8.89642E-50	1.06485E-48	positive
PRJNA729271	stage_5W_vs_E10	ordinary_muscle_development	ENSGALG00010028419	ENSGALG00010028419	6.155264821	0.359773696	17.10871276	1.27793E-65	2.40565E-64	positive
PRJNA729271	stage_5W_vs_E10	ordinary_muscle_development	ENSGALG00010028420	ENSGALG00010028420	6.180970104	0.825005157	7.492038147	6.78122E-14	2.00559E-13	positive
PRJNA729271	stage_5W_vs_E10	ordinary_muscle_development	ENSGALG00010028421	ZBTB7A	1.345024841	0.18943193	7.10030689	1.2448E-12	3.4546E-12	positive
PRJNA729271	stage_5W_vs_E10	ordinary_muscle_development	ENSGALG00010028422	RXRA	2.344901291	0.207826619	11.28296895	1.59278E-29	9.46591E-29	positive
PRJNA729271	stage_5W_vs_E10	ordinary_muscle_development	ENSGALG00010028425	DAP3	1.043835444	0.129222086	8.077840823	6.59236E-16	2.17075E-15	positive
PRJNA729271	stage_5W_vs_E10	ordinary_muscle_development	ENSGALG00010028426	GPR21	-0.429662946	0.154674435	-2.777853665	0.005471926	0.007693071	negative
PRJNA729271	stage_5W_vs_E10	ordinary_muscle_development	ENSGALG00010028427	ENSGALG00010028427	0.645452921	0.205287421	3.144142575	0.001665743	0.002467546	positive
PRJNA729271	stage_5W_vs_E10	ordinary_muscle_development	ENSGALG00010028428	RABGAP1	0.57538263	0.067055751	8.580660385	9.433E-18	3.40636E-17	positive
PRJNA729271	stage_5W_vs_E10	ordinary_muscle_development	ENSGALG00010028429	REXO1	-0.113702981	0.088675314	-1.282239399	0.199758679	0.232234685	negative
PRJNA729271	stage_5W_vs_E10	ordinary_muscle_development	ENSGALG00010028430	NR2C2AP	-0.127849223	0.117059217	-1.092175626	0.274755914	0.311943837	negative
PRJNA729271	stage_5W_vs_E10	ordinary_muscle_development	ENSGALG00010028431	ENSGALG00010028431	-0.013648225	0.269805377	-0.050585446	0.959655861	0.965182605	negative
PRJNA729271	stage_5W_vs_E10	ordinary_muscle_development	ENSGALG00010028432	RFXANK	0.826280492	0.13391815	6.170041113	6.82722E-10	1.60964E-09	positive
PRJNA729271	stage_5W_vs_E10	ordinary_muscle_development	ENSGALG00010028433	MSTO1	-0.652964702	0.096632572	-6.757190564	1.40693E-11	3.69581E-11	negative
PRJNA729271	stage_5W_vs_E10	ordinary_muscle_development	ENSGALG00010028434	COL5A1	0.166164976	0.195905369	0.848190009	0.39633216	0.437601666	positive
PRJNA729271	stage_5W_vs_E10	ordinary_muscle_development	ENSGALG00010028435	ATP8B3	1.281043637	0.345416561	3.708692	0.000208333	0.000334232	positive
PRJNA729271	stage_5W_vs_E10	ordinary_muscle_development	ENSGALG00010028436	S100A1	3.538832933	0.184811632	19.14832359	9.99529E-82	2.79694E-80	positive
PRJNA729271	stage_5W_vs_E10	ordinary_muscle_development	ENSGALG00010028437	ENSGALG00010028437	0.161511758	0.067312282	2.399439661	0.016420186	0.021938482	positive
PRJNA729271	stage_5W_vs_E10	ordinary_muscle_development	ENSGALG00010028438	MED27	-0.527214284	0.073843249	-7.139640987	9.3575E-13	2.61444E-12	negative
PRJNA729271	stage_5W_vs_E10	ordinary_muscle_development	ENSGALG00010028439	BORCS8	-0.323909936	0.122157125	-2.651584464	0.008011507	0.01107763	negative
PRJNA729271	stage_5W_vs_E10	ordinary_muscle_development	ENSGALG00010028440	PSMB7	-0.199609734	0.078637138	-2.538364684	0.011137186	0.015176602	negative
PRJNA729271	stage_5W_vs_E10	ordinary_muscle_development	ENSGALG00010028442	CHTOP	-0.601325223	0.091005908	-6.607540517	3.90757E-11	1.00018E-10	negative
PRJNA729271	stage_5W_vs_E10	ordinary_muscle_development	ENSGALG00010028443	ENSGALG00010028443	0.887507601	0.324691947	2.733383466	0.00626873	0.008764009	positive
PRJNA729271	stage_5W_vs_E10	ordinary_muscle_development	ENSGALG00010028444	MEF2B	2.176444745	0.309412009	7.034131462	2.00506E-12	5.50755E-12	positive
PRJNA729271	stage_5W_vs_E10	ordinary_muscle_development	ENSGALG00010028445	SYT11	-2.417573012	0.204580141	-11.81724188	3.17953E-32	2.11216E-31	negative
PRJNA729271	stage_5W_vs_E10	ordinary_muscle_development	ENSGALG00010028446	ENSGALG00010028446	-0.648245155	0.222237288	-2.916905449	0.003535229	0.005069563	negative
PRJNA729271	stage_5W_vs_E10	ordinary_muscle_development	ENSGALG00010028447	OLFM1	-4.068268719	0.166211718	-24.47642544	2.6336E-132	1.978E-130	negative
PRJNA729271	stage_5W_vs_E10	ordinary_muscle_development	ENSGALG00010028448	RIT1	-0.376160802	0.058052086	-6.479712027	9.18978E-11	2.29428E-10	negative
PRJNA729271	stage_5W_vs_E10	ordinary_muscle_development	ENSGALG00010028449	EFCAB12	-0.95204618	0.341750805	-2.785790602	0.005339735	0.007513782	negative
PRJNA729271	stage_5W_vs_E10	ordinary_muscle_development	ENSGALG00010028450	PTGDS	8.11553209	0.432934871	18.74538791	2.11127E-78	5.54399E-77	positive
PRJNA729271	stage_5W_vs_E10	ordinary_muscle_development	ENSGALG00010028451	TMEM59L	-0.580566844	0.305805569	-1.898483553	0.057632412	0.072108918	negative
PRJNA729271	stage_5W_vs_E10	ordinary_muscle_development	ENSGALG00010028452	GOLPH3L	-0.511633223	0.641442267	-0.797629419	0.425085574	0.466859781	negative
PRJNA729271	stage_5W_vs_E10	ordinary_muscle_development	ENSGALG00010028453	ENSGALG00010028453	-0.02768976	0.095233548	-0.290756363	0.771237662	0.798278342	negative
PRJNA729271	stage_5W_vs_E10	ordinary_muscle_development	ENSGALG00010028454	C8G	1.150890038	0.231395999	4.973681658	6.56932E-07	1.27867E-06	positive
PRJNA729271	stage_5W_vs_E10	ordinary_muscle_development	ENSGALG00010028455	ENSA	1.014494514	0.069502835	14.59644792	2.95864E-48	3.35043E-47	positive
PRJNA729271	stage_5W_vs_E10	ordinary_muscle_development	ENSGALG00010028456	FBXW5	0.494181218	0.093097339	5.308220679	1.10701E-07	2.27765E-07	positive
PRJNA729271	stage_5W_vs_E10	ordinary_muscle_development	ENSGALG00010028457	KANK3	1.230605556	0.104546647	11.77087544	5.51492E-32	3.61878E-31	positive
PRJNA729271	stage_5W_vs_E10	ordinary_muscle_development	ENSGALG00010028458	TRAF2	0.479164108	0.108722433	4.407223925	1.04704E-05	1.86333E-05	positive
PRJNA729271	stage_5W_vs_E10	ordinary_muscle_development	ENSGALG00010028459	MCL1	2.447729695	0.209826299	11.66550478	1.91265E-31	1.22618E-30	positive
PRJNA729271	stage_5W_vs_E10	ordinary_muscle_development	ENSGALG00010028460	CRLF1	-1.070795724	0.249171422	-4.297425901	1.72793E-05	3.02273E-05	negative
PRJNA729271	stage_5W_vs_E10	ordinary_muscle_development	ENSGALG00010028461	ENSGALG00010028461	2.722512758	0.315596094	8.626573048	6.32176E-18	2.3041E-17	positive
PRJNA729271	stage_5W_vs_E10	ordinary_muscle_development	ENSGALG00010028462	EDF1	0.891332311	0.10542905	8.454333115	2.80687E-17	9.89435E-17	positive
PRJNA729271	stage_5W_vs_E10	ordinary_muscle_development	ENSGALG00010028463	RPS28	1.70026748	0.169234836	10.04679369	9.49051E-24	4.4664E-23	positive
PRJNA729271	stage_5W_vs_E10	ordinary_muscle_development	ENSGALG00010028464	ENSGALG00010028464	0.195790665	0.112363223	1.742479968	0.081424494	0.100012544	positive
PRJNA729271	stage_5W_vs_E10	ordinary_muscle_development	ENSGALG00010028465	ENSGALG00010028465	0.440812934	0.136146769	3.237777425	0.001204648	0.001809843	positive
PRJNA729271	stage_5W_vs_E10	ordinary_muscle_development	ENSGALG00010028466	MAMDC4	0.481357401	0.67000565	0.718437824	0.472487372	0.514475996	positive
PRJNA729271	stage_5W_vs_E10	ordinary_muscle_development	ENSGALG00010028467	ENSGALG00010028467	1.799501283	0.233086402	7.720318588	1.16039E-14	3.57794E-14	positive
PRJNA729271	stage_5W_vs_E10	ordinary_muscle_development	ENSGALG00010028468	PHPT1	-0.857909493	0.105606661	-8.123630487	4.52442E-16	1.50451E-15	negative
PRJNA729271	stage_5W_vs_E10	ordinary_muscle_development	ENSGALG00010028470	AJM1	1.811918777	0.443237138	4.087921844	4.35255E-05	7.37207E-05	positive
PRJNA729271	stage_5W_vs_E10	ordinary_muscle_development	ENSGALG00010028471	TPGS1	-0.040192999	0.169401594	-0.237264586	0.812451534	0.834324122	negative
PRJNA729271	stage_5W_vs_E10	ordinary_muscle_development	ENSGALG00010028472	RPRD2	0.789295473	0.138459306	5.700559201	1.19415E-08	2.6238E-08	positive
PRJNA729271	stage_5W_vs_E10	ordinary_muscle_development	ENSGALG00010028473	CDC34	1.379634286	0.139265321	9.906517083	3.90005E-23	1.78953E-22	positive
PRJNA729271	stage_5W_vs_E10	ordinary_muscle_development	ENSGALG00010028474	PEX19	0.335320512	0.120865225	2.774334066	0.005531484	0.007775297	positive
PRJNA729271	stage_5W_vs_E10	ordinary_muscle_development	ENSGALG00010028475	AMBP	6.272565363	1.373212984	4.567802253			positive
PRJNA729271	stage_5W_vs_E10	ordinary_muscle_development	ENSGALG00010028476	ENSGALG00010028476	2.219642273	0.154788701	14.33982102	1.23373E-46	1.32761E-45	positive
PRJNA729271	stage_5W_vs_E10	ordinary_muscle_development	ENSGALG00010028477	NRARP	-0.446055249	0.21680021	-2.057448421	0.039643112	0.050681505	negative
PRJNA729271	stage_5W_vs_E10	ordinary_muscle_development	ENSGALG00010028478	PEA15	0.276214458	0.160869489	1.717009608	0.085977416	0.105345955	positive
PRJNA729271	stage_5W_vs_E10	ordinary_muscle_development	ENSGALG00010028479	CASQ1	8.525616437	0.314682757	27.09273465	1.1992E-161	1.3795E-159	positive
PRJNA729271	stage_5W_vs_E10	ordinary_muscle_development	ENSGALG00010028480	F11R	0.016206949	0.219756861	0.073749456	0.941209746	0.949000784	positive
PRJNA729271	stage_5W_vs_E10	ordinary_muscle_development	ENSGALG00010028481	ENSGALG00010028481	0.426296178	0.146455409	2.910757477	0.003605538	0.005165787	positive
PRJNA729271	stage_5W_vs_E10	ordinary_muscle_development	ENSGALG00010028482	CD320	0.247952253	0.178613491	1.388205622	0.165074441	0.19496081	positive
PRJNA729271	stage_5W_vs_E10	ordinary_muscle_development	ENSGALG00010028483	EXD3	0.410367011	0.092182453	4.451682464	8.52001E-06	1.5256E-05	positive
PRJNA729271	stage_5W_vs_E10	ordinary_muscle_development	ENSGALG00010028484	NOXA1	0.514069555	0.48087053	1.069039425	0.285051905	0.322723374	positive
PRJNA729271	stage_5W_vs_E10	ordinary_muscle_development	ENSGALG00010028485	USF1	0.53044214	0.181298129	2.925800415	0.003435713	0.004935156	positive
PRJNA729271	stage_5W_vs_E10	ordinary_muscle_development	ENSGALG00010028486	ARHGAP30	3.114271904	0.219073344	14.21565879	7.32588E-46	7.68369E-45	positive
PRJNA729271	stage_5W_vs_E10	ordinary_muscle_development	ENSGALG00010028488	PTGES	1.491714033	0.129983194	11.47620696	1.73736E-30	1.07071E-29	positive
PRJNA729271	stage_5W_vs_E10	ordinary_muscle_development	ENSGALG00010028489	ENSGALG00010028489	4.648283657	0.772226491	6.019326857	1.75144E-09	4.0329E-09	positive
PRJNA729271	stage_5W_vs_E10	ordinary_muscle_development	ENSGALG00010028490	NECTIN4	-2.244724304	0.36652327	-6.124370504	9.10428E-10	2.13021E-09	negative
PRJNA729271	stage_5W_vs_E10	ordinary_muscle_development	ENSGALG00010028491	TOR1B	0.062310362	0.098092072	0.63522322	0.525282841	0.565926474	positive
PRJNA729271	stage_5W_vs_E10	ordinary_muscle_development	ENSGALG00010028492	ENSGALG00010028492	1.930635191	0.252717452	7.639500865	2.18065E-14	6.62654E-14	positive
PRJNA729271	stage_5W_vs_E10	ordinary_muscle_development	ENSGALG00010028493	TOR1A	-0.373570702	0.087307904	-4.278772988	1.87926E-05	3.27915E-05	negative
PRJNA729271	stage_5W_vs_E10	ordinary_muscle_development	ENSGALG00010028494	ENSGALG00010028494	-1.180448039	0.205639101	-5.740387079	9.44604E-09	2.08879E-08	negative
PRJNA729271	stage_5W_vs_E10	ordinary_muscle_development	ENSGALG00010028495	LINGO3	-4.916798941	0.536117521	-9.171121529	4.68123E-20	1.87236E-19	negative
PRJNA729271	stage_5W_vs_E10	ordinary_muscle_development	ENSGALG00010028496	ENSGALG00010028496	2.042227848	0.138840068	14.70921097	5.62596E-49	6.58178E-48	positive
PRJNA729271	stage_5W_vs_E10	ordinary_muscle_development	ENSGALG00010028498	C9orf78	0.340044075	0.104004374	3.269517049	0.001077312	0.001628496	positive
PRJNA729271	stage_5W_vs_E10	ordinary_muscle_development	ENSGALG00010028500	VPS45	0.240364503	0.07056214	3.406423101	0.000658201	0.00101302	positive
PRJNA729271	stage_5W_vs_E10	ordinary_muscle_development	ENSGALG00010028501	USP20	0.495252781	0.087928183	5.632469172	1.77648E-08	3.86115E-08	positive
PRJNA729271	stage_5W_vs_E10	ordinary_muscle_development	ENSGALG00010028502	PLEKHO1	-0.440551146	0.150996606	-2.917622843	0.003527107	0.005058416	negative
PRJNA729271	stage_5W_vs_E10	ordinary_muscle_development	ENSGALG00010028503	NDOR1	-0.269622287	0.128530654	-2.097727495	0.035929225	0.046192953	negative
PRJNA729271	stage_5W_vs_E10	ordinary_muscle_development	ENSGALG00010028506	SHD	-6.120449802	0.538601912	-11.36358721	6.34832E-30	3.83253E-29	negative
PRJNA729271	stage_5W_vs_E10	ordinary_muscle_development	ENSGALG00010028507	ANP32E	-1.121452977	0.057083878	-19.64570397	6.29183E-86	1.93631E-84	negative
PRJNA729271	stage_5W_vs_E10	ordinary_muscle_development	ENSGALG00010028510	CILP2	-1.250580379	0.595637907	-2.099564794	0.035767142	0.046014443	negative
PRJNA729271	stage_5W_vs_E10	ordinary_muscle_development	ENSGALG00010028511	YJEFN3	-0.715522348	0.493380006	-1.450245934	0.14698995	0.174612303	negative
PRJNA729271	stage_5W_vs_E10	ordinary_muscle_development	ENSGALG00010028512	NDUFA13	0.69231458	0.122745837	5.640228598	1.69825E-08	3.69666E-08	positive
PRJNA729271	stage_5W_vs_E10	ordinary_muscle_development	ENSGALG00010028513	ENSGALG00010028513	-1.330880245	1.315732802	-1.011512552	0.311771176	0.350863603	negative
PRJNA729271	stage_5W_vs_E10	ordinary_muscle_development	ENSGALG00010028514	FNBP1	1.768029971	0.058309635	30.32140325	5.9877E-202	1.0332E-199	positive
PRJNA729271	stage_5W_vs_E10	ordinary_muscle_development	ENSGALG00010028515	ENSGALG00010028515	1.158336595	3.388812944	0.341811901	0.732492453	0.76192882	positive
PRJNA729271	stage_5W_vs_E10	ordinary_muscle_development	ENSGALG00010028516	GATAD2A	-0.713283682	0.064011636	-11.14303165	7.74376E-29	4.47729E-28	negative
PRJNA729271	stage_5W_vs_E10	ordinary_muscle_development	ENSGALG00010028517	RNF208	-2.328359907	0.340176728	-6.844559657	7.67115E-12	2.03987E-11	negative
PRJNA729271	stage_5W_vs_E10	ordinary_muscle_development	ENSGALG00010028518	MAU2	0.507166568	0.115508743	4.390720178	1.12976E-05	2.00562E-05	positive
PRJNA729271	stage_5W_vs_E10	ordinary_muscle_development	ENSGALG00010028519	ENSGALG00010028519	1.664963099	2.100649177	0.792594555	0.428014082	0.469862475	positive
PRJNA729271	stage_5W_vs_E10	ordinary_muscle_development	ENSGALG00010028523	SUGP1	-0.211145174	0.114394076	-1.845770174	0.064925583	0.080739218	negative
PRJNA729271	stage_5W_vs_E10	ordinary_muscle_development	ENSGALG00010028528	UHRF1	-2.907854951	0.185824164	-15.64842207	3.40559E-55	4.73289E-54	negative
PRJNA729271	stage_5W_vs_E10	ordinary_muscle_development	ENSGALG00010028534	ENSGALG00010028534	0.677765994	4.345747042	0.155960756	0.876063955	0.891188647	positive
PRJNA729271	stage_5W_vs_E10	ordinary_muscle_development	ENSGALG00010028535	TICAM1	0.527618991	0.106072994	4.974112375	6.55473E-07	1.276E-06	positive
PRJNA729271	stage_5W_vs_E10	ordinary_muscle_development	ENSGALG00010028536	GPR107	-0.282916759	0.064357148	-4.396042506	1.10242E-05	1.95973E-05	negative
PRJNA729271	stage_5W_vs_E10	ordinary_muscle_development	ENSGALG00010028537	ENSGALG00010028537	1.158589115	2.123141423	0.545695686	0.585275138	0.62344708	positive
PRJNA729271	stage_5W_vs_E10	ordinary_muscle_development	ENSGALG00010028538	FEM1A	2.056365836	0.116917403	17.58819287	3.034E-69	6.24685E-68	positive
PRJNA729271	stage_5W_vs_E10	ordinary_muscle_development	ENSGALG00010028539	ENSGALG00010028539	1.158327156	3.347138525	0.346064899	0.729293942	0.758983033	positive
PRJNA729271	stage_5W_vs_E10	ordinary_muscle_development	ENSGALG00010028541	ENSGALG00010028541	0.022893634	0.304368462	0.075216839	0.940042187	0.94795544	positive
PRJNA729271	stage_5W_vs_E10	ordinary_muscle_development	ENSGALG00010028542	ENSGALG00010028542	1.730496652	1.996452615	0.866785737	0.386059418	0.427366066	positive
PRJNA729271	stage_5W_vs_E10	ordinary_muscle_development	ENSGALG00010028543	ANP32B	-1.700183466	0.094145935	-18.05902153	6.69997E-73	1.5318E-71	negative
PRJNA729271	stage_5W_vs_E10	ordinary_muscle_development	ENSGALG00010028544	ZNF414	1.174356145	0.16149053	7.27198151	3.54252E-13	1.00842E-12	positive
PRJNA729271	stage_5W_vs_E10	ordinary_muscle_development	ENSGALG00010028545	ENSGALG00010028545	2.788292777	1.47822518	1.886243595	0.059262136	0.07403298	positive
PRJNA729271	stage_5W_vs_E10	ordinary_muscle_development	ENSGALG00010028546	ENSGALG00010028546	1.639063387	1.730374514	0.947230425	0.343521375	0.383350707	positive
PRJNA729271	stage_5W_vs_E10	ordinary_muscle_development	ENSGALG00010028547	ZNF618	-0.349761559	0.105816898	-3.305346939	0.000948589	0.00144143	negative
PRJNA729271	stage_5W_vs_E10	ordinary_muscle_development	ENSGALG00010028548	MYO1F	2.74146367	0.247390475	11.08152476	1.54223E-28	8.81842E-28	positive
PRJNA729271	stage_5W_vs_E10	ordinary_muscle_development	ENSGALG00010028549	ADAMTS10	-1.023316694	0.178794396	-5.723427119	1.04396E-08	2.30148E-08	negative
PRJNA729271	stage_5W_vs_E10	ordinary_muscle_development	ENSGALG00010028550	ZAP70	3.669032587	0.563882246	6.506735432	7.68015E-11	1.92902E-10	positive
PRJNA729271	stage_5W_vs_E10	ordinary_muscle_development	ENSGALG00010028551	NCS1	-2.082804587	0.222220161	-9.372707572	7.06953E-21	2.9345E-20	negative
PRJNA729271	stage_5W_vs_E10	ordinary_muscle_development	ENSGALG00010028552	ENSGALG00010028552	-2.744552544	0.721677173	-3.803019752	0.000142943	0.000232282	negative
PRJNA729271	stage_5W_vs_E10	ordinary_muscle_development	ENSGALG00010028553	NFILZ	6.440972283	0.913825987	7.048357536	1.81042E-12	4.9833E-12	positive
PRJNA729271	stage_5W_vs_E10	ordinary_muscle_development	ENSGALG00010028554	ENSGALG00010028554	-3.825516666	0.984656535	-3.885127991	0.000102276	0.00016835	negative
PRJNA729271	stage_5W_vs_E10	ordinary_muscle_development	ENSGALG00010028555	ENSGALG00010028555	1.595185008	1.262384129	1.263628852	0.206363273	0.239490443	positive

PRJNA729271	stage_5W_vs_E10	ordinary_muscle_development	ENSGALG00010028556	SPTAN1	-0.512025898	0.093448049	-5.479257213	4.27115E-08	9.03141E-08	negative
PRJNA729271	stage_5W_vs_E10	ordinary_muscle_development	ENSGALG00010028558	ENSGALG00010028558	3.235420296	1.447547214	2.235105193	0.025410444	0.033188356	positive
PRJNA729271	stage_5W_vs_E10	ordinary_muscle_development	ENSGALG00010028559	DYNC2I2	-0.877298083	0.128103932	-6.848330656	7.47167E-12	1.98792E-11	negative
PRJNA729271	stage_5W_vs_E10	ordinary_muscle_development	ENSGALG00010028560	ENSGALG00010028560	2.245974291	3.496491524	0.642350847	0.520645407	0.561263957	positive
PRJNA729271	stage_5W_vs_E10	ordinary_muscle_development	ENSGALG00010028561	SET	-1.22810434	0.051760356	-23.72673691	1.9105E-124	1.2531E-122	negative
PRJNA729271	stage_5W_vs_E10	ordinary_muscle_development	ENSGALG00010028562	ACSBG2	1.586624176	0.199489415	7.953425375	1.81424E-15	5.85428E-15	positive
PRJNA729271	stage_5W_vs_E10	ordinary_muscle_development	ENSGALG00010028563	ENSGALG00010028563	2.201017384	2.290414073	0.960969202	0.336567654	0.376373101	positive
PRJNA729271	stage_5W_vs_E10	ordinary_muscle_development	ENSGALG00010028565	PKN1	0.489657625	0.11967938	4.09141178	4.28755E-05	7.26453E-05	positive
PRJNA729271	stage_5W_vs_E10	ordinary_muscle_development	ENSGALG00010028566	ZDHHC12	0.498119215	0.125382749	3.972789057	7.1036E-05	0.000118666	positive
PRJNA729271	stage_5W_vs_E10	ordinary_muscle_development	ENSGALG00010028567	ENSGALG00010028567	-3.265885618	1.200756272	-2.719857221	0.006531011	0.009114867	negative
PRJNA729271	stage_5W_vs_E10	ordinary_muscle_development	ENSGALG00010028568	MLLT1	-1.174816426	0.066636248	-17.63029066	1.44224E-69	3.00795E-68	negative
PRJNA729271	stage_5W_vs_E10	ordinary_muscle_development	ENSGALG00010028569	ZER1	0.442771143	0.069114384	6.406353009	1.49042E-10	3.66722E-10	positive
PRJNA729271	stage_5W_vs_E10	ordinary_muscle_development	ENSGALG00010028572	TBC1D13	-0.30655194	0.069294634	-4.42389143	9.69387E-06	1.73045E-05	negative
PRJNA729271	stage_5W_vs_E10	ordinary_muscle_development	ENSGALG00010028573	KCNN1	-0.254398702	0.265806128	-0.957083661	0.33852503	0.378299307	negative
PRJNA729271	stage_5W_vs_E10	ordinary_muscle_development	ENSGALG00010028574	ENDOG	-0.359020521	0.122028642	-2.942100441	0.003259942	0.004694303	negative
PRJNA729271	stage_5W_vs_E10	ordinary_muscle_development	ENSGALG00010028575	SETDB1	-0.096361935	0.119344442	-0.807427082	0.419420469	0.461022196	negative
PRJNA729271	stage_5W_vs_E10	ordinary_muscle_development	ENSGALG00010028577	CERS2	0.087013685	0.132705426	0.655690487	0.512023291	0.552667928	positive
PRJNA729271	stage_5W_vs_E10	ordinary_muscle_development	ENSGALG00010028578	SPOUT1	-0.680103634	0.125786051	-5.40682872	6.41504E-08	1.34005E-07	negative
PRJNA729271	stage_5W_vs_E10	ordinary_muscle_development	ENSGALG00010028579	OCLN	0.25523452	0.273258069	0.934042022	0.350282217	0.390354481	positive
PRJNA729271	stage_5W_vs_E10	ordinary_muscle_development	ENSGALG00010028580	PRUNE1	0.929815622	0.151975262	6.11820376	9.4636E-10	2.21286E-09	positive
PRJNA729271	stage_5W_vs_E10	ordinary_muscle_development	ENSGALG00010028581	NR2F6	-0.159302559	0.12459142	-1.278599758	0.201038035	0.233647075	negative
PRJNA729271	stage_5W_vs_E10	ordinary_muscle_development	ENSGALG00010028582	KYAT1	1.854375227	0.206652816	8.973384754	2.8754E-19	1.11084E-18	positive
PRJNA729271	stage_5W_vs_E10	ordinary_muscle_development	ENSGALG00010028583	USHBP1	1.241463251	0.199787319	6.213924176	5.16775E-10	1.22878E-09	positive
PRJNA729271	stage_5W_vs_E10	ordinary_muscle_development	ENSGALG00010028584	LRRC8A	0.359178754	0.07570732	4.744306796	2.09222E-06	3.91868E-06	positive
PRJNA729271	stage_5W_vs_E10	ordinary_muscle_development	ENSGALG00010028585	MINDY1	1.029542586	0.121171313	8.496586852	1.95247E-17	6.93993E-17	positive
PRJNA729271	stage_5W_vs_E10	ordinary_muscle_development	ENSGALG00010028586	BABAM1	-0.379381416	0.121977101	-3.110267535	0.00186918	0.002755111	negative
PRJNA729271	stage_5W_vs_E10	ordinary_muscle_development	ENSGALG00010028587	ANKLE1	-1.844505907	0.215848379	-8.545377621	1.28117E-17	4.59894E-17	negative
PRJNA729271	stage_5W_vs_E10	ordinary_muscle_development	ENSGALG00010028588	ABHD8	-3.096591762	0.788610742	-3.926641618	8.61402E-05	0.000142877	negative
PRJNA729271	stage_5W_vs_E10	ordinary_muscle_development	ENSGALG00010028589	PHYHD1	0.017618932	0.192030079	0.091750899	0.926895956	0.936784053	positive
PRJNA729271	stage_5W_vs_E10	ordinary_muscle_development	ENSGALG00010028590	ENSGALG00010028590	1.295617484	0.228167312	5.678365892	1.35988E-08	2.98025E-08	positive
PRJNA729271	stage_5W_vs_E10	ordinary_muscle_development	ENSGALG00010028591	GABPB2	-1.267160858	0.128152778	-9.887892224	4.69815E-23	2.14555E-22	negative
PRJNA729271	stage_5W_vs_E10	ordinary_muscle_development	ENSGALG00010028592	SEMA6C	2.930066601	0.15977302	18.33893226	4.04667E-75	9.85823E-74	positive
PRJNA729271	stage_5W_vs_E10	ordinary_muscle_development	ENSGALG00010028593	DOLK	-0.321446753	0.121508623	-2.645464539	0.008157884	0.011270351	negative
PRJNA729271	stage_5W_vs_E10	ordinary_muscle_development	ENSGALG00010028594	ENSGALG00010028594	1.659229958	0.141968828	11.68728361	1.48046E-31	9.53323E-31	positive
PRJNA729271	stage_5W_vs_E10	ordinary_muscle_development	ENSGALG00010028595	SCNM1	0.665461837	0.112333458	5.923986021	3.1423E-09	7.13466E-09	positive
PRJNA729271	stage_5W_vs_E10	ordinary_muscle_development	ENSGALG00010028596	DDA1	0.044015138	0.088698111	0.496235345	0.619728377	0.656265548	positive
PRJNA729271	stage_5W_vs_E10	ordinary_muscle_development	ENSGALG00010028597	NUP188	-0.984769342	0.057534699	-17.11609436	1.1258E-65	2.12204E-64	negative
PRJNA729271	stage_5W_vs_E10	ordinary_muscle_development	ENSGALG00010028598	TMOD4	9.022889499	0.250361284	36.03947608	2.0159E-284	7.6897E-282	positive
PRJNA729271	stage_5W_vs_E10	ordinary_muscle_development	ENSGALG00010028599	VPS72	1.180841828	0.117879064	10.01740078	1.2782E-23	5.98048E-23	positive
PRJNA729271	stage_5W_vs_E10	ordinary_muscle_development	ENSGALG00010028600	PSMD4	1.38254395	0.131943626	10.47829282	1.08688E-25	5.53645E-25	positive
PRJNA729271	stage_5W_vs_E10	ordinary_muscle_development	ENSGALG00010028601	ANO8	6.448751943	0.22153823	29.10898015	2.7634E-186	4.046E-184	positive
PRJNA729271	stage_5W_vs_E10	ordinary_muscle_development	ENSGALG00010028602	ZNF687	0.672578373	0.169259737	3.973646558	7.07806E-05	0.000118253	positive
PRJNA729271	stage_5W_vs_E10	ordinary_muscle_development	ENSGALG00010028603	SH3GLB2	-0.021143125	0.338444894	-0.062471395	0.950187441	0.956788257	negative
PRJNA729271	stage_5W_vs_E10	ordinary_muscle_development	ENSGALG00010028604	PI4KB	-0.093216516	0.140646898	-0.662769793	0.507478002	0.548333481	negative
PRJNA729271	stage_5W_vs_E10	ordinary_muscle_development	ENSGALG00010028605	GTPBP3	1.936330137	0.213419578	9.072879622	1.15912E-19	4.56935E-19	positive
PRJNA729271	stage_5W_vs_E10	ordinary_muscle_development	ENSGALG00010028606	PRPF3	0.100577321	0.136202629	0.73843891	0.460247777	0.502205024	positive
PRJNA729271	stage_5W_vs_E10	ordinary_muscle_development	ENSGALG00010028607	ENSGALG00010028607	1.06265715	0.239132624	4.443798304	8.83845E-06	1.58126E-05	positive
PRJNA729271	stage_5W_vs_E10	ordinary_muscle_development	ENSGALG00010028608	MIGA2	0.092316973	0.16018194	0.576325725	0.564395038	0.603623263	positive
PRJNA729271	stage_5W_vs_E10	ordinary_muscle_development	ENSGALG00010028609	MRPS21	0.492283742	0.145276336	3.388602401	0.000702498	0.00107822	positive
PRJNA729271	stage_5W_vs_E10	ordinary_muscle_development	ENSGALG00010028610	ENSGALG00010028610	4.770459795	0.528979197	9.018237053	1.9114E-19	7.44379E-19	positive
PRJNA729271	stage_5W_vs_E10	ordinary_muscle_development	ENSGALG00010028612	DIRAS1	-0.979313061	0.647639308	-1.512127274	0.130501488	0.156138594	negative
PRJNA729271	stage_5W_vs_E10	ordinary_muscle_development	ENSGALG00010028613	ENSGALG00010028613	3.195082336	0.36165868	8.834524124	1.00525E-18	3.78963E-18	positive
PRJNA729271	stage_5W_vs_E10	ordinary_muscle_development	ENSGALG00010028618	DOLPP1	-1.762250038	0.111067103	-15.86653459	1.08044E-56	1.57397E-55	negative
PRJNA729271	stage_5W_vs_E10	ordinary_muscle_development	ENSGALG00010028626	CRAT	2.668701139	0.172908506	15.4341808	9.64168E-54	1.28217E-52	positive
PRJNA729271	stage_5W_vs_E10	ordinary_muscle_development	ENSGALG00010028642	APH1A	0.496612005	0.138978217	3.573308233	0.000352499	0.000554715	positive
PRJNA729271	stage_5W_vs_E10	ordinary_muscle_development	ENSGALG00010028649	ENSGALG00010028649	1.067935019	0.170534753	6.26227208	3.79408E-10	9.09613E-10	positive
PRJNA729271	stage_5W_vs_E10	ordinary_muscle_development	ENSGALG00010028658	PTPA	-0.528852187	0.082419446	-6.416594791	1.39356E-10	3.4359E-10	negative
PRJNA729271	stage_5W_vs_E10	ordinary_muscle_development	ENSGALG00010028662	FCRL4	0.150084211	0.228639405	0.656423205	0.511551864	0.552274502	positive
PRJNA729271	stage_5W_vs_E10	ordinary_muscle_development	ENSGALG00010028669	ZBTB34	0.082040785	0.111123105	0.738287371	0.460339839	0.502229863	positive
PRJNA729271	stage_5W_vs_E10	ordinary_muscle_development	ENSGALG00010028671	CADM3	3.719742747	0.212067124	17.54040269	7.04266E-69	1.44185E-67	positive
PRJNA729271	stage_5W_vs_E10	ordinary_muscle_development	ENSGALG00010028675	ZBTB43	-0.075454954	0.07153063	-1.05486215	0.291488375	0.329341648	negative
PRJNA729271	stage_5W_vs_E10	ordinary_muscle_development	ENSGALG00010028678	ENSGALG00010028678	1.219122563	0.364654763	3.343224019	0.00082811	0.001263921	positive
PRJNA729271	stage_5W_vs_E10	ordinary_muscle_development	ENSGALG00010028686	TAGLN2	0.592506838	0.119349976	4.964448755	6.88965E-07	1.3394E-06	positive
PRJNA729271	stage_5W_vs_E10	ordinary_muscle_development	ENSGALG00010028689	CFAP45	-0.101106013	0.648949448	-0.155799521	0.876191048	0.891255385	negative
PRJNA729271	stage_5W_vs_E10	ordinary_muscle_development	ENSGALG00010028692	RALGPS1	0.03811204	0.112104757	0.339968088	0.73388056	0.763263164	positive
PRJNA729271	stage_5W_vs_E10	ordinary_muscle_development	ENSGALG00010028695	VSIG8	-1.032321847	1.14247398	-0.903584558	0.366215732	0.406828406	negative
PRJNA729271	stage_5W_vs_E10	ordinary_muscle_development	ENSGALG00010028700	ANGPTL2	1.956734964	0.197551413	9.904940343	3.96206E-23	1.81741E-22	positive
PRJNA729271	stage_5W_vs_E10	ordinary_muscle_development	ENSGALG00010028707	ENSGALG00010028707	-4.182933245	1.23336907	-3.391469227	0.00069519	0.001067342	negative
PRJNA729271	stage_5W_vs_E10	ordinary_muscle_development	ENSGALG00010028712	FBXW2	0.020587896	0.085133195	0.241831592	0.808910658	0.831571631	positive
PRJNA729271	stage_5W_vs_E10	ordinary_muscle_development	ENSGALG00010028726	SLC2A8	-0.18777332	0.113276961	-1.657647931	0.097388549	0.118486404	negative
PRJNA729271	stage_5W_vs_E10	ordinary_muscle_development	ENSGALG00010028727	ENSGALG00010028727	-0.397630079	0.177116067	-2.245025452	0.024766499	0.032396932	negative
PRJNA729271	stage_5W_vs_E10	ordinary_muscle_development	ENSGALG00010028734	ENSGALG00010028734	0.857598857	0.362345166	2.366800878	0.017942583	0.023851591	positive
PRJNA729271	stage_5W_vs_E10	ordinary_muscle_development	ENSGALG00010028735	ENSGALG00010028735	-0.590247478	0.346236831	-1.704750696	0.08824093	0.107891378	negative
PRJNA729271	stage_5W_vs_E10	ordinary_muscle_development	ENSGALG00010028738	MIDN	-0.705130416	0.186047338	-3.790059146	0.000150611	0.000244114	negative
PRJNA729271	stage_5W_vs_E10	ordinary_muscle_development	ENSGALG00010028739	GARNL3	-0.434931414	0.078083046	-5.570113282	2.54574E-08	5.47728E-08	negative
PRJNA729271	stage_5W_vs_E10	ordinary_muscle_development	ENSGALG00010028740	DAZAP1	-0.174439829	0.106034995	-1.645115642	0.099945965	0.121373724	negative
PRJNA729271	stage_5W_vs_E10	ordinary_muscle_development	ENSGALG00010028742	ENSGALG00010028742	-0.176526196	0.149207195	-1.183094395	0.236771739	0.271885159	negative
PRJNA729271	stage_5W_vs_E10	ordinary_muscle_development	ENSGALG00010028743	ARRDC1	1.039337654	0.187109562	5.554700901	2.78088E-08	5.95843E-08	positive
PRJNA729271	stage_5W_vs_E10	ordinary_muscle_development	ENSGALG00010028744	HOMER3	0.879973293	0.208325127	4.224038201	2.39963E-05	4.14969E-05	positive
PRJNA729271	stage_5W_vs_E10	ordinary_muscle_development	ENSGALG00010028745	ENSGALG00010028745	0.612657378	0.138310048	4.429594139	9.44106E-06	1.68636E-05	positive
PRJNA729271	stage_5W_vs_E10	ordinary_muscle_development	ENSGALG00010028746	MYDGF	-0.905686562	0.125962838	-7.190109232	6.47395E-13	1.82355E-12	negative
PRJNA729271	stage_5W_vs_E10	ordinary_muscle_development	ENSGALG00010028747	ENSGALG00010028747	-4.492051458	0.193610097	-23.20153511	4.3936E-119	2.6316E-117	negative
PRJNA729271	stage_5W_vs_E10	ordinary_muscle_development	ENSGALG00010028748	PIAS3	0.967121115	0.199557086	4.846338127	1.25761E-06	2.39447E-06	positive
PRJNA729271	stage_5W_vs_E10	ordinary_muscle_development	ENSGALG00010028750	OTUD7B	0.499459144	0.120305846	4.151578341	3.3019E-05	5.64266E-05	positive
PRJNA729271	stage_5W_vs_E10	ordinary_muscle_development	ENSGALG00010028751	EHMT1	-0.7934564	0.096007325	-8.264540229	1.40234E-16	4.77607E-16	negative
PRJNA729271	stage_5W_vs_E10	ordinary_muscle_development	ENSGALG00010028752	MTMR11	2.292137401	0.131876412	17.38095056	1.15031E-67	2.26853E-66	positive
PRJNA729271	stage_5W_vs_E10	ordinary_muscle_development	ENSGALG00010028753	SV2A	2.489463314	0.632415089	3.936438828	8.26997E-05	0.000137422	positive
PRJNA729271	stage_5W_vs_E10	ordinary_muscle_development	ENSGALG00010028756	ENSGALG00010028756	-6.215593168	1.000450391	-6.212794978	5.20503E-10	1.23744E-09	negative
PRJNA729271	stage_5W_vs_E10	ordinary_muscle_development	ENSGALG00010028757	MAT2A	-3.007330132	1.377129337	-2.183767386	0.028979345	0.037646138	negative
PRJNA729271	stage_5W_vs_E10	ordinary_muscle_development	ENSGALG00010028760	SSR2	-0.264278937	0.097699359	-2.705022208	0.006829982	0.009511971	negative
PRJNA729271	stage_5W_vs_E10	ordinary_muscle_development	ENSGALG00010028761	CTSS	3.680710673	0.236300503	15.57639798	1.05325E-54	1.44572E-53	positive
PRJNA729271	stage_5W_vs_E10	ordinary_muscle_development	ENSGALG00010028762	CACNA1B	-5.148104722	0.98782122	-5.21157535	1.87244E-07	3.78905E-07	negative
PRJNA729271	stage_5W_vs_E10	ordinary_muscle_development	ENSGALG00010028763	CTSK	1.016452332	0.217505847	4.673218419	2.96516E-06	5.49827E-06	positive
PRJNA729271	stage_5W_vs_E10	ordinary_muscle_development	ENSGALG00010028764	ARNT	0.188869579	0.125599763	1.503741523	0.132647933	0.158549666	positive
PRJNA729271	stage_5W_vs_E10	ordinary_muscle_development	ENSGALG00010028768	NTNG2	-0.333283906	0.34885687	-0.955360019	0.339395665	0.379096877	negative
PRJNA729271	stage_5W_vs_E10	ordinary_muscle_development	ENSGALG00010028771	SETX	0.333400114	0.071843983	4.640612885	3.47377E-06	6.40371E-06	positive
PRJNA729271	stage_5W_vs_E10	ordinary_muscle_development	ENSGALG00010028772	TTF1	0.119165486	0.072408155	1.645746755	0.099815909	0.121246258	positive
PRJNA729271	stage_5W_vs_E10	ordinary_muscle_development	ENSGALG00010028782	MLLT11	0.720968943	0.251677458	2.864654423	0.004174646	0.005947659	positive
PRJNA729271	stage_5W_vs_E10	ordinary_muscle_development	ENSGALG00010028783	DDX31	-0.613467186	0.076874925	-7.980068768	1.46252E-15	4.74149E-15	negative
PRJNA729271	stage_5W_vs_E10	ordinary_muscle_development	ENSGALG00010028786	GTF3C4	-0.566655143	0.068734616	-8.244101421	1.66406E-16	5.64487E-16	negative
PRJNA729271	stage_5W_vs_E10	ordinary_muscle_development	ENSGALG00010028800	ENSGALG00010028800	-0.862152518	0.451945814	-1.907645765	0.056437016	0.070680365	negative
PRJNA729271	stage_5W_vs_E10	ordinary_muscle_development	ENSGALG00010028806	SPACA9	0.454584037	0.431438	1.053648581	0.292043838	0.329917811	positive
PRJNA729271	stage_5W_vs_E10	ordinary_muscle_development	ENSGALG00010028818	TSC1	0.169328894	0.117562682	1.440328602	0.149774453	0.177803497	positive
PRJNA729271	stage_5W_vs_E10	ordinary_muscle_development	ENSGALG00010028823	GFI1B	-0.054347729	0.332606015	-0.163399717	0.870203725	0.886471502	negative
PRJNA729271	stage_5W_vs_E10	ordinary_muscle_development	ENSGALG00010028826	CEL	6.099954376	2.730911967	2.233669357	0.025504837	0.03330564	positive
PRJNA729271	stage_5W_vs_E10	ordinary_muscle_development	ENSGALG00010028829	GTF3C5	-0.323997318	0.114423707	-2.831557601	0.004632189	0.006562758	negative
PRJNA729271	stage_5W_vs_E10	ordinary_muscle_development	ENSGALG00010028833	GBGT1	2.936524623	0.165906798	17.69984505	4.20462E-70	8.92328E-69	positive
PRJNA729271	stage_5W_vs_E10	ordinary_muscle_development	ENSGALG00010028849	ENSGALG00010028849	6.282382119	0.325405276	19.30633147	4.75148E-83	1.38021E-81	positive
PRJNA729271	stage_5W_vs_E10	ordinary_muscle_development	ENSGALG00010028853	ENSGALG00010028853	1.157451544	1.440009812	0.803780318	0.421523859	0.463263957	positive
PRJNA729271	stage_5W_vs_E10	ordinary_muscle_development	ENSGALG00010028854	RPL7A	-0.009193305	0.140355297	-0.065500232	0.947775721	0.954691389	negative
PRJNA729271	stage_5W_vs_E10	ordinary_muscle_development	ENSGALG00010028855	SURF6	-0.517068921	0.169901263	-3.043349486	0.002339604	0.003416883	negative
PRJNA729271	stage_5W_vs_E10	ordinary_muscle_development	ENSGALG00010028856	ETV3	-0.143239679	0.110099028	-1.30100766	0.193255833	0.225397755	negative
PRJNA729271	stage_5W_vs_E10	ordinary_muscle_development	ENSGALG00010028857	MED22	0.16445275	0.079248163	2.075161661	0.037971555	0.048656089	positive
PRJNA729271	stage_5W_vs_E10	ordinary_muscle_development	ENSGALG00010028858	ENSGALG00010028858	0.088870396	0.459716588	0.193315617	0.846711796	0.86539892	positive
PRJNA729271	stage_5W_vs_E10	ordinary_muscle_development	ENSGALG00010028859	SURF1	0.540960873	0.110320207	4.90355203	9.4119E-07	1.81176E-06	positive
PRJNA729271	stage_5W_vs_E10	ordinary_muscle_development	ENSGALG00010028860	SURF2	0.329327445	0.172626913	1.907741038	0.056424695	0.070671041	positive
PRJNA729271	stage_5W_vs_E10	ordinary_muscle_development	ENSGALG00010028861	SURF4	-0.535021509	0.074882162	-7.14484588	9.00969E-13	2.52018E-12	negative
PRJNA729271	stage_5W_vs_E10	ordinary_muscle_development	ENSGALG00010028862	ENSGALG00010028862	0.220333175	1.406930892	0.156605542	0.875555735	0.890885127	positive
PRJNA729271	stage_5W_vs_E10	ordinary_muscle_development	ENSGALG00010028865	STKLD1	0.933500026	0.168395341	5.543502677	2.9648E-08	6.3394E-08	positive
PRJNA729271	stage_5W_vs_E10	ordinary_muscle_development	ENSGALG00010028866	HORMAD1	-0.85550399	0.495251813	-1.727412133	0.084093673	0.103116301	negative
PRJNA729271	stage_5W_vs_E10	ordinary_muscle_development	ENSGALG00010028867	REXO4	-0.563170169	0.119095045	-4.728745589	2.25911E-06	4.222E-06	negative
PRJNA729271	stage_5W_vs_E10	ordinary_muscle_development	ENSGALG00010028868	ENSGALG00010028868	0.669421259	4.241825273	0.157814435	0.874603024	0.890139786	positive
PRJNA729271	stage_5W_vs_E10	ordinary_muscle_development	ENSGALG00010028869	ADAMTS13	3.650713852	0.162952047	22.40360835	3.6296E-111	1.9062E-109	positive
PRJNA729271	stage_5W_vs_E10	ordinary_muscle_development	ENSGALG00010028870	CACFD1	-2.072980625	0.228042244	-9.090336016	9.87334E-20	3.90382E-19	negative
PRJNA729271	stage_5W_vs_E10	ordinary_muscle_development	ENSGALG00010028871	PIP5K1A	-0.453314753	0.109261581	-4.148894336	3.34085E-05	5.70653E-05	negative
PRJNA729271	stage_5W_vs_E10	ordinary_muscle_development	ENSGALG00010028872	SLC2A6	3.448748388	0.327345613	10.53549598	5.927E-26	3.05193E-25	positive
PRJNA729271	stage_5W_vs_E10	ordinary_muscle_development	ENSGALG00010028873	TMEM8C	-0.902554887	0.280228722	-3.220779367	0.001278425	0.001916118	negative
PRJNA729271	stage_5W_vs_E10	ordinary_muscle_development	ENSGALG00010028874	CRPL1	0.538335663	0.536681025	1.003083094	0.315820771	0.35489744	positive
PRJNA729271	stage_5W_vs_E10	ordinary_muscle_development	ENSGALG00010028875	DUSP23	1.123590469	0.120236003	9.344875452	9.19977E-21	3.79376E-20	positive
PRJNA729271	stage_5W_vs_E10	ordinary_muscle_development	ENSGALG00010028876	ADAMTSL2	3.806654101	0.194501566	19.57132878	2.71509E-85	8.21614E-84	positive
PRJNA729271	stage_5W_vs_E10	ordinary_muscle_development	ENSGALG00010028877	FAM163B	-3.17107472	0.659398308	-4.809042854	1.51655E-06	2.87126E-06	negative

PRJNA729271	stage_5W_vs_E10	ordinary_muscle_development	ENSGALG00010028878	IGSF9	0.543105214	0.158011513	3.437124322	0.000587926	0.000908624	positive
PRJNA729271	stage_5W_vs_E10	ordinary_muscle_development	ENSGALG00010028881	SARDH	-1.375422285	0.231780819	-5.934150601	2.95371E-09	6.71065E-09	negative
PRJNA729271	stage_5W_vs_E10	ordinary_muscle_development	ENSGALG00010028884	VAV2	-1.5632865	0.131111142	-11.92336879	8.94183E-33	6.04251E-32	negative
PRJNA729271	stage_5W_vs_E10	ordinary_muscle_development	ENSGALG00010028885	BRD3	-0.455695703	0.141709851	-3.215695312	0.001301289	0.00194958	negative
PRJNA729271	stage_5W_vs_E10	ordinary_muscle_development	ENSGALG00010028886	ENSGALG00010028886	0.200343432	1.859824927	0.10772162	0.914216486	0.925776719	positive
PRJNA729271	stage_5W_vs_E10	ordinary_muscle_development	ENSGALG00010028887	SF3B4	-0.196073565	0.175712395	-1.115877826	0.264474434	0.301238168	negative
PRJNA729271	stage_5W_vs_E10	ordinary_muscle_development	ENSGALG00010028888	WDR5B	-1.221869908	0.082925773	-14.73450124	3.87044E-49	4.55374E-48	negative
PRJNA729271	stage_5W_vs_E10	ordinary_muscle_development	ENSGALG00010028889	FUBP3	-1.153459764	0.083842499	-13.75745929	4.59389E-43	4.39818E-42	negative
PRJNA729271	stage_5W_vs_E10	ordinary_muscle_development	ENSGALG00010028890	ENSGALG00010028890	-6.56267737	0.550430298	-11.92281274	9.00173E-33	6.08015E-32	negative
PRJNA729271	stage_5W_vs_E10	ordinary_muscle_development	ENSGALG00010028891	SLC31A2	2.576231587	0.21859985	11.78514804	4.65601E-32	3.06907E-31	positive
PRJNA729271	stage_5W_vs_E10	ordinary_muscle_development	ENSGALG00010028892	NIBAN2	0.421445559	0.140680062	2.9957732	0.0027375	0.003973569	positive
PRJNA729271	stage_5W_vs_E10	ordinary_muscle_development	ENSGALG00010028893	STXBP1	1.637516351	0.196528503	8.332207922	7.93484E-17	2.73651E-16	positive
PRJNA729271	stage_5W_vs_E10	ordinary_muscle_development	ENSGALG00010028894	AKNA	-0.930302882	0.157510765	-5.906281267	3.49916E-09	7.92878E-09	negative
PRJNA729271	stage_5W_vs_E10	ordinary_muscle_development	ENSGALG00010028895	WHRN	-4.184170833	0.838618246	-4.98936298	6.05787E-07	1.18213E-06	negative
PRJNA729271	stage_5W_vs_E10	ordinary_muscle_development	ENSGALG00010028896	ATP6V1G1	0.62043737	0.098901584	6.273280413	3.53519E-10	8.4895E-10	positive
PRJNA729271	stage_5W_vs_E10	ordinary_muscle_development	ENSGALG00010028897	TMEM268	0.825943798	0.279243998	2.957785322	0.003098578	0.004472159	positive
PRJNA729271	stage_5W_vs_E10	ordinary_muscle_development	ENSGALG00010028898	TNFSF15	-1.555689121	0.254495779	-6.112828787	9.78804E-10	2.28503E-09	negative
PRJNA729271	stage_5W_vs_E10	ordinary_muscle_development	ENSGALG00010028899	TNFSF8	4.036916301	0.517018236	7.808073337	5.80688E-15	1.81912E-14	positive
PRJNA729271	stage_5W_vs_E10	ordinary_muscle_development	ENSGALG00010028900	TNC	-1.668234628	0.54938058	-3.036573714	0.002392836	0.003493569	negative
PRJNA729271	stage_5W_vs_E10	ordinary_muscle_development	ENSGALG00010028901	PAPPA	-2.269711135	0.124613766	-18.21396794	3.99887E-74	9.48667E-73	negative
PRJNA729271	stage_5W_vs_E10	ordinary_muscle_development	ENSGALG00010028902	ENSGALG00010028902	-2.721887126	0.623266103	-4.36713486	1.25887E-05	2.22827E-05	negative
PRJNA729271	stage_5W_vs_E10	ordinary_muscle_development	ENSGALG00010028903	ASTN2	-4.26638442	0.259633177	-16.43235458	1.12216E-60	1.82966E-59	negative
PRJNA729271	stage_5W_vs_E10	ordinary_muscle_development	ENSGALG00010028904	TRIM32	-0.630849116	0.084758742	-7.44287963	9.85138E-14	2.88593E-13	negative
PRJNA729271	stage_5W_vs_E10	ordinary_muscle_development	ENSGALG00010028905	COLGALT1	0.131846334	0.086376629	1.526412122	0.126907261	0.152114508	positive
PRJNA729271	stage_5W_vs_E10	ordinary_muscle_development	ENSGALG00010028909	ENSGALG00010028909	-1.031836101	0.137648373	-7.496173588	6.57078E-14	1.94652E-13	negative
PRJNA729271	stage_5W_vs_E10	ordinary_muscle_development	ENSGALG00010028911	LMX1B	-5.696917898	0.704174558	-8.090206946	5.95634E-16	1.96489E-15	negative
PRJNA729271	stage_5W_vs_E10	ordinary_muscle_development	ENSGALG00010028912	EXFABP	1.560194717	0.497252853	3.137628485	0.001703206	0.002520982	positive
PRJNA729271	stage_5W_vs_E10	ordinary_muscle_development	ENSGALG00010028914	TLR4	2.395234048	0.227109121	10.54662196	5.26563E-26	2.72202E-25	positive
PRJNA729271	stage_5W_vs_E10	ordinary_muscle_development	ENSGALG00010028915	ADGRD2	2.768058224	0.4595058	6.02398974	1.70169E-09	3.92084E-09	positive
PRJNA729271	stage_5W_vs_E10	ordinary_muscle_development	ENSGALG00010028917	NR6A1	-0.432284099	0.131838699	-3.278886277	0.001042176	0.001577192	negative
PRJNA729271	stage_5W_vs_E10	ordinary_muscle_development	ENSGALG00010028918	AGPAT2	1.418763979	0.184722085	7.68053253	1.58429E-14	4.85091E-14	positive
PRJNA729271	stage_5W_vs_E10	ordinary_muscle_development	ENSGALG00010028919	EGFL7	1.2680589	0.169028775	7.502029737	6.28371E-14	1.86415E-13	positive
PRJNA729271	stage_5W_vs_E10	ordinary_muscle_development	ENSGALG00010028921	MRPL41	-0.158452578	0.122156866	-1.297123797	0.194588595	0.226824421	negative
PRJNA729271	stage_5W_vs_E10	ordinary_muscle_development	ENSGALG00010028923	ENSGALG00010028923	2.314544129	0.179655977	12.88320134	5.59651E-38	4.56765E-37	positive
PRJNA729271	stage_5W_vs_E10	ordinary_muscle_development	ENSGALG00010028924	TMEM203	0.271726735	0.120475697	2.255448541	0.024105189	0.031574617	positive
PRJNA729271	stage_5W_vs_E10	ordinary_muscle_development	ENSGALG00010028925	ENSGALG00010028925	2.132491065	0.320537189	6.652866301	2.87439E-11	7.40963E-11	positive
PRJNA729271	stage_5W_vs_E10	ordinary_muscle_development	ENSGALG00010028926	ENSGALG00010028926	-2.79727628	0.66603203	-4.199912547	2.67018E-05	4.59835E-05	negative
PRJNA729271	stage_5W_vs_E10	ordinary_muscle_development	ENSGALG00010029140	NPDC1	0.972715121	0.190267637	5.112351924	3.18172E-07	6.32809E-07	positive
PRJNA729271	stage_5W_vs_E10	ordinary_muscle_development	ENSGALG00010029141	C5	-1.130547126	0.574375316	-1.968307298	0.049032691	0.061877839	negative
PRJNA729271	stage_5W_vs_E10	ordinary_muscle_development	ENSGALG00010029142	ENSGALG00010029142	0.928758229	0.18212074	5.099684025	3.40221E-07	6.75271E-07	positive
PRJNA729271	stage_5W_vs_E10	ordinary_muscle_development	ENSGALG00010029143	SH2D3C	0.232526157	0.109080099	2.131701014	0.033031433	0.042650154	positive
PRJNA729271	stage_5W_vs_E10	ordinary_muscle_development	ENSGALG00010029144	ENSGALG00010029144	5.422730373	0.2187451	24.79017982	1.1441E-135	8.9642E-134	positive
PRJNA729271	stage_5W_vs_E10	ordinary_muscle_development	ENSGALG00010029145	RC3H2	1.014279905	0.133470206	7.599298293	2.97741E-14	9.00053E-14	positive
PRJNA729271	stage_5W_vs_E10	ordinary_muscle_development	ENSGALG00010029146	NEK6	-1.543212784	0.059262038	-26.04049452	1.7239E-149	1.7115E-147	negative
PRJNA729271	stage_5W_vs_E10	ordinary_muscle_development	ENSGALG00010029147	RABL6	-0.530565169	0.104058262	-5.098731817	3.41937E-07	6.78304E-07	negative
PRJNA729271	stage_5W_vs_E10	ordinary_muscle_development	ENSGALG00010029148	RALGDS	0.40874244	0.118691281	3.443744453	0.000573718	0.000887139	positive
PRJNA729271	stage_5W_vs_E10	ordinary_muscle_development	ENSGALG00010029149	ENSGALG00010029149	-0.995293519	0.223559936	-4.452020951	8.50659E-06	1.52339E-05	negative
PRJNA729271	stage_5W_vs_E10	ordinary_muscle_development	ENSGALG00010029150	NARF	0.001950224	0.123257919	0.015822301	0.987376157	0.989233001	positive
PRJNA729271	stage_5W_vs_E10	ordinary_muscle_development	ENSGALG00010029151	CYBC1	-0.88942796	0.114794226	-7.748020015	9.33364E-15	2.89641E-14	negative
PRJNA729271	stage_5W_vs_E10	ordinary_muscle_development	ENSGALG00010029152	HEXD	-0.361633779	0.1324797	-2.729729758	0.006338626	0.008857456	negative
PRJNA729271	stage_5W_vs_E10	ordinary_muscle_development	ENSGALG00010029153	OGFOD3	-1.089440823	0.081815842	-13.31576857	1.87434E-40	1.66168E-39	negative
PRJNA729271	stage_5W_vs_E10	ordinary_muscle_development	ENSGALG00010029154	UTS2R	-1.305243349	0.146025273	-8.938475668	3.94575E-19	1.51547E-18	negative
PRJNA729271	stage_5W_vs_E10	ordinary_muscle_development	ENSGALG00010029155	ENSGALG00010029155	-0.090331078	0.580565337	-0.155591579	0.876354963	0.891359567	negative
PRJNA729271	stage_5W_vs_E10	ordinary_muscle_development	ENSGALG00010029156	ENSGALG00010029156	-2.98051479	1.020824631	-2.919712848	0.00350354	0.005027553	negative
PRJNA729271	stage_5W_vs_E10	ordinary_muscle_development	ENSGALG00010029158	CD7	2.722034982	0.399956381	6.80582961	1.00469E-11	2.65941E-11	positive
PRJNA729271	stage_5W_vs_E10	ordinary_muscle_development	ENSGALG00010029159	SSC4D	-4.844326042	1.067842225	-4.536555987	5.71803E-06	1.03591E-05	negative
PRJNA729271	stage_5W_vs_E10	ordinary_muscle_development	ENSGALG00010029160	DUS1L	0.701294348	0.091835994	7.636377832	2.23418E-14	6.78777E-14	positive
PRJNA729271	stage_5W_vs_E10	ordinary_muscle_development	ENSGALG00010029162	ELN	0.624351547	0.310983135	2.007670118	0.044678358	0.056693758	positive
PRJNA729271	stage_5W_vs_E10	ordinary_muscle_development	ENSGALG00010029163	ANKFY1	-0.701726196	0.094604992	-7.417433081	1.19412E-13	3.48545E-13	negative
PRJNA729271	stage_5W_vs_E10	ordinary_muscle_development	ENSGALG00010029164	CYB5D2	0.546616115	0.082499791	6.625666672	3.45684E-11	8.87949E-11	positive
PRJNA729271	stage_5W_vs_E10	ordinary_muscle_development	ENSGALG00010029165	RAC3	-0.377828761	0.120922402	-3.124555536	0.001780739	0.002630369	negative
PRJNA729271	stage_5W_vs_E10	ordinary_muscle_development	ENSGALG00010029166	ZZEF1	2.00504157	0.123364258	16.25301853	2.12632E-59	3.34648E-58	positive
PRJNA729271	stage_5W_vs_E10	ordinary_muscle_development	ENSGALG00010029167	ENSGALG00010029167	1.663886694	0.126274383	13.17675569	1.19417E-39	1.03278E-38	positive
PRJNA729271	stage_5W_vs_E10	ordinary_muscle_development	ENSGALG00010029168	RFNG	0.708919025	0.080103379	8.850051467	8.74789E-19	3.30641E-18	positive
PRJNA729271	stage_5W_vs_E10	ordinary_muscle_development	ENSGALG00010029169	SBDS	1.441299946	0.077391161	18.62357321	2.06934E-77	5.33721E-76	positive
PRJNA729271	stage_5W_vs_E10	ordinary_muscle_development	ENSGALG00010029170	DCXR	-1.104877986	0.144244136	-7.65977748	1.86256E-14	5.67537E-14	negative
PRJNA729271	stage_5W_vs_E10	ordinary_muscle_development	ENSGALG00010029171	ENSGALG00010029171	0.343791704	0.146317668	2.349625356	0.018792315	0.024924019	positive
PRJNA729271	stage_5W_vs_E10	ordinary_muscle_development	ENSGALG00010029172	LRRC45	-0.122903932	0.074754269	-1.644105876	0.100154334	0.121616577	negative
PRJNA729271	stage_5W_vs_E10	ordinary_muscle_development	ENSGALG00010029173	ZNHIT3	0.114084842	0.135725604	0.840555052	0.400597249	0.441974206	positive
PRJNA729271	stage_5W_vs_E10	ordinary_muscle_development	ENSGALG00010029174	CALN1	-9.108514466	0.860555463	-10.58445953	3.51805E-26	1.83366E-25	negative
PRJNA729271	stage_5W_vs_E10	ordinary_muscle_development	ENSGALG00010029175	CENPX	-1.309493172	0.133584871	-9.802705673	1.0961E-22	4.9418E-22	negative
PRJNA729271	stage_5W_vs_E10	ordinary_muscle_development	ENSGALG00010029176	ENSGALG00010029176	0.962078217	0.4744807	2.027644575	0.042596536	0.054218194	positive
PRJNA729271	stage_5W_vs_E10	ordinary_muscle_development	ENSGALG00010029177	ASPSCR1	0.281816466	0.081008549	3.478848482	0.000503573	0.000781928	positive
PRJNA729271	stage_5W_vs_E10	ordinary_muscle_development	ENSGALG00010029178	NOTUM	0.455268983	0.206048684	2.20952143	0.027138394	0.035352837	positive
PRJNA729271	stage_5W_vs_E10	ordinary_muscle_development	ENSGALG00010029179	PIGW	0.079639083	0.152158956	0.523393986	0.600700108	0.638494395	positive
PRJNA729271	stage_5W_vs_E10	ordinary_muscle_development	ENSGALG00010029180	CA4	6.260684212	0.427603463	14.64133188	1.53041E-48	1.755E-47	positive
PRJNA729271	stage_5W_vs_E10	ordinary_muscle_development	ENSGALG00010029181	MYO19	-0.055792007	0.105540581	-0.52863085	0.597061556	0.635139237	negative
PRJNA729271	stage_5W_vs_E10	ordinary_muscle_development	ENSGALG00010029182	HEATR6	-0.420735593	0.068708608	-6.123477174	9.1555E-10	2.1415E-09	negative
PRJNA729271	stage_5W_vs_E10	ordinary_muscle_development	ENSGALG00010029183	ENSGALG00010029183	1.325012457	0.138960835	9.535150398	1.49668E-21	6.41468E-21	positive
PRJNA729271	stage_5W_vs_E10	ordinary_muscle_development	ENSGALG00010029184	DYNLL2	0.515410838	0.095685353	5.386517586	7.18359E-08	1.49592E-07	positive
PRJNA729271	stage_5W_vs_E10	ordinary_muscle_development	ENSGALG00010029186	ZNF302	0.403814762	0.140190148	2.880478896	0.003970715	0.005669377	positive
PRJNA729271	stage_5W_vs_E10	ordinary_muscle_development	ENSGALG00010029187	SRSF1	-1.566883044	0.053007471	-29.5596641	4.9339E-192	7.4496E-190	negative
PRJNA729271	stage_5W_vs_E10	ordinary_muscle_development	ENSGALG00010029188	ENSGALG00010029188	0.914079929	0.253317698	3.60843295	0.000308052	0.000487044	positive
PRJNA729271	stage_5W_vs_E10	ordinary_muscle_development	ENSGALG00010029189	ENSGALG00010029189	0.201566794	0.36346898	0.554563953	0.579192951	0.617854123	positive
PRJNA729271	stage_5W_vs_E10	ordinary_muscle_development	ENSGALG00010029190	DHRS7C	6.399762943	0.162882633	39.29064033	0	0	positive
PRJNA729271	stage_5W_vs_E10	ordinary_muscle_development	ENSGALG00010029192	DTX2	0.286928439	0.101573673	2.824830788	0.004730559	0.006696236	positive
PRJNA729271	stage_5W_vs_E10	ordinary_muscle_development	ENSGALG00010029194	DHRS11	-1.754005011	0.137422718	-12.76357383	2.61904E-37	2.10088E-36	negative
PRJNA729271	stage_5W_vs_E10	ordinary_muscle_development	ENSGALG00010029196	MRM1	-0.098092255	0.098518773	-0.995670699	0.319410171	0.358708486	negative
PRJNA729271	stage_5W_vs_E10	ordinary_muscle_development	ENSGALG00010029197	LHX1	0.812082178	0.704409971	1.152854461	0.248970126	0.284854525	positive
PRJNA729271	stage_5W_vs_E10	ordinary_muscle_development	ENSGALG00010029198	PPM1D	0.37340208	0.174735136	2.136960482	0.032601206	0.042117155	positive
PRJNA729271	stage_5W_vs_E10	ordinary_muscle_development	ENSGALG00010029199	APPBP2	-0.179252354	0.090058675	-1.990395209	0.046547417	0.058926185	negative
PRJNA729271	stage_5W_vs_E10	ordinary_muscle_development	ENSGALG00010029200	GAS7	1.486573131	0.12442984	11.94707904	6.72474E-33	4.572E-32	positive
PRJNA729271	stage_5W_vs_E10	ordinary_muscle_development	ENSGALG00010029201	PIGS	-0.665387988	0.112063699	-5.937587236	2.89247E-09	6.57359E-09	negative
PRJNA729271	stage_5W_vs_E10	ordinary_muscle_development	ENSGALG00010029202	PSMD12	0.901950523	0.142589974	6.325483467	2.52441E-10	6.12509E-10	positive
PRJNA729271	stage_5W_vs_E10	ordinary_muscle_development	ENSGALG00010029203	ALDOC	-1.783240381	0.173541631	-10.27557693	9.07995E-25	4.47666E-24	negative
PRJNA729271	stage_5W_vs_E10	ordinary_muscle_development	ENSGALG00010029204	HELZ	0.418390806	0.097919025	4.272824453	1.93012E-05	3.36345E-05	positive
PRJNA729271	stage_5W_vs_E10	ordinary_muscle_development	ENSGALG00010029205	CACNG1	5.458820967	0.113963778	47.89961385	0	0	positive
PRJNA729271	stage_5W_vs_E10	ordinary_muscle_development	ENSGALG00010029206	CACNG4	-2.46757494	0.363490818	-6.788548212	1.13268E-11	2.99001E-11	negative
PRJNA729271	stage_5W_vs_E10	ordinary_muscle_development	ENSGALG00010029207	SPAG5	-2.457761443	0.225549085	-10.89679191	1.19393E-27	6.60534E-27	negative
PRJNA729271	stage_5W_vs_E10	ordinary_muscle_development	ENSGALG00010029208	CACNG5	-4.352360902	0.533062935	-8.164816226	3.21925E-16	1.07767E-15	negative
PRJNA729271	stage_5W_vs_E10	ordinary_muscle_development	ENSGALG00010029209	PRKCA	0.53334525	0.096595841	5.521410087	3.3629E-08	7.15999E-08	positive
PRJNA729271	stage_5W_vs_E10	ordinary_muscle_development	ENSGALG00010029210	RSKR	-1.983299347	0.541527173	-3.662418887	0.000249845	0.000398142	negative
PRJNA729271	stage_5W_vs_E10	ordinary_muscle_development	ENSGALG00010029211	SDF2	-1.021241041	0.169408804	-6.02826427	1.6573E-09	3.81977E-09	negative
PRJNA729271	stage_5W_vs_E10	ordinary_muscle_development	ENSGALG00010029212	SUPT6H	0.304493088	0.113583451	2.680787432	0.007344916	0.010195801	positive
PRJNA729271	stage_5W_vs_E10	ordinary_muscle_development	ENSGALG00010029213	APOH	4.160511182	1.51743413	2.741806778	0.006110227	0.008550661	positive
PRJNA729271	stage_5W_vs_E10	ordinary_muscle_development	ENSGALG00010029214	ENSGALG00010029214	7.636105303	0.374942566	20.36606669	3.34489E-92	1.20308E-90	positive
PRJNA729271	stage_5W_vs_E10	ordinary_muscle_development	ENSGALG00010029215	ENSGALG00010029215	7.615298427	0.236918588	32.14310237	1.1027E-226	2.4974E-224	positive
PRJNA729271	stage_5W_vs_E10	ordinary_muscle_development	ENSGALG00010029216	RAB34	-1.426058162	0.136281586	-10.46405614	1.26329E-25	6.41606E-25	negative
PRJNA729271	stage_5W_vs_E10	ordinary_muscle_development	ENSGALG00010029217	CEP112	-0.767383211	0.093079722	-8.244365121	1.66039E-16	5.63376E-16	negative
PRJNA729271	stage_5W_vs_E10	ordinary_muscle_development	ENSGALG00010029218	RPL23A	0.31092235	0.150254455	2.069305359	0.038517441	0.049316342	positive
PRJNA729271	stage_5W_vs_E10	ordinary_muscle_development	ENSGALG00010029219	TLCD1	-0.133777609	0.204550963	-0.654006253	0.513107777	0.553664153	negative
PRJNA729271	stage_5W_vs_E10	ordinary_muscle_development	ENSGALG00010029220	NEK8	0.066128053	0.128931007	0.512894879	0.608024856	0.645144593	positive
PRJNA729271	stage_5W_vs_E10	ordinary_muscle_development	ENSGALG00010029221	AXIN2	-1.281830162	0.130925649	-9.790519841	1.23658E-22	5.5631E-22	negative
PRJNA729271	stage_5W_vs_E10	ordinary_muscle_development	ENSGALG00010029222	TRAF4	-0.931017791	0.24120247	-3.859901566	0.000113433	0.000185849	negative
PRJNA729271	stage_5W_vs_E10	ordinary_muscle_development	ENSGALG00010029223	NME2	-0.38285559	0.145918634	-2.623760796	0.008696484	0.011976773	negative
PRJNA729271	stage_5W_vs_E10	ordinary_muscle_development	ENSGALG00010029224	FAM222B	0.016417214	0.161501796	0.101653445	0.919031757	0.930133034	positive
PRJNA729271	stage_5W_vs_E10	ordinary_muscle_development	ENSGALG00010029226	MBTD1	-0.643315856	0.076693004	-8.388194755	4.93664E-17	1.72011E-16	negative
PRJNA729271	stage_5W_vs_E10	ordinary_muscle_development	ENSGALG00010029227	UTP18	-1.046897666	0.082627643	-12.67006569	8.66418E-37	6.82911E-36	negative
PRJNA729271	stage_5W_vs_E10	ordinary_muscle_development	ENSGALG00010029228	ERAL1	0.093084721	0.119136651	0.781327328	0.434610001	0.47659797	positive
PRJNA729271	stage_5W_vs_E10	ordinary_muscle_development	ENSGALG00010029229	CBX4	0.236690584	0.222939998	1.061678415	0.288381702	0.326085714	positive
PRJNA729271	stage_5W_vs_E10	ordinary_muscle_development	ENSGALG00010029230	PPM1E	1.079535738	0.164540191	6.560924298	5.34753E-11	1.35511E-10	positive
PRJNA729271	stage_5W_vs_E10	ordinary_muscle_development	ENSGALG00010029231	CBX8	-1.092521901	0.115856706	-9.429940955	4.1032E-21	1.72544E-20	negative
PRJNA729271	stage_5W_vs_E10	ordinary_muscle_development	ENSGALG00010029232	CA10	-5.979797013	0.482771509	-12.38639171	3.09666E-35	2.30421E-34	negative
PRJNA729271	stage_5W_vs_E10	ordinary_muscle_development	ENSGALG00010029233	CBX2	-4.080396623	0.194009527	-21.03193944	3.34685E-98	1.3551E-96	negative
PRJNA729271	stage_5W_vs_E10	ordinary_muscle_development	ENSGALG00010029234	TOM1L1	-2.113228574	0.242313066	-8.72106739	2.75591E-18	1.01854E-17	negative
PRJNA729271	stage_5W_vs_E10	ordinary_muscle_development	ENSGALG00010029235	TRIM37	-0.191735968	0.074560782	-2.571539131	0.010124757	0.013859601	negative
PRJNA729271	stage_5W_vs_E10	ordinary_muscle_development	ENSGALG00010029237	COX11	-0.502839677	0.107340625	-4.684523456	2.80612E-06	5.2107E-06	negative
PRJNA729271	stage_5W_vs_E10	ordinary_muscle_development	ENSGALG00010029238	CAMKK1	0.56649256	0.243600081	2.325502349	0.020045114	0.026483815	positive
PRJNA729271	stage_5W_vs_E10	ordinary_muscle_development	ENSGALG00010029239	RBFOX3	-3.322790641	0.722355975	-4.599940153	4.22622E-06	7.73767E-06	negative
PRJNA729271	stage_5W_vs_E10	ordinary_muscle_development	ENSGALG00010029240	MIS12	-2.840571191	0.25920625	-10.95872956	6.03405E-28	3.37306E-27	negative
PRJNA729271	stage_5W_vs_E10	ordinary_muscle_development	ENSGALG00010029241	STXBP4	-2.241233339	0.101949268	-21.98380995	4.1144E-107	1.949E-105	negative

PRJNA729271	stage_5W_vs_E10	ordinary_muscle_development	ENSGALG00010029242	DERL2	-0.317138227	0.099552853	-3.185626694	0.001444408	0.00215509	negative
PRJNA729271	stage_5W_vs_E10	ordinary_muscle_development	ENSGALG00010029243	NME1	-1.083606866	0.383438381	-2.826026087	0.004712943	0.006673254	negative
PRJNA729271	stage_5W_vs_E10	ordinary_muscle_development	ENSGALG00010029244	HLF	2.333778572	0.182646336	12.7775822	2.18765E-37	1.75873E-36	positive
PRJNA729271	stage_5W_vs_E10	ordinary_muscle_development	ENSGALG00010029245	SKA2	-1.38905456	0.116003262	-11.97427155	4.84676E-33	3.30295E-32	negative
PRJNA729271	stage_5W_vs_E10	ordinary_muscle_development	ENSGALG00010029246	DHX33	-1.657163038	0.138170386	-11.99361952	3.83762E-33	2.63507E-32	negative
PRJNA729271	stage_5W_vs_E10	ordinary_muscle_development	ENSGALG00010029247	CCDC92B	7.309084656	0.526220955	13.88976358	7.30761E-44	7.18615E-43	positive
PRJNA729271	stage_5W_vs_E10	ordinary_muscle_development	ENSGALG00010029248	C1QBP	-0.695588378	0.081964352	-8.486474472	2.13E-17	7.55245E-17	negative
PRJNA729271	stage_5W_vs_E10	ordinary_muscle_development	ENSGALG00010029249	PRR11	-2.080919181	0.293114037	-7.099350149	1.25345E-12	3.47794E-12	negative
PRJNA729271	stage_5W_vs_E10	ordinary_muscle_development	ENSGALG00010029250	SPAG9	0.673232107	0.087307397	7.711054616	1.24782E-14	3.84017E-14	positive
PRJNA729271	stage_5W_vs_E10	ordinary_muscle_development	ENSGALG00010029251	ENGASE	-0.957193468	0.199229765	-4.804470188	1.55162E-06	2.93344E-06	negative
PRJNA729271	stage_5W_vs_E10	ordinary_muscle_development	ENSGALG00010029252	RPAIN	1.411841729	0.137664358	10.25568094	1.11587E-24	5.47175E-24	positive
PRJNA729271	stage_5W_vs_E10	ordinary_muscle_development	ENSGALG00010029253	NUP88	-0.35712123	0.086033027	-4.150978334	3.31057E-05	5.65614E-05	negative
PRJNA729271	stage_5W_vs_E10	ordinary_muscle_development	ENSGALG00010029254	MMD	-0.077100819	0.118996649	-0.647924284	0.517033923	0.557495107	negative
PRJNA729271	stage_5W_vs_E10	ordinary_muscle_development	ENSGALG00010029255	RAP1GAP2	0.168746099	0.194044414	0.869626161	0.384504736	0.425710064	positive
PRJNA729271	stage_5W_vs_E10	ordinary_muscle_development	ENSGALG00010029256	RABEP1	0.362574351	0.096623133	3.752459069	0.000175108	0.000282336	positive
PRJNA729271	stage_5W_vs_E10	ordinary_muscle_development	ENSGALG00010029257	YPEL2	0.964893214	0.161174258	5.986645928	2.14212E-09	4.90445E-09	positive
PRJNA729271	stage_5W_vs_E10	ordinary_muscle_development	ENSGALG00010029258	TMEM100	0.356037673	0.235225703	1.513600206	0.130127269	0.155755141	positive
PRJNA729271	stage_5W_vs_E10	ordinary_muscle_development	ENSGALG00010029259	PTRH2	-0.34805073	0.108923473	-3.195369388	0.00139652	0.002086001	negative
PRJNA729271	stage_5W_vs_E10	ordinary_muscle_development	ENSGALG00010029260	DHX40	0.192926813	0.102791888	1.876868073	0.060536178	0.075526932	positive
PRJNA729271	stage_5W_vs_E10	ordinary_muscle_development	ENSGALG00010029261	IFT22	-1.644126833	0.102446336	-16.04866416	5.84077E-58	8.83736E-57	negative
PRJNA729271	stage_5W_vs_E10	ordinary_muscle_development	ENSGALG00010029262	CLTC	-0.343499143	0.065173103	-5.270566019	1.36004E-07	2.78246E-07	negative
PRJNA729271	stage_5W_vs_E10	ordinary_muscle_development	ENSGALG00010029263	TOB1	3.578817855	0.188196191	19.01642026	1.24713E-80	3.41079E-79	positive
PRJNA729271	stage_5W_vs_E10	ordinary_muscle_development	ENSGALG00010029264	COL26A1	-5.951072028	0.182333619	-32.63836954	1.1718E-233	2.7844E-231	negative
PRJNA729271	stage_5W_vs_E10	ordinary_muscle_development	ENSGALG00010029266	CANT1	0.225639782	0.111937142	2.015772221	0.0438238	0.055672741	positive
PRJNA729271	stage_5W_vs_E10	ordinary_muscle_development	ENSGALG00010029267	ENSGALG00010029267	4.688967993	0.272864535	17.1842339	3.4852E-66	6.64711E-65	positive
PRJNA729271	stage_5W_vs_E10	ordinary_muscle_development	ENSGALG00010029268	PCTP	1.403384043	0.179043898	7.838212066	4.57007E-15	1.43913E-14	positive
PRJNA729271	stage_5W_vs_E10	ordinary_muscle_development	ENSGALG00010029269	WSCD1	0.786542011	0.154394743	5.094357473	3.49926E-07	6.93394E-07	positive
PRJNA729271	stage_5W_vs_E10	ordinary_muscle_development	ENSGALG00010029270	TIMP2	1.614267999	0.149284128	10.81339335	2.97459E-27	1.61971E-26	positive
PRJNA729271	stage_5W_vs_E10	ordinary_muscle_development	ENSGALG00010029271	MYL10	-7.311220848	0.805027736	-9.081949003	1.06648E-19	4.211E-19	negative
PRJNA729271	stage_5W_vs_E10	ordinary_muscle_development	ENSGALG00010029273	SLC13A5	-3.417443888	0.49155593	-6.952299172	3.5938E-12	9.7314E-12	negative
PRJNA729271	stage_5W_vs_E10	ordinary_muscle_development	ENSGALG00010029274	VMP1	-0.072186427	0.147258522	-0.490202032	0.623990943	0.660345238	negative
PRJNA729271	stage_5W_vs_E10	ordinary_muscle_development	ENSGALG00010029275	PCYT2	0.32229273	0.071374802	4.51549734	6.31683E-06	1.14054E-05	positive
PRJNA729271	stage_5W_vs_E10	ordinary_muscle_development	ENSGALG00010029276	TUBD1	-0.023703868	0.177076192	-0.133862536	0.893511275	0.906756739	negative
PRJNA729271	stage_5W_vs_E10	ordinary_muscle_development	ENSGALG00010029277	WFIKKN2	3.394610739	0.737456783	4.60313176	4.16185E-06	7.62365E-06	positive
PRJNA729271	stage_5W_vs_E10	ordinary_muscle_development	ENSGALG00010029278	MED31	-0.562161644	0.071060549	-7.911023053	2.55282E-15	8.16126E-15	negative
PRJNA729271	stage_5W_vs_E10	ordinary_muscle_development	ENSGALG00010029279	ENSGALG00010029279	0.824227615	0.158580722	5.197527203	2.01957E-07	4.07882E-07	positive
PRJNA729271	stage_5W_vs_E10	ordinary_muscle_development	ENSGALG00010029280	USP36	0.665064085	0.182938114	3.635459396	0.000277486	0.000440253	positive
PRJNA729271	stage_5W_vs_E10	ordinary_muscle_development	ENSGALG00010029281	KIAA0753	-0.716226429	0.13433356	-5.331701413	9.72968E-08	2.00957E-07	negative
PRJNA729271	stage_5W_vs_E10	ordinary_muscle_development	ENSGALG00010029282	LUC7L3	-0.375279811	0.141350941	-2.654950932	0.007931995	0.010975015	negative
PRJNA729271	stage_5W_vs_E10	ordinary_muscle_development	ENSGALG00010029283	RPS6KB1	-0.004556506	0.077675652	-0.058660677	0.95322238	0.959644284	negative
PRJNA729271	stage_5W_vs_E10	ordinary_muscle_development	ENSGALG00010029284	CYTH1	0.967574095	0.09292665	10.41223476	2.18042E-25	1.09512E-24	positive
PRJNA729271	stage_5W_vs_E10	ordinary_muscle_development	ENSGALG00010029285	CUX1	-0.093022055	0.070093211	-1.327119337	0.18446921	0.21601884	negative
PRJNA729271	stage_5W_vs_E10	ordinary_muscle_development	ENSGALG00010029286	MAFG	1.659657429	0.182724099	9.082860118	1.05758E-19	4.17817E-19	positive
PRJNA729271	stage_5W_vs_E10	ordinary_muscle_development	ENSGALG00010029287	ANKRD40	0.574730664	0.125292371	4.587116186	4.49411E-06	8.20739E-06	positive
PRJNA729271	stage_5W_vs_E10	ordinary_muscle_development	ENSGALG00010029288	PYCR1	-2.002139823	0.294745296	-6.792779557	1.09993E-11	2.90675E-11	negative
PRJNA729271	stage_5W_vs_E10	ordinary_muscle_development	ENSGALG00010029289	PITPNM3	0.290772685	0.21937943	1.325432769	0.185027654	0.216599235	positive
PRJNA729271	stage_5W_vs_E10	ordinary_muscle_development	ENSGALG00010029290	ENSGALG00010029290	-1.681177684	0.198668798	-8.462212989	2.62351E-17	9.25925E-17	negative
PRJNA729271	stage_5W_vs_E10	ordinary_muscle_development	ENSGALG00010029291	RNFT1	-0.189378027	0.114276808	-1.657186888	0.097481697	0.118589777	negative
PRJNA729271	stage_5W_vs_E10	ordinary_muscle_development	ENSGALG00010029292	ENSGALG00010029292	-0.781011097	0.254022723	-3.074571788	0.00210805	0.003091479	negative
PRJNA729271	stage_5W_vs_E10	ordinary_muscle_development	ENSGALG00010029293	DNAH17	-1.563502198	0.145125941	-10.77341646	4.59623E-27	2.48128E-26	negative
PRJNA729271	stage_5W_vs_E10	ordinary_muscle_development	ENSGALG00010029294	PGS1	-0.823066128	0.077935577	-10.56085238	4.52534E-26	2.34854E-25	negative
PRJNA729271	stage_5W_vs_E10	ordinary_muscle_development	ENSGALG00010029295	SOCS3	1.922009152	0.250392371	7.675989259	1.64147E-14	5.01541E-14	positive
PRJNA729271	stage_5W_vs_E10	ordinary_muscle_development	ENSGALG00010029296	FBXO39	-1.331320648	0.161058494	-8.266069148	1.38448E-16	4.71968E-16	negative
PRJNA729271	stage_5W_vs_E10	ordinary_muscle_development	ENSGALG00010029297	SH2B2	2.930838604	0.252104723	11.62548073	3.05867E-31	1.94881E-30	positive
PRJNA729271	stage_5W_vs_E10	ordinary_muscle_development	ENSGALG00010029298	ENDOV	0.529546669	0.145257245	3.645578353	0.000266791	0.000423982	positive
PRJNA729271	stage_5W_vs_E10	ordinary_muscle_development	ENSGALG00010029299	PRKRIP1	-0.077378823	0.091593205	-0.844809637	0.398217133	0.439482058	negative
PRJNA729271	stage_5W_vs_E10	ordinary_muscle_development	ENSGALG00010029300	TEKT1	1.713317608	0.344602195	4.97187085	6.63099E-07	1.29032E-06	positive
PRJNA729271	stage_5W_vs_E10	ordinary_muscle_development	ENSGALG00010029301	RNF213	5.700104995	0.464125804	12.28137919	1.14037E-34	8.31057E-34	positive
PRJNA729271	stage_5W_vs_E10	ordinary_muscle_development	ENSGALG00010029303	SLC26A11	0.118755997	0.152371067	0.77938679	0.435751903	0.477705629	positive
PRJNA729271	stage_5W_vs_E10	ordinary_muscle_development	ENSGALG00010029304	ORAI3	0.224768802	0.116833024	1.92384648	0.054373829	0.068226165	positive
PRJNA729271	stage_5W_vs_E10	ordinary_muscle_development	ENSGALG00010029305	SMTNL2	2.290126051	0.18994346	12.05688285	1.78408E-33	1.23733E-32	positive
PRJNA729271	stage_5W_vs_E10	ordinary_muscle_development	ENSGALG00010029306	CEP95	-1.645580488	0.163722133	-10.051057	9.08873E-24	4.28148E-23	negative
PRJNA729271	stage_5W_vs_E10	ordinary_muscle_development	ENSGALG00010029307	SGSH	0.65276908	0.16434685	3.971898956	7.1302E-05	0.000119097	positive
PRJNA729271	stage_5W_vs_E10	ordinary_muscle_development	ENSGALG00010029308	GAA	0.273945552	0.138094306	1.983757048	0.04728293	0.059810287	positive
PRJNA729271	stage_5W_vs_E10	ordinary_muscle_development	ENSGALG00010029309	ALKBH4	2.461215729	0.163348729	15.06724752	2.65965E-51	3.34069E-50	positive
PRJNA729271	stage_5W_vs_E10	ordinary_muscle_development	ENSGALG00010029310	LRWD1	-1.159960669	0.092726396	-12.509498	6.62415E-36	5.06954E-35	negative
PRJNA729271	stage_5W_vs_E10	ordinary_muscle_development	ENSGALG00010029311	ENSGALG00010029311	0.427283363	0.167515578	2.550708225	0.010750428	0.014674401	positive
PRJNA729271	stage_5W_vs_E10	ordinary_muscle_development	ENSGALG00010029312	SMURF2	0.30795628	0.09434602	3.264115218	0.001098065	0.001658828	positive
PRJNA729271	stage_5W_vs_E10	ordinary_muscle_development	ENSGALG00010029313	CCDC40	-1.139762874	0.242392742	-4.702132847	2.57458E-06	4.79057E-06	negative
PRJNA729271	stage_5W_vs_E10	ordinary_muscle_development	ENSGALG00010029314	MED13	0.797339888	0.125551066	6.350721749	2.14307E-10	5.22169E-10	positive
PRJNA729271	stage_5W_vs_E10	ordinary_muscle_development	ENSGALG00010029315	TBC1D16	0.937212246	0.093868464	9.984314296	1.78531E-23	8.31291E-23	positive
PRJNA729271	stage_5W_vs_E10	ordinary_muscle_development	ENSGALG00010029316	INTS2	-0.455473906	0.095052798	-4.791799062	1.65292E-06	3.11887E-06	negative
PRJNA729271	stage_5W_vs_E10	ordinary_muscle_development	ENSGALG00010029317	KPNA2	-3.315843889	0.20621624	-16.07945081	3.55517E-58	5.43588E-57	negative
PRJNA729271	stage_5W_vs_E10	ordinary_muscle_development	ENSGALG00010029318	C17orf58	2.306699842	0.205951934	11.20018539	4.06881E-29	2.38389E-28	positive
PRJNA729271	stage_5W_vs_E10	ordinary_muscle_development	ENSGALG00010029319	BRIP1	-1.331464566	0.12708391	-10.47705067	1.10125E-25	5.6068E-25	negative
PRJNA729271	stage_5W_vs_E10	ordinary_muscle_development	ENSGALG00010029320	TBX4	-6.181062754	1.001705123	-6.170541221	6.80566E-10	1.60534E-09	negative
PRJNA729271	stage_5W_vs_E10	ordinary_muscle_development	ENSGALG00010029321	TBX2	-1.245510547	0.174569643	-7.134748784	9.69641E-13	2.70704E-12	negative
PRJNA729271	stage_5W_vs_E10	ordinary_muscle_development	ENSGALG00010029322	BPTF	-0.224563485	0.097026679	-2.314450896	0.020643002	0.027229076	negative
PRJNA729271	stage_5W_vs_E10	ordinary_muscle_development	ENSGALG00010029323	NOL11	0.043792172	0.09136199	0.479325941	0.63170677	0.667244016	positive
PRJNA729271	stage_5W_vs_E10	ordinary_muscle_development	ENSGALG00010029324	BCAS3	1.000158784	0.126317644	7.91780747	2.41735E-15	7.74012E-15	positive
PRJNA729271	stage_5W_vs_E10	ordinary_muscle_development	ENSGALG00010029325	LRRC75A	-1.060282366	0.170147993	-6.231530239	4.61901E-10	1.10131E-09	negative
PRJNA729271	stage_5W_vs_E10	ordinary_muscle_development	ENSGALG00010029326	NCBP3	-0.680442395	0.082193441	-8.278548539	1.24686E-16	4.25953E-16	negative
PRJNA729271	stage_5W_vs_E10	ordinary_muscle_development	ENSGALG00010029327	CRK	-0.550767401	0.054992084	-10.01539432	1.30441E-23	6.10112E-23	negative
PRJNA729271	stage_5W_vs_E10	ordinary_muscle_development	ENSGALG00010029328	ENSGALG00010029328	-0.360339057	0.083108953	-4.33574293	1.45269E-05	2.55977E-05	negative
PRJNA729271	stage_5W_vs_E10	ordinary_muscle_development	ENSGALG00010029329	ENSGALG00010029329	0.465889624	0.18253127	2.552382529	0.010698898	0.014608188	positive
PRJNA729271	stage_5W_vs_E10	ordinary_muscle_development	ENSGALG00010029330	AFMID	0.414782929	0.13132582	3.15842635	0.001586234	0.002355785	positive
PRJNA729271	stage_5W_vs_E10	ordinary_muscle_development	ENSGALG00010029331	TK1	-2.645416718	0.279119381	-9.477724954	2.59887E-21	1.10245E-20	negative
PRJNA729271	stage_5W_vs_E10	ordinary_muscle_development	ENSGALG00010029332	YWHAE	-1.005393213	0.148896342	-6.752302959	1.45517E-11	3.82044E-11	negative
PRJNA729271	stage_5W_vs_E10	ordinary_muscle_development	ENSGALG00010029333	ENSGALG00010029333	-1.291893703	0.176087035	-7.336677028	2.18962E-13	6.30234E-13	negative
PRJNA729271	stage_5W_vs_E10	ordinary_muscle_development	ENSGALG00010029335	RNF222	-0.76504041	1.507705825	-0.507420213	0.611860006	0.648928865	negative
PRJNA729271	stage_5W_vs_E10	ordinary_muscle_development	ENSGALG00010029336	SYNGR2	2.135989083	0.110255113	19.37315219	1.30047E-83	3.82358E-82	positive
PRJNA729271	stage_5W_vs_E10	ordinary_muscle_development	ENSGALG00010029337	MYO1C	1.479993564	0.133629327	11.07536492	1.65206E-28	9.42778E-28	positive
PRJNA729271	stage_5W_vs_E10	ordinary_muscle_development	ENSGALG00010029338	INPP5K	0.153172713	0.120301442	1.273240869	0.202932582	0.235773307	positive
PRJNA729271	stage_5W_vs_E10	ordinary_muscle_development	ENSGALG00010029339	PITPNA	0.72771392	0.126963893	5.731660434	9.94522E-09	2.19583E-08	positive
PRJNA729271	stage_5W_vs_E10	ordinary_muscle_development	ENSGALG00010029340	NDEL1	0.730221674	0.10395841	7.024171239	2.15341E-12	5.89715E-12	positive
PRJNA729271	stage_5W_vs_E10	ordinary_muscle_development	ENSGALG00010029341	SLC43A2	2.477087797	0.326247881	7.592655582	3.13415E-14	9.46053E-14	positive
PRJNA729271	stage_5W_vs_E10	ordinary_muscle_development	ENSGALG00010029342	TMC6	-0.417245127	0.130638839	-3.193882681	0.001403732	0.002096557	negative
PRJNA729271	stage_5W_vs_E10	ordinary_muscle_development	ENSGALG00010029343	SCARF1	1.786054751	0.13026772	13.71064718	8.76723E-43	8.33865E-42	positive
PRJNA729271	stage_5W_vs_E10	ordinary_muscle_development	ENSGALG00010029344	ENSGALG00010029344	1.781697173	0.164000447	10.86397754	1.71129E-27	9.39235E-27	positive
PRJNA729271	stage_5W_vs_E10	ordinary_muscle_development	ENSGALG00010029345	RILP	1.908429018	0.244362924	7.809814119	5.72724E-15	1.79495E-14	positive
PRJNA729271	stage_5W_vs_E10	ordinary_muscle_development	ENSGALG00010029346	ARL16	-1.145974611	0.13392535	-8.556816271	1.16027E-17	4.17324E-17	negative
PRJNA729271	stage_5W_vs_E10	ordinary_muscle_development	ENSGALG00010029347	PRPF8	-0.302375426	0.069987026	-4.320449725	1.55712E-05	2.73514E-05	negative
PRJNA729271	stage_5W_vs_E10	ordinary_muscle_development	ENSGALG00010029348	HGS	0.511011789	0.12358408	4.134932196	3.55059E-05	6.05196E-05	positive
PRJNA729271	stage_5W_vs_E10	ordinary_muscle_development	ENSGALG00010029349	MYH10	-2.351543006	0.107461541	-21.88264739	3.8012E-106	1.766E-104	negative
PRJNA729271	stage_5W_vs_E10	ordinary_muscle_development	ENSGALG00010029350	MRPL12	0.493721909	0.121702898	4.056780214	4.97539E-05	8.39951E-05	positive
PRJNA729271	stage_5W_vs_E10	ordinary_muscle_development	ENSGALG00010029351	ENSGALG00010029351	-0.869143528	0.199382625	-4.359173858	1.30554E-05	2.30863E-05	negative
PRJNA729271	stage_5W_vs_E10	ordinary_muscle_development	ENSGALG00010029352	AATF	-1.09800851	0.113384441	-9.683943435	3.52838E-22	1.55831E-21	negative
PRJNA729271	stage_5W_vs_E10	ordinary_muscle_development	ENSGALG00010029353	GCGR	3.741727104	0.889223448	4.207859244	2.57801E-05	4.44649E-05	positive
PRJNA729271	stage_5W_vs_E10	ordinary_muscle_development	ENSGALG00010029354	TLCD2	-0.97153125	0.290983984	-3.338779118	0.000841474	0.001283216	negative
PRJNA729271	stage_5W_vs_E10	ordinary_muscle_development	ENSGALG00010029355	MCRIP1	1.180904235	0.172411265	6.849345003	7.41889E-12	1.97424E-11	positive
PRJNA729271	stage_5W_vs_E10	ordinary_muscle_development	ENSGALG00010029357	ARHGDIA	-0.780131077	0.097567106	-7.99584112	1.28692E-15	4.18437E-15	negative
PRJNA729271	stage_5W_vs_E10	ordinary_muscle_development	ENSGALG00010029359	ENSGALG00010029359	-3.144802763	0.534363292	-5.885139961	3.97717E-09	8.97822E-09	negative
PRJNA729271	stage_5W_vs_E10	ordinary_muscle_development	ENSGALG00010029360	ACACA	-0.634456544	0.086073387	-7.371111628	1.69211E-13	4.89564E-13	negative
PRJNA729271	stage_5W_vs_E10	ordinary_muscle_development	ENSGALG00010029361	ALYREF	-2.002370026	0.136542681	-14.66479203	1.08349E-48	1.2514E-47	negative
PRJNA729271	stage_5W_vs_E10	ordinary_muscle_development	ENSGALG00010029362	TADA2A	-0.588752171	0.114806471	-5.12821416	2.92504E-07	5.82556E-07	negative
PRJNA729271	stage_5W_vs_E10	ordinary_muscle_development	ENSGALG00010029363	PIK3R6	2.999876145	0.189523423	15.82852448	1.97806E-56	2.86147E-55	positive
PRJNA729271	stage_5W_vs_E10	ordinary_muscle_development	ENSGALG00010029364	ANAPC11	-0.161691449	0.130392279	-1.24003852	0.214961147	0.248635997	negative
PRJNA729271	stage_5W_vs_E10	ordinary_muscle_development	ENSGALG00010029365	PIK3R5	2.763289748	0.182571507	15.13538339	9.46276E-52	1.21062E-50	positive
PRJNA729271	stage_5W_vs_E10	ordinary_muscle_development	ENSGALG00010029366	MGAT5B	3.03966337	0.338422944	8.981847786	2.66257E-19	1.03E-18	positive
PRJNA729271	stage_5W_vs_E10	ordinary_muscle_development	ENSGALG00010029367	DUSP14	-0.67969335	0.118590391	-5.731436962	9.95834E-09	2.19839E-08	negative
PRJNA729271	stage_5W_vs_E10	ordinary_muscle_development	ENSGALG00010029368	DDX52	-0.405665314	0.09299157	-4.36238804	1.2865E-05	2.27607E-05	negative
PRJNA729271	stage_5W_vs_E10	ordinary_muscle_development	ENSGALG00010029369	MFSD11	-0.173413308	0.085291537	-2.033183064	0.042034031	0.053530424	negative
PRJNA729271	stage_5W_vs_E10	ordinary_muscle_development	ENSGALG00010029370	SRSF2	-1.457551514	0.127553732	-11.42696093	3.06654E-30	1.86998E-29	negative
PRJNA729271	stage_5W_vs_E10	ordinary_muscle_development	ENSGALG00010029371	AUTS2	-2.304687859	0.179533909	-12.83706164	1.0166E-37	8.23681E-37	negative
PRJNA729271	stage_5W_vs_E10	ordinary_muscle_development	ENSGALG00010029372	METTL23	-0.754300799	0.15462291	-4.878324938	1.06991E-06	2.04973E-06	negative
PRJNA729271	stage_5W_vs_E10	ordinary_muscle_development	ENSGALG00010029373	GATSL2	1.55796048	0.168843645	9.227237905	2.777E-20	1.12218E-19	positive
PRJNA729271	stage_5W_vs_E10	ordinary_muscle_development	ENSGALG00010029374	RCC1L	-0.55309155	0.119542567	-4.626733091	3.71479E-06	6.83063E-06	negative
PRJNA729271	stage_5W_vs_E10	ordinary_muscle_development	ENSGALG00010029375	JMJD6	1.473138371	0.159818712	9.21755877	3.03941E-20	1.22719E-19	positive
PRJNA729271	stage_5W_vs_E10	ordinary_muscle_development	ENSGALG00010029376	ENSGALG00010029376	-0.033296736	0.206770121	-0.16103263	0.872067701	0.888005307	negative
PRJNA729271	stage_5W_vs_E10	ordinary_muscle_development	ENSGALG00010029377	NCF1C	2.126467558	0.287234754	7.403239081	1.32902E-13	3.86443E-13	positive
PRJNA729271	stage_5W_vs_E10	ordinary_muscle_development	ENSGALG00010029378	ST6GALNAC1	-2.917533777	0.377235858	-7.733977878	1.04237E-14	3.22708E-14	negative

PRJNA729271	stage_5W_vs_E10	ordinary_muscle_development	ENSGALG00010029379	GTF2I	0.044796853	0.112814888	0.397082815	0.691306392	0.72428523	positive
PRJNA729271	stage_5W_vs_E10	ordinary_muscle_development	ENSGALG00010029380	RGS9	0.067675816	0.149282704	0.453339968	0.650303957	0.685090185	positive
PRJNA729271	stage_5W_vs_E10	ordinary_muscle_development	ENSGALG00010029382	GTF2IRD1	0.012935397	0.206649117	0.062595948	0.950088256	0.956754847	positive
PRJNA729271	stage_5W_vs_E10	ordinary_muscle_development	ENSGALG00010029383	GNA13	0.308452835	0.078792264	3.914760408	9.0494E-05	0.000149722	positive
PRJNA729271	stage_5W_vs_E10	ordinary_muscle_development	ENSGALG00010029384	WDR81	-0.141567978	0.075385795	-1.877913177	0.060393043	0.075367814	negative
PRJNA729271	stage_5W_vs_E10	ordinary_muscle_development	ENSGALG00010029385	CLIP2	-0.780293535	0.122937537	-6.34707308	2.1945E-10	5.3425E-10	negative
PRJNA729271	stage_5W_vs_E10	ordinary_muscle_development	ENSGALG00010029386	SLC16A6	-1.396980448	0.240953104	-5.797727535	6.72195E-09	1.49784E-08	negative
PRJNA729271	stage_5W_vs_E10	ordinary_muscle_development	ENSGALG00010029387	SERPINF1	2.107394511	0.175911051	11.97988701	4.52941E-33	3.09541E-32	positive
PRJNA729271	stage_5W_vs_E10	ordinary_muscle_development	ENSGALG00010029388	ST6GALNAC2	1.612562422	0.32075147	5.027451383	4.97041E-07	9.74914E-07	positive
PRJNA729271	stage_5W_vs_E10	ordinary_muscle_development	ENSGALG00010029389	SMYD4	0.164684957	0.096130348	1.713142205	0.086686381	0.106169759	positive
PRJNA729271	stage_5W_vs_E10	ordinary_muscle_development	ENSGALG00010029390	RPA1	-0.929861678	0.080026519	-11.61941925	3.28361E-31	2.08845E-30	negative
PRJNA729271	stage_5W_vs_E10	ordinary_muscle_development	ENSGALG00010029391	ARSG	-0.122806453	0.143275681	-0.857133969	0.39137085	0.432684041	negative
PRJNA729271	stage_5W_vs_E10	ordinary_muscle_development	ENSGALG00010029392	RTN4RL1	3.630087775	0.49636326	7.313369201	2.60526E-13	7.47195E-13	positive
PRJNA729271	stage_5W_vs_E10	ordinary_muscle_development	ENSGALG00010029394	CYGB	-0.462900073	0.143066575	-3.235557105	0.001214056	0.001823222	negative
PRJNA729271	stage_5W_vs_E10	ordinary_muscle_development	ENSGALG00010029395	DPH1	-0.549786888	0.122145199	-4.501092912	6.76049E-06	1.21822E-05	negative
PRJNA729271	stage_5W_vs_E10	ordinary_muscle_development	ENSGALG00010029396	WIPI1	1.918447084	0.154167598	12.44390594	1.50938E-35	1.13773E-34	positive
PRJNA729271	stage_5W_vs_E10	ordinary_muscle_development	ENSGALG00010029397	OVCA2	-0.844423333	0.158665616	-5.322031036	1.02615E-07	2.1155E-07	negative
PRJNA729271	stage_5W_vs_E10	ordinary_muscle_development	ENSGALG00010029398	RHBDF2	0.650576284	0.123390395	5.272503453	1.34575E-07	2.75479E-07	positive
PRJNA729271	stage_5W_vs_E10	ordinary_muscle_development	ENSGALG00010029399	PRKAR1A	-0.762249125	0.053820755	-14.16273552	1.55809E-45	1.6178E-44	negative
PRJNA729271	stage_5W_vs_E10	ordinary_muscle_development	ENSGALG00010029400	AANAT	3.199084041	0.615505853	5.197487599	2.02E-07	4.07912E-07	positive
PRJNA729271	stage_5W_vs_E10	ordinary_muscle_development	ENSGALG00010029401	HIC1	-1.216873975	0.202554497	-6.007637407	1.88246E-09	4.32361E-09	negative
PRJNA729271	stage_5W_vs_E10	ordinary_muscle_development	ENSGALG00010029402	SMG6	-0.049747527	0.088977818	-0.559100323	0.576093255	0.614909553	negative
PRJNA729271	stage_5W_vs_E10	ordinary_muscle_development	ENSGALG00010029403	FAM20A	1.275837347	0.159356172	8.006199781	1.18307E-15	3.85363E-15	positive
PRJNA729271	stage_5W_vs_E10	ordinary_muscle_development	ENSGALG00010029404	SRR	-0.481460254	0.196333582	-2.452256257	0.014196352	0.019104644	negative
PRJNA729271	stage_5W_vs_E10	ordinary_muscle_development	ENSGALG00010029405	POLDIP2	0.697170801	0.079021235	8.822575342	1.11857E-18	4.20697E-18	positive
PRJNA729271	stage_5W_vs_E10	ordinary_muscle_development	ENSGALG00010029406	TMEM199	0.140362173	0.103381202	1.357714646	0.174554248	0.205153962	positive
PRJNA729271	stage_5W_vs_E10	ordinary_muscle_development	ENSGALG00010029407	UBE2O	0.270225203	0.188964885	1.43002867	0.152708791	0.181049724	positive
PRJNA729271	stage_5W_vs_E10	ordinary_muscle_development	ENSGALG00010029408	KIF12	1.93652589	0.470566011	4.115311865	3.86656E-05	6.57583E-05	positive
PRJNA729271	stage_5W_vs_E10	ordinary_muscle_development	ENSGALG00010029409	ENSGALG00010029409	2.790841678	0.189136192	14.75572522	2.82637E-49	3.34434E-48	positive
PRJNA729271	stage_5W_vs_E10	ordinary_muscle_development	ENSGALG00010029410	VTN	2.658463593	0.883367345	3.009465551	0.002617078	0.003806046	positive
PRJNA729271	stage_5W_vs_E10	ordinary_muscle_development	ENSGALG00010029411	SARM1	-3.077815049	0.249882291	-12.3170595	7.33192E-35	5.37291E-34	negative
PRJNA729271	stage_5W_vs_E10	ordinary_muscle_development	ENSGALG00010029412	SLC46A1	-0.194147403	0.113429677	-1.7116103	0.086968509	0.106452334	negative
PRJNA729271	stage_5W_vs_E10	ordinary_muscle_development	ENSGALG00010029413	B3GNTL1	-0.028034932	0.220547983	-0.127114889	0.898849468	0.911680291	negative
PRJNA729271	stage_5W_vs_E10	ordinary_muscle_development	ENSGALG00010029416	UNC119	-3.599743821	0.480959477	-7.48450544	7.18171E-14	2.12187E-13	negative
PRJNA729271	stage_5W_vs_E10	ordinary_muscle_development	ENSGALG00010029417	ENSGALG00010029417	3.498910523	0.209588417	16.69419796	1.44447E-62	2.48665E-61	positive
PRJNA729271	stage_5W_vs_E10	ordinary_muscle_development	ENSGALG00010029418	ABCA5	0.212184152	0.132775268	1.59806985	0.110027433	0.132915046	positive
PRJNA729271	stage_5W_vs_E10	ordinary_muscle_development	ENSGALG00010029419	RFC2	-0.965536116	0.13379013	-7.216796326	5.32266E-13	1.50364E-12	negative
PRJNA729271	stage_5W_vs_E10	ordinary_muscle_development	ENSGALG00010029420	LAT2	1.179324086	0.300891135	3.919437799	8.87558E-05	0.000147098	positive
PRJNA729271	stage_5W_vs_E10	ordinary_muscle_development	ENSGALG00010029421	TBCD	-0.216037887	0.093427292	-2.312363802	0.020757643	0.027367841	negative
PRJNA729271	stage_5W_vs_E10	ordinary_muscle_development	ENSGALG00010029422	EIF4H	-0.615566112	0.08742011	-7.041470312	1.90222E-12	5.22803E-12	negative
PRJNA729271	stage_5W_vs_E10	ordinary_muscle_development	ENSGALG00010029423	MYBBP1A	-1.356664782	0.122361953	-11.08730899	1.44572E-28	8.27632E-28	negative
PRJNA729271	stage_5W_vs_E10	ordinary_muscle_development	ENSGALG00010029424	MAP2K6	0.589826464	0.217815862	2.707915513	0.006770726	0.009435792	positive
PRJNA729271	stage_5W_vs_E10	ordinary_muscle_development	ENSGALG00010029425	SPNS2	2.631763704	0.162769339	16.16866988	8.389E-59	1.30191E-57	positive
PRJNA729271	stage_5W_vs_E10	ordinary_muscle_development	ENSGALG00010029426	ZNF750	-4.842163072	0.979379992	-4.944110674	7.64922E-07	1.48289E-06	negative
PRJNA729271	stage_5W_vs_E10	ordinary_muscle_development	ENSGALG00010029427	SPNS3	0.65396618	0.280310856	2.3330034	0.019647967	0.025989896	positive
PRJNA729271	stage_5W_vs_E10	ordinary_muscle_development	ENSGALG00010029428	FN3K	0.923995453	0.25608898	3.608103147	0.000308444	0.00048761	positive
PRJNA729271	stage_5W_vs_E10	ordinary_muscle_development	ENSGALG00010029429	UBE2G1	1.983336511	0.089396821	22.18576118	4.7139E-109	2.3162E-107	positive
PRJNA729271	stage_5W_vs_E10	ordinary_muscle_development	ENSGALG00010029430	FN3KRP	-1.06406984	0.099102906	-10.7370196	6.82109E-27	3.66056E-26	negative
PRJNA729271	stage_5W_vs_E10	ordinary_muscle_development	ENSGALG00010029432	ENSGALG00010029432	-21.69924661	5.492799762	-3.950489286			negative
PRJNA729271	stage_5W_vs_E10	ordinary_muscle_development	ENSGALG00010029436	WDR45B	-0.401258739	0.080408246	-4.990268527	6.02954E-07	1.17708E-06	negative
PRJNA729271	stage_5W_vs_E10	ordinary_muscle_development	ENSGALG00010029437	ENSGALG00010029437	-0.373634403	2.870557307	-0.130160928	0.896439106	0.909553748	negative
PRJNA729271	stage_5W_vs_E10	ordinary_muscle_development	ENSGALG00010029438	ENSGALG00010029438	-1.171781385	0.270800607	-4.327100283	1.51085E-05	2.65774E-05	negative
PRJNA729271	stage_5W_vs_E10	ordinary_muscle_development	ENSGALG00010029439	ENSGALG00010029439	-1.953596256	0.216345659	-9.029976677	1.71708E-19	6.70324E-19	negative
PRJNA729271	stage_5W_vs_E10	ordinary_muscle_development	ENSGALG00010029440	FOXK2	0.698861232	0.181553446	3.849341608	0.000118436	0.000193674	positive
PRJNA729271	stage_5W_vs_E10	ordinary_muscle_development	ENSGALG00010029442	ENSGALG00010029442	-0.727725927	0.283353238	-2.568264021	0.010220928	0.013980593	negative
PRJNA729271	stage_5W_vs_E10	ordinary_muscle_development	ENSGALG00010029443	ENSGALG00010029443	-0.898744675	0.318307572	-2.823510194	0.004750092	0.006722571	negative
PRJNA729271	stage_5W_vs_E10	ordinary_muscle_development	ENSGALG00010029445	SPHK1	0.048913947	0.138467378	0.353252499	0.723899153	0.754072456	positive
PRJNA729271	stage_5W_vs_E10	ordinary_muscle_development	ENSGALG00010029446	PRPSAP1	0.177371789	0.080703514	2.197819891	0.027961943	0.036386423	positive
PRJNA729271	stage_5W_vs_E10	ordinary_muscle_development	ENSGALG00010029449	UBALD2	1.519376572	0.181534192	8.36964406	5.77925E-17	2.00792E-16	positive
PRJNA729271	stage_5W_vs_E10	ordinary_muscle_development	ENSGALG00010029451	ENSGALG00010029451	-1.951076911	0.223532817	-8.728369012	2.58371E-18	9.56357E-18	negative
PRJNA729271	stage_5W_vs_E10	ordinary_muscle_development	ENSGALG00010029452	ENSGALG00010029452	-0.629578869	0.961895145	-0.65451923	0.512777342	0.553399418	negative
PRJNA729271	stage_5W_vs_E10	ordinary_muscle_development	ENSGALG00010029453	ENSGALG00010029453	-1.888828632	1.548487412	-1.219789465	0.222544696	0.256707415	negative
PRJNA729271	stage_5W_vs_E10	ordinary_muscle_development	ENSGALG00010029454	ENSGALG00010029454	-0.752243085	0.291043905	-2.58463782	0.009748134	0.013370477	negative
PRJNA729271	stage_5W_vs_E10	ordinary_muscle_development	ENSGALG00010029455	ENSGALG00010029455	-1.228218445	0.259553713	-4.732039582	2.22275E-06	4.15672E-06	negative
PRJNA729271	stage_5W_vs_E10	ordinary_muscle_development	ENSGALG00010029456	RNF157	-2.819768042	0.526910522	-5.351512111	8.72223E-08	1.80561E-07	negative
PRJNA729271	stage_5W_vs_E10	ordinary_muscle_development	ENSGALG00010029457	ENSGALG00010029457	-1.480113934	0.191978387	-7.709794615	1.2602E-14	3.8758E-14	negative
PRJNA729271	stage_5W_vs_E10	ordinary_muscle_development	ENSGALG00010029460	ENSGALG00010029460	-1.29886698	0.283904375	-4.575015721	4.76184E-06	8.67776E-06	negative
PRJNA729271	stage_5W_vs_E10	ordinary_muscle_development	ENSGALG00010029461	ENSGALG00010029461	-0.713477402	0.203289381	-3.509663892	0.000448673	0.000698928	negative
PRJNA729271	stage_5W_vs_E10	ordinary_muscle_development	ENSGALG00010029462	ENSGALG00010029462	2.049053357	1.804993115	1.135213946	0.256285688	0.292485713	positive
PRJNA729271	stage_5W_vs_E10	ordinary_muscle_development	ENSGALG00010029463	ENSGALG00010029463	-0.019280904	0.426963859	-0.045158163	0.963981241	0.968860636	negative
PRJNA729271	stage_5W_vs_E10	ordinary_muscle_development	ENSGALG00010029464	ENSGALG00010029464	-0.118635391	0.558210799	-0.21252794	0.831695183	0.85185286	negative
PRJNA729271	stage_5W_vs_E10	ordinary_muscle_development	ENSGALG00010029465	ENSGALG00010029465	-0.597647457	2.374732045	-0.251669429	0.801296589	0.824972942	negative
PRJNA729271	stage_5W_vs_E10	ordinary_muscle_development	ENSGALG00010029466	SLC39A11	-1.351779626	0.125258767	-10.79189635	3.75951E-27	2.04021E-26	negative
PRJNA729271	stage_5W_vs_E10	ordinary_muscle_development	ENSGALG00010029468	ENSGALG00010029468	-2.16874328	0.242707307	-8.935632431	4.04856E-19	1.5533E-18	negative
PRJNA729271	stage_5W_vs_E10	ordinary_muscle_development	ENSGALG00010029469	ENSGALG00010029469	-2.79270865	0.417355613	-6.691436666	2.2099E-11	5.73751E-11	negative
PRJNA729271	stage_5W_vs_E10	ordinary_muscle_development	ENSGALG00010029470	SSTR2	0.273871016	0.919914557	0.297713536	0.765921811	0.793512733	positive
PRJNA729271	stage_5W_vs_E10	ordinary_muscle_development	ENSGALG00010029472	ENSGALG00010029472	-1.901603033	0.848758836	-2.240451531	0.025061624	0.032762287	negative
PRJNA729271	stage_5W_vs_E10	ordinary_muscle_development	ENSGALG00010029473	COG1	1.113244635	0.100292221	11.10000982	1.2543E-28	7.19473E-28	positive
PRJNA729271	stage_5W_vs_E10	ordinary_muscle_development	ENSGALG00010029474	ENSGALG00010029474	-0.167175565	1.050358195	-0.159160528	0.873542401	0.88918519	negative
PRJNA729271	stage_5W_vs_E10	ordinary_muscle_development	ENSGALG00010029477	FAM104A	-0.090667386	0.058537139	-1.548886532	0.121408999	0.14577729	negative
PRJNA729271	stage_5W_vs_E10	ordinary_muscle_development	ENSGALG00010029479	ENSGALG00010029479	-2.113065006	0.558764413	-3.781674272	0.000155777	0.000252205	negative
PRJNA729271	stage_5W_vs_E10	ordinary_muscle_development	ENSGALG00010029480	ENSGALG00010029480	0.460950116	0.116427184	3.959127915	7.52239E-05	0.000125416	positive
PRJNA729271	stage_5W_vs_E10	ordinary_muscle_development	ENSGALG00010029481	ENSGALG00010029481	-1.634198997	0.281189163	-5.811742453	6.18259E-09	1.38169E-08	negative
PRJNA729271	stage_5W_vs_E10	ordinary_muscle_development	ENSGALG00010029482	DDX25	-0.652445509	0.189713221	-3.439114603	0.00058362	0.000902066	negative
PRJNA729271	stage_5W_vs_E10	ordinary_muscle_development	ENSGALG00010029484	ENSGALG00010029484	-2.507168499	0.655339446	-3.825755515	0.000130372	0.000212473	negative
PRJNA729271	stage_5W_vs_E10	ordinary_muscle_development	ENSGALG00010029487	CDC42EP4	-2.12323519	0.148366518	-14.31074352	1.87498E-46	1.9969E-45	negative
PRJNA729271	stage_5W_vs_E10	ordinary_muscle_development	ENSGALG00010029489	ENSGALG00010029489	-2.246175443	1.024239128	-2.193018585	0.02830604	0.036817664	negative
PRJNA729271	stage_5W_vs_E10	ordinary_muscle_development	ENSGALG00010029490	ENSGALG00010029490	-1.286868611	0.641282812	-2.006709967	0.044780554	0.056813491	negative
PRJNA729271	stage_5W_vs_E10	ordinary_muscle_development	ENSGALG00010029491	ENSGALG00010029491	-0.332495101	0.199640337	-1.665470544	0.095818916	0.116713881	negative
PRJNA729271	stage_5W_vs_E10	ordinary_muscle_development	ENSGALG00010029492	SDK2	-1.583650159	0.2306526	-6.865954067	6.60483E-12	1.76279E-11	negative
PRJNA729271	stage_5W_vs_E10	ordinary_muscle_development	ENSGALG00010029494	MYH7	-6.742772058	0.614193253	-10.97825811	4.86208E-28	2.72951E-27	negative
PRJNA729271	stage_5W_vs_E10	ordinary_muscle_development	ENSGALG00010029495	RPL38	-0.003109526	0.188657631	-0.016482376	0.986849562	0.9888279	negative
PRJNA729271	stage_5W_vs_E10	ordinary_muscle_development	ENSGALG00010029496	TTYH2	-1.038598543	0.14787688	-7.023400433	2.16532E-12	5.92867E-12	negative
PRJNA729271	stage_5W_vs_E10	ordinary_muscle_development	ENSGALG00010029497	ENSGALG00010029497	-0.16960205	0.571884679	-0.296566871	0.766797206	0.794306118	negative
PRJNA729271	stage_5W_vs_E10	ordinary_muscle_development	ENSGALG00010029498	ENSGALG00010029498	-0.346804481	0.149732298	-2.316163487	0.020549344	0.027115407	negative
PRJNA729271	stage_5W_vs_E10	ordinary_muscle_development	ENSGALG00010029501	ENSGALG00010029501	-1.158144184	0.39515666	-2.930848197	0.003380379	0.004859526	negative
PRJNA729271	stage_5W_vs_E10	ordinary_muscle_development	ENSGALG00010029502	BTBD17	-1.690348651	0.66155686	-2.555107131	0.010615512	0.014495699	negative
PRJNA729271	stage_5W_vs_E10	ordinary_muscle_development	ENSGALG00010029504	GPR142	-3.225246888	0.662361077	-4.869318264	1.11984E-06	2.14059E-06	negative
PRJNA729271	stage_5W_vs_E10	ordinary_muscle_development	ENSGALG00010029505	VEZF1	0.33480216	0.109798929	3.049229731	0.00229429	0.003353068	positive
PRJNA729271	stage_5W_vs_E10	ordinary_muscle_development	ENSGALG00010029506	OXLD1	-1.608042762	0.17729939	-9.06964634	1.19404E-19	4.70443E-19	negative
PRJNA729271	stage_5W_vs_E10	ordinary_muscle_development	ENSGALG00010029507	VPS37D	-0.516924015	0.176450608	-2.929567775	0.003394338	0.004879108	negative
PRJNA729271	stage_5W_vs_E10	ordinary_muscle_development	ENSGALG00010029508	ENSGALG00010029508	-1.578574486	0.156693116	-10.07430653	7.17652E-24	3.39171E-23	negative
PRJNA729271	stage_5W_vs_E10	ordinary_muscle_development	ENSGALG00010029509	DNAJC30	0.672437132	0.170813405	3.936676577	8.26178E-05	0.000137317	positive
PRJNA729271	stage_5W_vs_E10	ordinary_muscle_development	ENSGALG00010029510	PDE6G	-1.289280757	0.333047281	-3.871164341	0.000108317	0.000177728	negative
PRJNA729271	stage_5W_vs_E10	ordinary_muscle_development	ENSGALG00010029511	CUEDC1	-0.097619371	0.07406245	-1.318068339	0.187480772	0.219279738	negative
PRJNA729271	stage_5W_vs_E10	ordinary_muscle_development	ENSGALG00010029512	TSPAN10	-0.954743583	0.680923785	-1.402129876	0.160876467	0.19018876	negative
PRJNA729271	stage_5W_vs_E10	ordinary_muscle_development	ENSGALG00010029513	WBSCR22	-0.531667202	0.087484944	-6.077242321	1.22267E-09	2.84335E-09	negative
PRJNA729271	stage_5W_vs_E10	ordinary_muscle_development	ENSGALG00010029514	MRPS23	0.032076823	0.098642472	0.325182675	0.74504282	0.773484865	positive
PRJNA729271	stage_5W_vs_E10	ordinary_muscle_development	ENSGALG00010029515	NPLOC4	0.897961145	0.152399426	5.89215567	3.8119E-09	8.61854E-09	positive
PRJNA729271	stage_5W_vs_E10	ordinary_muscle_development	ENSGALG00010029516	ENSGALG00010029516	3.270495737	0.227698299	14.36328578	8.79562E-47	9.52859E-46	positive
PRJNA729271	stage_5W_vs_E10	ordinary_muscle_development	ENSGALG00010029517	MSI2	0.592582532	0.112057112	5.288218863	1.23513E-07	2.53371E-07	positive
PRJNA729271	stage_5W_vs_E10	ordinary_muscle_development	ENSGALG00010029518	FAAP100	-0.553612918	0.188201081	-2.941603288	0.003265179	0.004700444	negative
PRJNA729271	stage_5W_vs_E10	ordinary_muscle_development	ENSGALG00010029519	STX1A	-1.037845764	0.12109979	-8.570169815	1.03333E-17	3.72588E-17	negative
PRJNA729271	stage_5W_vs_E10	ordinary_muscle_development	ENSGALG00010029520	MKS1	-0.43950494	0.111502436	-3.941662234	8.09189E-05	0.000134586	negative
PRJNA729271	stage_5W_vs_E10	ordinary_muscle_development	ENSGALG00010029521	ACTG1	-0.651058862	0.098406178	-6.616036497	3.68957E-11	9.46388E-11	negative
PRJNA729271	stage_5W_vs_E10	ordinary_muscle_development	ENSGALG00010029522	AKAP1	0.776004556	0.112160429	6.918701727	4.55801E-12	1.22599E-11	positive
PRJNA729271	stage_5W_vs_E10	ordinary_muscle_development	ENSGALG00010029523	FSCN2	2.364377317	0.672833658	3.514059214	0.000441314	0.000688056	positive
PRJNA729271	stage_5W_vs_E10	ordinary_muscle_development	ENSGALG00010029524	ENSGALG00010029524	0.533164828	0.148346282	3.594055895	0.00032557	0.00051362	positive
PRJNA729271	stage_5W_vs_E10	ordinary_muscle_development	ENSGALG00010029525	SLC38A10	-1.116808062	0.13867913	-8.053180482	8.06698E-16	2.64789E-15	negative
PRJNA729271	stage_5W_vs_E10	ordinary_muscle_development	ENSGALG00010029526	BCL7B	0.837608824	0.11228282	7.459812837	8.66455E-14	2.54752E-13	positive
PRJNA729271	stage_5W_vs_E10	ordinary_muscle_development	ENSGALG00010029527	NDUFAF8	0.791503096	0.154031828	5.13568494	2.76839E-07	5.52649E-07	positive
PRJNA729271	stage_5W_vs_E10	ordinary_muscle_development	ENSGALG00010029528	TBL2	-0.431885071	0.097817361	-4.415219022	1.00908E-05	1.79865E-05	negative
PRJNA729271	stage_5W_vs_E10	ordinary_muscle_development	ENSGALG00010029529	TEPSIN	0.71787395	0.128940905	5.56746482	2.58472E-08	5.55704E-08	positive
PRJNA729271	stage_5W_vs_E10	ordinary_muscle_development	ENSGALG00010029530	ENSGALG00010029530	1.982690122	0.363580454	5.453236271	4.94613E-08	1.04222E-07	positive
PRJNA729271	stage_5W_vs_E10	ordinary_muscle_development	ENSGALG00010029531	CEP131	-0.938491074	0.172443436	-5.442312539	5.25933E-08	1.10516E-07	negative
PRJNA729271	stage_5W_vs_E10	ordinary_muscle_development	ENSGALG00010029532	EVI2A	4.274867762	0.447001015	9.563440839	1.13905E-21	4.90074E-21	positive
PRJNA729271	stage_5W_vs_E10	ordinary_muscle_development	ENSGALG00010029533	MLXIPL	-0.436751766	0.763726797	-0.571869113	0.56741065	0.606419223	negative
PRJNA729271	stage_5W_vs_E10	ordinary_muscle_development	ENSGALG00010029534	NF1	-0.191746823	0.090965023	-2.107918153	0.03503807	0.045112526	negative
PRJNA729271	stage_5W_vs_E10	ordinary_muscle_development	ENSGALG00010029536	BAZ1B	-0.599524483	0.077631755	-7.722670731	1.13917E-14	3.51325E-14	negative
PRJNA729271	stage_5W_vs_E10	ordinary_muscle_development	ENSGALG00010029537	MYH1B	4.687326898	0.206075165	22.74571465	1.5822E-114	8.7537E-113	positive
PRJNA729271	stage_5W_vs_E10	ordinary_muscle_development	ENSGALG00010029538	FZD9	0.521375151	0.172987183	3.013952491	0.002578682	0.003754318	positive

PRJNA729271	stage_5W_vs_E10	ordinary_muscle_development	ENSGALG00010029539	FKBP6	-1.922659582	0.405090688	-4.746244822	2.07228E-06	3.88234E-06	negative
PRJNA729271	stage_5W_vs_E10	ordinary_muscle_development	ENSGALG00010029541	ENSGALG00010029541	1.660626897	0.219807378	7.554918831	4.19121E-14	1.25572E-13	positive
PRJNA729271	stage_5W_vs_E10	ordinary_muscle_development	ENSGALG00010029542	AATK	1.631904531	0.14219439	11.47657462	1.72999E-30	1.06662E-29	positive
PRJNA729271	stage_5W_vs_E10	ordinary_muscle_development	ENSGALG00010029543	NSUN5	-0.06804706	0.135921887	-0.500633571	0.616629032	0.653365337	negative
PRJNA729271	stage_5W_vs_E10	ordinary_muscle_development	ENSGALG00010029544	ENSGALG00010029544	-0.728774073	0.178435516	-4.084243375	4.42207E-05	7.48719E-05	negative
PRJNA729271	stage_5W_vs_E10	ordinary_muscle_development	ENSGALG00010029545	OMG	4.272143618	0.290663802	14.69788667	6.6503E-49	7.76136E-48	positive
PRJNA729271	stage_5W_vs_E10	ordinary_muscle_development	ENSGALG00010029546	WSB1	0.093101832	0.165393512	0.562911029	0.573495469	0.612362197	positive
PRJNA729271	stage_5W_vs_E10	ordinary_muscle_development	ENSGALG00010029547	HIP1	-0.628681055	0.101194324	-6.212611827	5.21111E-10	1.23868E-09	negative
PRJNA729271	stage_5W_vs_E10	ordinary_muscle_development	ENSGALG00010029548	BAIAP2	1.27194797	0.219733122	5.788603722	7.09739E-09	1.58053E-08	positive
PRJNA729271	stage_5W_vs_E10	ordinary_muscle_development	ENSGALG00010029549	KSR1	1.43200535	0.124647169	11.48847072	1.50757E-30	9.33059E-30	positive
PRJNA729271	stage_5W_vs_E10	ordinary_muscle_development	ENSGALG00010029550	CHMP6	0.653238406	0.239499958	2.727509483	0.006381442	0.008914708	positive
PRJNA729271	stage_5W_vs_E10	ordinary_muscle_development	ENSGALG00010029552	NOS2	-0.116230166	0.1295411	-0.897245477	0.369587943	0.410323012	negative
PRJNA729271	stage_5W_vs_E10	ordinary_muscle_development	ENSGALG00010029553	MYH1C	6.568240029	0.194786865	33.72013838	2.9302E-249	8.4945E-247	positive
PRJNA729271	stage_5W_vs_E10	ordinary_muscle_development	ENSGALG00010029554	ENSGALG00010029554	9.517476385	0.364902644	26.08223464	5.7995E-150	5.7975E-148	positive
PRJNA729271	stage_5W_vs_E10	ordinary_muscle_development	ENSGALG00010029555	LYRM9	0.682913602	0.157021074	4.349184382	1.36645E-05	2.41339E-05	positive
PRJNA729271	stage_5W_vs_E10	ordinary_muscle_development	ENSGALG00010029556	NPTX1	2.226270218	0.56121068	3.966906365	7.28116E-05	0.000121562	positive
PRJNA729271	stage_5W_vs_E10	ordinary_muscle_development	ENSGALG00010029557	ENSGALG00010029557	3.707338803	0.553031643	6.703664878	2.03256E-11	5.28656E-11	positive
PRJNA729271	stage_5W_vs_E10	ordinary_muscle_development	ENSGALG00010029558	ENSGALG00010029558	-2.986426413	0.97499309	-3.063023157	0.002191131	0.003207154	negative
PRJNA729271	stage_5W_vs_E10	ordinary_muscle_development	ENSGALG00010029560	ENSGALG00010029560	4.385642563	0.166942054	26.27044812	4.1747E-152	4.2916E-150	positive
PRJNA729271	stage_5W_vs_E10	ordinary_muscle_development	ENSGALG00010029561	SCO1	-0.078811981	0.104525919	-0.753994622	0.450852451	0.492769286	negative
PRJNA729271	stage_5W_vs_E10	ordinary_muscle_development	ENSGALG00010029562	ENSGALG00010029562	4.732739335	0.675334837	7.007989336	2.41767E-12	6.60214E-12	positive
PRJNA729271	stage_5W_vs_E10	ordinary_muscle_development	ENSGALG00010029563	ADPRM	0.40086333	0.127229356	3.150714128	0.001628718	0.00241591	positive
PRJNA729271	stage_5W_vs_E10	ordinary_muscle_development	ENSGALG00010029564	NLK	0.454021158	0.087948839	5.16233258	2.43891E-07	4.88896E-07	positive
PRJNA729271	stage_5W_vs_E10	ordinary_muscle_development	ENSGALG00010029566	TMEM97	-0.666051303	0.147071866	-4.528747207	5.93344E-06	1.07372E-05	negative
PRJNA729271	stage_5W_vs_E10	ordinary_muscle_development	ENSGALG00010029567	IFT20	-1.16988217	0.121800908	-9.604872316	7.62524E-22	3.31517E-21	negative
PRJNA729271	stage_5W_vs_E10	ordinary_muscle_development	ENSGALG00010029568	ENSGALG00010029568	-1.731637439	1.066848643	-1.623133188	0.104560936	0.126670353	negative
PRJNA729271	stage_5W_vs_E10	ordinary_muscle_development	ENSGALG00010029569	TMEM220	0.83934448	0.204167684	4.111054526	3.93856E-05	6.69514E-05	positive
PRJNA729271	stage_5W_vs_E10	ordinary_muscle_development	ENSGALG00010029570	TNFAIP1	-0.787302706	0.079898141	-9.853830132	6.59806E-23	3.00279E-22	negative
PRJNA729271	stage_5W_vs_E10	ordinary_muscle_development	ENSGALG00010029571	EPX	-0.529181733	0.783164191	-0.675697049	0.49923301	0.540402582	negative
PRJNA729271	stage_5W_vs_E10	ordinary_muscle_development	ENSGALG00010029572	TSPOAP1	0.419459998	0.116955094	3.586504743	0.00033514	0.000528257	positive
PRJNA729271	stage_5W_vs_E10	ordinary_muscle_development	ENSGALG00010029573	MAP2K4	-0.406900116	0.061511077	-6.61507059	3.71374E-11	9.52419E-11	negative
PRJNA729271	stage_5W_vs_E10	ordinary_muscle_development	ENSGALG00010029574	SGSM2	-0.042128219	0.131819331	-0.319590603	0.749278699	0.777548306	negative
PRJNA729271	stage_5W_vs_E10	ordinary_muscle_development	ENSGALG00010029575	ANKFN1	-3.6828758	0.704400888	-5.228380404	1.71001E-07	3.47055E-07	negative
PRJNA729271	stage_5W_vs_E10	ordinary_muscle_development	ENSGALG00010029576	ATP2A3	2.068427248	0.129998515	15.91116057	5.30214E-57	7.82633E-56	positive
PRJNA729271	stage_5W_vs_E10	ordinary_muscle_development	ENSGALG00010029577	NOG	-7.013475644	0.712163408	-9.848126934	6.98332E-23	3.17513E-22	negative
PRJNA729271	stage_5W_vs_E10	ordinary_muscle_development	ENSGALG00010029578	C17orf67	-8.098101127	0.972045042	-8.330993706	8.01666E-17	2.76276E-16	negative
PRJNA729271	stage_5W_vs_E10	ordinary_muscle_development	ENSGALG00010029579	GALNT17	-0.19679833	0.18454543	-1.06639503	0.286245107	0.323998347	negative
PRJNA729271	stage_5W_vs_E10	ordinary_muscle_development	ENSGALG00010029580	P2RX1	2.197838402	0.326551891	6.730441513	1.69149E-11	4.42247E-11	positive
PRJNA729271	stage_5W_vs_E10	ordinary_muscle_development	ENSGALG00010029581	DGKE	0.004072811	0.105498108	0.038605534	0.96920489	0.973503214	positive
PRJNA729271	stage_5W_vs_E10	ordinary_muscle_development	ENSGALG00010029582	SCPEP1	0.51110571	0.14997371	3.407968711	0.000654484	0.00100762	positive
PRJNA729271	stage_5W_vs_E10	ordinary_muscle_development	ENSGALG00010029583	LIMK1	-1.051703255	0.206768929	-5.086369899	3.64982E-07	7.22241E-07	negative
PRJNA729271	stage_5W_vs_E10	ordinary_muscle_development	ENSGALG00010029584	TRIM25	1.592211636	0.232774363	6.840150342	7.91101E-12	2.10288E-11	positive
PRJNA729271	stage_5W_vs_E10	ordinary_muscle_development	ENSGALG00010029585	ENSGALG00010029585	1.316647198	0.347080814	3.793488847	0.000148545	0.0002409	positive
PRJNA729271	stage_5W_vs_E10	ordinary_muscle_development	ENSGALG00010029586	COIL	-0.280669565	0.189005282	-1.484982651	0.13754842	0.164072193	negative
PRJNA729271	stage_5W_vs_E10	ordinary_muscle_development	ENSGALG00010029588	RAB11FIP4	-2.91116435	0.151547545	-19.20957773	3.07759E-82	8.69584E-81	negative
PRJNA729271	stage_5W_vs_E10	ordinary_muscle_development	ENSGALG00010029590	UTP6	-0.399150861	0.098848263	-4.038015922	5.39052E-05	9.07814E-05	negative
PRJNA729271	stage_5W_vs_E10	ordinary_muscle_development	ENSGALG00010029591	SUZ12	-1.197054827	0.081494368	-14.68880439	7.60437E-49	8.83924E-48	negative
PRJNA729271	stage_5W_vs_E10	ordinary_muscle_development	ENSGALG00010029592	MNT	0.469506993	0.167050075	2.81057636	0.004945285	0.006987222	positive
PRJNA729271	stage_5W_vs_E10	ordinary_muscle_development	ENSGALG00010029593	PAFAH1B1	0.297731305	0.078961103	3.770607206	0.000162851	0.000263392	positive
PRJNA729271	stage_5W_vs_E10	ordinary_muscle_development	ENSGALG00010029594	CRLF3	-0.829516187	0.068690452	-12.07614971	1.41177E-33	9.83828E-33	negative
PRJNA729271	stage_5W_vs_E10	ordinary_muscle_development	ENSGALG00010029595	CLUH	0.903420463	0.135072536	6.688409733	2.25609E-11	5.85113E-11	positive
PRJNA729271	stage_5W_vs_E10	ordinary_muscle_development	ENSGALG00010029596	ATAD5	-1.910126089	0.132903204	-14.37231027	7.7212E-47	8.38343E-46	negative
PRJNA729271	stage_5W_vs_E10	ordinary_muscle_development	ENSGALG00010029597	ADAP2	1.734366388	0.204401392	8.485100688	2.15532E-17	7.64034E-17	positive
PRJNA729271	stage_5W_vs_E10	ordinary_muscle_development	ENSGALG00010029598	SUPT4H1	-0.338062255	0.150214776	-2.250525973	0.024415577	0.03196674	negative
PRJNA729271	stage_5W_vs_E10	ordinary_muscle_development	ENSGALG00010029599	RNF43	-3.098546701	0.42377424	-7.311786338	2.63614E-13	7.55753E-13	negative
PRJNA729271	stage_5W_vs_E10	ordinary_muscle_development	ENSGALG00010029600	RHOT1	0.959790132	0.108838546	8.818476259	1.16028E-18	4.35705E-18	positive
PRJNA729271	stage_5W_vs_E10	ordinary_muscle_development	ENSGALG00010029601	HSF5	-2.79499411	0.547211448	-5.107704018	3.26097E-07	6.48036E-07	negative
PRJNA729271	stage_5W_vs_E10	ordinary_muscle_development	ENSGALG00010029602	MTMR4	-0.87562899	0.088206531	-9.927031207	3.1757E-23	1.46225E-22	negative
PRJNA729271	stage_5W_vs_E10	ordinary_muscle_development	ENSGALG00010029603	RHBDL3	-0.087692157	0.43405638	-0.202029416	0.839893728	0.859217982	negative
PRJNA729271	stage_5W_vs_E10	ordinary_muscle_development	ENSGALG00010029604	ENSGALG00010029604	0.38486878	0.11853315	3.246929501	0.001166573	0.001756099	positive
PRJNA729271	stage_5W_vs_E10	ordinary_muscle_development	ENSGALG00010029605	C17orf75	-0.894691435	0.094591386	-9.458487401	3.1243E-21	1.31954E-20	negative
PRJNA729271	stage_5W_vs_E10	ordinary_muscle_development	ENSGALG00010029606	ABHD11	0.67896815	0.213724695	3.176835274	0.001488916	0.002216249	positive
PRJNA729271	stage_5W_vs_E10	ordinary_muscle_development	ENSGALG00010029607	ZNF207	-0.572861373	0.065309944	-8.771426459	1.76417E-18	6.58723E-18	negative
PRJNA729271	stage_5W_vs_E10	ordinary_muscle_development	ENSGALG00010029608	ENSGALG00010029608	-3.351629139	0.377121	-8.887410505	6.25496E-19	2.3803E-18	negative
PRJNA729271	stage_5W_vs_E10	ordinary_muscle_development	ENSGALG00010029609	METTL27	-1.224262886	0.159424079	-7.679284653	1.59979E-14	4.8922E-14	negative
PRJNA729271	stage_5W_vs_E10	ordinary_muscle_development	ENSGALG00010029610	ERN1	-0.144638265	0.080352714	-1.800042062	0.071853997	0.08892791	negative
PRJNA729271	stage_5W_vs_E10	ordinary_muscle_development	ENSGALG00010029611	YWHAG	0.871200538	0.130683004	6.666517561	2.61944E-11	6.76686E-11	positive
PRJNA729271	stage_5W_vs_E10	ordinary_muscle_development	ENSGALG00010029612	HSPB1	2.671047798	0.195430306	13.66752096	1.58711E-42	1.49288E-41	positive
PRJNA729271	stage_5W_vs_E10	ordinary_muscle_development	ENSGALG00010029614	MDH2	1.301027495	0.12854387	10.1212722	4.44599E-24	2.12338E-23	positive
PRJNA729271	stage_5W_vs_E10	ordinary_muscle_development	ENSGALG00010029615	ENSGALG00010029615	-0.829124719	0.29947722	-2.768573576	0.005630227	0.007907193	negative
PRJNA729271	stage_5W_vs_E10	ordinary_muscle_development	ENSGALG00010029616	TMEM120A	0.746993593	0.136314257	5.479937381	4.25476E-08	8.99939E-08	positive
PRJNA729271	stage_5W_vs_E10	ordinary_muscle_development	ENSGALG00010029617	POR	-0.243606224	0.09532355	-2.555572311	0.010601333	0.014479066	negative
PRJNA729271	stage_5W_vs_E10	ordinary_muscle_development	ENSGALG00010029618	TEX2	1.223818394	0.112592689	10.86943035	1.61204E-27	8.86103E-27	positive
PRJNA729271	stage_5W_vs_E10	ordinary_muscle_development	ENSGALG00010029619	ENSGALG00010029619	0.446068682	0.104152063	4.282859789	1.84506E-05	3.22258E-05	positive
PRJNA729271	stage_5W_vs_E10	ordinary_muscle_development	ENSGALG00010029620	ENSGALG00010029620	0.558049282	0.39673957	1.406588415	0.15954947	0.188681535	positive
PRJNA729271	stage_5W_vs_E10	ordinary_muscle_development	ENSGALG00010029621	TAF15	-2.031188252	0.123093409	-16.5011942	3.59708E-61	5.99996E-60	negative
PRJNA729271	stage_5W_vs_E10	ordinary_muscle_development	ENSGALG00010029622	MMP28	0.016390427	0.158208585	0.10360011	0.9174867	0.928699003	positive
PRJNA729271	stage_5W_vs_E10	ordinary_muscle_development	ENSGALG00010029623	ENSGALG00010029623	0.667144979	0.389585085	1.712449998	0.086813772	0.106289858	positive
PRJNA729271	stage_5W_vs_E10	ordinary_muscle_development	ENSGALG00010029624	RASL10B	-2.841382902	0.354481055	-8.015612856	1.09589E-15	3.57528E-15	negative
PRJNA729271	stage_5W_vs_E10	ordinary_muscle_development	ENSGALG00010029625	PECAM1	2.448736515	0.174308413	14.0482979	7.89078E-45	8.00959E-44	positive
PRJNA729271	stage_5W_vs_E10	ordinary_muscle_development	ENSGALG00010029626	AP2B1	-0.50126657	0.109237039	-4.588796775	4.45808E-06	8.14365E-06	negative
PRJNA729271	stage_5W_vs_E10	ordinary_muscle_development	ENSGALG00010029627	PEX12	-0.405666776	0.14878744	-2.72648535	0.006401279	0.008940696	negative
PRJNA729271	stage_5W_vs_E10	ordinary_muscle_development	ENSGALG00010029628	UNC45B	1.815718682	0.160479438	11.31433846	1.1144E-29	6.65562E-29	positive
PRJNA729271	stage_5W_vs_E10	ordinary_muscle_development	ENSGALG00010029629	ENSGALG00010029629	2.516359083	0.25537545	9.853566919	6.61536E-23	3.00972E-22	positive
PRJNA729271	stage_5W_vs_E10	ordinary_muscle_development	ENSGALG00010029630	RAD51D	0.022472237	0.121678949	0.184684675	0.853476367	0.871176332	positive
PRJNA729271	stage_5W_vs_E10	ordinary_muscle_development	ENSGALG00010029631	LIG3	-0.575828097	0.087974361	-6.54540815	5.93331E-11	1.49832E-10	negative
PRJNA729271	stage_5W_vs_E10	ordinary_muscle_development	ENSGALG00010029632	TMEM132E	2.850034239	0.224423153	12.69937707	5.96022E-37	4.72612E-36	positive
PRJNA729271	stage_5W_vs_E10	ordinary_muscle_development	ENSGALG00010029633	POLG2	-0.596973681	0.178801305	-3.338754603	0.000841549	0.001283216	negative
PRJNA729271	stage_5W_vs_E10	ordinary_muscle_development	ENSGALG00010029634	DDX5	-0.533313291	0.091219804	-5.846463889	5.02133E-09	1.12879E-08	negative
PRJNA729271	stage_5W_vs_E10	ordinary_muscle_development	ENSGALG00010029635	ENSGALG00010029635	4.088245382	0.574039968	7.121882809	1.06463E-12	2.96625E-12	positive
PRJNA729271	stage_5W_vs_E10	ordinary_muscle_development	ENSGALG00010029636	SPATA20	0.671124709	0.076032137	8.826855769	1.07659E-18	4.05542E-18	positive
PRJNA729271	stage_5W_vs_E10	ordinary_muscle_development	ENSGALG00010029638	EPN3	2.232458285	0.195244362	11.43417544	2.82205E-30	1.72307E-29	positive
PRJNA729271	stage_5W_vs_E10	ordinary_muscle_development	ENSGALG00010029639	ENSGALG00010029639	-0.404012834	0.760469297	-0.531267779	0.59523323	0.633426744	negative
PRJNA729271	stage_5W_vs_E10	ordinary_muscle_development	ENSGALG00010029641	ENSGALG00010029641	-0.291926229	0.722902893	-0.403824955	0.686341444	0.719551514	negative
PRJNA729271	stage_5W_vs_E10	ordinary_muscle_development	ENSGALG00010029642	NAT9	1.26388637	0.144461678	8.74893874	2.15369E-18	8.00658E-18	positive
PRJNA729271	stage_5W_vs_E10	ordinary_muscle_development	ENSGALG00010029644	MYCBPAP	0.020755151	0.169146543	0.122705142	0.902340592	0.914773177	positive
PRJNA729271	stage_5W_vs_E10	ordinary_muscle_development	ENSGALG00010029645	ENSGALG00010029645	-3.7501573	0.42563104	-8.810817224	1.24237E-18	4.66049E-18	negative
PRJNA729271	stage_5W_vs_E10	ordinary_muscle_development	ENSGALG00010029646	MRPS17	0.234017505	0.108558489	2.155681309	0.031108559	0.040307371	positive
PRJNA729271	stage_5W_vs_E10	ordinary_muscle_development	ENSGALG00010029647	RSAD1	0.6322316	0.156064233	4.051098628	5.09777E-05	8.59311E-05	positive
PRJNA729271	stage_5W_vs_E10	ordinary_muscle_development	ENSGALG00010029648	NIPSNAP2	0.872861497	0.121947657	7.157673352	8.20578E-13	2.29974E-12	positive
PRJNA729271	stage_5W_vs_E10	ordinary_muscle_development	ENSGALG00010029649	PSPH	3.848638689	0.257690267	14.93513409	1.94682E-50	2.37936E-49	positive
PRJNA729271	stage_5W_vs_E10	ordinary_muscle_development	ENSGALG00010029650	ACSF2	-0.207708862	0.135388197	-1.534172598	0.124987197	0.149862637	negative
PRJNA729271	stage_5W_vs_E10	ordinary_muscle_development	ENSGALG00010029651	ENSGALG00010029651	4.593198489	0.274606183	16.72649333	8.4043E-63	1.45893E-61	positive
PRJNA729271	stage_5W_vs_E10	ordinary_muscle_development	ENSGALG00010029652	LRRC59	-1.174369501	0.104001628	-11.29183763	1.43994E-29	8.57517E-29	negative
PRJNA729271	stage_5W_vs_E10	ordinary_muscle_development	ENSGALG00010029653	CCT6A	-0.559533885	0.090363815	-6.192012646	5.94008E-10	1.40689E-09	negative
PRJNA729271	stage_5W_vs_E10	ordinary_muscle_development	ENSGALG00010029654	EME1	-2.609147722	0.137478715	-18.97855771	2.56542E-80	6.96363E-79	negative
PRJNA729271	stage_5W_vs_E10	ordinary_muscle_development	ENSGALG00010029655	PHKG1	5.47333181	0.128236589	42.68151435	0	0	positive
PRJNA729271	stage_5W_vs_E10	ordinary_muscle_development	ENSGALG00010029656	MRPL27	0.434522296	0.11333686	3.83390097	0.000126127	0.000205787	positive
PRJNA729271	stage_5W_vs_E10	ordinary_muscle_development	ENSGALG00010029657	CHCHD2	2.066828639	0.146742048	14.08477439	4.71161E-45	4.80949E-44	positive
PRJNA729271	stage_5W_vs_E10	ordinary_muscle_development	ENSGALG00010029658	XYLT2	0.6173827	0.124755535	4.948739937	7.46955E-07	1.44883E-06	positive
PRJNA729271	stage_5W_vs_E10	ordinary_muscle_development	ENSGALG00010029659	VKORC1L1	-0.760180281	0.146586131	-5.185894986	2.1498E-07	4.33218E-07	negative
PRJNA729271	stage_5W_vs_E10	ordinary_muscle_development	ENSGALG00010029660	CD300LG	1.898787882	0.303590108	6.254445827	3.9893E-10	9.5531E-10	positive
PRJNA729271	stage_5W_vs_E10	ordinary_muscle_development	ENSGALG00010029661	KRABZFP	5.773629795	0.570399613	10.12207874	4.40949E-24	2.10665E-23	positive
PRJNA729271	stage_5W_vs_E10	ordinary_muscle_development	ENSGALG00010029663	GUSB	0.161293963	0.153970819	1.047561897	0.2948405	0.332973358	positive
PRJNA729271	stage_5W_vs_E10	ordinary_muscle_development	ENSGALG00010029664	RAB37	2.663695146	0.370458179	7.190272196	6.46623E-13	1.82173E-12	positive
PRJNA729271	stage_5W_vs_E10	ordinary_muscle_development	ENSGALG00010029665	ASL1	1.405556294	0.189637317	7.411812818	1.24585E-13	3.63351E-13	positive
PRJNA729271	stage_5W_vs_E10	ordinary_muscle_development	ENSGALG00010029666	SLC9A3R1	0.193193747	0.198398787	0.973764761	0.330173353	0.369793135	positive
PRJNA729271	stage_5W_vs_E10	ordinary_muscle_development	ENSGALG00010029667	TMEM104	0.182088155	0.092935872	1.959288177	0.050079048	0.063137844	positive
PRJNA729271	stage_5W_vs_E10	ordinary_muscle_development	ENSGALG00010029668	CRCP	-0.544846695	0.182510272	-2.98529332	0.002833065	0.004106939	negative
PRJNA729271	stage_5W_vs_E10	ordinary_muscle_development	ENSGALG00010029669	HID1	0.619731809	0.074117218	8.361509293	6.19209E-17	2.14826E-16	positive
PRJNA729271	stage_5W_vs_E10	ordinary_muscle_development	ENSGALG00010029670	TPST1	0.135277799	0.170052801	0.795504681	0.426319989	0.468109101	positive
PRJNA729271	stage_5W_vs_E10	ordinary_muscle_development	ENSGALG00010029671	GRIN2C	2.388019411	0.204785147	11.66109675	2.01431E-31	1.29078E-30	positive
PRJNA729271	stage_5W_vs_E10	ordinary_muscle_development	ENSGALG00010029672	KCTD7	-1.589251664	0.238145862	-6.673438074	2.49879E-11	6.46093E-11	negative
PRJNA729271	stage_5W_vs_E10	ordinary_muscle_development	ENSGALG00010029673	TMEM248	-0.466164805	0.090585545	-5.146127935	2.65918E-07	5.31433E-07	negative
PRJNA729271	stage_5W_vs_E10	ordinary_muscle_development	ENSGALG00010029674	FDXR	-0.989191924	0.116295046	-8.505881888	1.8022E-17	6.41841E-17	negative
PRJNA729271	stage_5W_vs_E10	ordinary_muscle_development	ENSGALG00010029675	DHRS13	0.348267902	0.223409974	1.558873566	0.119026308	0.143070432	positive
PRJNA729271	stage_5W_vs_E10	ordinary_muscle_development	ENSGALG00010029676	FADS6	-0.917202124	1.083882881	-0.846218848	0.397430669	0.438647495	negative
PRJNA729271	stage_5W_vs_E10	ordinary_muscle_development	ENSGALG00010029677	PHF12	0.08799654	0.10058271	0.874867462	0.381646025	0.422803358	positive
PRJNA729271	stage_5W_vs_E10	ordinary_muscle_development	ENSGALG00010029682	CDR2L	-1.566845117	0.133838623	-11.70697283	1.17397E-31	7.59671E-31	negative
PRJNA729271	stage_5W_vs_E10	ordinary_muscle_development	ENSGALG00010029683	MRPL58	0.241558245	0.110994928	2.176299855	0.029532846	0.038337687	positive
PRJNA729271	stage_5W_vs_E10	ordinary_muscle_development	ENSGALG00010029684	ENSGALG00010029684	-0.749262316	0.648477028	-1.155418439	0.247919114	0.283764021	negative

PRJNA729271	stage_5W_vs_E10	ordinary_muscle_development	ENSGALG00010029685	ATP5PD	0.551940388	0.115166	4.792563685	1.64663E-06	3.1074E-06	positive
PRJNA729271	stage_5W_vs_E10	ordinary_muscle_development	ENSGALG00010029686	KCTD2	-0.147269128	0.060007783	-2.45416713	0.014121133	0.019005184	negative
PRJNA729271	stage_5W_vs_E10	ordinary_muscle_development	ENSGALG00010029687	SLC16A5	-2.079949935	0.532800675	-3.903804992	9.46921E-05	0.000156328	negative
PRJNA729271	stage_5W_vs_E10	ordinary_muscle_development	ENSGALG00010029689	ENSGALG00010029689	1.787317787	0.175745519	10.16991951	2.70132E-24	1.30215E-23	positive
PRJNA729271	stage_5W_vs_E10	ordinary_muscle_development	ENSGALG00010029690	ENSGALG00010029690	1.684020498	0.160631601	10.48374345	1.026E-25	5.23475E-25	positive
PRJNA729271	stage_5W_vs_E10	ordinary_muscle_development	ENSGALG00010029691	NUFIP2	0.991630441	0.140378025	7.064000491	1.61776E-12	4.46062E-12	positive
PRJNA729271	stage_5W_vs_E10	ordinary_muscle_development	ENSGALG00010029692	CRYBA1	-2.13144151	0.690951119	-3.084793482	0.002036935	0.002990215	negative
PRJNA729271	stage_5W_vs_E10	ordinary_muscle_development	ENSGALG00010029693	HN1	-0.97090516	0.126891169	-7.651479369	1.9868E-14	6.0438E-14	negative
PRJNA729271	stage_5W_vs_E10	ordinary_muscle_development	ENSGALG00010029694	ARMC7	-0.093837211	0.093272803	-1.006051154	0.314390964	0.35342772	negative
PRJNA729271	stage_5W_vs_E10	ordinary_muscle_development	ENSGALG00010029695	TAOK1	0.180988032	0.086539758	2.09138593	0.036493482	0.046886459	positive
PRJNA729271	stage_5W_vs_E10	ordinary_muscle_development	ENSGALG00010029696	SUMO2	-0.432956407	0.108268183	-3.998925572	6.36307E-05	0.000106768	negative
PRJNA729271	stage_5W_vs_E10	ordinary_muscle_development	ENSGALG00010029697	GIT1	1.92156981	0.164330138	11.69334994	1.3784E-31	8.88393E-31	positive
PRJNA729271	stage_5W_vs_E10	ordinary_muscle_development	ENSGALG00010029698	NUP85	-0.82811405	0.086552665	-9.567747554	1.0926E-21	4.70786E-21	negative
PRJNA729271	stage_5W_vs_E10	ordinary_muscle_development	ENSGALG00010029699	ANKRD13B	-0.955368869	0.178167275	-5.362201717	8.22137E-08	1.70509E-07	negative
PRJNA729271	stage_5W_vs_E10	ordinary_muscle_development	ENSGALG00010029700	GGA3	0.269991025	0.079867787	3.380474613	0.000723608	0.001109445	positive
PRJNA729271	stage_5W_vs_E10	ordinary_muscle_development	ENSGALG00010029701	MRPS7	0.159675554	0.06642528	2.403837135	0.016224	0.02168236	positive
PRJNA729271	stage_5W_vs_E10	ordinary_muscle_development	ENSGALG00010029702	CORO6	5.343193438	0.169586546	31.50717777	6.9269E-218	1.4344E-215	positive
PRJNA729271	stage_5W_vs_E10	ordinary_muscle_development	ENSGALG00010029703	MIF4GD	-0.365877909	0.115952201	-3.155420132	0.001602671	0.002379466	negative
PRJNA729271	stage_5W_vs_E10	ordinary_muscle_development	ENSGALG00010029704	SLC25A19	1.141880144	0.099472318	11.47937604	1.67485E-30	1.0335E-29	positive
PRJNA729271	stage_5W_vs_E10	ordinary_muscle_development	ENSGALG00010029705	GRB2	-0.71071826	0.068160544	-10.42712134	1.86452E-25	9.3906E-25	negative
PRJNA729271	stage_5W_vs_E10	ordinary_muscle_development	ENSGALG00010029706	SSH2	1.414266843	0.217603113	6.499295094	8.06972E-11	2.02336E-10	positive
PRJNA729271	stage_5W_vs_E10	ordinary_muscle_development	ENSGALG00010029707	NSRP1	0.305528543	0.223160761	1.369096172	0.170969215	0.201348616	positive
PRJNA729271	stage_5W_vs_E10	ordinary_muscle_development	ENSGALG00010029708	TMEM94	0.063894771	0.08494061	0.752228769	0.451913496	0.493854502	positive
PRJNA729271	stage_5W_vs_E10	ordinary_muscle_development	ENSGALG00010029709	SLC6A4	1.376852203	0.195801578	7.031874893	2.03776E-12	5.59315E-12	positive
PRJNA729271	stage_5W_vs_E10	ordinary_muscle_development	ENSGALG00010029710	BLMH	-0.229769655	0.08297736	-2.769064422	0.005621752	0.007897586	negative
PRJNA729271	stage_5W_vs_E10	ordinary_muscle_development	ENSGALG00010029711	CASKIN2	1.014911983	0.111048615	9.139348449	6.28297E-20	2.50472E-19	positive
PRJNA729271	stage_5W_vs_E10	ordinary_muscle_development	ENSGALG00010029713	CPD	0.309113831	0.116629606	2.650389056	0.008039913	0.011114786	positive
PRJNA729271	stage_5W_vs_E10	ordinary_muscle_development	ENSGALG00010029716	GOSR1	0.015602796	0.063724383	0.244848126	0.806574029	0.829463678	positive
PRJNA729271	stage_5W_vs_E10	ordinary_muscle_development	ENSGALG00010029717	TRARG1	-1.337429085	0.49219018	-2.717301441	0.006581663	0.009180255	negative
PRJNA729271	stage_5W_vs_E10	ordinary_muscle_development	ENSGALG00010029718	ABR	0.619737317	0.110257642	5.620810544	1.90064E-08	4.12236E-08	positive
PRJNA729271	stage_5W_vs_E10	ordinary_muscle_development	ENSGALG00010029720	TIMM22	0.067265738	0.108392177	0.620577427	0.534877695	0.574973426	positive
PRJNA729271	stage_5W_vs_E10	ordinary_muscle_development	ENSGALG00010029721	EXOC7	0.052352623	0.105474693	0.496352455	0.619645763	0.656226005	positive
PRJNA729271	stage_5W_vs_E10	ordinary_muscle_development	ENSGALG00010029722	SPECC1	0.39690359	0.142266918	2.78985161	0.00527322	0.007425953	positive
PRJNA729271	stage_5W_vs_E10	ordinary_muscle_development	ENSGALG00010029723	ADORA2B	1.766055001	0.17817657	9.911825129	3.69828E-23	1.69856E-22	positive
PRJNA729271	stage_5W_vs_E10	ordinary_muscle_development	ENSGALG00010029724	CACNA1G	-1.862241633	0.216404523	-8.605372965	7.6069E-18	2.76138E-17	negative
PRJNA729271	stage_5W_vs_E10	ordinary_muscle_development	ENSGALG00010029725	TSEN54	-0.410587165	0.069756755	-5.885984274	3.95692E-09	8.93667E-09	negative
PRJNA729271	stage_5W_vs_E10	ordinary_muscle_development	ENSGALG00010029726	SEPTIN9	-1.20216309	0.125606539	-9.570863911	1.06016E-21	4.5708E-21	negative
PRJNA729271	stage_5W_vs_E10	ordinary_muscle_development	ENSGALG00010029727	ZSWIM7	-0.227253778	0.14425067	-1.575408817	0.11516211	0.138828575	negative
PRJNA729271	stage_5W_vs_E10	ordinary_muscle_development	ENSGALG00010029728	TNRC6C	0.282099692	0.103636428	2.722012893	0.006488561	0.009057366	positive
PRJNA729271	stage_5W_vs_E10	ordinary_muscle_development	ENSGALG00010029729	RASL10A	-0.462856599	1.119494816	-0.413451311	0.679275988	0.712711215	negative
PRJNA729271	stage_5W_vs_E10	ordinary_muscle_development	ENSGALG00010029730	TTC19	1.323008821	0.132121732	10.01355947	1.32884E-23	6.21138E-23	positive
PRJNA729271	stage_5W_vs_E10	ordinary_muscle_development	ENSGALG00010029731	NCOR1	0.262864515	0.184752433	1.422793249	0.154796099	0.183314498	positive
PRJNA729271	stage_5W_vs_E10	ordinary_muscle_development	ENSGALG00010029732	PIGL	0.346775367	0.181245486	1.91329105	0.055710803	0.069819133	positive
PRJNA729271	stage_5W_vs_E10	ordinary_muscle_development	ENSGALG00010029733	ENSGALG00010029733	2.476471363	0.164384724	15.06509431	2.74773E-51	3.44536E-50	positive
PRJNA729271	stage_5W_vs_E10	ordinary_muscle_development	ENSGALG00010029734	SEC14L1	-0.17035465	0.084058615	-2.026617383	0.042701557	0.054328015	negative
PRJNA729271	stage_5W_vs_E10	ordinary_muscle_development	ENSGALG00010029735	SRP68	-0.604410625	0.114908469	-5.259931052	1.44109E-07	2.94165E-07	negative
PRJNA729271	stage_5W_vs_E10	ordinary_muscle_development	ENSGALG00010029736	ENSGALG00010029736	1.789985814	0.133064232	13.4520433	2.99481E-41	2.71779E-40	positive
PRJNA729271	stage_5W_vs_E10	ordinary_muscle_development	ENSGALG00010029737	ENSGALG00010029737	-6.05324013	0.578442086	-10.4647298	1.25434E-25	6.37282E-25	negative
PRJNA729271	stage_5W_vs_E10	ordinary_muscle_development	ENSGALG00010029738	TRPV2	0.853948262	0.166915958	5.116037284	3.12021E-07	6.20831E-07	positive
PRJNA729271	stage_5W_vs_E10	ordinary_muscle_development	ENSGALG00010029739	P2RX5	0.768972649	0.204371509	3.762621576	0.000168142	0.000271646	positive
PRJNA729271	stage_5W_vs_E10	ordinary_muscle_development	ENSGALG00010029740	HASPIN	-2.723118686	0.229191805	-11.88139641	1.47876E-32	9.93265E-32	negative
PRJNA729271	stage_5W_vs_E10	ordinary_muscle_development	ENSGALG00010029741	EMC6	0.739588083	0.163130122	4.533730952	5.79508E-06	1.04908E-05	positive
PRJNA729271	stage_5W_vs_E10	ordinary_muscle_development	ENSGALG00010029742	TAX1BP3	-1.424940867	0.119537623	-11.92043837	9.26199E-33	6.25012E-32	negative
PRJNA729271	stage_5W_vs_E10	ordinary_muscle_development	ENSGALG00010029743	MYOCD	4.187369064	0.253530516	16.51623297	2.80372E-61	4.70369E-60	positive
PRJNA729271	stage_5W_vs_E10	ordinary_muscle_development	ENSGALG00010029744	CTNS	0.901436232	0.089089344	10.1183395	4.58122E-24	2.18652E-23	positive
PRJNA729271	stage_5W_vs_E10	ordinary_muscle_development	ENSGALG00010029745	SHPK	0.935453221	0.168187838	5.561955192	2.66769E-08	5.72946E-08	positive
PRJNA729271	stage_5W_vs_E10	ordinary_muscle_development	ENSGALG00010029746	TRPV1	-0.086121223	0.326680022	-0.263625619	0.792068415	0.816806465	negative
PRJNA729271	stage_5W_vs_E10	ordinary_muscle_development	ENSGALG00010029747	TRPV3	-0.580989809	0.348451253	-1.667348887	0.095445051	0.11629758	negative
PRJNA729271	stage_5W_vs_E10	ordinary_muscle_development	ENSGALG00010029749	ASPA	3.130998889	0.28343288	11.04670315	2.27414E-28	1.29218E-27	positive
PRJNA729271	stage_5W_vs_E10	ordinary_muscle_development	ENSGALG00010029750	TEN1	-2.81779695	0.269865493	-10.44148667	1.60281E-25	8.08937E-25	negative
PRJNA729271	stage_5W_vs_E10	ordinary_muscle_development	ENSGALG00010029752	ENSGALG00010029752	0.098386516	0.172302008	0.571012011	0.567991501	0.606977057	positive
PRJNA729271	stage_5W_vs_E10	ordinary_muscle_development	ENSGALG00010029753	AKAP10	-0.056056618	0.07518901	-0.745542709	0.455943702	0.497920889	negative
PRJNA729271	stage_5W_vs_E10	ordinary_muscle_development	ENSGALG00010029754	NLE1	-1.116897758	0.181752066	-6.145172266	7.98768E-10	1.87409E-09	negative
PRJNA729271	stage_5W_vs_E10	ordinary_muscle_development	ENSGALG00010029755	ACOX1	0.553621426	0.119265611	4.641920028	3.45186E-06	6.36899E-06	positive
PRJNA729271	stage_5W_vs_E10	ordinary_muscle_development	ENSGALG00010029756	ELAC2	-0.353255061	0.08460632	-4.17527985	2.9762E-05	5.10714E-05	negative
PRJNA729271	stage_5W_vs_E10	ordinary_muscle_development	ENSGALG00010029757	ULK2	0.794723976	0.209204153	3.798796358	0.0001454	0.00023617	positive
PRJNA729271	stage_5W_vs_E10	ordinary_muscle_development	ENSGALG00010029758	DNAH9	0.991352591	0.304201099	3.258872481	0.001118559	0.001687326	positive
PRJNA729271	stage_5W_vs_E10	ordinary_muscle_development	ENSGALG00010029759	ALDH3A2	0.419311795	0.107816931	3.889109	0.000100613	0.000165688	positive
PRJNA729271	stage_5W_vs_E10	ordinary_muscle_development	ENSGALG00010029760	SHISA6	-5.634654416	0.835483604	-6.744183113	1.5389E-11	4.03225E-11	negative
PRJNA729271	stage_5W_vs_E10	ordinary_muscle_development	ENSGALG00010029761	MRPL38	-0.239114523	0.120221706	-1.988946355	0.046707124	0.059117012	negative
PRJNA729271	stage_5W_vs_E10	ordinary_muscle_development	ENSGALG00010029762	SLC47A2	3.760707762	0.471603765	7.974295451	1.53253E-15	4.9629E-15	positive
PRJNA729271	stage_5W_vs_E10	ordinary_muscle_development	ENSGALG00010029763	GEMIN4	-0.555392234	0.098941626	-5.613332381	1.98467E-08	4.3014E-08	negative
PRJNA729271	stage_5W_vs_E10	ordinary_muscle_development	ENSGALG00010029764	NXN	-1.30636443	0.126765558	-10.30535781	6.66432E-25	3.29916E-24	negative
PRJNA729271	stage_5W_vs_E10	ordinary_muscle_development	ENSGALG00010029765	KCNJ16	-1.360018488	0.307958393	-4.416241023	1.00432E-05	1.79105E-05	negative
PRJNA729271	stage_5W_vs_E10	ordinary_muscle_development	ENSGALG00010029766	ENSGALG00010029766	0.290255419	0.146983758	1.974744856	0.04829711	0.061002667	positive
PRJNA729271	stage_5W_vs_E10	ordinary_muscle_development	ENSGALG00010029767	GLOD4	-0.359627188	0.087644009	-4.103271769	4.07348E-05	6.91638E-05	negative
PRJNA729271	stage_5W_vs_E10	ordinary_muscle_development	ENSGALG00010029768	KCNJ2	0.439132816	0.175800272	2.497907492	0.012492879	0.01691586	positive
PRJNA729271	stage_5W_vs_E10	ordinary_muscle_development	ENSGALG00010029770	TLCD3A	-1.250282219	0.233961709	-5.343960875	9.09372E-08	1.8809E-07	negative
PRJNA729271	stage_5W_vs_E10	ordinary_muscle_development	ENSGALG00010029771	SOX9	-3.600628097	0.261588052	-13.76449755	4.16772E-43	3.99808E-42	negative
PRJNA729271	stage_5W_vs_E10	ordinary_muscle_development	ENSGALG00010029772	VPS53	-0.11219222	0.069678818	-1.610133796	0.10736865	0.129876374	negative
PRJNA729271	stage_5W_vs_E10	ordinary_muscle_development	ENSGALG00010029773	TRIM65	0.981804181	0.133663282	7.345354448	2.05215E-13	5.91604E-13	positive
PRJNA729271	stage_5W_vs_E10	ordinary_muscle_development	ENSGALG00010029774	RFLNB	-0.445320383	0.125358682	-3.552369694	0.000381778	0.000598515	negative
PRJNA729271	stage_5W_vs_E10	ordinary_muscle_development	ENSGALG00010029775	ENSGALG00010029775	0.247589222	0.141953679	1.744155024	0.081132061	0.099678694	positive
PRJNA729271	stage_5W_vs_E10	ordinary_muscle_development	ENSGALG00010029776	WBP2	1.165922413	0.136240643	8.557816404	1.15025E-17	4.13926E-17	positive
PRJNA729271	stage_5W_vs_E10	ordinary_muscle_development	ENSGALG00010029777	ENSGALG00010029777	-0.624762624	0.142123693	-4.395907606	1.10311E-05	1.96071E-05	negative
PRJNA729271	stage_5W_vs_E10	ordinary_muscle_development	ENSGALG00010029778	DOC2B	-1.53349743	0.364348227	-4.208878529	2.56641E-05	4.42806E-05	negative
PRJNA729271	stage_5W_vs_E10	ordinary_muscle_development	ENSGALG00010029780	TEX14	0.150052399	0.257287548	0.583208942	0.559752652	0.599498647	positive
PRJNA729271	stage_5W_vs_E10	ordinary_muscle_development	ENSGALG00010029781	UNC13D	1.868647354	0.243739994	7.666560262	1.7667E-14	5.38556E-14	positive
PRJNA729271	stage_5W_vs_E10	ordinary_muscle_development	ENSGALG00010029782	UNK	-0.054283842	0.1224832	-0.443194179	0.657625289	0.692199445	negative
PRJNA729271	stage_5W_vs_E10	ordinary_muscle_development	ENSGALG00010029783	RAD51C	-0.582768737	0.123221822	-4.729428015	2.25153E-06	4.20946E-06	negative
PRJNA729271	stage_5W_vs_E10	ordinary_muscle_development	ENSGALG00010029784	CLDN3	-5.104859594	0.790310027	-6.459312695	1.0518E-10	2.61461E-10	negative
PRJNA729271	stage_5W_vs_E10	ordinary_muscle_development	ENSGALG00010029785	ENSGALG00010029785	2.525494983	0.244292247	10.33800708	4.74298E-25	2.36172E-24	positive
PRJNA729271	stage_5W_vs_E10	ordinary_muscle_development	ENSGALG00010029787	H3-3B	-1.406547514	0.064438368	-21.82779556	1.2637E-105	5.815E-104	negative
PRJNA729271	stage_5W_vs_E10	ordinary_muscle_development	ENSGALG00010029791	GALK1	-0.550536198	0.115984131	-4.746651081	2.06812E-06	3.87506E-06	negative
PRJNA729271	stage_5W_vs_E10	ordinary_muscle_development	ENSGALG00010029804	ITGB4	1.110590994	0.226337063	4.906801302	9.25737E-07	1.78249E-06	positive
PRJNA729271	stage_5W_vs_E10	ordinary_muscle_development	ENSGALG00010029812	SAP30BP	-0.315798064	0.115975605	-2.722969755	0.006469798	0.009032045	negative
PRJNA729271	stage_5W_vs_E10	ordinary_muscle_development	ENSGALG00010029835	RECQL5	-0.552509703	0.111164447	-4.970201524	6.68833E-07	1.30131E-06	negative
PRJNA729271	stage_5W_vs_E10	ordinary_muscle_development	ENSGALG00010029841	SMIM5	0.029147581	0.134547269	0.216634504	0.828493194	0.848993202	positive
PRJNA729271	stage_5W_vs_E10	ordinary_muscle_development	ENSGALG00010029859	CSNK1D	-0.612223767	0.053470786	-11.44968698	2.35996E-30	1.44519E-29	negative
PRJNA729271	stage_5W_vs_E10	ordinary_muscle_development	ENSGALG00010029864	SLC16A3	0.806001973	0.339403752	2.374758579	0.017560432	0.023373596	positive
PRJNA729271	stage_5W_vs_E10	ordinary_muscle_development	ENSGALG00010029879	ENSGALG00010029879	-0.769242348	0.342132823	-2.24837343	0.024552388	0.032140058	negative
PRJNA729271	stage_5W_vs_E10	ordinary_muscle_development	ENSGALG00010029895	FASN	-1.632576355	0.168507056	-9.688474726	3.37532E-22	1.49162E-21	negative
PRJNA729271	stage_5W_vs_E10	ordinary_muscle_development	ENSGALG00010029907	NTN1	-0.438285019	0.272925712	-1.605876617	0.108301022	0.130938637	negative
PRJNA729271	stage_5W_vs_E10	ordinary_muscle_development	ENSGALG00010029921	STX8	-0.098412971	0.105464012	-0.933142686	0.350746302	0.390841609	negative
PRJNA729271	stage_5W_vs_E10	ordinary_muscle_development	ENSGALG00010029925	GGNBP2	0.491089415	0.125362577	3.91735259	8.95268E-05	0.000148223	positive
PRJNA729271	stage_5W_vs_E10	ordinary_muscle_development	ENSGALG00010029926	BLTP2	0.267710916	0.132532042	2.019971255	0.043386369	0.055146038	positive
PRJNA729271	stage_5W_vs_E10	ordinary_muscle_development	ENSGALG00010029927	ENSGALG00010029927	-0.460373889	0.708106818	-0.650147517	0.515596939	0.556028393	negative
PRJNA729271	stage_5W_vs_E10	ordinary_muscle_development	ENSGALG00010029928	USP43	-0.544184941	0.14150225	-3.84576882	0.000120175	0.000196385	negative
PRJNA729271	stage_5W_vs_E10	ordinary_muscle_development	ENSGALG00010029929	GDPD1	0.536215655	0.093816863	5.715557269	1.09345E-08	2.40765E-08	positive
PRJNA729271	stage_5W_vs_E10	ordinary_muscle_development	ENSGALG00010029930	ARHGAP44	-1.050835106	0.291828697	-3.600862827	0.000317163	0.000500902	negative
PRJNA729271	stage_5W_vs_E10	ordinary_muscle_development	ENSGALG00010029932	RASA4B	2.829882529	0.263675804	10.7324316	7.16846E-27	3.84413E-26	positive
PRJNA729271	stage_5W_vs_E10	ordinary_muscle_development	ENSGALG00010029933	TVP23B	0.02319129	0.09454461	0.245294679	0.806228271	0.829166936	positive
PRJNA729271	stage_5W_vs_E10	ordinary_muscle_development	ENSGALG00010029934	TEKT3	1.493454773	0.493701861	3.025013457	0.002486221	0.00362335	positive
PRJNA729271	stage_5W_vs_E10	ordinary_muscle_development	ENSGALG00010029935	SYNRG	0.029602245	0.073550928	0.402472757	0.687336134	0.720438012	positive
PRJNA729271	stage_5W_vs_E10	ordinary_muscle_development	ENSGALG00010029936	SERPINF2	3.566654766	0.499076355	7.146511205			positive
PRJNA729271	stage_5W_vs_E10	ordinary_muscle_development	ENSGALG00010029937	PMP22	1.517894098	0.124218465	12.21955284	2.44425E-34	1.75307E-33	positive
PRJNA729271	stage_5W_vs_E10	ordinary_muscle_development	ENSGALG00010029938	ENSGALG00010029938	-1.088683821	0.313541308	-3.472218146	0.000516177	0.000800983	negative
PRJNA729271	stage_5W_vs_E10	ordinary_muscle_development	ENSGALG00010029939	ENSGALG00010029939	4.666537766	1.476952084	3.159572891	0.001580006	0.002347257	positive
PRJNA729271	stage_5W_vs_E10	ordinary_muscle_development	ENSGALG00010029941	COX10	1.256331543	0.172452103	7.285104238	3.21422E-13	9.17489E-13	positive
PRJNA729271	stage_5W_vs_E10	ordinary_muscle_development	ENSGALG00010029942	ENSGALG00010029942	-1.88798555	1.532712168	-1.231793933	0.21802607	0.251916133	negative
PRJNA729271	stage_5W_vs_E10	ordinary_muscle_development	ENSGALG00010029943	HS3ST3A1	-1.6991972	0.202672905	-8.383938637	5.11855E-17	1.78221E-16	negative
PRJNA729271	stage_5W_vs_E10	ordinary_muscle_development	ENSGALG00010029945	CCL4	5.151270296	0.682220949	7.550736024	4.32805E-14	1.29591E-13	positive
PRJNA729271	stage_5W_vs_E10	ordinary_muscle_development	ENSGALG00010029946	METRNL	-0.758043004	0.137214233	-5.524521671	3.30384E-08	7.03736E-08	negative
PRJNA729271	stage_5W_vs_E10	ordinary_muscle_development	ENSGALG00010029947	CCL5	8.768864045	0.97556111	8.988533839	2.5055E-19	9.69754E-19	positive
PRJNA729271	stage_5W_vs_E10	ordinary_muscle_development	ENSGALG00010029948	MYADML2	1.240650208	0.219172842	5.660601902	1.50843E-08	3.29784E-08	positive
PRJNA729271	stage_5W_vs_E10	ordinary_muscle_development	ENSGALG00010029950	RFFL	0.342048298	0.103801283	3.295222257	0.000983439	0.001492195	positive
PRJNA729271	stage_5W_vs_E10	ordinary_muscle_development	ENSGALG00010029956	CCLI5	7.264297588	0.181181793	40.09397123	0	0	positive
PRJNA729271	stage_5W_vs_E10	ordinary_muscle_development	ENSGALG00010029962	ENSGALG00010029962	-0.419536453	0.764189433	-0.54899536	0.583008634	0.621421439	negative
PRJNA729271	stage_5W_vs_E10	ordinary_muscle_development	ENSGALG00010029966	RABGEF1	0.306758163	0.080801536	3.796439769	0.000146789	0.000238292	positive
PRJNA729271	stage_5W_vs_E10	ordinary_muscle_development	ENSGALG00010030004	FLOT2	0.406183234	0.070920525	5.727301596	1.02041E-08	2.25195E-08	positive
PRJNA729271	stage_5W_vs_E10	ordinary_muscle_development	ENSGALG00010030014	ENSGALG00010030014	-1.43799462	0.313477031	-4.587240777	4.49143E-06	8.20353E-06	negative

PRJNA729271	stage_5W_vs_E10	ordinary_muscle_development	ENSGALG00010030102	GPS1	0.325823517	0.083159626	3.918049316	8.92684E-05	0.000147857	positive
PRJNA729271	stage_5W_vs_E10	ordinary_muscle_development	ENSGALG00010030103	C1QTNF1	2.401717749	0.208831498	11.5007447	1.30782E-30	8.12902E-30	positive
PRJNA729271	stage_5W_vs_E10	ordinary_muscle_development	ENSGALG00010030104	P4HB	-1.48979274	0.110228012	-13.51555488	1.26598E-41	1.15994E-40	negative
PRJNA729271	stage_5W_vs_E10	ordinary_muscle_development	ENSGALG00010030105	RAB40B	1.495287202	0.191579311	7.805055763	5.94752E-15	1.86157E-14	positive
PRJNA729271	stage_5W_vs_E10	ordinary_muscle_development	ENSGALG00010030106	GPRC5C	-2.111690218	0.216856148	-9.737746597	2.08118E-22	9.27065E-22	negative
PRJNA729271	stage_5W_vs_E10	ordinary_muscle_development	ENSGALG00010030107	RPTOR	0.860789898	0.138191572	6.228960912	4.69539E-10	1.11922E-09	positive
PRJNA729271	stage_5W_vs_E10	ordinary_muscle_development	ENSGALG00010030108	LLGL2	0.514227758	0.119144526	4.315999848	1.58882E-05	2.78813E-05	positive
PRJNA729271	stage_5W_vs_E10	ordinary_muscle_development	ENSGALG00010030109	FBF1	-0.04635696	0.090087512	-0.514576975	0.606848658	0.644132345	negative
PRJNA729271	ed_Lushi_vs_Arbor_Acr	breed_growth_background	ENSGALG00010000007	ND1	0.20945657	0.13020703	1.608642559	0.107694523	0.226993181	positive
PRJNA729271	ed_Lushi_vs_Arbor_Acr	breed_growth_background	ENSGALG00010000011	ND2	-0.05446627	0.135510041	-0.401935307	0.687731638	0.798132113	negative
PRJNA729271	ed_Lushi_vs_Arbor_Acr	breed_growth_background	ENSGALG00010000017	COX1	0.13300923	0.145614181	0.913435968	0.361013296	0.524389992	positive
PRJNA729271	ed_Lushi_vs_Arbor_Acr	breed_growth_background	ENSGALG00010000020	COX2	0.332275624	0.127758752	2.600805174	0.009300526	0.039743844	positive
PRJNA729271	ed_Lushi_vs_Arbor_Acr	breed_growth_background	ENSGALG00010000022	ATP8	-0.074431431	0.148653874	-0.500702938	0.616580204	0.745000268	negative
PRJNA729271	ed_Lushi_vs_Arbor_Acr	breed_growth_background	ENSGALG00010000023	ATP6	0.151572184	0.106338249	1.425377839	0.15404801	0.290518594	positive
PRJNA729271	ed_Lushi_vs_Arbor_Acr	breed_growth_background	ENSGALG00010000024	COX3	0.262889376	0.118396044	2.220423653	0.026390023	0.083012887	positive
PRJNA729271	ed_Lushi_vs_Arbor_Acr	breed_growth_background	ENSGALG00010000026	ND3	0.334413822	0.169995975	1.967186706	0.04916169	0.131082191	positive
PRJNA729271	ed_Lushi_vs_Arbor_Acr	breed_growth_background	ENSGALG00010000028	ND4L	0.153296419	0.149393361	1.026126051	0.304832173	0.467372788	positive
PRJNA729271	ed_Lushi_vs_Arbor_Acr	breed_growth_background	ENSGALG00010000029	ND4	0.137481866	0.13659828	1.006468496	0.314190259	0.477828958	positive
PRJNA729271	ed_Lushi_vs_Arbor_Acr	breed_growth_background	ENSGALG00010000033	ND5	0.052878372	0.124389763	0.425102282	0.670762115	0.787008002	positive
PRJNA729271	ed_Lushi_vs_Arbor_Acr	breed_growth_background	ENSGALG00010000034	CYTB	0.10558575	0.117256102	0.900471261	0.367869513	0.53114349	positive
PRJNA729271	ed_Lushi_vs_Arbor_Acr	breed_growth_background	ENSGALG00010000037	ND6	-0.041935597	0.102387695	-0.409576531	0.682116616	0.794541976	negative
PRJNA729271	ed_Lushi_vs_Arbor_Acr	breed_growth_background	ENSGALG00010000039	PDIA3	-0.067809069	0.035358991	-1.917731991	0.055145003	0.142228971	negative
PRJNA729271	ed_Lushi_vs_Arbor_Acr	breed_growth_background	ENSGALG00010000042	CKMT1B	-0.486134815	0.209619013	-2.319135116	0.020387711	0.069192197	negative
PRJNA729271	ed_Lushi_vs_Arbor_Acr	breed_growth_background	ENSGALG00010000043	PPIP5K1	0.042385272	0.065622277	0.645897611	0.518345667	0.666614508	positive
PRJNA729271	ed_Lushi_vs_Arbor_Acr	breed_growth_background	ENSGALG00010000044	MAP1A	-0.148294567	0.070918246	-2.091063646	0.036522359	0.105267766	negative
PRJNA729271	ed_Lushi_vs_Arbor_Acr	breed_growth_background	ENSGALG00010000045	TP53BP1	0.011487438	0.04042119	0.284193469	0.77626212	0.861212243	positive
PRJNA729271	ed_Lushi_vs_Arbor_Acr	breed_growth_background	ENSGALG00010000046	TUBGCP4	0.075307985	0.034503396	2.182625272	0.029063417	0.088820205	positive
PRJNA729271	ed_Lushi_vs_Arbor_Acr	breed_growth_background	ENSGALG00010000047	IQGAP1	-0.188291296	0.052149254	-3.610623007	0.000305462	0.003514075	negative
PRJNA729271	ed_Lushi_vs_Arbor_Acr	breed_growth_background	ENSGALG00010000048	MAN2A2	0.095496781	0.079020008	1.208513934	0.226849639	0.380049182	positive
PRJNA729271	ed_Lushi_vs_Arbor_Acr	breed_growth_background	ENSGALG00010000049	FES	-0.251555625	0.102548282	-2.45304573	0.014165233	0.053303492	negative
PRJNA729271	ed_Lushi_vs_Arbor_Acr	breed_growth_background	ENSGALG00010000050	CIB1	0.116082065	0.069876217	1.661252869	0.096662671	0.20978561	positive
PRJNA729271	ed_Lushi_vs_Arbor_Acr	breed_growth_background	ENSGALG00010000051	GDPGP1	0.219400278	0.1038604	2.11245361	0.034647561	0.101151338	positive
PRJNA729271	ed_Lushi_vs_Arbor_Acr	breed_growth_background	ENSGALG00010000052	TTLL13	-0.037485084	0.071013792	-0.527856391	0.597599015	0.730959358	negative
PRJNA729271	ed_Lushi_vs_Arbor_Acr	breed_growth_background	ENSGALG00010000053	HDDC3	0.115418728	0.062663133	1.841892088	0.065490936	0.160380321	positive
PRJNA729271	ed_Lushi_vs_Arbor_Acr	breed_growth_background	ENSGALG00010000054	VPS33B	0.014369077	0.05329686	0.269604579	0.787464477	0.867922978	positive
PRJNA729271	ed_Lushi_vs_Arbor_Acr	breed_growth_background	ENSGALG00010000055	PRC1	-0.213642117	0.115192593	-1.854651515	0.063646007	0.157142129	negative
PRJNA729271	ed_Lushi_vs_Arbor_Acr	breed_growth_background	ENSGALG00010000056	UNC45A	-0.077515584	0.044527709	-1.740839245	0.081711761	0.186697978	negative
PRJNA729271	ed_Lushi_vs_Arbor_Acr	breed_growth_background	ENSGALG00010000058	FURIN	0.282282622	0.092495893	3.051839515	0.002274437	0.014592066	positive
PRJNA729271	ed_Lushi_vs_Arbor_Acr	breed_growth_background	ENSGALG00010000059	MFAP1	0.166787614	0.059715178	2.793052272	0.005221325	0.026315407	positive
PRJNA729271	ed_Lushi_vs_Arbor_Acr	breed_growth_background	ENSGALG00010000060	HYPK	0.324614302	0.132054301	2.458188033	0.013964004	0.052834309	positive
PRJNA729271	ed_Lushi_vs_Arbor_Acr	breed_growth_background	ENSGALG00010000061	SERINC4	0.253625887	0.169493111	1.496378734	0.134554988	0.264375827	positive
PRJNA729271	ed_Lushi_vs_Arbor_Acr	breed_growth_background	ENSGALG00010000063	SERF2	-0.027037823	0.068186383	-0.396528188	0.691715418	0.800767908	negative
PRJNA729271	ed_Lushi_vs_Arbor_Acr	breed_growth_background	ENSGALG00010000069	ENSGALG00010000069	-0.052565378	0.124530727	-0.422107694	0.672946412	0.788422102	negative
PRJNA729271	ed_Lushi_vs_Arbor_Acr	breed_growth_background	ENSGALG00010000076	ENSGALG00010000076	-0.190257043	0.104156048	-1.826653826	0.067751835	0.164306985	negative
PRJNA729271	ed_Lushi_vs_Arbor_Acr	breed_growth_background	ENSGALG00010000078	ENSGALG00010000078	-0.352905316	0.250319059	-1.409822	0.158592249	0.297001892	negative
PRJNA729271	ed_Lushi_vs_Arbor_Acr	breed_growth_background	ENSGALG00010000081	ENSGALG00010000081	0.639965268	0.155542092	4.114418553	3.88157E-05	0.000805513	positive
PRJNA729271	ed_Lushi_vs_Arbor_Acr	breed_growth_background	ENSGALG00010000084	ENSGALG00010000084	0.383595181	0.136626304	2.80762321	0.004990858	0.025530466	positive
PRJNA729271	ed_Lushi_vs_Arbor_Acr	breed_growth_background	ENSGALG00010000085	ENSGALG00010000085	-0.034471876	0.082861228	-0.416019369	0.67739583	0.791587597	negative
PRJNA729271	ed_Lushi_vs_Arbor_Acr	breed_growth_background	ENSGALG00010000089	ENSGALG00010000089	0.290300023	0.151258409	1.919232287	0.054954943	0.141864987	positive
PRJNA729271	ed_Lushi_vs_Arbor_Acr	breed_growth_background	ENSGALG00010000091	ENSGALG00010000091	0.444946421	0.441789804	1.007145067	0.313865067	0.477384485	positive
PRJNA729271	ed_Lushi_vs_Arbor_Acr	breed_growth_background	ENSGALG00010000092	INPPL1	0.231189173	0.091028247	2.539752	0.01109311	0.045078392	positive
PRJNA729271	ed_Lushi_vs_Arbor_Acr	breed_growth_background	ENSGALG00010000093	ENSGALG00010000093	-0.50965332	0.175049285	-2.911484729	0.003597155	0.020038724	negative
PRJNA729271	ed_Lushi_vs_Arbor_Acr	breed_growth_background	ENSGALG00010000098	ENSGALG00010000098	-0.19068502	0.103241178	-1.846986096	0.064749155	0.158927103	negative
PRJNA729271	ed_Lushi_vs_Arbor_Acr	breed_growth_background	ENSGALG00010000105	CACNA1F	-0.388938632	0.159171698	-2.443516256	0.014544914	0.054214129	negative
PRJNA729271	ed_Lushi_vs_Arbor_Acr	breed_growth_background	ENSGALG00010000107	ENSGALG00010000107	0.477520258	0.303446444	1.573655803	0.115567045	0.238183466	positive
PRJNA729271	ed_Lushi_vs_Arbor_Acr	breed_growth_background	ENSGALG00010000121	ENSGALG00010000121	-0.106697496	0.074641476	-1.429466599	0.152870168	0.289362527	negative
PRJNA729271	ed_Lushi_vs_Arbor_Acr	breed_growth_background	ENSGALG00010000123	ENSGALG00010000123	0.170506465	0.139278325	1.22421392	0.220871539	0.373317689	positive
PRJNA729271	ed_Lushi_vs_Arbor_Acr	breed_growth_background	ENSGALG00010000124	ENSGALG00010000124	-0.287052971	0.097128243	-2.955401672	0.003122622	0.018252167	negative
PRJNA729271	ed_Lushi_vs_Arbor_Acr	breed_growth_background	ENSGALG00010000126	ENSGALG00010000126	0.054416909	0.109245921	0.498113876	0.618403781	0.74660693	positive
PRJNA729271	ed_Lushi_vs_Arbor_Acr	breed_growth_background	ENSGALG00010000127	ENSGALG00010000127	0.322274508	0.110322034	2.921216146	0.003486678	0.019690988	positive
PRJNA729271	ed_Lushi_vs_Arbor_Acr	breed_growth_background	ENSGALG00010000133	USP39	0.28276831	0.076429379	3.699733189	0.000215826	0.002751453	positive
PRJNA729271	ed_Lushi_vs_Arbor_Acr	breed_growth_background	ENSGALG00010000134	ENSGALG00010000134	0.351948746	0.068167179	5.163023515	2.42993E-07	1.6472E-05	positive
PRJNA729271	ed_Lushi_vs_Arbor_Acr	breed_growth_background	ENSGALG00010000137	ENSGALG00010000137	-1.41286379	0.293178483	-4.819125112	1.44189E-06	6.5825E-05	negative
PRJNA729271	ed_Lushi_vs_Arbor_Acr	breed_growth_background	ENSGALG00010000140	SEPHS3	0.117611931	0.068701138	1.711935709	0.086908517	0.195096632	positive
PRJNA729271	ed_Lushi_vs_Arbor_Acr	breed_growth_background	ENSGALG00010000141	ENSGALG00010000141	0.022102531	0.074920341	0.295013755	0.767983372	0.855117451	positive
PRJNA729271	ed_Lushi_vs_Arbor_Acr	breed_growth_background	ENSGALG00010000143	SCARF2	-0.180086695	0.086396461	-2.084422139	0.037121786	0.106571655	negative
PRJNA729271	ed_Lushi_vs_Arbor_Acr	breed_growth_background	ENSGALG00010000145	ENSGALG00010000145	-0.177223236	0.150301367	-1.179119253	0.238350693	0.393639996	negative
PRJNA729271	ed_Lushi_vs_Arbor_Acr	breed_growth_background	ENSGALG00010000146	ACADM	-0.089680435	0.085121863	-1.053553483	0.292087396	0.453492588	negative
PRJNA729271	ed_Lushi_vs_Arbor_Acr	breed_growth_background	ENSGALG00010000147	ENSGALG00010000147	-0.170851213	0.059583769	-2.867411972	0.004138439	0.022159836	negative
PRJNA729271	ed_Lushi_vs_Arbor_Acr	breed_growth_background	ENSGALG00010000148	ENSGALG00010000148	0.092921716	0.155463487	0.597707652	0.550035019	0.69326276	positive
PRJNA729271	ed_Lushi_vs_Arbor_Acr	breed_growth_background	ENSGALG00010000149	ENSGALG00010000149	0.063755873	0.076430623	0.834166602	0.404187108	0.564965491	positive
PRJNA729271	ed_Lushi_vs_Arbor_Acr	breed_growth_background	ENSGALG00010000150	B3GAT3	0.015725955	0.067676467	0.232369623	0.81625094	0.886694805	positive
PRJNA729271	ed_Lushi_vs_Arbor_Acr	breed_growth_background	ENSGALG00010000151	TBX5	0.137130125	0.138910248	0.987185089	0.32355191	0.487514027	positive
PRJNA729271	ed_Lushi_vs_Arbor_Acr	breed_growth_background	ENSGALG00010000153	NF2	0.111321652	0.050040301	2.224639959	0.026105418	0.082421702	positive
PRJNA729271	ed_Lushi_vs_Arbor_Acr	breed_growth_background	ENSGALG00010000154	ENSGALG00010000154	5.829113457	2.129079182	2.737856585	0.006184104	0.029642389	positive
PRJNA729271	ed_Lushi_vs_Arbor_Acr	breed_growth_background	ENSGALG00010000155	MAZ	-0.166940941	0.090114268	-1.852547266	0.063947277	0.157721632	negative
PRJNA729271	ed_Lushi_vs_Arbor_Acr	breed_growth_background	ENSGALG00010000156	ENSGALG00010000156	0.035989489	0.267438152	0.134571259	0.892950867	0.937432231	positive
PRJNA729271	ed_Lushi_vs_Arbor_Acr	breed_growth_background	ENSGALG00010000157	ENSGALG00010000157	7.318783122	2.85394904	2.564440717			positive
PRJNA729271	ed_Lushi_vs_Arbor_Acr	breed_growth_background	ENSGALG00010000161	CDC42EP2	-0.292193414	0.17447211	-1.674728496	0.093987514	0.205886204	negative
PRJNA729271	ed_Lushi_vs_Arbor_Acr	breed_growth_background	ENSGALG00010000166	PFDN6	0.1329606	0.069460188	1.914198689	0.055594773	0.143007318	positive
PRJNA729271	ed_Lushi_vs_Arbor_Acr	breed_growth_background	ENSGALG00010000171	DPF2	0.335887799	0.108469163	3.096620187	0.001957405	0.013086986	positive
PRJNA729271	ed_Lushi_vs_Arbor_Acr	breed_growth_background	ENSGALG00010000172	ENSGALG00010000172	-0.552227619	0.601759919	-0.917687604	0.358782439	0.522323865	negative
PRJNA729271	ed_Lushi_vs_Arbor_Acr	breed_growth_background	ENSGALG00010000173	NIPSNAP1	-0.156677227	0.070708294	-2.215825304	0.026703469	0.083726321	negative
PRJNA729271	ed_Lushi_vs_Arbor_Acr	breed_growth_background	ENSGALG00010000174	ENSGALG00010000174	0.038065356	0.052388859	0.726592583	0.467475573	0.622456222	positive
PRJNA729271	ed_Lushi_vs_Arbor_Acr	breed_growth_background	ENSGALG00010000175	ENSGALG00010000175	0.107531144	0.527069042	0.204017188	0.838340068	0.901798833	positive
PRJNA729271	ed_Lushi_vs_Arbor_Acr	breed_growth_background	ENSGALG00010000176	ENSGALG00010000176	0.564172908	0.277260978	2.034808189	0.041870177	0.116377414	positive
PRJNA729271	ed_Lushi_vs_Arbor_Acr	breed_growth_background	ENSGALG00010000177	ENSGALG00010000177	0.183009938	0.112640119	1.624731392	0.104219805	0.221550282	positive
PRJNA729271	ed_Lushi_vs_Arbor_Acr	breed_growth_background	ENSGALG00010000184	ENSGALG00010000184	1.271564085	1.818417836	0.699269475	0.48438364	0.636549489	positive
PRJNA729271	ed_Lushi_vs_Arbor_Acr	breed_growth_background	ENSGALG00010000186	THOC5	0.016332524	0.043084193	0.379083913	0.704625552	0.808993851	positive
PRJNA729271	ed_Lushi_vs_Arbor_Acr	breed_growth_background	ENSGALG00010000188	ENSGALG00010000188	-0.943749561	0.716769948	-1.316670103	0.187949229	0.333740546	negative
PRJNA729271	ed_Lushi_vs_Arbor_Acr	breed_growth_background	ENSGALG00010000189	ENSGALG00010000189	-0.249284708	0.309979286	-0.804197955	0.42128266	0.5803547	negative
PRJNA729271	ed_Lushi_vs_Arbor_Acr	breed_growth_background	ENSGALG00010000191	NEFH	-0.097664818	0.191438103	-0.510163945	0.60993661	0.740454871	negative
PRJNA729271	ed_Lushi_vs_Arbor_Acr	breed_growth_background	ENSGALG00010000192	ENSGALG00010000192	0.240286751	0.370969821	0.647725872	0.517162268	0.665565753	positive
PRJNA729271	ed_Lushi_vs_Arbor_Acr	breed_growth_background	ENSGALG00010000196	KSR2	0.234391302	0.522982239	0.448182145	0.654021748	0.774638962	positive
PRJNA729271	ed_Lushi_vs_Arbor_Acr	breed_growth_background	ENSGALG00010000197	ENSGALG00010000197	-0.135122023	0.280236517	-0.482171363	0.629684218	0.755756624	negative
PRJNA729271	ed_Lushi_vs_Arbor_Acr	breed_growth_background	ENSGALG00010000200	GAL3ST1	0.352347443	0.153941846	2.288834721	0.022088957	0.073317938	positive
PRJNA729271	ed_Lushi_vs_Arbor_Acr	breed_growth_background	ENSGALG00010000202	ENSGALG00010000202	-0.62756269	0.16468943	-3.810582681	0.00013864	0.002025788	negative
PRJNA729271	ed_Lushi_vs_Arbor_Acr	breed_growth_background	ENSGALG00010000205	RNF215	0.090645371	0.062209725	1.457093245	0.145090643	0.27896125	positive
PRJNA729271	ed_Lushi_vs_Arbor_Acr	breed_growth_background	ENSGALG00010000210	CCDC157	0.135059987	0.304160904	0.444041245	0.657012765	0.776596267	positive
PRJNA729271	ed_Lushi_vs_Arbor_Acr	breed_growth_background	ENSGALG00010000215	SF3A1	0.021242186	0.04867793	0.436382278	0.662559395	0.781196255	positive
PRJNA729271	ed_Lushi_vs_Arbor_Acr	breed_growth_background	ENSGALG00010000221	TBC1D10A	0.010506341	0.047613557	0.220658595	0.825358273	0.89328695	positive
PRJNA729271	ed_Lushi_vs_Arbor_Acr	breed_growth_background	ENSGALG00010000223	CASTOR1	0.12270715	0.084221793	1.456952471	0.145129501	0.278961956	positive
PRJNA729271	ed_Lushi_vs_Arbor_Acr	breed_growth_background	ENSGALG00010000224	MYBPC2	0.399631716	0.115953894	3.44647085	0.00056796	0.00541617	positive
PRJNA729271	ed_Lushi_vs_Arbor_Acr	breed_growth_background	ENSGALG00010000225	LIF	0.327278922	0.212184266	1.542427851	0.122969658	0.247940631	positive
PRJNA729271	ed_Lushi_vs_Arbor_Acr	breed_growth_background	ENSGALG00010000226	ENSGALG00010000226	0.242188025	0.234466386	1.032932819	0.301635339	0.464091311	positive
PRJNA729271	ed_Lushi_vs_Arbor_Acr	breed_growth_background	ENSGALG00010000227	ENSGALG00010000227	-0.103808998	0.15575339	-0.666495915	0.505094174	0.655130385	negative
PRJNA729271	ed_Lushi_vs_Arbor_Acr	breed_growth_background	ENSGALG00010000231	ENSGALG00010000231	0.370007418	0.22395438	1.652155311	0.098502894	0.21269171	positive
PRJNA729271	ed_Lushi_vs_Arbor_Acr	breed_growth_background	ENSGALG00010000232	ENSGALG00010000232	0.149537944	0.086925308	1.720303858	0.085377222	0.192844152	positive
PRJNA729271	ed_Lushi_vs_Arbor_Acr	breed_growth_background	ENSGALG00010000233	HORMAD2	-0.210404493	0.28174785	-0.746782957	0.455194585	0.611238119	negative
PRJNA729271	ed_Lushi_vs_Arbor_Acr	breed_growth_background	ENSGALG00010000234	ENSGALG00010000234	1.970151197	2.451100878	0.803782176			positive
PRJNA729271	ed_Lushi_vs_Arbor_Acr	breed_growth_background	ENSGALG00010000235	ENSGALG00010000235	0.086492832	0.131962073	0.655437053	0.512186402	0.660863619	positive
PRJNA729271	ed_Lushi_vs_Arbor_Acr	breed_growth_background	ENSGALG00010000239	ENSGALG00010000239	3.825211957	2.484486368	1.53963894	0.123648388	0.248997414	positive
PRJNA729271	ed_Lushi_vs_Arbor_Acr	breed_growth_background	ENSGALG00010000242	ENSGALG00010000242	0.098762561	0.101634209	0.971745264	0.331177292	0.49550112	positive
PRJNA729271	ed_Lushi_vs_Arbor_Acr	breed_growth_background	ENSGALG00010000246	ENSGALG00010000246	3.9212194	2.279762715	1.720012075	0.085430247	0.192883399	positive
PRJNA729271	ed_Lushi_vs_Arbor_Acr	breed_growth_background	ENSGALG00010000247	ENSGALG00010000247	0.387904053	0.117593124	3.298696725	0.000971348	0.007983799	positive
PRJNA729271	ed_Lushi_vs_Arbor_Acr	breed_growth_background	ENSGALG00010000249	ENSGALG00010000249	4.438307633	2.373157401	1.870212078			positive
PRJNA729271	ed_Lushi_vs_Arbor_Acr	breed_growth_background	ENSGALG00010000254	ENSGALG00010000254	-0.310130795	0.357184508	-0.868264967	0.385249296	0.547094008	negative
PRJNA729271	ed_Lushi_vs_Arbor_Acr	breed_growth_background	ENSGALG00010000255	ENSGALG00010000255	0.034881098	0.077480575	0.450191519	0.652572351	0.773428775	positive
PRJNA729271	ed_Lushi_vs_Arbor_Acr	breed_growth_background	ENSGALG00010000256	ENSGALG00010000256	0.232393573	0.232041807	1.001515958	0.316577429	0.47989854	positive
PRJNA729271	ed_Lushi_vs_Arbor_Acr	breed_growth_background	ENSGALG00010000262	HNRNPKL	2.343776629	1.873802212	1.250813247	0.211002621	0.361053357	positive
PRJNA729271	ed_Lushi_vs_Arbor_Acr	breed_growth_background	ENSGALG00010000263	MTMR3	0.004168225	0.0489037	0.08523333	0.932075894	0.962674736	positive
PRJNA729271	ed_Lushi_vs_Arbor_Acr	breed_growth_background	ENSGALG00010000267	ENSGALG00010000267	0.075529705	0.066411376	1.137300706	0.2554126	0.412318257	positive
PRJNA729271	ed_Lushi_vs_Arbor_Acr	breed_growth_background	ENSGALG00010000269	ENSGALG00010000269	0.176122227	0.28331092	0.621657036	0.534167409	0.679606476	positive
PRJNA729271	ed_Lushi_vs_Arbor_Acr	breed_growth_background	ENSGALG00010000271	RTN4R	0.423215583	0.237886239	1.77906711	0.07522876	0.176618218	positive
PRJNA729271	ed_Lushi_vs_Arbor_Acr	breed_growth_background	ENSGALG00010000272	ENSGALG00010000272	-0.602008328	0.19503913	-3.086602809	0.002024579	0.013369602	negative
PRJNA729271	ed_Lushi_vs_Arbor_Acr	breed_growth_background	ENSGALG00010000277	ENSGALG00010000277	0.111456254	0.304114643	0.366494205	0.713996336	0.816325674	positive
PRJNA729271	ed_Lushi_vs_Arbor_Acr	breed_growth_background	ENSGALG00010000279	ENSGALG00010000279	0.088623974	0.075780104	1.169488674	0.242206802	0.39795824	positive
PRJNA729271	ed_Lushi_vs_Arbor_Acr	breed_growth_background	ENSGALG00010000281	OSBP2	0.27153543	0.108196833	2.509643054	0.012085325	0.047758122	positive
PRJNA729271	ed_Lushi_vs_Arbor_Acr	breed_growth_background	ENSGALG00010000282	ENSGALG00010000282	5.048505623	2.656241914	1.900619667	0.057351848	0.146075388	positive
PRJNA729271	ed_Lushi_vs_Arbor_Acr	breed_growth_background	ENSGALG00010000284	ENSGALG00010000284	0.241782706	0.084177907	2.872282232	0.004075188	0.02190206	positive
PRJNA729271	ed_Lushi_vs_Arbor_Acr	breed_growth_background	ENSGALG00010000285	MYLPF	0.069609014	0.07782105	0.894475383	0.371067595	0.53417666	positive

PRJNA729271	eed_Lushi_vs_Arbor_Acr	breed_growth_background	ENSGALG00010000286	TCN2	-0.051120623	0.072376893	-0.706311373	0.479994523	0.632847063	negative
PRJNA729271	eed_Lushi_vs_Arbor_Acr	breed_growth_background	ENSGALG00010000287	IQCD	-0.03143955	0.125908344	-0.249701876	0.802817907	0.878395942	negative
PRJNA729271	eed_Lushi_vs_Arbor_Acr	breed_growth_background	ENSGALG00010000288	VARS2	0.160203028	0.083552299	1.917398198	0.055187363	0.142312903	positive
PRJNA729271	eed_Lushi_vs_Arbor_Acr	breed_growth_background	ENSGALG00010000289	QPRT	0.02010656	0.255707918	0.078630962	0.937326159	0.965501896	positive
PRJNA729271	eed_Lushi_vs_Arbor_Acr	breed_growth_background	ENSGALG00010000292	ABHD16A	-0.005062497	0.074904363	-0.06758614	0.946115089	0.970470493	negative
PRJNA729271	eed_Lushi_vs_Arbor_Acr	breed_growth_background	ENSGALG00010000293	ENSGALG00010000293	-0.384216594	0.190518017	-2.016694279	0.043727427	0.120175811	negative
PRJNA729271	eed_Lushi_vs_Arbor_Acr	breed_growth_background	ENSGALG00010000294	DUSP21	-0.606777526	0.166365954	-3.647245787	0.000265066	0.003185851	negative
PRJNA729271	eed_Lushi_vs_Arbor_Acr	breed_growth_background	ENSGALG00010000298	SLC35E4	0.199524873	0.119514369	1.669463465	0.095025565	0.207470337	positive
PRJNA729271	eed_Lushi_vs_Arbor_Acr	breed_growth_background	ENSGALG00010000301	UQCR10	-0.107554593	0.082659582	-1.301175143	0.193198512	0.340186155	negative
PRJNA729271	eed_Lushi_vs_Arbor_Acr	breed_growth_background	ENSGALG00010000302	ENSGALG00010000302	-0.064682805	0.125948148	-0.513566942	0.607554797	0.7383673	negative
PRJNA729271	eed_Lushi_vs_Arbor_Acr	breed_growth_background	ENSGALG00010000303	ENSGALG00010000303	-0.119710317	0.060874223	-1.966519027	0.049238687	0.131113581	negative
PRJNA729271	eed_Lushi_vs_Arbor_Acr	breed_growth_background	ENSGALG00010000306	ENSGALG00010000306	0.198250515	0.109790918	1.805709603	0.070963712	0.169320001	positive
PRJNA729271	eed_Lushi_vs_Arbor_Acr	breed_growth_background	ENSGALG00010000307	ZMAT5	0.24370806	0.06014676	4.051890069	5.08055E-05	0.000980594	positive
PRJNA729271	eed_Lushi_vs_Arbor_Acr	breed_growth_background	ENSGALG00010000308	ENSGALG00010000308	0.241267023	0.189146913	1.275553586	0.20211337	0.351190758	positive
PRJNA729271	eed_Lushi_vs_Arbor_Acr	breed_growth_background	ENSGALG00010000309	ENSGALG00010000309	4.209648596	2.31068952	1.8218149	0.068483076	0.165416129	positive
PRJNA729271	eed_Lushi_vs_Arbor_Acr	breed_growth_background	ENSGALG00010000312	CABP7	-0.520494882	0.307123884	-1.694739189	0.090124908	0.200423526	negative
PRJNA729271	eed_Lushi_vs_Arbor_Acr	breed_growth_background	ENSGALG00010000313	ENSGALG00010000313	6.020598153	2.053421754	2.931983233	0.003368049	0.019273539	positive
PRJNA729271	eed_Lushi_vs_Arbor_Acr	breed_growth_background	ENSGALG00010000318	ENSGALG00010000318	4.277390437	2.545400912	1.680438794	0.092871974	0.204338078	positive
PRJNA729271	eed_Lushi_vs_Arbor_Acr	breed_growth_background	ENSGALG00010000323	AP1B1	0.193735643	0.077511163	2.499454734	0.012438459	0.048781239	positive
PRJNA729271	eed_Lushi_vs_Arbor_Acr	breed_growth_background	ENSGALG00010000324	ENSGALG00010000324	0.35593969	0.095809065	3.715094084	0.000203128	0.002624191	positive
PRJNA729271	eed_Lushi_vs_Arbor_Acr	breed_growth_background	ENSGALG00010000326	ENSGALG00010000326	2.062893683	0.972739616	2.120704914	0.033946643	0.099626764	positive
PRJNA729271	eed_Lushi_vs_Arbor_Acr	breed_growth_background	ENSGALG00010000331	ENSGALG00010000331	-0.131848726	0.096013226	-1.373235031	0.169679292	0.311814454	negative
PRJNA729271	eed_Lushi_vs_Arbor_Acr	breed_growth_background	ENSGALG00010000338	ENSGALG00010000338	0.197996345	0.104991677	1.885828958	0.059318007	0.149637055	positive
PRJNA729271	eed_Lushi_vs_Arbor_Acr	breed_growth_background	ENSGALG00010000340	NAA10	0.181946378	0.072005748	2.526831305	0.011509677	0.046214064	positive
PRJNA729271	eed_Lushi_vs_Arbor_Acr	breed_growth_background	ENSGALG00010000341	EWSR1	0.225023707	0.079548158	2.828773313	0.004672678	0.02422406	positive
PRJNA729271	eed_Lushi_vs_Arbor_Acr	breed_growth_background	ENSGALG00010000346	ENSGALG00010000346	0.181733795	0.061891502	2.93632874	0.003321222	0.019078395	positive
PRJNA729271	eed_Lushi_vs_Arbor_Acr	breed_growth_background	ENSGALG00010000349	CHIR-IG1-5	-0.476918856	0.236388738	-2.017519361	0.043641343	0.119988858	negative
PRJNA729271	eed_Lushi_vs_Arbor_Acr	breed_growth_background	ENSGALG00010000350	ENSGALG00010000350	0.201546659	0.088709483	2.271985502	0.023087386	0.075610407	positive
PRJNA729271	eed_Lushi_vs_Arbor_Acr	breed_growth_background	ENSGALG00010000353	ENSGALG00010000353	-0.135644802	0.059073346	-2.296209912	0.021663882	0.072237858	negative
PRJNA729271	eed_Lushi_vs_Arbor_Acr	breed_growth_background	ENSGALG00010000356	ENSGALG00010000356	-0.060129243	0.065060726	-0.924201842	0.355381237	0.519122345	negative
PRJNA729271	eed_Lushi_vs_Arbor_Acr	breed_growth_background	ENSGALG00010000366	EMID1	-0.028193561	0.121740741	-0.231586904	0.81685888	0.887046157	negative
PRJNA729271	eed_Lushi_vs_Arbor_Acr	breed_growth_background	ENSGALG00010000367	HSD17B10	-0.038501334	0.074417653	-0.517368295	0.604899088	0.736869676	negative
PRJNA729271	eed_Lushi_vs_Arbor_Acr	breed_growth_background	ENSGALG00010000373	ENSGALG00010000373	-0.670550776	0.248403162	-2.699445415	0.006945515	0.03220577	negative
PRJNA729271	eed_Lushi_vs_Arbor_Acr	breed_growth_background	ENSGALG00010000376	ASCC2	-0.007407718	0.04688105	-0.158010923	0.874448192	0.925799908	negative
PRJNA729271	eed_Lushi_vs_Arbor_Acr	breed_growth_background	ENSGALG00010000377	ENSGALG00010000377	-0.269321398	0.132675627	-2.029923691	0.042364297	0.117480482	negative
PRJNA729271	eed_Lushi_vs_Arbor_Acr	breed_growth_background	ENSGALG00010000381	ENSGALG00010000381	0.306017698	0.16823342	1.819006578	0.068910427	0.166088565	positive
PRJNA729271	eed_Lushi_vs_Arbor_Acr	breed_growth_background	ENSGALG00010000382	ENSGALG00010000382	0.327997692	0.299992122	1.093354352	0.274238248	0.43310998	positive
PRJNA729271	eed_Lushi_vs_Arbor_Acr	breed_growth_background	ENSGALG00010000384	ENSGALG00010000384	0.176681611	0.079949762	2.209907904	0.027111555	0.084523818	positive
PRJNA729271	eed_Lushi_vs_Arbor_Acr	breed_growth_background	ENSGALG00010000386	ENSGALG00010000386	0.116519099	0.199585359	0.583805843	0.559350946	0.700336181	positive
PRJNA729271	eed_Lushi_vs_Arbor_Acr	breed_growth_background	ENSGALG00010000387	DTX1	0.056186964	0.279320203	0.201156103	0.840576514	0.903266111	positive
PRJNA729271	eed_Lushi_vs_Arbor_Acr	breed_growth_background	ENSGALG00010000388	ENSGALG00010000388	0.220526765	0.099691269	2.212097086	0.026959957	0.084184529	positive
PRJNA729271	eed_Lushi_vs_Arbor_Acr	breed_growth_background	ENSGALG00010000391	ENSGALG00010000391	-0.160761893	0.334366938	-0.480794823	0.63066233	0.756492095	negative
PRJNA729271	eed_Lushi_vs_Arbor_Acr	breed_growth_background	ENSGALG00010000402	PFKFB1	0.27916427	0.144116213	1.937077477	0.052735873	0.137681283	positive
PRJNA729271	eed_Lushi_vs_Arbor_Acr	breed_growth_background	ENSGALG00010000405	ENSGALG00010000405	-0.886857415	0.201949394	-4.391483422	1.1258E-05	0.000318765	negative
PRJNA729271	eed_Lushi_vs_Arbor_Acr	breed_growth_background	ENSGALG00010000406	NSMCE3	0.004098199	0.053339809	0.076831905	0.938757269	0.96653972	positive
PRJNA729271	eed_Lushi_vs_Arbor_Acr	breed_growth_background	ENSGALG00010000407	WSB2	-0.065000339	0.045642651	-1.424114013	0.154413471	0.290905024	negative
PRJNA729271	eed_Lushi_vs_Arbor_Acr	breed_growth_background	ENSGALG00010000408	ENSGALG00010000408	0.979454313	0.16595371	5.901972998	3.5918E-09	5.91627E-07	positive
PRJNA729271	eed_Lushi_vs_Arbor_Acr	breed_growth_background	ENSGALG00010000411	ENSGALG00010000411	-1.041685129	0.387355421	-2.689223056	0.007161855	0.032903671	negative
PRJNA729271	eed_Lushi_vs_Arbor_Acr	breed_growth_background	ENSGALG00010000412	RFC5	-0.000737864	0.057902538	-0.012743208	0.989832666	0.993946969	negative
PRJNA729271	eed_Lushi_vs_Arbor_Acr	breed_growth_background	ENSGALG00010000422	ITIH6	-0.510250055	0.32967092	-1.547755731	0.121681118	0.246345837	negative
PRJNA729271	eed_Lushi_vs_Arbor_Acr	breed_growth_background	ENSGALG00010000423	ENSGALG00010000423	-0.329489889	0.106492456	-3.094020941	0.001974635	0.013159697	negative
PRJNA729271	eed_Lushi_vs_Arbor_Acr	breed_growth_background	ENSGALG00010000428	GNL3L	0.331968587	0.081205493	4.088006527	4.35096E-05	0.000874718	positive
PRJNA729271	eed_Lushi_vs_Arbor_Acr	breed_growth_background	ENSGALG00010000431	AIFM3	0.136923487	0.648797261	0.211042024	0.83285448	0.898568792	positive
PRJNA729271	eed_Lushi_vs_Arbor_Acr	breed_growth_background	ENSGALG00010000432	ENSGALG00010000432	0.264618402	0.09789878	2.702979553	0.006872097	0.031988135	positive
PRJNA729271	eed_Lushi_vs_Arbor_Acr	breed_growth_background	ENSGALG00010000434	TP53	-0.021328429	0.119334669	-0.178727854	0.858151397	0.914805541	negative
PRJNA729271	eed_Lushi_vs_Arbor_Acr	breed_growth_background	ENSGALG00010000435	P2RX6	0.191752256	0.173934603	1.102438807	0.270270948	0.428968544	positive
PRJNA729271	eed_Lushi_vs_Arbor_Acr	breed_growth_background	ENSGALG00010000436	ENSGALG00010000436	-0.119924283	0.201450915	-0.595302747	0.55164112	0.694401914	negative
PRJNA729271	eed_Lushi_vs_Arbor_Acr	breed_growth_background	ENSGALG00010000440	ENSGALG00010000440	0.070876888	0.264738633	0.267724007	0.78891177	0.869007987	positive
PRJNA729271	eed_Lushi_vs_Arbor_Acr	breed_growth_background	ENSGALG00010000441	SLC7A4	0.233748912	0.118310952	1.975716598	0.048186884	0.128916368	positive
PRJNA729271	eed_Lushi_vs_Arbor_Acr	breed_growth_background	ENSGALG00010000442	DGCR6	0.20528191	0.070737727	2.902014488	0.003707714	0.020512715	positive
PRJNA729271	eed_Lushi_vs_Arbor_Acr	breed_growth_background	ENSGALG00010000443	TBX3	0.008202949	0.084928302	0.096586753	0.923054577	0.95712684	positive
PRJNA729271	eed_Lushi_vs_Arbor_Acr	breed_growth_background	ENSGALG00010000444	RBM19	0.170493287	0.064052879	2.661758395	0.007773365	0.034937962	positive
PRJNA729271	eed_Lushi_vs_Arbor_Acr	breed_growth_background	ENSGALG00010000449	PLBD2	-0.12478349	0.05736135	-2.175393169	0.029600666	0.090082228	negative
PRJNA729271	eed_Lushi_vs_Arbor_Acr	breed_growth_background	ENSGALG00010000450	ENSGALG00010000450	-0.046259796	0.102251597	-0.452411478	0.65097258	0.772173962	negative
PRJNA729271	eed_Lushi_vs_Arbor_Acr	breed_growth_background	ENSGALG00010000452	SLC8B1	-0.08013779	0.118830821	-0.674385561	0.50006622	0.651254255	negative
PRJNA729271	eed_Lushi_vs_Arbor_Acr	breed_growth_background	ENSGALG00010000453	ACHE	0.168131431	0.133056673	1.263607663	0.206370882	0.35557521	positive
PRJNA729271	eed_Lushi_vs_Arbor_Acr	breed_growth_background	ENSGALG00010000457	TPCN1	-0.121267992	0.044135824	-2.747609105	0.006003153	0.02905899	negative
PRJNA729271	eed_Lushi_vs_Arbor_Acr	breed_growth_background	ENSGALG00010000458	ENSGALG00010000458	0.202004299	0.106917419	1.88934882	0.058845104	0.14865106	positive
PRJNA729271	eed_Lushi_vs_Arbor_Acr	breed_growth_background	ENSGALG00010000462	ENSGALG00010000462	0.06817263	0.126074244	0.540733995	0.588690943	0.724369714	positive
PRJNA729271	eed_Lushi_vs_Arbor_Acr	breed_growth_background	ENSGALG00010000465	ENSGALG00010000465	0.214898059	0.102665477	2.093187173	0.036332449	0.104803752	positive
PRJNA729271	eed_Lushi_vs_Arbor_Acr	breed_growth_background	ENSGALG00010000466	FBXO21	0.05561744	0.04404755	1.262668194	0.206708449	0.355721117	positive
PRJNA729271	eed_Lushi_vs_Arbor_Acr	breed_growth_background	ENSGALG00010000467	RNASEK	-0.031737286	0.106535786	-0.297902587	0.765777513	0.853185631	negative
PRJNA729271	eed_Lushi_vs_Arbor_Acr	breed_growth_background	ENSGALG00010000468	TESC	-0.402819125	0.178409975	-2.257828491	0.023956354	0.077597174	negative
PRJNA729271	eed_Lushi_vs_Arbor_Acr	breed_growth_background	ENSGALG00010000469	ENSGALG00010000469	0.267670574	0.153312995	1.745909231	0.080826726	0.185377118	positive
PRJNA729271	eed_Lushi_vs_Arbor_Acr	breed_growth_background	ENSGALG00010000470	FBXW8	0.081666884	0.05898894	1.384443996	0.166222545	0.306812147	positive
PRJNA729271	eed_Lushi_vs_Arbor_Acr	breed_growth_background	ENSGALG00010000471	ENSGALG00010000471	-0.018107217	0.112425925	-0.161059092	0.872046859	0.924270198	negative
PRJNA729271	eed_Lushi_vs_Arbor_Acr	breed_growth_background	ENSGALG00010000475	RNFT2	0.024039533	0.060870164	0.394931296	0.692893593	0.801747635	positive
PRJNA729271	eed_Lushi_vs_Arbor_Acr	breed_growth_background	ENSGALG00010000476	ENSGALG00010000476	0.498229092	0.11372035	4.38117797	1.18039E-05	0.000327774	positive
PRJNA729271	eed_Lushi_vs_Arbor_Acr	breed_growth_background	ENSGALG00010000479	C12orf49	-0.218021271	0.061613199	-3.538548177	0.000402334	0.004253705	negative
PRJNA729271	eed_Lushi_vs_Arbor_Acr	breed_growth_background	ENSGALG00010000483	ENSGALG00010000483	-0.010176609	0.13269143	-0.076693791	0.938867144	0.96653972	negative
PRJNA729271	eed_Lushi_vs_Arbor_Acr	breed_growth_background	ENSGALG00010000485	MED13L	0.011508229	0.067343004	0.170889748	0.864310458	0.918555619	positive
PRJNA729271	eed_Lushi_vs_Arbor_Acr	breed_growth_background	ENSGALG00010000491	ENSGALG00010000491	0.237427775	0.083617634	2.839446243	0.004519191	0.023622673	positive
PRJNA729271	eed_Lushi_vs_Arbor_Acr	breed_growth_background	ENSGALG00010000495	ENSGALG00010000495	-0.890470596	0.30076231	-2.960712057	0.003069287	0.018026467	negative
PRJNA729271	eed_Lushi_vs_Arbor_Acr	breed_growth_background	ENSGALG00010000501	ENSGALG00010000501	0.23865259	0.087584581	2.724824259	0.006433572	0.030545243	positive
PRJNA729271	eed_Lushi_vs_Arbor_Acr	breed_growth_background	ENSGALG00010000507	TRPT1	-0.106648749	0.151458311	-0.704145898	0.481341919	0.634164332	negative
PRJNA729271	eed_Lushi_vs_Arbor_Acr	breed_growth_background	ENSGALG00010000509	ENSGALG00010000509	0.654059796	0.60192576	1.08661207	0.277208288	0.436418744	positive
PRJNA729271	eed_Lushi_vs_Arbor_Acr	breed_growth_background	ENSGALG00010000511	FERMT3	-0.155096994	0.086489369	-1.793249216	0.072933084	0.172824105	negative
PRJNA729271	eed_Lushi_vs_Arbor_Acr	breed_growth_background	ENSGALG00010000525	ENSGALG00010000525	0.750594714	0.304750743	2.46297911	0.013778794	0.052448431	positive
PRJNA729271	eed_Lushi_vs_Arbor_Acr	breed_growth_background	ENSGALG00010000526	POLD1	-0.158270807	0.09039743	-1.750833042	0.079974673	0.184112914	negative
PRJNA729271	eed_Lushi_vs_Arbor_Acr	breed_growth_background	ENSGALG00010000529	STIP1	0.159767139	0.103250751	1.547370234	0.121773995	0.246450566	positive
PRJNA729271	eed_Lushi_vs_Arbor_Acr	breed_growth_background	ENSGALG00010000532	ENSGALG00010000532	-0.761724009	0.249146192	-3.057337556	0.002233126	0.014399096	negative
PRJNA729271	eed_Lushi_vs_Arbor_Acr	breed_growth_background	ENSGALG00010000546	FLRT1	0.102791871	0.360976197	0.284760802	0.775827405	0.860941527	positive
PRJNA729271	eed_Lushi_vs_Arbor_Acr	breed_growth_background	ENSGALG00010000555	OTUB1	0.043186941	0.055392156	0.779658059	0.435592172	0.593468233	positive
PRJNA729271	eed_Lushi_vs_Arbor_Acr	breed_growth_background	ENSGALG00010000561	ENSGALG00010000561	0.152264662	0.149145156	1.020915911	0.30729428	0.469852136	positive
PRJNA729271	eed_Lushi_vs_Arbor_Acr	breed_growth_background	ENSGALG00010000564	NAA40	-0.095869578	0.049381608	-1.941402501	0.052209475	0.136717843	negative
PRJNA729271	eed_Lushi_vs_Arbor_Acr	breed_growth_background	ENSGALG00010000569	SIPA1	0.038509185	0.125022682	0.308017587	0.75806895	0.84717942	positive
PRJNA729271	eed_Lushi_vs_Arbor_Acr	breed_growth_background	ENSGALG00010000574	ENSGALG00010000574	0.378111668	0.088389861	4.277771962	1.88773E-05	0.000459877	positive
PRJNA729271	eed_Lushi_vs_Arbor_Acr	breed_growth_background	ENSGALG00010000575	ENSGALG00010000575	0.184507368	0.084821681	2.175238294	0.029612264	0.090085386	positive
PRJNA729271	eed_Lushi_vs_Arbor_Acr	breed_growth_background	ENSGALG00010000576	NOS1	0.190896538	0.217756422	0.8766517	0.380675849	0.542352705	positive
PRJNA729271	eed_Lushi_vs_Arbor_Acr	breed_growth_background	ENSGALG00010000577	ENSGALG00010000577	-0.324379964	0.148528898	-2.183951863	0.028965785	0.088648258	negative
PRJNA729271	eed_Lushi_vs_Arbor_Acr	breed_growth_background	ENSGALG00010000578	ENSGALG00010000578	0.556266783	0.207523796	2.680496374	0.007351306	0.033529636	positive
PRJNA729271	eed_Lushi_vs_Arbor_Acr	breed_growth_background	ENSGALG00010000579	ENSGALG00010000579	-0.889221185	0.508235752	-1.749623442	0.080183312	0.184390772	negative
PRJNA729271	eed_Lushi_vs_Arbor_Acr	breed_growth_background	ENSGALG00010000580	ENSGALG00010000580	-0.138840572	0.281648268	-0.492957309	0.622042752	0.748932569	negative
PRJNA729271	eed_Lushi_vs_Arbor_Acr	breed_growth_background	ENSGALG00010000581	ENSGALG00010000581	0.185572056	0.070458065	2.633794379	0.008443661	0.036895153	positive
PRJNA729271	eed_Lushi_vs_Arbor_Acr	breed_growth_background	ENSGALG00010000583	CCS	0.29170122	0.075559761	3.860536582	0.000113138	0.001752072	positive
PRJNA729271	eed_Lushi_vs_Arbor_Acr	breed_growth_background	ENSGALG00010000584	RBM14	0.314691758	0.113499894	2.772617196	0.005560749	0.027458945	positive
PRJNA729271	eed_Lushi_vs_Arbor_Acr	breed_growth_background	ENSGALG00010000585	SPTBN2	-0.141214587	0.321126569	-0.439747441	0.660120038	0.779188921	negative
PRJNA729271	eed_Lushi_vs_Arbor_Acr	breed_growth_background	ENSGALG00010000586	ENSGALG00010000586	0.355150104	0.115946536	3.063050576	0.00219093	0.014171144	positive
PRJNA729271	eed_Lushi_vs_Arbor_Acr	breed_growth_background	ENSGALG00010000589	ENSGALG00010000589	-0.01213522	0.169552661	-0.071571983	0.942942537	0.969134715	negative
PRJNA729271	eed_Lushi_vs_Arbor_Acr	breed_growth_background	ENSGALG00010000590	DNAH5	-0.08651724	0.240996026	-0.358998619	0.719596122	0.820398442	negative
PRJNA729271	eed_Lushi_vs_Arbor_Acr	breed_growth_background	ENSGALG00010000591	SBK2	-0.66874642	0.157319297	-4.250886134	2.12926E-05	0.000501034	negative
PRJNA729271	eed_Lushi_vs_Arbor_Acr	breed_growth_background	ENSGALG00010000592	CCT5	0.133546334	0.063899414	2.089946145	0.036622638	0.105451954	positive
PRJNA729271	eed_Lushi_vs_Arbor_Acr	breed_growth_background	ENSGALG00010000593	ATPSCKMT	0.118693412	0.081186206	1.461989878	0.143743984	0.277070351	positive
PRJNA729271	eed_Lushi_vs_Arbor_Acr	breed_growth_background	ENSGALG00010000595	MYO10	-0.017606978	0.075259847	-0.233949162	0.815024446	0.886255015	negative
PRJNA729271	eed_Lushi_vs_Arbor_Acr	breed_growth_background	ENSGALG00010000596	ENSGALG00010000596	-0.318709469	0.392448307	-0.812105603	0.416731038	0.576880565	negative
PRJNA729271	eed_Lushi_vs_Arbor_Acr	breed_growth_background	ENSGALG00010000597	CDH9	0.135470075	0.406387062	0.333352332	0.738868341	0.832625901	positive
PRJNA729271	eed_Lushi_vs_Arbor_Acr	breed_growth_background	ENSGALG00010000598	ENSGALG00010000598	-0.18375554	0.300442749	-0.611615827	0.540791964	0.685627527	negative
PRJNA729271	eed_Lushi_vs_Arbor_Acr	breed_growth_background	ENSGALG00010000599	RETREG1	0.309070597	0.199793502	1.546950192	0.121875257	0.246524982	positive
PRJNA729271	eed_Lushi_vs_Arbor_Acr	breed_growth_background	ENSGALG00010000601	ENSGALG00010000601	0.803724113	0.196406723	4.092141562	4.27408E-05	0.000862851	positive
PRJNA729271	eed_Lushi_vs_Arbor_Acr	breed_growth_background	ENSGALG00010000603	ZNF622	0.148087082	0.05906164	2.507331015	0.012164673	0.0480324	positive
PRJNA729271	eed_Lushi_vs_Arbor_Acr	breed_growth_background	ENSGALG00010000604	ENSGALG00010000604	-0.130322366	0.073212884	-1.780046879	0.075068289	0.176470135	negative
PRJNA729271	eed_Lushi_vs_Arbor_Acr	breed_growth_background	ENSGALG00010000605	MARCHF11	-0.6532516	0.411026447	-1.589317681	0.111988689	0.233296356	negative
PRJNA729271	eed_Lushi_vs_Arbor_Acr	breed_growth_background	ENSGALG00010000606	UBE2QL1	-0.272491026	0.08472264	-3.216271659	0.001298678	0.009842037	negative
PRJNA729271	eed_Lushi_vs_Arbor_Acr	breed_growth_background	ENSGALG00010000607	ENSGALG00010000607	-0.074646019	0.152576918	-0.489235336	0.624675096	0.751237494	negative
PRJNA729271	eed_Lushi_vs_Arbor_Acr	breed_growth_background	ENSGALG00010000608	ENSGALG00010000608	0.682162456	0.578438438	1.179317298	0.238271853	0.393589801	positive
PRJNA729271	eed_Lushi_vs_Arbor_Acr	breed_growth_background	ENSGALG00010000610	ENSGALG00010000610	0.177145164	0.043360047	4.085446738	4.39921E-05	0.000880753	positive
PRJNA729271	eed_Lushi_vs_Arbor_Acr	breed_growth_background	ENSGALG00010000611	C7orf57	-0.109580843	0.170972312	-0.640927421	0.521569839	0.668803505	negative
PRJNA729271	eed_Lushi_vs_Arbor_Acr	breed_growth_background	ENSGALG00010000612	ENSGALG00010000612	0.098499498	0.227335051	0.433278977	0.664812109	0.782560624	positive
PRJNA729271	eed_Lushi_vs_Arbor_Acr	breed_growth_background	ENSGALG00010000613	ENSGALG00010000613	-0.152134111	0.095394652	-1.59478658	0.110759969	0.231418036	negative
PRJNA729271	eed_Lushi_vs_Arbor_Acr	breed_growth_background	ENSGALG00010000614	KAT8	-0.059116947	0.097393655	-0.606989718	0.543857736	0.688250208	negative
PRJNA729271	eed_Lushi_vs_Arbor_Acr	breed_growth_background	ENSGALG00010000615	ENSGALG00010000615	-0.324520525	0.338189687	-0.959581375	0.337265946	0.501453472	negative

PRJNA729271	eed_Lushi_vs_Arbor_Acr	breed_growth_background	ENSGALG00010000616	PSENEN	-0.685510467	0.272470917	-2.515903256	0.011872777	0.047201289	negative
PRJNA729271	eed_Lushi_vs_Arbor_Acr	breed_growth_background	ENSGALG00010000619	ENSGALG00010000619	0.072368253	0.171092938	0.42297627	0.672312575	0.78799796	positive
PRJNA729271	eed_Lushi_vs_Arbor_Acr	breed_growth_background	ENSGALG00010000621	UPP1	-0.298906595	0.129866165	-2.301651055	0.021354858	0.071470486	negative
PRJNA729271	eed_Lushi_vs_Arbor_Acr	breed_growth_background	ENSGALG00010000622	ENSGALG00010000622	-0.021411232	0.248828192	-0.086048258	0.931428055	0.962367215	negative
PRJNA729271	eed_Lushi_vs_Arbor_Acr	breed_growth_background	ENSGALG00010000623	ENSGALG00010000623	-0.092490633	0.135003478	-0.685098151	0.493281987	0.644925186	negative
PRJNA729271	eed_Lushi_vs_Arbor_Acr	breed_growth_background	ENSGALG00010000625	ENSGALG00010000625	-0.476087552	0.180611909	-2.635969875	0.008389718	0.036755715	negative
PRJNA729271	eed_Lushi_vs_Arbor_Acr	breed_growth_background	ENSGALG00010000626	ABCA13	1.822975031	0.380571882	4.790093849	1.66703E-06	7.32232E-05	positive
PRJNA729271	eed_Lushi_vs_Arbor_Acr	breed_growth_background	ENSGALG00010000627	ENSGALG00010000627	0.021902311	0.125341037	0.174741741	0.861282567	0.916713909	positive
PRJNA729271	eed_Lushi_vs_Arbor_Acr	breed_growth_background	ENSGALG00010000630	ENSGALG00010000630	0.184434631	0.207332053	0.88956159	0.373701338	0.536051548	positive
PRJNA729271	eed_Lushi_vs_Arbor_Acr	breed_growth_background	ENSGALG00010000632	ENSGALG00010000632	-0.091167519	0.218769275	-0.416729079	0.676876582	0.791390582	negative
PRJNA729271	eed_Lushi_vs_Arbor_Acr	breed_growth_background	ENSGALG00010000633	VWC2	-0.133956721	0.294435805	-0.454960703	0.64913751	0.771060248	negative
PRJNA729271	eed_Lushi_vs_Arbor_Acr	breed_growth_background	ENSGALG00010000634	ENSGALG00010000634	0.133558455	0.101485969	1.316028767	0.188164387	0.333999853	positive
PRJNA729271	eed_Lushi_vs_Arbor_Acr	breed_growth_background	ENSGALG00010000635	ENSGALG00010000635	0.69484059	0.072188153	9.625410309	6.24572E-22	1.50886E-18	positive
PRJNA729271	eed_Lushi_vs_Arbor_Acr	breed_growth_background	ENSGALG00010000636	ENSGALG00010000636	-0.172545076	0.078666842	-2.193364735	0.028281111	0.087172385	negative
PRJNA729271	eed_Lushi_vs_Arbor_Acr	breed_growth_background	ENSGALG00010000639	SCN1B	0.348596197	0.140358476	2.483613439	0.01300569	0.050298154	positive
PRJNA729271	eed_Lushi_vs_Arbor_Acr	breed_growth_background	ENSGALG00010000641	ENSGALG00010000641	0.238123997	0.237427872	1.002931941	0.3158937	0.479330391	positive
PRJNA729271	eed_Lushi_vs_Arbor_Acr	breed_growth_background	ENSGALG00010000643	VKORC1	0.389685229	0.113184221	3.442928929	0.000575451	0.00546961	positive
PRJNA729271	eed_Lushi_vs_Arbor_Acr	breed_growth_background	ENSGALG00010000645	ENSGALG00010000645	-0.229705576	0.30184188	-0.76101294	0.446649338	0.603428293	negative
PRJNA729271	eed_Lushi_vs_Arbor_Acr	breed_growth_background	ENSGALG00010000646	ENSGALG00010000646	1.318742835	0.283670338	4.64885699	3.3378E-06	0.000123509	positive
PRJNA729271	eed_Lushi_vs_Arbor_Acr	breed_growth_background	ENSGALG00010000649	PAPD7	-0.060857857	0.045061923	-1.350538375	0.176843353	0.320899399	negative
PRJNA729271	eed_Lushi_vs_Arbor_Acr	breed_growth_background	ENSGALG00010000650	BCKDK	0.15420209	0.089796982	1.717230199	0.08593712	0.193695934	positive
PRJNA729271	eed_Lushi_vs_Arbor_Acr	breed_growth_background	ENSGALG00010000651	ENSGALG00010000651	0.375246303	0.348656704	1.07626298	0.28180966	0.4416511	positive
PRJNA729271	eed_Lushi_vs_Arbor_Acr	breed_growth_background	ENSGALG00010000652	SRD5A1	-0.161050812	0.123032361	-1.309011796	0.190530355	0.337126159	negative
PRJNA729271	eed_Lushi_vs_Arbor_Acr	breed_growth_background	ENSGALG00010000653	NSUN2	-0.073887606	0.050324381	-1.468226814	0.142042623	0.274856986	negative
PRJNA729271	eed_Lushi_vs_Arbor_Acr	breed_growth_background	ENSGALG00010000654	ENSGALG00010000654	-0.087303474	0.095688226	-0.91237426	0.361571735	0.52496204	negative
PRJNA729271	eed_Lushi_vs_Arbor_Acr	breed_growth_background	ENSGALG00010000655	ENSGALG00010000655	2.202543491	0.540984528	4.071361339	4.67392E-05	0.000918779	positive
PRJNA729271	eed_Lushi_vs_Arbor_Acr	breed_growth_background	ENSGALG00010000656	ENSGALG00010000656	-0.156878101	0.221022444	-0.709783577	0.477838355	0.630979954	negative
PRJNA729271	eed_Lushi_vs_Arbor_Acr	breed_growth_background	ENSGALG00010000659	ENSGALG00010000659	-0.53093259	0.290542015	-1.827386618	0.06764166	0.164162244	negative
PRJNA729271	eed_Lushi_vs_Arbor_Acr	breed_growth_background	ENSGALG00010000660	ENSGALG00010000660	0.302157571	0.092051606	3.28248016	0.001028982	0.008323157	positive
PRJNA729271	eed_Lushi_vs_Arbor_Acr	breed_growth_background	ENSGALG00010000661	ENSGALG00010000661	0.643694034	0.384511058	1.674058578	0.094119087	0.206112128	positive
PRJNA729271	eed_Lushi_vs_Arbor_Acr	breed_growth_background	ENSGALG00010000670	ENSGALG00010000670	0.338849357	0.335968971	1.00857337	0.313179284	0.476591467	positive
PRJNA729271	eed_Lushi_vs_Arbor_Acr	breed_growth_background	ENSGALG00010000674	GRB10	0.168958715	0.09049363	1.867078546	0.06189062	0.154221168	positive
PRJNA729271	eed_Lushi_vs_Arbor_Acr	breed_growth_background	ENSGALG00010000684	COBL	0.19146805	0.072019158	2.658571084	0.007847279	0.035139424	positive
PRJNA729271	eed_Lushi_vs_Arbor_Acr	breed_growth_background	ENSGALG00010000686	ENSGALG00010000686	-0.284220056	0.072011332	-3.946879564	7.91764E-05	0.001339161	negative
PRJNA729271	eed_Lushi_vs_Arbor_Acr	breed_growth_background	ENSGALG00010000687	IKZF1	0.145143031	0.15816686	0.917657665	0.358798118	0.522323865	positive
PRJNA729271	eed_Lushi_vs_Arbor_Acr	breed_growth_background	ENSGALG00010000688	ENSGALG00010000688	0.274997601	0.150088297	1.832238799	0.066915851	0.163043412	positive
PRJNA729271	eed_Lushi_vs_Arbor_Acr	breed_growth_background	ENSGALG00010000695	FIGNL1	-0.108130429	0.082363712	-1.312840644	0.189236651	0.33536927	negative
PRJNA729271	eed_Lushi_vs_Arbor_Acr	breed_growth_background	ENSGALG00010000697	ENSGALG00010000697	-0.204705914	0.133833258	-1.529559366	0.126125834	0.252757357	negative
PRJNA729271	eed_Lushi_vs_Arbor_Acr	breed_growth_background	ENSGALG00010000702	DDC	-0.094044591	0.242029533	-0.388566591	0.697596785	0.805133004	negative
PRJNA729271	eed_Lushi_vs_Arbor_Acr	breed_growth_background	ENSGALG00010000704	ENSGALG00010000704	-0.136414768	0.051917957	-2.627506474	0.008601321	0.037350551	negative
PRJNA729271	eed_Lushi_vs_Arbor_Acr	breed_growth_background	ENSGALG00010000705	ENSGALG00010000705	-0.543308806	0.407200022	-1.334255347	0.182120186	0.326873712	negative
PRJNA729271	eed_Lushi_vs_Arbor_Acr	breed_growth_background	ENSGALG00010000710	ENSGALG00010000710	-0.359052202	0.272787561	-1.31623378	0.188095589	0.333918624	negative
PRJNA729271	eed_Lushi_vs_Arbor_Acr	breed_growth_background	ENSGALG00010000711	SPRY2	-0.177247957	0.059434416	-2.982244467	0.002861434	0.017131965	negative
PRJNA729271	eed_Lushi_vs_Arbor_Acr	breed_growth_background	ENSGALG00010000714	FBXL7	-0.083763095	0.053804942	-1.556791849	0.119519913	0.243662608	negative
PRJNA729271	eed_Lushi_vs_Arbor_Acr	breed_growth_background	ENSGALG00010000715	PCDH20	0.160245175	0.138425019	1.157631589	0.247014413	0.403070349	positive
PRJNA729271	eed_Lushi_vs_Arbor_Acr	breed_growth_background	ENSGALG00010000717	NDFIP2	-0.094496895	0.036143865	-2.614465708	0.008936713	0.038518482	negative
PRJNA729271	eed_Lushi_vs_Arbor_Acr	breed_growth_background	ENSGALG00010000718	EEF1G	-0.055091978	0.080702724	-0.682653258	0.494825972	0.646404332	negative
PRJNA729271	eed_Lushi_vs_Arbor_Acr	breed_growth_background	ENSGALG00010000720	RBM26	0.034707669	0.027826422	1.247291818	0.212290516	0.362529725	positive
PRJNA729271	eed_Lushi_vs_Arbor_Acr	breed_growth_background	ENSGALG00010000721	ENSGALG00010000721	-0.219678876	0.084303278	-2.605816529	0.009165554	0.039271271	negative
PRJNA729271	eed_Lushi_vs_Arbor_Acr	breed_growth_background	ENSGALG00010000722	ANKH	0.300955026	0.091356811	3.29428119	0.000986737	0.008077329	positive
PRJNA729271	eed_Lushi_vs_Arbor_Acr	breed_growth_background	ENSGALG00010000724	OBI1	-0.282936069	0.080000062	-3.536698121	0.000405162	0.004277371	negative
PRJNA729271	eed_Lushi_vs_Arbor_Acr	breed_growth_background	ENSGALG00010000725	ENSGALG00010000725	0.259394973	0.379716423	0.683128139	0.494525876	0.646097077	positive
PRJNA729271	eed_Lushi_vs_Arbor_Acr	breed_growth_background	ENSGALG00010000727	ENSGALG00010000727	0.018771459	0.080614135	0.232855678	0.815873476	0.886680308	positive
PRJNA729271	eed_Lushi_vs_Arbor_Acr	breed_growth_background	ENSGALG00010000728	OTULIN	0.014509559	0.16215206	0.089481186	0.928699505	0.960438023	positive
PRJNA729271	eed_Lushi_vs_Arbor_Acr	breed_growth_background	ENSGALG00010000729	EDNRB	0.168371175	0.084418684	1.99447762	0.046099883	0.124923875	positive
PRJNA729271	eed_Lushi_vs_Arbor_Acr	breed_growth_background	ENSGALG00010000730	INTS5	0.142548789	0.119036076	1.197525939	0.231101624	0.385036556	positive
PRJNA729271	eed_Lushi_vs_Arbor_Acr	breed_growth_background	ENSGALG00010000731	ENSGALG00010000731	-1.434693515	0.244494054	-5.868009833	4.41057E-09	6.88659E-07	negative
PRJNA729271	eed_Lushi_vs_Arbor_Acr	breed_growth_background	ENSGALG00010000732	ENSGALG00010000732	0.201699451	0.07903016	2.552183249	0.01070502	0.043957298	positive
PRJNA729271	eed_Lushi_vs_Arbor_Acr	breed_growth_background	ENSGALG00010000733	ENSGALG00010000733	-0.173620006	0.258051401	-0.672811719	0.501067081	0.652147557	negative
PRJNA729271	eed_Lushi_vs_Arbor_Acr	breed_growth_background	ENSGALG00010000734	SLAIN1	0.076262352	0.182399904	0.418105219	0.67587019	0.790569593	positive
PRJNA729271	eed_Lushi_vs_Arbor_Acr	breed_growth_background	ENSGALG00010000735	ENSGALG00010000735	-0.204766213	0.286194368	-0.715479534	0.474312783	0.627868839	negative
PRJNA729271	eed_Lushi_vs_Arbor_Acr	breed_growth_background	ENSGALG00010000737	ENSGALG00010000737	0.2277587	0.476384981	0.478097986	0.632580466	0.757789574	positive
PRJNA729271	eed_Lushi_vs_Arbor_Acr	breed_growth_background	ENSGALG00010000741	FAM105A	-0.111087077	0.10635946	-1.044449427	0.296277506	0.457951961	negative
PRJNA729271	eed_Lushi_vs_Arbor_Acr	breed_growth_background	ENSGALG00010000753	TRIO	-0.195286833	0.036653519	-5.327915028	9.93466E-08	8.18198E-06	negative
PRJNA729271	eed_Lushi_vs_Arbor_Acr	breed_growth_background	ENSGALG00010000757	SCEL	0.098685759	0.275522812	0.358176364	0.720211335	0.82080679	positive
PRJNA729271	eed_Lushi_vs_Arbor_Acr	breed_growth_background	ENSGALG00010000760	CDH10	0.559748073	0.173897078	3.21884692	0.001287072	0.009788093	positive
PRJNA729271	eed_Lushi_vs_Arbor_Acr	breed_growth_background	ENSGALG00010000762	ENSGALG00010000762	-0.161810398	0.1664719	-0.971998265	0.331051412	0.495415054	negative
PRJNA729271	eed_Lushi_vs_Arbor_Acr	breed_growth_background	ENSGALG00010000768	ENSGALG00010000768	-0.421941093	0.204348072	-2.064815635	0.038940448	0.110285618	negative
PRJNA729271	eed_Lushi_vs_Arbor_Acr	breed_growth_background	ENSGALG00010000770	ENSGALG00010000770	0.418561779	0.363436148	1.151678999	0.249453005	0.405452042	positive
PRJNA729271	eed_Lushi_vs_Arbor_Acr	breed_growth_background	ENSGALG00010000777	MYCBP2	-0.079796763	0.044243363	-1.803587194	0.071296045	0.169917161	negative
PRJNA729271	eed_Lushi_vs_Arbor_Acr	breed_growth_background	ENSGALG00010000783	DAP	-0.043964134	0.087366068	-0.503217501	0.614811362	0.743938078	negative
PRJNA729271	eed_Lushi_vs_Arbor_Acr	breed_growth_background	ENSGALG00010000784	FBXL3	0.043695588	0.051350593	0.850926656	0.394810097	0.556347402	positive
PRJNA729271	eed_Lushi_vs_Arbor_Acr	breed_growth_background	ENSGALG00010000785	CLN5	-0.167844178	0.050719794	-3.309244055	0.000935483	0.007757334	negative
PRJNA729271	eed_Lushi_vs_Arbor_Acr	breed_growth_background	ENSGALG00010000786	ANKRD33B	-0.073591306	0.162055129	-0.454112783	0.649747653	0.771407505	negative
PRJNA729271	eed_Lushi_vs_Arbor_Acr	breed_growth_background	ENSGALG00010000787	SNRNP48	0.074306994	0.057646793	1.289004835	0.197396406	0.345938931	positive
PRJNA729271	eed_Lushi_vs_Arbor_Acr	breed_growth_background	ENSGALG00010000788	GATD3AL1	-0.127213299	0.055895185	-2.275925871	0.022850447	0.075122982	negative
PRJNA729271	eed_Lushi_vs_Arbor_Acr	breed_growth_background	ENSGALG00010000789	ROPN1L	0.260629567	0.123544376	2.10960285	0.034892579	0.101723236	positive
PRJNA729271	eed_Lushi_vs_Arbor_Acr	breed_growth_background	ENSGALG00010000790	MARCHF6	-0.018657645	0.053090933	-0.351428083	0.725267217	0.824053245	negative
PRJNA729271	eed_Lushi_vs_Arbor_Acr	breed_growth_background	ENSGALG00010000791	ACOD1	0.472107901	0.278002539	1.698214351	0.089467313	0.199389407	positive
PRJNA729271	eed_Lushi_vs_Arbor_Acr	breed_growth_background	ENSGALG00010000792	ENSGALG00010000792	0.783378001	0.986530498	0.79407378	0.427152481	0.58632341	positive
PRJNA729271	eed_Lushi_vs_Arbor_Acr	breed_growth_background	ENSGALG00010000793	ENSGALG00010000793	-1.036381949	0.597045814	-1.735849955	0.082590368	0.188112412	negative
PRJNA729271	eed_Lushi_vs_Arbor_Acr	breed_growth_background	ENSGALG00010000795	ENSGALG00010000795	-0.125134887	0.131368285	-0.952550205	0.340818017	0.50482449	negative
PRJNA729271	eed_Lushi_vs_Arbor_Acr	breed_growth_background	ENSGALG00010000796	ENSGALG00010000796	-0.080450622	0.112102667	-0.717651283	0.472972329	0.626724006	negative
PRJNA729271	eed_Lushi_vs_Arbor_Acr	breed_growth_background	ENSGALG00010000797	ENSGALG00010000797	-0.144841006	0.213220463	-0.679301617	0.496946772	0.648356747	negative
PRJNA729271	eed_Lushi_vs_Arbor_Acr	breed_growth_background	ENSGALG00010000798	ENSGALG00010000798	-0.284432196	0.318301881	-0.893592572	0.371539922	0.534463681	negative
PRJNA729271	eed_Lushi_vs_Arbor_Acr	breed_growth_background	ENSGALG00010000799	ENSGALG00010000799	-0.284407628	0.161509572	-1.76093358	0.078249646	0.181070979	negative
PRJNA729271	eed_Lushi_vs_Arbor_Acr	breed_growth_background	ENSGALG00010000800	LMO7	-0.199743135	0.081040998	-2.464717128	0.013712146	0.052304619	negative
PRJNA729271	eed_Lushi_vs_Arbor_Acr	breed_growth_background	ENSGALG00010000802	SEMA5A	-0.082831885	0.068483408	-1.209517566	0.226464067	0.379617499	negative
PRJNA729271	eed_Lushi_vs_Arbor_Acr	breed_growth_background	ENSGALG00010000803	MTRR	-0.058864181	0.053864839	-1.092812711	0.274476039	0.433296688	negative
PRJNA729271	eed_Lushi_vs_Arbor_Acr	breed_growth_background	ENSGALG00010000804	ENSGALG00010000804	-0.153117642	0.262097104	-0.58420196	0.559084443	0.700117509	negative
PRJNA729271	eed_Lushi_vs_Arbor_Acr	breed_growth_background	ENSGALG00010000805	FASTKD3	0.015263588	0.070359266	0.216937851	0.828256779	0.895288222	positive
PRJNA729271	eed_Lushi_vs_Arbor_Acr	breed_growth_background	ENSGALG00010000806	CFAP90	-1.395371322	0.239663338	-5.822214327	5.8073E-09	8.33434E-07	negative
PRJNA729271	eed_Lushi_vs_Arbor_Acr	breed_growth_background	ENSGALG00010000807	ENSGALG00010000807	-0.261444198	0.413144067	-0.632816054	0.526853772	0.67295959	negative
PRJNA729271	eed_Lushi_vs_Arbor_Acr	breed_growth_background	ENSGALG00010000808	ADCY2	-0.041754035	0.096328858	-0.43345303	0.664685682	0.78255942	negative
PRJNA729271	eed_Lushi_vs_Arbor_Acr	breed_growth_background	ENSGALG00010000810	UCHL3	-0.174540099	0.074147536	-2.35395685	0.018574767	0.064908691	negative
PRJNA729271	eed_Lushi_vs_Arbor_Acr	breed_growth_background	ENSGALG00010000811	ENSGALG00010000811	1.954130876	0.532647249	3.668714857	0.000243773	0.002988837	positive
PRJNA729271	eed_Lushi_vs_Arbor_Acr	breed_growth_background	ENSGALG00010000812	ENSGALG00010000812	0.016596629	0.051992912	0.319209447	0.749567694	0.840747792	positive
PRJNA729271	eed_Lushi_vs_Arbor_Acr	breed_growth_background	ENSGALG00010000813	ENSGALG00010000813	0.250704153	0.253375127	0.989458418	0.322438905	0.48629195	positive
PRJNA729271	eed_Lushi_vs_Arbor_Acr	breed_growth_background	ENSGALG00010000814	CDH18	-0.418756225	0.160522254	-2.60871135	0.009088387	0.038998274	negative
PRJNA729271	eed_Lushi_vs_Arbor_Acr	breed_growth_background	ENSGALG00010000815	ENSGALG00010000815	0.038296948	0.096128587	0.398392919	0.690340577	0.799752495	positive
PRJNA729271	eed_Lushi_vs_Arbor_Acr	breed_growth_background	ENSGALG00010000816	TBC1D4	-0.086891009	0.055369914	-1.569281974	0.116582251	0.23966242	negative
PRJNA729271	eed_Lushi_vs_Arbor_Acr	breed_growth_background	ENSGALG00010000817	ENSGALG00010000817	0.038575404	0.06173329	0.624871981	0.532055083	0.677655883	positive
PRJNA729271	eed_Lushi_vs_Arbor_Acr	breed_growth_background	ENSGALG00010000818	KLF12	-0.112825909	0.044275473	-2.548271126	0.01082583	0.044302767	negative
PRJNA729271	eed_Lushi_vs_Arbor_Acr	breed_growth_background	ENSGALG00010000820	KLF5	0.227476151	0.103301444	2.202061671	0.027660952	0.08572707	positive
PRJNA729271	eed_Lushi_vs_Arbor_Acr	breed_growth_background	ENSGALG00010000821	ENSGALG00010000821	-0.502184373	0.078898079	-6.364975952	1.9532E-10	5.55131E-08	negative
PRJNA729271	eed_Lushi_vs_Arbor_Acr	breed_growth_background	ENSGALG00010000822	BASP1	-0.109436661	0.089234956	-1.2263878	0.220052775	0.372624413	negative
PRJNA729271	eed_Lushi_vs_Arbor_Acr	breed_growth_background	ENSGALG00010000823	PIBF1	-0.075822752	0.086172655	-0.879893413	0.378917053	0.540963527	negative
PRJNA729271	eed_Lushi_vs_Arbor_Acr	breed_growth_background	ENSGALG00010000843	DIS3	-0.034270895	0.045863945	-0.747229549	0.45492501	0.611020944	negative
PRJNA729271	eed_Lushi_vs_Arbor_Acr	breed_growth_background	ENSGALG00010000853	ENSGALG00010000853	-0.153130211	0.16325009	-0.938009962	0.348239291	0.512303716	negative
PRJNA729271	eed_Lushi_vs_Arbor_Acr	breed_growth_background	ENSGALG00010000857	ENSGALG00010000857	-0.231128062	0.419245659	-0.551295063			negative
PRJNA729271	eed_Lushi_vs_Arbor_Acr	breed_growth_background	ENSGALG00010000859	BORA	-0.170042879	0.106613367	-1.594948958	0.11072365	0.231418036	negative
PRJNA729271	eed_Lushi_vs_Arbor_Acr	breed_growth_background	ENSGALG00010000864	MZT1	0.075594652	0.069582327	1.086405912	0.277299447	0.436470354	positive
PRJNA729271	eed_Lushi_vs_Arbor_Acr	breed_growth_background	ENSGALG00010000870	DACH1	-0.140248149	0.077534129	-1.808856963	0.070473228	0.168565914	negative
PRJNA729271	eed_Lushi_vs_Arbor_Acr	breed_growth_background	ENSGALG00010000901	PCDH9	-0.153219822	0.132828613	-1.153515191	0.248698986	0.404816598	negative
PRJNA729271	eed_Lushi_vs_Arbor_Acr	breed_growth_background	ENSGALG00010001066	SLC33A1	-0.043928889	0.048587155	-0.904125564	0.365928824	0.529302296	negative
PRJNA729271	eed_Lushi_vs_Arbor_Acr	breed_growth_background	ENSGALG00010001070	C3orf33	0.208993386	0.099356687	2.103465733	0.035425078	0.102903106	positive
PRJNA729271	eed_Lushi_vs_Arbor_Acr	breed_growth_background	ENSGALG00010001082	P2RY1	0.337111623	0.095106725	3.544561365	0.000393267	0.004191474	positive
PRJNA729271	eed_Lushi_vs_Arbor_Acr	breed_growth_background	ENSGALG00010001104	MBNL1	0.056692145	0.065544497	0.864941335	0.387070989	0.549035521	positive
PRJNA729271	eed_Lushi_vs_Arbor_Acr	breed_growth_background	ENSGALG00010001111	SUCNR1	0.481099935	0.186876424	2.574428198	0.010040592	0.041953989	positive
PRJNA729271	eed_Lushi_vs_Arbor_Acr	breed_growth_background	ENSGALG00010001112	TSC22D2	0.066962803	0.048655112	1.376274758	0.168736585	0.310543016	positive
PRJNA729271	eed_Lushi_vs_Arbor_Acr	breed_growth_background	ENSGALG00010001113	IGSF10	-0.155124933	0.16122331	-0.962174345	0.335962034	0.500181767	negative
PRJNA729271	eed_Lushi_vs_Arbor_Acr	breed_growth_background	ENSGALG00010001114	MED12L	0.150262002	0.054575652	2.753279098	0.005900157	0.028669718	positive
PRJNA729271	eed_Lushi_vs_Arbor_Acr	breed_growth_background	ENSGALG00010001115	PFN2	-0.12325801	0.051364644	-2.399666396	0.01641002	0.059391569	negative
PRJNA729271	eed_Lushi_vs_Arbor_Acr	breed_growth_background	ENSGALG00010001116	RNF13	-0.092777512	0.037795136	-2.454747408	0.014098361	0.053176099	negative
PRJNA729271	eed_Lushi_vs_Arbor_Acr	breed_growth_background	ENSGALG00010001117	P2RY13	0.00715406	0.172166389	0.041553173	0.966854903	0.98113985	positive
PRJNA729271	eed_Lushi_vs_Arbor_Acr	breed_growth_background	ENSGALG00010001118	P2RY12	0.013892366	0.180976882	0.076763206	0.938811922	0.96653972	positive
PRJNA729271	eed_Lushi_vs_Arbor_Acr	breed_growth_background	ENSGALG00010001119	COMMD2	-0.046058963	0.053481135	-0.861218888	0.389117499	0.550967876	negative
PRJNA729271	eed_Lushi_vs_Arbor_Acr	breed_growth_background	ENSGALG00010001120	GPR87	0.134066445	0.577063286	0.232325377	0.816285304	0.886694805	positive
PRJNA729271	eed_Lushi_vs_Arbor_Acr	breed_growth_background	ENSGALG00010001121	WWTR1	-0.042410111	0.049016663	-0.865218249	0.386919011	0.549016741	negative
PRJNA729271	eed_Lushi_vs_Arbor_Acr	breed_growth_background	ENSGALG00010001122	P2RY14	0.592763032	0.221203353	2.679719924	0.007368378	0.033586366	positive
PRJNA729271	eed_Lushi_vs_Arbor_Acr	breed_growth_background	ENSGALG00010001123	CP	-0.463481295	0.083265223	-5.566325019	2.60168E-08	3.01691E-06	negative

PRJNA729271	ed_Lushi_vs_Arbor_Acr	breed_growth_background	ENSGALG00010001124	GPR171	0. 614622857	0. 303913241	2. 022362879	0. 043138877	0. 118945791	positive
PRJNA729271	ed_Lushi_vs_Arbor_Acr	breed_growth_background	ENSGALG00010001125	CLRN1	-0. 193991665	0. 144599635	-1. 341577832	0. 179732918	0. 324033413	negative
PRJNA729271	ed_Lushi_vs_Arbor_Acr	breed_growth_background	ENSGALG00010001126	MINDY4B	0. 185433767	0. 627010066	0. 295742887	0. 767426443	0. 854694298	positive
PRJNA729271	ed_Lushi_vs_Arbor_Acr	breed_growth_background	ENSGALG00010001127	SIAH2	0. 094250723	0. 054589625	1. 726531782	0. 084251787	0. 190936468	positive
PRJNA729271	ed_Lushi_vs_Arbor_Acr	breed_growth_background	ENSGALG00010001129	TM4SF4	-1. 592140762	0. 559596463	-2. 84515873	0. 00443893	0. 023346258	negative
PRJNA729271	ed_Lushi_vs_Arbor_Acr	breed_growth_background	ENSGALG00010001131	HPS3	0. 043172548	0. 053381904	0. 808748745	0. 418659684	0. 578555831	positive
PRJNA729271	ed_Lushi_vs_Arbor_Acr	breed_growth_background	ENSGALG00010001132	SELENOT	0. 073367431	0. 050742326	1. 445882294	0. 148210219	0. 283156336	positive
PRJNA729271	ed_Lushi_vs_Arbor_Acr	breed_growth_background	ENSGALG00010001133	EIF2A	-0. 072291459	0. 040125658	-1. 801626747	0. 07160415	0. 170486963	negative
PRJNA729271	ed_Lushi_vs_Arbor_Acr	breed_growth_background	ENSGALG00010001134	ENSGALG00010001134	0. 134125966	0. 365028813	0. 367439396	0. 71329129	0. 815844447	positive
PRJNA729271	ed_Lushi_vs_Arbor_Acr	breed_growth_background	ENSGALG00010001135	GYG1	-0. 0468625	0. 085038245	-0. 551075574	0. 581581869	0. 717815837	negative
PRJNA729271	ed_Lushi_vs_Arbor_Acr	breed_growth_background	ENSGALG00010001136	SERP1	-0. 010369468	0. 068102523	-0. 152262605	0. 878979818	0. 928157096	negative
PRJNA729271	ed_Lushi_vs_Arbor_Acr	breed_growth_background	ENSGALG00010001138	SPTSSB	-0. 122945386	0. 483230361	-0. 254423967	0. 799168037	0. 875647494	negative
PRJNA729271	ed_Lushi_vs_Arbor_Acr	breed_growth_background	ENSGALG00010001140	NMD3	0. 189309946	0. 065082139	2. 908784937	0. 003628364	0. 020158348	positive
PRJNA729271	ed_Lushi_vs_Arbor_Acr	breed_growth_background	ENSGALG00010001141	MTAP	0. 104708666	0. 061406052	1. 705184779	0. 088159968	0. 197110464	positive
PRJNA729271	ed_Lushi_vs_Arbor_Acr	breed_growth_background	ENSGALG00010001142	PPM1L	0. 309983514	0. 077793911	3. 984675808	6. 75723E-05	0. 00119047	positive
PRJNA729271	ed_Lushi_vs_Arbor_Acr	breed_growth_background	ENSGALG00010001143	CDKN2A	-0. 74844396	0. 228063575	-3. 281733871	0. 001031709	0. 008340561	negative
PRJNA729271	ed_Lushi_vs_Arbor_Acr	breed_growth_background	ENSGALG00010001144	CDKN2B	-0. 73038604	0. 698640103	-1. 045439614	0. 295819836	0. 457425701	negative
PRJNA729271	ed_Lushi_vs_Arbor_Acr	breed_growth_background	ENSGALG00010001145	TRIM36	0. 079349643	0. 119420809	0. 664454068	0. 506399737	0. 655899691	positive
PRJNA729271	ed_Lushi_vs_Arbor_Acr	breed_growth_background	ENSGALG00010001146	DIPK1C	-0. 383929439	0. 127863466	-3. 002651594	0. 002676386	0. 016306944	negative
PRJNA729271	ed_Lushi_vs_Arbor_Acr	breed_growth_background	ENSGALG00010001147	PGGT1B	0. 197081802	0. 061279431	3. 216116705	0. 00129938	0. 009842037	positive
PRJNA729271	ed_Lushi_vs_Arbor_Acr	breed_growth_background	ENSGALG00010001149	CIDEA	0. 260794321	0. 331526413	0. 786647188	0. 431488428	0. 589761882	positive
PRJNA729271	ed_Lushi_vs_Arbor_Acr	breed_growth_background	ENSGALG00010001150	ENSGALG00010001150	0. 240919314	0. 343786267	0. 700782253	0. 483438915	0. 635653762	positive
PRJNA729271	ed_Lushi_vs_Arbor_Acr	breed_growth_background	ENSGALG00010001151	CCDC112	0. 112069419	0. 115044823	0. 974137001	0. 32998852	0. 494079495	positive
PRJNA729271	ed_Lushi_vs_Arbor_Acr	breed_growth_background	ENSGALG00010001152	FEM1C	0. 238342989	0. 105357275	2. 262235699	0. 023682845	0. 077021053	positive
PRJNA729271	ed_Lushi_vs_Arbor_Acr	breed_growth_background	ENSGALG00010001153	TSHZ1	-0. 037602294	0. 063760526	-0. 589742531	0. 555363276	0. 697196692	negative
PRJNA729271	ed_Lushi_vs_Arbor_Acr	breed_growth_background	ENSGALG00010001154	PTGR3	-0. 082692353	0. 046338434	-1. 784530602	0. 074337487	0. 17518721	negative
PRJNA729271	ed_Lushi_vs_Arbor_Acr	breed_growth_background	ENSGALG00010001155	CYB5A	-0. 010823054	0. 050774551	-0. 213159021	0. 83120293	0. 897317827	negative
PRJNA729271	ed_Lushi_vs_Arbor_Acr	breed_growth_background	ENSGALG00010001156	ALDH7A1	-0. 654326254	0. 096875147	-6. 754325287	1. 43501E-11	5. 33347E-09	negative
PRJNA729271	ed_Lushi_vs_Arbor_Acr	breed_growth_background	ENSGALG00010001157	TIMM21	0. 064456197	0. 048571685	1. 327032347	0. 184497983	0. 329879447	positive
PRJNA729271	ed_Lushi_vs_Arbor_Acr	breed_growth_background	ENSGALG00010001158	FRMPD1	0. 080564452	0. 20505312	0. 392895516	0. 694396653	0. 803166254	positive
PRJNA729271	ed_Lushi_vs_Arbor_Acr	breed_growth_background	ENSGALG00010001159	RTTN	-0. 222209327	0. 062176949	-3. 573821684	0. 000351809	0. 003874973	negative
PRJNA729271	ed_Lushi_vs_Arbor_Acr	breed_growth_background	ENSGALG00010001160	TRMT10B	-0. 219815845	0. 069355789	-3. 169394336	0. 00152757	0. 011026955	negative
PRJNA729271	ed_Lushi_vs_Arbor_Acr	breed_growth_background	ENSGALG00010001161	B3GALNT1	-0. 068811624	0. 053359732	-1. 289579644	0. 197196647	0. 345672439	negative
PRJNA729271	ed_Lushi_vs_Arbor_Acr	breed_growth_background	ENSGALG00010001162	ENSGALG00010001162	0. 183732405	0. 227839283	0. 80641232	0. 420005146	0. 579310552	positive
PRJNA729271	ed_Lushi_vs_Arbor_Acr	breed_growth_background	ENSGALG00010001163	ARL14	-0. 611884197	0. 269066134	-2. 27410335	0. 022959773	0. 075379822	negative
PRJNA729271	ed_Lushi_vs_Arbor_Acr	breed_growth_background	ENSGALG00010001164	KPNA4	0. 110743042	0. 039923087	2. 773909777	0. 005538704	0. 027391167	positive
PRJNA729271	ed_Lushi_vs_Arbor_Acr	breed_growth_background	ENSGALG00010001165	ENSGALG00010001165	0. 063288432	0. 084889083	0. 74554265	0. 455943738	0. 611920636	positive
PRJNA729271	ed_Lushi_vs_Arbor_Acr	breed_growth_background	ENSGALG00010001166	TRIM59	-0. 184433979	0. 051983302	-3. 547946621	0. 000388247	0. 004159378	negative
PRJNA729271	ed_Lushi_vs_Arbor_Acr	breed_growth_background	ENSGALG00010001167	SMC4	-0. 263913983	0. 082787387	-3. 187852539	0. 001433336	0. 0105947	negative
PRJNA729271	ed_Lushi_vs_Arbor_Acr	breed_growth_background	ENSGALG00010001168	DOK6	0. 000270644	0. 089010142	0. 003040595	0. 99757396	0. 998883293	positive
PRJNA729271	ed_Lushi_vs_Arbor_Acr	breed_growth_background	ENSGALG00010001169	ENSGALG00010001169	0. 076074278	0. 055856189	1. 361966847	0. 173208348	0. 316715896	positive
PRJNA729271	ed_Lushi_vs_Arbor_Acr	breed_growth_background	ENSGALG00010001170	DCAF10	0. 046795928	0. 038150035	1. 226628703	0. 219962176	0. 372514516	positive
PRJNA729271	ed_Lushi_vs_Arbor_Acr	breed_growth_background	ENSGALG00010001171	POLR1E	-0. 08045091	0. 0685289	-1. 173970554	0. 24040682	0. 396125022	negative
PRJNA729271	ed_Lushi_vs_Arbor_Acr	breed_growth_background	ENSGALG00010001172	TMEM200C	-0. 036790667	0. 161894213	-0. 227251278	0. 820228356	0. 889578004	negative
PRJNA729271	ed_Lushi_vs_Arbor_Acr	breed_growth_background	ENSGALG00010001173	IFT80	-0. 089932874	0. 059603676	-1. 508847775	0. 131337689	0. 259683508	negative
PRJNA729271	ed_Lushi_vs_Arbor_Acr	breed_growth_background	ENSGALG00010001174	ENSGALG00010001174	-0. 260872975	0. 077998999	-3. 344568229	0. 000824108	0. 007089281	negative
PRJNA729271	ed_Lushi_vs_Arbor_Acr	breed_growth_background	ENSGALG00010001177	SEH1L	-0. 0179907	0. 039191427	-0. 45904684	0. 64620053	0. 768921315	negative
PRJNA729271	ed_Lushi_vs_Arbor_Acr	breed_growth_background	ENSGALG00010001178	ZBTB5	-0. 100937769	0. 035181292	-2. 869075115	0. 00411674	0. 022068102	negative
PRJNA729271	ed_Lushi_vs_Arbor_Acr	breed_growth_background	ENSGALG00010001179	GRHPR	-0. 281273735	0. 099630739	-2. 823162187	0. 004755251	0. 024564277	negative
PRJNA729271	ed_Lushi_vs_Arbor_Acr	breed_growth_background	ENSGALG00010001180	SPIRE1	-0. 072732855	0. 092079466	-0. 789892236	0. 429590706	0. 588166363	negative
PRJNA729271	ed_Lushi_vs_Arbor_Acr	breed_growth_background	ENSGALG00010001181	MFSD1	-0. 026059049	0. 056046877	-0. 464950952	0. 641966582	0. 765427786	negative
PRJNA729271	ed_Lushi_vs_Arbor_Acr	breed_growth_background	ENSGALG00010001182	ZCCHC7	-0. 108028837	0. 046145432	-2. 341051583	0. 01922951	0. 066538971	negative
PRJNA729271	ed_Lushi_vs_Arbor_Acr	breed_growth_background	ENSGALG00010001183	RARRES1	-0. 060943172	0. 101093206	-0. 602841427	0. 546614185	0. 690337619	negative
PRJNA729271	ed_Lushi_vs_Arbor_Acr	breed_growth_background	ENSGALG00010001185	PTPN2	0. 046567711	0. 037245673	1. 250285144	0. 211195403	0. 361255295	positive
PRJNA729271	ed_Lushi_vs_Arbor_Acr	breed_growth_background	ENSGALG00010001186	ENSGALG00010001186	-0. 052127012	0. 209812118	-0. 248446146	0. 803789234	0. 878850791	negative
PRJNA729271	ed_Lushi_vs_Arbor_Acr	breed_growth_background	ENSGALG00010001187	GFM1	0. 190560895	0. 097274979	1. 958991887	0. 050113737	0. 132772549	positive
PRJNA729271	ed_Lushi_vs_Arbor_Acr	breed_growth_background	ENSGALG00010001188	PSMG2	0. 082227792	0. 061422491	1. 338724473	0. 180660386	0. 325220107	positive
PRJNA729271	ed_Lushi_vs_Arbor_Acr	breed_growth_background	ENSGALG00010001190	LXN	0. 424915232	0. 24046642	1. 767046027	0. 07722052	0. 179491892	positive
PRJNA729271	ed_Lushi_vs_Arbor_Acr	breed_growth_background	ENSGALG00010001191	TOMM5	-0. 102879212	0. 080858455	-1. 272337097	0. 203253373	0. 352326913	negative
PRJNA729271	ed_Lushi_vs_Arbor_Acr	breed_growth_background	ENSGALG00010001192	ENSGALG00010001192	0. 380369794	0. 118028464	3. 222695462	0. 001269905	0. 009708477	positive
PRJNA729271	ed_Lushi_vs_Arbor_Acr	breed_growth_background	ENSGALG00010001193	PRELID3A	-0. 005919297	0. 126300643	-0. 046866725	0. 962619449	0. 978835813	negative
PRJNA729271	ed_Lushi_vs_Arbor_Acr	breed_growth_background	ENSGALG00010001194	POT1	-0. 040225248	0. 060870075	-0. 660837835	0. 508716316	0. 657674189	negative
PRJNA729271	ed_Lushi_vs_Arbor_Acr	breed_growth_background	ENSGALG00010001195	ZNF608	-0. 311590566	0. 077027625	-4. 045179474	5. 22831E-05	0. 000999793	negative
PRJNA729271	ed_Lushi_vs_Arbor_Acr	breed_growth_background	ENSGALG00010001196	LSMEM1	-0. 170204582	0. 105444821	-1. 614157814	0. 1064932	0. 225115784	negative
PRJNA729271	ed_Lushi_vs_Arbor_Acr	breed_growth_background	ENSGALG00010001197	CEP76	0. 254587505	0. 079699915	3. 194325919	0. 001401578	0. 010434448	positive
PRJNA729271	ed_Lushi_vs_Arbor_Acr	breed_growth_background	ENSGALG00010001198	IFRD1	0. 27089614	0. 094308277	2. 872453482	0. 00407298	0. 021898313	positive
PRJNA729271	ed_Lushi_vs_Arbor_Acr	breed_growth_background	ENSGALG00010001199	AFG3L2	0. 315628481	0. 048447673	6. 5148326	7. 27708E-11	2. 17075E-08	positive
PRJNA729271	ed_Lushi_vs_Arbor_Acr	breed_growth_background	ENSGALG00010001200	CTTNBP2	0. 259177904	0. 068792355	3. 767539347	0. 000164865	0. 002286806	positive
PRJNA729271	ed_Lushi_vs_Arbor_Acr	breed_growth_background	ENSGALG00010001201	TUBB6	-0. 362589277	0. 088735881	-4. 086163016	4. 38566E-05	0. 000879254	negative
PRJNA729271	ed_Lushi_vs_Arbor_Acr	breed_growth_background	ENSGALG00010001202	CFTR	0. 038911937	0. 130924073	0. 297209947	0. 766306229	0. 853643466	positive
PRJNA729271	ed_Lushi_vs_Arbor_Acr	breed_growth_background	ENSGALG00010001203	TMX3	-0. 105651669	0. 054043667	-1. 954931531	0. 050591152	0. 133495486	negative
PRJNA729271	ed_Lushi_vs_Arbor_Acr	breed_growth_background	ENSGALG00010001204	SNX2	0. 003668178	0. 043852787	0. 083647548	0. 933336661	0. 963515056	positive
PRJNA729271	ed_Lushi_vs_Arbor_Acr	breed_growth_background	ENSGALG00010001205	CEP192	0. 069424549	0. 056515622	1. 228413433	0. 219291808	0. 371683203	positive
PRJNA729271	ed_Lushi_vs_Arbor_Acr	breed_growth_background	ENSGALG00010001206	RSRC1	0. 084856324	0. 041726695	2. 033621969	0. 041989725	0. 116611551	positive
PRJNA729271	ed_Lushi_vs_Arbor_Acr	breed_growth_background	ENSGALG00010001208	MOV10L1	0. 562252341	0. 407793461	1. 378767427	0. 167966473	0. 309361375	positive
PRJNA729271	ed_Lushi_vs_Arbor_Acr	breed_growth_background	ENSGALG00010001209	CDH7	-0. 139372204	0. 132711322	-1. 050190758	0. 293630418	0. 455011001	negative
PRJNA729271	ed_Lushi_vs_Arbor_Acr	breed_growth_background	ENSGALG00010001210	PANX2	-0. 062926825	0. 139696148	-0. 450454974	0. 652382413	0. 773428775	negative
PRJNA729271	ed_Lushi_vs_Arbor_Acr	breed_growth_background	ENSGALG00010001213	PPIC	-0. 071241153	0. 117152745	-0. 608104851	0. 543117935	0. 687677997	negative
PRJNA729271	ed_Lushi_vs_Arbor_Acr	breed_growth_background	ENSGALG00010001214	TES	-0. 339919278	0. 093239595	-3. 645653753	0. 000266713	0. 003200334	negative
PRJNA729271	ed_Lushi_vs_Arbor_Acr	breed_growth_background	ENSGALG00010001215	PRDM6	0. 438396809	0. 39116342	1. 120751037	0. 262393851	0. 420636902	positive
PRJNA729271	ed_Lushi_vs_Arbor_Acr	breed_growth_background	ENSGALG00010001216	SHOX2	-0. 289900663	0. 283809012	-1. 021463912	0. 307034699	0. 469755959	negative
PRJNA729271	ed_Lushi_vs_Arbor_Acr	breed_growth_background	ENSGALG00010001218	ENSGALG00010001218	-0. 170447663	0. 060949543	-2. 796537191	0. 005165347	0. 026160621	negative
PRJNA729271	ed_Lushi_vs_Arbor_Acr	breed_growth_background	ENSGALG00010001219	TFEC	-0. 133481767	0. 223893967	-0. 596182955	0. 551053011	0. 694023234	negative
PRJNA729271	ed_Lushi_vs_Arbor_Acr	breed_growth_background	ENSGALG00010001220	PLXNB2	0. 040296509	0. 044410434	0. 907365797	0. 36421339	0. 527610743	positive
PRJNA729271	ed_Lushi_vs_Arbor_Acr	breed_growth_background	ENSGALG00010001221	GRAMD2B	-0. 189073538	0. 084234388	-2. 244612246	0. 024793036	0. 079557724	negative
PRJNA729271	ed_Lushi_vs_Arbor_Acr	breed_growth_background	ENSGALG00010001222	ZNF800	0. 068633299	0. 054520204	1. 258859906	0. 208080938	0. 357319417	positive
PRJNA729271	ed_Lushi_vs_Arbor_Acr	breed_growth_background	ENSGALG00010001223	GRM8	1. 03040802	0. 323581394	3. 184385879	0. 001450615	0. 010678854	positive
PRJNA729271	ed_Lushi_vs_Arbor_Acr	breed_growth_background	ENSGALG00010001224	DNAJB9	0. 199681268	0. 10099344	1. 977170675	0. 048022341	0. 128621324	positive
PRJNA729271	ed_Lushi_vs_Arbor_Acr	breed_growth_background	ENSGALG00010001225	SOCS6	0. 059185977	0. 052478983	1. 127803431	0. 259402944	0. 417133977	positive
PRJNA729271	ed_Lushi_vs_Arbor_Acr	breed_growth_background	ENSGALG00010001226	VEPH1	-0. 092912963	0. 319299894	-0. 290989645	0. 771059239	0. 857250922	negative
PRJNA729271	ed_Lushi_vs_Arbor_Acr	breed_growth_background	ENSGALG00010001227	ENSGALG00010001227	-1. 337289995	0. 451028169	-2. 964981097	0. 003027015	0. 017828763	negative
PRJNA729271	ed_Lushi_vs_Arbor_Acr	breed_growth_background	ENSGALG00010001228	THAP5	0. 072187152	0. 060190694	1. 19930752	0. 230408392	0. 384234888	positive
PRJNA729271	ed_Lushi_vs_Arbor_Acr	breed_growth_background	ENSGALG00010001229	ZNF407	0. 165600374	0. 05549638	2. 983985151	0. 002845206	0. 017067799	positive
PRJNA729271	ed_Lushi_vs_Arbor_Acr	breed_growth_background	ENSGALG00010001230	PTX3	-0. 590549177	0. 315531528	-1. 871601173	0. 061261801	0. 153154503	negative
PRJNA729271	ed_Lushi_vs_Arbor_Acr	breed_growth_background	ENSGALG00010001231	CNDP1	-0. 140365173	0. 277424547	-0. 505958015	0. 612886124	0. 742729234	negative
PRJNA729271	ed_Lushi_vs_Arbor_Acr	breed_growth_background	ENSGALG00010001232	TSPAN12	-0. 095746414	0. 05512332	-1. 73694932	0. 082396117	0. 187788005	negative
PRJNA729271	ed_Lushi_vs_Arbor_Acr	breed_growth_background	ENSGALG00010001233	CNDP2	-0. 102154723	0. 051948046	-1. 966478632	0. 049243348	0. 131113581	negative
PRJNA729271	ed_Lushi_vs_Arbor_Acr	breed_growth_background	ENSGALG00010001234	AASS	0. 167782405	0. 052651742	3. 186644886	0. 001439334	0. 01061527	positive
PRJNA729271	ed_Lushi_vs_Arbor_Acr	breed_growth_background	ENSGALG00010001235	CCDC102B	0. 297964312	0. 168088057	1. 772667957	0. 076283744	0. 178200301	positive
PRJNA729271	ed_Lushi_vs_Arbor_Acr	breed_growth_background	ENSGALG00010001237	MELK	-0. 745743992	0. 149170897	-4. 999259295	5. 7551E-07	3. 14901E-05	negative
PRJNA729271	ed_Lushi_vs_Arbor_Acr	breed_growth_background	ENSGALG00010001238	GOLIM4	-0. 044231052	0. 06550617	-0. 675219641	0. 49953623	0. 650739431	negative
PRJNA729271	ed_Lushi_vs_Arbor_Acr	breed_growth_background	ENSGALG00010001239	ENSGALG00010001239	0. 04716443	0. 135078122	0. 349164092	0. 726966122	0. 824527408	positive
PRJNA729271	ed_Lushi_vs_Arbor_Acr	breed_growth_background	ENSGALG00010001242	ENSGALG00010001242	-2. 719897371	0. 358791575	-7. 580716949	3. 43651E-14	2. 76734E-11	negative
PRJNA729271	ed_Lushi_vs_Arbor_Acr	breed_growth_background	ENSGALG00010001244	ENSGALG00010001244	-0. 06940999	0. 083140808	-0. 834848637	0. 403802937	0. 564646303	negative
PRJNA729271	ed_Lushi_vs_Arbor_Acr	breed_growth_background	ENSGALG00010001245	SERPINI1	-0. 255924386	0. 121569397	-2. 105171143	0. 035276413	0. 102574044	negative
PRJNA729271	ed_Lushi_vs_Arbor_Acr	breed_growth_background	ENSGALG00010001248	PTPRZ1	0. 301182648	0. 134178294	2. 244645079	0. 024790926	0. 079557724	positive
PRJNA729271	ed_Lushi_vs_Arbor_Acr	breed_growth_background	ENSGALG00010001249	PDCD10	0. 08868072	0. 074520753	1. 190013746	0. 234040989	0. 388466603	positive
PRJNA729271	ed_Lushi_vs_Arbor_Acr	breed_growth_background	ENSGALG00010001250	ENSGALG00010001250	-1. 544412227	0. 386586866	-3. 994994043	6. 46959E-05	0. 001154885	negative
PRJNA729271	ed_Lushi_vs_Arbor_Acr	breed_growth_background	ENSGALG00010001251	ZNF277	0. 14925666	0. 067533261	2. 21012073	0. 027096785	0. 084520743	positive
PRJNA729271	ed_Lushi_vs_Arbor_Acr	breed_growth_background	ENSGALG00010001252	SNCAIP	-0. 418918466	0. 066621102	-6. 288074696	3. 21427E-10	7. 86851E-08	negative
PRJNA729271	ed_Lushi_vs_Arbor_Acr	breed_growth_background	ENSGALG00010001253	ENSGALG00010001253	0. 936270769	0. 532156822	1. 75938883	0. 078511488	0. 181531985	positive
PRJNA729271	ed_Lushi_vs_Arbor_Acr	breed_growth_background	ENSGALG00010001254	NETO1	-0. 472260466	0. 228175141	-2. 069727948	0. 038477828	0. 109531838	negative
PRJNA729271	ed_Lushi_vs_Arbor_Acr	breed_growth_background	ENSGALG00010001258	ENSGALG00010001258	-0. 083114131	0. 070687965	-1. 175788984	0. 23967921	0. 395279676	negative
PRJNA729271	ed_Lushi_vs_Arbor_Acr	breed_growth_background	ENSGALG00010001259	ENSGALG00010001259	-0. 097721545	0. 092820082	-1. 052806063	0. 292429885	0. 453829892	negative
PRJNA729271	ed_Lushi_vs_Arbor_Acr	breed_growth_background	ENSGALG00010001260	ENSGALG00010001260	-0. 26058499	0. 554174307	-0. 470222069	0. 638196368	0. 762602412	negative
PRJNA729271	ed_Lushi_vs_Arbor_Acr	breed_growth_background	ENSGALG00010001262	ENSGALG00010001262	-0. 103397831	0. 077867084	-1. 327875997	0. 184219075	0. 329620479	negative
PRJNA729271	ed_Lushi_vs_Arbor_Acr	breed_growth_background	ENSGALG00010001264	FAM3C	0. 195169873	0. 06059741	3. 220762616	0. 0012785	0. 009743353	positive
PRJNA729271	ed_Lushi_vs_Arbor_Acr	breed_growth_background	ENSGALG00010001265	OPN1LW	0. 565131117	0. 324473193	1. 741688158	0. 081563026	0. 186535208	positive
PRJNA729271	ed_Lushi_vs_Arbor_Acr	breed_growth_background	ENSGALG00010001267	BCHE	-0. 494949019	0. 133982785	-3. 694123977	0. 000220646	0. 002793245	negative
PRJNA729271	ed_Lushi_vs_Arbor_Acr	breed_growth_background	ENSGALG00010001268	CBLN2	-0. 994447799	0. 231700679	-4. 291950294	1. 77111E-05	0. 000438092	negative
PRJNA729271	ed_Lushi_vs_Arbor_Acr	breed_growth_background	ENSGALG00010001270	DRAP1	0. 049375876	0. 059396297	0. 831295523	0. 405806703	0. 566682867	positive
PRJNA729271	ed_Lushi_vs_Arbor_Acr	breed_growth_background	ENSGALG00010001271	ENSGALG00010001271	0. 139784026	0. 099306452	1. 407602672	0. 159248756	0. 297731293	positive
PRJNA729271	ed_Lushi_vs_Arbor_Acr	breed_growth_background	ENSGALG00010001273	ENSGALG00010001273	0. 459785245	0. 310284713	1. 481817268	0. 138388923	0. 269906814	positive
PRJNA729271	ed_Lushi_vs_Arbor_Acr	breed_growth_background	ENSGALG00010001274	DLGAP1	0. 05767072	0. 256461068	0. 224871248	0. 822079437	0. 890918986	positive
PRJNA729271	ed_Lushi_vs_Arbor_Acr	breed_growth_background	ENSGALG00010001276	ENSGALG00010001276	3. 106212913	0. 638957378	4. 86137733			positive
PRJNA729271	ed_Lushi_vs_Arbor_Acr	breed_growth_background	ENSGALG00010001277	ENSGALG00010001277	0. 255883696	0. 087051902	2. 939438331	0. 003288077	0. 018943035	positive
PRJNA729271	ed_Lushi_vs_Arbor_Acr	breed_growth_background	ENSGALG00010001279	LPIN2	-0. 172737144	0. 074628948	-2. 314613134	0. 020634113	0. 069718292	negative
PRJNA729271	ed_Lushi_vs_Arbor_Acr	breed_growth_background	ENSGALG00010001281	LRRN3	0. 092155707	0. 079097045	1. 165096707	0. 24397985	0. 399693481	positive
PRJNA729271	ed_Lushi_vs_Arbor_Acr	breed_growth_background	ENSGALG00010001283	ENSGALG00010001283	0. 091404664	0. 095307471	0. 959050356	0. 337533378	0. 501755381	positive
PRJNA729271	ed_Lushi_vs_Arbor_Acr	breed_growth_background	ENSGALG00010001285	ENSGALG00010001285	0. 215202554	0. 114890606	1. 873108349	0. 061053425	0. 152786722	positive
PRJNA729271	ed_Lushi_vs_Arbor_Acr	breed_growth_background	ENSGALG00010001286	SRFBP1	0. 087708486	0. 061327323	1. 430169801	0. 15266829	0. 289158091	positive

PRJNA729271	eed_Lushi_vs_Arbor_Acr	breed_growth_background	ENSGALG00010001287	PLPP4	0.258572809	0.23657935	1.092964406	0.274409428	0.433285911	positive
PRJNA729271	eed_Lushi_vs_Arbor_Acr	breed_growth_background	ENSGALG00010001288	TGIF1	0.106842126	0.08375055	1.275718507	0.202055044	0.351131503	positive
PRJNA729271	eed_Lushi_vs_Arbor_Acr	breed_growth_background	ENSGALG00010001289	ENSGALG00010001289	0.351173833	0.189120874	1.856875051	0.063328933	0.156727486	positive
PRJNA729271	eed_Lushi_vs_Arbor_Acr	breed_growth_background	ENSGALG00010001291	ENSGALG00010001291	0.156170108	0.076681666	2.036602962	0.041689846	0.115940079	positive
PRJNA729271	eed_Lushi_vs_Arbor_Acr	breed_growth_background	ENSGALG00010001292	LOX	-0.074545481	0.075953402	-0.981463356	0.326364296	0.489918234	negative
PRJNA729271	eed_Lushi_vs_Arbor_Acr	breed_growth_background	ENSGALG00010001295	ENSGALG00010001295	0.231976162	0.068255085	3.398664915	0.000677156	0.00616544	positive
PRJNA729271	eed_Lushi_vs_Arbor_Acr	breed_growth_background	ENSGALG00010001297	MYL12A	-0.489067024	0.119174252	-4.103797734	4.06423E-05	0.000834433	negative
PRJNA729271	eed_Lushi_vs_Arbor_Acr	breed_growth_background	ENSGALG00010001298	DOCK4	0.197734173	0.070782592	2.793542403	0.005213419	0.0262847	positive
PRJNA729271	eed_Lushi_vs_Arbor_Acr	breed_growth_background	ENSGALG00010001300	MRPL49	-0.026400736	0.097144438	-0.271767865	0.785800516	0.867312702	negative
PRJNA729271	eed_Lushi_vs_Arbor_Acr	breed_growth_background	ENSGALG00010001301	GMPS	0.058786566	0.041884267	1.403547703	0.160453577	0.299250464	positive
PRJNA729271	eed_Lushi_vs_Arbor_Acr	breed_growth_background	ENSGALG00010001302	ENSGALG00010001302	-0.046055754	0.232741851	-0.197883425	0.843136273	0.90506146	negative
PRJNA729271	eed_Lushi_vs_Arbor_Acr	breed_growth_background	ENSGALG00010001304	SYVN1	0.337840876	0.095438462	3.539881816	0.000400306	0.004244652	positive
PRJNA729271	eed_Lushi_vs_Arbor_Acr	breed_growth_background	ENSGALG00010001305	WNT16	-0.178283663	0.212034955	-0.840822038	0.400447639	0.561404993	negative
PRJNA729271	eed_Lushi_vs_Arbor_Acr	breed_growth_background	ENSGALG00010001306	CEP120	0.07986335	0.04664623	1.712107279	0.0868769	0.195055866	positive
PRJNA729271	eed_Lushi_vs_Arbor_Acr	breed_growth_background	ENSGALG00010001307	CALU	-0.281339234	0.083943689	-3.351523336	0.000803683	0.006975677	negative
PRJNA729271	eed_Lushi_vs_Arbor_Acr	breed_growth_background	ENSGALG00010001308	MGMT	-0.011993991	0.075352876	-0.159170985	0.873534163	0.925372922	negative
PRJNA729271	eed_Lushi_vs_Arbor_Acr	breed_growth_background	ENSGALG00010001313	EMILIN2	-0.28782901	0.094342456	-3.050895879	0.002281597	0.014620577	negative
PRJNA729271	eed_Lushi_vs_Arbor_Acr	breed_growth_background	ENSGALG00010001320	CPED1	0.101515343	0.106788769	0.950618163	0.341798245	0.505702313	positive
PRJNA729271	eed_Lushi_vs_Arbor_Acr	breed_growth_background	ENSGALG00010001330	ENSGALG00010001330	0.002825012	0.076717194	0.036823714	0.970625566	0.98289909	positive
PRJNA729271	eed_Lushi_vs_Arbor_Acr	breed_growth_background	ENSGALG00010001333	PLCH1	0.276970209	0.163714639	1.691786456	0.090686697	0.201271424	positive
PRJNA729271	eed_Lushi_vs_Arbor_Acr	breed_growth_background	ENSGALG00010001334	EBF3	-0.178135017	0.085532323	-2.082663157	0.037281938	0.107010236	negative
PRJNA729271	eed_Lushi_vs_Arbor_Acr	breed_growth_background	ENSGALG00010001343	ENSGALG00010001343	0.052325493	0.165350385	0.3164522	0.751659301	0.842702573	positive
PRJNA729271	eed_Lushi_vs_Arbor_Acr	breed_growth_background	ENSGALG00010001347	MYOM1	-0.124410499	0.088063237	-1.412740473	0.157732047	0.295581903	negative
PRJNA729271	eed_Lushi_vs_Arbor_Acr	breed_growth_background	ENSGALG00010001349	ING3	0.088793338	0.055966878	1.586533693	0.112618295	0.234036156	positive
PRJNA729271	eed_Lushi_vs_Arbor_Acr	breed_growth_background	ENSGALG00010001351	CAPN1	0.078639735	0.083681159	0.939754363	0.347343573	0.511349288	positive
PRJNA729271	eed_Lushi_vs_Arbor_Acr	breed_growth_background	ENSGALG00010001353	GLRX3	-0.011588733	0.042413241	-0.273233849	0.784673462	0.866703955	negative
PRJNA729271	eed_Lushi_vs_Arbor_Acr	breed_growth_background	ENSGALG00010001358	TCERG1L	0.024604637	0.286920213	0.085754284	0.931661749	0.962498809	positive
PRJNA729271	eed_Lushi_vs_Arbor_Acr	breed_growth_background	ENSGALG00010001363	HIBADH	-0.159755672	0.112348649	-1.421963438	0.155036865	0.291863563	negative
PRJNA729271	eed_Lushi_vs_Arbor_Acr	breed_growth_background	ENSGALG00010001364	SMCHD1	-0.056589156	0.098651503	-0.573626898	0.566220306	0.705716947	negative
PRJNA729271	eed_Lushi_vs_Arbor_Acr	breed_growth_background	ENSGALG00010001365	ENSGALG00010001365	-0.203774032	0.140637004	-1.448936099	0.147355429	0.282155474	negative
PRJNA729271	eed_Lushi_vs_Arbor_Acr	breed_growth_background	ENSGALG00010001367	ENSGALG00010001367	0.593418617	0.234190514	2.533913982	0.011279642	0.04561489	positive
PRJNA729271	eed_Lushi_vs_Arbor_Acr	breed_growth_background	ENSGALG00010001368	WIPF3	-0.006152515	0.074892358	-0.082151436	0.934526292	0.964262428	negative
PRJNA729271	eed_Lushi_vs_Arbor_Acr	breed_growth_background	ENSGALG00010001369	PPP2R2D	0.123929172	0.066515756	1.863155117	0.062440446	0.155104428	positive
PRJNA729271	eed_Lushi_vs_Arbor_Acr	breed_growth_background	ENSGALG00010001372	ENSGALG00010001372	0.092677008	0.158641468	0.584191568	0.559091434	0.700117509	positive
PRJNA729271	eed_Lushi_vs_Arbor_Acr	breed_growth_background	ENSGALG00010001373	IMPA2	0.114361067	0.07475502	1.529811201	0.126063468	0.25270225	positive
PRJNA729271	eed_Lushi_vs_Arbor_Acr	breed_growth_background	ENSGALG00010001378	POLA2	-0.041189537	0.07391225	-0.557276191	0.577338741	0.714751649	negative
PRJNA729271	eed_Lushi_vs_Arbor_Acr	breed_growth_background	ENSGALG00010001380	SCRN1	-0.299401323	0.096451337	-3.104169765	0.001908138	0.012840511	negative
PRJNA729271	eed_Lushi_vs_Arbor_Acr	breed_growth_background	ENSGALG00010001381	MDFIC	-0.223319712	0.069964337	-3.191907796	0.001413364	0.010495245	negative
PRJNA729271	eed_Lushi_vs_Arbor_Acr	breed_growth_background	ENSGALG00010001382	BNIP3	0.252513085	0.103053744	2.450304804	0.014273533	0.053558079	positive
PRJNA729271	eed_Lushi_vs_Arbor_Acr	breed_growth_background	ENSGALG00010001385	MME	0.173139352	0.135223248	1.280396349	0.200405777	0.34918641	positive
PRJNA729271	eed_Lushi_vs_Arbor_Acr	breed_growth_background	ENSGALG00010001386	FKBP14	0.011923871	0.099544254	0.119784628	0.90465376	0.945214175	positive
PRJNA729271	eed_Lushi_vs_Arbor_Acr	breed_growth_background	ENSGALG00010001388	MPPE1	-0.5209577	0.088453911	-5.88959488	3.87143E-09	6.30522E-07	negative
PRJNA729271	eed_Lushi_vs_Arbor_Acr	breed_growth_background	ENSGALG00010001395	GNAL	-0.054552981	0.057555934	-0.947825474	0.34321831	0.507078728	negative
PRJNA729271	eed_Lushi_vs_Arbor_Acr	breed_growth_background	ENSGALG00010001397	KLHL7	0.058704684	0.034828112	1.685554577	0.091881624	0.20268211	positive
PRJNA729271	eed_Lushi_vs_Arbor_Acr	breed_growth_background	ENSGALG00010001398	WDR11	0.103598086	0.05234926	1.978979019	0.047818369	0.128238159	positive
PRJNA729271	eed_Lushi_vs_Arbor_Acr	breed_growth_background	ENSGALG00010001400	SNX24	-0.234424306	0.085220949	-2.750782636	0.005945308	0.028831461	negative
PRJNA729271	eed_Lushi_vs_Arbor_Acr	breed_growth_background	ENSGALG00010001402	NUP42	0.047592857	0.073785968	0.645012311	0.518919208	0.666965268	positive
PRJNA729271	eed_Lushi_vs_Arbor_Acr	breed_growth_background	ENSGALG00010001406	EML3	0.144549122	0.08558853	1.68888427	0.09124161	0.201853959	positive
PRJNA729271	eed_Lushi_vs_Arbor_Acr	breed_growth_background	ENSGALG00010001407	FOXP2	0.189649397	0.082481784	2.299288249	0.021488577	0.071835083	positive
PRJNA729271	eed_Lushi_vs_Arbor_Acr	breed_growth_background	ENSGALG00010001409	GPNMB	3.440287778	0.543367816	6.331416169	2.42921E-10	6.52063E-08	positive
PRJNA729271	eed_Lushi_vs_Arbor_Acr	breed_growth_background	ENSGALG00010001411	FGFR2	-0.423680175	0.19992712	-2.119173107	0.03407584	0.099945225	negative
PRJNA729271	eed_Lushi_vs_Arbor_Acr	breed_growth_background	ENSGALG00010001412	ENSGALG00010001412	-0.141578825	0.133273583	-1.06231724	0.288091691	0.449116913	negative
PRJNA729271	eed_Lushi_vs_Arbor_Acr	breed_growth_background	ENSGALG00010001414	CCDC126	0.189053879	0.092350036	2.04714462	0.040643887	0.113666437	positive
PRJNA729271	eed_Lushi_vs_Arbor_Acr	breed_growth_background	ENSGALG00010001415	MC5R	-0.419002464	0.143455153	-2.920790605	0.003491444	0.019707353	negative
PRJNA729271	eed_Lushi_vs_Arbor_Acr	breed_growth_background	ENSGALG00010001416	AP4M1	-0.158537584	0.050276245	-3.153329841	0.001614193	0.011439265	negative
PRJNA729271	eed_Lushi_vs_Arbor_Acr	breed_growth_background	ENSGALG00010001418	RNMT	-0.016483821	0.03713167	-0.443928893	0.657093994	0.77662895	negative
PRJNA729271	eed_Lushi_vs_Arbor_Acr	breed_growth_background	ENSGALG00010001419	KCND2	-0.18983874	0.147902033	-1.283543818	0.199301618	0.347972244	negative
PRJNA729271	eed_Lushi_vs_Arbor_Acr	breed_growth_background	ENSGALG00010001420	FAM221A	0.223778199	0.309415487	0.723228823	0.469539314	0.624229327	positive
PRJNA729271	eed_Lushi_vs_Arbor_Acr	breed_growth_background	ENSGALG00010001422	AP1S1	0.021656429	0.076461436	0.283233357	0.77699796	0.86149992	positive
PRJNA729271	eed_Lushi_vs_Arbor_Acr	breed_growth_background	ENSGALG00010001423	FAM210A	0.005898101	0.073042311	0.080749099	0.935641489	0.964536447	positive
PRJNA729271	eed_Lushi_vs_Arbor_Acr	breed_growth_background	ENSGALG00010001426	ATE1	-0.082976297	0.048833742	-1.699159117	0.089289207	0.19914557	negative
PRJNA729271	eed_Lushi_vs_Arbor_Acr	breed_growth_background	ENSGALG00010001427	ASB11	0.715360715	0.174578236	4.097651182	4.17364E-05	0.000850814	positive
PRJNA729271	eed_Lushi_vs_Arbor_Acr	breed_growth_background	ENSGALG00010001432	NSMCE4A	0.091998253	0.061611916	1.493189293	0.135387644	0.265518049	positive
PRJNA729271	eed_Lushi_vs_Arbor_Acr	breed_growth_background	ENSGALG00010001436	ENSGALG00010001436	-1.661816021	0.731029672	-2.273253857	0.023010886	0.075479249	negative
PRJNA729271	eed_Lushi_vs_Arbor_Acr	breed_growth_background	ENSGALG00010001440	CDCA7L	-0.120241237	0.081213291	-1.48056107	0.138723576	0.270377603	negative
PRJNA729271	eed_Lushi_vs_Arbor_Acr	breed_growth_background	ENSGALG00010001441	HMGCLL1	-0.616503002	0.16776678	-3.674762091	0.000238071	0.002939388	negative
PRJNA729271	eed_Lushi_vs_Arbor_Acr	breed_growth_background	ENSGALG00010001442	LDLRAD4	-0.170333219	0.060934362	-2.795355742	0.005184263	0.026192366	negative
PRJNA729271	eed_Lushi_vs_Arbor_Acr	breed_growth_background	ENSGALG00010001443	ANKRD26	0.046882543	0.078425715	0.597795547	0.549976362	0.69326276	positive
PRJNA729271	eed_Lushi_vs_Arbor_Acr	breed_growth_background	ENSGALG00010001448	GPR37	-0.489562779	0.188646412	-2.595134325	0.009455396	0.040263209	negative
PRJNA729271	eed_Lushi_vs_Arbor_Acr	breed_growth_background	ENSGALG00010001450	GPR149	-0.05584476	0.232745191	-0.23993948	0.810377174	0.883255668	negative
PRJNA729271	eed_Lushi_vs_Arbor_Acr	breed_growth_background	ENSGALG00010001453	TAX1BP1	0.036195664	0.039420091	0.918203452	0.358512362	0.522142208	positive
PRJNA729271	eed_Lushi_vs_Arbor_Acr	breed_growth_background	ENSGALG00010001454	VEGFD	0.233054653	0.090177741	2.584392242	0.009755078	0.041068794	positive
PRJNA729271	eed_Lushi_vs_Arbor_Acr	breed_growth_background	ENSGALG00010001456	LSM8	0.089698968	0.063856559	1.404694661	0.160112094	0.298882782	positive
PRJNA729271	eed_Lushi_vs_Arbor_Acr	breed_growth_background	ENSGALG00010001457	NDC80	-0.508301131	0.122605019	-4.145842759	3.38566E-05	0.0007292	negative
PRJNA729271	eed_Lushi_vs_Arbor_Acr	breed_growth_background	ENSGALG00010001458	JAZF1	-0.22919717	0.086089147	-2.662323612	0.007760323	0.034900987	negative
PRJNA729271	eed_Lushi_vs_Arbor_Acr	breed_growth_background	ENSGALG00010001460	MALSU1	0.219080264	0.0575118	3.809309821	0.000139355	0.002027751	positive
PRJNA729271	eed_Lushi_vs_Arbor_Acr	breed_growth_background	ENSGALG00010001461	ASB9	-0.108968884	0.127029307	-0.857824755	0.390989238	0.552538657	negative
PRJNA729271	eed_Lushi_vs_Arbor_Acr	breed_growth_background	ENSGALG00010001462	DHX36	0.086189423	0.058632173	1.470002205	0.141561157	0.274315699	positive
PRJNA729271	eed_Lushi_vs_Arbor_Acr	breed_growth_background	ENSGALG00010001463	DSEL	-0.171454973	0.085051671	-2.01589187	0.043811284	0.120296374	negative
PRJNA729271	eed_Lushi_vs_Arbor_Acr	breed_growth_background	ENSGALG00010001465	TACC2	0.184807016	0.083979434	2.20062232	0.027762772	0.085968937	positive
PRJNA729271	eed_Lushi_vs_Arbor_Acr	breed_growth_background	ENSGALG00010001466	PRKX	-0.188405989	0.070208947	-2.683503981	0.007285511	0.033344123	negative
PRJNA729271	eed_Lushi_vs_Arbor_Acr	breed_growth_background	ENSGALG00010001468	ANOS1	-0.444417887	0.082117693	-5.411962626	6.23377E-08	5.68292E-06	negative
PRJNA729271	eed_Lushi_vs_Arbor_Acr	breed_growth_background	ENSGALG00010001469	ARHGEF26	-0.075337713	0.068683743	-1.096878383	0.27269456	0.43161254	negative
PRJNA729271	eed_Lushi_vs_Arbor_Acr	breed_growth_background	ENSGALG00010001470	GPR85	0.379737255	0.180864884	2.099563199	0.035767283	0.103585767	positive
PRJNA729271	eed_Lushi_vs_Arbor_Acr	breed_growth_background	ENSGALG00010001471	BMP5	-0.806500389	0.199428778	-4.044052212	5.25352E-05	0.001003291	negative
PRJNA729271	eed_Lushi_vs_Arbor_Acr	breed_growth_background	ENSGALG00010001472	ENSGALG00010001472	0.034728133	0.099208529	0.350051891	0.726299755	0.824474505	positive
PRJNA729271	eed_Lushi_vs_Arbor_Acr	breed_growth_background	ENSGALG00010001475	BMT2	0.039565818	0.074508641	0.531023215	0.595402692	0.72916205	positive
PRJNA729271	eed_Lushi_vs_Arbor_Acr	breed_growth_background	ENSGALG00010001476	IGF2BP3	0.193752927	0.103873202	1.865283089	0.062141734	0.154660789	positive
PRJNA729271	eed_Lushi_vs_Arbor_Acr	breed_growth_background	ENSGALG00010001477	NLGN4	0.025082696	0.1234732	0.203142832	0.839023395	0.902332995	positive
PRJNA729271	eed_Lushi_vs_Arbor_Acr	breed_growth_background	ENSGALG00010001479	TMEM168	-0.00686571	0.083250435	-0.082470556	0.934272531	0.96406922	negative
PRJNA729271	eed_Lushi_vs_Arbor_Acr	breed_growth_background	ENSGALG00010001480	JAKMIP3	0.003063671	0.16577752	0.01848062	0.985255438	0.991204718	positive
PRJNA729271	eed_Lushi_vs_Arbor_Acr	breed_growth_background	ENSGALG00010001482	TRA2A	-0.054860108	0.03555047	-1.543161231	0.122791661	0.247696504	negative
PRJNA729271	eed_Lushi_vs_Arbor_Acr	breed_growth_background	ENSGALG00010001483	ENSGALG00010001483	0.241771599	0.181451482	1.332431103	0.182718567	0.327744788	positive
PRJNA729271	eed_Lushi_vs_Arbor_Acr	breed_growth_background	ENSGALG00010001486	WNT2	-0.069229464	0.14032075	-0.493365837	0.621754117	0.74878093	negative
PRJNA729271	eed_Lushi_vs_Arbor_Acr	breed_growth_background	ENSGALG00010001487	CDH19	0.452743277	0.126097845	3.590412488	0.000330155	0.003695442	positive
PRJNA729271	eed_Lushi_vs_Arbor_Acr	breed_growth_background	ENSGALG00010001488	GRPR	-0.388428635	0.249886173	-1.554422281	0.120083722	0.244433864	negative
PRJNA729271	eed_Lushi_vs_Arbor_Acr	breed_growth_background	ENSGALG00010001491	PHF3	0.035880074	0.047310339	0.758398159	0.44821266	0.60477939	positive
PRJNA729271	eed_Lushi_vs_Arbor_Acr	breed_growth_background	ENSGALG00010001492	ENSGALG00010001492	0.239849284	0.146357133	1.638794628	0.101256038	0.217052097	positive
PRJNA729271	eed_Lushi_vs_Arbor_Acr	breed_growth_background	ENSGALG00010001495	CCNL1	0.157928597	0.058346637	2.706730079	0.006794948	0.031735173	positive
PRJNA729271	eed_Lushi_vs_Arbor_Acr	breed_growth_background	ENSGALG00010001496	PTP4A1	0.282543001	0.101751298	2.776799972	0.005489695	0.027260408	positive
PRJNA729271	eed_Lushi_vs_Arbor_Acr	breed_growth_background	ENSGALG00010001497	LEKR1	0.167276802	0.156087817	1.071683905	0.283862032	0.444051388	positive
PRJNA729271	eed_Lushi_vs_Arbor_Acr	breed_growth_background	ENSGALG00010001499	TIPARP	-0.178974624	0.099657375	-1.795899442	0.072510511	0.172047775	negative
PRJNA729271	eed_Lushi_vs_Arbor_Acr	breed_growth_background	ENSGALG00010001501	CAPZA2	0.061231039	0.056888486	1.076334481	0.281777693	0.441648752	positive
PRJNA729271	eed_Lushi_vs_Arbor_Acr	breed_growth_background	ENSGALG00010001502	LGSN	-4.746531463	0.569062134	-8.340972246	7.36822E-17	1.06802E-13	negative
PRJNA729271	eed_Lushi_vs_Arbor_Acr	breed_growth_background	ENSGALG00010001504	RBMS3	-0.181350481	0.053324094	-3.400910709	0.000671618	0.006122703	negative
PRJNA729271	eed_Lushi_vs_Arbor_Acr	breed_growth_background	ENSGALG00010001505	SSR3	-0.109660012	0.052002718	-2.108736189	0.034967359	0.101797926	negative
PRJNA729271	eed_Lushi_vs_Arbor_Acr	breed_growth_background	ENSGALG00010001506	DPYSL4	0.480223965	0.148023822	3.244234337	0.001177668	0.009207282	positive
PRJNA729271	eed_Lushi_vs_Arbor_Acr	breed_growth_background	ENSGALG00010001507	METTL4	-0.225610961	0.054211295	-4.161696547	3.15892E-05	0.00069167	negative
PRJNA729271	eed_Lushi_vs_Arbor_Acr	breed_growth_background	ENSGALG00010001508	CD109	0.070224476	0.156712796	0.448109392	0.65407425	0.774638962	positive
PRJNA729271	eed_Lushi_vs_Arbor_Acr	breed_growth_background	ENSGALG00010001510	AP1S2	-0.08839759	0.052920328	-1.670390072	0.094842213	0.207288581	negative
PRJNA729271	eed_Lushi_vs_Arbor_Acr	breed_growth_background	ENSGALG00010001512	PLCL2	0.284918353	0.092134334	3.092423195	0.001985296	0.013188298	positive
PRJNA729271	eed_Lushi_vs_Arbor_Acr	breed_growth_background	ENSGALG00010001513	ZRSR2	0.151765508	0.050344318	3.014550861	0.002573601	0.01591139	positive
PRJNA729271	eed_Lushi_vs_Arbor_Acr	breed_growth_background	ENSGALG00010001514	MET	0.066098473	0.080913783	0.816900046	0.413985552	0.574341556	positive
PRJNA729271	eed_Lushi_vs_Arbor_Acr	breed_growth_background	ENSGALG00010001516	CLTRN	0.224505367	0.321334327	0.698665995	0.48476079	0.636583407	positive
PRJNA729271	eed_Lushi_vs_Arbor_Acr	breed_growth_background	ENSGALG00010001517	CAV2	-0.262530183	0.087151719	-3.012335101	0.002592462	0.015970141	negative
PRJNA729271	eed_Lushi_vs_Arbor_Acr	breed_growth_background	ENSGALG00010001518	SLC17A5	-0.242348102	0.089851543	-2.697205795	0.006992404	0.032367036	negative
PRJNA729271	eed_Lushi_vs_Arbor_Acr	breed_growth_background	ENSGALG00010001520	KCNAB1	-0.042881841	0.116753645	-0.367284815	0.713406579	0.815844447	negative
PRJNA729271	eed_Lushi_vs_Arbor_Acr	breed_growth_background	ENSGALG00010001521	TBC1D5	0.007941501	0.040275041	0.197181711	0.843685342	0.905130563	positive
PRJNA729271	eed_Lushi_vs_Arbor_Acr	breed_growth_background	ENSGALG00010001524	ACE2	-0.630523996	0.777164609	-0.811313315	0.417185765	0.577344631	negative
PRJNA729271	eed_Lushi_vs_Arbor_Acr	breed_growth_background	ENSGALG00010001525	EEF1A1	-0.245563539	0.094455723	-2.599774072	0.009328515	0.039825823	negative
PRJNA729271	eed_Lushi_vs_Arbor_Acr	breed_growth_background	ENSGALG00010001526	PPP1R3A	0.175760025	0.078330102	2.243837558	0.024842855	0.079667518	positive
PRJNA729271	eed_Lushi_vs_Arbor_Acr	breed_growth_background	ENSGALG00010001527	STK32C	0.048256747	0.121890034	0.395903962	0.692175878	0.801236972	positive
PRJNA729271	eed_Lushi_vs_Arbor_Acr	breed_growth_background	ENSGALG00010001528	NR1D2	0.038486688	0.096539586	0.39866225	0.690142087	0.799649045	positive
PRJNA729271	eed_Lushi_vs_Arbor_Acr	breed_growth_background	ENSGALG00010001531	ENSGALG00010001531	-10.21429184	2.934397675	-3.480881931			negative
PRJNA729271	eed_Lushi_vs_Arbor_Acr	breed_growth_background	ENSGALG00010001532	ENSGALG00010001532	-0.336008316	0.148231671	-2.266778177	0.02340378	0.076370507	negative
PRJNA729271	eed_Lushi_vs_Arbor_Acr	breed_growth_background	ENSGALG00010001533	ENSGALG00010001533	-0.088934741	0.080398397	-1.106175559	0.268650539	0.427170861	negative
PRJNA729271	eed_Lushi_vs_Arbor_Acr	breed_growth_background	ENSGALG00010001534	SLITRK3	0.359545872	0.180706765	1.989664709	0.046627883	0.125943071	positive
PRJNA729271	eed_Lushi_vs_Arbor_Acr	breed_growth_background	ENSGALG00010001535	RAP2B	0.041895919	0.057047558	0.734403366	0.462702936	0.617746989	positive
PRJNA729271	eed_Lushi_vs_Arbor_Acr	breed_growth_background	ENSGALG00010001540	TRABD	0.041991642	0.070563595	0.595089323	0.551783765	0.69452116	positive
PRJNA729271	eed_Lushi_vs_Arbor_Acr	breed_growth_background	ENSGALG00010001542	MTO1	0.098448978	0.068699559	1.433036537	0.151847396	0.287834275	positive
PRJNA729271	eed_Lushi_vs_Arbor_Acr	breed_growth_background	ENSGALG00010001545	SELENOO	0.095300331	0.070178506	1.357970361	0.174473089	0.318338822	positive

PRJNA729271	ed_Lushi_vs_Arbor_Acr	breed_growth_background	ENSGALG00010001546	CGAS	-0.089315472	0.092611428	-0.964410915	0.334839952	0.499177733	negative
PRJNA729271	ed_Lushi_vs_Arbor_Acr	breed_growth_background	ENSGALG00010001547	PWWP2B	0.213499345	0.058519535	3.648343155	0.000263937	0.003177547	positive
PRJNA729271	ed_Lushi_vs_Arbor_Acr	breed_growth_background	ENSGALG00010001548	AKAP17A	0.158905091	0.051698952	3.073661726	0.00211449	0.013799882	positive
PRJNA729271	ed_Lushi_vs_Arbor_Acr	breed_growth_background	ENSGALG00010001549	ENSGALG00010001549	0.875482789	0.291963172	2.998606921	0.00271217	0.016469585	positive
PRJNA729271	ed_Lushi_vs_Arbor_Acr	breed_growth_background	ENSGALG00010001551	FHIP1B	0.077263151	0.085398898	0.904732408	0.365607168	0.528942598	positive
PRJNA729271	ed_Lushi_vs_Arbor_Acr	breed_growth_background	ENSGALG00010001552	TUBGCP6	-0.011619147	0.058685625	-0.197989661	0.843053153	0.90506146	negative
PRJNA729271	ed_Lushi_vs_Arbor_Acr	breed_growth_background	ENSGALG00010001553	CNGA4	-0.057081186	0.22022295	-0.259197266	0.795483041	0.873373925	negative
PRJNA729271	ed_Lushi_vs_Arbor_Acr	breed_growth_background	ENSGALG00010001554	ENSGALG00010001554	0.090312032	0.098650757	0.915472266	0.359943756	0.523409251	positive
PRJNA729271	ed_Lushi_vs_Arbor_Acr	breed_growth_background	ENSGALG00010001556	EPB41L3	-0.081569586	0.062981539	-1.295134854	0.195273713	0.34317319	negative
PRJNA729271	ed_Lushi_vs_Arbor_Acr	breed_growth_background	ENSGALG00010001557	THRB	-0.362830679	0.185377381	-1.95725432	0.050317575	0.133166561	negative
PRJNA729271	ed_Lushi_vs_Arbor_Acr	breed_growth_background	ENSGALG00010001558	CCKBR	-0.410434631	0.360316584	-1.13909448	0.25466375	0.411292596	negative
PRJNA729271	ed_Lushi_vs_Arbor_Acr	breed_growth_background	ENSGALG00010001559	HDAC10	-0.066967445	0.071537918	-0.936111183	0.349215946	0.513167593	negative
PRJNA729271	ed_Lushi_vs_Arbor_Acr	breed_growth_background	ENSGALG00010001567	INPP5A	-0.085072053	0.044721544	-1.902261089	0.05713703	0.145681838	negative
PRJNA729271	ed_Lushi_vs_Arbor_Acr	breed_growth_background	ENSGALG00010001570	SMPD1	0.14865094	0.086603863	1.716446995	0.086080261	0.193928099	positive
PRJNA729271	ed_Lushi_vs_Arbor_Acr	breed_growth_background	ENSGALG00010001585	ENSGALG00010001585	-0.052645462	0.074387622	-0.707718034	0.479120376	0.631981969	negative
PRJNA729271	ed_Lushi_vs_Arbor_Acr	breed_growth_background	ENSGALG00010001586	ZBTB14	0.229048124	0.051914208	4.412050861	1.02396E-05	0.000297441	positive
PRJNA729271	ed_Lushi_vs_Arbor_Acr	breed_growth_background	ENSGALG00010001588	ENSGALG00010001588	0.599521204	0.310171913	1.932867481	0.053252525	0.138605736	positive
PRJNA729271	ed_Lushi_vs_Arbor_Acr	breed_growth_background	ENSGALG00010001595	AKAIN1	0.227189361	0.139831205	1.624740062	0.104217957	0.221550282	positive
PRJNA729271	ed_Lushi_vs_Arbor_Acr	breed_growth_background	ENSGALG00010001599	TBLX1	0.089873876	0.040340872	2.227861487	0.025889753	0.081955006	positive
PRJNA729271	ed_Lushi_vs_Arbor_Acr	breed_growth_background	ENSGALG00010001604	ENSGALG00010001604	0.469364685	0.175532328	2.673950095	0.007496361	0.033998669	positive
PRJNA729271	ed_Lushi_vs_Arbor_Acr	breed_growth_background	ENSGALG00010001605	APBB1	-0.018534791	0.061099829	-0.303352577	0.761621182	0.850133304	negative
PRJNA729271	ed_Lushi_vs_Arbor_Acr	breed_growth_background	ENSGALG00010001606	PIGA	-0.003663029	0.060207825	-0.06083975	0.951486833	0.973322003	negative
PRJNA729271	ed_Lushi_vs_Arbor_Acr	breed_growth_background	ENSGALG00010001608	ENSGALG00010001608	0.455580987	0.269424464	1.690941427	0.09084799	0.201450683	positive
PRJNA729271	ed_Lushi_vs_Arbor_Acr	breed_growth_background	ENSGALG00010001609	ENSGALG00010001609	0.272175517	0.246568605	1.103853093	0.269656869	0.428254226	positive
PRJNA729271	ed_Lushi_vs_Arbor_Acr	breed_growth_background	ENSGALG00010001611	MAPK12	-0.297780303	0.089012805	-3.345364797	0.000821744	0.007081558	negative
PRJNA729271	ed_Lushi_vs_Arbor_Acr	breed_growth_background	ENSGALG00010001612	HPX	-0.314814178	0.494659519	-0.636425998	0.5244988	0.671312151	negative
PRJNA729271	ed_Lushi_vs_Arbor_Acr	breed_growth_background	ENSGALG00010001616	OPNVA	-0.174945385	0.331899491	-0.527103504	0.598121713	0.731317945	negative
PRJNA729271	ed_Lushi_vs_Arbor_Acr	breed_growth_background	ENSGALG00010001617	TRIM3	-0.068904875	0.050737195	-1.358074188	0.174440145	0.318331852	negative
PRJNA729271	ed_Lushi_vs_Arbor_Acr	breed_growth_background	ENSGALG00010001620	MOSPD2	-0.021025202	0.076883241	-0.273469248	0.784492528	0.866642725	negative
PRJNA729271	ed_Lushi_vs_Arbor_Acr	breed_growth_background	ENSGALG00010001621	DLGAP2	-0.237361052	0.136615109	-1.737443632	0.082308895	0.187648229	negative
PRJNA729271	ed_Lushi_vs_Arbor_Acr	breed_growth_background	ENSGALG00010001627	ARFIP2	0.226263462	0.064861678	3.488399783	0.000485921	0.004873213	positive
PRJNA729271	ed_Lushi_vs_Arbor_Acr	breed_growth_background	ENSGALG00010001628	MAPK11	-0.126969331	0.114709599	-1.106876258	0.268347432	0.42687621	negative
PRJNA729271	ed_Lushi_vs_Arbor_Acr	breed_growth_background	ENSGALG00010001632	FANCB	-0.644336956	0.14734225	-4.373063102	1.22515E-05	0.000335701	negative
PRJNA729271	ed_Lushi_vs_Arbor_Acr	breed_growth_background	ENSGALG00010001633	B3GAT2	-0.664058641	0.222617942	-2.982952019	0.002854827	0.017106542	negative
PRJNA729271	ed_Lushi_vs_Arbor_Acr	breed_growth_background	ENSGALG00010001635	TIMM10B	0.042974621	0.080875877	0.53136513	0.59516578	0.728995097	positive
PRJNA729271	ed_Lushi_vs_Arbor_Acr	breed_growth_background	ENSGALG00010001639	ENSGALG00010001639	-0.312366821	0.086617527	-3.606277284	0.000310621	0.00354524	negative
PRJNA729271	ed_Lushi_vs_Arbor_Acr	breed_growth_background	ENSGALG00010001642	ENSGALG00010001642	0.101869417	0.06421914	1.58627811	0.112676236	0.234076555	positive
PRJNA729271	ed_Lushi_vs_Arbor_Acr	breed_growth_background	ENSGALG00010001647	ARL8BL	-0.169062084	0.082794852	-2.041939569	0.041157526	0.114696424	negative
PRJNA729271	ed_Lushi_vs_Arbor_Acr	breed_growth_background	ENSGALG00010001649	UBE2E2	-0.187009413	0.124856814	-1.497791001	0.13418756	0.264093507	negative
PRJNA729271	ed_Lushi_vs_Arbor_Acr	breed_growth_background	ENSGALG00010001654	RPS11	-0.067674147	0.09055065	-0.74736236	0.454844859	0.611020944	negative
PRJNA729271	ed_Lushi_vs_Arbor_Acr	breed_growth_background	ENSGALG00010001657	SMAP1	-0.059388425	0.053095108	-1.118529121	0.263341076	0.421456211	negative
PRJNA729271	ed_Lushi_vs_Arbor_Acr	breed_growth_background	ENSGALG00010001661	TCTN3	-0.086229757	0.056832792	-1.517253566	0.129202677	0.256652432	negative
PRJNA729271	ed_Lushi_vs_Arbor_Acr	breed_growth_background	ENSGALG00010001664	SPAM1	-0.863993459	1.071145702	-0.806606848	0.419893027	0.579310552	negative
PRJNA729271	ed_Lushi_vs_Arbor_Acr	breed_growth_background	ENSGALG00010001666	PIEZO2	-0.158142077	0.098876665	-1.599387254	0.109734583	0.230101646	negative
PRJNA729271	ed_Lushi_vs_Arbor_Acr	breed_growth_background	ENSGALG00010001668	UBE2E1	-0.064373419	0.046381336	-1.387916431	0.165162494	0.305244211	negative
PRJNA729271	ed_Lushi_vs_Arbor_Acr	breed_growth_background	ENSGALG00010001670	SHOX	-0.303597324	0.089008004	-3.410899144	0.00064749	0.005987563	negative
PRJNA729271	ed_Lushi_vs_Arbor_Acr	breed_growth_background	ENSGALG00010001672	ENSGALG00010001672	0.289228846	0.277769068	1.041256495	0.297756522	0.459847406	positive
PRJNA729271	ed_Lushi_vs_Arbor_Acr	breed_growth_background	ENSGALG00010001674	ENSGALG00010001674	0.074938195	0.056892301	1.317193958	0.187773619	0.33359218	positive
PRJNA729271	ed_Lushi_vs_Arbor_Acr	breed_growth_background	ENSGALG00010001677	SDHAF4	-0.025978781	0.057713883	-0.450130527	0.652616327	0.773428775	negative
PRJNA729271	ed_Lushi_vs_Arbor_Acr	breed_growth_background	ENSGALG00010001678	SYAP1	0.081516383	0.031605523	2.579181551	0.009903472	0.041512869	positive
PRJNA729271	ed_Lushi_vs_Arbor_Acr	breed_growth_background	ENSGALG00010001681	NAPG	-0.006368019	0.04937818	-0.128964234	0.89738595	0.940413702	negative
PRJNA729271	ed_Lushi_vs_Arbor_Acr	breed_growth_background	ENSGALG00010001686	WASL	0.003406385	0.046475149	0.073294756	0.941571564	0.968223597	positive
PRJNA729271	ed_Lushi_vs_Arbor_Acr	breed_growth_background	ENSGALG00010001688	ENSGALG00010001688	0.222219529	0.08950729	2.482697532	0.013039175	0.050347055	positive
PRJNA729271	ed_Lushi_vs_Arbor_Acr	breed_growth_background	ENSGALG00010001689	ILK	0.031332802	0.036265968	0.863972589	0.387602949	0.549574953	positive
PRJNA729271	ed_Lushi_vs_Arbor_Acr	breed_growth_background	ENSGALG00010001691	CTPS2	-0.007487066	0.07153806	-0.104658496	0.916646796	0.953054582	negative
PRJNA729271	ed_Lushi_vs_Arbor_Acr	breed_growth_background	ENSGALG00010001692	ALDH18A1	-0.183002585	0.136813967	-1.337601625	0.181026334	0.325555424	negative
PRJNA729271	ed_Lushi_vs_Arbor_Acr	breed_growth_background	ENSGALG00010001693	NKIRAS1	0.093114311	0.104575033	0.890406713	0.373247539	0.535718693	positive
PRJNA729271	ed_Lushi_vs_Arbor_Acr	breed_growth_background	ENSGALG00010001694	ENSGALG00010001694	0.113576395	0.118564038	0.957932914	0.338096588	0.502430802	positive
PRJNA729271	ed_Lushi_vs_Arbor_Acr	breed_growth_background	ENSGALG00010001695	LMOD2	-0.877522348	0.251179452	-3.493607223	0.000476542	0.004800749	negative
PRJNA729271	ed_Lushi_vs_Arbor_Acr	breed_growth_background	ENSGALG00010001696	FAM135A	0.047362153	0.048747556	0.971580058	0.331259507	0.495572976	positive
PRJNA729271	ed_Lushi_vs_Arbor_Acr	breed_growth_background	ENSGALG00010001697	TAF10	-0.1036896	0.064360642	-1.611071572	0.107164124	0.226046088	negative
PRJNA729271	ed_Lushi_vs_Arbor_Acr	breed_growth_background	ENSGALG00010001698	RPL15	-0.178351214	0.094313043	-1.891055654	0.058616915	0.148203766	negative
PRJNA729271	ed_Lushi_vs_Arbor_Acr	breed_growth_background	ENSGALG00010001699	ASB15	-0.034782485	0.108923108	-0.319330632	0.749475806	0.840709783	negative
PRJNA729271	ed_Lushi_vs_Arbor_Acr	breed_growth_background	ENSGALG00010001700	ENSGALG00010001700	0.033038579	0.10633439	0.310704557	0.756025233	0.84582305	positive
PRJNA729271	ed_Lushi_vs_Arbor_Acr	breed_growth_background	ENSGALG00010001701	NDUFA5	-0.254734936	0.090064054	-2.828375194	0.004678494	0.024236873	negative
PRJNA729271	ed_Lushi_vs_Arbor_Acr	breed_growth_background	ENSGALG00010001702	PLEKHA1	-0.006666203	0.044916636	-0.148412791	0.882017006	0.930144525	negative
PRJNA729271	ed_Lushi_vs_Arbor_Acr	breed_growth_background	ENSGALG00010001704	ENSGALG00010001704	-0.180087123	0.078645931	-2.289846666	0.022030207	0.073156437	negative
PRJNA729271	ed_Lushi_vs_Arbor_Acr	breed_growth_background	ENSGALG00010001705	HTRA1	-0.00766985	0.082508172	-0.092958676	0.925936391	0.958537922	negative
PRJNA729271	ed_Lushi_vs_Arbor_Acr	breed_growth_background	ENSGALG00010001706	IQUB	0.431019626	0.181980744	2.368490288	0.017860849	0.063212986	positive
PRJNA729271	ed_Lushi_vs_Arbor_Acr	breed_growth_background	ENSGALG00010001707	ENSGALG00010001707	-0.70600005	0.54836334	-1.287467631	0.197931345	0.346309987	negative
PRJNA729271	ed_Lushi_vs_Arbor_Acr	breed_growth_background	ENSGALG00010001708	AADAC	0.252729107	0.132894396	1.901728844	0.057206613	0.145807958	positive
PRJNA729271	ed_Lushi_vs_Arbor_Acr	breed_growth_background	ENSGALG00010001710	LCMT2	-0.050549439	0.044737233	-1.129918761	0.258510458	0.41580611	negative
PRJNA729271	ed_Lushi_vs_Arbor_Acr	breed_growth_background	ENSGALG00010001711	CREB5	-0.194958641	0.094764634	-2.057293232	0.039658028	0.111793682	negative
PRJNA729271	ed_Lushi_vs_Arbor_Acr	breed_growth_background	ENSGALG00010001712	ATP6AP1	0.033308886	0.084901671	0.392323093	0.694819503	0.803462999	positive
PRJNA729271	ed_Lushi_vs_Arbor_Acr	breed_growth_background	ENSGALG00010001713	PNPLA4	0.071689991	0.122907573	0.583283758	0.559702295	0.700715561	positive
PRJNA729271	ed_Lushi_vs_Arbor_Acr	breed_growth_background	ENSGALG00010001716	TRIL	-0.216668148	0.081755988	-2.650180771	0.008044871	0.035759095	negative
PRJNA729271	ed_Lushi_vs_Arbor_Acr	breed_growth_background	ENSGALG00010001717	STARD10	0.101764547	0.28504243	0.357015435	0.721080251	0.821263427	positive
PRJNA729271	ed_Lushi_vs_Arbor_Acr	breed_growth_background	ENSGALG00010001721	TM4SF1	-0.445698955	0.086872024	-5.130523421	2.88938E-07	1.86971E-05	negative
PRJNA729271	ed_Lushi_vs_Arbor_Acr	breed_growth_background	ENSGALG00010001725	MTA2	0.098709586	0.100435757	0.982813182	0.325699393	0.489325529	positive
PRJNA729271	ed_Lushi_vs_Arbor_Acr	breed_growth_background	ENSGALG00010001727	COL9A1	0.563762212	0.432985931	1.302033557	0.192904915	0.339916939	positive
PRJNA729271	ed_Lushi_vs_Arbor_Acr	breed_growth_background	ENSGALG00010001728	STS	-0.122366673	0.173535599	-0.705138737	0.480723902	0.633578192	negative
PRJNA729271	ed_Lushi_vs_Arbor_Acr	breed_growth_background	ENSGALG00010001729	ENSGALG00010001729	0.110840719	0.126518077	0.876086023	0.380983271	0.542630688	positive
PRJNA729271	ed_Lushi_vs_Arbor_Acr	breed_growth_background	ENSGALG00010001731	ENSGALG00010001731	0.334414241	0.108397706	3.085067504	0.00203506	0.013432691	positive
PRJNA729271	ed_Lushi_vs_Arbor_Acr	breed_growth_background	ENSGALG00010001738	PUDP	0.062034651	0.065740898	0.943623424	0.345362118	0.509259807	positive
PRJNA729271	ed_Lushi_vs_Arbor_Acr	breed_growth_background	ENSGALG00010001741	SATB1	0.334929895	0.107518016	3.115104852	0.001838795	0.012525065	positive
PRJNA729271	ed_Lushi_vs_Arbor_Acr	breed_growth_background	ENSGALG00010001745	CADPS2	0.005520979	0.060933635	0.090606425	0.927805326	0.959924211	positive
PRJNA729271	ed_Lushi_vs_Arbor_Acr	breed_growth_background	ENSGALG00010001749	VAPA	0.077303719	0.056668722	1.364133796	0.17252546	0.315712226	positive
PRJNA729271	ed_Lushi_vs_Arbor_Acr	breed_growth_background	ENSGALG00010001751	AVPR2C	-0.147488218	0.236278144	-0.624214393	0.532486796	0.67788478	negative
PRJNA729271	ed_Lushi_vs_Arbor_Acr	breed_growth_background	ENSGALG00010001752	ARRB1	-0.029040554	0.055552859	-0.522755349	0.601144516	0.733837776	negative
PRJNA729271	ed_Lushi_vs_Arbor_Acr	breed_growth_background	ENSGALG00010001753	PNPLA8	0.334399334	0.109350932	3.058038268	0.002227911	0.014391075	positive
PRJNA729271	ed_Lushi_vs_Arbor_Acr	breed_growth_background	ENSGALG00010001754	APCDD1	-0.093928716	0.109494389	-0.857840456	0.390980567	0.552538657	negative
PRJNA729271	ed_Lushi_vs_Arbor_Acr	breed_growth_background	ENSGALG00010001755	CUZD1	-1.717329437	0.809129605	-2.122440493	0.033800765	0.09935174	negative
PRJNA729271	ed_Lushi_vs_Arbor_Acr	breed_growth_background	ENSGALG00010001756	ENSGALG00010001756	0.423274325	0.117547637	3.600874815	0.000317148	0.003596097	positive
PRJNA729271	ed_Lushi_vs_Arbor_Acr	breed_growth_background	ENSGALG00010001758	COL19A1	-0.189542545	0.149081233	-1.27140446	0.203584797	0.352774851	negative
PRJNA729271	ed_Lushi_vs_Arbor_Acr	breed_growth_background	ENSGALG00010001759	C10orf88	0.108864154	0.048945631	2.224185313	0.026135979	0.082431303	positive
PRJNA729271	ed_Lushi_vs_Arbor_Acr	breed_growth_background	ENSGALG00010001760	RAB5A	-0.342831582	0.05154797	-6.650729002	2.91645E-11	9.3942E-09	negative
PRJNA729271	ed_Lushi_vs_Arbor_Acr	breed_growth_background	ENSGALG00010001761	ELOB	-0.035837282	0.080253509	-0.446550967	0.655199305	0.775238902	negative
PRJNA729271	ed_Lushi_vs_Arbor_Acr	breed_growth_background	ENSGALG00010001763	ENSGALG00010001763	0.522727488	0.14401142	3.62976414	0.00028368	0.003345766	positive
PRJNA729271	ed_Lushi_vs_Arbor_Acr	breed_growth_background	ENSGALG00010001764	ENSGALG00010001764	0.424327944	0.241226979	1.759040158	0.078570688	0.181639892	positive
PRJNA729271	ed_Lushi_vs_Arbor_Acr	breed_growth_background	ENSGALG00010001767	ENSGALG00010001767	0.073188279	0.094138853	0.777450292	0.436893151	0.5943469	positive
PRJNA729271	ed_Lushi_vs_Arbor_Acr	breed_growth_background	ENSGALG00010001771	ENSGALG00010001771	-0.005592833	0.073394034	-0.076202822	0.939257737	0.966697969	negative
PRJNA729271	ed_Lushi_vs_Arbor_Acr	breed_growth_background	ENSGALG00010001773	NRCAM	0.563878879	0.133700849	4.217466722	2.47062E-05	0.000562193	positive
PRJNA729271	ed_Lushi_vs_Arbor_Acr	breed_growth_background	ENSGALG00010001776	LMBRD1	0.145086872	0.070219247	2.066198061	0.038809782	0.11006609	positive
PRJNA729271	ed_Lushi_vs_Arbor_Acr	breed_growth_background	ENSGALG00010001778	KAT2B	0.170006281	0.078305325	2.171069221	0.02992594	0.090691303	positive
PRJNA729271	ed_Lushi_vs_Arbor_Acr	breed_growth_background	ENSGALG00010001779	ENSGALG00010001779	0.867266884	0.326491307	2.656324584	0.007899754	0.035298068	positive
PRJNA729271	ed_Lushi_vs_Arbor_Acr	breed_growth_background	ENSGALG00010001780	ENSGALG00010001780	0.155606311	0.127090288	1.224376099	0.220810382	0.373317689	positive
PRJNA729271	ed_Lushi_vs_Arbor_Acr	breed_growth_background	ENSGALG00010001783	SGO1	-0.440341099	0.13580404	-3.242474229	0.001184967	0.009254084	negative
PRJNA729271	ed_Lushi_vs_Arbor_Acr	breed_growth_background	ENSGALG00010001785	CNTN1	-0.411101325	0.113943531	-3.607939132	0.000308639	0.003533744	negative
PRJNA729271	ed_Lushi_vs_Arbor_Acr	breed_growth_background	ENSGALG00010001786	P2RY8	-0.442210978	0.112781084	-3.920967609	8.81941E-05	0.001460999	negative
PRJNA729271	ed_Lushi_vs_Arbor_Acr	breed_growth_background	ENSGALG00010001788	HOXA1	0.050034545	0.232148279	0.215528389	0.829355381	0.895856671	positive
PRJNA729271	ed_Lushi_vs_Arbor_Acr	breed_growth_background	ENSGALG00010001790	HOXA2	0.190404191	0.140502847	1.355162513	0.175365785	0.319377693	positive
PRJNA729271	ed_Lushi_vs_Arbor_Acr	breed_growth_background	ENSGALG00010001793	IKZF5	0.12495907	0.055479843	2.2523328	0.024301249	0.078346664	positive
PRJNA729271	ed_Lushi_vs_Arbor_Acr	breed_growth_background	ENSGALG00010001794	ENSGALG00010001794	0.412541429	0.343186609	1.202090693	0.229328392	0.382918447	positive
PRJNA729271	ed_Lushi_vs_Arbor_Acr	breed_growth_background	ENSGALG00010001795	ENSGALG00010001795	-0.36142955	0.250289138	-1.444048084	0.14872545	0.283654658	negative
PRJNA729271	ed_Lushi_vs_Arbor_Acr	breed_growth_background	ENSGALG00010001796	SLCO2B1	-0.104782999	0.08257846	-1.268890199	0.204480229	0.353861141	negative
PRJNA729271	ed_Lushi_vs_Arbor_Acr	breed_growth_background	ENSGALG00010001798	PSTK	0.108020706	0.069860891	1.546225701	0.122050068	0.24672737	positive
PRJNA729271	ed_Lushi_vs_Arbor_Acr	breed_growth_background	ENSGALG00010001800	ENSGALG00010001800	0.099518179	0.099965654	0.995523716	0.319481616	0.482786282	positive
PRJNA729271	ed_Lushi_vs_Arbor_Acr	breed_growth_background	ENSGALG00010001801	HOXA3	0.115310567	0.082826367	1.392196366	0.163862943	0.30369433	positive
PRJNA729271	ed_Lushi_vs_Arbor_Acr	breed_growth_background	ENSGALG00010001803	PPP4R1	-0.12688595	0.052264572	-2.427762168	0.015192302	0.056162311	negative
PRJNA729271	ed_Lushi_vs_Arbor_Acr	breed_growth_background	ENSGALG00010001805	ENSGALG00010001805	-0.025711789	0.137662929	-0.18677351	0.851838217	0.910642006	negative
PRJNA729271	ed_Lushi_vs_Arbor_Acr	breed_growth_background	ENSGALG00010001806	ENSGALG00010001806	-0.489827725	0.466448289	-1.050122246	0.293661912	0.455011161	negative
PRJNA729271	ed_Lushi_vs_Arbor_Acr	breed_growth_background	ENSGALG00010001807	ASMTL	0.04527709	0.056241676	0.80504518	0.420793609	0.579901441	positive
PRJNA729271	ed_Lushi_vs_Arbor_Acr	breed_growth_background	ENSGALG00010001808	HOXA4	-0.057969523	0.084862925	-0.683095984	0.494546193	0.646097077	negative
PRJNA729271	ed_Lushi_vs_Arbor_Acr	breed_growth_background	ENSGALG00010001811	ACADSB	0.126322143	0.079822025	1.58254746	0.113524647	0.235144292	positive
PRJNA729271	ed_Lushi_vs_Arbor_Acr	breed_growth_background	ENSGALG00010001813	HOXA5	0.002725125	0.056750683	0.048019252	0.961700899	0.978276808	positive
PRJNA729271	ed_Lushi_vs_Arbor_Acr	breed_growth_background	ENSGALG00010001817	SLC25A6	-0.109954558	0.060450766	-1.818910903	0.068925024	0.166096131	negative
PRJNA729271	ed_Lushi_vs_Arbor_Acr	breed_growth_background	ENSGALG00010001818	ADGRB3	0.553634714	0.139205664	3.977099037	6.97611E-05	0.001218298	positive
PRJNA729271	ed_Lushi_vs_Arbor_Acr	breed_growth_background	ENSGALG00010001819	HOXA6	-0.014123928	0.076592281	-0.184404066	0.85369648	0.911714438	negative
PRJNA729271	ed_Lushi_vs_Arbor_Acr	breed_growth_background	ENSGALG00010001820	PGM2L1	0.318810841	0.08298585	3.841749443	0.000122161	0.001850278	positive
PRJNA729271	ed_Lushi_vs_Arbor_Acr	breed_growth_background	ENSGALG00010001822	KCNE3	-0.232214541	0.162539874	-1.428662004	0.153101404	0.289565385	negative
PRJNA729271	ed_Lushi_vs_Arbor_Acr	breed_growth_background	ENSGALG00010001823	RALBP1	0.069534743	0.041125814	1.69078094	0.090878649	0.201450683	positive

PRJNA729271	ed_Lushi_vs_Arbor_Acr	breed_growth_background	ENSGALG00010001825	LIPT2	0.112297298	0.075833994	1.48083058	0.138651726	0.270273906	positive
PRJNA729271	ed_Lushi_vs_Arbor_Acr	breed_growth_background	ENSGALG00010001826	HOXA7	0.081639289	0.071032973	1.149315389	0.250425955	0.406304479	positive
PRJNA729271	ed_Lushi_vs_Arbor_Acr	breed_growth_background	ENSGALG00010001827	PDZRN4	0.102276031	0.107685636	0.949764841	0.342231754	0.505938073	positive
PRJNA729271	ed_Lushi_vs_Arbor_Acr	breed_growth_background	ENSGALG00010001828	ENSGALG00010001828	3.591356965	0.656879214	5.467301886	4.56937E-08	4.64009E-06	positive
PRJNA729271	ed_Lushi_vs_Arbor_Acr	breed_growth_background	ENSGALG00010001829	POLD3	0.171579331	0.065229164	2.630408242	0.008528239	0.037146343	positive
PRJNA729271	ed_Lushi_vs_Arbor_Acr	breed_growth_background	ENSGALG00010001831	CSF2RA	-0.113379804	0.135487313	-0.83682967	0.402688318	0.563740309	negative
PRJNA729271	ed_Lushi_vs_Arbor_Acr	breed_growth_background	ENSGALG00010001832	BUB3	-0.160109617	0.047063674	-3.401978731	0.000668998	0.006102664	negative
PRJNA729271	ed_Lushi_vs_Arbor_Acr	breed_growth_background	ENSGALG00010001834	ZCRB1	0.010612609	0.045598945	0.232738044	0.815964825	0.886680308	positive
PRJNA729271	ed_Lushi_vs_Arbor_Acr	breed_growth_background	ENSGALG00010001835	MECP2	0.213694872	0.102446593	2.085914868	0.036986335	0.106245923	positive
PRJNA729271	ed_Lushi_vs_Arbor_Acr	breed_growth_background	ENSGALG00010001836	TWSG1	-0.204784748	0.075243164	-2.721639247	0.006495901	0.03072042	negative
PRJNA729271	ed_Lushi_vs_Arbor_Acr	breed_growth_background	ENSGALG00010001837	TAB3	0.186769925	0.074629802	2.502618514	0.012327834	0.048478556	positive
PRJNA729271	ed_Lushi_vs_Arbor_Acr	breed_growth_background	ENSGALG00010001838	PPHLN1	0.030771123	0.036451141	0.844174476	0.398571915	0.559761643	positive
PRJNA729271	ed_Lushi_vs_Arbor_Acr	breed_growth_background	ENSGALG00010001839	ENSGALG00010001839	0.817517125	0.316239614	2.585119284	0.009734531	0.041009543	positive
PRJNA729271	ed_Lushi_vs_Arbor_Acr	breed_growth_background	ENSGALG00010001840	HOXA9	0.157643389	0.077779341	2.026802851	0.042682579	0.118091999	positive
PRJNA729271	ed_Lushi_vs_Arbor_Acr	breed_growth_background	ENSGALG00010001842	GPR26	-0.047400569	0.287453945	-0.16489796	0.869024298	0.921985721	negative
PRJNA729271	ed_Lushi_vs_Arbor_Acr	breed_growth_background	ENSGALG00010001843	PRICKLE1	-0.032084373	0.066201544	-0.484646893	0.627926844	0.754084474	negative
PRJNA729271	ed_Lushi_vs_Arbor_Acr	breed_growth_background	ENSGALG00010001844	HOXA10	0.153105926	0.091129144	1.680098367	0.092938179	0.204432158	positive
PRJNA729271	ed_Lushi_vs_Arbor_Acr	breed_growth_background	ENSGALG00010001845	ANKRD12	0.203151215	0.071507887	2.840962342	0.004497762	0.023536125	positive
PRJNA729271	ed_Lushi_vs_Arbor_Acr	breed_growth_background	ENSGALG00010001848	HOXA11	-0.028876324	0.198946398	-0.14514625	0.884595403	0.931933811	negative
PRJNA729271	ed_Lushi_vs_Arbor_Acr	breed_growth_background	ENSGALG00010001849	NDUFV2	-0.060705802	0.050607092	-1.199551292	0.230313653	0.384165293	negative
PRJNA729271	ed_Lushi_vs_Arbor_Acr	breed_growth_background	ENSGALG00010001850	MYOM2	-0.238398175	0.100329003	-2.376164108	0.017493681	0.062092038	negative
PRJNA729271	ed_Lushi_vs_Arbor_Acr	breed_growth_background	ENSGALG00010001852	CPXM2	0.546376754	0.20775489	2.629910444	0.008540736	0.037146343	positive
PRJNA729271	ed_Lushi_vs_Arbor_Acr	breed_growth_background	ENSGALG00010001853	ENSGALG00010001853	0.169607917	0.101115759	1.677363829	0.093471358	0.205065436	positive
PRJNA729271	ed_Lushi_vs_Arbor_Acr	breed_growth_background	ENSGALG00010001854	CHRDL2	-0.063068241	0.157487584	-0.400464847	0.68881417	0.798748912	negative
PRJNA729271	ed_Lushi_vs_Arbor_Acr	breed_growth_background	ENSGALG00010001856	CHST15	-0.206385831	0.06346991	-3.251711402	0.001147124	0.009014916	negative
PRJNA729271	ed_Lushi_vs_Arbor_Acr	breed_growth_background	ENSGALG00010001857	HNRNPA2B1	0.178710878	0.100414962	1.779723611	0.075121204	0.176508649	positive
PRJNA729271	ed_Lushi_vs_Arbor_Acr	breed_growth_background	ENSGALG00010001858	ADAMTS20	0.037895384	0.085795644	0.441693566	0.658710966	0.777969157	positive
PRJNA729271	ed_Lushi_vs_Arbor_Acr	breed_growth_background	ENSGALG00010001860	RNF169	-0.03219603	0.069456711	-0.463540948	0.642976671	0.766316958	negative
PRJNA729271	ed_Lushi_vs_Arbor_Acr	breed_growth_background	ENSGALG00010001861	CBX3	-0.016137503	0.040067786	-0.402755037	0.687128441	0.797989263	negative
PRJNA729271	ed_Lushi_vs_Arbor_Acr	breed_growth_background	ENSGALG00010001864	ENSGALG00010001864	0.444957631	0.106295751	4.186034022	2.83871E-05	0.00063206	positive
PRJNA729271	ed_Lushi_vs_Arbor_Acr	breed_growth_background	ENSGALG00010001865	CYBB	0.56205044	0.171506462	3.277138561	0.001048649	0.00845393	positive
PRJNA729271	ed_Lushi_vs_Arbor_Acr	breed_growth_background	ENSGALG00010001866	PUS7L	0.205564749	0.065335352	3.146301981	0.001653492	0.011617724	positive
PRJNA729271	ed_Lushi_vs_Arbor_Acr	breed_growth_background	ENSGALG00010001868	ENSGALG00010001868	0.053159553	0.067120528	0.792001412	0.428359853	0.587424415	positive
PRJNA729271	ed_Lushi_vs_Arbor_Acr	breed_growth_background	ENSGALG00010001869	CRLF2	-0.934848583	0.2669745	-3.501639977	0.000462404	0.004691226	negative
PRJNA729271	ed_Lushi_vs_Arbor_Acr	breed_growth_background	ENSGALG00010001870	PLP2	-0.154939068	0.087164657	-1.777544635	0.075478674	0.176962042	negative
PRJNA729271	ed_Lushi_vs_Arbor_Acr	breed_growth_background	ENSGALG00010001871	ENSGALG00010001871	0.183505504	0.066803	2.746965009	0.006014955	0.029081646	positive
PRJNA729271	ed_Lushi_vs_Arbor_Acr	breed_growth_background	ENSGALG00010001873	ENSGALG00010001873	-0.054987641	0.117173349	-0.469284543	0.638866262	0.763049313	negative
PRJNA729271	ed_Lushi_vs_Arbor_Acr	breed_growth_background	ENSGALG00010001877	OAT	-0.337918808	0.073129213	-4.620845654	3.82179E-06	0.000139188	negative
PRJNA729271	ed_Lushi_vs_Arbor_Acr	breed_growth_background	ENSGALG00010001878	XK	0.309556419	0.108574808	2.851088797	0.00435698	0.02298196	positive
PRJNA729271	ed_Lushi_vs_Arbor_Acr	breed_growth_background	ENSGALG00010001879	IRAK4	-0.167584048	0.08934629	-1.875668795	0.060700773	0.152066663	negative
PRJNA729271	ed_Lushi_vs_Arbor_Acr	breed_growth_background	ENSGALG00010001880	MTCL1	0.313729394	0.076898978	4.079760227	4.50822E-05	0.000896387	positive
PRJNA729271	ed_Lushi_vs_Arbor_Acr	breed_growth_background	ENSGALG00010001883	KBTBD11	0.025375643	0.048272826	0.525671371	0.599116559	0.73210205	positive
PRJNA729271	ed_Lushi_vs_Arbor_Acr	breed_growth_background	ENSGALG00010001885	SMPX	-0.540287148	0.197944933	-2.729482081	0.00634339	0.030176381	negative
PRJNA729271	ed_Lushi_vs_Arbor_Acr	breed_growth_background	ENSGALG00010001886	NEU3	0.044811891	0.046270329	0.968480066	0.33280467	0.497217449	positive
PRJNA729271	ed_Lushi_vs_Arbor_Acr	breed_growth_background	ENSGALG00010001888	SNX10	-0.310552436	0.10658387	-2.913690741	0.003571836	0.019978922	negative
PRJNA729271	ed_Lushi_vs_Arbor_Acr	breed_growth_background	ENSGALG00010001890	KLHL34	-0.004856882	0.128428833	-0.037817689	0.969833041	0.982586499	negative
PRJNA729271	ed_Lushi_vs_Arbor_Acr	breed_growth_background	ENSGALG00010001892	RAB12	0.040034559	0.03770896	1.061672326	0.288384467	0.449331704	positive
PRJNA729271	ed_Lushi_vs_Arbor_Acr	breed_growth_background	ENSGALG00010001895	P4HA3	-0.204954909	0.128818988	-1.591030268	0.111602764	0.232560026	negative
PRJNA729271	ed_Lushi_vs_Arbor_Acr	breed_growth_background	ENSGALG00010001896	ARHGEF10	-0.124314203	0.07175621	-1.732452191	0.083193082	0.189162417	negative
PRJNA729271	ed_Lushi_vs_Arbor_Acr	breed_growth_background	ENSGALG00010001897	TLR7	-0.373831131	0.094565795	-3.953132659	7.71346E-05	0.001312285	negative
PRJNA729271	ed_Lushi_vs_Arbor_Acr	breed_growth_background	ENSGALG00010001898	LHPP	0.004256435	0.071426248	0.059592029	0.952480567	0.973433394	positive
PRJNA729271	ed_Lushi_vs_Arbor_Acr	breed_growth_background	ENSGALG00010001899	TWF1	-0.131216992	0.06061508	-2.164758221	0.030406206	0.091820407	negative
PRJNA729271	ed_Lushi_vs_Arbor_Acr	breed_growth_background	ENSGALG00010001901	PRPS2	0.010844624	0.09692407	0.111887835	0.910912342	0.949563063	positive
PRJNA729271	ed_Lushi_vs_Arbor_Acr	breed_growth_background	ENSGALG00010001902	WDR45	0.018437635	0.076435667	0.24121769	0.809386401	0.882640377	positive
PRJNA729271	ed_Lushi_vs_Arbor_Acr	breed_growth_background	ENSGALG00010001903	NARS2	0.013341178	0.064212438	0.207766253	0.835411484	0.899984352	positive
PRJNA729271	ed_Lushi_vs_Arbor_Acr	breed_growth_background	ENSGALG00010001906	SKAP2	-0.176280675	0.068690037	-2.56632087	0.01027837	0.042652439	negative
PRJNA729271	ed_Lushi_vs_Arbor_Acr	breed_growth_background	ENSGALG00010001908	FAM83B	0.726725911	0.220485701	3.296022865	0.00098064	0.00804805	positive
PRJNA729271	ed_Lushi_vs_Arbor_Acr	breed_growth_background	ENSGALG00010001910	FAM53B	0.033120681	0.052129876	0.635349306	0.525200623	0.671795184	positive
PRJNA729271	ed_Lushi_vs_Arbor_Acr	breed_growth_background	ENSGALG00010001911	USP35	0.574904469	0.218091725	2.636067326	0.008387309	0.036755715	positive
PRJNA729271	ed_Lushi_vs_Arbor_Acr	breed_growth_background	ENSGALG00010001912	TMEM117	0.085671869	0.10578943	0.809833924	0.418035632	0.578134385	positive
PRJNA729271	ed_Lushi_vs_Arbor_Acr	breed_growth_background	ENSGALG00010001914	ENSGALG00010001914	0.295170152	0.0838316	3.520989141	0.00042994	0.004460976	positive
PRJNA729271	ed_Lushi_vs_Arbor_Acr	breed_growth_background	ENSGALG00010001915	EEF1AKMT2	0.620319476	0.0921891	6.728772468	1.711E-11	6.20025E-09	positive
PRJNA729271	ed_Lushi_vs_Arbor_Acr	breed_growth_background	ENSGALG00010001918	CNKSR2	-0.301077242	0.077744826	-3.872633795	0.000107666	0.001681694	negative
PRJNA729271	ed_Lushi_vs_Arbor_Acr	breed_growth_background	ENSGALG00010001921	ENSGALG00010001921	-0.201079763	0.158372613	-1.269662467	0.204204888	0.353595729	negative
PRJNA729271	ed_Lushi_vs_Arbor_Acr	breed_growth_background	ENSGALG00010001923	RAPGEF5	-0.108649688	0.053763017	-2.02090014	0.043290103	0.119181396	negative
PRJNA729271	ed_Lushi_vs_Arbor_Acr	breed_growth_background	ENSGALG00010001925	FRMPD4	-0.134654938	0.108990221	-1.235477248	0.216652939	0.368252452	negative
PRJNA729271	ed_Lushi_vs_Arbor_Acr	breed_growth_background	ENSGALG00010001928	KCTD21	0.092339845	0.093124565	0.991573439	0.321405651	0.485036431	positive
PRJNA729271	ed_Lushi_vs_Arbor_Acr	breed_growth_background	ENSGALG00010001929	POLR2I	0.039202315	0.06790385	0.577320951	0.563722663	0.704106851	positive
PRJNA729271	ed_Lushi_vs_Arbor_Acr	breed_growth_background	ENSGALG00010001935	ABRAXAS2	0.327463754	0.069869324	4.686802972	2.77506E-06	0.00010698	positive
PRJNA729271	ed_Lushi_vs_Arbor_Acr	breed_growth_background	ENSGALG00010001938	ALG8	0.166973611	0.058006805	2.878517621	0.00399549	0.021650326	positive
PRJNA729271	ed_Lushi_vs_Arbor_Acr	breed_growth_background	ENSGALG00010001940	PTPRM	-0.066361163	0.059225686	-1.12047944	0.262509509	0.42072925	negative
PRJNA729271	ed_Lushi_vs_Arbor_Acr	breed_growth_background	ENSGALG00010001947	TINAG	-0.1801614	0.127934763	-1.408228657	0.159063373	0.297615024	negative
PRJNA729271	ed_Lushi_vs_Arbor_Acr	breed_growth_background	ENSGALG00010001948	EMX2	-0.306763113	0.14884341	-2.060978801	0.039305063	0.111039142	negative
PRJNA729271	ed_Lushi_vs_Arbor_Acr	breed_growth_background	ENSGALG00010001952	ENSGALG00010001952	0.098917348	0.03932573	2.515334029	0.011891965	0.047238706	positive
PRJNA729271	ed_Lushi_vs_Arbor_Acr	breed_growth_background	ENSGALG00010001957	NDUFC2	0.005284647	0.059648761	0.088596097	0.92940291	0.960822707	positive
PRJNA729271	ed_Lushi_vs_Arbor_Acr	breed_growth_background	ENSGALG00010001958	RAB11FIP2	-0.079795964	0.060150966	-1.326594883	0.184642729	0.330093286	negative
PRJNA729271	ed_Lushi_vs_Arbor_Acr	breed_growth_background	ENSGALG00010001966	ARHGAP6	-0.219285496	0.075740502	-2.895221053	0.003788916	0.020850546	negative
PRJNA729271	ed_Lushi_vs_Arbor_Acr	breed_growth_background	ENSGALG00010001967	LAMA1	0.412463395	0.130215661	3.167540602	0.001537342	0.011069935	positive
PRJNA729271	ed_Lushi_vs_Arbor_Acr	breed_growth_background	ENSGALG00010001969	PLEKHA8	0.037339309	0.069462453	0.537546653	0.590890068	0.725906847	positive
PRJNA729271	ed_Lushi_vs_Arbor_Acr	breed_growth_background	ENSGALG00010001971	MLIP	-0.074866652	0.10431922	-0.717668825	0.47296151	0.626724006	negative
PRJNA729271	ed_Lushi_vs_Arbor_Acr	breed_growth_background	ENSGALG00010001972	FAM204A	-0.320911407	0.085111531	-3.770480959	0.000162933	0.00226808	negative
PRJNA729271	ed_Lushi_vs_Arbor_Acr	breed_growth_background	ENSGALG00010001974	ENSGALG00010001974	-0.208530461	0.212973178	-0.979139547	0.327511034	0.491385202	negative
PRJNA729271	ed_Lushi_vs_Arbor_Acr	breed_growth_background	ENSGALG00010001976	THRSP	0.190000435	0.682793713	0.278269163	0.780805746	0.864348502	positive
PRJNA729271	ed_Lushi_vs_Arbor_Acr	breed_growth_background	ENSGALG00010001982	DMD	-0.010060189	0.065025955	-0.154710365	0.877049658	0.927199679	negative
PRJNA729271	ed_Lushi_vs_Arbor_Acr	breed_growth_background	ENSGALG00010001987	KCTD14	-0.310072812	0.277104348	-1.118974905	0.263150845	0.421305734	negative
PRJNA729271	ed_Lushi_vs_Arbor_Acr	breed_growth_background	ENSGALG00010001991	TOMM7	-0.036475402	0.069673484	-0.523519129	0.600613043	0.733353922	negative
PRJNA729271	ed_Lushi_vs_Arbor_Acr	breed_growth_background	ENSGALG00010001995	LRRC1	-0.038078973	0.077182044	-0.493365705	0.62175421	0.74878093	negative
PRJNA729271	ed_Lushi_vs_Arbor_Acr	breed_growth_background	ENSGALG00010001997	INTS4	0.053672895	0.042207844	1.271633187	0.203503479	0.352693382	positive
PRJNA729271	ed_Lushi_vs_Arbor_Acr	breed_growth_background	ENSGALG00010002005	COLQ	-0.479156967	0.11691145	-4.098460552	4.15907E-05	0.000849095	negative
PRJNA729271	ed_Lushi_vs_Arbor_Acr	breed_growth_background	ENSGALG00010002006	ENSGALG00010002006	-0.046478864	0.206012971	-0.225611346	0.821503715	0.890494792	negative
PRJNA729271	ed_Lushi_vs_Arbor_Acr	breed_growth_background	ENSGALG00010002012	MID1	-0.192571442	0.077527421	-2.483913933	0.012994721	0.050269144	negative
PRJNA729271	ed_Lushi_vs_Arbor_Acr	breed_growth_background	ENSGALG00010002017	ARHGAP28	0.031120238	0.063772047	0.487991839	0.625555622	0.751989283	positive
PRJNA729271	ed_Lushi_vs_Arbor_Acr	breed_growth_background	ENSGALG00010002022	ERNI	1.513769849	0.40204032	3.765218995	0.000166403	0.00230154	positive
PRJNA729271	ed_Lushi_vs_Arbor_Acr	breed_growth_background	ENSGALG00010002023	CLCN4	-0.19621231	0.130774918	-1.50038183	0.133515524	0.263056615	negative
PRJNA729271	ed_Lushi_vs_Arbor_Acr	breed_growth_background	ENSGALG00010002024	KLHL31	0.133753495	0.088552469	1.510443437	0.130930312	0.259062549	positive
PRJNA729271	ed_Lushi_vs_Arbor_Acr	breed_growth_background	ENSGALG00010002027	ENSGALG00010002027	-0.49992651	0.211403389	-2.364798943	0.018039862	0.063629859	negative
PRJNA729271	ed_Lushi_vs_Arbor_Acr	breed_growth_background	ENSGALG00010002034	WWC3	0.021748113	0.053643134	0.405422112	0.68516726	0.796623039	positive
PRJNA729271	ed_Lushi_vs_Arbor_Acr	breed_growth_background	ENSGALG00010002039	AAMDC	0.230331761	0.066384692	3.469651712	0.000521134	0.005114308	positive
PRJNA729271	ed_Lushi_vs_Arbor_Acr	breed_growth_background	ENSGALG00010002041	GCLC	0.134267983	0.074164387	1.810410476	0.070232156	0.168239151	positive
PRJNA729271	ed_Lushi_vs_Arbor_Acr	breed_growth_background	ENSGALG00010002046	FAM126A	-0.144521543	0.047529177	-3.040691032	0.002360359	0.015012464	negative
PRJNA729271	ed_Lushi_vs_Arbor_Acr	breed_growth_background	ENSGALG00010002050	ENSGALG00010002050	1.509226262	0.588723755	2.563555909	0.010360601	0.04290769	positive
PRJNA729271	ed_Lushi_vs_Arbor_Acr	breed_growth_background	ENSGALG00010002054	RSF1	0.084756323	0.053878714	1.573094776	0.115696874	0.238349374	positive
PRJNA729271	ed_Lushi_vs_Arbor_Acr	breed_growth_background	ENSGALG00010002056	PRLHR	0.096382197	0.270281311	0.356599563	0.721391605	0.821393372	positive
PRJNA729271	ed_Lushi_vs_Arbor_Acr	breed_growth_background	ENSGALG00010002062	GSTA4	0.655697627	0.197877635	3.313652036	0.00092086	0.007666783	positive
PRJNA729271	ed_Lushi_vs_Arbor_Acr	breed_growth_background	ENSGALG00010002065	CLNS1A	0.044559775	0.03427416	1.300098212	0.193567311	0.340711374	positive
PRJNA729271	ed_Lushi_vs_Arbor_Acr	breed_growth_background	ENSGALG00010002072	NEURL4	0.085525465	0.086543355	0.988238379	0.323035916	0.486995449	positive
PRJNA729271	ed_Lushi_vs_Arbor_Acr	breed_growth_background	ENSGALG00010002079	ARX	0.240317435	0.099788993	2.408255935	0.01602894	0.058376754	positive
PRJNA729271	ed_Lushi_vs_Arbor_Acr	breed_growth_background	ENSGALG00010002084	HACL1	-0.116738772	0.050454805	-2.313729526	0.020682563	0.06983316	negative
PRJNA729271	ed_Lushi_vs_Arbor_Acr	breed_growth_background	ENSGALG00010002087	POLA1	-0.097311978	0.040439915	-2.406334909	0.016113485	0.058625745	negative
PRJNA729271	ed_Lushi_vs_Arbor_Acr	breed_growth_background	ENSGALG00010002088	OZFL1	0.215337035	0.075059393	2.868888584	0.004119169	0.022072958	positive
PRJNA729271	ed_Lushi_vs_Arbor_Acr	breed_growth_background	ENSGALG00010002090	AQP11	0.078067733	0.164995423	0.473150899	0.636105525	0.760754917	positive
PRJNA729271	ed_Lushi_vs_Arbor_Acr	breed_growth_background	ENSGALG00010002097	PCYT1B	-0.352312923	0.265458603	-1.327185933	0.184447184	0.329865754	negative
PRJNA729271	ed_Lushi_vs_Arbor_Acr	breed_growth_background	ENSGALG00010002098	ENSGALG00010002098	0.099225859	0.239721878	0.413920748	0.678932148	0.792623996	positive
PRJNA729271	ed_Lushi_vs_Arbor_Acr	breed_growth_background	ENSGALG00010002100	BTD	0.085577843	0.063174346	1.354629647	0.175535583	0.319646768	positive
PRJNA729271	ed_Lushi_vs_Arbor_Acr	breed_growth_background	ENSGALG00010002102	PAK1	0.690194975	0.152966549	4.512064761	6.41996E-06	0.000210062	positive
PRJNA729271	ed_Lushi_vs_Arbor_Acr	breed_growth_background	ENSGALG00010002106	ENSGALG00010002106	-2.935006787	0.464471058	-6.319030516	2.63209E-10	6.81289E-08	negative
PRJNA729271	ed_Lushi_vs_Arbor_Acr	breed_growth_background	ENSGALG00010002113	GDPD4	-0.273760227	0.162884365	-1.680702917	0.092820634	0.204287137	negative
PRJNA729271	ed_Lushi_vs_Arbor_Acr	breed_growth_background	ENSGALG00010002118	ENSGALG00010002118	-0.101730337	0.140590664	-0.723592409	0.469316003	0.624046919	negative
PRJNA729271	ed_Lushi_vs_Arbor_Acr	breed_growth_background	ENSGALG00010002122	ENSGALG00010002122	-0.667443675	0.276361888	-2.415107522	0.015730565	0.057652223	negative
PRJNA729271	ed_Lushi_vs_Arbor_Acr	breed_growth_background	ENSGALG00010002126	ENSGALG00010002126	0.113013351	0.156939841	0.720106188	0.471459618	0.625728411	positive
PRJNA729271	ed_Lushi_vs_Arbor_Acr	breed_growth_background	ENSGALG00010002134	ANKRD28	0.033989553	0.064759485	0.524858296	0.599681702	0.732681409	positive
PRJNA729271	ed_Lushi_vs_Arbor_Acr	breed_growth_background	ENSGALG00010002135	ELOVL5	-0.297776468	0.090213859	-3.300784063	0.000964151	0.007936162	negative
PRJNA729271	ed_Lushi_vs_Arbor_Acr	breed_growth_background	ENSGALG00010002136	ACAP1	0.053954721	0.139067896	0.387973949	0.698035313	0.805308638	positive
PRJNA729271	ed_Lushi_vs_Arbor_Acr	breed_growth_background	ENSGALG00010002137	PDK3	0.364034272	0.070254573	5.181645191	2.19937E-07	1.54009E-05	positive
PRJNA729271	ed_Lushi_vs_Arbor_Acr	breed_growth_background	ENSGALG00010002138	MYO7A	0.040865626	0.093651053	0.436360563	0.662575147	0.781196255	positive
PRJNA729271	ed_Lushi_vs_Arbor_Acr	breed_growth_background	ENSGALG00010002140	ENSGALG00010002140	-0.162291054	0.237454371	-0.683462061	0.494314916	0.64601044	negative
PRJNA729271	ed_Lushi_vs_Arbor_Acr	breed_growth_background	ENSGALG00010002142	CLDN34	-0.00214077	0.140589382	-0.015227111	0.987850993	0.992713543	negative
PRJNA729271	ed_Lushi_vs_Arbor_Acr	breed_growth_background	ENSGALG00010002145	ENSGALG00010002145	-0.36093218	0.252496756	-1.429452742	0.152874149	0.289362527	negative
PRJNA729271	ed_Lushi_vs_Arbor_Acr	breed_growth_background	ENSGALG00010002146	CACUL1	0.094828275	0.047809407	1.983464795	0.047315536	0.127172018	positive
PRJNA729271	ed_Lushi_vs_Arbor_Acr	breed_growth_background	ENSGALG00010002148	POLR2A	0.223183543	0.09513842	2.345882387	0.018982096	0.065966311	positive
PRJNA729271	ed_Lushi_vs_Arbor_Acr	breed_growth_background	ENSGALG00010002149	ENSGALG00010002149	-0.280493497	0.207112366	-1.354305885	0.175638809	0.319715342	negative

PRJNA729271	ed_Lushi_vs_Arbor_Acr	breed_growth_background	ENSGALG00010002150	EIF3A	0.094783374	0.070841268	1.337968358	0.180906752	0.325474425	positive
PRJNA729271	ed_Lushi_vs_Arbor_Acr	breed_growth_background	ENSGALG00010002159	GALNT15	0.075097813	0.10117686	0.742242971	0.457940138	0.61376258	positive
PRJNA729271	ed_Lushi_vs_Arbor_Acr	breed_growth_background	ENSGALG00010002160	CAPN5	-0.031039097	0.088888031	-0.349193211	0.726944262	0.824527408	negative
PRJNA729271	ed_Lushi_vs_Arbor_Acr	breed_growth_background	ENSGALG00010002161	CDKL5	-0.431720236	0.093712437	-4.606861712	4.08792E-06	0.000148021	negative
PRJNA729271	ed_Lushi_vs_Arbor_Acr	breed_growth_background	ENSGALG00010002173	GCM1	-0.049783599	0.506174598	-0.098352622	0.921652295	0.956327938	negative
PRJNA729271	ed_Lushi_vs_Arbor_Acr	breed_growth_background	ENSGALG00010002174	ENSGALG00010002174	-0.512571615	0.248092241	-2.066052582	0.038823515	0.110083499	negative
PRJNA729271	ed_Lushi_vs_Arbor_Acr	breed_growth_background	ENSGALG00010002177	FBXO9	-0.098878826	0.083048854	-1.190610366	0.23380658	0.388249098	negative
PRJNA729271	ed_Lushi_vs_Arbor_Acr	breed_growth_background	ENSGALG00010002181	FIS1	0.319781698	0.139997945	2.284188515	0.022360454	0.073982847	positive
PRJNA729271	ed_Lushi_vs_Arbor_Acr	breed_growth_background	ENSGALG00010002185	ENSGALG00010002185	0.750518089	0.182188984	4.119448247	3.79781E-05	0.000794091	positive
PRJNA729271	ed_Lushi_vs_Arbor_Acr	breed_growth_background	ENSGALG00010002186	CILK1	-0.013707735	0.040414351	-0.339179887	0.73447422	0.829919225	negative
PRJNA729271	ed_Lushi_vs_Arbor_Acr	breed_growth_background	ENSGALG00010002192	ENSGALG00010002192	-0.154548405	0.104470712	-1.479346722	0.139047672	0.270687701	negative
PRJNA729271	ed_Lushi_vs_Arbor_Acr	breed_growth_background	ENSGALG00010002194	SHROOM2	-0.191631508	0.073008725	-2.624775451	0.008670613	0.037561426	negative
PRJNA729271	ed_Lushi_vs_Arbor_Acr	breed_growth_background	ENSGALG00010002196	ENSGALG00010002196	-0.106483595	0.055273423	-1.926488151	0.05404345	0.140010689	negative
PRJNA729271	ed_Lushi_vs_Arbor_Acr	breed_growth_background	ENSGALG00010002197	GSTA2	-0.350783064	0.101848926	-3.444150843	0.000572856	0.005448522	negative
PRJNA729271	ed_Lushi_vs_Arbor_Acr	breed_growth_background	ENSGALG00010002202	SFXN4	-0.044102412	0.05458129	-0.808013361	0.419082891	0.578975077	negative
PRJNA729271	ed_Lushi_vs_Arbor_Acr	breed_growth_background	ENSGALG00010002206	DPH3P1	0.030128622	0.058781583	0.512552076	0.608264684	0.739014307	positive
PRJNA729271	ed_Lushi_vs_Arbor_Acr	breed_growth_background	ENSGALG00010002210	GPR143	0.374938292	0.648188759	0.578439979	0.562967109	0.703400028	positive
PRJNA729271	ed_Lushi_vs_Arbor_Acr	breed_growth_background	ENSGALG00010002211	ACER3	-0.335796395	0.093645232	-3.585835473	0.000336001	0.003746408	negative
PRJNA729271	ed_Lushi_vs_Arbor_Acr	breed_growth_background	ENSGALG00010002215	YLEC18	-0.296049557	0.189095458	-1.565609031	0.117440175	0.240879486	negative
PRJNA729271	ed_Lushi_vs_Arbor_Acr	breed_growth_background	ENSGALG00010002225	PRDX3	-0.000533155	0.056422739	-0.009449299	0.992460662	0.995758102	negative
PRJNA729271	ed_Lushi_vs_Arbor_Acr	breed_growth_background	ENSGALG00010002226	OXNAD1	-0.079720549	0.072738599	-1.095986871	0.27308452	0.431968757	negative
PRJNA729271	ed_Lushi_vs_Arbor_Acr	breed_growth_background	ENSGALG00010002227	ENSGALG00010002227	-1.523620985	0.619529327	-2.459320195	0.013920041	0.052750585	negative
PRJNA729271	ed_Lushi_vs_Arbor_Acr	breed_growth_background	ENSGALG00010002231	TMEM14A	-0.08209765	0.074476794	-1.102325245	0.270320298	0.428978839	negative
PRJNA729271	ed_Lushi_vs_Arbor_Acr	breed_growth_background	ENSGALG00010002235	MXRA5	-0.464000523	0.107600138	-4.312266977	1.61589E-05	0.000409482	negative
PRJNA729271	ed_Lushi_vs_Arbor_Acr	breed_growth_background	ENSGALG00010002238	ENSGALG00010002238	0.306619531	0.229755636	1.334546285	0.182024888	0.326743128	positive
PRJNA729271	ed_Lushi_vs_Arbor_Acr	breed_growth_background	ENSGALG00010002239	AGPAT5	0.096368799	0.068382824	1.409254457	0.15875994	0.29726165	positive
PRJNA729271	ed_Lushi_vs_Arbor_Acr	breed_growth_background	ENSGALG00010002243	ARSH	-0.418137151	0.09702796	-4.309450101	1.63661E-05	0.000413287	negative
PRJNA729271	ed_Lushi_vs_Arbor_Acr	breed_growth_background	ENSGALG00010002247	RFTN1	-0.087494826	0.099582625	-0.87861538	0.379609854	0.541525916	negative
PRJNA729271	ed_Lushi_vs_Arbor_Acr	breed_growth_background	ENSGALG00010002251	GRK5	-0.229462947	0.082022157	-2.79757268	0.005148818	0.026122548	negative
PRJNA729271	ed_Lushi_vs_Arbor_Acr	breed_growth_background	ENSGALG00010002253	ENSGALG00010002253	-0.415022152	0.172731246	-2.402704555	0.01627433	0.059021435	negative
PRJNA729271	ed_Lushi_vs_Arbor_Acr	breed_growth_background	ENSGALG00010002254	ENSGALG00010002254	0.239948353	0.196235183	1.222759085	0.221420703	0.373892485	positive
PRJNA729271	ed_Lushi_vs_Arbor_Acr	breed_growth_background	ENSGALG00010002259	TASL	-0.300712563	0.280486895	-1.072109139	0.283671014	0.443848376	negative
PRJNA729271	ed_Lushi_vs_Arbor_Acr	breed_growth_background	ENSGALG00010002261	NR0B1	0.496277256	0.225311083	2.202631356	0.027620742	0.085684971	positive
PRJNA729271	ed_Lushi_vs_Arbor_Acr	breed_growth_background	ENSGALG00010002269	MCPH1	-0.246806697	0.08153909	-3.026851241	0.002471155	0.015464503	negative
PRJNA729271	ed_Lushi_vs_Arbor_Acr	breed_growth_background	ENSGALG00010002270	IMMP2L	-0.066791403	0.136642028	-0.488805707	0.624979257	0.751540927	negative
PRJNA729271	ed_Lushi_vs_Arbor_Acr	breed_growth_background	ENSGALG00010002271	ENSGALG00010002271	-0.194409767	0.335346025	-0.579728855	0.562097481	0.702771033	negative
PRJNA729271	ed_Lushi_vs_Arbor_Acr	breed_growth_background	ENSGALG00010002274	ENSGALG00010002274	-0.420961933	0.406039435	-1.036751349	0.299851754	0.462377785	negative
PRJNA729271	ed_Lushi_vs_Arbor_Acr	breed_growth_background	ENSGALG00010002275	IL1RAPL1	0.128552666	0.165212764	0.77810372	0.43650787	0.594045778	positive
PRJNA729271	ed_Lushi_vs_Arbor_Acr	breed_growth_background	ENSGALG00010002277	RGS10	-0.209361466	0.096293298	-2.174206005	0.029689668	0.090220489	negative
PRJNA729271	ed_Lushi_vs_Arbor_Acr	breed_growth_background	ENSGALG00010002279	ENSGALG00010002279	0.457746214	0.384097857	1.191743733	0.233361745	0.387776968	positive
PRJNA729271	ed_Lushi_vs_Arbor_Acr	breed_growth_background	ENSGALG00010002280	ST7	0.049810381	0.075411033	0.660518491	0.508921156	0.657880331	positive
PRJNA729271	ed_Lushi_vs_Arbor_Acr	breed_growth_background	ENSGALG00010002281	TGFBR2	-0.334077182	0.082639646	-4.042577624	5.28668E-05	0.001008295	negative
PRJNA729271	ed_Lushi_vs_Arbor_Acr	breed_growth_background	ENSGALG00010002282	TSKU	-0.364570688	0.088997986	-4.096392558	4.19638E-05	0.000853107	negative
PRJNA729271	ed_Lushi_vs_Arbor_Acr	breed_growth_background	ENSGALG00010002283	ENSGALG00010002283	1.49444113	0.389041747	3.841338732	0.000122365	0.001851443	positive
PRJNA729271	ed_Lushi_vs_Arbor_Acr	breed_growth_background	ENSGALG00010002285	CAV1	-0.269903254	0.083982922	-3.213787376	0.001309966	0.009889564	negative
PRJNA729271	ed_Lushi_vs_Arbor_Acr	breed_growth_background	ENSGALG00010002288	ANGPT2	-0.48240946	0.095416903	-5.055807136	4.28574E-07	2.52528E-05	negative
PRJNA729271	ed_Lushi_vs_Arbor_Acr	breed_growth_background	ENSGALG00010002289	ENSGALG00010002289	-0.093204198	0.501050141	-0.186017706	0.852430877	0.910982566	negative
PRJNA729271	ed_Lushi_vs_Arbor_Acr	breed_growth_background	ENSGALG00010002291	RNASEH1	-0.029121051	0.062941496	-0.462668553	0.643601962	0.766999132	negative
PRJNA729271	ed_Lushi_vs_Arbor_Acr	breed_growth_background	ENSGALG00010002293	GYG2	-0.328344487	0.204514519	-1.605482533	0.108387654	0.228089292	negative
PRJNA729271	ed_Lushi_vs_Arbor_Acr	breed_growth_background	ENSGALG00010002294	DAZL	-0.224778004	0.445009614	-0.505108197	0.613482845	0.743174212	negative
PRJNA729271	ed_Lushi_vs_Arbor_Acr	breed_growth_background	ENSGALG00010002295	YAF2	0.247686205	0.061341661	4.037813823	5.39516E-05	0.001023598	positive
PRJNA729271	ed_Lushi_vs_Arbor_Acr	breed_growth_background	ENSGALG00010002298	ENSGALG00010002298	-0.646835293	0.24588866	-2.630602372	0.008523369	0.037146343	negative
PRJNA729271	ed_Lushi_vs_Arbor_Acr	breed_growth_background	ENSGALG00010002303	ENSGALG00010002303	2.370354766	0.434563374	5.454566368	4.90925E-08	4.84079E-06	positive
PRJNA729271	ed_Lushi_vs_Arbor_Acr	breed_growth_background	ENSGALG00010002308	GXYLT1	-0.158285075	0.056647585	-2.794206949	0.005202717	0.026267289	negative
PRJNA729271	ed_Lushi_vs_Arbor_Acr	breed_growth_background	ENSGALG00010002309	GADL1	-0.215054725	0.172107371	-1.249538142	0.211468312	0.361515094	negative
PRJNA729271	ed_Lushi_vs_Arbor_Acr	breed_growth_background	ENSGALG00010002310	PRRG1	0.126525626	0.057042509	2.21809361	0.02654845	0.083437738	positive
PRJNA729271	ed_Lushi_vs_Arbor_Acr	breed_growth_background	ENSGALG00010002312	ENSGALG00010002312	-0.25740103	0.103184124	-2.494579799	0.012610637	0.049177073	negative
PRJNA729271	ed_Lushi_vs_Arbor_Acr	breed_growth_background	ENSGALG00010002315	ENSGALG00010002315	-0.094252584	0.096394721	-0.977777438	0.328184412	0.491988112	negative
PRJNA729271	ed_Lushi_vs_Arbor_Acr	breed_growth_background	ENSGALG00010002324	SNTG2	0.087610334	0.217963358	0.401949827	0.687720952	0.798132113	positive
PRJNA729271	ed_Lushi_vs_Arbor_Acr	breed_growth_background	ENSGALG00010002328	ENSGALG00010002328	-0.079887709	0.142248224	-0.561607775	0.574383282	0.712023063	negative
PRJNA729271	ed_Lushi_vs_Arbor_Acr	breed_growth_background	ENSGALG00010002329	STT3B	0.131865495	0.038919455	3.38816394	0.000703622	0.00635056	positive
PRJNA729271	ed_Lushi_vs_Arbor_Acr	breed_growth_background	ENSGALG00010002330	KHDRBS2	0.313939899	0.161067107	1.949124844	0.051280517	0.134902196	positive
PRJNA729271	ed_Lushi_vs_Arbor_Acr	breed_growth_background	ENSGALG00010002331	ENSGALG00010002331	-0.495038598	0.179033941	-2.76505447	0.005691329	0.027907921	negative
PRJNA729271	ed_Lushi_vs_Arbor_Acr	breed_growth_background	ENSGALG00010002332	ENSGALG00010002332	-0.455783503	0.341881697	-1.333161463	0.182478823	0.327355265	negative
PRJNA729271	ed_Lushi_vs_Arbor_Acr	breed_growth_background	ENSGALG00010002333	ENSGALG00010002333	0.993483611	0.375079335	2.648729266	0.008079503	0.035836105	positive
PRJNA729271	ed_Lushi_vs_Arbor_Acr	breed_growth_background	ENSGALG00010002334	OSBPL10	-0.009520535	0.09101992	-0.104598362	0.916694515	0.953054582	negative
PRJNA729271	ed_Lushi_vs_Arbor_Acr	breed_growth_background	ENSGALG00010002335	TMEM47	-0.014853501	0.071720422	-0.207102807	0.83592957	0.900341739	negative
PRJNA729271	ed_Lushi_vs_Arbor_Acr	breed_growth_background	ENSGALG00010002336	PRIM2	-0.090041524	0.066704382	-1.349859208	0.177061148	0.321053459	negative
PRJNA729271	ed_Lushi_vs_Arbor_Acr	breed_growth_background	ENSGALG00010002338	CD99	-0.414735143	0.07929526	-5.230264012	1.69268E-07	1.27126E-05	negative
PRJNA729271	ed_Lushi_vs_Arbor_Acr	breed_growth_background	ENSGALG00010002339	ZNFY4	-0.134180885	0.168127338	-0.79809082	0.424817788	0.584003589	negative
PRJNA729271	ed_Lushi_vs_Arbor_Acr	breed_growth_background	ENSGALG00010002340	RAB23	-0.026628384	0.074643443	-0.3567411	0.721285634	0.821355587	negative
PRJNA729271	ed_Lushi_vs_Arbor_Acr	breed_growth_background	ENSGALG00010002341	FAM49A	-0.143966826	0.059629662	-2.414349177	0.015763348	0.05774317	negative
PRJNA729271	ed_Lushi_vs_Arbor_Acr	breed_growth_background	ENSGALG00010002342	BAG2	0.12497163	0.065633643	1.904078838	0.056899917	0.145281716	positive
PRJNA729271	ed_Lushi_vs_Arbor_Acr	breed_growth_background	ENSGALG00010002343	GPD1L	-0.145801675	0.043918764	-3.319803697	0.000900808	0.007548369	negative
PRJNA729271	ed_Lushi_vs_Arbor_Acr	breed_growth_background	ENSGALG00010002345	SCML2	0.063605672	0.060241001	1.055853501	0.291035151	0.452440424	positive
PRJNA729271	ed_Lushi_vs_Arbor_Acr	breed_growth_background	ENSGALG00010002346	FBXO15	-0.4281978	0.204462038	-2.094265536	0.036236333	0.104621183	negative
PRJNA729271	ed_Lushi_vs_Arbor_Acr	breed_growth_background	ENSGALG00010002347	ZNF451	0.000294304	0.033972468	0.008663012	0.993088003	0.996042804	positive
PRJNA729271	ed_Lushi_vs_Arbor_Acr	breed_growth_background	ENSGALG00010002348	THAP12	0.251606675	0.084321445	2.983899008	0.002846007	0.017067799	positive
PRJNA729271	ed_Lushi_vs_Arbor_Acr	breed_growth_background	ENSGALG00010002349	ZBED1	0.013590114	0.069549738	0.195401366	0.845078747	0.905887919	positive
PRJNA729271	ed_Lushi_vs_Arbor_Acr	breed_growth_background	ENSGALG00010002350	BEND6	-0.056968645	0.133099846	-0.428014359	0.668640666	0.785281677	negative
PRJNA729271	ed_Lushi_vs_Arbor_Acr	breed_growth_background	ENSGALG00010002352	ENSGALG00010002352	-0.133756852	0.057295367	-2.334514247	0.019568811	0.06724749	negative
PRJNA729271	ed_Lushi_vs_Arbor_Acr	breed_growth_background	ENSGALG00010002353	CMTM8	-0.094076125	0.07456799	-1.261615409	0.207087209	0.356226329	negative
PRJNA729271	ed_Lushi_vs_Arbor_Acr	breed_growth_background	ENSGALG00010002355	UFM1	-0.099317097	0.054338258	-1.827756355	0.067586126	0.164069821	negative
PRJNA729271	ed_Lushi_vs_Arbor_Acr	breed_growth_background	ENSGALG00010002356	WNT11	-0.432764539	0.178765793	-2.420846476	0.015484415	0.057038526	negative
PRJNA729271	ed_Lushi_vs_Arbor_Acr	breed_growth_background	ENSGALG00010002357	RAB31	-0.277223328	0.076745871	-3.612224654	0.000303581	0.003504145	negative
PRJNA729271	ed_Lushi_vs_Arbor_Acr	breed_growth_background	ENSGALG00010002358	FREM2	0.027134952	0.12546594	0.216273455	0.8287746	0.895563428	positive
PRJNA729271	ed_Lushi_vs_Arbor_Acr	breed_growth_background	ENSGALG00010002359	CMTM6	-0.130672095	0.062520935	-2.090053426	0.036613001	0.105445151	negative
PRJNA729271	ed_Lushi_vs_Arbor_Acr	breed_growth_background	ENSGALG00010002360	DHRSX	-0.24341546	0.086998311	-2.797933156	0.005143076	0.026102549	negative
PRJNA729271	ed_Lushi_vs_Arbor_Acr	breed_growth_background	ENSGALG00010002361	ENSGALG00010002361	0.126242179	0.053234119	2.371452419	0.017718328	0.062763236	positive
PRJNA729271	ed_Lushi_vs_Arbor_Acr	breed_growth_background	ENSGALG00010002362	TRPC4	0.263750592	0.199920785	1.319275487	0.18707703	0.332792468	positive
PRJNA729271	ed_Lushi_vs_Arbor_Acr	breed_growth_background	ENSGALG00010002364	VSNL1	-0.93436319	0.213184073	-4.382893982	1.17113E-05	0.00032665	negative
PRJNA729271	ed_Lushi_vs_Arbor_Acr	breed_growth_background	ENSGALG00010002365	RAI2	0.046174485	0.073724986	0.626307143	0.531113502	0.676849852	positive
PRJNA729271	ed_Lushi_vs_Arbor_Acr	breed_growth_background	ENSGALG00010002366	ENSGALG00010002366	0.042910151	0.404475942	0.106088265	0.915512322	0.952192002	positive
PRJNA729271	ed_Lushi_vs_Arbor_Acr	breed_growth_background	ENSGALG00010002367	STK31	0.335561824	0.449361738	0.746752105	0.455213211	0.611238119	positive
PRJNA729271	ed_Lushi_vs_Arbor_Acr	breed_growth_background	ENSGALG00010002368	UVRAG	-0.049149071	0.051150218	-0.960877053	0.33661399	0.500895163	negative
PRJNA729271	ed_Lushi_vs_Arbor_Acr	breed_growth_background	ENSGALG00010002369	GEMIN8	0.005305107	0.065966726	0.08042095	0.935902465	0.964536447	positive
PRJNA729271	ed_Lushi_vs_Arbor_Acr	breed_growth_background	ENSGALG00010002370	ENSGALG00010002370	-0.189196805	0.084732557	-2.232870227	0.025557503	0.081240354	negative
PRJNA729271	ed_Lushi_vs_Arbor_Acr	breed_growth_background	ENSGALG00010002371	BAG3	0.282866156	0.08251897	3.427892488	0.000608286	0.005677469	positive
PRJNA729271	ed_Lushi_vs_Arbor_Acr	breed_growth_background	ENSGALG00010002372	SMC6	0.22748684	0.068559523	3.318092506	0.000906345	0.007577248	positive
PRJNA729271	ed_Lushi_vs_Arbor_Acr	breed_growth_background	ENSGALG00010002373	POSTN	-0.454978951	0.136631865	-3.329962226	0.000868578	0.007366901	negative
PRJNA729271	ed_Lushi_vs_Arbor_Acr	breed_growth_background	ENSGALG00010002374	DST	0.106346786	0.061066631	1.741487697	0.081598128	0.186535208	positive
PRJNA729271	ed_Lushi_vs_Arbor_Acr	breed_growth_background	ENSGALG00010002375	NPY	0.066473076	0.297179477	0.223679901	0.823006386	0.891790204	positive
PRJNA729271	ed_Lushi_vs_Arbor_Acr	breed_growth_background	ENSGALG00010002376	GEN1	-0.053548788	0.103752962	-0.516118164	0.605771883	0.73704479	negative
PRJNA729271	ed_Lushi_vs_Arbor_Acr	breed_growth_background	ENSGALG00010002377	ART1A	0.075075756	0.115128076	0.652106404	0.514332533	0.662924602	positive
PRJNA729271	ed_Lushi_vs_Arbor_Acr	breed_growth_background	ENSGALG00010002378	ENSGALG00010002378	-0.14694817	0.239213333	-0.614297575	0.539018705	0.684337052	negative
PRJNA729271	ed_Lushi_vs_Arbor_Acr	breed_growth_background	ENSGALG00010002379	NHS	0.119253061	0.074808451	1.594112149	0.110910918	0.231617024	positive
PRJNA729271	ed_Lushi_vs_Arbor_Acr	breed_growth_background	ENSGALG00010002380	CMPK2	0.571420251	0.398961016	1.432270893	0.15206631	0.288130871	positive
PRJNA729271	ed_Lushi_vs_Arbor_Acr	breed_growth_background	ENSGALG00010002381	TDRD3	0.231439538	0.051715386	4.475254979	7.63202E-06	0.000236381	positive
PRJNA729271	ed_Lushi_vs_Arbor_Acr	breed_growth_background	ENSGALG00010002382	ART1D	-0.060888736	0.317977727	-0.191487425	0.848143731	0.908434448	negative
PRJNA729271	ed_Lushi_vs_Arbor_Acr	breed_growth_background	ENSGALG00010002383	INPP5F	-0.030262186	0.066984282	-0.45178041	0.651427184	0.772598355	negative
PRJNA729271	ed_Lushi_vs_Arbor_Acr	breed_growth_background	ENSGALG00010002384	ENSGALG00010002384	0.00024481	0.167836828	0.001458619	0.998836191	0.99912554	positive
PRJNA729271	ed_Lushi_vs_Arbor_Acr	breed_growth_background	ENSGALG00010002385	PALS2	-0.514834228	0.068005384	-7.570492151	3.71813E-14	2.83654E-11	negative
PRJNA729271	ed_Lushi_vs_Arbor_Acr	breed_growth_background	ENSGALG00010002386	MHCY2B2	0.121101143	0.22665769	0.534290909	0.593140281	0.72761115	positive
PRJNA729271	ed_Lushi_vs_Arbor_Acr	breed_growth_background	ENSGALG00010002387	MCMBP	0.168239151	0.058503542	2.875708792	0.004031214	0.021748495	positive
PRJNA729271	ed_Lushi_vs_Arbor_Acr	breed_growth_background	ENSGALG00010002388	ENSGALG00010002388	-0.020287411	0.322376986	-0.062930706	0.949821684	0.972423971	negative
PRJNA729271	ed_Lushi_vs_Arbor_Acr	breed_growth_background	ENSGALG00010002389	COL21A1	-0.669800557	0.172953344	-3.872723958	0.000107626	0.001681694	negative
PRJNA729271	ed_Lushi_vs_Arbor_Acr	breed_growth_background	ENSGALG00010002391	REPS2	-0.211673495	0.086306529	-2.452577992	0.014183663	0.053358991	negative
PRJNA729271	ed_Lushi_vs_Arbor_Acr	breed_growth_background	ENSGALG00010002392	DFNA5	-0.049656336	0.112128312	-0.442852795	0.657872213	0.777405413	negative
PRJNA729271	ed_Lushi_vs_Arbor_Acr	breed_growth_background	ENSGALG00010002393	GPM6B	-0.055755932	0.05743013	-0.970848079	0.331623937	0.495913439	negative
PRJNA729271	ed_Lushi_vs_Arbor_Acr	breed_growth_background	ENSGALG00010002395	CLN8	-0.122091193	0.04892821	-2.495312878	0.012584611	0.049102002	negative
PRJNA729271	ed_Lushi_vs_Arbor_Acr	breed_growth_background	ENSGALG00010002396	SEC23IP	0.081504167	0.047655105	1.710292477	0.087211802	0.19550496	positive
PRJNA729271	ed_Lushi_vs_Arbor_Acr	breed_growth_background	ENSGALG00010002398	ENSGALG00010002398	-0.525268888	0.245809924	-2.136890485	0.0326069	0.0969313	negative
PRJNA729271	ed_Lushi_vs_Arbor_Acr	breed_growth_background	ENSGALG00010002399	CSMD1	-0.19677227	0.213836598	-0.920199216	0.357468664	0.521173635	negative
PRJNA729271	ed_Lushi_vs_Arbor_Acr	breed_growth_background	ENSGALG00010002400	RBBP7	-0.230208178	0.086340838	-2.666272226	0.007669756	0.034590265	negative
PRJNA729271	ed_Lushi_vs_Arbor_Acr	breed_growth_background	ENSGALG00010002401	ZRANB1	0.269476711	0.086494632	3.115531058	0.00183614	0.012520963	positive
PRJNA729271	ed_Lushi_vs_Arbor_Acr	breed_growth_background	ENSGALG00010002402	TXLNG	-0.027381988	0.067721959	-0.404329534	0.68597041	0.797278152	negative
PRJNA729271	ed_Lushi_vs_Arbor_Acr	breed_growth_background	ENSGALG00010002403	ZNFY3	0.031725251	0.143418087	0.221208158	0.824930357	0.893205943	positive
PRJNA729271	ed_Lushi_vs_Arbor_Acr	breed_growth_background	ENSGALG00010002404	OSBPL3	-0.292493712	0.137739608	-2.123526529	0.033709755	0.099152373	negative
PRJNA729271	ed_Lushi_vs_Arbor_Acr	breed_growth_background	ENSGALG00010002405	KCNQ5	0.312777617	0.106259888	2.943515396	0.003245077	0.018777401	positive
PRJNA729271	ed_Lushi_vs_Arbor_Acr	breed_growth_background	ENSGALG00010002406	DIAPH3	-0.38665566	0.133963406	-2.886278215	0.003898274	0.021297407	negative
PRJNA729271	ed_Lushi_vs_Arbor_Acr	breed_growth_background	ENSGALG00010002408	OFD1	0.23043215	0.062891658	3.663954139	0.000248351	0.003032346	positive

PRJNA729271	ed_Lushi_vs_Arbor_Acr	breed_growth_background	ENSGALG00010002409	CYCS	-0.063617212	0.089177189	-0.713379876	0.475610722	0.628901833	negative
PRJNA729271	ed_Lushi_vs_Arbor_Acr	breed_growth_background	ENSGALG00010002410	RNF121	0.153112704	0.056272189	2.720930314	0.006509848	0.030756275	positive
PRJNA729271	ed_Lushi_vs_Arbor_Acr	breed_growth_background	ENSGALG00010002411	ENSGALG00010002411	-0.000652433	0.103477147	-0.006305093	0.994969297	0.997172872	negative
PRJNA729271	ed_Lushi_vs_Arbor_Acr	breed_growth_background	ENSGALG00010002413	TSSC1	0.00400879	0.046815383	0.085629753	0.931760746	0.962498809	positive
PRJNA729271	ed_Lushi_vs_Arbor_Acr	breed_growth_background	ENSGALG00010002414	ENSGALG00010002414	0.488596409	0.19441275	2.513191182	0.011964448	0.047474588	positive
PRJNA729271	ed_Lushi_vs_Arbor_Acr	breed_growth_background	ENSGALG00010002415	PCDH17	-0.070004011	0.205801026	-0.340153848	0.733740672	0.829451058	negative
PRJNA729271	ed_Lushi_vs_Arbor_Acr	breed_growth_background	ENSGALG00010002416	SH3BGRL2	-0.602537886	0.152146663	-3.96024385	7.48733E-05	0.001285886	negative
PRJNA729271	ed_Lushi_vs_Arbor_Acr	breed_growth_background	ENSGALG00010002418	TRAPPC2	0.065747247	0.045421882	1.447479582	0.147762651	0.282623401	positive
PRJNA729271	ed_Lushi_vs_Arbor_Acr	breed_growth_background	ENSGALG00010002419	CTBP2	-0.254791715	0.062462379	-4.079122773	4.5206E-05	0.000896389	negative
PRJNA729271	ed_Lushi_vs_Arbor_Acr	breed_growth_background	ENSGALG00010002420	TMEM272	-1.25540503	0.449771005	-2.791209342	0.00525115	0.026427325	negative
PRJNA729271	ed_Lushi_vs_Arbor_Acr	breed_growth_background	ENSGALG00010002421	ENSGALG00010002421	-0.361497184	0.10266392	-3.521170665	0.000429646	0.004460976	negative
PRJNA729271	ed_Lushi_vs_Arbor_Acr	breed_growth_background	ENSGALG00010002422	TRAPPC12	0.033670469	0.055863842	0.602723828	0.546692427	0.690337619	positive
PRJNA729271	ed_Lushi_vs_Arbor_Acr	breed_growth_background	ENSGALG00010002423	ENSGALG00010002423	0.012112143	0.304078666	0.039832267	0.968226852	0.981910601	positive
PRJNA729271	ed_Lushi_vs_Arbor_Acr	breed_growth_background	ENSGALG00010002424	CASK	0.047948354	0.030698617	1.561906	0.118310135	0.242115686	positive
PRJNA729271	ed_Lushi_vs_Arbor_Acr	breed_growth_background	ENSGALG00010002425	FDX2	0.051640875	0.07202046	0.717030611	0.473355209	0.627059382	positive
PRJNA729271	ed_Lushi_vs_Arbor_Acr	breed_growth_background	ENSGALG00010002427	CYB5R4	0.061180506	0.061376217	0.996811281	0.318856121	0.482142654	positive
PRJNA729271	ed_Lushi_vs_Arbor_Acr	breed_growth_background	ENSGALG00010002428	IBSP	-0.281246716	0.403822069	-0.696461975	0.486139563	0.63784036	negative
PRJNA729271	ed_Lushi_vs_Arbor_Acr	breed_growth_background	ENSGALG00010002429	ENSGALG00010002429	-1.275143458	0.291799438	-4.369931162	1.24286E-05	0.000338632	negative
PRJNA729271	ed_Lushi_vs_Arbor_Acr	breed_growth_background	ENSGALG00010002430	NUMA1	0.343348777	0.09315444	3.685801506	0.000227984	0.002843914	positive
PRJNA729271	ed_Lushi_vs_Arbor_Acr	breed_growth_background	ENSGALG00010002431	BCKDHB	0.214690254	0.078753956	2.726088523	0.006408981	0.030448435	positive
PRJNA729271	ed_Lushi_vs_Arbor_Acr	breed_growth_background	ENSGALG00010002432	RAVER1	0.124820355	0.098016268	1.273465696	0.202852838	0.352095783	positive
PRJNA729271	ed_Lushi_vs_Arbor_Acr	breed_growth_background	ENSGALG00010002433	GRHL1	-0.331155956	0.178555789	-1.854635789	0.063648254	0.157142129	negative
PRJNA729271	ed_Lushi_vs_Arbor_Acr	breed_growth_background	ENSGALG00010002434	GPR34	-0.125300097	0.130836958	-0.957681218	0.33822353	0.502516408	negative
PRJNA729271	ed_Lushi_vs_Arbor_Acr	breed_growth_background	ENSGALG00010002435	ATP7B	-0.941312656	0.389614326	-2.416011407	0.01569157	0.057582102	negative
PRJNA729271	ed_Lushi_vs_Arbor_Acr	breed_growth_background	ENSGALG00010002436	ENSGALG00010002436	-1.136939578	0.466054227	-2.439500626	0.014707577	0.054719284	negative
PRJNA729271	ed_Lushi_vs_Arbor_Acr	breed_growth_background	ENSGALG00010002437	TTK	-0.262646852	0.106469854	-2.466865904	0.013630139	0.052032885	negative
PRJNA729271	ed_Lushi_vs_Arbor_Acr	breed_growth_background	ENSGALG00010002438	RARB	-0.064386834	0.050955606	-1.263586855	0.206378354	0.35557521	negative
PRJNA729271	ed_Lushi_vs_Arbor_Acr	breed_growth_background	ENSGALG00010002439	GPR82	0.58352959	0.44135631	1.322128124	0.18612549	0.331613191	positive
PRJNA729271	ed_Lushi_vs_Arbor_Acr	breed_growth_background	ENSGALG00010002440	LRRC51	0.242992201	0.145299379	1.672355399	0.094454262	0.206658796	positive
PRJNA729271	ed_Lushi_vs_Arbor_Acr	breed_growth_background	ENSGALG00010002441	USO1	-0.000375482	0.036594466	-0.010260625	0.99181335	0.995521742	negative
PRJNA729271	ed_Lushi_vs_Arbor_Acr	breed_growth_background	ENSGALG00010002442	ENSGALG00010002442	-0.627835038	0.246442177	-2.547595734	0.010846809	0.044351538	negative
PRJNA729271	ed_Lushi_vs_Arbor_Acr	breed_growth_background	ENSGALG00010002443	TYK2	0.052431642	0.074060572	0.707956207	0.478972454	0.63184435	positive
PRJNA729271	ed_Lushi_vs_Arbor_Acr	breed_growth_background	ENSGALG00010002444	KLF11	0.256660317	0.09699127	2.646220821	0.008139666	0.036036794	positive
PRJNA729271	ed_Lushi_vs_Arbor_Acr	breed_growth_background	ENSGALG00010002445	EDRF1	-0.018659463	0.059636377	-0.312887274	0.754366307	0.844757387	negative
PRJNA729271	ed_Lushi_vs_Arbor_Acr	breed_growth_background	ENSGALG00010002446	RIMS1	-0.051494111	0.167206431	-0.307967284	0.758107227	0.84717942	negative
PRJNA729271	ed_Lushi_vs_Arbor_Acr	breed_growth_background	ENSGALG00010002447	YLEC12	-1.223503905	0.305529795	-4.004532207	6.21403E-05	0.001125905	negative
PRJNA729271	ed_Lushi_vs_Arbor_Acr	breed_growth_background	ENSGALG00010002448	RAB9A	0.098121324	0.072966769	1.344739872	0.178709254	0.323031629	positive
PRJNA729271	ed_Lushi_vs_Arbor_Acr	breed_growth_background	ENSGALG00010002449	ALG11	-0.299484184	0.076631129	-3.90812701	9.30144E-05	0.001516585	negative
PRJNA729271	ed_Lushi_vs_Arbor_Acr	breed_growth_background	ENSGALG00010002453	TOP2B	0.020168522	0.033989279	0.593378947	0.552927575	0.695357036	positive
PRJNA729271	ed_Lushi_vs_Arbor_Acr	breed_growth_background	ENSGALG00010002454	NEK5	0.014984594	0.391197661	0.038304405	0.969444978	0.982470096	positive
PRJNA729271	ed_Lushi_vs_Arbor_Acr	breed_growth_background	ENSGALG00010002455	NYX	0.462976727	0.192171295	2.409187734	0.015988071	0.05834519	positive
PRJNA729271	ed_Lushi_vs_Arbor_Acr	breed_growth_background	ENSGALG00010002456	ELOVL4	-0.04570254	0.310454444	-0.147211743	0.882964892	0.931076394	negative
PRJNA729271	ed_Lushi_vs_Arbor_Acr	breed_growth_background	ENSGALG00010002457	ENSGALG00010002457	0.048875755	0.236738634	0.206454497	0.836435905	0.900753228	positive
PRJNA729271	ed_Lushi_vs_Arbor_Acr	breed_growth_background	ENSGALG00010002458	ENSGALG00010002458	0.099709261	0.105924448	0.941324345	0.34653867	0.510371675	positive
PRJNA729271	ed_Lushi_vs_Arbor_Acr	breed_growth_background	ENSGALG00010002459	G3BP2	-0.025547782	0.0403879	-0.632560291	0.527020825	0.673113654	negative
PRJNA729271	ed_Lushi_vs_Arbor_Acr	breed_growth_background	ENSGALG00010002460	LAMTOR1	0.132015406	0.097315106	1.3565767	0.174915749	0.318838504	positive
PRJNA729271	ed_Lushi_vs_Arbor_Acr	breed_growth_background	ENSGALG00010002461	TOMT	-0.070657974	0.18257087	-0.387016689	0.698743852	0.805494842	negative
PRJNA729271	ed_Lushi_vs_Arbor_Acr	breed_growth_background	ENSGALG00010002462	RRM2	-0.507569457	0.148384277	-3.420641779	0.000624736	0.005797403	negative
PRJNA729271	ed_Lushi_vs_Arbor_Acr	breed_growth_background	ENSGALG00010002463	ASRGL1	-0.158926355	0.055484146	-2.864356159	0.004178579	0.022284217	negative
PRJNA729271	ed_Lushi_vs_Arbor_Acr	breed_growth_background	ENSGALG00010002464	NGLY1	0.069087738	0.044565075	1.550266404	0.121077587	0.245938849	positive
PRJNA729271	ed_Lushi_vs_Arbor_Acr	breed_growth_background	ENSGALG00010002466	UROS	-0.105895042	0.096623522	-1.095955095	0.273098426	0.431968757	negative
PRJNA729271	ed_Lushi_vs_Arbor_Acr	breed_growth_background	ENSGALG00010002467	NEK3	-0.253355637	0.123273858	-2.05522599	0.039857182	0.11205001	negative
PRJNA729271	ed_Lushi_vs_Arbor_Acr	breed_growth_background	ENSGALG00010002468	ENSGALG00010002468	-0.650755338	0.295044497	-2.205617613	0.027410785	0.085136803	negative
PRJNA729271	ed_Lushi_vs_Arbor_Acr	breed_growth_background	ENSGALG00010002469	MEPE	-0.218702263	0.761821212	-0.287078201	0.774052443	0.859781567	negative
PRJNA729271	ed_Lushi_vs_Arbor_Acr	breed_growth_background	ENSGALG00010002470	OXSM	0.040178323	0.088104524	0.456030189	0.64836827	0.770525381	positive
PRJNA729271	ed_Lushi_vs_Arbor_Acr	breed_growth_background	ENSGALG00010002471	OGFRL1	-0.219815811	0.082498271	-2.664489919	0.007710518	0.034725981	negative
PRJNA729271	ed_Lushi_vs_Arbor_Acr	breed_growth_background	ENSGALG00010002472	CDC37	0.265718233	0.11493421	2.31191594	0.020782316	0.070055738	positive
PRJNA729271	ed_Lushi_vs_Arbor_Acr	breed_growth_background	ENSGALG00010002473	ENSGALG00010002473	1.736879207	0.339297948	5.11903834	3.07098E-07	1.95236E-05	positive
PRJNA729271	ed_Lushi_vs_Arbor_Acr	breed_growth_background	ENSGALG00010002474	HPCAL1	-0.22685281	0.076376724	-2.970182496	0.002976229	0.017607832	negative
PRJNA729271	ed_Lushi_vs_Arbor_Acr	breed_growth_background	ENSGALG00010002475	ENSGALG00010002475	0.017678639	0.104193138	0.169671819	0.865268238	0.91896711	positive
PRJNA729271	ed_Lushi_vs_Arbor_Acr	breed_growth_background	ENSGALG00010002476	DDX3X	0.199763713	0.077494248	2.577787607	0.00994351	0.041644372	positive
PRJNA729271	ed_Lushi_vs_Arbor_Acr	breed_growth_background	ENSGALG00010002478	PPME1	-0.025291217	0.031330553	-0.807238104	0.419529317	0.579132152	negative
PRJNA729271	ed_Lushi_vs_Arbor_Acr	breed_growth_background	ENSGALG00010002481	CKAP2	-0.509790235	0.132070105	-3.859997213	0.000113388	0.0017522	negative
PRJNA729271	ed_Lushi_vs_Arbor_Acr	breed_growth_background	ENSGALG00010002483	COQ2	0.124504562	0.07358993	1.691869553	0.090670848	0.201267067	positive
PRJNA729271	ed_Lushi_vs_Arbor_Acr	breed_growth_background	ENSGALG00010002487	BCCIP	-0.178875289	0.064118267	-2.789771093	0.005274532	0.026482964	negative
PRJNA729271	ed_Lushi_vs_Arbor_Acr	breed_growth_background	ENSGALG00010002492	RPL8	-0.023223811	0.069262185	-0.335302891	0.737396613	0.83173013	negative
PRJNA729271	ed_Lushi_vs_Arbor_Acr	breed_growth_background	ENSGALG00010002500	OSR1	-0.106354071	0.108769899	-0.977789556	0.328178418	0.491988112	negative
PRJNA729271	ed_Lushi_vs_Arbor_Acr	breed_growth_background	ENSGALG00010002501	TCEANC	0.008143766	0.051878803	0.156976741	0.875263179	0.926392097	positive
PRJNA729271	ed_Lushi_vs_Arbor_Acr	breed_growth_background	ENSGALG00010002504	ENSGALG00010002504	0.087993942	0.035156394	2.502928534	0.012317041	0.048449257	positive
PRJNA729271	ed_Lushi_vs_Arbor_Acr	breed_growth_background	ENSGALG00010002507	C2CD3	-0.015231848	0.060406285	-0.252156674	0.800919965	0.877102969	negative
PRJNA729271	ed_Lushi_vs_Arbor_Acr	breed_growth_background	ENSGALG00010002511	VPS36	-0.00337078	0.041868322	-0.080509066	0.935832386	0.964536447	negative
PRJNA729271	ed_Lushi_vs_Arbor_Acr	breed_growth_background	ENSGALG00010002512	DAXX	0.216857708	0.123970989	1.749261743	0.080245786	0.184394842	positive
PRJNA729271	ed_Lushi_vs_Arbor_Acr	breed_growth_background	ENSGALG00010002518	MAST1	0.086672966	0.201239183	0.430696275	0.666689228	0.783942594	positive
PRJNA729271	ed_Lushi_vs_Arbor_Acr	breed_growth_background	ENSGALG00010002520	TTC32	-0.137704341	0.08742303	-1.57514949	0.115221943	0.2377826	negative
PRJNA729271	ed_Lushi_vs_Arbor_Acr	breed_growth_background	ENSGALG00010002522	HPSE	-0.056645743	0.163286457	-0.346910237	0.728658756	0.825471564	negative
PRJNA729271	ed_Lushi_vs_Arbor_Acr	breed_growth_background	ENSGALG00010002523	DNAJB13	0.61996571	0.220469641	2.812023036	0.004923098	0.02527818	positive
PRJNA729271	ed_Lushi_vs_Arbor_Acr	breed_growth_background	ENSGALG00010002526	ZBTB22	0.159874968	0.058110484	2.751224173	0.0059373	0.028818972	positive
PRJNA729271	ed_Lushi_vs_Arbor_Acr	breed_growth_background	ENSGALG00010002527	LRRC3B	0.0676815	0.097811873	0.691955869	0.488965043	0.640827152	positive
PRJNA729271	ed_Lushi_vs_Arbor_Acr	breed_growth_background	ENSGALG00010002528	MED14	0.041973946	0.063094873	0.665251289	0.505889781	0.65550588	positive
PRJNA729271	ed_Lushi_vs_Arbor_Acr	breed_growth_background	ENSGALG00010002530	STAG3	-0.18325557	0.065708744	-2.78890693	0.005288626	0.026534661	negative
PRJNA729271	ed_Lushi_vs_Arbor_Acr	breed_growth_background	ENSGALG00010002533	LPAR2	-0.139110167	0.07969227	-1.745591718	0.080881922	0.18543342	negative
PRJNA729271	ed_Lushi_vs_Arbor_Acr	breed_growth_background	ENSGALG00010002536	RGL2	0.138378073	0.074886545	1.847836242	0.064626036	0.158879646	positive
PRJNA729271	ed_Lushi_vs_Arbor_Acr	breed_growth_background	ENSGALG00010002539	SNAP91	0.122875128	0.109370454	1.123476436	0.261235203	0.419284926	positive
PRJNA729271	ed_Lushi_vs_Arbor_Acr	breed_growth_background	ENSGALG00010002543	UCP3	1.123706695	0.249866265	4.497232531	6.88437E-06	0.000221261	positive
PRJNA729271	ed_Lushi_vs_Arbor_Acr	breed_growth_background	ENSGALG00010002544	THSD1	-0.120399343	0.067418244	-1.785857009	0.074122413	0.174927447	negative
PRJNA729271	ed_Lushi_vs_Arbor_Acr	breed_growth_background	ENSGALG00010002546	ATP6AP2	-0.058093832	0.034019185	-1.70767854	0.087696006	0.196347484	negative
PRJNA729271	ed_Lushi_vs_Arbor_Acr	breed_growth_background	ENSGALG00010002547	PBX4	0.180639196	0.058740805	3.075191015	0.002103678	0.013747885	positive
PRJNA729271	ed_Lushi_vs_Arbor_Acr	breed_growth_background	ENSGALG00010002551	WDR35	-0.308322621	0.060436821	-5.101569122	3.36849E-07	2.0848E-05	negative
PRJNA729271	ed_Lushi_vs_Arbor_Acr	breed_growth_background	ENSGALG00010002553	ENSGALG00010002553	-0.064879153	0.123240719	-0.526442503	0.598580791	0.731570705	negative
PRJNA729271	ed_Lushi_vs_Arbor_Acr	breed_growth_background	ENSGALG00010002554	EGFL6	-0.716311693	0.156956255	-4.563766478	5.0244E-06	0.00017517	negative
PRJNA729271	ed_Lushi_vs_Arbor_Acr	breed_growth_background	ENSGALG00010002556	ENSGALG00010002556	-0.385399737	0.116002069	-3.322352268	0.000892619	0.007526769	negative
PRJNA729271	ed_Lushi_vs_Arbor_Acr	breed_growth_background	ENSGALG00010002557	ENSGALG00010002557	-0.255248705	0.218741274	-1.166897771	0.243251656	0.39892893	negative
PRJNA729271	ed_Lushi_vs_Arbor_Acr	breed_growth_background	ENSGALG00010002559	NEK10	0.247880649	0.312684534	0.79274995	0.427923522	0.58704092	positive
PRJNA729271	ed_Lushi_vs_Arbor_Acr	breed_growth_background	ENSGALG00010002561	PAAF1	-0.20159632	0.045741324	-4.407312792	1.04661E-05	0.000302203	negative
PRJNA729271	ed_Lushi_vs_Arbor_Acr	breed_growth_background	ENSGALG00010002565	MRPS18C	0.122267952	0.06224673	1.964246997	0.049501455	0.13153503	positive
PRJNA729271	ed_Lushi_vs_Arbor_Acr	breed_growth_background	ENSGALG00010002566	TMSB4X	-0.174173589	0.061912889	-2.81320404	0.004905052	0.025203376	negative
PRJNA729271	ed_Lushi_vs_Arbor_Acr	breed_growth_background	ENSGALG00010002567	COA4	0.141871565	0.127209878	1.115255883	0.264740785	0.422949154	positive
PRJNA729271	ed_Lushi_vs_Arbor_Acr	breed_growth_background	ENSGALG00010002569	GTPBP6	-0.061085386	0.064018698	-0.954180376	0.339992346	0.50398788	negative
PRJNA729271	ed_Lushi_vs_Arbor_Acr	breed_growth_background	ENSGALG00010002570	ADAM12	-0.253829753	0.144924289	-1.751464535	0.079865925	0.183959413	negative
PRJNA729271	ed_Lushi_vs_Arbor_Acr	breed_growth_background	ENSGALG00010002571	BCOR	0.081668768	0.075542778	1.081093005	0.279655745	0.43941572	positive
PRJNA729271	ed_Lushi_vs_Arbor_Acr	breed_growth_background	ENSGALG00010002573	MVB12A	-0.060067914	0.050371151	-1.19250627	0.233062794	0.387505604	negative
PRJNA729271	ed_Lushi_vs_Arbor_Acr	breed_growth_background	ENSGALG00010002575	BGV	-0.535068564	0.214628723	-2.492996072	0.012667025	0.04935713	negative
PRJNA729271	ed_Lushi_vs_Arbor_Acr	breed_growth_background	ENSGALG00010002576	HELQ	0.091994657	0.059349055	1.550061024	0.12112687	0.245998297	positive
PRJNA729271	ed_Lushi_vs_Arbor_Acr	breed_growth_background	ENSGALG00010002577	MRPL48	0.147293353	0.05558678	2.649791048	0.008054157	0.035789393	positive
PRJNA729271	ed_Lushi_vs_Arbor_Acr	breed_growth_background	ENSGALG00010002579	MATN3	-0.172862724	0.307845047	-0.561525109	0.574439618	0.712032005	negative
PRJNA729271	ed_Lushi_vs_Arbor_Acr	breed_growth_background	ENSGALG00010002581	ATP13A1	-0.034606875	0.041722008	-0.829463304	0.406842293	0.567582198	negative
PRJNA729271	ed_Lushi_vs_Arbor_Acr	breed_growth_background	ENSGALG00010002582	ENSGALG00010002582	-0.448949363	0.25319149	-1.773161346	0.076201975	0.178066682	negative
PRJNA729271	ed_Lushi_vs_Arbor_Acr	breed_growth_background	ENSGALG00010002584	ENSGALG00010002584	-0.558789853	0.237435281	-2.353440694	0.018600575	0.064964396	negative
PRJNA729271	ed_Lushi_vs_Arbor_Acr	breed_growth_background	ENSGALG00010002586	GPR63	-0.035734585	0.079908535	-0.447193589	0.65473529	0.775189999	negative
PRJNA729271	ed_Lushi_vs_Arbor_Acr	breed_growth_background	ENSGALG00010002587	DOCK1	-0.132179113	0.035912181	-3.680620546	0.000232667	0.002889896	negative
PRJNA729271	ed_Lushi_vs_Arbor_Acr	breed_growth_background	ENSGALG00010002588	FAM175A	-0.045546482	0.048645005	-0.936303375	0.349117011	0.513074224	negative
PRJNA729271	ed_Lushi_vs_Arbor_Acr	breed_growth_background	ENSGALG00010002589	SLC4A7	-0.152985486	0.077428236	-1.975835875	0.048173369	0.128904002	negative
PRJNA729271	ed_Lushi_vs_Arbor_Acr	breed_growth_background	ENSGALG00010002590	ATP6V1C2	0.144334326	0.141877548	1.017316185	0.30900304	0.47177828	positive
PRJNA729271	ed_Lushi_vs_Arbor_Acr	breed_growth_background	ENSGALG00010002591	ENSGALG00010002591	-0.360279776	0.114522014	-3.145943417	0.00165552	0.011620955	negative
PRJNA729271	ed_Lushi_vs_Arbor_Acr	breed_growth_background	ENSGALG00010002592	RPS6KA3	0.007208422	0.054103428	0.133234104	0.89400824	0.938080892	positive
PRJNA729271	ed_Lushi_vs_Arbor_Acr	breed_growth_background	ENSGALG00010002597	ENSGALG00010002597	-0.150184061	0.064301422	-2.335625805	0.019510752	0.067143485	negative
PRJNA729271	ed_Lushi_vs_Arbor_Acr	breed_growth_background	ENSGALG00010002598	NMT2	0.103551933	0.054986414	1.883227629	0.059669526	0.150254993	positive
PRJNA729271	ed_Lushi_vs_Arbor_Acr	breed_growth_background	ENSGALG00010002599	KIFC1	0.486197289	0.117822712	4.126515845	3.68301E-05	0.000781628	positive
PRJNA729271	ed_Lushi_vs_Arbor_Acr	breed_growth_background	ENSGALG00010002600	TRPA1	0.360184127	0.319457102	1.127488247	0.259536107	0.417255531	positive
PRJNA729271	ed_Lushi_vs_Arbor_Acr	breed_growth_background	ENSGALG00010002601	PLCXD1	-0.007565391	0.099438084	-0.07608142	0.939354322	0.966697969	negative
PRJNA729271	ed_Lushi_vs_Arbor_Acr	breed_growth_background	ENSGALG00010002603	INSYN2A	0.210852711	0.140883507	1.496645808	0.134485444	0.264285047	positive
PRJNA729271	ed_Lushi_vs_Arbor_Acr	breed_growth_background	ENSGALG00010002604	BLEC3	1.042626171	0.211996585	4.918127199	8.73761E-07	4.37689E-05	positive
PRJNA729271	ed_Lushi_vs_Arbor_Acr	breed_growth_background	ENSGALG00010002605	EOMES	0.90145081	0.429815406	2.097297577	0.035967241	0.10406091	positive
PRJNA729271	ed_Lushi_vs_Arbor_Acr	breed_growth_background	ENSGALG00010002608	RPP38	0.144135435	0.071536749	2.014844639	0.04392093	0.120528944	positive
PRJNA729271	ed_Lushi_vs_Arbor_Acr	breed_growth_background	ENSGALG00010002609	PDIA6	-0.046893111	0.067247788	-0.697318275	0.485603633	0.637343776	negative
PRJNA729271	ed_Lushi_vs_Arbor_Acr	breed_growth_background	ENSGALG00010002611	NPS	-0.682170101	0.233644108	-2.919697423	0.003503714	0.019753533	negative
PRJNA729271	ed_Lushi_vs_Arbor_Acr	breed_growth_background	ENSGALG00010002612	JPH1	0.317619296	0.114423827	2.7758143	0.005506365	0.027283131	positive
PRJNA729271	ed_Lushi_vs_Arbor_Acr	breed_growth_background	ENSGALG00010002613	ENSGALG00010002613	0.172295934	0.05680038	3.033358842	0.002418478	0.015238839	positive
PRJNA729271	ed_Lushi_vs_Arbor_Acr	breed_growth_background	ENSGALG00010002614	GPAT3	-0.119091748	0.077465274	-1.537356574	0.124206015	0.249877333	negative
PRJNA729271	ed_Lushi_vs_Arbor_Acr	breed_growth_background	ENSGALG00010002616	ZNFB3	0.35125507	0.092197692	3.809803288	0.000139077	0.002027751	positive
PRJNA729271	ed_Lushi_vs_Arbor_Acr	breed_growth_background	ENSGALG00010002619	RAB6A	-0.123261191	0.036903316	-3.340111536	0.000837447	0.007178475	negative

PRJNA729271	:ed_Lushi_vs_Arbor_Acr	breed_growth_background	ENSGALG00010002621	SLC25A15	-0.097091318	0.088497964	-1.097102273	0.272596687	0.431504748	negative
PRJNA729271	:ed_Lushi_vs_Arbor_Acr	breed_growth_background	ENSGALG00010002622	AZI2	0.02588744	0.043366207	0.596949591	0.550541036	0.693619498	positive
PRJNA729271	:ed_Lushi_vs_Arbor_Acr	breed_growth_background	ENSGALG00010002623	MID1IP1	0.276994023	0.072871639	3.801122437	0.000144042	0.002073162	positive
PRJNA729271	:ed_Lushi_vs_Arbor_Acr	breed_growth_background	ENSGALG00010002625	FHL5	-0.645333442	0.117271585	-5.502896926	3.73601E-08	4.0413E-06	negative
PRJNA729271	:ed_Lushi_vs_Arbor_Acr	breed_growth_background	ENSGALG00010002626	CHD7	0.077945737	0.072679645	1.072456206	0.283515174	0.44374824	positive
PRJNA729271	:ed_Lushi_vs_Arbor_Acr	breed_growth_background	ENSGALG00010002627	MRPS31	-0.20141853	0.078253763	-2.573914943	0.010055499	0.042004167	negative
PRJNA729271	:ed_Lushi_vs_Arbor_Acr	breed_growth_background	ENSGALG00010002628	TRIM7.2	0.140122834	0.102862048	1.362240369	0.17312204	0.316604083	positive
PRJNA729271	:ed_Lushi_vs_Arbor_Acr	breed_growth_background	ENSGALG00010002632	HPGD	0.415362672	0.145300328	2.858649226	0.004254489	0.022556264	positive
PRJNA729271	:ed_Lushi_vs_Arbor_Acr	breed_growth_background	ENSGALG00010002633	ENSGALG00010002633	0.15118598	0.071678577	2.109221294	0.034925485	0.101778227	positive
PRJNA729271	:ed_Lushi_vs_Arbor_Acr	breed_growth_background	ENSGALG00010002634	RGN	-0.181886715	0.098328366	-1.849788857	0.064343988	0.158331755	negative
PRJNA729271	:ed_Lushi_vs_Arbor_Acr	breed_growth_background	ENSGALG00010002638	FABP9	0.462738267	0.236664532	1.955249746	0.0505536	0.133495486	positive
PRJNA729271	:ed_Lushi_vs_Arbor_Acr	breed_growth_background	ENSGALG00010002644	KCNF1	1.13016264	0.271217797	4.166992921	3.08644E-05	0.000680943	positive
PRJNA729271	:ed_Lushi_vs_Arbor_Acr	breed_growth_background	ENSGALG00010002646	FOXO1	0.277139138	0.090631223	3.057877051	0.00222911	0.014392406	positive
PRJNA729271	:ed_Lushi_vs_Arbor_Acr	breed_growth_background	ENSGALG00010002647	FAM168A	0.196437892	0.053116944	3.698215256	0.000217121	0.002761229	positive
PRJNA729271	:ed_Lushi_vs_Arbor_Acr	breed_growth_background	ENSGALG00010002649	UFL1	0.14955319	0.042491243	3.519623773	0.000432159	0.00446801	positive
PRJNA729271	:ed_Lushi_vs_Arbor_Acr	breed_growth_background	ENSGALG00010002653	EIF1AX	-0.04475781	0.034251568	-1.306737536	0.191301866	0.338202287	negative
PRJNA729271	:ed_Lushi_vs_Arbor_Acr	breed_growth_background	ENSGALG00010002655	FABP4	-0.271166413	0.502608163	-0.539518522	0.58952912	0.725094939	negative
PRJNA729271	:ed_Lushi_vs_Arbor_Acr	breed_growth_background	ENSGALG00010002656	GLRA3	-0.179706583	0.26295475	-0.683412578	0.494346175	0.64601044	negative
PRJNA729271	:ed_Lushi_vs_Arbor_Acr	breed_growth_background	ENSGALG00010002658	FBXL12	-0.109860957	0.081771474	-1.343512007	0.179106241	0.323305724	negative
PRJNA729271	:ed_Lushi_vs_Arbor_Acr	breed_growth_background	ENSGALG00010002661	ACBD7	0.393878726	0.113929964	3.457200475	0.000545819	0.005289773	positive
PRJNA729271	:ed_Lushi_vs_Arbor_Acr	breed_growth_background	ENSGALG00010002664	ZNFB1	0.040801356	0.075549089	0.540064173	0.589152777	0.72481493	positive
PRJNA729271	:ed_Lushi_vs_Arbor_Acr	breed_growth_background	ENSGALG00010002667	ENSGALG00010002667	-1.877441395	0.845746955	-2.219861845			negative
PRJNA729271	:ed_Lushi_vs_Arbor_Acr	breed_growth_background	ENSGALG00010002669	IL4I1	0.415821021	0.526800743	0.789332639	0.429917615	0.588354685	positive
PRJNA729271	:ed_Lushi_vs_Arbor_Acr	breed_growth_background	ENSGALG00010002673	SLC66A3	-0.129118543	0.082107205	-1.572560449	0.115820632	0.238502636	negative
PRJNA729271	:ed_Lushi_vs_Arbor_Acr	breed_growth_background	ENSGALG00010002678	CCNI	0.308266672	0.092322922	3.339004714	0.000840791	0.007202879	positive
PRJNA729271	:ed_Lushi_vs_Arbor_Acr	breed_growth_background	ENSGALG00010002681	PLEKHB1	-0.082256827	0.08619549	-0.954305465	0.339929043	0.50396558	negative
PRJNA729271	:ed_Lushi_vs_Arbor_Acr	breed_growth_background	ENSGALG00010002682	UBL5	0.141628375	0.089175919	1.588190811	0.112243198	0.233524495	positive
PRJNA729271	:ed_Lushi_vs_Arbor_Acr	breed_growth_background	ENSGALG00010002685	CA8	-0.269483674	0.095979474	-2.807721946	0.004989329	0.025530466	negative
PRJNA729271	:ed_Lushi_vs_Arbor_Acr	breed_growth_background	ENSGALG00010002689	COG6	-0.008519613	0.033855388	-0.251647192	0.801313778	0.87731434	negative
PRJNA729271	:ed_Lushi_vs_Arbor_Acr	breed_growth_background	ENSGALG00010002693	MSC	0.045001636	0.090652913	0.49641688	0.619600318	0.747457756	positive
PRJNA729271	:ed_Lushi_vs_Arbor_Acr	breed_growth_background	ENSGALG00010002700	LHFPL6	-0.40002166	0.091485014	-4.372537558	1.22811E-05	0.000335876	negative
PRJNA729271	:ed_Lushi_vs_Arbor_Acr	breed_growth_background	ENSGALG00010002701	TMEM196	-0.678284425	0.280812349	-2.415436596	0.015716358	0.057629298	negative
PRJNA729271	:ed_Lushi_vs_Arbor_Acr	breed_growth_background	ENSGALG00010002703	PIN1	0.018163433	0.052026298	0.349120221	0.726999056	0.824527408	positive
PRJNA729271	:ed_Lushi_vs_Arbor_Acr	breed_growth_background	ENSGALG00010002704	KCNB2	-0.388525202	0.138744375	-2.800295161	0.00510559	0.025994214	negative
PRJNA729271	:ed_Lushi_vs_Arbor_Acr	breed_growth_background	ENSGALG00010002705	JADE3	-0.016100002	0.030241021	-0.53238949	0.594456263	0.728680214	negative
PRJNA729271	:ed_Lushi_vs_Arbor_Acr	breed_growth_background	ENSGALG00010002706	ROCK2	0.130346491	0.077107172	1.690458718	0.09094023	0.20149478	positive
PRJNA729271	:ed_Lushi_vs_Arbor_Acr	breed_growth_background	ENSGALG00010002707	RELT	-0.240521573	0.149828499	-1.605312574	0.108425033	0.228101721	negative
PRJNA729271	:ed_Lushi_vs_Arbor_Acr	breed_growth_background	ENSGALG00010002712	PTPRE	-0.0757605	0.065738555	-1.152451556	0.249135566	0.40520871	negative
PRJNA729271	:ed_Lushi_vs_Arbor_Acr	breed_growth_background	ENSGALG00010002713	OLFM2	0.093876857	0.0965415	0.972398979	0.330852099	0.495167907	positive
PRJNA729271	:ed_Lushi_vs_Arbor_Acr	breed_growth_background	ENSGALG00010002716	NHLRC3	-0.033663101	0.060531565	-0.556124745	0.57812558	0.715255231	negative
PRJNA729271	:ed_Lushi_vs_Arbor_Acr	breed_growth_background	ENSGALG00010002722	TRIM7.1	-0.028055568	0.078781323	-0.356119535	0.721751048	0.821439161	negative
PRJNA729271	:ed_Lushi_vs_Arbor_Acr	breed_growth_background	ENSGALG00010002725	TSPAN7	0.058376941	0.095989493	0.6081597	0.54308156	0.687677997	positive
PRJNA729271	:ed_Lushi_vs_Arbor_Acr	breed_growth_background	ENSGALG00010002728	RP2	-0.162061858	0.060360786	-2.684886502	0.007255444	0.033259855	negative
PRJNA729271	:ed_Lushi_vs_Arbor_Acr	breed_growth_background	ENSGALG00010002730	E2F6	0.146298765	0.054510313	2.68387311	0.007277472	0.033318686	positive
PRJNA729271	:ed_Lushi_vs_Arbor_Acr	breed_growth_background	ENSGALG00010002733	RDH8	0.037224663	0.197260636	0.188708015	0.85032167	0.910016284	positive
PRJNA729271	:ed_Lushi_vs_Arbor_Acr	breed_growth_background	ENSGALG00010002739	HEP21	-0.574552152	0.43276624	-1.327627017	0.184301355	0.329686306	negative
PRJNA729271	:ed_Lushi_vs_Arbor_Acr	breed_growth_background	ENSGALG00010002741	PDK4	0.443274201	0.306051976	1.448362486	0.147515701	0.282387755	positive
PRJNA729271	:ed_Lushi_vs_Arbor_Acr	breed_growth_background	ENSGALG00010002742	PROSER1	0.072644935	0.065504013	1.109015035	0.267423696	0.425780588	positive
PRJNA729271	:ed_Lushi_vs_Arbor_Acr	breed_growth_background	ENSGALG00010002747	FUT9	-0.420249328	0.288204384	-1.458164245	0.144795277	0.278467233	negative
PRJNA729271	:ed_Lushi_vs_Arbor_Acr	breed_growth_background	ENSGALG00010002750	ENSGALG00010002750	-0.31123289	0.125763179	-2.474753684	0.013332809	0.051167348	negative
PRJNA729271	:ed_Lushi_vs_Arbor_Acr	breed_growth_background	ENSGALG00010002752	NDUFA4	0.00219063	0.082380832	0.026591501	0.978785552	0.987465271	positive
PRJNA729271	:ed_Lushi_vs_Arbor_Acr	breed_growth_background	ENSGALG00010002753	SLC9A7	-0.063448041	0.083053152	-0.763945004	0.444900017	0.601741266	negative
PRJNA729271	:ed_Lushi_vs_Arbor_Acr	breed_growth_background	ENSGALG00010002758	GREB1	-0.201223901	0.191492161	-1.050820568	0.293341008	0.454741887	negative
PRJNA729271	:ed_Lushi_vs_Arbor_Acr	breed_growth_background	ENSGALG00010002760	RAB2A	0.110256812	0.049511837	2.226877853	0.025955438	0.082127064	positive
PRJNA729271	:ed_Lushi_vs_Arbor_Acr	breed_growth_background	ENSGALG00010002762	PPAN	0.179946934	0.080786755	2.22743113	0.025918474	0.082028008	positive
PRJNA729271	:ed_Lushi_vs_Arbor_Acr	breed_growth_background	ENSGALG00010002763	SEPTIN11	-0.293583825	0.062179464	-4.72155603	2.34047E-06	9.51363E-05	negative
PRJNA729271	:ed_Lushi_vs_Arbor_Acr	breed_growth_background	ENSGALG00010002765	MAP7D2	-0.231157364	0.242112845	-0.954750517	0.339703876	0.50378634	negative
PRJNA729271	:ed_Lushi_vs_Arbor_Acr	breed_growth_background	ENSGALG00010002766	NANOS1	0.247948955	0.182455707	1.358954232	0.174161092	0.317982747	positive
PRJNA729271	:ed_Lushi_vs_Arbor_Acr	breed_growth_background	ENSGALG00010002768	TRIM39.2	0.145990203	0.346679388	0.421110133	0.673674662	0.789009546	positive
PRJNA729271	:ed_Lushi_vs_Arbor_Acr	breed_growth_background	ENSGALG00010002774	TBX18	0.111511593	0.063364747	1.759836462	0.078435539	0.181419151	positive
PRJNA729271	:ed_Lushi_vs_Arbor_Acr	breed_growth_background	ENSGALG00010002779	FBXO8	0.053332169	0.046126784	1.156208289	0.24759597	0.403792033	positive
PRJNA729271	:ed_Lushi_vs_Arbor_Acr	breed_growth_background	ENSGALG00010002781	EIF3G	0.042805358	0.073601463	0.581582978	0.560847611	0.701664606	positive
PRJNA729271	:ed_Lushi_vs_Arbor_Acr	breed_growth_background	ENSGALG00010002782	GSTA3	-0.084098691	0.079293969	-1.060593792	0.288874545	0.449853516	negative
PRJNA729271	:ed_Lushi_vs_Arbor_Acr	breed_growth_background	ENSGALG00010002784	TRIM27.2	0.141492945	0.300132205	0.471435398	0.637329843	0.761904831	positive
PRJNA729271	:ed_Lushi_vs_Arbor_Acr	breed_growth_background	ENSGALG00010002787	CEP44	0.051499567	0.071521065	0.720061529	0.471487113	0.625728411	positive
PRJNA729271	:ed_Lushi_vs_Arbor_Acr	breed_growth_background	ENSGALG00010002788	ENSGALG00010002788	-0.307596696	0.333238414	-0.923052933	0.355979621	0.519666017	negative
PRJNA729271	:ed_Lushi_vs_Arbor_Acr	breed_growth_background	ENSGALG00010002790	BCLAF3	0.142768658	0.054519398	2.618676326	0.008827166	0.038171172	positive
PRJNA729271	:ed_Lushi_vs_Arbor_Acr	breed_growth_background	ENSGALG00010002792	ENSGALG00010002792	-0.437916852	0.123896271	-3.534544255	0.000408479	0.004307821	negative
PRJNA729271	:ed_Lushi_vs_Arbor_Acr	breed_growth_background	ENSGALG00010002796	PRDM8	-0.669976068	0.368433616	-1.818444459	0.068996228	0.166238382	negative
PRJNA729271	:ed_Lushi_vs_Arbor_Acr	breed_growth_background	ENSGALG00010002800	TUBGCP5	-0.120639798	0.040914032	-2.948616699	0.003191996	0.018537701	negative
PRJNA729271	:ed_Lushi_vs_Arbor_Acr	breed_growth_background	ENSGALG00010002801	FGF5	-0.488296832	0.237342903	-2.057347511	0.03965281	0.111793682	negative
PRJNA729271	:ed_Lushi_vs_Arbor_Acr	breed_growth_background	ENSGALG00010002805	FAM171A1	-0.088725397	0.053954624	-1.64444473	0.100084372	0.215176946	negative
PRJNA729271	:ed_Lushi_vs_Arbor_Acr	breed_growth_background	ENSGALG00010002807	P2RY6	0.011032556	0.114651879	0.096226561	0.923340636	0.957149576	positive
PRJNA729271	:ed_Lushi_vs_Arbor_Acr	breed_growth_background	ENSGALG00010002809	SH3KBP1	0.012866335	0.059314493	0.216917222	0.828272856	0.895288222	positive
PRJNA729271	:ed_Lushi_vs_Arbor_Acr	breed_growth_background	ENSGALG00010002810	TRIM27.1	-0.447338126	0.167203675	-2.675408464	0.007463825	0.033882915	negative
PRJNA729271	:ed_Lushi_vs_Arbor_Acr	breed_growth_background	ENSGALG00010002812	TRIM13	-0.102477549	0.056020468	-1.829287622	0.067356532	0.163786769	negative
PRJNA729271	:ed_Lushi_vs_Arbor_Acr	breed_growth_background	ENSGALG00010002814	TERF1	-0.055635847	0.084412426	-0.659095466	0.509834467	0.658566838	negative
PRJNA729271	:ed_Lushi_vs_Arbor_Acr	breed_growth_background	ENSGALG00010002817	SHFM1	0.11316595	0.049143445	2.302767941	0.021291903	0.071380652	positive
PRJNA729271	:ed_Lushi_vs_Arbor_Acr	breed_growth_background	ENSGALG00010002819	BMP3	-0.210316469	0.188478922	-1.115862008	0.264481206	0.42267421	negative
PRJNA729271	:ed_Lushi_vs_Arbor_Acr	breed_growth_background	ENSGALG00010002824	NXPH1	-0.510378858	0.324465097	-1.572985396	0.1157222	0.238367669	negative
PRJNA729271	:ed_Lushi_vs_Arbor_Acr	breed_growth_background	ENSGALG00010002825	TRIM41	0.120013406	0.064971104	1.847181271	0.064720873	0.158927103	positive
PRJNA729271	:ed_Lushi_vs_Arbor_Acr	breed_growth_background	ENSGALG00010002827	SBSPON	0.10796246	0.188839671	0.571714935	0.567515115	0.706590928	positive
PRJNA729271	:ed_Lushi_vs_Arbor_Acr	breed_growth_background	ENSGALG00010002832	LPIN1	0.039837905	0.108618211	0.366770039	0.713790558	0.81615478	positive
PRJNA729271	:ed_Lushi_vs_Arbor_Acr	breed_growth_background	ENSGALG00010002834	MRPL53	0.031558854	0.061108042	0.516443545	0.605544659	0.73704479	positive
PRJNA729271	:ed_Lushi_vs_Arbor_Acr	breed_growth_background	ENSGALG00010002835	CYFIP1	-0.188403992	0.045404668	-4.149440991	3.33288E-05	0.000718901	negative
PRJNA729271	:ed_Lushi_vs_Arbor_Acr	breed_growth_background	ENSGALG00010002837	DNMT1	0.145003855	0.069961015	2.072637941	0.038205987	0.108907726	positive
PRJNA729271	:ed_Lushi_vs_Arbor_Acr	breed_growth_background	ENSGALG00010002838	ENSGALG00010002838	0.508373528	0.144565421	3.516563803	0.000437172	0.004506972	positive
PRJNA729271	:ed_Lushi_vs_Arbor_Acr	breed_growth_background	ENSGALG00010002840	SRPX	-0.426519806	0.090825027	-4.696060297	2.65227E-06	0.000103346	negative
PRJNA729271	:ed_Lushi_vs_Arbor_Acr	breed_growth_background	ENSGALG00010002848	TRIB2	0.125976744	0.057850442	2.177628033	0.029433741	0.089668363	positive
PRJNA729271	:ed_Lushi_vs_Arbor_Acr	breed_growth_background	ENSGALG00010002853	CEP162	0.455636904	0.090738217	5.021444299	5.12844E-07	2.84815E-05	positive
PRJNA729271	:ed_Lushi_vs_Arbor_Acr	breed_growth_background	ENSGALG00010002855	RACK1	-0.139412502	0.0669742	-2.081585186	0.037380376	0.107229081	negative
PRJNA729271	:ed_Lushi_vs_Arbor_Acr	breed_growth_background	ENSGALG00010002857	DCAF15	0.10537339	0.080968151	1.301417763	0.193115497	0.340122615	positive
PRJNA729271	:ed_Lushi_vs_Arbor_Acr	breed_growth_background	ENSGALG00010002860	NIPA2	-0.136649509	0.058485901	-2.336452151	0.019467689	0.06707491	negative
PRJNA729271	:ed_Lushi_vs_Arbor_Acr	breed_growth_background	ENSGALG00010002864	RNASEH2B	-0.370594242	0.050959244	-7.272365314	3.53247E-13	2.22622E-10	negative
PRJNA729271	:ed_Lushi_vs_Arbor_Acr	breed_growth_background	ENSGALG00010002866	ENSGALG00010002866	-0.202885782	0.134201978	-1.511794272	0.130586207	0.258661487	negative
PRJNA729271	:ed_Lushi_vs_Arbor_Acr	breed_growth_background	ENSGALG00010002868	PPP1R9A	-0.065037952	0.035882958	-1.812502524	0.069908584	0.167787717	negative
PRJNA729271	:ed_Lushi_vs_Arbor_Acr	breed_growth_background	ENSGALG00010002869	ENSGALG00010002869	-0.132777467	0.168451815	-0.788222243	0.430566721	0.588779681	negative
PRJNA729271	:ed_Lushi_vs_Arbor_Acr	breed_growth_background	ENSGALG00010002870	FABP5	-0.252329787	0.115235547	-2.18968707	0.028546939	0.087677394	negative
PRJNA729271	:ed_Lushi_vs_Arbor_Acr	breed_growth_background	ENSGALG00010002878	P2RY2	-0.057386975	0.128702249	-0.445889451	0.655677102	0.775566395	negative
PRJNA729271	:ed_Lushi_vs_Arbor_Acr	breed_growth_background	ENSGALG00010002880	RASGEF1B	-0.20726693	0.11190808	-1.852117655	0.06400893	0.157817561	negative
PRJNA729271	:ed_Lushi_vs_Arbor_Acr	breed_growth_background	ENSGALG00010002881	ENSGALG00010002881	-0.462212793	0.153344086	-3.014219892	0.00257641	0.015918611	negative
PRJNA729271	:ed_Lushi_vs_Arbor_Acr	breed_growth_background	ENSGALG00010002883	MAP3K15	1.426476588	0.21755217	6.556940298	5.4923E-11	1.69385E-08	positive
PRJNA729271	:ed_Lushi_vs_Arbor_Acr	breed_growth_background	ENSGALG00010002884	ADHFE1	0.189772629	0.126965886	1.494674157	0.134999502	0.264966886	positive
PRJNA729271	:ed_Lushi_vs_Arbor_Acr	breed_growth_background	ENSGALG00010002886	HERC2	0.171845179	0.056583662	3.037010556	0.002389371	0.01511079	positive
PRJNA729271	:ed_Lushi_vs_Arbor_Acr	breed_growth_background	ENSGALG00010002888	ENSGALG00010002888	0.6404926	0.224757961	2.849699281	0.004376058	0.023065805	positive
PRJNA729271	:ed_Lushi_vs_Arbor_Acr	breed_growth_background	ENSGALG00010002890	VXN	0.007216468	0.088361919	0.081669438	0.934909582	0.964451953	positive
PRJNA729271	:ed_Lushi_vs_Arbor_Acr	breed_growth_background	ENSGALG00010002894	FCHSD2	0.010650585	0.039457917	0.269922646	0.787219765	0.867849953	positive
PRJNA729271	:ed_Lushi_vs_Arbor_Acr	breed_growth_background	ENSGALG00010002896	OCA2	0.361234781	0.153227968	2.357498998	0.018398505	0.064459034	positive
PRJNA729271	:ed_Lushi_vs_Arbor_Acr	breed_growth_background	ENSGALG00010002904	PON1	0.274781116	0.095284323	2.883801942	0.003929058	0.021394964	positive
PRJNA729271	:ed_Lushi_vs_Arbor_Acr	breed_growth_background	ENSGALG00010002907	GABRG3	-0.414714257	0.204375829	-2.02917468	0.042440502	0.117646791	negative
PRJNA729271	:ed_Lushi_vs_Arbor_Acr	breed_growth_background	ENSGALG00010002908	S1PR2	-0.076505456	0.098970467	-0.773012988	0.439514694	0.596904852	negative
PRJNA729271	:ed_Lushi_vs_Arbor_Acr	breed_growth_background	ENSGALG00010002911	MYBL1	-0.172005395	0.126013793	-1.364972769	0.172261609	0.315428502	negative
PRJNA729271	:ed_Lushi_vs_Arbor_Acr	breed_growth_background	ENSGALG00010002912	RPS3	-0.205034654	0.102789887	-1.994696746	0.046075965	0.124923875	negative
PRJNA729271	:ed_Lushi_vs_Arbor_Acr	breed_growth_background	ENSGALG00010002913	NXNL1	0.867575917	0.304275807	2.851281299	0.004354343	0.022976411	positive
PRJNA729271	:ed_Lushi_vs_Arbor_Acr	breed_growth_background	ENSGALG00010002914	SLC27A1	0.566968955	0.162995011	3.478443622	0.000504335	0.004994066	positive
PRJNA729271	:ed_Lushi_vs_Arbor_Acr	breed_growth_background	ENSGALG00010002916	SDCBP	-0.089040985	0.065855429	-1.352067496	0.176353724	0.320278054	negative
PRJNA729271	:ed_Lushi_vs_Arbor_Acr	breed_growth_background	ENSGALG00010002917	PGLS	-0.009602962	0.051740895	-0.18559714	0.852760697	0.911100026	negative
PRJNA729271	:ed_Lushi_vs_Arbor_Acr	breed_growth_background	ENSGALG00010002918	LRATD1	0.054609355	0.223128282	0.244744209	0.806654495	0.881053191	positive
PRJNA729271	:ed_Lushi_vs_Arbor_Acr	breed_growth_background	ENSGALG00010002919	ENSGALG00010002919	0.316917967	0.108696482	2.91562303	0.003549791	0.019912625	positive
PRJNA729271	:ed_Lushi_vs_Arbor_Acr	breed_growth_background	ENSGALG00010002920	VCPIP1	0.125941864	0.054729203	2.301182138	0.021381338	0.071542589	positive
PRJNA729271	:ed_Lushi_vs_Arbor_Acr	breed_growth_background	ENSGALG00010002922	TMEM106B	0.04080221	0.049223244	0.828921598	0.407148773	0.567791174	positive
PRJNA729271	:ed_Lushi_vs_Arbor_Acr	breed_growth_background	ENSGALG00010002923	SERPINH1	-0.015104035	0.084759555	-0.178198608	0.858567003	0.915021992	negative
PRJNA729271	:ed_Lushi_vs_Arbor_Acr	breed_growth_background	ENSGALG00010002925	INTS6	-0.039773155	0.065680838	-0.605551885	0.544812361	0.688856872	negative
PRJNA729271	:ed_Lushi_vs_Arbor_Acr	breed_growth_background	ENSGALG00010002926	GALNTL6	-0.607106272	0.200755258	-3.024111441	0.002493646	0.015553095	negative
PRJNA729271	:ed_Lushi_vs_Arbor_Acr	breed_growth_background	ENSGALG00010002927	GABRA5	0.055164001	0.393220187	0.140287815	0.888432591	0.934462696	positive
PRJNA729271	:ed_Lushi_vs_Arbor_Acr	breed_growth_background	ENSGALG00010002928	ASB4	0.086009854	0.162187013	0.53031283	0.595895053	0.729641729	positive
PRJNA729271	:ed_Lushi_vs_Arbor_Acr	breed_growth_background	ENSGALG00010002929	PDHA2	0.060755333	0.060455798	1.004954613	0.314918705	0.47813414	positive
PRJNA729271	:ed_Lushi_vs_Arbor_Acr	breed_growth_background	ENSGALG00010002930	SERAF	0.915476038	0.154815498	5.913335888	3.35248E-09	5.71696E-07	positive
PRJNA729271	:ed_Lushi_vs_Arbor_Acr	breed_growth_background	ENSGALG00010002934	VWDE	-0.367969303	0.453164105	-0.81200011	0.416791568	0.576909261	negative
PRJNA729271	:ed_Lushi_vs_Arbor_Acr	breed_growth_background	ENSGALG00010002935	SYTL5	0.484070714	0.395969251	1.222495716	0.221520223	0.374016963	positive
PRJNA729271	:ed_Lushi_vs_Arbor_Acr	breed_growth_background	ENSGALG00010002937	GALNT7	-0.144516592	0.058422501	-2.473646124	0.01337421	0.051299066	negative

PRJNA729271	ed_Lushi_vs_Arbor_Acr	breed_growth_background	ENSGALG00010002939	HMGB2	-0.149151939	0.092395995	-1.614268445	0.106469212	0.225107032	negative
PRJNA729271	ed_Lushi_vs_Arbor_Acr	breed_growth_background	ENSGALG00010002940	BG1	-0.683083931	0.224612527	-3.041165778	0.00235664	0.015001298	negative
PRJNA729271	ed_Lushi_vs_Arbor_Acr	breed_growth_background	ENSGALG00010002942	GABRB3	-0.427130441	0.331715255	-1.287641838	0.197870668	0.346267698	negative
PRJNA729271	ed_Lushi_vs_Arbor_Acr	breed_growth_background	ENSGALG00010002943	SAP30	0.125807333	0.080807655	1.556873948	0.119500416	0.243657129	positive
PRJNA729271	ed_Lushi_vs_Arbor_Acr	breed_growth_background	ENSGALG00010002946	GDPD5	-0.001317312	0.063713959	-0.020675401	0.983504592	0.990199282	negative
PRJNA729271	ed_Lushi_vs_Arbor_Acr	breed_growth_background	ENSGALG00010002947	ENSGALG00010002947	1.211718067	0.394800171	3.069193367	0.002146376	0.013951884	positive
PRJNA729271	ed_Lushi_vs_Arbor_Acr	breed_growth_background	ENSGALG00010002948	DLX6	0.732303454	0.90797891	0.806520334	0.419942889	0.579310552	positive
PRJNA729271	ed_Lushi_vs_Arbor_Acr	breed_growth_background	ENSGALG00010002949	ENSGALG00010002949	4.877867169	0.632374853	7.713569165	1.22347E-14	1.10839E-11	positive
PRJNA729271	ed_Lushi_vs_Arbor_Acr	breed_growth_background	ENSGALG00010002951	ENSGALG00010002951	-0.41409283	2.316228874	-0.178778891	0.858111321	0.914805541	negative
PRJNA729271	ed_Lushi_vs_Arbor_Acr	breed_growth_background	ENSGALG00010002952	NBAS	0.051390493	0.034742698	1.479173937	0.139093833	0.270687701	positive
PRJNA729271	ed_Lushi_vs_Arbor_Acr	breed_growth_background	ENSGALG00010002953	WDFY2	-0.235695205	0.072056283	-3.270987559	0.001071726	0.008597454	negative
PRJNA729271	ed_Lushi_vs_Arbor_Acr	breed_growth_background	ENSGALG00010002954	CHST7	-0.027565383	0.066612886	-0.413814574	0.67900991	0.792623996	negative
PRJNA729271	ed_Lushi_vs_Arbor_Acr	breed_growth_background	ENSGALG00010002955	BLEC1	0.32962667	0.261632215	1.259885634	0.207710622	0.356937221	positive
PRJNA729271	ed_Lushi_vs_Arbor_Acr	breed_growth_background	ENSGALG00010002956	SCRG1	0.430249458	0.203280155	2.116534487	0.034299375	0.100417985	positive
PRJNA729271	ed_Lushi_vs_Arbor_Acr	breed_growth_background	ENSGALG00010002957	ADGRG2	-0.746448599	0.158207716	-4.718155466	2.37993E-06	9.58251E-05	negative
PRJNA729271	ed_Lushi_vs_Arbor_Acr	breed_growth_background	ENSGALG00010002958	ENSGALG00010002958	0.195337888	0.096541277	2.023361343	0.043035907	0.118729628	positive
PRJNA729271	ed_Lushi_vs_Arbor_Acr	breed_growth_background	ENSGALG00010002959	DLX5	0.662821385	0.408483798	1.622638129	0.104666785	0.222097064	positive
PRJNA729271	ed_Lushi_vs_Arbor_Acr	breed_growth_background	ENSGALG00010002960	BLEC2	-1.176035168	0.396496015	-2.966070584	0.003016313	0.017787409	negative
PRJNA729271	ed_Lushi_vs_Arbor_Acr	breed_growth_background	ENSGALG00010002965	PRSS35	-0.380572937	0.113111996	-3.364567423	0.000766638	0.006743395	negative
PRJNA729271	ed_Lushi_vs_Arbor_Acr	breed_growth_background	ENSGALG00010002967	OLFM4	0.622164149	0.482799724	1.288658875	0.197516706	0.346066076	positive
PRJNA729271	ed_Lushi_vs_Arbor_Acr	breed_growth_background	ENSGALG00010002972	ME1	0.008175465	0.08169461	0.100073488	0.920285983	0.955805912	positive
PRJNA729271	ed_Lushi_vs_Arbor_Acr	breed_growth_background	ENSGALG00010002978	DYNLT3	-0.013251215	0.054391945	-0.243624587	0.807521585	0.881734491	negative
PRJNA729271	ed_Lushi_vs_Arbor_Acr	breed_growth_background	ENSGALG00010002981	KLHL35	-0.480050535	0.173021335	-2.774516415	0.005528384	0.027358802	negative
PRJNA729271	ed_Lushi_vs_Arbor_Acr	breed_growth_background	ENSGALG00010002982	HAND2	0.003908191	0.168760552	0.023158205	0.981524077	0.989068549	positive
PRJNA729271	ed_Lushi_vs_Arbor_Acr	breed_growth_background	ENSGALG00010002983	NSMAF	0.082911816	0.059677031	1.38934217	0.164728726	0.304714509	positive
PRJNA729271	ed_Lushi_vs_Arbor_Acr	breed_growth_background	ENSGALG00010002985	ENSGALG00010002985	-0.171416039	0.119367539	-1.436035632	0.150992202	0.286807999	negative
PRJNA729271	ed_Lushi_vs_Arbor_Acr	breed_growth_background	ENSGALG00010002987	PCDH8	0.566352025	0.211737054	2.674789385	0.007477621	0.033934914	positive
PRJNA729271	ed_Lushi_vs_Arbor_Acr	breed_growth_background	ENSGALG00010002991	BLB1	0.424833044	0.164584389	2.581247512	0.009844397	0.041360736	positive
PRJNA729271	ed_Lushi_vs_Arbor_Acr	breed_growth_background	ENSGALG00010002992	ENSGALG00010002992	0.501823182	0.134778719	3.723311712	0.000196627	0.002563294	positive
PRJNA729271	ed_Lushi_vs_Arbor_Acr	breed_growth_background	ENSGALG00010002994	DUSP4	-0.111170419	0.095528799	-1.163737211	0.244530525	0.40041459	negative
PRJNA729271	ed_Lushi_vs_Arbor_Acr	breed_growth_background	ENSGALG00010002995	RWDD2A	0.023576625	0.0704892	0.334471449	0.738023832	0.832113833	positive
PRJNA729271	ed_Lushi_vs_Arbor_Acr	breed_growth_background	ENSGALG00010002998	LECT1	0.266627678	0.157147611	1.696670258	0.089759019	0.199901772	positive
PRJNA729271	ed_Lushi_vs_Arbor_Acr	breed_growth_background	ENSGALG00010002999	CMTM7	0.017428115	0.115643696	0.150705276	0.880208211	0.928845226	positive
PRJNA729271	ed_Lushi_vs_Arbor_Acr	breed_growth_background	ENSGALG00010003001	MAP6	0.517356547	0.177770439	2.910250708	0.003611389	0.020102569	positive
PRJNA729271	ed_Lushi_vs_Arbor_Acr	breed_growth_background	ENSGALG00010003002	PHKA2	-0.347937879	0.101341841	-3.433309225	0.000596262	0.005590435	negative
PRJNA729271	ed_Lushi_vs_Arbor_Acr	breed_growth_background	ENSGALG00010003006	CYP7B1	-0.47108314	0.107718887	-4.373264078	1.22403E-05	0.000335701	negative
PRJNA729271	ed_Lushi_vs_Arbor_Acr	breed_growth_background	ENSGALG00010003009	TNKS	-0.044978433	0.031214723	-1.440936478	0.149602629	0.284728219	negative
PRJNA729271	ed_Lushi_vs_Arbor_Acr	breed_growth_background	ENSGALG00010003017	PGM3	-0.010597136	0.084156469	-0.125921825	0.899793806	0.941833566	negative
PRJNA729271	ed_Lushi_vs_Arbor_Acr	breed_growth_background	ENSGALG00010003018	MOGAT2	-0.356487812	0.533864916	-0.667749089	0.504293769	0.654597749	negative
PRJNA729271	ed_Lushi_vs_Arbor_Acr	breed_growth_background	ENSGALG00010003019	TAPBP	0.069921966	0.13466789	0.519217804	0.603608865	0.73560707	positive
PRJNA729271	ed_Lushi_vs_Arbor_Acr	breed_growth_background	ENSGALG00010003022	BLB2	0.437038891	0.141333849	3.092245029	0.001986488	0.013190171	positive
PRJNA729271	ed_Lushi_vs_Arbor_Acr	breed_growth_background	ENSGALG00010003024	SUGT1	-0.038836659	0.038858198	-0.999445702	0.31757883	0.480963864	negative
PRJNA729271	ed_Lushi_vs_Arbor_Acr	breed_growth_background	ENSGALG00010003025	MFHAS1	0.120741147	0.065621695	1.839957759	0.065774439	0.161020181	positive
PRJNA729271	ed_Lushi_vs_Arbor_Acr	breed_growth_background	ENSGALG00010003027	SCIN	-0.177516963	0.137359357	-1.292354357	0.196234456	0.344527427	negative
PRJNA729271	ed_Lushi_vs_Arbor_Acr	breed_growth_background	ENSGALG00010003030	PPEF1	-0.28503578	0.393385639	-0.724575458	0.46871252	0.623530468	negative
PRJNA729271	ed_Lushi_vs_Arbor_Acr	breed_growth_background	ENSGALG00010003034	ERI1	0.023977145	0.034738681	0.690214619	0.490059231	0.641854934	positive
PRJNA729271	ed_Lushi_vs_Arbor_Acr	breed_growth_background	ENSGALG00010003035	DGAT2	0.959298786	0.242762448	3.951594633	7.76322E-05	0.001317388	positive
PRJNA729271	ed_Lushi_vs_Arbor_Acr	breed_growth_background	ENSGALG00010003042	TOX	0.37391211	0.172613914	2.166175953	0.030297744	0.091588279	positive
PRJNA729271	ed_Lushi_vs_Arbor_Acr	breed_growth_background	ENSGALG00010003052	ENSGALG00010003052	0.211995535	0.050286326	4.21576903	2.48928E-05	0.000564666	positive
PRJNA729271	ed_Lushi_vs_Arbor_Acr	breed_growth_background	ENSGALG00010003057	DDX1	0.060569066	0.048158122	1.257712359	0.208495801	0.357989414	positive
PRJNA729271	ed_Lushi_vs_Arbor_Acr	breed_growth_background	ENSGALG00010003061	ENSGALG00010003061	0.576670156	0.179319876	3.215874171	0.001300478	0.009843569	positive
PRJNA729271	ed_Lushi_vs_Arbor_Acr	breed_growth_background	ENSGALG00010003063	GGH	-0.164092103	0.047515927	-3.453412653	0.000553542	0.005331285	negative
PRJNA729271	ed_Lushi_vs_Arbor_Acr	breed_growth_background	ENSGALG00010003064	VAV1	-0.013252874	0.132627304	-0.099925686	0.920403324	0.955805912	negative
PRJNA729271	ed_Lushi_vs_Arbor_Acr	breed_growth_background	ENSGALG00010003068	BRD2	0.120683076	0.067639294	1.784215482	0.074388658	0.175266544	positive
PRJNA729271	ed_Lushi_vs_Arbor_Acr	breed_growth_background	ENSGALG00010003072	ELF1	-0.070979231	0.039829529	-1.782075577	0.074736908	0.175862253	negative
PRJNA729271	ed_Lushi_vs_Arbor_Acr	breed_growth_background	ENSGALG00010003074	TTPA	-1.07725602	0.184392981	-5.842174776	5.15237E-09	7.77954E-07	negative
PRJNA729271	ed_Lushi_vs_Arbor_Acr	breed_growth_background	ENSGALG00010003077	EIF2S3	-0.048019154	0.0437396	-1.097841632	0.272273649	0.431087552	negative
PRJNA729271	ed_Lushi_vs_Arbor_Acr	breed_growth_background	ENSGALG00010003081	DOP1A	0.01752176	0.055921445	0.313328093	0.754031409	0.844512847	positive
PRJNA729271	ed_Lushi_vs_Arbor_Acr	breed_growth_background	ENSGALG00010003087	TWISTNB	0.179574136	0.096853769	1.854074841	0.063728454	0.15731334	positive
PRJNA729271	ed_Lushi_vs_Arbor_Acr	breed_growth_background	ENSGALG00010003090	DMA	-0.175606082	0.104291339	-1.68380312	0.092219724	0.203211116	negative
PRJNA729271	ed_Lushi_vs_Arbor_Acr	breed_growth_background	ENSGALG00010003092	ART1F	0.324560405	0.728950949	0.445243133	0.656144059	0.775885799	positive
PRJNA729271	ed_Lushi_vs_Arbor_Acr	breed_growth_background	ENSGALG00010003097	TFPI2	-0.091502286	0.072524481	-1.261674478	0.207065944	0.356226329	negative
PRJNA729271	ed_Lushi_vs_Arbor_Acr	breed_growth_background	ENSGALG00010003101	KLHL15	0.087308767	0.03946197	2.212478691	0.026933607	0.084163309	positive
PRJNA729271	ed_Lushi_vs_Arbor_Acr	breed_growth_background	ENSGALG00010003103	YTHDF3	-0.09558908	0.04613873	-2.071793229	0.038284728	0.109110723	negative
PRJNA729271	ed_Lushi_vs_Arbor_Acr	breed_growth_background	ENSGALG00010003107	GNG11	-0.033886065	0.096849493	-0.349883765	0.726425932	0.824527408	negative
PRJNA729271	ed_Lushi_vs_Arbor_Acr	breed_growth_background	ENSGALG00010003108	PPP1R3B	0.109025274	0.098074373	1.111659149	0.26628473	0.424526745	positive
PRJNA729271	ed_Lushi_vs_Arbor_Acr	breed_growth_background	ENSGALG00010003109	ART5	0.390507117	0.261132783	1.495435051	0.134800939	0.264653881	positive
PRJNA729271	ed_Lushi_vs_Arbor_Acr	breed_growth_background	ENSGALG00010003110	BF1	0.607624921	0.136073608	4.465413478	7.99143E-06	0.000244179	positive
PRJNA729271	ed_Lushi_vs_Arbor_Acr	breed_growth_background	ENSGALG00010003113	WBP4	-0.026576607	0.063281604	-0.419973662	0.674504694	0.789585995	negative
PRJNA729271	ed_Lushi_vs_Arbor_Acr	breed_growth_background	ENSGALG00010003115	UBE3D	-0.129838149	0.085405347	-1.520257842	0.128446185	0.255733531	negative
PRJNA729271	ed_Lushi_vs_Arbor_Acr	breed_growth_background	ENSGALG00010003117	ID2	-0.224943452	0.068463759	-3.285584321	0.001017711	0.008255018	negative
PRJNA729271	ed_Lushi_vs_Arbor_Acr	breed_growth_background	ENSGALG00010003121	ARMC1	0.067781562	0.044437406	1.525326714	0.127177628	0.254092311	positive
PRJNA729271	ed_Lushi_vs_Arbor_Acr	breed_growth_background	ENSGALG00010003125	APOO	0.007439066	0.057226126	0.129994233	0.896570989	0.940021446	positive
PRJNA729271	ed_Lushi_vs_Arbor_Acr	breed_growth_background	ENSGALG00010003126	ENSGALG00010003126	-0.361391259	0.280158949	-1.289950795	0.197067743	0.345530051	negative
PRJNA729271	ed_Lushi_vs_Arbor_Acr	breed_growth_background	ENSGALG00010003131	TAP1	0.547844429	0.186077491	2.944173567	0.003238183	0.018744996	positive
PRJNA729271	ed_Lushi_vs_Arbor_Acr	breed_growth_background	ENSGALG00010003132	TPBG	0.312501726	0.083507474	3.742200686	0.000182416	0.002430254	positive
PRJNA729271	ed_Lushi_vs_Arbor_Acr	breed_growth_background	ENSGALG00010003133	SAT1	0.049482299	0.089314245	0.554024714	0.579561932	0.716481894	positive
PRJNA729271	ed_Lushi_vs_Arbor_Acr	breed_growth_background	ENSGALG00010003136	NUP98	0.128712273	0.056297129	2.286302606	0.022236562	0.073723458	positive
PRJNA729271	ed_Lushi_vs_Arbor_Acr	breed_growth_background	ENSGALG00010003152	ENSGALG00010003152	-0.051646363	0.067529944	-0.764792033	0.444395393	0.601167636	negative
PRJNA729271	ed_Lushi_vs_Arbor_Acr	breed_growth_background	ENSGALG00010003154	MTFR1	-0.079440892	0.044425814	-1.788169664	0.073748639	0.174271388	negative
PRJNA729271	ed_Lushi_vs_Arbor_Acr	breed_growth_background	ENSGALG00010003159	TAP2	0.51600261	0.14067974	3.667924112	0.000244528	0.002993606	positive
PRJNA729271	ed_Lushi_vs_Arbor_Acr	breed_growth_background	ENSGALG00010003160	KIDINS220	0.259184009	0.08103496	3.198422117	0.001381819	0.010313831	positive
PRJNA729271	ed_Lushi_vs_Arbor_Acr	breed_growth_background	ENSGALG00010003164	C3	-0.41636735	0.244091195	-1.705786029	0.088047925	0.197013689	negative
PRJNA729271	ed_Lushi_vs_Arbor_Acr	breed_growth_background	ENSGALG00010003165	BMP2K	-0.012486682	0.058693019	-0.212745601	0.831525396	0.897599093	negative
PRJNA729271	ed_Lushi_vs_Arbor_Acr	breed_growth_background	ENSGALG00010003166	PGAP2	0.216408308	0.059753895	3.621660284	0.000292718	0.003417045	positive
PRJNA729271	ed_Lushi_vs_Arbor_Acr	breed_growth_background	ENSGALG00010003169	MTRF1	-0.008266093	0.058266243	-0.141867616	0.887184574	0.93356713	negative
PRJNA729271	ed_Lushi_vs_Arbor_Acr	breed_growth_background	ENSGALG00010003170	C4	0.62391984	0.21781124	2.864497899	0.004176709	0.022282444	positive
PRJNA729271	ed_Lushi_vs_Arbor_Acr	breed_growth_background	ENSGALG00010003171	PAQR3	0.030932116	0.059459274	0.520223569	0.60290776	0.735140042	positive
PRJNA729271	ed_Lushi_vs_Arbor_Acr	breed_growth_background	ENSGALG00010003176	PRAG1	-0.45555909	0.14100841	-3.230722828	0.001234776	0.009520256	negative
PRJNA729271	ed_Lushi_vs_Arbor_Acr	breed_growth_background	ENSGALG00010003178	CENPA	-0.048005535	0.129877954	-0.369620355	0.711665383	0.814495833	negative
PRJNA729271	ed_Lushi_vs_Arbor_Acr	breed_growth_background	ENSGALG00010003180	IBTK	-0.056386309	0.067916927	-0.830224685	0.406411761	0.567309175	negative
PRJNA729271	ed_Lushi_vs_Arbor_Acr	breed_growth_background	ENSGALG00010003183	NAA16	0.112902197	0.046878401	2.408405444	0.016022376	0.058376754	positive
PRJNA729271	ed_Lushi_vs_Arbor_Acr	breed_growth_background	ENSGALG00010003184	BET1	0.054834705	0.043970236	1.2470869	0.212365636	0.362529725	positive
PRJNA729271	ed_Lushi_vs_Arbor_Acr	breed_growth_background	ENSGALG00010003186	CYP21A1	0.18260021	0.141610296	1.289455746	0.197239692	0.345706086	positive
PRJNA729271	ed_Lushi_vs_Arbor_Acr	breed_growth_background	ENSGALG00010003187	RHOG	-0.033810414	0.134204145	-0.251932712	0.801093075	0.877160003	negative
PRJNA729271	ed_Lushi_vs_Arbor_Acr	breed_growth_background	ENSGALG00010003189	ANTXR2	-0.111801692	0.067753036	-1.650135522	0.098915221	0.213264335	negative
PRJNA729271	ed_Lushi_vs_Arbor_Acr	breed_growth_background	ENSGALG00010003191	CC2D1A	0.251724277	0.085480797	2.944805	0.003231583	0.018714259	positive
PRJNA729271	ed_Lushi_vs_Arbor_Acr	breed_growth_background	ENSGALG00010003192	RGCC	-0.279232788	0.164775754	-1.694623029	0.090146956	0.200424457	negative
PRJNA729271	ed_Lushi_vs_Arbor_Acr	breed_growth_background	ENSGALG00010003193	ACOT9	-0.123539224	0.073241674	-1.686734042	0.091654503	0.202396713	negative
PRJNA729271	ed_Lushi_vs_Arbor_Acr	breed_growth_background	ENSGALG00010003198	PRKG2	-0.310483752	0.186206705	-1.667414455	0.095432022	0.20804439	negative
PRJNA729271	ed_Lushi_vs_Arbor_Acr	breed_growth_background	ENSGALG00010003201	MBOAT2	-0.044248939	0.069515756	-0.636531072	0.524430335	0.671283796	negative
PRJNA729271	ed_Lushi_vs_Arbor_Acr	breed_growth_background	ENSGALG00010003202	FAM46A	-0.061729915	0.054966085	-1.123054604	0.261414304	0.419437718	negative
PRJNA729271	ed_Lushi_vs_Arbor_Acr	breed_growth_background	ENSGALG00010003204	PKD2	-0.202425529	0.076815853	-2.635205118	0.008408645	0.036789409	negative
PRJNA729271	ed_Lushi_vs_Arbor_Acr	breed_growth_background	ENSGALG00010003205	ENSGALG00010003205	0.105237168	0.139760217	0.752983721	0.451459696	0.607809976	positive
PRJNA729271	ed_Lushi_vs_Arbor_Acr	breed_growth_background	ENSGALG00010003206	ENSGALG00010003206	0.016234769	0.362912936	0.044734612	0.964318845	0.979609321	positive
PRJNA729271	ed_Lushi_vs_Arbor_Acr	breed_growth_background	ENSGALG00010003210	EYA1	0.056072612	0.107778365	0.520258515	0.602883406	0.735140042	positive
PRJNA729271	ed_Lushi_vs_Arbor_Acr	breed_growth_background	ENSGALG00010003212	LTB4R	-0.762750105	0.366391953	-2.081787271	0.037361905	0.10719731	negative
PRJNA729271	ed_Lushi_vs_Arbor_Acr	breed_growth_background	ENSGALG00010003214	PRDX4	0.037021075	0.051125707	0.72411859	0.468992933	0.62373177	positive
PRJNA729271	ed_Lushi_vs_Arbor_Acr	breed_growth_background	ENSGALG00010003218	MAP3K7	0.032772339	0.032606607	1.005082779	0.314856992	0.47813414	positive
PRJNA729271	ed_Lushi_vs_Arbor_Acr	breed_growth_background	ENSGALG00010003220	SPP1	0.882858748	0.513720856	1.71855734	0.085695005	0.193270437	positive
PRJNA729271	ed_Lushi_vs_Arbor_Acr	breed_growth_background	ENSGALG00010003222	ITGA8	-0.311959941	0.166303039	-1.875852321	0.060675561	0.152029777	negative
PRJNA729271	ed_Lushi_vs_Arbor_Acr	breed_growth_background	ENSGALG00010003223	AREG	-0.398964112	0.463104122	-0.861499806	0.388962828	0.550902931	negative
PRJNA729271	ed_Lushi_vs_Arbor_Acr	breed_growth_background	ENSGALG00010003229	STIM1	0.034077322	0.056437019	0.603811517	0.545968962	0.689777748	positive
PRJNA729271	ed_Lushi_vs_Arbor_Acr	breed_growth_background	ENSGALG00010003239	RRM1	-0.347606754	0.064223506	-5.412453683	6.21669E-08	5.68292E-06	negative
PRJNA729271	ed_Lushi_vs_Arbor_Acr	breed_growth_background	ENSGALG00010003240	PLACL2	0.915126768	0.462933502	1.976799614	0.048064285	0.128683379	positive
PRJNA729271	ed_Lushi_vs_Arbor_Acr	breed_growth_background	ENSGALG00010003242	ASAP2	-0.163146844	0.069748104	-2.339086448	0.01933096	0.066723435	negative
PRJNA729271	ed_Lushi_vs_Arbor_Acr	breed_growth_background	ENSGALG00010003243	VPS50	-0.03361705	0.050107776	-0.670894886	0.502287495	0.653325308	negative
PRJNA729271	ed_Lushi_vs_Arbor_Acr	breed_growth_background	ENSGALG00010003245	ENSGALG00010003245	-0.263761692	0.238777725	-1.104632738	0.26931876	0.427904792	negative
PRJNA729271	ed_Lushi_vs_Arbor_Acr	breed_growth_background	ENSGALG00010003246	VWA8	0.024938032	0.058224665	0.428307015	0.668427612	0.785095069	positive
PRJNA729271	ed_Lushi_vs_Arbor_Acr	breed_growth_background	ENSGALG00010003247	ENSGALG00010003247	-0.358252818	0.117396243	-3.05165488	0.002275836	0.014592066	negative
PRJNA729271	ed_Lushi_vs_Arbor_Acr	breed_growth_background	ENSGALG00010003248	MRI1	0.01171587	0.051731831	0.226473125	0.820833459	0.88996791	positive
PRJNA729271	ed_Lushi_vs_Arbor_Acr	breed_growth_background	ENSGALG00010003249	YJU2B	0.030653012	0.163489212	0.187492565	0.851274453	0.910572119	positive
PRJNA729271	ed_Lushi_vs_Arbor_Acr	breed_growth_background	ENSGALG00010003250	CD1G	0.891903137	0.40297765	2.213281895	0.026878217	0.084056042	positive
PRJNA729271	ed_Lushi_vs_Arbor_Acr	breed_growth_background	ENSGALG00010003251	ELOF1	-0.23293511	0.083533564	-2.788521167	0.005294929	0.026557091	negative
PRJNA729271	ed_Lushi_vs_Arbor_Acr	breed_growth_background	ENSGALG00010003252	PHEX	0.202934958	0.106676002	1.902348735	0.057125578	0.145678265	positive
PRJNA729271	ed_Lushi_vs_Arbor_Acr	breed_growth_background	ENSGALG00010003253	ITGB1BP1	0.025027136	0.062155868	0.402651218	0.687204825	0.797989263	positive
PRJNA729271	ed_Lushi_vs_Arbor_Acr	breed_growth_background	ENSGALG00010003254	DGKH	-0.063084628	0.090445142	-0.697490514	0.485495874	0.637343776	negative
PRJNA729271	ed_Lushi_vs_Arbor_Acr	breed_growth_background	ENSGALG00010003255	CALCR	0.223680444	0.183318098	1.220176549	0.222397955	0.375018422	positive
PRJNA729271	ed_Lushi_vs_Arbor_Acr	breed_growth_background	ENSGALG00010003256	ENSGALG00010003256	0.826011795	0.727030167	1.136145145	0.255895824	0.412960361	positive
PRJNA729271	ed_Lushi_vs_Arbor_Acr	breed_growth_background	ENSGALG00010003257	ENSGALG00010003257	-0.733563792	0.424024309	-1.730004098	0.083629543	0.189793366	negative
PRJNA729271	ed_Lushi_vs_Arbor_Acr	breed_growth_background	ENSGALG00010003259	VEGFC	0.16481998	0.075051355	2.196096	0.028085073	0.086726274	positive

PRJNA729271	:ed_Lushi_vs_Arbor_Acr	breed_growth_background	ENSGALG00010003260	ACP5	-0.207960014	0.144317748	-1.440987106	0.149588326	0.284728219	negative
PRJNA729271	:ed_Lushi_vs_Arbor_Acr	breed_growth_background	ENSGALG00010003261	PEX2	-0.275135021	0.093012165	-2.958054161	0.003095877	0.018160559	negative
PRJNA729271	:ed_Lushi_vs_Arbor_Acr	breed_growth_background	ENSGALG00010003262	ENSGALG00010003262	0.896609051	0.326534269	2.745834469	0.006035721	0.029158615	positive
PRJNA729271	:ed_Lushi_vs_Arbor_Acr	breed_growth_background	ENSGALG00010003263	ENSGALG00010003263	0.16281086	0.41265746	0.394542389	0.693180638	0.802015753	positive
PRJNA729271	:ed_Lushi_vs_Arbor_Acr	breed_growth_background	ENSGALG00010003264	ENSGALG00010003264	0.395229314	0.212349997	1.861216487	0.062713611	0.155576552	positive
PRJNA729271	:ed_Lushi_vs_Arbor_Acr	breed_growth_background	ENSGALG00010003268	SMS	0.098611351	0.034636133	2.847065867	0.004412423	0.023215272	positive
PRJNA729271	:ed_Lushi_vs_Arbor_Acr	breed_growth_background	ENSGALG00010003269	ENSGALG00010003269	-0.667374817	0.223200286	-2.990026714	0.002789531	0.016840585	negative
PRJNA729271	:ed_Lushi_vs_Arbor_Acr	breed_growth_background	ENSGALG00010003271	AKAP11	0.059500113	0.045102322	1.319225061	0.187093882	0.332792468	positive
PRJNA729271	:ed_Lushi_vs_Arbor_Acr	breed_growth_background	ENSGALG00010003272	CPSF3	-0.050785213	0.036322223	-1.398185686	0.162057314	0.301388346	negative
PRJNA729271	:ed_Lushi_vs_Arbor_Acr	breed_growth_background	ENSGALG00010003273	NFIX	-0.047092511	0.10588129	-0.444767071	0.656488093	0.776152258	negative
PRJNA729271	:ed_Lushi_vs_Arbor_Acr	breed_growth_background	ENSGALG00010003274	BACH2	0.074408193	0.079219356	0.939267834	0.347593249	0.511573575	positive
PRJNA729271	:ed_Lushi_vs_Arbor_Acr	breed_growth_background	ENSGALG00010003275	MBTPS2	-0.064343792	0.069361274	-0.927661619	0.353583124	0.517277693	negative
PRJNA729271	:ed_Lushi_vs_Arbor_Acr	breed_growth_background	ENSGALG00010003276	TNFSF11	-0.013841298	0.214972301	-0.064386425	0.948662539	0.971711451	negative
PRJNA729271	:ed_Lushi_vs_Arbor_Acr	breed_growth_background	ENSGALG00010003278	ENSGALG00010003278	-0.494716695	0.273124935	-1.81132014	0.07009131	0.168040611	negative
PRJNA729271	:ed_Lushi_vs_Arbor_Acr	breed_growth_background	ENSGALG00010003279	ENSGALG00010003279	0.010683676	0.192179846	0.055592071	0.955666781	0.975259256	positive
PRJNA729271	:ed_Lushi_vs_Arbor_Acr	breed_growth_background	ENSGALG00010003280	ENSGALG00010003280	-0.3212301	0.089310192	-3.596791037	0.000322167	0.003631269	negative
PRJNA729271	:ed_Lushi_vs_Arbor_Acr	breed_growth_background	ENSGALG00010003287	ENSGALG00010003287	-2.903795266	1.038714415	-2.795566544	0.005180883	0.026192366	negative
PRJNA729271	:ed_Lushi_vs_Arbor_Acr	breed_growth_background	ENSGALG00010003292	GADD45GIP1	0.064170561	0.06650009	0.964969541	0.334560068	0.498965756	positive
PRJNA729271	:ed_Lushi_vs_Arbor_Acr	breed_growth_background	ENSGALG00010003294	ENSGALG00010003294	0.100099524	2.376445175	0.042121537	0.966401811	0.980988414	positive
PRJNA729271	:ed_Lushi_vs_Arbor_Acr	breed_growth_background	ENSGALG00010003295	IAH1	-0.048592861	0.072291086	-0.672183308	0.501467006	0.65255088	negative
PRJNA729271	:ed_Lushi_vs_Arbor_Acr	breed_growth_background	ENSGALG00010003296	ENSGALG00010003296	0.28096379	0.285363565	0.984581859	0.324829504	0.488864643	positive
PRJNA729271	:ed_Lushi_vs_Arbor_Acr	breed_growth_background	ENSGALG00010003298	IL7	0.661184553	0.332948279	1.985847635	0.047050245	0.126730702	positive
PRJNA729271	:ed_Lushi_vs_Arbor_Acr	breed_growth_background	ENSGALG00010003299	RAD23A	0.121933801	0.064299533	1.896340376	0.057915051	0.146993286	positive
PRJNA729271	:ed_Lushi_vs_Arbor_Acr	breed_growth_background	ENSGALG00010003300	CASP8AP2	0.080286797	0.060032885	1.337380293	0.181098534	0.325604471	positive
PRJNA729271	:ed_Lushi_vs_Arbor_Acr	breed_growth_background	ENSGALG00010003303	MTHFD2L	-0.173448874	0.105600757	-1.642496497	0.100487152	0.215882802	negative
PRJNA729271	:ed_Lushi_vs_Arbor_Acr	breed_growth_background	ENSGALG00010003304	ENSGALG00010003304	0.14154881	0.377647571	0.374817214	0.707796411	0.811711832	positive
PRJNA729271	:ed_Lushi_vs_Arbor_Acr	breed_growth_background	ENSGALG00010003305	CALR	0.01707277	0.041902189	0.4074434	0.683682357	0.795785187	positive
PRJNA729271	:ed_Lushi_vs_Arbor_Acr	breed_growth_background	ENSGALG00010003307	COL1A2	-0.042450051	0.117800528	-0.360355352	0.718581411	0.819922757	negative
PRJNA729271	:ed_Lushi_vs_Arbor_Acr	breed_growth_background	ENSGALG00010003308	MDN1	0.119823767	0.053433816	2.242470714	0.024930966	0.079861735	positive
PRJNA729271	:ed_Lushi_vs_Arbor_Acr	breed_growth_background	ENSGALG00010003311	ENSGALG00010003311	-0.075876298	0.627738971	-0.120872371	0.903792126	0.94479063	negative
PRJNA729271	:ed_Lushi_vs_Arbor_Acr	breed_growth_background	ENSGALG00010003315	EPGN	-0.564382498	0.19576403	-2.88297343	0.003939407	0.021427056	negative
PRJNA729271	:ed_Lushi_vs_Arbor_Acr	breed_growth_background	ENSGALG00010003317	LYRM2	-0.153709223	0.095349311	-1.612064328	0.106947942	0.225714971	negative
PRJNA729271	:ed_Lushi_vs_Arbor_Acr	breed_growth_background	ENSGALG00010003320	OR51E2	0.014418839	0.252741369	0.057049777	0.954505543	0.974678256	positive
PRJNA729271	:ed_Lushi_vs_Arbor_Acr	breed_growth_background	ENSGALG00010003323	STMN2	0.193015021	0.274282338	0.703709258	0.481613853	0.63434737	positive
PRJNA729271	:ed_Lushi_vs_Arbor_Acr	breed_growth_background	ENSGALG00010003324	ENSGALG00010003324	-0.204375859	0.31380635	-0.651280191	0.514865633	0.663257852	negative
PRJNA729271	:ed_Lushi_vs_Arbor_Acr	breed_growth_background	ENSGALG00010003330	HEY1	-0.050350739	0.064460781	-0.781106554	0.434739828	0.592920004	negative
PRJNA729271	:ed_Lushi_vs_Arbor_Acr	breed_growth_background	ENSGALG00010003331	EPSTI1	0.237861911	0.330768299	0.719119432	0.472067335	0.626104411	positive
PRJNA729271	:ed_Lushi_vs_Arbor_Acr	breed_growth_background	ENSGALG00010003333	ENSGALG00010003333	-0.075867928	1.406061568	-0.053957757	0.95696882	0.975818716	negative
PRJNA729271	:ed_Lushi_vs_Arbor_Acr	breed_growth_background	ENSGALG00010003334	TAC1	-0.502370124	0.570660476	-0.880331028	0.378680008	0.540731625	negative
PRJNA729271	:ed_Lushi_vs_Arbor_Acr	breed_growth_background	ENSGALG00010003339	ENSGALG00010003339	-0.971858558	0.262379392	-3.704020169	0.000212209	0.002719695	negative
PRJNA729271	:ed_Lushi_vs_Arbor_Acr	breed_growth_background	ENSGALG00010003344	ZFAND1	0.125191475	0.102230766	1.224596853	0.220727154	0.373317642	positive
PRJNA729271	:ed_Lushi_vs_Arbor_Acr	breed_growth_background	ENSGALG00010003346	ENSGALG00010003346	-0.090156449	2.926320011	-0.030808814	0.975422011	0.985621614	negative
PRJNA729271	:ed_Lushi_vs_Arbor_Acr	breed_growth_background	ENSGALG00010003347	GPM6A	1.333367648	0.195072624	6.835237148	8.18695E-12	3.59605E-09	positive
PRJNA729271	:ed_Lushi_vs_Arbor_Acr	breed_growth_background	ENSGALG00010003348	ADAM17	0.001889738	0.045002742	0.041991614	0.966505383	0.980988414	positive
PRJNA729271	:ed_Lushi_vs_Arbor_Acr	breed_growth_background	ENSGALG00010003351	ENSGALG00010003351	2.08489098	0.874283659	2.384684831	0.017093759	0.06116899	positive
PRJNA729271	:ed_Lushi_vs_Arbor_Acr	breed_growth_background	ENSGALG00010003352	FARSA	0.040478383	0.054686459	0.740190235	0.459184572	0.614774289	positive
PRJNA729271	:ed_Lushi_vs_Arbor_Acr	breed_growth_background	ENSGALG00010003356	ENSGALG00010003356	-0.139975594	0.592198183	-0.236366132	0.813148575	0.885027629	negative
PRJNA729271	:ed_Lushi_vs_Arbor_Acr	breed_growth_background	ENSGALG00010003358	DNAJC15	-0.11117108	0.072223073	-1.53927374	0.123737482	0.249142214	negative
PRJNA729271	:ed_Lushi_vs_Arbor_Acr	breed_growth_background	ENSGALG00010003363	HBE1	-0.873238559	0.282566029	-3.090387625	0.001998954	0.013254731	negative
PRJNA729271	:ed_Lushi_vs_Arbor_Acr	breed_growth_background	ENSGALG00010003364	ANKRD6	0.034357538	0.090862604	0.378126269	0.705336795	0.809489853	positive
PRJNA729271	:ed_Lushi_vs_Arbor_Acr	breed_growth_background	ENSGALG00010003366	CASD1	-0.086944439	0.058257375	-1.49241943	0.135589224	0.265769548	negative
PRJNA729271	:ed_Lushi_vs_Arbor_Acr	breed_growth_background	ENSGALG00010003371	ENSGALG00010003371	-0.438120124	0.204204005	-2.145502109	0.031912723	0.095460462	negative
PRJNA729271	:ed_Lushi_vs_Arbor_Acr	breed_growth_background	ENSGALG00010003372	ENOX1	-0.259858216	0.077108456	-3.370035253	0.000751586	0.006655	negative
PRJNA729271	:ed_Lushi_vs_Arbor_Acr	breed_growth_background	ENSGALG00010003374	SYCE2	0.660204837	0.249285331	2.648390237	0.008087611	0.035850128	positive
PRJNA729271	:ed_Lushi_vs_Arbor_Acr	breed_growth_background	ENSGALG00010003375	YWHAQ	-0.203531074	0.046163163	-4.408949962	1.03873E-05	0.000300527	negative
PRJNA729271	:ed_Lushi_vs_Arbor_Acr	breed_growth_background	ENSGALG00010003379	ENSGALG00010003379	-0.518577816	0.259865193	-1.995564739	0.045981321	0.124778339	negative
PRJNA729271	:ed_Lushi_vs_Arbor_Acr	breed_growth_background	ENSGALG00010003382	ASNS	-0.684492105	0.320622581	-2.134884269	0.032770465	0.097178372	negative
PRJNA729271	:ed_Lushi_vs_Arbor_Acr	breed_growth_background	ENSGALG00010003383	UBE2J1	-0.084094059	0.046551584	-1.806470393	0.070844895	0.169162888	negative
PRJNA729271	:ed_Lushi_vs_Arbor_Acr	breed_growth_background	ENSGALG00010003388	PXDN	-0.52167553	0.099572703	-5.239141993	1.61325E-07	1.25048E-05	negative
PRJNA729271	:ed_Lushi_vs_Arbor_Acr	breed_growth_background	ENSGALG00010003389	ENSGALG00010003389	-0.728813034	0.22017239	-3.310192679	0.000932318	0.007739946	negative
PRJNA729271	:ed_Lushi_vs_Arbor_Acr	breed_growth_background	ENSGALG00010003395	ENSGALG00010003395	0.480584474	0.53817438	0.89299025	0.371862394	0.534514311	positive
PRJNA729271	:ed_Lushi_vs_Arbor_Acr	breed_growth_background	ENSGALG00010003397	SPATA4	0.2114838	0.201907546	1.047428905	0.294901806	0.456298215	positive
PRJNA729271	:ed_Lushi_vs_Arbor_Acr	breed_growth_background	ENSGALG00010003400	PKIA	0.011343634	0.06112336	0.185585902	0.85276951	0.911100026	positive
PRJNA729271	:ed_Lushi_vs_Arbor_Acr	breed_growth_background	ENSGALG00010003401	ENSGALG00010003401	0.271144319	0.924624767	0.293247951	0.769332635	0.856298601	positive
PRJNA729271	:ed_Lushi_vs_Arbor_Acr	breed_growth_background	ENSGALG00010003403	GCDH	-0.056158535	0.079874387	-0.70308565	0.482002374	0.63467461	negative
PRJNA729271	:ed_Lushi_vs_Arbor_Acr	breed_growth_background	ENSGALG00010003405	GABRR2	-0.078156015	0.138466645	-0.564439293	0.572455204	0.71078792	negative
PRJNA729271	:ed_Lushi_vs_Arbor_Acr	breed_growth_background	ENSGALG00010003406	C1GALT1	-0.20360945	0.057317657	-3.552298909	0.000381881	0.004115511	negative
PRJNA729271	:ed_Lushi_vs_Arbor_Acr	breed_growth_background	ENSGALG00010003407	CHMP4C	0.019419221	0.114270459	0.169940866	0.865056642	0.918877036	positive
PRJNA729271	:ed_Lushi_vs_Arbor_Acr	breed_growth_background	ENSGALG00010003408	ENSGALG00010003408	2.576663018	1.972986107	1.305971192	0.191562354	0.338373913	positive
PRJNA729271	:ed_Lushi_vs_Arbor_Acr	breed_growth_background	ENSGALG00010003412	ASB5	0.002730219	0.078192237	0.034916751	0.972146123	0.983408337	positive
PRJNA729271	:ed_Lushi_vs_Arbor_Acr	breed_growth_background	ENSGALG00010003419	ENSGALG00010003419	-0.362113608	0.55127548	-0.656865073	0.511267678	0.659913178	negative
PRJNA729271	:ed_Lushi_vs_Arbor_Acr	breed_growth_background	ENSGALG00010003420	ENSGALG00010003420	-0.113407483	0.33760035	-0.335922289	0.736929468	0.831591486	negative
PRJNA729271	:ed_Lushi_vs_Arbor_Acr	breed_growth_background	ENSGALG00010003428	SNX16	0.179581354	0.083786848	2.143311971	0.032088056	0.095781791	positive
PRJNA729271	:ed_Lushi_vs_Arbor_Acr	breed_growth_background	ENSGALG00010003431	PM20D2	0.353131757	0.152381987	2.317411415	0.020481331	0.069344457	positive
PRJNA729271	:ed_Lushi_vs_Arbor_Acr	breed_growth_background	ENSGALG00010003432	LACC1	-0.079197491	0.163556811	-0.484220074	0.628229692	0.754260696	negative
PRJNA729271	:ed_Lushi_vs_Arbor_Acr	breed_growth_background	ENSGALG00010003437	PNRC1	0.193550876	0.095627044	2.024018188	0.04296828	0.118633376	positive
PRJNA729271	:ed_Lushi_vs_Arbor_Acr	breed_growth_background	ENSGALG00010003438	KCNS3	0.485838883	0.09563931	5.079907887	3.77618E-07	2.2783E-05	positive
PRJNA729271	:ed_Lushi_vs_Arbor_Acr	breed_growth_background	ENSGALG00010003439	GMIP	0.1619349	0.128164765	1.263490012	0.206413134	0.355592866	positive
PRJNA729271	:ed_Lushi_vs_Arbor_Acr	breed_growth_background	ENSGALG00010003442	SPCS3	0.080549325	0.048085407	1.675130343	0.093908661	0.205744564	positive
PRJNA729271	:ed_Lushi_vs_Arbor_Acr	breed_growth_background	ENSGALG00010003447	ZC2HC1A	-0.063778469	0.067516977	-0.944628628	0.344848506	0.508761232	negative
PRJNA729271	:ed_Lushi_vs_Arbor_Acr	breed_growth_background	ENSGALG00010003449	BORCS6	0.07978851	0.058489878	1.364142174	0.172522824	0.315712226	positive
PRJNA729271	:ed_Lushi_vs_Arbor_Acr	breed_growth_background	ENSGALG00010003454	BPNT2	0.10980307	0.065177798	1.68466984	0.092052289	0.202904185	positive
PRJNA729271	:ed_Lushi_vs_Arbor_Acr	breed_growth_background	ENSGALG00010003456	CDS1	-0.303271045	0.217756817	-1.392705174	0.163708963	0.30352538	negative
PRJNA729271	:ed_Lushi_vs_Arbor_Acr	breed_growth_background	ENSGALG00010003466	TSC22D1	-0.066680806	0.054056418	-1.233540966	0.217374003	0.369327443	negative
PRJNA729271	:ed_Lushi_vs_Arbor_Acr	breed_growth_background	ENSGALG00010003468	ILVBL	0.173995459	0.072312442	2.406162122	0.016121109	0.058638763	positive
PRJNA729271	:ed_Lushi_vs_Arbor_Acr	breed_growth_background	ENSGALG00010003470	METTL3	0.158819376	0.099952353	1.588950845	0.112071491	0.233365185	positive
PRJNA729271	:ed_Lushi_vs_Arbor_Acr	breed_growth_background	ENSGALG00010003471	ADI1	0.083875568	0.071648226	1.170657992	0.241736277	0.397500548	positive
PRJNA729271	:ed_Lushi_vs_Arbor_Acr	breed_growth_background	ENSGALG00010003472	FAM110B	-0.063265081	0.071011984	-0.890907111	0.372979005	0.535492342	negative
PRJNA729271	:ed_Lushi_vs_Arbor_Acr	breed_growth_background	ENSGALG00010003473	ENSGALG00010003473	-0.220077977	0.141028382	-1.560522601	0.118636433	0.242509533	negative
PRJNA729271	:ed_Lushi_vs_Arbor_Acr	breed_growth_background	ENSGALG00010003474	RPS7	-0.121489666	0.088800673	-1.368116494	0.171275615	0.314099194	negative
PRJNA729271	:ed_Lushi_vs_Arbor_Acr	breed_growth_background	ENSGALG00010003475	ENSGALG00010003475	0.611827505	1.135775339	0.538687084	0.590102787	0.725368038	positive
PRJNA729271	:ed_Lushi_vs_Arbor_Acr	breed_growth_background	ENSGALG00010003478	COLEC11	-0.245350664	0.095438597	-2.570769805	0.010147275	0.042241457	negative
PRJNA729271	:ed_Lushi_vs_Arbor_Acr	breed_growth_background	ENSGALG00010003479	SYDE1	0.01058093	0.108443373	0.097571012	0.922272943	0.956658531	positive
PRJNA729271	:ed_Lushi_vs_Arbor_Acr	breed_growth_background	ENSGALG00010003485	RNGTT	-0.063346785	0.056218129	-1.12680352	0.259825562	0.417628246	negative
PRJNA729271	:ed_Lushi_vs_Arbor_Acr	breed_growth_background	ENSGALG00010003489	SGCE	-0.2862133	0.064265901	-4.453579484	8.44504E-06	0.000255555	negative
PRJNA729271	:ed_Lushi_vs_Arbor_Acr	breed_growth_background	ENSGALG00010003503	NUFIP1	0.053127833	0.047518205	1.118052205	0.263544698	0.421642428	positive
PRJNA729271	:ed_Lushi_vs_Arbor_Acr	breed_growth_background	ENSGALG00010003506	DYNC1I1	-0.122349696	0.098126239	-1.246860142	0.212448784	0.362628959	negative
PRJNA729271	:ed_Lushi_vs_Arbor_Acr	breed_growth_background	ENSGALG00010003507	WDFY3	-0.048185014	0.052212364	-0.92286598	0.356077052	0.519666017	negative
PRJNA729271	:ed_Lushi_vs_Arbor_Acr	breed_growth_background	ENSGALG00010003509	GPALPP1	0.116532162	0.040723802	2.861524648	0.004216087	0.022393618	positive
PRJNA729271	:ed_Lushi_vs_Arbor_Acr	breed_growth_background	ENSGALG00010003511	UBXN2B	-0.294764811	0.077910861	-3.783359692	0.000154726	0.002185913	negative
PRJNA729271	:ed_Lushi_vs_Arbor_Acr	breed_growth_background	ENSGALG00010003512	ENSGALG00010003512	-0.724977065	0.254548077	-2.848094846	0.004398181	0.023148744	negative
PRJNA729271	:ed_Lushi_vs_Arbor_Acr	breed_growth_background	ENSGALG00010003517	GTF2F2	-0.056727719	0.048567988	-1.168006358	0.2428042	0.398488098	negative
PRJNA729271	:ed_Lushi_vs_Arbor_Acr	breed_growth_background	ENSGALG00010003519	KCTD4	-0.039231087	0.388934544	-0.100868096	0.91965517	0.955435422	negative
PRJNA729271	:ed_Lushi_vs_Arbor_Acr	breed_growth_background	ENSGALG00010003520	ENSGALG00010003520	-0.185938831	0.889508243	-0.209035534	0.834420499	0.899384677	negative
PRJNA729271	:ed_Lushi_vs_Arbor_Acr	breed_growth_background	ENSGALG00010003522	ENSGALG00010003522	-0.397494304	0.34108907	-1.165368047	0.243870046	0.399603924	negative
PRJNA729271	:ed_Lushi_vs_Arbor_Acr	breed_growth_background	ENSGALG00010003525	NOL10	0.071806229	0.037179424	1.931343205	0.053440624	0.138949628	positive
PRJNA729271	:ed_Lushi_vs_Arbor_Acr	breed_growth_background	ENSGALG00010003528	GDAP1	0.231544194	0.112738892	2.053809373	0.039994145	0.112326126	positive
PRJNA729271	:ed_Lushi_vs_Arbor_Acr	breed_growth_background	ENSGALG00010003531	PPM1K	-0.246800139	0.080821676	-3.053637958	0.002260847	0.014526145	negative
PRJNA729271	:ed_Lushi_vs_Arbor_Acr	breed_growth_background	ENSGALG00010003539	ENSGALG00010003539	0.081214289	0.114572876	0.70884394	0.478421327	0.631404638	positive
PRJNA729271	:ed_Lushi_vs_Arbor_Acr	breed_growth_background	ENSGALG00010003541	CNR1	-0.298300952	0.117224533	-2.5446973	0.01093725	0.044595062	negative
PRJNA729271	:ed_Lushi_vs_Arbor_Acr	breed_growth_background	ENSGALG00010003543	SOX11	-0.035292778	0.044335179	-0.796044552	0.426006141	0.585193235	negative
PRJNA729271	:ed_Lushi_vs_Arbor_Acr	breed_growth_background	ENSGALG00010003552	PI15	-0.334476018	0.208262863	-1.606028136	0.108267728	0.227903096	negative
PRJNA729271	:ed_Lushi_vs_Arbor_Acr	breed_growth_background	ENSGALG00010003556	ENSGALG00010003556	-0.323624895	1.002751073	-0.322737022	0.746894412	0.838661641	negative
PRJNA729271	:ed_Lushi_vs_Arbor_Acr	breed_growth_background	ENSGALG00010003558	TPT1	0.234623562	0.098680767	2.377601722	0.017425636	0.061907989	positive
PRJNA729271	:ed_Lushi_vs_Arbor_Acr	breed_growth_background	ENSGALG00010003560	ADGRL3	-0.198756606	0.075558854	-2.630487307	0.008526255	0.037146343	negative
PRJNA729271	:ed_Lushi_vs_Arbor_Acr	breed_growth_background	ENSGALG00010003571	RPL7	-0.305299475	0.084166996	-3.627306319	0.000286393	0.003364079	negative
PRJNA729271	:ed_Lushi_vs_Arbor_Acr	breed_growth_background	ENSGALG00010003577	ENSGALG00010003577	-0.212785713	0.852738041	-0.249532334	0.802949033	0.878395942	negative
PRJNA729271	:ed_Lushi_vs_Arbor_Acr	breed_growth_background	ENSGALG00010003582	RDH10	-0.218217424	0.076520538	-2.851749729	0.004347932	0.022954768	negative
PRJNA729271	:ed_Lushi_vs_Arbor_Acr	breed_growth_background	ENSGALG00010003583	RSAD2	0.479463296	0.430675906	1.113280983	0.265587771	0.423834088	positive
PRJNA729271	:ed_Lushi_vs_Arbor_Acr	breed_growth_background	ENSGALG00010003585	PNPLA6	-0.053886123	0.076092804	-0.70816319	0.478843922	0.631789792	negative
PRJNA729271	:ed_Lushi_vs_Arbor_Acr	breed_growth_background	ENSGALG00010003589	SLC25A30	0.304238592	0.207466908	1.466443953	0.14252738	0.275494648	positive
PRJNA729271	:ed_Lushi_vs_Arbor_Acr	breed_growth_background	ENSGALG00010003595	SNX13	-0.130396844	0.05043797	-2.585291294	0.009729676	0.041009494	negative
PRJNA729271	:ed_Lushi_vs_Arbor_Acr	breed_growth_background	ENSGALG00010003596	CRISPLD1	-0.203735408	0.083026316	-2.453865446	0.014132985	0.053215572	negative
PRJNA729271	:ed_Lushi_vs_Arbor_Acr	breed_growth_background	ENSGALG00010003597	RNF144A	-0.219833251	0.077905171	-2.82180565	0.004775411	0.024624539	negative
PRJNA729271	:ed_Lushi_vs_Arbor_Acr	breed_growth_background	ENSGALG00010003604	ODC	-0.247756689	0.115883514	-2.137980462	0.032518329	0.096747368	negative
PRJNA729271	:ed_Lushi_vs_Arbor_Acr	breed_growth_background	ENSGALG00010003617	COG3	0.061287049	0.045112856	1.358527351	0.17429641	0.318149661	positive
PRJNA729271	:ed_Lushi_vs_Arbor_Acr	breed_growth_background	ENSGALG00010003619	HNF4G	-0.153625873	0.291005207	-0.527914517	0.597558669	0.730959358	negative
PRJNA729271	:ed_Lushi_vs_Arbor_Acr	breed_growth_background	ENSGALG00010003627	MCOLN1	-0.037250102	0.064692727	-0.575800467	0.564750058	0.704723648	negative
PRJNA729271	:ed_Lushi_vs_Arbor_Acr	breed_growth_background	ENSGALG00010003632	TECRL	0.771470719	0.19985627	3.860127671	0.000113328	0.0017522	positive
PRJNA729271	:ed_Lushi_vs_Arbor_Acr	breed_growth_background	ENSGALG00010003634	CYP4F11	-0.74637955	0.162193497	-4.601784693	4.18886E-06	0.00014995	negative
PRJNA729271	:ed_Lushi_vs_Arbor_Acr	breed_growth_background	ENSGALG00010003639	ENSGALG00010003639	0.899283093	0.43360088	2.073988165	0.03808041	0.108677996	positive

PRJNA729271	ed_Lushi_vs_Arbor_Acr	breed_growth_background	ENSGALG00010003641	ENSGALG00010003641	0.414193741	1.331468674	0.31108035	0.755739539	0.845641857	positive
PRJNA729271	ed_Lushi_vs_Arbor_Acr	breed_growth_background	ENSGALG00010003650	SPACA1	-0.14315616	0.32065826	-0.446444635	0.655276097	0.775238902	negative
PRJNA729271	ed_Lushi_vs_Arbor_Acr	breed_growth_background	ENSGALG00010003652	MYCN	-0.042391734	0.076487834	-0.554228456	0.579422506	0.716370626	negative
PRJNA729271	ed_Lushi_vs_Arbor_Acr	breed_growth_background	ENSGALG00010003660	IGFBP7	-0.191826271	0.087838819	-2.183843925	0.028973719	0.088648258	negative
PRJNA729271	ed_Lushi_vs_Arbor_Acr	breed_growth_background	ENSGALG00010003665	RDH14	0.083941273	0.049782647	1.686155294	0.091765892	0.202550115	positive
PRJNA729271	ed_Lushi_vs_Arbor_Acr	breed_growth_background	ENSGALG00010003666	SLC25A13	-0.154230237	0.042027316	-3.66976173	0.000242777	0.002979719	negative
PRJNA729271	ed_Lushi_vs_Arbor_Acr	breed_growth_background	ENSGALG00010003669	ENSGALG00010003669	-0.324262191	0.808925546	-0.400855421	0.688526572	0.798539286	negative
PRJNA729271	ed_Lushi_vs_Arbor_Acr	breed_growth_background	ENSGALG00010003671	ENSGALG00010003671	1.164927103	0.226041391	5.15360085	2.55532E-07	1.69905E-05	positive
PRJNA729271	ed_Lushi_vs_Arbor_Acr	breed_growth_background	ENSGALG00010003674	AKIRIN2	0.168082769	0.07150445	2.350661669	0.018740065	0.065297413	positive
PRJNA729271	ed_Lushi_vs_Arbor_Acr	breed_growth_background	ENSGALG00010003676	POLR2B	0.122289924	0.042035774	2.909186935	0.003623701	0.02014789	positive
PRJNA729271	ed_Lushi_vs_Arbor_Acr	breed_growth_background	ENSGALG00010003678	ZNFY1	0.804875755	0.239475766	3.360990414	0.000776635	0.006800554	positive
PRJNA729271	ed_Lushi_vs_Arbor_Acr	breed_growth_background	ENSGALG00010003680	TRAPPC5	0.014286382	0.057359032	0.249069437	0.803307071	0.878522408	positive
PRJNA729271	ed_Lushi_vs_Arbor_Acr	breed_growth_background	ENSGALG00010003681	NT5C1B	0.084460441	0.071075287	1.188323608	0.23470594	0.389118449	positive
PRJNA729271	ed_Lushi_vs_Arbor_Acr	breed_growth_background	ENSGALG00010003683	ENSGALG00010003683	0.564062429	0.852557214	0.661612404	0.508219656	0.657231031	positive
PRJNA729271	ed_Lushi_vs_Arbor_Acr	breed_growth_background	ENSGALG00010003691	ERICH1	0.107164659	0.082164352	1.304271941	0.192140879	0.338993581	positive
PRJNA729271	ed_Lushi_vs_Arbor_Acr	breed_growth_background	ENSGALG00010003692	ZNFY2	0.19637251	0.141447457	1.388307108	0.165043549	0.305218964	positive
PRJNA729271	ed_Lushi_vs_Arbor_Acr	breed_growth_background	ENSGALG00010003695	NOA1	0.133529624	0.054289381	2.459590095	0.013909578	0.052739635	positive
PRJNA729271	ed_Lushi_vs_Arbor_Acr	breed_growth_background	ENSGALG00010003698	MACC1	-0.980836016	0.696067912	-1.409109655	0.158802746	0.297280874	negative
PRJNA729271	ed_Lushi_vs_Arbor_Acr	breed_growth_background	ENSGALG00010003700	REST	-0.00853052	0.036496399	-0.233735926	0.815189995	0.886306539	negative
PRJNA729271	ed_Lushi_vs_Arbor_Acr	breed_growth_background	ENSGALG00010003701	SARAF	-0.009488457	0.043614008	-0.217555268	0.827775641	0.894989313	negative
PRJNA729271	ed_Lushi_vs_Arbor_Acr	breed_growth_background	ENSGALG00010003703	TDRP	-0.265593204	0.124521401	-2.132912103	0.032931937	0.097448847	negative
PRJNA729271	ed_Lushi_vs_Arbor_Acr	breed_growth_background	ENSGALG00010003713	SPERT	0.151031944	0.140589159	1.074278735	0.282697773	0.442660065	positive
PRJNA729271	ed_Lushi_vs_Arbor_Acr	breed_growth_background	ENSGALG00010003714	ORC3	0.127217534	0.060714335	2.095345917	0.036140254	0.104394774	positive
PRJNA729271	ed_Lushi_vs_Arbor_Acr	breed_growth_background	ENSGALG00010003716	YLEC8	-0.067093141	0.368518246	-0.182061924	0.855534129	0.913178734	negative
PRJNA729271	ed_Lushi_vs_Arbor_Acr	breed_growth_background	ENSGALG00010003717	LEPROTL1	-0.045124949	0.052445229	-0.860420474	0.389557304	0.551323379	negative
PRJNA729271	ed_Lushi_vs_Arbor_Acr	breed_growth_background	ENSGALG00010003719	RPGR	0.010828101	0.062057338	0.17448542	0.861483988	0.916828958	positive
PRJNA729271	ed_Lushi_vs_Arbor_Acr	breed_growth_background	ENSGALG00010003720	ENSGALG00010003720	-0.032887266	1.423086958	-0.023109808	0.981562682	0.989068549	negative
PRJNA729271	ed_Lushi_vs_Arbor_Acr	breed_growth_background	ENSGALG00010003722	MHCY11	0.969149546	0.348383998	2.781842887	0.00540512	0.026932696	positive
PRJNA729271	ed_Lushi_vs_Arbor_Acr	breed_growth_background	ENSGALG00010003724	ENSGALG00010003724	-0.266670343	0.116703411	-2.285026122	0.022311296	0.073886735	negative
PRJNA729271	ed_Lushi_vs_Arbor_Acr	breed_growth_background	ENSGALG00010003726	ENPEP	-0.362917856	0.268382492	-1.352241174	0.176298176	0.320230835	negative
PRJNA729271	ed_Lushi_vs_Arbor_Acr	breed_growth_background	ENSGALG00010003727	FBXO25	-0.258056238	0.082067033	-3.144456797	0.001663955	0.011662971	negative
PRJNA729271	ed_Lushi_vs_Arbor_Acr	breed_growth_background	ENSGALG00010003729	RARS2	-0.099601346	0.056597149	-1.759829742	0.078436679	0.181419151	negative
PRJNA729271	ed_Lushi_vs_Arbor_Acr	breed_growth_background	ENSGALG00010003731	ENSGALG00010003731	0.080171157	0.308304744	0.260038674	0.794833942	0.872944233	positive
PRJNA729271	ed_Lushi_vs_Arbor_Acr	breed_growth_background	ENSGALG00010003733	STAU2	0.034256585	0.037999046	0.901511719	0.367316306	0.53072666	positive
PRJNA729271	ed_Lushi_vs_Arbor_Acr	breed_growth_background	ENSGALG00010003734	ZC3H13	0.256825297	0.076070345	3.376155291	0.000735064	0.006532652	positive
PRJNA729271	ed_Lushi_vs_Arbor_Acr	breed_growth_background	ENSGALG00010003736	ENSGALG00010003736	0.139572386	1.630876814	0.085581195	0.931799348	0.962498809	positive
PRJNA729271	ed_Lushi_vs_Arbor_Acr	breed_growth_background	ENSGALG00010003737	SRP72	0.034901659	0.030604323	1.140415982	0.254113038	0.410732789	positive
PRJNA729271	ed_Lushi_vs_Arbor_Acr	breed_growth_background	ENSGALG00010003740	PITX2	0.665171761	0.193248552	3.442053011	0.000577317	0.005480165	positive
PRJNA729271	ed_Lushi_vs_Arbor_Acr	breed_growth_background	ENSGALG00010003742	ZFHX4	-0.166995054	0.089268291	-1.870709669	0.061385334	0.153410416	negative
PRJNA729271	ed_Lushi_vs_Arbor_Acr	breed_growth_background	ENSGALG00010003743	ARL9	-0.00073291	0.095432891	-0.007679845	0.99387243	0.996451492	negative
PRJNA729271	ed_Lushi_vs_Arbor_Acr	breed_growth_background	ENSGALG00010003746	HOPX	-0.189565142	0.216693451	-0.874807892	0.381678442	0.543140489	negative
PRJNA729271	ed_Lushi_vs_Arbor_Acr	breed_growth_background	ENSGALG00010003749	MHCY15	0.589840721	0.502899774	1.172879271	0.240844223	0.396618611	positive
PRJNA729271	ed_Lushi_vs_Arbor_Acr	breed_growth_background	ENSGALG00010003753	SLCO5A1	0.367366307	0.086963058	4.224394979	2.39584E-05	0.000551232	positive
PRJNA729271	ed_Lushi_vs_Arbor_Acr	breed_growth_background	ENSGALG00010003754	FAM110C	-0.121330967	0.133388987	-0.909602582	0.363032136	0.526268217	negative
PRJNA729271	ed_Lushi_vs_Arbor_Acr	breed_growth_background	ENSGALG00010003755	SLC35A1	-0.064447382	0.050574049	-1.274317226	0.202551014	0.35174038	negative
PRJNA729271	ed_Lushi_vs_Arbor_Acr	breed_growth_background	ENSGALG00010003756	MHCY7	0.170135871	0.202374334	0.840698853	0.400516663	0.561404993	positive
PRJNA729271	ed_Lushi_vs_Arbor_Acr	breed_growth_background	ENSGALG00010003761	UGT8	0.681073091	0.173888882	3.916714416	8.9764E-05	0.001476877	positive
PRJNA729271	ed_Lushi_vs_Arbor_Acr	breed_growth_background	ENSGALG00010003762	EVI5L	-0.034606248	0.053450664	-0.647442799	0.517345404	0.665637338	negative
PRJNA729271	ed_Lushi_vs_Arbor_Acr	breed_growth_background	ENSGALG00010003764	HDAC9	-0.273017428	0.103359088	-2.641445786	0.008255302	0.036371003	negative
PRJNA729271	ed_Lushi_vs_Arbor_Acr	breed_growth_background	ENSGALG00010003765	ENSGALG00010003765	-1.08177842	0.471763336	-2.293053186	0.021844943	0.072693436	negative
PRJNA729271	ed_Lushi_vs_Arbor_Acr	breed_growth_background	ENSGALG00010003767	ENSGALG00010003767	-0.013394501	0.222199547	-0.060281403	0.951931513	0.973433394	negative
PRJNA729271	ed_Lushi_vs_Arbor_Acr	breed_growth_background	ENSGALG00010003768	ARSJ	-0.046529339	0.072208256	-0.644376993	0.519331	0.66723124	negative
PRJNA729271	ed_Lushi_vs_Arbor_Acr	breed_growth_background	ENSGALG00010003769	C6orf163	-0.632939402	0.278447494	-2.273101444	0.023020067	0.07549228	negative
PRJNA729271	ed_Lushi_vs_Arbor_Acr	breed_growth_background	ENSGALG00010003772	ENSGALG00010003772	0.275423435	0.236579867	1.164187968	0.244347845	0.400160661	positive
PRJNA729271	ed_Lushi_vs_Arbor_Acr	breed_growth_background	ENSGALG00010003775	NCOA2	0.255023324	0.061237442	4.164499957	3.12036E-05	0.000685297	positive
PRJNA729271	ed_Lushi_vs_Arbor_Acr	breed_growth_background	ENSGALG00010003777	ENSGALG00010003777	1.004430996	0.412989923	2.432095651	0.015011742	0.055622496	positive
PRJNA729271	ed_Lushi_vs_Arbor_Acr	breed_growth_background	ENSGALG00010003778	LCA5	0.213623451	0.06865483	3.111557489	0.001861032	0.012617242	positive
PRJNA729271	ed_Lushi_vs_Arbor_Acr	breed_growth_background	ENSGALG00010003785	ENSGALG00010003785	0.673209216	0.526584789	1.278444099	0.201092883	0.34994349	positive
PRJNA729271	ed_Lushi_vs_Arbor_Acr	breed_growth_background	ENSGALG00010003786	ENSGALG00010003786	-1.617562389	0.328455773	-4.924749461	8.44685E-07	4.28102E-05	negative
PRJNA729271	ed_Lushi_vs_Arbor_Acr	breed_growth_background	ENSGALG00010003787	SMIM8	-0.004171807	0.100854026	-0.041364808	0.967005068	0.981220753	negative
PRJNA729271	ed_Lushi_vs_Arbor_Acr	breed_growth_background	ENSGALG00010003788	NUDT9	0.203395655	0.068364388	2.975169693	0.002928265	0.017438457	positive
PRJNA729271	ed_Lushi_vs_Arbor_Acr	breed_growth_background	ENSGALG00010003790	ELOVL6	-0.263260818	0.097877065	-2.689708949	0.007151436	0.032899432	negative
PRJNA729271	ed_Lushi_vs_Arbor_Acr	breed_growth_background	ENSGALG00010003791	ITGB8	0.223893024	0.095059051	2.355304654	0.018507525	0.064755446	positive
PRJNA729271	ed_Lushi_vs_Arbor_Acr	breed_growth_background	ENSGALG00010003793	TRAM1L1	-0.038410476	0.045619658	-0.841972024	0.399803613	0.560726886	negative
PRJNA729271	ed_Lushi_vs_Arbor_Acr	breed_growth_background	ENSGALG00010003795	LCP1	0.204193644	0.107634788	1.897097094	0.057815125	0.146828243	positive
PRJNA729271	ed_Lushi_vs_Arbor_Acr	breed_growth_background	ENSGALG00010003796	ENSGALG00010003796	0.451484352	0.160822654	2.807343006	0.004995202	0.025539843	positive
PRJNA729271	ed_Lushi_vs_Arbor_Acr	breed_growth_background	ENSGALG00010003797	ENSGALG00010003797	0.002017795	0.093326548	0.021620807	0.982750436	0.989816659	positive
PRJNA729271	ed_Lushi_vs_Arbor_Acr	breed_growth_background	ENSGALG00010003803	LACTB2	-0.255352587	0.070659064	-3.613868816	0.000301662	0.003486911	negative
PRJNA729271	ed_Lushi_vs_Arbor_Acr	breed_growth_background	ENSGALG00010003804	PSME1	0.192803664	0.131243486	1.469053213	0.141818356	0.274693342	positive
PRJNA729271	ed_Lushi_vs_Arbor_Acr	breed_growth_background	ENSGALG00010003806	EGF	-0.436815081	0.166072225	-2.630271752	0.008531664	0.037146343	negative
PRJNA729271	ed_Lushi_vs_Arbor_Acr	breed_growth_background	ENSGALG00010003808	TWIST1	-0.15070842	0.0913748	-1.649343358	0.099077312	0.21336951	negative
PRJNA729271	ed_Lushi_vs_Arbor_Acr	breed_growth_background	ENSGALG00010003810	HMGN2P46	-0.006779218	0.048741567	-0.139084952	0.889383019	0.935190922	negative
PRJNA729271	ed_Lushi_vs_Arbor_Acr	breed_growth_background	ENSGALG00010003812	BG8	-0.528069968	0.216601057	-2.437984268	0.014769417	0.054892999	negative
PRJNA729271	ed_Lushi_vs_Arbor_Acr	breed_growth_background	ENSGALG00010003813	LRIT3	-0.29398842	0.301216855	-0.976002554	0.329063199	0.492846773	negative
PRJNA729271	ed_Lushi_vs_Arbor_Acr	breed_growth_background	ENSGALG00010003814	TPP1	-0.020039651	0.070650599	-0.283644452	0.776682867	0.861363287	negative
PRJNA729271	ed_Lushi_vs_Arbor_Acr	breed_growth_background	ENSGALG00010003815	RASAL3	0.4225665	0.278467037	1.517474039	0.129147043	0.256612254	positive
PRJNA729271	ed_Lushi_vs_Arbor_Acr	breed_growth_background	ENSGALG00010003816	KLHL8	0.022675262	0.098034179	0.231299559	0.817082089	0.887094965	positive
PRJNA729271	ed_Lushi_vs_Arbor_Acr	breed_growth_background	ENSGALG00010003817	BF2	0.533502117	0.149544589	3.567512013	0.000360387	0.003927675	positive
PRJNA729271	ed_Lushi_vs_Arbor_Acr	breed_growth_background	ENSGALG00010003818	GAB2	-0.176509371	0.085500902	-2.064415301	0.038978357	0.110307844	negative
PRJNA729271	ed_Lushi_vs_Arbor_Acr	breed_growth_background	ENSGALG00010003819	PHIP	0.127130556	0.050566879	2.51410723	0.011933414	0.047390368	positive
PRJNA729271	ed_Lushi_vs_Arbor_Acr	breed_growth_background	ENSGALG00010003820	SH3YL1	-0.572528377	0.163120221	-3.509855324	0.000448351	0.004583722	negative
PRJNA729271	ed_Lushi_vs_Arbor_Acr	breed_growth_background	ENSGALG00010003821	RUBCNL	-0.275186109	0.083882053	-3.28063154	0.00103575	0.008363894	negative
PRJNA729271	ed_Lushi_vs_Arbor_Acr	breed_growth_background	ENSGALG00010003822	ENSGALG00010003822	-0.041202429	0.091621423	-0.449703006	0.652924603	0.773719925	negative
PRJNA729271	ed_Lushi_vs_Arbor_Acr	breed_growth_background	ENSGALG00010003823	PTPN13	-0.259699891	0.07981266	-3.253868399	0.00113845	0.008978147	negative
PRJNA729271	ed_Lushi_vs_Arbor_Acr	breed_growth_background	ENSGALG00010003824	SP4	0.246115182	0.085065389	2.893246986	0.003812813	0.0209423	positive
PRJNA729271	ed_Lushi_vs_Arbor_Acr	breed_growth_background	ENSGALG00010003826	ENSGALG00010003826	0.105351829	0.057167822	1.842851899	0.065350637	0.160172046	positive
PRJNA729271	ed_Lushi_vs_Arbor_Acr	breed_growth_background	ENSGALG00010003827	RRH	-0.03663006	0.407117563	-0.089974158	0.928307751	0.960231941	negative
PRJNA729271	ed_Lushi_vs_Arbor_Acr	breed_growth_background	ENSGALG00010003829	ENSGALG00010003829	-0.062373602	0.073464882	-0.849026089	0.395866775	0.557312442	negative
PRJNA729271	ed_Lushi_vs_Arbor_Acr	breed_growth_background	ENSGALG00010003830	DMB2	0.53228864	0.127793636	4.165220241	3.11052E-05	0.000685213	positive
PRJNA729271	ed_Lushi_vs_Arbor_Acr	breed_growth_background	ENSGALG00010003831	IRAK1BP1	0.216420268	0.091525112	2.364599874	0.01804956	0.063629859	positive
PRJNA729271	ed_Lushi_vs_Arbor_Acr	breed_growth_background	ENSGALG00010003832	ART1B	-0.141752483	0.301512467	-0.470138049	0.638256392	0.762602412	negative
PRJNA729271	ed_Lushi_vs_Arbor_Acr	breed_growth_background	ENSGALG00010003833	ACP1	-0.218749404	0.08435779	-2.593114436	0.009511112	0.0404055	negative
PRJNA729271	ed_Lushi_vs_Arbor_Acr	breed_growth_background	ENSGALG00010003834	MAPK10	-0.758274903	0.235528913	-3.219455706	0.001284342	0.009782731	negative
PRJNA729271	ed_Lushi_vs_Arbor_Acr	breed_growth_background	ENSGALG00010003835	ENSGALG00010003835	0.185003592	0.087334545	2.118332338	0.034146932	0.100052512	positive
PRJNA729271	ed_Lushi_vs_Arbor_Acr	breed_growth_background	ENSGALG00010003836	CD1F	-0.322490024	0.16269353	-1.982193296	0.047457611	0.127482963	negative
PRJNA729271	ed_Lushi_vs_Arbor_Acr	breed_growth_background	ENSGALG00010003837	ALKAL2	-0.009829769	0.13070544	-0.075205506	0.940051204	0.96719605	negative
PRJNA729271	ed_Lushi_vs_Arbor_Acr	breed_growth_background	ENSGALG00010003838	GAR1	0.088900601	0.100972818	0.880440927	0.378620493	0.540699906	positive
PRJNA729271	ed_Lushi_vs_Arbor_Acr	breed_growth_background	ENSGALG00010003839	ENSGALG00010003839	-0.391527425	0.370887695	-1.055649542	0.291128358	0.452488264	negative
PRJNA729271	ed_Lushi_vs_Arbor_Acr	breed_growth_background	ENSGALG00010003841	UBE2W	0.049899	0.076551561	0.65183517	0.514507511	0.66296271	positive
PRJNA729271	ed_Lushi_vs_Arbor_Acr	breed_growth_background	ENSGALG00010003842	ENSGALG00010003842	0.168554028	0.07753766	2.173834353	0.029717578	0.090282784	positive
PRJNA729271	ed_Lushi_vs_Arbor_Acr	breed_growth_background	ENSGALG00010003843	LRCH1	0.112221781	0.05796684	1.935965115	0.052871974	0.137887596	positive
PRJNA729271	ed_Lushi_vs_Arbor_Acr	breed_growth_background	ENSGALG00010003844	TMEM18	-0.071260721	0.06420794	-1.109842821	0.267066763	0.425445954	negative
PRJNA729271	ed_Lushi_vs_Arbor_Acr	breed_growth_background	ENSGALG00010003845	CFI	-0.759629609	0.468677861	-1.6207926			negative
PRJNA729271	ed_Lushi_vs_Arbor_Acr	breed_growth_background	ENSGALG00010003848	ESD	0.078000885	0.06627558	1.176917416	0.23922847	0.394765104	positive
PRJNA729271	ed_Lushi_vs_Arbor_Acr	breed_growth_background	ENSGALG00010003850	PLA2G12A	-0.082165401	0.05359596	-1.533052139	0.125263009	0.251445411	negative
PRJNA729271	ed_Lushi_vs_Arbor_Acr	breed_growth_background	ENSGALG00010003851	PHF14	0.038767494	0.035186273	1.101778915	0.270557799	0.429167793	positive
PRJNA729271	ed_Lushi_vs_Arbor_Acr	breed_growth_background	ENSGALG00010003852	TCEB1	0.054959475	0.057714171	0.952270013	0.340960062	0.50482449	positive
PRJNA729271	ed_Lushi_vs_Arbor_Acr	breed_growth_background	ENSGALG00010003853	ENSGALG00010003853	-0.034436547	0.124133551	-0.277415304	0.781461232	0.864774709	negative
PRJNA729271	ed_Lushi_vs_Arbor_Acr	breed_growth_background	ENSGALG00010003858	SUCLA2	0.131993175	0.066399172	1.987873803	0.046825649	0.1263238	positive
PRJNA729271	ed_Lushi_vs_Arbor_Acr	breed_growth_background	ENSGALG00010003860	TMEM70	0.167764167	0.061099136	2.745769857	0.00603691	0.029158615	positive
PRJNA729271	ed_Lushi_vs_Arbor_Acr	breed_growth_background	ENSGALG00010003861	NUDT15	-0.091866923	0.057437123	-1.599434629	0.109724063	0.230101646	negative
PRJNA729271	ed_Lushi_vs_Arbor_Acr	breed_growth_background	ENSGALG00010003862	PCK2	-0.114593497	0.078098097	-1.467302034	0.142293911	0.275190158	negative
PRJNA729271	ed_Lushi_vs_Arbor_Acr	breed_growth_background	ENSGALG00010003863	HTR1B	0.341535882	0.18229464	1.873537705	0.060994171	0.152748879	positive
PRJNA729271	ed_Lushi_vs_Arbor_Acr	breed_growth_background	ENSGALG00010003865	AKAP8L	0.160741277	0.102387762	1.569926653	0.116432176	0.239387857	positive
PRJNA729271	ed_Lushi_vs_Arbor_Acr	breed_growth_background	ENSGALG00010003868	HBBA	-0.406135693	0.213273856	-1.904291978	0.056872168	0.145262041	negative
PRJNA729271	ed_Lushi_vs_Arbor_Acr	breed_growth_background	ENSGALG00010003869	LY96	0.009645717	0.149572124	0.064488735	0.948581077	0.971711451	positive
PRJNA729271	ed_Lushi_vs_Arbor_Acr	breed_growth_background	ENSGALG00010003870	MED4	0.11842935	0.053694755	2.205603699	0.02741176	0.085136803	positive
PRJNA729271	ed_Lushi_vs_Arbor_Acr	breed_growth_background	ENSGALG00010003871	ENSGALG00010003871	0.014791541	0.10993414	0.134549109	0.892968381	0.937432231	positive
PRJNA729271	ed_Lushi_vs_Arbor_Acr	breed_growth_background	ENSGALG00010003874	YES1	-0.006907132	0.052582306	-0.131358488	0.895491726	0.939025723	negative
PRJNA729271	ed_Lushi_vs_Arbor_Acr	breed_growth_background	ENSGALG00010003875	AKAP8	0.090566965	0.060285325	1.502305309	0.133018278	0.262290837	positive
PRJNA729271	ed_Lushi_vs_Arbor_Acr	breed_growth_background	ENSGALG00010003878	ARHGAP24	-0.02781446	0.047592435	-0.584430285	0.558930856	0.700112579	negative
PRJNA729271	ed_Lushi_vs_Arbor_Acr	breed_growth_background	ENSGALG00010003879	CASP6	-0.239881212	0.065136159	-3.682765719	0.000230717	0.002873063	negative
PRJNA729271	ed_Lushi_vs_Arbor_Acr	breed_growth_background	ENSGALG00010003882	ENSGALG00010003882	0.046415003	0.065377384	0.709955039	0.477732018	0.630897011	positive
PRJNA729271	ed_Lushi_vs_Arbor_Acr	breed_growth_background	ENSGALG00010003884	TYMS	-0.123857471	0.105591087	-1.17299172	0.240799126	0.396589403	negative
PRJNA729271	ed_Lushi_vs_Arbor_Acr	breed_growth_background	ENSGALG00010003887	GIPC1	0.157123191	0.078363504	2.00505572	0.044957091	0.122629475	positive
PRJNA729271	ed_Lushi_vs_Arbor_Acr	breed_growth_background	ENSGALG00010003889	ITM2B	-0.145172693	0.046928184	-3.093507604	0.001978055	0.013175592	negative
PRJNA729271	ed_Lushi_vs_Arbor_Acr	breed_growth_background	ENSGALG00010003892	OPRK1	0.428969537	1.414079446	0.303356037	0.761618546	0.850133304	positive
PRJNA729271	ed_Lushi_vs_Arbor_Acr	breed_growth_background	ENSGALG00010003894	COPS4	-0.120796	0.066698588	-1.811072818	0.07012958	0.168104559	negative
PRJNA729271	ed_Lushi_vs_Arbor_Acr	breed_growth_background	ENSGALG00010003895	THSD7A	-0.077895545	0.086916755	-0.896208616	0.370141355	0.533518842	negative
PRJNA729271	ed_Lushi_vs_Arbor_Acr	breed_growth_background	ENSGALG00010003896	MCUB	-0.28613408	0.073145488	-3.911848658	9.15923E-05	0.001498454	negative
PRJNA729271	ed_Lushi_vs_Arbor_Acr	breed_growth_background	ENSGALG00010003898	NDST4	-0.012406144	0.329635405	-0.037635957	0.969977939	0.982586499	negative

PRJNA729271	ed_Lushi_vs_Arbor_Acr	breed_growth_background	ENSGALG00010003899	LIN54	0.214977366	0.088796014	2.421024958	0.015476815	0.05702502	positive
PRJNA729271	ed_Lushi_vs_Arbor_Acr	breed_growth_background	ENSGALG00010003900	RB1	-0.061394045	0.047941446	-1.280604763	0.200332526	0.349142715	negative
PRJNA729271	ed_Lushi_vs_Arbor_Acr	breed_growth_background	ENSGALG00010003902	ENSGALG00010003902	0.116073094	0.066737151	1.739257557	0.081989469	0.187065231	positive
PRJNA729271	ed_Lushi_vs_Arbor_Acr	breed_growth_background	ENSGALG00010003903	COL28A1	-0.998892895	0.140003723	-7.134759509	9.69565E-13	5.40533E-10	negative
PRJNA729271	ed_Lushi_vs_Arbor_Acr	breed_growth_background	ENSGALG00010003909	DNAJB1	0.264562132	0.084789855	3.120209753	0.001807223	0.012391532	positive
PRJNA729271	ed_Lushi_vs_Arbor_Acr	breed_growth_background	ENSGALG00010003910	THAP9	0.101087319	0.078922872	1.280836794	0.200250997	0.349042593	positive
PRJNA729271	ed_Lushi_vs_Arbor_Acr	breed_growth_background	ENSGALG00010003913	PSMB5	0.102281509	0.062607314	1.633699041	0.10232206	0.218659775	positive
PRJNA729271	ed_Lushi_vs_Arbor_Acr	breed_growth_background	ENSGALG00010003914	CAMK2D	0.211061343	0.056686835	3.723286799	0.000196646	0.002563294	positive
PRJNA729271	ed_Lushi_vs_Arbor_Acr	breed_growth_background	ENSGALG00010003915	MIOS	0.103139252	0.049960391	2.064420428	0.038977871	0.110307844	positive
PRJNA729271	ed_Lushi_vs_Arbor_Acr	breed_growth_background	ENSGALG00010003918	HTR1E	-0.179934364	0.378574552	-0.475294397	0.634577152	0.759481605	negative
PRJNA729271	ed_Lushi_vs_Arbor_Acr	breed_growth_background	ENSGALG00010003920	LPAR6	-0.050487397	0.082406156	-0.612665358	0.540097633	0.684926963	negative
PRJNA729271	ed_Lushi_vs_Arbor_Acr	breed_growth_background	ENSGALG00010003921	COLEC12	-0.356688198	0.090055194	-3.960773191	7.47075E-05	0.001284561	negative
PRJNA729271	ed_Lushi_vs_Arbor_Acr	breed_growth_background	ENSGALG00010003923	ENSGALG00010003923	-0.113032249	0.065843877	-1.716670636	0.086039368	0.193866103	negative
PRJNA729271	ed_Lushi_vs_Arbor_Acr	breed_growth_background	ENSGALG00010003925	SEC31A	-0.030126571	0.04613929	-0.652948316	0.513789599	0.662401515	negative
PRJNA729271	ed_Lushi_vs_Arbor_Acr	breed_growth_background	ENSGALG00010003928	ENSGALG00010003928	-0.301723323	0.083371055	-3.619041678	0.000295696	0.003442662	negative
PRJNA729271	ed_Lushi_vs_Arbor_Acr	breed_growth_background	ENSGALG00010003929	RCBTB2	-0.026008849	0.052755294	-0.493009266	0.62200604	0.748932569	negative
PRJNA729271	ed_Lushi_vs_Arbor_Acr	breed_growth_background	ENSGALG00010003933	TECR	0.173792562	0.110569326	1.57179725	0.115997579	0.238765253	positive
PRJNA729271	ed_Lushi_vs_Arbor_Acr	breed_growth_background	ENSGALG00010003937	ENSGALG00010003937	-0.104231014	0.110324841	-0.944764684	0.344779025	0.508761232	negative
PRJNA729271	ed_Lushi_vs_Arbor_Acr	breed_growth_background	ENSGALG00010003948	SCD5	-0.29297909	0.079763965	-3.673075809	0.000239648	0.002954987	negative
PRJNA729271	ed_Lushi_vs_Arbor_Acr	breed_growth_background	ENSGALG00010003958	CYSLTR2	0.166148057	0.190156641	0.873743122	0.382258161	0.543645217	positive
PRJNA729271	ed_Lushi_vs_Arbor_Acr	breed_growth_background	ENSGALG00010003960	ASPH	-0.301255315	0.104770368	-2.875386619	0.00403533	0.021752367	negative
PRJNA729271	ed_Lushi_vs_Arbor_Acr	breed_growth_background	ENSGALG00010003962	UMAD1	-0.297151638	0.109434181	-2.71534576	0.006620661	0.031147835	negative
PRJNA729271	ed_Lushi_vs_Arbor_Acr	breed_growth_background	ENSGALG00010003965	SNTG1	0.573931302	0.292971498	1.95900047	0.050112732	0.132772549	positive
PRJNA729271	ed_Lushi_vs_Arbor_Acr	breed_growth_background	ENSGALG00010003968	DSN1	0.142861392	0.092747807	1.540320962	0.123482137	0.248697175	positive
PRJNA729271	ed_Lushi_vs_Arbor_Acr	breed_growth_background	ENSGALG00010003972	PENK	-0.265189058	0.128977805	-2.056082897	0.039774526	0.111969656	negative
PRJNA729271	ed_Lushi_vs_Arbor_Acr	breed_growth_background	ENSGALG00010003977	MYO6	-0.05536717	0.065654607	-0.843309737	0.399055238	0.560277575	negative
PRJNA729271	ed_Lushi_vs_Arbor_Acr	breed_growth_background	ENSGALG00010003978	ANK2	0.003622292	0.064218072	0.056406115	0.955018285	0.974885706	positive
PRJNA729271	ed_Lushi_vs_Arbor_Acr	breed_growth_background	ENSGALG00010003980	CHCHD7	-0.109419141	0.065173317	-1.678894779	0.093172552	0.204626689	negative
PRJNA729271	ed_Lushi_vs_Arbor_Acr	breed_growth_background	ENSGALG00010003981	TMEM150C	-0.043366801	0.08962275	-0.483881612	0.628469891	0.754486589	negative
PRJNA729271	ed_Lushi_vs_Arbor_Acr	breed_growth_background	ENSGALG00010003982	GLCCI1	-0.025277726	0.06593265	-0.383387084	0.701432779	0.80733032	negative
PRJNA729271	ed_Lushi_vs_Arbor_Acr	breed_growth_background	ENSGALG00010003985	PRMT5	0.18596512	0.07205962	2.580711904	0.009859682	0.041400955	positive
PRJNA729271	ed_Lushi_vs_Arbor_Acr	breed_growth_background	ENSGALG00010003986	FNDC3A	-0.094071954	0.034421708	-2.732925202	0.006277459	0.029931501	negative
PRJNA729271	ed_Lushi_vs_Arbor_Acr	breed_growth_background	ENSGALG00010003988	ENOPH1	0.055891658	0.04861869	1.149592012	0.25031195	0.406287174	positive
PRJNA729271	ed_Lushi_vs_Arbor_Acr	breed_growth_background	ENSGALG00010003990	ICA1	-0.087145656	0.096147736	-0.906372419	0.364738766	0.528107923	negative
PRJNA729271	ed_Lushi_vs_Arbor_Acr	breed_growth_background	ENSGALG00010003993	ENSGALG00010003993	-0.082927666	0.071816851	-1.154710405	0.248209037	0.404382375	negative
PRJNA729271	ed_Lushi_vs_Arbor_Acr	breed_growth_background	ENSGALG00010003994	SDR16C5	-0.036731035	0.044780788	-0.820240916	0.412078781	0.572361531	negative
PRJNA729271	ed_Lushi_vs_Arbor_Acr	breed_growth_background	ENSGALG00010003995	MLNR	-0.461563154	0.146947283	-3.141011833	0.001683652	0.011744244	negative
PRJNA729271	ed_Lushi_vs_Arbor_Acr	breed_growth_background	ENSGALG00010004000	ENSGALG00010004000	0.228806023	0.093435249	2.448819108	0.014332541	0.053696092	positive
PRJNA729271	ed_Lushi_vs_Arbor_Acr	breed_growth_background	ENSGALG00010004001	LARP7	0.217888557	0.061455686	3.545458055	0.000391931	0.004183788	positive
PRJNA729271	ed_Lushi_vs_Arbor_Acr	breed_growth_background	ENSGALG00010004002	CDADC1	-0.109492634	0.066092244	-1.656663883	0.097587449	0.211120251	negative
PRJNA729271	ed_Lushi_vs_Arbor_Acr	breed_growth_background	ENSGALG00010004003	HNRNPDL	0.12059154	0.036603308	3.294553067	0.000985783	0.008077329	positive
PRJNA729271	ed_Lushi_vs_Arbor_Acr	breed_growth_background	ENSGALG00010004006	NKAIN3	0.176703184	0.1839591	0.960556906	0.336775005	0.50103189	positive
PRJNA729271	ed_Lushi_vs_Arbor_Acr	breed_growth_background	ENSGALG00010004007	ENSGALG00010004007	0.252537559	0.088887234	2.841100444	0.004495815	0.02353443	positive
PRJNA729271	ed_Lushi_vs_Arbor_Acr	breed_growth_background	ENSGALG00010004016	ENSGALG00010004016	0.345087002	0.26848411	1.285316295	0.198681778	0.347309741	positive
PRJNA729271	ed_Lushi_vs_Arbor_Acr	breed_growth_background	ENSGALG00010004018	HNRNPD	0.153997632	0.086986133	1.770369916	0.076665538	0.178733945	positive
PRJNA729271	ed_Lushi_vs_Arbor_Acr	breed_growth_background	ENSGALG00010004029	ARL4A	-0.17924752	0.10424886	-1.719419484	0.085538017	0.193006469	negative
PRJNA729271	ed_Lushi_vs_Arbor_Acr	breed_growth_background	ENSGALG00010004034	NEDD8	0.053128798	0.060703237	0.875221824	0.381453221	0.542893196	positive
PRJNA729271	ed_Lushi_vs_Arbor_Acr	breed_growth_background	ENSGALG00010004036	ADCYAP1	-0.602122984	0.322466614	-1.867241312	0.061867897	0.154191053	negative
PRJNA729271	ed_Lushi_vs_Arbor_Acr	breed_growth_background	ENSGALG00010004038	SENP6	0.145264562	0.047047025	3.087646054	0.002017486	0.013328833	positive
PRJNA729271	ed_Lushi_vs_Arbor_Acr	breed_growth_background	ENSGALG00010004039	ALPK1	0.297262015	0.110750877	2.684060131	0.007273402	0.033318686	positive
PRJNA729271	ed_Lushi_vs_Arbor_Acr	breed_growth_background	ENSGALG00010004040	LGALS1B	-0.282596885	0.121070542	-2.334105655	0.019587835	0.06729691	negative
PRJNA729271	ed_Lushi_vs_Arbor_Acr	breed_growth_background	ENSGALG00010004042	PLAG1	0.0487639	0.066741693	0.730636239	0.465001371	0.620244305	positive
PRJNA729271	ed_Lushi_vs_Arbor_Acr	breed_growth_background	ENSGALG00010004043	CAB39L	-0.303799804	0.107620301	-2.822885651	0.004759354	0.024576716	negative
PRJNA729271	ed_Lushi_vs_Arbor_Acr	breed_growth_background	ENSGALG00010004044	RNF138	-0.076422785	0.072898188	-1.048349576	0.294477576	0.455804099	negative
PRJNA729271	ed_Lushi_vs_Arbor_Acr	breed_growth_background	ENSGALG00010004046	ENSGALG00010004046	-0.041658216	0.0649407	-0.641480866	0.521210308	0.668506095	negative
PRJNA729271	ed_Lushi_vs_Arbor_Acr	breed_growth_background	ENSGALG00010004047	PDE7A	0.037099282	0.065497488	0.566422983	0.571106275	0.70953848	positive
PRJNA729271	ed_Lushi_vs_Arbor_Acr	breed_growth_background	ENSGALG00010004049	SETDB2	-0.174890596	0.074577807	-2.345075606	0.019023222	0.066068426	negative
PRJNA729271	ed_Lushi_vs_Arbor_Acr	breed_growth_background	ENSGALG00010004055	FILIP1	0.137138888	0.093545109	1.466018794	0.142643167	0.275644941	positive
PRJNA729271	ed_Lushi_vs_Arbor_Acr	breed_growth_background	ENSGALG00010004056	SHROOM3	-0.378771479	0.099882191	-3.792182298	0.000149329	0.002128345	negative
PRJNA729271	ed_Lushi_vs_Arbor_Acr	breed_growth_background	ENSGALG00010004057	ENSGALG00010004057	0.208914318	0.224497123	0.930587952	0.352066749	0.516070027	positive
PRJNA729271	ed_Lushi_vs_Arbor_Acr	breed_growth_background	ENSGALG00010004058	TMEM30A	-0.076669289	0.033330437	-2.300278546	0.021432444	0.071680498	negative
PRJNA729271	ed_Lushi_vs_Arbor_Acr	breed_growth_background	ENSGALG00010004060	ETV1	-0.190748316	0.050742312	-3.759156964	0.000170487	0.002325274	negative
PRJNA729271	ed_Lushi_vs_Arbor_Acr	breed_growth_background	ENSGALG00010004063	ENSGALG00010004063	-0.251041425	0.213980932	-1.173195307	0.240717494	0.396524429	negative
PRJNA729271	ed_Lushi_vs_Arbor_Acr	breed_growth_background	ENSGALG00010004065	TIFA	0.032869844	0.1509753	0.2177167	0.827649851	0.894989313	positive
PRJNA729271	ed_Lushi_vs_Arbor_Acr	breed_growth_background	ENSGALG00010004070	ARL11	-0.278358619	0.220188386	-1.264183932	0.206164017	0.355459429	negative
PRJNA729271	ed_Lushi_vs_Arbor_Acr	breed_growth_background	ENSGALG00010004072	SGK3	-0.058506856	0.073028883	-0.801146806	0.423046662	0.582231425	negative
PRJNA729271	ed_Lushi_vs_Arbor_Acr	breed_growth_background	ENSGALG00010004076	COX7A2	-0.099837263	0.073783349	-1.353113735	0.176019298	0.320026796	negative
PRJNA729271	ed_Lushi_vs_Arbor_Acr	breed_growth_background	ENSGALG00010004079	AP1AR	0.05406231	0.081101915	0.666597209	0.505029453	0.655130385	positive
PRJNA729271	ed_Lushi_vs_Arbor_Acr	breed_growth_background	ENSGALG00010004081	RPS20	-0.045285324	0.089772326	-0.504446371	0.613947739	0.743456348	negative
PRJNA729271	ed_Lushi_vs_Arbor_Acr	breed_growth_background	ENSGALG00010004082	FAM241A	-0.122687323	0.065496402	-1.873191799	0.061041904	0.152786722	negative
PRJNA729271	ed_Lushi_vs_Arbor_Acr	breed_growth_background	ENSGALG00010004083	PHF11	0.039588091	0.100024228	0.395785013	0.692263634	0.801274564	positive
PRJNA729271	ed_Lushi_vs_Arbor_Acr	breed_growth_background	ENSGALG00010004085	RB1CC1	0.27237849	0.07520947	3.621598298	0.000292789	0.003417045	positive
PRJNA729271	ed_Lushi_vs_Arbor_Acr	breed_growth_background	ENSGALG00010004086	UTP3	0.365470057	0.138385359	2.640958984	0.008267173	0.036410802	positive
PRJNA729271	ed_Lushi_vs_Arbor_Acr	breed_growth_background	ENSGALG00010004087	NCOA7	-0.20332485	0.059327587	-3.427155213	0.00060994	0.005685585	negative
PRJNA729271	ed_Lushi_vs_Arbor_Acr	breed_growth_background	ENSGALG00010004094	DGKB	0.256515749	0.104213519	2.46144409	0.013837896	0.052618129	positive
PRJNA729271	ed_Lushi_vs_Arbor_Acr	breed_growth_background	ENSGALG00010004095	ANKRD50	-0.237461665	0.060899954	-3.899209287	9.65073E-05	0.00154914	negative
PRJNA729271	ed_Lushi_vs_Arbor_Acr	breed_growth_background	ENSGALG00010004096	COL12A1	-0.08695921	0.221463729	-0.392656671	0.694573077	0.803306212	negative
PRJNA729271	ed_Lushi_vs_Arbor_Acr	breed_growth_background	ENSGALG00010004097	ENSGALG00010004097	-0.151135394	0.06552449	-2.306548205	0.02108002	0.07077945	negative
PRJNA729271	ed_Lushi_vs_Arbor_Acr	breed_growth_background	ENSGALG00010004101	ARID1B	0.224680865	0.088962673	2.525563329	0.011551296	0.046329838	positive
PRJNA729271	ed_Lushi_vs_Arbor_Acr	breed_growth_background	ENSGALG00010004102	PCMTD1	0.241696607	0.100181164	2.412595319	0.015839395	0.057933897	positive
PRJNA729271	ed_Lushi_vs_Arbor_Acr	breed_growth_background	ENSGALG00010004104	SCARB2	-0.123710824	0.050574792	-2.446096563	0.014441232	0.053922116	negative
PRJNA729271	ed_Lushi_vs_Arbor_Acr	breed_growth_background	ENSGALG00010004108	PERPB	-0.071133479	0.129205085	-0.550547053	0.581944213	0.718201905	negative
PRJNA729271	ed_Lushi_vs_Arbor_Acr	breed_growth_background	ENSGALG00010004109	AGMO	0.60598802	0.282554772	2.144674518	0.03197888	0.095593702	positive
PRJNA729271	ed_Lushi_vs_Arbor_Acr	breed_growth_background	ENSGALG00010004113	ECHDC1	0.27181031	0.101669954	2.673457605	0.007507376	0.034027336	positive
PRJNA729271	ed_Lushi_vs_Arbor_Acr	breed_growth_background	ENSGALG00010004116	SPRY1	-0.096314421	0.051271571	-1.878515103	0.060310733	0.151403546	negative
PRJNA729271	ed_Lushi_vs_Arbor_Acr	breed_growth_background	ENSGALG00010004117	STBD1	-0.291580695	0.100323645	-2.906400525	0.003656131	0.020273765	negative
PRJNA729271	ed_Lushi_vs_Arbor_Acr	breed_growth_background	ENSGALG00010004122	TRIM55	0.326091711	0.104054062	3.133868169	0.001725183	0.011982048	positive
PRJNA729271	ed_Lushi_vs_Arbor_Acr	breed_growth_background	ENSGALG00010004123	NDUFAF4	0.223973131	0.060072716	3.728366997	0.000192725	0.002525807	positive
PRJNA729271	ed_Lushi_vs_Arbor_Acr	breed_growth_background	ENSGALG00010004125	MANEA	0.009898712	0.050099694	0.19758029	0.843373457	0.90506146	positive
PRJNA729271	ed_Lushi_vs_Arbor_Acr	breed_growth_background	ENSGALG00010004127	EPHA7	-0.07775525	0.107470104	-0.723505857	0.469369157	0.62406035	negative
PRJNA729271	ed_Lushi_vs_Arbor_Acr	breed_growth_background	ENSGALG00010004131	NUDT6	-0.012858018	0.084602925	-0.151980779	0.879202096	0.92824647	negative
PRJNA729271	ed_Lushi_vs_Arbor_Acr	breed_growth_background	ENSGALG00010004133	CRH	-0.954836341	0.499121076	-1.913035508	0.055743507	0.143282278	negative
PRJNA729271	ed_Lushi_vs_Arbor_Acr	breed_growth_background	ENSGALG00010004135	NUP54	0.029863434	0.036280314	0.823130536	0.410433765	0.570944091	positive
PRJNA729271	ed_Lushi_vs_Arbor_Acr	breed_growth_background	ENSGALG00010004137	CROT	-0.040698004	0.045520464	-0.894059516	0.371290048	0.534337694	negative
PRJNA729271	ed_Lushi_vs_Arbor_Acr	breed_growth_background	ENSGALG00010004139	PPEF2	-0.022845802	0.339013733	-0.067389017	0.946272013	0.970509646	negative
PRJNA729271	ed_Lushi_vs_Arbor_Acr	breed_growth_background	ENSGALG00010004141	KPNA3	0.153435223	0.075648792	2.028257404	0.042533984	0.117815803	positive
PRJNA729271	ed_Lushi_vs_Arbor_Acr	breed_growth_background	ENSGALG00010004142	RBBP8	-0.092154581	0.063528664	-1.450598454	0.146891706	0.281453441	negative
PRJNA729271	ed_Lushi_vs_Arbor_Acr	breed_growth_background	ENSGALG00010004143	TCF24	-0.694421995	0.23630467	-2.938672327	0.003296214	0.018982366	negative
PRJNA729271	ed_Lushi_vs_Arbor_Acr	breed_growth_background	ENSGALG00010004146	NAAA	0.000412348	0.090859142	0.004538323	0.996378955	0.998171223	positive
PRJNA729271	ed_Lushi_vs_Arbor_Acr	breed_growth_background	ENSGALG00010004147	SPRYD7	0.074466943	0.049417806	1.506884831	0.13184018	0.260570413	positive
PRJNA729271	ed_Lushi_vs_Arbor_Acr	breed_growth_background	ENSGALG00010004148	ENSGALG00010004148	-0.089827578	0.082890184	-1.083693788	0.278500591	0.437835737	negative
PRJNA729271	ed_Lushi_vs_Arbor_Acr	breed_growth_background	ENSGALG00010004149	SDAD1	0.120703837	0.066571346	1.813150014	0.069808687	0.167668089	positive
PRJNA729271	ed_Lushi_vs_Arbor_Acr	breed_growth_background	ENSGALG00010004154	COPS5	0.176390895	0.069516118	2.537410045	0.011167606	0.045266906	positive
PRJNA729271	ed_Lushi_vs_Arbor_Acr	breed_growth_background	ENSGALG00010004158	JCHAIN	0.276769238	0.249781326	1.108046157	0.267841882	0.426250921	positive
PRJNA729271	ed_Lushi_vs_Arbor_Acr	breed_growth_background	ENSGALG00010004172	ST8SIA6	-0.008572174	0.068817637	-0.124563626	0.900869026	0.942711173	negative
PRJNA729271	ed_Lushi_vs_Arbor_Acr	breed_growth_background	ENSGALG00010004177	CSPP1	0.218156204	0.069030794	3.160273696	0.00157621	0.01123093	positive
PRJNA729271	ed_Lushi_vs_Arbor_Acr	breed_growth_background	ENSGALG00010004181	BBS12	-0.171069197	0.072074507	-2.373504927	0.017620158	0.062461284	negative
PRJNA729271	ed_Lushi_vs_Arbor_Acr	breed_growth_background	ENSGALG00010004187	ARFGEF1	0.203457765	0.050395261	4.037240016	5.40837E-05	0.001024763	positive
PRJNA729271	ed_Lushi_vs_Arbor_Acr	breed_growth_background	ENSGALG00010004188	BMI1	0.147132425	0.06229661	2.361804691	0.018186221	0.063982832	positive
PRJNA729271	ed_Lushi_vs_Arbor_Acr	breed_growth_background	ENSGALG00010004191	CABLES1	-0.017651875	0.068463844	-0.257827691	0.796539893	0.873692451	negative
PRJNA729271	ed_Lushi_vs_Arbor_Acr	breed_growth_background	ENSGALG00010004200	ABCB1	0.07297699	0.09742312	0.749072597	0.45381345	0.610126592	positive
PRJNA729271	ed_Lushi_vs_Arbor_Acr	breed_growth_background	ENSGALG00010004201	CPA6	0.296082449	0.307296535	0.963507281	0.335293012	0.499544887	positive
PRJNA729271	ed_Lushi_vs_Arbor_Acr	breed_growth_background	ENSGALG00010004210	SYNCRIP	-0.004918329	0.037995353	-0.129445541	0.897005115	0.9402177	negative
PRJNA729271	ed_Lushi_vs_Arbor_Acr	breed_growth_background	ENSGALG00010004211	RNF146	0.026884159	0.040766155	0.659472525	0.509592384	0.658395722	positive
PRJNA729271	ed_Lushi_vs_Arbor_Acr	breed_growth_background	ENSGALG00010004212	RSPO3	-0.29215017	0.207966649	-1.404793369	0.160082731	0.298882782	negative
PRJNA729271	ed_Lushi_vs_Arbor_Acr	breed_growth_background	ENSGALG00010004214	REPS1	0.16497056	0.067891143	2.429927579	0.01510184	0.055899176	positive
PRJNA729271	ed_Lushi_vs_Arbor_Acr	breed_growth_background	ENSGALG00010004216	SNX14	-0.053390558	0.039793436	-1.341692575	0.179695696	0.324006606	negative
PRJNA729271	ed_Lushi_vs_Arbor_Acr	breed_growth_background	ENSGALG00010004217	CCDC28A	0.118335609	0.0612002	1.933582075	0.053164533	0.138451294	positive
PRJNA729271	ed_Lushi_vs_Arbor_Acr	breed_growth_background	ENSGALG00010004219	FGF2	-0.227327528	0.097526576	-2.330929023	0.019757103	0.067622008	negative
PRJNA729271	ed_Lushi_vs_Arbor_Acr	breed_growth_background	ENSGALG00010004222	SPAG6	-0.404925932	0.141400419	-2.863682688	0.004187473	0.02229883	negative
PRJNA729271	ed_Lushi_vs_Arbor_Acr	breed_growth_background	ENSGALG00010004224	NT5E	-0.419569998	0.137898498	-3.042600215	0.002345437	0.014950356	negative
PRJNA729271	ed_Lushi_vs_Arbor_Acr	breed_growth_background	ENSGALG00010004225	CETN4	-0.133780751	0.070257708	-1.904143382	0.056891513	0.145281716	negative
PRJNA729271	ed_Lushi_vs_Arbor_Acr	breed_growth_background	ENSGALG00010004226	RUNDC3B	0.076992725	0.108000293	0.712893667	0.475911559	0.628960067	positive
PRJNA729271	ed_Lushi_vs_Arbor_Acr	breed_growth_background	ENSGALG00010004230	TAF1B	0.080376087	0.063281631	1.27013298	0.204037266	0.353389911	positive
PRJNA729271	ed_Lushi_vs_Arbor_Acr	breed_growth_background	ENSGALG00010004231	ARMC3	-0.35324382	0.221658754	-1.593638032	0.111017131	0.231705301	negative
PRJNA729271	ed_Lushi_vs_Arbor_Acr	breed_growth_background	ENSGALG00010004233	NHSL1	-0.132591157	0.067499469	-1.964328906	0.049491962	0.13153503	negative
PRJNA729271	ed_Lushi_vs_Arbor_Acr	breed_growth_background	ENSGALG00010004235	ADAD1	0.530747925	0.257576195	2.060547271	0.039346252	0.111130927	positive
PRJNA729271	ed_Lushi_vs_Arbor_Acr	breed_growth_background	ENSGALG00010004236	DBF4	-0.093871822	0.069208269	-1.356367142	0.174982382	0.318919858	negative
PRJNA729271	ed_Lushi_vs_Arbor_Acr	breed_growth_background	ENSGALG00010004237	RUFY3	0.001128038	0.049054522	0.02299559	0.98165379	0.989091595	positive
PRJNA729271	ed_Lushi_vs_Arbor_Acr	breed_growth_background	ENSGALG00010004238	CCNC	-0.064024681	0.067226687	-0.95237001	0.340909364	0.50482449	negative
PRJNA729271	ed_Lushi_vs_Arbor_Acr	breed_growth_background	ENSGALG00010004240	ENSGALG00010004240	-0.30681736	0.094167836	-3.258196991	0.001121225	0.00889068	negative

PRJNA729271	ed_Lushi_vs_Arbor_Acr	breed_growth_background	ENSGALG00010004241	PREX2	-0.208390425	0.061647639	-3.380347241	0.000723943	0.00646553	negative
PRJNA729271	ed_Lushi_vs_Arbor_Acr	breed_growth_background	ENSGALG00010004242	ADAM22	-0.024879137	0.096084902	-0.25892868	0.795690269	0.873418163	negative
PRJNA729271	ed_Lushi_vs_Arbor_Acr	breed_growth_background	ENSGALG00010004243	BLTP1	0.077645376	0.060460801	1.284226716	0.19906264	0.347723905	positive
PRJNA729271	ed_Lushi_vs_Arbor_Acr	breed_growth_background	ENSGALG00010004244	TMEM241	-0.053369733	0.044320457	-1.204178309	0.228520668	0.382053873	negative
PRJNA729271	ed_Lushi_vs_Arbor_Acr	breed_growth_background	ENSGALG00010004245	ARFGEF3	0.219987274	0.123972062	1.774490734	0.075982013	0.177704179	positive
PRJNA729271	ed_Lushi_vs_Arbor_Acr	breed_growth_background	ENSGALG00010004246	C8orf34	-0.250462623	0.195095115	-1.283797514	0.199212814	0.347860466	negative
PRJNA729271	ed_Lushi_vs_Arbor_Acr	breed_growth_background	ENSGALG00010004247	MEOX2	-0.366248819	0.056297035	-6.50565027	7.7358E-11	2.24261E-08	negative
PRJNA729271	ed_Lushi_vs_Arbor_Acr	breed_growth_background	ENSGALG00010004248	GRSF1	0.410662134	0.085463933	4.805092878	1.5468E-06	6.94143E-05	positive
PRJNA729271	ed_Lushi_vs_Arbor_Acr	breed_growth_background	ENSGALG00010004249	USP45	0.111940101	0.054116603	2.068498308	0.03859319	0.109676486	positive
PRJNA729271	ed_Lushi_vs_Arbor_Acr	breed_growth_background	ENSGALG00010004250	MOB1B	-0.05998614	0.047771047	-1.255700755	0.209224485	0.358995622	negative
PRJNA729271	ed_Lushi_vs_Arbor_Acr	breed_growth_background	ENSGALG00010004251	CCN2	-0.530857565	0.142776869	-3.718092214	0.000200733	0.002597879	negative
PRJNA729271	ed_Lushi_vs_Arbor_Acr	breed_growth_background	ENSGALG00010004255	RPS12	-0.088106859	0.080764088	-1.090916278	0.275309724	0.434225232	negative
PRJNA729271	ed_Lushi_vs_Arbor_Acr	breed_growth_background	ENSGALG00010004256	ENSGALG00010004256	-0.385861239	0.315155003	-1.224353845	0.220818773	0.373317689	negative
PRJNA729271	ed_Lushi_vs_Arbor_Acr	breed_growth_background	ENSGALG00010004257	ENSGALG00010004257	-0.668019371	0.350318701	-1.906890411	0.05653478	0.144706275	negative
PRJNA729271	ed_Lushi_vs_Arbor_Acr	breed_growth_background	ENSGALG00010004265	PIP4K2A	-0.083990682	0.049563976	-1.694591287	0.090152981	0.200424457	negative
PRJNA729271	ed_Lushi_vs_Arbor_Acr	breed_growth_background	ENSGALG00010004275	ADARB2	0.498372287	0.415747682	1.198737381	0.230630078	0.384516101	positive
PRJNA729271	ed_Lushi_vs_Arbor_Acr	breed_growth_background	ENSGALG00010004281	VIM	-0.290157782	0.091745651	-3.162632559	0.001563495	0.011180496	negative
PRJNA729271	ed_Lushi_vs_Arbor_Acr	breed_growth_background	ENSGALG00010004283	DCK	-0.204817974	0.071240211	-2.875033223	0.00403985	0.021768633	negative
PRJNA729271	ed_Lushi_vs_Arbor_Acr	breed_growth_background	ENSGALG00010004285	SLC18B1	0.075765669	0.065076451	1.164256321	0.244320152	0.40016052	positive
PRJNA729271	ed_Lushi_vs_Arbor_Acr	breed_growth_background	ENSGALG00010004296	ENSGALG00010004296	-0.128746826	0.430274175	-0.299220436	0.764771858	0.852392778	negative
PRJNA729271	ed_Lushi_vs_Arbor_Acr	breed_growth_background	ENSGALG00010004297	SULF1	-0.0871378	0.099657398	-0.874373616	0.381914821	0.543370173	negative
PRJNA729271	ed_Lushi_vs_Arbor_Acr	breed_growth_background	ENSGALG00010004300	VNN2	-0.016464531	0.12889601	-0.12773499	0.898358699	0.941162567	negative
PRJNA729271	ed_Lushi_vs_Arbor_Acr	breed_growth_background	ENSGALG00010004302	CRPPA	0.207591947	0.068921635	3.011999726	0.002595328	0.015981004	positive
PRJNA729271	ed_Lushi_vs_Arbor_Acr	breed_growth_background	ENSGALG00010004306	PNISR	0.092577264	0.077184797	1.199423563	0.23036329	0.384203876	positive
PRJNA729271	ed_Lushi_vs_Arbor_Acr	breed_growth_background	ENSGALG00010004307	NPC2	0.025329775	0.075167106	0.336979522	0.736132337	0.830886017	positive
PRJNA729271	ed_Lushi_vs_Arbor_Acr	breed_growth_background	ENSGALG00010004308	DSG2	-0.529700395	0.269602052	-1.964749117	0.049443282	0.131452746	negative
PRJNA729271	ed_Lushi_vs_Arbor_Acr	breed_growth_background	ENSGALG00010004309	MRPS28	-0.074078905	0.073645015	-1.005891634	0.314467701	0.477940308	negative
PRJNA729271	ed_Lushi_vs_Arbor_Acr	breed_growth_background	ENSGALG00010004312	PDLIM5	-0.014609251	0.096916608	-0.150740427	0.880180482	0.928845226	negative
PRJNA729271	ed_Lushi_vs_Arbor_Acr	breed_growth_background	ENSGALG00010004319	TAAR1	-0.10722157	0.257892829	-0.415760183	0.677585498	0.791617779	negative
PRJNA729271	ed_Lushi_vs_Arbor_Acr	breed_growth_background	ENSGALG00010004323	TRDMT1	-0.197239714	0.085809888	-2.298566276	0.02152958	0.071877868	negative
PRJNA729271	ed_Lushi_vs_Arbor_Acr	breed_growth_background	ENSGALG00010004325	LTBP2	-0.213680344	0.060537506	-3.529718325	0.000416002	0.004366367	negative
PRJNA729271	ed_Lushi_vs_Arbor_Acr	breed_growth_background	ENSGALG00010004326	ENSGALG00010004326	-1.093173433	0.512824441	-2.131671865	0.033033831	0.09763976	negative
PRJNA729271	ed_Lushi_vs_Arbor_Acr	breed_growth_background	ENSGALG00010004328	ENSGALG00010004328	-0.954781443	0.378006023	-2.525836589	0.011542316	0.046319453	negative
PRJNA729271	ed_Lushi_vs_Arbor_Acr	breed_growth_background	ENSGALG00010004329	COQ3	-0.07032143	0.064302099	-1.093610174	0.274125985	0.433066223	negative
PRJNA729271	ed_Lushi_vs_Arbor_Acr	breed_growth_background	ENSGALG00010004330	STX7	-0.010224189	0.055454262	-0.184371562	0.853721977	0.911714438	negative
PRJNA729271	ed_Lushi_vs_Arbor_Acr	breed_growth_background	ENSGALG00010004332	ENSGALG00010004332	-0.574385408	0.485947928	-1.181989623	0.237209818	0.39231764	negative
PRJNA729271	ed_Lushi_vs_Arbor_Acr	breed_growth_background	ENSGALG00010004333	DIO2	-0.331976735	0.175752169	-1.888891249	0.058906402	0.148779979	negative
PRJNA729271	ed_Lushi_vs_Arbor_Acr	breed_growth_background	ENSGALG00010004334	ENSGALG00010004334	-0.057807375	0.124549804	-0.464130599	0.64255418	0.765939379	negative
PRJNA729271	ed_Lushi_vs_Arbor_Acr	breed_growth_background	ENSGALG00010004336	ENSGALG00010004336	0.094948735	0.31103512	0.305266927	0.760162863	0.848952978	positive
PRJNA729271	ed_Lushi_vs_Arbor_Acr	breed_growth_background	ENSGALG00010004337	BMPR1B	0.151339285	0.129943934	1.16465063	0.244160441	0.399944128	positive
PRJNA729271	ed_Lushi_vs_Arbor_Acr	breed_growth_background	ENSGALG00010004338	ENSGALG00010004338	-0.137387194	0.088146967	-1.558615096	0.119087508	0.243140562	negative
PRJNA729271	ed_Lushi_vs_Arbor_Acr	breed_growth_background	ENSGALG00010004339	B4GALT6	-0.233879397	0.072669176	-3.218412663	0.001289022	0.009797785	negative
PRJNA729271	ed_Lushi_vs_Arbor_Acr	breed_growth_background	ENSGALG00010004340	SLC4A4	-0.110650342	0.090670329	-1.220358888	0.222328855	0.374945521	negative
PRJNA729271	ed_Lushi_vs_Arbor_Acr	breed_growth_background	ENSGALG00010004341	ENSGALG00010004341	-0.086554597	0.092440363	-0.936329044	0.349103799	0.513074224	negative
PRJNA729271	ed_Lushi_vs_Arbor_Acr	breed_growth_background	ENSGALG00010004342	ZNF438	-0.134033365	0.070619912	-1.897954284	0.057702104	0.146607341	negative
PRJNA729271	ed_Lushi_vs_Arbor_Acr	breed_growth_background	ENSGALG00010004344	SOSTDC1	-0.648114652	0.226198891	-2.865242401	0.004166901	0.022253504	negative
PRJNA729271	ed_Lushi_vs_Arbor_Acr	breed_growth_background	ENSGALG00010004345	FAXC	-0.522636596	0.105617945	-4.948369296	7.48378E-07	3.84672E-05	negative
PRJNA729271	ed_Lushi_vs_Arbor_Acr	breed_growth_background	ENSGALG00010004346	CEP128	-0.007185718	0.056607563	-0.126939181	0.898988536	0.941398557	negative
PRJNA729271	ed_Lushi_vs_Arbor_Acr	breed_growth_background	ENSGALG00010004350	MOXD1	-0.017837419	0.12926971	-0.137986064	0.890251431	0.935832511	negative
PRJNA729271	ed_Lushi_vs_Arbor_Acr	breed_growth_background	ENSGALG00010004352	CHMP2A	-0.058969303	0.030297229	-1.946359613	0.051611557	0.135489897	negative
PRJNA729271	ed_Lushi_vs_Arbor_Acr	breed_growth_background	ENSGALG00010004355	ANKMY2	0.039773056	0.066148956	0.601265061	0.547663457	0.691255817	positive
PRJNA729271	ed_Lushi_vs_Arbor_Acr	breed_growth_background	ENSGALG00010004356	TRAPPC8	0.083562755	0.041653797	2.00612575	0.044842833	0.122433013	positive
PRJNA729271	ed_Lushi_vs_Arbor_Acr	breed_growth_background	ENSGALG00010004358	GC	-2.747018304	1.102172839	-2.492366177	0.012689514	0.04941819	negative
PRJNA729271	ed_Lushi_vs_Arbor_Acr	breed_growth_background	ENSGALG00010004362	FBXL4	-0.125424497	0.064481125	-1.945135071	0.051758724	0.135741397	negative
PRJNA729271	ed_Lushi_vs_Arbor_Acr	breed_growth_background	ENSGALG00010004364	TSHR	-0.473978523	0.24138254	-1.963599034	0.049576609	0.131710585	negative
PRJNA729271	ed_Lushi_vs_Arbor_Acr	breed_growth_background	ENSGALG00010004365	TPD52	-0.047931599	0.084256074	-0.568880049	0.569437545	0.708376006	negative
PRJNA729271	ed_Lushi_vs_Arbor_Acr	breed_growth_background	ENSGALG00010004366	UNC5C	-0.310252584	0.207137231	-1.497811777	0.13418216	0.264093507	negative
PRJNA729271	ed_Lushi_vs_Arbor_Acr	breed_growth_background	ENSGALG00010004367	TSPAN13	-0.221101701	0.096278647	-2.296477023	0.021648621	0.072203582	negative
PRJNA729271	ed_Lushi_vs_Arbor_Acr	breed_growth_background	ENSGALG00010004368	GJD4	-0.009141779	0.230761447	-0.039615714	0.9683995	0.982016982	negative
PRJNA729271	ed_Lushi_vs_Arbor_Acr	breed_growth_background	ENSGALG00010004369	UBE2M	0.210454849	0.12633062	1.665905298	0.095732278	0.208419674	positive
PRJNA729271	ed_Lushi_vs_Arbor_Acr	breed_growth_background	ENSGALG00010004370	ATP6V1H	-0.153319051	0.040739413	-3.763408453	0.000167613	0.002313859	negative
PRJNA729271	ed_Lushi_vs_Arbor_Acr	breed_growth_background	ENSGALG00010004372	SYNJ2	-0.270219811	0.100682613	-2.683877623	0.007277374	0.033318686	negative
PRJNA729271	ed_Lushi_vs_Arbor_Acr	breed_growth_background	ENSGALG00010004373	MMS22L	0.025046148	0.069618014	0.359765331	0.719022631	0.82014807	positive
PRJNA729271	ed_Lushi_vs_Arbor_Acr	breed_growth_background	ENSGALG00010004374	ILDR1	-0.305609154	0.397930816	-0.767995696	0.442489742	0.599540924	negative
PRJNA729271	ed_Lushi_vs_Arbor_Acr	breed_growth_background	ENSGALG00010004375	ENSGALG00010004375	0.166728899	0.119774264	1.392026079	0.163914501	0.303727027	positive
PRJNA729271	ed_Lushi_vs_Arbor_Acr	breed_growth_background	ENSGALG00010004377	AGR2	-0.486660984	0.461074249	-1.055493741	0.29119957	0.452548661	negative
PRJNA729271	ed_Lushi_vs_Arbor_Acr	breed_growth_background	ENSGALG00010004378	TRIM28	0.190183639	0.083742221	2.271060369	0.023143324	0.075725164	positive
PRJNA729271	ed_Lushi_vs_Arbor_Acr	breed_growth_background	ENSGALG00010004379	ZBTB10	-0.192896956	0.032534584	-5.928981701	3.04819E-09	5.25994E-07	negative
PRJNA729271	ed_Lushi_vs_Arbor_Acr	breed_growth_background	ENSGALG00010004380	ISCA2	0.063447965	0.045519962	1.393849245	0.163363134	0.303194446	positive
PRJNA729271	ed_Lushi_vs_Arbor_Acr	breed_growth_background	ENSGALG00010004381	ENSGALG00010004381	0.191477589	0.252049495	0.759682493	0.447444396	0.604052018	positive
PRJNA729271	ed_Lushi_vs_Arbor_Acr	breed_growth_background	ENSGALG00010004383	ENSGALG00010004383	0.03748426	0.282085323	0.132882701	0.894286148	0.938112726	positive
PRJNA729271	ed_Lushi_vs_Arbor_Acr	breed_growth_background	ENSGALG00010004384	ENSGALG00010004384	0.178656003	0.271297815	0.658523562	0.510201762	0.658786517	positive
PRJNA729271	ed_Lushi_vs_Arbor_Acr	breed_growth_background	ENSGALG00010004385	ADAMTS3	-0.039302828	0.112201866	-0.350286759	0.726123501	0.824474505	negative
PRJNA729271	ed_Lushi_vs_Arbor_Acr	breed_growth_background	ENSGALG00010004386	CFAP44	-0.119022278	0.219291429	-0.542758461	0.587296117	0.72308309	negative
PRJNA729271	ed_Lushi_vs_Arbor_Acr	breed_growth_background	ENSGALG00010004389	ENSGALG00010004389	0.011313158	0.0735043	0.153911514	0.877679505	0.927662566	positive
PRJNA729271	ed_Lushi_vs_Arbor_Acr	breed_growth_background	ENSGALG00010004390	PNLDC1	0.154855883	0.299913211	0.51633565	0.605620001	0.73704479	positive
PRJNA729271	ed_Lushi_vs_Arbor_Acr	breed_growth_background	ENSGALG00010004393	ZBTB45	0.189627733	0.112136763	1.691039825	0.090829197	0.201450683	positive
PRJNA729271	ed_Lushi_vs_Arbor_Acr	breed_growth_background	ENSGALG00010004394	CCNY	-0.116920756	0.048785134	-2.396647221	0.016545845	0.059702583	negative
PRJNA729271	ed_Lushi_vs_Arbor_Acr	breed_growth_background	ENSGALG00010004395	BOC	-0.040027415	0.103140782	-0.388085243	0.697952952	0.805308638	negative
PRJNA729271	ed_Lushi_vs_Arbor_Acr	breed_growth_background	ENSGALG00010004397	ENSGALG00010004397	-0.067179913	0.092077399	-0.729602639	0.465633109	0.620687136	negative
PRJNA729271	ed_Lushi_vs_Arbor_Acr	breed_growth_background	ENSGALG00010004398	COX18	-0.047719525	0.069033014	-0.691256582	0.489404312	0.641259841	negative
PRJNA729271	ed_Lushi_vs_Arbor_Acr	breed_growth_background	ENSGALG00010004399	POU3F2	0.193483608	0.328034545	0.589826929	0.555306687	0.697196692	positive
PRJNA729271	ed_Lushi_vs_Arbor_Acr	breed_growth_background	ENSGALG00010004404	RGS20	-0.081841917	0.236200778	-0.346493003	0.728972242	0.825762164	negative
PRJNA729271	ed_Lushi_vs_Arbor_Acr	breed_growth_background	ENSGALG00010004406	ENSGALG00010004406	-0.167290652	0.081370323	-2.055917269	0.039790491	0.111971107	negative
PRJNA729271	ed_Lushi_vs_Arbor_Acr	breed_growth_background	ENSGALG00010004408	SERAC1	-0.051843495	0.060049188	-0.863350473	0.387944801	0.550005858	negative
PRJNA729271	ed_Lushi_vs_Arbor_Acr	breed_growth_background	ENSGALG00010004416	STXBP6	-0.294567813	0.114632171	-2.569678392	0.010179297	0.042301865	negative
PRJNA729271	ed_Lushi_vs_Arbor_Acr	breed_growth_background	ENSGALG00010004420	ENSGALG00010004420	0.139506434	0.146601725	0.951601588	0.341299076	0.505248701	positive
PRJNA729271	ed_Lushi_vs_Arbor_Acr	breed_growth_background	ENSGALG00010004421	RAP1GDS1	-0.107842954	0.040887271	-2.637567916	0.00835029	0.03670026	negative
PRJNA729271	ed_Lushi_vs_Arbor_Acr	breed_growth_background	ENSGALG00010004424	MRPL18	0.097072177	0.077779883	1.248037066	0.212017484	0.362148648	positive
PRJNA729271	ed_Lushi_vs_Arbor_Acr	breed_growth_background	ENSGALG00010004425	CREM	0.223330591	0.128410777	1.739188846	0.082001551	0.187065231	positive
PRJNA729271	ed_Lushi_vs_Arbor_Acr	breed_growth_background	ENSGALG00010004429	ENSGALG00010004429	-0.90804947	0.186921743	-4.857912489	1.1863E-06	5.63783E-05	negative
PRJNA729271	ed_Lushi_vs_Arbor_Acr	breed_growth_background	ENSGALG00010004430	TCEA1	0.021212358	0.051288618	0.413588027	0.679175843	0.792633988	positive
PRJNA729271	ed_Lushi_vs_Arbor_Acr	breed_growth_background	ENSGALG00010004433	AHR1A	-0.124015326	0.085679468	-1.447433435	0.147775567	0.282623401	negative
PRJNA729271	ed_Lushi_vs_Arbor_Acr	breed_growth_background	ENSGALG00010004434	TCP1	0.022847648	0.046504532	0.491299382	0.623214717	0.749916763	positive
PRJNA729271	ed_Lushi_vs_Arbor_Acr	breed_growth_background	ENSGALG00010004436	NOVA1	-0.312796542	0.081471591	-3.839332703	0.000123369	0.001860807	negative
PRJNA729271	ed_Lushi_vs_Arbor_Acr	breed_growth_background	ENSGALG00010004440	GTF2H5	-0.022512616	0.056171282	-0.40078516	0.688578306	0.798539286	negative
PRJNA729271	ed_Lushi_vs_Arbor_Acr	breed_growth_background	ENSGALG00010004443	ANKRD17	0.072135852	0.050427104	1.430497636	0.152574244	0.289017729	positive
PRJNA729271	ed_Lushi_vs_Arbor_Acr	breed_growth_background	ENSGALG00010004445	EPHA6	0.062421587	0.318936121	0.195718149	0.844830778	0.905734415	positive
PRJNA729271	ed_Lushi_vs_Arbor_Acr	breed_growth_background	ENSGALG00010004446	ACAT2	-0.252962268	0.070160583	-3.605475568	0.000311582	0.003550612	negative
PRJNA729271	ed_Lushi_vs_Arbor_Acr	breed_growth_background	ENSGALG00010004451	CLDND1	-0.103212853	0.045812519	-2.252939914	0.024262938	0.078273733	negative
PRJNA729271	ed_Lushi_vs_Arbor_Acr	breed_growth_background	ENSGALG00010004454	SRI	-0.196892039	0.087954229	-2.238573871	0.025183656	0.08042236	negative
PRJNA729271	ed_Lushi_vs_Arbor_Acr	breed_growth_background	ENSGALG00010004457	AGA	-0.271862648	0.068132891	-3.99018218	6.60226E-05	0.001175672	negative
PRJNA729271	ed_Lushi_vs_Arbor_Acr	breed_growth_background	ENSGALG00010004460	ENSGALG00010004460	-0.218891004	0.384222722	-0.569698228	0.568882392	0.707806891	negative
PRJNA729271	ed_Lushi_vs_Arbor_Acr	breed_growth_background	ENSGALG00010004468	LYPLA1	0.065554007	0.065555092	0.999983441	0.317318522	0.480634474	positive
PRJNA729271	ed_Lushi_vs_Arbor_Acr	breed_growth_background	ENSGALG00010004471	C1H3ORF52	-0.047772636	0.143192951	-0.333624217	0.738663142	0.832575602	negative
PRJNA729271	ed_Lushi_vs_Arbor_Acr	breed_growth_background	ENSGALG00010004472	NEIL3	-0.31818433	0.11879699	-2.678387139	0.007397766	0.033699125	negative
PRJNA729271	ed_Lushi_vs_Arbor_Acr	breed_growth_background	ENSGALG00010004473	JDP2	-0.074678366	0.072910587	-1.024245849	0.305719168	0.468279164	negative
PRJNA729271	ed_Lushi_vs_Arbor_Acr	breed_growth_background	ENSGALG00010004477	TULP4	0.175871321	0.068014773	2.585781202	0.009715858	0.040966741	positive
PRJNA729271	ed_Lushi_vs_Arbor_Acr	breed_growth_background	ENSGALG00010004481	ZNF704	-0.133409193	0.042926742	-3.107834096	0.001884638	0.012723723	negative
PRJNA729271	ed_Lushi_vs_Arbor_Acr	breed_growth_background	ENSGALG00010004483	STEAP4	-0.301463836	0.349731197	-0.86198726	0.388694529	0.55069174	negative
PRJNA729271	ed_Lushi_vs_Arbor_Acr	breed_growth_background	ENSGALG00010004487	ABHD10	0.09826837	0.054181418	1.813691364	0.069725255	0.167523218	positive
PRJNA729271	ed_Lushi_vs_Arbor_Acr	breed_growth_background	ENSGALG00010004489	LIG1	-0.162090311	0.143054955	-1.133063243	0.257187696	0.414445319	negative
PRJNA729271	ed_Lushi_vs_Arbor_Acr	breed_growth_background	ENSGALG00010004494	WTAP	0.070000792	0.031071934	2.252862385	0.024267827	0.078273733	positive
PRJNA729271	ed_Lushi_vs_Arbor_Acr	breed_growth_background	ENSGALG00010004495	MRPL15	-0.054033749	0.060875697	-0.887607901	0.374751702	0.537113438	negative
PRJNA729271	ed_Lushi_vs_Arbor_Acr	breed_growth_background	ENSGALG00010004501	TAGLN3	0.318763921	0.189336243	1.683586384	0.092261632	0.203211116	positive
PRJNA729271	ed_Lushi_vs_Arbor_Acr	breed_growth_background	ENSGALG00010004503	ENSGALG00010004503	-0.130315255	0.098965881	-1.316769505	0.187915898	0.333722241	negative
PRJNA729271	ed_Lushi_vs_Arbor_Acr	breed_growth_background	ENSGALG00010004504	ZNF804B	-0.502968111	0.396730895	-1.267781553	0.204875972	0.354292199	negative
PRJNA729271	ed_Lushi_vs_Arbor_Acr	breed_growth_background	ENSGALG00010004505	ENSGALG00010004505	0.173264195	0.069965304	2.476430262	0.013270355	0.05099199	positive
PRJNA729271	ed_Lushi_vs_Arbor_Acr	breed_growth_background	ENSGALG00010004507	CUL2	-0.017385853	0.048338207	-0.359671025	0.719093163	0.82014807	negative
PRJNA729271	ed_Lushi_vs_Arbor_Acr	breed_growth_background	ENSGALG00010004515	ENSGALG00010004515	-0.000840693	0.138779928	-0.006057745	0.995166648	0.997299542	negative
PRJNA729271	ed_Lushi_vs_Arbor_Acr	breed_growth_background	ENSGALG00010004516	ENSGALG00010004516	0.097694206	0.155284956	0.629128592	0.529264873	0.67514691	positive
PRJNA729271	ed_Lushi_vs_Arbor_Acr	breed_growth_background	ENSGALG00010004522	TSPAN5	-0.009371361	0.03444237	-0.272088168	0.785554227	0.867250796	negative
PRJNA729271	ed_Lushi_vs_Arbor_Acr	breed_growth_background	ENSGALG00010004533	PAG1	-0.356615829	0.060775937	-5.867714201	4.41844E-09	6.88659E-07	negative
PRJNA729271	ed_Lushi_vs_Arbor_Acr	breed_growth_background	ENSGALG00010004534	CUBN	-0.378453358	0.113571535	-3.3322906	0.000861343	0.007316166	negative
PRJNA729271	ed_Lushi_vs_Arbor_Acr	breed_growth_background	ENSGALG00010004542	ERMARD	0.008403449	0.070882295	0.11855498	0.905627934	0.945822963	positive
PRJNA729271	ed_Lushi_vs_Arbor_Acr	breed_growth_background	ENSGALG00010004545	TMPRSS7	0.113692675	0.325691961	0.349080385	0.727028962	0.824527408	positive
PRJNA729271	ed_Lushi_vs_Arbor_Acr	breed_growth_background	ENSGALG00010004553	BATF	0.076217766	0.198085201	0.384772639	0.700405872	0.806705055	positive
PRJNA729271	ed_Lushi_vs_Arbor_Acr	breed_growth_background	ENSGALG00010004565	ENSGALG00010004565	0.113539281	0.187076917	0.606912299	0.543909116	0.68825514	positive
PRJNA729271	ed_Lushi_vs_Arbor_Acr	breed_growth_background	ENSGALG00010004568	RSU1	-0.123575376	0.036532843	-3.382583083	0.000718076	0.006440907	negative
PRJNA729271	ed_Lushi_vs_Arbor_Acr	breed_growth_background	ENSGALG00010004570	ENSGALG00010004570	-0.699710657	0.356047334	-1.96521808	0.049389002	0.131388369	negative
PRJNA729271	ed_Lushi_vs_Arbor_Acr	breed_growth_background	ENSGALG00010004576	STEAP2	-0.186753769	0.113080896	-1.651505917	0.098635314	0.212840426	negative

PRJNA729271	ed_Lushi_vs_Arbor_Acr	breed_growth_background	ENSGALG00010004577	ZPLD1	-0.180009062	0.256114401	-0.70284631	0.482151534	0.634709516	negative
PRJNA729271	ed_Lushi_vs_Arbor_Acr	breed_growth_background	ENSGALG00010004583	XKR4	-0.055633103	0.061472894	-0.905002191	0.365464227	0.528797399	negative
PRJNA729271	ed_Lushi_vs_Arbor_Acr	breed_growth_background	ENSGALG00010004584	EIF4E	0.053273862	0.035870889	1.485155878	0.137502537	0.268684183	positive
PRJNA729271	ed_Lushi_vs_Arbor_Acr	breed_growth_background	ENSGALG00010004586	ENSGALG00010004586	0.280627346	0.22531141	1.245508809	0.21294478	0.36326169	positive
PRJNA729271	ed_Lushi_vs_Arbor_Acr	breed_growth_background	ENSGALG00010004588	MINDY3	-0.056411582	0.083443438	-0.676045747	0.499011601	0.650321282	negative
PRJNA729271	ed_Lushi_vs_Arbor_Acr	breed_growth_background	ENSGALG00010004591	RRS1	0.129762128	0.086961717	1.492175315	0.135653192	0.26585898	positive
PRJNA729271	ed_Lushi_vs_Arbor_Acr	breed_growth_background	ENSGALG00010004602	DYNLT2	-0.254365748	0.196239798	-1.296198579	0.194907079	0.342736638	negative
PRJNA729271	ed_Lushi_vs_Arbor_Acr	breed_growth_background	ENSGALG00010004609	CFAP69	0.519954806	0.366174533	1.419964414	0.155618041	0.292641867	positive
PRJNA729271	ed_Lushi_vs_Arbor_Acr	breed_growth_background	ENSGALG00010004610	TGS1	0.181881276	0.066938694	2.717132113	0.006585032	0.031050759	positive
PRJNA729271	ed_Lushi_vs_Arbor_Acr	breed_growth_background	ENSGALG00010004622	ENSGALG00010004622	0.242331654	0.119745012	2.023730678	0.04299787	0.118669865	positive
PRJNA729271	ed_Lushi_vs_Arbor_Acr	breed_growth_background	ENSGALG00010004623	MBIP	0.005409264	0.069370781	0.077976119	0.937847049	0.965969799	positive
PRJNA729271	ed_Lushi_vs_Arbor_Acr	breed_growth_background	ENSGALG00010004624	PHF10	0.003241277	0.04096386	0.079125288	0.93693297	0.965234072	positive
PRJNA729271	ed_Lushi_vs_Arbor_Acr	breed_growth_background	ENSGALG00010004630	ENSGALG00010004630	0.167792174	0.161897575	1.036409433	0.300011171	0.462429238	positive
PRJNA729271	ed_Lushi_vs_Arbor_Acr	breed_growth_background	ENSGALG00010004639	HBS1L	0.005361657	0.042464135	0.126263194	0.89952359	0.941686728	positive
PRJNA729271	ed_Lushi_vs_Arbor_Acr	breed_growth_background	ENSGALG00010004641	METAP1	-0.073322242	0.044140032	-1.66112798	0.096687746	0.20978561	negative
PRJNA729271	ed_Lushi_vs_Arbor_Acr	breed_growth_background	ENSGALG00010004650	GTPBP10	0.04373499	0.069317134	0.63094055	0.528079397	0.673871895	positive
PRJNA729271	ed_Lushi_vs_Arbor_Acr	breed_growth_background	ENSGALG00010004666	C6orf120	0.090820331	0.063249911	1.435896599	0.151031766	0.286807999	positive
PRJNA729271	ed_Lushi_vs_Arbor_Acr	breed_growth_background	ENSGALG00010004669	ELMOD1	-0.790173193	0.218494006	-3.616452491	0.000298668	0.003457823	negative
PRJNA729271	ed_Lushi_vs_Arbor_Acr	breed_growth_background	ENSGALG00010004673	RAB18	0.022630727	0.045532362	0.497025111	0.619171344	0.747222432	positive
PRJNA729271	ed_Lushi_vs_Arbor_Acr	breed_growth_background	ENSGALG00010004689	BBX	0.099849911	0.045169215	2.21057442	0.027065322	0.084447347	positive
PRJNA729271	ed_Lushi_vs_Arbor_Acr	breed_growth_background	ENSGALG00010004705	ENSGALG00010004705	0.138928437	0.120208555	1.15572837	0.24779228	0.403900197	positive
PRJNA729271	ed_Lushi_vs_Arbor_Acr	breed_growth_background	ENSGALG00010004707	OTUD1	0.071118768	0.158242884	0.449427908	0.653123003	0.773891762	positive
PRJNA729271	ed_Lushi_vs_Arbor_Acr	breed_growth_background	ENSGALG00010004715	WDR27	0.029672071	0.187193651	0.158510031	0.874054917	0.925586354	positive
PRJNA729271	ed_Lushi_vs_Arbor_Acr	breed_growth_background	ENSGALG00010004716	ADH6	-0.480561457	0.528032428	-0.910098378	0.36277063	0.526099078	negative
PRJNA729271	ed_Lushi_vs_Arbor_Acr	breed_growth_background	ENSGALG00010004717	ALKBH8	-0.074856828	0.09502921	-0.787724401	0.430857929	0.589011194	negative
PRJNA729271	ed_Lushi_vs_Arbor_Acr	breed_growth_background	ENSGALG00010004721	LAMA2	-0.05694485	0.084838497	-0.671214743	0.502083738	0.65316178	negative
PRJNA729271	ed_Lushi_vs_Arbor_Acr	breed_growth_background	ENSGALG00010004728	ENSGALG00010004728	0.309345565	0.116305015	2.659778384	0.007819208	0.035067891	positive
PRJNA729271	ed_Lushi_vs_Arbor_Acr	breed_growth_background	ENSGALG00010004729	ENSGALG00010004729	1.705762091	0.461732866	3.694261806	0.000220527	0.002793245	positive
PRJNA729271	ed_Lushi_vs_Arbor_Acr	breed_growth_background	ENSGALG00010004730	CLDN12	0.024738569	0.034395164	0.719245557	0.471989634	0.626104411	positive
PRJNA729271	ed_Lushi_vs_Arbor_Acr	breed_growth_background	ENSGALG00010004735	ENSGALG00010004735	-0.310974733	0.086934571	-3.577112421	0.000347411	0.003838201	negative
PRJNA729271	ed_Lushi_vs_Arbor_Acr	breed_growth_background	ENSGALG00010004736	CD47	-0.225097136	0.054188803	-4.153941828	3.26796E-05	0.000707214	negative
PRJNA729271	ed_Lushi_vs_Arbor_Acr	breed_growth_background	ENSGALG00010004739	ENSGALG00010004739	-0.246526193	0.39467514	-0.624630659	0.532213493	0.677655883	negative
PRJNA729271	ed_Lushi_vs_Arbor_Acr	breed_growth_background	ENSGALG00010004746	LYN	-0.173513796	0.07621004	-2.276783961	0.02279913	0.074988288	negative
PRJNA729271	ed_Lushi_vs_Arbor_Acr	breed_growth_background	ENSGALG00010004751	PTER	0.334869181	0.067170099	4.985390629	6.18368E-07	3.33526E-05	positive
PRJNA729271	ed_Lushi_vs_Arbor_Acr	breed_growth_background	ENSGALG00010004755	PAX9	0.310087106	0.433962612	0.714547977	0.4748884	0.628401256	positive
PRJNA729271	ed_Lushi_vs_Arbor_Acr	breed_growth_background	ENSGALG00010004757	TRMT10A	-0.013408655	0.040219002	-0.333391044	0.738839123	0.832625901	negative
PRJNA729271	ed_Lushi_vs_Arbor_Acr	breed_growth_background	ENSGALG00010004758	FLT3	0.292038339	0.353568972	0.825972759	0.408819545	0.569177299	positive
PRJNA729271	ed_Lushi_vs_Arbor_Acr	breed_growth_background	ENSGALG00010004760	THBS2	-0.180433237	0.128922058	-1.399552876	0.161647254	0.300857338	negative
PRJNA729271	ed_Lushi_vs_Arbor_Acr	breed_growth_background	ENSGALG00010004762	IFT57	0.332335658	0.062251925	5.338560343	9.36875E-08	7.89535E-06	positive
PRJNA729271	ed_Lushi_vs_Arbor_Acr	breed_growth_background	ENSGALG00010004765	ENSGALG00010004765	-0.057834548	0.105329908	-0.549080019	0.582950537	0.719076506	negative
PRJNA729271	ed_Lushi_vs_Arbor_Acr	breed_growth_background	ENSGALG00010004769	HTR2A	-0.091194171	0.200847488	-0.45404686	0.649795099	0.771407505	negative
PRJNA729271	ed_Lushi_vs_Arbor_Acr	breed_growth_background	ENSGALG00010004777	CDK14	-0.172303631	0.067197732	-2.564128674	0.010343519	0.042869859	negative
PRJNA729271	ed_Lushi_vs_Arbor_Acr	breed_growth_background	ENSGALG00010004778	ENSGALG00010004778	0.395681837	0.266817251	1.482969469	0.138082523	0.26949053	positive
PRJNA729271	ed_Lushi_vs_Arbor_Acr	breed_growth_background	ENSGALG00010004780	ENSGALG00010004780	0.392213107	0.138999961	2.821677821	0.004777314	0.024625595	positive
PRJNA729271	ed_Lushi_vs_Arbor_Acr	breed_growth_background	ENSGALG00010004781	ENSGALG00010004781	-0.148974044	0.045549759	-3.27057807	0.001073279	0.008604635	negative
PRJNA729271	ed_Lushi_vs_Arbor_Acr	breed_growth_background	ENSGALG00010004787	RIOK3	-0.188605769	0.061726093	-3.055527411	0.002246651	0.014466993	negative
PRJNA729271	ed_Lushi_vs_Arbor_Acr	breed_growth_background	ENSGALG00010004791	FZD1	-0.040655332	0.063525125	-0.639988219	0.522180258	0.669349385	negative
PRJNA729271	ed_Lushi_vs_Arbor_Acr	breed_growth_background	ENSGALG00010004798	PTPRK	0.007883321	0.063414446	0.124314273	0.901066447	0.942711173	positive
PRJNA729271	ed_Lushi_vs_Arbor_Acr	breed_growth_background	ENSGALG00010004800	ZC3H12C	-0.178923725	0.1509838	-1.185052464	0.235996704	0.390737679	negative
PRJNA729271	ed_Lushi_vs_Arbor_Acr	breed_growth_background	ENSGALG00010004803	RMC1	0.067237259	0.075078211	0.895562878	0.370486269	0.533657803	positive
PRJNA729271	ed_Lushi_vs_Arbor_Acr	breed_growth_background	ENSGALG00010004808	THEMIS	0.395741911	0.402962546	0.982081126	0.326059883	0.489664111	positive
PRJNA729271	ed_Lushi_vs_Arbor_Acr	breed_growth_background	ENSGALG00010004824	ATM	0.03960758	0.059822189	0.662088447	0.507914539	0.656958809	positive
PRJNA729271	ed_Lushi_vs_Arbor_Acr	breed_growth_background	ENSGALG00010004832	C6orf58	-1.24188915	0.978701757	-1.268914806	0.204471451	0.353861141	negative
PRJNA729271	ed_Lushi_vs_Arbor_Acr	breed_growth_background	ENSGALG00010004835	HHLA2	0.556887471	0.220762334	2.522565609	0.011650222	0.046584817	positive
PRJNA729271	ed_Lushi_vs_Arbor_Acr	breed_growth_background	ENSGALG00010004837	CENPC	-0.166228122	0.076538246	-2.171830833	0.029868425	0.090611725	negative
PRJNA729271	ed_Lushi_vs_Arbor_Acr	breed_growth_background	ENSGALG00010004841	AKAP9	0.336331779	0.104059659	3.232105341	0.001228817	0.009494975	positive
PRJNA729271	ed_Lushi_vs_Arbor_Acr	breed_growth_background	ENSGALG00010004846	TTLL2	-0.651406253	0.197502204	-3.298222705	0.000972989	0.00799064	negative
PRJNA729271	ed_Lushi_vs_Arbor_Acr	breed_growth_background	ENSGALG00010004851	MTCL3	0.480152387	0.284725667	1.686368471	0.09172485	0.202521204	positive
PRJNA729271	ed_Lushi_vs_Arbor_Acr	breed_growth_background	ENSGALG00010004852	ENSGALG00010004852	0.019663747	0.116980191	0.168094674	0.866508799	0.919959516	positive
PRJNA729271	ed_Lushi_vs_Arbor_Acr	breed_growth_background	ENSGALG00010004855	ZEB1	-0.057732387	0.049200372	-1.173413625	0.240629976	0.396445954	negative
PRJNA729271	ed_Lushi_vs_Arbor_Acr	breed_growth_background	ENSGALG00010004859	NPC1	0.206446181	0.102239525	2.019240413	0.043462238	0.119587156	positive
PRJNA729271	ed_Lushi_vs_Arbor_Acr	breed_growth_background	ENSGALG00010004866	SLC25A21	-0.447884327	0.169938862	-2.635561536	0.008399819	0.036761891	negative
PRJNA729271	ed_Lushi_vs_Arbor_Acr	breed_growth_background	ENSGALG00010004868	NPAT	0.098428796	0.06159187	1.598080707	0.110024957	0.230447288	positive
PRJNA729271	ed_Lushi_vs_Arbor_Acr	breed_growth_background	ENSGALG00010004871	C11orf87	-0.64296184	0.171637553	-3.746044077	0.000179645	0.002411069	negative
PRJNA729271	ed_Lushi_vs_Arbor_Acr	breed_growth_background	ENSGALG00010004872	KIAA0408	-0.096012674	0.137168515	-0.699961459	0.483951374	0.636096769	negative
PRJNA729271	ed_Lushi_vs_Arbor_Acr	breed_growth_background	ENSGALG00010004876	ENSGALG00010004876	-1.136605015	0.394659734	-2.879961944	0.003977232	0.021599841	negative
PRJNA729271	ed_Lushi_vs_Arbor_Acr	breed_growth_background	ENSGALG00010004879	ENSGALG00010004879	-0.326142249	0.339111327	-0.961755692	0.33617234	0.50044347	negative
PRJNA729271	ed_Lushi_vs_Arbor_Acr	breed_growth_background	ENSGALG00010004880	STAP1	0.138605534	0.329950687	0.420079544	0.674427346	0.789582778	positive
PRJNA729271	ed_Lushi_vs_Arbor_Acr	breed_growth_background	ENSGALG00010004881	MIPOL1	0.005625272	0.084674667	0.066433946	0.947032345	0.970898153	positive
PRJNA729271	ed_Lushi_vs_Arbor_Acr	breed_growth_background	ENSGALG00010004883	KRIT1	0.011283741	0.039939657	0.282519729	0.777545021	0.86192376	positive
PRJNA729271	ed_Lushi_vs_Arbor_Acr	breed_growth_background	ENSGALG00010004887	MYH15	-2.041991538	0.39493518	-5.170447304	2.33534E-07	1.61966E-05	negative
PRJNA729271	ed_Lushi_vs_Arbor_Acr	breed_growth_background	ENSGALG00010004890	ACAT1	-0.062301535	0.05287608	-1.178255541	0.238694746	0.39410545	negative
PRJNA729271	ed_Lushi_vs_Arbor_Acr	breed_growth_background	ENSGALG00010004891	ANKIB1	-0.035113225	0.041940624	-0.837212736	0.402473	0.5634933	negative
PRJNA729271	ed_Lushi_vs_Arbor_Acr	breed_growth_background	ENSGALG00010004892	HEY2	0.235820324	0.175450948	1.344081215	0.178922126	0.323229119	positive
PRJNA729271	ed_Lushi_vs_Arbor_Acr	breed_growth_background	ENSGALG00010004896	UBA6	0.014839185	0.044686582	0.332072514	0.739834504	0.833377459	positive
PRJNA729271	ed_Lushi_vs_Arbor_Acr	breed_growth_background	ENSGALG00010004899	GATAD1	0.141892253	0.057779163	2.455768571	0.014058366	0.053067435	positive
PRJNA729271	ed_Lushi_vs_Arbor_Acr	breed_growth_background	ENSGALG00010004901	CUL5	0.06803296	0.049599107	1.371656945	0.170170258	0.312507017	positive
PRJNA729271	ed_Lushi_vs_Arbor_Acr	breed_growth_background	ENSGALG00010004902	ANKRD29	-0.184683651	0.163077392	-1.132490829	0.257428138	0.414710096	negative
PRJNA729271	ed_Lushi_vs_Arbor_Acr	breed_growth_background	ENSGALG00010004903	ENSGALG00010004903	0.015871572	0.241511541	0.065717656	0.947602615	0.971255827	positive
PRJNA729271	ed_Lushi_vs_Arbor_Acr	breed_growth_background	ENSGALG00010004907	ENSGALG00010004907	0.036058476	0.06350233	0.567829183	0.570150962	0.70877295	positive
PRJNA729271	ed_Lushi_vs_Arbor_Acr	breed_growth_background	ENSGALG00010004917	ENSGALG00010004917	-0.181218856	0.050109228	-3.616476666	0.00029864	0.003457823	negative
PRJNA729271	ed_Lushi_vs_Arbor_Acr	breed_growth_background	ENSGALG00010004921	RRAGD	0.134279895	0.070867356	1.894806059	0.0581181	0.147328063	positive
PRJNA729271	ed_Lushi_vs_Arbor_Acr	breed_growth_background	ENSGALG00010004924	ENSGALG00010004924	-0.297373023	0.186914522	-1.5909573	0.111619186	0.232560026	negative
PRJNA729271	ed_Lushi_vs_Arbor_Acr	breed_growth_background	ENSGALG00010004927	KIF25	-0.07253427	0.140355194	-0.516790779	0.605302218	0.73704479	negative
PRJNA729271	ed_Lushi_vs_Arbor_Acr	breed_growth_background	ENSGALG00010004928	HDDC2	0.171840666	0.066421511	2.587123712	0.009678084	0.04087524	positive
PRJNA729271	ed_Lushi_vs_Arbor_Acr	breed_growth_background	ENSGALG00010004929	ENSGALG00010004929	0.423665969	0.083614258	5.066910619	4.04324E-07	2.40192E-05	positive
PRJNA729271	ed_Lushi_vs_Arbor_Acr	breed_growth_background	ENSGALG00010004933	SPTLC2	-0.066762985	0.049453686	-1.3500103	0.177012679	0.321005728	negative
PRJNA729271	ed_Lushi_vs_Arbor_Acr	breed_growth_background	ENSGALG00010004934	CIP2A	-0.24272582	0.090046081	-2.695573401	0.007026759	0.032479118	negative
PRJNA729271	ed_Lushi_vs_Arbor_Acr	breed_growth_background	ENSGALG00010004935	SVIL	-0.048080517	0.036970689	-1.300503657	0.193428404	0.340508223	negative
PRJNA729271	ed_Lushi_vs_Arbor_Acr	breed_growth_background	ENSGALG00010004939	DDX10	0.05385418	0.059686188	0.902288818	0.366903464	0.530341615	positive
PRJNA729271	ed_Lushi_vs_Arbor_Acr	breed_growth_background	ENSGALG00010004942	GABRR1	-0.331749758	0.291489261	-1.138119999	0.255070378	0.41185754	negative
PRJNA729271	ed_Lushi_vs_Arbor_Acr	breed_growth_background	ENSGALG00010004944	ALKBH1	0.115953922	0.053328563	2.174330509	0.029680323	0.090211004	positive
PRJNA729271	ed_Lushi_vs_Arbor_Acr	breed_growth_background	ENSGALG00010004946	SH2D1B	0.493541076	0.230249653	2.143504108	0.032072642	0.095755497	positive
PRJNA729271	ed_Lushi_vs_Arbor_Acr	breed_growth_background	ENSGALG00010004949	RAB39A	0.429284766	0.18021581	2.382059406	0.01721612	0.061404443	positive
PRJNA729271	ed_Lushi_vs_Arbor_Acr	breed_growth_background	ENSGALG00010004950	SLIRP	-0.096484755	0.05952981	-1.620780501	0.105064722	0.222757596	negative
PRJNA729271	ed_Lushi_vs_Arbor_Acr	breed_growth_background	ENSGALG00010004951	AFDN	-0.0591023	0.034427924	-1.716696616	0.086034619	0.193866103	negative
PRJNA729271	ed_Lushi_vs_Arbor_Acr	breed_growth_background	ENSGALG00010004952	PEX1	0.283563688	0.065114187	4.354867974	1.33147E-05	0.000357401	positive
PRJNA729271	ed_Lushi_vs_Arbor_Acr	breed_growth_background	ENSGALG00010004957	ENSGALG00010004957	0.238508821	0.078201606	3.049922272	0.002289006	0.014643477	positive
PRJNA729271	ed_Lushi_vs_Arbor_Acr	breed_growth_background	ENSGALG00010004958	TPD52L1	0.410331227	0.244624621	1.677391364	0.093465977	0.205065436	positive
PRJNA729271	ed_Lushi_vs_Arbor_Acr	breed_growth_background	ENSGALG00010004962	ENSGALG00010004962	0.101309578	0.051678883	1.960367042	0.049952905	0.132467501	positive
PRJNA729271	ed_Lushi_vs_Arbor_Acr	breed_growth_background	ENSGALG00010004963	ENSGALG00010004963	0.185771507	0.13274545	1.399456684	0.161676079	0.300872354	positive
PRJNA729271	ed_Lushi_vs_Arbor_Acr	breed_growth_background	ENSGALG00010004965	JCAD	-0.186574715	0.053902782	-3.461318862	0.000537536	0.005249789	negative
PRJNA729271	ed_Lushi_vs_Arbor_Acr	breed_growth_background	ENSGALG00010004966	LAMA3	-0.035438794	0.113035721	-0.313518541	0.753886737	0.844416061	negative
PRJNA729271	ed_Lushi_vs_Arbor_Acr	breed_growth_background	ENSGALG00010004967	SLC35F2	-0.355662286	0.178672565	-1.990581411	0.046526926	0.125811693	negative
PRJNA729271	ed_Lushi_vs_Arbor_Acr	breed_growth_background	ENSGALG00010004968	TRAT1	0.443913865	0.335386093	1.323590555	0.185639064	0.33118009	positive
PRJNA729271	ed_Lushi_vs_Arbor_Acr	breed_growth_background	ENSGALG00010004969	YTHDC1	0.019874447	0.041714461	0.476440222	0.633760785	0.758827241	positive
PRJNA729271	ed_Lushi_vs_Arbor_Acr	breed_growth_background	ENSGALG00010004970	RBM48	0.246445158	0.090017753	2.737739495	0.006186306	0.029643141	positive
PRJNA729271	ed_Lushi_vs_Arbor_Acr	breed_growth_background	ENSGALG00010004973	SLN	-0.189826275	0.081592491	-2.326516486	0.019991014	0.068245348	negative
PRJNA729271	ed_Lushi_vs_Arbor_Acr	breed_growth_background	ENSGALG00010004974	MTPAP	0.011847493	0.033247698	0.356340251	0.721585769	0.821439161	positive
PRJNA729271	ed_Lushi_vs_Arbor_Acr	breed_growth_background	ENSGALG00010004975	SNW1	0.048815355	0.037449203	1.303508518	0.19240121	0.339196828	positive
PRJNA729271	ed_Lushi_vs_Arbor_Acr	breed_growth_background	ENSGALG00010004976	ENSGALG00010004976	0.267401091	0.119240029	2.242544675	0.024926191	0.079861735	positive
PRJNA729271	ed_Lushi_vs_Arbor_Acr	breed_growth_background	ENSGALG00010004977	RNF217	0.232510554	0.072292048	3.216267336	0.001298698	0.009842037	positive
PRJNA729271	ed_Lushi_vs_Arbor_Acr	breed_growth_background	ENSGALG00010004980	FNDC1	-0.663816053	0.201801766	-3.2894462	0.001003847	0.008160835	negative
PRJNA729271	ed_Lushi_vs_Arbor_Acr	breed_growth_background	ENSGALG00010004981	ENSGALG00010004981	0.038643546	0.110476674	0.349789191	0.726496911	0.824527408	positive
PRJNA729271	ed_Lushi_vs_Arbor_Acr	breed_growth_background	ENSGALG00010004983	CLXN	-0.31776315	0.149751033	-2.121942958	0.033842528	0.099421859	negative
PRJNA729271	ed_Lushi_vs_Arbor_Acr	breed_growth_background	ENSGALG00010004987	MAP3K8	0.012411235	0.139752961	0.088808385	0.929234193	0.960716807	positive
PRJNA729271	ed_Lushi_vs_Arbor_Acr	breed_growth_background	ENSGALG00010004988	HJURP	-0.254153943	0.118673975	-2.141614817	0.03222449	0.096109874	negative
PRJNA729271	ed_Lushi_vs_Arbor_Acr	breed_growth_background	ENSGALG00010004990	ENSGALG00010004990	-2.032542172	1.300303666	-1.563128848	0.118022292	0.241765562	negative
PRJNA729271	ed_Lushi_vs_Arbor_Acr	breed_growth_background	ENSGALG00010004991	FAM133B	-0.0086303	0.034302519	-0.251593765	0.801355078	0.87731434	negative
PRJNA729271	ed_Lushi_vs_Arbor_Acr	breed_growth_background	ENSGALG00010004993	SULT1B	-0.346641796	0.118463169	-2.926156704	0.00343178	0.019469141	negative
PRJNA729271	ed_Lushi_vs_Arbor_Acr	breed_growth_background	ENSGALG00010004995	PIT54	-2.687347258	1.138289632	-2.360864215	0.018232405	0.064114195	negative
PRJNA729271	ed_Lushi_vs_Arbor_Acr	breed_growth_background	ENSGALG00010004996	GPR158	-0.116518892	0.118488806	-0.983374684	0.325423065	0.489113162	negative
PRJNA729271	ed_Lushi_vs_Arbor_Acr	breed_growth_background	ENSGALG00010004999	ADCK1	0.250234175	0.105087096	2.381207453	0.017255991	0.061455919	positive
PRJNA729271	ed_Lushi_vs_Arbor_Acr	breed_growth_background	ENSGALG00010005003	EXPH5	-0.76930525	0.235810574	-3.262386578	0.001104784	0.008798813	negative
PRJNA729271	ed_Lushi_vs_Arbor_Acr	breed_growth_background	ENSGALG00010005004	CDK6	-0.176099981	0.05255591	-3.350716994	0.000806026	0.006991832	negative
PRJNA729271	ed_Lushi_vs_Arbor_Acr	breed_growth_background	ENSGALG00010005010	NKAIN2	-0.434901144	0.184398148	-2.35848976	0.018349466	0.064369678	negative
PRJNA729271	ed_Lushi_vs_Arbor_Acr	breed_growth_background	ENSGALG00010005012	ENSGALG00010005012	0.993802667	0.527856047	1.882715321	0.059738958	0.15038489	positive
PRJNA729271	ed_Lushi_vs_Arbor_Acr	breed_growth_background	ENSGALG00010005014	SULT1B1	-0.001495296	0.081438083	-0.01836114	0.985350753	0.991231811	negative
PRJNA729271	ed_Lushi_vs_Arbor_Acr	breed_growth_background	ENSGALG00010005015	NRXN3	0.496953086	0.223033761	2.228151846	0.025870391	0.081929499	positive
PRJNA729271	ed_Lushi_vs_Arbor_Acr	breed_growth_background	ENSGALG00010005016	STX11	-0.119883238	0.093909813	-1.276578394	0.201751134	0.350813662	negative
PRJNA729271	ed_Lushi_vs_Arbor_Acr	breed_growth_background	ENSGALG00010005017	ENSGALG00010005017	0.033330078	0.091437954	0.364510328	0.715476963	0.817438211	positive

PRJNA729271	ed_Lushi_vs_Arbor_Acr	breed_growth_background	ENSGALG00010005023	POGLUT3	-0.143566805	0.09674057	-1.484039276	0.137798501	0.269081136	negative
PRJNA729271	ed_Lushi_vs_Arbor_Acr	breed_growth_background	ENSGALG00010005025	THOC1	-0.011339938	0.03242934	-0.349681443	0.726577782	0.824527408	negative
PRJNA729271	ed_Lushi_vs_Arbor_Acr	breed_growth_background	ENSGALG00010005029	FAT4	-0.255460437	0.10581729	-2.41416536	0.015771303	0.057745321	negative
PRJNA729271	ed_Lushi_vs_Arbor_Acr	breed_growth_background	ENSGALG00010005033	USP14	0.174509824	0.072509939	2.406702127	0.016097293	0.058581539	positive
PRJNA729271	ed_Lushi_vs_Arbor_Acr	breed_growth_background	ENSGALG00010005035	NECTIN3	-0.176414205	0.052715796	-3.346515065	0.000818342	0.007065766	negative
PRJNA729271	ed_Lushi_vs_Arbor_Acr	breed_growth_background	ENSGALG00010005037	THNSL1	0.221852451	0.075924677	2.922007167	0.003477835	0.019668835	positive
PRJNA729271	ed_Lushi_vs_Arbor_Acr	breed_growth_background	ENSGALG00010005042	SLC46A3	0.097313884	0.106574279	0.913108546	0.361185456	0.524534935	positive
PRJNA729271	ed_Lushi_vs_Arbor_Acr	breed_growth_background	ENSGALG00010005045	UTRN	-0.045337783	0.053176361	-0.852592801	0.393885156	0.555331713	negative
PRJNA729271	ed_Lushi_vs_Arbor_Acr	breed_growth_background	ENSGALG00010005048	ROCK1	-0.078171239	0.061474459	-1.27160514	0.203513449	0.352693382	negative
PRJNA729271	ed_Lushi_vs_Arbor_Acr	breed_growth_background	ENSGALG00010005057	FLVCR2	-0.350673421	0.158894114	-2.206962936	0.027316649	0.084944094	negative
PRJNA729271	ed_Lushi_vs_Arbor_Acr	breed_growth_background	ENSGALG00010005060	POMP	0.056815089	0.0740425	0.767330783	0.44288487	0.599908064	positive
PRJNA729271	ed_Lushi_vs_Arbor_Acr	breed_growth_background	ENSGALG00010005065	ENKUR	0.036868778	0.240493838	0.15330446	0.878158183	0.9277626	positive
PRJNA729271	ed_Lushi_vs_Arbor_Acr	breed_growth_background	ENSGALG00010005068	PLCXD2	-0.096399634	0.137365721	-0.701773581	0.482820377	0.63501328	negative
PRJNA729271	ed_Lushi_vs_Arbor_Acr	breed_growth_background	ENSGALG00010005070	ALB	-2.167656471	0.749495834	-2.892152795	0.003826118	0.020983573	negative
PRJNA729271	ed_Lushi_vs_Arbor_Acr	breed_growth_background	ENSGALG00010005075	TRDN	0.016485268	0.087997633	0.187337625	0.851395924	0.910572119	positive
PRJNA729271	ed_Lushi_vs_Arbor_Acr	breed_growth_background	ENSGALG00010005077	ERG28	0.033501701	0.056955869	0.588204548	0.556395005	0.69796154	positive
PRJNA729271	ed_Lushi_vs_Arbor_Acr	breed_growth_background	ENSGALG00010005081	GREB1L	-0.456622128	0.096146251	-4.749245279	2.04177E-06	8.5784E-05	negative
PRJNA729271	ed_Lushi_vs_Arbor_Acr	breed_growth_background	ENSGALG00010005082	ENSGALG00010005082	0.086684441	0.047982627	1.806579728	0.070827833	0.169162866	positive
PRJNA729271	ed_Lushi_vs_Arbor_Acr	breed_growth_background	ENSGALG00010005087	ENPP1	-0.089131721	0.114665099	-0.777322149	0.436968731	0.594393933	negative
PRJNA729271	ed_Lushi_vs_Arbor_Acr	breed_growth_background	ENSGALG00010005092	ESCO1	0.426186423	0.074869557	5.692386043	1.25276E-08	1.6508E-06	positive
PRJNA729271	ed_Lushi_vs_Arbor_Acr	breed_growth_background	ENSGALG00010005104	ENPP3	0.18143088	0.107422555	1.688945864	0.091229805	0.201853959	positive
PRJNA729271	ed_Lushi_vs_Arbor_Acr	breed_growth_background	ENSGALG00010005107	TTLL5	-0.078699493	0.040347245	-1.950554323	0.051110085	0.134600415	negative
PRJNA729271	ed_Lushi_vs_Arbor_Acr	breed_growth_background	ENSGALG00010005109	ABHD3	0.287864944	0.204557314	1.407258137	0.159350858	0.29784535	positive
PRJNA729271	ed_Lushi_vs_Arbor_Acr	breed_growth_background	ENSGALG00010005111	RASSF6	0.150751351	0.227236953	0.663410369	0.507067767	0.65653839	positive
PRJNA729271	ed_Lushi_vs_Arbor_Acr	breed_growth_background	ENSGALG00010005114	PHLDB2	-0.188050248	0.053827438	-3.493576031	0.000476597	0.004800749	negative
PRJNA729271	ed_Lushi_vs_Arbor_Acr	breed_growth_background	ENSGALG00010005118	PRTFDC1	-0.136685701	0.075116497	-1.819649559	0.068812389	0.165990279	negative
PRJNA729271	ed_Lushi_vs_Arbor_Acr	breed_growth_background	ENSGALG00010005119	MIB1	-0.037538865	0.031217435	-1.202496777	0.229171114	0.382780705	negative
PRJNA729271	ed_Lushi_vs_Arbor_Acr	breed_growth_background	ENSGALG00010005122	IL8L1	0.114713238	0.565920849	0.202701911	0.839368029	0.902502751	positive
PRJNA729271	ed_Lushi_vs_Arbor_Acr	breed_growth_background	ENSGALG00010005123	MTIF3	0.235009533	0.055595256	4.22715083	2.36669E-05	0.000545729	positive
PRJNA729271	ed_Lushi_vs_Arbor_Acr	breed_growth_background	ENSGALG00010005126	MED23	0.019405436	0.031843668	0.609397009	0.54226132	0.687189879	positive
PRJNA729271	ed_Lushi_vs_Arbor_Acr	breed_growth_background	ENSGALG00010005127	GATA6	0.087410798	0.176925098	0.494055389	0.621267062	0.7485052	positive
PRJNA729271	ed_Lushi_vs_Arbor_Acr	breed_growth_background	ENSGALG00010005131	IL8L2	0.801847762	0.337754881	2.374052334	0.017594057	0.062384014	positive
PRJNA729271	ed_Lushi_vs_Arbor_Acr	breed_growth_background	ENSGALG00010005132	LNX2	0.140945115	0.052338505	2.692952658	0.007082232	0.032662093	positive
PRJNA729271	ed_Lushi_vs_Arbor_Acr	breed_growth_background	ENSGALG00010005138	ENSGALG00010005138	0.282861406	0.302022706	0.936556757	0.348986605	0.512986597	positive
PRJNA729271	ed_Lushi_vs_Arbor_Acr	breed_growth_background	ENSGALG00010005140	DCT	1.694749296	0.53571724	3.163514575	0.001558765	0.011168713	positive
PRJNA729271	ed_Lushi_vs_Arbor_Acr	breed_growth_background	ENSGALG00010005141	CCDC170	-0.691123787	0.173448546	-3.984604088	6.75927E-05	0.00119047	negative
PRJNA729271	ed_Lushi_vs_Arbor_Acr	breed_growth_background	ENSGALG00010005143	GTF3A	0.238965604	0.074023699	3.228231079	0.001245583	0.009586056	positive
PRJNA729271	ed_Lushi_vs_Arbor_Acr	breed_growth_background	ENSGALG00010005145	AKAP7	-0.297915405	0.093121048	-3.199227354	0.001377965	0.010300978	negative
PRJNA729271	ed_Lushi_vs_Arbor_Acr	breed_growth_background	ENSGALG00010005146	TGFB3	-0.480779205	0.109483231	-4.391350177	1.12649E-05	0.000318765	negative
PRJNA729271	ed_Lushi_vs_Arbor_Acr	breed_growth_background	ENSGALG00010005148	TTC39C	-0.061692781	0.0473461	-1.303017148	0.192568907	0.339407382	negative
PRJNA729271	ed_Lushi_vs_Arbor_Acr	breed_growth_background	ENSGALG00010005150	ALCAM	0.130885796	0.101049026	1.295270241	0.195227021	0.343132736	positive
PRJNA729271	ed_Lushi_vs_Arbor_Acr	breed_growth_background	ENSGALG00010005154	CWF19L2	0.238691503	0.080798698	2.95415037	0.003135312	0.018310373	positive
PRJNA729271	ed_Lushi_vs_Arbor_Acr	breed_growth_background	ENSGALG00010005157	IMPA1	-0.049574094	0.059249772	-0.836696787	0.402763025	0.563790445	negative
PRJNA729271	ed_Lushi_vs_Arbor_Acr	breed_growth_background	ENSGALG00010005158	IFT43	0.038633481	0.110264613	0.350370625	0.726060569	0.824474505	positive
PRJNA729271	ed_Lushi_vs_Arbor_Acr	breed_growth_background	ENSGALG00010005159	ARHGAP21	-0.138676703	0.052601926	-2.636342701	0.008380505	0.036751005	negative
PRJNA729271	ed_Lushi_vs_Arbor_Acr	breed_growth_background	ENSGALG00010005163	GPC6	-0.936807532	0.215584793	-4.345424921	1.39006E-05	0.000368354	negative
PRJNA729271	ed_Lushi_vs_Arbor_Acr	breed_growth_background	ENSGALG00010005165	METTL21C	-1.008272661	0.297686932	-3.387023586	0.000706553	0.00636918	negative
PRJNA729271	ed_Lushi_vs_Arbor_Acr	breed_growth_background	ENSGALG00010005166	CBLB	-0.077566948	0.067172971	-1.154734521	0.248199159	0.404382375	negative
PRJNA729271	ed_Lushi_vs_Arbor_Acr	breed_growth_background	ENSGALG00010005167	GUCY1A2	-0.258323984	0.09542071	-2.707210881	0.006785114	0.031705426	negative
PRJNA729271	ed_Lushi_vs_Arbor_Acr	breed_growth_background	ENSGALG00010005170	GPATCH2L	0.0659743	0.03296187	2.001533882	0.045334886	0.123450906	positive
PRJNA729271	ed_Lushi_vs_Arbor_Acr	breed_growth_background	ENSGALG00010005172	OSBPL1A	0.00947428	0.042922196	0.220731496	0.825301506	0.89328695	positive
PRJNA729271	ed_Lushi_vs_Arbor_Acr	breed_growth_background	ENSGALG00010005174	TPP2	-0.237635902	0.098948647	-2.401608402	0.016323172	0.05913631	negative
PRJNA729271	ed_Lushi_vs_Arbor_Acr	breed_growth_background	ENSGALG00010005176	BAMBI	-0.109582453	0.172082936	-0.636800225	0.52425498	0.671118602	negative
PRJNA729271	ed_Lushi_vs_Arbor_Acr	breed_growth_background	ENSGALG00010005177	AASDHPPT	0.125377148	0.068746499	1.82376048	0.068188291	0.164979015	positive
PRJNA729271	ed_Lushi_vs_Arbor_Acr	breed_growth_background	ENSGALG00010005178	EPHA5	-0.209809389	0.107547375	-1.950855512	0.051074236	0.134530447	negative
PRJNA729271	ed_Lushi_vs_Arbor_Acr	breed_growth_background	ENSGALG00010005180	WAC	0.231425181	0.061823472	3.743322306	0.000181603	0.002428354	positive
PRJNA729271	ed_Lushi_vs_Arbor_Acr	breed_growth_background	ENSGALG00010005181	IMPACT	-0.030275751	0.070768997	-0.427810937	0.668788773	0.78532836	negative
PRJNA729271	ed_Lushi_vs_Arbor_Acr	breed_growth_background	ENSGALG00010005182	NEPRO	-0.072429094	0.049501727	-1.463162973	0.14342279	0.2766109	negative
PRJNA729271	ed_Lushi_vs_Arbor_Acr	breed_growth_background	ENSGALG00010005184	FGF14	-0.225955234	0.183358249	-1.23231562	0.217831207	0.369856313	negative
PRJNA729271	ed_Lushi_vs_Arbor_Acr	breed_growth_background	ENSGALG00010005186	ESR1	0.177374376	0.10352878	1.713285675	0.086659996	0.194774713	positive
PRJNA729271	ed_Lushi_vs_Arbor_Acr	breed_growth_background	ENSGALG00010005187	KBTBD3	0.148489452	0.134981845	1.10006981	0.271301706	0.430065424	positive
PRJNA729271	ed_Lushi_vs_Arbor_Acr	breed_growth_background	ENSGALG00010005190	ENSGALG00010005190	-0.006087494	0.144717593	-0.042064643	0.966447166	0.980988414	negative
PRJNA729271	ed_Lushi_vs_Arbor_Acr	breed_growth_background	ENSGALG00010005191	MSANTD4	0.059300314	0.057377494	1.033511763	0.301364469	0.463773009	positive
PRJNA729271	ed_Lushi_vs_Arbor_Acr	breed_growth_background	ENSGALG00010005193	EPB41L2	-0.140760009	0.052594318	-2.67633489	0.007443223	0.033831771	negative
PRJNA729271	ed_Lushi_vs_Arbor_Acr	breed_growth_background	ENSGALG00010005194	ENSGALG00010005194	-0.651997526	0.58423045	-1.115993742	0.264424814	0.42267421	negative
PRJNA729271	ed_Lushi_vs_Arbor_Acr	breed_growth_background	ENSGALG00010005198	TRPC3	0.175871586	0.273637765	0.642716789	0.520407885	0.668019523	positive
PRJNA729271	ed_Lushi_vs_Arbor_Acr	breed_growth_background	ENSGALG00010005203	ENSGALG00010005203	-0.137726471	0.056596817	-2.433466708	0.014955011	0.055483204	negative
PRJNA729271	ed_Lushi_vs_Arbor_Acr	breed_growth_background	ENSGALG00010005204	ITGBL1	0.254913563	0.16703128	1.526142664	0.126974339	0.253826099	positive
PRJNA729271	ed_Lushi_vs_Arbor_Acr	breed_growth_background	ENSGALG00010005208	ENSGALG00010005208	-0.006519529	0.06001472	-0.108632163	0.91349425	0.951296727	negative
PRJNA729271	ed_Lushi_vs_Arbor_Acr	breed_growth_background	ENSGALG00010005211	ESRRB	0.272736797	0.172173279	1.584083188	0.11317479	0.234707218	positive
PRJNA729271	ed_Lushi_vs_Arbor_Acr	breed_growth_background	ENSGALG00010005212	TAF13	0.044146931	0.054696718	0.807122124	0.419596127	0.579132152	positive
PRJNA729271	ed_Lushi_vs_Arbor_Acr	breed_growth_background	ENSGALG00010005213	ENSGALG00010005213	-0.086177509	0.08760212	-0.983737713	0.325244492	0.488946163	negative
PRJNA729271	ed_Lushi_vs_Arbor_Acr	breed_growth_background	ENSGALG00010005214	BBS7	0.028495034	0.065455764	0.435332691	0.66332096	0.781503479	positive
PRJNA729271	ed_Lushi_vs_Arbor_Acr	breed_growth_background	ENSGALG00010005215	VASH1	-0.255796782	0.076213951	-3.356298674	0.000789932	0.00688933	negative
PRJNA729271	ed_Lushi_vs_Arbor_Acr	breed_growth_background	ENSGALG00010005216	ENSGALG00010005216	0.032179851	0.089550474	0.359348756	0.719334204	0.820228862	positive
PRJNA729271	ed_Lushi_vs_Arbor_Acr	breed_growth_background	ENSGALG00010005217	CCNA2	-0.110141434	0.07784302	-1.414917289	0.157092748	0.294574306	negative
PRJNA729271	ed_Lushi_vs_Arbor_Acr	breed_growth_background	ENSGALG00010005218	GTPBP8	0.132996115	0.06473219	2.054559172	0.039921603	0.112181212	positive
PRJNA729271	ed_Lushi_vs_Arbor_Acr	breed_growth_background	ENSGALG00010005219	NALCN	-0.01129118	0.099281237	-0.113729247	0.909452427	0.948586956	negative
PRJNA729271	ed_Lushi_vs_Arbor_Acr	breed_growth_background	ENSGALG00010005220	ANGEL1	0.066850405	0.04590283	1.456346057	0.145296979	0.279172815	positive
PRJNA729271	ed_Lushi_vs_Arbor_Acr	breed_growth_background	ENSGALG00010005221	ENSGALG00010005221	0.319678325	0.162600197	1.966038982	0.049294108	0.131200531	positive
PRJNA729271	ed_Lushi_vs_Arbor_Acr	breed_growth_background	ENSGALG00010005222	ABCB1	-1.133361145	0.461256585	-2.457116454	0.014005728	0.052923103	negative
PRJNA729271	ed_Lushi_vs_Arbor_Acr	breed_growth_background	ENSGALG00010005223	GRIA4	0.056995277	0.254133022	0.224273399	0.822544573	0.891356429	positive
PRJNA729271	ed_Lushi_vs_Arbor_Acr	breed_growth_background	ENSGALG00010005224	ENSGALG00010005224	-0.136324628	0.211641393	-0.64413027	0.519490963	0.66731846	negative
PRJNA729271	ed_Lushi_vs_Arbor_Acr	breed_growth_background	ENSGALG00010005226	MYCT1	-0.408678359	0.089272597	-4.577870187	4.69734E-06	0.000165664	negative
PRJNA729271	ed_Lushi_vs_Arbor_Acr	breed_growth_background	ENSGALG00010005227	EXOSC9	-0.005281135	0.056915178	-0.092789579	0.92607073	0.958608529	negative
PRJNA729271	ed_Lushi_vs_Arbor_Acr	breed_growth_background	ENSGALG00010005228	ZNF521	-0.180172419	0.070984179	-2.538205308	0.01114226	0.045214741	negative
PRJNA729271	ed_Lushi_vs_Arbor_Acr	breed_growth_background	ENSGALG00010005229	CIPC	0.013640384	0.040245453	0.338929817	0.734662602	0.83006738	positive
PRJNA729271	ed_Lushi_vs_Arbor_Acr	breed_growth_background	ENSGALG00010005231	ENSGALG00010005231	-0.027471096	0.229181742	-0.119865989	0.904589307	0.945214175	negative
PRJNA729271	ed_Lushi_vs_Arbor_Acr	breed_growth_background	ENSGALG00010005232	GATA3	0.033695322	0.2707269	0.124462408	0.900949163	0.942711173	positive
PRJNA729271	ed_Lushi_vs_Arbor_Acr	breed_growth_background	ENSGALG00010005236	TMTC4	0.049738956	0.049983156	0.99511437	0.319680642	0.482955643	positive
PRJNA729271	ed_Lushi_vs_Arbor_Acr	breed_growth_background	ENSGALG00010005237	RAP2A	-0.061076561	0.061168259	-0.998500882	0.318036537	0.481421596	negative
PRJNA729271	ed_Lushi_vs_Arbor_Acr	breed_growth_background	ENSGALG00010005238	TMEM63C	-0.196557137	0.092166915	-2.132621416	0.032955795	0.097448847	negative
PRJNA729271	ed_Lushi_vs_Arbor_Acr	breed_growth_background	ENSGALG00010005239	ENSGALG00010005239	0.196105813	0.107756016	1.819905935	0.068773331	0.165923674	positive
PRJNA729271	ed_Lushi_vs_Arbor_Acr	breed_growth_background	ENSGALG00010005240	VIP	-0.032776248	0.225851626	-0.145122924	0.884613819	0.931933811	negative
PRJNA729271	ed_Lushi_vs_Arbor_Acr	breed_growth_background	ENSGALG00010005241	C4orf54	0.222450198	0.100010248	2.224274045	0.026130012	0.082431303	positive
PRJNA729271	ed_Lushi_vs_Arbor_Acr	breed_growth_background	ENSGALG00010005242	CCDC80	-0.329856049	0.125067326	-2.637427849	0.008353739	0.03670429	negative
PRJNA729271	ed_Lushi_vs_Arbor_Acr	breed_growth_background	ENSGALG00010005243	NGB	0.130827099	0.30949363	0.422713381	0.672504391	0.788031621	positive
PRJNA729271	ed_Lushi_vs_Arbor_Acr	breed_growth_background	ENSGALG00010005244	FBXO5	0.095372948	0.053199128	1.792753972	0.073012272	0.172898691	positive
PRJNA729271	ed_Lushi_vs_Arbor_Acr	breed_growth_background	ENSGALG00010005245	KIAA1217	-0.03102349	0.072234213	-0.429484714	0.667570516	0.784724242	negative
PRJNA729271	ed_Lushi_vs_Arbor_Acr	breed_growth_background	ENSGALG00010005246	ENSGALG00010005246	-0.186603143	0.106947392	-1.744812468	0.081017518	0.185581907	negative
PRJNA729271	ed_Lushi_vs_Arbor_Acr	breed_growth_background	ENSGALG00010005247	THAP7	0.144860705	0.062910964	2.302630501	0.021299642	0.071380652	positive
PRJNA729271	ed_Lushi_vs_Arbor_Acr	breed_growth_background	ENSGALG00010005248	TMEM200A	-0.404218214	0.12940091	-3.123766391	0.001785522	0.012271757	negative
PRJNA729271	ed_Lushi_vs_Arbor_Acr	breed_growth_background	ENSGALG00010005249	PDGFD	-0.417152669	0.122410513	-3.407817331	0.000654847	0.006011406	negative
PRJNA729271	ed_Lushi_vs_Arbor_Acr	breed_growth_background	ENSGALG00010005250	SLC35A5	-0.154549258	0.04994207	-3.09457052	0.001970981	0.013141383	negative
PRJNA729271	ed_Lushi_vs_Arbor_Acr	breed_growth_background	ENSGALG00010005251	MTRF1L	-0.028803211	0.0565139	-0.509665953	0.61028551	0.740584881	negative
PRJNA729271	ed_Lushi_vs_Arbor_Acr	breed_growth_background	ENSGALG00010005252	KLHL14	0.068738758	0.12865065	0.534305567	0.593130141	0.72761115	positive
PRJNA729271	ed_Lushi_vs_Arbor_Acr	breed_growth_background	ENSGALG00010005253	POMT2	0.134387715	0.052969093	2.537096762	0.011177605	0.04529477	positive
PRJNA729271	ed_Lushi_vs_Arbor_Acr	breed_growth_background	ENSGALG00010005254	ENSGALG00010005254	-0.772726715	0.411683044	-1.87699427	0.060518879	0.151759356	negative
PRJNA729271	ed_Lushi_vs_Arbor_Acr	breed_growth_background	ENSGALG00010005255	BZW2	0.084085779	0.049773382	1.689372433	0.09114808	0.201801042	positive
PRJNA729271	ed_Lushi_vs_Arbor_Acr	breed_growth_background	ENSGALG00010005256	OPRM1	-0.123725219	0.317156815	-0.390107394	0.696457135	0.804450019	negative
PRJNA729271	ed_Lushi_vs_Arbor_Acr	breed_growth_background	ENSGALG00010005258	ITIH5	-0.196205702	0.148757236	-1.318965762	0.187180559	0.332801341	negative
PRJNA729271	ed_Lushi_vs_Arbor_Acr	breed_growth_background	ENSGALG00010005259	SLC27A5	-1.305959782	0.363835696	-3.589421809	0.000331412	0.003703793	negative
PRJNA729271	ed_Lushi_vs_Arbor_Acr	breed_growth_background	ENSGALG00010005260	ANXA5	-0.334416053	0.080231045	-4.168162757	3.07065E-05	0.000679078	negative
PRJNA729271	ed_Lushi_vs_Arbor_Acr	breed_growth_background	ENSGALG00010005261	ATG3	0.263185849	0.059539608	4.420349023	9.85416E-06	0.000288558	positive
PRJNA729271	ed_Lushi_vs_Arbor_Acr	breed_growth_background	ENSGALG00010005262	ENSGALG00010005262	-0.117142959	0.067012769	-1.748069238	0.080452039	0.184693111	negative
PRJNA729271	ed_Lushi_vs_Arbor_Acr	breed_growth_background	ENSGALG00010005263	ENSGALG00010005263	-0.590540262	0.171703113	-3.439310159	0.000583199	0.005507143	negative
PRJNA729271	ed_Lushi_vs_Arbor_Acr	breed_growth_background	ENSGALG00010005264	ITIH2	-1.476596617	0.555781257	-2.656794557			negative
PRJNA729271	ed_Lushi_vs_Arbor_Acr	breed_growth_background	ENSGALG00010005265	DYNC2H1	-0.040526708	0.051529543	-0.786475202	0.431589141	0.589843919	negative
PRJNA729271	ed_Lushi_vs_Arbor_Acr	breed_growth_background	ENSGALG00010005266	SLC19A2	-0.281494172	0.147233808	-1.91188543	0.055890891	0.14353977	negative
PRJNA729271	ed_Lushi_vs_Arbor_Acr	breed_growth_background	ENSGALG00010005267	CYP51A1	-0.138446587	0.099057646	-1.397636554	0.162222236	0.301617664	negative
PRJNA729271	ed_Lushi_vs_Arbor_Acr	breed_growth_background	ENSGALG00010005268	MTTP	-1.072054232	0.45304617	-2.366324458	0.017965692	0.063468853	negative
PRJNA729271	ed_Lushi_vs_Arbor_Acr	breed_growth_background	ENSGALG00010005269	KIN	0.254094236	0.057433225	4.424168001	9.68147E-06	0.000284651	positive
PRJNA729271	ed_Lushi_vs_Arbor_Acr	breed_growth_background	ENSGALG00010005270	GSTZ1	-0.367022982	0.116301616	-3.155785737	0.001600664	0.011362206	negative
PRJNA729271	ed_Lushi_vs_Arbor_Acr	breed_growth_background	ENSGALG00010005272	MBNL2	0.075211053	0.049957459	1.505501962	0.132195073	0.261059216	positive
PRJNA729271	ed_Lushi_vs_Arbor_Acr	breed_growth_background	ENSGALG00010005273	ENSGALG00010005273	-0.091381761	0.318206881	-0.287177199	0.773976644	0.859781567	negative
PRJNA729271	ed_Lushi_vs_Arbor_Acr	breed_growth_background	ENSGALG00010005275	L3MBTL3	0.326709987	0.091873736	3.556075981	0.000376435	0.00407805	positive
PRJNA729271	ed_Lushi_vs_Arbor_Acr	breed_growth_background	ENSGALG00010005277	ENSGALG00010005277	-0.1769199	0.107854881	-1.640351349	0.100932135	0.216489002	negative
PRJNA729271	ed_Lushi_vs_Arbor_Acr	breed_growth_background	ENSGALG00010005278	DAPP1	0.088286153	0.070655627	1.249527557	0.211472181	0.361515094	positive
PRJNA729271	ed_Lushi_vs_Arbor_Acr	breed_growth_background	ENSGALG00010005279	CCDC181	0.008153732	0.177287905	0.045991473	0.963317046	0.979054872	positive
PRJNA729271	ed_Lushi_vs_Arbor_Acr	breed_growth_background	ENSGALG00010005280	TMEM244	-0.150763869	0.346186065	-0.435499532	0.663199878	0.781503479	negative
PRJNA729271	ed_Lushi_vs_Arbor_Acr	breed_growth_background	ENSGALG00010005281	TMED8	0.008316978	0.038074651	0.218438728	0.827087294	0.894673904	positive

PRJNA729271	ed_Lushi_vs_Arbor_Acr	breed_growth_background	ENSGALG00010005282	FLT1	0.035770393	0.066595882	0.537126204	0.591180441	0.726077867	positive
PRJNA729271	ed_Lushi_vs_Arbor_Acr	breed_growth_background	ENSGALG00010005283	ATP5C1	-0.088472695	0.070607358	-1.25302373	0.210197071	0.360106575	negative
PRJNA729271	ed_Lushi_vs_Arbor_Acr	breed_growth_background	ENSGALG00010005284	ENSGALG00010005284	-0.094369004	0.116940288	-0.806984535	0.419675393	0.579186406	negative
PRJNA729271	ed_Lushi_vs_Arbor_Acr	breed_growth_background	ENSGALG00010005285	MPP7	0.055461764	0.068409927	0.810726846	0.417522553	0.577590132	positive
PRJNA729271	ed_Lushi_vs_Arbor_Acr	breed_growth_background	ENSGALG00010005286	ENSGALG00010005286	-0.92522465	0.328916907	-2.812943418	0.004909029	0.025214873	negative
PRJNA729271	ed_Lushi_vs_Arbor_Acr	breed_growth_background	ENSGALG00010005287	ENSGALG00010005287	-0.164158683	0.223429106	-0.734723805	0.46250772	0.617543239	negative
PRJNA729271	ed_Lushi_vs_Arbor_Acr	breed_growth_background	ENSGALG00010005288	ENSGALG00010005288	-0.044706834	0.051039346	-0.875928815	0.381068734	0.542699086	negative
PRJNA729271	ed_Lushi_vs_Arbor_Acr	breed_growth_background	ENSGALG00010005291	IFNGR1	-0.150377189	0.051478397	-2.921170761	0.003487186	0.019690988	negative
PRJNA729271	ed_Lushi_vs_Arbor_Acr	breed_growth_background	ENSGALG00010005292	TAF3	0.167372707	0.053110528	3.15140354	0.001624878	0.011478474	positive
PRJNA729271	ed_Lushi_vs_Arbor_Acr	breed_growth_background	ENSGALG00010005293	BLZF1	0.196961158	0.052347644	3.762560169	0.000168183	0.002314101	positive
PRJNA729271	ed_Lushi_vs_Arbor_Acr	breed_growth_background	ENSGALG00010005295	ENSGALG00010005295	0.071301421	0.299714331	0.237897938	0.811960255	0.884249729	positive
PRJNA729271	ed_Lushi_vs_Arbor_Acr	breed_growth_background	ENSGALG00010005296	PAN3	-0.014095169	0.054004704	-0.260998916	0.794093343	0.872445923	negative
PRJNA729271	ed_Lushi_vs_Arbor_Acr	breed_growth_background	ENSGALG00010005297	IL22RA2	0.346259071	0.340063563	1.018218677	0.308574046	0.471364822	positive
PRJNA729271	ed_Lushi_vs_Arbor_Acr	breed_growth_background	ENSGALG00010005299	ODAD2	1.449933486	0.386558541	3.750876853	0.000176217	0.002372056	positive
PRJNA729271	ed_Lushi_vs_Arbor_Acr	breed_growth_background	ENSGALG00010005300	QRFPR	-1.588942873	0.490062163	-3.242329227	0.00118557	0.009254084	negative
PRJNA729271	ed_Lushi_vs_Arbor_Acr	breed_growth_background	ENSGALG00010005301	ENSGALG00010005301	-0.272365157	0.196951894	-1.382901942	0.166694935	0.307449177	negative
PRJNA729271	ed_Lushi_vs_Arbor_Acr	breed_growth_background	ENSGALG00010005302	PSPC1	-0.091797432	0.043394021	-2.115439639	0.034392494	0.100609325	negative
PRJNA729271	ed_Lushi_vs_Arbor_Acr	breed_growth_background	ENSGALG00010005303	ENSGALG00010005303	-0.260671217	0.148224873	-1.758619936	0.078642085	0.181775955	negative
PRJNA729271	ed_Lushi_vs_Arbor_Acr	breed_growth_background	ENSGALG00010005304	ENSGALG00010005304	-0.323604033	0.112259881	-2.882632955	0.003943667	0.021441654	negative
PRJNA729271	ed_Lushi_vs_Arbor_Acr	breed_growth_background	ENSGALG00010005305	SCAF8	0.137491937	0.046455973	2.95961806	0.003080207	0.01808327	positive
PRJNA729271	ed_Lushi_vs_Arbor_Acr	breed_growth_background	ENSGALG00010005307	TIAM2	-0.252476743	0.085166057	-2.964523119	0.003031525	0.017840824	negative
PRJNA729271	ed_Lushi_vs_Arbor_Acr	breed_growth_background	ENSGALG00010005308	ENSGALG00010005308	0.959611236	0.29975453	3.201323552	0.001367978	0.010247465	positive
PRJNA729271	ed_Lushi_vs_Arbor_Acr	breed_growth_background	ENSGALG00010005309	ASXL3	-0.140657598	0.078357224	-1.795081441	0.072640725	0.172300329	negative
PRJNA729271	ed_Lushi_vs_Arbor_Acr	breed_growth_background	ENSGALG00010005310	LAMTOR3	0.144479874	0.040893996	3.533033926	0.00041082	0.00432764	positive
PRJNA729271	ed_Lushi_vs_Arbor_Acr	breed_growth_background	ENSGALG00010005311	YME1L1	0.125788183	0.041959851	2.997822406	0.002719161	0.016498215	positive
PRJNA729271	ed_Lushi_vs_Arbor_Acr	breed_growth_background	ENSGALG00010005313	MPHOSPH8	0.240730737	0.076883944	3.131092463	0.001741573	0.012043942	positive
PRJNA729271	ed_Lushi_vs_Arbor_Acr	breed_growth_background	ENSGALG00010005314	IL20RA	0.132542097	0.346140941	0.382913668	0.701783777	0.80748274	positive
PRJNA729271	ed_Lushi_vs_Arbor_Acr	breed_growth_background	ENSGALG00010005315	ENSGALG00010005315	-0.584796962	0.270090721	-2.165187165	0.030373355	0.091759437	negative
PRJNA729271	ed_Lushi_vs_Arbor_Acr	breed_growth_background	ENSGALG00010005316	PARP4	-0.175122664	0.05773369	-3.033283735	0.00241908	0.015238839	negative
PRJNA729271	ed_Lushi_vs_Arbor_Acr	breed_growth_background	ENSGALG00010005317	CHCHD3	-0.013561245	0.058596039	-0.231436212	0.816975936	0.887046157	negative
PRJNA729271	ed_Lushi_vs_Arbor_Acr	breed_growth_background	ENSGALG00010005318	SLC35D3	0.013546266	0.232790806	0.058190727	0.953596707	0.974074966	positive
PRJNA729271	ed_Lushi_vs_Arbor_Acr	breed_growth_background	ENSGALG00010005319	NOL4	0.281513936	0.453900007	0.620211351	0.53511865	0.680219664	positive
PRJNA729271	ed_Lushi_vs_Arbor_Acr	breed_growth_background	ENSGALG00010005321	HS6ST3	-0.371957501	0.174088528	-2.136599722	0.032630562	0.096941996	negative
PRJNA729271	ed_Lushi_vs_Arbor_Acr	breed_growth_background	ENSGALG00010005322	TFB1M	0.155896757	0.065970381	2.363132594	0.018121185	0.063800479	positive
PRJNA729271	ed_Lushi_vs_Arbor_Acr	breed_growth_background	ENSGALG00010005323	DNAJB14	0.060795884	0.034300764	1.77243527	0.076322331	0.178261713	positive
PRJNA729271	ed_Lushi_vs_Arbor_Acr	breed_growth_background	ENSGALG00010005324	CENPJ	-0.085616177	0.107219294	-0.79851465	0.424571895	0.583720916	negative
PRJNA729271	ed_Lushi_vs_Arbor_Acr	breed_growth_background	ENSGALG00010005326	MASTL	-0.026442718	0.102631298	-0.257647699	0.796678814	0.873728929	negative
PRJNA729271	ed_Lushi_vs_Arbor_Acr	breed_growth_background	ENSGALG00010005329	VIPAS39	0.216123124	0.063624126	3.396873735	0.000681604	0.006194264	positive
PRJNA729271	ed_Lushi_vs_Arbor_Acr	breed_growth_background	ENSGALG00010005331	ENSGALG00010005331	-0.014846547	0.439244801	-0.033800165	0.973036504	0.984110627	negative
PRJNA729271	ed_Lushi_vs_Arbor_Acr	breed_growth_background	ENSGALG00010005332	ENSGALG00010005332	-0.118344825	0.378452593	-0.312707132	0.754503176	0.844845388	negative
PRJNA729271	ed_Lushi_vs_Arbor_Acr	breed_growth_background	ENSGALG00010005333	PLXNA4	-0.032173611	0.084313398	-0.381595477	0.70276144	0.808005637	negative
PRJNA729271	ed_Lushi_vs_Arbor_Acr	breed_growth_background	ENSGALG00010005334	NME7	0.041872257	0.072335445	0.578862224	0.562682141	0.703265572	positive
PRJNA729271	ed_Lushi_vs_Arbor_Acr	breed_growth_background	ENSGALG00010005335	AHSA1	0.16786221	0.071854026	2.336155958	0.019483115	0.067096163	positive
PRJNA729271	ed_Lushi_vs_Arbor_Acr	breed_growth_background	ENSGALG00010005336	PEX7	0.051546068	0.068565033	0.751783604	0.452181203	0.608406808	positive
PRJNA729271	ed_Lushi_vs_Arbor_Acr	breed_growth_background	ENSGALG00010005337	DCTN1	0.139790129	0.082807766	1.688128246	0.091386613	0.202020583	positive
PRJNA729271	ed_Lushi_vs_Arbor_Acr	breed_growth_background	ENSGALG00010005338	PCGF1	0.044105061	0.061207884	0.720578108	0.471169127	0.625420924	positive
PRJNA729271	ed_Lushi_vs_Arbor_Acr	breed_growth_background	ENSGALG00010005341	UGGT2	-0.323500139	0.063929522	-5.060262107	4.18681E-07	2.47705E-05	negative
PRJNA729271	ed_Lushi_vs_Arbor_Acr	breed_growth_background	ENSGALG00010005342	MAP3K5	-0.119266787	0.077944454	-1.530151043	0.125979346	0.252568551	negative
PRJNA729271	ed_Lushi_vs_Arbor_Acr	breed_growth_background	ENSGALG00010005343	RNF17	0.342781533	1.001876021	0.342139672	0.732245783	0.828240196	positive
PRJNA729271	ed_Lushi_vs_Arbor_Acr	breed_growth_background	ENSGALG00010005344	ENSGALG00010005344	-0.845604594	0.392039966	-2.156934667	0.031010755	0.093257448	negative
PRJNA729271	ed_Lushi_vs_Arbor_Acr	breed_growth_background	ENSGALG00010005345	NOX3	0.138517808	0.293265014	0.472329807	0.636691398	0.761329963	positive
PRJNA729271	ed_Lushi_vs_Arbor_Acr	breed_growth_background	ENSGALG00010005346	FBXO30	0.308123892	0.117683485	2.61824241	0.008838399	0.03820835	positive
PRJNA729271	ed_Lushi_vs_Arbor_Acr	breed_growth_background	ENSGALG00010005347	DOK1	-0.189251065	0.082335371	-2.298539048	0.021531128	0.071877868	negative
PRJNA729271	ed_Lushi_vs_Arbor_Acr	breed_growth_background	ENSGALG00010005349	ACBD5	0.105948211	0.055383745	1.912983879	0.055750116	0.143282278	positive
PRJNA729271	ed_Lushi_vs_Arbor_Acr	breed_growth_background	ENSGALG00010005350	ARHGAP20	0.1515641	0.152558832	0.993479685	0.320476246	0.483885748	positive
PRJNA729271	ed_Lushi_vs_Arbor_Acr	breed_growth_background	ENSGALG00010005351	DTNA	0.062621807	0.086862153	0.720933159	0.47095064	0.625245423	positive
PRJNA729271	ed_Lushi_vs_Arbor_Acr	breed_growth_background	ENSGALG00010005352	ENSGALG00010005352	-0.160637473	0.315468086	-0.509203562	0.610609547	0.740775476	negative
PRJNA729271	ed_Lushi_vs_Arbor_Acr	breed_growth_background	ENSGALG00010005353	ENSGALG00010005353	-0.112666253	0.05626975	-2.002252598	0.045257571	0.123273008	negative
PRJNA729271	ed_Lushi_vs_Arbor_Acr	breed_growth_background	ENSGALG00010005354	M1AP	0.447127174	0.191973187	2.329112632	0.0198531	0.067838444	positive
PRJNA729271	ed_Lushi_vs_Arbor_Acr	breed_growth_background	ENSGALG00010005355	FDX1	0.06492314	0.055961068	1.160148336	0.245988418	0.401905991	positive
PRJNA729271	ed_Lushi_vs_Arbor_Acr	breed_growth_background	ENSGALG00010005356	DNAJC3	0.011435406	0.045019636	0.254009283	0.799488386	0.875866084	positive
PRJNA729271	ed_Lushi_vs_Arbor_Acr	breed_growth_background	ENSGALG00010005357	TNIP3	-0.064677063	0.291138752	-0.222152023	0.824195541	0.892745058	negative
PRJNA729271	ed_Lushi_vs_Arbor_Acr	breed_growth_background	ENSGALG00010005358	SHPRH	0.060787055	0.048216142	1.260720015	0.20740974	0.356546986	positive
PRJNA729271	ed_Lushi_vs_Arbor_Acr	breed_growth_background	ENSGALG00010005359	MEIS2	-0.026715526	0.074277354	-0.35967256	0.719092014	0.82014807	negative
PRJNA729271	ed_Lushi_vs_Arbor_Acr	breed_growth_background	ENSGALG00010005360	CAMK1D	-0.346498899	0.081475651	-4.25279082	2.11123E-05	0.000500037	negative
PRJNA729271	ed_Lushi_vs_Arbor_Acr	breed_growth_background	ENSGALG00010005361	ENSGALG00010005361	-0.568451221	0.229221652	-2.479919402	0.013141208	0.050646587	negative
PRJNA729271	ed_Lushi_vs_Arbor_Acr	breed_growth_background	ENSGALG00010005362	ATP1B1	-0.142922134	0.093073652	-1.535581023	0.124641171	0.250502855	negative
PRJNA729271	ed_Lushi_vs_Arbor_Acr	breed_growth_background	ENSGALG00010005363	RDX	0.058019801	0.045689479	1.269872254	0.204130138	0.353508525	positive
PRJNA729271	ed_Lushi_vs_Arbor_Acr	breed_growth_background	ENSGALG00010005364	MAP7	-0.084859381	0.204379821	-0.415204304	0.677992348	0.791965435	negative
PRJNA729271	ed_Lushi_vs_Arbor_Acr	breed_growth_background	ENSGALG00010005366	AUP1	0.206462038	0.060956851	3.38701945	0.000706564	0.00636918	positive
PRJNA729271	ed_Lushi_vs_Arbor_Acr	breed_growth_background	ENSGALG00010005367	H2AZ1	0.197247692	0.051408316	3.836882958	0.000124606	0.001870492	positive
PRJNA729271	ed_Lushi_vs_Arbor_Acr	breed_growth_background	ENSGALG00010005368	CCDC3	0.214194603	0.128151591	1.671415873	0.094639563	0.206891146	positive
PRJNA729271	ed_Lushi_vs_Arbor_Acr	breed_growth_background	ENSGALG00010005369	DPT	-0.183255652	0.166798612	-1.098664133	0.271914593	0.430660186	negative
PRJNA729271	ed_Lushi_vs_Arbor_Acr	breed_growth_background	ENSGALG00010005371	GRM1	-0.617304399	0.716779118	-0.861219842	0.389116974	0.550967876	negative
PRJNA729271	ed_Lushi_vs_Arbor_Acr	breed_growth_background	ENSGALG00010005372	CDIN1	-0.100520064	0.059866324	-1.679075255	0.093137378	0.204580436	negative
PRJNA729271	ed_Lushi_vs_Arbor_Acr	breed_growth_background	ENSGALG00010005376	HTRA2	0.138791475	0.051237314	2.708796856	0.006752767	0.031593275	positive
PRJNA729271	ed_Lushi_vs_Arbor_Acr	breed_growth_background	ENSGALG00010005377	EMCN	-0.260508872	0.074881306	-3.47895736	0.000503369	0.004994065	negative
PRJNA729271	ed_Lushi_vs_Arbor_Acr	breed_growth_background	ENSGALG00010005378	ENSGALG00010005378	0.167225017	0.479906721	0.348453168	0.727499879	0.824738835	positive
PRJNA729271	ed_Lushi_vs_Arbor_Acr	breed_growth_background	ENSGALG00010005379	DZIP1	-0.007271412	0.09054027	-0.080311353	0.935989629	0.964536447	negative
PRJNA729271	ed_Lushi_vs_Arbor_Acr	breed_growth_background	ENSGALG00010005380	XCL1	1.203201357	0.481905097	2.496759972	0.012533376	0.048968002	positive
PRJNA729271	ed_Lushi_vs_Arbor_Acr	breed_growth_background	ENSGALG00010005381	FGF9	0.177930357	0.122903626	1.447722595	0.147694648	0.282581036	positive
PRJNA729271	ed_Lushi_vs_Arbor_Acr	breed_growth_background	ENSGALG00010005382	RAB32	0.046879492	0.069414997	0.675351057	0.499452753	0.650689165	positive
PRJNA729271	ed_Lushi_vs_Arbor_Acr	breed_growth_background	ENSGALG00010005383	OPTN	0.143214876	0.074143338	1.931594659	0.053409556	0.138922777	positive
PRJNA729271	ed_Lushi_vs_Arbor_Acr	breed_growth_background	ENSGALG00010005384	MAPRE2	0.106317182	0.092604333	1.148079991	0.250935543	0.406903535	positive
PRJNA729271	ed_Lushi_vs_Arbor_Acr	breed_growth_background	ENSGALG00010005385	PDSS1	-0.031822727	0.053749852	-0.592052355	0.55381553	0.695872179	negative
PRJNA729271	ed_Lushi_vs_Arbor_Acr	breed_growth_background	ENSGALG00010005386	CPOX	-0.293191736	0.076268978	-3.844180729	0.000120956	0.001837793	negative
PRJNA729271	ed_Lushi_vs_Arbor_Acr	breed_growth_background	ENSGALG00010005387	CLDN10	0.022873694	0.355637872	0.064317373	0.94871752	0.971711451	positive
PRJNA729271	ed_Lushi_vs_Arbor_Acr	breed_growth_background	ENSGALG00010005388	ENSGALG00010005388	0.106776417	0.246762022	0.432710091	0.665225398	0.782957065	positive
PRJNA729271	ed_Lushi_vs_Arbor_Acr	breed_growth_background	ENSGALG00010005389	MCM10	0.298097961	0.102249383	2.915401078	0.003552317	0.019916301	positive
PRJNA729271	ed_Lushi_vs_Arbor_Acr	breed_growth_background	ENSGALG00010005391	ADGB	-0.047235194	0.2131462	-0.221609364	0.824617992	0.893003836	negative
PRJNA729271	ed_Lushi_vs_Arbor_Acr	breed_growth_background	ENSGALG00010005392	ENSGALG00010005392	0.039004307	0.058281918	0.669235124	0.503345504	0.654055856	positive
PRJNA729271	ed_Lushi_vs_Arbor_Acr	breed_growth_background	ENSGALG00010005394	PHYH	0.266260191	0.071934685	3.701415966	0.0002144	0.002742915	positive
PRJNA729271	ed_Lushi_vs_Arbor_Acr	breed_growth_background	ENSGALG00010005395	ENSGALG00010005395	-0.267938743	0.196156862	-1.365941215	0.171957415	0.315053886	negative
PRJNA729271	ed_Lushi_vs_Arbor_Acr	breed_growth_background	ENSGALG00010005396	MICU2	0.097460716	0.065497778	1.488000357	0.1367508	0.267575977	positive
PRJNA729271	ed_Lushi_vs_Arbor_Acr	breed_growth_background	ENSGALG00010005398	SEPHS1	0.103504527	0.046507024	2.225567636	0.026043156	0.08227889	positive
PRJNA729271	ed_Lushi_vs_Arbor_Acr	breed_growth_background	ENSGALG00010005399	NDNF	0.789367989	0.145616514	5.420868599	5.93101E-08	5.54646E-06	positive
PRJNA729271	ed_Lushi_vs_Arbor_Acr	breed_growth_background	ENSGALG00010005401	BCLAF1	-0.10612107	0.046908443	-2.262302096	0.023678745	0.077021053	negative
PRJNA729271	ed_Lushi_vs_Arbor_Acr	breed_growth_background	ENSGALG00010005402	ABCC4	0.434989073	0.091764386	4.740282035	2.13421E-06	8.864E-05	positive
PRJNA729271	ed_Lushi_vs_Arbor_Acr	breed_growth_background	ENSGALG00010005403	ENSGALG00010005403	0.055339394	0.079204095	0.698693597	0.484743536	0.636583407	positive
PRJNA729271	ed_Lushi_vs_Arbor_Acr	breed_growth_background	ENSGALG00010005404	BEND7	0.27301149	0.099800591	2.735569864	0.006227237	0.029790031	positive
PRJNA729271	ed_Lushi_vs_Arbor_Acr	breed_growth_background	ENSGALG00010005405	CEBPD	0.78748008	0.252470923	3.119092175	0.001814092	0.012425613	positive
PRJNA729271	ed_Lushi_vs_Arbor_Acr	breed_growth_background	ENSGALG00010005407	DPH6	-0.004067387	0.065888681	-0.061731192	0.9507769	0.972858334	negative
PRJNA729271	ed_Lushi_vs_Arbor_Acr	breed_growth_background	ENSGALG00010005408	APBB1IP	-0.217542027	0.112770208	-1.929073558	0.053721731	0.139354145	negative
PRJNA729271	ed_Lushi_vs_Arbor_Acr	breed_growth_background	ENSGALG00010005409	PRKCQ	-0.54222656	0.116564447	-4.65173193	3.29159E-06	0.000122706	negative
PRJNA729271	ed_Lushi_vs_Arbor_Acr	breed_growth_background	ENSGALG00010005411	STXBP5	-0.140196855	0.051813772	-2.705783601	0.006814344	0.031780537	negative
PRJNA729271	ed_Lushi_vs_Arbor_Acr	breed_growth_background	ENSGALG00010005412	MTFR2	-0.284786011	0.091640583	-3.107640746	0.001885872	0.012726122	negative
PRJNA729271	ed_Lushi_vs_Arbor_Acr	breed_growth_background	ENSGALG00010005413	GAD2	0.380272826	0.172388265	2.205909007	0.027390371	0.085106846	positive
PRJNA729271	ed_Lushi_vs_Arbor_Acr	breed_growth_background	ENSGALG00010005414	ENSGALG00010005414	-0.107598796	0.124896859	-0.861501211	0.388962055	0.550902931	negative
PRJNA729271	ed_Lushi_vs_Arbor_Acr	breed_growth_background	ENSGALG00010005415	PRKDC	-0.015316482	0.047387432	-0.323218238	0.74652997	0.838573459	negative
PRJNA729271	ed_Lushi_vs_Arbor_Acr	breed_growth_background	ENSGALG00010005416	PPP3CA	0.268389611	0.086804412	3.091889049	0.001988872	0.013199952	positive
PRJNA729271	ed_Lushi_vs_Arbor_Acr	breed_growth_background	ENSGALG00010005417	LOXL3	-0.246584141	0.088195249	-2.795889154	0.005175715	0.026192366	negative
PRJNA729271	ed_Lushi_vs_Arbor_Acr	breed_growth_background	ENSGALG00010005420	DHRS4	-0.037813783	0.063790049	-0.592784977	0.553325063	0.695615506	negative
PRJNA729271	ed_Lushi_vs_Arbor_Acr	breed_growth_background	ENSGALG00010005421	ENSGALG00010005421	-0.257146779	0.175996302	-1.461091946	0.14399021	0.277360545	negative
PRJNA729271	ed_Lushi_vs_Arbor_Acr	breed_growth_background	ENSGALG00010005423	ST3GAL6	-0.237685833	0.080115757	-2.966780109	0.003009361	0.017760866	negative
PRJNA729271	ed_Lushi_vs_Arbor_Acr	breed_growth_background	ENSGALG00010005424	ENSGALG00010005424	0.274300264	0.073636313	3.725067901	0.000195263	0.002556759	positive
PRJNA729271	ed_Lushi_vs_Arbor_Acr	breed_growth_background	ENSGALG00010005425	ELAPOR2	0.016884783	0.138425649	0.121977275	0.902917016	0.944095603	positive
PRJNA729271	ed_Lushi_vs_Arbor_Acr	breed_growth_background	ENSGALG00010005426	ENSGALG00010005426	-1.000097093	0.447792623	-2.233393411	0.025523012	0.081151376	negative
PRJNA729271	ed_Lushi_vs_Arbor_Acr	breed_growth_background	ENSGALG00010005427	ENSGALG00010005427	0.19425524	0.075209256	2.582863458	0.009798409	0.041215306	positive
PRJNA729271	ed_Lushi_vs_Arbor_Acr	breed_growth_background	ENSGALG00010005428	ZNF770	0.066053367	0.05525046	1.195526091	0.231881548	0.385981057	positive
PRJNA729271	ed_Lushi_vs_Arbor_Acr	breed_growth_background	ENSGALG00010005429	ZDHHC20	-0.086929081	0.064379376	-1.350262869	0.176931678	0.320939141	negative
PRJNA729271	ed_Lushi_vs_Arbor_Acr	breed_growth_background	ENSGALG00010005430	MCM4	-0.021019421	0.073344052	-0.286586574	0.774428896	0.860006248	negative
PRJNA729271	ed_Lushi_vs_Arbor_Acr	breed_growth_background	ENSGALG00010005431	GRM3	0.059387093	0.498775688	0.119065733	0.905223278	0.945468469	positive
PRJNA729271	ed_Lushi_vs_Arbor_Acr	breed_growth_background	ENSGALG00010005433	GPC5	0.067202874	0.082523931	0.81434408	0.415447864	0.575599005	positive
PRJNA729271	ed_Lushi_vs_Arbor_Acr	breed_growth_background	ENSGALG00010005434	PRDM5	-0.339885521	0.119786791	-2.837420717	0.004547964	0.023738833	negative
PRJNA729271	ed_Lushi_vs_Arbor_Acr	breed_growth_background	ENSGALG00010005435	MAD2L1	-0.125677638	0.094354982	-1.331966101	0.182871328	0.327897069	negative
PRJNA729271	ed_Lushi_vs_Arbor_Acr	breed_growth_background	ENSGALG00010005436	AQR	0.060335982	0.030160247	2.000513522	0.045444841	0.123634192	positive
PRJNA729271	ed_Lushi_vs_Arbor_Acr	breed_growth_background	ENSGALG00010005437	PDE7B	-0.094426682	0.08405324	-1.123415135	0.261261225	0.419284926	negative
PRJNA729271	ed_Lushi_vs_Arbor_Acr	breed_growth_background	ENSGALG00010005438	GPR180	0.176892709	0.076057074	2.325789037	0.020029808	0.068345589	positive
PRJNA729271	ed_Lushi_vs_Arbor_Acr	breed_growth_background	ENSGALG00010005439	TBX19	-0.523992157	0.293107465	-1.787713446	0.073822251	0.174361012	negative
PRJNA729271	ed_Lushi_vs_Arbor_Acr	breed_growth_background	ENSGALG00010005440	ENSGALG00010005440	-0.426238333	0.228267754	-1.867273521	0.061863401	0.154191053	negative
PRJNA729271	ed_Lushi_vs_Arbor_Acr	breed_growth_background	ENSGALG00010005441	UBE2V2	0.054236133	0.051568784	1.051724094	0.292926153	0.454316489	positive

PRJNA729271	ed_Lushi_vs_Arbor_Acr	breed_growth_background	ENSGALG00010005442	SASH1	-0.141352017	0.05228496	-2.703492863	0.006861492	0.031969569	negative
PRJNA729271	ed_Lushi_vs_Arbor_Acr	breed_growth_background	ENSGALG00010005443	BANK1	-0.028090168	0.237375048	-0.118336648	0.90580092	0.945935471	negative
PRJNA729271	ed_Lushi_vs_Arbor_Acr	breed_growth_background	ENSGALG00010005444	ACTC1	-0.827299688	0.228020869	-3.628175322	0.000285431	0.003360578	negative
PRJNA729271	ed_Lushi_vs_Arbor_Acr	breed_growth_background	ENSGALG00010005445	SFT2D2	0.330380271	0.058223772	5.674319263	1.39241E-08	1.77044E-06	positive
PRJNA729271	ed_Lushi_vs_Arbor_Acr	breed_growth_background	ENSGALG00010005446	ENSGALG00010005446	-0.652149351	0.205211461	-3.177938246	0.001483263	0.010832846	negative
PRJNA729271	ed_Lushi_vs_Arbor_Acr	breed_growth_background	ENSGALG00010005447	MYO3A	0.395707222	0.217106169	1.822643839	0.068357351	0.165205664	positive
PRJNA729271	ed_Lushi_vs_Arbor_Acr	breed_growth_background	ENSGALG00010005448	ENSGALG00010005448	-0.288386995	0.078986543	-3.651090232	0.000261129	0.003153469	negative
PRJNA729271	ed_Lushi_vs_Arbor_Acr	breed_growth_background	ENSGALG00010005449	TIPRL	0.031819	0.0463529	0.68645112	0.492428678	0.644383742	positive
PRJNA729271	ed_Lushi_vs_Arbor_Acr	breed_growth_background	ENSGALG00010005450	ENSGALG00010005450	-0.557635371	0.212414477	-2.625223003	0.008659223	0.037534522	negative
PRJNA729271	ed_Lushi_vs_Arbor_Acr	breed_growth_background	ENSGALG00010005453	UST	-0.083730807	0.061441182	-1.362779888	0.172951891	0.316372749	negative
PRJNA729271	ed_Lushi_vs_Arbor_Acr	breed_growth_background	ENSGALG00010005454	GPR161	-0.129051036	0.053715131	-2.402508096	0.016283074	0.059021435	negative
PRJNA729271	ed_Lushi_vs_Arbor_Acr	breed_growth_background	ENSGALG00010005455	ENSGALG00010005455	-0.946413639	0.369799281	-2.559263058	0.010489433	0.043292807	negative
PRJNA729271	ed_Lushi_vs_Arbor_Acr	breed_growth_background	ENSGALG00010005456	RPL7L1	0.00493792	0.038143094	0.12945778	0.896995431	0.9402177	positive
PRJNA729271	ed_Lushi_vs_Arbor_Acr	breed_growth_background	ENSGALG00010005457	SEMA3D	0.000872599	0.085759296	0.010174977	0.991881683	0.995521742	positive
PRJNA729271	ed_Lushi_vs_Arbor_Acr	breed_growth_background	ENSGALG00010005458	PRKD1	-0.151703926	0.093543097	-1.621754365	0.104855953	0.222465902	negative
PRJNA729271	ed_Lushi_vs_Arbor_Acr	breed_growth_background	ENSGALG00010005459	ZC3H12D	-0.089421402	0.235716287	-0.379360304	0.704420324	0.808849041	negative
PRJNA729271	ed_Lushi_vs_Arbor_Acr	breed_growth_background	ENSGALG00010005460	TGDS	0.151535275	0.056322731	2.690481684	0.007134895	0.032852702	positive
PRJNA729271	ed_Lushi_vs_Arbor_Acr	breed_growth_background	ENSGALG00010005462	AHI1	0.195004745	0.080665328	2.417454312	0.015629496	0.057429076	positive
PRJNA729271	ed_Lushi_vs_Arbor_Acr	breed_growth_background	ENSGALG00010005463	SKA3	-0.473521234	0.138832835	-3.410729414	0.000647894	0.005987563	negative
PRJNA729271	ed_Lushi_vs_Arbor_Acr	breed_growth_background	ENSGALG00010005464	SLC39A8	-0.132482395	0.181796212	-0.728741228	0.466159968	0.621160943	negative
PRJNA729271	ed_Lushi_vs_Arbor_Acr	breed_growth_background	ENSGALG00010005465	ENSGALG00010005465	-0.186952056	0.050223361	-3.72241225	0.000197328	0.002567573	negative
PRJNA729271	ed_Lushi_vs_Arbor_Acr	breed_growth_background	ENSGALG00010005466	LATS2	-0.029185952	0.047977113	-0.60833072	0.54296815	0.687664774	negative
PRJNA729271	ed_Lushi_vs_Arbor_Acr	breed_growth_background	ENSGALG00010005467	ENSGALG00010005467	-0.131926489	0.134071616	-0.984000145	0.325115442	0.488946163	negative
PRJNA729271	ed_Lushi_vs_Arbor_Acr	breed_growth_background	ENSGALG00010005468	PPIL4	0.135718345	0.056760492	2.391070624	0.016799321	0.060333538	positive
PRJNA729271	ed_Lushi_vs_Arbor_Acr	breed_growth_background	ENSGALG00010005470	SEMA3A	-0.168750327	0.075949907	-2.221863521	0.02629253	0.082760093	negative
PRJNA729271	ed_Lushi_vs_Arbor_Acr	breed_growth_background	ENSGALG00010005472	PFKP	-0.221878903	0.061970676	-3.580385417	0.000343088	0.003799127	negative
PRJNA729271	ed_Lushi_vs_Arbor_Acr	breed_growth_background	ENSGALG00010005473	ENSGALG00010005473	-0.081369968	0.37002215	-0.219905668	0.825944622	0.893637926	negative
PRJNA729271	ed_Lushi_vs_Arbor_Acr	breed_growth_background	ENSGALG00010005474	COL8A1	-0.450073829	0.185034848	-2.432373329	0.015000237	0.055594078	negative
PRJNA729271	ed_Lushi_vs_Arbor_Acr	breed_growth_background	ENSGALG00010005475	TMEM181	-0.084132904	0.049991922	-1.682929977	0.092388648	0.203459959	negative
PRJNA729271	ed_Lushi_vs_Arbor_Acr	breed_growth_background	ENSGALG00010005476	NFKB1	0.052665045	0.061972709	0.849810285	0.395430571	0.556914703	positive
PRJNA729271	ed_Lushi_vs_Arbor_Acr	breed_growth_background	ENSGALG00010005477	TMEM161B	0.067384166	0.086865492	0.775729978	0.437908436	0.595342503	positive
PRJNA729271	ed_Lushi_vs_Arbor_Acr	breed_growth_background	ENSGALG00010005479	ENSGALG00010005479	0.364551982	0.336417741	1.083628886	0.278529378	0.437835737	positive
PRJNA729271	ed_Lushi_vs_Arbor_Acr	breed_growth_background	ENSGALG00010005481	ENSGALG00010005481	0.733069683	0.304736457	2.405585766	0.016146561	0.058657746	positive
PRJNA729271	ed_Lushi_vs_Arbor_Acr	breed_growth_background	ENSGALG00010005482	XPO4	0.076271427	0.041233211	1.84975715	0.064348559	0.158331755	positive
PRJNA729271	ed_Lushi_vs_Arbor_Acr	breed_growth_background	ENSGALG00010005484	GINM1	-0.133153538	0.065323573	-2.038368861	0.041513058	0.115517715	negative
PRJNA729271	ed_Lushi_vs_Arbor_Acr	breed_growth_background	ENSGALG00010005485	PITRM1	0.089780185	0.052455921	1.711535763	0.086982255	0.195171484	positive
PRJNA729271	ed_Lushi_vs_Arbor_Acr	breed_growth_background	ENSGALG00010005486	IRF5	-0.008676122	0.170390933	-0.050918919	0.95939013	0.977517846	negative
PRJNA729271	ed_Lushi_vs_Arbor_Acr	breed_growth_background	ENSGALG00010005487	ENSGALG00010005487	-0.35450667	0.114062081	-3.108015115	0.001883484	0.012721857	negative
PRJNA729271	ed_Lushi_vs_Arbor_Acr	breed_growth_background	ENSGALG00010005489	G2E3	-0.031216345	0.046737404	-0.667909249	0.504191523	0.654597749	negative
PRJNA729271	ed_Lushi_vs_Arbor_Acr	breed_growth_background	ENSGALG00010005490	DCBLD2	-0.114587829	0.061668198	-1.858134884	0.063149862	0.156417849	negative
PRJNA729271	ed_Lushi_vs_Arbor_Acr	breed_growth_background	ENSGALG00010005491	GGACT	-0.000800515	0.094039241	-0.008512563	0.99320804	0.996094274	negative
PRJNA729271	ed_Lushi_vs_Arbor_Acr	breed_growth_background	ENSGALG00010005492	TNPO3	-0.029478602	0.051906025	-0.567922551	0.570087558	0.70877295	negative
PRJNA729271	ed_Lushi_vs_Arbor_Acr	breed_growth_background	ENSGALG00010005493	KLF6	-0.185652786	0.096857756	-1.916757057	0.055268803	0.142400325	negative
PRJNA729271	ed_Lushi_vs_Arbor_Acr	breed_growth_background	ENSGALG00010005494	PPDPFL	-0.260623986	0.234326314	-1.112226713	0.266040685	0.424230993	negative
PRJNA729271	ed_Lushi_vs_Arbor_Acr	breed_growth_background	ENSGALG00010005495	TXN	-0.119967661	0.078452112	-1.529183322	0.126219004	0.252829155	negative
PRJNA729271	ed_Lushi_vs_Arbor_Acr	breed_growth_background	ENSGALG00010005497	MKX	0.031142465	0.110413189	0.282053854	0.777902217	0.862253776	positive
PRJNA729271	ed_Lushi_vs_Arbor_Acr	breed_growth_background	ENSGALG00010005498	SMO	0.143595308	0.073422812	1.955731513	0.05049679	0.133470273	positive
PRJNA729271	ed_Lushi_vs_Arbor_Acr	breed_growth_background	ENSGALG00010005499	PDE5A	-0.135369383	0.060436322	-2.239867984	0.025099495	0.080224295	negative
PRJNA729271	ed_Lushi_vs_Arbor_Acr	breed_growth_background	ENSGALG00010005500	KATNA1	0.176280962	0.070082336	2.515340855	0.011891735	0.047238706	positive
PRJNA729271	ed_Lushi_vs_Arbor_Acr	breed_growth_background	ENSGALG00010005502	SCFD1	0.098589859	0.039340494	2.506065596	0.012208297	0.048112905	positive
PRJNA729271	ed_Lushi_vs_Arbor_Acr	breed_growth_background	ENSGALG00010005503	DYNLT1	0.116595397	0.083032631	1.404211764	0.1602558	0.299035507	positive
PRJNA729271	ed_Lushi_vs_Arbor_Acr	breed_growth_background	ENSGALG00010005504	LATS1	0.025044502	0.043068468	0.581504368	0.560900575	0.701670306	positive
PRJNA729271	ed_Lushi_vs_Arbor_Acr	breed_growth_background	ENSGALG00010005505	FABP2	-0.992856403	0.562052001	-1.766484953	0.077314524	0.179652777	negative
PRJNA729271	ed_Lushi_vs_Arbor_Acr	breed_growth_background	ENSGALG00010005507	USP53	-0.148171195	0.064029819	-2.314096725	0.020662417	0.069781392	negative
PRJNA729271	ed_Lushi_vs_Arbor_Acr	breed_growth_background	ENSGALG00010005508	SS18	0.032103143	0.035224669	0.911382386	0.362093931	0.525396209	positive
PRJNA729271	ed_Lushi_vs_Arbor_Acr	breed_growth_background	ENSGALG00010005509	COCH	0.823488693	0.241644746	3.407848532	0.000654772	0.006011406	positive
PRJNA729271	ed_Lushi_vs_Arbor_Acr	breed_growth_background	ENSGALG00010005510	MYOZ2	-0.612550107	0.170248027	-3.597986522	0.00032069	0.003625901	negative
PRJNA729271	ed_Lushi_vs_Arbor_Acr	breed_growth_background	ENSGALG00010005511	AHCYL2	0.069644693	0.053035819	1.31316333	0.189127918	0.335340571	positive
PRJNA729271	ed_Lushi_vs_Arbor_Acr	breed_growth_background	ENSGALG00010005512	SMARCAD1	0.092048362	0.061174847	1.504676628	0.132407235	0.261406002	positive
PRJNA729271	ed_Lushi_vs_Arbor_Acr	breed_growth_background	ENSGALG00010005513	TMEM30CP	0.038975747	0.141601521	0.275249491	0.783124566	0.865725335	positive
PRJNA729271	ed_Lushi_vs_Arbor_Acr	breed_growth_background	ENSGALG00010005514	EEF1AKMT1	-0.154598593	0.07712978	-2.004395611	0.045027699	0.122775865	negative
PRJNA729271	ed_Lushi_vs_Arbor_Acr	breed_growth_background	ENSGALG00010005515	PCCA	-0.074363299	0.052054775	-1.428558657	0.153131124	0.289577481	negative
PRJNA729271	ed_Lushi_vs_Arbor_Acr	breed_growth_background	ENSGALG00010005516	LARP4B	0.190475653	0.070391303	2.705954352	0.006810841	0.031780537	positive
PRJNA729271	ed_Lushi_vs_Arbor_Acr	breed_growth_background	ENSGALG00010005518	HPGDS	-0.39443853	0.351543276	-1.122019838	0.261854005	0.420003741	negative
PRJNA729271	ed_Lushi_vs_Arbor_Acr	breed_growth_background	ENSGALG00010005519	PTBP3	0.068133028	0.140066912	0.486431998	0.626660908	0.75289116	positive
PRJNA729271	ed_Lushi_vs_Arbor_Acr	breed_growth_background	ENSGALG00010005521	SYTL3	0.303193177	0.209990175	1.443844585	0.148782697	0.283726509	positive
PRJNA729271	ed_Lushi_vs_Arbor_Acr	breed_growth_background	ENSGALG00010005522	SYNPO2	0.191937919	0.095181338	2.016549928	0.043742503	0.120175811	positive
PRJNA729271	ed_Lushi_vs_Arbor_Acr	breed_growth_background	ENSGALG00010005523	ENSGALG00010005523	-0.690229105	0.315224911	-2.189640102	0.028550348	0.087677394	negative
PRJNA729271	ed_Lushi_vs_Arbor_Acr	breed_growth_background	ENSGALG00010005524	STRIP2	0.164306067	0.14639914	1.122315793	0.261728194	0.419848403	positive
PRJNA729271	ed_Lushi_vs_Arbor_Acr	breed_growth_background	ENSGALG00010005526	STRN3	0.079798232	0.037403575	2.133438638	0.03288876	0.0974137	positive
PRJNA729271	ed_Lushi_vs_Arbor_Acr	breed_growth_background	ENSGALG00010005527	NUP43	0.199694251	0.052296065	3.818533046	0.000134248	0.001979572	positive
PRJNA729271	ed_Lushi_vs_Arbor_Acr	breed_growth_background	ENSGALG00010005529	ENSGALG00010005529	0.30883461	0.199447356	1.548451763	0.121513567	0.246237823	positive
PRJNA729271	ed_Lushi_vs_Arbor_Acr	breed_growth_background	ENSGALG00010005530	IL17D	-0.352163071	0.112947396	-3.117938818	0.001821206	0.012451102	negative
PRJNA729271	ed_Lushi_vs_Arbor_Acr	breed_growth_background	ENSGALG00010005531	SUSD1	-0.447180028	0.137306577	-3.256799766	0.001126759	0.008924626	negative
PRJNA729271	ed_Lushi_vs_Arbor_Acr	breed_growth_background	ENSGALG00010005532	GTPBP4	0.039518507	0.052262223	0.756158172	0.449554368	0.605810259	positive
PRJNA729271	ed_Lushi_vs_Arbor_Acr	breed_growth_background	ENSGALG00010005533	OPN4-1	0.084044129	0.453435028	0.185349882	0.852954615	0.911163472	positive
PRJNA729271	ed_Lushi_vs_Arbor_Acr	breed_growth_background	ENSGALG00010005534	ENSGALG00010005534	-0.176223029	0.088547539	-1.99015164	0.046574234	0.125856361	negative
PRJNA729271	ed_Lushi_vs_Arbor_Acr	breed_growth_background	ENSGALG00010005535	AP4S1	-0.004736289	0.083823078	-0.056503407	0.954940782	0.974885706	negative
PRJNA729271	ed_Lushi_vs_Arbor_Acr	breed_growth_background	ENSGALG00010005536	TAF4B	-0.38493768	0.115884469	-3.321736576	0.000894591	0.007530254	negative
PRJNA729271	ed_Lushi_vs_Arbor_Acr	breed_growth_background	ENSGALG00010005537	GRIN3A	0.024748982	0.184342321	0.13425556	0.893200493	0.93750479	positive
PRJNA729271	ed_Lushi_vs_Arbor_Acr	breed_growth_background	ENSGALG00010005538	ENSGALG00010005538	-0.849034872	0.17321688	-4.901571213	9.50732E-07	4.67148E-05	negative
PRJNA729271	ed_Lushi_vs_Arbor_Acr	breed_growth_background	ENSGALG00010005539	IFT88	-0.094084129	0.072288747	-1.30150449	0.193085829	0.340122615	negative
PRJNA729271	ed_Lushi_vs_Arbor_Acr	breed_growth_background	ENSGALG00010005540	PCMT1	0.092361121	0.073325926	1.259597058	0.207814757	0.356976032	positive
PRJNA729271	ed_Lushi_vs_Arbor_Acr	breed_growth_background	ENSGALG00010005542	KCTD1	0.109532445	0.074680782	1.466675117	0.142464455	0.275446482	positive
PRJNA729271	ed_Lushi_vs_Arbor_Acr	breed_growth_background	ENSGALG00010005543	SEC24D	-0.231432511	0.108325937	-2.136445963	0.032643081	0.09694429	negative
PRJNA729271	ed_Lushi_vs_Arbor_Acr	breed_growth_background	ENSGALG00010005544	AQP4	0.284282249	0.15833693	1.795426046	0.072585846	0.172198336	positive
PRJNA729271	ed_Lushi_vs_Arbor_Acr	breed_growth_background	ENSGALG00010005545	RPL34	-0.071084423	0.08921703	-0.796758459	0.425591326	0.584734243	negative
PRJNA729271	ed_Lushi_vs_Arbor_Acr	breed_growth_background	ENSGALG00010005546	NRF1	0.26407581	0.081590922	3.236583237	0.001209699	0.00940697	positive
PRJNA729271	ed_Lushi_vs_Arbor_Acr	breed_growth_background	ENSGALG00010005547	LRP11	-0.433508311	0.117821813	-3.679355304	0.000233824	0.002896823	negative
PRJNA729271	ed_Lushi_vs_Arbor_Acr	breed_growth_background	ENSGALG00010005548	EZR	-0.28391935	0.096236405	-2.950228155	0.003175393	0.018490252	negative
PRJNA729271	ed_Lushi_vs_Arbor_Acr	breed_growth_background	ENSGALG00010005549	RNF20	0.012432984	0.061191491	0.203181578	0.838993111	0.902332995	positive
PRJNA729271	ed_Lushi_vs_Arbor_Acr	breed_growth_background	ENSGALG00010005550	ENSGALG00010005550	0.055571255	0.294586893	0.1886413	0.850373961	0.910016284	positive
PRJNA729271	ed_Lushi_vs_Arbor_Acr	breed_growth_background	ENSGALG00010005551	METTL14	-0.087898315	0.046297806	-1.898541716	0.057624758	0.146522531	negative
PRJNA729271	ed_Lushi_vs_Arbor_Acr	breed_growth_background	ENSGALG00010005552	WDR37	-0.172719107	0.047034588	-3.672172215	0.000240498	0.002957703	negative
PRJNA729271	ed_Lushi_vs_Arbor_Acr	breed_growth_background	ENSGALG00010005553	PPP1R14C	0.007410449	0.087968165	0.084240128	0.932865514	0.963190242	positive
PRJNA729271	ed_Lushi_vs_Arbor_Acr	breed_growth_background	ENSGALG00010005554	CRYL1	0.131638311	0.08686467	1.51544133	0.129660678	0.257280153	positive
PRJNA729271	ed_Lushi_vs_Arbor_Acr	breed_growth_background	ENSGALG00010005555	PRSS12	-0.368588405	0.097991922	-3.761416239	0.000168954	0.002314734	negative
PRJNA729271	ed_Lushi_vs_Arbor_Acr	breed_growth_background	ENSGALG00010005556	IYD	-1.237808787	0.463376356	-2.671281717	0.00755622	0.034218827	negative
PRJNA729271	ed_Lushi_vs_Arbor_Acr	breed_growth_background	ENSGALG00010005557	CMSS1	0.015443846	0.091283432	0.169185645	0.865650621	0.919171178	positive
PRJNA729271	ed_Lushi_vs_Arbor_Acr	breed_growth_background	ENSGALG00010005558	TAGAP	0.107312567	0.112963024	0.949979591	0.342122623	0.505938073	positive
PRJNA729271	ed_Lushi_vs_Arbor_Acr	breed_growth_background	ENSGALG00010005559	FILIP1L	-0.03051963	0.07008677	-0.435454943	0.663232237	0.781503479	negative
PRJNA729271	ed_Lushi_vs_Arbor_Acr	breed_growth_background	ENSGALG00010005560	RSPH3	-0.557487072	0.214708549	-2.59648288	0.00941836	0.040152684	negative
PRJNA729271	ed_Lushi_vs_Arbor_Acr	breed_growth_background	ENSGALG00010005561	CLYBL	0.672629149	0.105906902	6.351136115	2.1373E-10	5.84533E-08	positive
PRJNA729271	ed_Lushi_vs_Arbor_Acr	breed_growth_background	ENSGALG00010005562	HECTD1	0.078442465	0.045709917	1.716092926	0.086145036	0.194013719	positive
PRJNA729271	ed_Lushi_vs_Arbor_Acr	breed_growth_background	ENSGALG00010005564	TM9SF2	-0.069811367	0.031130208	-2.242560276	0.024925184	0.079861735	negative
PRJNA729271	ed_Lushi_vs_Arbor_Acr	breed_growth_background	ENSGALG00010005565	PGAP4	-0.228145926	0.096360971	-2.367617557	0.017903031	0.063309207	negative
PRJNA729271	ed_Lushi_vs_Arbor_Acr	breed_growth_background	ENSGALG00010005567	NDST3	-0.244732427	0.130748869	-1.871774711	0.061237778	0.153140498	negative
PRJNA729271	ed_Lushi_vs_Arbor_Acr	breed_growth_background	ENSGALG00010005568	PLEKHG1	0.006926993	0.070901113	0.097699357	0.922171026	0.956621271	positive
PRJNA729271	ed_Lushi_vs_Arbor_Acr	breed_growth_background	ENSGALG00010005572	ENSGALG00010005572	-0.211818226	0.082367197	-2.571633292	0.010122004	0.042184719	negative
PRJNA729271	ed_Lushi_vs_Arbor_Acr	breed_growth_background	ENSGALG00010005573	CHST9	0.048877729	0.151613997	0.322382695	0.747162792	0.838829267	positive
PRJNA729271	ed_Lushi_vs_Arbor_Acr	breed_growth_background	ENSGALG00010005575	ENSGALG00010005575	-0.235680239	0.222072523	-1.061275997	0.288564491	0.449533423	negative
PRJNA729271	ed_Lushi_vs_Arbor_Acr	breed_growth_background	ENSGALG00010005578	HEATR5A	-0.098301281	0.064473368	-1.524680411	0.12733883	0.254239166	negative
PRJNA729271	ed_Lushi_vs_Arbor_Acr	breed_growth_background	ENSGALG00010005581	GR42L5	0.143757736	0.417858522	0.344034472	0.730820361	0.827442114	positive
PRJNA729271	ed_Lushi_vs_Arbor_Acr	breed_growth_background	ENSGALG00010005583	ALDOB	-1.145246151	0.541904295	-2.113373453			negative
PRJNA729271	ed_Lushi_vs_Arbor_Acr	breed_growth_background	ENSGALG00010005587	LEF1	0.08943105	0.125210535	0.714245417	0.475075438	0.628533997	positive
PRJNA729271	ed_Lushi_vs_Arbor_Acr	breed_growth_background	ENSGALG00010005588	PLAGL1	0.046340357	0.06828603	0.678621341	0.497377817	0.648743945	positive
PRJNA729271	ed_Lushi_vs_Arbor_Acr	breed_growth_background	ENSGALG00010005590	TBC1D23	0.004235139	0.030916008	0.136988541	0.891039853	0.936321783	positive
PRJNA729271	ed_Lushi_vs_Arbor_Acr	breed_growth_background	ENSGALG00010005593	ENSGALG00010005593	-0.801033141	0.591739704	-1.353691727	0.175834749	0.319882257	negative
PRJNA729271	ed_Lushi_vs_Arbor_Acr	breed_growth_background	ENSGALG00010005594	MRPL50	-0.136278408	0.080577176	-1.69127804	0.090783712	0.201450683	negative
PRJNA729271	ed_Lushi_vs_Arbor_Acr	breed_growth_background	ENSGALG00010005596	PARD3	0.013105704	0.029289559	0.447453114	0.654547933	0.775136625	positive
PRJNA729271	ed_Lushi_vs_Arbor_Acr	breed_growth_background	ENSGALG00010005597	ENSGALG00010005597	-0.654149622	0.254696083	-2.568353679	0.010218284	0.042417058	negative
PRJNA729271	ed_Lushi_vs_Arbor_Acr	breed_growth_background	ENSGALG00010005601	TMEM215	-0.169735587	0.237780502	-0.713833075	0.475330405	0.628756545	negative
PRJNA729271	ed_Lushi_vs_Arbor_Acr	breed_growth_background	ENSGALG00010005609	GJB6	0.234134627	0.301534849	0.77647618	0.43746788	0.594961243	positive
PRJNA729271	ed_Lushi_vs_Arbor_Acr	breed_growth_background	ENSGALG00010005610	MTHFD1L	-0.187244614	0.102155789	-1.832931981	0.066812687	0.162874184	negative
PRJNA729271	ed_Lushi_vs_Arbor_Acr	breed_growth_background	ENSGALG00010005617	DTD2	0.003475842	0.111626315	0.031138199	0.975159326	0.985493581	positive
PRJNA729271	ed_Lushi_vs_Arbor_Acr	breed_growth_background	ENSGALG00010005618	PHACTR2	-0.061963578	0.036958984	-1.6765498	0.09363055	0.205244769	negative
PRJNA729271	ed_Lushi_vs_Arbor_Acr	breed_growth_background	ENSGALG00010005619	CELF2	-0.053181515	0.041200231	-1.290806252	0.196770869	0.34517654	negative
PRJNA729271	ed_Lushi_vs_Arbor_Acr	breed_growth_background	ENSGALG00010005621	CDH2	-0.191040511	0.077697346	-2.458777826	0.013941086	0.052799794	negative
PRJNA729271	ed_Lushi_vs_Arbor_Acr	breed_growth_background	ENSGALG00010005629	NRP1	-0.327196739	0.092616918	-3.53279668	0.000411189	0.004328379	negative
PRJNA729271	ed_Lushi_vs_Arbor_Acr	breed_growth_background	ENSGALG00010005630	NIT2	-0.022204082	0.088936517	-0.249662149	0.802848632	0.878395942	negative
PRJNA729271	ed_Lushi_vs_Arbor_Acr	breed_growth_background	ENSGALG00010005632	ITGB1	-0.233096498	0.049971203	-4.66461646	3.09193E-06	0.000116712	negative

PRJNA729271	ed_Lushi_vs_Arbor_Acr	breed_growth_background	ENSGALG00010005634	HADH	0.052701608	0.04642201	1.13527201	0.256261366	0.413366181	positive
PRJNA729271	ed_Lushi_vs_Arbor_Acr	breed_growth_background	ENSGALG00010005636	ENSGALG00010005636	-0.527630322	0.300654994	-1.754936165	0.079270227	0.182936148	negative
PRJNA729271	ed_Lushi_vs_Arbor_Acr	breed_growth_background	ENSGALG00010005637	TOPORS	0.063957835	0.039285479	1.628027392	0.103519078	0.220468562	positive
PRJNA729271	ed_Lushi_vs_Arbor_Acr	breed_growth_background	ENSGALG00010005643	ENSGALG00010005643	0.467426902	0.70088378	0.666910713	0.504829167	0.654985569	positive
PRJNA729271	ed_Lushi_vs_Arbor_Acr	breed_growth_background	ENSGALG00010005645	FUCA2	0.035430979	0.084468244	0.419459169	0.67488059	0.789732392	positive
PRJNA729271	ed_Lushi_vs_Arbor_Acr	breed_growth_background	ENSGALG00010005649	TOMM70	-0.055242987	0.04244036	-1.301661613	0.193032087	0.340099685	negative
PRJNA729271	ed_Lushi_vs_Arbor_Acr	breed_growth_background	ENSGALG00010005651	USP6NL	0.208428752	0.065094048	3.20196331	0.001364944	0.010230773	positive
PRJNA729271	ed_Lushi_vs_Arbor_Acr	breed_growth_background	ENSGALG00010005656	CYP2U1	0.074659811	0.071390174	1.045799548	0.29565359	0.45721741	positive
PRJNA729271	ed_Lushi_vs_Arbor_Acr	breed_growth_background	ENSGALG00010005660	UBAC2	0.063326246	0.054013114	1.172423536	0.241027055	0.396784459	positive
PRJNA729271	ed_Lushi_vs_Arbor_Acr	breed_growth_background	ENSGALG00010005668	LNP1	0.051898213	0.203290393	0.255291025	0.798498332	0.875112152	positive
PRJNA729271	ed_Lushi_vs_Arbor_Acr	breed_growth_background	ENSGALG00010005670	SGMS2	0.033792842	0.111488868	0.303105079	0.761809784	0.850202711	positive
PRJNA729271	ed_Lushi_vs_Arbor_Acr	breed_growth_background	ENSGALG00010005675	GJA3	0.06921082	0.156800477	0.441394189	0.658927648	0.777971513	positive
PRJNA729271	ed_Lushi_vs_Arbor_Acr	breed_growth_background	ENSGALG00010005679	TMEM45A	0.038345936	0.112106419	0.342049422	0.732313699	0.828240196	positive
PRJNA729271	ed_Lushi_vs_Arbor_Acr	breed_growth_background	ENSGALG00010005680	ENSGALG00010005680	0.1272919	0.232569824	0.547327671	0.584153637	0.720009096	positive
PRJNA729271	ed_Lushi_vs_Arbor_Acr	breed_growth_background	ENSGALG00010005681	EPC1	0.042063372	0.035776474	1.17572716	0.239703922	0.395279676	positive
PRJNA729271	ed_Lushi_vs_Arbor_Acr	breed_growth_background	ENSGALG00010005688	ENSGALG00010005688	-0.794356409	0.268037004	-2.963607255	0.003040561	0.017879484	negative
PRJNA729271	ed_Lushi_vs_Arbor_Acr	breed_growth_background	ENSGALG00010005692	SEC24B	0.296710121	0.077112966	3.847733239	0.000119216	0.001819696	positive
PRJNA729271	ed_Lushi_vs_Arbor_Acr	breed_growth_background	ENSGALG00010005694	ENSGALG00010005694	-0.020910093	0.040097076	-0.521486725	0.602027751	0.734482977	negative
PRJNA729271	ed_Lushi_vs_Arbor_Acr	breed_growth_background	ENSGALG00010005695	KIF5B	0.0737462	0.044887234	1.642921484	0.100399179	0.215757762	positive
PRJNA729271	ed_Lushi_vs_Arbor_Acr	breed_growth_background	ENSGALG00010005696	NUBPL	-0.217062344	0.07725605	-2.809648494	0.004959563	0.025429385	negative
PRJNA729271	ed_Lushi_vs_Arbor_Acr	breed_growth_background	ENSGALG00010005699	PRPF18	0.18741498	0.049876784	3.757559466	0.000171579	0.002326569	positive
PRJNA729271	ed_Lushi_vs_Arbor_Acr	breed_growth_background	ENSGALG00010005704	AKAP12	-0.112331371	0.050162133	-2.239365913	0.025132117	0.080293154	negative
PRJNA729271	ed_Lushi_vs_Arbor_Acr	breed_growth_background	ENSGALG00010005705	ECHDC3	-0.090152776	0.066308051	-1.359605286	0.173954864	0.317766321	negative
PRJNA729271	ed_Lushi_vs_Arbor_Acr	breed_growth_background	ENSGALG00010005712	ENSGALG00010005712	0.021410756	0.05917179	0.361840605	0.717471147	0.818941985	positive
PRJNA729271	ed_Lushi_vs_Arbor_Acr	breed_growth_background	ENSGALG00010005714	ARHGAP5	-0.018562471	0.036700855	-0.50577762	0.613012771	0.742820608	negative
PRJNA729271	ed_Lushi_vs_Arbor_Acr	breed_growth_background	ENSGALG00010005716	ZBTB2	0.025647016	0.060519523	0.423780866	0.671725634	0.787778446	positive
PRJNA729271	ed_Lushi_vs_Arbor_Acr	breed_growth_background	ENSGALG00010005720	COL25A1	0.013365124	0.11312222	0.118147647	0.905950671	0.945955552	positive
PRJNA729271	ed_Lushi_vs_Arbor_Acr	breed_growth_background	ENSGALG00010005724	PROSER2	-0.066401319	0.065013383	-1.021348461	0.307089374	0.469790024	negative
PRJNA729271	ed_Lushi_vs_Arbor_Acr	breed_growth_background	ENSGALG00010005738	ELAVL2	0.75680203	0.234278208	3.230356058	0.001236361	0.00952741	positive
PRJNA729271	ed_Lushi_vs_Arbor_Acr	breed_growth_background	ENSGALG00010005740	ENSGALG00010005740	-0.258119767	0.175232928	-1.473009501	0.140748474	0.2731489	negative
PRJNA729271	ed_Lushi_vs_Arbor_Acr	breed_growth_background	ENSGALG00010005742	GPR183	-0.272700107	0.113500839	-2.402626361	0.01627781	0.059021435	negative
PRJNA729271	ed_Lushi_vs_Arbor_Acr	breed_growth_background	ENSGALG00010005743	ETNPPL	-0.165042572	0.117578603	-1.403678619	0.160414572	0.299216887	negative
PRJNA729271	ed_Lushi_vs_Arbor_Acr	breed_growth_background	ENSGALG00010005748	AIG1	-0.273731094	0.095897029	-2.854427264	0.00431145	0.022816529	negative
PRJNA729271	ed_Lushi_vs_Arbor_Acr	breed_growth_background	ENSGALG00010005752	RMND1	0.133822427	0.053439344	2.504192912	0.01227311	0.04829567	positive
PRJNA729271	ed_Lushi_vs_Arbor_Acr	breed_growth_background	ENSGALG00010005755	ZMYM2	0.046426655	0.052199618	0.889406041	0.3737849	0.536065313	positive
PRJNA729271	ed_Lushi_vs_Arbor_Acr	breed_growth_background	ENSGALG00010005756	TET2	-0.013198965	0.087845334	-0.150252321	0.880565548	0.929121364	negative
PRJNA729271	ed_Lushi_vs_Arbor_Acr	breed_growth_background	ENSGALG00010005757	ARHGAP12	-0.095809578	0.071352634	-1.342761606	0.179349179	0.323583066	negative
PRJNA729271	ed_Lushi_vs_Arbor_Acr	breed_growth_background	ENSGALG00010005758	HIVEP2	-0.0381521	0.056361016	-0.676923575	0.498454446	0.649972759	negative
PRJNA729271	ed_Lushi_vs_Arbor_Acr	breed_growth_background	ENSGALG00010005760	GPR18	0.238124814	0.195313958	1.219189945	0.222772107	0.375372259	positive
PRJNA729271	ed_Lushi_vs_Arbor_Acr	breed_growth_background	ENSGALG00010005763	AKAP6	-0.247311072	0.076022027	-3.253150185	0.001141332	0.008991697	negative
PRJNA729271	ed_Lushi_vs_Arbor_Acr	breed_growth_background	ENSGALG00010005768	CXXC4	-0.107024124	0.383622914	-0.278982615	0.780258167	0.863940274	negative
PRJNA729271	ed_Lushi_vs_Arbor_Acr	breed_growth_background	ENSGALG00010005769	TFG	0.064832271	0.056760756	1.142202384	0.253369905	0.409979546	positive
PRJNA729271	ed_Lushi_vs_Arbor_Acr	breed_growth_background	ENSGALG00010005772	SVEP1	-0.287424815	0.075188713	-3.822712258	0.000131992	0.001951615	negative
PRJNA729271	ed_Lushi_vs_Arbor_Acr	breed_growth_background	ENSGALG00010005777	ENSGALG00010005777	2.341032429	0.538123112	4.350365883	1.35911E-05	0.000362804	positive
PRJNA729271	ed_Lushi_vs_Arbor_Acr	breed_growth_background	ENSGALG00010005779	ARMT1	-0.049561512	0.059812387	-0.828616193	0.407321622	0.567846171	negative
PRJNA729271	ed_Lushi_vs_Arbor_Acr	breed_growth_background	ENSGALG00010005784	RASL11A	-0.65397389	0.245402886	-2.664898944	0.007701146	0.034699445	negative
PRJNA729271	ed_Lushi_vs_Arbor_Acr	breed_growth_background	ENSGALG00010005785	RPL21	-0.06571967	0.074743438	-0.879270105	0.379254841	0.541125989	negative
PRJNA729271	ed_Lushi_vs_Arbor_Acr	breed_growth_background	ENSGALG00010005789	RNASET2	-0.265135239	0.083282021	-3.183583146	0.001454643	0.010692218	negative
PRJNA729271	ed_Lushi_vs_Arbor_Acr	breed_growth_background	ENSGALG00010005790	MUSK	-0.143595878	0.080721009	-1.778915785	0.07525357	0.176647853	negative
PRJNA729271	ed_Lushi_vs_Arbor_Acr	breed_growth_background	ENSGALG00010005792	ADGRG6	-0.246010105	0.086210828	-2.853587073	0.004322868	0.022851922	negative
PRJNA729271	ed_Lushi_vs_Arbor_Acr	breed_growth_background	ENSGALG00010005799	UFSP2	0.109269989	0.053185616	2.054502624	0.03992707	0.112181212	positive
PRJNA729271	ed_Lushi_vs_Arbor_Acr	breed_growth_background	ENSGALG00010005800	FRMD4A	-0.185994646	0.058809231	-3.16267775	0.001563253	0.011180496	negative
PRJNA729271	ed_Lushi_vs_Arbor_Acr	breed_growth_background	ENSGALG00010005814	UPF2	0.06598991	0.06675961	0.988470577	0.322922237	0.486919571	positive
PRJNA729271	ed_Lushi_vs_Arbor_Acr	breed_growth_background	ENSGALG00010005817	VTA1	0.029366734	0.037761711	0.777685478	0.436754456	0.594322561	positive
PRJNA729271	ed_Lushi_vs_Arbor_Acr	breed_growth_background	ENSGALG00010005821	DOCK9	-0.069870259	0.08228893	-0.849084545	0.39583425	0.557312442	negative
PRJNA729271	ed_Lushi_vs_Arbor_Acr	breed_growth_background	ENSGALG00010005824	NMBR	0.113968179	0.759620877	0.150032973	0.880738601	0.929121364	positive
PRJNA729271	ed_Lushi_vs_Arbor_Acr	breed_growth_background	ENSGALG00010005826	LRP2BP	-0.394170699	0.112180826	-3.513708288	0.000441898	0.004539553	negative
PRJNA729271	ed_Lushi_vs_Arbor_Acr	breed_growth_background	ENSGALG00010005828	LPAR1	-0.025216428	0.084099055	-0.299841986	0.764297688	0.852060836	negative
PRJNA729271	ed_Lushi_vs_Arbor_Acr	breed_growth_background	ENSGALG00010005829	NPAS3	-0.06521879	0.073664886	-0.885344341	0.375970942	0.53845458	negative
PRJNA729271	ed_Lushi_vs_Arbor_Acr	breed_growth_background	ENSGALG00010005830	USP12	-0.15126393	0.10071546	-1.501893856	0.133124524	0.26241802	negative
PRJNA729271	ed_Lushi_vs_Arbor_Acr	breed_growth_background	ENSGALG00010005831	SEC61A2	0.036755019	0.056392018	0.651776988	0.514545049	0.66296271	positive
PRJNA729271	ed_Lushi_vs_Arbor_Acr	breed_growth_background	ENSGALG00010005836	NEBL	0.024040011	0.132249929	0.181777117	0.855757643	0.913282804	positive
PRJNA729271	ed_Lushi_vs_Arbor_Acr	breed_growth_background	ENSGALG00010005839	RPS6KA2	-0.326852596	0.101051565	-3.234512954	0.001218504	0.009455147	negative
PRJNA729271	ed_Lushi_vs_Arbor_Acr	breed_growth_background	ENSGALG00010005841	ENSGALG00010005841	-0.398118594	0.168935169	-2.356635374	0.018441345	0.064566978	negative
PRJNA729271	ed_Lushi_vs_Arbor_Acr	breed_growth_background	ENSGALG00010005843	GPR12	-0.299094289	0.236357311	-1.265432781	0.20571623	0.355069764	negative
PRJNA729271	ed_Lushi_vs_Arbor_Acr	breed_growth_background	ENSGALG00010005848	WASF3	-0.20769275	0.103006461	-2.016307982	0.043767781	0.120199694	negative
PRJNA729271	ed_Lushi_vs_Arbor_Acr	breed_growth_background	ENSGALG00010005849	SNX25	-0.031598765	0.052967236	-0.5965719	0.550793236	0.693842471	negative
PRJNA729271	ed_Lushi_vs_Arbor_Acr	breed_growth_background	ENSGALG00010005851	SLC15A1	-0.616487775	0.534861353	-1.152612302	0.249069552	0.405183203	negative
PRJNA729271	ed_Lushi_vs_Arbor_Acr	breed_growth_background	ENSGALG00010005853	COMMD3	0.100845728	0.076957324	1.310411058	0.190056815	0.336493652	positive
PRJNA729271	ed_Lushi_vs_Arbor_Acr	breed_growth_background	ENSGALG00010005858	CFAP97	-0.110382766	0.061818747	-1.785587243	0.074166114	0.174981644	negative
PRJNA729271	ed_Lushi_vs_Arbor_Acr	breed_growth_background	ENSGALG00010005859	ENSGALG00010005859	-0.313011901	0.298109521	-1.049989611	0.293722889	0.455051304	negative
PRJNA729271	ed_Lushi_vs_Arbor_Acr	breed_growth_background	ENSGALG00010005860	ABI3BP	-0.223114477	0.113552984	-1.964849088	0.049431707	0.131452746	negative
PRJNA729271	ed_Lushi_vs_Arbor_Acr	breed_growth_background	ENSGALG00010005861	MPC1L	0.2055312	0.070480717	2.916133806	0.003543985	0.019901078	positive
PRJNA729271	ed_Lushi_vs_Arbor_Acr	breed_growth_background	ENSGALG00010005866	DNAJC1	0.280967018	0.079398709	3.538684966	0.000402125	0.004253705	positive
PRJNA729271	ed_Lushi_vs_Arbor_Acr	breed_growth_background	ENSGALG00010005871	EGLN3	-0.056087366	0.104421411	-0.537125156	0.591181165	0.726077867	negative
PRJNA729271	ed_Lushi_vs_Arbor_Acr	breed_growth_background	ENSGALG00010005872	STK24	0.04758103	0.072781629	0.653750555	0.513272527	0.661799138	positive
PRJNA729271	ed_Lushi_vs_Arbor_Acr	breed_growth_background	ENSGALG00010005875	CDK8	0.040695659	0.084225085	0.483177416	0.628969769	0.755024166	positive
PRJNA729271	ed_Lushi_vs_Arbor_Acr	breed_growth_background	ENSGALG00010005879	FAM107B	-0.075057125	0.067198103	-1.116953037	0.264014406	0.422114362	negative
PRJNA729271	ed_Lushi_vs_Arbor_Acr	breed_growth_background	ENSGALG00010005880	SLC25A4	0.225593135	0.096034911	2.349074237	0.018820154	0.065505157	positive
PRJNA729271	ed_Lushi_vs_Arbor_Acr	breed_growth_background	ENSGALG00010005881	CORO2A	-0.023480782	0.153804289	-0.152666625	0.878661181	0.928012768	negative
PRJNA729271	ed_Lushi_vs_Arbor_Acr	breed_growth_background	ENSGALG00010005884	MYB	-0.207343963	0.164049097	-1.263914075	0.206260869	0.355485014	negative
PRJNA729271	ed_Lushi_vs_Arbor_Acr	breed_growth_background	ENSGALG00010005885	SLITRK5	0.629974014	0.187111156	3.366843693	0.000760338	0.006716085	positive
PRJNA729271	ed_Lushi_vs_Arbor_Acr	breed_growth_background	ENSGALG00010005887	CCNH	-0.030526648	0.046049455	-0.662910087	0.507388141	0.656777202	negative
PRJNA729271	ed_Lushi_vs_Arbor_Acr	breed_growth_background	ENSGALG00010005888	IMPG2	-0.00665015	0.122894582	-0.05411264	0.956845422	0.97576153	negative
PRJNA729271	ed_Lushi_vs_Arbor_Acr	breed_growth_background	ENSGALG00010005890	SNX32	-0.08480553	0.04805106	-1.764904447	0.077579826	0.179951925	negative
PRJNA729271	ed_Lushi_vs_Arbor_Acr	breed_growth_background	ENSGALG00010005891	RNF6	0.187526386	0.063488422	2.95370998	0.003139789	0.018329136	positive
PRJNA729271	ed_Lushi_vs_Arbor_Acr	breed_growth_background	ENSGALG00010005892	ENSGALG00010005892	0.358521934	0.471725257	0.760022764	0.447240978	0.60394615	positive
PRJNA729271	ed_Lushi_vs_Arbor_Acr	breed_growth_background	ENSGALG00010005893	SFT2D1	0.047864906	0.036069328	1.327025169	0.184500357	0.329879447	positive
PRJNA729271	ed_Lushi_vs_Arbor_Acr	breed_growth_background	ENSGALG00010005895	MLLT10	0.065125792	0.053297656	1.221926003	0.221735611	0.374162711	positive
PRJNA729271	ed_Lushi_vs_Arbor_Acr	breed_growth_background	ENSGALG00010005899	ENSGALG00010005899	0.042673104	0.047702474	0.894567935	0.371018099	0.534158457	positive
PRJNA729271	ed_Lushi_vs_Arbor_Acr	breed_growth_background	ENSGALG00010005900	SPTSSA	0.04704473	0.047094615	0.998940742	0.317823398	0.48128397	positive
PRJNA729271	ed_Lushi_vs_Arbor_Acr	breed_growth_background	ENSGALG00010005901	ENSGALG00010005901	0.206157821	0.133486966	1.544404127	0.122490461	0.247284015	positive
PRJNA729271	ed_Lushi_vs_Arbor_Acr	breed_growth_background	ENSGALG00010005902	EAPP	0.021400631	0.055927725	0.382647981	0.701980789	0.807492385	positive
PRJNA729271	ed_Lushi_vs_Arbor_Acr	breed_growth_background	ENSGALG00010005903	PRR18	-0.180656659	0.149427032	-1.20899583	0.226664447	0.37987064	negative
PRJNA729271	ed_Lushi_vs_Arbor_Acr	breed_growth_background	ENSGALG00010005905	CITED2	0.243106784	0.108661771	2.23727979	0.025268059	0.080603107	positive
PRJNA729271	ed_Lushi_vs_Arbor_Acr	breed_growth_background	ENSGALG00010005909	BRMS1L	0.035373412	0.055758066	0.634408881	0.525814015	0.672164578	positive
PRJNA729271	ed_Lushi_vs_Arbor_Acr	breed_growth_background	ENSGALG00010005910	ENSGALG00010005910	-0.204879022	0.227571425	-0.900284479	0.367968879	0.531192999	negative
PRJNA729271	ed_Lushi_vs_Arbor_Acr	breed_growth_background	ENSGALG00010005911	TBXT	0.213581701	0.262943486	0.812272265	0.416635421	0.576803289	positive
PRJNA729271	ed_Lushi_vs_Arbor_Acr	breed_growth_background	ENSGALG00010005913	NFKBIA	-0.025120984	0.075775089	-0.33152035	0.740251472	0.833782352	negative
PRJNA729271	ed_Lushi_vs_Arbor_Acr	breed_growth_background	ENSGALG00010005915	PAPSS1	-0.168931015	0.054873104	-3.078575895	0.002079925	0.013641864	negative
PRJNA729271	ed_Lushi_vs_Arbor_Acr	breed_growth_background	ENSGALG00010005926	NUDT5	-0.487882285	0.126233239	-3.864927233	0.000111122	0.001726386	negative
PRJNA729271	ed_Lushi_vs_Arbor_Acr	breed_growth_background	ENSGALG00010005934	SLITRK6	0.18253582	0.173088512	1.05458079	0.291617094	0.453044292	positive
PRJNA729271	ed_Lushi_vs_Arbor_Acr	breed_growth_background	ENSGALG00010005935	ATP8A2	-0.210798602	0.111046924	-1.898284024	0.057658677	0.146573576	negative
PRJNA729271	ed_Lushi_vs_Arbor_Acr	breed_growth_background	ENSGALG00010005941	TXLNB	-0.151066211	0.095659148	-1.57921344	0.114287108	0.236288875	negative
PRJNA729271	ed_Lushi_vs_Arbor_Acr	breed_growth_background	ENSGALG00010005943	PROX2	-0.358617986	0.484492323	-0.74019333	0.459182694	0.614774289	negative
PRJNA729271	ed_Lushi_vs_Arbor_Acr	breed_growth_background	ENSGALG00010005949	SLITRK1	-0.103365637	0.190614874	-0.542274769	0.587629234	0.723363093	negative
PRJNA729271	ed_Lushi_vs_Arbor_Acr	breed_growth_background	ENSGALG00010005956	TXNL4B	0.123998517	0.06028407	2.056903525	0.039695507	0.111854757	positive
PRJNA729271	ed_Lushi_vs_Arbor_Acr	breed_growth_background	ENSGALG00010005963	UGCG	-0.007771629	0.05510237	-0.141039838	0.88783847	0.934041126	negative
PRJNA729271	ed_Lushi_vs_Arbor_Acr	breed_growth_background	ENSGALG00010005964	HSPA14	0.017297991	0.062126473	0.278431886	0.780680845	0.864276243	positive
PRJNA729271	ed_Lushi_vs_Arbor_Acr	breed_growth_background	ENSGALG00010005966	PLXDC2	0.191751568	0.082306673	2.329720787	0.019820913	0.067776394	positive
PRJNA729271	ed_Lushi_vs_Arbor_Acr	breed_growth_background	ENSGALG00010005967	GJD2	-0.231926559	0.154797499	-1.498257794	0.134066285	0.263998207	negative
PRJNA729271	ed_Lushi_vs_Arbor_Acr	breed_growth_background	ENSGALG00010005969	HECA	0.075204218	0.03544757	2.121562001	0.033874536	0.099475568	positive
PRJNA729271	ed_Lushi_vs_Arbor_Acr	breed_growth_background	ENSGALG00010005970	SHISA2	-0.228753338	0.066158256	-3.457668807	0.000544871	0.005289773	negative
PRJNA729271	ed_Lushi_vs_Arbor_Acr	breed_growth_background	ENSGALG00010005975	ABRACL	-0.081810667	0.059364393	-1.378110059	0.16816931	0.309695611	negative
PRJNA729271	ed_Lushi_vs_Arbor_Acr	breed_growth_background	ENSGALG00010005978	VPS16	-0.051599062	0.061293276	-0.841838868	0.399878152	0.560726886	negative
PRJNA729271	ed_Lushi_vs_Arbor_Acr	breed_growth_background	ENSGALG00010005979	SUV39H2	0.056640163	0.049846247	1.136297435	0.255832104	0.412903501	positive
PRJNA729271	ed_Lushi_vs_Arbor_Acr	breed_growth_background	ENSGALG00010005981	AREL1	0.040537257	0.054356678	0.745764061	0.455809953	0.611920636	positive
PRJNA729271	ed_Lushi_vs_Arbor_Acr	breed_growth_background	ENSGALG00010005984	IDH3B	0.115420054	0.0627676	1.838847654	0.065937597	0.161365096	positive
PRJNA729271	ed_Lushi_vs_Arbor_Acr	breed_growth_background	ENSGALG00010005985	ENSGALG00010005985	0.551785752	0.17396345	3.171848746	0.001514719	0.010981812	positive
PRJNA729271	ed_Lushi_vs_Arbor_Acr	breed_growth_background	ENSGALG00010005988	NUP58	0.137150203	0.048466548	2.829791041	0.004657841	0.024181738	positive
PRJNA729271	ed_Lushi_vs_Arbor_Acr	breed_growth_background	ENSGALG00010005989	FCF1	0.02094489	0.054634498	0.383363826	0.701450021	0.80733032	positive
PRJNA729271	ed_Lushi_vs_Arbor_Acr	breed_growth_background	ENSGALG00010005990	SLC2A12	0.140557315	0.080303242	1.750331752	0.080061085	0.184204036	positive
PRJNA729271	ed_Lushi_vs_Arbor_Acr	breed_growth_background	ENSGALG00010005991	FARP1	-0.032893743	0.049277475	-0.667520878	0.504439478	0.654655765	negative
PRJNA729271	ed_Lushi_vs_Arbor_Acr	breed_growth_background	ENSGALG00010005997	ENSGALG00010005997	0.291922604	0.124616632	2.34256535	0.01915168	0.066364714	positive
PRJNA729271	ed_Lushi_vs_Arbor_Acr	breed_growth_background	ENSGALG00010005998	YLPM1	0.185269359	0.071464308	2.592473976	0.009528839	0.040457095	positive
PRJNA729271	ed_Lushi_vs_Arbor_Acr	breed_growth_background	ENSGALG00010005999	PDE10A	0.396173501	0.156908319	2.524872512	0.011574027	0.046408166	positive
PRJNA729271	ed_Lushi_vs_Arbor_Acr	breed_growth_background	ENSGALG00010006001	NOP56	0.221053173	0.075379924	2.932520511	0.003362227	0.019247821	positive
PRJNA729271	ed_Lushi_vs_Arbor_Acr	breed_growth_background	ENSGALG00010006004	DCLRE1C	-0.000150606	0.084707348	-0.001777952	0.9985814	0.999063873	negative
PRJNA729271	ed_Lushi_vs_Arbor_Acr	breed_growth_background	ENSGALG00010006009	MALRD1	-0.132323556	0.345693565	-0.382777029	0.701885095	0.807492385	negative
PRJNA729271	ed_Lushi_vs_Arbor_Acr	breed_growth_background	ENSGALG00010006010	IPO5	0.017879686	0.043301884	0.412907817	0.679674153	0.792972809	positive
PRJNA729271	ed_Lushi_vs_Arbor_Acr	breed_growth_background	ENSGALG00010006013	TRMT10C	-0.083415505	0.057391294	-1.453452233	0.146098233	0.280410509	negative

PRJNA729271	ed_Lushi_vs_Arbor_Acr	breed_growth_background	ENSGALG00010006015	CDC123	-0.198292933	0.060622045	-3.270970679	0.00107179	0.008597454	negative
PRJNA729271	ed_Lushi_vs_Arbor_Acr	breed_growth_background	ENSGALG00010006016	MTMR6	0.034590768	0.041484337	0.833827196	0.404378368	0.565178328	positive
PRJNA729271	ed_Lushi_vs_Arbor_Acr	breed_growth_background	ENSGALG00010006019	SLC10A2	0.905314388	0.638493364	1.417891617	0.15622241	0.293487696	positive
PRJNA729271	ed_Lushi_vs_Arbor_Acr	breed_growth_background	ENSGALG00010006021	ENSGALG00010006021	0.178037699	0.067343728	2.643716102	0.00820014	0.036249171	positive
PRJNA729271	ed_Lushi_vs_Arbor_Acr	breed_growth_background	ENSGALG00010006022	AFG2A	0.23675846	0.050166441	4.719458932	2.36473E-06	9.54784E-05	positive
PRJNA729271	ed_Lushi_vs_Arbor_Acr	breed_growth_background	ENSGALG00010006024	AMER2	-0.417543077	0.22089612	-1.890223683	0.058728051	0.148432972	negative
PRJNA729271	ed_Lushi_vs_Arbor_Acr	breed_growth_background	ENSGALG00010006025	TNFAIP3	0.027756022	0.079984002	0.347019667	0.728576544	0.825443768	positive
PRJNA729271	ed_Lushi_vs_Arbor_Acr	breed_growth_background	ENSGALG00010006028	ARL5B	-0.039069457	0.068786929	-0.567977914	0.570049965	0.70877295	negative
PRJNA729271	ed_Lushi_vs_Arbor_Acr	breed_growth_background	ENSGALG00010006029	HSDL2	-0.109238094	0.072293622	-1.51103363	0.130779883	0.258898443	negative
PRJNA729271	ed_Lushi_vs_Arbor_Acr	breed_growth_background	ENSGALG00010006031	ERCC5	0.297543033	0.052389156	5.679477559	1.35107E-08	1.73307E-06	positive
PRJNA729271	ed_Lushi_vs_Arbor_Acr	breed_growth_background	ENSGALG00010006033	NSUN6	0.064013733	0.099161212	0.645552146	0.518569437	0.66666643	positive
PRJNA729271	ed_Lushi_vs_Arbor_Acr	breed_growth_background	ENSGALG00010006035	ADH1C	-0.413540682	0.373752928	-1.106454696	0.268529763	0.427072516	negative
PRJNA729271	ed_Lushi_vs_Arbor_Acr	breed_growth_background	ENSGALG00010006037	METTL21EP	0.802624478	0.32429949	2.474948321	0.013325546	0.051153015	positive
PRJNA729271	ed_Lushi_vs_Arbor_Acr	breed_growth_background	ENSGALG00010006038	ENSGALG00010006038	0.165036155	0.105995504	1.557010902	0.119467897	0.243625094	positive
PRJNA729271	ed_Lushi_vs_Arbor_Acr	breed_growth_background	ENSGALG00010006039	PCNP	0.060792949	0.067861808	0.895834505	0.370341158	0.533607862	positive
PRJNA729271	ed_Lushi_vs_Arbor_Acr	breed_growth_background	ENSGALG00010006041	QKI	0.05463189	0.045960971	1.188658306	0.234574154	0.389033451	positive
PRJNA729271	ed_Lushi_vs_Arbor_Acr	breed_growth_background	ENSGALG00010006047	ZBTB11	0.160878514	0.072682778	2.213433745	0.026867756	0.084056042	positive
PRJNA729271	ed_Lushi_vs_Arbor_Acr	breed_growth_background	ENSGALG00010006049	AIMP1	-0.090553123	0.066729814	-1.357011477	0.174777564	0.318666766	negative
PRJNA729271	ed_Lushi_vs_Arbor_Acr	breed_growth_background	ENSGALG00010006052	RALGAPA1	-0.087277987	0.043911913	-1.98756969	0.046859301	0.126367548	negative
PRJNA729271	ed_Lushi_vs_Arbor_Acr	breed_growth_background	ENSGALG00010006053	TMEM243	-0.163831817	0.135373016	-1.210225064	0.226192543	0.379386821	negative
PRJNA729271	ed_Lushi_vs_Arbor_Acr	breed_growth_background	ENSGALG00010006054	ENSGALG00010006054	-1.967049596	1.175808298	-1.67293393	0.094340303	0.206502975	negative
PRJNA729271	ed_Lushi_vs_Arbor_Acr	breed_growth_background	ENSGALG00010006055	ENSGALG00010006055	0.374622707	0.301887551	1.240934598	0.21462991	0.36540528	positive
PRJNA729271	ed_Lushi_vs_Arbor_Acr	breed_growth_background	ENSGALG00010006059	BIVM	0.038895247	0.03061488	1.27046871	0.203917722	0.353252474	positive
PRJNA729271	ed_Lushi_vs_Arbor_Acr	breed_growth_background	ENSGALG00010006060	ENSGALG00010006060	0.575527063	0.201318122	2.858794109	0.004252547	0.022554214	positive
PRJNA729271	ed_Lushi_vs_Arbor_Acr	breed_growth_background	ENSGALG00010006061	RPL24	-0.112646018	0.085017016	-1.324982027	0.185177112	0.330681563	negative
PRJNA729271	ed_Lushi_vs_Arbor_Acr	breed_growth_background	ENSGALG00010006062	ENSGALG00010006062	-0.766754567	0.349642365	-2.192968144	0.028309674	0.0871784	negative
PRJNA729271	ed_Lushi_vs_Arbor_Acr	breed_growth_background	ENSGALG00010006063	POGLUT2	-0.125641579	0.055132226	-2.278913573	0.022672203	0.074723417	negative
PRJNA729271	ed_Lushi_vs_Arbor_Acr	breed_growth_background	ENSGALG00010006064	DMTF1	0.01910556	0.044818388	0.426288421	0.669897694	0.786491924	positive
PRJNA729271	ed_Lushi_vs_Arbor_Acr	breed_growth_background	ENSGALG00010006065	WDR54	-0.111077067	0.087750627	-1.265826472	0.205575215	0.354950893	negative
PRJNA729271	ed_Lushi_vs_Arbor_Acr	breed_growth_background	ENSGALG00010006066	ARHGAP18	-0.053262349	0.064798156	-0.821973222	0.411092137	0.571476027	negative
PRJNA729271	ed_Lushi_vs_Arbor_Acr	breed_growth_background	ENSGALG00010006068	PACRG	-0.047212975	0.144724391	-0.326226802	0.744252765	0.836988426	negative
PRJNA729271	ed_Lushi_vs_Arbor_Acr	breed_growth_background	ENSGALG00010006069	TTR	-2.398180206	0.742135389	-3.231459168	0.001231599	0.009507227	negative
PRJNA729271	ed_Lushi_vs_Arbor_Acr	breed_growth_background	ENSGALG00010006070	TMEM242	-0.147689744	0.061107689	-2.416876596	0.015654323	0.057474523	negative
PRJNA729271	ed_Lushi_vs_Arbor_Acr	breed_growth_background	ENSGALG00010006071	TEX30	-0.259030621	0.13048333	-1.985162563	0.047126388	0.126801477	negative
PRJNA729271	ed_Lushi_vs_Arbor_Acr	breed_growth_background	ENSGALG00010006072	ENSGALG00010006072	0.45850554	0.124768162	3.674860109	0.00023798	0.002939388	positive
PRJNA729271	ed_Lushi_vs_Arbor_Acr	breed_growth_background	ENSGALG00010006073	CEP97	0.16618464	0.057422324	2.894077227	0.003802746	0.020910776	positive
PRJNA729271	ed_Lushi_vs_Arbor_Acr	breed_growth_background	ENSGALG00010006075	SOX21	0.121281931	0.227338203	0.533486803	0.593696644	0.728056925	positive
PRJNA729271	ed_Lushi_vs_Arbor_Acr	breed_growth_background	ENSGALG00010006076	PODXL	-0.006760776	0.087023143	-0.077689403	0.938075124	0.965998787	negative
PRJNA729271	ed_Lushi_vs_Arbor_Acr	breed_growth_background	ENSGALG00010006077	TBCK	-0.048701231	0.0551368	-0.883279969	0.377085024	0.539517069	negative
PRJNA729271	ed_Lushi_vs_Arbor_Acr	breed_growth_background	ENSGALG00010006078	SPATA13	0.130898589	0.091666929	1.427980531	0.153297462	0.289781784	positive
PRJNA729271	ed_Lushi_vs_Arbor_Acr	breed_growth_background	ENSGALG00010006079	ENSGALG00010006079	1.133376155	0.453578912	2.498740849	0.012463542	0.048839967	positive
PRJNA729271	ed_Lushi_vs_Arbor_Acr	breed_growth_background	ENSGALG00010006080	ENSGALG00010006080	-0.118192051	0.112119239	-1.054163867	0.2918079	0.45325284	negative
PRJNA729271	ed_Lushi_vs_Arbor_Acr	breed_growth_background	ENSGALG00010006081	ZDHHC14	-0.025019926	0.042616121	-0.587100016	0.557136537	0.698621901	negative
PRJNA729271	ed_Lushi_vs_Arbor_Acr	breed_growth_background	ENSGALG00010006083	CACNB2	-0.190755319	0.094182119	-2.025387847	0.042827554	0.11838013	negative
PRJNA729271	ed_Lushi_vs_Arbor_Acr	breed_growth_background	ENSGALG00010006084	TMEM68	-0.189303179	0.082811474	-2.285953497	0.022256979	0.073757411	negative
PRJNA729271	ed_Lushi_vs_Arbor_Acr	breed_growth_background	ENSGALG00010006085	DLST	0.053272172	0.048176514	1.105770588	0.268825828	0.427355822	positive
PRJNA729271	ed_Lushi_vs_Arbor_Acr	breed_growth_background	ENSGALG00010006086	ENSGALG00010006086	-0.114968573	0.163511464	-0.703122403	0.481979472	0.63467461	negative
PRJNA729271	ed_Lushi_vs_Arbor_Acr	breed_growth_background	ENSGALG00010006087	ENSGALG00010006087	-0.31081973	0.315935057	-0.98380893	0.325209468	0.488946163	negative
PRJNA729271	ed_Lushi_vs_Arbor_Acr	breed_growth_background	ENSGALG00010006091	RTKN	-0.013121476	0.055201156	-0.237702924	0.812111516	0.884281582	negative
PRJNA729271	ed_Lushi_vs_Arbor_Acr	breed_growth_background	ENSGALG00010006096	EDIL3	-0.107002744	0.126679008	-0.844676206	0.398291648	0.559530668	negative
PRJNA729271	ed_Lushi_vs_Arbor_Acr	breed_growth_background	ENSGALG00010006100	RPS6KL1	-0.142699765	0.112489135	-1.268564872	0.2045963	0.353977485	negative
PRJNA729271	ed_Lushi_vs_Arbor_Acr	breed_growth_background	ENSGALG00010006101	ENSGALG00010006101	0.140563415	0.062022252	2.266338451	0.023430669	0.076406647	positive
PRJNA729271	ed_Lushi_vs_Arbor_Acr	breed_growth_background	ENSGALG00010006103	NXPE3	0.015831768	0.066044458	0.239713796	0.810552139	0.883379944	positive
PRJNA729271	ed_Lushi_vs_Arbor_Acr	breed_growth_background	ENSGALG00010006106	SHOC1	-0.141579115	0.198516458	-0.713185779	0.475730805	0.628932047	negative
PRJNA729271	ed_Lushi_vs_Arbor_Acr	breed_growth_background	ENSGALG00010006107	PGF	-0.176513647	0.097996972	-1.801215311	0.07166895	0.1705559	negative
PRJNA729271	ed_Lushi_vs_Arbor_Acr	breed_growth_background	ENSGALG00010006108	MKLN1	0.062024125	0.03298867	1.880164451	0.060085669	0.150969279	positive
PRJNA729271	ed_Lushi_vs_Arbor_Acr	breed_growth_background	ENSGALG00010006111	WBP1	0.063273987	0.045585962	1.388014725	0.165132561	0.305244211	positive
PRJNA729271	ed_Lushi_vs_Arbor_Acr	breed_growth_background	ENSGALG00010006113	EIF2B2	0.119699158	0.090451053	1.323358369	0.185716229	0.331236218	positive
PRJNA729271	ed_Lushi_vs_Arbor_Acr	breed_growth_background	ENSGALG00010006118	NPNT	0.09713863	0.114219455	0.850456083	0.395071567	0.556517237	positive
PRJNA729271	ed_Lushi_vs_Arbor_Acr	breed_growth_background	ENSGALG00010006120	MOGS	0.212625938	0.072287541	2.941391226	0.003267416	0.018861486	positive
PRJNA729271	ed_Lushi_vs_Arbor_Acr	breed_growth_background	ENSGALG00010006121	ENSGALG00010006121	-0.139924828	0.174935604	-0.799864779	0.423789146	0.58303186	negative
PRJNA729271	ed_Lushi_vs_Arbor_Acr	breed_growth_background	ENSGALG00010006123	MLH3	-0.07281346	0.055618629	-1.30915597	0.190481523	0.337126159	negative
PRJNA729271	ed_Lushi_vs_Arbor_Acr	breed_growth_background	ENSGALG00010006126	ENSGALG00010006126	-0.031511517	0.14625017	-0.215463115	0.829406267	0.895856671	negative
PRJNA729271	ed_Lushi_vs_Arbor_Acr	breed_growth_background	ENSGALG00010006128	ACYP1	0.083413432	0.064965533	1.28396441	0.199154409	0.34780038	positive
PRJNA729271	ed_Lushi_vs_Arbor_Acr	breed_growth_background	ENSGALG00010006131	ENSGALG00010006131	-0.143826748	0.067023785	-2.145906102	0.031880471	0.0953979	negative
PRJNA729271	ed_Lushi_vs_Arbor_Acr	breed_growth_background	ENSGALG00010006132	MIPEP	-0.025690881	0.072142437	-0.356113297	0.72175572	0.821439161	negative
PRJNA729271	ed_Lushi_vs_Arbor_Acr	breed_growth_background	ENSGALG00010006134	K123	-0.738841856	0.378955581	-1.94967931	0.051214354	0.134801536	negative
PRJNA729271	ed_Lushi_vs_Arbor_Acr	breed_growth_background	ENSGALG00010006135	ZC2HC1C	-0.009150182	0.083378822	-0.109742279	0.912613769	0.950653006	negative
PRJNA729271	ed_Lushi_vs_Arbor_Acr	breed_growth_background	ENSGALG00010006139	GAREM1	-0.182148365	0.071608535	-2.543668375	0.010969517	0.044688914	negative
PRJNA729271	ed_Lushi_vs_Arbor_Acr	breed_growth_background	ENSGALG00010006146	CCDC142	-0.081273493	0.097908129	-0.830099537	0.406482509	0.567353295	negative
PRJNA729271	ed_Lushi_vs_Arbor_Acr	breed_growth_background	ENSGALG00010006147	ENSGALG00010006147	0.109000133	0.114572913	0.951360403	0.341421454	0.505351166	positive
PRJNA729271	ed_Lushi_vs_Arbor_Acr	breed_growth_background	ENSGALG00010006151	ENSGALG00010006151	0.084670702	0.089017037	0.95117412	0.341515994	0.505439486	positive
PRJNA729271	ed_Lushi_vs_Arbor_Acr	breed_growth_background	ENSGALG00010006155	TNFRSF19	-0.420396312	0.104996588	-4.003904504	6.23055E-05	0.001127489	negative
PRJNA729271	ed_Lushi_vs_Arbor_Acr	breed_growth_background	ENSGALG00010006156	NFKBIZ	-0.046910427	0.105345941	-0.445298855	0.656103795	0.775885799	negative
PRJNA729271	ed_Lushi_vs_Arbor_Acr	breed_growth_background	ENSGALG00010006157	SNX9	-0.09877892	0.039446117	-2.504148119	0.012274664	0.04829567	negative
PRJNA729271	ed_Lushi_vs_Arbor_Acr	breed_growth_background	ENSGALG00010006163	ENSGALG00010006163	-0.179973087	0.26596395	-0.676682261	0.498607574	0.649997013	negative
PRJNA729271	ed_Lushi_vs_Arbor_Acr	breed_growth_background	ENSGALG00010006172	ENSGALG00010006172	0.112085054	0.10267189	1.091681997	0.274972901	0.433844802	positive
PRJNA729271	ed_Lushi_vs_Arbor_Acr	breed_growth_background	ENSGALG00010006173	TBPL1	0.167696598	0.045102222	3.718144901	0.000200691	0.002597879	positive
PRJNA729271	ed_Lushi_vs_Arbor_Acr	breed_growth_background	ENSGALG00010006175	IL2RA	-0.371854608	0.226602841	-1.640997111	0.100798015	0.216294186	negative
PRJNA729271	ed_Lushi_vs_Arbor_Acr	breed_growth_background	ENSGALG00010006176	MMR1L3	-0.488364483	0.114556049	-4.263105162	2.01606E-05	0.000485427	negative
PRJNA729271	ed_Lushi_vs_Arbor_Acr	breed_growth_background	ENSGALG00010006177	NEK9	0.062439004	0.060981566	1.023899653	0.305882674	0.468339428	positive
PRJNA729271	ed_Lushi_vs_Arbor_Acr	breed_growth_background	ENSGALG00010006178	RPS23	-0.136270082	0.075311091	-1.80942912	0.070384363	0.168464553	negative
PRJNA729271	ed_Lushi_vs_Arbor_Acr	breed_growth_background	ENSGALG00010006180	CD200L	1.302825498	0.531182454	2.452689256			positive
PRJNA729271	ed_Lushi_vs_Arbor_Acr	breed_growth_background	ENSGALG00010006183	PRKN	-0.062715321	0.072251185	-0.868017886	0.385384541	0.547225564	negative
PRJNA729271	ed_Lushi_vs_Arbor_Acr	breed_growth_background	ENSGALG00010006184	LBX2	0.348396614	0.157778734	2.208134165	0.027234922	0.084751008	positive
PRJNA729271	ed_Lushi_vs_Arbor_Acr	breed_growth_background	ENSGALG00010006187	TCF21	-0.56748991	0.343041616	-1.654288819	0.098068844	0.21186493	negative
PRJNA729271	ed_Lushi_vs_Arbor_Acr	breed_growth_background	ENSGALG00010006188	TMED10	-0.082053011	0.040081017	-2.047178841	0.040640528	0.113666437	negative
PRJNA729271	ed_Lushi_vs_Arbor_Acr	breed_growth_background	ENSGALG00010006193	RBM17	0.123127094	0.053868271	2.285707178	0.022271395	0.073788313	positive
PRJNA729271	ed_Lushi_vs_Arbor_Acr	breed_growth_background	ENSGALG00010006197	ENSGALG00010006197	-0.075698116	0.255923795	-0.295783812	0.767395186	0.854694298	negative
PRJNA729271	ed_Lushi_vs_Arbor_Acr	breed_growth_background	ENSGALG00010006198	ENSGALG00010006198	-0.036624658	0.36709496	-0.099768892	0.920527805	0.955805912	negative
PRJNA729271	ed_Lushi_vs_Arbor_Acr	breed_growth_background	ENSGALG00010006200	GSTCD	0.182132701	0.057422523	3.171798998	0.001514978	0.010981812	positive
PRJNA729271	ed_Lushi_vs_Arbor_Acr	breed_growth_background	ENSGALG00010006201	SSTR1	-0.477034978	0.210424115	-2.267016682	0.023389207	0.076340137	negative
PRJNA729271	ed_Lushi_vs_Arbor_Acr	breed_growth_background	ENSGALG00010006202	SACS	-0.021985883	0.107104365	-0.205275327	0.83735702	0.901343383	negative
PRJNA729271	ed_Lushi_vs_Arbor_Acr	breed_growth_background	ENSGALG00010006203	MMR1L4	-0.136651776	0.09286326	-1.471537574	0.141145794	0.273736725	negative
PRJNA729271	ed_Lushi_vs_Arbor_Acr	breed_growth_background	ENSGALG00010006205	CGGBP1	-0.054390979	0.040165675	-1.354165682	0.175683525	0.319715342	negative
PRJNA729271	ed_Lushi_vs_Arbor_Acr	breed_growth_background	ENSGALG00010006207	ENSGALG00010006207	0.611815813	0.112038379	5.46076993	4.74074E-08	4.70665E-06	positive
PRJNA729271	ed_Lushi_vs_Arbor_Acr	breed_growth_background	ENSGALG00010006212	FOS	-0.412292665	0.194625711	-2.118387451	0.034142268	0.100052512	negative
PRJNA729271	ed_Lushi_vs_Arbor_Acr	breed_growth_background	ENSGALG00010006219	CLEC14A	-0.308312274	0.098519746	-3.12944651	0.00175136	0.012100351	negative
PRJNA729271	ed_Lushi_vs_Arbor_Acr	breed_growth_background	ENSGALG00010006220	SGCG	-0.272467668	0.091309616	-2.983997514	0.002845091	0.017067799	negative
PRJNA729271	ed_Lushi_vs_Arbor_Acr	breed_growth_background	ENSGALG00010006223	ATG10	-0.132366713	0.061521886	-2.151538591	0.031433713	0.094314153	negative
PRJNA729271	ed_Lushi_vs_Arbor_Acr	breed_growth_background	ENSGALG00010006227	AGPAT4	-0.235331665	0.080451753	-2.925127887	0.003443147	0.019503093	negative
PRJNA729271	ed_Lushi_vs_Arbor_Acr	breed_growth_background	ENSGALG00010006229	INTS12	0.263772452	0.072744206	3.62602695	0.000287815	0.003373118	positive
PRJNA729271	ed_Lushi_vs_Arbor_Acr	breed_growth_background	ENSGALG00010006232	ZNF654	0.156486429	0.055891581	2.799821133	0.005113093	0.026014139	positive
PRJNA729271	ed_Lushi_vs_Arbor_Acr	breed_growth_background	ENSGALG00010006236	C3orf38	0.101023172	0.046720978	2.162265764	0.030597697	0.092302522	positive
PRJNA729271	ed_Lushi_vs_Arbor_Acr	breed_growth_background	ENSGALG00010006237	ENSGALG00010006237	-0.294573795	0.160462173	-1.835783411	0.066389696	0.162142989	negative
PRJNA729271	ed_Lushi_vs_Arbor_Acr	breed_growth_background	ENSGALG00010006239	EYA4	0.302325578	0.086805459	3.482794544	0.000496209	0.004946733	positive
PRJNA729271	ed_Lushi_vs_Arbor_Acr	breed_growth_background	ENSGALG00010006241	MRC1	0.081025137	0.131767656	0.614909145	0.538614723	0.683943969	positive
PRJNA729271	ed_Lushi_vs_Arbor_Acr	breed_growth_background	ENSGALG00010006242	ENSGALG00010006242	-0.60824092	0.300346513	-2.025130618	0.042853954	0.118385374	negative
PRJNA729271	ed_Lushi_vs_Arbor_Acr	breed_growth_background	ENSGALG00010006244	EPHA3	-0.188203101	0.086937427	-2.16481103	0.03040216	0.091820407	negative
PRJNA729271	ed_Lushi_vs_Arbor_Acr	breed_growth_background	ENSGALG00010006245	PFKFB3	1.212266936	0.27230198	4.451921126	8.51054E-06	0.000257001	positive
PRJNA729271	ed_Lushi_vs_Arbor_Acr	breed_growth_background	ENSGALG00010006246	SUPT20H	0.218960662	0.054082466	4.048644215	5.15152E-05	0.000987715	positive
PRJNA729271	ed_Lushi_vs_Arbor_Acr	breed_growth_background	ENSGALG00010006249	C9orf72	-0.230719656	0.056424434	-4.08900259	4.33232E-05	0.00087218	negative
PRJNA729271	ed_Lushi_vs_Arbor_Acr	breed_growth_background	ENSGALG00010006251	SEMA3E	-0.085704804	0.204733863	-0.418615672	0.675497034	0.790304289	negative
PRJNA729271	ed_Lushi_vs_Arbor_Acr	breed_growth_background	ENSGALG00010006252	COX7C	-0.053385416	0.073013978	-0.731167067	0.464677113	0.619982949	negative
PRJNA729271	ed_Lushi_vs_Arbor_Acr	breed_growth_background	ENSGALG00010006254	PROS1	0.071917236	0.073269634	0.981542173	0.326325448	0.489910654	positive
PRJNA729271	ed_Lushi_vs_Arbor_Acr	breed_growth_background	ENSGALG00010006259	PPA2	0.053393	0.046871527	1.139135063	0.254646825	0.411292596	positive
PRJNA729271	ed_Lushi_vs_Arbor_Acr	breed_growth_background	ENSGALG00010006260	MAP3K4	-0.024569758	0.085740894	-0.286558222	0.774450607	0.860006248	negative
PRJNA729271	ed_Lushi_vs_Arbor_Acr	breed_growth_background	ENSGALG00010006261	PCLO	0.266555888	0.298187138	0.89392148	0.371363904	0.5343491	positive
PRJNA729271	ed_Lushi_vs_Arbor_Acr	breed_growth_background	ENSGALG00010006263	EXOSC8	0.086245734	0.081031021	1.064354523	0.287168127	0.448062649	positive
PRJNA729271	ed_Lushi_vs_Arbor_Acr	breed_growth_background	ENSGALG00010006266	CENPW	-0.258717869	0.121235191	-2.134016257	0.03284145	0.097309243	negative
PRJNA729271	ed_Lushi_vs_Arbor_Acr	breed_growth_background	ENSGALG00010006269	ENSGALG00010006269	-2.109977748	0.657381856	-3.209668367	0.001328882	0.010014133	negative
PRJNA729271	ed_Lushi_vs_Arbor_Acr	breed_growth_background	ENSGALG00010006270	STAM	-0.027959909	0.049898001	-0.56034127	0.575246683	0.712698396	negative
PRJNA729271	ed_Lushi_vs_Arbor_Acr	breed_growth_background	ENSGALG00010006273	ARL13B	0.104082179	0.053551191	1.943601551	0.051943519	0.136127518	positive
PRJNA729271	ed_Lushi_vs_Arbor_Acr	breed_growth_background	ENSGALG00010006277	GNG10	-0.239898627	0.088420268	-2.713163298	0.006664426	0.031302932	negative
PRJNA729271	ed_Lushi_vs_Arbor_Acr	breed_growth_background	ENSGALG00010006279	HACD1	-0.215552056	0.075262329	-2.864009895	0.004183149	0.022292188	negative
PRJNA729271	ed_Lushi_vs_Arbor_Acr	breed_growth_background	ENSGALG00010006281	TRMT11	-0.002218862	0.043638611	-0.050846307	0.959447991	0.977517846	negative
PRJNA729271	ed_Lushi_vs_Arbor_Acr	breed_growth_background	ENSGALG00010006283	DNAJC25	0.164808825	0.057938022	2.844571128	0.004447126	0.02338088	positive
PRJNA729271	ed_Lushi_vs_Arbor_Acr	breed_growth_background	ENSGALG00010006285	FZD8	-0.127926157	0.083695319	-1.528474457	0.126394781	0.252967135	negative
PRJNA729271	ed_Lushi_vs_Arbor_Acr	breed_growth_background	ENSGALG00010006287	ECPAS	0.243247893	0.065511119	3.713077981	0.000204754	0.002642837	positive
PRJNA729271	ed_Lushi_vs_Arbor_Acr	breed_growth_background	ENSGALG00010006288	ARF5	-0.037381022	0.102968831	-0.363032402	0.71658068	0.818312215	negative
PRJNA729271	ed_Lushi_vs_Arbor_Acr	breed_growth_background	ENSGALG00010006289	SKIDA1	-0.073680633	0.084928301	-0.867562774	0.385633733	0.547479036	negative

PRJNA729271	:ed_Lushi_vs_Arbor_Acr	breed_growth_background	ENSGALG00010006291	ENSGALG00010006291	-0.066022628	0.10723545	-0.615679126	0.538106317	0.683418125	negative
PRJNA729271	:ed_Lushi_vs_Arbor_Acr	breed_growth_background	ENSGALG00010006293	SLC22A3	-0.503492429	0.209540034	-2.402845984	0.016268037	0.059021435	negative
PRJNA729271	:ed_Lushi_vs_Arbor_Acr	breed_growth_background	ENSGALG00010006300	GCC1	0.074690532	0.064929632	1.150330427	0.250007803	0.405944115	positive
PRJNA729271	:ed_Lushi_vs_Arbor_Acr	breed_growth_background	ENSGALG00010006301	ENSGALG00010006301	-0.171893408	0.079576016	-2.160115777	0.030763708	0.092657786	negative
PRJNA729271	:ed_Lushi_vs_Arbor_Acr	breed_growth_background	ENSGALG00010006310	ALG5	-0.034043488	0.059960863	-0.567761811	0.570196714	0.70877295	negative
PRJNA729271	:ed_Lushi_vs_Arbor_Acr	breed_growth_background	ENSGALG00010006311	ABCD4	-0.091641749	0.063316277	-1.447364786	0.147794783	0.282623401	negative
PRJNA729271	:ed_Lushi_vs_Arbor_Acr	breed_growth_background	ENSGALG00010006323	ANAPC10	0.262602722	0.057821978	4.541572763	5.58361E-06	0.000187783	positive
PRJNA729271	:ed_Lushi_vs_Arbor_Acr	breed_growth_background	ENSGALG00010006327	DENND6B	-0.026972039	0.060367167	-0.446799812	0.655019607	0.775238902	negative
PRJNA729271	:ed_Lushi_vs_Arbor_Acr	breed_growth_background	ENSGALG00010006328	SYNDIG1L	-0.850744291	0.251249874	-3.386048624	0.000709068	0.006385686	negative
PRJNA729271	:ed_Lushi_vs_Arbor_Acr	breed_growth_background	ENSGALG00010006330	STX19	1.079057287	0.225081247	4.794079042	1.63424E-06	7.2887E-05	positive
PRJNA729271	:ed_Lushi_vs_Arbor_Acr	breed_growth_background	ENSGALG00010006331	SMAD9	-0.140647861	0.086931185	-1.617921811	0.105679449	0.223793519	negative
PRJNA729271	:ed_Lushi_vs_Arbor_Acr	breed_growth_background	ENSGALG00010006336	ALDH8A1	-0.90103103	0.453359198	-1.987455055	0.046871992	0.126378259	negative
PRJNA729271	:ed_Lushi_vs_Arbor_Acr	breed_growth_background	ENSGALG00010006339	ENSGALG00010006339	0.838650661	0.393366452	2.131983185	0.033008229	0.097583985	positive
PRJNA729271	:ed_Lushi_vs_Arbor_Acr	breed_growth_background	ENSGALG00010006341	HHIP	-0.128088126	0.119466149	-1.072170881	0.283643287	0.443848376	negative
PRJNA729271	:ed_Lushi_vs_Arbor_Acr	breed_growth_background	ENSGALG00010006342	RFXAP	0.098326258	0.046027253	2.136261726	0.032658087	0.09694429	positive
PRJNA729271	:ed_Lushi_vs_Arbor_Acr	breed_growth_background	ENSGALG00010006343	SEC23A	0.061329844	0.050805104	1.207159091	0.227370882	0.380482675	positive
PRJNA729271	:ed_Lushi_vs_Arbor_Acr	breed_growth_background	ENSGALG00010006344	HINT3	0.043421686	0.056675396	0.766147027	0.443588824	0.600468809	positive
PRJNA729271	:ed_Lushi_vs_Arbor_Acr	breed_growth_background	ENSGALG00010006345	SMC2	-0.472477398	0.13394801	-3.527319282	0.00041979	0.004387376	negative
PRJNA729271	:ed_Lushi_vs_Arbor_Acr	breed_growth_background	ENSGALG00010006346	NSUN3	0.076363122	0.071988642	1.06076625	0.288796144	0.449779747	positive
PRJNA729271	:ed_Lushi_vs_Arbor_Acr	breed_growth_background	ENSGALG00010006347	PPP6R2	0.179839229	0.072427594	2.483020887	0.013027345	0.050328189	positive
PRJNA729271	:ed_Lushi_vs_Arbor_Acr	breed_growth_background	ENSGALG00010006351	IGF2R	0.268258898	0.093049633	2.882965676	0.003939504	0.021427056	positive
PRJNA729271	:ed_Lushi_vs_Arbor_Acr	breed_growth_background	ENSGALG00010006352	GEMIN2	0.167773232	0.052546467	3.192854663	0.001408738	0.010466254	positive
PRJNA729271	:ed_Lushi_vs_Arbor_Acr	breed_growth_background	ENSGALG00010006353	ABCE1	0.015845177	0.043462852	0.364568274	0.715433701	0.817438211	positive
PRJNA729271	:ed_Lushi_vs_Arbor_Acr	breed_growth_background	ENSGALG00010006355	PTGR1	-0.225227533	0.081918062	-2.749424573	0.00597	0.028922176	negative
PRJNA729271	:ed_Lushi_vs_Arbor_Acr	breed_growth_background	ENSGALG00010006359	SGK1	-0.221650507	0.087571478	-2.531081033	0.011371157	0.045797424	negative
PRJNA729271	:ed_Lushi_vs_Arbor_Acr	breed_growth_background	ENSGALG00010006361	SERTM1	-0.218767579	0.143472256	-1.524807547	0.127307107	0.254210844	negative
PRJNA729271	:ed_Lushi_vs_Arbor_Acr	breed_growth_background	ENSGALG00010006363	TRAPPC6A	0.020858371	0.056112884	0.371721592	0.710100147	0.813025405	positive
PRJNA729271	:ed_Lushi_vs_Arbor_Acr	breed_growth_background	ENSGALG00010006372	SBF1	0.091466456	0.060861248	1.502868571	0.132872939	0.262075555	positive
PRJNA729271	:ed_Lushi_vs_Arbor_Acr	breed_growth_background	ENSGALG00010006378	CAAP1	-0.253227319	0.057316773	-4.418031664	9.96038E-06	0.000290494	negative
PRJNA729271	:ed_Lushi_vs_Arbor_Acr	breed_growth_background	ENSGALG00010006381	SPART	-0.117494965	0.083640727	-1.404757815	0.160093307	0.298882782	negative
PRJNA729271	:ed_Lushi_vs_Arbor_Acr	breed_growth_background	ENSGALG00010006382	PNN	0.334341258	0.105487367	3.169490975	0.001527062	0.011026955	positive
PRJNA729271	:ed_Lushi_vs_Arbor_Acr	breed_growth_background	ENSGALG00010006387	OTUD4	0.033072807	0.073209527	0.451755507	0.651445126	0.772598355	positive
PRJNA729271	:ed_Lushi_vs_Arbor_Acr	breed_growth_background	ENSGALG00010006404	SMAD1	0.009795797	0.058643418	0.167040003	0.867338573	0.92058965	positive
PRJNA729271	:ed_Lushi_vs_Arbor_Acr	breed_growth_background	ENSGALG00010006408	ENSGALG00010006408	-0.305042271	0.369843232	-0.824788031	0.409491943	0.569962139	negative
PRJNA729271	:ed_Lushi_vs_Arbor_Acr	breed_growth_background	ENSGALG00010006411	RGS17	0.141870194	0.23141396	0.613058062	0.539837949	0.684926963	positive
PRJNA729271	:ed_Lushi_vs_Arbor_Acr	breed_growth_background	ENSGALG00010006421	LMF2	-0.071956834	0.061036643	-1.178912052	0.238433198	0.393721714	negative
PRJNA729271	:ed_Lushi_vs_Arbor_Acr	breed_growth_background	ENSGALG00010006434	MMAA	0.078562426	0.063046876	1.246095469	0.212729348	0.363022357	positive
PRJNA729271	:ed_Lushi_vs_Arbor_Acr	breed_growth_background	ENSGALG00010006446	LSAMP	-0.243024228	0.099961518	-2.431177855	0.015049825	0.055735108	negative
PRJNA729271	:ed_Lushi_vs_Arbor_Acr	breed_growth_background	ENSGALG00010006454	SOD2	0.040372516	0.056400936	0.715812866	0.474106907	0.627710963	positive
PRJNA729271	:ed_Lushi_vs_Arbor_Acr	breed_growth_background	ENSGALG00010006457	NCAPH2	-0.359363618	0.113693994	-3.160796859	0.001573382	0.011223509	negative
PRJNA729271	:ed_Lushi_vs_Arbor_Acr	breed_growth_background	ENSGALG00010006463	SCO2	-0.104823638	0.073148419	-1.433026713	0.151850204	0.287834275	negative
PRJNA729271	:ed_Lushi_vs_Arbor_Acr	breed_growth_background	ENSGALG00010006465	PLAA	0.098849831	0.062503175	1.58151695	0.113759887	0.235530575	positive
PRJNA729271	:ed_Lushi_vs_Arbor_Acr	breed_growth_background	ENSGALG00010006472	DCLK1	-0.709495534	0.175545287	-4.041666662	5.30726E-05	0.001010891	negative
PRJNA729271	:ed_Lushi_vs_Arbor_Acr	breed_growth_background	ENSGALG00010006478	ENSGALG00010006478	0.227718285	0.071215472	3.197595675	0.001385784	0.010337713	positive
PRJNA729271	:ed_Lushi_vs_Arbor_Acr	breed_growth_background	ENSGALG00010006480	IFT74	0.062825018	0.065124142	0.964696283	0.334696958	0.499067216	positive
PRJNA729271	:ed_Lushi_vs_Arbor_Acr	breed_growth_background	ENSGALG00010006486	CEP43	0.064097216	0.0610107	1.05058975	0.293447051	0.454775473	positive
PRJNA729271	:ed_Lushi_vs_Arbor_Acr	breed_growth_background	ENSGALG00010006492	GAP43	0.318140377	0.267099525	1.191093006	0.233617075	0.388023319	positive
PRJNA729271	:ed_Lushi_vs_Arbor_Acr	breed_growth_background	ENSGALG00010006496	UBE2H	0.080095437	0.060888436	1.315445797	0.188360122	0.334265423	positive
PRJNA729271	:ed_Lushi_vs_Arbor_Acr	breed_growth_background	ENSGALG00010006499	CCR6	-0.273891502	0.423016797	-0.647471931	0.517326555	0.665637338	negative
PRJNA729271	:ed_Lushi_vs_Arbor_Acr	breed_growth_background	ENSGALG00010006503	LRRC19	0.353810926	0.42701132	0.82857505	0.407344911	0.567846171	positive
PRJNA729271	:ed_Lushi_vs_Arbor_Acr	breed_growth_background	ENSGALG00010006506	ZNF827	-0.008590776	0.056866284	-0.151069758	0.879920689	0.928677034	negative
PRJNA729271	:ed_Lushi_vs_Arbor_Acr	breed_growth_background	ENSGALG00010006515	FBXO33	0.047573061	0.056175613	0.846863238	0.397071355	0.558303356	positive
PRJNA729271	:ed_Lushi_vs_Arbor_Acr	breed_growth_background	ENSGALG00010006516	NBEA	0.038894012	0.068398466	0.568638663	0.569601381	0.708458213	positive
PRJNA729271	:ed_Lushi_vs_Arbor_Acr	breed_growth_background	ENSGALG00010006517	ZBTB20	-0.079774403	0.073968007	-1.07849875	0.280811239	0.440705816	negative
PRJNA729271	:ed_Lushi_vs_Arbor_Acr	breed_growth_background	ENSGALG00010006521	ZNF410	0.04217185	0.056985742	0.740042134	0.459274428	0.614802205	positive
PRJNA729271	:ed_Lushi_vs_Arbor_Acr	breed_growth_background	ENSGALG00010006524	TAB2	0.094329957	0.049333172	1.912099976	0.055863372	0.143519953	positive
PRJNA729271	:ed_Lushi_vs_Arbor_Acr	breed_growth_background	ENSGALG00010006526	LSM6	-0.095672494	0.086853453	-1.101539328	0.270661998	0.429239131	negative
PRJNA729271	:ed_Lushi_vs_Arbor_Acr	breed_growth_background	ENSGALG00010006528	ZC3HC1	0.031889287	0.045407845	0.702285854	0.482500914	0.634858631	positive
PRJNA729271	:ed_Lushi_vs_Arbor_Acr	breed_growth_background	ENSGALG00010006531	FAM161B	-0.108274108	0.079795542	-1.356894188	0.174814834	0.318694632	negative
PRJNA729271	:ed_Lushi_vs_Arbor_Acr	breed_growth_background	ENSGALG00010006532	KLHDC10	0.079939632	0.055971569	1.428218529	0.153228969	0.289690088	positive
PRJNA729271	:ed_Lushi_vs_Arbor_Acr	breed_growth_background	ENSGALG00010006537	DRD3	0.088208267	0.178518475	0.494112818	0.621226506	0.7485052	positive
PRJNA729271	:ed_Lushi_vs_Arbor_Acr	breed_growth_background	ENSGALG00010006538	TEK	-0.181371651	0.056262943	-3.223643144	0.00126571	0.009686348	negative
PRJNA729271	:ed_Lushi_vs_Arbor_Acr	breed_growth_background	ENSGALG00010006542	RFC3	-0.215276543	0.076920258	-2.798697605	0.005130916	0.026059087	negative
PRJNA729271	:ed_Lushi_vs_Arbor_Acr	breed_growth_background	ENSGALG00010006544	MOB3B	-0.035414681	0.087184901	-0.406201994	0.684594189	0.796342332	negative
PRJNA729271	:ed_Lushi_vs_Arbor_Acr	breed_growth_background	ENSGALG00010006546	SMOC2	-0.508991722	0.111688218	-4.557255274	5.18264E-06	0.000178863	negative
PRJNA729271	:ed_Lushi_vs_Arbor_Acr	breed_growth_background	ENSGALG00010006548	TMEM209	0.029324947	0.044531029	0.658528388	0.510198662	0.658786517	positive
PRJNA729271	:ed_Lushi_vs_Arbor_Acr	breed_growth_background	ENSGALG00010006549	ZDHHC23	0.377925628	0.101476728	3.724259094	0.00019589	0.002562654	positive
PRJNA729271	:ed_Lushi_vs_Arbor_Acr	breed_growth_background	ENSGALG00010006552	CPA2	-2.344706484	0.856053703	-2.738971254	0.006163176	0.029571413	negative
PRJNA729271	:ed_Lushi_vs_Arbor_Acr	breed_growth_background	ENSGALG00010006557	COQ6	-0.103405854	0.058558888	-1.765843866	0.077422047	0.179791712	negative
PRJNA729271	:ed_Lushi_vs_Arbor_Acr	breed_growth_background	ENSGALG00010006558	USP10	0.041098413	0.047761095	0.860499804	0.389513592	0.551323379	positive
PRJNA729271	:ed_Lushi_vs_Arbor_Acr	breed_growth_background	ENSGALG00010006564	SLC10A7	-0.191302916	0.077657363	-2.463422778	0.013761754	0.052397326	negative
PRJNA729271	:ed_Lushi_vs_Arbor_Acr	breed_growth_background	ENSGALG00010006566	CPA5	-1.734937527	1.486380171	-1.167223272	0.243120215	0.398826111	negative
PRJNA729271	:ed_Lushi_vs_Arbor_Acr	breed_growth_background	ENSGALG00010006568	ENTPD5	0.502922134	0.107076883	4.696832044	2.64228E-06	0.000103234	positive
PRJNA729271	:ed_Lushi_vs_Arbor_Acr	breed_growth_background	ENSGALG00010006570	ENSGALG00010006570	0.406893188	0.257830608	1.578141523	0.114533101	0.236658203	positive
PRJNA729271	:ed_Lushi_vs_Arbor_Acr	breed_growth_background	ENSGALG00010006574	CPA1	-1.777907789	1.346341271	-1.320547641	0.186652241	0.332210736	negative
PRJNA729271	:ed_Lushi_vs_Arbor_Acr	breed_growth_background	ENSGALG00010006575	SSBP2	0.162909969	0.063123848	2.580799074	0.009857193	0.041400955	positive
PRJNA729271	:ed_Lushi_vs_Arbor_Acr	breed_growth_background	ENSGALG00010006576	CCDC191	0.014941002	0.061895103	0.24139232	0.809251064	0.882565732	positive
PRJNA729271	:ed_Lushi_vs_Arbor_Acr	breed_growth_background	ENSGALG00010006580	ALDH6A1	-0.044803756	0.074156153	-0.604181238	0.545723154	0.689527294	negative
PRJNA729271	:ed_Lushi_vs_Arbor_Acr	breed_growth_background	ENSGALG00010006581	CEP41	-0.075660909	0.059018893	-1.281977767	0.199850446	0.348721826	negative
PRJNA729271	:ed_Lushi_vs_Arbor_Acr	breed_growth_background	ENSGALG00010006585	TTC29	0.080954919	0.103408333	0.782866485	0.433705521	0.591736607	positive
PRJNA729271	:ed_Lushi_vs_Arbor_Acr	breed_growth_background	ENSGALG00010006588	ACOT12	-0.133945567	0.426943364	-0.313731464	0.753725002	0.844357459	negative
PRJNA729271	:ed_Lushi_vs_Arbor_Acr	breed_growth_background	ENSGALG00010006592	STARD13	-0.198327627	0.061306447	-3.235020711	0.001216339	0.009444292	negative
PRJNA729271	:ed_Lushi_vs_Arbor_Acr	breed_growth_background	ENSGALG00010006598	ENSGALG00010006598	0.061298047	0.081235483	0.754572326	0.450505634	0.606771897	positive
PRJNA729271	:ed_Lushi_vs_Arbor_Acr	breed_growth_background	ENSGALG00010006602	EDNRA	-0.255761099	0.063609716	-4.020786703	5.80041E-05	0.00107791	negative
PRJNA729271	:ed_Lushi_vs_Arbor_Acr	breed_growth_background	ENSGALG00010006603	MEST	-0.420848083	0.126282035	-3.332604548	0.000860371	0.007316166	negative
PRJNA729271	:ed_Lushi_vs_Arbor_Acr	breed_growth_background	ENSGALG00010006606	SULT	-1.549404228	0.658434937	-2.353162236			negative
PRJNA729271	:ed_Lushi_vs_Arbor_Acr	breed_growth_background	ENSGALG00010006608	KL	-0.206954341	0.209912687	-0.985906777	0.324178861	0.488247675	negative
PRJNA729271	:ed_Lushi_vs_Arbor_Acr	breed_growth_background	ENSGALG00010006614	TMEM184C	0.072031767	0.030263192	2.380177485	0.017304301	0.061567463	positive
PRJNA729271	:ed_Lushi_vs_Arbor_Acr	breed_growth_background	ENSGALG00010006615	GRAMD1C	-0.068452977	0.05591627	-1.224204997	0.220874904	0.373317689	negative
PRJNA729271	:ed_Lushi_vs_Arbor_Acr	breed_growth_background	ENSGALG00010006616	COPG2	-0.078362573	0.037470932	-2.091289679	0.036502104	0.105230311	negative
PRJNA729271	:ed_Lushi_vs_Arbor_Acr	breed_growth_background	ENSGALG00010006619	LIN52	0.173867124	0.054913716	3.166187552	0.001544512	0.011088508	positive
PRJNA729271	:ed_Lushi_vs_Arbor_Acr	breed_growth_background	ENSGALG00010006621	DACT2	-0.330253481	0.11315566	-2.918576768	0.003516333	0.019791686	negative
PRJNA729271	:ed_Lushi_vs_Arbor_Acr	breed_growth_background	ENSGALG00010006623	ENSGALG00010006623	0.002507892	0.100679139	0.024909745	0.980126954	0.988308884	positive
PRJNA729271	:ed_Lushi_vs_Arbor_Acr	breed_growth_background	ENSGALG00010006627	SF3B5	0.077328936	0.059856685	1.291901419	0.196391286	0.344719265	positive
PRJNA729271	:ed_Lushi_vs_Arbor_Acr	breed_growth_background	ENSGALG00010006633	FBXO18	-0.028396504	0.050634325	-0.560815284	0.574923466	0.712509888	negative
PRJNA729271	:ed_Lushi_vs_Arbor_Acr	breed_growth_background	ENSGALG00010006634	PRMT9	0.22221427	0.051413682	4.322084324	1.54562E-05	0.000399878	positive
PRJNA729271	:ed_Lushi_vs_Arbor_Acr	breed_growth_background	ENSGALG00010006635	ENSGALG00010006635	0.780245941	0.259181223	3.010426185	0.002608813	0.016030901	positive
PRJNA729271	:ed_Lushi_vs_Arbor_Acr	breed_growth_background	ENSGALG00010006641	ENSGALG00010006641	-0.487015378	0.305539153	-1.593954077	0.110946321	0.231656435	negative
PRJNA729271	:ed_Lushi_vs_Arbor_Acr	breed_growth_background	ENSGALG00010006651	ENSGALG00010006651	-0.038013297	0.061263852	-0.620484934	0.53493857	0.680219664	negative
PRJNA729271	:ed_Lushi_vs_Arbor_Acr	breed_growth_background	ENSGALG00010006653	CFL2	-0.09681339	0.055945321	-1.730500217	0.083540942	0.189681385	negative
PRJNA729271	:ed_Lushi_vs_Arbor_Acr	breed_growth_background	ENSGALG00010006657	PDS5B	-0.162158923	0.047523876	-3.412156936	0.00064451	0.005965627	negative
PRJNA729271	:ed_Lushi_vs_Arbor_Acr	breed_growth_background	ENSGALG00010006664	ENSGALG00010006664	0.392513063	0.68220417	0.575360105	0.565047779	0.704850909	positive
PRJNA729271	:ed_Lushi_vs_Arbor_Acr	breed_growth_background	ENSGALG00010006670	ANKRD16	-0.134355356	0.102223015	-1.314335678	0.188733266	0.334763667	negative
PRJNA729271	:ed_Lushi_vs_Arbor_Acr	breed_growth_background	ENSGALG00010006673	BAZ1A	-0.059706385	0.044602239	-1.338640986	0.180687576	0.325228663	negative
PRJNA729271	:ed_Lushi_vs_Arbor_Acr	breed_growth_background	ENSGALG00010006678	ENSGALG00010006678	-1.813035245	0.719903033	-2.518443682	0.011787473	0.046978121	negative
PRJNA729271	:ed_Lushi_vs_Arbor_Acr	breed_growth_background	ENSGALG00010006681	CRISPLD2	-0.146106982	0.115981244	-1.259746629	0.207760778	0.35693878	negative
PRJNA729271	:ed_Lushi_vs_Arbor_Acr	breed_growth_background	ENSGALG00010006682	ARHGAP10	-0.351940492	0.065707301	-5.356185473	8.49973E-08	7.33354E-06	negative
PRJNA729271	:ed_Lushi_vs_Arbor_Acr	breed_growth_background	ENSGALG00010006685	ENSGALG00010006685	0.016796704	0.064781395	0.259282846	0.795417014	0.873373925	positive
PRJNA729271	:ed_Lushi_vs_Arbor_Acr	breed_growth_background	ENSGALG00010006694	ENSGALG00010006694	0.242612587	0.420990294	0.576290214	0.564419036	0.704562618	positive
PRJNA729271	:ed_Lushi_vs_Arbor_Acr	breed_growth_background	ENSGALG00010006701	LINGO2	0.101987453	0.219545231	0.464539598	0.642261196	0.765716074	positive
PRJNA729271	:ed_Lushi_vs_Arbor_Acr	breed_growth_background	ENSGALG00010006715	IRF8	0.110465822	0.086548705	1.276342858	0.201834346	0.350874173	positive
PRJNA729271	:ed_Lushi_vs_Arbor_Acr	breed_growth_background	ENSGALG00010006716	ENSGALG00010006716	-0.0049876	0.052019818	-0.095878852	0.92361679	0.957367375	negative
PRJNA729271	:ed_Lushi_vs_Arbor_Acr	breed_growth_background	ENSGALG00010006720	SRP54	0.056968006	0.049104844	1.160130059	0.245995857	0.401905991	positive
PRJNA729271	:ed_Lushi_vs_Arbor_Acr	breed_growth_background	ENSGALG00010006728	ENSGALG00010006728	-0.294244665	0.0734216	-4.007603563	6.1338E-05	0.001116952	negative
PRJNA729271	:ed_Lushi_vs_Arbor_Acr	breed_growth_background	ENSGALG00010006730	HAPLN1	0.39429448	0.280210781	1.407135293	0.159387274	0.297875006	positive
PRJNA729271	:ed_Lushi_vs_Arbor_Acr	breed_growth_background	ENSGALG00010006735	NR3C2	-0.021109631	0.061196785	-0.344946739	0.730134412	0.82688478	negative
PRJNA729271	:ed_Lushi_vs_Arbor_Acr	breed_growth_background	ENSGALG00010006739	VCAN	-0.537901377	0.135754914	-3.962297657	7.42319E-05	0.0012779	negative
PRJNA729271	:ed_Lushi_vs_Arbor_Acr	breed_growth_background	ENSGALG00010006740	BRCA2	0.049332472	0.08256545	0.597495344	0.550176715	0.693341287	positive
PRJNA729271	:ed_Lushi_vs_Arbor_Acr	breed_growth_background	ENSGALG00010006748	FAM177A1	0.032620265	0.041308662	0.789671297	0.429719758	0.588187903	positive
PRJNA729271	:ed_Lushi_vs_Arbor_Acr	breed_growth_background	ENSGALG00010006752	ZAR1L	1.243447349	0.251969036	4.934921242	8.01831E-07	4.07808E-05	positive
PRJNA729271	:ed_Lushi_vs_Arbor_Acr	breed_growth_background	ENSGALG00010006760	TASOR2	0.041961551	0.055181226	0.760431654	0.446996608	0.603728646	positive
PRJNA729271	:ed_Lushi_vs_Arbor_Acr	breed_growth_background	ENSGALG00010006761	PPP2R3C	0.042297339	0.051870004	0.815448917	0.414815394	0.575162534	positive
PRJNA729271	:ed_Lushi_vs_Arbor_Acr	breed_growth_background	ENSGALG00010006767	ENSGALG00010006767	-0.103604625	0.111458614	-0.929534483	0.352612163	0.51637839	negative
PRJNA729271	:ed_Lushi_vs_Arbor_Acr	breed_growth_background	ENSGALG00010006794	PERPA	-0.43325721	0.320234354	-1.352937952	0.176075454	0.320026796	negative
PRJNA729271	:ed_Lushi_vs_Arbor_Acr	breed_growth_background	ENSGALG00010006803	COX4I1	-0.050120293	0.055487769	-0.903267399	0.366383995	0.529854934	negative
PRJNA729271	:ed_Lushi_vs_Arbor_Acr	breed_growth_background	ENSGALG00010006804	PSMA6	0.109158458	0.054739103	1.99415868	0.046134716	0.124994899	positive
PRJNA729271	:ed_Lushi_vs_Arbor_Acr	breed_growth_background	ENSGALG00010006814	ENSGALG00010006814	-0.370924839	0.212036337	-1.749345628	0.080231293	0.184390772	negative
PRJNA729271	:ed_Lushi_vs_Arbor_Acr	breed_growth_background	ENSGALG00010006816	ENSGALG00010006816	-0.140184693	0.230209398	-0.608944264	0.542561383	0.687358576	negative
PRJNA729271	:ed_Lushi_vs_Arbor_Acr	breed_growth_background	ENSGALG00010006819	EMC8	0.119748369	0.070724503	1.69316664	0.090423753	0.200903172	positive
PRJNA729271	:ed_Lushi_vs_Arbor_Acr	breed_growth_background	ENSGALG00010006821	XRCC4	-0.133855045	0.070158068	-1.907906644	0.056403283	0.144512484	negative
PRJNA729271	:ed_Lushi_vs_Arbor_Acr	breed_growth_background	ENSGALG00010006828	GTF2A1	0.254021257	0.056865775	4.467032335	7.93121E-06	0.000243051	positive

PRJNA729271	ed_Lushi_vs_Arbor_Acr	breed_growth_background	ENSGALG00010006833	LTV1	-0.006706122	0.047274555	-0.141854794	0.887194702	0.93356713	negative
PRJNA729271	ed_Lushi_vs_Arbor_Acr	breed_growth_background	ENSGALG00010006835	FRY	0.078849809	0.081448706	0.968091612	0.332998619	0.497404676	positive
PRJNA729271	ed_Lushi_vs_Arbor_Acr	breed_growth_background	ENSGALG00010006838	ASB13	0.240012219	0.065998428	3.636635371	0.000276223	0.00328993	positive
PRJNA729271	ed_Lushi_vs_Arbor_Acr	breed_growth_background	ENSGALG00010006840	ENSGALG00010006840	-0.436787079	0.158391028	-2.757650385	0.005821843	0.028356052	negative
PRJNA729271	ed_Lushi_vs_Arbor_Acr	breed_growth_background	ENSGALG00010006843	STON2	0.059419245	0.103772176	0.572593228	0.566920146	0.706085057	positive
PRJNA729271	ed_Lushi_vs_Arbor_Acr	breed_growth_background	ENSGALG00010006845	CALML3	-0.568756805	0.396817116	-1.433297057	0.151772963	0.287800771	negative
PRJNA729271	ed_Lushi_vs_Arbor_Acr	breed_growth_background	ENSGALG00010006847	SEL1L	-0.140943156	0.046598738	-3.024613174	0.002489513	0.015540694	negative
PRJNA729271	ed_Lushi_vs_Arbor_Acr	breed_growth_background	ENSGALG00010006850	IRF2BPL	0.051447247	0.085803342	0.599594906	0.548776243	0.692178179	positive
PRJNA729271	ed_Lushi_vs_Arbor_Acr	breed_growth_background	ENSGALG00010006851	TMEM167A	-0.072612343	0.056367679	-1.288191107	0.197679447	0.346105929	negative
PRJNA729271	ed_Lushi_vs_Arbor_Acr	breed_growth_background	ENSGALG00010006853	NET1	-0.092387727	0.044996207	-2.053233664	0.040049921	0.11241239	negative
PRJNA729271	ed_Lushi_vs_Arbor_Acr	breed_growth_background	ENSGALG00010006855	DCLK2	0.212742055	0.066843366	3.182695113	0.001459112	0.010708772	positive
PRJNA729271	ed_Lushi_vs_Arbor_Acr	breed_growth_background	ENSGALG00010006865	EFCAB2	-0.182109662	0.19656724	-0.926449706	0.354212323	0.517900047	negative
PRJNA729271	ed_Lushi_vs_Arbor_Acr	breed_growth_background	ENSGALG00010006877	GSE1	0.386936209	0.128920736	3.001349668	0.002687857	0.016356209	positive
PRJNA729271	ed_Lushi_vs_Arbor_Acr	breed_growth_background	ENSGALG00010006883	LRBA	0.009385092	0.038371135	0.244587304	0.806775995	0.881119504	positive
PRJNA729271	ed_Lushi_vs_Arbor_Acr	breed_growth_background	ENSGALG00010006885	RHOU	0.294961868	0.082003106	3.596959711	0.000321958	0.003631269	positive
PRJNA729271	ed_Lushi_vs_Arbor_Acr	breed_growth_background	ENSGALG00010006901	TUBAL3	-0.718409257	0.140839012	-5.100925139	3.37997E-07	2.0848E-05	negative
PRJNA729271	ed_Lushi_vs_Arbor_Acr	breed_growth_background	ENSGALG00010006903	B3GLCT	-0.252911295	0.078986841	-3.201942139	0.001365044	0.010230773	negative
PRJNA729271	ed_Lushi_vs_Arbor_Acr	breed_growth_background	ENSGALG00010006912	ENSGALG00010006912	-0.086491668	0.099203217	-0.871863546	0.383282823	0.544728358	negative
PRJNA729271	ed_Lushi_vs_Arbor_Acr	breed_growth_background	ENSGALG00010006913	HSPH1	0.136991846	0.100773779	1.359399704	0.174019965	0.317845185	positive
PRJNA729271	ed_Lushi_vs_Arbor_Acr	breed_growth_background	ENSGALG00010006922	SMYD3	0.148905829	0.087121282	1.709178582	0.087417875	0.195845765	positive
PRJNA729271	ed_Lushi_vs_Arbor_Acr	breed_growth_background	ENSGALG00010006923	ENSGALG00010006923	-0.354207529	0.123092506	-2.877571833	0.004007487	0.021691008	negative
PRJNA729271	ed_Lushi_vs_Arbor_Acr	breed_growth_background	ENSGALG00010006926	ID4	-0.1680747	0.125097346	-1.343551286	0.179093531	0.323305724	negative
PRJNA729271	ed_Lushi_vs_Arbor_Acr	breed_growth_background	ENSGALG00010006927	UCN3	1.408472789	0.513961644	2.740423931	0.006135998	0.02948982	positive
PRJNA729271	ed_Lushi_vs_Arbor_Acr	breed_growth_background	ENSGALG00010006939	FBXO31	0.027092866	0.068099451	0.397842645	0.69074618	0.800093166	positive
PRJNA729271	ed_Lushi_vs_Arbor_Acr	breed_growth_background	ENSGALG00010006942	ALOX5AP	0.05164843	0.102464286	0.504062754	0.61421728	0.74359916	positive
PRJNA729271	ed_Lushi_vs_Arbor_Acr	breed_growth_background	ENSGALG00010006944	MAB21L2	0.326264763	0.300853976	1.084462194	0.278159921	0.437571737	positive
PRJNA729271	ed_Lushi_vs_Arbor_Acr	breed_growth_background	ENSGALG00010006946	PLPPR1	0.436253965	0.200825428	2.17230442	0.029832709	0.090560234	positive
PRJNA729271	ed_Lushi_vs_Arbor_Acr	breed_growth_background	ENSGALG00010006948	ENSGALG00010006948	0.304652875	0.176999442	1.721208115	0.085213066	0.192562883	positive
PRJNA729271	ed_Lushi_vs_Arbor_Acr	breed_growth_background	ENSGALG00010006953	MAP1LC3B	0.201414101	0.068492344	2.940680533	0.003274921	0.018889765	positive
PRJNA729271	ed_Lushi_vs_Arbor_Acr	breed_growth_background	ENSGALG00010006957	PALM2AKAP2	-0.088856013	0.049394827	-1.798893093	0.072035593	0.171060932	negative
PRJNA729271	ed_Lushi_vs_Arbor_Acr	breed_growth_background	ENSGALG00010006958	KIF26B	0.205037048	0.134880249	1.520141387	0.128475445	0.255733531	positive
PRJNA729271	ed_Lushi_vs_Arbor_Acr	breed_growth_background	ENSGALG00010006961	ZCCHC14	0.030885112	0.062744274	0.492237942	0.622551145	0.749242681	positive
PRJNA729271	ed_Lushi_vs_Arbor_Acr	breed_growth_background	ENSGALG00010006963	ENSGALG00010006963	-0.314792441	0.232448743	-1.354244542	0.175658373	0.319715342	negative
PRJNA729271	ed_Lushi_vs_Arbor_Acr	breed_growth_background	ENSGALG00010006966	PGBD5	-0.077090253	0.138282037	-0.557485663	0.577195652	0.714714759	negative
PRJNA729271	ed_Lushi_vs_Arbor_Acr	breed_growth_background	ENSGALG00010006983	GTPBP2	0.065110316	0.066576435	0.977978408	0.328085003	0.491987666	positive
PRJNA729271	ed_Lushi_vs_Arbor_Acr	breed_growth_background	ENSGALG00010006988	USPL1	0.020401673	0.056092307	0.363716068	0.716070044	0.817922402	positive
PRJNA729271	ed_Lushi_vs_Arbor_Acr	breed_growth_background	ENSGALG00010006992	SLC35B4	-0.019361223	0.057265077	-0.338098265	0.735289138	0.830503295	negative
PRJNA729271	ed_Lushi_vs_Arbor_Acr	breed_growth_background	ENSGALG00010006998	POLH	0.167635103	0.065378763	2.564060489	0.010345551	0.042869859	positive
PRJNA729271	ed_Lushi_vs_Arbor_Acr	breed_growth_background	ENSGALG00010007000	LRGUK	0.016040067	0.342753273	0.046797705	0.962674458	0.978835813	positive
PRJNA729271	ed_Lushi_vs_Arbor_Acr	breed_growth_background	ENSGALG00010007001	SNX30	0.004800593	0.050834952	0.09443488	0.924763709	0.957871228	positive
PRJNA729271	ed_Lushi_vs_Arbor_Acr	breed_growth_background	ENSGALG00010007002	EXOC4	0.077447897	0.064393564	1.202727292	0.229081869	0.38268315	positive
PRJNA729271	ed_Lushi_vs_Arbor_Acr	breed_growth_background	ENSGALG00010007004	ENSGALG00010007004	-0.491087975	0.324840028	-1.511784054	0.130588807	0.258661487	negative
PRJNA729271	ed_Lushi_vs_Arbor_Acr	breed_growth_background	ENSGALG00010007007	ACSL1	0.029960413	0.115939078	0.258415137	0.796086535	0.873593259	positive
PRJNA729271	ed_Lushi_vs_Arbor_Acr	breed_growth_background	ENSGALG00010007010	ENSGALG00010007010	0.494026864	0.547487249	0.902353186	0.366869281	0.530341615	positive
PRJNA729271	ed_Lushi_vs_Arbor_Acr	breed_growth_background	ENSGALG00010007011	HMGB1	0.084076033	0.054569289	1.540720712	0.123384774	0.248570159	positive
PRJNA729271	ed_Lushi_vs_Arbor_Acr	breed_growth_background	ENSGALG00010007014	ENSGALG00010007014	0.571788904	0.329810141	1.733691092	0.082972908	0.188776063	positive
PRJNA729271	ed_Lushi_vs_Arbor_Acr	breed_growth_background	ENSGALG00010007017	SRD5A2	-0.252742833	0.190827966	-1.324453847	0.185352361	0.330872226	negative
PRJNA729271	ed_Lushi_vs_Arbor_Acr	breed_growth_background	ENSGALG00010007021	SULT6B1	0.327022676	0.13951871	2.343934209	0.019081537	0.066200785	positive
PRJNA729271	ed_Lushi_vs_Arbor_Acr	breed_growth_background	ENSGALG00010007025	ENSGALG00010007025	-0.257287489	0.398471847	-0.645685488	0.518483061	0.666614508	negative
PRJNA729271	ed_Lushi_vs_Arbor_Acr	breed_growth_background	ENSGALG00010007028	KATNAL1	-0.080176531	0.076835142	-1.043487764	0.296722445	0.458430169	negative
PRJNA729271	ed_Lushi_vs_Arbor_Acr	breed_growth_background	ENSGALG00010007033	CEBPZOS	-0.621962336	0.099110454	-6.275446341	3.48633E-10	8.28431E-08	negative
PRJNA729271	ed_Lushi_vs_Arbor_Acr	breed_growth_background	ENSGALG00010007038	CENPU	0.046010178	0.111955369	0.410968928	0.681095319	0.793926549	positive
PRJNA729271	ed_Lushi_vs_Arbor_Acr	breed_growth_background	ENSGALG00010007041	NAE1	-0.049907768	0.043681664	-1.142533599	0.253232289	0.409878764	negative
PRJNA729271	ed_Lushi_vs_Arbor_Acr	breed_growth_background	ENSGALG00010007049	ENSGALG00010007049	0.806788147	0.290680394	2.775516216	0.005511415	0.027293461	positive
PRJNA729271	ed_Lushi_vs_Arbor_Acr	breed_growth_background	ENSGALG00010007051	E2F3	-0.079597967	0.086225721	-0.923134844	0.355936939	0.519666017	negative
PRJNA729271	ed_Lushi_vs_Arbor_Acr	breed_growth_background	ENSGALG00010007066	INIP	0.066631643	0.083766553	0.795444485	0.426354992	0.585505978	positive
PRJNA729271	ed_Lushi_vs_Arbor_Acr	breed_growth_background	ENSGALG00010007067	CASP3	-0.165275531	0.05833963	-2.83298904	0.004611497	0.02400993	negative
PRJNA729271	ed_Lushi_vs_Arbor_Acr	breed_growth_background	ENSGALG00010007068	NCAM2	0.413435495	0.14744134	2.80406767	0.005046231	0.025755325	positive
PRJNA729271	ed_Lushi_vs_Arbor_Acr	breed_growth_background	ENSGALG00010007070	UBL3	0.015557399	0.028086266	0.553914815	0.579637146	0.716513767	positive
PRJNA729271	ed_Lushi_vs_Arbor_Acr	breed_growth_background	ENSGALG00010007085	PRIMPOL	-0.147940691	0.090831132	-1.628744331	0.103367154	0.220222434	negative
PRJNA729271	ed_Lushi_vs_Arbor_Acr	breed_growth_background	ENSGALG00010007090	CEBPZ	0.116789915	0.050508172	2.312297415	0.020761299	0.07000117	positive
PRJNA729271	ed_Lushi_vs_Arbor_Acr	breed_growth_background	ENSGALG00010007092	DYNC1LI2	-0.045233481	0.033015152	-1.370082483	0.170661155	0.313130816	negative
PRJNA729271	ed_Lushi_vs_Arbor_Acr	breed_growth_background	ENSGALG00010007097	ZCCHC9	-0.270646086	0.090543667	-2.989122213	0.002797802	0.016869442	negative
PRJNA729271	ed_Lushi_vs_Arbor_Acr	breed_growth_background	ENSGALG00010007098	NDUFAF7	0.229650682	0.069574678	3.300779668	0.000964166	0.007936162	positive
PRJNA729271	ed_Lushi_vs_Arbor_Acr	breed_growth_background	ENSGALG00010007102	CMTM3	-0.260390554	0.088717936	-2.935038443	0.003335064	0.019122527	negative
PRJNA729271	ed_Lushi_vs_Arbor_Acr	breed_growth_background	ENSGALG00010007106	SLC7A1	-0.415486353	0.129566564	-3.206740546	0.00134248	0.01009821	negative
PRJNA729271	ed_Lushi_vs_Arbor_Acr	breed_growth_background	ENSGALG00010007108	MBOAT1	-0.427400311	0.103494266	-4.129700411	3.63236E-05	0.00077201	negative
PRJNA729271	ed_Lushi_vs_Arbor_Acr	breed_growth_background	ENSGALG00010007110	ARL6	-0.096649949	0.055077875	-1.754787191	0.079295715	0.182965838	negative
PRJNA729271	ed_Lushi_vs_Arbor_Acr	breed_growth_background	ENSGALG00010007114	CKLF	-0.089909259	0.082564534	-1.088957379	0.276172681	0.435216679	negative
PRJNA729271	ed_Lushi_vs_Arbor_Acr	breed_growth_background	ENSGALG00010007115	ENSGALG00010007115	-0.008043998	0.148623175	-0.054123443	0.956836815	0.97576153	negative
PRJNA729271	ed_Lushi_vs_Arbor_Acr	breed_growth_background	ENSGALG00010007118	ENSGALG00010007118	-0.033736384	0.072123753	-0.467756905	0.639958442	0.76391317	negative
PRJNA729271	ed_Lushi_vs_Arbor_Acr	breed_growth_background	ENSGALG00010007123	ENSGALG00010007123	-0.308426162	0.128790154	-2.394796132	0.016629609	0.059954106	negative
PRJNA729271	ed_Lushi_vs_Arbor_Acr	breed_growth_background	ENSGALG00010007126	ENSGALG00010007126	0.865467168	0.573966972	1.507869286	0.131587985	0.260142913	positive
PRJNA729271	ed_Lushi_vs_Arbor_Acr	breed_growth_background	ENSGALG00010007130	IRF2	0.100578469	0.065046165	1.546262849	0.1220411	0.24672737	positive
PRJNA729271	ed_Lushi_vs_Arbor_Acr	breed_growth_background	ENSGALG00010007133	BLVRA	0.037850473	0.100275502	0.37746181	0.705828211	0.809925579	positive
PRJNA729271	ed_Lushi_vs_Arbor_Acr	breed_growth_background	ENSGALG00010007135	TK2	-0.103953922	0.063741403	-1.63086968	0.102917821	0.219529124	negative
PRJNA729271	ed_Lushi_vs_Arbor_Acr	breed_growth_background	ENSGALG00010007137	ENSGALG00010007137	-0.308580172	0.192215735	-1.605384549	0.108409203	0.228101523	negative
PRJNA729271	ed_Lushi_vs_Arbor_Acr	breed_growth_background	ENSGALG00010007141	PRKD3	-0.138343275	0.044887949	-3.081969188	0.002056361	0.013530166	negative
PRJNA729271	ed_Lushi_vs_Arbor_Acr	breed_growth_background	ENSGALG00010007143	CKMT2	-0.103667072	0.155091943	-0.668423324	0.503863411	0.6545528	negative
PRJNA729271	ed_Lushi_vs_Arbor_Acr	breed_growth_background	ENSGALG00010007146	ENSGALG00010007146	0.229961316	0.076267736	3.015184778	0.002568227	0.015895156	positive
PRJNA729271	ed_Lushi_vs_Arbor_Acr	breed_growth_background	ENSGALG00010007155	FOXL1	0.309917396	0.397105144	0.780441654	0.435130958	0.593247111	positive
PRJNA729271	ed_Lushi_vs_Arbor_Acr	breed_growth_background	ENSGALG00010007160	ENSGALG00010007160	0.734299381	0.157287181	4.668526569	3.03368E-06	0.000115113	positive
PRJNA729271	ed_Lushi_vs_Arbor_Acr	breed_growth_background	ENSGALG00010007162	ENSGALG00010007162	-0.264136135	0.223583442	-1.181376099	0.237453349	0.392595676	negative
PRJNA729271	ed_Lushi_vs_Arbor_Acr	breed_growth_background	ENSGALG00010007165	ENSGALG00010007165	-0.392174542	0.175711311	-2.231925415	0.025619893	0.081408964	negative
PRJNA729271	ed_Lushi_vs_Arbor_Acr	breed_growth_background	ENSGALG00010007166	RASGRF2	0.099910653	0.096240075	1.038139811	0.299204969	0.461527725	positive
PRJNA729271	ed_Lushi_vs_Arbor_Acr	breed_growth_background	ENSGALG00010007168	MINA	0.003479452	0.058172708	0.059812454	0.952305006	0.973433394	positive
PRJNA729271	ed_Lushi_vs_Arbor_Acr	breed_growth_background	ENSGALG00010007169	QPCT	-0.386605891	0.117107631	-3.301286916	0.000962424	0.007930834	negative
PRJNA729271	ed_Lushi_vs_Arbor_Acr	breed_growth_background	ENSGALG00010007175	FOXC2	-0.027824887	0.2619461	-0.106223713	0.915404857	0.952192002	negative
PRJNA729271	ed_Lushi_vs_Arbor_Acr	breed_growth_background	ENSGALG00010007181	GJB2	0.550431181	0.330425255	1.665826607	0.095747955	0.208419674	positive
PRJNA729271	ed_Lushi_vs_Arbor_Acr	breed_growth_background	ENSGALG00010007182	ENSGALG00010007182	-0.780002771	0.157183996	-4.962354872	6.96436E-07	3.65593E-05	negative
PRJNA729271	ed_Lushi_vs_Arbor_Acr	breed_growth_background	ENSGALG00010007183	VOPP1	-0.068264889	0.171765525	-0.397430679	0.691049896	0.800381002	negative
PRJNA729271	ed_Lushi_vs_Arbor_Acr	breed_growth_background	ENSGALG00010007184	VGLL3	-0.001625691	0.050137739	-0.032424502	0.974133523	0.984850683	negative
PRJNA729271	ed_Lushi_vs_Arbor_Acr	breed_growth_background	ENSGALG00010007187	ENPP6	-0.505701812	0.14988889	-3.373844537	0.000741262	0.006583698	negative
PRJNA729271	ed_Lushi_vs_Arbor_Acr	breed_growth_background	ENSGALG00010007188	MAB21L1	0.299304004	0.118887989	2.517529376	0.011818111	0.04707434	positive
PRJNA729271	ed_Lushi_vs_Arbor_Acr	breed_growth_background	ENSGALG00010007190	ENSGALG00010007190	-0.0181656	0.08034001	-0.226109006	0.82111664	0.890152517	negative
PRJNA729271	ed_Lushi_vs_Arbor_Acr	breed_growth_background	ENSGALG00010007201	ENSGALG00010007201	0.028246381	0.137035456	0.206124618	0.83669357	0.900861581	positive
PRJNA729271	ed_Lushi_vs_Arbor_Acr	breed_growth_background	ENSGALG00010007203	SLC30A6	0.124362549	0.056493813	2.201348121	0.027711389	0.085844662	positive
PRJNA729271	ed_Lushi_vs_Arbor_Acr	breed_growth_background	ENSGALG00010007204	LANCL2	-0.159578479	0.047200656	-3.380852983	0.000722612	0.00646553	negative
PRJNA729271	ed_Lushi_vs_Arbor_Acr	breed_growth_background	ENSGALG00010007207	EPM2A	0.189773637	0.055005523	3.450083308	0.000560414	0.005378874	positive
PRJNA729271	ed_Lushi_vs_Arbor_Acr	breed_growth_background	ENSGALG00010007214	MTHFSD	0.240862041	0.075741854	3.180038884	0.001472553	0.010791029	positive
PRJNA729271	ed_Lushi_vs_Arbor_Acr	breed_growth_background	ENSGALG00010007217	MSH3	-0.146465904	0.076967924	-1.902947317	0.05704742	0.145504549	negative
PRJNA729271	ed_Lushi_vs_Arbor_Acr	breed_growth_background	ENSGALG00010007223	FOXF1	-0.062267732	0.27128181	-0.229531543	0.818455807	0.888320249	negative
PRJNA729271	ed_Lushi_vs_Arbor_Acr	breed_growth_background	ENSGALG00010007224	DHFR	-0.455356173	0.117625009	-3.871253045	0.000108277	0.001689429	negative
PRJNA729271	ed_Lushi_vs_Arbor_Acr	breed_growth_background	ENSGALG00010007226	ENSGALG00010007226	-1.755521855	0.81260893	-2.16035265	0.03074538	0.09263236	negative
PRJNA729271	ed_Lushi_vs_Arbor_Acr	breed_growth_background	ENSGALG00010007230	ENSGALG00010007230	0.522591545	0.188270334	2.775750883	0.005507439	0.027283131	positive
PRJNA729271	ed_Lushi_vs_Arbor_Acr	breed_growth_background	ENSGALG00010007243	BEAN1	0.27624922	0.101868001	2.711835092	0.006691187	0.031373036	positive
PRJNA729271	ed_Lushi_vs_Arbor_Acr	breed_growth_background	ENSGALG00010007251	SPAST	-0.127584425	0.034530739	-3.69480725	0.000220054	0.002790619	negative
PRJNA729271	ed_Lushi_vs_Arbor_Acr	breed_growth_background	ENSGALG00010007252	NFATC1	0.04577718	0.082056308	0.557875212	0.576929599	0.714446351	positive
PRJNA729271	ed_Lushi_vs_Arbor_Acr	breed_growth_background	ENSGALG00010007259	ANKRD34B	-0.025889332	0.05233315	-0.494702347	0.620810245	0.748203583	negative
PRJNA729271	ed_Lushi_vs_Arbor_Acr	breed_growth_background	ENSGALG00010007265	CD200R1A	0.563919097	0.162883006	3.462111313	0.000535955	0.005238486	positive
PRJNA729271	ed_Lushi_vs_Arbor_Acr	breed_growth_background	ENSGALG00010007270	CDH5	-0.287411206	0.057981293	-4.956964396	7.16031E-07	3.70674E-05	negative
PRJNA729271	ed_Lushi_vs_Arbor_Acr	breed_growth_background	ENSGALG00010007271	CADM2	-0.037488132	0.175346815	-0.213794198	0.83070755	0.896916643	negative
PRJNA729271	ed_Lushi_vs_Arbor_Acr	breed_growth_background	ENSGALG00010007275	DPY30	0.068828981	0.054045996	1.273526013	0.202831448	0.352095783	positive
PRJNA729271	ed_Lushi_vs_Arbor_Acr	breed_growth_background	ENSGALG00010007277	FAM151B	-0.344281826	0.102691578	-3.352580947	0.000800618	0.006961585	negative
PRJNA729271	ed_Lushi_vs_Arbor_Acr	breed_growth_background	ENSGALG00010007283	STOX2	0.077279023	0.066102957	1.169070594	0.24237519	0.398144752	positive
PRJNA729271	ed_Lushi_vs_Arbor_Acr	breed_growth_background	ENSGALG00010007284	ENSGALG00010007284	-0.02197457	0.044408738	-0.494825367	0.620723397	0.74816112	negative
PRJNA729271	ed_Lushi_vs_Arbor_Acr	breed_growth_background	ENSGALG00010007287	ENSGALG00010007287	-0.116406	0.331642073	-0.350998892	0.72558918	0.824192339	negative
PRJNA729271	ed_Lushi_vs_Arbor_Acr	breed_growth_background	ENSGALG00010007294	TRAPPC11	0.188810945	0.034886117	5.412208701	6.22521E-08	5.68292E-06	positive
PRJNA729271	ed_Lushi_vs_Arbor_Acr	breed_growth_background	ENSGALG00010007300	CCDC125	-0.183885942	0.147088544	-1.250171744	0.211236816	0.361283499	negative
PRJNA729271	ed_Lushi_vs_Arbor_Acr	breed_growth_background	ENSGALG00010007302	CDH11	-0.258352289	0.093851802	-2.75276856	0.005909366	0.028695563	negative
PRJNA729271	ed_Lushi_vs_Arbor_Acr	breed_growth_background	ENSGALG00010007304	F5	0.15318842	0.190177434	0.805502612	0.4205297	0.579688731	positive
PRJNA729271	ed_Lushi_vs_Arbor_Acr	breed_growth_background	ENSGALG00010007318	RMDN2	-0.013060913	0.066130063	-0.197503412	0.843433611	0.90506146	negative
PRJNA729271	ed_Lushi_vs_Arbor_Acr	breed_growth_background	ENSGALG00010007319	ATP9B	-0.060340334	0.054775022	-1.101603101	0.27063426	0.429239131	negative
PRJNA729271	ed_Lushi_vs_Arbor_Acr	breed_growth_background	ENSGALG00010007320	ENSGALG00010007320	0.710453342	0.161869192	4.389058423	1.13842E-05	0.00032104	positive
PRJNA729271	ed_Lushi_vs_Arbor_Acr	breed_growth_background	ENSGALG00010007321	CD200	-0.360373639	0.078231481	-4.606504121	4.09495E-06	0.000148021	negative
PRJNA729271	ed_Lushi_vs_Arbor_Acr	breed_growth_background	ENSGALG00010007322	AK6	0.011612497	0.058834398	0.197375979	0.843533326	0.905101455	positive
PRJNA729271	ed_Lushi_vs_Arbor_Acr	breed_growth_background	ENSGALG00010007324	ENSGALG00010007324	0.073661502	0.076462266	0.963370648	0.335361551	0.49959565	positive
PRJNA729271	ed_Lushi_vs_Arbor_Acr	breed_growth_background	ENSGALG00010007326	RWDD4	-0.07444537	0.062052408	-1.199717675	0.230249006	0.384101663	negative
PRJNA729271	ed_Lushi_vs_Arbor_Acr	breed_growth_background	ENSGALG00010007328	NOVA1	-0.187673961	0.073946106	-2.537983012	0.01114934	0.045218153	negative
PRJNA729271	ed_Lushi_vs_Arbor_Acr	breed_growth_background	ENSGALG00010007330	MMP13	-0.097507081	0.38134499	-0.25569257	0.798188233	0.875036941	negative
PRJNA729271	ed_Lushi_vs_Arbor_Acr	breed_growth_background	ENSGALG00010007333	QTRT2	-0.054525011	0.041795377	-1.304570398	0.192039174	0.338968317	negative

PRJNA729271	ed_Lushi_vs_Arbor_Acr	breed_growth_background	ENSGALG00010007337	PRORP	-0.12934914	0.055169442	-2.344579466	0.019048551	0.066125064	negative
PRJNA729271	ed_Lushi_vs_Arbor_Acr	breed_growth_background	ENSGALG00010007339	ENSGALG00010007339	0.646768211	0.119717289	5.4024629	6.57321E-08	5.91793E-06	positive
PRJNA729271	ed_Lushi_vs_Arbor_Acr	breed_growth_background	ENSGALG00010007344	RAD17	0.055415594	0.058236235	0.951565529	0.34131737	0.505248701	positive
PRJNA729271	ed_Lushi_vs_Arbor_Acr	breed_growth_background	ENSGALG00010007353	ING2	0.062221985	0.070525443	0.882262933	0.377634636	0.539930366	positive
PRJNA729271	ed_Lushi_vs_Arbor_Acr	breed_growth_background	ENSGALG00010007357	NOX4	0.238024212	0.063327021	3.758651657	0.000170831	0.002325274	positive
PRJNA729271	ed_Lushi_vs_Arbor_Acr	breed_growth_background	ENSGALG00010007360	ROBO1	-0.634637735	0.1004864	-6.315657971	2.69014E-10	6.84099E-08	negative
PRJNA729271	ed_Lushi_vs_Arbor_Acr	breed_growth_background	ENSGALG00010007365	MEMO1	0.039754424	0.046489464	0.855127603	0.39248051	0.553944011	positive
PRJNA729271	ed_Lushi_vs_Arbor_Acr	breed_growth_background	ENSGALG00010007369	ENSGALG00010007369	0.316671536	0.113949896	2.779041901	0.005451949	0.027119425	positive
PRJNA729271	ed_Lushi_vs_Arbor_Acr	breed_growth_background	ENSGALG00010007374	CMIP	-0.112675784	0.072428311	-1.555687026	0.119782533	0.244043896	negative
PRJNA729271	ed_Lushi_vs_Arbor_Acr	breed_growth_background	ENSGALG00010007375	CHORDC1	-0.074189904	0.04054368	-1.829875941	0.067268492	0.163710005	negative
PRJNA729271	ed_Lushi_vs_Arbor_Acr	breed_growth_background	ENSGALG00010007376	CDKN2AIP	0.015807832	0.033402571	0.473251973	0.636033422	0.760731451	positive
PRJNA729271	ed_Lushi_vs_Arbor_Acr	breed_growth_background	ENSGALG00010007381	EGFR	-0.150413577	0.052607983	-2.859139791	0.004247915	0.022537897	negative
PRJNA729271	ed_Lushi_vs_Arbor_Acr	breed_growth_background	ENSGALG00010007382	EIF2AK2	-0.250924744	0.137941441	-1.819067149	0.068901186	0.166088565	negative
PRJNA729271	ed_Lushi_vs_Arbor_Acr	breed_growth_background	ENSGALG00010007387	ENSGALG00010007387	0.5500574	0.292328079	1.881644079	0.059884357	0.150583962	positive
PRJNA729271	ed_Lushi_vs_Arbor_Acr	breed_growth_background	ENSGALG00010007388	CYP1B1	0.074162798	0.11099721	0.668150115	0.504037775	0.654597749	positive
PRJNA729271	ed_Lushi_vs_Arbor_Acr	breed_growth_background	ENSGALG00010007391	NANP	-0.072464578	0.074674452	-0.970406565	0.331843877	0.49619115	negative
PRJNA729271	ed_Lushi_vs_Arbor_Acr	breed_growth_background	ENSGALG00010007392	NAALAD2	-0.210793225	0.106406092	-1.9810259	0.047588369	0.12776318	negative
PRJNA729271	ed_Lushi_vs_Arbor_Acr	breed_growth_background	ENSGALG00010007393	PLCG2	0.040523032	0.069504253	0.583029534	0.559873418	0.700869263	positive
PRJNA729271	ed_Lushi_vs_Arbor_Acr	breed_growth_background	ENSGALG00010007399	WWC2	0.224012624	0.059541318	3.762305435	0.000168354	0.002314101	positive
PRJNA729271	ed_Lushi_vs_Arbor_Acr	breed_growth_background	ENSGALG00010007401	SEC61G	-0.16704859	0.117896873	-1.416904334	0.156510899	0.293901475	negative
PRJNA729271	ed_Lushi_vs_Arbor_Acr	breed_growth_background	ENSGALG00010007403	SDR42E1	0.064013771	0.127977098	0.500197085	0.616936311	0.745269758	positive
PRJNA729271	ed_Lushi_vs_Arbor_Acr	breed_growth_background	ENSGALG00010007404	FOLH1	0.364436892	0.091085074	4.001060502	6.30592E-05	0.0011372	positive
PRJNA729271	ed_Lushi_vs_Arbor_Acr	breed_growth_background	ENSGALG00010007408	HCLS1	0.44410038	0.132710444	3.346386067	0.000818723	0.007065766	positive
PRJNA729271	ed_Lushi_vs_Arbor_Acr	breed_growth_background	ENSGALG00010007411	ENSGALG00010007411	-0.01488998	0.310335684	-0.047980238	0.961731992	0.978276808	negative
PRJNA729271	ed_Lushi_vs_Arbor_Acr	breed_growth_background	ENSGALG00010007413	GPATCH11	0.205749702	0.054339507	3.786374083	0.000152861	0.002168851	positive
PRJNA729271	ed_Lushi_vs_Arbor_Acr	breed_growth_background	ENSGALG00010007414	ROBO2	-0.43188598	0.129175674	-3.343400247	0.000827584	0.007110751	negative
PRJNA729271	ed_Lushi_vs_Arbor_Acr	breed_growth_background	ENSGALG00010007416	DCTD	-0.439337062	0.135760059	-3.236128983	0.001211626	0.009416902	negative
PRJNA729271	ed_Lushi_vs_Arbor_Acr	breed_growth_background	ENSGALG00010007418	NME6	0.154723888	0.078487654	1.971314991	0.048687858	0.130112555	positive
PRJNA729271	ed_Lushi_vs_Arbor_Acr	breed_growth_background	ENSGALG00010007419	RABL2A	0.021361299	0.086579533	0.246724577	0.805121385	0.879909106	positive
PRJNA729271	ed_Lushi_vs_Arbor_Acr	breed_growth_background	ENSGALG00010007423	HEATR5B	-0.057932359	0.036160431	-1.602092576	0.109135139	0.229196442	negative
PRJNA729271	ed_Lushi_vs_Arbor_Acr	breed_growth_background	ENSGALG00010007425	ARSA	0.119160431	0.071082667	1.676364115	0.093666893	0.205276931	positive
PRJNA729271	ed_Lushi_vs_Arbor_Acr	breed_growth_background	ENSGALG00010007429	C21orf91	0.003354793	0.111885321	0.029984211	0.976079645	0.985805076	positive
PRJNA729271	ed_Lushi_vs_Arbor_Acr	breed_growth_background	ENSGALG00010007430	MEF2C	-0.022846762	0.078563423	-0.290806599	0.771199238	0.857315205	negative
PRJNA729271	ed_Lushi_vs_Arbor_Acr	breed_growth_background	ENSGALG00010007432	NAT	0.116412229	0.101282757	1.149378556	0.250399918	0.406304479	positive
PRJNA729271	ed_Lushi_vs_Arbor_Acr	breed_growth_background	ENSGALG00010007433	RNF144B	0.449365601	0.141163147	3.183306768	0.001456033	0.010693382	positive
PRJNA729271	ed_Lushi_vs_Arbor_Acr	breed_growth_background	ENSGALG00010007434	ENSGALG00010007434	0.438225507	0.134712045	3.253053642	0.001141719	0.008991697	positive
PRJNA729271	ed_Lushi_vs_Arbor_Acr	breed_growth_background	ENSGALG00010007436	PNAT3	-0.025605613	0.141921015	-0.180421577	0.856821616	0.913895307	negative
PRJNA729271	ed_Lushi_vs_Arbor_Acr	breed_growth_background	ENSGALG00010007437	TUSC1	0.080390016	0.074607486	1.077506026	0.28125426	0.441216963	positive
PRJNA729271	ed_Lushi_vs_Arbor_Acr	breed_growth_background	ENSGALG00010007439	HSD17B2	0.173782015	0.196886636	0.882650133	0.377425333	0.539749119	positive
PRJNA729271	ed_Lushi_vs_Arbor_Acr	breed_growth_background	ENSGALG00010007441	STRN	0.13155162	0.072477963	1.815056801	0.069515181	0.1671293	positive
PRJNA729271	ed_Lushi_vs_Arbor_Acr	breed_growth_background	ENSGALG00010007455	TRPC6	-0.084350271	0.111434393	-0.756950069	0.449079777	0.605470316	negative
PRJNA729271	ed_Lushi_vs_Arbor_Acr	breed_growth_background	ENSGALG00010007457	SHANK3	0.066818188	0.096279364	0.69400321	0.487680196	0.639432333	positive
PRJNA729271	ed_Lushi_vs_Arbor_Acr	breed_growth_background	ENSGALG00010007465	PNAT10	0.153828378	0.106488862	1.444548979	0.148584613	0.283563468	positive
PRJNA729271	ed_Lushi_vs_Arbor_Acr	breed_growth_background	ENSGALG00010007467	CHODL	-0.37917164	0.178019459	-2.129944912	0.033176162	0.097885129	negative
PRJNA729271	ed_Lushi_vs_Arbor_Acr	breed_growth_background	ENSGALG00010007479	NFX1	-0.030902935	0.052418408	-0.589543567	0.555496695	0.697196692	negative
PRJNA729271	ed_Lushi_vs_Arbor_Acr	breed_growth_background	ENSGALG00010007480	TENM3	0.133867187	0.05692686	2.351564562	0.018694645	0.065170728	positive
PRJNA729271	ed_Lushi_vs_Arbor_Acr	breed_growth_background	ENSGALG00010007485	CTSC	-0.248283152	0.083362356	-2.97836054	0.002897949	0.017291542	negative
PRJNA729271	ed_Lushi_vs_Arbor_Acr	breed_growth_background	ENSGALG00010007500	MPHOSPH6	-0.016131859	0.06233899	-0.258776393	0.795807774	0.873418163	negative
PRJNA729271	ed_Lushi_vs_Arbor_Acr	breed_growth_background	ENSGALG00010007506	RAB38	0.964631372	0.316208207	3.050620924	0.002283687	0.014627373	positive
PRJNA729271	ed_Lushi_vs_Arbor_Acr	breed_growth_background	ENSGALG00010007513	GALNT12	0.155142299	0.070320398	2.206220449	0.027368568	0.085068816	positive
PRJNA729271	ed_Lushi_vs_Arbor_Acr	breed_growth_background	ENSGALG00010007514	VIT	0.779401279	0.211262653	3.689252546	0.000224914	0.00282262	positive
PRJNA729271	ed_Lushi_vs_Arbor_Acr	breed_growth_background	ENSGALG00010007521	SLC12A7	-0.14401753	0.074318363	-1.937845825	0.052642035	0.137510596	negative
PRJNA729271	ed_Lushi_vs_Arbor_Acr	breed_growth_background	ENSGALG00010007529	CDH13	-0.178551271	0.071707235	-2.490003548	0.012774182	0.049654537	negative
PRJNA729271	ed_Lushi_vs_Arbor_Acr	breed_growth_background	ENSGALG00010007532	DCUN1D5	-0.66231416	0.142472648	-4.648710972	3.34016E-06	0.000123509	negative
PRJNA729271	ed_Lushi_vs_Arbor_Acr	breed_growth_background	ENSGALG00010007536	GABBR2	0.296381553	0.112142553	2.642900005	0.008219931	0.036301721	positive
PRJNA729271	ed_Lushi_vs_Arbor_Acr	breed_growth_background	ENSGALG00010007539	RPS3A	-0.149429563	0.093734296	-1.594182375	0.110895193	0.231617024	negative
PRJNA729271	ed_Lushi_vs_Arbor_Acr	breed_growth_background	ENSGALG00010007542	MRPL36	-0.175775976	0.058813293	-2.988711714	0.002801563	0.016884434	negative
PRJNA729271	ed_Lushi_vs_Arbor_Acr	breed_growth_background	ENSGALG00010007544	MLYCD	0.124602743	0.055969855	2.226247371	0.025997617	0.082178344	positive
PRJNA729271	ed_Lushi_vs_Arbor_Acr	breed_growth_background	ENSGALG00010007547	ENSGALG00010007547	0.579803305	0.32139831	1.804002346	0.071230939	0.169865179	positive
PRJNA729271	ed_Lushi_vs_Arbor_Acr	breed_growth_background	ENSGALG00010007548	HSBP1	0.071945945	0.047098156	1.527574548	0.126618205	0.253288833	positive
PRJNA729271	ed_Lushi_vs_Arbor_Acr	breed_growth_background	ENSGALG00010007549	SH3D19	-0.321597481	0.084530393	-3.804518956	0.00014208	0.002054462	negative
PRJNA729271	ed_Lushi_vs_Arbor_Acr	breed_growth_background	ENSGALG00010007551	FEZ2	0.181996019	0.059820318	3.042377961	0.00234717	0.014954824	positive
PRJNA729271	ed_Lushi_vs_Arbor_Acr	breed_growth_background	ENSGALG00010007555	NDUFS6	0.0811824	0.069119918	1.174515277	0.240188697	0.39594395	positive
PRJNA729271	ed_Lushi_vs_Arbor_Acr	breed_growth_background	ENSGALG00010007564	IRX4	0.256279182	0.422184491	0.607031256	0.54383017	0.688250208	positive
PRJNA729271	ed_Lushi_vs_Arbor_Acr	breed_growth_background	ENSGALG00010007566	FHIP1A	-0.003635277	0.09390082	-0.038714003	0.969118409	0.982402359	negative
PRJNA729271	ed_Lushi_vs_Arbor_Acr	breed_growth_background	ENSGALG00010007567	TRIP13	-0.17780871	0.077818359	-2.284919796	0.022317531	0.073890501	negative
PRJNA729271	ed_Lushi_vs_Arbor_Acr	breed_growth_background	ENSGALG00010007569	GPR89A	0.01383955	0.044625763	0.310124673	0.75646615	0.845997751	positive
PRJNA729271	ed_Lushi_vs_Arbor_Acr	breed_growth_background	ENSGALG00010007574	ENSGALG00010007574	0.04371104	0.101572515	0.430343192	0.666946012	0.784145344	positive
PRJNA729271	ed_Lushi_vs_Arbor_Acr	breed_growth_background	ENSGALG00010007575	MMP27	-0.332732147	0.192588671	-1.727682868	0.084045097	0.190587246	negative
PRJNA729271	ed_Lushi_vs_Arbor_Acr	breed_growth_background	ENSGALG00010007577	WFDC1	-0.252930072	0.063939491	-3.955772385	7.62878E-05	0.001307082	negative
PRJNA729271	ed_Lushi_vs_Arbor_Acr	breed_growth_background	ENSGALG00010007579	GATB	-0.1061951	0.095395008	-1.113214445	0.26561634	0.423834088	negative
PRJNA729271	ed_Lushi_vs_Arbor_Acr	breed_growth_background	ENSGALG00010007580	BRD9	-0.03688777	0.05565303	-0.662816912	0.507447821	0.656783633	negative
PRJNA729271	ed_Lushi_vs_Arbor_Acr	breed_growth_background	ENSGALG00010007581	LPCAT1	-0.277274374	0.064157223	-4.3217951	1.54765E-05	0.000399878	negative
PRJNA729271	ed_Lushi_vs_Arbor_Acr	breed_growth_background	ENSGALG00010007584	ELP2	0.1064521	0.063263631	1.682674529	0.092438115	0.203537973	positive
PRJNA729271	ed_Lushi_vs_Arbor_Acr	breed_growth_background	ENSGALG00010007585	KCNG4	1.122224938	0.388216469	2.890719555	0.003843609	0.021039698	positive
PRJNA729271	ed_Lushi_vs_Arbor_Acr	breed_growth_background	ENSGALG00010007586	MMP7	0.429791131	0.865011622	0.496861684	0.619286593	0.747259049	positive
PRJNA729271	ed_Lushi_vs_Arbor_Acr	breed_growth_background	ENSGALG00010007588	TTC27	-0.05578661	0.056343271	-0.99012019	0.322115376	0.485854566	negative
PRJNA729271	ed_Lushi_vs_Arbor_Acr	breed_growth_background	ENSGALG00010007590	FBXW7	0.126023836	0.075098609	1.678111442	0.093325345	0.204869133	positive
PRJNA729271	ed_Lushi_vs_Arbor_Acr	breed_growth_background	ENSGALG00010007593	SEC61B	-0.063812711	0.06412773	-0.995087631	0.319693646	0.482955643	negative
PRJNA729271	ed_Lushi_vs_Arbor_Acr	breed_growth_background	ENSGALG00010007595	ATP2C2	-0.462436919	0.085512351	-5.407837711	6.37902E-08	5.77899E-06	negative
PRJNA729271	ed_Lushi_vs_Arbor_Acr	breed_growth_background	ENSGALG00010007596	ENSGALG00010007596	0.993726565	0.279259557	3.558433501	0.000373073	0.004050709	positive
PRJNA729271	ed_Lushi_vs_Arbor_Acr	breed_growth_background	ENSGALG00010007597	SMKR1	-0.718630168	0.43264249	-1.661025407	0.096708344	0.20978561	negative
PRJNA729271	ed_Lushi_vs_Arbor_Acr	breed_growth_background	ENSGALG00010007601	TMEM154	0.357932508	0.174498798	2.051203287	0.040247154	0.112857597	positive
PRJNA729271	ed_Lushi_vs_Arbor_Acr	breed_growth_background	ENSGALG00010007603	ALG2	0.030211161	0.057352288	0.526764704	0.598356997	0.731420532	positive
PRJNA729271	ed_Lushi_vs_Arbor_Acr	breed_growth_background	ENSGALG00010007604	ENSGALG00010007604	0.125657813	0.307528181	0.408605848	0.682828938	0.79524389	positive
PRJNA729271	ed_Lushi_vs_Arbor_Acr	breed_growth_background	ENSGALG00010007610	TLDC1	-0.162314389	0.085510409	-1.898182816	0.057672004	0.146581745	negative
PRJNA729271	ed_Lushi_vs_Arbor_Acr	breed_growth_background	ENSGALG00010007614	BIRC6	0.071468663	0.047110422	1.517045688	0.129255149	0.256720521	positive
PRJNA729271	ed_Lushi_vs_Arbor_Acr	breed_growth_background	ENSGALG00010007624	TPPP	-0.570334455	0.132064495	-4.31860549	1.57018E-05	0.000402828	negative
PRJNA729271	ed_Lushi_vs_Arbor_Acr	breed_growth_background	ENSGALG00010007626	DLK2	-0.403903679	0.207346918	-1.947960856	0.051419647	0.135219119	negative
PRJNA729271	ed_Lushi_vs_Arbor_Acr	breed_growth_background	ENSGALG00010007633	ENSGALG00010007633	-0.026319244	0.0559545	-0.470368675	0.63809164	0.762563964	negative
PRJNA729271	ed_Lushi_vs_Arbor_Acr	breed_growth_background	ENSGALG00010007640	TMEM123	-0.103121465	0.117535446	-0.877364823	0.380288514	0.542032629	negative
PRJNA729271	ed_Lushi_vs_Arbor_Acr	breed_growth_background	ENSGALG00010007642	ZNF318	0.14829954	0.057294644	2.588366533	0.009643231	0.040775564	positive
PRJNA729271	ed_Lushi_vs_Arbor_Acr	breed_growth_background	ENSGALG00010007643	COTL1	-0.288681137	0.08773536	-3.290362494	0.001000584	0.008154066	negative
PRJNA729271	ed_Lushi_vs_Arbor_Acr	breed_growth_background	ENSGALG00010007650	KLHL36	-0.12783279	0.047892344	-2.669169633	0.007603904	0.034357414	negative
PRJNA729271	ed_Lushi_vs_Arbor_Acr	breed_growth_background	ENSGALG00010007651	CTNNAL1	0.108220608	0.074282535	1.456878221	0.145149999	0.278964364	positive
PRJNA729271	ed_Lushi_vs_Arbor_Acr	breed_growth_background	ENSGALG00010007658	BIRC2	0.187098043	0.063835678	2.930932168	0.003379466	0.01930077	positive
PRJNA729271	ed_Lushi_vs_Arbor_Acr	breed_growth_background	ENSGALG00010007660	FAM98A	0.016286426	0.05218583	0.312085207	0.754975769	0.845048167	positive
PRJNA729271	ed_Lushi_vs_Arbor_Acr	breed_growth_background	ENSGALG00010007663	ARFIP1	-0.09334962	0.059287027	-1.574537043	0.115363345	0.237966654	negative
PRJNA729271	ed_Lushi_vs_Arbor_Acr	breed_growth_background	ENSGALG00010007671	FAM206A	0.08675423	0.055390823	1.566220261	0.117297061	0.240673499	positive
PRJNA729271	ed_Lushi_vs_Arbor_Acr	breed_growth_background	ENSGALG00010007673	FHDC1	0.036700409	0.182418384	0.201188105	0.840551492	0.903266111	positive
PRJNA729271	ed_Lushi_vs_Arbor_Acr	breed_growth_background	ENSGALG00010007678	CDH8	-0.088656551	0.153167292	-0.578821692	0.562709493	0.703265572	negative
PRJNA729271	ed_Lushi_vs_Arbor_Acr	breed_growth_background	ENSGALG00010007679	TMEM135	-0.224347895	0.075713849	-2.963102491	0.003045551	0.017901569	negative
PRJNA729271	ed_Lushi_vs_Arbor_Acr	breed_growth_background	ENSGALG00010007680	CHMP2B	-0.049257049	0.067567241	-0.729007841	0.465996866	0.621000697	negative
PRJNA729271	ed_Lushi_vs_Arbor_Acr	breed_growth_background	ENSGALG00010007695	RASGRP3	0.126715764	0.07616183	1.663769933	0.096158415	0.209014842	positive
PRJNA729271	ed_Lushi_vs_Arbor_Acr	breed_growth_background	ENSGALG00010007698	ADAMTS18	-0.5626352	0.216549554	-2.598182214	0.009371875	0.039966262	negative
PRJNA729271	ed_Lushi_vs_Arbor_Acr	breed_growth_background	ENSGALG00010007699	SLC39A6	-0.119085072	0.058157323	-2.047646753	0.040595605	0.113597162	negative
PRJNA729271	ed_Lushi_vs_Arbor_Acr	breed_growth_background	ENSGALG00010007707	ENSGALG00010007707	0.039542336	0.318340958	0.124213787	0.901146007	0.942711173	positive
PRJNA729271	ed_Lushi_vs_Arbor_Acr	breed_growth_background	ENSGALG00010007713	SCLT1	-0.255540941	0.115316047	-2.216005045	0.026691157	0.083705823	negative
PRJNA729271	ed_Lushi_vs_Arbor_Acr	breed_growth_background	ENSGALG00010007719	LTBP1	-0.164985832	0.102326695	-1.612343998	0.106887104	0.225652283	negative
PRJNA729271	ed_Lushi_vs_Arbor_Acr	breed_growth_background	ENSGALG00010007724	RBM11	-0.265517415	0.185693406	-1.429869918	0.152754357	0.289245514	negative
PRJNA729271	ed_Lushi_vs_Arbor_Acr	breed_growth_background	ENSGALG00010007730	COG2	0.050648614	0.035561277	1.424263069	0.154370334	0.290899375	positive
PRJNA729271	ed_Lushi_vs_Arbor_Acr	breed_growth_background	ENSGALG00010007731	ENSGALG00010007731	-0.151246299	0.14622874	-1.034313086	0.300989821	0.463393251	negative
PRJNA729271	ed_Lushi_vs_Arbor_Acr	breed_growth_background	ENSGALG00010007741	ENSGALG00010007741	-0.314505285	0.112299031	-2.800605517	0.005100683	0.025987486	negative
PRJNA729271	ed_Lushi_vs_Arbor_Acr	breed_growth_background	ENSGALG00010007748	CSGALNACT1	-0.143709463	0.148442025	-0.968118448	0.332985217	0.497404676	negative
PRJNA729271	ed_Lushi_vs_Arbor_Acr	breed_growth_background	ENSGALG00010007751	HSPA13	0.073980228	0.054111351	1.367185021	0.171567319	0.314497727	positive
PRJNA729271	ed_Lushi_vs_Arbor_Acr	breed_growth_background	ENSGALG00010007753	PRSS23	-0.247252055	0.121705905	-2.031553483	0.04219888	0.117111385	negative
PRJNA729271	ed_Lushi_vs_Arbor_Acr	breed_growth_background	ENSGALG00010007758	DEK	-0.015740448	0.041282444	-0.381286727	0.702990502	0.808169717	negative
PRJNA729271	ed_Lushi_vs_Arbor_Acr	breed_growth_background	ENSGALG00010007759	AGT	-1.898095013	0.693153377	-2.738347783			negative
PRJNA729271	ed_Lushi_vs_Arbor_Acr	breed_growth_background	ENSGALG00010007761	ENSGALG00010007761	0.00671161	0.061907909	0.108412811	0.913668239	0.95134122	positive
PRJNA729271	ed_Lushi_vs_Arbor_Acr	breed_growth_background	ENSGALG00010007762	SH2D4A	-0.345160211	0.190855272	-1.808491887	0.070529978	0.168618182	negative
PRJNA729271	ed_Lushi_vs_Arbor_Acr	breed_growth_background	ENSGALG00010007763	FZD4	-0.26056923	0.053335564	-4.885468746	1.03183E-06	5.00214E-05	negative
PRJNA729271	ed_Lushi_vs_Arbor_Acr	breed_growth_background	ENSGALG00010007765	NUDT7	0.181656867	0.051898367	3.500242408	0.000464835	0.004711136	positive
PRJNA729271	ed_Lushi_vs_Arbor_Acr	breed_growth_background	ENSGALG00010007766	ENSGALG00010007766	-0.045935414	0.068511534	-0.670477089	0.502553707	0.653484116	negative
PRJNA729271	ed_Lushi_vs_Arbor_Acr	breed_growth_background	ENSGALG00010007767	VAT1L	-0.498678575	0.175418422	-2.84279478	0.004471986	0.023452037	negative
PRJNA729271	ed_Lushi_vs_Arbor_Acr	breed_growth_background	ENSGALG00010007770	ENSGALG00010007770	0.230621624	0.238796944	0.96576455	0.334162011	0.498525972	positive
PRJNA729271	ed_Lushi_vs_Arbor_Acr	breed_growth_background	ENSGALG00010007771	CAPN9	0.501598668	0.280574247	1.787757334	0.073815167	0.174361012	positive
PRJNA729271	ed_Lushi_vs_Arbor_Acr	breed_growth_background	ENSGALG00010007772	CLEC3A	0.310468477	0.356043015	0.871997103	0.383209958	0.544678206	positive
PRJNA729271	ed_Lushi_vs_Arbor_Acr	breed_growth_background	ENSGALG00010007774	ENSGALG00010007774	0.21977569	0.152182082	1.44416272	0.148693209	0.283630486	positive
PRJNA729271	ed_Lushi_vs_Arbor_Acr	breed_growth_background	ENSGALG00010007777	KIF13A	0.132544294	0.066769757	1.985094731	0.047133933	0.126801477	positive
PRJNA729271	ed_Lushi_vs_Arbor_Acr	breed_growth_background	ENSGALG00010007780	SIRT5	0.128121656	0.076799237	1.668267305	0.095262675	0.207862785	positive
PRJNA729271	ed_Lushi_vs_Arbor_Acr	breed_growth_background	ENSGALG00010007781	MBP	0.54606929	0.125423502	4.353803561	1.33796E-05	0.000358478	positive

PRJNA729271	ed_Lushi_vs_Arbor_Acr	breed_growth_background	ENSGALG00010007783	NOL7	-0.099515885	0.05972102	-1.666346039	0.095644512	0.208310848	negative
PRJNA729271	ed_Lushi_vs_Arbor_Acr	breed_growth_background	ENSGALG00010007784	INTS10	0.008376958	0.051285861	0.163338544	0.870251888	0.922876636	positive
PRJNA729271	ed_Lushi_vs_Arbor_Acr	breed_growth_background	ENSGALG00010007786	SAMSN1	-0.112153534	0.122289073	-0.917118196	0.359080707	0.522472882	negative
PRJNA729271	ed_Lushi_vs_Arbor_Acr	breed_growth_background	ENSGALG00010007787	C1orf198	-0.252793315	0.064515704	-3.918322193	8.91675E-05	0.001472076	negative
PRJNA729271	ed_Lushi_vs_Arbor_Acr	breed_growth_background	ENSGALG00010007788	PALM2AKAP2	0.260205312	0.090716821	2.868324859	0.004126516	0.022104157	positive
PRJNA729271	ed_Lushi_vs_Arbor_Acr	breed_growth_background	ENSGALG00010007789	WWOX	-0.053125465	0.061766106	-0.860107073	0.389730023	0.551450285	negative
PRJNA729271	ed_Lushi_vs_Arbor_Acr	breed_growth_background	ENSGALG00010007792	ENSGALG00010007792	0.043332179	0.12132545	0.35715655	0.720974611	0.821259488	positive
PRJNA729271	ed_Lushi_vs_Arbor_Acr	breed_growth_background	ENSGALG00010007795	ZNF236	0.061348467	0.062444294	0.982451129	0.325877648	0.489443956	positive
PRJNA729271	ed_Lushi_vs_Arbor_Acr	breed_growth_background	ENSGALG00010007796	MCUR1	-0.071175921	0.056164053	-1.267286049	0.205053027	0.354344734	negative
PRJNA729271	ed_Lushi_vs_Arbor_Acr	breed_growth_background	ENSGALG00010007798	HGSNAT	-0.006657243	0.052269032	-0.127364952	0.898651555	0.941317697	negative
PRJNA729271	ed_Lushi_vs_Arbor_Acr	breed_growth_background	ENSGALG00010007800	MAF	0.067859957	0.061182535	1.10913934	0.267370076	0.425741981	positive
PRJNA729271	ed_Lushi_vs_Arbor_Acr	breed_growth_background	ENSGALG00010007801	TTC13	0.082187563	0.086735045	0.947570422	0.34334819	0.507167228	positive
PRJNA729271	ed_Lushi_vs_Arbor_Acr	breed_growth_background	ENSGALG00010007802	ENSGALG00010007802	-0.058937635	0.361106719	-0.163213896	0.870350025	0.922876636	negative
PRJNA729271	ed_Lushi_vs_Arbor_Acr	breed_growth_background	ENSGALG00010007803	ME3	-0.255770991	0.094954895	-2.693605126	0.007068385	0.032618988	negative
PRJNA729271	ed_Lushi_vs_Arbor_Acr	breed_growth_background	ENSGALG00010007805	ENSGALG00010007805	-0.151617422	0.7037257	-0.215449602	0.829416801	0.895856671	negative
PRJNA729271	ed_Lushi_vs_Arbor_Acr	breed_growth_background	ENSGALG00010007806	DYNLRB2	-0.436906227	0.21510532	-2.031127014	0.042242112	0.117208922	negative
PRJNA729271	ed_Lushi_vs_Arbor_Acr	breed_growth_background	ENSGALG00010007809	ARV1	-0.007472366	0.065646662	-0.113827054	0.909374892	0.948586956	negative
PRJNA729271	ed_Lushi_vs_Arbor_Acr	breed_growth_background	ENSGALG00010007811	PRCP	-0.316057881	0.09790695	-3.228145509	0.001245956	0.009586056	negative
PRJNA729271	ed_Lushi_vs_Arbor_Acr	breed_growth_background	ENSGALG00010007812	POMK	0.114493717	0.06533763	1.752339602	0.07971543	0.18365933	positive
PRJNA729271	ed_Lushi_vs_Arbor_Acr	breed_growth_background	ENSGALG00010007817	CDYL2	0.026412582	0.076701676	0.344354695	0.730579556	0.827259641	positive
PRJNA729271	ed_Lushi_vs_Arbor_Acr	breed_growth_background	ENSGALG00010007818	ENSGALG00010007818	-0.056148493	0.119936643	-0.468151281	0.639676409	0.763702294	negative
PRJNA729271	ed_Lushi_vs_Arbor_Acr	breed_growth_background	ENSGALG00010007819	NRIP1	0.039736857	0.088963923	0.446662595	0.655118693	0.775238902	positive
PRJNA729271	ed_Lushi_vs_Arbor_Acr	breed_growth_background	ENSGALG00010007821	FAM89A	-0.170656749	0.068300797	-2.49860553	0.012468301	0.048845059	negative
PRJNA729271	ed_Lushi_vs_Arbor_Acr	breed_growth_background	ENSGALG00010007826	ENSGALG00010007826	-0.518822055	0.60989708	-0.850671486	0.394951867	0.556402693	negative
PRJNA729271	ed_Lushi_vs_Arbor_Acr	breed_growth_background	ENSGALG00010007830	SMIM19	0.037059156	0.069590618	0.532530916	0.594358335	0.728621792	positive
PRJNA729271	ed_Lushi_vs_Arbor_Acr	breed_growth_background	ENSGALG00010007835	GUCY1A1	-0.19000867	0.099247151	-1.9145	0.055556299	0.14295909	negative
PRJNA729271	ed_Lushi_vs_Arbor_Acr	breed_growth_background	ENSGALG00010007845	TFAP2B	0.27889669	0.562023323	0.496236861	0.619727307	0.747457756	positive
PRJNA729271	ed_Lushi_vs_Arbor_Acr	breed_growth_background	ENSGALG00010007846	ENSGALG00010007846	-0.147632658	0.080275017	-1.839085966	0.065902543	0.161306545	negative
PRJNA729271	ed_Lushi_vs_Arbor_Acr	breed_growth_background	ENSGALG00010007847	CMC2	0.366171778	0.063381567	5.77725976	7.5927E-09	1.07898E-06	positive
PRJNA729271	ed_Lushi_vs_Arbor_Acr	breed_growth_background	ENSGALG00010007849	CXADR	-0.088224015	0.172374217	-0.511816771	0.608779252	0.739545362	negative
PRJNA729271	ed_Lushi_vs_Arbor_Acr	breed_growth_background	ENSGALG00010007850	ENSGALG00010007850	0.090060714	0.157740794	0.570941173	0.56803952	0.707122367	positive
PRJNA729271	ed_Lushi_vs_Arbor_Acr	breed_growth_background	ENSGALG00010007851	AvBD7	1.103878394	0.483783698	2.281760211	0.022503501	0.074285641	positive
PRJNA729271	ed_Lushi_vs_Arbor_Acr	breed_growth_background	ENSGALG00010007852	SLC20A2	0.218081697	0.110877767	1.966865887	0.049198674	0.131113581	positive
PRJNA729271	ed_Lushi_vs_Arbor_Acr	breed_growth_background	ENSGALG00010007853	C1orf131	0.198719009	0.060621356	3.278036363	0.001045319	0.008436472	positive
PRJNA729271	ed_Lushi_vs_Arbor_Acr	breed_growth_background	ENSGALG00010007854	FAM181B	0.032802692	0.063001726	0.520663379	0.602601289	0.73493527	positive
PRJNA729271	ed_Lushi_vs_Arbor_Acr	breed_growth_background	ENSGALG00010007855	RNF182	0.062088823	0.126563906	0.490572902	0.623728556	0.750348196	positive
PRJNA729271	ed_Lushi_vs_Arbor_Acr	breed_growth_background	ENSGALG00010007856	PDCD2	0.167303987	0.069744489	2.398813011	0.016448312	0.05947076	positive
PRJNA729271	ed_Lushi_vs_Arbor_Acr	breed_growth_background	ENSGALG00010007857	ENSGALG00010007857	-0.510538569	0.183888338	-2.776350989	0.005497283	0.027269178	negative
PRJNA729271	ed_Lushi_vs_Arbor_Acr	breed_growth_background	ENSGALG00010007859	CENPN	-0.082807706	0.08861227	-0.934494808	0.350048713	0.513974483	negative
PRJNA729271	ed_Lushi_vs_Arbor_Acr	breed_growth_background	ENSGALG00010007860	DEFB4A	0.839411299	0.479818881	1.749433656	0.080216087	0.184390772	positive
PRJNA729271	ed_Lushi_vs_Arbor_Acr	breed_growth_background	ENSGALG00010007861	INTU	-0.044295156	0.098639741	-0.449059941	0.653388417	0.774016435	negative
PRJNA729271	ed_Lushi_vs_Arbor_Acr	breed_growth_background	ENSGALG00010007862	ATMIN	0.122874987	0.046165952	2.661593246	0.00777718	0.034944271	positive
PRJNA729271	ed_Lushi_vs_Arbor_Acr	breed_growth_background	ENSGALG00010007863	AvBD1	0.686507817	0.448918416	1.529248507	0.12620285	0.252829155	positive
PRJNA729271	ed_Lushi_vs_Arbor_Acr	breed_growth_background	ENSGALG00010007864	TYR	2.726976669	0.607006413	4.49250059	7.03917E-06	0.000223267	positive
PRJNA729271	ed_Lushi_vs_Arbor_Acr	breed_growth_background	ENSGALG00010007865	GABRR3	0.176783439	0.230347811	0.767463073	0.44280624	0.599857612	positive
PRJNA729271	ed_Lushi_vs_Arbor_Acr	breed_growth_background	ENSGALG00010007866	TBP	0.081324312	0.046758744	1.739232188	0.08199393	0.187065231	positive
PRJNA729271	ed_Lushi_vs_Arbor_Acr	breed_growth_background	ENSGALG00010007867	ZNF516	0.028113591	0.057611449	0.487986186	0.625559626	0.751989283	positive
PRJNA729271	ed_Lushi_vs_Arbor_Acr	breed_growth_background	ENSGALG00010007868	INHBA	0.063114302	0.163019094	0.387158956	0.698638533	0.805494842	positive
PRJNA729271	ed_Lushi_vs_Arbor_Acr	breed_growth_background	ENSGALG00010007869	GCSH	-0.084513536	0.067447657	-1.253024046	0.210196956	0.360106575	negative
PRJNA729271	ed_Lushi_vs_Arbor_Acr	breed_growth_background	ENSGALG00010007870	AvBD3	0.417334758	0.355049547	1.175426812	0.239824002	0.395432705	positive
PRJNA729271	ed_Lushi_vs_Arbor_Acr	breed_growth_background	ENSGALG00010007871	HSPA4L	0.040821282	0.047696892	0.855847835	0.392081952	0.553651037	positive
PRJNA729271	ed_Lushi_vs_Arbor_Acr	breed_growth_background	ENSGALG00010007872	KLHL2	-0.017907292	0.042375137	-0.422589606	0.672594712	0.788073749	negative
PRJNA729271	ed_Lushi_vs_Arbor_Acr	breed_growth_background	ENSGALG00010007873	ENSGALG00010007873	4.949964034	0.410131923	12.06919958	1.53621E-33	7.42246E-30	positive
PRJNA729271	ed_Lushi_vs_Arbor_Acr	breed_growth_background	ENSGALG00010007874	ENSGALG00010007874	0.341223545	0.324301614	1.052179611	0.292717152	0.454129843	positive
PRJNA729271	ed_Lushi_vs_Arbor_Acr	breed_growth_background	ENSGALG00010007875	AvBD4	0.734796058	0.69709791	1.054078699	0.291846888	0.453264827	positive
PRJNA729271	ed_Lushi_vs_Arbor_Acr	breed_growth_background	ENSGALG00010007877	MSMO1	-0.164056016	0.101057606	-1.62339108	0.10450583	0.221885456	negative
PRJNA729271	ed_Lushi_vs_Arbor_Acr	breed_growth_background	ENSGALG00010007878	PSMB1	0.060545412	0.065486757	0.924544364	0.355202965	0.518914228	positive
PRJNA729271	ed_Lushi_vs_Arbor_Acr	breed_growth_background	ENSGALG00010007879	PLK4	-0.34256542	0.105893685	-3.23499385	0.001216454	0.009444292	negative
PRJNA729271	ed_Lushi_vs_Arbor_Acr	breed_growth_background	ENSGALG00010007880	BCO1	0.162549484	0.151799045	1.070820204	0.284250281	0.444514815	positive
PRJNA729271	ed_Lushi_vs_Arbor_Acr	breed_growth_background	ENSGALG00010007881	AvBD14	0.212238701	0.431903005	0.491403621	0.623141004	0.749890315	positive
PRJNA729271	ed_Lushi_vs_Arbor_Acr	breed_growth_background	ENSGALG00010007882	CPE	-0.282771035	0.12168338	-2.323826271	0.020134807	0.068590841	negative
PRJNA729271	ed_Lushi_vs_Arbor_Acr	breed_growth_background	ENSGALG00010007883	DHTKD1	0.246454907	0.064486215	3.821823106	0.000132469	0.00195533	positive
PRJNA729271	ed_Lushi_vs_Arbor_Acr	breed_growth_background	ENSGALG00010007884	GLI3	-0.189669144	0.062092069	-3.054643632	0.002253281	0.014490376	negative
PRJNA729271	ed_Lushi_vs_Arbor_Acr	breed_growth_background	ENSGALG00010007885	USP38	0.187330301	0.055601618	3.369151947	0.000753999	0.006668217	positive
PRJNA729271	ed_Lushi_vs_Arbor_Acr	breed_growth_background	ENSGALG00010007886	GRM5	1.51093905	0.474039646	3.187368527	0.001435737	0.010596237	positive
PRJNA729271	ed_Lushi_vs_Arbor_Acr	breed_growth_background	ENSGALG00010007887	CDT1	-0.189496814	0.06852943	-2.765188838	0.005688985	0.027905868	negative
PRJNA729271	ed_Lushi_vs_Arbor_Acr	breed_growth_background	ENSGALG00010007889	DCAF6	-0.072093776	0.051791517	-1.391999687	0.163922493	0.303727027	negative
PRJNA729271	ed_Lushi_vs_Arbor_Acr	breed_growth_background	ENSGALG00010007890	ENSGALG00010007890	-0.232825832	0.067646282	-3.44181267	0.00057783	0.005481446	negative
PRJNA729271	ed_Lushi_vs_Arbor_Acr	breed_growth_background	ENSGALG00010007891	GDI1	-0.102564663	0.030139953	-3.402946993	0.000666632	0.006088741	negative
PRJNA729271	ed_Lushi_vs_Arbor_Acr	breed_growth_background	ENSGALG00010007892	ENSGALG00010007892	1.431186262	0.255026073	5.611921341	2.00092E-08	2.45791E-06	positive
PRJNA729271	ed_Lushi_vs_Arbor_Acr	breed_growth_background	ENSGALG00010007893	MFSD8	-0.025799619	0.080949696	-0.318711751	0.749945103	0.841105496	negative
PRJNA729271	ed_Lushi_vs_Arbor_Acr	breed_growth_background	ENSGALG00010007894	CD88	-0.051828182	0.139906122	-0.370449705	0.711047444	0.813852867	negative
PRJNA729271	ed_Lushi_vs_Arbor_Acr	breed_growth_background	ENSGALG00010007895	GOLGB1	-0.037671375	0.060168911	-0.626093675	0.5312535	0.676968746	negative
PRJNA729271	ed_Lushi_vs_Arbor_Acr	breed_growth_background	ENSGALG00010007896	TIMMDC1	-0.089379625	0.056539576	-1.580832965	0.113916235	0.235747917	negative
PRJNA729271	ed_Lushi_vs_Arbor_Acr	breed_growth_background	ENSGALG00010007897	EFHC1	0.155180393	0.214898305	0.722110829	0.470226341	0.624586688	positive
PRJNA729271	ed_Lushi_vs_Arbor_Acr	breed_growth_background	ENSGALG00010007898	GAB1	0.107570164	0.047984915	2.241749577	0.024977562	0.079993318	positive
PRJNA729271	ed_Lushi_vs_Arbor_Acr	breed_growth_background	ENSGALG00010007899	APRT	-0.221774056	0.083209634	-2.665244938	0.007693227	0.03467454	negative
PRJNA729271	ed_Lushi_vs_Arbor_Acr	breed_growth_background	ENSGALG00010007900	FHOD3	-0.329484258	0.106970694	-3.080135741	0.002069063	0.013589064	negative
PRJNA729271	ed_Lushi_vs_Arbor_Acr	breed_growth_background	ENSGALG00010007901	FSTL5	0.087605082	0.169688251	0.51627076	0.605665316	0.73704479	positive
PRJNA729271	ed_Lushi_vs_Arbor_Acr	breed_growth_background	ENSGALG00010007902	C7orf25	0.265495366	0.076796307	3.457137165	0.000545947	0.005289773	positive
PRJNA729271	ed_Lushi_vs_Arbor_Acr	breed_growth_background	ENSGALG00010007903	PSMA2	0.17840679	0.055672436	3.204580269	0.001352596	0.010153223	positive
PRJNA729271	ed_Lushi_vs_Arbor_Acr	breed_growth_background	ENSGALG00010007904	ENSGALG00010007904	0.162633194	0.067274932	2.417441204	0.015630059	0.057429076	positive
PRJNA729271	ed_Lushi_vs_Arbor_Acr	breed_growth_background	ENSGALG00010007905	MRPL32	-0.016023802	0.084581125	-0.189448914	0.84974099	0.909674716	negative
PRJNA729271	ed_Lushi_vs_Arbor_Acr	breed_growth_background	ENSGALG00010007906	NAF1	0.010179153	0.061764919	0.16480476	0.869097657	0.921985721	positive
PRJNA729271	ed_Lushi_vs_Arbor_Acr	breed_growth_background	ENSGALG00010007907	IRX2	0.549886474	0.470460243	1.16882666	0.242473477	0.398200184	positive
PRJNA729271	ed_Lushi_vs_Arbor_Acr	breed_growth_background	ENSGALG00010007908	IRX1	-0.033166573	0.39855446	-0.083217167	0.933678863	0.96379968	negative
PRJNA729271	ed_Lushi_vs_Arbor_Acr	breed_growth_background	ENSGALG00010007909	GALNS	-0.367786435	0.058464158	-6.290801848	3.1583E-10	7.86851E-08	negative
PRJNA729271	ed_Lushi_vs_Arbor_Acr	breed_growth_background	ENSGALG00010007910	ANKS6	0.007099183	0.079766649	0.088999386	0.929082398	0.96068726	positive
PRJNA729271	ed_Lushi_vs_Arbor_Acr	breed_growth_background	ENSGALG00010007911	IFI6	0.260673531	0.429758113	0.60655872	0.544143802	0.688371885	positive
PRJNA729271	ed_Lushi_vs_Arbor_Acr	breed_growth_background	ENSGALG00010007912	TRAPPC2L	0.103646044	0.062426811	1.660280933	0.09685795	0.209984442	positive
PRJNA729271	ed_Lushi_vs_Arbor_Acr	breed_growth_background	ENSGALG00010007914	PAQR8	-0.120476708	0.068228634	-1.765779264	0.077432889	0.179791712	negative
PRJNA729271	ed_Lushi_vs_Arbor_Acr	breed_growth_background	ENSGALG00010007915	HECW1	-0.280980929	0.073304538	-3.833063239	0.000126557	0.001891185	negative
PRJNA729271	ed_Lushi_vs_Arbor_Acr	breed_growth_background	ENSGALG00010007916	MCM3	-0.329653827	0.110586228	-2.980966363	0.002873404	0.017189428	negative
PRJNA729271	ed_Lushi_vs_Arbor_Acr	breed_growth_background	ENSGALG00010007917	POGLUT1	-0.073446331	0.045132056	-1.627365049	0.10365959	0.220702961	negative
PRJNA729271	ed_Lushi_vs_Arbor_Acr	breed_growth_background	ENSGALG00010007918	NPY1R	0.331420452	0.397145064	0.834507293	0.40399518	0.564806147	positive
PRJNA729271	ed_Lushi_vs_Arbor_Acr	breed_growth_background	ENSGALG00010007920	FAT3	-0.201278734	0.098553697	-2.042325544	0.04111925	0.114686074	negative
PRJNA729271	ed_Lushi_vs_Arbor_Acr	breed_growth_background	ENSGALG00010007921	FAM120B	-0.040774426	0.055109867	-0.739875235	0.459375702	0.614802205	negative
PRJNA729271	ed_Lushi_vs_Arbor_Acr	breed_growth_background	ENSGALG00010007922	IL17A	0.22971439	0.25776404	0.891180902	0.372832128	0.535387527	positive
PRJNA729271	ed_Lushi_vs_Arbor_Acr	breed_growth_background	ENSGALG00010007923	YAP1	-0.055247442	0.049374194	-1.118953784	0.263159856	0.421305734	negative
PRJNA729271	ed_Lushi_vs_Arbor_Acr	breed_growth_background	ENSGALG00010007924	ABHD18	-0.09098366	0.061984193	-1.467852603	0.142144265	0.275010828	negative
PRJNA729271	ed_Lushi_vs_Arbor_Acr	breed_growth_background	ENSGALG00010007925	STK17A	0.078109428	0.127426132	0.612978101	0.539890819	0.684926963	positive
PRJNA729271	ed_Lushi_vs_Arbor_Acr	breed_growth_background	ENSGALG00010007926	EIF3E	0.009321129	0.070248378	0.132688174	0.894439996	0.938194352	positive
PRJNA729271	ed_Lushi_vs_Arbor_Acr	breed_growth_background	ENSGALG00010007927	DLL1	-0.045058951	0.090551937	-0.49760339	0.618763614	0.746807523	negative
PRJNA729271	ed_Lushi_vs_Arbor_Acr	breed_growth_background	ENSGALG00010007928	C11orf70	-0.009169763	0.396465443	-0.023128784	0.981547546	0.989068549	negative
PRJNA729271	ed_Lushi_vs_Arbor_Acr	breed_growth_background	ENSGALG00010007929	LRRC14B	0.283424713	0.099478468	2.849106124	0.004384225	0.023100453	positive
PRJNA729271	ed_Lushi_vs_Arbor_Acr	breed_growth_background	ENSGALG00010007930	CBFA2T3	0.063525877	0.105249198	0.603575876	0.546125655	0.689875076	positive
PRJNA729271	ed_Lushi_vs_Arbor_Acr	breed_growth_background	ENSGALG00010007931	GBE1	-0.041718801	0.101086024	-0.412705928	0.679822081	0.793060497	negative
PRJNA729271	ed_Lushi_vs_Arbor_Acr	breed_growth_background	ENSGALG00010007932	NPY5R	0.085057214	0.096997971	0.876896833	0.380542676	0.542216268	positive
PRJNA729271	ed_Lushi_vs_Arbor_Acr	breed_growth_background	ENSGALG00010007933	CEP126	-0.331903063	0.25104155	-1.3221041	0.186133489	0.331613191	negative
PRJNA729271	ed_Lushi_vs_Arbor_Acr	breed_growth_background	ENSGALG00010007934	PKHD1	-0.4024031	0.19262115	-2.089090939	0.036699538	0.105610443	negative
PRJNA729271	ed_Lushi_vs_Arbor_Acr	breed_growth_background	ENSGALG00010007935	LARP1B	0.146027424	0.065585332	2.226525663	0.025978992	0.082178344	positive
PRJNA729271	ed_Lushi_vs_Arbor_Acr	breed_growth_background	ENSGALG00010007936	ENSGALG00010007936	-0.091529385	0.074207006	-1.2334332	0.217414185	0.369327443	negative
PRJNA729271	ed_Lushi_vs_Arbor_Acr	breed_growth_background	ENSGALG00010007937	ANGPTL5	-0.287524467	0.214376779	-1.341210872	0.179851997	0.324091691	negative
PRJNA729271	ed_Lushi_vs_Arbor_Acr	breed_growth_background	ENSGALG00010007938	BCL9	0.031823306	0.057300737	0.555373411	0.578639275	0.715707509	positive
PRJNA729271	ed_Lushi_vs_Arbor_Acr	breed_growth_background	ENSGALG00010007940	GLIPR2	-0.745894168	0.146090437	-5.105701545	3.2957E-07	2.0591E-05	negative
PRJNA729271	ed_Lushi_vs_Arbor_Acr	breed_growth_background	ENSGALG00010007941	MRPL19	0.075204485	0.040785866	1.843885931	0.065199766	0.159937486	positive
PRJNA729271	ed_Lushi_vs_Arbor_Acr	breed_growth_background	ENSGALG00010007942	PGRMC2	-0.077401022	0.046789111	-1.654252883	0.098076142	0.21186493	negative
PRJNA729271	ed_Lushi_vs_Arbor_Acr	breed_growth_background	ENSGALG00010007943	BLOC1S4	-0.101209841	0.054283342	-1.864473294	0.062255269	0.154757352	negative
PRJNA729271	ed_Lushi_vs_Arbor_Acr	breed_growth_background	ENSGALG00010007944	ACSF3	-0.113322741	0.075033874	-1.510287756	0.130970015	0.259062549	negative
PRJNA729271	ed_Lushi_vs_Arbor_Acr	breed_growth_background	ENSGALG00010007945	ENSGALG00010007945	-0.066453336	0.067254109	-0.988093315	0.32310695	0.486995449	negative
PRJNA729271	ed_Lushi_vs_Arbor_Acr	breed_growth_background	ENSGALG00010007946	ENSGALG00010007946	-0.081659205	0.086818529	-0.940573466	0.346923485	0.510826201	negative
PRJNA729271	ed_Lushi_vs_Arbor_Acr	breed_growth_background	ENSGALG00010007948	ENSGALG00010007948	-0.610331897	0.269677436	-2.263192303	0.023623838	0.076915437	negative
PRJNA729271	ed_Lushi_vs_Arbor_Acr	breed_growth_background	ENSGALG00010007950	ARC	-0.484921024	0.312395643	-1.552265646	0.120598674	0.245145699	negative
PRJNA729271	ed_Lushi_vs_Arbor_Acr	breed_growth_background	ENSGALG00010007951	TGFBR1	-0.014853977	0.061172904	-0.242819546	0.808145193	0.881950352	negative
PRJNA729271	ed_Lushi_vs_Arbor_Acr	breed_growth_background	ENSGALG00010007952	EMC2	-0.064435712	0.058949005	-1.09307548	0.274360661	0.433256104	negative
PRJNA729271	ed_Lushi_vs_Arbor_Acr	breed_growth_background	ENSGALG00010007953	SCGN	-0.080382551	0.30250239	-0.265725341	0.790450746	0.86990992	negative
PRJNA729271	ed_Lushi_vs_Arbor_Acr	breed_growth_background	ENSGALG00010007954	PSCA	1.445171334	0.331569007	4.358583892	1.30907E-05	0.000352879	positive
PRJNA729271	ed_Lushi_vs_Arbor_Acr	breed_growth_background	ENSGALG00010007955	TMA16	-0.016032145	0.066097944	-0.242551334	0.808352985	0.882110707	negative
PRJNA729271	ed_Lushi_vs_Arbor_Acr	breed_growth_background	ENSGALG00010007956	HS1BP3	-0.523574502	0.07095084	-7.379398193	1.59007E-13	1.04764E-10	negative
PRJNA729271	ed_Lushi_vs_Arbor_Acr	breed_growth_background	ENSGALG00010007957	CDH15	0.051820691	0.087142835	0.594663814	0.552068215	0.694779257	positive
PRJNA729271	ed_Lushi_vs_Arbor_Acr	breed_growth_background	ENSGALG00010007958	ENSGALG00010007958	-1.180260824	0.300292502	-3.93037061	8.4815E-05	0.001417986	negative

PRJNA729271	eed_Lushi_vs_Arbor_Acr	breed_growth_background	ENSGALG00010007960	TSNAX	-0.08511653	0.034612684	-2.459113839	0.013928045	0.052767121	negative
PRJNA729271	eed_Lushi_vs_Arbor_Acr	breed_growth_background	ENSGALG00010007962	ADCY8	0.421396299	0.269682818	1.56256265	0.118155499	0.241872972	positive
PRJNA729271	eed_Lushi_vs_Arbor_Acr	breed_growth_background	ENSGALG00010007963	LDAH	-0.005530406	0.045964031	-0.120320298	0.904229425	0.945179601	negative
PRJNA729271	eed_Lushi_vs_Arbor_Acr	breed_growth_background	ENSGALG00010007964	RECQL4	-0.146280523	0.092350498	-1.583971139	0.113200287	0.234707218	negative
PRJNA729271	eed_Lushi_vs_Arbor_Acr	breed_growth_background	ENSGALG00010007965	EGLN1	0.344891406	0.084218904	4.095178033	4.21844E-05	0.000853999	positive
PRJNA729271	eed_Lushi_vs_Arbor_Acr	breed_growth_background	ENSGALG00010007966	TMEM74	0.115874461	0.124810392	0.928403955	0.353198068	0.516923061	positive
PRJNA729271	eed_Lushi_vs_Arbor_Acr	breed_growth_background	ENSGALG00010007968	SLC2A31	-0.000172426	0.11497325	-0.001499706	0.998803408	0.99912554	negative
PRJNA729271	eed_Lushi_vs_Arbor_Acr	breed_growth_background	ENSGALG00010007970	SPRTN	0.313738859	0.057387962	5.466980295	4.57767E-08	4.64009E-06	positive
PRJNA729271	eed_Lushi_vs_Arbor_Acr	breed_growth_background	ENSGALG00010007972	EXOC8	0.016497397	0.061057441	0.270194701	0.787010469	0.867770938	positive
PRJNA729271	eed_Lushi_vs_Arbor_Acr	breed_growth_background	ENSGALG00010007973	CARMIL1	-0.198401136	0.072393231	-2.740603402	0.006132648	0.02948982	negative
PRJNA729271	eed_Lushi_vs_Arbor_Acr	breed_growth_background	ENSGALG00010007975	ANKRD11	0.204979164	0.094243398	2.174997594	0.029630297	0.090096738	positive
PRJNA729271	eed_Lushi_vs_Arbor_Acr	breed_growth_background	ENSGALG00010007976	ENSGALG00010007976	-0.487088391	0.201763804	-2.414151509	0.015771902	0.057745321	negative
PRJNA729271	eed_Lushi_vs_Arbor_Acr	breed_growth_background	ENSGALG00010007977	APOB	-1.956449542	0.736387968	-2.656818998	0.007888178	0.035278971	negative
PRJNA729271	eed_Lushi_vs_Arbor_Acr	breed_growth_background	ENSGALG00010007978	JADE1	0.185468143	0.062278481	2.978045399	0.00290093	0.01729699	positive
PRJNA729271	eed_Lushi_vs_Arbor_Acr	breed_growth_background	ENSGALG00010007979	RAB30	-0.110425241	0.048316657	-2.285448687	0.022286532	0.073821591	negative
PRJNA729271	eed_Lushi_vs_Arbor_Acr	breed_growth_background	ENSGALG00010007980	MARCHF1	0.113076348	0.122786965	0.920914924	0.357094846	0.52070497	positive
PRJNA729271	eed_Lushi_vs_Arbor_Acr	breed_growth_background	ENSGALG00010007981	TDRD15	0.854318992	0.38192118	2.236898702	0.025292961	0.080629311	positive
PRJNA729271	eed_Lushi_vs_Arbor_Acr	breed_growth_background	ENSGALG00010007982	RECK	-0.14442181	0.07856088	-1.838342581	0.06601194	0.161465251	negative
PRJNA729271	eed_Lushi_vs_Arbor_Acr	breed_growth_background	ENSGALG00010007983	RCHY1	-0.120237185	0.047019324	-2.557186565	0.010552261	0.043440789	negative
PRJNA729271	eed_Lushi_vs_Arbor_Acr	breed_growth_background	ENSGALG00010007984	EIF3H	-0.04674163	0.065276728	-0.716053503	0.473958313	0.627628882	negative
PRJNA729271	eed_Lushi_vs_Arbor_Acr	breed_growth_background	ENSGALG00010007985	APELA	-0.144615006	0.205932981	-0.702243056	0.482527598	0.634858631	negative
PRJNA729271	eed_Lushi_vs_Arbor_Acr	breed_growth_background	ENSGALG00010007986	MYC	0.143978159	0.102538525	1.404137221	0.160277992	0.299038421	positive
PRJNA729271	eed_Lushi_vs_Arbor_Acr	breed_growth_background	ENSGALG00010007987	ENSGALG00010007987	0.322968505	0.158213876	2.041341203	0.041216924	0.114825931	positive
PRJNA729271	eed_Lushi_vs_Arbor_Acr	breed_growth_background	ENSGALG00010007988	TARBP1	-0.097032789	0.066540477	-1.458252096	0.144771069	0.278457623	negative
PRJNA729271	eed_Lushi_vs_Arbor_Acr	breed_growth_background	ENSGALG00010007989	SPG7	0.20045052	0.052097046	3.847636983	0.000119263	0.001819696	positive
PRJNA729271	eed_Lushi_vs_Arbor_Acr	breed_growth_background	ENSGALG00010007990	TRHR	-0.34217914	0.605134232	-0.565459895	0.571760995	0.710108442	negative
PRJNA729271	eed_Lushi_vs_Arbor_Acr	breed_growth_background	ENSGALG00010007991	PCF11	0.153961826	0.058837352	2.616736167	0.008877493	0.038343045	positive
PRJNA729271	eed_Lushi_vs_Arbor_Acr	breed_growth_background	ENSGALG00010007992	LAPTM4A	-0.061624127	0.053013837	-1.162415905	0.245066566	0.400926922	negative
PRJNA729271	eed_Lushi_vs_Arbor_Acr	breed_growth_background	ENSGALG00010007993	LRRC14	0.317795637	0.093610424	3.39487446	0.000686601	0.006224064	positive
PRJNA729271	eed_Lushi_vs_Arbor_Acr	breed_growth_background	ENSGALG00010007994	FAM65B	-0.193178873	0.088715892	-2.177500205	0.029443267	0.089678535	negative
PRJNA729271	eed_Lushi_vs_Arbor_Acr	breed_growth_background	ENSGALG00010007995	KIAA0319	-0.452374258	0.451119426	-1.002781597	0.31596625	0.479373121	negative
PRJNA729271	eed_Lushi_vs_Arbor_Acr	breed_growth_background	ENSGALG00010007996	NUDCD1	-0.039048606	0.041425886	-0.94261367	0.345878545	0.509710198	negative
PRJNA729271	eed_Lushi_vs_Arbor_Acr	breed_growth_background	ENSGALG00010007997	TMEM192	0.002530052	0.075063336	0.033705563	0.973111943	0.984110627	positive
PRJNA729271	eed_Lushi_vs_Arbor_Acr	breed_growth_background	ENSGALG00010007998	RPL13	-0.114058502	0.077417576	-1.473289505	0.140672989	0.273141994	negative
PRJNA729271	eed_Lushi_vs_Arbor_Acr	breed_growth_background	ENSGALG00010007999	SDC1	-0.293785803	0.111642692	-2.631482603	0.008501322	0.037105288	negative
PRJNA729271	eed_Lushi_vs_Arbor_Acr	breed_growth_background	ENSGALG00010008000	C8orf82	-0.253245917	0.093535653	-2.707480082	0.006779614	0.03168994	negative
PRJNA729271	eed_Lushi_vs_Arbor_Acr	breed_growth_background	ENSGALG00010008001	ALDH5A1	-0.20167248	0.073304957	-2.751143836	0.005938756	0.028818972	negative
PRJNA729271	eed_Lushi_vs_Arbor_Acr	breed_growth_background	ENSGALG00010008002	GJA8	0.008838902	0.09338637	0.094648736	0.924593838	0.957813766	positive
PRJNA729271	eed_Lushi_vs_Arbor_Acr	breed_growth_background	ENSGALG00010008004	ENY2	-0.249185622	0.092209766	-2.702377756	0.00688455	0.032035809	negative
PRJNA729271	eed_Lushi_vs_Arbor_Acr	breed_growth_background	ENSGALG00010008005	ANKRD42	-0.134082686	0.19151317	-0.700122535	0.483850783	0.636022228	negative
PRJNA729271	eed_Lushi_vs_Arbor_Acr	breed_growth_background	ENSGALG00010008006	CPNE7	-0.683201482	0.307834282	-2.219380758	0.026460832	0.083199513	negative
PRJNA729271	eed_Lushi_vs_Arbor_Acr	breed_growth_background	ENSGALG00010008007	CBR4	0.09797936	0.065687524	1.491597706	0.13580464	0.266047879	positive
PRJNA729271	eed_Lushi_vs_Arbor_Acr	breed_growth_background	ENSGALG00010008008	BTC	-0.035456834	0.093419505	-0.379544226	0.704283769	0.808793633	negative
PRJNA729271	eed_Lushi_vs_Arbor_Acr	breed_growth_background	ENSGALG00010008009	GPLD1	-1.21224202	0.730888372	-1.658587093	0.097199024	0.210566412	negative
PRJNA729271	eed_Lushi_vs_Arbor_Acr	breed_growth_background	ENSGALG00010008010	CERK	0.044383558	0.049964688	0.888298525	0.374380193	0.536706646	positive
PRJNA729271	eed_Lushi_vs_Arbor_Acr	breed_growth_background	ENSGALG00010008011	ADNP2	-0.095776746	0.054375406	-1.761398251	0.078171021	0.180946814	negative
PRJNA729271	eed_Lushi_vs_Arbor_Acr	breed_growth_background	ENSGALG00010008012	CCDC90B	0.11851064	0.043455424	2.727177159	0.006387873	0.030358106	positive
PRJNA729271	eed_Lushi_vs_Arbor_Acr	breed_growth_background	ENSGALG00010008013	ENSGALG00010008013	-0.071015375	0.126227814	-0.562596883	0.573709415	0.711491955	negative
PRJNA729271	eed_Lushi_vs_Arbor_Acr	breed_growth_background	ENSGALG00010008015	ARHGAP39	-0.046842124	0.084707499	-0.552986737	0.580272492	0.716871198	negative
PRJNA729271	eed_Lushi_vs_Arbor_Acr	breed_growth_background	ENSGALG00010008016	GSR	-0.362708067	0.077404503	-4.685878087	2.78762E-06	0.000107179	negative
PRJNA729271	eed_Lushi_vs_Arbor_Acr	breed_growth_background	ENSGALG00010008017	GMNN	-0.379317422	0.073995007	-5.126256961	2.95559E-07	1.88728E-05	negative
PRJNA729271	eed_Lushi_vs_Arbor_Acr	breed_growth_background	ENSGALG00010008018	ENSGALG00010008018	-0.067111642	0.111228619	-0.60336668	0.546264783	0.689971073	negative
PRJNA729271	eed_Lushi_vs_Arbor_Acr	breed_growth_background	ENSGALG00010008019	PPP2CB	-0.044574264	0.054110088	-0.823769935	0.410070294	0.570547985	negative
PRJNA729271	eed_Lushi_vs_Arbor_Acr	breed_growth_background	ENSGALG00010008020	COA6	-0.018683528	0.070487471	-0.265061684	0.790961943	0.870340345	negative
PRJNA729271	eed_Lushi_vs_Arbor_Acr	breed_growth_background	ENSGALG00010008022	SLC35F3	0.166796485	0.211661307	0.788034843	0.430676326	0.588859819	positive
PRJNA729271	eed_Lushi_vs_Arbor_Acr	breed_growth_background	ENSGALG00010008023	ATAD2	-0.001881911	0.042029862	-0.044775564	0.964286202	0.979609321	negative
PRJNA729271	eed_Lushi_vs_Arbor_Acr	breed_growth_background	ENSGALG00010008024	MRS2	0.019046379	0.051215179	0.371889335	0.709975246	0.813025405	positive
PRJNA729271	eed_Lushi_vs_Arbor_Acr	breed_growth_background	ENSGALG00010008025	TBC1D22A	-0.004267989	0.052323724	-0.081568916	0.934989521	0.964465777	negative
PRJNA729271	eed_Lushi_vs_Arbor_Acr	breed_growth_background	ENSGALG00010008026	PURG	0.075088541	0.065693083	1.143020509	0.253030076	0.409658321	positive
PRJNA729271	eed_Lushi_vs_Arbor_Acr	breed_growth_background	ENSGALG00010008027	C6orf62	-0.183655478	0.057220799	-3.209593045	0.00132923	0.010014133	negative
PRJNA729271	eed_Lushi_vs_Arbor_Acr	breed_growth_background	ENSGALG00010008028	KCNK1	-0.332434868	0.092750151	-3.584197603	0.000338116	0.003766357	negative
PRJNA729271	eed_Lushi_vs_Arbor_Acr	breed_growth_background	ENSGALG00010008029	LY6E	0.15983653	0.168109013	0.950790958	0.341710504	0.505675727	positive
PRJNA729271	eed_Lushi_vs_Arbor_Acr	breed_growth_background	ENSGALG00010008030	GJA5	-0.199243394	0.081329693	-2.449823506	0.014292625	0.05361584	negative
PRJNA729271	eed_Lushi_vs_Arbor_Acr	breed_growth_background	ENSGALG00010008031	MAP3K21	-0.107344501	0.102163763	-1.050710136	0.293391739	0.454741887	negative
PRJNA729271	eed_Lushi_vs_Arbor_Acr	breed_growth_background	ENSGALG00010008032	PUM2	-0.008074567	0.029373491	-0.274892992	0.783398452	0.865763995	negative
PRJNA729271	eed_Lushi_vs_Arbor_Acr	breed_growth_background	ENSGALG00010008033	ACOT13	0.008638159	0.064264977	0.13441472	0.893074643	0.937440578	positive
PRJNA729271	eed_Lushi_vs_Arbor_Acr	breed_growth_background	ENSGALG00010008034	DPEP1	0.442077191	0.196002225	2.255470264	0.024103827	0.077952916	positive
PRJNA729271	eed_Lushi_vs_Arbor_Acr	breed_growth_background	ENSGALG00010008035	TDP2	-0.075877767	0.057523491	-1.319074453	0.187144223	0.332801341	negative
PRJNA729271	eed_Lushi_vs_Arbor_Acr	breed_growth_background	ENSGALG00010008036	SPOCK3	0.188756155	0.100272502	1.882431888	0.0597774	0.150455532	positive
PRJNA729271	eed_Lushi_vs_Arbor_Acr	breed_growth_background	ENSGALG00010008037	ENSGALG00010008037	0.334483087	0.45715182	0.731667408	0.464371594	0.619689399	positive
PRJNA729271	eed_Lushi_vs_Arbor_Acr	breed_growth_background	ENSGALG00010008038	WRN	-0.035964421	0.041359116	-0.869564553	0.384538416	0.546352121	negative
PRJNA729271	eed_Lushi_vs_Arbor_Acr	breed_growth_background	ENSGALG00010008039	CHMP1A	0.013248202	0.049551187	0.267363967	0.78918894	0.86924724	positive
PRJNA729271	eed_Lushi_vs_Arbor_Acr	breed_growth_background	ENSGALG00010008040	ENSGALG00010008040	0.013616491	0.145014665	0.093897341	0.925190706	0.957971233	positive
PRJNA729271	eed_Lushi_vs_Arbor_Acr	breed_growth_background	ENSGALG00010008041	ENSGALG00010008041	0.155021694	0.117726628	1.316793803	0.187907751	0.333722241	positive
PRJNA729271	eed_Lushi_vs_Arbor_Acr	breed_growth_background	ENSGALG00010008042	ENSGALG00010008042	-0.747713837	0.447188558	-1.672032578	0.094517898	0.206735617	negative
PRJNA729271	eed_Lushi_vs_Arbor_Acr	breed_growth_background	ENSGALG00010008043	IL17REL	0.414515612	0.215339719	1.924938023	0.054237112	0.140412028	positive
PRJNA729271	eed_Lushi_vs_Arbor_Acr	breed_growth_background	ENSGALG00010008045	DCDC2	-1.548343026	0.467159921	-3.314374708	0.000918483	0.007651388	negative
PRJNA729271	eed_Lushi_vs_Arbor_Acr	breed_growth_background	ENSGALG00010008046	RHOB	-0.262279041	0.090401897	-2.901255944	0.003716702	0.020546755	negative
PRJNA729271	eed_Lushi_vs_Arbor_Acr	breed_growth_background	ENSGALG00010008047	ASAP1	-0.184769176	0.071360115	-2.589249974	0.009618524	0.040730503	negative
PRJNA729271	eed_Lushi_vs_Arbor_Acr	breed_growth_background	ENSGALG00010008048	ENSGALG00010008048	0.107721853	0.369948257	0.291180865	0.770912996	0.857194222	positive
PRJNA729271	eed_Lushi_vs_Arbor_Acr	breed_growth_background	ENSGALG00010008050	CDK10	-0.088293484	0.085197447	-1.036339551	0.300043761	0.462429238	negative
PRJNA729271	eed_Lushi_vs_Arbor_Acr	breed_growth_background	ENSGALG00010008051	ENSGALG00010008051	-0.000394032	0.042670374	-0.009234322	0.992632182	0.995792337	negative
PRJNA729271	eed_Lushi_vs_Arbor_Acr	breed_growth_background	ENSGALG00010008052	RBFA	0.181216317	0.066226474	2.73631234	0.006213202	0.029732706	positive
PRJNA729271	eed_Lushi_vs_Arbor_Acr	breed_growth_background	ENSGALG00010008054	CRISP3	-0.822778424	0.463196633	-1.776304845	0.075682685	0.177253275	negative
PRJNA729271	eed_Lushi_vs_Arbor_Acr	breed_growth_background	ENSGALG00010008056	GTF2E2	0.121891051	0.039126082	3.115340105	0.001837329	0.012520963	positive
PRJNA729271	eed_Lushi_vs_Arbor_Acr	breed_growth_background	ENSGALG00010008057	BAG1	-0.087239179	0.072168369	-1.208828466	0.226728753	0.379934483	negative
PRJNA729271	eed_Lushi_vs_Arbor_Acr	breed_growth_background	ENSGALG00010008058	USP25	0.000957444	0.07374779	0.012982681	0.98964161	0.993884212	positive
PRJNA729271	eed_Lushi_vs_Arbor_Acr	breed_growth_background	ENSGALG00010008059	ENSGALG00010008059	0.039382581	0.050166359	0.785039662	0.432430315	0.590659387	positive
PRJNA729271	eed_Lushi_vs_Arbor_Acr	breed_growth_background	ENSGALG00010008062	VPS9D1	0.145470812	0.076804196	1.894047724	0.058218676	0.147531417	positive
PRJNA729271	eed_Lushi_vs_Arbor_Acr	breed_growth_background	ENSGALG00010008063	KCNV1	-0.615508943	0.155767222	-3.951466395	7.76738E-05	0.001317388	negative
PRJNA729271	eed_Lushi_vs_Arbor_Acr	breed_growth_background	ENSGALG00010008064	DDX60	-0.36001819	0.347382015	-1.036375443	0.300027022	0.462429238	negative
PRJNA729271	eed_Lushi_vs_Arbor_Acr	breed_growth_background	ENSGALG00010008065	CHMP5	0.011304081	0.046761886	0.241737054	0.808983916	0.882536092	positive
PRJNA729271	eed_Lushi_vs_Arbor_Acr	breed_growth_background	ENSGALG00010008066	PCNX2	0.30282249	0.107286486	2.822559487	0.004764198	0.024584213	positive
PRJNA729271	eed_Lushi_vs_Arbor_Acr	breed_growth_background	ENSGALG00010008067	MPC2	-0.04773448	0.077870392	-0.612999091	0.53987694	0.684926963	negative
PRJNA729271	eed_Lushi_vs_Arbor_Acr	breed_growth_background	ENSGALG00010008069	DLG2	-0.239469508	0.122153851	-1.96039262	0.049949918	0.132467501	negative
PRJNA729271	eed_Lushi_vs_Arbor_Acr	breed_growth_background	ENSGALG00010008071	SMIM18	-0.314689188	0.197327996	-1.594751859	0.110767736	0.231418036	negative
PRJNA729271	eed_Lushi_vs_Arbor_Acr	breed_growth_background	ENSGALG00010008072	NRSN1	0.450398587	0.106758447	4.218856662	2.45544E-05	0.000562193	positive
PRJNA729271	eed_Lushi_vs_Arbor_Acr	breed_growth_background	ENSGALG00010008073	ZNF276	0.179370639	0.045794329	3.916874486	8.97044E-05	0.001476877	positive
PRJNA729271	eed_Lushi_vs_Arbor_Acr	breed_growth_background	ENSGALG00010008074	RHAG	-0.65607386	0.235500491	-2.785870449	0.00533842	0.026701751	negative
PRJNA729271	eed_Lushi_vs_Arbor_Acr	breed_growth_background	ENSGALG00010008075	SDHA	0.083282223	0.072962653	1.141436343	0.253688388	0.41031167	positive
PRJNA729271	eed_Lushi_vs_Arbor_Acr	breed_growth_background	ENSGALG00010008076	ENSGALG00010008076	-1.423796396	0.977505252	-1.456561377	0.145237496	0.279095519	negative
PRJNA729271	eed_Lushi_vs_Arbor_Acr	breed_growth_background	ENSGALG00010008077	ENSGALG00010008077	2.550506247	0.80427954	3.171168878	0.001518268	0.01098717	positive
PRJNA729271	eed_Lushi_vs_Arbor_Acr	breed_growth_background	ENSGALG00010008078	GUCY1B1	-0.255909503	0.075734233	-3.379046603	0.000727377	0.00648421	negative
PRJNA729271	eed_Lushi_vs_Arbor_Acr	breed_growth_background	ENSGALG00010008079	CCDC127	0.125973014	0.058434112	2.155812925	0.031098276	0.093481856	positive
PRJNA729271	eed_Lushi_vs_Arbor_Acr	breed_growth_background	ENSGALG00010008081	TOP1MT	-0.053037369	0.076367415	-0.694502611	0.487367064	0.639195222	negative
PRJNA729271	eed_Lushi_vs_Arbor_Acr	breed_growth_background	ENSGALG00010008083	FANCA	-0.49563983	0.142934189	-3.467608644	0.000525111	0.005139426	negative
PRJNA729271	eed_Lushi_vs_Arbor_Acr	breed_growth_background	ENSGALG00010008084	CYP2AC1	-1.935431714	0.682269296	-2.836756286			negative
PRJNA729271	eed_Lushi_vs_Arbor_Acr	breed_growth_background	ENSGALG00010008086	RHPN1	-0.027455599	0.112897996	-0.243189432	0.807858653	0.881865544	negative
PRJNA729271	eed_Lushi_vs_Arbor_Acr	breed_growth_background	ENSGALG00010008087	CPNE8	0.198438684	0.099386293	1.996640361	0.045864265	0.124542696	positive
PRJNA729271	eed_Lushi_vs_Arbor_Acr	breed_growth_background	ENSGALG00010008088	SLC35A4	0.043935528	0.038136781	1.152051312	0.249299989	0.405294228	positive
PRJNA729271	eed_Lushi_vs_Arbor_Acr	breed_growth_background	ENSGALG00010008091	ENSGALG00010008091	0.185063563	0.145381215	1.272953747	0.203034455	0.352215918	positive
PRJNA729271	eed_Lushi_vs_Arbor_Acr	breed_growth_background	ENSGALG00010008092	SPIRE2	-1.077907421	0.357952724	-3.011312246	0.002601212	0.015998854	negative
PRJNA729271	eed_Lushi_vs_Arbor_Acr	breed_growth_background	ENSGALG00010008093	EFR3A	-0.139437305	0.049643405	-2.808778012	0.004972992	0.025487936	negative
PRJNA729271	eed_Lushi_vs_Arbor_Acr	breed_growth_background	ENSGALG00010008094	PIM3	-0.098228921	0.076262055	-1.288044502	0.197730473	0.346105929	negative
PRJNA729271	eed_Lushi_vs_Arbor_Acr	breed_growth_background	ENSGALG00010008096	CPSF1	-0.137198172	0.08285861	-1.65581068	0.097760164	0.211380635	negative
PRJNA729271	eed_Lushi_vs_Arbor_Acr	breed_growth_background	ENSGALG00010008098	ENSGALG00010008098	0.207204613	0.090396668	2.292170922	0.021895782	0.07279625	positive
PRJNA729271	eed_Lushi_vs_Arbor_Acr	breed_growth_background	ENSGALG00010008099	PANK3	-0.074500062	0.041366465	-1.800977238	0.071706468	0.1705559	negative
PRJNA729271	eed_Lushi_vs_Arbor_Acr	breed_growth_background	ENSGALG00010008100	ENSGALG00010008100	-0.038311473	0.125162422	-0.306094057	0.759533033	0.848314941	negative
PRJNA729271	eed_Lushi_vs_Arbor_Acr	breed_growth_background	ENSGALG00010008101	CENPQ	-0.423718053	0.114287801	-3.707465272	0.000209344	0.002685348	negative
PRJNA729271	eed_Lushi_vs_Arbor_Acr	breed_growth_background	ENSGALG00010008103	RBPMS	-0.047303174	0.05860518	-0.807150038	0.419580047	0.579132152	negative
PRJNA729271	eed_Lushi_vs_Arbor_Acr	breed_growth_background	ENSGALG00010008104	TCF25	0.147217957	0.050872314	2.893871829	0.003805235	0.020916524	positive
PRJNA729271	eed_Lushi_vs_Arbor_Acr	breed_growth_background	ENSGALG00010008105	MMUT	0.186770729	0.051496665	3.626850941	0.000286899	0.003367286	positive
PRJNA729271	eed_Lushi_vs_Arbor_Acr	breed_growth_background	ENSGALG00010008106	ENSGALG00010008106	-0.788741523	0.201986498	-3.904922017	9.42558E-05	0.001523119	negative
PRJNA729271	eed_Lushi_vs_Arbor_Acr	breed_growth_background	ENSGALG00010008107	TUBB3	-0.030473616	0.112383186	-0.271158139	0.786269407	0.867374086	negative
PRJNA729271	eed_Lushi_vs_Arbor_Acr	breed_growth_background	ENSGALG00010008108	ENSGALG00010008108	0.22668038	0.173390396	1.307341036	0.191096913	0.337945536	positive
PRJNA729271	eed_Lushi_vs_Arbor_Acr	breed_growth_background	ENSGALG00010008110	DEF8	0.255598462	0.083081936	3.076462504	0.002094726	0.013695562	positive
PRJNA729271	eed_Lushi_vs_Arbor_Acr	breed_growth_background	ENSGALG00010008111	SYTL2	-0.176522405	0.1406427	-1.255112463	0.209437937	0.359181602	negative
PRJNA729271	eed_Lushi_vs_Arbor_Acr	breed_growth_background	ENSGALG00010008113	DBNDD1	0.061684353	0.101238481	0.609297492	0.542327269	0.687213372	positive
PRJNA729271	eed_Lushi_vs_Arbor_Acr	breed_growth_background	ENSGALG00010008115	ENSGALG00010008115	0.360323931	0.109234674	3.29862228	0.000971606	0.007983799	positive
PRJNA729271	eed_Lushi_vs_Arbor_Acr	breed_growth_background	ENSGALG00010008116	URAH	1.207331026	0.216171214	5.585068433	2.33608E-08	2.78703E-06	positive
PRJNA729271	eed_Lushi_vs_Arbor_Acr	breed_growth_background	ENSGALG00010008117	ENSGALG00010008117	-0.234151769	0.09199343	-2.545309701	0.010918086	0.044529446	negative

PRJNA729271	:ed_Lushi_vs_Arbor_Acr	breed_growth_background	ENSGALG00010008118	ENSGALG00010008118	-1.187557302	0.334548647	-3.549729804	0.000385627	0.004137424	negative
PRJNA729271	:ed_Lushi_vs_Arbor_Acr	breed_growth_background	ENSGALG00010008119	CCDC83	-0.606456973	0.311781338	-1.945135581	0.051758663	0.135741397	negative
PRJNA729271	:ed_Lushi_vs_Arbor_Acr	breed_growth_background	ENSGALG00010008120	CDC5L	0.082414978	0.034811076	2.367492997	0.017909059	0.063315076	positive
PRJNA729271	:ed_Lushi_vs_Arbor_Acr	breed_growth_background	ENSGALG00010008121	MPZL1	-0.29477821	0.072666417	-4.056594799	4.97934E-05	0.000962511	negative
PRJNA729271	:ed_Lushi_vs_Arbor_Acr	breed_growth_background	ENSGALG00010008123	ENSGALG00010008123	-0.20972973	0.089626529	-2.340040746	0.019281636	0.066623913	negative
PRJNA729271	:ed_Lushi_vs_Arbor_Acr	breed_growth_background	ENSGALG00010008124	ENSGALG00010008124	0.257436023	0.117630564	2.188513044	0.028632252	0.087873067	positive
PRJNA729271	:ed_Lushi_vs_Arbor_Acr	breed_growth_background	ENSGALG00010008126	CDH3	0.135997716	0.178197683	0.763184536	0.44535335	0.602070212	positive
PRJNA729271	:ed_Lushi_vs_Arbor_Acr	breed_growth_background	ENSGALG00010008127	TBC1D2	-0.266409527	0.073897318	-3.605131181	0.000311995	0.003552531	negative
PRJNA729271	:ed_Lushi_vs_Arbor_Acr	breed_growth_background	ENSGALG00010008128	RARS	-0.028956089	0.037968667	-0.762631176	0.445683386	0.602460196	negative
PRJNA729271	:ed_Lushi_vs_Arbor_Acr	breed_growth_background	ENSGALG00010008129	TANGO6	0.221038098	0.062796701	3.519899873	0.00043171	0.004466547	positive
PRJNA729271	:ed_Lushi_vs_Arbor_Acr	breed_growth_background	ENSGALG00010008130	RCSD1	-0.015328468	0.063795901	-0.240273549	0.810118199	0.883106212	negative
PRJNA729271	:ed_Lushi_vs_Arbor_Acr	breed_growth_background	ENSGALG00010008131	OC90	0.111341052	0.362764502	0.306923779	0.758901389	0.847740107	positive
PRJNA729271	:ed_Lushi_vs_Arbor_Acr	breed_growth_background	ENSGALG00010008132	TDO2	-1.110999371	0.344214881	-3.227633181	0.001248189	0.009598046	negative
PRJNA729271	:ed_Lushi_vs_Arbor_Acr	breed_growth_background	ENSGALG00010008133	CREG1	0.263869505	0.089271331	2.955814635	0.003118444	0.018248628	positive
PRJNA729271	:ed_Lushi_vs_Arbor_Acr	breed_growth_background	ENSGALG00010008134	SLC2A13	-0.07743559	0.043206649	-1.792214662	0.073098587	0.172995863	negative
PRJNA729271	:ed_Lushi_vs_Arbor_Acr	breed_growth_background	ENSGALG00010008135	WWC1	0.087138343	0.070926376	1.22857458	0.219231351	0.371624188	positive
PRJNA729271	:ed_Lushi_vs_Arbor_Acr	breed_growth_background	ENSGALG00010008136	ELP1	-0.05029736	0.060717368	-0.82838505	0.407452471	0.567941491	negative
PRJNA729271	:ed_Lushi_vs_Arbor_Acr	breed_growth_background	ENSGALG00010008137	HHLA1	0.636588675	0.291284363	2.185454335	0.028855551	0.088408625	positive
PRJNA729271	:ed_Lushi_vs_Arbor_Acr	breed_growth_background	ENSGALG00010008139	ENSGALG00010008139	0.55988659	0.211027249	2.653148314	0.007974482	0.035533391	positive
PRJNA729271	:ed_Lushi_vs_Arbor_Acr	breed_growth_background	ENSGALG00010008141	APTX	-0.012990371	0.083538749	-0.155501139	0.876426256	0.926804252	negative
PRJNA729271	:ed_Lushi_vs_Arbor_Acr	breed_growth_background	ENSGALG00010008142	CD247	0.471024285	0.171780185	2.742017562	0.006106308	0.029405625	positive
PRJNA729271	:ed_Lushi_vs_Arbor_Acr	breed_growth_background	ENSGALG00010008143	DCTN6	0.058574189	0.061672955	0.949754861	0.342236827	0.505938073	positive
PRJNA729271	:ed_Lushi_vs_Arbor_Acr	breed_growth_background	ENSGALG00010008144	NTPCR	0.052891902	0.071930338	0.735321183	0.46214391	0.617182224	positive
PRJNA729271	:ed_Lushi_vs_Arbor_Acr	breed_growth_background	ENSGALG00010008145	ENSGALG00010008145	0.168447098	0.111253032	1.514089954	0.130003026	0.257747759	positive
PRJNA729271	:ed_Lushi_vs_Arbor_Acr	breed_growth_background	ENSGALG00010008146	SOX4	-0.146215789	0.064334462	-2.272744393	0.023041588	0.075528678	negative
PRJNA729271	:ed_Lushi_vs_Arbor_Acr	breed_growth_background	ENSGALG00010008147	CDKAL1	0.010815975	0.060442092	0.178947722	0.857978751	0.914805541	positive
PRJNA729271	:ed_Lushi_vs_Arbor_Acr	breed_growth_background	ENSGALG00010008148	ABCD2	-0.040417203	0.074083654	-0.545561683	0.58536727	0.721259654	negative
PRJNA729271	:ed_Lushi_vs_Arbor_Acr	breed_growth_background	ENSGALG00010008149	DNAJA1	0.030271401	0.150552091	0.201069285	0.840644399	0.903272095	positive
PRJNA729271	:ed_Lushi_vs_Arbor_Acr	breed_growth_background	ENSGALG00010008150	TERT	0.793453768	0.363719795	2.181497346	0.029146651	0.089037028	positive
PRJNA729271	:ed_Lushi_vs_Arbor_Acr	breed_growth_background	ENSGALG00010008151	SIPA1L2	0.026956795	0.065210936	0.413378442	0.679329367	0.792696762	positive
PRJNA729271	:ed_Lushi_vs_Arbor_Acr	breed_growth_background	ENSGALG00010008152	ENSGALG00010008152	-0.309161323	0.089806599	-3.442523432	0.000576314	0.005474227	negative
PRJNA729271	:ed_Lushi_vs_Arbor_Acr	breed_growth_background	ENSGALG00010008153	SMU1	0.009066476	0.033337732	0.271958377	0.785654024	0.867264875	positive
PRJNA729271	:ed_Lushi_vs_Arbor_Acr	breed_growth_background	ENSGALG00010008154	KCNQ3	-0.286621543	0.122405339	-2.341577132	0.019202458	0.066477102	negative
PRJNA729271	:ed_Lushi_vs_Arbor_Acr	breed_growth_background	ENSGALG00010008155	MAP10	-0.077260162	0.120234574	-0.642578581	0.520497585	0.668019523	negative
PRJNA729271	:ed_Lushi_vs_Arbor_Acr	breed_growth_background	ENSGALG00010008156	HAS3	2.63804E-05	0.623454922	4.23132E-05	0.999966239	0.999966239	positive
PRJNA729271	:ed_Lushi_vs_Arbor_Acr	breed_growth_background	ENSGALG00010008157	CTSO	-0.25036419	0.084212492	-2.97300535	0.002948993	0.017518711	negative
PRJNA729271	:ed_Lushi_vs_Arbor_Acr	breed_growth_background	ENSGALG00010008158	CLPTM1L	-0.005721805	0.040956454	-0.13970461	0.888893384	0.934871371	negative
PRJNA729271	:ed_Lushi_vs_Arbor_Acr	breed_growth_background	ENSGALG00010008159	SUPT3H	0.154259326	0.078267456	1.97092551	0.048732397	0.130188213	positive
PRJNA729271	:ed_Lushi_vs_Arbor_Acr	breed_growth_background	ENSGALG00010008160	TXNL4A	0.028622804	0.050116504	0.571125322	0.567914695	0.707027699	positive
PRJNA729271	:ed_Lushi_vs_Arbor_Acr	breed_growth_background	ENSGALG00010008161	TMEFF1	0.131035486	0.205057768	0.639017418	0.522811601	0.669757753	positive
PRJNA729271	:ed_Lushi_vs_Arbor_Acr	breed_growth_background	ENSGALG00010008162	HSBP1L1	-0.343400143	0.106830471	-3.214440025	0.001306992	0.009877399	negative
PRJNA729271	:ed_Lushi_vs_Arbor_Acr	breed_growth_background	ENSGALG00010008163	CHTF8	0.010706396	0.129213175	0.082858393	0.933964138	0.963956863	positive
PRJNA729271	:ed_Lushi_vs_Arbor_Acr	breed_growth_background	ENSGALG00010008164	TG	0.232904063	0.155946524	1.493486721	0.135309828	0.265401347	positive
PRJNA729271	:ed_Lushi_vs_Arbor_Acr	breed_growth_background	ENSGALG00010008165	MRPL1	0.023159598	0.045611568	0.507757115	0.611623688	0.741571339	positive
PRJNA729271	:ed_Lushi_vs_Arbor_Acr	breed_growth_background	ENSGALG00010008166	RUNX2	0.079833374	0.229952412	0.347173458	0.72846101	0.825441083	positive
PRJNA729271	:ed_Lushi_vs_Arbor_Acr	breed_growth_background	ENSGALG00010008167	UTP4	0.208257257	0.093666514	2.223390693	0.026189467	0.082521209	positive
PRJNA729271	:ed_Lushi_vs_Arbor_Acr	breed_growth_background	ENSGALG00010008168	CAVIN4	0.114250447	0.066160615	1.726864949	0.084191921	0.190860477	positive
PRJNA729271	:ed_Lushi_vs_Arbor_Acr	breed_growth_background	ENSGALG00010008169	HTR4	-0.450802978	0.199067758	-2.264570531	0.023539047	0.076691051	negative
PRJNA729271	:ed_Lushi_vs_Arbor_Acr	breed_growth_background	ENSGALG00010008170	PICALM	-0.070663275	0.038353436	-1.842423585	0.065413215	0.160271221	negative
PRJNA729271	:ed_Lushi_vs_Arbor_Acr	breed_growth_background	ENSGALG00010008171	KIF21A	0.429726578	0.097620326	4.402019516	1.07248E-05	0.000306619	positive
PRJNA729271	:ed_Lushi_vs_Arbor_Acr	breed_growth_background	ENSGALG00010008172	SNTB2	0.083724124	0.07252329	1.154444641	0.248317922	0.404423403	positive
PRJNA729271	:ed_Lushi_vs_Arbor_Acr	breed_growth_background	ENSGALG00010008174	SLC26A2	-0.012867374	0.067549458	-0.19048819	0.848926597	0.909004286	negative
PRJNA729271	:ed_Lushi_vs_Arbor_Acr	breed_growth_background	ENSGALG00010008175	ENSGALG00010008175	-0.406341138	0.244436583	-1.662358119	0.096440991	0.209456423	negative
PRJNA729271	:ed_Lushi_vs_Arbor_Acr	breed_growth_background	ENSGALG00010008176	SLA	0.137498377	0.162869444	0.84422451	0.398543961	0.559761643	positive
PRJNA729271	:ed_Lushi_vs_Arbor_Acr	breed_growth_background	ENSGALG00010008177	NREP	-0.247983454	0.09872038	-2.511978323	0.012005646	0.04755655	negative
PRJNA729271	:ed_Lushi_vs_Arbor_Acr	breed_growth_background	ENSGALG00010008178	PGR	0.518100193	0.16397579	3.159613941	0.001579783	0.011247032	positive
PRJNA729271	:ed_Lushi_vs_Arbor_Acr	breed_growth_background	ENSGALG00010008179	SEMA6A	-0.102749826	0.052332022	-1.963421659	0.049597199	0.13174114	negative
PRJNA729271	:ed_Lushi_vs_Arbor_Acr	breed_growth_background	ENSGALG00010008180	POU2F1	0.240572654	0.08465309	2.841865007	0.004485048	0.023495037	positive
PRJNA729271	:ed_Lushi_vs_Arbor_Acr	breed_growth_background	ENSGALG00010008181	DISC1	-0.191523989	0.070302649	-2.724278404	0.006444216	0.030585759	negative
PRJNA729271	:ed_Lushi_vs_Arbor_Acr	breed_growth_background	ENSGALG00010008182	CLIC5	-0.656725151	0.146062441	-4.496194543	6.91804E-06	0.000221788	negative
PRJNA729271	:ed_Lushi_vs_Arbor_Acr	breed_growth_background	ENSGALG00010008183	ALG10	0.017059581	0.048846135	0.349251406	0.726900576	0.824527408	positive
PRJNA729271	:ed_Lushi_vs_Arbor_Acr	breed_growth_background	ENSGALG00010008184	SLC66A2	-0.185515561	0.046643853	-3.977277768	6.97087E-05	0.001218298	negative
PRJNA729271	:ed_Lushi_vs_Arbor_Acr	breed_growth_background	ENSGALG00010008185	PDGFC	-0.17847691	0.049474298	-3.607467226	0.000309201	0.003537382	negative
PRJNA729271	:ed_Lushi_vs_Arbor_Acr	breed_growth_background	ENSGALG00010008186	NOP53	0.011582282	0.064989958	0.17821649	0.85855296	0.915021992	positive
PRJNA729271	:ed_Lushi_vs_Arbor_Acr	breed_growth_background	ENSGALG00010008187	ENSGALG00010008187	-0.073056956	0.199542789	-0.366121756	0.714274224	0.81651458	negative
PRJNA729271	:ed_Lushi_vs_Arbor_Acr	breed_growth_background	ENSGALG00010008188	VPS4A	0.187086539	0.060421968	3.096333078	0.001959301	0.013093626	positive
PRJNA729271	:ed_Lushi_vs_Arbor_Acr	breed_growth_background	ENSGALG00010008189	EXOC3	0.01852143	0.061417574	0.301565644	0.762983201	0.851053598	positive
PRJNA729271	:ed_Lushi_vs_Arbor_Acr	breed_growth_background	ENSGALG00010008190	TSNARE1	-0.233933203	0.091804904	-2.548155835	0.010829408	0.044304904	negative
PRJNA729271	:ed_Lushi_vs_Arbor_Acr	breed_growth_background	ENSGALG00010008192	HNRNPU	0.047805929	0.044413459	1.076383836	0.281755628	0.441648752	positive
PRJNA729271	:ed_Lushi_vs_Arbor_Acr	breed_growth_background	ENSGALG00010008193	CNOT6L	0.136545029	0.069434425	1.966532134	0.049237174	0.131113581	positive
PRJNA729271	:ed_Lushi_vs_Arbor_Acr	breed_growth_background	ENSGALG00010008194	PDF	0.287359222	0.088352834	3.252405263	0.001144327	0.009000014	positive
PRJNA729271	:ed_Lushi_vs_Arbor_Acr	breed_growth_background	ENSGALG00010008195	ENSGALG00010008195	0.475785476	0.198527983	2.396566319	0.016549498	0.059702583	positive
PRJNA729271	:ed_Lushi_vs_Arbor_Acr	breed_growth_background	ENSGALG00010008196	PDCD6	0.056576564	0.07889032	0.717154704	0.473278645	0.627015259	positive
PRJNA729271	:ed_Lushi_vs_Arbor_Acr	breed_growth_background	ENSGALG00010008197	STARD4	-0.253242863	0.085595259	-2.958608514	0.003090314	0.018135265	negative
PRJNA729271	:ed_Lushi_vs_Arbor_Acr	breed_growth_background	ENSGALG00010008198	GLRB	0.460150719	0.217786014	2.112857065	0.034613004	0.101070807	positive
PRJNA729271	:ed_Lushi_vs_Arbor_Acr	breed_growth_background	ENSGALG00010008199	TPRKB	0.237244437	0.061186937	3.877370712	0.000105591	0.001654646	positive
PRJNA729271	:ed_Lushi_vs_Arbor_Acr	breed_growth_background	ENSGALG00010008200	COG8	0.147891091	0.069271895	2.134936404	0.032766205	0.097178372	positive
PRJNA729271	:ed_Lushi_vs_Arbor_Acr	breed_growth_background	ENSGALG00010008201	ENPP4	0.103060045	0.128902943	0.799516618	0.423990915	0.583198739	positive
PRJNA729271	:ed_Lushi_vs_Arbor_Acr	breed_growth_background	ENSGALG00010008202	KCNG2	-0.174075211	0.103233847	-1.68622226	0.091752998	0.202550115	negative
PRJNA729271	:ed_Lushi_vs_Arbor_Acr	breed_growth_background	ENSGALG00010008203	CXCL13	1.265875534	0.709316879	1.784640366	0.074319669	0.17518721	positive
PRJNA729271	:ed_Lushi_vs_Arbor_Acr	breed_growth_background	ENSGALG00010008204	ENSGALG00010008204	0.066399455	0.082534266	0.80450773	0.421103807	0.580163452	positive
PRJNA729271	:ed_Lushi_vs_Arbor_Acr	breed_growth_background	ENSGALG00010008205	ENSGALG00010008205	-0.016329182	0.097777684	-0.167003158	0.867367564	0.92058965	negative
PRJNA729271	:ed_Lushi_vs_Arbor_Acr	breed_growth_background	ENSGALG00010008206	CXCL13L2	0.96701596	0.461476558	2.095482304	0.03612814	0.104380584	positive
PRJNA729271	:ed_Lushi_vs_Arbor_Acr	breed_growth_background	ENSGALG00010008207	CAMK4	-0.091922398	0.086457698	-1.06320663	0.287688256	0.448729288	negative
PRJNA729271	:ed_Lushi_vs_Arbor_Acr	breed_growth_background	ENSGALG00010008208	ILDR2	-0.089822049	0.080849732	-1.110975224	0.266579014	0.424855723	negative
PRJNA729271	:ed_Lushi_vs_Arbor_Acr	breed_growth_background	ENSGALG00010008209	PPARGC1B	0.28275556	0.122023448	2.317223164	0.020491578	0.069344457	positive
PRJNA729271	:ed_Lushi_vs_Arbor_Acr	breed_growth_background	ENSGALG00010008210	CTDP1	0.047281859	0.045982584	1.0282558	0.303829516	0.466378147	positive
PRJNA729271	:ed_Lushi_vs_Arbor_Acr	breed_growth_background	ENSGALG00010008211	ADGRB1	-0.373152372	0.127710244	-2.921867187	0.003479399	0.019670002	negative
PRJNA729271	:ed_Lushi_vs_Arbor_Acr	breed_growth_background	ENSGALG00010008212	GTSE1	-0.353201987	0.124642731	-2.833715084	0.004601033	0.023966169	negative
PRJNA729271	:ed_Lushi_vs_Arbor_Acr	breed_growth_background	ENSGALG00010008213	ISCA1	-0.04272376	0.0626426	-0.682024049	0.495223744	0.646702902	negative
PRJNA729271	:ed_Lushi_vs_Arbor_Acr	breed_growth_background	ENSGALG00010008214	CCNG2	-0.1269176	0.072862491	-1.741878418	0.081529721	0.186535208	negative
PRJNA729271	:ed_Lushi_vs_Arbor_Acr	breed_growth_background	ENSGALG00010008215	SELENOW	0.18111479	0.068290165	2.652135769	0.007998437	0.035629179	positive
PRJNA729271	:ed_Lushi_vs_Arbor_Acr	breed_growth_background	ENSGALG00010008217	ARHGEF37	0.144368868	0.079201698	1.822800152	0.068333665	0.165205664	positive
PRJNA729271	:ed_Lushi_vs_Arbor_Acr	breed_growth_background	ENSGALG00010008218	ARHGAP42	-0.114116801	0.055495791	-2.05631454	0.039752208	0.111928565	negative
PRJNA729271	:ed_Lushi_vs_Arbor_Acr	breed_growth_background	ENSGALG00010008219	NIP7	-0.10239178	0.064305989	-1.592258859	0.111326553	0.232190429	negative
PRJNA729271	:ed_Lushi_vs_Arbor_Acr	breed_growth_background	ENSGALG00010008221	ENSGALG00010008221	0.232314626	0.278181688	0.83511833	0.403651087	0.564488423	positive
PRJNA729271	:ed_Lushi_vs_Arbor_Acr	breed_growth_background	ENSGALG00010008222	STYXL2	0.422233762	0.146783281	2.876579403	0.00402011	0.021723142	positive
PRJNA729271	:ed_Lushi_vs_Arbor_Acr	breed_growth_background	ENSGALG00010008223	ENSGALG00010008223	-0.160022628	0.082771893	-1.933296708	0.053199657	0.138493001	negative
PRJNA729271	:ed_Lushi_vs_Arbor_Acr	breed_growth_background	ENSGALG00010008224	ENSGALG00010008224	0.370072226	0.113573175	3.258447476	0.001120236	0.00889068	positive
PRJNA729271	:ed_Lushi_vs_Arbor_Acr	breed_growth_background	ENSGALG00010008226	COX20	-0.101930052	0.08944628	-1.139567254	0.254466634	0.411065849	negative
PRJNA729271	:ed_Lushi_vs_Arbor_Acr	breed_growth_background	ENSGALG00010008227	DUSP1	-0.21762445	0.093820066	-2.319593863	0.020362858	0.069124035	negative
PRJNA729271	:ed_Lushi_vs_Arbor_Acr	breed_growth_background	ENSGALG00010008228	TMED6	-0.061188508	0.306898116	-0.199377268	0.841967641	0.90437053	negative
PRJNA729271	:ed_Lushi_vs_Arbor_Acr	breed_growth_background	ENSGALG00010008229	PIGY	-0.046864797	0.065694484	-0.713374916	0.47561379	0.628901833	negative
PRJNA729271	:ed_Lushi_vs_Arbor_Acr	breed_growth_background	ENSGALG00010008230	TRMU	0.059562479	0.062158512	0.958235283	0.337944129	0.502255732	positive
PRJNA729271	:ed_Lushi_vs_Arbor_Acr	breed_growth_background	ENSGALG00010008231	ZCCHC6	-0.003968698	0.059169407	-0.067073488	0.9465232	0.970698584	negative
PRJNA729271	:ed_Lushi_vs_Arbor_Acr	breed_growth_background	ENSGALG00010008232	PYURF	0.007302511	0.068300111	0.106917991	0.91485404	0.951960468	positive
PRJNA729271	:ed_Lushi_vs_Arbor_Acr	breed_growth_background	ENSGALG00010008233	CYRIB	-0.067480726	0.03876718	-1.740666339	0.081742082	0.186737821	negative
PRJNA729271	:ed_Lushi_vs_Arbor_Acr	breed_growth_background	ENSGALG00010008234	COMMD10	0.080016868	0.065859953	1.214954829	0.22438332	0.377137782	positive
PRJNA729271	:ed_Lushi_vs_Arbor_Acr	breed_growth_background	ENSGALG00010008236	ENSGALG00010008236	-0.683118341	0.354169061	-1.928791688	0.053756728	0.139392446	negative
PRJNA729271	:ed_Lushi_vs_Arbor_Acr	breed_growth_background	ENSGALG00010008237	GRIA2	-0.063177564	0.241166202	-0.261966906	0.793346957	0.871971803	negative
PRJNA729271	:ed_Lushi_vs_Arbor_Acr	breed_growth_background	ENSGALG00010008238	NTAQ1	-0.148079369	0.090409244	-1.637878628	0.101447015	0.217305484	negative
PRJNA729271	:ed_Lushi_vs_Arbor_Acr	breed_growth_background	ENSGALG00010008240	RCAN2	-0.126705995	0.075194652	-1.685039974	0.091980859	0.202777575	negative
PRJNA729271	:ed_Lushi_vs_Arbor_Acr	breed_growth_background	ENSGALG00010008241	HERC3	-0.026715164	0.045116694	-0.592134795	0.553760329	0.695872179	negative
PRJNA729271	:ed_Lushi_vs_Arbor_Acr	breed_growth_background	ENSGALG00010008242	CNTN5	-0.438760679	0.722682105	-0.607128191			negative
PRJNA729271	:ed_Lushi_vs_Arbor_Acr	breed_growth_background	ENSGALG00010008243	YTHDC2	-0.028670919	0.055777802	-0.514020241	0.607237839	0.738105868	negative
PRJNA729271	:ed_Lushi_vs_Arbor_Acr	breed_growth_background	ENSGALG00010008245	FBXO32	0.513797046	0.262794345	1.955129768	0.050567756	0.133495486	positive
PRJNA729271	:ed_Lushi_vs_Arbor_Acr	breed_growth_background	ENSGALG00010008246	TERF2	-0.00844476	0.032369487	-0.260886452	0.794180073	0.872445923	negative
PRJNA729271	:ed_Lushi_vs_Arbor_Acr	breed_growth_background	ENSGALG00010008247	GASK1B	-0.157094961	0.086191737	-1.822622053	0.068360653	0.165205664	negative
PRJNA729271	:ed_Lushi_vs_Arbor_Acr	breed_growth_background	ENSGALG00010008248	TPK1	-0.072188327	0.088416176	-0.816460633	0.41423673	0.574580039	negative
PRJNA729271	:ed_Lushi_vs_Arbor_Acr	breed_growth_background	ENSGALG00010008250	KLHL38	0.511519353	0.314905253	1.624359543	0.104299096	0.221658869	positive
PRJNA729271	:ed_Lushi_vs_Arbor_Acr	breed_growth_background	ENSGALG00010008251	AHRR	0.92209639	0.165876646	5.558928364	2.71436E-08	3.098E-06	positive
PRJNA729271	:ed_Lushi_vs_Arbor_Acr	breed_growth_background	ENSGALG00010008252	ZFP92	-0.237403396	0.070811184	-3.352625703	0.000800489	0.006961585	negative
PRJNA729271	:ed_Lushi_vs_Arbor_Acr	breed_growth_background	ENSGALG00010008253	ENSGALG00010008253	0.474742713	0.388574668	1.221754148	0.221800613	0.37422883	positive
PRJNA729271	:ed_Lushi_vs_Arbor_Acr	breed_growth_background	ENSGALG00010008255	MSANTD3	-0.001688345	0.073819783	-0.022871169	0.981753039	0.98912284	negative
PRJNA729271	:ed_Lushi_vs_Arbor_Acr	breed_growth_background	ENSGALG00010008257	DESI2	-0.039742882	0.032513187	-1.222361905	0.221570799	0.374058785	negative
PRJNA729271	:ed_Lushi_vs_Arbor_Acr	breed_growth_background	ENSGALG00010008258	CCDC82	-0.190880836	0.070524771	-2.706578618	0.006798048	0.031735173	negative
PRJNA729271	:ed_Lushi_vs_Arbor_Acr	breed_growth_background	ENSGALG00010008259	MCC	0.030956531	0.058738669	0.527021321	0.598178782	0.731326033	positive
PRJNA729271	:ed_Lushi_vs_Arbor_Acr	breed_growth_background	ENSGALG00010008261	CRX	-0.485407315	0.224553978	-2.161650928	0.030645093	0.092426263	negative
PRJNA729271	:ed_Lushi_vs_Arbor_Acr	breed_growth_background	ENSGALG00010008262	CYB5B	0.001464362	0.03935193	0.037211948	0.970316012	0.982722932	positive
PRJNA729271	:ed_Lushi_vs_Arbor_Acr	breed_growth_background	ENSGALG00010008263	C1orf101	-0.329252313	0.305571698	-1.077496101	0.281258692	0.441216963	negative
PRJNA729271	:ed_Lushi_vs_Arbor_Acr	breed_growth_background	ENSGALG00010008264	CNTNAP2	-0.166594018	0.183087752	-0.909913504	0.362868127	0.526135191	negative

PRJNA729271	ed_Lushi_vs_Arbor_Acr	breed_growth_background	ENSGALG00010008265	ENSGALG00010008265	0.391669117	0.419189813	0.934347891	0.350124468	0.514016172	positive
PRJNA729271	ed_Lushi_vs_Arbor_Acr	breed_growth_background	ENSGALG00010008266	ECSCR	-0.074800597	0.100504855	-0.744248591	0.456726096	0.612588578	negative
PRJNA729271	ed_Lushi_vs_Arbor_Acr	breed_growth_background	ENSGALG00010008267	MAML2	-0.090811194	0.064506142	-1.407791427	0.15919284	0.297731293	negative
PRJNA729271	ed_Lushi_vs_Arbor_Acr	breed_growth_background	ENSGALG00010008268	ENSGALG00010008268	-0.498841647	0.128245887	-3.88972823	0.000100357	0.001589801	negative
PRJNA729271	ed_Lushi_vs_Arbor_Acr	breed_growth_background	ENSGALG00010008269	ENSGALG00010008269	0.130978079	0.167760719	0.780743431	0.434953411	0.593099689	positive
PRJNA729271	ed_Lushi_vs_Arbor_Acr	breed_growth_background	ENSGALG00010008270	ENSGALG00010008270	0.022730143	0.086405995	0.263062102	0.792502714	0.871506474	positive
PRJNA729271	ed_Lushi_vs_Arbor_Acr	breed_growth_background	ENSGALG00010008272	DCP2	0.059556642	0.047007124	1.26697057	0.205165813	0.354491834	positive
PRJNA729271	ed_Lushi_vs_Arbor_Acr	breed_growth_background	ENSGALG00010008273	TMEM144	-0.081496244	0.064905383	-1.255616097	0.209255192	0.358995622	negative
PRJNA729271	ed_Lushi_vs_Arbor_Acr	breed_growth_background	ENSGALG00010008274	NFAT5	0.005727405	0.059211589	0.096727772	0.922942585	0.957079179	positive
PRJNA729271	ed_Lushi_vs_Arbor_Acr	breed_growth_background	ENSGALG00010008275	CELSR1	-0.363231382	0.093370934	-3.890197585	0.000100163	0.001588465	negative
PRJNA729271	ed_Lushi_vs_Arbor_Acr	breed_growth_background	ENSGALG00010008276	DNAJC18	-0.082310598	0.049899813	-1.649517155	0.099041732	0.21334944	negative
PRJNA729271	ed_Lushi_vs_Arbor_Acr	breed_growth_background	ENSGALG00010008277	MZB1	0.753726047	0.416199456	1.810973169	0.070145005	0.168110965	positive
PRJNA729271	ed_Lushi_vs_Arbor_Acr	breed_growth_background	ENSGALG00010008278	CYP39A1	-0.013523044	0.086013192	-0.157220578	0.875071011	0.926323984	negative
PRJNA729271	ed_Lushi_vs_Arbor_Acr	breed_growth_background	ENSGALG00010008279	MAPK15	-0.037329326	0.095398097	-0.391300532	0.695575106	0.80388783	negative
PRJNA729271	ed_Lushi_vs_Arbor_Acr	breed_growth_background	ENSGALG00010008282	FAM83H	-0.157979637	0.160766434	-0.982665552	0.32577207	0.489383994	negative
PRJNA729271	ed_Lushi_vs_Arbor_Acr	breed_growth_background	ENSGALG00010008283	NQO1	-0.473788232	0.107759286	-4.39672764	1.09895E-05	0.000313569	negative
PRJNA729271	ed_Lushi_vs_Arbor_Acr	breed_growth_background	ENSGALG00010008284	BCKDHA	0.203788293	0.068377454	2.980343388	0.002879254	0.017210223	positive
PRJNA729271	ed_Lushi_vs_Arbor_Acr	breed_growth_background	ENSGALG00010008285	ADSS2	0.028330089	0.049087776	0.577131246	0.563850798	0.704145543	positive
PRJNA729271	ed_Lushi_vs_Arbor_Acr	breed_growth_background	ENSGALG00010008289	MTMR2	0.051507237	0.03848125	1.338502173	0.180732793	0.325229323	positive
PRJNA729271	ed_Lushi_vs_Arbor_Acr	breed_growth_background	ENSGALG00010008290	FAM13A	0.095084579	0.087440799	1.087416625	0.276852727	0.436051318	positive
PRJNA729271	ed_Lushi_vs_Arbor_Acr	breed_growth_background	ENSGALG00010008291	PTGFRN	-0.408281608	0.086144248	-4.739510938	2.14235E-06	8.87093E-05	negative
PRJNA729271	ed_Lushi_vs_Arbor_Acr	breed_growth_background	ENSGALG00010008292	REEP5	-0.112953794	0.060745176	-1.859469371	0.062960637	0.156082509	negative
PRJNA729271	ed_Lushi_vs_Arbor_Acr	breed_growth_background	ENSGALG00010008293	PLA2G7	0.140650711	0.0615864	2.283794966	0.022383584	0.073990888	positive
PRJNA729271	ed_Lushi_vs_Arbor_Acr	breed_growth_background	ENSGALG00010008294	NOB1	0.111592242	0.082736997	1.348758679	0.177414491	0.321573471	positive
PRJNA729271	ed_Lushi_vs_Arbor_Acr	breed_growth_background	ENSGALG00010008295	DMAC2	0.249155951	0.071379816	3.490565874	0.000481999	0.004844978	positive
PRJNA729271	ed_Lushi_vs_Arbor_Acr	breed_growth_background	ENSGALG00010008296	PALLD	-0.172134961	0.081897964	-2.101822232	0.035568851	0.103237984	negative
PRJNA729271	ed_Lushi_vs_Arbor_Acr	breed_growth_background	ENSGALG00010008297	ENSGALG00010008297	0.360228391	0.164688066	2.187337543	0.028717892	0.088054684	positive
PRJNA729271	ed_Lushi_vs_Arbor_Acr	breed_growth_background	ENSGALG00010008298	ENSGALG00010008298	-0.259180994	0.071367294	-3.631649437	0.000281616	0.003329541	negative
PRJNA729271	ed_Lushi_vs_Arbor_Acr	breed_growth_background	ENSGALG00010008299	ANKRD66	-0.136863823	0.153565836	-0.891238745	0.372801103	0.535387527	negative
PRJNA729271	ed_Lushi_vs_Arbor_Acr	breed_growth_background	ENSGALG00010008300	CUL1	0.059680749	0.045780681	1.30362301	0.192362151	0.339196828	positive
PRJNA729271	ed_Lushi_vs_Arbor_Acr	breed_growth_background	ENSGALG00010008301	FAM91A1	-0.126592399	0.056985718	-2.221475914	0.026318744	0.08282462	negative
PRJNA729271	ed_Lushi_vs_Arbor_Acr	breed_growth_background	ENSGALG00010008302	ENSGALG00010008302	0.13130667	0.077679488	1.690364766	0.090958191	0.20149478	positive
PRJNA729271	ed_Lushi_vs_Arbor_Acr	breed_growth_background	ENSGALG00010008303	TEX10	-0.020303946	0.039281035	-0.516889282	0.60523345	0.73704479	negative
PRJNA729271	ed_Lushi_vs_Arbor_Acr	breed_growth_background	ENSGALG00010008306	ENSGALG00010008306	0.236434774	0.16832811	1.404606599	0.160138293	0.298893196	positive
PRJNA729271	ed_Lushi_vs_Arbor_Acr	breed_growth_background	ENSGALG00010008307	ENSGALG00010008307	-1.287923717	0.535316363	-2.405911358	0.016132178	0.058654727	negative
PRJNA729271	ed_Lushi_vs_Arbor_Acr	breed_growth_background	ENSGALG00010008308	WWP2	0.157999543	0.068938759	2.291882607	0.021912418	0.072832034	positive
PRJNA729271	ed_Lushi_vs_Arbor_Acr	breed_growth_background	ENSGALG00010008309	ENSGALG00010008309	-0.502764302	0.148763646	-3.379618036	0.000725866	0.006478714	negative
PRJNA729271	ed_Lushi_vs_Arbor_Acr	breed_growth_background	ENSGALG00010008310	EXOSC5	0.115076579	0.097547479	1.179698133	0.238120297	0.393429123	positive
PRJNA729271	ed_Lushi_vs_Arbor_Acr	breed_growth_background	ENSGALG00010008311	SRP19	0.079328594	0.062260992	1.274129948	0.202617367	0.351813456	positive
PRJNA729271	ed_Lushi_vs_Arbor_Acr	breed_growth_background	ENSGALG00010008312	ZBTB18	0.099465531	0.116111289	0.856639624	0.39164408	0.553299686	positive
PRJNA729271	ed_Lushi_vs_Arbor_Acr	breed_growth_background	ENSGALG00010008313	PSMD7	0.285483076	0.095337327	2.994452285	0.002749381	0.016639779	positive
PRJNA729271	ed_Lushi_vs_Arbor_Acr	breed_growth_background	ENSGALG00010008314	INVS	0.056496351	0.057752387	0.97825137	0.327950015	0.491840193	positive
PRJNA729271	ed_Lushi_vs_Arbor_Acr	breed_growth_background	ENSGALG00010008316	ADGRF5	0.037030065	0.071220582	0.519934887	0.603108959	0.73524511	positive
PRJNA729271	ed_Lushi_vs_Arbor_Acr	breed_growth_background	ENSGALG00010008317	CEP57	0.205919142	0.060916361	3.38035858	0.000723913	0.00646553	positive
PRJNA729271	ed_Lushi_vs_Arbor_Acr	breed_growth_background	ENSGALG00010008318	ADPRH	0.183865301	0.071058546	2.587518475	0.009667001	0.04085224	positive
PRJNA729271	ed_Lushi_vs_Arbor_Acr	breed_growth_background	ENSGALG00010008319	ERP44	0.072947309	0.035053756	2.081012657	0.037432748	0.10733683	positive
PRJNA729271	ed_Lushi_vs_Arbor_Acr	breed_growth_background	ENSGALG00010008320	GPRIN3	-0.200943634	0.061492483	-3.267775559	0.001083963	0.008666322	negative
PRJNA729271	ed_Lushi_vs_Arbor_Acr	breed_growth_background	ENSGALG00010008321	ZFHX3	0.042014491	0.055113487	0.762326845	0.445864955	0.602649434	positive
PRJNA729271	ed_Lushi_vs_Arbor_Acr	breed_growth_background	ENSGALG00010008323	AKT3	-0.166332481	0.055980632	-2.971250532	0.002965897	0.017575912	negative
PRJNA729271	ed_Lushi_vs_Arbor_Acr	breed_growth_background	ENSGALG00010008324	SCRIB	-0.079907449	0.05561816	-1.436715089	0.150798966	0.28659119	negative
PRJNA729271	ed_Lushi_vs_Arbor_Acr	breed_growth_background	ENSGALG00010008325	AADAT	-0.132869563	0.071751579	-1.851799851	0.064054569	0.157824909	negative
PRJNA729271	ed_Lushi_vs_Arbor_Acr	breed_growth_background	ENSGALG00010008327	TGFB1	-0.092175042	0.068739445	-1.340933758	0.179941961	0.324208666	negative
PRJNA729271	ed_Lushi_vs_Arbor_Acr	breed_growth_background	ENSGALG00010008328	ENSGALG00010008328	0.22318893	0.25961146	0.859703687	0.389952402	0.551665046	positive
PRJNA729271	ed_Lushi_vs_Arbor_Acr	breed_growth_background	ENSGALG00010008330	EZH2	-0.183272116	0.044267284	-4.140125646	3.47116E-05	0.0007421	negative
PRJNA729271	ed_Lushi_vs_Arbor_Acr	breed_growth_background	ENSGALG00010008331	CCDC97	0.361818783	0.142658777	2.536253232	0.011204568	0.045378656	positive
PRJNA729271	ed_Lushi_vs_Arbor_Acr	breed_growth_background	ENSGALG00010008332	DHX38	0.075263553	0.050446085	1.491960238	0.135709569	0.265931616	positive
PRJNA729271	ed_Lushi_vs_Arbor_Acr	breed_growth_background	ENSGALG00010008333	APC	-0.056836665	0.049456763	-1.149219274	0.250465575	0.40632325	negative
PRJNA729271	ed_Lushi_vs_Arbor_Acr	breed_growth_background	ENSGALG00010008334	STX17	0.001567772	0.071901475	0.021804455	0.98260394	0.98977374	positive
PRJNA729271	ed_Lushi_vs_Arbor_Acr	breed_growth_background	ENSGALG00010008335	HNRNPUL1	0.192999658	0.103071346	1.872486058	0.061139389	0.152953994	positive
PRJNA729271	ed_Lushi_vs_Arbor_Acr	breed_growth_background	ENSGALG00010008336	PAIP2B	-0.083366394	0.05833602	-1.429072377	0.152983432	0.289452401	negative
PRJNA729271	ed_Lushi_vs_Arbor_Acr	breed_growth_background	ENSGALG00010008339	FAM76B	-0.071790158	0.062374412	-1.150955253	0.249750645	0.405708349	negative
PRJNA729271	ed_Lushi_vs_Arbor_Acr	breed_growth_background	ENSGALG00010008340	PDIA4	-0.170454466	0.056663122	-3.008208139	0.002627931	0.016113307	negative
PRJNA729271	ed_Lushi_vs_Arbor_Acr	breed_growth_background	ENSGALG00010008341	DHODH	-0.124760105	0.061243415	-2.0371187	0.041638148	0.115821332	negative
PRJNA729271	ed_Lushi_vs_Arbor_Acr	breed_growth_background	ENSGALG00010008342	PLD3	0.22497268	0.079341799	2.8354875	0.004575581	0.023874385	positive
PRJNA729271	ed_Lushi_vs_Arbor_Acr	breed_growth_background	ENSGALG00010008343	TNFRSF21	0.266760494	0.119655926	2.229396428	0.02578754	0.081792208	positive
PRJNA729271	ed_Lushi_vs_Arbor_Acr	breed_growth_background	ENSGALG00010008344	MFAP3L	0.204877163	0.155372156	1.318622124	0.187295474	0.332905934	positive
PRJNA729271	ed_Lushi_vs_Arbor_Acr	breed_growth_background	ENSGALG00010008346	NR4A3	-0.295254163	0.280673424	-1.051949127	0.292822891	0.45424527	negative
PRJNA729271	ed_Lushi_vs_Arbor_Acr	breed_growth_background	ENSGALG00010008347	PUF60	-0.001340408	0.029893256	-0.044839806	0.964234996	0.979609321	negative
PRJNA729271	ed_Lushi_vs_Arbor_Acr	breed_growth_background	ENSGALG00010008348	ENSGALG00010008348	0.046710118	0.05685032	0.82163332	0.41128562	0.571543795	positive
PRJNA729271	ed_Lushi_vs_Arbor_Acr	breed_growth_background	ENSGALG00010008350	LRRC6	0.118879208	0.098198217	1.210604555	0.226046998	0.379274364	positive
PRJNA729271	ed_Lushi_vs_Arbor_Acr	breed_growth_background	ENSGALG00010008351	IST1	-0.029384326	0.037018248	-0.793779492	0.427323813	0.586474019	negative
PRJNA729271	ed_Lushi_vs_Arbor_Acr	breed_growth_background	ENSGALG00010008352	ENSGALG00010008352	0.014315411	0.082559316	0.173395459	0.862340594	0.917201857	positive
PRJNA729271	ed_Lushi_vs_Arbor_Acr	breed_growth_background	ENSGALG00010008354	TMEM71	0.006389091	0.114962565	0.055575403	0.95568006	0.975259256	positive
PRJNA729271	ed_Lushi_vs_Arbor_Acr	breed_growth_background	ENSGALG00010008355	MAFA	0.150839266	0.092928693	1.623172152	0.104552609	0.221919764	positive
PRJNA729271	ed_Lushi_vs_Arbor_Acr	breed_growth_background	ENSGALG00010008356	ENSGALG00010008356	-0.112508236	0.072361565	-1.554806555	0.119992148	0.244384738	negative
PRJNA729271	ed_Lushi_vs_Arbor_Acr	breed_growth_background	ENSGALG00010008358	HPF1	-0.044849289	0.063454971	-0.706789208	0.479697482	0.632570506	negative
PRJNA729271	ed_Lushi_vs_Arbor_Acr	breed_growth_background	ENSGALG00010008360	ZC3H3	0.046862894	0.033880027	1.383201189	0.166603185	0.307319059	positive
PRJNA729271	ed_Lushi_vs_Arbor_Acr	breed_growth_background	ENSGALG00010008361	MATR3	0.130106991	0.030642475	4.245968736	2.17651E-05	0.000508028	positive
PRJNA729271	ed_Lushi_vs_Arbor_Acr	breed_growth_background	ENSGALG00010008362	RAMP3	-0.509962902	0.140380231	-3.632725909	0.000280443	0.00332381	negative
PRJNA729271	ed_Lushi_vs_Arbor_Acr	breed_growth_background	ENSGALG00010008364	ENSGALG00010008364	1.020700809	0.333899819	3.056907346	0.002236334	0.014413366	positive
PRJNA729271	ed_Lushi_vs_Arbor_Acr	breed_growth_background	ENSGALG00010008366	RPRD1A	-0.024120118	0.058277004	-0.413887399	0.678956573	0.792623996	negative
PRJNA729271	ed_Lushi_vs_Arbor_Acr	breed_growth_background	ENSGALG00010008367	ENSGALG00010008367	0.431017626	0.345606917	1.247132521	0.21234891	0.362529725	positive
PRJNA729271	ed_Lushi_vs_Arbor_Acr	breed_growth_background	ENSGALG00010008368	SNCA	0.790881573	0.173784227	4.550939903	5.34068E-06	0.000182578	positive
PRJNA729271	ed_Lushi_vs_Arbor_Acr	breed_growth_background	ENSGALG00010008372	CLCN3	0.008180273	0.037469961	0.218315493	0.827183304	0.894710991	positive
PRJNA729271	ed_Lushi_vs_Arbor_Acr	breed_growth_background	ENSGALG00010008373	MACIR	-0.028978618	0.089377003	-0.324229024	0.74576465	0.837908581	negative
PRJNA729271	ed_Lushi_vs_Arbor_Acr	breed_growth_background	ENSGALG00010008374	ZNF821	0.059014896	0.047221804	1.249738264	0.211395175	0.361469041	positive
PRJNA729271	ed_Lushi_vs_Arbor_Acr	breed_growth_background	ENSGALG00010008375	ENSGALG00010008375	-0.185121198	0.05316894	-3.481754559	0.00049814	0.004960945	negative
PRJNA729271	ed_Lushi_vs_Arbor_Acr	breed_growth_background	ENSGALG00010008376	MMRN1	0.166254263	0.140864638	1.1802413	0.237904257	0.393161807	positive
PRJNA729271	ed_Lushi_vs_Arbor_Acr	breed_growth_background	ENSGALG00010008377	AKT2	0.181327681	0.062539508	2.899410072	0.003738656	0.020652369	positive
PRJNA729271	ed_Lushi_vs_Arbor_Acr	breed_growth_background	ENSGALG00010008380	SIL1	0.011583628	0.091428185	0.126696462	0.899180646	0.94153171	positive
PRJNA729271	ed_Lushi_vs_Arbor_Acr	breed_growth_background	ENSGALG00010008381	C18orf21	0.220329132	0.060904528	3.617614947	0.00029733	0.003453367	positive
PRJNA729271	ed_Lushi_vs_Arbor_Acr	breed_growth_background	ENSGALG00010008382	ENSGALG00010008382	0.182140254	0.069257988	2.629880818	0.008541481	0.037146343	positive
PRJNA729271	ed_Lushi_vs_Arbor_Acr	breed_growth_background	ENSGALG00010008383	PLD5	-0.202185514	0.241301788	-0.837894804	0.402089787	0.563119948	negative
PRJNA729271	ed_Lushi_vs_Arbor_Acr	breed_growth_background	ENSGALG00010008385	CTNNA1	-0.266227087	0.049005049	-5.43264602	5.55245E-08	5.32998E-06	negative
PRJNA729271	ed_Lushi_vs_Arbor_Acr	breed_growth_background	ENSGALG00010008386	SESN3	-0.422196231	0.122081162	-3.458324151	0.000543547	0.005280639	negative
PRJNA729271	ed_Lushi_vs_Arbor_Acr	breed_growth_background	ENSGALG00010008387	PHF20L1	0.209153055	0.060210389	3.473703772	0.000513327	0.005061688	positive
PRJNA729271	ed_Lushi_vs_Arbor_Acr	breed_growth_background	ENSGALG00010008388	ADCY1	-0.357521661	0.229176334	-1.560028709	0.118753097	0.242648979	negative
PRJNA729271	ed_Lushi_vs_Arbor_Acr	breed_growth_background	ENSGALG00010008389	GALNT1	-0.134142874	0.047649398	-2.815206079	0.004874596	0.025073554	negative
PRJNA729271	ed_Lushi_vs_Arbor_Acr	breed_growth_background	ENSGALG00010008391	ENSGALG00010008391	-0.132234503	0.040120982	-3.295893994	0.00098109	0.00804805	negative
PRJNA729271	ed_Lushi_vs_Arbor_Acr	breed_growth_background	ENSGALG00010008392	CD36	-0.510035678	0.197131857	-2.587281861	0.009673643	0.040868391	negative
PRJNA729271	ed_Lushi_vs_Arbor_Acr	breed_growth_background	ENSGALG00010008394	CD2AP	-0.156041423	0.059340781	-2.629581545	0.008549003	0.037167904	negative
PRJNA729271	ed_Lushi_vs_Arbor_Acr	breed_growth_background	ENSGALG00010008395	ENDOD1	-0.197051834	0.077896386	-2.529665934	0.011417117	0.045905996	negative
PRJNA729271	ed_Lushi_vs_Arbor_Acr	breed_growth_background	ENSGALG00010008396	LRRTM2	-0.273206554	0.218039634	-1.253013268	0.210200878	0.360106575	negative
PRJNA729271	ed_Lushi_vs_Arbor_Acr	breed_growth_background	ENSGALG00010008397	ATXN1L	-0.053744062	0.039402262	-1.363984185	0.172572543	0.315758522	negative
PRJNA729271	ed_Lushi_vs_Arbor_Acr	breed_growth_background	ENSGALG00010008399	INO80C	-0.039327315	0.061615045	-0.638274548	0.523294977	0.670244826	negative
PRJNA729271	ed_Lushi_vs_Arbor_Acr	breed_growth_background	ENSGALG00010008400	MAP1LC3C	-0.282664236	0.144962758	-1.949909338	0.051186926	0.134753812	negative
PRJNA729271	ed_Lushi_vs_Arbor_Acr	breed_growth_background	ENSGALG00010008401	PSMD8	0.334231449	0.083824873	3.987258621	6.68412E-05	0.001184428	positive
PRJNA729271	ed_Lushi_vs_Arbor_Acr	breed_growth_background	ENSGALG00010008403	GNAT3	-1.292215887	0.24682721	-5.235305641	1.64712E-07	1.25396E-05	negative
PRJNA729271	ed_Lushi_vs_Arbor_Acr	breed_growth_background	ENSGALG00010008405	LRATD2	0.076924693	0.078028882	0.985848962	0.324207235	0.488247675	positive
PRJNA729271	ed_Lushi_vs_Arbor_Acr	breed_growth_background	ENSGALG00010008407	GNAI1	-0.269519303	0.104113783	-2.588699564	0.009633911	0.04074804	negative
PRJNA729271	ed_Lushi_vs_Arbor_Acr	breed_growth_background	ENSGALG00010008408	SMARCA4	0.139546584	0.082653749	1.688327337	0.09134841	0.201966932	positive
PRJNA729271	ed_Lushi_vs_Arbor_Acr	breed_growth_background	ENSGALG00010008409	CWC15	0.082253896	0.045533949	1.806430105	0.070851183	0.169162888	positive
PRJNA729271	ed_Lushi_vs_Arbor_Acr	breed_growth_background	ENSGALG00010008410	NAPRT	-0.264840958	0.071614175	-3.698163919	0.000217165	0.002761229	negative
PRJNA729271	ed_Lushi_vs_Arbor_Acr	breed_growth_background	ENSGALG00010008411	ENSGALG00010008411	0.022611579	0.220759194	0.102426442	0.918418196	0.954367464	positive
PRJNA729271	ed_Lushi_vs_Arbor_Acr	breed_growth_background	ENSGALG00010008412	NEK1	-0.02287243	0.057023934	-0.401102283	0.68834482	0.798539286	negative
PRJNA729271	ed_Lushi_vs_Arbor_Acr	breed_growth_background	ENSGALG00010008415	ENSGALG00010008415	-0.177482634	0.189397332	-0.937091524	0.348711481	0.512790192	negative
PRJNA729271	ed_Lushi_vs_Arbor_Acr	breed_growth_background	ENSGALG00010008416	MSX2	0.071736116	0.262238115	0.273553356	0.784427883	0.866637361	positive
PRJNA729271	ed_Lushi_vs_Arbor_Acr	breed_growth_background	ENSGALG00010008417	IGFBP1	-0.94600291	0.495563041	-1.908945649	0.056269103	0.144281028	negative
PRJNA729271	ed_Lushi_vs_Arbor_Acr	breed_growth_background	ENSGALG00010008418	TMEM245	-0.131954924	0.047597131	-2.772329338	0.00556567	0.027458945	negative
PRJNA729271	ed_Lushi_vs_Arbor_Acr	breed_growth_background	ENSGALG00010008422	EXO1	-0.471595681	0.128689502	-3.664601025	0.000247724	0.003027627	negative
PRJNA729271	ed_Lushi_vs_Arbor_Acr	breed_growth_background	ENSGALG00010008424	PPIP5K2	-0.076044324	0.065396409	-1.162821099	0.244902095	0.400796643	negative
PRJNA729271	ed_Lushi_vs_Arbor_Acr	breed_growth_background	ENSGALG00010008425	MAGI2	-0.098162971	0.058851417	-1.667979739	0.095319749	0.207897002	negative
PRJNA729271	ed_Lushi_vs_Arbor_Acr	breed_growth_background	ENSGALG00010008426	AP1G1	0.036861434	0.030375498	1.213525275	0.224929057	0.377835981	positive
PRJNA729271	ed_Lushi_vs_Arbor_Acr	breed_growth_background	ENSGALG00010008429	IGFBP3	-0.089549704	0.063372058	-1.413078687	0.157632589	0.295433719	negative
PRJNA729271	ed_Lushi_vs_Arbor_Acr	breed_growth_background	ENSGALG00010008430	ENSGALG00010008430	-0.002412162	0.225075042	-0.010717144	0.99144912	0.995225415	negative
PRJNA729271	ed_Lushi_vs_Arbor_Acr	breed_growth_background	ENSGALG00010008431	SH3RF1	0.062767382	0.068392208	0.917756339	0.358746445	0.522323865	positive
PRJNA729271	ed_Lushi_vs_Arbor_Acr	breed_growth_background	ENSGALG00010008433	EBAG9	0.019543641	0.051386557	0.380325949	0.703703476	0.808511563	positive
PRJNA729271	ed_Lushi_vs_Arbor_Acr	breed_growth_background	ENSGALG00010008434	PTCHD4	-0.59455302	0.311633345	-1.907860727	0.056409219	0.144512484	negative
PRJNA729271	ed_Lushi_vs_Arbor_Acr	breed_growth_background	ENSGALG00010008437	SHKBP1	-0.06153665	0.087619145	-0.702319681	0.482479822	0.634858631	negative
PRJNA729271	ed_Lushi_vs_Arbor_Acr	breed_growth_background	ENSGALG00010008438	SYBU	-0.238730398	0.142120793	-1.679771076	0.093001865	0.204458738	negative
PRJNA729271	ed_Lushi_vs_Arbor_Acr	breed_growth_background	ENSGALG00010008440	ENSGALG00010008440	-0.139430659	0.171044941	-0.815169731	0.414975162	0.575239875	negative

PRJNA729271	ed_Lushi_vs_Arbor_Acr	breed_growth_background	ENSGALG00010008441	PHLPP2	0.10064861	0.047098301	2.136990242	0.032598785	0.096927055	positive
PRJNA729271	ed_Lushi_vs_Arbor_Acr	breed_growth_background	ENSGALG00010008443	RSFR	-0.250195458	0.232661186	-1.075363973	0.282211805	0.442090145	negative
PRJNA729271	ed_Lushi_vs_Arbor_Acr	breed_growth_background	ENSGALG00010008444	SLC7A11	-0.122268697	0.264899147	-0.46156697	0.644391885	0.767309651	negative
PRJNA729271	ed_Lushi_vs_Arbor_Acr	breed_growth_background	ENSGALG00010008447	FRRS1L	-0.268810293	0.083896931	-3.204053967	0.001355071	0.010166537	negative
PRJNA729271	ed_Lushi_vs_Arbor_Acr	breed_growth_background	ENSGALG00010008448	TNS3	-0.112272807	0.043647716	-2.572249329	0.01010401	0.042139346	negative
PRJNA729271	ed_Lushi_vs_Arbor_Acr	breed_growth_background	ENSGALG00010008449	SRA1	-0.071478014	0.058870248	-1.214161914	0.224685901	0.377515027	negative
PRJNA729271	ed_Lushi_vs_Arbor_Acr	breed_growth_background	ENSGALG00010008450	GIN1	-0.035415419	0.098484719	-0.359603186	0.7191439	0.82014807	negative
PRJNA729271	ed_Lushi_vs_Arbor_Acr	breed_growth_background	ENSGALG00010008451	OPN3	-0.311399286	0.168209791	-1.851255417	0.064132817	0.157988643	negative
PRJNA729271	ed_Lushi_vs_Arbor_Acr	breed_growth_background	ENSGALG00010008452	ENSGALG00010008452	-0.19798813	0.228894607	-0.864975072	0.387052471	0.549035521	negative
PRJNA729271	ed_Lushi_vs_Arbor_Acr	breed_growth_background	ENSGALG00010008453	AMOTL1	0.094892544	0.064032956	1.481932889	0.138358153	0.269906814	positive
PRJNA729271	ed_Lushi_vs_Arbor_Acr	breed_growth_background	ENSGALG00010008455	FKBP1B	0.182063149	0.080515517	2.261218155	0.023745751	0.07710454	positive
PRJNA729271	ed_Lushi_vs_Arbor_Acr	breed_growth_background	ENSGALG00010008456	NHLRC1	0.2568333	0.069594444	3.690428233	0.000223877	0.002816923	positive
PRJNA729271	ed_Lushi_vs_Arbor_Acr	breed_growth_background	ENSGALG00010008457	MARVELD3	-0.114100105	0.064300973	-1.774469355	0.075985547	0.177704179	negative
PRJNA729271	ed_Lushi_vs_Arbor_Acr	breed_growth_background	ENSGALG00010008459	EPB41L4B	0.142160861	0.128634967	1.105149432	0.269094844	0.427736568	positive
PRJNA729271	ed_Lushi_vs_Arbor_Acr	breed_growth_background	ENSGALG00010008464	SF3B6	0.064193795	0.060121448	1.067735343	0.285639911	0.44615846	positive
PRJNA729271	ed_Lushi_vs_Arbor_Acr	breed_growth_background	ENSGALG00010008466	C11orf97	-0.445461652	0.199676797	-2.230913454	0.025686862	0.0815264	negative
PRJNA729271	ed_Lushi_vs_Arbor_Acr	breed_growth_background	ENSGALG00010008468	EPPK1	-0.610409824	0.162430249	-3.757981221	0.00017129	0.002326569	negative
PRJNA729271	ed_Lushi_vs_Arbor_Acr	breed_growth_background	ENSGALG00010008469	NOCT	0.413841158	0.148352914	2.789572159	0.005277773	0.026489379	positive
PRJNA729271	ed_Lushi_vs_Arbor_Acr	breed_growth_background	ENSGALG00010008473	CSMD3	-0.123183752	0.247281112	-0.498152693	0.618376423	0.74660693	negative
PRJNA729271	ed_Lushi_vs_Arbor_Acr	breed_growth_background	ENSGALG00010008474	ENSGALG00010008474	-0.017610625	0.073912232	-0.238264017	0.811676328	0.884139804	negative
PRJNA729271	ed_Lushi_vs_Arbor_Acr	breed_growth_background	ENSGALG00010008476	RNF166	0.059781793	0.072107448	0.829065443	0.407067377	0.567732284	positive
PRJNA729271	ed_Lushi_vs_Arbor_Acr	breed_growth_background	ENSGALG00010008486	CTU2	0.055866244	0.075016334	0.744721061	0.456440365	0.612318657	positive
PRJNA729271	ed_Lushi_vs_Arbor_Acr	breed_growth_background	ENSGALG00010008488	CCSER1	-0.155130974	0.084664655	-1.832299129	0.066906867	0.163043412	negative
PRJNA729271	ed_Lushi_vs_Arbor_Acr	breed_growth_background	ENSGALG00010008492	ANKRD49	0.072303375	0.067240603	1.075293377	0.2822434	0.442091862	positive
PRJNA729271	ed_Lushi_vs_Arbor_Acr	breed_growth_background	ENSGALG00010008497	PAM	-0.045613032	0.06970568	-0.654366081	0.512875981	0.661517828	negative
PRJNA729271	ed_Lushi_vs_Arbor_Acr	breed_growth_background	ENSGALG00010008498	ENSGALG00010008498	0.221356308	0.07884253	2.807574913	0.004991607	0.025530466	positive
PRJNA729271	ed_Lushi_vs_Arbor_Acr	breed_growth_background	ENSGALG00010008500	ENSGALG00010008500	0.161463087	0.080308309	2.010540226	0.044374043	0.121463607	positive
PRJNA729271	ed_Lushi_vs_Arbor_Acr	breed_growth_background	ENSGALG00010008501	ENSGALG00010008501	-0.032577684	0.238835017	-0.136402461	0.891503128	0.936401293	negative
PRJNA729271	ed_Lushi_vs_Arbor_Acr	breed_growth_background	ENSGALG00010008504	RNF139	0.024154049	0.046922283	0.514767134	0.606715754	0.737698976	positive
PRJNA729271	ed_Lushi_vs_Arbor_Acr	breed_growth_background	ENSGALG00010008505	PTPN3	-0.49905782	0.146475117	-3.407116729	0.00065653	0.006023043	negative
PRJNA729271	ed_Lushi_vs_Arbor_Acr	breed_growth_background	ENSGALG00010008509	MGARP	0.569696414	0.191809308	2.970118709	0.002976847	0.017607832	positive
PRJNA729271	ed_Lushi_vs_Arbor_Acr	breed_growth_background	ENSGALG00010008514	PIEZO1	-0.042380834	0.088621715	-0.478221783	0.63249236	0.757789574	negative
PRJNA729271	ed_Lushi_vs_Arbor_Acr	breed_growth_background	ENSGALG00010008515	TAFA5	-0.077748767	0.117970263	-0.659053945	0.509861129	0.658566838	negative
PRJNA729271	ed_Lushi_vs_Arbor_Acr	breed_growth_background	ENSGALG00010008516	MRE11	-0.060066091	0.043490062	-1.381145205	0.167234319	0.308208703	negative
PRJNA729271	ed_Lushi_vs_Arbor_Acr	breed_growth_background	ENSGALG00010008522	NUDT12	-0.265363181	0.08056572	-3.293748042	0.000988611	0.008085384	negative
PRJNA729271	ed_Lushi_vs_Arbor_Acr	breed_growth_background	ENSGALG00010008527	CHUK	0.019975879	0.0297929	0.670491262	0.502544675	0.653484116	positive
PRJNA729271	ed_Lushi_vs_Arbor_Acr	breed_growth_background	ENSGALG00010008529	GPR83	0.39190477	0.283128109	1.384195908	0.166298476	0.306865985	positive
PRJNA729271	ed_Lushi_vs_Arbor_Acr	breed_growth_background	ENSGALG00010008533	ENSGALG00010008533	0.058085638	0.326005977	0.178173536	0.858586693	0.915021992	positive
PRJNA729271	ed_Lushi_vs_Arbor_Acr	breed_growth_background	ENSGALG00010008534	RAB33B	0.078249609	0.038836089	2.014868401	0.04391844	0.120528944	positive
PRJNA729271	ed_Lushi_vs_Arbor_Acr	breed_growth_background	ENSGALG00010008542	MOCOS	0.113978457	0.082120406	1.387943179	0.165154348	0.305244211	positive
PRJNA729271	ed_Lushi_vs_Arbor_Acr	breed_growth_background	ENSGALG00010008561	EEF1D	0.068067243	0.061308681	1.110238264	0.266896368	0.425227371	positive
PRJNA729271	ed_Lushi_vs_Arbor_Acr	breed_growth_background	ENSGALG00010008580	PANX1	-0.201241093	0.136515074	-1.474130931	0.140446342	0.272818243	negative
PRJNA729271	ed_Lushi_vs_Arbor_Acr	breed_growth_background	ENSGALG00010008588	PYCR3	-0.133061738	0.098835928	-1.346289155	0.178209282	0.322449575	negative
PRJNA729271	ed_Lushi_vs_Arbor_Acr	breed_growth_background	ENSGALG00010008592	KIAA0513	-0.166905509	0.074092126	-2.252675397	0.024279623	0.078294358	negative
PRJNA729271	ed_Lushi_vs_Arbor_Acr	breed_growth_background	ENSGALG00010008596	ENSGALG00010008596	0.0592607	0.085196387	0.69557762	0.486693386	0.638484988	positive
PRJNA729271	ed_Lushi_vs_Arbor_Acr	breed_growth_background	ENSGALG00010008618	EIF4EBP3	0.205495869	0.06249791	3.288043834	0.001008861	0.008192406	positive
PRJNA729271	ed_Lushi_vs_Arbor_Acr	breed_growth_background	ENSGALG00010008623	TRPS1	-0.167432617	0.071560967	-2.339719881	0.019298208	0.066665283	negative
PRJNA729271	ed_Lushi_vs_Arbor_Acr	breed_growth_background	ENSGALG00010008636	AGTPBP1	0.546235762	0.127492786	4.284444489	1.83196E-05	0.000447796	positive
PRJNA729271	ed_Lushi_vs_Arbor_Acr	breed_growth_background	ENSGALG00010008637	RGS7	0.602907067	0.490078001	1.230226752	0.218612205	0.370791471	positive
PRJNA729271	ed_Lushi_vs_Arbor_Acr	breed_growth_background	ENSGALG00010008653	ENSGALG00010008653	-0.302253372	0.087425878	-3.457252932	0.000545712	0.005289773	negative
PRJNA729271	ed_Lushi_vs_Arbor_Acr	breed_growth_background	ENSGALG00010008656	CCDC6	0.109442653	0.03868482	2.829085222	0.004668127	0.024209122	positive
PRJNA729271	ed_Lushi_vs_Arbor_Acr	breed_growth_background	ENSGALG00010008658	TSTA3	-0.19455137	0.065353018	-2.97693013	0.002911504	0.017345764	negative
PRJNA729271	ed_Lushi_vs_Arbor_Acr	breed_growth_background	ENSGALG00010008666	SLC16A9	-0.004839424	0.100888218	-0.047968181	0.961741602	0.978276808	negative
PRJNA729271	ed_Lushi_vs_Arbor_Acr	breed_growth_background	ENSGALG00010008668	ENSGALG00010008668	0.012103527	0.176419987	0.068606326	0.945302983	0.970336705	positive
PRJNA729271	ed_Lushi_vs_Arbor_Acr	breed_growth_background	ENSGALG00010008674	RXFP1	-0.195883632	0.146204028	-1.339796409	0.180311543	0.324793814	negative
PRJNA729271	ed_Lushi_vs_Arbor_Acr	breed_growth_background	ENSGALG00010008686	ENSGALG00010008686	-0.524852389	0.234838432	-2.234951001	0.025420566	0.080956272	negative
PRJNA729271	ed_Lushi_vs_Arbor_Acr	breed_growth_background	ENSGALG00010008688	HBEGF	-0.2969283	0.123879628	-2.396909845	0.016533991	0.05969814	negative
PRJNA729271	ed_Lushi_vs_Arbor_Acr	breed_growth_background	ENSGALG00010008690	YIF1B	0.140584207	0.067069361	2.096101787	0.036073163	0.104263308	positive
PRJNA729271	ed_Lushi_vs_Arbor_Acr	breed_growth_background	ENSGALG00010008693	C4orf46	0.122356694	0.057177332	2.1399511	0.032358719	0.096430845	positive
PRJNA729271	ed_Lushi_vs_Arbor_Acr	breed_growth_background	ENSGALG00010008696	PFN4	0.070289973	0.115319097	0.609525872	0.54217593	0.687141742	positive
PRJNA729271	ed_Lushi_vs_Arbor_Acr	breed_growth_background	ENSGALG00010008699	NAA35	0.13859686	0.053736793	2.57917994	0.009903518	0.041512869	positive
PRJNA729271	ed_Lushi_vs_Arbor_Acr	breed_growth_background	ENSGALG00010008700	ENSGALG00010008700	-0.024921491	0.14099185	-0.176758383	0.85969818	0.915667949	negative
PRJNA729271	ed_Lushi_vs_Arbor_Acr	breed_growth_background	ENSGALG00010008708	ZC3H4	0.394780985	0.131455268	3.003158343	0.002671934	0.01630037	positive
PRJNA729271	ed_Lushi_vs_Arbor_Acr	breed_growth_background	ENSGALG00010008716	TATDN1	0.13246255	0.060896349	2.175213332	0.029614134	0.090085386	positive
PRJNA729271	ed_Lushi_vs_Arbor_Acr	breed_growth_background	ENSGALG00010008729	TMEM254	-0.292181955	0.064535398	-4.527468106	5.96946E-06	0.000197551	negative
PRJNA729271	ed_Lushi_vs_Arbor_Acr	breed_growth_background	ENSGALG00010008731	NDUFB9	-0.046413401	0.046858186	-0.990507857	0.321925951	0.485716621	negative
PRJNA729271	ed_Lushi_vs_Arbor_Acr	breed_growth_background	ENSGALG00010008739	VSTM5	-0.198757024	0.144738479	-1.373214815	0.169685575	0.311814454	negative
PRJNA729271	ed_Lushi_vs_Arbor_Acr	breed_growth_background	ENSGALG00010008745	GRAMD4	0.298324757	0.081839351	3.645248309	0.000267134	0.003202733	positive
PRJNA729271	ed_Lushi_vs_Arbor_Acr	breed_growth_background	ENSGALG00010008750	PFDN1	-0.304799248	0.088730138	-3.435126493	0.000592277	0.005563875	negative
PRJNA729271	ed_Lushi_vs_Arbor_Acr	breed_growth_background	ENSGALG00010008752	GOLM1	-0.527208541	0.092688904	-5.687935872	1.28584E-08	1.66458E-06	negative
PRJNA729271	ed_Lushi_vs_Arbor_Acr	breed_growth_background	ENSGALG00010008756	ITSN2	0.065492164	0.038396791	1.705667638	0.088069978	0.197032619	positive
PRJNA729271	ed_Lushi_vs_Arbor_Acr	breed_growth_background	ENSGALG00010008762	SAE1	0.105539245	0.055418686	1.904398173	0.056858347	0.145262041	positive
PRJNA729271	ed_Lushi_vs_Arbor_Acr	breed_growth_background	ENSGALG00010008764	ETFDH	0.144042136	0.066263829	2.173767178	0.029722625	0.090282784	positive
PRJNA729271	ed_Lushi_vs_Arbor_Acr	breed_growth_background	ENSGALG00010008768	PRXL2C	-0.095595636	0.076076744	-1.256568445	0.208909947	0.358615547	negative
PRJNA729271	ed_Lushi_vs_Arbor_Acr	breed_growth_background	ENSGALG00010008775	MED17	0.088427178	0.048510732	1.822837424	0.068328018	0.165205664	positive
PRJNA729271	ed_Lushi_vs_Arbor_Acr	breed_growth_background	ENSGALG00010008780	KARS1	-0.04017576	0.030750348	-1.306513967	0.191377832	0.338295327	negative
PRJNA729271	ed_Lushi_vs_Arbor_Acr	breed_growth_background	ENSGALG00010008782	CCDC9	0.241235173	0.15463583	1.560021201	0.118754871	0.242648979	positive
PRJNA729271	ed_Lushi_vs_Arbor_Acr	breed_growth_background	ENSGALG00010008790	GPN1	0.020771194	0.051569537	0.402780302	0.687109853	0.797989263	positive
PRJNA729271	ed_Lushi_vs_Arbor_Acr	breed_growth_background	ENSGALG00010008793	C11orf54	0.082154839	0.061711743	1.331267524	0.183101	0.328224121	positive
PRJNA729271	ed_Lushi_vs_Arbor_Acr	breed_growth_background	ENSGALG00010008799	ENSGALG00010008799	-0.073460931	0.093658488	-0.784348893	0.432835418	0.591045631	negative
PRJNA729271	ed_Lushi_vs_Arbor_Acr	breed_growth_background	ENSGALG00010008807	BRD1	0.055574349	0.033438771	1.661973427	0.096518103	0.209561099	positive
PRJNA729271	ed_Lushi_vs_Arbor_Acr	breed_growth_background	ENSGALG00010008811	TERF2IP	-0.055105764	0.050566134	-1.089776089	0.275811789	0.434742484	negative
PRJNA729271	ed_Lushi_vs_Arbor_Acr	breed_growth_background	ENSGALG00010008814	ENSGALG00010008814	0.00569536	0.1277177	0.044593352	0.964431442	0.979637964	positive
PRJNA729271	ed_Lushi_vs_Arbor_Acr	breed_growth_background	ENSGALG00010008821	ENSGALG00010008821	-0.033450737	0.074315527	-0.450117734	0.65262555	0.773428775	negative
PRJNA729271	ed_Lushi_vs_Arbor_Acr	breed_growth_background	ENSGALG00010008824	GREM2	0.223529414	0.167923814	1.331135882	0.183144304	0.328224121	positive
PRJNA729271	ed_Lushi_vs_Arbor_Acr	breed_growth_background	ENSGALG00010008825	PPID	-0.058762162	0.058421194	-1.005836378	0.314494285	0.477940308	negative
PRJNA729271	ed_Lushi_vs_Arbor_Acr	breed_growth_background	ENSGALG00010008830	DHX34	0.020023762	0.10710907	0.186947398	0.851701876	0.91064135	positive
PRJNA729271	ed_Lushi_vs_Arbor_Acr	breed_growth_background	ENSGALG00010008832	MTSS1	-0.207443053	0.066430291	-3.122717817	0.001791895	0.01230389	negative
PRJNA729271	ed_Lushi_vs_Arbor_Acr	breed_growth_background	ENSGALG00010008837	ENSGALG00010008837	0.13482526	0.053670091	2.512111641	0.012001112	0.04755655	positive
PRJNA729271	ed_Lushi_vs_Arbor_Acr	breed_growth_background	ENSGALG00010008839	ZNF512	0.105110251	0.04012925	2.619292706	0.008811231	0.038120224	positive
PRJNA729271	ed_Lushi_vs_Arbor_Acr	breed_growth_background	ENSGALG00010008846	SLC35D2	-0.138253416	0.154960821	-0.892183036	0.372294833	0.53488092	negative
PRJNA729271	ed_Lushi_vs_Arbor_Acr	breed_growth_background	ENSGALG00010008850	CYSTM1	0.002732484	0.064743756	0.042204601	0.966335595	0.980988414	positive
PRJNA729271	ed_Lushi_vs_Arbor_Acr	breed_growth_background	ENSGALG00010008852	KHDRBS3	-0.02015744	0.044758816	-0.450356857	0.652453148	0.773428775	negative
PRJNA729271	ed_Lushi_vs_Arbor_Acr	breed_growth_background	ENSGALG00010008859	ENSGALG00010008859	-0.217334022	0.119100927	-1.824788659	0.068032928	0.164766958	negative
PRJNA729271	ed_Lushi_vs_Arbor_Acr	breed_growth_background	ENSGALG00010008860	SQLE	-0.292753199	0.134593692	-2.175088548	0.029623482	0.090094915	negative
PRJNA729271	ed_Lushi_vs_Arbor_Acr	breed_growth_background	ENSGALG00010008862	ZNF367	-0.238871833	0.079079124	-3.020668674	0.002522172	0.015683754	negative
PRJNA729271	ed_Lushi_vs_Arbor_Acr	breed_growth_background	ENSGALG00010008864	ADAT1	0.072965422	0.073937641	0.986850819	0.323715777	0.48765955	positive
PRJNA729271	ed_Lushi_vs_Arbor_Acr	breed_growth_background	ENSGALG00010008866	WASHC5	0.059705137	0.040138428	1.487480706	0.136887895	0.267771936	positive
PRJNA729271	ed_Lushi_vs_Arbor_Acr	breed_growth_background	ENSGALG00010008871	PRR7	-0.218904325	0.107016617	-2.045517144	0.040803901	0.114003959	negative
PRJNA729271	ed_Lushi_vs_Arbor_Acr	breed_growth_background	ENSGALG00010008877	FNDC4	0.215584473	0.135208871	1.594455101	0.110834139	0.231523397	positive
PRJNA729271	ed_Lushi_vs_Arbor_Acr	breed_growth_background	ENSGALG00010008879	HABP4	-0.133895414	0.096072664	-1.393688997	0.16341154	0.303206641	negative
PRJNA729271	ed_Lushi_vs_Arbor_Acr	breed_growth_background	ENSGALG00010008880	FMN2	0.089886246	0.156387739	0.574765305	0.565450034	0.705196223	positive
PRJNA729271	ed_Lushi_vs_Arbor_Acr	breed_growth_background	ENSGALG00010008884	FAM135B	-0.213517291	0.275792069	-0.774196632	0.43881452	0.596121505	negative
PRJNA729271	ed_Lushi_vs_Arbor_Acr	breed_growth_background	ENSGALG00010008888	FBXO46	0.109162828	0.076006683	1.4362267	0.150937844	0.286807999	positive
PRJNA729271	ed_Lushi_vs_Arbor_Acr	breed_growth_background	ENSGALG00010008892	ENSGALG00010008892	0.041319151	0.06820445	0.605813126	0.544638852	0.688757648	positive
PRJNA729271	ed_Lushi_vs_Arbor_Acr	breed_growth_background	ENSGALG00010008893	ENSGALG00010008893	-0.272899715	0.071733688	-3.804345224	0.00014218	0.002054462	negative
PRJNA729271	ed_Lushi_vs_Arbor_Acr	breed_growth_background	ENSGALG00010008897	NSMCE2	-0.040473363	0.088221697	-0.45876881	0.646400195	0.768998501	negative
PRJNA729271	ed_Lushi_vs_Arbor_Acr	breed_growth_background	ENSGALG00010008903	ENSGALG00010008903	0.523263734	0.215823969	2.424493143	0.015329773	0.056583921	positive
PRJNA729271	ed_Lushi_vs_Arbor_Acr	breed_growth_background	ENSGALG00010008905	GABARAPL2	0.018657679	0.050507853	0.369401548	0.711828445	0.814618136	positive
PRJNA729271	ed_Lushi_vs_Arbor_Acr	breed_growth_background	ENSGALG00010008906	DBN1	-0.500474962	0.115397429	-4.336968055	1.44462E-05	0.000381291	negative
PRJNA729271	ed_Lushi_vs_Arbor_Acr	breed_growth_background	ENSGALG00010008910	TRIB1	0.238981265	0.139452515	1.713710694	0.086581872	0.194695041	positive
PRJNA729271	ed_Lushi_vs_Arbor_Acr	breed_growth_background	ENSGALG00010008918	PLAC9	-0.302060154	0.131624651	-2.294860064	0.021741145	0.072445494	negative
PRJNA729271	ed_Lushi_vs_Arbor_Acr	breed_growth_background	ENSGALG00010008921	OPA3	0.011702833	0.0678789	0.172407522	0.863117159	0.917659101	positive
PRJNA729271	ed_Lushi_vs_Arbor_Acr	breed_growth_background	ENSGALG00010008922	ENSGALG00010008922	1.413258333	0.593721705	2.380337994	0.017296765	0.061555759	positive
PRJNA729271	ed_Lushi_vs_Arbor_Acr	breed_growth_background	ENSGALG00010008928	CFC1	0.19247354	0.351186552	0.548066374	0.583646328	0.719624132	positive
PRJNA729271	ed_Lushi_vs_Arbor_Acr	breed_growth_background	ENSGALG00010008931	FNIP2	-0.114947349	0.070934365	-1.620474773	0.105130329	0.222819728	negative
PRJNA729271	ed_Lushi_vs_Arbor_Acr	breed_growth_background	ENSGALG00010008932	VASP	0.043721828	0.063786206	0.685443308	0.493064223	0.644837536	positive
PRJNA729271	ed_Lushi_vs_Arbor_Acr	breed_growth_background	ENSGALG00010008936	IFT172	-0.115742754	0.051465442	-2.248941209	0.024516237	0.078881876	negative
PRJNA729271	ed_Lushi_vs_Arbor_Acr	breed_growth_background	ENSGALG00010008938	ZBED4	0.124450673	0.048881748	2.545953834	0.01089796	0.044484914	positive
PRJNA729271	ed_Lushi_vs_Arbor_Acr	breed_growth_background	ENSGALG00010008939	RANBP9	-0.026399804	0.055242841	-0.477886419	0.632731048	0.757844698	negative
PRJNA729271	ed_Lushi_vs_Arbor_Acr	breed_growth_background	ENSGALG00010008943	ENSGALG00010008943	0.156290387	0.196707913	0.794530248	0.426886807	0.586014231	positive
PRJNA729271	ed_Lushi_vs_Arbor_Acr	breed_growth_background	ENSGALG00010008945	FER1L6	-0.244452201	0.298028531	-0.820230869	0.412084508	0.572361531	negative
PRJNA729271	ed_Lushi_vs_Arbor_Acr	breed_growth_background	ENSGALG00010008946	COL22A1	-0.179356819	0.333144694	-0.538375132	0.590318091	0.725509643	negative
PRJNA729271	ed_Lushi_vs_Arbor_Acr	breed_growth_background	ENSGALG00010008955	KRTCAP3	-0.342159968	0.219497747	-1.558831344	0.119036303	0.243086957	negative
PRJNA729271	ed_Lushi_vs_Arbor_Acr	breed_growth_background	ENSGALG00010008956	LVRN	0.063486279	0.259307057	0.244830509	0.80658767	0.881046592	positive
PRJNA729271	ed_Lushi_vs_Arbor_Acr	breed_growth_background	ENSGALG00010008960	LRRC2	0.227060329	0.151613075	1.497630267	0.134229338	0.264104013	positive
PRJNA729271	ed_Lushi_vs_Arbor_Acr	breed_growth_background	ENSGALG00010008966	NRBP1	-0.03879891	0.062538006	-0.620405289	0.534990991	0.680219664	negative
PRJNA729271	ed_Lushi_vs_Arbor_Acr	breed_growth_background	ENSGALG00010008969	PPP1R37	0.117692207	0.100812458	1.167437125	0.243033886	0.398774754	positive
PRJNA729271	ed_Lushi_vs_Arbor_Acr	breed_growth_background	ENSGALG00010008971	ANXA11	0.032163355	0.055533352	0.579171868	0.562473211	0.703152151	positive
PRJNA729271	ed_Lushi_vs_Arbor_Acr	breed_growth_background	ENSGALG00010008973	KCNK9	-0.191068807	0.350914262	-0.544488578	0.586105307	0.72184313	negative
PRJNA729271	ed_Lushi_vs_Arbor_Acr	breed_growth_background	ENSGALG00010008974	SMCO4	0.266797401	0.055869396	4.775376497	1.79371E-06	7.73806E-05	positive

PRJNA729271	eed_Lushi_vs_Arbor_Acr	breed_growth_background	ENSGALG00010008980	ENSGALG00010008980	-0.014274598	0.149222984	-0.095659511	0.923790999	0.95747948	negative
PRJNA729271	eed_Lushi_vs_Arbor_Acr	breed_growth_background	ENSGALG00010008983	ENSGALG00010008983	-0.474778658	0.190539559	-2.491758986	0.012711226	0.04947616	negative
PRJNA729271	eed_Lushi_vs_Arbor_Acr	breed_growth_background	ENSGALG00010008988	PPM1G	-0.015257891	0.043786562	-0.348460595	0.727494302	0.824738835	negative
PRJNA729271	eed_Lushi_vs_Arbor_Acr	breed_growth_background	ENSGALG00010008991	ENSGALG00010008991	0.066789783	0.071787013	0.930388105	0.352170175	0.5161483	positive
PRJNA729271	eed_Lushi_vs_Arbor_Acr	breed_growth_background	ENSGALG00010008995	ECD	0.072714743	0.054877605	1.32503492	0.185159569	0.330681563	positive
PRJNA729271	eed_Lushi_vs_Arbor_Acr	breed_growth_background	ENSGALG00010008996	CHRM3	-0.111508774	0.130844608	-0.852222919	0.394090378	0.555512986	negative
PRJNA729271	eed_Lushi_vs_Arbor_Acr	breed_growth_background	ENSGALG00010008997	CHST6	0.000956887	0.072220999	0.013249425	0.989428798	0.993747951	positive
PRJNA729271	eed_Lushi_vs_Arbor_Acr	breed_growth_background	ENSGALG00010008999	TRAPPC9	-0.055493575	0.035648276	-1.556697321	0.119542365	0.243674108	negative
PRJNA729271	eed_Lushi_vs_Arbor_Acr	breed_growth_background	ENSGALG00010009000	FAM149B1	0.075431759	0.056092952	1.34476359	0.178701592	0.323031629	positive
PRJNA729271	eed_Lushi_vs_Arbor_Acr	breed_growth_background	ENSGALG00010009002	ENSGALG00010009002	-0.029794569	0.05159936	-0.57742129	0.563654896	0.704082878	negative
PRJNA729271	eed_Lushi_vs_Arbor_Acr	breed_growth_background	ENSGALG00010009003	ALG12	-0.188555725	0.073524868	-2.564516339	0.010331971	0.04283808	negative
PRJNA729271	eed_Lushi_vs_Arbor_Acr	breed_growth_background	ENSGALG00010009007	TMEM65	-0.020090151	0.053686971	-0.374209055	0.708248787	0.811867629	negative
PRJNA729271	eed_Lushi_vs_Arbor_Acr	breed_growth_background	ENSGALG00010009011	ACP6	-0.075156657	0.068649549	-1.094787344	0.273609812	0.432631565	negative
PRJNA729271	eed_Lushi_vs_Arbor_Acr	breed_growth_background	ENSGALG00010009012	DEUP1	-0.436418653	0.511484653	-0.853238998	0.393526781	0.554988392	negative
PRJNA729271	eed_Lushi_vs_Arbor_Acr	breed_growth_background	ENSGALG00010009013	LIPG	0.15860971	0.120198791	1.319561602	0.18698143	0.332674092	positive
PRJNA729271	eed_Lushi_vs_Arbor_Acr	breed_growth_background	ENSGALG00010009015	TMEM231	-0.132982019	0.050049141	-2.657029018	0.007883266	0.035267882	negative
PRJNA729271	eed_Lushi_vs_Arbor_Acr	breed_growth_background	ENSGALG00010009016	DNAJC9	-0.053920158	0.0607507	-0.887564389	0.374775116	0.537113438	negative
PRJNA729271	eed_Lushi_vs_Arbor_Acr	breed_growth_background	ENSGALG00010009019	ZNF513	0.317083318	0.072480082	4.374764898	1.21563E-05	0.000335701	positive
PRJNA729271	eed_Lushi_vs_Arbor_Acr	breed_growth_background	ENSGALG00010009020	ARHGAP35	0.15161566	0.097855286	1.54938651	0.121288835	0.246036921	positive
PRJNA729271	eed_Lushi_vs_Arbor_Acr	breed_growth_background	ENSGALG00010009021	HNF4beta	-0.709269631	0.252425165	-2.809821405	0.0049569	0.025424721	negative
PRJNA729271	eed_Lushi_vs_Arbor_Acr	breed_growth_background	ENSGALG00010009024	PMAIP1	-0.167748003	0.138929941	-1.20742873	0.227267077	0.380352878	negative
PRJNA729271	eed_Lushi_vs_Arbor_Acr	breed_growth_background	ENSGALG00010009025	UTP23	-0.013145498	0.052652495	-0.249665238	0.802846243	0.878395942	negative
PRJNA729271	eed_Lushi_vs_Arbor_Acr	breed_growth_background	ENSGALG00010009026	RAPGEF2	0.014170776	0.073438394	0.192961407	0.846989193	0.907398991	positive
PRJNA729271	eed_Lushi_vs_Arbor_Acr	breed_growth_background	ENSGALG00010009028	PDLIM7	-0.072960115	0.061853986	-1.179553964	0.238177662	0.393479053	negative
PRJNA729271	eed_Lushi_vs_Arbor_Acr	breed_growth_background	ENSGALG00010009029	IGSF3	-0.487767059	0.087341838	-5.584575127	2.34272E-08	2.78703E-06	negative
PRJNA729271	eed_Lushi_vs_Arbor_Acr	breed_growth_background	ENSGALG00010009030	ZDHHC7	-0.079981602	0.049645408	-1.611057407	0.107167211	0.226046088	negative
PRJNA729271	eed_Lushi_vs_Arbor_Acr	breed_growth_background	ENSGALG00010009031	SPIN1	-0.098935226	0.042722577	-2.315759802	0.020571387	0.069571222	negative
PRJNA729271	eed_Lushi_vs_Arbor_Acr	breed_growth_background	ENSGALG00010009034	SNX17	0.148225416	0.063754208	2.324951093	0.020074575	0.068466111	positive
PRJNA729271	eed_Lushi_vs_Arbor_Acr	breed_growth_background	ENSGALG00010009035	ENSGALG00010009035	-0.113983913	0.044968176	-2.534768437	0.011252168	0.045545985	negative
PRJNA729271	eed_Lushi_vs_Arbor_Acr	breed_growth_background	ENSGALG00010009037	ENSGALG00010009037	-0.38841218	0.096714473	-4.016070895	5.91765E-05	0.001092691	negative
PRJNA729271	eed_Lushi_vs_Arbor_Acr	breed_growth_background	ENSGALG00010009038	RAD21	-0.055127918	0.066605176	-0.827682194	0.407850505	0.568439171	negative
PRJNA729271	eed_Lushi_vs_Arbor_Acr	breed_growth_background	ENSGALG00010009039	SLC36A4	-0.12555554	0.094585177	-1.327433581	0.184365297	0.329759993	negative
PRJNA729271	eed_Lushi_vs_Arbor_Acr	breed_growth_background	ENSGALG00010009040	CRELD2	-0.176232282	0.078210533	-2.25330623	0.024239847	0.078253137	negative
PRJNA729271	eed_Lushi_vs_Arbor_Acr	breed_growth_background	ENSGALG00010009043	TFAM	0.035087365	0.045008929	0.779564527	0.435647243	0.59348748	positive
PRJNA729271	eed_Lushi_vs_Arbor_Acr	breed_growth_background	ENSGALG00010009047	SUPT5H	0.179445751	0.059154438	3.033512922	0.002417244	0.015238839	positive
PRJNA729271	eed_Lushi_vs_Arbor_Acr	breed_growth_background	ENSGALG00010009050	PIK3CG	0.172831311	0.048977436	3.528794597	0.000417457	0.0043753	positive
PRJNA729271	eed_Lushi_vs_Arbor_Acr	breed_growth_background	ENSGALG00010009051	TROJANZ	-0.113474033	0.126416747	-0.897618674	0.36938888	0.532748149	negative
PRJNA729271	eed_Lushi_vs_Arbor_Acr	breed_growth_background	ENSGALG00010009054	RYR2	-0.157930258	0.273726227	-0.57696429	0.563963579	0.704225713	negative
PRJNA729271	eed_Lushi_vs_Arbor_Acr	breed_growth_background	ENSGALG00010009057	NXNL2	-0.117881576	0.193958035	-0.60776846	0.543341051	0.687776485	negative
PRJNA729271	eed_Lushi_vs_Arbor_Acr	breed_growth_background	ENSGALG00010009059	MED29	-0.034694451	0.067186879	-0.516387305	0.605583931	0.73704479	negative
PRJNA729271	eed_Lushi_vs_Arbor_Acr	breed_growth_background	ENSGALG00010009065	EIF2B4	0.031031655	0.051238518	0.605631384	0.544759557	0.688850194	positive
PRJNA729271	eed_Lushi_vs_Arbor_Acr	breed_growth_background	ENSGALG00010009067	ENSGALG00010009067	6.694761497	0.867462987	7.717633603	1.18509E-14	1.10839E-11	positive
PRJNA729271	eed_Lushi_vs_Arbor_Acr	breed_growth_background	ENSGALG00010009068	AGO2	0.072824117	0.043952989	1.656863805	0.097547014	0.211099427	positive
PRJNA729271	eed_Lushi_vs_Arbor_Acr	breed_growth_background	ENSGALG00010009069	GRID2	0.013521889	0.179961647	0.075137616	0.940105219	0.96719605	positive
PRJNA729271	eed_Lushi_vs_Arbor_Acr	breed_growth_background	ENSGALG00010009070	ENSGALG00010009070	-0.013101562	0.674347851	-0.019428492	0.984499281	0.990787828	negative
PRJNA729271	eed_Lushi_vs_Arbor_Acr	breed_growth_background	ENSGALG00010009073	PRKAR2B	-0.397123496	0.216017001	-1.838390006	0.066004957	0.161465251	negative
PRJNA729271	eed_Lushi_vs_Arbor_Acr	breed_growth_background	ENSGALG00010009076	TMEM214	0.073301302	0.063195795	1.159907906	0.246086304	0.401917856	positive
PRJNA729271	eed_Lushi_vs_Arbor_Acr	breed_growth_background	ENSGALG00010009078	PTTG2	-0.30250211	0.092778383	-3.260480524	0.001112236	0.008841001	negative
PRJNA729271	eed_Lushi_vs_Arbor_Acr	breed_growth_background	ENSGALG00010009079	KCTD15	0.073806493	0.052627687	1.402427062	0.160787757	0.299758011	positive
PRJNA729271	eed_Lushi_vs_Arbor_Acr	breed_growth_background	ENSGALG00010009080	TLL1	-0.075969347	0.09246133	-0.821633728	0.411285387	0.571543795	negative
PRJNA729271	eed_Lushi_vs_Arbor_Acr	breed_growth_background	ENSGALG00010009081	HBP1	0.096568622	0.065178347	1.481605869	0.138445197	0.26998024	positive
PRJNA729271	eed_Lushi_vs_Arbor_Acr	breed_growth_background	ENSGALG00010009083	MED30	0.000312243	0.068836096	0.004536033	0.996380781	0.998171223	positive
PRJNA729271	eed_Lushi_vs_Arbor_Acr	breed_growth_background	ENSGALG00010009085	PAF1	0.078827252	0.047786967	1.649555458	0.099033892	0.21334944	positive
PRJNA729271	eed_Lushi_vs_Arbor_Acr	breed_growth_background	ENSGALG00010009086	UBE2D1	0.21840672	0.091246066	2.393601487	0.016683865	0.060053606	positive
PRJNA729271	eed_Lushi_vs_Arbor_Acr	breed_growth_background	ENSGALG00010009088	ENSGALG00010009088	0.023762864	0.076242299	0.311675597	0.755287076	0.845200816	positive
PRJNA729271	eed_Lushi_vs_Arbor_Acr	breed_growth_background	ENSGALG00010009089	AGBL5	0.119024218	0.084107326	1.415146864	0.15702544	0.294524295	positive
PRJNA729271	eed_Lushi_vs_Arbor_Acr	breed_growth_background	ENSGALG00010009091	EXT1	-0.072991666	0.060214318	-1.21219783	0.225436662	0.378476346	negative
PRJNA729271	eed_Lushi_vs_Arbor_Acr	breed_growth_background	ENSGALG00010009092	EED	0.046863087	0.065564811	0.714759725	0.474757526	0.628296403	positive
PRJNA729271	eed_Lushi_vs_Arbor_Acr	breed_growth_background	ENSGALG00010009093	SLU7	0.23596157	0.070594063	3.34251297	0.000830235	0.007129297	positive
PRJNA729271	eed_Lushi_vs_Arbor_Acr	breed_growth_background	ENSGALG00010009094	DARS	-0.076987204	0.051110443	-1.506291071	0.131992469	0.260835834	negative
PRJNA729271	eed_Lushi_vs_Arbor_Acr	breed_growth_background	ENSGALG00010009096	ENSGALG00010009096	-0.969993075	0.619745568	-1.565147254	0.117548386	0.241033223	negative
PRJNA729271	eed_Lushi_vs_Arbor_Acr	breed_growth_background	ENSGALG00010009097	C1QTNF2	-0.095691838	0.097136658	-0.985125898	0.324562234	0.488680749	negative
PRJNA729271	eed_Lushi_vs_Arbor_Acr	breed_growth_background	ENSGALG00010009098	CAD	-0.176785984	0.105992671	-1.66790762	0.095334067	0.207897002	negative
PRJNA729271	eed_Lushi_vs_Arbor_Acr	breed_growth_background	ENSGALG00010009099	CXCR4	0.039194604	0.102907278	0.380873	0.703297488	0.808237302	positive
PRJNA729271	eed_Lushi_vs_Arbor_Acr	breed_growth_background	ENSGALG00010009100	ENSGALG00010009100	0.220468963	0.083035449	2.655118589	0.007928053	0.03540269	positive
PRJNA729271	eed_Lushi_vs_Arbor_Acr	breed_growth_background	ENSGALG00010009101	HIKESHI	0.001711645	0.052688119	0.032486365	0.974084189	0.984850683	positive
PRJNA729271	eed_Lushi_vs_Arbor_Acr	breed_growth_background	ENSGALG00010009102	HTRA3	-0.254377517	0.115984784	-2.193197313	0.028293166	0.087172385	negative
PRJNA729271	eed_Lushi_vs_Arbor_Acr	breed_growth_background	ENSGALG00010009103	AP3S1	-0.040926938	0.066782924	-0.612835375	0.539985197	0.684926963	negative
PRJNA729271	eed_Lushi_vs_Arbor_Acr	breed_growth_background	ENSGALG00010009104	MTR	-0.274214724	0.066014225	-4.153873252	3.26894E-05	0.000707214	negative
PRJNA729271	eed_Lushi_vs_Arbor_Acr	breed_growth_background	ENSGALG00010009106	THSD7B	0.045280973	0.127630785	0.354780961	0.722753693	0.822187629	positive
PRJNA729271	eed_Lushi_vs_Arbor_Acr	breed_growth_background	ENSGALG00010009109	ENSGALG00010009109	-0.183761533	0.122048376	-1.505645054	0.132158316	0.261056799	negative
PRJNA729271	eed_Lushi_vs_Arbor_Acr	breed_growth_background	ENSGALG00010009111	LRFN1	0.290404746	0.13197482	2.200455702	0.02777458	0.085968937	positive
PRJNA729271	eed_Lushi_vs_Arbor_Acr	breed_growth_background	ENSGALG00010009112	ENSGALG00010009112	0.033216449	0.136605807	0.243155466	0.807884964	0.881865544	positive
PRJNA729271	eed_Lushi_vs_Arbor_Acr	breed_growth_background	ENSGALG00010009113	RPLP1	-0.120445864	0.09427484	-1.277603482	0.201389272	0.350310513	negative
PRJNA729271	eed_Lushi_vs_Arbor_Acr	breed_growth_background	ENSGALG00010009114	SETD7	0.107364898	0.080185751	1.338952329	0.180586192	0.325167312	positive
PRJNA729271	eed_Lushi_vs_Arbor_Acr	breed_growth_background	ENSGALG00010009115	COG5	0.178197525	0.061868648	2.880255662	0.003973528	0.021587815	positive
PRJNA729271	eed_Lushi_vs_Arbor_Acr	breed_growth_background	ENSGALG00010009116	S1PR3	-0.231249435	0.099138295	-2.332594434	0.019669441	0.06749729	negative
PRJNA729271	eed_Lushi_vs_Arbor_Acr	breed_growth_background	ENSGALG00010009117	ERGIC1	-0.057516529	0.036057504	-1.595133414	0.110682405	0.231373155	negative
PRJNA729271	eed_Lushi_vs_Arbor_Acr	breed_growth_background	ENSGALG00010009118	ACTN2	-0.033081786	0.076892176	-0.430236044	0.667023945	0.784145344	negative
PRJNA729271	eed_Lushi_vs_Arbor_Acr	breed_growth_background	ENSGALG00010009119	CISD1	-0.179038845	0.081854414	-2.187283922	0.028721804	0.088054684	negative
PRJNA729271	eed_Lushi_vs_Arbor_Acr	breed_growth_background	ENSGALG00010009120	ATG12	0.019937742	0.048378552	0.412119441	0.68025188	0.793358386	positive
PRJNA729271	eed_Lushi_vs_Arbor_Acr	breed_growth_background	ENSGALG00010009121	SYCN	-1.215873972	0.417634343	-2.911336178	0.003598866	0.020040553	negative
PRJNA729271	eed_Lushi_vs_Arbor_Acr	breed_growth_background	ENSGALG00010009123	ENSGALG00010009123	-0.282682937	0.073069355	-3.868693459	0.00010942	0.001701764	negative
PRJNA729271	eed_Lushi_vs_Arbor_Acr	breed_growth_background	ENSGALG00010009125	CD72L1	-0.558536835	0.29193897	-1.913197248	0.055722806	0.143282278	negative
PRJNA729271	eed_Lushi_vs_Arbor_Acr	breed_growth_background	ENSGALG00010009126	JPH3	-0.039347755	0.074564862	-0.527698349	0.597708719	0.730997965	negative
PRJNA729271	eed_Lushi_vs_Arbor_Acr	breed_growth_background	ENSGALG00010009127	SCOC	-0.014633865	0.040695433	-0.35959478	0.719150188	0.82014807	negative
PRJNA729271	eed_Lushi_vs_Arbor_Acr	breed_growth_background	ENSGALG00010009129	ENSGALG00010009129	0.308544303	0.210508224	1.465711392	0.142726929	0.27577004	positive
PRJNA729271	eed_Lushi_vs_Arbor_Acr	breed_growth_background	ENSGALG00010009130	HEATR1	-0.020473472	0.039852187	-0.513735218	0.607437126	0.738286193	negative
PRJNA729271	eed_Lushi_vs_Arbor_Acr	breed_growth_background	ENSGALG00010009131	CDKL2	-0.254587803	0.173779978	-1.465000777	0.142920706	0.276034062	negative
PRJNA729271	eed_Lushi_vs_Arbor_Acr	breed_growth_background	ENSGALG00010009133	BOD1L1	0.187807117	0.069163174	2.715420726	0.006619162	0.031147835	positive
PRJNA729271	eed_Lushi_vs_Arbor_Acr	breed_growth_background	ENSGALG00010009135	TENM4	-0.151213809	0.099835144	-1.514635054	0.12986485	0.25754426	negative
PRJNA729271	eed_Lushi_vs_Arbor_Acr	breed_growth_background	ENSGALG00010009136	KHK	-0.599246166	0.216253206	-2.771039454	0.005587766	0.027549205	negative
PRJNA729271	eed_Lushi_vs_Arbor_Acr	breed_growth_background	ENSGALG00010009137	IPMK	-0.057747732	0.049439538	-1.168047571	0.242787577	0.398488098	negative
PRJNA729271	eed_Lushi_vs_Arbor_Acr	breed_growth_background	ENSGALG00010009138	ENSGALG00010009138	0.048851671	0.057974863	0.842635381	0.399432397	0.56058991	positive
PRJNA729271	eed_Lushi_vs_Arbor_Acr	breed_growth_background	ENSGALG00010009139	CKS2	-0.377739969	0.164912228	-2.290551605	0.021989361	0.07303753	negative
PRJNA729271	eed_Lushi_vs_Arbor_Acr	breed_growth_background	ENSGALG00010009141	MAML3	0.10831052	0.062980291	1.719752631	0.085477416	0.192959843	positive
PRJNA729271	eed_Lushi_vs_Arbor_Acr	breed_growth_background	ENSGALG00010009142	LGALS8	-0.138813072	0.073676582	-1.884086759	0.059553241	0.150157815	negative
PRJNA729271	eed_Lushi_vs_Arbor_Acr	breed_growth_background	ENSGALG00010009143	MCCC2	0.27731237	0.072375679	3.831568486	0.000127329	0.001898799	positive
PRJNA729271	eed_Lushi_vs_Arbor_Acr	breed_growth_background	ENSGALG00010009144	CREBZF	0.255151343	0.084088223	3.034329104	0.002410713	0.015212575	positive
PRJNA729271	eed_Lushi_vs_Arbor_Acr	breed_growth_background	ENSGALG00010009147	HNMT	-0.23095316	0.120647886	-1.914274403	0.055585103	0.143007318	negative
PRJNA729271	eed_Lushi_vs_Arbor_Acr	breed_growth_background	ENSGALG00010009149	FUT4	0.155110049	0.115469143	1.343303031	0.179173871	0.323387531	positive
PRJNA729271	eed_Lushi_vs_Arbor_Acr	breed_growth_background	ENSGALG00010009150	ENSGALG00010009150	-0.439287129	0.216393246	-2.030040847	0.042352387	0.11746993	negative
PRJNA729271	eed_Lushi_vs_Arbor_Acr	breed_growth_background	ENSGALG00010009151	CCNJL	0.186338387	0.196802652	0.946828635	0.343726108	0.507570286	positive
PRJNA729271	eed_Lushi_vs_Arbor_Acr	breed_growth_background	ENSGALG00010009152	RPP25L	0.136264376	0.066102404	2.061413325	0.039263625	0.110984056	positive
PRJNA729271	eed_Lushi_vs_Arbor_Acr	breed_growth_background	ENSGALG00010009153	SECISBP2	-0.048496736	0.066771618	-0.726307625	0.467650206	0.622575388	negative
PRJNA729271	eed_Lushi_vs_Arbor_Acr	breed_growth_background	ENSGALG00010009155	PAK4	0.167528083	0.119216378	1.405243858	0.159948776	0.298731802	positive
PRJNA729271	eed_Lushi_vs_Arbor_Acr	breed_growth_background	ENSGALG00010009157	ABHD1	0.202500912	0.073930211	2.739082026	0.0061611	0.02957124	positive
PRJNA729271	eed_Lushi_vs_Arbor_Acr	breed_growth_background	ENSGALG00010009159	PTK2	-0.248107332	0.053027752	-4.678820496	2.8853E-06	0.000110349	negative
PRJNA729271	eed_Lushi_vs_Arbor_Acr	breed_growth_background	ENSGALG00010009160	ENSGALG00010009160	-0.482328716	0.210753904	-2.288587334	0.02210334	0.073348883	negative
PRJNA729271	eed_Lushi_vs_Arbor_Acr	breed_growth_background	ENSGALG00010009163	SAMD12	0.10783482	0.096789528	1.114116603	0.265229167	0.423438766	positive
PRJNA729271	eed_Lushi_vs_Arbor_Acr	breed_growth_background	ENSGALG00010009164	TMEM128	0.030038412	0.054382593	0.552353433	0.580706229	0.717223652	positive
PRJNA729271	eed_Lushi_vs_Arbor_Acr	breed_growth_background	ENSGALG00010009166	CLGN	0.009038683	0.127484322	0.07090035	0.943477065	0.969225493	positive
PRJNA729271	eed_Lushi_vs_Arbor_Acr	breed_growth_background	ENSGALG00010009167	DCTN3	-0.078995061	0.059566722	-1.326160948	0.184786392	0.330146524	negative
PRJNA729271	eed_Lushi_vs_Arbor_Acr	breed_growth_background	ENSGALG00010009168	MRPS12	-0.180884451	0.093843895	-1.927503655	0.053916893	0.139757755	negative
PRJNA729271	eed_Lushi_vs_Arbor_Acr	breed_growth_background	ENSGALG00010009170	RPL26L1	-0.122615693	0.102249616	-1.199179978	0.230457971	0.384273356	negative
PRJNA729271	eed_Lushi_vs_Arbor_Acr	breed_growth_background	ENSGALG00010009172	SPOPL	0.083778897	0.070894975	1.181732509	0.237311855	0.392406495	positive
PRJNA729271	eed_Lushi_vs_Arbor_Acr	breed_growth_background	ENSGALG00010009173	LYAR	0.295852958	0.077138306	3.835357234	0.000125382	0.001877491	positive
PRJNA729271	eed_Lushi_vs_Arbor_Acr	breed_growth_background	ENSGALG00010009178	ENSGALG00010009178	0.491976345	0.137893405	3.567801834	0.000359989	0.003926286	positive
PRJNA729271	eed_Lushi_vs_Arbor_Acr	breed_growth_background	ENSGALG00010009180	NFKBIB	0.174626509	0.078516036	2.224087166	0.02614258	0.082431303	positive
PRJNA729271	eed_Lushi_vs_Arbor_Acr	breed_growth_background	ENSGALG00010009181	ARID3C	0.227517296	0.362377514	0.627846063	0.530104787	0.675861456	positive
PRJNA729271	eed_Lushi_vs_Arbor_Acr	breed_growth_background	ENSGALG00010009186	PREB	-0.022182999	0.053118779	-0.417611236	0.67623138	0.790800634	negative
PRJNA729271	eed_Lushi_vs_Arbor_Acr	breed_growth_background	ENSGALG00010009188	ENSGALG00010009188	-0.395782664	0.273286617	-1.448232875	0.147551933	0.282399005	negative
PRJNA729271	eed_Lushi_vs_Arbor_Acr	breed_growth_background	ENSGALG00010009189	ENSGALG00010009189	0.089573558	0.131709807	0.680082672	0.496452116	0.647944662	positive
PRJNA729271	eed_Lushi_vs_Arbor_Acr	breed_growth_background	ENSGALG00010009190	ZBTB49	-0.004842626	0.074943009	-0.064617451	0.94847859	0.971711451	negative
PRJNA729271	eed_Lushi_vs_Arbor_Acr	breed_growth_background	ENSGALG00010009192	LSM14A	0.055315854	0.032047984	1.726032242	0.084341612	0.191034455	positive
PRJNA729271	eed_Lushi_vs_Arbor_Acr	breed_growth_background	ENSGALG00010009193	EDARADD	-0.161518228	0.12202556	-1.323642589	0.185621774	0.33118009	negative
PRJNA729271	eed_Lushi_vs_Arbor_Acr	breed_growth_background	ENSGALG00010009195	ENSGALG00010009195	0.146566819	0.331744341	0.441806539	0.658629206	0.77793598	positive
PRJNA729271	eed_Lushi_vs_Arbor_Acr	breed_growth_background	ENSGALG00010009197	FH	0.043506732	0.054811885	0.793746327	0.427343125	0.586474019	positive
PRJNA729271	eed_Lushi_vs_Arbor_Acr	breed_growth_background	ENSGALG00010009198	KCNN2	0.206847813	0.095057743	2.17602276	0.029553559	0.089957756	positive
PRJNA729271	eed_Lushi_vs_Arbor_Acr	breed_growth_background	ENSGALG00010009199	SEMA4D	-0.003004605	0.083878293	-0.03582101	0.97142508	0.983216541	negative
PRJNA729271	eed_Lushi_vs_Arbor_Acr	breed_growth_background	ENSGALG00010009200	ADGRA3	-0.023722959	0.050979427	-0.465343773	0.641685293	0.765265521	negative

PRJNA729271	:ed_Lushi_vs_Arbor_Acr	breed_growth_background	ENSGALG00010009202	FABP6	0.11958469	0.156978701	0.761789266	0.446185788	0.602970632	positive
PRJNA729271	:ed_Lushi_vs_Arbor_Acr	breed_growth_background	ENSGALG00010009205	SIRT2	-0.018371011	0.05257119	-0.349450165	0.726751378	0.824527408	negative
PRJNA729271	:ed_Lushi_vs_Arbor_Acr	breed_growth_background	ENSGALG00010009208	SLC5A6	0.029301667	0.1045986	0.280134408	0.779374387	0.863291437	positive
PRJNA729271	:ed_Lushi_vs_Arbor_Acr	breed_growth_background	ENSGALG00010009209	SLC6A19	-1.061156937	0.87865735	-1.207702795	0.227161603	0.380335933	negative
PRJNA729271	:ed_Lushi_vs_Arbor_Acr	breed_growth_background	ENSGALG00010009210	PCDH15	0.169198167	0.174243959	0.971041792	0.331527468	0.495871493	positive
PRJNA729271	:ed_Lushi_vs_Arbor_Acr	breed_growth_background	ENSGALG00010009212	NXPH2	0.927794892	0.150763581	6.153972224	7.55659E-10	1.65959E-07	positive
PRJNA729271	:ed_Lushi_vs_Arbor_Acr	breed_growth_background	ENSGALG00010009213	DUS4L	-0.029790004	0.053506158	-0.556758408	0.577692504	0.714841459	negative
PRJNA729271	:ed_Lushi_vs_Arbor_Acr	breed_growth_background	ENSGALG00010009214	PCDH10	-0.644282053	0.157195475	-4.098604311	4.15649E-05	0.000849095	negative
PRJNA729271	:ed_Lushi_vs_Arbor_Acr	breed_growth_background	ENSGALG00010009216	NSG1	0.192257405	0.121047221	1.588284329	0.112222059	0.233514033	positive
PRJNA729271	:ed_Lushi_vs_Arbor_Acr	breed_growth_background	ENSGALG00010009219	TNFRSF11B	0.068357561	0.097825165	0.698772771	0.484694048	0.636583407	positive
PRJNA729271	:ed_Lushi_vs_Arbor_Acr	breed_growth_background	ENSGALG00010009220	GADD45G	-0.191331337	0.101573695	-1.883670142	0.059609608	0.150214989	negative
PRJNA729271	:ed_Lushi_vs_Arbor_Acr	breed_growth_background	ENSGALG00010009223	ENSGALG00010009223	-0.176996752	0.058450315	-3.02815736	0.002460499	0.015419341	negative
PRJNA729271	:ed_Lushi_vs_Arbor_Acr	breed_growth_background	ENSGALG00010009224	ENSGALG00010009224	0.550634839	0.172619003	3.189885411	0.001423292	0.0105312	positive
PRJNA729271	:ed_Lushi_vs_Arbor_Acr	breed_growth_background	ENSGALG00010009225	HNRNPL	0.12308382	0.067270913	1.829673689	0.067298748	0.163756143	positive
PRJNA729271	:ed_Lushi_vs_Arbor_Acr	breed_growth_background	ENSGALG00010009227	ENSGALG00010009227	0.081985241	0.092883154	0.882670729	0.377414201	0.539749119	positive
PRJNA729271	:ed_Lushi_vs_Arbor_Acr	breed_growth_background	ENSGALG00010009228	ATP6V0E1	-4.48726E-05	0.053677006	-0.000835974	0.999332989	0.999539862	negative
PRJNA729271	:ed_Lushi_vs_Arbor_Acr	breed_growth_background	ENSGALG00010009232	ECH1	-0.050016485	0.088081323	-0.567844388	0.570140636	0.70877295	negative
PRJNA729271	:ed_Lushi_vs_Arbor_Acr	breed_growth_background	ENSGALG00010009234	PCDH18	-0.072344346	0.069166933	-1.045938335	0.295589504	0.457167078	negative
PRJNA729271	:ed_Lushi_vs_Arbor_Acr	breed_growth_background	ENSGALG00010009235	KLHDC4	0.100423917	0.070107755	1.432422381	0.152022977	0.288086423	positive
PRJNA729271	:ed_Lushi_vs_Arbor_Acr	breed_growth_background	ENSGALG00010009236	DTWD2	0.268489104	0.063771181	4.210194967	2.5515E-05	0.000577876	positive
PRJNA729271	:ed_Lushi_vs_Arbor_Acr	breed_growth_background	ENSGALG00010009237	DENND3	0.080151766	0.096148929	0.833621005	0.404494585	0.565286253	positive
PRJNA729271	:ed_Lushi_vs_Arbor_Acr	breed_growth_background	ENSGALG00010009239	STX18	0.096760671	0.044147444	2.191761573	0.028396727	0.087363525	positive
PRJNA729271	:ed_Lushi_vs_Arbor_Acr	breed_growth_background	ENSGALG00010009243	BCAP29	-0.233976322	0.053619477	-4.363644202	1.27914E-05	0.000346562	negative
PRJNA729271	:ed_Lushi_vs_Arbor_Acr	breed_growth_background	ENSGALG00010009246	RUSC2	0.156528681	0.075005423	2.086898193	0.036897338	0.106053325	positive
PRJNA729271	:ed_Lushi_vs_Arbor_Acr	breed_growth_background	ENSGALG00010009250	ERO1B	-0.132293013	0.058627449	-2.256502963	0.02403915	0.077813193	negative
PRJNA729271	:ed_Lushi_vs_Arbor_Acr	breed_growth_background	ENSGALG00010009252	SIRT1	0.069539811	0.05184034	1.341422737	0.17978324	0.324043529	positive
PRJNA729271	:ed_Lushi_vs_Arbor_Acr	breed_growth_background	ENSGALG00010009253	SLC45A4	0.003619701	0.077085722	0.046956828	0.962547636	0.978835813	positive
PRJNA729271	:ed_Lushi_vs_Arbor_Acr	breed_growth_background	ENSGALG00010009254	SLC26A4	0.52759894	0.430231933	1.226312832	0.220080974	0.372628633	positive
PRJNA729271	:ed_Lushi_vs_Arbor_Acr	breed_growth_background	ENSGALG00010009256	CREBRF	0.112464713	0.092239084	1.21927396	0.222740229	0.375372259	positive
PRJNA729271	:ed_Lushi_vs_Arbor_Acr	breed_growth_background	ENSGALG00010009257	ENSGALG00010009257	-0.25938902	0.098290279	-2.639009913	0.008314855	0.036566694	negative
PRJNA729271	:ed_Lushi_vs_Arbor_Acr	breed_growth_background	ENSGALG00010009258	DNAJC12	0.194921784	0.090881602	2.144788172	0.031969787	0.095586235	positive
PRJNA729271	:ed_Lushi_vs_Arbor_Acr	breed_growth_background	ENSGALG00010009259	MGAT4D	-0.003133023	0.404506377	-0.0077453	0.993820207	0.996451492	negative
PRJNA729271	:ed_Lushi_vs_Arbor_Acr	breed_growth_background	ENSGALG00010009263	COLEC10	-0.594522066	0.119263556	-4.984943317	6.198E-07	3.33526E-05	negative
PRJNA729271	:ed_Lushi_vs_Arbor_Acr	breed_growth_background	ENSGALG00010009266	PCDH7	-0.287880831	0.135094365	-2.130961054	0.033092351	0.097713103	negative
PRJNA729271	:ed_Lushi_vs_Arbor_Acr	breed_growth_background	ENSGALG00010009268	PWWP2A	0.134902989	0.072074421	1.871717956	0.061245634	0.153140498	positive
PRJNA729271	:ed_Lushi_vs_Arbor_Acr	breed_growth_background	ENSGALG00010009269	ENSGALG00010009269	-0.187677834	0.286649043	-0.654730371	0.512641368	0.661274061	negative
PRJNA729271	:ed_Lushi_vs_Arbor_Acr	breed_growth_background	ENSGALG00010009273	MAL2	-0.169487264	0.26836697	-0.631550385	0.527680716	0.67360035	negative
PRJNA729271	:ed_Lushi_vs_Arbor_Acr	breed_growth_background	ENSGALG00010009275	CBLL1	0.085118843	0.077795067	1.094141896	0.27389275	0.432941703	positive
PRJNA729271	:ed_Lushi_vs_Arbor_Acr	breed_growth_background	ENSGALG00010009276	ENSGALG00010009276	0.033481736	0.180041753	0.185966508	0.852471026	0.910982566	positive
PRJNA729271	:ed_Lushi_vs_Arbor_Acr	breed_growth_background	ENSGALG00010009277	ACTN4	-0.138514043	0.068854652	-2.011687502	0.044252889	0.121210436	negative
PRJNA729271	:ed_Lushi_vs_Arbor_Acr	breed_growth_background	ENSGALG00010009279	ELMOD2	-0.203717064	0.061297744	-3.323402285	0.000889266	0.007511601	negative
PRJNA729271	:ed_Lushi_vs_Arbor_Acr	breed_growth_background	ENSGALG00010009280	HSD17B4	-0.048527928	0.044750279	-1.084416192	0.278180307	0.437571737	negative
PRJNA729271	:ed_Lushi_vs_Arbor_Acr	breed_growth_background	ENSGALG00010009284	CCN3	-0.836464982	0.364924944	-2.292156225	0.02189663	0.07279625	negative
PRJNA729271	:ed_Lushi_vs_Arbor_Acr	breed_growth_background	ENSGALG00010009286	ENSGALG00010009286	-0.273108924	0.099166765	-2.754036852	0.005886514	0.028613353	negative
PRJNA729271	:ed_Lushi_vs_Arbor_Acr	breed_growth_background	ENSGALG00010009289	GPR137B	-0.174134907	0.079745634	-2.183629356	0.028989495	0.08866907	negative
PRJNA729271	:ed_Lushi_vs_Arbor_Acr	breed_growth_background	ENSGALG00010009294	ACOX3	-0.144852618	0.075309389	-1.923433713	0.054425604	0.140774291	negative
PRJNA729271	:ed_Lushi_vs_Arbor_Acr	breed_growth_background	ENSGALG00010009297	CTNNA3	0.138359825	0.105196687	1.315248886	0.18842627	0.334341876	positive
PRJNA729271	:ed_Lushi_vs_Arbor_Acr	breed_growth_background	ENSGALG00010009299	MAP4K1	-0.041749317	0.15185808	-0.274923251	0.783375203	0.865763995	negative
PRJNA729271	:ed_Lushi_vs_Arbor_Acr	breed_growth_background	ENSGALG00010009303	TTC1	0.039336697	0.052025034	0.756110945	0.44958268	0.605810259	positive
PRJNA729271	:ed_Lushi_vs_Arbor_Acr	breed_growth_background	ENSGALG00010009306	TBC1D9	0.163718299	0.058538505	2.796762566	0.005161745	0.026160621	positive
PRJNA729271	:ed_Lushi_vs_Arbor_Acr	breed_growth_background	ENSGALG00010009307	DIRAS2	-0.21062421	0.164665336	-1.279104727	0.200860179	0.349767936	negative
PRJNA729271	:ed_Lushi_vs_Arbor_Acr	breed_growth_background	ENSGALG00010009309	TRIM54	0.082112723	0.07912375	1.037775924	0.299374388	0.461739919	positive
PRJNA729271	:ed_Lushi_vs_Arbor_Acr	breed_growth_background	ENSGALG00010009311	BNIP1	0.001056789	0.060990519	0.017327108	0.98617566	0.991827433	positive
PRJNA729271	:ed_Lushi_vs_Arbor_Acr	breed_growth_background	ENSGALG00010009313	UNC13B	0.4050447	0.099394774	4.075110624	4.59925E-05	0.000907022	positive
PRJNA729271	:ed_Lushi_vs_Arbor_Acr	breed_growth_background	ENSGALG00010009317	LRRTM3	0.327238442	0.304080138	1.076158555	0.281856352	0.441676521	positive
PRJNA729271	:ed_Lushi_vs_Arbor_Acr	breed_growth_background	ENSGALG00010009320	GPR20	0.027969449	0.119719779	0.233624292	0.815276667	0.886329805	positive
PRJNA729271	:ed_Lushi_vs_Arbor_Acr	breed_growth_background	ENSGALG00010009322	ENSGALG00010009322	0.118290658	0.087527828	1.351463422	0.176547029	0.320522193	positive
PRJNA729271	:ed_Lushi_vs_Arbor_Acr	breed_growth_background	ENSGALG00010009323	RNF150	0.01849435	0.057771041	0.320131853	0.74886838	0.840288525	positive
PRJNA729271	:ed_Lushi_vs_Arbor_Acr	breed_growth_background	ENSGALG00010009326	DLD	-0.031200938	0.070503141	-0.442546784	0.658093583	0.777493397	negative
PRJNA729271	:ed_Lushi_vs_Arbor_Acr	breed_growth_background	ENSGALG00010009327	NID1	-0.313851535	0.087141176	-3.60164449	0.000316211	0.003590476	negative
PRJNA729271	:ed_Lushi_vs_Arbor_Acr	breed_growth_background	ENSGALG00010009328	ENSGALG00010009328	-0.084350731	0.074505128	-1.132146652	0.257572784	0.414881377	negative
PRJNA729271	:ed_Lushi_vs_Arbor_Acr	breed_growth_background	ENSGALG00010009330	EIF3K	-0.107296876	0.07567489	-1.417866283	0.156229808	0.293487696	negative
PRJNA729271	:ed_Lushi_vs_Arbor_Acr	breed_growth_background	ENSGALG00010009332	ENSGALG00010009332	0.509097163	0.087431649	5.822801789	5.78692E-09	8.33434E-07	positive
PRJNA729271	:ed_Lushi_vs_Arbor_Acr	breed_growth_background	ENSGALG00010009333	ADRA1B	-0.451199539	0.164580153	-2.741518527	0.006115591	0.029430775	negative
PRJNA729271	:ed_Lushi_vs_Arbor_Acr	breed_growth_background	ENSGALG00010009334	SS18L2	0.232678615	0.088342753	2.633816673	0.008443106	0.036895153	positive
PRJNA729271	:ed_Lushi_vs_Arbor_Acr	breed_growth_background	ENSGALG00010009335	PTP4A3	0.425313338	0.104260152	4.079347014	4.51624E-05	0.000896389	positive
PRJNA729271	:ed_Lushi_vs_Arbor_Acr	breed_growth_background	ENSGALG00010009336	LAMB1	-0.082632238	0.058749255	-1.406524012	0.159568579	0.298175397	negative
PRJNA729271	:ed_Lushi_vs_Arbor_Acr	breed_growth_background	ENSGALG00010009337	ZNF330	0.329792752	0.055065075	5.989145556	2.10946E-09	3.87046E-07	positive
PRJNA729271	:ed_Lushi_vs_Arbor_Acr	breed_growth_background	ENSGALG00010009338	KYNU	0.280300688	0.307881838	0.910416444	0.362602929	0.52590849	positive
PRJNA729271	:ed_Lushi_vs_Arbor_Acr	breed_growth_background	ENSGALG00010009340	SYK	-0.1336122	0.075074459	-1.77972912	0.075120302	0.176508649	negative
PRJNA729271	:ed_Lushi_vs_Arbor_Acr	breed_growth_background	ENSGALG00010009344	ENPP2	0.235222951	0.13873154	1.69552613	0.089975658	0.200183754	positive
PRJNA729271	:ed_Lushi_vs_Arbor_Acr	breed_growth_background	ENSGALG00010009345	ZFAT	0.079860085	0.067485673	1.183363539	0.236665101	0.391591185	positive
PRJNA729271	:ed_Lushi_vs_Arbor_Acr	breed_growth_background	ENSGALG00010009346	STC2	0.056465793	0.164451351	0.343358643	0.731328669	0.827784558	positive
PRJNA729271	:ed_Lushi_vs_Arbor_Acr	breed_growth_background	ENSGALG00010009347	TESK1	0.025926728	0.073516945	0.352663298	0.724340877	0.823436302	positive
PRJNA729271	:ed_Lushi_vs_Arbor_Acr	breed_growth_background	ENSGALG00010009348	ERGIC2	0.008304728	0.044444913	0.186854418	0.851774779	0.91064135	positive
PRJNA729271	:ed_Lushi_vs_Arbor_Acr	breed_growth_background	ENSGALG00010009351	GTF3C2	0.124092758	0.065477179	1.895206239	0.058065084	0.14722672	positive
PRJNA729271	:ed_Lushi_vs_Arbor_Acr	breed_growth_background	ENSGALG00010009352	HSF1	0.182553856	0.096003231	1.901538665	0.057231493	0.145845726	positive
PRJNA729271	:ed_Lushi_vs_Arbor_Acr	breed_growth_background	ENSGALG00010009355	GARRE1	-0.12059578	0.038827595	-3.105929658	0.001896818	0.0127762	negative
PRJNA729271	:ed_Lushi_vs_Arbor_Acr	breed_growth_background	ENSGALG00010009357	LYST	0.079662748	0.062680253	1.27093851	0.203750525	0.353019825	positive
PRJNA729271	:ed_Lushi_vs_Arbor_Acr	breed_growth_background	ENSGALG00010009359	REEP3	-0.207508108	0.059659205	-3.478224527	0.000504747	0.004994066	negative
PRJNA729271	:ed_Lushi_vs_Arbor_Acr	breed_growth_background	ENSGALG00010009360	TNFAIP8	-0.073538152	0.070103564	-1.048993065	0.29418131	0.455475122	negative
PRJNA729271	:ed_Lushi_vs_Arbor_Acr	breed_growth_background	ENSGALG00010009361	LRRK2	0.035096216	0.046993865	0.746825486	0.45516891	0.611238119	positive
PRJNA729271	:ed_Lushi_vs_Arbor_Acr	breed_growth_background	ENSGALG00010009362	IL15	0.441076141	0.162652002	2.711778126	0.006692337	0.031373036	positive
PRJNA729271	:ed_Lushi_vs_Arbor_Acr	breed_growth_background	ENSGALG00010009363	TAF2	0.061224238	0.041679448	1.468931107	0.141851476	0.274701022	positive
PRJNA729271	:ed_Lushi_vs_Arbor_Acr	breed_growth_background	ENSGALG00010009365	CA5B	-0.212360961	0.086522739	-2.454394803	0.014112195	0.053203541	negative
PRJNA729271	:ed_Lushi_vs_Arbor_Acr	breed_growth_background	ENSGALG00010009367	DMXL1	0.009355516	0.063819744	0.146592811	0.883453429	0.93134085	positive
PRJNA729271	:ed_Lushi_vs_Arbor_Acr	breed_growth_background	ENSGALG00010009368	DSCC1	0.09401083	0.107291972	0.87621495	0.380913191	0.542630688	positive
PRJNA729271	:ed_Lushi_vs_Arbor_Acr	breed_growth_background	ENSGALG00010009370	ENSGALG00010009370	0.540047304	0.241980511	2.231780161	0.025629496	0.081415636	positive
PRJNA729271	:ed_Lushi_vs_Arbor_Acr	breed_growth_background	ENSGALG00010009371	PHTF2	-0.289159441	0.082337245	-3.511891124	0.00044493	0.004558246	negative
PRJNA729271	:ed_Lushi_vs_Arbor_Acr	breed_growth_background	ENSGALG00010009372	RAB28	-0.027159534	0.053333094	-0.509243543	0.610581525	0.740775476	negative
PRJNA729271	:ed_Lushi_vs_Arbor_Acr	breed_growth_background	ENSGALG00010009373	ARHGAP15	-0.040775852	0.118877905	-0.343006146	0.731593836	0.827890753	negative
PRJNA729271	:ed_Lushi_vs_Arbor_Acr	breed_growth_background	ENSGALG00010009376	CDO1	-0.259693429	0.134084823	-1.936784668	0.052771671	0.137725129	negative
PRJNA729271	:ed_Lushi_vs_Arbor_Acr	breed_growth_background	ENSGALG00010009378	ENSGALG00010009378	0.123236518	0.045894765	2.685197714	0.007248691	0.033239411	positive
PRJNA729271	:ed_Lushi_vs_Arbor_Acr	breed_growth_background	ENSGALG00010009379	RYR1	0.526927147	0.117256882	4.493784392	6.99685E-06	0.000223267	positive
PRJNA729271	:ed_Lushi_vs_Arbor_Acr	breed_growth_background	ENSGALG00010009381	INPP4B	-0.178584692	0.063433424	-2.815309043	0.004873035	0.025073554	negative
PRJNA729271	:ed_Lushi_vs_Arbor_Acr	breed_growth_background	ENSGALG00010009383	ASXL2	-0.000166618	0.059651931	-0.002793174	0.997771372	0.998913967	negative
PRJNA729271	:ed_Lushi_vs_Arbor_Acr	breed_growth_background	ENSGALG00010009385	GPI	-0.09973739	0.095139008	-1.048333305	0.29448507	0.455804099	negative
PRJNA729271	:ed_Lushi_vs_Arbor_Acr	breed_growth_background	ENSGALG00010009386	TMEM60	0.166378244	0.060202613	2.763638229	0.005716088	0.027972549	positive
PRJNA729271	:ed_Lushi_vs_Arbor_Acr	breed_growth_background	ENSGALG00010009387	DEPTOR	0.034432669	0.073391645	0.469163331	0.638952894	0.763089906	positive
PRJNA729271	:ed_Lushi_vs_Arbor_Acr	breed_growth_background	ENSGALG00010009388	TMED7	-0.011476871	0.039585642	-0.289925088	0.771873548	0.857867434	negative
PRJNA729271	:ed_Lushi_vs_Arbor_Acr	breed_growth_background	ENSGALG00010009389	CPEB4	-0.074452859	0.06612823	-1.12588616	0.260213708	0.418059136	negative
PRJNA729271	:ed_Lushi_vs_Arbor_Acr	breed_growth_background	ENSGALG00010009391	RSBN1L	0.028400372	0.057524887	0.493705824	0.621513951	0.748615982	positive
PRJNA729271	:ed_Lushi_vs_Arbor_Acr	breed_growth_background	ENSGALG00010009392	MAPRE3	0.256380168	0.057125955	4.487980398	7.19015E-06	0.000225587	positive
PRJNA729271	:ed_Lushi_vs_Arbor_Acr	breed_growth_background	ENSGALG00010009393	MARVELD2	0.13679294	0.072700926	1.881584574	0.059892442	0.150583962	positive
PRJNA729271	:ed_Lushi_vs_Arbor_Acr	breed_growth_background	ENSGALG00010009394	BANP	0.196044699	0.051581799	3.800656526	0.000144313	0.002073162	positive
PRJNA729271	:ed_Lushi_vs_Arbor_Acr	breed_growth_background	ENSGALG00010009396	AUH	0.107767076	0.059652011	1.806595846	0.070825318	0.169162866	positive
PRJNA729271	:ed_Lushi_vs_Arbor_Acr	breed_growth_background	ENSGALG00010009398	WTIP	-0.222778682	0.074909963	-2.973952634	0.002939905	0.017486219	negative
PRJNA729271	:ed_Lushi_vs_Arbor_Acr	breed_growth_background	ENSGALG00010009400	CNTFR	0.444941938	0.151579259	2.935374818	0.00333145	0.019116931	positive
PRJNA729271	:ed_Lushi_vs_Arbor_Acr	breed_growth_background	ENSGALG00010009404	GNG4	-0.469956266	0.219486528	-2.141162242	0.032260957	0.096198841	negative
PRJNA729271	:ed_Lushi_vs_Arbor_Acr	breed_growth_background	ENSGALG00010009407	PTPN12	-0.028508727	0.04710511	-0.605215161	0.545036044	0.689020186	negative
PRJNA729271	:ed_Lushi_vs_Arbor_Acr	breed_growth_background	ENSGALG00010009408	ENSGALG00010009408	-0.03051867	0.050253193	-0.607298136	0.543653075	0.688111362	negative
PRJNA729271	:ed_Lushi_vs_Arbor_Acr	breed_growth_background	ENSGALG00010009410	JMJD1C	-0.073914752	0.057921992	-1.2761086	0.201917132	0.350933912	negative
PRJNA729271	:ed_Lushi_vs_Arbor_Acr	breed_growth_background	ENSGALG00010009411	SMARCA5	-0.011267576	0.053118131	-0.212122975	0.832011096	0.897922778	negative
PRJNA729271	:ed_Lushi_vs_Arbor_Acr	breed_growth_background	ENSGALG00010009415	HMP19	0.086362201	0.248354073	0.34773821	0.728036798	0.825153912	positive
PRJNA729271	:ed_Lushi_vs_Arbor_Acr	breed_growth_background	ENSGALG00010009416	COL14A1	-0.540487069	0.253387499	-2.133045519	0.032920992	0.097445331	negative
PRJNA729271	:ed_Lushi_vs_Arbor_Acr	breed_growth_background	ENSGALG00010009424	ENSGALG00010009424	-0.678639488	0.505378333	-1.342834553	0.179325552	0.323580715	negative
PRJNA729271	:ed_Lushi_vs_Arbor_Acr	breed_growth_background	ENSGALG00010009431	SMN	0.219571452	0.080682366	2.721430503	0.006500005	0.030726931	positive
PRJNA729271	:ed_Lushi_vs_Arbor_Acr	breed_growth_background	ENSGALG00010009432	BOP1	0.221248639	0.098996798	2.234907029	0.025423453	0.080956272	positive
PRJNA729271	:ed_Lushi_vs_Arbor_Acr	breed_growth_background	ENSGALG00010009435	GTDC1	0.122206571	0.049520074	2.467818852	0.013593909	0.051935613	positive
PRJNA729271	:ed_Lushi_vs_Arbor_Acr	breed_growth_background	ENSGALG00010009437	DPYSL5	0.06967051	0.109011769	0.639109985	0.522751385	0.669757753	positive
PRJNA729271	:ed_Lushi_vs_Arbor_Acr	breed_growth_background	ENSGALG00010009439	B3GALNT2	0.130216643	0.053408696	2.438116854	0.014764001	0.054886942	positive
PRJNA729271	:ed_Lushi_vs_Arbor_Acr	breed_growth_background	ENSGALG00010009440	DOK3	0.035876588	0.121503266	0.295272621	0.76778563	0.854962949	positive
PRJNA729271	:ed_Lushi_vs_Arbor_Acr	breed_growth_background	ENSGALG00010009441	ZNF518B	-0.065098292	0.053118012	-1.225540834	0.220371515	0.37294085	negative
PRJNA729271	:ed_Lushi_vs_Arbor_Acr	breed_growth_background	ENSGALG00010009444	NFIL3	0.296442798	0.118256787	2.506771954	0.012183929	0.048082236	positive
PRJNA729271	:ed_Lushi_vs_Arbor_Acr	breed_growth_background	ENSGALG00010009446	ENSGALG00010009446	-0.304205476	0.133484659	-2.278954576	0.022669766	0.074723417	negative
PRJNA729271	:ed_Lushi_vs_Arbor_Acr	breed_growth_background	ENSGALG00010009447	DDX41	-0.01927732	0.057581643	-0.334782387	0.737789247	0.832028969	negative
PRJNA729271	:ed_Lushi_vs_Arbor_Acr	breed_growth_background	ENSGALG00010009451	ROR2	0.158722126	0.076734424	2.068460507	0.038596741	0.109676486	positive
PRJNA729271	:ed_Lushi_vs_Arbor_Acr	breed_growth_background	ENSGALG00010009452	TBCE	0.152452903	0.047972939	3.177893788	0.001483491	0.010832846	positive
PRJNA729271	:ed_Lushi_vs_Arbor_Acr	breed_growth_background	ENSGALG00010009453	GSAP	-0.002983195	0.061621647	-0.048411486	0.961388306	0.978276808	negative
PRJNA729271	:ed_Lushi_vs_Arbor_Acr	breed_growth_background	ENSGALG00010009457	BDP1	0.163870125	0.069632137	2.353369162	0.018604154	0.064964396	positive
PRJNA729271	:ed_Lushi_vs_Arbor_Acr	breed_growth_background	ENSGALG00010009458	SCX	0.074276756	0.254312693	0.292068617	0.770234161	0.856833781	positive

PRJNA729271	ed_Lushi_vs_Arbor_Acr	breed_growth_background	ENSGALG00010009461	ZEB2	-0.047387089	0.067185953	-0.705312455	0.480615811	0.633493333	negative
PRJNA729271	ed_Lushi_vs_Arbor_Acr	breed_growth_background	ENSGALG00010009462	NRBF2	0.298287371	0.064316894	4.637776335	3.52178E-06	0.000129236	positive
PRJNA729271	ed_Lushi_vs_Arbor_Acr	breed_growth_background	ENSGALG00010009463	MRPL13	-0.027039622	0.05856101	-0.461734216	0.644271931	0.767308993	negative
PRJNA729271	ed_Lushi_vs_Arbor_Acr	breed_growth_background	ENSGALG00010009464	SPTLC1	0.038580786	0.07931672	0.486414287	0.626673463	0.75289116	positive
PRJNA729271	ed_Lushi_vs_Arbor_Acr	breed_growth_background	ENSGALG00010009465	GGPS1	0.032634723	0.054639258	0.5972761	0.550323059	0.693465421	positive
PRJNA729271	ed_Lushi_vs_Arbor_Acr	breed_growth_background	ENSGALG00010009466	FAM193B	0.112262093	0.060296982	1.861819424	0.062628548	0.155423311	positive
PRJNA729271	ed_Lushi_vs_Arbor_Acr	breed_growth_background	ENSGALG00010009467	EGR2	-0.260123033	0.162624371	-1.59953291	0.109702242	0.230101646	negative
PRJNA729271	ed_Lushi_vs_Arbor_Acr	breed_growth_background	ENSGALG00010009469	DNMT3A	0.343968624	0.112716925	3.051614678	0.002276141	0.014592066	positive
PRJNA729271	ed_Lushi_vs_Arbor_Acr	breed_growth_background	ENSGALG00010009470	ZNF469	0.044586578	0.113748429	0.391975329	0.695076443	0.80351363	positive
PRJNA729271	ed_Lushi_vs_Arbor_Acr	breed_growth_background	ENSGALG00010009472	ADO	0.136605012	0.045957374	2.972428584	0.002954539	0.017537284	positive
PRJNA729271	ed_Lushi_vs_Arbor_Acr	breed_growth_background	ENSGALG00010009474	ACVR2A	0.085382604	0.051478288	1.658613894	0.09719362	0.210566412	positive
PRJNA729271	ed_Lushi_vs_Arbor_Acr	breed_growth_background	ENSGALG00010009477	ZNF365	0.543574409	0.130587406	4.162533181	3.14736E-05	0.000690182	positive
PRJNA729271	ed_Lushi_vs_Arbor_Acr	breed_growth_background	ENSGALG00010009479	MTBP	-0.038338837	0.077012545	-0.497825864	0.618606785	0.746727627	negative
PRJNA729271	ed_Lushi_vs_Arbor_Acr	breed_growth_background	ENSGALG00010009482	ARID4B	0.08299871	0.057939628	1.432503329	0.151999826	0.288080214	positive
PRJNA729271	ed_Lushi_vs_Arbor_Acr	breed_growth_background	ENSGALG00010009484	RTKN2	-0.059355738	0.072992456	-0.813176329	0.416116969	0.576305701	negative
PRJNA729271	ed_Lushi_vs_Arbor_Acr	breed_growth_background	ENSGALG00010009486	B4GALT1	-0.093199769	0.068821366	-1.35422726	0.175663885	0.319715342	negative
PRJNA729271	ed_Lushi_vs_Arbor_Acr	breed_growth_background	ENSGALG00010009489	MROH1	0.055872196	0.074344414	0.751531862	0.452332633	0.608554067	positive
PRJNA729271	ed_Lushi_vs_Arbor_Acr	breed_growth_background	ENSGALG00010009490	SNTB1	-0.00246948	0.085463897	-0.028895012	0.976948324	0.986338787	negative
PRJNA729271	ed_Lushi_vs_Arbor_Acr	breed_growth_background	ENSGALG00010009491	RBM34	0.271130898	0.055123517	4.918606673	8.71624E-07	4.37689E-05	positive
PRJNA729271	ed_Lushi_vs_Arbor_Acr	breed_growth_background	ENSGALG00010009492	CDC42SE2	-0.249553973	0.068973501	-3.618113762	0.000296758	0.003451345	negative
PRJNA729271	ed_Lushi_vs_Arbor_Acr	breed_growth_background	ENSGALG00010009493	ENSGALG00010009493	-0.535971946	0.219871517	-2.437659748	0.014782681	0.054928213	negative
PRJNA729271	ed_Lushi_vs_Arbor_Acr	breed_growth_background	ENSGALG00010009494	UBA2	-0.031741094	0.040458508	-0.784534464	0.432726567	0.591008348	negative
PRJNA729271	ed_Lushi_vs_Arbor_Acr	breed_growth_background	ENSGALG00010009497	HS3ST1	-0.243623388	0.099859724	-2.439656145	0.014701248	0.054709778	negative
PRJNA729271	ed_Lushi_vs_Arbor_Acr	breed_growth_background	ENSGALG00010009498	ORC4	-0.112512935	0.064377494	-1.747706024	0.080514945	0.184749744	negative
PRJNA729271	ed_Lushi_vs_Arbor_Acr	breed_growth_background	ENSGALG00010009499	MXD3	-0.442051555	0.132507886	-3.336039598	0.000849811	0.007258694	negative
PRJNA729271	ed_Lushi_vs_Arbor_Acr	breed_growth_background	ENSGALG00010009500	CCDC146	-0.077253765	0.336751461	-0.22940885	0.818551159	0.888357221	negative
PRJNA729271	ed_Lushi_vs_Arbor_Acr	breed_growth_background	ENSGALG00010009501	HGH1	-0.070031222	0.060554449	-1.156500041	0.247476683	0.403642907	negative
PRJNA729271	ed_Lushi_vs_Arbor_Acr	breed_growth_background	ENSGALG00010009502	ARID5B	-0.069755117	0.052295515	-1.333864219	0.18224836	0.327022775	negative
PRJNA729271	ed_Lushi_vs_Arbor_Acr	breed_growth_background	ENSGALG00010009503	IL11RA	0.501923417	0.074079361	6.775482555	1.23992E-11	4.92795E-09	positive
PRJNA729271	ed_Lushi_vs_Arbor_Acr	breed_growth_background	ENSGALG00010009506	HAS2	-0.366013873	0.139571428	-2.622412603	0.008730966	0.037811577	negative
PRJNA729271	ed_Lushi_vs_Arbor_Acr	breed_growth_background	ENSGALG00010009508	TOMM20	-0.087490036	0.048090818	-1.81926696	0.068870711	0.166048064	negative
PRJNA729271	ed_Lushi_vs_Arbor_Acr	breed_growth_background	ENSGALG00010009511	ZFPM1	0.272128472	0.145411437	1.871437882	0.061284412	0.15318461	positive
PRJNA729271	ed_Lushi_vs_Arbor_Acr	breed_growth_background	ENSGALG00010009514	PRELID1	0.024038147	0.049568348	0.484949531	0.627712147	0.753951571	positive
PRJNA729271	ed_Lushi_vs_Arbor_Acr	breed_growth_background	ENSGALG00010009515	MBD5	0.052599137	0.051406549	1.023199157	0.306213691	0.468697725	positive
PRJNA729271	ed_Lushi_vs_Arbor_Acr	breed_growth_background	ENSGALG00010009518	FGL2	-0.291427076	0.150599918	-1.9351078	0.052977069	0.138087144	negative
PRJNA729271	ed_Lushi_vs_Arbor_Acr	breed_growth_background	ENSGALG00010009520	SPINK4	-0.789710557	0.334768416	-2.358975694	0.018325456	0.064332159	negative
PRJNA729271	ed_Lushi_vs_Arbor_Acr	breed_growth_background	ENSGALG00010009522	TAF5L	0.117381156	0.043684339	2.687030608	0.007209035	0.033099448	positive
PRJNA729271	ed_Lushi_vs_Arbor_Acr	breed_growth_background	ENSGALG00010009524	FAM185A	0.132518315	0.085793156	1.544625709	0.122436824	0.247237806	positive
PRJNA729271	ed_Lushi_vs_Arbor_Acr	breed_growth_background	ENSGALG00010009525	ENSGALG00010009525	0.028693893	0.083415248	0.343988582	0.730854873	0.827442114	positive
PRJNA729271	ed_Lushi_vs_Arbor_Acr	breed_growth_background	ENSGALG00010009526	ENSGALG00010009526	0.089341469	0.13774967	0.648578459	0.516610882	0.664915178	positive
PRJNA729271	ed_Lushi_vs_Arbor_Acr	breed_growth_background	ENSGALG00010009527	ENSGALG00010009527	-0.23060564	0.297406795	-0.775387934	0.438110463	0.595444085	negative
PRJNA729271	ed_Lushi_vs_Arbor_Acr	breed_growth_background	ENSGALG00010009530	KIF3C	-0.231244804	0.087151175	-2.653375616	0.007969114	0.035520388	negative
PRJNA729271	ed_Lushi_vs_Arbor_Acr	breed_growth_background	ENSGALG00010009531	FBXL13	-0.332836053	0.261472777	-1.272928128	0.203043547	0.352215918	negative
PRJNA729271	ed_Lushi_vs_Arbor_Acr	breed_growth_background	ENSGALG00010009534	XPA	-0.307167378	0.07031556	-4.368412613	1.25153E-05	0.000340355	negative
PRJNA729271	ed_Lushi_vs_Arbor_Acr	breed_growth_background	ENSGALG00010009538	CRIM1	-0.037818175	0.039669837	-0.953323174	0.340426354	0.504444898	negative
PRJNA729271	ed_Lushi_vs_Arbor_Acr	breed_growth_background	ENSGALG00010009540	RAB10	0.007520053	0.048195498	0.156032275	0.876007581	0.926645502	positive
PRJNA729271	ed_Lushi_vs_Arbor_Acr	breed_growth_background	ENSGALG00010009541	PMPCB	-0.001407696	0.049731487	-0.028305929	0.977418152	0.986744401	negative
PRJNA729271	ed_Lushi_vs_Arbor_Acr	breed_growth_background	ENSGALG00010009542	WDR1	-0.152978458	0.076591464	-1.997330389	0.045789304	0.124407866	negative
PRJNA729271	ed_Lushi_vs_Arbor_Acr	breed_growth_background	ENSGALG00010009543	MAF1	0.176919464	0.069200088	2.556636408	0.010568962	0.043497192	positive
PRJNA729271	ed_Lushi_vs_Arbor_Acr	breed_growth_background	ENSGALG00010009546	GAREM2	-0.106836737	0.364333425	-0.293238912	0.769339543	0.856298601	negative
PRJNA729271	ed_Lushi_vs_Arbor_Acr	breed_growth_background	ENSGALG00010009548	PIK3C3	-0.029124395	0.044204543	-0.658855252	0.509988723	0.658672952	negative
PRJNA729271	ed_Lushi_vs_Arbor_Acr	breed_growth_background	ENSGALG00010009551	HADHA	-0.059325339	0.069570621	-0.852735511	0.393805994	0.555322347	negative
PRJNA729271	ed_Lushi_vs_Arbor_Acr	breed_growth_background	ENSGALG00010009554	ABCB10	0.100377229	0.055107559	1.82147841	0.068534166	0.16548438	positive
PRJNA729271	ed_Lushi_vs_Arbor_Acr	breed_growth_background	ENSGALG00010009555	EPC2	0.070433415	0.041773951	1.686060625	0.091784123	0.20255951	positive
PRJNA729271	ed_Lushi_vs_Arbor_Acr	breed_growth_background	ENSGALG00010009557	CIDEC	-0.354195133	0.555633348	-0.637461978	0.523823969	0.670666978	negative
PRJNA729271	ed_Lushi_vs_Arbor_Acr	breed_growth_background	ENSGALG00010009558	TMEM26	0.000923311	0.109643609	0.008421015	0.993281081	0.996098607	positive
PRJNA729271	ed_Lushi_vs_Arbor_Acr	breed_growth_background	ENSGALG00010009560	RAB24	0.197055259	0.076916679	2.561931448	0.010409186	0.043022855	positive
PRJNA729271	ed_Lushi_vs_Arbor_Acr	breed_growth_background	ENSGALG00010009562	HADHB	0.027926066	0.101275635	0.27574318	0.782745328	0.865636189	positive
PRJNA729271	ed_Lushi_vs_Arbor_Acr	breed_growth_background	ENSGALG00010009565	TMEM8B	-0.026226417	0.122383768	-0.214296532	0.830315823	0.89662727	negative
PRJNA729271	ed_Lushi_vs_Arbor_Acr	breed_growth_background	ENSGALG00010009567	NSD1	0.057033877	0.057120756	0.998479019	0.318047134	0.481421596	positive
PRJNA729271	ed_Lushi_vs_Arbor_Acr	breed_growth_background	ENSGALG00010009569	ZC3H18	0.187654699	0.077450296	2.42290488	0.015396958	0.056793713	positive
PRJNA729271	ed_Lushi_vs_Arbor_Acr	breed_growth_background	ENSGALG00010009570	SHARPIN	0.342452853	0.071963765	4.758684517	1.94859E-06	8.32823E-05	positive
PRJNA729271	ed_Lushi_vs_Arbor_Acr	breed_growth_background	ENSGALG00010009571	DNAJC2	0.362513383	0.099633461	3.638470234	0.000274262	0.00326927	positive
PRJNA729271	ed_Lushi_vs_Arbor_Acr	breed_growth_background	ENSGALG00010009574	TAF1C	-0.046757114	0.07990187	-0.585181718	0.558425537	0.699600532	negative
PRJNA729271	ed_Lushi_vs_Arbor_Acr	breed_growth_background	ENSGALG00010009575	RHOBTB1	-0.148246385	0.136778746	-1.083840801	0.278435391	0.437835737	negative
PRJNA729271	ed_Lushi_vs_Arbor_Acr	breed_growth_background	ENSGALG00010009578	KIF5C	-0.220054235	0.086670494	-2.538975189	0.011117771	0.045153289	negative
PRJNA729271	ed_Lushi_vs_Arbor_Acr	breed_growth_background	ENSGALG00010009582	TIGD5	0.091824895	0.075324038	1.219064964	0.222819537	0.375379961	positive
PRJNA729271	ed_Lushi_vs_Arbor_Acr	breed_growth_background	ENSGALG00010009583	LYPD6B	-0.337585738	0.175782496	-1.920474139	0.054798037	0.141556807	negative
PRJNA729271	ed_Lushi_vs_Arbor_Acr	breed_growth_background	ENSGALG00010009591	LYPD6	-0.689681598	0.26927888	-2.561216824	0.010430624	0.04309917	negative
PRJNA729271	ed_Lushi_vs_Arbor_Acr	breed_growth_background	ENSGALG00010009593	NCBP1	0.01236755	0.034166448	0.361979375	0.717367443	0.8188881	positive
PRJNA729271	ed_Lushi_vs_Arbor_Acr	breed_growth_background	ENSGALG00010009594	HINT2	-0.103925388	0.085531208	-1.215058099	0.224343933	0.377115309	negative
PRJNA729271	ed_Lushi_vs_Arbor_Acr	breed_growth_background	ENSGALG00010009595	ENSGALG00010009595	-0.114400988	0.099464227	-1.150172194	0.250072956	0.406004425	negative
PRJNA729271	ed_Lushi_vs_Arbor_Acr	breed_growth_background	ENSGALG00010009596	URB2	0.099762491	0.054013852	1.846979773	0.064750072	0.158927103	positive
PRJNA729271	ed_Lushi_vs_Arbor_Acr	breed_growth_background	ENSGALG00010009597	CDK1	-0.524837761	0.139594638	-3.759727233	0.000170099	0.002323827	negative
PRJNA729271	ed_Lushi_vs_Arbor_Acr	breed_growth_background	ENSGALG00010009600	LRRC17	-0.299788974	0.116399927	-2.575508268	0.010009288	0.041859385	negative
PRJNA729271	ed_Lushi_vs_Arbor_Acr	breed_growth_background	ENSGALG00010009601	ZHX2	0.105701446	0.073271664	1.442596501	0.14913417	0.284013715	positive
PRJNA729271	ed_Lushi_vs_Arbor_Acr	breed_growth_background	ENSGALG00010009602	TSTD2	-0.048434138	0.043916822	-1.102860719	0.270087655	0.428844403	negative
PRJNA729271	ed_Lushi_vs_Arbor_Acr	breed_growth_background	ENSGALG00010009603	HINT1	-0.201551984	0.094736877	-2.127492366	0.033379195	0.098339722	negative
PRJNA729271	ed_Lushi_vs_Arbor_Acr	breed_growth_background	ENSGALG00010009605	ENSGALG00010009605	-0.282745444	0.091490172	-3.090446092	0.001998561	0.013254731	negative
PRJNA729271	ed_Lushi_vs_Arbor_Acr	breed_growth_background	ENSGALG00010009606	CHSY3	-0.521074342	0.116074426	-4.48913995	7.15113E-06	0.00022485	negative
PRJNA729271	ed_Lushi_vs_Arbor_Acr	breed_growth_background	ENSGALG00010009608	AFAP1	-0.258787336	0.057504208	-4.500319978	6.78512E-06	0.000218556	negative
PRJNA729271	ed_Lushi_vs_Arbor_Acr	breed_growth_background	ENSGALG00010009609	MMADHC	0.09908265	0.038924656	2.545498407	0.010912186	0.044517911	positive
PRJNA729271	ed_Lushi_vs_Arbor_Acr	breed_growth_background	ENSGALG00010009612	DERL1	-0.057604773	0.034305939	-1.679148673	0.093123072	0.204580014	negative
PRJNA729271	ed_Lushi_vs_Arbor_Acr	breed_growth_background	ENSGALG00010009615	ENSGALG00010009615	-0.193277862	0.211294435	-0.914732381	0.360332141	0.523610464	negative
PRJNA729271	ed_Lushi_vs_Arbor_Acr	breed_growth_background	ENSGALG00010009616	ARMC10	-0.091616894	0.098538612	-0.92975629	0.352497282	0.51637839	negative
PRJNA729271	ed_Lushi_vs_Arbor_Acr	breed_growth_background	ENSGALG00010009617	SLC25A46	0.098661142	0.052257888	1.887966519	0.059030445	0.149041335	positive
PRJNA729271	ed_Lushi_vs_Arbor_Acr	breed_growth_background	ENSGALG00010009619	ENSGALG00010009619	-0.080651869	0.102225663	-0.788959122	0.430135899	0.588468131	negative
PRJNA729271	ed_Lushi_vs_Arbor_Acr	breed_growth_background	ENSGALG00010009620	TMOD1	-0.102062782	0.124239653	-0.821499254	0.411361949	0.571543795	negative
PRJNA729271	ed_Lushi_vs_Arbor_Acr	breed_growth_background	ENSGALG00010009623	XPO5	0.064759575	0.059959209	1.080060543	0.280115221	0.439959615	positive
PRJNA729271	ed_Lushi_vs_Arbor_Acr	breed_growth_background	ENSGALG00010009629	ENSGALG00010009629	0.19322014	0.306722589	0.629950798	0.528726776	0.674539357	positive
PRJNA729271	ed_Lushi_vs_Arbor_Acr	breed_growth_background	ENSGALG00010009634	TBC1D31	0.155892963	0.057667698	2.703297825	0.00686552	0.031977184	positive
PRJNA729271	ed_Lushi_vs_Arbor_Acr	breed_growth_background	ENSGALG00010009635	TDRD7	-0.080240388	0.075091881	-1.068562775	0.28526673	0.445719656	negative
PRJNA729271	ed_Lushi_vs_Arbor_Acr	breed_growth_background	ENSGALG00010009637	CYBA	-0.134506372	0.072826374	-1.846945892	0.064754983	0.158927103	negative
PRJNA729271	ed_Lushi_vs_Arbor_Acr	breed_growth_background	ENSGALG00010009638	RND3	-0.112583012	0.080887138	-1.391853083	0.163966891	0.303731641	negative
PRJNA729271	ed_Lushi_vs_Arbor_Acr	breed_growth_background	ENSGALG00010009640	NPR2	0.267745597	0.100524201	2.663493913	0.007733381	0.034801415	positive
PRJNA729271	ed_Lushi_vs_Arbor_Acr	breed_growth_background	ENSGALG00010009643	PSMC2	-0.046981164	0.058158898	-0.807806986	0.419201703	0.578987226	negative
PRJNA729271	ed_Lushi_vs_Arbor_Acr	breed_growth_background	ENSGALG00010009644	SLC27A6	-0.434081827	0.122207709	-3.55200037	0.000382314	0.004116185	negative
PRJNA729271	ed_Lushi_vs_Arbor_Acr	breed_growth_background	ENSGALG00010009646	MVD	0.148344651	0.090581246	1.637697173	0.101484881	0.217336124	positive
PRJNA729271	ed_Lushi_vs_Arbor_Acr	breed_growth_background	ENSGALG00010009647	GALNT2	-0.016657864	0.042652063	-0.390552364	0.696128142	0.804270478	negative
PRJNA729271	ed_Lushi_vs_Arbor_Acr	breed_growth_background	ENSGALG00010009649	TENM2	0.327525196	0.169142817	1.936382529	0.052820868	0.137828709	positive
PRJNA729271	ed_Lushi_vs_Arbor_Acr	breed_growth_background	ENSGALG00010009650	KLHL29	0.301849577	0.103599474	2.91362075	0.003572636	0.019978922	positive
PRJNA729271	ed_Lushi_vs_Arbor_Acr	breed_growth_background	ENSGALG00010009652	CIAO2B	-0.099935277	0.044067341	-2.26778552	0.023342282	0.076290053	negative
PRJNA729271	ed_Lushi_vs_Arbor_Acr	breed_growth_background	ENSGALG00010009653	ABLIM2	0.305929675	0.09696214	3.155145645	0.00160418	0.011381591	positive
PRJNA729271	ed_Lushi_vs_Arbor_Acr	breed_growth_background	ENSGALG00010009654	NDUFA12	0.028793041	0.074596789	0.385982314	0.699509767	0.806185424	positive
PRJNA729271	ed_Lushi_vs_Arbor_Acr	breed_growth_background	ENSGALG00010009657	MSMP	-0.04574263	0.352198192	-0.129877528	0.896663324	0.940050259	negative
PRJNA729271	ed_Lushi_vs_Arbor_Acr	breed_growth_background	ENSGALG00010009658	NAPEPLD	0.017597028	0.065838935	0.267273889	0.789258289	0.869257572	positive
PRJNA729271	ed_Lushi_vs_Arbor_Acr	breed_growth_background	ENSGALG00010009659	APOLD1	-0.532691356	0.136872374	-3.891883672	9.94689E-05	0.001582659	negative
PRJNA729271	ed_Lushi_vs_Arbor_Acr	breed_growth_background	ENSGALG00010009663	FER	-0.15342056	0.065801246	-2.331575301	0.019723044	0.067602248	negative
PRJNA729271	ed_Lushi_vs_Arbor_Acr	breed_growth_background	ENSGALG00010009665	DGKQ	-0.007979353	0.093684511	-0.085172598	0.932124175	0.962674736	negative
PRJNA729271	ed_Lushi_vs_Arbor_Acr	breed_growth_background	ENSGALG00010009666	RRAD	-0.422097862	0.19482857	-2.16650906	0.030272308	0.091539839	negative
PRJNA729271	ed_Lushi_vs_Arbor_Acr	breed_growth_background	ENSGALG00010009668	TFAP2A	0.284512253	0.4245939	0.670080877	0.502806235	0.653648105	positive
PRJNA729271	ed_Lushi_vs_Arbor_Acr	breed_growth_background	ENSGALG00010009671	MINAR2	-1.561568203	0.4160824	-3.753026329	0.000174712	0.002360492	negative
PRJNA729271	ed_Lushi_vs_Arbor_Acr	breed_growth_background	ENSGALG00010009672	DDX47	0.176319806	0.065003421	2.712469652	0.00667839	0.031348205	positive
PRJNA729271	ed_Lushi_vs_Arbor_Acr	breed_growth_background	ENSGALG00010009676	ACO1	-0.297191506	0.051908545	-5.725290578	1.03257E-08	1.41199E-06	negative
PRJNA729271	ed_Lushi_vs_Arbor_Acr	breed_growth_background	ENSGALG00010009677	SLC26A5	0.270084207	0.086890404	3.108331804	0.001881467	0.012714156	positive
PRJNA729271	ed_Lushi_vs_Arbor_Acr	breed_growth_background	ENSGALG00010009678	JARID2	-0.010090916	0.054608496	-0.184786564	0.853396447	0.911501105	negative
PRJNA729271	ed_Lushi_vs_Arbor_Acr	breed_growth_background	ENSGALG00010009679	ANK3	-0.098827479	0.054871077	-1.801085089	0.07168947	0.1705559	negative
PRJNA729271	ed_Lushi_vs_Arbor_Acr	breed_growth_background	ENSGALG00010009683	ADAMTS19	-0.523914058	0.127068771	-4.123074883	3.73848E-05	0.000791084	negative
PRJNA729271	ed_Lushi_vs_Arbor_Acr	breed_growth_background	ENSGALG00010009686	MAT2B	0.117526692	0.046552169	2.524623332	0.011582236	0.046428237	positive
PRJNA729271	ed_Lushi_vs_Arbor_Acr	breed_growth_background	ENSGALG00010009689	ENSGALG00010009689	-0.475670135	0.29312108	-1.622776961	0.104637093	0.222066568	negative
PRJNA729271	ed_Lushi_vs_Arbor_Acr	breed_growth_background	ENSGALG00010009690	RBM43	-0.410443006	0.100583572	-4.080616712	4.49164E-05	0.000894317	negative
PRJNA729271	ed_Lushi_vs_Arbor_Acr	breed_growth_background	ENSGALG00010009695	ATAD2B	0.078055066	0.041230125	1.893156159	0.058337106	0.147739021	positive
PRJNA729271	ed_Lushi_vs_Arbor_Acr	breed_growth_background	ENSGALG00010009697	ENSGALG00010009697	0.404851546	0.15254387	2.654000748	0.007954365	0.035481811	positive
PRJNA729271	ed_Lushi_vs_Arbor_Acr	breed_growth_background	ENSGALG00010009699	POLR1C	-0.121120367	0.060118125	-2.014706337	0.043935428	0.120545907	negative
PRJNA729271	ed_Lushi_vs_Arbor_Acr	breed_growth_background	ENSGALG00010009700	SH3TC1	-0.282494522	0.110449718	-2.557675361	0.010537441	0.043392106	negative
PRJNA729271	ed_Lushi_vs_Arbor_Acr	breed_growth_background	ENSGALG00010009702	ENSGALG00010009702	0.394344195	0.06753519	5.839092141	5.2486E-09	7.84315E-07	positive
PRJNA729271	ed_Lushi_vs_Arbor_Acr	breed_growth_background	ENSGALG00010009704	RGP1	0.178366502	0.07027711	2.538045486	0.01114735	0.045218153	positive
PRJNA729271	ed_Lushi_vs_Arbor_Acr	breed_growth_background	ENSGALG00010009705	DTNBP1	0.156630599	0.039797764	3.935663269	8.29672E-05	0.001393523	positive
PRJNA729271	ed_Lushi_vs_Arbor_Acr	breed_growth_background	ENSGALG00010009710	ENSGALG00010009710	0.303837592	0.061213619	4.963561954	6.92119E-07	3.65593E-05	positive
PRJNA729271	ed_Lushi_vs_Arbor_Acr	breed_growth_background	ENSGALG00010009712	UBXN2A	0.144188546	0.094987124	1.517979902	0.129019465	0.256464228	positive
PRJNA729271	ed_Lushi_vs_Arbor_Acr	breed_growth_background	ENSGALG00010009715	NUDCD2	-0.052351114	0.050611486	-1.034372189	0.300962201	0.463393251	negative

PRJNA729271	ed_Lushi_vs_Arbor_Acr	breed_growth_background	ENSGALG00010009717	EDN1	-0.103762778	0.133347956	-0.778135498	0.436489138	0.594045778	negative
PRJNA729271	ed_Lushi_vs_Arbor_Acr	breed_growth_background	ENSGALG00010009718	ENSGALG00010009718	0.340413219	0.10033758	3.392679192	0.000692126	0.006262405	positive
PRJNA729271	ed_Lushi_vs_Arbor_Acr	breed_growth_background	ENSGALG00010009719	YIPF3	-0.043832123	0.044541965	-0.984063532	0.325084276	0.488946163	negative
PRJNA729271	ed_Lushi_vs_Arbor_Acr	breed_growth_background	ENSGALG00010009720	RELN	0.091495132	0.090790889	1.007756757	0.31357125	0.477087779	positive
PRJNA729271	ed_Lushi_vs_Arbor_Acr	breed_growth_background	ENSGALG00010009721	ZHX1	-0.042953159	0.083438444	-0.514788591	0.606700759	0.737698976	negative
PRJNA729271	ed_Lushi_vs_Arbor_Acr	breed_growth_background	ENSGALG00010009722	GBA2	-0.035915499	0.052664038	-0.681973896	0.495255457	0.646702902	negative
PRJNA729271	ed_Lushi_vs_Arbor_Acr	breed_growth_background	ENSGALG00010009724	ISOC1	-0.154153942	0.077162664	-1.997778911	0.045740635	0.124345555	negative
PRJNA729271	ed_Lushi_vs_Arbor_Acr	breed_growth_background	ENSGALG00010009726	PRR16	0.016126824	0.080099009	0.201336127	0.840435755	0.903248778	positive
PRJNA729271	ed_Lushi_vs_Arbor_Acr	breed_growth_background	ENSGALG00010009727	CDH20	-0.113114929	0.110450878	-1.024119781	0.305778702	0.468279164	negative
PRJNA729271	ed_Lushi_vs_Arbor_Acr	breed_growth_background	ENSGALG00010009728	CCDC149	-0.084493378	0.057079983	-1.480262842	0.138803115	0.270459895	negative
PRJNA729271	ed_Lushi_vs_Arbor_Acr	breed_growth_background	ENSGALG00010009730	C8orf76	0.028275741	0.06330502	0.446658754	0.655121466	0.775238902	positive
PRJNA729271	ed_Lushi_vs_Arbor_Acr	breed_growth_background	ENSGALG00010009731	NMI	-0.096430512	0.130016447	-0.741679336	0.458281643	0.614038466	negative
PRJNA729271	ed_Lushi_vs_Arbor_Acr	breed_growth_background	ENSGALG00010009732	HMMR	-0.327388556	0.132257922	-2.475379546	0.013309465	0.05110482	negative
PRJNA729271	ed_Lushi_vs_Arbor_Acr	breed_growth_background	ENSGALG00010009736	WISP1	-0.269635598	0.178244096	-1.512732283	0.130347676	0.258289755	negative
PRJNA729271	ed_Lushi_vs_Arbor_Acr	breed_growth_background	ENSGALG00010009738	ENSGALG00010009738	-0.064704379	0.140423839	-0.460779163	0.644957053	0.767604276	negative
PRJNA729271	ed_Lushi_vs_Arbor_Acr	breed_growth_background	ENSGALG00010009740	LRRC73	-0.452604373	0.191018927	-2.369421605	0.017815932	0.063078146	negative
PRJNA729271	ed_Lushi_vs_Arbor_Acr	breed_growth_background	ENSGALG00010009741	C2H6orf52	0.10016247	0.038459049	2.60439277	0.009203722	0.039399865	positive
PRJNA729271	ed_Lushi_vs_Arbor_Acr	breed_growth_background	ENSGALG00010009743	CCNG1	0.116995847	0.092368654	1.266618504	0.205291733	0.354588139	positive
PRJNA729271	ed_Lushi_vs_Arbor_Acr	breed_growth_background	ENSGALG00010009744	DAPK1	-0.13479753	0.056742021	-2.37562088	0.017519453	0.062165109	negative
PRJNA729271	ed_Lushi_vs_Arbor_Acr	breed_growth_background	ENSGALG00010009749	NDRG1	-0.193680628	0.089291973	-2.169070998	0.030077294	0.091035786	negative
PRJNA729271	ed_Lushi_vs_Arbor_Acr	breed_growth_background	ENSGALG00010009751	ENSGALG00010009751	0.012094216	0.114422361	0.105698007	0.915821961	0.952352345	positive
PRJNA729271	ed_Lushi_vs_Arbor_Acr	breed_growth_background	ENSGALG00010009761	PDP2	0.060713147	0.062128886	0.97721286	0.328463783	0.492254191	positive
PRJNA729271	ed_Lushi_vs_Arbor_Acr	breed_growth_background	ENSGALG00010009762	MAN2A1	0.016509806	0.055056932	0.299867889	0.764277929	0.852060836	positive
PRJNA729271	ed_Lushi_vs_Arbor_Acr	breed_growth_background	ENSGALG00010009765	STX10	-0.083508867	0.053625269	-1.557267106	0.119407082	0.24356961	negative
PRJNA729271	ed_Lushi_vs_Arbor_Acr	breed_growth_background	ENSGALG00010009769	TMCC3	-0.217097903	0.123126089	-1.763216095	0.07786405	0.180466806	negative
PRJNA729271	ed_Lushi_vs_Arbor_Acr	breed_growth_background	ENSGALG00010009772	ENSGALG00010009772	0.155901149	0.31464044	0.495489865	0.620254374	0.74781131	positive
PRJNA729271	ed_Lushi_vs_Arbor_Acr	breed_growth_background	ENSGALG00010009774	PIAS2	-0.006969005	0.033999453	-0.204974041	0.837592407	0.901461422	negative
PRJNA729271	ed_Lushi_vs_Arbor_Acr	breed_growth_background	ENSGALG00010009775	KIAA1614	0.113247148	0.077606951	1.459239744	0.144499133	0.278002906	positive
PRJNA729271	ed_Lushi_vs_Arbor_Acr	breed_growth_background	ENSGALG00010009777	ST3GAL1	-0.349727826	0.103027185	-3.394519871	0.000687491	0.006228234	negative
PRJNA729271	ed_Lushi_vs_Arbor_Acr	breed_growth_background	ENSGALG00010009778	ENSGALG00010009778	0.092178311	0.06000976	1.536055326	0.124524812	0.250379684	positive
PRJNA729271	ed_Lushi_vs_Arbor_Acr	breed_growth_background	ENSGALG00010009779	PPP1R3E	-0.279916692	0.175669	-1.593432487	0.111063203	0.231768085	negative
PRJNA729271	ed_Lushi_vs_Arbor_Acr	breed_growth_background	ENSGALG00010009780	MFSD2B	-0.630622261	0.142086718	-4.43829143	9.06758E-06	0.000269886	negative
PRJNA729271	ed_Lushi_vs_Arbor_Acr	breed_growth_background	ENSGALG00010009784	ENSGALG00010009784	-0.067068919	0.097029909	-0.691219018	0.489427914	0.641259841	negative
PRJNA729271	ed_Lushi_vs_Arbor_Acr	breed_growth_background	ENSGALG00010009786	DIPK2B	-0.114237132	0.086161846	-1.325843591	0.184891511	0.33025292	negative
PRJNA729271	ed_Lushi_vs_Arbor_Acr	breed_growth_background	ENSGALG00010009788	PAK1IP1	0.093378921	0.049636523	1.881254258	0.05993734	0.15064882	positive
PRJNA729271	ed_Lushi_vs_Arbor_Acr	breed_growth_background	ENSGALG00010009791	GABRG2	-0.201390048	0.16613778	-1.21218695	0.225440826	0.378476346	negative
PRJNA729271	ed_Lushi_vs_Arbor_Acr	breed_growth_background	ENSGALG00010009793	TNFAIP6	-0.112903924	0.157529269	-0.716717121	0.473548659	0.627143702	negative
PRJNA729271	ed_Lushi_vs_Arbor_Acr	breed_growth_background	ENSGALG00010009795	NDUFA6	0.018452584	0.061285601	0.30109168	0.763344586	0.851352263	positive
PRJNA729271	ed_Lushi_vs_Arbor_Acr	breed_growth_background	ENSGALG00010009796	ENSGALG00010009796	0.216609645	0.106855129	2.027133811	0.04264873	0.118020874	positive
PRJNA729271	ed_Lushi_vs_Arbor_Acr	breed_growth_background	ENSGALG00010009798	YIPF4	0.197730838	0.041628418	4.749900369	2.03517E-06	8.57552E-05	positive
PRJNA729271	ed_Lushi_vs_Arbor_Acr	breed_growth_background	ENSGALG00010009801	ENSGALG00010009801	-0.665265062	0.337777817	-1.969534495	0.048891745	0.130561137	negative
PRJNA729271	ed_Lushi_vs_Arbor_Acr	breed_growth_background	ENSGALG00010009804	XPR1	0.164642591	0.062952185	2.615359443	0.00891336	0.038440688	positive
PRJNA729271	ed_Lushi_vs_Arbor_Acr	breed_growth_background	ENSGALG00010009806	WDCP	0.098622962	0.093789392	1.051536422	0.29301229	0.454316489	positive
PRJNA729271	ed_Lushi_vs_Arbor_Acr	breed_growth_background	ENSGALG00010009809	SMDT1	-0.153069551	0.048317389	-3.168001334	0.001534908	0.011063398	negative
PRJNA729271	ed_Lushi_vs_Arbor_Acr	breed_growth_background	ENSGALG00010009811	ENSGALG00010009811	0.01785966	0.046934066	0.380526585	0.703554566	0.808468641	positive
PRJNA729271	ed_Lushi_vs_Arbor_Acr	breed_growth_background	ENSGALG00010009822	ENSGALG00010009822	0.024790203	0.109969973	0.22542702	0.821647094	0.890583611	positive
PRJNA729271	ed_Lushi_vs_Arbor_Acr	breed_growth_background	ENSGALG00010009830	RNASEL	-0.030209255	0.108432529	-0.278599558	0.780552152	0.864199774	negative
PRJNA729271	ed_Lushi_vs_Arbor_Acr	breed_growth_background	ENSGALG00010009831	N6AMT1	-0.025275271	0.066110736	-0.382317186	0.702226109	0.80764639	negative
PRJNA729271	ed_Lushi_vs_Arbor_Acr	breed_growth_background	ENSGALG00010009833	SOD3	0.300521617	0.164837404	1.823139713	0.068282233	0.165151171	positive
PRJNA729271	ed_Lushi_vs_Arbor_Acr	breed_growth_background	ENSGALG00010009840	ENSGALG00010009840	-0.107678904	0.096133978	-1.120092044	0.262674541	0.420900671	negative
PRJNA729271	ed_Lushi_vs_Arbor_Acr	breed_growth_background	ENSGALG00010009841	GABRA1	0.277350728	0.129211677	2.146483466	0.031834427	0.095279788	positive
PRJNA729271	ed_Lushi_vs_Arbor_Acr	breed_growth_background	ENSGALG00010009842	PHETA2	0.055741736	0.048260781	1.155011051	0.248085901	0.40427264	positive
PRJNA729271	ed_Lushi_vs_Arbor_Acr	breed_growth_background	ENSGALG00010009847	ENSGALG00010009847	-0.178059586	0.064468517	-2.761961895	0.005745518	0.028069191	negative
PRJNA729271	ed_Lushi_vs_Arbor_Acr	breed_growth_background	ENSGALG00010009850	RIF1	-0.179563243	0.057082361	-3.145687018	0.001656972	0.011625272	negative
PRJNA729271	ed_Lushi_vs_Arbor_Acr	breed_growth_background	ENSGALG00010009852	ENSGALG00010009852	-0.32010724	0.141519364	-2.261932445	0.023701577	0.07702198	negative
PRJNA729271	ed_Lushi_vs_Arbor_Acr	breed_growth_background	ENSGALG00010009857	ORC5	0.286325167	0.076622274	3.73683987	0.000186348	0.002460025	positive
PRJNA729271	ed_Lushi_vs_Arbor_Acr	breed_growth_background	ENSGALG00010009858	ST8SIA5	0.027663779	0.135506599	0.204150789	0.838235666	0.901798833	positive
PRJNA729271	ed_Lushi_vs_Arbor_Acr	breed_growth_background	ENSGALG00010009866	NAGA	-0.121716092	0.061933978	-1.965255528	0.04938467	0.131388369	negative
PRJNA729271	ed_Lushi_vs_Arbor_Acr	breed_growth_background	ENSGALG00010009868	LTN1	0.112038206	0.050875028	2.202223962	0.027649492	0.085709878	positive
PRJNA729271	ed_Lushi_vs_Arbor_Acr	breed_growth_background	ENSGALG00010009876	SELENOI	0.029476268	0.059822781	0.492726481	0.622205864	0.749013703	positive
PRJNA729271	ed_Lushi_vs_Arbor_Acr	breed_growth_background	ENSGALG00010009883	ENSGALG00010009883	-0.116564952	0.109042068	-1.068990658	0.285073879	0.445523254	negative
PRJNA729271	ed_Lushi_vs_Arbor_Acr	breed_growth_background	ENSGALG00010009884	GFOD1	0.041455183	0.061137215	0.678067903	0.49772864	0.649143121	positive
PRJNA729271	ed_Lushi_vs_Arbor_Acr	breed_growth_background	ENSGALG00010009910	ENSGALG00010009910	-0.195270344	0.095514066	-2.04441452	0.040912614	0.11426365	negative
PRJNA729271	ed_Lushi_vs_Arbor_Acr	breed_growth_background	ENSGALG00010009914	RWDD2B	0.177239633	0.068369782	2.592367981	0.009531776	0.040457713	positive
PRJNA729271	ed_Lushi_vs_Arbor_Acr	breed_growth_background	ENSGALG00010009941	CACNA1E	-0.39949807	0.240468186	-1.661334402	0.096646304	0.20978561	negative
PRJNA729271	ed_Lushi_vs_Arbor_Acr	breed_growth_background	ENSGALG00010009943	DHX15	-0.018633395	0.037217664	-0.500659993	0.616610433	0.745000268	negative
PRJNA729271	ed_Lushi_vs_Arbor_Acr	breed_growth_background	ENSGALG00010009948	WBP2NL	0.055483921	0.034984502	1.585957145	0.112749031	0.23417355	positive
PRJNA729271	ed_Lushi_vs_Arbor_Acr	breed_growth_background	ENSGALG00010009972	PTK2B	0.020230751	0.144885387	0.139632795	0.888950128	0.934871371	positive
PRJNA729271	ed_Lushi_vs_Arbor_Acr	breed_growth_background	ENSGALG00010009985	USP16	-0.105297761	0.062935976	-1.673093325	0.094308925	0.206465469	negative
PRJNA729271	ed_Lushi_vs_Arbor_Acr	breed_growth_background	ENSGALG00010009989	TBC1D7	-0.182619479	0.073896403	-2.471290488	0.013462641	0.051529174	negative
PRJNA729271	ed_Lushi_vs_Arbor_Acr	breed_growth_background	ENSGALG00010009991	LHFPL3	0.526190548	0.317025401	1.659774094	0.096959907	0.210142622	positive
PRJNA729271	ed_Lushi_vs_Arbor_Acr	breed_growth_background	ENSGALG00010009993	ENSGALG00010009993	-0.242637631	0.201726889	-1.202802622	0.22905271	0.382678542	negative
PRJNA729271	ed_Lushi_vs_Arbor_Acr	breed_growth_background	ENSGALG00010009995	SEPTIN3	1.060906787	0.191017396	5.55397995	2.79238E-08	3.16215E-06	positive
PRJNA729271	ed_Lushi_vs_Arbor_Acr	breed_growth_background	ENSGALG00010009999	GABRB2	0.208113882	0.126191695	1.649188418	0.09910904	0.213396544	positive
PRJNA729271	ed_Lushi_vs_Arbor_Acr	breed_growth_background	ENSGALG00010010006	PPARGC1A	0.176214151	0.141714572	1.243444122	0.21370422	0.364214306	positive
PRJNA729271	ed_Lushi_vs_Arbor_Acr	breed_growth_background	ENSGALG00010010007	CT1F	0.087948617	0.051466212	1.708861265	0.087476651	0.195947157	positive
PRJNA729271	ed_Lushi_vs_Arbor_Acr	breed_growth_background	ENSGALG00010010009	ELF2	0.122775513	0.047445192	2.587733532	0.009660968	0.040838652	positive
PRJNA729271	ed_Lushi_vs_Arbor_Acr	breed_growth_background	ENSGALG00010010011	EPB41L4A	-0.236933722	0.066897834	-3.541724867	0.00039752	0.004224377	negative
PRJNA729271	ed_Lushi_vs_Arbor_Acr	breed_growth_background	ENSGALG00010010014	GLUL	0.476722101	0.209734627	2.272977565	0.023027532	0.075499678	positive
PRJNA729271	ed_Lushi_vs_Arbor_Acr	breed_growth_background	ENSGALG00010010017	CCDC59	0.100264055	0.05813969	1.724537158	0.084610917	0.191540722	positive
PRJNA729271	ed_Lushi_vs_Arbor_Acr	breed_growth_background	ENSGALG00010010022	KMT2E	-0.062008836	0.054334792	-1.141236287	0.253771608	0.410400474	negative
PRJNA729271	ed_Lushi_vs_Arbor_Acr	breed_growth_background	ENSGALG00010010035	ATP10B	0.237958658	0.169918824	1.400425527	0.16138593	0.30046714	positive
PRJNA729271	ed_Lushi_vs_Arbor_Acr	breed_growth_background	ENSGALG00010010037	NAA15	0.236656186	0.073988395	3.198558207	0.001381166	0.010313831	positive
PRJNA729271	ed_Lushi_vs_Arbor_Acr	breed_growth_background	ENSGALG00010010042	METTL25	-0.146628975	0.06516739	-2.250036042	0.024446658	0.078675466	negative
PRJNA729271	ed_Lushi_vs_Arbor_Acr	breed_growth_background	ENSGALG00010010049	ZBTB7C	-0.308458753	0.160741311	-1.918976222	0.054987343	0.141898084	negative
PRJNA729271	ed_Lushi_vs_Arbor_Acr	breed_growth_background	ENSGALG00010010052	ZNF346	0.128016414	0.061953539	2.066329317	0.038797395	0.11006609	positive
PRJNA729271	ed_Lushi_vs_Arbor_Acr	breed_growth_background	ENSGALG00010010053	CHRNA2	0.737193181	0.461033307	1.599002002	0.109820158	0.230101646	positive
PRJNA729271	ed_Lushi_vs_Arbor_Acr	breed_growth_background	ENSGALG00010010059	ENSGALG00010010059	0.023422676	0.072640004	0.322448717	0.747112783	0.838829267	positive
PRJNA729271	ed_Lushi_vs_Arbor_Acr	breed_growth_background	ENSGALG00010010061	CYP2D6	0.242600339	0.108309396	2.239882659	0.025098542	0.080224295	positive
PRJNA729271	ed_Lushi_vs_Arbor_Acr	breed_growth_background	ENSGALG00010010062	RGS7BP	-0.060999904	0.0945384	-0.645239432	0.518772037	0.666867743	negative
PRJNA729271	ed_Lushi_vs_Arbor_Acr	breed_growth_background	ENSGALG00010010064	ENSGALG00010010064	0.036856904	0.047934112	0.768907619	0.441948154	0.599143144	positive
PRJNA729271	ed_Lushi_vs_Arbor_Acr	breed_growth_background	ENSGALG00010010071	GLT8D2	-0.439400265	0.144639874	-3.037891638	0.002382396	0.015079839	negative
PRJNA729271	ed_Lushi_vs_Arbor_Acr	breed_growth_background	ENSGALG00010010077	CCT8	-0.111422981	0.061797716	-1.803027502	0.071383896	0.170070606	negative
PRJNA729271	ed_Lushi_vs_Arbor_Acr	breed_growth_background	ENSGALG00010010080	CDC42EP3	0.195337087	0.063370758	3.082448348	0.002053054	0.013514537	positive
PRJNA729271	ed_Lushi_vs_Arbor_Acr	breed_growth_background	ENSGALG00010010084	HK3	0.594263601	0.118031995	5.034767049	4.7843E-07	2.71955E-05	positive
PRJNA729271	ed_Lushi_vs_Arbor_Acr	breed_growth_background	ENSGALG00010010085	TDG	0.030262544	0.056153739	0.538923053	0.589939949	0.725330808	positive
PRJNA729271	ed_Lushi_vs_Arbor_Acr	breed_growth_background	ENSGALG00010010094	SLC2A9	-0.646367554	0.128701204	-5.02223392	5.10739E-07	2.84815E-05	negative
PRJNA729271	ed_Lushi_vs_Arbor_Acr	breed_growth_background	ENSGALG00010010095	C12orf73	0.070441155	0.053252107	1.322786243	0.185906473	0.331412413	positive
PRJNA729271	ed_Lushi_vs_Arbor_Acr	breed_growth_background	ENSGALG00010010100	IRF2BP2	0.123689119	0.086742602	1.425932776	0.153887748	0.290405273	positive
PRJNA729271	ed_Lushi_vs_Arbor_Acr	breed_growth_background	ENSGALG00010010106	C7orf73	-0.414634317	0.110861571	-3.740108632	0.000183941	0.00243833	negative
PRJNA729271	ed_Lushi_vs_Arbor_Acr	breed_growth_background	ENSGALG00010010107	MGST2	0.078061462	0.173831873	0.449062996	0.653386214	0.774016435	positive
PRJNA729271	ed_Lushi_vs_Arbor_Acr	breed_growth_background	ENSGALG00010010111	CNOT4	0.078062815	0.072420747	1.077906787	0.281075356	0.441024821	positive
PRJNA729271	ed_Lushi_vs_Arbor_Acr	breed_growth_background	ENSGALG00010010115	NEB	0.11065132	0.091758744	1.205894022	0.227858356	0.381122418	positive
PRJNA729271	ed_Lushi_vs_Arbor_Acr	breed_growth_background	ENSGALG00010010116	DRD5	0.71395206	0.341385408	2.091337367	0.036497832	0.105230311	positive
PRJNA729271	ed_Lushi_vs_Arbor_Acr	breed_growth_background	ENSGALG00010010119	SRPK2	-0.240523319	0.067639916	-3.555937564	0.000376634	0.00407805	negative
PRJNA729271	ed_Lushi_vs_Arbor_Acr	breed_growth_background	ENSGALG00010010122	THBS4	-0.338663983	0.13361883	-2.534552824	0.011259095	0.045551903	negative
PRJNA729271	ed_Lushi_vs_Arbor_Acr	breed_growth_background	ENSGALG00010010124	ENSGALG00010010124	-0.38604324	0.079837738	-4.835347886	1.32913E-06	6.19477E-05	negative
PRJNA729271	ed_Lushi_vs_Arbor_Acr	breed_growth_background	ENSGALG00010010125	AHCTF1	0.066364556	0.035899889	1.848600618	0.064515503	0.158634813	positive
PRJNA729271	ed_Lushi_vs_Arbor_Acr	breed_growth_background	ENSGALG00010010127	FAM13C	0.118747618	0.178638859	0.664735646	0.50621959	0.655733061	positive
PRJNA729271	ed_Lushi_vs_Arbor_Acr	breed_growth_background	ENSGALG00010010136	EPHX2	0.156854293	0.063735687	2.461012022	0.013854572	0.052640112	positive
PRJNA729271	ed_Lushi_vs_Arbor_Acr	breed_growth_background	ENSGALG00010010142	ENSGALG00010010142	-0.296732351	0.162572239	-1.825233835	0.06796575	0.164660462	negative
PRJNA729271	ed_Lushi_vs_Arbor_Acr	breed_growth_background	ENSGALG00010010146	PHACTR1	-0.519754015	0.199546119	-2.604681151	0.009195979	0.039378352	negative
PRJNA729271	ed_Lushi_vs_Arbor_Acr	breed_growth_background	ENSGALG00010010150	NTRK2	-0.140875472	0.159430823	-0.88361503	0.376904063	0.539344823	negative
PRJNA729271	ed_Lushi_vs_Arbor_Acr	breed_growth_background	ENSGALG00010010153	ENSGALG00010010153	0.354427076	0.238983583	1.483060349	0.138058378	0.269479691	positive
PRJNA729271	ed_Lushi_vs_Arbor_Acr	breed_growth_background	ENSGALG00010010154	IQSEC3	-0.054173329	0.193681523	-0.279703133	0.779705274	0.863591958	negative
PRJNA729271	ed_Lushi_vs_Arbor_Acr	breed_growth_background	ENSGALG00010010155	BRINP3	0.427577965	0.23089545	1.851824994	0.064050957	0.157824909	positive
PRJNA729271	ed_Lushi_vs_Arbor_Acr	breed_growth_background	ENSGALG00010010156	ENSGALG00010010156	-0.130868918	0.05271999	-2.482339564	0.013052283	0.050370831	negative
PRJNA729271	ed_Lushi_vs_Arbor_Acr	breed_growth_background	ENSGALG00010010161	SEPSECS	0.035334589	0.034927078	1.011667486	0.311697066	0.474934193	positive
PRJNA729271	ed_Lushi_vs_Arbor_Acr	breed_growth_background	ENSGALG00010010164	ENSGALG00010010164	0.267826067	0.215644017	1.241982369	0.214243067	0.364881679	positive
PRJNA729271	ed_Lushi_vs_Arbor_Acr	breed_growth_background	ENSGALG00010010167	PUS7	-0.160490372	0.038947669	-4.120666898	3.77777E-05	0.000794091	negative
PRJNA729271	ed_Lushi_vs_Arbor_Acr	breed_growth_background	ENSGALG00010010168	SNCB	0.186510564	0.170705682	1.092585567	0.274575803	0.433406976	positive
PRJNA729271	ed_Lushi_vs_Arbor_Acr	breed_growth_background	ENSGALG00010010171	ENSGALG00010010171	0.630058567	0.208015124	3.028907486	0.002454398	0.015409523	positive
PRJNA729271	ed_Lushi_vs_Arbor_Acr	breed_growth_background	ENSGALG00010010178	LGI2	0.028295944	0.10983239	0.257628405	0.796693705	0.873728929	positive
PRJNA729271	ed_Lushi_vs_Arbor_Acr	breed_growth_background	ENSGALG00010010179	CDHR2	0.088273137	0.480437203	0.183735015	0.854221339	0.912180514	positive
PRJNA729271	ed_Lushi_vs_Arbor_Acr	breed_growth_background	ENSGALG00010010183	NKX3-2	0.199232893	0.256007358	0.778231121	0.436432773	0.594045778	positive
PRJNA729271	ed_Lushi_vs_Arbor_Acr	breed_growth_background	ENSGALG00010010185	PHYHIPL	-0.155216446	0.189888919	-0.817406551	0.413696133	0.574104878	negative
PRJNA729271	ed_Lushi_vs_Arbor_Acr	breed_growth_background	ENSGALG00010010186	TNPO1	0.00388452	0.038613972	0.100598828	0.919868927	0.955493617	positive
PRJNA729271	ed_Lushi_vs_Arbor_Acr	breed_growth_background	ENSGALG00010010188	DSP	-0.233813957	0.192469984	-1.214807383	0.224439564	0.377188578	negative
PRJNA729271	ed_Lushi_vs_Arbor_Acr	breed_growth_background	ENSGALG00010010190	RNF44	0.275094465	0.098565727	2.79097485	0.005254956	0.026429764	positive
PRJNA729271	ed_Lushi_vs_Arbor_Acr	breed_growth_background	ENSGALG00010010191	RINT1	0.025061781	0.051118829	0.490265163	0.623946275	0.750485541	positive
PRJNA729271	ed_Lushi_vs_Arbor_Acr	breed_growth_background	ENSGALG00010010195	ENSGALG00010010195	-0.006732866	0.139915269	-0.04812102	0.961619794	0.978276808	negative

PRJNA729271	eed_Lushi_vs_Arbor_Acr	breed_growth_background	ENSGALG00010010196	EFCAB10	-0.515622043	0.191710734	-2.689583589	0.007154123	0.032899432	negative
PRJNA729271	eed_Lushi_vs_Arbor_Acr	breed_growth_background	ENSGALG00010010197	SCCPDH	-0.011282794	0.065288098	-0.17281548	0.862796469	0.917552077	negative
PRJNA729271	eed_Lushi_vs_Arbor_Acr	breed_growth_background	ENSGALG00010010201	RIOK1	0.139534012	0.054295294	2.56990988	0.010172497	0.042297863	positive
PRJNA729271	eed_Lushi_vs_Arbor_Acr	breed_growth_background	ENSGALG00010010205	ARL5A	-0.12631264	0.044676119	-2.827296587	0.004694283	0.02429199	negative
PRJNA729271	eed_Lushi_vs_Arbor_Acr	breed_growth_background	ENSGALG00010010206	SCARA5	-0.072621598	0.084439	-0.86004806	0.389762551	0.551450285	negative
PRJNA729271	eed_Lushi_vs_Arbor_Acr	breed_growth_background	ENSGALG00010010214	FAF2	0.060292295	0.043491504	1.386300514	0.16565516	0.305998668	positive
PRJNA729271	eed_Lushi_vs_Arbor_Acr	breed_growth_background	ENSGALG00010010215	ENSGALG00010010215	-0.194764604	0.066991147	-2.907318554	0.003645418	0.020229835	negative
PRJNA729271	eed_Lushi_vs_Arbor_Acr	breed_growth_background	ENSGALG00010010216	ATXN7L1	0.214644126	0.068736064	3.122729381	0.001791824	0.01230389	positive
PRJNA729271	eed_Lushi_vs_Arbor_Acr	breed_growth_background	ENSGALG00010010218	PBK	-0.439591573	0.121502133	-3.617974137	0.000296918	0.003451345	negative
PRJNA729271	eed_Lushi_vs_Arbor_Acr	breed_growth_background	ENSGALG00010010220	LHX9	0.419593348	0.377398304	1.111805072	0.266221971	0.424473376	positive
PRJNA729271	eed_Lushi_vs_Arbor_Acr	breed_growth_background	ENSGALG00010010222	PAH	-1.784106561	0.573613757	-3.11029249	0.001869022	0.012647745	negative
PRJNA729271	eed_Lushi_vs_Arbor_Acr	breed_growth_background	ENSGALG00010010223	ENSGALG00010010223	-0.179326258	0.107499814	-1.668154112	0.095285138	0.207880504	negative
PRJNA729271	eed_Lushi_vs_Arbor_Acr	breed_growth_background	ENSGALG00010010226	BICC1	-0.151529304	0.087844969	-1.724962798	0.084534177	0.191396891	negative
PRJNA729271	eed_Lushi_vs_Arbor_Acr	breed_growth_background	ENSGALG00010010231	C1orf53	-0.308465873	0.130379926	-2.365900046	0.0179863	0.063510698	negative
PRJNA729271	eed_Lushi_vs_Arbor_Acr	breed_growth_background	ENSGALG00010010234	ENSGALG00010010234	-0.341866584	0.143427367	-2.383551975	0.017146463	0.061246422	negative
PRJNA729271	eed_Lushi_vs_Arbor_Acr	breed_growth_background	ENSGALG00010010243	ESCO2	-0.0120409	0.061229631	-0.196651528	0.844100244	0.905307661	negative
PRJNA729271	eed_Lushi_vs_Arbor_Acr	breed_growth_background	ENSGALG00010010246	CLTB	-0.007489545	0.035596349	-0.210402046	0.833353896	0.898832166	negative
PRJNA729271	eed_Lushi_vs_Arbor_Acr	breed_growth_background	ENSGALG00010010252	WDR7	0.046130215	0.051390353	0.897643479	0.369375651	0.532748149	positive
PRJNA729271	eed_Lushi_vs_Arbor_Acr	breed_growth_background	ENSGALG00010010253	IL10RB	-0.109586282	0.0663526	-1.65157481	0.098621259	0.212840426	negative
PRJNA729271	eed_Lushi_vs_Arbor_Acr	breed_growth_background	ENSGALG00010010254	WDR41	-0.027732185	0.060953572	-0.454972259	0.649129197	0.771060248	negative
PRJNA729271	eed_Lushi_vs_Arbor_Acr	breed_growth_background	ENSGALG00010010255	CACNB4	-0.355615624	0.094521199	-3.762284312	0.000168368	0.002314101	negative
PRJNA729271	eed_Lushi_vs_Arbor_Acr	breed_growth_background	ENSGALG00010010257	ENSGALG00010010257	-0.139120592	0.073304079	-1.897856087	0.057715042	0.14661401	negative
PRJNA729271	eed_Lushi_vs_Arbor_Acr	breed_growth_background	ENSGALG00010010259	IGF1	-0.775476778	0.211897828	-3.659673094	0.000252537	0.003068338	negative
PRJNA729271	eed_Lushi_vs_Arbor_Acr	breed_growth_background	ENSGALG00010010262	HSPA9	0.130841727	0.077900697	1.679596362	0.093035876	0.204458738	positive
PRJNA729271	eed_Lushi_vs_Arbor_Acr	breed_growth_background	ENSGALG00010010263	RAB4A	0.187765315	0.074282713	2.527712135	0.011480844	0.046136634	positive
PRJNA729271	eed_Lushi_vs_Arbor_Acr	breed_growth_background	ENSGALG00010010266	EFNA5	0.257109091	0.127843204	2.011128346	0.044311902	0.121326221	positive
PRJNA729271	eed_Lushi_vs_Arbor_Acr	breed_growth_background	ENSGALG00010010267	CCSER2	0.033718296	0.057428042	0.587139918	0.55710974	0.698621901	positive
PRJNA729271	eed_Lushi_vs_Arbor_Acr	breed_growth_background	ENSGALG00010010270	SSR1	-0.041960439	0.041728633	-1.005555103	0.31462963	0.478010639	negative
PRJNA729271	eed_Lushi_vs_Arbor_Acr	breed_growth_background	ENSGALG00010010271	HUNK	-0.191829171	0.062225336	-3.082814531	0.002050529	0.013510191	negative
PRJNA729271	eed_Lushi_vs_Arbor_Acr	breed_growth_background	ENSGALG00010010274	MIS18A	-0.115747673	0.05417936	-2.136379494	0.032648494	0.09694429	negative
PRJNA729271	eed_Lushi_vs_Arbor_Acr	breed_growth_background	ENSGALG00010010275	PDE8B	1.157041415	0.506146484	2.285981333	0.022255351	0.073757411	positive
PRJNA729271	eed_Lushi_vs_Arbor_Acr	breed_growth_background	ENSGALG00010010279	CCSAP	-0.512356388	0.128516399	-3.986700455	6.69985E-05	0.001185768	negative
PRJNA729271	eed_Lushi_vs_Arbor_Acr	breed_growth_background	ENSGALG00010010280	HSP90B1	0.17052893	0.072511521	2.35174946	0.018685356	0.065169449	positive
PRJNA729271	eed_Lushi_vs_Arbor_Acr	breed_growth_background	ENSGALG00010010284	CCDC25	0.190891044	0.050739178	3.762202129	0.000168424	0.002314101	positive
PRJNA729271	eed_Lushi_vs_Arbor_Acr	breed_growth_background	ENSGALG00010010285	ENSGALG00010010285	0.171241126	0.314527826	0.544438717	0.586139609	0.72184313	positive
PRJNA729271	eed_Lushi_vs_Arbor_Acr	breed_growth_background	ENSGALG00010010287	STAM2	0.092279408	0.05217523	1.768644004	0.076953302	0.17917114	positive
PRJNA729271	eed_Lushi_vs_Arbor_Acr	breed_growth_background	ENSGALG00010010288	ENSGALG00010010288	0.089097116	0.087510938	1.018125489	0.308618324	0.471382782	positive
PRJNA729271	eed_Lushi_vs_Arbor_Acr	breed_growth_background	ENSGALG00010010290	ENSGALG00010010290	0.219248344	0.088285675	2.483396601	0.013013611	0.050306652	positive
PRJNA729271	eed_Lushi_vs_Arbor_Acr	breed_growth_background	ENSGALG00010010291	TCF20	0.218555842	0.063022584	3.467897221	0.000524548	0.005137692	positive
PRJNA729271	eed_Lushi_vs_Arbor_Acr	breed_growth_background	ENSGALG00010010293	ENSGALG00010010293	0.164736003	0.32229369	0.511136298	0.609255621	0.739971286	positive
PRJNA729271	eed_Lushi_vs_Arbor_Acr	breed_growth_background	ENSGALG00010010295	ENSGALG00010010295	-0.021372991	0.096868707	-0.220638753	0.825373724	0.89328695	negative
PRJNA729271	eed_Lushi_vs_Arbor_Acr	breed_growth_background	ENSGALG00010010298	KCNIP4	-0.040535641	0.237957285	-0.170348393	0.864736155	0.918680641	negative
PRJNA729271	eed_Lushi_vs_Arbor_Acr	breed_growth_background	ENSGALG00010010300	DENND1B	0.146734553	0.048796245	3.007087	0.002637643	0.016152357	positive
PRJNA729271	eed_Lushi_vs_Arbor_Acr	breed_growth_background	ENSGALG00010010301	ENSGALG00010010301	0.461296167	0.295972308	1.558578811	0.119096101	0.243140562	positive
PRJNA729271	eed_Lushi_vs_Arbor_Acr	breed_growth_background	ENSGALG00010010302	WDR36	0.181818321	0.056080246	3.242109898	0.001186483	0.009254529	positive
PRJNA729271	eed_Lushi_vs_Arbor_Acr	breed_growth_background	ENSGALG00010010303	ETF1	0.352168771	0.098685166	3.568608986	0.000358882	0.003918806	positive
PRJNA729271	eed_Lushi_vs_Arbor_Acr	breed_growth_background	ENSGALG00010010305	URB1	-0.009185498	0.055559316	-0.165327765	0.868686006	0.921786505	negative
PRJNA729271	eed_Lushi_vs_Arbor_Acr	breed_growth_background	ENSGALG00010010308	PACRGL	0.06960378	0.124982072	0.55691011	0.577588847	0.714782786	positive
PRJNA729271	eed_Lushi_vs_Arbor_Acr	breed_growth_background	ENSGALG00010010310	NT5DC3	-0.176708932	0.063393742	-2.787482269	0.005311936	0.026606055	negative
PRJNA729271	eed_Lushi_vs_Arbor_Acr	breed_growth_background	ENSGALG00010010311	CTSV	0.209055584	0.101282135	2.06409141	0.03900905	0.110350543	positive
PRJNA729271	eed_Lushi_vs_Arbor_Acr	breed_growth_background	ENSGALG00010010313	GRID1	0.037953503	0.118256505	0.320942202	0.748254192	0.839794387	positive
PRJNA729271	eed_Lushi_vs_Arbor_Acr	breed_growth_background	ENSGALG00010010314	CRB1	0.321356099	0.152624672	2.105531796	0.035245043	0.102503389	positive
PRJNA729271	eed_Lushi_vs_Arbor_Acr	breed_growth_background	ENSGALG00010010317	ENSGALG00010010317	-0.025712061	0.0582257	-0.441592985	0.658783761	0.777971513	negative
PRJNA729271	eed_Lushi_vs_Arbor_Acr	breed_growth_background	ENSGALG00010010320	SYPL1	0.45098889	0.331144869	1.361908131	0.17322688	0.316715896	positive
PRJNA729271	eed_Lushi_vs_Arbor_Acr	breed_growth_background	ENSGALG00010010321	NUP133	-0.077518049	0.039666862	-1.954226882	0.050674392	0.133622941	negative
PRJNA729271	eed_Lushi_vs_Arbor_Acr	breed_growth_background	ENSGALG00010010322	ENSGALG00010010322	0.044844072	0.036184842	1.239305468	0.215232395	0.366301933	positive
PRJNA729271	eed_Lushi_vs_Arbor_Acr	breed_growth_background	ENSGALG00010010323	FBP2	0.052542462	0.124085036	0.423439146	0.671974889	0.787780808	positive
PRJNA729271	eed_Lushi_vs_Arbor_Acr	breed_growth_background	ENSGALG00010010327	FBP1	-0.7220078	0.310073352	-2.328506452	0.019885227	0.06793221	negative
PRJNA729271	eed_Lushi_vs_Arbor_Acr	breed_growth_background	ENSGALG00010010330	ALPK2	0.221153156	0.08199886	2.697027205	0.006996155	0.032368105	positive
PRJNA729271	eed_Lushi_vs_Arbor_Acr	breed_growth_background	ENSGALG00010010333	WAPL	0.067760792	0.033981166	1.994069098	0.046144504	0.124998053	positive
PRJNA729271	eed_Lushi_vs_Arbor_Acr	breed_growth_background	ENSGALG00010010334	ZBTB41	0.002776273	0.04644468	0.059775912	0.952334111	0.973433394	positive
PRJNA729271	eed_Lushi_vs_Arbor_Acr	breed_growth_background	ENSGALG00010010336	FMNL2	-0.281104808	0.048216535	-5.830049928	5.54108E-09	8.19571E-07	negative
PRJNA729271	eed_Lushi_vs_Arbor_Acr	breed_growth_background	ENSGALG00010010337	SCARA3	-0.170060105	0.08364655	-2.033079722	0.042044469	0.116727558	negative
PRJNA729271	eed_Lushi_vs_Arbor_Acr	breed_growth_background	ENSGALG00010010338	CNST	0.143134756	0.083564793	1.712859582	0.086738376	0.194805199	positive
PRJNA729271	eed_Lushi_vs_Arbor_Acr	breed_growth_background	ENSGALG00010010345	ENSGALG00010010345	0.119951782	0.075765855	1.583190492	0.113378053	0.234974961	positive
PRJNA729271	eed_Lushi_vs_Arbor_Acr	breed_growth_background	ENSGALG00010010346	TFB2M	-0.013128266	0.075306546	-0.174331004	0.861605335	0.916890781	negative
PRJNA729271	eed_Lushi_vs_Arbor_Acr	breed_growth_background	ENSGALG00010010347	FBXW11	0.096381463	0.045120237	2.136102766	0.032671038	0.096962879	positive
PRJNA729271	eed_Lushi_vs_Arbor_Acr	breed_growth_background	ENSGALG00010010349	RREB1	-0.007809359	0.043898894	-0.17789421	0.858806058	0.915188488	negative
PRJNA729271	eed_Lushi_vs_Arbor_Acr	breed_growth_background	ENSGALG00010010351	NAMPTP1	0.40122159	0.113631188	3.530910809	0.000414131	0.00434988	positive
PRJNA729271	eed_Lushi_vs_Arbor_Acr	breed_growth_background	ENSGALG00010010352	MALT1	0.077847327	0.062004025	1.255520551	0.209289852	0.359012593	positive
PRJNA729271	eed_Lushi_vs_Arbor_Acr	breed_growth_background	ENSGALG00010010356	STAB2	-0.281569024	0.152743613	-1.843409484	0.065269246	0.160028894	negative
PRJNA729271	eed_Lushi_vs_Arbor_Acr	breed_growth_background	ENSGALG00010010358	ASPM	-0.294817729	0.09743662	-3.025738469	0.002480267	0.015503008	negative
PRJNA729271	eed_Lushi_vs_Arbor_Acr	breed_growth_background	ENSGALG00010010361	PIGM	0.214394448	0.091553786	2.341732197	0.019194482	0.066465365	positive
PRJNA729271	eed_Lushi_vs_Arbor_Acr	breed_growth_background	ENSGALG00010010366	CLU	-0.120706776	0.125888044	-0.958842255	0.337638218	0.501852541	negative
PRJNA729271	eed_Lushi_vs_Arbor_Acr	breed_growth_background	ENSGALG00010010368	ENSGALG00010010368	-0.012021399	0.043039276	-0.279312295	0.780005172	0.863792113	negative
PRJNA729271	eed_Lushi_vs_Arbor_Acr	breed_growth_background	ENSGALG00010010369	ENSGALG00010010369	-0.128840552	0.137844615	-0.934679623	0.349953431	0.513886636	negative
PRJNA729271	eed_Lushi_vs_Arbor_Acr	breed_growth_background	ENSGALG00010010371	SOCS1L	-0.138026304	0.116682591	-1.182921145	0.236840401	0.391805707	negative
PRJNA729271	eed_Lushi_vs_Arbor_Acr	breed_growth_background	ENSGALG00010010374	CMTM4	0.136864323	0.060508493	2.261902702	0.023703415	0.07702198	positive
PRJNA729271	eed_Lushi_vs_Arbor_Acr	breed_growth_background	ENSGALG00010010379	SLC35F6	0.195211227	0.100786548	1.936877807	0.052760282	0.137720203	positive
PRJNA729271	eed_Lushi_vs_Arbor_Acr	breed_growth_background	ENSGALG00010010381	MAST4	0.174902974	0.070432617	2.483266732	0.013018357	0.050306873	positive
PRJNA729271	eed_Lushi_vs_Arbor_Acr	breed_growth_background	ENSGALG00010010382	SEC11C	0.249843475	0.081670843	3.059151425	0.002219649	0.0143441	positive
PRJNA729271	eed_Lushi_vs_Arbor_Acr	breed_growth_background	ENSGALG00010010383	SLIT2	-0.350694827	0.086451431	-4.056553183	4.98022E-05	0.000962511	negative
PRJNA729271	eed_Lushi_vs_Arbor_Acr	breed_growth_background	ENSGALG00010010386	PRPF40A	0.261448964	0.078402289	3.33471083	0.000853881	0.00728488	positive
PRJNA729271	eed_Lushi_vs_Arbor_Acr	breed_growth_background	ENSGALG00010010390	KCNK3	0.352259194	0.230385336	1.529000067	0.126264427	0.252860303	positive
PRJNA729271	eed_Lushi_vs_Arbor_Acr	breed_growth_background	ENSGALG00010010393	FANCC	-0.012517329	0.081115447	-0.154314979	0.877361387	0.927461589	negative
PRJNA729271	eed_Lushi_vs_Arbor_Acr	breed_growth_background	ENSGALG00010010394	MRPL42	-0.004514433	0.0668569	-0.067523811	0.946164707	0.970470493	negative
PRJNA729271	eed_Lushi_vs_Arbor_Acr	breed_growth_background	ENSGALG00010010395	GRP	0.484155492	0.475903586	1.01733945	0.308991976	0.47177828	positive
PRJNA729271	eed_Lushi_vs_Arbor_Acr	breed_growth_background	ENSGALG00010010397	PICK1	0.06149741	0.050514211	1.217427898	0.223441454	0.37611903	positive
PRJNA729271	eed_Lushi_vs_Arbor_Acr	breed_growth_background	ENSGALG00010010399	FGF18	-0.114899445	0.096430534	-1.191525548	0.233447333	0.387830269	negative
PRJNA729271	eed_Lushi_vs_Arbor_Acr	breed_growth_background	ENSGALG00010010403	ENSGALG00010010403	0.209071939	0.257819712	0.810923018	0.417409881	0.577489379	positive
PRJNA729271	eed_Lushi_vs_Arbor_Acr	breed_growth_background	ENSGALG00010010404	COP1	-0.234436353	0.062633032	-3.743014577	0.000181826	0.00242909	negative
PRJNA729271	eed_Lushi_vs_Arbor_Acr	breed_growth_background	ENSGALG00010010405	EVA1C	-0.384071889	0.124083252	-3.095275823	0.001966299	0.013116204	negative
PRJNA729271	eed_Lushi_vs_Arbor_Acr	breed_growth_background	ENSGALG00010010406	PTCH1	0.117010982	0.051932881	2.253119412	0.024251621	0.078256288	positive
PRJNA729271	eed_Lushi_vs_Arbor_Acr	breed_growth_background	ENSGALG00010010407	SOX10	0.755745933	0.161710751	4.673442737	2.96192E-06	0.000112982	positive
PRJNA729271	eed_Lushi_vs_Arbor_Acr	breed_growth_background	ENSGALG00010010408	GAS8	-0.042952146	0.064786809	-0.662976717	0.507345466	0.656777202	negative
PRJNA729271	eed_Lushi_vs_Arbor_Acr	breed_growth_background	ENSGALG00010010410	CACNA2D1	-0.038732748	0.084439546	-0.458703883	0.646446825	0.768998501	negative
PRJNA729271	eed_Lushi_vs_Arbor_Acr	breed_growth_background	ENSGALG00010010411	CD180	0.24255668	0.239893001	1.011103611	0.311966842	0.475196894	positive
PRJNA729271	eed_Lushi_vs_Arbor_Acr	breed_growth_background	ENSGALG00010010416	SLC35B3	-0.098997581	0.069357655	-1.427349011	0.15347932	0.289952686	negative
PRJNA729271	eed_Lushi_vs_Arbor_Acr	breed_growth_background	ENSGALG00010010417	OTOF	-0.619699623	0.361782803	-1.71290514	0.086729993	0.194805199	negative
PRJNA729271	eed_Lushi_vs_Arbor_Acr	breed_growth_background	ENSGALG00010010418	BRINP2	-0.313341851	0.29145298	-1.075102579	0.282328805	0.442177853	negative
PRJNA729271	eed_Lushi_vs_Arbor_Acr	breed_growth_background	ENSGALG00010010420	NPM1	0.022362417	0.060076996	0.372229283	0.709722145	0.812977912	positive
PRJNA729271	eed_Lushi_vs_Arbor_Acr	breed_growth_background	ENSGALG00010010421	POLR2F	0.128663878	0.048627984	2.645881371	0.008147838	0.03606196	positive
PRJNA729271	eed_Lushi_vs_Arbor_Acr	breed_growth_background	ENSGALG00010010422	CDH1	-0.839033346	0.492624681	-1.70318983	0.088532549	0.197731788	negative
PRJNA729271	eed_Lushi_vs_Arbor_Acr	breed_growth_background	ENSGALG00010010423	C21orf59	0.237173628	0.053565812	4.427705282	9.52409E-06	0.000281691	positive
PRJNA729271	eed_Lushi_vs_Arbor_Acr	breed_growth_background	ENSGALG00010010425	BLOC1S5	0.111154576	0.042303954	2.627522124	0.008600925	0.037350551	positive
PRJNA729271	eed_Lushi_vs_Arbor_Acr	breed_growth_background	ENSGALG00010010430	ASTN1	0.32796549	0.272749974	1.202440042	0.229193083	0.382780705	positive
PRJNA729271	eed_Lushi_vs_Arbor_Acr	breed_growth_background	ENSGALG00010010431	SOCS2	0.198414548	0.136879122	1.449560352	0.147181159	0.281896261	positive
PRJNA729271	eed_Lushi_vs_Arbor_Acr	breed_growth_background	ENSGALG00010010433	ENSGALG00010010433	4.209148206	0.892954429	4.713732378	2.4322E-06	9.71205E-05	positive
PRJNA729271	eed_Lushi_vs_Arbor_Acr	breed_growth_background	ENSGALG00010010434	PROM1	-0.1268712	0.082445568	-1.538848038	0.123841399	0.249316817	negative
PRJNA729271	eed_Lushi_vs_Arbor_Acr	breed_growth_background	ENSGALG00010010435	GAS1	-0.286124024	0.08883626	-3.220802237	0.001278323	0.009743353	negative
PRJNA729271	eed_Lushi_vs_Arbor_Acr	breed_growth_background	ENSGALG00010010438	LDB3	-0.023629781	0.098786818	-0.239199739	0.810950705	0.883681436	negative
PRJNA729271	eed_Lushi_vs_Arbor_Acr	breed_growth_background	ENSGALG00010010439	HGF	-0.557279059	0.128374389	-4.341045464	1.41806E-05	0.000375088	negative
PRJNA729271	eed_Lushi_vs_Arbor_Acr	breed_growth_background	ENSGALG00010010444	TXNDC5	-0.03431212	0.075069915	-0.457068851	0.647621558	0.769953616	negative
PRJNA729271	eed_Lushi_vs_Arbor_Acr	breed_growth_background	ENSGALG00010010445	LMAN1L	-0.01206746	0.044729042	-0.269790256	0.78732162	0.867849953	negative
PRJNA729271	eed_Lushi_vs_Arbor_Acr	breed_growth_background	ENSGALG00010010446	C22orf23	-0.293091284	0.331692225	-0.88362422	0.3768991	0.539344823	negative
PRJNA729271	eed_Lushi_vs_Arbor_Acr	breed_growth_background	ENSGALG00010010447	BTF3	-0.107086293	0.051846895	-2.065433114	0.038882038	0.110206324	negative
PRJNA729271	eed_Lushi_vs_Arbor_Acr	breed_growth_background	ENSGALG00010010448	GALNT13	-0.184937117	0.204318423	-0.905141663	0.365390342	0.528787242	negative
PRJNA729271	eed_Lushi_vs_Arbor_Acr	breed_growth_background	ENSGALG00010010449	PAPPA2	-1.506376849	0.429790497	-3.504909626	0.000456762	0.004655956	negative
PRJNA729271	eed_Lushi_vs_Arbor_Acr	breed_growth_background	ENSGALG00010010451	RANBP17	0.032033467	0.273425656	0.117156038	0.906736403	0.946503253	positive
PRJNA729271	eed_Lushi_vs_Arbor_Acr	breed_growth_background	ENSGALG00010010453	DRC1	0.023172838	0.240130393	0.096501064	0.923122629	0.957127674	positive
PRJNA729271	eed_Lushi_vs_Arbor_Acr	breed_growth_background	ENSGALG00010010456	BMP6	-0.15830942	0.095307068	-1.661045953	0.096704218	0.20978561	negative
PRJNA729271	eed_Lushi_vs_Arbor_Acr	breed_growth_background	ENSGALG00010010457	AGGF1	0.025986062	0.049543144	0.524513793	0.599921229	0.732838211	positive
PRJNA729271	eed_Lushi_vs_Arbor_Acr	breed_growth_background	ENSGALG00010010460	SEMA3C	-0.146536703	0.075929584	-1.929902627	0.053618904	0.139239701	negative
PRJNA729271	eed_Lushi_vs_Arbor_Acr	breed_growth_background	ENSGALG00010010461	SLC7A5	-0.301462376	0.140199941	-2.150231829	0.031536882	0.094584544	negative
PRJNA729271	eed_Lushi_vs_Arbor_Acr	breed_growth_background	ENSGALG00010010463	ACAA2	-0.145020059	0.06873199	-2.109935385	0.034863922	0.101669253	negative
PRJNA729271	eed_Lushi_vs_Arbor_Acr	breed_growth_background	ENSGALG00010010465	BMPR1A	-0.03462749	0.052584922	-0.658506066	0.510213001	0.658786517	negative
PRJNA729271	eed_Lushi_vs_Arbor_Acr	breed_growth_background	ENSGALG00010010466	CRADD	-0.104545188	0.067340727	-1.552480841	0.120547213	0.245101958	negative
PRJNA729271	eed_Lushi_vs_Arbor_Acr	breed_growth_background	ENSGALG00010010467	FGFBP2	0.685835763	0.323375179	2.120867057	0.033932992	0.099606868	positive
PRJNA729271	eed_Lushi_vs_Arbor_Acr	breed_growth_background	ENSGALG00010010468	ENSGALG00010010468	0.81460256	0.668055639	1.219363347	0.222706315	0.375372259	positive
PRJNA729271	eed_Lushi_vs_Arbor_Acr	breed_growth_background	ENSGALG00010010471	KCNJ3	0.352563822	0.087061387	4.049600339	5.13052E-05	0.000984991	positive
PRJNA729271	eed_Lushi_vs_Arbor_Acr	breed_growth_background	ENSGALG00010010472	MMRN2	-0.179358246	0.072925017	-2.459488577	0.013913513	0.052739635	negative

PRJNA729271	ed_Lushi_vs_Arbor_Acr	breed_growth_background	ENSGALG00010010473	TRIM35	−0.128028143	0.09722517	−1.316820975	0.18789864	0.333722241	negative
PRJNA729271	ed_Lushi_vs_Arbor_Acr	breed_growth_background	ENSGALG00010010475	CCDC71L	0.077345444	0.058518915	1.321716973	0.186262415	0.331740209	positive
PRJNA729271	ed_Lushi_vs_Arbor_Acr	breed_growth_background	ENSGALG00010010478	ENSGALG00010010478	0.031253779	0.114881308	0.272052778	0.785581438	0.867250796	positive
PRJNA729271	ed_Lushi_vs_Arbor_Acr	breed_growth_background	ENSGALG00010010483	FGFBP1	−0.227095565	0.533455485	−0.425706682	0.670321592	0.786745869	negative
PRJNA729271	ed_Lushi_vs_Arbor_Acr	breed_growth_background	ENSGALG00010010486	ENSGALG00010010486	−0.492571899	0.257489811	−1.912976273	0.05575109	0.143282278	negative
PRJNA729271	ed_Lushi_vs_Arbor_Acr	breed_growth_background	ENSGALG00010010494	ENSGALG00010010494	−0.35522758	0.14081762	−2.52260747	0.011648835	0.046584817	negative
PRJNA729271	ed_Lushi_vs_Arbor_Acr	breed_growth_background	ENSGALG00010010496	MRPL17	−0.037442659	0.076267408	−0.490939191	0.623469457	0.750098753	negative
PRJNA729271	ed_Lushi_vs_Arbor_Acr	breed_growth_background	ENSGALG00010010499	NR4A2	0.147140305	0.237226614	0.620252097	0.535091827	0.680219664	positive
PRJNA729271	ed_Lushi_vs_Arbor_Acr	breed_growth_background	ENSGALG00010010505	CD38	0.409920707	0.107944814	3.797502571	0.000146161	0.002089356	positive
PRJNA729271	ed_Lushi_vs_Arbor_Acr	breed_growth_background	ENSGALG00010010511	GABRP	0.072505913	0.247543485	0.292901722	0.769597272	0.856433612	positive
PRJNA729271	ed_Lushi_vs_Arbor_Acr	breed_growth_background	ENSGALG00010010512	PLXNC1	−0.121081201	0.107111651	−1.130420453	0.2582991	0.415686568	negative
PRJNA729271	ed_Lushi_vs_Arbor_Acr	breed_growth_background	ENSGALG00010010515	RXRG	−0.251546461	0.21024927	−1.196420138	0.231532647	0.385575311	negative
PRJNA729271	ed_Lushi_vs_Arbor_Acr	breed_growth_background	ENSGALG00010010517	STMN4	0.16274227	0.178053155	0.914009472	0.360711869	0.524057185	positive
PRJNA729271	ed_Lushi_vs_Arbor_Acr	breed_growth_background	ENSGALG00010010521	SNCG	0.030177831	0.206810141	0.145920458	0.883984181	0.931676776	positive
PRJNA729271	ed_Lushi_vs_Arbor_Acr	breed_growth_background	ENSGALG00010010526	ENSGALG00010010526	0.137405597	0.136020477	1.010183169	0.312407541	0.475666735	positive
PRJNA729271	ed_Lushi_vs_Arbor_Acr	breed_growth_background	ENSGALG00010010528	GPD2	0.020665169	0.066525117	0.31063709	0.756076527	0.84582305	positive
PRJNA729271	ed_Lushi_vs_Arbor_Acr	breed_growth_background	ENSGALG00010010534	SYNJ1	0.175986495	0.065163241	2.700702017	0.00691933	0.032135755	positive
PRJNA729271	ed_Lushi_vs_Arbor_Acr	breed_growth_background	ENSGALG00010010535	LMX1A	0.669367012	0.233708882	2.864106003	0.00418188	0.022292188	positive
PRJNA729271	ed_Lushi_vs_Arbor_Acr	breed_growth_background	ENSGALG00010010537	ENSGALG00010010537	−0.086739015	0.11942483	−0.726306376	0.467650971	0.622575388	negative
PRJNA729271	ed_Lushi_vs_Arbor_Acr	breed_growth_background	ENSGALG00010010544	ENSGALG00010010544	0.052700027	0.090444252	0.582679677	0.560108957	0.70104303	positive
PRJNA729271	ed_Lushi_vs_Arbor_Acr	breed_growth_background	ENSGALG00010010546	MICALL1	0.090738408	0.090195561	1.006018551	0.314406646	0.477907334	positive
PRJNA729271	ed_Lushi_vs_Arbor_Acr	breed_growth_background	ENSGALG00010010547	BST1	−0.212981643	0.088135501	−2.416525006	0.01566945	0.057515492	negative
PRJNA729271	ed_Lushi_vs_Arbor_Acr	breed_growth_background	ENSGALG00010010567	ERMN	0.71659994	0.312814829	2.290811925	0.021974294	0.073004214	positive
PRJNA729271	ed_Lushi_vs_Arbor_Acr	breed_growth_background	ENSGALG00010010572	AQP3	−0.318405466	0.07801115	−4.08153792	4.47387E-05	0.000892004	negative
PRJNA729271	ed_Lushi_vs_Arbor_Acr	breed_growth_background	ENSGALG00010010573	CEP83	−0.332683127	0.087571448	−3.798990835	0.000145286	0.002078902	negative
PRJNA729271	ed_Lushi_vs_Arbor_Acr	breed_growth_background	ENSGALG00010010575	PAXBP1	0.250258929	0.065959883	3.79410815	0.000148175	0.002116057	positive
PRJNA729271	ed_Lushi_vs_Arbor_Acr	breed_growth_background	ENSGALG00010010577	PBX1	0.235462422	0.07808849	3.015328158	0.002567014	0.01589443	positive
PRJNA729271	ed_Lushi_vs_Arbor_Acr	breed_growth_background	ENSGALG00010010578	GLUD2	−0.190638734	0.059588961	−3.199229023	0.001377957	0.010300978	negative
PRJNA729271	ed_Lushi_vs_Arbor_Acr	breed_growth_background	ENSGALG00010010585	FBXL5	0.351820136	0.087440891	4.023519589	5.73348E-05	0.001072346	positive
PRJNA729271	ed_Lushi_vs_Arbor_Acr	breed_growth_background	ENSGALG00010010586	CYTIP	0.454817251	0.149584812	3.040530965	0.002361614	0.015013858	positive
PRJNA729271	ed_Lushi_vs_Arbor_Acr	breed_growth_background	ENSGALG00010010588	SLC25A3	−0.050527505	0.054006673	−0.935578935	0.349490024	0.513466238	negative
PRJNA729271	ed_Lushi_vs_Arbor_Acr	breed_growth_background	ENSGALG00010010593	EIF3L	0.074915582	0.055481809	1.350272867	0.176928472	0.320939141	positive
PRJNA729271	ed_Lushi_vs_Arbor_Acr	breed_growth_background	ENSGALG00010010596	SHLD2	0.174296845	0.071967828	2.421871699	0.015440801	0.056935744	positive
PRJNA729271	ed_Lushi_vs_Arbor_Acr	breed_growth_background	ENSGALG00010010599	F2RL1	−0.23534403	0.11102033	−2.119828236	0.034020533	0.099823407	negative
PRJNA729271	ed_Lushi_vs_Arbor_Acr	breed_growth_background	ENSGALG00010010604	HIVEP1	−0.011222454	0.053787618	−0.208643818	0.834726302	0.899446755	negative
PRJNA729271	ed_Lushi_vs_Arbor_Acr	breed_growth_background	ENSGALG00010010605	NUF2	−0.354612203	0.144449463	−2.454922268	0.014091506	0.053164074	negative
PRJNA729271	ed_Lushi_vs_Arbor_Acr	breed_growth_background	ENSGALG00010010614	IKBIP	0.222853814	0.077277278	2.883820691	0.003928824	0.021394964	positive
PRJNA729271	ed_Lushi_vs_Arbor_Acr	breed_growth_background	ENSGALG00010010618	APAF1	0.128319142	0.073558979	1.744438869	0.081082592	0.185699506	positive
PRJNA729271	ed_Lushi_vs_Arbor_Acr	breed_growth_background	ENSGALG00010010619	LRRC52	−0.313152653	0.150760697	−2.077150474	0.037787673	0.108097952	negative
PRJNA729271	ed_Lushi_vs_Arbor_Acr	breed_growth_background	ENSGALG00010010620	ANKRD54	0.110877032	0.059379286	1.867267858	0.061864192	0.154191053	positive
PRJNA729271	ed_Lushi_vs_Arbor_Acr	breed_growth_background	ENSGALG00010010623	SMAD2	0.008526275	0.041776582	0.204092224	0.838281431	0.901798833	positive
PRJNA729271	ed_Lushi_vs_Arbor_Acr	breed_growth_background	ENSGALG00010010625	FAM169A	0.187058819	0.177769974	1.05225205	0.292683924	0.454126898	positive
PRJNA729271	ed_Lushi_vs_Arbor_Acr	breed_growth_background	ENSGALG00010010626	ADTRP	−0.082996426	0.315763625	−0.26284353	0.792671183	0.871559493	negative
PRJNA729271	ed_Lushi_vs_Arbor_Acr	breed_growth_background	ENSGALG00010010629	ENSGALG00010010629	0.067506673	0.190569624	0.354236273	0.723161822	0.822458267	positive
PRJNA729271	ed_Lushi_vs_Arbor_Acr	breed_growth_background	ENSGALG00010010633	EPCIP	0.171119813	0.177578249	0.963630479	0.33523122	0.499504167	positive
PRJNA729271	ed_Lushi_vs_Arbor_Acr	breed_growth_background	ENSGALG00010010635	ALDH9A1	0.023694705	0.041399461	0.572343327	0.567089402	0.706122715	positive
PRJNA729271	ed_Lushi_vs_Arbor_Acr	breed_growth_background	ENSGALG00010010638	ACVR1C	−0.142174111	0.231386253	−0.614444935	0.53892135	0.684273385	negative
PRJNA729271	ed_Lushi_vs_Arbor_Acr	breed_growth_background	ENSGALG00010010642	CC2D2A	−0.120324496	0.064826195	−1.856109191	0.063437996	0.156873916	negative
PRJNA729271	ed_Lushi_vs_Arbor_Acr	breed_growth_background	ENSGALG00010010645	FAM219A	−0.016438375	0.046473548	−0.353714647	0.723552745	0.822838305	negative
PRJNA729271	ed_Lushi_vs_Arbor_Acr	breed_growth_background	ENSGALG00010010646	MCM3AP	0.051440378	0.031545641	1.630665144	0.102960995	0.219570344	positive
PRJNA729271	ed_Lushi_vs_Arbor_Acr	breed_growth_background	ENSGALG00010010648	CENPM	−0.117783646	0.143180554	−0.822623202	0.4107223	0.571196221	negative
PRJNA729271	ed_Lushi_vs_Arbor_Acr	breed_growth_background	ENSGALG00010010651	NUAK1	−0.308721109	0.071708111	−4.305246712	1.668E-05	0.000418298	negative
PRJNA729271	ed_Lushi_vs_Arbor_Acr	breed_growth_background	ENSGALG00010010655	C1QTNF7	−0.227265149	0.135287384	−1.679869498	0.09298271	0.204458738	negative
PRJNA729271	ed_Lushi_vs_Arbor_Acr	breed_growth_background	ENSGALG00010010657	TMEM170B	−0.28368823	0.097954568	−2.89612047	0.003778073	0.020822499	negative
PRJNA729271	ed_Lushi_vs_Arbor_Acr	breed_growth_background	ENSGALG00010010659	ADAMTS1	0.288750649	0.081382699	3.548059395	0.000388081	0.004159378	positive
PRJNA729271	ed_Lushi_vs_Arbor_Acr	breed_growth_background	ENSGALG00010010661	CPEB2	−0.021159393	0.100344582	−0.21086732	0.832990806	0.898645559	negative
PRJNA729271	ed_Lushi_vs_Arbor_Acr	breed_growth_background	ENSGALG00010010662	MGST3	−0.081946889	0.082397296	−0.994533712	0.319963101	0.483312333	negative
PRJNA729271	ed_Lushi_vs_Arbor_Acr	breed_growth_background	ENSGALG00010010663	EFR3B	0.00390093	0.093708703	0.041628256	0.966795048	0.98113985	positive
PRJNA729271	ed_Lushi_vs_Arbor_Acr	breed_growth_background	ENSGALG00010010666	C12orf75	0.042461927	0.203459458	0.208699697	0.834682677	0.899446755	positive
PRJNA729271	ed_Lushi_vs_Arbor_Acr	breed_growth_background	ENSGALG00010010671	SIGMAR1	0.138437462	0.077194602	1.793356757	0.072915897	0.172824105	positive
PRJNA729271	ed_Lushi_vs_Arbor_Acr	breed_growth_background	ENSGALG00010010678	KCNIP1	−0.162883643	0.207442402	−0.785199365	0.432336688	0.590587154	negative
PRJNA729271	ed_Lushi_vs_Arbor_Acr	breed_growth_background	ENSGALG00010010680	ENSGALG00010010680	0.537115182	0.347382456	1.546178203	0.122061536	0.24672737	positive
PRJNA729271	ed_Lushi_vs_Arbor_Acr	breed_growth_background	ENSGALG00010010682	SMIM13	−0.10764951	0.056921052	−1.891207339	0.058596672	0.148195608	negative
PRJNA729271	ed_Lushi_vs_Arbor_Acr	breed_growth_background	ENSGALG00010010685	STIM2	−0.011854402	0.05852077	−0.202567428	0.839473151	0.902548829	negative
PRJNA729271	ed_Lushi_vs_Arbor_Acr	breed_growth_background	ENSGALG00010010688	IQGAP2	−0.138608395	0.091080572	−1.521821738	0.128053753	0.255314876	negative
PRJNA729271	ed_Lushi_vs_Arbor_Acr	breed_growth_background	ENSGALG00010010689	POFUT2	−0.251333339	0.080373553	−3.127065185	0.001765608	0.012163728	negative
PRJNA729271	ed_Lushi_vs_Arbor_Acr	breed_growth_background	ENSGALG00010010690	MTPN	−0.312295714	0.072438628	−4.311176543	1.62388E-05	0.000410788	negative
PRJNA729271	ed_Lushi_vs_Arbor_Acr	breed_growth_background	ENSGALG00010010693	KCNMB1	0.158267281	0.183578447	0.862123436	0.388619596	0.550639398	positive
PRJNA729271	ed_Lushi_vs_Arbor_Acr	breed_growth_background	ENSGALG00010010697	NEDD9	0.122837742	0.075037363	1.637021033	0.101626075	0.21747574	positive
PRJNA729271	ed_Lushi_vs_Arbor_Acr	breed_growth_background	ENSGALG00010010698	GPRIN2	−0.159401753	0.109044728	−1.461801552	0.143795599	0.277132988	negative
PRJNA729271	ed_Lushi_vs_Arbor_Acr	breed_growth_background	ENSGALG00010010699	DNAJC27	0.073620537	0.082364518	0.893838013	0.371408567	0.5343491	positive
PRJNA729271	ed_Lushi_vs_Arbor_Acr	breed_growth_background	ENSGALG00010010706	SREBF2	−0.162879927	0.074330378	−2.191296886	0.028430315	0.087438451	negative
PRJNA729271	ed_Lushi_vs_Arbor_Acr	breed_growth_background	ENSGALG00010010710	UCK2	−0.191892257	0.054608981	−3.513932209	0.000441525	0.004538945	negative
PRJNA729271	ed_Lushi_vs_Arbor_Acr	breed_growth_background	ENSGALG00010010711	ENSGALG00010010711	−0.935247968	0.238622935	−3.91935489	8.87863E-05	0.001467454	negative
PRJNA729271	ed_Lushi_vs_Arbor_Acr	breed_growth_background	ENSGALG00010010712	ADAMTS5	0.018333015	0.077741643	0.235819755	0.813572538	0.885406858	positive
PRJNA729271	ed_Lushi_vs_Arbor_Acr	breed_growth_background	ENSGALG00010010713	NSA2	0.006170839	0.048579872	0.127024595	0.898920932	0.941398557	positive
PRJNA729271	ed_Lushi_vs_Arbor_Acr	breed_growth_background	ENSGALG00010010719	SH3BP4	−0.219886401	0.08856135	−2.482870916	0.013032831	0.050335966	negative
PRJNA729271	ed_Lushi_vs_Arbor_Acr	breed_growth_background	ENSGALG00010010722	TBC1D19	−0.102886261	0.091459879	−1.124933279	0.260617309	0.418529569	negative
PRJNA729271	ed_Lushi_vs_Arbor_Acr	breed_growth_background	ENSGALG00010010724	ADCY3	−0.185843827	0.080202437	−2.317184287	0.020493695	0.069344457	negative
PRJNA729271	ed_Lushi_vs_Arbor_Acr	breed_growth_background	ENSGALG00010010728	ELOVL2	0.134853947	0.509919384	0.264461307	0.791424476	0.870651015	positive
PRJNA729271	ed_Lushi_vs_Arbor_Acr	breed_growth_background	ENSGALG00010010732	CCKAR	−0.473186046	0.631730495	−0.749031509	0.453838214	0.610126592	negative
PRJNA729271	ed_Lushi_vs_Arbor_Acr	breed_growth_background	ENSGALG00010010733	GFM2	0.022765098	0.068807221	0.330853326	0.740755279	0.834220167	positive
PRJNA729271	ed_Lushi_vs_Arbor_Acr	breed_growth_background	ENSGALG00010010734	AMN1	−0.096142451	0.076780374	−1.252174829	0.210506166	0.360544355	negative
PRJNA729271	ed_Lushi_vs_Arbor_Acr	breed_growth_background	ENSGALG00010010740	CCDC134	0.010492004	0.051235451	0.204780165	0.837743885	0.901464309	positive
PRJNA729271	ed_Lushi_vs_Arbor_Acr	breed_growth_background	ENSGALG00010010742	LCP2	0.133785679	0.139866829	0.956521861	0.338808645	0.503024819	positive
PRJNA729271	ed_Lushi_vs_Arbor_Acr	breed_growth_background	ENSGALG00010010743	KLHL20	0.048781866	0.045748109	1.066314361	0.28628156	0.446919894	positive
PRJNA729271	ed_Lushi_vs_Arbor_Acr	breed_growth_background	ENSGALG00010010745	ATVR1	−0.084328643	0.034344366	−2.455385065	0.014073375	0.053109494	negative
PRJNA729271	ed_Lushi_vs_Arbor_Acr	breed_growth_background	ENSGALG00010010748	SF3B1	−0.019849738	0.038446591	−0.516293831	0.605649204	0.73704479	negative
PRJNA729271	ed_Lushi_vs_Arbor_Acr	breed_growth_background	ENSGALG00010010749	NCOA4	−0.091274629	0.052713505	−1.731522684	0.083358583	0.189415685	negative
PRJNA729271	ed_Lushi_vs_Arbor_Acr	breed_growth_background	ENSGALG00010010751	SLC30A5	0.002103966	0.042555247	0.049440808	0.960568008	0.977975225	positive
PRJNA729271	ed_Lushi_vs_Arbor_Acr	breed_growth_background	ENSGALG00010010752	ENSGALG00010010752	−0.455382798	0.143675446	−3.169524155	0.001526888	0.011026955	negative
PRJNA729271	ed_Lushi_vs_Arbor_Acr	breed_growth_background	ENSGALG00010010753	ENSGALG00010010753	4.316214737	0.621863112	6.940779498			positive
PRJNA729271	ed_Lushi_vs_Arbor_Acr	breed_growth_background	ENSGALG00010010754	DNAI1	0.093128571	0.163872863	0.568297698	0.569832842	0.708685289	positive
PRJNA729271	ed_Lushi_vs_Arbor_Acr	breed_growth_background	ENSGALG00010010756	CENPL	−0.339992553	0.112329204	−3.02675119	0.002471973	0.015464503	negative
PRJNA729271	ed_Lushi_vs_Arbor_Acr	breed_growth_background	ENSGALG00010010759	ENSGALG00010010759	−0.123915409	0.158883361	−0.779914325	0.435441306	0.593318456	negative
PRJNA729271	ed_Lushi_vs_Arbor_Acr	breed_growth_background	ENSGALG00010010760	FOXI1	0.067853323	0.361968063	0.187456656	0.851302604	0.910572119	positive
PRJNA729271	ed_Lushi_vs_Arbor_Acr	breed_growth_background	ENSGALG00010010761	MEI1	0.138914152	0.145181966	0.956827874	0.338654141	0.502942842	positive
PRJNA729271	ed_Lushi_vs_Arbor_Acr	breed_growth_background	ENSGALG00010010762	MRPL39	0.218424691	0.059955603	3.643107236	0.000269367	0.003224169	positive
PRJNA729271	ed_Lushi_vs_Arbor_Acr	breed_growth_background	ENSGALG00010010765	ENSGALG00010010765	−0.047835974	0.355081258	−0.134718386	0.892834536	0.937432231	negative
PRJNA729271	ed_Lushi_vs_Arbor_Acr	breed_growth_background	ENSGALG00010010766	CENPO	−0.216772902	0.136753433	−1.585136822	0.112935251	0.234424353	negative
PRJNA729271	ed_Lushi_vs_Arbor_Acr	breed_growth_background	ENSGALG00010010768	ENSGALG00010010768	−0.063309601	0.049188773	−1.287074206	0.198068425	0.346446461	negative
PRJNA729271	ed_Lushi_vs_Arbor_Acr	breed_growth_background	ENSGALG00010010769	HEXB	−0.152521747	0.065884692	−2.31498005	0.020614024	0.06967925	negative
PRJNA729271	ed_Lushi_vs_Arbor_Acr	breed_growth_background	ENSGALG00010010771	ENSGALG00010010771	−0.102216347	0.038811513	−2.633660493	0.00844699	0.036895153	negative
PRJNA729271	ed_Lushi_vs_Arbor_Acr	breed_growth_background	ENSGALG00010010774	KPTN	−0.317901319	0.074452756	−4.269839481	1.95614E-05	0.000473359	negative
PRJNA729271	ed_Lushi_vs_Arbor_Acr	breed_growth_background	ENSGALG00010010776	GCAT	0.260917826	0.104074398	2.507031796	0.012174976	0.048059988	positive
PRJNA729271	ed_Lushi_vs_Arbor_Acr	breed_growth_background	ENSGALG00010010778	UPP2	0.196290552	0.190817487	1.028682195	0.303629039	0.466317326	positive
PRJNA729271	ed_Lushi_vs_Arbor_Acr	breed_growth_background	ENSGALG00010010779	DOCK2	0.125280138	0.101113574	1.23900415	0.215343961	0.36641506	positive
PRJNA729271	ed_Lushi_vs_Arbor_Acr	breed_growth_background	ENSGALG00010010780	PARG	0.047156799	0.02709754	1.740261272	0.081813151	0.186870726	positive
PRJNA729271	ed_Lushi_vs_Arbor_Acr	breed_growth_background	ENSGALG00010010781	NAPA	0.014345131	0.052289877	0.274338598	0.783824425	0.866102687	positive
PRJNA729271	ed_Lushi_vs_Arbor_Acr	breed_growth_background	ENSGALG00010010783	F2RL2	−0.33157837	0.080408677	−4.123664059	3.72893E-05	0.000790216	negative
PRJNA729271	ed_Lushi_vs_Arbor_Acr	breed_growth_background	ENSGALG00010010784	CCDC148	−0.040746964	0.154830062	−0.263172172	0.792417879	0.871479298	negative
PRJNA729271	ed_Lushi_vs_Arbor_Acr	breed_growth_background	ENSGALG00010010786	NABP1	0.219166777	0.082861807	2.644967402	0.008169879	0.036148471	positive
PRJNA729271	ed_Lushi_vs_Arbor_Acr	breed_growth_background	ENSGALG00010010789	ENSGALG00010010789	0.050521724	0.154022359	0.328015522	0.742899926	0.83598161	positive
PRJNA729271	ed_Lushi_vs_Arbor_Acr	breed_growth_background	ENSGALG00010010791	H-10	−0.144149032	0.157998733	−0.912342966	0.361588203	0.52496204	negative
PRJNA729271	ed_Lushi_vs_Arbor_Acr	breed_growth_background	ENSGALG00010010792	XRCC6	−0.153989541	0.081078168	−1.89927258	0.057528646	0.146448494	negative
PRJNA729271	ed_Lushi_vs_Arbor_Acr	breed_growth_background	ENSGALG00010010796	CAVIN2	−0.130320398	0.077923685	−1.672410622	0.094443379	0.206658796	negative
PRJNA729271	ed_Lushi_vs_Arbor_Acr	breed_growth_background	ENSGALG00010010799	DARS2	0.040604951	0.057968556	0.700465116	0.483636883	0.635856383	positive
PRJNA729271	ed_Lushi_vs_Arbor_Acr	breed_growth_background	ENSGALG00010010800	BICRA	0.064555889	0.102198849	0.631669433	0.527602906	0.673560342	positive
PRJNA729271	ed_Lushi_vs_Arbor_Acr	breed_growth_background	ENSGALG00010010805	PTRHD1	−0.01282409	0.092666879	−0.138389145	0.889932875	0.935632281	negative
PRJNA729271	ed_Lushi_vs_Arbor_Acr	breed_growth_background	ENSGALG00010010806	INSYN2B	0.170971318	0.450283528	0.37969703	0.704170325	0.808727427	positive
PRJNA729271	ed_Lushi_vs_Arbor_Acr	breed_growth_background	ENSGALG00010010809	CENPH	−0.254209619	0.087041645	−2.920551637	0.003494123	0.019714796	negative
PRJNA729271	ed_Lushi_vs_Arbor_Acr	breed_growth_background	ENSGALG00010010810	GALT	0.114888826	0.061057853	1.881638825	0.059885071	0.150583962	positive
PRJNA729271	ed_Lushi_vs_Arbor_Acr	breed_growth_background	ENSGALG00010010813	EHD2	−0.119642336	0.078666929	−1.520872071	0.128291944	0.255504975	negative
PRJNA729271	ed_Lushi_vs_Arbor_Acr	breed_growth_background	ENSGALG00010010816	AP2S1	−0.147188592	0.061418941	−2.39646905	0.016553891	0.059703572	negative
PRJNA729271	ed_Lushi_vs_Arbor_Acr	breed_growth_background	ENSGALG00010010821	RGS8	−0.018833704	0.247851746	−0.075987779	0.939428821	0.966706003	negative
PRJNA729271	ed_Lushi_vs_Arbor_Acr	breed_growth_background	ENSGALG00010010822	OGDHL	−0.358455601	0.212460924	−1.687160138	0.091572564	0.202277402	negative
PRJNA729271	ed_Lushi_vs_Arbor_Acr	breed_growth_background	ENSGALG00010010823	TMEFF2	0.038405403	0.096766488	0.396887433	0.691450472	0.800634364	positive
PRJNA729271	ed_Lushi_vs_Arbor_Acr	breed_growth_background	ENSGALG00010010824	PKP4	−0.160209021	0.059068603	−2.712253424	0.006682748	0.031358508	negative
PRJNA729271	ed_Lushi_vs_Arbor_Acr	breed_growth_background	ENSGALG00010010825	MAK	0.901864083	0.198226218	4.54967104	5.37298E-06	0.000183158	positive
PRJNA729271	ed_Lushi_vs_Arbor_Acr	breed_growth_background	ENSGALG00010010826	NCOA1	0.179861576	0.086896549	2.069835669	0.038467736	0.109524618	positive
PRJNA729271	ed_Lushi_vs_Arbor_Acr	breed_growth_background	ENSGALG00010010828	SPDL1	0.058816055	0.068606603	0.857294387	0.39128221	0.552898775	positive

PRJNA729271	:ed_Lushi_vs_Arbor_Acr	breed_growth_background	ENSGALG00010010829	LAMB4	-0.364894925	0.141860747	-2.57220502	0.010105303	0.042139346	negative
PRJNA729271	:ed_Lushi_vs_Arbor_Acr	breed_growth_background	ENSGALG00010010831	DESI1	0.014619118	0.049977073	0.292516499	0.769891747	0.856557109	positive
PRJNA729271	:ed_Lushi_vs_Arbor_Acr	breed_growth_background	ENSGALG00010010834	KGD4	-0.004392298	0.066939045	-0.065616381	0.947683247	0.971269792	negative
PRJNA729271	:ed_Lushi_vs_Arbor_Acr	breed_growth_background	ENSGALG00010010836	DIP2A	-0.15044102	0.056909574	-2.643509877	0.008205137	0.036260202	negative
PRJNA729271	:ed_Lushi_vs_Arbor_Acr	breed_growth_background	ENSGALG00010010837	RBPJ	-0.039769145	0.066044664	-0.602155314	0.547070758	0.690748313	negative
PRJNA729271	:ed_Lushi_vs_Arbor_Acr	breed_growth_background	ENSGALG00010010838	JAM2	-0.140533881	0.093075377	-1.50989322	0.131070673	0.259201834	negative
PRJNA729271	:ed_Lushi_vs_Arbor_Acr	breed_growth_background	ENSGALG00010010840	RGS16	-0.138822912	0.317313005	-0.437495186	0.661752267	0.780657871	negative
PRJNA729271	:ed_Lushi_vs_Arbor_Acr	breed_growth_background	ENSGALG00010010841	PMM1	0.222394892	0.07108009	3.128793079	0.001755259	0.012115464	positive
PRJNA729271	:ed_Lushi_vs_Arbor_Acr	breed_growth_background	ENSGALG00010010843	ZNF395	-0.034994967	0.096123689	-0.364061837	0.715811833	0.817711654	negative
PRJNA729271	:ed_Lushi_vs_Arbor_Acr	breed_growth_background	ENSGALG00010010844	CDC14B	-0.170310869	0.093576411	-1.820019239	0.068756075	0.165909657	negative
PRJNA729271	:ed_Lushi_vs_Arbor_Acr	breed_growth_background	ENSGALG00010010845	FKRP	0.482415517	0.135673211	3.555716799	0.00037695	0.00407805	positive
PRJNA729271	:ed_Lushi_vs_Arbor_Acr	breed_growth_background	ENSGALG00010010848	RGR	0.391488444	0.304245171	1.286753188	0.198180328	0.346600368	positive
PRJNA729271	:ed_Lushi_vs_Arbor_Acr	breed_growth_background	ENSGALG00010010849	ACBD6	0.097452168	0.059691795	1.632589015	0.102555463	0.21901391	positive
PRJNA729271	:ed_Lushi_vs_Arbor_Acr	breed_growth_background	ENSGALG00010010850	ENSGALG00010010850	0.039403149	0.056502745	0.697366987	0.485573156	0.637343776	positive
PRJNA729271	:ed_Lushi_vs_Arbor_Acr	breed_growth_background	ENSGALG00010010851	PNOC	-0.492745532	0.297543631	-1.656044626	0.097712782	0.211331957	negative
PRJNA729271	:ed_Lushi_vs_Arbor_Acr	breed_growth_background	ENSGALG00010010853	SMIM20	-0.120610646	0.078159343	-1.543137924	0.122797315	0.247696504	negative
PRJNA729271	:ed_Lushi_vs_Arbor_Acr	breed_growth_background	ENSGALG00010010854	LRIT1	-0.622928069	0.294240881	-2.117068396	0.034254043	0.100305527	negative
PRJNA729271	:ed_Lushi_vs_Arbor_Acr	breed_growth_background	ENSGALG00010010855	ATP5PF	-0.141200949	0.07939474	-1.778467307	0.075327137	0.176734679	negative
PRJNA729271	:ed_Lushi_vs_Arbor_Acr	breed_growth_background	ENSGALG00010010856	CSDC2	0.187115364	0.348618011	0.536734642	0.591450923	0.726253486	positive
PRJNA729271	:ed_Lushi_vs_Arbor_Acr	breed_growth_background	ENSGALG00010010857	SEL1L3	0.029067728	0.03283703	0.885211855	0.37604238	0.538503684	positive
PRJNA729271	:ed_Lushi_vs_Arbor_Acr	breed_growth_background	ENSGALG00010010859	ENSGALG00010010859	-0.570341871	0.149130711	-3.824442767	0.000131068	0.00194257	negative
PRJNA729271	:ed_Lushi_vs_Arbor_Acr	breed_growth_background	ENSGALG00010010861	ENSGALG00010010861	-0.011137373	0.128861489	-0.086429029	0.931125372	0.96226465	negative
PRJNA729271	:ed_Lushi_vs_Arbor_Acr	breed_growth_background	ENSGALG00010010862	STRN4	0.112385515	0.082805308	1.357225966	0.174709423	0.318582599	positive
PRJNA729271	:ed_Lushi_vs_Arbor_Acr	breed_growth_background	ENSGALG00010010865	ENSGALG00010010865	0.208955344	0.083759732	2.494699281	0.012606392	0.049173748	positive
PRJNA729271	:ed_Lushi_vs_Arbor_Acr	breed_growth_background	ENSGALG00010010867	QSOX1	-0.455424627	0.08752834	-5.203167622	1.9592E-07	1.40587E-05	negative
PRJNA729271	:ed_Lushi_vs_Arbor_Acr	breed_growth_background	ENSGALG00010010868	SLC39A10	-0.185046535	0.060259956	-3.070804325	0.00213483	0.013907578	negative
PRJNA729271	:ed_Lushi_vs_Arbor_Acr	breed_growth_background	ENSGALG00010010869	ENSGALG00010010869	0.220920518	0.091702954	2.40908835	0.015992426	0.058346392	positive
PRJNA729271	:ed_Lushi_vs_Arbor_Acr	breed_growth_background	ENSGALG00010010870	NOL6	-0.047761485	0.06697467	-0.713127589	0.475766809	0.628932047	negative
PRJNA729271	:ed_Lushi_vs_Arbor_Acr	breed_growth_background	ENSGALG00010010871	GCNT2	0.018319988	0.583992487	0.031370245	0.97497427	0.985375265	positive
PRJNA729271	:ed_Lushi_vs_Arbor_Acr	breed_growth_background	ENSGALG00010010874	ENSGALG00010010874	-0.360783937	0.143640516	-2.511714289	0.012014632	0.04755655	negative
PRJNA729271	:ed_Lushi_vs_Arbor_Acr	breed_growth_background	ENSGALG00010010876	ENSGALG00010010876	0.16302104	0.086543326	1.883692794	0.059606542	0.150214989	positive
PRJNA729271	:ed_Lushi_vs_Arbor_Acr	breed_growth_background	ENSGALG00010010880	ELP3	0.128710182	0.057356846	2.244024737	0.02483081	0.079646512	positive
PRJNA729271	:ed_Lushi_vs_Arbor_Acr	breed_growth_background	ENSGALG00010010882	CEP350	0.08884696	0.052494867	1.692488523	0.090552867	0.201111735	positive
PRJNA729271	:ed_Lushi_vs_Arbor_Acr	breed_growth_background	ENSGALG00010010884	DNAH7	-0.014942349	0.172896483	-0.086423673	0.931129629	0.96226465	negative
PRJNA729271	:ed_Lushi_vs_Arbor_Acr	breed_growth_background	ENSGALG00010010885	SNRPD2	0.167798888	0.148365864	1.130980422	0.258063332	0.415398266	positive
PRJNA729271	:ed_Lushi_vs_Arbor_Acr	breed_growth_background	ENSGALG00010010887	GABPA	0.06013746	0.035633554	1.687663812	0.091475782	0.202125223	positive
PRJNA729271	:ed_Lushi_vs_Arbor_Acr	breed_growth_background	ENSGALG00010010888	GNPAT	0.007325817	0.06357925	0.115223396	0.908268055	0.947760813	positive
PRJNA729271	:ed_Lushi_vs_Arbor_Acr	breed_growth_background	ENSGALG00010010889	CCL19	0.368912819	0.355122215	1.038833401	0.298882224	0.461226216	positive
PRJNA729271	:ed_Lushi_vs_Arbor_Acr	breed_growth_background	ENSGALG00010010890	NOL12	0.239325075	0.073513149	3.255541071	0.001131765	0.00894002	positive
PRJNA729271	:ed_Lushi_vs_Arbor_Acr	breed_growth_background	ENSGALG00010010891	POLR3H	-0.142836685	0.058374034	-2.446921628	0.014408217	0.053840448	negative
PRJNA729271	:ed_Lushi_vs_Arbor_Acr	breed_growth_background	ENSGALG00010010893	CCL21	0.10210227	0.272441136	0.374768184	0.707832878	0.811711832	positive
PRJNA729271	:ed_Lushi_vs_Arbor_Acr	breed_growth_background	ENSGALG00010010894	SPAG1	-0.219781229	0.079207177	-2.774764081	0.005524176	0.027347315	negative
PRJNA729271	:ed_Lushi_vs_Arbor_Acr	breed_growth_background	ENSGALG00010010896	LY86	-0.672392857	0.155685324	-4.318922561	1.56793E-05	0.000402828	negative
PRJNA729271	:ed_Lushi_vs_Arbor_Acr	breed_growth_background	ENSGALG00010010897	CEP170	0.018212792	0.048814926	0.373098833	0.709074888	0.81249332	positive
PRJNA729271	:ed_Lushi_vs_Arbor_Acr	breed_growth_background	ENSGALG00010010898	PHF24	-0.046446891	1.941684582	-0.023920925	0.980915683	0.98876028	negative
PRJNA729271	:ed_Lushi_vs_Arbor_Acr	breed_growth_background	ENSGALG00010010900	SLC34A2	-1.176374714	0.569332794	-2.06623389	0.0388064	0.11006609	negative
PRJNA729271	:ed_Lushi_vs_Arbor_Acr	breed_growth_background	ENSGALG00010010904	TOR1AIP1	-0.078491349	0.044655699	-1.757700598	0.078798466	0.18207935	negative
PRJNA729271	:ed_Lushi_vs_Arbor_Acr	breed_growth_background	ENSGALG00010010905	ENC1	-0.477004374	0.0792846	-6.016355976	1.78387E-09	3.35808E-07	negative
PRJNA729271	:ed_Lushi_vs_Arbor_Acr	breed_growth_background	ENSGALG00010010906	ATP6V1C1	0.248689404	0.074840352	3.322932044	0.000890766	0.007515515	positive
PRJNA729271	:ed_Lushi_vs_Arbor_Acr	breed_growth_background	ENSGALG00010010908	STK17B	-0.120523586	0.066591214	-1.809902221	0.070310952	0.168316638	negative
PRJNA729271	:ed_Lushi_vs_Arbor_Acr	breed_growth_background	ENSGALG00010010909	SLIT3	-0.256336335	0.17647564	-1.452530984	0.14635402	0.280720064	negative
PRJNA729271	:ed_Lushi_vs_Arbor_Acr	breed_growth_background	ENSGALG00010010911	ENSGALG00010010911	0.106388933	0.209680012	0.507387098	0.611883237	0.74176194	positive
PRJNA729271	:ed_Lushi_vs_Arbor_Acr	breed_growth_background	ENSGALG00010010912	BAALC	-0.527362983	0.19456492	-2.710473112	0.00671873	0.031466231	negative
PRJNA729271	:ed_Lushi_vs_Arbor_Acr	breed_growth_background	ENSGALG00010010913	IFT56	-0.270776825	0.081380446	-3.327295893	0.000876932	0.007424725	negative
PRJNA729271	:ed_Lushi_vs_Arbor_Acr	breed_growth_background	ENSGALG00010010915	APP	-0.194457102	0.059029461	-3.294238133	0.000986889	0.008077329	negative
PRJNA729271	:ed_Lushi_vs_Arbor_Acr	breed_growth_background	ENSGALG00010010916	ENSGALG00010010916	0.404830136	0.369823551	1.094657533	0.2736667	0.432631565	positive
PRJNA729271	:ed_Lushi_vs_Arbor_Acr	breed_growth_background	ENSGALG00010010917	TANC1	0.093650379	0.036984491	2.532152694	0.011336461	0.045746661	positive
PRJNA729271	:ed_Lushi_vs_Arbor_Acr	breed_growth_background	ENSGALG00010010918	TOR1AIP2	0.004376662	0.064236027	0.068134067	0.945678912	0.970470493	positive
PRJNA729271	:ed_Lushi_vs_Arbor_Acr	breed_growth_background	ENSGALG00010010919	CDHR1	-0.319872629	0.317507551	-1.007448888	0.313719108	0.477262644	negative
PRJNA729271	:ed_Lushi_vs_Arbor_Acr	breed_growth_background	ENSGALG00010010920	UBN2	0.187552213	0.061061169	3.071546414	0.002129531	0.013885535	positive
PRJNA729271	:ed_Lushi_vs_Arbor_Acr	breed_growth_background	ENSGALG00010010921	ENSGALG00010010921	0.073187865	0.070712118	1.035011634	0.300663478	0.463136782	positive
PRJNA729271	:ed_Lushi_vs_Arbor_Acr	breed_growth_background	ENSGALG00010010922	DYM	0.246512922	0.054498606	4.523288569	6.08861E-06	0.000200913	positive
PRJNA729271	:ed_Lushi_vs_Arbor_Acr	breed_growth_background	ENSGALG00010010923	F13A1	-0.236352004	0.123281964	-1.917166108	0.055216832	0.142333389	negative
PRJNA729271	:ed_Lushi_vs_Arbor_Acr	breed_growth_background	ENSGALG00010010924	ENSGALG00010010924	-0.035440611	0.115211351	-0.30761388	0.758376157	0.847384708	negative
PRJNA729271	:ed_Lushi_vs_Arbor_Acr	breed_growth_background	ENSGALG00010010928	HECW2	-0.248617691	0.062654563	-3.968069993	7.24571E-05	0.001254797	negative
PRJNA729271	:ed_Lushi_vs_Arbor_Acr	breed_growth_background	ENSGALG00010010929	FMC1	0.057324208	0.079744899	0.718844823	0.472236537	0.626216185	positive
PRJNA729271	:ed_Lushi_vs_Arbor_Acr	breed_growth_background	ENSGALG00010010930	FAM163A	0.142178585	0.301727992	0.471214435	0.637487611	0.762030589	positive
PRJNA729271	:ed_Lushi_vs_Arbor_Acr	breed_growth_background	ENSGALG00010010931	CYYR1	0.109114011	0.107219515	1.01766932	0.308835132	0.471664233	positive
PRJNA729271	:ed_Lushi_vs_Arbor_Acr	breed_growth_background	ENSGALG00010010932	GHITM	0.154233673	0.047163829	3.270168624	0.001074834	0.008607579	positive
PRJNA729271	:ed_Lushi_vs_Arbor_Acr	breed_growth_background	ENSGALG00010010933	ANAPC4	-0.013985563	0.06010641	-0.232680057	0.816009857	0.886680308	negative
PRJNA729271	:ed_Lushi_vs_Arbor_Acr	breed_growth_background	ENSGALG00010010934	ENSGALG00010010934	0.180936847	0.065466879	2.76379218	0.005713392	0.027968798	positive
PRJNA729271	:ed_Lushi_vs_Arbor_Acr	breed_growth_background	ENSGALG00010010935	LGALS1A	-0.018722426	0.121911517	-0.153573891	0.877945725	0.92767337	negative
PRJNA729271	:ed_Lushi_vs_Arbor_Acr	breed_growth_background	ENSGALG00010010937	CDK7	-0.080328271	0.062383181	-1.287659096	0.197864658	0.346267698	negative
PRJNA729271	:ed_Lushi_vs_Arbor_Acr	breed_growth_background	ENSGALG00010010938	FZD6	-0.164261242	0.065146805	-2.521401326	0.011688845	0.046670309	negative
PRJNA729271	:ed_Lushi_vs_Arbor_Acr	breed_growth_background	ENSGALG00010010939	ZCCHC4	0.045917753	0.07598408	0.604307553	0.545639186	0.6894813	positive
PRJNA729271	:ed_Lushi_vs_Arbor_Acr	breed_growth_background	ENSGALG00010010940	NRG3	0.131575052	0.192599836	0.683152461	0.494510509	0.646097077	positive
PRJNA729271	:ed_Lushi_vs_Arbor_Acr	breed_growth_background	ENSGALG00010010941	RIPK4	-0.336042545	0.432235043	-0.777453263	0.436891398	0.5943469	negative
PRJNA729271	:ed_Lushi_vs_Arbor_Acr	breed_growth_background	ENSGALG00010010942	NRN1	-0.079462707	0.179455882	-0.442798007	0.657911845	0.777405413	negative
PRJNA729271	:ed_Lushi_vs_Arbor_Acr	breed_growth_background	ENSGALG00010010943	WDSUB1	-0.325338218	0.055830634	-5.827234906	5.63533E-09	8.25091E-07	negative
PRJNA729271	:ed_Lushi_vs_Arbor_Acr	breed_growth_background	ENSGALG00010010945	PI4K2B	0.134074989	0.054716185	2.45037166	0.014270883	0.053558079	positive
PRJNA729271	:ed_Lushi_vs_Arbor_Acr	breed_growth_background	ENSGALG00010010946	SH2D4B	0.508356376	0.170596765	2.979871131	0.002883697	0.017229672	positive
PRJNA729271	:ed_Lushi_vs_Arbor_Acr	breed_growth_background	ENSGALG00010010947	LUC7L2	0.09588125	0.038027911	2.521338874	0.01169092	0.046670309	positive
PRJNA729271	:ed_Lushi_vs_Arbor_Acr	breed_growth_background	ENSGALG00010010948	PRDM15	0.113877446	0.046632967	2.441994467	0.01460637	0.054412578	positive
PRJNA729271	:ed_Lushi_vs_Arbor_Acr	breed_growth_background	ENSGALG00010010949	FZD3	0.057492474	0.127000118	0.452696224	0.650767499	0.772054003	positive
PRJNA729271	:ed_Lushi_vs_Arbor_Acr	breed_growth_background	ENSGALG00010010951	ENSGALG00010010951	-0.120271203	0.0742994	-1.618737193	0.105503821	0.223545956	negative
PRJNA729271	:ed_Lushi_vs_Arbor_Acr	breed_growth_background	ENSGALG00010010952	ENSGALG00010010952	0.980033739	0.26988465	3.631305959	0.000281991	0.003331259	positive
PRJNA729271	:ed_Lushi_vs_Arbor_Acr	breed_growth_background	ENSGALG00010010953	EXTL3	-0.031106264	0.059318167	-0.524396922	0.600002497	0.732875722	negative
PRJNA729271	:ed_Lushi_vs_Arbor_Acr	breed_growth_background	ENSGALG00010010954	MTERF2	0.30257253	0.120911564	2.502428395	0.012334457	0.048491445	positive
PRJNA729271	:ed_Lushi_vs_Arbor_Acr	breed_growth_background	ENSGALG00010010955	GTF3C3	-0.124274964	0.045096219	-2.755773459	0.005855354	0.028490551	negative
PRJNA729271	:ed_Lushi_vs_Arbor_Acr	breed_growth_background	ENSGALG00010010956	AXDND1	0.045667237	0.258188802	0.176875357	0.859606295	0.915637364	positive
PRJNA729271	:ed_Lushi_vs_Arbor_Acr	breed_growth_background	ENSGALG00010010957	ZMAT2	0.029302328	0.040485718	0.723769501	0.469207256	0.623959558	positive
PRJNA729271	:ed_Lushi_vs_Arbor_Acr	breed_growth_background	ENSGALG00010010958	LCORL	0.105275965	0.085928991	1.225150706	0.220518443	0.373107836	positive
PRJNA729271	:ed_Lushi_vs_Arbor_Acr	breed_growth_background	ENSGALG00010010959	TMEM263	-0.096419484	0.048528279	-1.986872098	0.046936572	0.126528848	negative
PRJNA729271	:ed_Lushi_vs_Arbor_Acr	breed_growth_background	ENSGALG00010010960	POLDIP3	0.09431063	0.037892789	2.488880636	0.012814598	0.049749738	positive
PRJNA729271	:ed_Lushi_vs_Arbor_Acr	breed_growth_background	ENSGALG00010010961	C2orf66	-0.237739777	0.203455304	-1.168511084	0.242600671	0.39828282	negative
PRJNA729271	:ed_Lushi_vs_Arbor_Acr	breed_growth_background	ENSGALG00010010962	ANKDD1B	-0.533666328	0.149805708	-3.562389814	0.000367494	0.00399612	negative
PRJNA729271	:ed_Lushi_vs_Arbor_Acr	breed_growth_background	ENSGALG00010010963	TSPAN14	-0.115064627	0.039962968	-2.879281295	0.003985826	0.021625501	negative
PRJNA729271	:ed_Lushi_vs_Arbor_Acr	breed_growth_background	ENSGALG00010010964	INTS9	0.059514963	0.050853977	1.170310905	0.241875875	0.397594784	positive
PRJNA729271	:ed_Lushi_vs_Arbor_Acr	breed_growth_background	ENSGALG00010010965	BAZ2B	0.164645808	0.070815753	2.324988447	0.020072578	0.068466111	positive
PRJNA729271	:ed_Lushi_vs_Arbor_Acr	breed_growth_background	ENSGALG00010010966	ENSGALG00010010966	-0.3079419	0.235868832	-1.305564188	0.191700805	0.338542146	negative
PRJNA729271	:ed_Lushi_vs_Arbor_Acr	breed_growth_background	ENSGALG00010010967	PRXL2A	-0.175299339	0.208920417	-0.839072319	0.401428728	0.562508666	negative
PRJNA729271	:ed_Lushi_vs_Arbor_Acr	breed_growth_background	ENSGALG00010010968	RRP7A	-0.075558475	0.131972854	-0.572530432	0.566962675	0.706085057	negative
PRJNA729271	:ed_Lushi_vs_Arbor_Acr	breed_growth_background	ENSGALG00010010970	TPM2	-0.13763327	0.188411545	-0.730492764	0.465089035	0.620247084	negative
PRJNA729271	:ed_Lushi_vs_Arbor_Acr	breed_growth_background	ENSGALG00010010971	LCORL	0.00348545	0.067135566	0.051916598	0.958595149	0.977133381	positive
PRJNA729271	:ed_Lushi_vs_Arbor_Acr	breed_growth_background	ENSGALG00010010972	EXOSC3	0.230071829	0.090187193	2.551047682	0.010739963	0.044050867	positive
PRJNA729271	:ed_Lushi_vs_Arbor_Acr	breed_growth_background	ENSGALG00010010973	PGAP1	-0.188601197	0.060867612	-3.098547638	0.001944717	0.013020172	negative
PRJNA729271	:ed_Lushi_vs_Arbor_Acr	breed_growth_background	ENSGALG00010010974	NCAPG	-0.274019706	0.103889406	-2.637609708	0.008349261	0.03670026	negative
PRJNA729271	:ed_Lushi_vs_Arbor_Acr	breed_growth_background	ENSGALG00010010975	ATP8B1	-0.152504185	0.532689531	-0.286290938	0.774655298	0.860145825	negative
PRJNA729271	:ed_Lushi_vs_Arbor_Acr	breed_growth_background	ENSGALG00010010976	DYDC2	0.497628364	0.367632816	1.353601592	0.175863519	0.319882257	positive
PRJNA729271	:ed_Lushi_vs_Arbor_Acr	breed_growth_background	ENSGALG00010010977	FARS2	-0.032864764	0.044347031	-0.741081497	0.458644028	0.61436514	negative
PRJNA729271	:ed_Lushi_vs_Arbor_Acr	breed_growth_background	ENSGALG00010010978	SERHL	0.059501231	0.073355126	0.811139372	0.417285638	0.577427715	positive
PRJNA729271	:ed_Lushi_vs_Arbor_Acr	breed_growth_background	ENSGALG00010010979	ENSGALG00010010979	0.006739985	0.227423797	0.029636233	0.976357169	0.986016663	positive
PRJNA729271	:ed_Lushi_vs_Arbor_Acr	breed_growth_background	ENSGALG00010010980	POLK	0.034402539	0.067321395	0.511019405	0.609337469	0.73997542	positive
PRJNA729271	:ed_Lushi_vs_Arbor_Acr	breed_growth_background	ENSGALG00010010981	RIC8B	-0.136254014	0.036003125	-3.784505245	0.000154015	0.00218012	negative
PRJNA729271	:ed_Lushi_vs_Arbor_Acr	breed_growth_background	ENSGALG00010010982	ZBTB21	-0.043082457	0.064060387	-0.67252883	0.501247093	0.652323273	negative
PRJNA729271	:ed_Lushi_vs_Arbor_Acr	breed_growth_background	ENSGALG00010010983	MED28	0.013076075	0.048171436	0.271448723	0.786045933	0.867374086	positive
PRJNA729271	:ed_Lushi_vs_Arbor_Acr	breed_growth_background	ENSGALG00010010984	MAT1A	-0.303736883	0.208835709	-1.454429827	0.145827176	0.280005949	negative
PRJNA729271	:ed_Lushi_vs_Arbor_Acr	breed_growth_background	ENSGALG00010010985	CERT1	0.138595442	0.057903247	2.393569439	0.016685323	0.060053606	positive
PRJNA729271	:ed_Lushi_vs_Arbor_Acr	breed_growth_background	ENSGALG00010010986	NARS	0.235819363	0.069759225	3.380475663	0.000723605	0.00646553	positive
PRJNA729271	:ed_Lushi_vs_Arbor_Acr	breed_growth_background	ENSGALG00010010987	MARCHF7	-0.047969345	0.04353253	-1.101919534	0.270496656	0.429117766	negative
PRJNA729271	:ed_Lushi_vs_Arbor_Acr	breed_growth_background	ENSGALG00010010988	ANKRD44	0.089457827	0.046787652	1.911996513	0.055876641	0.143528605	positive
PRJNA729271	:ed_Lushi_vs_Arbor_Acr	breed_growth_background	ENSGALG00010010989	TRIQK	-0.139509334	0.09773098	-1.427483223	0.153440657	0.289939034	negative
PRJNA729271	:ed_Lushi_vs_Arbor_Acr	breed_growth_background	ENSGALG00010010990	MED18	0.001751256	0.064043494	0.027344792	0.978184731	0.987037082	positive
PRJNA729271	:ed_Lushi_vs_Arbor_Acr	breed_growth_background	ENSGALG00010010991	C2CD2	-0.511805478	0.087170903	-5.871288025	4.32422E-09	6.88659E-07	negative
PRJNA729271	:ed_Lushi_vs_Arbor_Acr	breed_growth_background	ENSGALG00010010992	PCDHA6	-0.242314117	0.156881366	-1.544569145	0.122450514	0.247237806	negative
PRJNA729271	:ed_Lushi_vs_Arbor_Acr	breed_growth_background	ENSGALG00010010993	ENSGALG00010010993	-0.216181798	0.188566661	-1.146447611	0.251609993	0.407769101	negative
PRJNA729271	:ed_Lushi_vs_Arbor_Acr	breed_growth_background	ENSGALG00010010994	NAA20	-0.28952079	0.080226016	-3.60881426	0.0003076	0.003532834	negative
PRJNA729271	:ed_Lushi_vs_Arbor_Acr	breed_growth_background	ENSGALG00010010995	HEBP1	-0.164520173	0.098521764	-1.669886599	0.094941802	0.207349921	negative
PRJNA729271	:ed_Lushi_vs_Arbor_Acr	breed_growth_background	ENSGALG00010010996	RFX4	-0.408789959	0.343159867	-1.191252236	0.233554579	0.387963972	negative
PRJNA729271	:ed_Lushi_vs_Arbor_Acr	breed_growth_background	ENSGALG00010010997	LAP3	0.154282013	0.080516721	1.916148748	0.055346165	0.142570226	positive
PRJNA729271	:ed_Lushi_vs_Arbor_Acr	breed_growth_background	ENSGALG00010010998	SOAT1	-0.207836313	0.088904646	-2.337744105	0.019400527	0.066891207	negative

PRJNA729271	ed_Lushi_vs_Arbor_Acr	breed_growth_background	ENSGALG00010011001	ANGPT1	-0.310850158	0.138258264	-2.248329677	0.024555176	0.078954586	negative
PRJNA729271	ed_Lushi_vs_Arbor_Acr	breed_growth_background	ENSGALG00010011002	RPRM	-0.307476028	0.365723355	-0.840733914	0.400497017	0.561404993	negative
PRJNA729271	ed_Lushi_vs_Arbor_Acr	breed_growth_background	ENSGALG00010011003	FECH	-0.022694791	0.060704328	-0.373857866	0.708510065	0.812102909	negative
PRJNA729271	ed_Lushi_vs_Arbor_Acr	breed_growth_background	ENSGALG00010011004	UMODL1	-1.043235264	0.222224734	-4.694505619	2.67252E-06	0.000103856	negative
PRJNA729271	ed_Lushi_vs_Arbor_Acr	breed_growth_background	ENSGALG00010011006	TMEM64	-0.150071568	0.054278212	-2.764858351	0.005694752	0.027915262	negative
PRJNA729271	ed_Lushi_vs_Arbor_Acr	breed_growth_background	ENSGALG00010011007	ENSGALG00010011007	0.063481702	0.096575544	0.657326892	0.510970749	0.659588655	positive
PRJNA729271	ed_Lushi_vs_Arbor_Acr	breed_growth_background	ENSGALG00010011009	SFTPA1	0.865862135	0.640212258	1.352461039	0.176227874	0.320183384	positive
PRJNA729271	ed_Lushi_vs_Arbor_Acr	breed_growth_background	ENSGALG00010011013	ENSGALG00010011013	-0.218393645	0.146979997	-1.485873243	0.137312652	0.268421698	negative
PRJNA729271	ed_Lushi_vs_Arbor_Acr	breed_growth_background	ENSGALG00010011015	LYRM4	-0.153417612	0.09883567	-1.552249436	0.120602551	0.245145699	negative
PRJNA729271	ed_Lushi_vs_Arbor_Acr	breed_growth_background	ENSGALG00010011018	QDPR	-0.07130052	0.056523339	-1.26143504	0.207152151	0.356273187	negative
PRJNA729271	ed_Lushi_vs_Arbor_Acr	breed_growth_background	ENSGALG00010011019	NECAB1	-0.163658291	0.126851844	-1.290153033	0.19699753	0.345454754	negative
PRJNA729271	ed_Lushi_vs_Arbor_Acr	breed_growth_background	ENSGALG00010011020	SFTPA2	0.137442276	0.478934522	0.286975086	0.774131397	0.85978351	positive
PRJNA729271	ed_Lushi_vs_Arbor_Acr	breed_growth_background	ENSGALG00010011021	PPP1R3G	-0.121896712	0.094290255	-1.292781647	0.19608659	0.344351238	negative
PRJNA729271	ed_Lushi_vs_Arbor_Acr	breed_growth_background	ENSGALG00010011022	C5orf22	-0.514918933	0.104683218	-4.918829831	8.70631E-07	4.37689E-05	negative
PRJNA729271	ed_Lushi_vs_Arbor_Acr	breed_growth_background	ENSGALG00010011025	POLR3B	0.040253838	0.049673919	0.810361639	0.417732359	0.57777009	positive
PRJNA729271	ed_Lushi_vs_Arbor_Acr	breed_growth_background	ENSGALG00010011027	FAM234B	0.022728309	0.0545439	0.416697544	0.676899651	0.791390582	positive
PRJNA729271	ed_Lushi_vs_Arbor_Acr	breed_growth_background	ENSGALG00010011028	HMBOX1	-0.011936205	0.047038103	-0.253756086	0.799684001	0.876014178	negative
PRJNA729271	ed_Lushi_vs_Arbor_Acr	breed_growth_background	ENSGALG00010011029	HMGCR	-0.174034334	0.067587426	-2.574951363	0.010025418	0.04191475	negative
PRJNA729271	ed_Lushi_vs_Arbor_Acr	breed_growth_background	ENSGALG00010011033	DROSHA	0.044755036	0.055409058	0.807720568	0.419251461	0.578987226	positive
PRJNA729271	ed_Lushi_vs_Arbor_Acr	breed_growth_background	ENSGALG00010011034	FAM180A	0.197593375	0.21596242	0.914943324	0.360221384	0.523554494	positive
PRJNA729271	ed_Lushi_vs_Arbor_Acr	breed_growth_background	ENSGALG00010011038	GSG1	-0.289555597	0.265579881	-1.090276852	0.275591209	0.434489294	negative
PRJNA729271	ed_Lushi_vs_Arbor_Acr	breed_growth_background	ENSGALG00010011040	CDH6	0.360231829	0.122427921	2.9423993	0.003256797	0.018807678	positive
PRJNA729271	ed_Lushi_vs_Arbor_Acr	breed_growth_background	ENSGALG00010011042	SLC13A4	0.204954811	0.278475771	0.735987947	0.461738033	0.616970206	positive
PRJNA729271	ed_Lushi_vs_Arbor_Acr	breed_growth_background	ENSGALG00010011044	MBL2	-0.485293916	0.32994886	-1.470815555	0.141341005	0.274005331	negative
PRJNA729271	ed_Lushi_vs_Arbor_Acr	breed_growth_background	ENSGALG00010011045	ENSGALG00010011045	0.387195568	0.227354101	1.703050729	0.088558575	0.197759444	positive
PRJNA729271	ed_Lushi_vs_Arbor_Acr	breed_growth_background	ENSGALG00010011049	ABL2	0.050009495	0.069848622	0.715969668	0.474010078	0.627640092	positive
PRJNA729271	ed_Lushi_vs_Arbor_Acr	breed_growth_background	ENSGALG00010011050	SYT10	-0.425385803	0.19226931	-2.212447751	0.026935743	0.084163309	negative
PRJNA729271	ed_Lushi_vs_Arbor_Acr	breed_growth_background	ENSGALG00010011055	TOR3A	-0.037309224	0.073206089	-0.509646464	0.610299165	0.740584881	negative
PRJNA729271	ed_Lushi_vs_Arbor_Acr	breed_growth_background	ENSGALG00010011056	ENSGALG00010011056	-0.601984515	0.290411595	-2.072866667	0.03818469	0.108883113	negative
PRJNA729271	ed_Lushi_vs_Arbor_Acr	breed_growth_background	ENSGALG00010011059	ENSGALG00010011059	-0.209040101	0.111870862	-1.868583979	0.061680716	0.153936292	negative
PRJNA729271	ed_Lushi_vs_Arbor_Acr	breed_growth_background	ENSGALG00010011061	KIF13B	0.172015023	0.08279584	2.077580503	0.037748013	0.108048471	positive
PRJNA729271	ed_Lushi_vs_Arbor_Acr	breed_growth_background	ENSGALG00010011062	TMEM55A	-0.181456931	0.066266258	-2.738300544	0.006175761	0.02962199	negative
PRJNA729271	ed_Lushi_vs_Arbor_Acr	breed_growth_background	ENSGALG00010011064	MYLIP	-0.032493802	0.052015362	-0.624696247	0.532170436	0.677655883	negative
PRJNA729271	ed_Lushi_vs_Arbor_Acr	breed_growth_background	ENSGALG00010011067	TMTC2	-0.042351363	0.055438009	-0.763940905	0.44490246	0.601741266	negative
PRJNA729271	ed_Lushi_vs_Arbor_Acr	breed_growth_background	ENSGALG00010011069	ENSGALG00010011069	-0.057196869	0.295594873	-0.1934975	0.846569364	0.90715036	negative
PRJNA729271	ed_Lushi_vs_Arbor_Acr	breed_growth_background	ENSGALG00010011076	PKP2	-0.512368545	0.165436317	-3.097074186	0.00195441	0.013079024	negative
PRJNA729271	ed_Lushi_vs_Arbor_Acr	breed_growth_background	ENSGALG00010011077	RASGEF1A	-0.024032164	0.077893654	-0.308525306	0.757682647	0.846954991	negative
PRJNA729271	ed_Lushi_vs_Arbor_Acr	breed_growth_background	ENSGALG00010011080	EMP1	-0.233632976	0.137328903	-1.701265877	0.088893073	0.198445262	negative
PRJNA729271	ed_Lushi_vs_Arbor_Acr	breed_growth_background	ENSGALG00010011085	UGT1A1	-1.847235658	0.714600627	-2.584990256	0.009738175	0.041009543	negative
PRJNA729271	ed_Lushi_vs_Arbor_Acr	breed_growth_background	ENSGALG00010011086	OTUD6A	0.025699765	0.060681907	0.423516116	0.671918743	0.787780808	positive
PRJNA729271	ed_Lushi_vs_Arbor_Acr	breed_growth_background	ENSGALG00010011090	GRIN2B	-0.471673081	0.205663092	-2.29342599	0.021823492	0.072669771	negative
PRJNA729271	ed_Lushi_vs_Arbor_Acr	breed_growth_background	ENSGALG00010011091	YARS2	0.050070634	0.054222871	0.923422773	0.355786928	0.519610229	positive
PRJNA729271	ed_Lushi_vs_Arbor_Acr	breed_growth_background	ENSGALG00010011094	CSGALNACT2	-0.118749686	0.049154949	-2.415823598	0.015699665	0.057597228	negative
PRJNA729271	ed_Lushi_vs_Arbor_Acr	breed_growth_background	ENSGALG00010011095	TNN	-0.19330187	0.266571003	-0.725142151	0.468364828	0.623239528	negative
PRJNA729271	ed_Lushi_vs_Arbor_Acr	breed_growth_background	ENSGALG00010011096	LDB2	-0.173324007	0.062033809	-2.79402489	0.005205647	0.02627293	negative
PRJNA729271	ed_Lushi_vs_Arbor_Acr	breed_growth_background	ENSGALG00010011099	CCBE1	-0.208856099	0.107080177	-1.950464643	0.051120764	0.134604081	negative
PRJNA729271	ed_Lushi_vs_Arbor_Acr	breed_growth_background	ENSGALG00010011101	ENSGALG00010011101	-0.77578449	0.452259266	-1.715353446	0.086280445	0.194258318	negative
PRJNA729271	ed_Lushi_vs_Arbor_Acr	breed_growth_background	ENSGALG00010011104	ABCG1	-0.272996239	0.136661416	-1.997610201	0.045758936	0.124371983	negative
PRJNA729271	ed_Lushi_vs_Arbor_Acr	breed_growth_background	ENSGALG00010011106	USP40	0.063000506	0.062284678	1.011492832	0.31178061	0.475011556	positive
PRJNA729271	ed_Lushi_vs_Arbor_Acr	breed_growth_background	ENSGALG00010011107	ENSGALG00010011107	-0.015585414	0.102571185	-0.151947294	0.879228506	0.92824647	negative
PRJNA729271	ed_Lushi_vs_Arbor_Acr	breed_growth_background	ENSGALG00010011109	ENSGALG00010011109	-1.233213502	0.676296337	-1.823480971	0.068230577	0.165053773	negative
PRJNA729271	ed_Lushi_vs_Arbor_Acr	breed_growth_background	ENSGALG00010011111	MRPS14	-0.272258201	0.0625665	-4.351501207	1.35209E-05	0.000361596	negative
PRJNA729271	ed_Lushi_vs_Arbor_Acr	breed_growth_background	ENSGALG00010011116	TAPT1	0.120934502	0.068174185	1.773904641	0.076078925	0.177836481	positive
PRJNA729271	ed_Lushi_vs_Arbor_Acr	breed_growth_background	ENSGALG00010011117	SLC6A15	0.853614554	0.313586512	2.722102264	0.006486807	0.030687422	positive
PRJNA729271	ed_Lushi_vs_Arbor_Acr	breed_growth_background	ENSGALG00010011118	GMPR2	0.174514925	0.072125487	2.419601333	0.015537531	0.057173428	positive
PRJNA729271	ed_Lushi_vs_Arbor_Acr	breed_growth_background	ENSGALG00010011119	ENSGALG00010011119	-0.41864176	0.159132981	-2.630766775	0.008519248	0.037146343	negative
PRJNA729271	ed_Lushi_vs_Arbor_Acr	breed_growth_background	ENSGALG00010011120	ENSGALG00010011120	0.283996284	1.636714177	0.173516114	0.862245763	0.917201857	positive
PRJNA729271	ed_Lushi_vs_Arbor_Acr	breed_growth_background	ENSGALG00010011121	NUP35	0.155133914	0.057749836	2.686309144	0.007224621	0.03315001	positive
PRJNA729271	ed_Lushi_vs_Arbor_Acr	breed_growth_background	ENSGALG00010011124	CACYBP	0.097493215	0.036498521	2.671155251	0.007559068	0.034218827	positive
PRJNA729271	ed_Lushi_vs_Arbor_Acr	breed_growth_background	ENSGALG00010011126	ATXN1	-0.145943891	0.075333723	-1.937298256	0.052708895	0.13763564	negative
PRJNA729271	ed_Lushi_vs_Arbor_Acr	breed_growth_background	ENSGALG00010011128	GCNT4	0.004316246	0.133374309	0.032361897	0.974183449	0.984850683	positive
PRJNA729271	ed_Lushi_vs_Arbor_Acr	breed_growth_background	ENSGALG00010011129	ENSGALG00010011129	0.093696809	0.063537782	1.474662892	0.140303197	0.27261325	positive
PRJNA729271	ed_Lushi_vs_Arbor_Acr	breed_growth_background	ENSGALG00010011130	DNM1L	-0.008345371	0.061759775	-0.135126321	0.892512	0.937189325	negative
PRJNA729271	ed_Lushi_vs_Arbor_Acr	breed_growth_background	ENSGALG00010011131	RET	-0.681780766	0.265109783	-2.571692221	0.010120282	0.042184719	negative
PRJNA729271	ed_Lushi_vs_Arbor_Acr	breed_growth_background	ENSGALG00010011133	CDC37L1	0.092866651	0.0645697	1.438238838	0.150366299	0.285993899	positive
PRJNA729271	ed_Lushi_vs_Arbor_Acr	breed_growth_background	ENSGALG00010011136	ENSGALG00010011136	-0.541516005	0.219891011	-2.462656395	0.013791201	0.0524681	negative
PRJNA729271	ed_Lushi_vs_Arbor_Acr	breed_growth_background	ENSGALG00010011139	UBASH3A	0.417847427	0.23381368	1.787095721	0.073922019	0.174568209	positive
PRJNA729271	ed_Lushi_vs_Arbor_Acr	breed_growth_background	ENSGALG00010011141	MSX1	0.416843585	0.507434931	0.821471996	0.411377469	0.571543795	positive
PRJNA729271	ed_Lushi_vs_Arbor_Acr	breed_growth_background	ENSGALG00010011142	DUSP19	0.450282534	0.081465641	5.527269241	3.25254E-08	3.57163E-06	positive
PRJNA729271	ed_Lushi_vs_Arbor_Acr	breed_growth_background	ENSGALG00010011143	AK3	0.197782702	0.087699756	2.255225225	0.024119195	0.077985219	positive
PRJNA729271	ed_Lushi_vs_Arbor_Acr	breed_growth_background	ENSGALG00010011144	ATF7IP	0.10006235	0.033672216	2.9716592	0.002961953	0.0175669	positive
PRJNA729271	ed_Lushi_vs_Arbor_Acr	breed_growth_background	ENSGALG00010011146	BMS1	0.139884575	0.069361993	2.016732357	0.043723451	0.120175811	positive
PRJNA729271	ed_Lushi_vs_Arbor_Acr	breed_growth_background	ENSGALG00010011148	ENSGALG00010011148	0.38060799	0.098844122	3.850588018	0.000117835	0.001809335	positive
PRJNA729271	ed_Lushi_vs_Arbor_Acr	breed_growth_background	ENSGALG00010011150	FGD4	-0.166522023	0.088417517	-1.883360088	0.059651585	0.15024322	negative
PRJNA729271	ed_Lushi_vs_Arbor_Acr	breed_growth_background	ENSGALG00010011152	SLC18A3	0.762146703	0.297848207	2.558842676	0.010502126	0.043322608	positive
PRJNA729271	ed_Lushi_vs_Arbor_Acr	breed_growth_background	ENSGALG00010011154	SLC26A7	0.297620419	0.314476862	0.946398463	0.343945389	0.507790631	positive
PRJNA729271	ed_Lushi_vs_Arbor_Acr	breed_growth_background	ENSGALG00010011157	ENSGALG00010011157	0.53987125	0.902144577	0.598430965	0.549552409	0.692855717	positive
PRJNA729271	ed_Lushi_vs_Arbor_Acr	breed_growth_background	ENSGALG00010011161	ENSGALG00010011161	-0.054977707	0.050906897	-1.079965789	0.280157415	0.439959615	negative
PRJNA729271	ed_Lushi_vs_Arbor_Acr	breed_growth_background	ENSGALG00010011166	SOX7	-0.127916176	0.075542424	-1.69330251	0.090397901	0.200876525	negative
PRJNA729271	ed_Lushi_vs_Arbor_Acr	breed_growth_background	ENSGALG00010011170	RBM24	0.141292249	0.076298453	1.851836355	0.064049325	0.157824909	positive
PRJNA729271	ed_Lushi_vs_Arbor_Acr	breed_growth_background	ENSGALG00010011175	GPR52	-0.062228572	0.105216131	-0.591435663	0.554228551	0.696207891	negative
PRJNA729271	ed_Lushi_vs_Arbor_Acr	breed_growth_background	ENSGALG00010011178	RSPO2	0.524177118	0.17539239	2.988596696	0.002802618	0.016884434	positive
PRJNA729271	ed_Lushi_vs_Arbor_Acr	breed_growth_background	ENSGALG00010011181	ENSGALG00010011181	-0.180362186	0.155212617	-1.162033024	0.245222051	0.401048587	negative
PRJNA729271	ed_Lushi_vs_Arbor_Acr	breed_growth_background	ENSGALG00010011184	FAM8A1	0.068579652	0.072765682	0.942472465	0.345950802	0.509764855	positive
PRJNA729271	ed_Lushi_vs_Arbor_Acr	breed_growth_background	ENSGALG00010011187	STK32B	0.379127269	0.299931471	1.264046309	0.206213406	0.355460021	positive
PRJNA729271	ed_Lushi_vs_Arbor_Acr	breed_growth_background	ENSGALG00010011190	ENSGALG00010011190	-0.444477584	0.116277979	-3.822543067	0.000132082	0.001951615	negative
PRJNA729271	ed_Lushi_vs_Arbor_Acr	breed_growth_background	ENSGALG00010011192	RPL17	-0.043599635	0.066022817	-0.660372233	0.509014986	0.657888698	negative
PRJNA729271	ed_Lushi_vs_Arbor_Acr	breed_growth_background	ENSGALG00010011193	FAM20B	-0.028538265	0.043653574	-0.653744064	0.51327671	0.661799138	negative
PRJNA729271	ed_Lushi_vs_Arbor_Acr	breed_growth_background	ENSGALG00010011198	PINX1	0.059264656	0.036911816	1.605574104	0.108367519	0.228080033	positive
PRJNA729271	ed_Lushi_vs_Arbor_Acr	breed_growth_background	ENSGALG00010011199	ENSGALG00010011199	0.402879468	0.971578476	0.414664875	0.678387249	0.792299023	positive
PRJNA729271	ed_Lushi_vs_Arbor_Acr	breed_growth_background	ENSGALG00010011200	PCDHB4	-0.347064664	0.327033607	-1.061250758	0.288575957	0.449533423	negative
PRJNA729271	ed_Lushi_vs_Arbor_Acr	breed_growth_background	ENSGALG00010011203	ENSGALG00010011203	-0.042698384	0.056316878	-0.75818095	0.448342664	0.60477939	negative
PRJNA729271	ed_Lushi_vs_Arbor_Acr	breed_growth_background	ENSGALG00010011205	CYTL1	0.822688546	0.510269703	1.612262185	0.106904899	0.225656983	positive
PRJNA729271	ed_Lushi_vs_Arbor_Acr	breed_growth_background	ENSGALG00010011210	NUP153	0.053542911	0.052892677	1.012293456	0.311397763	0.474611543	positive
PRJNA729271	ed_Lushi_vs_Arbor_Acr	breed_growth_background	ENSGALG00010011211	SLC37A1	0.059293726	0.080274273	0.738639215	0.460126105	0.615326866	positive
PRJNA729271	ed_Lushi_vs_Arbor_Acr	breed_growth_background	ENSGALG00010011212	NCKAP1	-0.118499942	0.033989946	-3.486323323	0.000489709	0.004898777	negative
PRJNA729271	ed_Lushi_vs_Arbor_Acr	breed_growth_background	ENSGALG00010011213	XKR6	0.135664472	0.099141544	1.368391763	0.171189481	0.314020695	positive
PRJNA729271	ed_Lushi_vs_Arbor_Acr	breed_growth_background	ENSGALG00010011218	ENSGALG00010011218	-0.280135361	0.3255806	-0.860417853	0.389558748	0.551323379	negative
PRJNA729271	ed_Lushi_vs_Arbor_Acr	breed_growth_background	ENSGALG00010011222	RCL1	-0.090098459	0.048395011	-1.861730292	0.062641117	0.155423311	negative
PRJNA729271	ed_Lushi_vs_Arbor_Acr	breed_growth_background	ENSGALG00010011231	PLBD1	-0.064952232	0.110278568	-0.588983277	0.555872492	0.697547551	negative
PRJNA729271	ed_Lushi_vs_Arbor_Acr	breed_growth_background	ENSGALG00010011235	ENSGALG00010011235	-0.126096633	0.050751766	-2.484576278	0.012970572	0.050210567	negative
PRJNA729271	ed_Lushi_vs_Arbor_Acr	breed_growth_background	ENSGALG00010011237	ENSGALG00010011237	-0.06415751	0.14282621	-0.449199835	0.653287506	0.774016435	negative
PRJNA729271	ed_Lushi_vs_Arbor_Acr	breed_growth_background	ENSGALG00010011239	RALGPS2	0.026426742	0.041819085	0.631930195	0.527432492	0.673402094	positive
PRJNA729271	ed_Lushi_vs_Arbor_Acr	breed_growth_background	ENSGALG00010011243	ALX1	1.227620396	0.356265439	3.445802658	0.000569366	0.005419051	positive
PRJNA729271	ed_Lushi_vs_Arbor_Acr	breed_growth_background	ENSGALG00010011244	PDE9A	-0.399403836	0.093427572	-4.275010311	1.91128E-05	0.000464833	negative
PRJNA729271	ed_Lushi_vs_Arbor_Acr	breed_growth_background	ENSGALG00010011246	DNAJB5	-0.445906047	0.124139092	-3.591987348	0.000328166	0.003681424	negative
PRJNA729271	ed_Lushi_vs_Arbor_Acr	breed_growth_background	ENSGALG00010011247	ENSGALG00010011247	-0.041334362	0.052297527	-0.790369341	0.429312102	0.588076607	negative
PRJNA729271	ed_Lushi_vs_Arbor_Acr	breed_growth_background	ENSGALG00010011250	ENSGALG00010011250	0.0746306	0.053452714	1.396198514	0.162654724	0.302150484	positive
PRJNA729271	ed_Lushi_vs_Arbor_Acr	breed_growth_background	ENSGALG00010011251	FRZB	-0.481051166	0.093790485	-5.128997533	2.91289E-07	1.87655E-05	negative
PRJNA729271	ed_Lushi_vs_Arbor_Acr	breed_growth_background	ENSGALG00010011253	KDM4C	0.082597443	0.058018465	1.423640609	0.154550534	0.291125405	positive
PRJNA729271	ed_Lushi_vs_Arbor_Acr	breed_growth_background	ENSGALG00010011262	ENSGALG00010011262	0.440624357	0.111765322	3.94240671	8.0668E-05	0.001361214	positive
PRJNA729271	ed_Lushi_vs_Arbor_Acr	breed_growth_background	ENSGALG00010011267	EVC2	-0.2882696	0.089668762	-3.214827457	0.00130523	0.009869224	negative
PRJNA729271	ed_Lushi_vs_Arbor_Acr	breed_growth_background	ENSGALG00010011271	ANGPTL1	-0.264640584	0.087405074	-3.027748529	0.00246383	0.015426877	negative
PRJNA729271	ed_Lushi_vs_Arbor_Acr	breed_growth_background	ENSGALG00010011273	RUNX1T1	0.000533241	0.053381201	0.009989303	0.992029822	0.99553152	positive
PRJNA729271	ed_Lushi_vs_Arbor_Acr	breed_growth_background	ENSGALG00010011274	ENSGALG00010011274	-1.018529444	0.295305616	-3.449068993	0.000562523	0.005385581	negative
PRJNA729271	ed_Lushi_vs_Arbor_Acr	breed_growth_background	ENSGALG00010011275	WDR4	0.001373939	0.056492904	0.024320557	0.980596916	0.988576456	positive
PRJNA729271	ed_Lushi_vs_Arbor_Acr	breed_growth_background	ENSGALG00010011278	ENSGALG00010011278	0.283037909	0.280309242	1.009734488	0.312622515	0.475844098	positive
PRJNA729271	ed_Lushi_vs_Arbor_Acr	breed_growth_background	ENSGALG00010011280	FAM199X	0.171266446	0.044088559	3.884600622	0.000102498	0.001614904	positive
PRJNA729271	ed_Lushi_vs_Arbor_Acr	breed_growth_background	ENSGALG00010011281	ENSGALG00010011281	-0.789565505	0.346619902	-2.277900087	0.022732531	0.074856639	negative
PRJNA729271	ed_Lushi_vs_Arbor_Acr	breed_growth_background	ENSGALG00010011282	CRY1	0.220250083	0.067945899	3.24155079	0.001188812	0.009264428	positive
PRJNA729271	ed_Lushi_vs_Arbor_Acr	breed_growth_background	ENSGALG00010011283	LONRF3	0.10610415	0.076515795	1.386696028	0.165534472	0.305814705	positive
PRJNA729271	ed_Lushi_vs_Arbor_Acr	breed_growth_background	ENSGALG00010011285	ENSGALG00010011285	0.194677166	0.139358537	1.396951849	0.162428051	0.301806769	positive
PRJNA729271	ed_Lushi_vs_Arbor_Acr	breed_growth_background	ENSGALG00010011287	ENSGALG00010011287	0.032472301	0.063624772	0.510371972	0.609790889	0.740339972	positive
PRJNA729271	ed_Lushi_vs_Arbor_Acr	breed_growth_background	ENSGALG00010011288	ENSGALG00010011288	-0.346816987	0.162723886	-2.131321933	0.033062629	0.097685041	negative
PRJNA729271	ed_Lushi_vs_Arbor_Acr	breed_growth_background	ENSGALG00010011293	NDUFV3	0.037245084	0.089466742	0.416300885	0.677189845	0.791538328	positive
PRJNA729271	ed_Lushi_vs_Arbor_Acr	breed_growth_background	ENSGALG00010011295	FAM167A	0.422762961	0.210095821	2.012238789	0.044194771	0.121097015	positive
PRJNA729271	ed_Lushi_vs_Arbor_Acr	breed_growth_background	ENSGALG00010011297	OSR2	0.245548649	0.099309148	2.472568289	0.013414608	0.051385539	positive
PRJNA729271	ed_Lushi_vs_Arbor_Acr	breed_growth_background	ENSGALG00010011300	EVC	-0.164416857	0.087385099	-1.881520516	0.059901147	0.150583962	negative
PRJNA729271	ed_Lushi_vs_Arbor_Acr	breed_growth_background	ENSGALG00010011304	COMMD5	-0.085290669	0.050653298	-1.68381276	0.092217861	0.203211116	negative
PRJNA729271	ed_Lushi_vs_Arbor_Acr	breed_growth_background	ENSGALG00010011306	ECI2	-0.148447843	0.073956442	-2.007233428	0.044724814	0.12217983	negative
PRJNA729271	ed_Lushi_vs_Arbor_Acr	breed_growth_background	ENSGALG00010011307	ENSGALG00010011307	0.709424623	0.256649664	2.764175155	0.00570669	0.027948642	positive

PRJNA729271	:ed_Lushi_vs_Arbor_Acr	breed_growth_background	ENSGALG00010011308	SPIC	2.018344307	0.72674434	2.77724118	0.005482248	0.027232758	positive
PRJNA729271	:ed_Lushi_vs_Arbor_Acr	breed_growth_background	ENSGALG00010011311	DOCK8	0.166447381	0.103096733	1.614477744	0.106423841	0.225067635	positive
PRJNA729271	:ed_Lushi_vs_Arbor_Acr	breed_growth_background	ENSGALG00010011312	VPS13B	-0.030023086	0.028551889	-1.051527134	0.293016554	0.454316489	negative
PRJNA729271	:ed_Lushi_vs_Arbor_Acr	breed_growth_background	ENSGALG00010011313	RASSF9	-0.088008636	0.137050546	-0.642161875	0.520768084	0.668189199	negative
PRJNA729271	:ed_Lushi_vs_Arbor_Acr	breed_growth_background	ENSGALG00010011316	PARPBP	0.109214859	0.117668811	0.928154695	0.353327331	0.517060037	positive
PRJNA729271	:ed_Lushi_vs_Arbor_Acr	breed_growth_background	ENSGALG00010011317	BLK	-0.151819862	0.205779847	-0.737778089	0.46064931	0.615856092	negative
PRJNA729271	:ed_Lushi_vs_Arbor_Acr	breed_growth_background	ENSGALG00010011318	PRP4K	0.105398959	0.052562756	2.005202317	0.044941422	0.12260981	positive
PRJNA729271	:ed_Lushi_vs_Arbor_Acr	breed_growth_background	ENSGALG00010011321	MAP2	-0.295251761	0.084289662	-3.502822919	0.000460355	0.004682703	negative
PRJNA729271	:ed_Lushi_vs_Arbor_Acr	breed_growth_background	ENSGALG00010011322	GATA4	-1.13257812	0.71152672	-1.591757677	0.111439163	0.232351938	negative
PRJNA729271	:ed_Lushi_vs_Arbor_Acr	breed_growth_background	ENSGALG00010011323	GUCY2C	-0.989405941	0.484021113	-2.044137983	0.040939918	0.114295861	negative
PRJNA729271	:ed_Lushi_vs_Arbor_Acr	breed_growth_background	ENSGALG00010011326	COX6C	-0.006784976	0.072483779	-0.093606821	0.925421491	0.958073317	negative
PRJNA729271	:ed_Lushi_vs_Arbor_Acr	breed_growth_background	ENSGALG00010011329	PCDHGC5	-0.159409488	0.062665404	-2.543819681	0.010964767	0.04468212	negative
PRJNA729271	:ed_Lushi_vs_Arbor_Acr	breed_growth_background	ENSGALG00010011333	ENSGALG00010011333	-0.131756651	0.070769628	-1.861768309	0.062635755	0.155423311	negative
PRJNA729271	:ed_Lushi_vs_Arbor_Acr	breed_growth_background	ENSGALG00010011335	VCP	0.194250451	0.092909151	2.090756929	0.036549859	0.105326084	positive
PRJNA729271	:ed_Lushi_vs_Arbor_Acr	breed_growth_background	ENSGALG00010011339	ENSGALG00010011339	-0.558517075	0.166852476	-3.347370609	0.000815821	0.007059893	negative
PRJNA729271	:ed_Lushi_vs_Arbor_Acr	breed_growth_background	ENSGALG00010011340	PKNOX1	0.003021867	0.058635586	0.051536403	0.958898095	0.977373454	positive
PRJNA729271	:ed_Lushi_vs_Arbor_Acr	breed_growth_background	ENSGALG00010011342	RASAL2	-0.063400641	0.035918607	-1.765119698	0.077543651	0.179925599	negative
PRJNA729271	:ed_Lushi_vs_Arbor_Acr	breed_growth_background	ENSGALG00010011343	PXDC1	-0.393601494	0.076236908	-5.162873253	2.43188E-07	1.6472E-05	negative
PRJNA729271	:ed_Lushi_vs_Arbor_Acr	breed_growth_background	ENSGALG00010011345	CRMP1	-0.634688133	0.125718283	-5.048495074	4.45304E-07	2.60269E-05	negative
PRJNA729271	:ed_Lushi_vs_Arbor_Acr	breed_growth_background	ENSGALG00010011347	RGS22	0.515405475	0.232986715	2.212166792	0.026955142	0.084184529	positive
PRJNA729271	:ed_Lushi_vs_Arbor_Acr	breed_growth_background	ENSGALG00010011348	SLC22A23	0.245260702	0.088073473	2.784728401	0.005357258	0.026767821	positive
PRJNA729271	:ed_Lushi_vs_Arbor_Acr	breed_growth_background	ENSGALG00010011349	ENSGALG00010011349	-9.74006E-05	0.049721602	-0.001958919	0.99843701	0.999025066	negative
PRJNA729271	:ed_Lushi_vs_Arbor_Acr	breed_growth_background	ENSGALG00010011351	KANK1	-0.051973184	0.059879852	-0.867957792	0.38541744	0.547225564	negative
PRJNA729271	:ed_Lushi_vs_Arbor_Acr	breed_growth_background	ENSGALG00010011352	NTS	-0.887313169	0.321820297	-2.757169689	0.005830409	0.028378701	negative
PRJNA729271	:ed_Lushi_vs_Arbor_Acr	breed_growth_background	ENSGALG00010011353	ENSGALG00010011353	-0.390679661	0.118056906	-3.30924869	0.000935467	0.007757334	negative
PRJNA729271	:ed_Lushi_vs_Arbor_Acr	breed_growth_background	ENSGALG00010011357	UNC80	-0.631383509	0.229255278	-2.754063137	0.005886041	0.028613353	negative
PRJNA729271	:ed_Lushi_vs_Arbor_Acr	breed_growth_background	ENSGALG00010011359	RNF19A	-0.119638844	0.046560786	-2.569519411	0.010183969	0.04230915	negative
PRJNA729271	:ed_Lushi_vs_Arbor_Acr	breed_growth_background	ENSGALG00010011360	TCF4	-0.036260704	0.092786005	-0.390799286	0.695945602	0.80418778	negative
PRJNA729271	:ed_Lushi_vs_Arbor_Acr	breed_growth_background	ENSGALG00010011361	NUP37	0.014601427	0.050018031	0.291923257	0.770345301	0.856891654	positive
PRJNA729271	:ed_Lushi_vs_Arbor_Acr	breed_growth_background	ENSGALG00010011363	FDFT1	0.156026078	0.07856334	1.9859909	0.047034334	0.126721687	positive
PRJNA729271	:ed_Lushi_vs_Arbor_Acr	breed_growth_background	ENSGALG00010011365	PSMG4	0.101198743	0.096140694	1.052610914	0.292519353	0.45392014	positive
PRJNA729271	:ed_Lushi_vs_Arbor_Acr	breed_growth_background	ENSGALG00010011367	IER3IP1	0.245219838	0.060415832	4.05886716	4.93114E-05	0.000961027	positive
PRJNA729271	:ed_Lushi_vs_Arbor_Acr	breed_growth_background	ENSGALG00010011368	ENSGALG00010011368	-0.139456455	0.160438894	-0.8692185	0.38472763	0.546460265	negative
PRJNA729271	:ed_Lushi_vs_Arbor_Acr	breed_growth_background	ENSGALG00010011370	MGAT4C	0.407410547	0.895874221	0.45476311	0.649279673	0.771097909	positive
PRJNA729271	:ed_Lushi_vs_Arbor_Acr	breed_growth_background	ENSGALG00010011371	ENSGALG00010011371	-0.0707862	0.106269722	-0.666099417	0.505347557	0.655130385	negative
PRJNA729271	:ed_Lushi_vs_Arbor_Acr	breed_growth_background	ENSGALG00010011372	VMA21	-0.050088083	0.049840135	-1.00497488	0.314908946	0.47813414	negative
PRJNA729271	:ed_Lushi_vs_Arbor_Acr	breed_growth_background	ENSGALG00010011374	ENSGALG00010011374	-0.099761975	0.050170626	-1.988453847	0.046761518	0.126197768	negative
PRJNA729271	:ed_Lushi_vs_Arbor_Acr	breed_growth_background	ENSGALG00010011375	C12orf50	-0.196609431	0.073968876	-2.658002138	0.007860539	0.035187931	negative
PRJNA729271	:ed_Lushi_vs_Arbor_Acr	breed_growth_background	ENSGALG00010011377	SEC16B	0.023515762	0.160944651	0.146110862	0.883833872	0.931586095	positive
PRJNA729271	:ed_Lushi_vs_Arbor_Acr	breed_growth_background	ENSGALG00010011378	TUBB2B	-0.183876055	0.104505556	-1.759485928	0.078495008	0.181522838	negative
PRJNA729271	:ed_Lushi_vs_Arbor_Acr	breed_growth_background	ENSGALG00010011379	CELF4	-0.05084959	0.319835336	-0.158986779	0.87367929	0.9253914	negative
PRJNA729271	:ed_Lushi_vs_Arbor_Acr	breed_growth_background	ENSGALG00010011381	RLIG1	0.174362597	0.066118275	2.637131679	0.008361037	0.036712669	positive
PRJNA729271	:ed_Lushi_vs_Arbor_Acr	breed_growth_background	ENSGALG00010011382	ENSGALG00010011382	-0.210869565	0.106270912	-1.984264181	0.047226397	0.126979526	negative
PRJNA729271	:ed_Lushi_vs_Arbor_Acr	breed_growth_background	ENSGALG00010011384	PUM3	0.087190092	0.044551045	1.957082969	0.050337714	0.13317123	positive
PRJNA729271	:ed_Lushi_vs_Arbor_Acr	breed_growth_background	ENSGALG00010011386	CTSB	-0.137629651	0.0673944	-2.042152615	0.041136395	0.114689757	negative
PRJNA729271	:ed_Lushi_vs_Arbor_Acr	breed_growth_background	ENSGALG00010011388	GPR50	0.02153322	0.089207106	0.241384579	0.809257064	0.882565732	positive
PRJNA729271	:ed_Lushi_vs_Arbor_Acr	breed_growth_background	ENSGALG00010011389	RC3H1	0.081551184	0.045966277	1.774152485	0.076037931	0.177798002	positive
PRJNA729271	:ed_Lushi_vs_Arbor_Acr	breed_growth_background	ENSGALG00010011394	SERPINC1	-0.746712213	0.477763682	-1.562932136	0.118068558	0.241791996	negative
PRJNA729271	:ed_Lushi_vs_Arbor_Acr	breed_growth_background	ENSGALG00010011398	ENSGALG00010011398	-0.131677975	0.10044707	-1.310919022	0.189885123	0.336312911	negative
PRJNA729271	:ed_Lushi_vs_Arbor_Acr	breed_growth_background	ENSGALG00010011399	JAKMIP1	-0.37749759	0.141780746	-2.662544811	0.007755224	0.034888881	negative
PRJNA729271	:ed_Lushi_vs_Arbor_Acr	breed_growth_background	ENSGALG00010011400	BPHL	0.141981191	0.106831099	1.329024899	0.183839755	0.329144918	positive
PRJNA729271	:ed_Lushi_vs_Arbor_Acr	breed_growth_background	ENSGALG00010011401	ANKRD46	-0.049936171	0.037128953	-1.34493885	0.178644983	0.322996012	negative
PRJNA729271	:ed_Lushi_vs_Arbor_Acr	breed_growth_background	ENSGALG00010011403	SV2C	-0.263325164	0.32321736	-0.814699941	0.415244087	0.575426677	negative
PRJNA729271	:ed_Lushi_vs_Arbor_Acr	breed_growth_background	ENSGALG00010011406	UBE2R2	0.025290479	0.062373171	0.40547047	0.68513172	0.796623039	positive
PRJNA729271	:ed_Lushi_vs_Arbor_Acr	breed_growth_background	ENSGALG00010011407	CEP290	0.174576307	0.097541757	1.78975971	0.073492549	0.17375216	positive
PRJNA729271	:ed_Lushi_vs_Arbor_Acr	breed_growth_background	ENSGALG00010011411	ENSGALG00010011411	-0.233716713	0.121132551	-1.929429469	0.053677568	0.139311791	negative
PRJNA729271	:ed_Lushi_vs_Arbor_Acr	breed_growth_background	ENSGALG00010011412	KANSL1L	0.117961661	0.06447835	1.82947703	0.067328177	0.163786769	positive
PRJNA729271	:ed_Lushi_vs_Arbor_Acr	breed_growth_background	ENSGALG00010011413	ZBTB37	0.042583037	0.055552346	0.766538945	0.443355688	0.600377494	positive
PRJNA729271	:ed_Lushi_vs_Arbor_Acr	breed_growth_background	ENSGALG00010011414	PABPC1	0.088117685	0.061061261	1.44310293	0.148991478	0.283900549	positive
PRJNA729271	:ed_Lushi_vs_Arbor_Acr	breed_growth_background	ENSGALG00010011416	ENSGALG00010011416	0.356238316	0.234699207	1.517850529	0.129052084	0.256493892	positive
PRJNA729271	:ed_Lushi_vs_Arbor_Acr	breed_growth_background	ENSGALG00010011417	WFS1	-0.041349942	0.077717621	-0.532053621	0.594688858	0.728842056	negative
PRJNA729271	:ed_Lushi_vs_Arbor_Acr	breed_growth_background	ENSGALG00010011418	RIPK1	-0.119316506	0.047788125	-2.496781484	0.012532616	0.048968002	negative
PRJNA729271	:ed_Lushi_vs_Arbor_Acr	breed_growth_background	ENSGALG00010011419	RNF180	-0.007310262	0.080333526	-0.090998896	0.927493468	0.959738565	negative
PRJNA729271	:ed_Lushi_vs_Arbor_Acr	breed_growth_background	ENSGALG00010011420	HMGB3	0.035518069	0.053627955	0.662305107	0.507775704	0.656958809	positive
PRJNA729271	:ed_Lushi_vs_Arbor_Acr	breed_growth_background	ENSGALG00010011421	ENSGALG00010011421	0.102322609	0.062492131	1.637367893	0.101553623	0.217400644	positive
PRJNA729271	:ed_Lushi_vs_Arbor_Acr	breed_growth_background	ENSGALG00010011422	KATNAL2	-0.099705838	0.133267338	-0.748164103	0.45436118	0.610603125	negative
PRJNA729271	:ed_Lushi_vs_Arbor_Acr	breed_growth_background	ENSGALG00010011423	GDF6	-0.666374062	0.225380531	-2.956662045	0.003109888	0.018213262	negative
PRJNA729271	:ed_Lushi_vs_Arbor_Acr	breed_growth_background	ENSGALG00010011424	ACADL	0.148165309	0.05190952	2.854299364	0.004313187	0.022817387	positive
PRJNA729271	:ed_Lushi_vs_Arbor_Acr	breed_growth_background	ENSGALG00010011425	WASHC3	0.123602434	0.043392501	2.848474561	0.004392937	0.023129537	positive
PRJNA729271	:ed_Lushi_vs_Arbor_Acr	breed_growth_background	ENSGALG00010011426	NQO2	-0.042306962	0.068164705	-0.620657888	0.534824743	0.680144292	negative
PRJNA729271	:ed_Lushi_vs_Arbor_Acr	breed_growth_background	ENSGALG00010011427	TMTC3	-0.073443997	0.047403608	-1.549333486	0.121301574	0.246036921	negative
PRJNA729271	:ed_Lushi_vs_Arbor_Acr	breed_growth_background	ENSGALG00010011428	UQCRB	-0.030131693	0.087107786	-0.345912744	0.729408291	0.826191543	negative
PRJNA729271	:ed_Lushi_vs_Arbor_Acr	breed_growth_background	ENSGALG00010011429	UBAP2	0.080165016	0.057502161	1.3941218	0.163280827	0.303080495	positive
PRJNA729271	:ed_Lushi_vs_Arbor_Acr	breed_growth_background	ENSGALG00010011430	ENSGALG00010011430	-0.238955303	0.183692814	-1.300841864	0.193312589	0.340345679	negative
PRJNA729271	:ed_Lushi_vs_Arbor_Acr	breed_growth_background	ENSGALG00010011431	DRAM1	0.111664752	0.222429335	0.502023492	0.615650996	0.744275329	positive
PRJNA729271	:ed_Lushi_vs_Arbor_Acr	breed_growth_background	ENSGALG00010011433	RNASE6	0.140288702	0.173369312	0.809189932	0.418405905	0.578370551	positive
PRJNA729271	:ed_Lushi_vs_Arbor_Acr	breed_growth_background	ENSGALG00010011434	NPL	-0.219621905	0.096273697	-2.28122438	0.022535173	0.074356324	negative
PRJNA729271	:ed_Lushi_vs_Arbor_Acr	breed_growth_background	ENSGALG00010011436	UBAP2L2	2.423448297	1.225435909	1.977621416			positive
PRJNA729271	:ed_Lushi_vs_Arbor_Acr	breed_growth_background	ENSGALG00010011437	DMRT1	0.377276249	0.303625668	1.242570341	0.214026205	0.364677306	positive
PRJNA729271	:ed_Lushi_vs_Arbor_Acr	breed_growth_background	ENSGALG00010011438	ENSGALG00010011438	1.136644128	0.233535995	4.867104665	1.13245E-06	5.41745E-05	positive
PRJNA729271	:ed_Lushi_vs_Arbor_Acr	breed_growth_background	ENSGALG00010011439	ENSGALG00010011439	-0.465377949	0.182329131	-2.552405895	0.010698181	0.043941663	negative
PRJNA729271	:ed_Lushi_vs_Arbor_Acr	breed_growth_background	ENSGALG00010011440	FUNDC1	-0.027400438	0.060026464	-0.45647263	0.648050148	0.770293025	negative
PRJNA729271	:ed_Lushi_vs_Arbor_Acr	breed_growth_background	ENSGALG00010011441	CD99L2	0.091069305	0.042692607	2.133139922	0.03291325	0.097445331	positive
PRJNA729271	:ed_Lushi_vs_Arbor_Acr	breed_growth_background	ENSGALG00010011442	ENSGALG00010011442	1.60907146	1.112652589	1.446158016	0.148132887	0.283120542	positive
PRJNA729271	:ed_Lushi_vs_Arbor_Acr	breed_growth_background	ENSGALG00010011443	ENSGALG00010011443	-0.621249086	0.092878937	-6.688804862	2.25001E-11	7.58461E-09	negative
PRJNA729271	:ed_Lushi_vs_Arbor_Acr	breed_growth_background	ENSGALG00010011445	MTERF3	0.062665184	0.043955516	1.425650074	0.153969375	0.290408079	positive
PRJNA729271	:ed_Lushi_vs_Arbor_Acr	breed_growth_background	ENSGALG00010011446	ENSGALG00010011446	-0.369701364	0.122395817	-3.020539211	0.00252325	0.015683754	negative
PRJNA729271	:ed_Lushi_vs_Arbor_Acr	breed_growth_background	ENSGALG00010011447	LAMC1	-0.089412097	0.069630651	-1.284091058	0.199110096	0.347764893	negative
PRJNA729271	:ed_Lushi_vs_Arbor_Acr	breed_growth_background	ENSGALG00010011448	AvBD10	-0.606954234	1.39207538	-0.43600673	0.662831847	0.781371797	negative
PRJNA729271	:ed_Lushi_vs_Arbor_Acr	breed_growth_background	ENSGALG00010011449	DUSP22	0.071961365	0.077673973	0.92645403	0.354210076	0.517900047	positive
PRJNA729271	:ed_Lushi_vs_Arbor_Acr	breed_growth_background	ENSGALG00010011450	PTDSS1	-0.019047866	0.037839592	-0.503384548	0.614693933	0.743862796	negative
PRJNA729271	:ed_Lushi_vs_Arbor_Acr	breed_growth_background	ENSGALG00010011451	CRHBP	-0.454271534	0.386158445	-1.176386377	0.239440513	0.394980111	negative
PRJNA729271	:ed_Lushi_vs_Arbor_Acr	breed_growth_background	ENSGALG00010011452	KITLG	-0.088735647	0.101141605	-0.877340703	0.38030161	0.542032629	negative
PRJNA729271	:ed_Lushi_vs_Arbor_Acr	breed_growth_background	ENSGALG00010011453	GNPTAB	-0.218817303	0.066637485	-3.283696917	0.00102455	0.008297858	negative
PRJNA729271	:ed_Lushi_vs_Arbor_Acr	breed_growth_background	ENSGALG00010011456	KIAA1328	-0.033446437	0.0535038	-0.625122639	0.531890571	0.677542299	negative
PRJNA729271	:ed_Lushi_vs_Arbor_Acr	breed_growth_background	ENSGALG00010011457	ENSGALG00010011457	-0.237068587	0.144585241	-1.639645834	0.101078827	0.216768398	negative
PRJNA729271	:ed_Lushi_vs_Arbor_Acr	breed_growth_background	ENSGALG00010011458	TPGS2	0.089360485	0.072550274	1.231704306	0.218059561	0.370027316	positive
PRJNA729271	:ed_Lushi_vs_Arbor_Acr	breed_growth_background	ENSGALG00010011459	ENSGALG00010011459	-0.030659778	0.151575882	-0.202273461	0.839702946	0.902661985	negative
PRJNA729271	:ed_Lushi_vs_Arbor_Acr	breed_growth_background	ENSGALG00010011460	LAMC2	-0.755465629	0.348834106	-2.165687403	0.030335082	0.09168203	negative
PRJNA729271	:ed_Lushi_vs_Arbor_Acr	breed_growth_background	ENSGALG00010011461	IRF4	0.188067686	0.198814253	0.945946697	0.344175774	0.507975547	positive
PRJNA729271	:ed_Lushi_vs_Arbor_Acr	breed_growth_background	ENSGALG00010011463	IMP3	0.090092138	0.07179279	1.254891158	0.209518274	0.359257796	positive
PRJNA729271	:ed_Lushi_vs_Arbor_Acr	breed_growth_background	ENSGALG00010011464	FANCG	-0.185474857	0.089296903	-2.077058117	0.037796196	0.108100997	negative
PRJNA729271	:ed_Lushi_vs_Arbor_Acr	breed_growth_background	ENSGALG00010011465	PPP2R2C	0.456624688	0.185588381	2.460416358	0.013877592	0.052699945	positive
PRJNA729271	:ed_Lushi_vs_Arbor_Acr	breed_growth_background	ENSGALG00010011466	MTMR1	0.136733905	0.036151539	3.782242963	0.000155422	0.002189344	positive
PRJNA729271	:ed_Lushi_vs_Arbor_Acr	breed_growth_background	ENSGALG00010011467	ENSGALG00010011467	3.560547125	2.142545565	1.661830294			positive
PRJNA729271	:ed_Lushi_vs_Arbor_Acr	breed_growth_background	ENSGALG00010011468	EFHC2	-0.228127324	0.159836994	-1.42724984	0.153507893	0.289952686	negative
PRJNA729271	:ed_Lushi_vs_Arbor_Acr	breed_growth_background	ENSGALG00010011469	CPS1	-0.774467574	0.1770828	-4.373477107	1.22283E-05	0.000335701	negative
PRJNA729271	:ed_Lushi_vs_Arbor_Acr	breed_growth_background	ENSGALG00010011471	NMNAT2	-0.435860702	0.233402102	-1.867424068	0.061842391	0.154191053	negative
PRJNA729271	:ed_Lushi_vs_Arbor_Acr	breed_growth_background	ENSGALG00010011475	NDP	0.167247399	0.181531596	0.92131289	0.356887093	0.520535159	positive
PRJNA729271	:ed_Lushi_vs_Arbor_Acr	breed_growth_background	ENSGALG00010011477	SDC2	-0.260836787	0.089453359	-2.915897084	0.003546675	0.019902845	negative
PRJNA729271	:ed_Lushi_vs_Arbor_Acr	breed_growth_background	ENSGALG00010011478	HDAC3	0.100137045	0.041970954	2.385865321	0.01703899	0.061027961	positive
PRJNA729271	:ed_Lushi_vs_Arbor_Acr	breed_growth_background	ENSGALG00010011480	BLTP3B	0.109328939	0.035627368	3.06867856	0.002150078	0.013962984	positive
PRJNA729271	:ed_Lushi_vs_Arbor_Acr	breed_growth_background	ENSGALG00010011482	DMRT2	-0.032519972	0.084470636	-0.384985529	0.700248137	0.806702465	negative
PRJNA729271	:ed_Lushi_vs_Arbor_Acr	breed_growth_background	ENSGALG00010011484	CHPT1	0.089658455	0.041902934	2.139670123	0.032381435	0.096478706	positive
PRJNA729271	:ed_Lushi_vs_Arbor_Acr	breed_growth_background	ENSGALG00010011485	MTM1	0.074144105	0.041112122	1.803460914	0.071315859	0.169936441	positive
PRJNA729271	:ed_Lushi_vs_Arbor_Acr	breed_growth_background	ENSGALG00010011487	MAN2B2	-0.131909862	0.085342242	-1.545657339	0.122187345	0.24687839	negative
PRJNA729271	:ed_Lushi_vs_Arbor_Acr	breed_growth_background	ENSGALG00010011493	ENSGALG00010011493	0.193526928	0.14101027	1.372431441	0.169929168	0.312182927	positive
PRJNA729271	:ed_Lushi_vs_Arbor_Acr	breed_growth_background	ENSGALG00010011496	ACTR6	0.13689228	0.047653761	2.872643757	0.004070528	0.021896896	positive
PRJNA729271	:ed_Lushi_vs_Arbor_Acr	breed_growth_background	ENSGALG00010011497	CPQ	-0.03316754	0.133246792	-0.24891811	0.803424126	0.878584135	negative
PRJNA729271	:ed_Lushi_vs_Arbor_Acr	breed_growth_background	ENSGALG00010011499	SMG7	0.206918114	0.078947902	2.620945073	0.008768639	0.037952051	positive
PRJNA729271	:ed_Lushi_vs_Arbor_Acr	breed_growth_background	ENSGALG00010011503	NCF2	-0.093992644	0.209625325	-0.448384011	0.653876079	0.77453083	negative
PRJNA729271	:ed_Lushi_vs_Arbor_Acr	breed_growth_background	ENSGALG00010011505	MYL1	-0.023510333	0.081326444	-0.289085959	0.772515598	0.858515188	negative
PRJNA729271	:ed_Lushi_vs_Arbor_Acr	breed_growth_background	ENSGALG00010011509	EXOC2	0.048660739	0.036410541	1.336446488	0.18140338	0.326031245	positive
PRJNA729271	:ed_Lushi_vs_Arbor_Acr	breed_growth_background	ENSGALG00010011510	ENSGALG00010011510	0.146990215	0.142626307	1.030596795	0.302729939	0.465281569	positive
PRJNA729271	:ed_Lushi_vs_Arbor_Acr	breed_growth_background	ENSGALG00010011515	SERINC5	-0.17589794	0.046169874	-3.809798971	0.00013908	0.002027751	negative
PRJNA729271	:ed_Lushi_vs_Arbor_Acr	breed_growth_background	ENSGALG00010011523	PIGO	0.103970301	0.065297929	1.592245007	0.111329664	0.232190429	positive
PRJNA729271	:ed_Lushi_vs_Arbor_Acr	breed_growth_background	ENSGALG00010011525	ENSGALG00010011525	0.023355564	0.143310852	0.162971358	0.870540986	0.923006541	positive
PRJNA729271	:ed_Lushi_vs_Arbor_Acr	breed_growth_background	ENSGALG00010011529	SCYL2	0.192281647	0.044209743	4.34930481	1.3657E-05	0.000363893	positive
PRJNA729271	:ed_Lushi_vs_Arbor_Acr	breed_growth_background	ENSGALG00010011530	ARPC5	-0.204317601	0.064314934	-3.176829814	0.001488944	0.010856258	negative
PRJNA729271	:ed_Lushi_vs_Arbor_Acr	breed_growth_background	ENSGALG00010011531	MTDH	0.12039832	0.038497869	3.127402167	0.001763585	0.012161354	positive
PRJNA729271	:ed_Lushi_vs_Arbor_Acr	breed_growth_background	ENSGALG00010011533	ENSGALG00010011533	-0.155966345	0.147629611	-1.056470603	0.290753265	0.452196198	negative

PRJNA729271	ed_Lushi_vs_Arbor_Acr	breed_growth_background	ENSGALG00010011536	FCHSD1	-0.035124052	0.102354023	-0.343162399	0.731476291	0.827856943	negative
PRJNA729271	ed_Lushi_vs_Arbor_Acr	breed_growth_background	ENSGALG00010011538	ENSGALG00010011538	-0.062149713	0.135901724	-0.45731365	0.64744562	0.769807585	negative
PRJNA729271	ed_Lushi_vs_Arbor_Acr	breed_growth_background	ENSGALG00010011539	LANCL1	0.040384105	0.05729573	0.70483621	0.480912171	0.633768699	positive
PRJNA729271	ed_Lushi_vs_Arbor_Acr	breed_growth_background	ENSGALG00010011544	STOML2	0.135099371	0.049857834	2.709691944	0.006734573	0.031520061	positive
PRJNA729271	ed_Lushi_vs_Arbor_Acr	breed_growth_background	ENSGALG00010011545	USP12-like	0.131456208	0.041441943	3.1720571	0.001513632	0.010981812	positive
PRJNA729271	ed_Lushi_vs_Arbor_Acr	breed_growth_background	ENSGALG00010011546	MAOA	-0.391294798	0.066822042	-5.855774343	4.74793E-09	7.3214E-07	negative
PRJNA729271	ed_Lushi_vs_Arbor_Acr	breed_growth_background	ENSGALG00010011549	KIAA0232	0.124063062	0.076885367	1.613610846	0.106611863	0.225268069	positive
PRJNA729271	ed_Lushi_vs_Arbor_Acr	breed_growth_background	ENSGALG00010011551	GMDS	-0.057664172	0.084943969	-0.678849504	0.497233224	0.648672089	negative
PRJNA729271	ed_Lushi_vs_Arbor_Acr	breed_growth_background	ENSGALG00010011555	ENSGALG00010011555	1.523523304	2.275369969	0.669571685	0.503130869	0.653894194	positive
PRJNA729271	ed_Lushi_vs_Arbor_Acr	breed_growth_background	ENSGALG00010011556	LAPTM4B	-0.049606137	0.077140199	-0.643064668	0.520182139	0.667851206	negative
PRJNA729271	ed_Lushi_vs_Arbor_Acr	breed_growth_background	ENSGALG00010011558	MYBPC1	-0.363304653	0.299287851	-1.213897094			negative
PRJNA729271	ed_Lushi_vs_Arbor_Acr	breed_growth_background	ENSGALG00010011559	MTX3	0.101250842	0.06847386	1.478678756	0.139226193	0.270881998	positive
PRJNA729271	ed_Lushi_vs_Arbor_Acr	breed_growth_background	ENSGALG00010011562	ENSGALG00010011562	0.265546671	0.320209071	0.829291532	0.406939461	0.567608496	positive
PRJNA729271	ed_Lushi_vs_Arbor_Acr	breed_growth_background	ENSGALG00010011564	ARAP3	-0.2025516	0.064941556	-3.118982855	0.001814765	0.012425613	negative
PRJNA729271	ed_Lushi_vs_Arbor_Acr	breed_growth_background	ENSGALG00010011570	RGL1	-0.195432546	0.073348648	-2.664432814	0.007711827	0.034725981	negative
PRJNA729271	ed_Lushi_vs_Arbor_Acr	breed_growth_background	ENSGALG00010011571	ATOSB	0.056188668	0.061801775	0.909175635	0.363257423	0.526488986	positive
PRJNA729271	ed_Lushi_vs_Arbor_Acr	breed_growth_background	ENSGALG00010011572	PTCD2	-0.081965959	0.054957509	-1.491442391	0.135845385	0.266091738	negative
PRJNA729271	ed_Lushi_vs_Arbor_Acr	breed_growth_background	ENSGALG00010011573	CMAS	-0.076739901	0.096306316	-0.796831451	0.425548926	0.584731414	negative
PRJNA729271	ed_Lushi_vs_Arbor_Acr	breed_growth_background	ENSGALG00010011574	GAS2L3	0.064499368	0.080394774	0.802283093	0.42238922	0.581547468	positive
PRJNA729271	ed_Lushi_vs_Arbor_Acr	breed_growth_background	ENSGALG00010011575	ENSGALG00010011575	-1.304184549	0.283870837	-4.594288601	4.34228E-06	0.000154643	negative
PRJNA729271	ed_Lushi_vs_Arbor_Acr	breed_growth_background	ENSGALG00010011577	ENSGALG00010011577	-0.033328505	0.085859374	-0.388175495	0.697886167	0.805308638	negative
PRJNA729271	ed_Lushi_vs_Arbor_Acr	breed_growth_background	ENSGALG00010011578	MYLK4	0.094194513	0.175842666	0.535674957	0.592183215	0.726815893	positive
PRJNA729271	ed_Lushi_vs_Arbor_Acr	breed_growth_background	ENSGALG00010011584	ENSGALG00010011584	-0.978953155	0.55950689	-1.749671313	0.080175047	0.184390772	negative
PRJNA729271	ed_Lushi_vs_Arbor_Acr	breed_growth_background	ENSGALG00010011586	ENSGALG00010011586	-0.272507605	0.135278844	-2.014414057	0.04396608	0.120561545	negative
PRJNA729271	ed_Lushi_vs_Arbor_Acr	breed_growth_background	ENSGALG00010011588	COLGALT2	-0.157695912	0.107031373	-1.473361569	0.140653567	0.273141994	negative
PRJNA729271	ed_Lushi_vs_Arbor_Acr	breed_growth_background	ENSGALG00010011589	RERG	-0.235104776	0.120794397	-1.946321863	0.051616089	0.135489897	negative
PRJNA729271	ed_Lushi_vs_Arbor_Acr	breed_growth_background	ENSGALG00010011593	WRNIP1	-0.017930959	0.056813555	-0.315610576	0.752298109	0.843027529	negative
PRJNA729271	ed_Lushi_vs_Arbor_Acr	breed_growth_background	ENSGALG00010011595	ENSGALG00010011595	-0.009802057	0.087936828	-0.111467028	0.911246011	0.949705991	negative
PRJNA729271	ed_Lushi_vs_Arbor_Acr	breed_growth_background	ENSGALG00010011597	TBC1D14	-0.119795886	0.0699989	-1.711396701	0.087007906	0.195198823	negative
PRJNA729271	ed_Lushi_vs_Arbor_Acr	breed_growth_background	ENSGALG00010011601	ANO4	0.772363645	0.349713615	2.208560408	0.027205232	0.084700253	positive
PRJNA729271	ed_Lushi_vs_Arbor_Acr	breed_growth_background	ENSGALG00010011603	ENSGALG00010011603	-0.023739049	0.150214702	-0.158034127	0.874429907	0.925799908	negative
PRJNA729271	ed_Lushi_vs_Arbor_Acr	breed_growth_background	ENSGALG00010011605	APPL2	-0.304185023	0.076347496	-3.984217411	6.77029E-05	0.001190962	negative
PRJNA729271	ed_Lushi_vs_Arbor_Acr	breed_growth_background	ENSGALG00010011607	TSEN15	0.130712422	0.090075856	1.451137166	0.146741669	0.281240314	positive
PRJNA729271	ed_Lushi_vs_Arbor_Acr	breed_growth_background	ENSGALG00010011609	ENSGALG00010011609	0.006029107	0.467101507	0.012907487	0.989701601	0.993884212	positive
PRJNA729271	ed_Lushi_vs_Arbor_Acr	breed_growth_background	ENSGALG00010011612	ENSGALG00010011612	0.157452366	0.265217151	0.593673391	0.552730583	0.69519148	positive
PRJNA729271	ed_Lushi_vs_Arbor_Acr	breed_growth_background	ENSGALG00010011617	MAMLD1	-0.135670002	0.051473582	-2.635721029	0.008395873	0.036755715	negative
PRJNA729271	ed_Lushi_vs_Arbor_Acr	breed_growth_background	ENSGALG00010011619	PCDH1	-0.272487858	0.085680001	-3.180297109	0.001471241	0.010791029	negative
PRJNA729271	ed_Lushi_vs_Arbor_Acr	breed_growth_background	ENSGALG00010011621	WASHC4	0.10874509	0.047237105	2.302111639	0.021328877	0.071449521	positive
PRJNA729271	ed_Lushi_vs_Arbor_Acr	breed_growth_background	ENSGALG00010011622	BACH1	0.095741564	0.085607585	1.118377122	0.263405961	0.421466984	positive
PRJNA729271	ed_Lushi_vs_Arbor_Acr	breed_growth_background	ENSGALG00010011625	ERBB4	0.40016034	0.115294399	3.470770003	0.000518968	0.005099963	positive
PRJNA729271	ed_Lushi_vs_Arbor_Acr	breed_growth_background	ENSGALG00010011626	SERPINB1	0.402215195	0.321459185	1.251216993	0.21085532	0.360960183	positive
PRJNA729271	ed_Lushi_vs_Arbor_Acr	breed_growth_background	ENSGALG00010011628	MATN2	-0.10256108	0.102356552	-1.001998193	0.316344466	0.479694946	negative
PRJNA729271	ed_Lushi_vs_Arbor_Acr	breed_growth_background	ENSGALG00010011630	DELE1	0.28495201	0.054055902	5.271431976	1.35363E-07	1.07218E-05	positive
PRJNA729271	ed_Lushi_vs_Arbor_Acr	breed_growth_background	ENSGALG00010011633	TADA2B	-0.015591491	0.047516858	-0.328125469	0.742816797	0.835957257	negative
PRJNA729271	ed_Lushi_vs_Arbor_Acr	breed_growth_background	ENSGALG00010011634	AVD	0.514690029	0.463953131	1.109357808	0.267275856	0.42563871	positive
PRJNA729271	ed_Lushi_vs_Arbor_Acr	breed_growth_background	ENSGALG00010011635	PCDH12	-0.279282083	0.10603525	-2.633860743	0.00844201	0.036895153	negative
PRJNA729271	ed_Lushi_vs_Arbor_Acr	breed_growth_background	ENSGALG00010011636	GRIK1	-0.421522571	0.215494284	-1.956073097	0.050456544	0.133388219	negative
PRJNA729271	ed_Lushi_vs_Arbor_Acr	breed_growth_background	ENSGALG00010011637	CECR2	0.225847157	0.083894975	2.692022443	0.007102017	0.032742917	positive
PRJNA729271	ed_Lushi_vs_Arbor_Acr	breed_growth_background	ENSGALG00010011638	GRPEL1	0.089015121	0.044044928	2.021007292	0.04327901	0.11917349	positive
PRJNA729271	ed_Lushi_vs_Arbor_Acr	breed_growth_background	ENSGALG00010011639	ENSGALG00010011639	-0.212354167	0.078396846	-2.708708054	0.006754575	0.031593275	negative
PRJNA729271	ed_Lushi_vs_Arbor_Acr	breed_growth_background	ENSGALG00010011641	ENSGALG00010011641	0.937480244	0.331537497	2.827674858	0.00468874	0.024281275	positive
PRJNA729271	ed_Lushi_vs_Arbor_Acr	breed_growth_background	ENSGALG00010011643	C1orf21	0.026108248	0.043138846	0.60521433	0.545036595	0.689020186	positive
PRJNA729271	ed_Lushi_vs_Arbor_Acr	breed_growth_background	ENSGALG00010011647	FBXO38	0.109196734	0.044773951	2.43884517	0.01473428	0.054804564	positive
PRJNA729271	ed_Lushi_vs_Arbor_Acr	breed_growth_background	ENSGALG00010011648	ENSGALG00010011648	-0.541974557	0.12008029	-4.513434791	6.37861E-06	0.000209181	negative
PRJNA729271	ed_Lushi_vs_Arbor_Acr	breed_growth_background	ENSGALG00010011649	MRPS27	0.215019169	0.073480975	2.926188281	0.003431432	0.019469141	positive
PRJNA729271	ed_Lushi_vs_Arbor_Acr	breed_growth_background	ENSGALG00010011650	RPL30	-0.132978256	0.083046402	-1.601252467	0.109321012	0.229487049	negative
PRJNA729271	ed_Lushi_vs_Arbor_Acr	breed_growth_background	ENSGALG00010011651	SERPINB6	-0.233835104	0.06906947	-3.385505963	0.000710472	0.006392481	negative
PRJNA729271	ed_Lushi_vs_Arbor_Acr	breed_growth_background	ENSGALG00010011653	NR2C1	0.032865526	0.048254794	0.681083119	0.495818898	0.647293067	positive
PRJNA729271	ed_Lushi_vs_Arbor_Acr	breed_growth_background	ENSGALG00010011654	CLDN8	-0.415769965	0.736163626	-0.564779283	0.5722239	0.710561589	negative
PRJNA729271	ed_Lushi_vs_Arbor_Acr	breed_growth_background	ENSGALG00010011655	SORCS2	-0.391378107	0.09904888	-3.951363281	7.77073E-05	0.001317388	negative
PRJNA729271	ed_Lushi_vs_Arbor_Acr	breed_growth_background	ENSGALG00010011657	ALDH1L2	-0.099215133	0.076057701	-1.304471898	0.192072735	0.338974975	negative
PRJNA729271	ed_Lushi_vs_Arbor_Acr	breed_growth_background	ENSGALG00010011658	TENM1	0.286862281	0.174366586	1.64516773	0.099935226	0.214920045	positive
PRJNA729271	ed_Lushi_vs_Arbor_Acr	breed_growth_background	ENSGALG00010011659	CD14	-0.06268797	0.082618184	-0.758767227	0.447991813	0.604622098	negative
PRJNA729271	ed_Lushi_vs_Arbor_Acr	breed_growth_background	ENSGALG00010011660	ENSGALG00010011660	0.146950799	0.047662112	3.083178511	0.002048023	0.013505954	positive
PRJNA729271	ed_Lushi_vs_Arbor_Acr	breed_growth_background	ENSGALG00010011662	RIDA	-0.293487534	0.093087344	-3.152818849	0.001617021	0.011439265	negative
PRJNA729271	ed_Lushi_vs_Arbor_Acr	breed_growth_background	ENSGALG00010011663	UTP20	0.136432545	0.073646191	1.852540412	0.06394826	0.157721632	positive
PRJNA729271	ed_Lushi_vs_Arbor_Acr	breed_growth_background	ENSGALG00010011664	SERPINB10B	-0.611629972	0.274438102	-2.228662739	0.025836353	0.081857473	negative
PRJNA729271	ed_Lushi_vs_Arbor_Acr	breed_growth_background	ENSGALG00010011667	ENSGALG00010011667	1.615711891	0.223154531	7.240327528	4.47602E-13	2.70333E-10	positive
PRJNA729271	ed_Lushi_vs_Arbor_Acr	breed_growth_background	ENSGALG00010011669	ENSGALG00010011669	-0.526221188	0.430849633	-1.221356939	0.221950904	0.374395246	negative
PRJNA729271	ed_Lushi_vs_Arbor_Acr	breed_growth_background	ENSGALG00010011670	TMCO6	-0.200704501	0.09209258	-2.179377546	0.029303633	0.089365907	negative
PRJNA729271	ed_Lushi_vs_Arbor_Acr	breed_growth_background	ENSGALG00010011671	SLC41A2	-0.387948514	0.105916092	-3.662791054	0.000249482	0.003039454	negative
PRJNA729271	ed_Lushi_vs_Arbor_Acr	breed_growth_background	ENSGALG00010011673	POP1	0.054047825	0.069052054	0.782711329	0.433796648	0.591800697	positive
PRJNA729271	ed_Lushi_vs_Arbor_Acr	breed_growth_background	ENSGALG00010011674	ENSGALG00010011674	0.41634125	0.283569644	1.468215162	0.142045787	0.274856986	positive
PRJNA729271	ed_Lushi_vs_Arbor_Acr	breed_growth_background	ENSGALG00010011677	EDEM3	-0.034573024	0.043340532	-0.797706498	0.425040832	0.584199399	negative
PRJNA729271	ed_Lushi_vs_Arbor_Acr	breed_growth_background	ENSGALG00010011679	OVAL	-0.778463288	0.655396057	-1.18777536	0.234921924	0.389342933	negative
PRJNA729271	ed_Lushi_vs_Arbor_Acr	breed_growth_background	ENSGALG00010011681	MAP1B	-0.632337718	0.19198845	-3.293623752	0.000989048	0.008085384	negative
PRJNA729271	ed_Lushi_vs_Arbor_Acr	breed_growth_background	ENSGALG00010011682	USP15	0.070754878	0.065513875	1.079998361	0.28014291	0.439959615	positive
PRJNA729271	ed_Lushi_vs_Arbor_Acr	breed_growth_background	ENSGALG00010011683	NIPAL2	0.727673481	0.154443277	4.711590526	2.45791E-06	9.74871E-05	positive
PRJNA729271	ed_Lushi_vs_Arbor_Acr	breed_growth_background	ENSGALG00010011685	NDUFA2	0.012912973	0.059566951	0.216780826	0.828379156	0.895336355	positive
PRJNA729271	ed_Lushi_vs_Arbor_Acr	breed_growth_background	ENSGALG00010011688	PDE3A	-0.205935357	0.079389939	-2.593972982	0.009487394	0.040328381	negative
PRJNA729271	ed_Lushi_vs_Arbor_Acr	breed_growth_background	ENSGALG00010011692	ARL1	-0.113680331	0.048274797	-2.35485883	0.018529744	0.06481386	negative
PRJNA729271	ed_Lushi_vs_Arbor_Acr	breed_growth_background	ENSGALG00010011696	ZFC3H1	0.000291302	0.05848245	0.004981019	0.996025738	0.997974018	positive
PRJNA729271	ed_Lushi_vs_Arbor_Acr	breed_growth_background	ENSGALG00010011699	TIAM1	-0.07430374	0.078798149	-0.942963012	0.345699823	0.509654081	negative
PRJNA729271	ed_Lushi_vs_Arbor_Acr	breed_growth_background	ENSGALG00010011702	NUP205	0.0718905	0.044969048	1.598666274	0.109894775	0.23022471	positive
PRJNA729271	ed_Lushi_vs_Arbor_Acr	breed_growth_background	ENSGALG00010011703	YWHAZ	-0.168294492	0.050632558	-3.32383945	0.000887873	0.007504268	negative
PRJNA729271	ed_Lushi_vs_Arbor_Acr	breed_growth_background	ENSGALG00010011708	IK	0.087895556	0.049127528	1.789130439	0.073593811	0.173934826	positive
PRJNA729271	ed_Lushi_vs_Arbor_Acr	breed_growth_background	ENSGALG00010011712	DCAF12L2	-0.002622432	0.040950646	-0.064038855	0.948939289	0.971869921	negative
PRJNA729271	ed_Lushi_vs_Arbor_Acr	breed_growth_background	ENSGALG00010011713	AVPR1A	-0.329663097	0.422523797	-0.780223739	0.435259192	0.593293397	negative
PRJNA729271	ed_Lushi_vs_Arbor_Acr	breed_growth_background	ENSGALG00010011715	SLCO1B3	-2.228773692	0.961729842	-2.317463382	0.020478503	0.069344457	negative
PRJNA729271	ed_Lushi_vs_Arbor_Acr	breed_growth_background	ENSGALG00010011720	IKZF2	0.200637531	0.096478545	2.079607771	0.037561524	0.10762093	positive
PRJNA729271	ed_Lushi_vs_Arbor_Acr	breed_growth_background	ENSGALG00010011723	SPRY3	-0.128508439	0.08294679	-1.549287672	0.121312582	0.246036921	negative
PRJNA729271	ed_Lushi_vs_Arbor_Acr	breed_growth_background	ENSGALG00010011724	ARHGEF28	-0.19387961	0.083136618	-2.332060339	0.019697517	0.067545661	negative
PRJNA729271	ed_Lushi_vs_Arbor_Acr	breed_growth_background	ENSGALG00010011726	WDR55	0.085556543	0.047738297	1.792199306	0.073101046	0.172995863	positive
PRJNA729271	ed_Lushi_vs_Arbor_Acr	breed_growth_background	ENSGALG00010011729	FAM129A	-0.501872339	0.124388362	-4.034721006	5.46672E-05	0.001033118	negative
PRJNA729271	ed_Lushi_vs_Arbor_Acr	breed_growth_background	ENSGALG00010011734	ENSGALG00010011734	-0.276441448	0.163027321	-1.695675586	0.089947334	0.200183754	negative
PRJNA729271	ed_Lushi_vs_Arbor_Acr	breed_growth_background	ENSGALG00010011738	FGD6	-0.115323527	0.062904699	-1.833305436	0.066757161	0.162766199	negative
PRJNA729271	ed_Lushi_vs_Arbor_Acr	breed_growth_background	ENSGALG00010011745	AFAP1L1	-0.230630499	0.102731648	-2.244980047	0.024769414	0.079519967	negative
PRJNA729271	ed_Lushi_vs_Arbor_Acr	breed_growth_background	ENSGALG00010011746	SOD1	0.100059077	0.076404035	1.309604615	0.190329626	0.336935507	positive
PRJNA729271	ed_Lushi_vs_Arbor_Acr	breed_growth_background	ENSGALG00010011747	UTP15	0.105067625	0.063151692	1.663734128	0.096165573	0.209014842	positive
PRJNA729271	ed_Lushi_vs_Arbor_Acr	breed_growth_background	ENSGALG00010011751	ZNF706	0.17993465	0.0584613	3.077842085	0.002085054	0.013650792	positive
PRJNA729271	ed_Lushi_vs_Arbor_Acr	breed_growth_background	ENSGALG00010011752	RNF2	0.019795119	0.070594981	0.280404061	0.779167521	0.863194224	positive
PRJNA729271	ed_Lushi_vs_Arbor_Acr	breed_growth_background	ENSGALG00010011758	SERPINB11	-0.180874817	0.265754062	-0.680609795	0.496118428	0.647625775	negative
PRJNA729271	ed_Lushi_vs_Arbor_Acr	breed_growth_background	ENSGALG00010011759	CREB3	0.288641728	0.0701232	4.116208729	3.85156E-05	0.00080098	positive
PRJNA729271	ed_Lushi_vs_Arbor_Acr	breed_growth_background	ENSGALG00010011764	PCYOX1L	-0.093653078	0.071870746	-1.303076461	0.192548659	0.339407382	negative
PRJNA729271	ed_Lushi_vs_Arbor_Acr	breed_growth_background	ENSGALG00010011770	CHST11	0.241937705	0.170905596	1.415621907	0.156886234	0.294339368	positive
PRJNA729271	ed_Lushi_vs_Arbor_Acr	breed_growth_background	ENSGALG00010011772	GRHL2	-0.547180739	0.25994922	-2.10495242	0.03529545	0.102608814	negative
PRJNA729271	ed_Lushi_vs_Arbor_Acr	breed_growth_background	ENSGALG00010011774	ANKRA2	0.003615556	0.050813313	0.071153725	0.94327541	0.969219329	positive
PRJNA729271	ed_Lushi_vs_Arbor_Acr	breed_growth_background	ENSGALG00010011775	VEZT	0.010411198	0.043329351	0.240280503	0.810112808	0.883106212	positive
PRJNA729271	ed_Lushi_vs_Arbor_Acr	breed_growth_background	ENSGALG00010011776	IL17RA	-0.106492657	0.074689286	-1.425808998	0.153923483	0.290408079	negative
PRJNA729271	ed_Lushi_vs_Arbor_Acr	breed_growth_background	ENSGALG00010011780	SERPINB5	0.074641306	0.470588875	0.15861256	0.873974133	0.925568426	positive
PRJNA729271	ed_Lushi_vs_Arbor_Acr	breed_growth_background	ENSGALG00010011784	GRPEL2	0.031404212	0.071750138	0.437688529	0.661612086	0.780568711	positive
PRJNA729271	ed_Lushi_vs_Arbor_Acr	breed_growth_background	ENSGALG00010011789	TXNRD1	-0.356144654	0.073732265	-4.830241582	1.36367E-06	6.29505E-05	negative
PRJNA729271	ed_Lushi_vs_Arbor_Acr	breed_growth_background	ENSGALG00010011790	AMY2A	-1.018799447	0.595442351	-1.710995943	0.087081862	0.195304284	negative
PRJNA729271	ed_Lushi_vs_Arbor_Acr	breed_growth_background	ENSGALG00010011792	HDHD5	0.213662626	0.07550299	2.829856498	0.004656889	0.024181738	positive
PRJNA729271	ed_Lushi_vs_Arbor_Acr	breed_growth_background	ENSGALG00010011793	TLN1	-0.122896858	0.061027774	-2.013785668	0.044032041	0.120696754	negative
PRJNA729271	ed_Lushi_vs_Arbor_Acr	breed_growth_background	ENSGALG00010011796	METAP2	0.110011791	0.047948956	2.294352198	0.021770277	0.072516387	positive
PRJNA729271	ed_Lushi_vs_Arbor_Acr	breed_growth_background	ENSGALG00010011797	SCAF4	0.217560502	0.083941337	2.591816003	0.009547082	0.040498961	positive
PRJNA729271	ed_Lushi_vs_Arbor_Acr	breed_growth_background	ENSGALG00010011802	VPS4B	-0.079598666	0.050029172	-1.591045026	0.111599443	0.232560026	negative
PRJNA729271	ed_Lushi_vs_Arbor_Acr	breed_growth_background	ENSGALG00010011808	SMARCA2	0.204182748	0.065593732	3.112839314	0.001852969	0.012574336	positive
PRJNA729271	ed_Lushi_vs_Arbor_Acr	breed_growth_background	ENSGALG00010011810	FGFR4	-0.020196198	0.067481668	-0.299284219	0.764723194	0.852392778	negative
PRJNA729271	ed_Lushi_vs_Arbor_Acr	breed_growth_background	ENSGALG00010011814	ENSGALG00010011814	0.134407725	0.278602221	0.482435941	0.629496294	0.755593639	positive
PRJNA729271	ed_Lushi_vs_Arbor_Acr	breed_growth_background	ENSGALG00010011817	KDSR	0.088696646	0.048453433	1.830554393	0.067167081	0.16354558	positive
PRJNA729271	ed_Lushi_vs_Arbor_Acr	breed_growth_background	ENSGALG00010011819	USP44	0.131818622	0.075202364	1.752852106	0.079627396	0.183555838	positive
PRJNA729271	ed_Lushi_vs_Arbor_Acr	breed_growth_background	ENSGALG00010011823	ENSGALG00010011823	-0.09006218	0.372723239	-0.241632854	0.809064663	0.882536092	negative
PRJNA729271	ed_Lushi_vs_Arbor_Acr	breed_growth_background	ENSGALG00010011825	TAFA2	0.53372908	0.243246682	2.194188533	0.028221859	0.087065688	positive
PRJNA729271	ed_Lushi_vs_Arbor_Acr	breed_growth_background	ENSGALG00010011837	VLDLR	0.162820376	0.071806774	2.267479323	0.023360961	0.076316683	positive
PRJNA729271	ed_Lushi_vs_Arbor_Acr	breed_growth_background	ENSGALG00010011839	CBS	1.144326754	0.246355703	4.645018331	3.40046E-06	0.000125419	positive
PRJNA729271	ed_Lushi_vs_Arbor_Acr	breed_growth_background	ENSGALG00010011841	KCNMB4	-0.305335904	0.144110029	-2.11876929	0.034109969	0.100025087	negative
PRJNA729271	ed_Lushi_vs_Arbor_Acr	breed_growth_background	ENSGALG00010011843	BCL2	0.05952338	0.059725349	0.996618372	0.318949785	0.48219851	positive
PRJNA729271	ed_Lushi_vs_Arbor_Acr	breed_growth_background	ENSGALG00010011846	UBAP1	0.0973357	0.056714913	1.716227616	0.086120391	0.193988355	positive
PRJNA729271	ed_Lushi_vs_Arbor_Acr	breed_growth_background	ENSGALG00010011850	SPAG16	-0.968434526	0.200955027	-4.81916048	1.44164E-06	6.5825E-05	negative

PRJNA729271	eed_Lushi_vs_Arbor_Acr	breed_growth_background	ENSGALG00010011856	NFYB	0.220831645	0.069279352	3.18755354	0.001434819	0.010596237	positive
PRJNA729271	eed_Lushi_vs_Arbor_Acr	breed_growth_background	ENSGALG00010011857	ENSGALG00010011857	0.08215057	0.130824803	0.627943389	0.530041026	0.675839608	positive
PRJNA729271	eed_Lushi_vs_Arbor_Acr	breed_growth_background	ENSGALG00010011859	NTN4	-0.191146338	0.105552894	-1.810905709	0.070155449	0.168110965	negative
PRJNA729271	eed_Lushi_vs_Arbor_Acr	breed_growth_background	ENSGALG00010011860	CECR1	-0.219309086	0.083802625	-2.616971549	0.008871374	0.038328036	negative
PRJNA729271	eed_Lushi_vs_Arbor_Acr	breed_growth_background	ENSGALG00010011861	ENSGALG00010011861	0.377273917	0.258876994	1.457348185	0.145020292	0.278862979	positive
PRJNA729271	eed_Lushi_vs_Arbor_Acr	breed_growth_background	ENSGALG00010011866	KLF8	0.242486676	0.100256696	2.418658159	0.015577872	0.057282981	positive
PRJNA729271	eed_Lushi_vs_Arbor_Acr	breed_growth_background	ENSGALG00010011867	ENSGALG00010011867	0.062347365	0.148582773	0.419613684	0.67476769	0.789726094	positive
PRJNA729271	eed_Lushi_vs_Arbor_Acr	breed_growth_background	ENSGALG00010011868	SNRPF	-0.048841135	0.067723168	-0.721187989	0.470793861	0.625094533	negative
PRJNA729271	eed_Lushi_vs_Arbor_Acr	breed_growth_background	ENSGALG00010011870	AvBD6	1.40179436	0.704247168	1.990486329	0.046537388	0.125811693	positive
PRJNA729271	eed_Lushi_vs_Arbor_Acr	breed_growth_background	ENSGALG00010011871	CCT2	0.048568019	0.052673741	0.922053729	0.356500551	0.520128383	positive
PRJNA729271	eed_Lushi_vs_Arbor_Acr	breed_growth_background	ENSGALG00010011873	RFX3	0.124451954	0.104492456	1.191013778	0.233648176	0.388030512	positive
PRJNA729271	eed_Lushi_vs_Arbor_Acr	breed_growth_background	ENSGALG00010011874	KCNS2	-0.376107976	0.447760833	-0.839975156	0.400922316	0.561913142	negative
PRJNA729271	eed_Lushi_vs_Arbor_Acr	breed_growth_background	ENSGALG00010011875	PSD2	-0.285939824	0.158882063	-1.799698581	0.071908245	0.170870494	negative
PRJNA729271	eed_Lushi_vs_Arbor_Acr	breed_growth_background	ENSGALG00010011876	AMDHD1	-0.273101861	0.248179964	-1.100418651	0.271149756	0.429871564	negative
PRJNA729271	eed_Lushi_vs_Arbor_Acr	breed_growth_background	ENSGALG00010011880	U2AF1	0.267726338	0.083849047	3.192956263	0.001408243	0.010466254	positive
PRJNA729271	eed_Lushi_vs_Arbor_Acr	breed_growth_background	ENSGALG00010011881	RIOK2	0.009292909	0.052688416	0.176374801	0.8599995	0.915854291	positive
PRJNA729271	eed_Lushi_vs_Arbor_Acr	breed_growth_background	ENSGALG00010011882	ENSGALG00010011882	0.257008525	0.158581488	1.620671672	0.105088072	0.222762738	positive
PRJNA729271	eed_Lushi_vs_Arbor_Acr	breed_growth_background	ENSGALG00010011883	HCFC2	0.132627813	0.057209243	2.318293424	0.02043338	0.069298511	positive
PRJNA729271	eed_Lushi_vs_Arbor_Acr	breed_growth_background	ENSGALG00010011885	NCALD	-0.393421954	0.114464936	-3.437052133	0.000588082	0.00553523	negative
PRJNA729271	eed_Lushi_vs_Arbor_Acr	breed_growth_background	ENSGALG00010011886	NTNG1	-0.056288391	0.10664387	-0.527816472	0.597626723	0.730959358	negative
PRJNA729271	eed_Lushi_vs_Arbor_Acr	breed_growth_background	ENSGALG00010011887	PGM5	0.031241642	0.07062322	0.442370691	0.658220984	0.777580535	positive
PRJNA729271	eed_Lushi_vs_Arbor_Acr	breed_growth_background	ENSGALG00010011888	CAND1	-0.074794183	0.038100743	-1.963063654	0.049638778	0.131825017	negative
PRJNA729271	eed_Lushi_vs_Arbor_Acr	breed_growth_background	ENSGALG00010011891	HAL	-0.777631943	0.308373936	-2.521717478	0.011678346	0.046658662	negative
PRJNA729271	eed_Lushi_vs_Arbor_Acr	breed_growth_background	ENSGALG00010011893	LIX1	0.973231777	0.289369675	3.363281853	0.000770217	0.006754244	positive
PRJNA729271	eed_Lushi_vs_Arbor_Acr	breed_growth_background	ENSGALG00010011894	VWC2L	-0.62681621	0.194260842	-3.226672971	0.001252385	0.009615107	negative
PRJNA729271	eed_Lushi_vs_Arbor_Acr	breed_growth_background	ENSGALG00010011895	PHF21B	0.836533313	0.447758861	1.868267467	0.061724799	0.15401979	positive
PRJNA729271	eed_Lushi_vs_Arbor_Acr	breed_growth_background	ENSGALG00010011896	SIK1	0.668835034	0.159157255	4.202353427	2.64154E-05	0.000596304	positive
PRJNA729271	eed_Lushi_vs_Arbor_Acr	breed_growth_background	ENSGALG00010011898	ENSGALG00010011898	0.032601535	0.059119636	0.551450189	0.581325103	0.717621136	positive
PRJNA729271	eed_Lushi_vs_Arbor_Acr	breed_growth_background	ENSGALG00010011899	PHLPP1	0.035409549	0.045893893	0.771552517	0.440379506	0.597743323	positive
PRJNA729271	eed_Lushi_vs_Arbor_Acr	breed_growth_background	ENSGALG00010011902	TP53I3	0.188990331	0.141603998	1.334639795	0.181994267	0.326728622	positive
PRJNA729271	eed_Lushi_vs_Arbor_Acr	breed_growth_background	ENSGALG00010011903	LTA4H	-0.325487054	0.06224284	-5.229309195	1.70145E-07	1.27126E-05	negative
PRJNA729271	eed_Lushi_vs_Arbor_Acr	breed_growth_background	ENSGALG00010011904	KIF24	-0.632109744	0.181394745	-3.484719163	0.000492654	0.004921445	negative
PRJNA729271	eed_Lushi_vs_Arbor_Acr	breed_growth_background	ENSGALG00010011906	ENSGALG00010011906	-0.899500821	0.522515429	-1.721481837	0.085163425	0.192520875	negative
PRJNA729271	eed_Lushi_vs_Arbor_Acr	breed_growth_background	ENSGALG00010011907	BIK	0.055385038	0.134372287	0.41217605	0.68021039	0.793358386	positive
PRJNA729271	eed_Lushi_vs_Arbor_Acr	breed_growth_background	ENSGALG00010011908	ENSGALG00010011908	-0.016051264	0.113750358	-0.141109565	0.887783387	0.934041126	negative
PRJNA729271	eed_Lushi_vs_Arbor_Acr	breed_growth_background	ENSGALG00010011909	C8orf48	-0.236758353	0.115164561	-2.055826477	0.039799245	0.111974002	negative
PRJNA729271	eed_Lushi_vs_Arbor_Acr	breed_growth_background	ENSGALG00010011911	NUDT2	-0.142282584	0.074856448	-1.900739199	0.057336181	0.146061151	negative
PRJNA729271	eed_Lushi_vs_Arbor_Acr	breed_growth_background	ENSGALG00010011912	TCP11X2	0.055601676	0.067003473	0.829832748	0.406633353	0.567419558	positive
PRJNA729271	eed_Lushi_vs_Arbor_Acr	breed_growth_background	ENSGALG00010011913	CXXC5	-0.012905693	0.059643359	-0.216381055	0.828690733	0.895563428	negative
PRJNA729271	eed_Lushi_vs_Arbor_Acr	breed_growth_background	ENSGALG00010011915	RRM2B	0.12498322	0.065464208	1.909184031	0.056238356	0.144253223	positive
PRJNA729271	eed_Lushi_vs_Arbor_Acr	breed_growth_background	ENSGALG00010011916	MCAT	0.067756997	0.052757813	1.284302594	0.1990361	0.347719448	positive
PRJNA729271	eed_Lushi_vs_Arbor_Acr	breed_growth_background	ENSGALG00010011917	HSF2BP	0.019691113	0.096914646	0.20317995	0.838994384	0.902332995	positive
PRJNA729271	eed_Lushi_vs_Arbor_Acr	breed_growth_background	ENSGALG00010011921	MEGF10	-0.064638096	0.089499746	-0.722215412	0.470162049	0.624586688	negative
PRJNA729271	eed_Lushi_vs_Arbor_Acr	breed_growth_background	ENSGALG00010011922	PIK3R1	-0.337886139	0.092552376	-3.650755949	0.00026147	0.003153469	negative
PRJNA729271	eed_Lushi_vs_Arbor_Acr	breed_growth_background	ENSGALG00010011923	BARD1	-0.103733076	0.090328193	-1.148401987	0.250802654	0.40673355	negative
PRJNA729271	eed_Lushi_vs_Arbor_Acr	breed_growth_background	ENSGALG00010011925	RRP1B	-0.072436012	0.2389237	-0.303176335	0.761755483	0.850202711	negative
PRJNA729271	eed_Lushi_vs_Arbor_Acr	breed_growth_background	ENSGALG00010011926	DYRK2	0.205100365	0.10415733	1.969139998	0.048937017	0.130609843	positive
PRJNA729271	eed_Lushi_vs_Arbor_Acr	breed_growth_background	ENSGALG00010011927	ENSGALG00010011927	0.617706985	0.280181049	2.204670831	0.027477201	0.085303499	positive
PRJNA729271	eed_Lushi_vs_Arbor_Acr	breed_growth_background	ENSGALG00010011929	ELK3	-0.328652818	0.073194077	-4.490155902	7.11711E-06	0.000224755	negative
PRJNA729271	eed_Lushi_vs_Arbor_Acr	breed_growth_background	ENSGALG00010011930	TSPO	-0.024722317	0.081327189	-0.303985882	0.761138647	0.849780844	negative
PRJNA729271	eed_Lushi_vs_Arbor_Acr	breed_growth_background	ENSGALG00010011931	ENSGALG00010011931	1.690410303	0.695747722	2.429631099			positive
PRJNA729271	eed_Lushi_vs_Arbor_Acr	breed_growth_background	ENSGALG00010011932	VAV3	-0.180765532	0.089911012	-2.010493802	0.044378952	0.121463607	negative
PRJNA729271	eed_Lushi_vs_Arbor_Acr	breed_growth_background	ENSGALG00010011935	ST8SIA4	-0.424808189	0.068020268	-6.245317755	4.22939E-10	9.73096E-08	negative
PRJNA729271	eed_Lushi_vs_Arbor_Acr	breed_growth_background	ENSGALG00010011936	CKAP4	-0.17775582	0.093968235	-1.891658594	0.058536484	0.14810374	negative
PRJNA729271	eed_Lushi_vs_Arbor_Acr	breed_growth_background	ENSGALG00010011937	SLC1A1	0.137958518	0.178126991	0.774495302	0.438637946	0.595993347	positive
PRJNA729271	eed_Lushi_vs_Arbor_Acr	breed_growth_background	ENSGALG00010011941	ENSGALG00010011941	-0.306390508	0.133264511	-2.299115542	0.021498379	0.071845334	negative
PRJNA729271	eed_Lushi_vs_Arbor_Acr	breed_growth_background	ENSGALG00010011943	TTLL12	-0.009898315	0.030204076	-0.327714538	0.74312751	0.836047288	negative
PRJNA729271	eed_Lushi_vs_Arbor_Acr	breed_growth_background	ENSGALG00010011945	ENSGALG00010011945	0.075566596	0.264747778	0.285428632	0.775315775	0.860505525	positive
PRJNA729271	eed_Lushi_vs_Arbor_Acr	breed_growth_background	ENSGALG00010011947	THAP1	0.017279385	0.063221338	0.273315711	0.784610539	0.866703955	positive
PRJNA729271	eed_Lushi_vs_Arbor_Acr	breed_growth_background	ENSGALG00010011949	ABCA12	-0.613818183	0.240808269	-2.5489913	0.0108035	0.044223871	negative
PRJNA729271	eed_Lushi_vs_Arbor_Acr	breed_growth_background	ENSGALG00010011950	PDXK	0.024242627	0.088249179	0.274706544	0.783541703	0.865856293	positive
PRJNA729271	eed_Lushi_vs_Arbor_Acr	breed_growth_background	ENSGALG00010011952	UBE2D2	0.164971449	0.092885752	1.77606839	0.075721645	0.177315873	positive
PRJNA729271	eed_Lushi_vs_Arbor_Acr	breed_growth_background	ENSGALG00010011953	CDK17	-0.016778629	0.031724469	-0.528886061	0.596884492	0.730544686	negative
PRJNA729271	eed_Lushi_vs_Arbor_Acr	breed_growth_background	ENSGALG00010011954	ENSGALG00010011954	0.371074402	0.234611235	1.581656573	0.113727992	0.235498177	positive
PRJNA729271	eed_Lushi_vs_Arbor_Acr	breed_growth_background	ENSGALG00010011957	ENSGALG00010011957	0.213376276	0.111582137	1.912279894	0.055840303	0.143486119	positive
PRJNA729271	eed_Lushi_vs_Arbor_Acr	breed_growth_background	ENSGALG00010011960	CFAP54	-0.695143426	0.162039305	-4.289967947	1.78699E-05	0.000441268	negative
PRJNA729271	eed_Lushi_vs_Arbor_Acr	breed_growth_background	ENSGALG00010011961	AGPAT3	-0.151408527	0.055247543	-2.740547719	0.006133687	0.02948982	negative
PRJNA729271	eed_Lushi_vs_Arbor_Acr	breed_growth_background	ENSGALG00010011962	CPSF6	-0.015592973	0.032588554	-0.478480061	0.632308563	0.757776984	negative
PRJNA729271	eed_Lushi_vs_Arbor_Acr	breed_growth_background	ENSGALG00010011963	ZCCHC2	-0.014433861	0.05423976	-0.266112188	0.790152809	0.869780146	negative
PRJNA729271	eed_Lushi_vs_Arbor_Acr	breed_growth_background	ENSGALG00010011964	ENSGALG00010011964	1.805708567	2.328004777	0.775646418	0.437957785	0.595348222	positive
PRJNA729271	eed_Lushi_vs_Arbor_Acr	breed_growth_background	ENSGALG00010011965	ATIC	-0.301663591	0.078591836	-3.838357882	0.00012386	0.001866267	negative
PRJNA729271	eed_Lushi_vs_Arbor_Acr	breed_growth_background	ENSGALG00010011966	SLC25A24	-0.33547473	0.074042861	-4.53081804	5.87557E-06	0.000194889	negative
PRJNA729271	eed_Lushi_vs_Arbor_Acr	breed_growth_background	ENSGALG00010011967	MTMR9	0.253974731	0.073624451	3.449597628	0.000561423	0.005378874	positive
PRJNA729271	eed_Lushi_vs_Arbor_Acr	breed_growth_background	ENSGALG00010011968	ENSGALG00010011968	0.084347396	0.090415716	0.932884235	0.350879743	0.514829626	positive
PRJNA729271	eed_Lushi_vs_Arbor_Acr	breed_growth_background	ENSGALG00010011969	TMEM173	0.21796642	0.155580107	1.400991583	0.16121659	0.300325726	positive
PRJNA729271	eed_Lushi_vs_Arbor_Acr	breed_growth_background	ENSGALG00010011971	RNF170	0.082411714	0.049470082	1.665889978	0.09573533	0.208419674	positive
PRJNA729271	eed_Lushi_vs_Arbor_Acr	breed_growth_background	ENSGALG00010011972	IPO11	0.012859023	0.044195229	0.290959531	0.771082271	0.857250922	positive
PRJNA729271	eed_Lushi_vs_Arbor_Acr	breed_growth_background	ENSGALG00010011973	TRAPPC10	0.057557791	0.027250392	2.112182086	0.034670834	0.101198902	positive
PRJNA729271	eed_Lushi_vs_Arbor_Acr	breed_growth_background	ENSGALG00010011976	TNFRSF11A	0.169737071	0.048753737	3.481519172	0.000498578	0.004960945	positive
PRJNA729271	eed_Lushi_vs_Arbor_Acr	breed_growth_background	ENSGALG00010011977	GATD3A	-0.056410505	0.063440643	-0.88918558	0.373903351	0.53618214	negative
PRJNA729271	eed_Lushi_vs_Arbor_Acr	breed_growth_background	ENSGALG00010011978	ENSGALG00010011978	1.616458631	2.922193266	0.55316623	0.580149589	0.716871198	positive
PRJNA729271	eed_Lushi_vs_Arbor_Acr	breed_growth_background	ENSGALG00010011979	NEDD1	-0.009758783	0.041815117	-0.233379294	0.81546689	0.886440088	negative
PRJNA729271	eed_Lushi_vs_Arbor_Acr	breed_growth_background	ENSGALG00010011980	AvBD9	-3.117160676	1.199102481	-2.599578206	0.009333841	0.039827501	negative
PRJNA729271	eed_Lushi_vs_Arbor_Acr	breed_growth_background	ENSGALG00010011981	SCUBE1	0.080613859	0.19064917	0.422838764	0.672412903	0.788031621	positive
PRJNA729271	eed_Lushi_vs_Arbor_Acr	breed_growth_background	ENSGALG00010011982	STK3	-0.101860637	0.056635062	-1.798543744	0.072090883	0.171164185	negative
PRJNA729271	eed_Lushi_vs_Arbor_Acr	breed_growth_background	ENSGALG00010011983	LYZ	1.105668693	0.286380069	3.860843734	0.000112996	0.001751743	positive
PRJNA729271	eed_Lushi_vs_Arbor_Acr	breed_growth_background	ENSGALG00010011984	FN1	-0.413082999	0.12843021	-3.216400546	0.001298095	0.009842037	negative
PRJNA729271	eed_Lushi_vs_Arbor_Acr	breed_growth_background	ENSGALG00010011987	H2BC32	-0.666947323	0.20087859	-3.320151362	0.000899687	0.00754685	negative
PRJNA729271	eed_Lushi_vs_Arbor_Acr	breed_growth_background	ENSGALG00010011988	HINTW	4.093082644	1.698912719	2.409236565	0.015985932	0.05834519	positive
PRJNA729271	eed_Lushi_vs_Arbor_Acr	breed_growth_background	ENSGALG00010011989	H1-010	-0.257145817	0.073266155	-3.509749041	0.00044853	0.004583722	negative
PRJNA729271	eed_Lushi_vs_Arbor_Acr	breed_growth_background	ENSGALG00010011991	CSNK1A1	-0.064752751	0.030214785	-2.143081674	0.032106541	0.095817234	negative
PRJNA729271	eed_Lushi_vs_Arbor_Acr	breed_growth_background	ENSGALG00010011992	YEATS4	0.054527609	0.048055279	1.134685109	0.256507279	0.413624764	positive
PRJNA729271	eed_Lushi_vs_Arbor_Acr	breed_growth_background	ENSGALG00010011993	RELCH	-0.114707452	0.028527664	-4.020919933	5.79713E-05	0.00107791	negative
PRJNA729271	eed_Lushi_vs_Arbor_Acr	breed_growth_background	ENSGALG00010011996	LRRC70	-0.341587705	0.117069961	-2.917808317	0.00352501	0.019811949	negative
PRJNA729271	eed_Lushi_vs_Arbor_Acr	breed_growth_background	ENSGALG00010011997	DENND5B	0.074835939	0.072460632	1.032780652	0.30170656	0.464151622	positive
PRJNA729271	eed_Lushi_vs_Arbor_Acr	breed_growth_background	ENSGALG00010011998	MPPED1	0.008274921	0.041333978	0.200196578	0.841326843	0.903938373	positive
PRJNA729271	eed_Lushi_vs_Arbor_Acr	breed_growth_background	ENSGALG00010012001	EEIG2	-0.957328554	0.136110333	-7.033474462	2.01453E-12	1.00692E-09	negative
PRJNA729271	eed_Lushi_vs_Arbor_Acr	breed_growth_background	ENSGALG00010012004	LGR5	0.087239672	0.112422343	0.775999411	0.437749334	0.595232326	positive
PRJNA729271	eed_Lushi_vs_Arbor_Acr	breed_growth_background	ENSGALG00010012007	MREG	-0.272076228	0.115307642	-2.359568045	0.018296225	0.064276244	negative
PRJNA729271	eed_Lushi_vs_Arbor_Acr	breed_growth_background	ENSGALG00010012008	ZNF366	-0.101936462	0.088394842	-1.15319468	0.248830487	0.404985168	negative
PRJNA729271	eed_Lushi_vs_Arbor_Acr	breed_growth_background	ENSGALG00010012010	HENMT1	0.086117344	0.097892233	0.8797158	0.379013288	0.541017706	positive
PRJNA729271	eed_Lushi_vs_Arbor_Acr	breed_growth_background	ENSGALG00010012011	HOOK3	0.097899283	0.036680845	2.668948458	0.007608913	0.034358625	positive
PRJNA729271	eed_Lushi_vs_Arbor_Acr	breed_growth_background	ENSGALG00010012013	FOXD1	0.032870329	0.495072354	0.066395001	0.94706335	0.970898153	positive
PRJNA729271	eed_Lushi_vs_Arbor_Acr	breed_growth_background	ENSGALG00010012015	ENSGALG00010012015	-0.321396489	0.115139791	-2.791358976	0.005248722	0.026425923	negative
PRJNA729271	eed_Lushi_vs_Arbor_Acr	breed_growth_background	ENSGALG00010012016	WDR91	-0.03866468	0.035167503	-1.099443428	0.271574697	0.430309929	negative
PRJNA729271	eed_Lushi_vs_Arbor_Acr	breed_growth_background	ENSGALG00010012017	N4BP3	-0.065745728	0.070699136	-0.929936797	0.35240381	0.516360799	negative
PRJNA729271	eed_Lushi_vs_Arbor_Acr	breed_growth_background	ENSGALG00010012019	ENSGALG00010012019	0.236586524	0.153818221	1.53809167	0.124026203	0.249586525	positive
PRJNA729271	eed_Lushi_vs_Arbor_Acr	breed_growth_background	ENSGALG00010012021	ENSGALG00010012021	0.276570494	0.070104375	3.945124606	7.97585E-05	0.001347435	positive
PRJNA729271	eed_Lushi_vs_Arbor_Acr	breed_growth_background	ENSGALG00010012024	UBR5	0.055668508	0.04059352	1.371364383	0.170261395	0.312595176	positive
PRJNA729271	eed_Lushi_vs_Arbor_Acr	breed_growth_background	ENSGALG00010012026	SYT1	0.561124855	0.147634372	3.800773801	0.000144245	0.002073162	positive
PRJNA729271	eed_Lushi_vs_Arbor_Acr	breed_growth_background	ENSGALG00010012028	TMEM140	-0.122131465	0.086233071	-1.416294977	0.156689158	0.294121904	negative
PRJNA729271	eed_Lushi_vs_Arbor_Acr	breed_growth_background	ENSGALG00010012031	GRK6	-0.032356742	0.0595059	-0.543756876	0.586608788	0.722352333	negative
PRJNA729271	eed_Lushi_vs_Arbor_Acr	breed_growth_background	ENSGALG00010012034	ARHGEF9	-0.239003729	0.080470847	-2.970066022	0.002977358	0.017607832	negative
PRJNA729271	eed_Lushi_vs_Arbor_Acr	breed_growth_background	ENSGALG00010012035	ENSGALG00010012035	-0.004226085	0.276950058	-0.015259376	0.987825252	0.992713543	negative
PRJNA729271	eed_Lushi_vs_Arbor_Acr	breed_growth_background	ENSGALG00010012039	ODF1	0.156044795	0.372809305	0.418564646	0.675534332	0.790304289	positive
PRJNA729271	eed_Lushi_vs_Arbor_Acr	breed_growth_background	ENSGALG00010012040	B4GALT7	0.105251401	0.08170068	1.288256116	0.197656824	0.346105929	positive
PRJNA729271	eed_Lushi_vs_Arbor_Acr	breed_growth_background	ENSGALG00010012041	ICOSLG	-0.079587308	0.082299719	-0.967042277	0.3335229	0.497982533	negative
PRJNA729271	eed_Lushi_vs_Arbor_Acr	breed_growth_background	ENSGALG00010012042	FNDC7	-0.507007785	0.212990532	-2.38042405	0.017292725	0.061555759	negative
PRJNA729271	eed_Lushi_vs_Arbor_Acr	breed_growth_background	ENSGALG00010012043	NUP50	0.148274554	0.051561604	2.875677704	0.004031611	0.021748495	positive
PRJNA729271	eed_Lushi_vs_Arbor_Acr	breed_growth_background	ENSGALG00010012044	ENSGALG00010012044	0.261307491	0.304097506	0.859288506	0.390181364	0.551782232	positive
PRJNA729271	eed_Lushi_vs_Arbor_Acr	breed_growth_background	ENSGALG00010012045	ENSGALG00010012045	-1.527962624	0.160818258	-9.501176337	2.07532E-21	4.2974E-18	negative
PRJNA729271	eed_Lushi_vs_Arbor_Acr	breed_growth_background	ENSGALG00010012046	LOXHD1	0.343944411	0.148687691	2.313200301	0.020711629	0.069893238	positive
PRJNA729271	eed_Lushi_vs_Arbor_Acr	breed_growth_background	ENSGALG00010012047	LMAN2	0.001765252	0.048823024	0.036156142	0.971157856	0.983094708	positive
PRJNA729271	eed_Lushi_vs_Arbor_Acr	breed_growth_background	ENSGALG00010012048	PDE6H	-0.642072145	0.276329483	-2.323574519	0.020148309	0.068620711	negative
PRJNA729271	eed_Lushi_vs_Arbor_Acr	breed_growth_background	ENSGALG00010012049	IFNAR1	-0.223676741	0.077745992	-2.877019578	0.004014507	0.021720895	negative
PRJNA729271	eed_Lushi_vs_Arbor_Acr	breed_growth_background	ENSGALG00010012051	ARK2C	-0.106308713	0.112053528	-0.948731509	0.342757184	0.506577486	negative
PRJNA729271	eed_Lushi_vs_Arbor_Acr	breed_growth_background	ENSGALG00010012052	KLF10	0.205357492	0.065541549	3.133241363	0.001728872	0.012001916	positive
PRJNA729271	eed_Lushi_vs_Arbor_Acr	breed_growth_background	ENSGALG00010012054	FAM60A	-0.124814991	0.049650107	-2.513891692	0.01194071	0.047406351	negative
PRJNA729271	eed_Lushi_vs_Arbor_Acr	breed_growth_background	ENSGALG00010012055	ENSGALG00010012055	-0.050680915	0.083575017	-0.606412267	0.544241025	0.688434737	negative
PRJNA729271	eed_Lushi_vs_Arbor_Acr	breed_growth_background	ENSGALG00010012058	ENSGALG00010012058	0.096237182	0.175148371	0.549460903	0.582689189	0.718876482	positive
PRJNA729271	eed_Lushi_vs_Arbor_Acr	breed_growth_background	ENSGALG00010012059	ARHGDIB	-0.152939062	0.078500862	-1.948246911	0.051385426	0.135153647	negative

PRJNA729271	ed_Lushi_vs_Arbor_Acr	breed_growth_background	ENSGALG00010012060	ENSGALG00010012060	0.164934105	0.066461779	2.481638436	0.01307799	0.050456605	positive
PRJNA729271	ed_Lushi_vs_Arbor_Acr	breed_growth_background	ENSGALG00010012061	AZIN1	0.132866026	0.064341899	2.065000075	0.038922993	0.110279278	positive
PRJNA729271	ed_Lushi_vs_Arbor_Acr	breed_growth_background	ENSGALG00010012062	HMGA2	-0.075648453	0.090813809	-0.833006054	0.404841315	0.565661737	negative
PRJNA729271	ed_Lushi_vs_Arbor_Acr	breed_growth_background	ENSGALG00010012063	PTPRD	-0.51029495	0.105608053	-4.831970052	1.35189E-06	6.26057E-05	negative
PRJNA729271	ed_Lushi_vs_Arbor_Acr	breed_growth_background	ENSGALG00010012064	RGS14	-0.098237748	0.097790277	-1.004575822	0.315101144	0.478354217	negative
PRJNA729271	ed_Lushi_vs_Arbor_Acr	breed_growth_background	ENSGALG00010012066	STXBP3	-0.010203244	0.067201535	-0.151830516	0.879320613	0.92824647	negative
PRJNA729271	ed_Lushi_vs_Arbor_Acr	breed_growth_background	ENSGALG00010012067	ENSGALG00010012067	-0.016956	0.045305522	-0.374259012	0.708211624	0.811867629	negative
PRJNA729271	ed_Lushi_vs_Arbor_Acr	breed_growth_background	ENSGALG00010012069	ERP27	0.17839102	0.252140061	0.707507643	0.479251065	0.632096832	positive
PRJNA729271	ed_Lushi_vs_Arbor_Acr	breed_growth_background	ENSGALG00010012070	DNAJC28	-0.194421142	0.086758654	-2.240942368	0.025029808	0.080107545	negative
PRJNA729271	ed_Lushi_vs_Arbor_Acr	breed_growth_background	ENSGALG00010012071	ENSGALG00010012071	-0.407678562	0.082900287	-4.917697826	8.75679E-07	4.37689E-05	negative
PRJNA729271	ed_Lushi_vs_Arbor_Acr	breed_growth_background	ENSGALG00010012072	CAPRIN2	-0.050564897	0.082481571	-0.61304478	0.539846731	0.684926963	negative
PRJNA729271	ed_Lushi_vs_Arbor_Acr	breed_growth_background	ENSGALG00010012074	KIAA0930	-0.036398648	0.047995292	-0.758379554	0.448223794	0.60477939	negative
PRJNA729271	ed_Lushi_vs_Arbor_Acr	breed_growth_background	ENSGALG00010012075	PIGN	-0.05631361	0.052955778	-1.063408241	0.287596857	0.448635003	negative
PRJNA729271	ed_Lushi_vs_Arbor_Acr	breed_growth_background	ENSGALG00010012077	IFNGR2	0.019156406	0.036961756	0.518276411	0.604265429	0.736221517	positive
PRJNA729271	ed_Lushi_vs_Arbor_Acr	breed_growth_background	ENSGALG00010012078	TMBIM4	0.06026358	0.045050205	1.337698235	0.180994827	0.32553915	positive
PRJNA729271	ed_Lushi_vs_Arbor_Acr	breed_growth_background	ENSGALG00010012079	ENSGALG00010012079	-1.439178972	0.203802054	-7.061650975	1.64536E-12	8.51767E-10	negative
PRJNA729271	ed_Lushi_vs_Arbor_Acr	breed_growth_background	ENSGALG00010012081	CHIC1	-0.088245674	0.067596071	-1.305485261	0.191727663	0.338542146	negative
PRJNA729271	ed_Lushi_vs_Arbor_Acr	breed_growth_background	ENSGALG00010012082	HAUS1	-0.069804912	0.116509554	-0.599134663	0.549083088	0.69238446	negative
PRJNA729271	ed_Lushi_vs_Arbor_Acr	breed_growth_background	ENSGALG00010012083	MGP	-0.584292843	0.148334601	-3.939019198	8.18154E-05	0.001377368	negative
PRJNA729271	ed_Lushi_vs_Arbor_Acr	breed_growth_background	ENSGALG00010012084	TMEM50B	-0.100106927	0.056780024	-1.763065966	0.077889365	0.180496617	negative
PRJNA729271	ed_Lushi_vs_Arbor_Acr	breed_growth_background	ENSGALG00010012085	IPO8	-0.025459917	0.051957256	-0.490016576	0.624122169	0.75063482	negative
PRJNA729271	ed_Lushi_vs_Arbor_Acr	breed_growth_background	ENSGALG00010012086	CIBAR1	-0.12963961	0.073344723	-1.767538341	0.077138114	0.179350524	negative
PRJNA729271	ed_Lushi_vs_Arbor_Acr	breed_growth_background	ENSGALG00010012087	TMEM171	0.300239601	0.0967142	3.104400401	0.001906651	0.012836464	positive
PRJNA729271	ed_Lushi_vs_Arbor_Acr	breed_growth_background	ENSGALG00010012088	ATP5F1AZ	-0.064638042	0.090817395	-0.71173636	0.476628048	0.629553814	negative
PRJNA729271	ed_Lushi_vs_Arbor_Acr	breed_growth_background	ENSGALG00010012089	ENSGALG00010012089	0.306654149	0.149656327	2.049055687	0.04045667	0.113317764	positive
PRJNA729271	ed_Lushi_vs_Arbor_Acr	breed_growth_background	ENSGALG00010012090	HELB	0.142785215	0.119984401	1.190031487	0.234034016	0.388466603	positive
PRJNA729271	ed_Lushi_vs_Arbor_Acr	breed_growth_background	ENSGALG00010012091	ENSGALG00010012091	-0.234682956	0.238386185	-0.984465418	0.324886727	0.488864643	negative
PRJNA729271	ed_Lushi_vs_Arbor_Acr	breed_growth_background	ENSGALG00010012092	OC3	0.141551326	0.316636922	0.447046178	0.654841719	0.775231192	positive
PRJNA729271	ed_Lushi_vs_Arbor_Acr	breed_growth_background	ENSGALG00010012093	ENSGALG00010012093	-0.01744683	0.037363003	-0.46695471	0.640532281	0.764409305	negative
PRJNA729271	ed_Lushi_vs_Arbor_Acr	breed_growth_background	ENSGALG00010012094	ENSGALG00010012094	0.001279759	0.1912289	0.006692287	0.994660368	0.997061176	positive
PRJNA729271	ed_Lushi_vs_Arbor_Acr	breed_growth_background	ENSGALG00010012095	RNF38	-0.092343788	0.047189526	-1.95687042	0.050362705	0.133209951	negative
PRJNA729271	ed_Lushi_vs_Arbor_Acr	breed_growth_background	ENSGALG00010012096	ENSGALG00010012096	-0.356550849	0.090175776	-3.953953759	7.68703E-05	0.001310864	negative
PRJNA729271	ed_Lushi_vs_Arbor_Acr	breed_growth_background	ENSGALG00010012097	GART	0.232793181	0.103099587	2.257944857	0.023949098	0.077597174	positive
PRJNA729271	ed_Lushi_vs_Arbor_Acr	breed_growth_background	ENSGALG00010012098	ART4	0.537453162	0.282566669	1.902040198	0.0571659	0.145729812	positive
PRJNA729271	ed_Lushi_vs_Arbor_Acr	breed_growth_background	ENSGALG00010012099	UPK3A	-0.144514209	0.286742266	-0.503986424	0.614270917	0.74359916	negative
PRJNA729271	ed_Lushi_vs_Arbor_Acr	breed_growth_background	ENSGALG00010012100	UBE2F	0.020372428	0.059570554	0.341988229	0.732359751	0.828240196	positive
PRJNA729271	ed_Lushi_vs_Arbor_Acr	breed_growth_background	ENSGALG00010012101	ENSGALG00010012101	-0.175173027	0.074621665	-2.347482155	0.018900779	0.065715228	negative
PRJNA729271	ed_Lushi_vs_Arbor_Acr	breed_growth_background	ENSGALG00010012102	SON	0.152249975	0.056084887	2.714634622	0.006634893	0.031184426	positive
PRJNA729271	ed_Lushi_vs_Arbor_Acr	breed_growth_background	ENSGALG00010012103	ENSGALG00010012103	0.124858106	0.111018614	1.12465921	0.260733473	0.418623359	positive
PRJNA729271	ed_Lushi_vs_Arbor_Acr	breed_growth_background	ENSGALG00010012105	DONSON	-0.046481785	0.115065388	-0.403959744	0.686242322	0.797489173	negative
PRJNA729271	ed_Lushi_vs_Arbor_Acr	breed_growth_background	ENSGALG00010012106	TMTC1	0.037953003	0.074678623	0.508217764	0.611300634	0.74155182	positive
PRJNA729271	ed_Lushi_vs_Arbor_Acr	breed_growth_background	ENSGALG00010012107	CLCC1	-0.16025857	0.045942046	-3.488276735	0.000486145	0.004873213	negative
PRJNA729271	ed_Lushi_vs_Arbor_Acr	breed_growth_background	ENSGALG00010012108	PSTPIP2	0.30799124	0.187672076	1.641113835	0.100773787	0.216274215	positive
PRJNA729271	ed_Lushi_vs_Arbor_Acr	breed_growth_background	ENSGALG00010012110	ENSGALG00010012110	-0.085472324	0.060528236	-1.412106641	0.157918564	0.295893172	negative
PRJNA729271	ed_Lushi_vs_Arbor_Acr	breed_growth_background	ENSGALG00010012111	DRD1	0.252627328	0.18914083	1.335657284	0.181661317	0.326332977	positive
PRJNA729271	ed_Lushi_vs_Arbor_Acr	breed_growth_background	ENSGALG00010012112	MBD2	-0.153746766	0.101138037	-1.520167595	0.128468859	0.255733531	negative
PRJNA729271	ed_Lushi_vs_Arbor_Acr	breed_growth_background	ENSGALG00010012113	WDR47	-0.145454475	0.05198172	-2.798185124	0.005139065	0.026091329	negative
PRJNA729271	ed_Lushi_vs_Arbor_Acr	breed_growth_background	ENSGALG00010012114	TAL2	0.663804823	0.167866559	3.954360103	7.67398E-05	0.00131018	positive
PRJNA729271	ed_Lushi_vs_Arbor_Acr	breed_growth_background	ENSGALG00010012116	RBM12B	0.078210751	0.042001815	1.862080272	0.062591776	0.155407297	positive
PRJNA729271	ed_Lushi_vs_Arbor_Acr	breed_growth_background	ENSGALG00010012118	ENSGALG00010012118	0.460511687	0.409432208	1.124756866	0.260692077	0.418603263	positive
PRJNA729271	ed_Lushi_vs_Arbor_Acr	breed_growth_background	ENSGALG00010012119	EPG5	0.125211427	0.065741972	1.904588843	0.056833538	0.14524015	positive
PRJNA729271	ed_Lushi_vs_Arbor_Acr	breed_growth_background	ENSGALG00010012120	ENSGALG00010012120	0.272112278	0.101908533	2.670161866	0.007581469	0.034277415	positive
PRJNA729271	ed_Lushi_vs_Arbor_Acr	breed_growth_background	ENSGALG00010012121	SREK1	-0.013790321	0.053921724	-0.255747033	0.798146175	0.875036941	negative
PRJNA729271	ed_Lushi_vs_Arbor_Acr	breed_growth_background	ENSGALG00010012122	GRIP1	-0.016729697	0.150286291	-0.11131885	0.911363508	0.949760159	negative
PRJNA729271	ed_Lushi_vs_Arbor_Acr	breed_growth_background	ENSGALG00010012123	ENSGALG00010012123	0.013192659	0.114046159	0.115678242	0.90790755	0.947477673	positive
PRJNA729271	ed_Lushi_vs_Arbor_Acr	breed_growth_background	ENSGALG00010012124	ERAP1	-0.21391604	0.078073587	-2.739928413	0.006145257	0.029524527	negative
PRJNA729271	ed_Lushi_vs_Arbor_Acr	breed_growth_background	ENSGALG00010012125	RNF152	-0.261751048	0.079171735	-3.306117349	0.000945985	0.00783098	negative
PRJNA729271	ed_Lushi_vs_Arbor_Acr	breed_growth_background	ENSGALG00010012127	ENSGALG00010012127	0.074576577	0.062706087	1.189303634	0.234320206	0.388681632	positive
PRJNA729271	ed_Lushi_vs_Arbor_Acr	breed_growth_background	ENSGALG00010012128	ENSGALG00010012128	0.632649061	0.40659144	1.555982245	0.119712314	0.243951919	positive
PRJNA729271	ed_Lushi_vs_Arbor_Acr	breed_growth_background	ENSGALG00010012129	CRYZL1	-0.11176448	0.0541429	-2.064249972	0.038994022	0.110329562	negative
PRJNA729271	ed_Lushi_vs_Arbor_Acr	breed_growth_background	ENSGALG00010012130	TMEM67	-0.29911144	0.092711365	-3.226265097	0.001254171	0.009623723	negative
PRJNA729271	ed_Lushi_vs_Arbor_Acr	breed_growth_background	ENSGALG00010012131	SLC14A1	-0.256334199	0.136854168	-1.873046346	0.061061985	0.152786722	negative
PRJNA729271	ed_Lushi_vs_Arbor_Acr	breed_growth_background	ENSGALG00010012132	MC4R	-0.653496189	0.610229659	-1.070902043	0.284213477	0.444505217	negative
PRJNA729271	ed_Lushi_vs_Arbor_Acr	breed_growth_background	ENSGALG00010012133	RAB3IP	0.444793796	0.131535278	3.381555144	0.000720768	0.006453074	positive
PRJNA729271	ed_Lushi_vs_Arbor_Acr	breed_growth_background	ENSGALG00010012135	CAMSAP2	-0.002485215	0.049606166	-0.050098905	0.960043572	0.977647294	negative
PRJNA729271	ed_Lushi_vs_Arbor_Acr	breed_growth_background	ENSGALG00010012136	ENSGALG00010012136	-0.151150007	0.095001925	-1.591020463	0.111604971	0.232560026	negative
PRJNA729271	ed_Lushi_vs_Arbor_Acr	breed_growth_background	ENSGALG00010012137	ENSGALG00010012137	0.328718593	0.564602061	0.582212881	0.560423298	0.701315351	positive
PRJNA729271	ed_Lushi_vs_Arbor_Acr	breed_growth_background	ENSGALG00010012138	DDX11	-0.122533316	0.097283402	-1.25955007	0.207831717	0.356976032	negative
PRJNA729271	ed_Lushi_vs_Arbor_Acr	breed_growth_background	ENSGALG00010012140	GLOD5	-1.027957068	0.340823395	-3.016098903	0.002560497	0.015865402	negative
PRJNA729271	ed_Lushi_vs_Arbor_Acr	breed_growth_background	ENSGALG00010012141	ENSGALG00010012141	0.110476237	0.050993697	2.166468499	0.030275405	0.091539839	positive
PRJNA729271	ed_Lushi_vs_Arbor_Acr	breed_growth_background	ENSGALG00010012144	H1-01	0.094324439	0.084307563	1.118813497	0.263219713	0.421355023	positive
PRJNA729271	ed_Lushi_vs_Arbor_Acr	breed_growth_background	ENSGALG00010012145	ENSGALG00010012145	0.348062444	0.220408213	1.579171845	0.114296646	0.236288875	positive
PRJNA729271	ed_Lushi_vs_Arbor_Acr	breed_growth_background	ENSGALG00010012147	ARFGAP3	-0.017772246	0.066022862	-0.269183205	0.787788704	0.86816675	negative
PRJNA729271	ed_Lushi_vs_Arbor_Acr	breed_growth_background	ENSGALG00010012148	SETBP1	0.135525153	0.058748104	2.306885571	0.0210612	0.070732645	positive
PRJNA729271	ed_Lushi_vs_Arbor_Acr	breed_growth_background	ENSGALG00010012149	SFXN1	-0.036468901	0.093100486	-0.391715471	0.695268457	0.803597503	negative
PRJNA729271	ed_Lushi_vs_Arbor_Acr	breed_growth_background	ENSGALG00010012151	MYRFL	-0.523606728	0.295169941	-1.773916159	0.076077019	0.177836481	negative
PRJNA729271	ed_Lushi_vs_Arbor_Acr	breed_growth_background	ENSGALG00010012154	CWC27	0.073831461	0.061378323	1.202891464	0.229018324	0.3826652	positive
PRJNA729271	ed_Lushi_vs_Arbor_Acr	breed_growth_background	ENSGALG00010012156	ENSGALG00010012156	-0.326754223	0.329300605	-0.992267303	0.321067149	0.484626505	negative
PRJNA729271	ed_Lushi_vs_Arbor_Acr	breed_growth_background	ENSGALG00010012157	H2BC32	-0.313601612	0.106408553	-2.947146663	0.00320721	0.018595406	negative
PRJNA729271	ed_Lushi_vs_Arbor_Acr	breed_growth_background	ENSGALG00010012159	WASHC1	0.075390354	0.055144355	1.367145452	0.171579719	0.314497727	positive
PRJNA729271	ed_Lushi_vs_Arbor_Acr	breed_growth_background	ENSGALG00010012160	SYT4	0.46844749	0.263975543	1.77458671	0.075966153	0.177704179	positive
PRJNA729271	ed_Lushi_vs_Arbor_Acr	breed_growth_background	ENSGALG00010012163	PDP1	0.316955567	0.118144603	2.682776507	0.007301377	0.033364897	positive
PRJNA729271	ed_Lushi_vs_Arbor_Acr	breed_growth_background	ENSGALG00010012168	ENSGALG00010012168	1.487081756	0.319020003	4.661405999	3.14057E-06	0.000117934	positive
PRJNA729271	ed_Lushi_vs_Arbor_Acr	breed_growth_background	ENSGALG00010012169	H3C33	-0.58329613	0.122317198	-4.768717243	1.85403E-06	7.95092E-05	negative
PRJNA729271	ed_Lushi_vs_Arbor_Acr	breed_growth_background	ENSGALG00010012171	CDH17	-0.623357672	0.213225902	-2.923461298	0.003461633	0.019592489	negative
PRJNA729271	ed_Lushi_vs_Arbor_Acr	breed_growth_background	ENSGALG00010012172	CNOT2	0.019326521	0.048199394	0.400970195	0.688442068	0.798539286	positive
PRJNA729271	ed_Lushi_vs_Arbor_Acr	breed_growth_background	ENSGALG00010012175	PACSIN2	-0.135772844	0.082723354	-1.64128795	0.100737656	0.216228688	negative
PRJNA729271	ed_Lushi_vs_Arbor_Acr	breed_growth_background	ENSGALG00010012179	ENSGALG00010012179	0.012906399	0.096423301	0.133851453	0.89352004	0.937704386	positive
PRJNA729271	ed_Lushi_vs_Arbor_Acr	breed_growth_background	ENSGALG00010012180	DDX59	0.276833145	0.058634628	4.721325133	2.34313E-06	9.51363E-05	positive
PRJNA729271	ed_Lushi_vs_Arbor_Acr	breed_growth_background	ENSGALG00010012186	KIF20A	-0.421044059	0.13321582	-3.160616052	0.001574359	0.011224953	negative
PRJNA729271	ed_Lushi_vs_Arbor_Acr	breed_growth_background	ENSGALG00010012187	ENSGALG00010012187	0.82492132	0.460475381	1.79145586	0.073220172	0.173221216	positive
PRJNA729271	ed_Lushi_vs_Arbor_Acr	breed_growth_background	ENSGALG00010012188	ADAMTS6	-0.21328444	0.099820494	-2.136679872	0.032624038	0.096941996	negative
PRJNA729271	ed_Lushi_vs_Arbor_Acr	breed_growth_background	ENSGALG00010012190	CAST	-0.19719371	0.070946952	-2.779452861	0.005445056	0.027094432	negative
PRJNA729271	ed_Lushi_vs_Arbor_Acr	breed_growth_background	ENSGALG00010012191	BEST3	0.774323179	0.192448228	4.023540183	5.73298E-05	0.001072346	positive
PRJNA729271	ed_Lushi_vs_Arbor_Acr	breed_growth_background	ENSGALG00010012193	TTLL1	0.069369369	0.125716772	0.551790887	0.581091632	0.717394021	positive
PRJNA729271	ed_Lushi_vs_Arbor_Acr	breed_growth_background	ENSGALG00010012194	LRRFIP1	-0.121231279	0.085185357	-1.423146929	0.154693567	0.291356971	negative
PRJNA729271	ed_Lushi_vs_Arbor_Acr	breed_growth_background	ENSGALG00010012195	ITSN1	0.06479378	0.038643628	1.676700235	0.093601115	0.205244769	positive
PRJNA729271	ed_Lushi_vs_Arbor_Acr	breed_growth_background	ENSGALG00010012196	CENPK	-0.460557644	0.142551462	-3.230816703	0.001234371	0.009520256	negative
PRJNA729271	ed_Lushi_vs_Arbor_Acr	breed_growth_background	ENSGALG00010012198	TMEM5	-0.06996458	0.054356938	-1.287132472	0.198048119	0.346446461	negative
PRJNA729271	ed_Lushi_vs_Arbor_Acr	breed_growth_background	ENSGALG00010012199	ENSGALG00010012199	-0.075502283	0.154575033	-0.4884507	0.625230638	0.751779952	negative
PRJNA729271	ed_Lushi_vs_Arbor_Acr	breed_growth_background	ENSGALG00010012200	GEM	-0.479644121	0.127611425	-3.75862993	0.000170846	0.002325274	negative
PRJNA729271	ed_Lushi_vs_Arbor_Acr	breed_growth_background	ENSGALG00010012205	SLC6A13	-0.217071905	0.17350498	-1.25109899	0.210898364	0.360960183	negative
PRJNA729271	ed_Lushi_vs_Arbor_Acr	breed_growth_background	ENSGALG00010012206	RIBC2	-0.39220558	0.457964413	-0.856410605	0.391770701	0.553319333	negative
PRJNA729271	ed_Lushi_vs_Arbor_Acr	breed_growth_background	ENSGALG00010012207	GRIA3	-0.289558221	0.146275631	-1.979538348	0.047755427	0.128093062	negative
PRJNA729271	ed_Lushi_vs_Arbor_Acr	breed_growth_background	ENSGALG00010012209	KIF14	-0.38264181	0.123344577	-3.102218359	0.001920762	0.01289182	negative
PRJNA729271	ed_Lushi_vs_Arbor_Acr	breed_growth_background	ENSGALG00010012210	PPWD1	0.078840637	0.041967536	1.878610082	0.060297753	0.151397183	positive
PRJNA729271	ed_Lushi_vs_Arbor_Acr	breed_growth_background	ENSGALG00010012211	ENSGALG00010012211	2.194106295	2.160255042	1.015670026	0.309786546	0.472768581	positive
PRJNA729271	ed_Lushi_vs_Arbor_Acr	breed_growth_background	ENSGALG00010012212	RAD54B	-0.099214631	0.107093577	-0.926429327	0.354222909	0.517900047	negative
PRJNA729271	ed_Lushi_vs_Arbor_Acr	breed_growth_background	ENSGALG00010012215	RAB17	-0.3319041	0.317075047	-1.046768275	0.295206466	0.456672115	negative
PRJNA729271	ed_Lushi_vs_Arbor_Acr	breed_growth_background	ENSGALG00010012216	BRD8	0.007117998	0.031356687	0.227000942	0.82042301	0.889722544	positive
PRJNA729271	ed_Lushi_vs_Arbor_Acr	breed_growth_background	ENSGALG00010012219	KDM5A	0.091131183	0.041929654	2.173430338	0.029747944	0.090340759	positive
PRJNA729271	ed_Lushi_vs_Arbor_Acr	breed_growth_background	ENSGALG00010012226	SLCO4C1	0.215625158	0.290128626	0.743205388	0.457357342	0.613208276	positive
PRJNA729271	ed_Lushi_vs_Arbor_Acr	breed_growth_background	ENSGALG00010012228	NHP2	-0.107712604	0.063656205	-1.692099047	0.09062709	0.201200746	negative
PRJNA729271	ed_Lushi_vs_Arbor_Acr	breed_growth_background	ENSGALG00010012229	FSBP	0.127131228	0.108610791	1.170521147	0.241791309	0.397545942	positive
PRJNA729271	ed_Lushi_vs_Arbor_Acr	breed_growth_background	ENSGALG00010012230	PTPRQ	0.264877508	0.220536435	1.20106008	0.229727897	0.38340885	positive
PRJNA729271	ed_Lushi_vs_Arbor_Acr	breed_growth_background	ENSGALG00010012232	PCSK1	-0.176328204	0.23430356	-0.752563063	0.451712521	0.608001949	negative
PRJNA729271	ed_Lushi_vs_Arbor_Acr	breed_growth_background	ENSGALG00010012234	C1GALT1C1	-0.112966062	0.03566995	-3.166981236	0.001540303	0.011079355	negative
PRJNA729271	ed_Lushi_vs_Arbor_Acr	breed_growth_background	ENSGALG00010012236	PRLH	-0.331267799	0.339205061	-0.976600403	0.328767019	0.492657701	negative
PRJNA729271	ed_Lushi_vs_Arbor_Acr	breed_growth_background	ENSGALG00010012240	TRIM14	-0.233139964	0.136799484	-1.704245926	0.088335153	0.197382154	negative
PRJNA729271	ed_Lushi_vs_Arbor_Acr	breed_growth_background	ENSGALG00010012242	CCDC77	0.083277381	0.079529731	1.047122623	0.295043027	0.456467998	positive
PRJNA729271	ed_Lushi_vs_Arbor_Acr	breed_growth_background	ENSGALG00010012243	ENSGALG00010012243	0.624659047	0.866762154	0.720681036	0.471105783	0.625394113	positive
PRJNA729271	ed_Lushi_vs_Arbor_Acr	breed_growth_background	ENSGALG00010012244	ENSGALG00010012244	-0.013185412	0.045820159	-0.287764429	0.773527068	0.859375707	negative
PRJNA729271	ed_Lushi_vs_Arbor_Acr	breed_growth_background	ENSGALG00010012245	ENSGALG00010012245	0.010525178	0.042781593	0.246021183	0.805665834	0.880238658	positive
PRJNA729271	ed_Lushi_vs_Arbor_Acr	breed_growth_background	ENSGALG00010012247	TRIM23	0.113896418	0.067168121	1.695691606	0.089944299	0.200183754	positive
PRJNA729271	ed_Lushi_vs_Arbor_Acr	breed_growth_background	ENSGALG00010012248	H2AC39	-0.420125283	0.112257646	-3.742509285	0.000182192	0.002430254	negative
PRJNA729271	ed_Lushi_vs_Arbor_Acr	breed_growth_background	ENSGALG00010012250	NANS	0.223354193	0.06035668	3.700571235	0.000215115	0.002744966	positive
PRJNA729271	ed_Lushi_vs_Arbor_Acr	breed_growth_background	ENSGALG00010012253	MYF6	0.376962241	0.154100481	2.44621067	0.014436662	0.053918941	positive
PRJNA729271	ed_Lushi_vs_Arbor_Acr	breed_growth_background	ENSGALG00010012259	IL1RAPL2	-0.074226935	0.124195846	-0.597660368	0.550066575	0.69326276	negative
PRJNA729271	ed_Lushi_vs_Arbor_Acr	breed_growth_background	ENSGALG00010012260	ENSGALG00010012260	0.251098088	0.161117564	1.558477439	0.119120112	0.243155334	positive
PRJNA729271	ed_Lushi_vs_Arbor_Acr	breed_growth_background	ENSGALG00010012263	SHLD3	0.206447122	0.069631507	2.96485214	0.003028284	0.017828994	positive
PRJNA729271	ed_Lushi_vs_Arbor_Acr	breed_growth_background	ENSGALG00010012264	MYF5	0.011278136	0.082136913	0.137308979	0.890786574	0.936191371	positive

PRJNA729271	eed_Lushi_vs_Arbor_Acr	breed_growth_background	ENSGALG00010012266	H3C33	-0.365518265	0.108668239	-3.363616344	0.000769284	0.00675395	negative
PRJNA729271	eed_Lushi_vs_Arbor_Acr	breed_growth_background	ENSGALG00010012268	ENSGALG00010012268	-0.335711105	0.091021855	-3.688247249	0.000225804	0.002826452	negative
PRJNA729271	eed_Lushi_vs_Arbor_Acr	breed_growth_background	ENSGALG00010012271	ENSGALG00010012271	0.281295231	0.152552792	1.843920569	0.065194717	0.159937486	positive
PRJNA729271	eed_Lushi_vs_Arbor_Acr	breed_growth_background	ENSGALG00010012274	B4GALNT3	0.579661806	0.148363114	3.907047981	9.34306E-05	0.00152092	positive
PRJNA729271	eed_Lushi_vs_Arbor_Acr	breed_growth_background	ENSGALG00010012275	VIRMA	0.090438312	0.039696888	2.27822169	0.022713373	0.074825076	positive
PRJNA729271	eed_Lushi_vs_Arbor_Acr	breed_growth_background	ENSGALG00010012277	H3C33	-0.351570082	0.112441788	-3.126685259	0.001767891	0.012173671	negative
PRJNA729271	eed_Lushi_vs_Arbor_Acr	breed_growth_background	ENSGALG00010012282	ENSGALG00010012282	1.138443281	0.813488787	1.399457865			positive
PRJNA729271	eed_Lushi_vs_Arbor_Acr	breed_growth_background	ENSGALG00010012283	CUPIN1A	1.853827307	0.422012971	4.392820681	1.11889E-05	0.000318007	positive
PRJNA729271	eed_Lushi_vs_Arbor_Acr	breed_growth_background	ENSGALG00010012285	FBLN1	-0.345955897	0.106838844	-3.238109717	0.001203245	0.009366831	negative
PRJNA729271	eed_Lushi_vs_Arbor_Acr	breed_growth_background	ENSGALG00010012286	ENSGALG00010012286	1.735691528	1.44630749	1.200084726	0.230106437	0.383908011	positive
PRJNA729271	eed_Lushi_vs_Arbor_Acr	breed_growth_background	ENSGALG00010012288	MCTS1	0.095164917	0.061104944	1.557401269	0.119375245	0.243538941	positive
PRJNA729271	eed_Lushi_vs_Arbor_Acr	breed_growth_background	ENSGALG00010012290	ENSGALG00010012290	0.094027698	0.05251101	1.790628242	0.073352972	0.173478761	positive
PRJNA729271	eed_Lushi_vs_Arbor_Acr	breed_growth_background	ENSGALG00010012291	NR5A2	-0.292603419	0.10611404	-2.757443034	0.005825536	0.028364511	negative
PRJNA729271	eed_Lushi_vs_Arbor_Acr	breed_growth_background	ENSGALG00010012293	SRGAP1	-0.026176002	0.057679906	-0.453814916	0.649962047	0.771407505	negative
PRJNA729271	eed_Lushi_vs_Arbor_Acr	breed_growth_background	ENSGALG00010012294	ENSGALG00010012294	-0.12225553	0.279487504	-0.437427534	0.661801319	0.780657871	negative
PRJNA729271	eed_Lushi_vs_Arbor_Acr	breed_growth_background	ENSGALG00010012295	CUL4B	0.103079521	0.035401924	2.911692586	0.003594762	0.020033095	positive
PRJNA729271	eed_Lushi_vs_Arbor_Acr	breed_growth_background	ENSGALG00010012297	ABLIM3	-0.111820882	0.103942023	-1.075800512	0.282016483	0.44183192	negative
PRJNA729271	eed_Lushi_vs_Arbor_Acr	breed_growth_background	ENSGALG00010012300	NINJ2	0.321622496	0.123613312	2.601843533	0.009272415	0.039658793	positive
PRJNA729271	eed_Lushi_vs_Arbor_Acr	breed_growth_background	ENSGALG00010012303	CA9	0.390611091	0.112936649	3.458674347	0.000542841	0.005278964	positive
PRJNA729271	eed_Lushi_vs_Arbor_Acr	breed_growth_background	ENSGALG00010012304	ESRP1	-0.329535286	0.416370267	-0.791447689	0.428682791	0.587700468	negative
PRJNA729271	eed_Lushi_vs_Arbor_Acr	breed_growth_background	ENSGALG00010012307	ENSGALG00010012307	-0.037186554	0.092603328	-0.401568229	0.688001816	0.798317829	negative
PRJNA729271	eed_Lushi_vs_Arbor_Acr	breed_growth_background	ENSGALG00010012308	TRABD2A	0.24046624	0.18159927	1.324158628	0.185450368	0.330965659	positive
PRJNA729271	eed_Lushi_vs_Arbor_Acr	breed_growth_background	ENSGALG00010012309	TRAPPC13	-0.128494538	0.067392049	-1.90667207	0.056563066	0.144753114	negative
PRJNA729271	eed_Lushi_vs_Arbor_Acr	breed_growth_background	ENSGALG00010012311	ENSGALG00010012311	0.450668493	0.29257342	1.540360343	0.123472542	0.248697175	positive
PRJNA729271	eed_Lushi_vs_Arbor_Acr	breed_growth_background	ENSGALG00010012312	SH3TC2	-0.005068999	0.095311344	-0.053183588	0.957585632	0.976310314	negative
PRJNA729271	eed_Lushi_vs_Arbor_Acr	breed_growth_background	ENSGALG00010012314	ADRB2	0.419478684	0.134684757	3.114522342	0.00184243	0.01254136	positive
PRJNA729271	eed_Lushi_vs_Arbor_Acr	breed_growth_background	ENSGALG00010012315	ATP6V1E1	-0.024819582	0.054518434	-0.455251182	0.648928543	0.77094019	negative
PRJNA729271	eed_Lushi_vs_Arbor_Acr	breed_growth_background	ENSGALG00010012316	ENSGALG00010012316	-0.378313936	0.526052788	-0.719155842	0.472044904	0.626104411	negative
PRJNA729271	eed_Lushi_vs_Arbor_Acr	breed_growth_background	ENSGALG00010012317	ENSGALG00010012317	1.541812933	0.616633673	2.500370965	0.012406332	0.048694769	positive
PRJNA729271	eed_Lushi_vs_Arbor_Acr	breed_growth_background	ENSGALG00010012318	POLR1D	-0.062787723	0.077926838	-0.805726557	0.420400533	0.579635283	negative
PRJNA729271	eed_Lushi_vs_Arbor_Acr	breed_growth_background	ENSGALG00010012320	DPY19L4	-0.058357027	0.033228858	-1.756215232	0.079051665	0.182518936	negative
PRJNA729271	eed_Lushi_vs_Arbor_Acr	breed_growth_background	ENSGALG00010012322	ENSGALG00010012322	0.19843013	0.149376162	1.328392211	0.184048571	0.32939672	positive
PRJNA729271	eed_Lushi_vs_Arbor_Acr	breed_growth_background	ENSGALG00010012323	SMAD7B	1.607729352	1.916269514	0.838989161	0.401475392	0.562508666	positive
PRJNA729271	eed_Lushi_vs_Arbor_Acr	breed_growth_background	ENSGALG00010012324	ENSGALG00010012324	-0.401931164	0.087896537	-4.572775901	4.81305E-06	0.000169333	negative
PRJNA729271	eed_Lushi_vs_Arbor_Acr	breed_growth_background	ENSGALG00010012325	MLPH	1.362019375	0.32464417	4.195422253	2.72364E-05	0.000610187	positive
PRJNA729271	eed_Lushi_vs_Arbor_Acr	breed_growth_background	ENSGALG00010012326	SGTB	-0.040725234	0.063491667	-0.641426434	0.521245663	0.668506095	negative
PRJNA729271	eed_Lushi_vs_Arbor_Acr	breed_growth_background	ENSGALG00010012327	UBTD2	-0.022215999	0.048093418	-0.461934281	0.644128449	0.767308993	negative
PRJNA729271	eed_Lushi_vs_Arbor_Acr	breed_growth_background	ENSGALG00010012329	ATXN10	-0.037447494	0.058600634	-0.639028823	0.522804181	0.669757753	negative
PRJNA729271	eed_Lushi_vs_Arbor_Acr	breed_growth_background	ENSGALG00010012331	ENSGALG00010012331	-0.415468394	0.143464428	-2.895968009	0.003779909	0.0208247	negative
PRJNA729271	eed_Lushi_vs_Arbor_Acr	breed_growth_background	ENSGALG00010012334	ARHGEF39	-0.366780436	0.128618321	-2.851696662	0.004348658	0.022954768	negative
PRJNA729271	eed_Lushi_vs_Arbor_Acr	breed_growth_background	ENSGALG00010012336	ENSGALG00010012336	3.941319134	2.212856383	1.781100285	0.074896069	0.176122408	positive
PRJNA729271	eed_Lushi_vs_Arbor_Acr	breed_growth_background	ENSGALG00010012337	ACSS3	0.069676795	0.082088134	0.848804713	0.395989967	0.557329416	positive
PRJNA729271	eed_Lushi_vs_Arbor_Acr	breed_growth_background	ENSGALG00010012338	H3C33	-0.457002286	0.120815692	-3.782640152	0.000155174	0.002187979	negative
PRJNA729271	eed_Lushi_vs_Arbor_Acr	breed_growth_background	ENSGALG00010012339	WNK1	0.238417106	0.082878778	2.876696698	0.004018617	0.021723142	positive
PRJNA729271	eed_Lushi_vs_Arbor_Acr	breed_growth_background	ENSGALG00010012340	ENHO	0.11749106	0.088679849	1.324890171	0.185207581	0.330695232	positive
PRJNA729271	eed_Lushi_vs_Arbor_Acr	breed_growth_background	ENSGALG00010012341	GOPC	-0.041149135	0.043470921	-0.946589923	0.343847781	0.507698236	negative
PRJNA729271	eed_Lushi_vs_Arbor_Acr	breed_growth_background	ENSGALG00010012342	ENSGALG00010012342	1.027441943	0.872941653	1.176988107	0.239200253	0.394763483	positive
PRJNA729271	eed_Lushi_vs_Arbor_Acr	breed_growth_background	ENSGALG00010012343	BCL2L13	0.253720187	0.091095501	2.785210939	0.005349291	0.026737233	positive
PRJNA729271	eed_Lushi_vs_Arbor_Acr	breed_growth_background	ENSGALG00010012345	H2BC32	-0.137384586	0.08842743	-1.553642196	0.120269788	0.244709515	negative
PRJNA729271	eed_Lushi_vs_Arbor_Acr	breed_growth_background	ENSGALG00010012346	NLN	-0.078081098	0.076478645	-1.020952945	0.307276733	0.469852136	negative
PRJNA729271	eed_Lushi_vs_Arbor_Acr	breed_growth_background	ENSGALG00010012347	ENSGALG00010012347	-0.325992527	0.270906927	-1.203337733	0.228845653	0.382553078	negative
PRJNA729271	eed_Lushi_vs_Arbor_Acr	breed_growth_background	ENSGALG00010012350	H2AC32	-0.331419885	0.102852368	-3.22228737	0.001271715	0.009717191	negative
PRJNA729271	eed_Lushi_vs_Arbor_Acr	breed_growth_background	ENSGALG00010012352	PTPRC	0.199316959	0.104067435	1.915267332	0.05545842	0.142732562	positive
PRJNA729271	eed_Lushi_vs_Arbor_Acr	breed_growth_background	ENSGALG00010012353	PIGG	0.025262913	0.081814318	0.308783518	0.757486208	0.846877176	positive
PRJNA729271	eed_Lushi_vs_Arbor_Acr	breed_growth_background	ENSGALG00010012354	DCBLD1	0.31234452	0.080349547	3.887321476	0.000101356	0.001600395	positive
PRJNA729271	eed_Lushi_vs_Arbor_Acr	breed_growth_background	ENSGALG00010012358	STK10	0.00780635	0.079853614	0.097758252	0.922124258	0.956621271	positive
PRJNA729271	eed_Lushi_vs_Arbor_Acr	breed_growth_background	ENSGALG00010012360	ROS1	0.231084178	0.196821169	1.174081928	0.240362211	0.396125022	positive
PRJNA729271	eed_Lushi_vs_Arbor_Acr	breed_growth_background	ENSGALG00010012361	WIF1	0.635374464	0.238117735	2.668320621	0.007623147	0.034412183	positive
PRJNA729271	eed_Lushi_vs_Arbor_Acr	breed_growth_background	ENSGALG00010012362	COL6A3	-0.407648976	0.156310749	-2.607939502	0.009108905	0.039063189	negative
PRJNA729271	eed_Lushi_vs_Arbor_Acr	breed_growth_background	ENSGALG00010012366	INTS8	-0.004096976	0.036923566	-0.110958308	0.911649408	0.949989803	negative
PRJNA729271	eed_Lushi_vs_Arbor_Acr	breed_growth_background	ENSGALG00010012367	RAD52	-0.190947519	0.082405678	-2.317164593	0.020494767	0.069344457	negative
PRJNA729271	eed_Lushi_vs_Arbor_Acr	breed_growth_background	ENSGALG00010012371	ATP6V1G3	-0.471629298	0.272438286	-1.731141775	0.083426481	0.189510554	negative
PRJNA729271	eed_Lushi_vs_Arbor_Acr	breed_growth_background	ENSGALG00010012372	LEMD3	-0.004992598	0.039709366	-0.125728479	0.899946858	0.941925749	negative
PRJNA729271	eed_Lushi_vs_Arbor_Acr	breed_growth_background	ENSGALG00010012373	WNT7B	0.299928052	0.410577347	0.730503167	0.465082678	0.620247084	positive
PRJNA729271	eed_Lushi_vs_Arbor_Acr	breed_growth_background	ENSGALG00010012376	VGLL2	-0.05812528	0.09921994	-0.585822574	0.557994752	0.699302606	negative
PRJNA729271	eed_Lushi_vs_Arbor_Acr	breed_growth_background	ENSGALG00010012378	RPS19BP1	0.024001168	0.049835825	0.481604714	0.630086777	0.756114565	positive
PRJNA729271	eed_Lushi_vs_Arbor_Acr	breed_growth_background	ENSGALG00010012382	SH3PXD2B	-0.222505276	0.072289903	-3.077957871	0.002084244	0.013650792	negative
PRJNA729271	eed_Lushi_vs_Arbor_Acr	breed_growth_background	ENSGALG00010012384	NRK	-0.245411466	0.065290526	-3.758760719	0.000170757	0.002325274	negative
PRJNA729271	eed_Lushi_vs_Arbor_Acr	breed_growth_background	ENSGALG00010012387	ERBIN	0.029849136	0.047781273	0.624703661	0.53216557	0.677655883	positive
PRJNA729271	eed_Lushi_vs_Arbor_Acr	breed_growth_background	ENSGALG00010012390	MMP16	-0.293089538	0.093604438	-3.131150012	0.001741232	0.012043942	negative
PRJNA729271	eed_Lushi_vs_Arbor_Acr	breed_growth_background	ENSGALG00010012391	NEURL1B	-0.25549714	0.084918389	-3.008737492	0.002623357	0.016102793	negative
PRJNA729271	eed_Lushi_vs_Arbor_Acr	breed_growth_background	ENSGALG00010012393	ENSGALG00010012393	1.62654647	0.865651733	1.878984825	0.060246565	0.151321082	positive
PRJNA729271	eed_Lushi_vs_Arbor_Acr	breed_growth_background	ENSGALG00010012398	ENSGALG00010012398	0.634386046	0.397677222	1.595228519	0.110661143	0.231362076	positive
PRJNA729271	eed_Lushi_vs_Arbor_Acr	breed_growth_background	ENSGALG00010012399	ERC1	0.057872087	0.046670515	1.240013906	0.214970251	0.365898753	positive
PRJNA729271	eed_Lushi_vs_Arbor_Acr	breed_growth_background	ENSGALG00010012401	RIPK2	-0.082716665	0.071442001	-1.157815632	0.246939283	0.403002549	negative
PRJNA729271	eed_Lushi_vs_Arbor_Acr	breed_growth_background	ENSGALG00010012404	VAMP7	0.04841547	0.028634758	1.690793754	0.0908762	0.201450683	positive
PRJNA729271	eed_Lushi_vs_Arbor_Acr	breed_growth_background	ENSGALG00010012405	MSRB3	0.009704051	0.066616178	0.145671091	0.884181044	0.931748763	positive
PRJNA729271	eed_Lushi_vs_Arbor_Acr	breed_growth_background	ENSGALG00010012406	ENSGALG00010012406	0.553634795	0.119564353	4.630433577	3.64901E-06	0.00013323	positive
PRJNA729271	eed_Lushi_vs_Arbor_Acr	breed_growth_background	ENSGALG00010012407	ATF4	0.20693724	0.068863863	3.005019336	0.00265564	0.016221448	positive
PRJNA729271	eed_Lushi_vs_Arbor_Acr	breed_growth_background	ENSGALG00010012410	CACNA1I	-0.270044869	0.231466643	-1.166668618	0.243344221	0.39901295	negative
PRJNA729271	eed_Lushi_vs_Arbor_Acr	breed_growth_background	ENSGALG00010012412	PURA	0.111453343	0.07033048	1.584708974	0.113032472	0.234560584	positive
PRJNA729271	eed_Lushi_vs_Arbor_Acr	breed_growth_background	ENSGALG00010012413	NEK7	-0.001994202	0.039287125	-0.050759675	0.959517024	0.977517846	negative
PRJNA729271	eed_Lushi_vs_Arbor_Acr	breed_growth_background	ENSGALG00010012414	ENSGALG00010012414	0.0873067	0.108344983	0.805821346	0.420345868	0.579615046	positive
PRJNA729271	eed_Lushi_vs_Arbor_Acr	breed_growth_background	ENSGALG00010012415	BID	-0.200951718	0.062847331	-3.197458283	0.001386445	0.010337713	negative
PRJNA729271	eed_Lushi_vs_Arbor_Acr	breed_growth_background	ENSGALG00010012418	ENSGALG00010012418	-0.001151187	0.433312566	-0.002656714	0.997880252	0.998913967	negative
PRJNA729271	eed_Lushi_vs_Arbor_Acr	breed_growth_background	ENSGALG00010012419	HACE1	0.175793523	0.052372522	3.356598376	0.000789076	0.00688601	positive
PRJNA729271	eed_Lushi_vs_Arbor_Acr	breed_growth_background	ENSGALG00010012426	MIEF1	-0.061048539	0.044302321	-1.377998654	0.168203704	0.309719599	negative
PRJNA729271	eed_Lushi_vs_Arbor_Acr	breed_growth_background	ENSGALG00010012427	C7	-0.418844875	0.241137765	-1.736952625	0.082395533	0.187788005	negative
PRJNA729271	eed_Lushi_vs_Arbor_Acr	breed_growth_background	ENSGALG00010012428	CCNE2	-0.289132138	0.125268993	-2.308090224	0.020994119	0.070540045	negative
PRJNA729271	eed_Lushi_vs_Arbor_Acr	breed_growth_background	ENSGALG00010012429	CXCR7	0.28241567	0.054035412	5.226492427	1.72756E-07	1.2776E-05	positive
PRJNA729271	eed_Lushi_vs_Arbor_Acr	breed_growth_background	ENSGALG00010012431	LAMP2	-0.047165385	0.042534929	-1.108862448	0.267489526	0.42579186	negative
PRJNA729271	eed_Lushi_vs_Arbor_Acr	breed_growth_background	ENSGALG00010012433	ENSGALG00010012433	-0.789087785	0.414367349	-1.904319409	0.056868598	0.145262041	negative
PRJNA729271	eed_Lushi_vs_Arbor_Acr	breed_growth_background	ENSGALG00010012436	ENSGALG00010012436	-0.72426436	0.106260024	-6.815962694	9.36346E-12	3.99186E-09	negative
PRJNA729271	eed_Lushi_vs_Arbor_Acr	breed_growth_background	ENSGALG00010012437	ENSGALG00010012437	4.186853091	2.048980651	2.04338342	0.041014497	0.114482022	positive
PRJNA729271	eed_Lushi_vs_Arbor_Acr	breed_growth_background	ENSGALG00010012439	TENT2	-0.124714636	0.055069295	-2.264685561	0.023531982	0.07668527	negative
PRJNA729271	eed_Lushi_vs_Arbor_Acr	breed_growth_background	ENSGALG00010012441	FST	-0.387007365	0.127964942	-3.024323384	0.002491899	0.015548893	negative
PRJNA729271	eed_Lushi_vs_Arbor_Acr	breed_growth_background	ENSGALG00010012442	CPLX1	0.69468241	0.220424534	3.151565742	0.001623976	0.011478474	positive
PRJNA729271	eed_Lushi_vs_Arbor_Acr	breed_growth_background	ENSGALG00010012445	SOBP	0.012432896	0.089863433	0.138353223	0.889961264	0.935632281	positive
PRJNA729271	eed_Lushi_vs_Arbor_Acr	breed_growth_background	ENSGALG00010012447	COPS8	0.113132867	0.062718417	1.803822098	0.0712592	0.169885216	positive
PRJNA729271	eed_Lushi_vs_Arbor_Acr	breed_growth_background	ENSGALG00010012449	SUB1	0.01298376	0.058829901	0.220700022	0.825326015	0.89328695	positive
PRJNA729271	eed_Lushi_vs_Arbor_Acr	breed_growth_background	ENSGALG00010012450	PPFIA2	-0.164821099	0.150319166	-1.096474279	0.272871273	0.431797938	negative
PRJNA729271	eed_Lushi_vs_Arbor_Acr	breed_growth_background	ENSGALG00010012454	IQCA1	0.570856421	0.210181978	2.716010322	0.006607386	0.03112579	positive
PRJNA729271	eed_Lushi_vs_Arbor_Acr	breed_growth_background	ENSGALG00010012456	ENSGALG00010012456	0.00073996	0.050013841	0.014795095	0.988195653	0.992922223	positive
PRJNA729271	eed_Lushi_vs_Arbor_Acr	breed_growth_background	ENSGALG00010012458	MRPS30	0.082117921	0.058086687	1.413713284	0.157446103	0.295160536	positive
PRJNA729271	eed_Lushi_vs_Arbor_Acr	breed_growth_background	ENSGALG00010012464	ENSGALG00010012464	-0.139502451	0.127552204	-1.093689069	0.27409137	0.433066223	negative
PRJNA729271	eed_Lushi_vs_Arbor_Acr	breed_growth_background	ENSGALG00010012466	PDSS2	0.005903103	0.064988585	0.09083292	0.927625352	0.959806516	positive
PRJNA729271	eed_Lushi_vs_Arbor_Acr	breed_growth_background	ENSGALG00010012469	CETN2	-0.13289621	0.07120531	-1.86638061	0.061988134	0.154358014	negative
PRJNA729271	eed_Lushi_vs_Arbor_Acr	breed_growth_background	ENSGALG00010012470	CHD1	0.1027456	0.056713931	1.811646588	0.070040821	0.167975133	positive
PRJNA729271	eed_Lushi_vs_Arbor_Acr	breed_growth_background	ENSGALG00010012474	ASB18	0.521868296	0.507507824	1.028296062	0.303810583	0.466378147	positive
PRJNA729271	eed_Lushi_vs_Arbor_Acr	breed_growth_background	ENSGALG00010012477	ENSGALG00010012477	-0.326823053	0.14082706	-2.320740447	0.020300856	0.068965906	negative
PRJNA729271	eed_Lushi_vs_Arbor_Acr	breed_growth_background	ENSGALG00010012483	ENSGALG00010012483	-0.057552958	0.135366668	-0.42516344	0.670717533	0.787008002	negative
PRJNA729271	eed_Lushi_vs_Arbor_Acr	breed_growth_background	ENSGALG00010012485	C6	-1.941508194	0.632470082	-3.069723373	0.002142571	0.013951738	negative
PRJNA729271	eed_Lushi_vs_Arbor_Acr	breed_growth_background	ENSGALG00010012486	RADX	0.180449999	0.057606765	3.132444594	0.001733571	0.012018611	positive
PRJNA729271	eed_Lushi_vs_Arbor_Acr	breed_growth_background	ENSGALG00010012489	GBX2	-0.567382401	0.545720808	-1.039693544	0.298482301	0.460707161	negative
PRJNA729271	eed_Lushi_vs_Arbor_Acr	breed_growth_background	ENSGALG00010012497	PLCXD3	-0.345786294	0.231089854	-1.496328327	0.134568117	0.264375827	negative
PRJNA729271	eed_Lushi_vs_Arbor_Acr	breed_growth_background	ENSGALG00010012498	KCNT2	0.117596465	0.116876953	1.006156145	0.314340464	0.477906968	positive
PRJNA729271	eed_Lushi_vs_Arbor_Acr	breed_growth_background	ENSGALG00010012506	TP53INP1	-0.026789614	0.06861513	-0.390433044	0.696216357	0.804308289	negative
PRJNA729271	eed_Lushi_vs_Arbor_Acr	breed_growth_background	ENSGALG00010012508	MDM1	-0.000353454	0.037943057	-0.00931537	0.992567518	0.995792337	negative
PRJNA729271	eed_Lushi_vs_Arbor_Acr	breed_growth_background	ENSGALG00010012509	MICAL3	0.199609394	0.050028944	3.989878197	6.61072E-05	0.001175735	positive
PRJNA729271	eed_Lushi_vs_Arbor_Acr	breed_growth_background	ENSGALG00010012511	OXCT1	-0.312808272	0.065429936	-4.780812746	1.74588E-06	7.5768E-05	negative
PRJNA729271	eed_Lushi_vs_Arbor_Acr	breed_growth_background	ENSGALG00010012517	MGAT3	-0.022425471	0.09388473	-0.238861753	0.811212784	0.883798705	negative
PRJNA729271	eed_Lushi_vs_Arbor_Acr	breed_growth_background	ENSGALG00010012519	ENSGALG00010012519	1.015117365	0.922885038	1.099939128	0.271358645	0.430072246	positive
PRJNA729271	eed_Lushi_vs_Arbor_Acr	breed_growth_background	ENSGALG00010012521	HOMER1	-0.069073106	0.069990303	-0.986895379	0.32369393	0.48765955	negative
PRJNA729271	eed_Lushi_vs_Arbor_Acr	breed_growth_background	ENSGALG00010012529	BEND3	-0.227689766	0.067804428	-3.358036813	0.000784982	0.006858534	negative
PRJNA729271	eed_Lushi_vs_Arbor_Acr	breed_growth_background	ENSGALG00010012532	FAM174A	0.020212082	0.081677634	0.247461653	0.804550965	0.879484634	positive
PRJNA729271	eed_Lushi_vs_Arbor_Acr	breed_growth_background	ENSGALG00010012535	JMY	0.068602206	0.056127938	1.222247045	0.221614218	0.374088516	positive
PRJNA729271	eed_Lushi_vs_Arbor_Acr	breed_growth_background	ENSGALG00010012536	FBXO4	-0.132592962	0.072966621	-1.817172851	0.069190651	0.166625433	negative
PRJNA729271	eed_Lushi_vs_Arbor_Acr	breed_growth_background	ENSGALG00010012538	RAP1B	-0.147453597	0.04733117	-3.115359236	0.00183721	0.012520963	negative
PRJNA729271	eed_Lushi_vs_Arbor_Acr	breed_growth_background	ENSGALG00010012547	ENSGALG00010012547	-0.023186252	0.232242889	-0.099836218	0.920474354	0.955805912	negative
PRJNA729271	eed_Lushi_vs_Arbor_Acr	breed_growth_background	ENSGALG00010012550	TAB1	-0.048209452	0.051832916	-0.930093382	0.352322737	0.516319692	negative
PRJNA729271	eed_Lushi_vs_Arbor_Acr	breed_growth_background	ENSGALG00010012551	PCGF3	-0.001686717	0.08377615	-0.020133615	0.983936784	0.990496819	negative

PRJNA729271	ed_Lushi_vs_Arbor_Acr	breed_growth_background	ENSGALG00010012553	ENSGALG00010012553	0.364687019	0.120447408	3.027769747	0.002463657	0.015426877	positive
PRJNA729271	ed_Lushi_vs_Arbor_Acr	breed_growth_background	ENSGALG00010012555	ENSGALG00010012555	-0.09029746	0.113904211	-0.792749096	0.42792402	0.58704092	negative
PRJNA729271	ed_Lushi_vs_Arbor_Acr	breed_growth_background	ENSGALG00010012561	OSGIN2	0.067531425	0.070587885	0.956699932	0.338718733	0.502942842	positive
PRJNA729271	ed_Lushi_vs_Arbor_Acr	breed_growth_background	ENSGALG00010012565	SYNGR1	0.206906545	0.065316505	3.167752856	0.001536221	0.011067354	positive
PRJNA729271	ed_Lushi_vs_Arbor_Acr	breed_growth_background	ENSGALG00010012569	C6orf203	0.09534499	0.064921336	1.46862334	0.141934981	0.274789307	positive
PRJNA729271	ed_Lushi_vs_Arbor_Acr	breed_growth_background	ENSGALG00010012571	AGAP1	-0.22465016	0.044151808	-5.088130427	3.61611E-07	2.20233E-05	negative
PRJNA729271	ed_Lushi_vs_Arbor_Acr	breed_growth_background	ENSGALG00010012574	PHAX	0.019021421	0.056658201	0.335722295	0.737080292	0.831696935	positive
PRJNA729271	ed_Lushi_vs_Arbor_Acr	breed_growth_background	ENSGALG00010012578	NDUFS4	-0.032112203	0.081815868	-0.392493579	0.694693555	0.803381448	negative
PRJNA729271	ed_Lushi_vs_Arbor_Acr	breed_growth_background	ENSGALG00010012582	CD24	-0.194449918	0.122578762	-1.586326335	0.112665301	0.234076555	negative
PRJNA729271	ed_Lushi_vs_Arbor_Acr	breed_growth_background	ENSGALG00010012583	NDUFAF6	0.023155043	0.056210104	0.411937378	0.680385323	0.793417961	positive
PRJNA729271	ed_Lushi_vs_Arbor_Acr	breed_growth_background	ENSGALG00010012586	CDC73	0.156766463	0.071639443	2.188270278	0.028649921	0.087908679	positive
PRJNA729271	ed_Lushi_vs_Arbor_Acr	breed_growth_background	ENSGALG00010012587	MBLAC2	0.296524281	0.078842376	3.760975975	0.000169252	0.002316622	positive
PRJNA729271	ed_Lushi_vs_Arbor_Acr	breed_growth_background	ENSGALG00010012588	PEX26	0.076483707	0.056775818	1.347117672	0.177942337	0.32215023	positive
PRJNA729271	ed_Lushi_vs_Arbor_Acr	breed_growth_background	ENSGALG00010012593	TUBA8B	-1.317021078	0.508398432	-2.590529388	0.009582844	0.040606644	negative
PRJNA729271	ed_Lushi_vs_Arbor_Acr	breed_growth_background	ENSGALG00010012599	DMGDH	-0.255342583	0.106700664	-2.393073985	0.016707872	0.060109357	negative
PRJNA729271	ed_Lushi_vs_Arbor_Acr	breed_growth_background	ENSGALG00010012600	NUP107	0.068959866	0.054408951	1.267436063	0.204999412	0.35433657	positive
PRJNA729271	ed_Lushi_vs_Arbor_Acr	breed_growth_background	ENSGALG00010012601	TUBA8A	0.09789687	0.127445926	0.768144368	0.44240142	0.599533338	positive
PRJNA729271	ed_Lushi_vs_Arbor_Acr	breed_growth_background	ENSGALG00010012604	ENSGALG00010012604	1.612561049	1.158309106	1.392168153			positive
PRJNA729271	ed_Lushi_vs_Arbor_Acr	breed_growth_background	ENSGALG00010012606	ATP5PO	-0.04766942	0.061092231	-0.780286126	0.435222477	0.593293397	negative
PRJNA729271	ed_Lushi_vs_Arbor_Acr	breed_growth_background	ENSGALG00010012607	LMNB1	-0.226625248	0.088860858	-2.550338284	0.010761844	0.044115647	negative
PRJNA729271	ed_Lushi_vs_Arbor_Acr	breed_growth_background	ENSGALG00010012610	RPL3	-0.193202309	0.082880642	-2.331090876	0.019748568	0.067608933	negative
PRJNA729271	ed_Lushi_vs_Arbor_Acr	breed_growth_background	ENSGALG00010012614	LHFPL2	-0.211829004	0.077908686	-2.718939521	0.006549158	0.030901708	negative
PRJNA729271	ed_Lushi_vs_Arbor_Acr	breed_growth_background	ENSGALG00010012618	B3GALT2	0.100719965	0.176712619	0.56996476	0.5687016	0.707642689	positive
PRJNA729271	ed_Lushi_vs_Arbor_Acr	breed_growth_background	ENSGALG00010012624	PDGFB	-0.245121451	0.09847578	-2.489154689	0.012804724	0.049733246	negative
PRJNA729271	ed_Lushi_vs_Arbor_Acr	breed_growth_background	ENSGALG00010012629	NUS1	-0.106851792	0.059332501	-1.800898149	0.071718935	0.1705559	negative
PRJNA729271	ed_Lushi_vs_Arbor_Acr	breed_growth_background	ENSGALG00010012631	GLRX2	-0.123956855	0.083250533	-1.4889617	0.136497455	0.267188468	negative
PRJNA729271	ed_Lushi_vs_Arbor_Acr	breed_growth_background	ENSGALG00010012633	USP18	-0.100834562	0.29057386	-0.347018696	0.728577273	0.825443768	negative
PRJNA729271	ed_Lushi_vs_Arbor_Acr	breed_growth_background	ENSGALG00010012640	PRRC1	-0.021741303	0.040094801	-0.54224742	0.587648072	0.723363093	negative
PRJNA729271	ed_Lushi_vs_Arbor_Acr	breed_growth_background	ENSGALG00010012648	MRPS6	0.132808586	0.071003314	1.870456152	0.061420501	0.153471842	positive
PRJNA729271	ed_Lushi_vs_Arbor_Acr	breed_growth_background	ENSGALG00010012650	DSE	-0.099523882	0.057015862	-1.745547265	0.080889653	0.18543342	negative
PRJNA729271	ed_Lushi_vs_Arbor_Acr	breed_growth_background	ENSGALG00010012653	CBX7	0.295107436	0.133142354	2.216480545	0.026658609	0.083639943	positive
PRJNA729271	ed_Lushi_vs_Arbor_Acr	breed_growth_background	ENSGALG00010012654	TENT5B	0.118491315	0.164774818	0.719110576	0.472072791	0.626104411	positive
PRJNA729271	ed_Lushi_vs_Arbor_Acr	breed_growth_background	ENSGALG00010012662	SLC35E3	-0.073260562	0.058829569	-1.245301695	0.213020873	0.363348736	negative
PRJNA729271	ed_Lushi_vs_Arbor_Acr	breed_growth_background	ENSGALG00010012667	SCAMP1	-0.098927424	0.048230492	-2.051138598	0.040253451	0.112857597	negative
PRJNA729271	ed_Lushi_vs_Arbor_Acr	breed_growth_background	ENSGALG00010012669	ERCC8	-0.032256954	0.080173333	-0.402340191	0.68743368	0.798105822	negative
PRJNA729271	ed_Lushi_vs_Arbor_Acr	breed_growth_background	ENSGALG00010012670	ENSGALG00010012670	0.170702201	0.120569101	1.415803874	0.156832935	0.294282698	positive
PRJNA729271	ed_Lushi_vs_Arbor_Acr	breed_growth_background	ENSGALG00010012671	NBN	0.262929131	0.099488364	2.642812901	0.008222046	0.036301721	positive
PRJNA729271	ed_Lushi_vs_Arbor_Acr	breed_growth_background	ENSGALG00010012672	RO60	-0.085554823	0.04999582	-1.71123954	0.087036902	0.195233658	negative
PRJNA729271	ed_Lushi_vs_Arbor_Acr	breed_growth_background	ENSGALG00010012673	ENSGALG00010012673	0.086522355	0.180550149	0.479215086	0.631785623	0.757338125	positive
PRJNA729271	ed_Lushi_vs_Arbor_Acr	breed_growth_background	ENSGALG00010012674	ENSGALG00010012674	-0.046037345	0.072907908	-0.63144516	0.527749496	0.673628825	negative
PRJNA729271	ed_Lushi_vs_Arbor_Acr	breed_growth_background	ENSGALG00010012676	ENSGALG00010012676	-0.484961786	0.111903864	-4.333735842	1.466E-05	0.000383568	negative
PRJNA729271	ed_Lushi_vs_Arbor_Acr	breed_growth_background	ENSGALG00010012680	TRPM8	-0.086360451	0.161038391	-0.536272439	0.591770276	0.726555154	negative
PRJNA729271	ed_Lushi_vs_Arbor_Acr	breed_growth_background	ENSGALG00010012682	ENSGALG00010012682	-0.06850372	0.060103357	-1.139765281	0.254384102	0.41102414	negative
PRJNA729271	ed_Lushi_vs_Arbor_Acr	breed_growth_background	ENSGALG00010012683	MDM2	0.027445039	0.046044374	0.596056288	0.551137625	0.694069493	positive
PRJNA729271	ed_Lushi_vs_Arbor_Acr	breed_growth_background	ENSGALG00010012685	NPTXR	0.197011354	0.137915325	1.428495014	0.153149429	0.289577481	positive
PRJNA729271	ed_Lushi_vs_Arbor_Acr	breed_growth_background	ENSGALG00010012688	NDUFAF2	-0.079550841	0.060851137	-1.307302464	0.191110008	0.337945536	negative
PRJNA729271	ed_Lushi_vs_Arbor_Acr	breed_growth_background	ENSGALG00010012690	NT5DC1	-0.534592827	0.180244278	-2.965935073	0.003017642	0.017788011	negative
PRJNA729271	ed_Lushi_vs_Arbor_Acr	breed_growth_background	ENSGALG00010012693	UCHL5	-0.019290991	0.042033618	-0.45894196	0.646275846	0.768921315	negative
PRJNA729271	ed_Lushi_vs_Arbor_Acr	breed_growth_background	ENSGALG00010012696	DNAL4	-0.078649623	0.052184136	-1.507155807	0.131770725	0.260468657	negative
PRJNA729271	ed_Lushi_vs_Arbor_Acr	breed_growth_background	ENSGALG00010012699	RNF128	0.117078718	0.077741102	1.506007964	0.132065129	0.26090828	positive
PRJNA729271	ed_Lushi_vs_Arbor_Acr	breed_growth_background	ENSGALG00010012702	PTPRO	-0.381077721	0.199945324	-1.90590964	0.05666193	0.144910883	negative
PRJNA729271	ed_Lushi_vs_Arbor_Acr	breed_growth_background	ENSGALG00010012704	SMIM15	0.049853106	0.036846362	1.352999394	0.176055824	0.320026796	positive
PRJNA729271	ed_Lushi_vs_Arbor_Acr	breed_growth_background	ENSGALG00010012705	CPM	0.157557274	0.098342379	1.602129972	0.109126871	0.229196442	positive
PRJNA729271	ed_Lushi_vs_Arbor_Acr	breed_growth_background	ENSGALG00010012706	TBC1D8B	0.030639823	0.055724885	0.549840932	0.582428481	0.718616006	positive
PRJNA729271	ed_Lushi_vs_Arbor_Acr	breed_growth_background	ENSGALG00010012708	SPP2	-1.968891234	0.602999965	-3.265159785	0.001094023	0.008729463	negative
PRJNA729271	ed_Lushi_vs_Arbor_Acr	breed_growth_background	ENSGALG00010012709	PPP1R8	-0.096016421	0.031865953	-3.013135136	0.002585638	0.015948432	negative
PRJNA729271	ed_Lushi_vs_Arbor_Acr	breed_growth_background	ENSGALG00010012711	ELL2	0.132850063	0.069139639	1.92147464	0.054671897	0.141260096	positive
PRJNA729271	ed_Lushi_vs_Arbor_Acr	breed_growth_background	ENSGALG00010012712	EEF1E1	-0.037761203	0.071369929	-0.529091224	0.596742169	0.730432169	negative
PRJNA729271	ed_Lushi_vs_Arbor_Acr	breed_growth_background	ENSGALG00010012714	RGS2	0.052968926	0.092254354	0.574161804	0.565858314	0.705583401	positive
PRJNA729271	ed_Lushi_vs_Arbor_Acr	breed_growth_background	ENSGALG00010012719	COL10A1	0.733719913	0.426177316	1.721630612	0.085136454	0.192501774	positive
PRJNA729271	ed_Lushi_vs_Arbor_Acr	breed_growth_background	ENSGALG00010012720	ACO2	0.156838822	0.064083491	2.447413824	0.014388553	0.053795426	positive
PRJNA729271	ed_Lushi_vs_Arbor_Acr	breed_growth_background	ENSGALG00010012722	ENSGALG00010012722	-0.001651397	0.054887162	-0.030087135	0.975997561	0.985790861	negative
PRJNA729271	ed_Lushi_vs_Arbor_Acr	breed_growth_background	ENSGALG00010012725	ENSGALG00010012725	0.12439218	0.141117113	0.881481897	0.378057048	0.540321131	positive
PRJNA729271	ed_Lushi_vs_Arbor_Acr	breed_growth_background	ENSGALG00010012726	IL7R	-0.16108469	0.183534728	-0.877679622	0.380117606	0.541972552	negative
PRJNA729271	ed_Lushi_vs_Arbor_Acr	breed_growth_background	ENSGALG00010012730	CAP2	0.03425845	0.089663154	0.382079468	0.702402421	0.807720991	positive
PRJNA729271	ed_Lushi_vs_Arbor_Acr	breed_growth_background	ENSGALG00010012732	MGST1	-0.244127206	0.094303924	-2.588727964	0.009633116	0.04074804	negative
PRJNA729271	ed_Lushi_vs_Arbor_Acr	breed_growth_background	ENSGALG00010012733	ENSGALG00010012733	0.591775034	0.61375069	0.96419449	0.334948426	0.499254093	positive
PRJNA729271	ed_Lushi_vs_Arbor_Acr	breed_growth_background	ENSGALG00010012734	TMEM19	-0.081932366	0.078918546	-1.038188989	0.299182078	0.461527725	negative
PRJNA729271	ed_Lushi_vs_Arbor_Acr	breed_growth_background	ENSGALG00010012738	RAB21	0.051489714	0.036882326	1.396053871	0.162698273	0.302192654	positive
PRJNA729271	ed_Lushi_vs_Arbor_Acr	breed_growth_background	ENSGALG00010012741	ENSGALG00010012741	0.068346432	0.081179086	0.841921679	0.399831795	0.560726886	positive
PRJNA729271	ed_Lushi_vs_Arbor_Acr	breed_growth_background	ENSGALG00010012742	ENSGALG00010012742	-0.384002155	0.093400938	-4.111330806	3.93385E-05	0.000813426	negative
PRJNA729271	ed_Lushi_vs_Arbor_Acr	breed_growth_background	ENSGALG00010012743	ENSGALG00010012743	0.105198085	0.274336643	0.383463486	0.701376139	0.80733032	positive
PRJNA729271	ed_Lushi_vs_Arbor_Acr	breed_growth_background	ENSGALG00010012744	ARL4C	-0.046319853	0.126111134	-0.367293926	0.713399784	0.815844447	negative
PRJNA729271	ed_Lushi_vs_Arbor_Acr	breed_growth_background	ENSGALG00010012745	MARCHF3	-0.27587243	0.17175425	-1.606204384	0.10822901	0.227887784	negative
PRJNA729271	ed_Lushi_vs_Arbor_Acr	breed_growth_background	ENSGALG00010012747	TOB2	0.342644864	0.077676512	4.411177292	1.0281E-05	0.000298046	positive
PRJNA729271	ed_Lushi_vs_Arbor_Acr	breed_growth_background	ENSGALG00010012748	ENSGALG00010012748	-0.008796038	0.115523216	-0.076140871	0.939307024	0.966697969	negative
PRJNA729271	ed_Lushi_vs_Arbor_Acr	breed_growth_background	ENSGALG00010012749	RGS4	-0.097073388	0.093976445	-1.032954466	0.301625208	0.464091311	negative
PRJNA729271	ed_Lushi_vs_Arbor_Acr	breed_growth_background	ENSGALG00010012751	AP3B1	-0.081414385	0.040502418	-2.010111728	0.044419365	0.121469898	negative
PRJNA729271	ed_Lushi_vs_Arbor_Acr	breed_growth_background	ENSGALG00010012753	GTPBP1	0.009533864	0.028824136	0.330759768	0.740825952	0.834234942	positive
PRJNA729271	ed_Lushi_vs_Arbor_Acr	breed_growth_background	ENSGALG00010012755	CLVS2	0.34497522	0.222178736	1.552692331	0.120496655	0.245039499	positive
PRJNA729271	ed_Lushi_vs_Arbor_Acr	breed_growth_background	ENSGALG00010012756	RPA2	-0.025611592	0.057455356	-0.445765098	0.655766936	0.775566395	negative
PRJNA729271	ed_Lushi_vs_Arbor_Acr	breed_growth_background	ENSGALG00010012758	NAB1	-0.186431205	0.069173509	-2.695124311	0.007036237	0.032512038	negative
PRJNA729271	ed_Lushi_vs_Arbor_Acr	breed_growth_background	ENSGALG00010012759	ATP1B4	-0.055084698	0.103566166	-0.531879278	0.59480961	0.728928415	negative
PRJNA729271	ed_Lushi_vs_Arbor_Acr	breed_growth_background	ENSGALG00010012760	PLEKHF2	0.03732158	0.056312289	0.662760849	0.507483732	0.656783633	positive
PRJNA729271	ed_Lushi_vs_Arbor_Acr	breed_growth_background	ENSGALG00010012761	SMPDL3A	-0.625368691	0.12110196	-5.163984881	2.41747E-07	1.6472E-05	negative
PRJNA729271	ed_Lushi_vs_Arbor_Acr	breed_growth_background	ENSGALG00010012763	SMPDL3B	-0.074479882	0.074578321	-0.998680055	0.317949706	0.481421596	negative
PRJNA729271	ed_Lushi_vs_Arbor_Acr	breed_growth_background	ENSGALG00010012765	PHF5A	0.118471098	0.057473551	2.061315097	0.039272989	0.110988882	positive
PRJNA729271	ed_Lushi_vs_Arbor_Acr	breed_growth_background	ENSGALG00010012767	JOSD1	-0.048593167	0.03856638	-1.259987764	0.207673776	0.356916219	negative
PRJNA729271	ed_Lushi_vs_Arbor_Acr	breed_growth_background	ENSGALG00010012768	EPS8	-0.188558553	0.062830865	-3.00104977	0.002690506	0.016365456	negative
PRJNA729271	ed_Lushi_vs_Arbor_Acr	breed_growth_background	ENSGALG00010012769	NEMP2	0.041134948	0.046335641	0.887760414	0.374669641	0.537068477	positive
PRJNA729271	ed_Lushi_vs_Arbor_Acr	breed_growth_background	ENSGALG00010012771	ITGA2	0.067725534	0.104769642	0.646423264	0.518005278	0.666295724	positive
PRJNA729271	ed_Lushi_vs_Arbor_Acr	breed_growth_background	ENSGALG00010012772	TOMM22	-0.094524216	0.040203382	-2.351150863	0.018715444	0.065227305	negative
PRJNA729271	ed_Lushi_vs_Arbor_Acr	breed_growth_background	ENSGALG00010012773	XKR8	-0.270247978	0.070947133	-3.809145879	0.000139448	0.002027751	negative
PRJNA729271	ed_Lushi_vs_Arbor_Acr	breed_growth_background	ENSGALG00010012775	KCNE2	0.01939413	0.077710893	0.249567719	0.802921665	0.878395942	positive
PRJNA729271	ed_Lushi_vs_Arbor_Acr	breed_growth_background	ENSGALG00010012778	TBC1D15	0.087363464	0.081152713	1.076531638	0.281689559	0.441648752	positive
PRJNA729271	ed_Lushi_vs_Arbor_Acr	breed_growth_background	ENSGALG00010012780	FABP7	0.172221885	0.137844597	1.249391624	0.21152187	0.361515094	positive
PRJNA729271	ed_Lushi_vs_Arbor_Acr	breed_growth_background	ENSGALG00010012781	SPIN1L	1.090240686	1.727785616	0.631004609	0.528037511	0.673871895	positive
PRJNA729271	ed_Lushi_vs_Arbor_Acr	breed_growth_background	ENSGALG00010012786	TEF	-0.131915786	0.047030059	-2.804924954	0.00503283	0.025705027	negative
PRJNA729271	ed_Lushi_vs_Arbor_Acr	breed_growth_background	ENSGALG00010012788	RGS1	-0.160000004	0.354387081	-0.451483737	0.651640944	0.772767363	negative
PRJNA729271	ed_Lushi_vs_Arbor_Acr	breed_growth_background	ENSGALG00010012790	PITX1	1.079996751	0.284117452	3.801233416	0.000143978	0.002073162	positive
PRJNA729271	ed_Lushi_vs_Arbor_Acr	breed_growth_background	ENSGALG00010012791	CFAP418	0.041950625	0.103195986	0.406514113	0.684364889	0.796201065	positive
PRJNA729271	ed_Lushi_vs_Arbor_Acr	breed_growth_background	ENSGALG00010012792	CBY1	0.154798857	0.079769229	1.940583583	0.052308807	0.136913766	positive
PRJNA729271	ed_Lushi_vs_Arbor_Acr	breed_growth_background	ENSGALG00010012795	TBCA	0.112753409	0.073482706	1.534421036	0.124926106	0.250977672	positive
PRJNA729271	ed_Lushi_vs_Arbor_Acr	breed_growth_background	ENSGALG00010012796	MOCS2	-0.26388698	0.065636835	-4.020409858	5.8097E-05	0.001078227	negative
PRJNA729271	ed_Lushi_vs_Arbor_Acr	breed_growth_background	ENSGALG00010012797	ZC3H7B	-0.05989676	0.037777398	-1.585518414	0.112848596	0.234313194	negative
PRJNA729271	ed_Lushi_vs_Arbor_Acr	breed_growth_background	ENSGALG00010012798	MFSD6	-0.102780433	0.052468663	-1.958891774	0.050125463	0.132779346	negative
PRJNA729271	ed_Lushi_vs_Arbor_Acr	breed_growth_background	ENSGALG00010012801	DMC1	-0.328573253	0.223168952	-1.47230719	0.140937943	0.273406783	negative
PRJNA729271	ed_Lushi_vs_Arbor_Acr	breed_growth_background	ENSGALG00010012803	NUP155	0.002181751	0.048734355	0.044768232	0.964292047	0.979609321	positive
PRJNA729271	ed_Lushi_vs_Arbor_Acr	breed_growth_background	ENSGALG00010012804	ENSGALG00010012804	-0.03321937	0.086023616	-0.386165701	0.699373954	0.806092991	negative
PRJNA729271	ed_Lushi_vs_Arbor_Acr	breed_growth_background	ENSGALG00010012808	EYA3	0.148052023	0.050301304	2.943303897	0.003247295	0.018782737	positive
PRJNA729271	ed_Lushi_vs_Arbor_Acr	breed_growth_background	ENSGALG00010012809	RHOBTB3	0.456134802	0.126461917	3.606894567	0.000309883	0.003539607	positive
PRJNA729271	ed_Lushi_vs_Arbor_Acr	breed_growth_background	ENSGALG00010012812	DECR1	0.023911367	0.128136357	0.18660876	0.851967398	0.910666043	positive
PRJNA729271	ed_Lushi_vs_Arbor_Acr	breed_growth_background	ENSGALG00010012818	RGS18	-0.151707121	0.104950467	-1.445511633	0.148314228	0.283243048	negative
PRJNA729271	ed_Lushi_vs_Arbor_Acr	breed_growth_background	ENSGALG00010012819	RANGAP1	-0.004465195	0.037275541	-0.11978887	0.904650399	0.945214175	negative
PRJNA729271	ed_Lushi_vs_Arbor_Acr	breed_growth_background	ENSGALG00010012821	ZBTB33	0.045113206	0.028020563	1.610003531	0.107397085	0.22646505	positive
PRJNA729271	ed_Lushi_vs_Arbor_Acr	breed_growth_background	ENSGALG00010012824	HIBCH	-0.045082551	0.062794414	-0.717938886	0.47279497	0.626604063	negative
PRJNA729271	ed_Lushi_vs_Arbor_Acr	breed_growth_background	ENSGALG00010012827	ENSGALG00010012827	0.192110808	0.101881187	1.885635749	0.059344057	0.149676718	positive
PRJNA729271	ed_Lushi_vs_Arbor_Acr	breed_growth_background	ENSGALG00010012829	RGS5	-0.187830538	0.095533401	-1.966124274	0.049284257	0.131198403	negative
PRJNA729271	ed_Lushi_vs_Arbor_Acr	breed_growth_background	ENSGALG00010012830	ARRDC3	-0.006469979	0.079778919	-0.081098859	0.935363333	0.964536447	negative
PRJNA729271	ed_Lushi_vs_Arbor_Acr	breed_growth_background	ENSGALG00010012831	ENSGALG00010012831	-1.561953033	0.205541178	-7.599221944	2.97916E-14	2.54018E-11	negative
PRJNA729271	ed_Lushi_vs_Arbor_Acr	breed_growth_background	ENSGALG00010012832	CHADL	0.542136509	0.199778325	2.71369033	0.006653833	0.031263311	positive
PRJNA729271	ed_Lushi_vs_Arbor_Acr	breed_growth_background	ENSGALG00010012833	JAK2	-0.022236658	0.056486947	-0.393660113	0.693831994	0.802577189	negative
PRJNA729271	ed_Lushi_vs_Arbor_Acr	breed_growth_background	ENSGALG00010012834	TPH2	0.408138051	0.45679696	0.893478037	0.371601229	0.534463681	positive
PRJNA729271	ed_Lushi_vs_Arbor_Acr	breed_growth_background	ENSGALG00010012836	ENSGALG00010012836	3.689012239	0.616259688	5.986132651			positive
PRJNA729271	ed_Lushi_vs_Arbor_Acr	breed_growth_background	ENSGALG00010012837	PKIB	-0.35456821	0.144467716	-2.45430758	0.014115619	0.053203541	negative
PRJNA729271	ed_Lushi_vs_Arbor_Acr	breed_growth_background	ENSGALG00010012838	C2orf88	-0.594453916	0.12654772	-4.697468403	2.63406E-06	0.000103191	negative
PRJNA729271	ed_Lushi_vs_Arbor_Acr	breed_growth_background	ENSGALG00010012839	TGFBI	-0.136782376	0.096355248	-1.419563317	0.155734851	0.29274759	negative
PRJNA729271	ed_Lushi_vs_Arbor_Acr	breed_growth_background	ENSGALG00010012841	L3MBTL2	0.065203088	0.05162124	1.263105791	0.206551163	0.355721117	positive
PRJNA729271	ed_Lushi_vs_Arbor_Acr	breed_growth_background	ENSGALG00010012842	DDX17	-0.032589193	0.026317349	-1.238315945	0.215598934	0.366753497	negative
PRJNA729271	ed_Lushi_vs_Arbor_Acr	breed_growth_background	ENSGALG00010012843	ENSGALG00010012843	0.131165825	0.085821937	1.528348462	0.126426044	0.252974255	positive
PRJNA729271	ed_Lushi_vs_Arbor_Acr	breed_growth_background	ENSGALG00010012845	HDHD2	0.027821111	0.055107566	0.504851017	0.61366348	0.743174212	positive

PRJNA729271	ed_Lushi_vs_Arbor_Acr	breed_growth_background	ENSGALG00010012847	RLN3	0.597479125	0.432145852	1.38258674	0.166791617	0.307549229	positive
PRJNA729271	ed_Lushi_vs_Arbor_Acr	breed_growth_background	ENSGALG00010012848	SERINC1	-0.037993214	0.049468331	-0.768031043	0.442468742	0.599540924	negative
PRJNA729271	ed_Lushi_vs_Arbor_Acr	breed_growth_background	ENSGALG00010012849	TRHDE	-0.358901596	0.19431563	-1.847003231	0.064746672	0.158927103	negative
PRJNA729271	ed_Lushi_vs_Arbor_Acr	breed_growth_background	ENSGALG00010012850	CALB1	-0.361373682	0.589178248	-0.613352042	0.539643589	0.684926963	negative
PRJNA729271	ed_Lushi_vs_Arbor_Acr	breed_growth_background	ENSGALG00010012851	ENSGALG00010012851	0.324132963	0.140663747	2.304310599	0.021205214	0.071179689	positive
PRJNA729271	ed_Lushi_vs_Arbor_Acr	breed_growth_background	ENSGALG00010012852	MSTN	-0.662868605	0.224138044	-2.957412287	0.00310233	0.018191049	negative
PRJNA729271	ed_Lushi_vs_Arbor_Acr	breed_growth_background	ENSGALG00010012854	LECT2	0.659308258	0.400153601	1.647637949	0.099426988	0.213954007	positive
PRJNA729271	ed_Lushi_vs_Arbor_Acr	breed_growth_background	ENSGALG00010012856	AVR2	1.727954216	0.622035506	2.77790287	0.005471097	0.027186682	positive
PRJNA729271	ed_Lushi_vs_Arbor_Acr	breed_growth_background	ENSGALG00010012857	NSDHL	-0.126662374	0.12414347	-1.020290267	0.307590818	0.470209756	negative
PRJNA729271	ed_Lushi_vs_Arbor_Acr	breed_growth_background	ENSGALG00010012859	FBXL21	0.025914227	0.059555539	0.435127075	0.663470192	0.781552249	positive
PRJNA729271	ed_Lushi_vs_Arbor_Acr	breed_growth_background	ENSGALG00010012860	ENSGALG00010012860	0.015995088	0.140545174	0.11380745	0.909390433	0.948586956	positive
PRJNA729271	ed_Lushi_vs_Arbor_Acr	breed_growth_background	ENSGALG00010012861	WDR70	-0.068730112	0.044996347	-1.527459801	0.126646716	0.253310908	negative
PRJNA729271	ed_Lushi_vs_Arbor_Acr	breed_growth_background	ENSGALG00010012863	KCNE1	-0.774626701	0.247384822	-3.131262036	0.001740568	0.012043942	negative
PRJNA729271	ed_Lushi_vs_Arbor_Acr	breed_growth_background	ENSGALG00010012865	EP300	0.03930567	0.066100033	0.594639182	0.552084683	0.694779257	positive
PRJNA729271	ed_Lushi_vs_Arbor_Acr	breed_growth_background	ENSGALG00010012867	A4GALT	-0.29905187	0.054639239	-5.473207029	4.41963E-08	4.55817E-06	negative
PRJNA729271	ed_Lushi_vs_Arbor_Acr	breed_growth_background	ENSGALG00010012868	CTHRC1	-0.561162717	0.323184256	-1.736355365	0.082501019	0.187967978	negative
PRJNA729271	ed_Lushi_vs_Arbor_Acr	breed_growth_background	ENSGALG00010012869	ENSGALG00010012869	0.083163679	0.118305144	0.70295911	0.482081232	0.63467461	positive
PRJNA729271	ed_Lushi_vs_Arbor_Acr	breed_growth_background	ENSGALG00010012870	KCNC2	-0.052281086	0.115303052	-0.453423261	0.65024399	0.771678945	negative
PRJNA729271	ed_Lushi_vs_Arbor_Acr	breed_growth_background	ENSGALG00010012871	ENSGALG00010012871	0.136380396	0.193009143	0.706600705	0.479814651	0.632667458	positive
PRJNA729271	ed_Lushi_vs_Arbor_Acr	breed_growth_background	ENSGALG00010012873	PTAFR	-0.167720289	0.119016925	-1.409213762	0.158771969	0.29726165	negative
PRJNA729271	ed_Lushi_vs_Arbor_Acr	breed_growth_background	ENSGALG00010012875	HSF2	-0.199942953	0.072032036	-2.775750419	0.005507447	0.027283131	negative
PRJNA729271	ed_Lushi_vs_Arbor_Acr	breed_growth_background	ENSGALG00010012876	KDELR3	-0.126156994	0.087679799	-1.438837622	0.150196534	0.285708499	negative
PRJNA729271	ed_Lushi_vs_Arbor_Acr	breed_growth_background	ENSGALG00010012877	SLC25A32	0.042256491	0.051737271	0.816751462	0.414070475	0.574404397	positive
PRJNA729271	ed_Lushi_vs_Arbor_Acr	breed_growth_background	ENSGALG00010012878	GDNF	0.068802653	0.171077072	0.40217343	0.687556395	0.798120441	positive
PRJNA729271	ed_Lushi_vs_Arbor_Acr	breed_growth_background	ENSGALG00010012879	RCAN1	-0.234974564	0.092791143	-2.532295168	0.011331856	0.045746661	negative
PRJNA729271	ed_Lushi_vs_Arbor_Acr	breed_growth_background	ENSGALG00010012880	CLK4	0.137939084	0.074584334	1.849437761	0.064394627	0.158418215	positive
PRJNA729271	ed_Lushi_vs_Arbor_Acr	breed_growth_background	ENSGALG00010012882	ENSGALG00010012882	-0.654610787	0.316322416	-2.069441667	0.03850466	0.109585318	negative
PRJNA729271	ed_Lushi_vs_Arbor_Acr	breed_growth_background	ENSGALG00010012883	DCAF13	0.004685708	0.036226771	0.129343794	0.89708562	0.940220973	positive
PRJNA729271	ed_Lushi_vs_Arbor_Acr	breed_growth_background	ENSGALG00010012884	ENSGALG00010012884	0.400302539	0.115863692	3.454943755	0.000550408	0.005315229	positive
PRJNA729271	ed_Lushi_vs_Arbor_Acr	breed_growth_background	ENSGALG00010012885	PMS1	-0.093766187	0.069530458	-1.348562775	0.177477444	0.321607145	negative
PRJNA729271	ed_Lushi_vs_Arbor_Acr	breed_growth_background	ENSGALG00010012887	KCNJ4	-0.513219148	0.232227684	-2.20998263	0.027106368	0.084523818	negative
PRJNA729271	ed_Lushi_vs_Arbor_Acr	breed_growth_background	ENSGALG00010012888	ENSGALG00010012888	1.944160571	1.947513764	0.998278219	0.318144467	0.481518643	positive
PRJNA729271	ed_Lushi_vs_Arbor_Acr	breed_growth_background	ENSGALG00010012889	RIC1	-0.087991493	0.056182244	-1.566179766	0.117306538	0.240673499	negative
PRJNA729271	ed_Lushi_vs_Arbor_Acr	breed_growth_background	ENSGALG00010012890	MFAP3	0.036263193	0.037722084	0.961325293	0.336388635	0.50066262	positive
PRJNA729271	ed_Lushi_vs_Arbor_Acr	breed_growth_background	ENSGALG00010012891	CAPS2	-0.175059768	0.190841203	-0.917305934	0.358982349	0.522397622	negative
PRJNA729271	ed_Lushi_vs_Arbor_Acr	breed_growth_background	ENSGALG00010012892	ORMDL1	-0.136326887	0.088162553	-1.54631283	0.122029035	0.24672737	negative
PRJNA729271	ed_Lushi_vs_Arbor_Acr	breed_growth_background	ENSGALG00010012893	ENSGALG00010012893	-0.335363294	0.101049436	-3.318804218	0.000904038	0.007570208	negative
PRJNA729271	ed_Lushi_vs_Arbor_Acr	breed_growth_background	ENSGALG00010012894	CLIC6	-0.667166846	0.366080139	-1.822461189	0.068385038	0.165206853	negative
PRJNA729271	ed_Lushi_vs_Arbor_Acr	breed_growth_background	ENSGALG00010012896	STK38L	0.03859487	0.05337951	0.723027795	0.469662808	0.624278991	positive
PRJNA729271	ed_Lushi_vs_Arbor_Acr	breed_growth_background	ENSGALG00010012897	ENSGALG00010012897	0.147290854	0.164951434	0.892934672	0.371892158	0.534514311	positive
PRJNA729271	ed_Lushi_vs_Arbor_Acr	breed_growth_background	ENSGALG00010012898	FAM114A2	-0.010835164	0.061729731	-0.175525855	0.860666457	0.916307481	negative
PRJNA729271	ed_Lushi_vs_Arbor_Acr	breed_growth_background	ENSGALG00010012899	ENSGALG00010012899	0.116246923	0.071508765	1.625631803	0.104028006	0.221292331	positive
PRJNA729271	ed_Lushi_vs_Arbor_Acr	breed_growth_background	ENSGALG00010012900	RBX1	-0.29460689	0.092328097	-3.190869283	0.001418454	0.010511501	negative
PRJNA729271	ed_Lushi_vs_Arbor_Acr	breed_growth_background	ENSGALG00010012903	RGMB	-0.174213623	0.049539901	-3.516632451	0.000437059	0.004506972	negative
PRJNA729271	ed_Lushi_vs_Arbor_Acr	breed_growth_background	ENSGALG00010012904	ERMP1	-0.252846054	0.074190556	-3.408062522	0.000654259	0.006011406	negative
PRJNA729271	ed_Lushi_vs_Arbor_Acr	breed_growth_background	ENSGALG00010012905	NKAIN1	-0.007793789	0.135186135	-0.057652278	0.954025607	0.974325454	negative
PRJNA729271	ed_Lushi_vs_Arbor_Acr	breed_growth_background	ENSGALG00010012908	STRAP	0.07607041	0.044783413	1.698629125	0.089389085	0.199306997	positive
PRJNA729271	ed_Lushi_vs_Arbor_Acr	breed_growth_background	ENSGALG00010012910	XPNPEP3	-0.137609666	0.053879068	-2.554046894	0.010647892	0.043784734	negative
PRJNA729271	ed_Lushi_vs_Arbor_Acr	breed_growth_background	ENSGALG00010012911	CSNK1E	-0.002354401	0.046112817	-0.05105742	0.959279766	0.977517846	negative
PRJNA729271	ed_Lushi_vs_Arbor_Acr	breed_growth_background	ENSGALG00010012912	NKAPL	0.04480598	0.054214298	0.826460576	0.408542873	0.569051413	positive
PRJNA729271	ed_Lushi_vs_Arbor_Acr	breed_growth_background	ENSGALG00010012913	EGFLAM	-0.030977148	0.083299051	-0.371878764	0.709983117	0.813025405	negative
PRJNA729271	ed_Lushi_vs_Arbor_Acr	breed_growth_background	ENSGALG00010012915	ENSGALG00010012915	0.248020561	0.142011526	1.74648191	0.080727248	0.185178265	positive
PRJNA729271	ed_Lushi_vs_Arbor_Acr	breed_growth_background	ENSGALG00010012917	RUNX1	-0.27050279	0.140688536	-1.922706688	0.054516896	0.140960117	negative
PRJNA729271	ed_Lushi_vs_Arbor_Acr	breed_growth_background	ENSGALG00010012918	GLRX	0.226503479	0.151305668	1.496992696	0.134395159	0.264179255	positive
PRJNA729271	ed_Lushi_vs_Arbor_Acr	breed_growth_background	ENSGALG00010012921	EBF1	-0.37690256	0.075403631	-4.99846697	5.77879E-07	3.14901E-05	negative
PRJNA729271	ed_Lushi_vs_Arbor_Acr	breed_growth_background	ENSGALG00010012922	SNRNP40	0.04370773	0.06999777	0.624416045	0.532354391	0.677775748	positive
PRJNA729271	ed_Lushi_vs_Arbor_Acr	breed_growth_background	ENSGALG00010012923	MLANA	1.629611539	0.505789363	3.221917379	0.001273358	0.009724621	positive
PRJNA729271	ed_Lushi_vs_Arbor_Acr	breed_growth_background	ENSGALG00010012924	GLIPR1	0.1820623	0.109007122	1.670187207	0.09488233	0.207343819	positive
PRJNA729271	ed_Lushi_vs_Arbor_Acr	breed_growth_background	ENSGALG00010012925	SETD4	-0.423556299	0.107660304	-3.934191919	8.34771E-05	0.001400463	negative
PRJNA729271	ed_Lushi_vs_Arbor_Acr	breed_growth_background	ENSGALG00010012926	OSGEPL1	0.138889113	0.088332625	1.57234219	0.115871214	0.238572903	positive
PRJNA729271	ed_Lushi_vs_Arbor_Acr	breed_growth_background	ENSGALG00010012927	ENSGALG00010012927	3.821496257	1.993576517	1.916904731			positive
PRJNA729271	ed_Lushi_vs_Arbor_Acr	breed_growth_background	ENSGALG00010012930	IL6ST	-0.174785832	0.048637662	-3.593631427	0.000326101	0.003667056	negative
PRJNA729271	ed_Lushi_vs_Arbor_Acr	breed_growth_background	ENSGALG00010012932	ATF6	0.153819746	0.063328085	2.42893412	0.015143284	0.056023966	positive
PRJNA729271	ed_Lushi_vs_Arbor_Acr	breed_growth_background	ENSGALG00010012933	ZCCHC17	0.041806494	0.079879355	0.523370459	0.600716477	0.733377018	positive
PRJNA729271	ed_Lushi_vs_Arbor_Acr	breed_growth_background	ENSGALG00010012934	NDUFA1	-0.074887966	0.081041952	-0.924064192	0.355452895	0.519174699	negative
PRJNA729271	ed_Lushi_vs_Arbor_Acr	breed_growth_background	ENSGALG00010012938	UPF3B	-0.026297799	0.080710273	-0.325829632	0.744553258	0.837066584	negative
PRJNA729271	ed_Lushi_vs_Arbor_Acr	breed_growth_background	ENSGALG00010012939	GJA1	-0.505343941	0.111120435	-4.54771385	5.42318E-06	0.000183238	negative
PRJNA729271	ed_Lushi_vs_Arbor_Acr	breed_growth_background	ENSGALG00010012940	FBN2	-0.311001029	0.120134956	-2.588763831	0.009632113	0.04074804	negative
PRJNA729271	ed_Lushi_vs_Arbor_Acr	breed_growth_background	ENSGALG00010012943	ASNSD1	-0.1530273	0.066698477	-2.294314749	0.021772426	0.072516387	negative
PRJNA729271	ed_Lushi_vs_Arbor_Acr	breed_growth_background	ENSGALG00010012944	NCK1	-0.049402225	0.028622409	-1.725998173	0.084347742	0.191034455	negative
PRJNA729271	ed_Lushi_vs_Arbor_Acr	breed_growth_background	ENSGALG00010012945	CLINT1	0.032277233	0.033831108	0.954069638	0.340048394	0.50398788	positive
PRJNA729271	ed_Lushi_vs_Arbor_Acr	breed_growth_background	ENSGALG00010012948	ST13P5	-0.096195159	0.051068296	-1.883657107	0.059611372	0.150214989	negative
PRJNA729271	ed_Lushi_vs_Arbor_Acr	breed_growth_background	ENSGALG00010012949	ENSGALG00010012949	-0.152950533	0.096423673	-1.586234249	0.112686181	0.234076555	negative
PRJNA729271	ed_Lushi_vs_Arbor_Acr	breed_growth_background	ENSGALG00010012952	BRD10	-0.099370735	0.099833293	-0.995366697	0.319557949	0.482851296	negative
PRJNA729271	ed_Lushi_vs_Arbor_Acr	breed_growth_background	ENSGALG00010012954	RIMS2	-0.159965396	0.187646121	-0.852484429	0.393945278	0.555362459	negative
PRJNA729271	ed_Lushi_vs_Arbor_Acr	breed_growth_background	ENSGALG00010012955	RPL39L	0.002754874	0.082963418	0.033205885	0.973510405	0.984380341	positive
PRJNA729271	ed_Lushi_vs_Arbor_Acr	breed_growth_background	ENSGALG00010012956	FABP3	-0.018467335	0.089609425	-0.206086977	0.836722971	0.900861581	negative
PRJNA729271	ed_Lushi_vs_Arbor_Acr	breed_growth_background	ENSGALG00010012957	DERA	-0.390000406	0.122674515	-3.179147743	0.001477088	0.010813327	negative
PRJNA729271	ed_Lushi_vs_Arbor_Acr	breed_growth_background	ENSGALG00010012958	OLFML2B	0.017047445	0.109470486	0.155726407	0.876248681	0.926758455	positive
PRJNA729271	ed_Lushi_vs_Arbor_Acr	breed_growth_background	ENSGALG00010012959	TBC1D32	0.044454228	0.079242366	0.56099067	0.574803897	0.712422616	positive
PRJNA729271	ed_Lushi_vs_Arbor_Acr	breed_growth_background	ENSGALG00010012962	SLC25A17	0.318761793	0.113369978	2.811694926	0.004928122	0.025295017	positive
PRJNA729271	ed_Lushi_vs_Arbor_Acr	breed_growth_background	ENSGALG00010012963	UIMC1	0.074556098	0.049974127	1.491893957	0.135726947	0.265931616	positive
PRJNA729271	ed_Lushi_vs_Arbor_Acr	breed_growth_background	ENSGALG00010012964	CBR3	-0.000922783	0.039181045	-0.023551778	0.981210137	0.988988313	negative
PRJNA729271	ed_Lushi_vs_Arbor_Acr	breed_growth_background	ENSGALG00010012965	ENSGALG00010012965	-0.081507063	0.046106373	-1.767804703	0.077093558	0.179340576	negative
PRJNA729271	ed_Lushi_vs_Arbor_Acr	breed_growth_background	ENSGALG00010012966	GLIPR1L	-0.407681602	0.145301215	-2.805768702	0.005019671	0.025655899	negative
PRJNA729271	ed_Lushi_vs_Arbor_Acr	breed_growth_background	ENSGALG00010012967	ECRG4	-0.316223171	0.112247956	-2.817184215	0.004844673	0.024937335	negative
PRJNA729271	ed_Lushi_vs_Arbor_Acr	breed_growth_background	ENSGALG00010012968	LSM11	-0.12533739	0.042385412	-2.957087912	0.003105596	0.018202834	negative
PRJNA729271	ed_Lushi_vs_Arbor_Acr	breed_growth_background	ENSGALG00010012969	SLC12A2	0.020756594	0.076505754	0.271307622	0.786154444	0.867374086	positive
PRJNA729271	ed_Lushi_vs_Arbor_Acr	breed_growth_background	ENSGALG00010012970	UNC5A	-0.107699869	0.102735028	-1.04832667	0.294488126	0.455804099	negative
PRJNA729271	ed_Lushi_vs_Arbor_Acr	breed_growth_background	ENSGALG00010012971	ENSGALG00010012971	0.148013183	0.246404307	0.600692353	0.548044913	0.691567579	positive
PRJNA729271	ed_Lushi_vs_Arbor_Acr	breed_growth_background	ENSGALG00010012973	GLDC	0.001185588	0.18811724	0.006302388	0.994971455	0.997172872	positive
PRJNA729271	ed_Lushi_vs_Arbor_Acr	breed_growth_background	ENSGALG00010012974	SLC49A3	-0.485022461	0.120590331	-4.022067576	5.76895E-05	0.001076202	negative
PRJNA729271	ed_Lushi_vs_Arbor_Acr	breed_growth_background	ENSGALG00010012975	KRR1	0.303219838	0.050558213	5.997439814	2.00453E-09	3.72508E-07	positive
PRJNA729271	ed_Lushi_vs_Arbor_Acr	breed_growth_background	ENSGALG00010012976	SLC9A2	0.685213281	0.114752652	5.971219568	2.35487E-09	4.21405E-07	positive
PRJNA729271	ed_Lushi_vs_Arbor_Acr	breed_growth_background	ENSGALG00010012977	NKRF	0.140926299	0.054078487	2.605958625	0.009161753	0.03926659	positive
PRJNA729271	ed_Lushi_vs_Arbor_Acr	breed_growth_background	ENSGALG00010012978	HSD17B7	-0.261375583	0.114517539	-2.282406559	0.022465348	0.074210398	negative
PRJNA729271	ed_Lushi_vs_Arbor_Acr	breed_growth_background	ENSGALG00010012979	HTR1A	0.309762527	0.281621822	1.099923737	0.271365351	0.430072246	positive
PRJNA729271	ed_Lushi_vs_Arbor_Acr	breed_growth_background	ENSGALG00010012980	KICS2	-0.077140387	0.042194497	-1.828209656	0.067518092	0.163990154	negative
PRJNA729271	ed_Lushi_vs_Arbor_Acr	breed_growth_background	ENSGALG00010012982	DDR2	-0.06501682	0.130883366	-0.496753885	0.619362618	0.747266618	negative
PRJNA729271	ed_Lushi_vs_Arbor_Acr	breed_growth_background	ENSGALG00010012985	ARNTL2	-0.115700079	0.091732214	-1.261280788	0.2072077	0.356287489	negative
PRJNA729271	ed_Lushi_vs_Arbor_Acr	breed_growth_background	ENSGALG00010012986	TMEM184B	0.237438682	0.074988484	3.166335291	0.001543727	0.011088369	positive
PRJNA729271	ed_Lushi_vs_Arbor_Acr	breed_growth_background	ENSGALG00010012988	RPL18A	0.01986662	0.072124987	0.275447122	0.782972746	0.865693523	positive
PRJNA729271	ed_Lushi_vs_Arbor_Acr	breed_growth_background	ENSGALG00010012991	MFSD9	-0.012795189	0.055033051	-0.232500093	0.816149615	0.886680308	negative
PRJNA729271	ed_Lushi_vs_Arbor_Acr	breed_growth_background	ENSGALG00010012992	HARS1	-0.071183299	0.068335685	-1.041670977	0.297564249	0.459632757	negative
PRJNA729271	ed_Lushi_vs_Arbor_Acr	breed_growth_background	ENSGALG00010012993	DOP1B	-0.120418706	0.053551261	-2.248662365	0.024533985	0.078921464	negative
PRJNA729271	ed_Lushi_vs_Arbor_Acr	breed_growth_background	ENSGALG00010012995	SERINC2	0.005823326	0.079870218	0.072909855	0.941877852	0.96841306	positive
PRJNA729271	ed_Lushi_vs_Arbor_Acr	breed_growth_background	ENSGALG00010012997	UBE2A	0.023337689	0.03109306	0.75057551	0.452908164	0.609271818	positive
PRJNA729271	ed_Lushi_vs_Arbor_Acr	breed_growth_background	ENSGALG00010012998	MAFF	-0.087567549	0.107177937	-0.817029614	0.413911505	0.574293793	negative
PRJNA729271	ed_Lushi_vs_Arbor_Acr	breed_growth_background	ENSGALG00010013001	ADGRV1	-0.743611434	0.190973479	-3.893794258	9.86884E-05	0.001571965	negative
PRJNA729271	ed_Lushi_vs_Arbor_Acr	breed_growth_background	ENSGALG00010013004	UAP1	-0.022797547	0.082812787	-0.275290185	0.783093305	0.865725335	negative
PRJNA729271	ed_Lushi_vs_Arbor_Acr	breed_growth_background	ENSGALG00010013005	SLC40A1	-0.15059299	0.048684792	-3.093224487	0.001979943	0.013175592	negative
PRJNA729271	ed_Lushi_vs_Arbor_Acr	breed_growth_background	ENSGALG00010013006	GRIK2	0.422350516	0.643640625	0.65618996	0.511701908	0.66035605	positive
PRJNA729271	ed_Lushi_vs_Arbor_Acr	breed_growth_background	ENSGALG00010013008	STEEP1	-0.093855248	0.059778888	-1.570040057	0.116405793	0.239367565	negative
PRJNA729271	ed_Lushi_vs_Arbor_Acr	breed_growth_background	ENSGALG00010013012	TMEM182	0.019460292	0.080879031	0.240609851	0.809857514	0.882954846	positive
PRJNA729271	ed_Lushi_vs_Arbor_Acr	breed_growth_background	ENSGALG00010013013	DCSTAMP	0.474574917	0.5161734	0.919409868	0.35788123	0.52161774	positive
PRJNA729271	ed_Lushi_vs_Arbor_Acr	breed_growth_background	ENSGALG00010013015	PLA2G6	0.001388068	0.080347282	0.017275861	0.986216543	0.991827433	positive
PRJNA729271	ed_Lushi_vs_Arbor_Acr	breed_growth_background	ENSGALG00010013016	QRSL1	0.096979452	0.065830791	1.473162485	0.140707228	0.273141994	positive
PRJNA729271	ed_Lushi_vs_Arbor_Acr	breed_growth_background	ENSGALG00010013017	SCMH1	0.242962986	0.084463292	2.876551225	0.004020469	0.021723142	positive
PRJNA729271	ed_Lushi_vs_Arbor_Acr	breed_growth_background	ENSGALG00010013018	ID1	-0.191529068	0.103588433	-1.848942616	0.064466099	0.158567132	negative
PRJNA729271	ed_Lushi_vs_Arbor_Acr	breed_growth_background	ENSGALG00010013019	DPYS	-0.88166779	0.525031585	-1.679266191	0.093100177	0.204560719	negative
PRJNA729271	ed_Lushi_vs_Arbor_Acr	breed_growth_background	ENSGALG00010013021	SLC25A5	0.036336599	0.088453797	0.410797506	0.681221022	0.794009225	positive
PRJNA729271	ed_Lushi_vs_Arbor_Acr	breed_growth_background	ENSGALG00010013027	UHMK1	0.018269807	0.067231089	0.271746401	0.78581702	0.867312702	positive
PRJNA729271	ed_Lushi_vs_Arbor_Acr	breed_growth_background	ENSGALG00010013029	MYLK2	0.370542112	0.127506255	2.906070071	0.003659995	0.020287428	positive
PRJNA729271	ed_Lushi_vs_Arbor_Acr	breed_growth_background	ENSGALG00010013032	BAIAP2L2	0.267697035	0.202301582	1.323257245	0.185749845	0.331255414	positive
PRJNA729271	ed_Lushi_vs_Arbor_Acr	breed_growth_background	ENSGALG00010013033	MAP3K1	-0.236309915	0.067289025	-3.511864148	0.000444975	0.004558246	negative
PRJNA729271	ed_Lushi_vs_Arbor_Acr	breed_growth_background	ENSGALG00010013036	ENSGALG00010013036	-0.194293876	0.222000388	-0.87519611	0.38146721	0.542893196	negative
PRJNA729271	ed_Lushi_vs_Arbor_Acr	breed_growth_background	ENSGALG00010013037	MORC4	-0.020425664	0.047930408	-0.426152527	0.669996707	0.786491924	negative
PRJNA729271	ed_Lushi_vs_Arbor_Acr	breed_growth_background	ENSGALG00010013040	SLC25A43	0.477742147	0.108912861	4.386462212	1.15209E-05	0.000324263	positive
PRJNA729271	ed_Lushi_vs_Arbor_Acr	breed_growth_background	ENSGALG00010013041	LRP12	0.040455048	0.040354043	1.002502971	0.316100733	0.47948595	positive
PRJNA729271	ed_Lushi_vs_Arbor_Acr	breed_growth_background	ENSGALG00010013042	SLC25A48	-0.634394133	0.173729045	-3.651629648	0.000260581	0.003152862	negative
PRJNA729271	ed_Lushi_vs_Arbor_Acr	breed_growth_background	ENSGALG00010013044	ENSGALG00010013044	-0.109513853	0.06714555	-1.630991967	0.102892014	0.219529124	negative

PRJNA729271	ɔed_Lushi_vs_Arbor_Acr	breed_growth_background	ENSGALG00010013045	FSD1L	-0.093430295	0.077236463	-1.209665646	0.226407217	0.379612741	negative
PRJNA729271	ɔed_Lushi_vs_Arbor_Acr	breed_growth_background	ENSGALG00010013046	SLC12A5	-0.689411834	0.203009495	-3.395958569	0.000683887	0.006211118	negative
PRJNA729271	ɔed_Lushi_vs_Arbor_Acr	breed_growth_background	ENSGALG00010013047	CTPS1	0.046488386	0.05440856	0.854431478	0.392865961	0.554326108	positive
PRJNA729271	ɔed_Lushi_vs_Arbor_Acr	breed_growth_background	ENSGALG00010013048	WDR75	-0.095353199	0.048417865	-1.969380487	0.048909415	0.130584264	negative
PRJNA729271	ɔed_Lushi_vs_Arbor_Acr	breed_growth_background	ENSGALG00010013051	XPOT	0.167538209	0.042204569	3.969669926	7.19723E-05	0.001249387	positive
PRJNA729271	ɔed_Lushi_vs_Arbor_Acr	breed_growth_background	ENSGALG00010013053	MRTFA	0.077066881	0.093265862	0.826313929	0.408626034	0.569084778	positive
PRJNA729271	ɔed_Lushi_vs_Arbor_Acr	breed_growth_background	ENSGALG00010013056	ENSGALG00010013056	-0.138348098	0.249588293	-0.554305238	0.579369966	0.716366771	negative
PRJNA729271	ɔed_Lushi_vs_Arbor_Acr	breed_growth_background	ENSGALG00010013058	ENSGALG00010013058	0.074932177	0.14822158	0.505541613	0.613178479	0.742959295	positive
PRJNA729271	ɔed_Lushi_vs_Arbor_Acr	breed_growth_background	ENSGALG00010013062	ENSGALG00010013062	0.085110118	0.059696808	1.425706334	0.153953127	0.290408079	positive
PRJNA729271	ɔed_Lushi_vs_Arbor_Acr	breed_growth_background	ENSGALG00010013063	PARP12	0.213253569	0.11682568	1.82539977	0.067940724	0.164635087	positive
PRJNA729271	ɔed_Lushi_vs_Arbor_Acr	breed_growth_background	ENSGALG00010013065	FKTN	0.139312382	0.056298605	2.474526374	0.013341297	0.051186368	positive
PRJNA729271	ɔed_Lushi_vs_Arbor_Acr	breed_growth_background	ENSGALG00010013071	ENSGALG00010013071	-0.346336573	0.120801787	-2.866982197	0.004144063	0.022181757	negative
PRJNA729271	ɔed_Lushi_vs_Arbor_Acr	breed_growth_background	ENSGALG00010013076	RTN4IP1	0.083443789	0.045327065	1.840926382	0.065632348	0.160699473	positive
PRJNA729271	ɔed_Lushi_vs_Arbor_Acr	breed_growth_background	ENSGALG00010013078	COL5A2	-0.369143883	0.12937723	-2.853236868	0.004327635	0.022868783	negative
PRJNA729271	ɔed_Lushi_vs_Arbor_Acr	breed_growth_background	ENSGALG00010013079	ENSGALG00010013079	0.383747106	0.110901799	3.460242389	0.000539689	0.005257256	positive
PRJNA729271	ɔed_Lushi_vs_Arbor_Acr	breed_growth_background	ENSGALG00010013080	ARHGEF7	-0.070306764	0.053500355	-1.314136405	0.188800305	0.334841603	negative
PRJNA729271	ɔed_Lushi_vs_Arbor_Acr	breed_growth_background	ENSGALG00010013081	NCOA5	0.169976376	0.055135536	3.082882474	0.002050061	0.013510191	positive
PRJNA729271	ɔed_Lushi_vs_Arbor_Acr	breed_growth_background	ENSGALG00010013082	CHAF1B	0.096313961	0.063014451	1.528442445	0.126402723	0.252967135	positive
PRJNA729271	ɔed_Lushi_vs_Arbor_Acr	breed_growth_background	ENSGALG00010013084	SGSM3	0.170759544	0.06280238	2.718997947	0.006548001	0.030901708	positive
PRJNA729271	ɔed_Lushi_vs_Arbor_Acr	breed_growth_background	ENSGALG00010013085	LYSMD3	0.047795395	0.048649321	0.982447331	0.325879518	0.489443956	positive
PRJNA729271	ɔed_Lushi_vs_Arbor_Acr	breed_growth_background	ENSGALG00010013086	CXCL14	-0.653205203	0.276878083	-2.359179882	0.018315375	0.064312346	negative
PRJNA729271	ɔed_Lushi_vs_Arbor_Acr	breed_growth_background	ENSGALG00010013087	GPN2	0.00376602	0.050496415	0.074579957	0.940548921	0.967515195	positive
PRJNA729271	ɔed_Lushi_vs_Arbor_Acr	breed_growth_background	ENSGALG00010013089	TBK1	0.021786401	0.0414005	0.526235218	0.598724787	0.731685001	positive
PRJNA729271	ɔed_Lushi_vs_Arbor_Acr	breed_growth_background	ENSGALG00010013093	GPATCH3	0.169266722	0.086256472	1.96236546	0.049719951	0.131970462	positive
PRJNA729271	ɔed_Lushi_vs_Arbor_Acr	breed_growth_background	ENSGALG00010013095	ADSL	0.086990844	0.068035422	1.27861107	0.20103405	0.34994349	positive
PRJNA729271	ɔed_Lushi_vs_Arbor_Acr	breed_growth_background	ENSGALG00010013096	CLDN14	0.086482899	0.243088156	0.355767635	0.722014589	0.821669268	positive
PRJNA729271	ɔed_Lushi_vs_Arbor_Acr	breed_growth_background	ENSGALG00010013097	ENSGALG00010013097	0.321479979	0.237848045	1.351619178	0.176497172	0.320471816	positive
PRJNA729271	ɔed_Lushi_vs_Arbor_Acr	breed_growth_background	ENSGALG00010013099	SIM2	-0.026234539	0.108020411	-0.242866501	0.808108817	0.881950352	negative
PRJNA729271	ɔed_Lushi_vs_Arbor_Acr	breed_growth_background	ENSGALG00010013100	ZFPM2	-0.129529095	0.091431409	-1.416680507	0.156576359	0.293986311	negative
PRJNA729271	ɔed_Lushi_vs_Arbor_Acr	breed_growth_background	ENSGALG00010013103	AIRIM	0.212632409	0.079646715	2.669694634	0.007592026	0.034314441	positive
PRJNA729271	ɔed_Lushi_vs_Arbor_Acr	breed_growth_background	ENSGALG00010013104	HLCS	-0.284152108	0.058967175	-4.818818383	1.44411E-06	6.5825E-05	negative
PRJNA729271	ɔed_Lushi_vs_Arbor_Acr	breed_growth_background	ENSGALG00010013106	SETD9	0.086596951	0.09748162	0.888341327	0.374357176	0.536706646	positive
PRJNA729271	ɔed_Lushi_vs_Arbor_Acr	breed_growth_background	ENSGALG00010013109	ENSGALG00010013109	0.12808579	0.807270923	0.158665184	0.87393267	0.925568426	positive
PRJNA729271	ɔed_Lushi_vs_Arbor_Acr	breed_growth_background	ENSGALG00010013110	ANKRD10	-0.047321692	0.08297021	-0.570345572	0.568443337	0.707382055	negative
PRJNA729271	ɔed_Lushi_vs_Arbor_Acr	breed_growth_background	ENSGALG00010013111	RFESD	0.203924586	0.143599298	1.42009459	0.155580145	0.292641867	positive
PRJNA729271	ɔed_Lushi_vs_Arbor_Acr	breed_growth_background	ENSGALG00010013112	CD40	0.003845682	0.087220767	0.044091354	0.964831585	0.979838424	positive
PRJNA729271	ɔed_Lushi_vs_Arbor_Acr	breed_growth_background	ENSGALG00010013114	PIGP	-0.269458754	0.067059722	-4.018190717	5.86467E-05	0.001085676	negative
PRJNA729271	ɔed_Lushi_vs_Arbor_Acr	breed_growth_background	ENSGALG00010013115	ENSGALG00010013115	0.132962987	0.210374906	0.632028743	0.527368096	0.673379188	positive
PRJNA729271	ɔed_Lushi_vs_Arbor_Acr	breed_growth_background	ENSGALG00010013116	TARS	-0.225671514	0.090085059	-2.505093706	0.012241896	0.048192905	negative
PRJNA729271	ɔed_Lushi_vs_Arbor_Acr	breed_growth_background	ENSGALG00010013117	ARSK	-0.245452209	0.071230794	-3.445872137	0.00056922	0.005419051	negative
PRJNA729271	ɔed_Lushi_vs_Arbor_Acr	breed_growth_background	ENSGALG00010013119	RNF114	-0.044611753	0.042372194	-1.052854462	0.292407699	0.453829892	negative
PRJNA729271	ɔed_Lushi_vs_Arbor_Acr	breed_growth_background	ENSGALG00010013121	PCBD2	-0.275615411	0.089447853	-3.081297115	0.002061009	0.013548485	negative
PRJNA729271	ɔed_Lushi_vs_Arbor_Acr	breed_growth_background	ENSGALG00010013123	PPFIBP1	-0.237283042	0.057911471	-4.097340921	4.17923E-05	0.000850814	negative
PRJNA729271	ɔed_Lushi_vs_Arbor_Acr	breed_growth_background	ENSGALG00010013125	POU3F3	-0.005489964	0.279294717	-0.019656527	0.984317371	0.990707268	negative
PRJNA729271	ɔed_Lushi_vs_Arbor_Acr	breed_growth_background	ENSGALG00010013129	RASSF3	-0.053370752	0.059494877	-0.897064666	0.369684412	0.533032483	negative
PRJNA729271	ɔed_Lushi_vs_Arbor_Acr	breed_growth_background	ENSGALG00010013131	SPATA2	0.091803499	0.063049308	1.456058791	0.145376368	0.279288331	positive
PRJNA729271	ɔed_Lushi_vs_Arbor_Acr	breed_growth_background	ENSGALG00010013132	SKIC3	-0.116786857	0.048388139	-2.41354304	0.015798262	0.057812625	negative
PRJNA729271	ɔed_Lushi_vs_Arbor_Acr	breed_growth_background	ENSGALG00010013133	TXNDC15	0.03858137	0.065518632	0.588861033	0.555954499	0.69755094	positive
PRJNA729271	ɔed_Lushi_vs_Arbor_Acr	breed_growth_background	ENSGALG00010013134	ENSGALG00010013134	2.052253187	2.893512215	0.709260247	0.478162993	0.631272364	positive
PRJNA729271	ɔed_Lushi_vs_Arbor_Acr	breed_growth_background	ENSGALG00010013135	TTC3	-0.019880774	0.04601229	-0.432075287	0.665686695	0.783335659	negative
PRJNA729271	ɔed_Lushi_vs_Arbor_Acr	breed_growth_background	ENSGALG00010013137	TMEM121	-0.0898077	0.11118141	-0.807758237	0.419229771	0.578987226	negative
PRJNA729271	ɔed_Lushi_vs_Arbor_Acr	breed_growth_background	ENSGALG00010013139	TMEM185A	-0.011654151	0.044523272	-0.261754153	0.793510987	0.872085962	negative
PRJNA729271	ɔed_Lushi_vs_Arbor_Acr	breed_growth_background	ENSGALG00010013143	ENSGALG00010013143	-0.268350869	0.231348565	-1.159941795	0.246072505	0.401917856	negative
PRJNA729271	ɔed_Lushi_vs_Arbor_Acr	breed_growth_background	ENSGALG00010013144	TEDC1	-0.294583678	0.095230899	-3.093362355	0.001979023	0.013175592	negative
PRJNA729271	ɔed_Lushi_vs_Arbor_Acr	breed_growth_background	ENSGALG00010013145	ENSGALG00010013145	-0.173670615	0.160008224	-1.085385558	0.277750926	0.437038609	negative
PRJNA729271	ɔed_Lushi_vs_Arbor_Acr	breed_growth_background	ENSGALG00010013146	TNRC6B	0.15127273	0.063906645	2.367089199	0.017928611	0.063353797	positive
PRJNA729271	ɔed_Lushi_vs_Arbor_Acr	breed_growth_background	ENSGALG00010013149	MCTP1	-0.115233071	0.107889593	-1.068064752	0.285491304	0.4459744	negative
PRJNA729271	ɔed_Lushi_vs_Arbor_Acr	breed_growth_background	ENSGALG00010013150	GNS	0.071516433	0.055133662	1.29714643	0.194580809	0.342245944	positive
PRJNA729271	ɔed_Lushi_vs_Arbor_Acr	breed_growth_background	ENSGALG00010013152	TBXAS1	-0.332955654	0.122239434	-2.723799059	0.006453576	0.030587703	negative
PRJNA729271	ɔed_Lushi_vs_Arbor_Acr	breed_growth_background	ENSGALG00010013154	ENSGALG00010013154	-0.004560096	0.223494388	-0.020403626	0.983721392	0.990348769	negative
PRJNA729271	ɔed_Lushi_vs_Arbor_Acr	breed_growth_background	ENSGALG00010013156	ABCC9	-0.130120987	0.069295571	-1.877767726	0.060412947	0.151633882	negative
PRJNA729271	ɔed_Lushi_vs_Arbor_Acr	breed_growth_background	ENSGALG00010013158	CRIP1	-0.18279119	0.098181745	-1.861763503	0.062636433	0.155423311	negative
PRJNA729271	ɔed_Lushi_vs_Arbor_Acr	breed_growth_background	ENSGALG00010013159	ENSGALG00010013159	0.190072108	0.778824557	0.244049968	0.807192121	0.881507556	positive
PRJNA729271	ɔed_Lushi_vs_Arbor_Acr	breed_growth_background	ENSGALG00010013160	CRYBG1	-0.756564883	0.32851099	-2.303012396	0.021278146	0.071380652	negative
PRJNA729271	ɔed_Lushi_vs_Arbor_Acr	breed_growth_background	ENSGALG00010013162	IDS	-0.009402871	0.060036284	-0.156619797	0.8755445	0.926486898	negative
PRJNA729271	ɔed_Lushi_vs_Arbor_Acr	breed_growth_background	ENSGALG00010013163	ENSGALG00010013163	-0.263968537	0.264610855	-0.997572594	0.318486656	0.481746816	negative
PRJNA729271	ɔed_Lushi_vs_Arbor_Acr	breed_growth_background	ENSGALG00010013164	FAM83F	-0.264235536	0.268746499	-0.983214801	0.325501732	0.489129961	negative
PRJNA729271	ɔed_Lushi_vs_Arbor_Acr	breed_growth_background	ENSGALG00010013165	NUDC	0.076580688	0.043734235	1.751046717	0.079937863	0.184066613	positive
PRJNA729271	ɔed_Lushi_vs_Arbor_Acr	breed_growth_background	ENSGALG00010013168	VPS26C	-0.101094782	0.074047596	-1.365267577	0.172168966	0.315298693	negative
PRJNA729271	ɔed_Lushi_vs_Arbor_Acr	breed_growth_background	ENSGALG00010013170	COL3A1	-0.195095404	0.125560339	-1.55379801	0.120232605	0.244668204	negative
PRJNA729271	ɔed_Lushi_vs_Arbor_Acr	breed_growth_background	ENSGALG00010013171	SLC9A8	-0.075134721	0.073189196	-1.026582125	0.304617274	0.467142128	negative
PRJNA729271	ɔed_Lushi_vs_Arbor_Acr	breed_growth_background	ENSGALG00010013172	TNFSF13B	0.888530834	0.231335677	3.840872471	0.000122598	0.001853029	positive
PRJNA729271	ɔed_Lushi_vs_Arbor_Acr	breed_growth_background	ENSGALG00010013173	KCNJ8	-0.26456208	0.083505121	-3.168213818	0.001533787	0.011060815	negative
PRJNA729271	ɔed_Lushi_vs_Arbor_Acr	breed_growth_background	ENSGALG00010013174	ADAMTS12	-0.257981741	0.088390109	-2.918672059	0.003515258	0.019791686	negative
PRJNA729271	ɔed_Lushi_vs_Arbor_Acr	breed_growth_background	ENSGALG00010013175	HIPK2	0.273917993	0.087176708	3.142100684	0.001677403	0.011711294	positive
PRJNA729271	ɔed_Lushi_vs_Arbor_Acr	breed_growth_background	ENSGALG00010013177	OXR1	0.0750707	0.037340886	2.010415625	0.044387218	0.121463607	positive
PRJNA729271	ɔed_Lushi_vs_Arbor_Acr	breed_growth_background	ENSGALG00010013179	TBC1D30	-0.093125412	0.101981827	-0.913156934	0.36116001	0.524534935	negative
PRJNA729271	ɔed_Lushi_vs_Arbor_Acr	breed_growth_background	ENSGALG00010013180	C5orf24	-0.035361456	0.032086053	-1.10208182	0.270426102	0.429099765	negative
PRJNA729271	ɔed_Lushi_vs_Arbor_Acr	breed_growth_background	ENSGALG00010013181	DYRK1A	0.0139189	0.042238254	0.329533045	0.741752821	0.83508405	positive
PRJNA729271	ɔed_Lushi_vs_Arbor_Acr	breed_growth_background	ENSGALG00010013183	CLTA	-0.213133693	0.074095559	-2.876470529	0.004021497	0.021723142	negative
PRJNA729271	ɔed_Lushi_vs_Arbor_Acr	breed_growth_background	ENSGALG00010013184	LDHB	-0.287169365	0.061486852	-4.670419084	3.00586E-06	0.000114357	negative
PRJNA729271	ɔed_Lushi_vs_Arbor_Acr	breed_growth_background	ENSGALG00010013185	ENSGALG00010013185	-0.099073214	0.177294236	-0.55880674	0.576293622	0.713780744	negative
PRJNA729271	ɔed_Lushi_vs_Arbor_Acr	breed_growth_background	ENSGALG00010013186	DDX46	0.092731334	0.043360782	2.138599203	0.032468142	0.096624277	positive
PRJNA729271	ɔed_Lushi_vs_Arbor_Acr	breed_growth_background	ENSGALG00010013187	KDF1	-0.078381117	0.152065127	-0.515444391	0.606242519	0.737514504	negative
PRJNA729271	ɔed_Lushi_vs_Arbor_Acr	breed_growth_background	ENSGALG00010013188	ENSGALG00010013188	-0.312012295	0.195323699	-1.597411353	0.110174044	0.230685935	negative
PRJNA729271	ɔed_Lushi_vs_Arbor_Acr	breed_growth_background	ENSGALG00010013190	SLC45A2	0.543551771	0.284536188	1.910308056	0.056093561	0.14395031	positive
PRJNA729271	ɔed_Lushi_vs_Arbor_Acr	breed_growth_background	ENSGALG00010013191	ABRA	0.206903608	0.219438655	0.942876761	0.345743943	0.509662861	positive
PRJNA729271	ɔed_Lushi_vs_Arbor_Acr	breed_growth_background	ENSGALG00010013192	B4GALT5	-0.045627141	0.044104759	-1.03451741	0.300894342	0.463393251	negative
PRJNA729271	ɔed_Lushi_vs_Arbor_Acr	breed_growth_background	ENSGALG00010013194	GRAP2	0.350997147	0.220926836	1.588748351	0.112117218	0.233365185	positive
PRJNA729271	ɔed_Lushi_vs_Arbor_Acr	breed_growth_background	ENSGALG00010013196	MRPS9	0.044472201	0.061524766	0.72283413	0.469781796	0.624365565	positive
PRJNA729271	ɔed_Lushi_vs_Arbor_Acr	breed_growth_background	ENSGALG00010013198	CAMLG	0.01321249	0.047255623	0.279596151	0.77978736	0.863616885	positive
PRJNA729271	ɔed_Lushi_vs_Arbor_Acr	breed_growth_background	ENSGALG00010013199	RALYL	0.349935999	0.376048792	0.930560093	0.352081165	0.516070027	positive
PRJNA729271	ɔed_Lushi_vs_Arbor_Acr	breed_growth_background	ENSGALG00010013202	ENSGALG00010013202	0.011478773	0.375805584	0.030544444	0.975632849	0.985697229	positive
PRJNA729271	ɔed_Lushi_vs_Arbor_Acr	breed_growth_background	ENSGALG00010013203	AMACR	-0.120309852	0.064521274	-1.864654016	0.062229916	0.154747407	negative
PRJNA729271	ɔed_Lushi_vs_Arbor_Acr	breed_growth_background	ENSGALG00010013204	ENSGALG00010013204	0.006019702	0.186904874	0.032207303	0.974306733	0.984906625	positive
PRJNA729271	ɔed_Lushi_vs_Arbor_Acr	breed_growth_background	ENSGALG00010013205	KCNJ6	-0.568254993	0.200447623	-2.834930064	0.004583572	0.023907475	negative
PRJNA729271	ɔed_Lushi_vs_Arbor_Acr	breed_growth_background	ENSGALG00010013206	AFF2	0.100082106	0.060928534	1.642614695	0.100462678	0.215862218	positive
PRJNA729271	ɔed_Lushi_vs_Arbor_Acr	breed_growth_background	ENSGALG00010013207	C1QTNF3	-0.177184372	0.106880543	-1.65777948	0.097361984	0.210793423	negative
PRJNA729271	ɔed_Lushi_vs_Arbor_Acr	breed_growth_background	ENSGALG00010013208	ENSGALG00010013208	0.071341782	0.168505631	0.423379216	0.672018606	0.787780808	positive
PRJNA729271	ɔed_Lushi_vs_Arbor_Acr	breed_growth_background	ENSGALG00010013209	KIAA1549	0.044429434	0.056247375	0.789893478	0.42958998	0.588166363	positive
PRJNA729271	ɔed_Lushi_vs_Arbor_Acr	breed_growth_background	ENSGALG00010013212	ENSGALG00010013212	-0.095807551	0.081704528	-1.172610049	0.240952218	0.39675138	negative
PRJNA729271	ɔed_Lushi_vs_Arbor_Acr	breed_growth_background	ENSGALG00010013213	SH2D1A	0.791869951	0.19061079	4.154381567	3.26169E-05	0.000707214	positive
PRJNA729271	ɔed_Lushi_vs_Arbor_Acr	breed_growth_background	ENSGALG00010013214	RAI14	-0.357858049	0.090575527	-3.95093533	7.78464E-05	0.001318204	negative
PRJNA729271	ɔed_Lushi_vs_Arbor_Acr	breed_growth_background	ENSGALG00010013215	SF3A3	0.108081411	0.044699092	2.417977773	0.01560703	0.057373548	positive
PRJNA729271	ɔed_Lushi_vs_Arbor_Acr	breed_growth_background	ENSGALG00010013217	TGFBRAP1	-0.034971522	0.045146764	-0.774618583	0.438565073	0.595950196	negative
PRJNA729271	ɔed_Lushi_vs_Arbor_Acr	breed_growth_background	ENSGALG00010013220	CSTF1	0.150908433	0.04676274	3.227108431	0.001250481	0.009605573	positive
PRJNA729271	ɔed_Lushi_vs_Arbor_Acr	breed_growth_background	ENSGALG00010013221	C2orf49	-0.053173552	0.064033648	-0.830400164	0.406312573	0.567225344	negative
PRJNA729271	ɔed_Lushi_vs_Arbor_Acr	breed_growth_background	ENSGALG00010013224	ATP6V0A4	0.371951536	0.393965985	0.944120938	0.345107851	0.508950202	positive
PRJNA729271	ɔed_Lushi_vs_Arbor_Acr	breed_growth_background	ENSGALG00010013225	GNE	-0.050701733	0.052960278	-0.957353977	0.33838862	0.502710161	negative
PRJNA729271	ɔed_Lushi_vs_Arbor_Acr	breed_growth_background	ENSGALG00010013226	DUSP6	-0.186687829	0.061610103	-3.03014958	0.002444326	0.015371155	negative
PRJNA729271	ɔed_Lushi_vs_Arbor_Acr	breed_growth_background	ENSGALG00010013227	CITED4	-0.257801787	0.086050937	-2.995920732	0.002736176	0.01658141	negative
PRJNA729271	ɔed_Lushi_vs_Arbor_Acr	breed_growth_background	ENSGALG00010013228	RTFDC1	0.117635942	0.045493915	2.585751124	0.009716706	0.040966741	positive
PRJNA729271	ɔed_Lushi_vs_Arbor_Acr	breed_growth_background	ENSGALG00010013230	ENSGALG00010013230	0.234011247	0.18123083	1.291233103	0.19662286	0.345083951	positive
PRJNA729271	ɔed_Lushi_vs_Arbor_Acr	breed_growth_background	ENSGALG00010013232	FHL2	0.978409651	0.225614687	4.336639892	1.44677E-05	0.000381291	positive
PRJNA729271	ɔed_Lushi_vs_Arbor_Acr	breed_growth_background	ENSGALG00010013233	LRRCC1	-0.019476666	0.071477088	-0.272488248	0.785246626	0.867080252	negative
PRJNA729271	ɔed_Lushi_vs_Arbor_Acr	breed_growth_background	ENSGALG00010013235	GCNT7	-0.173242297	0.136588546	-1.268351571	0.204672428	0.354024683	negative
PRJNA729271	ɔed_Lushi_vs_Arbor_Acr	breed_growth_background	ENSGALG00010013236	MON2	0.082500237	0.044406505	1.857841265	0.063191559	0.156453626	positive
PRJNA729271	ɔed_Lushi_vs_Arbor_Acr	breed_growth_background	ENSGALG00010013238	RAD1	0.047007674	0.072992709	0.644005065	0.51957215	0.667335282	positive
PRJNA729271	ɔed_Lushi_vs_Arbor_Acr	breed_growth_background	ENSGALG00010013241	SEC24A	0.336595644	0.072605588	4.635946822	3.55307E-06	0.000130055	positive
PRJNA729271	ɔed_Lushi_vs_Arbor_Acr	breed_growth_background	ENSGALG00010013242	SULT4A1	-0.033201077	0.087672068	-0.378696181	0.70491349	0.809181857	negative
PRJNA729271	ɔed_Lushi_vs_Arbor_Acr	breed_growth_background	ENSGALG00010013244	TRIM24	0.088585541	0.079644607	1.112260384	0.266026212	0.424230993	positive
PRJNA729271	ɔed_Lushi_vs_Arbor_Acr	breed_growth_background	ENSGALG00010013246	LNPEP	-0.029193828	0.093396483	-0.312579525	0.754600136	0.844888689	negative
PRJNA729271	ɔed_Lushi_vs_Arbor_Acr	breed_growth_background	ENSGALG00010013247	ATG5	0.027595545	0.050183933	0.549888057	0.582396156	0.718616006	positive
PRJNA729271	ɔed_Lushi_vs_Arbor_Acr	breed_growth_background	ENSGALG00010013248	ENSGALG00010013248	0.560564238	0.368781312	1.520045131	0.128499633	0.255746558	positive
PRJNA729271	ɔed_Lushi_vs_Arbor_Acr	breed_growth_background	ENSGALG00010013249	AURKA	-0.356488028	0.125440376	-2.841892205	0.004484665	0.023495037	negative
PRJNA729271	ɔed_Lushi_vs_Arbor_Acr	breed_growth_background	ENSGALG00010013250	E2F5	0.215399294	0.052931889	4.069367247	4.7141E-05	0.00092339	positive
PRJNA729271	ɔed_Lushi_vs_Arbor_Acr	breed_growth_background	ENSGALG00010013251	MTA1	0.017869026	0.044562746	0.400985751	0.688430614	0.798539286	positive
PRJNA729271	ɔed_Lushi_vs_Arbor_Acr	breed_growth_background	ENSGALG00010013252	PPM1H	-0.176506849	0.107328693	-1.644544844	0.100063709	0.215164436	negative
PRJNA729271	ɔed_Lushi_vs_Arbor_Acr	breed_growth_background	ENSGALG00010013253	SHB	0.206503918	0.05299486	3.896678236	9.7521E-05	0.001561953	positive
PRJNA729271	ɔed_Lushi_vs_Arbor_Acr	breed_growth_background	ENSGALG00010013254	SAR1B	0.321665827	0.075748399	4.246503328	2.17133E-05	0.000507635	positive
PRJNA729271	ɔed_Lushi_vs_Arbor_Acr	breed_growth_background	ENSGALG00010013256	BRIX1	0.141819589	0.063465111	2.234607132	0.025443152	0.080983419	positive

PRJNA729271	ed_Lushi_vs_Arbor_Acr	breed_growth_background	ENSGALG00010013257	STX12	−0.057127529	0.048657398	−1.174076948	0.240364206	0.396125022	negative
PRJNA729271	ed_Lushi_vs_Arbor_Acr	breed_growth_background	ENSGALG00010013258	RBIS	0.067665578	0.078153011	0.86580897	0.38659493	0.548628696	positive
PRJNA729271	ed_Lushi_vs_Arbor_Acr	breed_growth_background	ENSGALG00010013259	PTPRB	−0.144397475	0.074267605	−1.944286126	0.051860957	0.135965767	negative
PRJNA729271	ed_Lushi_vs_Arbor_Acr	breed_growth_background	ENSGALG00010013260	FAM210B	0.271690165	0.045077741	6.027146848	1.66879E-09	3.22972E-07	positive
PRJNA729271	ed_Lushi_vs_Arbor_Acr	breed_growth_background	ENSGALG00010013261	JADE2	0.296545502	0.105492298	2.811063052	0.004937811	0.025335776	positive
PRJNA729271	ed_Lushi_vs_Arbor_Acr	breed_growth_background	ENSGALG00010013262	CA13	−0.12834378	0.141395586	−0.907692974	0.364040457	0.527412936	negative
PRJNA729271	ed_Lushi_vs_Arbor_Acr	breed_growth_background	ENSGALG00010013263	ATP11A	−0.113879068	0.060974873	−1.867639267	0.061812369	0.154158688	negative
PRJNA729271	ed_Lushi_vs_Arbor_Acr	breed_growth_background	ENSGALG00010013264	DNAJC21	0.172229787	0.065985684	2.610108376	0.009051354	0.038885412	positive
PRJNA729271	ed_Lushi_vs_Arbor_Acr	breed_growth_background	ENSGALG00010013265	PACS2	0.11950972	0.065120935	1.835196627	0.066476561	0.162300446	positive
PRJNA729271	ed_Lushi_vs_Arbor_Acr	breed_growth_background	ENSGALG00010013266	KIAA1755	−0.485237765	0.099769078	−4.863608787	1.15265E-06	5.49592E-05	negative
PRJNA729271	ed_Lushi_vs_Arbor_Acr	breed_growth_background	ENSGALG00010013267	PNPLA3	0.07587148	0.09607489	0.789711857	0.429696065	0.588187903	positive
PRJNA729271	ed_Lushi_vs_Arbor_Acr	breed_growth_background	ENSGALG00010013268	PTPRR	−0.33043006	0.140085262	−2.35877819	0.018335211	0.064349095	negative
PRJNA729271	ed_Lushi_vs_Arbor_Acr	breed_growth_background	ENSGALG00010013269	INPP5B	0.184367648	0.054463945	3.385132103	0.00071144	0.006397223	positive
PRJNA729271	ed_Lushi_vs_Arbor_Acr	breed_growth_background	ENSGALG00010013271	GULP1	−0.11574976	0.074511333	−1.553451731	0.120315252	0.244767661	negative
PRJNA729271	ed_Lushi_vs_Arbor_Acr	breed_growth_background	ENSGALG00010013273	STAG2	0.298586247	0.062171582	4.802616222	1.56606E-06	7.00618E-05	positive
PRJNA729271	ed_Lushi_vs_Arbor_Acr	breed_growth_background	ENSGALG00010013274	MTF1	0.243098508	0.064102965	3.792312984	0.000149251	0.002128345	positive
PRJNA729271	ed_Lushi_vs_Arbor_Acr	breed_growth_background	ENSGALG00010013275	MAPRE1	−0.15391714	0.041108253	−3.744190744	0.000180976	0.002423367	negative
PRJNA729271	ed_Lushi_vs_Arbor_Acr	breed_growth_background	ENSGALG00010013276	POC1B	−0.06241083	0.060507796	−1.031451053	0.30232935	0.464813739	negative
PRJNA729271	ed_Lushi_vs_Arbor_Acr	breed_growth_background	ENSGALG00010013277	YRDC	0.07540532	0.051621715	1.460728675	0.144089916	0.277441995	positive
PRJNA729271	ed_Lushi_vs_Arbor_Acr	breed_growth_background	ENSGALG00010013278	SAMM50	−0.015587431	0.055045504	−0.283173547	0.777043805	0.86149992	negative
PRJNA729271	ed_Lushi_vs_Arbor_Acr	breed_growth_background	ENSGALG00010013280	FRS2	−0.033828176	0.060702783	−0.557275532	0.577339191	0.714751649	negative
PRJNA729271	ed_Lushi_vs_Arbor_Acr	breed_growth_background	ENSGALG00010013281	BIRC8	0.176911462	0.073913122	2.393505478	0.016688232	0.060053606	positive
PRJNA729271	ed_Lushi_vs_Arbor_Acr	breed_growth_background	ENSGALG00010013282	SEC22B	−0.131719547	0.048767475	−2.700971217	0.006913732	0.032120047	negative
PRJNA729271	ed_Lushi_vs_Arbor_Acr	breed_growth_background	ENSGALG00010013283	PAWR	−0.358674818	0.152359963	−2.354127763	0.018566228	0.064895918	negative
PRJNA729271	ed_Lushi_vs_Arbor_Acr	breed_growth_background	ENSGALG00010013284	MANEAL	−0.312180453	0.182229699	−1.713115121	0.086691363	0.194790157	negative
PRJNA729271	ed_Lushi_vs_Arbor_Acr	breed_growth_background	ENSGALG00010013285	ATP2B1	−0.100973957	0.058045017	−1.739580115	0.081932773	0.187026071	negative
PRJNA729271	ed_Lushi_vs_Arbor_Acr	breed_growth_background	ENSGALG00010013286	RNF14	0.183302214	0.064036698	2.862455727	0.00420372	0.022344305	positive
PRJNA729271	ed_Lushi_vs_Arbor_Acr	breed_growth_background	ENSGALG00010013288	CA3A	1.84451969	0.412034371	4.476616078	7.58354E-06	0.000235382	positive
PRJNA729271	ed_Lushi_vs_Arbor_Acr	breed_growth_background	ENSGALG00010013289	TM9SF4	−0.072615337	0.040815463	−1.779113408	0.075221171	0.176618218	negative
PRJNA729271	ed_Lushi_vs_Arbor_Acr	breed_growth_background	ENSGALG00010013290	AKR1D1	−0.000504842	0.181469326	−0.002781971	0.997780312	0.998913967	negative
PRJNA729271	ed_Lushi_vs_Arbor_Acr	breed_growth_background	ENSGALG00010013292	PRDM1	−0.102145323	0.160612738	−0.635972744	0.524794187	0.67157162	negative
PRJNA729271	ed_Lushi_vs_Arbor_Acr	breed_growth_background	ENSGALG00010013293	TUBGCP3	−0.069000037	0.032060522	−2.152180702	0.031383125	0.094220877	negative
PRJNA729271	ed_Lushi_vs_Arbor_Acr	breed_growth_background	ENSGALG00010013295	CA3B	0.168581813	0.218387206	0.77193997	0.440149983	0.597543692	positive
PRJNA729271	ed_Lushi_vs_Arbor_Acr	breed_growth_background	ENSGALG00010013296	AGXT2	−0.249950437	0.24476405	−1.021189334	0.307164745	0.469852136	negative
PRJNA729271	ed_Lushi_vs_Arbor_Acr	breed_growth_background	ENSGALG00010013297	FCHO2	−0.04549497	0.067353283	−0.675467746	0.499378637	0.650651087	negative
PRJNA729271	ed_Lushi_vs_Arbor_Acr	breed_growth_background	ENSGALG00010013298	EPYC	−0.461562769	0.224526344	−2.055717656	0.039809739	0.111981791	negative
PRJNA729271	ed_Lushi_vs_Arbor_Acr	breed_growth_background	ENSGALG00010013299	PLAGL2	0.026042188	0.061240301	0.425245922	0.670657411	0.787008002	positive
PRJNA729271	ed_Lushi_vs_Arbor_Acr	breed_growth_background	ENSGALG00010013300	GNPDA1	−0.173256963	0.053616381	−3.231418466	0.001231775	0.009507227	negative
PRJNA729271	ed_Lushi_vs_Arbor_Acr	breed_growth_background	ENSGALG00010013301	PARVB	−0.339928907	0.075900633	−4.478604358	7.51326E-06	0.000234179	negative
PRJNA729271	ed_Lushi_vs_Arbor_Acr	breed_growth_background	ENSGALG00010013302	ENSGALG00010013302	−0.247736618	0.318583267	−0.77761968	0.436793256	0.594322561	negative
PRJNA729271	ed_Lushi_vs_Arbor_Acr	breed_growth_background	ENSGALG00010013303	TFPI	−0.053519246	0.12593024	−0.42499122	0.670843075	0.787039285	negative
PRJNA729271	ed_Lushi_vs_Arbor_Acr	breed_growth_background	ENSGALG00010013304	KCNJ15	−0.407908949	0.127089314	−3.209624275	0.001329086	0.010014133	negative
PRJNA729271	ed_Lushi_vs_Arbor_Acr	breed_growth_background	ENSGALG00010013305	THOC2	0.077686467	0.048895339	1.588831756	0.112098382	0.233365185	positive
PRJNA729271	ed_Lushi_vs_Arbor_Acr	breed_growth_background	ENSGALG00010013306	ARSB	−0.106110288	0.080823704	−1.312860992	0.189229793	0.33536927	negative
PRJNA729271	ed_Lushi_vs_Arbor_Acr	breed_growth_background	ENSGALG00010013307	POFUT1	−0.152634008	0.048545292	−3.144156796	0.001665662	0.011669292	negative
PRJNA729271	ed_Lushi_vs_Arbor_Acr	breed_growth_background	ENSGALG00010013309	ENSGALG00010013309	0.29280378	0.154446233	1.895829858	0.057982546	0.147125192	positive
PRJNA729271	ed_Lushi_vs_Arbor_Acr	breed_growth_background	ENSGALG00010013310	MYO16	−0.344139872	0.161357279	−2.132781825	0.032942628	0.097448847	negative
PRJNA729271	ed_Lushi_vs_Arbor_Acr	breed_growth_background	ENSGALG00010013311	RSPO1	0.262359868	0.151053134	1.736871391	0.082409874	0.187789833	positive
PRJNA729271	ed_Lushi_vs_Arbor_Acr	breed_growth_background	ENSGALG00010013312	ENSGALG00010013312	−0.119419093	0.349002477	−0.342172623	0.732220987	0.828240196	negative
PRJNA729271	ed_Lushi_vs_Arbor_Acr	breed_growth_background	ENSGALG00010013313	GAK	−0.049694655	0.050925796	−0.975824794	0.329151296	0.492927785	negative
PRJNA729271	ed_Lushi_vs_Arbor_Acr	breed_growth_background	ENSGALG00010013314	KIT	−0.003899873	0.110993357	−0.035136096	0.971971219	0.983408337	negative
PRJNA729271	ed_Lushi_vs_Arbor_Acr	breed_growth_background	ENSGALG00010013316	KDM7A	0.162425328	0.069303082	2.343695598	0.019093748	0.066227297	positive
PRJNA729271	ed_Lushi_vs_Arbor_Acr	breed_growth_background	ENSGALG00010013317	PREP	−0.171298337	0.058893829	−2.908595699	0.00363056	0.020162825	negative
PRJNA729271	ed_Lushi_vs_Arbor_Acr	breed_growth_background	ENSGALG00010013318	KERA	−1.065910792	0.262736626	−4.056955465	4.97166E-05	0.000962511	negative
PRJNA729271	ed_Lushi_vs_Arbor_Acr	breed_growth_background	ENSGALG00010013319	ENSGALG00010013319	1.70485412	0.841859747	2.025104687			positive
PRJNA729271	ed_Lushi_vs_Arbor_Acr	breed_growth_background	ENSGALG00010013320	CALCRL	−0.054722162	0.066248582	−0.826012584	0.408796954	0.569177299	negative
PRJNA729271	ed_Lushi_vs_Arbor_Acr	breed_growth_background	ENSGALG00010013322	IRS2	0.763169009	0.274081863	2.784456438	0.005361752	0.026781047	positive
PRJNA729271	ed_Lushi_vs_Arbor_Acr	breed_growth_background	ENSGALG00010013323	NOTCH2	−0.277316576	0.058282117	−4.758176092	1.9535E-06	8.32823E-05	negative
PRJNA729271	ed_Lushi_vs_Arbor_Acr	breed_growth_background	ENSGALG00010013324	PARVG	−0.121486891	0.079467369	−1.528764484	0.12632284	0.252942335	negative
PRJNA729271	ed_Lushi_vs_Arbor_Acr	breed_growth_background	ENSGALG00010013325	CA2	0.288888698	0.169346886	1.705899092	0.088026869	0.196996984	positive
PRJNA729271	ed_Lushi_vs_Arbor_Acr	breed_growth_background	ENSGALG00010013326	FGF20	0.06094252	0.546357681	0.111543266	0.911185558	0.949705991	positive
PRJNA729271	ed_Lushi_vs_Arbor_Acr	breed_growth_background	ENSGALG00010013327	SLF1	−0.009771426	0.054093439	−0.180639759	0.856650346	0.91386933	negative
PRJNA729271	ed_Lushi_vs_Arbor_Acr	breed_growth_background	ENSGALG00010013328	SLC37A3	0.017158349	0.042786608	0.401021474	0.688404314	0.798539286	positive
PRJNA729271	ed_Lushi_vs_Arbor_Acr	breed_growth_background	ENSGALG00010013329	LIFR	−0.177818891	0.065696381	−2.706677114	0.006796032	0.031735173	negative
PRJNA729271	ed_Lushi_vs_Arbor_Acr	breed_growth_background	ENSGALG00010013330	ERG	−0.185993433	0.069780882	−2.665392386	0.007689854	0.034670119	negative
PRJNA729271	ed_Lushi_vs_Arbor_Acr	breed_growth_background	ENSGALG00010013331	KIF3B	0.204359764	0.081321559	2.512983852	0.011971481	0.047489497	positive
PRJNA729271	ed_Lushi_vs_Arbor_Acr	breed_growth_background	ENSGALG00010013332	LUM	−0.505007785	0.171442911	−2.945632356	0.003222952	0.01867174	negative
PRJNA729271	ed_Lushi_vs_Arbor_Acr	breed_growth_background	ENSGALG00010013333	PPP1R12A	−0.021315584	0.05255066	−0.405619726	0.685022033	0.796582548	negative
PRJNA729271	ed_Lushi_vs_Arbor_Acr	breed_growth_background	ENSGALG00010013334	SH3BGRL3	−0.124602204	0.064840746	−1.921665192	0.0546479	0.141248364	negative
PRJNA729271	ed_Lushi_vs_Arbor_Acr	breed_growth_background	ENSGALG00010013335	POPDC3	0.499823615	0.093739547	5.332046397	9.71121E-08	8.04366E-06	positive
PRJNA729271	ed_Lushi_vs_Arbor_Acr	breed_growth_background	ENSGALG00010013336	MTNR1A	−0.104763356	0.366736055	−0.285664184	0.775135339	0.860371142	negative
PRJNA729271	ed_Lushi_vs_Arbor_Acr	breed_growth_background	ENSGALG00010013337	RAB19	−0.071064221	0.136795317	−0.519493083	0.603416935	0.735435001	negative
PRJNA729271	ed_Lushi_vs_Arbor_Acr	breed_growth_background	ENSGALG00010013339	ATP6V0D2	0.278466767	0.311190165	0.894844371	0.370870288	0.534051741	positive
PRJNA729271	ed_Lushi_vs_Arbor_Acr	breed_growth_background	ENSGALG00010013340	LIN28B	0.089159654	0.248054583	0.359435627	0.719269227	0.820219293	positive
PRJNA729271	ed_Lushi_vs_Arbor_Acr	breed_growth_background	ENSGALG00010013343	SHISAL1	−0.007561312	0.245155034	−0.030842982	0.975394762	0.985621614	negative
PRJNA729271	ed_Lushi_vs_Arbor_Acr	breed_growth_background	ENSGALG00010013344	OSMR	−0.116287634	0.065892479	−1.764808922	0.077595885	0.179960376	negative
PRJNA729271	ed_Lushi_vs_Arbor_Acr	breed_growth_background	ENSGALG00010013345	DCN	−0.266286643	0.114272723	−2.330273013	0.019791726	0.06769256	negative
PRJNA729271	ed_Lushi_vs_Arbor_Acr	breed_growth_background	ENSGALG00010013346	HMGCS2	−1.403151734	0.374090592	−3.750834063	0.000176247	0.002372056	negative
PRJNA729271	ed_Lushi_vs_Arbor_Acr	breed_growth_background	ENSGALG00010013347	ENSGALG00010013347	0.181698094	0.16938565	1.072688824	0.283410756	0.443632712	positive
PRJNA729271	ed_Lushi_vs_Arbor_Acr	breed_growth_background	ENSGALG00010013348	MKRN4P	0.028652111	0.037647184	0.761069155	0.446615762	0.603428293	positive
PRJNA729271	ed_Lushi_vs_Arbor_Acr	breed_growth_background	ENSGALG00010013349	ETS2	−0.013828356	0.054309745	−0.254620163	0.799016485	0.875547622	negative
PRJNA729271	ed_Lushi_vs_Arbor_Acr	breed_growth_background	ENSGALG00010013351	EDAR	0.225780195	0.235960128	0.956857404	0.338639234	0.502942842	positive
PRJNA729271	ed_Lushi_vs_Arbor_Acr	breed_growth_background	ENSGALG00010013352	ASXL1	0.011405912	0.048259075	0.236347515	0.813163019	0.885027629	positive
PRJNA729271	ed_Lushi_vs_Arbor_Acr	breed_growth_background	ENSGALG00010013353	NDFIP1	0.054919154	0.045480653	1.207527824	0.227228936	0.380335933	positive
PRJNA729271	ed_Lushi_vs_Arbor_Acr	breed_growth_background	ENSGALG00010013354	WWP1	0.244780848	0.077634104	3.153006677	0.001615981	0.011439265	positive
PRJNA729271	ed_Lushi_vs_Arbor_Acr	breed_growth_background	ENSGALG00010013355	FAM171B	−0.090434353	0.082676896	−1.093828602	0.274030157	0.433017239	negative
PRJNA729271	ed_Lushi_vs_Arbor_Acr	breed_growth_background	ENSGALG00010013356	AMER1	0.036574268	0.035567519	1.028305279	0.303806248	0.466378147	positive
PRJNA729271	ed_Lushi_vs_Arbor_Acr	breed_growth_background	ENSGALG00010013357	RICTOR	−0.10440145	0.0588348	−1.774484679	0.075983014	0.177704179	negative
PRJNA729271	ed_Lushi_vs_Arbor_Acr	breed_growth_background	ENSGALG00010013358	RPS16	−0.170273942	0.093096723	−1.829000376	0.067399552	0.163848368	negative
PRJNA729271	ed_Lushi_vs_Arbor_Acr	breed_growth_background	ENSGALG00010013359	BTG1	−0.051548234	0.070850828	−0.727560076	0.466882927	0.621856316	negative
PRJNA729271	ed_Lushi_vs_Arbor_Acr	breed_growth_background	ENSGALG00010013360	PRR5	−0.293363419	0.061904523	−4.738965828	2.14812E-06	8.87093E-05	negative
PRJNA729271	ed_Lushi_vs_Arbor_Acr	breed_growth_background	ENSGALG00010013361	ENSGALG00010013361	0.146441488	0.125571986	1.166195518	0.243535404	0.399145826	positive
PRJNA729271	ed_Lushi_vs_Arbor_Acr	breed_growth_background	ENSGALG00010013362	ENSGALG00010013362	−0.265106909	0.182446107	−1.453069698	0.146204403	0.280507323	negative
PRJNA729271	ed_Lushi_vs_Arbor_Acr	breed_growth_background	ENSGALG00010013363	HS3ST5	−0.245480388	0.120203143	−2.042212719	0.041130435	0.114689757	negative
PRJNA729271	ed_Lushi_vs_Arbor_Acr	breed_growth_background	ENSGALG00010013364	OTOGL	−0.006668561	0.174733193	−0.038164251	0.969556724	0.982503126	negative
PRJNA729271	ed_Lushi_vs_Arbor_Acr	breed_growth_background	ENSGALG00010013365	CEP85	0.241548128	0.121407955	1.989557675	0.046639683	0.125943071	positive
PRJNA729271	ed_Lushi_vs_Arbor_Acr	breed_growth_background	ENSGALG00010013367	NOL4L	0.014396089	0.096334506	0.149438553	0.881207592	0.929426184	positive
PRJNA729271	ed_Lushi_vs_Arbor_Acr	breed_growth_background	ENSGALG00010013369	MTMR8	−0.075319016	0.03424851	−2.199191021	0.027864343	0.086173169	negative
PRJNA729271	ed_Lushi_vs_Arbor_Acr	breed_growth_background	ENSGALG00010013370	CNKSR1	0.378900068	0.121266881	3.124514005	0.00178099	0.012246421	positive
PRJNA729271	ed_Lushi_vs_Arbor_Acr	breed_growth_background	ENSGALG00010013371	FAT1	−0.071985861	0.083780237	−0.859222464	0.390217792	0.551782232	negative
PRJNA729271	ed_Lushi_vs_Arbor_Acr	breed_growth_background	ENSGALG00010013372	SPRY4	−0.153993546	0.163359446	−0.942666922	0.345851298	0.509710198	negative
PRJNA729271	ed_Lushi_vs_Arbor_Acr	breed_growth_background	ENSGALG00010013373	ENSGALG00010013373	1.016099176	0.307629381	3.302997822	0.000956571	0.007896071	positive
PRJNA729271	ed_Lushi_vs_Arbor_Acr	breed_growth_background	ENSGALG00010013374	RMDN1	0.057080411	0.088963443	0.641616486	0.521122226	0.668466077	positive
PRJNA729271	ed_Lushi_vs_Arbor_Acr	breed_growth_background	ENSGALG00010013375	PSMG1	0.13779447	0.047968703	2.872591136	0.004071206	0.021896896	positive
PRJNA729271	ed_Lushi_vs_Arbor_Acr	breed_growth_background	ENSGALG00010013376	ARHGAP8	−0.13091428	0.220478241	−0.593774152	0.55266318	0.69519148	negative
PRJNA729271	ed_Lushi_vs_Arbor_Acr	breed_growth_background	ENSGALG00010013378	SH3RF3	−0.230931109	0.07769642	−2.972223297	0.002956516	0.017541832	negative
PRJNA729271	ed_Lushi_vs_Arbor_Acr	breed_growth_background	ENSGALG00010013379	ZNF593	−0.013442403	0.070459751	−0.190781305	0.848696936	0.908825509	negative
PRJNA729271	ed_Lushi_vs_Arbor_Acr	breed_growth_background	ENSGALG00010013380	PHGDH	−0.92632266	0.326452941	−2.837538111	0.004546292	0.023738653	negative
PRJNA729271	ed_Lushi_vs_Arbor_Acr	breed_growth_background	ENSGALG00010013381	DENND2A	−0.159055202	0.048004139	−3.31336435	0.000921808	0.007666792	negative
PRJNA729271	ed_Lushi_vs_Arbor_Acr	breed_growth_background	ENSGALG00010013382	ASB12	0.198533551	0.117635192	1.687705414	0.091467792	0.202125223	positive
PRJNA729271	ed_Lushi_vs_Arbor_Acr	breed_growth_background	ENSGALG00010013383	SEPTIN10	−0.008943856	0.074497273	−0.120056148	0.90443867	0.945214175	negative
PRJNA729271	ed_Lushi_vs_Arbor_Acr	breed_growth_background	ENSGALG00010013385	PGRMC1	−0.115517253	0.083345804	−1.385999621	0.165747019	0.306129338	negative
PRJNA729271	ed_Lushi_vs_Arbor_Acr	breed_growth_background	ENSGALG00010013386	ITGAV	−0.250929857	0.065189724	−3.849224113	0.000118493	0.001811761	negative
PRJNA729271	ed_Lushi_vs_Arbor_Acr	breed_growth_background	ENSGALG00010013387	NDST1	−0.020793045	0.050205355	−0.41415991	0.678757	0.792623996	negative
PRJNA729271	ed_Lushi_vs_Arbor_Acr	breed_growth_background	ENSGALG00010013388	SOWAHC	0.080212474	0.118005574	0.679734624	0.496672508	0.648173945	positive
PRJNA729271	ed_Lushi_vs_Arbor_Acr	breed_growth_background	ENSGALG00010013389	NAP1L1	0.077284318	0.033540013	2.304242354	0.021209043	0.071179689	positive
PRJNA729271	ed_Lushi_vs_Arbor_Acr	breed_growth_background	ENSGALG00010013390	SLC16A2	−0.15604018	0.073355216	−2.127185882	0.033404642	0.098394692	negative
PRJNA729271	ed_Lushi_vs_Arbor_Acr	breed_growth_background	ENSGALG00010013391	HMGN1	0.101053966	0.04710837	2.145138218	0.031941798	0.095522252	positive
PRJNA729271	ed_Lushi_vs_Arbor_Acr	breed_growth_background	ENSGALG00010013392	HDAC2	−0.053049263	0.037324798	−1.421287362	0.155233236	0.292183581	negative
PRJNA729271	ed_Lushi_vs_Arbor_Acr	breed_growth_background	ENSGALG00010013393	ADCK2	−0.421491883	0.089347612	−4.717438715	2.38832E-06	9.58968E-05	negative
PRJNA729271	ed_Lushi_vs_Arbor_Acr	breed_growth_background	ENSGALG00010013394	DNMT3B	−0.166846574	0.094236486	−1.770509298	0.076642337	0.178721151	negative
PRJNA729271	ed_Lushi_vs_Arbor_Acr	breed_growth_background	ENSGALG00010013396	FYB1	0.075036817	0.100567967	0.746130396	0.455588649	0.611685575	positive
PRJNA729271	ed_Lushi_vs_Arbor_Acr	breed_growth_background	ENSGALG00010013397	MATN1	0.516023156	0.267269933	1.93071907	0.053517803	0.139071452	positive
PRJNA729271	ed_Lushi_vs_Arbor_Acr	breed_growth_background	ENSGALG00010013398	CHAMP1	0.119831253	0.083245132	1.439498619	0.150009301	0.285464726	positive
PRJNA729271	ed_Lushi_vs_Arbor_Acr	breed_growth_background	ENSGALG00010013399	FGF1	0.629154249	0.164310521	3.829056385	0.000128636	0.001912382	positive
PRJNA729271	ed_Lushi_vs_Arbor_Acr	breed_growth_background	ENSGALG00010013400	BBS10	0.109350947	0.089315632	1.22432036	0.220831399	0.373317689	positive
PRJNA729271	ed_Lushi_vs_Arbor_Acr	breed_growth_background	ENSGALG00010013402	MARCKS	−0.264040841	0.082946726	−3.183258163	0.001456277	0.010693382	negative
PRJNA729271	ed_Lushi_vs_Arbor_Acr	breed_growth_background	ENSGALG00010013403	CPNE3	−0.038822412	0.055000623	−0.70585405	0.480278905	0.633164414	negative
PRJNA729271	ed_Lushi_vs_Arbor_Acr	breed_growth_background	ENSGALG00010013404	ENSGALG00010013404	0.093568043	0.124761515	0.749975204	0.453269639	0.609531814	positive
PRJNA729271	ed_Lushi_vs_Arbor_Acr	breed_growth_background	ENSGALG00010013405	RPS14	−0.168370735	0.093742938	−1.796089807	0.072480235	0.172041294	negative
PRJNA729271	ed_Lushi_vs_Arbor_Acr	breed_growth_background	ENSGALG00010013406	ENSGALG00010013406	−0.111517001	0.14963366	−0.745266813	0.45611044	0.612045994	negative

PRJNA729271	ed_Lushi_vs_Arbor_Acr	breed_growth_background	ENSGALG00010013407	ZC3H15	0.16512533	0.060282911	2.739173146	0.006159393	0.02957124	positive
PRJNA729271	ed_Lushi_vs_Arbor_Acr	breed_growth_background	ENSGALG00010013408	RLIM	0.061894981	0.035119907	1.762390244	0.078003386	0.180703065	positive
PRJNA729271	ed_Lushi_vs_Arbor_Acr	breed_growth_background	ENSGALG00010013409	DAB2	-0.055728895	0.079353548	-0.702286119	0.482500748	0.634858631	negative
PRJNA729271	ed_Lushi_vs_Arbor_Acr	breed_growth_background	ENSGALG00010013410	KIAA0825	0.03868344	0.056413795	0.685708879	0.492896706	0.644755686	positive
PRJNA729271	ed_Lushi_vs_Arbor_Acr	breed_growth_background	ENSGALG00010013411	ENSGALG00010013411	0.413248779	0.238764773	1.730777845	0.083491395	0.189598585	positive
PRJNA729271	ed_Lushi_vs_Arbor_Acr	breed_growth_background	ENSGALG00010013412	COMMD7	-0.21698487	0.05553897	-3.906894057	9.34901E-05	0.00152092	negative
PRJNA729271	ed_Lushi_vs_Arbor_Acr	breed_growth_background	ENSGALG00010013413	WRB	-0.003131299	0.045695129	-0.06852588	0.945367019	0.970336705	negative
PRJNA729271	ed_Lushi_vs_Arbor_Acr	breed_growth_background	ENSGALG00010013414	LAPTM5	-0.068700756	0.093021716	-0.738545345	0.460183123	0.615346343	negative
PRJNA729271	ed_Lushi_vs_Arbor_Acr	breed_growth_background	ENSGALG00010013416	NDUFB2	-0.151042976	0.059087458	-2.556261193	0.010580367	0.043531767	negative
PRJNA729271	ed_Lushi_vs_Arbor_Acr	breed_growth_background	ENSGALG00010013417	AKR1B1	0.021613766	0.047351888	0.456449932	0.648066467	0.770293025	positive
PRJNA729271	ed_Lushi_vs_Arbor_Acr	breed_growth_background	ENSGALG00010013418	HCN1	0.796545347	0.433550688	1.837260022	0.066171519	0.16171913	positive
PRJNA729271	ed_Lushi_vs_Arbor_Acr	breed_growth_background	ENSGALG00010013419	ENSGALG00010013419	0.388490662	0.177057917	2.19414454	0.02822502	0.087065688	positive
PRJNA729271	ed_Lushi_vs_Arbor_Acr	breed_growth_background	ENSGALG00010013420	ENSGALG00010013420	3.213833825	2.264725526	1.419083146			positive
PRJNA729271	ed_Lushi_vs_Arbor_Acr	breed_growth_background	ENSGALG00010013423	ZNF804A	0.024765954	0.225306643	0.109921102	0.912471947	0.950627252	positive
PRJNA729271	ed_Lushi_vs_Arbor_Acr	breed_growth_background	ENSGALG00010013425	ENSGALG00010013425	0.423669863	0.120324327	3.52106572	0.000429816	0.004460976	positive
PRJNA729271	ed_Lushi_vs_Arbor_Acr	breed_growth_background	ENSGALG00010013427	NEXMIF	0.25458255	0.104869354	2.427616277	0.015198414	0.056170579	positive
PRJNA729271	ed_Lushi_vs_Arbor_Acr	breed_growth_background	ENSGALG00010013428	BPGM	0.073116624	0.081754126	0.894347817	0.371135823	0.53418404	positive
PRJNA729271	ed_Lushi_vs_Arbor_Acr	breed_growth_background	ENSGALG00010013429	PDE4DIP	0.133627626	0.100188127	1.333767084	0.182280202	0.327039426	positive
PRJNA729271	ed_Lushi_vs_Arbor_Acr	breed_growth_background	ENSGALG00010013431	TMEM175	-0.080710546	0.047548808	-1.697425222	0.089616297	0.199629358	negative
PRJNA729271	ed_Lushi_vs_Arbor_Acr	breed_growth_background	ENSGALG00010013434	CORIN	0.371231726	0.236816448	1.567592661	0.116976226	0.240249112	positive
PRJNA729271	ed_Lushi_vs_Arbor_Acr	breed_growth_background	ENSGALG00010013435	SDC3	0.064332627	0.09741952	0.660366908	0.509018403	0.657888698	positive
PRJNA729271	ed_Lushi_vs_Arbor_Acr	breed_growth_background	ENSGALG00010013436	NPAS2	0.218670162	0.115372812	1.8953353	0.058047994	0.14722672	positive
PRJNA729271	ed_Lushi_vs_Arbor_Acr	breed_growth_background	ENSGALG00010013437	ENSGALG00010013437	0.007978036	0.06695883	0.119148382	0.905157801	0.945468208	positive
PRJNA729271	ed_Lushi_vs_Arbor_Acr	breed_growth_background	ENSGALG00010013438	RPL37	-0.065736349	0.10022304	-0.655900572	0.5118881	0.660537525	negative
PRJNA729271	ed_Lushi_vs_Arbor_Acr	breed_growth_background	ENSGALG00010013440	EEA1	-0.023130249	0.052885888	-0.437361461	0.661849229	0.780657871	negative
PRJNA729271	ed_Lushi_vs_Arbor_Acr	breed_growth_background	ENSGALG00010013442	ENSGALG00010013442	0.03175568	0.055659763	0.570532074	0.568316874	0.707337319	positive
PRJNA729271	ed_Lushi_vs_Arbor_Acr	breed_growth_background	ENSGALG00010013444	OSBPL8	-0.037530684	0.055519072	-0.675996242	0.499043031	0.650321282	negative
PRJNA729271	ed_Lushi_vs_Arbor_Acr	breed_growth_background	ENSGALG00010013446	ATP5I	-0.099383619	0.077626832	-1.280274057	0.200448768	0.349219339	negative
PRJNA729271	ed_Lushi_vs_Arbor_Acr	breed_growth_background	ENSGALG00010013449	LCA5L	0.449268878	0.127214154	3.531595073	0.000413061	0.004341787	positive
PRJNA729271	ed_Lushi_vs_Arbor_Acr	breed_growth_background	ENSGALG00010013450	CD74	0.347074339	0.101444562	3.421320294	0.000623179	0.005790371	positive
PRJNA729271	ed_Lushi_vs_Arbor_Acr	breed_growth_background	ENSGALG00010013451	PRKAB2	0.073961864	0.088470177	0.836009003	0.403149838	0.564197894	positive
PRJNA729271	ed_Lushi_vs_Arbor_Acr	breed_growth_background	ENSGALG00010013452	LSS	-0.351008646	0.122226117	-2.871797407	0.004081445	0.021924968	negative
PRJNA729271	ed_Lushi_vs_Arbor_Acr	breed_growth_background	ENSGALG00010013453	PUM1	0.132731254	0.04786479	2.77304578	0.005553431	0.027445271	positive
PRJNA729271	ed_Lushi_vs_Arbor_Acr	breed_growth_background	ENSGALG00010013457	SH3BGR	-0.017910786	0.078636307	-0.22776738	0.819827087	0.889400161	negative
PRJNA729271	ed_Lushi_vs_Arbor_Acr	breed_growth_background	ENSGALG00010013458	LAMA4	-0.18816606	0.076782818	-2.450627176	0.014260758	0.053537861	negative
PRJNA729271	ed_Lushi_vs_Arbor_Acr	breed_growth_background	ENSGALG00010013459	ATP10D	-0.158326187	0.079065233	-2.002475446	0.045233621	0.123244613	negative
PRJNA729271	ed_Lushi_vs_Arbor_Acr	breed_growth_background	ENSGALG00010013461	S100B	1.002253718	0.294814313	3.399610104	0.00067482	0.00614803	positive
PRJNA729271	ed_Lushi_vs_Arbor_Acr	breed_growth_background	ENSGALG00010013463	ENSGALG00010013463	0.222592948	0.11183616	1.990348633	0.046552544	0.125821206	positive
PRJNA729271	ed_Lushi_vs_Arbor_Acr	breed_growth_background	ENSGALG00010013464	ABCB7	0.02235092	0.040502313	0.551843043	0.581055894	0.717394021	positive
PRJNA729271	ed_Lushi_vs_Arbor_Acr	breed_growth_background	ENSGALG00010013465	CALD1	-0.168184169	0.084209909	-1.997201654	0.045803281	0.12442252	negative
PRJNA729271	ed_Lushi_vs_Arbor_Acr	breed_growth_background	ENSGALG00010013466	BRAF	-0.046189617	0.033191425	-1.391612939	0.164039639	0.303818645	negative
PRJNA729271	ed_Lushi_vs_Arbor_Acr	breed_growth_background	ENSGALG00010013468	DEPDC1B	-0.20502772	0.150552298	-1.361837203	0.173249268	0.316716879	negative
PRJNA729271	ed_Lushi_vs_Arbor_Acr	breed_growth_background	ENSGALG00010013470	RPL31	-0.130501009	0.078247782	-1.667791804	0.095357064	0.20791225	negative
PRJNA729271	ed_Lushi_vs_Arbor_Acr	breed_growth_background	ENSGALG00010013472	ENSGALG00010013472	-0.043945713	0.123174514	-0.356776023	0.721259487	0.821355587	negative
PRJNA729271	ed_Lushi_vs_Arbor_Acr	breed_growth_background	ENSGALG00010013476	ZDHHC17	-0.06187874	0.070358314	-0.87948015	0.379140991	0.541047703	negative
PRJNA729271	ed_Lushi_vs_Arbor_Acr	breed_growth_background	ENSGALG00010013477	RFC1	-0.039000289	0.048567674	-0.803009198	0.421969418	0.58113508	negative
PRJNA729271	ed_Lushi_vs_Arbor_Acr	breed_growth_background	ENSGALG00010013480	TBC1D8	0.294813811	0.081171152	3.632002296	0.000281231	0.003329541	positive
PRJNA729271	ed_Lushi_vs_Arbor_Acr	breed_growth_background	ENSGALG00010013482	ENSGALG00010013482	0.006369417	0.127038459	0.050137709	0.96001265	0.977647294	positive
PRJNA729271	ed_Lushi_vs_Arbor_Acr	breed_growth_background	ENSGALG00010013483	ENSGALG00010013483	-0.114398495	0.112654325	-1.015482498	0.309875885	0.472838911	negative
PRJNA729271	ed_Lushi_vs_Arbor_Acr	breed_growth_background	ENSGALG00010013484	TCOF1	0.014891829	0.054959574	0.270959689	0.786422036	0.867374086	positive
PRJNA729271	ed_Lushi_vs_Arbor_Acr	breed_growth_background	ENSGALG00010013486	PLPP6	0.223775525	0.086499659	2.587010499	0.009681264	0.040876762	positive
PRJNA729271	ed_Lushi_vs_Arbor_Acr	breed_growth_background	ENSGALG00010013487	ENSGALG00010013487	0.015937586	0.050433158	0.316014038	0.751991853	0.842756653	positive
PRJNA729271	ed_Lushi_vs_Arbor_Acr	breed_growth_background	ENSGALG00010013488	UPRT	0.136554218	0.065873255	2.072984219	0.038173748	0.108880063	positive
PRJNA729271	ed_Lushi_vs_Arbor_Acr	breed_growth_background	ENSGALG00010013491	CNOT11	0.212108386	0.059653341	3.555683245	0.000376998	0.00407805	positive
PRJNA729271	ed_Lushi_vs_Arbor_Acr	breed_growth_background	ENSGALG00010013492	B3GALT5	-0.057503373	0.348649825	-0.164931598	0.868997821	0.921985721	negative
PRJNA729271	ed_Lushi_vs_Arbor_Acr	breed_growth_background	ENSGALG00010013493	ENSGALG00010013493	0.147723917	0.068009818	2.172096938	0.029848352	0.090577439	positive
PRJNA729271	ed_Lushi_vs_Arbor_Acr	breed_growth_background	ENSGALG00010013494	ASB1	0.091341589	0.078015438	1.170814294	0.241673431	0.397500548	positive
PRJNA729271	ed_Lushi_vs_Arbor_Acr	breed_growth_background	ENSGALG00010013497	FAM172A	-0.232763556	0.058032179	-4.010939433	6.04776E-05	0.001106847	negative
PRJNA729271	ed_Lushi_vs_Arbor_Acr	breed_growth_background	ENSGALG00010013498	KDR	-0.114730264	0.066989919	-1.712649678	0.086777008	0.194861771	negative
PRJNA729271	ed_Lushi_vs_Arbor_Acr	breed_growth_background	ENSGALG00010013501	AKR1B10	-0.172515388	0.074923172	-2.302563865	0.021303394	0.071380652	negative
PRJNA729271	ed_Lushi_vs_Arbor_Acr	breed_growth_background	ENSGALG00010013502	PAFAH2	-0.135877394	0.108749028	-1.24945847	0.211497434	0.361515094	negative
PRJNA729271	ed_Lushi_vs_Arbor_Acr	breed_growth_background	ENSGALG00010013504	IGSF5	-0.695719053	0.444115254	-1.566528163	0.117225021	0.240574356	negative
PRJNA729271	ed_Lushi_vs_Arbor_Acr	breed_growth_background	ENSGALG00010013506	RNF149	0.191216008	0.056946861	3.357797144	0.000785662	0.006860348	positive
PRJNA729271	ed_Lushi_vs_Arbor_Acr	breed_growth_background	ENSGALG00010013508	TUBE1	-0.001028508	0.104411335	-0.00985054	0.992140533	0.99553152	negative
PRJNA729271	ed_Lushi_vs_Arbor_Acr	breed_growth_background	ENSGALG00010013509	ENSGALG00010013509	-0.328284317	0.104351832	-3.145937268	0.001655555	0.011620955	negative
PRJNA729271	ed_Lushi_vs_Arbor_Acr	breed_growth_background	ENSGALG00010013510	MRPS33	-0.077521189	0.047550224	-1.63030124	0.103037846	0.219669594	negative
PRJNA729271	ed_Lushi_vs_Arbor_Acr	breed_growth_background	ENSGALG00010013511	FAM78B	0.243074579	0.121351935	2.00305482	0.045171405	0.123144538	positive
PRJNA729271	ed_Lushi_vs_Arbor_Acr	breed_growth_background	ENSGALG00010013512	ENSGALG00010013512	3.865760553	1.858046733	2.080550766			positive
PRJNA729271	ed_Lushi_vs_Arbor_Acr	breed_growth_background	ENSGALG00010013514	LMBRD2	0.195201318	0.073740711	2.647130935	0.008117792	0.035961916	positive
PRJNA729271	ed_Lushi_vs_Arbor_Acr	breed_growth_background	ENSGALG00010013516	CSRP2	-0.220565126	0.072073564	-3.060277794	0.002211318	0.014296633	negative
PRJNA729271	ed_Lushi_vs_Arbor_Acr	breed_growth_background	ENSGALG00010013517	STMN1	-0.299422526	0.116582174	-2.568338849	0.010218721	0.042417058	negative
PRJNA729271	ed_Lushi_vs_Arbor_Acr	breed_growth_background	ENSGALG00010013520	CREG2	0.193263945	0.104632006	1.847082489	0.064735186	0.158927103	positive
PRJNA729271	ed_Lushi_vs_Arbor_Acr	breed_growth_background	ENSGALG00010013523	TMEM178B	-0.048177401	0.107704839	-0.447309534	0.654651584	0.775189999	negative
PRJNA729271	ed_Lushi_vs_Arbor_Acr	breed_growth_background	ENSGALG00010013524	ZDHHC15	-0.099427862	0.066203801	-1.501845207	0.13313709	0.26241802	negative
PRJNA729271	ed_Lushi_vs_Arbor_Acr	breed_growth_background	ENSGALG00010013526	SIRPA	-0.693017202	0.131984588	-5.250743366	1.51487E-07	1.19337E-05	negative
PRJNA729271	ed_Lushi_vs_Arbor_Acr	breed_growth_background	ENSGALG00010013527	ADARB1	-0.004366103	0.054356083	-0.080324092	0.935979498	0.964536447	negative
PRJNA729271	ed_Lushi_vs_Arbor_Acr	breed_growth_background	ENSGALG00010013528	PRDX6	0.156795429	0.070369697	2.22816689	0.025869388	0.081929499	positive
PRJNA729271	ed_Lushi_vs_Arbor_Acr	breed_growth_background	ENSGALG00010013531	ARSI	-0.205888174	0.137004567	-1.502783289	0.132894937	0.26208328	negative
PRJNA729271	ed_Lushi_vs_Arbor_Acr	breed_growth_background	ENSGALG00010013532	WDR19	0.005847086	0.041301427	0.141571041	0.887418842	0.9337352	positive
PRJNA729271	ed_Lushi_vs_Arbor_Acr	breed_growth_background	ENSGALG00010013533	SKP2	-0.082433323	0.044446561	-1.854661436	0.063644589	0.157142129	negative
PRJNA729271	ed_Lushi_vs_Arbor_Acr	breed_growth_background	ENSGALG00010013537	LMO3	0.362506432	0.238023716	1.522984509	0.127762581	0.254804432	positive
PRJNA729271	ed_Lushi_vs_Arbor_Acr	breed_growth_background	ENSGALG00010013538	E2F7	-0.252445738	0.145779059	-1.73170097	0.083326818	0.189402889	negative
PRJNA729271	ed_Lushi_vs_Arbor_Acr	breed_growth_background	ENSGALG00010013539	NSFL1C	0.026958532	0.049449526	0.545172715	0.585634735	0.721466555	positive
PRJNA729271	ed_Lushi_vs_Arbor_Acr	breed_growth_background	ENSGALG00010013544	ENSGALG00010013544	0.367492368	0.156275779	2.351563181	0.018694714	0.065170728	positive
PRJNA729271	ed_Lushi_vs_Arbor_Acr	breed_growth_background	ENSGALG00010013546	IDUA	-0.428500284	0.07112298	-6.024779699	1.6934E-09	3.22972E-07	negative
PRJNA729271	ed_Lushi_vs_Arbor_Acr	breed_growth_background	ENSGALG00010013549	PCP4	0.278535049	0.185139701	1.504458779	0.13246328	0.261481035	positive
PRJNA729271	ed_Lushi_vs_Arbor_Acr	breed_growth_background	ENSGALG00010013550	KLHL5	-0.07631604	0.067238751	-1.135000859	0.256374959	0.41345739	negative
PRJNA729271	ed_Lushi_vs_Arbor_Acr	breed_growth_background	ENSGALG00010013551	UBE2N	-0.002219452	0.05988831	-0.037059858	0.970437278	0.98277708	negative
PRJNA729271	ed_Lushi_vs_Arbor_Acr	breed_growth_background	ENSGALG00010013552	FGF16	-0.764319221	0.217296452	-3.517403133	0.000435791	0.004499142	negative
PRJNA729271	ed_Lushi_vs_Arbor_Acr	breed_growth_background	ENSGALG00010013553	TADA1	0.216273458	0.060344302	3.583991403	0.000338383	0.003766357	positive
PRJNA729271	ed_Lushi_vs_Arbor_Acr	breed_growth_background	ENSGALG00010013554	ENSGALG00010013554	2.158322029	2.910266175	0.741623583	0.458315431	0.614038466	positive
PRJNA729271	ed_Lushi_vs_Arbor_Acr	breed_growth_background	ENSGALG00010013556	RASL11B	0.326006966	0.095023058	3.430819548	0.000601761	0.005631067	positive
PRJNA729271	ed_Lushi_vs_Arbor_Acr	breed_growth_background	ENSGALG00010013558	SNAPC3	0.008134409	0.047355094	0.171774742	0.863614624	0.918085366	positive
PRJNA729271	ed_Lushi_vs_Arbor_Acr	breed_growth_background	ENSGALG00010013561	AGK	0.023264719	0.058575896	0.397172219	0.691240467	0.800512354	positive
PRJNA729271	ed_Lushi_vs_Arbor_Acr	breed_growth_background	ENSGALG00010013562	RERGL	-0.149847024	0.084878535	-1.765428967	0.077491699	0.179891443	negative
PRJNA729271	ed_Lushi_vs_Arbor_Acr	breed_growth_background	ENSGALG00010013563	FKBP1A	-0.116074564	0.052907081	-2.193932471	0.028240265	0.087094178	negative
PRJNA729271	ed_Lushi_vs_Arbor_Acr	breed_growth_background	ENSGALG00010013565	ENSGALG00010013565	1.46245102	2.831062997	0.516573111	0.60545419	0.73704479	positive
PRJNA729271	ed_Lushi_vs_Arbor_Acr	breed_growth_background	ENSGALG00010013569	ENSGALG00010013569	-0.809228699	0.298012036	-2.715422873	0.006619119	0.031147835	negative
PRJNA729271	ed_Lushi_vs_Arbor_Acr	breed_growth_background	ENSGALG00010013572	DENND11	0.106632015	0.056516846	1.88672976	0.059196683	0.149409005	positive
PRJNA729271	ed_Lushi_vs_Arbor_Acr	breed_growth_background	ENSGALG00010013573	ENSGALG00010013573	-0.796579273	0.261942422	-3.041047215	0.002357569	0.015001298	negative
PRJNA729271	ed_Lushi_vs_Arbor_Acr	breed_growth_background	ENSGALG00010013574	NR2F1	-0.054144651	0.085830509	-0.630832227	0.528150229	0.673902955	negative
PRJNA729271	ed_Lushi_vs_Arbor_Acr	breed_growth_background	ENSGALG00010013576	CAMK2A	-0.283354638	0.099660891	-2.843187891	0.004466473	0.023431706	negative
PRJNA729271	ed_Lushi_vs_Arbor_Acr	breed_growth_background	ENSGALG00010013579	H2AC32	-0.280209986	0.094849211	-2.954267967	0.003134117	0.018310373	negative
PRJNA729271	ed_Lushi_vs_Arbor_Acr	breed_growth_background	ENSGALG00010013580	RANBP3L	-0.261942256	0.167790538	-1.561126509	0.118493904	0.242354896	negative
PRJNA729271	ed_Lushi_vs_Arbor_Acr	breed_growth_background	ENSGALG00010013582	MAP4K4	-0.170058891	0.057702158	-2.947184227	0.003206821	0.018595406	negative
PRJNA729271	ed_Lushi_vs_Arbor_Acr	breed_growth_background	ENSGALG00010013583	FASLG	0.150724833	0.1850598	0.814465557	0.415378296	0.575557633	positive
PRJNA729271	ed_Lushi_vs_Arbor_Acr	breed_growth_background	ENSGALG00010013587	SLC26A1	-0.321124012	0.266900116	-1.203161755	0.228913732	0.382578639	negative
PRJNA729271	ed_Lushi_vs_Arbor_Acr	breed_growth_background	ENSGALG00010013589	DSCAM	0.400958159	0.348797181	1.149545296	0.2503312	0.406287174	positive
PRJNA729271	ed_Lushi_vs_Arbor_Acr	breed_growth_background	ENSGALG00010013592	H3C33	-0.158065412	0.098623281	-1.602719062	0.108996693	0.229071635	negative
PRJNA729271	ed_Lushi_vs_Arbor_Acr	breed_growth_background	ENSGALG00010013596	SDCBP2	0.09796156	0.065890492	1.48673286	0.13708538	0.268049722	positive
PRJNA729271	ed_Lushi_vs_Arbor_Acr	breed_growth_background	ENSGALG00010013598	PAQR7	0.045139624	0.121295687	0.372145334	0.709784645	0.812985257	positive
PRJNA729271	ed_Lushi_vs_Arbor_Acr	breed_growth_background	ENSGALG00010013604	FYN	-0.16777015	0.060016589	-2.795396299	0.005183613	0.026192366	negative
PRJNA729271	ed_Lushi_vs_Arbor_Acr	breed_growth_background	ENSGALG00010013609	PIGC	0.308425012	0.086315898	3.573212103	0.000352629	0.003878115	positive
PRJNA729271	ed_Lushi_vs_Arbor_Acr	breed_growth_background	ENSGALG00010013612	TWIST2	0.144581338	0.158127122	0.914336117	0.360540258	0.523860369	positive
PRJNA729271	ed_Lushi_vs_Arbor_Acr	breed_growth_background	ENSGALG00010013614	ATRX	0.015823025	0.046549809	0.339915998	0.733919789	0.829486732	positive
PRJNA729271	ed_Lushi_vs_Arbor_Acr	breed_growth_background	ENSGALG00010013617	PIK3C2G	-0.177713251	0.088414648	-2.009997837	0.044431418	0.121469898	negative
PRJNA729271	ed_Lushi_vs_Arbor_Acr	breed_growth_background	ENSGALG00010013619	IL1R2	-0.098435971	0.106007895	-0.928572069	0.353110902	0.516899872	negative
PRJNA729271	ed_Lushi_vs_Arbor_Acr	breed_growth_background	ENSGALG00010013621	PDGFRA	-0.27288	0.088051874	-3.099082257	0.001941211	0.013002706	negative
PRJNA729271	ed_Lushi_vs_Arbor_Acr	breed_growth_background	ENSGALG00010013624	SST1	0.391046089	0.989828665	0.395064421	0.692795345	0.801697951	positive
PRJNA729271	ed_Lushi_vs_Arbor_Acr	breed_growth_background	ENSGALG00010013625	SSBP1	0.057948065	0.060852693	0.952267882	0.340961142	0.50482449	positive
PRJNA729271	ed_Lushi_vs_Arbor_Acr	breed_growth_background	ENSGALG00010013627	MYCBP	-0.073454293	0.079204593	-0.927399408	0.353719198	0.517424541	negative
PRJNA729271	ed_Lushi_vs_Arbor_Acr	breed_growth_background	ENSGALG00010013632	SLC1A3	-0.205982393	0.334732827	-0.615363586	0.538314634	0.683622798	negative
PRJNA729271	ed_Lushi_vs_Arbor_Acr	breed_growth_background	ENSGALG00010013635	BCL6	-0.083246529	0.085126525	-0.977915272	0.328116231	0.491987666	negative
PRJNA729271	ed_Lushi_vs_Arbor_Acr	breed_growth_background	ENSGALG00010013638	COMMD8	-0.010088306	0.064637182	-0.156075896	0.875973197	0.926645502	negative
PRJNA729271	ed_Lushi_vs_Arbor_Acr	breed_growth_background	ENSGALG00010013639	ENSGALG00010013639	-0.1138438	0.243760864	-0.467030671	0.640477935	0.764407383	negative
PRJNA729271	ed_Lushi_vs_Arbor_Acr	breed_growth_background	ENSGALG00010013640	SNPH	-0.379410624	0.391359505	-0.969468273	0.332311603	0.496736793	negative
PRJNA729271	ed_Lushi_vs_Arbor_Acr	breed_growth_background	ENSGALG00010013645	RRAGC	0.104833619	0.065998225	1.588430888	0.112188938	0.233478629	positive
PRJNA729271	ed_Lushi_vs_Arbor_Acr	breed_growth_background	ENSGALG00010013646	CLDN1	0.476623383	0.179996975	2.647952191	0.008098098	0.035885639	positive
PRJNA729271	ed_Lushi_vs_Arbor_Acr	breed_growth_background	ENSGALG00010013648	NAV3	-0.350235426	0.063748661	-5.494004413	3.92921E-08	4.18778E-06	negative
PRJNA729271	ed_Lushi_vs_Arbor_Acr	breed_growth_background	ENSGALG00010013650	BACE2	-0.276482046	0.087372157	-3.164418247	0.001553933	0.011150624	negative
PRJNA729271	ed_Lushi_vs_Arbor_Acr	breed_growth_background	ENSGALG00010013651	ENSGALG00010013651	0.098513388	0.087604372	1.124525932	0.260789976	0.418667704	positive

PRJNA729271	ed_Lushi_vs_Arbor_Acr	breed_growth_background	ENSGALG00010013654	SLC6A7	0.306465176	0.298209087	1.02768557	0.304097758	0.466592251	positive
PRJNA729271	ed_Lushi_vs_Arbor_Acr	breed_growth_background	ENSGALG00010013657	MAGT1	-0.088309137	0.056331127	-1.567679235	0.11695601	0.240249112	negative
PRJNA729271	ed_Lushi_vs_Arbor_Acr	breed_growth_background	ENSGALG00010013661	GABRA4	-0.814824114	0.209163833	-3.89562622	9.79453E-05	0.001567017	negative
PRJNA729271	ed_Lushi_vs_Arbor_Acr	breed_growth_background	ENSGALG00010013663	OPA1	0.08205471	0.057058585	1.438078233	0.150411858	0.286043018	positive
PRJNA729271	ed_Lushi_vs_Arbor_Acr	breed_growth_background	ENSGALG00010013664	REV3L	0.090349273	0.051560517	1.752295707	0.079722974	0.18365933	positive
PRJNA729271	ed_Lushi_vs_Arbor_Acr	breed_growth_background	ENSGALG00010013666	COX7B	-0.079398162	0.076689776	-1.035316138	0.300521296	0.46298844	negative
PRJNA729271	ed_Lushi_vs_Arbor_Acr	breed_growth_background	ENSGALG00010013667	SOWAHA	-0.176991448	0.315884799	-0.560303782	0.575272248	0.712698396	negative
PRJNA729271	ed_Lushi_vs_Arbor_Acr	breed_growth_background	ENSGALG00010013669	NPR3	0.042461883	0.139667309	0.304021631	0.761111411	0.849780844	positive
PRJNA729271	ed_Lushi_vs_Arbor_Acr	breed_growth_background	ENSGALG00010013672	ENSGALG00010013672	-0.223356247	0.276382717	-0.80814115	0.419009331	0.57892863	negative
PRJNA729271	ed_Lushi_vs_Arbor_Acr	breed_growth_background	ENSGALG00010013675	CHRNA6	-0.685811525	0.204819287	-3.348373748	0.000812873	0.007042795	negative
PRJNA729271	ed_Lushi_vs_Arbor_Acr	breed_growth_background	ENSGALG00010013677	GABRG1	0.465821009	0.272411867	1.709987946	0.087268102	0.195600919	positive
PRJNA729271	ed_Lushi_vs_Arbor_Acr	breed_growth_background	ENSGALG00010013678	ATP7A	0.003231362	0.054603249	0.059178931	0.952809591	0.97363236	positive
PRJNA729271	ed_Lushi_vs_Arbor_Acr	breed_growth_background	ENSGALG00010013679	MFSD4B	0.208158538	0.11458825	1.816578382	0.069281696	0.166739329	positive
PRJNA729271	ed_Lushi_vs_Arbor_Acr	breed_growth_background	ENSGALG00010013686	UQCRQ	0.041262108	0.073626948	0.560421284	0.575192118	0.712698396	positive
PRJNA729271	ed_Lushi_vs_Arbor_Acr	breed_growth_background	ENSGALG00010013690	CREB3L2	-0.144005701	0.054750045	-2.630238952	0.008532487	0.037146343	negative
PRJNA729271	ed_Lushi_vs_Arbor_Acr	breed_growth_background	ENSGALG00010013696	CETN3	0.144807336	0.060704628	2.385441465	0.017058637	0.061068151	positive
PRJNA729271	ed_Lushi_vs_Arbor_Acr	breed_growth_background	ENSGALG00010013697	HDAC4	0.000998208	0.036160156	0.027605196	0.977977037	0.987020774	positive
PRJNA729271	ed_Lushi_vs_Arbor_Acr	breed_growth_background	ENSGALG00010013698	TFAP2E	-0.591862497	0.405244359	-1.460507675	0.1441506	0.277485119	negative
PRJNA729271	ed_Lushi_vs_Arbor_Acr	breed_growth_background	ENSGALG00010013709	PSMF1	0.048329662	0.053914915	0.896406158	0.37003588	0.533433126	positive
PRJNA729271	ed_Lushi_vs_Arbor_Acr	breed_growth_background	ENSGALG00010013715	IL1R1	-0.267441686	0.125131158	-2.137290912	0.032574338	0.096874235	negative
PRJNA729271	ed_Lushi_vs_Arbor_Acr	breed_growth_background	ENSGALG00010013719	NCDN	-0.002698184	0.07556547	-0.035706571	0.971516331	0.983216541	negative
PRJNA729271	ed_Lushi_vs_Arbor_Acr	breed_growth_background	ENSGALG00010013720	SLC16A10	0.055815395	0.101865242	0.547933661	0.583737455	0.719624132	positive
PRJNA729271	ed_Lushi_vs_Arbor_Acr	breed_growth_background	ENSGALG00010013732	FNTA	0.037141221	0.041190907	0.90168496	0.367224245	0.530646539	positive
PRJNA729271	ed_Lushi_vs_Arbor_Acr	breed_growth_background	ENSGALG00010013737	AFF4	0.15229055	0.045343718	3.358580968	0.000783438	0.006849173	positive
PRJNA729271	ed_Lushi_vs_Arbor_Acr	breed_growth_background	ENSGALG00010013740	GNPDA2	0.032247829	0.066183729	0.487247093	0.626083236	0.752435045	positive
PRJNA729271	ed_Lushi_vs_Arbor_Acr	breed_growth_background	ENSGALG00010013742	RSPO4	1.101798599	0.671575927	1.640616578	0.100877032	0.216431701	positive
PRJNA729271	ed_Lushi_vs_Arbor_Acr	breed_growth_background	ENSGALG00010013744	ENSGALG00010013744	-0.134161893	0.170816206	-0.785416652	0.43220932	0.590571577	negative
PRJNA729271	ed_Lushi_vs_Arbor_Acr	breed_growth_background	ENSGALG00010013746	ENSGALG00010013746	0.012538858	0.046518001	0.269548501	0.787507624	0.867922978	positive
PRJNA729271	ed_Lushi_vs_Arbor_Acr	breed_growth_background	ENSGALG00010013749	NELL2	-0.730511278	0.24932334	-2.929975505	0.003389887	0.019323579	negative
PRJNA729271	ed_Lushi_vs_Arbor_Acr	breed_growth_background	ENSGALG00010013751	FUT10	0.111094187	0.092068747	1.206643844	0.227569334	0.38073399	positive
PRJNA729271	ed_Lushi_vs_Arbor_Acr	breed_growth_background	ENSGALG00010013753	PLEKHA5	0.083900285	0.066436985	1.262855104	0.206641257	0.355721117	positive
PRJNA729271	ed_Lushi_vs_Arbor_Acr	breed_growth_background	ENSGALG00010013755	ANGPT4	-0.167603535	0.081491702	-2.056694491	0.039715622	0.111854757	negative
PRJNA729271	ed_Lushi_vs_Arbor_Acr	breed_growth_background	ENSGALG00010013757	DGKI	0.028998462	0.139405128	0.208015748	0.835216671	0.89984136	positive
PRJNA729271	ed_Lushi_vs_Arbor_Acr	breed_growth_background	ENSGALG00010013758	FAM3B	-0.017661895	0.363086184	-0.04864381	0.961203156	0.978276808	negative
PRJNA729271	ed_Lushi_vs_Arbor_Acr	breed_growth_background	ENSGALG00010013760	RPF2	0.111855131	0.062908055	1.778073278	0.075391821	0.176829197	positive
PRJNA729271	ed_Lushi_vs_Arbor_Acr	breed_growth_background	ENSGALG00010013766	DNM3	0.076303607	0.059789526	1.276203564	0.201883569	0.350917656	positive
PRJNA729271	ed_Lushi_vs_Arbor_Acr	breed_growth_background	ENSGALG00010013767	PGK2	0.151645013	0.091919186	1.649764525	0.098991107	0.21334944	positive
PRJNA729271	ed_Lushi_vs_Arbor_Acr	breed_growth_background	ENSGALG00010013768	FAM110A	-0.309106457	0.126205084	-2.449239337	0.014315829	0.0536751	negative
PRJNA729271	ed_Lushi_vs_Arbor_Acr	breed_growth_background	ENSGALG00010013771	PELO	0.064893707	0.055790419	1.163169387	0.244760784	0.400655852	positive
PRJNA729271	ed_Lushi_vs_Arbor_Acr	breed_growth_background	ENSGALG00010013772	KIAA0319L	-0.160992603	0.07662222	-2.101121627	0.035630291	0.103354226	negative
PRJNA729271	ed_Lushi_vs_Arbor_Acr	breed_growth_background	ENSGALG00010013775	DBX2	0.28667711	0.218225009	1.313676701	0.188955025	0.335075004	positive
PRJNA729271	ed_Lushi_vs_Arbor_Acr	breed_growth_background	ENSGALG00010013777	CPLANE1	-0.193188902	0.051885426	-3.723375068	0.000196577	0.002563294	negative
PRJNA729271	ed_Lushi_vs_Arbor_Acr	breed_growth_background	ENSGALG00010013778	ENSGALG00010013778	-0.799333033	0.373769398	-2.138572706	0.03247029	0.096624277	negative
PRJNA729271	ed_Lushi_vs_Arbor_Acr	breed_growth_background	ENSGALG00010013779	SLC52A3	0.119575534	0.140899393	0.848658975	0.39607108	0.557329416	positive
PRJNA729271	ed_Lushi_vs_Arbor_Acr	breed_growth_background	ENSGALG00010013783	NDUFA10	-0.051222637	0.051183752	-1.000759719	0.316942988	0.480402448	negative
PRJNA729271	ed_Lushi_vs_Arbor_Acr	breed_growth_background	ENSGALG00010013784	ANO6	-0.168952846	0.050219508	-3.364287134	0.000767417	0.006743395	negative
PRJNA729271	ed_Lushi_vs_Arbor_Acr	breed_growth_background	ENSGALG00010013787	ZCCHC10	0.018500017	0.056613683	0.326776436	0.743836983	0.836650661	positive
PRJNA729271	ed_Lushi_vs_Arbor_Acr	breed_growth_background	ENSGALG00010013791	AEBP2	0.086484142	0.035098852	2.464016271	0.013738987	0.052351899	positive
PRJNA729271	ed_Lushi_vs_Arbor_Acr	breed_growth_background	ENSGALG00010013792	FGF10	0.518676188	0.12664457	4.095526473	4.2121E-05	0.000853908	positive
PRJNA729271	ed_Lushi_vs_Arbor_Acr	breed_growth_background	ENSGALG00010013793	METTL13	-0.142584056	0.071878712	-1.983675719	0.047292002	0.127132339	negative
PRJNA729271	ed_Lushi_vs_Arbor_Acr	breed_growth_background	ENSGALG00010013794	GTF3C6	-0.185345545	0.097265812	-1.905556947	0.056707712	0.144969716	negative
PRJNA729271	ed_Lushi_vs_Arbor_Acr	breed_growth_background	ENSGALG00010013797	IL1RL1	-0.171283233	0.169248707	-1.012020925	0.311528049	0.474726563	negative
PRJNA729271	ed_Lushi_vs_Arbor_Acr	breed_growth_background	ENSGALG00010013802	PYROXD1	-0.027044508	0.076895664	-0.351703936	0.725060308	0.824004405	negative
PRJNA729271	ed_Lushi_vs_Arbor_Acr	breed_growth_background	ENSGALG00010013805	HSPA4	0.143361527	0.077770029	1.843403291	0.06527015	0.160028894	positive
PRJNA729271	ed_Lushi_vs_Arbor_Acr	breed_growth_background	ENSGALG00010013808	PLAAT1	-0.019063909	0.072599191	-0.262591208	0.792865678	0.871707221	negative
PRJNA729271	ed_Lushi_vs_Arbor_Acr	breed_growth_background	ENSGALG00010013809	MX1	-0.538701037	0.660499112	-0.815596914	0.414730715	0.575100136	negative
PRJNA729271	ed_Lushi_vs_Arbor_Acr	breed_growth_background	ENSGALG00010013814	AMD1	-0.169612294	0.059251973	-2.862559455	0.004202344	0.022344305	negative
PRJNA729271	ed_Lushi_vs_Arbor_Acr	breed_growth_background	ENSGALG00010013815	SRXN1	0.015119114	0.134660987	0.112275382	0.91060506	0.949311015	positive
PRJNA729271	ed_Lushi_vs_Arbor_Acr	breed_growth_background	ENSGALG00010013820	MASP1	-0.120625684	0.27013373	-0.446540623	0.655206775	0.775238902	negative
PRJNA729271	ed_Lushi_vs_Arbor_Acr	breed_growth_background	ENSGALG00010013823	GUF1	0.022073269	0.060935828	0.362237946	0.717174225	0.818732015	positive
PRJNA729271	ed_Lushi_vs_Arbor_Acr	breed_growth_background	ENSGALG00010013828	PTN	0.373231127	0.147917894	2.523231753	0.011628174	0.046527416	positive
PRJNA729271	ed_Lushi_vs_Arbor_Acr	breed_growth_background	ENSGALG00010013831	TCERG1	0.144455091	0.060658736	2.381439184	0.017245138	0.061455919	positive
PRJNA729271	ed_Lushi_vs_Arbor_Acr	breed_growth_background	ENSGALG00010013833	TMPRSS2	-0.746577497	0.706960383	-1.056038662	0.290950552	0.452357422	negative
PRJNA729271	ed_Lushi_vs_Arbor_Acr	breed_growth_background	ENSGALG00010013836	IL18R1	-0.536576147	0.140631243	-3.815483209	0.000135917	0.001996061	negative
PRJNA729271	ed_Lushi_vs_Arbor_Acr	breed_growth_background	ENSGALG00010013837	IFI27L2	-0.377413279	0.179636195	-2.100986822	0.035642123	0.103367862	negative
PRJNA729271	ed_Lushi_vs_Arbor_Acr	breed_growth_background	ENSGALG00010013842	CDK19	0.182045083	0.059396701	3.064902272	0.002177411	0.014108886	positive
PRJNA729271	ed_Lushi_vs_Arbor_Acr	breed_growth_background	ENSGALG00010013846	YIPF7	-0.024630218	0.128774238	-0.191266659	0.848316681	0.90848543	negative
PRJNA729271	ed_Lushi_vs_Arbor_Acr	breed_growth_background	ENSGALG00010013848	ITPA	0.033749795	0.056674624	0.595501002	0.551508629	0.694391707	positive
PRJNA729271	ed_Lushi_vs_Arbor_Acr	breed_growth_background	ENSGALG00010013857	IL18RAP	0.509008753	0.384939857	1.322307222	0.186065869	0.331574228	positive
PRJNA729271	ed_Lushi_vs_Arbor_Acr	breed_growth_background	ENSGALG00010013859	MYOC	0.407264221	0.132241804	3.079693465	0.002072137	0.013603094	positive
PRJNA729271	ed_Lushi_vs_Arbor_Acr	breed_growth_background	ENSGALG00010013861	TCF15	-0.014301775	0.082468135	-0.173421834	0.862319864	0.917201857	negative
PRJNA729271	ed_Lushi_vs_Arbor_Acr	breed_growth_background	ENSGALG00010013866	KCTD8	-0.929732893	0.232122953	-4.005346646	6.19266E-05	0.001123437	negative
PRJNA729271	ed_Lushi_vs_Arbor_Acr	breed_growth_background	ENSGALG00010013867	RECQL	-0.414586735	0.070234505	-5.902892496	3.57183E-09	5.91627E-07	negative
PRJNA729271	ed_Lushi_vs_Arbor_Acr	breed_growth_background	ENSGALG00010013871	ENSGALG00010013871	-0.292779492	0.123366893	-2.373241999	0.017632707	0.062490486	negative
PRJNA729271	ed_Lushi_vs_Arbor_Acr	breed_growth_background	ENSGALG00010013872	GLS2	-0.150692653	0.105445173	-1.429109063	0.152972889	0.289452401	negative
PRJNA729271	ed_Lushi_vs_Arbor_Acr	breed_growth_background	ENSGALG00010013878	ARID2	-0.089053249	0.044054288	-2.021443365	0.043233889	0.119125952	negative
PRJNA729271	ed_Lushi_vs_Arbor_Acr	breed_growth_background	ENSGALG00010013880	ZMYM4	0.094257275	0.048827981	1.930394683	0.053557953	0.139125902	positive
PRJNA729271	ed_Lushi_vs_Arbor_Acr	breed_growth_background	ENSGALG00010013889	ENSGALG00010013889	0.48052685	0.225497271	2.130965259	0.033092004	0.097713103	positive
PRJNA729271	ed_Lushi_vs_Arbor_Acr	breed_growth_background	ENSGALG00010013890	SFPQ	0.054870447	0.038945362	1.408908368	0.158862265	0.297315498	positive
PRJNA729271	ed_Lushi_vs_Arbor_Acr	breed_growth_background	ENSGALG00010013893	CSNK2A1	0.099532022	0.042519512	2.340855215	0.019239627	0.066558088	positive
PRJNA729271	ed_Lushi_vs_Arbor_Acr	breed_growth_background	ENSGALG00010013897	AMOT	-0.093405225	0.041105154	-2.272348246	0.023065485	0.075589917	negative
PRJNA729271	ed_Lushi_vs_Arbor_Acr	breed_growth_background	ENSGALG00010013898	EMB	-0.133783865	0.160139386	-0.83542137	0.403480502	0.564358764	negative
PRJNA729271	ed_Lushi_vs_Arbor_Acr	breed_growth_background	ENSGALG00010013899	WNT5B	0.135459682	0.076207737	1.777505632	0.075485085	0.176962042	positive
PRJNA729271	ed_Lushi_vs_Arbor_Acr	breed_growth_background	ENSGALG00010013901	NFXL1	0.149637681	0.066161898	2.261689671	0.023716583	0.077027084	positive
PRJNA729271	ed_Lushi_vs_Arbor_Acr	breed_growth_background	ENSGALG00010013908	TBC1D20	0.181163245	0.061766707	2.933024169	0.003356778	0.019224216	positive
PRJNA729271	ed_Lushi_vs_Arbor_Acr	breed_growth_background	ENSGALG00010013910	ADIPOR2	0.266066018	0.11120054	2.392668398	0.01672635	0.060145981	positive
PRJNA729271	ed_Lushi_vs_Arbor_Acr	breed_growth_background	ENSGALG00010013911	TNIK	0.072054419	0.079221327	0.9095331	0.363068794	0.526268217	positive
PRJNA729271	ed_Lushi_vs_Arbor_Acr	breed_growth_background	ENSGALG00010013914	NRG1	0.364116684	0.122537556	2.971470094	0.002963777	0.017570533	positive
PRJNA729271	ed_Lushi_vs_Arbor_Acr	breed_growth_background	ENSGALG00010013915	ENSGALG00010013915	-0.234766921	0.101164325	-2.320649307	0.020305779	0.068965906	negative
PRJNA729271	ed_Lushi_vs_Arbor_Acr	breed_growth_background	ENSGALG00010013917	TRPC5	-0.350240922	0.32739738	-1.069773135	0.284721438	0.445107554	negative
PRJNA729271	ed_Lushi_vs_Arbor_Acr	breed_growth_background	ENSGALG00010013921	PPP2R2B	-0.168599242	0.056159603	-3.00214446	0.002680849	0.0163247	negative
PRJNA729271	ed_Lushi_vs_Arbor_Acr	breed_growth_background	ENSGALG00010013922	ENSGALG00010013922	0.11150552	0.530223369	0.210299143	0.833434204	0.898832166	positive
PRJNA729271	ed_Lushi_vs_Arbor_Acr	breed_growth_background	ENSGALG00010013923	FMR1NB	0.247713572	0.269376164	0.919582373	0.357791041	0.521538731	positive
PRJNA729271	ed_Lushi_vs_Arbor_Acr	breed_growth_background	ENSGALG00010013928	CCNO	-0.205069924	0.246419668	-0.832197875	0.405297263	0.56613509	negative
PRJNA729271	ed_Lushi_vs_Arbor_Acr	breed_growth_background	ENSGALG00010013930	SLC22A16	-0.049693979	0.161862531	-0.307013479	0.758833112	0.847729169	negative
PRJNA729271	ed_Lushi_vs_Arbor_Acr	breed_growth_background	ENSGALG00010013934	STAT1	0.096367935	0.15118604	0.637412916	0.523855918	0.670666978	positive
PRJNA729271	ed_Lushi_vs_Arbor_Acr	breed_growth_background	ENSGALG00010013936	DLGAP3	-0.395103049	0.229677139	-1.720254143	0.085386255	0.192844152	negative
PRJNA729271	ed_Lushi_vs_Arbor_Acr	breed_growth_background	ENSGALG00010013938	ENSGALG00010013938	0.021109605	0.222752644	0.094767022	0.924499882	0.957812249	positive
PRJNA729271	ed_Lushi_vs_Arbor_Acr	breed_growth_background	ENSGALG00010013940	UPF3A	-0.02941632	0.134805791	-0.218212585	0.82726348	0.894730947	negative
PRJNA729271	ed_Lushi_vs_Arbor_Acr	breed_growth_background	ENSGALG00010013942	CACNA2D4	0.145306913	0.24280421	0.59845302	0.549537697	0.692855717	positive
PRJNA729271	ed_Lushi_vs_Arbor_Acr	breed_growth_background	ENSGALG00010013944	METTL24	0.396019092	0.126427732	3.132375205	0.001733981	0.012018611	positive
PRJNA729271	ed_Lushi_vs_Arbor_Acr	breed_growth_background	ENSGALG00010013945	SCAF11	0.058832474	0.041374734	1.421942035	0.155043079	0.291863563	positive
PRJNA729271	ed_Lushi_vs_Arbor_Acr	breed_growth_background	ENSGALG00010013947	ENSGALG00010013947	0.217828236	0.098658345	2.207904826	0.027250908	0.08476436	positive
PRJNA729271	ed_Lushi_vs_Arbor_Acr	breed_growth_background	ENSGALG00010013949	PRRC2C	0.028886925	0.067571903	0.427499054	0.669015874	0.785531397	positive
PRJNA729271	ed_Lushi_vs_Arbor_Acr	breed_growth_background	ENSGALG00010013953	DHX29	0.080220132	0.044315145	1.81021932	0.070261783	0.168254509	positive
PRJNA729271	ed_Lushi_vs_Arbor_Acr	breed_growth_background	ENSGALG00010013954	LPL	-0.657227685	0.17855982	-3.680714317	0.000232581	0.002889896	negative
PRJNA729271	ed_Lushi_vs_Arbor_Acr	breed_growth_background	ENSGALG00010013958	TUSC3	-0.144909688	0.094371498	-1.535523873	0.124655198	0.250502855	negative
PRJNA729271	ed_Lushi_vs_Arbor_Acr	breed_growth_background	ENSGALG00010013959	STK32A	-1.059854901	0.266205547	-3.981340414	6.85277E-05	0.001204011	negative
PRJNA729271	ed_Lushi_vs_Arbor_Acr	breed_growth_background	ENSGALG00010013964	SLC38A1	-0.210231304	0.086831968	-2.421127944	0.01547243	0.057023361	negative
PRJNA729271	ed_Lushi_vs_Arbor_Acr	breed_growth_background	ENSGALG00010013965	SLC2A2	-1.28093639	0.541478602	-2.365626978			negative
PRJNA729271	ed_Lushi_vs_Arbor_Acr	breed_growth_background	ENSGALG00010013967	MLLT3	0.148019665	0.083602723	1.770512494	0.076641805	0.178721151	positive
PRJNA729271	ed_Lushi_vs_Arbor_Acr	breed_growth_background	ENSGALG00010013971	ENSGALG00010013971	-0.044365337	0.2157099	-0.205671305	0.837047675	0.901077315	negative
PRJNA729271	ed_Lushi_vs_Arbor_Acr	breed_growth_background	ENSGALG00010013973	ENSGALG00010013973	-0.392142327	0.129036792	-3.038996261	0.002373678	0.015055655	negative
PRJNA729271	ed_Lushi_vs_Arbor_Acr	breed_growth_background	ENSGALG00010013976	CDC40	0.098579088	0.041030573	2.402576457	0.016280031	0.059021435	positive
PRJNA729271	ed_Lushi_vs_Arbor_Acr	breed_growth_background	ENSGALG00010013977	SLC38A2	-0.258188848	0.103041205	-2.505685442	0.012221429	0.048138483	negative
PRJNA729271	ed_Lushi_vs_Arbor_Acr	breed_growth_background	ENSGALG00010013980	FMO4	0.005793484	0.209734104	0.027622994	0.977962842	0.987020774	positive
PRJNA729271	ed_Lushi_vs_Arbor_Acr	breed_growth_background	ENSGALG00010013981	RLF	0.143847367	0.057500382	2.501676717	0.012360674	0.048541849	positive
PRJNA729271	ed_Lushi_vs_Arbor_Acr	breed_growth_background	ENSGALG00010013989	ENSGALG00010013989	-0.002628516	0.107264932	-0.024504898	0.980449877	0.988565732	negative
PRJNA729271	ed_Lushi_vs_Arbor_Acr	breed_growth_background	ENSGALG00010013994	CHRM2	-0.694473297	0.271020498	-2.562438273	0.010394006	0.043000515	negative
PRJNA729271	ed_Lushi_vs_Arbor_Acr	breed_growth_background	ENSGALG00010013995	REM1	-0.231677762	0.112180203	-2.065228583	0.038901377	0.110239582	negative
PRJNA729271	ed_Lushi_vs_Arbor_Acr	breed_growth_background	ENSGALG00010014000	STAT4	0.154546686	0.302984597	0.510080999	0.609994717	0.740463397	positive
PRJNA729271	ed_Lushi_vs_Arbor_Acr	breed_growth_background	ENSGALG00010014001	CDC16	-0.151887446	0.047485235	-3.198624717	0.001380848	0.010313831	negative
PRJNA729271	ed_Lushi_vs_Arbor_Acr	breed_growth_background	ENSGALG00010014002	ZDHHC18	0.073896662	0.14833382	0.498178111	0.618358509	0.74660693	positive
PRJNA729271	ed_Lushi_vs_Arbor_Acr	breed_growth_background	ENSGALG00010014010	EIF5A2	-0.005240601	0.054912564	-0.095435372	0.923969022	0.957557608	negative
PRJNA729271	ed_Lushi_vs_Arbor_Acr	breed_growth_background	ENSGALG00010014011	SFN	0.061130596	0.325102673	0.188034738	0.85084942	0.910323467	positive
PRJNA729271	ed_Lushi_vs_Arbor_Acr	breed_growth_background	ENSGALG00010014014	PARP8	-0.330162936	0.109518569	-3.014675401	0.002572544	0.01591139	negative
PRJNA729271	ed_Lushi_vs_Arbor_Acr	breed_growth_background	ENSGALG00010014018	DPYSL3	-0.57906791	0.128030513	-4.522889855	6.1001E-06	0.000200913	negative
PRJNA729271	ed_Lushi_vs_Arbor_Acr	breed_growth_background	ENSGALG00010014019	IRAG2	0.004623113	0.128928231	0.035858036	0.971395557	0.983216541	positive
PRJNA729271	ed_Lushi_vs_Arbor_Acr	breed_growth_background	ENSGALG00010014020	PDXP	0.036839587	0.057996401	0.635204703	0.525294916	0.671844513	positive
PRJNA729271	ed_Lushi_vs_Arbor_Acr	breed_growth_background	ENSGALG00010014026	SH3BP1	0.150679405	0.175599319	0.858086498	0.390844703	0.552442122	positive
PRJNA729271	ed_Lushi_vs_Arbor_Acr	breed_growth_background	ENSGALG00010014030	WASF1	0.236662577	0.202098585	1.171025401	0.241588567	0.397438007	positive

PRJNA729271	eed_Lushi_vs_Arbor_Acr	breed_growth_background	ENSGALG00010014031	RPL22L1	-0.233732392	0.100831205	-2.318056126	0.020446271	0.069326011	negative
PRJNA729271	eed_Lushi_vs_Arbor_Acr	breed_growth_background	ENSGALG00010014032	PSD3	-0.007298905	0.042345555	-0.172365313	0.86315034	0.917659101	negative
PRJNA729271	eed_Lushi_vs_Arbor_Acr	breed_growth_background	ENSGALG00010014033	SLC38A4	-0.08449055	0.253651787	-0.333096607	0.739061361	0.832700694	negative
PRJNA729271	eed_Lushi_vs_Arbor_Acr	breed_growth_background	ENSGALG00010014035	PSIP1	0.117965792	0.064187161	1.837840936	0.066085847	0.161618755	positive
PRJNA729271	eed_Lushi_vs_Arbor_Acr	breed_growth_background	ENSGALG00010014041	SKIV2L2	-0.005187162	0.052784602	-0.098270366	0.921717609	0.956327938	negative
PRJNA729271	eed_Lushi_vs_Arbor_Acr	breed_growth_background	ENSGALG00010014042	SGCZ	-0.336240934	0.192793178	-1.744049958	0.081150379	0.185825394	negative
PRJNA729271	eed_Lushi_vs_Arbor_Acr	breed_growth_background	ENSGALG00010014045	GOLT1B	-0.085901119	0.060166568	-1.427721763	0.15337196	0.289884803	negative
PRJNA729271	eed_Lushi_vs_Arbor_Acr	breed_growth_background	ENSGALG00010014047	HM13	0.041832191	0.089625802	0.466742725	0.640683958	0.764526221	positive
PRJNA729271	eed_Lushi_vs_Arbor_Acr	breed_growth_background	ENSGALG00010014056	FMO3	-0.244699462	0.134138907	-1.82422436	0.06811816	0.164864374	negative
PRJNA729271	eed_Lushi_vs_Arbor_Acr	breed_growth_background	ENSGALG00010014062	GGA1	0.115125547	0.055675512	2.06779504	0.0386593	0.10976818	positive
PRJNA729271	eed_Lushi_vs_Arbor_Acr	breed_growth_background	ENSGALG00010014065	CHUNK-1	-0.345374047	0.424595057	-0.813419849	0.415977383	0.576222493	negative
PRJNA729271	eed_Lushi_vs_Arbor_Acr	breed_growth_background	ENSGALG00010014066	BCL2L1	-0.117712533	0.063080706	-1.86606237	0.06203264	0.154415785	negative
PRJNA729271	eed_Lushi_vs_Arbor_Acr	breed_growth_background	ENSGALG00010014076	PRRX1	-0.174266379	0.133392499	-1.306418131	0.191410403	0.338311644	negative
PRJNA729271	eed_Lushi_vs_Arbor_Acr	breed_growth_background	ENSGALG00010014077	SLITRK2	-0.014530999	0.053590666	-0.271147938	0.786277253	0.867374086	negative
PRJNA729271	eed_Lushi_vs_Arbor_Acr	breed_growth_background	ENSGALG00010014080	MYO1B	-0.312221005	0.08605885	-3.627994163	0.000285632	0.003360578	negative
PRJNA729271	eed_Lushi_vs_Arbor_Acr	breed_growth_background	ENSGALG00010014082	HNRNPR	-0.050440277	0.023337614	-2.161329644	0.030669885	0.092462558	negative
PRJNA729271	eed_Lushi_vs_Arbor_Acr	breed_growth_background	ENSGALG00010014083	ENSGALG00010014083	-0.168906699	0.078782194	-2.143970478	0.032035252	0.095683285	negative
PRJNA729271	eed_Lushi_vs_Arbor_Acr	breed_growth_background	ENSGALG00010014089	FIG4	-0.019244357	0.063614869	-0.302513506	0.762260639	0.850509426	negative
PRJNA729271	eed_Lushi_vs_Arbor_Acr	breed_growth_background	ENSGALG00010014091	CHIC2	-0.029758683	0.052759709	-0.564041824	0.572725669	0.710909624	negative
PRJNA729271	eed_Lushi_vs_Arbor_Acr	breed_growth_background	ENSGALG00010014092	TYRP1	2.582685951	0.546330356	4.727333788	2.27487E-06	9.28852E-05	positive
PRJNA729271	eed_Lushi_vs_Arbor_Acr	breed_growth_background	ENSGALG00010014093	PLD1	-0.124853237	0.04995085	-2.499521761	0.012436106	0.048781239	negative
PRJNA729271	eed_Lushi_vs_Arbor_Acr	breed_growth_background	ENSGALG00010014096	JAKMIP2	-0.264104338	0.088607024	-2.98062531	0.002876605	0.017201482	negative
PRJNA729271	eed_Lushi_vs_Arbor_Acr	breed_growth_background	ENSGALG00010014099	TPX2	-0.272966836	0.077345111	-3.529206081	0.000416808	0.004371663	negative
PRJNA729271	eed_Lushi_vs_Arbor_Acr	breed_growth_background	ENSGALG00010014102	TRIM2	0.163538734	0.073858692	2.214211095	0.026814261	0.083978007	positive
PRJNA729271	eed_Lushi_vs_Arbor_Acr	breed_growth_background	ENSGALG00010014104	ALDH1A1	1.054473398	0.288916863	3.6497468	0.000262499	0.003162861	positive
PRJNA729271	eed_Lushi_vs_Arbor_Acr	breed_growth_background	ENSGALG00010014105	AHR1B	-0.145861572	0.315685036	-0.462047785	0.644047053	0.767277228	negative
PRJNA729271	eed_Lushi_vs_Arbor_Acr	breed_growth_background	ENSGALG00010014109	AMIGO2	-0.018503453	0.089292339	-0.207223293	0.835835477	0.900307292	negative
PRJNA729271	eed_Lushi_vs_Arbor_Acr	breed_growth_background	ENSGALG00010014111	GORAB	-0.027802004	0.044268785	-0.628027276	0.529986072	0.675828988	negative
PRJNA729271	eed_Lushi_vs_Arbor_Acr	breed_growth_background	ENSGALG00010014114	ANXA1	-0.384714744	0.177430357	-2.168257731	0.030139083	0.091203758	negative
PRJNA729271	eed_Lushi_vs_Arbor_Acr	breed_growth_background	ENSGALG00010014120	SOX5	-0.066081362	0.074788902	-0.883571763	0.376927427	0.539344823	negative
PRJNA729271	eed_Lushi_vs_Arbor_Acr	breed_growth_background	ENSGALG00010014122	ABCA1	-0.185804209	0.075300571	-2.467500684	0.013605996	0.051956165	negative
PRJNA729271	eed_Lushi_vs_Arbor_Acr	breed_growth_background	ENSGALG00010014125	PLPP1	-0.338258003	0.073633885	-4.593781838	4.35284E-06	0.000154643	negative
PRJNA729271	eed_Lushi_vs_Arbor_Acr	breed_growth_background	ENSGALG00010014128	COL4A1	-0.158574905	0.077227942	-2.05333589	0.040040012	0.112411385	negative
PRJNA729271	eed_Lushi_vs_Arbor_Acr	breed_growth_background	ENSGALG00010014131	CDC23	-0.100073983	0.064329331	-1.555650923	0.119791123	0.244043896	negative
PRJNA729271	eed_Lushi_vs_Arbor_Acr	breed_growth_background	ENSGALG00010014134	RPAP3	0.122877682	0.061606347	1.994562058	0.046090665	0.124923875	positive
PRJNA729271	eed_Lushi_vs_Arbor_Acr	breed_growth_background	ENSGALG00010014135	ZBTB24	0.301955594	0.083423869	3.61953477	0.000295133	0.003438871	positive
PRJNA729271	eed_Lushi_vs_Arbor_Acr	breed_growth_background	ENSGALG00010014142	PPIL6	-0.900313957	0.271163355	-3.320190355	0.000899561	0.00754685	negative
PRJNA729271	eed_Lushi_vs_Arbor_Acr	breed_growth_background	ENSGALG00010014143	NFATC2	0.051200616	0.083447718	0.613565207	0.539502681	0.684831541	positive
PRJNA729271	eed_Lushi_vs_Arbor_Acr	breed_growth_background	ENSGALG00010014145	NTMT2	0.117790082	0.130266909	0.904221058	0.365878196	0.529281882	positive
PRJNA729271	eed_Lushi_vs_Arbor_Acr	breed_growth_background	ENSGALG00010014147	ENSGALG00010014147	0.199459724	0.202022512	0.987314341	0.323488562	0.48746925	positive
PRJNA729271	eed_Lushi_vs_Arbor_Acr	breed_growth_background	ENSGALG00010014148	KCNG1	0.304271201	0.201286349	1.511633561	0.130627109	0.258666659	positive
PRJNA729271	eed_Lushi_vs_Arbor_Acr	breed_growth_background	ENSGALG00010014149	LGALS2	0.40997284	0.267310935	1.533692737	0.125105261	0.25126014	positive
PRJNA729271	eed_Lushi_vs_Arbor_Acr	breed_growth_background	ENSGALG00010014151	ATP23	-0.05070645	0.049139065	-1.031896914	0.302120412	0.464582231	negative
PRJNA729271	eed_Lushi_vs_Arbor_Acr	breed_growth_background	ENSGALG00010014153	FOCAD	-0.005753738	0.071032183	-0.081001849	0.935440482	0.964536447	negative
PRJNA729271	eed_Lushi_vs_Arbor_Acr	breed_growth_background	ENSGALG00010014154	DNAI7	0.335748614	0.25687437	1.307053772	0.19119445	0.338053617	positive
PRJNA729271	eed_Lushi_vs_Arbor_Acr	breed_growth_background	ENSGALG00010014155	ENSGALG00010014155	-0.495618767	0.798942327	-0.62034361	0.535031589	0.680219664	negative
PRJNA729271	eed_Lushi_vs_Arbor_Acr	breed_growth_background	ENSGALG00010014156	CD164	-0.011622697	0.04935287	-0.235501953	0.813819163	0.885537337	negative
PRJNA729271	eed_Lushi_vs_Arbor_Acr	breed_growth_background	ENSGALG00010014159	CDC42EP1	-0.158271238	0.080419105	-1.968080066	0.049058826	0.130886745	negative
PRJNA729271	eed_Lushi_vs_Arbor_Acr	breed_growth_background	ENSGALG00010014161	GFRA3	-0.096202181	0.133900196	-0.71846184	0.472472569	0.626348078	negative
PRJNA729271	eed_Lushi_vs_Arbor_Acr	breed_growth_background	ENSGALG00010014162	COL18A1	-0.346482386	0.100552254	-3.445794324	0.000569384	0.005419051	negative
PRJNA729271	eed_Lushi_vs_Arbor_Acr	breed_growth_background	ENSGALG00010014165	LUZP1	-0.222812018	0.064862347	-3.435151966	0.000592222	0.005563875	negative
PRJNA729271	eed_Lushi_vs_Arbor_Acr	breed_growth_background	ENSGALG00010014166	FGF12	0.092217728	0.112104402	0.822605773	0.410732215	0.571196221	positive
PRJNA729271	eed_Lushi_vs_Arbor_Acr	breed_growth_background	ENSGALG00010014167	SLC38A9	0.010444781	0.058959514	0.177151745	0.859389198	0.915473391	positive
PRJNA729271	eed_Lushi_vs_Arbor_Acr	breed_growth_background	ENSGALG00010014170	CEMIP2	-0.181478529	0.058333452	-3.111054161	0.001864207	0.012628957	negative
PRJNA729271	eed_Lushi_vs_Arbor_Acr	breed_growth_background	ENSGALG00010014171	DLC1	-0.203004503	0.071045539	-2.857385638	0.004271465	0.022637985	negative
PRJNA729271	eed_Lushi_vs_Arbor_Acr	breed_growth_background	ENSGALG00010014173	MND1	-0.428274208	0.110139358	-3.888475635	0.000100876	0.001594542	negative
PRJNA729271	eed_Lushi_vs_Arbor_Acr	breed_growth_background	ENSGALG00010014176	ABHD17B	-0.059708626	0.049879225	-1.19706404	0.231281595	0.385274665	negative
PRJNA729271	eed_Lushi_vs_Arbor_Acr	breed_growth_background	ENSGALG00010014178	LRTM2	0.25730404	0.216311	1.189509731	0.234239144	0.388633847	positive
PRJNA729271	eed_Lushi_vs_Arbor_Acr	breed_growth_background	ENSGALG00010014179	CTBPL	-0.006279264	0.023215748	-0.270474346	0.786795351	0.867599742	negative
PRJNA729271	eed_Lushi_vs_Arbor_Acr	breed_growth_background	ENSGALG00010014180	SLC19A1	0.225131217	0.07800321	2.886178883	0.003899505	0.021297407	positive
PRJNA729271	eed_Lushi_vs_Arbor_Acr	breed_growth_background	ENSGALG00010014184	SLC44A1	-0.086219166	0.070763881	-1.218406402	0.223069573	0.37568029	negative
PRJNA729271	eed_Lushi_vs_Arbor_Acr	breed_growth_background	ENSGALG00010014186	MOCS3	-0.001561237	0.059933914	-0.026049304	0.979218013	0.987735915	negative
PRJNA729271	eed_Lushi_vs_Arbor_Acr	breed_growth_background	ENSGALG00010014189	DDX4	-0.500473438	0.320487035	-1.561602759	0.118381599	0.242227736	negative
PRJNA729271	eed_Lushi_vs_Arbor_Acr	breed_growth_background	ENSGALG00010014190	HACD4	-0.13580057	0.117862068	-1.152199117	0.24923926	0.405272594	negative
PRJNA729271	eed_Lushi_vs_Arbor_Acr	breed_growth_background	ENSGALG00010014192	KDM1A	-0.012231061	0.033743312	-0.362473654	0.716998108	0.81859543	negative
PRJNA729271	eed_Lushi_vs_Arbor_Acr	breed_growth_background	ENSGALG00010014193	CARD10	-0.086028778	0.216627835	-0.397127071	0.691273758	0.800512354	negative
PRJNA729271	eed_Lushi_vs_Arbor_Acr	breed_growth_background	ENSGALG00010014194	LRIG3	-0.09120306	0.050550743	-1.804188325	0.071201789	0.169832143	negative
PRJNA729271	eed_Lushi_vs_Arbor_Acr	breed_growth_background	ENSGALG00010014197	SH3GL2	-0.329249944	0.199299681	-1.652034474	0.098527523	0.212713204	negative
PRJNA729271	eed_Lushi_vs_Arbor_Acr	breed_growth_background	ENSGALG00010014198	KDM3B	0.003863432	0.02485671	0.155428141	0.876483801	0.926804252	positive
PRJNA729271	eed_Lushi_vs_Arbor_Acr	breed_growth_background	ENSGALG00010014199	LNX1	0.449599645	0.128387641	3.501891938	0.000461967	0.004691226	positive
PRJNA729271	eed_Lushi_vs_Arbor_Acr	breed_growth_background	ENSGALG00010014200	DCP1B	0.113880014	0.048158344	2.364699528	0.018044704	0.063629859	positive
PRJNA729271	eed_Lushi_vs_Arbor_Acr	breed_growth_background	ENSGALG00010014203	TMEM38B	-0.071861678	0.090379778	-0.795107919	0.426550728	0.585663624	negative
PRJNA729271	eed_Lushi_vs_Arbor_Acr	breed_growth_background	ENSGALG00010014204	KIFAP3	0.054839971	0.045691024	1.200235094	0.23004805	0.383854781	positive
PRJNA729271	eed_Lushi_vs_Arbor_Acr	breed_growth_background	ENSGALG00010014205	DPM1	0.004979788	0.046529496	0.107024321	0.914769685	0.951941029	positive
PRJNA729271	eed_Lushi_vs_Arbor_Acr	breed_growth_background	ENSGALG00010014209	CCDC50	-0.081506202	0.057552337	-1.416210114	0.156713996	0.294130437	negative
PRJNA729271	eed_Lushi_vs_Arbor_Acr	breed_growth_background	ENSGALG00010014210	ENSGALG00010014210	-0.151300901	0.136616214	-1.107488605	0.268082736	0.426548766	negative
PRJNA729271	eed_Lushi_vs_Arbor_Acr	breed_growth_background	ENSGALG00010014213	ETFRF1	0.184452731	0.08112704	2.273628277	0.022988346	0.075429519	positive
PRJNA729271	eed_Lushi_vs_Arbor_Acr	breed_growth_background	ENSGALG00010014214	REEP2	-0.075501559	0.073007219	-1.034165657	0.301058726	0.46345011	negative
PRJNA729271	eed_Lushi_vs_Arbor_Acr	breed_growth_background	ENSGALG00010014217	RASA3	-0.201938577	0.058751005	-3.437193585	0.000587775	0.00553523	negative
PRJNA729271	eed_Lushi_vs_Arbor_Acr	breed_growth_background	ENSGALG00010014218	IL31RA	-0.310698473	0.088986286	-3.491532069	0.000480259	0.004830917	negative
PRJNA729271	eed_Lushi_vs_Arbor_Acr	breed_growth_background	ENSGALG00010014220	C9orf85	0.137943836	0.072333784	1.907045758	0.056514662	0.14468033	positive
PRJNA729271	eed_Lushi_vs_Arbor_Acr	breed_growth_background	ENSGALG00010014221	SCYL3	0.304304786	0.070229472	4.33300692	1.47086E-05	0.000384147	positive
PRJNA729271	eed_Lushi_vs_Arbor_Acr	breed_growth_background	ENSGALG00010014222	TBX22	-0.330619456	0.233571788	-1.415493968	0.156923716	0.294371588	negative
PRJNA729271	eed_Lushi_vs_Arbor_Acr	breed_growth_background	ENSGALG00010014224	NMUR2	0.645388208	0.429322782	1.50327035	0.132769343	0.26194251	positive
PRJNA729271	eed_Lushi_vs_Arbor_Acr	breed_growth_background	ENSGALG00010014225	ZMPSTE24	-0.051873958	0.041340904	-1.254785272	0.209556721	0.359257796	negative
PRJNA729271	eed_Lushi_vs_Arbor_Acr	breed_growth_background	ENSGALG00010014226	SLC16A7	-0.296099111	0.139095719	-2.12874352	0.033275486	0.098113949	negative
PRJNA729271	eed_Lushi_vs_Arbor_Acr	breed_growth_background	ENSGALG00010014234	GLRA1	0.106948357	0.387964389	0.275665397	0.782805075	0.86563622	positive
PRJNA729271	eed_Lushi_vs_Arbor_Acr	breed_growth_background	ENSGALG00010014236	WDR44	0.0499019	0.044447269	1.122721399	0.261555837	0.419618357	positive
PRJNA729271	eed_Lushi_vs_Arbor_Acr	breed_growth_background	ENSGALG00010014238	PHLDA1	-0.336276433	0.066678392	-5.043259509	4.57668E-07	2.66422E-05	negative
PRJNA729271	eed_Lushi_vs_Arbor_Acr	breed_growth_background	ENSGALG00010014240	PIGV	-0.071307892	0.070030038	-1.018247224	0.308560482	0.471364822	negative
PRJNA729271	eed_Lushi_vs_Arbor_Acr	breed_growth_background	ENSGALG00010014241	ENSGALG00010014241	-2.168395066	0.490597163	-4.41990951	9.87422E-06	0.000288562	negative
PRJNA729271	eed_Lushi_vs_Arbor_Acr	breed_growth_background	ENSGALG00010014243	ENSGALG00010014243	0.009865777	0.077297335	0.127634123	0.898438526	0.941162567	positive
PRJNA729271	eed_Lushi_vs_Arbor_Acr	breed_growth_background	ENSGALG00010014244	SESN1	0.330323085	0.197606211	1.671622987	0.094598689	0.20688037	positive
PRJNA729271	eed_Lushi_vs_Arbor_Acr	breed_growth_background	ENSGALG00010014246	KRAS	0.095765199	0.06187437	1.547736145	0.121685836	0.246345837	positive
PRJNA729271	eed_Lushi_vs_Arbor_Acr	breed_growth_background	ENSGALG00010014249	ENSGALG00010014249	0.169114569	0.054232248	3.118339626	0.001818731	0.012446355	positive
PRJNA729271	eed_Lushi_vs_Arbor_Acr	breed_growth_background	ENSGALG00010014250	ENSGALG00010014250	0.036847255	0.133460261	0.276091584	0.782477724	0.865538356	positive
PRJNA729271	eed_Lushi_vs_Arbor_Acr	breed_growth_background	ENSGALG00010014257	NOP16	-0.147639377	0.068314067	-2.161185573	0.030681007	0.092476856	negative
PRJNA729271	eed_Lushi_vs_Arbor_Acr	breed_growth_background	ENSGALG00010014258	ENSGALG00010014258	-0.000812933	0.328011962	-0.002478364	0.998022554	0.998918445	negative
PRJNA729271	eed_Lushi_vs_Arbor_Acr	breed_growth_background	ENSGALG00010014260	ADNP	-0.051702236	0.040605323	-1.273287135	0.20291617	0.352136359	negative
PRJNA729271	eed_Lushi_vs_Arbor_Acr	breed_growth_background	ENSGALG00010014263	ZNF131	0.008854207	0.064807368	0.136623457	0.891328434	0.936368897	positive
PRJNA729271	eed_Lushi_vs_Arbor_Acr	breed_growth_background	ENSGALG00010014265	ZNF462	-0.276499317	0.074911001	-3.691037525	0.000223341	0.002812624	negative
PRJNA729271	eed_Lushi_vs_Arbor_Acr	breed_growth_background	ENSGALG00010014267	SCFD2	0.087740175	0.041707883	2.103683243	0.035406087	0.102871342	positive
PRJNA729271	eed_Lushi_vs_Arbor_Acr	breed_growth_background	ENSGALG00010014272	PARD6B	0.628161752	0.138542911	4.534059129	5.78608E-06	0.000193247	positive
PRJNA729271	eed_Lushi_vs_Arbor_Acr	breed_growth_background	ENSGALG00010014274	FIRRM	-0.006922074	0.082199713	-0.084210436	0.932889121	0.963190242	negative
PRJNA729271	eed_Lushi_vs_Arbor_Acr	breed_growth_background	ENSGALG00010014278	G3BP1	-0.030902012	0.074639846	-0.414014952	0.678863157	0.792623996	negative
PRJNA729271	eed_Lushi_vs_Arbor_Acr	breed_growth_background	ENSGALG00010014284	TMEM131L	-0.141001699	0.046231652	-3.049895317	0.002289211	0.014643477	negative
PRJNA729271	eed_Lushi_vs_Arbor_Acr	breed_growth_background	ENSGALG00010014288	ARMC2	-0.328543904	0.137717089	-2.385643687	0.017049261	0.061049663	negative
PRJNA729271	eed_Lushi_vs_Arbor_Acr	breed_growth_background	ENSGALG00010014291	OSTN	-0.666294849	0.300401529	-2.218014177	0.026553866	0.083437738	negative
PRJNA729271	eed_Lushi_vs_Arbor_Acr	breed_growth_background	ENSGALG00010014292	NIM1K	0.310192954	0.237684924	1.305059445	0.191872607	0.338715557	positive
PRJNA729271	eed_Lushi_vs_Arbor_Acr	breed_growth_background	ENSGALG00010014294	TMPO	0.064812907	0.054652848	1.185901736	0.235661107	0.390368533	positive
PRJNA729271	eed_Lushi_vs_Arbor_Acr	breed_growth_background	ENSGALG00010014295	CACNA1C	0.310871953	0.095752221	3.246629163	0.001167804	0.009144962	positive
PRJNA729271	eed_Lushi_vs_Arbor_Acr	breed_growth_background	ENSGALG00010014298	METTL18	-0.438182061	0.121994925	-3.591805661	0.000328395	0.003681424	negative
PRJNA729271	eed_Lushi_vs_Arbor_Acr	breed_growth_background	ENSGALG00010014300	ELFN2	-0.420938722	0.266379898	-1.580219546	0.114056597	0.235942682	negative
PRJNA729271	eed_Lushi_vs_Arbor_Acr	breed_growth_background	ENSGALG00010014301	ARID1A	0.058031053	0.064060619	0.905877181	0.365000863	0.528381855	positive
PRJNA729271	eed_Lushi_vs_Arbor_Acr	breed_growth_background	ENSGALG00010014302	FOXO3	0.291367507	0.124654825	2.337394536	0.019418679	0.066937872	positive
PRJNA729271	eed_Lushi_vs_Arbor_Acr	breed_growth_background	ENSGALG00010014303	GABRA2	-0.190950654	0.209522319	-0.911361875	0.362104734	0.525396209	negative
PRJNA729271	eed_Lushi_vs_Arbor_Acr	breed_growth_background	ENSGALG00010014305	ARL10	0.008007923	0.172054523	0.046542937	0.962877513	0.978835813	positive
PRJNA729271	eed_Lushi_vs_Arbor_Acr	breed_growth_background	ENSGALG00010014306	ENSGALG00010014306	0.122008692	0.177782332	0.686281314	0.49253573	0.644399802	positive
PRJNA729271	eed_Lushi_vs_Arbor_Acr	breed_growth_background	ENSGALG00010014308	ENSGALG00010014308	0.440086441	0.107284133	4.102064566	4.0948E-05	0.000839521	positive
PRJNA729271	eed_Lushi_vs_Arbor_Acr	breed_growth_background	ENSGALG00010014310	COL6A1	-0.33600493	0.12706378	-2.644380095	0.00818407	0.036189167	negative
PRJNA729271	eed_Lushi_vs_Arbor_Acr	breed_growth_background	ENSGALG00010014312	KIAA1191	0.073901844	0.045439794	1.626368362	0.103871317	0.22102389	positive
PRJNA729271	eed_Lushi_vs_Arbor_Acr	breed_growth_background	ENSGALG00010014314	ENSGALG00010014314	-0.023471117	0.088825766	-0.264237711	0.791596754	0.870774452	negative
PRJNA729271	eed_Lushi_vs_Arbor_Acr	breed_growth_background	ENSGALG00010014317	RAD23B	0.234740162	0.078251151	2.999830159	0.002701302	0.016417346	positive
PRJNA729271	eed_Lushi_vs_Arbor_Acr	breed_growth_background	ENSGALG00010014318	ENSGALG00010014318	-0.785286197	0.253576109	-3.096846157	0.001955914	0.013083049	negative
PRJNA729271	eed_Lushi_vs_Arbor_Acr	breed_growth_background	ENSGALG00010014319	ENSGALG00010014319	-0.002210892	0.061990644	-0.035664929	0.971549536	0.983216541	negative
PRJNA729271	eed_Lushi_vs_Arbor_Acr	breed_growth_background	ENSGALG00010014320	UTS2B	-1.178834127	0.582563866	-2.023527712	0.043018769	0.118704942	negative
PRJNA729271	eed_Lushi_vs_Arbor_Acr	breed_growth_background	ENSGALG00010014323	KLF4	-0.11144536	0.095197104	-1.170680148	0.241727368	0.397500548	negative
PRJNA729271	eed_Lushi_vs_Arbor_Acr	breed_growth_background	ENSGALG00010014324	COL4A2	-0.17137261	0.075961311	-2.256051237	0.024067423	0.077869931	negative
PRJNA729271	eed_Lushi_vs_Arbor_Acr	breed_growth_background	ENSGALG00010014328	RASSF8	-0.087117133	0.049617082	-1.755789121	0.079124423	0.182657829	negative
PRJNA729271	eed_Lushi_vs_Arbor_Acr	breed_growth_background	ENSGALG00010014329	IER5	0.182211769	0.08296441	2.196264257	0.028073035	0.086707572	positive
PRJNA729271	eed_Lushi_vs_Arbor_Acr	breed_growth_background	ENSGALG00010014334	HMGCS1	-0.053039352	0.084823533	-0.625290525	0.531780398	0.677521039	negative

PRJNA729271	:ed_Lushi_vs_Arbor_Acr	breed_growth_background	ENSGALG00010014338	BHLHE41	0.186451739	0.117710067	1.583991445	0.113195666	0.234707218	positive
PRJNA729271	:ed_Lushi_vs_Arbor_Acr	breed_growth_background	ENSGALG00010014340	EFNB1	0.034052967	0.07409397	0.459591615	0.645809379	0.768555578	positive
PRJNA729271	:ed_Lushi_vs_Arbor_Acr	breed_growth_background	ENSGALG00010014344	LSG1	0.063522651	0.04777011	1.329757268	0.183598259	0.328793769	positive
PRJNA729271	:ed_Lushi_vs_Arbor_Acr	breed_growth_background	ENSGALG00010014346	AFG1L	-0.125397719	0.085764347	-1.462119446	0.143708481	0.277038759	negative
PRJNA729271	:ed_Lushi_vs_Arbor_Acr	breed_growth_background	ENSGALG00010014347	KIAA0040	0.294061242	0.17865538	1.645969134	0.099770114	0.214596795	positive
PRJNA729271	:ed_Lushi_vs_Arbor_Acr	breed_growth_background	ENSGALG00010014350	PTGER4	-0.274054753	0.097087216	-2.822768675	0.004761091	0.024576928	negative
PRJNA729271	:ed_Lushi_vs_Arbor_Acr	breed_growth_background	ENSGALG00010014355	RAB20	0.144626461	0.088323508	1.637462821	0.101533801	0.217390318	positive
PRJNA729271	:ed_Lushi_vs_Arbor_Acr	breed_growth_background	ENSGALG00010014359	TMEM271	0.096447189	0.224879324	0.4288842	0.668007499	0.78486007	positive
PRJNA729271	:ed_Lushi_vs_Arbor_Acr	breed_growth_background	ENSGALG00010014361	SSPN	0.08060107	0.079460624	1.014352341	0.310414657	0.473328471	positive
PRJNA729271	:ed_Lushi_vs_Arbor_Acr	breed_growth_background	ENSGALG00010014362	NAXD	0.071425038	0.057008521	1.252883555	0.210248087	0.360144886	positive
PRJNA729271	:ed_Lushi_vs_Arbor_Acr	breed_growth_background	ENSGALG00010014367	OPHN1	-0.170578177	0.058727836	-2.904554091	0.003677766	0.020370357	negative
PRJNA729271	:ed_Lushi_vs_Arbor_Acr	breed_growth_background	ENSGALG00010014371	TTC33	-0.007660749	0.055868074	-0.137122117	0.890934271	0.936278711	negative
PRJNA729271	:ed_Lushi_vs_Arbor_Acr	breed_growth_background	ENSGALG00010014372	CCDC171	0.043706858	0.068687038	0.636318868	0.52456861	0.671342221	positive
PRJNA729271	:ed_Lushi_vs_Arbor_Acr	breed_growth_background	ENSGALG00010014375	H2BC35	-0.12694159	0.132100297	-0.960948561	0.336578032	0.500893078	negative
PRJNA729271	:ed_Lushi_vs_Arbor_Acr	breed_growth_background	ENSGALG00010014379	CARS2	-0.19261161	0.049325222	-3.904931405	9.42521E-05	0.001523119	negative
PRJNA729271	:ed_Lushi_vs_Arbor_Acr	breed_growth_background	ENSGALG00010014380	SNX3	-0.065894029	0.062864966	-1.048183638	0.294554007	0.455857392	negative
PRJNA729271	:ed_Lushi_vs_Arbor_Acr	breed_growth_background	ENSGALG00010014388	MFNG	-0.030292034	0.117286245	-0.258274398	0.796195144	0.873593259	negative
PRJNA729271	:ed_Lushi_vs_Arbor_Acr	breed_growth_background	ENSGALG00010014389	COL6A2	-0.448974118	0.141358171	-3.176145499	0.001492461	0.010870964	negative
PRJNA729271	:ed_Lushi_vs_Arbor_Acr	breed_growth_background	ENSGALG00010014390	SPARC	-0.290847752	0.08500306	-3.421615066	0.000622504	0.005787808	negative
PRJNA729271	:ed_Lushi_vs_Arbor_Acr	breed_growth_background	ENSGALG00010014393	ATP8A1	0.087985363	0.077288755	1.138397992	0.254954332	0.411716025	positive
PRJNA729271	:ed_Lushi_vs_Arbor_Acr	breed_growth_background	ENSGALG00010014396	ING1	0.028153141	0.063300406	0.444754515	0.656497168	0.776152258	positive
PRJNA729271	:ed_Lushi_vs_Arbor_Acr	breed_growth_background	ENSGALG00010014397	PRKAA1	-0.020105671	0.041484936	-0.484649917	0.627924699	0.754084474	negative
PRJNA729271	:ed_Lushi_vs_Arbor_Acr	breed_growth_background	ENSGALG00010014399	GAS6	-0.148246659	0.101172816	-1.465281528	0.142844124	0.275959693	negative
PRJNA729271	:ed_Lushi_vs_Arbor_Acr	breed_growth_background	ENSGALG00010014400	RIPOR3	0.074443173	0.055374013	1.344370192	0.178828708	0.323126667	positive
PRJNA729271	:ed_Lushi_vs_Arbor_Acr	breed_growth_background	ENSGALG00010014403	H2BC37	0.189351644	0.100763371	1.879171387	0.060221095	0.151283323	positive
PRJNA729271	:ed_Lushi_vs_Arbor_Acr	breed_growth_background	ENSGALG00010014407	YIPF6	0.103221465	0.04923449	2.096527567	0.036035417	0.104195765	positive
PRJNA729271	:ed_Lushi_vs_Arbor_Acr	breed_growth_background	ENSGALG00010014408	ENSGALG00010014408	-0.337904011	0.18988799	-1.779491224	0.075159262	0.176540838	negative
PRJNA729271	:ed_Lushi_vs_Arbor_Acr	breed_growth_background	ENSGALG00010014411	ATP13A3	-0.023746502	0.043813744	-0.541987497	0.587827119	0.723491049	negative
PRJNA729271	:ed_Lushi_vs_Arbor_Acr	breed_growth_background	ENSGALG00010014414	TMEM255B	-0.09330857	0.265844414	-0.350989394	0.725596305	0.824192339	negative
PRJNA729271	:ed_Lushi_vs_Arbor_Acr	breed_growth_background	ENSGALG00010014416	H2BC32	-0.436114031	0.144340508	-3.021425088	0.002515879	0.015653557	negative
PRJNA729271	:ed_Lushi_vs_Arbor_Acr	breed_growth_background	ENSGALG00010014418	RAC2	0.027110215	0.159031004	0.170471253	0.86463954	0.918680641	positive
PRJNA729271	:ed_Lushi_vs_Arbor_Acr	breed_growth_background	ENSGALG00010014427	TMEM267	-0.159128055	0.074690101	-2.130510653	0.033129477	0.09778289	negative
PRJNA729271	:ed_Lushi_vs_Arbor_Acr	breed_growth_background	ENSGALG00010014431	ENSGALG00010014431	-0.101269942	0.084557944	-1.197639596	0.231057354	0.385036556	negative
PRJNA729271	:ed_Lushi_vs_Arbor_Acr	breed_growth_background	ENSGALG00010014439	ENSGALG00010014439	1.277857321	0.449589249	2.842277308			positive
PRJNA729271	:ed_Lushi_vs_Arbor_Acr	breed_growth_background	ENSGALG00010014442	SHISA3	0.052111451	0.470012603	0.110872454	0.911717489	0.949992453	positive
PRJNA729271	:ed_Lushi_vs_Arbor_Acr	breed_growth_background	ENSGALG00010014449	ENSGALG00010014449	-0.18508392	0.082871675	-2.233379737	0.025523913	0.081151376	negative
PRJNA729271	:ed_Lushi_vs_Arbor_Acr	breed_growth_background	ENSGALG00010014453	NR2E1	-0.450512613	0.188529367	-2.389615043	0.016866041	0.060528165	negative
PRJNA729271	:ed_Lushi_vs_Arbor_Acr	breed_growth_background	ENSGALG00010014455	C4orf19	0.14949804	0.145511226	1.027398673	0.304232776	0.466704108	positive
PRJNA729271	:ed_Lushi_vs_Arbor_Acr	breed_growth_background	ENSGALG00010014465	SIMC1	0.10873636	0.080472737	1.351219853	0.176625017	0.320623622	positive
PRJNA729271	:ed_Lushi_vs_Arbor_Acr	breed_growth_background	ENSGALG00010014469	TMCO3	-0.218815852	0.069532277	-3.146968022	0.00164973	0.011608173	negative
PRJNA729271	:ed_Lushi_vs_Arbor_Acr	breed_growth_background	ENSGALG00010014470	RPS6KA1	-0.115278782	0.07019869	-1.642178523	0.100553012	0.215960278	negative
PRJNA729271	:ed_Lushi_vs_Arbor_Acr	breed_growth_background	ENSGALG00010014474	PTPN1	-0.038599623	0.041986335	-0.919337769	0.357918928	0.521620236	negative
PRJNA729271	:ed_Lushi_vs_Arbor_Acr	breed_growth_background	ENSGALG00010014476	C5orf34	-0.227276837	0.096765269	-2.348743918	0.018836857	0.065540144	negative
PRJNA729271	:ed_Lushi_vs_Arbor_Acr	breed_growth_background	ENSGALG00010014477	ITPR2	-0.133160514	0.052903817	-2.517030354	0.011834863	0.047115172	negative
PRJNA729271	:ed_Lushi_vs_Arbor_Acr	breed_growth_background	ENSGALG00010014482	THOC3	-0.016624872	0.047755162	-0.348127234	0.727744632	0.824951783	negative
PRJNA729271	:ed_Lushi_vs_Arbor_Acr	breed_growth_background	ENSGALG00010014487	OSTM1	-0.057324476	0.045638374	-1.256058668	0.2090947	0.358847701	negative
PRJNA729271	:ed_Lushi_vs_Arbor_Acr	breed_growth_background	ENSGALG00010014488	ENSGALG00010014488	0.685991703	0.219220119	3.129236976	0.001752609	0.012102938	positive
PRJNA729271	:ed_Lushi_vs_Arbor_Acr	breed_growth_background	ENSGALG00010014492	CPLX2	-0.422194213	0.191862211	-2.200507392	0.027770916	0.085968937	negative
PRJNA729271	:ed_Lushi_vs_Arbor_Acr	breed_growth_background	ENSGALG00010014494	KLHL9	-0.015372079	0.036934492	-0.416198469	0.67726478	0.791555888	negative
PRJNA729271	:ed_Lushi_vs_Arbor_Acr	breed_growth_background	ENSGALG00010014504	SEC63	0.025808942	0.025086267	1.028807612	0.303570089	0.466276194	positive
PRJNA729271	:ed_Lushi_vs_Arbor_Acr	breed_growth_background	ENSGALG00010014505	CYTH4	-0.132547064	0.112586467	-1.177291263	0.239079273	0.394608752	negative
PRJNA729271	:ed_Lushi_vs_Arbor_Acr	breed_growth_background	ENSGALG00010014512	HMGN4	-0.039725602	0.059542401	-0.667181725	0.504656063	0.654878213	negative
PRJNA729271	:ed_Lushi_vs_Arbor_Acr	breed_growth_background	ENSGALG00010014515	ETNK1	-0.106444868	0.061135864	-1.741119876	0.081662568	0.186644422	negative
PRJNA729271	:ed_Lushi_vs_Arbor_Acr	breed_growth_background	ENSGALG00010014517	DCUN1D2	0.141481982	0.067377695	2.099834097	0.035743438	0.103564251	positive
PRJNA729271	:ed_Lushi_vs_Arbor_Acr	breed_growth_background	ENSGALG00010014522	FAT2	0.064361497	0.271213369	0.237309456	0.812416727	0.884481032	positive
PRJNA729271	:ed_Lushi_vs_Arbor_Acr	breed_growth_background	ENSGALG00010014537	GKAP1	0.088217612	0.064236166	1.373332458	0.169649017	0.311814454	positive
PRJNA729271	:ed_Lushi_vs_Arbor_Acr	breed_growth_background	ENSGALG00010014539	PAIP1	0.060601176	0.045992261	1.317638537	0.187624679	0.333409307	positive
PRJNA729271	:ed_Lushi_vs_Arbor_Acr	breed_growth_background	ENSGALG00010014547	DHDDS	-0.099057093	0.057607917	-1.719504868	0.085522482	0.19300146	negative
PRJNA729271	:ed_Lushi_vs_Arbor_Acr	breed_growth_background	ENSGALG00010014550	SCML4	-0.125071011	0.471317773	-0.265364514	0.790728671	0.870149718	negative
PRJNA729271	:ed_Lushi_vs_Arbor_Acr	breed_growth_background	ENSGALG00010014555	VGLL1	-1.066012614	0.482564277	-2.209058284	0.027170587	0.084623477	negative
PRJNA729271	:ed_Lushi_vs_Arbor_Acr	breed_growth_background	ENSGALG00010014567	UTP11	-0.117590558	0.063056435	-1.864846292	0.062202952	0.154706897	negative
PRJNA729271	:ed_Lushi_vs_Arbor_Acr	breed_growth_background	ENSGALG00010014570	KIF27	0.020715387	0.090977094	0.227698936	0.8198803	0.889400161	positive
PRJNA729271	:ed_Lushi_vs_Arbor_Acr	breed_growth_background	ENSGALG00010014572	IL1RAP	-0.126843932	0.067845667	-1.869595177	0.061540056	0.153638151	negative
PRJNA729271	:ed_Lushi_vs_Arbor_Acr	breed_growth_background	ENSGALG00010014573	CEBPB	0.784614102	0.184305844	4.257130884	2.07067E-05	0.000492919	positive
PRJNA729271	:ed_Lushi_vs_Arbor_Acr	breed_growth_background	ENSGALG00010014576	HRH2	-0.160261814	0.144451984	-1.109446958	0.267237414	0.425624252	negative
PRJNA729271	:ed_Lushi_vs_Arbor_Acr	breed_growth_background	ENSGALG00010014578	ENSGALG00010014578	0.597598984	0.13564164	4.405719244	1.05433E-05	0.000303226	positive
PRJNA729271	:ed_Lushi_vs_Arbor_Acr	breed_growth_background	ENSGALG00010014579	NNT	0.139187537	0.077348242	1.799491916	0.071940902	0.170920074	positive
PRJNA729271	:ed_Lushi_vs_Arbor_Acr	breed_growth_background	ENSGALG00010014592	QNG1	-0.1138383	0.050493578	-2.254510449	0.024164074	0.078060676	negative
PRJNA729271	:ed_Lushi_vs_Arbor_Acr	breed_growth_background	ENSGALG00010014599	SSTR3	0.180769821	0.223061456	0.810403662	0.417708214	0.57777009	positive
PRJNA729271	:ed_Lushi_vs_Arbor_Acr	breed_growth_background	ENSGALG00010014602	FRK	-1.000891776	0.723980384	-1.382484661	0.166822937	0.307567855	negative
PRJNA729271	:ed_Lushi_vs_Arbor_Acr	breed_growth_background	ENSGALG00010014603	ENSGALG00010014603	0.415025512	0.117141153	3.542952261	0.000395674	0.004210939	positive
PRJNA729271	:ed_Lushi_vs_Arbor_Acr	breed_growth_background	ENSGALG00010014611	TMEM189	0.032431063	0.05594498	0.579695677	0.562119859	0.702771033	positive
PRJNA729271	:ed_Lushi_vs_Arbor_Acr	breed_growth_background	ENSGALG00010014614	GHR	0.341443371	0.048239699	7.078057637	1.46189E-12	7.84818E-10	positive
PRJNA729271	:ed_Lushi_vs_Arbor_Acr	breed_growth_background	ENSGALG00010014617	GRTP1	0.249729663	0.107595573	2.321003129	0.020286675	0.068946156	positive
PRJNA729271	:ed_Lushi_vs_Arbor_Acr	breed_growth_background	ENSGALG00010014624	GPRC6A	0.110361369	0.239362572	0.461063601	0.644752975	0.76754764	positive
PRJNA729271	:ed_Lushi_vs_Arbor_Acr	breed_growth_background	ENSGALG00010014626	C2CD5	0.131236704	0.049511699	2.650620093	0.008034416	0.035734537	positive
PRJNA729271	:ed_Lushi_vs_Arbor_Acr	breed_growth_background	ENSGALG00010014632	NWD2	0.062611178	0.094363908	0.663507683	0.507005461	0.656516362	positive
PRJNA729271	:ed_Lushi_vs_Arbor_Acr	breed_growth_background	ENSGALG00010014634	CCDC152	-0.280478177	0.230822905	-1.215122808	0.224319256	0.377115309	negative
PRJNA729271	:ed_Lushi_vs_Arbor_Acr	breed_growth_background	ENSGALG00010014641	DOCK11	-0.155984995	0.051798575	-3.011376208	0.002600664	0.015998854	negative
PRJNA729271	:ed_Lushi_vs_Arbor_Acr	breed_growth_background	ENSGALG00010014645	SLC36A1	0.236018477	0.06784764	3.478654181	0.000503938	0.004994066	positive
PRJNA729271	:ed_Lushi_vs_Arbor_Acr	breed_growth_background	ENSGALG00010014648	C1QTNF6	0.001991944	0.14709702	0.013541702	0.989195615	0.993582596	positive
PRJNA729271	:ed_Lushi_vs_Arbor_Acr	breed_growth_background	ENSGALG00010014653	FAM162B	0.479466054	0.264649565	1.81170165	0.070032309	0.167975133	positive
PRJNA729271	:ed_Lushi_vs_Arbor_Acr	breed_growth_background	ENSGALG00010014654	LAMP1	0.091820244	0.055853834	1.643938074	0.100188995	0.215369936	positive
PRJNA729271	:ed_Lushi_vs_Arbor_Acr	breed_growth_background	ENSGALG00010014655	FHL3	0.009626414	0.072074129	0.133562678	0.893748398	0.937876133	positive
PRJNA729271	:ed_Lushi_vs_Arbor_Acr	breed_growth_background	ENSGALG00010014658	SELENOP1	-0.105812894	0.092683768	-1.141655073	0.253597422	0.410210316	negative
PRJNA729271	:ed_Lushi_vs_Arbor_Acr	breed_growth_background	ENSGALG00010014668	GP5	0.139162942	0.086423442	1.610245307	0.107344314	0.226386706	positive
PRJNA729271	:ed_Lushi_vs_Arbor_Acr	breed_growth_background	ENSGALG00010014672	ISL1	-0.036543961	0.668509518	-0.054664833	0.956405487	0.97569799	negative
PRJNA729271	:ed_Lushi_vs_Arbor_Acr	breed_growth_background	ENSGALG00010014676	UBE2V1	-0.069241657	0.031878284	-2.172063486	0.029850875	0.090577439	negative
PRJNA729271	:ed_Lushi_vs_Arbor_Acr	breed_growth_background	ENSGALG00010014677	ENSGALG00010014677	-0.00857075	0.048296502	-0.177461085	0.859146229	0.915349107	negative
PRJNA729271	:ed_Lushi_vs_Arbor_Acr	breed_growth_background	ENSGALG00010014682	IL13RA1	-0.147021381	0.068137825	-2.15770579	0.030950714	0.093134854	negative
PRJNA729271	:ed_Lushi_vs_Arbor_Acr	breed_growth_background	ENSGALG00010014687	CUL4A	0.222684808	0.061341791	3.630229967	0.000283169	0.003342453	positive
PRJNA729271	:ed_Lushi_vs_Arbor_Acr	breed_growth_background	ENSGALG00010014690	CSF3R	-0.133023649	0.192595039	-0.690690943	0.489759782	0.641578675	negative
PRJNA729271	:ed_Lushi_vs_Arbor_Acr	breed_growth_background	ENSGALG00010014691	PABIR2	-0.013978101	0.029123942	-0.479952224	0.631261365	0.756897468	negative
PRJNA729271	:ed_Lushi_vs_Arbor_Acr	breed_growth_background	ENSGALG00010014696	SPINK5	-0.496995036	0.338891698	-1.466530573	0.142503798	0.275485804	negative
PRJNA729271	:ed_Lushi_vs_Arbor_Acr	breed_growth_background	ENSGALG00010014697	ENSGALG00010014697	-0.890290689	0.382196784	-2.329403922	0.019837677	0.06780173	negative
PRJNA729271	:ed_Lushi_vs_Arbor_Acr	breed_growth_background	ENSGALG00010014702	ENSGALG00010014702	0.362559049	0.146327608	2.477721422	0.013222434	0.050864165	positive
PRJNA729271	:ed_Lushi_vs_Arbor_Acr	breed_growth_background	ENSGALG00010014703	PCID2	-0.054853968	0.043542497	-1.259780008	0.207748734	0.35693878	negative
PRJNA729271	:ed_Lushi_vs_Arbor_Acr	breed_growth_background	ENSGALG00010014705	ARAP2	0.102745	0.118577593	0.866479053	0.386227509	0.548214624	positive
PRJNA729271	:ed_Lushi_vs_Arbor_Acr	breed_growth_background	ENSGALG00010014707	KPNA5	0.129036039	0.069239303	1.863624189	0.062374498	0.155015495	positive
PRJNA729271	:ed_Lushi_vs_Arbor_Acr	breed_growth_background	ENSGALG00010014710	NPEPL1	0.023725193	0.053392478	0.444354599	0.656786233	0.776391816	positive
PRJNA729271	:ed_Lushi_vs_Arbor_Acr	breed_growth_background	ENSGALG00010014713	ST8SIA1	-0.1768084	0.076527253	-2.310397844	0.020866138	0.070223979	negative
PRJNA729271	:ed_Lushi_vs_Arbor_Acr	breed_growth_background	ENSGALG00010014716	TMEM35A	-0.142873313	0.073390374	-1.946758199	0.051563731	0.135468188	negative
PRJNA729271	:ed_Lushi_vs_Arbor_Acr	breed_growth_background	ENSGALG00010014717	ELOVL7	0.017513939	0.096935565	0.180676089	0.856621828	0.91386933	positive
PRJNA729271	:ed_Lushi_vs_Arbor_Acr	breed_growth_background	ENSGALG00010014719	ENSGALG00010014719	1.289516764	0.441895644	2.918147716	0.003521175	0.019805755	positive
PRJNA729271	:ed_Lushi_vs_Arbor_Acr	breed_growth_background	ENSGALG00010014722	KCTD17	0.00081624	0.235879111	0.003460417	0.997238992	0.998754867	positive
PRJNA729271	:ed_Lushi_vs_Arbor_Acr	breed_growth_background	ENSGALG00010014726	PWP2	-0.059130762	0.035091296	-1.685054966	0.091977967	0.202777575	negative
PRJNA729271	:ed_Lushi_vs_Arbor_Acr	breed_growth_background	ENSGALG00010014728	INTS13	0.197374971	0.060008466	3.289118755	0.001005016	0.008165756	positive
PRJNA729271	:ed_Lushi_vs_Arbor_Acr	breed_growth_background	ENSGALG00010014729	MRPS15	-0.076037993	0.051222301	-1.48447048	0.137684149	0.268930298	negative
PRJNA729271	:ed_Lushi_vs_Arbor_Acr	breed_growth_background	ENSGALG00010014734	ZUP1	0.128895728	0.054549032	2.362933381	0.018130928	0.063813813	positive
PRJNA729271	:ed_Lushi_vs_Arbor_Acr	breed_growth_background	ENSGALG00010014735	STX16	0.200252879	0.05635397	3.553483065	0.000380166	0.004103128	positive
PRJNA729271	:ed_Lushi_vs_Arbor_Acr	breed_growth_background	ENSGALG00010014736	KIF2A	0.032989055	0.048750079	0.676697461	0.498597928	0.649997013	positive
PRJNA729271	:ed_Lushi_vs_Arbor_Acr	breed_growth_background	ENSGALG00010014737	RMI1	0.142299224	0.062625797	2.272214178	0.023073577	0.075599345	positive
PRJNA729271	:ed_Lushi_vs_Arbor_Acr	breed_growth_background	ENSGALG00010014738	BRWD1	0.093596606	0.046467956	2.014218281	0.043986621	0.120595059	positive
PRJNA729271	:ed_Lushi_vs_Arbor_Acr	breed_growth_background	ENSGALG00010014739	TRMT2B	-0.24290826	0.0952633	-2.549861907	0.01077656	0.044156902	negative
PRJNA729271	:ed_Lushi_vs_Arbor_Acr	breed_growth_background	ENSGALG00010014740	TRMT9B	0.13326033	0.279852178	0.476181143	0.633945333	0.758958209	positive
PRJNA729271	:ed_Lushi_vs_Arbor_Acr	breed_growth_background	ENSGALG00010014742	PROZ	-4.431132963	1.521225988	-2.912869619	0.003581241	0.019991535	negative
PRJNA729271	:ed_Lushi_vs_Arbor_Acr	breed_growth_background	ENSGALG00010014744	IL2RB	0.711488992	0.226325601	3.143652288	0.001668536	0.011678139	positive
PRJNA729271	:ed_Lushi_vs_Arbor_Acr	breed_growth_background	ENSGALG00010014745	LSM10	-0.090737375	0.098474092	-0.92143398	0.356823895	0.520535159	negative
PRJNA729271	:ed_Lushi_vs_Arbor_Acr	breed_growth_background	ENSGALG00010014748	FGFR1OP2	0.068775046	0.058655145	1.172532184	0.240983459	0.39675775	positive
PRJNA729271	:ed_Lushi_vs_Arbor_Acr	breed_growth_background	ENSGALG00010014750	LONRF1	-0.048176689	0.097734545	-0.492934087	0.622059161	0.748932569	negative
PRJNA729271	:ed_Lushi_vs_Arbor_Acr	breed_growth_background	ENSGALG00010014753	ENSGALG00010014753	0.827307543	0.272354588	3.037611916	0.002384608	0.015087254	positive
PRJNA729271	:ed_Lushi_vs_Arbor_Acr	breed_growth_background	ENSGALG00010014754	DIMT1	0.147810148	0.077414529	1.909333436	0.056219092	0.144229333	positive
PRJNA729271	:ed_Lushi_vs_Arbor_Acr	breed_growth_background	ENSGALG00010014758	FXN	-0.144669964	0.055781854	-2.593495092	0.00950059	0.040372631	negative
PRJNA729271	:ed_Lushi_vs_Arbor_Acr	breed_growth_background	ENSGALG00010014762	STK40	0.132937745	0.05659177	2.349064962	0.018820623	0.065505157	positive
PRJNA729271	:ed_Lushi_vs_Arbor_Acr	breed_growth_background	ENSGALG00010014764	ENSGALG00010014764	-3.909547015	1.18346937	-3.303462779	0.000954986	0.007887481	negative
PRJNA729271	:ed_Lushi_vs_Arbor_Acr	breed_growth_background	ENSGALG00010014766	F10	-0.112795127	0.117537471	-0.959652499	0.337230138	0.501451666	negative
PRJNA729271	:ed_Lushi_vs_Arbor_Acr	breed_growth_background	ENSGALG00010014767	LRRC15	-0.213709602	0.132153927	-1.61712638	0.105851004	0.224085044	negative
PRJNA729271	:ed_Lushi_vs_Arbor_Acr	breed_growth_background	ENSGALG00010014769	CCDC69	0.899754275	0.171776577	5.237933422	1.62385E-07	1.252E-05	positive
PRJNA729271	:ed_Lushi_vs_Arbor_Acr	breed_growth_background	ENSGALG00010014777	APCDD1L	0.366145546	0.124908151	2.931318282	0.003375268	0.019287888	positive
PRJNA729271	:ed_Lushi_vs_Arbor_Acr	breed_growth_background	ENSGALG00010014780	EVA1B	-0.211108385	0.067402352	-3.132062603	0.001735829	0.012021422	negative
PRJNA729271	:ed_Lushi_vs_Arbor_Acr	breed_growth_background	ENSGALG00010014783	ENSGALG00010014783	0.098439707	0.064193358	1.533487419	0.125155804	0.251299817	positive

PRJNA729271	ed_Lushi_vs_Arbor_Acr	breed_growth_background	ENSGALG00010014789	F7	-0.904067407	0.510347177	-1.771475279	0.0764817	0.178476796	negative
PRJNA729271	ed_Lushi_vs_Arbor_Acr	breed_growth_background	ENSGALG00010014792	ENSGALG00010014792	-0.240785238	0.145219606	-1.658076648	0.097301997	0.210726496	negative
PRJNA729271	ed_Lushi_vs_Arbor_Acr	breed_growth_background	ENSGALG00010014793	TLR2A	-0.141611997	0.094063096	-1.505500065	0.13219556	0.261059216	negative
PRJNA729271	ed_Lushi_vs_Arbor_Acr	breed_growth_background	ENSGALG00010014795	THRAP3	0.012032095	0.051141285	0.235271654	0.813997894	0.885537337	positive
PRJNA729271	ed_Lushi_vs_Arbor_Acr	breed_growth_background	ENSGALG00010014796	PRLR	-0.437367158	0.135320166	-3.23209149	0.001228877	0.009494975	negative
PRJNA729271	ed_Lushi_vs_Arbor_Acr	breed_growth_background	ENSGALG00010014797	ZDHHC9	-0.072379168	0.049469374	-1.463110664	0.1434371	0.2766109	negative
PRJNA729271	ed_Lushi_vs_Arbor_Acr	breed_growth_background	ENSGALG00010014801	MPST	-0.11647065	0.047346265	-2.459975475	0.013894651	0.052737095	negative
PRJNA729271	ed_Lushi_vs_Arbor_Acr	breed_growth_background	ENSGALG00010014802	ENSGALG00010014802	-0.478890617	0.234526997	-2.041942387	0.041157247	0.114696424	negative
PRJNA729271	ed_Lushi_vs_Arbor_Acr	breed_growth_background	ENSGALG00010014805	SPEF2	0.358394062	0.207344398	1.728496483	0.083899251	0.190345851	positive
PRJNA729271	ed_Lushi_vs_Arbor_Acr	breed_growth_background	ENSGALG00010014806	MAP7D1	0.301264231	0.120854217	2.492790394	0.012674364	0.049372456	positive
PRJNA729271	ed_Lushi_vs_Arbor_Acr	breed_growth_background	ENSGALG00010014807	HES1	0.03272418	0.120751319	0.271004741	0.786387386	0.867374086	positive
PRJNA729271	ed_Lushi_vs_Arbor_Acr	breed_growth_background	ENSGALG00010014810	ENTREP1	-0.445276032	0.113739152	-3.914887911	9.04462E-05	0.001484731	negative
PRJNA729271	ed_Lushi_vs_Arbor_Acr	breed_growth_background	ENSGALG00010014812	RWDD1	0.081729369	0.046166225	1.770328188	0.076672485	0.178733945	positive
PRJNA729271	ed_Lushi_vs_Arbor_Acr	breed_growth_background	ENSGALG00010014813	SASH3	-0.112604369	0.138413116	-0.813538287	0.415909504	0.576183528	negative
PRJNA729271	ed_Lushi_vs_Arbor_Acr	breed_growth_background	ENSGALG00010014816	TST	0.16459524	0.058229796	2.82664976	0.004703774	0.02432437	positive
PRJNA729271	ed_Lushi_vs_Arbor_Acr	breed_growth_background	ENSGALG00010014818	ENSGALG00010014818	-0.362987927	0.233533603	-1.554328468	0.120106086	0.244445061	negative
PRJNA729271	ed_Lushi_vs_Arbor_Acr	breed_growth_background	ENSGALG00010014821	C3AR1L	-0.078421154	0.122106116	-0.642237723	0.520718843	0.668185166	negative
PRJNA729271	ed_Lushi_vs_Arbor_Acr	breed_growth_background	ENSGALG00010014824	ANXA6	-0.206255592	0.048508672	-4.251932349	2.11934E-05	0.000500323	negative
PRJNA729271	ed_Lushi_vs_Arbor_Acr	breed_growth_background	ENSGALG00010014829	ENSGALG00010014829	0.155832017	0.203672188	0.765111911	0.444204908	0.601022136	positive
PRJNA729271	ed_Lushi_vs_Arbor_Acr	breed_growth_background	ENSGALG00010014830	TRAPPC3L	0.117892287	0.268275056	0.439445579	0.660338707	0.779383564	positive
PRJNA729271	ed_Lushi_vs_Arbor_Acr	breed_growth_background	ENSGALG00010014831	VAPB	0.056839984	0.03248515	1.74972208	0.080166282	0.184390772	positive
PRJNA729271	ed_Lushi_vs_Arbor_Acr	breed_growth_background	ENSGALG00010014837	APBA1	-0.321891066	0.061798066	-5.208756303	1.90111E-07	1.37783E-05	negative
PRJNA729271	ed_Lushi_vs_Arbor_Acr	breed_growth_background	ENSGALG00010014839	TM7SF3	0.054937127	0.059000911	0.93112336	0.351789756	0.515792799	positive
PRJNA729271	ed_Lushi_vs_Arbor_Acr	breed_growth_background	ENSGALG00010014840	NCF4	-0.052770436	0.115113714	-0.458420067	0.646650677	0.769114759	negative
PRJNA729271	ed_Lushi_vs_Arbor_Acr	breed_growth_background	ENSGALG00010014847	PAICS	-0.246849478	0.049758134	-4.960987484	7.01357E-07	3.65593E-05	negative
PRJNA729271	ed_Lushi_vs_Arbor_Acr	breed_growth_background	ENSGALG00010014851	CALHM4	0.673502179	0.249404439	2.700441829	0.006924745	0.032145293	positive
PRJNA729271	ed_Lushi_vs_Arbor_Acr	breed_growth_background	ENSGALG00010014856	MMGT1	-0.031036402	0.03842202	-0.807776426	0.419219298	0.578987226	negative
PRJNA729271	ed_Lushi_vs_Arbor_Acr	breed_growth_background	ENSGALG00010014859	TRAPPC3	0.22931185	0.068898122	3.328274317	0.000873858	0.00740302	positive
PRJNA729271	ed_Lushi_vs_Arbor_Acr	breed_growth_background	ENSGALG00010014861	MED21	0.162721314	0.0536772	3.031479163	0.002433587	0.015310263	positive
PRJNA729271	ed_Lushi_vs_Arbor_Acr	breed_growth_background	ENSGALG00010014862	ENSGALG00010014862	-0.518647847	0.179631887	-2.887281631	0.003885862	0.021238905	negative
PRJNA729271	ed_Lushi_vs_Arbor_Acr	breed_growth_background	ENSGALG00010014865	TLR2	0.311489188	0.199565804	1.560834483	0.118562809	0.242461613	positive
PRJNA729271	ed_Lushi_vs_Arbor_Acr	breed_growth_background	ENSGALG00010014866	FAM26E	-0.202844076	0.080078782	-2.533056461	0.011307274	0.045692482	negative
PRJNA729271	ed_Lushi_vs_Arbor_Acr	breed_growth_background	ENSGALG00010014867	TP63	0.681773772	0.146869734	4.6420304	3.45002E-06	0.000126924	positive
PRJNA729271	ed_Lushi_vs_Arbor_Acr	breed_growth_background	ENSGALG00010014870	ENSGALG00010014870	-0.860538313	1.017475794	-0.84575802	0.397687749	0.558953158	negative
PRJNA729271	ed_Lushi_vs_Arbor_Acr	breed_growth_background	ENSGALG00010014873	CALHM6	0.238109729	0.391655359	0.607957287	0.543215803	0.687677997	positive
PRJNA729271	ed_Lushi_vs_Arbor_Acr	breed_growth_background	ENSGALG00010014876	PCNT	0.086793068	0.055469032	1.564712156	0.117650417	0.241174204	positive
PRJNA729271	ed_Lushi_vs_Arbor_Acr	breed_growth_background	ENSGALG00010014877	ENSGALG00010014877	-0.148249284	0.048999576	-3.025521804	0.002482045	0.015507433	negative
PRJNA729271	ed_Lushi_vs_Arbor_Acr	breed_growth_background	ENSGALG00010014878	TMCO1	-0.010102643	0.069721595	-0.144899776	0.884790003	0.932051679	negative
PRJNA729271	ed_Lushi_vs_Arbor_Acr	breed_growth_background	ENSGALG00010014880	SFRP2	-0.489289862	0.156993984	-3.116615364	0.001829401	0.012490422	negative
PRJNA729271	ed_Lushi_vs_Arbor_Acr	breed_growth_background	ENSGALG00010014881	ENSGALG00010014881	0.556340316	0.227972612	2.440382249	0.014671728	0.054613945	positive
PRJNA729271	ed_Lushi_vs_Arbor_Acr	breed_growth_background	ENSGALG00010014882	XPNPEP2	-0.882792605	0.339485747	-2.600381941	0.009312005	0.039769452	negative
PRJNA729271	ed_Lushi_vs_Arbor_Acr	breed_growth_background	ENSGALG00010014883	PPAT	-0.177271698	0.066379031	-2.670597838	0.00757163	0.034243613	negative
PRJNA729271	ed_Lushi_vs_Arbor_Acr	breed_growth_background	ENSGALG00010014886	RABGAP1L	-0.112377949	0.037717991	-2.979425612	0.002887893	0.017240533	negative
PRJNA729271	ed_Lushi_vs_Arbor_Acr	breed_growth_background	ENSGALG00010014887	MTMR12	-0.202545629	0.070937661	-2.855262291	0.00430013	0.022773249	negative
PRJNA729271	ed_Lushi_vs_Arbor_Acr	breed_growth_background	ENSGALG00010014889	PVALB	0.404994589	0.23099923	1.753229175	0.079562677	0.183523393	positive
PRJNA729271	ed_Lushi_vs_Arbor_Acr	breed_growth_background	ENSGALG00010014893	SLC9A6	0.021002451	0.048713978	0.431138077	0.666367976	0.783819185	positive
PRJNA729271	ed_Lushi_vs_Arbor_Acr	breed_growth_background	ENSGALG00010014894	RAB22A	-0.147743518	0.043729389	-3.378586351	0.000728595	0.006485902	negative
PRJNA729271	ed_Lushi_vs_Arbor_Acr	breed_growth_background	ENSGALG00010014897	COL8A2	-0.04597334	0.136244617	-0.33743234	0.735791009	0.830630114	negative
PRJNA729271	ed_Lushi_vs_Arbor_Acr	breed_growth_background	ENSGALG00010014898	ENSGALG00010014898	-0.182950807	0.301799553	-0.606199727	0.544382134	0.688553144	negative
PRJNA729271	ed_Lushi_vs_Arbor_Acr	breed_growth_background	ENSGALG00010014900	UBLCP1	0.095671413	0.045886284	2.084967556	0.037072246	0.106450515	positive
PRJNA729271	ed_Lushi_vs_Arbor_Acr	breed_growth_background	ENSGALG00010014902	AASDH	0.140028174	0.068279972	2.050794246	0.040286988	0.112907945	positive
PRJNA729271	ed_Lushi_vs_Arbor_Acr	breed_growth_background	ENSGALG00010014903	MCF2L	-0.180533647	0.082370864	-2.191717294	0.028399926	0.087363525	negative
PRJNA729271	ed_Lushi_vs_Arbor_Acr	breed_growth_background	ENSGALG00010014906	IFT27	-0.061239242	0.059316213	-1.03241995	0.30187543	0.464362131	negative
PRJNA729271	ed_Lushi_vs_Arbor_Acr	breed_growth_background	ENSGALG00010014907	ENSGALG00010014907	-0.025432211	0.038412025	-0.662089834	0.507913651	0.656958809	negative
PRJNA729271	ed_Lushi_vs_Arbor_Acr	breed_growth_background	ENSGALG00010014908	ENSGALG00010014908	-0.028616959	0.05486753	-0.521564558	0.601973545	0.734478665	negative
PRJNA729271	ed_Lushi_vs_Arbor_Acr	breed_growth_background	ENSGALG00010014909	FBXL14	0.106674305	0.05327024	2.002512183	0.045229674	0.123244613	positive
PRJNA729271	ed_Lushi_vs_Arbor_Acr	breed_growth_background	ENSGALG00010014912	ENSGALG00010014912	0.155420456	0.166269646	0.934749422	0.349917451	0.513885861	positive
PRJNA729271	ed_Lushi_vs_Arbor_Acr	breed_growth_background	ENSGALG00010014914	PTAR1	0.105019518	0.097252096	1.079868939	0.280200547	0.439959615	positive
PRJNA729271	ed_Lushi_vs_Arbor_Acr	breed_growth_background	ENSGALG00010014916	DYNLT2B	-0.122490847	0.086254281	-1.420113237	0.155574717	0.292641867	negative
PRJNA729271	ed_Lushi_vs_Arbor_Acr	breed_growth_background	ENSGALG00010014917	EIF3D	0.028086309	0.056162182	0.50009292	0.617009651	0.745296241	positive
PRJNA729271	ed_Lushi_vs_Arbor_Acr	breed_growth_background	ENSGALG00010014918	PLRG1	0.07817502	0.045414572	1.721364216	0.085184754	0.192539062	positive
PRJNA729271	ed_Lushi_vs_Arbor_Acr	breed_growth_background	ENSGALG00010014922	MAN1A1	-0.08254266	0.088551272	-0.932145392	0.351261392	0.515181006	negative
PRJNA729271	ed_Lushi_vs_Arbor_Acr	breed_growth_background	ENSGALG00010014923	CIMIP1	-0.092526492	0.237267563	-0.389966885	0.696561034	0.804450019	negative
PRJNA729271	ed_Lushi_vs_Arbor_Acr	breed_growth_background	ENSGALG00010014928	SUCO	-0.000691793	0.043762108	-0.015808032	0.987387541	0.992713543	negative
PRJNA729271	ed_Lushi_vs_Arbor_Acr	breed_growth_background	ENSGALG00010014929	FHL1	-0.315703104	0.152240131	-2.073718027	0.038105506	0.108728211	negative
PRJNA729271	ed_Lushi_vs_Arbor_Acr	breed_growth_background	ENSGALG00010014931	RNF145	0.177865069	0.058507585	3.040034357	0.002365512	0.015032043	positive
PRJNA729271	ed_Lushi_vs_Arbor_Acr	breed_growth_background	ENSGALG00010014934	MAMDC2	-0.290940459	0.109383403	-2.659822694	0.00781818	0.035067891	negative
PRJNA729271	ed_Lushi_vs_Arbor_Acr	breed_growth_background	ENSGALG00010014935	PCYT1A	-0.073463023	0.070610027	-1.04040497	0.298151794	0.460344083	negative
PRJNA729271	ed_Lushi_vs_Arbor_Acr	breed_growth_background	ENSGALG00010014936	FOXRED2	-0.080695455	0.092687287	-0.870620538	0.383961378	0.545585745	negative
PRJNA729271	ed_Lushi_vs_Arbor_Acr	breed_growth_background	ENSGALG00010014940	VAMP4	-0.047558997	0.046663888	-1.019182037	0.308116553	0.470864463	negative
PRJNA729271	ed_Lushi_vs_Arbor_Acr	breed_growth_background	ENSGALG00010014942	PMEPA1	-0.250401834	0.079421677	-3.152814724	0.001617044	0.011439265	negative
PRJNA729271	ed_Lushi_vs_Arbor_Acr	breed_growth_background	ENSGALG00010014943	APLN	-0.221371317	0.163040898	-1.357765564	0.174538085	0.318390328	negative
PRJNA729271	ed_Lushi_vs_Arbor_Acr	breed_growth_background	ENSGALG00010014944	FGB	-1.201660079	0.491853687	-2.443125079			negative
PRJNA729271	ed_Lushi_vs_Arbor_Acr	breed_growth_background	ENSGALG00010014945	TXN2	0.076888223	0.055952143	1.374178342	0.169386323	0.311461975	positive
PRJNA729271	ed_Lushi_vs_Arbor_Acr	breed_growth_background	ENSGALG00010014946	PCK1	-1.633200673	0.537303058	-3.039626611	0.002368716	0.015040642	negative
PRJNA729271	ed_Lushi_vs_Arbor_Acr	breed_growth_background	ENSGALG00010014948	RBM38	0.271209032	0.138262629	1.961549795	0.049814922	0.132149945	positive
PRJNA729271	ed_Lushi_vs_Arbor_Acr	breed_growth_background	ENSGALG00010014949	AGO3	0.037396833	0.059009067	0.633747236	0.526245792	0.672565849	positive
PRJNA729271	ed_Lushi_vs_Arbor_Acr	breed_growth_background	ENSGALG00010014952	C21orf58	0.020245396	0.33637613	0.060186781	0.952006874	0.973433394	positive
PRJNA729271	ed_Lushi_vs_Arbor_Acr	breed_growth_background	ENSGALG00010014953	ENSGALG00010014953	0.120834339	0.190877197	0.633047538	0.5267026	0.672885086	positive
PRJNA729271	ed_Lushi_vs_Arbor_Acr	breed_growth_background	ENSGALG00010014956	FGG	-1.500892707	0.820925957	-1.828292423	0.067505676	0.163990154	negative
PRJNA729271	ed_Lushi_vs_Arbor_Acr	breed_growth_background	ENSGALG00010014965	ABHD13	0.037316911	0.053363061	0.699302299	0.48436313	0.636549489	positive
PRJNA729271	ed_Lushi_vs_Arbor_Acr	breed_growth_background	ENSGALG00010014967	HNRNPH1	0.101734027	0.057648682	1.764724251	0.077610121	0.179964598	positive
PRJNA729271	ed_Lushi_vs_Arbor_Acr	breed_growth_background	ENSGALG00010014970	SLC51A	-0.161272261	0.281632974	-0.572632738	0.566893388	0.706085057	negative
PRJNA729271	ed_Lushi_vs_Arbor_Acr	breed_growth_background	ENSGALG00010014971	SMC5	0.111064335	0.067429762	1.647111484	0.099535133	0.214154929	positive
PRJNA729271	ed_Lushi_vs_Arbor_Acr	breed_growth_background	ENSGALG00010014972	FGA	-2.391477899	0.902222647	-2.650651596	0.008033667	0.035734537	negative
PRJNA729271	ed_Lushi_vs_Arbor_Acr	breed_growth_background	ENSGALG00010014974	LIG4	0.132385228	0.060761738	2.178759736	0.029349522	0.089468205	positive
PRJNA729271	ed_Lushi_vs_Arbor_Acr	breed_growth_background	ENSGALG00010014976	ITGB2	-0.00904009	0.112978411	-0.080016086	0.936224462	0.964709858	negative
PRJNA729271	ed_Lushi_vs_Arbor_Acr	breed_growth_background	ENSGALG00010014980	ENSGALG00010014980	2.687483325	0.388832394	6.911675486	4.78963E-12	2.23954E-09	positive
PRJNA729271	ed_Lushi_vs_Arbor_Acr	breed_growth_background	ENSGALG00010014982	ENSGALG00010014982	-0.242637775	0.18891866	-1.2843505	0.199019344	0.347719448	negative
PRJNA729271	ed_Lushi_vs_Arbor_Acr	breed_growth_background	ENSGALG00010014984	LPP	-0.132214588	0.049305656	-2.681529835	0.007328638	0.033463824	negative
PRJNA729271	ed_Lushi_vs_Arbor_Acr	breed_growth_background	ENSGALG00010014985	ENSGALG00010014985	-0.021124735	0.058028821	-0.36403868	0.715829125	0.817711654	negative
PRJNA729271	ed_Lushi_vs_Arbor_Acr	breed_growth_background	ENSGALG00010014987	KLF9	0.795112375	0.142410535	5.583241243	2.36077E-08	2.78703E-06	positive
PRJNA729271	ed_Lushi_vs_Arbor_Acr	breed_growth_background	ENSGALG00010014990	AGO1	-0.003571319	0.026407692	-0.13523783	0.892423838	0.937189325	negative
PRJNA729271	ed_Lushi_vs_Arbor_Acr	breed_growth_background	ENSGALG00010014992	ARL15	-0.047202826	0.052877413	-0.892684105	0.372026365	0.53465419	negative
PRJNA729271	ed_Lushi_vs_Arbor_Acr	breed_growth_background	ENSGALG00010014994	ENSGALG00010014994	-0.110383905	0.063933132	-1.726552454	0.084248071	0.190936468	negative
PRJNA729271	ed_Lushi_vs_Arbor_Acr	breed_growth_background	ENSGALG00010014995	LRAT	-0.003449234	0.141328287	-0.024405828	0.9805289	0.988576456	negative
PRJNA729271	ed_Lushi_vs_Arbor_Acr	breed_growth_background	ENSGALG00010014997	RAE1	0.076682103	0.048440258	1.583024246	0.113415938	0.235018202	positive
PRJNA729271	ed_Lushi_vs_Arbor_Acr	breed_growth_background	ENSGALG00010014998	NALF1	-0.678428367	0.273681141	-2.478900685	0.0131788	0.050737503	negative
PRJNA729271	ed_Lushi_vs_Arbor_Acr	breed_growth_background	ENSGALG00010015002	ADAM19	-0.300479376	0.107655598	-2.791117051	0.005252647	0.026427325	negative
PRJNA729271	ed_Lushi_vs_Arbor_Acr	breed_growth_background	ENSGALG00010015006	HSPB3	-0.582404384	0.173104962	-3.364458056	0.000766941	0.006743395	negative
PRJNA729271	ed_Lushi_vs_Arbor_Acr	breed_growth_background	ENSGALG00010015009	AGO4	0.123226878	0.055948504	2.202505305	0.027629635	0.085684971	positive
PRJNA729271	ed_Lushi_vs_Arbor_Acr	breed_growth_background	ENSGALG00010015013	SNX18	0.009233749	0.06052761	0.152554326	0.878749746	0.928012768	positive
PRJNA729271	ed_Lushi_vs_Arbor_Acr	breed_growth_background	ENSGALG00010015016	ARGLU1	0.0712801	0.042866664	1.662832942	0.096345881	0.209312581	positive
PRJNA729271	ed_Lushi_vs_Arbor_Acr	breed_growth_background	ENSGALG00010015017	FAM184A	0.103139662	0.103140853	0.999988456	0.317316094	0.480634474	positive
PRJNA729271	ed_Lushi_vs_Arbor_Acr	breed_growth_background	ENSGALG00010015018	MAP7D3	0.059560377	0.037172116	1.602286438	0.109092283	0.229196442	positive
PRJNA729271	ed_Lushi_vs_Arbor_Acr	breed_growth_background	ENSGALG00010015020	NIPAL4	-0.632956173	0.441503403	-1.433638264	0.151675518	0.287653622	negative
PRJNA729271	ed_Lushi_vs_Arbor_Acr	breed_growth_background	ENSGALG00010015021	RBM46	-0.602061178	0.52507243	-1.146625006	0.251536637	0.407695802	negative
PRJNA729271	ed_Lushi_vs_Arbor_Acr	breed_growth_background	ENSGALG00010015024	CRACD	0.243467905	0.211241383	1.152557807	0.24909193	0.405183203	positive
PRJNA729271	ed_Lushi_vs_Arbor_Acr	breed_growth_background	ENSGALG00010015025	TRAF3IP1	0.193654179	0.104649572	1.850501402	0.064241316	0.158202154	positive
PRJNA729271	ed_Lushi_vs_Arbor_Acr	breed_growth_background	ENSGALG00010015027	CLSPN	0.017785808	0.100196246	0.177509719	0.859108032	0.915349107	positive
PRJNA729271	ed_Lushi_vs_Arbor_Acr	breed_growth_background	ENSGALG00010015028	MCM9	-0.810273077	0.147600693	-5.489629213	4.02778E-08	4.25473E-06	negative
PRJNA729271	ed_Lushi_vs_Arbor_Acr	breed_growth_background	ENSGALG00010015029	TFRC	0.007883915	0.097800599	0.080612134	0.935750416	0.964536447	positive
PRJNA729271	ed_Lushi_vs_Arbor_Acr	breed_growth_background	ENSGALG00010015031	MYH9	-0.251132885	0.058588964	-4.286351362	1.81632E-05	0.000444721	negative
PRJNA729271	ed_Lushi_vs_Arbor_Acr	breed_growth_background	ENSGALG00010015032	C1orf216	-0.145034673	0.142387074	-1.018594375	0.308395576	0.471241184	negative
PRJNA729271	ed_Lushi_vs_Arbor_Acr	breed_growth_background	ENSGALG00010015033	BMP7	0.469406937	0.174730443	2.686463387	0.007221286	0.033145201	positive
PRJNA729271	ed_Lushi_vs_Arbor_Acr	breed_growth_background	ENSGALG00010015035	SLC35F1	0.243186812	0.06908482	3.520119335	0.000431353	0.004466547	positive
PRJNA729271	ed_Lushi_vs_Arbor_Acr	breed_growth_background	ENSGALG00010015037	CYFIP2	0.530142187	0.104074915	5.093851729	3.50861E-07	2.14588E-05	positive
PRJNA729271	ed_Lushi_vs_Arbor_Acr	breed_growth_background	ENSGALG00010015038	PCSK5	-0.014571868	0.110776889	-0.131542496	0.895346172	0.938941018	negative
PRJNA729271	ed_Lushi_vs_Arbor_Acr	breed_growth_background	ENSGALG00010015039	FAM114A1	-0.105691459	0.06836074	-1.546084179	0.122084239	0.246738852	negative
PRJNA729271	ed_Lushi_vs_Arbor_Acr	breed_growth_background	ENSGALG00010015040	ZC4H2	0.04170461	0.048478762	0.860265569	0.389642668	0.551388311	positive
PRJNA729271	ed_Lushi_vs_Arbor_Acr	breed_growth_background	ENSGALG00010015041	TFAP2C	0.030739766	0.326877022	0.094040768	0.925076772	0.95792169	positive
PRJNA729271	ed_Lushi_vs_Arbor_Acr	breed_growth_background	ENSGALG00010015042	EFNB2	-0.244246244	0.071650646	-3.408849153	0.000652375	0.006003925	negative
PRJNA729271	ed_Lushi_vs_Arbor_Acr	breed_growth_background	ENSGALG00010015043	COQ10B	0.084635658	0.118601294	0.713614959	0.475465305	0.628820219	positive
PRJNA729271	ed_Lushi_vs_Arbor_Acr	breed_growth_background	ENSGALG00010015044	ZC3H12B	0.023145048	0.071070607	0.325662727	0.744679547	0.837143636	positive
PRJNA729271	ed_Lushi_vs_Arbor_Acr	breed_growth_background	ENSGALG00010015046	PSMB2	0.117045506	0.050427275	2.321075359	0.020282777	0.068946156	positive
PRJNA729271	ed_Lushi_vs_Arbor_Acr	breed_growth_background	ENSGALG00010015047	NPY2R	-0.43184069	0.117628155	-3.671235779	0.000241381	0.002965094	negative
PRJNA729271	ed_Lushi_vs_Arbor_Acr	breed_growth_background	ENSGALG00010015049	DCK2	-0.435124292	0.134535111	-3.234280538	0.001219496	0.009456299	negative
PRJNA729271	ed_Lushi_vs_Arbor_Acr	breed_growth_background	ENSGALG00010015050	HIVEP3	-0.093305063	0.096641037	-0.965480772	0.334304062	0.498635252	negative
PRJNA729271	ed_Lushi_vs_Arbor_Acr	breed_growth_background	ENSGALG00010015052	ENSGALG00010015052	0.073164926	0.05873293	1.245722398	0.212866328	0.363192898	positive
PRJNA729271	ed_Lushi_vs_Arbor_Acr	breed_growth_background	ENSGALG00010015054	ENSGALG00010015054	-0.050775213	0.095199633	-0.533355133	0.593787769	0.728107074	negative
PRJNA729271	ed_Lushi_vs_Arbor_Acr	breed_growth_background	ENSGALG00010015055	ZNF335	0.313295863	0.09747442	3.214134156	0.001308385	0.009882775	positive

PRJNA729271	ed_Lushi_vs_Arbor_Acr	breed_growth_background	ENSGALG00010015056	ENSGALG00010015056	0.103934276	0.27321616	0.380410429	0.703640774	0.808503609	positive
PRJNA729271	ed_Lushi_vs_Arbor_Acr	breed_growth_background	ENSGALG00010015057	LAS1L	-0.044136007	0.06091076	-0.724601147	0.468696755	0.623530468	negative
PRJNA729271	ed_Lushi_vs_Arbor_Acr	breed_growth_background	ENSGALG00010015059	ENSGALG00010015059	0.262926866	0.088919475	2.956909782	0.00310739	0.018205991	positive
PRJNA729271	ed_Lushi_vs_Arbor_Acr	breed_growth_background	ENSGALG00010015061	MMP9	-0.145892772	0.259444079	-0.562328394	0.573892296	0.711596991	negative
PRJNA729271	ed_Lushi_vs_Arbor_Acr	breed_growth_background	ENSGALG00010015062	ENSGALG00010015062	-0.003785564	0.12872436	-0.029408296	0.976538956	0.986062847	negative
PRJNA729271	ed_Lushi_vs_Arbor_Acr	breed_growth_background	ENSGALG00010015063	EEF1AKMT4	-0.03921113	0.129561008	-0.302646068	0.762159602	0.850462158	negative
PRJNA729271	ed_Lushi_vs_Arbor_Acr	breed_growth_background	ENSGALG00010015064	RFK	0.160729767	0.080627748	1.993479557	0.04620896	0.125149266	positive
PRJNA729271	ed_Lushi_vs_Arbor_Acr	breed_growth_background	ENSGALG00010015065	F11	-0.853851304	0.551306206	-1.548778689	0.12143493	0.246147296	negative
PRJNA729271	ed_Lushi_vs_Arbor_Acr	breed_growth_background	ENSGALG00010015066	RUFY1	0.046076541	0.03866092	1.191811805	0.233335046	0.387776968	positive
PRJNA729271	ed_Lushi_vs_Arbor_Acr	breed_growth_background	ENSGALG00010015068	ESM1	-0.156250961	0.177960424	-0.8780096	0.379938509	0.541834779	negative
PRJNA729271	ed_Lushi_vs_Arbor_Acr	breed_growth_background	ENSGALG00010015069	HSPD1	0.076266902	0.055059261	1.385178453	0.165997908	0.306475566	positive
PRJNA729271	ed_Lushi_vs_Arbor_Acr	breed_growth_background	ENSGALG00010015070	EDN2	-0.578268431	0.239437897	-2.415108212	0.015730536	0.057652223	negative
PRJNA729271	ed_Lushi_vs_Arbor_Acr	breed_growth_background	ENSGALG00010015071	SNAI1	-0.174117468	0.070680592	-2.463441015	0.013761054	0.052397326	negative
PRJNA729271	ed_Lushi_vs_Arbor_Acr	breed_growth_background	ENSGALG00010015072	MAP9	-0.036572473	0.076237576	-0.479717159	0.631428525	0.757035274	negative
PRJNA729271	ed_Lushi_vs_Arbor_Acr	breed_growth_background	ENSGALG00010015075	ENSGALG00010015075	0.138379755	0.08916213	1.552001448	0.120661877	0.24519752	positive
PRJNA729271	ed_Lushi_vs_Arbor_Acr	breed_growth_background	ENSGALG00010015076	ITK	0.393880819	0.356534306	1.104748722	0.269268485	0.427872933	positive
PRJNA729271	ed_Lushi_vs_Arbor_Acr	breed_growth_background	ENSGALG00010015080	ASF1A	-0.004598529	0.043322804	-0.106145693	0.915466758	0.952192002	negative
PRJNA729271	ed_Lushi_vs_Arbor_Acr	breed_growth_background	ENSGALG00010015083	CEP135	-0.161973942	0.106351694	-1.523002931	0.127757972	0.254804432	negative
PRJNA729271	ed_Lushi_vs_Arbor_Acr	breed_growth_background	ENSGALG00010015084	FOXO6	-0.062142454	0.108325911	-0.573661956	0.566196577	0.705716947	negative
PRJNA729271	ed_Lushi_vs_Arbor_Acr	breed_growth_background	ENSGALG00010015085	PSMD2	0.134699544	0.087596812	1.537721987	0.124116605	0.24973212	positive
PRJNA729271	ed_Lushi_vs_Arbor_Acr	breed_growth_background	ENSGALG00010015086	FOXN3	0.047995139	0.051613137	0.929901601	0.352422034	0.516360799	positive
PRJNA729271	ed_Lushi_vs_Arbor_Acr	breed_growth_background	ENSGALG00010015087	GABRA3	0.145924372	0.323490636	0.451093032	0.6519225	0.773020986	positive
PRJNA729271	ed_Lushi_vs_Arbor_Acr	breed_growth_background	ENSGALG00010015088	ENSGALG00010015088	2.409251043	0.578848221	4.162146406			positive
PRJNA729271	ed_Lushi_vs_Arbor_Acr	breed_growth_background	ENSGALG00010015090	GZMA	0.60185233	0.305227338	1.971816594	0.048630547	0.130007337	positive
PRJNA729271	ed_Lushi_vs_Arbor_Acr	breed_growth_background	ENSGALG00010015091	MSN	-0.321326842	0.064902596	-4.950908918	7.38677E-07	3.81036E-05	negative
PRJNA729271	ed_Lushi_vs_Arbor_Acr	breed_growth_background	ENSGALG00010015092	HSPE1	-0.089427374	0.065730738	-1.360510721	0.173668363	0.317322944	negative
PRJNA729271	ed_Lushi_vs_Arbor_Acr	breed_growth_background	ENSGALG00010015094	CDK5RAP1	-0.036336919	0.114039015	-0.318635858	0.750002659	0.841105496	negative
PRJNA729271	ed_Lushi_vs_Arbor_Acr	breed_growth_background	ENSGALG00010015095	RBFOX2	0.160441123	0.071205938	2.253198636	0.024246627	0.078256288	positive
PRJNA729271	ed_Lushi_vs_Arbor_Acr	breed_growth_background	ENSGALG00010015096	GABRE	-0.355941694	0.664974224	-0.535271415	0.59246219	0.727035171	negative
PRJNA729271	ed_Lushi_vs_Arbor_Acr	breed_growth_background	ENSGALG00010015097	MFSD2A	-0.16143435	0.089309375	-1.807585724	0.070671002	0.168927469	negative
PRJNA729271	ed_Lushi_vs_Arbor_Acr	breed_growth_background	ENSGALG00010015098	HAVCR1	-0.418827565	0.115324073	-3.631744469	0.000281512	0.003329541	negative
PRJNA729271	ed_Lushi_vs_Arbor_Acr	breed_growth_background	ENSGALG00010015100	MOB4	0.029837281	0.047630297	0.626434904	0.531029721	0.676802586	positive
PRJNA729271	ed_Lushi_vs_Arbor_Acr	breed_growth_background	ENSGALG00010015101	CAP1	-0.170826733	0.053551788	-3.189935159	0.001423047	0.0105312	negative
PRJNA729271	ed_Lushi_vs_Arbor_Acr	breed_growth_background	ENSGALG00010015103	ITPK1	-0.115567375	0.050488176	-2.288998801	0.022079422	0.073303074	negative
PRJNA729271	ed_Lushi_vs_Arbor_Acr	breed_growth_background	ENSGALG00010015104	CNGA2	0.438881254	0.151571766	2.895534335	0.003785136	0.020837656	positive
PRJNA729271	ed_Lushi_vs_Arbor_Acr	breed_growth_background	ENSGALG00010015105	ENSGALG00010015105	0.756588265	0.319059339	2.371308948	0.017725208	0.062772267	positive
PRJNA729271	ed_Lushi_vs_Arbor_Acr	breed_growth_background	ENSGALG00010015107	MB	0.342373418	0.308743279	1.108925899	0.26746215	0.42579186	positive
PRJNA729271	ed_Lushi_vs_Arbor_Acr	breed_growth_background	ENSGALG00010015108	ENSGALG00010015108	0.098020616	0.048466716	2.022431551	0.043131788	0.118945791	positive
PRJNA729271	ed_Lushi_vs_Arbor_Acr	breed_growth_background	ENSGALG00010015109	PPT1	-0.1566418	0.058076903	-2.697144507	0.006993691	0.032367036	negative
PRJNA729271	ed_Lushi_vs_Arbor_Acr	breed_growth_background	ENSGALG00010015110	ENSGALG00010015110	0.694488449	0.319834357	2.171400393	0.029900919	0.090672348	positive
PRJNA729271	ed_Lushi_vs_Arbor_Acr	breed_growth_background	ENSGALG00010015111	ADAMTS2	-0.031808982	0.075780323	-0.419752525	0.67466625	0.789671131	negative
PRJNA729271	ed_Lushi_vs_Arbor_Acr	breed_growth_background	ENSGALG00010015112	CBLN4	0.459235209	0.226479832	2.027709063	0.04258995	0.117903233	positive
PRJNA729271	ed_Lushi_vs_Arbor_Acr	breed_growth_background	ENSGALG00010015115	CLDN2	0.433061382	0.231580273	1.870027082	0.061480057	0.153541252	positive
PRJNA729271	ed_Lushi_vs_Arbor_Acr	breed_growth_background	ENSGALG00010015117	RASD2	-0.418934907	0.11123017	-3.766378366	0.000165633	0.002293072	negative
PRJNA729271	ed_Lushi_vs_Arbor_Acr	breed_growth_background	ENSGALG00010015118	GPX8	-0.365377531	0.100200313	-3.646470965	0.000265867	0.003192821	negative
PRJNA729271	ed_Lushi_vs_Arbor_Acr	breed_growth_background	ENSGALG00010015119	TLR1A	-0.271092609	0.119097496	-2.276224263	0.02283259	0.075081306	negative
PRJNA729271	ed_Lushi_vs_Arbor_Acr	breed_growth_background	ENSGALG00010015121	UNC79	0.167316783	0.169929948	0.984622106	0.324809727	0.488864643	positive
PRJNA729271	ed_Lushi_vs_Arbor_Acr	breed_growth_background	ENSGALG00010015122	ENSGALG00010015122	0.296787815	0.115473218	2.570187446	0.01016435	0.042280991	positive
PRJNA729271	ed_Lushi_vs_Arbor_Acr	breed_growth_background	ENSGALG00010015123	CEP85L	0.478609003	0.134070768	3.569823682	0.000357222	0.003907869	positive
PRJNA729271	ed_Lushi_vs_Arbor_Acr	breed_growth_background	ENSGALG00010015124	UXS1	0.017288096	0.051674042	0.334560556	0.737956603	0.832102759	positive
PRJNA729271	ed_Lushi_vs_Arbor_Acr	breed_growth_background	ENSGALG00010015126	MCM5	-0.334284318	0.098134963	-3.406373302	0.000658321	0.006032421	negative
PRJNA729271	ed_Lushi_vs_Arbor_Acr	breed_growth_background	ENSGALG00010015127	RPL11	-0.051805292	0.081024627	-0.639377112	0.522577635	0.669681091	negative
PRJNA729271	ed_Lushi_vs_Arbor_Acr	breed_growth_background	ENSGALG00010015128	OCRL	0.226802288	0.057346306	3.954958973	7.65478E-05	0.001308444	positive
PRJNA729271	ed_Lushi_vs_Arbor_Acr	breed_growth_background	ENSGALG00010015129	PRRG3	0.042655426	0.075333871	0.566218434	0.5712453	0.709589564	positive
PRJNA729271	ed_Lushi_vs_Arbor_Acr	breed_growth_background	ENSGALG00010015131	DOK5	0.0428808	0.12906219	0.332249126	0.739701151	0.833292002	positive
PRJNA729271	ed_Lushi_vs_Arbor_Acr	breed_growth_background	ENSGALG00010015132	GCNT1	0.964156898	0.158672182	6.076407895	1.22905E-09	2.545E-07	positive
PRJNA729271	ed_Lushi_vs_Arbor_Acr	breed_growth_background	ENSGALG00010015133	RFTN2	-0.214476753	0.068691421	-3.122322248	0.001794304	0.012314602	negative
PRJNA729271	ed_Lushi_vs_Arbor_Acr	breed_growth_background	ENSGALG00010015134	PLN	-0.004568531	0.276250762	-0.016537624	0.986805487	0.992213203	negative
PRJNA729271	ed_Lushi_vs_Arbor_Acr	breed_growth_background	ENSGALG00010015136	TCEB3	0.126004026	0.048612546	2.592006341	0.009541801	0.04048841	positive
PRJNA729271	ed_Lushi_vs_Arbor_Acr	breed_growth_background	ENSGALG00010015137	AMN	0.206345737	0.216272711	0.954099739	0.340033158	0.50398788	positive
PRJNA729271	ed_Lushi_vs_Arbor_Acr	breed_growth_background	ENSGALG00010015138	ENSGALG00010015138	-0.248462193	0.255225686	-0.973499953	0.330304883	0.494502095	negative
PRJNA729271	ed_Lushi_vs_Arbor_Acr	breed_growth_background	ENSGALG00010015140	ASCC3	0.020792417	0.03948441	0.526598155	0.598472674	0.731500245	positive
PRJNA729271	ed_Lushi_vs_Arbor_Acr	breed_growth_background	ENSGALG00010015141	PFDN4	0.033015838	0.072711961	0.454063374	0.649783214	0.771407505	positive
PRJNA729271	ed_Lushi_vs_Arbor_Acr	breed_growth_background	ENSGALG00010015142	HNRNPAB	0.09238594	0.06886867	1.341479944	0.179764677	0.324043529	positive
PRJNA729271	ed_Lushi_vs_Arbor_Acr	breed_growth_background	ENSGALG00010015143	EXOC1	-0.041113959	0.043259768	-0.95039712	0.341910507	0.505765159	negative
PRJNA729271	ed_Lushi_vs_Arbor_Acr	breed_growth_background	ENSGALG00010015145	CLIC2	-0.105073204	0.066380487	-1.582892942	0.113445868	0.235018202	negative
PRJNA729271	ed_Lushi_vs_Arbor_Acr	breed_growth_background	ENSGALG00010015146	ENSGALG00010015146	0.157627734	0.068741786	2.293041014	0.021845644	0.072693436	positive
PRJNA729271	ed_Lushi_vs_Arbor_Acr	breed_growth_background	ENSGALG00010015148	ENSGALG00010015148	0.051660938	0.041364207	1.248928513	0.211691222	0.361676598	positive
PRJNA729271	ed_Lushi_vs_Arbor_Acr	breed_growth_background	ENSGALG00010015149	ENSGALG00010015149	-0.046903144	0.073940572	-0.634335693	0.525861768	0.672166342	negative
PRJNA729271	ed_Lushi_vs_Arbor_Acr	breed_growth_background	ENSGALG00010015151	ENSGALG00010015151	0.084546922	0.234816603	0.360055126	0.718805909	0.820078052	positive
PRJNA729271	ed_Lushi_vs_Arbor_Acr	breed_growth_background	ENSGALG00010015152	BOLL	0.10280289	0.140096697	0.733799524	0.463070931	0.618181354	positive
PRJNA729271	ed_Lushi_vs_Arbor_Acr	breed_growth_background	ENSGALG00010015153	ERCC6L	-0.454012683	0.134953518	-3.364215235	0.000767617	0.006743395	negative
PRJNA729271	ed_Lushi_vs_Arbor_Acr	breed_growth_background	ENSGALG00010015154	CYP24A1	0.098968771	0.416253704	0.237760696	0.812066704	0.884281582	positive
PRJNA729271	ed_Lushi_vs_Arbor_Acr	breed_growth_background	ENSGALG00010015155	HMOX1	0.09568334	0.096239178	0.994224415	0.320113624	0.483489318	positive
PRJNA729271	ed_Lushi_vs_Arbor_Acr	breed_growth_background	ENSGALG00010015156	CNOT6	-0.090052651	0.039846717	-2.25997666	0.023822699	0.077319755	negative
PRJNA729271	ed_Lushi_vs_Arbor_Acr	breed_growth_background	ENSGALG00010015157	CDC42BPB	-0.148697812	0.037613098	-3.953351939	7.7064E-05	0.001312285	negative
PRJNA729271	ed_Lushi_vs_Arbor_Acr	breed_growth_background	ENSGALG00010015158	PIN4	0.059363826	0.088534449	0.670516701	0.502528464	0.653484116	positive
PRJNA729271	ed_Lushi_vs_Arbor_Acr	breed_growth_background	ENSGALG00010015160	SIM1	1.544856358	0.322834591	4.785287574	1.70743E-06	7.45456E-05	positive
PRJNA729271	ed_Lushi_vs_Arbor_Acr	breed_growth_background	ENSGALG00010015161	GFPT2	-0.134719017	0.107690162	-1.250987223	0.210939139	0.360987346	negative
PRJNA729271	ed_Lushi_vs_Arbor_Acr	breed_growth_background	ENSGALG00010015163	PLK2	-0.27964619	0.112278436	-2.490649133	0.012750997	0.049604322	negative
PRJNA729271	ed_Lushi_vs_Arbor_Acr	breed_growth_background	ENSGALG00010015165	KLF3	-0.030788516	0.063445742	-0.485273168	0.627482586	0.75380077	negative
PRJNA729271	ed_Lushi_vs_Arbor_Acr	breed_growth_background	ENSGALG00010015166	YTHDF2	0.278787555	0.097246228	2.866821285	0.004146171	0.022184845	positive
PRJNA729271	ed_Lushi_vs_Arbor_Acr	breed_growth_background	ENSGALG00010015167	RAB3C	0.382832393	0.249484211	1.534495476	0.124907805	0.250975692	positive
PRJNA729271	ed_Lushi_vs_Arbor_Acr	breed_growth_background	ENSGALG00010015169	ENSGALG00010015169	0.284393799	0.332674396	0.854871317	0.392622391	0.554036367	positive
PRJNA729271	ed_Lushi_vs_Arbor_Acr	breed_growth_background	ENSGALG00010015170	EXOC1L	-0.767466134	0.423431429	-1.812492132	0.069910189	0.167787717	negative
PRJNA729271	ed_Lushi_vs_Arbor_Acr	breed_growth_background	ENSGALG00010015171	NHSL2	-0.172653314	0.104290516	-1.655503506	0.097822406	0.211474388	negative
PRJNA729271	ed_Lushi_vs_Arbor_Acr	breed_growth_background	ENSGALG00010015172	PPP4R3A	0.091406714	0.041297701	2.213360812	0.02687278	0.084056042	positive
PRJNA729271	ed_Lushi_vs_Arbor_Acr	breed_growth_background	ENSGALG00010015173	ENS-1	0.312292886	0.373599687	0.83590243	0.403209795	0.564197894	positive
PRJNA729271	ed_Lushi_vs_Arbor_Acr	breed_growth_background	ENSGALG00010015174	ST6GAL2	-0.135700437	0.120560118	-1.125583141	0.260342007	0.418180119	negative
PRJNA729271	ed_Lushi_vs_Arbor_Acr	breed_growth_background	ENSGALG00010015175	MAPK9	-0.011746889	0.037131478	-0.316359324	0.751729787	0.842716416	negative
PRJNA729271	ed_Lushi_vs_Arbor_Acr	breed_growth_background	ENSGALG00010015177	TC2N	0.150548292	0.086277296	1.744935217	0.080996146	0.185581907	positive
PRJNA729271	ed_Lushi_vs_Arbor_Acr	breed_growth_background	ENSGALG00010015180	PITHD1	0.079544107	0.066701127	1.192545166	0.233047552	0.387505604	positive
PRJNA729271	ed_Lushi_vs_Arbor_Acr	breed_growth_background	ENSGALG00010015181	VPS13A	0.069379948	0.064184627	1.080943384	0.279722299	0.439425026	positive
PRJNA729271	ed_Lushi_vs_Arbor_Acr	breed_growth_background	ENSGALG00010015182	PHYKPL	-0.760651658	0.179020578	-4.248962151	2.14763E-05	0.000503781	negative
PRJNA729271	ed_Lushi_vs_Arbor_Acr	breed_growth_background	ENSGALG00010015183	SLC5A7	0.178729521	0.457530838	0.390639288	0.696063881	0.804260339	positive
PRJNA729271	ed_Lushi_vs_Arbor_Acr	breed_growth_background	ENSGALG00010015186	ENSGALG00010015186	-0.200359748	0.152644249	-1.312592837	0.189320184	0.335476293	negative
PRJNA729271	ed_Lushi_vs_Arbor_Acr	breed_growth_background	ENSGALG00010015188	SULT1C3	-1.615186622	0.657162272	-2.457820071	0.013978319	0.052860874	negative
PRJNA729271	ed_Lushi_vs_Arbor_Acr	breed_growth_background	ENSGALG00010015190	FBLN5	-0.172585374	0.102819366	-1.678529831	0.093243712	0.204720934	negative
PRJNA729271	ed_Lushi_vs_Arbor_Acr	breed_growth_background	ENSGALG00010015191	TOM1	0.213761362	0.099633386	2.145479238	0.03191455	0.095460462	positive
PRJNA729271	ed_Lushi_vs_Arbor_Acr	breed_growth_background	ENSGALG00010015192	ENSGALG00010015192	0.501861056	0.116264005	4.316564317	1.58477E-05	0.000405135	positive
PRJNA729271	ed_Lushi_vs_Arbor_Acr	breed_growth_background	ENSGALG00010015193	LYPLA2	0.058791044	0.057830434	1.016610806	0.309338611	0.472134692	positive
PRJNA729271	ed_Lushi_vs_Arbor_Acr	breed_growth_background	ENSGALG00010015194	TMLHE	0.220081422	0.06095392	3.610619628	0.000305466	0.003514075	positive
PRJNA729271	ed_Lushi_vs_Arbor_Acr	breed_growth_background	ENSGALG00010015196	PDE4D	-0.059345853	0.116823799	-0.507994548	0.611457167	0.741565902	negative
PRJNA729271	ed_Lushi_vs_Arbor_Acr	breed_growth_background	ENSGALG00010015198	NME5	-0.008600129	0.119467725	-0.07198705	0.942612214	0.968910565	negative
PRJNA729271	ed_Lushi_vs_Arbor_Acr	breed_growth_background	ENSGALG00010015199	ENSGALG00010015199	-0.159188737	0.095315884	-1.67011762	0.094896095	0.207343819	negative
PRJNA729271	ed_Lushi_vs_Arbor_Acr	breed_growth_background	ENSGALG00010015200	TBC1D1	0.023329277	0.046366442	0.503150035	0.614858791	0.743938078	positive
PRJNA729271	ed_Lushi_vs_Arbor_Acr	breed_growth_background	ENSGALG00010015201	GCC2	0.112896773	0.058444478	1.931692712	0.053397445	0.138922777	positive
PRJNA729271	ed_Lushi_vs_Arbor_Acr	breed_growth_background	ENSGALG00010015203	HMGXB4	0.086444347	0.035980959	2.402502578	0.01628332	0.059021435	positive
PRJNA729271	ed_Lushi_vs_Arbor_Acr	breed_growth_background	ENSGALG00010015204	ENSGALG00010015204	0.031771553	0.075310084	0.421876483	0.673115176	0.788556088	positive
PRJNA729271	ed_Lushi_vs_Arbor_Acr	breed_growth_background	ENSGALG00010015207	AKR1E2	0.170344147	0.096240008	1.769993066	0.076728296	0.178835287	positive
PRJNA729271	ed_Lushi_vs_Arbor_Acr	breed_growth_background	ENSGALG00010015208	GNA14	0.992268952	0.345662965	2.870625591	0.004096604	0.021986682	positive
PRJNA729271	ed_Lushi_vs_Arbor_Acr	breed_growth_background	ENSGALG00010015212	GNAQ	-0.220264619	0.052517092	-4.194151064	2.73895E-05	0.000612672	negative
PRJNA729271	ed_Lushi_vs_Arbor_Acr	breed_growth_background	ENSGALG00010015213	RUBCN	-0.047478069	0.063823749	-0.743893441	0.456940944	0.612820039	negative
PRJNA729271	ed_Lushi_vs_Arbor_Acr	breed_growth_background	ENSGALG00010015218	PLCL1	-0.141285839	0.08761363	-1.612601127	0.106831194	0.225632836	negative
PRJNA729271	ed_Lushi_vs_Arbor_Acr	breed_growth_background	ENSGALG00010015227	FYTTD1	-0.089920143	0.041062088	-2.189857996	0.028534537	0.087665984	negative
PRJNA729271	ed_Lushi_vs_Arbor_Acr	breed_growth_background	ENSGALG00010015233	ENSGALG00010015233	0.247741394	0.372458741	0.665151242	0.505953762	0.65550588	positive
PRJNA729271	ed_Lushi_vs_Arbor_Acr	breed_growth_background	ENSGALG00010015234	TRIP11	0.178680082	0.07695099	2.321998476	0.020233018	0.068828348	positive
PRJNA729271	ed_Lushi_vs_Arbor_Acr	breed_growth_background	ENSGALG00010015241	ENSGALG00010015241	-0.071166317	0.08687763	-0.819155835	0.412697509	0.572993333	negative
PRJNA729271	ed_Lushi_vs_Arbor_Acr	breed_growth_background	ENSGALG00010015248	ENSGALG00010015248	-0.078592933	0.073442492	-1.070128906	0.28456129	0.444929984	negative
PRJNA729271	ed_Lushi_vs_Arbor_Acr	breed_growth_background	ENSGALG00010015250	RORB	0.243784191	0.28462994	0.85649525	0.391723899	0.553307144	positive
PRJNA729271	ed_Lushi_vs_Arbor_Acr	breed_growth_background	ENSGALG00010015253	ENSGALG00010015253	-0.102390468	0.039654159	-2.582086522	0.009820496	0.041284249	negative
PRJNA729271	ed_Lushi_vs_Arbor_Acr	breed_growth_background	ENSGALG00010015260	RASGEF1C	-0.07811242	0.075360233	-1.03652041	0.299959423	0.462429238	negative
PRJNA729271	ed_Lushi_vs_Arbor_Acr	breed_growth_background	ENSGALG00010015261	IQCG	0.078704455	0.232453272	0.338581833	0.73492477	0.830298873	positive
PRJNA729271	ed_Lushi_vs_Arbor_Acr	breed_growth_background	ENSGALG00010015283	ENSGALG00010015283	0.571782173	0.345153992	1.656600202	0.097600331	0.211120251	positive
PRJNA729271	ed_Lushi_vs_Arbor_Acr	breed_growth_background	ENSGALG00010015285	ALG13	0.047654165	0.225808506	0.211037953	0.832857656	0.898568792	positive
PRJNA729271	ed_Lushi_vs_Arbor_Acr	breed_growth_background	ENSGALG00010015291	ZFP64	0.122906633	0.049810417	2.467488541	0.013606458	0.051956165	positive
PRJNA729271	ed_Lushi_vs_Arbor_Acr	breed_growth_background	ENSGALG00010015296	RPL35A	-0.168882837	0.104452204	-1.616843203	0.105912131	0.224148976	negative
PRJNA729271	ed_Lushi_vs_Arbor_Acr	breed_growth_background	ENSGALG00010015298	ENSGALG00010015298	0.504939006	0.116807703	4.322822826	1.54045E-05	0.000399443	positive
PRJNA729271	ed_Lushi_vs_Arbor_Acr	breed_growth_background	ENSGALG00010015299	GALE	0.031238335	0.051683081	0.604420921	0.54556383	0.689446183	positive
PRJNA729271	ed_Lushi_vs_Arbor_Acr	breed_growth_background	ENSGALG00010015300	ATXN3	-0.099863651	0.067786698	-1.47320424	0.140695972	0.273141994	negative
PRJNA729271	ed_Lushi_vs_Arbor_Acr	breed_growth_background	ENSGALG00010015306	BCAT1	-0.024055484	0.048472527	-0.496270468	0.619703599	0.747457756	negative

PRJNA729271	ed_Lushi_vs_Arbor_Acr	breed_growth_background	ENSGALG00010015310	PGM2	-0.06930637	0.051922853	-1.334795124	0.18194341	0.326677781	negative
PRJNA729271	ed_Lushi_vs_Arbor_Acr	breed_growth_background	ENSGALG00010015314	GMEB1	0.038075871	0.037868877	1.005466081	0.314672475	0.478010639	positive
PRJNA729271	ed_Lushi_vs_Arbor_Acr	breed_growth_background	ENSGALG00010015318	SALL4	-0.124544281	0.096481255	-1.290865059	0.196750473	0.34517654	negative
PRJNA729271	ed_Lushi_vs_Arbor_Acr	breed_growth_background	ENSGALG00010015320	TRPM6	-0.023749454	0.239964662	-0.098970631	0.921161589	0.956121535	negative
PRJNA729271	ed_Lushi_vs_Arbor_Acr	breed_growth_background	ENSGALG00010015327	LARGE1	-0.239960239	0.067938262	-3.532033835	0.000412377	0.004337735	negative
PRJNA729271	ed_Lushi_vs_Arbor_Acr	breed_growth_background	ENSGALG00010015328	ENSGALG00010015328	0.093249653	0.217423808	0.428884278	0.668007442	0.78486007	positive
PRJNA729271	ed_Lushi_vs_Arbor_Acr	breed_growth_background	ENSGALG00010015333	MED7	0.072543015	0.037646029	1.926976555	0.053982552	0.139877921	positive
PRJNA729271	ed_Lushi_vs_Arbor_Acr	breed_growth_background	ENSGALG00010015339	ENSGALG00010015339	1.479573275	0.426141763	3.472021291	0.000516555	0.005079695	positive
PRJNA729271	ed_Lushi_vs_Arbor_Acr	breed_growth_background	ENSGALG00010015348	CPSF2	-0.048371464	0.033446886	-1.446217233	0.148116282	0.283120542	negative
PRJNA729271	ed_Lushi_vs_Arbor_Acr	breed_growth_background	ENSGALG00010015353	RELL1	-0.100701068	0.048980042	-2.055961235	0.039786253	0.111971107	negative
PRJNA729271	ed_Lushi_vs_Arbor_Acr	breed_growth_background	ENSGALG00010015356	ENSGALG00010015356	0.041020797	0.046844504	0.87568003	0.381204004	0.54273176	positive
PRJNA729271	ed_Lushi_vs_Arbor_Acr	breed_growth_background	ENSGALG00010015362	ATP9A	-0.237493035	0.071246925	-3.333379455	0.000857978	0.007311227	negative
PRJNA729271	ed_Lushi_vs_Arbor_Acr	breed_growth_background	ENSGALG00010015364	CLOCK	0.329257501	0.108039492	3.047566158	0.002307027	0.01474346	positive
PRJNA729271	ed_Lushi_vs_Arbor_Acr	breed_growth_background	ENSGALG00010015370	ENSGALG00010015370	0.113799196	0.07607542	1.49587338	0.134686654	0.264536999	positive
PRJNA729271	ed_Lushi_vs_Arbor_Acr	breed_growth_background	ENSGALG00010015374	LIMS1	-0.331396462	0.081261484	-4.078149282	4.53956E-05	0.000896471	negative
PRJNA729271	ed_Lushi_vs_Arbor_Acr	breed_growth_background	ENSGALG00010015384	TAF12	0.132453298	0.062684625	2.113010932	0.034599832	0.101052704	positive
PRJNA729271	ed_Lushi_vs_Arbor_Acr	breed_growth_background	ENSGALG00010015386	SLC24A4	-0.046880757	0.138841191	-0.33765741	0.735621374	0.830503295	negative
PRJNA729271	ed_Lushi_vs_Arbor_Acr	breed_growth_background	ENSGALG00010015388	PSMD1	0.077776679	0.065519823	1.187070957	0.235199633	0.389758624	positive
PRJNA729271	ed_Lushi_vs_Arbor_Acr	breed_growth_background	ENSGALG00010015390	CCNB3	-0.4071313	0.113207163	-3.596338702	0.000322728	0.00363476	negative
PRJNA729271	ed_Lushi_vs_Arbor_Acr	breed_growth_background	ENSGALG00010015391	SYN3	-0.300280008	0.248135969	-1.210143009	0.226224022	0.379395719	negative
PRJNA729271	ed_Lushi_vs_Arbor_Acr	breed_growth_background	ENSGALG00010015392	TMEM165	-0.08265055	0.04445768	-1.859083727	0.063015271	0.156164534	negative
PRJNA729271	ed_Lushi_vs_Arbor_Acr	breed_growth_background	ENSGALG00010015393	DCX	-0.642409603	0.202362551	-3.174547859	0.001500701	0.010903592	negative
PRJNA729271	ed_Lushi_vs_Arbor_Acr	breed_growth_background	ENSGALG00010015397	SRD5A3	0.126306045	0.046963841	2.689431722	0.007157379	0.032901908	positive
PRJNA729271	ed_Lushi_vs_Arbor_Acr	breed_growth_background	ENSGALG00010015404	BEND4	0.079829454	0.170748425	0.46752674	0.640123065	0.764046758	positive
PRJNA729271	ed_Lushi_vs_Arbor_Acr	breed_growth_background	ENSGALG00010015407	TIMP3	-0.246417254	0.093858744	-2.625405389	0.008654586	0.037525642	negative
PRJNA729271	ed_Lushi_vs_Arbor_Acr	breed_growth_background	ENSGALG00010015409	FAM13B	0.265009564	0.070667469	3.750092766	0.000176769	0.002376873	positive
PRJNA729271	ed_Lushi_vs_Arbor_Acr	breed_growth_background	ENSGALG00010015410	HTR2B	-0.193259033	0.123463403	-1.565314322	0.117509227	0.240987017	negative
PRJNA729271	ed_Lushi_vs_Arbor_Acr	breed_growth_background	ENSGALG00010015413	CARNMT1	0.057419209	0.05728048	1.002421927	0.316139856	0.47948595	positive
PRJNA729271	ed_Lushi_vs_Arbor_Acr	breed_growth_background	ENSGALG00010015416	CAPN6	-0.06675183	0.086675054	-0.770138891	0.441217508	0.598600504	negative
PRJNA729271	ed_Lushi_vs_Arbor_Acr	breed_growth_background	ENSGALG00010015422	ENSGALG00010015422	0.102147727	0.292841668	0.348815547	0.727227791	0.824587756	positive
PRJNA729271	ed_Lushi_vs_Arbor_Acr	breed_growth_background	ENSGALG00010015430	HMGCL	0.110998709	0.069922886	1.587444615	0.112411982	0.233723868	positive
PRJNA729271	ed_Lushi_vs_Arbor_Acr	breed_growth_background	ENSGALG00010015431	RIN3	-0.187950332	0.086103603	-2.182839351	0.029047643	0.088809445	negative
PRJNA729271	ed_Lushi_vs_Arbor_Acr	breed_growth_background	ENSGALG00010015436	SLC30A9	0.086996798	0.053260621	1.633416883	0.102381349	0.218720361	positive
PRJNA729271	ed_Lushi_vs_Arbor_Acr	breed_growth_background	ENSGALG00010015440	ENSGALG00010015440	0.205320302	0.20455759	1.003728594	0.31550945	0.478830435	positive
PRJNA729271	ed_Lushi_vs_Arbor_Acr	breed_growth_background	ENSGALG00010015443	PAK3	0.170679999	0.054805392	3.114292104	0.001843868	0.01254194	positive
PRJNA729271	ed_Lushi_vs_Arbor_Acr	breed_growth_background	ENSGALG00010015449	HDX	0.000736692	0.05091885	0.01446796	0.988456641	0.993047113	positive
PRJNA729271	ed_Lushi_vs_Arbor_Acr	breed_growth_background	ENSGALG00010015466	NMRK1	0.022374888	0.067547004	0.331249149	0.740456298	0.833948255	positive
PRJNA729271	ed_Lushi_vs_Arbor_Acr	breed_growth_background	ENSGALG00010015469	TMEM33	0.132625641	0.039899089	3.324026765	0.000887277	0.007504268	positive
PRJNA729271	ed_Lushi_vs_Arbor_Acr	breed_growth_background	ENSGALG00010015472	LGMN	-0.167945416	0.071900826	-2.335792589	0.019502054	0.067129488	negative
PRJNA729271	ed_Lushi_vs_Arbor_Acr	breed_growth_background	ENSGALG00010015477	ARMC9	-0.073961798	0.058100638	-1.272994589	0.203019962	0.352215918	negative
PRJNA729271	ed_Lushi_vs_Arbor_Acr	breed_growth_background	ENSGALG00010015478	FBXO7	-0.099147881	0.033834022	-2.930419591	0.003385046	0.019316766	negative
PRJNA729271	ed_Lushi_vs_Arbor_Acr	breed_growth_background	ENSGALG00010015481	GOLGA5	0.066958727	0.038697565	1.730308551	0.083575163	0.189699652	positive
PRJNA729271	ed_Lushi_vs_Arbor_Acr	breed_growth_background	ENSGALG00010015483	SGCD	-0.22413189	0.086674956	-2.58588988	0.009712796	0.040966741	negative
PRJNA729271	ed_Lushi_vs_Arbor_Acr	breed_growth_background	ENSGALG00010015485	BCAS1	0.424008568	0.099892354	4.2446549	2.1893E-05	0.000510192	positive
PRJNA729271	ed_Lushi_vs_Arbor_Acr	breed_growth_background	ENSGALG00010015486	MTFR1L	-0.025749277	0.063792812	-0.403639163	0.686478082	0.797699198	negative
PRJNA729271	ed_Lushi_vs_Arbor_Acr	breed_growth_background	ENSGALG00010015487	RPS6KA6	0.044530667	0.063941108	0.696432516	0.486158006	0.63784036	positive
PRJNA729271	ed_Lushi_vs_Arbor_Acr	breed_growth_background	ENSGALG00010015490	RANBP2	0.032247882	0.044431589	0.725787289	0.467969181	0.622884598	positive
PRJNA729271	ed_Lushi_vs_Arbor_Acr	breed_growth_background	ENSGALG00010015493	OSTF1	-0.040689137	0.076164108	-0.534229816	0.593182543	0.72761115	negative
PRJNA729271	ed_Lushi_vs_Arbor_Acr	breed_growth_background	ENSGALG00010015494	CHRDL1	0.021213582	0.129936396	0.16326128	0.870312719	0.922876636	positive
PRJNA729271	ed_Lushi_vs_Arbor_Acr	breed_growth_background	ENSGALG00010015498	MID2	-0.1657815	0.071117591	-2.33108993	0.019748618	0.067608933	negative
PRJNA729271	ed_Lushi_vs_Arbor_Acr	breed_growth_background	ENSGALG00010015502	BPIFB6	1.262615364	0.691843323	1.825001878	0.068000746	0.164717716	positive
PRJNA729271	ed_Lushi_vs_Arbor_Acr	breed_growth_background	ENSGALG00010015505	CHGA	-0.185986128	0.267947466	-0.694114151	0.487610625	0.639398951	negative
PRJNA729271	ed_Lushi_vs_Arbor_Acr	breed_growth_background	ENSGALG00010015507	AMMECR1	-0.047444121	0.039144937	-1.212011697	0.225507903	0.378535953	negative
PRJNA729271	ed_Lushi_vs_Arbor_Acr	breed_growth_background	ENSGALG00010015508	FUCA1	-0.076652318	0.055902688	-1.37117411	0.170320687	0.312624839	negative
PRJNA729271	ed_Lushi_vs_Arbor_Acr	breed_growth_background	ENSGALG00010015511	BPIFB4	0.548291179	0.226298067	2.422871685	0.015398365	0.056793713	positive
PRJNA729271	ed_Lushi_vs_Arbor_Acr	breed_growth_background	ENSGALG00010015516	SELENON	-0.261397049	0.060684108	-4.307504173	1.65107E-05	0.000415491	negative
PRJNA729271	ed_Lushi_vs_Arbor_Acr	breed_growth_background	ENSGALG00010015520	UBE3A	0.096354856	0.042061148	2.290827986	0.021973364	0.073004214	positive
PRJNA729271	ed_Lushi_vs_Arbor_Acr	breed_growth_background	ENSGALG00010015521	DKC1	0.06378286	0.044208017	1.442789424	0.149079799	0.283994177	positive
PRJNA729271	ed_Lushi_vs_Arbor_Acr	breed_growth_background	ENSGALG00010015523	MRPL22	0.115742286	0.07840677	1.476177196	0.13989633	0.271895588	positive
PRJNA729271	ed_Lushi_vs_Arbor_Acr	breed_growth_background	ENSGALG00010015532	NXT2	0.19217013	0.060041135	3.200641221	0.001371222	0.010261155	positive
PRJNA729271	ed_Lushi_vs_Arbor_Acr	breed_growth_background	ENSGALG00010015533	PSAT1	-0.539392001	0.148915346	-3.622138462	0.000292178	0.003415415	negative
PRJNA729271	ed_Lushi_vs_Arbor_Acr	breed_growth_background	ENSGALG00010015540	ACSL4	0.120378614	0.082014926	1.467764711	0.142168146	0.275020323	positive
PRJNA729271	ed_Lushi_vs_Arbor_Acr	breed_growth_background	ENSGALG00010015541	RASEF	-0.041615756	0.191317854	-0.217521551	0.827801914	0.894989313	negative
PRJNA729271	ed_Lushi_vs_Arbor_Acr	breed_growth_background	ENSGALG00010015542	CNGA3	-0.377038438	0.165163372	-2.282821138	0.022440906	0.074146555	negative
PRJNA729271	ed_Lushi_vs_Arbor_Acr	breed_growth_background	ENSGALG00010015544	RTCB	0.128032366	0.039587364	3.234172558	0.001219957	0.009456299	positive
PRJNA729271	ed_Lushi_vs_Arbor_Acr	breed_growth_background	ENSGALG00010015551	VWA3B	-0.567295487	0.24295366	-2.334994614	0.019543702	0.067209006	negative
PRJNA729271	ed_Lushi_vs_Arbor_Acr	breed_growth_background	ENSGALG00010015553	ASCL4	-0.479633299	0.556751826	-0.861484913	0.388971027	0.550902931	negative
PRJNA729271	ed_Lushi_vs_Arbor_Acr	breed_growth_background	ENSGALG00010015555	PNRC2	0.131686727	0.054885206	2.399311885	0.016425917	0.059404609	positive
PRJNA729271	ed_Lushi_vs_Arbor_Acr	breed_growth_background	ENSGALG00010015557	IRS4	0.355501876	0.102788087	3.458590243	0.00054301	0.005278964	positive
PRJNA729271	ed_Lushi_vs_Arbor_Acr	breed_growth_background	ENSGALG00010015558	MPP1	0.257334873	0.138934204	1.852206767	0.063996137	0.157812863	positive
PRJNA729271	ed_Lushi_vs_Arbor_Acr	breed_growth_background	ENSGALG00010015562	IRF9	0.42070671	0.143717145	2.927324437	0.00341892	0.019433	positive
PRJNA729271	ed_Lushi_vs_Arbor_Acr	breed_growth_background	ENSGALG00010015563	B3GNT7	-0.259424692	0.175047262	-1.482026558	0.138333229	0.269906814	negative
PRJNA729271	ed_Lushi_vs_Arbor_Acr	breed_growth_background	ENSGALG00010015564	PKD2L2	-0.141190097	0.170598539	-0.827616097	0.407887949	0.568439171	negative
PRJNA729271	ed_Lushi_vs_Arbor_Acr	breed_growth_background	ENSGALG00010015565	ENSGALG00010015565	0.070285864	0.243495751	0.288653347	0.772846667	0.858685608	positive
PRJNA729271	ed_Lushi_vs_Arbor_Acr	breed_growth_background	ENSGALG00010015567	ENSGALG00010015567	-0.027351592	0.172854017	-0.158235212	0.874271458	0.92574803	negative
PRJNA729271	ed_Lushi_vs_Arbor_Acr	breed_growth_background	ENSGALG00010015570	LIMCH1	0.311290515	0.068292596	4.558188338	5.15968E-06	0.000178495	positive
PRJNA729271	ed_Lushi_vs_Arbor_Acr	breed_growth_background	ENSGALG00010015571	DUSP15	0.849550055	0.163371681	5.200105966	1.99175E-07	1.42219E-05	positive
PRJNA729271	ed_Lushi_vs_Arbor_Acr	breed_growth_background	ENSGALG00010015573	MAN1C1	-0.206247668	0.06619989	-3.115528865	0.001836154	0.012520963	negative
PRJNA729271	ed_Lushi_vs_Arbor_Acr	breed_growth_background	ENSGALG00010015574	C14orf132	-0.602063236	0.139933831	-4.302485195	1.68893E-05	0.000422014	negative
PRJNA729271	ed_Lushi_vs_Arbor_Acr	breed_growth_background	ENSGALG00010015580	GEMIN5	0.012729304	0.03891005	0.32714695	0.743556743	0.836465269	positive
PRJNA729271	ed_Lushi_vs_Arbor_Acr	breed_growth_background	ENSGALG00010015583	PRDM4	0.322926534	0.073046633	4.420827107	9.83238E-06	0.000288503	positive
PRJNA729271	ed_Lushi_vs_Arbor_Acr	breed_growth_background	ENSGALG00010015584	TTLL9	0.051710971	0.124537125	0.415225349	0.677976943	0.791965435	positive
PRJNA729271	ed_Lushi_vs_Arbor_Acr	breed_growth_background	ENSGALG00010015585	BDKRB1	-0.316523775	0.161519789	-1.959659419	0.050035611	0.132662555	negative
PRJNA729271	ed_Lushi_vs_Arbor_Acr	breed_growth_background	ENSGALG00010015588	BDKRB2	0.454354115	0.254921	1.782333014	0.074694942	0.175792042	positive
PRJNA729271	ed_Lushi_vs_Arbor_Acr	breed_growth_background	ENSGALG00010015589	FRMD3	0.022328853	0.095694857	0.233333889	0.815502145	0.886440088	positive
PRJNA729271	ed_Lushi_vs_Arbor_Acr	breed_growth_background	ENSGALG00010015596	UCHL1	-0.646805644	0.271351189	-2.383647721	0.017142003	0.061246422	negative
PRJNA729271	ed_Lushi_vs_Arbor_Acr	breed_growth_background	ENSGALG00010015605	LDLRAP1	-0.067023017	0.032325585	-2.073373671	0.038137517	0.108798133	negative
PRJNA729271	ed_Lushi_vs_Arbor_Acr	breed_growth_background	ENSGALG00010015606	TMEM131	0.117229824	0.058138572	2.016386372	0.043759589	0.120199694	positive
PRJNA729271	ed_Lushi_vs_Arbor_Acr	breed_growth_background	ENSGALG00010015610	ENSGALG00010015610	-0.065660375	0.153124067	-0.428805063	0.668065094	0.78486007	negative
PRJNA729271	ed_Lushi_vs_Arbor_Acr	breed_growth_background	ENSGALG00010015613	PWP1	-0.017005618	0.043987375	-0.386602247	0.699050694	0.805784478	negative
PRJNA729271	ed_Lushi_vs_Arbor_Acr	breed_growth_background	ENSGALG00010015614	ATG2B	0.140675696	0.057395195	2.451001265	0.014245945	0.053509971	positive
PRJNA729271	ed_Lushi_vs_Arbor_Acr	breed_growth_background	ENSGALG00010015616	NADK2	-0.125224919	0.063931884	-1.958724061	0.050145111	0.132807123	negative
PRJNA729271	ed_Lushi_vs_Arbor_Acr	breed_growth_background	ENSGALG00010015617	SRSF10	0.08154303	0.03021006	2.699201142	0.006950615	0.032219114	positive
PRJNA729271	ed_Lushi_vs_Arbor_Acr	breed_growth_background	ENSGALG00010015618	NCL	0.027220735	0.054145939	0.502729024	0.615154801	0.7440478	positive
PRJNA729271	ed_Lushi_vs_Arbor_Acr	breed_growth_background	ENSGALG00010015620	PDRG1	0.175051093	0.07672304	2.281597455	0.022513117	0.074300463	positive
PRJNA729271	ed_Lushi_vs_Arbor_Acr	breed_growth_background	ENSGALG00010015621	IDNK	0.011307553	0.087364421	0.12942972	0.897017632	0.9402177	positive
PRJNA729271	ed_Lushi_vs_Arbor_Acr	breed_growth_background	ENSGALG00010015624	ITGA1	-0.196043179	0.069054565	-2.838960449	0.004526077	0.023650137	negative
PRJNA729271	ed_Lushi_vs_Arbor_Acr	breed_growth_background	ENSGALG00010015626	XKR7	0.209380289	0.292626031	0.715521749	0.474286707	0.627868839	positive
PRJNA729271	ed_Lushi_vs_Arbor_Acr	breed_growth_background	ENSGALG00010015628	UBQLN1	0.159563107	0.063501194	2.5127576	0.011979161	0.047493967	positive
PRJNA729271	ed_Lushi_vs_Arbor_Acr	breed_growth_background	ENSGALG00010015631	GSK1P	0.061424334	0.058533245	1.049392255	0.293997621	0.455287983	positive
PRJNA729271	ed_Lushi_vs_Arbor_Acr	breed_growth_background	ENSGALG00010015632	COL4A5	-0.108153142	0.086397169	-1.251813499	0.21063783	0.360727239	negative
PRJNA729271	ed_Lushi_vs_Arbor_Acr	breed_growth_background	ENSGALG00010015633	CCM2L	-0.088801037	0.099697261	-0.890706884	0.373086441	0.535593539	negative
PRJNA729271	ed_Lushi_vs_Arbor_Acr	breed_growth_background	ENSGALG00010015634	ENSGALG00010015634	-5.640428114	0.612093003	-9.21498544	3.11321E-20	5.01399E-17	negative
PRJNA729271	ed_Lushi_vs_Arbor_Acr	breed_growth_background	ENSGALG00010015635	ABTB3	-0.176521552	0.071011617	-2.485812307	0.012925612	0.050099726	negative
PRJNA729271	ed_Lushi_vs_Arbor_Acr	breed_growth_background	ENSGALG00010015636	APBB2	-0.195287927	0.066493288	-2.936956966	0.003314501	0.019049838	negative
PRJNA729271	ed_Lushi_vs_Arbor_Acr	breed_growth_background	ENSGALG00010015642	ENSGALG00010015642	-1.949217349	0.476308249	-4.092344299	4.27034E-05	0.000862851	negative
PRJNA729271	ed_Lushi_vs_Arbor_Acr	breed_growth_background	ENSGALG00010015643	ENSGALG00010015643	-0.054190804	0.065505109	-0.827275991	0.408080649	0.568598385	negative
PRJNA729271	ed_Lushi_vs_Arbor_Acr	breed_growth_background	ENSGALG00010015645	MACO1	0.09600979	0.050375351	1.905888263	0.056664704	0.144910883	positive
PRJNA729271	ed_Lushi_vs_Arbor_Acr	breed_growth_background	ENSGALG00010015647	PDZD2	-0.112821315	0.07437244	-1.516977459	0.129272375	0.256720521	negative
PRJNA729271	ed_Lushi_vs_Arbor_Acr	breed_growth_background	ENSGALG00010015650	ENSGALG00010015650	-6.471284676	0.688358286	-9.401041303	5.40257E-21	9.78878E-18	negative
PRJNA729271	ed_Lushi_vs_Arbor_Acr	breed_growth_background	ENSGALG00010015653	HCK	1.517032712	0.278395884	5.449192312	5.05991E-08	4.95563E-06	positive
PRJNA729271	ed_Lushi_vs_Arbor_Acr	breed_growth_background	ENSGALG00010015655	ENSGALG00010015655	0.209875956	0.15754672	1.332150591	0.182810709	0.32786949	positive
PRJNA729271	ed_Lushi_vs_Arbor_Acr	breed_growth_background	ENSGALG00010015657	NMUR1	-0.041745677	0.645243945	-0.064697511	0.948414845	0.971711451	negative
PRJNA729271	ed_Lushi_vs_Arbor_Acr	breed_growth_background	ENSGALG00010015660	KLHL3	-0.191098372	0.067615588	-2.826247269	0.004709689	0.024339557	negative
PRJNA729271	ed_Lushi_vs_Arbor_Acr	breed_growth_background	ENSGALG00010015668	ZNF217	-0.178160062	0.072307812	-2.463911673	0.013742997	0.052353416	negative
PRJNA729271	ed_Lushi_vs_Arbor_Acr	breed_growth_background	ENSGALG00010015669	INPP4A	-0.127903663	0.069784585	-1.832835483	0.066827041	0.162881781	negative
PRJNA729271	ed_Lushi_vs_Arbor_Acr	breed_growth_background	ENSGALG00010015670	ENSGALG00010015670	-0.068556395	0.070908288	-0.966831914	0.333628068	0.498059534	negative
PRJNA729271	ed_Lushi_vs_Arbor_Acr	breed_growth_background	ENSGALG00010015674	RFKL	-0.124221406	0.103856168	-1.196090792	0.23166113	0.385702744	negative
PRJNA729271	ed_Lushi_vs_Arbor_Acr	breed_growth_background	ENSGALG00010015676	TGM2	0.249656913	0.102515233	2.435315267	0.01487882	0.055242954	positive
PRJNA729271	ed_Lushi_vs_Arbor_Acr	breed_growth_background	ENSGALG00010015677	GRHL3	-0.059787116	0.146411602	-0.408349582	0.683017043	0.795271269	negative
PRJNA729271	ed_Lushi_vs_Arbor_Acr	breed_growth_background	ENSGALG00010015680	ENSGALG00010015680	0.175694861	0.056283475	3.121606489	0.001798672	0.012338736	positive
PRJNA729271	ed_Lushi_vs_Arbor_Acr	breed_growth_background	ENSGALG00010015684	PDE6D	-0.083843678	0.05478692	-1.530359391	0.125927795	0.252500123	negative
PRJNA729271	ed_Lushi_vs_Arbor_Acr	breed_growth_background	ENSGALG00010015686	CNOT8	0.017556566	0.029738913	0.590356684	0.554951544	0.696874524	positive
PRJNA729271	ed_Lushi_vs_Arbor_Acr	breed_growth_background	ENSGALG00010015691	EXOC3L4	-0.606402008	0.480231028	-1.262729755	0.206686317	0.355721117	negative
PRJNA729271	ed_Lushi_vs_Arbor_Acr	breed_growth_background	ENSGALG00010015693	COA5	-0.181552273	0.058317426	-3.113173628	0.001850871	0.012565983	negative
PRJNA729271	ed_Lushi_vs_Arbor_Acr	breed_growth_background	ENSGALG00010015695	ASAH1	0.061363835	0.050435733	1.216673788	0.223728356	0.376342407	positive
PRJNA729271	ed_Lushi_vs_Arbor_Acr	breed_growth_background	ENSGALG00010015697	FAXDC2	-0.011784303	0.187645193	-0.062800986	0.949924981	0.972423971	negative
PRJNA729271	ed_Lushi_vs_Arbor_Acr	breed_growth_background	ENSGALG00010015698	TNFAIP2	0.144374666	0.108045455	1.336240066	0.181470819	0.32603902	positive
PRJNA729271	ed_Lushi_vs_Arbor_Acr	breed_growth_background	ENSGALG00010015701	UNC50	0.015949569	0.047649998	0.334723383	0.737833761	0.832028969	positive
PRJNA729271	ed_Lushi_vs_Arbor_Acr	breed_growth_background	ENSGALG00010015703	GK5	0.019240193	0.101152084	0.19021055	0.849144144	0.909157054	positive
PRJNA729271	ed_Lushi_vs_Arbor_Acr	breed_growth_background	ENSGALG00010015705	NPY6R	0.201546914	0.616548472	0.326895489	0.743746933	0.836614294	positive

PRJNA729271	ed_Lushi_vs_Arbor_Acr	breed_growth_background	ENSGALG00010015706	ENSGALG00010015706	-0.124295932	0.088062633	-1.411449199	0.158112205	0.296179427	negative
PRJNA729271	ed_Lushi_vs_Arbor_Acr	breed_growth_background	ENSGALG00010015709	HNRNPA0	0.022965433	0.061338467	0.374405067	0.708102974	0.811828886	positive
PRJNA729271	ed_Lushi_vs_Arbor_Acr	breed_growth_background	ENSGALG00010015711	LBFABP	-1.260705479	0.366350597	-3.441254063	0.000579025	0.005482013	negative
PRJNA729271	ed_Lushi_vs_Arbor_Acr	breed_growth_background	ENSGALG00010015716	MGAT4A	-0.060361797	0.071061749	-0.849427399	0.395643513	0.557106345	negative
PRJNA729271	ed_Lushi_vs_Arbor_Acr	breed_growth_background	ENSGALG00010015719	XRN1	0.157400152	0.053258828	2.955381461	0.003122827	0.018252167	positive
PRJNA729271	ed_Lushi_vs_Arbor_Acr	breed_growth_background	ENSGALG00010015729	RHCE	-0.374998261	0.106439743	-3.523103791	0.000426524	0.004444622	negative
PRJNA729271	ed_Lushi_vs_Arbor_Acr	breed_growth_background	ENSGALG00010015731	PHF6	-0.027784748	0.038422538	-0.723136706	0.4695959	0.624247301	negative
PRJNA729271	ed_Lushi_vs_Arbor_Acr	breed_growth_background	ENSGALG00010015733	COL4A6	-0.320504695	0.081609477	-3.927297503	8.59057E-05	0.001429625	negative
PRJNA729271	ed_Lushi_vs_Arbor_Acr	breed_growth_background	ENSGALG00010015734	TSHZ2	-0.200913446	0.090662082	-2.216069175	0.026686765	0.083705823	negative
PRJNA729271	ed_Lushi_vs_Arbor_Acr	breed_growth_background	ENSGALG00010015738	CRACDL	-0.091930657	0.260696875	-0.35263429	0.724362627	0.823436302	negative
PRJNA729271	ed_Lushi_vs_Arbor_Acr	breed_growth_background	ENSGALG00010015742	HPRT1	-0.07654359	0.045866143	-1.66884732	0.095147642	0.207660301	negative
PRJNA729271	ed_Lushi_vs_Arbor_Acr	breed_growth_background	ENSGALG00010015746	PAPOLB	0.1549149	0.038734185	3.99943619	6.34936E-05	0.00114186	positive
PRJNA729271	ed_Lushi_vs_Arbor_Acr	breed_growth_background	ENSGALG00010015749	ATR	0.239909104	0.07265783	3.301902974	0.000960313	0.007919582	positive
PRJNA729271	ed_Lushi_vs_Arbor_Acr	breed_growth_background	ENSGALG00010015751	MYOM3	-0.918963356	0.289459607	-3.174755073	0.00149963	0.010901274	negative
PRJNA729271	ed_Lushi_vs_Arbor_Acr	breed_growth_background	ENSGALG00010015753	RPRD1B	0.069188833	0.042352982	1.633623626	0.102337904	0.218659775	positive
PRJNA729271	ed_Lushi_vs_Arbor_Acr	breed_growth_background	ENSGALG00010015756	PCM1	0.150195613	0.055361663	2.712989523	0.006667921	0.031309207	positive
PRJNA729271	ed_Lushi_vs_Arbor_Acr	breed_growth_background	ENSGALG00010015758	PCDH19	-0.398935252	0.091007433	-4.383545809	1.16763E-05	0.00032665	negative
PRJNA729271	ed_Lushi_vs_Arbor_Acr	breed_growth_background	ENSGALG00010015759	EIF5	0.141836574	0.049035315	2.892539286	0.003821414	0.020973644	positive
PRJNA729271	ed_Lushi_vs_Arbor_Acr	breed_growth_background	ENSGALG00010015764	TTI1	0.150105082	0.052437901	2.862530311	0.004202731	0.022344305	positive
PRJNA729271	ed_Lushi_vs_Arbor_Acr	breed_growth_background	ENSGALG00010015765	ENSGALG00010015765	-0.649696281	0.203771732	-3.188353328	0.001430856	0.010581763	negative
PRJNA729271	ed_Lushi_vs_Arbor_Acr	breed_growth_background	ENSGALG00010015766	TLE1	-0.052265296	0.055518721	-0.941399502	0.346500168	0.510366827	negative
PRJNA729271	ed_Lushi_vs_Arbor_Acr	breed_growth_background	ENSGALG00010015769	TMEM50A	-0.026986426	0.053649039	-0.503017885	0.614951698	0.743988387	negative
PRJNA729271	ed_Lushi_vs_Arbor_Acr	breed_growth_background	ENSGALG00010015774	PLS1	-0.2271474	0.209855324	-1.082399986	0.279074834	0.438550491	negative
PRJNA729271	ed_Lushi_vs_Arbor_Acr	breed_growth_background	ENSGALG00010015776	SPOCK1	0.057152668	0.123812271	0.461607461	0.644362843	0.767309651	positive
PRJNA729271	ed_Lushi_vs_Arbor_Acr	breed_growth_background	ENSGALG00010015778	PABIR3	-0.067779592	0.047416681	-1.429446149	0.152876042	0.289362527	negative
PRJNA729271	ed_Lushi_vs_Arbor_Acr	breed_growth_background	ENSGALG00010015782	ATG4A	0.134993306	0.044362266	3.042975855	0.002342511	0.014938276	positive
PRJNA729271	ed_Lushi_vs_Arbor_Acr	breed_growth_background	ENSGALG00010015785	FGL1	-0.119275454	0.101929847	-1.170172016	0.241931752	0.397603761	negative
PRJNA729271	ed_Lushi_vs_Arbor_Acr	breed_growth_background	ENSGALG00010015788	TRPC1	-0.106828039	0.069017813	-1.547832854	0.121662544	0.246345837	negative
PRJNA729271	ed_Lushi_vs_Arbor_Acr	breed_growth_background	ENSGALG00010015792	MARK3	0.138153716	0.043747645	3.157969203	0.001588723	0.011299581	positive
PRJNA729271	ed_Lushi_vs_Arbor_Acr	breed_growth_background	ENSGALG00010015793	RSRP1	0.508880674	0.124407491	4.090434331	4.30566E-05	0.000868019	positive
PRJNA729271	ed_Lushi_vs_Arbor_Acr	breed_growth_background	ENSGALG00010015795	PSMD10	-0.294880336	0.072418298	-4.071903694	4.66305E-05	0.000918354	negative
PRJNA729271	ed_Lushi_vs_Arbor_Acr	breed_growth_background	ENSGALG00010015796	LARP1	0.18474303	0.07555432	2.445168304	0.014478456	0.05400259	positive
PRJNA729271	ed_Lushi_vs_Arbor_Acr	breed_growth_background	ENSGALG00010015797	PIP5K1B	-0.423852853	0.089098243	-4.757140428	1.96355E-06	8.34637E-05	negative
PRJNA729271	ed_Lushi_vs_Arbor_Acr	breed_growth_background	ENSGALG00010015798	IL22RA1	-0.377076396	0.157502431	-2.394098893	0.016661256	0.060016129	negative
PRJNA729271	ed_Lushi_vs_Arbor_Acr	breed_growth_background	ENSGALG00010015800	BVES	0.406259063	0.096737791	4.199590052	2.67399E-05	0.000601353	positive
PRJNA729271	ed_Lushi_vs_Arbor_Acr	breed_growth_background	ENSGALG00010015801	VSTM2L	-0.125293543	0.142809877	-0.877345085	0.380299231	0.542032629	negative
PRJNA729271	ed_Lushi_vs_Arbor_Acr	breed_growth_background	ENSGALG00010015802	TRPC7	0.405213128	0.54022474	0.750082509	0.453205013	0.609531814	positive
PRJNA729271	ed_Lushi_vs_Arbor_Acr	breed_growth_background	ENSGALG00010015803	SYF2	0.257161377	0.085860029	2.995123348	0.002743339	0.016610149	positive
PRJNA729271	ed_Lushi_vs_Arbor_Acr	breed_growth_background	ENSGALG00010015804	CKB	-0.376381393	0.086356326	-4.358469289	1.30975E-05	0.000352879	negative
PRJNA729271	ed_Lushi_vs_Arbor_Acr	breed_growth_background	ENSGALG00010015807	ENSGALG00010015807	0.384757108	0.503944969	0.763490325	0.44517103	0.601928171	positive
PRJNA729271	ed_Lushi_vs_Arbor_Acr	breed_growth_background	ENSGALG00010015809	PCOLCE2	0.148354302	0.126457187	1.173158327	0.24073232	0.396524429	positive
PRJNA729271	ed_Lushi_vs_Arbor_Acr	breed_growth_background	ENSGALG00010015810	LIPT1	0.181060123	0.057175991	3.166715973	0.001541708	0.011079355	positive
PRJNA729271	ed_Lushi_vs_Arbor_Acr	breed_growth_background	ENSGALG00010015811	CTNNBL1	-0.008932541	0.030761065	-0.290384659	0.771521979	0.857542449	negative
PRJNA729271	ed_Lushi_vs_Arbor_Acr	breed_growth_background	ENSGALG00010015813	ENSGALG00010015813	0.01376122	0.074384326	0.185001604	0.853227778	0.911388109	positive
PRJNA729271	ed_Lushi_vs_Arbor_Acr	breed_growth_background	ENSGALG00010015814	PAQR9	-0.200999024	0.229849119	-0.874482464	0.381855566	0.5433392	negative
PRJNA729271	ed_Lushi_vs_Arbor_Acr	breed_growth_background	ENSGALG00010015816	MITD1	-0.122406572	0.117199416	-1.044429878	0.296286546	0.457951961	negative
PRJNA729271	ed_Lushi_vs_Arbor_Acr	breed_growth_background	ENSGALG00010015820	ENSGALG00010015820	0.429525703	0.281518769	1.525744465	0.127073516	0.253989329	positive
PRJNA729271	ed_Lushi_vs_Arbor_Acr	breed_growth_background	ENSGALG00010015822	WFDC2	0.343114976	0.299605207	1.145223673	0.252116514	0.408544313	positive
PRJNA729271	ed_Lushi_vs_Arbor_Acr	breed_growth_background	ENSGALG00010015825	RUNX3	0.598383569	0.331343225	1.805932711	0.070928851	0.169292556	positive
PRJNA729271	ed_Lushi_vs_Arbor_Acr	breed_growth_background	ENSGALG00010015827	ENSGALG00010015827	-0.279193575	0.07818966	-3.570722468	0.000355998	0.003899885	negative
PRJNA729271	ed_Lushi_vs_Arbor_Acr	breed_growth_background	ENSGALG00010015829	SMAD5	0.013205972	0.02576703	0.512514301	0.608291114	0.739014307	positive
PRJNA729271	ed_Lushi_vs_Arbor_Acr	breed_growth_background	ENSGALG00010015831	LYG2	0.626545739	0.319378797	1.961763729	0.049789998	0.132108004	positive
PRJNA729271	ed_Lushi_vs_Arbor_Acr	breed_growth_background	ENSGALG00010015832	RAB39B	-0.104052666	0.046893127	-2.218932121	0.026491343	0.083277383	negative
PRJNA729271	ed_Lushi_vs_Arbor_Acr	breed_growth_background	ENSGALG00010015834	STARD8	-0.149868772	0.071346529	-2.100575499	0.035678247	0.103431237	negative
PRJNA729271	ed_Lushi_vs_Arbor_Acr	breed_growth_background	ENSGALG00010015837	GDA	-0.209680567	0.083984564	-2.496656021	0.01253705	0.048969158	negative
PRJNA729271	ed_Lushi_vs_Arbor_Acr	breed_growth_background	ENSGALG00010015838	IFNLR1	-1.333458629	0.681322803	-1.957161309	0.050328506	0.133171175	negative
PRJNA729271	ed_Lushi_vs_Arbor_Acr	breed_growth_background	ENSGALG00010015839	U2SURP	0.056578568	0.038408479	1.473074937	0.140730831	0.2731489	positive
PRJNA729271	ed_Lushi_vs_Arbor_Acr	breed_growth_background	ENSGALG00010015840	MTUS1	-0.0785892	0.078024383	-1.007238977	0.313819947	0.477365949	negative
PRJNA729271	ed_Lushi_vs_Arbor_Acr	breed_growth_background	ENSGALG00010015843	ENSGALG00010015843	0.064877651	0.247960594	0.261645005	0.793595143	0.872112328	positive
PRJNA729271	ed_Lushi_vs_Arbor_Acr	breed_growth_background	ENSGALG00010015845	SAP30L	0.0787393	0.06701507	1.174949153	0.240015059	0.395702716	positive
PRJNA729271	ed_Lushi_vs_Arbor_Acr	breed_growth_background	ENSGALG00010015848	UBE2B	0.344614142	0.086132177	4.000991883	6.30775E-05	0.0011372	positive
PRJNA729271	ed_Lushi_vs_Arbor_Acr	breed_growth_background	ENSGALG00010015852	ZFAND5	0.035994295	0.152786485	0.235585594	0.813754253	0.885537337	positive
PRJNA729271	ed_Lushi_vs_Arbor_Acr	breed_growth_background	ENSGALG00010015855	NIPAL3	-0.050966854	0.044030386	-1.157538218	0.247052534	0.403087177	negative
PRJNA729271	ed_Lushi_vs_Arbor_Acr	breed_growth_background	ENSGALG00010015856	TXNDC9	-0.045166274	0.071363836	-0.632901438	0.526798009	0.672947664	negative
PRJNA729271	ed_Lushi_vs_Arbor_Acr	breed_growth_background	ENSGALG00010015858	CHST2	0.022896605	0.049934456	0.458533177	0.646569433	0.769081235	positive
PRJNA729271	ed_Lushi_vs_Arbor_Acr	breed_growth_background	ENSGALG00010015861	PPP2CA	-0.070975519	0.03645936	-1.946702304	0.051570435	0.135468188	negative
PRJNA729271	ed_Lushi_vs_Arbor_Acr	breed_growth_background	ENSGALG00010015862	PDGFRL	-0.203191478	0.112017028	-1.813933849	0.069687911	0.167461251	negative
PRJNA729271	ed_Lushi_vs_Arbor_Acr	breed_growth_background	ENSGALG00010015863	ENSGALG00010015863	0.422262368	0.312862784	1.349672734	0.177120982	0.321121781	positive
PRJNA729271	ed_Lushi_vs_Arbor_Acr	breed_growth_background	ENSGALG00010015865	VRK1	0.025968558	0.077913525	0.333299752	0.738908026	0.832625901	positive
PRJNA729271	ed_Lushi_vs_Arbor_Acr	breed_growth_background	ENSGALG00010015866	ARHGEF6	-0.097113213	0.038868415	-2.49851232	0.012471581	0.048845059	negative
PRJNA729271	ed_Lushi_vs_Arbor_Acr	breed_growth_background	ENSGALG00010015867	PICALML	0.103278918	0.030526196	3.38328821	0.000716234	0.006432352	positive
PRJNA729271	ed_Lushi_vs_Arbor_Acr	breed_growth_background	ENSGALG00010015869	SLC7A2	0.286360278	0.102907418	2.782698104	0.005390894	0.026889543	positive
PRJNA729271	ed_Lushi_vs_Arbor_Acr	breed_growth_background	ENSGALG00010015870	EIF5B	0.160508899	0.083670423	1.918346939	0.055067034	0.142061999	positive
PRJNA729271	ed_Lushi_vs_Arbor_Acr	breed_growth_background	ENSGALG00010015872	TMC1	-0.195801617	0.209569897	-0.934302201	0.350148029	0.514016172	negative
PRJNA729271	ed_Lushi_vs_Arbor_Acr	breed_growth_background	ENSGALG00010015874	CLIC4	-0.276851476	0.061416607	-4.507762481	6.55149E-06	0.000213017	negative
PRJNA729271	ed_Lushi_vs_Arbor_Acr	breed_growth_background	ENSGALG00010015875	DNTTIP1	-0.009263386	0.074935961	-0.123617358	0.901618248	0.943062239	negative
PRJNA729271	ed_Lushi_vs_Arbor_Acr	breed_growth_background	ENSGALG00010015876	SKP1	-0.057281498	0.041581583	-1.377568961	0.16833641	0.309924585	negative
PRJNA729271	ed_Lushi_vs_Arbor_Acr	breed_growth_background	ENSGALG00010015877	REV1	0.01975364	0.056229446	0.351304194	0.725360149	0.824053245	positive
PRJNA729271	ed_Lushi_vs_Arbor_Acr	breed_growth_background	ENSGALG00010015879	TRMT61A	-0.092022244	0.067698051	-1.359304183	0.174050219	0.317860391	negative
PRJNA729271	ed_Lushi_vs_Arbor_Acr	breed_growth_background	ENSGALG00010015881	GAB3	-0.015620644	0.093490355	-0.167082945	0.867304785	0.92058965	negative
PRJNA729271	ed_Lushi_vs_Arbor_Acr	breed_growth_background	ENSGALG00010015886	AFF3	0.072388334	0.114279002	0.633435125	0.526449533	0.672680358	positive
PRJNA729271	ed_Lushi_vs_Arbor_Acr	breed_growth_background	ENSGALG00010015887	SLC9A9	-0.214248328	0.096873217	-2.211636354	0.026991801	0.084265811	negative
PRJNA729271	ed_Lushi_vs_Arbor_Acr	breed_growth_background	ENSGALG00010015889	SH3BP5	0.072457379	0.088180299	0.821695766	0.41125007	0.571543795	positive
PRJNA729271	ed_Lushi_vs_Arbor_Acr	breed_growth_background	ENSGALG00010015892	UBE2C	-0.354619644	0.121734675	-2.913053692	0.003579131	0.019991535	negative
PRJNA729271	ed_Lushi_vs_Arbor_Acr	breed_growth_background	ENSGALG00010015893	TCF7	0.616426639	0.243419165	2.532366915	0.011329537	0.045746661	positive
PRJNA729271	ed_Lushi_vs_Arbor_Acr	breed_growth_background	ENSGALG00010015894	CEP78	-0.058407272	0.066596119	-0.877037173	0.380466448	0.542170887	negative
PRJNA729271	ed_Lushi_vs_Arbor_Acr	breed_growth_background	ENSGALG00010015895	RBMXL1	-0.030572952	0.045840773	-0.666937975	0.504811753	0.654985569	negative
PRJNA729271	ed_Lushi_vs_Arbor_Acr	breed_growth_background	ENSGALG00010015896	LONRF2	0.087643051	0.042142872	2.07966489	0.037556281	0.10762093	positive
PRJNA729271	ed_Lushi_vs_Arbor_Acr	breed_growth_background	ENSGALG00010015898	ENSGALG00010015898	0.164959022	0.116574412	1.415053434	0.15705283	0.294537556	positive
PRJNA729271	ed_Lushi_vs_Arbor_Acr	breed_growth_background	ENSGALG00010015899	SMARCA1	-0.05891427	0.062255546	-0.946329668	0.343980466	0.507790697	negative
PRJNA729271	ed_Lushi_vs_Arbor_Acr	breed_growth_background	ENSGALG00010015900	ENSGALG00010015900	0.347468336	0.086191762	4.031340458	5.54596E-05	0.001045367	positive
PRJNA729271	ed_Lushi_vs_Arbor_Acr	breed_growth_background	ENSGALG00010015902	ENSGALG00010015902	0.241797613	0.259304106	0.932486633	0.351085091	0.515078785	positive
PRJNA729271	ed_Lushi_vs_Arbor_Acr	breed_growth_background	ENSGALG00010015904	MTMR7	0.390229456	0.139326798	2.800821239	0.005097275	0.025979253	positive
PRJNA729271	ed_Lushi_vs_Arbor_Acr	breed_growth_background	ENSGALG00010015905	ANPEP	-0.120392512	0.116220274	-1.035899394	0.30024908	0.462673128	negative
PRJNA729271	ed_Lushi_vs_Arbor_Acr	breed_growth_background	ENSGALG00010015906	BAG5	0.152198765	0.053833274	2.827224743	0.004695336	0.02429199	positive
PRJNA729271	ed_Lushi_vs_Arbor_Acr	breed_growth_background	ENSGALG00010015908	CHRD	-0.00848413	0.073361533	-0.115648217	0.907931347	0.947477673	negative
PRJNA729271	ed_Lushi_vs_Arbor_Acr	breed_growth_background	ENSGALG00010015909	TNNC2	-0.013619144	0.056926281	-0.23924176	0.810918123	0.883681436	negative
PRJNA729271	ed_Lushi_vs_Arbor_Acr	breed_growth_background	ENSGALG00010015910	C5orf15	-0.015406401	0.049907038	-0.30870198	0.757548238	0.846881205	negative
PRJNA729271	ed_Lushi_vs_Arbor_Acr	breed_growth_background	ENSGALG00010015911	TM9SF2L	-0.080481127	0.042009091	-1.915802628	0.055390223	0.142636251	negative
PRJNA729271	ed_Lushi_vs_Arbor_Acr	breed_growth_background	ENSGALG00010015912	ENSGALG00010015912	-0.015725875	0.144947401	-0.108493665	0.913604105	0.95134122	negative
PRJNA729271	ed_Lushi_vs_Arbor_Acr	breed_growth_background	ENSGALG00010015913	CHST10	-0.152317746	0.056556358	-2.693202883	0.007076919	0.032647976	negative
PRJNA729271	ed_Lushi_vs_Arbor_Acr	breed_growth_background	ENSGALG00010015914	SRRM1	0.061976627	0.063288074	0.97927814	0.327442568	0.491333336	positive
PRJNA729271	ed_Lushi_vs_Arbor_Acr	breed_growth_background	ENSGALG00010015916	COA8	0.462136895	0.077382246	5.972130788	2.34175E-09	4.21405E-07	positive
PRJNA729271	ed_Lushi_vs_Arbor_Acr	breed_growth_background	ENSGALG00010015917	TMEM63A	-0.025189866	0.058220026	-0.432666692	0.665256931	0.782957065	negative
PRJNA729271	ed_Lushi_vs_Arbor_Acr	breed_growth_background	ENSGALG00010015918	BICD1	0.149765704	0.105403244	1.420883252	0.155350703	0.29232876	positive
PRJNA729271	ed_Lushi_vs_Arbor_Acr	breed_growth_background	ENSGALG00010015919	ENSGALG00010015919	-0.27658005	0.12340579	-2.241224256	0.025011552	0.080066796	negative
PRJNA729271	ed_Lushi_vs_Arbor_Acr	breed_growth_background	ENSGALG00010015921	SNX21	0.28265582	0.057830466	4.887662832	1.0204E-06	4.96333E-05	positive
PRJNA729271	ed_Lushi_vs_Arbor_Acr	breed_growth_background	ENSGALG00010015923	RCAN3	-0.163054736	0.065436158	-2.491814008	0.012709257	0.04947616	negative
PRJNA729271	ed_Lushi_vs_Arbor_Acr	breed_growth_background	ENSGALG00010015927	GLA	0.035219735	0.089447647	0.393746911	0.693767903	0.8025671	positive
PRJNA729271	ed_Lushi_vs_Arbor_Acr	breed_growth_background	ENSGALG00010015928	CAPN7	0.067372077	0.065771617	1.024333596	0.305677735	0.468279164	positive
PRJNA729271	ed_Lushi_vs_Arbor_Acr	breed_growth_background	ENSGALG00010015929	SLC6A12	-0.866279106	0.516715298	-1.676511435	0.093638058	0.205244769	negative
PRJNA729271	ed_Lushi_vs_Arbor_Acr	breed_growth_background	ENSGALG00010015931	RBM47	-0.252214871	0.149436858	-1.687768827	0.091455614	0.202125223	negative
PRJNA729271	ed_Lushi_vs_Arbor_Acr	breed_growth_background	ENSGALG00010015932	ACOT8	-0.13923293	0.078339757	-1.777295918	0.075519565	0.176983395	negative
PRJNA729271	ed_Lushi_vs_Arbor_Acr	breed_growth_background	ENSGALG00010015933	ENSGALG00010015933	0.14820385	0.264967414	0.55932859	0.575937487	0.713400605	positive
PRJNA729271	ed_Lushi_vs_Arbor_Acr	breed_growth_background	ENSGALG00010015934	TMC2	0.609557988	0.650213585	0.937473474	0.348515062	0.512605361	positive
PRJNA729271	ed_Lushi_vs_Arbor_Acr	breed_growth_background	ENSGALG00010015935	EPHX1	-0.071892475	0.059891323	-1.200382164	0.229990952	0.383803691	negative
PRJNA729271	ed_Lushi_vs_Arbor_Acr	breed_growth_background	ENSGALG00010015936	RPL36AL	-0.034166646	0.074608551	-0.45794544	0.64699164	0.769330912	negative
PRJNA729271	ed_Lushi_vs_Arbor_Acr	breed_growth_background	ENSGALG00010015937	ENSGALG00010015937	0.162583417	0.10242051	1.587410739	0.112419649	0.233723868	positive
PRJNA729271	ed_Lushi_vs_Arbor_Acr	breed_growth_background	ENSGALG00010015939	SUN2	-0.003851312	0.042840596	-0.089898665	0.928367742	0.960231941	negative
PRJNA729271	ed_Lushi_vs_Arbor_Acr	breed_growth_background	ENSGALG00010015942	AP3S2	-0.129377539	0.070488908	-1.835431163	0.06644183	0.162242979	negative
PRJNA729271	ed_Lushi_vs_Arbor_Acr	breed_growth_background	ENSGALG00010015946	TLE4	0.035003987	0.052871767	0.662054415	0.507936349	0.656958809	positive
PRJNA729271	ed_Lushi_vs_Arbor_Acr	breed_growth_background	ENSGALG00010015954	CTSA	0.004634462	0.051725298	0.089597596	0.928606995	0.960410874	positive
PRJNA729271	ed_Lushi_vs_Arbor_Acr	breed_growth_background	ENSGALG00010015957	ENSGALG00010015957	0.061334805	0.114416329	0.536066885	0.591912326	0.726606467	positive
PRJNA729271	ed_Lushi_vs_Arbor_Acr	breed_growth_background	ENSGALG00010015959	TRNAU1AP	0.09479144	0.069276233	1.368311135	0.171214707	0.314027227	positive
PRJNA729271	ed_Lushi_vs_Arbor_Acr	breed_growth_background	ENSGALG00010015960	CHRNA9	-0.877363072	0.48310751	-1.816082452	0.069357725	0.166833758	negative
PRJNA729271	ed_Lushi_vs_Arbor_Acr	breed_growth_background	ENSGALG00010015963	DIPK2A	-0.170618935	0.058454393	-2.918838554	0.003513381	0.019791686	negative
PRJNA729271	ed_Lushi_vs_Arbor_Acr	breed_growth_background	ENSGALG00010015964	KLC1	-0.116013187	0.047269718	-2.454281353	0.014116648	0.053203541	negative
PRJNA729271	ed_Lushi_vs_Arbor_Acr	breed_growth_background	ENSGALG00010015967	ZSWIM1	0.055911922	0.122043236	0.458132087	0.646857547	0.769234568	positive
PRJNA729271	ed_Lushi_vs_Arbor_Acr	breed_growth_background	ENSGALG00010015970	THPO	-0.08652488	0.207926982	-0.416131083	0.677314086	0.791555888	negative
PRJNA729271	ed_Lushi_vs_Arbor_Acr	breed_growth_background	ENSGALG00010015971	ENSGALG00010015971	-0.137804741	0.087266053	-1.579133425	0.114305456	0.236288875	negative
PRJNA729271	ed_Lushi_vs_Arbor_Acr	breed_growth_background	ENSGALG00010015972	FSTL4	-0.063614233	0.166144635	-0.382884665	0.701805282	0.80748274	negative

PRJNA729271	₂ed_Lushi_vs_Arbor_Acr	breed_growth_background	ENSGALG00010015974	VPS37A	0.154736105	0.087927035	1.759823981	0.078437656	0.181419151	positive
PRJNA729271	₂ed_Lushi_vs_Arbor_Acr	breed_growth_background	ENSGALG00010015975	BTK	-0.259654079	0.093778949	-2.768788545	0.005626514	0.027697901	negative
PRJNA729271	₂ed_Lushi_vs_Arbor_Acr	breed_growth_background	ENSGALG00010015979	ENSGALG00010015979	-0.265667573	0.105611231	-2.515523875	0.011885563	0.047238706	negative
PRJNA729271	₂ed_Lushi_vs_Arbor_Acr	breed_growth_background	ENSGALG00010015980	EXOSC7	0.056102051	0.051302767	1.09354824	0.274153161	0.433066223	positive
PRJNA729271	₂ed_Lushi_vs_Arbor_Acr	breed_growth_background	ENSGALG00010015983	CNOT7	-0.00231452	0.035034135	-0.066064722	0.947326297	0.971041275	negative
PRJNA729271	₂ed_Lushi_vs_Arbor_Acr	breed_growth_background	ENSGALG00010015984	PDCL3	0.069921976	0.057693412	1.211957733	0.225528561	0.378535953	positive
PRJNA729271	₂ed_Lushi_vs_Arbor_Acr	breed_growth_background	ENSGALG00010015985	TIMM8A	-0.040124123	0.059993906	-0.66880331	0.503620955	0.654296472	negative
PRJNA729271	₂ed_Lushi_vs_Arbor_Acr	breed_growth_background	ENSGALG00010015986	NEURL2	0.194796208	0.117072336	1.663896143	0.096133186	0.209007129	positive
PRJNA729271	₂ed_Lushi_vs_Arbor_Acr	breed_growth_background	ENSGALG00010015987	POLR2H	0.04165603	0.05262209	0.791607277	0.428589703	0.587628428	positive
PRJNA729271	₂ed_Lushi_vs_Arbor_Acr	breed_growth_background	ENSGALG00010015989	ENAH	-0.245420739	0.087379374	-2.808680454	0.004974499	0.025487936	negative
PRJNA729271	₂ed_Lushi_vs_Arbor_Acr	breed_growth_background	ENSGALG00010015992	ATP10A	0.063596986	0.08558065	0.743123433	0.457406954	0.613218072	positive
PRJNA729271	₂ed_Lushi_vs_Arbor_Acr	breed_growth_background	ENSGALG00010015994	ENSGALG00010015994	-0.090289602	0.059759861	-1.510873699	0.130820633	0.258943749	negative
PRJNA729271	₂ed_Lushi_vs_Arbor_Acr	breed_growth_background	ENSGALG00010016004	XRCC3	0.063555872	0.092596926	0.686371298	0.492478999	0.644383742	positive
PRJNA729271	₂ed_Lushi_vs_Arbor_Acr	breed_growth_background	ENSGALG00010016006	PLTP	-0.384965094	0.172259537	-2.23479698	0.02543068	0.0809615	negative
PRJNA729271	₂ed_Lushi_vs_Arbor_Acr	breed_growth_background	ENSGALG00010016008	TAF7	-0.063364249	0.052114331	-1.215869962	0.224034462	0.376769872	negative
PRJNA729271	₂ed_Lushi_vs_Arbor_Acr	breed_growth_background	ENSGALG00010016011	ADD1	-0.015586495	0.043396921	-0.359161304	0.719474423	0.820324216	negative
PRJNA729271	₂ed_Lushi_vs_Arbor_Acr	breed_growth_background	ENSGALG00010016015	ZDHHC3	-0.03890385	0.044560459	-0.873057667	0.382631646	0.544069621	negative
PRJNA729271	₂ed_Lushi_vs_Arbor_Acr	breed_growth_background	ENSGALG00010016016	ZDHHC2	-0.099031835	0.149073622	-0.664314947	0.506488756	0.655905881	negative
PRJNA729271	₂ed_Lushi_vs_Arbor_Acr	breed_growth_background	ENSGALG00010016017	RAPGEF6	0.007393457	0.038332082	0.192879093	0.847053659	0.90740099	positive
PRJNA729271	₂ed_Lushi_vs_Arbor_Acr	breed_growth_background	ENSGALG00010016022	EPHB3	0.012924532	0.090710322	0.142481385	0.886699782	0.933419695	positive
PRJNA729271	₂ed_Lushi_vs_Arbor_Acr	breed_growth_background	ENSGALG00010016025	PCIF1	0.217793169	0.057862389	3.763985074	0.000167227	0.002310728	positive
PRJNA729271	₂ed_Lushi_vs_Arbor_Acr	breed_growth_background	ENSGALG00010016027	COPS2	-0.006505807	0.080291696	-0.081027147	0.935420363	0.964536447	negative
PRJNA729271	₂ed_Lushi_vs_Arbor_Acr	breed_growth_background	ENSGALG00010016030	TGM4	1.250920682	0.388800187	3.21738704	0.00129364	0.009827728	positive
PRJNA729271	₂ed_Lushi_vs_Arbor_Acr	breed_growth_background	ENSGALG00010016040	RPSA2	-0.033230325	0.056627034	-0.586827935	0.557319273	0.698663836	negative
PRJNA729271	₂ed_Lushi_vs_Arbor_Acr	breed_growth_background	ENSGALG00010016041	ENSGALG00010016041	0.072221413	0.049328674	1.464085832	0.143170498	0.276295615	positive
PRJNA729271	₂ed_Lushi_vs_Arbor_Acr	breed_growth_background	ENSGALG00010016043	ADAMTSL1	-0.098479612	0.103095455	-0.955227489	0.339462667	0.503613951	negative
PRJNA729271	₂ed_Lushi_vs_Arbor_Acr	breed_growth_background	ENSGALG00010016047	SRP9	0.017003605	0.047840543	0.355422504	0.722273092	0.821834404	positive
PRJNA729271	₂ed_Lushi_vs_Arbor_Acr	breed_growth_background	ENSGALG00010016049	ZFYVE21	-0.001702773	0.065592437	-0.025959904	0.97928932	0.987739106	negative
PRJNA729271	₂ed_Lushi_vs_Arbor_Acr	breed_growth_background	ENSGALG00010016053	DRP2	1.281451681	0.250947985	5.106443387	3.28279E-07	2.0591E-05	positive
PRJNA729271	₂ed_Lushi_vs_Arbor_Acr	breed_growth_background	ENSGALG00010016057	SOHO1	0.536293079	0.37934876	1.413720396	0.157444014	0.295160536	positive
PRJNA729271	₂ed_Lushi_vs_Arbor_Acr	breed_growth_background	ENSGALG00010016059	EPDR1	-0.077114724	0.107933124	-0.714467639	0.47493806	0.628409601	negative
PRJNA729271	₂ed_Lushi_vs_Arbor_Acr	breed_growth_background	ENSGALG00010016060	ENSGALG00010016060	-0.175383857	0.730355522	-0.24013491	0.810225672	0.883156949	negative
PRJNA729271	₂ed_Lushi_vs_Arbor_Acr	breed_growth_background	ENSGALG00010016066	C14orf2	-0.059093734	0.079912104	-0.739484151	0.45961306	0.61486768	negative
PRJNA729271	₂ed_Lushi_vs_Arbor_Acr	breed_growth_background	ENSGALG00010016067	AHSG	-1.287540858	0.440677261	-2.921732004	0.003480909	0.019670869	negative
PRJNA729271	₂ed_Lushi_vs_Arbor_Acr	breed_growth_background	ENSGALG00010016069	GALK2	0.015413027	0.077587562	0.198653319	0.842533942	0.904698829	positive
PRJNA729271	₂ed_Lushi_vs_Arbor_Acr	breed_growth_background	ENSGALG00010016073	SAXO1	-0.672510692	0.448253662	-1.500290461	0.133539179	0.263067465	negative
PRJNA729271	₂ed_Lushi_vs_Arbor_Acr	breed_growth_background	ENSGALG00010016076	RAB3GAP2	0.009176871	0.048712473	0.188388528	0.850572092	0.910161116	positive
PRJNA729271	₂ed_Lushi_vs_Arbor_Acr	breed_growth_background	ENSGALG00010016083	CDH22	-0.76615114	0.242226954	-3.162947501	0.001561805	0.011179438	negative
PRJNA729271	₂ed_Lushi_vs_Arbor_Acr	breed_growth_background	ENSGALG00010016086	TDRD9	0.148142106	0.193599929	0.76519711	0.44415418	0.601009601	positive
PRJNA729271	₂ed_Lushi_vs_Arbor_Acr	breed_growth_background	ENSGALG00010016087	IARS2	0.033099237	0.048397036	0.68391043	0.494031728	0.645715951	positive
PRJNA729271	₂ed_Lushi_vs_Arbor_Acr	breed_growth_background	ENSGALG00010016091	HMX1	0.5322389	0.478899777	1.111378467	0.266405477	0.424672538	positive
PRJNA729271	₂ed_Lushi_vs_Arbor_Acr	breed_growth_background	ENSGALG00010016094	FGF7	0.15778239	0.099167563	1.591068552	0.111594149	0.232560026	positive
PRJNA729271	₂ed_Lushi_vs_Arbor_Acr	breed_growth_background	ENSGALG00010016097	FNIP1	0.142884587	0.058892711	2.426184591	0.015258507	0.056378297	positive
PRJNA729271	₂ed_Lushi_vs_Arbor_Acr	breed_growth_background	ENSGALG00010016100	SFRP4	-0.870202672	0.234715035	-3.707485853	0.000209327	0.002685348	negative
PRJNA729271	₂ed_Lushi_vs_Arbor_Acr	breed_growth_background	ENSGALG00010016103	BPNT1	0.119208435	0.050415068	2.364539802	0.018052487	0.063629859	positive
PRJNA729271	₂ed_Lushi_vs_Arbor_Acr	breed_growth_background	ENSGALG00010016107	UVSSA	0.040482299	0.050198134	0.806450271	0.41998327	0.579310552	positive
PRJNA729271	₂ed_Lushi_vs_Arbor_Acr	breed_growth_background	ENSGALG00010016109	MLH1	-0.000750574	0.055318498	-0.013568234	0.989174448	0.993582596	negative
PRJNA729271	₂ed_Lushi_vs_Arbor_Acr	breed_growth_background	ENSGALG00010016111	EPB41	0.087864139	0.055046299	1.596186127	0.110447244	0.231081525	positive
PRJNA729271	₂ed_Lushi_vs_Arbor_Acr	breed_growth_background	ENSGALG00010016113	DTWD1	0.120485169	0.079798289	1.509871583	0.131076195	0.259201834	positive
PRJNA729271	₂ed_Lushi_vs_Arbor_Acr	breed_growth_background	ENSGALG00010016114	HAUS6	-0.167883846	0.081524405	-2.059307849	0.039464757	0.111383448	negative
PRJNA729271	₂ed_Lushi_vs_Arbor_Acr	breed_growth_background	ENSGALG00010016115	RD3L	-0.584472211	0.253136189	-2.30892396	0.020947801	0.070400737	negative
PRJNA729271	₂ed_Lushi_vs_Arbor_Acr	breed_growth_background	ENSGALG00010016117	ENSGALG00010016117	0.071211879	0.128752812	0.553089891	0.580201859	0.716871198	positive
PRJNA729271	₂ed_Lushi_vs_Arbor_Acr	breed_growth_background	ENSGALG00010016118	EPRS	0.038406127	0.046844772	0.819859407	0.41229626	0.572545918	positive
PRJNA729271	₂ed_Lushi_vs_Arbor_Acr	breed_growth_background	ENSGALG00010016119	FETUB	-1.795522836	0.762549951	-2.354629797			negative
PRJNA729271	₂ed_Lushi_vs_Arbor_Acr	breed_growth_background	ENSGALG00010016121	SLC35C2	-0.048710024	0.054739845	-0.889845851	0.373548663	0.535938619	negative
PRJNA729271	₂ed_Lushi_vs_Arbor_Acr	breed_growth_background	ENSGALG00010016122	AFG2B	-0.098418333	0.071606453	-1.37443385	0.169307033	0.311421529	negative
PRJNA729271	₂ed_Lushi_vs_Arbor_Acr	breed_growth_background	ENSGALG00010016127	ENSGALG00010016127	0.21282195	0.093874878	2.267080972	0.02338528	0.076340137	positive
PRJNA729271	₂ed_Lushi_vs_Arbor_Acr	breed_growth_background	ENSGALG00010016129	CENPI	-0.495384992	0.128645485	-3.850776342	0.000117744	0.001809335	negative
PRJNA729271	₂ed_Lushi_vs_Arbor_Acr	breed_growth_background	ENSGALG00010016131	SMYD1	-0.272508489	0.116320635	-2.342735575	0.019142945	0.066350307	negative
PRJNA729271	₂ed_Lushi_vs_Arbor_Acr	breed_growth_background	ENSGALG00010016137	STARD3NL	-0.043557741	0.075925822	-0.5736881	0.566178882	0.705716947	negative
PRJNA729271	₂ed_Lushi_vs_Arbor_Acr	breed_growth_background	ENSGALG00010016139	LURAP1L	0.23156503	0.10622291	2.179991389	0.0292581	0.089264609	positive
PRJNA729271	₂ed_Lushi_vs_Arbor_Acr	breed_growth_background	ENSGALG00010016141	RRP15	0.038360374	0.096364602	0.398075364	0.690574633	0.799958388	positive
PRJNA729271	₂ed_Lushi_vs_Arbor_Acr	breed_growth_background	ENSGALG00010016147	FABP1	-1.152922127	0.42352996	-2.722173724	0.006485404	0.030687422	negative
PRJNA729271	₂ed_Lushi_vs_Arbor_Acr	breed_growth_background	ENSGALG00010016150	POLG	0.162273012	0.051522794	3.149538274	0.001635287	0.011520759	positive
PRJNA729271	₂ed_Lushi_vs_Arbor_Acr	breed_growth_background	ENSGALG00010016159	ASPG	-0.129480288	0.107007815	-1.210007776	0.226275909	0.379438836	negative
PRJNA729271	₂ed_Lushi_vs_Arbor_Acr	breed_growth_background	ENSGALG00010016165	KK34	0.9598209	0.454073613	2.113800214	0.034532335	0.100880899	positive
PRJNA729271	₂ed_Lushi_vs_Arbor_Acr	breed_growth_background	ENSGALG00010016168	PLIN2	-0.04724442	0.122595491	-0.38536833	0.699964543	0.806517174	negative
PRJNA729271	₂ed_Lushi_vs_Arbor_Acr	breed_growth_background	ENSGALG00010016169	RHOH	0.033409241	0.066488897	0.502478499	0.615330973	0.744136697	positive
PRJNA729271	₂ed_Lushi_vs_Arbor_Acr	breed_growth_background	ENSGALG00010016173	ENSGALG00010016173	-0.315376617	0.199342696	-1.582082633	0.113630708	0.235330349	negative
PRJNA729271	₂ed_Lushi_vs_Arbor_Acr	breed_growth_background	ENSGALG00010016177	ENSGALG00010016177	-1.78630239	0.837596211	-2.132653379	0.032953171	0.097448847	negative
PRJNA729271	₂ed_Lushi_vs_Arbor_Acr	breed_growth_background	ENSGALG00010016181	FANCI	-0.200388928	0.09731047	-2.059274075	0.039467991	0.111383448	negative
PRJNA729271	₂ed_Lushi_vs_Arbor_Acr	breed_growth_background	ENSGALG00010016182	ELMO2	-0.009260542	0.033682537	-0.274935992	0.783365415	0.865763995	negative
PRJNA729271	₂ed_Lushi_vs_Arbor_Acr	breed_growth_background	ENSGALG00010016190	THNSL2	0.105439546	0.082275154	1.281547834	0.20000131	0.348847618	positive
PRJNA729271	₂ed_Lushi_vs_Arbor_Acr	breed_growth_background	ENSGALG00010016193	TCAIM	0.26668851	0.072738458	3.666403147	0.000245986	0.003008918	positive
PRJNA729271	₂ed_Lushi_vs_Arbor_Acr	breed_growth_background	ENSGALG00010016197	SNRPN	-0.328245192	0.062977504	-5.212102276	1.86713E-07	1.36687E-05	negative
PRJNA729271	₂ed_Lushi_vs_Arbor_Acr	breed_growth_background	ENSGALG00010016199	RLBP1	0.466705033	0.262720094	1.776434475	0.075661333	0.177253275	positive
PRJNA729271	₂ed_Lushi_vs_Arbor_Acr	breed_growth_background	ENSGALG00010016200	MICU3	-0.023105904	0.062901047	-0.367337343	0.713367403	0.815844447	negative
PRJNA729271	₂ed_Lushi_vs_Arbor_Acr	breed_growth_background	ENSGALG00010016205	CD40LG	0.355763569	0.419720077	0.847621042	0.396649052	0.557926056	positive
PRJNA729271	₂ed_Lushi_vs_Arbor_Acr	breed_growth_background	ENSGALG00010016206	KNG1	-1.894437084	0.620705987	-3.052068327	0.002272704	0.014589388	negative
PRJNA729271	₂ed_Lushi_vs_Arbor_Acr	breed_growth_background	ENSGALG00010016209	ADAMTS7	-0.443976497	0.089500046	-4.960628708	7.02654E-07	3.65593E-05	negative
PRJNA729271	₂ed_Lushi_vs_Arbor_Acr	breed_growth_background	ENSGALG00010016211	TACC3	-0.337156856	0.086808283	-3.883924944	0.000102784	0.001617643	negative
PRJNA729271	₂ed_Lushi_vs_Arbor_Acr	breed_growth_background	ENSGALG00010016215	SPATA17	-0.444455167	0.210026409	-2.116187054	0.034328902	0.100463847	negative
PRJNA729271	₂ed_Lushi_vs_Arbor_Acr	breed_growth_background	ENSGALG00010016218	N4BP2	0.077171292	0.040638698	1.89896075	0.057569637	0.146522531	positive
PRJNA729271	₂ed_Lushi_vs_Arbor_Acr	breed_growth_background	ENSGALG00010016221	TMEM200B	-0.24696071	0.108444029	-2.277310352	0.022767699	0.074901906	negative
PRJNA729271	₂ed_Lushi_vs_Arbor_Acr	breed_growth_background	ENSGALG00010016224	MOSPD1	0.153791009	0.061658818	2.494225695	0.012623225	0.049212923	positive
PRJNA729271	₂ed_Lushi_vs_Arbor_Acr	breed_growth_background	ENSGALG00010016225	P4HA2	0.201141875	0.08351905	2.408335283	0.016025456	0.058376754	positive
PRJNA729271	₂ed_Lushi_vs_Arbor_Acr	breed_growth_background	ENSGALG00010016229	ENSGALG00010016229	-0.001088141	0.119050675	-0.009140149	0.992707318	0.995798794	negative
PRJNA729271	₂ed_Lushi_vs_Arbor_Acr	breed_growth_background	ENSGALG00010016235	DDX27	0.169620165	0.066583158	2.547493535	0.010849986	0.044351538	positive
PRJNA729271	₂ed_Lushi_vs_Arbor_Acr	breed_growth_background	ENSGALG00010016236	GPATCH2	-0.045958351	0.036109345	-1.272755064	0.203104971	0.352280311	negative
PRJNA729271	₂ed_Lushi_vs_Arbor_Acr	breed_growth_background	ENSGALG00010016240	EIF4A2	0.034853848	0.053628945	0.649907393	0.515752042	0.664045644	positive
PRJNA729271	₂ed_Lushi_vs_Arbor_Acr	breed_growth_background	ENSGALG00010016241	TOPAZ1	0.537574345	0.441613646	1.217295594	0.22349177	0.37611903	positive
PRJNA729271	₂ed_Lushi_vs_Arbor_Acr	breed_growth_background	ENSGALG00010016242	PDS5A	0.212062311	0.049458739	4.287661097	1.80564E-05	0.000442985	positive
PRJNA729271	₂ed_Lushi_vs_Arbor_Acr	breed_growth_background	ENSGALG00010016245	FAM96A	-0.007234483	0.057376481	-0.126087952	0.899662304	0.941763927	negative
PRJNA729271	₂ed_Lushi_vs_Arbor_Acr	breed_growth_background	ENSGALG00010016247	KIF26A	-0.195646607	0.090097578	-2.17149685	0.029893635	0.090669228	negative
PRJNA729271	₂ed_Lushi_vs_Arbor_Acr	breed_growth_background	ENSGALG00010016251	MPDZ	0.034373285	0.039387415	0.872697151	0.382828171	0.544295669	positive
PRJNA729271	₂ed_Lushi_vs_Arbor_Acr	breed_growth_background	ENSGALG00010016253	EDN3	-0.222632821	0.113469219	-1.962054763	0.049756109	0.132042255	negative
PRJNA729271	₂ed_Lushi_vs_Arbor_Acr	breed_growth_background	ENSGALG00010016257	ENSGALG00010016257	-0.097801088	0.061635057	-1.586776944	0.112563172	0.233955146	negative
PRJNA729271	₂ed_Lushi_vs_Arbor_Acr	breed_growth_background	ENSGALG00010016258	CTNNB1	-0.183204664	0.04139912	-4.425327497	9.62961E-06	0.000283702	negative
PRJNA729271	₂ed_Lushi_vs_Arbor_Acr	breed_growth_background	ENSGALG00010016260	INTS6L	0.184829814	0.077276838	2.391787998	0.016766524	0.060260542	positive
PRJNA729271	₂ed_Lushi_vs_Arbor_Acr	breed_growth_background	ENSGALG00010016262	SPG11	0.020387028	0.032686216	0.62371941	0.532811874	0.678239054	positive
PRJNA729271	₂ed_Lushi_vs_Arbor_Acr	breed_growth_background	ENSGALG00010016263	HGFAC	-1.771376688	0.737556956	-2.401681218			negative
PRJNA729271	₂ed_Lushi_vs_Arbor_Acr	breed_growth_background	ENSGALG00010016266	PDLIM4	-0.054703403	0.064978458	-0.841869831	0.399860819	0.560726886	negative
PRJNA729271	₂ed_Lushi_vs_Arbor_Acr	breed_growth_background	ENSGALG00010016270	RFC4	-0.238574748	0.06072399	-3.928838455	8.53572E-05	0.001425405	negative
PRJNA729271	₂ed_Lushi_vs_Arbor_Acr	breed_growth_background	ENSGALG00010016274	ZNF831	0.055494503	0.274254437	0.202346782	0.839645629	0.902661985	positive
PRJNA729271	₂ed_Lushi_vs_Arbor_Acr	breed_growth_background	ENSGALG00010016276	ACTR2	-0.120132154	0.057956145	-2.072811334	0.038189841	0.108883113	negative
PRJNA729271	₂ed_Lushi_vs_Arbor_Acr	breed_growth_background	ENSGALG00010016279	PRELID3B	0.138211831	0.057178768	2.417187996	0.015640937	0.057442543	positive
PRJNA729271	₂ed_Lushi_vs_Arbor_Acr	breed_growth_background	ENSGALG00010016281	SLC22A4	-0.190125324	0.122100282	-1.557124356	0.119440963	0.243604441	negative
PRJNA729271	₂ed_Lushi_vs_Arbor_Acr	breed_growth_background	ENSGALG00010016286	ULK4	-0.144012559	0.078372062	-1.837549694	0.066128787	0.161641952	negative
PRJNA729271	₂ed_Lushi_vs_Arbor_Acr	breed_growth_background	ENSGALG00010016287	BCL11B	0.392358927	0.200510267	1.95680218	0.050370731	0.133209951	positive
PRJNA729271	₂ed_Lushi_vs_Arbor_Acr	breed_growth_background	ENSGALG00010016289	IRF1	0.269540312	0.136224232	1.978651733	0.047855231	0.128313276	positive
PRJNA729271	₂ed_Lushi_vs_Arbor_Acr	breed_growth_background	ENSGALG00010016292	PHKA1	0.237874432	0.079146467	3.005496548	0.002651476	0.016209678	positive
PRJNA729271	₂ed_Lushi_vs_Arbor_Acr	breed_growth_background	ENSGALG00010016295	UBE2K	0.123217161	0.050327817	2.448291393	0.014353552	0.053747026	positive
PRJNA729271	₂ed_Lushi_vs_Arbor_Acr	breed_growth_background	ENSGALG00010016299	SRSF4	0.017979135	0.059568304	0.301823849	0.762786349	0.850927178	positive
PRJNA729271	₂ed_Lushi_vs_Arbor_Acr	breed_growth_background	ENSGALG00010016303	ENSGALG00010016303	-0.154669165	0.108727323	-1.422541824	0.154869019	0.29164953	negative
PRJNA729271	₂ed_Lushi_vs_Arbor_Acr	breed_growth_background	ENSGALG00010016304	EIF3J	0.14287374	0.053368461	2.677119362	0.007425818	0.033763245	positive
PRJNA729271	₂ed_Lushi_vs_Arbor_Acr	breed_growth_background	ENSGALG00010016305	DENND4C	-0.110991865	0.051463484	-2.156711038	0.031028186	0.093290513	negative
PRJNA729271	₂ed_Lushi_vs_Arbor_Acr	breed_growth_background	ENSGALG00010016307	RAD50	0.193282922	0.069282926	2.789762712	0.005274668	0.026482964	positive
PRJNA729271	₂ed_Lushi_vs_Arbor_Acr	breed_growth_background	ENSGALG00010016308	RPS6	-0.160634832	0.075224755	-2.135398541	0.03272847	0.097113443	negative
PRJNA729271	₂ed_Lushi_vs_Arbor_Acr	breed_growth_background	ENSGALG00010016310	HDAC8	0.084627282	0.050265945	1.683590794	0.092260779	0.203211116	positive
PRJNA729271	₂ed_Lushi_vs_Arbor_Acr	breed_growth_background	ENSGALG00010016312	MECR	-0.14927836	0.08166928	-1.827839804	0.067573598	0.164066884	negative
PRJNA729271	₂ed_Lushi_vs_Arbor_Acr	breed_growth_background	ENSGALG00010016314	ACER2	0.116360876	0.085527096	1.360514744	0.17366709	0.317322944	positive
PRJNA729271	₂ed_Lushi_vs_Arbor_Acr	breed_growth_background	ENSGALG00010016315	RPS4Y1	-0.100768172	0.072933416	-1.38164613	0.167080383	0.307964159	negative
PRJNA729271	₂ed_Lushi_vs_Arbor_Acr	breed_growth_background	ENSGALG00010016317	CTDSPL2	0.062625646	0.030893275	2.027161144	0.042645935	0.118020874	positive
PRJNA729271	₂ed_Lushi_vs_Arbor_Acr	breed_growth_background	ENSGALG00010016319	RBM41	0.347871863	0.100655652	3.456058921	0.000548135	0.005303888	positive
PRJNA729271	₂ed_Lushi_vs_Arbor_Acr	breed_growth_background	ENSGALG00010016322	EBF4	-0.379829421	0.07242442	-5.244493777	1.56712E-07	1.22786E-05	negative
PRJNA729271	₂ed_Lushi_vs_Arbor_Acr	breed_growth_background	ENSGALG00010016324	TUBB1	0.028440134	0.115168628	0.246943416	0.804952016	0.879790338	positive
PRJNA729271	₂ed_Lushi_vs_Arbor_Acr	breed_growth_background	ENSGALG00010016328	USH2A	-0.728924142	0.335743711	-2.171073107	0.029925647	0.090691303	negative
PRJNA729271	₂ed_Lushi_vs_Arbor_Acr	breed_growth_background	ENSGALG00010016332	ENSGALG00010016332	0.277821984	0.132027571	2.104272482	0.035354685	0.102760409	positive
PRJNA729271	₂ed_Lushi_vs_Arbor_Acr	breed_growth_background	ENSGALG00010016335	ABHD4	0.264734578	0.097612601	2.712094293	0.006685957	0.031363414	positive
PRJNA729271	₂ed_Lushi_vs_Arbor_Acr	breed_growth_background	ENSGALG00010016336	SLC24A2	-0.96160996	0.20216532	-4.756552514	1.96927E-06	8.34637E-05	negative
PRJNA729271	₂ed_Lushi_vs_Arbor_Acr	breed_growth_background	ENSGALG00010016338	PTPRU	-0.131475607	0.081711486	-1.609022339	0.107611457	0.226851086	negative

PRJNA729271	ed_Lushi_vs_Arbor_Acr	breed_growth_background	ENSGALG00010016339	SETD3	-0.203920446	0.065089572	-3.13292037	0.001730764	0.012009298	negative
PRJNA729271	ed_Lushi_vs_Arbor_Acr	breed_growth_background	ENSGALG00010016340	RGS12	0.049483451	0.055854303	0.885938034	0.375650919	0.538102587	positive
PRJNA729271	ed_Lushi_vs_Arbor_Acr	breed_growth_background	ENSGALG00010016341	SLX4	-0.017781094	0.035433638	-0.501813969	0.615798386	0.744391427	negative
PRJNA729271	ed_Lushi_vs_Arbor_Acr	breed_growth_background	ENSGALG00010016344	SMIM14	-0.076745032	0.060054786	-1.277917012	0.201278689	0.350180486	negative
PRJNA729271	ed_Lushi_vs_Arbor_Acr	breed_growth_background	ENSGALG00010016345	CTSZ	0.154922654	0.059529122	2.602468298	0.009255537	0.039598292	positive
PRJNA729271	ed_Lushi_vs_Arbor_Acr	breed_growth_background	ENSGALG00010016346	TRAP1	-0.144046911	0.055853305	-2.579022135	0.009908044	0.041519831	negative
PRJNA729271	ed_Lushi_vs_Arbor_Acr	breed_growth_background	ENSGALG00010016347	ANO10	-0.103479792	0.048686394	-2.125435547	0.033550287	0.098723388	negative
PRJNA729271	ed_Lushi_vs_Arbor_Acr	breed_growth_background	ENSGALG00010016350	CASC4	0.145584751	0.033738324	4.315115046	1.5952E-05	0.000407084	positive
PRJNA729271	ed_Lushi_vs_Arbor_Acr	breed_growth_background	ENSGALG00010016353	C1orf232	0.235556432	0.078368133	3.005768076	0.00264911	0.016202044	positive
PRJNA729271	ed_Lushi_vs_Arbor_Acr	breed_growth_background	ENSGALG00010016355	MCF2L2	-0.324326075	0.122739608	-2.642391329	0.008232288	0.036321486	negative
PRJNA729271	ed_Lushi_vs_Arbor_Acr	breed_growth_background	ENSGALG00010016361	NELFCD	-0.026507479	0.045324151	-0.584842261	0.558653785	0.699825997	negative
PRJNA729271	ed_Lushi_vs_Arbor_Acr	breed_growth_background	ENSGALG00010016362	NUP62	0.223981395	0.091268916	2.454081889	0.014124481	0.053215572	positive
PRJNA729271	ed_Lushi_vs_Arbor_Acr	breed_growth_background	ENSGALG00010016366	KIF3A	0.014971698	0.053193874	0.281455297	0.77836121	0.862608136	positive
PRJNA729271	ed_Lushi_vs_Arbor_Acr	breed_growth_background	ENSGALG00010016368	CCNK	-0.06657099	0.081690058	-0.814921568	0.415117207	0.575360863	negative
PRJNA729271	ed_Lushi_vs_Arbor_Acr	breed_growth_background	ENSGALG00010016369	MSANTD1	0.218806001	0.17825358	1.227498491	0.219635289	0.372134834	positive
PRJNA729271	ed_Lushi_vs_Arbor_Acr	breed_growth_background	ENSGALG00010016370	TMEM255A	0.393511987	0.09849428	3.995277553	6.46185E-05	0.001154885	positive
PRJNA729271	ed_Lushi_vs_Arbor_Acr	breed_growth_background	ENSGALG00010016372	DNAAF6	-0.380806903	0.109107477	-3.490199892	0.000482659	0.004844978	negative
PRJNA729271	ed_Lushi_vs_Arbor_Acr	breed_growth_background	ENSGALG00010016373	SRL	-0.138565783	0.07027778	-1.971686957	0.048645353	0.130022939	negative
PRJNA729271	ed_Lushi_vs_Arbor_Acr	breed_growth_background	ENSGALG00010016374	FRMD5	0.561518265	0.17283455	3.248877415	0.001158614	0.009087722	positive
PRJNA729271	ed_Lushi_vs_Arbor_Acr	breed_growth_background	ENSGALG00010016375	CCNI2	-0.012036113	0.077528106	-0.155248384	0.876625506	0.926886476	negative
PRJNA729271	ed_Lushi_vs_Arbor_Acr	breed_growth_background	ENSGALG00010016377	FRMPD3	-0.107482511	0.144562274	-0.743503184	0.457177095	0.61302331	negative
PRJNA729271	ed_Lushi_vs_Arbor_Acr	breed_growth_background	ENSGALG00010016378	WDR76	0.34886333	0.09338447	3.735774597	0.000187138	0.002468215	positive
PRJNA729271	ed_Lushi_vs_Arbor_Acr	breed_growth_background	ENSGALG00010016379	TFAP4	0.213520372	0.084331562	2.531915292	0.011344139	0.045756154	positive
PRJNA729271	ed_Lushi_vs_Arbor_Acr	breed_growth_background	ENSGALG00010016382	UGDH	-0.267377556	0.099610393	-2.684233524	0.007269631	0.033314354	negative
PRJNA729271	ed_Lushi_vs_Arbor_Acr	breed_growth_background	ENSGALG00010016383	FAM110D	-0.19791741	0.093608787	-2.114303748	0.034489332	0.10083156	negative
PRJNA729271	ed_Lushi_vs_Arbor_Acr	breed_growth_background	ENSGALG00010016384	GNAS	-0.003577417	0.040078153	-0.089261035	0.92887446	0.960550425	negative
PRJNA729271	ed_Lushi_vs_Arbor_Acr	breed_growth_background	ENSGALG00010016385	HTT	0.048308047	0.057165613	0.845054298	0.398080523	0.559342496	positive
PRJNA729271	ed_Lushi_vs_Arbor_Acr	breed_growth_background	ENSGALG00010016387	CCDC85C	-0.032858074	0.088051842	-0.373167372	0.709023879	0.81249332	negative
PRJNA729271	ed_Lushi_vs_Arbor_Acr	breed_growth_background	ENSGALG00010016390	ENSGALG00010016390	0.096595971	0.157826568	0.612038726	0.540512136	0.685392652	positive
PRJNA729271	ed_Lushi_vs_Arbor_Acr	breed_growth_background	ENSGALG00010016392	STMN3	0.407093807	0.172187757	2.364243629	0.018066927	0.063655836	positive
PRJNA729271	ed_Lushi_vs_Arbor_Acr	breed_growth_background	ENSGALG00010016393	BLM	-0.132445279	0.068824434	-1.924393295	0.054305304	0.140513278	negative
PRJNA729271	ed_Lushi_vs_Arbor_Acr	breed_growth_background	ENSGALG00010016394	PDIK1L	0.060552824	0.05169075	1.171444102	0.241420314	0.397206294	positive
PRJNA729271	ed_Lushi_vs_Arbor_Acr	breed_growth_background	ENSGALG00010016395	ENSGALG00010016395	-0.472697481	0.088145502	-5.362695431	8.19892E-08	7.11637E-06	negative
PRJNA729271	ed_Lushi_vs_Arbor_Acr	breed_growth_background	ENSGALG00010016397	LIAS	0.062774826	0.05596095	1.121761275	0.261963956	0.420133607	positive
PRJNA729271	ed_Lushi_vs_Arbor_Acr	breed_growth_background	ENSGALG00010016398	SEPTIN6	0.008289731	0.037133628	0.223240522	0.823348315	0.891960675	positive
PRJNA729271	ed_Lushi_vs_Arbor_Acr	breed_growth_background	ENSGALG00010016399	ACSM5	-0.048521632	0.129424342	-0.374903444	0.707732278	0.811711832	negative
PRJNA729271	ed_Lushi_vs_Arbor_Acr	breed_growth_background	ENSGALG00010016400	ENSGALG00010016400	0.118206111	0.046365015	2.549467741	0.010788749	0.044175966	positive
PRJNA729271	ed_Lushi_vs_Arbor_Acr	breed_growth_background	ENSGALG00010016402	SEPTIN8	-0.096129402	0.04231499	-2.271757655	0.023101152	0.075636477	negative
PRJNA729271	ed_Lushi_vs_Arbor_Acr	breed_growth_background	ENSGALG00010016403	GRK4	0.031908466	0.073266105	0.435514704	0.663188869	0.781503479	positive
PRJNA729271	ed_Lushi_vs_Arbor_Acr	breed_growth_background	ENSGALG00010016404	ESRRG	-0.059136394	0.079925049	-0.739898127	0.459361811	0.614802205	negative
PRJNA729271	ed_Lushi_vs_Arbor_Acr	breed_growth_background	ENSGALG00010016407	RPL9	-0.236061657	0.096608052	-2.443498772	0.014545619	0.054214129	negative
PRJNA729271	ed_Lushi_vs_Arbor_Acr	breed_growth_background	ENSGALG00010016409	GMEB2	0.256035987	0.110072471	2.326067409	0.020014955	0.068310989	positive
PRJNA729271	ed_Lushi_vs_Arbor_Acr	breed_growth_background	ENSGALG00010016410	TRAK1	-0.003366956	0.051823161	-0.064970108	0.9481978	0.971621434	negative
PRJNA729271	ed_Lushi_vs_Arbor_Acr	breed_growth_background	ENSGALG00010016411	CTSH	0.00822002	0.066209462	0.124151742	0.901195131	0.942711173	positive
PRJNA729271	ed_Lushi_vs_Arbor_Acr	breed_growth_background	ENSGALG00010016412	TMSB15B	-0.392138097	0.115545974	-3.393784182	0.00068934	0.006241088	negative
PRJNA729271	ed_Lushi_vs_Arbor_Acr	breed_growth_background	ENSGALG00010016415	RASGRF1	0.372841716	0.253894077	1.468493161	0.141970313	0.274793471	positive
PRJNA729271	ed_Lushi_vs_Arbor_Acr	breed_growth_background	ENSGALG00010016416	CYP46A1	-0.014902968	0.060309314	-0.247108894	0.804823951	0.879716701	negative
PRJNA729271	ed_Lushi_vs_Arbor_Acr	breed_growth_background	ENSGALG00010016417	NOP14	0.127987996	0.058607793	2.183805075	0.028976575	0.088648258	positive
PRJNA729271	ed_Lushi_vs_Arbor_Acr	breed_growth_background	ENSGALG00010016418	NFIB	-0.176780643	0.052562129	-3.363270183	0.000770249	0.006754244	negative
PRJNA729271	ed_Lushi_vs_Arbor_Acr	breed_growth_background	ENSGALG00010016421	ENSGALG00010016421	0.111617928	0.270487756	0.41265427	0.679859933	0.793060497	positive
PRJNA729271	ed_Lushi_vs_Arbor_Acr	breed_growth_background	ENSGALG00010016422	KCTD3	-0.068893677	0.048660794	-1.415794346	0.156835726	0.294282698	negative
PRJNA729271	ed_Lushi_vs_Arbor_Acr	breed_growth_background	ENSGALG00010016423	TRMT44	0.129431346	0.061372521	2.1089462	0.034949226	0.10178977	positive
PRJNA729271	ed_Lushi_vs_Arbor_Acr	breed_growth_background	ENSGALG00010016425	TRIM63	0.341271486	0.220996091	1.544242181	0.122529674	0.247294294	positive
PRJNA729271	ed_Lushi_vs_Arbor_Acr	breed_growth_background	ENSGALG00010016426	HELZ2	-0.063825921	0.197766311	-0.322734041	0.746896669	0.838661641	negative
PRJNA729271	ed_Lushi_vs_Arbor_Acr	breed_growth_background	ENSGALG00010016428	ADRA1D	-0.021206612	0.107505338	-0.197261016	0.843623285	0.905130563	negative
PRJNA729271	ed_Lushi_vs_Arbor_Acr	breed_growth_background	ENSGALG00010016429	ENSGALG00010016429	0.349217314	0.233248118	1.497192424	0.134343196	0.264126891	positive
PRJNA729271	ed_Lushi_vs_Arbor_Acr	breed_growth_background	ENSGALG00010016431	B3GNT5	-0.25707344	0.087456102	-2.939456892	0.00328788	0.018943035	negative
PRJNA729271	ed_Lushi_vs_Arbor_Acr	breed_growth_background	ENSGALG00010016433	TMED3	-0.030351813	0.078734457	-0.385495937	0.699870016	0.806472365	negative
PRJNA729271	ed_Lushi_vs_Arbor_Acr	breed_growth_background	ENSGALG00010016434	GPR139	0.020386341	0.325216326	0.062685479	0.950016961	0.972423971	positive
PRJNA729271	ed_Lushi_vs_Arbor_Acr	breed_growth_background	ENSGALG00010016436	SLC30A2	0.254516595	0.284341144	0.895109975	0.370728302	0.533953372	positive
PRJNA729271	ed_Lushi_vs_Arbor_Acr	breed_growth_background	ENSGALG00010016441	ENSGALG00010016441	0.114561828	0.356220446	0.321603742	0.747752908	0.83936176	positive
PRJNA729271	ed_Lushi_vs_Arbor_Acr	breed_growth_background	ENSGALG00010016445	ZDHHC21	-0.157982129	0.076753683	-2.058300288	0.039561317	0.111571866	negative
PRJNA729271	ed_Lushi_vs_Arbor_Acr	breed_growth_background	ENSGALG00010016447	ID3	-0.245668816	0.060991371	-4.027927435	5.62707E-05	0.001056533	negative
PRJNA729271	ed_Lushi_vs_Arbor_Acr	breed_growth_background	ENSGALG00010016449	FMR1	0.117832776	0.057226773	2.059049827	0.039489465	0.111405177	positive
PRJNA729271	ed_Lushi_vs_Arbor_Acr	breed_growth_background	ENSGALG00010016450	ENSGALG00010016450	-0.251063077	0.147977293	-1.696632454	0.08976617	0.199901772	negative
PRJNA729271	ed_Lushi_vs_Arbor_Acr	breed_growth_background	ENSGALG00010016451	USP46	0.215808303	0.07948932	2.714934562	0.006628887	0.031176416	positive
PRJNA729271	ed_Lushi_vs_Arbor_Acr	breed_growth_background	ENSGALG00010016452	GLCE	-0.258507831	0.061846749	-4.179812746	2.91749E-05	0.000646622	negative
PRJNA729271	ed_Lushi_vs_Arbor_Acr	breed_growth_background	ENSGALG00010016453	MYCL	-0.156397819	0.099152219	-1.57735066	0.114714862	0.236932448	negative
PRJNA729271	ed_Lushi_vs_Arbor_Acr	breed_growth_background	ENSGALG00010016454	ANKS3	-0.018828264	0.051495728	-0.365627681	0.714642917	0.816807213	negative
PRJNA729271	ed_Lushi_vs_Arbor_Acr	breed_growth_background	ENSGALG00010016455	RBM27	0.171269331	0.049647949	3.449675839	0.00056126	0.005378874	positive
PRJNA729271	ed_Lushi_vs_Arbor_Acr	breed_growth_background	ENSGALG00010016456	LAMP3	-0.031620234	0.198815256	-0.159043299	0.873634761	0.9253914	negative
PRJNA729271	ed_Lushi_vs_Arbor_Acr	breed_growth_background	ENSGALG00010016457	SMOX	0.050797812	0.096088079	0.528658832	0.59704214	0.730675939	positive
PRJNA729271	ed_Lushi_vs_Arbor_Acr	breed_growth_background	ENSGALG00010016458	ZC3H12A	-0.018481018	0.047527171	-0.388851627	0.697385907	0.804953713	negative
PRJNA729271	ed_Lushi_vs_Arbor_Acr	breed_growth_background	ENSGALG00010016459	PAQR5	-0.144208331	0.141258805	-1.020880298	0.307311155	0.469852136	negative
PRJNA729271	ed_Lushi_vs_Arbor_Acr	breed_growth_background	ENSGALG00010016460	LBR	-0.106091426	0.037488829	-2.829947746	0.004655561	0.024181738	negative
PRJNA729271	ed_Lushi_vs_Arbor_Acr	breed_growth_background	ENSGALG00010016461	HPCAL4	-0.025252003	0.17385811	-0.145244894	0.884517521	0.931933811	negative
PRJNA729271	ed_Lushi_vs_Arbor_Acr	breed_growth_background	ENSGALG00010016462	RNF24	-0.066348504	0.08631354	-0.76869173	0.442076335	0.599156055	negative
PRJNA729271	ed_Lushi_vs_Arbor_Acr	breed_growth_background	ENSGALG00010016463	FREM1	-0.112743191	0.077059356	-1.46306947	0.143448371	0.2766109	negative
PRJNA729271	ed_Lushi_vs_Arbor_Acr	breed_growth_background	ENSGALG00010016464	PANK2	-0.049791674	0.049257668	-1.010841079	0.312092499	0.475286906	negative
PRJNA729271	ed_Lushi_vs_Arbor_Acr	breed_growth_background	ENSGALG00010016465	STK35	-0.15619885	0.117388294	-1.330616919	0.183315092	0.328408388	negative
PRJNA729271	ed_Lushi_vs_Arbor_Acr	breed_growth_background	ENSGALG00010016466	TMEM164	0.353420628	0.126253101	2.799302559	0.005121313	0.02602855	positive
PRJNA729271	ed_Lushi_vs_Arbor_Acr	breed_growth_background	ENSGALG00010016468	TTC39B	-0.170077266	0.089340534	-1.903696557	0.056949715	0.145383255	negative
PRJNA729271	ed_Lushi_vs_Arbor_Acr	breed_growth_background	ENSGALG00010016469	MAVS	0.135420192	0.054851949	2.468831012	0.013555522	0.051843611	positive
PRJNA729271	ed_Lushi_vs_Arbor_Acr	breed_growth_background	ENSGALG00010016470	TNIP1	0.208061615	0.084549233	2.460833853	0.013861454	0.052652457	positive
PRJNA729271	ed_Lushi_vs_Arbor_Acr	breed_growth_background	ENSGALG00010016471	POMGNT2	0.097461259	0.073676579	1.322825515	0.18589341	0.331412413	positive
PRJNA729271	ed_Lushi_vs_Arbor_Acr	breed_growth_background	ENSGALG00010016472	TFDP1	-0.223193852	0.065866577	-3.388575257	0.000702567	0.006344994	negative
PRJNA729271	ed_Lushi_vs_Arbor_Acr	breed_growth_background	ENSGALG00010016473	RAB9B	0.201640694	0.086756147	2.324223716	0.020113507	0.068550501	positive
PRJNA729271	ed_Lushi_vs_Arbor_Acr	breed_growth_background	ENSGALG00010016474	MAPK8IP3	0.101037096	0.059698541	1.692455028	0.090559248	0.201111735	positive
PRJNA729271	ed_Lushi_vs_Arbor_Acr	breed_growth_background	ENSGALG00010016475	MCCC1	-0.00315582	0.044924735	-0.070246814	0.943997214	0.969479922	negative
PRJNA729271	ed_Lushi_vs_Arbor_Acr	breed_growth_background	ENSGALG00010016477	CNIH3	0.020127388	0.107655885	0.186960402	0.85169168	0.91064135	positive
PRJNA729271	ed_Lushi_vs_Arbor_Acr	breed_growth_background	ENSGALG00010016479	ENSGALG00010016479	0.233404493	0.251548514	0.927870688	0.353474651	0.517223407	positive
PRJNA729271	ed_Lushi_vs_Arbor_Acr	breed_growth_background	ENSGALG00010016480	GPX3	0.078201392	0.070837468	1.103955219	0.269612563	0.428230781	positive
PRJNA729271	ed_Lushi_vs_Arbor_Acr	breed_growth_background	ENSGALG00010016482	EML1	-0.036049274	0.074945431	-0.481006954	0.630511556	0.756436435	negative
PRJNA729271	ed_Lushi_vs_Arbor_Acr	breed_growth_background	ENSGALG00010016483	WDR26	0.067939653	0.055286812	1.228858201	0.219124976	0.371530766	positive
PRJNA729271	ed_Lushi_vs_Arbor_Acr	breed_growth_background	ENSGALG00010016489	CCK	-0.106497094	0.343318748	-0.310198888	0.756409716	0.845997751	negative
PRJNA729271	ed_Lushi_vs_Arbor_Acr	breed_growth_background	ENSGALG00010016491	DNAJC8	-0.017269884	0.029378939	-0.587832115	0.556644986	0.698154285	negative
PRJNA729271	ed_Lushi_vs_Arbor_Acr	breed_growth_background	ENSGALG00010016496	PLP1	0.727395548	0.170655616	4.262359288	2.0228E-05	0.000486243	positive
PRJNA729271	ed_Lushi_vs_Arbor_Acr	breed_growth_background	ENSGALG00010016498	KIF23	-0.334769817	0.111933601	-2.990789293	0.002782574	0.016805589	negative
PRJNA729271	ed_Lushi_vs_Arbor_Acr	breed_growth_background	ENSGALG00010016500	ENSGALG00010016500	0.012498454	0.05709506	0.218906046	0.826723236	0.89434684	positive
PRJNA729271	ed_Lushi_vs_Arbor_Acr	breed_growth_background	ENSGALG00010016502	YIPF5	-0.157056258	0.071761381	-2.188590241	0.028626636	0.087873067	negative
PRJNA729271	ed_Lushi_vs_Arbor_Acr	breed_growth_background	ENSGALG00010016504	SNRK	-0.162971327	0.045362004	-3.592683605	0.00032729	0.003674723	negative
PRJNA729271	ed_Lushi_vs_Arbor_Acr	breed_growth_background	ENSGALG00010016505	KCTD16	-0.356465937	0.188211396	-1.893965742	0.058229557	0.147533199	negative
PRJNA729271	ed_Lushi_vs_Arbor_Acr	breed_growth_background	ENSGALG00010016507	SPATA18	-0.086976364	0.076872339	-1.131439022	0.257870355	0.415268392	negative
PRJNA729271	ed_Lushi_vs_Arbor_Acr	breed_growth_background	ENSGALG00010016508	CREBBP	0.058346126	0.064026075	0.911286941	0.362144205	0.525400886	positive
PRJNA729271	ed_Lushi_vs_Arbor_Acr	breed_growth_background	ENSGALG00010016510	THG1L	-0.183950849	0.061597189	-2.986351352	0.002823281	0.016973422	negative
PRJNA729271	ed_Lushi_vs_Arbor_Acr	breed_growth_background	ENSGALG00010016514	GASK1A	-0.016141649	0.134984161	-0.119581798	0.90481444	0.945313918	negative
PRJNA729271	ed_Lushi_vs_Arbor_Acr	breed_growth_background	ENSGALG00010016518	TMEM42	0.270872708	0.0785233	3.449583852	0.000561451	0.005378874	positive
PRJNA729271	ed_Lushi_vs_Arbor_Acr	breed_growth_background	ENSGALG00010016519	MEX3B	-0.102997326	0.097456868	-1.056850367	0.290579883	0.452072062	negative
PRJNA729271	ed_Lushi_vs_Arbor_Acr	breed_growth_background	ENSGALG00010016520	CHMP1B	0.022375687	0.044569459	0.502040806	0.615638817	0.744275329	positive
PRJNA729271	ed_Lushi_vs_Arbor_Acr	breed_growth_background	ENSGALG00010016526	EIF1B	0.112389065	0.06166805	1.822484488	0.068381505	0.165206853	positive
PRJNA729271	ed_Lushi_vs_Arbor_Acr	breed_growth_background	ENSGALG00010016527	MRPS34	0.014506015	0.0532356	0.272487115	0.785247497	0.867080252	positive
PRJNA729271	ed_Lushi_vs_Arbor_Acr	breed_growth_background	ENSGALG00010016529	DCUN1D1	0.142008422	0.052590504	2.700267372	0.006928377	0.032145293	positive
PRJNA729271	ed_Lushi_vs_Arbor_Acr	breed_growth_background	ENSGALG00010016533	CNIH4	0.114073312	0.059315312	1.923168042	0.054458949	0.14083541	positive
PRJNA729271	ed_Lushi_vs_Arbor_Acr	breed_growth_background	ENSGALG00010016534	TRIT1	0.105934712	0.07883859	1.343691101	0.179048296	0.323281646	positive
PRJNA729271	ed_Lushi_vs_Arbor_Acr	breed_growth_background	ENSGALG00010016535	BNC2	-0.083412796	0.071525056	-1.166203853	0.243532035	0.399145826	negative
PRJNA729271	ed_Lushi_vs_Arbor_Acr	breed_growth_background	ENSGALG00010016541	UBE2J2	0.110187274	0.042716337	2.579511331	0.009894021	0.04150907	positive
PRJNA729271	ed_Lushi_vs_Arbor_Acr	breed_growth_background	ENSGALG00010016542	ENOX2	0.166716313	0.041728616	3.995251428	6.46256E-05	0.001154885	positive
PRJNA729271	ed_Lushi_vs_Arbor_Acr	breed_growth_background	ENSGALG00010016543	TCEA3	0.196393514	0.089603641	2.191802824	0.028393747	0.087363525	positive
PRJNA729271	ed_Lushi_vs_Arbor_Acr	breed_growth_background	ENSGALG00010016544	ADCY9	0.163586606	0.072421195	2.258822227	0.023894445	0.077505962	positive
PRJNA729271	ed_Lushi_vs_Arbor_Acr	breed_growth_background	ENSGALG00010016546	C1QTNF12	-0.10492908	0.233100387	-0.450145456	0.652605562	0.773428775	negative
PRJNA729271	ed_Lushi_vs_Arbor_Acr	breed_growth_background	ENSGALG00010016547	SGCB	-0.27296684	0.076426196	-3.571639736	0.000354753	0.003889673	negative
PRJNA729271	ed_Lushi_vs_Arbor_Acr	breed_growth_background	ENSGALG00010016548	EVL	-0.216595413	0.058344494	-3.71235395	0.000205341	0.002648053	negative
PRJNA729271	ed_Lushi_vs_Arbor_Acr	breed_growth_background	ENSGALG00010016549	PTPRA	-0.181774435	0.055752052	-3.260407991	0.001112521	0.008841001	negative
PRJNA729271	ed_Lushi_vs_Arbor_Acr	breed_growth_background	ENSGALG00010016550	FBLIM1	-0.207788509	0.080417586	-2.583869019	0.009769889	0.041119205	negative
PRJNA729271	ed_Lushi_vs_Arbor_Acr	breed_growth_background	ENSGALG00010016552	ENSGALG00010016552	0.519383051	0.224800356	2.31041917	0.020864959	0.070223979	positive
PRJNA729271	ed_Lushi_vs_Arbor_Acr	breed_growth_background	ENSGALG00010016553	SLC25A14	0.08135963	0.052225104	1.557864388	0.119265399	0.243349093	positive
PRJNA729271	ed_Lushi_vs_Arbor_Acr	breed_growth_background	ENSGALG00010016556	ASAP3	-0.088372727	0.105403985	-0.83841922	0.401795299	0.562816279	negative
PRJNA729271	ed_Lushi_vs_Arbor_Acr	breed_growth_background	ENSGALG00010016558	NR2F2	-0.130843021	0.059020908	-2.21689272	0.026630423	0.083569601	negative
PRJNA729271	ed_Lushi_vs_Arbor_Acr	breed_growth_background	ENSGALG00010016559	ROGDI	0.044790287	0.063801488	0.702025739	0.482663112	0.634921663	positive
PRJNA729271	ed_Lushi_vs_Arbor_Acr	breed_growth_background	ENSGALG00010016560	DCUN1D4	-0.18043369	0.090420212	-1.99550174	0.045988185	0.124778339	negative
PRJNA729271	ed_Lushi_vs_Arbor_Acr	breed_growth_background	ENSGALG00010016561	DEGS2	0.12343612	0.298222735	0.413905801	0.678943095	0.792623996	positive

PRJNA729271	:ed_Lushi_vs_Arbor_Acr	breed_growth_background	ENSGALG00010016562	GLIS2	0.285322329	0.118466737	2.40845942	0.016020007	0.058376754	positive
PRJNA729271	:ed_Lushi_vs_Arbor_Acr	breed_growth_background	ENSGALG00010016564	SPEN	0.162374643	0.088716991	1.830254178	0.06721194	0.163617219	positive
PRJNA729271	:ed_Lushi_vs_Arbor_Acr	breed_growth_background	ENSGALG00010016565	NVL	0.140729779	0.053268961	2.641872041	0.00824492	0.036347358	positive
PRJNA729271	:ed_Lushi_vs_Arbor_Acr	breed_growth_background	ENSGALG00010016566	CNTLN	-0.410618529	0.115778992	-3.54657199	0.000390278	0.004174968	negative
PRJNA729271	:ed_Lushi_vs_Arbor_Acr	breed_growth_background	ENSGALG00010016567	E2F2	-0.500602359	0.148719193	-3.366091142	0.000762415	0.006730333	negative
PRJNA729271	:ed_Lushi_vs_Arbor_Acr	breed_growth_background	ENSGALG00010016568	PAM16	-0.010807338	0.071403701	-0.151355432	0.879695345	0.92850681	negative
PRJNA729271	:ed_Lushi_vs_Arbor_Acr	breed_growth_background	ENSGALG00010016569	YY1	0.111066245	0.046628672	2.381930238	0.01722216	0.061410875	positive
PRJNA729271	:ed_Lushi_vs_Arbor_Acr	breed_growth_background	ENSGALG00010016572	RAB33A	0.227389831	0.061793888	3.679811044	0.000233407	0.002894125	positive
PRJNA729271	:ed_Lushi_vs_Arbor_Acr	breed_growth_background	ENSGALG00010016573	DEGS1	-0.372168023	0.069044947	-5.390228213	7.03683E-08	6.21944E-06	negative
PRJNA729271	:ed_Lushi_vs_Arbor_Acr	breed_growth_background	ENSGALG00010016575	CWH43	0.16422369	0.2135478	0.769025348	0.44187821	0.599104354	positive
PRJNA729271	:ed_Lushi_vs_Arbor_Acr	breed_growth_background	ENSGALG00010016576	MRPS26	0.180089644	0.057178998	3.149576803	0.001635071	0.011520759	positive
PRJNA729271	:ed_Lushi_vs_Arbor_Acr	breed_growth_background	ENSGALG00010016577	ZBTB17	0.31544652	0.099347234	3.175191791	0.001497375	0.010895808	positive
PRJNA729271	:ed_Lushi_vs_Arbor_Acr	breed_growth_background	ENSGALG00010016580	MARS2	0.157425488	0.060174576	2.61614619	0.008892847	0.038386487	positive
PRJNA729271	:ed_Lushi_vs_Arbor_Acr	breed_growth_background	ENSGALG00010016581	H2AFYMACROH2A1	-0.022157989	0.04138319	-0.535434519	0.592349426	0.726958338	negative
PRJNA729271	:ed_Lushi_vs_Arbor_Acr	breed_growth_background	ENSGALG00010016583	VPS41	-0.175412616	0.044882376	-3.90827381	9.29579E-05	0.001516585	negative
PRJNA729271	:ed_Lushi_vs_Arbor_Acr	breed_growth_background	ENSGALG00010016584	EHBP1	0.115516484	0.086047878	1.342467538	0.179444449	0.323714659	positive
PRJNA729271	:ed_Lushi_vs_Arbor_Acr	breed_growth_background	ENSGALG00010016588	ATP11B	-0.122585009	0.053827104	-2.277384455	0.022763278	0.074901906	negative
PRJNA729271	:ed_Lushi_vs_Arbor_Acr	breed_growth_background	ENSGALG00010016589	FBXO28	0.0041782	0.042447096	0.098433131	0.921588368	0.956327938	positive
PRJNA729271	:ed_Lushi_vs_Arbor_Acr	breed_growth_background	ENSGALG00010016591	HOMER2	-0.12300105	0.112434769	-1.093976986	0.273965072	0.432990283	negative
PRJNA729271	:ed_Lushi_vs_Arbor_Acr	breed_growth_background	ENSGALG00010016592	TMEM129	0.149560409	0.056091375	2.666370867	0.007667506	0.034590265	positive
PRJNA729271	:ed_Lushi_vs_Arbor_Acr	breed_growth_background	ENSGALG00010016597	FRYL	-0.119050533	0.067135493	-1.773287527	0.076181075	0.178046546	negative
PRJNA729271	:ed_Lushi_vs_Arbor_Acr	breed_growth_background	ENSGALG00010016598	AIFM1	0.090774999	0.047554568	1.908859727	0.056280189	0.144283931	positive
PRJNA729271	:ed_Lushi_vs_Arbor_Acr	breed_growth_background	ENSGALG00010016599	ENSGALG00010016599	0.190728193	0.152546896	1.250292197	0.211192828	0.361255295	positive
PRJNA729271	:ed_Lushi_vs_Arbor_Acr	breed_growth_background	ENSGALG00010016600	GNL2	0.170128966	0.05924293	2.871717617	0.004082476	0.021924968	positive
PRJNA729271	:ed_Lushi_vs_Arbor_Acr	breed_growth_background	ENSGALG00010016602	HSPB7	0.084570023	0.273374587	0.309355832	0.757050865	0.846495913	positive
PRJNA729271	:ed_Lushi_vs_Arbor_Acr	breed_growth_background	ENSGALG00010016603	ENSGALG00010016603	0.120258908	0.068246496	1.762125767	0.078048051	0.180748761	positive
PRJNA729271	:ed_Lushi_vs_Arbor_Acr	breed_growth_background	ENSGALG00010016604	TP53BP2	-0.152479934	0.034303891	-4.44497495	8.79021E-06	0.000264768	negative
PRJNA729271	:ed_Lushi_vs_Arbor_Acr	breed_growth_background	ENSGALG00010016605	WDPCP	-0.103897754	0.090756288	-1.144799512	0.252292218	0.408737643	negative
PRJNA729271	:ed_Lushi_vs_Arbor_Acr	breed_growth_background	ENSGALG00010016606	VASN	0.177309074	0.097282801	1.822614813	0.06836175	0.165205664	positive
PRJNA729271	:ed_Lushi_vs_Arbor_Acr	breed_growth_background	ENSGALG00010016607	FAM103A1	-0.11753619	0.061869123	-1.899755227	0.057465249	0.146312802	negative
PRJNA729271	:ed_Lushi_vs_Arbor_Acr	breed_growth_background	ENSGALG00010016609	SOX2	0.18515075	0.301904361	0.61327617	0.539693748	0.684926963	positive
PRJNA729271	:ed_Lushi_vs_Arbor_Acr	breed_growth_background	ENSGALG00010016611	DNALI1	-0.142191284	0.115069228	-1.235702075	0.216569326	0.368188175	negative
PRJNA729271	:ed_Lushi_vs_Arbor_Acr	breed_growth_background	ENSGALG00010016612	GRIA1	0.275547248	0.184183919	1.496044001	0.134642189	0.264485504	positive
PRJNA729271	:ed_Lushi_vs_Arbor_Acr	breed_growth_background	ENSGALG00010016613	SNIP1	0.095325061	0.042166629	2.260675386	0.023779366	0.077196396	positive
PRJNA729271	:ed_Lushi_vs_Arbor_Acr	breed_growth_background	ENSGALG00010016614	DNAJC19	-0.125503969	0.074653832	-1.681145698	0.092734618	0.204128821	negative
PRJNA729271	:ed_Lushi_vs_Arbor_Acr	breed_growth_background	ENSGALG00010016615	BCORL1	0.085149574	0.066499517	1.280454025	0.200385504	0.34918641	positive
PRJNA729271	:ed_Lushi_vs_Arbor_Acr	breed_growth_background	ENSGALG00010016616	SLBP	-0.411844002	0.131164458	-3.139905478	0.001690024	0.011778497	negative
PRJNA729271	:ed_Lushi_vs_Arbor_Acr	breed_growth_background	ENSGALG00010016618	TRPM3	0.554905816	0.294137289	1.886553786	0.059220368	0.149436264	positive
PRJNA729271	:ed_Lushi_vs_Arbor_Acr	breed_growth_background	ENSGALG00010016619	SLC25A47	-0.246162696	0.176395287	-1.395517415	0.162859867	0.302415292	negative
PRJNA729271	:ed_Lushi_vs_Arbor_Acr	breed_growth_background	ENSGALG00010016620	ENSGALG00010016620	-0.107926985	0.065746689	-1.641557712	0.100681697	0.216203701	negative
PRJNA729271	:ed_Lushi_vs_Arbor_Acr	breed_growth_background	ENSGALG00010016622	KIAA1143	0.111090727	0.072429873	1.533769469	0.125086377	0.25126014	positive
PRJNA729271	:ed_Lushi_vs_Arbor_Acr	breed_growth_background	ENSGALG00010016623	DNAJA3	0.171876666	0.075470482	2.277402505	0.022762201	0.074901906	positive
PRJNA729271	:ed_Lushi_vs_Arbor_Acr	breed_growth_background	ENSGALG00010016624	MDH1	-0.100840133	0.082713797	-1.21914525	0.222789068	0.375372259	negative
PRJNA729271	:ed_Lushi_vs_Arbor_Acr	breed_growth_background	ENSGALG00010016626	CAPN2	-0.253867805	0.124024124	-2.046922775	0.040665668	0.113705411	negative
PRJNA729271	:ed_Lushi_vs_Arbor_Acr	breed_growth_background	ENSGALG00010016629	LARS1	0.001541073	0.038544293	0.03998188	0.968107573	0.981858331	positive
PRJNA729271	:ed_Lushi_vs_Arbor_Acr	breed_growth_background	ENSGALG00010016630	ENSGALG00010016630	-0.011541534	0.179327021	-0.06436026	0.948683372	0.971711451	negative
PRJNA729271	:ed_Lushi_vs_Arbor_Acr	breed_growth_background	ENSGALG00010016631	FAM53A	0.041889546	0.048670941	0.860668494	0.38942065	0.551323379	positive
PRJNA729271	:ed_Lushi_vs_Arbor_Acr	breed_growth_background	ENSGALG00010016637	POU6F2	0.361549706	0.389693638	0.927779338	0.353522044	0.51724054	positive
PRJNA729271	:ed_Lushi_vs_Arbor_Acr	breed_growth_background	ENSGALG00010016640	OCIAD1	0.000927214	0.055469954	0.016715611	0.986663493	0.992139253	positive
PRJNA729271	:ed_Lushi_vs_Arbor_Acr	breed_growth_background	ENSGALG00010016643	BTBD1	-0.035671684	0.092676545	-0.384905202	0.700307652	0.806702465	negative
PRJNA729271	:ed_Lushi_vs_Arbor_Acr	breed_growth_background	ENSGALG00010016644	NMRAL1	-0.048260833	0.113888656	-0.42375452	0.671744849	0.787778446	negative
PRJNA729271	:ed_Lushi_vs_Arbor_Acr	breed_growth_background	ENSGALG00010016647	EGR1	-0.620440107	0.108921436	-5.696216735	1.22495E-08	1.62896E-06	negative
PRJNA729271	:ed_Lushi_vs_Arbor_Acr	breed_growth_background	ENSGALG00010016648	ADRA2C	0.129205879	0.156754939	0.824253961	0.409795272	0.570258262	positive
PRJNA729271	:ed_Lushi_vs_Arbor_Acr	breed_growth_background	ENSGALG00010016651	ENSGALG00010016651	0.265712536	0.09190506	2.891163299	0.003838186	0.021017949	positive
PRJNA729271	:ed_Lushi_vs_Arbor_Acr	breed_growth_background	ENSGALG00010016652	FXR1	0.022015757	0.052820813	0.416800797	0.676824119	0.791390582	positive
PRJNA729271	:ed_Lushi_vs_Arbor_Acr	breed_growth_background	ENSGALG00010016653	EPHA2	0.048808249	0.113021122	0.431850688	0.665849937	0.783464148	positive
PRJNA729271	:ed_Lushi_vs_Arbor_Acr	breed_growth_background	ENSGALG00010016654	SMIM3	0.124477952	0.097446565	1.277397023	0.201462115	0.350353133	positive
PRJNA729271	:ed_Lushi_vs_Arbor_Acr	breed_growth_background	ENSGALG00010016655	TRANK1	-0.135575559	0.173160879	-0.782945663	0.433659021	0.591736607	negative
PRJNA729271	:ed_Lushi_vs_Arbor_Acr	breed_growth_background	ENSGALG00010016656	CAPN8	-0.11228072	0.659167519	-0.170337155	0.864744993	0.918680641	negative
PRJNA729271	:ed_Lushi_vs_Arbor_Acr	breed_growth_background	ENSGALG00010016657	SLC25A29	0.078773566	0.14923797	0.527838634	0.59761134	0.730959358	positive
PRJNA729271	:ed_Lushi_vs_Arbor_Acr	breed_growth_background	ENSGALG00010016658	ZAR1	0.497116303	0.326725068	1.521512584	0.128131256	0.255348244	positive
PRJNA729271	:ed_Lushi_vs_Arbor_Acr	breed_growth_background	ENSGALG00010016661	UBE4B	0.192659366	0.06915529	2.785894849	0.005338018	0.026701751	positive
PRJNA729271	:ed_Lushi_vs_Arbor_Acr	breed_growth_background	ENSGALG00010016664	TM6SF1	-0.17200894	0.1321939	-1.301186667	0.193194568	0.340186155	negative
PRJNA729271	:ed_Lushi_vs_Arbor_Acr	breed_growth_background	ENSGALG00010016665	ENSGALG00010016665	0.338586879	0.356464453	0.949847528	0.342189732	0.505938073	positive
PRJNA729271	:ed_Lushi_vs_Arbor_Acr	breed_growth_background	ENSGALG00010016667	WARS1	-0.069506377	0.077160563	-0.900801837	0.367693691	0.531073155	negative
PRJNA729271	:ed_Lushi_vs_Arbor_Acr	breed_growth_background	ENSGALG00010016671	CASS4	-0.032615956	0.133759475	-0.243840344	0.807354473	0.881618433	negative
PRJNA729271	:ed_Lushi_vs_Arbor_Acr	breed_growth_background	ENSGALG00010016672	DCTN4	0.126527971	0.043363086	2.917872837	0.00352428	0.019811949	positive
PRJNA729271	:ed_Lushi_vs_Arbor_Acr	breed_growth_background	ENSGALG00010016673	LRPAP1	0.063317727	0.042892931	1.476180915	0.139895331	0.271895588	positive
PRJNA729271	:ed_Lushi_vs_Arbor_Acr	breed_growth_background	ENSGALG00010016674	SLC10A4	-0.1697425	0.194830114	-0.871233384	0.383626735	0.545163679	negative
PRJNA729271	:ed_Lushi_vs_Arbor_Acr	breed_growth_background	ENSGALG00010016675	SKIL	0.096397508	0.075212044	1.281676478	0.19995616	0.348822305	positive
PRJNA729271	:ed_Lushi_vs_Arbor_Acr	breed_growth_background	ENSGALG00010016676	NLRC3	0.382393582	0.239665032	1.595533479	0.110592989	0.231290127	positive
PRJNA729271	:ed_Lushi_vs_Arbor_Acr	breed_growth_background	ENSGALG00010016677	MEAF6	0.106581915	0.047167966	2.259624983	0.023844536	0.077373303	positive
PRJNA729271	:ed_Lushi_vs_Arbor_Acr	breed_growth_background	ENSGALG00010016678	NOX1	0.463158604	0.150920603	3.068889164	0.002148563	0.013959399	positive
PRJNA729271	:ed_Lushi_vs_Arbor_Acr	breed_growth_background	ENSGALG00010016680	BPIFB3	0.321902857	0.486186668	0.662097253	0.507908896	0.656958809	positive
PRJNA729271	:ed_Lushi_vs_Arbor_Acr	breed_growth_background	ENSGALG00010016681	SUSD4	0.169335517	0.148073203	1.14359326	0.252792358	0.409456389	positive
PRJNA729271	:ed_Lushi_vs_Arbor_Acr	breed_growth_background	ENSGALG00010016682	GPR141	-0.357525755	0.246499555	-1.450411361	0.146943841	0.281478918	negative
PRJNA729271	:ed_Lushi_vs_Arbor_Acr	breed_growth_background	ENSGALG00010016683	RBM22	0.062655387	0.039990744	1.56674724	0.117173784	0.240537318	positive
PRJNA729271	:ed_Lushi_vs_Arbor_Acr	breed_growth_background	ENSGALG00010016691	ENSGALG00010016691	-0.167015553	0.162306738	-1.02901183	0.303474116	0.466178181	negative
PRJNA729271	:ed_Lushi_vs_Arbor_Acr	breed_growth_background	ENSGALG00010016692	PRKCI	0.008726394	0.040606074	0.21490367	0.829842423	0.896239391	positive
PRJNA729271	:ed_Lushi_vs_Arbor_Acr	breed_growth_background	ENSGALG00010016696	DOK7	-0.033621726	0.069004028	-0.487242948	0.626086173	0.752435045	negative
PRJNA729271	:ed_Lushi_vs_Arbor_Acr	breed_growth_background	ENSGALG00010016699	SYNPO	0.104850059	0.077987644	1.344444512	0.178804688	0.323123545	positive
PRJNA729271	:ed_Lushi_vs_Arbor_Acr	breed_growth_background	ENSGALG00010016700	CLUAP1	-0.078172309	0.069028287	-1.132467747	0.257437837	0.414710096	negative
PRJNA729271	:ed_Lushi_vs_Arbor_Acr	breed_growth_background	ENSGALG00010016702	WDR25	0.011680094	0.053105141	0.219942806	0.825915698	0.893637926	positive
PRJNA729271	:ed_Lushi_vs_Arbor_Acr	breed_growth_background	ENSGALG00010016704	CSTF2	0.023441486	0.040056312	0.585213282	0.558404315	0.699600532	positive
PRJNA729271	:ed_Lushi_vs_Arbor_Acr	breed_growth_background	ENSGALG00010016706	RTN4	-0.257218012	0.067038042	-3.836896256	0.000124599	0.001870492	negative
PRJNA729271	:ed_Lushi_vs_Arbor_Acr	breed_growth_background	ENSGALG00010016707	KIF1B	0.23923526	0.069701952	3.432260539	0.000598572	0.005608471	positive
PRJNA729271	:ed_Lushi_vs_Arbor_Acr	breed_growth_background	ENSGALG00010016712	ENSGALG00010016712	-0.105489885	0.086894385	-1.214001166	0.224747279	0.377574387	negative
PRJNA729271	:ed_Lushi_vs_Arbor_Acr	breed_growth_background	ENSGALG00010016720	INTS15	0.139829466	0.054911312	2.546460127	0.010882164	0.044445469	positive
PRJNA729271	:ed_Lushi_vs_Arbor_Acr	breed_growth_background	ENSGALG00010016724	PGD	-0.234400886	0.06001095	-3.90596857	9.38487E-05	0.001523119	negative
PRJNA729271	:ed_Lushi_vs_Arbor_Acr	breed_growth_background	ENSGALG00010016729	ELMO1	-0.159079211	0.050664154	-3.139876992	0.001690188	0.011778497	negative
PRJNA729271	:ed_Lushi_vs_Arbor_Acr	breed_growth_background	ENSGALG00010016731	C4orf48	-0.460362557	0.149247901	-3.084549613	0.002038606	0.013449975	negative
PRJNA729271	:ed_Lushi_vs_Arbor_Acr	breed_growth_background	ENSGALG00010016732	ARHGAP26	-0.142091382	0.041542862	-3.420356109	0.000625392	0.005799782	negative
PRJNA729271	:ed_Lushi_vs_Arbor_Acr	breed_growth_background	ENSGALG00010016733	PHC3	-0.042639935	0.083660416	-0.509678739	0.610276551	0.740584881	negative
PRJNA729271	:ed_Lushi_vs_Arbor_Acr	breed_growth_background	ENSGALG00010016735	ENSGALG00010016735	-0.050597611	0.185764772	-0.272374628	0.785333979	0.867109691	negative
PRJNA729271	:ed_Lushi_vs_Arbor_Acr	breed_growth_background	ENSGALG00010016736	CENPS	-0.376388014	0.146975764	-2.560884885	0.010440594	0.043128075	negative
PRJNA729271	:ed_Lushi_vs_Arbor_Acr	breed_growth_background	ENSGALG00010016738	NAA60	0.275511557	0.079738182	3.455202401	0.00054988	0.005313672	positive
PRJNA729271	:ed_Lushi_vs_Arbor_Acr	breed_growth_background	ENSGALG00010016741	NELFA	-0.018863081	0.048262633	-0.390842354	0.695913766	0.80418778	negative
PRJNA729271	:ed_Lushi_vs_Arbor_Acr	breed_growth_background	ENSGALG00010016742	TRMT12	0.007255552	0.076570789	0.094756131	0.924508533	0.957812249	positive
PRJNA729271	:ed_Lushi_vs_Arbor_Acr	breed_growth_background	ENSGALG00010016744	CRYBG2	-0.225268454	0.496246761	-0.453944432	0.649868822	0.771407505	negative
PRJNA729271	:ed_Lushi_vs_Arbor_Acr	breed_growth_background	ENSGALG00010016745	BEGAIN	-0.626788024	0.200435289	-3.127134089	0.001765194	0.012163728	negative
PRJNA729271	:ed_Lushi_vs_Arbor_Acr	breed_growth_background	ENSGALG00010016746	HDGFL3	-0.268643724	0.06712437	-4.002178677	6.27619E-05	0.001134331	negative
PRJNA729271	:ed_Lushi_vs_Arbor_Acr	breed_growth_background	ENSGALG00010016750	SYTL4	-0.21739726	0.06859159	-3.169444813	0.001527305	0.011026955	negative
PRJNA729271	:ed_Lushi_vs_Arbor_Acr	breed_growth_background	ENSGALG00010016751	DFFA	-0.08091264	0.049801472	-1.624703788	0.10422569	0.221550282	negative
PRJNA729271	:ed_Lushi_vs_Arbor_Acr	breed_growth_background	ENSGALG00010016752	ENSGALG00010016752	-0.151590085	0.091910567	-1.649321613	0.099081764	0.21336951	negative
PRJNA729271	:ed_Lushi_vs_Arbor_Acr	breed_growth_background	ENSGALG00010016754	ENSGALG00010016754	-0.11737441	0.099137099	-1.183960502	0.236428698	0.391347948	negative
PRJNA729271	:ed_Lushi_vs_Arbor_Acr	breed_growth_background	ENSGALG00010016756	NUDT16L1	-0.083872617	0.057397405	-1.461261478	0.143943697	0.27732494	negative
PRJNA729271	:ed_Lushi_vs_Arbor_Acr	breed_growth_background	ENSGALG00010016757	SRPX2	-0.462821104	0.150796143	-3.069183967	0.002146444	0.013951884	negative
PRJNA729271	:ed_Lushi_vs_Arbor_Acr	breed_growth_background	ENSGALG00010016758	NR3C1	-0.011434492	0.036665286	-0.311861528	0.755145762	0.845107923	negative
PRJNA729271	:ed_Lushi_vs_Arbor_Acr	breed_growth_background	ENSGALG00010016760	DLK1	-0.351604827	0.13962983	-2.518121146	0.011798273	0.047008238	negative
PRJNA729271	:ed_Lushi_vs_Arbor_Acr	breed_growth_background	ENSGALG00010016761	GPR160	0.012849865	0.190202805	0.067558758	0.946136887	0.970470493	positive
PRJNA729271	:ed_Lushi_vs_Arbor_Acr	breed_growth_background	ENSGALG00010016762	EML6	0.12169905	0.067857885	1.793440071	0.072902585	0.172824105	positive
PRJNA729271	:ed_Lushi_vs_Arbor_Acr	breed_growth_background	ENSGALG00010016763	UBALD1	0.176472575	0.092068904	1.916744611	0.055270385	0.142400325	positive
PRJNA729271	:ed_Lushi_vs_Arbor_Acr	breed_growth_background	ENSGALG00010016766	AOAH	0.072234249	0.186274436	0.38778402	0.698175872	0.805308638	positive
PRJNA729271	:ed_Lushi_vs_Arbor_Acr	breed_growth_background	ENSGALG00010016767	SLAIN2	0.057038313	0.04988511	1.143393553	0.252875228	0.409544853	positive
PRJNA729271	:ed_Lushi_vs_Arbor_Acr	breed_growth_background	ENSGALG00010016768	GALNT10	-0.043330963	0.05394825	-0.803194961	0.421862058	0.581042429	negative
PRJNA729271	:ed_Lushi_vs_Arbor_Acr	breed_growth_background	ENSGALG00010016769	BNC1	0.175690094	0.189150819	0.92883602	0.352974074	0.51680396	positive
PRJNA729271	:ed_Lushi_vs_Arbor_Acr	breed_growth_background	ENSGALG00010016772	TSPAN6	-0.300999959	0.06831727	-4.405913166	1.05339E-05	0.000303226	negative
PRJNA729271	:ed_Lushi_vs_Arbor_Acr	breed_growth_background	ENSGALG00010016773	RPS27A	-0.033531162	0.086216484	-0.388918228	0.697336638	0.804953713	negative
PRJNA729271	:ed_Lushi_vs_Arbor_Acr	breed_growth_background	ENSGALG00010016774	PEX14	0.144800608	0.065204626	2.220710647	0.026370566	0.082969688	positive
PRJNA729271	:ed_Lushi_vs_Arbor_Acr	breed_growth_background	ENSGALG00010016777	TNMD	0.122514564	0.261433704	0.468625743	0.639337173	0.763423044	positive
PRJNA729271	:ed_Lushi_vs_Arbor_Acr	breed_growth_background	ENSGALG00010016778	MTIF2	0.160361042	0.058514712	2.740525189	0.006134108	0.02948982	positive
PRJNA729271	:ed_Lushi_vs_Arbor_Acr	breed_growth_background	ENSGALG00010016779	CTNNA2	0.009134616	0.083676254	0.109166163	0.913070698	0.951037921	positive
PRJNA729271	:ed_Lushi_vs_Arbor_Acr	breed_growth_background	ENSGALG00010016780	PRORSD1P	-0.13728596	0.076840447	-1.786636659	0.073996233	0.174686546	negative
PRJNA729271	:ed_Lushi_vs_Arbor_Acr	breed_growth_background	ENSGALG00010016781	CDIP1	0.168967854	0.07642009	2.21103972	0.027033087	0.084376527	positive
PRJNA729271	:ed_Lushi_vs_Arbor_Acr	breed_growth_background	ENSGALG00010016783	CLBA1	-0.197120017	0.168701737	-1.168452802	0.242624167	0.39828282	negative
PRJNA729271	:ed_Lushi_vs_Arbor_Acr	breed_growth_background	ENSGALG00010016785	PDGFRB	-0.408833626	0.103985723	-3.931632302	8.4371E-05	0.001413825	negative
PRJNA729271	:ed_Lushi_vs_Arbor_Acr	breed_growth_background	ENSGALG00010016786	DISP1	-0.195443789	0.060106465	-3.251626742	0.001147466	0.009014916	negative
PRJNA729271	:ed_Lushi_vs_Arbor_Acr	breed_growth_background	ENSGALG00010016787	PPP2R5C	-0.007704384	0.040780776	-0.188921948	0.850153991	0.90991524	negative
PRJNA729271	:ed_Lushi_vs_Arbor_Acr	breed_growth_background	ENSGALG00010016788	ANLN	-0.469143422	0.154903812	-3.028611216	0.002456806	0.015409523	negative
PRJNA729271	:ed_Lushi_vs_Arbor_Acr	breed_growth_background	ENSGALG00010016789	DISP3	-0.132411586	0.059793106	-2.214495873	0.026794686	0.083939697	negative
PRJNA729271	:ed_Lushi_vs_Arbor_Acr	breed_growth_background	ENSGALG00010016791	LRRTM1	0.144064274	0.290451578	0.496001005	0.619893702	0.74759624	positive

PRJNA729271	ed_Lushi_vs_Arbor_Acr	breed_growth_background	ENSGALG00010016792	HMOX2	-0.009521223	0.066661025	-0.142830429	0.886424104	0.933298278	negative
PRJNA729271	ed_Lushi_vs_Arbor_Acr	breed_growth_background	ENSGALG00010016793	SLC9A1	-0.046188723	0.083414845	-0.553723059	0.579768392	0.716587236	negative
PRJNA729271	ed_Lushi_vs_Arbor_Acr	breed_growth_background	ENSGALG00010016795	GUCA2B	-1.109509698	0.682744673	-1.625072653	0.104147079	0.221498259	negative
PRJNA729271	ed_Lushi_vs_Arbor_Acr	breed_growth_background	ENSGALG00010016796	BROX	-0.021157486	0.046737409	-0.452688473	0.650773081	0.772054003	negative
PRJNA729271	ed_Lushi_vs_Arbor_Acr	breed_growth_background	ENSGALG00010016797	SEC62	0.109185302	0.05870855	1.859785371	0.062915898	0.156024968	positive
PRJNA729271	ed_Lushi_vs_Arbor_Acr	breed_growth_background	ENSGALG00010016799	WDTC1	0.237897246	0.070600043	3.369647322	0.000752644	0.006660306	positive
PRJNA729271	ed_Lushi_vs_Arbor_Acr	breed_growth_background	ENSGALG00010016800	RPL14	-0.136934795	0.090386382	-1.514993657	0.12977401	0.257399327	negative
PRJNA729271	ed_Lushi_vs_Arbor_Acr	breed_growth_background	ENSGALG00010016802	TMEM222	-0.090945414	0.04658046	-1.952436997	0.050886344	0.134101264	negative
PRJNA729271	ed_Lushi_vs_Arbor_Acr	breed_growth_background	ENSGALG00010016804	CSF1R	-0.175452715	0.072541744	-2.418644854	0.015578442	0.057282981	negative
PRJNA729271	ed_Lushi_vs_Arbor_Acr	breed_growth_background	ENSGALG00010016805	ENSGALG00010016805	0.361967265	0.522791626	0.692373878	0.488702566	0.640587927	positive
PRJNA729271	ed_Lushi_vs_Arbor_Acr	breed_growth_background	ENSGALG00010016807	VSIG4	-0.206206447	0.117783093	-1.750730442	0.079992353	0.184112914	negative
PRJNA729271	ed_Lushi_vs_Arbor_Acr	breed_growth_background	ENSGALG00010016808	ENTPD3	-0.345806069	0.350470416	-0.986691183	0.323794054	0.487726781	negative
PRJNA729271	ed_Lushi_vs_Arbor_Acr	breed_growth_background	ENSGALG00010016809	SYTL1	0.26288758	0.283723091	0.926563921	0.354152995	0.517900047	positive
PRJNA729271	ed_Lushi_vs_Arbor_Acr	breed_growth_background	ENSGALG00010016810	SH3GL3	-0.252082843	0.109145621	-2.309601072	0.02091025	0.070339772	negative
PRJNA729271	ed_Lushi_vs_Arbor_Acr	breed_growth_background	ENSGALG00010016812	LRRC31	-0.153478816	0.072209651	-2.125461275	0.033548142	0.098723388	negative
PRJNA729271	ed_Lushi_vs_Arbor_Acr	breed_growth_background	ENSGALG00010016813	ENSGALG00010016813	-0.4373205	0.283073712	-1.544899727	0.122370519	0.247179582	negative
PRJNA729271	ed_Lushi_vs_Arbor_Acr	breed_growth_background	ENSGALG00010016814	HMGXB3	0.163958893	0.053940977	3.039598153	0.00236894	0.015040642	positive
PRJNA729271	ed_Lushi_vs_Arbor_Acr	breed_growth_background	ENSGALG00010016816	MAP3K6	0.009531565	0.138063525	0.069037531	0.944959745	0.970104686	positive
PRJNA729271	ed_Lushi_vs_Arbor_Acr	breed_growth_background	ENSGALG00010016817	GGCT	0.096808952	0.05894894	1.642250943	0.100538009	0.215960054	positive
PRJNA729271	ed_Lushi_vs_Arbor_Acr	breed_growth_background	ENSGALG00010016820	EIF2AK3	-0.076886077	0.058893715	-1.305505628	0.191720732	0.338542146	negative
PRJNA729271	ed_Lushi_vs_Arbor_Acr	breed_growth_background	ENSGALG00010016823	HSF3	0.078086106	0.042239432	1.848654242	0.064507755	0.158634813	positive
PRJNA729271	ed_Lushi_vs_Arbor_Acr	breed_growth_background	ENSGALG00010016825	ENSGALG00010016825	0.179974738	0.156334331	1.15121698	0.249642978	0.405624366	positive
PRJNA729271	ed_Lushi_vs_Arbor_Acr	breed_growth_background	ENSGALG00010016828	LACTBL1	-0.139562793	0.170812508	-0.817052532	0.413898408	0.574293793	negative
PRJNA729271	ed_Lushi_vs_Arbor_Acr	breed_growth_background	ENSGALG00010016831	AIDA	-0.101346213	0.036634091	-2.766445407	0.005667107	0.027826802	negative
PRJNA729271	ed_Lushi_vs_Arbor_Acr	breed_growth_background	ENSGALG00010016832	DYNC1H1	0.048686479	0.050371286	0.966552234	0.333767923	0.498059534	positive
PRJNA729271	ed_Lushi_vs_Arbor_Acr	breed_growth_background	ENSGALG00010016834	ENSGALG00010016834	0.073441193	0.039560501	1.856427257	0.063392683	0.156858473	positive
PRJNA729271	ed_Lushi_vs_Arbor_Acr	breed_growth_background	ENSGALG00010016835	CCDC88A	-0.295048111	0.081681501	-3.612177862	0.000303636	0.003504145	negative
PRJNA729271	ed_Lushi_vs_Arbor_Acr	breed_growth_background	ENSGALG00010016836	HEPH	-0.094110529	0.07951748	-1.183520008	0.236603122	0.391559861	negative
PRJNA729271	ed_Lushi_vs_Arbor_Acr	breed_growth_background	ENSGALG00010016840	LRRC34	0.077621695	0.118467766	0.655213632	0.512330219	0.660990344	positive
PRJNA729271	ed_Lushi_vs_Arbor_Acr	breed_growth_background	ENSGALG00010016842	NPY7R	-0.616040905	0.234564742	-2.626315019	0.008631489	0.037447901	negative
PRJNA729271	ed_Lushi_vs_Arbor_Acr	breed_growth_background	ENSGALG00010016845	MIA3	0.279955273	0.073592438	3.804130961	0.000142303	0.002054462	positive
PRJNA729271	ed_Lushi_vs_Arbor_Acr	breed_growth_background	ENSGALG00010016847	EPHB2	-0.048283122	0.057485806	-0.839913803	0.400956718	0.561913142	negative
PRJNA729271	ed_Lushi_vs_Arbor_Acr	breed_growth_background	ENSGALG00010016848	ENSGALG00010016848	0.134315692	0.092305693	1.45511817	0.14563655	0.27971403	positive
PRJNA729271	ed_Lushi_vs_Arbor_Acr	breed_growth_background	ENSGALG00010016849	WASF2	0.197785675	0.081690334	2.421163749	0.015470907	0.057023361	positive
PRJNA729271	ed_Lushi_vs_Arbor_Acr	breed_growth_background	ENSGALG00010016851	TEC	-0.116603157	0.073743959	-1.581189276	0.113834767	0.235651948	negative
PRJNA729271	ed_Lushi_vs_Arbor_Acr	breed_growth_background	ENSGALG00010016852	AHDC1	0.052187438	0.090980035	0.573614181	0.566228913	0.705716947	positive
PRJNA729271	ed_Lushi_vs_Arbor_Acr	breed_growth_background	ENSGALG00010016854	TAF1A	0.062356188	0.057079073	1.092452701	0.274634169	0.433451904	positive
PRJNA729271	ed_Lushi_vs_Arbor_Acr	breed_growth_background	ENSGALG00010016856	NOD1	0.153810085	0.076677931	2.005923772	0.044864382	0.122468778	positive
PRJNA729271	ed_Lushi_vs_Arbor_Acr	breed_growth_background	ENSGALG00010016858	ENSGALG00010016858	-0.353126298	0.103710478	-3.404924005	0.000661825	0.006056281	negative
PRJNA729271	ed_Lushi_vs_Arbor_Acr	breed_growth_background	ENSGALG00010016859	RPIA	0.458033498	0.069740274	6.567704306	5.10969E-11	1.61011E-08	positive
PRJNA729271	ed_Lushi_vs_Arbor_Acr	breed_growth_background	ENSGALG00010016860	VSX1	0.33345727	0.274287758	1.215720573	0.224091384	0.376821881	positive
PRJNA729271	ed_Lushi_vs_Arbor_Acr	breed_growth_background	ENSGALG00010016861	FGR	-0.111239136	0.065066322	-1.709626938	0.087334881	0.195720331	negative
PRJNA729271	ed_Lushi_vs_Arbor_Acr	breed_growth_background	ENSGALG00010016862	TXK	0.043200714	0.198816057	0.217289863	0.827982456	0.895107824	positive
PRJNA729271	ed_Lushi_vs_Arbor_Acr	breed_growth_background	ENSGALG00010016863	MYNN	0.02735998	0.045106899	0.606558653	0.544143847	0.688371885	positive
PRJNA729271	ed_Lushi_vs_Arbor_Acr	breed_growth_background	ENSGALG00010016864	ENSGALG00010016864	-0.159849603	0.143417614	-1.114574415	0.265032839	0.423322425	negative
PRJNA729271	ed_Lushi_vs_Arbor_Acr	breed_growth_background	ENSGALG00010016865	CFAP36	-0.252361936	0.075722868	-3.332704396	0.000860063	0.007316166	negative
PRJNA729271	ed_Lushi_vs_Arbor_Acr	breed_growth_background	ENSGALG00010016866	ZNRF2	0.027780859	0.055389425	0.501555289	0.615980377	0.744487248	positive
PRJNA729271	ed_Lushi_vs_Arbor_Acr	breed_growth_background	ENSGALG00010016867	FLT4	-0.151353356	0.064465911	-2.347804504	0.01888443	0.0656899	negative
PRJNA729271	ed_Lushi_vs_Arbor_Acr	breed_growth_background	ENSGALG00010016868	NIPAL1	-0.547592857	0.263747224	-2.076203299	0.037875152	0.108262736	negative
PRJNA729271	ed_Lushi_vs_Arbor_Acr	breed_growth_background	ENSGALG00010016869	HSP90AA1	0.301693721	0.083603588	3.608621678	0.000307828	0.003532834	positive
PRJNA729271	ed_Lushi_vs_Arbor_Acr	breed_growth_background	ENSGALG00010016872	EDA2R	-0.258270146	0.149630632	-1.72605129	0.084338186	0.191034455	negative
PRJNA729271	ed_Lushi_vs_Arbor_Acr	breed_growth_background	ENSGALG00010016873	ENTPD6	-0.17926895	0.075359686	-2.378844162	0.017367016	0.061729991	negative
PRJNA729271	ed_Lushi_vs_Arbor_Acr	breed_growth_background	ENSGALG00010016874	ADAMTSL3	-0.211076147	0.077751503	-2.714753265	0.006632516	0.031183369	negative
PRJNA729271	ed_Lushi_vs_Arbor_Acr	breed_growth_background	ENSGALG00010016875	SESN2	0.126383087	0.115646388	1.092840766	0.274463719	0.433296688	positive
PRJNA729271	ed_Lushi_vs_Arbor_Acr	breed_growth_background	ENSGALG00010016876	MTURN	-0.186588279	0.104679044	-1.782479781	0.074671026	0.175764293	negative
PRJNA729271	ed_Lushi_vs_Arbor_Acr	breed_growth_background	ENSGALG00010016877	UBE2D3	0.211311046	0.072529004	2.913469572	0.003574367	0.019980889	positive
PRJNA729271	ed_Lushi_vs_Arbor_Acr	breed_growth_background	ENSGALG00010016878	SUCLG1	0.07740203	0.067040388	1.154558199	0.248271393	0.40439306	positive
PRJNA729271	ed_Lushi_vs_Arbor_Acr	breed_growth_background	ENSGALG00010016879	RNF130	-0.082960602	0.054502621	-1.522139699	0.127974081	0.255191127	negative
PRJNA729271	ed_Lushi_vs_Arbor_Acr	breed_growth_background	ENSGALG00010016880	AR	0.446789308	0.107478682	4.157003986	3.22448E-05	0.000703899	positive
PRJNA729271	ed_Lushi_vs_Arbor_Acr	breed_growth_background	ENSGALG00010016881	C1QB	-0.115630664	0.155255258	-0.744777766	0.456406078	0.612318657	negative
PRJNA729271	ed_Lushi_vs_Arbor_Acr	breed_growth_background	ENSGALG00010016882	ENSGALG00010016882	0.014978661	0.027104601	0.552624284	0.58052071	0.717116728	positive
PRJNA729271	ed_Lushi_vs_Arbor_Acr	breed_growth_background	ENSGALG00010016883	TMEM251	-0.011727967	0.042294785	-0.277291094	0.781556597	0.864783426	negative
PRJNA729271	ed_Lushi_vs_Arbor_Acr	breed_growth_background	ENSGALG00010016884	SBK1	0.299307441	0.146969089	2.036533273	0.041696835	0.115940079	positive
PRJNA729271	ed_Lushi_vs_Arbor_Acr	breed_growth_background	ENSGALG00010016885	CDCA4	-0.270787195	0.082268854	-3.291491045	0.000996578	0.008129092	negative
PRJNA729271	ed_Lushi_vs_Arbor_Acr	breed_growth_background	ENSGALG00010016887	TJP2	-0.129726551	0.074619923	-1.73849752	0.082123187	0.187283762	negative
PRJNA729271	ed_Lushi_vs_Arbor_Acr	breed_growth_background	ENSGALG00010016888	ENSGALG00010016888	0.028340138	0.046955546	0.603552523	0.546141186	0.689875076	positive
PRJNA729271	ed_Lushi_vs_Arbor_Acr	breed_growth_background	ENSGALG00010016889	FUNDC2	-0.035189181	0.046493882	-0.756856171	0.449136036	0.605489847	negative
PRJNA729271	ed_Lushi_vs_Arbor_Acr	breed_growth_background	ENSGALG00010016890	UBR7	-0.118847212	0.040808779	-2.912295242	0.003587833	0.020017569	negative
PRJNA729271	ed_Lushi_vs_Arbor_Acr	breed_growth_background	ENSGALG00010016891	ENSGALG00010016891	3.275130767	0.62334393	5.254131159			positive
PRJNA729271	ed_Lushi_vs_Arbor_Acr	breed_growth_background	ENSGALG00010016892	MAL	0.25478287	0.236129546	1.078996145	0.280589446	0.440500815	positive
PRJNA729271	ed_Lushi_vs_Arbor_Acr	breed_growth_background	ENSGALG00010016893	TBC1D9B	-0.076697753	0.027515123	-2.787476339	0.005312033	0.026606055	negative
PRJNA729271	ed_Lushi_vs_Arbor_Acr	breed_growth_background	ENSGALG00010016894	BTBD7	0.094834586	0.049192209	1.927837528	0.053875338	0.139675018	positive
PRJNA729271	ed_Lushi_vs_Arbor_Acr	breed_growth_background	ENSGALG00010016895	ENSGALG00010016895	0.168647533	0.086272345	1.954827268	0.050603462	0.133495486	positive
PRJNA729271	ed_Lushi_vs_Arbor_Acr	breed_growth_background	ENSGALG00010016896	SPTBN1	-0.142376554	0.052818757	-2.695568046	0.007026872	0.032479118	negative
PRJNA729271	ed_Lushi_vs_Arbor_Acr	breed_growth_background	ENSGALG00010016898	ENSGALG00010016898	-1.138662438	0.392595488	-2.900345198	0.003727519	0.020598699	negative
PRJNA729271	ed_Lushi_vs_Arbor_Acr	breed_growth_background	ENSGALG00010016899	SAXO2	0.277174641	0.322518407	0.859407201	0.390115898	0.551782232	positive
PRJNA729271	ed_Lushi_vs_Arbor_Acr	breed_growth_background	ENSGALG00010016900	CISD2	0.133838332	0.049544182	2.701393549	0.006904958	0.032089569	positive
PRJNA729271	ed_Lushi_vs_Arbor_Acr	breed_growth_background	ENSGALG00010016901	MECOM	-0.080747241	0.103625549	-0.779221357	0.435849331	0.59359542	negative
PRJNA729271	ed_Lushi_vs_Arbor_Acr	breed_growth_background	ENSGALG00010016902	MRNIP	-0.320670878	0.079148168	-4.051526209	5.08846E-05	0.000980815	negative
PRJNA729271	ed_Lushi_vs_Arbor_Acr	breed_growth_background	ENSGALG00010016903	PPM1A	-0.014682606	0.078558808	-0.186899557	0.851739386	0.91064135	negative
PRJNA729271	ed_Lushi_vs_Arbor_Acr	breed_growth_background	ENSGALG00010016904	EAF1	-0.029305362	0.037632556	-0.778723671	0.436142509	0.593883106	negative
PRJNA729271	ed_Lushi_vs_Arbor_Acr	breed_growth_background	ENSGALG00010016905	C1QC	-0.191463814	0.197804472	-0.967944818	0.333071929	0.497462918	negative
PRJNA729271	ed_Lushi_vs_Arbor_Acr	breed_growth_background	ENSGALG00010016907	SQSTM1	0.042907676	0.084980315	0.504913121	0.613619858	0.743174212	positive
PRJNA729271	ed_Lushi_vs_Arbor_Acr	breed_growth_background	ENSGALG00010016908	CMC4	0.411530654	0.085292425	4.824937911	1.40047E-06	6.44438E-05	positive
PRJNA729271	ed_Lushi_vs_Arbor_Acr	breed_growth_background	ENSGALG00010016909	MRPS5	0.29310719	0.072243588	4.057207003	4.96631E-05	0.000962511	positive
PRJNA729271	ed_Lushi_vs_Arbor_Acr	breed_growth_background	ENSGALG00010016910	NSD2	0.0365259	0.059797472	0.610826827	0.541314231	0.686169635	positive
PRJNA729271	ed_Lushi_vs_Arbor_Acr	breed_growth_background	ENSGALG00010016911	AXIN1	0.172024827	0.054239467	3.171580339	0.001516119	0.010982583	positive
PRJNA729271	ed_Lushi_vs_Arbor_Acr	breed_growth_background	ENSGALG00010016912	CCDC88C	0.101121897	0.085443036	1.183500748	0.23661075	0.391559861	positive
PRJNA729271	ed_Lushi_vs_Arbor_Acr	breed_growth_background	ENSGALG00010016913	BRCC3	0.048827748	0.060935709	0.801299411	0.422958332	0.582165134	positive
PRJNA729271	ed_Lushi_vs_Arbor_Acr	breed_growth_background	ENSGALG00010016915	PLOD2	-0.230490763	0.090049387	-2.559603906	0.010479152	0.043275018	negative
PRJNA729271	ed_Lushi_vs_Arbor_Acr	breed_growth_background	ENSGALG00010016916	ACYP2	0.185276243	0.083840925	2.209854467	0.027115264	0.084523818	positive
PRJNA729271	ed_Lushi_vs_Arbor_Acr	breed_growth_background	ENSGALG00010016917	METTL6	-0.006840916	0.091314004	-0.074916399	0.940281228	0.967308474	negative
PRJNA729271	ed_Lushi_vs_Arbor_Acr	breed_growth_background	ENSGALG00010016918	RCOR1	0.045207356	0.040632468	1.112591935	0.265883726	0.424101315	positive
PRJNA729271	ed_Lushi_vs_Arbor_Acr	breed_growth_background	ENSGALG00010016919	ARHGDIG	-0.161720957	0.106159872	-1.523371812	0.12766571	0.25468132	negative
PRJNA729271	ed_Lushi_vs_Arbor_Acr	breed_growth_background	ENSGALG00010016920	C1QA	-0.03552192	0.169743131	-0.209268672	0.834238506	0.899260093	negative
PRJNA729271	ed_Lushi_vs_Arbor_Acr	breed_growth_background	ENSGALG00010016921	EFL1	0.072366433	0.040587449	1.782975653	0.074590267	0.17563124	positive
PRJNA729271	ed_Lushi_vs_Arbor_Acr	breed_growth_background	ENSGALG00010016922	ENSGALG00010016922	-0.235973195	0.104472314	-2.258715118	0.023901111	0.077505962	negative
PRJNA729271	ed_Lushi_vs_Arbor_Acr	breed_growth_background	ENSGALG00010016923	ENSGALG00010016923	-0.221880386	0.095582644	-2.321345972	0.020268179	0.068931782	negative
PRJNA729271	ed_Lushi_vs_Arbor_Acr	breed_growth_background	ENSGALG00010016924	LETM1	0.119233409	0.062822116	1.897952777	0.057702303	0.146607341	positive
PRJNA729271	ed_Lushi_vs_Arbor_Acr	breed_growth_background	ENSGALG00010016925	PHACTR4	0.098776144	0.072740568	1.357923725	0.174487888	0.318338822	positive
PRJNA729271	ed_Lushi_vs_Arbor_Acr	breed_growth_background	ENSGALG00010016926	ENSGALG00010016926	0.172225004	0.098769118	1.743713089	0.081209132	0.185901195	positive
PRJNA729271	ed_Lushi_vs_Arbor_Acr	breed_growth_background	ENSGALG00010016927	MGAT4B	0.030575349	0.061913787	0.493837485	0.621420957	0.748566175	positive
PRJNA729271	ed_Lushi_vs_Arbor_Acr	breed_growth_background	ENSGALG00010016928	RGS11	-0.12782223	0.17774188	-0.719145258	0.472051424	0.626104411	negative
PRJNA729271	ed_Lushi_vs_Arbor_Acr	breed_growth_background	ENSGALG00010016930	C10H15ORF48	0.47304086	0.186291506	2.539250822	0.011109015	0.045130373	positive
PRJNA729271	ed_Lushi_vs_Arbor_Acr	breed_growth_background	ENSGALG00010016931	SLC30A4	-0.088107479	0.044260473	-1.9906583	0.046518466	0.125811693	negative
PRJNA729271	ed_Lushi_vs_Arbor_Acr	breed_growth_background	ENSGALG00010016932	OGT	0.04138212	0.049326161	0.838948733	0.401498079	0.562508666	positive
PRJNA729271	ed_Lushi_vs_Arbor_Acr	breed_growth_background	ENSGALG00010016934	SLC25A38	0.021891576	0.076480027	0.286239123	0.774694981	0.860145825	positive
PRJNA729271	ed_Lushi_vs_Arbor_Acr	breed_growth_background	ENSGALG00010016935	PRIMA1	0.065251522	0.141158637	0.462256677	0.643897264	0.767218258	positive
PRJNA729271	ed_Lushi_vs_Arbor_Acr	breed_growth_background	ENSGALG00010016936	RCC1	0.197949569	0.088725966	2.231021839	0.025679682	0.081521462	positive
PRJNA729271	ed_Lushi_vs_Arbor_Acr	breed_growth_background	ENSGALG00010016937	DHRS7	-0.146881789	0.092102914	-1.594757234	0.110766534	0.231418036	negative
PRJNA729271	ed_Lushi_vs_Arbor_Acr	breed_growth_background	ENSGALG00010016938	PSME4	0.07140303	0.089013351	0.802160904	0.422459888	0.581589522	positive
PRJNA729271	ed_Lushi_vs_Arbor_Acr	breed_growth_background	ENSGALG00010016939	FAM234A	-0.22989315	0.042957165	-5.351683498	8.71397E-08	7.47391E-06	negative
PRJNA729271	ed_Lushi_vs_Arbor_Acr	breed_growth_background	ENSGALG00010016940	FGFR3	0.219732221	0.12005871	1.830206419	0.067219078	0.163617219	positive
PRJNA729271	ed_Lushi_vs_Arbor_Acr	breed_growth_background	ENSGALG00010016941	SLC8A1	-0.350783416	0.092549369	-3.790230226	0.000150508	0.002140932	negative
PRJNA729271	ed_Lushi_vs_Arbor_Acr	breed_growth_background	ENSGALG00010016942	ZBTB40	0.307344947	0.081173326	3.786280088	0.000152919	0.002168851	positive
PRJNA729271	ed_Lushi_vs_Arbor_Acr	breed_growth_background	ENSGALG00010016943	PCNX4	-0.098689623	0.059321242	-1.663647294	0.096182935	0.209021235	negative
PRJNA729271	ed_Lushi_vs_Arbor_Acr	breed_growth_background	ENSGALG00010016944	OPRD1	0.426538617	0.317212525	1.344646201	0.178739516	0.323046045	positive
PRJNA729271	ed_Lushi_vs_Arbor_Acr	breed_growth_background	ENSGALG00010016945	BDH2	-0.090408128	0.078227649	-1.155705548	0.247801618	0.403900197	negative
PRJNA729271	ed_Lushi_vs_Arbor_Acr	breed_growth_background	ENSGALG00010016946	TAF1	0.03763342	0.057039374	0.659779683	0.509395223	0.658258337	positive
PRJNA729271	ed_Lushi_vs_Arbor_Acr	breed_growth_background	ENSGALG00010016947	CANX	-0.184621902	0.038362136	-4.812607508	1.48974E-06	6.74408E-05	negative
PRJNA729271	ed_Lushi_vs_Arbor_Acr	breed_growth_background	ENSGALG00010016948	CCR8	0.36595551	0.31556194	1.159694702	0.246173128	0.402014363	positive
PRJNA729271	ed_Lushi_vs_Arbor_Acr	breed_growth_background	ENSGALG00010016949	LUC7L	0.163865408	0.051469612	3.183731175	0.0014539	0.010692174	positive
PRJNA729271	ed_Lushi_vs_Arbor_Acr	breed_growth_background	ENSGALG00010016950	ENSGALG00010016950	0.509355133	0.521710443	0.976317686	0.328907058	0.492765665	positive
PRJNA729271	ed_Lushi_vs_Arbor_Acr	breed_growth_background	ENSGALG00010016952	GINS1	-0.298149032	0.121209087	-2.45979109	0.013901791	0.052739635	negative
PRJNA729271	ed_Lushi_vs_Arbor_Acr	breed_growth_background	ENSGALG00010016953	GJA4	0.128478872	0.085961239	1.494614013	0.135015207	0.264966886	positive
PRJNA729271	ed_Lushi_vs_Arbor_Acr	breed_growth_background	ENSGALG00010016955	ENSGALG00010016955	-0.096404862	0.144795769	-0.665798889	0.505539655	0.655203621	negative
PRJNA729271	ed_Lushi_vs_Arbor_Acr	breed_growth_background	ENSGALG00010016956	CENPE	-0.331748841	0.137601035	-2.410947288	0.015911148	0.058152317	negative
PRJNA729271	ed_Lushi_vs_Arbor_Acr	breed_growth_background	ENSGALG00010016957	WNT4	0.53092189	0.121117656	4.383521833	1.16776E-05	0.00032665	positive
PRJNA729271	ed_Lushi_vs_Arbor_Acr	breed_growth_background	ENSGALG00010016958	TLR15	0.39098178	0.304286407	1.284913725	0.198822434	0.347513708	positive
PRJNA729271	ed_Lushi_vs_Arbor_Acr	breed_growth_background	ENSGALG00010016959	PERCC1	-0.577160745	0.657173783	-0.878246759	0.379809822	0.541757859	negative
PRJNA729271	ed_Lushi_vs_Arbor_Acr	breed_growth_background	ENSGALG00010016960	GJB3	0.031953328	0.529601299	0.060334687	0.951889076	0.973433394	positive

PRJNA729271	ed_Lushi_vs_Arbor_Acr	breed_growth_background	ENSGALG00010016961	EIF4A3	0.037766099	0.040199976	0.939455753	0.3474968	0.511522912	positive
PRJNA729271	ed_Lushi_vs_Arbor_Acr	breed_growth_background	ENSGALG00010016962	SQOR	-0.002725264	0.072380422	-0.037651955	0.969965183	0.982586499	negative
PRJNA729271	ed_Lushi_vs_Arbor_Acr	breed_growth_background	ENSGALG00010016963	CD8A	0.874675401	0.354348627	2.468403524	0.013571723	0.051878198	positive
PRJNA729271	ed_Lushi_vs_Arbor_Acr	breed_growth_background	ENSGALG00010016964	MAML1	0.012477483	0.045931747	0.271652696	0.785889077	0.867326189	positive
PRJNA729271	ed_Lushi_vs_Arbor_Acr	breed_growth_background	ENSGALG00010016965	SLC25A33	0.049259543	0.181839325	0.270895986	0.786471032	0.867374086	positive
PRJNA729271	ed_Lushi_vs_Arbor_Acr	breed_growth_background	ENSGALG00010016966	KDM3A	0.197808866	0.084752579	2.333956895	0.019597979	0.067315807	positive
PRJNA729271	ed_Lushi_vs_Arbor_Acr	breed_growth_background	ENSGALG00010016967	ERLEC1	0.07158036	0.040421505	1.770848473	0.076585903	0.178647033	positive
PRJNA729271	ed_Lushi_vs_Arbor_Acr	breed_growth_background	ENSGALG00010016968	C15orf61	0.07305296	0.079513061	0.918754218	0.358224144	0.521960089	positive
PRJNA729271	ed_Lushi_vs_Arbor_Acr	breed_growth_background	ENSGALG00010016969	CHMP3	0.17387192	0.044703413	3.889455126	0.00010047	0.001589854	positive
PRJNA729271	ed_Lushi_vs_Arbor_Acr	breed_growth_background	ENSGALG00010016970	SYNE3	-0.353760507	0.052810857	-6.698632195	2.10379E-11	7.26059E-09	negative
PRJNA729271	ed_Lushi_vs_Arbor_Acr	breed_growth_background	ENSGALG00010016972	ASB3	-0.08190829	0.063036568	-1.299377384	0.193814449	0.341104959	negative
PRJNA729271	ed_Lushi_vs_Arbor_Acr	breed_growth_background	ENSGALG00010016973	PLSCR1	-0.070136285	0.064593791	-1.085805378	0.277565106	0.436841066	negative
PRJNA729271	ed_Lushi_vs_Arbor_Acr	breed_growth_background	ENSGALG00010016974	CFAP96	0.310670297	0.401986612	0.772837421	0.439618604	0.596990038	positive
PRJNA729271	ed_Lushi_vs_Arbor_Acr	breed_growth_background	ENSGALG00010016975	MALL	0.273687389	0.12238141	2.236347735	0.025329002	0.080708701	positive
PRJNA729271	ed_Lushi_vs_Arbor_Acr	breed_growth_background	ENSGALG00010016976	ENSGALG00010016976	0.64216831	0.265971798	2.414422564	0.015760173	0.05774317	positive
PRJNA729271	ed_Lushi_vs_Arbor_Acr	breed_growth_background	ENSGALG00010016977	CLCN7	0.144799022	0.06471294	2.237559024	0.025249826	0.080563645	positive
PRJNA729271	ed_Lushi_vs_Arbor_Acr	breed_growth_background	ENSGALG00010016978	FAM181A	-0.217805932	0.182606631	-1.192760256	0.23296328	0.387505604	negative
PRJNA729271	ed_Lushi_vs_Arbor_Acr	breed_growth_background	ENSGALG00010016979	PLSCR5	0.584779497	0.261347615	2.237554364	0.02525013	0.080563645	positive
PRJNA729271	ed_Lushi_vs_Arbor_Acr	breed_growth_background	ENSGALG00010016980	NPHP1	-0.025456399	0.055123901	-0.461803293	0.644222389	0.767308993	negative
PRJNA729271	ed_Lushi_vs_Arbor_Acr	breed_growth_background	ENSGALG00010016981	ENSGALG00010016981	0.592520313	0.262423902	2.25787479	0.023953467	0.077597174	positive
PRJNA729271	ed_Lushi_vs_Arbor_Acr	breed_growth_background	ENSGALG00010016982	GLRX5	-0.027065708	0.075726936	-0.3574119	0.720783469	0.82115647	negative
PRJNA729271	ed_Lushi_vs_Arbor_Acr	breed_growth_background	ENSGALG00010016983	ZFAND2A	0.408677227	0.154258783	2.649296324	0.008065957	0.03579783	positive
PRJNA729271	ed_Lushi_vs_Arbor_Acr	breed_growth_background	ENSGALG00010016984	RNF103	0.238932459	0.061469585	3.887002933	0.00010149	0.001600752	positive
PRJNA729271	ed_Lushi_vs_Arbor_Acr	breed_growth_background	ENSGALG00010016985	ENSGALG00010016985	-0.128762509	0.097873788	-1.31559748	0.18830918	0.334215938	negative
PRJNA729271	ed_Lushi_vs_Arbor_Acr	breed_growth_background	ENSGALG00010016986	GFER	0.099048927	0.047286929	2.094636502	0.036203318	0.104556105	positive
PRJNA729271	ed_Lushi_vs_Arbor_Acr	breed_growth_background	ENSGALG00010016987	ASB2	-0.147882609	0.169474851	-0.872593234	0.38288483	0.544322833	negative
PRJNA729271	ed_Lushi_vs_Arbor_Acr	breed_growth_background	ENSGALG00010016988	ECI1	-0.11415276	0.058619583	-1.947348542	0.051492963	0.135338259	negative
PRJNA729271	ed_Lushi_vs_Arbor_Acr	breed_growth_background	ENSGALG00010016989	RHOG2	-0.078861024	0.05008086	-1.574673928	0.115331729	0.237935299	negative
PRJNA729271	ed_Lushi_vs_Arbor_Acr	breed_growth_background	ENSGALG00010016991	PDLIM3	-0.015329635	0.106387921	-0.144091879	0.885427917	0.932384865	negative
PRJNA729271	ed_Lushi_vs_Arbor_Acr	breed_growth_background	ENSGALG00010016993	ENSGALG00010016993	0.149330547	0.096148887	1.553117782	0.120394999	0.244895524	positive
PRJNA729271	ed_Lushi_vs_Arbor_Acr	breed_growth_background	ENSGALG00010016994	ATL2	0.017436424	0.061842956	0.28194681	0.777984296	0.862278817	positive
PRJNA729271	ed_Lushi_vs_Arbor_Acr	breed_growth_background	ENSGALG00010016998	DNASE1L2	-0.28899318	0.186771383	-1.547309733	0.121788576	0.246450566	negative
PRJNA729271	ed_Lushi_vs_Arbor_Acr	breed_growth_background	ENSGALG00010016999	ITGB1BP2	-0.092205892	0.091410447	-1.008701899	0.313117621	0.47654766	negative
PRJNA729271	ed_Lushi_vs_Arbor_Acr	breed_growth_background	ENSGALG00010017001	TPCN3	-0.167721919	0.066519674	-2.521388169	0.011689282	0.046670309	negative
PRJNA729271	ed_Lushi_vs_Arbor_Acr	breed_growth_background	ENSGALG00010017003	CPZ	0.061429605	0.21276591	0.288719209	0.772796262	0.858685608	positive
PRJNA729271	ed_Lushi_vs_Arbor_Acr	breed_growth_background	ENSGALG00010017004	CX3CR1	0.703334105	0.257849198	2.727695533	0.006377845	0.030320386	positive
PRJNA729271	ed_Lushi_vs_Arbor_Acr	breed_growth_background	ENSGALG00010017006	DNAH3	0.164998161	0.13156097	1.254157376	0.209784809	0.359606292	positive
PRJNA729271	ed_Lushi_vs_Arbor_Acr	breed_growth_background	ENSGALG00010017008	RTN1	0.019507932	0.094636587	0.206135199	0.836685304	0.900861581	positive
PRJNA729271	ed_Lushi_vs_Arbor_Acr	breed_growth_background	ENSGALG00010017010	NONO	-0.047337983	0.029859653	-1.585349392	0.112886973	0.234359306	negative
PRJNA729271	ed_Lushi_vs_Arbor_Acr	breed_growth_background	ENSGALG00010017012	SYNGR3	-0.028745845	0.096134603	-0.299016631	0.764927356	0.852481815	negative
PRJNA729271	ed_Lushi_vs_Arbor_Acr	breed_growth_background	ENSGALG00010017016	ZIC1	0.686121898	0.526642116	1.30282383	0.192634913	0.33948244	positive
PRJNA729271	ed_Lushi_vs_Arbor_Acr	breed_growth_background	ENSGALG00010017017	ENSGALG00010017017	0.39165056	0.144947444	2.702017699	0.006892009	0.032049944	positive
PRJNA729271	ed_Lushi_vs_Arbor_Acr	breed_growth_background	ENSGALG00010017018	HP1BP3	-0.127777122	0.045018724	-2.838310576	0.004535303	0.023689808	negative
PRJNA729271	ed_Lushi_vs_Arbor_Acr	breed_growth_background	ENSGALG00010017020	GJB5	0.39874296	0.230162462	1.732441324	0.083195016	0.189162417	positive
PRJNA729271	ed_Lushi_vs_Arbor_Acr	breed_growth_background	ENSGALG00010017022	CCR4	-0.2304638	0.342476184	-0.672933799	0.500989408	0.652105017	negative
PRJNA729271	ed_Lushi_vs_Arbor_Acr	breed_growth_background	ENSGALG00010017024	BUB1	-0.365654865	0.11839843	-3.088342165	0.002012766	0.01330633	negative
PRJNA729271	ed_Lushi_vs_Arbor_Acr	breed_growth_background	ENSGALG00010017033	LDAF1	-0.04695416	0.059587773	-0.787983128	0.430706575	0.588859819	negative
PRJNA729271	ed_Lushi_vs_Arbor_Acr	breed_growth_background	ENSGALG00010017034	ZMYM3	0.132693304	0.048952192	2.710671341	0.006714715	0.031457592	positive
PRJNA729271	ed_Lushi_vs_Arbor_Acr	breed_growth_background	ENSGALG00010017035	PIK3CD	-0.074613991	0.109551957	-0.681083143	0.495818883	0.647293067	negative
PRJNA729271	ed_Lushi_vs_Arbor_Acr	breed_growth_background	ENSGALG00010017038	ZNF598	0.245397214	0.070620477	3.474873336	0.000511095	0.005046537	positive
PRJNA729271	ed_Lushi_vs_Arbor_Acr	breed_growth_background	ENSGALG00010017039	MAP2K5	-0.175333658	0.042584353	-4.117325892	3.83294E-05	0.000798254	negative
PRJNA729271	ed_Lushi_vs_Arbor_Acr	breed_growth_background	ENSGALG00010017041	JKAMP	0.029792677	0.049250034	0.604927039	0.545227477	0.689081199	positive
PRJNA729271	ed_Lushi_vs_Arbor_Acr	breed_growth_background	ENSGALG00010017042	HNRNPLL	-0.12706152	0.038274168	-3.31977224	0.000900909	0.007548369	negative
PRJNA729271	ed_Lushi_vs_Arbor_Acr	breed_growth_background	ENSGALG00010017045	GLB1	-0.303382775	0.053338373	-5.687889608	1.28619E-08	1.66458E-06	negative
PRJNA729271	ed_Lushi_vs_Arbor_Acr	breed_growth_background	ENSGALG00010017049	TRAF3	-0.030904591	0.049768324	-0.620969104	0.534619952	0.680003177	negative
PRJNA729271	ed_Lushi_vs_Arbor_Acr	breed_growth_background	ENSGALG00010017050	L3HYPDH	-0.352976256	0.084415393	-4.181420501	2.89694E-05	0.000644035	negative
PRJNA729271	ed_Lushi_vs_Arbor_Acr	breed_growth_background	ENSGALG00010017054	GALM	-0.354888166	0.082064231	-4.324517045	1.52866E-05	0.000397096	negative
PRJNA729271	ed_Lushi_vs_Arbor_Acr	breed_growth_background	ENSGALG00010017055	GPR135	0.332923237	0.180646582	1.842953421	0.065335812	0.160162792	positive
PRJNA729271	ed_Lushi_vs_Arbor_Acr	breed_growth_background	ENSGALG00010017056	AGTR1	-0.087589604	0.107796053	-0.812549267	0.416476529	0.57674857	negative
PRJNA729271	ed_Lushi_vs_Arbor_Acr	breed_growth_background	ENSGALG00010017057	NDUFAB1	0.100695984	0.064967445	1.549945274	0.121154652	0.245998297	positive
PRJNA729271	ed_Lushi_vs_Arbor_Acr	breed_growth_background	ENSGALG00010017060	CLSTN1	-0.023590424	0.055008665	-0.428849241	0.668032942	0.78486007	negative
PRJNA729271	ed_Lushi_vs_Arbor_Acr	breed_growth_background	ENSGALG00010017063	TMPPE	-0.129647798	0.059265436	-2.187578586	0.028700313	0.088026034	negative
PRJNA729271	ed_Lushi_vs_Arbor_Acr	breed_growth_background	ENSGALG00010017065	VRK2	0.046702501	0.073702885	0.633659059	0.526303349	0.672565849	positive
PRJNA729271	ed_Lushi_vs_Arbor_Acr	breed_growth_background	ENSGALG00010017068	ZP2	-0.717433536	0.625178906	-1.147565168	0.251148116	0.407202678	negative
PRJNA729271	ed_Lushi_vs_Arbor_Acr	breed_growth_background	ENSGALG00010017070	BLOC1S6	0.074842978	0.057102817	1.310670509	0.189969106	0.336420549	positive
PRJNA729271	ed_Lushi_vs_Arbor_Acr	breed_growth_background	ENSGALG00010017077	CTNNBIP1	-0.021579583	0.056789219	-0.379994369	0.703949595	0.808602059	negative
PRJNA729271	ed_Lushi_vs_Arbor_Acr	breed_growth_background	ENSGALG00010017080	PALB2	-0.023370191	0.057018286	-0.409871867	0.681899944	0.794479296	negative
PRJNA729271	ed_Lushi_vs_Arbor_Acr	breed_growth_background	ENSGALG00010017083	ENSGALG00010017083	0.124853273	0.226140327	0.552105301	0.580876211	0.717372469	positive
PRJNA729271	ed_Lushi_vs_Arbor_Acr	breed_growth_background	ENSGALG00010017085	ENSGALG00010017085	-0.060386481	0.065217581	-0.92592335	0.354485816	0.518136641	negative
PRJNA729271	ed_Lushi_vs_Arbor_Acr	breed_growth_background	ENSGALG00010017089	CRTAP	-0.110160502	0.084230983	-1.307838252	0.190928177	0.337706397	negative
PRJNA729271	ed_Lushi_vs_Arbor_Acr	breed_growth_background	ENSGALG00010017094	SORBS2	0.047065253	0.079534031	0.591762451	0.554009671	0.695993255	positive
PRJNA729271	ed_Lushi_vs_Arbor_Acr	breed_growth_background	ENSGALG00010017096	DCTN5	-0.065819098	0.049560689	-1.328050498	0.184161425	0.329558007	negative
PRJNA729271	ed_Lushi_vs_Arbor_Acr	breed_growth_background	ENSGALG00010017098	DAAM1	0.040967501	0.067296948	0.608757189	0.542685394	0.687426792	positive
PRJNA729271	ed_Lushi_vs_Arbor_Acr	breed_growth_background	ENSGALG00010017100	SUSD5	-0.014035225	0.331673302	-0.042316416	0.966246459	0.980988414	negative
PRJNA729271	ed_Lushi_vs_Arbor_Acr	breed_growth_background	ENSGALG00010017101	CSMD2	0.126645805	0.131031859	0.966526814	0.333780636	0.498059534	positive
PRJNA729271	ed_Lushi_vs_Arbor_Acr	breed_growth_background	ENSGALG00010017104	FANCL	-0.298946114	0.098464264	-3.036087435	0.002396699	0.015143917	negative
PRJNA729271	ed_Lushi_vs_Arbor_Acr	breed_growth_background	ENSGALG00010017105	PLK1	-0.496774618	0.127211638	-3.905103542	9.4185E-05	0.001523119	negative
PRJNA729271	ed_Lushi_vs_Arbor_Acr	breed_growth_background	ENSGALG00010017106	CRYM	0.048841229	0.063606353	0.767867141	0.442566121	0.599588365	positive
PRJNA729271	ed_Lushi_vs_Arbor_Acr	breed_growth_background	ENSGALG00010017109	ITGB1BP3	0.000520404	0.068157287	0.007635332	0.993907946	0.996451492	positive
PRJNA729271	ed_Lushi_vs_Arbor_Acr	breed_growth_background	ENSGALG00010017110	BRF1	0.054021807	0.039465558	1.368834244	0.171051094	0.313806557	positive
PRJNA729271	ed_Lushi_vs_Arbor_Acr	breed_growth_background	ENSGALG00010017111	GJB1	-0.096562622	0.256557469	-0.376378138	0.706635797	0.81072391	negative
PRJNA729271	ed_Lushi_vs_Arbor_Acr	breed_growth_background	ENSGALG00010017112	LZIC	-0.060948503	0.034335833	-1.775069859	0.075886353	0.177644168	negative
PRJNA729271	ed_Lushi_vs_Arbor_Acr	breed_growth_background	ENSGALG00010017115	LGALSL	-0.203993517	0.102337044	-1.99334971	0.046223167	0.125164356	negative
PRJNA729271	ed_Lushi_vs_Arbor_Acr	breed_growth_background	ENSGALG00010017116	ENSGALG00010017116	-0.151407743	0.157732858	-0.959899827	0.337105633	0.501369398	negative
PRJNA729271	ed_Lushi_vs_Arbor_Acr	breed_growth_background	ENSGALG00010017119	BTBD6	0.073544841	0.081248589	0.905182995	0.365368449	0.528787242	positive
PRJNA729271	ed_Lushi_vs_Arbor_Acr	breed_growth_background	ENSGALG00010017120	ERN2	-0.031025217	0.075693131	-0.409881538	0.681892849	0.794479296	negative
PRJNA729271	ed_Lushi_vs_Arbor_Acr	breed_growth_background	ENSGALG00010017122	NCBP2	-0.161813256	0.103538666	-1.562829253	0.118092762	0.2418074	negative
PRJNA729271	ed_Lushi_vs_Arbor_Acr	breed_growth_background	ENSGALG00010017127	SOX8	-0.069560957	0.072824479	-0.955186479	0.339483402	0.503613951	negative
PRJNA729271	ed_Lushi_vs_Arbor_Acr	breed_growth_background	ENSGALG00010017131	TLR3	-0.30590105	0.201114626	-1.521028366	0.128252719	0.255465599	negative
PRJNA729271	ed_Lushi_vs_Arbor_Acr	breed_growth_background	ENSGALG00010017132	TMEM108	0.016833797	0.157678278	0.106760404	0.914979061	0.952022215	positive
PRJNA729271	ed_Lushi_vs_Arbor_Acr	breed_growth_background	ENSGALG00010017133	WDR20	0.060389861	0.060367292	1.000373856	0.317129617	0.480562474	positive
PRJNA729271	ed_Lushi_vs_Arbor_Acr	breed_growth_background	ENSGALG00010017135	ENSGALG00010017135	-0.006851767	0.071062084	-0.09641945	0.923187445	0.957127674	negative
PRJNA729271	ed_Lushi_vs_Arbor_Acr	breed_growth_background	ENSGALG00010017136	ENSGALG00010017136	0.081637273	0.224603363	0.363473067	0.716251528	0.818065236	positive
PRJNA729271	ed_Lushi_vs_Arbor_Acr	breed_growth_background	ENSGALG00010017139	COL9A2	0.121347526	0.318423843	0.381088066	0.703137902	0.808237302	positive
PRJNA729271	ed_Lushi_vs_Arbor_Acr	breed_growth_background	ENSGALG00010017140	UQCRC2	0.045450661	0.061492151	0.739129473	0.459828382	0.615098966	positive
PRJNA729271	ed_Lushi_vs_Arbor_Acr	breed_growth_background	ENSGALG00010017141	AFTPH	0.017546946	0.040889034	0.429135743	0.667824442	0.78486007	positive
PRJNA729271	ed_Lushi_vs_Arbor_Acr	breed_growth_background	ENSGALG00010017142	RBP7	0.141387518	0.371153491	0.380940829	0.703247156	0.808237302	positive
PRJNA729271	ed_Lushi_vs_Arbor_Acr	breed_growth_background	ENSGALG00010017143	FAM149A	-0.079152327	0.148157424	-0.534244761	0.593172205	0.72761115	negative
PRJNA729271	ed_Lushi_vs_Arbor_Acr	breed_growth_background	ENSGALG00010017144	CPB1	-0.658414024	0.389756109	-1.689297506	0.091162431	0.201801991	negative
PRJNA729271	ed_Lushi_vs_Arbor_Acr	breed_growth_background	ENSGALG00010017147	SRSF7L	0.008875196	0.038432777	0.230927793	0.817370898	0.887342059	positive
PRJNA729271	ed_Lushi_vs_Arbor_Acr	breed_growth_background	ENSGALG00010017148	MOK	0.015765166	0.269684219	0.058457874	0.953383917	0.974074966	positive
PRJNA729271	ed_Lushi_vs_Arbor_Acr	breed_growth_background	ENSGALG00010017152	CYP4V2	-0.242958829	0.10224915	-2.376145215	0.017494576	0.062092038	negative
PRJNA729271	ed_Lushi_vs_Arbor_Acr	breed_growth_background	ENSGALG00010017153	SLC35F4	0.673329582	0.325192725	2.070555488	0.038400356	0.109354254	positive
PRJNA729271	ed_Lushi_vs_Arbor_Acr	breed_growth_background	ENSGALG00010017154	PIGZ	0.177575386	0.10402257	1.707085155	0.087806226	0.196533546	positive
PRJNA729271	ed_Lushi_vs_Arbor_Acr	breed_growth_background	ENSGALG00010017162	BCL11A	0.201138941	0.119865498	1.678038675	0.093339549	0.204869286	positive
PRJNA729271	ed_Lushi_vs_Arbor_Acr	breed_growth_background	ENSGALG00010017163	CDV3	-0.378905431	0.066878975	-5.665538812	1.46563E-08	1.84733E-06	negative
PRJNA729271	ed_Lushi_vs_Arbor_Acr	breed_growth_background	ENSGALG00010017167	SMAP2	-0.017167648	0.052378314	-0.327762512	0.743091234	0.836047288	negative
PRJNA729271	ed_Lushi_vs_Arbor_Acr	breed_growth_background	ENSGALG00010017169	CCDC198	0.32606604	0.127270065	2.562001043	0.010407101	0.043022855	positive
PRJNA729271	ed_Lushi_vs_Arbor_Acr	breed_growth_background	ENSGALG00010017174	DHX57	0.059297034	0.053703398	1.104157947	0.269524628	0.428165889	positive
PRJNA729271	ed_Lushi_vs_Arbor_Acr	breed_growth_background	ENSGALG00010017180	ENSGALG00010017180	0.23387992	0.316270082	0.739494292	0.459606904	0.61486768	positive
PRJNA729271	ed_Lushi_vs_Arbor_Acr	breed_growth_background	ENSGALG00010017183	NFYC	0.077414077	0.050193506	1.542312599	0.122997649	0.247962576	positive
PRJNA729271	ed_Lushi_vs_Arbor_Acr	breed_growth_background	ENSGALG00010017184	CARD11	-0.034129088	0.113427869	-0.300888033	0.763499876	0.851367851	negative
PRJNA729271	ed_Lushi_vs_Arbor_Acr	breed_growth_background	ENSGALG00010017185	NAA30	0.193477046	0.034655104	5.582930714	2.36499E-08	2.78703E-06	positive
PRJNA729271	ed_Lushi_vs_Arbor_Acr	breed_growth_background	ENSGALG00010017187	ZNF839	0.318862447	0.074042462	4.306480898	1.65872E-05	0.000416693	positive
PRJNA729271	ed_Lushi_vs_Arbor_Acr	breed_growth_background	ENSGALG00010017188	ENSGALG00010017188	-0.113632544	0.084010513	-1.352599097	0.176183741	0.320183384	negative
PRJNA729271	ed_Lushi_vs_Arbor_Acr	breed_growth_background	ENSGALG00010017190	STK26	0.079088767	0.03675661	2.15168826	0.031421915	0.094314153	positive
PRJNA729271	ed_Lushi_vs_Arbor_Acr	breed_growth_background	ENSGALG00010017198	TOPBP1	-0.086626395	0.037913958	-2.284815415	0.022323653	0.073893893	negative
PRJNA729271	ed_Lushi_vs_Arbor_Acr	breed_growth_background	ENSGALG00010017200	KCNQ4	-0.695328042	0.164405322	-4.229352397	2.34365E-05	0.000541805	negative
PRJNA729271	ed_Lushi_vs_Arbor_Acr	breed_growth_background	ENSGALG00010017201	AP5M1	-0.120661065	0.085863271	-1.405269847	0.159941051	0.298731802	negative
PRJNA729271	ed_Lushi_vs_Arbor_Acr	breed_growth_background	ENSGALG00010017207	ENSGALG00010017207	0.861288505	0.137016484	6.286021046	3.25706E-10	7.86851E-08	positive
PRJNA729271	ed_Lushi_vs_Arbor_Acr	breed_growth_background	ENSGALG00010017208	CINP	-0.043894611	0.094526872	-0.464361195	0.642388987	0.765805442	negative
PRJNA729271	ed_Lushi_vs_Arbor_Acr	breed_growth_background	ENSGALG00010017211	UBFD1	0.147896131	0.06864775	2.154420663	0.031207198	0.093770383	positive
PRJNA729271	ed_Lushi_vs_Arbor_Acr	breed_growth_background	ENSGALG00010017212	TINAGL1	-0.125318601	0.070744091	-1.77143558	0.076488296	0.178476796	negative
PRJNA729271	ed_Lushi_vs_Arbor_Acr	breed_growth_background	ENSGALG00010017215	FRMD7	0.170985239	0.317813903	0.538004278	0.590574095	0.725639697	positive
PRJNA729271	ed_Lushi_vs_Arbor_Acr	breed_growth_background	ENSGALG00010017219	EARS2	-0.145911863	0.08097982	-1.801829926	0.071572168	0.170463124	negative
PRJNA729271	ed_Lushi_vs_Arbor_Acr	breed_growth_background	ENSGALG00010017221	SPPL2A	-0.072260817	0.040479852	-1.785105749	0.074244166	0.17507226	negative
PRJNA729271	ed_Lushi_vs_Arbor_Acr	breed_growth_background	ENSGALG00010017223	NOC2L	0.029338851	0.042068079	0.697413618	0.485543982	0.637343776	positive
PRJNA729271	ed_Lushi_vs_Arbor_Acr	breed_growth_background	ENSGALG00010017226	PEF1	0.133120231	0.041016819	3.245503493	0.001172431	0.009171285	positive
PRJNA729271	ed_Lushi_vs_Arbor_Acr	breed_growth_background	ENSGALG00010017228	RAP2C	-0.053566802	0.038904818	-1.376868066	0.168553042	0.310284016	negative
PRJNA729271	ed_Lushi_vs_Arbor_Acr	breed_growth_background	ENSGALG00010017240	MELTF	-0.046509478	0.129080835	-0.360312804	0.718613225	0.819922757	negative
PRJNA729271	ed_Lushi_vs_Arbor_Acr	breed_growth_background	ENSGALG00010017246	GGA2	0.097340435	0.071290896	1.365397831	0.172128045	0.315263586	positive

PRJNA729271	ed_Lushi_vs_Arbor_Acr	breed_growth_background	ENSGALG00010017248	TECPR2	0.114682369	0.045287505	2.532318078	0.011331115	0.045746661	positive
PRJNA729271	ed_Lushi_vs_Arbor_Acr	breed_growth_background	ENSGALG00010017250	COL16A1	0.007249109	0.090963819	0.079692223	0.936482045	0.964898329	positive
PRJNA729271	ed_Lushi_vs_Arbor_Acr	breed_growth_background	ENSGALG00010017252	EXOC5	-0.103496717	0.047457414	-2.180833494	0.029195734	0.089149393	negative
PRJNA729271	ed_Lushi_vs_Arbor_Acr	breed_growth_background	ENSGALG00010017256	CARHSP1	-0.070069468	0.069083769	-1.01426817	0.310454808	0.473339901	negative
PRJNA729271	ed_Lushi_vs_Arbor_Acr	breed_growth_background	ENSGALG00010017263	COG7	-0.06668558	0.039098758	-1.705567744	0.088088589	0.197043843	negative
PRJNA729271	ed_Lushi_vs_Arbor_Acr	breed_growth_background	ENSGALG00010017264	PIAS1	0.040736822	0.046157236	0.882566319	0.377470633	0.539749119	positive
PRJNA729271	ed_Lushi_vs_Arbor_Acr	breed_growth_background	ENSGALG00010017266	PMM2	-0.148948146	0.059529982	-2.502069406	0.012346972	0.048527482	negative
PRJNA729271	ed_Lushi_vs_Arbor_Acr	breed_growth_background	ENSGALG00010017272	NRXN1	-0.30238003	0.071611782	-4.222489957	2.41618E-05	0.000555033	negative
PRJNA729271	ed_Lushi_vs_Arbor_Acr	breed_growth_background	ENSGALG00010017278	CCR5	-0.043367658	0.124049024	-0.349600965	0.726638187	0.824527408	negative
PRJNA729271	ed_Lushi_vs_Arbor_Acr	breed_growth_background	ENSGALG00010017280	SOS1	0.17239427	0.083366972	2.067896507	0.038649756	0.10976818	positive
PRJNA729271	ed_Lushi_vs_Arbor_Acr	breed_growth_background	ENSGALG00010017283	INF2	-0.394473436	0.073943821	-5.334772121	9.56646E-08	7.9693E-06	negative
PRJNA729271	ed_Lushi_vs_Arbor_Acr	breed_growth_background	ENSGALG00010017284	ENSGALG00010017284	-0.315593454	0.107010706	-2.949176451	0.00318622	0.018525575	negative
PRJNA729271	ed_Lushi_vs_Arbor_Acr	breed_growth_background	ENSGALG00010017285	IL21R	1.240376764	0.389557565	3.184065402	0.001452222	0.010685258	positive
PRJNA729271	ed_Lushi_vs_Arbor_Acr	breed_growth_background	ENSGALG00010017289	ADGRB2	0.127627356	0.180140441	0.708488081	0.478642211	0.631583576	positive
PRJNA729271	ed_Lushi_vs_Arbor_Acr	breed_growth_background	ENSGALG00010017290	DACT1	-0.205937148	0.084158612	-2.447012155	0.014404598	0.053840448	negative
PRJNA729271	ed_Lushi_vs_Arbor_Acr	breed_growth_background	ENSGALG00010017296	SCNN1G	0.211482161	0.118766781	1.780650782	0.074969518	0.176266532	positive
PRJNA729271	ed_Lushi_vs_Arbor_Acr	breed_growth_background	ENSGALG00010017299	USP31	0.04334098	0.058942651	0.7353076	0.462152181	0.617182224	positive
PRJNA729271	ed_Lushi_vs_Arbor_Acr	breed_growth_background	ENSGALG00010017300	TRPM7	-0.050918438	0.056595923	-0.899683852	0.368288519	0.53154855	negative
PRJNA729271	ed_Lushi_vs_Arbor_Acr	breed_growth_background	ENSGALG00010017301	LHCGR	0.199819951	0.283224621	0.705517585	0.480488192	0.633382716	positive
PRJNA729271	ed_Lushi_vs_Arbor_Acr	breed_growth_background	ENSGALG00010017304	ANKRD9	0.154892138	0.159507827	0.971062929	0.331516943	0.495871493	positive
PRJNA729271	ed_Lushi_vs_Arbor_Acr	breed_growth_background	ENSGALG00010017305	HS3ST2	-0.857880341	0.206569193	-4.152992646	3.28155E-05	0.000708884	negative
PRJNA729271	ed_Lushi_vs_Arbor_Acr	breed_growth_background	ENSGALG00010017307	TMEM186	0.003779002	0.084515873	0.044713515	0.964335661	0.979609321	positive
PRJNA729271	ed_Lushi_vs_Arbor_Acr	breed_growth_background	ENSGALG00010017310	SAMD11	-0.020422615	0.09711066	-0.210302505	0.83343158	0.898832166	negative
PRJNA729271	ed_Lushi_vs_Arbor_Acr	breed_growth_background	ENSGALG00010017312	PTP4A2	0.298424946	0.082693301	3.608816456	0.000307597	0.003532834	positive
PRJNA729271	ed_Lushi_vs_Arbor_Acr	breed_growth_background	ENSGALG00010017313	MAP4K3	-0.037004527	0.044333074	-0.834693453	0.403890329	0.564714027	negative
PRJNA729271	ed_Lushi_vs_Arbor_Acr	breed_growth_background	ENSGALG00010017314	CALML4	-0.607346559	0.118726724	-5.115500021	3.12911E-07	1.97202E-05	negative
PRJNA729271	ed_Lushi_vs_Arbor_Acr	breed_growth_background	ENSGALG00010017315	HS3ST4	-1.977776965	0.558114211	-3.543677844	0.000394587	0.004202454	negative
PRJNA729271	ed_Lushi_vs_Arbor_Acr	breed_growth_background	ENSGALG00010017318	ADSS1	0.828354082	0.065915507	12.56690761	3.21037E-36	2.32672E-32	positive
PRJNA729271	ed_Lushi_vs_Arbor_Acr	breed_growth_background	ENSGALG00010017322	KHDRBS1	0.054944334	0.038213738	1.43781624	0.1504862	0.286146855	positive
PRJNA729271	ed_Lushi_vs_Arbor_Acr	breed_growth_background	ENSGALG00010017323	ENSGALG00010017323	-0.108050957	0.055881041	-1.933588817	0.053163703	0.138451294	negative
PRJNA729271	ed_Lushi_vs_Arbor_Acr	breed_growth_background	ENSGALG00010017326	STON1	-0.029666714	0.06650609	-0.446075146	0.655542966	0.775491332	negative
PRJNA729271	ed_Lushi_vs_Arbor_Acr	breed_growth_background	ENSGALG00010017327	CLN6	-0.019154071	0.071852968	-0.266573135	0.789797842	0.869521474	negative
PRJNA729271	ed_Lushi_vs_Arbor_Acr	breed_growth_background	ENSGALG00010017332	FGFR1	0.286015078	0.084993969	3.365122019	0.000765098	0.006738256	positive
PRJNA729271	ed_Lushi_vs_Arbor_Acr	breed_growth_background	ENSGALG00010017334	KDM8	-0.295839029	0.086796526	-3.408420178	0.000653402	0.006009557	negative
PRJNA729271	ed_Lushi_vs_Arbor_Acr	breed_growth_background	ENSGALG00010017335	THUMPD2	0.163766519	0.074231221	2.206167652	0.027372263	0.085068816	positive
PRJNA729271	ed_Lushi_vs_Arbor_Acr	breed_growth_background	ENSGALG00010017337	ABAT	0.242212041	0.109392817	2.214149409	0.026818503	0.083978007	positive
PRJNA729271	ed_Lushi_vs_Arbor_Acr	breed_growth_background	ENSGALG00010017338	MBNL3	-0.025697036	0.040214391	-0.639000999	0.522822282	0.669757753	negative
PRJNA729271	ed_Lushi_vs_Arbor_Acr	breed_growth_background	ENSGALG00010017340	SIVA1	-0.050130543	0.05801459	-0.864102343	0.387531672	0.549527645	negative
PRJNA729271	ed_Lushi_vs_Arbor_Acr	breed_growth_background	ENSGALG00010017341	TA3	0.049070048	0.043961442	1.116206531	0.26433374	0.422578312	positive
PRJNA729271	ed_Lushi_vs_Arbor_Acr	breed_growth_background	ENSGALG00010017343	DLG1	0.22026895	0.053235851	4.137605502	3.50949E-05	0.000749191	positive
PRJNA729271	ed_Lushi_vs_Arbor_Acr	breed_growth_background	ENSGALG00010017344	RERE	0.158325774	0.081801073	1.935497531	0.052929271	0.137987372	positive
PRJNA729271	ed_Lushi_vs_Arbor_Acr	breed_growth_background	ENSGALG00010017346	GM2A	-0.045255625	0.086232144	-0.524811542	0.599714207	0.732681409	negative
PRJNA729271	ed_Lushi_vs_Arbor_Acr	breed_growth_background	ENSGALG00010017347	TMEM39B	-0.104211456	0.066297873	-1.571867256	0.11598134	0.238765253	negative
PRJNA729271	ed_Lushi_vs_Arbor_Acr	breed_growth_background	ENSGALG00010017349	ENSGALG00010017349	-0.125504418	0.06068986	-2.067963561	0.03864345	0.10976818	negative
PRJNA729271	ed_Lushi_vs_Arbor_Acr	breed_growth_background	ENSGALG00010017351	CCR2	0.098619212	0.109432025	0.901191513	0.367486503	0.530866739	positive
PRJNA729271	ed_Lushi_vs_Arbor_Acr	breed_growth_background	ENSGALG00010017352	GPCPD1	0.334473082	0.127428985	2.624780238	0.008670491	0.037561426	positive
PRJNA729271	ed_Lushi_vs_Arbor_Acr	breed_growth_background	ENSGALG00010017354	FEM1B	0.163804477	0.056455368	2.9014863	0.00371397	0.020539488	positive
PRJNA729271	ed_Lushi_vs_Arbor_Acr	breed_growth_background	ENSGALG00010017356	NSMCE1	-0.112748151	0.064987897	-1.734909981	0.082756751	0.188461761	negative
PRJNA729271	ed_Lushi_vs_Arbor_Acr	breed_growth_background	ENSGALG00010017359	EHD3	0.004442493	0.051062903	0.087000398	0.930671196	0.961928051	positive
PRJNA729271	ed_Lushi_vs_Arbor_Acr	breed_growth_background	ENSGALG00010017361	METTL22	0.162302791	0.081483296	1.991853534	0.04638713	0.125514552	positive
PRJNA729271	ed_Lushi_vs_Arbor_Acr	breed_growth_background	ENSGALG00010017362	ENO1	-0.140475948	0.069588103	-2.01867766	0.043520733	0.119702662	negative
PRJNA729271	ed_Lushi_vs_Arbor_Acr	breed_growth_background	ENSGALG00010017363	TIMM9	0.070618679	0.076303614	0.925495867	0.354708033	0.518400175	positive
PRJNA729271	ed_Lushi_vs_Arbor_Acr	breed_growth_background	ENSGALG00010017364	AKT1	-0.0217581	0.040019092	-0.543692995	0.586652754	0.722352333	negative
PRJNA729271	ed_Lushi_vs_Arbor_Acr	breed_growth_background	ENSGALG00010017367	TIMD4	-0.498604244	0.170191573	-2.929664703	0.003393279	0.019333956	negative
PRJNA729271	ed_Lushi_vs_Arbor_Acr	breed_growth_background	ENSGALG00010017368	ERLIN2	-0.029259471	0.027137495	-1.078193489	0.280947417	0.440871799	negative
PRJNA729271	ed_Lushi_vs_Arbor_Acr	breed_growth_background	ENSGALG00010017369	CAPN14	-0.377454735	0.278376046	-1.35591672	0.175125667	0.319060652	negative
PRJNA729271	ed_Lushi_vs_Arbor_Acr	breed_growth_background	ENSGALG00010017371	TMEM114	-1.245442018	0.704284807	-1.768378367	0.076997671	0.179220526	negative
PRJNA729271	ed_Lushi_vs_Arbor_Acr	breed_growth_background	ENSGALG00010017372	ENSGALG00010017372	-0.036751169	0.059422213	-0.618475262	0.536262099	0.681374398	negative
PRJNA729271	ed_Lushi_vs_Arbor_Acr	breed_growth_background	ENSGALG00010017373	RP1-2705.3	-0.006137893	0.057918235	-0.105975138	0.915602078	0.952192002	negative
PRJNA729271	ed_Lushi_vs_Arbor_Acr	breed_growth_background	ENSGALG00010017375	BDH1A	0.105111156	0.086698217	1.212379675	0.225367078	0.378468518	positive
PRJNA729271	ed_Lushi_vs_Arbor_Acr	breed_growth_background	ENSGALG00010017376	GALNT14	-0.049320434	0.156074549	-0.316005618	0.751998244	0.842756653	negative
PRJNA729271	ed_Lushi_vs_Arbor_Acr	breed_growth_background	ENSGALG00010017377	USP8	0.144499562	0.040469752	3.570557156	0.000356223	0.003899885	positive
PRJNA729271	ed_Lushi_vs_Arbor_Acr	breed_growth_background	ENSGALG00010017378	ENSGALG00010017378	-1.179659356	0.315561951	-3.738281343	0.000185283	0.002450429	negative
PRJNA729271	ed_Lushi_vs_Arbor_Acr	breed_growth_background	ENSGALG00010017380	MARCKSL1	-0.207092998	0.072542248	-2.854791583	0.004306508	0.022798697	negative
PRJNA729271	ed_Lushi_vs_Arbor_Acr	breed_growth_background	ENSGALG00010017381	CA6	0.797846441	0.379555001	2.10205751	0.035548238	0.103207759	positive
PRJNA729271	ed_Lushi_vs_Arbor_Acr	breed_growth_background	ENSGALG00010017383	ENSGALG00010017383	-0.087784646	0.08229926	-1.0666517	0.286129145	0.44677504	negative
PRJNA729271	ed_Lushi_vs_Arbor_Acr	breed_growth_background	ENSGALG00010017384	ARID4A	0.044617731	0.050013649	0.892111085	0.372333394	0.534883305	positive
PRJNA729271	ed_Lushi_vs_Arbor_Acr	breed_growth_background	ENSGALG00010017386	ENSGALG00010017386	-0.82547029	0.323737289	-2.549815289	0.010778001	0.044156902	negative
PRJNA729271	ed_Lushi_vs_Arbor_Acr	breed_growth_background	ENSGALG00010017387	HS6ST2	-0.018601096	0.061804209	-0.300968114	0.763438809	0.851365251	negative
PRJNA729271	ed_Lushi_vs_Arbor_Acr	breed_growth_background	ENSGALG00010017388	PLPBP	-0.036556301	0.044353373	-0.824205646	0.409822719	0.570258262	negative
PRJNA729271	ed_Lushi_vs_Arbor_Acr	breed_growth_background	ENSGALG00010017390	DDX24	-0.2789831	0.055970265	-4.984487729	6.21262E-07	3.33526E-05	negative
PRJNA729271	ed_Lushi_vs_Arbor_Acr	breed_growth_background	ENSGALG00010017391	CRAMP1	0.151073967	0.062922372	2.400957893	0.016352218	0.059211941	positive
PRJNA729271	ed_Lushi_vs_Arbor_Acr	breed_growth_background	ENSGALG00010017393	VDAC1	0.115899211	0.087241983	1.32847979	0.184019656	0.32938564	positive
PRJNA729271	ed_Lushi_vs_Arbor_Acr	breed_growth_background	ENSGALG00010017395	BRF2	0.235289192	0.069808823	3.370479275	0.000750376	0.006648345	positive
PRJNA729271	ed_Lushi_vs_Arbor_Acr	breed_growth_background	ENSGALG00010017396	CCR8L	-0.24215802	0.140157685	-1.72775414	0.084032312	0.190587246	negative
PRJNA729271	ed_Lushi_vs_Arbor_Acr	breed_growth_background	ENSGALG00010017398	SLC22A5	0.232919829	0.098695741	2.359978534	0.018275993	0.06422073	positive
PRJNA729271	ed_Lushi_vs_Arbor_Acr	breed_growth_background	ENSGALG00010017399	CPXM1	-0.496648844	0.15553516	-3.193161235	0.001407243	0.010466254	negative
PRJNA729271	ed_Lushi_vs_Arbor_Acr	breed_growth_background	ENSGALG00010017400	GPC4	-0.209165165	0.063815203	-3.277669834	0.001046677	0.008442732	negative
PRJNA729271	ed_Lushi_vs_Arbor_Acr	breed_growth_background	ENSGALG00010017402	SLC2A5	-0.024632195	0.120050531	-0.205181892	0.837430017	0.901355023	negative
PRJNA729271	ed_Lushi_vs_Arbor_Acr	breed_growth_background	ENSGALG00010017403	ITGA11	-0.423736923	0.157078101	-2.697619344	0.006983725	0.032349687	negative
PRJNA729271	ed_Lushi_vs_Arbor_Acr	breed_growth_background	ENSGALG00010017405	XCR1	-0.775287249	0.378254401	-2.049645019	0.040399084	0.113200217	negative
PRJNA729271	ed_Lushi_vs_Arbor_Acr	breed_growth_background	ENSGALG00010017406	IFT140	-0.122681964	0.068125019	-1.800835656	0.071728787	0.1705559	negative
PRJNA729271	ed_Lushi_vs_Arbor_Acr	breed_growth_background	ENSGALG00010017407	EPN2	0.061537533	0.045666612	1.34753883	0.177806756	0.322002616	positive
PRJNA729271	ed_Lushi_vs_Arbor_Acr	breed_growth_background	ENSGALG00010017409	GPC3	-0.128634951	0.187961265	-0.684369469	0.49374189	0.64539532	negative
PRJNA729271	ed_Lushi_vs_Arbor_Acr	breed_growth_background	ENSGALG00010017411	RAB11FIP1	0.274082018	0.074359507	3.68590418	0.000227892	0.002843914	positive
PRJNA729271	ed_Lushi_vs_Arbor_Acr	breed_growth_background	ENSGALG00010017413	ZIC3	-0.018817716	0.497688863	-0.037810201	0.969839011	0.982586499	negative
PRJNA729271	ed_Lushi_vs_Arbor_Acr	breed_growth_background	ENSGALG00010017414	ENSGALG00010017414	-0.312529493	0.105739815	-2.9556463	0.003120147	0.018251222	negative
PRJNA729271	ed_Lushi_vs_Arbor_Acr	breed_growth_background	ENSGALG00010017415	HDAC1	-0.023540417	0.035337122	-0.666166788	0.505304499	0.655130385	negative
PRJNA729271	ed_Lushi_vs_Arbor_Acr	breed_growth_background	ENSGALG00010017418	CHGB	-0.084114922	0.155354868	-0.541437307	0.588206198	0.723896149	negative
PRJNA729271	ed_Lushi_vs_Arbor_Acr	breed_growth_background	ENSGALG00010017419	B9D1	0.046432617	0.056456175	0.822454173	0.410818458	0.571205137	positive
PRJNA729271	ed_Lushi_vs_Arbor_Acr	breed_growth_background	ENSGALG00010017421	GPR157	0.200345938	0.139515471	1.436012339	0.15099883	0.286807999	positive
PRJNA729271	ed_Lushi_vs_Arbor_Acr	breed_growth_background	ENSGALG00010017422	ENSGALG00010017422	-0.373177925	0.125752939	-2.967548347	0.003001851	0.017723758	negative
PRJNA729271	ed_Lushi_vs_Arbor_Acr	breed_growth_background	ENSGALG00010017423	TMEM204	-0.225334737	0.071262333	-3.162045452	0.001566651	0.01119202	negative
PRJNA729271	ed_Lushi_vs_Arbor_Acr	breed_growth_background	ENSGALG00010017424	APOD	0.375176958	0.253637019	1.479188485	0.139089946	0.270687701	positive
PRJNA729271	ed_Lushi_vs_Arbor_Acr	breed_growth_background	ENSGALG00010017425	H6PD	-0.112215912	0.066810678	-1.67961043	0.093033137	0.204458738	negative
PRJNA729271	ed_Lushi_vs_Arbor_Acr	breed_growth_background	ENSGALG00010017428	PSMA3	0.027468885	0.057989777	0.473684962	0.635724578	0.76048756	positive
PRJNA729271	ed_Lushi_vs_Arbor_Acr	breed_growth_background	ENSGALG00010017429	TELO2	0.208287409	0.056866542	3.662740889	0.000249531	0.003039454	positive
PRJNA729271	ed_Lushi_vs_Arbor_Acr	breed_growth_background	ENSGALG00010017430	CACNA1H	-0.099047796	0.116681337	-0.848874363	0.395951206	0.557329416	negative
PRJNA729271	ed_Lushi_vs_Arbor_Acr	breed_growth_background	ENSGALG00010017431	FYCO1	0.031435429	0.062264361	0.504870338	0.613649909	0.743174212	positive
PRJNA729271	ed_Lushi_vs_Arbor_Acr	breed_growth_background	ENSGALG00010017433	RNPS1	0.087728594	0.042866013	2.046576923	0.040699643	0.113778461	positive
PRJNA729271	ed_Lushi_vs_Arbor_Acr	breed_growth_background	ENSGALG00010017434	FGF13	-0.172743127	0.078722758	-2.19432261	0.028212225	0.087065688	negative
PRJNA729271	ed_Lushi_vs_Arbor_Acr	breed_growth_background	ENSGALG00010017435	ACTR10	-0.04559091	0.048941706	-0.931534957	0.351576911	0.515591595	negative
PRJNA729271	ed_Lushi_vs_Arbor_Acr	breed_growth_background	ENSGALG00010017437	DRG2	0.111502661	0.07485903	1.489501812	0.136355277	0.266946209	positive
PRJNA729271	ed_Lushi_vs_Arbor_Acr	breed_growth_background	ENSGALG00010017438	ADRB3	0.297753764	0.390760441	0.761985435	0.446068697	0.602868603	positive
PRJNA729271	ed_Lushi_vs_Arbor_Acr	breed_growth_background	ENSGALG00010017439	PPP4R4	-0.200578853	0.154971583	-1.294294409	0.195563747	0.343557934	negative
PRJNA729271	ed_Lushi_vs_Arbor_Acr	breed_growth_background	ENSGALG00010017441	SPSB1	0.168188862	0.137346643	1.224557505	0.220741987	0.373317642	positive
PRJNA729271	ed_Lushi_vs_Arbor_Acr	breed_growth_background	ENSGALG00010017442	LCK	0.204649606	0.133270707	1.535593308	0.124638156	0.250502855	positive
PRJNA729271	ed_Lushi_vs_Arbor_Acr	breed_growth_background	ENSGALG00010017445	F9	-2.130618305	0.565030411	-3.770802888	0.000162723	0.002267954	negative
PRJNA729271	ed_Lushi_vs_Arbor_Acr	breed_growth_background	ENSGALG00010017447	CAPN13	-0.238468607	0.301590875	-0.790702329	0.429117717	0.587963069	negative
PRJNA729271	ed_Lushi_vs_Arbor_Acr	breed_growth_background	ENSGALG00010017449	TRMT6	-0.052413911	0.047544543	-1.102416973	0.270280436	0.428968544	negative
PRJNA729271	ed_Lushi_vs_Arbor_Acr	breed_growth_background	ENSGALG00010017450	ENSGALG00010017450	0.133576241	0.112048571	1.192128026	0.23321105	0.38761543	positive
PRJNA729271	ed_Lushi_vs_Arbor_Acr	breed_growth_background	ENSGALG00010017451	GABPB1	0.033489843	0.042084987	0.795766977	0.426167489	0.585359401	positive
PRJNA729271	ed_Lushi_vs_Arbor_Acr	breed_growth_background	ENSGALG00010017452	ARMH4	-1.98085165	0.254777135	-7.774840745	7.55424E-15	8.42298E-12	negative
PRJNA729271	ed_Lushi_vs_Arbor_Acr	breed_growth_background	ENSGALG00010017453	PPP1R2	0.105101036	0.074204817	1.416364059	0.156668941	0.294121904	positive
PRJNA729271	ed_Lushi_vs_Arbor_Acr	breed_growth_background	ENSGALG00010017454	ENSGALG00010017454	-0.290356692	0.084878491	-3.420851255	0.000624255	0.00579665	negative
PRJNA729271	ed_Lushi_vs_Arbor_Acr	breed_growth_background	ENSGALG00010017455	CCR9	0.215641871	0.332481059	0.648583927	0.516607348	0.664915178	positive
PRJNA729271	ed_Lushi_vs_Arbor_Acr	breed_growth_background	ENSGALG00010017457	ZMAT4	-0.272156234	0.146358922	-1.859512421	0.06295454	0.156082509	negative
PRJNA729271	ed_Lushi_vs_Arbor_Acr	breed_growth_background	ENSGALG00010017458	ENSGALG00010017458	0.337413632	0.092423751	3.650724282	0.000261502	0.003153469	positive
PRJNA729271	ed_Lushi_vs_Arbor_Acr	breed_growth_background	ENSGALG00010017459	FAM167B	-0.223990436	0.150309803	-1.490191797	0.136173813	0.266662985	negative
PRJNA729271	ed_Lushi_vs_Arbor_Acr	breed_growth_background	ENSGALG00010017462	MCM8	0.035718692	0.064253438	0.555903203	0.578277029	0.715381542	positive
PRJNA729271	ed_Lushi_vs_Arbor_Acr	breed_growth_background	ENSGALG00010017463	PPP1R36	-0.217712556	0.179873963	-1.210361701	0.226140131	0.379342808	negative
PRJNA729271	ed_Lushi_vs_Arbor_Acr	breed_growth_background	ENSGALG00010017464	SH3BP2	-0.21906916	0.070233197	-3.119168271	0.001813624	0.012425613	negative
PRJNA729271	ed_Lushi_vs_Arbor_Acr	breed_growth_background	ENSGALG00010017465	TMEM234	-0.35814447	0.130442837	-2.74560473	0.006039949	0.029163576	negative
PRJNA729271	ed_Lushi_vs_Arbor_Acr	breed_growth_background	ENSGALG00010017466	SPIA4	-1.664219937	1.43028231	-1.163560456			negative
PRJNA729271	ed_Lushi_vs_Arbor_Acr	breed_growth_background	ENSGALG00010017467	SFRP1	-0.052924305	0.063430335	-0.834368997	0.404073082	0.564860577	negative
PRJNA729271	ed_Lushi_vs_Arbor_Acr	breed_growth_background	ENSGALG00010017468	AMDHD2	-0.11787026	0.069429125	-1.697706261	0.089563215	0.199541778	negative
PRJNA729271	ed_Lushi_vs_Arbor_Acr	breed_growth_background	ENSGALG00010017470	HSPA2	-0.277479989	0.090755566	-3.057443206	0.002232339	0.014399096	negative
PRJNA729271	ed_Lushi_vs_Arbor_Acr	breed_growth_background	ENSGALG00010017471	CRLS1	0.147454298	0.048042698	3.069234349	0.002146082	0.013951884	positive
PRJNA729271	ed_Lushi_vs_Arbor_Acr	breed_growth_background	ENSGALG00010017472	LZTFL1	-0.147067964	0.093319728	-1.575957919	0.115035502	0.237459356	negative
PRJNA729271	ed_Lushi_vs_Arbor_Acr	breed_growth_background	ENSGALG00010017473	EIF3I	-0.127500482	0.055928616	-2.279700263	0.022625472	0.074603324	negative
PRJNA729271	ed_Lushi_vs_Arbor_Acr	breed_growth_background	ENSGALG00010017475	STUM	-0.321368588	0.131047701	-2.452302381	0.014194532	0.053386027	negative

PRJNA729271	:ed_Lushi_vs_Arbor_Acr	breed_growth_background	ENSGALG00010017476	ATP6V0C	0.061532016	0.060035455	1.024927957	0.305397182	0.468090531	positive
PRJNA729271	:ed_Lushi_vs_Arbor_Acr	breed_growth_background	ENSGALG00010017477	MCF2	-0.190055023	0.097155521	-1.95619374	0.050442336	0.133374983	negative
PRJNA729271	:ed_Lushi_vs_Arbor_Acr	breed_growth_background	ENSGALG00010017479	SSTR5	0.827252084	0.273350658	3.026340197	0.002475336	0.015478861	positive
PRJNA729271	:ed_Lushi_vs_Arbor_Acr	breed_growth_background	ENSGALG00010017480	DCDC2B	0.050267163	0.108094922	0.465027977	0.641911422	0.76542498	positive
PRJNA729271	:ed_Lushi_vs_Arbor_Acr	breed_growth_background	ENSGALG00010017481	SPIA1	-2.070594389	1.30788211	-1.583165924			negative
PRJNA729271	:ed_Lushi_vs_Arbor_Acr	breed_growth_background	ENSGALG00010017482	CORO2B	0.010701533	0.078380997	0.13653224	0.891400539	0.936368897	positive
PRJNA729271	:ed_Lushi_vs_Arbor_Acr	breed_growth_background	ENSGALG00010017484	IQCC	-0.093102535	0.073525266	-1.26626587	0.205417912	0.354745732	negative
PRJNA729271	:ed_Lushi_vs_Arbor_Acr	breed_growth_background	ENSGALG00010017485	LBH	0.003660837	0.057910088	0.063215873	0.949594605	0.972266285	positive
PRJNA729271	:ed_Lushi_vs_Arbor_Acr	breed_growth_background	ENSGALG00010017487	C1QTNF8	-0.222757038	0.276288487	-0.80624799	0.420099873	0.579386076	negative
PRJNA729271	:ed_Lushi_vs_Arbor_Acr	breed_growth_background	ENSGALG00010017489	TNFRSF1B	-0.350062536	0.095329003	-3.672151439	0.000240517	0.002957703	negative
PRJNA729271	:ed_Lushi_vs_Arbor_Acr	breed_growth_background	ENSGALG00010017491	CCDC28B	0.005900144	0.105941992	0.055692212	0.955587004	0.975259256	positive
PRJNA729271	:ed_Lushi_vs_Arbor_Acr	breed_growth_background	ENSGALG00010017493	YPEL5	0.183128403	0.072083541	2.540502315	0.011069336	0.045019648	positive
PRJNA729271	:ed_Lushi_vs_Arbor_Acr	breed_growth_background	ENSGALG00010017494	TBC1D24	-0.226028986	0.052310992	-4.320869775	1.55415E-05	0.000400844	negative
PRJNA729271	:ed_Lushi_vs_Arbor_Acr	breed_growth_background	ENSGALG00010017495	NT5M	-0.114781843	0.072703596	-1.578764327	0.114390124	0.236430179	negative
PRJNA729271	:ed_Lushi_vs_Arbor_Acr	breed_growth_background	ENSGALG00010017496	PARP1	-0.145693614	0.055796054	-2.611181318	0.009023004	0.038786608	negative
PRJNA729271	:ed_Lushi_vs_Arbor_Acr	breed_growth_background	ENSGALG00010017499	SNRPD1	7.6806E-05	0.053280842	0.001441532	0.998849824	0.99912554	positive
PRJNA729271	:ed_Lushi_vs_Arbor_Acr	breed_growth_background	ENSGALG00010017500	ACAP2	-0.167051784	0.054018288	-3.092504247	0.001984754	0.013188298	negative
PRJNA729271	:ed_Lushi_vs_Arbor_Acr	breed_growth_background	ENSGALG00010017502	NAT8B	0.024535837	0.062963068	0.389686179	0.696768618	0.804497461	positive
PRJNA729271	:ed_Lushi_vs_Arbor_Acr	breed_growth_background	ENSGALG00010017503	MED9	-0.064349229	0.067333177	-0.955683838	0.33923199	0.503498638	negative
PRJNA729271	:ed_Lushi_vs_Arbor_Acr	breed_growth_background	ENSGALG00010017505	TNFRSF8	-0.07363938	0.167145872	-0.440569543	0.659524657	0.778612958	negative
PRJNA729271	:ed_Lushi_vs_Arbor_Acr	breed_growth_background	ENSGALG00010017509	RASD1	-0.663907435	0.161685863	-4.106156365	4.02297E-05	0.000827134	negative
PRJNA729271	:ed_Lushi_vs_Arbor_Acr	breed_growth_background	ENSGALG00010017510	HDC	-0.781052145	0.176417197	-4.427301633	9.54193E-06	0.000281691	negative
PRJNA729271	:ed_Lushi_vs_Arbor_Acr	breed_growth_background	ENSGALG00010017513	TNIP2	0.042998673	0.077891802	0.552030787	0.58092726	0.717374394	positive
PRJNA729271	:ed_Lushi_vs_Arbor_Acr	breed_growth_background	ENSGALG00010017514	TXLNA	-0.043244484	0.052270612	-0.827319274	0.408056122	0.568598385	negative
PRJNA729271	:ed_Lushi_vs_Arbor_Acr	breed_growth_background	ENSGALG00010017516	AAK1	-0.022645995	0.04333425	-0.522588822	0.601260421	0.733917457	negative
PRJNA729271	:ed_Lushi_vs_Arbor_Acr	breed_growth_background	ENSGALG00010017517	LIN9	-0.043154365	0.056100247	-0.769236633	0.441752847	0.5991025	negative
PRJNA729271	:ed_Lushi_vs_Arbor_Acr	breed_growth_background	ENSGALG00010017518	PEMT	0.373015646	0.078754173	4.736455642	2.17488E-06	8.90535E-05	positive
PRJNA729271	:ed_Lushi_vs_Arbor_Acr	breed_growth_background	ENSGALG00010017519	NTN3	-0.161498213	0.114246556	-1.413593711	0.157481229	0.295188208	negative
PRJNA729271	:ed_Lushi_vs_Arbor_Acr	breed_growth_background	ENSGALG00010017520	ZBTB1	-0.083733848	0.043833861	-1.910254911	0.0561004	0.14395031	negative
PRJNA729271	:ed_Lushi_vs_Arbor_Acr	breed_growth_background	ENSGALG00010017521	MIIP	0.114877284	0.06318468	1.818119248	0.069045909	0.166304494	positive
PRJNA729271	:ed_Lushi_vs_Arbor_Acr	breed_growth_background	ENSGALG00010017522	XXYLT1	-0.157166746	0.057800709	-2.719114517	0.006545694	0.030901708	negative
PRJNA729271	:ed_Lushi_vs_Arbor_Acr	breed_growth_background	ENSGALG00010017524	ACBD3	-0.09906753	0.032574331	-3.041275943	0.002355778	0.015001298	negative
PRJNA729271	:ed_Lushi_vs_Arbor_Acr	breed_growth_background	ENSGALG00010017525	ENSGALG00010017525	-0.273498523	0.363724886	-0.75193789	0.452088411	0.608338425	negative
PRJNA729271	:ed_Lushi_vs_Arbor_Acr	breed_growth_background	ENSGALG00010017526	PLEKHA2	-0.164815939	0.091203229	-1.807128335	0.070742272	0.169069947	negative
PRJNA729271	:ed_Lushi_vs_Arbor_Acr	breed_growth_background	ENSGALG00010017528	RAI1	0.124973759	0.095463932	1.309120163	0.19049365	0.337126159	positive
PRJNA729271	:ed_Lushi_vs_Arbor_Acr	breed_growth_background	ENSGALG00010017529	MFN2	0.035100543	0.063546092	0.552363517	0.580699321	0.717223652	positive
PRJNA729271	:ed_Lushi_vs_Arbor_Acr	breed_growth_background	ENSGALG00010017530	TLR1B	-0.18101116	0.114917749	-1.575136658	0.115224904	0.2377826	negative
PRJNA729271	:ed_Lushi_vs_Arbor_Acr	breed_growth_background	ENSGALG00010017531	SACM1L	-0.083257107	0.060647476	-1.372804161	0.169813237	0.31200949	negative
PRJNA729271	:ed_Lushi_vs_Arbor_Acr	breed_growth_background	ENSGALG00010017532	MIXL1	-0.056750125	0.175684109	-0.323023664	0.74667732	0.838619176	negative
PRJNA729271	:ed_Lushi_vs_Arbor_Acr	breed_growth_background	ENSGALG00010017533	FAM193A	0.055166282	0.042388282	1.301451237	0.193104045	0.340122615	positive
PRJNA729271	:ed_Lushi_vs_Arbor_Acr	breed_growth_background	ENSGALG00010017534	ATP11C	-0.119269103	0.04563069	-2.613791341	0.00895437	0.038571649	negative
PRJNA729271	:ed_Lushi_vs_Arbor_Acr	breed_growth_background	ENSGALG00010017535	LIMD1	-0.056653677	0.04875345	-1.162044473	0.245217401	0.401048587	negative
PRJNA729271	:ed_Lushi_vs_Arbor_Acr	breed_growth_background	ENSGALG00010017536	GSC	0.193788183	0.273681604	0.708078951	0.47889623	0.631801298	positive
PRJNA729271	:ed_Lushi_vs_Arbor_Acr	breed_growth_background	ENSGALG00010017537	MANBA	-0.23985225	0.100237356	-2.39284295	0.016718396	0.060132294	negative
PRJNA729271	:ed_Lushi_vs_Arbor_Acr	breed_growth_background	ENSGALG00010017538	H3-3A	-0.040100267	0.040744164	-0.984196586	0.325018864	0.488946163	negative
PRJNA729271	:ed_Lushi_vs_Arbor_Acr	breed_growth_background	ENSGALG00010017539	KPNA6	0.192304697	0.074074724	2.596090627	0.009429119	0.040175542	positive
PRJNA729271	:ed_Lushi_vs_Arbor_Acr	breed_growth_background	ENSGALG00010017540	EIF4G1	0.093256003	0.088076205	1.058810419	0.289686127	0.450875166	positive
PRJNA729271	:ed_Lushi_vs_Arbor_Acr	breed_growth_background	ENSGALG00010017541	SREBF1	0.100970009	0.121739937	0.829391006	0.406883189	0.567584624	positive
PRJNA729271	:ed_Lushi_vs_Arbor_Acr	breed_growth_background	ENSGALG00010017542	PLOD1	-0.204310818	0.067050456	-3.04712047	0.002310451	0.014753298	negative
PRJNA729271	:ed_Lushi_vs_Arbor_Acr	breed_growth_background	ENSGALG00010017543	ZBTB25	-0.100623329	0.095838193	-1.049929326	0.293750607	0.455051304	negative
PRJNA729271	:ed_Lushi_vs_Arbor_Acr	breed_growth_background	ENSGALG00010017544	SDE2	0.205938654	0.036938888	5.575117796	2.47362E-08	2.89155E-06	positive
PRJNA729271	:ed_Lushi_vs_Arbor_Acr	breed_growth_background	ENSGALG00010017545	DICER1	0.07785016	0.065036791	1.197017229	0.231299839	0.385274665	positive
PRJNA729271	:ed_Lushi_vs_Arbor_Acr	breed_growth_background	ENSGALG00010017546	NFU1	-0.036250741	0.057779578	-0.627397125	0.530398952	0.676117562	negative
PRJNA729271	:ed_Lushi_vs_Arbor_Acr	breed_growth_background	ENSGALG00010017547	RNF4	0.151838815	0.035667211	4.257098093	2.07098E-05	0.000492919	positive
PRJNA729271	:ed_Lushi_vs_Arbor_Acr	breed_growth_background	ENSGALG00010017550	TM2D2	-0.020656048	0.041116512	-0.50237841	0.615401363	0.744159736	negative
PRJNA729271	:ed_Lushi_vs_Arbor_Acr	breed_growth_background	ENSGALG00010017551	NALF2	-0.095502134	0.153169314	-0.623506966	0.532951426	0.678357123	negative
PRJNA729271	:ed_Lushi_vs_Arbor_Acr	breed_growth_background	ENSGALG00010017552	LARS2	0.099653562	0.055600505	1.792313964	0.073082688	0.172995863	positive
PRJNA729271	:ed_Lushi_vs_Arbor_Acr	breed_growth_background	ENSGALG00010017553	EDA	-0.033442928	0.058382793	-0.572821655	0.566765455	0.706085057	negative
PRJNA729271	:ed_Lushi_vs_Arbor_Acr	breed_growth_background	ENSGALG00010017554	FAM131A	-0.456066518	0.195331695	-2.33483111	0.019552245	0.067210674	negative
PRJNA729271	:ed_Lushi_vs_Arbor_Acr	breed_growth_background	ENSGALG00010017555	CELA2A	-2.270745526	0.659898273	-3.441053899	0.000579453	0.005482489	negative
PRJNA729271	:ed_Lushi_vs_Arbor_Acr	breed_growth_background	ENSGALG00010017556	TOM1L2	0.081974415	0.061890658	1.324503848	0.185335766	0.330872226	positive
PRJNA729271	:ed_Lushi_vs_Arbor_Acr	breed_growth_background	ENSGALG00010017558	ENSGALG00010017558	-0.104529563	0.040564427	-2.576877606	0.009969725	0.041730048	negative
PRJNA729271	:ed_Lushi_vs_Arbor_Acr	breed_growth_background	ENSGALG00010017559	DUSP10	-0.098026172	0.079971433	-1.225764846	0.22028718	0.372847112	negative
PRJNA729271	:ed_Lushi_vs_Arbor_Acr	breed_growth_background	ENSGALG00010017561	CFAP99	-0.721407733	0.367265369	-1.964268326	0.049498983	0.13153503	negative
PRJNA729271	:ed_Lushi_vs_Arbor_Acr	breed_growth_background	ENSGALG00010017562	SLC30A10	-0.404414171	0.330927936	-1.222061139	0.221684507	0.374162711	negative
PRJNA729271	:ed_Lushi_vs_Arbor_Acr	breed_growth_background	ENSGALG00010017563	ALK	0.029053157	0.081079935	0.358327327	0.72009837	0.820777375	positive
PRJNA729271	:ed_Lushi_vs_Arbor_Acr	breed_growth_background	ENSGALG00010017565	ENSGALG00010017565	-0.386541502	0.102162567	-3.783592287	0.000154581	0.002185913	negative
PRJNA729271	:ed_Lushi_vs_Arbor_Acr	breed_growth_background	ENSGALG00010017568	CDCP1	0.459513068	0.138488433	3.318061002	0.000906447	0.007577248	positive
PRJNA729271	:ed_Lushi_vs_Arbor_Acr	breed_growth_background	ENSGALG00010017569	CLMN	-0.545041639	0.132224207	-4.122101777	3.75431E-05	0.000792276	negative
PRJNA729271	:ed_Lushi_vs_Arbor_Acr	breed_growth_background	ENSGALG00010017570	DRC3	0.333126062	0.218511679	1.524522918	0.127378136	0.254282618	positive
PRJNA729271	:ed_Lushi_vs_Arbor_Acr	breed_growth_background	ENSGALG00010017571	ADAM9	-0.042114711	0.080253486	-0.524771108	0.599742319	0.732681409	negative
PRJNA729271	:ed_Lushi_vs_Arbor_Acr	breed_growth_background	ENSGALG00010017573	ENSGALG00010017573	-0.06745717	0.123577041	-0.545871384	0.58515435	0.721119903	negative
PRJNA729271	:ed_Lushi_vs_Arbor_Acr	breed_growth_background	ENSGALG00010017574	DYNC1LI1	-0.046438174	0.030587368	-1.518214141	0.128960424	0.256417194	negative
PRJNA729271	:ed_Lushi_vs_Arbor_Acr	breed_growth_background	ENSGALG00010017575	CLCN2	0.288478597	0.377843465	0.763487061	0.445172976	0.601928171	positive
PRJNA729271	:ed_Lushi_vs_Arbor_Acr	breed_growth_background	ENSGALG00010017576	ENSGALG00010017576	-0.425343263	0.163840248	-2.596085321	0.009429265	0.040175542	negative
PRJNA729271	:ed_Lushi_vs_Arbor_Acr	breed_growth_background	ENSGALG00010017577	CLPX	0.224713357	0.073536392	3.055811543	0.002244523	0.014459715	positive
PRJNA729271	:ed_Lushi_vs_Arbor_Acr	breed_growth_background	ENSGALG00010017578	BSDC1	0.092818291	0.053880652	1.722664581	0.084949199	0.192186458	positive
PRJNA729271	:ed_Lushi_vs_Arbor_Acr	breed_growth_background	ENSGALG00010017579	P3H2	-0.086981249	0.07675458	-1.133238554	0.257114088	0.414418848	negative
PRJNA729271	:ed_Lushi_vs_Arbor_Acr	breed_growth_background	ENSGALG00010017580	LYPLAL1	-0.148785757	0.083593352	-1.779875476	0.075096342	0.176507455	negative
PRJNA729271	:ed_Lushi_vs_Arbor_Acr	breed_growth_background	ENSGALG00010017581	EFCAB11	0.349344656	0.254980335	1.370084704	0.170660462	0.313130816	positive
PRJNA729271	:ed_Lushi_vs_Arbor_Acr	breed_growth_background	ENSGALG00010017582	CLCN6	0.139852484	0.055823286	2.505271458	0.012235744	0.048181776	positive
PRJNA729271	:ed_Lushi_vs_Arbor_Acr	breed_growth_background	ENSGALG00010017583	MTHFD1	-0.156879894	0.05759579	-2.723808329	0.006453395	0.030587703	negative
PRJNA729271	:ed_Lushi_vs_Arbor_Acr	breed_growth_background	ENSGALG00010017584	WNT11B	0.153558883	0.424800357	0.361484826	0.717737046	0.819180983	positive
PRJNA729271	:ed_Lushi_vs_Arbor_Acr	breed_growth_background	ENSGALG00010017585	ENSGALG00010017585	0.042130092	0.065753985	0.640723023	0.521702653	0.668855471	positive
PRJNA729271	:ed_Lushi_vs_Arbor_Acr	breed_growth_background	ENSGALG00010017586	ZFYVE28	-0.028045336	0.086617106	-0.323785184	0.746100674	0.838156186	negative
PRJNA729271	:ed_Lushi_vs_Arbor_Acr	breed_growth_background	ENSGALG00010017587	MB21D2	-0.097158535	0.079855752	-1.216675469	0.223727716	0.376342407	negative
PRJNA729271	:ed_Lushi_vs_Arbor_Acr	breed_growth_background	ENSGALG00010017588	NOX5	-0.645509727	0.290204508	-2.224327014	0.02612645	0.082431303	negative
PRJNA729271	:ed_Lushi_vs_Arbor_Acr	breed_growth_background	ENSGALG00010017589	ATPAF2	0.206346966	0.07905577	2.610144289	0.009050404	0.038885412	positive
PRJNA729271	:ed_Lushi_vs_Arbor_Acr	breed_growth_background	ENSGALG00010017590	ENSGALG00010017590	0.097282558	0.104133531	0.934209738	0.350195714	0.514034113	positive
PRJNA729271	:ed_Lushi_vs_Arbor_Acr	breed_growth_background	ENSGALG00010017591	ESR2	-0.049740752	0.154974559	-0.320960759	0.748240128	0.839794387	negative
PRJNA729271	:ed_Lushi_vs_Arbor_Acr	breed_growth_background	ENSGALG00010017592	GID4	0.030536069	0.060445838	0.505180673	0.613431943	0.743174212	positive
PRJNA729271	:ed_Lushi_vs_Arbor_Acr	breed_growth_background	ENSGALG00010017593	MTHFR	0.228242904	0.071772704	3.180079477	0.001472347	0.010791029	positive
PRJNA729271	:ed_Lushi_vs_Arbor_Acr	breed_growth_background	ENSGALG00010017594	CEP68	-0.029446529	0.054486535	-0.540436801	0.588895834	0.724560319	negative
PRJNA729271	:ed_Lushi_vs_Arbor_Acr	breed_growth_background	ENSGALG00010017595	CCNF	-0.106337039	0.070153047	-1.515786457	0.129573358	0.257182234	negative
PRJNA729271	:ed_Lushi_vs_Arbor_Acr	breed_growth_background	ENSGALG00010017596	TDP1	-0.054045362	0.054645907	-0.989010247	0.322658128	0.486571948	negative
PRJNA729271	:ed_Lushi_vs_Arbor_Acr	breed_growth_background	ENSGALG00010017597	CNOT10	0.143451572	0.073364302	1.955332058	0.05054389	0.133495486	positive
PRJNA729271	:ed_Lushi_vs_Arbor_Acr	breed_growth_background	ENSGALG00010017598	ENSGALG00010017598	0.151591208	0.063303375	2.394678136	0.016634961	0.059954106	positive
PRJNA729271	:ed_Lushi_vs_Arbor_Acr	breed_growth_background	ENSGALG00010017599	KCNK13	0.071255356	0.12433464	0.573093353	0.566581487	0.705974268	positive
PRJNA729271	:ed_Lushi_vs_Arbor_Acr	breed_growth_background	ENSGALG00010017600	MXD4	-0.003664809	0.055255957	-0.066324235	0.947119689	0.970898153	negative
PRJNA729271	:ed_Lushi_vs_Arbor_Acr	breed_growth_background	ENSGALG00010017601	ENSGALG00010017601	-0.014414017	0.061251089	-0.235326707	0.813955168	0.885537337	negative
PRJNA729271	:ed_Lushi_vs_Arbor_Acr	breed_growth_background	ENSGALG00010017603	CYP3A5	-2.146196578	0.732501873	-2.929953706	0.003390125	0.019323579	negative
PRJNA729271	:ed_Lushi_vs_Arbor_Acr	breed_growth_background	ENSGALG00010017604	GATM	-0.310674703	0.128814941	-2.411790901	0.015874383	0.058047219	negative
PRJNA729271	:ed_Lushi_vs_Arbor_Acr	breed_growth_background	ENSGALG00010017605	GFPT1	0.057651843	0.038991404	1.478578271	0.139253064	0.270881998	positive
PRJNA729271	:ed_Lushi_vs_Arbor_Acr	breed_growth_background	ENSGALG00010017606	ENSGALG00010017606	0.539321239	0.137481759	3.922856696	8.75052E-05	0.001451245	positive
PRJNA729271	:ed_Lushi_vs_Arbor_Acr	breed_growth_background	ENSGALG00010017608	RBBP4	0.033573134	0.043583262	0.770321732	0.441109069	0.598509403	positive
PRJNA729271	:ed_Lushi_vs_Arbor_Acr	breed_growth_background	ENSGALG00010017609	TRIM71	0.098915186	0.111787921	0.884846811	0.376239258	0.538679183	positive
PRJNA729271	:ed_Lushi_vs_Arbor_Acr	breed_growth_background	ENSGALG00010017610	PSMC1	0.13843717	0.063927458	2.165535362	0.03034671	0.091698054	positive
PRJNA729271	:ed_Lushi_vs_Arbor_Acr	breed_growth_background	ENSGALG00010017612	CLDN11	-0.041377484	0.227827061	-0.181617952	0.85588256	0.91334887	negative
PRJNA729271	:ed_Lushi_vs_Arbor_Acr	breed_growth_background	ENSGALG00010017613	HAUS3	-0.221348737	0.066261324	-3.340541997	0.00083615	0.007171597	negative
PRJNA729271	:ed_Lushi_vs_Arbor_Acr	breed_growth_background	ENSGALG00010017615	AGTRAP	0.065740941	0.073477492	0.894708552	0.370942906	0.53410325	positive
PRJNA729271	:ed_Lushi_vs_Arbor_Acr	breed_growth_background	ENSGALG00010017616	PDCD7	0.26332753	0.083244632	3.163297412	0.001559929	0.011171526	positive
PRJNA729271	:ed_Lushi_vs_Arbor_Acr	breed_growth_background	ENSGALG00010017619	POLN	0.679417247	0.181033508	3.752991672	0.000174737	0.002360492	positive
PRJNA729271	:ed_Lushi_vs_Arbor_Acr	breed_growth_background	ENSGALG00010017620	UBAP1L	0.211361374	0.18083672	1.168796765	0.242485524	0.398200184	positive
PRJNA729271	:ed_Lushi_vs_Arbor_Acr	breed_growth_background	ENSGALG00010017621	SYNE2	0.135174881	0.083355964	1.621658186	0.104876557	0.222477051	positive
PRJNA729271	:ed_Lushi_vs_Arbor_Acr	breed_growth_background	ENSGALG00010017622	RAB1B	0.134927032	0.060219076	2.240602812	0.025051814	0.080124901	positive
PRJNA729271	:ed_Lushi_vs_Arbor_Acr	breed_growth_background	ENSGALG00010017625	YBX1	0.084239059	0.048800365	1.726197327	0.084311919	0.191034455	positive
PRJNA729271	:ed_Lushi_vs_Arbor_Acr	breed_growth_background	ENSGALG00010017626	COPS7B	0.241448048	0.075966433	3.178351784	0.001481149	0.010832118	positive
PRJNA729271	:ed_Lushi_vs_Arbor_Acr	breed_growth_background	ENSGALG00010017628	NRDE2	0.030496597	0.056740831	0.537471801	0.590941759	0.725906847	positive
PRJNA729271	:ed_Lushi_vs_Arbor_Acr	breed_growth_background	ENSGALG00010017629	ENSGALG00010017629	0.257339332	0.077227782	3.332211851	0.000861586	0.007316166	positive
PRJNA729271	:ed_Lushi_vs_Arbor_Acr	breed_growth_background	ENSGALG00010017631	NAT8L	-0.371665272	0.147060365	-2.527297351	0.011494414	0.046178361	negative
PRJNA729271	:ed_Lushi_vs_Arbor_Acr	breed_growth_background	ENSGALG00010017632	AWAT1	-0.577311309	0.318358101	-1.813402289	0.069769797	0.167602454	negative
PRJNA729271	:ed_Lushi_vs_Arbor_Acr	breed_growth_background	ENSGALG00010017633	HLX	-0.058924576	0.082142032	-0.717349867	0.473158245	0.626913049	negative
PRJNA729271	:ed_Lushi_vs_Arbor_Acr	breed_growth_background	ENSGALG00010017634	PPIH	0.078455387	0.053041738	1.479125508	0.139106774	0.270687701	positive
PRJNA729271	:ed_Lushi_vs_Arbor_Acr	breed_growth_background	ENSGALG00010017636	ZBTB80S	-0.0347365	0.085921569	-0.404281492	0.686005734	0.797278152	negative
PRJNA729271	:ed_Lushi_vs_Arbor_Acr	breed_growth_background	ENSGALG00010017638	GFRA2	-0.243488313	0.138903612	-1.752930037	0.079614017	0.183555838	negative
PRJNA729271	:ed_Lushi_vs_Arbor_Acr	breed_growth_background	ENSGALG00010017639	ENSGALG00010017639	-0.100213482	0.075704289	-1.323749068	0.185586396	0.331167649	negative
PRJNA729271	:ed_Lushi_vs_Arbor_Acr	breed_growth_background	ENSGALG00010017641	GPRC5B	0.198603795	0.085037413	2.33548727	0.01951798	0.067152414	positive
PRJNA729271	:ed_Lushi_vs_Arbor_Acr	breed_growth_background	ENSGALG00010017642	MTARC1	0.221591677	0.064974995	3.410414678	0.000648642	0.005988575	positive
PRJNA729271	:ed_Lushi_vs_Arbor_Acr	breed_growth_background	ENSGALG00010017643	CALM1	0.024427774	0.032670298	0.747705878	0.454637585	0.610861309	positive
PRJNA729271	:ed_Lushi_vs_Arbor_Acr	breed_growth_background	ENSGALG00010017644	RBBP6	0.176911978	0.052165155	3.391382191	0.000695411	0.006284274	positive
PRJNA729271	:ed_Lushi_vs_Arbor_Acr	breed_growth_background	ENSGALG00010017645	SYNC	0.132462996	0.143517102	0.922977085	0.356019147	0.519666017	positive

PRJNA729271	ed_Lushi_vs_Arbor_Acr	breed_growth_background	ENSGALG00010017646	MAEA	-0.121430489	0.046808869	-2.594176937	0.009481768	0.040319652	negative
PRJNA729271	ed_Lushi_vs_Arbor_Acr	breed_growth_background	ENSGALG00010017647	ITGA9	-0.424716845	0.089494259	-4.745744023	2.07741E-06	8.70292E-05	negative
PRJNA729271	ed_Lushi_vs_Arbor_Acr	breed_growth_background	ENSGALG00010017648	ENSGALG00010017648	-0.074759016	0.086620714	-0.863061644	0.388103575	0.550177147	negative
PRJNA729271	ed_Lushi_vs_Arbor_Acr	breed_growth_background	ENSGALG00010017649	ENSGALG00010017649	-0.102868523	0.061598031	-1.669996928	0.094919971	0.207349921	negative
PRJNA729271	ed_Lushi_vs_Arbor_Acr	breed_growth_background	ENSGALG00010017651	PPCS	0.02918241	0.067246954	0.433958837	0.664318332	0.782233487	positive
PRJNA729271	ed_Lushi_vs_Arbor_Acr	breed_growth_background	ENSGALG00010017652	C1orf115	-0.084268547	0.090287718	-0.933333447	0.350647831	0.514541437	negative
PRJNA729271	ed_Lushi_vs_Arbor_Acr	breed_growth_background	ENSGALG00010017653	ENSGALG00010017653	0.01407573	0.091642991	0.153593084	0.87793059	0.92767337	positive
PRJNA729271	ed_Lushi_vs_Arbor_Acr	breed_growth_background	ENSGALG00010017654	KBTBD13	-0.091187675	0.103478005	-0.881227608	0.378194639	0.540411208	negative
PRJNA729271	ed_Lushi_vs_Arbor_Acr	breed_growth_background	ENSGALG00010017655	ENSGALG00010017655	0.152149394	0.061356556	2.479757712	0.013147168	0.050656089	positive
PRJNA729271	ed_Lushi_vs_Arbor_Acr	breed_growth_background	ENSGALG00010017656	FSD2	0.09261462	0.092829689	0.997683195	0.318433005	0.481746816	positive
PRJNA729271	ed_Lushi_vs_Arbor_Acr	breed_growth_background	ENSGALG00010017657	DIS3L2	0.127001368	0.051046705	2.487944506	0.012848378	0.049862715	positive
PRJNA729271	ed_Lushi_vs_Arbor_Acr	breed_growth_background	ENSGALG00010017658	WHAMM	0.258985365	0.086211575	3.004067201	0.002663965	0.016265446	positive
PRJNA729271	ed_Lushi_vs_Arbor_Acr	breed_growth_background	ENSGALG00010017659	TNRC6A	0.025784481	0.042411154	0.60796463	0.543210933	0.687677997	positive
PRJNA729271	ed_Lushi_vs_Arbor_Acr	breed_growth_background	ENSGALG00010017660	CLIP4	0.237901731	0.131414143	1.810320602	0.070246084	0.16824471	positive
PRJNA729271	ed_Lushi_vs_Arbor_Acr	breed_growth_background	ENSGALG00010017661	NHSL3	0.001815621	0.092565165	0.019614515	0.984350884	0.990707268	positive
PRJNA729271	ed_Lushi_vs_Arbor_Acr	breed_growth_background	ENSGALG00010017662	GOLGA4	0.190509578	0.088483772	2.153045399	0.031315111	0.094055642	positive
PRJNA729271	ed_Lushi_vs_Arbor_Acr	breed_growth_background	ENSGALG00010017663	ZMYND12	-0.112364854	0.121248234	-0.926733943	0.35406469	0.517877666	negative
PRJNA729271	ed_Lushi_vs_Arbor_Acr	breed_growth_background	ENSGALG00010017664	ENSGALG00010017664	-0.015690764	0.040012125	-0.39215024	0.694947208	0.80351363	negative
PRJNA729271	ed_Lushi_vs_Arbor_Acr	breed_growth_background	ENSGALG00010017665	CTBP1	0.112854603	0.033860716	3.33290653	0.000859438	0.007316166	positive
PRJNA729271	ed_Lushi_vs_Arbor_Acr	breed_growth_background	ENSGALG00010017667	SLC2A1	-0.114036316	0.060766723	-1.876624397	0.060569591	0.151841007	negative
PRJNA729271	ed_Lushi_vs_Arbor_Acr	breed_growth_background	ENSGALG00010017668	ENSGALG00010017668	0.600172161	0.30966108	1.938158199	0.052603925	0.137435813	positive
PRJNA729271	ed_Lushi_vs_Arbor_Acr	breed_growth_background	ENSGALG00010017669	ENSGALG00010017669	-0.315803514	0.094373551	-3.346313765	0.000818937	0.007065766	negative
PRJNA729271	ed_Lushi_vs_Arbor_Acr	breed_growth_background	ENSGALG00010017670	FAM179A	-0.218506748	0.170463398	-1.281839681	0.199898891	0.348764375	negative
PRJNA729271	ed_Lushi_vs_Arbor_Acr	breed_growth_background	ENSGALG00010017671	ANTXR1	-0.27227015	0.099138154	-2.746370993	0.006025858	0.029124645	negative
PRJNA729271	ed_Lushi_vs_Arbor_Acr	breed_growth_background	ENSGALG00010017672	SPRED2	-0.097486055	0.04252389	-2.292500859	0.021876758	0.072780264	negative
PRJNA729271	ed_Lushi_vs_Arbor_Acr	breed_growth_background	ENSGALG00010017673	WDR89	-0.012778074	0.048998874	-0.260783018	0.794259841	0.872445923	negative
PRJNA729271	ed_Lushi_vs_Arbor_Acr	breed_growth_background	ENSGALG00010017675	ENSGALG00010017675	0.10182317	0.265409414	0.383645661	0.701241092	0.807282156	positive
PRJNA729271	ed_Lushi_vs_Arbor_Acr	breed_growth_background	ENSGALG00010017676	SPON2	0.253683255	0.140259607	1.808669376	0.070502383	0.168580014	positive
PRJNA729271	ed_Lushi_vs_Arbor_Acr	breed_growth_background	ENSGALG00010017677	WDR43	0.056579754	0.059138852	0.956727306	0.338704912	0.502942842	positive
PRJNA729271	ed_Lushi_vs_Arbor_Acr	breed_growth_background	ENSGALG00010017678	YARS	-0.041299436	0.074117004	-0.557219449	0.577377503	0.714751649	negative
PRJNA729271	ed_Lushi_vs_Arbor_Acr	breed_growth_background	ENSGALG00010017679	RASL12	-0.104354015	0.082011094	-1.272437781	0.203217617	0.352326913	negative
PRJNA729271	ed_Lushi_vs_Arbor_Acr	breed_growth_background	ENSGALG00010017681	ENSGALG00010017681	-1.243358039	0.624658809	-1.990459467	0.046540345	0.125811693	negative
PRJNA729271	ed_Lushi_vs_Arbor_Acr	breed_growth_background	ENSGALG00010017684	TRMT61B	0.198262253	0.052552736	3.772634256	0.000161533	0.002262242	positive
PRJNA729271	ed_Lushi_vs_Arbor_Acr	breed_growth_background	ENSGALG00010017685	PHC2	0.341832193	0.098448277	3.472200855	0.00051621	0.005079695	positive
PRJNA729271	ed_Lushi_vs_Arbor_Acr	breed_growth_background	ENSGALG00010017687	TTC7B	-0.068226057	0.052310411	-1.304253881	0.192147034	0.338993581	negative
PRJNA729271	ed_Lushi_vs_Arbor_Acr	breed_growth_background	ENSGALG00010017688	SPDYA	-0.50869918	0.322270404	-1.578485564	0.114454102	0.236528687	negative
PRJNA729271	ed_Lushi_vs_Arbor_Acr	breed_growth_background	ENSGALG00010017691	LZTS1	-0.133595015	0.14105277	-0.947127909	0.343573605	0.507448482	negative
PRJNA729271	ed_Lushi_vs_Arbor_Acr	breed_growth_background	ENSGALG00010017692	EPCAM	-0.743035291	0.544522868	-1.36456214	0.172390711	0.315585167	negative
PRJNA729271	ed_Lushi_vs_Arbor_Acr	breed_growth_background	ENSGALG00010017693	MTFMT	0.12662706	0.06339769	1.997345032	0.045787715	0.124407866	positive
PRJNA729271	ed_Lushi_vs_Arbor_Acr	breed_growth_background	ENSGALG00010017694	ATP6V1B2	0.023486877	0.03832917	0.612767688	0.540029959	0.684926963	positive
PRJNA729271	ed_Lushi_vs_Arbor_Acr	breed_growth_background	ENSGALG00010017695	FNDC5	-0.363608633	0.111484591	-3.261514691	0.001108187	0.00881975	negative
PRJNA729271	ed_Lushi_vs_Arbor_Acr	breed_growth_background	ENSGALG00010017698	ENSGALG00010017698	0.493054808	0.427926458	1.152195193	0.249240873	0.405272594	positive
PRJNA729271	ed_Lushi_vs_Arbor_Acr	breed_growth_background	ENSGALG00010017699	RNF19B	-0.146253361	0.133437119	-1.096047053	0.273058183	0.431968757	negative
PRJNA729271	ed_Lushi_vs_Arbor_Acr	breed_growth_background	ENSGALG00010017700	VPS35L	-0.078975858	0.042566334	-1.855359632	0.063544888	0.157020653	negative
PRJNA729271	ed_Lushi_vs_Arbor_Acr	breed_growth_background	ENSGALG00010017701	PPP2R5E	0.071873125	0.053487016	1.343749025	0.179029558	0.323281646	positive
PRJNA729271	ed_Lushi_vs_Arbor_Acr	breed_growth_background	ENSGALG00010017703	RPS6KA5	-0.094097775	0.074903733	-1.256249473	0.209025535	0.358771477	negative
PRJNA729271	ed_Lushi_vs_Arbor_Acr	breed_growth_background	ENSGALG00010017704	ZNF362	0.161160865	0.108385804	1.486918573	0.137036318	0.268026101	positive
PRJNA729271	ed_Lushi_vs_Arbor_Acr	breed_growth_background	ENSGALG00010017705	MAK16	0.113655135	0.055511961	2.047399012	0.040618923	0.113640473	positive
PRJNA729271	ed_Lushi_vs_Arbor_Acr	breed_growth_background	ENSGALG00010017706	FGFRL1	0.048384664	0.077769163	0.622157444	0.533838345	0.679247438	positive
PRJNA729271	ed_Lushi_vs_Arbor_Acr	breed_growth_background	ENSGALG00010017707	AK2	-0.123351271	0.051562474	-2.392268304	0.016744597	0.060196659	negative
PRJNA729271	ed_Lushi_vs_Arbor_Acr	breed_growth_background	ENSGALG00010017708	MEIS1	0.160217842	0.056586615	2.831373514	0.004634856	0.024114228	positive
PRJNA729271	ed_Lushi_vs_Arbor_Acr	breed_growth_background	ENSGALG00010017710	RAB41	-0.008696777	0.063602367	-0.136736686	0.891238931	0.936368897	negative
PRJNA729271	ed_Lushi_vs_Arbor_Acr	breed_growth_background	ENSGALG00010017711	LRRFIP2	0.001530645	0.071811432	0.021314788	0.982994547	0.989816659	positive
PRJNA729271	ed_Lushi_vs_Arbor_Acr	breed_growth_background	ENSGALG00010017712	MSH2	-0.05914376	0.040396298	-1.464088598	0.143169742	0.276295615	negative
PRJNA729271	ed_Lushi_vs_Arbor_Acr	breed_growth_background	ENSGALG00010017713	ALKBH5	0.188950517	0.079789026	2.368126629	0.017878416	0.063253023	positive
PRJNA729271	ed_Lushi_vs_Arbor_Acr	breed_growth_background	ENSGALG00010017714	ATAD3A	0.042374177	0.084280366	0.502776384	0.615121499	0.7440478	positive
PRJNA729271	ed_Lushi_vs_Arbor_Acr	breed_growth_background	ENSGALG00010017715	CILP	-0.051691171	0.090033575	-0.574132156	0.565878375	0.705583401	negative
PRJNA729271	ed_Lushi_vs_Arbor_Acr	breed_growth_background	ENSGALG00010017717	LLGL1	0.202468066	0.080435197	2.517157584	0.01183059	0.0471111	positive
PRJNA729271	ed_Lushi_vs_Arbor_Acr	breed_growth_background	ENSGALG00010017718	ATOH8	-0.354340006	0.160005811	-2.214544611	0.026791337	0.083939697	negative
PRJNA729271	ed_Lushi_vs_Arbor_Acr	breed_growth_background	ENSGALG00010017719	ENSGALG00010017719	-1.163088154	0.94980996	-1.224548281	0.220745465	0.373317642	negative
PRJNA729271	ed_Lushi_vs_Arbor_Acr	breed_growth_background	ENSGALG00010017720	ENSGALG00010017720	-0.194050199	0.200735149	-0.966697661	0.333695197	0.498059534	negative
PRJNA729271	ed_Lushi_vs_Arbor_Acr	breed_growth_background	ENSGALG00010017721	VWA1	-0.11476633	0.064091718	-1.790657717	0.073348239	0.173478761	negative
PRJNA729271	ed_Lushi_vs_Arbor_Acr	breed_growth_background	ENSGALG00010017723	KCNK12	0.058121227	0.22522223	0.258061682	0.796359303	0.873626588	positive
PRJNA729271	ed_Lushi_vs_Arbor_Acr	breed_growth_background	ENSGALG00010017724	FLII	0.140598241	0.068415137	2.055075047	0.039871757	0.112069248	positive
PRJNA729271	ed_Lushi_vs_Arbor_Acr	breed_growth_background	ENSGALG00010017725	ENSGALG00010017725	0.146921246	0.21435899	0.685398106	0.493092739	0.644837536	positive
PRJNA729271	ed_Lushi_vs_Arbor_Acr	breed_growth_background	ENSGALG00010017726	TTI2	0.013088636	0.066231793	0.197618628	0.84334346	0.90506146	positive
PRJNA729271	ed_Lushi_vs_Arbor_Acr	breed_growth_background	ENSGALG00010017727	AZIN2	0.056909485	0.081441923	0.698773837	0.484693381	0.636583407	positive
PRJNA729271	ed_Lushi_vs_Arbor_Acr	breed_growth_background	ENSGALG00010017729	MIEF2	0.019203478	0.074380438	0.258179143	0.796268654	0.873593259	positive
PRJNA729271	ed_Lushi_vs_Arbor_Acr	breed_growth_background	ENSGALG00010017730	TARP	0.296823663	0.253271936	1.171956386	0.241214567	0.396957896	positive
PRJNA729271	ed_Lushi_vs_Arbor_Acr	breed_growth_background	ENSGALG00010017732	RHOJ	-0.016360648	0.043039417	-0.380131733	0.703847631	0.808549011	negative
PRJNA729271	ed_Lushi_vs_Arbor_Acr	breed_growth_background	ENSGALG00010017733	TRIM62	0.050862516	0.071371519	0.712644441	0.476065804	0.629040459	positive
PRJNA729271	ed_Lushi_vs_Arbor_Acr	breed_growth_background	ENSGALG00010017734	TOP3A	0.143284542	0.064407608	2.22465242	0.026104581	0.082421702	positive
PRJNA729271	ed_Lushi_vs_Arbor_Acr	breed_growth_background	ENSGALG00010017736	ENSGALG00010017736	-0.591637546	2.302941932	-0.256905108	0.797252027	0.87427509	negative
PRJNA729271	ed_Lushi_vs_Arbor_Acr	breed_growth_background	ENSGALG00010017737	ENSGALG00010017737	-0.142304128	0.109584247	-1.298581981	0.194087425	0.341461006	negative
PRJNA729271	ed_Lushi_vs_Arbor_Acr	breed_growth_background	ENSGALG00010017738	NDUFS5	0.129832214	0.059900523	2.167463793	0.030199508	0.091359621	positive
PRJNA729271	ed_Lushi_vs_Arbor_Acr	breed_growth_background	ENSGALG00010017739	MRPL20	0.058525358	0.05380342	1.087762789	0.276699841	0.435876331	positive
PRJNA729271	ed_Lushi_vs_Arbor_Acr	breed_growth_background	ENSGALG00010017741	SMCR8	0.156563804	0.074224238	2.109335295	0.03491565	0.101770028	positive
PRJNA729271	ed_Lushi_vs_Arbor_Acr	breed_growth_background	ENSGALG00010017742	DGLUCY	-0.604987354	0.129786446	-4.661406261	3.14056E-06	0.000117934	negative
PRJNA729271	ed_Lushi_vs_Arbor_Acr	breed_growth_background	ENSGALG00010017743	KCNH5	0.517017625	0.205919519	2.51077522	0.012046638	0.047643754	positive
PRJNA729271	ed_Lushi_vs_Arbor_Acr	breed_growth_background	ENSGALG00010017744	ETAA1	-0.098478821	0.053678182	-1.834615436	0.06656269	0.162432515	negative
PRJNA729271	ed_Lushi_vs_Arbor_Acr	breed_growth_background	ENSGALG00010017745	SHMT1	0.126646149	0.101323713	1.249916184	0.211330167	0.361400515	positive
PRJNA729271	ed_Lushi_vs_Arbor_Acr	breed_growth_background	ENSGALG00010017746	MSH6	-0.014530661	0.048264934	-0.301060412	0.763368428	0.851352263	negative
PRJNA729271	ed_Lushi_vs_Arbor_Acr	breed_growth_background	ENSGALG00010017747	DUSP26	0.216724725	0.064063543	3.382965004	0.000717078	0.006435939	positive
PRJNA729271	ed_Lushi_vs_Arbor_Acr	breed_growth_background	ENSGALG00010017748	ENSGALG00010017748	-0.030901322	0.296445615	-0.10423943	0.916979344	0.953145614	negative
PRJNA729271	ed_Lushi_vs_Arbor_Acr	breed_growth_background	ENSGALG00010017751	ST3GAL5	-0.050537066	0.068720763	-0.735397328	0.462097549	0.617182224	negative
PRJNA729271	ed_Lushi_vs_Arbor_Acr	breed_growth_background	ENSGALG00010017753	GNRHR	-0.036509562	0.183237532	-0.199247185	0.842069391	0.904400994	negative
PRJNA729271	ed_Lushi_vs_Arbor_Acr	breed_growth_background	ENSGALG00010017754	PDZD11	0.123995892	0.08733312	1.419803769	0.155664818	0.292655577	positive
PRJNA729271	ed_Lushi_vs_Arbor_Acr	breed_growth_background	ENSGALG00010017755	C1D	0.014951022	0.075900884	0.196980864	0.843842513	0.905225337	positive
PRJNA729271	ed_Lushi_vs_Arbor_Acr	breed_growth_background	ENSGALG00010017756	EIF2B5	0.178990726	0.063891917	2.801461186	0.005087176	0.025936903	positive
PRJNA729271	ed_Lushi_vs_Arbor_Acr	breed_growth_background	ENSGALG00010017757	ENSGALG00010017757	-0.153822083	0.074033945	-2.077723703	0.037734815	0.108032024	negative
PRJNA729271	ed_Lushi_vs_Arbor_Acr	breed_growth_background	ENSGALG00010017759	ENSGALG00010017759	1.288624919	0.418045555	3.082498797	0.002052706	0.013514537	positive
PRJNA729271	ed_Lushi_vs_Arbor_Acr	breed_growth_background	ENSGALG00010017761	PARP16	0.0892438	0.057099786	1.562944551	0.118065638	0.241791996	positive
PRJNA729271	ed_Lushi_vs_Arbor_Acr	breed_growth_background	ENSGALG00010017762	FBXO11	-0.04603329	0.030189207	-1.524826088	0.127302481	0.254210844	negative
PRJNA729271	ed_Lushi_vs_Arbor_Acr	breed_growth_background	ENSGALG00010017763	POLR1A	0.049688155	0.046905839	1.059317053	0.289455411	0.450586221	positive
PRJNA729271	ed_Lushi_vs_Arbor_Acr	breed_growth_background	ENSGALG00010017764	IGDCC3	0.02577304	0.133060406	0.193694281	0.84641527	0.907052295	positive
PRJNA729271	ed_Lushi_vs_Arbor_Acr	breed_growth_background	ENSGALG00010017765	SYT16	-0.372877293	0.16836813	-2.214654825	0.026783766	0.083939697	negative
PRJNA729271	ed_Lushi_vs_Arbor_Acr	breed_growth_background	ENSGALG00010017766	DNAAF10	0.238277909	0.082391391	2.892024362	0.003827683	0.020984213	positive
PRJNA729271	ed_Lushi_vs_Arbor_Acr	breed_growth_background	ENSGALG00010017767	KIF4B	-0.334447762	0.119675044	-2.794632455	0.005195875	0.026241885	negative
PRJNA729271	ed_Lushi_vs_Arbor_Acr	breed_growth_background	ENSGALG00010017768	FOXN2	0.018750872	0.046028581	0.407374541	0.683732923	0.795785187	positive
PRJNA729271	ed_Lushi_vs_Arbor_Acr	breed_growth_background	ENSGALG00010017770	PTCD3	-0.037159954	0.037981374	-0.978373098	0.327889829	0.491800814	negative
PRJNA729271	ed_Lushi_vs_Arbor_Acr	breed_growth_background	ENSGALG00010017771	PNO1	0.252593971	0.084773543	2.979632127	0.002885947	0.017236015	positive
PRJNA729271	ed_Lushi_vs_Arbor_Acr	breed_growth_background	ENSGALG00010017772	ENSGALG00010017772	-0.441558199	0.174425023	-2.531507192	0.011357349	0.045767243	negative
PRJNA729271	ed_Lushi_vs_Arbor_Acr	breed_growth_background	ENSGALG00010017773	DVL3	-0.008314243	0.045715635	-0.181868704	0.855685765	0.913273335	negative
PRJNA729271	ed_Lushi_vs_Arbor_Acr	breed_growth_background	ENSGALG00010017774	ASH2L	0.388483314	0.087523843	4.438599842	9.0546E-06	0.000269886	positive
PRJNA729271	ed_Lushi_vs_Arbor_Acr	breed_growth_background	ENSGALG00010017775	PRPSAP2	-0.133688328	0.046926222	-2.848904581	0.004387004	0.023106692	negative
PRJNA729271	ed_Lushi_vs_Arbor_Acr	breed_growth_background	ENSGALG00010017776	GPR68	-0.418101578	0.197768763	-2.114093106	0.034507316	0.100863792	negative
PRJNA729271	ed_Lushi_vs_Arbor_Acr	breed_growth_background	ENSGALG00010017778	PPP3R1	0.014216008	0.042024045	0.338282714	0.735150149	0.830424049	positive
PRJNA729271	ed_Lushi_vs_Arbor_Acr	breed_growth_background	ENSGALG00010017779	SNAPC1	-0.018797145	0.067081369	-0.280214097	0.779313252	0.86328969	negative
PRJNA729271	ed_Lushi_vs_Arbor_Acr	breed_growth_background	ENSGALG00010017780	TAF9	0.156164439	0.074168203	2.10554434	0.035243952	0.102503389	positive
PRJNA729271	ed_Lushi_vs_Arbor_Acr	breed_growth_background	ENSGALG00010017781	CYP26C1	-0.023292946	0.159113699	-0.146391832	0.883612075	0.931420044	negative
PRJNA729271	ed_Lushi_vs_Arbor_Acr	breed_growth_background	ENSGALG00010017782	IGDCC4	-0.038202632	0.06252493	-0.610998389	0.541200647	0.686085654	negative
PRJNA729271	ed_Lushi_vs_Arbor_Acr	breed_growth_background	ENSGALG00010017783	STAR	-0.02663431	0.218388257	-0.121958528	0.902931863	0.944095603	negative
PRJNA729271	ed_Lushi_vs_Arbor_Acr	breed_growth_background	ENSGALG00010017786	KAZALD1	-0.206159765	0.091102217	-2.26295002	0.02363877	0.076929497	negative
PRJNA729271	ed_Lushi_vs_Arbor_Acr	breed_growth_background	ENSGALG00010017787	CNRIP1	-0.315718041	0.125680712	-2.512064387	0.012002719	0.04755655	negative
PRJNA729271	ed_Lushi_vs_Arbor_Acr	breed_growth_background	ENSGALG00010017789	IMMT	0.123044656	0.069594751	1.768016333	0.077058173	0.179287034	positive
PRJNA729271	ed_Lushi_vs_Arbor_Acr	breed_growth_background	ENSGALG00010017790	R3HCC1L	0.283019992	0.095351648	2.968170967	0.002995776	0.017702316	positive
PRJNA729271	ed_Lushi_vs_Arbor_Acr	breed_growth_background	ENSGALG00010017791	CCNL2	-0.09569773	0.059908773	-1.597390924	0.110178594	0.230685935	negative
PRJNA729271	ed_Lushi_vs_Arbor_Acr	breed_growth_background	ENSGALG00010017792	LSM1	-0.022484096	0.068270941	-0.329336259	0.74190154	0.835186613	negative
PRJNA729271	ed_Lushi_vs_Arbor_Acr	breed_growth_background	ENSGALG00010017793	FAM83G	0.144836026	0.244623062	0.59207838	0.553798104	0.695872179	positive
PRJNA729271	ed_Lushi_vs_Arbor_Acr	breed_growth_background	ENSGALG00010017795	MACF1	-0.125036867	0.061125911	-2.045562419	0.040799442	0.114003959	negative
PRJNA729271	ed_Lushi_vs_Arbor_Acr	breed_growth_background	ENSGALG00010017796	MXI1	-0.003593617	0.070526012	-0.050954485	0.959361789	0.977517846	negative
PRJNA729271	ed_Lushi_vs_Arbor_Acr	breed_growth_background	ENSGALG00010017797	PPP1R21	-0.035524064	0.050089587	-0.70921056	0.478193822	0.631272364	negative
PRJNA729271	ed_Lushi_vs_Arbor_Acr	breed_growth_background	ENSGALG00010017798	BAG4	0.283125932	0.085254266	3.320959099	0.000897087	0.00754685	positive
PRJNA729271	ed_Lushi_vs_Arbor_Acr	breed_growth_background	ENSGALG00010017799	GRAPL	0.142110009	0.180833504	0.78586106	0.43194889	0.590279925	positive
PRJNA729271	ed_Lushi_vs_Arbor_Acr	breed_growth_background	ENSGALG00010017801	SMNDC1	0.094390078	0.03878135	2.433903894	0.01493696	0.055430425	positive
PRJNA729271	ed_Lushi_vs_Arbor_Acr	breed_growth_background	ENSGALG00010017803	ENSGALG00010017803	0.002894385	0.063238101	0.045769633	0.963493863	0.979165922	positive
PRJNA729271	ed_Lushi_vs_Arbor_Acr	breed_growth_background	ENSGALG00010017804	DUSP5	-0.32829953	0.134458644	-2.441639451	0.01462074	0.054452115	negative
PRJNA729271	ed_Lushi_vs_Arbor_Acr	breed_growth_background	ENSGALG00010017805	DPP8	0.060843991	0.057423422	1.059567489	0.28934141	0.450483753	positive
PRJNA729271	ed_Lushi_vs_Arbor_Acr	breed_growth_background	ENSGALG00010017806	ENSGALG00010017806	-0.623960266	0.396523603	-1.573576606	0.115585365	0.238187357	negative
PRJNA729271	ed_Lushi_vs_Arbor_Acr	breed_growth_background	ENSGALG00010017807	MRPL35	-0.135373489	0.078703481	-1.7200445	0.085424353	0.192883399	negative
PRJNA729271	ed_Lushi_vs_Arbor_Acr	breed_growth_background	ENSGALG00010017808	SFRP5	0.709931234	0.176473263	4.022882694	5.74901E-05	0.001073865	positive

PRJNA729271	ed_Lushi_vs_Arbor_Acr	breed_growth_background	ENSGALG00010017809	MOSMO	0.122826739	0.056222268	2.184663536	0.028913526	0.088530113	positive
PRJNA729271	ed_Lushi_vs_Arbor_Acr	breed_growth_background	ENSGALG00010017810	HIF1A	-0.106314072	0.042526458	-2.499951235	0.01242104	0.048739301	negative
PRJNA729271	ed_Lushi_vs_Arbor_Acr	breed_growth_background	ENSGALG00010017811	HACD3	-0.011541846	0.036135552	-0.319404159	0.749420057	0.840709783	negative
PRJNA729271	ed_Lushi_vs_Arbor_Acr	breed_growth_background	ENSGALG00010017812	ENSGALG00010017812	-1.494150714	0.912073419	-1.638191272	0.1013818	0.217225305	negative
PRJNA729271	ed_Lushi_vs_Arbor_Acr	breed_growth_background	ENSGALG00010017813	DDHD2	0.235292816	0.087411415	2.691785913	0.007107055	0.032751623	positive
PRJNA729271	ed_Lushi_vs_Arbor_Acr	breed_growth_background	ENSGALG00010017814	ENSGALG00010017814	0.268890316	0.141756953	1.896840405	0.057849005	0.146877093	positive
PRJNA729271	ed_Lushi_vs_Arbor_Acr	breed_growth_background	ENSGALG00010017815	VWA9	0.066308537	0.047265384	1.40289853	0.160647099	0.299534307	positive
PRJNA729271	ed_Lushi_vs_Arbor_Acr	breed_growth_background	ENSGALG00010017816	PRKCH	0.410478347	0.159669175	2.570805211	0.010146238	0.042241457	positive
PRJNA729271	ed_Lushi_vs_Arbor_Acr	breed_growth_background	ENSGALG00010017817	REEP1	0.157706248	0.062117075	2.538855035	0.01112159	0.045156146	positive
PRJNA729271	ed_Lushi_vs_Arbor_Acr	breed_growth_background	ENSGALG00010017818	SLC38A6	-0.026759525	0.074567536	-0.358862941	0.719697624	0.820449631	negative
PRJNA729271	ed_Lushi_vs_Arbor_Acr	breed_growth_background	ENSGALG00010017820	FLRT2	-0.050803014	0.072335489	-0.702324889	0.482476575	0.634858631	negative
PRJNA729271	ed_Lushi_vs_Arbor_Acr	breed_growth_background	ENSGALG00010017821	ENSGALG00010017821	-0.346107162	0.227075239	-1.52419596	0.127459767	0.254380091	negative
PRJNA729271	ed_Lushi_vs_Arbor_Acr	breed_growth_background	ENSGALG00010017822	VWA3A	0.116211644	0.143601021	0.809267535	0.418361276	0.578364015	positive
PRJNA729271	ed_Lushi_vs_Arbor_Acr	breed_growth_background	ENSGALG00010017823	PPIE	0.024336649	0.04887124	0.497974873	0.618501753	0.746663022	positive
PRJNA729271	ed_Lushi_vs_Arbor_Acr	breed_growth_background	ENSGALG00010017824	YAE1	0.063106614	0.067973085	0.928405925	0.353197046	0.516923061	positive
PRJNA729271	ed_Lushi_vs_Arbor_Acr	breed_growth_background	ENSGALG00010017825	PLPP5	-0.073287744	0.083334539	-0.879440194	0.379162647	0.541047703	negative
PRJNA729271	ed_Lushi_vs_Arbor_Acr	breed_growth_background	ENSGALG00010017826	PABPC4	-0.018643645	0.07447157	-0.250345802	0.802319939	0.878171677	negative
PRJNA729271	ed_Lushi_vs_Arbor_Acr	breed_growth_background	ENSGALG00010017828	ENSGALG00010017828	0.269446388	0.126528256	2.129535299	0.033209998	0.097960707	positive
PRJNA729271	ed_Lushi_vs_Arbor_Acr	breed_growth_background	ENSGALG00010017830	DENND4A	0.235112942	0.068439893	3.435320109	0.000591854	0.005563875	positive
PRJNA729271	ed_Lushi_vs_Arbor_Acr	breed_growth_background	ENSGALG00010017831	PLEK	-0.062930198	0.098018588	-0.642023103	0.520858183	0.66824565	negative
PRJNA729271	ed_Lushi_vs_Arbor_Acr	breed_growth_background	ENSGALG00010017832	POU3F1	0.663985989	0.261204431	2.542016563	0.011021495	0.044862839	positive
PRJNA729271	ed_Lushi_vs_Arbor_Acr	breed_growth_background	ENSGALG00010017834	ENSGALG00010017834	-0.338148568	0.189642221	-1.783086941	0.074572152	0.175617115	negative
PRJNA729271	ed_Lushi_vs_Arbor_Acr	breed_growth_background	ENSGALG00010017838	B3GNT2	-0.047028028	0.068377679	-0.68776871	0.491598443	0.643580151	negative
PRJNA729271	ed_Lushi_vs_Arbor_Acr	breed_growth_background	ENSGALG00010017841	GALC	-0.108788555	0.145429219	-0.748051567	0.454429054	0.610637725	negative
PRJNA729271	ed_Lushi_vs_Arbor_Acr	breed_growth_background	ENSGALG00010017843	NSD3	0.052890377	0.038457174	1.375305867	0.169036638	0.310976783	positive
PRJNA729271	ed_Lushi_vs_Arbor_Acr	breed_growth_background	ENSGALG00010017848	CYP3A4	-0.224354858	0.105195296	-2.132746103	0.03294556	0.097448847	negative
PRJNA729271	ed_Lushi_vs_Arbor_Acr	breed_growth_background	ENSGALG00010017849	FBX048	0.141430702	0.091743995	1.541579937	0.123175704	0.248217966	positive
PRJNA729271	ed_Lushi_vs_Arbor_Acr	breed_growth_background	ENSGALG00010017850	TRMT5	-0.040840881	0.052299621	-0.780902043	0.43486011	0.593028253	negative
PRJNA729271	ed_Lushi_vs_Arbor_Acr	breed_growth_background	ENSGALG00010017852	LETM2	0.116189834	0.062617012	1.855563388	0.063515816	0.156975577	positive
PRJNA729271	ed_Lushi_vs_Arbor_Acr	breed_growth_background	ENSGALG00010017855	SDR42E2	-0.216965065	0.147304265	-1.472904162	0.14077688	0.273167453	negative
PRJNA729271	ed_Lushi_vs_Arbor_Acr	breed_growth_background	ENSGALG00010017856	FASTKD5	0.199851501	0.062752546	3.184755255	0.001448765	0.010670653	positive
PRJNA729271	ed_Lushi_vs_Arbor_Acr	breed_growth_background	ENSGALG00010017858	GPR65	-0.196231059	0.150541165	-1.303504316	0.192402644	0.339196828	negative
PRJNA729271	ed_Lushi_vs_Arbor_Acr	breed_growth_background	ENSGALG00010017859	EXOC6	0.086417983	0.071451869	1.209457287	0.226487211	0.379617499	positive
PRJNA729271	ed_Lushi_vs_Arbor_Acr	breed_growth_background	ENSGALG00010017860	CYSLTR1	-0.461804939	0.112754433	-4.0956699	4.20949E-05	0.000853908	negative
PRJNA729271	ed_Lushi_vs_Arbor_Acr	breed_growth_background	ENSGALG00010017861	PRSS56	0.558416868	0.280193046	1.992971905	0.046264524	0.125252945	positive
PRJNA729271	ed_Lushi_vs_Arbor_Acr	breed_growth_background	ENSGALG00010017865	UBOX5	0.164150477	0.073853998	2.222634963	0.026240425	0.082631969	positive
PRJNA729271	ed_Lushi_vs_Arbor_Acr	breed_growth_background	ENSGALG00010017866	RALA	-0.04118481	0.051380611	-0.801563253	0.422805641	0.582010234	negative
PRJNA729271	ed_Lushi_vs_Arbor_Acr	breed_growth_background	ENSGALG00010017867	APLF	0.044136262	0.058618573	0.752939899	0.451486031	0.607809976	positive
PRJNA729271	ed_Lushi_vs_Arbor_Acr	breed_growth_background	ENSGALG00010017868	CHRND	0.049636246	0.128677953	0.385740092	0.699689167	0.806328071	positive
PRJNA729271	ed_Lushi_vs_Arbor_Acr	breed_growth_background	ENSGALG00010017870	RAB11A	0.116556005	0.03961039	2.942561406	0.003255092	0.018805326	positive
PRJNA729271	ed_Lushi_vs_Arbor_Acr	breed_growth_background	ENSGALG00010017873	HSPG2	-0.082130878	0.05858011	-1.402026708	0.160907271	0.299942245	negative
PRJNA729271	ed_Lushi_vs_Arbor_Acr	breed_growth_background	ENSGALG00010017876	COMMD1	-0.291695249	0.074040445	-3.939674449	8.15923E-05	0.001375209	negative
PRJNA729271	ed_Lushi_vs_Arbor_Acr	breed_growth_background	ENSGALG00010017878	CHRNG	0.123611626	0.132976826	0.929572694	0.352592371	0.51637839	positive
PRJNA729271	ed_Lushi_vs_Arbor_Acr	breed_growth_background	ENSGALG00010017880	CDK13	0.127239555	0.045385546	2.803525917	0.005054717	0.025789554	positive
PRJNA729271	ed_Lushi_vs_Arbor_Acr	breed_growth_background	ENSGALG00010017881	KCNK10	0.234172431	0.28922532	0.809653978	0.418139076	0.578206431	positive
PRJNA729271	ed_Lushi_vs_Arbor_Acr	breed_growth_background	ENSGALG00010017882	LZTS3	0.019869314	0.105020984	0.189193756	0.849940961	0.909754411	positive
PRJNA729271	ed_Lushi_vs_Arbor_Acr	breed_growth_background	ENSGALG00010017886	DDRGK1	0.014637062	0.055042935	0.265920817	0.790300193	0.869810273	positive
PRJNA729271	ed_Lushi_vs_Arbor_Acr	breed_growth_background	ENSGALG00010017887	PROKR1	0.458248011	0.195651156	2.342168687	0.019172047	0.066419414	positive
PRJNA729271	ed_Lushi_vs_Arbor_Acr	breed_growth_background	ENSGALG00010017888	MPLKIP	0.132736995	0.061898752	2.144421175	0.031999155	0.095614874	positive
PRJNA729271	ed_Lushi_vs_Arbor_Acr	breed_growth_background	ENSGALG00010017890	ANAPC1	-0.051976193	0.035569513	-1.461256826	0.143944973	0.27732494	negative
PRJNA729271	ed_Lushi_vs_Arbor_Acr	breed_growth_background	ENSGALG00010017891	GOLGA7B	0.462741759	0.146857614	3.150955188	0.001627375	0.011485754	positive
PRJNA729271	ed_Lushi_vs_Arbor_Acr	breed_growth_background	ENSGALG00010017894	KAT6A	0.063926304	0.043512934	1.46913338	0.141796615	0.274693342	positive
PRJNA729271	ed_Lushi_vs_Arbor_Acr	breed_growth_background	ENSGALG00010017895	MNAT1	0.07678623	0.043804851	1.75291613	0.079616404	0.183555838	positive
PRJNA729271	ed_Lushi_vs_Arbor_Acr	breed_growth_background	ENSGALG00010017898	NHERF2	-0.05495949	0.061335302	-0.89604988	0.370226124	0.533548187	negative
PRJNA729271	ed_Lushi_vs_Arbor_Acr	breed_growth_background	ENSGALG00010017901	HTR7L	-0.07041661	0.43098725	-0.163384438	0.870215755	0.922876636	negative
PRJNA729271	ed_Lushi_vs_Arbor_Acr	breed_growth_background	ENSGALG00010017903	ENSGALG00010017903	0.042138511	0.23214081	0.181521341	0.855958383	0.913362542	positive
PRJNA729271	ed_Lushi_vs_Arbor_Acr	breed_growth_background	ENSGALG00010017904	MEGF11	0.317891244	0.178175063	1.784151158	0.074399106	0.175266544	positive
PRJNA729271	ed_Lushi_vs_Arbor_Acr	breed_growth_background	ENSGALG00010017905	EIF4E2	0.131710622	0.047399769	2.778718633	0.005457377	0.027127807	positive
PRJNA729271	ed_Lushi_vs_Arbor_Acr	breed_growth_background	ENSGALG00010017909	NTHL1	0.080600008	0.076905183	1.048043901	0.294618381	0.455908341	positive
PRJNA729271	ed_Lushi_vs_Arbor_Acr	breed_growth_background	ENSGALG00010017915	BFSP1	0.377601851	0.199459641	1.893124084	0.05834137	0.147739021	positive
PRJNA729271	ed_Lushi_vs_Arbor_Acr	breed_growth_background	ENSGALG00010017918	SIX4	0.2843521	0.107829943	2.637042112	0.008363245	0.036712669	positive
PRJNA729271	ed_Lushi_vs_Arbor_Acr	breed_growth_background	ENSGALG00010017920	AP3M2	-0.119906578	0.044480352	-2.695720061	0.007023667	0.032479118	negative
PRJNA729271	ed_Lushi_vs_Arbor_Acr	breed_growth_background	ENSGALG00010017921	SPATA7	-0.010582856	0.107568155	-0.098382798	0.921628334	0.956327938	negative
PRJNA729271	ed_Lushi_vs_Arbor_Acr	breed_growth_background	ENSGALG00010017922	MERTK	-0.20887205	0.070304953	-2.970943593	0.002968863	0.017586297	negative
PRJNA729271	ed_Lushi_vs_Arbor_Acr	breed_growth_background	ENSGALG00010017927	PCSK2	0.07107735	0.069106072	1.028525397	0.30370275	0.466378147	positive
PRJNA729271	ed_Lushi_vs_Arbor_Acr	breed_growth_background	ENSGALG00010017930	TSC2	0.088687934	0.042316719	2.095813131	0.036098771	0.104316524	positive
PRJNA729271	ed_Lushi_vs_Arbor_Acr	breed_growth_background	ENSGALG00010017933	SIX1	0.312584004	0.104775848	2.983359338	0.002851031	0.017090855	positive
PRJNA729271	ed_Lushi_vs_Arbor_Acr	breed_growth_background	ENSGALG00010017937	PTPN21	-0.010336945	0.063457119	-0.162896545	0.870599891	0.923006541	negative
PRJNA729271	ed_Lushi_vs_Arbor_Acr	breed_growth_background	ENSGALG00010017941	DIS3L	0.209863402	0.073140293	2.869326793	0.004113466	0.022058706	positive
PRJNA729271	ed_Lushi_vs_Arbor_Acr	breed_growth_background	ENSGALG00010017946	PTGDR	-0.311619112	0.136966696	-2.275145132	0.022897225	0.075225584	negative
PRJNA729271	ed_Lushi_vs_Arbor_Acr	breed_growth_background	ENSGALG00010017948	PLAT	0.177090293	0.084584131	2.093658597	0.036290404	0.1047176	positive
PRJNA729271	ed_Lushi_vs_Arbor_Acr	breed_growth_background	ENSGALG00010017949	SFXN3	0.167918469	0.07406996	2.267025257	0.023388683	0.076340137	positive
PRJNA729271	ed_Lushi_vs_Arbor_Acr	breed_growth_background	ENSGALG00010017950	FBLN7	-0.869644111	0.172681509	-5.03611601	4.75073E-07	2.71109E-05	negative
PRJNA729271	ed_Lushi_vs_Arbor_Acr	breed_growth_background	ENSGALG00010017954	PTGER2	0.006756341	0.140585753	0.048058502	0.961669619	0.978276808	positive
PRJNA729271	ed_Lushi_vs_Arbor_Acr	breed_growth_background	ENSGALG00010017955	IKBKB	-0.073605662	0.034532432	-2.131493755	0.033048486	0.097663162	negative
PRJNA729271	ed_Lushi_vs_Arbor_Acr	breed_growth_background	ENSGALG00010017956	TIPIN	-0.102564121	0.044254683	-2.317587996	0.020471723	0.069344457	negative
PRJNA729271	ed_Lushi_vs_Arbor_Acr	breed_growth_background	ENSGALG00010017957	THUMPD1	0.057436963	0.05335832	1.076438735	0.281731087	0.441648752	positive
PRJNA729271	ed_Lushi_vs_Arbor_Acr	breed_growth_background	ENSGALG00010017958	CAPN10	0.014298707	0.0518085	0.275991532	0.78255457	0.865541166	positive
PRJNA729271	ed_Lushi_vs_Arbor_Acr	breed_growth_background	ENSGALG00010017959	PDZD7	0.325682522	0.149407661	2.179824776	0.029270453	0.089283504	positive
PRJNA729271	ed_Lushi_vs_Arbor_Acr	breed_growth_background	ENSGALG00010017962	TXNDC16	-0.085757945	0.081186608	-1.056306538	0.29082819	0.452264201	negative
PRJNA729271	ed_Lushi_vs_Arbor_Acr	breed_growth_background	ENSGALG00010017963	LPAR4	-0.297786393	0.091448187	-3.256340056	0.001128585	0.008924626	negative
PRJNA729271	ed_Lushi_vs_Arbor_Acr	breed_growth_background	ENSGALG00010017970	AMPH	-1.488903748	0.374057691	-3.980412072	6.87959E-05	0.00120726	negative
PRJNA729271	ed_Lushi_vs_Arbor_Acr	breed_growth_background	ENSGALG00010017972	RNPEPL1	0.005735589	0.092098638	0.062276589	0.950342572	0.972551227	positive
PRJNA729271	ed_Lushi_vs_Arbor_Acr	breed_growth_background	ENSGALG00010017973	PKD1	-0.268584395	0.068964722	-3.89451862	9.83939E-05	0.001570693	negative
PRJNA729271	ed_Lushi_vs_Arbor_Acr	breed_growth_background	ENSGALG00010017974	MXRA8	-0.269000729	0.098515694	-2.730536813	0.006323127	0.030109634	negative
PRJNA729271	ed_Lushi_vs_Arbor_Acr	breed_growth_background	ENSGALG00010017976	GPR137C	-0.088435614	0.059525966	-1.485664489	0.137367888	0.268493465	negative
PRJNA729271	ed_Lushi_vs_Arbor_Acr	breed_growth_background	ENSGALG00010017979	ENSGALG00010017979	-0.141981841	0.092908264	-1.528193881	0.126464408	0.253016092	negative
PRJNA729271	ed_Lushi_vs_Arbor_Acr	breed_growth_background	ENSGALG00010017983	SLC4A11	-0.033800379	0.108196444	-0.31239824	0.754737888	0.844912394	negative
PRJNA729271	ed_Lushi_vs_Arbor_Acr	breed_growth_background	ENSGALG00010017985	ABCC5	0.456668074	0.143080697	3.191681925	0.00141447	0.010498076	positive
PRJNA729271	ed_Lushi_vs_Arbor_Acr	breed_growth_background	ENSGALG00010017987	ERI2	-0.275674113	0.129172607	-2.134153051	0.032830254	0.097295959	negative
PRJNA729271	ed_Lushi_vs_Arbor_Acr	breed_growth_background	ENSGALG00010017991	RAB26	-0.49435053	0.13173225	-3.752691757	0.000174946	0.002361118	negative
PRJNA729271	ed_Lushi_vs_Arbor_Acr	breed_growth_background	ENSGALG00010017992	ENSGALG00010017992	0.509936182	0.283930217	1.795991238	0.072495911	0.172041294	positive
PRJNA729271	ed_Lushi_vs_Arbor_Acr	breed_growth_background	ENSGALG00010017993	REXO5	-0.114917178	0.052696818	-2.180723305	0.029203888	0.08915551	negative
PRJNA729271	ed_Lushi_vs_Arbor_Acr	breed_growth_background	ENSGALG00010017996	ZC3H14	0.105153332	0.04981207	2.111001037	0.034772223	0.101453979	positive
PRJNA729271	ed_Lushi_vs_Arbor_Acr	breed_growth_background	ENSGALG00010017997	ENSGALG00010017997	0.22288291	0.205111608	1.086642105	0.277195009	0.436418744	positive
PRJNA729271	ed_Lushi_vs_Arbor_Acr	breed_growth_background	ENSGALG00010017999	GPR174	0.353174684	0.31174638	1.132891051	0.257260008	0.414515765	positive
PRJNA729271	ed_Lushi_vs_Arbor_Acr	breed_growth_background	ENSGALG00010018000	MAP2K1	-0.014587296	0.039352385	-0.370683913	0.710872973	0.813717425	negative
PRJNA729271	ed_Lushi_vs_Arbor_Acr	breed_growth_background	ENSGALG00010018001	TRAF7	-0.093027214	0.046383574	-2.005606859	0.044898209	0.122514975	negative
PRJNA729271	ed_Lushi_vs_Arbor_Acr	breed_growth_background	ENSGALG00010018002	ZC3H8	0.068609962	0.04290697	1.599040017	0.109811711	0.230101646	positive
PRJNA729271	ed_Lushi_vs_Arbor_Acr	breed_growth_background	ENSGALG00010018004	CDC42BPA	-0.133335042	0.033279344	-4.006540575	6.16145E-05	0.001119176	negative
PRJNA729271	ed_Lushi_vs_Arbor_Acr	breed_growth_background	ENSGALG00010018006	DCUN1D3	0.129048964	0.056833409	2.270653237	0.023167978	0.075788725	positive
PRJNA729271	ed_Lushi_vs_Arbor_Acr	breed_growth_background	ENSGALG00010018007	DNAAF9	-0.361862248	0.096062907	-3.76693001	0.000165267	0.0022902	negative
PRJNA729271	ed_Lushi_vs_Arbor_Acr	breed_growth_background	ENSGALG00010018008	ABCF3	0.122221214	0.0705906	1.731409202	0.083378807	0.189431944	positive
PRJNA729271	ed_Lushi_vs_Arbor_Acr	breed_growth_background	ENSGALG00010018010	ENSGALG00010018010	-0.157697051	0.064265777	-2.453826248	0.014134526	0.053215572	negative
PRJNA729271	ed_Lushi_vs_Arbor_Acr	breed_growth_background	ENSGALG00010018011	LIPA	-0.27568174	0.099562899	-2.768920374	0.005624238	0.027697901	negative
PRJNA729271	ed_Lushi_vs_Arbor_Acr	breed_growth_background	ENSGALG00010018012	DVL1	0.283748827	0.073783177	3.845711711	0.000120203	0.001828272	positive
PRJNA729271	ed_Lushi_vs_Arbor_Acr	breed_growth_background	ENSGALG00010018014	LYRM1	-0.009775795	0.052467545	-0.186320801	0.852193196	0.910819966	negative
PRJNA729271	ed_Lushi_vs_Arbor_Acr	breed_growth_background	ENSGALG00010018016	IFIT5	0.54444169	0.458283379	1.188002261	0.234832519	0.389283779	positive
PRJNA729271	ed_Lushi_vs_Arbor_Acr	breed_growth_background	ENSGALG00010018019	NAPB	-0.077226229	0.190119075	-0.406199269	0.684596191	0.796342332	negative
PRJNA729271	ed_Lushi_vs_Arbor_Acr	breed_growth_background	ENSGALG00010018020	SNAPC5	-0.304050097	0.056330248	-5.397634591	6.75252E-08	6.04184E-06	negative
PRJNA729271	ed_Lushi_vs_Arbor_Acr	breed_growth_background	ENSGALG00010018021	ATRN	0.012246553	0.037913651	0.323011701	0.746686381	0.838619176	positive
PRJNA729271	ed_Lushi_vs_Arbor_Acr	breed_growth_background	ENSGALG00010018022	XPO6	0.016368562	0.063469471	0.257896619	0.796486694	0.873692451	positive
PRJNA729271	ed_Lushi_vs_Arbor_Acr	breed_growth_background	ENSGALG00010018025	CPTP	-0.250184131	0.072995543	-3.427389118	0.000609415	0.005684345	negative
PRJNA729271	ed_Lushi_vs_Arbor_Acr	breed_growth_background	ENSGALG00010018026	CCDC39	0.152573804	0.430234152	0.354629689	0.722867031	0.82225203	positive
PRJNA729271	ed_Lushi_vs_Arbor_Acr	breed_growth_background	ENSGALG00010018027	EML5	0.017855484	0.167432897	0.106642627	0.9150725	0.952051097	positive
PRJNA729271	ed_Lushi_vs_Arbor_Acr	breed_growth_background	ENSGALG00010018030	POLB	-0.076436205	0.05452462	-1.401865903	0.160955294	0.299993184	negative
PRJNA729271	ed_Lushi_vs_Arbor_Acr	breed_growth_background	ENSGALG00010018032	ERO1A	-0.25438903	0.066685344	-3.814766697	0.000136312	0.001997813	negative
PRJNA729271	ed_Lushi_vs_Arbor_Acr	breed_growth_background	ENSGALG00010018033	COQ8A	0.511710407	0.113900443	4.49261122	7.03552E-06	0.000223267	positive
PRJNA729271	ed_Lushi_vs_Arbor_Acr	breed_growth_background	ENSGALG00010018035	ITM2A	-0.150053424	0.046158431	-3.250834603	0.001150668	0.009030281	negative
PRJNA729271	ed_Lushi_vs_Arbor_Acr	breed_growth_background	ENSGALG00010018036	RPL4	-0.012088348	0.057226896	-0.211235435	0.832703561	0.8985363	negative
PRJNA729271	ed_Lushi_vs_Arbor_Acr	breed_growth_background	ENSGALG00010018037	GZF1	0.124604747	0.045300022	2.750655297	0.005947619	0.028833024	positive
PRJNA729271	ed_Lushi_vs_Arbor_Acr	breed_growth_background	ENSGALG00010018039	TTC14	0.135753328	0.070627494	1.922103133	0.054592782	0.141131153	positive
PRJNA729271	ed_Lushi_vs_Arbor_Acr	breed_growth_background	ENSGALG00010018042	ZWILCH	-0.067697845	0.075797729	-0.893138166	0.371783187	0.534463681	negative
PRJNA729271	ed_Lushi_vs_Arbor_Acr	breed_growth_background	ENSGALG00010018043	PSMC6	0.090137662	0.048784063	1.847686647	0.064647687	0.158905922	positive
PRJNA729271	ed_Lushi_vs_Arbor_Acr	breed_growth_background	ENSGALG00010018044	INTS11	-0.013890108	0.033040762	-0.420393096	0.674198311	0.789379264	negative
PRJNA729271	ed_Lushi_vs_Arbor_Acr	breed_growth_background	ENSGALG00010018045	PSEN2	0.078826289	0.039389971	2.001176628	0.045373359	0.123514102	positive
PRJNA729271	ed_Lushi_vs_Arbor_Acr	breed_growth_background	ENSGALG00010018046	CAPN15	-0.045874245	0.055729137	-0.823164468	0.410414472	0.570944091	negative
PRJNA729271	ed_Lushi_vs_Arbor_Acr	breed_growth_background	ENSGALG00010018048	GFRA4	0.18377298	0.092140234	1.994492206	0.046098291	0.124923875	positive
PRJNA729271	ed_Lushi_vs_Arbor_Acr	breed_growth_background	ENSGALG00010018049	SUGCT	-0.408155087	0.097132622	-4.202039213	2.64521E-05	0.000596304	negative
PRJNA729271	ed_Lushi_vs_Arbor_Acr	breed_growth_background	ENSGALG00010018051	GNPNAT1	0.099531672	0.073592285	1.352474261	0.176223647	0.320183384	positive
PRJNA729271	ed_Lushi_vs_Arbor_Acr	breed_growth_background	ENSGALG00010018052	PPP1RL	0.131173544	0.07062488	1.857327682	0.063264548	0.156594882	positive

PRJNA729271	eed_Lushi_vs_Arbor_Acr	breed_growth_background	ENSGALG00010018053	DIAPH2	-0.069659487	0.048832768	-1.426490668	0.153726759	0.290214817	negative
PRJNA729271	eed_Lushi_vs_Arbor_Acr	breed_growth_background	ENSGALG00010018054	SMAD6	-0.029165004	0.087514095	-0.333260655	0.738937536	0.832625901	negative
PRJNA729271	eed_Lushi_vs_Arbor_Acr	breed_growth_background	ENSGALG00010018055	PEX5L	-0.000166741	0.267608123	-0.000623079	0.999502855	0.999571815	negative
PRJNA729271	eed_Lushi_vs_Arbor_Acr	breed_growth_background	ENSGALG00010018057	SSTR4	-0.039007887	0.096429217	-0.404523527	0.685827781	0.797199173	negative
PRJNA729271	eed_Lushi_vs_Arbor_Acr	breed_growth_background	ENSGALG00010018058	ITPKB	-0.087917774	0.05002602	-1.757440903	0.078842687	0.182152493	negative
PRJNA729271	eed_Lushi_vs_Arbor_Acr	breed_growth_background	ENSGALG00010018060	CASKIN1	-0.067840813	0.202318213	-0.335317382	0.737385683	0.83173013	negative
PRJNA729271	eed_Lushi_vs_Arbor_Acr	breed_growth_background	ENSGALG00010018061	NDUFAF5	0.096456781	0.072835026	1.32431862	0.185397249	0.330911603	positive
PRJNA729271	eed_Lushi_vs_Arbor_Acr	breed_growth_background	ENSGALG00010018062	GOLGA7	-0.003122434	0.051185659	-0.061002129	0.951357513	0.973315017	negative
PRJNA729271	eed_Lushi_vs_Arbor_Acr	breed_growth_background	ENSGALG00010018063	DECR2	0.01696386	0.053032494	0.319876707	0.749061796	0.840440494	positive
PRJNA729271	eed_Lushi_vs_Arbor_Acr	breed_growth_background	ENSGALG00010018064	TTC8	0.304590951	0.087705744	3.472873447	0.000514918	0.005073921	positive
PRJNA729271	eed_Lushi_vs_Arbor_Acr	breed_growth_background	ENSGALG00010018067	ENSGALG00010018067	0.059682002	0.095531032	0.624739426	0.532142092	0.677655883	positive
PRJNA729271	eed_Lushi_vs_Arbor_Acr	breed_growth_background	ENSGALG00010018068	ADAM33	-0.172902017	0.07442885	-2.323051023	0.020176412	0.068684144	negative
PRJNA729271	eed_Lushi_vs_Arbor_Acr	breed_growth_background	ENSGALG00010018069	ENSGALG00010018069	1.042849508	0.212675197	4.903484398	9.41514E-07	4.64192E-05	positive
PRJNA729271	eed_Lushi_vs_Arbor_Acr	breed_growth_background	ENSGALG00010018071	GPAT4	-0.090053294	0.043006224	-2.09395955	0.036263584	0.104667592	negative
PRJNA729271	eed_Lushi_vs_Arbor_Acr	breed_growth_background	ENSGALG00010018075	MLST8	0.157247243	0.05489505	2.864506796	0.004176592	0.022282444	positive
PRJNA729271	eed_Lushi_vs_Arbor_Acr	breed_growth_background	ENSGALG00010018076	FOXA2	-0.824697597	0.840442911	-0.981265457	0.326461852	0.490013933	negative
PRJNA729271	eed_Lushi_vs_Arbor_Acr	breed_growth_background	ENSGALG00010018078	NKX6-3	0.360749363	0.181770159	1.984645693	0.047183905	0.126888813	positive
PRJNA729271	eed_Lushi_vs_Arbor_Acr	breed_growth_background	ENSGALG00010018080	BRICD5	0.036158404	0.172484139	0.209633208	0.833953959	0.899096503	positive
PRJNA729271	eed_Lushi_vs_Arbor_Acr	breed_growth_background	ENSGALG00010018081	DIO3	0.122702693	0.142822064	0.859129816	0.390268899	0.55178967	positive
PRJNA729271	eed_Lushi_vs_Arbor_Acr	breed_growth_background	ENSGALG00010018082	RAB11FIP3	0.140889926	0.095401646	1.476808127	0.139727077	0.27167592	positive
PRJNA729271	eed_Lushi_vs_Arbor_Acr	breed_growth_background	ENSGALG00010018083	PAX1	1.170984826	0.57828186	2.02493785	0.042873746	0.118417484	positive
PRJNA729271	eed_Lushi_vs_Arbor_Acr	breed_growth_background	ENSGALG00010018085	PGP	0.077918607	0.085281234	0.913666505	0.360892109	0.524266498	positive
PRJNA729271	eed_Lushi_vs_Arbor_Acr	breed_growth_background	ENSGALG00010018087	ENSGALG00010018087	-0.031678605	0.030758151	-1.029925529	0.303044966	0.465666996	negative
PRJNA729271	eed_Lushi_vs_Arbor_Acr	breed_growth_background	ENSGALG00010018089	TASP1	-0.101047143	0.051003045	-1.981198228	0.047569048	0.127758634	negative
PRJNA729271	eed_Lushi_vs_Arbor_Acr	breed_growth_background	ENSGALG00010018092	ENSGALG00010018092	-0.201900244	0.117758443	-1.714528818	0.086431649	0.194508113	negative
PRJNA729271	eed_Lushi_vs_Arbor_Acr	breed_growth_background	ENSGALG00010018095	E4F1	0.130792428	0.038094935	3.433328602	0.000596219	0.005590435	positive
PRJNA729271	eed_Lushi_vs_Arbor_Acr	breed_growth_background	ENSGALG00010018099	SMAD3	-0.168783639	0.070269818	-2.401936462	0.016308541	0.059098075	negative
PRJNA729271	eed_Lushi_vs_Arbor_Acr	breed_growth_background	ENSGALG00010018102	ZDHHC4	-0.10712835	0.091043784	-1.176668472	0.239327857	0.394839208	negative
PRJNA729271	eed_Lushi_vs_Arbor_Acr	breed_growth_background	ENSGALG00010018103	BTRC	0.14881202	0.058014165	2.565098033	0.010314666	0.042778564	positive
PRJNA729271	eed_Lushi_vs_Arbor_Acr	breed_growth_background	ENSGALG00010018111	NKX2-2	0.580212613	0.370879647	1.564422901	0.117718286	0.241245095	positive
PRJNA729271	eed_Lushi_vs_Arbor_Acr	breed_growth_background	ENSGALG00010018115	KLHL4	-0.247511086	0.107088558	-2.311274804	0.020817681	0.070109731	negative
PRJNA729271	eed_Lushi_vs_Arbor_Acr	breed_growth_background	ENSGALG00010018119	GRID2IP	0.121133836	0.351571778	0.344549373	0.730433172	0.827158502	positive
PRJNA729271	eed_Lushi_vs_Arbor_Acr	breed_growth_background	ENSGALG00010018121	FERMT2	0.021061322	0.085488688	0.246363846	0.805400591	0.880147886	positive
PRJNA729271	eed_Lushi_vs_Arbor_Acr	breed_growth_background	ENSGALG00010018123	USP13	0.034498315	0.074834777	0.460993092	0.644803561	0.76754764	positive
PRJNA729271	eed_Lushi_vs_Arbor_Acr	breed_growth_background	ENSGALG00010018128	SEPTIN12	0.099919993	0.105824709	0.944202855	0.345065997	0.508950202	positive
PRJNA729271	eed_Lushi_vs_Arbor_Acr	breed_growth_background	ENSGALG00010018137	NDUFB5	0.017636832	0.06813751	0.25884175	0.795757343	0.873418163	positive
PRJNA729271	eed_Lushi_vs_Arbor_Acr	breed_growth_background	ENSGALG00010018140	CHPF	-0.074089097	0.057253936	-1.294043736	0.195650314	0.343658409	negative
PRJNA729271	eed_Lushi_vs_Arbor_Acr	breed_growth_background	ENSGALG00010018142	DDHD1	-0.057677765	0.078321397	-0.736424109	0.461472637	0.61668718	negative
PRJNA729271	eed_Lushi_vs_Arbor_Acr	breed_growth_background	ENSGALG00010018143	MRPL47	0.191070875	0.066428534	2.876337389	0.004023194	0.021723142	positive
PRJNA729271	eed_Lushi_vs_Arbor_Acr	breed_growth_background	ENSGALG00010018145	ENSGALG00010018145	-0.320017241	0.198245335	-1.614248529	0.10647353	0.225107032	negative
PRJNA729271	eed_Lushi_vs_Arbor_Acr	breed_growth_background	ENSGALG00010018147	ISM1	-0.017617062	0.153877422	-0.114487637	0.908851246	0.94816459	negative
PRJNA729271	eed_Lushi_vs_Arbor_Acr	breed_growth_background	ENSGALG00010018148	XRN2	0.005882478	0.029874064	0.196909213	0.843898584	0.905225337	positive
PRJNA729271	eed_Lushi_vs_Arbor_Acr	breed_growth_background	ENSGALG00010018149	ENSGALG00010018149	0.734381474	0.157380317	4.666285398	3.06694E-06	0.000116071	positive
PRJNA729271	eed_Lushi_vs_Arbor_Acr	breed_growth_background	ENSGALG00010018150	AAGAB	0.043374205	0.044219575	0.980882454	0.326650711	0.49024664	positive
PRJNA729271	eed_Lushi_vs_Arbor_Acr	breed_growth_background	ENSGALG00010018154	SLC16A12	0.259270498	0.133741154	1.93859922	0.052550159	0.137335919	positive
PRJNA729271	eed_Lushi_vs_Arbor_Acr	breed_growth_background	ENSGALG00010018157	FBXL2	0.125013738	0.082491614	1.515472084	0.129652895	0.257280153	positive
PRJNA729271	eed_Lushi_vs_Arbor_Acr	breed_growth_background	ENSGALG00010018160	ENSGALG00010018160	0.095654676	0.088582438	1.079837921	0.280214361	0.439959615	positive
PRJNA729271	eed_Lushi_vs_Arbor_Acr	breed_growth_background	ENSGALG00010018162	ENSGALG00010018162	0.398933012	0.197634851	2.018535751	0.043535495	0.119720546	positive
PRJNA729271	eed_Lushi_vs_Arbor_Acr	breed_growth_background	ENSGALG00010018163	CCT7	-0.021085405	0.043531588	-0.484370229	0.628123143	0.754243029	negative
PRJNA729271	eed_Lushi_vs_Arbor_Acr	breed_growth_background	ENSGALG00010018165	DACH2	-0.131437892	0.09692909	-1.356021108	0.175092452	0.319040238	negative
PRJNA729271	eed_Lushi_vs_Arbor_Acr	breed_growth_background	ENSGALG00010018166	MSS51	0.094243973	0.111222698	0.847344787	0.39680297	0.558037543	positive
PRJNA729271	eed_Lushi_vs_Arbor_Acr	breed_growth_background	ENSGALG00010018168	ENSGALG00010018168	0.223004981	0.143914566	1.549565049	0.121245947	0.246019401	positive
PRJNA729271	eed_Lushi_vs_Arbor_Acr	breed_growth_background	ENSGALG00010018172	ACTL6A	-0.048889404	0.051334669	-0.952366211	0.340911289	0.50482449	negative
PRJNA729271	eed_Lushi_vs_Arbor_Acr	breed_growth_background	ENSGALG00010018174	BMP4	0.126004348	0.109506739	1.150653822	0.249874681	0.405864355	positive
PRJNA729271	eed_Lushi_vs_Arbor_Acr	breed_growth_background	ENSGALG00010018177	KIF20B	-0.19686961	0.101974868	-1.930569903	0.053536263	0.139094485	negative
PRJNA729271	eed_Lushi_vs_Arbor_Acr	breed_growth_background	ENSGALG00010018179	PRADC1	0.073465527	0.063737805	1.152620924	0.249066011	0.405183203	positive
PRJNA729271	eed_Lushi_vs_Arbor_Acr	breed_growth_background	ENSGALG00010018183	CYP17A1	0.345912989	0.165749505	2.086962429	0.036891531	0.106053325	positive
PRJNA729271	eed_Lushi_vs_Arbor_Acr	breed_growth_background	ENSGALG00010018185	GNB4	-0.087382663	0.03740851	-2.335903303	0.019496282	0.067125559	negative
PRJNA729271	eed_Lushi_vs_Arbor_Acr	breed_growth_background	ENSGALG00010018186	SPTLC3	0.143060623	0.355096343	0.402878333	0.687037731	0.797989263	positive
PRJNA729271	eed_Lushi_vs_Arbor_Acr	breed_growth_background	ENSGALG00010018188	UBP1	0.021958854	0.028212356	0.778341719	0.436367587	0.594045778	positive
PRJNA729271	eed_Lushi_vs_Arbor_Acr	breed_growth_background	ENSGALG00010018189	MIS18BP1	-0.071983304	0.065488389	-1.099176581	0.271691052	0.430400196	negative
PRJNA729271	eed_Lushi_vs_Arbor_Acr	breed_growth_background	ENSGALG00010018192	IQCH	0.12061013	0.123405008	0.977351993	0.328394921	0.49220188	positive
PRJNA729271	eed_Lushi_vs_Arbor_Acr	breed_growth_background	ENSGALG00010018193	ANK1	0.091917059	0.131529792	0.69883072	0.484657828	0.636583407	positive
PRJNA729271	eed_Lushi_vs_Arbor_Acr	breed_growth_background	ENSGALG00010018198	BTBD3	0.116065091	0.055118611	2.105733215	0.035227533	0.102493595	positive
PRJNA729271	eed_Lushi_vs_Arbor_Acr	breed_growth_background	ENSGALG00010018199	BORCS7	-0.120999649	0.065128174	-1.857869509	0.063187547	0.156453626	negative
PRJNA729271	eed_Lushi_vs_Arbor_Acr	breed_growth_background	ENSGALG00010018206	KIZ	0.034080264	0.068749547	0.495716198	0.620094657	0.747714171	positive
PRJNA729271	eed_Lushi_vs_Arbor_Acr	breed_growth_background	ENSGALG00010018209	GMPPA	0.098897401	0.070556634	1.401674024	0.161012611	0.300061429	positive
PRJNA729271	eed_Lushi_vs_Arbor_Acr	breed_growth_background	ENSGALG00010018215	CFAP70	0.112056588	0.132964002	0.842758837	0.399363334	0.560547257	positive
PRJNA729271	eed_Lushi_vs_Arbor_Acr	breed_growth_background	ENSGALG00010018216	FANCM	0.052195747	0.046139531	1.131258725	0.257946211	0.415344405	positive
PRJNA729271	eed_Lushi_vs_Arbor_Acr	breed_growth_background	ENSGALG00010018217	ENSGALG00010018217	0.000188944	0.258796789	0.000730087	0.999417475	0.999555392	positive
PRJNA729271	eed_Lushi_vs_Arbor_Acr	breed_growth_background	ENSGALG00010018219	SLC24A5	0.416660206	0.361107788	1.15383888	0.248566231	0.404691398	positive
PRJNA729271	eed_Lushi_vs_Arbor_Acr	breed_growth_background	ENSGALG00010018226	JAG1	-0.028520855	0.055135787	-0.517283898	0.604957993	0.736879504	negative
PRJNA729271	eed_Lushi_vs_Arbor_Acr	breed_growth_background	ENSGALG00010018229	AS3MT	-0.16589801	0.100930419	-1.643686925	0.100240888	0.215449536	negative
PRJNA729271	eed_Lushi_vs_Arbor_Acr	breed_growth_background	ENSGALG00010018231	SMYD5	-0.209899958	0.043798457	-4.792405331	1.64794E-06	7.32232E-05	negative
PRJNA729271	eed_Lushi_vs_Arbor_Acr	breed_growth_background	ENSGALG00010018233	RASSF2	-0.018009324	0.100171719	-0.17978452	0.857321736	0.914278882	negative
PRJNA729271	eed_Lushi_vs_Arbor_Acr	breed_growth_background	ENSGALG00010018240	SOUL	0.246886074	0.388476947	0.635523102	0.525087305	0.671709512	positive
PRJNA729271	eed_Lushi_vs_Arbor_Acr	breed_growth_background	ENSGALG00010018241	RALGAPA2	-0.023967479	0.049487465	-0.484314141	0.628162942	0.754243029	negative
PRJNA729271	eed_Lushi_vs_Arbor_Acr	breed_growth_background	ENSGALG00010018244	CHM	-0.116432356	0.043945612	-2.649464891	0.008061935	0.03579783	negative
PRJNA729271	eed_Lushi_vs_Arbor_Acr	breed_growth_background	ENSGALG00010018251	USP48	0.164198219	0.089119953	1.842440604	0.065410727	0.160271221	positive
PRJNA729271	eed_Lushi_vs_Arbor_Acr	breed_growth_background	ENSGALG00010018255	MFN1	0.24061611	0.060375001	3.985359942	6.73779E-05	0.001189577	positive
PRJNA729271	eed_Lushi_vs_Arbor_Acr	breed_growth_background	ENSGALG00010018258	OSCP1	-1.086918306	0.155929104	-6.970592896	3.15608E-12	1.52491E-09	negative
PRJNA729271	eed_Lushi_vs_Arbor_Acr	breed_growth_background	ENSGALG00010018259	WDR90	-0.132816385	0.070572237	-1.881992006	0.059837101	0.150532536	negative
PRJNA729271	eed_Lushi_vs_Arbor_Acr	breed_growth_background	ENSGALG00010018261	PRNP	0.073855661	0.104169137	0.708997534	0.478326007	0.631336321	positive
PRJNA729271	eed_Lushi_vs_Arbor_Acr	breed_growth_background	ENSGALG00010018263	SLX4IP	-0.126458602	0.063462841	-1.992640096	0.046300871	0.125327942	negative
PRJNA729271	eed_Lushi_vs_Arbor_Acr	breed_growth_background	ENSGALG00010018264	CFAP61	0.344047061	0.200209569	1.718434653	0.085717364	0.193290789	positive
PRJNA729271	eed_Lushi_vs_Arbor_Acr	breed_growth_background	ENSGALG00010018265	PDCD4	-0.029597799	0.035423231	-0.835547688	0.403409408	0.564313779	negative
PRJNA729271	eed_Lushi_vs_Arbor_Acr	breed_growth_background	ENSGALG00010018267	ADPRHL2	0.102587358	0.041708682	2.459616412	0.013908558	0.052739635	positive
PRJNA729271	eed_Lushi_vs_Arbor_Acr	breed_growth_background	ENSGALG00010018273	PROM2	-0.668158261	0.525129349	-1.272368915	0.203242073	0.352326913	negative
PRJNA729271	eed_Lushi_vs_Arbor_Acr	breed_growth_background	ENSGALG00010018274	ZNF711	-0.007343486	0.052291389	-0.140433942	0.888317142	0.934409069	negative
PRJNA729271	eed_Lushi_vs_Arbor_Acr	breed_growth_background	ENSGALG00010018275	ZNF639	0.020879795	0.045291712	0.461006972	0.644793603	0.76754764	positive
PRJNA729271	eed_Lushi_vs_Arbor_Acr	breed_growth_background	ENSGALG00010018276	ARHGAP17	0.045877897	0.038873984	1.180169676	0.237932737	0.393164048	positive
PRJNA729271	eed_Lushi_vs_Arbor_Acr	breed_growth_background	ENSGALG00010018278	PUSL1	0.068677126	0.081555623	0.842089398	0.399737915	0.560726886	positive
PRJNA729271	eed_Lushi_vs_Arbor_Acr	breed_growth_background	ENSGALG00010018283	POF1B	0.072829336	0.093985714	0.774897938	0.438399971	0.595781697	positive
PRJNA729271	eed_Lushi_vs_Arbor_Acr	breed_growth_background	ENSGALG00010018284	ENSGALG00010018284	0.449063054	0.089092043	5.0404395	4.64464E-07	2.68289E-05	positive
PRJNA729271	eed_Lushi_vs_Arbor_Acr	breed_growth_background	ENSGALG00010018285	BBIP1	-0.076392084	0.103275842	-0.739689769	0.459488258	0.614814206	negative
PRJNA729271	eed_Lushi_vs_Arbor_Acr	breed_growth_background	ENSGALG00010018287	MKKS	-0.148538065	0.055394269	-2.681469886	0.007329951	0.033463824	negative
PRJNA729271	eed_Lushi_vs_Arbor_Acr	breed_growth_background	ENSGALG00010018288	CRNKL1	0.083950422	0.033620322	2.497014206	0.012524394	0.048959301	positive
PRJNA729271	eed_Lushi_vs_Arbor_Acr	breed_growth_background	ENSGALG00010018289	HEYL	-0.260422601	0.115297075	-2.258709531	0.023901459	0.077505962	negative
PRJNA729271	eed_Lushi_vs_Arbor_Acr	breed_growth_background	ENSGALG00010018290	RHOT2	0.186865301	0.073381086	2.546504973	0.010880766	0.044445469	positive
PRJNA729271	eed_Lushi_vs_Arbor_Acr	breed_growth_background	ENSGALG00010018294	FKBP3	0.251087626	0.09503569	2.642035062	0.008240952	0.03634092	positive
PRJNA729271	eed_Lushi_vs_Arbor_Acr	breed_growth_background	ENSGALG00010018296	PIK3CA	0.059173921	0.051766287	1.143097655	0.252998048	0.409652224	positive
PRJNA729271	eed_Lushi_vs_Arbor_Acr	breed_growth_background	ENSGALG00010018298	ENSGALG00010018298	0.19518078	0.106697345	1.829293696	0.067355622	0.163786769	positive
PRJNA729271	eed_Lushi_vs_Arbor_Acr	breed_growth_background	ENSGALG00010018299	SHOC2	0.040935807	0.045451547	0.900647171	0.367775946	0.531073155	positive
PRJNA729271	eed_Lushi_vs_Arbor_Acr	breed_growth_background	ENSGALG00010018301	ENSGALG00010018301	-0.170368827	0.173174886	-0.98379639	0.325215635	0.488946163	negative
PRJNA729271	eed_Lushi_vs_Arbor_Acr	breed_growth_background	ENSGALG00010018302	RAB11FIP5	0.120344858	0.054053902	2.226386146	0.025988328	0.082178344	positive
PRJNA729271	eed_Lushi_vs_Arbor_Acr	breed_growth_background	ENSGALG00010018306	DES	-0.192083545	0.080602657	-2.383091975	0.017167904	0.061292802	negative
PRJNA729271	eed_Lushi_vs_Arbor_Acr	breed_growth_background	ENSGALG00010018308	ACAP3	-0.229771803	0.09550429	-2.405879378	0.016133591	0.058654727	negative
PRJNA729271	eed_Lushi_vs_Arbor_Acr	breed_growth_background	ENSGALG00010018309	DPYSL2	-0.353160676	0.066610729	-5.301858709	1.1463E-07	9.28243E-06	negative
PRJNA729271	eed_Lushi_vs_Arbor_Acr	breed_growth_background	ENSGALG00010018310	APOOL	-0.072782733	0.06022101	-1.208593681	0.226818985	0.380041752	negative
PRJNA729271	eed_Lushi_vs_Arbor_Acr	breed_growth_background	ENSGALG00010018311	NKX2-3	-0.128960077	0.368195377	-0.350249039	0.726151807	0.824474505	negative
PRJNA729271	eed_Lushi_vs_Arbor_Acr	breed_growth_background	ENSGALG00010018312	PRPF39	0.041388248	0.042679395	0.969747768	0.332172234	0.496630896	positive
PRJNA729271	eed_Lushi_vs_Arbor_Acr	breed_growth_background	ENSGALG00010018313	MYEF2	0.098599075	0.044347568	2.223325404	0.026193866	0.082521209	positive
PRJNA729271	eed_Lushi_vs_Arbor_Acr	breed_growth_background	ENSGALG00010018314	SLC24A3	-0.144056215	0.070581043	-2.041004317	0.041250398	0.114875028	negative
PRJNA729271	eed_Lushi_vs_Arbor_Acr	breed_growth_background	ENSGALG00010018315	CLASP2	-0.217333493	0.062651409	-3.468932275	0.000522531	0.005124553	negative
PRJNA729271	eed_Lushi_vs_Arbor_Acr	breed_growth_background	ENSGALG00010018317	ZMAT3	-0.060387739	0.077216221	-0.782060268	0.434179155	0.592211053	negative
PRJNA729271	eed_Lushi_vs_Arbor_Acr	breed_growth_background	ENSGALG00010018319	STUB1	0.353402109	0.107920479	3.274652894	0.001057919	0.008509729	positive
PRJNA729271	eed_Lushi_vs_Arbor_Acr	breed_growth_background	ENSGALG00010018322	NT5C1A	0.303878305	0.124125638	2.448151006	0.014359146	0.053754087	positive
PRJNA729271	eed_Lushi_vs_Arbor_Acr	breed_growth_background	ENSGALG00010018323	GOT1	0.173045719	0.076090869	2.274198222	0.022954071	0.075378174	positive
PRJNA729271	eed_Lushi_vs_Arbor_Acr	breed_growth_background	ENSGALG00010018325	ENSGALG00010018325	0.076364918	0.104626497	0.729881252	0.465462773	0.620574218	positive
PRJNA729271	eed_Lushi_vs_Arbor_Acr	breed_growth_background	ENSGALG00010018326	LCMT1	-0.106493165	0.060944259	-1.747386326	0.080570348	0.184847609	negative
PRJNA729271	eed_Lushi_vs_Arbor_Acr	breed_growth_background	ENSGALG00010018327	SNAP25	-0.21618986	0.285302154	-0.757757545	0.448596142	0.604930792	negative
PRJNA729271	eed_Lushi_vs_Arbor_Acr	breed_growth_background	ENSGALG00010018328	ENSGALG00010018328	0.052784271	0.275954015	0.191279227	0.848306835	0.90848543	positive
PRJNA729271	eed_Lushi_vs_Arbor_Acr	breed_growth_background	ENSGALG00010018329	ENSGALG00010018329	-0.103095971	0.121143436	-0.851023995	0.394756024	0.556343078	negative
PRJNA729271	eed_Lushi_vs_Arbor_Acr	breed_growth_background	ENSGALG00010018333	ENSGALG00010018333	0.133337236	0.114755397	1.161925625	0.245265677	0.401074683	positive
PRJNA729271	eed_Lushi_vs_Arbor_Acr	breed_growth_background	ENSGALG00010018336	JMJD8	-0.173474165	0.082068215	-2.113780169	0.034534048	0.100880899	negative
PRJNA729271	eed_Lushi_vs_Arbor_Acr	breed_growth_background	ENSGALG00010018337	TMEM151B	0.140104265	0.178612082	0.784405304	0.432802327	0.591045631	positive
PRJNA729271	eed_Lushi_vs_Arbor_Acr	breed_growth_background	ENSGALG00010018338	PDCD6IP	-0.139461545	0.045318374	-3.077373112	0.002088337	0.013666117	negative
PRJNA729271	eed_Lushi_vs_Arbor_Acr	breed_growth_background	ENSGALG00010018339	FAM76A	0.181830651	0.062302983	2.918490288	0.003517308	0.019791686	positive
PRJNA729271	eed_Lushi_vs_Arbor_Acr	breed_growth_background	ENSGALG00010018340	DNPEP	0.014844038	0.052096197	0.284935162	0.775693817	0.860859189	positive
PRJNA729271	eed_Lushi_vs_Arbor_Acr	breed_growth_background	ENSGALG00010018342	BNIP3L	0.029835248	0.056925481	0.524110606	0.600201612	0.733057159	positive
PRJNA729271	eed_Lushi_vs_Arbor_Acr	breed_growth_background	ENSGALG00010018344	SFXN5	0.091992419	0.108907548	0.844683593	0.398287522	0.559530668	positive

PRJNA729271	ed_Lushi_vs_Arbor_Acr	breed_growth_background	ENSGALG00010018345	WDR24	-0.049175492	0.09211042	-0.53387545	0.593427708	0.727850282	negative
PRJNA729271	ed_Lushi_vs_Arbor_Acr	breed_growth_background	ENSGALG00010018347	CTXN2	0.568687833	0.436236124	1.303623892	0.192361851	0.339196828	positive
PRJNA729271	ed_Lushi_vs_Arbor_Acr	breed_growth_background	ENSGALG00010018348	TOGARAM1	0.222803602	0.057871625	3.84996275	0.000118136	0.001810125	positive
PRJNA729271	ed_Lushi_vs_Arbor_Acr	breed_growth_background	ENSGALG00010018349	KCNMB2	-0.437660861	0.106283267	-4.117871744	3.82387E-05	0.000797512	negative
PRJNA729271	ed_Lushi_vs_Arbor_Acr	breed_growth_background	ENSGALG00010018350	CNNM1	0.396486821	0.154969439	2.558483941	0.010512968	0.043328253	positive
PRJNA729271	ed_Lushi_vs_Arbor_Acr	breed_growth_background	ENSGALG00010018351	RIN2	-0.200519622	0.046417294	-4.319933423	1.56076E-05	0.000401834	negative
PRJNA729271	ed_Lushi_vs_Arbor_Acr	breed_growth_background	ENSGALG00010018354	AARS2	0.091091698	0.063563089	1.433091111	0.151831801	0.287834275	positive
PRJNA729271	ed_Lushi_vs_Arbor_Acr	breed_growth_background	ENSGALG00010018355	PRKCB	-0.057912949	0.08412713	-0.68839801	0.491202177	0.643121469	negative
PRJNA729271	ed_Lushi_vs_Arbor_Acr	breed_growth_background	ENSGALG00010018356	ADRA1A	0.034357961	0.221840364	0.154876958	0.876918319	0.927128449	positive
PRJNA729271	ed_Lushi_vs_Arbor_Acr	breed_growth_background	ENSGALG00010018359	CACNG3	0.124278799	0.387577596	0.320655271	0.748471648	0.839973408	positive
PRJNA729271	ed_Lushi_vs_Arbor_Acr	breed_growth_background	ENSGALG00010018361	ASIC4	-0.172179862	0.128840939	-1.336375405	0.181426601	0.326032554	negative
PRJNA729271	ed_Lushi_vs_Arbor_Acr	breed_growth_background	ENSGALG00010018363	TCTE1	0.211632032	0.21798515	0.97085527	0.331620355	0.495913439	positive
PRJNA729271	ed_Lushi_vs_Arbor_Acr	breed_growth_background	ENSGALG00010018365	HPSE2	0.356010335	0.147283861	2.417171393	0.01564165	0.057442543	positive
PRJNA729271	ed_Lushi_vs_Arbor_Acr	breed_growth_background	ENSGALG00010018367	PAK5	-0.373959129	0.118737487	-3.149461376	0.001635717	0.011520759	negative
PRJNA729271	ed_Lushi_vs_Arbor_Acr	breed_growth_background	ENSGALG00010018370	ZBTB8A	0.135133139	0.060937087	2.217584477	0.026583177	0.083488573	positive
PRJNA729271	ed_Lushi_vs_Arbor_Acr	breed_growth_background	ENSGALG00010018371	ENSGALG00010018371	-0.083062071	0.073162534	-1.135308833	0.256245943	0.413366181	negative
PRJNA729271	ed_Lushi_vs_Arbor_Acr	breed_growth_background	ENSGALG00010018372	NLGN3	-0.482686451	0.255375722	-1.89010313	0.058744169	0.148447825	negative
PRJNA729271	ed_Lushi_vs_Arbor_Acr	breed_growth_background	ENSGALG00010018374	HPS1	-0.104699269	0.073009736	-1.434045317	0.151559331	0.287508508	negative
PRJNA729271	ed_Lushi_vs_Arbor_Acr	breed_growth_background	ENSGALG00010018376	PYGB	0.224997671	0.077770704	2.893090311	0.003814716	0.020944813	positive
PRJNA729271	ed_Lushi_vs_Arbor_Acr	breed_growth_background	ENSGALG00010018377	RAP1GAP	-0.203693848	0.140717018	-1.447542383	0.147745075	0.282623401	negative
PRJNA729271	ed_Lushi_vs_Arbor_Acr	breed_growth_background	ENSGALG00010018378	ARPP21	0.368197292	0.077719249	4.737530236	2.16339E-06	8.88336E-05	positive
PRJNA729271	ed_Lushi_vs_Arbor_Acr	breed_growth_background	ENSGALG00010018379	SRBD1	0.009839944	0.067477837	0.14582483	0.884059674	0.931688597	positive
PRJNA729271	ed_Lushi_vs_Arbor_Acr	breed_growth_background	ENSGALG00010018380	SLC20A1	-0.063334607	0.07937547	-0.797911587	0.424921799	0.584091178	negative
PRJNA729271	ed_Lushi_vs_Arbor_Acr	breed_growth_background	ENSGALG00010018381	HAPSTR1	0.052510737	0.036178466	1.451436249	0.146658422	0.281155116	positive
PRJNA729271	ed_Lushi_vs_Arbor_Acr	breed_growth_background	ENSGALG00010018383	METRN	-0.210756896	0.089008095	-2.367839647	0.017892289	0.063286658	negative
PRJNA729271	ed_Lushi_vs_Arbor_Acr	breed_growth_background	ENSGALG00010018384	LAMP5	-0.075478575	0.130051113	-0.580376233	0.561660929	0.702439618	negative
PRJNA729271	ed_Lushi_vs_Arbor_Acr	breed_growth_background	ENSGALG00010018385	SPR	-0.189270936	0.078555993	-2.409376149	0.015979819	0.058344452	negative
PRJNA729271	ed_Lushi_vs_Arbor_Acr	breed_growth_background	ENSGALG00010018387	NT5DC4	-0.305163973	0.18420327	-1.656669689	0.097586274	0.211120251	negative
PRJNA729271	ed_Lushi_vs_Arbor_Acr	breed_growth_background	ENSGALG00010018388	SMC3	-0.034715784	0.035677461	-0.973045267	0.330530805	0.494738126	negative
PRJNA729271	ed_Lushi_vs_Arbor_Acr	breed_growth_background	ENSGALG00010018391	STAC	-0.093559973	0.12582517	-0.7435712	0.457135932	0.61302331	negative
PRJNA729271	ed_Lushi_vs_Arbor_Acr	breed_growth_background	ENSGALG00010018393	PRKCE	0.092104712	0.041999061	2.193018379	0.028306055	0.0871784	positive
PRJNA729271	ed_Lushi_vs_Arbor_Acr	breed_growth_background	ENSGALG00010018394	KLHL28	0.064411959	0.052989407	1.215562932	0.224151462	0.376879184	positive
PRJNA729271	ed_Lushi_vs_Arbor_Acr	breed_growth_background	ENSGALG00010018395	MED12	0.091128762	0.042628065	2.137764442	0.032535866	0.09677968	positive
PRJNA729271	ed_Lushi_vs_Arbor_Acr	breed_growth_background	ENSGALG00010018396	TBL1XR1	0.042855361	0.040249485	1.064743097	0.286992202	0.447836362	positive
PRJNA729271	ed_Lushi_vs_Arbor_Acr	breed_growth_background	ENSGALG00010018397	FAM173A	0.150974918	0.084728055	1.781876349	0.074769398	0.175910149	positive
PRJNA729271	ed_Lushi_vs_Arbor_Acr	breed_growth_background	ENSGALG00010018398	LRFN5	-0.130354171	0.102275311	-1.274541915	0.202471428	0.351644302	negative
PRJNA729271	ed_Lushi_vs_Arbor_Acr	breed_growth_background	ENSGALG00010018399	ABHD12	-0.263262613	0.087171994	-3.020036618	0.002527441	0.015696342	negative
PRJNA729271	ed_Lushi_vs_Arbor_Acr	breed_growth_background	ENSGALG00010018401	ENSGALG00010018401	-0.093818388	0.055794752	-1.681491268	0.092667531	0.20401213	negative
PRJNA729271	ed_Lushi_vs_Arbor_Acr	breed_growth_background	ENSGALG00010018404	IL2RG	0.090498855	0.136658725	0.662225229	0.507826888	0.656958809	positive
PRJNA729271	ed_Lushi_vs_Arbor_Acr	breed_growth_background	ENSGALG00010018405	DCLK3	-0.236114048	0.105290461	-2.24250181	0.024928958	0.079861735	negative
PRJNA729271	ed_Lushi_vs_Arbor_Acr	breed_growth_background	ENSGALG00010018406	TMEM260	-0.056961428	0.070704157	-0.805630549	0.420455906	0.579656491	negative
PRJNA729271	ed_Lushi_vs_Arbor_Acr	breed_growth_background	ENSGALG00010018410	EXOC6B	-0.066933159	0.053882025	-1.24221685	0.214156564	0.364813656	negative
PRJNA729271	ed_Lushi_vs_Arbor_Acr	breed_growth_background	ENSGALG00010018413	SLC15A2	-0.331538962	0.193412386	-1.714155795	0.086500117	0.19457157	negative
PRJNA729271	ed_Lushi_vs_Arbor_Acr	breed_growth_background	ENSGALG00010018414	EPAS1	-0.018132829	0.073681091	-0.246098809	0.805605745	0.880238658	negative
PRJNA729271	ed_Lushi_vs_Arbor_Acr	breed_growth_background	ENSGALG00010018415	PELI2	0.063121757	0.070950791	0.889655443	0.373650926	0.536032282	positive
PRJNA729271	ed_Lushi_vs_Arbor_Acr	breed_growth_background	ENSGALG00010018416	DUT	-0.203372871	0.072642377	-2.799645034	0.005115883	0.026019201	negative
PRJNA729271	ed_Lushi_vs_Arbor_Acr	breed_growth_background	ENSGALG00010018418	PLCB4	-0.017110228	0.064339951	-0.265934743	0.790289467	0.869810273	negative
PRJNA729271	ed_Lushi_vs_Arbor_Acr	breed_growth_background	ENSGALG00010018419	USP7	0.20259505	0.071569746	2.83073591	0.004644105	0.024149372	positive
PRJNA729271	ed_Lushi_vs_Arbor_Acr	breed_growth_background	ENSGALG00010018420	RHOQ	0.104479308	0.070440809	1.483221301	0.138015624	0.26943252	positive
PRJNA729271	ed_Lushi_vs_Arbor_Acr	breed_growth_background	ENSGALG00010018422	ENSGALG00010018422	0.415353829	0.114311169	3.633536709	0.000279563	0.003318805	positive
PRJNA729271	ed_Lushi_vs_Arbor_Acr	breed_growth_background	ENSGALG00010018425	CYP26B1	-0.155936574	0.127046604	-1.227396639	0.219673549	0.372156158	negative
PRJNA729271	ed_Lushi_vs_Arbor_Acr	breed_growth_background	ENSGALG00010018426	ENSGALG00010018426	0.531099026	0.18133201	2.928876296	0.003401898	0.019367835	positive
PRJNA729271	ed_Lushi_vs_Arbor_Acr	breed_growth_background	ENSGALG00010018427	PIGF	-0.10938543	0.078483349	-1.393740594	0.163395953	0.303206641	negative
PRJNA729271	ed_Lushi_vs_Arbor_Acr	breed_growth_background	ENSGALG00010018428	PPP2R5D	-0.048812417	0.034923613	-1.397690931	0.162205899	0.301617664	negative
PRJNA729271	ed_Lushi_vs_Arbor_Acr	breed_growth_background	ENSGALG00010018430	LOXL4	0.158985767	0.145767291	1.090682044	0.275412814	0.434255275	positive
PRJNA729271	ed_Lushi_vs_Arbor_Acr	breed_growth_background	ENSGALG00010018434	HSPBAP1	0.189526581	0.085229786	2.223712989	0.026167761	0.082480931	positive
PRJNA729271	ed_Lushi_vs_Arbor_Acr	breed_growth_background	ENSGALG00010018436	FOXO4	0.164821638	0.077464949	2.127693102	0.033362537	0.098310627	positive
PRJNA729271	ed_Lushi_vs_Arbor_Acr	breed_growth_background	ENSGALG00010018438	PPP2R2A	0.166564649	0.052747214	3.157790436	0.001589698	0.011300967	positive
PRJNA729271	ed_Lushi_vs_Arbor_Acr	breed_growth_background	ENSGALG00010018440	MEA1	-0.003763117	0.054371425	-0.069211293	0.944821434	0.970051473	negative
PRJNA729271	ed_Lushi_vs_Arbor_Acr	breed_growth_background	ENSGALG00010018441	CRIPT	-0.006279802	0.050313087	-0.124814492	0.900670414	0.942614993	negative
PRJNA729271	ed_Lushi_vs_Arbor_Acr	breed_growth_background	ENSGALG00010018443	PLCB1	-0.336404461	0.078207889	-4.301413391	1.69712E-05	0.000422014	negative
PRJNA729271	ed_Lushi_vs_Arbor_Acr	breed_growth_background	ENSGALG00010018444	NUBP2	0.028057769	0.055067418	0.509516706	0.610390091	0.740633214	positive
PRJNA729271	ed_Lushi_vs_Arbor_Acr	breed_growth_background	ENSGALG00010018446	FBN1	-0.406040973	0.124043716	-3.273369952	0.001062733	0.008543712	negative
PRJNA729271	ed_Lushi_vs_Arbor_Acr	breed_growth_background	ENSGALG00010018448	KLHDC3	0.383634886	0.087367888	4.391028476	1.12816E-05	0.000318765	positive
PRJNA729271	ed_Lushi_vs_Arbor_Acr	breed_growth_background	ENSGALG00010018451	PIK3R4	0.18166621	0.060633143	2.996153613	0.002734087	0.01658141	positive
PRJNA729271	ed_Lushi_vs_Arbor_Acr	breed_growth_background	ENSGALG00010018452	SPSB3	0.285394771	0.064216841	4.444235609	8.82049E-06	0.000264768	positive
PRJNA729271	ed_Lushi_vs_Arbor_Acr	breed_growth_background	ENSGALG00010018453	TMX4	-0.166103797	0.049662497	-3.344652567	0.000823857	0.007089281	negative
PRJNA729271	ed_Lushi_vs_Arbor_Acr	breed_growth_background	ENSGALG00010018455	RBM20	0.240864148	0.131132958	1.836793371	0.066240405	0.161860194	positive
PRJNA729271	ed_Lushi_vs_Arbor_Acr	breed_growth_background	ENSGALG00010018458	SEMA5B	-0.198709086	0.058275775	-3.409805986	0.000650091	0.005995338	negative
PRJNA729271	ed_Lushi_vs_Arbor_Acr	breed_growth_background	ENSGALG00010018459	SNX12	-0.034742667	0.054941397	-0.632358635	0.527152557	0.673163273	negative
PRJNA729271	ed_Lushi_vs_Arbor_Acr	breed_growth_background	ENSGALG00010018462	KTN1	0.23723831	0.062321689	3.806673327	0.000140849	0.002039561	positive
PRJNA729271	ed_Lushi_vs_Arbor_Acr	breed_growth_background	ENSGALG00010018464	ENSGALG00010018464	-0.312825758	0.615631691	-0.50813784	0.61135668	0.741557747	negative
PRJNA729271	ed_Lushi_vs_Arbor_Acr	breed_growth_background	ENSGALG00010018465	NAALADL2	-0.161698386	0.112477877	-1.437601699	0.150547099	0.286187567	negative
PRJNA729271	ed_Lushi_vs_Arbor_Acr	breed_growth_background	ENSGALG00010018466	DYSF	0.218203941	0.097432027	2.239550461	0.025120122	0.080272524	positive
PRJNA729271	ed_Lushi_vs_Arbor_Acr	breed_growth_background	ENSGALG00010018467	TLX1	0.157961178	0.550282236	0.287054838	0.774070331	0.859781567	positive
PRJNA729271	ed_Lushi_vs_Arbor_Acr	breed_growth_background	ENSGALG00010018469	KLHDC7A	-0.035804254	0.109069657	-0.328269616	0.742707815	0.835899509	negative
PRJNA729271	ed_Lushi_vs_Arbor_Acr	breed_growth_background	ENSGALG00010018470	IQCB1	0.00493261	0.084280355	0.058526218	0.95332948	0.974074966	positive
PRJNA729271	ed_Lushi_vs_Arbor_Acr	breed_growth_background	ENSGALG00010018471	HHEX	-0.081759807	0.096245778	-0.849489805	0.395608801	0.557106345	negative
PRJNA729271	ed_Lushi_vs_Arbor_Acr	breed_growth_background	ENSGALG00010018472	LMF1	-0.285374493	0.075946847	-3.757555485	0.000171581	0.002326569	negative
PRJNA729271	ed_Lushi_vs_Arbor_Acr	breed_growth_background	ENSGALG00010018476	ENSGALG00010018476	-0.63291321	0.201428963	-3.142116214	0.001677314	0.011711294	negative
PRJNA729271	ed_Lushi_vs_Arbor_Acr	breed_growth_background	ENSGALG00010018480	EME2	-0.174750322	0.131622714	-1.327660834	0.184290178	0.329686306	negative
PRJNA729271	ed_Lushi_vs_Arbor_Acr	breed_growth_background	ENSGALG00010018482	TWNK	-0.041417283	0.068892221	-0.60118954	0.54771375	0.691259104	negative
PRJNA729271	ed_Lushi_vs_Arbor_Acr	breed_growth_background	ENSGALG00010018484	NLGN1	-0.218370583	0.141081953	-1.547827895	0.121663739	0.246345837	negative
PRJNA729271	ed_Lushi_vs_Arbor_Acr	breed_growth_background	ENSGALG00010018485	ATP2C1	-0.176271622	0.03604425	-4.890422843	1.0062E-06	4.91071E-05	negative
PRJNA729271	ed_Lushi_vs_Arbor_Acr	breed_growth_background	ENSGALG00010018486	ENSGALG00010018486	0.066567133	0.053993372	1.232876007	0.217622028	0.369619151	positive
PRJNA729271	ed_Lushi_vs_Arbor_Acr	breed_growth_background	ENSGALG00010018487	ENSGALG00010018487	-0.00182681	0.071703084	-0.025477423	0.979674156	0.987989765	negative
PRJNA729271	ed_Lushi_vs_Arbor_Acr	breed_growth_background	ENSGALG00010018488	ENSGALG00010018488	0.07421551	0.082423413	0.900417821	0.36789794	0.53114349	positive
PRJNA729271	ed_Lushi_vs_Arbor_Acr	breed_growth_background	ENSGALG00010018489	SLC7A3	-0.01119746	0.112090975	-0.099896179	0.92042675	0.955805912	negative
PRJNA729271	ed_Lushi_vs_Arbor_Acr	breed_growth_background	ENSGALG00010018490	NME3	0.104528662	0.065361477	1.599239592	0.109767376	0.230101646	positive
PRJNA729271	ed_Lushi_vs_Arbor_Acr	breed_growth_background	ENSGALG00010018491	TIA1	0.133745644	0.064421637	2.076098193	0.03788487	0.108269163	positive
PRJNA729271	ed_Lushi_vs_Arbor_Acr	breed_growth_background	ENSGALG00010018492	ENSGALG00010018492	0.183745196	0.092780199	1.980435465	0.047654618	0.127869991	positive
PRJNA729271	ed_Lushi_vs_Arbor_Acr	breed_growth_background	ENSGALG00010018494	HAO1	-0.515423234	0.153957798	-3.347821548	0.000814494	0.007052626	negative
PRJNA729271	ed_Lushi_vs_Arbor_Acr	breed_growth_background	ENSGALG00010018495	ENSGALG00010018495	1.163710603	0.353488572	3.292074183	0.000994514	0.008116823	positive
PRJNA729271	ed_Lushi_vs_Arbor_Acr	breed_growth_background	ENSGALG00010018496	GNG13	0.1000775	0.286934093	0.348782187	0.727252837	0.824587756	positive
PRJNA729271	ed_Lushi_vs_Arbor_Acr	breed_growth_background	ENSGALG00010018497	ASTE1	0.051202916	0.062846448	0.81473047	0.415226608	0.575426677	positive
PRJNA729271	ed_Lushi_vs_Arbor_Acr	breed_growth_background	ENSGALG00010018498	ENSGALG00010018498	0.383963755	0.199417054	1.925430888	0.054175474	0.14027751	positive
PRJNA729271	ed_Lushi_vs_Arbor_Acr	breed_growth_background	ENSGALG00010018501	ATG14	0.24524029	0.072733411	3.371769408	0.00074687	0.006625382	positive
PRJNA729271	ed_Lushi_vs_Arbor_Acr	breed_growth_background	ENSGALG00010018502	ARHGEF10L	0.074108858	0.065292392	1.13503052	0.256362531	0.41345739	positive
PRJNA729271	ed_Lushi_vs_Arbor_Acr	breed_growth_background	ENSGALG00010018503	BMP2	0.081293329	0.109832355	0.740158298	0.459203948	0.614774289	positive
PRJNA729271	ed_Lushi_vs_Arbor_Acr	breed_growth_background	ENSGALG00010018504	SLCO3A1	0.302796791	0.097468428	3.106614072	0.001892433	0.012763138	positive
PRJNA729271	ed_Lushi_vs_Arbor_Acr	breed_growth_background	ENSGALG00010018505	CHTF18	-0.116045804	0.07891574	-1.47050264	0.141425671	0.274132803	negative
PRJNA729271	ed_Lushi_vs_Arbor_Acr	breed_growth_background	ENSGALG00010018506	ENSGALG00010018506	-0.121506265	0.077563993	-1.566529257	0.117224765	0.240574356	negative
PRJNA729271	ed_Lushi_vs_Arbor_Acr	breed_growth_background	ENSGALG00010018508	ENSGALG00010018508	0.081607481	0.055093842	1.481245051	0.138541285	0.270131279	positive
PRJNA729271	ed_Lushi_vs_Arbor_Acr	breed_growth_background	ENSGALG00010018509	ENSGALG00010018509	-0.110636356	0.06066364	-1.823767175	0.068187278	0.164979015	negative
PRJNA729271	ed_Lushi_vs_Arbor_Acr	breed_growth_background	ENSGALG00010018510	ENSGALG00010018510	0.063693392	0.067721763	0.940515867	0.346953015	0.510826201	positive
PRJNA729271	ed_Lushi_vs_Arbor_Acr	breed_growth_background	ENSGALG00010018511	SOCS5	-0.039234849	0.054181943	-0.724131439	0.468985045	0.62373177	negative
PRJNA729271	ed_Lushi_vs_Arbor_Acr	breed_growth_background	ENSGALG00010018512	FERMT1	0.079928416	0.094528941	0.845544396	0.397806956	0.559066495	positive
PRJNA729271	ed_Lushi_vs_Arbor_Acr	breed_growth_background	ENSGALG00010018513	RPUSD1	0.053124157	0.085356622	0.622378855	0.533692781	0.679121838	positive
PRJNA729271	ed_Lushi_vs_Arbor_Acr	breed_growth_background	ENSGALG00010018514	EAF2	-0.532484647	0.132603973	-4.015600992	5.92945E-05	0.001093478	negative
PRJNA729271	ed_Lushi_vs_Arbor_Acr	breed_growth_background	ENSGALG00010018515	SEMA4G	-0.082266774	0.096159333	-0.855525632	0.39226022	0.553745828	negative
PRJNA729271	ed_Lushi_vs_Arbor_Acr	breed_growth_background	ENSGALG00010018516	LRRN4	0.008019166	0.082900387	0.096732556	0.922938785	0.957079179	positive
PRJNA729271	ed_Lushi_vs_Arbor_Acr	breed_growth_background	ENSGALG00010018517	ECT2	-0.37791703	0.108550857	-3.481474406	0.000498661	0.004960945	negative
PRJNA729271	ed_Lushi_vs_Arbor_Acr	breed_growth_background	ENSGALG00010018518	ENSGALG00010018518	0.891292078	0.491948592	1.811758569	0.070023509	0.167975133	positive
PRJNA729271	ed_Lushi_vs_Arbor_Acr	breed_growth_background	ENSGALG00010018519	OGDH	0.158807922	0.072807127	2.181213948	0.029167596	0.089082237	positive
PRJNA729271	ed_Lushi_vs_Arbor_Acr	breed_growth_background	ENSGALG00010018520	ENSGALG00010018520	-0.05253477	0.067495166	-0.77834863	0.436363514	0.594045778	negative
PRJNA729271	ed_Lushi_vs_Arbor_Acr	breed_growth_background	ENSGALG00010018521	ENSGALG00010018521	0.364729908	0.085044209	4.288709491	1.79714E-05	0.000442268	positive
PRJNA729271	ed_Lushi_vs_Arbor_Acr	breed_growth_background	ENSGALG00010018522	NEK11	-0.220204589	0.147397577	-1.493949857	0.135188726	0.265235595	negative
PRJNA729271	ed_Lushi_vs_Arbor_Acr	breed_growth_background	ENSGALG00010018523	TEX11	0.1740271	0.095238504	1.827276711	0.067658175	0.164162244	positive
PRJNA729271	ed_Lushi_vs_Arbor_Acr	breed_growth_background	ENSGALG00010018524	SEC22A	-0.172727262	0.062914613	-2.745423568	0.006043284	0.029163644	negative
PRJNA729271	ed_Lushi_vs_Arbor_Acr	breed_growth_background	ENSGALG00010018525	ST8SIA2	-0.737441088	0.156226326	-4.720338175	2.35453E-06	9.53321E-05	negative
PRJNA729271	ed_Lushi_vs_Arbor_Acr	breed_growth_background	ENSGALG00010018526	ENSGALG00010018526	0.02065252	0.054206976	0.380993773	0.703207869	0.808237302	positive
PRJNA729271	ed_Lushi_vs_Arbor_Acr	breed_growth_background	ENSGALG00010018527	CCT4	0.045512339	0.043827791	1.038435608	0.299067299	0.461462689	positive
PRJNA729271	ed_Lushi_vs_Arbor_Acr	breed_growth_background	ENSGALG00010018528	ENSGALG00010018528	-0.467370441	0.369516216	-1.264817132	0.205936888	0.355321414	negative
PRJNA729271	ed_Lushi_vs_Arbor_Acr	breed_growth_background	ENSGALG00010018529	UBR2	0.087012471	0.049759966	1.748644091	0.08035256	0.184552425	positive
PRJNA729271	ed_Lushi_vs_Arbor_Acr	breed_growth_background	ENSGALG00010018530	FAM161A	0.332612447	0.127205038	2.614774155	0.008928647	0.038495164	positive
PRJNA729271	ed_Lushi_vs_Arbor_Acr	breed_growth_background	ENSGALG00010018531	ADCY5	-0.091610418	0.072457892	-1.26432629	0.206112937	0.355413636	negative
PRJNA729271	ed_Lushi_vs_Arbor_Acr	breed_growth_background	ENSGALG00010018532	MFSD13A	-0.143170018	0.07210069	-1.985695538	0.047067141	0.126730702	negative
PRJNA729271	ed_Lushi_vs_Arbor_Acr	breed_growth_background	ENSGALG00010018533	PCYOX1	0.208512186	0.092517179	2.253767224	0.024210816	0.078176827	positive
PRJNA729271	ed_Lushi_vs_Arbor_Acr	breed_growth_background	ENSGALG00010018534	NCEH1	-0.034013803	0.10461408	-0.325135995	0.745078147	0.837267055	negative
PRJNA729271	ed_Lushi_vs_Arbor_Acr	breed_growth_background	ENSGALG00010018535	FAM174B	-0.138695318	0.055530852	-2.497626328	0.012502791	0.048914427	negative
PRJNA729271	ed_Lushi_vs_Arbor_Acr	breed_growth_background	ENSGALG00010018536	ENSGALG00010018536	0.158390277	0.12990808	1.219248848	0.222749757	0.375372259	positive

PRJNA729271	ed_Lushi_vs_Arbor_Acr	breed_growth_background	ENSGALG00010018537	ENSGALG00010018537	-0.517226778	0.37729687	-1.370874815	0.170413983	0.312756481	negative
PRJNA729271	ed_Lushi_vs_Arbor_Acr	breed_growth_background	ENSGALG00010018539	CHD2	0.081329141	0.047476757	1.713030667	0.086706898	0.194794867	positive
PRJNA729271	ed_Lushi_vs_Arbor_Acr	breed_growth_background	ENSGALG00010018540	TNFSF10	-0.071130281	0.058209373	-1.221972987	0.221717843	0.374162711	negative
PRJNA729271	ed_Lushi_vs_Arbor_Acr	breed_growth_background	ENSGALG00010018542	ZMIZ2	0.56753226	0.139827938	4.058790171	4.93276E-05	0.000961027	positive
PRJNA729271	ed_Lushi_vs_Arbor_Acr	breed_growth_background	ENSGALG00010018543	MRPL3	-0.024434253	0.041964575	-0.582259036	0.560392213	0.701315351	negative
PRJNA729271	ed_Lushi_vs_Arbor_Acr	breed_growth_background	ENSGALG00010018544	HACD2	-0.037917994	0.055802834	-0.679499434	0.496821466	0.648251612	negative
PRJNA729271	ed_Lushi_vs_Arbor_Acr	breed_growth_background	ENSGALG00010018546	SNRPGP15	0.194329856	0.0731471	2.65669939	0.007890977	0.035280603	positive
PRJNA729271	ed_Lushi_vs_Arbor_Acr	breed_growth_background	ENSGALG00010018548	RGMA	-0.07375568	0.066975046	-1.101241201	0.270791694	0.429397835	negative
PRJNA729271	ed_Lushi_vs_Arbor_Acr	breed_growth_background	ENSGALG00010018549	GHSR	0.345666403	0.280867707	1.230708955	0.218431738	0.370572102	positive
PRJNA729271	ed_Lushi_vs_Arbor_Acr	breed_growth_background	ENSGALG00010018551	FBXO34	0.209409075	0.059443522	3.522824158	0.000426975	0.004446118	positive
PRJNA729271	ed_Lushi_vs_Arbor_Acr	breed_growth_background	ENSGALG00010018552	DTD1	0.081600592	0.055416873	1.472486397	0.140889578	0.273349542	positive
PRJNA729271	ed_Lushi_vs_Arbor_Acr	breed_growth_background	ENSGALG00010018553	ENSGALG00010018553	-0.371479046	0.268377511	-1.384166075	0.166307609	0.306865985	negative
PRJNA729271	ed_Lushi_vs_Arbor_Acr	breed_growth_background	ENSGALG00010018554	PPIA	-0.102766798	0.066209872	-1.552137091	0.120629425	0.245165944	negative
PRJNA729271	ed_Lushi_vs_Arbor_Acr	breed_growth_background	ENSGALG00010018555	UBN1	0.038406229	0.041532755	0.924721454	0.355110817	0.518831901	positive
PRJNA729271	ed_Lushi_vs_Arbor_Acr	breed_growth_background	ENSGALG00010018556	MRPL2	0.105596811	0.067137062	1.572854211	0.11575258	0.238396369	positive
PRJNA729271	ed_Lushi_vs_Arbor_Acr	breed_growth_background	ENSGALG00010018557	TBCC	0.174232912	0.055295908	3.150918736	0.001627578	0.011485754	positive
PRJNA729271	ed_Lushi_vs_Arbor_Acr	breed_growth_background	ENSGALG00010018558	ENSGALG00010018558	-0.303300503	0.1247338	-2.431582327	0.015033031	0.055687143	negative
PRJNA729271	ed_Lushi_vs_Arbor_Acr	breed_growth_background	ENSGALG00010018559	DLG3	0.120251462	0.075479039	1.593176896	0.111120513	0.231820931	positive
PRJNA729271	ed_Lushi_vs_Arbor_Acr	breed_growth_background	ENSGALG00010018560	ENSGALG00010018560	0.089646231	0.0429146	2.088944806	0.036712692	0.105627326	positive
PRJNA729271	ed_Lushi_vs_Arbor_Acr	breed_growth_background	ENSGALG00010018561	ACTR1A	0.01766026	0.031711727	0.556899969	0.577595776	0.714782786	positive
PRJNA729271	ed_Lushi_vs_Arbor_Acr	breed_growth_background	ENSGALG00010018562	ENSGALG00010018562	-0.87489728	0.453359655	-1.929808421	0.05363058	0.139239701	negative
PRJNA729271	ed_Lushi_vs_Arbor_Acr	breed_growth_background	ENSGALG00010018564	DLGAP5	-0.427814462	0.083234239	-5.13988556	2.74906E-07	1.79494E-05	negative
PRJNA729271	ed_Lushi_vs_Arbor_Acr	breed_growth_background	ENSGALG00010018566	ENSGALG00010018566	0.221789662	0.076102611	2.914350222	0.003564298	0.019961394	positive
PRJNA729271	ed_Lushi_vs_Arbor_Acr	breed_growth_background	ENSGALG00010018568	FAM136A	-0.09932966	0.10514902	-0.944656063	0.344834495	0.508761232	negative
PRJNA729271	ed_Lushi_vs_Arbor_Acr	breed_growth_background	ENSGALG00010018573	SEC23B	-0.038156206	0.043231995	-0.882591847	0.377456835	0.539749119	negative
PRJNA729271	ed_Lushi_vs_Arbor_Acr	breed_growth_background	ENSGALG00010018574	ENSGALG00010018574	-0.158349374	0.129634901	-1.221502646	0.221895765	0.374345799	negative
PRJNA729271	ed_Lushi_vs_Arbor_Acr	breed_growth_background	ENSGALG00010018576	PTK7	-0.081780056	0.062082622	-1.317277753	0.18774554	0.333583182	negative
PRJNA729271	ed_Lushi_vs_Arbor_Acr	breed_growth_background	ENSGALG00010018577	NARFL	0.147146602	0.043197775	3.406346786	0.000658385	0.006032421	positive
PRJNA729271	ed_Lushi_vs_Arbor_Acr	breed_growth_background	ENSGALG00010018579	FNDC3B	-0.130315511	0.054309713	-2.399488098	0.016418014	0.059404609	negative
PRJNA729271	ed_Lushi_vs_Arbor_Acr	breed_growth_background	ENSGALG00010018581	SUFU	-0.000781033	0.049862246	-0.015663812	0.987502597	0.992713543	negative
PRJNA729271	ed_Lushi_vs_Arbor_Acr	breed_growth_background	ENSGALG00010018582	RCC2	-0.228721104	0.060461553	-3.782918091	0.000155	0.002187664	negative
PRJNA729271	ed_Lushi_vs_Arbor_Acr	breed_growth_background	ENSGALG00010018583	PPL	-0.058775621	0.113680026	-0.517026809	0.605137445	0.737036153	negative
PRJNA729271	ed_Lushi_vs_Arbor_Acr	breed_growth_background	ENSGALG00010018585	GDPD2	0.080229202	0.291818572	0.274928363	0.783371276	0.865763995	positive
PRJNA729271	ed_Lushi_vs_Arbor_Acr	breed_growth_background	ENSGALG00010018587	SRF	-0.034188949	0.092725089	-0.368713041	0.712341627	0.815012383	negative
PRJNA729271	ed_Lushi_vs_Arbor_Acr	breed_growth_background	ENSGALG00010018588	POLR3F	0.102091738	0.055095804	1.852985704	0.063884408	0.157644619	positive
PRJNA729271	ed_Lushi_vs_Arbor_Acr	breed_growth_background	ENSGALG00010018590	TGFA	0.155515032	0.313882741	0.495455824	0.620278398	0.74781131	positive
PRJNA729271	ed_Lushi_vs_Arbor_Acr	breed_growth_background	ENSGALG00010018593	LGALS3	0.500856981	0.189927003	2.637102531	0.008361755	0.036712669	positive
PRJNA729271	ed_Lushi_vs_Arbor_Acr	breed_growth_background	ENSGALG00010018594	BICRAL	-0.002761282	0.056214098	-0.049120805	0.960823023	0.978128797	negative
PRJNA729271	ed_Lushi_vs_Arbor_Acr	breed_growth_background	ENSGALG00010018598	ENSGALG00010018598	0.082098231	0.04228158	1.941702061	0.052173179	0.136655264	positive
PRJNA729271	ed_Lushi_vs_Arbor_Acr	breed_growth_background	ENSGALG00010018601	CPNE4	-0.505902431	0.19036753	-2.657503786	0.007872171	0.035229118	negative
PRJNA729271	ed_Lushi_vs_Arbor_Acr	breed_growth_background	ENSGALG00010018602	MCTP2	-0.066940912	0.121037739	-0.553058179	0.580223572	0.716871198	negative
PRJNA729271	ed_Lushi_vs_Arbor_Acr	breed_growth_background	ENSGALG00010018604	FAM43A	0.004033531	0.066313783	0.060824925	0.95149864	0.973322003	positive
PRJNA729271	ed_Lushi_vs_Arbor_Acr	breed_growth_background	ENSGALG00010018606	DZANK1	0.18245464	0.138320844	1.319068291	0.187146283	0.332801341	positive
PRJNA729271	ed_Lushi_vs_Arbor_Acr	breed_growth_background	ENSGALG00010018607	HAGHL	-0.231203656	0.062678021	-3.688751673	0.000225357	0.002825736	negative
PRJNA729271	ed_Lushi_vs_Arbor_Acr	breed_growth_background	ENSGALG00010018608	TRIM8	0.008580596	0.067894391	0.12638152	0.89942993	0.941656687	positive
PRJNA729271	ed_Lushi_vs_Arbor_Acr	breed_growth_background	ENSGALG00010018609	ENSGALG00010018609	0.113178217	0.067350327	1.680440483	0.092871645	0.204338078	positive
PRJNA729271	ed_Lushi_vs_Arbor_Acr	breed_growth_background	ENSGALG00010018611	NUDCD3	0.097487357	0.060461297	1.612392747	0.106876502	0.225652283	positive
PRJNA729271	ed_Lushi_vs_Arbor_Acr	breed_growth_background	ENSGALG00010018613	PADI3	0.185985331	0.190511838	0.97624029	0.328945402	0.492772179	positive
PRJNA729271	ed_Lushi_vs_Arbor_Acr	breed_growth_background	ENSGALG00010018615	HTR2C	0.460303973	0.144842695	3.177957806	0.001483163	0.010832846	positive
PRJNA729271	ed_Lushi_vs_Arbor_Acr	breed_growth_background	ENSGALG00010018616	ENSGALG00010018616	6.98482304	0.45422782	15.37735634	2.32238E-53	3.36629E-49	positive
PRJNA729271	ed_Lushi_vs_Arbor_Acr	breed_growth_background	ENSGALG00010018618	FAHD1	-0.036751274	0.091136866	-0.40325365	0.686761635	0.797900762	negative
PRJNA729271	ed_Lushi_vs_Arbor_Acr	breed_growth_background	ENSGALG00010018619	MAPK1IP1L	-0.083463696	0.041711587	-2.000971489	0.045395463	0.123546232	negative
PRJNA729271	ed_Lushi_vs_Arbor_Acr	breed_growth_background	ENSGALG00010018623	ARRDC4	0.131589403	0.070250097	1.873156182	0.061046821	0.152786722	positive
PRJNA729271	ed_Lushi_vs_Arbor_Acr	breed_growth_background	ENSGALG00010018624	ENSGALG00010018624	-0.313517292	0.207394354	-1.511696373	0.130611122	0.258666659	negative
PRJNA729271	ed_Lushi_vs_Arbor_Acr	breed_growth_background	ENSGALG00010018627	MYLK	-0.151621723	0.096478262	-1.571563582	0.116051798	0.23880903	negative
PRJNA729271	ed_Lushi_vs_Arbor_Acr	breed_growth_background	ENSGALG00010018628	XDH	-0.430320351	0.321238697	-1.339565733	0.180386571	0.324888586	negative
PRJNA729271	ed_Lushi_vs_Arbor_Acr	breed_growth_background	ENSGALG00010018631	KAT14	-0.012656216	0.028860572	-0.43852963	0.661002396	0.77997637	negative
PRJNA729271	ed_Lushi_vs_Arbor_Acr	breed_growth_background	ENSGALG00010018633	ENSGALG00010018633	0.286531865	0.097128979	2.950014182	0.003177593	0.018490252	positive
PRJNA729271	ed_Lushi_vs_Arbor_Acr	breed_growth_background	ENSGALG00010018638	SEC14L5	0.3931376	0.086327716	4.554013669	5.26319E-06	0.000181211	positive
PRJNA729271	ed_Lushi_vs_Arbor_Acr	breed_growth_background	ENSGALG00010018641	ENSGALG00010018641	-0.672014097	0.441662042	-1.521557285	0.128120047	0.255348244	negative
PRJNA729271	ed_Lushi_vs_Arbor_Acr	breed_growth_background	ENSGALG00010018644	ESF1	0.205659037	0.052801312	3.89496072	9.82146E-05	0.001569593	positive
PRJNA729271	ed_Lushi_vs_Arbor_Acr	breed_growth_background	ENSGALG00010018649	HAGH	-0.21057787	0.055829919	-3.771774617	0.000162091	0.002265674	negative
PRJNA729271	ed_Lushi_vs_Arbor_Acr	breed_growth_background	ENSGALG00010018654	ENSGALG00010018654	-0.040875844	0.120191584	-0.340089073	0.733789451	0.829451058	negative
PRJNA729271	ed_Lushi_vs_Arbor_Acr	breed_growth_background	ENSGALG00010018655	BIRC5	-0.262800409	0.114475876	-2.29568377	0.021693969	0.072321546	negative
PRJNA729271	ed_Lushi_vs_Arbor_Acr	breed_growth_background	ENSGALG00010018662	PADI1	-0.789077512	0.411598775	-1.917103644	0.055224766	0.142333389	negative
PRJNA729271	ed_Lushi_vs_Arbor_Acr	breed_growth_background	ENSGALG00010018665	IL13RA2	-0.574257349	0.179968304	-3.19088048	0.001418399	0.010511501	negative
PRJNA729271	ed_Lushi_vs_Arbor_Acr	breed_growth_background	ENSGALG00010018668	OVOL2	0.007536963	0.486660108	0.015487119	0.987643561	0.992713543	positive
PRJNA729271	ed_Lushi_vs_Arbor_Acr	breed_growth_background	ENSGALG00010018670	MRAS	0.359567427	0.113845672	3.158375912	0.001586508	0.011289366	positive
PRJNA729271	ed_Lushi_vs_Arbor_Acr	breed_growth_background	ENSGALG00010018672	PKDCC	0.046068383	0.053478326	0.861440244	0.388995619	0.550902931	positive
PRJNA729271	ed_Lushi_vs_Arbor_Acr	breed_growth_background	ENSGALG00010018673	NAGPA	-0.17223608	0.050296902	-3.424387424	0.000616187	0.005736437	negative
PRJNA729271	ed_Lushi_vs_Arbor_Acr	breed_growth_background	ENSGALG00010018684	PADI2	1.208332919	0.424944345	2.843508644	0.00446198	0.02342499	positive
PRJNA729271	ed_Lushi_vs_Arbor_Acr	breed_growth_background	ENSGALG00010018685	ARL3	-0.273682515	0.057116606	-4.791645238	1.65419E-06	7.32232E-05	negative
PRJNA729271	ed_Lushi_vs_Arbor_Acr	breed_growth_background	ENSGALG00010018693	ENSGALG00010018693	0.386176332	0.088814038	4.348145196	1.37294E-05	0.000365151	positive
PRJNA729271	ed_Lushi_vs_Arbor_Acr	breed_growth_background	ENSGALG00010018694	MGME1	0.090146736	0.057504085	1.567657951	0.11696098	0.240249112	positive
PRJNA729271	ed_Lushi_vs_Arbor_Acr	breed_growth_background	ENSGALG00010018698	MRPS16	-0.054105897	0.061161845	-0.88463481	0.376353626	0.538789709	negative
PRJNA729271	ed_Lushi_vs_Arbor_Acr	breed_growth_background	ENSGALG00010018701	SFXN2	-0.017160345	0.071229339	-0.240916813	0.809619591	0.882761845	negative
PRJNA729271	ed_Lushi_vs_Arbor_Acr	breed_growth_background	ENSGALG00010018704	PGPEP1L	-0.052683284	0.224700374	-0.234460154	0.814627764	0.886011499	negative
PRJNA729271	ed_Lushi_vs_Arbor_Acr	breed_growth_background	ENSGALG00010018707	SLC45A1	-0.041911315	0.053516213	-0.783151736	0.433538013	0.591736607	negative
PRJNA729271	ed_Lushi_vs_Arbor_Acr	breed_growth_background	ENSGALG00010018709	SNX5	-0.124316462	0.061880215	-2.008985629	0.044538658	0.121740118	negative
PRJNA729271	ed_Lushi_vs_Arbor_Acr	breed_growth_background	ENSGALG00010018713	ENSGALG00010018713	-0.116039346	0.07495412	-1.548138338	0.121588993	0.246345837	negative
PRJNA729271	ed_Lushi_vs_Arbor_Acr	breed_growth_background	ENSGALG00010018715	DNAJC13	-0.014783502	0.055410506	-0.26679962	0.789623446	0.869461551	negative
PRJNA729271	ed_Lushi_vs_Arbor_Acr	breed_growth_background	ENSGALG00010018717	MEIOB	0.524006986	0.431978557	1.213039346	0.225114778	0.378104137	positive
PRJNA729271	ed_Lushi_vs_Arbor_Acr	breed_growth_background	ENSGALG00010018719	ERRFI1	-0.115073802	0.048383583	-2.378364625	0.017389621	0.061795184	negative
PRJNA729271	ed_Lushi_vs_Arbor_Acr	breed_growth_background	ENSGALG00010018721	LRCH2	-0.189741034	0.101090402	-1.876944097	0.060525756	0.151759356	negative
PRJNA729271	ed_Lushi_vs_Arbor_Acr	breed_growth_background	ENSGALG00010018724	RRBP1	-0.189697917	0.075961989	-2.497274235	0.012515213	0.048936979	negative
PRJNA729271	ed_Lushi_vs_Arbor_Acr	breed_growth_background	ENSGALG00010018726	EBF2	-0.2451506	0.083740253	-2.927512065	0.003416858	0.019433	negative
PRJNA729271	ed_Lushi_vs_Arbor_Acr	breed_growth_background	ENSGALG00010018727	PARK7	-0.036789018	0.070790546	-0.519688298	0.603280844	0.735392804	negative
PRJNA729271	ed_Lushi_vs_Arbor_Acr	breed_growth_background	ENSGALG00010018729	HS3ST6	0.547252712	0.305200098	1.79309481	0.072957765	0.172826084	positive
PRJNA729271	ed_Lushi_vs_Arbor_Acr	breed_growth_background	ENSGALG00010018730	IGF1R	-0.033184825	0.047137359	-0.70400263	0.481431136	0.634164332	negative
PRJNA729271	ed_Lushi_vs_Arbor_Acr	breed_growth_background	ENSGALG00010018732	ENSGALG00010018732	0.058436474	0.115965291	0.503913482	0.614322176	0.74359916	positive
PRJNA729271	ed_Lushi_vs_Arbor_Acr	breed_growth_background	ENSGALG00010018736	EEF2KMT	0.180092587	0.071775637	2.509104699	0.01210376	0.047804906	positive
PRJNA729271	ed_Lushi_vs_Arbor_Acr	breed_growth_background	ENSGALG00010018747	FLRT3	0.006786437	0.068406052	0.099208131	0.920973019	0.956061319	positive
PRJNA729271	ed_Lushi_vs_Arbor_Acr	breed_growth_background	ENSGALG00010018750	SOCS4	-0.045686272	0.06770127	-0.674821496	0.499789182	0.651010441	negative
PRJNA729271	ed_Lushi_vs_Arbor_Acr	breed_growth_background	ENSGALG00010018752	PLS3	-0.305623401	0.07546615	-4.049807794	5.12597E-05	0.000984991	negative
PRJNA729271	ed_Lushi_vs_Arbor_Acr	breed_growth_background	ENSGALG00010018753	SYNM	-0.298207147	0.082192558	-3.628152663	0.000285456	0.003360578	negative
PRJNA729271	ed_Lushi_vs_Arbor_Acr	breed_growth_background	ENSGALG00010018756	EML4	-0.076849916	0.034334485	-2.238271972	0.025203325	0.080467443	negative
PRJNA729271	ed_Lushi_vs_Arbor_Acr	breed_growth_background	ENSGALG00010018758	ENSGALG00010018758	-0.123507749	0.076328327	-1.618111572	0.105638555	0.223766017	negative
PRJNA729271	ed_Lushi_vs_Arbor_Acr	breed_growth_background	ENSGALG00010018764	TMEM177	0.067676536	0.050002912	1.353451904	0.175911305	0.31992903	positive
PRJNA729271	ed_Lushi_vs_Arbor_Acr	breed_growth_background	ENSGALG00010018767	NDUFB6	0.101072433	0.090294882	1.119359492	0.262986805	0.421229946	positive
PRJNA729271	ed_Lushi_vs_Arbor_Acr	breed_growth_background	ENSGALG00010018774	MSRB1	0.204234097	0.066094117	3.090049565	0.002001231	0.01326376	positive
PRJNA729271	ed_Lushi_vs_Arbor_Acr	breed_growth_background	ENSGALG00010018780	WDHD1	-0.42387758	0.113293212	-3.741420809	0.000182983	0.002433336	negative
PRJNA729271	ed_Lushi_vs_Arbor_Acr	breed_growth_background	ENSGALG00010018783	ENSGALG00010018783	-0.031987605	0.095736273	-0.334122101	0.738287423	0.832346286	negative
PRJNA729271	ed_Lushi_vs_Arbor_Acr	breed_growth_background	ENSGALG00010018786	DSTN	-0.232038532	0.061534975	-3.770839799	0.000162699	0.002267954	negative
PRJNA729271	ed_Lushi_vs_Arbor_Acr	breed_growth_background	ENSGALG00010018796	PER3	-0.146411256	0.16626001	-0.880616192	0.378525592	0.540617643	negative
PRJNA729271	ed_Lushi_vs_Arbor_Acr	breed_growth_background	ENSGALG00010018799	ENSGALG00010018799	0.433851177	0.19781992	2.193162227	0.028295693	0.087172385	positive
PRJNA729271	ed_Lushi_vs_Arbor_Acr	breed_growth_background	ENSGALG00010018806	GCH1	0.492198238	0.169974218	2.895722917	0.003782862	0.020833052	positive
PRJNA729271	ed_Lushi_vs_Arbor_Acr	breed_growth_background	ENSGALG00010018807	RD3	0.011223574	0.147030804	0.076334844	0.939152705	0.966697969	positive
PRJNA729271	ed_Lushi_vs_Arbor_Acr	breed_growth_background	ENSGALG00010018808	MATCAP2	0.019126285	0.049975983	0.382709523	0.701935153	0.807492385	positive
PRJNA729271	ed_Lushi_vs_Arbor_Acr	breed_growth_background	ENSGALG00010018811	VAMP3	0.016553944	0.065967441	0.250941122	0.80185963	0.877734129	positive
PRJNA729271	ed_Lushi_vs_Arbor_Acr	breed_growth_background	ENSGALG00010018815	ENSGALG00010018815	-0.054668157	0.091010551	-0.600679329	0.54805359	0.691567579	negative
PRJNA729271	ed_Lushi_vs_Arbor_Acr	breed_growth_background	ENSGALG00010018819	LRRC28	-0.035542267	0.04266056	-0.833141119	0.404765145	0.565609832	negative
PRJNA729271	ed_Lushi_vs_Arbor_Acr	breed_growth_background	ENSGALG00010018822	TRAF5	-0.066406008	0.107717117	-0.616485198	0.537574338	0.682802316	negative
PRJNA729271	ed_Lushi_vs_Arbor_Acr	breed_growth_background	ENSGALG00010018823	RAB40C	0.072931554	0.041876288	1.741595485	0.081579252	0.186535208	positive
PRJNA729271	ed_Lushi_vs_Arbor_Acr	breed_growth_background	ENSGALG00010018829	PTPN4	0.275505871	0.073758427	3.735245993	0.000187532	0.002471157	positive
PRJNA729271	ed_Lushi_vs_Arbor_Acr	breed_growth_background	ENSGALG00010018831	WBP1L	0.685249932	0.128347114	5.339036541	9.34418E-08	7.89535E-06	positive
PRJNA729271	ed_Lushi_vs_Arbor_Acr	breed_growth_background	ENSGALG00010018832	EEPD1	0.359826221	0.098296535	3.660619601	0.000251606	0.003059589	positive
PRJNA729271	ed_Lushi_vs_Arbor_Acr	breed_growth_background	ENSGALG00010018834	SAMD4A	0.048758571	0.091743372	0.531466962	0.595095229	0.728970282	positive
PRJNA729271	ed_Lushi_vs_Arbor_Acr	breed_growth_background	ENSGALG00010018835	CAMK2B	0.054928478	0.094010578	0.584279755	0.559032111	0.700117509	positive
PRJNA729271	ed_Lushi_vs_Arbor_Acr	breed_growth_background	ENSGALG00010018837	RCOR3	-0.201151634	0.051372644	-3.915539806	9.02022E-05	0.001482404	negative
PRJNA729271	ed_Lushi_vs_Arbor_Acr	breed_growth_background	ENSGALG00010018838	WFIKKN1	-0.333637622	0.146848297	-2.271988357	0.023087214	0.075610407	negative
PRJNA729271	ed_Lushi_vs_Arbor_Acr	breed_growth_background	ENSGALG00010018839	RPL3L	0.326764036	0.12709789	2.570963494	0.010141602	0.042241457	positive
PRJNA729271	ed_Lushi_vs_Arbor_Acr	breed_growth_background	ENSGALG00010018843	MCRIP2	0.602792589	0.125897367	4.787968179	1.68478E-06	7.37793E-05	positive
PRJNA729271	ed_Lushi_vs_Arbor_Acr	breed_growth_background	ENSGALG00010018847	CGRRF1	-0.081112368	0.086534352	-0.937342984	0.348582159	0.512652029	negative
PRJNA729271	ed_Lushi_vs_Arbor_Acr	breed_growth_background	ENSGALG00010018848	MEF2A	0.153534303	0.061185073	2.509342468	0.012095615	0.047785757	positive
PRJNA729271	ed_Lushi_vs_Arbor_Acr	breed_growth_background	ENSGALG00010018849	KCNH1	0.078145342	0.200529398	0.389695192	0.696761952	0.804497461	positive
PRJNA729271	ed_Lushi_vs_Arbor_Acr	breed_growth_background	ENSGALG00010018852	NDUFB10	0.164017972	0.061855325	2.65163866	0.008010222	0.03567071	positive
PRJNA729271	ed_Lushi_vs_Arbor_Acr	breed_growth_background	ENSGALG00010018854	LYSMD4	0.15311506	0.091867329	1.666697634	0.095574544	0.208231947	positive
PRJNA729271	ed_Lushi_vs_Arbor_Acr	breed_growth_background	ENSGALG00010018855	SYNDIG1	0.724867654	0.375320311	1.931330738	0.053442164	0.138949628	positive
PRJNA729271	ed_Lushi_vs_Arbor_Acr	breed_growth_background	ENSGALG00010018857	ENSGALG00010018857	0.055766101	0.044215525	1.261233482	0.207224739	0.356287489	positive
PRJNA729271	ed_Lushi_vs_Arbor_Acr	breed_growth_background	ENSGALG00010018858	EPB41L5	0.000192985	0.063377681	0.003044999	0.997570446	0.998883293	positive
PRJNA729271	ed_Lushi_vs_Arbor_Acr	breed_growth_background	ENSGALG00010018860	AGTR2	-0.263764832	0.114827359	-2.297055642	0.021615596	0.072121268	negative

PRJNA729271	ed_Lushi_vs_Arbor_Acr	breed_growth_background	ENSGALG00010018864	ABCA3	0.238630945	0.063735698	3.744070453	0.000181063	0.002423367	positive
PRJNA729271	ed_Lushi_vs_Arbor_Acr	breed_growth_background	ENSGALG00010018865	ENSGALG00010018865	-0.356194163	0.125870787	-2.829839812	0.004657131	0.024181738	negative
PRJNA729271	ed_Lushi_vs_Arbor_Acr	breed_growth_background	ENSGALG00010018867	RPS2	-0.140951562	0.083357105	-1.690936391	0.090848952	0.201450683	negative
PRJNA729271	ed_Lushi_vs_Arbor_Acr	breed_growth_background	ENSGALG00010018872	KCTD9	-0.084274675	0.054377192	-1.549816615	0.121185538	0.245998297	negative
PRJNA729271	ed_Lushi_vs_Arbor_Acr	breed_growth_background	ENSGALG00010018874	ENSGALG00010018874	0.018157325	0.063980984	0.283792529	0.77656938	0.861342825	positive
PRJNA729271	ed_Lushi_vs_Arbor_Acr	breed_growth_background	ENSGALG00010018876	GMFB	-0.185730323	0.052885731	-3.51191751	0.000444886	0.004558246	negative
PRJNA729271	ed_Lushi_vs_Arbor_Acr	breed_growth_background	ENSGALG00010018877	CST7	0.722567394	0.320979659	2.251131414	0.024377215	0.078556633	positive
PRJNA729271	ed_Lushi_vs_Arbor_Acr	breed_growth_background	ENSGALG00010018880	TMEM17	-0.145264262	0.139619349	-1.040430734	0.298139829	0.460344083	negative
PRJNA729271	ed_Lushi_vs_Arbor_Acr	breed_growth_background	ENSGALG00010018881	ENSGALG00010018881	1.137757986	0.340842649	3.338074005	0.000843613	0.00722278	positive
PRJNA729271	ed_Lushi_vs_Arbor_Acr	breed_growth_background	ENSGALG00010018882	HHAT	0.170137771	0.088594552	1.920408948	0.054806264	0.141556807	positive
PRJNA729271	ed_Lushi_vs_Arbor_Acr	breed_growth_background	ENSGALG00010018883	TBL3	-0.027837277	0.04169369	-0.66766162	0.504349614	0.654597749	negative
PRJNA729271	ed_Lushi_vs_Arbor_Acr	breed_growth_background	ENSGALG00010018885	EFEMP1	-0.179323572	0.11083669	-1.61790804	0.105682417	0.223793519	negative
PRJNA729271	ed_Lushi_vs_Arbor_Acr	breed_growth_background	ENSGALG00010018887	ADAMTS17	-0.158089991	0.110610773	-1.429245876	0.152933576	0.289433632	negative
PRJNA729271	ed_Lushi_vs_Arbor_Acr	breed_growth_background	ENSGALG00010018891	GNRH1	-0.454886421	0.20861976	-2.18045702	0.029223601	0.089186038	negative
PRJNA729271	ed_Lushi_vs_Arbor_Acr	breed_growth_background	ENSGALG00010018892	APMAP	-0.135566811	0.066635019	-2.034467969	0.041904435	0.116450304	negative
PRJNA729271	ed_Lushi_vs_Arbor_Acr	breed_growth_background	ENSGALG00010018894	SERTAD4	-0.163900336	0.050206086	-3.264551175	0.001096377	0.008741462	negative
PRJNA729271	ed_Lushi_vs_Arbor_Acr	breed_growth_background	ENSGALG00010018895	SEPTIN7	-0.181776096	0.04818743	-3.772272022	0.000161768	0.002263343	negative
PRJNA729271	ed_Lushi_vs_Arbor_Acr	breed_growth_background	ENSGALG00010018897	CCDC85A	-0.228473329	0.090144355	-2.534527308	0.011259915	0.045551903	negative
PRJNA729271	ed_Lushi_vs_Arbor_Acr	breed_growth_background	ENSGALG00010018899	ACSS1	-0.240134497	0.053792241	-4.464110272	8.04022E-06	0.000244838	negative
PRJNA729271	ed_Lushi_vs_Arbor_Acr	breed_growth_background	ENSGALG00010018901	TCF7L2	-0.137844552	0.079569994	-1.732368497	0.083207973	0.189162417	negative
PRJNA729271	ed_Lushi_vs_Arbor_Acr	breed_growth_background	ENSGALG00010018902	NOXO1	-0.912687162	0.318478902	-2.865769619	0.004159969	0.022234051	negative
PRJNA729271	ed_Lushi_vs_Arbor_Acr	breed_growth_background	ENSGALG00010018903	CAMTA1	-0.032492316	0.052520184	-0.618663406	0.536138122	0.681325769	negative
PRJNA729271	ed_Lushi_vs_Arbor_Acr	breed_growth_background	ENSGALG00010018904	UGP2	0.480856255	0.144945589	3.317494908	0.000908286	0.007588243	positive
PRJNA729271	ed_Lushi_vs_Arbor_Acr	breed_growth_background	ENSGALG00010018908	ENSGALG00010018908	-0.211799704	0.129891663	-1.630587367	0.102977417	0.219573059	negative
PRJNA729271	ed_Lushi_vs_Arbor_Acr	breed_growth_background	ENSGALG00010018909	TTBK1	0.339026385	0.072007872	4.708185064	2.49932E-06	9.8713E-05	positive
PRJNA729271	ed_Lushi_vs_Arbor_Acr	breed_growth_background	ENSGALG00010018911	CNIH1	-0.093275312	0.050409226	-1.850361912	0.064261405	0.158224743	negative
PRJNA729271	ed_Lushi_vs_Arbor_Acr	breed_growth_background	ENSGALG00010018913	ENSGALG00010018913	-0.178238089	0.118490127	-1.50424422	0.132518496	0.261554411	negative
PRJNA729271	ed_Lushi_vs_Arbor_Acr	breed_growth_background	ENSGALG00010018915	DOCK5	-0.288711758	0.103469407	-2.79031037	0.005265754	0.026474887	negative
PRJNA729271	ed_Lushi_vs_Arbor_Acr	breed_growth_background	ENSGALG00010018916	CERS3	0.113253998	0.182991646	0.618902558	0.535980554	0.681195802	positive
PRJNA729271	ed_Lushi_vs_Arbor_Acr	breed_growth_background	ENSGALG00010018917	DNAJC11	0.229632719	0.056853699	4.039011056	5.3677E-05	0.001020214	positive
PRJNA729271	ed_Lushi_vs_Arbor_Acr	breed_growth_background	ENSGALG00010018919	RALB	0.05697163	0.057681973	0.987685185	0.323306852	0.487246083	positive
PRJNA729271	ed_Lushi_vs_Arbor_Acr	breed_growth_background	ENSGALG00010018920	ACAD11	-0.221231672	0.06346577	-3.485842422	0.00049059	0.004904207	negative
PRJNA729271	ed_Lushi_vs_Arbor_Acr	breed_growth_background	ENSGALG00010018922	EEF2K	0.081753401	0.060830627	1.343951302	0.178964135	0.323250484	positive
PRJNA729271	ed_Lushi_vs_Arbor_Acr	breed_growth_background	ENSGALG00010018923	VPS54	0.017604091	0.050235428	0.35043179	0.726014672	0.824474505	positive
PRJNA729271	ed_Lushi_vs_Arbor_Acr	breed_growth_background	ENSGALG00010018925	LINS1	0.088847901	0.072947495	1.21797056	0.22323516	0.375905396	positive
PRJNA729271	ed_Lushi_vs_Arbor_Acr	breed_growth_background	ENSGALG00010018926	SLC22A7	-0.518521271	0.527078463	-0.983764862	0.32523114	0.488946163	negative
PRJNA729271	ed_Lushi_vs_Arbor_Acr	breed_growth_background	ENSGALG00010018932	CDKN3	-0.555130541	0.12869011	-4.313700091	1.60545E-05	0.000408413	negative
PRJNA729271	ed_Lushi_vs_Arbor_Acr	breed_growth_background	ENSGALG00010018933	PHF13	-0.010316694	0.047688672	-0.216334274	0.828727195	0.895563428	negative
PRJNA729271	ed_Lushi_vs_Arbor_Acr	breed_growth_background	ENSGALG00010018936	SYT14	-0.404997218	0.114501386	-3.53705078	0.000404622	0.004274777	negative
PRJNA729271	ed_Lushi_vs_Arbor_Acr	breed_growth_background	ENSGALG00010018937	INHBB	0.273071708	0.205072173	1.33158831	0.182995508	0.328079146	positive
PRJNA729271	ed_Lushi_vs_Arbor_Acr	breed_growth_background	ENSGALG00010018941	KLHL21	-0.02483604	0.076620705	-0.32414267	0.745830023	0.837917082	negative
PRJNA729271	ed_Lushi_vs_Arbor_Acr	breed_growth_background	ENSGALG00010018943	ACKR4	0.174932831	0.182403968	0.959040714	0.337538235	0.501755381	positive
PRJNA729271	ed_Lushi_vs_Arbor_Acr	breed_growth_background	ENSGALG00010018944	NEFL	-0.331753312	0.098104112	-3.381645339	0.000720531	0.006453074	negative
PRJNA729271	ed_Lushi_vs_Arbor_Acr	breed_growth_background	ENSGALG00010018945	HABP2	-0.819992002	0.374037919	-2.192269718	0.028360037	0.087296398	negative
PRJNA729271	ed_Lushi_vs_Arbor_Acr	breed_growth_background	ENSGALG00010018947	ENSGALG00010018947	-0.180198437	0.119716495	-1.50520976	0.132270157	0.261170947	negative
PRJNA729271	ed_Lushi_vs_Arbor_Acr	breed_growth_background	ENSGALG00010018948	POLR3E	0.127109019	0.058199466	2.184023797	0.028960499	0.088648258	positive
PRJNA729271	ed_Lushi_vs_Arbor_Acr	breed_growth_background	ENSGALG00010018950	TTL	0.172695432	0.051956645	3.323837268	0.00088788	0.007504268	positive
PRJNA729271	ed_Lushi_vs_Arbor_Acr	breed_growth_background	ENSGALG00010018951	YKT6	-0.0336311	0.049551429	-0.678711016	0.497320985	0.648728192	negative
PRJNA729271	ed_Lushi_vs_Arbor_Acr	breed_growth_background	ENSGALG00010018955	ZBTB48	0.251981784	0.075851602	3.322036415	0.00089363	0.007530254	positive
PRJNA729271	ed_Lushi_vs_Arbor_Acr	breed_growth_background	ENSGALG00010018958	PELI3	-0.202575537	0.063493001	-3.190517588	0.001420182	0.010518926	negative
PRJNA729271	ed_Lushi_vs_Arbor_Acr	breed_growth_background	ENSGALG00010018959	UBA5	-0.07416841	0.063778934	-1.162898241	0.244870791	0.400790664	negative
PRJNA729271	ed_Lushi_vs_Arbor_Acr	breed_growth_background	ENSGALG00010018960	RBFOX1	0.503503807	0.1188748	4.235580697	2.27962E-05	0.000529536	positive
PRJNA729271	ed_Lushi_vs_Arbor_Acr	breed_growth_background	ENSGALG00010018961	ASB7	0.065471656	0.053727965	1.218576885	0.223004827	0.375648456	positive
PRJNA729271	ed_Lushi_vs_Arbor_Acr	breed_growth_background	ENSGALG00010018968	GLI2	-0.374199739	0.106814776	-3.503258202	0.000459604	0.00467834	negative
PRJNA729271	ed_Lushi_vs_Arbor_Acr	breed_growth_background	ENSGALG00010018969	DIEXF	0.078162164	0.04924398	1.587243024	0.112457614	0.233769269	positive
PRJNA729271	ed_Lushi_vs_Arbor_Acr	breed_growth_background	ENSGALG00010018971	POLR1B	0.121135388	0.053448254	2.266404973	0.023426599	0.076406647	positive
PRJNA729271	ed_Lushi_vs_Arbor_Acr	breed_growth_background	ENSGALG00010018973	NEFM	0.282343808	0.300631464	0.939169188	0.347643886	0.511583566	positive
PRJNA729271	ed_Lushi_vs_Arbor_Acr	breed_growth_background	ENSGALG00010018974	TAS1R1	-0.058787847	0.15787564	-0.372368064	0.709618828	0.812923806	negative
PRJNA729271	ed_Lushi_vs_Arbor_Acr	breed_growth_background	ENSGALG00010018977	NPHP3	-0.013378407	0.060329217	-0.221756678	0.824503306	0.893003836	negative
PRJNA729271	ed_Lushi_vs_Arbor_Acr	breed_growth_background	ENSGALG00010018983	MDGA2	0.330534058	0.109398281	3.02138255	0.002516232	0.015653557	positive
PRJNA729271	ed_Lushi_vs_Arbor_Acr	breed_growth_background	ENSGALG00010018985	NOL9	0.056266534	0.048285804	1.165281086	0.243905232	0.399616406	positive
PRJNA729271	ed_Lushi_vs_Arbor_Acr	breed_growth_background	ENSGALG00010018986	CTDSP1	0.39213999	0.10221218	3.836528966	0.000124785	0.001870492	positive
PRJNA729271	ed_Lushi_vs_Arbor_Acr	breed_growth_background	ENSGALG00010018987	MRPS10	0.143624027	0.058448992	2.457254133	0.014000361	0.052923103	positive
PRJNA729271	ed_Lushi_vs_Arbor_Acr	breed_growth_background	ENSGALG00010018990	GCK	1.100199875	0.233932449	4.703066546	2.56283E-06	0.000100946	positive
PRJNA729271	ed_Lushi_vs_Arbor_Acr	breed_growth_background	ENSGALG00010018991	RYBP	0.21002168	0.072625355	2.891850661	0.003829799	0.02098788	positive
PRJNA729271	ed_Lushi_vs_Arbor_Acr	breed_growth_background	ENSGALG00010018993	ENSGALG00010018993	-0.025669141	0.26646835	-0.09633092	0.923257755	0.95713211	negative
PRJNA729271	ed_Lushi_vs_Arbor_Acr	breed_growth_background	ENSGALG00010018998	RPS29	-0.436475038	0.235155738	-1.856110521	0.063437806	0.156873916	negative
PRJNA729271	ed_Lushi_vs_Arbor_Acr	breed_growth_background	ENSGALG00010018999	THUMPD3	0.0578641	0.039516912	1.464286975	0.143115554	0.276295615	positive
PRJNA729271	ed_Lushi_vs_Arbor_Acr	breed_growth_background	ENSGALG00010019000	TRERF1	0.024225233	0.064679379	0.374543373	0.708000094	0.811828886	positive
PRJNA729271	ed_Lushi_vs_Arbor_Acr	breed_growth_background	ENSGALG00010019001	ALDH1A3	-0.141033525	0.085483607	-1.649831236	0.098977458	0.21334944	negative
PRJNA729271	ed_Lushi_vs_Arbor_Acr	breed_growth_background	ENSGALG00010019004	ECE2	0.031361845	0.113205899	0.277033668	0.781754254	0.864936105	positive
PRJNA729271	ed_Lushi_vs_Arbor_Acr	breed_growth_background	ENSGALG00010019005	ENSGALG00010019005	-0.298916194	0.118085201	-2.531360339	0.011362106	0.045773685	negative
PRJNA729271	ed_Lushi_vs_Arbor_Acr	breed_growth_background	ENSGALG00010019006	ENSGALG00010019006	-0.014099596	0.043353096	-0.32522696	0.745009306	0.837262036	negative
PRJNA729271	ed_Lushi_vs_Arbor_Acr	breed_growth_background	ENSGALG00010019008	HERPUD2	-0.057853509	0.024381817	-2.372813691	0.017653166	0.0625477	negative
PRJNA729271	ed_Lushi_vs_Arbor_Acr	breed_growth_background	ENSGALG00010019009	NRAP	-0.259882142	0.116567033	-2.229465186	0.025782969	0.081792208	negative
PRJNA729271	ed_Lushi_vs_Arbor_Acr	breed_growth_background	ENSGALG00010019011	VIL1	0.190359216	0.206284582	0.922799046	0.356111938	0.519666017	positive
PRJNA729271	ed_Lushi_vs_Arbor_Acr	breed_growth_background	ENSGALG00010019012	ADAM28	-0.487489539	0.197162738	-2.472523683	0.013416282	0.051385539	negative
PRJNA729271	ed_Lushi_vs_Arbor_Acr	breed_growth_background	ENSGALG00010019013	LRRK1	-0.062438159	0.056352638	-1.107989998	0.267866136	0.426250921	negative
PRJNA729271	ed_Lushi_vs_Arbor_Acr	breed_growth_background	ENSGALG00010019015	GSG1L	0.924244721	0.55275551	1.672067857	0.094510942	0.206735617	positive
PRJNA729271	ed_Lushi_vs_Arbor_Acr	breed_growth_background	ENSGALG00010019016	PLEKHG5	-0.139746857	0.077567465	-1.801616899	0.071605701	0.170486963	negative
PRJNA729271	ed_Lushi_vs_Arbor_Acr	breed_growth_background	ENSGALG00010019017	IRAK2	-0.049444078	0.044373113	-1.114280121	0.265159032	0.423430668	negative
PRJNA729271	ed_Lushi_vs_Arbor_Acr	breed_growth_background	ENSGALG00010019018	SH3BGRL	-0.224107924	0.059280445	-3.780469655	0.000156533	0.002202857	negative
PRJNA729271	ed_Lushi_vs_Arbor_Acr	breed_growth_background	ENSGALG00010019019	TBX20	-0.888877806	0.172624456	-5.149199751	2.616E-07	1.73146E-05	negative
PRJNA729271	ed_Lushi_vs_Arbor_Acr	breed_growth_background	ENSGALG00010019020	KCNK2	-0.88863396	0.235726013	-3.769774695	0.000163395	0.002270767	negative
PRJNA729271	ed_Lushi_vs_Arbor_Acr	breed_growth_background	ENSGALG00010019021	LRR1	-0.101222382	0.084658039	-1.195661788	0.231828569	0.385937189	negative
PRJNA729271	ed_Lushi_vs_Arbor_Acr	breed_growth_background	ENSGALG00010019023	AP2M1	-0.091065373	0.038356875	-2.374160388	0.017588908	0.062381019	negative
PRJNA729271	ed_Lushi_vs_Arbor_Acr	breed_growth_background	ENSGALG00010019024	CNPY3	0.040010503	0.055606961	0.719523286	0.47181856	0.626104411	positive
PRJNA729271	ed_Lushi_vs_Arbor_Acr	breed_growth_background	ENSGALG00010019025	DNAAF2	0.151447898	0.136005408	1.1135432	0.265475205	0.423702169	positive
PRJNA729271	ed_Lushi_vs_Arbor_Acr	breed_growth_background	ENSGALG00010019026	CHSY1	-0.059617381	0.067976884	-0.877024331	0.380473422	0.542170887	negative
PRJNA729271	ed_Lushi_vs_Arbor_Acr	breed_growth_background	ENSGALG00010019027	GNLY	0.283323538	0.171411551	1.652884748	0.098354322	0.212434197	positive
PRJNA729271	ed_Lushi_vs_Arbor_Acr	breed_growth_background	ENSGALG00010019028	TNFRSF25	0.028493465	0.186492571	0.152786058	0.878566993	0.927991588	positive
PRJNA729271	ed_Lushi_vs_Arbor_Acr	breed_growth_background	ENSGALG00010019029	CENPF	-0.320660357	0.139525863	-2.298214473	0.021549585	0.07192292	negative
PRJNA729271	ed_Lushi_vs_Arbor_Acr	breed_growth_background	ENSGALG00010019030	CDR2	-0.126915573	0.083632555	-1.517537901	0.129130932	0.256612254	negative
PRJNA729271	ed_Lushi_vs_Arbor_Acr	breed_growth_background	ENSGALG00010019032	GNMT	0.005932836	0.102162971	0.058072274	0.953691059	0.974074966	positive
PRJNA729271	ed_Lushi_vs_Arbor_Acr	breed_growth_background	ENSGALG00010019033	CYP2AB4	-0.185303034	0.608115637	-0.304716773	0.760581874	0.849315923	negative
PRJNA729271	ed_Lushi_vs_Arbor_Acr	breed_growth_background	ENSGALG00010019034	CASP7	-0.229587335	0.091902291	-2.498167697	0.012483712	0.048852972	negative
PRJNA729271	ed_Lushi_vs_Arbor_Acr	breed_growth_background	ENSGALG00010019035	ENSGALG00010019035	-0.373116431	0.286039651	-1.304422063	0.192089717	0.338974975	negative
PRJNA729271	ed_Lushi_vs_Arbor_Acr	breed_growth_background	ENSGALG00010019036	REL	0.002754954	0.059761162	0.04609941	0.963231016	0.979036083	positive
PRJNA729271	ed_Lushi_vs_Arbor_Acr	breed_growth_background	ENSGALG00010019038	PLEKHS1	0.427929964	0.285211044	1.500397596	0.133511442	0.263056615	positive
PRJNA729271	ed_Lushi_vs_Arbor_Acr	breed_growth_background	ENSGALG00010019040	HMGN5	-0.049092944	0.044979022	-1.091463136	0.275069144	0.433949417	negative
PRJNA729271	ed_Lushi_vs_Arbor_Acr	breed_growth_background	ENSGALG00010019041	KATNIP	0.063201497	0.049390392	1.279631426	0.200674792	0.349507714	positive
PRJNA729271	ed_Lushi_vs_Arbor_Acr	breed_growth_background	ENSGALG00010019042	PEX6	0.015686798	0.056680668	0.276757459	0.781966348	0.865104734	positive
PRJNA729271	ed_Lushi_vs_Arbor_Acr	breed_growth_background	ENSGALG00010019043	METTL9	-0.140739487	0.056904524	-2.473256569	0.013388798	0.051314287	negative
PRJNA729271	ed_Lushi_vs_Arbor_Acr	breed_growth_background	ENSGALG00010019045	LBX1	0.289490044	0.141336943	2.048226295	0.040537831	0.113457398	positive
PRJNA729271	ed_Lushi_vs_Arbor_Acr	breed_growth_background	ENSGALG00010019046	SELENOS	0.05138334	0.043505862	1.181067067	0.237576082	0.392732961	positive
PRJNA729271	ed_Lushi_vs_Arbor_Acr	breed_growth_background	ENSGALG00010019047	GHRL	-0.444208921	0.249387704	-1.781198165	0.074880083	0.176113388	negative
PRJNA729271	ed_Lushi_vs_Arbor_Acr	breed_growth_background	ENSGALG00010019049	POLD2	0.172111979	0.087898671	1.958072602	0.050221494	0.132963253	positive
PRJNA729271	ed_Lushi_vs_Arbor_Acr	breed_growth_background	ENSGALG00010019050	TNS1	-0.081077869	0.071490617	-1.134105037	0.256750491	0.413924854	negative
PRJNA729271	ed_Lushi_vs_Arbor_Acr	breed_growth_background	ENSGALG00010019051	IGSF6	-0.31373279	0.135526878	-2.314911951	0.020617751	0.06967925	negative
PRJNA729271	ed_Lushi_vs_Arbor_Acr	breed_growth_background	ENSGALG00010019059	BRWD3	0.088052452	0.04170306	2.111414666	0.034736686	0.101370698	positive
PRJNA729271	ed_Lushi_vs_Arbor_Acr	breed_growth_background	ENSGALG00010019062	PUS10	-0.065217829	0.055886895	-1.166961044	0.243226102	0.39892893	negative
PRJNA729271	ed_Lushi_vs_Arbor_Acr	breed_growth_background	ENSGALG00010019064	ENSGALG00010019064	-0.256391223	0.092266409	-2.778814357	0.00545577	0.027127807	negative
PRJNA729271	ed_Lushi_vs_Arbor_Acr	breed_growth_background	ENSGALG00010019065	CAND2	0.140508782	0.064374632	2.182673155	0.029059888	0.088820205	positive
PRJNA729271	ed_Lushi_vs_Arbor_Acr	breed_growth_background	ENSGALG00010019067	GTF3C1	-0.068796461	0.03170226	-2.170080652	0.030000737	0.090860986	negative
PRJNA729271	ed_Lushi_vs_Arbor_Acr	breed_growth_background	ENSGALG00010019068	SNRPA1	0.249011867	0.087581966	2.843186531	0.004466492	0.023431706	positive
PRJNA729271	ed_Lushi_vs_Arbor_Acr	breed_growth_background	ENSGALG00010019072	AEBP1	-0.218351473	0.112937312	-1.933386482	0.053188605	0.138489102	negative
PRJNA729271	ed_Lushi_vs_Arbor_Acr	breed_growth_background	ENSGALG00010019076	ESPN	0.461946246	0.227368263	2.031709436	0.04218308	0.117089955	positive
PRJNA729271	ed_Lushi_vs_Arbor_Acr	breed_growth_background	ENSGALG00010019079	CAPG	-0.369307185	0.131743912	-2.803220119	0.005059512	0.025804938	negative
PRJNA729271	ed_Lushi_vs_Arbor_Acr	breed_growth_background	ENSGALG00010019081	PCSK6	-0.386313824	0.073045103	-5.288702604	1.23187E-07	9.86517E-06	negative
PRJNA729271	ed_Lushi_vs_Arbor_Acr	breed_growth_background	ENSGALG00010019082	POLR3K	-0.042021648	0.095451896	-0.440239007	0.659764011	0.778832099	negative
PRJNA729271	ed_Lushi_vs_Arbor_Acr	breed_growth_background	ENSGALG00010019083	OTOA	0.020558367	0.128598228	0.159865085	0.872987353	0.924996467	positive
PRJNA729271	ed_Lushi_vs_Arbor_Acr	breed_growth_background	ENSGALG00010019087	PEX13	0.131746488	0.046712128	2.820391511	0.004796509	0.024715747	positive
PRJNA729271	ed_Lushi_vs_Arbor_Acr	breed_growth_background	ENSGALG00010019093	RPL32	-0.058143593	0.081124667	-0.716719026	0.473547484	0.627143702	negative
PRJNA729271	ed_Lushi_vs_Arbor_Acr	breed_growth_background	ENSGALG00010019094	SNRNP25	-0.144662961	0.090209075	-1.603640892	0.108793232	0.228677188	negative
PRJNA729271	ed_Lushi_vs_Arbor_Acr	breed_growth_background	ENSGALG00010019097	STC1	-0.479030024	0.153642486	-3.117822653	0.001821924	0.012451102	negative
PRJNA729271	ed_Lushi_vs_Arbor_Acr	breed_growth_background	ENSGALG00010019101	POLE2	-0.259850527	0.066416002	-3.912468663	9.13574E-05	0.0014963	negative
PRJNA729271	ed_Lushi_vs_Arbor_Acr	breed_growth_background	ENSGALG00010019104	POLL	0.132151767	0.065165219	2.027949409	0.042565412	0.117857811	positive
PRJNA729271	ed_Lushi_vs_Arbor_Acr	breed_growth_background	ENSGALG00010019108	KDELR2	-0.030582228	0.053409142	-0.572602868	0.566913618	0.706085057	negative
PRJNA729271	ed_Lushi_vs_Arbor_Acr	breed_growth_background	ENSGALG00010019110	MBD4	0.232750525	0.059261393	3.927523709	8.5825E-05	0.001429625	positive
PRJNA729271	ed_Lushi_vs_Arbor_Acr	breed_growth_background	ENSGALG00010019113	KLHDC1	0.128934775	0.071117554	1.812980998	0.069834752	0.167702905	positive
PRJNA729271	ed_Lushi_vs_Arbor_Acr	breed_growth_background	ENSGALG00010019114	TENT5D	0.280210635	0.196505774	1.425966423	0.153878034	0.290405273	positive

PRJNA729271	ɛed_Lushi_vs_Arbor_Acr	breed_growth_background	ENSGALG00010019115	DBNL	-0.087789844	0.055954557	-1.568948959	0.116659833	0.2397879	negative
PRJNA729271	ɛed_Lushi_vs_Arbor_Acr	breed_growth_background	ENSGALG00010019120	DAGLB	0.036235977	0.043254439	0.83774006	0.40217671	0.563187267	positive
PRJNA729271	ɛed_Lushi_vs_Arbor_Acr	breed_growth_background	ENSGALG00010019123	PLEKHO2	-0.049770043	0.062979859	-0.79025333	0.429379836	0.588076607	negative
PRJNA729271	ɛed_Lushi_vs_Arbor_Acr	breed_growth_background	ENSGALG00010019124	BMPER	-0.167457469	0.134535308	-1.244710188	0.2132383	0.363553846	negative
PRJNA729271	ɛed_Lushi_vs_Arbor_Acr	breed_growth_background	ENSGALG00010019125	ENSGALG00010019125	-0.37509613	0.105605407	-3.551864808	0.000382511	0.004116185	negative
PRJNA729271	ɛed_Lushi_vs_Arbor_Acr	breed_growth_background	ENSGALG00010019127	TMBIM1	0.164516799	0.065201822	2.523193268	0.011629447	0.046527416	positive
PRJNA729271	ɛed_Lushi_vs_Arbor_Acr	breed_growth_background	ENSGALG00010019130	RPS6KC1	-0.001254995	0.045124142	-0.027812056	0.97781205	0.987004573	negative
PRJNA729271	ɛed_Lushi_vs_Arbor_Acr	breed_growth_background	ENSGALG00010019132	ENSGALG00010019132	0.14192097	0.082305492	1.724319572	0.084650168	0.191599654	positive
PRJNA729271	ɛed_Lushi_vs_Arbor_Acr	breed_growth_background	ENSGALG00010019135	KIAA1841	-0.066538937	0.112044161	-0.593863493	0.552603418	0.69519148	negative
PRJNA729271	ɛed_Lushi_vs_Arbor_Acr	breed_growth_background	ENSGALG00010019139	DPCD	-0.07244242	0.074870682	-0.967567258	0.333260536	0.497693331	negative
PRJNA729271	ɛed_Lushi_vs_Arbor_Acr	breed_growth_background	ENSGALG00010019141	ACOT7	0.435700799	0.083567131	5.213781976	1.85029E-07	1.36142E-05	positive
PRJNA729271	ɛed_Lushi_vs_Arbor_Acr	breed_growth_background	ENSGALG00010019144	IFT122	0.05063955	0.040803224	1.241067379	0.214580859	0.365364684	positive
PRJNA729271	ɛed_Lushi_vs_Arbor_Acr	breed_growth_background	ENSGALG00010019145	RAC1	-0.140325753	0.041692104	-3.365763257	0.000763322	0.006732233	negative
PRJNA729271	ɛed_Lushi_vs_Arbor_Acr	breed_growth_background	ENSGALG00010019146	KLHDC2	-0.019226605	0.045734936	-0.420392078	0.674199054	0.789379264	negative
PRJNA729271	ɛed_Lushi_vs_Arbor_Acr	breed_growth_background	ENSGALG00010019148	ENSGALG00010019148	-0.006948934	0.116531212	-0.059631524	0.952449111	0.973433394	negative
PRJNA729271	ɛed_Lushi_vs_Arbor_Acr	breed_growth_background	ENSGALG00010019149	COX5B	0.227065115	0.096239611	2.359372737	0.018305858	0.064294504	positive
PRJNA729271	ɛed_Lushi_vs_Arbor_Acr	breed_growth_background	ENSGALG00010019158	ENSGALG00010019158	-0.162929443	0.128762877	-1.265344844	0.205747738	0.355079588	negative
PRJNA729271	ɛed_Lushi_vs_Arbor_Acr	breed_growth_background	ENSGALG00010019166	PNPT1	0.28129277	0.059334412	4.74080315	2.12873E-06	8.864E-05	positive
PRJNA729271	ɛed_Lushi_vs_Arbor_Acr	breed_growth_background	ENSGALG00010019169	GPR153	0.389075362	0.12803711	3.038770262	0.002375459	0.015055655	positive
PRJNA729271	ɛed_Lushi_vs_Arbor_Acr	breed_growth_background	ENSGALG00010019172	NEMF	0.083987511	0.046819652	1.79385169	0.072836844	0.172737247	positive
PRJNA729271	ɛed_Lushi_vs_Arbor_Acr	breed_growth_background	ENSGALG00010019173	RHO	-0.137465791	0.471233615	-0.291714738	0.77050474	0.857003238	negative
PRJNA729271	ɛed_Lushi_vs_Arbor_Acr	breed_growth_background	ENSGALG00010019174	ENSGALG00010019174	0.070815683	0.24986018	0.283421244	0.776853945	0.861421201	positive
PRJNA729271	ɛed_Lushi_vs_Arbor_Acr	breed_growth_background	ENSGALG00010019183	ANGEL2	0.019703101	0.04292919	0.458967444	0.646257545	0.768921315	positive
PRJNA729271	ɛed_Lushi_vs_Arbor_Acr	breed_growth_background	ENSGALG00010019184	PNKD	0.280111078	0.103839618	2.697535711	0.006985479	0.032349687	positive
PRJNA729271	ɛed_Lushi_vs_Arbor_Acr	breed_growth_background	ENSGALG00010019185	ANKDD1A	-0.05489995	0.117678992	-0.466522946	0.640841227	0.764526221	negative
PRJNA729271	ɛed_Lushi_vs_Arbor_Acr	breed_growth_background	ENSGALG00010019188	ENSGALG00010019188	-0.402904634	0.199145443	-2.023167731	0.043055857	0.118762065	negative
PRJNA729271	ɛed_Lushi_vs_Arbor_Acr	breed_growth_background	ENSGALG00010019189	ICMT	0.075961962	0.051260565	1.481879149	0.138372454	0.269906814	positive
PRJNA729271	ɛed_Lushi_vs_Arbor_Acr	breed_growth_background	ENSGALG00010019193	ENSGALG00010019193	1.069980469	0.475360274	2.250883227	0.024392934	0.078572352	positive
PRJNA729271	ɛed_Lushi_vs_Arbor_Acr	breed_growth_background	ENSGALG00010019194	ENSGALG00010019194	-0.279969401	0.101072715	-2.76998003	0.005605973	0.027629574	negative
PRJNA729271	ɛed_Lushi_vs_Arbor_Acr	breed_growth_background	ENSGALG00010019204	ARF6	0.006643886	0.123341962	0.053865578	0.957042262	0.975824957	positive
PRJNA729271	ɛed_Lushi_vs_Arbor_Acr	breed_growth_background	ENSGALG00010019205	ENSGALG00010019205	0.148134979	0.143676902	1.031028484	0.302527463	0.465068997	positive
PRJNA729271	ɛed_Lushi_vs_Arbor_Acr	breed_growth_background	ENSGALG00010019206	RNF207	0.615846633	0.151002991	4.078373752	4.53518E-05	0.000896471	positive
PRJNA729271	ɛed_Lushi_vs_Arbor_Acr	breed_growth_background	ENSGALG00010019208	PLXND1	-0.328751321	0.084492769	-3.890881127	9.98809E-05	0.00158747	negative
PRJNA729271	ɛed_Lushi_vs_Arbor_Acr	breed_growth_background	ENSGALG00010019209	VCPKMT	-0.008356046	0.04626954	-0.180594964	0.856685509	0.91386933	negative
PRJNA729271	ɛed_Lushi_vs_Arbor_Acr	breed_growth_background	ENSGALG00010019212	RPL22	-0.014259842	0.060923165	-0.234062724	0.814936284	0.886225631	negative
PRJNA729271	ɛed_Lushi_vs_Arbor_Acr	breed_growth_background	ENSGALG00010019213	RHBDF1	-0.006871595	0.059352224	-0.115776539	0.907829645	0.947477673	negative
PRJNA729271	ɛed_Lushi_vs_Arbor_Acr	breed_growth_background	ENSGALG00010019215	ENSGALG00010019215	-0.022739893	0.055182965	-0.412081754	0.680279502	0.793358386	negative
PRJNA729271	ɛed_Lushi_vs_Arbor_Acr	breed_growth_background	ENSGALG00010019218	RMND5A	0.271401666	0.073671202	3.68395869	0.000229639	0.002862102	positive
PRJNA729271	ɛed_Lushi_vs_Arbor_Acr	breed_growth_background	ENSGALG00010019221	VASH2	-0.499679243	0.124473786	-4.014333131	5.96141E-05	0.001096582	negative
PRJNA729271	ɛed_Lushi_vs_Arbor_Acr	breed_growth_background	ENSGALG00010019223	AAMP	-0.024755826	0.067827913	-0.364979912	0.715126401	0.817230935	negative
PRJNA729271	ɛed_Lushi_vs_Arbor_Acr	breed_growth_background	ENSGALG00010019225	PROK2	0.331298523	0.367526433	0.901427745	0.367360935	0.53073824	positive
PRJNA729271	ɛed_Lushi_vs_Arbor_Acr	breed_growth_background	ENSGALG00010019227	CLCN5	-0.088059192	0.03958814	-2.224383172	0.026122675	0.082431303	negative
PRJNA729271	ɛed_Lushi_vs_Arbor_Acr	breed_growth_background	ENSGALG00010019230	SPG21	-0.068340459	0.052953669	-1.290570798	0.196852548	0.345278035	negative
PRJNA729271	ɛed_Lushi_vs_Arbor_Acr	breed_growth_background	ENSGALG00010019231	ENSGALG00010019231	0.514846949	0.166085954	3.099882539	0.001935974	0.012979622	positive
PRJNA729271	ɛed_Lushi_vs_Arbor_Acr	breed_growth_background	ENSGALG00010019232	SOS2	-0.003005137	0.050240028	-0.059815593	0.952302506	0.973433394	negative
PRJNA729271	ɛed_Lushi_vs_Arbor_Acr	breed_growth_background	ENSGALG00010019233	ARPC2	-0.113490771	0.029122861	-3.896965084	9.74057E-05	0.001561831	negative
PRJNA729271	ɛed_Lushi_vs_Arbor_Acr	breed_growth_background	ENSGALG00010019236	MPG	0.178646946	0.112772246	1.584139302	0.113162022	0.234707218	positive
PRJNA729271	ɛed_Lushi_vs_Arbor_Acr	breed_growth_background	ENSGALG00010019237	SORCS3	0.309863471	0.192722182	1.607824631	0.107873592	0.227238442	positive
PRJNA729271	ɛed_Lushi_vs_Arbor_Acr	breed_growth_background	ENSGALG00010019239	CYTH3	-0.032232744	0.043503485	-0.740923262	0.45873997	0.614372268	negative
PRJNA729271	ɛed_Lushi_vs_Arbor_Acr	breed_growth_background	ENSGALG00010019240	ENSGALG00010019240	0.207751805	0.110058763	1.887644379	0.059073708	0.14912459	positive
PRJNA729271	ɛed_Lushi_vs_Arbor_Acr	breed_growth_background	ENSGALG00010019243	SERTAD2	0.007789554	0.082656551	0.094240013	0.9249185	0.95789466	positive
PRJNA729271	ɛed_Lushi_vs_Arbor_Acr	breed_growth_background	ENSGALG00010019246	IL17RC	-0.037650627	0.072059598	-0.522492877	0.601327205	0.73393717	negative
PRJNA729271	ɛed_Lushi_vs_Arbor_Acr	breed_growth_background	ENSGALG00010019247	HBZ	0.647143435	0.296524194	2.182430469	0.029077778	0.08884536	positive
PRJNA729271	ɛed_Lushi_vs_Arbor_Acr	breed_growth_background	ENSGALG00010019248	RMI2	-0.035352058	0.091327619	-0.38709055	0.698689174	0.805494842	negative
PRJNA729271	ɛed_Lushi_vs_Arbor_Acr	breed_growth_background	ENSGALG00010019250	CXCR1	-0.238600645	0.138592245	-1.721601698	0.085141695	0.192501774	negative
PRJNA729271	ɛed_Lushi_vs_Arbor_Acr	breed_growth_background	ENSGALG00010019252	TATDN3	-0.204095268	0.063360594	-3.221170369	0.001276682	0.009743353	negative
PRJNA729271	ɛed_Lushi_vs_Arbor_Acr	breed_growth_background	ENSGALG00010019253	HBAD	-0.422307563	0.228239895	-1.850279346	0.064273298	0.158227149	negative
PRJNA729271	ɛed_Lushi_vs_Arbor_Acr	breed_growth_background	ENSGALG00010019255	ENSGALG00010019255	-0.234903839	0.105436374	-2.227920324	0.025885829	0.081955006	negative
PRJNA729271	ɛed_Lushi_vs_Arbor_Acr	breed_growth_background	ENSGALG00010019256	BBS9	-0.156549229	0.058193844	-2.690133848	0.007142337	0.032876522	negative
PRJNA729271	ɛed_Lushi_vs_Arbor_Acr	breed_growth_background	ENSGALG00010019257	SLC1A4	-0.207632765	0.085256711	-2.435383228	0.014876025	0.055242954	negative
PRJNA729271	ɛed_Lushi_vs_Arbor_Acr	breed_growth_background	ENSGALG00010019259	L2HGDH	0.157112338	0.058609218	2.680676248	0.007347356	0.033529636	positive
PRJNA729271	ɛed_Lushi_vs_Arbor_Acr	breed_growth_background	ENSGALG00010019260	TRAIL-LIKE	-0.147134306	0.094655286	-1.554422504	0.120083669	0.244433864	negative
PRJNA729271	ɛed_Lushi_vs_Arbor_Acr	breed_growth_background	ENSGALG00010019262	TMC3	-0.174260897	0.292693219	-0.595370461	0.551595866	0.694401914	negative
PRJNA729271	ɛed_Lushi_vs_Arbor_Acr	breed_growth_background	ENSGALG00010019263	SNRPB2	-0.076665909	0.055229282	-1.388138794	0.165094786	0.305244211	negative
PRJNA729271	ɛed_Lushi_vs_Arbor_Acr	breed_growth_background	ENSGALG00010019264	HK2	0.318120872	0.114740181	2.772532421	0.005562198	0.027458945	positive
PRJNA729271	ɛed_Lushi_vs_Arbor_Acr	breed_growth_background	ENSGALG00010019265	RUFY4	0.730356878	0.334547549	2.1831183	0.029027099	0.088765359	positive
PRJNA729271	ɛed_Lushi_vs_Arbor_Acr	breed_growth_background	ENSGALG00010019266	ATP5S	-0.101889702	0.08715446	-1.169070438	0.242375253	0.398144752	negative
PRJNA729271	ɛed_Lushi_vs_Arbor_Acr	breed_growth_background	ENSGALG00010019267	STARD5	-0.005290835	0.105841406	-0.049988326	0.960131691	0.977668343	negative
PRJNA729271	ɛed_Lushi_vs_Arbor_Acr	breed_growth_background	ENSGALG00010019268	NSL1	-0.107277839	0.051412662	-2.086603458	0.036923994	0.106087869	negative
PRJNA729271	ɛed_Lushi_vs_Arbor_Acr	breed_growth_background	ENSGALG00010019269	SOCS1	0.02225273	0.138405638	0.16077907	0.872267411	0.924436362	positive
PRJNA729271	ɛed_Lushi_vs_Arbor_Acr	breed_growth_background	ENSGALG00010019270	ENSGALG00010019270	0.103873765	0.120374432	0.862922162	0.388180265	0.550178248	positive
PRJNA729271	ɛed_Lushi_vs_Arbor_Acr	breed_growth_background	ENSGALG00010019271	CDKL1	0.179387877	0.108566741	1.652328105	0.098467683	0.212647358	positive
PRJNA729271	ɛed_Lushi_vs_Arbor_Acr	breed_growth_background	ENSGALG00010019274	ENSGALG00010019274	0.041903287	0.415709075	0.100799548	0.919709586	0.955435422	positive
PRJNA729271	ɛed_Lushi_vs_Arbor_Acr	breed_growth_background	ENSGALG00010019275	CTAGE1	0.910876205	0.314442005	2.896801923	0.003769877	0.020785229	positive
PRJNA729271	ɛed_Lushi_vs_Arbor_Acr	breed_growth_background	ENSGALG00010019277	IGFBP5	-0.00413553	0.071086813	-0.058175767	0.953608623	0.974074966	negative
PRJNA729271	ɛed_Lushi_vs_Arbor_Acr	breed_growth_background	ENSGALG00010019278	JPT2	-0.092145781	0.039772247	-2.31683618	0.020512657	0.069388791	negative
PRJNA729271	ɛed_Lushi_vs_Arbor_Acr	breed_growth_background	ENSGALG00010019280	TLNRD1	0.099313336	0.062741957	1.582885525	0.113447559	0.235018202	positive
PRJNA729271	ɛed_Lushi_vs_Arbor_Acr	breed_growth_background	ENSGALG00010019281	ENSGALG00010019281	-1.378144832	0.674163443	-2.04422955	0.040930875	0.114292629	negative
PRJNA729271	ɛed_Lushi_vs_Arbor_Acr	breed_growth_background	ENSGALG00010019282	IGFBP2	0.206862284	0.084385089	2.451408016	0.014229855	0.053477249	positive
PRJNA729271	ɛed_Lushi_vs_Arbor_Acr	breed_growth_background	ENSGALG00010019283	NT5C3A	-0.110159429	0.136662451	-0.806069462	0.420202798	0.579472891	negative
PRJNA729271	ɛed_Lushi_vs_Arbor_Acr	breed_growth_background	ENSGALG00010019284	CHD5	0.32183328	0.070713552	4.551224903	5.33345E-06	0.000182578	positive
PRJNA729271	ɛed_Lushi_vs_Arbor_Acr	breed_growth_background	ENSGALG00010019285	BATF3	0.00333139	0.14957731	0.022272025	0.982230964	0.989466803	positive
PRJNA729271	ɛed_Lushi_vs_Arbor_Acr	breed_growth_background	ENSGALG00010019287	AHSA2	0.117038392	0.053908017	2.171075819	0.029925442	0.090691303	positive
PRJNA729271	ɛed_Lushi_vs_Arbor_Acr	breed_growth_background	ENSGALG00010019288	RPL37A	0.048213174	0.083773704	0.575516796	0.564941835	0.704779404	positive
PRJNA729271	ɛed_Lushi_vs_Arbor_Acr	breed_growth_background	ENSGALG00010019289	FKBP9	-0.357383066	0.089170305	-4.007870853	6.12686E-05	0.001116952	negative
PRJNA729271	ɛed_Lushi_vs_Arbor_Acr	breed_growth_background	ENSGALG00010019291	ADD2	-0.108197198	0.149838893	-0.722090213	0.470239015	0.624586688	negative
PRJNA729271	ɛed_Lushi_vs_Arbor_Acr	breed_growth_background	ENSGALG00010019292	ENSGALG00010019292	0.050734298	0.052726424	0.962217708	0.335940256	0.500181767	positive
PRJNA729271	ɛed_Lushi_vs_Arbor_Acr	breed_growth_background	ENSGALG00010019294	SMARCAL1	0.241682553	0.053321059	4.532591016	5.82646E-06	0.000193703	positive
PRJNA729271	ɛed_Lushi_vs_Arbor_Acr	breed_growth_background	ENSGALG00010019295	MAP4K5	0.041454152	0.069893773	0.593102221	0.553112744	0.695529559	positive
PRJNA729271	ɛed_Lushi_vs_Arbor_Acr	breed_growth_background	ENSGALG00010019296	ENSGALG00010019296	0.019533401	0.055782043	0.350173633	0.726208394	0.824474505	positive
PRJNA729271	ɛed_Lushi_vs_Arbor_Acr	breed_growth_background	ENSGALG00010019297	SLC25A26	0.076080529	0.079011336	0.962906504	0.335594446	0.499801315	positive
PRJNA729271	ɛed_Lushi_vs_Arbor_Acr	breed_growth_background	ENSGALG00010019298	PGAP6	0.036511974	0.050199992	0.72733028	0.467023653	0.621911608	positive
PRJNA729271	ɛed_Lushi_vs_Arbor_Acr	breed_growth_background	ENSGALG00010019300	MESDC2	-0.043735816	0.035709488	-1.224767359	0.220662887	0.373308653	negative
PRJNA729271	ɛed_Lushi_vs_Arbor_Acr	breed_growth_background	ENSGALG00010019302	USP34	-0.001222752	0.050844593	-0.02404882	0.980813667	0.988726205	negative
PRJNA729271	ɛed_Lushi_vs_Arbor_Acr	breed_growth_background	ENSGALG00010019305	SNRNP27	0.253797211	0.064031947	3.96360292	7.3827E-05	0.001272441	positive
PRJNA729271	ɛed_Lushi_vs_Arbor_Acr	breed_growth_background	ENSGALG00010019307	ATF3	-0.185689363	0.259724258	-0.714948093	0.474641118	0.628246097	negative
PRJNA729271	ɛed_Lushi_vs_Arbor_Acr	breed_growth_background	ENSGALG00010019310	CRELD1	0.022695016	0.05166554	0.439267965	0.660467383	0.77944254	positive
PRJNA729271	ɛed_Lushi_vs_Arbor_Acr	breed_growth_background	ENSGALG00010019312	KBTBD2	0.073919866	0.039298923	1.880964169	0.059976793	0.150721847	positive
PRJNA729271	ɛed_Lushi_vs_Arbor_Acr	breed_growth_background	ENSGALG00010019313	MRPL28	-0.021754486	0.053632801	-0.405619059	0.685022523	0.796582548	negative
PRJNA729271	ɛed_Lushi_vs_Arbor_Acr	breed_growth_background	ENSGALG00010019314	GKN2	-0.610014102	0.685180601	-0.890296809	0.373306534	0.535750318	negative
PRJNA729271	ɛed_Lushi_vs_Arbor_Acr	breed_growth_background	ENSGALG00010019316	CLEC16A	0.074390376	0.047364111	1.570606405	0.116274101	0.239164623	positive
PRJNA729271	ɛed_Lushi_vs_Arbor_Acr	breed_growth_background	ENSGALG00010019318	NOG2	-0.091895932	0.149989259	-0.61268342	0.540085687	0.684926963	negative
PRJNA729271	ɛed_Lushi_vs_Arbor_Acr	breed_growth_background	ENSGALG00010019319	ACTR3	-0.059123259	0.036289267	-1.629221636	0.103266108	0.220091493	negative
PRJNA729271	ɛed_Lushi_vs_Arbor_Acr	breed_growth_background	ENSGALG00010019322	EGR3	-0.390084882	0.1672437	-2.332433945	0.019677874	0.067510245	negative
PRJNA729271	ɛed_Lushi_vs_Arbor_Acr	breed_growth_background	ENSGALG00010019323	LRIG1	0.132499103	0.064044153	2.068871173	0.038558177	0.109632069	positive
PRJNA729271	ɛed_Lushi_vs_Arbor_Acr	breed_growth_background	ENSGALG00010019325	SLITRK4	-0.349715669	0.117936214	-2.965295033	0.003023928	0.017817818	negative
PRJNA729271	ɛed_Lushi_vs_Arbor_Acr	breed_growth_background	ENSGALG00010019331	CEMIP	-0.129774824	0.171918737	-0.754861429	0.450332131	0.606594577	negative
PRJNA729271	ɛed_Lushi_vs_Arbor_Acr	breed_growth_background	ENSGALG00010019334	ATL1	-0.123131871	0.070705868	-1.741466075	0.081601915	0.186535208	negative
PRJNA729271	ɛed_Lushi_vs_Arbor_Acr	breed_growth_background	ENSGALG00010019335	HIPK3	0.058755316	0.061509019	0.955230914	0.339460936	0.503613951	positive
PRJNA729271	ɛed_Lushi_vs_Arbor_Acr	breed_growth_background	ENSGALG00010019336	DEXI	0.13680797	0.122458563	1.117177657	0.263918372	0.422007369	positive
PRJNA729271	ɛed_Lushi_vs_Arbor_Acr	breed_growth_background	ENSGALG00010019337	XPO1	0.053660287	0.045092521	1.190004144	0.234044763	0.388466603	positive
PRJNA729271	ɛed_Lushi_vs_Arbor_Acr	breed_growth_background	ENSGALG00010019344	DNAJC24	0.214408078	0.04756598	4.507593034	6.55672E-06	0.000213017	positive
PRJNA729271	ɛed_Lushi_vs_Arbor_Acr	breed_growth_background	ENSGALG00010019345	ENSGALG00010019345	0.141044876	0.10012149	1.40873729	0.158912864	0.297371801	positive
PRJNA729271	ɛed_Lushi_vs_Arbor_Acr	breed_growth_background	ENSGALG00010019346	POU3F4	0.67493607	0.288552816	2.339038236	0.019333455	0.066723435	positive
PRJNA729271	ɛed_Lushi_vs_Arbor_Acr	breed_growth_background	ENSGALG00010019350	SAV1	0.136096016	0.047596423	2.859374842	0.004244769	0.022529449	positive
PRJNA729271	ɛed_Lushi_vs_Arbor_Acr	breed_growth_background	ENSGALG00010019355	ENTPD4	-0.050660267	0.05293562	-0.957016596	0.338558878	0.502911554	negative
PRJNA729271	ɛed_Lushi_vs_Arbor_Acr	breed_growth_background	ENSGALG00010019356	AVL9	-0.033879632	0.0519607	-0.65202416	0.514385587	0.662932785	negative
PRJNA729271	ɛed_Lushi_vs_Arbor_Acr	breed_growth_background	ENSGALG00010019365	ENSGALG00010019365	-0.074942642	0.096079705	-0.780004913	0.435387983	0.593301572	negative
PRJNA729271	ɛed_Lushi_vs_Arbor_Acr	breed_growth_background	ENSGALG00010019371	TMEM87A	0.056539035	0.041006108	1.378795435	0.167957835	0.309361375	positive
PRJNA729271	ɛed_Lushi_vs_Arbor_Acr	breed_growth_background	ENSGALG00010019373	DENND5A	-0.001561787	0.033201914	-0.047039071	0.962482088	0.978835813	negative
PRJNA729271	ɛed_Lushi_vs_Arbor_Acr	breed_growth_background	ENSGALG00010019376	ENSGALG00010019376	0.287472233	0.180376519	1.593734228	0.110995575	0.231693671	positive
PRJNA729271	ɛed_Lushi_vs_Arbor_Acr	breed_growth_background	ENSGALG00010019388	GPR75	-0.013436358	0.07485312	-0.179502975	0.857542782	0.914447335	negative
PRJNA729271	ɛed_Lushi_vs_Arbor_Acr	breed_growth_background	ENSGALG00010019390	TVP23A	0.207698559	0.127561948	1.628217208	0.103478837	0.220415246	positive
PRJNA729271	ɛed_Lushi_vs_Arbor_Acr	breed_growth_background	ENSGALG00010019391	GANC	0.229238366	0.070445914	3.254104524	0.001137504	0.008978147	positive
PRJNA729271	ɛed_Lushi_vs_Arbor_Acr	breed_growth_background	ENSGALG00010019392	NIN	-0.199599115	0.059211661	-3.370942684	0.000749114	0.006641231	negative
PRJNA729271	ɛed_Lushi_vs_Arbor_Acr	breed_growth_background	ENSGALG00010019398	SLC35F5	-0.012827142	0.06428569	-0.199533399	0.84184552	0.904361581	negative
PRJNA729271	ɛed_Lushi_vs_Arbor_Acr	breed_growth_background	ENSGALG00010019399	CD93	-0.207275002	0.081039445	-2.557705093	0.010536541	0.043392106	negative
PRJNA729271	ɛed_Lushi_vs_Arbor_Acr	breed_growth_background	ENSGALG00010019403	PACC1	0.089374688	0.058095879	1.538399801	0.123950891	0.249502592	positive
PRJNA729271	ɛed_Lushi_vs_Arbor_Acr	breed_growth_background	ENSGALG00010019411	LSM5	0.088120217	0.085394382	1.031920541	0.302109343	0.464582231	positive
PRJNA729271	ɛed_Lushi_vs_Arbor_Acr	breed_growth_background	ENSGALG00010019416	GPR39	0.265502752	0.244085556	1.087744626	0.276707861	0.435876331	positive
PRJNA729271	ɛed_Lushi_vs_Arbor_Acr	breed_growth_background	ENSGALG00010019418	ZNF703	0.168799852	0.125437933	1.345684255	0.178404366	0.322632206	positive

PRJNA729271	ed_Lushi_vs_Arbor_Acr	breed_growth_background	ENSGALG00010019422	IMMP1L	0.307585523	0.074999963	4.101142303	4.11116E-05	0.000841684	positive
PRJNA729271	ed_Lushi_vs_Arbor_Acr	breed_growth_background	ENSGALG00010019423	LYPD1	-0.435957673	0.097676776	-4.463268468	8.07188E-06	0.000245287	negative
PRJNA729271	ed_Lushi_vs_Arbor_Acr	breed_growth_background	ENSGALG00010019426	GRM7	0.527961788	0.143776914	3.672090138	0.000240575	0.002957703	positive
PRJNA729271	ed_Lushi_vs_Arbor_Acr	breed_growth_background	ENSGALG00010019430	ENSGALG00010019430	0.003124413	0.215960048	0.014467551	0.988456967	0.993047113	positive
PRJNA729271	ed_Lushi_vs_Arbor_Acr	breed_growth_background	ENSGALG00010019441	LMCD1	-0.065141725	0.073420363	-0.887243293	0.374947929	0.537254866	negative
PRJNA729271	ed_Lushi_vs_Arbor_Acr	breed_growth_background	ENSGALG00010019443	NCKAP5	0.123952798	0.07215755	1.717807748	0.085831688	0.19351477	positive
PRJNA729271	ed_Lushi_vs_Arbor_Acr	breed_growth_background	ENSGALG00010019451	CAV3	-0.169080536	0.074956012	-2.255730156	0.024087536	0.077917615	negative
PRJNA729271	ed_Lushi_vs_Arbor_Acr	breed_growth_background	ENSGALG00010019458	ELP4	0.063334208	0.055536729	1.140402202	0.254118776	0.410732789	positive
PRJNA729271	ed_Lushi_vs_Arbor_Acr	breed_growth_background	ENSGALG00010019461	MGAT5	-0.098936498	0.037887971	-2.611290481	0.009020124	0.038785731	negative
PRJNA729271	ed_Lushi_vs_Arbor_Acr	breed_growth_background	ENSGALG00010019470	TGFB2	-0.5443432	0.107351789	-5.070648622	3.96462E-07	2.37468E-05	negative
PRJNA729271	ed_Lushi_vs_Arbor_Acr	breed_growth_background	ENSGALG00010019471	NUBP1	0.095528485	0.050315013	1.898607973	0.057616039	0.146522531	positive
PRJNA729271	ed_Lushi_vs_Arbor_Acr	breed_growth_background	ENSGALG00010019472	CAPN3	0.423270754	0.131145717	3.227484389	0.001248839	0.009598046	positive
PRJNA729271	ed_Lushi_vs_Arbor_Acr	breed_growth_background	ENSGALG00010019473	OXTR	-0.535922969	0.254297241	-2.107466704	0.035077145	0.102076534	negative
PRJNA729271	ed_Lushi_vs_Arbor_Acr	breed_growth_background	ENSGALG00010019474	TMEM163	-0.737241036	0.309432837	-2.382555916	0.017192921	0.061344199	negative
PRJNA729271	ed_Lushi_vs_Arbor_Acr	breed_growth_background	ENSGALG00010019477	NCAPH	-0.335516369	0.097127504	-3.454390951	0.000551537	0.00531905	negative
PRJNA729271	ed_Lushi_vs_Arbor_Acr	breed_growth_background	ENSGALG00010019481	PYGL	-0.258469762	0.05310259	-4.867366413	1.13095E-06	5.41745E-05	negative
PRJNA729271	ed_Lushi_vs_Arbor_Acr	breed_growth_background	ENSGALG00010019483	SCUBE2	-0.781426062	0.142787928	-5.472633931	4.43395E-08	4.55817E-06	negative
PRJNA729271	ed_Lushi_vs_Arbor_Acr	breed_growth_background	ENSGALG00010019488	TEKT5	-0.796673948	0.711769477	-1.119286474	0.263017944	0.421229946	negative
PRJNA729271	ed_Lushi_vs_Arbor_Acr	breed_growth_background	ENSGALG00010019495	EMP2	-0.326696621	0.105331585	-3.101601664	0.001924768	0.012910462	negative
PRJNA729271	ed_Lushi_vs_Arbor_Acr	breed_growth_background	ENSGALG00010019498	ENSGALG00010019498	-0.008009183	0.155740888	-0.051426337	0.958985799	0.977394118	negative
PRJNA729271	ed_Lushi_vs_Arbor_Acr	breed_growth_background	ENSGALG00010019509	ZNF106	-0.090400682	0.118753756	-0.761244825	0.446510849	0.603353665	negative
PRJNA729271	ed_Lushi_vs_Arbor_Acr	breed_growth_background	ENSGALG00010019515	ENSGALG00010019515	-0.058864548	0.05885502	-1.000161897	0.317232166	0.480634474	negative
PRJNA729271	ed_Lushi_vs_Arbor_Acr	breed_growth_background	ENSGALG00010019520	CD6	0.554875659	0.43522399	1.274919746	0.202337649	0.351538323	positive
PRJNA729271	ed_Lushi_vs_Arbor_Acr	breed_growth_background	ENSGALG00010019524	HHIPL2	0.449311265	0.201029149	2.235055303	0.025413718	0.080956272	positive
PRJNA729271	ed_Lushi_vs_Arbor_Acr	breed_growth_background	ENSGALG00010019529	SNAP23	-0.131352104	0.036448718	-3.603751024	0.000313658	0.003565856	negative
PRJNA729271	ed_Lushi_vs_Arbor_Acr	breed_growth_background	ENSGALG00010019534	PPP2R5A	-0.010235264	0.055825709	-0.1833432	0.85452874	0.912441553	negative
PRJNA729271	ed_Lushi_vs_Arbor_Acr	breed_growth_background	ENSGALG00010019540	UNC5D	-0.073066051	0.207816737	-0.351588867	0.725146616	0.824004405	negative
PRJNA729271	ed_Lushi_vs_Arbor_Acr	breed_growth_background	ENSGALG00010019546	HSD17B12	-0.073056315	0.042445985	-1.721159624	0.085221862	0.192562883	negative
PRJNA729271	ed_Lushi_vs_Arbor_Acr	breed_growth_background	ENSGALG00010019557	TRIM9	-0.300047099	0.193725025	-1.548829843	0.12142263	0.246147296	negative
PRJNA729271	ed_Lushi_vs_Arbor_Acr	breed_growth_background	ENSGALG00010019572	NFKBIE	-0.185536174	0.102728385	-1.806084791	0.070905096	0.16926373	negative
PRJNA729271	ed_Lushi_vs_Arbor_Acr	breed_growth_background	ENSGALG00010019577	MXD1	0.061859196	0.103038589	0.600349794	0.54827314	0.691724186	positive
PRJNA729271	ed_Lushi_vs_Arbor_Acr	breed_growth_background	ENSGALG00010019580	CD5	-0.290474456	0.239708365	-1.211782727	0.225595562	0.37860457	negative
PRJNA729271	ed_Lushi_vs_Arbor_Acr	breed_growth_background	ENSGALG00010019581	SLC35B2	-0.042766532	0.072208526	-0.592264298	0.553673619	0.695872179	negative
PRJNA729271	ed_Lushi_vs_Arbor_Acr	breed_growth_background	ENSGALG00010019584	RAD18	-0.02274985	0.064362296	-0.353465478	0.723739505	0.822986124	negative
PRJNA729271	ed_Lushi_vs_Arbor_Acr	breed_growth_background	ENSGALG00010019585	PDE1C	-0.591354052	0.115355946	-5.126342196	2.95425E-07	1.88728E-05	negative
PRJNA729271	ed_Lushi_vs_Arbor_Acr	breed_growth_background	ENSGALG00010019586	GLYR1	0.001302386	0.048643879	0.026773896	0.978640074	0.987427806	positive
PRJNA729271	ed_Lushi_vs_Arbor_Acr	breed_growth_background	ENSGALG00010019587	CCNT2	-0.056635391	0.068849249	-0.822599983	0.410735508	0.571196221	negative
PRJNA729271	ed_Lushi_vs_Arbor_Acr	breed_growth_background	ENSGALG00010019588	CHMP7	-0.038865475	0.049642703	-0.782904076	0.433683444	0.591736607	negative
PRJNA729271	ed_Lushi_vs_Arbor_Acr	breed_growth_background	ENSGALG00010019591	ENSGALG00010019591	-0.433848967	0.121565683	-3.568844073	0.00035856	0.003918806	negative
PRJNA729271	ed_Lushi_vs_Arbor_Acr	breed_growth_background	ENSGALG00010019592	ALKBH3	-0.090955795	0.071256413	-1.276457674	0.20179378	0.350845728	negative
PRJNA729271	ed_Lushi_vs_Arbor_Acr	breed_growth_background	ENSGALG00010019594	TMX1	-0.344900456	0.17922281	-1.924422768	0.054301613	0.140513278	negative
PRJNA729271	ed_Lushi_vs_Arbor_Acr	breed_growth_background	ENSGALG00010019595	GRIN2A	-0.156444028	0.125436573	-1.247196287	0.212325534	0.362529725	negative
PRJNA729271	ed_Lushi_vs_Arbor_Acr	breed_growth_background	ENSGALG00010019598	KCNAB2	0.015789934	0.075129731	0.210168917	0.833535838	0.898832166	positive
PRJNA729271	ed_Lushi_vs_Arbor_Acr	breed_growth_background	ENSGALG00010019600	MAP3K19	0.008404021	0.132226245	0.063557889	0.949322263	0.97207424	positive
PRJNA729271	ed_Lushi_vs_Arbor_Acr	breed_growth_background	ENSGALG00010019602	SLC29A1	0.206191924	0.103538134	1.991458756	0.046430475	0.125608387	positive
PRJNA729271	ed_Lushi_vs_Arbor_Acr	breed_growth_background	ENSGALG00010019610	CORO7	-0.304693129	0.054524914	-5.588145076	2.29508E-08	2.78703E-06	negative
PRJNA729271	ed_Lushi_vs_Arbor_Acr	breed_growth_background	ENSGALG00010019613	FRMD6	-0.224674051	0.06731621	-3.337592121	0.000845077	0.007231046	negative
PRJNA729271	ed_Lushi_vs_Arbor_Acr	breed_growth_background	ENSGALG00010019614	MICALL2	-0.201103404	0.084274089	-2.386301735	0.017018781	0.060970646	negative
PRJNA729271	ed_Lushi_vs_Arbor_Acr	breed_growth_background	ENSGALG00010019617	ANO3	-0.042474348	0.078736312	-0.539450569	0.589575996	0.725094939	negative
PRJNA729271	ed_Lushi_vs_Arbor_Acr	breed_growth_background	ENSGALG00010019618	NPHP4	0.033326609	0.057830861	0.57627723	0.564427811	0.704562618	positive
PRJNA729271	ed_Lushi_vs_Arbor_Acr	breed_growth_background	ENSGALG00010019621	ENSGALG00010019621	0.00280035	0.080837427	0.034641754	0.972365406	0.983561519	positive
PRJNA729271	ed_Lushi_vs_Arbor_Acr	breed_growth_background	ENSGALG00010019622	C2orf42	0.205383355	0.075929462	2.704923091	0.006832021	0.03185273	positive
PRJNA729271	ed_Lushi_vs_Arbor_Acr	breed_growth_background	ENSGALG00010019624	SRGAP3	-0.061177013	0.069852652	-0.875800866	0.381138299	0.54273176	negative
PRJNA729271	ed_Lushi_vs_Arbor_Acr	breed_growth_background	ENSGALG00010019625	INTS1	0.07646374	0.041146033	1.858350224	0.063119296	0.156368859	positive
PRJNA729271	ed_Lushi_vs_Arbor_Acr	breed_growth_background	ENSGALG00010019630	AJAP1	-0.157238106	0.208639516	-0.75363531	0.451068236	0.607416767	negative
PRJNA729271	ed_Lushi_vs_Arbor_Acr	breed_growth_background	ENSGALG00010019634	GNG2	-0.158165488	0.159509493	-0.99157414	0.321405309	0.485036431	negative
PRJNA729271	ed_Lushi_vs_Arbor_Acr	breed_growth_background	ENSGALG00010019635	LRRC57	0.067187508	0.042013366	1.59919367	0.109777576	0.230101646	positive
PRJNA729271	ed_Lushi_vs_Arbor_Acr	breed_growth_background	ENSGALG00010019640	MGRN1	0.143535813	0.062848755	2.283829051	0.02238158	0.073990888	positive
PRJNA729271	ed_Lushi_vs_Arbor_Acr	breed_growth_background	ENSGALG00010019641	CAPN11	-0.135396423	0.043738508	-3.095588503	0.001964227	0.013116204	negative
PRJNA729271	ed_Lushi_vs_Arbor_Acr	breed_growth_background	ENSGALG00010019643	RAB3GAP1	0.001420421	0.038152508	0.037230085	0.970301551	0.982722932	positive
PRJNA729271	ed_Lushi_vs_Arbor_Acr	breed_growth_background	ENSGALG00010019645	ENSGALG00010019645	0.028315346	0.086014988	0.329190837	0.742011448	0.835210343	positive
PRJNA729271	ed_Lushi_vs_Arbor_Acr	breed_growth_background	ENSGALG00010019646	VPS37C	0.251674227	0.067803342	3.711826282	0.000205769	0.002651222	positive
PRJNA729271	ed_Lushi_vs_Arbor_Acr	breed_growth_background	ENSGALG00010019648	PRDM16	0.042465246	0.107328835	0.39565552	0.692359173	0.801321161	positive
PRJNA729271	ed_Lushi_vs_Arbor_Acr	breed_growth_background	ENSGALG00010019649	TMEM63B	-0.014860065	0.041724724	-0.356145325	0.721731735	0.821439161	negative
PRJNA729271	ed_Lushi_vs_Arbor_Acr	breed_growth_background	ENSGALG00010019650	KBTBD8	0.028067611	0.046736156	0.600554536	0.548136727	0.691612278	positive
PRJNA729271	ed_Lushi_vs_Arbor_Acr	breed_growth_background	ENSGALG00010019652	TNFRSF10B	0.002706292	0.17397614	0.015555537	0.987588978	0.992713543	positive
PRJNA729271	ed_Lushi_vs_Arbor_Acr	breed_growth_background	ENSGALG00010019655	RTRAF	0.008944528	0.052791741	0.169430443	0.86545808	0.919101389	positive
PRJNA729271	ed_Lushi_vs_Arbor_Acr	breed_growth_background	ENSGALG00010019657	PPP1R17	-0.278886208	0.149405837	-1.866635291	0.061952536	0.154322393	negative
PRJNA729271	ed_Lushi_vs_Arbor_Acr	breed_growth_background	ENSGALG00010019659	MRPL14	0.03579978	0.067081408	0.533676633	0.593565278	0.727957417	positive
PRJNA729271	ed_Lushi_vs_Arbor_Acr	breed_growth_background	ENSGALG00010019660	ENSGALG00010019660	0.29471257	0.261342841	1.127685645	0.259452702	0.417167712	positive
PRJNA729271	ed_Lushi_vs_Arbor_Acr	breed_growth_background	ENSGALG00010019667	ITPRID1	-1.485519879	0.32752287	-4.53562183	5.7434E-06	0.000192265	negative
PRJNA729271	ed_Lushi_vs_Arbor_Acr	breed_growth_background	ENSGALG00010019670	ENSGALG00010019670	-0.013699188	0.067815006	-0.202008208	0.839910307	0.902817939	negative
PRJNA729271	ed_Lushi_vs_Arbor_Acr	breed_growth_background	ENSGALG00010019673	NID2	-0.167779983	0.070955008	-2.364596778	0.018049711	0.063629859	negative
PRJNA729271	ed_Lushi_vs_Arbor_Acr	breed_growth_background	ENSGALG00010019675	MAFK	0.036482882	0.073169896	0.498605084	0.618057623	0.746375509	positive
PRJNA729271	ed_Lushi_vs_Arbor_Acr	breed_growth_background	ENSGALG00010019679	RHOBTB2	-0.328031864	0.095316301	-3.441508532	0.00057848	0.005482013	negative
PRJNA729271	ed_Lushi_vs_Arbor_Acr	breed_growth_background	ENSGALG00010019680	CCDC34	0.091483037	0.109367548	0.836473333	0.402888673	0.563911869	positive
PRJNA729271	ed_Lushi_vs_Arbor_Acr	breed_growth_background	ENSGALG00010019682	TMEM158	0.244209942	0.200643927	1.217130991	0.223554381	0.376137058	positive
PRJNA729271	ed_Lushi_vs_Arbor_Acr	breed_growth_background	ENSGALG00010019683	TMEM184A	-0.09605402	0.154596435	-0.621321058	0.534388401	0.679768309	negative
PRJNA729271	ed_Lushi_vs_Arbor_Acr	breed_growth_background	ENSGALG00010019684	PIGQ	-0.067038964	0.070379204	-0.952539392	0.340823498	0.50482449	negative
PRJNA729271	ed_Lushi_vs_Arbor_Acr	breed_growth_background	ENSGALG00010019690	ENSGALG00010019690	0.074525317	0.049116085	1.517330154	0.129183349	0.25664921	positive
PRJNA729271	ed_Lushi_vs_Arbor_Acr	breed_growth_background	ENSGALG00010019692	MMEL1	0.144760408	0.16561507	0.87407751	0.382076044	0.543492861	positive
PRJNA729271	ed_Lushi_vs_Arbor_Acr	breed_growth_background	ENSGALG00010019693	HAUS2	-0.096465193	0.064963707	-1.484909003	0.137567931	0.268775732	negative
PRJNA729271	ed_Lushi_vs_Arbor_Acr	breed_growth_background	ENSGALG00010019695	PGA5	-2.858104258	1.272043128	-2.246861128	0.024648904	0.079181797	negative
PRJNA729271	ed_Lushi_vs_Arbor_Acr	breed_growth_background	ENSGALG00010019697	EIF4EBP1	0.258471063	0.077563133	3.332395859	0.000861017	0.007316166	positive
PRJNA729271	ed_Lushi_vs_Arbor_Acr	breed_growth_background	ENSGALG00010019700	HES5B	0.233655337	0.144611538	1.615744775	0.106149506	0.224618553	positive
PRJNA729271	ed_Lushi_vs_Arbor_Acr	breed_growth_background	ENSGALG00010019706	NPRL3	0.081119296	0.053318726	1.521403494	0.128158613	0.255348328	positive
PRJNA729271	ed_Lushi_vs_Arbor_Acr	breed_growth_background	ENSGALG00010019713	NUDT14	-0.024636609	0.06145818	-0.400867865	0.68851741	0.798539286	negative
PRJNA729271	ed_Lushi_vs_Arbor_Acr	breed_growth_background	ENSGALG00010019716	VEGFA	-0.025216007	0.069533385	-0.362646036	0.716869317	0.818577339	negative
PRJNA729271	ed_Lushi_vs_Arbor_Acr	breed_growth_background	ENSGALG00010019717	ZRANB3	0.028817739	0.077553795	0.371583867	0.710202702	0.813078601	positive
PRJNA729271	ed_Lushi_vs_Arbor_Acr	breed_growth_background	ENSGALG00010019724	HES5A	-0.314001946	0.174358416	-1.800899277	0.071718757	0.1705559	negative
PRJNA729271	ed_Lushi_vs_Arbor_Acr	breed_growth_background	ENSGALG00010019726	PEBP4	0.05742574	0.108778958	0.527912206	0.597560272	0.730959358	positive
PRJNA729271	ed_Lushi_vs_Arbor_Acr	breed_growth_background	ENSGALG00010019731	PANK4	0.239015153	0.071798133	3.328988407	0.00087162	0.007388384	positive
PRJNA729271	ed_Lushi_vs_Arbor_Acr	breed_growth_background	ENSGALG00010019732	HBA1	-0.241814674	0.215778042	-1.120663952	0.262430932	0.420649824	negative
PRJNA729271	ed_Lushi_vs_Arbor_Acr	breed_growth_background	ENSGALG00010019735	ENSGALG00010019735	-0.122545863	0.091624436	-1.337480123	0.181065966	0.325586302	negative
PRJNA729271	ed_Lushi_vs_Arbor_Acr	breed_growth_background	ENSGALG00010019738	ADRA2A	-0.560025551	0.193258816	-2.897800791	0.003757892	0.020734924	negative
PRJNA729271	ed_Lushi_vs_Arbor_Acr	breed_growth_background	ENSGALG00010019743	KCNU1	-0.894795978	0.24712212	-3.620865579	0.000293619	0.003423981	negative
PRJNA729271	ed_Lushi_vs_Arbor_Acr	breed_growth_background	ENSGALG00010019744	KBP	0.359286438	0.256828089	1.398937477	0.161831733	0.301084709	positive
PRJNA729271	ed_Lushi_vs_Arbor_Acr	breed_growth_background	ENSGALG00010019752	GPAM	-0.137545549	0.090170901	-1.52538732	0.12716252	0.254092311	negative
PRJNA729271	ed_Lushi_vs_Arbor_Acr	breed_growth_background	ENSGALG00010019761	MRPS18A	0.065662201	0.047195302	1.391286785	0.164138479	0.303924439	positive
PRJNA729271	ed_Lushi_vs_Arbor_Acr	breed_growth_background	ENSGALG00010019767	SUCLG2	0.013787103	0.042389663	0.325246825	0.744994272	0.837262036	positive
PRJNA729271	ed_Lushi_vs_Arbor_Acr	breed_growth_background	ENSGALG00010019771	VWCE	-0.216698594	0.213404409	-1.015436352	0.309897872	0.472838911	negative
PRJNA729271	ed_Lushi_vs_Arbor_Acr	breed_growth_background	ENSGALG00010019772	ENSGALG00010019772	0.28289698	0.089456807	3.162386274	0.001564818	0.011184439	positive
PRJNA729271	ed_Lushi_vs_Arbor_Acr	breed_growth_background	ENSGALG00010019778	ABHD17C	0.197944114	0.072043891	2.747548929	0.006004255	0.02905899	positive
PRJNA729271	ed_Lushi_vs_Arbor_Acr	breed_growth_background	ENSGALG00010019785	ACSL5	-0.106557533	0.119207018	-0.893886414	0.371382668	0.5343491	negative
PRJNA729271	ed_Lushi_vs_Arbor_Acr	breed_growth_background	ENSGALG00010019786	R3HDM1	0.145640762	0.046992388	3.099241584	0.001940167	0.013001723	positive
PRJNA729271	ed_Lushi_vs_Arbor_Acr	breed_growth_background	ENSGALG00010019789	ARNT2	-0.234551618	0.0952998	-2.461197373	0.013847416	0.052626718	negative
PRJNA729271	ed_Lushi_vs_Arbor_Acr	breed_growth_background	ENSGALG00010019794	RSPH9	-0.44648285	0.273749266	-1.630991951	0.102892018	0.219529124	negative
PRJNA729271	ed_Lushi_vs_Arbor_Acr	breed_growth_background	ENSGALG00010019799	DOK2	-0.210645426	0.147215783	-1.430861702	0.152469855	0.288857738	negative
PRJNA729271	ed_Lushi_vs_Arbor_Acr	breed_growth_background	ENSGALG00010019800	EXT2	-0.103200355	0.032686971	-3.157232088	0.001592745	0.011317079	negative
PRJNA729271	ed_Lushi_vs_Arbor_Acr	breed_growth_background	ENSGALG00010019801	FIBIN	-0.017485854	0.101281214	-0.172646567	0.862929247	0.917558644	negative
PRJNA729271	ed_Lushi_vs_Arbor_Acr	breed_growth_background	ENSGALG00010019803	FAH	-0.901096147	0.273694341	-3.292344822	0.000993557	0.008113583	negative
PRJNA729271	ed_Lushi_vs_Arbor_Acr	breed_growth_background	ENSGALG00010019804	ELFN1	0.070222854	0.161142314	0.435781592	0.662995201	0.781500808	positive
PRJNA729271	ed_Lushi_vs_Arbor_Acr	breed_growth_background	ENSGALG00010019807	ZDHHC6	0.070816156	0.042435187	1.668807422	0.095155551	0.207660301	positive
PRJNA729271	ed_Lushi_vs_Arbor_Acr	breed_growth_background	ENSGALG00010019810	ZFAND6	0.121414065	0.066843626	1.816389563	0.069310635	0.166775839	positive
PRJNA729271	ed_Lushi_vs_Arbor_Acr	breed_growth_background	ENSGALG00010019811	UBXN4	0.152081576	0.059091855	2.573646985	0.010063289	0.042017831	positive
PRJNA729271	ed_Lushi_vs_Arbor_Acr	breed_growth_background	ENSGALG00010019813	ENSGALG00010019813	-0.051101359	0.111503043	-0.458295646	0.646740052	0.769157947	negative
PRJNA729271	ed_Lushi_vs_Arbor_Acr	breed_growth_background	ENSGALG00010019816	ENSGALG00010019816	0.621700833	0.327122353	1.900514677	0.057365611	0.146084773	positive
PRJNA729271	ed_Lushi_vs_Arbor_Acr	breed_growth_background	ENSGALG00010019820	BBOX1	0.135223715	0.082903253	1.631102636	0.102868665	0.219529124	positive
PRJNA729271	ed_Lushi_vs_Arbor_Acr	breed_growth_background	ENSGALG00010019823	LCT	-0.242810519	0.115838101	-2.096119649	0.036071578	0.104263308	negative
PRJNA729271	ed_Lushi_vs_Arbor_Acr	breed_growth_background	ENSGALG00010019825	BCL2A1	0.026963955	0.14946486	0.180403306	0.856835959	0.913895307	positive
PRJNA729271	ed_Lushi_vs_Arbor_Acr	breed_growth_background	ENSGALG00010019830	JAG2	-0.007585529	0.052557855	-0.144327223	0.885242083	0.932273147	negative
PRJNA729271	ed_Lushi_vs_Arbor_Acr	breed_growth_background	ENSGALG00010019832	MAD2L1BP	-0.139206663	0.078818783	-1.766161027	0.077368837	0.179750168	negative
PRJNA729271	ed_Lushi_vs_Arbor_Acr	breed_growth_background	ENSGALG00010019833	MTHFS	-0.018097163	0.071158163	-0.254323077	0.799245973	0.875666695	negative
PRJNA729271	ed_Lushi_vs_Arbor_Acr	breed_growth_background	ENSGALG00010019834	PLCH2	-0.073405789	0.131788732	-0.556995945	0.5775302	0.714782786	negative
PRJNA729271	ed_Lushi_vs_Arbor_Acr	breed_growth_background	ENSGALG00010019836	MINAR1	-0.417310916	0.260963675	-1.599114959	0.109795061	0.230101646	negative
PRJNA729271	ed_Lushi_vs_Arbor_Acr	breed_growth_background	ENSGALG00010019838	ALX4	0.164619833	0.111425926	1.477392548	0.139570442	0.271407776	positive
PRJNA729271	ed_Lushi_vs_Arbor_Acr	breed_growth_background	ENSGALG00010019843	MCM6	-0.069679225	0.060045786	-1.160434884	0.245871791	0.401793868	negative
PRJNA729271	ed_Lushi_vs_Arbor_Acr	breed_growth_background	ENSGALG00010019846	ENSGALG00010019846	0.090547373	0.039147719	2.312966771	0.020724466	0.069893238	positive
PRJNA729271	ed_Lushi_vs_Arbor_Acr	breed_growth_background	ENSGALG00010019848	MAD1L1	0.1191496	0.068809233	1.731593212	0.083346016	0.189415685	positive
PRJNA729271	ed_Lushi_vs_Arbor_Acr	breed_growth_background	ENSGALG00010019854	CCDC14	0.153267763	0.081867262	1.872149603	0.061185909	0.153043961	positive
PRJNA729271	ed_Lushi_vs_Arbor_Acr	breed_growth_background	ENSGALG00010019855	TTC7A	-0.023172955	0.06778188	-0.341875365	0.73244469	0.828271632	negative

PRJNA729271	ed_Lushi_vs_Arbor_Acr	breed_growth_background	ENSGALG00010019859	BIN3	-0.06567078	0.073509793	-0.893360972	0.371663896	0.534463681	negative
PRJNA729271	ed_Lushi_vs_Arbor_Acr	breed_growth_background	ENSGALG00010019865	ENSGALG00010019865	0.033173752	0.073242001	0.452933453	0.650596661	0.771971071	positive
PRJNA729271	ed_Lushi_vs_Arbor_Acr	breed_growth_background	ENSGALG00010019867	PEX11A	-0.230777664	0.204246717	-1.129896566	0.258519811	0.41580611	negative
PRJNA729271	ed_Lushi_vs_Arbor_Acr	breed_growth_background	ENSGALG00010019871	TAFA1	-0.233337523	0.357904467	-0.651954767	0.514430353	0.662932785	negative
PRJNA729271	ed_Lushi_vs_Arbor_Acr	breed_growth_background	ENSGALG00010019872	VTI1A	-0.133959988	0.058863948	-2.275756082	0.022860613	0.075124069	negative
PRJNA729271	ed_Lushi_vs_Arbor_Acr	breed_growth_background	ENSGALG00010019873	LGR4	-0.069002101	0.053325004	-1.293991496	0.195668358	0.343658409	negative
PRJNA729271	ed_Lushi_vs_Arbor_Acr	breed_growth_background	ENSGALG00010019875	PLEKHH1	-0.304930645	0.161846779	-1.884069901	0.059555521	0.150157815	negative
PRJNA729271	ed_Lushi_vs_Arbor_Acr	breed_growth_background	ENSGALG00010019876	MRM2	0.219836866	0.119805317	1.834950828	0.066512976	0.162362004	positive
PRJNA729271	ed_Lushi_vs_Arbor_Acr	breed_growth_background	ENSGALG00010019878	MCFD2	-0.138213058	0.080256219	-1.722147643	0.085042776	0.19236814	negative
PRJNA729271	ed_Lushi_vs_Arbor_Acr	breed_growth_background	ENSGALG00010019891	PIGH	-0.058859577	0.058213318	-1.011101555	0.311967826	0.475196894	negative
PRJNA729271	ed_Lushi_vs_Arbor_Acr	breed_growth_background	ENSGALG00010019896	DDB1	0.082573753	0.048751888	1.693754979	0.090311852	0.200716084	positive
PRJNA729271	ed_Lushi_vs_Arbor_Acr	breed_growth_background	ENSGALG00010019898	NUDT1	0.4898362	0.102247959	4.790669715	1.66226E-06	7.32232E-05	positive
PRJNA729271	ed_Lushi_vs_Arbor_Acr	breed_growth_background	ENSGALG00010019899	ENSGALG00010019899	-0.46678752	0.244861062	-1.906336254	0.056606593	0.144838936	negative
PRJNA729271	ed_Lushi_vs_Arbor_Acr	breed_growth_background	ENSGALG00010019905	ENSGALG00010019905	-0.22978038	0.066815767	-3.43901434	0.000583836	0.005509575	negative
PRJNA729271	ed_Lushi_vs_Arbor_Acr	breed_growth_background	ENSGALG00010019906	ENSGALG00010019906	0.313104739	0.123898094	2.52711505	0.011500382	0.046189537	positive
PRJNA729271	ed_Lushi_vs_Arbor_Acr	breed_growth_background	ENSGALG00010019910	ENSGALG00010019910	-0.072634544	0.136988008	-0.530225568	0.595955546	0.729654163	negative
PRJNA729271	ed_Lushi_vs_Arbor_Acr	breed_growth_background	ENSGALG00010019912	ARG2	-0.362273158	0.127499111	-2.841377915	0.004491905	0.023522456	negative
PRJNA729271	ed_Lushi_vs_Arbor_Acr	breed_growth_background	ENSGALG00010019916	NOC3L	0.243076062	0.061201436	3.971737879	7.13502E-05	0.001241562	positive
PRJNA729271	ed_Lushi_vs_Arbor_Acr	breed_growth_background	ENSGALG00010019919	LIN7C	0.122869198	0.057687041	2.129927219	0.033177623	0.097885129	positive
PRJNA729271	ed_Lushi_vs_Arbor_Acr	breed_growth_background	ENSGALG00010019921	CALM2	0.038423277	0.060244499	0.637788971	0.52361106	0.67053117	positive
PRJNA729271	ed_Lushi_vs_Arbor_Acr	breed_growth_background	ENSGALG00010019925	RDH11	0.00675805	0.081760822	0.082656335	0.934124806	0.964054045	positive
PRJNA729271	ed_Lushi_vs_Arbor_Acr	breed_growth_background	ENSGALG00010019926	KIF7	-0.085598558	0.055259322	-1.549033804	0.121373594	0.246091796	negative
PRJNA729271	ed_Lushi_vs_Arbor_Acr	breed_growth_background	ENSGALG00010019931	ZFYVE26	0.084054298	0.071991681	1.167555701	0.242986028	0.398741364	positive
PRJNA729271	ed_Lushi_vs_Arbor_Acr	breed_growth_background	ENSGALG00010019932	KALRN	0.076453053	0.063293054	1.207921695	0.227077384	0.380298865	positive
PRJNA729271	ed_Lushi_vs_Arbor_Acr	breed_growth_background	ENSGALG00010019934	TKFC	0.081868031	0.082844322	0.988215361	0.323047187	0.486995449	positive
PRJNA729271	ed_Lushi_vs_Arbor_Acr	breed_growth_background	ENSGALG00010019935	ENSGALG00010019935	0.519684885	0.590762068	0.879685601	0.379029652	0.541017706	positive
PRJNA729271	ed_Lushi_vs_Arbor_Acr	breed_growth_background	ENSGALG00010019938	TAFA4	0.259813644	0.24488318	1.060969739	0.288703654	0.449684017	positive
PRJNA729271	ed_Lushi_vs_Arbor_Acr	breed_growth_background	ENSGALG00010019947	ENSGALG00010019947	0.286786213	0.161355434	1.777357021	0.075509517	0.176983395	positive
PRJNA729271	ed_Lushi_vs_Arbor_Acr	breed_growth_background	ENSGALG00010019948	TICRR	-0.064842854	0.070869584	-0.914960287	0.360212479	0.523554494	negative
PRJNA729271	ed_Lushi_vs_Arbor_Acr	breed_growth_background	ENSGALG00010019954	CYB561A3	0.216326201	0.068204139	3.17174592	0.001515255	0.010981812	positive
PRJNA729271	ed_Lushi_vs_Arbor_Acr	breed_growth_background	ENSGALG00010019956	SLC29A3	-0.203412336	0.098966052	-2.055374865	0.039842811	0.112031338	negative
PRJNA729271	ed_Lushi_vs_Arbor_Acr	breed_growth_background	ENSGALG00010019961	HTATSF1	0.005394244	0.037862208	0.142470403	0.886708456	0.933419695	positive
PRJNA729271	ed_Lushi_vs_Arbor_Acr	breed_growth_background	ENSGALG00010019962	SNX8	-0.122158569	0.076249143	-1.602097608	0.109134027	0.229196442	negative
PRJNA729271	ed_Lushi_vs_Arbor_Acr	breed_growth_background	ENSGALG00010019967	CEP170B	-0.11435988	0.0586604	-1.949524392	0.051232833	0.134807775	negative
PRJNA729271	ed_Lushi_vs_Arbor_Acr	breed_growth_background	ENSGALG00010019968	RHCG	-0.278952434	0.406172574	-0.686783037	0.492219461	0.644276782	negative
PRJNA729271	ed_Lushi_vs_Arbor_Acr	breed_growth_background	ENSGALG00010019972	PPP1CB	-0.140687213	0.048939113	-2.874739704	0.004043607	0.021772691	negative
PRJNA729271	ed_Lushi_vs_Arbor_Acr	breed_growth_background	ENSGALG00010019975	ENSGALG00010019975	-0.019216553	0.107551355	-0.178673273	0.858194257	0.914805541	negative
PRJNA729271	ed_Lushi_vs_Arbor_Acr	breed_growth_background	ENSGALG00010019977	ZMIZ1	-0.011092019	0.047680689	-0.232631269	0.816047745	0.886680308	negative
PRJNA729271	ed_Lushi_vs_Arbor_Acr	breed_growth_background	ENSGALG00010019979	ENSGALG00010019979	0.11787893	0.181565936	0.649234832	0.516186601	0.664546121	positive
PRJNA729271	ed_Lushi_vs_Arbor_Acr	breed_growth_background	ENSGALG00010019982	PLCE1	-0.04389119	0.060443198	-0.726155989	0.467743149	0.622640917	negative
PRJNA729271	ed_Lushi_vs_Arbor_Acr	breed_growth_background	ENSGALG00010019985	UMPS	-0.215409034	0.048195132	-4.469518519	7.83959E-06	0.000241776	negative
PRJNA729271	ed_Lushi_vs_Arbor_Acr	breed_growth_background	ENSGALG00010019989	ENSGALG00010019989	-0.826100734	0.146941501	-5.621970176	1.88792E-08	2.33892E-06	negative
PRJNA729271	ed_Lushi_vs_Arbor_Acr	breed_growth_background	ENSGALG00010019990	MARCHF8	-0.088262264	0.057203271	-1.542958342	0.122840884	0.247715444	negative
PRJNA729271	ed_Lushi_vs_Arbor_Acr	breed_growth_background	ENSGALG00010019991	EIF3B	0.042280484	0.040240102	1.0507052	0.293394007	0.454741887	positive
PRJNA729271	ed_Lushi_vs_Arbor_Acr	breed_growth_background	ENSGALG00010019992	CHAC2	-0.030259409	0.079011492	-0.38297478	0.701738464	0.80748274	negative
PRJNA729271	ed_Lushi_vs_Arbor_Acr	breed_growth_background	ENSGALG00010019994	PLD4	0.696902025	0.356610407	1.954239165	0.05067294	0.133622941	positive
PRJNA729271	ed_Lushi_vs_Arbor_Acr	breed_growth_background	ENSGALG00010019996	ITGB5	-0.074247108	0.066704358	-1.113077322	0.265675223	0.42383473	negative
PRJNA729271	ed_Lushi_vs_Arbor_Acr	breed_growth_background	ENSGALG00010019997	FSHR	-0.235895689	0.224006673	-1.053074385	0.292306902	0.45373619	negative
PRJNA729271	ed_Lushi_vs_Arbor_Acr	breed_growth_background	ENSGALG00010019998	PLEK2	-0.16744418	0.435952647	-0.384087999	0.700913226	0.807058487	negative
PRJNA729271	ed_Lushi_vs_Arbor_Acr	breed_growth_background	ENSGALG00010019999	ERLIN1	0.068188078	0.034918277	1.952790467	0.050844429	0.134022548	positive
PRJNA729271	ed_Lushi_vs_Arbor_Acr	breed_growth_background	ENSGALG00010020003	ENSGALG00010020003	-0.198486971	0.099925702	-1.986345524	0.046994971	0.126662719	negative
PRJNA729271	ed_Lushi_vs_Arbor_Acr	breed_growth_background	ENSGALG00010020005	EOGT	-0.239843172	0.087749504	-2.733270962	0.006270872	0.029931501	negative
PRJNA729271	ed_Lushi_vs_Arbor_Acr	breed_growth_background	ENSGALG00010020006	PLB1	-0.640702818	0.208145173	-3.078153625	0.002082875	0.013650792	negative
PRJNA729271	ed_Lushi_vs_Arbor_Acr	breed_growth_background	ENSGALG00010020008	EIF2S1	0.091322545	0.042659666	2.140723372	0.032296353	0.096284581	positive
PRJNA729271	ed_Lushi_vs_Arbor_Acr	breed_growth_background	ENSGALG00010020009	MACROD2	0.233861615	0.064335507	3.635031821	0.000277946	0.003305545	positive
PRJNA729271	ed_Lushi_vs_Arbor_Acr	breed_growth_background	ENSGALG00010020010	SORBS3	-0.02897156	0.107113885	-0.270474365	0.786795336	0.867599742	negative
PRJNA729271	ed_Lushi_vs_Arbor_Acr	breed_growth_background	ENSGALG00010020011	TLE3	0.008861945	0.109198399	0.081154533	0.935319057	0.964536447	positive
PRJNA729271	ed_Lushi_vs_Arbor_Acr	breed_growth_background	ENSGALG00010020013	ADAM8	0.110982387	0.324971178	0.341514555	0.73271625	0.828514084	positive
PRJNA729271	ed_Lushi_vs_Arbor_Acr	breed_growth_background	ENSGALG00010020015	ATP6V1D	0.000785281	0.041967934	0.018711453	0.985071292	0.991099088	positive
PRJNA729271	ed_Lushi_vs_Arbor_Acr	breed_growth_background	ENSGALG00010020017	ENSGALG00010020017	0.574575067	0.144411719	3.978728813	6.92847E-05	0.001212901	positive
PRJNA729271	ed_Lushi_vs_Arbor_Acr	breed_growth_background	ENSGALG00010020018	ENSGALG00010020018	-0.212011124	0.109534123	-1.935571468	0.052920208	0.137987372	negative
PRJNA729271	ed_Lushi_vs_Arbor_Acr	breed_growth_background	ENSGALG00010020019	PEX10	0.106149049	0.052859845	2.008122596	0.044630266	0.121944524	positive
PRJNA729271	ed_Lushi_vs_Arbor_Acr	breed_growth_background	ENSGALG00010020023	FOSL2	-0.365554962	0.112265382	-3.256168164	0.001129268	0.008925162	negative
PRJNA729271	ed_Lushi_vs_Arbor_Acr	breed_growth_background	ENSGALG00010020025	LOXL2	-0.105014902	0.075289534	-1.394814092	0.163071908	0.302759432	negative
PRJNA729271	ed_Lushi_vs_Arbor_Acr	breed_growth_background	ENSGALG00010020027	RER1	0.033909945	0.038103604	0.889940635	0.373497763	0.535918638	positive
PRJNA729271	ed_Lushi_vs_Arbor_Acr	breed_growth_background	ENSGALG00010020030	MRPL33	0.037381476	0.072596855	0.514918677	0.606609849	0.737698976	positive
PRJNA729271	ed_Lushi_vs_Arbor_Acr	breed_growth_background	ENSGALG00010020032	CHST12	-0.104935405	0.067018843	-1.565759724	0.117404879	0.240841171	negative
PRJNA729271	ed_Lushi_vs_Arbor_Acr	breed_growth_background	ENSGALG00010020036	TMF1	0.195041585	0.044993622	4.334871804	1.45845E-05	0.000382283	positive
PRJNA729271	ed_Lushi_vs_Arbor_Acr	breed_growth_background	ENSGALG00010020037	MPP5	-0.004575387	0.048538607	-0.094262849	0.924900361	0.95789466	negative
PRJNA729271	ed_Lushi_vs_Arbor_Acr	breed_growth_background	ENSGALG00010020038	SKI	-0.184442662	0.048939184	-3.768813568	0.000164025	0.002277344	negative
PRJNA729271	ed_Lushi_vs_Arbor_Acr	breed_growth_background	ENSGALG00010020041	CDH23	-0.214491465	0.082048953	-2.61418893	0.008943956	0.038538242	negative
PRJNA729271	ed_Lushi_vs_Arbor_Acr	breed_growth_background	ENSGALG00010020046	BDNF	-0.214673918	0.13087126	-1.64034425	0.10093361	0.216489002	negative
PRJNA729271	ed_Lushi_vs_Arbor_Acr	breed_growth_background	ENSGALG00010020048	ENSGALG00010020048	-0.316087513	0.143816783	-2.19784859	0.027959898	0.086431801	negative
PRJNA729271	ed_Lushi_vs_Arbor_Acr	breed_growth_background	ENSGALG00010020053	LFNG	-0.152468812	0.072534343	-2.102022359	0.035551317	0.103207759	negative
PRJNA729271	ed_Lushi_vs_Arbor_Acr	breed_growth_background	ENSGALG00010020055	R3HCC1	-0.031560363	0.045586582	-0.692316938	0.488738315	0.640587927	negative
PRJNA729271	ed_Lushi_vs_Arbor_Acr	breed_growth_background	ENSGALG00010020056	UACA	-0.205991991	0.089057993	-2.313009585	0.020722112	0.069893238	negative
PRJNA729271	ed_Lushi_vs_Arbor_Acr	breed_growth_background	ENSGALG00010020057	RBKS	0.04530434	0.050241833	0.901725451	0.367202729	0.530646539	positive
PRJNA729271	ed_Lushi_vs_Arbor_Acr	breed_growth_background	ENSGALG00010020059	PRKCZ	0.185089367	0.076473624	2.420303331	0.015507565	0.057109288	positive
PRJNA729271	ed_Lushi_vs_Arbor_Acr	breed_growth_background	ENSGALG00010020061	UBA3	0.040862964	0.039042455	1.046628976	0.295270732	0.45672279	positive
PRJNA729271	ed_Lushi_vs_Arbor_Acr	breed_growth_background	ENSGALG00010020063	ENSGALG00010020063	-0.193094757	0.068606082	-2.814542837	0.004884667	0.02511644	negative
PRJNA729271	ed_Lushi_vs_Arbor_Acr	breed_growth_background	ENSGALG00010020064	TMEM138	-0.022577505	0.072658283	-0.310735454	0.756001742	0.84582305	negative
PRJNA729271	ed_Lushi_vs_Arbor_Acr	breed_growth_background	ENSGALG00010020065	TTYH3	-0.231203927	0.060727746	-3.807220608	0.000140537	0.002037089	negative
PRJNA729271	ed_Lushi_vs_Arbor_Acr	breed_growth_background	ENSGALG00010020066	ENSGALG00010020066	0.03461486	0.189386524	0.182773619	0.854975649	0.912779746	positive
PRJNA729271	ed_Lushi_vs_Arbor_Acr	breed_growth_background	ENSGALG00010020069	TMEM216	-0.130665343	0.114447948	-1.141701058	0.253578301	0.410210316	negative
PRJNA729271	ed_Lushi_vs_Arbor_Acr	breed_growth_background	ENSGALG00010020070	ARL6IP5	-0.190337184	0.061185968	-3.110797931	0.001865826	0.012632015	negative
PRJNA729271	ed_Lushi_vs_Arbor_Acr	breed_growth_background	ENSGALG00010020071	ENSGALG00010020071	0.022605714	0.077292095	0.292471224	0.769926359	0.856557109	positive
PRJNA729271	ed_Lushi_vs_Arbor_Acr	breed_growth_background	ENSGALG00010020074	ENSGALG00010020074	-0.275148047	0.111586602	-2.465780328	0.013671515	0.052177096	negative
PRJNA729271	ed_Lushi_vs_Arbor_Acr	breed_growth_background	ENSGALG00010020075	CPSF7	0.046724842	0.079313037	0.589119306	0.555781244	0.697493431	positive
PRJNA729271	ed_Lushi_vs_Arbor_Acr	breed_growth_background	ENSGALG00010020080	SLC4A1AP	0.122972071	0.055907528	2.199561951	0.027837989	0.086110041	positive
PRJNA729271	ed_Lushi_vs_Arbor_Acr	breed_growth_background	ENSGALG00010020083	CFAP74	-0.42870477	0.253553092	-1.690788964	0.090877116	0.201450683	negative
PRJNA729271	ed_Lushi_vs_Arbor_Acr	breed_growth_background	ENSGALG00010020084	ENSGALG00010020084	-0.017563998	0.047334967	-0.371057585	0.710594642	0.813463065	negative
PRJNA729271	ed_Lushi_vs_Arbor_Acr	breed_growth_background	ENSGALG00010020087	ENSGALG00010020087	-0.106725441	0.268942017	-0.396834389	0.69148959	0.800634364	negative
PRJNA729271	ed_Lushi_vs_Arbor_Acr	breed_growth_background	ENSGALG00010020088	LMOD3	-0.181090661	0.103327629	-1.752587022	0.079672921	0.183606436	negative
PRJNA729271	ed_Lushi_vs_Arbor_Acr	breed_growth_background	ENSGALG00010020090	MORF4L2	-0.003459869	0.029032362	-0.119172851	0.905138415	0.945468208	negative
PRJNA729271	ed_Lushi_vs_Arbor_Acr	breed_growth_background	ENSGALG00010020091	SUPT7L	-0.02739057	0.045526891	-0.601634984	0.547417137	0.691125459	negative
PRJNA729271	ed_Lushi_vs_Arbor_Acr	breed_growth_background	ENSGALG00010020092	SDHAF2	-0.144118385	0.073143633	-1.970347656	0.04879854	0.130336252	negative
PRJNA729271	ed_Lushi_vs_Arbor_Acr	breed_growth_background	ENSGALG00010020094	CLEC3B	0.021195243	0.121321246	0.174703471	0.861312641	0.916713909	positive
PRJNA729271	ed_Lushi_vs_Arbor_Acr	breed_growth_background	ENSGALG00010020095	LRFN2	-0.367028383	0.177745792	-2.064906177	0.038931878	0.110282896	negative
PRJNA729271	ed_Lushi_vs_Arbor_Acr	breed_growth_background	ENSGALG00010020096	GPHN	0.222890388	0.062034608	3.59300066	0.000326892	0.003673098	positive
PRJNA729271	ed_Lushi_vs_Arbor_Acr	breed_growth_background	ENSGALG00010020097	RETSAT	0.121061417	0.122240489	0.990354484	0.322000884	0.48573242	positive
PRJNA729271	ed_Lushi_vs_Arbor_Acr	breed_growth_background	ENSGALG00010020098	GABRD	-0.136535682	0.181309641	-0.753052516	0.451418357	0.607809976	negative
PRJNA729271	ed_Lushi_vs_Arbor_Acr	breed_growth_background	ENSGALG00010020100	MOCS1	0.308721524	0.084296618	3.662323954	0.000249938	0.003041851	positive
PRJNA729271	ed_Lushi_vs_Arbor_Acr	breed_growth_background	ENSGALG00010020101	KIF15	-0.322577301	0.112089753	-2.877848265	0.004003977	0.021680106	negative
PRJNA729271	ed_Lushi_vs_Arbor_Acr	breed_growth_background	ENSGALG00010020102	TMEM127	0.13400793	0.111902942	1.197537152	0.231097256	0.385036556	positive
PRJNA729271	ed_Lushi_vs_Arbor_Acr	breed_growth_background	ENSGALG00010020103	PSAP	-0.025625049	0.056160167	-0.456285138	0.64818495	0.770370683	negative
PRJNA729271	ed_Lushi_vs_Arbor_Acr	breed_growth_background	ENSGALG00010020104	BRAT1	-0.018857406	0.061336343	-0.307442618	0.758506493	0.847429599	negative
PRJNA729271	ed_Lushi_vs_Arbor_Acr	breed_growth_background	ENSGALG00010020106	ENSGALG00010020106	0.073041362	0.086327725	0.84609391	0.397500358	0.558798146	positive
PRJNA729271	ed_Lushi_vs_Arbor_Acr	breed_growth_background	ENSGALG00010020108	RPAP1	0.032656814	0.063446086	0.514717547	0.60675041	0.737698976	positive
PRJNA729271	ed_Lushi_vs_Arbor_Acr	breed_growth_background	ENSGALG00010020109	WDFY4	0.108795349	0.194117251	0.560462032	0.575164331	0.712698396	positive
PRJNA729271	ed_Lushi_vs_Arbor_Acr	breed_growth_background	ENSGALG00010020111	ADRA2B	0.154536075	0.064431752	2.398445961	0.016464806	0.059500713	positive
PRJNA729271	ed_Lushi_vs_Arbor_Acr	breed_growth_background	ENSGALG00010020113	CIAO1	-0.130685414	0.056595579	-2.309109966	0.02093748	0.070382368	negative
PRJNA729271	ed_Lushi_vs_Arbor_Acr	breed_growth_background	ENSGALG00010020116	GNA12	-0.021730643	0.039100655	-0.555761613	0.578373831	0.715440235	negative
PRJNA729271	ed_Lushi_vs_Arbor_Acr	breed_growth_background	ENSGALG00010020117	AKAP13	-0.124534085	0.054942135	-2.26664081	0.023412177	0.076380712	negative
PRJNA729271	ed_Lushi_vs_Arbor_Acr	breed_growth_background	ENSGALG00010020118	GNB1	-0.117445996	0.050082775	-2.345037731	0.019025154	0.066068426	negative
PRJNA729271	ed_Lushi_vs_Arbor_Acr	breed_growth_background	ENSGALG00010020120	DAAM2	-0.056427166	0.055877005	-1.009845932	0.31256911	0.475812776	negative
PRJNA729271	ed_Lushi_vs_Arbor_Acr	breed_growth_background	ENSGALG00010020121	ENSGALG00010020121	0.29897349	0.144859873	2.063880655	0.039029033	0.110385529	positive
PRJNA729271	ed_Lushi_vs_Arbor_Acr	breed_growth_background	ENSGALG00010020123	ENSGALG00010020123	0.059447715	0.083501362	0.711937067	0.476503748	0.62944699	positive
PRJNA729271	ed_Lushi_vs_Arbor_Acr	breed_growth_background	ENSGALG00010020125	NAV2	0.143892393	0.069534524	2.0693662	0.038511736	0.109585318	positive
PRJNA729271	ed_Lushi_vs_Arbor_Acr	breed_growth_background	ENSGALG00010020126	NADK	-0.030723848	0.056307028	-0.545648549	0.585307545	0.721247374	negative
PRJNA729271	ed_Lushi_vs_Arbor_Acr	breed_growth_background	ENSGALG00010020127	FRMD4B	-0.055883481	0.075960972	-0.735686755	0.461921352	0.617158264	negative
PRJNA729271	ed_Lushi_vs_Arbor_Acr	breed_growth_background	ENSGALG00010020128	KIF6	0.333939496	0.284846249	1.172349983	0.241056572	0.396787986	positive
PRJNA729271	ed_Lushi_vs_Arbor_Acr	breed_growth_background	ENSGALG00010020129	MGAT2	-0.02613916	0.063305324	-0.412906187	0.679675348	0.792972809	negative
PRJNA729271	ed_Lushi_vs_Arbor_Acr	breed_growth_background	ENSGALG00010020130	PDXDC1	0.072758544	0.040143707	1.81245208	0.069916372	0.167787717	positive
PRJNA729271	ed_Lushi_vs_Arbor_Acr	breed_growth_background	ENSGALG00010020131	SNRNP200	0.155381097	0.0668822	2.32320552	0.020168114	0.068672027	positive
PRJNA729271	ed_Lushi_vs_Arbor_Acr	breed_growth_background	ENSGALG00010020132	SHC4	-0.107348729	0.124987424	-0.858876236	0.390408802	0.551933637	negative
PRJNA729271	ed_Lushi_vs_Arbor_Acr	breed_growth_background	ENSGALG00010020133	ENSGALG00010020133	-0.443911577	0.192843648	-2.301924806	0.021339413	0.071468297	negative
PRJNA729271	ed_Lushi_vs_Arbor_Acr	breed_growth_background	ENSGALG00010020136	KCNK17	0.144171549	0.160877569	0.896156931	0.370168955	0.533518842	positive
PRJNA729271	ed_Lushi_vs_Arbor_Acr	breed_growth_background	ENSGALG00010020137	NTAN1	-0.041807583	0.046107106	-0.906749224	0.364539426	0.527924766	negative
PRJNA729271	ed_Lushi_vs_Arbor_Acr	breed_growth_background	ENSGALG00010020138	ENSGALG00010020138	0.127220602	0.045996767	2.765859635	0.005677297	0.02785796	positive
PRJNA729271	ed_Lushi_vs_Arbor_Acr	breed_growth_background	ENSGALG00010020140	SECISBP2L	0.182479242	0.050685684	3.600212658	0.000317957	0.003600615	positive
PRJNA729271	ed_Lushi_vs_Arbor_Acr	breed_growth_background	ENSGALG00010020141	THOC7	-0.062128402	0.068509832	-0.906853798	0.364484116	0.527897409	negative

PRJNA729271	eed_Lushi_vs_Arbor_Acr	breed_growth_background	ENSGALG00010020142	SLC35E2B	-0.067802591	0.038398559	-1.765758737	0.077436334	0.179791712	negative
PRJNA729271	eed_Lushi_vs_Arbor_Acr	breed_growth_background	ENSGALG00010020143	KCNK5	-0.018337355	0.10036934	-0.182698766	0.855034384	0.912779746	negative
PRJNA729271	eed_Lushi_vs_Arbor_Acr	breed_growth_background	ENSGALG00010020147	ABHD2	0.018920618	0.078321343	0.241576782	0.809108115	0.882536092	positive
PRJNA729271	eed_Lushi_vs_Arbor_Acr	breed_growth_background	ENSGALG00010020149	FAHD2A	0.193672563	0.092256644	2.099280376	0.035792192	0.103627542	positive
PRJNA729271	eed_Lushi_vs_Arbor_Acr	breed_growth_background	ENSGALG00010020152	ATXN7	0.071103926	0.057782602	1.230542141	0.218494157	0.370634617	positive
PRJNA729271	eed_Lushi_vs_Arbor_Acr	breed_growth_background	ENSGALG00010020153	AXL	-0.151000348	0.065384977	-2.309404333	0.020921155	0.070360125	negative
PRJNA729271	eed_Lushi_vs_Arbor_Acr	breed_growth_background	ENSGALG00010020155	MFGE8	0.241322996	0.071832774	3.359511017	0.000780805	0.00683028	positive
PRJNA729271	eed_Lushi_vs_Arbor_Acr	breed_growth_background	ENSGALG00010020160	SYT7	-0.092385486	0.178099157	-0.518730621	0.603948605	0.735959229	negative
PRJNA729271	eed_Lushi_vs_Arbor_Acr	breed_growth_background	ENSGALG00010020163	CDC2L1	0.07128539	0.051814842	1.37577164	0.168892344	0.310750765	positive
PRJNA729271	eed_Lushi_vs_Arbor_Acr	breed_growth_background	ENSGALG00010020164	C10orf71	-0.173916215	0.078445462	-2.217033468	0.026620805	0.083557506	negative
PRJNA729271	eed_Lushi_vs_Arbor_Acr	breed_growth_background	ENSGALG00010020166	HAPLN3	-0.218757816	0.095151808	-2.299040042	0.021502666	0.071845334	negative
PRJNA729271	eed_Lushi_vs_Arbor_Acr	breed_growth_background	ENSGALG00010020168	COX7A2L	-0.119003123	0.061360147	-1.93942042	0.052450166	0.137157705	negative
PRJNA729271	eed_Lushi_vs_Arbor_Acr	breed_growth_background	ENSGALG00010020170	SORCS1	-0.171059875	0.167736177	-1.019815032	0.307816192	0.470504662	negative
PRJNA729271	eed_Lushi_vs_Arbor_Acr	breed_growth_background	ENSGALG00010020171	RRN3	0.083228791	0.041401113	2.010303215	0.044399107	0.121469898	positive
PRJNA729271	eed_Lushi_vs_Arbor_Acr	breed_growth_background	ENSGALG00010020175	ACAN	-0.083730166	0.297626257	-0.281326543	0.778459954	0.862608136	negative
PRJNA729271	eed_Lushi_vs_Arbor_Acr	breed_growth_background	ENSGALG00010020176	ENSGALG00010020176	-0.223027964	0.136041436	-1.639412018	0.10112748	0.216840655	negative
PRJNA729271	eed_Lushi_vs_Arbor_Acr	breed_growth_background	ENSGALG00010020177	KCNG3	-0.364662614	0.146752922	-2.484874647	0.012959706	0.050205011	negative
PRJNA729271	eed_Lushi_vs_Arbor_Acr	breed_growth_background	ENSGALG00010020179	PSMD6	0.198644315	0.07238416	2.744306424	0.006063891	0.029249948	positive
PRJNA729271	eed_Lushi_vs_Arbor_Acr	breed_growth_background	ENSGALG00010020180	TCF7L1	0.08678505	0.114854769	0.755606849	0.449884948	0.606104872	positive
PRJNA729271	eed_Lushi_vs_Arbor_Acr	breed_growth_background	ENSGALG00010020181	LEUTX	-0.155605778	0.106212468	-1.465042486	0.142909327	0.276034062	negative
PRJNA729271	eed_Lushi_vs_Arbor_Acr	breed_growth_background	ENSGALG00010020183	XPNPEP1	0.068432277	0.037436506	1.82795577	0.06755619	0.164052098	positive
PRJNA729271	eed_Lushi_vs_Arbor_Acr	breed_growth_background	ENSGALG00010020184	RSL1D1	0.019406009	0.03864384	0.502176005	0.61554372	0.744269789	positive
PRJNA729271	eed_Lushi_vs_Arbor_Acr	breed_growth_background	ENSGALG00010020186	MGA	0.061049403	0.05081068	1.201507296	0.229554478	0.383167419	positive
PRJNA729271	eed_Lushi_vs_Arbor_Acr	breed_growth_background	ENSGALG00010020187	MMP23B	0.027483568	0.11169634	0.246056122	0.805638789	0.880238658	positive
PRJNA729271	eed_Lushi_vs_Arbor_Acr	breed_growth_background	ENSGALG00010020193	VSTM4	-0.417828721	0.097121603	-4.302119302	1.69172E-05	0.000422014	negative
PRJNA729271	eed_Lushi_vs_Arbor_Acr	breed_growth_background	ENSGALG00010020194	PRICKLE2	0.004105367	0.08459241	0.04853115	0.961292939	0.978276808	positive
PRJNA729271	eed_Lushi_vs_Arbor_Acr	breed_growth_background	ENSGALG00010020199	DET1	-0.041361811	0.045991941	-0.899327363	0.368478317	0.531716595	negative
PRJNA729271	eed_Lushi_vs_Arbor_Acr	breed_growth_background	ENSGALG00010020202	MAPKBP1	0.21613011	0.048524558	4.454035609	8.42711E-06	0.000255546	positive
PRJNA729271	eed_Lushi_vs_Arbor_Acr	breed_growth_background	ENSGALG00010020204	GSPT2	0.034248248	0.046507293	0.736405969	0.461483673	0.61668718	positive
PRJNA729271	eed_Lushi_vs_Arbor_Acr	breed_growth_background	ENSGALG00010020206	MRPS11	0.071882503	0.043921266	1.63662185	0.101709507	0.217605802	positive
PRJNA729271	eed_Lushi_vs_Arbor_Acr	breed_growth_background	ENSGALG00010020211	ADD3	-0.098067185	0.044644843	-2.196607234	0.028048509	0.086650575	negative
PRJNA729271	eed_Lushi_vs_Arbor_Acr	breed_growth_background	ENSGALG00010020212	JMJD7	-0.041881952	0.076251892	-0.549257881	0.582828488	0.718987143	negative
PRJNA729271	eed_Lushi_vs_Arbor_Acr	breed_growth_background	ENSGALG00010020215	PRMT3	-0.060532937	0.044365523	-1.364413915	0.172437331	0.315630649	negative
PRJNA729271	eed_Lushi_vs_Arbor_Acr	breed_growth_background	ENSGALG00010020224	MRPL46	0.018555948	0.047332252	0.392036029	0.695031593	0.80351363	positive
PRJNA729271	eed_Lushi_vs_Arbor_Acr	breed_growth_background	ENSGALG00010020226	TCIM	-0.3308463	0.105552736	-3.134417093	0.001721959	0.011965387	negative
PRJNA729271	eed_Lushi_vs_Arbor_Acr	breed_growth_background	ENSGALG00010020228	TM9SF3	-0.109072325	0.044586983	-2.446281769	0.014433815	0.053918941	negative
PRJNA729271	eed_Lushi_vs_Arbor_Acr	breed_growth_background	ENSGALG00010020230	ENSGALG00010020230	0.242698388	0.116841927	2.077151547	0.037787574	0.108097952	positive
PRJNA729271	eed_Lushi_vs_Arbor_Acr	breed_growth_background	ENSGALG00010020236	SLC35G1	0.48514253	0.160175251	3.028823281	0.002455082	0.015409523	positive
PRJNA729271	eed_Lushi_vs_Arbor_Acr	breed_growth_background	ENSGALG00010020237	MIB2	0.104250584	0.041001398	2.542610463	0.011002782	0.044799247	positive
PRJNA729271	eed_Lushi_vs_Arbor_Acr	breed_growth_background	ENSGALG00010020240	SV2B	-0.013773214	0.211091475	-0.065247607	0.947976857	0.971502018	negative
PRJNA729271	eed_Lushi_vs_Arbor_Acr	breed_growth_background	ENSGALG00010020244	SLC25A37	-0.097453304	0.117028109	-0.832734163	0.404994673	0.565821473	negative
PRJNA729271	eed_Lushi_vs_Arbor_Acr	breed_growth_background	ENSGALG00010020246	ADAMTS9	-0.131978113	0.069751559	-1.892117025	0.05847539	0.148000835	negative
PRJNA729271	eed_Lushi_vs_Arbor_Acr	breed_growth_background	ENSGALG00010020248	NOLC1	0.003232164	0.043533051	0.0742462	0.940814485	0.967719697	positive
PRJNA729271	eed_Lushi_vs_Arbor_Acr	breed_growth_background	ENSGALG00010020250	ENSGALG00010020250	0.470758997	0.131738206	3.573443198	0.000352318	0.003877635	positive
PRJNA729271	eed_Lushi_vs_Arbor_Acr	breed_growth_background	ENSGALG00010020252	DMTN	-0.297735705	0.100805777	-2.953557966	0.003141336	0.018330784	negative
PRJNA729271	eed_Lushi_vs_Arbor_Acr	breed_growth_background	ENSGALG00010020254	ENSGALG00010020254	-0.497128226	0.073311929	-6.781000474	1.19346E-11	4.92795E-09	negative
PRJNA729271	eed_Lushi_vs_Arbor_Acr	breed_growth_background	ENSGALG00010020257	ZNF710	0.255652941	0.086617425	2.951518611	0.003162155	0.018437424	positive
PRJNA729271	eed_Lushi_vs_Arbor_Acr	breed_growth_background	ENSGALG00010020259	ENSGALG00010020259	0.018126136	0.113017359	0.160383649	0.872578869	0.924665971	positive
PRJNA729271	eed_Lushi_vs_Arbor_Acr	breed_growth_background	ENSGALG00010020261	SSU72	0.098097557	0.04904676	2.000082302	0.045491377	0.123737571	positive
PRJNA729271	eed_Lushi_vs_Arbor_Acr	breed_growth_background	ENSGALG00010020265	ENSGALG00010020265	-0.427719225	0.123076321	-3.475235698	0.000510405	0.00504316	negative
PRJNA729271	eed_Lushi_vs_Arbor_Acr	breed_growth_background	ENSGALG00010020268	CPLANE2	0.089889288	0.053601655	1.676987195	0.093544986	0.205164861	positive
PRJNA729271	eed_Lushi_vs_Arbor_Acr	breed_growth_background	ENSGALG00010020269	ENSGALG00010020269	0.187841439	0.399750114	0.469897148	0.638428502	0.762715004	positive
PRJNA729271	eed_Lushi_vs_Arbor_Acr	breed_growth_background	ENSGALG00010020272	ENSGALG00010020272	-0.099656945	0.050429522	-1.976162791	0.048136343	0.128828709	negative
PRJNA729271	eed_Lushi_vs_Arbor_Acr	breed_growth_background	ENSGALG00010020275	ELOVL3	-0.007195005	0.320260163	-0.022466125	0.982076133	0.989379591	negative
PRJNA729271	eed_Lushi_vs_Arbor_Acr	breed_growth_background	ENSGALG00010020277	IDH2	0.109868435	0.066646744	1.648519167	0.099246181	0.213628362	positive
PRJNA729271	eed_Lushi_vs_Arbor_Acr	breed_growth_background	ENSGALG00010020282	MAGI1	0.015173387	0.074240574	0.204381326	0.838055522	0.901693497	positive
PRJNA729271	eed_Lushi_vs_Arbor_Acr	breed_growth_background	ENSGALG00010020283	FBXO42	0.207968302	0.120184691	1.730405933	0.083557774	0.189689889	positive
PRJNA729271	eed_Lushi_vs_Arbor_Acr	breed_growth_background	ENSGALG00010020284	HEG1	-0.116972801	0.062808533	-1.862371165	0.062550791	0.155332142	negative
PRJNA729271	eed_Lushi_vs_Arbor_Acr	breed_growth_background	ENSGALG00010020285	ADAMTS14	-0.231468606	0.147025538	-1.574342862	0.115408206	0.237997561	negative
PRJNA729271	eed_Lushi_vs_Arbor_Acr	breed_growth_background	ENSGALG00010020287	GALNT16	-0.063965783	0.086458435	-0.739844327	0.459394459	0.614802205	negative
PRJNA729271	eed_Lushi_vs_Arbor_Acr	breed_growth_background	ENSGALG00010020290	NELL1	-0.341366329	0.337664607	-1.010962717	0.312034275	0.475248168	negative
PRJNA729271	eed_Lushi_vs_Arbor_Acr	breed_growth_background	ENSGALG00010020291	SNX29	-0.141919135	0.0784481	-1.809083134	0.070438089	0.168551381	negative
PRJNA729271	eed_Lushi_vs_Arbor_Acr	breed_growth_background	ENSGALG00010020292	NPM2	0.347462522	0.136057704	2.55378793	0.010655814	0.043804883	positive
PRJNA729271	eed_Lushi_vs_Arbor_Acr	breed_growth_background	ENSGALG00010020293	SEMA4B	0.154158921	0.060667841	2.541031909	0.011052583	0.04496413	positive
PRJNA729271	eed_Lushi_vs_Arbor_Acr	breed_growth_background	ENSGALG00010020295	LGI1	-0.149188376	0.081334067	-1.834266774	0.066614404	0.162500131	negative
PRJNA729271	eed_Lushi_vs_Arbor_Acr	breed_growth_background	ENSGALG00010020298	ENSGALG00010020298	-0.351084295	0.093504628	-3.754726386	0.000173531	0.00234926	negative
PRJNA729271	eed_Lushi_vs_Arbor_Acr	breed_growth_background	ENSGALG00010020300	EHD4	-0.240052471	0.06892019	-3.483050039	0.000495736	0.004945416	negative
PRJNA729271	eed_Lushi_vs_Arbor_Acr	breed_growth_background	ENSGALG00010020302	HAAO	-1.581248537	0.792631709	-1.994934746	0.046049997	0.124882079	negative
PRJNA729271	eed_Lushi_vs_Arbor_Acr	breed_growth_background	ENSGALG00010020303	EXD2	-0.074811056	0.041000434	-1.824640613	0.068055281	0.164767211	negative
PRJNA729271	eed_Lushi_vs_Arbor_Acr	breed_growth_background	ENSGALG00010020304	ZFP36L2	-0.053136014	0.082957379	-0.640521852	0.521833387	0.668963912	negative
PRJNA729271	eed_Lushi_vs_Arbor_Acr	breed_growth_background	ENSGALG00010020305	XPO7	0.234625488	0.074153552	3.164049231	0.001555905	0.011153729	positive
PRJNA729271	eed_Lushi_vs_Arbor_Acr	breed_growth_background	ENSGALG00010020307	SHQ1	-0.098312612	0.123014576	-0.799194818	0.424177456	0.583324402	negative
PRJNA729271	eed_Lushi_vs_Arbor_Acr	breed_growth_background	ENSGALG00010020309	GXYLT2	-0.14164316	0.085485468	-1.656926769	0.097534282	0.211099427	negative
PRJNA729271	eed_Lushi_vs_Arbor_Acr	breed_growth_background	ENSGALG00010020311	SGPL1	-0.06978738	0.072391004	-0.964033869	0.335028946	0.499254093	negative
PRJNA729271	eed_Lushi_vs_Arbor_Acr	breed_growth_background	ENSGALG00010020313	FRA10AC1	0.094541831	0.06190175	1.527288505	0.126689287	0.253361094	positive
PRJNA729271	eed_Lushi_vs_Arbor_Acr	breed_growth_background	ENSGALG00010020314	THADA	-0.045799457	0.043643498	-1.04939932	0.293994371	0.455287983	negative
PRJNA729271	eed_Lushi_vs_Arbor_Acr	breed_growth_background	ENSGALG00010020315	ANO5	-0.100752069	0.058078197	-1.734765794	0.082782297	0.188490323	negative
PRJNA729271	eed_Lushi_vs_Arbor_Acr	breed_growth_background	ENSGALG00010020316	PDPK1	0.114484902	0.060290415	1.898890586	0.057578863	0.146522531	positive
PRJNA729271	eed_Lushi_vs_Arbor_Acr	breed_growth_background	ENSGALG00010020319	NTRK3	-0.202913063	0.087065559	-2.330577856	0.01977563	0.067653472	negative
PRJNA729271	eed_Lushi_vs_Arbor_Acr	breed_growth_background	ENSGALG00010020321	SZRD1	0.168552729	0.09067804	1.858804284	0.063054885	0.156235993	positive
PRJNA729271	eed_Lushi_vs_Arbor_Acr	breed_growth_background	ENSGALG00010020323	ARHGAP25	-0.043811008	0.080443326	-0.544619549	0.586015207	0.72184313	negative
PRJNA729271	eed_Lushi_vs_Arbor_Acr	breed_growth_background	ENSGALG00010020324	PLEKHH2	-0.165309386	0.099562605	-1.660356171	0.096842822	0.209983052	negative
PRJNA729271	eed_Lushi_vs_Arbor_Acr	breed_growth_background	ENSGALG00010020329	PPP4R2	0.131499717	0.056924591	2.310068707	0.020884351	0.070268956	positive
PRJNA729271	eed_Lushi_vs_Arbor_Acr	breed_growth_background	ENSGALG00010020334	CABP4	-0.153377961	0.278713371	-0.550307151	0.58210872	0.718343768	negative
PRJNA729271	eed_Lushi_vs_Arbor_Acr	breed_growth_background	ENSGALG00010020336	OSBP	0.247971455	0.072089697	3.439762736	0.000582224	0.005501527	positive
PRJNA729271	eed_Lushi_vs_Arbor_Acr	breed_growth_background	ENSGALG00010020338	NECAP2	-0.048739996	0.090423875	-0.539016885	0.589875204	0.725330808	negative
PRJNA729271	eed_Lushi_vs_Arbor_Acr	breed_growth_background	ENSGALG00010020340	DCAF5	0.044638931	0.051953557	0.85920836	0.390225572	0.551782232	positive
PRJNA729271	eed_Lushi_vs_Arbor_Acr	breed_growth_background	ENSGALG00010020341	DYNC2LI1	0.030472741	0.071641785	0.425348707	0.670582492	0.786988359	positive
PRJNA729271	eed_Lushi_vs_Arbor_Acr	breed_growth_background	ENSGALG00010020342	CPPED1	0.218439123	0.058086445	3.760586894	0.000169515	0.00231804	positive
PRJNA729271	eed_Lushi_vs_Arbor_Acr	breed_growth_background	ENSGALG00010020343	PATL1	0.2610903	0.094044223	2.776250262	0.005498986	0.027269178	positive
PRJNA729271	eed_Lushi_vs_Arbor_Acr	breed_growth_background	ENSGALG00010020345	AGBL1	0.680896132	0.243549403	2.795720805	0.005178411	0.026192366	positive
PRJNA729271	eed_Lushi_vs_Arbor_Acr	breed_growth_background	ENSGALG00010020346	PCBD1	-0.171838716	0.150490543	-1.141857244	0.253513364	0.41016589	negative
PRJNA729271	eed_Lushi_vs_Arbor_Acr	breed_growth_background	ENSGALG00010020350	PDZRN3	0.107932056	0.057887644	1.864509399	0.062250203	0.154757352	positive
PRJNA729271	eed_Lushi_vs_Arbor_Acr	breed_growth_background	ENSGALG00010020351	CROCC	0.01158529	0.082208352	0.140925953	0.887928439	0.934067982	positive
PRJNA729271	eed_Lushi_vs_Arbor_Acr	breed_growth_background	ENSGALG00010020352	ABCG5	-0.394563809	0.352982216	-1.117800816	0.263652073	0.421721121	negative
PRJNA729271	eed_Lushi_vs_Arbor_Acr	breed_growth_background	ENSGALG00010020356	STX3	0.262645167	0.215088399	1.221103357	0.222046889	0.374513574	positive
PRJNA729271	eed_Lushi_vs_Arbor_Acr	breed_growth_background	ENSGALG00010020357	RBP4	-0.84134642	0.391960508	-2.146508137	0.03183246	0.095279788	negative
PRJNA729271	eed_Lushi_vs_Arbor_Acr	breed_growth_background	ENSGALG00010020362	CDS2	-0.23532112	0.068181795	-3.451377617	0.000557733	0.005364522	negative
PRJNA729271	eed_Lushi_vs_Arbor_Acr	breed_growth_background	ENSGALG00010020364	MRPL16	0.167189824	0.059063522	2.830678183	0.004644943	0.024149372	positive
PRJNA729271	eed_Lushi_vs_Arbor_Acr	breed_growth_background	ENSGALG00010020365	UNC5B	-0.077999802	0.076328666	-1.021893959	0.306831092	0.469494002	negative
PRJNA729271	eed_Lushi_vs_Arbor_Acr	breed_growth_background	ENSGALG00010020369	CNTN3	-0.661403566	0.181046778	-3.653219209	0.000258973	0.003136018	negative
PRJNA729271	eed_Lushi_vs_Arbor_Acr	breed_growth_background	ENSGALG00010020370	CEP55	-0.237081663	0.122298482	-1.938549519	0.052556216	0.137335919	negative
PRJNA729271	eed_Lushi_vs_Arbor_Acr	breed_growth_background	ENSGALG00010020373	MS4A4A	-0.261643471	0.117574383	-2.2253442	0.02605814	0.08230829	negative
PRJNA729271	eed_Lushi_vs_Arbor_Acr	breed_growth_background	ENSGALG00010020374	PCNA	-0.078421386	0.051922243	-1.510362067	0.130951062	0.259062549	negative
PRJNA729271	eed_Lushi_vs_Arbor_Acr	breed_growth_background	ENSGALG00010020375	LRPPRC	-0.00593882	0.054482093	-0.109004983	0.913198538	0.951057106	negative
PRJNA729271	eed_Lushi_vs_Arbor_Acr	breed_growth_background	ENSGALG00010020377	TMEM230	-0.086820304	0.055054107	-1.576999591	0.114795619	0.237065465	negative
PRJNA729271	eed_Lushi_vs_Arbor_Acr	breed_growth_background	ENSGALG00010020379	COL13A1	0.02504756	0.107381655	0.233257352	0.815561573	0.886440088	positive
PRJNA729271	eed_Lushi_vs_Arbor_Acr	breed_growth_background	ENSGALG00010020382	MFAP2	-0.021782913	0.084311125	-0.258363451	0.796126421	0.873593259	negative
PRJNA729271	eed_Lushi_vs_Arbor_Acr	breed_growth_background	ENSGALG00010020383	BAAT	0.080591804	0.057671782	1.397421769	0.162286777	0.301698964	positive
PRJNA729271	eed_Lushi_vs_Arbor_Acr	breed_growth_background	ENSGALG00010020385	SLC12A8	-0.076374338	0.240631939	-0.317390693	0.750947168	0.842099582	negative
PRJNA729271	eed_Lushi_vs_Arbor_Acr	breed_growth_background	ENSGALG00010020386	PPM1B	0.019111812	0.038409079	0.497585798	0.618776016	0.746807523	positive
PRJNA729271	eed_Lushi_vs_Arbor_Acr	breed_growth_background	ENSGALG00010020388	MYOF	-0.106750729	0.089229965	-1.196355161	0.231557991	0.385575311	negative
PRJNA729271	eed_Lushi_vs_Arbor_Acr	breed_growth_background	ENSGALG00010020389	ACTN1	-0.231188552	0.084524174	-2.735176718	0.006234679	0.029805963	negative
PRJNA729271	eed_Lushi_vs_Arbor_Acr	breed_growth_background	ENSGALG00010020390	KLHL25	0.396525406	0.118856619	3.336165948	0.000849424	0.007258694	positive
PRJNA729271	eed_Lushi_vs_Arbor_Acr	breed_growth_background	ENSGALG00010020392	SLC23A2	0.001523367	0.045136864	0.033749943	0.973076553	0.984110627	positive
PRJNA729271	eed_Lushi_vs_Arbor_Acr	breed_growth_background	ENSGALG00010020393	SHISA9	0.081774779	0.172489555	0.474085398	0.635439011	0.760271437	positive
PRJNA729271	eed_Lushi_vs_Arbor_Acr	breed_growth_background	ENSGALG00010020396	CHL1	0.249702204	0.107104399	2.33139074	0.019732765	0.067602248	positive
PRJNA729271	eed_Lushi_vs_Arbor_Acr	breed_growth_background	ENSGALG00010020399	ATP13A2	-0.06581302	0.071315536	-0.922842685	0.356089193	0.519666017	negative
PRJNA729271	eed_Lushi_vs_Arbor_Acr	breed_growth_background	ENSGALG00010020402	SLC3A1	0.052873373	0.149894563	0.352737097	0.724285545	0.823436302	positive
PRJNA729271	eed_Lushi_vs_Arbor_Acr	breed_growth_background	ENSGALG00010020405	UBE2I	-0.005834062	0.044176808	-0.132061642	0.894935536	0.938646208	negative
PRJNA729271	eed_Lushi_vs_Arbor_Acr	breed_growth_background	ENSGALG00010020413	CYP26A1	0.247112393	0.169995167	1.453643637	0.146045132	0.280350177	positive
PRJNA729271	eed_Lushi_vs_Arbor_Acr	breed_growth_background	ENSGALG00010020415	TSR3	-0.079558382	0.050772185	-1.566967845	0.117122208	0.240465496	negative
PRJNA729271	eed_Lushi_vs_Arbor_Acr	breed_growth_background	ENSGALG00010020416	ENSGALG00010020416	-0.578552724	0.533786962	-1.083864473	0.278424894	0.437835737	negative
PRJNA729271	eed_Lushi_vs_Arbor_Acr	breed_growth_background	ENSGALG00010020417	ENSGALG00010020417	-0.278180319	0.402429762	-0.691251856	0.489407281	0.641259841	negative
PRJNA729271	eed_Lushi_vs_Arbor_Acr	breed_growth_background	ENSGALG00010020422	ERCC4	0.100414159	0.048408033	2.074328412	0.038048821	0.108629506	positive
PRJNA729271	eed_Lushi_vs_Arbor_Acr	breed_growth_background	ENSGALG00010020428	SEMA4C	0.05548319	0.081360944	0.681938868	0.495277607	0.646702902	positive
PRJNA729271	eed_Lushi_vs_Arbor_Acr	breed_growth_background	ENSGALG00010020429	ZNF148	0.070899872	0.055243399	1.283408934	0.199348846	0.347972244	positive
PRJNA729271	eed_Lushi_vs_Arbor_Acr	breed_growth_background	ENSGALG00010020433	GNPTG	0.04113648	0.039402587	1.044004564	0.296483278	0.458116608	positive
PRJNA729271	eed_Lushi_vs_Arbor_Acr	breed_growth_background	ENSGALG00010020436	CNTN4	-0.345409728	0.146440827	-2.358698288	0.018339159	0.064349095	negative
PRJNA729271	eed_Lushi_vs_Arbor_Acr	breed_growth_background	ENSGALG00010020437	SNX4	-0.110299811	0.046010347	-2.397282726	0.016517174	0.059660212	negative
PRJNA729271	eed_Lushi_vs_Arbor_Acr	breed_growth_background	ENSGALG00010020438	HTR7	0.063713948	0.155479384	0.409790332	0.681959759	0.794479296	positive

PRJNA729271	ed_Lushi_vs_Arbor_Acr	breed_growth_background	ENSGALG00010020440	PAX7	-0.028409073	0.10007693	-0.283872344	0.776508212	0.861342825	negative
PRJNA729271	ed_Lushi_vs_Arbor_Acr	breed_growth_background	ENSGALG00010020443	PREPL	0.110355	0.07367222	1.49791875	0.134154362	0.264093507	positive
PRJNA729271	ed_Lushi_vs_Arbor_Acr	breed_growth_background	ENSGALG00010020444	ENSGALG00010020444	-0.844225459	0.581464092	-1.45189612	0.146530492	0.281010983	negative
PRJNA729271	ed_Lushi_vs_Arbor_Acr	breed_growth_background	ENSGALG00010020449	PRPF19	0.019467916	0.052354318	0.371849298	0.710005057	0.813025405	positive
PRJNA729271	ed_Lushi_vs_Arbor_Acr	breed_growth_background	ENSGALG00010020450	ANKRD39	-0.064568066	0.096374191	-0.669972583	0.502875268	0.653679222	negative
PRJNA729271	ed_Lushi_vs_Arbor_Acr	breed_growth_background	ENSGALG00010020453	ENSGALG00010020453	-0.08466279	0.077895202	-1.086880688	0.277089542	0.436329485	negative
PRJNA729271	ed_Lushi_vs_Arbor_Acr	breed_growth_background	ENSGALG00010020455	ENSGALG00010020455	-0.829231849	0.396435824	-2.091717749	0.036463772	0.105161634	negative
PRJNA729271	ed_Lushi_vs_Arbor_Acr	breed_growth_background	ENSGALG00010020457	IL5RA	-1.297079856	0.556313568	-2.331562503	0.019723718	0.067602248	negative
PRJNA729271	ed_Lushi_vs_Arbor_Acr	breed_growth_background	ENSGALG00010020460	MAPK8	0.156778145	0.075476739	2.077171666	0.037785718	0.108097952	positive
PRJNA729271	ed_Lushi_vs_Arbor_Acr	breed_growth_background	ENSGALG00010020462	CNNM4	0.278238134	0.097828744	2.844134796	0.00445322	0.023395951	positive
PRJNA729271	ed_Lushi_vs_Arbor_Acr	breed_growth_background	ENSGALG00010020467	ALDH4A1	0.045995287	0.070509212	0.652330182	0.514188193	0.662856445	positive
PRJNA729271	ed_Lushi_vs_Arbor_Acr	breed_growth_background	ENSGALG00010020468	ENSGALG00010020468	0.291742363	0.267181214	1.091926932	0.274865219	0.433722115	positive
PRJNA729271	ed_Lushi_vs_Arbor_Acr	breed_growth_background	ENSGALG00010020473	TMEM109	0.320726858	0.083466831	3.842566629	0.000121754	0.001846056	positive
PRJNA729271	ed_Lushi_vs_Arbor_Acr	breed_growth_background	ENSGALG00010020476	TMEM132A	-0.189749353	0.06108396	-3.10636955	0.001893999	0.012763138	negative
PRJNA729271	ed_Lushi_vs_Arbor_Acr	breed_growth_background	ENSGALG00010020478	ENSGALG00010020478	0.584594291	0.402390002	1.452805211	0.146277845	0.280611085	positive
PRJNA729271	ed_Lushi_vs_Arbor_Acr	breed_growth_background	ENSGALG00010020481	RPP30	0.00354534	0.067392196	0.05260758	0.958044578	0.976640843	positive
PRJNA729271	ed_Lushi_vs_Arbor_Acr	breed_growth_background	ENSGALG00010020484	TRNT1	0.303236068	0.067217652	4.511256493	6.44447E-06	0.000210389	positive
PRJNA729271	ed_Lushi_vs_Arbor_Acr	breed_growth_background	ENSGALG00010020485	LMAN2L	0.040328744	0.071178173	0.566588639	0.570993696	0.709459422	positive
PRJNA729271	ed_Lushi_vs_Arbor_Acr	breed_growth_background	ENSGALG00010020486	MRTFB	-0.001834415	0.059897347	-0.030625987	0.975567817	0.985697229	negative
PRJNA729271	ed_Lushi_vs_Arbor_Acr	breed_growth_background	ENSGALG00010020489	IFFO2	-0.039461666	0.104384353	-0.378041968	0.705399418	0.809497629	negative
PRJNA729271	ed_Lushi_vs_Arbor_Acr	breed_growth_background	ENSGALG00010020493	DEPDC7	0.115941903	0.083008717	1.396743695	0.162490659	0.301884402	positive
PRJNA729271	ed_Lushi_vs_Arbor_Acr	breed_growth_background	ENSGALG00010020497	OSBPL11	-0.114490167	0.038053495	-3.008663662	0.002623994	0.016102793	negative
PRJNA729271	ed_Lushi_vs_Arbor_Acr	breed_growth_background	ENSGALG00010020498	FRMPD2	-0.295616843	0.330319094	-0.89494325	0.370817425	0.534028671	negative
PRJNA729271	ed_Lushi_vs_Arbor_Acr	breed_growth_background	ENSGALG00010020507	ANKRD1	-0.729708332	0.312812424	-2.332734492	0.019662085	0.067488022	negative
PRJNA729271	ed_Lushi_vs_Arbor_Acr	breed_growth_background	ENSGALG00010020509	CRBN	0.023042389	0.055158324	0.417749988	0.67612992	0.790773647	positive
PRJNA729271	ed_Lushi_vs_Arbor_Acr	breed_growth_background	ENSGALG00010020514	CAMKMT	-0.197927353	0.076565951	-2.585057083	0.009736288	0.041009543	negative
PRJNA729271	ed_Lushi_vs_Arbor_Acr	breed_growth_background	ENSGALG00010020517	UBR4	0.081207495	0.050363085	1.612440823	0.106866047	0.225652283	positive
PRJNA729271	ed_Lushi_vs_Arbor_Acr	breed_growth_background	ENSGALG00010020520	ARHGAP22	-0.14903601	0.108667166	-1.371490721	0.170222034	0.312562501	negative
PRJNA729271	ed_Lushi_vs_Arbor_Acr	breed_growth_background	ENSGALG00010020521	ENSGALG00010020521	-0.107796324	0.383079184	-0.281394366	0.778407939	0.862608136	negative
PRJNA729271	ed_Lushi_vs_Arbor_Acr	breed_growth_background	ENSGALG00010020524	ENSGALG00010020524	0.31380952	0.219888635	1.427129331	0.15354262	0.289970116	positive
PRJNA729271	ed_Lushi_vs_Arbor_Acr	breed_growth_background	ENSGALG00010020525	ENSGALG00010020525	-0.558118258	0.38637714	-1.44449089	0.148600941	0.283563468	negative
PRJNA729271	ed_Lushi_vs_Arbor_Acr	breed_growth_background	ENSGALG00010020530	SRSF5A	-0.024407712	0.037277356	-0.65475974	0.512622456	0.661274061	negative
PRJNA729271	ed_Lushi_vs_Arbor_Acr	breed_growth_background	ENSGALG00010020533	ENSGALG00010020533	-0.190253608	0.053014348	-3.588719176	0.000332307	0.003710927	negative
PRJNA729271	ed_Lushi_vs_Arbor_Acr	breed_growth_background	ENSGALG00010020535	LMLN	0.242438698	0.084066983	2.883875343	0.003928142	0.021394964	positive
PRJNA729271	ed_Lushi_vs_Arbor_Acr	breed_growth_background	ENSGALG00010020537	CCDC167	0.031539745	0.05419924	0.581922276	0.560619035	0.701499734	positive
PRJNA729271	ed_Lushi_vs_Arbor_Acr	breed_growth_background	ENSGALG00010020540	PCGF5	-0.061469028	0.086620656	-0.709634756	0.477930661	0.631044355	negative
PRJNA729271	ed_Lushi_vs_Arbor_Acr	breed_growth_background	ENSGALG00010020544	BMERB1	-0.107186317	0.087973687	-1.218390638	0.223075561	0.37568029	negative
PRJNA729271	ed_Lushi_vs_Arbor_Acr	breed_growth_background	ENSGALG00010020545	STYX	0.192743859	0.074993236	2.570149907	0.010165452	0.042280991	positive
PRJNA729271	ed_Lushi_vs_Arbor_Acr	breed_growth_background	ENSGALG00010020546	LRRC20	0.213116231	0.125120206	1.703291891	0.088513457	0.197719612	positive
PRJNA729271	ed_Lushi_vs_Arbor_Acr	breed_growth_background	ENSGALG00010020548	CMTR1	0.001511274	0.099247041	0.015227394	0.987850767	0.992713543	positive
PRJNA729271	ed_Lushi_vs_Arbor_Acr	breed_growth_background	ENSGALG00010020550	HECTD2	-0.064961013	0.114404248	-0.567819939	0.570157239	0.70877295	negative
PRJNA729271	ed_Lushi_vs_Arbor_Acr	breed_growth_background	ENSGALG00010020554	ZFP36L1	0.189306318	0.112763763	1.678786815	0.093193599	0.204641906	positive
PRJNA729271	ed_Lushi_vs_Arbor_Acr	breed_growth_background	ENSGALG00010020556	EMC1	0.125934528	0.046166007	2.727862714	0.006374613	0.030314967	positive
PRJNA729271	ed_Lushi_vs_Arbor_Acr	breed_growth_background	ENSGALG00010020557	ENSGALG00010020557	0.209009262	0.139207048	1.501427296	0.133245078	0.262595161	positive
PRJNA729271	ed_Lushi_vs_Arbor_Acr	breed_growth_background	ENSGALG00010020558	EIF4EBP2	0.054880274	0.053586529	1.024143103	0.305767688	0.468279164	positive
PRJNA729271	ed_Lushi_vs_Arbor_Acr	breed_growth_background	ENSGALG00010020562	LRRN1	0.709883735	0.131963081	5.379411649	7.47297E-08	6.56489E-06	positive
PRJNA729271	ed_Lushi_vs_Arbor_Acr	breed_growth_background	ENSGALG00010020568	PLA2G4F	0.273596178	0.224726953	1.217460453	0.223429074	0.37611903	positive
PRJNA729271	ed_Lushi_vs_Arbor_Acr	breed_growth_background	ENSGALG00010020569	DTX3L	0.035288273	0.134215428	0.262922624	0.792610219	0.871558574	positive
PRJNA729271	ed_Lushi_vs_Arbor_Acr	breed_growth_background	ENSGALG00010020571	MRTO4	0.164185081	0.044607421	3.680667381	0.000232624	0.002889896	positive
PRJNA729271	ed_Lushi_vs_Arbor_Acr	breed_growth_background	ENSGALG00010020572	PPP1R3C	-0.120839514	0.14559904	-0.82994719	0.406568643	0.567418879	negative
PRJNA729271	ed_Lushi_vs_Arbor_Acr	breed_growth_background	ENSGALG00010020574	DTL	-0.325762748	0.101520634	-3.208832887	0.001332749	0.010035429	negative
PRJNA729271	ed_Lushi_vs_Arbor_Acr	breed_growth_background	ENSGALG00010020576	AKR7L	-0.215627682	0.056327824	-3.828084738	0.000129144	0.001916015	negative
PRJNA729271	ed_Lushi_vs_Arbor_Acr	breed_growth_background	ENSGALG00010020578	TNKS2	-0.064377826	0.033985258	-1.894286806	0.058186951	0.147476807	negative
PRJNA729271	ed_Lushi_vs_Arbor_Acr	breed_growth_background	ENSGALG00010020580	ENSGALG00010020580	-0.104693222	0.062638687	-1.671382765	0.094646098	0.206891146	negative
PRJNA729271	ed_Lushi_vs_Arbor_Acr	breed_growth_background	ENSGALG00010020582	KANSL3	0.274970642	0.079456026	3.460664439	0.000538844	0.005252552	positive
PRJNA729271	ed_Lushi_vs_Arbor_Acr	breed_growth_background	ENSGALG00010020584	PARP9	0.119124799	0.154285013	0.772108689	0.440050056	0.597463991	positive
PRJNA729271	ed_Lushi_vs_Arbor_Acr	breed_growth_background	ENSGALG00010020586	C11orf16	-0.003644205	0.052402268	-0.069542885	0.944557498	0.969849184	negative
PRJNA729271	ed_Lushi_vs_Arbor_Acr	breed_growth_background	ENSGALG00010020588	PALD1	-0.129834686	0.058951345	-2.202404131	0.027636774	0.085688779	negative
PRJNA729271	ed_Lushi_vs_Arbor_Acr	breed_growth_background	ENSGALG00010020590	INTS7	0.027929365	0.043205542	0.646430156	0.518000816	0.666295724	positive
PRJNA729271	ed_Lushi_vs_Arbor_Acr	breed_growth_background	ENSGALG00010020594	PQLC2	-0.207566916	0.083155734	-2.496122717	0.012555916	0.049016428	negative
PRJNA729271	ed_Lushi_vs_Arbor_Acr	breed_growth_background	ENSGALG00010020595	ZFYVE27	0.018679663	0.070599552	0.264586144	0.791328295	0.870651015	positive
PRJNA729271	ed_Lushi_vs_Arbor_Acr	breed_growth_background	ENSGALG00010020596	SUMF1	0.087147906	0.065135577	1.337946333	0.180913932	0.325474425	positive
PRJNA729271	ed_Lushi_vs_Arbor_Acr	breed_growth_background	ENSGALG00010020597	ARID5A	0.201779987	0.127636179	1.580899624	0.11390099	0.235747917	positive
PRJNA729271	ed_Lushi_vs_Arbor_Acr	breed_growth_background	ENSGALG00010020599	PARN	-0.011584865	0.037454067	-0.3093086	0.75708679	0.846495913	negative
PRJNA729271	ed_Lushi_vs_Arbor_Acr	breed_growth_background	ENSGALG00010020601	AKIP1	-0.014879481	0.095397973	-0.155972718	0.876054526	0.926645502	negative
PRJNA729271	ed_Lushi_vs_Arbor_Acr	breed_growth_background	ENSGALG00010020602	DENND2B	-0.175209134	0.052851673	-3.315110449	0.000916069	0.007640059	negative
PRJNA729271	ed_Lushi_vs_Arbor_Acr	breed_growth_background	ENSGALG00010020603	VPS39	0.085375667	0.05026583	1.698483188	0.089416603	0.199315177	positive
PRJNA729271	ed_Lushi_vs_Arbor_Acr	breed_growth_background	ENSGALG00010020605	STAMBP	0.034030802	0.048474371	0.702037001	0.482656088	0.634921663	positive
PRJNA729271	ed_Lushi_vs_Arbor_Acr	breed_growth_background	ENSGALG00010020607	ENSGALG00010020607	0.523035593	0.127127932	4.114246041	3.88447E-05	0.000805513	positive
PRJNA729271	ed_Lushi_vs_Arbor_Acr	breed_growth_background	ENSGALG00010020608	CAPZB	0.043727939	0.053178781	0.822281709	0.410916585	0.571286773	positive
PRJNA729271	ed_Lushi_vs_Arbor_Acr	breed_growth_background	ENSGALG00010020609	AVPI1	0.150423219	0.12693635	1.185028707	0.236006097	0.390737679	positive
PRJNA729271	ed_Lushi_vs_Arbor_Acr	breed_growth_background	ENSGALG00010020611	LPGAT1	-0.070354747	0.053775916	-1.308294731	0.190773362	0.337473746	negative
PRJNA729271	ed_Lushi_vs_Arbor_Acr	breed_growth_background	ENSGALG00010020612	RPL27A	-0.308367609	0.103054195	-2.992285855	0.002768969	0.016737364	negative
PRJNA729271	ed_Lushi_vs_Arbor_Acr	breed_growth_background	ENSGALG00010020613	ACTG2	0.136204077	0.13560488	1.004418699	0.315176841	0.478375739	positive
PRJNA729271	ed_Lushi_vs_Arbor_Acr	breed_growth_background	ENSGALG00010020614	FAIM	0.074779066	0.060703507	1.2318739	0.217996193	0.370006418	positive
PRJNA729271	ed_Lushi_vs_Arbor_Acr	breed_growth_background	ENSGALG00010020616	PI4K2A	0.129524623	0.070114123	1.847339987	0.064697881	0.158927103	positive
PRJNA729271	ed_Lushi_vs_Arbor_Acr	breed_growth_background	ENSGALG00010020617	DGUOK	0.025911006	0.061158408	0.423670383	0.671806218	0.787780808	positive
PRJNA729271	ed_Lushi_vs_Arbor_Acr	breed_growth_background	ENSGALG00010020618	ENSGALG00010020618	-0.096050036	0.178241444	-0.538875997	0.58997242	0.725330808	negative
PRJNA729271	ed_Lushi_vs_Arbor_Acr	breed_growth_background	ENSGALG00010020619	QARS	0.000475882	0.053738771	0.008855474	0.992934447	0.995957706	positive
PRJNA729271	ed_Lushi_vs_Arbor_Acr	breed_growth_background	ENSGALG00010020620	MARF1	0.053605761	0.05545836	0.966594764	0.333746653	0.498059534	positive
PRJNA729271	ed_Lushi_vs_Arbor_Acr	breed_growth_background	ENSGALG00010020622	ABCG2	-0.149943751	0.062257167	-2.408457677	0.016020084	0.058376754	negative
PRJNA729271	ed_Lushi_vs_Arbor_Acr	breed_growth_background	ENSGALG00010020623	NEK2	-0.395890349	0.121778745	-3.250898594	0.001150409	0.009030281	negative
PRJNA729271	ed_Lushi_vs_Arbor_Acr	breed_growth_background	ENSGALG00010020624	MINOS1	-0.137412965	0.072608829	-1.892510427	0.058423006	0.147910808	negative
PRJNA729271	ed_Lushi_vs_Arbor_Acr	breed_growth_background	ENSGALG00010020625	SLC30A1	-0.185174919	0.041445674	-4.467894992	7.89931E-06	0.000242586	negative
PRJNA729271	ed_Lushi_vs_Arbor_Acr	breed_growth_background	ENSGALG00010020626	MORN4	-0.019428863	0.064987828	-0.298961572	0.764969366	0.852481815	negative
PRJNA729271	ed_Lushi_vs_Arbor_Acr	breed_growth_background	ENSGALG00010020629	TRIM66	0.002472706	0.141618907	0.017460278	0.986069421	0.991817102	positive
PRJNA729271	ed_Lushi_vs_Arbor_Acr	breed_growth_background	ENSGALG00010020630	QRICH1	0.258737996	0.06765862	3.824168969	0.000131214	0.001942743	positive
PRJNA729271	ed_Lushi_vs_Arbor_Acr	breed_growth_background	ENSGALG00010020632	BLOC1S2	0.01229558	0.061596616	0.199614533	0.841782061	0.904360433	positive
PRJNA729271	ed_Lushi_vs_Arbor_Acr	breed_growth_background	ENSGALG00010020633	HOGA1	-0.135534186	0.200000771	-0.677668319	0.497982018	0.649415146	negative
PRJNA729271	ed_Lushi_vs_Arbor_Acr	breed_growth_background	ENSGALG00010020635	NDE1	-0.31339443	0.072430344	-4.326838885	1.51265E-05	0.000393641	negative
PRJNA729271	ed_Lushi_vs_Arbor_Acr	breed_growth_background	ENSGALG00010020636	NBL1	-0.346702066	0.09557285	-3.627620864	0.000286045	0.003362709	negative
PRJNA729271	ed_Lushi_vs_Arbor_Acr	breed_growth_background	ENSGALG00010020638	IMPDH2	0.09054221	0.05969796	1.516671756	0.129349579	0.25683865	positive
PRJNA729271	ed_Lushi_vs_Arbor_Acr	breed_growth_background	ENSGALG00010020640	PTPN14	0.030859162	0.050006187	0.61710689	0.537164224	0.6823412	positive
PRJNA729271	ed_Lushi_vs_Arbor_Acr	breed_growth_background	ENSGALG00010020642	ANKRD2	0.077371497	0.335338221	0.230726747	0.817527093	0.887445159	positive
PRJNA729271	ed_Lushi_vs_Arbor_Acr	breed_growth_background	ENSGALG00010020646	CEP70	0.001240627	0.06309442	0.019663024	0.984312188	0.990707268	positive
PRJNA729271	ed_Lushi_vs_Arbor_Acr	breed_growth_background	ENSGALG00010020648	TET3	0.098784834	0.081336938	1.214513806	0.224551581	0.377333082	positive
PRJNA729271	ed_Lushi_vs_Arbor_Acr	breed_growth_background	ENSGALG00010020649	MYH11	0.014318326	0.112575484	0.127188667	0.898791075	0.941395811	positive
PRJNA729271	ed_Lushi_vs_Arbor_Acr	breed_growth_background	ENSGALG00010020651	LMO1	-0.538212645	0.102663561	-5.242489546	1.58424E-07	1.2346E-05	negative
PRJNA729271	ed_Lushi_vs_Arbor_Acr	breed_growth_background	ENSGALG00010020653	NDUFAF3	0.019633563	0.062831308	0.312480567	0.754675329	0.844907616	positive
PRJNA729271	ed_Lushi_vs_Arbor_Acr	breed_growth_background	ENSGALG00010020654	SAYSD1	-0.160813694	0.080732029	-1.991944161	0.046377185	0.125511071	negative
PRJNA729271	ed_Lushi_vs_Arbor_Acr	breed_growth_background	ENSGALG00010020655	RIC3	-0.04597566	0.076627945	-0.599985551	0.548515865	0.69190997	negative
PRJNA729271	ed_Lushi_vs_Arbor_Acr	breed_growth_background	ENSGALG00010020657	DALRD3	-0.003636707	0.050334045	-0.072251438	0.942401811	0.968871143	negative
PRJNA729271	ed_Lushi_vs_Arbor_Acr	breed_growth_background	ENSGALG00010020659	RAD51B	-0.252165637	0.076371138	-3.301844711	0.000960512	0.007919582	negative
PRJNA729271	ed_Lushi_vs_Arbor_Acr	breed_growth_background	ENSGALG00010020662	BFAR	-0.019341102	0.047572475	-0.40656077	0.684330615	0.796201065	negative
PRJNA729271	ed_Lushi_vs_Arbor_Acr	breed_growth_background	ENSGALG00010020664	ESYT3	-0.122377067	0.23599889	-0.518549333	0.604075049	0.736051433	negative
PRJNA729271	ed_Lushi_vs_Arbor_Acr	breed_growth_background	ENSGALG00010020666	TMCO4	0.09180327	0.111956492	0.81999059	0.412221472	0.572496908	positive
PRJNA729271	ed_Lushi_vs_Arbor_Acr	breed_growth_background	ENSGALG00010020667	UBTD1	0.101491574	0.087315664	1.162352427	0.245092339	0.400926922	positive
PRJNA729271	ed_Lushi_vs_Arbor_Acr	breed_growth_background	ENSGALG00010020670	ENSGALG00010020670	0.026666554	0.072829859	0.366148639	0.714254165	0.81651458	positive
PRJNA729271	ed_Lushi_vs_Arbor_Acr	breed_growth_background	ENSGALG00010020673	MMS19	0.141998518	0.069172173	2.052827208	0.040089339	0.112484507	positive
PRJNA729271	ed_Lushi_vs_Arbor_Acr	breed_growth_background	ENSGALG00010020674	TRPV4	0.317370554	0.401056277	0.79133671	0.428747532	0.587733637	positive
PRJNA729271	ed_Lushi_vs_Arbor_Acr	breed_growth_background	ENSGALG00010020675	SMYD2	-0.60331764	0.098373258	-6.13294355	8.62678E-10	1.86635E-07	negative
PRJNA729271	ed_Lushi_vs_Arbor_Acr	breed_growth_background	ENSGALG00010020676	CYP2C23a	-1.518387045	0.63149271	-2.404441128	0.016197215	0.058812282	negative
PRJNA729271	ed_Lushi_vs_Arbor_Acr	breed_growth_background	ENSGALG00010020677	CYR61	-0.128891148	0.125819957	-1.024409416	0.305641936	0.468279164	negative
PRJNA729271	ed_Lushi_vs_Arbor_Acr	breed_growth_background	ENSGALG00010020679	OTUD3	0.076268602	0.05918291	1.288692988	0.197504841	0.346066076	positive
PRJNA729271	ed_Lushi_vs_Arbor_Acr	breed_growth_background	ENSGALG00010020680	CFAP221	-0.059085183	0.315358898	-0.18735854	0.851379527	0.910572119	negative
PRJNA729271	ed_Lushi_vs_Arbor_Acr	breed_growth_background	ENSGALG00010020681	BCL10	0.047250585	0.047993751	0.984515363	0.324862181	0.488864643	positive
PRJNA729271	ed_Lushi_vs_Arbor_Acr	breed_growth_background	ENSGALG00010020682	ZDHHC16	0.062481539	0.090689403	0.68896185	0.490847276	0.642770915	positive
PRJNA729271	ed_Lushi_vs_Arbor_Acr	breed_growth_background	ENSGALG00010020684	P4HTM	-0.154934164	0.064729653	-2.393557777	0.016685853	0.060053606	negative
PRJNA729271	ed_Lushi_vs_Arbor_Acr	breed_growth_background	ENSGALG00010020685	RPL6	0.072692783	0.062039146	1.171724426	0.241307713	0.397066103	positive
PRJNA729271	ed_Lushi_vs_Arbor_Acr	breed_growth_background	ENSGALG00010020686	C1orf52	0.189468326	0.06747435	2.808005196	0.004984942	0.025530466	positive
PRJNA729271	ed_Lushi_vs_Arbor_Acr	breed_growth_background	ENSGALG00010020691	ENSGALG00010020691	0.193767337	0.087519581	2.213988396	0.026829577	0.083979254	positive
PRJNA729271	ed_Lushi_vs_Arbor_Acr	breed_growth_background	ENSGALG00010020692	TUB	-0.118240591	0.082854973	-1.42707898	0.153557131	0.289970116	negative
PRJNA729271	ed_Lushi_vs_Arbor_Acr	breed_growth_background	ENSGALG00010020693	PTGFR	-0.250589522	0.103008801	-2.432700126	0.014986707	0.055558138	negative
PRJNA729271	ed_Lushi_vs_Arbor_Acr	breed_growth_background	ENSGALG00010020696	MOB1A	-0.067063448	0.055145483	-1.216118605	0.223939744	0.376654281	negative
PRJNA729271	ed_Lushi_vs_Arbor_Acr	breed_growth_background	ENSGALG00010020699	PLA2G10	0.692997214	0.224301361	3.089580963	0.002004391	0.01327863	positive
PRJNA729271	ed_Lushi_vs_Arbor_Acr	breed_growth_background	ENSGALG00010020700	MTHFD2	0.252738088	0.069701897	3.625985812	0.000287861	0.003373118	positive
PRJNA729271	ed_Lushi_vs_Arbor_Acr	breed_growth_background	ENSGALG00010020701	PTPN11	0.025182665	0.045956343	0.547969291	0.583712989	0.719624132	positive
PRJNA729271	ed_Lushi_vs_Arbor_Acr	breed_growth_background	ENSGALG00010020704	PROX1	-0.07341677	0.129505439	-0.566901057	0.570781406	0.70925645	negative
PRJNA729271	ed_Lushi_vs_Arbor_Acr	breed_growth_background	ENSGALG00010020705	EXOSC1	0.219873059	0.093099574	2.361697804	0.018191464	0.06398575	positive
PRJNA729271	ed_Lushi_vs_Arbor_Acr	breed_growth_background	ENSGALG00010020707	SLC4A5	-0.2182719	0.265693766	-0.821516827	0.411351943	0.571543795	negative
PRJNA729271	ed_Lushi_vs_Arbor_Acr	breed_growth_background	ENSGALG00010020708	FAM214A	0.153946457	0.071981037	2.138708502	0.032459284	0.096624277	positive

PRJNA729271	ed_Lushi_vs_Arbor_Acr	breed_growth_background	ENSGALG00010020712	ZC3H7A	0.004307847	0.038597022	0.111610874	0.911131949	0.949705991	positive
PRJNA729271	ed_Lushi_vs_Arbor_Acr	breed_growth_background	ENSGALG00010020713	SCTR	2.09124427	0.678765463	3.080952678	0.002063394	0.013557979	positive
PRJNA729271	ed_Lushi_vs_Arbor_Acr	breed_growth_background	ENSGALG00010020714	PGAM1	0.115256972	0.077208574	1.49280016	0.135489505	0.265645933	positive
PRJNA729271	ed_Lushi_vs_Arbor_Acr	breed_growth_background	ENSGALG00010020715	ENSGALG00010020715	-0.38927586	0.455009052	-0.855534322	0.392255412	0.553745828	negative
PRJNA729271	ed_Lushi_vs_Arbor_Acr	breed_growth_background	ENSGALG00010020716	RRAS	0.258775263	0.096059025	2.693919326	0.007061725	0.032605234	positive
PRJNA729271	ed_Lushi_vs_Arbor_Acr	breed_growth_background	ENSGALG00010020719	RPH3A	0.033808655	0.354704137	0.095315085	0.924064561	0.957557608	positive
PRJNA729271	ed_Lushi_vs_Arbor_Acr	breed_growth_background	ENSGALG00010020720	ENSGALG00010020720	-0.526267575	0.208570298	-2.52321438	0.011628748	0.046527416	negative
PRJNA729271	ed_Lushi_vs_Arbor_Acr	breed_growth_background	ENSGALG00010020721	PTDSS2	-0.281371242	0.091077865	-3.089348247	0.002005962	0.01328082	negative
PRJNA729271	ed_Lushi_vs_Arbor_Acr	breed_growth_background	ENSGALG00010020723	RPS24	-0.096362263	0.101628814	-0.948178562	0.343038559	0.506916496	negative
PRJNA729271	ed_Lushi_vs_Arbor_Acr	breed_growth_background	ENSGALG00010020724	TACR1	-0.551740658	0.1532297	-3.600742279	0.00031731	0.003596097	negative
PRJNA729271	ed_Lushi_vs_Arbor_Acr	breed_growth_background	ENSGALG00010020725	FOPNL	0.080805085	0.060020219	1.346297728	0.178206518	0.322449575	positive
PRJNA729271	ed_Lushi_vs_Arbor_Acr	breed_growth_background	ENSGALG00010020728	TMEM37	-0.221684949	0.111095576	-1.995443535	0.045994527	0.124778339	negative
PRJNA729271	ed_Lushi_vs_Arbor_Acr	breed_growth_background	ENSGALG00010020730	RRP12	-0.381543962	0.247067019	-1.54429338	0.122517276	0.247294294	negative
PRJNA729271	ed_Lushi_vs_Arbor_Acr	breed_growth_background	ENSGALG00010020732	ARIH2	0.274910589	0.076777763	3.580601685	0.000342804	0.003799127	positive
PRJNA729271	ed_Lushi_vs_Arbor_Acr	breed_growth_background	ENSGALG00010020733	TMEM229B	-0.297729496	0.060616527	-4.911688446	9.02954E-07	4.4823E-05	negative
PRJNA729271	ed_Lushi_vs_Arbor_Acr	breed_growth_background	ENSGALG00010020736	LRRTM4	0.070573123	0.122729803	0.575028405	0.565272087	0.705070037	positive
PRJNA729271	ed_Lushi_vs_Arbor_Acr	breed_growth_background	ENSGALG00010020737	POLR3A	0.215589427	0.062674254	3.439840326	0.000582057	0.005501527	positive
PRJNA729271	ed_Lushi_vs_Arbor_Acr	breed_growth_background	ENSGALG00010020739	PKP3	0.482561822	0.42176928	1.144136959	0.252566842	0.409136833	positive
PRJNA729271	ed_Lushi_vs_Arbor_Acr	breed_growth_background	ENSGALG00010020742	THAP10	-0.106713657	0.11778037	-0.906039409	0.364914994	0.528310311	negative
PRJNA729271	ed_Lushi_vs_Arbor_Acr	breed_growth_background	ENSGALG00010020743	PURB	0.195818329	0.089906686	2.178017432	0.029404739	0.089617681	positive
PRJNA729271	ed_Lushi_vs_Arbor_Acr	breed_growth_background	ENSGALG00010020744	CHST3	-0.383586989	0.064544189	-5.943013574	2.79829E-09	4.94649E-07	negative
PRJNA729271	ed_Lushi_vs_Arbor_Acr	breed_growth_background	ENSGALG00010020745	ABCC1	-0.136626587	0.089239709	-1.531006648	0.12576775	0.252214102	negative
PRJNA729271	ed_Lushi_vs_Arbor_Acr	breed_growth_background	ENSGALG00010020747	DBI	-0.02595142	0.064846097	-0.400200188	0.689009076	0.798847109	negative
PRJNA729271	ed_Lushi_vs_Arbor_Acr	breed_growth_background	ENSGALG00010020750	AIFM2	0.116110693	0.157552456	0.7369653	0.461143452	0.616403019	positive
PRJNA729271	ed_Lushi_vs_Arbor_Acr	breed_growth_background	ENSGALG00010020753	SYDE2	0.082842276	0.050978804	1.625033724	0.104155373	0.221498259	positive
PRJNA729271	ed_Lushi_vs_Arbor_Acr	breed_growth_background	ENSGALG00010020754	SLC25A20	-0.213507304	0.061598785	-3.466096022	0.000528075	0.005164941	negative
PRJNA729271	ed_Lushi_vs_Arbor_Acr	breed_growth_background	ENSGALG00010020756	MACROH2A2	-0.249532711	0.090183704	-2.766937916	0.005658553	0.027802494	negative
PRJNA729271	ed_Lushi_vs_Arbor_Acr	breed_growth_background	ENSGALG00010020759	ARHGAP19	-0.160736929	0.094354621	-1.703540619	0.088466943	0.197646169	negative
PRJNA729271	ed_Lushi_vs_Arbor_Acr	breed_growth_background	ENSGALG00010020760	TXNDC11	-0.076540973	0.052340817	-1.462357244	0.14364334	0.276950014	negative
PRJNA729271	ed_Lushi_vs_Arbor_Acr	breed_growth_background	ENSGALG00010020762	TTBK2	-0.125334771	0.076945468	-1.628877875	0.103338875	0.2202142	negative
PRJNA729271	ed_Lushi_vs_Arbor_Acr	breed_growth_background	ENSGALG00010020764	PRKAR2A	-0.008203779	0.053344507	-0.153788644	0.877776388	0.92767337	negative
PRJNA729271	ed_Lushi_vs_Arbor_Acr	breed_growth_background	ENSGALG00010020765	GLT1D1	0.241434793	0.126289554	1.911755837	0.055907519	0.143549969	positive
PRJNA729271	ed_Lushi_vs_Arbor_Acr	breed_growth_background	ENSGALG00010020766	C2orf76	-0.418368228	0.065768761	-6.361199781	2.00184E-10	5.58013E-08	negative
PRJNA729271	ed_Lushi_vs_Arbor_Acr	breed_growth_background	ENSGALG00010020767	UBXN10	0.01546657	0.096459857	0.160342037	0.872611646	0.924665971	positive
PRJNA729271	ed_Lushi_vs_Arbor_Acr	breed_growth_background	ENSGALG00010020768	DNAH8	0.528841406	0.365122915	1.448392814	0.147507223	0.282387755	positive
PRJNA729271	ed_Lushi_vs_Arbor_Acr	breed_growth_background	ENSGALG00010020770	B4GALNT4	-0.215878531	0.110418723	-1.955089898	0.05057246	0.133495486	negative
PRJNA729271	ed_Lushi_vs_Arbor_Acr	breed_growth_background	ENSGALG00010020771	LINGO1	0.211073082	0.106543826	1.981091629	0.047580999	0.12776318	positive
PRJNA729271	ed_Lushi_vs_Arbor_Acr	breed_growth_background	ENSGALG00010020773	ZCCHC24	0.021131541	0.047810841	0.441982216	0.658502075	0.777849204	positive
PRJNA729271	ed_Lushi_vs_Arbor_Acr	breed_growth_background	ENSGALG00010020775	UBR1	-0.096570465	0.043419336	-2.224135015	0.026139362	0.082431303	negative
PRJNA729271	ed_Lushi_vs_Arbor_Acr	breed_growth_background	ENSGALG00010020776	MCOLN3	-0.494170583	0.248383714	-1.98954503	0.046641077	0.125943071	negative
PRJNA729271	ed_Lushi_vs_Arbor_Acr	breed_growth_background	ENSGALG00010020778	SLIT1	0.27956438	0.122393668	2.284140871	0.022363253	0.073982847	positive
PRJNA729271	ed_Lushi_vs_Arbor_Acr	breed_growth_background	ENSGALG00010020779	SLC15A4	-0.010451439	0.035857472	-0.291471715	0.770690574	0.857012648	negative
PRJNA729271	ed_Lushi_vs_Arbor_Acr	breed_growth_background	ENSGALG00010020781	SNN	0.059456551	0.074309249	0.80012315	0.423639449	0.582881236	positive
PRJNA729271	ed_Lushi_vs_Arbor_Acr	breed_growth_background	ENSGALG00010020782	PPIF	-0.12218837	0.084632679	-1.443749295	0.14880951	0.283740311	negative
PRJNA729271	ed_Lushi_vs_Arbor_Acr	breed_growth_background	ENSGALG00010020786	DDX19	0.186806119	0.070514326	2.649193858	0.008068404	0.03579783	positive
PRJNA729271	ed_Lushi_vs_Arbor_Acr	breed_growth_background	ENSGALG00010020787	TMEM132C	-0.427282918	0.167509378	-2.550799979	0.010747599	0.044069716	negative
PRJNA729271	ed_Lushi_vs_Arbor_Acr	breed_growth_background	ENSGALG00010020790	PRPH2L	0.582661029	0.168847843	3.450805295	0.000558917	0.005372345	positive
PRJNA729271	ed_Lushi_vs_Arbor_Acr	breed_growth_background	ENSGALG00010020794	STEAP3	0.328478209	0.073351018	4.478168393	7.52862E-06	0.000234179	positive
PRJNA729271	ed_Lushi_vs_Arbor_Acr	breed_growth_background	ENSGALG00010020797	DGCR8	-0.051890729	0.036147234	-1.435538033	0.151133839	0.286964238	negative
PRJNA729271	ed_Lushi_vs_Arbor_Acr	breed_growth_background	ENSGALG00010020798	C1QL2	0.502759757	0.325423735	1.544938808	0.122361065	0.247179582	positive
PRJNA729271	ed_Lushi_vs_Arbor_Acr	breed_growth_background	ENSGALG00010020803	ABCC6	-0.161027002	0.082762826	-1.945644071	0.051697509	0.135629936	negative
PRJNA729271	ed_Lushi_vs_Arbor_Acr	breed_growth_background	ENSGALG00010020806	HTD2	0.036189832	0.093052482	0.388918507	0.697336431	0.804953713	positive
PRJNA729271	ed_Lushi_vs_Arbor_Acr	breed_growth_background	ENSGALG00010020809	ENSGALG00010020809	0.263357617	0.118762029	2.217523728	0.026587323	0.083488573	positive
PRJNA729271	ed_Lushi_vs_Arbor_Acr	breed_growth_background	ENSGALG00010020810	IFITM5	-0.003017083	0.129697061	-0.023262536	0.981440855	0.989068549	negative
PRJNA729271	ed_Lushi_vs_Arbor_Acr	breed_growth_background	ENSGALG00010020811	MCOLN2	-0.076224162	0.367477662	-0.207425295	0.83567773	0.900204272	negative
PRJNA729271	ed_Lushi_vs_Arbor_Acr	breed_growth_background	ENSGALG00010020813	NPY4R	-0.520777089	0.294434606	-1.768736008	0.07693794	0.17917114	negative
PRJNA729271	ed_Lushi_vs_Arbor_Acr	breed_growth_background	ENSGALG00010020816	TRMT2A	0.039193984	0.048711254	0.804618669	0.421039766	0.580130361	positive
PRJNA729271	ed_Lushi_vs_Arbor_Acr	breed_growth_background	ENSGALG00010020818	TMEM62	0.045538043	0.106319418	0.428313506	0.668422887	0.785095069	positive
PRJNA729271	ed_Lushi_vs_Arbor_Acr	breed_growth_background	ENSGALG00010020820	VWA5B1	0.128378827	0.261800245	0.490369392	0.623872532	0.750459116	positive
PRJNA729271	ed_Lushi_vs_Arbor_Acr	breed_growth_background	ENSGALG00010020827	ENSGALG00010020827	0.024594658	0.049502579	0.496835878	0.619304792	0.747259049	positive
PRJNA729271	ed_Lushi_vs_Arbor_Acr	breed_growth_background	ENSGALG00010020828	CAMK2N1	0.0292187	0.122765215	0.238004712	0.81187744	0.884225974	positive
PRJNA729271	ed_Lushi_vs_Arbor_Acr	breed_growth_background	ENSGALG00010020831	MUL1	0.224289118	0.077405463	2.897587726	0.003760446	0.020741118	positive
PRJNA729271	ed_Lushi_vs_Arbor_Acr	breed_growth_background	ENSGALG00010020833	RANBP1	-0.071136431	0.045190049	-1.5741614	0.115450141	0.238044067	negative
PRJNA729271	ed_Lushi_vs_Arbor_Acr	breed_growth_background	ENSGALG00010020835	PTGR2	0.026556309	0.061846781	0.429388694	0.66764038	0.784742727	positive
PRJNA729271	ed_Lushi_vs_Arbor_Acr	breed_growth_background	ENSGALG00010020839	PGGHG	-0.284752312	0.08011654	-3.554226294	0.000379093	0.004094599	negative
PRJNA729271	ed_Lushi_vs_Arbor_Acr	breed_growth_background	ENSGALG00010020840	LPAR3	-0.025383434	0.152434841	-0.166519899	0.867747824	0.920858387	negative
PRJNA729271	ed_Lushi_vs_Arbor_Acr	breed_growth_background	ENSGALG00010020843	HMG20A	-0.175294868	0.045564505	-3.847180301	0.000119485	0.001821173	negative
PRJNA729271	ed_Lushi_vs_Arbor_Acr	breed_growth_background	ENSGALG00010020845	RAB35	0.05664138	0.050047686	1.131748233	0.257740297	0.415105068	positive
PRJNA729271	ed_Lushi_vs_Arbor_Acr	breed_growth_background	ENSGALG00010020847	MIDEAS	0.041947821	0.048537226	0.864240198	0.387455954	0.549474029	positive
PRJNA729271	ed_Lushi_vs_Arbor_Acr	breed_growth_background	ENSGALG00010020854	ENSGALG00010020854	0.197795594	0.128998599	1.533315828	0.125198057	0.251349838	positive
PRJNA729271	ed_Lushi_vs_Arbor_Acr	breed_growth_background	ENSGALG00010020855	DNAL1	-0.073312059	0.054224024	-1.352021731	0.176368364	0.320278054	negative
PRJNA729271	ed_Lushi_vs_Arbor_Acr	breed_growth_background	ENSGALG00010020856	VTG2	-0.151455546	0.426832097	-0.354836357	0.722712189	0.822187629	negative
PRJNA729271	ed_Lushi_vs_Arbor_Acr	breed_growth_background	ENSGALG00010020861	CDA	0.106608948	0.096664547	1.102875366	0.270081293	0.428844403	positive
PRJNA729271	ed_Lushi_vs_Arbor_Acr	breed_growth_background	ENSGALG00010020865	BICDL1	0.388129526	0.479890698	0.80878735	0.418637474	0.578555831	positive
PRJNA729271	ed_Lushi_vs_Arbor_Acr	breed_growth_background	ENSGALG00010020866	LCOR	0.001474395	0.053544634	0.027535805	0.978032382	0.987020774	positive
PRJNA729271	ed_Lushi_vs_Arbor_Acr	breed_growth_background	ENSGALG00010020870	RIOX1	0.282200381	0.07984304	3.534439349	0.000408641	0.004307821	positive
PRJNA729271	ed_Lushi_vs_Arbor_Acr	breed_growth_background	ENSGALG00010020873	PINK1	0.131830599	0.072884975	1.808748627	0.070490065	0.168578368	positive
PRJNA729271	ed_Lushi_vs_Arbor_Acr	breed_growth_background	ENSGALG00010020875	ENSGALG00010020875	0.164472986	0.092461492	1.778826865	0.075268151	0.176653474	positive
PRJNA729271	ed_Lushi_vs_Arbor_Acr	breed_growth_background	ENSGALG00010020878	XYLT1	-0.306593297	0.067434864	-4.546510201	5.45427E-06	0.00018386	negative
PRJNA729271	ed_Lushi_vs_Arbor_Acr	breed_growth_background	ENSGALG00010020880	PXK	0.06331907	0.088085856	0.718833569	0.472243471	0.626216185	positive
PRJNA729271	ed_Lushi_vs_Arbor_Acr	breed_growth_background	ENSGALG00010020886	GBF1	0.095286681	0.04640095	2.053550228	0.040019244	0.112374842	positive
PRJNA729271	ed_Lushi_vs_Arbor_Acr	breed_growth_background	ENSGALG00010020887	ARPP19	-0.091060452	0.038250145	-2.380656398	0.017281823	0.0615328	negative
PRJNA729271	ed_Lushi_vs_Arbor_Acr	breed_growth_background	ENSGALG00010020889	PPRC1	0.440940617	0.151539251	2.909745265	0.003617234	0.020119652	positive
PRJNA729271	ed_Lushi_vs_Arbor_Acr	breed_growth_background	ENSGALG00010020890	RPS15A	-0.176513872	0.100694106	-1.752971236	0.079606945	0.183555838	negative
PRJNA729271	ed_Lushi_vs_Arbor_Acr	breed_growth_background	ENSGALG00010020894	PSMD13	0.060202997	0.064660482	0.93106322	0.351820863	0.515792799	positive
PRJNA729271	ed_Lushi_vs_Arbor_Acr	breed_growth_background	ENSGALG00010020896	MARCO	0.466497077	0.207928254	2.243548279	0.02486148	0.079709612	positive
PRJNA729271	ed_Lushi_vs_Arbor_Acr	breed_growth_background	ENSGALG00010020898	ENSGALG00010020898	-0.210659802	0.069226881	-3.043034707	0.002342053	0.014938276	negative
PRJNA729271	ed_Lushi_vs_Arbor_Acr	breed_growth_background	ENSGALG00010020900	PDHB	-0.046599378	0.062128842	-0.750044213	0.453228077	0.609531814	negative
PRJNA729271	ed_Lushi_vs_Arbor_Acr	breed_growth_background	ENSGALG00010020903	AGMAT	-0.316357803	0.206619545	-1.531112673	0.125741548	0.252214102	negative
PRJNA729271	ed_Lushi_vs_Arbor_Acr	breed_growth_background	ENSGALG00010020905	KCTD6	0.094691983	0.048620864	1.947558633	0.051467798	0.135321191	positive
PRJNA729271	ed_Lushi_vs_Arbor_Acr	breed_growth_background	ENSGALG00010020906	SIRT3	0.085895681	0.058810761	1.460543621	0.144140728	0.277485119	positive
PRJNA729271	ed_Lushi_vs_Arbor_Acr	breed_growth_background	ENSGALG00010020912	PDE8A	-0.268883747	0.076686735	-3.506261479	0.000454449	0.004637535	negative
PRJNA729271	ed_Lushi_vs_Arbor_Acr	breed_growth_background	ENSGALG00010020913	TMEM132B	-0.039557897	0.145975331	-0.270990292	0.786398499	0.867374086	negative
PRJNA729271	ed_Lushi_vs_Arbor_Acr	breed_growth_background	ENSGALG00010020915	EN1	0.832681201	0.220543068	3.775594522	0.000159626	0.00223987	positive
PRJNA729271	ed_Lushi_vs_Arbor_Acr	breed_growth_background	ENSGALG00010020916	ARL6IP1	-0.136990618	0.052665935	-2.601123828	0.009291891	0.039730371	negative
PRJNA729271	ed_Lushi_vs_Arbor_Acr	breed_growth_background	ENSGALG00010020918	ENSGALG00010020918	-0.419545643	0.197675982	-2.122390585	0.033804952	0.09935174	negative
PRJNA729271	ed_Lushi_vs_Arbor_Acr	breed_growth_background	ENSGALG00010020920	ACOX2	-0.058246343	0.109563419	-0.531622173	0.594987704	0.728950415	negative
PRJNA729271	ed_Lushi_vs_Arbor_Acr	breed_growth_background	ENSGALG00010020921	LDB1	0.194091293	0.082406763	2.355283551	0.018508576	0.064755446	positive
PRJNA729271	ed_Lushi_vs_Arbor_Acr	breed_growth_background	ENSGALG00010020922	INSIG2	-0.10699215	0.04317104	-2.478331541	0.013199843	0.050805024	negative
PRJNA729271	ed_Lushi_vs_Arbor_Acr	breed_growth_background	ENSGALG00010020924	DNAJC16	0.125516607	0.064485436	1.946433412	0.051602699	0.135489897	positive
PRJNA729271	ed_Lushi_vs_Arbor_Acr	breed_growth_background	ENSGALG00010020925	RIC8A	0.044946721	0.055670288	0.807373598	0.419451274	0.579117679	positive
PRJNA729271	ed_Lushi_vs_Arbor_Acr	breed_growth_background	ENSGALG00010020927	ENSGALG00010020927	-0.249634253	0.063724526	-3.917396772	8.95104E-05	0.001476055	negative
PRJNA729271	ed_Lushi_vs_Arbor_Acr	breed_growth_background	ENSGALG00010020928	AACS	-0.172778517	0.081929905	-2.108857771	0.03495686	0.10178977	negative
PRJNA729271	ed_Lushi_vs_Arbor_Acr	breed_growth_background	ENSGALG00010020932	CASP9	-0.128214039	0.067160855	-1.909059052	0.056254474	0.144269038	negative
PRJNA729271	ed_Lushi_vs_Arbor_Acr	breed_growth_background	ENSGALG00010020934	ABCD3	0.085233207	0.082363172	1.034846102	0.300740789	0.463206645	positive
PRJNA729271	ed_Lushi_vs_Arbor_Acr	breed_growth_background	ENSGALG00010020935	BRI3BP	-0.132647867	0.035262547	-3.761721049	0.000168748	0.002314101	negative
PRJNA729271	ed_Lushi_vs_Arbor_Acr	breed_growth_background	ENSGALG00010020936	BET1L	0.178556688	0.06799973	2.625844065	0.00864344	0.037488531	positive
PRJNA729271	ed_Lushi_vs_Arbor_Acr	breed_growth_background	ENSGALG00010020938	SMG1	0.027084775	0.042898878	0.631363251	0.527803039	0.673637849	positive
PRJNA729271	ed_Lushi_vs_Arbor_Acr	breed_growth_background	ENSGALG00010020939	CTRC	-0.963396921	1.093646767	-0.880903185	0.378370223	0.540448994	negative
PRJNA729271	ed_Lushi_vs_Arbor_Acr	breed_growth_background	ENSGALG00010020940	ENSGALG00010020940	0.084954294	0.073607431	1.154153761	0.248437136	0.404526655	positive
PRJNA729271	ed_Lushi_vs_Arbor_Acr	breed_growth_background	ENSGALG00010020941	ENSGALG00010020941	0.053325891	0.092916007	0.573915009	0.566025316	0.705705914	positive
PRJNA729271	ed_Lushi_vs_Arbor_Acr	breed_growth_background	ENSGALG00010020943	FAM107A	-0.344210186	0.190809115	-1.803950438	0.071239077	0.169865179	negative
PRJNA729271	ed_Lushi_vs_Arbor_Acr	breed_growth_background	ENSGALG00010020944	MYO5A	-0.287516436	0.084680195	-3.395320904	0.000685482	0.006217811	negative
PRJNA729271	ed_Lushi_vs_Arbor_Acr	breed_growth_background	ENSGALG00010020945	NUMB	-0.067603441	0.046150486	-1.464847875	0.142962427	0.276077855	negative
PRJNA729271	ed_Lushi_vs_Arbor_Acr	breed_growth_background	ENSGALG00010020946	CTBS	-0.22712142	0.115458282	-1.967129733	0.049168256	0.131082191	negative
PRJNA729271	ed_Lushi_vs_Arbor_Acr	breed_growth_background	ENSGALG00010020949	HPS6	0.191989692	0.059394722	3.232436898	0.001227392	0.009493625	positive
PRJNA729271	ed_Lushi_vs_Arbor_Acr	breed_growth_background	ENSGALG00010020950	EFHD2	-0.051218438	0.151586294	-0.337883042	0.735451327	0.830503295	negative
PRJNA729271	ed_Lushi_vs_Arbor_Acr	breed_growth_background	ENSGALG00010020951	ENSGALG00010020951	0.054407387	0.162208076	0.335417251	0.737310357	0.83173013	positive
PRJNA729271	ed_Lushi_vs_Arbor_Acr	breed_growth_background	ENSGALG00010020952	GNG5	-0.012907917	0.053568142	-0.240962575	0.809584123	0.882761845	negative
PRJNA729271	ed_Lushi_vs_Arbor_Acr	breed_growth_background	ENSGALG00010020954	PLIN1	-1.08621755	0.668721069	-1.624320813	0.104307358	0.221658869	negative
PRJNA729271	ed_Lushi_vs_Arbor_Acr	breed_growth_background	ENSGALG00010020955	FHAD1	0.602800089	0.520818168	1.157409871	0.247104943	0.40312731	positive
PRJNA729271	ed_Lushi_vs_Arbor_Acr	breed_growth_background	ENSGALG00010020957	RPF1	-0.023829719	0.055259931	-0.431229618	0.66630142	0.783804503	negative
PRJNA729271	ed_Lushi_vs_Arbor_Acr	breed_growth_background	ENSGALG00010020959	ENSGALG00010020959	-0.153860517	0.199627318	-0.770738789	0.440861776	0.598341895	negative
PRJNA729271	ed_Lushi_vs_Arbor_Acr	breed_growth_background	ENSGALG00010020960	CLEC19A	0.626818011	0.194349845	3.225204579	0.001258827	0.00964923	positive
PRJNA729271	ed_Lushi_vs_Arbor_Acr	breed_growth_background	ENSGALG00010020962	MYO5C	-0.309297	0.203497296	-1.519907172	0.128534308	0.255780449	negative
PRJNA729271	ed_Lushi_vs_Arbor_Acr	breed_growth_background	ENSGALG00010020963	CCDC93	0.156172113	0.053165703	2.937459753	0.003309131	0.01903407	positive
PRJNA729271	ed_Lushi_vs_Arbor_Acr	breed_growth_background	ENSGALG00010020965	DHX37	-0.024660556	0.051445239	-0.479355455	0.631685778	0.757281064	negative
PRJNA729271	ed_Lushi_vs_Arbor_Acr	breed_growth_background	ENSGALG00010020966	PAPLN	0.264848146	0.13157311	2.012935207	0.044121446	0.120918957	positive
PRJNA729271	ed_Lushi_vs_Arbor_Acr	breed_growth_background	ENSGALG00010020967	VPS51	0.078643799	0.041470806	1.896365327	0.057911753	0.146993286	positive
PRJNA729271	ed_Lushi_vs_Arbor_Acr	breed_growth_background	ENSGALG00010020969	RBP3	-0.331482992	0.205015379	-1.616868912	0.10590658	0.224148976	negative
PRJNA729271	ed_Lushi_vs_Arbor_Acr	breed_growth_background	ENSGALG00010020970	ARMH3	0.165281618	0.042387753	3.899277696	9.64801E-05	0.00154914	positive

PRJNA729271	ed_Lushi_vs_Arbor_Acr	breed_growth_background	ENSGALG00010020971	TMEM51	−0.156415171	0.158798387	−0.984992189	0.324627908	0.488728866	negative
PRJNA729271	ed_Lushi_vs_Arbor_Acr	breed_growth_background	ENSGALG00010020973	GNB5	−0.37052844	0.089434804	−4.143000514	3.42791E-05	0.000736112	negative
PRJNA729271	ed_Lushi_vs_Arbor_Acr	breed_growth_background	ENSGALG00010020975	HCCS	−0.037122634	0.069836759	−0.531562965	0.595028721	0.728950415	negative
PRJNA729271	ed_Lushi_vs_Arbor_Acr	breed_growth_background	ENSGALG00010020976	GDF10	−0.115962015	0.173607078	−0.667956721	0.504161219	0.654597749	negative
PRJNA729271	ed_Lushi_vs_Arbor_Acr	breed_growth_background	ENSGALG00010020978	BCL2L10	−0.146553286	0.061501854	−2.382908438	0.017176466	0.061308267	negative
PRJNA729271	ed_Lushi_vs_Arbor_Acr	breed_growth_background	ENSGALG00010020979	ENSGALG00010020979	−0.091531182	0.131718943	−0.694897637	0.487119454	0.638986107	negative
PRJNA729271	ed_Lushi_vs_Arbor_Acr	breed_growth_background	ENSGALG00010020981	CIT	−0.454857346	0.127866729	−3.557276769	0.000374719	0.004065536	negative
PRJNA729271	ed_Lushi_vs_Arbor_Acr	breed_growth_background	ENSGALG00010020982	TLCD4	0.289026169	0.081511635	3.545827177	0.000391383	0.004183697	positive
PRJNA729271	ed_Lushi_vs_Arbor_Acr	breed_growth_background	ENSGALG00010020984	PSEN1	−0.059018381	0.032911095	−1.793267018	0.072930238	0.172824105	negative
PRJNA729271	ed_Lushi_vs_Arbor_Acr	breed_growth_background	ENSGALG00010020986	CFAP20DC	0.419979165	0.188659104	2.226127214	0.026005662	0.082178344	positive
PRJNA729271	ed_Lushi_vs_Arbor_Acr	breed_growth_background	ENSGALG00010020987	NADSYN1	−0.029143202	0.050551473	−0.576505503	0.564273551	0.704491398	negative
PRJNA729271	ed_Lushi_vs_Arbor_Acr	breed_growth_background	ENSGALG00010020989	MAPK4	0.126736725	0.081775921	1.549804926	0.121188344	0.245998297	positive
PRJNA729271	ed_Lushi_vs_Arbor_Acr	breed_growth_background	ENSGALG00010020990	UBB	−0.052341081	0.057160851	−0.915680584	0.359834452	0.52335946	negative
PRJNA729271	ed_Lushi_vs_Arbor_Acr	breed_growth_background	ENSGALG00010020991	ALG14	−0.109072886	0.074963444	−1.455014342	0.145665291	0.279732167	negative
PRJNA729271	ed_Lushi_vs_Arbor_Acr	breed_growth_background	ENSGALG00010020992	KCNH7	0.734845254	0.239420712	3.0692635	0.002145872	0.013951884	positive
PRJNA729271	ed_Lushi_vs_Arbor_Acr	breed_growth_background	ENSGALG00010020994	DHCR7	−0.207105085	0.079404522	−2.608227857	0.009101235	0.039041847	negative
PRJNA729271	ed_Lushi_vs_Arbor_Acr	breed_growth_background	ENSGALG00010020998	SYT17	−0.277387452	0.159269232	−1.741626103	0.081573891	0.186535208	negative
PRJNA729271	ed_Lushi_vs_Arbor_Acr	breed_growth_background	ENSGALG00010020999	IFIH1	0.054694544	0.180551744	0.302930025	0.761943189	0.850286129	positive
PRJNA729271	ed_Lushi_vs_Arbor_Acr	breed_growth_background	ENSGALG00010021000	NFKB2	0.163579197	0.061247238	2.67080122	0.007567044	0.034233554	positive
PRJNA729271	ed_Lushi_vs_Arbor_Acr	breed_growth_background	ENSGALG00010021002	SLC44A3	−0.325697276	0.087083704	−3.740048503	0.000183985	0.00243833	negative
PRJNA729271	ed_Lushi_vs_Arbor_Acr	breed_growth_background	ENSGALG00010021003	KAZN	−0.042851616	0.066052592	−0.648749954	0.51650001	0.664890555	negative
PRJNA729271	ed_Lushi_vs_Arbor_Acr	breed_growth_background	ENSGALG00010021004	PSD	0.125685632	0.091456508	1.374266694	0.169358902	0.311451064	positive
PRJNA729271	ed_Lushi_vs_Arbor_Acr	breed_growth_background	ENSGALG00010021010	LEO1	0.112470172	0.061005477	1.843607785	0.065240321	0.16000989	positive
PRJNA729271	ed_Lushi_vs_Arbor_Acr	breed_growth_background	ENSGALG00010021011	SCARB1	−0.148610973	0.058435338	−2.543169559	0.010985191	0.044740191	negative
PRJNA729271	ed_Lushi_vs_Arbor_Acr	breed_growth_background	ENSGALG00010021012	KCNIP2	−0.297853659	0.456726638	−0.652148647	0.514305284	0.662924602	negative
PRJNA729271	ed_Lushi_vs_Arbor_Acr	breed_growth_background	ENSGALG00010021013	FAP	−0.441389853	0.158628157	−2.782544161	0.005393452	0.026893049	negative
PRJNA729271	ed_Lushi_vs_Arbor_Acr	breed_growth_background	ENSGALG00010021014	FBXL15	0.042475903	0.049244503	0.862551149	0.388384299	0.550413612	positive
PRJNA729271	ed_Lushi_vs_Arbor_Acr	breed_growth_background	ENSGALG00010021015	RBM25	0.181858519	0.072692186	2.501761605	0.012357711	0.048541849	positive
PRJNA729271	ed_Lushi_vs_Arbor_Acr	breed_growth_background	ENSGALG00010021016	FHIT	−0.342021229	0.11931043	−2.866649873	0.004148417	0.022188672	negative
PRJNA729271	ed_Lushi_vs_Arbor_Acr	breed_growth_background	ENSGALG00010021017	COQ7	−0.000825799	0.084106964	−0.009818433	0.99216615	0.99553152	negative
PRJNA729271	ed_Lushi_vs_Arbor_Acr	breed_growth_background	ENSGALG00010021018	F3	−0.348201386	0.119585546	−2.91173471	0.003594278	0.020033095	negative
PRJNA729271	ed_Lushi_vs_Arbor_Acr	breed_growth_background	ENSGALG00010021019	VSIG10	−0.308158718	0.052208886	−5.902418913	3.5821E-09	5.91627E-07	negative
PRJNA729271	ed_Lushi_vs_Arbor_Acr	breed_growth_background	ENSGALG00010021020	ZDHHC13	−0.226690899	0.064365125	−3.521952284	0.000428381	0.004454366	negative
PRJNA729271	ed_Lushi_vs_Arbor_Acr	breed_growth_background	ENSGALG00010021021	GCG	−0.263820094	0.18940511	−1.392887942	0.163653679	0.303461696	negative
PRJNA729271	ed_Lushi_vs_Arbor_Acr	breed_growth_background	ENSGALG00010021022	ZFYVE1	0.135863726	0.041753929	3.25391474	0.001138264	0.008978147	positive
PRJNA729271	ed_Lushi_vs_Arbor_Acr	breed_growth_background	ENSGALG00010021023	MGEA5	0.031234685	0.052050687	0.600082096	0.548451524	0.69188902	positive
PRJNA729271	ed_Lushi_vs_Arbor_Acr	breed_growth_background	ENSGALG00010021024	ENSGALG00010021024	0.369309672	0.101060937	3.65432661	0.000257858	0.00312774	positive
PRJNA729271	ed_Lushi_vs_Arbor_Acr	breed_growth_background	ENSGALG00010021025	CUEDC2	−0.040909094	0.062537378	−0.654154285	0.51301241	0.661634922	negative
PRJNA729271	ed_Lushi_vs_Arbor_Acr	breed_growth_background	ENSGALG00010021026	ADGRA2	0.036507905	0.072429138	0.504049974	0.61422626	0.74359916	positive
PRJNA729271	ed_Lushi_vs_Arbor_Acr	breed_growth_background	ENSGALG00010021027	PLA2G4A	−0.276427467	0.089094783	−3.102622381	0.001918142	0.012883907	negative
PRJNA729271	ed_Lushi_vs_Arbor_Acr	breed_growth_background	ENSGALG00010021028	ENSGALG00010021028	−0.442758126	0.173491369	−2.552046992	0.010709207	0.043962039	negative
PRJNA729271	ed_Lushi_vs_Arbor_Acr	breed_growth_background	ENSGALG00010021029	PRDM2	−0.007305792	0.040834022	−0.178914347	0.858004957	0.914805541	negative
PRJNA729271	ed_Lushi_vs_Arbor_Acr	breed_growth_background	ENSGALG00010021030	CSRP3	−0.081417835	0.292694206	−0.278166884	0.780884255	0.864369399	negative
PRJNA729271	ed_Lushi_vs_Arbor_Acr	breed_growth_background	ENSGALG00010021031	DDX54	0.131001556	0.059466199	2.202958312	0.027597687	0.085640864	positive
PRJNA729271	ed_Lushi_vs_Arbor_Acr	breed_growth_background	ENSGALG00010021032	TMC7	−0.171085636	0.064783831	−2.640869391	0.008269359	0.036410802	negative
PRJNA729271	ed_Lushi_vs_Arbor_Acr	breed_growth_background	ENSGALG00010021033	RNF122	−0.390879681	0.12568603	−3.10996919	0.001871069	0.012655688	negative
PRJNA729271	ed_Lushi_vs_Arbor_Acr	breed_growth_background	ENSGALG00010021034	NPM3	−0.358614899	0.15039525	−2.384482881	0.017103144	0.061182149	negative
PRJNA729271	ed_Lushi_vs_Arbor_Acr	breed_growth_background	ENSGALG00010021036	E2F8	−0.326592696	0.109071157	−2.994308529	0.002750676	0.016640674	negative
PRJNA729271	ed_Lushi_vs_Arbor_Acr	breed_growth_background	ENSGALG00010021038	PTGS2	−0.404236642	0.161751862	−2.499115845	0.01245036	0.048814708	negative
PRJNA729271	ed_Lushi_vs_Arbor_Acr	breed_growth_background	ENSGALG00010021040	FGF8	0.303898457	0.334110792	0.909573903	0.363047267	0.526268217	positive
PRJNA729271	ed_Lushi_vs_Arbor_Acr	breed_growth_background	ENSGALG00010021041	GLTP	0.143622882	0.10579793	1.357520723	0.174615813	0.318451965	positive
PRJNA729271	ed_Lushi_vs_Arbor_Acr	breed_growth_background	ENSGALG00010021042	LRRC38	−0.41958893	0.05185278	−8.091927303	5.8728E-16	7.73874E-13	negative
PRJNA729271	ed_Lushi_vs_Arbor_Acr	breed_growth_background	ENSGALG00010021043	PTPRG	−0.241144201	0.048614437	−4.960341299	7.03694E-07	3.65593E-05	negative
PRJNA729271	ed_Lushi_vs_Arbor_Acr	breed_growth_background	ENSGALG00010021044	EIF4G2	0.040419813	0.036389383	1.110758422	0.266672347	0.424957748	positive
PRJNA729271	ed_Lushi_vs_Arbor_Acr	breed_growth_background	ENSGALG00010021045	DPP4	−0.005325927	0.138686193	−0.038402717	0.969366594	0.982470096	negative
PRJNA729271	ed_Lushi_vs_Arbor_Acr	breed_growth_background	ENSGALG00010021046	ENSGALG00010021046	−0.19676573	0.135754711	−1.449421003	0.147220047	0.281933489	negative
PRJNA729271	ed_Lushi_vs_Arbor_Acr	breed_growth_background	ENSGALG00010021047	TMC5	−0.115258462	0.286566667	−0.402204705	0.68753338	0.798120441	negative
PRJNA729271	ed_Lushi_vs_Arbor_Acr	breed_growth_background	ENSGALG00010021048	AP15	0.059538034	0.040665105	1.464106228	0.143164926	0.276295615	positive
PRJNA729271	ed_Lushi_vs_Arbor_Acr	breed_growth_background	ENSGALG00010021050	CTR9	0.024699483	0.050908814	0.485171061	0.627555008	0.753825296	positive
PRJNA729271	ed_Lushi_vs_Arbor_Acr	breed_growth_background	ENSGALG00010021051	GDE1	0.146393609	0.073090243	2.002915882	0.045186318	0.123162031	positive
PRJNA729271	ed_Lushi_vs_Arbor_Acr	breed_growth_background	ENSGALG00010021052	CEP15	−0.349931407	0.082966871	−4.217724531	2.4678E-05	0.000562193	negative
PRJNA729271	ed_Lushi_vs_Arbor_Acr	breed_growth_background	ENSGALG00010021053	ENSGALG00010021053	0.246129756	0.241102241	1.020852211	0.307324464	0.469852136	positive
PRJNA729271	ed_Lushi_vs_Arbor_Acr	breed_growth_background	ENSGALG00010021055	IRAG1	−0.280123068	0.120157248	−2.331303951	0.019737338	0.067602248	negative
PRJNA729271	ed_Lushi_vs_Arbor_Acr	breed_growth_background	ENSGALG00010021057	ZDHHC8	−0.118340855	0.043348884	−2.729963111	0.006334141	0.030142278	negative
PRJNA729271	ed_Lushi_vs_Arbor_Acr	breed_growth_background	ENSGALG00010021058	CCP110	0.132107741	0.051420003	2.569189673	0.010193665	0.042337298	positive
PRJNA729271	ed_Lushi_vs_Arbor_Acr	breed_growth_background	ENSGALG00010021059	ENSGALG00010021059	0.256150994	0.731663316	0.350094078	0.726268095	0.824474505	positive
PRJNA729271	ed_Lushi_vs_Arbor_Acr	breed_growth_background	ENSGALG00010021062	MYO9A	0.079319722	0.054674074	1.45077395	0.146842816	0.281396961	positive
PRJNA729271	ed_Lushi_vs_Arbor_Acr	breed_growth_background	ENSGALG00010021064	LYVE1	0.201530347	0.155169693	1.298773901	0.194021534	0.341427963	positive
PRJNA729271	ed_Lushi_vs_Arbor_Acr	breed_growth_background	ENSGALG00010021065	CNNM2	0.002239585	0.061527412	0.03639979	0.970963581	0.983017828	positive
PRJNA729271	ed_Lushi_vs_Arbor_Acr	breed_growth_background	ENSGALG00010021067	SLC4A10	0.07076319	0.137014138	0.516466342	0.605528741	0.73704479	positive
PRJNA729271	ed_Lushi_vs_Arbor_Acr	breed_growth_background	ENSGALG00010021069	PGAM5	0.031414427	0.038361095	0.818913738	0.412835631	0.573075318	positive
PRJNA729271	ed_Lushi_vs_Arbor_Acr	breed_growth_background	ENSGALG00010021070	RNF141	−0.027917431	0.046145125	−0.604991992	0.545184318	0.689081199	negative
PRJNA729271	ed_Lushi_vs_Arbor_Acr	breed_growth_background	ENSGALG00010021071	HIF1AN	0.040534473	0.051248435	0.790940695	0.428978599	0.58782802	positive
PRJNA729271	ed_Lushi_vs_Arbor_Acr	breed_growth_background	ENSGALG00010021072	ENSGALG00010021072	0.053023116	0.048116031	1.101984404	0.270468452	0.429117766	positive
PRJNA729271	ed_Lushi_vs_Arbor_Acr	breed_growth_background	ENSGALG00010021073	NDUFB8	0.065522623	0.057611131	1.137325765	0.255402128	0.412318257	positive
PRJNA729271	ed_Lushi_vs_Arbor_Acr	breed_growth_background	ENSGALG00010021074	TTC17	−0.086037638	0.037230377	−2.310952669	0.020835469	0.07015334	negative
PRJNA729271	ed_Lushi_vs_Arbor_Acr	breed_growth_background	ENSGALG00010021075	DHRS3	−0.133543321	0.072076456	−1.852800879	0.063910904	0.157683158	negative
PRJNA729271	ed_Lushi_vs_Arbor_Acr	breed_growth_background	ENSGALG00010021077	SEC31B	−0.062054452	0.04275886	−1.451265341	0.146705988	0.281209112	negative
PRJNA729271	ed_Lushi_vs_Arbor_Acr	breed_growth_background	ENSGALG00010021078	ENSGALG00010021078	0.042665399	0.13082052	0.3261369	0.74432078	0.836999977	positive
PRJNA729271	ed_Lushi_vs_Arbor_Acr	breed_growth_background	ENSGALG00010021080	TTLL7	0.269362363	0.092243562	2.920120997	0.003498955	0.019734379	positive
PRJNA729271	ed_Lushi_vs_Arbor_Acr	breed_growth_background	ENSGALG00010021081	KCNH6	−0.009436058	0.115375924	−0.081785332	0.93481742	0.964451953	negative
PRJNA729271	ed_Lushi_vs_Arbor_Acr	breed_growth_background	ENSGALG00010021082	SENP8	0.219442873	0.08814762	2.489492896	0.012792547	0.04969927	positive
PRJNA729271	ed_Lushi_vs_Arbor_Acr	breed_growth_background	ENSGALG00010021083	SCD	−1.03377297	0.303971091	−3.400892388			negative
PRJNA729271	ed_Lushi_vs_Arbor_Acr	breed_growth_background	ENSGALG00010021084	ENSGALG00010021084	−0.459715167	0.39340612	−1.168551131	0.242584527	0.39828282	negative
PRJNA729271	ed_Lushi_vs_Arbor_Acr	breed_growth_background	ENSGALG00010021085	AMPD3	−0.100655546	0.09823149	−1.024676972	0.305515632	0.468222573	negative
PRJNA729271	ed_Lushi_vs_Arbor_Acr	breed_growth_background	ENSGALG00010021086	PSMD14	0.068832012	0.058809764	1.170418089	0.241832759	0.397568997	positive
PRJNA729271	ed_Lushi_vs_Arbor_Acr	breed_growth_background	ENSGALG00010021087	THBS1	−0.482850133	0.190781139	−2.530911265	0.011376663	0.045806868	negative
PRJNA729271	ed_Lushi_vs_Arbor_Acr	breed_growth_background	ENSGALG00010021088	ANKLE2	0.146173162	0.047053983	3.106499258	0.001893168	0.012763138	positive
PRJNA729271	ed_Lushi_vs_Arbor_Acr	breed_growth_background	ENSGALG00010021090	ADM	0.320980889	0.130377772	2.461929547	0.013819181	0.052560752	positive
PRJNA729271	ed_Lushi_vs_Arbor_Acr	breed_growth_background	ENSGALG00010021091	TANK	0.024599046	0.044457605	0.553314687	0.580047945	0.716838176	positive
PRJNA729271	ed_Lushi_vs_Arbor_Acr	breed_growth_background	ENSGALG00010021092	VPS13D	0.135677717	0.050661459	2.678124923	0.00740356	0.033714924	positive
PRJNA729271	ed_Lushi_vs_Arbor_Acr	breed_growth_background	ENSGALG00010021093	GRAMD2	0.363962377	0.118244207	3.078056729	0.002083552	0.013650792	positive
PRJNA729271	ed_Lushi_vs_Arbor_Acr	breed_growth_background	ENSGALG00010021094	ENSGALG00010021094	0.055829358	0.166060815	0.336198269	0.736721358	0.83142137	positive
PRJNA729271	ed_Lushi_vs_Arbor_Acr	breed_growth_background	ENSGALG00010021095	NT5C2	−0.093995503	0.056483847	−1.664112963	0.096089857	0.208975615	negative
PRJNA729271	ed_Lushi_vs_Arbor_Acr	breed_growth_background	ENSGALG00010021096	ZFAND4	−0.034944998	0.057108814	−0.611902016	0.540602588	0.685447386	negative
PRJNA729271	ed_Lushi_vs_Arbor_Acr	breed_growth_background	ENSGALG00010021097	KATNBL1	−0.092168345	0.069514494	−1.325886735	0.184877217	0.33025292	negative
PRJNA729271	ed_Lushi_vs_Arbor_Acr	breed_growth_background	ENSGALG00010021098	SDK1	−0.332059561	0.076387235	−4.347055661	1.37977E-05	0.000366297	negative
PRJNA729271	ed_Lushi_vs_Arbor_Acr	breed_growth_background	ENSGALG00010021099	HMCN1	−0.451377684	0.141613909	−3.187382428	0.001435668	0.010596237	negative
PRJNA729271	ed_Lushi_vs_Arbor_Acr	breed_growth_background	ENSGALG00010021100	EMC7	−0.119620624	0.045851904	−2.60884747	0.009084773	0.038994308	negative
PRJNA729271	ed_Lushi_vs_Arbor_Acr	breed_growth_background	ENSGALG00010021101	TMEM240	−0.016290369	0.20087417	−0.081097379	0.93536451	0.964536447	negative
PRJNA729271	ed_Lushi_vs_Arbor_Acr	breed_growth_background	ENSGALG00010021102	GOLGA3	0.158811236	0.060121924	2.641486269	0.008254315	0.036371003	positive
PRJNA729271	ed_Lushi_vs_Arbor_Acr	breed_growth_background	ENSGALG00010021103	RBMS1	−0.140244752	0.039858606	−3.518556375	0.000433902	0.004482825	negative
PRJNA729271	ed_Lushi_vs_Arbor_Acr	breed_growth_background	ENSGALG00010021104	INA	−0.295369948	0.165357424	−1.786251514	0.074058544	0.174805177	negative
PRJNA729271	ed_Lushi_vs_Arbor_Acr	breed_growth_background	ENSGALG00010021105	ENSGALG00010021105	−0.01047994	0.211425928	−0.049567906	0.960466723	0.977940794	negative
PRJNA729271	ed_Lushi_vs_Arbor_Acr	breed_growth_background	ENSGALG00010021106	ITGB6	0.236600814	0.114030983	2.074881815	0.03799749	0.108505439	positive
PRJNA729271	ed_Lushi_vs_Arbor_Acr	breed_growth_background	ENSGALG00010021108	PCGF6	−0.181997704	0.057241408	−3.179476385	0.001475414	0.010806531	negative
PRJNA729271	ed_Lushi_vs_Arbor_Acr	breed_growth_background	ENSGALG00010021110	PKLR	0.252075884	0.090224457	2.793875326	0.005208055	0.026275935	positive
PRJNA729271	ed_Lushi_vs_Arbor_Acr	breed_growth_background	ENSGALG00010021111	AVEN	−0.09022163	0.042782384	−2.108849953	0.034957535	0.10178977	negative
PRJNA729271	ed_Lushi_vs_Arbor_Acr	breed_growth_background	ENSGALG00010021112	TAF5	−0.094656998	0.039483166	−2.397401442	0.016511822	0.059655749	negative
PRJNA729271	ed_Lushi_vs_Arbor_Acr	breed_growth_background	ENSGALG00010021113	SYP	0.707319493	0.166347563	4.252058033	2.11815E-05	0.000500323	positive
PRJNA729271	ed_Lushi_vs_Arbor_Acr	breed_growth_background	ENSGALG00010021114	SBF2	−0.12933141	0.052939485	−2.443004699	0.014565548	0.054274451	negative
PRJNA729271	ed_Lushi_vs_Arbor_Acr	breed_growth_background	ENSGALG00010021115	ALPL	−0.018526575	0.132899031	−0.139403383	0.889131399	0.934994169	negative
PRJNA729271	ed_Lushi_vs_Arbor_Acr	breed_growth_background	ENSGALG00010021116	FAM21C	0.036529605	0.03448511	1.059286337	0.289469396	0.450586221	positive
PRJNA729271	ed_Lushi_vs_Arbor_Acr	breed_growth_background	ENSGALG00010021118	USMG5	−0.140919382	0.096117292	−1.466118948	0.142615885	0.275628966	negative
PRJNA729271	ed_Lushi_vs_Arbor_Acr	breed_growth_background	ENSGALG00010021120	PDCD11	−0.077114802	0.047796547	−1.613396929	0.1066583	0.225333342	negative
PRJNA729271	ed_Lushi_vs_Arbor_Acr	breed_growth_background	ENSGALG00010021121	PLA2R1	0.080164029	0.048838339	1.641415958	0.1007111	0.216203701	positive
PRJNA729271	ed_Lushi_vs_Arbor_Acr	breed_growth_background	ENSGALG00010021122	CYP1A1	−0.692647461	0.503100124	−1.376758676	0.168586871	0.310306882	negative
PRJNA729271	ed_Lushi_vs_Arbor_Acr	breed_growth_background	ENSGALG00010021123	RYR3	−0.162162393	0.11509595	−1.408932231	0.158855208	0.297315498	negative
PRJNA729271	ed_Lushi_vs_Arbor_Acr	breed_growth_background	ENSGALG00010021124	CALHM2	0.042170122	0.071129358	0.592865207	0.553271364	0.695608329	positive
PRJNA729271	ed_Lushi_vs_Arbor_Acr	breed_growth_background	ENSGALG00010021125	LY75	0.182607786	0.066381662	2.750877001	0.005943596	0.028831461	positive
PRJNA729271	ed_Lushi_vs_Arbor_Acr	breed_growth_background	ENSGALG00010021126	ECE1	−0.197371441	0.055673722	−3.545145434	0.000392397	0.004185274	negative
PRJNA729271	ed_Lushi_vs_Arbor_Acr	breed_growth_background	ENSGALG00010021127	SWAP70	−0.12595415	0.042149858	−2.988246156	0.002805835	0.016893619	negative
PRJNA729271	ed_Lushi_vs_Arbor_Acr	breed_growth_background	ENSGALG00010021129	TXNRD3	0.040544596	0.046299543	0.875701864	0.381192132	0.54273176	positive
PRJNA729271	ed_Lushi_vs_Arbor_Acr	breed_growth_background	ENSGALG00010021130	ENSGALG00010021130	−1.461138063	0.580751344	−2.51594435	0.011871393	0.047201289	negative
PRJNA729271	ed_Lushi_vs_Arbor_Acr	breed_growth_background	ENSGALG00010021131	EDC3	0.033370215	0.062645824	0.532680605	0.594254694	0.728556356	positive
PRJNA729271	ed_Lushi_vs_Arbor_Acr	breed_growth_background	ENSGALG00010021133	ENSGALG00010021133	−0.194892141	0.055442693	−3.515199789	0.000439423	0.004526965	negative
PRJNA729271	ed_Lushi_vs_Arbor_Acr	breed_growth_background	ENSGALG00010021134	CHFR	−0.045472325	0.05067788	−0.897281505	0.369568723	0.532918687	negative
PRJNA729271	ed_Lushi_vs_Arbor_Acr	breed_growth_background	ENSGALG00010021135	CLK3	0.248436332	0.056694524	4.38201635	1.17586E-05	0.000327142	positive
PRJNA729271	ed_Lushi_vs_Arbor_Acr	breed_growth_background	ENSGALG00010021136	WEE1	−0.345069964	0.080997019	−4.26027981	2.04171E-05	0.000489167	negative
PRJNA729271	ed_Lushi_vs_Arbor_Acr	breed_growth_background	ENSGALG00010021137	PLAU	−0.164714425	0.105572508	−1.560201881	0.118712181	0.242630156	negative
PRJNA729271	ed_Lushi_vs_Arbor_Acr	breed_growth_background	ENSGALG00010021138	DDX18	0.110831239	0.054091578	2.048955553	0.040466461	0.113323292	positive

PRJNA729271	ed_Lushi_vs_Arbor_Acr	breed_growth_background	ENSGALG00010021139	ARHGAP29	0.02338185	0.051360566	0.455249069	0.648930062	0.77094019	positive
PRJNA729271	ed_Lushi_vs_Arbor_Acr	breed_growth_background	ENSGALG00010021140	SLC11A1	0.955250271	0.245526937	3.890612906	9.99913E-05	0.001587486	positive
PRJNA729271	ed_Lushi_vs_Arbor_Acr	breed_growth_background	ENSGALG00010021141	SGSM1	0.086478009	0.084485889	1.023579316	0.306034019	0.468521663	positive
PRJNA729271	ed_Lushi_vs_Arbor_Acr	breed_growth_background	ENSGALG00010021142	WNT6	0.198425243	0.327391012	0.606080301	0.544461431	0.688593355	positive
PRJNA729271	ed_Lushi_vs_Arbor_Acr	breed_growth_background	ENSGALG00010021143	ABCA4	0.151513418	0.199139004	0.760842503	0.446751144	0.603509584	positive
PRJNA729271	ed_Lushi_vs_Arbor_Acr	breed_growth_background	ENSGALG00010021144	FOXK1	0.073041629	0.058663151	1.245102391	0.213094115	0.363430898	positive
PRJNA729271	ed_Lushi_vs_Arbor_Acr	breed_growth_background	ENSGALG00010021145	EIF4G3	0.298113269	0.084545918	3.526051605	0.000421805	0.004402835	positive
PRJNA729271	ed_Lushi_vs_Arbor_Acr	breed_growth_background	ENSGALG00010021146	AP5Z1	0.033183368	0.046659618	0.711179595	0.476972951	0.629951975	positive
PRJNA729271	ed_Lushi_vs_Arbor_Acr	breed_growth_background	ENSGALG00010021147	ARID3B	0.034313396	0.046251278	0.741890757	0.458153527	0.613935044	positive
PRJNA729271	ed_Lushi_vs_Arbor_Acr	breed_growth_background	ENSGALG00010021148	GPATCH8	0.102733643	0.056468838	1.819297976	0.068865982	0.166048064	positive
PRJNA729271	ed_Lushi_vs_Arbor_Acr	breed_growth_background	ENSGALG00010021149	ENSGALG00010021149	-0.896527874	0.730038968	-1.228054822	0.219426389	0.371867825	negative
PRJNA729271	ed_Lushi_vs_Arbor_Acr	breed_growth_background	ENSGALG00010021150	ENSGALG00010021150	-0.09017809	0.134928171	-0.668341455	0.503915657	0.654562008	negative
PRJNA729271	ed_Lushi_vs_Arbor_Acr	breed_growth_background	ENSGALG00010021151	WNT10A	0.061647539	0.41106152	0.149971564	0.88078705	0.929121364	positive
PRJNA729271	ed_Lushi_vs_Arbor_Acr	breed_growth_background	ENSGALG00010021152	STK11IP	0.128166884	0.068423252	1.873148102	0.061047937	0.152786722	positive
PRJNA729271	ed_Lushi_vs_Arbor_Acr	breed_growth_background	ENSGALG00010021153	PTPRF	-0.137706719	0.086396888	-1.59388518	0.110961754	0.231656435	negative
PRJNA729271	ed_Lushi_vs_Arbor_Acr	breed_growth_background	ENSGALG00010021154	CHST13	0.147744845	0.115417648	1.280088857	0.200513887	0.349290806	positive
PRJNA729271	ed_Lushi_vs_Arbor_Acr	breed_growth_background	ENSGALG00010021155	RADIL	0.157934935	0.124662283	1.266902318	0.20519022	0.354491834	positive
PRJNA729271	ed_Lushi_vs_Arbor_Acr	breed_growth_background	ENSGALG00010021156	DDOST	-0.126169498	0.043520794	-2.899062403	0.003742804	0.020667407	negative
PRJNA729271	ed_Lushi_vs_Arbor_Acr	breed_growth_background	ENSGALG00010021157	ENSGALG00010021157	0.286285868	0.267905124	1.068609152	0.285245823	0.445719656	positive
PRJNA729271	ed_Lushi_vs_Arbor_Acr	breed_growth_background	ENSGALG00010021158	ACTG1L	-0.153212818	0.06937302	-2.208536087	0.027206925	0.084700253	negative
PRJNA729271	ed_Lushi_vs_Arbor_Acr	breed_growth_background	ENSGALG00010021159	MMD2	0.539092427	0.127242057	4.236747183	2.26781E-05	0.000527639	positive
PRJNA729271	ed_Lushi_vs_Arbor_Acr	breed_growth_background	ENSGALG00010021160	ZNF143	0.256654675	0.088269177	2.907636427	0.003641715	0.020217025	positive
PRJNA729271	ed_Lushi_vs_Arbor_Acr	breed_growth_background	ENSGALG00010021161	ENSGALG00010021161	-0.083786067	0.0602702	-1.390174026	0.164476039	0.304441346	negative
PRJNA729271	ed_Lushi_vs_Arbor_Acr	breed_growth_background	ENSGALG00010021162	WIPI2	0.183607581	0.050980075	3.601555765	0.000316319	0.003590476	positive
PRJNA729271	ed_Lushi_vs_Arbor_Acr	breed_growth_background	ENSGALG00010021163	INHA	-0.247346556	0.204486053	-1.209601104	0.226431994	0.379612741	negative
PRJNA729271	ed_Lushi_vs_Arbor_Acr	breed_growth_background	ENSGALG00010021164	SLC41A3	-0.442973219	0.11505345	-3.850151554	0.000118045	0.001810125	negative
PRJNA729271	ed_Lushi_vs_Arbor_Acr	breed_growth_background	ENSGALG00010021165	PTRF	-0.193552807	0.056244396	-3.441281625	0.000578966	0.005482013	negative
PRJNA729271	ed_Lushi_vs_Arbor_Acr	breed_growth_background	ENSGALG00010021166	NEURL1	0.152779214	0.164992298	0.925977852	0.35445749	0.518136641	positive
PRJNA729271	ed_Lushi_vs_Arbor_Acr	breed_growth_background	ENSGALG00010021167	KIAA1671	-0.176630708	0.067617287	-2.612212272	0.008995838	0.038704266	negative
PRJNA729271	ed_Lushi_vs_Arbor_Acr	breed_growth_background	ENSGALG00010021168	IVNS1ABP	0.049630134	0.056768889	0.874248812	0.381982769	0.543413508	positive
PRJNA729271	ed_Lushi_vs_Arbor_Acr	breed_growth_background	ENSGALG00010021169	CAMK2G	0.234501864	0.07479441	3.135285964	0.001716867	0.011935723	positive
PRJNA729271	ed_Lushi_vs_Arbor_Acr	breed_growth_background	ENSGALG00010021170	UBL7	0.141217181	0.075218732	1.877420396	0.060460498	0.151674441	positive
PRJNA729271	ed_Lushi_vs_Arbor_Acr	breed_growth_background	ENSGALG00010021171	ENSGALG00010021171	-0.290777569	0.114966105	-2.529246062	0.011430786	0.045948208	negative
PRJNA729271	ed_Lushi_vs_Arbor_Acr	breed_growth_background	ENSGALG00010021172	SLC29A4	-0.238068743	0.153896006	-1.546945563	0.121876373	0.246524982	negative
PRJNA729271	ed_Lushi_vs_Arbor_Acr	breed_growth_background	ENSGALG00010021173	KLF15	0.177156887	0.153374512	1.155060805	0.248065527	0.40427264	positive
PRJNA729271	ed_Lushi_vs_Arbor_Acr	breed_growth_background	ENSGALG00010021175	IPO7	-0.077575619	0.052187845	-1.486469104	0.137155083	0.268149842	negative
PRJNA729271	ed_Lushi_vs_Arbor_Acr	breed_growth_background	ENSGALG00010021176	ENSGALG00010021176	0.182461674	0.063280633	2.883373063	0.003934412	0.021415433	positive
PRJNA729271	ed_Lushi_vs_Arbor_Acr	breed_growth_background	ENSGALG00010021177	OBSL1	0.21074568	0.100857729	2.089534255	0.036659658	0.105537585	positive
PRJNA729271	ed_Lushi_vs_Arbor_Acr	breed_growth_background	ENSGALG00010021178	NDST2	-0.137613835	0.052695023	-2.611514878	0.009014207	0.038771788	negative
PRJNA729271	ed_Lushi_vs_Arbor_Acr	breed_growth_background	ENSGALG00010021180	TMEM198	0.357413323	0.078350073	4.56174841	5.07294E-06	0.000176336	positive
PRJNA729271	ed_Lushi_vs_Arbor_Acr	breed_growth_background	ENSGALG00010021181	DAGLA	0.010683954	0.069285393	0.154202112	0.877450377	0.927488019	positive
PRJNA729271	ed_Lushi_vs_Arbor_Acr	breed_growth_background	ENSGALG00010021182	ZSWIM8	0.046258457	0.037205893	1.243309949	0.213753639	0.364255702	positive
PRJNA729271	ed_Lushi_vs_Arbor_Acr	breed_growth_background	ENSGALG00010021183	CFAP100	0.424609687	0.247626166	1.7147206	0.086396465	0.194459124	positive
PRJNA729271	ed_Lushi_vs_Arbor_Acr	breed_growth_background	ENSGALG00010021184	DRAXIN	0.426202232	0.266257819	1.600712548	0.109440601	0.229704823	positive
PRJNA729271	ed_Lushi_vs_Arbor_Acr	breed_growth_background	ENSGALG00010021186	SWT1	0.090391733	0.048616586	1.859277653	0.062987793	0.156123129	positive
PRJNA729271	ed_Lushi_vs_Arbor_Acr	breed_growth_background	ENSGALG00010021187	SEMA7A	0.33837786	0.148365241	2.280708454	0.022565705	0.07444012	positive
PRJNA729271	ed_Lushi_vs_Arbor_Acr	breed_growth_background	ENSGALG00010021188	TNRC18	0.069110768	0.071465669	0.967048497	0.33351979	0.497982533	positive
PRJNA729271	ed_Lushi_vs_Arbor_Acr	breed_growth_background	ENSGALG00010021189	ENSGALG00010021189	0.352238605	0.161192513	2.185204498	0.028873856	0.088427327	positive
PRJNA729271	ed_Lushi_vs_Arbor_Acr	breed_growth_background	ENSGALG00010021190	ENSGALG00010021190	0.201558831	0.05530538	3.644470601	0.000267943	0.003209777	positive
PRJNA729271	ed_Lushi_vs_Arbor_Acr	breed_growth_background	ENSGALG00010021191	MYRF	0.424426241	0.117629515	3.6081611	0.000308375	0.003533514	positive
PRJNA729271	ed_Lushi_vs_Arbor_Acr	breed_growth_background	ENSGALG00010021192	ENSGALG00010021192	0.025127506	0.095906504	0.262000024	0.793321424	0.871971803	positive
PRJNA729271	ed_Lushi_vs_Arbor_Acr	breed_growth_background	ENSGALG00010021193	USP37	0.230227627	0.084526755	2.723724881	0.006455025	0.030587703	positive
PRJNA729271	ed_Lushi_vs_Arbor_Acr	breed_growth_background	ENSGALG00010021194	CYP11A1	-0.547972577	0.205923001	-2.661055712	0.007789607	0.034989262	negative
PRJNA729271	ed_Lushi_vs_Arbor_Acr	breed_growth_background	ENSGALG00010021195	GCLM	-0.037217747	0.048987626	-0.759737708	0.447411384	0.604052018	negative
PRJNA729271	ed_Lushi_vs_Arbor_Acr	breed_growth_background	ENSGALG00010021196	ATP6V0A1	-0.067196016	0.06403043	-1.049438763	0.293976226	0.455287983	negative
PRJNA729271	ed_Lushi_vs_Arbor_Acr	breed_growth_background	ENSGALG00010021197	CHCHD1	0.157199186	0.057202825	2.748101809	0.005994139	0.029029419	positive
PRJNA729271	ed_Lushi_vs_Arbor_Acr	breed_growth_background	ENSGALG00010021199	TMEM258	-0.110334497	0.074208591	-1.486815689	0.137063497	0.26804309	negative
PRJNA729271	ed_Lushi_vs_Arbor_Acr	breed_growth_background	ENSGALG00010021200	FTO	0.251436528	0.090428901	2.780488608	0.005427716	0.027026709	positive
PRJNA729271	ed_Lushi_vs_Arbor_Acr	breed_growth_background	ENSGALG00010021201	TRMT1L	0.062285152	0.048026883	1.296880997	0.194672136	0.342365034	positive
PRJNA729271	ed_Lushi_vs_Arbor_Acr	breed_growth_background	ENSGALG00010021202	UROC1	-0.643797632	0.321788683	-2.000684503	0.045426401	0.123607223	negative
PRJNA729271	ed_Lushi_vs_Arbor_Acr	breed_growth_background	ENSGALG00010021203	FUT11	0.080114826	0.04452652	1.799260902	0.07197742	0.170950794	positive
PRJNA729271	ed_Lushi_vs_Arbor_Acr	breed_growth_background	ENSGALG00010021204	NAGLU	-0.054930829	0.051371027	-1.069295914	0.284936351	0.445395494	negative
PRJNA729271	ed_Lushi_vs_Arbor_Acr	breed_growth_background	ENSGALG00010021206	HSD17B1	-0.186285732	0.237198541	-0.785357829	0.432243798	0.590571577	negative
PRJNA729271	ed_Lushi_vs_Arbor_Acr	breed_growth_background	ENSGALG00010021207	MAD2L2	0.07717588	0.066535627	1.159918119	0.246082145	0.401917856	positive
PRJNA729271	ed_Lushi_vs_Arbor_Acr	breed_growth_background	ENSGALG00010021210	ENSGALG00010021210	0.018022039	0.034512505	0.522188674	0.601538971	0.73413382	positive
PRJNA729271	ed_Lushi_vs_Arbor_Acr	breed_growth_background	ENSGALG00010021211	FEN1	-0.07800277	0.085080045	-0.916816277	0.359238922	0.522598171	negative
PRJNA729271	ed_Lushi_vs_Arbor_Acr	breed_growth_background	ENSGALG00010021212	MYO18B	-0.021459591	0.085895814	-0.249832797	0.802716655	0.878395942	negative
PRJNA729271	ed_Lushi_vs_Arbor_Acr	breed_growth_background	ENSGALG00010021213	FBXO44	0.090943309	0.113615611	0.800447294	0.423451689	0.582678207	positive
PRJNA729271	ed_Lushi_vs_Arbor_Acr	breed_growth_background	ENSGALG00010021214	FBXL18	0.036155668	0.06280005	0.57572673	0.564799905	0.704723648	positive
PRJNA729271	ed_Lushi_vs_Arbor_Acr	breed_growth_background	ENSGALG00010021215	SH3PXD2A	0.105458032	0.059745194	1.765129953	0.077541928	0.179925599	positive
PRJNA729271	ed_Lushi_vs_Arbor_Acr	breed_growth_background	ENSGALG00010021217	FBXO2	0.162668257	0.120488775	1.350069808	0.176993592	0.321005728	positive
PRJNA729271	ed_Lushi_vs_Arbor_Acr	breed_growth_background	ENSGALG00010021218	DPF3	0.34893306	0.094532136	3.691158103	0.000223235	0.002812624	positive
PRJNA729271	ed_Lushi_vs_Arbor_Acr	breed_growth_background	ENSGALG00010021219	STRA6	-0.063614689	0.172816151	-0.368106158	0.712794079	0.815401324	negative
PRJNA729271	ed_Lushi_vs_Arbor_Acr	breed_growth_background	ENSGALG00010021220	SYNPO2L	-0.051117044	0.10956541	-0.466543631	0.640826425	0.764526221	negative
PRJNA729271	ed_Lushi_vs_Arbor_Acr	breed_growth_background	ENSGALG00010021222	ENSGALG00010021222	-0.052065453	0.155530538	-0.33476032	0.737805895	0.832028969	negative
PRJNA729271	ed_Lushi_vs_Arbor_Acr	breed_growth_background	ENSGALG00010021223	DFFB	0.007958109	0.060309618	0.131954219	0.895020503	0.938667404	positive
PRJNA729271	ed_Lushi_vs_Arbor_Acr	breed_growth_background	ENSGALG00010021224	MYOZ1	-0.084202861	0.072930698	-1.154559927	0.248270685	0.40439306	negative
PRJNA729271	ed_Lushi_vs_Arbor_Acr	breed_growth_background	ENSGALG00010021227	CHCHD4	0.029545786	0.053077927	0.556649219	0.577767119	0.71487276	positive
PRJNA729271	ed_Lushi_vs_Arbor_Acr	breed_growth_background	ENSGALG00010021228	ESRP2	-0.776369938	0.536261283	-1.447745645	0.147688199	0.282581036	negative
PRJNA729271	ed_Lushi_vs_Arbor_Acr	breed_growth_background	ENSGALG00010021229	GLO1	-0.109798952	0.095325365	-1.151833542	0.249389482	0.405394251	negative
PRJNA729271	ed_Lushi_vs_Arbor_Acr	breed_growth_background	ENSGALG00010021230	C1orf174	0.048974936	0.032348719	1.513968334	0.130033871	0.257767015	positive
PRJNA729271	ed_Lushi_vs_Arbor_Acr	breed_growth_background	ENSGALG00010021231	STN1	-0.164604182	0.065219254	-2.523858722	0.011607457	0.046516473	negative
PRJNA729271	ed_Lushi_vs_Arbor_Acr	breed_growth_background	ENSGALG00010021232	ACTB	-0.055867883	0.058513894	-0.954779788	0.339689071	0.50378634	negative
PRJNA729271	ed_Lushi_vs_Arbor_Acr	breed_growth_background	ENSGALG00010021233	COASY	0.042604404	0.033320633	1.278619288	0.201031155	0.34994349	positive
PRJNA729271	ed_Lushi_vs_Arbor_Acr	breed_growth_background	ENSGALG00010021234	ISLR2	-0.413695812	0.179948151	-2.298972289	0.021506513	0.071845334	negative
PRJNA729271	ed_Lushi_vs_Arbor_Acr	breed_growth_background	ENSGALG00010021235	XRCC5	-0.153764382	0.075332867	-2.041132749	0.041237634	0.11486155	negative
PRJNA729271	ed_Lushi_vs_Arbor_Acr	breed_growth_background	ENSGALG00010021236	BTBD9	0.066362541	0.039863227	1.664755864	0.095961473	0.20875905	positive
PRJNA729271	ed_Lushi_vs_Arbor_Acr	breed_growth_background	ENSGALG00010021237	FSCN1	-0.483786043	0.120052719	-4.029779966	5.58291E-05	0.001050257	negative
PRJNA729271	ed_Lushi_vs_Arbor_Acr	breed_growth_background	ENSGALG00010021238	NDUFS8	0.271118644	0.078588932	3.449832391	0.000560935	0.005378874	positive
PRJNA729271	ed_Lushi_vs_Arbor_Acr	breed_growth_background	ENSGALG00010021239	USP54	-0.161006796	0.037786739	-4.260933903	2.03574E-05	0.000488545	negative
PRJNA729271	ed_Lushi_vs_Arbor_Acr	breed_growth_background	ENSGALG00010021240	SLK	0.069708161	0.042298305	1.648013108	0.099349981	0.213820042	positive
PRJNA729271	ed_Lushi_vs_Arbor_Acr	breed_growth_background	ENSGALG00010021241	TMEM43	-0.308892677	0.091908198	-3.360882739	0.000776938	0.006800554	negative
PRJNA729271	ed_Lushi_vs_Arbor_Acr	breed_growth_background	ENSGALG00010021242	BCAR3	0.31671392	0.118461854	2.673551949	0.007505265	0.034027336	positive
PRJNA729271	ed_Lushi_vs_Arbor_Acr	breed_growth_background	ENSGALG00010021243	DYL1	-0.167906528	0.051074605	-3.287475807	0.001010899	0.008204355	negative
PRJNA729271	ed_Lushi_vs_Arbor_Acr	breed_growth_background	ENSGALG00010021244	PPP3CB	0.003720026	0.04672301	0.079618724	0.936540503	0.964898329	positive
PRJNA729271	ed_Lushi_vs_Arbor_Acr	breed_growth_background	ENSGALG00010021245	ZFAND3	0.159675768	0.037702244	4.235179415	2.28369E-05	0.000529634	positive
PRJNA729271	ed_Lushi_vs_Arbor_Acr	breed_growth_background	ENSGALG00010021246	PECR	0.003797277	0.062060096	0.061187098	0.951210205	0.973233	positive
PRJNA729271	ed_Lushi_vs_Arbor_Acr	breed_growth_background	ENSGALG00010021247	GNAO1	0.309553082	0.122015765	2.536992507	0.011180935	0.045295598	positive
PRJNA729271	ed_Lushi_vs_Arbor_Acr	breed_growth_background	ENSGALG00010021248	ANXA7	-0.328440577	0.090214553	-3.640660692	0.000271939	0.003244247	negative
PRJNA729271	ed_Lushi_vs_Arbor_Acr	breed_growth_background	ENSGALG00010021249	ENSGALG00010021249	0.007845554	0.215802119	0.036355316	0.970999043	0.983017828	positive
PRJNA729271	ed_Lushi_vs_Arbor_Acr	breed_growth_background	ENSGALG00010021250	ISLR	-0.036387572	0.07820433	-0.465288459	0.641724899	0.765265521	negative
PRJNA729271	ed_Lushi_vs_Arbor_Acr	breed_growth_background	ENSGALG00010021251	RNF216	0.171387593	0.046327932	3.699444044	0.000216072	0.002752168	positive
PRJNA729271	ed_Lushi_vs_Arbor_Acr	breed_growth_background	ENSGALG00010021252	XPC	0.165715516	0.070449865	2.352247443	0.018660357	0.065129274	positive
PRJNA729271	ed_Lushi_vs_Arbor_Acr	breed_growth_background	ENSGALG00010021253	TMEM169	0.050596865	0.063640759	0.795038685	0.426591	0.585663624	positive
PRJNA729271	ed_Lushi_vs_Arbor_Acr	breed_growth_background	ENSGALG00010021254	FNBP1L	-0.080693358	0.058153509	-1.387592257	0.165261241	0.305387772	negative
PRJNA729271	ed_Lushi_vs_Arbor_Acr	breed_growth_background	ENSGALG00010021255	EIF3F	0.001229722	0.049128847	0.025030543	0.980030601	0.988280476	positive
PRJNA729271	ed_Lushi_vs_Arbor_Acr	breed_growth_background	ENSGALG00010021256	ENSGALG00010021256	0.026867515	0.157101628	0.171019964	0.864208068	0.918514147	positive
PRJNA729271	ed_Lushi_vs_Arbor_Acr	breed_growth_background	ENSGALG00010021257	RGS6	0.5920447	0.176056785	3.36280536	0.000771547	0.006761536	positive
PRJNA729271	ed_Lushi_vs_Arbor_Acr	breed_growth_background	ENSGALG00010021258	LSM3	-0.099123473	0.062092212	-1.596391402	0.110401434	0.231052381	negative
PRJNA729271	ed_Lushi_vs_Arbor_Acr	breed_growth_background	ENSGALG00010021259	CEP104	0.033053784	0.073283656	0.451038955	0.651961473	0.773020986	positive
PRJNA729271	ed_Lushi_vs_Arbor_Acr	breed_growth_background	ENSGALG00010021260	KDM4A	0.071738745	0.036430607	1.969188866	0.048931407	0.130609843	positive
PRJNA729271	ed_Lushi_vs_Arbor_Acr	breed_growth_background	ENSGALG00010021261	JUP	-0.189628134	0.068656353	-2.761989611	0.005745031	0.028069191	negative
PRJNA729271	ed_Lushi_vs_Arbor_Acr	breed_growth_background	ENSGALG00010021262	MDGA1	0.531960418	0.194488024	2.735183418	0.006234553	0.029805963	positive
PRJNA729271	ed_Lushi_vs_Arbor_Acr	breed_growth_background	ENSGALG00010021263	STOML1	-0.087855386	0.133075617	-0.660191462	0.509130971	0.657975519	negative
PRJNA729271	ed_Lushi_vs_Arbor_Acr	breed_growth_background	ENSGALG00010021264	CBFB	-0.414729565	0.091173614	-4.548789334	5.39554E-06	0.000183158	negative
PRJNA729271	ed_Lushi_vs_Arbor_Acr	breed_growth_background	ENSGALG00010021265	SIPA1L1	0.020496392	0.048326854	0.424120144	0.671478195	0.78765691	positive
PRJNA729271	ed_Lushi_vs_Arbor_Acr	breed_growth_background	ENSGALG00010021266	COQ5	-0.025556624	0.053560771	-0.477151902	0.633253958	0.758283034	negative
PRJNA729271	ed_Lushi_vs_Arbor_Acr	breed_growth_background	ENSGALG00010021267	LRRC47	0.021877983	0.042479774	0.515021168	0.606538228	0.737698976	positive
PRJNA729271	ed_Lushi_vs_Arbor_Acr	breed_growth_background	ENSGALG00010021269	NUDT13	-0.116785083	0.091625113	-1.274596875	0.202451964	0.351644302	negative
PRJNA729271	ed_Lushi_vs_Arbor_Acr	breed_growth_background	ENSGALG00010021271	ENSGALG00010021271	0.007698565	0.25583668	0.030091718	0.975993906	0.985790861	positive
PRJNA729271	ed_Lushi_vs_Arbor_Acr	breed_growth_background	ENSGALG00010021272	ENSGALG00010021272	-0.000853482	0.193947496	-0.004400581	0.996488856	0.998210502	negative
PRJNA729271	ed_Lushi_vs_Arbor_Acr	breed_growth_background	ENSGALG00010021275	MAP3K2	0.041060243	0.049714559	0.825919889	0.408849537	0.569177299	positive
PRJNA729271	ed_Lushi_vs_Arbor_Acr	breed_growth_background	ENSGALG00010021277	ENSGALG00010021277	0.183007558	0.133994613	1.365782957	0.172007097	0.315081875	positive
PRJNA729271	ed_Lushi_vs_Arbor_Acr	breed_growth_background	ENSGALG00010021287	COL17A1	0.197369862	0.258530263	0.7634304	0.445206755	0.601928171	positive
PRJNA729271	ed_Lushi_vs_Arbor_Acr	breed_growth_background	ENSGALG00010021290	ERCC3	0.063635393	0.037015641	1.719148771	0.085587286	0.193087582	positive
PRJNA729271	ed_Lushi_vs_Arbor_Acr	breed_growth_background	ENSGALG00010021293	PCNX1	-0.024061946	0.036998942	-0.650341447	0.51547169	0.66382447	negative
PRJNA729271	ed_Lushi_vs_Arbor_Acr	breed_growth_background	ENSGALG00010021296	GATC	-0.061822285	0.066004042	-0.936643931	0.348941746	0.512972679	negative
PRJNA729271	ed_Lushi_vs_Arbor_Acr	breed_growth_background	ENSGALG00010021298	RPGRIP1L	0.289873904	0.081541777	3.554912766	0.000378105	0.004086969	positive
PRJNA729271	ed_Lushi_vs_Arbor_Acr	breed_growth_background	ENSGALG00010021299	CYP27C1	-0.003694573	0.096457918	-0.038302436	0.969446548	0.982470096	negative
PRJNA729271	ed_Lushi_vs_Arbor_Acr	breed_growth_background	ENSGALG00010021301	SMURF1	-0.033662003	0.043698614	-0.770321978	0.441108923	0.598509403	negative
PRJNA729271	ed_Lushi_vs_Arbor_Acr	breed_growth_background	ENSGALG00010021302	P4HA1	0.141197262	0.032348603	4.364864267	1.27202E-05	0.000345279	positive

PRJNA729271	:ed_Lushi_vs_Arbor_Acr	breed_growth_background	ENSGALG00010021307	MAP3K9	0.145389787	0.076269721	1.906258278	0.056616705	0.14483924	positive
PRJNA729271	:ed_Lushi_vs_Arbor_Acr	breed_growth_background	ENSGALG00010021309	ST3GAL3	0.006146432	0.067971511	0.090426593	0.927948225	0.960003535	positive
PRJNA729271	:ed_Lushi_vs_Arbor_Acr	breed_growth_background	ENSGALG00010021311	TRIAP1	-0.024306912	0.064807347	-0.375064144	0.707612763	0.811652053	negative
PRJNA729271	:ed_Lushi_vs_Arbor_Acr	breed_growth_background	ENSGALG00010021313	KPNA7	-2.5986828	0.655043273	-3.967192561	7.27242E-05	0.001257808	negative
PRJNA729271	:ed_Lushi_vs_Arbor_Acr	breed_growth_background	ENSGALG00010021315	COX6A1	-0.103863531	0.072421235	-1.434158522	0.151527031	0.287484857	negative
PRJNA729271	:ed_Lushi_vs_Arbor_Acr	breed_growth_background	ENSGALG00010021318	TP73	0.555516403	0.15061589	3.688298775	0.000225758	0.002826452	positive
PRJNA729271	:ed_Lushi_vs_Arbor_Acr	breed_growth_background	ENSGALG00010021320	SLC6A6	-0.210966523	0.104368361	-2.021364719	0.043242024	0.119125952	negative
PRJNA729271	:ed_Lushi_vs_Arbor_Acr	breed_growth_background	ENSGALG00010021325	ARPC1A	-0.087375845	0.034814676	-2.509741696	0.01208195	0.047757805	negative
PRJNA729271	:ed_Lushi_vs_Arbor_Acr	breed_growth_background	ENSGALG00010021327	IGSF1	-0.17509396	0.224468346	-0.780038536	0.435368193	0.593301572	negative
PRJNA729271	:ed_Lushi_vs_Arbor_Acr	breed_growth_background	ENSGALG00010021329	PSMC3IP	-0.212375557	0.090690572	-2.341760027	0.019193051	0.066465365	negative
PRJNA729271	:ed_Lushi_vs_Arbor_Acr	breed_growth_background	ENSGALG00010021330	COMMD4	-0.085560211	0.070855956	-1.207523208	0.227230713	0.380335933	negative
PRJNA729271	:ed_Lushi_vs_Arbor_Acr	breed_growth_background	ENSGALG00010021332	OIT3	-0.065838508	0.067426045	-0.976455129	0.328838974	0.492714588	negative
PRJNA729271	:ed_Lushi_vs_Arbor_Acr	breed_growth_background	ENSGALG00010021335	NFATC3	0.051436487	0.054556351	0.9428139	0.345776101	0.509662861	positive
PRJNA729271	:ed_Lushi_vs_Arbor_Acr	breed_growth_background	ENSGALG00010021338	PDIA5	-0.291485067	0.084862968	-3.434773416	0.000593049	0.005567521	negative
PRJNA729271	:ed_Lushi_vs_Arbor_Acr	breed_growth_background	ENSGALG00010021341	L3MBTL1	0.217737749	0.086058379	2.530116781	0.011402457	0.04585977	positive
PRJNA729271	:ed_Lushi_vs_Arbor_Acr	breed_growth_background	ENSGALG00010021342	SFR1	-0.160141728	0.102159819	-1.567560804	0.116983665	0.240249112	negative
PRJNA729271	:ed_Lushi_vs_Arbor_Acr	breed_growth_background	ENSGALG00010021345	NEIL1	-0.317173708	0.083297694	-3.807712957	0.000140258	0.002035074	negative
PRJNA729271	:ed_Lushi_vs_Arbor_Acr	breed_growth_background	ENSGALG00010021346	RPS21	-0.257649048	0.148852524	-1.730901441	0.083469345	0.189578212	negative
PRJNA729271	:ed_Lushi_vs_Arbor_Acr	breed_growth_background	ENSGALG00010021348	SLC4A3	0.129236041	0.062304893	2.074251872	0.038055925	0.108629506	positive
PRJNA729271	:ed_Lushi_vs_Arbor_Acr	breed_growth_background	ENSGALG00010021350	CABLES2	-0.129157465	0.073868323	-1.748482443	0.080380523	0.184587402	negative
PRJNA729271	:ed_Lushi_vs_Arbor_Acr	breed_growth_background	ENSGALG00010021352	CFAP43	-0.000663795	0.127286095	-0.005214981	0.995839066	0.997904408	negative
PRJNA729271	:ed_Lushi_vs_Arbor_Acr	breed_growth_background	ENSGALG00010021353	IPO13	0.396107809	0.095198113	4.160878769	3.17025E-05	0.000693104	positive
PRJNA729271	:ed_Lushi_vs_Arbor_Acr	breed_growth_background	ENSGALG00010021354	ENSGALG00010021354	0.089870732	0.060307857	1.490199382	0.136171819	0.266662985	positive
PRJNA729271	:ed_Lushi_vs_Arbor_Acr	breed_growth_background	ENSGALG00010021358	MMP15	0.203897741	0.069143474	2.948907945	0.00318899	0.018534243	positive
PRJNA729271	:ed_Lushi_vs_Arbor_Acr	breed_growth_background	ENSGALG00010021360	PTPRN	-0.268537643	0.151548113	-1.771962958	0.076400706	0.178387281	negative
PRJNA729271	:ed_Lushi_vs_Arbor_Acr	breed_growth_background	ENSGALG00010021361	WRAP73	-0.08406582	0.050638371	-1.660120928	0.096890127	0.21002279	negative
PRJNA729271	:ed_Lushi_vs_Arbor_Acr	breed_growth_background	ENSGALG00010021362	ARPC1B	-0.258480514	0.064853801	-3.985587772	6.73133E-05	0.001189577	negative
PRJNA729271	:ed_Lushi_vs_Arbor_Acr	breed_growth_background	ENSGALG00010021363	ENSGALG00010021363	0.021075007	0.040224839	0.523930182	0.600327101	0.733086885	positive
PRJNA729271	:ed_Lushi_vs_Arbor_Acr	breed_growth_background	ENSGALG00010021367	GRIP2	0.033352478	0.096013045	0.347374437	0.728310035	0.825399059	positive
PRJNA729271	:ed_Lushi_vs_Arbor_Acr	breed_growth_background	ENSGALG00010021372	CD86	-0.00808385	0.27377236	-0.029527633	0.976443781	0.986035433	negative
PRJNA729271	:ed_Lushi_vs_Arbor_Acr	breed_growth_background	ENSGALG00010021373	CEP250	0.133158379	0.082988037	1.604549097	0.108593071	0.228396102	positive
PRJNA729271	:ed_Lushi_vs_Arbor_Acr	breed_growth_background	ENSGALG00010021375	TUBG1	0.057156328	0.06398667	0.893253673	0.371721341	0.534463681	positive
PRJNA729271	:ed_Lushi_vs_Arbor_Acr	breed_growth_background	ENSGALG00010021378	KIF11	-0.360589609	0.100458	-3.589456377	0.000331368	0.003703793	negative
PRJNA729271	:ed_Lushi_vs_Arbor_Acr	breed_growth_background	ENSGALG00010021381	DNAJB2	0.183748282	0.078841529	2.330602714	0.019774318	0.067653472	positive
PRJNA729271	:ed_Lushi_vs_Arbor_Acr	breed_growth_background	ENSGALG00010021382	TPRG1L	0.072595814	0.046492299	1.561458909	0.118415512	0.242262928	positive
PRJNA729271	:ed_Lushi_vs_Arbor_Acr	breed_growth_background	ENSGALG00010021383	MSI1	0.078370944	0.065151334	1.202906205	0.229012619	0.3826652	positive
PRJNA729271	:ed_Lushi_vs_Arbor_Acr	breed_growth_background	ENSGALG00010021384	ARTN	0.461477996	0.206828696	2.231208752	0.025667305	0.081515504	positive
PRJNA729271	:ed_Lushi_vs_Arbor_Acr	breed_growth_background	ENSGALG00010021385	GDF5	-0.035031376	0.273093437	-0.128276153	0.897930436	0.940834298	negative
PRJNA729271	:ed_Lushi_vs_Arbor_Acr	breed_growth_background	ENSGALG00010021387	SLC2A14	0.000700741	0.050343914	0.013919072	0.988894546	0.99341787	positive
PRJNA729271	:ed_Lushi_vs_Arbor_Acr	breed_growth_background	ENSGALG00010021388	DPH2	-0.234753829	0.08414057	-2.790019466	0.005270487	0.026480316	negative
PRJNA729271	:ed_Lushi_vs_Arbor_Acr	breed_growth_background	ENSGALG00010021390	GSTO2	-0.437276049	0.116898191	-3.7406571	0.00018354	0.00243833	negative
PRJNA729271	:ed_Lushi_vs_Arbor_Acr	breed_growth_background	ENSGALG00010021391	ENSGALG00010021391	0.014256639	0.198142978	0.071951269	0.942640689	0.968910565	positive
PRJNA729271	:ed_Lushi_vs_Arbor_Acr	breed_growth_background	ENSGALG00010021393	CCDC174	0.121519832	0.061782731	1.966889954	0.049195899	0.131113581	positive
PRJNA729271	:ed_Lushi_vs_Arbor_Acr	breed_growth_background	ENSGALG00010021394	MAN2C1	0.063278625	0.053071335	1.192331512	0.233131283	0.387571734	positive
PRJNA729271	:ed_Lushi_vs_Arbor_Acr	breed_growth_background	ENSGALG00010021396	MED6	0.020584265	0.042871268	0.48014126	0.631126951	0.756798904	positive
PRJNA729271	:ed_Lushi_vs_Arbor_Acr	breed_growth_background	ENSGALG00010021397	ENSGALG00010021397	-1.49121157	0.904294425	-1.649033246	0.099140824	0.213433275	negative
PRJNA729271	:ed_Lushi_vs_Arbor_Acr	breed_growth_background	ENSGALG00010021400	B4GALT2	0.097817028	0.049277904	1.985007889	0.047143594	0.126803932	positive
PRJNA729271	:ed_Lushi_vs_Arbor_Acr	breed_growth_background	ENSGALG00010021401	PDAP1	0.146463698	0.05098098	2.872908619	0.004067117	0.02189115	positive
PRJNA729271	:ed_Lushi_vs_Arbor_Acr	breed_growth_background	ENSGALG00010021402	ENSGALG00010021402	-0.003735736	0.047963051	-0.077887798	0.937917305	0.965973522	negative
PRJNA729271	:ed_Lushi_vs_Arbor_Acr	breed_growth_background	ENSGALG00010021404	UQCC1	-0.001388525	0.050708532	-0.027382466	0.978154683	0.987037082	negative
PRJNA729271	:ed_Lushi_vs_Arbor_Acr	breed_growth_background	ENSGALG00010021405	IDE	-0.028203516	0.061021394	-0.462190627	0.643944624	0.767218258	negative
PRJNA729271	:ed_Lushi_vs_Arbor_Acr	breed_growth_background	ENSGALG00010021407	SIN3A	0.060292683	0.060117831	1.002908487	0.315905018	0.479330391	positive
PRJNA729271	:ed_Lushi_vs_Arbor_Acr	breed_growth_background	ENSGALG00010021408	TTC9	-0.307855354	0.112269161	-2.742118597	0.00610443	0.029405625	negative
PRJNA729271	:ed_Lushi_vs_Arbor_Acr	breed_growth_background	ENSGALG00010021409	MEGF6	-0.223013268	0.141161174	-1.579848495	0.114141567	0.236084762	negative
PRJNA729271	:ed_Lushi_vs_Arbor_Acr	breed_growth_background	ENSGALG00010021411	MCU	-0.074645154	0.068919965	-1.083070117	0.278777302	0.438130434	negative
PRJNA729271	:ed_Lushi_vs_Arbor_Acr	breed_growth_background	ENSGALG00010021412	ZNF319	0.201480876	0.066875567	3.012772623	0.002588728	0.015960703	positive
PRJNA729271	:ed_Lushi_vs_Arbor_Acr	breed_growth_background	ENSGALG00010021413	SYNJ2BP	-0.052599165	0.081579148	-0.644762368	0.519081192	0.667028536	negative
PRJNA729271	:ed_Lushi_vs_Arbor_Acr	breed_growth_background	ENSGALG00010021414	BUD31	-0.029919034	0.059674855	-0.501367513	0.616112499	0.744584849	negative
PRJNA729271	:ed_Lushi_vs_Arbor_Acr	breed_growth_background	ENSGALG00010021415	SIRT4	0.073488311	0.08070186	0.910614835	0.362498351	0.525809427	positive
PRJNA729271	:ed_Lushi_vs_Arbor_Acr	breed_growth_background	ENSGALG00010021416	PLEKHH3	0.078285924	0.066158057	1.183316567	0.23668371	0.391591185	positive
PRJNA729271	:ed_Lushi_vs_Arbor_Acr	breed_growth_background	ENSGALG00010021417	STK16	-0.236292893	0.090540881	-2.609792282	0.009059722	0.038909826	negative
PRJNA729271	:ed_Lushi_vs_Arbor_Acr	breed_growth_background	ENSGALG00010021419	MANSC1	-0.247612861	0.108503646	-2.282069492	0.022485238	0.074259176	negative
PRJNA729271	:ed_Lushi_vs_Arbor_Acr	breed_growth_background	ENSGALG00010021420	PLXNB3	0.443978763	0.103150553	4.304182087	1.67604E-05	0.000419588	positive
PRJNA729271	:ed_Lushi_vs_Arbor_Acr	breed_growth_background	ENSGALG00010021421	GLB1L	0.288738638	0.090431747	3.192890193	0.001408565	0.010466254	positive
PRJNA729271	:ed_Lushi_vs_Arbor_Acr	breed_growth_background	ENSGALG00010021424	PTCD1	0.1538753	0.058708444	2.621007987	0.008767021	0.037952051	positive
PRJNA729271	:ed_Lushi_vs_Arbor_Acr	breed_growth_background	ENSGALG00010021425	PTPN9	-0.275166373	0.068588655	-4.011835111	6.02486E-05	0.001104049	negative
PRJNA729271	:ed_Lushi_vs_Arbor_Acr	breed_growth_background	ENSGALG00010021426	SLC38A7	-0.034091523	0.079045979	-0.431287254	0.666259517	0.783804503	negative
PRJNA729271	:ed_Lushi_vs_Arbor_Acr	breed_growth_background	ENSGALG00010021427	RPLP0	-0.048352653	0.07237864	-0.668051419	0.504100771	0.654597749	negative
PRJNA729271	:ed_Lushi_vs_Arbor_Acr	breed_growth_background	ENSGALG00010021429	MED8	-0.04823647	0.061427747	-0.785255399	0.43230384	0.590587154	negative
PRJNA729271	:ed_Lushi_vs_Arbor_Acr	breed_growth_background	ENSGALG00010021431	CPSF4	0.169772783	0.057909894	2.931671444	0.003371432	0.019285284	positive
PRJNA729271	:ed_Lushi_vs_Arbor_Acr	breed_growth_background	ENSGALG00010021432	SYT9	-0.125526687	0.163694226	-0.766836372	0.443178808	0.600250123	negative
PRJNA729271	:ed_Lushi_vs_Arbor_Acr	breed_growth_background	ENSGALG00010021433	ENSGALG00010021433	0.458290316	0.132899056	3.448409122	0.000563899	0.005391635	positive
PRJNA729271	:ed_Lushi_vs_Arbor_Acr	breed_growth_background	ENSGALG00010021434	ANKZF1	0.092211479	0.095576712	0.964790246	0.334649882	0.499048358	positive
PRJNA729271	:ed_Lushi_vs_Arbor_Acr	breed_growth_background	ENSGALG00010021435	GCN1	0.127071402	0.047698266	2.664067516	0.007720207	0.034752918	positive
PRJNA729271	:ed_Lushi_vs_Arbor_Acr	breed_growth_background	ENSGALG00010021438	GOT2	-0.068704559	0.058910699	-1.166249257	0.243513683	0.399145826	negative
PRJNA729271	:ed_Lushi_vs_Arbor_Acr	breed_growth_background	ENSGALG00010021439	ITPRIP	-0.261166353	0.099967055	-2.612524223	0.008987632	0.038702378	negative
PRJNA729271	:ed_Lushi_vs_Arbor_Acr	breed_growth_background	ENSGALG00010021441	ENSGALG00010021441	-0.127918209	0.175592854	-0.728493254	0.466311697	0.6212253	negative
PRJNA729271	:ed_Lushi_vs_Arbor_Acr	breed_growth_background	ENSGALG00010021443	SNUPN	-0.124921788	0.073930156	-1.689727108	0.091080175	0.201681505	negative
PRJNA729271	:ed_Lushi_vs_Arbor_Acr	breed_growth_background	ENSGALG00010021444	ATG9A	0.465811179	0.131627465	3.538860069	0.000401859	0.004253705	positive
PRJNA729271	:ed_Lushi_vs_Arbor_Acr	breed_growth_background	ENSGALG00010021446	HOXB8	0.016903166	0.349475358	0.048367261	0.961423551	0.978276808	positive
PRJNA729271	:ed_Lushi_vs_Arbor_Acr	breed_growth_background	ENSGALG00010021447	OCSTAMP	-0.200695469	0.37340418	-0.537475153	0.590939444	0.725906847	negative
PRJNA729271	:ed_Lushi_vs_Arbor_Acr	breed_growth_background	ENSGALG00010021448	SZT2	0.097065828	0.053930219	1.799841159	0.071885723	0.170844983	positive
PRJNA729271	:ed_Lushi_vs_Arbor_Acr	breed_growth_background	ENSGALG00010021450	OLFML1	0.103872411	0.124304775	0.835626872	0.403364846	0.564305902	positive
PRJNA729271	:ed_Lushi_vs_Arbor_Acr	breed_growth_background	ENSGALG00010021452	SLC25A1	-0.095351642	0.064537255	-1.477466637	0.139550595	0.271405592	negative
PRJNA729271	:ed_Lushi_vs_Arbor_Acr	breed_growth_background	ENSGALG00010021454	ATP5J2	-0.172005501	0.093126181	-1.847015508	0.064744893	0.158927103	negative
PRJNA729271	:ed_Lushi_vs_Arbor_Acr	breed_growth_background	ENSGALG00010021455	CFAP58	0.784068459	0.285930218	2.742167176	0.006103527	0.029405625	positive
PRJNA729271	:ed_Lushi_vs_Arbor_Acr	breed_growth_background	ENSGALG00010021457	USB1	0.046026373	0.060557077	0.760049455	0.447225024	0.60394615	positive
PRJNA729271	:ed_Lushi_vs_Arbor_Acr	breed_growth_background	ENSGALG00010021458	USP30	0.115297641	0.044350471	2.599693713	0.0093307	0.039825823	positive
PRJNA729271	:ed_Lushi_vs_Arbor_Acr	breed_growth_background	ENSGALG00010021459	ARHGEF16	-0.238163821	0.068677048	-3.46788085	0.00052458	0.005137692	negative
PRJNA729271	:ed_Lushi_vs_Arbor_Acr	breed_growth_background	ENSGALG00010021461	SNX33	0.165425694	0.059095071	2.799314589	0.005121122	0.02602855	positive
PRJNA729271	:ed_Lushi_vs_Arbor_Acr	breed_growth_background	ENSGALG00010021462	SETD5	0.107492403	0.051205461	2.099237093	0.035796006	0.103627542	positive
PRJNA729271	:ed_Lushi_vs_Arbor_Acr	breed_growth_background	ENSGALG00010021464	ENSGALG00010021464	-0.187186183	0.220561937	-0.848678541	0.39606019	0.557329416	negative
PRJNA729271	:ed_Lushi_vs_Arbor_Acr	breed_growth_background	ENSGALG00010021465	ABCB6	0.185559578	0.113860479	1.629710141	0.103162773	0.219903588	positive
PRJNA729271	:ed_Lushi_vs_Arbor_Acr	breed_growth_background	ENSGALG00010021466	HES4	-0.228598417	0.097769909	-2.338126514	0.019380686	0.066854604	negative
PRJNA729271	:ed_Lushi_vs_Arbor_Acr	breed_growth_background	ENSGALG00010021467	LHFPL4	0.011917635	0.139299695	0.085553924	0.931821027	0.962498809	positive
PRJNA729271	:ed_Lushi_vs_Arbor_Acr	breed_growth_background	ENSGALG00010021469	CSPG4	-0.320369784	0.086747189	-3.693143121	0.000221499	0.002799156	negative
PRJNA729271	:ed_Lushi_vs_Arbor_Acr	breed_growth_background	ENSGALG00010021470	ENSGALG00010021470	0.412492084	0.328517213	1.255617872	0.209254549	0.358995622	positive
PRJNA729271	:ed_Lushi_vs_Arbor_Acr	breed_growth_background	ENSGALG00010021473	SLC8A3	0.139043999	0.070030093	1.985489273	0.047090062	0.126730702	positive
PRJNA729271	:ed_Lushi_vs_Arbor_Acr	breed_growth_background	ENSGALG00010021474	PPFIBP2	0.383478164	0.096668757	3.966929691	7.28045E-05	0.001257808	positive
PRJNA729271	:ed_Lushi_vs_Arbor_Acr	breed_growth_background	ENSGALG00010021476	ALKBH2	0.056182655	0.066240287	0.848164418	0.39634641	0.557662708	positive
PRJNA729271	:ed_Lushi_vs_Arbor_Acr	breed_growth_background	ENSGALG00010021477	ZFAND2B	0.073861632	0.064057267	1.153056257	0.248887295	0.405032147	positive
PRJNA729271	:ed_Lushi_vs_Arbor_Acr	breed_growth_background	ENSGALG00010021478	SLC13A3	0.14698311	0.106157205	1.384579688	0.166181026	0.306774576	positive
PRJNA729271	:ed_Lushi_vs_Arbor_Acr	breed_growth_background	ENSGALG00010021479	SKAP1	0.626439591	0.255852909	2.448436456	0.014347774	0.053739271	positive
PRJNA729271	:ed_Lushi_vs_Arbor_Acr	breed_growth_background	ENSGALG00010021480	ENSGALG00010021480	0.039093209	0.040663013	0.961394814	0.336353691	0.50066202	positive
PRJNA729271	:ed_Lushi_vs_Arbor_Acr	breed_growth_background	ENSGALG00010021481	MICU1	0.029573483	0.093561033	0.316087607	0.751936013	0.842756653	positive
PRJNA729271	:ed_Lushi_vs_Arbor_Acr	breed_growth_background	ENSGALG00010021483	UNG	-0.472499405	0.085398573	-5.53287235	3.15029E-08	3.51257E-06	negative
PRJNA729271	:ed_Lushi_vs_Arbor_Acr	breed_growth_background	ENSGALG00010021484	MARCHF5	-0.016455114	0.038135812	-0.431487183	0.66611417	0.783711437	negative
PRJNA729271	:ed_Lushi_vs_Arbor_Acr	breed_growth_background	ENSGALG00010021485	SMOC1	0.154539828	0.130048511	1.188324467	0.234705602	0.389118449	positive
PRJNA729271	:ed_Lushi_vs_Arbor_Acr	breed_growth_background	ENSGALG00010021487	KLHL17	0.036401091	0.084958764	0.428455983	0.668319174	0.785094937	positive
PRJNA729271	:ed_Lushi_vs_Arbor_Acr	breed_growth_background	ENSGALG00010021488	HYI	-0.097705444	0.075474709	-1.294545488	0.195477068	0.343447284	negative
PRJNA729271	:ed_Lushi_vs_Arbor_Acr	breed_growth_background	ENSGALG00010021489	CNPPD1	-0.006367473	0.046038105	-0.138308757	0.889996405	0.935632281	negative
PRJNA729271	:ed_Lushi_vs_Arbor_Acr	breed_growth_background	ENSGALG00010021490	ACACB	0.227106028	0.170046527	1.33555229	0.181695653	0.326354212	positive
PRJNA729271	:ed_Lushi_vs_Arbor_Acr	breed_growth_background	ENSGALG00010021492	PARP6	0.110947387	0.084378128	1.314883249	0.188549143	0.334478011	positive
PRJNA729271	:ed_Lushi_vs_Arbor_Acr	breed_growth_background	ENSGALG00010021493	UNC93B1	-0.539905517	0.072078124	-7.490560034	6.85803E-14	4.73368E-11	negative
PRJNA729271	:ed_Lushi_vs_Arbor_Acr	breed_growth_background	ENSGALG00010021494	SLC2A10	-0.312636278	0.132617072	-2.357436142	0.01840162	0.064459034	negative
PRJNA729271	:ed_Lushi_vs_Arbor_Acr	breed_growth_background	ENSGALG00010021496	MTMR14	0.129886384	0.062139036	2.090254254	0.036594966	0.105414157	positive
PRJNA729271	:ed_Lushi_vs_Arbor_Acr	breed_growth_background	ENSGALG00010021498	B3GALT6	-0.003506362	0.06199463	-0.056559122	0.954896398	0.974885706	negative
PRJNA729271	:ed_Lushi_vs_Arbor_Acr	breed_growth_background	ENSGALG00010021501	SLC23A3	0.884934228	0.281589249	3.142642101	0.001674304	0.011707207	positive
PRJNA729271	:ed_Lushi_vs_Arbor_Acr	breed_growth_background	ENSGALG00010021502	DNAJB12	0.200295535	0.078261942	2.559296774	0.010488416	0.043292807	positive
PRJNA729271	:ed_Lushi_vs_Arbor_Acr	breed_growth_background	ENSGALG00010021503	KCNS1	-0.176442032	0.306567144	-0.575541233	0.564925313	0.704779404	negative
PRJNA729271	:ed_Lushi_vs_Arbor_Acr	breed_growth_background	ENSGALG00010021504	ODR4	-0.018101088	0.043105356	-0.419926649	0.674539039	0.789585995	negative
PRJNA729271	:ed_Lushi_vs_Arbor_Acr	breed_growth_background	ENSGALG00010021505	IHH	0.020882373	0.321599378	0.064932876	0.948227444	0.971621434	positive
PRJNA729271	:ed_Lushi_vs_Arbor_Acr	breed_growth_background	ENSGALG00010021506	MAX	0.314483347	0.138554592	2.269743228	0.023223167	0.075952122	positive
PRJNA729271	:ed_Lushi_vs_Arbor_Acr	breed_growth_background	ENSGALG00010021509	DDIT4	0.21814641	0.148404527	1.46994444	0.141576802	0.274315699	positive
PRJNA729271	:ed_Lushi_vs_Arbor_Acr	breed_growth_background	ENSGALG00010021510	NTSR1	0.446750984	0.216214954	2.066235361	0.038806261	0.11006609	positive
PRJNA729271	:ed_Lushi_vs_Arbor_Acr	breed_growth_background	ENSGALG00010021512	CPNE9	-0.376880845	0.207262559	-1.818373985	0.069006992	0.166238382	negative
PRJNA729271	:ed_Lushi_vs_Arbor_Acr	breed_growth_background	ENSGALG00010021513	SDF4	-0.028573977	0.052149239	-0.547927016	0.583742018	0.719624132	negative
PRJNA729271	:ed_Lushi_vs_Arbor_Acr	breed_growth_background	ENSGALG00010021514	TPR	0.144490224	0.042555381	3.395345578	0.00068542	0.006217811	positive
PRJNA729271	:ed_Lushi_vs_Arbor_Acr	breed_growth_background	ENSGALG00010021516	SRSF5	-0.179982873	0.081743122	-2.201810598	0.02767869	0.085763705	negative
PRJNA729271	:ed_Lushi_vs_Arbor_Acr	breed_growth_background	ENSGALG00010021518	CPEB3	0.189998422	0.077612103	2.44805147	0.014363114	0.053755056	positive
PRJNA729271	:ed_Lushi_vs_Arbor_Acr	breed_growth_background	ENSGALG00010021519	FNTB	0.020825952	0.053551718	0.388894178	0.697354429	0.804953713	positive

PRJNA729271	ed_Lushi_vs_Arbor_Acr	breed_growth_background	ENSGALG00010021521	ENSGALG00010021521	-0.05721598	0.142047805	-0.402793836	0.687099896	0.797989263	negative
PRJNA729271	ed_Lushi_vs_Arbor_Acr	breed_growth_background	ENSGALG00010021523	CELF6	0.247980789	0.087499635	2.83407798	0.004595812	0.023954077	positive
PRJNA729271	ed_Lushi_vs_Arbor_Acr	breed_growth_background	ENSGALG00010021525	ENSGALG00010021525	0.316445369	0.407215655	0.777095294	0.43710255	0.594520171	positive
PRJNA729271	ed_Lushi_vs_Arbor_Acr	breed_growth_background	ENSGALG00010021526	KCNJ12	-0.050079837	0.115140282	-0.434946275	0.663601425	0.781643316	negative
PRJNA729271	ed_Lushi_vs_Arbor_Acr	breed_growth_background	ENSGALG00010021528	BRPF1	0.305315494	0.089518679	3.410634495	0.000648119	0.005987563	positive
PRJNA729271	ed_Lushi_vs_Arbor_Acr	breed_growth_background	ENSGALG00010021529	BTAF1	0.032623683	0.040644312	0.802662933	0.422169583	0.581300285	positive
PRJNA729271	ed_Lushi_vs_Arbor_Acr	breed_growth_background	ENSGALG00010021530	MLF2	0.022841801	0.032901848	0.69424067	0.487531291	0.639352761	positive
PRJNA729271	ed_Lushi_vs_Arbor_Acr	breed_growth_background	ENSGALG00010021531	RAB15	-0.031913618	0.15007833	-0.212646409	0.83160277	0.897615768	negative
PRJNA729271	ed_Lushi_vs_Arbor_Acr	breed_growth_background	ENSGALG00010021532	DPP10	0.277055102	0.099229719	2.79205771	0.005237401	0.026387255	positive
PRJNA729271	ed_Lushi_vs_Arbor_Acr	breed_growth_background	ENSGALG00010021533	SSX2IP	-0.324151407	0.183397157	-1.767483273	0.077147328	0.179350524	negative
PRJNA729271	ed_Lushi_vs_Arbor_Acr	breed_growth_background	ENSGALG00010021534	KIFC3	0.130972262	0.090509109	1.447061664	0.147879654	0.282711103	positive
PRJNA729271	ed_Lushi_vs_Arbor_Acr	breed_growth_background	ENSGALG00010021535	SUSD6	0.001768236	0.046179932	0.038290143	0.96945635	0.982470096	positive
PRJNA729271	ed_Lushi_vs_Arbor_Acr	breed_growth_background	ENSGALG00010021538	TNFRSF18	0.025215825	0.222193074	0.113486096	0.909645185	0.948651483	positive
PRJNA729271	ed_Lushi_vs_Arbor_Acr	breed_growth_background	ENSGALG00010021539	HEXA	-0.175425856	0.064927615	-2.70186819	0.006895109	0.032054075	negative
PRJNA729271	ed_Lushi_vs_Arbor_Acr	breed_growth_background	ENSGALG00010021540	STK4	-0.082179384	0.042360841	-1.939984721	0.052381547	0.137077184	negative
PRJNA729271	ed_Lushi_vs_Arbor_Acr	breed_growth_background	ENSGALG00010021541	CYP27A1	0.595664718	0.146313126	4.071163895	4.67788E-05	0.000918779	positive
PRJNA729271	ed_Lushi_vs_Arbor_Acr	breed_growth_background	ENSGALG00010021543	PLEKHD1	0.64774388	0.141805054	4.567847617	4.92758E-06	0.000172525	positive
PRJNA729271	ed_Lushi_vs_Arbor_Acr	breed_growth_background	ENSGALG00010021545	CYB5R2	-0.10647741	0.078824905	-1.350809232	0.17675655	0.320782045	negative
PRJNA729271	ed_Lushi_vs_Arbor_Acr	breed_growth_background	ENSGALG00010021546	OGG1	-0.177261912	0.099366104	-1.783927362	0.074435469	0.175323712	negative
PRJNA729271	ed_Lushi_vs_Arbor_Acr	breed_growth_background	ENSGALG00010021549	ANAPC16	0.036672644	0.048985547	0.748642129	0.454072933	0.610328928	positive
PRJNA729271	ed_Lushi_vs_Arbor_Acr	breed_growth_background	ENSGALG00010021550	MAP2K3	0.141057863	0.092090437	1.531731931	0.125588599	0.25199429	positive
PRJNA729271	ed_Lushi_vs_Arbor_Acr	breed_growth_background	ENSGALG00010021552	ENSGALG00010021552	0.003575314	0.091762403	0.03896273	0.968920103	0.982376831	positive
PRJNA729271	ed_Lushi_vs_Arbor_Acr	breed_growth_background	ENSGALG00010021553	SLC39A9	-0.067697005	0.046869987	-1.444357242	0.148638512	0.283563468	negative
PRJNA729271	ed_Lushi_vs_Arbor_Acr	breed_growth_background	ENSGALG00010021554	SNX11	-0.011898752	0.079385301	-0.149886089	0.880854487	0.929121364	negative
PRJNA729271	ed_Lushi_vs_Arbor_Acr	breed_growth_background	ENSGALG00010021556	CAMK1	0.330156456	0.07737511	4.266959423	1.98155E-05	0.000477913	positive
PRJNA729271	ed_Lushi_vs_Arbor_Acr	breed_growth_background	ENSGALG00010021558	CHURC1	0.053994651	0.095948969	0.562743425	0.57360961	0.711482353	positive
PRJNA729271	ed_Lushi_vs_Arbor_Acr	breed_growth_background	ENSGALG00010021559	MYO1H	-0.955990394	0.328070925	-2.913974757	0.003568588	0.01997169	negative
PRJNA729271	ed_Lushi_vs_Arbor_Acr	breed_growth_background	ENSGALG00010021561	TGIF2	0.02809674	0.053834654	0.521908067	0.601734341	0.734248613	positive
PRJNA729271	ed_Lushi_vs_Arbor_Acr	breed_growth_background	ENSGALG00010021562	LRP6	-0.071403435	0.044269286	-1.612933941	0.10675886	0.225512924	negative
PRJNA729271	ed_Lushi_vs_Arbor_Acr	breed_growth_background	ENSGALG00010021564	PRKAG3	0.519668614	0.134938104	3.851162842	0.000117558	0.001808925	positive
PRJNA729271	ed_Lushi_vs_Arbor_Acr	breed_growth_background	ENSGALG00010021565	CBX1	-0.045095	0.057003124	-0.791096982	0.4288874	0.58781419	negative
PRJNA729271	ed_Lushi_vs_Arbor_Acr	breed_growth_background	ENSGALG00010021566	ENSGALG00010021566	-0.002918244	0.052794758	-0.055275265	0.955919167	0.975434589	negative
PRJNA729271	ed_Lushi_vs_Arbor_Acr	breed_growth_background	ENSGALG00010021568	TADA3	0.264396077	0.076137675	3.472605071	0.000515433	0.005075546	positive
PRJNA729271	ed_Lushi_vs_Arbor_Acr	breed_growth_background	ENSGALG00010021570	KATNB1	-0.101651161	0.05750672	-1.767639697	0.077121157	0.179350524	negative
PRJNA729271	ed_Lushi_vs_Arbor_Acr	breed_growth_background	ENSGALG00010021572	MYL9	-0.345568394	0.060577445	-5.704571944	1.16636E-08	1.5654E-06	negative
PRJNA729271	ed_Lushi_vs_Arbor_Acr	breed_growth_background	ENSGALG00010021573	NATD1	0.107411387	0.104851265	1.0244167	0.305638498	0.468279164	positive
PRJNA729271	ed_Lushi_vs_Arbor_Acr	breed_growth_background	ENSGALG00010021574	TTLL4	0.617317278	0.121504769	5.080601232	3.76242E-07	2.2783E-05	positive
PRJNA729271	ed_Lushi_vs_Arbor_Acr	breed_growth_background	ENSGALG00010021575	ADGRL2	-0.087151623	0.044566682	-1.955533096	0.050520181	0.133495486	negative
PRJNA729271	ed_Lushi_vs_Arbor_Acr	breed_growth_background	ENSGALG00010021576	ERH	-0.002646054	0.065864414	-0.040174258	0.9679542	0.981793645	negative
PRJNA729271	ed_Lushi_vs_Arbor_Acr	breed_growth_background	ENSGALG00010021577	ARIH1	0.064549096	0.041288669	1.563361012	0.117967705	0.241687899	positive
PRJNA729271	ed_Lushi_vs_Arbor_Acr	breed_growth_background	ENSGALG00010021578	ENSGALG00010021578	0.460149061	0.380165947	1.210390003	0.226129276	0.379342808	positive
PRJNA729271	ed_Lushi_vs_Arbor_Acr	breed_growth_background	ENSGALG00010021579	C20H20ORF24	0.050694805	0.060101458	0.843487107	0.398956074	0.560192608	positive
PRJNA729271	ed_Lushi_vs_Arbor_Acr	breed_growth_background	ENSGALG00010021580	KCTD10	0.125609862	0.059776603	2.101321523	0.035612752	0.103324026	positive
PRJNA729271	ed_Lushi_vs_Arbor_Acr	breed_growth_background	ENSGALG00010021582	STK36	-0.093862131	0.079479639	-1.180958187	0.237619334	0.392735718	negative
PRJNA729271	ed_Lushi_vs_Arbor_Acr	breed_growth_background	ENSGALG00010021583	NFE2L1	0.292943972	0.118051857	2.481485513	0.013083603	0.050464828	positive
PRJNA729271	ed_Lushi_vs_Arbor_Acr	breed_growth_background	ENSGALG00010021584	OVCH2	0.155997822	0.205780882	0.758077335	0.448404687	0.604785144	positive
PRJNA729271	ed_Lushi_vs_Arbor_Acr	breed_growth_background	ENSGALG00010021586	BBS4	-0.177616919	0.066912573	-2.654462535	0.007943486	0.035460681	negative
PRJNA729271	ed_Lushi_vs_Arbor_Acr	breed_growth_background	ENSGALG00010021588	RNF25	0.287598072	0.074330553	3.869177093	0.000109203	0.001700217	positive
PRJNA729271	ed_Lushi_vs_Arbor_Acr	breed_growth_background	ENSGALG00010021593	DCAF7	-0.024904924	0.031164698	-0.799138938	0.424209854	0.583324402	negative
PRJNA729271	ed_Lushi_vs_Arbor_Acr	breed_growth_background	ENSGALG00010021594	ADPGK	-0.100702244	0.04951985	-2.033573269	0.041994639	0.116611551	negative
PRJNA729271	ed_Lushi_vs_Arbor_Acr	breed_growth_background	ENSGALG00010021595	UBE3B	-0.016197502	0.045555992	-0.355551524	0.722176453	0.821788953	negative
PRJNA729271	ed_Lushi_vs_Arbor_Acr	breed_growth_background	ENSGALG00010021597	GGT7	-0.21258781	0.101393927	-2.096652288	0.036024367	0.104184597	negative
PRJNA729271	ed_Lushi_vs_Arbor_Acr	breed_growth_background	ENSGALG00010021599	ARPC4	-0.057250251	0.055609668	-1.029501743	0.303243961	0.465874003	negative
PRJNA729271	ed_Lushi_vs_Arbor_Acr	breed_growth_background	ENSGALG00010021600	FMN1	-0.153645937	0.071191516	-2.158205722	0.030911841	0.093056517	negative
PRJNA729271	ed_Lushi_vs_Arbor_Acr	breed_growth_background	ENSGALG00010021601	LPAR5	-0.102115514	0.207401345	-0.492357051	0.622466956	0.749203564	negative
PRJNA729271	ed_Lushi_vs_Arbor_Acr	breed_growth_background	ENSGALG00010021602	PLEKHG4	0.004011281	0.052557762	0.076321387	0.939163411	0.966697969	positive
PRJNA729271	ed_Lushi_vs_Arbor_Acr	breed_growth_background	ENSGALG00010021604	SPTB	0.328363336	0.101133883	3.246818224	0.001167029	0.00914383	positive
PRJNA729271	ed_Lushi_vs_Arbor_Acr	breed_growth_background	ENSGALG00010021605	MAP3K3	0.118999531	0.06074318	1.959059959	0.050105765	0.132772549	positive
PRJNA729271	ed_Lushi_vs_Arbor_Acr	breed_growth_background	ENSGALG00010021606	RNF8	0.055797229	0.051149693	1.090861462	0.275333847	0.434225232	positive
PRJNA729271	ed_Lushi_vs_Arbor_Acr	breed_growth_background	ENSGALG00010021607	SLC9A5	0.086538401	0.104295975	0.829738645	0.406686567	0.567419558	positive
PRJNA729271	ed_Lushi_vs_Arbor_Acr	breed_growth_background	ENSGALG00010021609	ENSGALG00010021609	0.191663246	0.108760988	1.762242593	0.078028319	0.180731939	positive
PRJNA729271	ed_Lushi_vs_Arbor_Acr	breed_growth_background	ENSGALG00010021610	TMEM11	0.037957251	0.048739799	0.778773229	0.43611331	0.593883106	positive
PRJNA729271	ed_Lushi_vs_Arbor_Acr	breed_growth_background	ENSGALG00010021611	GREM1	0.130421512	0.323926977	0.402626274	0.687223178	0.797989263	positive
PRJNA729271	ed_Lushi_vs_Arbor_Acr	breed_growth_background	ENSGALG00010021612	BCS1L	0.091411733	0.072092529	1.267977888	0.204805847	0.354213191	positive
PRJNA729271	ed_Lushi_vs_Arbor_Acr	breed_growth_background	ENSGALG00010021613	ADGRL4	-0.37062285	0.079688654	-4.650886053	3.30512E-06	0.00012284	negative
PRJNA729271	ed_Lushi_vs_Arbor_Acr	breed_growth_background	ENSGALG00010021615	FLVCR1	-0.19666263	0.098010167	-2.006553362	0.044797241	0.122331578	negative
PRJNA729271	ed_Lushi_vs_Arbor_Acr	breed_growth_background	ENSGALG00010021617	SORBS1	-0.062873472	0.059657235	-1.053911947	0.291923233	0.453297138	negative
PRJNA729271	ed_Lushi_vs_Arbor_Acr	breed_growth_background	ENSGALG00010021618	NEO1	-0.01805002	0.057877491	-0.311865979	0.755142379	0.845107923	negative
PRJNA729271	ed_Lushi_vs_Arbor_Acr	breed_growth_background	ENSGALG00010021619	FHOD1	0.063711078	0.094926389	0.671162979	0.50211671	0.65316178	positive
PRJNA729271	ed_Lushi_vs_Arbor_Acr	breed_growth_background	ENSGALG00010021621	ZNF518A	-0.021946026	0.073658005	-0.297944883	0.765745231	0.853185631	negative
PRJNA729271	ed_Lushi_vs_Arbor_Acr	breed_growth_background	ENSGALG00010021622	PTHLH	-0.43818088	0.144194681	-3.038814455	0.002375111	0.015055655	negative
PRJNA729271	ed_Lushi_vs_Arbor_Acr	breed_growth_background	ENSGALG00010021623	FAR1	0.152158099	0.069448875	2.19093685	0.028456363	0.087481437	positive
PRJNA729271	ed_Lushi_vs_Arbor_Acr	breed_growth_background	ENSGALG00010021624	HSD11B2	-0.324753321	0.198278063	-1.637868137	0.101449204	0.217305484	negative
PRJNA729271	ed_Lushi_vs_Arbor_Acr	breed_growth_background	ENSGALG00010021625	HCN4	-0.479959109	0.523430504	-0.916949062	0.359169333	0.522549381	negative
PRJNA729271	ed_Lushi_vs_Arbor_Acr	breed_growth_background	ENSGALG00010021626	ZNF142	0.470971857	0.114309673	4.120140018	3.78642E-05	0.000794091	positive
PRJNA729271	ed_Lushi_vs_Arbor_Acr	breed_growth_background	ENSGALG00010021627	NCOA6	0.124235365	0.05573536	2.229022386	0.025812415	0.081835293	positive
PRJNA729271	ed_Lushi_vs_Arbor_Acr	breed_growth_background	ENSGALG00010021628	REC114	-0.654805036	0.322839336	-2.028269055	0.042532795	0.117815803	negative
PRJNA729271	ed_Lushi_vs_Arbor_Acr	breed_growth_background	ENSGALG00010021629	DHRS7B	-0.001621044	0.053777369	-0.030143616	0.975952516	0.985790861	negative
PRJNA729271	ed_Lushi_vs_Arbor_Acr	breed_growth_background	ENSGALG00010021630	VTG1	-0.417580938	0.173385014	-2.408402708	0.016022496	0.058376754	negative
PRJNA729271	ed_Lushi_vs_Arbor_Acr	breed_growth_background	ENSGALG00010021632	LIMD2	-0.36570599	0.092458875	-3.955336793	7.64269E-05	0.00130792	negative
PRJNA729271	ed_Lushi_vs_Arbor_Acr	breed_growth_background	ENSGALG00010021633	CCNJ	-0.100430127	0.058979691	-1.702791682	0.08860706	0.197837236	negative
PRJNA729271	ed_Lushi_vs_Arbor_Acr	breed_growth_background	ENSGALG00010021634	PLCD4	0.422558484	0.121393052	3.480911605	0.00049971	0.004967971	positive
PRJNA729271	ed_Lushi_vs_Arbor_Acr	breed_growth_background	ENSGALG00010021635	FOXJ2	0.225717325	0.060967473	3.702258173	0.000213689	0.002736239	positive
PRJNA729271	ed_Lushi_vs_Arbor_Acr	breed_growth_background	ENSGALG00010021636	C1orf159	-0.029014053	0.106214869	-0.273163763	0.784727334	0.866703955	negative
PRJNA729271	ed_Lushi_vs_Arbor_Acr	breed_growth_background	ENSGALG00010021637	ATP6V0D1	0.027927008	0.055806367	0.500426914	0.616774506	0.745136395	positive
PRJNA729271	ed_Lushi_vs_Arbor_Acr	breed_growth_background	ENSGALG00010021638	SRC	-0.203439465	0.096468031	-2.108879634	0.034954973	0.10178977	negative
PRJNA729271	ed_Lushi_vs_Arbor_Acr	breed_growth_background	ENSGALG00010021639	SCG5	-0.451943886	0.190284324	-2.375097835	0.017544299	0.062238037	negative
PRJNA729271	ed_Lushi_vs_Arbor_Acr	breed_growth_background	ENSGALG00010021640	PLEKHG3	-0.065781632	0.05877553	-1.119201007	0.263054396	0.421229946	negative
PRJNA729271	ed_Lushi_vs_Arbor_Acr	breed_growth_background	ENSGALG00010021642	NPTN	-0.050085602	0.048707141	-1.028300994	0.303808264	0.466378147	negative
PRJNA729271	ed_Lushi_vs_Arbor_Acr	breed_growth_background	ENSGALG00010021643	ENSGALG00010021643	-0.032166023	0.070733977	-0.454746427	0.649291676	0.771097909	negative
PRJNA729271	ed_Lushi_vs_Arbor_Acr	breed_growth_background	ENSGALG00010021644	TARDBP	0.249423608	0.082613776	3.019152717	0.002534827	0.015722003	positive
PRJNA729271	ed_Lushi_vs_Arbor_Acr	breed_growth_background	ENSGALG00010021645	ASCC1	-0.098048146	0.074670896	-1.313070437	0.189159214	0.335355041	negative
PRJNA729271	ed_Lushi_vs_Arbor_Acr	breed_growth_background	ENSGALG00010021646	RPUSD3	-0.313553242	0.089584332	-3.500090212	0.000465101	0.004711136	negative
PRJNA729271	ed_Lushi_vs_Arbor_Acr	breed_growth_background	ENSGALG00010021647	ARHGAP11B	0.114509568	0.075262262	1.521473909	0.128140954	0.255348244	positive
PRJNA729271	ed_Lushi_vs_Arbor_Acr	breed_growth_background	ENSGALG00010021648	BRE	0.077958789	0.03635168	2.144571822	0.031987097	0.095598552	positive
PRJNA729271	ed_Lushi_vs_Arbor_Acr	breed_growth_background	ENSGALG00010021649	PXN	-0.142765903	0.051571104	-2.768331333	0.005634414	0.027722618	negative
PRJNA729271	ed_Lushi_vs_Arbor_Acr	breed_growth_background	ENSGALG00010021650	CD276	-0.176730393	0.060673257	-2.912821925	0.003581788	0.019991535	negative
PRJNA729271	ed_Lushi_vs_Arbor_Acr	breed_growth_background	ENSGALG00010021651	RNF113B	0.140590419	0.084437669	1.66502013	0.09590874	0.20867565	positive
PRJNA729271	ed_Lushi_vs_Arbor_Acr	breed_growth_background	ENSGALG00010021652	BORCS5	-0.018254899	0.052308092	-0.348988053	0.727098278	0.824541507	negative
PRJNA729271	ed_Lushi_vs_Arbor_Acr	breed_growth_background	ENSGALG00010021655	USP22	0.126075887	0.050910666	2.476414006	0.013270959	0.05099199	positive
PRJNA729271	ed_Lushi_vs_Arbor_Acr	breed_growth_background	ENSGALG00010021656	SETD6	-0.175689626	0.043764193	-4.014460586	5.95819E-05	0.001096582	negative
PRJNA729271	ed_Lushi_vs_Arbor_Acr	breed_growth_background	ENSGALG00010021657	JAGN1	0.056021677	0.076605102	0.731304786	0.464593008	0.619927796	positive
PRJNA729271	ed_Lushi_vs_Arbor_Acr	breed_growth_background	ENSGALG00010021658	STRADA	0.02351927	0.049263375	0.477418977	0.633063804	0.758180603	positive
PRJNA729271	ed_Lushi_vs_Arbor_Acr	breed_growth_background	ENSGALG00010021659	SPOCK2	-0.217236707	0.081176729	-2.676095851	0.007448534	0.033845298	negative
PRJNA729271	ed_Lushi_vs_Arbor_Acr	breed_growth_background	ENSGALG00010021660	RNF223	-0.369007663	0.239342696	-1.541754438	0.123133278	0.248166972	negative
PRJNA729271	ed_Lushi_vs_Arbor_Acr	breed_growth_background	ENSGALG00010021661	DUSP16	0.0814075	0.065314501	1.246392434	0.212620358	0.362879087	positive
PRJNA729271	ed_Lushi_vs_Arbor_Acr	breed_growth_background	ENSGALG00010021662	RASGRP1	-0.033448772	0.087455879	-0.38246453	0.702116834	0.807584789	negative
PRJNA729271	ed_Lushi_vs_Arbor_Acr	breed_growth_background	ENSGALG00010021663	CNOT9	0.161910106	0.074937326	2.160606916	0.030725717	0.092592362	positive
PRJNA729271	ed_Lushi_vs_Arbor_Acr	breed_growth_background	ENSGALG00010021664	TYSND1	0.326580583	0.090539837	3.607037454	0.000309713	0.003539607	positive
PRJNA729271	ed_Lushi_vs_Arbor_Acr	breed_growth_background	ENSGALG00010021665	CNOT1	0.120126797	0.048953123	2.453914899	0.014131042	0.053215572	positive
PRJNA729271	ed_Lushi_vs_Arbor_Acr	breed_growth_background	ENSGALG00010021666	CCDC47	-0.108604372	0.049309163	-2.202519081	0.027628663	0.085684971	negative
PRJNA729271	ed_Lushi_vs_Arbor_Acr	breed_growth_background	ENSGALG00010021667	TBX15	0.010179458	0.102556379	0.099257193	0.920934065	0.956061319	positive
PRJNA729271	ed_Lushi_vs_Arbor_Acr	breed_growth_background	ENSGALG00010021668	PYGO1	0.149788922	0.071341662	2.099599559	0.035764082	0.103585767	positive
PRJNA729271	ed_Lushi_vs_Arbor_Acr	breed_growth_background	ENSGALG00010021669	ENSGALG00010021669	-0.018493569	0.040054048	-0.461715352	0.64428546	0.767308993	negative
PRJNA729271	ed_Lushi_vs_Arbor_Acr	breed_growth_background	ENSGALG00010021670	BLCAP	0.115600108	0.058112196	1.989257255	0.046672815	0.125981835	positive
PRJNA729271	ed_Lushi_vs_Arbor_Acr	breed_growth_background	ENSGALG00010021671	TNFRSF13B	0.062234928	0.367674212	0.169266502	0.865587023	0.919170982	positive
PRJNA729271	ed_Lushi_vs_Arbor_Acr	breed_growth_background	ENSGALG00010021674	ING4	-0.146984404	0.068444917	-2.147484577	0.031754724	0.095100149	negative
PRJNA729271	ed_Lushi_vs_Arbor_Acr	breed_growth_background	ENSGALG00010021675	DDX42	0.015590288	0.032024655	0.486821414	0.626384894	0.752669239	positive
PRJNA729271	ed_Lushi_vs_Arbor_Acr	breed_growth_background	ENSGALG00010021676	TP53INP2	0.335461562	0.147966652	2.267143019	0.02338149	0.076340137	positive
PRJNA729271	ed_Lushi_vs_Arbor_Acr	breed_growth_background	ENSGALG00010021677	ENSGALG00010021677	0.243599316	0.083004168	2.934784142	0.003337798	0.019130637	positive
PRJNA729271	ed_Lushi_vs_Arbor_Acr	breed_growth_background	ENSGALG00010021678	DNAAF4	0.268317577	0.08539773	3.141975523	0.001678121	0.011711294	positive
PRJNA729271	ed_Lushi_vs_Arbor_Acr	breed_growth_background	ENSGALG00010021679	MASP2	-0.782691079	0.628916221	-1.244507699	0.213312768	0.363632667	negative
PRJNA729271	ed_Lushi_vs_Arbor_Acr	breed_growth_background	ENSGALG00010021680	PIGT	-0.095351946	0.063678349	-1.497399786	0.134289264	0.264114367	negative
PRJNA729271	ed_Lushi_vs_Arbor_Acr	breed_growth_background	ENSGALG00010021681	BTBD10	-0.014961295	0.044294753	-0.337766754	0.735538965	0.830503295	negative
PRJNA729271	ed_Lushi_vs_Arbor_Acr	breed_growth_background	ENSGALG00010021682	MYL4	-0.257724282	0.130279434	-1.978242263	0.047901383	0.128389524	negative
PRJNA729271	ed_Lushi_vs_Arbor_Acr	breed_growth_background	ENSGALG00010021683	HFM1	-0.196747555	0.236907192	-0.830483671	0.406265376	0.567214085	negative
PRJNA729271	ed_Lushi_vs_Arbor_Acr	breed_growth_background	ENSGALG00010021684	BIN1	-0.080867914	0.064742858	-1.249063072	0.211642006	0.361635138	negative
PRJNA729271	ed_Lushi_vs_Arbor_Acr	breed_growth_background	ENSGALG00010021685	DBNDD2	-0.317884355	0.074045476	-4.293096237	1.76199E-05	0.000436581	negative
PRJNA729271	ed_Lushi_vs_Arbor_Acr	breed_growth_background	ENSGALG00010021686	CCPG1	0.406493515	0.088160545	4.610832624	4.01059E-06	0.000145698	positive
PRJNA729271	ed_Lushi_vs_Arbor_Acr	breed_growth_background	ENSGALG00010021687	ZNF384	0.081783693	0.057670863	1.418111127	0.156158323	0.293429465	positive
PRJNA729271	ed_Lushi_vs_Arbor_Acr	breed_growth_background	ENSGALG00010021688	ITPR1	-0.046657165	0.062888799	-0.741899454	0.458148257	0.613935044	negative

PRJNA729271	ed_Lushi_vs_Arbor_Acr	breed_growth_background	ENSGALG00010021689	MPRIP	0.044034824	0.058546724	0.752131305	0.4519721	0.608294855	positive
PRJNA729271	ed_Lushi_vs_Arbor_Acr	breed_growth_background	ENSGALG00010021691	PIGB	-0.165263337	0.082704247	-1.998244861	0.04569012	0.124231531	negative
PRJNA729271	ed_Lushi_vs_Arbor_Acr	breed_growth_background	ENSGALG00010021693	PPA1	-0.025563034	0.049523082	-0.516184224	0.605725747	0.73704479	negative
PRJNA729271	ed_Lushi_vs_Arbor_Acr	breed_growth_background	ENSGALG00010021694	ENSGALG00010021694	-0.525533033	0.58237127	-0.902402058	0.366843328	0.530341615	negative
PRJNA729271	ed_Lushi_vs_Arbor_Acr	breed_growth_background	ENSGALG00010021695	FNBP4	0.106123369	0.038468122	2.758735383	0.00580255	0.02828109	positive
PRJNA729271	ed_Lushi_vs_Arbor_Acr	breed_growth_background	ENSGALG00010021697	PIANP	-0.175489436	0.136241999	-1.288071501	0.197721075	0.346105929	negative
PRJNA729271	ed_Lushi_vs_Arbor_Acr	breed_growth_background	ENSGALG00010021698	GYPC	-0.170655962	0.072290731	-2.360689384	0.018241002	0.064128868	negative
PRJNA729271	ed_Lushi_vs_Arbor_Acr	breed_growth_background	ENSGALG00010021699	SYS1	0.028243088	0.046859722	0.602715657	0.546697864	0.690337619	positive
PRJNA729271	ed_Lushi_vs_Arbor_Acr	breed_growth_background	ENSGALG00010021700	FLCN	0.103960706	0.042282726	2.458703977	0.013943954	0.052799794	positive
PRJNA729271	ed_Lushi_vs_Arbor_Acr	breed_growth_background	ENSGALG00010021701	DR1	0.104798993	0.041650939	2.516125607	0.011865289	0.047201289	positive
PRJNA729271	ed_Lushi_vs_Arbor_Acr	breed_growth_background	ENSGALG00010021702	PLD6	0.145894577	0.17582249	0.829783359	0.406661281	0.567419558	positive
PRJNA729271	ed_Lushi_vs_Arbor_Acr	breed_growth_background	ENSGALG00010021703	CD82	-0.233681449	0.093031325	-2.511857682	0.012009751	0.04755655	negative
PRJNA729271	ed_Lushi_vs_Arbor_Acr	breed_growth_background	ENSGALG00010021704	COPS7A	0.078869164	0.054554314	1.44569985	0.148261406	0.283179482	positive
PRJNA729271	ed_Lushi_vs_Arbor_Acr	breed_growth_background	ENSGALG00010021705	CCDC18	0.157126658	0.103748794	1.51449141	0.129901251	0.257581208	positive
PRJNA729271	ed_Lushi_vs_Arbor_Acr	breed_growth_background	ENSGALG00010021706	ENSGALG00010021706	0.134871928	0.084531363	1.5955253	0.110594817	0.231290127	positive
PRJNA729271	ed_Lushi_vs_Arbor_Acr	breed_growth_background	ENSGALG00010021707	DLX2	-0.299115275	0.575281689	-0.519945761	0.603101379	0.73524511	negative
PRJNA729271	ed_Lushi_vs_Arbor_Acr	breed_growth_background	ENSGALG00010021708	CNTNAP5	-0.281126105	0.210224762	-1.33726447	0.181136324	0.325630789	negative
PRJNA729271	ed_Lushi_vs_Arbor_Acr	breed_growth_background	ENSGALG00010021710	ENSGALG00010021710	-0.251211427	0.175110068	-1.434591571	0.151403518	0.287288125	negative
PRJNA729271	ed_Lushi_vs_Arbor_Acr	breed_growth_background	ENSGALG00010021711	ENSGALG00010021711	-0.123854269	0.119774462	-1.03406241	0.301106988	0.463475182	negative
PRJNA729271	ed_Lushi_vs_Arbor_Acr	breed_growth_background	ENSGALG00010021713	RAB27A	-0.072117398	0.043339181	-1.66402309	0.096107815	0.208983316	negative
PRJNA729271	ed_Lushi_vs_Arbor_Acr	breed_growth_background	ENSGALG00010021714	TSPAN18	-0.211321075	0.063046355	-3.351836535	0.000802774	0.006975677	negative
PRJNA729271	ed_Lushi_vs_Arbor_Acr	breed_growth_background	ENSGALG00010021715	MMAB	0.068463797	0.071095026	0.962989959	0.335552562	0.499801315	positive
PRJNA729271	ed_Lushi_vs_Arbor_Acr	breed_growth_background	ENSGALG00010021716	TFCP2L1	-0.523867889	0.209006877	-2.506462455	0.012194601	0.04810211	negative
PRJNA729271	ed_Lushi_vs_Arbor_Acr	breed_growth_background	ENSGALG00010021717	SDC4	-0.108049518	0.105587686	-1.023315517	0.306158689	0.468663027	negative
PRJNA729271	ed_Lushi_vs_Arbor_Acr	breed_growth_background	ENSGALG00010021718	SST7	-0.063972245	0.271751902	-0.235406798	0.813893011	0.885537337	negative
PRJNA729271	ed_Lushi_vs_Arbor_Acr	breed_growth_background	ENSGALG00010021719	BHLHE40	0.288339706	0.113093104	2.549578128	0.010785334	0.044174462	positive
PRJNA729271	ed_Lushi_vs_Arbor_Acr	breed_growth_background	ENSGALG00010021720	CACNB1	0.281361643	0.139311622	2.019656648	0.043419015	0.1195136	positive
PRJNA729271	ed_Lushi_vs_Arbor_Acr	breed_growth_background	ENSGALG00010021721	TP53I11	-0.462335138	0.076738032	-6.024850099	1.69266E-09	3.22972E-07	negative
PRJNA729271	ed_Lushi_vs_Arbor_Acr	breed_growth_background	ENSGALG00010021722	AARS1	-0.1288458	0.085040784	-1.51510598	0.129745567	0.257378131	negative
PRJNA729271	ed_Lushi_vs_Arbor_Acr	breed_growth_background	ENSGALG00010021723	COPS3	0.033366273	0.057241115	0.582907464	0.559955596	0.700911603	positive
PRJNA729271	ed_Lushi_vs_Arbor_Acr	breed_growth_background	ENSGALG00010021725	SH2D6	-0.064529306	0.114853376	-0.561840739	0.574224532	0.711887153	negative
PRJNA729271	ed_Lushi_vs_Arbor_Acr	breed_growth_background	ENSGALG00010021726	ARNTL	0.229097195	0.079225801	2.891699307	0.003831645	0.020990057	positive
PRJNA729271	ed_Lushi_vs_Arbor_Acr	breed_growth_background	ENSGALG00010021727	MVK	0.003791923	0.056073783	0.067623819	0.946085094	0.970470493	positive
PRJNA729271	ed_Lushi_vs_Arbor_Acr	breed_growth_background	ENSGALG00010021728	TMED5	0.024071164	0.034895685	0.68980343	0.490317811	0.642135587	positive
PRJNA729271	ed_Lushi_vs_Arbor_Acr	breed_growth_background	ENSGALG00010021729	RBPJL	-0.023434391	0.373467613	-0.062748121	0.949967078	0.972423971	negative
PRJNA729271	ed_Lushi_vs_Arbor_Acr	breed_growth_background	ENSGALG00010021730	ARL8B	-0.01979624	0.038697763	-0.511560321	0.608958762	0.739701437	negative
PRJNA729271	ed_Lushi_vs_Arbor_Acr	breed_growth_background	ENSGALG00010021731	SRM	0.021379639	0.064952929	0.329155891	0.742037861	0.835210343	positive
PRJNA729271	ed_Lushi_vs_Arbor_Acr	breed_growth_background	ENSGALG00010021732	CASR	0.115448428	0.149824245	0.770559051	0.440968341	0.598430493	positive
PRJNA729271	ed_Lushi_vs_Arbor_Acr	breed_growth_background	ENSGALG00010021733	ENSGALG00010021733	0.461012441	0.317839556	1.450456472	0.146931269	0.281478918	positive
PRJNA729271	ed_Lushi_vs_Arbor_Acr	breed_growth_background	ENSGALG00010021734	PARD3B	-0.084993423	0.034375816	-2.472477274	0.013418024	0.051385539	negative
PRJNA729271	ed_Lushi_vs_Arbor_Acr	breed_growth_background	ENSGALG00010021735	PIGBOS1	0.157495506	0.077416976	2.034379466	0.041913351	0.116452754	positive
PRJNA729271	ed_Lushi_vs_Arbor_Acr	breed_growth_background	ENSGALG00010021736	EDEM1	-0.014309172	0.044410585	-0.322201844	0.747299787	0.838918093	negative
PRJNA729271	ed_Lushi_vs_Arbor_Acr	breed_growth_background	ENSGALG00010021738	PRDM11	-0.048031151	0.039270489	-1.223085117	0.221297549	0.373771612	negative
PRJNA729271	ed_Lushi_vs_Arbor_Acr	breed_growth_background	ENSGALG00010021739	EXOSC10	0.148007378	0.059058118	2.506130936	0.012206041	0.048112905	positive
PRJNA729271	ed_Lushi_vs_Arbor_Acr	breed_growth_background	ENSGALG00010021740	PDLIM1	-0.344530313	0.085109219	-4.048096286	5.16359E-05	0.000988722	negative
PRJNA729271	ed_Lushi_vs_Arbor_Acr	breed_growth_background	ENSGALG00010021741	RPL19	-0.132415185	0.078060777	-1.696308831	0.089827409	0.200007418	negative
PRJNA729271	ed_Lushi_vs_Arbor_Acr	breed_growth_background	ENSGALG00010021742	MATN4	0.709008465	0.273288746	2.594356612	0.009476813	0.040319652	positive
PRJNA729271	ed_Lushi_vs_Arbor_Acr	breed_growth_background	ENSGALG00010021743	SYT13	-0.483672236	0.239518243	-2.019354476	0.04345039	0.119577254	negative
PRJNA729271	ed_Lushi_vs_Arbor_Acr	breed_growth_background	ENSGALG00010021744	LITAF	-0.417629995	0.181683463	-2.29866818	0.021523789	0.071877868	negative
PRJNA729271	ed_Lushi_vs_Arbor_Acr	breed_growth_background	ENSGALG00010021745	RSL24D1	0.210289549	0.067520731	3.114444208	0.001842918	0.01254136	positive
PRJNA729271	ed_Lushi_vs_Arbor_Acr	breed_growth_background	ENSGALG00010021746	MTOR	0.046151802	0.059212092	0.779432053	0.435725249	0.593537964	positive
PRJNA729271	ed_Lushi_vs_Arbor_Acr	breed_growth_background	ENSGALG00010021747	AKTIP	0.190396894	0.061926107	3.074582023	0.002107977	0.013769776	positive
PRJNA729271	ed_Lushi_vs_Arbor_Acr	breed_growth_background	ENSGALG00010021748	CDCA7	-0.314927518	0.095981623	-3.281123091	0.001033946	0.008353985	negative
PRJNA729271	ed_Lushi_vs_Arbor_Acr	breed_growth_background	ENSGALG00010021749	CLASP1	0.042947338	0.04871633	0.881579908	0.378004024	0.540321131	positive
PRJNA729271	ed_Lushi_vs_Arbor_Acr	breed_growth_background	ENSGALG00010021750	HELLS	-0.217405666	0.082270607	-2.642567894	0.008227997	0.036316934	negative
PRJNA729271	ed_Lushi_vs_Arbor_Acr	breed_growth_background	ENSGALG00010021751	MTCH2	-0.080355441	0.050365501	-1.595446098	0.110612515	0.231293768	negative
PRJNA729271	ed_Lushi_vs_Arbor_Acr	breed_growth_background	ENSGALG00010021752	CSTB	0.011036129	0.059441014	0.185665213	0.85270731	0.911100026	positive
PRJNA729271	ed_Lushi_vs_Arbor_Acr	breed_growth_background	ENSGALG00010021753	MTF2	-0.061960825	0.052952146	-1.170128686	0.241949186	0.397603761	negative
PRJNA729271	ed_Lushi_vs_Arbor_Acr	breed_growth_background	ENSGALG00010021754	SLC17A9	0.107653695	0.280040978	0.38442122	0.700666275	0.806940859	positive
PRJNA729271	ed_Lushi_vs_Arbor_Acr	breed_growth_background	ENSGALG00010021755	ENSGALG00010021755	0.065012515	0.082027174	0.792572903	0.428026701	0.587078637	positive
PRJNA729271	ed_Lushi_vs_Arbor_Acr	breed_growth_background	ENSGALG00010021756	USP42	-0.044957104	0.07078209	-0.63514801	0.525331887	0.671844513	negative
PRJNA729271	ed_Lushi_vs_Arbor_Acr	breed_growth_background	ENSGALG00010021757	ZP4	0.222706059	0.197374283	1.12834385	0.259174731	0.41681324	positive
PRJNA729271	ed_Lushi_vs_Arbor_Acr	breed_growth_background	ENSGALG00010021758	RASSF10	-0.076955868	0.242879523	-0.316847905	0.751359013	0.84249624	negative
PRJNA729271	ed_Lushi_vs_Arbor_Acr	breed_growth_background	ENSGALG00010021759	NIFK	-0.001530879	0.050626844	-0.030238475	0.975876864	0.985790861	negative
PRJNA729271	ed_Lushi_vs_Arbor_Acr	breed_growth_background	ENSGALG00010021760	CSTA	0.266791339	0.211642664	1.260574467	0.207462203	0.35659488	positive
PRJNA729271	ed_Lushi_vs_Arbor_Acr	breed_growth_background	ENSGALG00010021761	MAFB	0.003182886	0.091075554	0.034947754	0.972121402	0.983408337	positive
PRJNA729271	ed_Lushi_vs_Arbor_Acr	breed_growth_background	ENSGALG00010021762	LAMB2	0.116757537	0.105476054	1.106957765	0.268312189	0.426866994	positive
PRJNA729271	ed_Lushi_vs_Arbor_Acr	breed_growth_background	ENSGALG00010021764	FAM222A	-0.345239916	0.463829324	-0.744325334	0.456679678	0.612583004	negative
PRJNA729271	ed_Lushi_vs_Arbor_Acr	breed_growth_background	ENSGALG00010021765	EIF2AK1	0.228947657	0.056685674	4.038898017	5.37029E-05	0.001020214	positive
PRJNA729271	ed_Lushi_vs_Arbor_Acr	breed_growth_background	ENSGALG00010021766	AGRN	-0.191794369	0.080104904	-2.394289982	0.016652577	0.059999778	negative
PRJNA729271	ed_Lushi_vs_Arbor_Acr	breed_growth_background	ENSGALG00010021767	CCDC58	-0.086629774	0.114316974	-0.757803247	0.448568778	0.604930792	negative
PRJNA729271	ed_Lushi_vs_Arbor_Acr	breed_growth_background	ENSGALG00010021768	DIPK1A	0.003236282	0.080320799	0.040291951	0.967860371	0.981793645	positive
PRJNA729271	ed_Lushi_vs_Arbor_Acr	breed_growth_background	ENSGALG00010021769	ENSGALG00010021769	0.15786519	0.098277438	1.60632179	0.108203225	0.227866591	positive
PRJNA729271	ed_Lushi_vs_Arbor_Acr	breed_growth_background	ENSGALG00010021770	RALGAPB	0.157859967	0.05257105	3.002792721	0.002675146	0.016306239	positive
PRJNA729271	ed_Lushi_vs_Arbor_Acr	breed_growth_background	ENSGALG00010021771	ANKRD61	0.196299876	0.07732828	2.538526336	0.011132042	0.04518593	positive
PRJNA729271	ed_Lushi_vs_Arbor_Acr	breed_growth_background	ENSGALG00010021772	TSN	0.057723755	0.04514809	1.278542573	0.201058184	0.34994349	positive
PRJNA729271	ed_Lushi_vs_Arbor_Acr	breed_growth_background	ENSGALG00010021773	ENSGALG00010021773	1.248143353	0.542246443	2.301800904	0.021346403	0.071470486	positive
PRJNA729271	ed_Lushi_vs_Arbor_Acr	breed_growth_background	ENSGALG00010021774	FBXL20	0.045858002	0.038603231	1.187931706	0.234860317	0.38928534	positive
PRJNA729271	ed_Lushi_vs_Arbor_Acr	breed_growth_background	ENSGALG00010021775	CCDC36	0.600253978	0.169023984	3.551294689	0.000383341	0.004118997	positive
PRJNA729271	ed_Lushi_vs_Arbor_Acr	breed_growth_background	ENSGALG00010021776	PMS2	0.213556579	0.051402861	4.154566022	3.25906E-05	0.000707214	positive
PRJNA729271	ed_Lushi_vs_Arbor_Acr	breed_growth_background	ENSGALG00010021778	RBL2	0.134500643	0.055808219	2.410050788	0.0159503	0.058266028	positive
PRJNA729271	ed_Lushi_vs_Arbor_Acr	breed_growth_background	ENSGALG00010021779	UNC13C	-0.375498607	0.132720357	-2.829246504	0.004665775	0.024205585	negative
PRJNA729271	ed_Lushi_vs_Arbor_Acr	breed_growth_background	ENSGALG00010021781	RAPGEF4	-0.049452804	0.146337986	-0.337935524	0.735411776	0.830503295	negative
PRJNA729271	ed_Lushi_vs_Arbor_Acr	breed_growth_background	ENSGALG00010021782	ADAM11	-0.463330054	0.174172684	-2.660176339	0.007809975	0.03504817	negative
PRJNA729271	ed_Lushi_vs_Arbor_Acr	breed_growth_background	ENSGALG00010021783	GIT2	-0.071991345	0.026338382	-2.733324528	0.006269852	0.029931501	negative
PRJNA729271	ed_Lushi_vs_Arbor_Acr	breed_growth_background	ENSGALG00010021784	ANGPTL7	0.914372898	0.465805436	1.962993187	0.049646966	0.131825017	positive
PRJNA729271	ed_Lushi_vs_Arbor_Acr	breed_growth_background	ENSGALG00010021785	CCDC71	0.104980563	0.04843583	2.167415395	0.030203194	0.091359621	positive
PRJNA729271	ed_Lushi_vs_Arbor_Acr	breed_growth_background	ENSGALG00010021786	FAM162A	0.154435483	0.092965851	1.661206579	0.096671965	0.20978561	positive
PRJNA729271	ed_Lushi_vs_Arbor_Acr	breed_growth_background	ENSGALG00010021788	RPL5	-0.076951703	0.066238702	-1.161733268	0.245343828	0.401111976	negative
PRJNA729271	ed_Lushi_vs_Arbor_Acr	breed_growth_background	ENSGALG00010021789	CHD9	0.020053557	0.048735796	0.411474892	0.680724348	0.793621767	positive
PRJNA729271	ed_Lushi_vs_Arbor_Acr	breed_growth_background	ENSGALG00010021790	PHF21A	0.240307092	0.06871534	3.49713893	0.000470277	0.004760239	positive
PRJNA729271	ed_Lushi_vs_Arbor_Acr	breed_growth_background	ENSGALG00010021791	PLEKHM2	0.34370089	0.098354531	3.494509982	0.000474933	0.004797318	positive
PRJNA729271	ed_Lushi_vs_Arbor_Acr	breed_growth_background	ENSGALG00010021792	KLHDC8B	0.240692983	0.131750553	1.826884042	0.067717206	0.164250486	positive
PRJNA729271	ed_Lushi_vs_Arbor_Acr	breed_growth_background	ENSGALG00010021793	ENSGALG00010021793	-0.368099819	0.085852251	-4.287596573	1.80617E-05	0.000442985	negative
PRJNA729271	ed_Lushi_vs_Arbor_Acr	breed_growth_background	ENSGALG00010021794	ENSGALG00010021794	0.324062713	0.109846916	2.950130268	0.0031764	0.018490252	positive
PRJNA729271	ed_Lushi_vs_Arbor_Acr	breed_growth_background	ENSGALG00010021795	NDRG3	0.083185584	0.057649391	1.442956861	0.149032624	0.283941625	positive
PRJNA729271	ed_Lushi_vs_Arbor_Acr	breed_growth_background	ENSGALG00010021797	SLC25A34	0.28125158	0.090336256	3.113385379	0.001849543	0.012565983	positive
PRJNA729271	ed_Lushi_vs_Arbor_Acr	breed_growth_background	ENSGALG00010021798	RANBP10	0.270134038	0.097553252	2.769093107	0.005621257	0.027695486	positive
PRJNA729271	ed_Lushi_vs_Arbor_Acr	breed_growth_background	ENSGALG00010021799	ENSGALG00010021799	-0.003116711	0.121407074	-0.025671579	0.979519293	0.98790232	negative
PRJNA729271	ed_Lushi_vs_Arbor_Acr	breed_growth_background	ENSGALG00010021800	TMEM82	0.629126021	0.18360749	3.426472536	0.000611476	0.005696233	positive
PRJNA729271	ed_Lushi_vs_Arbor_Acr	breed_growth_background	ENSGALG00010021801	VAMP1	-0.010529073	0.1304046	-0.080741576	0.935647472	0.964536447	negative
PRJNA729271	ed_Lushi_vs_Arbor_Acr	breed_growth_background	ENSGALG00010021803	TLL2	0.125695936	0.092691991	1.356060372	0.17507996	0.319040238	positive
PRJNA729271	ed_Lushi_vs_Arbor_Acr	breed_growth_background	ENSGALG00010021805	FUT8	-0.077345929	0.05921966	-1.306085318	0.191523545	0.338346592	negative
PRJNA729271	ed_Lushi_vs_Arbor_Acr	breed_growth_background	ENSGALG00010021807	CDC42	0.107907327	0.044423817	2.429042225	0.015138769	0.056021562	positive
PRJNA729271	ed_Lushi_vs_Arbor_Acr	breed_growth_background	ENSGALG00010021810	ANKRD13A	-0.209380519	0.052394492	-3.996231511	6.43588E-05	0.001154555	negative
PRJNA729271	ed_Lushi_vs_Arbor_Acr	breed_growth_background	ENSGALG00010021812	TEAD1	0.089730256	0.044637653	2.010192092	0.044410862	0.121469898	positive
PRJNA729271	ed_Lushi_vs_Arbor_Acr	breed_growth_background	ENSGALG00010021817	EVI5	-0.05400949	0.054090947	-0.998494074	0.318039836	0.481421596	negative
PRJNA729271	ed_Lushi_vs_Arbor_Acr	breed_growth_background	ENSGALG00010021821	PPP1R19L	-0.170762496	0.064601363	-2.643326519	0.008209582	0.036268789	negative
PRJNA729271	ed_Lushi_vs_Arbor_Acr	breed_growth_background	ENSGALG00010021823	ENSGALG00010021823	0.200896098	0.139006126	1.445231977	0.148392737	0.283318325	positive
PRJNA729271	ed_Lushi_vs_Arbor_Acr	breed_growth_background	ENSGALG00010021826	FBXO40	0.093261929	0.106450052	0.876109748	0.380970374	0.542630688	positive
PRJNA729271	ed_Lushi_vs_Arbor_Acr	breed_growth_background	ENSGALG00010021827	CCZ1	-0.042277545	0.052043697	-0.812347066	0.416592511	0.576800409	negative
PRJNA729271	ed_Lushi_vs_Arbor_Acr	breed_growth_background	ENSGALG00010021829	ERCC6	-0.039592016	0.052360795	-0.75613856	0.449566125	0.605810259	negative
PRJNA729271	ed_Lushi_vs_Arbor_Acr	breed_growth_background	ENSGALG00010021831	ENSGALG00010021831	-0.350226266	0.198686021	-1.762712166	0.077949048	0.18060605	negative
PRJNA729271	ed_Lushi_vs_Arbor_Acr	breed_growth_background	ENSGALG00010021835	NUDT8	-0.228990271	0.093104697	-2.459492159	0.013913374	0.052739635	negative
PRJNA729271	ed_Lushi_vs_Arbor_Acr	breed_growth_background	ENSGALG00010021837	ENSGALG00010021837	-1.000353412	0.175033959	-5.715196183	1.09578E-08	1.48442E-06	negative
PRJNA729271	ed_Lushi_vs_Arbor_Acr	breed_growth_background	ENSGALG00010021839	C1orf50	0.008777619	0.0660625	0.132868401	0.894297458	0.938112726	positive
PRJNA729271	ed_Lushi_vs_Arbor_Acr	breed_growth_background	ENSGALG00010021840	ENSGALG00010021840	0.013379018	0.117853035	0.113522895	0.909616013	0.948651483	positive
PRJNA729271	ed_Lushi_vs_Arbor_Acr	breed_growth_background	ENSGALG00010021841	CENPT	-0.184455811	0.051533167	-3.57936103	0.000344435	0.003808231	negative
PRJNA729271	ed_Lushi_vs_Arbor_Acr	breed_growth_background	ENSGALG00010021842	ENSGALG00010021842	0.116157898	0.100970206	1.150417566	0.249971928	0.405931336	positive
PRJNA729271	ed_Lushi_vs_Arbor_Acr	breed_growth_background	ENSGALG00010021843	ENSGALG00010021843	-0.266448255	0.207218505	-1.285832339	0.198501581	0.347120331	negative
PRJNA729271	ed_Lushi_vs_Arbor_Acr	breed_growth_background	ENSGALG00010021847	P3H1	-0.089804415	0.081287577	-1.104774164	0.269257458	0.427872933	negative
PRJNA729271	ed_Lushi_vs_Arbor_Acr	breed_growth_background	ENSGALG00010021848	LARGE2	-0.244448421	0.072910346	-3.352726106	0.000800199	0.006961585	negative
PRJNA729271	ed_Lushi_vs_Arbor_Acr	breed_growth_background	ENSGALG00010021851	ENSGALG00010021851	0.150104954	0.116267645	1.291029449	0.196693466	0.345166076	positive
PRJNA729271	ed_Lushi_vs_Arbor_Acr	breed_growth_background	ENSGALG00010021853	ENSGALG00010021853	0.089999525	0.330051833	0.272683002	0.785096902	0.867046065	positive
PRJNA729271	ed_Lushi_vs_Arbor_Acr	breed_growth_background	ENSGALG00010021854	TAPBPL	0.114979912	0.082303126	1.397029711	0.162404637	0.30180195	positive
PRJNA729271	ed_Lushi_vs_Arbor_Acr	breed_growth_background	ENSGALG00010021860	ENSGALG00010021860	0.037668196	0.14039728	0.26829719	0.788470569	0.868633582	positive
PRJNA729271	ed_Lushi_vs_Arbor_Acr	breed_growth_background	ENSGALG00010021862	PDK1	-0.284242073	0.057106907	-4.977367631	6.44548E-07	3.43483E-05	negative
PRJNA729271	ed_Lushi_vs_Arbor_Acr	breed_growth_background	ENSGALG00010021863	CLDN19	1.042742839	0.155146479	6.721021611	1.80455E-11	6.37974E-09	positive
PRJNA729271	ed_Lushi_vs_Arbor_Acr	breed_growth_background	ENSGALG00010021864	GFI1	0.340977852	0.236046033	1.44453964	0.148587238	0.283563468	positive
PRJNA729271	ed_Lushi_vs_Arbor_Acr	breed_growth_background	ENSGALG00010021866	SCNN1A	0.021127764	0.19582619	0.107890392	0.914082638	0.951499307	positive
PRJNA729271	ed_Lushi_vs_Arbor_Acr	breed_growth_background	ENSGALG00010021867	CXCL12	-0.283693137	0.097424406	-2.9119309	0.003592021	0.020033095	negative

PRJNA729271	ɛed_Lushi_vs_Arbor_Acr	breed_growth_background	ENSGALG00010021869	GJC3	-0.20041174	0.0655495	-3.057410661	0.002232582	0.014399096	negative
PRJNA729271	ɛed_Lushi_vs_Arbor_Acr	breed_growth_background	ENSGALG00010021870	ENSGALG00010021870	-0.346064501	0.091077306	-3.799678708	0.000144884	0.002077241	negative
PRJNA729271	ɛed_Lushi_vs_Arbor_Acr	breed_growth_background	ENSGALG00010021871	ENSGALG00010021871	-0.09155325	0.118343576	-0.773622473	0.439154078	0.596470986	negative
PRJNA729271	ɛed_Lushi_vs_Arbor_Acr	breed_growth_background	ENSGALG00010021874	TMCC1	0.086450906	0.042117545	2.052610281	0.04011039	0.112521792	positive
PRJNA729271	ɛed_Lushi_vs_Arbor_Acr	breed_growth_background	ENSGALG00010021877	PLEKHN1	-0.21691826	0.078556437	-2.7613047	0.005757094	0.028106795	negative
PRJNA729271	ɛed_Lushi_vs_Arbor_Acr	breed_growth_background	ENSGALG00010021879	CREBL2	0.22033545	0.083164365	2.649397381	0.008063546	0.03579783	positive
PRJNA729271	ɛed_Lushi_vs_Arbor_Acr	breed_growth_background	ENSGALG00010021881	PHF20	0.09057402	0.026826578	3.376279343	0.000734733	0.006532652	positive
PRJNA729271	ɛed_Lushi_vs_Arbor_Acr	breed_growth_background	ENSGALG00010021887	NUTF2	0.092103396	0.059628014	1.544632968	0.122435067	0.247237806	positive
PRJNA729271	ɛed_Lushi_vs_Arbor_Acr	breed_growth_background	ENSGALG00010021892	NDUFV1	0.134498327	0.065113577	2.065595727	0.038866668	0.110184306	positive
PRJNA729271	ɛed_Lushi_vs_Arbor_Acr	breed_growth_background	ENSGALG00010021897	ENSGALG00010021897	-0.15809632	0.118824226	-1.330505781	0.183351683	0.328433347	negative
PRJNA729271	ɛed_Lushi_vs_Arbor_Acr	breed_growth_background	ENSGALG00010021902	LMTK2	0.135373901	0.063802355	2.121769647	0.033857087	0.099444472	positive
PRJNA729271	ɛed_Lushi_vs_Arbor_Acr	breed_growth_background	ENSGALG00010021903	MORC2	-0.130412236	0.042909524	-3.039237553	0.002371778	0.015052065	negative
PRJNA729271	ɛed_Lushi_vs_Arbor_Acr	breed_growth_background	ENSGALG00010021905	RBSN	-0.040404169	0.039716794	-1.017306911	0.30900745	0.47177828	negative
PRJNA729271	ɛed_Lushi_vs_Arbor_Acr	breed_growth_background	ENSGALG00010021908	EDC4	0.191897737	0.058326504	3.290060684	0.001001658	0.008154066	positive
PRJNA729271	ɛed_Lushi_vs_Arbor_Acr	breed_growth_background	ENSGALG00010021913	ENSGALG00010021913	-0.217112677	0.093642622	-2.31852411	0.020420854	0.069288455	negative
PRJNA729271	ɛed_Lushi_vs_Arbor_Acr	breed_growth_background	ENSGALG00010021916	MRPS25	-0.113670454	0.079814091	-1.424190293	0.154391394	0.290901242	negative
PRJNA729271	ɛed_Lushi_vs_Arbor_Acr	breed_growth_background	ENSGALG00010021919	PARVA	-0.184014745	0.066697669	-2.758938164	0.005798951	0.028273054	negative
PRJNA729271	ɛed_Lushi_vs_Arbor_Acr	breed_growth_background	ENSGALG00010021922	MRPL51	-0.049720463	0.074319433	-0.669010265	0.50348893	0.654183582	negative
PRJNA729271	ɛed_Lushi_vs_Arbor_Acr	breed_growth_background	ENSGALG00010021924	ENSGALG00010021924	0.263054341	0.271441048	0.969103026	0.332493788	0.496858838	positive
PRJNA729271	ɛed_Lushi_vs_Arbor_Acr	breed_growth_background	ENSGALG00010021928	ENSGALG00010021928	-3.911751336	0.506803423	-7.71847853	1.17726E-14	1.10839E-11	negative
PRJNA729271	ɛed_Lushi_vs_Arbor_Acr	breed_growth_background	ENSGALG00010021931	ITGA6	-0.336065364	0.065023705	-5.168351533	2.36168E-07	1.63012E-05	negative
PRJNA729271	ɛed_Lushi_vs_Arbor_Acr	breed_growth_background	ENSGALG00010021932	RPAP2	0.256589662	0.08702077	2.948602539	0.003192142	0.018537701	positive
PRJNA729271	ɛed_Lushi_vs_Arbor_Acr	breed_growth_background	ENSGALG00010021933	PRR14L	0.034629949	0.048235275	0.717938265	0.472795353	0.626604063	positive
PRJNA729271	ɛed_Lushi_vs_Arbor_Acr	breed_growth_background	ENSGALG00010021934	EFTUD2	-0.04091846	0.0363206	-1.126590984	0.259915453	0.41772641	negative
PRJNA729271	ɛed_Lushi_vs_Arbor_Acr	breed_growth_background	ENSGALG00010021935	CETP	0.071563971	0.144309687	0.495905524	0.619961069	0.747615282	positive
PRJNA729271	ɛed_Lushi_vs_Arbor_Acr	breed_growth_background	ENSGALG00010021938	CABP2	0.211708536	0.093075462	2.27459022	0.022930523	0.07531791	positive
PRJNA729271	ɛed_Lushi_vs_Arbor_Acr	breed_growth_background	ENSGALG00010021943	OCM3	-1.200464458	0.304784035	-3.938737988			negative
PRJNA729271	ɛed_Lushi_vs_Arbor_Acr	breed_growth_background	ENSGALG00010021945	C1QTNF4	-0.303168107	0.118829617	-2.551284054	0.010732681	0.04403346	negative
PRJNA729271	ɛed_Lushi_vs_Arbor_Acr	breed_growth_background	ENSGALG00010021947	CDK2AP2	0.29202768	0.132663056	2.20127358	0.027716662	0.085844662	positive
PRJNA729271	ɛed_Lushi_vs_Arbor_Acr	breed_growth_background	ENSGALG00010021949	ENSGALG00010021949	-0.16650263	0.134135887	-1.241298159	0.214495624	0.365262462	negative
PRJNA729271	ɛed_Lushi_vs_Arbor_Acr	breed_growth_background	ENSGALG00010021951	PSKH1	0.114068582	0.077429718	1.47318865	0.140700175	0.273141994	positive
PRJNA729271	ɛed_Lushi_vs_Arbor_Acr	breed_growth_background	ENSGALG00010021953	PITPNM1	0.134993748	0.096897893	1.393154624	0.163573038	0.303428613	positive
PRJNA729271	ɛed_Lushi_vs_Arbor_Acr	breed_growth_background	ENSGALG00010021958	BHLHA15	-0.127787312	0.217023753	-0.58881717	0.555983927	0.69755094	negative
PRJNA729271	ɛed_Lushi_vs_Arbor_Acr	breed_growth_background	ENSGALG00010021960	CDKN1B	0.127535496	0.077452538	1.646627715	0.09963459	0.214337101	positive
PRJNA729271	ɛed_Lushi_vs_Arbor_Acr	breed_growth_background	ENSGALG00010021965	PEX16	0.084630559	0.05473275	1.54625081	0.122044007	0.24672737	positive
PRJNA729271	ɛed_Lushi_vs_Arbor_Acr	breed_growth_background	ENSGALG00010021966	ADGRA1	-0.240303642	0.085026474	-2.826221413	0.004710069	0.024339557	negative
PRJNA729271	ɛed_Lushi_vs_Arbor_Acr	breed_growth_background	ENSGALG00010021969	CCDC103	0.373390721	0.155525284	2.400836131	0.01635766	0.059216853	positive
PRJNA729271	ɛed_Lushi_vs_Arbor_Acr	breed_growth_background	ENSGALG00010021970	ERGIC3	-0.171572238	0.048987602	-3.502360443	0.000461155	0.00468755	negative
PRJNA729271	ɛed_Lushi_vs_Arbor_Acr	breed_growth_background	ENSGALG00010021971	ICOS	0.504247373	0.222678447	2.264464204	0.023545579	0.076695094	positive
PRJNA729271	ɛed_Lushi_vs_Arbor_Acr	breed_growth_background	ENSGALG00010021972	PISD	0.057984373	0.098885422	0.586379389	0.557620592	0.698954555	positive
PRJNA729271	ɛed_Lushi_vs_Arbor_Acr	breed_growth_background	ENSGALG00010021974	GFAP	-0.413340415	0.303378394	-1.362458312	0.173053292	0.316518292	negative
PRJNA729271	ɛed_Lushi_vs_Arbor_Acr	breed_growth_background	ENSGALG00010021978	NR2C2	-0.034767152	0.039592213	-0.878131069	0.379872594	0.541794081	negative
PRJNA729271	ɛed_Lushi_vs_Arbor_Acr	breed_growth_background	ENSGALG00010021980	GLMN	0.062580786	0.072333305	0.865172493	0.386944121	0.549016741	positive
PRJNA729271	ɛed_Lushi_vs_Arbor_Acr	breed_growth_background	ENSGALG00010021983	CTLA4	-0.235385662	0.359918838	-0.653996505	0.513114057	0.661707141	negative
PRJNA729271	ɛed_Lushi_vs_Arbor_Acr	breed_growth_background	ENSGALG00010021988	FAM180B	-0.678156889	0.136711798	-4.960485483	7.03172E-07	3.65593E-05	negative
PRJNA729271	ɛed_Lushi_vs_Arbor_Acr	breed_growth_background	ENSGALG00010021989	NCAPD2	-0.226285856	0.081928808	-2.761981544	0.005745173	0.028069191	negative
PRJNA729271	ɛed_Lushi_vs_Arbor_Acr	breed_growth_background	ENSGALG00010021994	ENSGALG00010021994	-0.451345755	0.200875258	-2.246895706	0.024646693	0.079181797	negative
PRJNA729271	ɛed_Lushi_vs_Arbor_Acr	breed_growth_background	ENSGALG00010021995	CD28	-0.843640102	0.23451261	-3.597418933	0.000321391	0.003630988	negative
PRJNA729271	ɛed_Lushi_vs_Arbor_Acr	breed_growth_background	ENSGALG00010021999	ANKRD22	-0.337164802	0.288949627	-1.166863599	0.243265458	0.39892893	negative
PRJNA729271	ɛed_Lushi_vs_Arbor_Acr	breed_growth_background	ENSGALG00010022002	KIF18B	-0.361062178	0.120939093	-2.985487735	0.002831265	0.017007536	negative
PRJNA729271	ɛed_Lushi_vs_Arbor_Acr	breed_growth_background	ENSGALG00010022005	NLRC5	0.083917301	0.093605987	0.896495018	0.36998844	0.533417788	positive
PRJNA729271	ɛed_Lushi_vs_Arbor_Acr	breed_growth_background	ENSGALG00010022006	SFI1	-0.034964666	0.058612326	-0.596541183	0.550813751	0.693842471	negative
PRJNA729271	ɛed_Lushi_vs_Arbor_Acr	breed_growth_background	ENSGALG00010022012	ENSGALG00010022012	-0.073695484	1.593141828	-0.046257956	0.96310465	0.97897629	negative
PRJNA729271	ɛed_Lushi_vs_Arbor_Acr	breed_growth_background	ENSGALG00010022017	CNP1	-0.067048509	0.295673049	-0.22676571	0.820605928	0.889854338	negative
PRJNA729271	ɛed_Lushi_vs_Arbor_Acr	breed_growth_background	ENSGALG00010022019	BTBD8	-0.023287475	0.083472089	-0.278985172	0.780256204	0.863940274	negative
PRJNA729271	ɛed_Lushi_vs_Arbor_Acr	breed_growth_background	ENSGALG00010022021	TECPR1	0.074758681	0.041498389	1.801483961	0.071626633	0.170508794	positive
PRJNA729271	ɛed_Lushi_vs_Arbor_Acr	breed_growth_background	ENSGALG00010022022	STAMBPL1	-0.190526811	0.141273287	-1.34864004	0.177452613	0.321602354	negative
PRJNA729271	ɛed_Lushi_vs_Arbor_Acr	breed_growth_background	ENSGALG00010022024	MICAL2	0.032907735	0.097732017	0.336713966	0.736332534	0.831047269	positive
PRJNA729271	ɛed_Lushi_vs_Arbor_Acr	breed_growth_background	ENSGALG00010022027	EIF4ENIF1	0.022800969	0.036872519	0.618372974	0.536329509	0.681394725	positive
PRJNA729271	ɛed_Lushi_vs_Arbor_Acr	breed_growth_background	ENSGALG00010022028	FGD5	-0.160055817	0.061872901	-2.586848413	0.009685819	0.040884086	negative
PRJNA729271	ɛed_Lushi_vs_Arbor_Acr	breed_growth_background	ENSGALG00010022029	ENSGALG00010022029	-0.140426107	0.346172585	-0.405653461	0.684997242	0.796582548	negative
PRJNA729271	ɛed_Lushi_vs_Arbor_Acr	breed_growth_background	ENSGALG00010022030	ZNF341	0.09504023	0.08542542	1.112552094	0.265900845	0.424101315	positive
PRJNA729271	ɛed_Lushi_vs_Arbor_Acr	breed_growth_background	ENSGALG00010022032	KBTBD4	0.063142584	0.044751183	1.410970173	0.158253409	0.296405629	positive
PRJNA729271	ɛed_Lushi_vs_Arbor_Acr	breed_growth_background	ENSGALG00010022033	RAPH1	-0.280881541	0.062905464	-4.465137393	8.00174E-06	0.000244179	negative
PRJNA729271	ɛed_Lushi_vs_Arbor_Acr	breed_growth_background	ENSGALG00010022034	ENSGALG00010022034	-0.036916633	0.055965789	-0.659628571	0.509492215	0.658324983	negative
PRJNA729271	ɛed_Lushi_vs_Arbor_Acr	breed_growth_background	ENSGALG00010022037	DRG1	0.097766997	0.050661053	1.92982561	0.053628449	0.139239701	positive
PRJNA729271	ɛed_Lushi_vs_Arbor_Acr	breed_growth_background	ENSGALG00010022038	GAPDH	0.070091221	0.073324455	0.955905106	0.339120179	0.503384229	positive
PRJNA729271	ɛed_Lushi_vs_Arbor_Acr	breed_growth_background	ENSGALG00010022041	MAPK8IP1	0.040435318	0.085085442	0.475231922	0.634621677	0.759481605	positive
PRJNA729271	ɛed_Lushi_vs_Arbor_Acr	breed_growth_background	ENSGALG00010022042	AIP	-0.068686652	0.046375892	-1.481085301	0.138583844	0.270177918	negative
PRJNA729271	ɛed_Lushi_vs_Arbor_Acr	breed_growth_background	ENSGALG00010022044	PXMP4	0.133505029	0.032410472	4.119194215	3.802E-05	0.000794091	positive
PRJNA729271	ɛed_Lushi_vs_Arbor_Acr	breed_growth_background	ENSGALG00010022046	CRY2	0.17438659	0.055336432	3.151388428	0.001624962	0.011478474	positive
PRJNA729271	ɛed_Lushi_vs_Arbor_Acr	breed_growth_background	ENSGALG00010022049	EPHX4	-0.034803404	0.351099947	-0.099126771	0.921037617	0.956061319	negative
PRJNA729271	ɛed_Lushi_vs_Arbor_Acr	breed_growth_background	ENSGALG00010022050	ENSGALG00010022050	-0.210320213	0.108074591	-1.946065323	0.051646893	0.13554621	negative
PRJNA729271	ɛed_Lushi_vs_Arbor_Acr	breed_growth_background	ENSGALG00010022051	BAIAP2L1	0.183809996	0.061489277	2.989301633	0.002796159	0.016866555	positive
PRJNA729271	ɛed_Lushi_vs_Arbor_Acr	breed_growth_background	ENSGALG00010022052	IFFO1	-0.005789758	0.05598125	-0.103423162	0.91762713	0.953613798	negative
PRJNA729271	ɛed_Lushi_vs_Arbor_Acr	breed_growth_background	ENSGALG00010022054	ENSGALG00010022054	0.033076206	0.207598117	0.159328064	0.873410411	0.925309451	positive
PRJNA729271	ɛed_Lushi_vs_Arbor_Acr	breed_growth_background	ENSGALG00010022055	E2F1	-0.207412586	0.088297041	-2.349032137	0.018822282	0.065505157	negative
PRJNA729271	ɛed_Lushi_vs_Arbor_Acr	breed_growth_background	ENSGALG00010022058	NLRP3	-0.166627178	0.070499635	-2.36351832	0.018102332	0.063765078	negative
PRJNA729271	ɛed_Lushi_vs_Arbor_Acr	breed_growth_background	ENSGALG00010022059	NPTX2	-0.274728591	0.13448961	-2.042749549	0.041077237	0.114613003	negative
PRJNA729271	ɛed_Lushi_vs_Arbor_Acr	breed_growth_background	ENSGALG00010022062	MITF	0.307551066	0.093679225	3.283023164	0.001027002	0.00831178	positive
PRJNA729271	ɛed_Lushi_vs_Arbor_Acr	breed_growth_background	ENSGALG00010022065	PABPC1L	-0.197408468	0.137564496	-1.435024837	0.15128002	0.287166565	negative
PRJNA729271	ɛed_Lushi_vs_Arbor_Acr	breed_growth_background	ENSGALG00010022066	DAD1	-0.122111172	0.075206707	-1.623673949	0.104445414	0.221789668	negative
PRJNA729271	ɛed_Lushi_vs_Arbor_Acr	breed_growth_background	ENSGALG00010022067	TMEM130	-0.179535881	0.132441177	-1.355589589	0.175229786	0.319170112	negative
PRJNA729271	ɛed_Lushi_vs_Arbor_Acr	breed_growth_background	ENSGALG00010022069	NOP2	0.05650454	0.072870085	0.775414765	0.438094613	0.595444085	positive
PRJNA729271	ɛed_Lushi_vs_Arbor_Acr	breed_growth_background	ENSGALG00010022071	ACTA2	-0.328154369	0.106091786	-3.093117587	0.001980656	0.013175592	negative
PRJNA729271	ɛed_Lushi_vs_Arbor_Acr	breed_growth_background	ENSGALG00010022072	SMTN	0.620018193	0.167099603	3.71047078	0.000206874	0.002663092	positive
PRJNA729271	ɛed_Lushi_vs_Arbor_Acr	breed_growth_background	ENSGALG00010022073	PTPMT1	0.093704611	0.07315523	1.280901056	0.200228422	0.349042593	positive
PRJNA729271	ɛed_Lushi_vs_Arbor_Acr	breed_growth_background	ENSGALG00010022076	YWHAB	-0.069128766	0.037812451	-1.828201152	0.067519368	0.163990154	negative
PRJNA729271	ɛed_Lushi_vs_Arbor_Acr	breed_growth_background	ENSGALG00010022079	ABI2	0.082228984	0.041518327	1.98054666	0.047642136	0.127860166	positive
PRJNA729271	ɛed_Lushi_vs_Arbor_Acr	breed_growth_background	ENSGALG00010022081	FAS	0.060744795	0.096611948	0.628750335	0.529512519	0.675376279	positive
PRJNA729271	ɛed_Lushi_vs_Arbor_Acr	breed_growth_background	ENSGALG00010022082	TGFBR3	-0.242210448	0.077172364	-3.138564571	0.001697775	0.011814331	negative
PRJNA729271	ɛed_Lushi_vs_Arbor_Acr	breed_growth_background	ENSGALG00010022084	SELENOM	-0.113479543	0.108689027	-1.044075439	0.296450488	0.458116608	negative
PRJNA729271	ɛed_Lushi_vs_Arbor_Acr	breed_growth_background	ENSGALG00010022085	MLLT6	0.244216518	0.0949031	2.573324993	0.010072657	0.042039496	positive
PRJNA729271	ɛed_Lushi_vs_Arbor_Acr	breed_growth_background	ENSGALG00010022087	SLC35C1	-0.007914796	0.040621062	-0.194844627	0.845514585	0.906288095	negative
PRJNA729271	ɛed_Lushi_vs_Arbor_Acr	breed_growth_background	ENSGALG00010022088	DKK3	-0.797268214	0.144689694	-5.510193518	3.58439E-08	3.90645E-06	negative
PRJNA729271	ɛed_Lushi_vs_Arbor_Acr	breed_growth_background	ENSGALG00010022090	TRRAP	0.078063782	0.048924965	1.595581768	0.110582201	0.231290127	positive
PRJNA729271	ɛed_Lushi_vs_Arbor_Acr	breed_growth_background	ENSGALG00010022092	CTRL	-0.928300797	0.948239763	-0.978972653	0.327593492	0.491458048	negative
PRJNA729271	ɛed_Lushi_vs_Arbor_Acr	breed_growth_background	ENSGALG00010022093	CHD4	0.146003047	0.073124508	1.996636308	0.045864706	0.124542696	positive
PRJNA729271	ɛed_Lushi_vs_Arbor_Acr	breed_growth_background	ENSGALG00010022097	INPP5J	0.067123512	0.157587827	0.42594351	0.670149009	0.786607003	positive
PRJNA729271	ɛed_Lushi_vs_Arbor_Acr	breed_growth_background	ENSGALG00010022098	CDC7	-0.258890391	0.093644037	-2.764622265	0.005698875	0.027926027	negative
PRJNA729271	ɛed_Lushi_vs_Arbor_Acr	breed_growth_background	ENSGALG00010022100	ENTPD1	-0.265101281	0.089152679	-2.973564927	0.002943621	0.017493969	negative
PRJNA729271	ɛed_Lushi_vs_Arbor_Acr	breed_growth_background	ENSGALG00010022101	ENSGALG00010022101	-0.155745286	0.096156101	-1.619712991	0.105293944	0.223133878	negative
PRJNA729271	ɛed_Lushi_vs_Arbor_Acr	breed_growth_background	ENSGALG00010022104	EXOC3L1	-0.197395215	0.076199669	-2.590499637	0.009583672	0.040606644	negative
PRJNA729271	ɛed_Lushi_vs_Arbor_Acr	breed_growth_background	ENSGALG00010022105	DLG5	-0.025569226	0.046047647	-0.555277585	0.578704808	0.715727491	negative
PRJNA729271	ɛed_Lushi_vs_Arbor_Acr	breed_growth_background	ENSGALG00010022108	USP47	0.005548068	0.047150971	0.117666044	0.906332273	0.946217682	positive
PRJNA729271	ɛed_Lushi_vs_Arbor_Acr	breed_growth_background	ENSGALG00010022111	PCGF2	-0.018193444	0.100389106	-0.181229262	0.856187627	0.913539908	negative
PRJNA729271	ɛed_Lushi_vs_Arbor_Acr	breed_growth_background	ENSGALG00010022115	E2F4	0.353129059	0.10010731	3.527505222	0.000419495	0.004387376	positive
PRJNA729271	ɛed_Lushi_vs_Arbor_Acr	breed_growth_background	ENSGALG00010022116	CYP20A1	-0.07570189	0.056235282	-1.34616361	0.178249758	0.322473729	negative
PRJNA729271	ɛed_Lushi_vs_Arbor_Acr	breed_growth_background	ENSGALG00010022117	FAM20C	-0.230852643	0.065225118	-3.539321226	0.000401157	0.004250567	negative
PRJNA729271	ɛed_Lushi_vs_Arbor_Acr	breed_growth_background	ENSGALG00010022120	FOXL3	0.27599704	0.344638395	0.800830795	0.423229608	0.58242791	positive
PRJNA729271	ɛed_Lushi_vs_Arbor_Acr	breed_growth_background	ENSGALG00010022122	PSMB3	0.071746458	0.084680367	0.847262011	0.396849096	0.55804498	positive
PRJNA729271	ɛed_Lushi_vs_Arbor_Acr	breed_growth_background	ENSGALG00010022124	ENSGALG00010022124	-0.431720457	0.084612434	-5.102328789	3.35499E-07	2.0848E-05	negative
PRJNA729271	ɛed_Lushi_vs_Arbor_Acr	breed_growth_background	ENSGALG00010022125	CBFA2T2	0.380672746	0.100184464	3.799718354	0.000144861	0.002077241	positive
PRJNA729271	ɛed_Lushi_vs_Arbor_Acr	breed_growth_background	ENSGALG00010022129	ELMO3	-0.034781166	0.133340719	-0.260844298	0.794212582	0.872445923	negative
PRJNA729271	ɛed_Lushi_vs_Arbor_Acr	breed_growth_background	ENSGALG00010022134	CH25H	-0.25512245	0.131603828	-1.93856406	0.052554443	0.137335919	negative
PRJNA729271	ɛed_Lushi_vs_Arbor_Acr	breed_growth_background	ENSGALG00010022135	CHST1	-0.02272435	0.146075365	-0.155565929	0.876375183	0.926804252	negative
PRJNA729271	ɛed_Lushi_vs_Arbor_Acr	breed_growth_background	ENSGALG00010022137	PDDC1	-0.01707137	0.070245813	-0.243023302	0.807987346	0.881910887	negative
PRJNA729271	ɛed_Lushi_vs_Arbor_Acr	breed_growth_background	ENSGALG00010022138	SPAG17	-0.190574154	0.11207935	-1.700350273	0.089065059	0.198767981	negative
PRJNA729271	ɛed_Lushi_vs_Arbor_Acr	breed_growth_background	ENSGALG00010022142	TMEM208	-0.026225578	0.056207375	-0.466586073	0.640796053	0.764526221	negative
PRJNA729271	ɛed_Lushi_vs_Arbor_Acr	breed_growth_background	ENSGALG00010022143	MATCAP1	0.153301136	0.107798425	1.422109233	0.154994543	0.291848	positive
PRJNA729271	ɛed_Lushi_vs_Arbor_Acr	breed_growth_background	ENSGALG00010022144	SNTA1	0.18202567	0.099419927	1.830877119	0.067118886	0.163455688	positive
PRJNA729271	ɛed_Lushi_vs_Arbor_Acr	breed_growth_background	ENSGALG00010022148	TALDO1	-0.120138878	0.046751343	-2.569741759	0.010177435	0.042301865	negative
PRJNA729271	ɛed_Lushi_vs_Arbor_Acr	breed_growth_background	ENSGALG00010022149	NBEAL1	-0.042297631	0.057064978	-0.741218741	0.458560822	0.614310454	negative
PRJNA729271	ɛed_Lushi_vs_Arbor_Acr	breed_growth_background	ENSGALG00010022150	CPNE2	-0.101971809	0.071889167	-1.418458632	0.156056908	0.29327692	negative
PRJNA729271	ɛed_Lushi_vs_Arbor_Acr	breed_growth_background	ENSGALG00010022151	PIP4K2B	0.115382445	0.082805426	1.393416469	0.163493888	0.303320608	positive
PRJNA729271	ɛed_Lushi_vs_Arbor_Acr	breed_growth_background	ENSGALG00010022152	ZNF644	0.045608051	0.054001096	0.844576397	0.398347392	0.559554748	positive
PRJNA729271	ɛed_Lushi_vs_Arbor_Acr	breed_growth_background	ENSGALG00010022154	CPN1	0.019269126	0.105552395	0.182555085	0.855147129	0.912832877	positive
PRJNA729271	ɛed_Lushi_vs_Arbor_Acr	breed_growth_background	ENSGALG00010022157	EPS8L2	0.04271879	0.074844266	0.570769041	0.568156212	0.707206895	positive
PRJNA729271	ɛed_Lushi_vs_Arbor_Acr	breed_growth_background	ENSGALG00010022158	CWC25	0.095663704	0.044088034	2.169833731	0.030019444	0.090898651	positive
PRJNA729271	ɛed_Lushi_vs_Arbor_Acr	breed_growth_background	ENSGALG00010022159	KIAA2013	0.025253068	0.048193039	0.523998251	0.600279756	0.733086885	positive
PRJNA729271	ɛed_Lushi_vs_Arbor_Acr	breed_growth_background	ENSGALG00010022161	CELF1	0.047355875	0.050504134	0.937663339	0.34841745	0.512513795	positive

PRJNA729271	:ed_Lushi_vs_Arbor_Acr	breed_growth_background	ENSGALG00010022162	POLE	-0.117813797	0.086635678	-1.35987621	0.1738691	0.317649686	negative
PRJNA729271	:ed_Lushi_vs_Arbor_Acr	breed_growth_background	ENSGALG00010022164	PDGFA	-0.042130589	0.058338665	-0.722172665	0.470188327	0.624586688	negative
PRJNA729271	:ed_Lushi_vs_Arbor_Acr	breed_growth_background	ENSGALG00010022165	FAM192A	0.054711913	0.04816796	1.135856959	0.256016434	0.413109008	positive
PRJNA729271	:ed_Lushi_vs_Arbor_Acr	breed_growth_background	ENSGALG00010022166	SDHB	0.070960429	0.057627346	1.231367298	0.218185522	0.370197722	positive
PRJNA729271	:ed_Lushi_vs_Arbor_Acr	breed_growth_background	ENSGALG00010022167	WDR3	-0.006892409	0.039841797	-0.172994436	0.862655802	0.917469795	negative
PRJNA729271	:ed_Lushi_vs_Arbor_Acr	breed_growth_background	ENSGALG00010022169	TOX2	-0.170192725	0.139826512	-1.217170638	0.223539299	0.376137058	negative
PRJNA729271	:ed_Lushi_vs_Arbor_Acr	breed_growth_background	ENSGALG00010022170	RSPRY1	0.18665067	0.077881479	2.396598925	0.016548026	0.059702583	positive
PRJNA729271	:ed_Lushi_vs_Arbor_Acr	breed_growth_background	ENSGALG00010022171	FOXP1	0.019850193	0.051211362	0.387613074	0.698302393	0.805308638	positive
PRJNA729271	:ed_Lushi_vs_Arbor_Acr	breed_growth_background	ENSGALG00010022172	GIPC2	0.291773655	0.098222322	2.970543246	0.002972735	0.017602043	positive
PRJNA729271	:ed_Lushi_vs_Arbor_Acr	breed_growth_background	ENSGALG00010022173	RPL23	0.067124617	0.055629698	1.20663278	0.227573597	0.38073399	positive
PRJNA729271	:ed_Lushi_vs_Arbor_Acr	breed_growth_background	ENSGALG00010022174	KCNMA1	0.052141583	0.066102592	0.788797871	0.430230154	0.588497923	positive
PRJNA729271	:ed_Lushi_vs_Arbor_Acr	breed_growth_background	ENSGALG00010022176	ARL2BPL	0.317797961	0.079194837	4.0128621	5.9987E-05	0.001102042	positive
PRJNA729271	:ed_Lushi_vs_Arbor_Acr	breed_growth_background	ENSGALG00010022178	ENSGALG00010022178	-0.283597121	0.176534538	-1.606468198	0.108171077	0.227831991	negative
PRJNA729271	:ed_Lushi_vs_Arbor_Acr	breed_growth_background	ENSGALG00010022180	PLLP	0.902879786	0.206390378	4.374621514	1.21643E-05	0.000335701	positive
PRJNA729271	:ed_Lushi_vs_Arbor_Acr	breed_growth_background	ENSGALG00010022181	ENSGALG00010022181	-0.079634149	0.129709863	-0.613940587	0.539254588	0.684576568	negative
PRJNA729271	:ed_Lushi_vs_Arbor_Acr	breed_growth_background	ENSGALG00010022182	CNTNAP1	-0.748607384	0.162739943	-4.600022403	4.22446E-06	0.000150821	negative
PRJNA729271	:ed_Lushi_vs_Arbor_Acr	breed_growth_background	ENSGALG00010022184	PRKAR1B	-0.10679708	0.050616558	-2.109923811	0.034864919	0.101669253	negative
PRJNA729271	:ed_Lushi_vs_Arbor_Acr	breed_growth_background	ENSGALG00010022186	DNAJB4	-0.103495575	0.06018728	-1.719558934	0.085512646	0.19300146	negative
PRJNA729271	:ed_Lushi_vs_Arbor_Acr	breed_growth_background	ENSGALG00010022190	ACVR1B	0.225334783	0.087673873	2.570147477	0.010165523	0.042280991	positive
PRJNA729271	:ed_Lushi_vs_Arbor_Acr	breed_growth_background	ENSGALG00010022193	GDAP2	0.076522666	0.045035585	1.69916003	0.089289035	0.19914557	positive
PRJNA729271	:ed_Lushi_vs_Arbor_Acr	breed_growth_background	ENSGALG00010022194	ENSGALG00010022194	-0.162953961	0.049893949	-3.26600649	0.001090757	0.008711036	negative
PRJNA729271	:ed_Lushi_vs_Arbor_Acr	breed_growth_background	ENSGALG00010022196	PLXNA1	-0.128913058	0.05824745	-2.213196598	0.026884095	0.084056288	negative
PRJNA729271	:ed_Lushi_vs_Arbor_Acr	breed_growth_background	ENSGALG00010022199	RBM39	0.071635139	0.033206178	2.157283487	0.030983583	0.093195068	positive
PRJNA729271	:ed_Lushi_vs_Arbor_Acr	breed_growth_background	ENSGALG00010022200	DNMBP	-0.137927342	0.058925849	-2.340693332	0.01924797	0.066571063	negative
PRJNA729271	:ed_Lushi_vs_Arbor_Acr	breed_growth_background	ENSGALG00010022202	CX3CL1	-0.485287595	0.084757047	-5.725631237	1.0305E-08	1.41199E-06	negative
PRJNA729271	:ed_Lushi_vs_Arbor_Acr	breed_growth_background	ENSGALG00010022203	DNAAF5	0.18076915	0.071830025	2.516623812	0.011848526	0.047156614	positive
PRJNA729271	:ed_Lushi_vs_Arbor_Acr	breed_growth_background	ENSGALG00010022204	ENSGALG00010022204	0.860827707	0.179991196	4.782610073	1.73034E-06	7.5319E-05	positive
PRJNA729271	:ed_Lushi_vs_Arbor_Acr	breed_growth_background	ENSGALG00010022205	GALNT18	-0.276378162	0.058641515	-4.713011985	2.44082E-06	9.71969E-05	negative
PRJNA729271	:ed_Lushi_vs_Arbor_Acr	breed_growth_background	ENSGALG00010022207	ENSGALG00010022207	-0.061411468	0.163429425	-0.375767512	0.707089742	0.811116319	negative
PRJNA729271	:ed_Lushi_vs_Arbor_Acr	breed_growth_background	ENSGALG00010022208	CCL17	0.340210109	0.426754759	0.797202847	0.425333233	0.584514104	positive
PRJNA729271	:ed_Lushi_vs_Arbor_Acr	breed_growth_background	ENSGALG00010022209	ZFYVE19	-0.198422331	0.065822921	-3.014486883	0.002574143	0.01591139	negative
PRJNA729271	:ed_Lushi_vs_Arbor_Acr	breed_growth_background	ENSGALG00010022210	EZH1	0.108405799	0.044641451	2.428366413	0.015167011	0.05608312	positive
PRJNA729271	:ed_Lushi_vs_Arbor_Acr	breed_growth_background	ENSGALG00010022211	ENSGALG00010022211	-0.058414041	0.270225494	-0.216167764	0.82885698	0.895585682	negative
PRJNA729271	:ed_Lushi_vs_Arbor_Acr	breed_growth_background	ENSGALG00010022213	IDH1	0.009046735	0.043298837	0.208937137	0.834497313	0.899400591	positive
PRJNA729271	:ed_Lushi_vs_Arbor_Acr	breed_growth_background	ENSGALG00010022214	ENSGALG00010022214	0.215595927	0.151365046	1.424344213	0.154346855	0.290892947	positive
PRJNA729271	:ed_Lushi_vs_Arbor_Acr	breed_growth_background	ENSGALG00010022215	ENSGALG00010022215	-0.098377388	0.068605854	-1.433950355	0.151586431	0.287522286	negative
PRJNA729271	:ed_Lushi_vs_Arbor_Acr	breed_growth_background	ENSGALG00010022216	ROMO1	-0.056519043	0.077587858	-0.728452167	0.46633684	0.6212253	negative
PRJNA729271	:ed_Lushi_vs_Arbor_Acr	breed_growth_background	ENSGALG00010022218	ENSGALG00010022218	0.523779771	0.130662329	4.008651723	6.10664E-05	0.001114808	positive
PRJNA729271	:ed_Lushi_vs_Arbor_Acr	breed_growth_background	ENSGALG00010022219	EIF4E3	0.06213432	0.073776589	0.842195622	0.399678464	0.560726886	positive
PRJNA729271	:ed_Lushi_vs_Arbor_Acr	breed_growth_background	ENSGALG00010022221	CUTC	0.009155561	0.045105156	0.202982585	0.839148644	0.902400742	positive
PRJNA729271	:ed_Lushi_vs_Arbor_Acr	breed_growth_background	ENSGALG00010022222	ENSGALG00010022222	-0.148251412	0.059033517	-2.511309151	0.012028431	0.047585181	negative
PRJNA729271	:ed_Lushi_vs_Arbor_Acr	breed_growth_background	ENSGALG00010022223	DOK4	-0.306855182	0.098054349	-3.129439792	0.0017514	0.012100351	negative
PRJNA729271	:ed_Lushi_vs_Arbor_Acr	breed_growth_background	ENSGALG00010022224	NHEJ1	0.1462507	0.081908041	1.785547511	0.074172552	0.174981644	positive
PRJNA729271	:ed_Lushi_vs_Arbor_Acr	breed_growth_background	ENSGALG00010022225	ENSGALG00010022225	0.22766951	0.324503138	0.701594172	0.482932287	0.63510284	positive
PRJNA729271	:ed_Lushi_vs_Arbor_Acr	breed_growth_background	ENSGALG00010022226	FBRSL1	0.087542731	0.071590713	1.222822447	0.221396765	0.373892485	positive
PRJNA729271	:ed_Lushi_vs_Arbor_Acr	breed_growth_background	ENSGALG00010022227	TMEM80	0.308264971	0.085665255	3.598483076	0.000320079	0.003621811	positive
PRJNA729271	:ed_Lushi_vs_Arbor_Acr	breed_growth_background	ENSGALG00010022228	FUBP1	0.038110096	0.030082736	1.266842758	0.20521152	0.354491834	positive
PRJNA729271	:ed_Lushi_vs_Arbor_Acr	breed_growth_background	ENSGALG00010022229	RAPSN	0.231861782	0.095402866	2.430343975	0.015084499	0.055849251	positive
PRJNA729271	:ed_Lushi_vs_Arbor_Acr	breed_growth_background	ENSGALG00010022230	NFS1	0.065786279	0.046867092	1.403677413	0.160414931	0.299216887	positive
PRJNA729271	:ed_Lushi_vs_Arbor_Acr	breed_growth_background	ENSGALG00010022231	ZNF503	-0.124548652	0.114935427	-1.083640223	0.278524349	0.437835737	negative
PRJNA729271	:ed_Lushi_vs_Arbor_Acr	breed_growth_background	ENSGALG00010022232	GPR27	-0.217206788	0.111985561	-1.939596374	0.052428762	0.137157705	negative
PRJNA729271	:ed_Lushi_vs_Arbor_Acr	breed_growth_background	ENSGALG00010022233	FAM46C	0.153044544	0.087170083	1.755700328	0.079139591	0.182663753	positive
PRJNA729271	:ed_Lushi_vs_Arbor_Acr	breed_growth_background	ENSGALG00010022234	COX15	0.080806106	0.051915512	1.556492524	0.119591019	0.243739008	positive
PRJNA729271	:ed_Lushi_vs_Arbor_Acr	breed_growth_background	ENSGALG00010022235	RAMP2	0.068147787	0.060633079	1.123937434	0.261039569	0.419021989	positive
PRJNA729271	:ed_Lushi_vs_Arbor_Acr	breed_growth_background	ENSGALG00010022236	SHF	0.103007165	0.096956424	1.062406811	0.288051043	0.449101847	positive
PRJNA729271	:ed_Lushi_vs_Arbor_Acr	breed_growth_background	ENSGALG00010022237	SUN1	0.026855798	0.048669519	0.551799136	0.581085979	0.717394021	positive
PRJNA729271	:ed_Lushi_vs_Arbor_Acr	breed_growth_background	ENSGALG00010022238	COMTD1	0.031239685	0.09157772	0.341127567	0.733007549	0.828778816	positive
PRJNA729271	:ed_Lushi_vs_Arbor_Acr	breed_growth_background	ENSGALG00010022239	DEAF1	0.059868069	0.061741873	0.969650994	0.332220486	0.496651809	positive
PRJNA729271	:ed_Lushi_vs_Arbor_Acr	breed_growth_background	ENSGALG00010022240	ENSGALG00010022240	0.355086176	0.072510298	4.897044751	9.72887E-07	4.76419E-05	positive
PRJNA729271	:ed_Lushi_vs_Arbor_Acr	breed_growth_background	ENSGALG00010022241	POLR2C	0.11382927	0.050958323	2.233771902	0.025498085	0.081140449	positive
PRJNA729271	:ed_Lushi_vs_Arbor_Acr	breed_growth_background	ENSGALG00010022242	USP19	0.233855046	0.06757304	3.460774415	0.000538624	0.005252552	positive
PRJNA729271	:ed_Lushi_vs_Arbor_Acr	breed_growth_background	ENSGALG00010022243	ENSGALG00010022243	0.132905719	0.311827859	0.426215025	0.66995117	0.786491924	positive
PRJNA729271	:ed_Lushi_vs_Arbor_Acr	breed_growth_background	ENSGALG00010022244	ENTPD7	0.09584984	0.096429619	0.993987539	0.320228934	0.483613086	positive
PRJNA729271	:ed_Lushi_vs_Arbor_Acr	breed_growth_background	ENSGALG00010022245	PIKFYVE	-0.039483827	0.053702265	-0.735235795	0.462195903	0.61718375	negative
PRJNA729271	:ed_Lushi_vs_Arbor_Acr	breed_growth_background	ENSGALG00010022246	RHOV	0.585094307	0.254079277	2.302802155	0.021289977	0.071380652	positive
PRJNA729271	:ed_Lushi_vs_Arbor_Acr	breed_growth_background	ENSGALG00010022247	GALNT9	-0.10774677	0.121851075	-0.884249647	0.376561463	0.539034012	negative
PRJNA729271	:ed_Lushi_vs_Arbor_Acr	breed_growth_background	ENSGALG00010022248	FIGNL2	-0.082336055	0.124526256	-0.66119434	0.508487691	0.657495903	negative
PRJNA729271	:ed_Lushi_vs_Arbor_Acr	breed_growth_background	ENSGALG00010022250	SLC25A28	0.274160738	0.068197251	4.02011418	5.81699E-05	0.001078227	positive
PRJNA729271	:ed_Lushi_vs_Arbor_Acr	breed_growth_background	ENSGALG00010022251	WNK4	-0.083219731	0.083503796	-0.996598172	0.318959594	0.48219851	negative
PRJNA729271	:ed_Lushi_vs_Arbor_Acr	breed_growth_background	ENSGALG00010022252	COQ9	-0.024625489	0.069890438	-0.352344181	0.724580158	0.82355441	negative
PRJNA729271	:ed_Lushi_vs_Arbor_Acr	breed_growth_background	ENSGALG00010022253	ALPK3	0.271924261	0.116062605	2.342910192	0.019133988	0.066336081	positive
PRJNA729271	:ed_Lushi_vs_Arbor_Acr	breed_growth_background	ENSGALG00010022255	NOC4L	-0.149000667	0.050726883	-2.937311734	0.003310711	0.019035605	negative
PRJNA729271	:ed_Lushi_vs_Arbor_Acr	breed_growth_background	ENSGALG00010022256	PSMC3	0.063420122	0.049379057	1.284352635	0.199018598	0.347719448	positive
PRJNA729271	:ed_Lushi_vs_Arbor_Acr	breed_growth_background	ENSGALG00010022257	KCNK18	0.832671529	0.445269561	1.870039188	0.061478376	0.153541252	positive
PRJNA729271	:ed_Lushi_vs_Arbor_Acr	breed_growth_background	ENSGALG00010022258	ENSGALG00010022258	0.742505125	0.26710518	2.779823005	0.005438853	0.027072864	positive
PRJNA729271	:ed_Lushi_vs_Arbor_Acr	breed_growth_background	ENSGALG00010022259	GET4	0.032021314	0.04580206	0.699123882	0.484474615	0.636583407	positive
PRJNA729271	:ed_Lushi_vs_Arbor_Acr	breed_growth_background	ENSGALG00010022264	SCN8A	-0.475633805	0.140472023	-3.385968219	0.000709276	0.006385686	negative
PRJNA729271	:ed_Lushi_vs_Arbor_Acr	breed_growth_background	ENSGALG00010022266	COA3	0.110708829	0.078646509	1.407676336	0.159226932	0.297731293	positive
PRJNA729271	:ed_Lushi_vs_Arbor_Acr	breed_growth_background	ENSGALG00010022269	CNTD1	0.227049011	0.213434782	1.063786364	0.28742549	0.448415938	positive
PRJNA729271	:ed_Lushi_vs_Arbor_Acr	breed_growth_background	ENSGALG00010022271	SLC18A2	-0.057815961	0.134218863	-0.430758839	0.666643731	0.783942594	negative
PRJNA729271	:ed_Lushi_vs_Arbor_Acr	breed_growth_background	ENSGALG00010022274	CCDC91	0.074855833	0.093031132	0.804632074	0.421032027	0.580130361	positive
PRJNA729271	:ed_Lushi_vs_Arbor_Acr	breed_growth_background	ENSGALG00010022275	VDAC2	0.036374393	0.066285896	0.548750109	0.583176953	0.719294583	positive
PRJNA729271	:ed_Lushi_vs_Arbor_Acr	breed_growth_background	ENSGALG00010022276	BECN1	0.066410401	0.04799755	1.383620632	0.166474647	0.30712104	positive
PRJNA729271	:ed_Lushi_vs_Arbor_Acr	breed_growth_background	ENSGALG00010022279	EP400	0.146292779	0.079852424	1.832039294	0.066945566	0.163088401	positive
PRJNA729271	:ed_Lushi_vs_Arbor_Acr	breed_growth_background	ENSGALG00010022280	PDZD8	-0.041239462	0.036904555	-1.117462646	0.263796563	0.421859132	negative
PRJNA729271	:ed_Lushi_vs_Arbor_Acr	breed_growth_background	ENSGALG00010022282	TMT1A	0.236498452	0.118180673	2.001160134	0.045375136	0.123514102	positive
PRJNA729271	:ed_Lushi_vs_Arbor_Acr	breed_growth_background	ENSGALG00010022290	NEXN	0.100768441	0.117597822	0.856890367	0.391505477	0.553160336	positive
PRJNA729271	:ed_Lushi_vs_Arbor_Acr	breed_growth_background	ENSGALG00010022293	DNAJC10	0.076720417	0.034926591	2.19661912	0.02804766	0.086650575	positive
PRJNA729271	:ed_Lushi_vs_Arbor_Acr	breed_growth_background	ENSGALG00010022294	ENSGALG00010022294	-0.439904651	0.36973659	-1.189778513	0.234133457	0.388524838	negative
PRJNA729271	:ed_Lushi_vs_Arbor_Acr	breed_growth_background	ENSGALG00010022296	CIAPIN1	0.082196256	0.052350988	1.570099438	0.116391979	0.239367565	positive
PRJNA729271	:ed_Lushi_vs_Arbor_Acr	breed_growth_background	ENSGALG00010022297	VPS18	0.060346253	0.031956137	1.888408876	0.058971081	0.148917389	positive
PRJNA729271	:ed_Lushi_vs_Arbor_Acr	breed_growth_background	ENSGALG00010022299	MRGBP	0.04337867	0.043141168	1.005505235	0.31465363	0.478010639	positive
PRJNA729271	:ed_Lushi_vs_Arbor_Acr	breed_growth_background	ENSGALG00010022302	ENSGALG00010022302	-0.151944485	0.084409462	-1.800088298	0.071846697	0.170780235	negative
PRJNA729271	:ed_Lushi_vs_Arbor_Acr	breed_growth_background	ENSGALG00010022303	DUSP13A	0.049411304	0.096385656	0.512641677	0.608201994	0.739014307	positive
PRJNA729271	:ed_Lushi_vs_Arbor_Acr	breed_growth_background	ENSGALG00010022304	ADAP1	0.160610822	0.105795308	1.518128022	0.128982128	0.256425175	positive
PRJNA729271	:ed_Lushi_vs_Arbor_Acr	breed_growth_background	ENSGALG00010022307	ENSGALG00010022307	0.474607542	0.196148632	2.419632182	0.015536213	0.057173428	positive
PRJNA729271	:ed_Lushi_vs_Arbor_Acr	breed_growth_background	ENSGALG00010022308	OGFR	-0.158828035	0.09799635	-1.620754601	0.105070278	0.222757596	negative
PRJNA729271	:ed_Lushi_vs_Arbor_Acr	breed_growth_background	ENSGALG00010022309	WSCD2	0.28045877	0.19450005	1.441947035	0.149317316	0.284297188	positive
PRJNA729271	:ed_Lushi_vs_Arbor_Acr	breed_growth_background	ENSGALG00010022310	DCLRE1A	-0.093677602	0.056361998	-1.662070272	0.096498685	0.209550329	negative
PRJNA729271	:ed_Lushi_vs_Arbor_Acr	breed_growth_background	ENSGALG00010022311	DLL4	-0.251632187	0.075722356	-3.32308979	0.000890263	0.007515515	negative
PRJNA729271	:ed_Lushi_vs_Arbor_Acr	breed_growth_background	ENSGALG00010022312	ZNF592	0.041879372	0.030289344	1.382643746	0.166774128	0.307549229	positive
PRJNA729271	:ed_Lushi_vs_Arbor_Acr	breed_growth_background	ENSGALG00010022313	ENSGALG00010022313	-0.610221383	0.221642929	-2.753173247	0.005902065	0.028669718	negative
PRJNA729271	:ed_Lushi_vs_Arbor_Acr	breed_growth_background	ENSGALG00010022314	DUSP13B	0.059415517	0.226684239	0.262106961	0.79323898	0.871971803	positive
PRJNA729271	:ed_Lushi_vs_Arbor_Acr	breed_growth_background	ENSGALG00010022316	INO80	0.054985092	0.037476833	1.467175531	0.142328312	0.275219967	positive
PRJNA729271	:ed_Lushi_vs_Arbor_Acr	breed_growth_background	ENSGALG00010022317	PSME3	-0.079761648	0.040094494	-1.989341657	0.046663505	0.125980164	negative
PRJNA729271	:ed_Lushi_vs_Arbor_Acr	breed_growth_background	ENSGALG00010022320	COL9A3	0.121617231	0.304627695	0.399232351	0.689722008	0.799354003	positive
PRJNA729271	:ed_Lushi_vs_Arbor_Acr	breed_growth_background	ENSGALG00010022322	ENSGALG00010022322	-0.322129633	0.206619367	-1.559048585	0.118984881	0.243016184	negative
PRJNA729271	:ed_Lushi_vs_Arbor_Acr	breed_growth_background	ENSGALG00010022323	DUSP29	-0.165494958	0.200193325	-0.826675704	0.408420896	0.568963083	negative
PRJNA729271	:ed_Lushi_vs_Arbor_Acr	breed_growth_background	ENSGALG00010022325	MIGA1	0.054314788	0.059347345	0.915201643	0.360085782	0.523501446	positive
PRJNA729271	:ed_Lushi_vs_Arbor_Acr	breed_growth_background	ENSGALG00010022326	COX19	0.138712589	0.050755988	2.732930529	0.006277357	0.029931501	positive
PRJNA729271	:ed_Lushi_vs_Arbor_Acr	breed_growth_background	ENSGALG00010022328	ENSGALG00010022328	-0.245761803	0.141687262	-1.734537037	0.08282284	0.188523409	negative
PRJNA729271	:ed_Lushi_vs_Arbor_Acr	breed_growth_background	ENSGALG00010022332	KAT6B	0.015541491	0.03368898	0.461322697	0.644567103	0.767455245	positive
PRJNA729271	:ed_Lushi_vs_Arbor_Acr	breed_growth_background	ENSGALG00010022333	ENSGALG00010022333	-0.142777945	0.06941708	-2.056812898	0.039704227	0.111854757	negative
PRJNA729271	:ed_Lushi_vs_Arbor_Acr	breed_growth_background	ENSGALG00010022334	PDE1A	-0.201913862	0.066092411	-3.055023388	0.00225043	0.014484893	negative
PRJNA729271	:ed_Lushi_vs_Arbor_Acr	breed_growth_background	ENSGALG00010022340	GFOD2	0.087887744	0.051288814	1.713585043	0.086604962	0.194706768	positive
PRJNA729271	:ed_Lushi_vs_Arbor_Acr	breed_growth_background	ENSGALG00010022343	GATA5	0.207610361	0.35081772	0.591789835	0.553991331	0.695993255	positive
PRJNA729271	:ed_Lushi_vs_Arbor_Acr	breed_growth_background	ENSGALG00010022345	USP33	-0.11802142	0.065961505	-1.789246924	0.073575058	0.173918862	negative
PRJNA729271	:ed_Lushi_vs_Arbor_Acr	breed_growth_background	ENSGALG00010022347	SEC11A	-0.170280975	0.056984911	-2.98817657	0.002806474	0.016893619	negative
PRJNA729271	:ed_Lushi_vs_Arbor_Acr	breed_growth_background	ENSGALG00010022348	G6PC1	-0.746173008	0.319865956	-2.332767816	0.019660335	0.067488022	negative
PRJNA729271	:ed_Lushi_vs_Arbor_Acr	breed_growth_background	ENSGALG00010022353	SLC39A13	-0.070901564	0.051081133	-1.38801863	0.165131372	0.305244211	negative
PRJNA729271	:ed_Lushi_vs_Arbor_Acr	breed_growth_background	ENSGALG00010022354	FAR2	-0.221710184	0.073776476	-3.005160937	0.002654404	0.016220734	negative
PRJNA729271	:ed_Lushi_vs_Arbor_Acr	breed_growth_background	ENSGALG00010022356	TCFL5	0.355885466	0.124204977	2.865307615	0.004166043	0.022253504	positive
PRJNA729271	:ed_Lushi_vs_Arbor_Acr	breed_growth_background	ENSGALG00010022357	ENSGALG00010022357	-0.195681332	0.424554011	-0.460910335	0.644862937	0.767555285	negative
PRJNA729271	:ed_Lushi_vs_Arbor_Acr	breed_growth_background	ENSGALG00010022359	ZZZ3	-0.057205414	0.042883161	-1.333983151	0.182209379	0.326993307	negative
PRJNA729271	:ed_Lushi_vs_Arbor_Acr	breed_growth_background	ENSGALG00010022363	KIF18A	-0.270688344	0.110525414	-2.449104994	0.01432117	0.053681239	negative
PRJNA729271	:ed_Lushi_vs_Arbor_Acr	breed_growth_background	ENSGALG00010022365	PLCG1	-0.050614779	0.06008074	-0.842445996	0.399538356	0.560684333	negative
PRJNA729271	:ed_Lushi_vs_Arbor_Acr	breed_growth_background	ENSGALG00010022366	AARSD1	0.146767254	0.061010818	2.405593951	0.0161462	0.058657746	positive
PRJNA729271	:ed_Lushi_vs_Arbor_Acr	breed_growth_background	ENSGALG00010022373	AK5	-0.00968818	0.122658903	-0.078984728	0.937044771	0.965280645	negative
PRJNA729271	:ed_Lushi_vs_Arbor_Acr	breed_growth_background	ENSGALG00010022375	NMB	-0.327911588	0.235397479	-1.393012318	0.163616066	0.303461696	negative
PRJNA729271	:ed_Lushi_vs_Arbor_Acr	breed_growth_background	ENSGALG00010022377	ZHX3	-0.105681788	0.047927345	-2.205041552	0.027451179	0.085240968	negative
PRJNA729271	:ed_Lushi_vs_Arbor_Acr	breed_growth_background	ENSGALG00010022379	METTL15	0.067840403	0.066842072	1.014935677	0.31013649	0.473103391	positive

PRJNA729271	:ed_Lushi_vs_Arbor_Acr	breed_growth_background	ENSGALG00010022382	CALB2	-1.156866972	0.550023892	-2.103303129	0.03543928	0.102923736	negative
PRJNA729271	:ed_Lushi_vs_Arbor_Acr	breed_growth_background	ENSGALG00010022385	ENSGALG00010022385	0.804597279	0.310533	2.591020213	0.009569187	0.040577033	positive
PRJNA729271	:ed_Lushi_vs_Arbor_Acr	breed_growth_background	ENSGALG00010022386	SPI1	-0.006366792	0.130984332	-0.048607277	0.961232271	0.978276808	negative
PRJNA729271	:ed_Lushi_vs_Arbor_Acr	breed_growth_background	ENSGALG00010022388	CYP2W1	0.216398295	0.240253381	0.900708636	0.367743256	0.531073155	positive
PRJNA729271	:ed_Lushi_vs_Arbor_Acr	breed_growth_background	ENSGALG00010022391	ENSGALG00010022391	-0.033361889	0.095553973	-0.34914183	0.726982834	0.824527408	negative
PRJNA729271	:ed_Lushi_vs_Arbor_Acr	breed_growth_background	ENSGALG00010022392	EMILIN3	-0.043482944	0.103190696	-0.421384344	0.67347445	0.788849466	negative
PRJNA729271	:ed_Lushi_vs_Arbor_Acr	breed_growth_background	ENSGALG00010022395	PTGES3L	-0.001532891	0.071218211	-0.021523869	0.982827763	0.989816659	negative
PRJNA729271	:ed_Lushi_vs_Arbor_Acr	breed_growth_background	ENSGALG00010022396	PPP1R1C	0.087932193	0.180049268	0.488378512	0.625281759	0.751779952	positive
PRJNA729271	:ed_Lushi_vs_Arbor_Acr	breed_growth_background	ENSGALG00010022397	ATF1	-0.021556916	0.025787538	-0.835943153	0.403186884	0.564197894	negative
PRJNA729271	:ed_Lushi_vs_Arbor_Acr	breed_growth_background	ENSGALG00010022400	NHLRC2	-0.100144692	0.038763111	-2.583505036	0.009780204	0.041150669	negative
PRJNA729271	:ed_Lushi_vs_Arbor_Acr	breed_growth_background	ENSGALG00010022401	LCAT	0.39309033	0.233028949	1.686873374	0.091627703	0.202368361	positive
PRJNA729271	:ed_Lushi_vs_Arbor_Acr	breed_growth_background	ENSGALG00010022406	ITPRID2	-0.164243644	0.055906386	-2.937833308	0.003305147	0.019026252	negative
PRJNA729271	:ed_Lushi_vs_Arbor_Acr	breed_growth_background	ENSGALG00010022407	RUNDC1	0.130922221	0.051186627	2.557742718	0.010535401	0.043392106	positive
PRJNA729271	:ed_Lushi_vs_Arbor_Acr	breed_growth_background	ENSGALG00010022408	RAD51AP1	-0.031376725	0.074220937	-0.422747627	0.672479403	0.788031621	negative
PRJNA729271	:ed_Lushi_vs_Arbor_Acr	breed_growth_background	ENSGALG00010022409	CARNS1	0.374037381	0.159029247	2.352003718	0.018672588	0.065140594	positive
PRJNA729271	:ed_Lushi_vs_Arbor_Acr	breed_growth_background	ENSGALG00010022411	ADRB1	-0.184543576	0.156919397	-1.176040562	0.239578668	0.395163039	negative
PRJNA729271	:ed_Lushi_vs_Arbor_Acr	breed_growth_background	ENSGALG00010022412	CHAC1	0.57931134	0.348428665	1.662639725	0.096384574	0.209365264	positive
PRJNA729271	:ed_Lushi_vs_Arbor_Acr	breed_growth_background	ENSGALG00010022415	DIP2B	0.005404098	0.058763556	0.091963421	0.926727102	0.959082489	positive
PRJNA729271	:ed_Lushi_vs_Arbor_Acr	breed_growth_background	ENSGALG00010022416	CCDC186	0.065042067	0.046740408	1.391559664	0.16405578	0.303818645	positive
PRJNA729271	:ed_Lushi_vs_Arbor_Acr	breed_growth_background	ENSGALG00010022419	DUOX2	-0.282460547	0.341585592	-0.826910015	0.408288067	0.568832711	negative
PRJNA729271	:ed_Lushi_vs_Arbor_Acr	breed_growth_background	ENSGALG00010022421	CMKLR1	-0.283665607	0.093940494	-3.019630795	0.00253083	0.015710655	negative
PRJNA729271	:ed_Lushi_vs_Arbor_Acr	breed_growth_background	ENSGALG00010022422	RPS6KB2	0.104489915	0.082984613	1.259148075	0.207976853	0.357182995	positive
PRJNA729271	:ed_Lushi_vs_Arbor_Acr	breed_growth_background	ENSGALG00010022425	VWA2	-0.183430598	0.224777092	-0.816055571	0.414468353	0.57479131	negative
PRJNA729271	:ed_Lushi_vs_Arbor_Acr	breed_growth_background	ENSGALG00010022426	EXD1	-0.419776677	0.132160244	-3.176270446	0.001491818	0.010870964	negative
PRJNA729271	:ed_Lushi_vs_Arbor_Acr	breed_growth_background	ENSGALG00010022427	RPL27	-0.08184324	0.07412548	-1.104117505	0.269542169	0.428165889	negative
PRJNA729271	:ed_Lushi_vs_Arbor_Acr	breed_growth_background	ENSGALG00010022429	ADK	-0.195189547	0.070123286	-2.783519689	0.00537726	0.026840008	negative
PRJNA729271	:ed_Lushi_vs_Arbor_Acr	breed_growth_background	ENSGALG00010022431	LARP4	0.167861512	0.071216387	2.357063018	0.018420121	0.06450825	positive
PRJNA729271	:ed_Lushi_vs_Arbor_Acr	breed_growth_background	ENSGALG00010022433	LNPK	-0.009732814	0.057329425	-0.169769963	0.86519105	0.918952463	negative
PRJNA729271	:ed_Lushi_vs_Arbor_Acr	breed_growth_background	ENSGALG00010022435	PIGK	-0.200155191	0.087714723	-2.281888201	0.022495942	0.074277602	negative
PRJNA729271	:ed_Lushi_vs_Arbor_Acr	breed_growth_background	ENSGALG00010022436	AFAP1L2	-0.126603279	0.083372511	-1.518525443	0.128881991	0.256401929	negative
PRJNA729271	:ed_Lushi_vs_Arbor_Acr	breed_growth_background	ENSGALG00010022438	RPN2	0.026143391	0.054373855	0.480808122	0.630652877	0.756492095	positive
PRJNA729271	:ed_Lushi_vs_Arbor_Acr	breed_growth_background	ENSGALG00010022439	IFI35	0.13450391	0.109556432	1.227713501	0.219554536	0.372041502	positive
PRJNA729271	:ed_Lushi_vs_Arbor_Acr	breed_growth_background	ENSGALG00010022440	SORD	-0.320902325	0.066081333	-4.856172057	1.19677E-06	5.669E-05	negative
PRJNA729271	:ed_Lushi_vs_Arbor_Acr	breed_growth_background	ENSGALG00010022441	FICD	0.074714887	0.065484207	1.14096039	0.253886407	0.410448747	positive
PRJNA729271	:ed_Lushi_vs_Arbor_Acr	breed_growth_background	ENSGALG00010022448	FERRY3	0.097059593	0.065604566	1.479463987	0.13901635	0.270687701	positive
PRJNA729271	:ed_Lushi_vs_Arbor_Acr	breed_growth_background	ENSGALG00010022449	ENSGALG00010022449	0.02288673	0.055345252	0.413526532	0.679220887	0.792633988	positive
PRJNA729271	:ed_Lushi_vs_Arbor_Acr	breed_growth_background	ENSGALG00010022451	CHP1	-0.081568122	0.06505466	-1.253839795	0.209900242	0.359761618	negative
PRJNA729271	:ed_Lushi_vs_Arbor_Acr	breed_growth_background	ENSGALG00010022452	AP3M1	0.016680379	0.043653322	0.382110174	0.702379646	0.807720991	positive
PRJNA729271	:ed_Lushi_vs_Arbor_Acr	breed_growth_background	ENSGALG00010022453	ENSGALG00010022453	0.202247429	0.124554848	1.623761994	0.104426614	0.221785864	positive
PRJNA729271	:ed_Lushi_vs_Arbor_Acr	breed_growth_background	ENSGALG00010022454	ABLIM1	-0.247795091	0.074753252	-3.314840302	0.000916955	0.007643049	negative
PRJNA729271	:ed_Lushi_vs_Arbor_Acr	breed_growth_background	ENSGALG00010022455	C7orf50	0.073015902	0.084858179	0.860446255	0.389543098	0.551323379	positive
PRJNA729271	:ed_Lushi_vs_Arbor_Acr	breed_growth_background	ENSGALG00010022456	ATP5G3	-0.218298873	0.084638095	-2.579203539	0.009902842	0.041512869	negative
PRJNA729271	:ed_Lushi_vs_Arbor_Acr	breed_growth_background	ENSGALG00010022459	FGF6	-1.459674916	0.365302131	-3.995801811	6.44757E-05	0.001154885	negative
PRJNA729271	:ed_Lushi_vs_Arbor_Acr	breed_growth_background	ENSGALG00010022460	VAT1	0.036841948	0.063894277	0.576607941	0.564204333	0.704465657	positive
PRJNA729271	:ed_Lushi_vs_Arbor_Acr	breed_growth_background	ENSGALG00010022462	ITGA5	-0.169444933	0.069775255	-2.428438742	0.015163987	0.05608312	negative
PRJNA729271	:ed_Lushi_vs_Arbor_Acr	breed_growth_background	ENSGALG00010022463	DUSP12	0.053342044	0.062898075	0.84807116	0.396398342	0.557681643	positive
PRJNA729271	:ed_Lushi_vs_Arbor_Acr	breed_growth_background	ENSGALG00010022466	NEDD4	-0.088396669	0.073961054	-1.195178607	0.232017255	0.386162603	negative
PRJNA729271	:ed_Lushi_vs_Arbor_Acr	breed_growth_background	ENSGALG00010022467	SART3	0.093230286	0.062978458	1.480351991	0.138779335	0.270449914	positive
PRJNA729271	:ed_Lushi_vs_Arbor_Acr	breed_growth_background	ENSGALG00010022468	MYBPC3	-1.037928694	0.229505547	-4.522455805	6.11263E-06	0.000200913	negative
PRJNA729271	:ed_Lushi_vs_Arbor_Acr	breed_growth_background	ENSGALG00010022470	ENSGALG00010022470	0.197231583	0.095209458	2.071554528	0.038307004	0.109152748	positive
PRJNA729271	:ed_Lushi_vs_Arbor_Acr	breed_growth_background	ENSGALG00010022471	ST6GALNAC5	-0.020283145	0.084977031	-0.238689736	0.811346176	0.883846598	negative
PRJNA729271	:ed_Lushi_vs_Arbor_Acr	breed_growth_background	ENSGALG00010022472	QSER1	0.138854964	0.04712765	2.946358751	0.003215392	0.018635391	positive
PRJNA729271	:ed_Lushi_vs_Arbor_Acr	breed_growth_background	ENSGALG00010022473	ZNF385A	-0.040086979	0.226214812	-0.177207579	0.859345343	0.915473391	negative
PRJNA729271	:ed_Lushi_vs_Arbor_Acr	breed_growth_background	ENSGALG00010022474	VCL	-0.068736373	0.064057409	-1.073043292	0.283251693	0.443431611	negative
PRJNA729271	:ed_Lushi_vs_Arbor_Acr	breed_growth_background	ENSGALG00010022476	OIP5	0.070774431	0.062337793	1.135337456	0.256233954	0.413366181	positive
PRJNA729271	:ed_Lushi_vs_Arbor_Acr	breed_growth_background	ENSGALG00010022478	ATP6V1A	-0.013909589	0.05185435	-0.268243431	0.788511947	0.868633582	negative
PRJNA729271	:ed_Lushi_vs_Arbor_Acr	breed_growth_background	ENSGALG00010022479	ENSGALG00010022479	0.712475604	0.341113803	2.088674212	0.03673706	0.10567646	positive
PRJNA729271	:ed_Lushi_vs_Arbor_Acr	breed_growth_background	ENSGALG00010022480	PIF1	-0.122684685	0.145503544	-0.843173177	0.399131597	0.560330509	negative
PRJNA729271	:ed_Lushi_vs_Arbor_Acr	breed_growth_background	ENSGALG00010022481	CORO1B	0.010964828	0.081888752	0.133899067	0.893482388	0.937704386	positive
PRJNA729271	:ed_Lushi_vs_Arbor_Acr	breed_growth_background	ENSGALG00010022482	PRTG	0.142922094	0.169803894	0.841689139	0.399961979	0.560790181	positive
PRJNA729271	:ed_Lushi_vs_Arbor_Acr	breed_growth_background	ENSGALG00010022483	TOP2A	-0.40015807	0.128069187	-3.124546018	0.001780797	0.012246421	negative
PRJNA729271	:ed_Lushi_vs_Arbor_Acr	breed_growth_background	ENSGALG00010022485	ATF2	0.009299367	0.031433061	0.295846689	0.767347166	0.854694298	positive
PRJNA729271	:ed_Lushi_vs_Arbor_Acr	breed_growth_background	ENSGALG00010022486	FAM183A	0.335093207	0.375565967	0.892235285	0.372266833	0.53488092	positive
PRJNA729271	:ed_Lushi_vs_Arbor_Acr	breed_growth_background	ENSGALG00010022489	NUSAP1	-0.470337364	0.128729377	-3.65369099	0.000258497	0.003132875	negative
PRJNA729271	:ed_Lushi_vs_Arbor_Acr	breed_growth_background	ENSGALG00010022493	ENSGALG00010022493	-0.104988346	0.089726512	-1.170092796	0.241963627	0.397603761	negative
PRJNA729271	:ed_Lushi_vs_Arbor_Acr	breed_growth_background	ENSGALG00010022494	FHIP2A	0.108241914	0.035503942	3.048729491	0.002298113	0.014693935	positive
PRJNA729271	:ed_Lushi_vs_Arbor_Acr	breed_growth_background	ENSGALG00010022496	TUBGCP2	0.102645617	0.038881767	2.63994217	0.008292018	0.036488401	positive
PRJNA729271	:ed_Lushi_vs_Arbor_Acr	breed_growth_background	ENSGALG00010022497	CORO1C	0.027423943	0.080178699	0.342035266	0.732324352	0.828240196	positive
PRJNA729271	:ed_Lushi_vs_Arbor_Acr	breed_growth_background	ENSGALG00010022498	NAA50	0.013080646	0.060886519	0.214836494	0.829894799	0.896239391	positive
PRJNA729271	:ed_Lushi_vs_Arbor_Acr	breed_growth_background	ENSGALG00010022500	ENSGALG00010022500	0.015375369	0.230716414	0.066641852	0.946866827	0.970844921	positive
PRJNA729271	:ed_Lushi_vs_Arbor_Acr	breed_growth_background	ENSGALG00010022502	ENSGALG00010022502	-0.063913853	0.280503253	-0.227854231	0.819759565	0.889400161	negative
PRJNA729271	:ed_Lushi_vs_Arbor_Acr	breed_growth_background	ENSGALG00010022504	RAD9A	0.200729674	0.077969733	2.574456336	0.010039776	0.041953989	positive
PRJNA729271	:ed_Lushi_vs_Arbor_Acr	breed_growth_background	ENSGALG00010022505	ZNF511	0.081253873	0.064289777	1.263869268	0.206276954	0.355485014	positive
PRJNA729271	:ed_Lushi_vs_Arbor_Acr	breed_growth_background	ENSGALG00010022506	EBNA1BP2	0.071546491	0.047259364	1.51391143	0.130048305	0.257767015	positive
PRJNA729271	:ed_Lushi_vs_Arbor_Acr	breed_growth_background	ENSGALG00010022507	ENSGALG00010022507	-0.024899778	0.121015035	-0.205757722	0.836980167	0.901071563	negative
PRJNA729271	:ed_Lushi_vs_Arbor_Acr	breed_growth_background	ENSGALG00010022508	USF3	-0.028623798	0.065946038	-0.434048799	0.664253005	0.782233487	negative
PRJNA729271	:ed_Lushi_vs_Arbor_Acr	breed_growth_background	ENSGALG00010022511	IGFBP4	-0.27451043	0.14524168	-1.890025165	0.058754594	0.148448291	negative
PRJNA729271	:ed_Lushi_vs_Arbor_Acr	breed_growth_background	ENSGALG00010022512	PRAP1	-0.575369294	0.447986942	-1.284343896	0.199021654	0.347719448	negative
PRJNA729271	:ed_Lushi_vs_Arbor_Acr	breed_growth_background	ENSGALG00010022513	NDUFAF1	0.154179926	0.045809477	3.365677518	0.000763559	0.006732233	positive
PRJNA729271	:ed_Lushi_vs_Arbor_Acr	breed_growth_background	ENSGALG00010022515	CHRNA1	-0.065177134	0.092864899	-0.701848977	0.482773351	0.63500905	negative
PRJNA729271	:ed_Lushi_vs_Arbor_Acr	breed_growth_background	ENSGALG00010022516	TRUB1	0.070592273	0.082849604	0.85205323	0.394184549	0.5555917	positive
PRJNA729271	:ed_Lushi_vs_Arbor_Acr	breed_growth_background	ENSGALG00010022517	HNRNPA1	0.15485576	0.097012248	1.596249575	0.110433083	0.231081525	positive
PRJNA729271	:ed_Lushi_vs_Arbor_Acr	breed_growth_background	ENSGALG00010022518	ECHS1	0.11401042	0.060052472	1.898513364	0.057628489	0.146522531	positive
PRJNA729271	:ed_Lushi_vs_Arbor_Acr	breed_growth_background	ENSGALG00010022520	TMOD2	-0.185992954	0.116075383	-1.602346246	0.109079064	0.229196442	negative
PRJNA729271	:ed_Lushi_vs_Arbor_Acr	breed_growth_background	ENSGALG00010022521	RTF1	0.149283676	0.048899156	3.052888624	0.0022665	0.014556014	positive
PRJNA729271	:ed_Lushi_vs_Arbor_Acr	breed_growth_background	ENSGALG00010022522	TNS4	-0.165949495	0.204975997	-0.809604524	0.418167507	0.578206431	negative
PRJNA729271	:ed_Lushi_vs_Arbor_Acr	breed_growth_background	ENSGALG00010022524	SOX18	-0.090924652	0.122728022	-0.740863013	0.458776504	0.614372268	negative
PRJNA729271	:ed_Lushi_vs_Arbor_Acr	breed_growth_background	ENSGALG00010022525	ISCU	0.251567828	0.103974023	2.419525775	0.015540759	0.057173428	positive
PRJNA729271	:ed_Lushi_vs_Arbor_Acr	breed_growth_background	ENSGALG00010022526	PPP1CA	0.207808506	0.073503536	2.827190599	0.004695837	0.02429199	positive
PRJNA729271	:ed_Lushi_vs_Arbor_Acr	breed_growth_background	ENSGALG00010022528	SIDT1	-0.72606118	0.280229502	-2.590951968	0.009571085	0.040577033	negative
PRJNA729271	:ed_Lushi_vs_Arbor_Acr	breed_growth_background	ENSGALG00010022529	CCR7	0.104454834	0.202404415	0.516069939	0.605805562	0.73704479	positive
PRJNA729271	:ed_Lushi_vs_Arbor_Acr	breed_growth_background	ENSGALG00010022531	ENSGALG00010022531	0.434100949	0.229253549	1.893540802	0.058285988	0.147650365	positive
PRJNA729271	:ed_Lushi_vs_Arbor_Acr	breed_growth_background	ENSGALG00010022532	CBX5	-0.141059116	0.097545568	-1.446084313	0.148153555	0.283122713	negative
PRJNA729271	:ed_Lushi_vs_Arbor_Acr	breed_growth_background	ENSGALG00010022533	TMEM119	-0.169467946	0.071860939	-2.358276257	0.018360024	0.064391132	negative
PRJNA729271	:ed_Lushi_vs_Arbor_Acr	breed_growth_background	ENSGALG00010022534	CFAP57	-0.259542339	0.292666954	-0.886818055	0.375176865	0.537529768	negative
PRJNA729271	:ed_Lushi_vs_Arbor_Acr	breed_growth_background	ENSGALG00010022536	SMUG1	0.154421299	0.053958168	2.861870692	0.004211487	0.022377384	positive
PRJNA729271	:ed_Lushi_vs_Arbor_Acr	breed_growth_background	ENSGALG00010022538	WIPF1	-0.216544127	0.084721334	-2.55595746	0.010589606	0.043557419	negative
PRJNA729271	:ed_Lushi_vs_Arbor_Acr	breed_growth_background	ENSGALG00010022539	LYSMD2	-0.006550966	0.054519724	-0.120157726	0.904358205	0.945214175	negative
PRJNA729271	:ed_Lushi_vs_Arbor_Acr	breed_growth_background	ENSGALG00010022540	GPR146	0.304843188	0.127879614	2.383829447	0.017133541	0.061246422	positive
PRJNA729271	:ed_Lushi_vs_Arbor_Acr	breed_growth_background	ENSGALG00010022541	ITPKA	-0.204246745	0.069635915	-2.933066131	0.003356324	0.019224216	negative
PRJNA729271	:ed_Lushi_vs_Arbor_Acr	breed_growth_background	ENSGALG00010022542	ENSGALG00010022542	-0.226666861	0.395460619	-0.573171764	0.566528401	0.705968808	negative
PRJNA729271	:ed_Lushi_vs_Arbor_Acr	breed_growth_background	ENSGALG00010022544	SPICE1	-0.090185362	0.037941509	-2.376957676	0.017456091	0.061985801	negative
PRJNA729271	:ed_Lushi_vs_Arbor_Acr	breed_growth_background	ENSGALG00010022545	CSTF3	0.020028615	0.036668238	0.546211543	0.584920533	0.720893047	positive
PRJNA729271	:ed_Lushi_vs_Arbor_Acr	breed_growth_background	ENSGALG00010022546	MTG1	-0.02641116	0.084131594	-0.313926768	0.753576659	0.844264467	negative
PRJNA729271	:ed_Lushi_vs_Arbor_Acr	breed_growth_background	ENSGALG00010022547	SMARCE1	0.061328598	0.033371636	1.837746259	0.066099804	0.161625617	positive
PRJNA729271	:ed_Lushi_vs_Arbor_Acr	breed_growth_background	ENSGALG00010022548	ENSGALG00010022548	-0.244973382	0.062198048	-3.938602406	8.19576E-05	0.001378162	negative
PRJNA729271	:ed_Lushi_vs_Arbor_Acr	breed_growth_background	ENSGALG00010022550	TRIM45	-0.321595513	0.090329039	-3.560267175	0.000370478	0.004025543	negative
PRJNA729271	:ed_Lushi_vs_Arbor_Acr	breed_growth_background	ENSGALG00010022551	GPER1	-0.925231832	0.351782932	-2.630121439	0.008535437	0.037146343	negative
PRJNA729271	:ed_Lushi_vs_Arbor_Acr	breed_growth_background	ENSGALG00010022552	GPR155	0.071135683	0.056667263	1.255322356	0.209361763	0.359093451	positive
PRJNA729271	:ed_Lushi_vs_Arbor_Acr	breed_growth_background	ENSGALG00010022553	PRPF6	0.233250612	0.090722356	2.571037852	0.010139424	0.042241457	positive
PRJNA729271	:ed_Lushi_vs_Arbor_Acr	breed_growth_background	ENSGALG00010022554	SCG3	-0.526870791	0.191911411	-2.745385431	0.006043987	0.029163644	negative
PRJNA729271	:ed_Lushi_vs_Arbor_Acr	breed_growth_background	ENSGALG00010022557	WNT1	-0.098575237	0.22243888	-0.443156506	0.657652536	0.777225724	negative
PRJNA729271	:ed_Lushi_vs_Arbor_Acr	breed_growth_background	ENSGALG00010022558	NYAP2	0.096929424	0.3309885	0.292848313	0.769638098	0.856433612	positive
PRJNA729271	:ed_Lushi_vs_Arbor_Acr	breed_growth_background	ENSGALG00010022559	SSH1	-0.056994697	0.04704106	-1.211594665	0.225667578	0.378681586	negative
PRJNA729271	:ed_Lushi_vs_Arbor_Acr	breed_growth_background	ENSGALG00010022560	SAMD10	0.35485535	0.803895083	0.441419979	0.658908981	0.777971513	positive
PRJNA729271	:ed_Lushi_vs_Arbor_Acr	breed_growth_background	ENSGALG00010022562	GHRH	0.132620288	0.647770225	0.204733536	0.837780318	0.901464309	positive
PRJNA729271	:ed_Lushi_vs_Arbor_Acr	breed_growth_background	ENSGALG00010022565	ARF3	-0.299887968	0.094699224	-3.166741552	0.001541573	0.011079355	negative
PRJNA729271	:ed_Lushi_vs_Arbor_Acr	breed_growth_background	ENSGALG00010022567	PAOX	-0.31986539	0.067059302	-4.769888417	1.84328E-06	7.92829E-05	negative
PRJNA729271	:ed_Lushi_vs_Arbor_Acr	breed_growth_background	ENSGALG00010022568	SCRN3	0.401238033	0.089247585	4.495785875	6.93135E-06	0.000221788	positive
PRJNA729271	:ed_Lushi_vs_Arbor_Acr	breed_growth_background	ENSGALG00010022569	KIAA1549L	0.13881627	0.143603256	0.966665198	0.33371143	0.498059534	positive
PRJNA729271	:ed_Lushi_vs_Arbor_Acr	breed_growth_background	ENSGALG00010022571	RIPOR1	0.063765784	0.057467385	1.109599545	0.267171627	0.425566235	positive
PRJNA729271	:ed_Lushi_vs_Arbor_Acr	breed_growth_background	ENSGALG00010022572	ENSGALG00010022572	-0.355665771	0.226443648	-1.570659072	0.116261861	0.239164623	negative
PRJNA729271	:ed_Lushi_vs_Arbor_Acr	breed_growth_background	ENSGALG00010022573	AP4E1	0.030378301	0.04451616	0.682410645	0.494979326	0.646546394	positive
PRJNA729271	:ed_Lushi_vs_Arbor_Acr	breed_growth_background	ENSGALG00010022574	ITPRIPL2	-0.094509852	0.059847019	-1.579190637	0.114292337	0.236288875	negative
PRJNA729271	:ed_Lushi_vs_Arbor_Acr	breed_growth_background	ENSGALG00010022575	TMEM41B	0.101147395	0.059207242	1.708361875	0.087569217	0.196099432	positive
PRJNA729271	:ed_Lushi_vs_Arbor_Acr	breed_growth_background	ENSGALG00010022578	ZNF512B	0.093620105	0.072582171	1.289849889	0.197102783	0.345549689	positive
PRJNA729271	:ed_Lushi_vs_Arbor_Acr	breed_growth_background	ENSGALG00010022579	TNFAIP8L3	-0.496681707	0.079437705	-6.252467975	4.04017E-10	9.44552E-08	negative
PRJNA729271	:ed_Lushi_vs_Arbor_Acr	breed_growth_background	ENSGALG00010022581	TMEM125	-0.600792081	0.375472986	-1.600094023	0.109577727	0.229959338	negative
PRJNA729271	:ed_Lushi_vs_Arbor_Acr	breed_growth_background	ENSGALG00010022582	ENSGALG00010022582	0.348040644	0.117409797	2.964323695	0.00303349	0.017845146	positive
PRJNA729271	:ed_Lushi_vs_Arbor_Acr	breed_growth_background	ENSGALG00010022583	FKBP11	0.164320866	0.065310941	2.515977617	0.011870272	0.047201289	positive
PRJNA729271	:ed_Lushi_vs_Arbor_Acr	breed_growth_background	ENSGALG00010022585	CIR1	0.228933978	0.051087167	4.481242431	7.42098E-06	0.000232326	positive

PRJNA729271	ed_Lushi_vs_Arbor_Acr	breed_growth_background	ENSGALG00010022587	ENSGALG00010022587	-1.981778069	0.538902031	-3.677436632	0.00023559	0.002913712	negative
PRJNA729271	ed_Lushi_vs_Arbor_Acr	breed_growth_background	ENSGALG00010022588	TCP11L1	-0.080398375	0.042688767	-1.883361388	0.059651409	0.15024322	negative
PRJNA729271	ed_Lushi_vs_Arbor_Acr	breed_growth_background	ENSGALG00010022590	ENSGALG00010022590	-0.018815134	0.087854777	-0.214161761	0.830420914	0.896673954	negative
PRJNA729271	ed_Lushi_vs_Arbor_Acr	breed_growth_background	ENSGALG00010022592	CCDC65	-0.342609127	0.229391448	-1.493556668	0.135291532	0.265401347	negative
PRJNA729271	ed_Lushi_vs_Arbor_Acr	breed_growth_background	ENSGALG00010022593	MANBAL	-0.010070811	0.079649846	-0.126438543	0.899384794	0.941656687	negative
PRJNA729271	ed_Lushi_vs_Arbor_Acr	breed_growth_background	ENSGALG00010022597	SLC04A1	-0.091389177	0.11948462	-0.764861427	0.444354065	0.601167636	negative
PRJNA729271	ed_Lushi_vs_Arbor_Acr	breed_growth_background	ENSGALG00010022599	RNLS	-0.376453333	0.15720531	-2.394660409	0.016635765	0.059954106	negative
PRJNA729271	ed_Lushi_vs_Arbor_Acr	breed_growth_background	ENSGALG00010022603	ATRNL1	-0.202595414	0.090886977	-2.229091788	0.025807798	0.081835293	negative
PRJNA729271	ed_Lushi_vs_Arbor_Acr	breed_growth_background	ENSGALG00010022608	P3H4	-0.139361236	0.111612828	-1.248613077	0.211806627	0.361831121	negative
PRJNA729271	ed_Lushi_vs_Arbor_Acr	breed_growth_background	ENSGALG00010022609	RBBP8NL	-0.215526837	0.529879776	-0.406746676	0.684194055	0.796130114	negative
PRJNA729271	ed_Lushi_vs_Arbor_Acr	breed_growth_background	ENSGALG00010022614	DAO	-0.165171832	0.110298719	-1.497495472	0.134264383	0.264114367	negative
PRJNA729271	ed_Lushi_vs_Arbor_Acr	breed_growth_background	ENSGALG00010022616	GLDN	0.836386518	0.176543375	4.737569561	2.16297E-06	8.88336E-05	positive
PRJNA729271	ed_Lushi_vs_Arbor_Acr	breed_growth_background	ENSGALG00010022619	MADD	-0.109309789	0.071671008	-1.52516048	0.127219075	0.25414009	negative
PRJNA729271	ed_Lushi_vs_Arbor_Acr	breed_growth_background	ENSGALG00010022622	MPPED2	0.087734631	0.073977613	1.185961915	0.23563734	0.390368533	positive
PRJNA729271	ed_Lushi_vs_Arbor_Acr	breed_growth_background	ENSGALG00010022623	TOP1	0.181924722	0.067868531	2.680546048	0.007350215	0.033529636	positive
PRJNA729271	ed_Lushi_vs_Arbor_Acr	breed_growth_background	ENSGALG00010022625	ENSGALG00010022625	-0.103140701	0.066552045	-1.549775075	0.121195511	0.245998297	negative
PRJNA729271	ed_Lushi_vs_Arbor_Acr	breed_growth_background	ENSGALG00010022627	CTCF	0.072808949	0.052911964	1.376039449	0.16880942	0.310637622	positive
PRJNA729271	ed_Lushi_vs_Arbor_Acr	breed_growth_background	ENSGALG00010022628	SVOP	0.023928651	0.307784706	0.07774477	0.938031081	0.965998787	positive
PRJNA729271	ed_Lushi_vs_Arbor_Acr	breed_growth_background	ENSGALG00010022630	ENSGALG00010022630	-0.416841307	0.174354588	-2.39076764	0.01681319	0.060368389	negative
PRJNA729271	ed_Lushi_vs_Arbor_Acr	breed_growth_background	ENSGALG00010022637	DMXL2	-0.060021426	0.059874136	-1.002459998	0.316121477	0.47948595	negative
PRJNA729271	ed_Lushi_vs_Arbor_Acr	breed_growth_background	ENSGALG00010022642	SEC13	0.098587592	0.036906848	2.671254741	0.007556827	0.034218827	positive
PRJNA729271	ed_Lushi_vs_Arbor_Acr	breed_growth_background	ENSGALG00010022643	SP3	0.10716515	0.041310342	2.594148244	0.009482559	0.040319652	positive
PRJNA729271	ed_Lushi_vs_Arbor_Acr	breed_growth_background	ENSGALG00010022644	ARMC8	0.042026205	0.045534408	0.922954899	0.356030709	0.519666017	positive
PRJNA729271	ed_Lushi_vs_Arbor_Acr	breed_growth_background	ENSGALG00010022645	ENSGALG00010022645	-0.843135803	0.383601811	-2.197945313	0.027953003	0.086428921	negative
PRJNA729271	ed_Lushi_vs_Arbor_Acr	breed_growth_background	ENSGALG00010022647	SVIP	-0.222538213	0.072589505	-3.06570781	0.002171554	0.014077226	negative
PRJNA729271	ed_Lushi_vs_Arbor_Acr	breed_growth_background	ENSGALG00010022649	PATZ1	-0.120783112	0.050722472	-2.381254447	0.01725379	0.061455919	negative
PRJNA729271	ed_Lushi_vs_Arbor_Acr	breed_growth_background	ENSGALG00010022650	NR1H3	0.230359062	0.052875928	4.35659607	1.32101E-05	0.00035525	positive
PRJNA729271	ed_Lushi_vs_Arbor_Acr	breed_growth_background	ENSGALG00010022652	CARMIL2	0.045960081	0.148997732	0.308461618	0.757731102	0.846954991	positive
PRJNA729271	ed_Lushi_vs_Arbor_Acr	breed_growth_background	ENSGALG00010022655	NRIP3	0.49282942	0.326042756	1.511548442	0.130648776	0.258674227	positive
PRJNA729271	ed_Lushi_vs_Arbor_Acr	breed_growth_background	ENSGALG00010022656	FKBP10	-0.398342955	0.113416387	-3.512216948	0.000444385	0.004558246	negative
PRJNA729271	ed_Lushi_vs_Arbor_Acr	breed_growth_background	ENSGALG00010022657	MRPS35	0.075835207	0.059979123	1.264360066	0.206100819	0.355413636	positive
PRJNA729271	ed_Lushi_vs_Arbor_Acr	breed_growth_background	ENSGALG00010022659	TIE1	-0.062183484	0.080761731	-0.769962246	0.441322288	0.598686623	negative
PRJNA729271	ed_Lushi_vs_Arbor_Acr	breed_growth_background	ENSGALG00010022660	METAP1D	0.108298154	0.100780078	1.074598826	0.282554378	0.44248333	positive
PRJNA729271	ed_Lushi_vs_Arbor_Acr	breed_growth_background	ENSGALG00010022661	ENSGALG00010022661	0.009466385	0.219766929	0.043074655	0.965642023	0.980524072	positive
PRJNA729271	ed_Lushi_vs_Arbor_Acr	breed_growth_background	ENSGALG00010022662	GFRA1	-0.179426502	0.089036048	-2.015211881	0.043882453	0.12046897	negative
PRJNA729271	ed_Lushi_vs_Arbor_Acr	breed_growth_background	ENSGALG00010022664	TRPC4AP	0.098029074	0.052179879	1.878675743	0.060288782	0.151397183	positive
PRJNA729271	ed_Lushi_vs_Arbor_Acr	breed_growth_background	ENSGALG00010022666	ENSGALG00010022666	0.066832185	0.057181045	1.168782153	0.242491413	0.398200184	positive
PRJNA729271	ed_Lushi_vs_Arbor_Acr	breed_growth_background	ENSGALG00010022667	PTEN	0.008604899	0.022756677	0.378126341	0.705336742	0.809489853	positive
PRJNA729271	ed_Lushi_vs_Arbor_Acr	breed_growth_background	ENSGALG00010022669	MANSC4	-0.872440463	0.651618435	-1.338882414	0.180608956	0.325167906	negative
PRJNA729271	ed_Lushi_vs_Arbor_Acr	breed_growth_background	ENSGALG00010022671	DBR1	0.022030923	0.030903983	0.712882968	0.47591818	0.628960067	positive
PRJNA729271	ed_Lushi_vs_Arbor_Acr	breed_growth_background	ENSGALG00010022673	KLHL42	0.052055664	0.080601605	0.645839045	0.518383599	0.666614508	positive
PRJNA729271	ed_Lushi_vs_Arbor_Acr	breed_growth_background	ENSGALG00010022674	ENSGALG00010022674	-0.314184412	0.162373744	-1.934945903	0.052996934	0.138114089	negative
PRJNA729271	ed_Lushi_vs_Arbor_Acr	breed_growth_background	ENSGALG00010022681	PPP2R3A	-0.126621614	0.04985492	-2.53980177	0.011091531	0.045078392	negative
PRJNA729271	ed_Lushi_vs_Arbor_Acr	breed_growth_background	ENSGALG00010022682	EIF1	0.200516546	0.083353642	2.405612299	0.016145389	0.058657746	positive
PRJNA729271	ed_Lushi_vs_Arbor_Acr	breed_growth_background	ENSGALG00010022684	ENSGALG00010022684	0.82465513	0.288064602	2.862743722	0.004199901	0.022344305	positive
PRJNA729271	ed_Lushi_vs_Arbor_Acr	breed_growth_background	ENSGALG00010022685	ATAD1	0.002950068	0.058259087	0.050637037	0.95961475	0.977517846	positive
PRJNA729271	ed_Lushi_vs_Arbor_Acr	breed_growth_background	ENSGALG00010022689	MPL	0.155562943	0.231753134	0.671244183	0.502064986	0.65316178	positive
PRJNA729271	ed_Lushi_vs_Arbor_Acr	breed_growth_background	ENSGALG00010022691	ASIC2	-0.509991872	0.347165731	-1.469015594	0.14182856	0.274693342	negative
PRJNA729271	ed_Lushi_vs_Arbor_Acr	breed_growth_background	ENSGALG00010022695	PIK3IP1	0.373626879	0.10833363	3.448854043	0.000562971	0.005386312	positive
PRJNA729271	ed_Lushi_vs_Arbor_Acr	breed_growth_background	ENSGALG00010022698	SEMA6D	0.196600674	0.062568035	3.142190315	0.00167689	0.011711294	positive
PRJNA729271	ed_Lushi_vs_Arbor_Acr	breed_growth_background	ENSGALG00010022702	CDC20	-0.621752318	0.126725718	-4.906283647	9.28183E-07	4.59181E-05	negative
PRJNA729271	ed_Lushi_vs_Arbor_Acr	breed_growth_background	ENSGALG00010022707	SALL1	0.478503306	0.293220315	1.631890018	0.102702657	0.21927751	positive
PRJNA729271	ed_Lushi_vs_Arbor_Acr	breed_growth_background	ENSGALG00010022708	SPRED1	-0.321917118	0.070207599	-4.58521759	4.53514E-06	0.000160334	negative
PRJNA729271	ed_Lushi_vs_Arbor_Acr	breed_growth_background	ENSGALG00010022709	RNF5	0.056642764	0.046155001	1.227229171	0.219736468	0.372175755	positive
PRJNA729271	ed_Lushi_vs_Arbor_Acr	breed_growth_background	ENSGALG00010022711	LRRC49	0.007346109	0.104294828	0.070435982	0.943846653	0.969393979	positive
PRJNA729271	ed_Lushi_vs_Arbor_Acr	breed_growth_background	ENSGALG00010022712	DDX23	0.392321617	0.107967001	3.633717847	0.000279366	0.003318805	positive
PRJNA729271	ed_Lushi_vs_Arbor_Acr	breed_growth_background	ENSGALG00010022713	PNLIPRP1	-2.332090525	1.4813225	-1.57433005	0.115411166	0.237997561	negative
PRJNA729271	ed_Lushi_vs_Arbor_Acr	breed_growth_background	ENSGALG00010022717	ELOVL1	-0.090877528	0.091444445	-0.993800422	0.320320041	0.48370028	negative
PRJNA729271	ed_Lushi_vs_Arbor_Acr	breed_growth_background	ENSGALG00010022718	C12orf43	0.037276535	0.077424321	0.481457697	0.630191238	0.756177318	positive
PRJNA729271	ed_Lushi_vs_Arbor_Acr	breed_growth_background	ENSGALG00010022719	MSL2	0.05171471	0.052617143	0.982849064	0.32568173	0.489325529	positive
PRJNA729271	ed_Lushi_vs_Arbor_Acr	breed_growth_background	ENSGALG00010022724	LUZP2	-0.048285231	0.230165515	-0.209784821	0.833835621	0.899088546	negative
PRJNA729271	ed_Lushi_vs_Arbor_Acr	breed_growth_background	ENSGALG00010022725	KRT222	-0.68135527	0.373500337	-1.824242717	0.068115386	0.164864374	negative
PRJNA729271	ed_Lushi_vs_Arbor_Acr	breed_growth_background	ENSGALG00010022727	ACP2	0.059187362	0.045341278	1.305374809	0.191765252	0.338567275	positive
PRJNA729271	ed_Lushi_vs_Arbor_Acr	breed_growth_background	ENSGALG00010022729	ENSGALG00010022729	0.180280204	0.072435249	2.488846335	0.012815834	0.049749738	positive
PRJNA729271	ed_Lushi_vs_Arbor_Acr	breed_growth_background	ENSGALG00010022731	LRRC8B	0.467868962	0.097117273	4.817566907	1.45319E-06	6.60315E-05	positive
PRJNA729271	ed_Lushi_vs_Arbor_Acr	breed_growth_background	ENSGALG00010022733	PCCB	0.097328031	0.061572661	1.580702039	0.113946182	0.235747917	positive
PRJNA729271	ed_Lushi_vs_Arbor_Acr	breed_growth_background	ENSGALG00010022734	ENSGALG00010022734	0.049911387	0.063675712	0.78383712	0.43313569	0.591344243	positive
PRJNA729271	ed_Lushi_vs_Arbor_Acr	breed_growth_background	ENSGALG00010022737	CYLD	0.05452453	0.042667827	1.277883908	0.201290363	0.350180486	positive
PRJNA729271	ed_Lushi_vs_Arbor_Acr	breed_growth_background	ENSGALG00010022742	IFT46	-0.071907617	0.068230236	-1.05389665	0.291930237	0.453297138	negative
PRJNA729271	ed_Lushi_vs_Arbor_Acr	breed_growth_background	ENSGALG00010022743	ZNF326	0.29441631	0.082400032	3.573012077	0.000352898	0.003878138	positive
PRJNA729271	ed_Lushi_vs_Arbor_Acr	breed_growth_background	ENSGALG00010022745	PAPSS2	0.575858437	0.127874496	4.503309525	6.69033E-06	0.000215983	positive
PRJNA729271	ed_Lushi_vs_Arbor_Acr	breed_growth_background	ENSGALG00010022747	NT5C3B	0.055878184	0.07527133	0.7423568	0.457871186	0.61376258	positive
PRJNA729271	ed_Lushi_vs_Arbor_Acr	breed_growth_background	ENSGALG00010022748	ENSGALG00010022748	0.042640877	0.181396188	0.235070412	0.814154083	0.885640783	positive
PRJNA729271	ed_Lushi_vs_Arbor_Acr	breed_growth_background	ENSGALG00010022749	HSPA12A	-0.240680656	0.070311385	-3.423068041	0.000619186	0.005760653	negative
PRJNA729271	ed_Lushi_vs_Arbor_Acr	breed_growth_background	ENSGALG00010022751	SLC25A12	0.251452177	0.102284954	2.458349622	0.013957722	0.052833171	positive
PRJNA729271	ed_Lushi_vs_Arbor_Acr	breed_growth_background	ENSGALG00010022754	SNX20	0.009439004	0.100647217	0.093783059	0.925281489	0.957996799	positive
PRJNA729271	ed_Lushi_vs_Arbor_Acr	breed_growth_background	ENSGALG00010022755	MINPP1	-0.309137264	0.062487286	-4.947202613	7.52876E-07	3.85616E-05	negative
PRJNA729271	ed_Lushi_vs_Arbor_Acr	breed_growth_background	ENSGALG00010022759	THSD4	-0.065708758	0.064835171	-1.013473968	0.310833824	0.473818097	negative
PRJNA729271	ed_Lushi_vs_Arbor_Acr	breed_growth_background	ENSGALG00010022760	ELAVL4	0.153529848	0.081215462	1.890401718	0.058704254	0.148398702	positive
PRJNA729271	ed_Lushi_vs_Arbor_Acr	breed_growth_background	ENSGALG00010022764	MFAP5	-0.432413404	0.119018467	-3.633162272	0.000279969	0.003320907	negative
PRJNA729271	ed_Lushi_vs_Arbor_Acr	breed_growth_background	ENSGALG00010022767	CACNB3	-0.635649614	0.25932843	-2.451137397	0.014240558	0.053503601	negative
PRJNA729271	ed_Lushi_vs_Arbor_Acr	breed_growth_background	ENSGALG00010022768	ENSGALG00010022768	-0.25845136	0.345084659	-0.748950594	0.453886984	0.610135569	negative
PRJNA729271	ed_Lushi_vs_Arbor_Acr	breed_growth_background	ENSGALG00010022769	FXYD6	-0.050513609	0.051899097	-0.973304186	0.330402143	0.494596619	negative
PRJNA729271	ed_Lushi_vs_Arbor_Acr	breed_growth_background	ENSGALG00010022770	HAT1	0.003585698	0.050712125	0.070706922	0.943631011	0.969309865	positive
PRJNA729271	ed_Lushi_vs_Arbor_Acr	breed_growth_background	ENSGALG00010022771	SGMS1	0.208849893	0.117363739	1.779509534	0.075156263	0.176540838	positive
PRJNA729271	ed_Lushi_vs_Arbor_Acr	breed_growth_background	ENSGALG00010022776	CDH4	0.065582494	0.096159499	0.68201784	0.49522767	0.646702902	positive
PRJNA729271	ed_Lushi_vs_Arbor_Acr	breed_growth_background	ENSGALG00010022780	DYNC1I2	-0.085216316	0.031301224	-2.722459548	0.006479797	0.030684303	negative
PRJNA729271	ed_Lushi_vs_Arbor_Acr	breed_growth_background	ENSGALG00010022782	TMPRSS13	-0.296868146	0.131918006	-2.250398983	0.02442363	0.078635554	negative
PRJNA729271	ed_Lushi_vs_Arbor_Acr	breed_growth_background	ENSGALG00010022783	STAG1	0.01884832	0.029805979	0.632367085	0.527147037	0.673163273	positive
PRJNA729271	ed_Lushi_vs_Arbor_Acr	breed_growth_background	ENSGALG00010022784	DMRTA2	-0.210985016	0.428052681	-0.492894975	0.622086798	0.748932569	negative
PRJNA729271	ed_Lushi_vs_Arbor_Acr	breed_growth_background	ENSGALG00010022786	KRT40	-0.432837582	0.29621474	-1.461229048	0.143952594	0.27732494	negative
PRJNA729271	ed_Lushi_vs_Arbor_Acr	breed_growth_background	ENSGALG00010022787	ENO4	-0.974610609	0.186464383	-5.226792331	1.72476E-07	1.2776E-05	negative
PRJNA729271	ed_Lushi_vs_Arbor_Acr	breed_growth_background	ENSGALG00010022788	CYBRD1	-0.148232576	0.092383589	-1.604533633	0.108596477	0.228396102	negative
PRJNA729271	ed_Lushi_vs_Arbor_Acr	breed_growth_background	ENSGALG00010022789	ENSGALG00010022789	-0.820510724	0.194457838	-4.219478797	2.44868E-05	0.000561607	negative
PRJNA729271	ed_Lushi_vs_Arbor_Acr	breed_growth_background	ENSGALG00010022792	DDB2	0.013541587	0.068817832	0.196774398	0.844004087	0.905271514	positive
PRJNA729271	ed_Lushi_vs_Arbor_Acr	breed_growth_background	ENSGALG00010022795	TAF4	0.120887845	0.059664976	2.026110697	0.042753442	0.118198205	positive
PRJNA729271	ed_Lushi_vs_Arbor_Acr	breed_growth_background	ENSGALG00010022796	RIMKLB	-0.300392048	0.074413865	-4.036775247	5.41909E-05	0.001025454	negative
PRJNA729271	ed_Lushi_vs_Arbor_Acr	breed_growth_background	ENSGALG00010022797	EFHD1	-0.123528477	0.070559121	-1.750708836	0.079996077	0.184112914	negative
PRJNA729271	ed_Lushi_vs_Arbor_Acr	breed_growth_background	ENSGALG00010022799	DCAF17	-0.184875248	0.083524842	-2.21341633	0.026868956	0.084056042	negative
PRJNA729271	ed_Lushi_vs_Arbor_Acr	breed_growth_background	ENSGALG00010022800	KLHL10	-0.084737183	0.249202163	-0.340033897	0.733831001	0.829451058	negative
PRJNA729271	ed_Lushi_vs_Arbor_Acr	breed_growth_background	ENSGALG00010022801	LSM14B	0.176063104	0.055264507	3.185826032	0.001443414	0.010636645	positive
PRJNA729271	ed_Lushi_vs_Arbor_Acr	breed_growth_background	ENSGALG00010022803	ENSGALG00010022803	-0.23599858	0.088747557	-2.659212135	0.007832363	0.035093114	negative
PRJNA729271	ed_Lushi_vs_Arbor_Acr	breed_growth_background	ENSGALG00010022804	SNX19	-0.045686654	0.062814084	-0.727331368	0.467022986	0.621911608	negative
PRJNA729271	ed_Lushi_vs_Arbor_Acr	breed_growth_background	ENSGALG00010022805	ENSGALG00010022805	-0.217546707	0.148924609	-1.460784136	0.144074691	0.277441995	negative
PRJNA729271	ed_Lushi_vs_Arbor_Acr	breed_growth_background	ENSGALG00010022806	SPPL3	-0.059588377	0.034760562	-1.714252396	0.086482382	0.194561869	negative
PRJNA729271	ed_Lushi_vs_Arbor_Acr	breed_growth_background	ENSGALG00010022807	ADCY6	-0.213894823	0.080141195	-2.668974712	0.007608318	0.034358625	negative
PRJNA729271	ed_Lushi_vs_Arbor_Acr	breed_growth_background	ENSGALG00010022808	PSMA7	0.098247257	0.073283806	1.340640755	0.180037118	0.3243398	positive
PRJNA729271	ed_Lushi_vs_Arbor_Acr	breed_growth_background	ENSGALG00010022809	MYO1E	-0.17118492	0.083939468	-2.039385346	0.041411584	0.115279606	negative
PRJNA729271	ed_Lushi_vs_Arbor_Acr	breed_growth_background	ENSGALG00010022811	GIGYF2	0.100537748	0.052489805	1.915376672	0.055444484	0.142722038	positive
PRJNA729271	ed_Lushi_vs_Arbor_Acr	breed_growth_background	ENSGALG00010022813	USP24	0.117951401	0.065164676	1.810051217	0.070287845	0.168289117	positive
PRJNA729271	ed_Lushi_vs_Arbor_Acr	breed_growth_background	ENSGALG00010022815	LRRC8D	0.156974134	0.128036739	1.226008533	0.220195464	0.372735403	positive
PRJNA729271	ed_Lushi_vs_Arbor_Acr	breed_growth_background	ENSGALG00010022816	METTL8	0.137525739	0.079866154	1.721952703	0.085078087	0.192417985	positive
PRJNA729271	ed_Lushi_vs_Arbor_Acr	breed_growth_background	ENSGALG00010022817	KRT23	-1.099196669	0.488284275	-2.251140833	0.024376619	0.078556633	negative
PRJNA729271	ed_Lushi_vs_Arbor_Acr	breed_growth_background	ENSGALG00010022819	PACSIN3	-0.1604517	0.057570516	-2.787046417	0.005319086	0.026632176	negative
PRJNA729271	ed_Lushi_vs_Arbor_Acr	breed_growth_background	ENSGALG00010022820	SHTN1	-0.049134564	0.096392634	-0.509733595	0.610238113	0.740584881	negative
PRJNA729271	ed_Lushi_vs_Arbor_Acr	breed_growth_background	ENSGALG00010022821	ENSGALG00010022821	-1.895909903	0.725535848	-2.613116787			negative
PRJNA729271	ed_Lushi_vs_Arbor_Acr	breed_growth_background	ENSGALG00010022823	IGHMBP2	0.221164336	0.061916832	3.571958215	0.000354322	0.003887885	positive
PRJNA729271	ed_Lushi_vs_Arbor_Acr	breed_growth_background	ENSGALG00010022824	PUS1	0.180074752	0.046619202	3.862673418	0.000112153	0.001740531	positive
PRJNA729271	ed_Lushi_vs_Arbor_Acr	breed_growth_background	ENSGALG00010022825	SS18L1	0.624319292	0.1442048	4.329393268	1.49521E-05	0.000389803	positive
PRJNA729271	ed_Lushi_vs_Arbor_Acr	breed_growth_background	ENSGALG00010022826	SNORC	0.728012599	0.327944729	2.219924687	0.02642388	0.083101355	positive
PRJNA729271	ed_Lushi_vs_Arbor_Acr	breed_growth_background	ENSGALG00010022827	CMTR2	-0.102360244	0.050891353	-2.011348436	0.044288666	0.121285511	negative
PRJNA729271	ed_Lushi_vs_Arbor_Acr	breed_growth_background	ENSGALG00010022828	PRKG1	-0.1499111	0.091914076	-1.630991746	0.102892061	0.219529124	negative
PRJNA729271	ed_Lushi_vs_Arbor_Acr	breed_growth_background	ENSGALG00010022830	MTG2	0.23929295	0.071396534	3.351604566	0.000803447	0.006975677	positive
PRJNA729271	ed_Lushi_vs_Arbor_Acr	breed_growth_background	ENSGALG00010022831	LRRC8C	-0.221317228	0.04820635	-4.591038872	4.41045E-06	0.000156307	negative
PRJNA729271	ed_Lushi_vs_Arbor_Acr	breed_growth_background	ENSGALG00010022833	HRH3	-0.42599811	0.298566463	-1.426811657	0.15363419	0.290077841	negative
PRJNA729271	ed_Lushi_vs_Arbor_Acr	breed_growth_background	ENSGALG00010022834	CHRNA7	0.075389791	0.140688853	0.535861869	0.59205402	0.72671886	positive
PRJNA729271	ed_Lushi_vs_Arbor_Acr	breed_growth_background	ENSGALG00010022837	ITGB3	-0.300705196	0.082178501	-3.659171096	0.000253032	0.003071779	negative
PRJNA729271	ed_Lushi_vs_Arbor_Acr	breed_growth_background	ENSGALG00010022838	ARFGAP2	0.105533462	0.042594481	2.477632323	0.013225736	0.050864165	positive
PRJNA729271	ed_Lushi_vs_Arbor_Acr	breed_growth_background	ENSGALG00010022839	SYT12	-0.408476285	0.180008279	-2.26920832	0.023255661	0.076041236	negative
PRJNA729271	ed_Lushi_vs_Arbor_Acr	breed_growth_background	ENSGALG00010022840	MARVELD1	0.198630257	0.104256809	1.90520176	0.05675385	0.145062079	positive
PRJNA729271	ed_Lushi_vs_Arbor_Acr	breed_growth_background	ENSGALG00010022841	ENSGALG00010022841	0.129657535	0.228944674	0.566326934	0.571171554	0.709558765	positive

PRJNA729271	ed_Lushi_vs_Arbor_Acr	breed_growth_background	ENSGALG00010022842	PHC1	0.099271234	0.077503921	1.280854344	0.200244832	0.349042593	positive
PRJNA729271	ed_Lushi_vs_Arbor_Acr	breed_growth_background	ENSGALG00010022845	HYDIN	0.130971022	0.16506106	0.793470136	0.427503962	0.586639205	positive
PRJNA729271	ed_Lushi_vs_Arbor_Acr	breed_growth_background	ENSGALG00010022848	SGIP1	-0.426454735	0.452642493	-0.942144719	0.346118552	0.509960196	negative
PRJNA729271	ed_Lushi_vs_Arbor_Acr	breed_growth_background	ENSGALG00010022850	ULK1	0.441876026	0.133363094	3.313330641	0.000921919	0.007666792	positive
PRJNA729271	ed_Lushi_vs_Arbor_Acr	breed_growth_background	ENSGALG00010022858	HERC4	0.037381698	0.068175067	0.548319195	0.58347275	0.719598188	positive
PRJNA729271	ed_Lushi_vs_Arbor_Acr	breed_growth_background	ENSGALG00010022860	MOB3C	-0.183403648	0.07154799	-2.563365486	0.010366286	0.042918971	negative
PRJNA729271	ed_Lushi_vs_Arbor_Acr	breed_growth_background	ENSGALG00010022862	ACADS	0.00281765	0.065772664	0.042839231	0.965829691	0.980645935	positive
PRJNA729271	ed_Lushi_vs_Arbor_Acr	breed_growth_background	ENSGALG00010022863	FAM81A	-0.132458845	0.178780266	-0.740903055	0.458752223	0.614372268	negative
PRJNA729271	ed_Lushi_vs_Arbor_Acr	breed_growth_background	ENSGALG00010022864	CSTPP1	-0.228226365	0.114787285	-1.988254749	0.046783523	0.126233649	negative
PRJNA729271	ed_Lushi_vs_Arbor_Acr	breed_growth_background	ENSGALG00010022866	KLHL11	-0.126391989	0.05349076	-2.362875165	0.018133777	0.063813813	negative
PRJNA729271	ed_Lushi_vs_Arbor_Acr	breed_growth_background	ENSGALG00010022867	ATPAF1	0.141344896	0.072713084	1.943871549	0.051910943	0.136066748	positive
PRJNA729271	ed_Lushi_vs_Arbor_Acr	breed_growth_background	ENSGALG00010022868	VAC14	-0.036984797	0.055547741	-0.66582001	0.505526153	0.655203621	negative
PRJNA729271	ed_Lushi_vs_Arbor_Acr	breed_growth_background	ENSGALG00010022872	KRT13	0.388942632	0.262887464	1.479502396	0.139006091	0.270687701	positive
PRJNA729271	ed_Lushi_vs_Arbor_Acr	breed_growth_background	ENSGALG00010022873	NGEF	0.606224785	0.206880926	2.930307765	0.003386264	0.019316766	positive
PRJNA729271	ed_Lushi_vs_Arbor_Acr	breed_growth_background	ENSGALG00010022874	EFCAB14	-0.01943798	0.041022343	-0.473838857	0.635614823	0.760419021	negative
PRJNA729271	ed_Lushi_vs_Arbor_Acr	breed_growth_background	ENSGALG00010022875	SPON1	-0.092265737	0.131014525	-0.70424052	0.481283001	0.634164332	negative
PRJNA729271	ed_Lushi_vs_Arbor_Acr	breed_growth_background	ENSGALG00010022877	KYAT3	0.166348159	0.054924556	3.028666404	0.002456358	0.015409523	positive
PRJNA729271	ed_Lushi_vs_Arbor_Acr	breed_growth_background	ENSGALG00010022879	ENSGALG00010022879	-0.04752935	0.150335048	-0.316156148	0.75188399	0.842756653	negative
PRJNA729271	ed_Lushi_vs_Arbor_Acr	breed_growth_background	ENSGALG00010022881	CCNB1	-0.291757373	0.113852435	-2.562592301	0.010389397	0.043000515	negative
PRJNA729271	ed_Lushi_vs_Arbor_Acr	breed_growth_background	ENSGALG00010022885	M6PR	-0.134694573	0.053192644	-2.532203016	0.011334834	0.045746661	negative
PRJNA729271	ed_Lushi_vs_Arbor_Acr	breed_growth_background	ENSGALG00010022886	PLA2G15	0.357759329	0.150352851	2.379464888	0.017337795	0.06164124	positive
PRJNA729271	ed_Lushi_vs_Arbor_Acr	breed_growth_background	ENSGALG00010022888	OSBPL2	0.195136438	0.048576631	4.017084609	5.89226E-05	0.001089391	positive
PRJNA729271	ed_Lushi_vs_Arbor_Acr	breed_growth_background	ENSGALG00010022890	MYPN	0.190065942	0.087292677	2.177341193	0.02945512	0.08969579	positive
PRJNA729271	ed_Lushi_vs_Arbor_Acr	breed_growth_background	ENSGALG00010022891	NTM	0.315843162	0.116549073	2.709958588	0.006729161	0.031504906	positive
PRJNA729271	ed_Lushi_vs_Arbor_Acr	breed_growth_background	ENSGALG00010022900	MMP17	-0.264214083	0.106666162	-2.47701874	0.013248495	0.050938177	negative
PRJNA729271	ed_Lushi_vs_Arbor_Acr	breed_growth_background	ENSGALG00010022911	SMPD3	0.555337187	0.194720846	2.851965761	0.004344978	0.022952061	positive
PRJNA729271	ed_Lushi_vs_Arbor_Acr	breed_growth_background	ENSGALG00010022917	CYP4B7	-0.809954796	0.268170708	-3.020295551	0.002525281	0.01568965	negative
PRJNA729271	ed_Lushi_vs_Arbor_Acr	breed_growth_background	ENSGALG00010022919	ACLY	-0.16322878	0.067576826	-2.415454964	0.015715566	0.057629298	negative
PRJNA729271	ed_Lushi_vs_Arbor_Acr	breed_growth_background	ENSGALG00010022921	ENSGALG00010022921	-0.077747517	0.106015757	-0.733358129	0.46334003	0.618483629	negative
PRJNA729271	ed_Lushi_vs_Arbor_Acr	breed_growth_background	ENSGALG00010022922	ATOH7	-1.023384797	0.240321933	-4.258391177	2.05903E-05	0.000491692	negative
PRJNA729271	ed_Lushi_vs_Arbor_Acr	breed_growth_background	ENSGALG00010022923	CD63	0.064224912	0.067576263	0.950406391	0.341905798	0.505765159	positive
PRJNA729271	ed_Lushi_vs_Arbor_Acr	breed_growth_background	ENSGALG00010022928	RNF111	0.060725932	0.044870838	1.353349631	0.175943961	0.319948276	positive
PRJNA729271	ed_Lushi_vs_Arbor_Acr	breed_growth_background	ENSGALG00010022929	LRP4	0.196508485	0.062736733	3.132271548	0.001734594	0.012018611	positive
PRJNA729271	ed_Lushi_vs_Arbor_Acr	breed_growth_background	ENSGALG00010022930	GTF2B	0.109896711	0.063867197	1.720706657	0.085304068	0.192718588	positive
PRJNA729271	ed_Lushi_vs_Arbor_Acr	breed_growth_background	ENSGALG00010022931	RDH5	-0.129499186	0.065705265	-1.970910341	0.048734132	0.130188213	negative
PRJNA729271	ed_Lushi_vs_Arbor_Acr	breed_growth_background	ENSGALG00010022932	OPCML	0.171008222	0.11013745	1.552680056	0.120499589	0.245039499	positive
PRJNA729271	ed_Lushi_vs_Arbor_Acr	breed_growth_background	ENSGALG00010022933	UNC119B	-0.008784233	0.041781781	-0.21024076	0.833479768	0.898832166	negative
PRJNA729271	ed_Lushi_vs_Arbor_Acr	breed_growth_background	ENSGALG00010022937	BLOC1S1	0.098328613	0.086284445	1.139586781	0.254458495	0.411065849	positive
PRJNA729271	ed_Lushi_vs_Arbor_Acr	breed_growth_background	ENSGALG00010022942	NEU2	-0.320202721	0.271121445	-1.18103059	0.237590571	0.392732961	negative
PRJNA729271	ed_Lushi_vs_Arbor_Acr	breed_growth_background	ENSGALG00010022945	KCNA4	-0.485122686	0.24780446	-1.957683435	0.05026717	0.133057456	negative
PRJNA729271	ed_Lushi_vs_Arbor_Acr	breed_growth_background	ENSGALG00010022946	KRT17	0.161939845	0.28777553	0.562729727	0.573618939	0.711482353	positive
PRJNA729271	ed_Lushi_vs_Arbor_Acr	breed_growth_background	ENSGALG00010022949	INSC	-0.240803159	0.145818859	-1.651385566	0.09865987	0.212840426	negative
PRJNA729271	ed_Lushi_vs_Arbor_Acr	breed_growth_background	ENSGALG00010022950	ITGA7	-0.004495095	0.081945712	-0.054854546	0.956254344	0.97569799	negative
PRJNA729271	ed_Lushi_vs_Arbor_Acr	breed_growth_background	ENSGALG00010022953	ENSGALG00010022953	0.286229626	0.563625603	0.507836452	0.611568044	0.741565902	positive
PRJNA729271	ed_Lushi_vs_Arbor_Acr	breed_growth_background	ENSGALG00010022954	ADRM1	0.115927977	0.076971152	1.506122409	0.132035753	0.260885802	positive
PRJNA729271	ed_Lushi_vs_Arbor_Acr	breed_growth_background	ENSGALG00010022956	PBLD	-0.01147821	0.066454291	-0.172723378	0.862868867	0.917558644	negative
PRJNA729271	ed_Lushi_vs_Arbor_Acr	breed_growth_background	ENSGALG00010022957	SLTM	0.397997104	0.076696738	5.189231196	2.11164E-07	1.49308E-05	positive
PRJNA729271	ed_Lushi_vs_Arbor_Acr	breed_growth_background	ENSGALG00010022958	SFSWAP	0.049071206	0.052477508	0.935090251	0.34974179	0.513731987	positive
PRJNA729271	ed_Lushi_vs_Arbor_Acr	breed_growth_background	ENSGALG00010022959	ADAMTS8	-0.119753917	0.141495843	-0.846342295	0.39736182	0.558657573	negative
PRJNA729271	ed_Lushi_vs_Arbor_Acr	breed_growth_background	ENSGALG00010022967	ADAMTS15	-0.091144264	0.087303386	-1.043994606	0.296487885	0.458116608	negative
PRJNA729271	ed_Lushi_vs_Arbor_Acr	breed_growth_background	ENSGALG00010022969	TTN	0.152137445	0.307615545	0.494570082	0.620903625	0.748253911	positive
PRJNA729271	ed_Lushi_vs_Arbor_Acr	breed_growth_background	ENSGALG00010022971	CKAP5	-0.043817104	0.031515961	-1.390314716	0.164433331	0.304401167	negative
PRJNA729271	ed_Lushi_vs_Arbor_Acr	breed_growth_background	ENSGALG00010022972	ENSGALG00010022972	-0.884513233	0.589176256	-1.501271011			negative
PRJNA729271	ed_Lushi_vs_Arbor_Acr	breed_growth_background	ENSGALG00010022974	INPP5D	-0.199847838	0.082868134	-2.411636769	0.015881094	0.058057115	negative
PRJNA729271	ed_Lushi_vs_Arbor_Acr	breed_growth_background	ENSGALG00010022976	MINDY2	-0.093637458	0.035554706	-2.633616439	0.008448086	0.036895153	negative
PRJNA729271	ed_Lushi_vs_Arbor_Acr	breed_growth_background	ENSGALG00010022978	ARL14EP	-0.027606806	0.047028581	-0.587021876	0.557189015	0.698621901	negative
PRJNA729271	ed_Lushi_vs_Arbor_Acr	breed_growth_background	ENSGALG00010022979	LAMA5	0.059669634	0.0798125	0.747622676	0.454687783	0.610872131	positive
PRJNA729271	ed_Lushi_vs_Arbor_Acr	breed_growth_background	ENSGALG00010022981	HNRNPH3	0.152152069	0.069535175	2.188130974	0.028660063	0.087921189	positive
PRJNA729271	ed_Lushi_vs_Arbor_Acr	breed_growth_background	ENSGALG00010022983	PKN2	-0.019037615	0.033194443	-0.573518128	0.566293929	0.705737297	negative
PRJNA729271	ed_Lushi_vs_Arbor_Acr	breed_growth_background	ENSGALG00010022986	ALG9	-0.014055103	0.057396997	-0.244875241	0.806553033	0.881046592	negative
PRJNA729271	ed_Lushi_vs_Arbor_Acr	breed_growth_background	ENSGALG00010022987	F2	-1.871809152	0.745124426	-2.512075953			negative
PRJNA729271	ed_Lushi_vs_Arbor_Acr	breed_growth_background	ENSGALG00010022990	SLC25A22	-0.180395456	0.054840235	-3.289472672	0.001003753	0.008160835	negative
PRJNA729271	ed_Lushi_vs_Arbor_Acr	breed_growth_background	ENSGALG00010022991	TAL1	0.159188601	0.094208639	1.689745246	0.091076703	0.201681505	positive
PRJNA729271	ed_Lushi_vs_Arbor_Acr	breed_growth_background	ENSGALG00010022992	SLC7A6	-0.045422379	0.057567282	-0.789031146	0.430093803	0.588466082	negative
PRJNA729271	ed_Lushi_vs_Arbor_Acr	breed_growth_background	ENSGALG00010022994	LIPC	-1.705652875	0.483737629	-3.525987587	0.000421907	0.004402835	negative
PRJNA729271	ed_Lushi_vs_Arbor_Acr	breed_growth_background	ENSGALG00010022997	ARHGAP1	-0.246155582	0.051761803	-4.755544936	1.97912E-06	8.36365E-05	negative
PRJNA729271	ed_Lushi_vs_Arbor_Acr	breed_growth_background	ENSGALG00010022999	ENSGALG00010022999	0.251973609	0.250543466	1.005708162	0.314555976	0.477983947	positive
PRJNA729271	ed_Lushi_vs_Arbor_Acr	breed_growth_background	ENSGALG00010023002	ATG16L1	0.220803271	0.071896895	3.071109987	0.002132646	0.013899595	positive
PRJNA729271	ed_Lushi_vs_Arbor_Acr	breed_growth_background	ENSGALG00010023003	ENSGALG00010023003	-0.128162276	0.374131011	-0.342559884	0.731929586	0.828206038	negative
PRJNA729271	ed_Lushi_vs_Arbor_Acr	breed_growth_background	ENSGALG00010023005	IRF7	0.160833189	0.187994796	0.855519365	0.392263688	0.553745828	positive
PRJNA729271	ed_Lushi_vs_Arbor_Acr	breed_growth_background	ENSGALG00010023007	LMO4	0.129581688	0.076434953	1.69531977	0.090014776	0.200240053	positive
PRJNA729271	ed_Lushi_vs_Arbor_Acr	breed_growth_background	ENSGALG00010023008	CDHR5	-0.563709831	0.493405301	-1.142488396	0.253251067	0.409878764	negative
PRJNA729271	ed_Lushi_vs_Arbor_Acr	breed_growth_background	ENSGALG00010023012	ENSGALG00010023012	0.36867149	0.286146264	1.288402249	0.197605976	0.346105929	positive
PRJNA729271	ed_Lushi_vs_Arbor_Acr	breed_growth_background	ENSGALG00010023013	ENSGALG00010023013	-0.243945723	0.235500308	-1.035861586	0.300266721	0.462673128	negative
PRJNA729271	ed_Lushi_vs_Arbor_Acr	breed_growth_background	ENSGALG00010023015	METTL2B	0.036944052	0.055896541	0.660936284	0.508653176	0.657651216	positive
PRJNA729271	ed_Lushi_vs_Arbor_Acr	breed_growth_background	ENSGALG00010023017	BUB1B	-0.35482618	0.108961222	-3.256444587	0.001128169	0.008924626	negative
PRJNA729271	ed_Lushi_vs_Arbor_Acr	breed_growth_background	ENSGALG00010023019	CABP1	-0.012645539	0.177966154	-0.071055866	0.943353293	0.969225493	negative
PRJNA729271	ed_Lushi_vs_Arbor_Acr	breed_growth_background	ENSGALG00010023024	PRMT7	0.041020291	0.061555398	0.666396325	0.505157811	0.655130385	positive
PRJNA729271	ed_Lushi_vs_Arbor_Acr	breed_growth_background	ENSGALG00010023026	PHRF1	0.147545254	0.049046483	3.008273887	0.002627362	0.016113307	positive
PRJNA729271	ed_Lushi_vs_Arbor_Acr	breed_growth_background	ENSGALG00010023028	TMEM61	0.338762989	0.175141722	1.934222098	0.053085827	0.13829602	positive
PRJNA729271	ed_Lushi_vs_Arbor_Acr	breed_growth_background	ENSGALG00010023030	HS2ST1	-0.159779875	0.045994247	-3.473910011	0.000512933	0.005061242	negative
PRJNA729271	ed_Lushi_vs_Arbor_Acr	breed_growth_background	ENSGALG00010023041	CD151	-0.079731769	0.063728496	-1.251116441	0.210891998	0.360960183	negative
PRJNA729271	ed_Lushi_vs_Arbor_Acr	breed_growth_background	ENSGALG00010023042	ENSGALG00010023042	-0.124308657	0.437152922	-0.284359661	0.77613477	0.861212243	negative
PRJNA729271	ed_Lushi_vs_Arbor_Acr	breed_growth_background	ENSGALG00010023043	CNP	0.120156855	0.077442261	1.551566982	0.120765869	0.245340051	positive
PRJNA729271	ed_Lushi_vs_Arbor_Acr	breed_growth_background	ENSGALG00010023046	SLC7A6OS	-0.012877953	0.04124425	-0.312236333	0.754860922	0.844984868	negative
PRJNA729271	ed_Lushi_vs_Arbor_Acr	breed_growth_background	ENSGALG00010023048	ARFGAP1	0.122444372	0.045080537	2.716124937	0.006605099	0.031125132	positive
PRJNA729271	ed_Lushi_vs_Arbor_Acr	breed_growth_background	ENSGALG00010023050	RUFY2	-0.060018827	0.051263437	-1.170792091	0.241682358	0.397500548	negative
PRJNA729271	ed_Lushi_vs_Arbor_Acr	breed_growth_background	ENSGALG00010023052	RAD54L	-0.037181582	0.074206599	-0.501054934	0.616332462	0.744788582	negative
PRJNA729271	ed_Lushi_vs_Arbor_Acr	breed_growth_background	ENSGALG00010023053	MAN1A2	-0.000769088	0.026341612	-0.029196679	0.97670773	0.986164569	negative
PRJNA729271	ed_Lushi_vs_Arbor_Acr	breed_growth_background	ENSGALG00010023054	AQP9	-0.513817708	0.122544368	-4.192911663	2.75397E-05	0.000614135	negative
PRJNA729271	ed_Lushi_vs_Arbor_Acr	breed_growth_background	ENSGALG00010023056	SAG	-0.764596994	0.236529432	-3.232565973	0.001226838	0.009493625	negative
PRJNA729271	ed_Lushi_vs_Arbor_Acr	breed_growth_background	ENSGALG00010023057	PAFAH1B2	-0.146673706	0.040609802	-3.611780873	0.000304101	0.003506723	negative
PRJNA729271	ed_Lushi_vs_Arbor_Acr	breed_growth_background	ENSGALG00010023058	PPARA	0.027197589	0.109466751	0.248455251	0.803782189	0.878850791	positive
PRJNA729271	ed_Lushi_vs_Arbor_Acr	breed_growth_background	ENSGALG00010023059	SELENOF	-0.06284899	0.050144253	-1.253363779	0.210073347	0.360015744	negative
PRJNA729271	ed_Lushi_vs_Arbor_Acr	breed_growth_background	ENSGALG00010023060	ALDH1A2	-0.456479458	0.090041224	-5.069671846	3.98502E-07	2.37707E-05	negative
PRJNA729271	ed_Lushi_vs_Arbor_Acr	breed_growth_background	ENSGALG00010023062	STX2	0.011632139	0.055492499	0.209616428	0.833967057	0.899096503	positive
PRJNA729271	ed_Lushi_vs_Arbor_Acr	breed_growth_background	ENSGALG00010023063	ENSGALG00010023063	0.075556667	0.056256702	1.343069621	0.179249431	0.323483629	positive
PRJNA729271	ed_Lushi_vs_Arbor_Acr	breed_growth_background	ENSGALG00010023064	ATG13	-0.007344906	0.036841832	-0.199363214	0.841978634	0.90437053	negative
PRJNA729271	ed_Lushi_vs_Arbor_Acr	breed_growth_background	ENSGALG00010023065	DNA2	-0.512466495	0.124378944	-4.120202988	3.78539E-05	0.000794091	negative
PRJNA729271	ed_Lushi_vs_Arbor_Acr	breed_growth_background	ENSGALG00010023066	TLR21	-0.176966265	0.099496168	-1.778623925	0.075301439	0.176702988	negative
PRJNA729271	ed_Lushi_vs_Arbor_Acr	breed_growth_background	ENSGALG00010023068	CDPF1	0.077595811	0.068643751	1.130413333	0.258302099	0.415686568	positive
PRJNA729271	ed_Lushi_vs_Arbor_Acr	breed_growth_background	ENSGALG00010023069	ENSGALG00010023069	-0.693392091	0.153949391	-4.504026212	6.6678E-06	0.000215736	negative
PRJNA729271	ed_Lushi_vs_Arbor_Acr	breed_growth_background	ENSGALG00010023070	SIDT2	0.181892637	0.107588642	1.690630482	0.090907399	0.201483601	positive
PRJNA729271	ed_Lushi_vs_Arbor_Acr	breed_growth_background	ENSGALG00010023071	POLR2M	0.022543158	0.044481571	0.506797711	0.612296763	0.742077055	positive
PRJNA729271	ed_Lushi_vs_Arbor_Acr	breed_growth_background	ENSGALG00010023073	POMGNT1	0.073134706	0.046468824	1.573844563	0.115523389	0.238127349	positive
PRJNA729271	ed_Lushi_vs_Arbor_Acr	breed_growth_background	ENSGALG00010023074	CCNT1	0.291949996	0.089748571	3.252976537	0.001142029	0.008991697	positive
PRJNA729271	ed_Lushi_vs_Arbor_Acr	breed_growth_background	ENSGALG00010023075	PKDREJ	-0.092362453	0.165559479	-0.557880792	0.576925788	0.714446351	negative
PRJNA729271	ed_Lushi_vs_Arbor_Acr	breed_growth_background	ENSGALG00010023076	DNAJC7	-0.030392861	0.042858409	-0.709145806	0.478234	0.631272364	negative
PRJNA729271	ed_Lushi_vs_Arbor_Acr	breed_growth_background	ENSGALG00010023077	KANSL2	0.30543354	0.081918578	3.728501475	0.000192622	0.002525807	positive
PRJNA729271	ed_Lushi_vs_Arbor_Acr	breed_growth_background	ENSGALG00010023078	TAGLN	0.175547696	0.105407582	1.665418103	0.09582937	0.208534262	positive
PRJNA729271	ed_Lushi_vs_Arbor_Acr	breed_growth_background	ENSGALG00010023079	SLC25A16	0.069465191	0.051691758	1.343834934	0.17900177	0.323278178	positive
PRJNA729271	ed_Lushi_vs_Arbor_Acr	breed_growth_background	ENSGALG00010023080	MCRS1	0.091602149	0.059904624	1.529133181	0.126231431	0.252829155	positive
PRJNA729271	ed_Lushi_vs_Arbor_Acr	breed_growth_background	ENSGALG00010023081	PRLHR2	-1.522049886	0.404610846	-3.761762446	0.00016872	0.002314101	negative
PRJNA729271	ed_Lushi_vs_Arbor_Acr	breed_growth_background	ENSGALG00010023083	HARBI1	-0.124052928	0.086344577	-1.436719391	0.150797743	0.28659119	negative
PRJNA729271	ed_Lushi_vs_Arbor_Acr	breed_growth_background	ENSGALG00010023084	ENSGALG00010023084	-0.048985999	0.129328295	-0.37877248	0.704856825	0.809181857	negative
PRJNA729271	ed_Lushi_vs_Arbor_Acr	breed_growth_background	ENSGALG00010023086	PCSK7	0.113834177	0.050177316	2.268638236	0.023290334	0.076137437	positive
PRJNA729271	ed_Lushi_vs_Arbor_Acr	breed_growth_background	ENSGALG00010023087	SH3GLB1	0.155901426	0.063029313	2.473474923	0.013380619	0.051301465	positive
PRJNA729271	ed_Lushi_vs_Arbor_Acr	breed_growth_background	ENSGALG00010023089	ENSGALG00010023089	0.439882395	0.368981039	1.192154471	0.233200682	0.38761543	positive
PRJNA729271	ed_Lushi_vs_Arbor_Acr	breed_growth_background	ENSGALG00010023090	LRRC41	-0.113373401	0.050424047	-2.248399495	0.024550727	0.078954586	negative
PRJNA729271	ed_Lushi_vs_Arbor_Acr	breed_growth_background	ENSGALG00010023091	ENSGALG00010023091	0.036657016	0.070558564	0.519526103	0.603393915	0.735435001	positive
PRJNA729271	ed_Lushi_vs_Arbor_Acr	breed_growth_background	ENSGALG00010023093	POP5	-0.098405299	0.07082825	-1.389350983	0.164726048	0.304714509	negative
PRJNA729271	ed_Lushi_vs_Arbor_Acr	breed_growth_background	ENSGALG00010023094	CCDC32	-0.123940301	0.055062882	-2.250886554	0.024392724	0.078572352	negative
PRJNA729271	ed_Lushi_vs_Arbor_Acr	breed_growth_background	ENSGALG00010023096	SPATS2	0.201876336	0.091992981	2.194475429	0.028201249	0.087065688	positive
PRJNA729271	ed_Lushi_vs_Arbor_Acr	breed_growth_background	ENSGALG00010023097	LURAP1	-0.360576563	0.112518861	-3.204587733	0.001352561	0.010153223	negative
PRJNA729271	ed_Lushi_vs_Arbor_Acr	breed_growth_background	ENSGALG00010023101	CLCA2	-1.722094744	0.669268419	-2.573100261	0.0100792	0.042054695	negative
PRJNA729271	ed_Lushi_vs_Arbor_Acr	breed_growth_background	ENSGALG00010023102	RNF10	0.071090831	0.054327823	1.3085529	0.190685844	0.337360102	positive
PRJNA729271	ed_Lushi_vs_Arbor_Acr	breed_growth_background	ENSGALG00010023103	DGKD	-0.185861924	0.067128882	-2.76873262	0.00562748	0.027697901	negative
PRJNA729271	ed_Lushi_vs_Arbor_Acr	breed_growth_background	ENSGALG00010023104	ENSGALG00010023104	-0.229253454	0.08421395	-2.722274092	0.006483434	0.030687422	negative
PRJNA729271	ed_Lushi_vs_Arbor_Acr	breed_growth_background	ENSGALG00010023107	ZC3HAV1	0.00284151	0.156486141	0.018158224	0.98551263	0.991325855	positive
PRJNA729271	ed_Lushi_vs_Arbor_Acr	breed_growth_background	ENSGALG00010023108	TET1	-0.031626193	0.033069999	-0.956340914	0.338900026	0.503108959	negative
PRJNA729271	ed_Lushi_vs_Arbor_Acr	breed_growth_background	ENSGALG00010023110	LIMA1	-0.442095518	0.083118624	-5.318850319	1.04425E-07	8.5036E-06	negative

PRJNA729271	ed_Lushi_vs_Arbor_Acr	breed_growth_background	ENSGALG00010023112	PCMT1L	-0.007017742	0.069571998	-0.100870212	0.91965349	0.955435422	negative
PRJNA729271	ed_Lushi_vs_Arbor_Acr	breed_growth_background	ENSGALG00010023113	MYZAP	-0.117242544	0.071789784	-1.63313688	0.102440212	0.218813862	negative
PRJNA729271	ed_Lushi_vs_Arbor_Acr	breed_growth_background	ENSGALG00010023114	UQCRHL	-0.165866909	0.089466308	-1.853959475	0.063744958	0.157327289	negative
PRJNA729271	ed_Lushi_vs_Arbor_Acr	breed_growth_background	ENSGALG00010023115	RPLP2	-0.106671229	0.072899051	-1.463273206	0.143392636	0.2766109	negative
PRJNA729271	ed_Lushi_vs_Arbor_Acr	breed_growth_background	ENSGALG00010023116	PROC	-0.792814569	0.484962334	-1.634796174	0.102091783	0.218310585	negative
PRJNA729271	ed_Lushi_vs_Arbor_Acr	breed_growth_background	ENSGALG00010023117	RPUSD2	-0.068447288	0.057104147	-1.19863954	0.230668136	0.384535323	negative
PRJNA729271	ed_Lushi_vs_Arbor_Acr	breed_growth_background	ENSGALG00010023118	PIDD1	-0.066602372	0.075267532	-0.884875195	0.376223948	0.538679183	negative
PRJNA729271	ed_Lushi_vs_Arbor_Acr	breed_growth_background	ENSGALG00010023119	NDRG4	0.116972974	0.093976718	1.244701624	0.213241449	0.363553846	positive
PRJNA729271	ed_Lushi_vs_Arbor_Acr	breed_growth_background	ENSGALG00010023120	CHRM4	-0.514959031	0.134112749	-3.839747037	0.000123161	0.001859605	negative
PRJNA729271	ed_Lushi_vs_Arbor_Acr	breed_growth_background	ENSGALG00010023122	ENSGALG00010023122	-0.527885433	0.354804181	-1.487821909	0.136797866	0.267631944	negative
PRJNA729271	ed_Lushi_vs_Arbor_Acr	breed_growth_background	ENSGALG00010023123	RNF214	0.16436101	0.077627629	2.117300418	0.034234359	0.100268143	positive
PRJNA729271	ed_Lushi_vs_Arbor_Acr	breed_growth_background	ENSGALG00010023124	NSUN4	0.149737285	0.072099247	2.07682175	0.037818014	0.108120732	positive
PRJNA729271	ed_Lushi_vs_Arbor_Acr	breed_growth_background	ENSGALG00010023125	COL20A1	-0.603816622	0.150972946	-3.999502139	6.34759E-05	0.00114186	negative
PRJNA729271	ed_Lushi_vs_Arbor_Acr	breed_growth_background	ENSGALG00010023126	CGNL1	-0.080131871	0.073721419	-1.086955081	0.277056662	0.43632511	negative
PRJNA729271	ed_Lushi_vs_Arbor_Acr	breed_growth_background	ENSGALG00010023127	CERS5	-0.096732142	0.053012146	-1.824716587	0.068043809	0.164766958	negative
PRJNA729271	ed_Lushi_vs_Arbor_Acr	breed_growth_background	ENSGALG00010023128	IWS1	0.212252578	0.07357264	2.884938973	0.003914895	0.021365365	positive
PRJNA729271	ed_Lushi_vs_Arbor_Acr	breed_growth_background	ENSGALG00010023130	DYNLT5	-0.305056413	0.300999335	-1.013478693	0.310831569	0.473818097	negative
PRJNA729271	ed_Lushi_vs_Arbor_Acr	breed_growth_background	ENSGALG00010023131	SOX6	0.165787712	0.073258385	2.263054437	0.023632334	0.07692582	positive
PRJNA729271	ed_Lushi_vs_Arbor_Acr	breed_growth_background	ENSGALG00010023134	KNL1	-0.308679376	0.116821377	-2.642319273	0.00823404	0.036321486	negative
PRJNA729271	ed_Lushi_vs_Arbor_Acr	breed_growth_background	ENSGALG00010023135	GINS3	-0.080669444	0.038957464	-2.070705716	0.038386306	0.109335724	negative
PRJNA729271	ed_Lushi_vs_Arbor_Acr	breed_growth_background	ENSGALG00010023136	MDK	-0.640622801	0.223785889	-2.862659499	0.004201018	0.022344305	negative
PRJNA729271	ed_Lushi_vs_Arbor_Acr	breed_growth_background	ENSGALG00010023137	ETV6	-0.01273901	0.061544509	-0.206988573	0.836018782	0.900370923	negative
PRJNA729271	ed_Lushi_vs_Arbor_Acr	breed_growth_background	ENSGALG00010023140	CCAR1	0.172075647	0.045134992	3.812466581	0.000137587	0.002014466	positive
PRJNA729271	ed_Lushi_vs_Arbor_Acr	breed_growth_background	ENSGALG00010023142	TLK2	0.386345899	0.090071502	4.289324497	1.79217E-05	0.000441795	positive
PRJNA729271	ed_Lushi_vs_Arbor_Acr	breed_growth_background	ENSGALG00010023144	C11orf58	-0.181043181	0.057022325	-3.174952662	0.001498609	0.01089932	negative
PRJNA729271	ed_Lushi_vs_Arbor_Acr	breed_growth_background	ENSGALG00010023147	RAD51	-0.425547919	0.106440238	-3.997998566	6.38803E-05	0.001147392	negative
PRJNA729271	ed_Lushi_vs_Arbor_Acr	breed_growth_background	ENSGALG00010023149	ADGRD1	-0.194044758	0.099100274	-1.958064809	0.050222408	0.132963253	negative
PRJNA729271	ed_Lushi_vs_Arbor_Acr	breed_growth_background	ENSGALG00010023151	ENSGALG00010023151	0.020047231	0.28133726	0.071256937	0.943193267	0.96920363	positive
PRJNA729271	ed_Lushi_vs_Arbor_Acr	breed_growth_background	ENSGALG00010023155	CFAP263	0.152789839	0.188275445	0.811522919	0.417065437	0.577233219	positive
PRJNA729271	ed_Lushi_vs_Arbor_Acr	breed_growth_background	ENSGALG00010023157	STOX1	0.533021703	0.214997223	2.479202739	0.013167644	0.050708022	positive
PRJNA729271	ed_Lushi_vs_Arbor_Acr	breed_growth_background	ENSGALG00010023158	ENSGALG00010023158	0.14156789	0.274946926	0.514891702	0.6066287	0.737698976	positive
PRJNA729271	ed_Lushi_vs_Arbor_Acr	breed_growth_background	ENSGALG00010023159	DAW1	-0.97624558	0.286309207	-3.409759647	0.000650202	0.005995338	negative
PRJNA729271	ed_Lushi_vs_Arbor_Acr	breed_growth_background	ENSGALG00010023160	ENSGALG00010023160	0.133915425	0.059763432	2.240758628	0.025041714	0.080115664	positive
PRJNA729271	ed_Lushi_vs_Arbor_Acr	breed_growth_background	ENSGALG00010023161	ODF2L	0.095975202	0.240021912	0.399860169	0.689259511	0.798983817	positive
PRJNA729271	ed_Lushi_vs_Arbor_Acr	breed_growth_background	ENSGALG00010023162	CHRNA4	-0.668462799	0.152478035	-4.38399404	1.16523E-05	0.00032665	negative
PRJNA729271	ed_Lushi_vs_Arbor_Acr	breed_growth_background	ENSGALG00010023163	CRTAC1	-0.098435609	0.15511725	-0.634588408	0.525696891	0.672164578	negative
PRJNA729271	ed_Lushi_vs_Arbor_Acr	breed_growth_background	ENSGALG00010023164	NKIRAS2	0.249838335	0.071094223	3.514186174	0.000441104	0.004537825	positive
PRJNA729271	ed_Lushi_vs_Arbor_Acr	breed_growth_background	ENSGALG00010023165	CSNK2A2	0.266467975	0.06958026	3.829649036	0.000128326	0.001909741	positive
PRJNA729271	ed_Lushi_vs_Arbor_Acr	breed_growth_background	ENSGALG00010023166	KDM2A	0.105486872	0.061373053	1.718781564	0.085654154	0.193208366	positive
PRJNA729271	ed_Lushi_vs_Arbor_Acr	breed_growth_background	ENSGALG00010023167	GPD1	-0.057508597	0.099465622	-0.578175614	0.563145561	0.703507275	negative
PRJNA729271	ed_Lushi_vs_Arbor_Acr	breed_growth_background	ENSGALG00010023168	SPHKAP	-0.170629614	0.191829802	-0.889484385	0.373742812	0.536057991	negative
PRJNA729271	ed_Lushi_vs_Arbor_Acr	breed_growth_background	ENSGALG00010023169	RAN	-0.098449654	0.052022032	-1.892460768	0.058429616	0.147910808	negative
PRJNA729271	ed_Lushi_vs_Arbor_Acr	breed_growth_background	ENSGALG00010023170	TCF12	-0.08644172	0.03476413	-2.486520467	0.012899915	0.050035929	negative
PRJNA729271	ed_Lushi_vs_Arbor_Acr	breed_growth_background	ENSGALG00010023171	ENSGALG00010023171	0.096851465	0.0619829	1.56255135	0.118158159	0.241872972	positive
PRJNA729271	ed_Lushi_vs_Arbor_Acr	breed_growth_background	ENSGALG00010023172	DHX58	-0.248492488	0.172176317	-1.443244296	0.148951665	0.283900549	negative
PRJNA729271	ed_Lushi_vs_Arbor_Acr	breed_growth_background	ENSGALG00010023174	KCNQ2	-0.379249578	0.191912075	-1.976163186	0.048136298	0.128828709	negative
PRJNA729271	ed_Lushi_vs_Arbor_Acr	breed_growth_background	ENSGALG00010023175	GRK2	0.049836292	0.07446564	0.66925218	0.503334626	0.654055856	positive
PRJNA729271	ed_Lushi_vs_Arbor_Acr	breed_growth_background	ENSGALG00010023177	LZTS2	0.231696059	0.091807536	2.523715029	0.011612202	0.046522627	positive
PRJNA729271	ed_Lushi_vs_Arbor_Acr	breed_growth_background	ENSGALG00010023178	CFAP20	0.074649912	0.053315467	1.400154891	0.161466941	0.300560332	positive
PRJNA729271	ed_Lushi_vs_Arbor_Acr	breed_growth_background	ENSGALG00010023179	KAT2A	0.051754711	0.072060569	0.718211239	0.472627049	0.626495572	positive
PRJNA729271	ed_Lushi_vs_Arbor_Acr	breed_growth_background	ENSGALG00010023180	EEF1A2	-0.026537033	0.064767978	-0.409724583	0.682007995	0.794479296	negative
PRJNA729271	ed_Lushi_vs_Arbor_Acr	breed_growth_background	ENSGALG00010023181	PLEKHA7	0.226185971	0.088787584	2.547495486	0.010849926	0.044351538	positive
PRJNA729271	ed_Lushi_vs_Arbor_Acr	breed_growth_background	ENSGALG00010023182	EPHB6	-0.203764062	0.078961065	-2.580563763	0.009863914	0.041406728	negative
PRJNA729271	ed_Lushi_vs_Arbor_Acr	breed_growth_background	ENSGALG00010023183	ENSGALG00010023183	-0.80140657	0.276461758	-2.8987972	0.003745971	0.020677018	negative
PRJNA729271	ed_Lushi_vs_Arbor_Acr	breed_growth_background	ENSGALG00010023184	SMARCD1	-0.007521759	0.030110754	-0.249803078	0.802739639	0.878395942	negative
PRJNA729271	ed_Lushi_vs_Arbor_Acr	breed_growth_background	ENSGALG00010023185	FBXW4	0.042992665	0.061689525	0.696920022	0.485852847	0.637555406	positive
PRJNA729271	ed_Lushi_vs_Arbor_Acr	breed_growth_background	ENSGALG00010023186	MIER1	0.07935952	0.053648196	1.479257954	0.139071386	0.270687701	positive
PRJNA729271	ed_Lushi_vs_Arbor_Acr	breed_growth_background	ENSGALG00010023187	ENSGALG00010023187	-0.322476688	0.122345403	-2.635789171	0.008394187	0.036755715	negative
PRJNA729271	ed_Lushi_vs_Arbor_Acr	breed_growth_background	ENSGALG00010023188	KIF1BP	0.003276882	0.031463442	0.104148856	0.91705122	0.953151974	positive
PRJNA729271	ed_Lushi_vs_Arbor_Acr	breed_growth_background	ENSGALG00010023189	RMDN3	0.042709437	0.046689138	0.914761748	0.36031672	0.523610464	positive
PRJNA729271	ed_Lushi_vs_Arbor_Acr	breed_growth_background	ENSGALG00010023190	SF3B3	-0.017602002	0.030400449	-0.579004668	0.562586023	0.703232529	negative
PRJNA729271	ed_Lushi_vs_Arbor_Acr	breed_growth_background	ENSGALG00010023193	PPDPF	0.686260956	0.16682829	4.113576624	3.89576E-05	0.0008067	positive
PRJNA729271	ed_Lushi_vs_Arbor_Acr	breed_growth_background	ENSGALG00010023194	MRC2	-0.226289289	0.103333689	-2.189888803	0.028532302	0.087665984	negative
PRJNA729271	ed_Lushi_vs_Arbor_Acr	breed_growth_background	ENSGALG00010023195	ENSGALG00010023195	0.476115626	0.107457983	4.430714356	9.39214E-06	0.000278403	positive
PRJNA729271	ed_Lushi_vs_Arbor_Acr	breed_growth_background	ENSGALG00010023196	DGKZ	0.116315401	0.068458143	1.699073269	0.089305379	0.199150996	positive
PRJNA729271	ed_Lushi_vs_Arbor_Acr	breed_growth_background	ENSGALG00010023198	PTK6	0.386939116	0.290943147	1.329947516	0.183535564	0.328722106	positive
PRJNA729271	ed_Lushi_vs_Arbor_Acr	breed_growth_background	ENSGALG00010023199	RPS13	-0.057955564	0.105166511	-0.551083829	0.58157621	0.717815837	negative
PRJNA729271	ed_Lushi_vs_Arbor_Acr	breed_growth_background	ENSGALG00010023200	VPS26A	0.157844928	0.044569699	3.541530056	0.000397814	0.004224401	positive
PRJNA729271	ed_Lushi_vs_Arbor_Acr	breed_growth_background	ENSGALG00010023201	ZNF280D	-0.040596204	0.063337342	-0.640952118	0.521553793	0.668803505	negative
PRJNA729271	ed_Lushi_vs_Arbor_Acr	breed_growth_background	ENSGALG00010023202	PID1	0.003064022	0.084323656	0.036336443	0.971014091	0.983017828	positive
PRJNA729271	ed_Lushi_vs_Arbor_Acr	breed_growth_background	ENSGALG00010023203	COG4	0.011727378	0.034726946	0.337702549	0.735587354	0.830503295	positive
PRJNA729271	ed_Lushi_vs_Arbor_Acr	breed_growth_background	ENSGALG00010023204	SRGN	-0.168876021	0.142000268	-1.189265507	0.234335204	0.388681632	negative
PRJNA729271	ed_Lushi_vs_Arbor_Acr	breed_growth_background	ENSGALG00010023205	DNAJC17	-0.033577113	0.063264078	-0.530745318	0.595595277	0.72933628	negative
PRJNA729271	ed_Lushi_vs_Arbor_Acr	breed_growth_background	ENSGALG00010023206	HSPB9	-0.232256042	0.168278849	-1.380185583	0.167529512	0.308674243	negative
PRJNA729271	ed_Lushi_vs_Arbor_Acr	breed_growth_background	ENSGALG00010023207	MNS1	0.306272785	0.089267998	3.430935977	0.000601503	0.005631067	positive
PRJNA729271	ed_Lushi_vs_Arbor_Acr	breed_growth_background	ENSGALG00010023208	BACE1	0.020126643	0.04255638	0.472940675	0.636255505	0.760871508	positive
PRJNA729271	ed_Lushi_vs_Arbor_Acr	breed_growth_background	ENSGALG00010023209	PIK3C2A	-0.290577558	0.057668809	-5.038729974	4.68631E-07	2.6849E-05	negative
PRJNA729271	ed_Lushi_vs_Arbor_Acr	breed_growth_background	ENSGALG00010023210	ZNHIT6	0.101032868	0.077015507	1.31185099	0.189570416	0.335837593	positive
PRJNA729271	ed_Lushi_vs_Arbor_Acr	breed_growth_background	ENSGALG00010023211	TLK1	0.009378479	0.041205183	0.227604346	0.819953841	0.889413375	positive
PRJNA729271	ed_Lushi_vs_Arbor_Acr	breed_growth_background	ENSGALG00010023212	RIMBP2	0.004639747	0.114067218	0.040675549	0.967554554	0.981640881	positive
PRJNA729271	ed_Lushi_vs_Arbor_Acr	breed_growth_background	ENSGALG00010023213	FCSK	-0.000773067	0.061542885	-0.01256143	0.989977692	0.994023736	negative
PRJNA729271	ed_Lushi_vs_Arbor_Acr	breed_growth_background	ENSGALG00010023214	SUPV3L1	0.024715663	0.048692117	0.507590649	0.61174045	0.741650872	positive
PRJNA729271	ed_Lushi_vs_Arbor_Acr	breed_growth_background	ENSGALG00010023215	TRIP12	0.071870179	0.030150906	2.383682231	0.017140396	0.061246422	positive
PRJNA729271	ed_Lushi_vs_Arbor_Acr	breed_growth_background	ENSGALG00010023216	SLC35D1	0.112557403	0.067088241	1.677751603	0.093395601	0.204961277	positive
PRJNA729271	ed_Lushi_vs_Arbor_Acr	breed_growth_background	ENSGALG00010023217	CREB3L1	0.172830583	0.110740785	1.56067688	0.118600009	0.242502458	positive
PRJNA729271	ed_Lushi_vs_Arbor_Acr	breed_growth_background	ENSGALG00010023218	SSH3	-0.10141629	0.070213227	-1.444404336	0.148625272	0.283563468	negative
PRJNA729271	ed_Lushi_vs_Arbor_Acr	breed_growth_background	ENSGALG00010023219	TEX9	0.144316762	0.081179004	1.777759699	0.07544333	0.176921383	positive
PRJNA729271	ed_Lushi_vs_Arbor_Acr	breed_growth_background	ENSGALG00010023220	ST3GAL2	-0.122866179	0.047740921	-2.573603023	0.010064568	0.042017831	negative
PRJNA729271	ed_Lushi_vs_Arbor_Acr	breed_growth_background	ENSGALG00010023221	AMY1A	0.096410487	0.07221208	1.335101913	0.181842994	0.326578391	positive
PRJNA729271	ed_Lushi_vs_Arbor_Acr	breed_growth_background	ENSGALG00010023222	ENSGALG00010023222	0.636819753	0.328482193	1.938673593	0.052541096	0.137335919	positive
PRJNA729271	ed_Lushi_vs_Arbor_Acr	breed_growth_background	ENSGALG00010023223	CEP164	-0.180953121	0.055721433	-3.247459952	0.0011644	0.009128169	negative
PRJNA729271	ed_Lushi_vs_Arbor_Acr	breed_growth_background	ENSGALG00010023225	PDPR	0.125731985	0.065478441	1.920204327	0.054832095	0.141598293	positive
PRJNA729271	ed_Lushi_vs_Arbor_Acr	breed_growth_background	ENSGALG00010023226	ENSGALG00010023226	0.0592357	0.114468182	0.517486166	0.604816824	0.736831389	positive
PRJNA729271	ed_Lushi_vs_Arbor_Acr	breed_growth_background	ENSGALG00010023227	RNPC3	0.158887982	0.067123866	2.367086269	0.017928753	0.063353797	positive
PRJNA729271	ed_Lushi_vs_Arbor_Acr	breed_growth_background	ENSGALG00010023228	GORASP2	0.126167562	0.054447189	2.317246579	0.020490303	0.069344457	positive
PRJNA729271	ed_Lushi_vs_Arbor_Acr	breed_growth_background	ENSGALG00010023229	HKDC1	-0.323456985	0.244914443	-1.320693798	0.186603483	0.332164741	negative
PRJNA729271	ed_Lushi_vs_Arbor_Acr	breed_growth_background	ENSGALG00010023230	ENSGALG00010023230	0.006150837	0.044606013	0.137892562	0.890325329	0.935842324	positive
PRJNA729271	ed_Lushi_vs_Arbor_Acr	breed_growth_background	ENSGALG00010023231	ASIC1	-0.080592379	0.137968231	-0.584137221	0.559127995	0.700117509	negative
PRJNA729271	ed_Lushi_vs_Arbor_Acr	breed_growth_background	ENSGALG00010023233	SEZ6L	0.209602475	0.514455627	0.407425761	0.683695309	0.795785187	positive
PRJNA729271	ed_Lushi_vs_Arbor_Acr	breed_growth_background	ENSGALG00010023234	ENSGALG00010023234	0.130910852	0.05129878	2.551929137	0.010712831	0.043964462	positive
PRJNA729271	ed_Lushi_vs_Arbor_Acr	breed_growth_background	ENSGALG00010023235	ENSGALG00010023235	-0.120317434	0.200027805	-0.601503549	0.547504649	0.691129279	negative
PRJNA729271	ed_Lushi_vs_Arbor_Acr	breed_growth_background	ENSGALG00010023237	GAD1	-0.501063293	0.391943389	-1.278407309	0.201105848	0.34994349	negative
PRJNA729271	ed_Lushi_vs_Arbor_Acr	breed_growth_background	ENSGALG00010023238	NUCB2	0.045523497	0.041935121	1.0855697	0.277669411	0.43695778	positive
PRJNA729271	ed_Lushi_vs_Arbor_Acr	breed_growth_background	ENSGALG00010023239	RBPMS2	-0.211284981	0.108493558	-1.947442639	0.05148169	0.135333171	negative
PRJNA729271	ed_Lushi_vs_Arbor_Acr	breed_growth_background	ENSGALG00010023240	PIWIL1	0.654191772	0.255342824	2.562013536	0.010406726	0.043022855	positive
PRJNA729271	ed_Lushi_vs_Arbor_Acr	breed_growth_background	ENSGALG00010023243	HK1	-0.036612821	0.061081443	-0.599409892	0.548899582	0.692273509	negative
PRJNA729271	ed_Lushi_vs_Arbor_Acr	breed_growth_background	ENSGALG00010023244	PPIB	-0.154732889	0.063595053	-2.433096329	0.014970318	0.055525781	negative
PRJNA729271	ed_Lushi_vs_Arbor_Acr	breed_growth_background	ENSGALG00010023245	TANC2	-0.186676809	0.07941884	-2.350535581	0.018746415	0.065303843	negative
PRJNA729271	ed_Lushi_vs_Arbor_Acr	breed_growth_background	ENSGALG00010023246	SP5	0.426373293	0.435588739	0.9788437	0.327657214	0.491502775	positive
PRJNA729271	ed_Lushi_vs_Arbor_Acr	breed_growth_background	ENSGALG00010023248	PRDM10	0.069622478	0.065314801	1.06595253	0.286445101	0.447127045	positive
PRJNA729271	ed_Lushi_vs_Arbor_Acr	breed_growth_background	ENSGALG00010023249	IL12RB2	0.028358701	0.247270951	0.114686749	0.908693418	0.948136416	positive
PRJNA729271	ed_Lushi_vs_Arbor_Acr	breed_growth_background	ENSGALG00010023250	FZD10	-0.165403315	0.248292497	-0.666163165	0.505306814	0.655130385	negative
PRJNA729271	ed_Lushi_vs_Arbor_Acr	breed_growth_background	ENSGALG00010023251	ENSGALG00010023251	0.485625592	0.29585033	1.641456987	0.100702589	0.216203701	positive
PRJNA729271	ed_Lushi_vs_Arbor_Acr	breed_growth_background	ENSGALG00010023252	GLG1	-0.023090163	0.042803626	-0.53944408	0.589580473	0.725094939	negative
PRJNA729271	ed_Lushi_vs_Arbor_Acr	breed_growth_background	ENSGALG00010023254	MYO3B	-0.265656646	0.174318823	-1.523969936	0.127516221	0.254418118	negative
PRJNA729271	ed_Lushi_vs_Arbor_Acr	breed_growth_background	ENSGALG00010023255	SNX22	0.269936044	0.157693622	1.711775281	0.086938089	0.195132797	positive
PRJNA729271	ed_Lushi_vs_Arbor_Acr	breed_growth_background	ENSGALG00010023257	PAK6	-0.162687173	0.156687933	-1.038287827	0.299136074	0.461519681	negative
PRJNA729271	ed_Lushi_vs_Arbor_Acr	breed_growth_background	ENSGALG00010023258	ENSGALG00010023258	-0.181527659	0.199100777	-0.911737569	0.361906882	0.525223116	negative
PRJNA729271	ed_Lushi_vs_Arbor_Acr	breed_growth_background	ENSGALG00010023259	TSPAN15	0.501827816	0.153469739	3.269881219	0.001075927	0.008611571	positive
PRJNA729271	ed_Lushi_vs_Arbor_Acr	breed_growth_background	ENSGALG00010023260	ENSGALG00010023260	0.182008724	0.143411198	1.269138853	0.204391545	0.353792148	positive
PRJNA729271	ed_Lushi_vs_Arbor_Acr	breed_growth_background	ENSGALG00010023261	SNX1	-0.283118562	0.051582777	-5.488625783	4.05073E-08	4.25473E-06	negative
PRJNA729271	ed_Lushi_vs_Arbor_Acr	breed_growth_background	ENSGALG00010023262	POLD4	0.296693411	0.111575809	2.659119514	0.007834517	0.035093114	positive
PRJNA729271	ed_Lushi_vs_Arbor_Acr	breed_growth_background	ENSGALG00010023263	SRP14	0.044349689	0.039670866	1.117941034	0.263592178	0.421671849	positive
PRJNA729271	ed_Lushi_vs_Arbor_Acr	breed_growth_background	ENSGALG00010023264	RACGAP1	-0.308369912	0.124574478	-2.475385956	0.013309226	0.05110482	negative
PRJNA729271	ed_Lushi_vs_Arbor_Acr	breed_growth_background	ENSGALG00010023265	RAB5C	-0.076445853	0.038502288	-1.985488588	0.047090139	0.126730702	negative
PRJNA729271	ed_Lushi_vs_Arbor_Acr	breed_growth_background	ENSGALG00010023266	RFWD3	-0.027684205	0.077944639	-0.355177802	0.722456393	0.821978447	negative
PRJNA729271	ed_Lushi_vs_Arbor_Acr	breed_growth_background	ENSGALG00010023267	DSCAML1	-0.774384931	0.452632328	-1.710847599	0.08710925	0.195335486	negative
PRJNA729271	ed_Lushi_vs_Arbor_Acr	breed_growth_background	ENSGALG00010023268	KCNJ11	0.088766241	0.094507459	0.939251169	0.347601803	0.511573575	positive
PRJNA729271	ed_Lushi_vs_Arbor_Acr	breed_growth_background	ENSGALG00010023269	OPRL1	-0.048591483	0.316242361	-0.15365267	0.877883605	0.92767337	negative
PRJNA729271	ed_Lushi_vs_Arbor_Acr	breed_growth_background	ENSGALG00010023271	PATL2	-0.024318161	0.226054746	-0.107576424	0.9143317	0.951621885	negative
PRJNA729271	ed_Lushi_vs_Arbor_Acr	breed_growth_background	ENSGALG00010023273	HDAC7	-0.163592559	0.060726612	-2.693918755	0.007061738	0.032605234	negative

PRJNA729271	ed_Lushi_vs_Arbor_Acr	breed_growth_background	ENSGALG00010023274	ZBTB38	0.162089661	0.061388666	2.640384171	0.008281209	0.036451908	positive
PRJNA729271	ed_Lushi_vs_Arbor_Acr	breed_growth_background	ENSGALG00010023275	TWIST3	0.552181479	0.339028678	1.628716139	0.103373125	0.220222434	positive
PRJNA729271	ed_Lushi_vs_Arbor_Acr	breed_growth_background	ENSGALG00010023276	ABCC8	-0.465693642	0.086301794	-5.396106138	6.81027E-08	6.05612E-06	negative
PRJNA729271	ed_Lushi_vs_Arbor_Acr	breed_growth_background	ENSGALG00010023278	ENSGALG00010023278	-0.117667603	0.149011051	-0.789656889	0.429728175	0.588187903	negative
PRJNA729271	ed_Lushi_vs_Arbor_Acr	breed_growth_background	ENSGALG00010023279	FA2H	0.316206997	0.231500694	1.36590086	0.171970083	0.315053886	positive
PRJNA729271	ed_Lushi_vs_Arbor_Acr	breed_growth_background	ENSGALG00010023281	B2M	0.229355046	0.106294142	2.157739278	0.030948108	0.093134854	positive
PRJNA729271	ed_Lushi_vs_Arbor_Acr	breed_growth_background	ENSGALG00010023282	SERBP1	-0.001000459	0.046712092	-0.021417553	0.982912572	0.989816659	negative
PRJNA729271	ed_Lushi_vs_Arbor_Acr	breed_growth_background	ENSGALG00010023285	SLC48A1	0.118256092	0.058924603	2.006905193	0.044759759	0.122252252	positive
PRJNA729271	ed_Lushi_vs_Arbor_Acr	breed_growth_background	ENSGALG00010023286	HCRT	-0.10733611	0.31247768	-0.343500087	0.731222275	0.827728769	negative
PRJNA729271	ed_Lushi_vs_Arbor_Acr	breed_growth_background	ENSGALG00010023287	RGS19	-0.074621677	0.110871968	-0.673043678	0.500919504	0.652072582	negative
PRJNA729271	ed_Lushi_vs_Arbor_Acr	breed_growth_background	ENSGALG00010023289	NFRKB	0.150168047	0.047057434	3.19116522	0.001417002	0.010511488	positive
PRJNA729271	ed_Lushi_vs_Arbor_Acr	breed_growth_background	ENSGALG00010023290	SLC16A14	-0.301543431	0.144832068	-2.082021166	0.037340536	0.107157212	negative
PRJNA729271	ed_Lushi_vs_Arbor_Acr	breed_growth_background	ENSGALG00010023293	COL11A1	0.65998402	0.261985724	2.519160246	0.011763511	0.046921863	positive
PRJNA729271	ed_Lushi_vs_Arbor_Acr	breed_growth_background	ENSGALG00010023295	ENSGALG00010023295	-0.369511634	0.274305934	-1.347078529	0.177954942	0.32215023	negative
PRJNA729271	ed_Lushi_vs_Arbor_Acr	breed_growth_background	ENSGALG00010023299	PASK	-0.123642285	0.077208468	-1.60140833	0.109286509	0.229447849	negative
PRJNA729271	ed_Lushi_vs_Arbor_Acr	breed_growth_background	ENSGALG00010023304	ENSGALG00010023304	-0.007968055	0.094005351	-0.084761721	0.932450826	0.962874883	negative
PRJNA729271	ed_Lushi_vs_Arbor_Acr	breed_growth_background	ENSGALG00010023305	PLCB2	0.051082706	0.0860489	0.593647403	0.552747969	0.69519148	positive
PRJNA729271	ed_Lushi_vs_Arbor_Acr	breed_growth_background	ENSGALG00010023309	PIK3R3	-0.429921539	0.108620755	-3.958005433	7.55782E-05	0.001296457	negative
PRJNA729271	ed_Lushi_vs_Arbor_Acr	breed_growth_background	ENSGALG00010023311	ETS1	-0.209691916	0.071681281	-2.925337172	0.003440832	0.019503093	negative
PRJNA729271	ed_Lushi_vs_Arbor_Acr	breed_growth_background	ENSGALG00010023312	UBR3	0.07033455	0.061046255	1.152151748	0.249258722	0.405272594	positive
PRJNA729271	ed_Lushi_vs_Arbor_Acr	breed_growth_background	ENSGALG00010023317	RAPGEF3	-0.057124598	0.07949552	-0.718588891	0.47239426	0.626311596	negative
PRJNA729271	ed_Lushi_vs_Arbor_Acr	breed_growth_background	ENSGALG00010023319	EIF2AK4	0.017871219	0.033901252	0.527155133	0.598085863	0.731317945	positive
PRJNA729271	ed_Lushi_vs_Arbor_Acr	breed_growth_background	ENSGALG00010023320	ENSGALG00010023320	-0.077480532	0.075226074	-1.029969095	0.303024513	0.465666996	negative
PRJNA729271	ed_Lushi_vs_Arbor_Acr	breed_growth_background	ENSGALG00010023322	PRKAB1	-0.090451173	0.049673836	-1.820901709	0.068621799	0.165640796	negative
PRJNA729271	ed_Lushi_vs_Arbor_Acr	breed_growth_background	ENSGALG00010023324	LAG3	0.450733484	0.467536792	0.964059924	0.335015884	0.499254093	positive
PRJNA729271	ed_Lushi_vs_Arbor_Acr	breed_growth_background	ENSGALG00010023328	TCEA2	-0.151786109	0.060404503	-2.512827698	0.011976781	0.047493967	negative
PRJNA729271	ed_Lushi_vs_Arbor_Acr	breed_growth_background	ENSGALG00010023330	TSPAN1	-0.577626272	0.729189738	-0.792148109	0.428274321	0.587362692	negative
PRJNA729271	ed_Lushi_vs_Arbor_Acr	breed_growth_background	ENSGALG00010023331	CD4	0.176504185	0.185990034	0.948998077	0.34262159	0.506455226	positive
PRJNA729271	ed_Lushi_vs_Arbor_Acr	breed_growth_background	ENSGALG00010023332	PPP1R7	-0.026986861	0.040285027	-0.669898043	0.502922787	0.653682371	negative
PRJNA729271	ed_Lushi_vs_Arbor_Acr	breed_growth_background	ENSGALG00010023333	ENDOU	0.134947569	0.310076384	0.435207506	0.663411815	0.781546998	positive
PRJNA729271	ed_Lushi_vs_Arbor_Acr	breed_growth_background	ENSGALG00010023334	RPS17	-0.093710041	0.081058611	-1.156077564	0.247649432	0.403809431	negative
PRJNA729271	ed_Lushi_vs_Arbor_Acr	breed_growth_background	ENSGALG00010023335	WDR59	-0.042550007	0.037550336	-1.133145856	0.257153007	0.414435495	negative
PRJNA729271	ed_Lushi_vs_Arbor_Acr	breed_growth_background	ENSGALG00010023336	GPR162	-0.300886423	0.083688746	-3.5953033	0.000324014	0.003646414	negative
PRJNA729271	ed_Lushi_vs_Arbor_Acr	breed_growth_background	ENSGALG00010023337	FLI1	-0.23688253	0.062778433	-3.773310636	0.000161096	0.002258297	negative
PRJNA729271	ed_Lushi_vs_Arbor_Acr	breed_growth_background	ENSGALG00010023338	HDLBP	0.24741271	0.066327392	3.730173972	0.000191348	0.002512304	positive
PRJNA729271	ed_Lushi_vs_Arbor_Acr	breed_growth_background	ENSGALG00010023340	P3H3	-0.119227651	0.085632412	-1.392319196	0.163825761	0.30367038	negative
PRJNA729271	ed_Lushi_vs_Arbor_Acr	breed_growth_background	ENSGALG00010023342	FITM2	-0.187546197	0.072771837	-2.577181025	0.009960977	0.041705477	negative
PRJNA729271	ed_Lushi_vs_Arbor_Acr	breed_growth_background	ENSGALG00010023343	TMEM233	0.593255236	0.15479161	3.832605878	0.000126793	0.001892754	positive
PRJNA729271	ed_Lushi_vs_Arbor_Acr	breed_growth_background	ENSGALG00010023347	STAT5A	0.317306928	0.090366545	3.511331851	0.000445867	0.004564158	positive
PRJNA729271	ed_Lushi_vs_Arbor_Acr	breed_growth_background	ENSGALG00010023349	ENSGALG00010023349	-0.499041412	0.279533813	-1.785263139	0.074218645	0.175040556	negative
PRJNA729271	ed_Lushi_vs_Arbor_Acr	breed_growth_background	ENSGALG00010023350	ZNRF1	0.235659619	0.123556738	1.907298817	0.056481903	0.14464756	positive
PRJNA729271	ed_Lushi_vs_Arbor_Acr	breed_growth_background	ENSGALG00010023351	GNB3	-0.084500109	0.206076365	-0.410042701	0.681774624	0.794479296	negative
PRJNA729271	ed_Lushi_vs_Arbor_Acr	breed_growth_background	ENSGALG00010023354	SEPTIN2	-0.233568658	0.09255781	-2.523489462	0.011619654	0.046526765	negative
PRJNA729271	ed_Lushi_vs_Arbor_Acr	breed_growth_background	ENSGALG00010023356	ENSGALG00010023356	0.272889379	0.104327294	2.615704559	0.008904357	0.038413289	positive
PRJNA729271	ed_Lushi_vs_Arbor_Acr	breed_growth_background	ENSGALG00010023359	CDCA3	-0.365811194	0.148168559	-2.468885412	0.013553461	0.051843611	negative
PRJNA729271	ed_Lushi_vs_Arbor_Acr	breed_growth_background	ENSGALG00010023362	GADD45A	0.086932919	0.079500747	1.093485563	0.274180664	0.433066223	positive
PRJNA729271	ed_Lushi_vs_Arbor_Acr	breed_growth_background	ENSGALG00010023363	CPEB1	-0.059701646	0.130962679	-0.455867627	0.64848517	0.770601127	negative
PRJNA729271	ed_Lushi_vs_Arbor_Acr	breed_growth_background	ENSGALG00010023364	CTRB1	-1.733961903	1.146495972	-1.512401216	0.130431826	0.258421175	negative
PRJNA729271	ed_Lushi_vs_Arbor_Acr	breed_growth_background	ENSGALG00010023366	GDAP1L1	-0.282699112	0.263622203	-1.072364578	0.283556312	0.443764709	negative
PRJNA729271	ed_Lushi_vs_Arbor_Acr	breed_growth_background	ENSGALG00010023368	ENSGALG00010023368	0.041896438	0.125500734	0.333834208	0.738504668	0.832461713	positive
PRJNA729271	ed_Lushi_vs_Arbor_Acr	breed_growth_background	ENSGALG00010023369	ACE	0.981170199	0.254467882	3.855772247	0.000115365	0.001778951	positive
PRJNA729271	ed_Lushi_vs_Arbor_Acr	breed_growth_background	ENSGALG00010023374	ENSGALG00010023374	-0.234201463	0.116396155	-2.012106525	0.044208709	0.121112311	negative
PRJNA729271	ed_Lushi_vs_Arbor_Acr	breed_growth_background	ENSGALG00010023375	ST14	-0.18643747	0.08073865	-2.309147717	0.020935386	0.070382368	negative
PRJNA729271	ed_Lushi_vs_Arbor_Acr	breed_growth_background	ENSGALG00010023377	OSER1	0.091851196	0.080404793	1.142359713	0.25330453	0.409919522	positive
PRJNA729271	ed_Lushi_vs_Arbor_Acr	breed_growth_background	ENSGALG00010023379	CCDC60	-0.020735905	0.281052519	-0.073779468	0.941185864	0.968033003	negative
PRJNA729271	ed_Lushi_vs_Arbor_Acr	breed_growth_background	ENSGALG00010023380	USP5	0.075308733	0.052184781	1.443116772	0.14898758	0.283900549	positive
PRJNA729271	ed_Lushi_vs_Arbor_Acr	breed_growth_background	ENSGALG00010023381	HOXB7	-0.212595086	0.09061067	-2.34624781	0.018963495	0.065917471	negative
PRJNA729271	ed_Lushi_vs_Arbor_Acr	breed_growth_background	ENSGALG00010023383	HOXB6	-0.084979047	0.093566094	-0.908224799	0.363759465	0.527163911	negative
PRJNA729271	ed_Lushi_vs_Arbor_Acr	breed_growth_background	ENSGALG00010023384	AP3B2	-0.376642942	0.130391936	-2.888544743	0.00387029	0.021161768	negative
PRJNA729271	ed_Lushi_vs_Arbor_Acr	breed_growth_background	ENSGALG00010023387	ENSGALG00010023387	-0.131965083	0.097536815	-1.352977149	0.176062931	0.320026796	negative
PRJNA729271	ed_Lushi_vs_Arbor_Acr	breed_growth_background	ENSGALG00010023388	FARP2	0.043437524	0.046050925	0.943249753	0.34555317	0.509489696	positive
PRJNA729271	ed_Lushi_vs_Arbor_Acr	breed_growth_background	ENSGALG00010023389	GNG12	-0.058096887	0.059187319	-0.981576586	0.326308487	0.489910654	negative
PRJNA729271	ed_Lushi_vs_Arbor_Acr	breed_growth_background	ENSGALG00010023390	HSPB8	-0.115824281	0.133891422	-0.865061257	0.387005168	0.549035521	negative
PRJNA729271	ed_Lushi_vs_Arbor_Acr	breed_growth_background	ENSGALG00010023392	TPI1	0.11249846	0.079912352	1.407773106	0.159198266	0.297731293	positive
PRJNA729271	ed_Lushi_vs_Arbor_Acr	breed_growth_background	ENSGALG00010023394	HOXB5	-0.014531927	0.099147745	-0.146568405	0.883472693	0.93134085	negative
PRJNA729271	ed_Lushi_vs_Arbor_Acr	breed_growth_background	ENSGALG00010023396	ENSGALG00010023396	0.135890221	0.079686939	1.705301046	0.088138293	0.197094192	positive
PRJNA729271	ed_Lushi_vs_Arbor_Acr	breed_growth_background	ENSGALG00010023397	ENSGALG00010023397	0.439396579	0.13650134	3.218990966	0.001286425	0.009788093	positive
PRJNA729271	ed_Lushi_vs_Arbor_Acr	breed_growth_background	ENSGALG00010023401	LRRC23	0.070132297	0.191977538	0.365315118	0.714876195	0.817009417	positive
PRJNA729271	ed_Lushi_vs_Arbor_Acr	breed_growth_background	ENSGALG00010023405	DNAJA2	0.022316589	0.041362253	0.539539967	0.589514327	0.725094939	positive
PRJNA729271	ed_Lushi_vs_Arbor_Acr	breed_growth_background	ENSGALG00010023408	ZBTB44	0.219887745	0.068204008	3.223970998	0.001264262	0.009680654	positive
PRJNA729271	ed_Lushi_vs_Arbor_Acr	breed_growth_background	ENSGALG00010023411	ENSGALG00010023411	-0.0564511	0.09480143	-0.595466755	0.551531515	0.694391707	negative
PRJNA729271	ed_Lushi_vs_Arbor_Acr	breed_growth_background	ENSGALG00010023412	GSS	-0.048383352	0.058621349	-0.825353778	0.409170769	0.569569796	negative
PRJNA729271	ed_Lushi_vs_Arbor_Acr	breed_growth_background	ENSGALG00010023414	SUDS3	-0.067400067	0.041420258	-1.627224691	0.103689385	0.220733976	negative
PRJNA729271	ed_Lushi_vs_Arbor_Acr	breed_growth_background	ENSGALG00010023415	HOXB4	-0.063904003	0.089637865	-0.712913047	0.475899565	0.628960067	negative
PRJNA729271	ed_Lushi_vs_Arbor_Acr	breed_growth_background	ENSGALG00010023421	OLFM3	-0.415275736	0.135979317	-3.053962511	0.002258403	0.014516874	negative
PRJNA729271	ed_Lushi_vs_Arbor_Acr	breed_growth_background	ENSGALG00010023422	WLS	-0.061933511	0.083816884	-0.738914501	0.459958916	0.615216803	negative
PRJNA729271	ed_Lushi_vs_Arbor_Acr	breed_growth_background	ENSGALG00010023426	KCNJ1	0.260013489	0.262339601	0.991133205	0.321620541	0.485310196	positive
PRJNA729271	ed_Lushi_vs_Arbor_Acr	breed_growth_background	ENSGALG00010023428	HOXB3	-0.114591407	0.105463717	-1.08654815	0.27723655	0.436418744	negative
PRJNA729271	ed_Lushi_vs_Arbor_Acr	breed_growth_background	ENSGALG00010023429	BMF	0.174435612	0.078357483	2.2261513	0.026004049	0.082178344	positive
PRJNA729271	ed_Lushi_vs_Arbor_Acr	breed_growth_background	ENSGALG00010023431	BCAR1	0.247118387	0.093307813	2.648421177	0.008086871	0.035850128	positive
PRJNA729271	ed_Lushi_vs_Arbor_Acr	breed_growth_background	ENSGALG00010023432	TMOD3	-0.292079645	0.093668918	-3.118213083	0.001819512	0.012446355	negative
PRJNA729271	ed_Lushi_vs_Arbor_Acr	breed_growth_background	ENSGALG00010023434	ATN1	-0.236639105	0.102094364	-2.31784691	0.020457643	0.069344457	negative
PRJNA729271	ed_Lushi_vs_Arbor_Acr	breed_growth_background	ENSGALG00010023437	KCNJ5	0.04416908	0.136199729	0.32429639	0.745713652	0.837908581	positive
PRJNA729271	ed_Lushi_vs_Arbor_Acr	breed_growth_background	ENSGALG00010023438	HOXB2	0.344962445	0.134563288	2.563570269	0.010360173	0.04290769	positive
PRJNA729271	ed_Lushi_vs_Arbor_Acr	breed_growth_background	ENSGALG00010023439	ACSS2	0.150310754	0.070714041	2.12561397	0.033535415	0.098719709	positive
PRJNA729271	ed_Lushi_vs_Arbor_Acr	breed_growth_background	ENSGALG00010023441	ENSGALG00010023441	-0.005641903	0.079582075	-0.070894139	0.943482008	0.969225493	negative
PRJNA729271	ed_Lushi_vs_Arbor_Acr	breed_growth_background	ENSGALG00010023442	STK25	0.168420684	0.060362133	2.790171165	0.005268018	0.026477089	positive
PRJNA729271	ed_Lushi_vs_Arbor_Acr	breed_growth_background	ENSGALG00010023443	ENSGALG00010023443	-0.085409611	0.096887151	-0.881537025	0.378027224	0.540321131	negative
PRJNA729271	ed_Lushi_vs_Arbor_Acr	breed_growth_background	ENSGALG00010023447	C12orf57	0.17067231	0.061053056	2.795475312	0.005182346	0.026192366	positive
PRJNA729271	ed_Lushi_vs_Arbor_Acr	breed_growth_background	ENSGALG00010023448	FAM219B	0.043700006	0.065189882	0.670349521	0.502635006	0.653484116	positive
PRJNA729271	ed_Lushi_vs_Arbor_Acr	breed_growth_background	ENSGALG00010023453	ARHGAP32	-0.26862593	0.097308377	-2.760563272	0.005770178	0.028161189	negative
PRJNA729271	ed_Lushi_vs_Arbor_Acr	breed_growth_background	ENSGALG00010023454	HOXB1	0.058217385	0.212368861	0.274133338	0.783982155	0.866210941	positive
PRJNA729271	ed_Lushi_vs_Arbor_Acr	breed_growth_background	ENSGALG00010023455	CFDP1	0.102100828	0.052923943	1.929199168	0.053706141	0.139354145	positive
PRJNA729271	ed_Lushi_vs_Arbor_Acr	breed_growth_background	ENSGALG00010023456	MPI	0.060952697	0.077731668	0.784142403	0.432956557	0.591155359	positive
PRJNA729271	ed_Lushi_vs_Arbor_Acr	breed_growth_background	ENSGALG00010023469	ENSGALG00010023469	0.012809347	0.114049809	0.11231362	0.910574743	0.949311015	positive
PRJNA729271	ed_Lushi_vs_Arbor_Acr	breed_growth_background	ENSGALG00010023472	PTPN6	-0.079897053	0.105045761	-0.760592836	0.4469003	0.603654817	negative
PRJNA729271	ed_Lushi_vs_Arbor_Acr	breed_growth_background	ENSGALG00010023473	BOK	0.095725315	0.108609137	0.881374414	0.378115201	0.540350965	positive
PRJNA729271	ed_Lushi_vs_Arbor_Acr	breed_growth_background	ENSGALG00010023475	ENSGALG00010023475	-0.73419696	0.328033688	-2.238175493	0.025209613	0.080469796	negative
PRJNA729271	ed_Lushi_vs_Arbor_Acr	breed_growth_background	ENSGALG00010023476	SCAMP2	0.060428677	0.08105887	0.74549124	0.455974805	0.611920636	positive
PRJNA729271	ed_Lushi_vs_Arbor_Acr	breed_growth_background	ENSGALG00010023478	TAOK3	0.112504541	0.065899944	1.707202391	0.087784441	0.19651513	positive
PRJNA729271	ed_Lushi_vs_Arbor_Acr	breed_growth_background	ENSGALG00010023479	MCCC2L	-0.178970421	0.083677573	-2.138809903	0.032451067	0.096624277	negative
PRJNA729271	ed_Lushi_vs_Arbor_Acr	breed_growth_background	ENSGALG00010023482	MTCL2	-0.139685739	0.052984614	-2.636345321	0.00838044	0.036751005	negative
PRJNA729271	ed_Lushi_vs_Arbor_Acr	breed_growth_background	ENSGALG00010023485	BARX2	-0.725605617	0.170685099	-4.25113628	2.12689E-05	0.000501034	negative
PRJNA729271	ed_Lushi_vs_Arbor_Acr	breed_growth_background	ENSGALG00010023487	GRN	-0.186122375	0.078658714	-2.366201582	0.017971656	0.063474453	negative
PRJNA729271	ed_Lushi_vs_Arbor_Acr	breed_growth_background	ENSGALG00010023489	ETV5	0.225797775	0.116922055	1.931182052	0.053460543	0.138959114	positive
PRJNA729271	ed_Lushi_vs_Arbor_Acr	breed_growth_background	ENSGALG00010023490	PHB2	0.011496905	0.055049261	0.208847577	0.834567229	0.899409069	positive
PRJNA729271	ed_Lushi_vs_Arbor_Acr	breed_growth_background	ENSGALG00010023496	PLEKHA3	-0.142196647	0.057101134	-2.490259572	0.012764983	0.049632089	negative
PRJNA729271	ed_Lushi_vs_Arbor_Acr	breed_growth_background	ENSGALG00010023499	PEBP1	-0.188422929	0.074796049	-2.519156191	0.011763646	0.046921863	negative
PRJNA729271	ed_Lushi_vs_Arbor_Acr	breed_growth_background	ENSGALG00010023501	ENSGALG00010023501	-1.729492405	1.576865295	-1.09679147	0.27273256	0.431625555	negative
PRJNA729271	ed_Lushi_vs_Arbor_Acr	breed_growth_background	ENSGALG00010023506	RPE65	-0.111965647	0.330782803	-0.338486903	0.734996295	0.830314964	negative
PRJNA729271	ed_Lushi_vs_Arbor_Acr	breed_growth_background	ENSGALG00010023510	ASPHD2	-0.087190478	0.137189517	-0.635547671	0.525071286	0.671709512	negative
PRJNA729271	ed_Lushi_vs_Arbor_Acr	breed_growth_background	ENSGALG00010023513	CDCA9	-0.185479819	0.117904665	-1.573133845	0.11568783	0.238349374	negative
PRJNA729271	ed_Lushi_vs_Arbor_Acr	breed_growth_background	ENSGALG00010023514	ULK3	0.20715694	0.057858151	3.580427929	0.000343032	0.003799127	positive
PRJNA729271	ed_Lushi_vs_Arbor_Acr	breed_growth_background	ENSGALG00010023518	METTL5	0.092508987	0.064476069	1.434780212	0.151349738	0.287261288	positive
PRJNA729271	ed_Lushi_vs_Arbor_Acr	breed_growth_background	ENSGALG00010023519	DGKG	-0.057995234	0.133828554	-0.433354709	0.664757098	0.78255942	negative
PRJNA729271	ed_Lushi_vs_Arbor_Acr	breed_growth_background	ENSGALG00010023522	EMG1	-0.155201761	0.088371049	-1.756251207	0.079045525	0.182518936	negative
PRJNA729271	ed_Lushi_vs_Arbor_Acr	breed_growth_background	ENSGALG00010023526	KNSTRN	-0.350926593	0.104061981	-3.372284372	0.000745474	0.006617055	negative
PRJNA729271	ed_Lushi_vs_Arbor_Acr	breed_growth_background	ENSGALG00010023527	TMEM98	0.406064624	0.123293402	3.293482191	0.000989546	0.008085384	positive
PRJNA729271	ed_Lushi_vs_Arbor_Acr	breed_growth_background	ENSGALG00010023533	FKBP7	-0.018562233	0.08742692	-0.212317133	0.83185963	0.89782616	negative
PRJNA729271	ed_Lushi_vs_Arbor_Acr	breed_growth_background	ENSGALG00010023536	ENSGALG00010023536	-0.074203698	0.114091982	-0.650384858	0.515443656	0.66382447	negative
PRJNA729271	ed_Lushi_vs_Arbor_Acr	breed_growth_background	ENSGALG00010023541	PRSS2	-1.963157091	1.311249547	-1.497165124	0.134350298	0.264126891	negative
PRJNA729271	ed_Lushi_vs_Arbor_Acr	breed_growth_background	ENSGALG00010023543	ENSGALG00010023543	-0.4467018	0.409745332	-1.090193751	0.275627806	0.434499733	negative
PRJNA729271	ed_Lushi_vs_Arbor_Acr	breed_growth_background	ENSGALG00010023544	SSB	0.181505937	0.05545287	3.27315676	0.001063535	0.008545419	positive
PRJNA729271	ed_Lushi_vs_Arbor_Acr	breed_growth_background	ENSGALG00010023545	PTPRT	0.037378605	0.073715886	0.507063091	0.612110551	0.741913407	positive
PRJNA729271	ed_Lushi_vs_Arbor_Acr	breed_growth_background	ENSGALG00010023546	SLC11A2	-0.155187001	0.083216802	-1.864851768	0.062202185	0.154706897	negative
PRJNA729271	ed_Lushi_vs_Arbor_Acr	breed_growth_background	ENSGALG00010023551	ENSGALG00010023551	-0.169688177	0.095944352	-1.768610376	0.076958918	0.17917114	negative
PRJNA729271	ed_Lushi_vs_Arbor_Acr	breed_growth_background	ENSGALG00010023552	SGPP2	-0.249158195	0.192311994	-1.295593633	0.195115524	0.342978355	negative
PRJNA729271	ed_Lushi_vs_Arbor_Acr	breed_growth_background	ENSGALG00010023555	HPS4	-0.206267353	0.092417959	-2.231896874	0.02562178	0.081408964	negative
PRJNA729271	ed_Lushi_vs_Arbor_Acr	breed_growth_background	ENSGALG00010023556	IVD	0.373008056	0.065146519	5.725679043	1.03021E-08	1.41199E-06	positive
PRJNA729271	ed_Lushi_vs_Arbor_Acr	breed_growth_background	ENSGALG00010023559	PJVK	0.972466181	0.389635223	2.495837451	0.012566017	0.049042654	positive
PRJNA729271	ed_Lushi_vs_Arbor_Acr	breed_growth_background	ENSGALG00010023569	LPCAT3	-0.087788445	0.055964564	-1.568643436	0.116731045	0.239900255	negative

PRJNA729271	ed_Lushi_vs_Arbor_Acr	breed_growth_background	ENSGALG00010023571	APLP2	-0.147227093	0.052702021	-2.793575872	0.00521288	0.0262847	negative
PRJNA729271	ed_Lushi_vs_Arbor_Acr	breed_growth_background	ENSGALG00010023572	MYBL2	-0.112205111	0.069612015	-1.611864153	0.106991504	0.225774036	negative
PRJNA729271	ed_Lushi_vs_Arbor_Acr	breed_growth_background	ENSGALG00010023574	DEPDC1	-0.28249003	0.114701864	-2.462819874	0.013784915	0.052457953	negative
PRJNA729271	ed_Lushi_vs_Arbor_Acr	breed_growth_background	ENSGALG00010023578	SRRD	0.207131919	0.055963793	3.701177307	0.000214601	0.002743076	positive
PRJNA729271	ed_Lushi_vs_Arbor_Acr	breed_growth_background	ENSGALG00010023580	RDM1	-0.031324857	0.091293702	-0.343121781	0.731506846	0.827856943	negative
PRJNA729271	ed_Lushi_vs_Arbor_Acr	breed_growth_background	ENSGALG00010023585	TFIP11	0.175954388	0.05207624	3.378784401	0.000728071	0.006485902	positive
PRJNA729271	ed_Lushi_vs_Arbor_Acr	breed_growth_background	ENSGALG00010023588	FARSB	-0.249195386	0.072286627	-3.447323468	0.00056617	0.005402658	negative
PRJNA729271	ed_Lushi_vs_Arbor_Acr	breed_growth_background	ENSGALG00010023590	S1PR1	-0.023891531	0.061620038	-0.38772341	0.698220729	0.805308638	negative
PRJNA729271	ed_Lushi_vs_Arbor_Acr	breed_growth_background	ENSGALG00010023592	BAHD1	0.039971252	0.056844252	0.703171398	0.481948942	0.63467461	positive
PRJNA729271	ed_Lushi_vs_Arbor_Acr	breed_growth_background	ENSGALG00010023593	CSK	0.095065782	0.044745388	2.124593963	0.033620508	0.098909938	positive
PRJNA729271	ed_Lushi_vs_Arbor_Acr	breed_growth_background	ENSGALG00010023597	MOGAT1	-1.016358013	0.183494356	-5.538906117	3.04367E-08	3.42E-06	negative
PRJNA729271	ed_Lushi_vs_Arbor_Acr	breed_growth_background	ENSGALG00010023598	IL10RA	0.087356452	0.187297502	0.466404789	0.640925784	0.764564171	positive
PRJNA729271	ed_Lushi_vs_Arbor_Acr	breed_growth_background	ENSGALG00010023600	CHST14	-0.253100693	0.046606189	-5.430624042	5.61573E-08	5.35527E-06	negative
PRJNA729271	ed_Lushi_vs_Arbor_Acr	breed_growth_background	ENSGALG00010023601	TPST2	-0.06489926	0.056114054	-1.156559823	0.247452245	0.403642907	negative
PRJNA729271	ed_Lushi_vs_Arbor_Acr	breed_growth_background	ENSGALG00010023603	C1S	0.201538617	0.210956886	0.955354533	0.339398439	0.503613951	positive
PRJNA729271	ed_Lushi_vs_Arbor_Acr	breed_growth_background	ENSGALG00010023604	ENSGALG00010023604	-1.221605615	0.223541478	-5.464782769	4.63474E-08	4.66532E-06	negative
PRJNA729271	ed_Lushi_vs_Arbor_Acr	breed_growth_background	ENSGALG00010023605	INSYN1	0.162370912	0.163063076	0.995755238	0.319369084	0.482681674	positive
PRJNA729271	ed_Lushi_vs_Arbor_Acr	breed_growth_background	ENSGALG00010023606	LRRC7	0.234314603	0.176342526	1.328747003	0.183931452	0.32926842	positive
PRJNA729271	ed_Lushi_vs_Arbor_Acr	breed_growth_background	ENSGALG00010023612	GPT2	0.177104335	0.100156913	1.768268713	0.077015992	0.179220526	positive
PRJNA729271	ed_Lushi_vs_Arbor_Acr	breed_growth_background	ENSGALG00010023613	COPB1	0.097814675	0.027995285	3.493969594	0.000475895	0.004800349	positive
PRJNA729271	ed_Lushi_vs_Arbor_Acr	breed_growth_background	ENSGALG00010023615	PRSS3	-1.76191171	2.731836177	-0.644955113	0.518956275	0.666965268	negative
PRJNA729271	ed_Lushi_vs_Arbor_Acr	breed_growth_background	ENSGALG00010023617	ENSGALG00010023617	-0.275131076	0.161988187	-1.698463823	0.089420255	0.199315177	negative
PRJNA729271	ed_Lushi_vs_Arbor_Acr	breed_growth_background	ENSGALG00010023620	IRS1	0.309991118	0.075470775	4.107432545	4.00082E-05	0.000823747	positive
PRJNA729271	ed_Lushi_vs_Arbor_Acr	breed_growth_background	ENSGALG00010023621	CD79B	1.034961554	0.390522243	2.650198737	0.008044444	0.035759095	positive
PRJNA729271	ed_Lushi_vs_Arbor_Acr	breed_growth_background	ENSGALG00010023623	OSBPL6	0.632306231	0.118437425	5.338736704	9.35965E-08	7.89535E-06	positive
PRJNA729271	ed_Lushi_vs_Arbor_Acr	breed_growth_background	ENSGALG00010023624	PSMA1	0.001682012	0.038055893	0.044198457	0.964746212	0.979820371	positive
PRJNA729271	ed_Lushi_vs_Arbor_Acr	breed_growth_background	ENSGALG00010023626	LOXL1	-0.305878386	0.104345834	-2.931390484	0.003374483	0.019287888	negative
PRJNA729271	ed_Lushi_vs_Arbor_Acr	breed_growth_background	ENSGALG00010023627	IFT52	0.177914863	0.054877971	3.24200876	0.001186904	0.009254529	positive
PRJNA729271	ed_Lushi_vs_Arbor_Acr	breed_growth_background	ENSGALG00010023631	ENSGALG00010023631	0.175572994	0.197566006	0.888680183	0.374174984	0.536518587	positive
PRJNA729271	ed_Lushi_vs_Arbor_Acr	breed_growth_background	ENSGALG00010023635	SCN4A	0.276572842	0.133767442	2.067564706	0.038680974	0.109801897	positive
PRJNA729271	ed_Lushi_vs_Arbor_Acr	breed_growth_background	ENSGALG00010023636	PDE3B	-0.178957012	0.043831556	-4.082835048	4.44896E-05	0.000889485	negative
PRJNA729271	ed_Lushi_vs_Arbor_Acr	breed_growth_background	ENSGALG00010023637	RHBDD1	0.040560397	0.056925985	0.712511113	0.476148332	0.629092159	positive
PRJNA729271	ed_Lushi_vs_Arbor_Acr	breed_growth_background	ENSGALG00010023638	DCTN2	0.194275862	0.070284439	2.764137635	0.005707346	0.027948642	positive
PRJNA729271	ed_Lushi_vs_Arbor_Acr	breed_growth_background	ENSGALG00010023639	KLHL23	-0.334276217	0.115690214	-2.889407877	0.003859681	0.021119695	negative
PRJNA729271	ed_Lushi_vs_Arbor_Acr	breed_growth_background	ENSGALG00010023641	LRRC40	-0.139240621	0.061712877	-2.256265258	0.024054024	0.077843955	negative
PRJNA729271	ed_Lushi_vs_Arbor_Acr	breed_growth_background	ENSGALG00010023642	PAX6	0.405955777	0.274265574	1.480155788	0.138831676	0.270479186	positive
PRJNA729271	ed_Lushi_vs_Arbor_Acr	breed_growth_background	ENSGALG00010023643	SCN4B	0.187679234	0.070902589	2.647001123	0.008120908	0.035964732	positive
PRJNA729271	ed_Lushi_vs_Arbor_Acr	breed_growth_background	ENSGALG00010023646	DPH5	-0.059192001	0.077004464	-0.768682722	0.442081684	0.599156055	negative
PRJNA729271	ed_Lushi_vs_Arbor_Acr	breed_growth_background	ENSGALG00010023648	SCN2B	-0.062230985	0.189760842	-0.327944292	0.742953784	0.83598161	negative
PRJNA729271	ed_Lushi_vs_Arbor_Acr	breed_growth_background	ENSGALG00010023652	SLC30A7	-0.15032451	0.052289627	-2.874843832	0.004042274	0.021772691	negative
PRJNA729271	ed_Lushi_vs_Arbor_Acr	breed_growth_background	ENSGALG00010023655	C1R	-0.143766732	0.099272563	-1.448202082	0.147560542	0.282399005	negative
PRJNA729271	ed_Lushi_vs_Arbor_Acr	breed_growth_background	ENSGALG00010023658	ENSGALG00010023658	-0.250155406	0.169671079	-1.474355015	0.14038603	0.272737636	negative
PRJNA729271	ed_Lushi_vs_Arbor_Acr	breed_growth_background	ENSGALG00010023660	CRYBB1	-0.385729598	0.283833468	-1.358999701	0.174146684	0.317982747	negative
PRJNA729271	ed_Lushi_vs_Arbor_Acr	breed_growth_background	ENSGALG00010023663	PHOSPHO2	-0.039920027	0.049473895	-0.806890718	0.419729447	0.579205858	negative
PRJNA729271	ed_Lushi_vs_Arbor_Acr	breed_growth_background	ENSGALG00010023664	SLC6A9	-0.370803413	0.175028326	-2.118533734	0.034129891	0.100052512	negative
PRJNA729271	ed_Lushi_vs_Arbor_Acr	breed_growth_background	ENSGALG00010023665	C16orf87	-0.019088711	0.074805592	-0.25517759	0.798585939	0.875141996	negative
PRJNA729271	ed_Lushi_vs_Arbor_Acr	breed_growth_background	ENSGALG00010023666	ENSGALG00010023666	-0.283652703	0.133894339	-2.118481676	0.034134295	0.100052512	negative
PRJNA729271	ed_Lushi_vs_Arbor_Acr	breed_growth_background	ENSGALG00010023668	RCN1	-0.136630395	0.0862943	-1.5833073	0.11335144	0.234953399	negative
PRJNA729271	ed_Lushi_vs_Arbor_Acr	breed_growth_background	ENSGALG00010023670	EXTL2	-0.001442478	0.067921525	-0.021237414	0.983056269	0.989816659	negative
PRJNA729271	ed_Lushi_vs_Arbor_Acr	breed_growth_background	ENSGALG00010023671	ENSGALG00010023671	0.644823235	0.300127642	2.148496655	0.031674322	0.094925253	positive
PRJNA729271	ed_Lushi_vs_Arbor_Acr	breed_growth_background	ENSGALG00010023676	ENSGALG00010023676	0.140493677	0.147790967	0.950624251	0.341795153	0.505702313	positive
PRJNA729271	ed_Lushi_vs_Arbor_Acr	breed_growth_background	ENSGALG00010023677	MN1	0.299531212	0.086079297	3.479712562	0.000501952	0.004986836	positive
PRJNA729271	ed_Lushi_vs_Arbor_Acr	breed_growth_background	ENSGALG00010023680	MPZL3	-0.228824681	0.254500069	-0.899114416	0.36859172	0.531774359	negative
PRJNA729271	ed_Lushi_vs_Arbor_Acr	breed_growth_background	ENSGALG00010023686	MYLK3	0.349872113	0.119981701	2.916045611	0.003544987	0.019901078	positive
PRJNA729271	ed_Lushi_vs_Arbor_Acr	breed_growth_background	ENSGALG00010023689	WT1	-0.229522482	0.138409752	-1.658282594	0.097260441	0.210667975	negative
PRJNA729271	ed_Lushi_vs_Arbor_Acr	breed_growth_background	ENSGALG00010023693	SRSF11	0.053825854	0.037194147	1.44715924	0.14785233	0.28269615	positive
PRJNA729271	ed_Lushi_vs_Arbor_Acr	breed_growth_background	ENSGALG00010023694	PPCDC	-0.047089141	0.080432514	-0.585449073	0.558245801	0.699556746	negative
PRJNA729271	ed_Lushi_vs_Arbor_Acr	breed_growth_background	ENSGALG00010023695	CLSTN3	-0.193774369	0.07113402	-2.724074493	0.006448196	0.030587703	negative
PRJNA729271	ed_Lushi_vs_Arbor_Acr	breed_growth_background	ENSGALG00010023696	LRRC4C	-1.073924656	0.284280583	-3.777692603	0.000158288	0.002224478	negative
PRJNA729271	ed_Lushi_vs_Arbor_Acr	breed_growth_background	ENSGALG00010023698	ENSGALG00010023698	0.002901379	0.081670846	0.035525277	0.971660891	0.983260585	positive
PRJNA729271	ed_Lushi_vs_Arbor_Acr	breed_growth_background	ENSGALG00010023699	PPIG	0.364560118	0.109016689	3.344076221	0.000825571	0.007097654	positive
PRJNA729271	ed_Lushi_vs_Arbor_Acr	breed_growth_background	ENSGALG00010023702	COL4A4	0.380626998	0.139646851	2.725639684	0.006417701	0.030479876	positive
PRJNA729271	ed_Lushi_vs_Arbor_Acr	breed_growth_background	ENSGALG00010023703	PITPNB	0.063274171	0.043971342	1.438986578	0.150154326	0.285665698	positive
PRJNA729271	ed_Lushi_vs_Arbor_Acr	breed_growth_background	ENSGALG00010023704	ANKRD13C	0.102828829	0.037098769	2.77175854	0.005575438	0.027497778	positive
PRJNA729271	ed_Lushi_vs_Arbor_Acr	breed_growth_background	ENSGALG00010023706	CDK5RAP3	-0.059719852	0.069991933	-0.853239073	0.39352674	0.554988392	negative
PRJNA729271	ed_Lushi_vs_Arbor_Acr	breed_growth_background	ENSGALG00010023707	MPZL2	0.178890685	0.165837883	1.078708204	0.280717827	0.440654636	positive
PRJNA729271	ed_Lushi_vs_Arbor_Acr	breed_growth_background	ENSGALG00010023710	CDC14A	-0.058741958	0.064745737	-0.90727143	0.364263278	0.527630281	negative
PRJNA729271	ed_Lushi_vs_Arbor_Acr	breed_growth_background	ENSGALG00010023711	ENSGALG00010023711	0.083940875	0.193129844	0.434634408	0.663827816	0.781846444	positive
PRJNA729271	ed_Lushi_vs_Arbor_Acr	breed_growth_background	ENSGALG00010023712	ARHGAP40	-0.27548831	0.088755367	-3.103905943	0.00190984	0.012846003	negative
PRJNA729271	ed_Lushi_vs_Arbor_Acr	breed_growth_background	ENSGALG00010023714	EIF3M	0.090381509	0.059098232	1.5293437	0.126179262	0.252829155	positive
PRJNA729271	ed_Lushi_vs_Arbor_Acr	breed_growth_background	ENSGALG00010023715	CD3E	0.228010269	0.197527643	1.154320812	0.248368667	0.404460603	positive
PRJNA729271	ed_Lushi_vs_Arbor_Acr	breed_growth_background	ENSGALG00010023716	FASTKD1	-0.010344177	0.058825321	-0.17584566	0.860415197	0.916229671	negative
PRJNA729271	ed_Lushi_vs_Arbor_Acr	breed_growth_background	ENSGALG00010023720	SCAMP5	0.054081145	0.053811665	1.005007832	0.314893079	0.47813414	positive
PRJNA729271	ed_Lushi_vs_Arbor_Acr	breed_growth_background	ENSGALG00010023724	CYP2R1	0.275718818	0.069129689	3.988428435	6.65125E-05	0.001181493	positive
PRJNA729271	ed_Lushi_vs_Arbor_Acr	breed_growth_background	ENSGALG00010023726	PNPO	-0.163680925	0.102026778	-1.604293777	0.108649312	0.228440931	negative
PRJNA729271	ed_Lushi_vs_Arbor_Acr	breed_growth_background	ENSGALG00010023730	SP2	0.110925665	0.098161512	1.13003216	0.258462673	0.41580611	positive
PRJNA729271	ed_Lushi_vs_Arbor_Acr	breed_growth_background	ENSGALG00010023731	ACTR5	0.119762265	0.053119543	2.254580117	0.024159697	0.078060676	positive
PRJNA729271	ed_Lushi_vs_Arbor_Acr	breed_growth_background	ENSGALG00010023732	TM2D1	-0.053296232	0.042055338	-1.267288159	0.205052273	0.354344734	negative
PRJNA729271	ed_Lushi_vs_Arbor_Acr	breed_growth_background	ENSGALG00010023733	ORC6	0.071666736	0.104528804	0.685617106	0.492954592	0.644773218	positive
PRJNA729271	ed_Lushi_vs_Arbor_Acr	breed_growth_background	ENSGALG00010023736	KIF5A	0.241395955	0.342881156	0.704022227	0.481418932	0.634164332	positive
PRJNA729271	ed_Lushi_vs_Arbor_Acr	breed_growth_background	ENSGALG00010023738	ENSGALG00010023738	0.225493113	0.213650888	1.055427921	0.291229659	0.452548661	positive
PRJNA729271	ed_Lushi_vs_Arbor_Acr	breed_growth_background	ENSGALG00010023740	PPP1R16B	-0.035160556	0.055537455	-0.633096275	0.526670775	0.672885086	negative
PRJNA729271	ed_Lushi_vs_Arbor_Acr	breed_growth_background	ENSGALG00010023743	CD3D	0.013866261	0.121000052	0.114597147	0.908764441	0.948142271	positive
PRJNA729271	ed_Lushi_vs_Arbor_Acr	breed_growth_background	ENSGALG00010023744	MT4	-0.21573183	0.168707207	-1.278735111	0.200990351	0.34994349	negative
PRJNA729271	ed_Lushi_vs_Arbor_Acr	breed_growth_background	ENSGALG00010023746	TTC28	-0.092210801	0.048348395	-1.907215355	0.056492706	0.144649668	negative
PRJNA729271	ed_Lushi_vs_Arbor_Acr	breed_growth_background	ENSGALG00010023748	ENSGALG00010023748	0.078199838	0.095651894	0.817546156	0.413616384	0.574049165	positive
PRJNA729271	ed_Lushi_vs_Arbor_Acr	breed_growth_background	ENSGALG00010023749	RPP25	0.184937621	0.140113934	1.319908843	0.186865455	0.332549388	positive
PRJNA729271	ed_Lushi_vs_Arbor_Acr	breed_growth_background	ENSGALG00010023750	ENSGALG00010023750	-0.32745034	0.090531184	-3.616989484	0.000298049	0.003457823	negative
PRJNA729271	ed_Lushi_vs_Arbor_Acr	breed_growth_background	ENSGALG00010023751	PRRG4	-0.065255551	0.16241304	-0.401787632	0.687840325	0.798194341	negative
PRJNA729271	ed_Lushi_vs_Arbor_Acr	breed_growth_background	ENSGALG00010023752	C1QL1	-0.008998983	0.164473387	-0.054713913	0.956366385	0.97569799	negative
PRJNA729271	ed_Lushi_vs_Arbor_Acr	breed_growth_background	ENSGALG00010023759	CALCA	-0.596540931	0.162609553	-3.668547868	0.000243932	0.002988837	negative
PRJNA729271	ed_Lushi_vs_Arbor_Acr	breed_growth_background	ENSGALG00010023760	ZW10	0.15148486	0.050943397	2.97359164	0.002943365	0.017493969	positive
PRJNA729271	ed_Lushi_vs_Arbor_Acr	breed_growth_background	ENSGALG00010023762	BBS5	-0.22735207	0.081228079	-2.798934453	0.005127154	0.026049107	negative
PRJNA729271	ed_Lushi_vs_Arbor_Acr	breed_growth_background	ENSGALG00010023764	COX5A	0.111569273	0.0947581	1.177411463	0.239031317	0.394574529	positive
PRJNA729271	ed_Lushi_vs_Arbor_Acr	breed_growth_background	ENSGALG00010023766	ENSGALG00010023766	-0.73508003	0.178918055	-4.108473174	3.98283E-05	0.000821212	negative
PRJNA729271	ed_Lushi_vs_Arbor_Acr	breed_growth_background	ENSGALG00010023767	ENSGALG00010023767	-0.264668922	0.112450692	-2.353644217	0.018590395	0.064947644	negative
PRJNA729271	ed_Lushi_vs_Arbor_Acr	breed_growth_background	ENSGALG00010023770	SCRN2	-0.055906524	0.075786885	-0.737680716	0.460708493	0.615878411	negative
PRJNA729271	ed_Lushi_vs_Arbor_Acr	breed_growth_background	ENSGALG00010023773	OVOA	0.039743435	0.275407609	0.144307687	0.885257509	0.932273147	positive
PRJNA729271	ed_Lushi_vs_Arbor_Acr	breed_growth_background	ENSGALG00010023775	ENSGALG00010023775	0.114304693	0.07276235	1.57093186	0.116198477	0.239076924	positive
PRJNA729271	ed_Lushi_vs_Arbor_Acr	breed_growth_background	ENSGALG00010023777	OAZ2	0.103253889	0.070869377	1.45696059	0.145127259	0.278961956	positive
PRJNA729271	ed_Lushi_vs_Arbor_Acr	breed_growth_background	ENSGALG00010023778	FANCF	-0.097830803	0.123686361	-0.790958698	0.428968093	0.58782802	negative
PRJNA729271	ed_Lushi_vs_Arbor_Acr	breed_growth_background	ENSGALG00010023781	COL4A3	0.204436606	0.12222572	1.672615274	0.094403058	0.206609139	positive
PRJNA729271	ed_Lushi_vs_Arbor_Acr	breed_growth_background	ENSGALG00010023783	VPS35	-0.031490461	0.033887737	-0.929258314	0.352755233	0.516535721	negative
PRJNA729271	ed_Lushi_vs_Arbor_Acr	breed_growth_background	ENSGALG00010023786	ENSGALG00010023786	-0.021865788	0.207912653	-0.105168146	0.916242386	0.95272119	negative
PRJNA729271	ed_Lushi_vs_Arbor_Acr	breed_growth_background	ENSGALG00010023788	RTCA	0.157351034	0.057485447	2.737232492	0.006195849	0.029669253	positive
PRJNA729271	ed_Lushi_vs_Arbor_Acr	breed_growth_background	ENSGALG00010023791	VTI1B	0.165992809	0.044126069	3.761785531	0.000168705	0.002314101	positive
PRJNA729271	ed_Lushi_vs_Arbor_Acr	breed_growth_background	ENSGALG00010023792	ZNF609	0.278728493	0.094793627	2.940371658	0.003278188	0.018901087	positive
PRJNA729271	ed_Lushi_vs_Arbor_Acr	breed_growth_background	ENSGALG00010023793	RBM45	0.003122619	0.035539274	0.087863894	0.929984854	0.961355759	positive
PRJNA729271	ed_Lushi_vs_Arbor_Acr	breed_growth_background	ENSGALG00010023794	MT3	-0.25929986	0.24903115	-1.041234643	0.297766661	0.459847406	negative
PRJNA729271	ed_Lushi_vs_Arbor_Acr	breed_growth_background	ENSGALG00010023798	ARCN1	-0.000813755	0.030741259	-0.026471107	0.978881578	0.987465271	negative
PRJNA729271	ed_Lushi_vs_Arbor_Acr	breed_growth_background	ENSGALG00010023799	NMT1	0.057954519	0.067967981	0.85267384	0.393840202	0.555322347	positive
PRJNA729271	ed_Lushi_vs_Arbor_Acr	breed_growth_background	ENSGALG00010023800	PEX5	0.118476424	0.039016935	3.036538456	0.002393116	0.015127873	positive
PRJNA729271	ed_Lushi_vs_Arbor_Acr	breed_growth_background	ENSGALG00010023802	ENSGALG00010023802	-0.010176532	0.150716376	-0.067521074	0.946166886	0.970470493	negative
PRJNA729271	ed_Lushi_vs_Arbor_Acr	breed_growth_background	ENSGALG00010023805	GALNT6	0.182040795	0.187863398	0.969006188	0.332542102	0.49687638	positive
PRJNA729271	ed_Lushi_vs_Arbor_Acr	breed_growth_background	ENSGALG00010023806	PATJ	-0.371962851	0.095137451	-3.909741629	9.23949E-05	0.00150988	negative
PRJNA729271	ed_Lushi_vs_Arbor_Acr	breed_growth_background	ENSGALG00010023807	MRPL10	0.315350783	0.066868386	4.715992157	2.40536E-06	9.63139E-05	positive
PRJNA729271	ed_Lushi_vs_Arbor_Acr	breed_growth_background	ENSGALG00010023808	DHX35	-0.006908747	0.063954697	-0.108025633	0.913975359	0.951499307	negative
PRJNA729271	ed_Lushi_vs_Arbor_Acr	breed_growth_background	ENSGALG00010023809	CDON	0.053769991	0.053744414	1.000475897	0.317080257	0.480560259	positive
PRJNA729271	ed_Lushi_vs_Arbor_Acr	breed_growth_background	ENSGALG00010023811	MRPL44	0.094815061	0.045200081	2.097674613	0.035933899	0.1039852	positive
PRJNA729271	ed_Lushi_vs_Arbor_Acr	breed_growth_background	ENSGALG00010023813	CELA1	-1.32622393	2.818951461	-0.470467104	0.638021332	0.76254281	negative
PRJNA729271	ed_Lushi_vs_Arbor_Acr	breed_growth_background	ENSGALG00010023815	PLCD3	-0.264693626	0.07394586	-3.579559774	0.000344174	0.003808231	negative
PRJNA729271	ed_Lushi_vs_Arbor_Acr	breed_growth_background	ENSGALG00010023817	BBS2	-0.156098741	0.063527872	-2.457169382	0.014003664	0.052923103	negative
PRJNA729271	ed_Lushi_vs_Arbor_Acr	breed_growth_background	ENSGALG00010023818	ALOX5	0.095390685	0.098150721	0.971879616	0.331110442	0.495452241	positive
PRJNA729271	ed_Lushi_vs_Arbor_Acr	breed_growth_background	ENSGALG00010023819	BIN2	0.026342982	0.12588452	0.209263075	0.834242875	0.899260093	positive
PRJNA729271	ed_Lushi_vs_Arbor_Acr	breed_growth_background	ENSGALG00010023820	CYP1C1	-0.863098723	0.492474128	-1.752576782	0.079674679	0.183606436	negative
PRJNA729271	ed_Lushi_vs_Arbor_Acr	breed_growth_background	ENSGALG00010023821	KANK4	-0.166043809	0.066771384	-2.48675103	0.012891558	0.0500169	negative
PRJNA729271	ed_Lushi_vs_Arbor_Acr	breed_growth_background	ENSGALG00010023822	CHEK2	-0.018744307	0.072027736	-0.260237346	0.794680699	0.872842065	negative
PRJNA729271	ed_Lushi_vs_Arbor_Acr	breed_growth_background	ENSGALG00010023823	MUC2	0.194426716	0.27025972	0.719406932	0.471890227	0.626104411	positive
PRJNA729271	ed_Lushi_vs_Arbor_Acr	breed_growth_background	ENSGALG00010023826	DBT	-0.061940493	0.067902207	-0.912201475	0.361662668	0.525017563	negative
PRJNA729271	ed_Lushi_vs_Arbor_Acr	breed_growth_background	ENSGALG00010023828	CHEK1	-0.13218281	0.098044047	-1.348198223	0.177594636	0.321739063	negative
PRJNA729271	ed_Lushi_vs_Arbor_Acr	breed_growth_background	ENSGALG00010023829	USP1	-0.222195619	0.054598323	-4.069641808	4.70855E-05	0.00092339	negative

PRJNA729271	eed_Lushi_vs_Arbor_Acr	breed_growth_background	ENSGALG00010023830	AGFG1	0.139511618	0.060313395	2.313111658	0.020716501	0.069893238	positive
PRJNA729271	eed_Lushi_vs_Arbor_Acr	breed_growth_background	ENSGALG00010023831	SATB2	-0.158339969	0.298808045	-0.52990531	0.596177585	0.729864366	negative
PRJNA729271	eed_Lushi_vs_Arbor_Acr	breed_growth_background	ENSGALG00010023832	ENSGALG00010023832	-0.28188113	0.291908482	-0.965648988	0.334219854	0.498560953	negative
PRJNA729271	eed_Lushi_vs_Arbor_Acr	breed_growth_background	ENSGALG00010023833	PIP4K2C	0.089025248	0.058408359	1.524186766	0.127462063	0.254380091	positive
PRJNA729271	eed_Lushi_vs_Arbor_Acr	breed_growth_background	ENSGALG00010023835	INAFM2	-0.069756733	0.065307161	-1.068132989	0.285460527	0.445974384	negative
PRJNA729271	eed_Lushi_vs_Arbor_Acr	breed_growth_background	ENSGALG00010023836	NDUFS3	0.040722077	0.078844063	0.516488825	0.605513042	0.73704479	positive
PRJNA729271	eed_Lushi_vs_Arbor_Acr	breed_growth_background	ENSGALG00010023837	STT3A	0.046685575	0.050070375	0.932399143	0.351130288	0.515092958	positive
PRJNA729271	eed_Lushi_vs_Arbor_Acr	breed_growth_background	ENSGALG00010023840	SMAGP	0.220511587	0.373327582	0.590665136	0.554744813	0.696675278	positive
PRJNA729271	eed_Lushi_vs_Arbor_Acr	breed_growth_background	ENSGALG00010023841	OSBPL7	0.025387724	0.081100229	0.313041339	0.754249255	0.844691568	positive
PRJNA729271	eed_Lushi_vs_Arbor_Acr	breed_growth_background	ENSGALG00010023847	PIK3AP1	-0.053102102	0.099984169	-0.531105095	0.595345954	0.72915417	negative
PRJNA729271	eed_Lushi_vs_Arbor_Acr	breed_growth_background	ENSGALG00010023850	DAZAP2	0.096868523	0.10878811	0.890432999	0.37323343	0.535718693	positive
PRJNA729271	eed_Lushi_vs_Arbor_Acr	breed_growth_background	ENSGALG00010023851	HSCB	-0.220262817	0.080321258	-2.742273002	0.006101561	0.029405625	negative
PRJNA729271	eed_Lushi_vs_Arbor_Acr	breed_growth_background	ENSGALG00010023852	LRRC39	0.041944969	0.100690718	0.416572349	0.676991238	0.791395845	positive
PRJNA729271	eed_Lushi_vs_Arbor_Acr	breed_growth_background	ENSGALG00010023854	SHCBP1	-0.482131052	0.119521535	-4.033842525	5.48721E-05	0.001035639	negative
PRJNA729271	eed_Lushi_vs_Arbor_Acr	breed_growth_background	ENSGALG00010023857	ACBD4	0.222186475	0.082153849	2.704516921	0.006840379	0.031881443	positive
PRJNA729271	eed_Lushi_vs_Arbor_Acr	breed_growth_background	ENSGALG00010023859	ENSGALG00010023859	-0.214755874	0.337775503	-0.635794698	0.524910244	0.671601552	negative
PRJNA729271	eed_Lushi_vs_Arbor_Acr	breed_growth_background	ENSGALG00010023860	TRIP4	0.374519974	0.061835173	6.056746623	1.38902E-09	2.79637E-07	positive
PRJNA729271	eed_Lushi_vs_Arbor_Acr	breed_growth_background	ENSGALG00010023861	ENSGALG00010023861	-0.123110801	0.0848122	-1.451569483	0.146621349	0.281121225	negative
PRJNA729271	eed_Lushi_vs_Arbor_Acr	breed_growth_background	ENSGALG00010023862	TRMT13	-0.003559439	0.072519985	-0.049082177	0.960853807	0.978128797	negative
PRJNA729271	eed_Lushi_vs_Arbor_Acr	breed_growth_background	ENSGALG00010023863	EYA2	-0.021100073	0.130374695	-0.161841785	0.871430447	0.923751962	negative
PRJNA729271	eed_Lushi_vs_Arbor_Acr	breed_growth_background	ENSGALG00010023864	EPHA1	0.093953836	0.39339992	0.238825254	0.811241087	0.883798705	positive
PRJNA729271	eed_Lushi_vs_Arbor_Acr	breed_growth_background	ENSGALG00010023866	AMBRA1	0.094947277	0.040332392	2.354119626	0.018566635	0.064895918	positive
PRJNA729271	eed_Lushi_vs_Arbor_Acr	breed_growth_background	ENSGALG00010023867	ENSGALG00010023867	0.310827426	0.266443429	1.166579439	0.243380251	0.39902689	positive
PRJNA729271	eed_Lushi_vs_Arbor_Acr	breed_growth_background	ENSGALG00010023869	DOCK7	-0.045119264	0.025815055	-1.747788785	0.080500608	0.184749744	negative
PRJNA729271	eed_Lushi_vs_Arbor_Acr	breed_growth_background	ENSGALG00010023871	UQCRFS1	-0.027953631	0.075002532	-0.372702498	0.709369877	0.812767083	negative
PRJNA729271	eed_Lushi_vs_Arbor_Acr	breed_growth_background	ENSGALG00010023874	HEXIM1	0.385339824	0.119817037	3.216068723	0.001299597	0.009842037	positive
PRJNA729271	eed_Lushi_vs_Arbor_Acr	breed_growth_background	ENSGALG00010023878	ENSGALG00010023878	-2.689814984	0.771362754	-3.487094717	0.000488298	0.00489142	negative
PRJNA729271	eed_Lushi_vs_Arbor_Acr	breed_growth_background	ENSGALG00010023879	EI24	0.13164672	0.062674713	2.100475836	0.035687004	0.103435938	positive
PRJNA729271	eed_Lushi_vs_Arbor_Acr	breed_growth_background	ENSGALG00010023880	ENSGALG00010023880	-0.188548857	0.108764714	-1.733548034	0.082998308	0.188804217	negative
PRJNA729271	eed_Lushi_vs_Arbor_Acr	breed_growth_background	ENSGALG00010023881	VSTM2B	-0.407462424	0.125031309	-3.258883146	0.001118517	0.008883785	negative
PRJNA729271	eed_Lushi_vs_Arbor_Acr	breed_growth_background	ENSGALG00010023883	TBX21	0.648958419	0.139511698	4.651641594	3.29303E-06	0.000122706	positive
PRJNA729271	eed_Lushi_vs_Arbor_Acr	breed_growth_background	ENSGALG00010023884	C2orf69	0.190273828	0.068994818	2.757798844	0.0058192	0.028352705	positive
PRJNA729271	eed_Lushi_vs_Arbor_Acr	breed_growth_background	ENSGALG00010023887	RASA2	-0.027889921	0.069151495	-0.403316246	0.686715592	0.797900762	negative
PRJNA729271	eed_Lushi_vs_Arbor_Acr	breed_growth_background	ENSGALG00010023888	CTH	-0.485045294	0.138722705	-3.496509769	0.000471387	0.004768149	negative
PRJNA729271	eed_Lushi_vs_Arbor_Acr	breed_growth_background	ENSGALG00010023889	SAMD8	0.17161126	0.101634188	1.688519018	0.091311641	0.201947245	positive
PRJNA729271	eed_Lushi_vs_Arbor_Acr	breed_growth_background	ENSGALG00010023890	SASS6	-0.062682171	0.065014724	-0.9641227	0.334984413	0.499254093	negative
PRJNA729271	eed_Lushi_vs_Arbor_Acr	breed_growth_background	ENSGALG00010023891	ENSGALG00010023891	0.224806556	0.123724391	1.816994644	0.069217934	0.166635766	positive
PRJNA729271	eed_Lushi_vs_Arbor_Acr	breed_growth_background	ENSGALG00010023896	POP4	0.016713076	0.071303005	0.234395114	0.814678252	0.886011499	positive
PRJNA729271	eed_Lushi_vs_Arbor_Acr	breed_growth_background	ENSGALG00010023902	PTGER3	0.363259754	0.125135026	2.902942256	0.003696748	0.020459855	positive
PRJNA729271	eed_Lushi_vs_Arbor_Acr	breed_growth_background	ENSGALG00010023903	TBKBP1	-0.433413902	0.223473085	-1.939445647	0.052447097	0.137157705	negative
PRJNA729271	eed_Lushi_vs_Arbor_Acr	breed_growth_background	ENSGALG00010023909	FOXRED1	0.144355632	0.082980315	1.739637073	0.081922765	0.187026071	positive
PRJNA729271	eed_Lushi_vs_Arbor_Acr	breed_growth_background	ENSGALG00010023910	RNF7	0.053036142	0.053918526	0.983634867	0.325295075	0.488971494	positive
PRJNA729271	eed_Lushi_vs_Arbor_Acr	breed_growth_background	ENSGALG00010023911	TFCP2	-0.0812154	0.066377725	-1.223533946	0.221128091	0.373572456	negative
PRJNA729271	eed_Lushi_vs_Arbor_Acr	breed_growth_background	ENSGALG00010023913	ZMYND8	0.00610032	0.045142366	0.135135138	0.892505029	0.937189325	positive
PRJNA729271	eed_Lushi_vs_Arbor_Acr	breed_growth_background	ENSGALG00010023914	CCDC117	0.058246566	0.035790089	1.627449606	0.103641643	0.220697167	positive
PRJNA729271	eed_Lushi_vs_Arbor_Acr	breed_growth_background	ENSGALG00010023917	ZRANB2	0.10505869	0.042918274	2.447877829	0.014370037	0.053767085	positive
PRJNA729271	eed_Lushi_vs_Arbor_Acr	breed_growth_background	ENSGALG00010023918	MFSD14A	-0.083444543	0.04360563	-1.91361855	0.055668913	0.143172622	negative
PRJNA729271	eed_Lushi_vs_Arbor_Acr	breed_growth_background	ENSGALG00010023920	ENSGALG00010023920	-0.159435336	0.217522655	-0.732959683	0.46358302	0.618751001	negative
PRJNA729271	eed_Lushi_vs_Arbor_Acr	breed_growth_background	ENSGALG00010023921	GRK7	0.133131549	0.096902251	1.373874687	0.169480589	0.311595781	positive
PRJNA729271	eed_Lushi_vs_Arbor_Acr	breed_growth_background	ENSGALG00010023923	CSNK1G1	-0.051037933	0.053147592	-0.960305653	0.336901406	0.501168501	negative
PRJNA729271	eed_Lushi_vs_Arbor_Acr	breed_growth_background	ENSGALG00010023925	NCOA3	0.037490307	0.041661087	0.899887867	0.368179927	0.531444737	positive
PRJNA729271	eed_Lushi_vs_Arbor_Acr	breed_growth_background	ENSGALG00010023926	ZYX	-0.174939031	0.074717229	-2.34134793	0.019214252	0.066502048	negative
PRJNA729271	eed_Lushi_vs_Arbor_Acr	breed_growth_background	ENSGALG00010023927	KPNB1	0.132570547	0.06993905	1.895515405	0.058024153	0.147192876	positive
PRJNA729271	eed_Lushi_vs_Arbor_Acr	breed_growth_background	ENSGALG00010023930	SRPRA	0.150063814	0.070243775	2.136329003	0.032652606	0.09694429	positive
PRJNA729271	eed_Lushi_vs_Arbor_Acr	breed_growth_background	ENSGALG00010023931	CSRNP2	0.119511682	0.083230429	1.435913329	0.151027005	0.286807999	positive
PRJNA729271	eed_Lushi_vs_Arbor_Acr	breed_growth_background	ENSGALG00010023937	XBP1	0.073464338	0.059297768	1.238905611	0.215380456	0.366424848	positive
PRJNA729271	eed_Lushi_vs_Arbor_Acr	breed_growth_background	ENSGALG00010023940	ATP1B3	-0.011552831	0.038492614	-0.300131109	0.764077151	0.851946023	negative
PRJNA729271	eed_Lushi_vs_Arbor_Acr	breed_growth_background	ENSGALG00010023942	NEGR1	-0.198483152	0.117423889	-1.690313222	0.090968047	0.20149478	negative
PRJNA729271	eed_Lushi_vs_Arbor_Acr	breed_growth_background	ENSGALG00010023945	LETMD1	-0.049369509	0.113054672	-0.436687029	0.662338338	0.78104411	negative
PRJNA729271	eed_Lushi_vs_Arbor_Acr	breed_growth_background	ENSGALG00010023946	ZNRF3	-0.008165131	0.069333535	-0.11776597	0.906253094	0.946203169	negative
PRJNA729271	eed_Lushi_vs_Arbor_Acr	breed_growth_background	ENSGALG00010023947	ENSGALG00010023947	0.288503885	0.332888959	0.86666703	0.386124475	0.548122051	positive
PRJNA729271	eed_Lushi_vs_Arbor_Acr	breed_growth_background	ENSGALG00010023948	SULF2	-0.346688406	0.118375678	-2.928713158	0.003403684	0.019370394	negative
PRJNA729271	eed_Lushi_vs_Arbor_Acr	breed_growth_background	ENSGALG00010023953	SLC35A3	0.13754276	0.083274495	1.651679304	0.098599944	0.212837853	positive
PRJNA729271	eed_Lushi_vs_Arbor_Acr	breed_growth_background	ENSGALG00010023954	PLEKHF1	0.052578388	0.050857125	1.03384506	0.301208603	0.463582363	positive
PRJNA729271	eed_Lushi_vs_Arbor_Acr	breed_growth_background	ENSGALG00010023956	KREMEN1	0.104688732	0.071116625	1.472071161	0.141001662	0.27349379	positive
PRJNA729271	eed_Lushi_vs_Arbor_Acr	breed_growth_background	ENSGALG00010023957	YBX3	0.228206836	0.074167984	3.076891446	0.002091715	0.013682041	positive
PRJNA729271	eed_Lushi_vs_Arbor_Acr	breed_growth_background	ENSGALG00010023962	FPGT	-0.055370449	0.064161349	-0.862987615	0.388144277	0.550178248	negative
PRJNA729271	eed_Lushi_vs_Arbor_Acr	breed_growth_background	ENSGALG00010023966	PEAK1	0.033641406	0.052503374	0.640747513	0.521686739	0.668855471	positive
PRJNA729271	eed_Lushi_vs_Arbor_Acr	breed_growth_background	ENSGALG00010023967	PRPH	-0.306916078	0.19854845	-1.545799415	0.122153018	0.246843441	negative
PRJNA729271	eed_Lushi_vs_Arbor_Acr	breed_growth_background	ENSGALG00010023968	TYW5	0.060436848	0.062905013	0.960763616	0.336671037	0.500928626	positive
PRJNA729271	eed_Lushi_vs_Arbor_Acr	breed_growth_background	ENSGALG00010023972	ENSGALG00010023972	0.205691723	0.274348561	0.749745953	0.453407725	0.609660944	positive
PRJNA729271	eed_Lushi_vs_Arbor_Acr	breed_growth_background	ENSGALG00010023974	PREX1	-0.093255834	0.055652919	-1.675668338	0.093803175	0.205544523	negative
PRJNA729271	eed_Lushi_vs_Arbor_Acr	breed_growth_background	ENSGALG00010023978	TFDP2	0.405989988	0.074959714	5.416109052	6.091E-08	5.65955E-06	positive
PRJNA729271	eed_Lushi_vs_Arbor_Acr	breed_growth_background	ENSGALG00010023979	FAM118B	-0.155325961	0.063358778	-2.451530268	0.014225022	0.05347295	negative
PRJNA729271	eed_Lushi_vs_Arbor_Acr	breed_growth_background	ENSGALG00010023981	ENSGALG00010023981	-0.252039637	0.197947852	-1.273262802	0.202924802	0.352136359	negative
PRJNA729271	eed_Lushi_vs_Arbor_Acr	breed_growth_background	ENSGALG00010023984	FMNL1	-0.198058525	0.105239615	-1.881976918	0.05983915	0.150532536	negative
PRJNA729271	eed_Lushi_vs_Arbor_Acr	breed_growth_background	ENSGALG00010023986	SYCE3	-0.049450942	0.337203127	-0.146650307	0.883408044	0.93134085	negative
PRJNA729271	eed_Lushi_vs_Arbor_Acr	breed_growth_background	ENSGALG00010023987	TNNI3K	0.427305763	0.252497911	1.692314049	0.09058611	0.201140575	positive
PRJNA729271	eed_Lushi_vs_Arbor_Acr	breed_growth_background	ENSGALG00010023989	ENSGALG00010023989	0.181721893	0.054919995	3.308847597	0.000936808	0.007763885	positive
PRJNA729271	eed_Lushi_vs_Arbor_Acr	breed_growth_background	ENSGALG00010023991	TSPAN3	0.03388025	0.056897776	0.595458247	0.551537201	0.694391707	positive
PRJNA729271	eed_Lushi_vs_Arbor_Acr	breed_growth_background	ENSGALG00010023992	FAM131B	-0.241688876	0.076807302	-3.146691374	0.001651292	0.011613523	negative
PRJNA729271	eed_Lushi_vs_Arbor_Acr	breed_growth_background	ENSGALG00010023993	C19orf12	-0.137807847	0.094163851	-1.463489926	0.143333368	0.276573106	negative
PRJNA729271	eed_Lushi_vs_Arbor_Acr	breed_growth_background	ENSGALG00010023996	MYO7B	-0.140257147	0.200969005	-0.69790437	0.485237001	0.637093327	negative
PRJNA729271	eed_Lushi_vs_Arbor_Acr	breed_growth_background	ENSGALG00010023997	ARFGEF2	-0.078595424	0.034179672	-2.299478585	0.021477778	0.071815546	negative
PRJNA729271	eed_Lushi_vs_Arbor_Acr	breed_growth_background	ENSGALG00010023999	DGCR14	0.120242432	0.049961424	2.406705479	0.016097146	0.058581539	positive
PRJNA729271	eed_Lushi_vs_Arbor_Acr	breed_growth_background	ENSGALG00010024001	ENSGALG00010024001	-0.092401914	0.231093342	-0.399846717	0.689269419	0.798983817	negative
PRJNA729271	eed_Lushi_vs_Arbor_Acr	breed_growth_background	ENSGALG00010024002	DGCR2	0.002436338	0.033097837	0.073610189	0.941320564	0.96810285	positive
PRJNA729271	eed_Lushi_vs_Arbor_Acr	breed_growth_background	ENSGALG00010024003	ERICH3	0.466068067	0.166681748	2.796155386	0.005171453	0.0261824	positive
PRJNA729271	eed_Lushi_vs_Arbor_Acr	breed_growth_background	ENSGALG00010024004	EIF4B	0.19611186	0.066914229	2.930794577	0.003380963	0.019301715	positive
PRJNA729271	eed_Lushi_vs_Arbor_Acr	breed_growth_background	ENSGALG00010024005	STYK1	0.052649686	0.131999762	0.398861975	0.689994909	0.799552239	positive
PRJNA729271	eed_Lushi_vs_Arbor_Acr	breed_growth_background	ENSGALG00010024008	CCNE1	0.171050501	0.057637338	2.967703013	0.003000341	0.017722062	positive
PRJNA729271	eed_Lushi_vs_Arbor_Acr	breed_growth_background	ENSGALG00010024009	CSE1L	-0.029186289	0.033186969	-0.879450262	0.37915719	0.541047703	negative
PRJNA729271	eed_Lushi_vs_Arbor_Acr	breed_growth_background	ENSGALG00010024010	ENSGALG00010024010	0.014396761	0.047251085	0.304686364	0.760605036	0.849315923	positive
PRJNA729271	eed_Lushi_vs_Arbor_Acr	breed_growth_background	ENSGALG00010024011	AGPS	0.105753079	0.03770037	2.805093898	0.005030192	0.025700613	positive
PRJNA729271	eed_Lushi_vs_Arbor_Acr	breed_growth_background	ENSGALG00010024013	PTPRJ	0.030925006	0.065094579	0.475078052	0.634731342	0.759550136	positive
PRJNA729271	eed_Lushi_vs_Arbor_Acr	breed_growth_background	ENSGALG00010024016	CRYZ	-0.035707355	0.055961263	-0.638072717	0.523426346	0.670353851	negative
PRJNA729271	eed_Lushi_vs_Arbor_Acr	breed_growth_background	ENSGALG00010024017	TYW3	-0.242491905	0.074127745	-3.271270515	0.001070654	0.008597454	negative
PRJNA729271	eed_Lushi_vs_Arbor_Acr	breed_growth_background	ENSGALG00010024018	KRT18	-0.408928164	0.572810578	-0.713897716	0.47529043	0.628756545	negative
PRJNA729271	eed_Lushi_vs_Arbor_Acr	breed_growth_background	ENSGALG00010024019	MAP3K14	0.054022972	0.057432737	0.940630283	0.346894357	0.510826201	positive
PRJNA729271	eed_Lushi_vs_Arbor_Acr	breed_growth_background	ENSGALG00010024020	MAIP1	-0.04501288	0.065501565	-0.687203121	0.491954738	0.643988434	negative
PRJNA729271	eed_Lushi_vs_Arbor_Acr	breed_growth_background	ENSGALG00010024021	IGLL1	0.703353124	0.577764857	1.217369169	0.223463788	0.37611903	positive
PRJNA729271	eed_Lushi_vs_Arbor_Acr	breed_growth_background	ENSGALG00010024023	LHX8	0.41170679	0.29811145	1.381049906	0.167263617	0.308223509	positive
PRJNA729271	eed_Lushi_vs_Arbor_Acr	breed_growth_background	ENSGALG00010024024	AGL	0.010563871	0.065462205	0.16137359	0.871799166	0.924075241	positive
PRJNA729271	eed_Lushi_vs_Arbor_Acr	breed_growth_background	ENSGALG00010024025	CLCN1	0.022375869	0.155033426	0.144329318	0.885240428	0.932273147	positive
PRJNA729271	eed_Lushi_vs_Arbor_Acr	breed_growth_background	ENSGALG00010024026	TIRAP	0.096669669	0.056415309	1.71353613	0.086613952	0.194706768	positive
PRJNA729271	eed_Lushi_vs_Arbor_Acr	breed_growth_background	ENSGALG00010024029	SCAPER	0.02943654	0.078870349	0.373226949	0.708979541	0.81249332	positive
PRJNA729271	eed_Lushi_vs_Arbor_Acr	breed_growth_background	ENSGALG00010024030	STAU1	0.039174789	0.048051858	0.815260653	0.414923126	0.575239875	positive
PRJNA729271	eed_Lushi_vs_Arbor_Acr	breed_growth_background	ENSGALG00010024031	LIMS2	-0.334757019	0.061556855	-5.438176143	5.38287E-08	5.20165E-06	negative
PRJNA729271	eed_Lushi_vs_Arbor_Acr	breed_growth_background	ENSGALG00010024034	LRP2	0.067749228	0.133398613	0.507870562	0.611544121	0.741565902	positive
PRJNA729271	eed_Lushi_vs_Arbor_Acr	breed_growth_background	ENSGALG00010024035	OSBPL5	-0.254909904	0.073743103	-3.456728721	0.000546775	0.005294257	negative
PRJNA729271	eed_Lushi_vs_Arbor_Acr	breed_growth_background	ENSGALG00010024038	NKD1	0.183157945	0.090438912	2.025211722	0.042845629	0.118384938	positive
PRJNA729271	eed_Lushi_vs_Arbor_Acr	breed_growth_background	ENSGALG00010024039	MRPL45	-0.102639292	0.057819562	-1.775165509	0.075870563	0.177635893	negative
PRJNA729271	eed_Lushi_vs_Arbor_Acr	breed_growth_background	ENSGALG00010024040	ENSGALG00010024040	-0.104224408	0.466407642	-0.223462049	0.823175917	0.891840564	negative
PRJNA729271	eed_Lushi_vs_Arbor_Acr	breed_growth_background	ENSGALG00010024042	DCPS	0.324613003	0.070483817	4.605496921	4.11482E-06	0.000148369	positive
PRJNA729271	eed_Lushi_vs_Arbor_Acr	breed_growth_background	ENSGALG00010024045	PSTPIP1	0.14392166	0.13089232	1.099542436	0.271531535	0.430288575	positive
PRJNA729271	eed_Lushi_vs_Arbor_Acr	breed_growth_background	ENSGALG00010024046	ENSGALG00010024046	1.068492219	0.338039481	3.160850369	0.001573093	0.011223509	positive
PRJNA729271	eed_Lushi_vs_Arbor_Acr	breed_growth_background	ENSGALG00010024049	RCN2	-0.226975694	0.077172947	-2.941130326	0.003270169	0.018869865	negative
PRJNA729271	eed_Lushi_vs_Arbor_Acr	breed_growth_background	ENSGALG00010024051	ENSGALG00010024051	-0.261104125	0.414498734	-0.629927437	0.528742061	0.674539357	negative
PRJNA729271	eed_Lushi_vs_Arbor_Acr	breed_growth_background	ENSGALG00010024052	ENSGALG00010024052	0.077534953	0.092474421	0.838447572	0.401779381	0.562816279	positive
PRJNA729271	eed_Lushi_vs_Arbor_Acr	breed_growth_background	ENSGALG00010024053	ISL2	0.77070201	0.322286656	2.391355635	0.016786284	0.060301657	positive
PRJNA729271	eed_Lushi_vs_Arbor_Acr	breed_growth_background	ENSGALG00010024054	ENSGALG00010024054	0.062421728	0.485659395	0.128529847	0.89772968	0.940691948	positive
PRJNA729271	eed_Lushi_vs_Arbor_Acr	breed_growth_background	ENSGALG00010024057	HSD3B1	0.713701172	0.452360171	1.577727699	0.11462818	0.236820905	positive
PRJNA729271	eed_Lushi_vs_Arbor_Acr	breed_growth_background	ENSGALG00010024058	SLC44A5	0.119431537	0.056479647	2.114594245	0.034464545	0.100779418	positive
PRJNA729271	eed_Lushi_vs_Arbor_Acr	breed_growth_background	ENSGALG00010024060	FRRS1	0.050422874	0.086787933	0.580989455	0.56124756	0.702043785	positive
PRJNA729271	eed_Lushi_vs_Arbor_Acr	breed_growth_background	ENSGALG00010024061	ENSGALG00010024061	-0.903606115	0.696533812	-1.297289665	0.194531539	0.342200808	negative
PRJNA729271	eed_Lushi_vs_Arbor_Acr	breed_growth_background	ENSGALG00010024062	ST3GAL4	-0.240478584	0.071891902	-3.345002394	0.000822819	0.007086605	negative
PRJNA729271	eed_Lushi_vs_Arbor_Acr	breed_growth_background	ENSGALG00010024063	BRD7	0.28101384	0.055908583	5.026309442	5.00009E-07	2.82009E-05	positive
PRJNA729271	eed_Lushi_vs_Arbor_Acr	breed_growth_background	ENSGALG00010024064	GPR17	0.021023224	0.119102061	0.176514361	0.859889867	0.915804822	positive
PRJNA729271	eed_Lushi_vs_Arbor_Acr	breed_growth_background	ENSGALG00010024066	ENSGALG00010024066	0.552451367	1.15505079	0.478291839	0.632442504	0.757789574	positive
PRJNA729271	eed_Lushi_vs_Arbor_Acr	breed_growth_background	ENSGALG00010024068	CASP2	-0.063807287	0.047574682	-1.341202613	0.179854678	0.324091691	negative
PRJNA729271	eed_Lushi_vs_Arbor_Acr	breed_growth_background	ENSGALG00010024069	ENSGALG00010024069	-0.32538034	1.210190981	-0.268866935	0.788032082	0.86836894	negative

PRJNA729271	ed_Lushi_vs_Arbor_Acr	breed_growth_background	ENSGALG00010024073	RABGGTB	0.060143054	0.067128457	0.895939771	0.370284932	0.533579887	positive
PRJNA729271	ed_Lushi_vs_Arbor_Acr	breed_growth_background	ENSGALG00010024074	ADCY7	0.031592159	0.123289886	0.256242911	0.797763277	0.874703381	positive
PRJNA729271	ed_Lushi_vs_Arbor_Acr	breed_growth_background	ENSGALG00010024075	SPATS2L	0.032120139	0.07350876	0.436956622	0.662142807	0.780877064	positive
PRJNA729271	ed_Lushi_vs_Arbor_Acr	breed_growth_background	ENSGALG00010024077	SFT2D3	0.014907507	0.062814459	0.237326041	0.812403861	0.884481032	positive
PRJNA729271	ed_Lushi_vs_Arbor_Acr	breed_growth_background	ENSGALG00010024078	KIRREL3	-0.348144239	0.132166184	-2.634140059	0.008435069	0.03689388	negative
PRJNA729271	ed_Lushi_vs_Arbor_Acr	breed_growth_background	ENSGALG00010024080	TENT4B	0.039972107	0.047255945	0.845864089	0.397628568	0.558924175	positive
PRJNA729271	ed_Lushi_vs_Arbor_Acr	breed_growth_background	ENSGALG00010024081	CARS	-0.009752493	0.053276543	-0.183054161	0.854755522	0.912616477	negative
PRJNA729271	ed_Lushi_vs_Arbor_Acr	breed_growth_background	ENSGALG00010024083	PALMD	0.294721308	0.102005919	2.889256926	0.003861534	0.021121864	positive
PRJNA729271	ed_Lushi_vs_Arbor_Acr	breed_growth_background	ENSGALG00010024086	GABARAPL1	0.006677185	0.115035501	0.058044557	0.953713137	0.974074966	positive
PRJNA729271	ed_Lushi_vs_Arbor_Acr	breed_growth_background	ENSGALG00010024089	ENSGALG00010024089	0.601208847	0.959711213	0.626447663	0.531021355	0.676802586	positive
PRJNA729271	ed_Lushi_vs_Arbor_Acr	breed_growth_background	ENSGALG00010024091	ETFA	0.200884418	0.062475178	3.215427718	0.001302503	0.009853747	positive
PRJNA729271	ed_Lushi_vs_Arbor_Acr	breed_growth_background	ENSGALG00010024095	RBM12	-0.078791545	0.028953071	-2.721353603	0.006501518	0.030726931	negative
PRJNA729271	ed_Lushi_vs_Arbor_Acr	breed_growth_background	ENSGALG00010024096	MYO1D	-0.061090659	0.118830605	-0.514098695	0.60718299	0.738101094	negative
PRJNA729271	ed_Lushi_vs_Arbor_Acr	breed_growth_background	ENSGALG00010024099	ENSGALG00010024099	0.276314655	1.047269943	0.26384282	0.791901038	0.871043067	positive
PRJNA729271	ed_Lushi_vs_Arbor_Acr	breed_growth_background	ENSGALG00010024101	CNEP1R1	0.027047408	0.0487934	0.554325128	0.579356357	0.716366771	positive
PRJNA729271	ed_Lushi_vs_Arbor_Acr	breed_growth_background	ENSGALG00010024102	SGF29	0.132012007	0.048335844	2.731141046	0.006311545	0.030074244	positive
PRJNA729271	ed_Lushi_vs_Arbor_Acr	breed_growth_background	ENSGALG00010024104	PLPPR5	-0.17594787	0.089209699	-1.972295303	0.048575906	0.129909179	negative
PRJNA729271	ed_Lushi_vs_Arbor_Acr	breed_growth_background	ENSGALG00010024106	WDR33	0.03404679	0.03454735	0.985510925	0.324373168	0.488446817	positive
PRJNA729271	ed_Lushi_vs_Arbor_Acr	breed_growth_background	ENSGALG00010024107	NFE2L2	-6.42916E-05	0.033608641	-0.001912947	0.99847369	0.999025066	negative
PRJNA729271	ed_Lushi_vs_Arbor_Acr	breed_growth_background	ENSGALG00010024110	CPNE1	-0.350075502	0.067920529	-5.154192814	2.54726E-07	1.69905E-05	negative
PRJNA729271	ed_Lushi_vs_Arbor_Acr	breed_growth_background	ENSGALG00010024112	NAP1L4	-0.075847575	0.048477376	-1.564597362	0.117677348	0.241195299	negative
PRJNA729271	ed_Lushi_vs_Arbor_Acr	breed_growth_background	ENSGALG00010024113	ENSGALG00010024113	0.081174732	0.087670075	0.925911513	0.354491968	0.518136641	positive
PRJNA729271	ed_Lushi_vs_Arbor_Acr	breed_growth_background	ENSGALG00010024115	POLR2D	0.030983296	0.04056932	0.763712469	0.445038609	0.601869251	positive
PRJNA729271	ed_Lushi_vs_Arbor_Acr	breed_growth_background	ENSGALG00010024116	MCAM	-0.082832577	0.097589087	-0.848789342	0.395998522	0.557329416	negative
PRJNA729271	ed_Lushi_vs_Arbor_Acr	breed_growth_background	ENSGALG00010024118	TMEM266	0.035978056	0.074778767	0.481126625	0.630426506	0.756397004	positive
PRJNA729271	ed_Lushi_vs_Arbor_Acr	breed_growth_background	ENSGALG00010024119	AMMECR1L	-0.097450291	0.040891925	-2.383118226	0.01716668	0.061292802	negative
PRJNA729271	ed_Lushi_vs_Arbor_Acr	breed_growth_background	ENSGALG00010024121	NRG4	-0.048589537	0.091386529	-0.531692557	0.594938948	0.728950415	negative
PRJNA729271	ed_Lushi_vs_Arbor_Acr	breed_growth_background	ENSGALG00010024122	SOCS7	0.137542599	0.057192978	2.404886106	0.016177507	0.05875544	positive
PRJNA729271	ed_Lushi_vs_Arbor_Acr	breed_growth_background	ENSGALG00010024123	PLPPR4	-0.3551005	0.216726766	-1.638470902	0.101323499	0.21716459	negative
PRJNA729271	ed_Lushi_vs_Arbor_Acr	breed_growth_background	ENSGALG00010024124	DIDO1	0.101683562	0.036060524	2.819802652	0.004805319	0.024743553	positive
PRJNA729271	ed_Lushi_vs_Arbor_Acr	breed_growth_background	ENSGALG00010024125	ENSGALG00010024125	0.060726398	0.894550801	0.067884795	0.945877343	0.970470493	positive
PRJNA729271	ed_Lushi_vs_Arbor_Acr	breed_growth_background	ENSGALG00010024126	CDK5R1	0.101637877	0.157377122	0.64582371	0.518393531	0.666614508	positive
PRJNA729271	ed_Lushi_vs_Arbor_Acr	breed_growth_background	ENSGALG00010024128	PHLDA2	-0.08893748	0.127405683	-0.698065248	0.48513639	0.637018931	negative
PRJNA729271	ed_Lushi_vs_Arbor_Acr	breed_growth_background	ENSGALG00010024129	GID8	0.016220462	0.054333411	0.298535678	0.76529435	0.852778413	positive
PRJNA729271	ed_Lushi_vs_Arbor_Acr	breed_growth_background	ENSGALG00010024131	ENSGALG00010024131	-0.664897595	0.57410195	-1.158152477	0.246801818	0.402938538	negative
PRJNA729271	ed_Lushi_vs_Arbor_Acr	breed_growth_background	ENSGALG00010024132	ENSGALG00010024132	0.146458934	0.082771681	1.769432877	0.076821663	0.178995339	positive
PRJNA729271	ed_Lushi_vs_Arbor_Acr	breed_growth_background	ENSGALG00010024133	PSMD11	0.221208524	0.075512747	2.929419651	0.003395956	0.019341604	positive
PRJNA729271	ed_Lushi_vs_Arbor_Acr	breed_growth_background	ENSGALG00010024135	FBXO22	0.070947727	0.046975014	1.510329017	0.130959491	0.259062549	positive
PRJNA729271	ed_Lushi_vs_Arbor_Acr	breed_growth_background	ENSGALG00010024136	SAP130	0.113716003	0.094309049	1.2057804	0.227902175	0.381128963	positive
PRJNA729271	ed_Lushi_vs_Arbor_Acr	breed_growth_background	ENSGALG00010024137	ENSGALG00010024137	-0.046329374	0.387101109	-0.119682877			negative
PRJNA729271	ed_Lushi_vs_Arbor_Acr	breed_growth_background	ENSGALG00010024138	CBL	-0.133514865	0.080126829	-1.666294123	0.095654847	0.208310848	negative
PRJNA729271	ed_Lushi_vs_Arbor_Acr	breed_growth_background	ENSGALG00010024139	SLC22A18	-0.48753165	0.45862555	-1.063027669	0.287769404	0.44875928	negative
PRJNA729271	ed_Lushi_vs_Arbor_Acr	breed_growth_background	ENSGALG00010024140	KRT6A	-0.352411832	0.484389461	-0.72753819	0.466896329	0.621856316	negative
PRJNA729271	ed_Lushi_vs_Arbor_Acr	breed_growth_background	ENSGALG00010024141	YTHDF1	0.087427971	0.051868655	1.685564643	0.091879684	0.20268211	positive
PRJNA729271	ed_Lushi_vs_Arbor_Acr	breed_growth_background	ENSGALG00010024142	DNAJC6	-0.294540983	0.148998739	-1.97680185	0.048064033	0.128683379	negative
PRJNA729271	ed_Lushi_vs_Arbor_Acr	breed_growth_background	ENSGALG00010024143	UBE2Q2	0.312073459	0.062146293	5.021594104	5.12444E-07	2.84815E-05	positive
PRJNA729271	ed_Lushi_vs_Arbor_Acr	breed_growth_background	ENSGALG00010024144	HNRNPA3	-0.022577274	0.067859294	-0.33270718	0.739355329	0.832967161	negative
PRJNA729271	ed_Lushi_vs_Arbor_Acr	breed_growth_background	ENSGALG00010024145	RAP1GAP3	-0.159418591	0.098783886	-1.613811705	0.106568275	0.225241636	negative
PRJNA729271	ed_Lushi_vs_Arbor_Acr	breed_growth_background	ENSGALG00010024146	CDKN1C	0.654345858	0.130908635	4.998492703	5.77802E-07	3.14901E-05	positive
PRJNA729271	ed_Lushi_vs_Arbor_Acr	breed_growth_background	ENSGALG00010024147	ENSGALG00010024147	-0.318838821	0.149380268	-2.134410552	0.032809189	0.097273305	negative
PRJNA729271	ed_Lushi_vs_Arbor_Acr	breed_growth_background	ENSGALG00010024148	UGGT1	-0.03514482	0.031708707	-1.108364962	0.26770423	0.426086836	negative
PRJNA729271	ed_Lushi_vs_Arbor_Acr	breed_growth_background	ENSGALG00010024149	TCIRG1	-0.02360035	0.082522055	-0.285988391	0.774887013	0.860248042	negative
PRJNA729271	ed_Lushi_vs_Arbor_Acr	breed_growth_background	ENSGALG00010024150	NHERF4	-0.159154133	0.18406615	-0.86465726	0.387226935	0.549202976	negative
PRJNA729271	ed_Lushi_vs_Arbor_Acr	breed_growth_background	ENSGALG00010024151	LEPROT	-0.252791686	0.079330713	-3.186555088	0.001439781	0.01061527	negative
PRJNA729271	ed_Lushi_vs_Arbor_Acr	breed_growth_background	ENSGALG00010024152	ZNF423	-0.036399242	0.057894973	-0.62871162	0.529537869	0.675376279	negative
PRJNA729271	ed_Lushi_vs_Arbor_Acr	breed_growth_background	ENSGALG00010024153	KCNQ1	0.275801244	0.110770502	2.48984377	0.012779926	0.049663546	positive
PRJNA729271	ed_Lushi_vs_Arbor_Acr	breed_growth_background	ENSGALG00010024154	VPS29L	-0.621345509	0.260794343	-2.382511454	0.017194997	0.061344199	negative
PRJNA729271	ed_Lushi_vs_Arbor_Acr	breed_growth_background	ENSGALG00010024155	SNX7	-0.24810418	0.0893636	-2.776344951	0.005497385	0.027269178	negative
PRJNA729271	ed_Lushi_vs_Arbor_Acr	breed_growth_background	ENSGALG00010024157	CHRNB4	0.299893317	0.216663818	1.384141198	0.166315224	0.306865985	positive
PRJNA729271	ed_Lushi_vs_Arbor_Acr	breed_growth_background	ENSGALG00010024158	ENSGALG00010024158	-0.008473414	0.182160986	-0.046516076	0.962898921	0.978835813	negative
PRJNA729271	ed_Lushi_vs_Arbor_Acr	breed_growth_background	ENSGALG00010024159	NLRX1	-0.217313181	0.088191166	-2.46411508	0.0137352	0.052351899	negative
PRJNA729271	ed_Lushi_vs_Arbor_Acr	breed_growth_background	ENSGALG00010024162	SRCIN1	0.303620517	0.186175631	1.630828457	0.102926521	0.219529124	positive
PRJNA729271	ed_Lushi_vs_Arbor_Acr	breed_growth_background	ENSGALG00010024163	HS6ST1	-0.11805675	0.052516008	-2.24801456	0.024575261	0.079001645	negative
PRJNA729271	ed_Lushi_vs_Arbor_Acr	breed_growth_background	ENSGALG00010024164	SNAP29	-0.014624014	0.060060419	-0.243488378	0.807627087	0.881783266	negative
PRJNA729271	ed_Lushi_vs_Arbor_Acr	breed_growth_background	ENSGALG00010024165	LEPR	-0.167177624	0.150104813	-1.113739264	0.26539106	0.423614515	negative
PRJNA729271	ed_Lushi_vs_Arbor_Acr	breed_growth_background	ENSGALG00010024166	ENSGALG00010024166	-0.037792708	0.278430496	-0.135734802	0.892030934	0.93688779	negative
PRJNA729271	ed_Lushi_vs_Arbor_Acr	breed_growth_background	ENSGALG00010024167	CHRNA3	0.085085992	0.223174721	0.381252821	0.703015658	0.808169717	positive
PRJNA729271	ed_Lushi_vs_Arbor_Acr	breed_growth_background	ENSGALG00010024168	BIRC7	-0.379368607	0.214002517	-1.772729647	0.076273516	0.178200301	negative
PRJNA729271	ed_Lushi_vs_Arbor_Acr	breed_growth_background	ENSGALG00010024169	FAM168B	-0.028296903	0.039001389	-0.725535764	0.468123412	0.62302707	negative
PRJNA729271	ed_Lushi_vs_Arbor_Acr	breed_growth_background	ENSGALG00010024170	NUP160	-0.08186021	0.046813101	-1.748660256	0.080349764	0.184552425	negative
PRJNA729271	ed_Lushi_vs_Arbor_Acr	breed_growth_background	ENSGALG00010024171	ENSGALG00010024171	-0.180050112	0.281581442	-0.639424638	0.522546725	0.669681091	negative
PRJNA729271	ed_Lushi_vs_Arbor_Acr	breed_growth_background	ENSGALG00010024172	ABCG4	-0.023237657	0.245672595	-0.094587909	0.924642154	0.957813766	negative
PRJNA729271	ed_Lushi_vs_Arbor_Acr	breed_growth_background	ENSGALG00010024173	CBLN1	-1.068546907	0.225220869	-4.744440034	2.09084E-06	8.73392E-05	negative
PRJNA729271	ed_Lushi_vs_Arbor_Acr	breed_growth_background	ENSGALG00010024174	PLEKHB2	-0.220586142	0.054374204	-4.056816059	4.97462E-05	0.000962511	negative
PRJNA729271	ed_Lushi_vs_Arbor_Acr	breed_growth_background	ENSGALG00010024175	DHRS9	-0.88028729	0.171133935	-5.143850005	2.69164E-07	1.77343E-05	negative
PRJNA729271	ed_Lushi_vs_Arbor_Acr	breed_growth_background	ENSGALG00010024177	ENSGALG00010024177	-0.012007152	0.236458792	-0.050779045	0.959501589	0.977517846	negative
PRJNA729271	ed_Lushi_vs_Arbor_Acr	breed_growth_background	ENSGALG00010024178	N4BP1	0.171423822	0.064651133	2.651520767	0.008013019	0.035672206	positive
PRJNA729271	ed_Lushi_vs_Arbor_Acr	breed_growth_background	ENSGALG00010024179	ATG4C	0.32520169	0.063768103	5.099754847	3.40094E-07	2.08884E-05	positive
PRJNA729271	ed_Lushi_vs_Arbor_Acr	breed_growth_background	ENSGALG00010024180	HINFP	0.327259171	0.075873317	4.313231386	1.60886E-05	0.000408413	positive
PRJNA729271	ed_Lushi_vs_Arbor_Acr	breed_growth_background	ENSGALG00010024181	FOXD3	0.808296776	0.167245991	4.832981471	1.34503E-06	6.2488E-05	positive
PRJNA729271	ed_Lushi_vs_Arbor_Acr	breed_growth_background	ENSGALG00010024183	MTX2	-0.119080077	0.05700079	-2.089095203	0.036699154	0.105610443	negative
PRJNA729271	ed_Lushi_vs_Arbor_Acr	breed_growth_background	ENSGALG00010024184	ENSGALG00010024184	-0.435625516	0.122159383	-3.566042218	0.000362413	0.003946789	negative
PRJNA729271	ed_Lushi_vs_Arbor_Acr	breed_growth_background	ENSGALG00010024185	CHRNA5	0.168804505	0.083411694	2.023751075	0.04299577	0.118669865	positive
PRJNA729271	ed_Lushi_vs_Arbor_Acr	breed_growth_background	ENSGALG00010024186	HAO2	-0.701852872	0.402605534	-1.743276762	0.081285282	0.186046134	negative
PRJNA729271	ed_Lushi_vs_Arbor_Acr	breed_growth_background	ENSGALG00010024188	TSSC4	-0.145673707	0.045843268	-3.177646632	0.001484756	0.010836625	negative
PRJNA729271	ed_Lushi_vs_Arbor_Acr	breed_growth_background	ENSGALG00010024189	KAT7	0.152651763	0.075397781	2.024618756	0.042906527	0.118485446	positive
PRJNA729271	ed_Lushi_vs_Arbor_Acr	breed_growth_background	ENSGALG00010024190	WARS2	-0.123652003	0.061173495	-2.021332961	0.043245309	0.119125952	negative
PRJNA729271	ed_Lushi_vs_Arbor_Acr	breed_growth_background	ENSGALG00010024191	DPYD	-0.069171053	0.060525896	-1.142834014	0.253107513	0.409737928	negative
PRJNA729271	ed_Lushi_vs_Arbor_Acr	breed_growth_background	ENSGALG00010024192	ALG6	0.069395579	0.050898721	1.363405179	0.17275485	0.316052196	positive
PRJNA729271	ed_Lushi_vs_Arbor_Acr	breed_growth_background	ENSGALG00010024193	GSTK1	0.054030036	0.067575066	0.799555804	0.423968202	0.583198739	positive
PRJNA729271	ed_Lushi_vs_Arbor_Acr	breed_growth_background	ENSGALG00010024194	NKAIN4	-0.190179641	0.046261378	-4.110980927	3.93982E-05	0.000813499	negative
PRJNA729271	ed_Lushi_vs_Arbor_Acr	breed_growth_background	ENSGALG00010024195	PSMA4	0.120123456	0.064721562	1.856003657	0.063453037	0.156873916	positive
PRJNA729271	ed_Lushi_vs_Arbor_Acr	breed_growth_background	ENSGALG00010024196	MRPL21	-0.009870247	0.05888	-0.167633274	0.866871793	0.92026561	negative
PRJNA729271	ed_Lushi_vs_Arbor_Acr	breed_growth_background	ENSGALG00010024197	KRT7	-0.433305079	0.167116183	-2.592837336	0.009518778	0.040426219	negative
PRJNA729271	ed_Lushi_vs_Arbor_Acr	breed_growth_background	ENSGALG00010024198	PI4KA	0.124289088	0.034680347	3.583847858	0.000338569	0.003766357	positive
PRJNA729271	ed_Lushi_vs_Arbor_Acr	breed_growth_background	ENSGALG00010024199	SLC35B1	0.01030807	0.055066908	0.187191731	0.851510308	0.910627262	positive
PRJNA729271	ed_Lushi_vs_Arbor_Acr	breed_growth_background	ENSGALG00010024200	GCHFR	-0.262718417	0.405788507	-0.647426953	0.517355657	0.665637338	negative
PRJNA729271	ed_Lushi_vs_Arbor_Acr	breed_growth_background	ENSGALG00010024201	IREB2	0.100580129	0.037494265	2.682547063	0.007306387	0.033377271	positive
PRJNA729271	ed_Lushi_vs_Arbor_Acr	breed_growth_background	ENSGALG00010024203	SPOP	0.076074579	0.039323405	1.934587793	0.053040899	0.138203817	positive
PRJNA729271	ed_Lushi_vs_Arbor_Acr	breed_growth_background	ENSGALG00010024204	PTBP2	0.08130589	0.057954132	1.402935151	0.160636178	0.299534307	positive
PRJNA729271	ed_Lushi_vs_Arbor_Acr	breed_growth_background	ENSGALG00010024205	ANKRD13D	-0.260356785	0.09123342	-2.853743569	0.004320739	0.022849002	negative
PRJNA729271	ed_Lushi_vs_Arbor_Acr	breed_growth_background	ENSGALG00010024206	KRT80	-1.198032029	0.351400949	-3.409302197	0.000651293	0.006001183	negative
PRJNA729271	ed_Lushi_vs_Arbor_Acr	breed_growth_background	ENSGALG00010024207	KCTD18	-0.458957093	0.067797364	-6.769541829	1.29191E-11	4.92795E-09	negative
PRJNA729271	ed_Lushi_vs_Arbor_Acr	breed_growth_background	ENSGALG00010024208	SERPIND1	-0.800277096	0.30666092	-2.609648127	0.00906354	0.038914694	negative
PRJNA729271	ed_Lushi_vs_Arbor_Acr	breed_growth_background	ENSGALG00010024209	RWDD3	-0.204871302	0.077269667	-2.651380684	0.008016343	0.03567605	negative
PRJNA729271	ed_Lushi_vs_Arbor_Acr	breed_growth_background	ENSGALG00010024210	NXPH3	-0.648979591	0.698118016	-0.929613011	0.352571488	0.51637839	negative
PRJNA729271	ed_Lushi_vs_Arbor_Acr	breed_growth_background	ENSGALG00010024211	TRPM5	-0.974968907	0.385281265	-2.530538065	0.011388773	0.045830166	negative
PRJNA729271	ed_Lushi_vs_Arbor_Acr	breed_growth_background	ENSGALG00010024212	RIMS4	-0.450049667	0.108063045	-4.164695394	3.11769E-05	0.000685297	negative
PRJNA729271	ed_Lushi_vs_Arbor_Acr	breed_growth_background	ENSGALG00010024213	ENSGALG00010024213	-0.037057557	0.103471835	-0.35814149	0.720237431	0.82080679	negative
PRJNA729271	ed_Lushi_vs_Arbor_Acr	breed_growth_background	ENSGALG00010024214	CRABP1	-0.417297526	0.264687546	-1.576566529	0.114895299	0.237237516	negative
PRJNA729271	ed_Lushi_vs_Arbor_Acr	breed_growth_background	ENSGALG00010024215	ENSGALG00010024215	-0.112052397	0.085207063	-1.315059966	0.18848975	0.334413577	negative
PRJNA729271	ed_Lushi_vs_Arbor_Acr	breed_growth_background	ENSGALG00010024218	NGFR	-0.143900428	0.125867688	-1.143267425	0.252927575	0.409583868	negative
PRJNA729271	ed_Lushi_vs_Arbor_Acr	breed_growth_background	ENSGALG00010024220	SIAH1	-0.129180789	0.107079753	-1.206397906	0.227664103	0.380841451	negative
PRJNA729271	ed_Lushi_vs_Arbor_Acr	breed_growth_background	ENSGALG00010024222	ENSGALG00010024222	-0.077799907	0.16530519	-0.470644066	0.637894934	0.762454611	negative
PRJNA729271	ed_Lushi_vs_Arbor_Acr	breed_growth_background	ENSGALG00010024223	ENSGALG00010024223	-1.020479291	0.186839931	-5.461783702	4.71374E-08	4.70665E-06	negative
PRJNA729271	ed_Lushi_vs_Arbor_Acr	breed_growth_background	ENSGALG00010024225	BCDIN3D	0.018387017	0.097156533	0.189251472	0.849895728	0.909754411	positive
PRJNA729271	ed_Lushi_vs_Arbor_Acr	breed_growth_background	ENSGALG00010024226	POU2AF1	0.541762638	0.742495094	0.729651471	0.465603252	0.620687136	positive
PRJNA729271	ed_Lushi_vs_Arbor_Acr	breed_growth_background	ENSGALG00010024227	MUC6	0.582013756	0.220747173	2.636562673	0.008375073	0.036751005	positive
PRJNA729271	ed_Lushi_vs_Arbor_Acr	breed_growth_background	ENSGALG00010024230	LONP2	0.312278358	0.080554148	3.876626661	0.000105915	0.001657919	positive
PRJNA729271	ed_Lushi_vs_Arbor_Acr	breed_growth_background	ENSGALG00010024233	FAIM2	0.007526823	0.155560742	0.048385109	0.961409327	0.978276808	positive
PRJNA729271	ed_Lushi_vs_Arbor_Acr	breed_growth_background	ENSGALG00010024234	ENSGALG00010024234	-0.48827815	0.144526494	-3.378468093	0.000728909	0.006485902	negative
PRJNA729271	ed_Lushi_vs_Arbor_Acr	breed_growth_background	ENSGALG00010024238	ATF7	0.313443841	0.108330872	2.893393491	0.003811035	0.020940468	positive
PRJNA729271	ed_Lushi_vs_Arbor_Acr	breed_growth_background	ENSGALG00010024240	WDR61	-0.06285594	0.068222446	-0.921338125	0.356873922	0.520535159	negative
PRJNA729271	ed_Lushi_vs_Arbor_Acr	breed_growth_background	ENSGALG00010024242	KMT2A	0.009526408	0.067130772	0.141908216	0.887152504	0.93356713	positive
PRJNA729271	ed_Lushi_vs_Arbor_Acr	breed_growth_background	ENSGALG00010024243	MPP3	-0.470961525	0.110577877	-4.259093556	2.05258E-05	0.000490958	negative
PRJNA729271	ed_Lushi_vs_Arbor_Acr	breed_growth_background	ENSGALG00010024245	TARBP2	0.430859246	0.088952997	4.84367317	1.27461E-06	5.95981E-05	positive
PRJNA729271	ed_Lushi_vs_Arbor_Acr	breed_growth_background	ENSGALG00010024250	AP2A2	-0.077744827	0.028860098	-2.693851845	0.007063155	0.032605234	negative
PRJNA729271	ed_Lushi_vs_Arbor_Acr	breed_growth_background	ENSGALG00010024251	ENSGALG00010024251	-0.055701671	0.110468921	-0.50422934	0.614100225	0.743578879	negative
PRJNA729271	ed_Lushi_vs_Arbor_Acr	breed_growth_background	ENSGALG00010024254	ENSGALG00010024254	0.369635918	0.151166518	2.445223473	0.014476241	0.05400259	positive
PRJNA729271	ed_Lushi_vs_Arbor_Acr	breed_growth_background	ENSGALG00010024255	CFAP97D1	-1.012763049	0.365308012	-2.772353784	0.005565252	0.027458945	negative
PRJNA729271	ed_Lushi_vs_Arbor_Acr	breed_growth_background	ENSGALG00010024258	DNAJA4	0.263869658	0.08149362	3.237917999	0.001204054	0.009368097	positive

PRJNA729271	ed_Lushi_vs_Arbor_Acr	breed_growth_background	ENSGALG00010024259	ENSGALG00010024259	0.038329547	0.072025539	0.532166058	0.594610989	0.728808243	positive
PRJNA729271	ed_Lushi_vs_Arbor_Acr	breed_growth_background	ENSGALG00010024263	PHKB	0.133432105	0.070115203	1.903040983	0.057035198	0.145498978	positive
PRJNA729271	ed_Lushi_vs_Arbor_Acr	breed_growth_background	ENSGALG00010024264	AMER3	-0.011740199	0.163140852	-0.071963573	0.942630897	0.968910565	negative
PRJNA729271	ed_Lushi_vs_Arbor_Acr	breed_growth_background	ENSGALG00010024266	ENSGALG00010024266	-0.161088123	0.13331645	-1.208313922	0.226926535	0.380116692	negative
PRJNA729271	ed_Lushi_vs_Arbor_Acr	breed_growth_background	ENSGALG00010024267	MAP3K12	0.016841981	0.118239274	0.14243982	0.886732611	0.933419695	positive
PRJNA729271	ed_Lushi_vs_Arbor_Acr	breed_growth_background	ENSGALG00010024270	RAB18L	-0.867963728	0.413911643	-2.096978288	0.035995497	0.104121879	negative
PRJNA729271	ed_Lushi_vs_Arbor_Acr	breed_growth_background	ENSGALG00010024274	ACSBG1	-0.40100373	0.16968488	-2.363226059	0.018116615	0.063799887	negative
PRJNA729271	ed_Lushi_vs_Arbor_Acr	breed_growth_background	ENSGALG00010024277	ITGB3BP	-0.482911538	0.104892446	-4.603873372	4.14705E-06	0.00014916	negative
PRJNA729271	ed_Lushi_vs_Arbor_Acr	breed_growth_background	ENSGALG00010024280	ENSGALG00010024280	-0.195820257	0.06593896	-2.969720125	0.002980712	0.01762048	negative
PRJNA729271	ed_Lushi_vs_Arbor_Acr	breed_growth_background	ENSGALG00010024282	HIC2	-0.08481856	0.081550953	-1.040068284	0.298308178	0.460536483	negative
PRJNA729271	ed_Lushi_vs_Arbor_Acr	breed_growth_background	ENSGALG00010024283	UBE4A	0.15307057	0.059125269	2.588919631	0.009627756	0.04074804	positive
PRJNA729271	ed_Lushi_vs_Arbor_Acr	breed_growth_background	ENSGALG00010024285	CCND2	-0.094659932	0.052325891	-1.809045754	0.070443896	0.168551381	negative
PRJNA729271	ed_Lushi_vs_Arbor_Acr	breed_growth_background	ENSGALG00010024286	ADA	-0.269320638	0.096037301	-2.804333673	0.005042069	0.025743148	negative
PRJNA729271	ed_Lushi_vs_Arbor_Acr	breed_growth_background	ENSGALG00010024288	CHID1	-0.127348076	0.073118772	-1.741660473	0.081567873	0.186535208	negative
PRJNA729271	ed_Lushi_vs_Arbor_Acr	breed_growth_background	ENSGALG00010024290	SOST	-0.168391693	0.261398606	-0.64419507	0.519448947	0.66731846	negative
PRJNA729271	ed_Lushi_vs_Arbor_Acr	breed_growth_background	ENSGALG00010024291	ENSGALG00010024291	-0.181347058	0.221733904	-0.817858953	0.413437731	0.573856163	negative
PRJNA729271	ed_Lushi_vs_Arbor_Acr	breed_growth_background	ENSGALG00010024300	MEOX1	-0.575156816	0.151290746	-3.801665536	0.000143727	0.002072953	negative
PRJNA729271	ed_Lushi_vs_Arbor_Acr	breed_growth_background	ENSGALG00010024304	PKIG	-0.303450632	0.071313115	-4.255186925	2.08874E-05	0.000496334	negative
PRJNA729271	ed_Lushi_vs_Arbor_Acr	breed_growth_background	ENSGALG00010024305	ENSGALG00010024305	-0.207289209	0.304716204	-0.680269727	0.496333689	0.647848431	negative
PRJNA729271	ed_Lushi_vs_Arbor_Acr	breed_growth_background	ENSGALG00010024309	ABCB11	-0.636360403	0.21309836	-2.986228532	0.002824415	0.016973422	negative
PRJNA729271	ed_Lushi_vs_Arbor_Acr	breed_growth_background	ENSGALG00010024311	KLHL6	-0.280418177	0.06600291	-4.24857296	2.15137E-05	0.000503781	negative
PRJNA729271	ed_Lushi_vs_Arbor_Acr	breed_growth_background	ENSGALG00010024313	SERINC3	0.015266038	0.056573591	0.269843889	0.787280356	0.867849953	positive
PRJNA729271	ed_Lushi_vs_Arbor_Acr	breed_growth_background	ENSGALG00010024314	ITFG1	0.283107035	0.078131688	3.623459879	0.000290688	0.003400748	positive
PRJNA729271	ed_Lushi_vs_Arbor_Acr	breed_growth_background	ENSGALG00010024321	UBE2L3	0.044137075	0.044122259	1.000335804	0.317148025	0.480562474	positive
PRJNA729271	ed_Lushi_vs_Arbor_Acr	breed_growth_background	ENSGALG00010024322	IDH3A	0.097821916	0.084966199	1.151303906	0.249607231	0.405624366	positive
PRJNA729271	ed_Lushi_vs_Arbor_Acr	breed_growth_background	ENSGALG00010024323	TTPAL	0.15996108	0.056310802	2.840681947	0.004501718	0.023548325	positive
PRJNA729271	ed_Lushi_vs_Arbor_Acr	breed_growth_background	ENSGALG00010024325	PCBP2	0.28853169	0.0911113	3.166804673	0.001541238	0.011079355	positive
PRJNA729271	ed_Lushi_vs_Arbor_Acr	breed_growth_background	ENSGALG00010024326	KLHL24	0.144441786	0.095321402	1.515313278	0.129693087	0.257309239	positive
PRJNA729271	ed_Lushi_vs_Arbor_Acr	breed_growth_background	ENSGALG00010024327	PPP1R1B	0.04135844	0.212420202	0.194701068	0.845626976	0.906341542	positive
PRJNA729271	ed_Lushi_vs_Arbor_Acr	breed_growth_background	ENSGALG00010024328	YDJC	0.038967728	0.084259671	0.462471869	0.643742971	0.767104108	positive
PRJNA729271	ed_Lushi_vs_Arbor_Acr	breed_growth_background	ENSGALG00010024330	SDF2L1	-0.031199704	0.055998418	-0.557153314	0.577422684	0.714751649	negative
PRJNA729271	ed_Lushi_vs_Arbor_Acr	breed_growth_background	ENSGALG00010024332	EFCAB7	-0.679387636	0.09018122	-7.533582222	4.93669E-14	3.57787E-11	negative
PRJNA729271	ed_Lushi_vs_Arbor_Acr	breed_growth_background	ENSGALG00010024333	ETV4	-0.691828383	0.133405578	-5.185902959	2.14971E-07	1.51262E-05	negative
PRJNA729271	ed_Lushi_vs_Arbor_Acr	breed_growth_background	ENSGALG00010024334	USP28	0.099645802	0.065324534	1.525396302	0.127160281	0.254092311	positive
PRJNA729271	ed_Lushi_vs_Arbor_Acr	breed_growth_background	ENSGALG00010024336	TSPAN4	-0.1872712	0.062703617	-2.986609199	0.002820901	0.016969989	negative
PRJNA729271	ed_Lushi_vs_Arbor_Acr	breed_growth_background	ENSGALG00010024338	NDUFA9	0.023674824	0.061090784	0.387535114	0.698360095	0.805308638	positive
PRJNA729271	ed_Lushi_vs_Arbor_Acr	breed_growth_background	ENSGALG00010024339	TOP3B	0.150180122	0.04735453	3.171399256	0.001517065	0.010983943	positive
PRJNA729271	ed_Lushi_vs_Arbor_Acr	breed_growth_background	ENSGALG00010024340	NETO2	-0.235562165	0.20329272	-1.158733892	0.246564672	0.402608417	negative
PRJNA729271	ed_Lushi_vs_Arbor_Acr	breed_growth_background	ENSGALG00010024342	HNF4A	-1.648928059	0.735887914	-2.240732627	0.025043399	0.080115664	negative
PRJNA729271	ed_Lushi_vs_Arbor_Acr	breed_growth_background	ENSGALG00010024343	POLR2L	-0.109761175	0.067820546	-1.618405941	0.105575143	0.22366438	negative
PRJNA729271	ed_Lushi_vs_Arbor_Acr	breed_growth_background	ENSGALG00010024344	YEATS2	0.054161232	0.071373982	0.758837198	0.447949951	0.604621896	positive
PRJNA729271	ed_Lushi_vs_Arbor_Acr	breed_growth_background	ENSGALG00010024345	STARD3	0.035807617	0.049584003	0.722160684	0.470195692	0.624586688	positive
PRJNA729271	ed_Lushi_vs_Arbor_Acr	breed_growth_background	ENSGALG00010024346	PPM1F	0.001798896	0.04924332	0.036530771	0.970859143	0.983017828	positive
PRJNA729271	ed_Lushi_vs_Arbor_Acr	breed_growth_background	ENSGALG00010024347	OTOG	0.219959195	0.157069799	1.40039139	0.161396147	0.30046714	positive
PRJNA729271	ed_Lushi_vs_Arbor_Acr	breed_growth_background	ENSGALG00010024349	R3HDML	-0.074800626	0.291666704	-0.256459257	0.797596237	0.874586387	negative
PRJNA729271	ed_Lushi_vs_Arbor_Acr	breed_growth_background	ENSGALG00010024350	PGM1	0.072183772	0.072373496	0.997378544	0.318580802	0.48182687	positive
PRJNA729271	ed_Lushi_vs_Arbor_Acr	breed_growth_background	ENSGALG00010024351	SP1	0.072314336	0.082087858	0.880938274	0.37835123	0.540448994	positive
PRJNA729271	ed_Lushi_vs_Arbor_Acr	breed_growth_background	ENSGALG00010024354	C2CD2L	0.049290072	0.042979673	1.146822861	0.251454839	0.4076088	positive
PRJNA729271	ed_Lushi_vs_Arbor_Acr	breed_growth_background	ENSGALG00010024356	PARL	-0.22367661	0.06317123	-3.540798744	0.000398918	0.004233025	negative
PRJNA729271	ed_Lushi_vs_Arbor_Acr	breed_growth_background	ENSGALG00010024358	MAPK3	-0.058754478	0.033165353	-1.771561983	0.076467295	0.178476796	negative
PRJNA729271	ed_Lushi_vs_Arbor_Acr	breed_growth_background	ENSGALG00010024360	FTL	-0.439274518	0.132631002	-3.312004811	0.0009263	0.007698803	negative
PRJNA729271	ed_Lushi_vs_Arbor_Acr	breed_growth_background	ENSGALG00010024361	H2AX	-0.197547122	0.117187539	-1.685734877	0.091846876	0.202667144	negative
PRJNA729271	ed_Lushi_vs_Arbor_Acr	breed_growth_background	ENSGALG00010024362	DHX8	0.052498705	0.03164419	1.659031395	0.097109466	0.210435298	positive
PRJNA729271	ed_Lushi_vs_Arbor_Acr	breed_growth_background	ENSGALG00010024363	EDEM2	-0.105140528	0.062580895	-1.680073888	0.092942941	0.204432158	negative
PRJNA729271	ed_Lushi_vs_Arbor_Acr	breed_growth_background	ENSGALG00010024365	ENSGALG00010024365	-0.044021912	0.059866675	-0.735332494	0.462137023	0.617182224	negative
PRJNA729271	ed_Lushi_vs_Arbor_Acr	breed_growth_background	ENSGALG00010024366	PARD6A	0.63103079	0.249752244	2.526627107	0.011516371	0.046228134	positive
PRJNA729271	ed_Lushi_vs_Arbor_Acr	breed_growth_background	ENSGALG00010024367	ENSGALG00010024367	-0.66678517	0.127999036	-5.209298354	1.89556E-07	1.37783E-05	negative
PRJNA729271	ed_Lushi_vs_Arbor_Acr	breed_growth_background	ENSGALG00010024368	DPAGT1	-0.052266141	0.042934604	-1.217343045	0.223473723	0.37611903	negative
PRJNA729271	ed_Lushi_vs_Arbor_Acr	breed_growth_background	ENSGALG00010024369	MYOD1	0.347217833	0.147252392	2.357977543	0.018374805	0.0644118	positive
PRJNA729271	ed_Lushi_vs_Arbor_Acr	breed_growth_background	ENSGALG00010024370	HERPUD1	0.05156956	0.049956905	1.032280922	0.301940536	0.464412995	positive
PRJNA729271	ed_Lushi_vs_Arbor_Acr	breed_growth_background	ENSGALG00010024371	YPEL1	0.012974883	0.056043001	0.231516562	0.81691352	0.887046157	positive
PRJNA729271	ed_Lushi_vs_Arbor_Acr	breed_growth_background	ENSGALG00010024373	TCAP	-0.036025766	0.157226953	-0.229132255	0.818766125	0.888523993	negative
PRJNA729271	ed_Lushi_vs_Arbor_Acr	breed_growth_background	ENSGALG00010024374	ENSGALG00010024374	-0.730323685	0.31215608	-2.339610639	0.019303853	0.066668894	negative
PRJNA729271	ed_Lushi_vs_Arbor_Acr	breed_growth_background	ENSGALG00010024377	CIB2	0.09757305	0.092509155	1.054739399	0.291544527	0.45298938	positive
PRJNA729271	ed_Lushi_vs_Arbor_Acr	breed_growth_background	ENSGALG00010024378	ZDHHC1	0.113730305	0.098652122	1.152841947	0.248975263	0.405129821	positive
PRJNA729271	ed_Lushi_vs_Arbor_Acr	breed_growth_background	ENSGALG00010024382	ENSGALG00010024382	-0.087909143	0.231511066	-0.379718967	0.704154039	0.808727427	negative
PRJNA729271	ed_Lushi_vs_Arbor_Acr	breed_growth_background	ENSGALG00010024383	DUSP28	-0.011808764	0.10070873	-0.117256608	0.90665671	0.946488225	negative
PRJNA729271	ed_Lushi_vs_Arbor_Acr	breed_growth_background	ENSGALG00010024386	TPPP3	-0.619352828	0.143743332	-4.308741277	1.64186E-05	0.000413892	negative
PRJNA729271	ed_Lushi_vs_Arbor_Acr	breed_growth_background	ENSGALG00010024387	PPIL2	-0.071125729	0.035584438	-1.998787503	0.045631351	0.124095014	negative
PRJNA729271	ed_Lushi_vs_Arbor_Acr	breed_growth_background	ENSGALG00010024389	AAAS	0.035116378	0.057757941	0.607992216	0.543192637	0.687677997	positive
PRJNA729271	ed_Lushi_vs_Arbor_Acr	breed_growth_background	ENSGALG00010024391	TBC1D2B	-0.173040025	0.05960428	-2.903147652	0.003694324	0.020454251	negative
PRJNA729271	ed_Lushi_vs_Arbor_Acr	breed_growth_background	ENSGALG00010024392	CHMP4B	-0.062623102	0.045635907	-1.372233107	0.169990882	0.312256728	negative
PRJNA729271	ed_Lushi_vs_Arbor_Acr	breed_growth_background	ENSGALG00010024393	OGFOD1	-0.011318775	0.057820396	-0.195757482	0.84479999	0.905734415	negative
PRJNA729271	ed_Lushi_vs_Arbor_Acr	breed_growth_background	ENSGALG00010024396	ENSGALG00010024396	0.474715962	0.180501147	2.629988616	0.008538773	0.037146343	positive
PRJNA729271	ed_Lushi_vs_Arbor_Acr	breed_growth_background	ENSGALG00010024398	PHB	0.08395229	0.047369996	1.772267205	0.076350212	0.178298104	positive
PRJNA729271	ed_Lushi_vs_Arbor_Acr	breed_growth_background	ENSGALG00010024399	ROR1	0.478745323	0.090430346	5.294078194	1.19618E-07	9.63259E-06	positive
PRJNA729271	ed_Lushi_vs_Arbor_Acr	breed_growth_background	ENSGALG00010024400	KCNA1	0.056954357	0.230050027	0.247573791	0.804464191	0.879456102	positive
PRJNA729271	ed_Lushi_vs_Arbor_Acr	breed_growth_background	ENSGALG00010024401	KCNC1	0.074375261	0.102659838	0.724482546	0.468769539	0.623549093	positive
PRJNA729271	ed_Lushi_vs_Arbor_Acr	breed_growth_background	ENSGALG00010024402	GPC1	0.061851879	0.064255488	0.962592942	0.335751841	0.499971537	positive
PRJNA729271	ed_Lushi_vs_Arbor_Acr	breed_growth_background	ENSGALG00010024403	HMBS	-0.088292481	0.042236792	-2.090416326	0.036580418	0.105393193	negative
PRJNA729271	ed_Lushi_vs_Arbor_Acr	breed_growth_background	ENSGALG00010024406	NUDT21	0.034229807	0.036256435	0.944102948	0.345117043	0.508950202	positive
PRJNA729271	ed_Lushi_vs_Arbor_Acr	breed_growth_background	ENSGALG00010024407	HIRA	0.017637405	0.044144643	0.399536699	0.689497788	0.799158039	positive
PRJNA729271	ed_Lushi_vs_Arbor_Acr	breed_growth_background	ENSGALG00010024408	ENSGALG00010024408	-0.257540668	0.714157497	-0.360621668	0.718382289	0.819852868	negative
PRJNA729271	ed_Lushi_vs_Arbor_Acr	breed_growth_background	ENSGALG00010024410	MYG1	-0.005823457	0.05850714	-0.099534121	0.920714198	0.955930972	negative
PRJNA729271	ed_Lushi_vs_Arbor_Acr	breed_growth_background	ENSGALG00010024412	PIGU	-0.037559429	0.055566464	-0.675937001	0.499080644	0.650321282	negative
PRJNA729271	ed_Lushi_vs_Arbor_Acr	breed_growth_background	ENSGALG00010024413	AMFR	0.146808101	0.049350894	2.974780967	0.002931978	0.017453398	positive
PRJNA729271	ed_Lushi_vs_Arbor_Acr	breed_growth_background	ENSGALG00010024415	SIK2	0.079067106	0.032169142	2.457855597	0.013976936	0.052860874	positive
PRJNA729271	ed_Lushi_vs_Arbor_Acr	breed_growth_background	ENSGALG00010024416	VDR	-0.066529228	0.171342068	-0.388283092	0.697806548	0.805308638	negative
PRJNA729271	ed_Lushi_vs_Arbor_Acr	breed_growth_background	ENSGALG00010024419	PGAP3	0.003720728	0.068212126	0.054546435	0.956499814	0.97569799	positive
PRJNA729271	ed_Lushi_vs_Arbor_Acr	breed_growth_background	ENSGALG00010024420	TMEM106C	-0.135477121	0.088155643	-1.53679465	0.124343604	0.250084715	negative
PRJNA729271	ed_Lushi_vs_Arbor_Acr	breed_growth_background	ENSGALG00010024421	DAPK2	-0.380058396	0.139960669	-2.715465709	0.006618263	0.031147835	negative
PRJNA729271	ed_Lushi_vs_Arbor_Acr	breed_growth_background	ENSGALG00010024423	SERGEF	-0.170059102	0.064425541	-2.639622431	0.008299844	0.036511756	negative
PRJNA729271	ed_Lushi_vs_Arbor_Acr	breed_growth_background	ENSGALG00010024424	SAMHD1	-0.176491974	0.096475868	-1.829389856	0.067341226	0.163786769	negative
PRJNA729271	ed_Lushi_vs_Arbor_Acr	breed_growth_background	ENSGALG00010024425	MRPL40	0.017636466	0.047439543	0.371767194	0.710066191	0.813025405	positive
PRJNA729271	ed_Lushi_vs_Arbor_Acr	breed_growth_background	ENSGALG00010024426	PHAF1	0.174248373	0.056056402	3.108447347	0.001880732	0.012714156	positive
PRJNA729271	ed_Lushi_vs_Arbor_Acr	breed_growth_background	ENSGALG00010024427	C22orf39	-0.191784772	0.072998228	-2.627252416	0.008607746	0.037367257	negative
PRJNA729271	ed_Lushi_vs_Arbor_Acr	breed_growth_background	ENSGALG00010024428	SAAL1	-0.088947641	0.035415882	-2.511518429	0.012021301	0.047569958	negative
PRJNA729271	ed_Lushi_vs_Arbor_Acr	breed_growth_background	ENSGALG00010024430	ZNF652	-0.021843327	0.035143473	-0.621547182	0.534239661	0.679638747	negative
PRJNA729271	ed_Lushi_vs_Arbor_Acr	breed_growth_background	ENSGALG00010024431	FEZ1	-0.318518232	0.086319397	-3.689996046	0.000224258	0.002819265	negative
PRJNA729271	ed_Lushi_vs_Arbor_Acr	breed_growth_background	ENSGALG00010024433	ENSGALG00010024433	-0.313209989	0.090632674	-3.455817574	0.000548626	0.005305097	negative
PRJNA729271	ed_Lushi_vs_Arbor_Acr	breed_growth_background	ENSGALG00010024434	B3GNT9	-0.186731554	0.085640098	-2.180422011	0.029226194	0.089186038	negative
PRJNA729271	ed_Lushi_vs_Arbor_Acr	breed_growth_background	ENSGALG00010024437	HERC1	0.076740784	0.04367796	1.75696814	0.07892324	0.18230954	positive
PRJNA729271	ed_Lushi_vs_Arbor_Acr	breed_growth_background	ENSGALG00010024438	RBL1	-0.291309795	0.066152474	-4.403611503	1.06464E-05	0.000304978	negative
PRJNA729271	ed_Lushi_vs_Arbor_Acr	breed_growth_background	ENSGALG00010024439	UFD1	0.077854965	0.060129059	1.29479766	0.195390039	0.343335995	positive
PRJNA729271	ed_Lushi_vs_Arbor_Acr	breed_growth_background	ENSGALG00010024440	PKNOX2	0.194429818	0.109286044	1.779091005	0.075224843	0.176618218	positive
PRJNA729271	ed_Lushi_vs_Arbor_Acr	breed_growth_background	ENSGALG00010024441	SAA	0.146749536	0.411062481	0.357000561	0.721091386	0.821263427	positive
PRJNA729271	ed_Lushi_vs_Arbor_Acr	breed_growth_background	ENSGALG00010024446	GRM4	-0.909023234	0.302206587	-3.007953078	0.002630137	0.016117144	negative
PRJNA729271	ed_Lushi_vs_Arbor_Acr	breed_growth_background	ENSGALG00010024449	CACHD1	0.094191612	0.045558043	2.067507848	0.038686325	0.109801897	positive
PRJNA729271	ed_Lushi_vs_Arbor_Acr	breed_growth_background	ENSGALG00010024451	PHOSPHO1	0.336052742	0.144816421	2.320543068	0.020311518	0.068965906	positive
PRJNA729271	ed_Lushi_vs_Arbor_Acr	breed_growth_background	ENSGALG00010024452	SLC16A1	0.102202869	0.135191959	0.755983339	0.449659185	0.605857026	positive
PRJNA729271	ed_Lushi_vs_Arbor_Acr	breed_growth_background	ENSGALG00010024453	HTR3A	-0.210902767	0.274130646	-0.769351292	0.441684796	0.599066259	negative
PRJNA729271	ed_Lushi_vs_Arbor_Acr	breed_growth_background	ENSGALG00010024454	HPS5	0.019556611	0.064801772	0.301791306	0.762811159	0.850927178	positive
PRJNA729271	ed_Lushi_vs_Arbor_Acr	breed_growth_background	ENSGALG00010024457	CHD6	0.024630587	0.054169492	0.454694819	0.649328808	0.771097909	positive
PRJNA729271	ed_Lushi_vs_Arbor_Acr	breed_growth_background	ENSGALG00010024459	AHCYL1	-0.126315953	0.050834755	-2.484834521	0.012961167	0.050205011	negative
PRJNA729271	ed_Lushi_vs_Arbor_Acr	breed_growth_background	ENSGALG00010024460	IMP4	0.203097689	0.083729547	2.425639407	0.015281445	0.056448662	positive
PRJNA729271	ed_Lushi_vs_Arbor_Acr	breed_growth_background	ENSGALG00010024462	ZBTB32	0.000614596	0.224895241	0.002732812	0.997819534	0.998913967	positive
PRJNA729271	ed_Lushi_vs_Arbor_Acr	breed_growth_background	ENSGALG00010024463	FBXL22	-0.340659739	0.12248093	-2.781328816	0.005413687	0.026966116	negative
PRJNA729271	ed_Lushi_vs_Arbor_Acr	breed_growth_background	ENSGALG00010024465	SENP1	0.099585673	0.087352667	1.140041584	0.254268977	0.410929738	positive
PRJNA729271	ed_Lushi_vs_Arbor_Acr	breed_growth_background	ENSGALG00010024466	ADGRG3	-0.103404027	0.300810751	-0.343751102	0.731033477	0.827579682	negative
PRJNA729271	ed_Lushi_vs_Arbor_Acr	breed_growth_background	ENSGALG00010024467	ENSGALG00010024467	-0.27822906	0.15606172	-1.782814254	0.074616545	0.175664579	negative
PRJNA729271	ed_Lushi_vs_Arbor_Acr	breed_growth_background	ENSGALG00010024469	GTF2H1	0.18405975	0.068596637	2.683218256	0.007291739	0.033344123	positive
PRJNA729271	ed_Lushi_vs_Arbor_Acr	breed_growth_background	ENSGALG00010024471	CCDC61	-0.030399957	0.063555685	-0.47832003	0.632422442	0.757789574	negative
PRJNA729271	ed_Lushi_vs_Arbor_Acr	breed_growth_background	ENSGALG00010024472	LDHA	0.19870216	0.107551299	1.847510557	0.064673179	0.158927103	positive
PRJNA729271	ed_Lushi_vs_Arbor_Acr	breed_growth_background	ENSGALG00010024473	RAVER2	-0.076540299	0.033239098	-2.302718901	0.021294664	0.071380652	negative
PRJNA729271	ed_Lushi_vs_Arbor_Acr	breed_growth_background	ENSGALG00010024474	STRIP1	0.100019044	0.055936009	1.788097618	0.07376026	0.174271388	positive
PRJNA729271	ed_Lushi_vs_Arbor_Acr	breed_growth_background	ENSGALG00010024475	ADGRG1	-0.107176322	0.084432928	-1.2693664	0.204310414	0.353732948	negative
PRJNA729271	ed_Lushi_vs_Arbor_Acr	breed_growth_background	ENSGALG00010024476	KCNB1	-0.001665436	0.09783374	-0.017023127	0.986418166	0.991961379	negative
PRJNA729271	ed_Lushi_vs_Arbor_Acr	breed_growth_background	ENSGALG00010024479	ERBB2	0.139390003	0.068796882	2.026109321	0.042753583	0.118198205	positive
PRJNA729271	ed_Lushi_vs_Arbor_Acr	breed_growth_background	ENSGALG00010024480	CDC45	-0.320900093	0.117543429	-2.730055582	0.006332365	0.030142278	negative
PRJNA729271	ed_Lushi_vs_Arbor_Acr	breed_growth_background	ENSGALG00010024482	USP3	0.019240472	0.032270663	0.596221776	0.55102708	0.694023234	positive
PRJNA729271	ed_Lushi_vs_Arbor_Acr	breed_growth_background	ENSGALG00010024486	GPR37L1	0.505201319	0.17380219	2.90676038	0.003651928	0.020258209	positive

PRJNA729271	ed_Lushi_vs_Arbor_Acr	breed_growth_background	ENSGALG00010024488	ADGRG5	-0.01497912	0.239882337	-0.062443616	0.950209562	0.972527553	negative
PRJNA729271	ed_Lushi_vs_Arbor_Acr	breed_growth_background	ENSGALG00010024490	TGM3	-0.298027856	0.429083828	-0.694567907	0.487326131	0.639195222	negative
PRJNA729271	ed_Lushi_vs_Arbor_Acr	breed_growth_background	ENSGALG00010024492	NTF3	-0.462849689	0.138808412	-3.334449855	0.000854683	0.00728743	negative
PRJNA729271	ed_Lushi_vs_Arbor_Acr	breed_growth_background	ENSGALG00010024493	JAK1	0.045758777	0.032224636	1.419993579	0.155609551	0.292641867	positive
PRJNA729271	ed_Lushi_vs_Arbor_Acr	breed_growth_background	ENSGALG00010024494	DDAH1	-0.089118861	0.101043171	-0.881987965	0.377783316	0.54008967	negative
PRJNA729271	ed_Lushi_vs_Arbor_Acr	breed_growth_background	ENSGALG00010024495	CLDN5	-0.263194219	0.090934052	-2.894341694	0.003799544	0.020901099	negative
PRJNA729271	ed_Lushi_vs_Arbor_Acr	breed_growth_background	ENSGALG00010024496	UEVLD	-0.166573977	0.111251813	-1.497269771	0.134323077	0.264126891	negative
PRJNA729271	ed_Lushi_vs_Arbor_Acr	breed_growth_background	ENSGALG00010024497	ABI3	0.075075887	0.097612473	0.76912187	0.441820967	0.599104354	positive
PRJNA729271	ed_Lushi_vs_Arbor_Acr	breed_growth_background	ENSGALG00010024498	CCDC102A	-0.083858553	0.065340396	-1.283410552	0.199348279	0.347972244	negative
PRJNA729271	ed_Lushi_vs_Arbor_Acr	breed_growth_background	ENSGALG00010024499	CA12	1.648858614	0.367161555	4.490825886	7.09475E-06	0.000224538	positive
PRJNA729271	ed_Lushi_vs_Arbor_Acr	breed_growth_background	ENSGALG00010024501	ARL8A	-0.00168487	0.048227141	-0.03493614	0.972130663	0.983408337	negative
PRJNA729271	ed_Lushi_vs_Arbor_Acr	breed_growth_background	ENSGALG00010024502	VPS8	0.131874795	0.039405901	3.346574814	0.000818166	0.007065766	positive
PRJNA729271	ed_Lushi_vs_Arbor_Acr	breed_growth_background	ENSGALG00010024504	CASC3	0.33819262	0.099947047	3.383717981	0.000715114	0.006426274	positive
PRJNA729271	ed_Lushi_vs_Arbor_Acr	breed_growth_background	ENSGALG00010024505	DNASE2B	-0.336779976	0.112594587	-2.991084948	0.002779882	0.016796324	negative
PRJNA729271	ed_Lushi_vs_Arbor_Acr	breed_growth_background	ENSGALG00010024506	RAB8B	-0.105234598	0.043661016	-2.410264531	0.015940958	0.05824658	negative
PRJNA729271	ed_Lushi_vs_Arbor_Acr	breed_growth_background	ENSGALG00010024508	RPS27L	-0.079854594	0.10105249	-0.790228857	0.429394126	0.588076607	negative
PRJNA729271	ed_Lushi_vs_Arbor_Acr	breed_growth_background	ENSGALG00010024509	SEPTIN5	-0.113344654	0.057603958	-1.967653919	0.049107871	0.130993485	negative
PRJNA729271	ed_Lushi_vs_Arbor_Acr	breed_growth_background	ENSGALG00010024510	CNN3	-0.208598218	0.05414567	-3.852537371	0.0001169	0.001800709	negative
PRJNA729271	ed_Lushi_vs_Arbor_Acr	breed_growth_background	ENSGALG00010024511	ENSGALG00010024511	-0.198045224	0.091888221	-2.155284134	0.031139607	0.093586688	negative
PRJNA729271	ed_Lushi_vs_Arbor_Acr	breed_growth_background	ENSGALG00010024512	CCNDBP1	-0.172140484	0.061739554	-2.788171806	0.005300642	0.026576552	negative
PRJNA729271	ed_Lushi_vs_Arbor_Acr	breed_growth_background	ENSGALG00010024513	SPTY2D1	0.27761878	0.074571997	3.722828809	0.000197003	0.002565642	positive
PRJNA729271	ed_Lushi_vs_Arbor_Acr	breed_growth_background	ENSGALG00010024514	FAF1	-0.074894289	0.036656598	-2.043132551	0.041039318	0.114529249	negative
PRJNA729271	ed_Lushi_vs_Arbor_Acr	breed_growth_background	ENSGALG00010024515	C3orf70	-0.346373514	0.08475043	-4.086982352	4.3702E-05	0.00087737	negative
PRJNA729271	ed_Lushi_vs_Arbor_Acr	breed_growth_background	ENSGALG00010024516	EHHADH	-0.286820826	0.129903223	-2.207957736	0.02724722	0.08476436	negative
PRJNA729271	ed_Lushi_vs_Arbor_Acr	breed_growth_background	ENSGALG00010024517	LACTB	0.015940773	0.053921331	0.29563018	0.767512523	0.854724495	positive
PRJNA729271	ed_Lushi_vs_Arbor_Acr	breed_growth_background	ENSGALG00010024518	ENSGALG00010024518	-1.151205759	0.601028876	-1.915391763	0.055442561	0.142722038	negative
PRJNA729271	ed_Lushi_vs_Arbor_Acr	breed_growth_background	ENSGALG00010024519	MIEN1	-0.075977576	0.074881251	-1.014640856	0.310277057	0.473218217	negative
PRJNA729271	ed_Lushi_vs_Arbor_Acr	breed_growth_background	ENSGALG00010024520	MAP3K13	-0.154314557	0.076770867	-2.010066609	0.04442414	0.121469898	negative
PRJNA729271	ed_Lushi_vs_Arbor_Acr	breed_growth_background	ENSGALG00010024521	HEPACAM	0.620012782	0.270090298	2.295575912	0.021700141	0.072325488	positive
PRJNA729271	ed_Lushi_vs_Arbor_Acr	breed_growth_background	ENSGALG00010024522	DNTTIP2	0.126522391	0.040586189	3.117375466	0.00182469	0.01246413	positive
PRJNA729271	ed_Lushi_vs_Arbor_Acr	breed_growth_background	ENSGALG00010024523	TOX3	0.048993212	0.161512203	0.303340622	0.761630292	0.850133304	positive
PRJNA729271	ed_Lushi_vs_Arbor_Acr	breed_growth_background	ENSGALG00010024524	TMEM41A	0.21155827	0.071768592	2.947783482	0.003200611	0.018572003	positive
PRJNA729271	ed_Lushi_vs_Arbor_Acr	breed_growth_background	ENSGALG00010024525	GP1BB	-0.05559335	0.103693808	-0.536129896	0.59186878	0.726606467	negative
PRJNA729271	ed_Lushi_vs_Arbor_Acr	breed_growth_background	ENSGALG00010024526	TGM6	-0.398087726	0.753808563	-0.528101889	0.59742862	0.730959358	negative
PRJNA729271	ed_Lushi_vs_Arbor_Acr	breed_growth_background	ENSGALG00010024527	ATP6V0B	0.112251323	0.053414097	2.101529921	0.035594474	0.103291673	positive
PRJNA729271	ed_Lushi_vs_Arbor_Acr	breed_growth_background	ENSGALG00010024528	TBX1	0.088022998	0.12133195	0.725472543	0.468162183	0.62302707	positive
PRJNA729271	ed_Lushi_vs_Arbor_Acr	breed_growth_background	ENSGALG00010024529	GRB7	-0.034633893	0.614374564	-0.056372602	0.955044983	0.974885706	negative
PRJNA729271	ed_Lushi_vs_Arbor_Acr	breed_growth_background	ENSGALG00010024530	ENSGALG00010024530	0.045779939	0.298971409	0.153124805	0.878299855	0.927844646	positive
PRJNA729271	ed_Lushi_vs_Arbor_Acr	breed_growth_background	ENSGALG00010024531	CEP63	0.011258189	0.071871012	0.156644364	0.875525137	0.926486898	positive
PRJNA729271	ed_Lushi_vs_Arbor_Acr	breed_growth_background	ENSGALG00010024532	TMEM86A	0.095737121	0.115077824	0.831933714	0.405446361	0.566288784	positive
PRJNA729271	ed_Lushi_vs_Arbor_Acr	breed_growth_background	ENSGALG00010024533	CDKN2C	-0.450109926	0.116291909	-3.870517981	0.000108604	0.00169271	negative
PRJNA729271	ed_Lushi_vs_Arbor_Acr	breed_growth_background	ENSGALG00010024534	VWF	0.012730449	0.06695091	0.190146016	0.849194712	0.909157054	positive
PRJNA729271	ed_Lushi_vs_Arbor_Acr	breed_growth_background	ENSGALG00010024535	ENSGALG00010024535	-0.124533189	0.079914816	-1.558324171	0.119156422	0.243195204	negative
PRJNA729271	ed_Lushi_vs_Arbor_Acr	breed_growth_background	ENSGALG00010024536	URI1	-0.023428199	0.0335134	-0.699069609	0.48450853	0.636583407	negative
PRJNA729271	ed_Lushi_vs_Arbor_Acr	breed_growth_background	ENSGALG00010024537	EPB42	-0.067110747	0.097931318	-0.685283813	0.493164844	0.644873651	negative
PRJNA729271	ed_Lushi_vs_Arbor_Acr	breed_growth_background	ENSGALG00010024540	PTPN5	-0.250226226	0.167939399	-1.489979293	0.136229681	0.266736353	negative
PRJNA729271	ed_Lushi_vs_Arbor_Acr	breed_growth_background	ENSGALG00010024541	WIPF2	0.274456895	0.108832342	2.521832123	0.011674541	0.046656319	positive
PRJNA729271	ed_Lushi_vs_Arbor_Acr	breed_growth_background	ENSGALG00010024542	ZNFX1	0.119722953	0.227258127	0.526814836	0.598322179	0.731420532	positive
PRJNA729271	ed_Lushi_vs_Arbor_Acr	breed_growth_background	ENSGALG00010024544	ZNF536	-0.009046479	0.133738223	-0.067643186	0.946069677	0.970470493	negative
PRJNA729271	ed_Lushi_vs_Arbor_Acr	breed_growth_background	ENSGALG00010024545	PHACTR3	0.13100141	0.164652882	0.795621722	0.426251937	0.585419919	positive
PRJNA729271	ed_Lushi_vs_Arbor_Acr	breed_growth_background	ENSGALG00010024546	KY	0.696935316	0.210674077	3.308120895	0.000939243	0.007779612	positive
PRJNA729271	ed_Lushi_vs_Arbor_Acr	breed_growth_background	ENSGALG00010024547	PFKM	0.218908108	0.104251392	2.099809936	0.035745564	0.103564251	positive
PRJNA729271	ed_Lushi_vs_Arbor_Acr	breed_growth_background	ENSGALG00010024548	CDC6	-0.324215664	0.16485767	-1.966639855	0.049224745	0.131113581	negative
PRJNA729271	ed_Lushi_vs_Arbor_Acr	breed_growth_background	ENSGALG00010024549	GNB1L	0.101579578	0.072829215	1.394764156	0.163086971	0.302759432	positive
PRJNA729271	ed_Lushi_vs_Arbor_Acr	breed_growth_background	ENSGALG00010024551	RNF11	-0.087745186	0.067698792	-1.296111548	0.194937057	0.342747773	negative
PRJNA729271	ed_Lushi_vs_Arbor_Acr	breed_growth_background	ENSGALG00010024552	CD81	-0.223762202	0.03536931	-6.32645091	2.50864E-10	6.61141E-08	negative
PRJNA729271	ed_Lushi_vs_Arbor_Acr	breed_growth_background	ENSGALG00010024553	ASB8	0.049010485	0.061333537	0.799081345	0.424243246	0.583324402	positive
PRJNA729271	ed_Lushi_vs_Arbor_Acr	breed_growth_background	ENSGALG00010024554	ST6GALNAC3	-0.171330474	0.114272796	-1.499311116	0.13379294	0.263531548	negative
PRJNA729271	ed_Lushi_vs_Arbor_Acr	breed_growth_background	ENSGALG00010024555	EPHB1	0.05570747	0.087096725	0.639604649	0.522429659	0.66960986	positive
PRJNA729271	ed_Lushi_vs_Arbor_Acr	breed_growth_background	ENSGALG00010024556	ENSGALG00010024556	-0.209326764	0.115924888	-1.805710297	0.070963603	0.169320001	negative
PRJNA729271	ed_Lushi_vs_Arbor_Acr	breed_growth_background	ENSGALG00010024557	TSPAN32	-0.272037973	0.155651481	-1.747737774	0.080509445	0.184749744	negative
PRJNA729271	ed_Lushi_vs_Arbor_Acr	breed_growth_background	ENSGALG00010024558	PFDN5	0.042266576	0.057972257	0.729082815	0.465951006	0.620996674	positive
PRJNA729271	ed_Lushi_vs_Arbor_Acr	breed_growth_background	ENSGALG00010024559	ENSGALG00010024559	-0.254125211	0.178008104	-1.427604726	0.153405663	0.289910701	negative
PRJNA729271	ed_Lushi_vs_Arbor_Acr	breed_growth_background	ENSGALG00010024560	A4GNT	0.324112403	0.256674159	1.262738736	0.206683088	0.355721117	positive
PRJNA729271	ed_Lushi_vs_Arbor_Acr	breed_growth_background	ENSGALG00010024561	ROBO3	-0.12967812	0.297747651	-0.435530286	0.66317756	0.781503479	negative
PRJNA729271	ed_Lushi_vs_Arbor_Acr	breed_growth_background	ENSGALG00010024563	ESPL1	-0.244698233	0.089996708	-2.718968705	0.00654858	0.030901708	negative
PRJNA729271	ed_Lushi_vs_Arbor_Acr	breed_growth_background	ENSGALG00010024564	PPP1R3D	0.204185341	0.105384377	1.937529517	0.052680649	0.137586668	positive
PRJNA729271	ed_Lushi_vs_Arbor_Acr	breed_growth_background	ENSGALG00010024565	FAM217B	0.04636585	0.07417075	0.625123115	0.531890258	0.677542299	positive
PRJNA729271	ed_Lushi_vs_Arbor_Acr	breed_growth_background	ENSGALG00010024566	MTSS2	-0.174500235	0.117939983	-1.479568086	0.138988549	0.270687701	negative
PRJNA729271	ed_Lushi_vs_Arbor_Acr	breed_growth_background	ENSGALG00010024567	IKZF3	0.346811216	0.221254307	1.567477808	0.11700305	0.240254881	positive
PRJNA729271	ed_Lushi_vs_Arbor_Acr	breed_growth_background	ENSGALG00010024568	DZIP1L	-0.607228713	0.145271787	-4.179949361	2.91574E-05	0.000646622	negative
PRJNA729271	ed_Lushi_vs_Arbor_Acr	breed_growth_background	ENSGALG00010024569	ASCL2	-0.249965904	0.157335601	-1.588743441	0.112118327	0.233365185	negative
PRJNA729271	ed_Lushi_vs_Arbor_Acr	breed_growth_background	ENSGALG00010024570	TPM1	0.017161326	0.087708083	0.19566413	0.844873062	0.905734415	positive
PRJNA729271	ed_Lushi_vs_Arbor_Acr	breed_growth_background	ENSGALG00010024571	KCNA3	0.314448545	0.234925703	1.338502094	0.180732818	0.325229323	positive
PRJNA729271	ed_Lushi_vs_Arbor_Acr	breed_growth_background	ENSGALG00010024573	ENSGALG00010024573	-0.264138753	0.147549327	-1.790172537	0.073426179	0.173623566	negative
PRJNA729271	ed_Lushi_vs_Arbor_Acr	breed_growth_background	ENSGALG00010024575	TXNRD2	-0.173425007	0.067777847	-2.558727008	0.01050562	0.043322608	negative
PRJNA729271	ed_Lushi_vs_Arbor_Acr	breed_growth_background	ENSGALG00010024576	CSAD	-0.530760909	0.085653191	-6.196627393	5.76858E-10	1.28639E-07	negative
PRJNA729271	ed_Lushi_vs_Arbor_Acr	breed_growth_background	ENSGALG00010024578	ENSGALG00010024578	-0.779776511	0.291245715	-2.677383634	0.007419962	0.0337578	negative
PRJNA729271	ed_Lushi_vs_Arbor_Acr	breed_growth_background	ENSGALG00010024579	ZNF740	0.162090118	0.057287074	2.829436136	0.004663011	0.024199906	positive
PRJNA729271	ed_Lushi_vs_Arbor_Acr	breed_growth_background	ENSGALG00010024580	IGF2	0.023903297	0.10448616	0.228769979	0.819047702	0.888696492	positive
PRJNA729271	ed_Lushi_vs_Arbor_Acr	breed_growth_background	ENSGALG00010024581	RARA	0.207108893	0.092179715	2.246794666	0.024653153	0.079181797	positive
PRJNA729271	ed_Lushi_vs_Arbor_Acr	breed_growth_background	ENSGALG00010024582	TLN2	0.095813768	0.057482534	1.666832731	0.09554767	0.208231947	positive
PRJNA729271	ed_Lushi_vs_Arbor_Acr	breed_growth_background	ENSGALG00010024583	ITGB7	-0.153144236	0.271745941	-0.563556664	0.573055887	0.711107361	negative
PRJNA729271	ed_Lushi_vs_Arbor_Acr	breed_growth_background	ENSGALG00010024585	TBCCD1	-0.084606452	0.071363467	-1.185570934	0.235791786	0.390516676	negative
PRJNA729271	ed_Lushi_vs_Arbor_Acr	breed_growth_background	ENSGALG00010024586	DLGAP4	0.088796816	0.051534822	1.723044995	0.084880389	0.192060761	positive
PRJNA729271	ed_Lushi_vs_Arbor_Acr	breed_growth_background	ENSGALG00010024587	ENSGALG00010024587	-0.579025315	0.093337027	-6.203597162	5.51869E-10	1.2499E-07	negative
PRJNA729271	ed_Lushi_vs_Arbor_Acr	breed_growth_background	ENSGALG00010024588	TTC39A	-0.204558233	0.226033524	-0.904990681	0.365470324	0.528797399	negative
PRJNA729271	ed_Lushi_vs_Arbor_Acr	breed_growth_background	ENSGALG00010024589	ADORA1	-0.170146191	0.252391322	-0.674136456	0.500224564	0.651387403	negative
PRJNA729271	ed_Lushi_vs_Arbor_Acr	breed_growth_background	ENSGALG00010024590	MRPL23	-0.070428318	0.056287017	-1.251235577	0.210848542	0.360960183	negative
PRJNA729271	ed_Lushi_vs_Arbor_Acr	breed_growth_background	ENSGALG00010024592	COMT	-0.689849429	0.10590945	-6.513577655	7.33816E-11	2.17075E-08	negative
PRJNA729271	ed_Lushi_vs_Arbor_Acr	breed_growth_background	ENSGALG00010024593	SRSF6	-0.105318474	0.040209651	-2.619233739	0.008812754	0.038120224	negative
PRJNA729271	ed_Lushi_vs_Arbor_Acr	breed_growth_background	ENSGALG00010024594	TNNI2	-0.177808856	0.097853879	-1.817085403	0.069204038	0.166629987	negative
PRJNA729271	ed_Lushi_vs_Arbor_Acr	breed_growth_background	ENSGALG00010024595	MYBPHL	0.645790034	0.168615904	3.829947353	0.000128171	0.001909387	positive
PRJNA729271	ed_Lushi_vs_Arbor_Acr	breed_growth_background	ENSGALG00010024596	EPS15	0.02580955	0.035708644	0.722781575	0.469814089	0.624365565	positive
PRJNA729271	ed_Lushi_vs_Arbor_Acr	breed_growth_background	ENSGALG00010024597	DNAJB11	-0.049843861	0.055252236	-0.902114828	0.366995873	0.530422293	negative
PRJNA729271	ed_Lushi_vs_Arbor_Acr	breed_growth_background	ENSGALG00010024598	LRRC3C	-0.005124718	0.127196099	-0.040289902	0.967862004	0.981793645	negative
PRJNA729271	ed_Lushi_vs_Arbor_Acr	breed_growth_background	ENSGALG00010024599	ENSGALG00010024599	-0.0304381	0.117890398	-0.258189815	0.796260418	0.873593259	negative
PRJNA729271	ed_Lushi_vs_Arbor_Acr	breed_growth_background	ENSGALG00010024600	CD9	0.865855624	0.178513783	4.850357252	1.23239E-06	5.78108E-05	positive
PRJNA729271	ed_Lushi_vs_Arbor_Acr	breed_growth_background	ENSGALG00010024601	ENSGALG00010024601	0.660503448	0.276777225	2.386408229	0.017013853	0.06096806	positive
PRJNA729271	ed_Lushi_vs_Arbor_Acr	breed_growth_background	ENSGALG00010024602	PROCR	-0.199112476	0.133226817	-1.494537512	0.135035185	0.264970218	negative
PRJNA729271	ed_Lushi_vs_Arbor_Acr	breed_growth_background	ENSGALG00010024603	ENSGALG00010024603	-0.387693426	0.572878699	-0.676746101	0.498567061	0.649997013	negative
PRJNA729271	ed_Lushi_vs_Arbor_Acr	breed_growth_background	ENSGALG00010024604	MFSD5	0.159234567	0.091024096	1.749367198	0.080227567	0.184390772	positive
PRJNA729271	ed_Lushi_vs_Arbor_Acr	breed_growth_background	ENSGALG00010024605	LSP1P1	-0.375900023	0.104829017	-3.58583943	0.000335996	0.003746408	negative
PRJNA729271	ed_Lushi_vs_Arbor_Acr	breed_growth_background	ENSGALG00010024607	RARG	0.028304489	0.076720255	0.368931115	0.712179071	0.814955051	positive
PRJNA729271	ed_Lushi_vs_Arbor_Acr	breed_growth_background	ENSGALG00010024608	DENND2D	0.692422674	0.180041562	3.845904609	0.000120108	0.001828272	positive
PRJNA729271	ed_Lushi_vs_Arbor_Acr	breed_growth_background	ENSGALG00010024609	PRR33	0.348643044	0.127282054	2.739137476	0.006160061	0.02957124	positive
PRJNA729271	ed_Lushi_vs_Arbor_Acr	breed_growth_background	ENSGALG00010024610	PFKL	-0.160234	0.076273426	-2.100784079	0.035659925	0.103398801	negative
PRJNA729271	ed_Lushi_vs_Arbor_Acr	breed_growth_background	ENSGALG00010024611	ENSGALG00010024611	0.202422013	0.142816408	1.417358245	0.156378214	0.293728419	positive
PRJNA729271	ed_Lushi_vs_Arbor_Acr	breed_growth_background	ENSGALG00010024612	ENSGALG00010024612	0.226194963	0.347606609	0.650721125	0.515226524	0.663663775	positive
PRJNA729271	ed_Lushi_vs_Arbor_Acr	breed_growth_background	ENSGALG00010024613	ENSGALG00010024613	0.076854285	0.051626422	1.488661852	0.136576435	0.267306971	positive
PRJNA729271	ed_Lushi_vs_Arbor_Acr	breed_growth_background	ENSGALG00010024614	ENSGALG00010024614	-1.077909099	0.465749077	-2.314355847	0.020648211	0.069749665	negative
PRJNA729271	ed_Lushi_vs_Arbor_Acr	breed_growth_background	ENSGALG00010024615	SYT8	0.317787849	0.395892925	0.802711614	0.422141438	0.581300285	positive
PRJNA729271	ed_Lushi_vs_Arbor_Acr	breed_growth_background	ENSGALG00010024616	ARVCF	0.008144168	0.053241583	0.152966306	0.878424846	0.927909062	positive
PRJNA729271	ed_Lushi_vs_Arbor_Acr	breed_growth_background	ENSGALG00010024618	VPS13C	0.127256945	0.065897109	1.931146107	0.053464987	0.138959114	positive
PRJNA729271	ed_Lushi_vs_Arbor_Acr	breed_growth_background	ENSGALG00010024619	ENSGALG00010024619	0.125052502	0.121667848	1.027818805	0.304035069	0.46654545	positive
PRJNA729271	ed_Lushi_vs_Arbor_Acr	breed_growth_background	ENSGALG00010024620	MSANTD2	0.130082665	0.071758903	1.812773883	0.069866704	0.167751842	positive
PRJNA729271	ed_Lushi_vs_Arbor_Acr	breed_growth_background	ENSGALG00010024621	THRA	0.240228447	0.098744125	2.432837856	0.014981008	0.055551217	positive
PRJNA729271	ed_Lushi_vs_Arbor_Acr	breed_growth_background	ENSGALG00010024622	PRMT8	-0.12949756	0.11450082	-1.130974949	0.258065635	0.415398266	negative
PRJNA729271	ed_Lushi_vs_Arbor_Acr	breed_growth_background	ENSGALG00010024623	NRDC	0.183189277	0.058572064	3.127587849	0.001762471	0.012159459	positive
PRJNA729271	ed_Lushi_vs_Arbor_Acr	breed_growth_background	ENSGALG00010024624	CTSD	0.057522168	0.068868023	0.835252202	0.403575724	0.564437487	positive
PRJNA729271	ed_Lushi_vs_Arbor_Acr	breed_growth_background	ENSGALG00010024625	SPRYD3	-0.0248276	0.042926498	-0.578374679	0.563011185	0.703400028	negative
PRJNA729271	ed_Lushi_vs_Arbor_Acr	breed_growth_background	ENSGALG00010024626	TSHZ3	-0.018116453	0.055587057	-0.325911348	0.74449143	0.837061998	negative
PRJNA729271	ed_Lushi_vs_Arbor_Acr	breed_growth_background	ENSGALG00010024627	CEPT1	0.020259087	0.04260107	0.47555347	0.634392532	0.759332762	positive
PRJNA729271	ed_Lushi_vs_Arbor_Acr	breed_growth_background	ENSGALG00010024628	TANGO2	-0.063336869	0.059637928	-1.062023292	0.288225111	0.449179979	negative
PRJNA729271	ed_Lushi_vs_Arbor_Acr	breed_growth_background	ENSGALG00010024629	ENSGALG00010024629	-0.068906917	0.092071001	-0.74841064	0.454212507	0.610459924	negative
PRJNA729271	ed_Lushi_vs_Arbor_Acr	breed_growth_background	ENSGALG00010024630	CFAP410	-0.099464955	0.053601096	-1.855651495	0.063503249	0.156971281	negative
PRJNA729271	ed_Lushi_vs_Arbor_Acr	breed_growth_background	ENSGALG00010024631	EPB41L1	-0.142394666	0.080676354	-1.765011165	0.077561889	0.179939114	negative
PRJNA729271	ed_Lushi_vs_Arbor_Acr	breed_growth_background	ENSGALG00010024632	IFITM10	-0.176013014	0.217027812	-0.811015939	0.417356518	0.577470668	negative
PRJNA729271	ed_Lushi_vs_Arbor_Acr	breed_growth_background	ENSGALG00010024633	NRGN	-0.03866971	0.157555422	-0.245435604	0.806119164	0.880667567	negative
PRJNA729271	ed_Lushi_vs_Arbor_Acr	breed_growth_background	ENSGALG00010024634	ENSGALG00010024634	-0.12542662	0.086948183	-1.442544464	0.149148838	0.284013715	negative
PRJNA729271	ed_Lushi_vs_Arbor_Acr	breed_growth_background	ENSGALG00010024635	ENSGALG00010024635	0.007310519	0.104509108	0.069951026	0.944232639	0.969653001	positive

PRJNA729271	:ed_Lushi_vs_Arbor_Acr	breed_growth_background	ENSGALG00010024636	PANX3	0.408862598	0.171455816	2.384652837	0.017095245	0.06116899	positive
PRJNA729271	:ed_Lushi_vs_Arbor_Acr	breed_growth_background	ENSGALG00010024637	DUSP8	0.015824751	0.14784696	0.107034671	0.914761474	0.951941029	positive
PRJNA729271	:ed_Lushi_vs_Arbor_Acr	breed_growth_background	ENSGALG00010024638	ZNF507	0.25077637	0.069979762	3.583555619	0.000338949	0.003767683	positive
PRJNA729271	:ed_Lushi_vs_Arbor_Acr	breed_growth_background	ENSGALG00010024639	AAR2	-0.056213106	0.041507956	-1.354273033	0.175649286	0.319715342	negative
PRJNA729271	:ed_Lushi_vs_Arbor_Acr	breed_growth_background	ENSGALG00010024640	GSDMA	-0.465035324	0.162017275	-2.870282339	0.004101054	0.02200029	negative
PRJNA729271	:ed_Lushi_vs_Arbor_Acr	breed_growth_background	ENSGALG00010024641	TRA2B	-0.109347108	0.047821657	-2.286560388	0.022221496	0.073690363	negative
PRJNA729271	:ed_Lushi_vs_Arbor_Acr	breed_growth_background	ENSGALG00010024642	RAB3B	-0.343566655	0.121738835	-2.822161521	0.004770115	0.024605983	negative
PRJNA729271	:ed_Lushi_vs_Arbor_Acr	breed_growth_background	ENSGALG00010024643	ETV7	-0.361190184	0.201390576	-1.793481062	0.072896036	0.172824105	negative
PRJNA729271	:ed_Lushi_vs_Arbor_Acr	breed_growth_background	ENSGALG00010024644	EIF6	-0.16887966	0.040789536	-4.140269231	3.46898E-05	0.0007421	negative
PRJNA729271	:ed_Lushi_vs_Arbor_Acr	breed_growth_background	ENSGALG00010024645	PARP11	0.022541234	0.06393616	0.352558461	0.724419484	0.823436357	positive
PRJNA729271	:ed_Lushi_vs_Arbor_Acr	breed_growth_background	ENSGALG00010024646	RORA	0.419871375	0.102941655	4.078731557	4.52821E-05	0.000896471	positive
PRJNA729271	:ed_Lushi_vs_Arbor_Acr	breed_growth_background	ENSGALG00010024647	RPUSD4	-0.041832109	0.07415478	-0.564118847	0.572673252	0.710909624	negative
PRJNA729271	:ed_Lushi_vs_Arbor_Acr	breed_growth_background	ENSGALG00010024648	MMP24	-0.017768446	0.068587382	-0.259062904	0.795586706	0.873373925	negative
PRJNA729271	:ed_Lushi_vs_Arbor_Acr	breed_growth_background	ENSGALG00010024649	NCOR2	0.065953064	0.069438723	0.949802376	0.342212678	0.505938073	positive
PRJNA729271	:ed_Lushi_vs_Arbor_Acr	breed_growth_background	ENSGALG00010024650	PRKAG1	0.333330101	0.06453962	5.164736011	2.40778E-07	1.6472E-05	positive
PRJNA729271	:ed_Lushi_vs_Arbor_Acr	breed_growth_background	ENSGALG00010024651	PSMD3	0.099572458	0.068418914	1.45533526	0.14557647	0.279635692	positive
PRJNA729271	:ed_Lushi_vs_Arbor_Acr	breed_growth_background	ENSGALG00010024652	PUS3	0.154408509	0.069226593	2.230479673	0.025715615	0.081599791	positive
PRJNA729271	:ed_Lushi_vs_Arbor_Acr	breed_growth_background	ENSGALG00010024653	RFLNA	0.385645379	0.139774614	2.759051638	0.005796937	0.028272748	positive
PRJNA729271	:ed_Lushi_vs_Arbor_Acr	breed_growth_background	ENSGALG00010024654	CCDC92	0.33032196	0.150608416	2.193250342	0.028289347	0.087172385	positive
PRJNA729271	:ed_Lushi_vs_Arbor_Acr	breed_growth_background	ENSGALG00010024655	IGF2BP2	0.296845682	0.089643268	3.311410736	0.000928268	0.007710746	positive
PRJNA729271	:ed_Lushi_vs_Arbor_Acr	breed_growth_background	ENSGALG00010024656	HYLS1	0.016606522	0.102202318	0.162486749	0.870922562	0.923281105	positive
PRJNA729271	:ed_Lushi_vs_Arbor_Acr	breed_growth_background	ENSGALG00010024657	DPY19L3	-0.302133124	0.06907122	-4.374225962	1.21864E-05	0.000335701	negative
PRJNA729271	:ed_Lushi_vs_Arbor_Acr	breed_growth_background	ENSGALG00010024659	BEND5	0.223727723	0.05768287	3.878581678	0.000105067	0.00165	positive
PRJNA729271	:ed_Lushi_vs_Arbor_Acr	breed_growth_background	ENSGALG00010024660	KMT2D	0.175388106	0.084274091	2.081162831	0.037419004	0.107318652	positive
PRJNA729271	:ed_Lushi_vs_Arbor_Acr	breed_growth_background	ENSGALG00010024662	MOB2	-0.041928261	0.038546348	-1.087736259	0.276711556	0.435876331	negative
PRJNA729271	:ed_Lushi_vs_Arbor_Acr	breed_growth_background	ENSGALG00010024663	ICE2	-0.17996683	0.067207823	-2.677766081	0.007411496	0.033740463	negative
PRJNA729271	:ed_Lushi_vs_Arbor_Acr	breed_growth_background	ENSGALG00010024664	DNAH10	0.147554337	0.137144322	1.075905547	0.281969501	0.441806066	positive
PRJNA729271	:ed_Lushi_vs_Arbor_Acr	breed_growth_background	ENSGALG00010024665	MAP1LC3A	0.176249943	0.078723365	2.238851734	0.025165565	0.080382297	positive
PRJNA729271	:ed_Lushi_vs_Arbor_Acr	breed_growth_background	ENSGALG00010024666	MED24	-0.163354392	0.062863199	-2.598569514	0.009361309	0.039932952	negative
PRJNA729271	:ed_Lushi_vs_Arbor_Acr	breed_growth_background	ENSGALG00010024667	RBM7	0.140761378	0.072506985	1.941349205	0.052215935	0.136717843	positive
PRJNA729271	:ed_Lushi_vs_Arbor_Acr	breed_growth_background	ENSGALG00010024668	ENSGALG00010024668	-0.152243823	0.098625109	-1.54366189	0.122670263	0.247543571	negative
PRJNA729271	:ed_Lushi_vs_Arbor_Acr	breed_growth_background	ENSGALG00010024669	STIL	-0.228569415	0.100616861	-2.27168103	0.023105783	0.075636477	negative
PRJNA729271	:ed_Lushi_vs_Arbor_Acr	breed_growth_background	ENSGALG00010024670	SENP2	0.10460805	0.051304464	2.038965843	0.041453437	0.115373956	positive
PRJNA729271	:ed_Lushi_vs_Arbor_Acr	breed_growth_background	ENSGALG00010024671	KCTD20	0.2591065	0.149814611	1.729514218	0.083717104	0.189962339	positive
PRJNA729271	:ed_Lushi_vs_Arbor_Acr	breed_growth_background	ENSGALG00010024672	DYNLRB1	0.075080847	0.056822366	1.321325588	0.186392826	0.331912041	positive
PRJNA729271	:ed_Lushi_vs_Arbor_Acr	breed_growth_background	ENSGALG00010024673	REXO2	0.038426438	0.05264137	0.729966531	0.465410643	0.620565546	positive
PRJNA729271	:ed_Lushi_vs_Arbor_Acr	breed_growth_background	ENSGALG00010024674	DHH	0.45302604	0.163306896	2.774077832	0.005535843	0.027386364	positive
PRJNA729271	:ed_Lushi_vs_Arbor_Acr	breed_growth_background	ENSGALG00010024675	NR1D1	0.804827485	0.191390856	4.205151194	2.60908E-05	0.000589993	positive
PRJNA729271	:ed_Lushi_vs_Arbor_Acr	breed_growth_background	ENSGALG00010024676	ENSGALG00010024676	0.010076541	0.088992925	0.113228563	0.909849351	0.94879614	positive
PRJNA729271	:ed_Lushi_vs_Arbor_Acr	breed_growth_background	ENSGALG00010024677	LMBR1L	-0.159082173	0.207733896	-0.765797861	0.443796587	0.600693952	negative
PRJNA729271	:ed_Lushi_vs_Arbor_Acr	breed_growth_background	ENSGALG00010024678	AMOTL2	-0.026647977	0.048872112	-0.545259358	0.585575152	0.721454469	negative
PRJNA729271	:ed_Lushi_vs_Arbor_Acr	breed_growth_background	ENSGALG00010024679	MSL1	0.121379108	0.059837557	2.028476997	0.042511589	0.117798791	positive
PRJNA729271	:ed_Lushi_vs_Arbor_Acr	breed_growth_background	ENSGALG00010024680	BRSK1	0.297715111	0.145803755	2.041889186	0.041162525	0.114696424	positive
PRJNA729271	:ed_Lushi_vs_Arbor_Acr	breed_growth_background	ENSGALG00010024681	ATP6V0A2	-0.104286886	0.04602335	-2.265956011	0.023454077	0.076465776	negative
PRJNA729271	:ed_Lushi_vs_Arbor_Acr	breed_growth_background	ENSGALG00010024682	ENSGALG00010024682	-0.223300956	0.095924562	-2.327880901	0.019918429	0.0680296	negative
PRJNA729271	:ed_Lushi_vs_Arbor_Acr	breed_growth_background	ENSGALG00010024683	SHISA4	0.288286496	0.091108644	3.164205765	0.001555068	0.011153247	positive
PRJNA729271	:ed_Lushi_vs_Arbor_Acr	breed_growth_background	ENSGALG00010024684	ENSGALG00010024684	-0.360359423	0.181934309	-1.980711752	0.047623608	0.127834111	negative
PRJNA729271	:ed_Lushi_vs_Arbor_Acr	breed_growth_background	ENSGALG00010024685	ENSGALG00010024685	0.162555156	0.119681556	1.358230636	0.174390512	0.31828135	positive
PRJNA729271	:ed_Lushi_vs_Arbor_Acr	breed_growth_background	ENSGALG00010024686	CMPK1	-0.048468574	0.071902941	-0.674083333	0.500258335	0.651387403	negative
PRJNA729271	:ed_Lushi_vs_Arbor_Acr	breed_growth_background	ENSGALG00010024687	RYK	0.054013037	0.057648111	0.936943746	0.348787495	0.512849944	positive
PRJNA729271	:ed_Lushi_vs_Arbor_Acr	breed_growth_background	ENSGALG00010024688	CADM1	0.161395352	0.070853083	2.277887484	0.022733282	0.074856639	positive
PRJNA729271	:ed_Lushi_vs_Arbor_Acr	breed_growth_background	ENSGALG00010024689	FOXD2	0.352649816	0.16567048	2.128621931	0.033285553	0.09812367	positive
PRJNA729271	:ed_Lushi_vs_Arbor_Acr	breed_growth_background	ENSGALG00010024690	TUBA1C	0.470793443	0.347802586	1.353622604	0.175856812	0.319882257	positive
PRJNA729271	:ed_Lushi_vs_Arbor_Acr	breed_growth_background	ENSGALG00010024691	TCTN2	-0.421652574	0.082062813	-5.138168646	2.77429E-07	1.80329E-05	negative
PRJNA729271	:ed_Lushi_vs_Arbor_Acr	breed_growth_background	ENSGALG00010024692	DRAM2	0.17110922	0.055405927	3.088283681	0.002013162	0.01330633	positive
PRJNA729271	:ed_Lushi_vs_Arbor_Acr	breed_growth_background	ENSGALG00010024693	ANXA2	-0.321637558	0.10774774	-2.985097945	0.002834875	0.01702217	negative
PRJNA729271	:ed_Lushi_vs_Arbor_Acr	breed_growth_background	ENSGALG00010024694	GTF2H3	-0.192002969	0.053379011	-3.596975023	0.000321939	0.003631269	negative
PRJNA729271	:ed_Lushi_vs_Arbor_Acr	breed_growth_background	ENSGALG00010024695	TRABD2B	0.107367998	0.106318338	1.009872804	0.312556234	0.475812776	positive
PRJNA729271	:ed_Lushi_vs_Arbor_Acr	breed_growth_background	ENSGALG00010024696	TRADD	-0.153356948	0.062441606	-2.456005832	0.014049088	0.053059283	negative
PRJNA729271	:ed_Lushi_vs_Arbor_Acr	breed_growth_background	ENSGALG00010024697	IGF2BP1	-0.039696609	0.06715352	-0.591132216	0.554431835	0.696342557	negative
PRJNA729271	:ed_Lushi_vs_Arbor_Acr	breed_growth_background	ENSGALG00010024698	ENSGALG00010024698	0.256437801	0.197473719	1.298592047	0.194083969	0.341461006	positive
PRJNA729271	:ed_Lushi_vs_Arbor_Acr	breed_growth_background	ENSGALG00010024699	FBXL8	-0.157936246	0.073345578	-2.15331654	0.03129381	0.094011145	negative
PRJNA729271	:ed_Lushi_vs_Arbor_Acr	breed_growth_background	ENSGALG00010024700	TOLLIP	0.002285844	0.045197946	0.050574072	0.959664924	0.977517846	positive
PRJNA729271	:ed_Lushi_vs_Arbor_Acr	breed_growth_background	ENSGALG00010024701	EIF2B1	-0.015259757	0.054910835	-0.277900653	0.781088623	0.864463586	negative
PRJNA729271	:ed_Lushi_vs_Arbor_Acr	breed_growth_background	ENSGALG00010024702	INAVA	-0.116236851	0.44787126	-0.259531837	0.795224921	0.873307465	negative
PRJNA729271	:ed_Lushi_vs_Arbor_Acr	breed_growth_background	ENSGALG00010024703	HSF4	-0.507365093	0.331951121	-1.528433134	0.126405033	0.252967135	negative
PRJNA729271	:ed_Lushi_vs_Arbor_Acr	breed_growth_background	ENSGALG00010024705	SLCO2A1	0.129162094	0.126513254	1.020937255	0.307284167	0.469852136	positive
PRJNA729271	:ed_Lushi_vs_Arbor_Acr	breed_growth_background	ENSGALG00010024706	ENSGALG00010024706	0.189788535	0.509339522	0.372616942	0.709433562	0.812775804	positive
PRJNA729271	:ed_Lushi_vs_Arbor_Acr	breed_growth_background	ENSGALG00010024707	ENSGALG00010024707	0.382275421	0.201100869	1.900913818	0.057313302	0.146028531	positive
PRJNA729271	:ed_Lushi_vs_Arbor_Acr	breed_growth_background	ENSGALG00010024708	BNIP2	0.014508129	0.033552752	0.4323976	0.665452462	0.783123605	positive
PRJNA729271	:ed_Lushi_vs_Arbor_Acr	breed_growth_background	ENSGALG00010024709	TEAD4	0.101105937	0.051459033	1.964785002	0.049439127	0.131452746	positive
PRJNA729271	:ed_Lushi_vs_Arbor_Acr	breed_growth_background	ENSGALG00010024710	HSDL1	-0.168688588	0.070039341	-2.408483354	0.016018957	0.058376754	negative
PRJNA729271	:ed_Lushi_vs_Arbor_Acr	breed_growth_background	ENSGALG00010024711	DDX55	0.097845539	0.050096566	1.953138653	0.050803168	0.133938145	positive
PRJNA729271	:ed_Lushi_vs_Arbor_Acr	breed_growth_background	ENSGALG00010024712	LRIF1	0.010170048	0.052664488	0.193110159	0.846872697	0.907341247	positive
PRJNA729271	:ed_Lushi_vs_Arbor_Acr	breed_growth_background	ENSGALG00010024713	RND2	-0.029265098	0.237917703	-0.123005132	0.902103034	0.943501226	negative
PRJNA729271	:ed_Lushi_vs_Arbor_Acr	breed_growth_background	ENSGALG00010024714	SRPRB	0.164341778	0.093839289	1.751310984	0.079892357	0.183991057	positive
PRJNA729271	:ed_Lushi_vs_Arbor_Acr	breed_growth_background	ENSGALG00010024715	DNAAF1	-0.041781429	0.096981886	-0.43081683	0.666601561	0.783942594	negative
PRJNA729271	:ed_Lushi_vs_Arbor_Acr	breed_growth_background	ENSGALG00010024716	GTF2A2	0.027999166	0.042091563	0.665196624	0.50592474	0.65550588	positive
PRJNA729271	:ed_Lushi_vs_Arbor_Acr	breed_growth_background	ENSGALG00010024717	TF	-0.90436538	0.348582086	-2.594411518	0.0094753	0.040319652	negative
PRJNA729271	:ed_Lushi_vs_Arbor_Acr	breed_growth_background	ENSGALG00010024718	ENSGALG00010024718	0.041105524	0.10281121	0.399815585	0.689292351	0.798983817	positive
PRJNA729271	:ed_Lushi_vs_Arbor_Acr	breed_growth_background	ENSGALG00010024719	PYM1	0.05366997	0.074410501	0.72126877	0.470744168	0.625085811	positive
PRJNA729271	:ed_Lushi_vs_Arbor_Acr	breed_growth_background	ENSGALG00010024720	BUD13	0.067786006	0.058403837	1.160643023	0.245787102	0.401711808	positive
PRJNA729271	:ed_Lushi_vs_Arbor_Acr	breed_growth_background	ENSGALG00010024721	ENSGALG00010024721	-0.675389716	0.412753408	-1.636303184	0.10177615	0.217716248	negative
PRJNA729271	:ed_Lushi_vs_Arbor_Acr	breed_growth_background	ENSGALG00010024722	BRCA1	-0.388950983	0.141167844	-2.755237816	0.005864949	0.028527662	negative
PRJNA729271	:ed_Lushi_vs_Arbor_Acr	breed_growth_background	ENSGALG00010024723	ITCH	0.047249666	0.053838107	0.877624945	0.380147288	0.541972552	positive
PRJNA729271	:ed_Lushi_vs_Arbor_Acr	breed_growth_background	ENSGALG00010024724	HOXD3	0.117847005	0.137277381	0.858459007	0.390639057	0.552205298	positive
PRJNA729271	:ed_Lushi_vs_Arbor_Acr	breed_growth_background	ENSGALG00010024726	TMED2	-0.095132169	0.03148594	-3.021417437	0.002515942	0.015653557	negative
PRJNA729271	:ed_Lushi_vs_Arbor_Acr	breed_growth_background	ENSGALG00010024728	SLC5A9	-0.183778473	0.456695939	-0.402408818	0.687383181	0.798105822	negative
PRJNA729271	:ed_Lushi_vs_Arbor_Acr	breed_growth_background	ENSGALG00010024729	FOXM1	-0.235200086	0.091788658	-2.562409036	0.010394881	0.043000515	negative
PRJNA729271	:ed_Lushi_vs_Arbor_Acr	breed_growth_background	ENSGALG00010024731	AOX1	-0.030445611	0.104792595	-0.290532087	0.771409207	0.857482857	negative
PRJNA729271	:ed_Lushi_vs_Arbor_Acr	breed_growth_background	ENSGALG00010024732	AHCY	-0.029251605	0.120247926	-0.24326079	0.807803377	0.881865544	negative
PRJNA729271	:ed_Lushi_vs_Arbor_Acr	breed_growth_background	ENSGALG00010024733	MBTPS1	-0.043430148	0.047263676	-0.918890609	0.358152792	0.521908588	negative
PRJNA729271	:ed_Lushi_vs_Arbor_Acr	breed_growth_background	ENSGALG00010024734	ZPR1	0.224076053	0.061954912	3.616760096	0.000298314	0.003457823	positive
PRJNA729271	:ed_Lushi_vs_Arbor_Acr	breed_growth_background	ENSGALG00010024735	OTUD7A	0.060849225	0.094558393	0.643509511	0.519893545	0.667615111	positive
PRJNA729271	:ed_Lushi_vs_Arbor_Acr	breed_growth_background	ENSGALG00010024736	DGKA	-0.040588044	0.081967751	-0.49517089	0.620479497	0.747991543	negative
PRJNA729271	:ed_Lushi_vs_Arbor_Acr	breed_growth_background	ENSGALG00010024737	STK38	-0.060480215	0.034171498	-1.76990238	0.076743404	0.178841743	negative
PRJNA729271	:ed_Lushi_vs_Arbor_Acr	breed_growth_background	ENSGALG00010024738	NBR1	0.142950513	0.042143607	3.391985744	0.000693881	0.006274359	positive
PRJNA729271	:ed_Lushi_vs_Arbor_Acr	breed_growth_background	ENSGALG00010024739	ASIP	0.30196997	0.337650138	0.894327991	0.371146428	0.53418404	positive
PRJNA729271	:ed_Lushi_vs_Arbor_Acr	breed_growth_background	ENSGALG00010024741	RILPL2	0.196906328	0.085114253	2.31343543	0.020698711	0.069871406	positive
PRJNA729271	:ed_Lushi_vs_Arbor_Acr	breed_growth_background	ENSGALG00010024742	PAK2	-0.002869051	0.037829542	-0.075841556	0.939545154	0.966757082	negative
PRJNA729271	:ed_Lushi_vs_Arbor_Acr	breed_growth_background	ENSGALG00010024744	KLF13	0.188431433	0.065766859	2.865142661	0.004168214	0.022253504	positive
PRJNA729271	:ed_Lushi_vs_Arbor_Acr	breed_growth_background	ENSGALG00010024745	PMEL	2.790980246	0.351752549	7.934498998	2.11347E-15	2.55289E-12	positive
PRJNA729271	:ed_Lushi_vs_Arbor_Acr	breed_growth_background	ENSGALG00010024746	HOXD4	0.18188627	0.174922438	1.039810966	0.298427734	0.460671991	positive
PRJNA729271	:ed_Lushi_vs_Arbor_Acr	breed_growth_background	ENSGALG00010024747	TULP3	0.079836855	0.046733353	1.708348546	0.087571689	0.196099432	positive
PRJNA729271	:ed_Lushi_vs_Arbor_Acr	breed_growth_background	ENSGALG00010024748	TRPM1	-0.205210181	0.206790527	-0.992357742	0.321023045	0.484610398	negative
PRJNA729271	:ed_Lushi_vs_Arbor_Acr	breed_growth_background	ENSGALG00010024749	APOA5	-0.811385391	0.356122408	-2.278389036	0.022703409	0.074809255	negative
PRJNA729271	:ed_Lushi_vs_Arbor_Acr	breed_growth_background	ENSGALG00010024750	APOA4	-2.86712333	0.814447568	-3.520328924	0.000431012	0.004466547	negative
PRJNA729271	:ed_Lushi_vs_Arbor_Acr	breed_growth_background	ENSGALG00010024751	MTMR10	-0.005200613	0.030492127	-0.17055594	0.864572945	0.918680641	negative
PRJNA729271	:ed_Lushi_vs_Arbor_Acr	breed_growth_background	ENSGALG00010024752	MPP2	-0.235389456	0.120403941	-1.954997928	0.050583315	0.133495486	negative
PRJNA729271	:ed_Lushi_vs_Arbor_Acr	breed_growth_background	ENSGALG00010024753	HOXD8	-0.089838372	0.095413501	-0.941568756	0.346413472	0.510290984	negative
PRJNA729271	:ed_Lushi_vs_Arbor_Acr	breed_growth_background	ENSGALG00010024754	EIF2S2	0.352458533	0.077443886	4.551147319	5.33542E-06	0.000182578	positive
PRJNA729271	:ed_Lushi_vs_Arbor_Acr	breed_growth_background	ENSGALG00010024755	PIGX	-0.253993838	0.084606387	-3.002064564	0.002681553	0.0163247	negative
PRJNA729271	:ed_Lushi_vs_Arbor_Acr	breed_growth_background	ENSGALG00010024756	CDK2	-0.131334921	0.091454469	-1.436068932	0.150982727	0.286807999	negative
PRJNA729271	:ed_Lushi_vs_Arbor_Acr	breed_growth_background	ENSGALG00010024757	PPFIA1	0.000626272	0.040817352	0.015343284	0.987758311	0.992713543	positive
PRJNA729271	:ed_Lushi_vs_Arbor_Acr	breed_growth_background	ENSGALG00010024758	SPATA6	-0.112338643	0.057468338	-1.954791908	0.050607637	0.133495486	negative
PRJNA729271	:ed_Lushi_vs_Arbor_Acr	breed_growth_background	ENSGALG00010024759	APOC3	-1.478147016	0.508908885	-2.904541576			negative
PRJNA729271	:ed_Lushi_vs_Arbor_Acr	breed_growth_background	ENSGALG00010024760	ENSGALG00010024760	-0.253782272	0.095433152	-2.659267418	0.007831078	0.035093114	negative
PRJNA729271	:ed_Lushi_vs_Arbor_Acr	breed_growth_background	ENSGALG00010024761	NECAB2	0.010179311	0.125705158	0.080977671	0.93545971	0.964536447	positive
PRJNA729271	:ed_Lushi_vs_Arbor_Acr	breed_growth_background	ENSGALG00010024762	FAN1	0.065069262	0.049650497	1.310546033	0.190011182	0.336453956	positive
PRJNA729271	:ed_Lushi_vs_Arbor_Acr	breed_growth_background	ENSGALG00010024763	HOXD9	0.037861058	0.172025575	0.220089706	0.825801292	0.893616254	positive
PRJNA729271	:ed_Lushi_vs_Arbor_Acr	breed_growth_background	ENSGALG00010024764	ENSGALG00010024764	-0.141946873	0.141218328	-1.005158995	0.314820297	0.47813414	negative
PRJNA729271	:ed_Lushi_vs_Arbor_Acr	breed_growth_background	ENSGALG00010024765	SNRNP35	0.254012471	0.072447645	3.506152193	0.000454635	0.004637535	positive
PRJNA729271	:ed_Lushi_vs_Arbor_Acr	breed_growth_background	ENSGALG00010024767	CTTN	-0.052527469	0.037488936	-1.401145916	0.161170444	0.300278352	negative
PRJNA729271	:ed_Lushi_vs_Arbor_Acr	breed_growth_background	ENSGALG00010024769	RALY	0.611479196	0.108585828	5.631298394	1.78858E-08	2.23495E-06	positive
PRJNA729271	:ed_Lushi_vs_Arbor_Acr	breed_growth_background	ENSGALG00010024770	CDC27	0.034280166	0.03895811	0.879923752	0.378900616	0.540963527	positive
PRJNA729271	:ed_Lushi_vs_Arbor_Acr	breed_growth_background	ENSGALG00010024771	AOX2	0.172000355	0.136191457	1.262930572	0.206614132	0.355721117	positive
PRJNA729271	:ed_Lushi_vs_Arbor_Acr	breed_growth_background	ENSGALG00010024772	ELAPOR1	-0.218153169	0.130275514	-1.674552356	0.094022094	0.205930833	negative
PRJNA729271	:ed_Lushi_vs_Arbor_Acr	breed_growth_background	ENSGALG00010024773	APOA1	-0.027248676	0.141010693	-0.193238368	0.846772292	0.907300737	negative
PRJNA729271	:ed_Lushi_vs_Arbor_Acr	breed_growth_background	ENSGALG00010024775	ITFG2	0.152410848	0.050308574	3.02952032	0.002449424	0.015389859	positive
PRJNA729271	:ed_Lushi_vs_Arbor_Acr	breed_growth_background	ENSGALG00010024776	MPHOSPH10	0.190353651	0.058103321	3.276123448	0.001052425	0.008470243	positive
PRJNA729271	:ed_Lushi_vs_Arbor_Acr	breed_growth_background	ENSGALG00010024778	RAB5B	0.015004779	0.075404034	0.198991722	0.84226922	0.904548592	positive

PRJNA729271	ed_Lushi_vs_Arbor_Acr	breed_growth_background	ENSGALG00010024779	ENSGALG00010024779	-0.164260666	0.585009257	-0.28078302	0.778876828	0.862938135	negative
PRJNA729271	ed_Lushi_vs_Arbor_Acr	breed_growth_background	ENSGALG00010024780	HOXD11	0.115561668	0.487701543	0.236951615	0.812694327	0.884716806	positive
PRJNA729271	ed_Lushi_vs_Arbor_Acr	breed_growth_background	ENSGALG00010024781	CEP19	-0.205862453	0.089808296	-2.292243168	0.021891615	0.07279625	negative
PRJNA729271	ed_Lushi_vs_Arbor_Acr	breed_growth_background	ENSGALG00010024782	HOXD12	-0.541984303	1.213856685	-0.44649777	0.655237723	0.775238902	negative
PRJNA729271	ed_Lushi_vs_Arbor_Acr	breed_growth_background	ENSGALG00010024783	UBE2T	-0.33392737	0.131124214	-2.546649168	0.010876272	0.044445469	negative
PRJNA729271	ed_Lushi_vs_Arbor_Acr	breed_growth_background	ENSGALG00010024784	ENSGALG00010024784	0.296761573	0.074729668	3.97113466	7.15311E-05	0.001243218	positive
PRJNA729271	ed_Lushi_vs_Arbor_Acr	breed_growth_background	ENSGALG00010024785	MCEE	-0.079295481	0.10733427	-0.738771322	0.460045869	0.61527633	negative
PRJNA729271	ed_Lushi_vs_Arbor_Acr	breed_growth_background	ENSGALG00010024786	PDCD5	0.107085529	0.052156137	2.053172186	0.040055881	0.11241239	positive
PRJNA729271	ed_Lushi_vs_Arbor_Acr	breed_growth_background	ENSGALG00010024787	SARS1	-0.128058599	0.05332904	-2.401292044	0.016337292	0.059172676	negative
PRJNA729271	ed_Lushi_vs_Arbor_Acr	breed_growth_background	ENSGALG00010024788	SIK3	0.284218451	0.088901897	3.196989718	0.001388699	0.010349197	positive
PRJNA729271	ed_Lushi_vs_Arbor_Acr	breed_growth_background	ENSGALG00010024789	SUOX	0.011194621	0.093388187	0.119871918	0.90458461	0.945214175	positive
PRJNA729271	ed_Lushi_vs_Arbor_Acr	breed_growth_background	ENSGALG00010024791	SHANK2	0.055620152	0.211293891	0.263235967	0.79236871	0.871479298	positive
PRJNA729271	ed_Lushi_vs_Arbor_Acr	breed_growth_background	ENSGALG00010024792	BZW1	-0.050051304	0.037118388	-1.348423404	0.177522241	0.32164811	negative
PRJNA729271	ed_Lushi_vs_Arbor_Acr	breed_growth_background	ENSGALG00010024793	FKBP4	0.085892063	0.062864248	1.366310181	0.171841627	0.314937967	positive
PRJNA729271	ed_Lushi_vs_Arbor_Acr	breed_growth_background	ENSGALG00010024794	DDX6	-0.067506204	0.034869186	-1.935984507	0.052869599	0.137887596	negative
PRJNA729271	ed_Lushi_vs_Arbor_Acr	breed_growth_background	ENSGALG00010024795	NRROS	0.053624784	0.112645516	0.476048991	0.634039476	0.758972847	positive
PRJNA729271	ed_Lushi_vs_Arbor_Acr	breed_growth_background	ENSGALG00010024797	IKZF4	0.174625582	0.125896761	1.387053813	0.165425354	0.305652073	positive
PRJNA729271	ed_Lushi_vs_Arbor_Acr	breed_growth_background	ENSGALG00010024798	FBXO45	-0.11953528	0.04569722	-2.61581076	0.008901588	0.038412776	negative
PRJNA729271	ed_Lushi_vs_Arbor_Acr	breed_growth_background	ENSGALG00010024799	ENSGALG00010024799	0.205182226	0.09916032	2.069196905	0.038527613	0.10960898	positive
PRJNA729271	ed_Lushi_vs_Arbor_Acr	breed_growth_background	ENSGALG00010024800	ENSGALG00010024800	0.239008153	0.070207066	3.404331873	0.000663261	0.006065597	positive
PRJNA729271	ed_Lushi_vs_Arbor_Acr	breed_growth_background	ENSGALG00010024801	CXCR5	0.939069883	0.514452119	1.82537859	0.067943918	0.164635087	positive
PRJNA729271	ed_Lushi_vs_Arbor_Acr	breed_growth_background	ENSGALG00010024802	CD59	-0.305441931	0.087865843	-3.476230574	0.000508515	0.005027915	negative
PRJNA729271	ed_Lushi_vs_Arbor_Acr	breed_growth_background	ENSGALG00010024803	CLK1	0.18160453	0.074604588	2.434227365	0.014923618	0.055395093	positive
PRJNA729271	ed_Lushi_vs_Arbor_Acr	breed_growth_background	ENSGALG00010024804	WDR53	0.085134552	0.056935631	1.495277225	0.134842107	0.264698853	positive
PRJNA729271	ed_Lushi_vs_Arbor_Acr	breed_growth_background	ENSGALG00010024805	ENSGALG00010024805	-0.018280306	0.198682047	-0.09200784	0.926691811	0.959082489	negative
PRJNA729271	ed_Lushi_vs_Arbor_Acr	breed_growth_background	ENSGALG00010024806	ANKRD27	0.152162506	0.04065891	3.742414771	0.00018226	0.002430254	positive
PRJNA729271	ed_Lushi_vs_Arbor_Acr	breed_growth_background	ENSGALG00010024807	PPIL3	0.184427589	0.10631832	1.734673651	0.082798626	0.188497893	positive
PRJNA729271	ed_Lushi_vs_Arbor_Acr	breed_growth_background	ENSGALG00010024808	CELSR2	0.355982818	0.134358487	2.649500045	0.008061096	0.03579783	positive
PRJNA729271	ed_Lushi_vs_Arbor_Acr	breed_growth_background	ENSGALG00010024809	KANSL1	0.227461786	0.091622572	2.482595509	0.01304291	0.050348063	positive
PRJNA729271	ed_Lushi_vs_Arbor_Acr	breed_growth_background	ENSGALG00010024810	ADIPOR1	0.141800962	0.060823446	2.331353643	0.01973472	0.067602248	positive
PRJNA729271	ed_Lushi_vs_Arbor_Acr	breed_growth_background	ENSGALG00010024811	NIF3L1	0.11449133	0.077435276	1.478542287	0.139262687	0.270881998	positive
PRJNA729271	ed_Lushi_vs_Arbor_Acr	breed_growth_background	ENSGALG00010024812	SBNO1	0.192215349	0.059968086	3.205294043	0.001349246	0.010138578	positive
PRJNA729271	ed_Lushi_vs_Arbor_Acr	breed_growth_background	ENSGALG00010024813	RNF168	0.034984595	0.051713512	0.676507811	0.498718289	0.650082877	positive
PRJNA729271	ed_Lushi_vs_Arbor_Acr	breed_growth_background	ENSGALG00010024814	APBA2	0.004094867	0.090519522	0.045237395	0.963918088	0.979528371	positive
PRJNA729271	ed_Lushi_vs_Arbor_Acr	breed_growth_background	ENSGALG00010024816	NFIA	-0.106565272	0.065746045	-1.620862104	0.105047216	0.222757596	negative
PRJNA729271	ed_Lushi_vs_Arbor_Acr	breed_growth_background	ENSGALG00010024817	UCKL1	0.634673433	0.117005798	5.424290458	5.81852E-08	5.499E-06	positive
PRJNA729271	ed_Lushi_vs_Arbor_Acr	breed_growth_background	ENSGALG00010024818	FBXO3	0.041908705	0.041314983	1.014370632	0.310405932	0.473328471	positive
PRJNA729271	ed_Lushi_vs_Arbor_Acr	breed_growth_background	ENSGALG00010024820	NUDT19	0.13858698	0.075553808	1.834281861	0.066612165	0.162500131	positive
PRJNA729271	ed_Lushi_vs_Arbor_Acr	breed_growth_background	ENSGALG00010024821	NECAP1	-0.155984721	0.056996014	-2.736765442	0.006204652	0.029701593	negative
PRJNA729271	ed_Lushi_vs_Arbor_Acr	breed_growth_background	ENSGALG00010024822	RPS26	0.129351005	0.07331106	1.76441323	0.077662432	0.180057095	positive
PRJNA729271	ed_Lushi_vs_Arbor_Acr	breed_growth_background	ENSGALG00010024823	ASB10	0.021608854	0.158279724	0.136523199	0.891407686	0.936368897	positive
PRJNA729271	ed_Lushi_vs_Arbor_Acr	breed_growth_background	ENSGALG00010024824	DNAJC5	0.000139464	0.060384482	0.002309595	0.998157211	0.998984242	positive
PRJNA729271	ed_Lushi_vs_Arbor_Acr	breed_growth_background	ENSGALG00010024825	UBXN7	0.041003792	0.041125564	0.997039015	0.318745573	0.482025778	positive
PRJNA729271	ed_Lushi_vs_Arbor_Acr	breed_growth_background	ENSGALG00010024826	RBM33	0.148786858	0.059913449	2.483363268	0.013014829	0.050306652	positive
PRJNA729271	ed_Lushi_vs_Arbor_Acr	breed_growth_background	ENSGALG00010024827	ENSGALG00010024827	0.791785389	0.197605051	4.006908651	6.15186E-05	0.001118837	positive
PRJNA729271	ed_Lushi_vs_Arbor_Acr	breed_growth_background	ENSGALG00010024828	TIMM17B	-0.108647901	0.071290344	-1.524019868	0.127503748	0.254418118	negative
PRJNA729271	ed_Lushi_vs_Arbor_Acr	breed_growth_background	ENSGALG00010024829	ERBB3	0.337153464	0.114827265	2.936179535	0.003322819	0.019078395	positive
PRJNA729271	ed_Lushi_vs_Arbor_Acr	breed_growth_background	ENSGALG00010024830	ORC2	-0.004605273	0.062693431	-0.073457023	0.941442442	0.968159503	negative
PRJNA729271	ed_Lushi_vs_Arbor_Acr	breed_growth_background	ENSGALG00010024833	MAPT	0.186720254	0.10731382	1.739946024	0.081868496	0.186967678	positive
PRJNA729271	ed_Lushi_vs_Arbor_Acr	breed_growth_background	ENSGALG00010024834	INSIG1	-0.421510458	0.140695472	-2.995906359	0.002736304	0.01658141	negative
PRJNA729271	ed_Lushi_vs_Arbor_Acr	breed_growth_background	ENSGALG00010024835	ENTREP2	-0.015742916	0.079683212	-0.197568797	0.84338245	0.90506146	negative
PRJNA729271	ed_Lushi_vs_Arbor_Acr	breed_growth_background	ENSGALG00010024836	TXNDC12	-0.072488323	0.057344151	-1.264092695	0.206196758	0.355460021	negative
PRJNA729271	ed_Lushi_vs_Arbor_Acr	breed_growth_background	ENSGALG00010024837	TNFRSF1A	-0.33738222	0.083295371	-4.050431823	5.11232E-05	0.000984105	negative
PRJNA729271	ed_Lushi_vs_Arbor_Acr	breed_growth_background	ENSGALG00010024838	EN2	1.672720842	0.519632131	3.219048137	0.001286169	0.009788093	positive
PRJNA729271	ed_Lushi_vs_Arbor_Acr	breed_growth_background	ENSGALG00010024839	PA2G4	0.103637	0.053223054	1.94722009	0.051508354	0.135354168	positive
PRJNA729271	ed_Lushi_vs_Arbor_Acr	breed_growth_background	ENSGALG00010024840	BTF3L4	-0.171267395	0.054553652	-3.139430459	0.001692766	0.011790793	negative
PRJNA729271	ed_Lushi_vs_Arbor_Acr	breed_growth_background	ENSGALG00010024841	ENSGALG00010024841	0.06661111	0.11810335	0.564006949	0.572749403	0.710909624	positive
PRJNA729271	ed_Lushi_vs_Arbor_Acr	breed_growth_background	ENSGALG00010024842	ZFYVE9	0.143436012	0.041069004	3.492561273	0.000478412	0.00481568	positive
PRJNA729271	ed_Lushi_vs_Arbor_Acr	breed_growth_background	ENSGALG00010024843	BCL9L	-0.04947937	0.072235036	-0.68497745	0.493358151	0.644951876	negative
PRJNA729271	ed_Lushi_vs_Arbor_Acr	breed_growth_background	ENSGALG00010024844	TM4SF19	0.296861207	0.207748107	1.428947827	0.153019229	0.289482345	positive
PRJNA729271	ed_Lushi_vs_Arbor_Acr	breed_growth_background	ENSGALG00010024845	HYCC2	0.088328796	0.059584177	1.482420347	0.138228483	0.269739077	positive
PRJNA729271	ed_Lushi_vs_Arbor_Acr	breed_growth_background	ENSGALG00010024846	LMO2	-0.249570955	0.076636452	-3.256556736	0.001127724	0.008924626	negative
PRJNA729271	ed_Lushi_vs_Arbor_Acr	breed_growth_background	ENSGALG00010024847	PPIL1	0.043057114	0.080972936	0.531746979	0.594901249	0.728950415	positive
PRJNA729271	ed_Lushi_vs_Arbor_Acr	breed_growth_background	ENSGALG00010024848	ESYT1	-0.070956446	0.046266747	-1.533638102	0.125118709	0.25126014	negative
PRJNA729271	ed_Lushi_vs_Arbor_Acr	breed_growth_background	ENSGALG00010024849	CENATAC	0.132847388	0.106344641	1.249215628	0.211586218	0.361582436	positive
PRJNA729271	ed_Lushi_vs_Arbor_Acr	breed_growth_background	ENSGALG00010024850	CDK2AP1	-0.137885089	0.062956054	-2.190179986	0.028511186	0.087631392	negative
PRJNA729271	ed_Lushi_vs_Arbor_Acr	breed_growth_background	ENSGALG00010024851	CPNE5	0.359746211	0.482498643	0.745590099	0.455915066	0.611920636	positive
PRJNA729271	ed_Lushi_vs_Arbor_Acr	breed_growth_background	ENSGALG00010024852	CEP89	0.042065708	0.052085153	0.807633375	0.419301668	0.579001398	positive
PRJNA729271	ed_Lushi_vs_Arbor_Acr	breed_growth_background	ENSGALG00010024853	TJP1	-0.041461683	0.036209446	-1.145051561	0.252187799	0.408614146	negative
PRJNA729271	ed_Lushi_vs_Arbor_Acr	breed_growth_background	ENSGALG00010024854	C12orf65	-0.151521809	0.080319164	-1.886496335	0.059228102	0.149436264	negative
PRJNA729271	ed_Lushi_vs_Arbor_Acr	breed_growth_background	ENSGALG00010024855	CAPRIN1	0.05260184	0.038468229	1.367409987	0.171496834	0.314425323	positive
PRJNA729271	ed_Lushi_vs_Arbor_Acr	breed_growth_background	ENSGALG00010024856	SORT1	0.027639941	0.069902495	0.395407074	0.692542489	0.801469332	positive
PRJNA729271	ed_Lushi_vs_Arbor_Acr	breed_growth_background	ENSGALG00010024857	SLC37A4	-0.043504566	0.075695005	-0.574734966	0.565470556	0.705196223	negative
PRJNA729271	ed_Lushi_vs_Arbor_Acr	breed_growth_background	ENSGALG00010024858	SLC25A39	0.076963663	0.065932184	1.167315546	0.243082963	0.398810135	positive
PRJNA729271	ed_Lushi_vs_Arbor_Acr	breed_growth_background	ENSGALG00010024859	TARSL2	-0.196071049	0.055574315	-3.528087585	0.000418574	0.004383833	negative
PRJNA729271	ed_Lushi_vs_Arbor_Acr	breed_growth_background	ENSGALG00010024860	NEU4	-0.344409914	0.29739171	-1.158101934	0.246822441	0.402938538	negative
PRJNA729271	ed_Lushi_vs_Arbor_Acr	breed_growth_background	ENSGALG00010024861	ORC1	0.101379147	0.034355487	2.950886671	0.003168632	0.018467758	positive
PRJNA729271	ed_Lushi_vs_Arbor_Acr	breed_growth_background	ENSGALG00010024862	METTL16	0.107118276	0.064171423	1.669252013	0.095067446	0.207530516	positive
PRJNA729271	ed_Lushi_vs_Arbor_Acr	breed_growth_background	ENSGALG00010024863	LMOD1	0.143226487	0.159284383	0.899187259	0.368552926	0.531771318	positive
PRJNA729271	ed_Lushi_vs_Arbor_Acr	breed_growth_background	ENSGALG00010024864	MPHOSPH9	0.094390826	0.071337853	1.323152038	0.185784822	0.331277033	positive
PRJNA729271	ed_Lushi_vs_Arbor_Acr	breed_growth_background	ENSGALG00010024865	SNAP47	0.286772559	0.076677463	3.739984968	0.000184031	0.00243833	positive
PRJNA729271	ed_Lushi_vs_Arbor_Acr	breed_growth_background	ENSGALG00010024866	TRAPPC4	0.142414472	0.046429123	3.067352187	0.002159642	0.014012541	positive
PRJNA729271	ed_Lushi_vs_Arbor_Acr	breed_growth_background	ENSGALG00010024867	TM2D3	0.019392556	0.057527543	0.337100374	0.736041236	0.830847886	positive
PRJNA729271	ed_Lushi_vs_Arbor_Acr	breed_growth_background	ENSGALG00010024868	FAAP24	0.168796917	0.119915546	1.407631634	0.159240175	0.297731293	positive
PRJNA729271	ed_Lushi_vs_Arbor_Acr	breed_growth_background	ENSGALG00010024869	NDUFB3	-0.030259843	0.064966704	-0.465774647	0.641376814	0.764976296	negative
PRJNA729271	ed_Lushi_vs_Arbor_Acr	breed_growth_background	ENSGALG00010024870	TPD52L2	-0.070952241	0.044441458	-1.596532718	0.110369907	0.231019755	negative
PRJNA729271	ed_Lushi_vs_Arbor_Acr	breed_growth_background	ENSGALG00010024871	POLQ	-0.143904448	0.074971512	-1.919455074	0.054926767	0.141817507	negative
PRJNA729271	ed_Lushi_vs_Arbor_Acr	breed_growth_background	ENSGALG00010024872	RUNDC3A	-0.255155663	0.118765996	-2.148389866	0.031682797	0.094925253	negative
PRJNA729271	ed_Lushi_vs_Arbor_Acr	breed_growth_background	ENSGALG00010024873	HYOU1	0.022530369	0.058497279	0.385152422	0.700124491	0.80663735	positive
PRJNA729271	ed_Lushi_vs_Arbor_Acr	breed_growth_background	ENSGALG00010024874	JMJD4	0.05833534	0.044125542	1.322031143	0.186157781	0.33161571	positive
PRJNA729271	ed_Lushi_vs_Arbor_Acr	breed_growth_background	ENSGALG00010024876	CFLAR	-0.180279222	0.051484936	-3.501591642	0.000462488	0.004691226	negative
PRJNA729271	ed_Lushi_vs_Arbor_Acr	breed_growth_background	ENSGALG00010024877	PRPF38A	-0.059246872	0.044478463	-1.332035061	0.182848668	0.327896998	negative
PRJNA729271	ed_Lushi_vs_Arbor_Acr	breed_growth_background	ENSGALG00010024878	RHPN2	0.162823048	0.152921968	1.064745959	0.286990906	0.447836362	positive
PRJNA729271	ed_Lushi_vs_Arbor_Acr	breed_growth_background	ENSGALG00010024879	D2HGDH	0.235821576	0.080786137	2.919084701	0.003510608	0.019784707	positive
PRJNA729271	ed_Lushi_vs_Arbor_Acr	breed_growth_background	ENSGALG00010024880	NAT10	0.179984895	0.075341477	2.388921787	0.016897899	0.060612485	positive
PRJNA729271	ed_Lushi_vs_Arbor_Acr	breed_growth_background	ENSGALG00010024881	CASP10	0.261521999	0.15845458	1.650454018	0.098850111	0.213172381	positive
PRJNA729271	ed_Lushi_vs_Arbor_Acr	breed_growth_background	ENSGALG00010024882	MYL6	-0.010956491	0.088189765	-0.124237672	0.901127095	0.942711173	negative
PRJNA729271	ed_Lushi_vs_Arbor_Acr	breed_growth_background	ENSGALG00010024883	ING5	0.087541884	0.066264665	1.321094505	0.186469855	0.33200842	positive
PRJNA729271	ed_Lushi_vs_Arbor_Acr	breed_growth_background	ENSGALG00010024884	ZBTB46	0.282314913	0.071173227	3.966588625	7.29087E-05	0.001258109	positive
PRJNA729271	ed_Lushi_vs_Arbor_Acr	breed_growth_background	ENSGALG00010024885	SLC4A1	-0.210452165	0.127190257	-1.654624892	0.098000611	0.211764886	negative
PRJNA729271	ed_Lushi_vs_Arbor_Acr	breed_growth_background	ENSGALG00010024886	ABTB2	0.101039951	0.079719075	1.267450121	0.204994388	0.35433657	positive
PRJNA729271	ed_Lushi_vs_Arbor_Acr	breed_growth_background	ENSGALG00010024887	CASP18	0.026752023	0.135267726	0.197770925	0.843224295	0.90506146	positive
PRJNA729271	ed_Lushi_vs_Arbor_Acr	breed_growth_background	ENSGALG00010024888	PITPNM2	-0.139950892	0.06899178	-2.028515459	0.042507667	0.117798791	negative
PRJNA729271	ed_Lushi_vs_Arbor_Acr	breed_growth_background	ENSGALG00010024889	SLC2A4RG	-0.047062374	0.27509955	-0.171073978	0.864165597	0.918514147	negative
PRJNA729271	ed_Lushi_vs_Arbor_Acr	breed_growth_background	ENSGALG00010024890	SMARCC2	0.088105112	0.078176211	1.127006679	0.259739657	0.417536468	positive
PRJNA729271	ed_Lushi_vs_Arbor_Acr	breed_growth_background	ENSGALG00010024891	PKP1	0.234411794	0.256145636	0.915150448	0.360112654	0.523501446	positive
PRJNA729271	ed_Lushi_vs_Arbor_Acr	breed_growth_background	ENSGALG00010024893	ARL6IP4	-0.147706317	0.075548187	-1.955127223	0.050568056	0.133495486	negative
PRJNA729271	ed_Lushi_vs_Arbor_Acr	breed_growth_background	ENSGALG00010024894	TUT4	0.078533276	0.065362622	1.201501298	0.229556803	0.383167419	positive
PRJNA729271	ed_Lushi_vs_Arbor_Acr	breed_growth_background	ENSGALG00010024895	ENSGALG00010024895	0.01118722	0.221846814	0.05042768	0.959781579	0.977517846	positive
PRJNA729271	ed_Lushi_vs_Arbor_Acr	breed_growth_background	ENSGALG00010024896	RNF41	0.273442569	0.054250255	5.040392346	4.64578E-07	2.68289E-05	positive
PRJNA729271	ed_Lushi_vs_Arbor_Acr	breed_growth_background	ENSGALG00010024897	ADAL	-0.091852548	0.102911243	-0.892541435	0.372102794	0.534711014	negative
PRJNA729271	ed_Lushi_vs_Arbor_Acr	breed_growth_background	ENSGALG00010024898	NABP2	0.218390972	0.168955646	1.292593513	0.196151684	0.344423824	positive
PRJNA729271	ed_Lushi_vs_Arbor_Acr	breed_growth_background	ENSGALG00010024899	OGFOD2	-0.103371279	0.070393591	-1.46847572	0.141975047	0.274793471	negative
PRJNA729271	ed_Lushi_vs_Arbor_Acr	breed_growth_background	ENSGALG00010024900	DTYMK	-0.15724481	0.056831896	-2.766840805	0.005660239	0.027802494	negative
PRJNA729271	ed_Lushi_vs_Arbor_Acr	breed_growth_background	ENSGALG00010024901	ZGPAT	0.149082342	0.071208506	2.093603018	0.036295358	0.1047176	positive
PRJNA729271	ed_Lushi_vs_Arbor_Acr	breed_growth_background	ENSGALG00010024902	LARP6	-0.006588954	0.063191478	-0.104269656	0.916955357	0.953145614	negative
PRJNA729271	ed_Lushi_vs_Arbor_Acr	breed_growth_background	ENSGALG00010024903	CASP8	-0.185440325	0.062864393	-2.94984674	0.003179316	0.018492851	negative
PRJNA729271	ed_Lushi_vs_Arbor_Acr	breed_growth_background	ENSGALG00010024904	ALS2CL	0.018711393	0.085988866	0.217602509	0.827738829	0.894989313	positive
PRJNA729271	ed_Lushi_vs_Arbor_Acr	breed_growth_background	ENSGALG00010024905	GPX7	-0.24803396	0.112322492	-2.208230564	0.027228205	0.084748299	negative
PRJNA729271	ed_Lushi_vs_Arbor_Acr	breed_growth_background	ENSGALG00010024907	PHLDB1	-0.015032328	0.071336398	-0.210724514	0.833102245	0.898698894	negative
PRJNA729271	ed_Lushi_vs_Arbor_Acr	breed_growth_background	ENSGALG00010024908	ENSGALG00010024908	0.166081737	0.0680447	2.440774046	0.014655821	0.054568747	positive
PRJNA729271	ed_Lushi_vs_Arbor_Acr	breed_growth_background	ENSGALG00010024910	UBTF	0.094163543	0.066257151	1.421183093	0.155263539	0.292202674	positive
PRJNA729271	ed_Lushi_vs_Arbor_Acr	breed_growth_background	ENSGALG00010024911	CAT	0.104825697	0.081920818	1.279597783	0.20068663	0.349507714	positive
PRJNA729271	ed_Lushi_vs_Arbor_Acr	breed_growth_background	ENSGALG00010024915	STXBP5L	0.048943111	0.093885513	0.521306313	0.602153404	0.734574448	positive
PRJNA729271	ed_Lushi_vs_Arbor_Acr	breed_growth_background	ENSGALG00010024917	TRAK2	0.002480411	0.065728336	0.037737322	0.969897118	0.982586499	positive
PRJNA729271	ed_Lushi_vs_Arbor_Acr	breed_growth_background	ENSGALG00010024918	ATG4B	-0.125151696	0.040970708	-3.054662781	0.002253137	0.014490376	negative
PRJNA729271	ed_Lushi_vs_Arbor_Acr	breed_growth_background	ENSGALG00010024919	ANKRD52	0.026034562	0.066756408	0.389993447	0.696541393	0.804450019	positive
PRJNA729271	ed_Lushi_vs_Arbor_Acr	breed_growth_background	ENSGALG00010024920	VIPR2	-0.000806686	0.162791467	-0.004955335	0.996046231	0.997974018	negative
PRJNA729271	ed_Lushi_vs_Arbor_Acr	breed_growth_background	ENSGALG00010024921	STRADB	0.31595686	0.060357903	5.234722262	1.65233E-07	1.25396E-05	positive
PRJNA729271	ed_Lushi_vs_Arbor_Acr	breed_growth_background	ENSGALG00010024924	MNX1	0.37937788	0.347826964	1.090708654	0.275401102	0.434255275	positive
PRJNA729271	ed_Lushi_vs_Arbor_Acr	breed_growth_background	ENSGALG00010024925	GTF2E1	0.07753435	0.045725436	1.695650306	0.089952125	0.200183754	positive

PRJNA729271	ed_Lushi_vs_Arbor_Acr	breed_growth_background	ENSGALG00010024926	ELF5	0.212593721	0.449031806	0.473449137	0.63589278	0.760625999	positive
PRJNA729271	ed_Lushi_vs_Arbor_Acr	breed_growth_background	ENSGALG00010024927	NOM1	0.056497331	0.06222344	0.907975046	0.363891406	0.527249693	positive
PRJNA729271	ed_Lushi_vs_Arbor_Acr	breed_growth_background	ENSGALG00010024928	THAP4	0.08423765	0.04553821	1.849823473	0.064338996	0.158331755	positive
PRJNA729271	ed_Lushi_vs_Arbor_Acr	breed_growth_background	ENSGALG00010024930	TMEM237	0.357343971	0.053622501	6.664067583	2.66351E-11	8.77445E-09	positive
PRJNA729271	ed_Lushi_vs_Arbor_Acr	breed_growth_background	ENSGALG00010024931	UBE3C	0.132427042	0.047564692	2.784145876	0.005366889	0.02679747	positive
PRJNA729271	ed_Lushi_vs_Arbor_Acr	breed_growth_background	ENSGALG00010024932	ANKK1	-0.719466402	0.932128306	-0.7718534	0.44020126	0.597557339	negative
PRJNA729271	ed_Lushi_vs_Arbor_Acr	breed_growth_background	ENSGALG00010024933	CS	0.15549453	0.083969687	1.851793627	0.064055463	0.157824909	positive
PRJNA729271	ed_Lushi_vs_Arbor_Acr	breed_growth_background	ENSGALG00010024934	G6PC3	0.252642093	0.120997094	2.088001324	0.036797715	0.105813596	positive
PRJNA729271	ed_Lushi_vs_Arbor_Acr	breed_growth_background	ENSGALG00010024935	KLHL30	0.471593636	0.206559398	2.283089707	0.022425085	0.074111173	positive
PRJNA729271	ed_Lushi_vs_Arbor_Acr	breed_growth_background	ENSGALG00010024936	EHF	-0.264527829	0.298712901	-0.885558769	0.375855338	0.538342206	negative
PRJNA729271	ed_Lushi_vs_Arbor_Acr	breed_growth_background	ENSGALG00010024937	ILKAP	0.086925432	0.068823972	1.263010976	0.206585235	0.355721117	positive
PRJNA729271	ed_Lushi_vs_Arbor_Acr	breed_growth_background	ENSGALG00010024938	ENSGALG00010024938	-0.150338676	0.09740108	-1.543501117	0.122709237	0.247587748	negative
PRJNA729271	ed_Lushi_vs_Arbor_Acr	breed_growth_background	ENSGALG00010024939	COQ10A	0.356940076	0.108702126	3.283653119	0.00102471	0.008297858	positive
PRJNA729271	ed_Lushi_vs_Arbor_Acr	breed_growth_background	ENSGALG00010024940	RABL3	0.039297837	0.049730188	0.790220954	0.429398741	0.588076607	positive
PRJNA729271	ed_Lushi_vs_Arbor_Acr	breed_growth_background	ENSGALG00010024941	PDHX	-0.089226981	0.063403674	-1.407284071	0.159343171	0.29784535	negative
PRJNA729271	ed_Lushi_vs_Arbor_Acr	breed_growth_background	ENSGALG00010024942	DNAJB6	0.026749402	0.035542733	0.752598333	0.45169132	0.608001949	positive
PRJNA729271	ed_Lushi_vs_Arbor_Acr	breed_growth_background	ENSGALG00010024943	ABCB9	0.015834115	0.100964055	0.156829228	0.875379437	0.926447499	positive
PRJNA729271	ed_Lushi_vs_Arbor_Acr	breed_growth_background	ENSGALG00010024944	ENSGALG00010024944	-0.034513684	0.060269406	-0.572656777	0.566877108	0.706085057	negative
PRJNA729271	ed_Lushi_vs_Arbor_Acr	breed_growth_background	ENSGALG00010024945	APIP	0.075032378	0.044119889	1.700647484	0.089009202	0.198673912	positive
PRJNA729271	ed_Lushi_vs_Arbor_Acr	breed_growth_background	ENSGALG00010024946	KIF1A	-0.095120549	0.126484443	-0.752033581	0.452030864	0.608317462	negative
PRJNA729271	ed_Lushi_vs_Arbor_Acr	breed_growth_background	ENSGALG00010024947	MPP4	-0.174668112	0.167165624	-1.044880568	0.296078172	0.457727506	negative
PRJNA729271	ed_Lushi_vs_Arbor_Acr	breed_growth_background	ENSGALG00010024949	PAN2	-0.118654811	0.062729512	-1.891530904	0.05855351	0.148120964	negative
PRJNA729271	ed_Lushi_vs_Arbor_Acr	breed_growth_background	ENSGALG00010024950	NCAM1	-0.450977999	0.082917223	-5.438894146	5.36123E-08	5.20165E-06	negative
PRJNA729271	ed_Lushi_vs_Arbor_Acr	breed_growth_background	ENSGALG00010024953	NCAPG2	-0.360762447	0.120331185	-2.998079415	0.002716869	0.016491211	negative
PRJNA729271	ed_Lushi_vs_Arbor_Acr	breed_growth_background	ENSGALG00010024956	VPS37B	-0.120870655	0.074301903	-1.626750462	0.103790107	0.220915946	negative
PRJNA729271	ed_Lushi_vs_Arbor_Acr	breed_growth_background	ENSGALG00010024960	ARFRP1	0.123420537	0.056105448	2.19979592	0.027821378	0.086077026	positive
PRJNA729271	ed_Lushi_vs_Arbor_Acr	breed_growth_background	ENSGALG00010024961	LSM12	0.238202098	0.109549232	2.174384001	0.029676309	0.090211004	positive
PRJNA729271	ed_Lushi_vs_Arbor_Acr	breed_growth_background	ENSGALG00010024963	TNFRSF6B	-0.255009752	0.311259264	-0.819284055	0.412624368	0.572946662	negative
PRJNA729271	ed_Lushi_vs_Arbor_Acr	breed_growth_background	ENSGALG00010024964	ITM2C	0.173681106	0.093221638	1.863098622	0.062448392	0.155104428	positive
PRJNA729271	ed_Lushi_vs_Arbor_Acr	breed_growth_background	ENSGALG00010024965	STAT2	-0.075201515	0.117931994	-0.637668483	0.523689506	0.670572384	negative
PRJNA729271	ed_Lushi_vs_Arbor_Acr	breed_growth_background	ENSGALG00010024966	AGAP3	-0.051410292	0.062363668	-0.824362869	0.409733406	0.570243468	negative
PRJNA729271	ed_Lushi_vs_Arbor_Acr	breed_growth_background	ENSGALG00010024968	TMEM101	0.298831668	0.071160099	4.199427393	2.67591E-05	0.000601353	positive
PRJNA729271	ed_Lushi_vs_Arbor_Acr	breed_growth_background	ENSGALG00010024970	CAB39	0.117724358	0.041745354	2.820058905	0.004801483	0.024732588	positive
PRJNA729271	ed_Lushi_vs_Arbor_Acr	breed_growth_background	ENSGALG00010024973	RTEL1	-0.010770012	0.048611806	-0.221551371	0.824663142	0.893003836	negative
PRJNA729271	ed_Lushi_vs_Arbor_Acr	breed_growth_background	ENSGALG00010024974	CD44	-0.287483999	0.089521016	-3.211357652	0.001321094	0.00996838	negative
PRJNA729271	ed_Lushi_vs_Arbor_Acr	breed_growth_background	ENSGALG00010024977	LRP3	-0.239098094	0.055938399	-4.274310605	1.9173E-05	0.000465514	negative
PRJNA729271	ed_Lushi_vs_Arbor_Acr	breed_growth_background	ENSGALG00010024983	APOF	0.369924203	0.262736113	1.407968621	0.159140362	0.297720644	positive
PRJNA729271	ed_Lushi_vs_Arbor_Acr	breed_growth_background	ENSGALG00010024984	SHISAL2A	0.112583104	0.277463243	0.405758625	0.684919962	0.796582548	positive
PRJNA729271	ed_Lushi_vs_Arbor_Acr	breed_growth_background	ENSGALG00010024986	PTS	-0.196586873	0.092894	-2.1162494	0.034323602	0.100463847	negative
PRJNA729271	ed_Lushi_vs_Arbor_Acr	breed_growth_background	ENSGALG00010024989	TIMELESS	-0.045637869	0.089293918	-0.511097174	0.609283015	0.739971286	negative
PRJNA729271	ed_Lushi_vs_Arbor_Acr	breed_growth_background	ENSGALG00010024994	NDUFB4	0.066347831	0.055588364	1.193556105	0.232651653	0.387040711	positive
PRJNA729271	ed_Lushi_vs_Arbor_Acr	breed_growth_background	ENSGALG00010024996	COA7	0.151276256	0.067329805	2.24679482	0.024653143	0.079181797	positive
PRJNA729271	ed_Lushi_vs_Arbor_Acr	breed_growth_background	ENSGALG00010024997	HES6	0.153200803	0.136860715	1.119392097	0.262972901	0.421229946	positive
PRJNA729271	ed_Lushi_vs_Arbor_Acr	breed_growth_background	ENSGALG00010025003	ENSGALG00010025003	-0.38782367	0.122117443	-3.175825333	0.001494109	0.010877503	negative
PRJNA729271	ed_Lushi_vs_Arbor_Acr	breed_growth_background	ENSGALG00010025005	HIP1R	0.110612481	0.068215576	1.621513557	0.104907545	0.222510223	positive
PRJNA729271	ed_Lushi_vs_Arbor_Acr	breed_growth_background	ENSGALG00010025006	ENSGALG00010025006	0.377754334	0.161233325	2.342904817	0.019134264	0.066336081	positive
PRJNA729271	ed_Lushi_vs_Arbor_Acr	breed_growth_background	ENSGALG00010025007	ZYG11B	-0.031873078	0.054936902	-0.580176105	0.561795866	0.702487153	negative
PRJNA729271	ed_Lushi_vs_Arbor_Acr	breed_growth_background	ENSGALG00010025008	ENSGALG00010025008	0.732021005	0.329336317	2.222715711	0.026234976	0.082631969	positive
PRJNA729271	ed_Lushi_vs_Arbor_Acr	breed_growth_background	ENSGALG00010025012	ENSGALG00010025012	0.538675933	0.438063807	1.229674592	0.218818985	0.371055356	positive
PRJNA729271	ed_Lushi_vs_Arbor_Acr	breed_growth_background	ENSGALG00010025026	DENR	-0.074772203	0.062594921	-1.194541055	0.232266391	0.386488502	negative
PRJNA729271	ed_Lushi_vs_Arbor_Acr	breed_growth_background	ENSGALG00010025028	HES6	-0.187200986	0.073326215	-2.552988532	0.010680301	0.043893101	negative
PRJNA729271	ed_Lushi_vs_Arbor_Acr	breed_growth_background	ENSGALG00010025031	ECHDC2	0.10415947	0.065435813	1.591780784	0.111433969	0.232351938	positive
PRJNA729271	ed_Lushi_vs_Arbor_Acr	breed_growth_background	ENSGALG00010025032	FSTL1	-0.31836545	0.096904228	-3.285361818	0.001018515	0.00825692	negative
PRJNA729271	ed_Lushi_vs_Arbor_Acr	breed_growth_background	ENSGALG00010025039	CEBPA	-0.248781184	0.165481809	-1.503374817	0.132742417	0.261925038	negative
PRJNA729271	ed_Lushi_vs_Arbor_Acr	breed_growth_background	ENSGALG00010025042	BCO2	0.625325694	0.360828227	1.733028759	0.083090557	0.188984407	positive
PRJNA729271	ed_Lushi_vs_Arbor_Acr	breed_growth_background	ENSGALG00010025044	TMEM9	-0.149307568	0.114308182	-1.306184437	0.191489844	0.33832829	negative
PRJNA729271	ed_Lushi_vs_Arbor_Acr	breed_growth_background	ENSGALG00010025047	PER2	0.012941002	0.156803827	0.082529887	0.934225353	0.96406922	positive
PRJNA729271	ed_Lushi_vs_Arbor_Acr	breed_growth_background	ENSGALG00010025048	ESYT2	0.005384434	0.04405397	0.122223582	0.902721951	0.944012314	positive
PRJNA729271	ed_Lushi_vs_Arbor_Acr	breed_growth_background	ENSGALG00010025055	ALS2	-0.242755639	0.082484399	-2.943049138	0.003249968	0.018790702	negative
PRJNA729271	ed_Lushi_vs_Arbor_Acr	breed_growth_background	ENSGALG00010025056	RBMS2	0.006603509	0.094757108	0.069688798	0.944441358	0.969798632	positive
PRJNA729271	ed_Lushi_vs_Arbor_Acr	breed_growth_background	ENSGALG00010025057	SCP2	0.12740711	0.035972782	3.541764167	0.000397461	0.004224377	positive
PRJNA729271	ed_Lushi_vs_Arbor_Acr	breed_growth_background	ENSGALG00010025060	GPR156	-0.023474671	0.091906845	-0.255418089	0.798400201	0.875112152	negative
PRJNA729271	ed_Lushi_vs_Arbor_Acr	breed_growth_background	ENSGALG00010025064	GPN3	-0.00157267	0.044916392	-0.035013271	0.972069159	0.983408337	negative
PRJNA729271	ed_Lushi_vs_Arbor_Acr	breed_growth_background	ENSGALG00010025067	SLC1A2	-0.678555815	0.183641154	-3.695009542	0.000219879	0.002790619	negative
PRJNA729271	ed_Lushi_vs_Arbor_Acr	breed_growth_background	ENSGALG00010025069	PODN	-0.018056202	0.105220845	-0.171602904	0.863749724	0.91816165	negative
PRJNA729271	ed_Lushi_vs_Arbor_Acr	breed_growth_background	ENSGALG00010025071	CDK15	0.03851361	0.178696732	0.215524982	0.829358037	0.895856671	positive
PRJNA729271	ed_Lushi_vs_Arbor_Acr	breed_growth_background	ENSGALG00010025072	ENSGALG00010025072	-1.02348805	0.434660098	-2.354686006	0.018538363	0.064828365	negative
PRJNA729271	ed_Lushi_vs_Arbor_Acr	breed_growth_background	ENSGALG00010025073	IL18	-0.014355081	0.108028823	-0.132881949	0.894286743	0.938112726	negative
PRJNA729271	ed_Lushi_vs_Arbor_Acr	breed_growth_background	ENSGALG00010025080	BAZ2A	0.235534036	0.108515604	2.17050845	0.029968349	0.090781865	positive
PRJNA729271	ed_Lushi_vs_Arbor_Acr	breed_growth_background	ENSGALG00010025092	COL1A1	0.209185371	0.11810332	1.771206528	0.076526364	0.178536882	positive
PRJNA729271	ed_Lushi_vs_Arbor_Acr	breed_growth_background	ENSGALG00010025097	LMBR1	-0.226404159	0.064975116	-3.484474891	0.000493104	0.004922547	negative
PRJNA729271	ed_Lushi_vs_Arbor_Acr	breed_growth_background	ENSGALG00010025098	ATP5B	0.069064527	0.073879455	0.93482723	0.349877344	0.513879025	positive
PRJNA729271	ed_Lushi_vs_Arbor_Acr	breed_growth_background	ENSGALG00010025102	ARPC3	-0.027914079	0.046587709	-0.599172593	0.549057797	0.69238446	negative
PRJNA729271	ed_Lushi_vs_Arbor_Acr	breed_growth_background	ENSGALG00010025103	GSK3B	0.118777702	0.053759465	2.209428658	0.02714484	0.084579632	positive
PRJNA729271	ed_Lushi_vs_Arbor_Acr	breed_growth_background	ENSGALG00010025113	SDHD	-0.0998905	0.064255674	-1.554578659	0.12004645	0.244433864	negative
PRJNA729271	ed_Lushi_vs_Arbor_Acr	breed_growth_background	ENSGALG00010025114	CEBPG	0.130416701	0.052130094	2.501754594	0.012357955	0.048541849	positive
PRJNA729271	ed_Lushi_vs_Arbor_Acr	breed_growth_background	ENSGALG00010025115	MGAT4F	0.080364515	0.140874082	0.570470553	0.568358589	0.707337319	positive
PRJNA729271	ed_Lushi_vs_Arbor_Acr	breed_growth_background	ENSGALG00010025116	ANAPC7	0.018378744	0.038358074	0.479136252	0.631841702	0.757342716	positive
PRJNA729271	ed_Lushi_vs_Arbor_Acr	breed_growth_background	ENSGALG00010025121	DYNC2I1	0.134533378	0.084906835	1.584482304	0.113084006	0.234633934	positive
PRJNA729271	ed_Lushi_vs_Arbor_Acr	breed_growth_background	ENSGALG00010025125	PIK3CB	-0.102071792	0.07062063	-1.445353757	0.148358545	0.283290359	negative
PRJNA729271	ed_Lushi_vs_Arbor_Acr	breed_growth_background	ENSGALG00010025126	ENSGALG00010025126	0.239629447	0.07327303	3.270363539	0.001074094	0.008606404	positive
PRJNA729271	ed_Lushi_vs_Arbor_Acr	breed_growth_background	ENSGALG00010025127	PAMR1	-0.072757167	0.085052149	-0.855441839	0.39230659	0.553752461	negative
PRJNA729271	ed_Lushi_vs_Arbor_Acr	breed_growth_background	ENSGALG00010025130	ATP2A2	-0.250478263	0.067378646	-3.717472495	0.000201226	0.002601935	negative
PRJNA729271	ed_Lushi_vs_Arbor_Acr	breed_growth_background	ENSGALG00010025131	SUMO1	0.047386004	0.043485772	1.089689834	0.275849796	0.434755115	positive
PRJNA729271	ed_Lushi_vs_Arbor_Acr	breed_growth_background	ENSGALG00010025138	CFAP91	-0.485199523	0.156351954	-3.103252058	0.001914065	0.012862484	negative
PRJNA729271	ed_Lushi_vs_Arbor_Acr	breed_growth_background	ENSGALG00010025139	COPB2	0.046109461	0.025319573	1.821099435	0.068591743	0.165595822	positive
PRJNA729271	ed_Lushi_vs_Arbor_Acr	breed_growth_background	ENSGALG00010025144	PIH1D2	0.141235671	0.087526895	1.613625969	0.10660858	0.225268069	positive
PRJNA729271	ed_Lushi_vs_Arbor_Acr	breed_growth_background	ENSGALG00010025145	CPT2	0.13221199	0.051245598	2.579967755	0.009880954	0.041466251	positive
PRJNA729271	ed_Lushi_vs_Arbor_Acr	breed_growth_background	ENSGALG00010025147	COX17	-0.202045097	0.06055593	-3.336503878	0.000848392	0.007255132	negative
PRJNA729271	ed_Lushi_vs_Arbor_Acr	breed_growth_background	ENSGALG00010025149	IFT81	-0.190064297	0.077135337	-2.464036654	0.013738206	0.052351899	negative
PRJNA729271	ed_Lushi_vs_Arbor_Acr	breed_growth_background	ENSGALG00010025154	TMEM183A	0.172870666	0.065078891	2.6563247	0.007899751	0.035298068	positive
PRJNA729271	ed_Lushi_vs_Arbor_Acr	breed_growth_background	ENSGALG00010025155	CZIB	0.160152205	0.049449045	3.238731993	0.001200623	0.009351443	positive
PRJNA729271	ed_Lushi_vs_Arbor_Acr	breed_growth_background	ENSGALG00010025156	DLAT	-0.014013551	0.061621253	-0.227414251	0.82010164	0.889507129	negative
PRJNA729271	ed_Lushi_vs_Arbor_Acr	breed_growth_background	ENSGALG00010025157	NOP58	0.101782085	0.075507047	1.347981269	0.177664407	0.321825241	positive
PRJNA729271	ed_Lushi_vs_Arbor_Acr	breed_growth_background	ENSGALG00010025160	MRPS22	0.195308973	0.070181536	2.782911063	0.005387357	0.026881151	positive
PRJNA729271	ed_Lushi_vs_Arbor_Acr	breed_growth_background	ENSGALG00010025161	ENSGALG00010025161	-0.051579425	0.056797949	-0.908121254	0.363814162	0.527190471	negative
PRJNA729271	ed_Lushi_vs_Arbor_Acr	breed_growth_background	ENSGALG00010025163	LDLRAD3	-0.011613602	0.074473383	-0.155942995	0.876077955	0.926645502	negative
PRJNA729271	ed_Lushi_vs_Arbor_Acr	breed_growth_background	ENSGALG00010025164	C11orf52	-0.035284068	0.066181704	-0.533139309	0.59393715	0.72822864	negative
PRJNA729271	ed_Lushi_vs_Arbor_Acr	breed_growth_background	ENSGALG00010025167	PTGES3	0.190312667	0.101059603	1.883172521	0.059676992	0.150254993	positive
PRJNA729271	ed_Lushi_vs_Arbor_Acr	breed_growth_background	ENSGALG00010025168	POPDC2	0.152330597	0.112888211	1.349393315	0.177210667	0.321244199	positive
PRJNA729271	ed_Lushi_vs_Arbor_Acr	breed_growth_background	ENSGALG00010025169	RBP2	-1.508416938	1.137315623	-1.326295803	0.184741737	0.330146524	negative
PRJNA729271	ed_Lushi_vs_Arbor_Acr	breed_growth_background	ENSGALG00010025170	COMMD9	-0.081965082	0.052606156	-1.558089169	0.119212112	0.243274611	negative
PRJNA729271	ed_Lushi_vs_Arbor_Acr	breed_growth_background	ENSGALG00010025172	RBP1	1.10823964	0.333764535	3.320423602	0.00089881	0.00754685	positive
PRJNA729271	ed_Lushi_vs_Arbor_Acr	breed_growth_background	ENSGALG00010025182	PLA1A	-0.308455972	0.097831152	-3.152942249	0.001616338	0.011439265	negative
PRJNA729271	ed_Lushi_vs_Arbor_Acr	breed_growth_background	ENSGALG00010025186	TMEM120B	-0.319631893	0.113006604	-2.828435535	0.004677612	0.024236873	negative
PRJNA729271	ed_Lushi_vs_Arbor_Acr	breed_growth_background	ENSGALG00010025188	PPFIA4	0.511079927	0.21400828	2.388131554	0.016934279	0.060712929	positive
PRJNA729271	ed_Lushi_vs_Arbor_Acr	breed_growth_background	ENSGALG00010025190	PRR5L	-0.075820479	0.129210881	-0.586796396	0.557340458	0.698663836	negative
PRJNA729271	ed_Lushi_vs_Arbor_Acr	breed_growth_background	ENSGALG00010025192	CRYAB	0.083563384	0.097252185	0.859244291	0.390205752	0.551782232	positive
PRJNA729271	ed_Lushi_vs_Arbor_Acr	breed_growth_background	ENSGALG00010025195	P2RX7	0.049602152	0.197303658	0.251400065	0.801504818	0.877412003	positive
PRJNA729271	ed_Lushi_vs_Arbor_Acr	breed_growth_background	ENSGALG00010025200	PEPD	-0.07958475	0.04352867	-1.828329443	0.067500124	0.163990154	negative
PRJNA729271	ed_Lushi_vs_Arbor_Acr	breed_growth_background	ENSGALG00010025204	TRAF6	0.152391105	0.062833011	2.425335053	0.015294264	0.05648162	positive
PRJNA729271	ed_Lushi_vs_Arbor_Acr	breed_growth_background	ENSGALG00010025207	BMPR2	-0.013944998	0.052305406	-0.266607197	0.789771614	0.869521474	negative
PRJNA729271	ed_Lushi_vs_Arbor_Acr	breed_growth_background	ENSGALG00010025210	HSPB2	0.360600734	0.158329938	2.277527156	0.022754765	0.074901906	positive
PRJNA729271	ed_Lushi_vs_Arbor_Acr	breed_growth_background	ENSGALG00010025212	P2RX4	-0.200848561	0.096625172	-2.078636006	0.037650819	0.107834148	negative
PRJNA729271	ed_Lushi_vs_Arbor_Acr	breed_growth_background	ENSGALG00010025214	LRP8	-0.140102586	0.126192474	-1.110229334	0.266900215	0.425227371	negative
PRJNA729271	ed_Lushi_vs_Arbor_Acr	breed_growth_background	ENSGALG00010025215	MYOG	-0.338369079	0.11528004	-2.935192246	0.003333411	0.019120614	negative
PRJNA729271	ed_Lushi_vs_Arbor_Acr	breed_growth_background	ENSGALG00010025216	DPP6	0.183546955	0.144955836	1.266226733	0.205431919	0.354745732	positive
PRJNA729271	ed_Lushi_vs_Arbor_Acr	breed_growth_background	ENSGALG00010025219	NACA	-0.112450629	0.067273171	-1.671552372	0.094612623	0.20688037	negative
PRJNA729271	ed_Lushi_vs_Arbor_Acr	breed_growth_background	ENSGALG00010025222	FAM117B	-0.103702027	0.080380249	-1.290143136	0.197000965	0.345454754	negative
PRJNA729271	ed_Lushi_vs_Arbor_Acr	breed_growth_background	ENSGALG00010025225	DMRTB1	0.062484799	0.416155153	0.150147843	0.880647974	0.929121364	positive
PRJNA729271	ed_Lushi_vs_Arbor_Acr	breed_growth_background	ENSGALG00010025228	FDXACB1	0.005653882	0.059468907	0.095072915	0.924256911	0.957688464	positive
PRJNA729271	ed_Lushi_vs_Arbor_Acr	breed_growth_background	ENSGALG00010025229	ENSGALG00010025229	-0.356088652	0.089632856	-3.972746915	7.10485E-05	0.001237799	negative
PRJNA729271	ed_Lushi_vs_Arbor_Acr	breed_growth_background	ENSGALG00010025230	CHST8	0.238921357	0.160829486	1.485556929	0.137396355	0.268512898	positive
PRJNA729271	ed_Lushi_vs_Arbor_Acr	breed_growth_background	ENSGALG00010025232	PRIM1	-0.151041187	0.082355112	-1.834023211	0.066650549	0.162560946	negative
PRJNA729271	ed_Lushi_vs_Arbor_Acr	breed_growth_background	ENSGALG00010025233	WDR12	-0.07948966	0.041657091	-1.908190369	0.056366616	0.144454402	negative
PRJNA729271	ed_Lushi_vs_Arbor_Acr	breed_growth_background	ENSGALG00010025236	ENSGALG00010025236	0.109814299	0.133823165	0.820592601	0.411878364	0.572184865	positive
PRJNA729271	ed_Lushi_vs_Arbor_Acr	breed_growth_background	ENSGALG00010025238	IGSF9B	-0.33339861	0.164390447	-2.02808993	0.04255107	0.117840613	negative
PRJNA729271	ed_Lushi_vs_Arbor_Acr	breed_growth_background	ENSGALG00010025239	RAG2	-0.379658212	0.38048099	-0.997837533	0.318358147	0.481741449	negative
PRJNA729271	ed_Lushi_vs_Arbor_Acr	breed_growth_background	ENSGALG00010025242	CLSTN2	0.847776008	0.167745522	5.053941219	4.32785E-07	2.53976E-05	positive
PRJNA729271	ed_Lushi_vs_Arbor_Acr	breed_growth_background	ENSGALG00010025243	PAXIP1	0.068104459	0.04889277	1.39293518	0.163639393	0.303461696	positive
PRJNA729271	ed_Lushi_vs_Arbor_Acr	breed_growth_background	ENSGALG00010025245	ENSGALG00010025245	0.034619944	0.036449228	0.949812819	0.342207371	0.505938073	positive

PRJNA729271	ed_Lushi_vs_Arbor_Acr	breed_growth_background	ENSGALG00010025247	SGCA	-0.006157707	0.073505415	-0.083772155	0.933237588	0.963481398	negative
PRJNA729271	ed_Lushi_vs_Arbor_Acr	breed_growth_background	ENSGALG00010025248	C11orf74	-0.354958288	0.117329542	-3.025310428	0.002483781	0.015511589	negative
PRJNA729271	ed_Lushi_vs_Arbor_Acr	breed_growth_background	ENSGALG00010025250	GLIS1	0.026466012	0.273468652	0.096778962	0.922901931	0.957079179	positive
PRJNA729271	ed_Lushi_vs_Arbor_Acr	breed_growth_background	ENSGALG00010025252	PPP1R9B	0.069568084	0.078476529	0.886482679	0.375357484	0.537735395	positive
PRJNA729271	ed_Lushi_vs_Arbor_Acr	breed_growth_background	ENSGALG00010025256	ANAPC5	-0.02759226	0.067540963	-0.408526302	0.682887325	0.795247993	negative
PRJNA729271	ed_Lushi_vs_Arbor_Acr	breed_growth_background	ENSGALG00010025257	LPCAT2	0.049133349	0.089647542	0.548072458	0.583642151	0.719624132	positive
PRJNA729271	ed_Lushi_vs_Arbor_Acr	breed_growth_background	ENSGALG00010025259	SHH	0.19559902	0.504305902	0.387857884	0.698121207	0.805308638	positive
PRJNA729271	ed_Lushi_vs_Arbor_Acr	breed_growth_background	ENSGALG00010025262	CARF	0.030934394	0.061427641	0.503590797	0.614548961	0.743755645	positive
PRJNA729271	ed_Lushi_vs_Arbor_Acr	breed_growth_background	ENSGALG00010025263	SAMD14	0.463318094	0.479420399	0.966412975	0.333837574	0.49809322	positive
PRJNA729271	ed_Lushi_vs_Arbor_Acr	breed_growth_background	ENSGALG00010025268	ZBTB39	-0.038692006	0.06015664	-0.643187617	0.520102368	0.667807939	negative
PRJNA729271	ed_Lushi_vs_Arbor_Acr	breed_growth_background	ENSGALG00010025270	NDC1	-0.109303019	0.067021785	-1.630858079	0.102920269	0.219529124	negative
PRJNA729271	ed_Lushi_vs_Arbor_Acr	breed_growth_background	ENSGALG00010025271	RNF34	0.130447133	0.06305627	2.068741642	0.038570337	0.109644448	positive
PRJNA729271	ed_Lushi_vs_Arbor_Acr	breed_growth_background	ENSGALG00010025272	NCAPD3	-0.308567639	0.10125426	-3.047453397	0.002307893	0.01474346	negative
PRJNA729271	ed_Lushi_vs_Arbor_Acr	breed_growth_background	ENSGALG00010025274	PDK2	0.719609744	0.142978352	5.032997881	4.82868E-07	2.73405E-05	positive
PRJNA729271	ed_Lushi_vs_Arbor_Acr	breed_growth_background	ENSGALG00010025280	HSPB11	-0.208651431	0.084438214	-2.471054532	0.013471527	0.051549575	negative
PRJNA729271	ed_Lushi_vs_Arbor_Acr	breed_growth_background	ENSGALG00010025286	ITGA3	0.009932919	0.095832674	0.103648564	0.917448247	0.95349626	positive
PRJNA729271	ed_Lushi_vs_Arbor_Acr	breed_growth_background	ENSGALG00010025289	KDM2B	-0.041441924	0.040842933	-1.014665731	0.310265195	0.473218217	negative
PRJNA729271	ed_Lushi_vs_Arbor_Acr	breed_growth_background	ENSGALG00010025291	DUS2	0.102647834	0.051913129	1.97730009	0.04800772	0.128621324	positive
PRJNA729271	ed_Lushi_vs_Arbor_Acr	breed_growth_background	ENSGALG00010025292	ATP1A1	-0.121186106	0.068188293	-1.77722746	0.075530823	0.176983395	negative
PRJNA729271	ed_Lushi_vs_Arbor_Acr	breed_growth_background	ENSGALG00010025297	SPSB4	-0.218975688	0.080085686	-2.734267485	0.006251923	0.029868698	negative
PRJNA729271	ed_Lushi_vs_Arbor_Acr	breed_growth_background	ENSGALG00010025298	ENSGALG00010025298	-0.691567577	0.193797896	-3.568498893	0.000359032	0.003918806	negative
PRJNA729271	ed_Lushi_vs_Arbor_Acr	breed_growth_background	ENSGALG00010025301	MORN3	0.381665429	0.150822862	2.530554218	0.011388248	0.045830166	positive
PRJNA729271	ed_Lushi_vs_Arbor_Acr	breed_growth_background	ENSGALG00010025303	TMCC2	0.110938468	0.100244138	1.10668284	0.268431077	0.426962413	positive
PRJNA729271	ed_Lushi_vs_Arbor_Acr	breed_growth_background	ENSGALG00010025304	DDX28	0.052136904	0.05070975	1.028143594	0.303882287	0.466409757	positive
PRJNA729271	ed_Lushi_vs_Arbor_Acr	breed_growth_background	ENSGALG00010025305	GPR182	0.456580779	0.197910409	2.307007409	0.021054407	0.070726218	positive
PRJNA729271	ed_Lushi_vs_Arbor_Acr	breed_growth_background	ENSGALG00010025311	ENSGALG00010025311	-0.184973338	0.31451045	-0.588130977	0.556444382	0.697963077	negative
PRJNA729271	ed_Lushi_vs_Arbor_Acr	breed_growth_background	ENSGALG00010025312	PLEKHM3	0.078045081	0.061272196	1.273743816	0.202754223	0.352008918	positive
PRJNA729271	ed_Lushi_vs_Arbor_Acr	breed_growth_background	ENSGALG00010025313	DIO1	-1.192594324	0.500328714	-2.38362159	0.01714322	0.061246422	negative
PRJNA729271	ed_Lushi_vs_Arbor_Acr	breed_growth_background	ENSGALG00010025315	LRRC42	0.004364075	0.065333783	0.066796606	0.946743625	0.970833716	positive
PRJNA729271	ed_Lushi_vs_Arbor_Acr	breed_growth_background	ENSGALG00010025316	SLC25A36	-0.179348717	0.059133828	-3.032929249	0.002421924	0.015250124	negative
PRJNA729271	ed_Lushi_vs_Arbor_Acr	breed_growth_background	ENSGALG00010025318	NUAK2	-0.138767219	0.257701381	-0.538480696	0.590245229	0.725481607	negative
PRJNA729271	ed_Lushi_vs_Arbor_Acr	breed_growth_background	ENSGALG00010025319	LDLRAD1	-0.435740062	0.192649627	-2.261826659	0.023708115	0.07702198	negative
PRJNA729271	ed_Lushi_vs_Arbor_Acr	breed_growth_background	ENSGALG00010025320	JAM3	-0.260237126	0.07761881	-3.352758492	0.000800105	0.006961585	negative
PRJNA729271	ed_Lushi_vs_Arbor_Acr	breed_growth_background	ENSGALG00010025323	KLHDC8A	-0.198444011	0.096977917	-2.04628041	0.04072879	0.113837989	negative
PRJNA729271	ed_Lushi_vs_Arbor_Acr	breed_growth_background	ENSGALG00010025325	DPEP2	-0.255090655	0.132235351	-1.929065517	0.053722729	0.139354145	negative
PRJNA729271	ed_Lushi_vs_Arbor_Acr	breed_growth_background	ENSGALG00010025327	TMEM59	-0.08996402	0.03804797	-2.36448934	0.018054947	0.063629859	negative
PRJNA729271	ed_Lushi_vs_Arbor_Acr	breed_growth_background	ENSGALG00010025329	MYO1A	-0.403588781	0.111853436	-3.608192964	0.000308337	0.003533514	negative
PRJNA729271	ed_Lushi_vs_Arbor_Acr	breed_growth_background	ENSGALG00010025330	USH1C	-0.546146072	0.219707998	-2.485781473	0.012926732	0.050099726	negative
PRJNA729271	ed_Lushi_vs_Arbor_Acr	breed_growth_background	ENSGALG00010025331	ENSGALG00010025331	-0.194632083	0.18904101	-1.029575979	0.303209096	0.465869816	negative
PRJNA729271	ed_Lushi_vs_Arbor_Acr	breed_growth_background	ENSGALG00010025333	FZD5	-0.154099238	0.106774794	-1.443217371	0.148959248	0.283900549	negative
PRJNA729271	ed_Lushi_vs_Arbor_Acr	breed_growth_background	ENSGALG00010025334	RHOF	0.018105902	0.121513201	0.149003577	0.88155081	0.929720532	positive
PRJNA729271	ed_Lushi_vs_Arbor_Acr	breed_growth_background	ENSGALG00010025336	ADAM10	-0.083340935	0.034084952	-2.445094681	0.014481412	0.05400259	negative
PRJNA729271	ed_Lushi_vs_Arbor_Acr	breed_growth_background	ENSGALG00010025337	NEMP1	0.166638036	0.08158487	2.04251152	0.041100818	0.114656727	positive
PRJNA729271	ed_Lushi_vs_Arbor_Acr	breed_growth_background	ENSGALG00010025338	FADD	-0.21583791	0.053156992	-4.060386041	4.89916E-05	0.000958345	negative
PRJNA729271	ed_Lushi_vs_Arbor_Acr	breed_growth_background	ENSGALG00010025340	METTL21A	0.238843214	0.063332848	3.771237556	0.00016244	0.002267954	positive
PRJNA729271	ed_Lushi_vs_Arbor_Acr	breed_growth_background	ENSGALG00010025341	VPS26B	0.102682971	0.071425039	1.437632702	0.150538297	0.286187567	positive
PRJNA729271	ed_Lushi_vs_Arbor_Acr	breed_growth_background	ENSGALG00010025342	TCEANC2	0.26173549	0.066869752	3.914108882	9.07387E-05	0.001487848	positive
PRJNA729271	ed_Lushi_vs_Arbor_Acr	breed_growth_background	ENSGALG00010025343	NAB2	-0.060344013	0.157967507	-0.382002689	0.70245937	0.807722399	negative
PRJNA729271	ed_Lushi_vs_Arbor_Acr	breed_growth_background	ENSGALG00010025344	ENSGALG00010025344	0.840574565	0.286762005	2.931261987	0.003375879	0.019287888	positive
PRJNA729271	ed_Lushi_vs_Arbor_Acr	breed_growth_background	ENSGALG00010025345	CCM2	-0.103432517	0.048630417	-2.126909956	0.033427565	0.098422214	negative
PRJNA729271	ed_Lushi_vs_Arbor_Acr	breed_growth_background	ENSGALG00010025346	PXYLP1	-0.142429097	0.071816341	-1.983240803	0.047340538	0.127215629	negative
PRJNA729271	ed_Lushi_vs_Arbor_Acr	breed_growth_background	ENSGALG00010025348	HIPK1	0.053911234	0.050070124	1.076714617	0.28160778	0.441621202	positive
PRJNA729271	ed_Lushi_vs_Arbor_Acr	breed_growth_background	ENSGALG00010025349	SETD1A	0.070128422	0.06826769	1.027256409	0.304299743	0.466753944	positive
PRJNA729271	ed_Lushi_vs_Arbor_Acr	breed_growth_background	ENSGALG00010025351	CREB1	0.027821099	0.036300139	0.766418539	0.443427306	0.600418383	positive
PRJNA729271	ed_Lushi_vs_Arbor_Acr	breed_growth_background	ENSGALG00010025353	SLC12A4	-0.16052957	0.041867516	-3.834227213	0.00012596	0.001884195	negative
PRJNA729271	ed_Lushi_vs_Arbor_Acr	breed_growth_background	ENSGALG00010025354	ENSGALG00010025354	-0.667755715	0.907832713	-0.735549299	0.462005028	0.617182224	negative
PRJNA729271	ed_Lushi_vs_Arbor_Acr	breed_growth_background	ENSGALG00010025355	STAT6	-0.046394031	0.074997867	-0.618604673	0.536176823	0.681325769	negative
PRJNA729271	ed_Lushi_vs_Arbor_Acr	breed_growth_background	ENSGALG00010025357	OLFML3	-0.113345787	0.064928473	-1.745702336	0.080862689	0.18543026	negative
PRJNA729271	ed_Lushi_vs_Arbor_Acr	breed_growth_background	ENSGALG00010025358	INCENP	-0.348601908	0.110541383	-3.153587348	0.00161277	0.011436935	negative
PRJNA729271	ed_Lushi_vs_Arbor_Acr	breed_growth_background	ENSGALG00010025359	CDCP2	0.508144626	0.207341623	2.450760344	0.014255483	0.053531924	positive
PRJNA729271	ed_Lushi_vs_Arbor_Acr	breed_growth_background	ENSGALG00010025360	THYN1	-0.149928151	0.059493856	-2.520061069	0.011733448	0.046827181	negative
PRJNA729271	ed_Lushi_vs_Arbor_Acr	breed_growth_background	ENSGALG00010025362	G6PC2	-0.540300218	0.134374544	-4.020852489	5.79879E-05	0.00107791	negative
PRJNA729271	ed_Lushi_vs_Arbor_Acr	breed_growth_background	ENSGALG00010025364	EPHA4	0.003235968	0.102449105	0.031586106	0.974802124	0.98526998	positive
PRJNA729271	ed_Lushi_vs_Arbor_Acr	breed_growth_background	ENSGALG00010025365	SIKE1	0.00808327	0.049059094	0.164765989	0.869128175	0.921985721	positive
PRJNA729271	ed_Lushi_vs_Arbor_Acr	breed_growth_background	ENSGALG00010025368	LRP1	-0.085739504	0.074179094	-1.155844578	0.247744735	0.403898317	negative
PRJNA729271	ed_Lushi_vs_Arbor_Acr	breed_growth_background	ENSGALG00010025370	HPD	-0.518510685	0.261132028	-1.985626537	0.047074808	0.126730702	negative
PRJNA729271	ed_Lushi_vs_Arbor_Acr	breed_growth_background	ENSGALG00010025373	CYB5RL	-0.027928535	0.049263997	-0.566915731	0.570771436	0.70925645	negative
PRJNA729271	ed_Lushi_vs_Arbor_Acr	breed_growth_background	ENSGALG00010025377	MYO1G	0.223672464	0.152144639	1.470130438	0.14152643	0.27429143	positive
PRJNA729271	ed_Lushi_vs_Arbor_Acr	breed_growth_background	ENSGALG00010025378	PAX3	-0.555787052	0.14861832	-3.739694089	0.000184244	0.002438924	negative
PRJNA729271	ed_Lushi_vs_Arbor_Acr	breed_growth_background	ENSGALG00010025379	ACAD8	0.184675416	0.098947772	1.866392868	0.06198642	0.154358014	positive
PRJNA729271	ed_Lushi_vs_Arbor_Acr	breed_growth_background	ENSGALG00010025380	SPC25	-0.449061404	0.121608038	-3.692695082	0.00022189	0.002801651	negative
PRJNA729271	ed_Lushi_vs_Arbor_Acr	breed_growth_background	ENSGALG00010025381	MRPL37	0.060383238	0.065274855	0.925061238	0.354934054	0.518678205	positive
PRJNA729271	ed_Lushi_vs_Arbor_Acr	breed_growth_background	ENSGALG00010025382	MAB21L3	-0.168984038	0.17437242	-0.969098425	0.332496084	0.496858838	negative
PRJNA729271	ed_Lushi_vs_Arbor_Acr	breed_growth_background	ENSGALG00010025386	TPCN2	-0.211369655	0.054206736	-3.899324536	9.64614E-05	0.00154914	negative
PRJNA729271	ed_Lushi_vs_Arbor_Acr	breed_growth_background	ENSGALG00010025387	PSMD9	-0.029230384	0.037337906	-0.782860823	0.433708846	0.591736607	negative
PRJNA729271	ed_Lushi_vs_Arbor_Acr	breed_growth_background	ENSGALG00010025389	SNED1	-0.101351409	0.081801675	-1.2389894	0.215349424	0.36641506	negative
PRJNA729271	ed_Lushi_vs_Arbor_Acr	breed_growth_background	ENSGALG00010025390	BCL7A	-0.052704614	0.047984235	-1.098373543	0.27204141	0.430813967	negative
PRJNA729271	ed_Lushi_vs_Arbor_Acr	breed_growth_background	ENSGALG00010025392	SLC6A2	-0.138922331	0.131415535	-1.057122592	0.290455642	0.451975794	negative
PRJNA729271	ed_Lushi_vs_Arbor_Acr	breed_growth_background	ENSGALG00010025398	TSPAN2	0.353992298	0.077829713	4.548292488	5.40829E-06	0.000183162	positive
PRJNA729271	ed_Lushi_vs_Arbor_Acr	breed_growth_background	ENSGALG00010025400	SLC22A15	-0.109463536	0.086646837	-1.263329848	0.206470664	0.355649705	negative
PRJNA729271	ed_Lushi_vs_Arbor_Acr	breed_growth_background	ENSGALG00010025403	ENSGALG00010025403	0.169339819	0.069235643	2.445847429	0.014451214	0.053945492	positive
PRJNA729271	ed_Lushi_vs_Arbor_Acr	breed_growth_background	ENSGALG00010025404	CERS6	-0.694176295	0.154015205	-4.507193277	6.56908E-06	0.000213017	negative
PRJNA729271	ed_Lushi_vs_Arbor_Acr	breed_growth_background	ENSGALG00010025405	TAMALIN	0.331034467	0.08625418	3.837894761	0.000124094	0.001867848	positive
PRJNA729271	ed_Lushi_vs_Arbor_Acr	breed_growth_background	ENSGALG00010025406	ENSGALG00010025406	0.153623354	0.079839292	1.924157281	0.054334872	0.140564692	positive
PRJNA729271	ed_Lushi_vs_Arbor_Acr	breed_growth_background	ENSGALG00010025407	MLXIP	0.137519033	0.068263433	2.014534365	0.043953461	0.120549747	positive
PRJNA729271	ed_Lushi_vs_Arbor_Acr	breed_growth_background	ENSGALG00010025413	NECAB3	0.02391123	0.064829098	0.368834845	0.712250831	0.814972828	positive
PRJNA729271	ed_Lushi_vs_Arbor_Acr	breed_growth_background	ENSGALG00010025414	MMP2	-0.224322145	0.118364279	-1.895184481	0.058067965	0.14722672	negative
PRJNA729271	ed_Lushi_vs_Arbor_Acr	breed_growth_background	ENSGALG00010025415	ENSGALG00010025415	0.005197176	0.138905955	0.037415068	0.970154059	0.982696232	positive
PRJNA729271	ed_Lushi_vs_Arbor_Acr	breed_growth_background	ENSGALG00010025416	ENSGALG00010025416	0.025062656	0.354952293	0.070608519	0.94370933	0.969321623	positive
PRJNA729271	ed_Lushi_vs_Arbor_Acr	breed_growth_background	ENSGALG00010025417	B3GAT1	0.161324777	0.109934427	1.467463669	0.142249965	0.27514188	positive
PRJNA729271	ed_Lushi_vs_Arbor_Acr	breed_growth_background	ENSGALG00010025418	CASQ2	-0.232192982	0.111883411	-2.075311973	0.03795763	0.108455717	negative
PRJNA729271	ed_Lushi_vs_Arbor_Acr	breed_growth_background	ENSGALG00010025419	CCND1	-0.376984233	0.072080809	-5.230022246	1.6949E-07	1.27126E-05	negative
PRJNA729271	ed_Lushi_vs_Arbor_Acr	breed_growth_background	ENSGALG00010025420	STK39	-0.428574917	0.113094771	-3.789520179	0.000150939	0.002144955	negative
PRJNA729271	ed_Lushi_vs_Arbor_Acr	breed_growth_background	ENSGALG00010025421	NR4A1	0.281042758	0.191922385	1.464356324	0.143096615	0.276295615	positive
PRJNA729271	ed_Lushi_vs_Arbor_Acr	breed_growth_background	ENSGALG00010025422	SSBP3	0.277783627	0.084789085	3.27617201	0.001052245	0.008470243	positive
PRJNA729271	ed_Lushi_vs_Arbor_Acr	breed_growth_background	ENSGALG00010025423	NGF	-0.071700309	0.186727871	-0.383982896	0.700991124	0.807058487	negative
PRJNA729271	ed_Lushi_vs_Arbor_Acr	breed_growth_background	ENSGALG00010025424	IRX6	1.130575067	0.232938871	4.853526857	1.21285E-06	5.70787E-05	positive
PRJNA729271	ed_Lushi_vs_Arbor_Acr	breed_growth_background	ENSGALG00010025425	ENSGALG00010025425	-0.234162699	0.075245606	-3.111978382	0.001858381	0.012605163	negative
PRJNA729271	ed_Lushi_vs_Arbor_Acr	breed_growth_background	ENSGALG00010025426	ENSGALG00010025426	0.263726962	0.081813851	3.223500183	0.001266342	0.009686348	positive
PRJNA729271	ed_Lushi_vs_Arbor_Acr	breed_growth_background	ENSGALG00010025427	SLC35G2	-0.27909653	0.071969828	-3.877965759	0.000105334	0.001652391	negative
PRJNA729271	ed_Lushi_vs_Arbor_Acr	breed_growth_background	ENSGALG00010025428	VANGL1	0.112239391	0.061367624	1.828967535	0.067404472	0.163848368	positive
PRJNA729271	ed_Lushi_vs_Arbor_Acr	breed_growth_background	ENSGALG00010025429	SCN3B	0.667955052	0.142708974	4.680539922	2.8612E-06	0.000109717	positive
PRJNA729271	ed_Lushi_vs_Arbor_Acr	breed_growth_background	ENSGALG00010025430	IRX5	1.650892074	0.520766606	3.170118929	0.001523766	0.011021448	positive
PRJNA729271	ed_Lushi_vs_Arbor_Acr	breed_growth_background	ENSGALG00010025432	IRX3	0.805477039	0.423242168	1.903111507	0.057025997	0.145498978	positive
PRJNA729271	ed_Lushi_vs_Arbor_Acr	breed_growth_background	ENSGALG00010025433	CHPF2	-0.145446972	0.065189675	-2.231135092	0.025672182	0.081515504	negative
PRJNA729271	ed_Lushi_vs_Arbor_Acr	breed_growth_background	ENSGALG00010025434	SYT6	-0.448630777	0.130107964	-3.448142314	0.000564457	0.005393406	negative
PRJNA729271	ed_Lushi_vs_Arbor_Acr	breed_growth_background	ENSGALG00010025435	SCLY	-0.211009561	0.05499832	-3.836654658	0.000124722	0.001870492	negative
PRJNA729271	ed_Lushi_vs_Arbor_Acr	breed_growth_background	ENSGALG00010025438	EXOSC6	-0.037850484	0.065414934	-0.578621445	0.562844632	0.70337382	negative
PRJNA729271	ed_Lushi_vs_Arbor_Acr	breed_growth_background	ENSGALG00010025442	ABCF2	0.019849392	0.07875322	0.252045471	0.801005917	0.877130828	positive
PRJNA729271	ed_Lushi_vs_Arbor_Acr	breed_growth_background	ENSGALG00010025445	B3GNT4	0.13622489	0.160767573	0.847340589	0.396805309	0.558037543	positive
PRJNA729271	ed_Lushi_vs_Arbor_Acr	breed_growth_background	ENSGALG00010025448	CHD1L	-0.053210934	0.05941172	-0.895630251	0.370450274	0.533657803	negative
PRJNA729271	ed_Lushi_vs_Arbor_Acr	breed_growth_background	ENSGALG00010025449	ENSGALG00010025449	-0.249913224	0.063689715	-3.923918085	8.71204E-05	0.001446517	negative
PRJNA729271	ed_Lushi_vs_Arbor_Acr	breed_growth_background	ENSGALG00010025451	LRRC43	0.427030163	0.170085307	2.510682253	0.01204981	0.047643754	positive
PRJNA729271	ed_Lushi_vs_Arbor_Acr	breed_growth_background	ENSGALG00010025452	TRIM33	0.144492361	0.067703828	2.13418302	0.032827802	0.097295959	positive
PRJNA729271	ed_Lushi_vs_Arbor_Acr	breed_growth_background	ENSGALG00010025458	BCAS2	0.156037346	0.06225494	2.506425142	0.012195888	0.04810211	positive
PRJNA729271	ed_Lushi_vs_Arbor_Acr	breed_growth_background	ENSGALG00010025459	DIABLO	-0.024077149	0.042763695	-0.563027802	0.573415952	0.711371468	negative
PRJNA729271	ed_Lushi_vs_Arbor_Acr	breed_growth_background	ENSGALG00010025464	VPS33A	0.069461002	0.049648142	1.399065503	0.161793341	0.301051923	positive
PRJNA729271	ed_Lushi_vs_Arbor_Acr	breed_growth_background	ENSGALG00010025465	ENSGALG00010025465	0.070408825	0.045507285	1.547198979	0.121815272	0.24647018	positive
PRJNA729271	ed_Lushi_vs_Arbor_Acr	breed_growth_background	ENSGALG00010025470	ORAOV1	-0.259179269	0.066755161	-3.882535321	0.000103373	0.001625154	negative
PRJNA729271	ed_Lushi_vs_Arbor_Acr	breed_growth_background	ENSGALG00010025472	ZCCHC8	0.07706886	0.052472027	1.468760857	0.141897664	0.27475376	positive
PRJNA729271	ed_Lushi_vs_Arbor_Acr	breed_growth_background	ENSGALG00010025473	PARS2	-0.002144822	0.04252858	-0.050432488	0.959777747	0.977517846	negative
PRJNA729271	ed_Lushi_vs_Arbor_Acr	breed_growth_background	ENSGALG00010025477	ENSGALG00010025477	0.064163384	0.037742618	1.700024729	0.089126274	0.198873975	positive
PRJNA729271	ed_Lushi_vs_Arbor_Acr	breed_growth_background	ENSGALG00010025480	FGF19	-0.906451431	0.262380754	-3.454717685	0.000550869	0.005316146	negative
PRJNA729271	ed_Lushi_vs_Arbor_Acr	breed_growth_background	ENSGALG00010025481	TMEM39A	-0.012604273	0.049338926	-0.25546307	0.798365464	0.875112152	negative
PRJNA729271	ed_Lushi_vs_Arbor_Acr	breed_growth_background	ENSGALG00010025483	FGF4	-0.0169971	0.197258102	-0.086166805	0.931333818	0.962338444	negative
PRJNA729271	ed_Lushi_vs_Arbor_Acr	breed_growth_background	ENSGALG00010025485	DENND2C	-0.122327143	0.085263372	-1.434697456	0.151373329	0.287268448	negative
PRJNA729271	ed_Lushi_vs_Arbor_Acr	breed_growth_background	ENSGALG00010025486	B3GALT1	-0.065514907	0.110827865	-0.59114111	0.554425877	0.696342557	negative
PRJNA729271	ed_Lushi_vs_Arbor_Acr	breed_growth_background	ENSGALG00010025487	FGF3	-0.727096207	0.377296564	-1.927121199	0.053964527	0.139856218	negative
PRJNA729271	ed_Lushi_vs_Arbor_Acr	breed_growth_background	ENSGALG00010025488	SLC4A8	-0.129990828	0.10803459	-1.203233414	0.228886008	0.382576417	negative
PRJNA729271	ed_Lushi_vs_Arbor_Acr	breed_growth_background	ENSGALG00010025491	ZMYND11	0.041762534	0.034563831	1.208272705	0.226942384	0.380116692	positive
PRJNA729271	ed_Lushi_vs_Arbor_Acr	breed_growth_background	ENSGALG00010025496	ATG101	-0.16121209	0.059704262	-2.700177237	0.006930255	0.032145293	negative

PRJNA729271	eed_Lushi_vs_Arbor_Acr	breed_growth_background	ENSGALG00010025499	STAC3	0.306569931	0.082416003	3.719786446	0.000199391	0.002587446	positive
PRJNA729271	eed_Lushi_vs_Arbor_Acr	breed_growth_background	ENSGALG00010025504	AMPD1	0.066878114	0.105171401	0.635896391	0.524843955	0.671576018	positive
PRJNA729271	eed_Lushi_vs_Arbor_Acr	breed_growth_background	ENSGALG00010025505	ENSGALG00010025505	0.519565948	0.151564694	3.42801437	0.000608013	0.005677469	positive
PRJNA729271	eed_Lushi_vs_Arbor_Acr	breed_growth_background	ENSGALG00010025506	R3HDM2	0.267239239	0.095430335	2.800359452	0.005104573	0.025994214	positive
PRJNA729271	eed_Lushi_vs_Arbor_Acr	breed_growth_background	ENSGALG00010025508	ENSGALG00010025508	0.017755405	0.071704969	0.247617503	0.804430367	0.879456102	positive
PRJNA729271	eed_Lushi_vs_Arbor_Acr	breed_growth_background	ENSGALG00010025512	NRAS	0.048712033	0.042071062	1.157851284	0.246924731	0.403002549	positive
PRJNA729271	eed_Lushi_vs_Arbor_Acr	breed_growth_background	ENSGALG00010025513	RSRC2	0.167675256	0.047293308	3.54543305	0.000391969	0.004183788	positive
PRJNA729271	eed_Lushi_vs_Arbor_Acr	breed_growth_background	ENSGALG00010025520	ACOT11	0.15116467	0.158319641	0.954806805	0.339675405	0.50378634	positive
PRJNA729271	eed_Lushi_vs_Arbor_Acr	breed_growth_background	ENSGALG00010025522	SUMO3	0.085463912	0.057045401	1.498173567	0.134088161	0.26400542	positive
PRJNA729271	eed_Lushi_vs_Arbor_Acr	breed_growth_background	ENSGALG00010025523	B4GALT4	0.182026271	0.072859837	2.498307425	0.012478792	0.048852972	positive
PRJNA729271	eed_Lushi_vs_Arbor_Acr	breed_growth_background	ENSGALG00010025527	KNTC1	-0.493476862	0.129325247	-3.815781321	0.000135753	0.001995675	negative
PRJNA729271	eed_Lushi_vs_Arbor_Acr	breed_growth_background	ENSGALG00010025531	ENSGALG00010025531	0.117309499	0.052447036	2.236723137	0.025304441	0.080648168	positive
PRJNA729271	eed_Lushi_vs_Arbor_Acr	breed_growth_background	ENSGALG00010025533	INHBC	0.01914267	0.352801914	0.054258974	0.956728836	0.97576153	positive
PRJNA729271	eed_Lushi_vs_Arbor_Acr	breed_growth_background	ENSGALG00010025538	ANO1	-0.276849422	0.111170043	-2.490323976	0.01276267	0.049632089	negative
PRJNA729271	eed_Lushi_vs_Arbor_Acr	breed_growth_background	ENSGALG00010025541	TTC21B	-0.093169359	0.06899404	-1.350397217	0.176888603	0.320939141	negative
PRJNA729271	eed_Lushi_vs_Arbor_Acr	breed_growth_background	ENSGALG00010025544	CSDE1	0.037994799	0.045992301	0.826112164	0.408740468	0.569177299	positive
PRJNA729271	eed_Lushi_vs_Arbor_Acr	breed_growth_background	ENSGALG00010025548	ENSGALG00010025548	0.427142168	0.108826193	3.924994111	8.67319E-05	0.001441718	positive
PRJNA729271	eed_Lushi_vs_Arbor_Acr	breed_growth_background	ENSGALG00010025549	DHCR24	-0.243101121	0.133611242	-1.819466067	0.068840355	0.166030108	negative
PRJNA729271	eed_Lushi_vs_Arbor_Acr	breed_growth_background	ENSGALG00010025553	UBE2G2	0.042390689	0.052003965	0.815143405	0.414990229	0.575239875	positive
PRJNA729271	eed_Lushi_vs_Arbor_Acr	breed_growth_background	ENSGALG00010025554	DIP2C	-0.024667262	0.038275388	-0.644467972	0.51927202	0.667214602	negative
PRJNA729271	eed_Lushi_vs_Arbor_Acr	breed_growth_background	ENSGALG00010025557	VPS29	-0.081346655	0.042173329	-1.928864949	0.05374763	0.139392446	negative
PRJNA729271	eed_Lushi_vs_Arbor_Acr	breed_growth_background	ENSGALG00010025558	GALNT3	-0.122427522	0.129408198	-0.946056929	0.34411955	0.507944286	negative
PRJNA729271	eed_Lushi_vs_Arbor_Acr	breed_growth_background	ENSGALG00010025559	SLC6A17	-0.689135088	0.649828152	-1.060488202	0.288922554	0.449879947	negative
PRJNA729271	eed_Lushi_vs_Arbor_Acr	breed_growth_background	ENSGALG00010025562	GRAMD1B	-0.114271846	0.09835663	-1.161811318	0.245312116	0.401105372	negative
PRJNA729271	eed_Lushi_vs_Arbor_Acr	breed_growth_background	ENSGALG00010025563	TSPEAR	0.149482901	0.129109268	1.157801479	0.246945059	0.403002549	positive
PRJNA729271	eed_Lushi_vs_Arbor_Acr	breed_growth_background	ENSGALG00010025565	ENSGALG00010025565	0.2298131	0.130440239	1.76182673	0.078098577	0.180836881	positive
PRJNA729271	eed_Lushi_vs_Arbor_Acr	breed_growth_background	ENSGALG00010025568	MYL3	1.364357624	0.52225785	2.612421477	0.008990334	0.038702378	positive
PRJNA729271	eed_Lushi_vs_Arbor_Acr	breed_growth_background	ENSGALG00010025569	PPTC7	0.06794373	0.103240445	0.658111557	0.51046645	0.659055063	positive
PRJNA729271	eed_Lushi_vs_Arbor_Acr	breed_growth_background	ENSGALG00010025571	SRSF3	-0.112863365	0.12304058	-0.91728571	0.358992944	0.522397622	negative
PRJNA729271	eed_Lushi_vs_Arbor_Acr	breed_growth_background	ENSGALG00010025572	ENSGALG00010025572	0.063953883	0.060504658	1.057007596	0.290508121	0.452008932	positive
PRJNA729271	eed_Lushi_vs_Arbor_Acr	breed_growth_background	ENSGALG00010025573	CLMP	-0.283287027	0.09060871	-3.126487811	0.001769078	0.012176064	negative
PRJNA729271	eed_Lushi_vs_Arbor_Acr	breed_growth_background	ENSGALG00010025574	ARHGAP31	-0.098029111	0.053601876	-1.828837311	0.067423984	0.163868317	negative
PRJNA729271	eed_Lushi_vs_Arbor_Acr	breed_growth_background	ENSGALG00010025575	TRPM2	0.319342726	0.272019963	1.173967978	0.240407852	0.396125022	positive
PRJNA729271	eed_Lushi_vs_Arbor_Acr	breed_growth_background	ENSGALG00010025577	PTH2R	0.171146909	0.107791804	1.587754388	0.112341889	0.233629223	positive
PRJNA729271	eed_Lushi_vs_Arbor_Acr	breed_growth_background	ENSGALG00010025578	ORMDL2	0.017541974	0.07932858	0.221130571	0.824990767	0.893205943	positive
PRJNA729271	eed_Lushi_vs_Arbor_Acr	breed_growth_background	ENSGALG00010025579	CSRNP3	0.158367562	0.140022589	1.13101438	0.258049039	0.415398266	positive
PRJNA729271	eed_Lushi_vs_Arbor_Acr	breed_growth_background	ENSGALG00010025580	RASSF7	0.0955446	0.085732352	1.114452108	0.265085279	0.423359533	positive
PRJNA729271	eed_Lushi_vs_Arbor_Acr	breed_growth_background	ENSGALG00010025582	DMAP1	0.105824014	0.061825218	1.711664225	0.086958565	0.195148537	positive
PRJNA729271	eed_Lushi_vs_Arbor_Acr	breed_growth_background	ENSGALG00010025585	SARNP	0.081180945	0.052390501	1.549535581	0.121253025	0.246019401	positive
PRJNA729271	eed_Lushi_vs_Arbor_Acr	breed_growth_background	ENSGALG00010025586	TCTN1	-0.17285871	0.067382246	-2.565345031	0.010307325	0.042760355	negative
PRJNA729271	eed_Lushi_vs_Arbor_Acr	breed_growth_background	ENSGALG00010025587	LRRC56	0.056107043	0.146441405	0.38313647	0.70161858	0.807460207	positive
PRJNA729271	eed_Lushi_vs_Arbor_Acr	breed_growth_background	ENSGALG00010025588	ENSGALG00010025588	-0.188801633	0.169899771	-1.111253017	0.266459456	0.424711878	negative
PRJNA729271	eed_Lushi_vs_Arbor_Acr	breed_growth_background	ENSGALG00010025590	HRAS	-0.100653441	0.060950622	-1.651393181	0.098658316	0.212840426	negative
PRJNA729271	eed_Lushi_vs_Arbor_Acr	breed_growth_background	ENSGALG00010025591	OTOS	0.696870731	0.182988869	3.808268415	0.000139943	0.002032544	positive
PRJNA729271	eed_Lushi_vs_Arbor_Acr	breed_growth_background	ENSGALG00010025592	HSPA8	0.293454869	0.111334677	2.635790355	0.008394158	0.036755715	positive
PRJNA729271	eed_Lushi_vs_Arbor_Acr	breed_growth_background	ENSGALG00010025594	UPK1B	0.377024402	0.290912812	1.296004807	0.194973829	0.342770849	positive
PRJNA729271	eed_Lushi_vs_Arbor_Acr	breed_growth_background	ENSGALG00010025595	ERI3	0.012122391	0.050908709	0.238120182	0.811787882	0.884194871	positive
PRJNA729271	eed_Lushi_vs_Arbor_Acr	breed_growth_background	ENSGALG00010025597	SCN2A	0.891176028	0.385490577	2.311797177	0.020788863	0.070061514	positive
PRJNA729271	eed_Lushi_vs_Arbor_Acr	breed_growth_background	ENSGALG00010025598	ENSGALG00010025598	-0.321948758	0.199363828	-1.614880504	0.106336575	0.224948724	negative
PRJNA729271	eed_Lushi_vs_Arbor_Acr	breed_growth_background	ENSGALG00010025599	COPS9	0.051016461	0.076598478	0.666024477	0.505395455	0.655133887	positive
PRJNA729271	eed_Lushi_vs_Arbor_Acr	breed_growth_background	ENSGALG00010025600	ENSGALG00010025600	-0.029011694	0.061447008	-0.47214168	0.636825664	0.761364896	negative
PRJNA729271	eed_Lushi_vs_Arbor_Acr	breed_growth_background	ENSGALG00010025604	HVCN1	-0.334967395	0.073633079	-4.549142823	5.38649E-06	0.000183158	negative
PRJNA729271	eed_Lushi_vs_Arbor_Acr	breed_growth_background	ENSGALG00010025606	CCDC12	0.349611685	0.071677616	4.877557391	1.07408E-06	5.17234E-05	positive
PRJNA729271	eed_Lushi_vs_Arbor_Acr	breed_growth_background	ENSGALG00010025608	ENSGALG00010025608	-0.011301678	0.281508528	-0.04014684	0.967976059	0.981793645	negative
PRJNA729271	eed_Lushi_vs_Arbor_Acr	breed_growth_background	ENSGALG00010025610	RNH1	-0.310523746	0.07192695	-4.317209972	1.58014E-05	0.000404666	negative
PRJNA729271	eed_Lushi_vs_Arbor_Acr	breed_growth_background	ENSGALG00010025612	TMEM53	0.259192833	0.082469415	3.142896481	0.00167285	0.011702683	positive
PRJNA729271	eed_Lushi_vs_Arbor_Acr	breed_growth_background	ENSGALG00010025613	CDKN1A	0.382771446	0.146307805	2.616206601	0.008891274	0.038386487	positive
PRJNA729271	eed_Lushi_vs_Arbor_Acr	breed_growth_background	ENSGALG00010025618	ADIPOQ	-0.289588163	0.096233268	-3.009231302	0.002619096	0.016086357	negative
PRJNA729271	eed_Lushi_vs_Arbor_Acr	breed_growth_background	ENSGALG00010025621	PPP1CC	-0.094971907	0.063181776	-1.503153487	0.132799469	0.26196629	negative
PRJNA729271	eed_Lushi_vs_Arbor_Acr	breed_growth_background	ENSGALG00010025623	ENSGALG00010025623	0.113635376	0.218443838	0.520204083	0.60292134	0.735140042	positive
PRJNA729271	eed_Lushi_vs_Arbor_Acr	breed_growth_background	ENSGALG00010025624	ARMH1	0.093001866	0.199510009	0.46615138	0.641107148	0.764717587	positive
PRJNA729271	eed_Lushi_vs_Arbor_Acr	breed_growth_background	ENSGALG00010025628	ENSGALG00010025628	0.261317513	0.169483145	1.541849564	0.123110154	0.248154873	positive
PRJNA729271	eed_Lushi_vs_Arbor_Acr	breed_growth_background	ENSGALG00010025629	ST6GAL1	-0.030772106	0.076055722	-0.40459949	0.685771934	0.797198186	negative
PRJNA729271	eed_Lushi_vs_Arbor_Acr	breed_growth_background	ENSGALG00010025635	CHKA	-0.128897113	0.088335287	-1.459180331	0.144515481	0.278002906	negative
PRJNA729271	eed_Lushi_vs_Arbor_Acr	breed_growth_background	ENSGALG00010025637	C6orf89	-0.059690777	0.0683329	-0.87352911	0.382374747	0.543757673	negative
PRJNA729271	eed_Lushi_vs_Arbor_Acr	breed_growth_background	ENSGALG00010025639	MYL2	0.532908362	0.247743299	2.151050556	0.031472209	0.094410115	positive
PRJNA729271	eed_Lushi_vs_Arbor_Acr	breed_growth_background	ENSGALG00010025640	SLC38A11	0.836698924	0.412209494	2.029790521	0.042377837	0.117495552	positive
PRJNA729271	eed_Lushi_vs_Arbor_Acr	breed_growth_background	ENSGALG00010025642	RPS8	-0.064370517	0.062842676	-1.024312152	0.30568786	0.468279164	negative
PRJNA729271	eed_Lushi_vs_Arbor_Acr	breed_growth_background	ENSGALG00010025644	ENSGALG00010025644	0.086636644	0.150106289	0.577168652	0.563825532	0.704145543	positive
PRJNA729271	eed_Lushi_vs_Arbor_Acr	breed_growth_background	ENSGALG00010025645	NBEAL2	0.095041101	0.100517046	0.945522226	0.344392329	0.508243414	positive
PRJNA729271	eed_Lushi_vs_Arbor_Acr	breed_growth_background	ENSGALG00010025647	JHY	0.021619372	0.200875648	0.10762565	0.91429265	0.951621885	positive
PRJNA729271	eed_Lushi_vs_Arbor_Acr	breed_growth_background	ENSGALG00010025648	ENSGALG00010025648	0.241815691	0.27126371	0.891441362	0.372692436	0.535346037	positive
PRJNA729271	eed_Lushi_vs_Arbor_Acr	breed_growth_background	ENSGALG00010025650	PPP1R1A	0.79889141	0.240882634	3.316517242	0.00091147	0.007606075	positive
PRJNA729271	eed_Lushi_vs_Arbor_Acr	breed_growth_background	ENSGALG00010025652	KMT5B	0.007075312	0.04145804	0.170662002	0.864489543	0.918678587	positive
PRJNA729271	eed_Lushi_vs_Arbor_Acr	breed_growth_background	ENSGALG00010025653	IGSF11	0.437573512	0.109953769	3.979613597	6.90274E-05	0.001209857	positive
PRJNA729271	eed_Lushi_vs_Arbor_Acr	breed_growth_background	ENSGALG00010025654	CRTAM	0.56792211	0.37062703	1.532327823	0.125441559	0.251734098	positive
PRJNA729271	eed_Lushi_vs_Arbor_Acr	breed_growth_background	ENSGALG00010025658	PDE1B	0.172949315	0.219143848	0.789204519	0.429992481	0.588382991	positive
PRJNA729271	eed_Lushi_vs_Arbor_Acr	breed_growth_background	ENSGALG00010025659	COBLL1	-0.109322744	0.069706636	-1.568326202	0.116805024	0.240018263	negative
PRJNA729271	eed_Lushi_vs_Arbor_Acr	breed_growth_background	ENSGALG00010025660	KIF2C	-0.318897329	0.110653926	-2.881934152	0.003952423	0.021481205	negative
PRJNA729271	eed_Lushi_vs_Arbor_Acr	breed_growth_background	ENSGALG00010025661	SNRPE	0.079065361	0.070527493	1.121057297	0.262263474	0.420474401	positive
PRJNA729271	eed_Lushi_vs_Arbor_Acr	breed_growth_background	ENSGALG00010025664	GPR55	-0.314161352	0.331489621	-0.94772606	0.343268931	0.507101829	negative
PRJNA729271	eed_Lushi_vs_Arbor_Acr	breed_growth_background	ENSGALG00010025666	CUX2	0.057835725	0.217034683	0.266481489	0.789868414	0.869533125	positive
PRJNA729271	eed_Lushi_vs_Arbor_Acr	breed_growth_background	ENSGALG00010025674	GRB14	-0.617540703	0.224769704	-2.747437458	0.006006296	0.029059167	negative
PRJNA729271	eed_Lushi_vs_Arbor_Acr	breed_growth_background	ENSGALG00010025679	ENSGALG00010025679	-2.496367182	0.647313285	-3.856505406	0.00011502	0.001775515	negative
PRJNA729271	eed_Lushi_vs_Arbor_Acr	breed_growth_background	ENSGALG00010025685	NCKAP1L	0.066934631	0.10295312	0.650146702	0.515597465	0.663905592	positive
PRJNA729271	eed_Lushi_vs_Arbor_Acr	breed_growth_background	ENSGALG00010025688	BEST4	-0.496985139	0.254929082	-1.949503504	0.051235325	0.134807775	negative
PRJNA729271	eed_Lushi_vs_Arbor_Acr	breed_growth_background	ENSGALG00010025691	ENSGALG00010025691	-0.191584228	0.096759952	-1.979995071	0.047704083	0.127979026	negative
PRJNA729271	eed_Lushi_vs_Arbor_Acr	breed_growth_background	ENSGALG00010025692	ENSGALG00010025692	-0.216760699	0.514812241	-0.421048067	0.673719983	0.789009546	negative
PRJNA729271	eed_Lushi_vs_Arbor_Acr	breed_growth_background	ENSGALG00010025696	MTCH1	0.019319778	0.056958762	0.339188876	0.734467449	0.829919225	positive
PRJNA729271	eed_Lushi_vs_Arbor_Acr	breed_growth_background	ENSGALG00010025701	ENSGALG00010025701	-0.638839035	1.705988852	-0.37446847	0.70805581	0.811828886	negative
PRJNA729271	eed_Lushi_vs_Arbor_Acr	breed_growth_background	ENSGALG00010025702	GTSF2	0.047735443	0.100015662	0.47727968	0.633162978	0.758236729	positive
PRJNA729271	eed_Lushi_vs_Arbor_Acr	breed_growth_background	ENSGALG00010025703	UBASH3B	-0.311909555	0.076885506	-4.056805665	4.97484E-05	0.000962511	negative
PRJNA729271	eed_Lushi_vs_Arbor_Acr	breed_growth_background	ENSGALG00010025705	ACSL3	0.016485638	0.045955889	0.358727443	0.719798996	0.820500665	positive
PRJNA729271	eed_Lushi_vs_Arbor_Acr	breed_growth_background	ENSGALG00010025706	ENSGALG00010025706	-0.652997221	1.602657154	-0.407446608	0.683680001	0.795785187	negative
PRJNA729271	eed_Lushi_vs_Arbor_Acr	breed_growth_background	ENSGALG00010025708	HOXC8	-0.285885539	0.141411994	-2.02164987	0.043212536	0.119103577	negative
PRJNA729271	eed_Lushi_vs_Arbor_Acr	breed_growth_background	ENSGALG00010025710	ENSGALG00010025710	-0.480549852	1.060545283	-0.453115826	0.65046534	0.771878437	negative
PRJNA729271	eed_Lushi_vs_Arbor_Acr	breed_growth_background	ENSGALG00010025719	HOXC11	0.366011199	1.468852059	0.2491818	0.803220157	0.878493638	positive
PRJNA729271	eed_Lushi_vs_Arbor_Acr	breed_growth_background	ENSGALG00010025720	PHETA1	-0.138470561	0.148547316	-0.932164678	0.351251427	0.515181006	negative
PRJNA729271	eed_Lushi_vs_Arbor_Acr	breed_growth_background	ENSGALG00010025723	SETD2	0.073875639	0.065369301	1.130127427	0.258422534	0.41578806	positive
PRJNA729271	eed_Lushi_vs_Arbor_Acr	breed_growth_background	ENSGALG00010025727	GLI1	0.266668159	0.129475283	2.059606686	0.039436157	0.111341466	positive
PRJNA729271	eed_Lushi_vs_Arbor_Acr	breed_growth_background	ENSGALG00010025734	PLK3	-0.221554038	0.099519086	-2.226246722	0.02599766	0.082178344	negative
PRJNA729271	eed_Lushi_vs_Arbor_Acr	breed_growth_background	ENSGALG00010025736	KIF9	-0.129601246	0.170929735	-0.758213579	0.448323133	0.60477939	negative
PRJNA729271	eed_Lushi_vs_Arbor_Acr	breed_growth_background	ENSGALG00010025737	ENSGALG00010025737	0.06919004	0.081322436	0.850811202	0.394874238	0.556347402	positive
PRJNA729271	eed_Lushi_vs_Arbor_Acr	breed_growth_background	ENSGALG00010025738	SOX13	-0.090763812	0.082969978	-1.093935592	0.273983227	0.432990283	negative
PRJNA729271	eed_Lushi_vs_Arbor_Acr	breed_growth_background	ENSGALG00010025743	SH2B3	-0.084562097	0.059133564	-1.430018625	0.152711673	0.28920247	negative
PRJNA729271	eed_Lushi_vs_Arbor_Acr	breed_growth_background	ENSGALG00010025745	COX14	-0.057100223	0.121463296	-0.470102699	0.638281646	0.762602412	negative
PRJNA729271	eed_Lushi_vs_Arbor_Acr	breed_growth_background	ENSGALG00010025748	ETNK2	0.404259124	0.111503352	3.625533387	0.000288366	0.0033763	positive
PRJNA729271	eed_Lushi_vs_Arbor_Acr	breed_growth_background	ENSGALG00010025753	SORL1	-0.284741503	0.093540655	-3.044040071	0.00233424	0.014898642	negative
PRJNA729271	eed_Lushi_vs_Arbor_Acr	breed_growth_background	ENSGALG00010025755	ENSGALG00010025755	0.005885445	0.149687822	0.039318131	0.968636751	0.982188857	positive
PRJNA729271	eed_Lushi_vs_Arbor_Acr	breed_growth_background	ENSGALG00010025782	ENSGALG00010025782	-0.425144047	2.423428555	-0.175430815	0.860741129	0.916307481	negative
PRJNA729271	eed_Lushi_vs_Arbor_Acr	breed_growth_background	ENSGALG00010025784	ENSGALG00010025784	0.290202929	1.288598251	0.225208228	0.821817288	0.89070148	positive
PRJNA729271	eed_Lushi_vs_Arbor_Acr	breed_growth_background	ENSGALG00010025787	KCNE4	-0.366897039	0.154073489	-2.381311933	0.017251097	0.061455919	negative
PRJNA729271	eed_Lushi_vs_Arbor_Acr	breed_growth_background	ENSGALG00010025790	LRP5	0.005479659	0.041839989	0.130967028	0.895801391	0.939282491	positive
PRJNA729271	eed_Lushi_vs_Arbor_Acr	breed_growth_background	ENSGALG00010025791	FIGN	0.411012773	0.127110392	3.233510398	0.001222789	0.009473182	positive
PRJNA729271	eed_Lushi_vs_Arbor_Acr	breed_growth_background	ENSGALG00010025794	ENSGALG00010025794	-0.862292536	2.161944325	-0.398850482	0.690003378	0.799552239	negative
PRJNA729271	eed_Lushi_vs_Arbor_Acr	breed_growth_background	ENSGALG00010025799	SC5D	0.10728473	0.147264814	0.728515709	0.466297956	0.6212253	positive
PRJNA729271	eed_Lushi_vs_Arbor_Acr	breed_growth_background	ENSGALG00010025800	ARL2BP	0.310750428	0.075405203	4.121074084	3.7711E-05	0.000794091	positive
PRJNA729271	eed_Lushi_vs_Arbor_Acr	breed_growth_background	ENSGALG00010025801	PTPN7	-0.036210405	0.207928887	-0.174148025	0.861749131	0.916909173	negative
PRJNA729271	eed_Lushi_vs_Arbor_Acr	breed_growth_background	ENSGALG00010025804	ENSGALG00010025804	-0.809397763	1.151414834	-0.702959298	0.482081115	0.63467461	negative
PRJNA729271	eed_Lushi_vs_Arbor_Acr	breed_growth_background	ENSGALG00010025805	ATXN2	0.096580628	0.05053829	1.911038714	0.055999606	0.143742569	positive
PRJNA729271	eed_Lushi_vs_Arbor_Acr	breed_growth_background	ENSGALG00010025809	ENSGALG00010025809	-0.158029824	0.103219425	-1.531008569	0.125767275	0.252214102	negative
PRJNA729271	eed_Lushi_vs_Arbor_Acr	breed_growth_background	ENSGALG00010025822	KLF7	-0.158066222	0.053612268	-2.948321875	0.003195042	0.01854711	negative
PRJNA729271	eed_Lushi_vs_Arbor_Acr	breed_growth_background	ENSGALG00010025829	ENSGALG00010025829	-0.464351507	0.138479683	-3.353210362	0.0007988	0.006961585	negative
PRJNA729271	eed_Lushi_vs_Arbor_Acr	breed_growth_background	ENSGALG00010025830	TBCEL	-0.054607746	0.103419331	-0.528022615	0.597483639	0.730959358	negative
PRJNA729271	eed_Lushi_vs_Arbor_Acr	breed_growth_background	ENSGALG00010025831	BRAP	0.031953144	0.037956327	0.841839719	0.399877676	0.560726886	positive
PRJNA729271	eed_Lushi_vs_Arbor_Acr	breed_growth_background	ENSGALG00010025832	ENKD1	0.208378597	0.078521394	2.653781171	0.007959543	0.035488641	positive
PRJNA729271	eed_Lushi_vs_Arbor_Acr	breed_growth_background	ENSGALG00010025835	PPP6R3	0.129933313	0.063887424	2.03378543	0.041973234	0.116596786	positive
PRJNA729271	eed_Lushi_vs_Arbor_Acr	breed_growth_background	ENSGALG00010025836	ENSGALG00010025836	0.133560954	0.061117505	2.185314257	0.028865813	0.088421377	positive
PRJNA729271	eed_Lushi_vs_Arbor_Acr	breed_growth_background	ENSGALG00010025837	ENSGALG00010025837	0.256564838	0.092750927	2.766170056	0.005671895	0.027840879	positive
PRJNA729271	eed_Lushi_vs_Arbor_Acr	breed_growth_background	ENSGALG00010025846	GRIK4	-0.290385248	0.125132809	-2.320616394	0.020307557	0.068965906	negative
PRJNA729271	eed_Lushi_vs_Arbor_Acr	breed_growth_background	ENSGALG00010025847	DEF6	-0.0040794	0.068351238	-0.059682903	0.952408189	0.973433394	negative

PRJNA729271	ed_Lushi_vs_Arbor_Acr	breed_growth_background	ENSGALG00010025849	OBSCN	0.228887842	0.122405028	1.869921899	0.061494664	0.153551276	positive
PRJNA729271	ed_Lushi_vs_Arbor_Acr	breed_growth_background	ENSGALG00010025852	BTBD19	0.15255993	0.136057048	1.121293846	0.262162804	0.420406002	positive
PRJNA729271	ed_Lushi_vs_Arbor_Acr	breed_growth_background	ENSGALG00010025858	ENSGALG00010025858	-0.657325983	1.553428678	-0.42314526	0.672189282	0.787917163	negative
PRJNA729271	ed_Lushi_vs_Arbor_Acr	breed_growth_background	ENSGALG00010025862	PTCH2	0.393027717	0.170683369	2.302671424	0.021297337	0.071380652	positive
PRJNA729271	ed_Lushi_vs_Arbor_Acr	breed_growth_background	ENSGALG00010025865	LGR6	-0.746102534	0.334771002	-2.228695226	0.02583419	0.081857473	negative
PRJNA729271	ed_Lushi_vs_Arbor_Acr	breed_growth_background	ENSGALG00010025869	FASTKD2	0.117481382	0.050432239	2.329489726	0.019833137	0.06780173	positive
PRJNA729271	ed_Lushi_vs_Arbor_Acr	breed_growth_background	ENSGALG00010025870	ENSGALG00010025870	0.204373527	0.085266934	2.396867302	0.016535911	0.05969814	positive
PRJNA729271	ed_Lushi_vs_Arbor_Acr	breed_growth_background	ENSGALG00010025871	ENSGALG00010025871	-1.877912051	0.789126212	-2.379735995	0.017325045	0.061626143	negative
PRJNA729271	ed_Lushi_vs_Arbor_Acr	breed_growth_background	ENSGALG00010025874	MDH1B	-0.297938874	0.170842518	-1.743938667	0.081169785	0.185840473	negative
PRJNA729271	ed_Lushi_vs_Arbor_Acr	breed_growth_background	ENSGALG00010025876	ENSGALG00010025876	-0.386527584	0.866982062	-0.445831121	0.65571924	0.775566395	negative
PRJNA729271	ed_Lushi_vs_Arbor_Acr	breed_growth_background	ENSGALG00010025877	ENSGALG00010025877	-0.193189619	1.85281444	-0.104268196	0.916956516	0.953145614	negative
PRJNA729271	ed_Lushi_vs_Arbor_Acr	breed_growth_background	ENSGALG00010025881	GAL	0.121459075	0.320778971	0.378637896	0.704956777	0.809181857	positive
PRJNA729271	ed_Lushi_vs_Arbor_Acr	breed_growth_background	ENSGALG00010025882	LDHD	0.267908319	0.088968424	3.011274177	0.002601538	0.015998854	positive
PRJNA729271	ed_Lushi_vs_Arbor_Acr	breed_growth_background	ENSGALG00010025886	ALDH2	-0.124267048	0.058786233	-2.113880113	0.034525508	0.100880899	negative
PRJNA729271	ed_Lushi_vs_Arbor_Acr	breed_growth_background	ENSGALG00010025887	ZNF76	-0.075267007	0.06326117	-1.189782104	0.234132045	0.388524838	negative
PRJNA729271	ed_Lushi_vs_Arbor_Acr	breed_growth_background	ENSGALG00010025889	TESMIN	-0.39054883	0.29614541	-1.318773873	0.187244721	0.332856541	negative
PRJNA729271	ed_Lushi_vs_Arbor_Acr	breed_growth_background	ENSGALG00010025890	SCG2	-0.253138298	0.277647337	-0.911726009	0.361912969	0.525223116	negative
PRJNA729271	ed_Lushi_vs_Arbor_Acr	breed_growth_background	ENSGALG00010025891	NUP93	-0.317814742	0.059690059	-5.3244166	1.01278E-07	8.29389E-06	negative
PRJNA729271	ed_Lushi_vs_Arbor_Acr	breed_growth_background	ENSGALG00010025892	ZC3H11A	0.120428936	0.046218005	2.605671417	0.009169438	0.039276301	positive
PRJNA729271	ed_Lushi_vs_Arbor_Acr	breed_growth_background	ENSGALG00010025894	DYTN	0.378554306	0.288564663	1.311852606	0.189569871	0.335837593	positive
PRJNA729271	ed_Lushi_vs_Arbor_Acr	breed_growth_background	ENSGALG00010025897	MAPKAPK5	0.03499435	0.064960263	0.538703943	0.590091153	0.725368038	positive
PRJNA729271	ed_Lushi_vs_Arbor_Acr	breed_growth_background	ENSGALG00010025898	ENSGALG00010025898	-0.294837722	1.00574448	-0.293153706	0.769404667	0.856305333	negative
PRJNA729271	ed_Lushi_vs_Arbor_Acr	breed_growth_background	ENSGALG00010025899	ENSGALG00010025899	-0.090257256	0.090471278	-0.997634365	0.318456691	0.481746816	negative
PRJNA729271	ed_Lushi_vs_Arbor_Acr	breed_growth_background	ENSGALG00010025900	FAM237A	-0.018684394	0.325529006	-0.057397018	0.954228939	0.974464454	negative
PRJNA729271	ed_Lushi_vs_Arbor_Acr	breed_growth_background	ENSGALG00010025902	ENSGALG00010025902	-0.379809167	0.944746239	-0.402022418	0.687667528	0.798132113	negative
PRJNA729271	ed_Lushi_vs_Arbor_Acr	breed_growth_background	ENSGALG00010025904	RPL10A	-0.078870344	0.074218652	-1.062675515	0.287929129	0.44896006	negative
PRJNA729271	ed_Lushi_vs_Arbor_Acr	breed_growth_background	ENSGALG00010025905	CPT1A	-0.219500925	0.093581079	-2.3455695	0.018998036	0.066005881	negative
PRJNA729271	ed_Lushi_vs_Arbor_Acr	breed_growth_background	ENSGALG00010025907	ARHGEF12	-0.175451649	0.038406408	-4.568291035	4.91717E-06	0.000172525	negative
PRJNA729271	ed_Lushi_vs_Arbor_Acr	breed_growth_background	ENSGALG00010025910	HEATR3	-0.003875014	0.060989611	-0.063535646	0.949339975	0.97207424	negative
PRJNA729271	ed_Lushi_vs_Arbor_Acr	breed_growth_background	ENSGALG00010025913	EIF2B3	-0.014253956	0.042479079	-0.335552381	0.737208438	0.83173013	negative
PRJNA729271	ed_Lushi_vs_Arbor_Acr	breed_growth_background	ENSGALG00010025914	ENSGALG00010025914	0.067584503	0.993379912	0.0680349	0.945757852	0.970470493	positive
PRJNA729271	ed_Lushi_vs_Arbor_Acr	breed_growth_background	ENSGALG00010025917	SCUBE3	0.043556011	0.23148326	0.188160523	0.850750817	0.910285163	positive
PRJNA729271	ed_Lushi_vs_Arbor_Acr	breed_growth_background	ENSGALG00010025919	RGS9BP	0.203484877	0.181729682	1.11971184	0.262836578	0.421113761	positive
PRJNA729271	ed_Lushi_vs_Arbor_Acr	breed_growth_background	ENSGALG00010025920	GTSF1	-0.100056817	0.140278068	-0.713274842	0.475675702	0.628926325	negative
PRJNA729271	ed_Lushi_vs_Arbor_Acr	breed_growth_background	ENSGALG00010025924	TMEM116	-0.059163847	0.087838797	-0.67355029	0.500597266	0.651711637	negative
PRJNA729271	ed_Lushi_vs_Arbor_Acr	breed_growth_background	ENSGALG00010025925	KLHL18	-0.142938355	0.049071441	-2.912862363	0.003581324	0.019991535	negative
PRJNA729271	ed_Lushi_vs_Arbor_Acr	breed_growth_background	ENSGALG00010025926	ENSGALG00010025926	0.986922478	0.233479946	4.22701176	2.36815E-05	0.000545729	positive
PRJNA729271	ed_Lushi_vs_Arbor_Acr	breed_growth_background	ENSGALG00010025927	ENSGALG00010025927	-0.409883915	0.611386906	-0.670416574	0.502592272	0.653484116	negative
PRJNA729271	ed_Lushi_vs_Arbor_Acr	breed_growth_background	ENSGALG00010025929	NELFE	0.08139758	0.048802456	1.667899254	0.095335728	0.207897002	positive
PRJNA729271	ed_Lushi_vs_Arbor_Acr	breed_growth_background	ENSGALG00010025931	TMEM136	-0.08713829	0.110136558	-0.791184062	0.42883659	0.58780013	negative
PRJNA729271	ed_Lushi_vs_Arbor_Acr	breed_growth_background	ENSGALG00010025932	AP1S3	-0.144981227	0.174924212	-0.828823094	0.407204518	0.567814285	negative
PRJNA729271	ed_Lushi_vs_Arbor_Acr	breed_growth_background	ENSGALG00010025933	SKIC2	0.062679472	0.054987178	1.139892485	0.254331096	0.410984308	positive
PRJNA729271	ed_Lushi_vs_Arbor_Acr	breed_growth_background	ENSGALG00010025935	CATH3	0.358312285	0.202635367	1.76826134	0.077017224	0.179220526	positive
PRJNA729271	ed_Lushi_vs_Arbor_Acr	breed_growth_background	ENSGALG00010025936	GOSR2	-0.042795455	0.03722289	-1.14970802	0.25026415	0.406269331	negative
PRJNA729271	ed_Lushi_vs_Arbor_Acr	breed_growth_background	ENSGALG00010025938	ERP29	-0.143525649	0.048443558	-2.962739632	0.003049144	0.017915419	negative
PRJNA729271	ed_Lushi_vs_Arbor_Acr	breed_growth_background	ENSGALG00010025939	TMEM136-1	-0.349097016	0.375872385	-0.928764733	0.353011025	0.516805859	negative
PRJNA729271	ed_Lushi_vs_Arbor_Acr	breed_growth_background	ENSGALG00010025940	HECTD3	-0.010200387	0.047302824	-0.215640121	0.82926828	0.895856671	negative
PRJNA729271	ed_Lushi_vs_Arbor_Acr	breed_growth_background	ENSGALG00010025941	ENSGALG00010025941	-0.353044761	0.403248095	-0.875502613	0.381300489	0.542815811	negative
PRJNA729271	ed_Lushi_vs_Arbor_Acr	breed_growth_background	ENSGALG00010025942	ENSGALG00010025942	-0.50065152	0.661668749	-0.75664979	0.449259704	0.605600242	negative
PRJNA729271	ed_Lushi_vs_Arbor_Acr	breed_growth_background	ENSGALG00010025943	CATH1	0.203650451	0.397253942	0.512645513	0.60819931	0.739014307	positive
PRJNA729271	ed_Lushi_vs_Arbor_Acr	breed_growth_background	ENSGALG00010025946	KRT5	-0.199045322	0.645750688	-0.308238653	0.757900742	0.847079286	negative
PRJNA729271	ed_Lushi_vs_Arbor_Acr	breed_growth_background	ENSGALG00010025948	WNT9B	0.332579529	0.400169474	0.831096698	0.405919005	0.566785086	positive
PRJNA729271	ed_Lushi_vs_Arbor_Acr	breed_growth_background	ENSGALG00010025949	GPATCH1	0.199130598	0.065883758	3.022453526	0.002507346	0.015625098	positive
PRJNA729271	ed_Lushi_vs_Arbor_Acr	breed_growth_background	ENSGALG00010025951	TBRG4	0.025133968	0.061487395	0.408766194	0.682711251	0.795170718	positive
PRJNA729271	ed_Lushi_vs_Arbor_Acr	breed_growth_background	ENSGALG00010025953	POU2F3	0.407099576	0.516201921	0.788644054	0.430320075	0.588497923	positive
PRJNA729271	ed_Lushi_vs_Arbor_Acr	breed_growth_background	ENSGALG00010025954	TUBA1A	-0.12904654	0.084994213	-1.518297953	0.128939304	0.256417194	negative
PRJNA729271	ed_Lushi_vs_Arbor_Acr	breed_growth_background	ENSGALG00010025956	ENSGALG00010025956	0.73549563	0.268629758	2.737952918	0.006182293	0.029642389	positive
PRJNA729271	ed_Lushi_vs_Arbor_Acr	breed_growth_background	ENSGALG00010025957	ENSGALG00010025957	-0.264010807	0.062616511	-4.216312982	2.48329E-05	0.00056419	negative
PRJNA729271	ed_Lushi_vs_Arbor_Acr	breed_growth_background	ENSGALG00010025958	HOXC9	0.199121538	0.239402522	0.831743693	0.405553633	0.566384035	positive
PRJNA729271	ed_Lushi_vs_Arbor_Acr	breed_growth_background	ENSGALG00010025959	UROD	0.011268327	0.056712249	0.198692998	0.842502901	0.904698829	positive
PRJNA729271	ed_Lushi_vs_Arbor_Acr	breed_growth_background	ENSGALG00010025960	CMKLR2	-0.175435807	0.084409979	-2.078377565	0.037674597	0.107880934	negative
PRJNA729271	ed_Lushi_vs_Arbor_Acr	breed_growth_background	ENSGALG00010025962	ENSGALG00010025962	-0.223711377	0.101095372	-2.212874577	0.026906294	0.084107554	negative
PRJNA729271	ed_Lushi_vs_Arbor_Acr	breed_growth_background	ENSGALG00010025963	OAF	-0.398780105	0.131687844	-3.028222601	0.002459968	0.015419341	negative
PRJNA729271	ed_Lushi_vs_Arbor_Acr	breed_growth_background	ENSGALG00010025964	HOXC10	0.924975754	0.553912999	1.669893568	0.094940423	0.207349921	positive
PRJNA729271	ed_Lushi_vs_Arbor_Acr	breed_growth_background	ENSGALG00010025968	FADS2	-0.496350021	0.084549165	-5.870549037	4.34354E-09	6.88659E-07	negative
PRJNA729271	ed_Lushi_vs_Arbor_Acr	breed_growth_background	ENSGALG00010025970	KLHL12	0.169797932	0.059950043	2.832323783	0.004621103	0.024051306	positive
PRJNA729271	ed_Lushi_vs_Arbor_Acr	breed_growth_background	ENSGALG00010025971	TRAFD1	-0.082696629	0.106872062	-0.773790898	0.439054456	0.59639156	negative
PRJNA729271	ed_Lushi_vs_Arbor_Acr	breed_growth_background	ENSGALG00010025972	WNT3	-0.35099494	0.737168049	-0.476139655	0.633974887	0.758958209	negative
PRJNA729271	ed_Lushi_vs_Arbor_Acr	breed_growth_background	ENSGALG00010025974	EEF1B2	0.010089652	0.051072601	0.197555088	0.843393177	0.90506146	positive
PRJNA729271	ed_Lushi_vs_Arbor_Acr	breed_growth_background	ENSGALG00010025976	NAA25	0.0548496	0.053387123	1.027393832	0.304235055	0.466704108	positive
PRJNA729271	ed_Lushi_vs_Arbor_Acr	breed_growth_background	ENSGALG00010025978	TRIM29	-0.329259782	0.331767329	-0.992441849	0.320982033	0.484598955	negative
PRJNA729271	ed_Lushi_vs_Arbor_Acr	breed_growth_background	ENSGALG00010025980	HECTD4	-0.118331869	0.034745777	-3.405647514	0.000660073	0.006044071	negative
PRJNA729271	ed_Lushi_vs_Arbor_Acr	breed_growth_background	ENSGALG00010025982	SERPINE2	-0.34751172	0.106513232	-3.262615492	0.001103892	0.008796543	negative
PRJNA729271	ed_Lushi_vs_Arbor_Acr	breed_growth_background	ENSGALG00010025983	RNF26	-0.041969084	0.046029439	-0.911787872	0.361880396	0.525223116	negative
PRJNA729271	ed_Lushi_vs_Arbor_Acr	breed_growth_background	ENSGALG00010025984	NDUFS1	-0.004312077	0.062513791	-0.068978018	0.945007117	0.970104686	negative
PRJNA729271	ed_Lushi_vs_Arbor_Acr	breed_growth_background	ENSGALG00010025985	CHCHD10	0.152819053	0.170040183	0.898723176	0.368800129	0.532022081	positive
PRJNA729271	ed_Lushi_vs_Arbor_Acr	breed_growth_background	ENSGALG00010025986	RAB3IL1	-0.145011783	0.058045778	-2.49823137	0.01248147	0.048852972	negative
PRJNA729271	ed_Lushi_vs_Arbor_Acr	breed_growth_background	ENSGALG00010025988	NSF	-0.001544393	0.032621859	-0.047342262	0.962240445	0.978715547	negative
PRJNA729271	ed_Lushi_vs_Arbor_Acr	breed_growth_background	ENSGALG00010025990	FAM124B	0.06499949	0.12872814	0.504936141	0.613603689	0.743174212	positive
PRJNA729271	ed_Lushi_vs_Arbor_Acr	breed_growth_background	ENSGALG00010025992	MMP11	0.223422039	0.194200227	1.150472592	0.249949276	0.405931336	positive
PRJNA729271	ed_Lushi_vs_Arbor_Acr	breed_growth_background	ENSGALG00010025994	INO80D	0.169218372	0.05825881	2.904597143	0.003677261	0.020370357	positive
PRJNA729271	ed_Lushi_vs_Arbor_Acr	breed_growth_background	ENSGALG00010025996	CUL3	0.231160901	0.051674859	4.473372671	7.69954E-06	0.000237963	positive
PRJNA729271	ed_Lushi_vs_Arbor_Acr	breed_growth_background	ENSGALG00010025997	CCDC43	0.276383588	0.080409458	3.437202499	0.000587756	0.00553523	positive
PRJNA729271	ed_Lushi_vs_Arbor_Acr	breed_growth_background	ENSGALG00010025998	SMARCB1	0.179582625	0.077695586	2.311362005	0.020812868	0.070109731	positive
PRJNA729271	ed_Lushi_vs_Arbor_Acr	breed_growth_background	ENSGALG00010025999	DBF4B	0.172444281	0.090822413	1.898697416	0.057604272	0.146522531	positive
PRJNA729271	ed_Lushi_vs_Arbor_Acr	breed_growth_background	ENSGALG00010026001	ANKS1A	-0.110289163	0.049300733	-2.237069469	0.0252818	0.080611459	negative
PRJNA729271	ed_Lushi_vs_Arbor_Acr	breed_growth_background	ENSGALG00010026002	FTH1	0.042092054	0.075817583	0.555175367	0.578774716	0.71575288	positive
PRJNA729271	ed_Lushi_vs_Arbor_Acr	breed_growth_background	ENSGALG00010026003	NECTIN1	0.082092818	0.08360701	0.981889176	0.326154449	0.489755385	positive
PRJNA729271	ed_Lushi_vs_Arbor_Acr	breed_growth_background	ENSGALG00010026005	DERL3	-0.152024917	0.063569765	-2.391465792	0.016781248	0.060298509	negative
PRJNA729271	ed_Lushi_vs_Arbor_Acr	breed_growth_background	ENSGALG00010026006	ITGA2B	-0.645033836	0.169041253	-3.815836805	0.000135722	0.001995675	negative
PRJNA729271	ed_Lushi_vs_Arbor_Acr	breed_growth_background	ENSGALG00010026008	NRP2	-0.143955871	0.053989991	-2.666343689	0.007668126	0.034590265	negative
PRJNA729271	ed_Lushi_vs_Arbor_Acr	breed_growth_background	ENSGALG00010026009	ENSGALG00010026009	-0.434687301	0.166079617	-2.617342869	0.008861728	0.038297777	negative
PRJNA729271	ed_Lushi_vs_Arbor_Acr	breed_growth_background	ENSGALG00010026010	ZSWIM5	-0.112022913	0.069511221	-1.611580285	0.107053304	0.225871563	negative
PRJNA729271	ed_Lushi_vs_Arbor_Acr	breed_growth_background	ENSGALG00010026011	GJC2	-0.162085901	0.088514335	-1.831182492	0.067073309	0.163372141	negative
PRJNA729271	ed_Lushi_vs_Arbor_Acr	breed_growth_background	ENSGALG00010026013	RABIF	0.062439533	0.0625681	0.997945168	0.318305948	0.481712749	positive
PRJNA729271	ed_Lushi_vs_Arbor_Acr	breed_growth_background	ENSGALG00010026016	SLC2A11	-0.210439669	0.224648828	-0.936749461	0.348887448	0.512944878	negative
PRJNA729271	ed_Lushi_vs_Arbor_Acr	breed_growth_background	ENSGALG00010026017	PLEKHM1	0.201598174	0.077510106	2.6009276	0.009297207	0.03974138	positive
PRJNA729271	ed_Lushi_vs_Arbor_Acr	breed_growth_background	ENSGALG00010026023	THY1	0.156073196	0.162614582	0.95977368	0.337169132	0.50141239	positive
PRJNA729271	ed_Lushi_vs_Arbor_Acr	breed_growth_background	ENSGALG00010026030	IBA57	-0.128599044	0.045127026	-2.849712357	0.004375878	0.023065805	negative
PRJNA729271	ed_Lushi_vs_Arbor_Acr	breed_growth_background	ENSGALG00010026036	TUB5A	-0.006882966	0.111113568	-0.061945328	0.95060637	0.972752512	negative
PRJNA729271	ed_Lushi_vs_Arbor_Acr	breed_growth_background	ENSGALG00010026040	GUK1	-0.177806852	0.06694274	-2.656103594	0.007904933	0.035310323	negative
PRJNA729271	ed_Lushi_vs_Arbor_Acr	breed_growth_background	ENSGALG00010026051	TAF11	0.249018577	0.069550164	3.580416804	0.000343047	0.003799127	positive
PRJNA729271	ed_Lushi_vs_Arbor_Acr	breed_growth_background	ENSGALG00010026061	MPEG1	0.411855434	0.183009096	2.250464284	0.024419488	0.078635554	positive
PRJNA729271	ed_Lushi_vs_Arbor_Acr	breed_growth_background	ENSGALG00010026062	ITGA4	-0.119516693	0.086645155	-1.379381141	0.167777271	0.309091452	negative
PRJNA729271	ed_Lushi_vs_Arbor_Acr	breed_growth_background	ENSGALG00010026064	ENSGALG00010026064	0.299013013	0.080293452	3.724002475	0.000196089	0.002562949	positive
PRJNA729271	ed_Lushi_vs_Arbor_Acr	breed_growth_background	ENSGALG00010026066	SELENOP2	0.042586536	0.309683498	0.137516322	0.890622692	0.936087008	positive
PRJNA729271	ed_Lushi_vs_Arbor_Acr	breed_growth_background	ENSGALG00010026068	BLTP3A	0.097166109	0.05951807	1.632548046	0.102564086	0.21901391	positive
PRJNA729271	ed_Lushi_vs_Arbor_Acr	breed_growth_background	ENSGALG00010026073	HPDL	-0.269363762	0.138384865	-1.946482814	0.05159677	0.135489897	negative
PRJNA729271	ed_Lushi_vs_Arbor_Acr	breed_growth_background	ENSGALG00010026076	C1orf35	0.253082501	0.073619007	3.437733143	0.000586605	0.005532106	positive
PRJNA729271	ed_Lushi_vs_Arbor_Acr	breed_growth_background	ENSGALG00010026078	UBE2E3	0.200587464	0.057404345	3.494290606	0.000475323	0.004797919	positive
PRJNA729271	ed_Lushi_vs_Arbor_Acr	breed_growth_background	ENSGALG00010026081	KPNA1	0.052794462	0.058295135	0.905640958	0.365125922	0.52851011	positive
PRJNA729271	ed_Lushi_vs_Arbor_Acr	breed_growth_background	ENSGALG00010026083	MUTYH	-0.096736903	0.070891	-1.36458653	0.172383041	0.315585167	negative
PRJNA729271	ed_Lushi_vs_Arbor_Acr	breed_growth_background	ENSGALG00010026088	SNRPC	0.075144436	0.044259677	1.697808066	0.089543993	0.19952962	positive
PRJNA729271	ed_Lushi_vs_Arbor_Acr	breed_growth_background	ENSGALG00010026090	USP2	0.345842929	0.124063093	2.78763748	0.005309392	0.026606055	positive
PRJNA729271	ed_Lushi_vs_Arbor_Acr	breed_growth_background	ENSGALG00010026091	DTX4	0.289512932	0.087187083	3.320594308	0.00089826	0.00754685	positive
PRJNA729271	ed_Lushi_vs_Arbor_Acr	breed_growth_background	ENSGALG00010026093	TOE1	0.158488617	0.071464618	2.217721455	0.02657383	0.083482374	positive
PRJNA729271	ed_Lushi_vs_Arbor_Acr	breed_growth_background	ENSGALG00010026094	C6orf106	0.008101713	0.041634557	0.194591062	0.845713101	0.906366832	positive
PRJNA729271	ed_Lushi_vs_Arbor_Acr	breed_growth_background	ENSGALG00010026095	MIF	-0.00482007	0.109858071	-0.043875434	0.965003698	0.979944556	negative
PRJNA729271	ed_Lushi_vs_Arbor_Acr	breed_growth_background	ENSGALG00010026097	ARF1	-0.041833485	0.030069363	-1.391232807	0.164154842	0.303924439	negative
PRJNA729271	ed_Lushi_vs_Arbor_Acr	breed_growth_background	ENSGALG00010026098	STAT3	0.08397376	0.05778445	1.453224181	0.14616152	0.280462171	positive
PRJNA729271	ed_Lushi_vs_Arbor_Acr	breed_growth_background	ENSGALG00010026102	IPO9	-0.026410664	0.032735474	-0.806790318	0.419787298	0.579230547	negative
PRJNA729271	ed_Lushi_vs_Arbor_Acr	breed_growth_background	ENSGALG00010026104	DDX51	0.12484592	0.057515247	2.170657822	0.029957048	0.090766598	positive
PRJNA729271	ed_Lushi_vs_Arbor_Acr	breed_growth_background	ENSGALG00010026105	CNTF	-0.128691002	0.09552119	-1.347250829	0.177899463	0.322130258	negative
PRJNA729271	ed_Lushi_vs_Arbor_Acr	breed_growth_background	ENSGALG00010026106	TESK2	0.07410647	0.063850408	1.160626407	0.245793862	0.401711808	positive
PRJNA729271	ed_Lushi_vs_Arbor_Acr	breed_growth_background	ENSGALG00010026111	ENSGALG00010026111	-0.228095436	0.088560095	-2.575600628	0.010006615	0.041859385	negative
PRJNA729271	ed_Lushi_vs_Arbor_Acr	breed_growth_background	ENSGALG00010026112	ZFP91	0.164162434	0.064627125	2.54014755	0.011080571	0.045052701	positive
PRJNA729271	ed_Lushi_vs_Arbor_Acr	breed_growth_background	ENSGALG00010026113	MFRP	0.20664652	0.25858164	0.799153874	0.424201194	0.583324402	positive
PRJNA729271	ed_Lushi_vs_Arbor_Acr	breed_growth_background	ENSGALG00010026114	CWC22	0.105377244	0.045050492	2.339091958	0.019330675	0.066723435	positive
PRJNA729271	ed_Lushi_vs_Arbor_Acr	breed_growth_background	ENSGALG00010026115	MMACHC	0.17983116	0.096327147	1.866879338	0.06191844	0.154263971	positive
PRJNA729271	ed_Lushi_vs_Arbor_Acr	breed_growth_background	ENSGALG00010026116	DLX3	-0.00270899	0.393111248	-0.006891153	0.994501699	0.996977808	negative
PRJNA729271	ed_Lushi_vs_Arbor_Acr	breed_growth_background	ENSGALG00010026117	LZTR1	0.025774827	0.039211069	0.657335493	0.510965219	0.659588655	positive

PRJNA729271	ed_Lushi_vs_Arbor_Acr	breed_growth_background	ENSGALG00010026120	C1QTNF5	-0.472746889	0.105228497	-4.492574752	7.03672E-06	0.000223267	negative
PRJNA729271	ed_Lushi_vs_Arbor_Acr	breed_growth_background	ENSGALG00010026121	ENSGALG00010026121	-0.368017367	0.138309051	-2.660833581	0.007794747	0.035001506	negative
PRJNA729271	ed_Lushi_vs_Arbor_Acr	breed_growth_background	ENSGALG00010026124	PACSIN1	-0.3586653	0.200018751	-1.793158384	0.072947602	0.172826084	negative
PRJNA729271	ed_Lushi_vs_Arbor_Acr	breed_growth_background	ENSGALG00010026127	ENSGALG00010026127	0.332757028	0.345581789	0.962889361	0.335603049	0.499801315	positive
PRJNA729271	ed_Lushi_vs_Arbor_Acr	breed_growth_background	ENSGALG00010026128	PRDX1	-0.057395406	0.076070647	-0.754501353	0.450548233	0.606772892	negative
PRJNA729271	ed_Lushi_vs_Arbor_Acr	breed_growth_background	ENSGALG00010026132	DDTL	-0.205382384	0.099487144	-2.064411301	0.038978736	0.110307844	negative
PRJNA729271	ed_Lushi_vs_Arbor_Acr	breed_growth_background	ENSGALG00010026137	LPXN	0.068814891	0.184238858	0.373509107	0.708769569	0.812336119	positive
PRJNA729271	ed_Lushi_vs_Arbor_Acr	breed_growth_background	ENSGALG00010026141	WNT3A	0.147379971	0.452036052	0.326035878	0.74439721	0.837020988	positive
PRJNA729271	ed_Lushi_vs_Arbor_Acr	breed_growth_background	ENSGALG00010026143	RNPEP	-0.069691708	0.075974857	-0.917299622	0.358985655	0.522397622	negative
PRJNA729271	ed_Lushi_vs_Arbor_Acr	breed_growth_background	ENSGALG00010026148	RPS10	-0.022218602	0.071674907	-0.309991359	0.756567528	0.846045851	negative
PRJNA729271	ed_Lushi_vs_Arbor_Acr	breed_growth_background	ENSGALG00010026149	LASP1	-0.178707937	0.069203774	-2.582343826	0.009813177	0.041265447	negative
PRJNA729271	ed_Lushi_vs_Arbor_Acr	breed_growth_background	ENSGALG00010026154	ZNF385B	-0.385891986	0.097226646	-3.968994122	7.21767E-05	0.001251436	negative
PRJNA729271	ed_Lushi_vs_Arbor_Acr	breed_growth_background	ENSGALG00010026162	MED1	0.051781002	0.081617079	0.634438314	0.525794812	0.672164578	positive
PRJNA729271	ed_Lushi_vs_Arbor_Acr	breed_growth_background	ENSGALG00010026163	CTNND1	-0.301992985	0.044043128	-6.85675614	7.04417E-12	3.19079E-09	negative
PRJNA729271	ed_Lushi_vs_Arbor_Acr	breed_growth_background	ENSGALG00010026166	WNT9A	0.434503115	0.132890462	3.269633574	0.001076869	0.008614356	positive
PRJNA729271	ed_Lushi_vs_Arbor_Acr	breed_growth_background	ENSGALG00010026167	CABIN1	-0.002332068	0.032655721	-0.071413771	0.94306845	0.969144075	negative
PRJNA729271	ed_Lushi_vs_Arbor_Acr	breed_growth_background	ENSGALG00010026173	CDK12	0.121321074	0.087136648	1.392308264	0.16382907	0.30367038	positive
PRJNA729271	ed_Lushi_vs_Arbor_Acr	breed_growth_background	ENSGALG00010026183	SESTD1	-0.316646755	0.090095895	-3.514552514	0.000440496	0.004534789	negative
PRJNA729271	ed_Lushi_vs_Arbor_Acr	breed_growth_background	ENSGALG00010026184	NUDT3	-0.061824315	0.055900526	-1.105970201	0.268739417	0.427265312	negative
PRJNA729271	ed_Lushi_vs_Arbor_Acr	breed_growth_background	ENSGALG00010026192	SMIM29	0.240014066	0.0630107	3.809100127	0.000139473	0.002027751	positive
PRJNA729271	ed_Lushi_vs_Arbor_Acr	breed_growth_background	ENSGALG00010026199	TBX6	-0.10287012	0.242859311	-0.423579065	0.671872825	0.787780808	negative
PRJNA729271	ed_Lushi_vs_Arbor_Acr	breed_growth_background	ENSGALG00010026203	TMX2	0.061336464	0.055979934	1.095686599	0.273215949	0.432107494	positive
PRJNA729271	ed_Lushi_vs_Arbor_Acr	breed_growth_background	ENSGALG00010026205	ENSGALG00010026205	-1.231868623	0.70379127	-1.750332344	0.080060983	0.184204036	negative
PRJNA729271	ed_Lushi_vs_Arbor_Acr	breed_growth_background	ENSGALG00010026209	MED19	0.178130627	0.091241324	1.95230208	0.050902351	0.134102067	positive
PRJNA729271	ed_Lushi_vs_Arbor_Acr	breed_growth_background	ENSGALG00010026213	AKR1A1	0.153929327	0.066912239	2.300465943	0.021421837	0.071661556	positive
PRJNA729271	ed_Lushi_vs_Arbor_Acr	breed_growth_background	ENSGALG00010026218	FTSJ3	0.177808374	0.090307967	1.968911273	0.048963281	0.130655883	positive
PRJNA729271	ed_Lushi_vs_Arbor_Acr	breed_growth_background	ENSGALG00010026220	ENSGALG00010026220	0.186836146	0.05230424	3.572103233	0.000354126	0.003887885	positive
PRJNA729271	ed_Lushi_vs_Arbor_Acr	breed_growth_background	ENSGALG00010026221	CRKL	0.036864674	0.078500094	0.469613113	0.638631454	0.762894588	positive
PRJNA729271	ed_Lushi_vs_Arbor_Acr	breed_growth_background	ENSGALG00010026229	CRHR2	0.214876228	0.112902221	1.903206382	0.057013621	0.145495147	positive
PRJNA729271	ed_Lushi_vs_Arbor_Acr	breed_growth_background	ENSGALG00010026232	PSMC5	0.051901176	0.070165195	0.739699741	0.459482206	0.614814206	positive
PRJNA729271	ed_Lushi_vs_Arbor_Acr	breed_growth_background	ENSGALG00010026234	ZDHHC5	0.117149548	0.085658528	1.367634389	0.171426547	0.314336217	positive
PRJNA729271	ed_Lushi_vs_Arbor_Acr	breed_growth_background	ENSGALG00010026237	KLHL22	0.386767428	0.074284281	5.206585056	1.92348E-07	1.3871E-05	positive
PRJNA729271	ed_Lushi_vs_Arbor_Acr	breed_growth_background	ENSGALG00010026239	GIP	-0.801403991	0.604237582	-1.3263061	0.184738327	0.330146524	negative
PRJNA729271	ed_Lushi_vs_Arbor_Acr	breed_growth_background	ENSGALG00010026242	CCDC141	-0.152018172	0.135584306	-1.121207726	0.262199452	0.420418258	negative
PRJNA729271	ed_Lushi_vs_Arbor_Acr	breed_growth_background	ENSGALG00010026244	CLP1	0.105804564	0.071319113	1.483537305	0.137931713	0.26930498	positive
PRJNA729271	ed_Lushi_vs_Arbor_Acr	breed_growth_background	ENSGALG00010026249	YPEL4	-0.252871967	0.328796252	-0.769084092	0.441843391	0.599104354	negative
PRJNA729271	ed_Lushi_vs_Arbor_Acr	breed_growth_background	ENSGALG00010026252	ENO2	-0.037393794	0.184398517	-0.202787933	0.83930079	0.9024974	negative
PRJNA729271	ed_Lushi_vs_Arbor_Acr	breed_growth_background	ENSGALG00010026254	ENSGALG00010026254	0.17849557	0.355174516	0.502557369	0.615275508	0.744131705	positive
PRJNA729271	ed_Lushi_vs_Arbor_Acr	breed_growth_background	ENSGALG00010026259	MED15	0.112451327	0.048448281	2.321059164	0.020283651	0.068946156	positive
PRJNA729271	ed_Lushi_vs_Arbor_Acr	breed_growth_background	ENSGALG00010026260	ENSGALG00010026260	-3.390913834	1.683190823	-2.014574812	0.043949219	0.120549747	negative
PRJNA729271	ed_Lushi_vs_Arbor_Acr	breed_growth_background	ENSGALG00010026261	IFT70A	0.126630647	0.059071576	2.143681547	0.032058412	0.095732731	positive
PRJNA729271	ed_Lushi_vs_Arbor_Acr	breed_growth_background	ENSGALG00010026262	NAV1	-0.111253089	0.08636213	-1.288216132	0.197670738	0.346105929	negative
PRJNA729271	ed_Lushi_vs_Arbor_Acr	breed_growth_background	ENSGALG00010026264	FZD7	0.060179761	0.076302064	0.788704243	0.430284887	0.588497923	positive
PRJNA729271	ed_Lushi_vs_Arbor_Acr	breed_growth_background	ENSGALG00010026266	ENSGALG00010026266	-0.403790644	0.187669916	-2.151600283	0.03142885	0.094314153	negative
PRJNA729271	ed_Lushi_vs_Arbor_Acr	breed_growth_background	ENSGALG00010026268	GARS1	0.071704832	0.080796082	0.887479069	0.37482103	0.537126133	positive
PRJNA729271	ed_Lushi_vs_Arbor_Acr	breed_growth_background	ENSGALG00010026272	LRRC58	0.02031846	0.05123871	0.396545114	0.691702933	0.800767908	positive
PRJNA729271	ed_Lushi_vs_Arbor_Acr	breed_growth_background	ENSGALG00010026277	SMARCD2	0.049636002	0.066642943	0.744805081	0.456389563	0.612318657	positive
PRJNA729271	ed_Lushi_vs_Arbor_Acr	breed_growth_background	ENSGALG00010026287	NASP	-0.114471113	0.04951934	-2.31164457	0.020797278	0.070073582	negative
PRJNA729271	ed_Lushi_vs_Arbor_Acr	breed_growth_background	ENSGALG00010026291	SERPING1	0.16076378	0.090896627	1.76864406	0.076953293	0.17917114	positive
PRJNA729271	ed_Lushi_vs_Arbor_Acr	breed_growth_background	ENSGALG00010026295	SMPD4	0.11049583	0.072771083	1.518402991	0.128912838	0.256417194	positive
PRJNA729271	ed_Lushi_vs_Arbor_Acr	breed_growth_background	ENSGALG00010026298	SNF8	0.194229889	0.062611047	3.102166453	0.001921099	0.01289182	positive
PRJNA729271	ed_Lushi_vs_Arbor_Acr	breed_growth_background	ENSGALG00010026300	HMGA1	-0.024631875	0.059907832	-0.411162857	0.680953121	0.793824634	negative
PRJNA729271	ed_Lushi_vs_Arbor_Acr	breed_growth_background	ENSGALG00010026305	ATP5MC1	-0.120827992	0.089261975	-1.35363342	0.175853359	0.319882257	negative
PRJNA729271	ed_Lushi_vs_Arbor_Acr	breed_growth_background	ENSGALG00010026315	SYT2	0.27081865	0.18339563	1.476690851	0.139758526	0.271700621	positive
PRJNA729271	ed_Lushi_vs_Arbor_Acr	breed_growth_background	ENSGALG00010026316	ENSGALG00010026316	0.262914505	0.187003045	1.405937022	0.159742827	0.298462525	positive
PRJNA729271	ed_Lushi_vs_Arbor_Acr	breed_growth_background	ENSGALG00010026320	ENSGALG00010026320	-0.403106286	0.093715554	-4.301380817	1.69737E-05	0.000422014	negative
PRJNA729271	ed_Lushi_vs_Arbor_Acr	breed_growth_background	ENSGALG00010026321	GPBP1L1	0.032963307	0.025511317	1.292105271	0.196320691	0.344637085	positive
PRJNA729271	ed_Lushi_vs_Arbor_Acr	breed_growth_background	ENSGALG00010026325	TMEM69	0.020432803	0.074181755	0.275442439	0.782976343	0.865693523	positive
PRJNA729271	ed_Lushi_vs_Arbor_Acr	breed_growth_background	ENSGALG00010026329	ENSGALG00010026329	0.260403364	0.143349433	1.816563619	0.069283958	0.166739329	positive
PRJNA729271	ed_Lushi_vs_Arbor_Acr	breed_growth_background	ENSGALG00010026334	ENSGALG00010026334	-0.074216105	0.067955385	-1.092129849	0.274776031	0.433628587	negative
PRJNA729271	ed_Lushi_vs_Arbor_Acr	breed_growth_background	ENSGALG00010026335	IPP	-0.003062619	0.057992993	-0.052810153	0.957883172	0.976544983	negative
PRJNA729271	ed_Lushi_vs_Arbor_Acr	breed_growth_background	ENSGALG00010026344	TIMM10	-0.172697907	0.092029285	-1.876553819	0.060579272	0.151841007	negative
PRJNA729271	ed_Lushi_vs_Arbor_Acr	breed_growth_background	ENSGALG00010026346	FZD2	0.247874594	0.087281628	2.839940067	0.004512201	0.023594644	positive
PRJNA729271	ed_Lushi_vs_Arbor_Acr	breed_growth_background	ENSGALG00010026354	ENSGALG00010026354	-0.423553369	0.129868516	-3.261401477	0.00110863	0.00881975	negative
PRJNA729271	ed_Lushi_vs_Arbor_Acr	breed_growth_background	ENSGALG00010026355	PPP1R12B	0.076205328	0.073313657	1.039442466	0.298599003	0.460838218	positive
PRJNA729271	ed_Lushi_vs_Arbor_Acr	breed_growth_background	ENSGALG00010026360	CTDSPL	0.172317838	0.067687517	2.545784581	0.010903245	0.044493957	positive
PRJNA729271	ed_Lushi_vs_Arbor_Acr	breed_growth_background	ENSGALG00010026362	ENSGALG00010026362	0.152314563	0.216831549	0.702455726	0.482395003	0.634858631	positive
PRJNA729271	ed_Lushi_vs_Arbor_Acr	breed_growth_background	ENSGALG00010026367	RBP	-0.306837362	0.277351843	-1.106310883	0.268591983	0.427124607	negative
PRJNA729271	ed_Lushi_vs_Arbor_Acr	breed_growth_background	ENSGALG00010026377	ENSGALG00010026377	-0.220754448	0.119272863	-1.85083548	0.064193225	0.158110586	negative
PRJNA729271	ed_Lushi_vs_Arbor_Acr	breed_growth_background	ENSGALG00010026388	SNRPD3	0.073963863	0.043058733	1.717743603	0.085843392	0.19351477	positive
PRJNA729271	ed_Lushi_vs_Arbor_Acr	breed_growth_background	ENSGALG00010026393	SLC43A3	-0.280761403	0.066950218	-4.193584608	2.74581E-05	0.000613258	negative
PRJNA729271	ed_Lushi_vs_Arbor_Acr	breed_growth_background	ENSGALG00010026402	TFEB	0.34335687	0.087350347	3.930801459	8.46632E-05	0.001417081	positive
PRJNA729271	ed_Lushi_vs_Arbor_Acr	breed_growth_background	ENSGALG00010026403	GUCD1	0.336807732	0.062100847	5.423560964	5.84233E-08	5.499E-06	positive
PRJNA729271	ed_Lushi_vs_Arbor_Acr	breed_growth_background	ENSGALG00010026409	VILL	-0.405803662	0.146322925	-2.773343009	0.00554836	0.027429565	negative
PRJNA729271	ed_Lushi_vs_Arbor_Acr	breed_growth_background	ENSGALG00010026410	ENSGALG00010026410	0.381244329	0.169854059	2.244540592	0.02479764	0.079557724	positive
PRJNA729271	ed_Lushi_vs_Arbor_Acr	breed_growth_background	ENSGALG00010026416	ENSGALG00010026416	-0.447867666	0.795159356	-0.563242654	0.573269662	0.71131174	negative
PRJNA729271	ed_Lushi_vs_Arbor_Acr	breed_growth_background	ENSGALG00010026422	P2RX3	-0.131524416	0.194455479	-0.676372898	0.49880392	0.650136033	negative
PRJNA729271	ed_Lushi_vs_Arbor_Acr	breed_growth_background	ENSGALG00010026427	PGC	-0.335312383	0.566358464	-0.592049743	0.553817279	0.695872179	negative
PRJNA729271	ed_Lushi_vs_Arbor_Acr	breed_growth_background	ENSGALG00010026433	VPS11	-0.006781723	0.039098133	-0.173453877	0.862294679	0.917201857	negative
PRJNA729271	ed_Lushi_vs_Arbor_Acr	breed_growth_background	ENSGALG00010026434	ENSGALG00010026434	-0.099459548	0.078409357	-1.268465287	0.20463184	0.35399672	negative
PRJNA729271	ed_Lushi_vs_Arbor_Acr	breed_growth_background	ENSGALG00010026436	PLCD1	-0.207890618	0.041691782	-4.986369248	6.15245E-07	3.33526E-05	negative
PRJNA729271	ed_Lushi_vs_Arbor_Acr	breed_growth_background	ENSGALG00010026438	TOMM6	0.000429288	0.064901118	0.006614498	0.994722433	0.997061176	positive
PRJNA729271	ed_Lushi_vs_Arbor_Acr	breed_growth_background	ENSGALG00010026443	UPB1	-0.403806977	0.195176555	-2.068931774	0.038552489	0.109632069	negative
PRJNA729271	ed_Lushi_vs_Arbor_Acr	breed_growth_background	ENSGALG00010026448	ADORA2A	-0.04764927	0.062709451	-0.759841925	0.447349079	0.604035855	negative
PRJNA729271	ed_Lushi_vs_Arbor_Acr	breed_growth_background	ENSGALG00010026457	SSRP1	0.026347119	0.053339223	0.493953937	0.621338711	0.748529307	positive
PRJNA729271	ed_Lushi_vs_Arbor_Acr	breed_growth_background	ENSGALG00010026458	DLEC1	0.03388976	0.095064622	0.356491822	0.721472276	0.821393372	positive
PRJNA729271	ed_Lushi_vs_Arbor_Acr	breed_growth_background	ENSGALG00010026480	USP49	0.308579698	0.115004542	2.683195737	0.00729223	0.033344123	positive
PRJNA729271	ed_Lushi_vs_Arbor_Acr	breed_growth_background	ENSGALG00010026481	DIXDC1	-0.137517791	0.075268383	-1.827032624	0.067694864	0.164223776	negative
PRJNA729271	ed_Lushi_vs_Arbor_Acr	breed_growth_background	ENSGALG00010026484	TNKS1BP1	-0.069319078	0.060212267	-1.151245124	0.249631406	0.405624366	negative
PRJNA729271	ed_Lushi_vs_Arbor_Acr	breed_growth_background	ENSGALG00010026485	ACAA1	-0.111321777	0.056272046	-1.978278444	0.047897304	0.128389524	negative
PRJNA729271	ed_Lushi_vs_Arbor_Acr	breed_growth_background	ENSGALG00010026492	SPECC1L	0.037603927	0.040655086	0.924950121	0.354991854	0.518710375	positive
PRJNA729271	ed_Lushi_vs_Arbor_Acr	breed_growth_background	ENSGALG00010026498	ENSGALG00010026498	-0.107587597	0.047443799	-2.267685115	0.023348406	0.076292863	negative
PRJNA729271	ed_Lushi_vs_Arbor_Acr	breed_growth_background	ENSGALG00010026507	APLNR	-0.577438806	0.156987104	-3.67825631	0.000234834	0.002906847	negative
PRJNA729271	ed_Lushi_vs_Arbor_Acr	breed_growth_background	ENSGALG00010026508	CSRP1	-0.035902568	0.080381236	-0.446653589	0.655125197	0.775238902	negative
PRJNA729271	ed_Lushi_vs_Arbor_Acr	breed_growth_background	ENSGALG00010026516	ENSGALG00010026516	-0.148770261	0.089594267	-1.660488616	0.096816196	0.209983052	negative
PRJNA729271	ed_Lushi_vs_Arbor_Acr	breed_growth_background	ENSGALG00010026522	MED20	0.362447934	0.069228267	5.235548301	1.64496E-07	1.25396E-05	positive
PRJNA729271	ed_Lushi_vs_Arbor_Acr	breed_growth_background	ENSGALG00010026536	BCR	-0.084228101	0.052384077	-1.607895119	0.107858151	0.227238442	negative
PRJNA729271	ed_Lushi_vs_Arbor_Acr	breed_growth_background	ENSGALG00010026537	FJX1	0.152077445	0.100294612	1.516307232	0.129441684	0.256986332	positive
PRJNA729271	ed_Lushi_vs_Arbor_Acr	breed_growth_background	ENSGALG00010026539	ENSGALG00010026539	0.017913338	0.207525388	0.086318779	0.931213011	0.962282212	positive
PRJNA729271	ed_Lushi_vs_Arbor_Acr	breed_growth_background	ENSGALG00010026550	MYD88	-0.056717247	0.056592561	-1.002203224	0.316245451	0.479595921	negative
PRJNA729271	ed_Lushi_vs_Arbor_Acr	breed_growth_background	ENSGALG00010026553	PLPP3	-0.10862215	0.068355385	-1.589079632	0.112042416	0.233341209	negative
PRJNA729271	ed_Lushi_vs_Arbor_Acr	breed_growth_background	ENSGALG00010026559	RAB36	0.868470156	0.27590535	3.147710462	0.001645546	0.011584356	positive
PRJNA729271	ed_Lushi_vs_Arbor_Acr	breed_growth_background	ENSGALG00010026560	TNNI1	0.062519104	0.316044948	0.197817128	0.843188145	0.90506146	positive
PRJNA729271	ed_Lushi_vs_Arbor_Acr	breed_growth_background	ENSGALG00010026565	RSPH14	0.694885359	0.264513567	2.627031072	0.008613347	0.037380378	positive
PRJNA729271	ed_Lushi_vs_Arbor_Acr	breed_growth_background	ENSGALG00010026566	ENSGALG00010026566	-0.167238763	0.06674134	-2.505774723	0.012218344	0.048138483	negative
PRJNA729271	ed_Lushi_vs_Arbor_Acr	breed_growth_background	ENSGALG00010026568	BYSL	0.053101152	0.059583504	0.891205587	0.372818887	0.535387527	positive
PRJNA729271	ed_Lushi_vs_Arbor_Acr	breed_growth_background	ENSGALG00010026569	GNAZ	0.152086915	0.112568777	1.351057715	0.176676945	0.320677726	positive
PRJNA729271	ed_Lushi_vs_Arbor_Acr	breed_growth_background	ENSGALG00010026581	DNER	0.149627895	0.127155828	1.17672857	0.239303861	0.394839208	positive
PRJNA729271	ed_Lushi_vs_Arbor_Acr	breed_growth_background	ENSGALG00010026584	ENSGALG00010026584	-0.183438389	0.059862681	-3.064319657	0.002181657	0.014117461	negative
PRJNA729271	ed_Lushi_vs_Arbor_Acr	breed_growth_background	ENSGALG00010026588	CCND3	0.203185517	0.077314006	2.62805574	0.008587444	0.037312652	positive
PRJNA729271	ed_Lushi_vs_Arbor_Acr	breed_growth_background	ENSGALG00010026595	PRKAA2	0.173051285	0.139306322	1.242235691	0.214149615	0.364813656	positive
PRJNA729271	ed_Lushi_vs_Arbor_Acr	breed_growth_background	ENSGALG00010026601	ENSGALG00010026601	-0.511481843	0.257078647	-1.989592873	0.046635802	0.125943071	negative
PRJNA729271	ed_Lushi_vs_Arbor_Acr	breed_growth_background	ENSGALG00010026612	MFF	0.045031947	0.044677871	1.007925081	0.31349043	0.477014883	positive
PRJNA729271	ed_Lushi_vs_Arbor_Acr	breed_growth_background	ENSGALG00010026625	ENSGALG00010026625	-0.485843778	0.255902891	-1.898547435	0.057624005	0.146522531	negative
PRJNA729271	ed_Lushi_vs_Arbor_Acr	breed_growth_background	ENSGALG00010026628	FYB2	-0.47123603	0.506929578	-0.929588745	0.352584057	0.51637839	negative
PRJNA729271	ed_Lushi_vs_Arbor_Acr	breed_growth_background	ENSGALG00010026630	XIRP1	-0.444877072	0.130311438	-3.413952597	0.000640277	0.005930235	negative
PRJNA729271	ed_Lushi_vs_Arbor_Acr	breed_growth_background	ENSGALG00010026633	SLC5A1	-0.410531656	0.277374467	-1.48006289	0.138856464	0.270491123	negative
PRJNA729271	ed_Lushi_vs_Arbor_Acr	breed_growth_background	ENSGALG00010026635	C8A	-2.061818336	1.365179727	-1.510290766	0.130969247	0.259062549	negative
PRJNA729271	ed_Lushi_vs_Arbor_Acr	breed_growth_background	ENSGALG00010026638	LAD1	-0.540864729	0.276066065	-1.95918585	0.050091026	0.132772549	negative
PRJNA729271	ed_Lushi_vs_Arbor_Acr	breed_growth_background	ENSGALG00010026643	YWHAH	0.05801066	0.060141065	0.964576532	0.334756959	0.49910534	positive
PRJNA729271	ed_Lushi_vs_Arbor_Acr	breed_growth_background	ENSGALG00010026659	LIPH	0.230677061	0.140807523	1.638243871	0.101370831	0.217225305	positive
PRJNA729271	ed_Lushi_vs_Arbor_Acr	breed_growth_background	ENSGALG00010026666	ENSGALG00010026666	-0.442563347	0.250137415	-1.769280887	0.076847011	0.179025623	negative
PRJNA729271	ed_Lushi_vs_Arbor_Acr	breed_growth_background	ENSGALG00010026671	TAF8	0.210352952	0.083092348	2.531556232	0.011355761	0.045767243	positive
PRJNA729271	ed_Lushi_vs_Arbor_Acr	breed_growth_background	ENSGALG00010026673	DEPDC5	0.032208849	0.065389947	0.492565766	0.622319442	0.749088218	positive
PRJNA729271	ed_Lushi_vs_Arbor_Acr	breed_growth_background	ENSGALG00010026681	PLXDC1	-0.205381408	0.137156448	-1.497424366	0.134282872	0.264114367	negative
PRJNA729271	ed_Lushi_vs_Arbor_Acr	breed_growth_background	ENSGALG00010026686	ENSGALG00010026686	1.601546963	0.224205066	7.143223795	9.1167E-13	5.28586E-10	positive
PRJNA729271	ed_Lushi_vs_Arbor_Acr	breed_growth_background	ENSGALG00010026690	KRT14	0.040950123	0.245510537	0.166795784	0.867530736	0.920695418	positive
PRJNA729271	ed_Lushi_vs_Arbor_Acr	breed_growth_background	ENSGALG00010026692	NMNAT3	0.666151953	0.232946242	2.859681045	0.004240673	0.022515953	positive
PRJNA729271	ed_Lushi_vs_Arbor_Acr	breed_growth_background	ENSGALG00010026696	CSRNP1	-0.138757368	0.08116736	-1.709521764	0.087354344	0.195733686	negative
PRJNA729271	ed_Lushi_vs_Arbor_Acr	breed_growth_background	ENSGALG00010026697	CHN1	-0.160255014	0.063802797	-2.511723972	0.012014302	0.04755655	negative
PRJNA729271	ed_Lushi_vs_Arbor_Acr	breed_growth_background	ENSGALG00010026700	C8B	-1.356266176	0.559344204	-2.424743416	0.01531921	0.056559334	negative

PRJNA729271	eed_Lushi_vs_Arbor_Acr	breed_growth_background	ENSGALG00010026704	WDFY1	-0.11063765	0.037950379	-2.915324009	0.003553194	0.019916301	negative
PRJNA729271	eed_Lushi_vs_Arbor_Acr	breed_growth_background	ENSGALG00010026706	KRT24	0.205409226	0.326970814	0.628218843	0.529860588	0.67572842	positive
PRJNA729271	eed_Lushi_vs_Arbor_Acr	breed_growth_background	ENSGALG00010026707	GORASP1	0.032637845	0.044209663	0.738251381	0.460361705	0.615528357	positive
PRJNA729271	eed_Lushi_vs_Arbor_Acr	breed_growth_background	ENSGALG00010026715	WDR48	-0.067813995	0.036963817	-1.834604759	0.066564273	0.162432515	negative
PRJNA729271	eed_Lushi_vs_Arbor_Acr	breed_growth_background	ENSGALG00010026719	ENSGALG00010026719	0.393688407	0.48413116	0.813185432	0.41611175	0.576305701	positive
PRJNA729271	eed_Lushi_vs_Arbor_Acr	breed_growth_background	ENSGALG00010026724	OLA1	-0.004807494	0.052343388	-0.09184529	0.92682096	0.959111146	negative
PRJNA729271	eed_Lushi_vs_Arbor_Acr	breed_growth_background	ENSGALG00010026731	ENSGALG00010026731	-1.036042164	0.28735124	-3.605490489	0.000311564	0.003550612	negative
PRJNA729271	eed_Lushi_vs_Arbor_Acr	breed_growth_background	ENSGALG00010026737	SCN11A	-0.258252045	0.621089792	-0.415804684	0.677552931	0.791617779	negative
PRJNA729271	eed_Lushi_vs_Arbor_Acr	breed_growth_background	ENSGALG00010026740	DAB1	0.266094743	0.084638793	3.143886314	0.001667202	0.011674441	positive
PRJNA729271	eed_Lushi_vs_Arbor_Acr	breed_growth_background	ENSGALG00010026743	TNNT2	-0.692183037	0.314619006	-2.200067462	0.027802109	0.086035775	negative
PRJNA729271	eed_Lushi_vs_Arbor_Acr	breed_growth_background	ENSGALG00010026746	HOXD10	-0.167464001	0.260243318	-0.643490108	0.519906131	0.667615111	negative
PRJNA729271	eed_Lushi_vs_Arbor_Acr	breed_growth_background	ENSGALG00010026755	ENSGALG00010026755	-0.080578161	0.112406356	-0.71684702	0.473468496	0.627143702	negative
PRJNA729271	eed_Lushi_vs_Arbor_Acr	breed_growth_background	ENSGALG00010026759	OMA1	-0.089643715	0.060730352	-1.476094108	0.13991863	0.271902473	negative
PRJNA729271	eed_Lushi_vs_Arbor_Acr	breed_growth_background	ENSGALG00010026767	ENSGALG00010026767	-1.771884773	1.338896007	-1.32339238	0.185704925	0.331236218	negative
PRJNA729271	eed_Lushi_vs_Arbor_Acr	breed_growth_background	ENSGALG00010026770	ENSGALG00010026770	-0.178572572	0.32934156	-0.542210864	0.587673252	0.723363093	negative
PRJNA729271	eed_Lushi_vs_Arbor_Acr	breed_growth_background	ENSGALG00010026775	BTG2	0.239775103	0.195558383	1.226104956	0.220159181	0.372717511	positive
PRJNA729271	eed_Lushi_vs_Arbor_Acr	breed_growth_background	ENSGALG00010026780	MYSM1	0.076389437	0.042283843	1.806586893	0.070826715	0.169162866	positive
PRJNA729271	eed_Lushi_vs_Arbor_Acr	breed_growth_background	ENSGALG00010026782	HOXB9	0.134666791	0.354170164	0.38023189	0.703773289	0.808527686	positive
PRJNA729271	eed_Lushi_vs_Arbor_Acr	breed_growth_background	ENSGALG00010026786	ENSGALG00010026786	-0.079788103	0.304436168	-0.262084836	0.793256038	0.871971803	negative
PRJNA729271	eed_Lushi_vs_Arbor_Acr	breed_growth_background	ENSGALG00010026796	DCAKD	-0.423929485	0.120989277	-3.503859982	0.000458566	0.004671061	negative
PRJNA729271	eed_Lushi_vs_Arbor_Acr	breed_growth_background	ENSGALG00010026801	FMOD	0.412764665	0.261907161	1.575996102	0.115026702	0.237459356	positive
PRJNA729271	eed_Lushi_vs_Arbor_Acr	breed_growth_background	ENSGALG00010026822	PRELP	-0.139918918	0.107119597	-1.306193468	0.191486774	0.33832829	negative
PRJNA729271	eed_Lushi_vs_Arbor_Acr	breed_growth_background	ENSGALG00010026825	ADAM23	-0.133853002	0.073484038	-1.821524848	0.068527113	0.16548438	negative
PRJNA729271	eed_Lushi_vs_Arbor_Acr	breed_growth_background	ENSGALG00010026827	FAM117A	0.011915672	0.068499935	0.17395158	0.861903516	0.917006126	positive
PRJNA729271	eed_Lushi_vs_Arbor_Acr	breed_growth_background	ENSGALG00010026834	ENSGALG00010026834	-0.68148895	1.056720559	-0.644909332	0.518985944	0.666965268	negative
PRJNA729271	eed_Lushi_vs_Arbor_Acr	breed_growth_background	ENSGALG00010026836	ENSGALG00010026836	-0.364533707	0.267843522	-1.360995049	0.173515254	0.317136245	negative
PRJNA729271	eed_Lushi_vs_Arbor_Acr	breed_growth_background	ENSGALG00010026839	FGGY	-0.059157835	0.064114774	-0.922686474	0.356170616	0.519699324	negative
PRJNA729271	eed_Lushi_vs_Arbor_Acr	breed_growth_background	ENSGALG00010026846	MICAL1	0.021406766	0.122876256	0.174214017	0.86169727	0.916909173	positive
PRJNA729271	eed_Lushi_vs_Arbor_Acr	breed_growth_background	ENSGALG00010026851	ENSGALG00010026851	-0.600860871	0.565846996	-1.061878696	0.288290758	0.449233986	negative
PRJNA729271	eed_Lushi_vs_Arbor_Acr	breed_growth_background	ENSGALG00010026854	SMPD2	-0.019781062	0.08186638	-0.241626202	0.809069817	0.882536092	negative
PRJNA729271	eed_Lushi_vs_Arbor_Acr	breed_growth_background	ENSGALG00010026856	UBE2Z	0.024770775	0.042016114	0.589554157	0.555489594	0.697196692	positive
PRJNA729271	eed_Lushi_vs_Arbor_Acr	breed_growth_background	ENSGALG00010026874	TEAD3	0.223780613	0.085663211	2.612330432	0.008992729	0.038702378	positive
PRJNA729271	eed_Lushi_vs_Arbor_Acr	breed_growth_background	ENSGALG00010026885	TULP1	0.436710164	0.305978574	1.427257337	0.153505733	0.289952686	positive
PRJNA729271	eed_Lushi_vs_Arbor_Acr	breed_growth_background	ENSGALG00010026886	JUN	0.068496292	0.103024819	0.664852343	0.50614494	0.65569496	positive
PRJNA729271	eed_Lushi_vs_Arbor_Acr	breed_growth_background	ENSGALG00010026889	ENSGALG00010026889	-0.227290115	0.151330966	-1.501940557	0.133112461	0.26241802	negative
PRJNA729271	eed_Lushi_vs_Arbor_Acr	breed_growth_background	ENSGALG00010026893	FKBP5	0.485724884	0.206469437	2.352526807	0.018646346	0.065096045	positive
PRJNA729271	eed_Lushi_vs_Arbor_Acr	breed_growth_background	ENSGALG00010026894	IP6K1	0.06441672	0.036227068	1.77813782	0.075381222	0.176829197	positive
PRJNA729271	eed_Lushi_vs_Arbor_Acr	breed_growth_background	ENSGALG00010026897	ENSGALG00010026897	-0.223773475	0.124361493	-1.799379123	0.07195873	0.170934413	negative
PRJNA729271	eed_Lushi_vs_Arbor_Acr	breed_growth_background	ENSGALG00010026898	PNPLA2	0.235645647	0.145123982	1.623753997	0.104428322	0.221785864	positive
PRJNA729271	eed_Lushi_vs_Arbor_Acr	breed_growth_background	ENSGALG00010026899	HOOK1	0.25997783	0.090276777	2.879786347	0.003979447	0.021603779	positive
PRJNA729271	eed_Lushi_vs_Arbor_Acr	breed_growth_background	ENSGALG00010026901	FAM212A	-0.120420455	0.095244609	-1.26432831	0.206112212	0.355413636	negative
PRJNA729271	eed_Lushi_vs_Arbor_Acr	breed_growth_background	ENSGALG00010026907	ENSGALG00010026907	-0.347934495	0.072823828	-4.777756185	1.77262E-06	7.66989E-05	negative
PRJNA729271	eed_Lushi_vs_Arbor_Acr	breed_growth_background	ENSGALG00010026909	ENSGALG00010026909	-0.1403073	0.084631994	-1.657851747	0.097347394	0.210793318	negative
PRJNA729271	eed_Lushi_vs_Arbor_Acr	breed_growth_background	ENSGALG00010026916	ENSGALG00010026916	-0.264932973	0.114323046	-2.317406517	0.020481598	0.069344457	negative
PRJNA729271	eed_Lushi_vs_Arbor_Acr	breed_growth_background	ENSGALG00010026917	AMT	-0.334875432	0.080596839	-4.154944979	3.25366E-05	0.000707214	negative
PRJNA729271	eed_Lushi_vs_Arbor_Acr	breed_growth_background	ENSGALG00010026919	NICN1	-0.136727858	0.092947056	-1.471029453	0.141283152	0.273929813	negative
PRJNA729271	eed_Lushi_vs_Arbor_Acr	breed_growth_background	ENSGALG00010026920	GATA2	-0.151941544	0.105410447	-1.441427759	0.149463873	0.284538854	negative
PRJNA729271	eed_Lushi_vs_Arbor_Acr	breed_growth_background	ENSGALG00010026924	TPH1	-0.583355454	0.194256742	-3.003012657	0.002673213	0.016301314	negative
PRJNA729271	eed_Lushi_vs_Arbor_Acr	breed_growth_background	ENSGALG00010026942	CDK18	-0.373613968	0.100470605	-3.718639592	0.000200299	0.002596895	negative
PRJNA729271	eed_Lushi_vs_Arbor_Acr	breed_growth_background	ENSGALG00010026943	EEFSEC	-0.005608663	0.03901233	-0.14376642	0.88568492	0.932587747	negative
PRJNA729271	eed_Lushi_vs_Arbor_Acr	breed_growth_background	ENSGALG00010026950	LHFPL5	0.136848829	0.148750776	0.91998733	0.357579381	0.521282623	positive
PRJNA729271	eed_Lushi_vs_Arbor_Acr	breed_growth_background	ENSGALG00010026951	RUVBL1	0.031443034	0.057413354	0.547660643	0.583924941	0.719788419	positive
PRJNA729271	eed_Lushi_vs_Arbor_Acr	breed_growth_background	ENSGALG00010026954	MFSD4A	-0.245464353	0.093238221	-2.632658038	0.00847196	0.03698827	negative
PRJNA729271	eed_Lushi_vs_Arbor_Acr	breed_growth_background	ENSGALG00010026958	CLTCL1	-0.008866918	0.045125915	-0.196492807	0.844224462	0.905369525	negative
PRJNA729271	eed_Lushi_vs_Arbor_Acr	breed_growth_background	ENSGALG00010026960	SEC61A1	-0.192981949	0.054986377	-3.509631991	0.000448727	0.004583722	negative
PRJNA729271	eed_Lushi_vs_Arbor_Acr	breed_growth_background	ENSGALG00010026964	SRPK1	0.068116074	0.032525731	2.094221121	0.036240287	0.104621183	positive
PRJNA729271	eed_Lushi_vs_Arbor_Acr	breed_growth_background	ENSGALG00010026972	TNNT3	-0.128190479	0.084570825	-1.515776629	0.129575844	0.257182234	negative
PRJNA729271	eed_Lushi_vs_Arbor_Acr	breed_growth_background	ENSGALG00010026973	MGLL	-0.10751992	0.065815808	-1.633648867	0.102332601	0.218659775	negative
PRJNA729271	eed_Lushi_vs_Arbor_Acr	breed_growth_background	ENSGALG00010026977	ELK4	-0.035138718	0.052732938	-0.666352353	0.50518591	0.655130385	negative
PRJNA729271	eed_Lushi_vs_Arbor_Acr	breed_growth_background	ENSGALG00010026980	LIMK2	-0.114323956	0.065349974	-1.749410879	0.080220022	0.184390772	negative
PRJNA729271	eed_Lushi_vs_Arbor_Acr	breed_growth_background	ENSGALG00010026982	ABTB1	-0.010428606	0.045050128	-0.23148893	0.816934984	0.887046157	negative
PRJNA729271	eed_Lushi_vs_Arbor_Acr	breed_growth_background	ENSGALG00010026984	PGR2/3	0.699342601	0.490865076	1.42471452	0.15423974	0.29073192	positive
PRJNA729271	eed_Lushi_vs_Arbor_Acr	breed_growth_background	ENSGALG00010026999	MLEC	-0.128647711	0.044610599	-2.883792532	0.003929175	0.021394964	negative
PRJNA729271	eed_Lushi_vs_Arbor_Acr	breed_growth_background	ENSGALG00010027001	SLC26A8	-0.002968196	0.138948286	-0.021361878	0.982956984	0.989816659	negative
PRJNA729271	eed_Lushi_vs_Arbor_Acr	breed_growth_background	ENSGALG00010027003	SEPTIN2L	-0.031990085	0.044284574	-0.722375364	0.470063729	0.624586688	negative
PRJNA729271	eed_Lushi_vs_Arbor_Acr	breed_growth_background	ENSGALG00010027012	RILPL1	0.364162895	0.102647459	3.547704909	0.000388603	0.004160122	positive
PRJNA729271	eed_Lushi_vs_Arbor_Acr	breed_growth_background	ENSGALG00010027015	ENSGALG00010027015	-0.471153221	0.266670541	-1.766798912	0.077261911	0.179559308	negative
PRJNA729271	eed_Lushi_vs_Arbor_Acr	breed_growth_background	ENSGALG00010027025	ORAI1	-0.131016334	0.067828953	-1.931569456	0.053412669	0.138922777	negative
PRJNA729271	eed_Lushi_vs_Arbor_Acr	breed_growth_background	ENSGALG00010027026	SLC45A3	-0.423969329	0.108870404	-3.894256952	9.85002E-05	0.001570693	negative
PRJNA729271	eed_Lushi_vs_Arbor_Acr	breed_growth_background	ENSGALG00010027027	ARHGEF3	0.01306069	0.055188641	0.236655407	0.812924132	0.884900518	positive
PRJNA729271	eed_Lushi_vs_Arbor_Acr	breed_growth_background	ENSGALG00010027036	NUCKS1	-0.089682914	0.03542326	-2.531752103	0.01134942	0.045760734	negative
PRJNA729271	eed_Lushi_vs_Arbor_Acr	breed_growth_background	ENSGALG00010027040	CLIP1	0.122720523	0.05785472	2.121184291	0.033906297	0.099548667	positive
PRJNA729271	eed_Lushi_vs_Arbor_Acr	breed_growth_background	ENSGALG00010027041	MAPK14	-0.15856245	0.03940461	-4.023956825	5.72284E-05	0.001072346	negative
PRJNA729271	eed_Lushi_vs_Arbor_Acr	breed_growth_background	ENSGALG00010027043	IL17RD	-0.245523126	0.095240915	-2.577916481	0.009939802	0.041640875	negative
PRJNA729271	eed_Lushi_vs_Arbor_Acr	breed_growth_background	ENSGALG00010027045	MAPK13	-0.317683283	0.101229774	-3.138239576	0.001699659	0.011821764	negative
PRJNA729271	eed_Lushi_vs_Arbor_Acr	breed_growth_background	ENSGALG00010027046	HESX1	0.219270775	0.172596494	1.270424269	0.203933543	0.353252474	positive
PRJNA729271	eed_Lushi_vs_Arbor_Acr	breed_growth_background	ENSGALG00010027050	GSTT1	-0.409028134	0.080528215	-5.079314558	3.78799E-07	2.2783E-05	negative
PRJNA729271	eed_Lushi_vs_Arbor_Acr	breed_growth_background	ENSGALG00010027052	APPL1	0.023784272	0.045561914	0.522020929	0.601655758	0.734214533	positive
PRJNA729271	eed_Lushi_vs_Arbor_Acr	breed_growth_background	ENSGALG00010027059	ASB14	0.244237782	0.085286721	2.863725782	0.004186903	0.02229883	positive
PRJNA729271	eed_Lushi_vs_Arbor_Acr	breed_growth_background	ENSGALG00010027063	RAB7L1	-0.101217973	0.047758681	-2.119362814	0.034059817	0.099918447	negative
PRJNA729271	eed_Lushi_vs_Arbor_Acr	breed_growth_background	ENSGALG00010027073	DNAH12	-0.547351378	0.177211553	-3.088689013	0.002010418	0.013300321	negative
PRJNA729271	eed_Lushi_vs_Arbor_Acr	breed_growth_background	ENSGALG00010027074	BRPF3	0.004769272	0.043251055	0.11026949	0.912195653	0.950422369	positive
PRJNA729271	eed_Lushi_vs_Arbor_Acr	breed_growth_background	ENSGALG00010027075	SLC41A1	0.05650319	0.050443412	1.120130203	0.262658282	0.420900671	positive
PRJNA729271	eed_Lushi_vs_Arbor_Acr	breed_growth_background	ENSGALG00010027076	VDHAP	0.207197688	0.091448471	2.265731561	0.023467824	0.076493391	positive
PRJNA729271	eed_Lushi_vs_Arbor_Acr	breed_growth_background	ENSGALG00010027077	ARF4	0.043594608	0.056308916	0.774204356	0.438809953	0.596121505	positive
PRJNA729271	eed_Lushi_vs_Arbor_Acr	breed_growth_background	ENSGALG00010027078	PDE12	0.040296198	0.06748663	0.59709898	0.550441299	0.693554122	positive
PRJNA729271	eed_Lushi_vs_Arbor_Acr	breed_growth_background	ENSGALG00010027079	PM20D1	-0.069632684	0.050703607	-1.373328023	0.169650395	0.311814454	negative
PRJNA729271	eed_Lushi_vs_Arbor_Acr	breed_growth_background	ENSGALG00010027080	TBC1D22B	0.273337686	0.083715051	3.26509611	0.001094269	0.008729463	positive
PRJNA729271	eed_Lushi_vs_Arbor_Acr	breed_growth_background	ENSGALG00010027081	PDE4B	0.508670047	0.156119469	3.25821021	0.001121173	0.00889068	positive
PRJNA729271	eed_Lushi_vs_Arbor_Acr	breed_growth_background	ENSGALG00010027082	SLC26A9	-0.385522821	0.374059199	-1.030646545	0.302706601	0.465281569	negative
PRJNA729271	eed_Lushi_vs_Arbor_Acr	breed_growth_background	ENSGALG00010027083	DENND6A	-0.108617641	0.047768727	-2.273823236	0.022976617	0.075418038	negative
PRJNA729271	eed_Lushi_vs_Arbor_Acr	breed_growth_background	ENSGALG00010027084	OSBPL9	-0.08549739	0.050726442	-1.685460019	0.091899852	0.202689459	negative
PRJNA729271	eed_Lushi_vs_Arbor_Acr	breed_growth_background	ENSGALG00010027085	PIM1	0.238183048	0.082830249	2.875556343	0.004033162	0.021748764	positive
PRJNA729271	eed_Lushi_vs_Arbor_Acr	breed_growth_background	ENSGALG00010027086	CACNA1S	0.172242599	0.107102166	1.608208368	0.107789551	0.227160446	positive
PRJNA729271	eed_Lushi_vs_Arbor_Acr	breed_growth_background	ENSGALG00010027087	RAB7B	-0.010234716	0.187853817	-0.054482344	0.956550875	0.97569799	negative
PRJNA729271	eed_Lushi_vs_Arbor_Acr	breed_growth_background	ENSGALG00010027088	AGBL4	-0.108638785	0.141904335	-0.765577635	0.443927656	0.600779795	negative
PRJNA729271	eed_Lushi_vs_Arbor_Acr	breed_growth_background	ENSGALG00010027092	ENSGALG00010027092	-0.221183552	0.109680978	-2.016608135	0.043736423	0.120175811	negative
PRJNA729271	eed_Lushi_vs_Arbor_Acr	breed_growth_background	ENSGALG00010027093	SLMAP	0.011584673	0.040827608	0.28374606	0.776604994	0.861342825	positive
PRJNA729271	eed_Lushi_vs_Arbor_Acr	breed_growth_background	ENSGALG00010027095	SCN5A	-0.555440609	0.221614474	-2.506337239	0.012198921	0.04810211	negative
PRJNA729271	eed_Lushi_vs_Arbor_Acr	breed_growth_background	ENSGALG00010027096	YIPF1	0.175432272	0.058814741	2.982794273	0.002856299	0.017108288	positive
PRJNA729271	eed_Lushi_vs_Arbor_Acr	breed_growth_background	ENSGALG00010027097	ENSGALG00010027097	-0.137598184	0.080239837	-1.714836281	0.086375248	0.194441562	negative
PRJNA729271	eed_Lushi_vs_Arbor_Acr	breed_growth_background	ENSGALG00010027098	EXOG	0.093981871	0.078650042	1.194937319	0.232111521	0.386275143	positive
PRJNA729271	eed_Lushi_vs_Arbor_Acr	breed_growth_background	ENSGALG00010027099	GNA11	0.052123072	0.046297498	1.125829142	0.260237846	0.418059136	positive
PRJNA729271	eed_Lushi_vs_Arbor_Acr	breed_growth_background	ENSGALG00010027100	MIER2	0.064558909	0.050120899	1.28806366	0.197723804	0.346105929	positive
PRJNA729271	eed_Lushi_vs_Arbor_Acr	breed_growth_background	ENSGALG00010027101	RNF220	0.044774707	0.043204747	1.036337676	0.300044635	0.462429238	positive
PRJNA729271	eed_Lushi_vs_Arbor_Acr	breed_growth_background	ENSGALG00010027102	DUS3L	0.104415334	0.078725972	1.326313681	0.184735817	0.330146524	positive
PRJNA729271	eed_Lushi_vs_Arbor_Acr	breed_growth_background	ENSGALG00010027103	ACVR2B	0.088179751	0.09990919	0.882598995	0.377452972	0.539749119	positive
PRJNA729271	eed_Lushi_vs_Arbor_Acr	breed_growth_background	ENSGALG00010027105	CHERP	0.143127888	0.069426969	2.061560389	0.039249608	0.110966077	positive
PRJNA729271	eed_Lushi_vs_Arbor_Acr	breed_growth_background	ENSGALG00010027106	CYP2J23	0.025108148	0.079326283	0.316517386	0.751609831	0.842702573	positive
PRJNA729271	eed_Lushi_vs_Arbor_Acr	breed_growth_background	ENSGALG00010027107	KIF21B	0.175904582	0.110775596	1.587936223	0.112300761	0.233610726	positive
PRJNA729271	eed_Lushi_vs_Arbor_Acr	breed_growth_background	ENSGALG00010027108	ENSGALG00010027108	0.213184925	0.078476226	2.716554248	0.006596538	0.031094902	positive
PRJNA729271	eed_Lushi_vs_Arbor_Acr	breed_growth_background	ENSGALG00010027109	C19orf44	0.067877303	0.067458824	1.006203479	0.314317699	0.477906968	positive
PRJNA729271	eed_Lushi_vs_Arbor_Acr	breed_growth_background	ENSGALG00010027110	IKBKE	-0.045746205	0.117334503	-0.389878544	0.69662636	0.804461368	negative
PRJNA729271	eed_Lushi_vs_Arbor_Acr	breed_growth_background	ENSGALG00010027111	CALR3	0.314425216	0.437566739	0.718576592	0.47240184	0.626311596	positive
PRJNA729271	eed_Lushi_vs_Arbor_Acr	breed_growth_background	ENSGALG00010027112	XYLB	-0.312242805	0.103514901	-3.016404411	0.002557919	0.015858438	negative
PRJNA729271	eed_Lushi_vs_Arbor_Acr	breed_growth_background	ENSGALG00010027113	PIAS4	0.172575515	0.074294119	2.322869116	0.020186185	0.068690824	positive
PRJNA729271	eed_Lushi_vs_Arbor_Acr	breed_growth_background	ENSGALG00010027114	FLNB	-0.203371585	0.060874732	-3.340821028	0.00083531	0.007168636	negative
PRJNA729271	eed_Lushi_vs_Arbor_Acr	breed_growth_background	ENSGALG00010027115	C9ORF152	-1.451026636	0.361822679	-4.010325278	6.06352E-05	0.001108331	negative
PRJNA729271	eed_Lushi_vs_Arbor_Acr	breed_growth_background	ENSGALG00010027116	CCDC94	0.098106874	0.055559137	1.765809901	0.077427747	0.179791712	positive
PRJNA729271	eed_Lushi_vs_Arbor_Acr	breed_growth_background	ENSGALG00010027117	RASSF5	-0.115396878	0.125665701	-0.918284593	0.358469891	0.522142208	negative
PRJNA729271	eed_Lushi_vs_Arbor_Acr	breed_growth_background	ENSGALG00010027118	ABHD6	-0.207844842	0.086624616	-2.3993739	0.016423135	0.059404609	negative
PRJNA729271	eed_Lushi_vs_Arbor_Acr	breed_growth_background	ENSGALG00010027119	NUB1	-0.075921666	0.052293199	-1.451845874	0.146544465	0.281010983	negative
PRJNA729271	eed_Lushi_vs_Arbor_Acr	breed_growth_background	ENSGALG00010027121	ELF3	0.2601478	0.506002415	0.514123633	0.607165555	0.738101094	positive
PRJNA729271	eed_Lushi_vs_Arbor_Acr	breed_growth_background	ENSGALG00010027122	DNASE1L3	-0.143107223	0.130226383	-1.098911141	0.271806826	0.430536548	negative
PRJNA729271	eed_Lushi_vs_Arbor_Acr	breed_growth_background	ENSGALG00010027123	WDR86	-0.302885489	0.122157333	-2.479470385	0.013157766	0.050683447	negative
PRJNA729271	eed_Lushi_vs_Arbor_Acr	breed_growth_background	ENSGALG00010027124	IP6K2	-0.064063846	0.093062547	-0.688395574	0.49120371	0.643121469	negative
PRJNA729271	eed_Lushi_vs_Arbor_Acr	breed_growth_background	ENSGALG00010027125	CRYGN	-0.22984348	0.218584704	-1.05150761	0.293025516	0.454316489	negative
PRJNA729271	eed_Lushi_vs_Arbor_Acr	breed_growth_background	ENSGALG00010027126	ENSGALG00010027126	-0.430582657	0.169666261	-2.537821345	0.011154491	0.045226391	negative
PRJNA729271	eed_Lushi_vs_Arbor_Acr	breed_growth_background	ENSGALG00010027127	S1PR4	0.313971581	0.330949953	0.948698069	0.342774197	0.506577486	positive
PRJNA729271	eed_Lushi_vs_Arbor_Acr	breed_growth_background	ENSGALG00010027128	EIF2D	-0.011711508	0.048365088	-0.242147964	0.808665514	0.882385323	negative
PRJNA729271	eed_Lushi_vs_Arbor_Acr	breed_growth_background	ENSGALG00010027129	NCLN	0.242715778	0.06891229	3.522097138	0.000428147	0.004454366	positive

PRJNA729271	ed_Lushi_vs_Arbor_Acr	breed_growth_background	ENSGALG00010027130	NCKIPSD	-0.008281774	0.077955842	-0.106236731	0.915394529	0.952192002	negative
PRJNA729271	ed_Lushi_vs_Arbor_Acr	breed_growth_background	ENSGALG00010027131	UBA52	-0.106827125	0.081367845	-1.312891172	0.189219622	0.33536927	negative
PRJNA729271	ed_Lushi_vs_Arbor_Acr	breed_growth_background	ENSGALG00010027133	CELF5	-0.21548448	0.16207761	-1.32951418	0.183678391	0.328896637	negative
PRJNA729271	ed_Lushi_vs_Arbor_Acr	breed_growth_background	ENSGALG00010027134	DYRK3	0.063889784	0.079500555	0.803639472	0.42160522	0.580743862	positive
PRJNA729271	ed_Lushi_vs_Arbor_Acr	breed_growth_background	ENSGALG00010027135	PRKAG2	-0.087120696	0.074870945	-1.163611544	0.244581471	0.400452776	negative
PRJNA729271	ed_Lushi_vs_Arbor_Acr	breed_growth_background	ENSGALG00010027136	MAPKAPK2	-0.0260941	0.076814569	-0.33970249	0.734080586	0.829603781	negative
PRJNA729271	ed_Lushi_vs_Arbor_Acr	breed_growth_background	ENSGALG00010027137	FKBP8	-0.056587115	0.04828364	-1.171972839	0.241207961	0.396957896	negative
PRJNA729271	ed_Lushi_vs_Arbor_Acr	breed_growth_background	ENSGALG00010027139	HSD11B1L	-0.191600714	0.076624734	-2.500507388	0.012401555	0.048689202	negative
PRJNA729271	ed_Lushi_vs_Arbor_Acr	breed_growth_background	ENSGALG00010027141	PIGR	-0.390244479	0.69239246	-0.56361746	0.573014502	0.711107361	negative
PRJNA729271	ed_Lushi_vs_Arbor_Acr	breed_growth_background	ENSGALG00010027143	GALNT11	-0.128692986	0.066225972	-1.943240415	0.051987117	0.136217148	negative
PRJNA729271	ed_Lushi_vs_Arbor_Acr	breed_growth_background	ENSGALG00010027144	ENSGALG00010027144	-0.158402525	0.417586198	-0.379328927	0.704443621	0.808849041	negative
PRJNA729271	ed_Lushi_vs_Arbor_Acr	breed_growth_background	ENSGALG00010027145	CELSR3	-0.034086591	0.094024746	-0.362527866	0.716957604	0.81859543	negative
PRJNA729271	ed_Lushi_vs_Arbor_Acr	breed_growth_background	ENSGALG00010027146	KXD1	-0.02659213	0.066441938	-0.400231099	0.688986311	0.798847109	negative
PRJNA729271	ed_Lushi_vs_Arbor_Acr	breed_growth_background	ENSGALG00010027147	KMT2C	0.03719777	0.055361318	0.671909034	0.501641609	0.65271949	positive
PRJNA729271	ed_Lushi_vs_Arbor_Acr	breed_growth_background	ENSGALG00010027148	ELL	0.291788855	0.088422313	3.299945978	0.000967034	0.007955258	positive
PRJNA729271	ed_Lushi_vs_Arbor_Acr	breed_growth_background	ENSGALG00010027149	C1orf116	-0.359213154	0.12047126	-2.981733198	0.002866217	0.017153514	negative
PRJNA729271	ed_Lushi_vs_Arbor_Acr	breed_growth_background	ENSGALG00010027150	YOD1	-0.02037281	0.049491453	-0.411642992	0.680601115	0.793541921	negative
PRJNA729271	ed_Lushi_vs_Arbor_Acr	breed_growth_background	ENSGALG00010027151	MICOS13	0.005194644	0.076649963	0.06777099	0.945967937	0.970470493	positive
PRJNA729271	ed_Lushi_vs_Arbor_Acr	breed_growth_background	ENSGALG00010027152	C4BPS	-0.631874792	0.233524532	-2.70581761	0.006813646	0.031780537	negative
PRJNA729271	ed_Lushi_vs_Arbor_Acr	breed_growth_background	ENSGALG00010027153	ENSGALG00010027153	0.195687886	0.122454503	1.598045654	0.110032817	0.230447288	positive
PRJNA729271	ed_Lushi_vs_Arbor_Acr	breed_growth_background	ENSGALG00010027154	PLPP2	-0.098613984	0.074081582	-1.331153861	0.18313839	0.328224121	negative
PRJNA729271	ed_Lushi_vs_Arbor_Acr	breed_growth_background	ENSGALG00010027155	ENSGALG00010027155	-0.074256936	0.15644815	-0.474642467	0.635041832	0.759858952	negative
PRJNA729271	ed_Lushi_vs_Arbor_Acr	breed_growth_background	ENSGALG00010027156	ISYNA1	-0.136667192	0.08085138	-1.690350775	0.090960867	0.20149478	negative
PRJNA729271	ed_Lushi_vs_Arbor_Acr	breed_growth_background	ENSGALG00010027157	ENSGALG00010027157	-0.008382796	0.134361439	-0.062389892	0.950252344	0.972527553	negative
PRJNA729271	ed_Lushi_vs_Arbor_Acr	breed_growth_background	ENSGALG00010027158	XRCC2	-0.215266698	0.07386677	-2.91425628	0.003565371	0.019961394	negative
PRJNA729271	ed_Lushi_vs_Arbor_Acr	breed_growth_background	ENSGALG00010027159	TMUB1	0.153203351	0.088119368	1.738588855	0.082107108	0.187276559	positive
PRJNA729271	ed_Lushi_vs_Arbor_Acr	breed_growth_background	ENSGALG00010027160	FASTK	0.250154012	0.088888889	2.814232621	0.004889383	0.025131777	positive
PRJNA729271	ed_Lushi_vs_Arbor_Acr	breed_growth_background	ENSGALG00010027161	C4BPG	-0.252550094	0.108935418	-2.31834695	0.020430473	0.069298511	negative
PRJNA729271	ed_Lushi_vs_Arbor_Acr	breed_growth_background	ENSGALG00010027162	SLC4A2	0.033571403	0.070085281	0.479007891	0.631933015	0.757389537	positive
PRJNA729271	ed_Lushi_vs_Arbor_Acr	breed_growth_background	ENSGALG00010027163	ENSGALG00010027163	0.115642137	0.042857341	2.698304043	0.006969376	0.032295748	positive
PRJNA729271	ed_Lushi_vs_Arbor_Acr	breed_growth_background	ENSGALG00010027164	ENSGALG00010027164	0.852178433	0.246740519	3.453743371	0.000552863	0.005328293	positive
PRJNA729271	ed_Lushi_vs_Arbor_Acr	breed_growth_background	ENSGALG00010027165	ENSGALG00010027165	-0.075783295	0.123658081	-0.612845475	0.539978519	0.684926963	negative
PRJNA729271	ed_Lushi_vs_Arbor_Acr	breed_growth_background	ENSGALG00010027166	CDK5	-0.159458113	0.053760369	-2.966090399	0.003016118	0.017787409	negative
PRJNA729271	ed_Lushi_vs_Arbor_Acr	breed_growth_background	ENSGALG00010027167	ENSGALG00010027167	0.329823708	0.234842203	1.404448197	0.160185427	0.298942677	positive
PRJNA729271	ed_Lushi_vs_Arbor_Acr	breed_growth_background	ENSGALG00010027168	SSBP4	-0.03299074	0.099963372	-0.330028286	0.74137859	0.834792407	negative
PRJNA729271	ed_Lushi_vs_Arbor_Acr	breed_growth_background	ENSGALG00010027169	ABCB8	0.047863325	0.060041313	0.797173198	0.42535045	0.584514104	positive
PRJNA729271	ed_Lushi_vs_Arbor_Acr	breed_growth_background	ENSGALG00010027170	TLE2	-0.050223392	0.095943141	-0.52347037	0.600646965	0.733353922	negative
PRJNA729271	ed_Lushi_vs_Arbor_Acr	breed_growth_background	ENSGALG00010027171	ATG9B	0.600595561	0.14570288	4.122056883	3.75504E-05	0.000792276	positive
PRJNA729271	ed_Lushi_vs_Arbor_Acr	breed_growth_background	ENSGALG00010027172	ENSGALG00010027172	-0.070417372	0.277882404	-0.253407093	0.799953647	0.87624334	negative
PRJNA729271	ed_Lushi_vs_Arbor_Acr	breed_growth_background	ENSGALG00010027173	ENSGALG00010027173	-0.02135531	0.052323572	-0.408139382	0.683171349	0.795387044	negative
PRJNA729271	ed_Lushi_vs_Arbor_Acr	breed_growth_background	ENSGALG00010027174	SHISA5	-0.100887	0.071202172	-1.416909035	0.156509525	0.293901475	negative
PRJNA729271	ed_Lushi_vs_Arbor_Acr	breed_growth_background	ENSGALG00010027175	NOS3	-0.186802313	0.087676115	-2.130595245	0.033122502	0.097782212	negative
PRJNA729271	ed_Lushi_vs_Arbor_Acr	breed_growth_background	ENSGALG00010027176	ENSGALG00010027176	0.310892178	0.091342992	3.403569037	0.000665116	0.006078724	positive
PRJNA729271	ed_Lushi_vs_Arbor_Acr	breed_growth_background	ENSGALG00010027178	C4BPM	-0.075418332	0.043439598	-1.736165518	0.082534572	0.188014871	negative
PRJNA729271	ed_Lushi_vs_Arbor_Acr	breed_growth_background	ENSGALG00010027183	CD34	0.000920439	0.084037351	0.01095274	0.991261153	0.995105645	positive
PRJNA729271	ed_Lushi_vs_Arbor_Acr	breed_growth_background	ENSGALG00010027184	MPND	0.609739125	0.190498003	3.200763871	0.001370638	0.010261155	positive
PRJNA729271	ed_Lushi_vs_Arbor_Acr	breed_growth_background	ENSGALG00010027185	ATRIP	-0.01424411	0.045911529	-0.310251257	0.756369895	0.845997751	negative
PRJNA729271	ed_Lushi_vs_Arbor_Acr	breed_growth_background	ENSGALG00010027186	PLXNA2	-0.102991867	0.066458361	-1.549720226	0.121208681	0.245998297	negative
PRJNA729271	ed_Lushi_vs_Arbor_Acr	breed_growth_background	ENSGALG00010027187	ENSGALG00010027187	0.157050062	0.07526402	2.08665524	0.03691931	0.106087869	positive
PRJNA729271	ed_Lushi_vs_Arbor_Acr	breed_growth_background	ENSGALG00010027188	KCNH2	-0.150604482	0.136815946	-1.100781639	0.270991705	0.42966799	negative
PRJNA729271	ed_Lushi_vs_Arbor_Acr	breed_growth_background	ENSGALG00010027189	ENSGALG00010027189	0.102165776	0.16460799	0.620661097	0.534822631	0.680144292	positive
PRJNA729271	ed_Lushi_vs_Arbor_Acr	breed_growth_background	ENSGALG00010027190	IPPK	0.221524602	0.062344103	3.553256725	0.000380493	0.004103605	positive
PRJNA729271	ed_Lushi_vs_Arbor_Acr	breed_growth_background	ENSGALG00010027192	SH3GL1	-0.180760679	0.06296976	-2.870594984	0.004097001	0.021986682	negative
PRJNA729271	ed_Lushi_vs_Arbor_Acr	breed_growth_background	ENSGALG00010027193	CAMK1G	0.080485544	0.343210579	0.234507759	0.81459081	0.886011499	positive
PRJNA729271	ed_Lushi_vs_Arbor_Acr	breed_growth_background	ENSGALG00010027194	ENSGALG00010027194	-0.03579422	0.148034128	-0.24179708	0.808937401	0.882536092	negative
PRJNA729271	ed_Lushi_vs_Arbor_Acr	breed_growth_background	ENSGALG00010027195	CENPP	-0.450782815	0.11544863	-3.904618152	9.43743E-05	0.001523335	negative
PRJNA729271	ed_Lushi_vs_Arbor_Acr	breed_growth_background	ENSGALG00010027196	ENSGALG00010027196	-0.615619694	0.177957096	-3.459371437	0.000541438	0.005270745	negative
PRJNA729271	ed_Lushi_vs_Arbor_Acr	breed_growth_background	ENSGALG00010027197	ENSGALG00010027197	0.070969986	0.319074063	0.222424804	0.823983204	0.892581761	positive
PRJNA729271	ed_Lushi_vs_Arbor_Acr	breed_growth_background	ENSGALG00010027198	ECM2	-0.314202357	0.108634094	-2.892299702	0.003824329	0.020981702	negative
PRJNA729271	ed_Lushi_vs_Arbor_Acr	breed_growth_background	ENSGALG00010027199	LAMB3	0.08336487	0.17113317	0.487134493	0.626163024	0.752465017	positive
PRJNA729271	ed_Lushi_vs_Arbor_Acr	breed_growth_background	ENSGALG00010027200	G0S2	-0.371837964	0.167217074	-2.223684186	0.0261697	0.082480931	negative
PRJNA729271	ed_Lushi_vs_Arbor_Acr	breed_growth_background	ENSGALG00010027201	ASPN	-0.271747794	0.169446219	-1.603740678	0.108771225	0.228664092	negative
PRJNA729271	ed_Lushi_vs_Arbor_Acr	breed_growth_background	ENSGALG00010027202	CHAF1A	-0.017272337	0.047932624	-0.36034616	0.718588284	0.819922757	negative
PRJNA729271	ed_Lushi_vs_Arbor_Acr	breed_growth_background	ENSGALG00010027203	ENSGALG00010027203	-0.207412706	0.166607385	-1.244919038	0.213161512	0.363503073	negative
PRJNA729271	ed_Lushi_vs_Arbor_Acr	breed_growth_background	ENSGALG00010027204	OMD	1.451953216	0.491169127	2.956116612	0.003115393	0.018238133	positive
PRJNA729271	ed_Lushi_vs_Arbor_Acr	breed_growth_background	ENSGALG00010027205	OGN	-0.202832712	0.118594774	-1.710300597	0.087210302	0.19550496	negative
PRJNA729271	ed_Lushi_vs_Arbor_Acr	breed_growth_background	ENSGALG00010027206	ENSGALG00010027206	-1.33158462	0.360502051	-3.693694996	0.000221019	0.002795522	negative
PRJNA729271	ed_Lushi_vs_Arbor_Acr	breed_growth_background	ENSGALG00010027207	NOL8	0.091381587	0.066893071	1.366084484	0.171912448	0.31502793	positive
PRJNA729271	ed_Lushi_vs_Arbor_Acr	breed_growth_background	ENSGALG00010027208	UBXN6	0.060349142	0.05070369	1.190231737	0.233955322	0.388451592	positive
PRJNA729271	ed_Lushi_vs_Arbor_Acr	breed_growth_background	ENSGALG00010027209	TRAF3IP3	-0.035443356	0.130801569	-0.270970416	0.786413786	0.867374086	negative
PRJNA729271	ed_Lushi_vs_Arbor_Acr	breed_growth_background	ENSGALG00010027210	ENSGALG00010027210	0.269676692	0.147057589	1.833816909	0.066681177	0.162608287	positive
PRJNA729271	ed_Lushi_vs_Arbor_Acr	breed_growth_background	ENSGALG00010027211	HDGFRP2	0.186136105	0.054702485	3.402699251	0.000667237	0.006090427	positive
PRJNA729271	ed_Lushi_vs_Arbor_Acr	breed_growth_background	ENSGALG00010027212	IRF6	-0.672882683	0.314085363	-2.142356068	0.03216484	0.09595171	negative
PRJNA729271	ed_Lushi_vs_Arbor_Acr	breed_growth_background	ENSGALG00010027213	KCNC4	0.316809755	0.188166412	1.683667935	0.092245862	0.203211116	positive
PRJNA729271	ed_Lushi_vs_Arbor_Acr	breed_growth_background	ENSGALG00010027214	PLIN4	0.098718177	0.142282054	0.693820295	0.487794913	0.639524898	positive
PRJNA729271	ed_Lushi_vs_Arbor_Acr	breed_growth_background	ENSGALG00010027215	ENSGALG00010027215	-0.168992109	0.110005836	-1.536210393	0.124486788	0.250337957	negative
PRJNA729271	ed_Lushi_vs_Arbor_Acr	breed_growth_background	ENSGALG00010027216	ENSGALG00010027216	0.736495109	0.317066915	2.322838095	0.020187852	0.068690824	positive
PRJNA729271	ed_Lushi_vs_Arbor_Acr	breed_growth_background	ENSGALG00010027217	SEMA6B	0.370514037	0.117759555	3.146360706	0.00165316	0.011617724	positive
PRJNA729271	ed_Lushi_vs_Arbor_Acr	breed_growth_background	ENSGALG00010027218	IARS1	-0.142803321	0.087974167	-1.623241514	0.104537786	0.221919764	negative
PRJNA729271	ed_Lushi_vs_Arbor_Acr	breed_growth_background	ENSGALG00010027219	ENSGALG00010027219	0.139987413	0.073841056	1.89579376	0.057987321	0.147125192	positive
PRJNA729271	ed_Lushi_vs_Arbor_Acr	breed_growth_background	ENSGALG00010027220	ANKRD24	-0.000678275	0.346170832	-0.001959366	0.998436653	0.999025066	negative
PRJNA729271	ed_Lushi_vs_Arbor_Acr	breed_growth_background	ENSGALG00010027221	LRRC61	-0.113572502	0.151356065	-0.75036638	0.453034074	0.609384641	negative
PRJNA729271	ed_Lushi_vs_Arbor_Acr	breed_growth_background	ENSGALG00010027222	GUCA1B	0.157212642	0.4592355	0.342335559	0.732098378	0.828240196	positive
PRJNA729271	ed_Lushi_vs_Arbor_Acr	breed_growth_background	ENSGALG00010027223	SIRT6	0.060820662	0.045483671	1.337197733	0.181158102	0.325630789	positive
PRJNA729271	ed_Lushi_vs_Arbor_Acr	breed_growth_background	ENSGALG00010027224	RARRES2	-0.174696929	0.064920222	-2.690947804	0.007124934	0.032817261	negative
PRJNA729271	ed_Lushi_vs_Arbor_Acr	breed_growth_background	ENSGALG00010027225	CREB3L3	-0.568685283	0.396287495	-1.435032116	0.151277946	0.287166565	negative
PRJNA729271	ed_Lushi_vs_Arbor_Acr	breed_growth_background	ENSGALG00010027227	ENSGALG00010027227	0.504875882	0.098190618	5.14179351	2.72128E-07	1.78484E-05	positive
PRJNA729271	ed_Lushi_vs_Arbor_Acr	breed_growth_background	ENSGALG00010027229	MDM4	-0.052321052	0.043538408	-1.201721743	0.229471354	0.383113025	negative
PRJNA729271	ed_Lushi_vs_Arbor_Acr	breed_growth_background	ENSGALG00010027230	MAP2K2	-0.004356508	0.044772415	-0.097303395	0.92248546	0.956810501	negative
PRJNA729271	ed_Lushi_vs_Arbor_Acr	breed_growth_background	ENSGALG00010027231	ENSGALG00010027231	-0.038547503	0.084917335	-0.453941509	0.649870926	0.771407505	negative
PRJNA729271	ed_Lushi_vs_Arbor_Acr	breed_growth_background	ENSGALG00010027232	ARRDC2	0.379536846	0.174674126	2.172828079	0.029793259	0.090459424	positive
PRJNA729271	ed_Lushi_vs_Arbor_Acr	breed_growth_background	ENSGALG00010027233	ENSGALG00010027233	0.231926122	0.139683338	1.660370703	0.0968399	0.209983052	positive
PRJNA729271	ed_Lushi_vs_Arbor_Acr	breed_growth_background	ENSGALG00010027234	SLC6A11	-0.07583867	0.274210341	-0.276571151	0.782109419	0.86519698	negative
PRJNA729271	ed_Lushi_vs_Arbor_Acr	breed_growth_background	ENSGALG00010027236	PEX11G	0.142992209	0.151011083	0.946898773	0.343690363	0.507569212	positive
PRJNA729271	ed_Lushi_vs_Arbor_Acr	breed_growth_background	ENSGALG00010027237	CTC-575C13.4	-0.236091084	0.086379081	-2.733197466	0.006272272	0.029931501	negative
PRJNA729271	ed_Lushi_vs_Arbor_Acr	breed_growth_background	ENSGALG00010027239	ENSGALG00010027239	-0.006238724	0.045653987	-0.136652342	0.891305601	0.936368897	negative
PRJNA729271	ed_Lushi_vs_Arbor_Acr	breed_growth_background	ENSGALG00010027240	SLC6A1	0.532870483	0.182039658	2.927221958	0.003420047	0.019433	positive
PRJNA729271	ed_Lushi_vs_Arbor_Acr	breed_growth_background	ENSGALG00010027241	LRRN2	-0.223674835	0.147758751	-1.513784017	0.130080628	0.257795829	negative
PRJNA729271	ed_Lushi_vs_Arbor_Acr	breed_growth_background	ENSGALG00010027242	ENSGALG00010027242	0.179972874	0.103374002	1.740987768	0.081685723	0.186667909	positive
PRJNA729271	ed_Lushi_vs_Arbor_Acr	breed_growth_background	ENSGALG00010027243	HRH1	-0.261698805	0.109562378	-2.38858274	0.016913499	0.060653432	negative
PRJNA729271	ed_Lushi_vs_Arbor_Acr	breed_growth_background	ENSGALG00010027244	ARHGEF18	0.081492428	0.04870089	1.67332525	0.094263284	0.206396723	positive
PRJNA729271	ed_Lushi_vs_Arbor_Acr	breed_growth_background	ENSGALG00010027245	LRIG2	0.122670349	0.035778428	3.428612061	0.000606676	0.005673399	positive
PRJNA729271	ed_Lushi_vs_Arbor_Acr	breed_growth_background	ENSGALG00010027246	ZNF767	-0.016339023	0.054867026	-0.29779313	0.765861058	0.853213131	negative
PRJNA729271	ed_Lushi_vs_Arbor_Acr	breed_growth_background	ENSGALG00010027247	ATG7	0.003640421	0.043801771	0.083111267	0.933763067	0.963817963	positive
PRJNA729271	ed_Lushi_vs_Arbor_Acr	breed_growth_background	ENSGALG00010027248	ENSGALG00010027248	0.051700082	0.357506485	0.144612992	0.885016439	0.932222461	positive
PRJNA729271	ed_Lushi_vs_Arbor_Acr	breed_growth_background	ENSGALG00010027249	C2CD4C	0.180227627	0.374920226	0.480709266	0.630723144	0.756502439	positive
PRJNA729271	ed_Lushi_vs_Arbor_Acr	breed_growth_background	ENSGALG00010027250	ENSGALG00010027250	-0.096820566	0.094334876	-1.026349648	0.304726803	0.467260659	negative
PRJNA729271	ed_Lushi_vs_Arbor_Acr	breed_growth_background	ENSGALG00010027251	ENSGALG00010027251	0.169848613	0.108631947	1.5635236	0.117929489	0.241643758	positive
PRJNA729271	ed_Lushi_vs_Arbor_Acr	breed_growth_background	ENSGALG00010027252	MAGI3	-0.168675421	0.038905229	-4.33554627	1.45399E-05	0.000382283	negative
PRJNA729271	ed_Lushi_vs_Arbor_Acr	breed_growth_background	ENSGALG00010027253	VGLL4	-0.177832271	0.047467443	-3.746405085	0.000179387	0.002409835	negative
PRJNA729271	ed_Lushi_vs_Arbor_Acr	breed_growth_background	ENSGALG00010027254	INSR	0.08012112	0.071146426	1.126143991	0.260104576	0.417984017	positive
PRJNA729271	ed_Lushi_vs_Arbor_Acr	breed_growth_background	ENSGALG00010027255	ENSGALG00010027255	0.009161278	0.098951491	0.092583522	0.926234435	0.958709522	positive
PRJNA729271	ed_Lushi_vs_Arbor_Acr	breed_growth_background	ENSGALG00010027256	PHTF1	-0.020871935	0.050324076	-0.414750482	0.678324573	0.79228966	negative
PRJNA729271	ed_Lushi_vs_Arbor_Acr	breed_growth_background	ENSGALG00010027257	TAFA3	-0.218375875	0.138743406	-1.573954981	0.115497858	0.238108583	negative
PRJNA729271	ed_Lushi_vs_Arbor_Acr	breed_growth_background	ENSGALG00010027258	ENSGALG00010027258	-0.175148339	0.095379441	-1.836332195	0.066308541	0.161972076	negative
PRJNA729271	ed_Lushi_vs_Arbor_Acr	breed_growth_background	ENSGALG00010027259	ENSGALG00010027259	0.023761652	0.046512605	0.510864786	0.609445741	0.739982913	positive
PRJNA729271	ed_Lushi_vs_Arbor_Acr	breed_growth_background	ENSGALG00010027260	LEMD2	0.013447524	0.053945213	0.249281139	0.80314332	0.878475884	positive
PRJNA729271	ed_Lushi_vs_Arbor_Acr	breed_growth_background	ENSGALG00010027261	ENSGALG00010027261	0.108489997	0.060795488	1.784507376	0.074341257	0.17518721	positive
PRJNA729271	ed_Lushi_vs_Arbor_Acr	breed_growth_background	ENSGALG00010027262	USE1	-0.041286883	0.07322091	-0.563867391	0.572844384	0.710966636	negative
PRJNA729271	ed_Lushi_vs_Arbor_Acr	breed_growth_background	ENSGALG00010027263	TAMM41	0.031829073	0.069840344	0.4557405	0.648576595	0.77064659	positive
PRJNA729271	ed_Lushi_vs_Arbor_Acr	breed_growth_background	ENSGALG00010027264	ENSGALG00010027264	0.156673769	0.050361105	3.111007377	0.001864503	0.012628957	positive
PRJNA729271	ed_Lushi_vs_Arbor_Acr	breed_growth_background	ENSGALG00010027265	IP6K3	-0.009761755	0.109771768	-0.08892774	0.929139337	0.96068726	negative
PRJNA729271	ed_Lushi_vs_Arbor_Acr	breed_growth_background	ENSGALG00010027267	MYO9B	-0.084367131	0.051534003	-1.63711581	0.101606274	0.21747574	negative
PRJNA729271	ed_Lushi_vs_Arbor_Acr	breed_growth_background	ENSGALG00010027268	UQCRC1	-0.019738715	0.0676901	-0.291604159	0.770589295	0.857011454	negative
PRJNA729271	ed_Lushi_vs_Arbor_Acr	breed_growth_background	ENSGALG00010027269	CDC25A	-0.103138563	0.054537298	-1.891156434	0.058603465	0.148195608	negative
PRJNA729271	ed_Lushi_vs_Arbor_Acr	breed_growth_background	ENSGALG00010027270	ENSGALG00010027270	-0.164292514	0.234430326	-0.700815962	0.483417876	0.635653762	negative
PRJNA729271	ed_Lushi_vs_Arbor_Acr	breed_growth_background	ENSGALG00010027271	INKA2	-0.177327744	0.078567735	-2.257004673	0.024007783	0.077729018	negative
PRJNA729271	ed_Lushi_vs_Arbor_Acr	breed_growth_background	ENSGALG00010027272	DDX20	0.116342512	0.05596047	2.079012435	0.037616208	0.10775631	positive
PRJNA729271	ed_Lushi_vs_Arbor_Acr	breed_growth_background	ENSGALG00010027273	HAUS8	-0.251599213	0.073801221	-3.409147017	0.000651664	0.006001183	negative
PRJNA729271	ed_Lushi_vs_Arbor_Acr	breed_growth_background	ENSGALG00010027274	COPG1	0.045926887	0.032836844	1.398638898	0.161921296	0.301212677	positive
PRJNA729271	ed_Lushi_vs_Arbor_Acr	breed_growth_background	ENSGALG00010027275	CPAMD8	-0.328732657	0.121659379	-2.702074097	0.00689084	0.032049944	negative

PRJNA729271	ed_Lushi_vs_Arbor_Acr	breed_growth_background	ENSGALG00010027276	ENSGALG00010027276	0.032163405	0.074106179	0.434017859	0.664275472	0.782233487	positive
PRJNA729271	ed_Lushi_vs_Arbor_Acr	breed_growth_background	ENSGALG00010027277	KCND3	-0.020570553	0.203377761	-0.101144555	0.91943571	0.955356316	negative
PRJNA729271	ed_Lushi_vs_Arbor_Acr	breed_growth_background	ENSGALG00010027278	H1-10	0.10365583	0.0692587	1.496647063	0.134485117	0.264285047	positive
PRJNA729271	ed_Lushi_vs_Arbor_Acr	breed_growth_background	ENSGALG00010027279	F2RL3	0.175302777	0.192037544	0.912856795	0.361317863	0.524674657	positive
PRJNA729271	ed_Lushi_vs_Arbor_Acr	breed_growth_background	ENSGALG00010027280	HMCES	0.136953251	0.068618538	1.995863737	0.045948757	0.124747561	positive
PRJNA729271	ed_Lushi_vs_Arbor_Acr	breed_growth_background	ENSGALG00010027281	ENSGALG00010027281	-0.023897699	0.14054613	-0.170034554	0.864982962	0.918866107	negative
PRJNA729271	ed_Lushi_vs_Arbor_Acr	breed_growth_background	ENSGALG00010027282	ENSGALG00010027282	0.063556463	0.069186505	0.91862514	0.358291678	0.522006017	positive
PRJNA729271	ed_Lushi_vs_Arbor_Acr	breed_growth_background	ENSGALG00010027283	RAB7A	0.111760008	0.033658042	3.320454839	0.000898709	0.00754685	positive
PRJNA729271	ed_Lushi_vs_Arbor_Acr	breed_growth_background	ENSGALG00010027284	CTTNBP2NL	-0.157092508	0.048085309	-3.266954342	0.001087112	0.008686709	negative
PRJNA729271	ed_Lushi_vs_Arbor_Acr	breed_growth_background	ENSGALG00010027285	PTPRS	-0.156454966	0.059997702	-2.607682637	0.009115743	0.039080949	negative
PRJNA729271	ed_Lushi_vs_Arbor_Acr	breed_growth_background	ENSGALG00010027286	RPN1	0.033808969	0.033656953	1.004516629	0.31512966	0.478354217	positive
PRJNA729271	ed_Lushi_vs_Arbor_Acr	breed_growth_background	ENSGALG00010027287	DHX30	0.065741617	0.048304888	1.360972356	0.173522426	0.317136245	positive
PRJNA729271	ed_Lushi_vs_Arbor_Acr	breed_growth_background	ENSGALG00010027288	DAG1	0.166974189	0.089598546	1.863581466	0.062380502	0.155015495	positive
PRJNA729271	ed_Lushi_vs_Arbor_Acr	breed_growth_background	ENSGALG00010027289	SMARCC1	0.082463356	0.074802918	1.102408274	0.270284216	0.428968544	positive
PRJNA729271	ed_Lushi_vs_Arbor_Acr	breed_growth_background	ENSGALG00010027290	CSPG5	-0.066683945	0.151065674	-0.441423545	0.6589064	0.777971513	negative
PRJNA729271	ed_Lushi_vs_Arbor_Acr	breed_growth_background	ENSGALG00010027291	MST1	-0.004676161	0.147255314	-0.031755464	0.974667064	0.985202168	negative
PRJNA729271	ed_Lushi_vs_Arbor_Acr	breed_growth_background	ENSGALG00010027292	KDM4B	0.063689406	0.03275751	1.944268863	0.051863037	0.135965767	positive
PRJNA729271	ed_Lushi_vs_Arbor_Acr	breed_growth_background	ENSGALG00010027293	ELP6	-0.043904754	0.081795995	-0.536759215	0.591433947	0.726253486	negative
PRJNA729271	ed_Lushi_vs_Arbor_Acr	breed_growth_background	ENSGALG00010027295	ENSGALG00010027295	0.189736988	0.121473703	1.56195936	0.118297563	0.242115686	positive
PRJNA729271	ed_Lushi_vs_Arbor_Acr	breed_growth_background	ENSGALG00010027297	ENSGALG00010027297	-3.815518789	1.123170603	-3.397096379	0.00068105	0.006193109	negative
PRJNA729271	ed_Lushi_vs_Arbor_Acr	breed_growth_background	ENSGALG00010027299	PGPEP1	0.039117085	0.087477293	0.447168445	0.654753443	0.775189999	positive
PRJNA729271	ed_Lushi_vs_Arbor_Acr	breed_growth_background	ENSGALG00010027301	SCAP	-0.074868776	0.035282425	-2.121984978	0.033839	0.099421859	negative
PRJNA729271	ed_Lushi_vs_Arbor_Acr	breed_growth_background	ENSGALG00010027303	APEH	-0.037873761	0.075335625	-0.502733751	0.615151477	0.7440478	negative
PRJNA729271	ed_Lushi_vs_Arbor_Acr	breed_growth_background	ENSGALG00010027304	PTPN23	0.098447765	0.073675945	1.336226703	0.181475186	0.32603902	positive
PRJNA729271	ed_Lushi_vs_Arbor_Acr	breed_growth_background	ENSGALG00010027305	LSM4	-0.041273292	0.070517323	-0.585292948	0.558350756	0.699600532	negative
PRJNA729271	ed_Lushi_vs_Arbor_Acr	breed_growth_background	ENSGALG00010027306	ENSGALG00010027306	-0.579572056	0.166575294	-3.479339833	0.000502651	0.004990358	negative
PRJNA729271	ed_Lushi_vs_Arbor_Acr	breed_growth_background	ENSGALG00010027307	JUND	-0.055476866	0.094225973	-0.588764053	0.556019563	0.69755094	negative
PRJNA729271	ed_Lushi_vs_Arbor_Acr	breed_growth_background	ENSGALG00010027309	ENSGALG00010027309	1.236056838	0.341853113	3.61575423	0.000299474	0.003464391	positive
PRJNA729271	ed_Lushi_vs_Arbor_Acr	breed_growth_background	ENSGALG00010027310	ARHGEF2	-0.166136231	0.07175281	-2.315396873	0.020591222	0.06962206	negative
PRJNA729271	ed_Lushi_vs_Arbor_Acr	breed_growth_background	ENSGALG00010027311	NFASC	-0.11720237	0.128014322	-0.915541081	0.359907646	0.523409251	negative
PRJNA729271	ed_Lushi_vs_Arbor_Acr	breed_growth_background	ENSGALG00010027312	ENSGALG00010027312	-0.019941059	0.336933927	-0.059183884	0.952805646	0.97363236	negative
PRJNA729271	ed_Lushi_vs_Arbor_Acr	breed_growth_background	ENSGALG00010027313	BSN	0.07675	0.133299893	0.575769401	0.564771059	0.704723648	positive
PRJNA729271	ed_Lushi_vs_Arbor_Acr	breed_growth_background	ENSGALG00010027314	ENSGALG00010027314	0.706339234	0.186595137	3.785410728	0.000153455	0.00217432	positive
PRJNA729271	ed_Lushi_vs_Arbor_Acr	breed_growth_background	ENSGALG00010027315	RAB3A	0.376150441	0.123949099	3.034717036	0.002407615	0.01519964	positive
PRJNA729271	ed_Lushi_vs_Arbor_Acr	breed_growth_background	ENSGALG00010027316	TRAIP	-0.353881847	0.120930065	-2.926334704	0.003429817	0.019469141	negative
PRJNA729271	ed_Lushi_vs_Arbor_Acr	breed_growth_background	ENSGALG00010027317	MPV17L2	0.208730671	0.078486035	2.659462574	0.007826543	0.035089927	positive
PRJNA729271	ed_Lushi_vs_Arbor_Acr	breed_growth_background	ENSGALG00010027319	MST1R	0.22371729	0.168691176	1.326194381	0.18477532	0.330146524	positive
PRJNA729271	ed_Lushi_vs_Arbor_Acr	breed_growth_background	ENSGALG00010027320	MON1A	0.328849829	0.086290785	3.810949574	0.000138434	0.002024824	positive
PRJNA729271	ed_Lushi_vs_Arbor_Acr	breed_growth_background	ENSGALG00010027321	IFI30	-0.191709299	0.108848177	-1.761254104	0.078195404	0.180974355	negative
PRJNA729271	ed_Lushi_vs_Arbor_Acr	breed_growth_background	ENSGALG00010027322	CRABP2	0.010570464	0.112327168	0.094104251	0.925026343	0.95792169	positive
PRJNA729271	ed_Lushi_vs_Arbor_Acr	breed_growth_background	ENSGALG00010027323	ISG20L2	0.171756039	0.071286069	2.409391354	0.015979153	0.058344452	positive
PRJNA729271	ed_Lushi_vs_Arbor_Acr	breed_growth_background	ENSGALG00010027324	PIK3R2	0.039785777	0.05247716	0.758154163	0.448358698	0.60477939	positive
PRJNA729271	ed_Lushi_vs_Arbor_Acr	breed_growth_background	ENSGALG00010027325	METTL25B	-0.310341025	0.160004844	-1.939572688	0.052431643	0.137157705	negative
PRJNA729271	ed_Lushi_vs_Arbor_Acr	breed_growth_background	ENSGALG00010027326	MRPL24	0.06684228	0.063292778	1.056080691	0.290931351	0.452357422	positive
PRJNA729271	ed_Lushi_vs_Arbor_Acr	breed_growth_background	ENSGALG00010027327	RBM6	0.151531768	0.048012575	3.156084987	0.001599023	0.011356116	positive
PRJNA729271	ed_Lushi_vs_Arbor_Acr	breed_growth_background	ENSGALG00010027328	RBM15	0.19617098	0.070655455	2.776444942	0.005495694	0.027269178	positive
PRJNA729271	ed_Lushi_vs_Arbor_Acr	breed_growth_background	ENSGALG00010027329	MINDY4	-0.002584094	0.066547883	-0.038830595	0.969025452	0.982376831	negative
PRJNA729271	ed_Lushi_vs_Arbor_Acr	breed_growth_background	ENSGALG00010027330	ENSGALG00010027330	-0.025101829	0.109652006	-0.228922659	0.818929029	0.888634247	negative
PRJNA729271	ed_Lushi_vs_Arbor_Acr	breed_growth_background	ENSGALG00010027331	SLC16A4	-0.370371028	0.089124005	-4.155682055	3.24319E-05	0.000706917	negative
PRJNA729271	ed_Lushi_vs_Arbor_Acr	breed_growth_background	ENSGALG00010027332	HDGF	0.118865438	0.094555238	1.257100516	0.20871724	0.358327182	positive
PRJNA729271	ed_Lushi_vs_Arbor_Acr	breed_growth_background	ENSGALG00010027333	PROK1	-0.163374298	0.16400732	-0.99614028	0.319181991	0.482484405	negative
PRJNA729271	ed_Lushi_vs_Arbor_Acr	breed_growth_background	ENSGALG00010027334	PRCC	-0.022134876	0.061898663	-0.357598613	0.720643716	0.821140686	negative
PRJNA729271	ed_Lushi_vs_Arbor_Acr	breed_growth_background	ENSGALG00010027335	MAST3	-0.055158903	0.096374038	-0.572341937	0.567090344	0.706122715	negative
PRJNA729271	ed_Lushi_vs_Arbor_Acr	breed_growth_background	ENSGALG00010027336	RAD54L2	0.252059201	0.074537658	3.381635662	0.000720556	0.006453074	positive
PRJNA729271	ed_Lushi_vs_Arbor_Acr	breed_growth_background	ENSGALG00010027337	AQP1	-0.212909217	0.14215834	-1.49769066	0.13421364	0.264104013	negative
PRJNA729271	ed_Lushi_vs_Arbor_Acr	breed_growth_background	ENSGALG00010027339	LAMTOR5	-0.020735776	0.05816981	-0.356469729	0.721488818	0.821393372	negative
PRJNA729271	ed_Lushi_vs_Arbor_Acr	breed_growth_background	ENSGALG00010027340	ENSGALG00010027340	0.275318409	0.397149987	0.693235348	0.488161869	0.639948113	positive
PRJNA729271	ed_Lushi_vs_Arbor_Acr	breed_growth_background	ENSGALG00010027341	ENSGALG00010027341	-0.257798007	0.30846117	-0.835755136	0.40329267	0.564259388	negative
PRJNA729271	ed_Lushi_vs_Arbor_Acr	breed_growth_background	ENSGALG00010027346	SIN3B	0.154655978	0.04768652	3.243180177	0.001182034	0.009236436	positive
PRJNA729271	ed_Lushi_vs_Arbor_Acr	breed_growth_background	ENSGALG00010027348	ENSGALG00010027348	-0.071312768	0.11103693	-0.642243696	0.520714966	0.668185166	negative
PRJNA729271	ed_Lushi_vs_Arbor_Acr	breed_growth_background	ENSGALG00010027350	INSRR	-0.290669075	0.174500292	-1.665722577	0.095768683	0.208433493	negative
PRJNA729271	ed_Lushi_vs_Arbor_Acr	breed_growth_background	ENSGALG00010027351	TMEM38A	0.011822339	0.078853227	0.149928411	0.880821096	0.929121364	positive
PRJNA729271	ed_Lushi_vs_Arbor_Acr	breed_growth_background	ENSGALG00010027354	ENSGALG00010027354	0.710070791	0.372060106	1.908484087	0.056328678	0.144382703	positive
PRJNA729271	ed_Lushi_vs_Arbor_Acr	breed_growth_background	ENSGALG00010027355	ADCYAP1R1	-0.054678114	0.14207614	-0.384850786	0.70034797	0.806702465	negative
PRJNA729271	ed_Lushi_vs_Arbor_Acr	breed_growth_background	ENSGALG00010027356	ENSGALG00010027356	-0.151228814	0.118636021	-1.274729318	0.202405066	0.351613307	negative
PRJNA729271	ed_Lushi_vs_Arbor_Acr	breed_growth_background	ENSGALG00010027358	RNF126	0.088461316	0.054382569	1.626648355	0.103811803	0.220929685	positive
PRJNA729271	ed_Lushi_vs_Arbor_Acr	breed_growth_background	ENSGALG00010027359	ENSGALG00010027359	-0.07490594	0.062306335	-1.202220301	0.229278186	0.382878722	negative
PRJNA729271	ed_Lushi_vs_Arbor_Acr	breed_growth_background	ENSGALG00010027360	SMIM7	0.051550609	0.060815111	0.847661179	0.396626692	0.557926056	positive
PRJNA729271	ed_Lushi_vs_Arbor_Acr	breed_growth_background	ENSGALG00010027361	FANCD2	0.146408703	0.10318547	1.418888764	0.155931449	0.293079143	positive
PRJNA729271	ed_Lushi_vs_Arbor_Acr	breed_growth_background	ENSGALG00010027362	ENSGALG00010027362	-0.020007263	0.077229343	-0.259062973	0.795586653	0.873373925	negative
PRJNA729271	ed_Lushi_vs_Arbor_Acr	breed_growth_background	ENSGALG00010027363	VIPR1	-0.358144186	0.140764542	-2.544278416	0.010950376	0.044636025	negative
PRJNA729271	ed_Lushi_vs_Arbor_Acr	breed_growth_background	ENSGALG00010027364	FSTL3	0.651934633	0.12344007	5.281385788	1.2821E-07	1.0211E-05	positive
PRJNA729271	ed_Lushi_vs_Arbor_Acr	breed_growth_background	ENSGALG00010027365	PLEKHA6	-0.233393144	0.090580398	-2.576640759	0.009976558	0.041746594	negative
PRJNA729271	ed_Lushi_vs_Arbor_Acr	breed_growth_background	ENSGALG00010027366	USP4	0.068434721	0.044175598	1.54915213	0.121345154	0.246068551	positive
PRJNA729271	ed_Lushi_vs_Arbor_Acr	breed_growth_background	ENSGALG00010027367	SEC22C	-0.067632324	0.051872323	-1.303822928	0.192293963	0.33917779	negative
PRJNA729271	ed_Lushi_vs_Arbor_Acr	breed_growth_background	ENSGALG00010027368	EMC3	-0.01623474	0.047613818	-0.340966993	0.73312843	0.828850838	negative
PRJNA729271	ed_Lushi_vs_Arbor_Acr	breed_growth_background	ENSGALG00010027369	ENSGALG00010027369	0.076273783	0.05454577	1.398344589	0.162009614	0.301338298	positive
PRJNA729271	ed_Lushi_vs_Arbor_Acr	breed_growth_background	ENSGALG00010027370	NUP210L	0.000501151	0.196148297	0.002554961	0.997961438	0.998918445	positive
PRJNA729271	ed_Lushi_vs_Arbor_Acr	breed_growth_background	ENSGALG00010027372	PIK3C2B	-0.185542127	0.092668192	-2.002220219	0.045261052	0.123273008	negative
PRJNA729271	ed_Lushi_vs_Arbor_Acr	breed_growth_background	ENSGALG00010027373	MLN	0.399260798	0.474175562	0.842010491	0.399782081	0.560726886	positive
PRJNA729271	ed_Lushi_vs_Arbor_Acr	breed_growth_background	ENSGALG00010027374	NKTR	0.098427866	0.063556747	1.54866117	0.121463193	0.246170159	positive
PRJNA729271	ed_Lushi_vs_Arbor_Acr	breed_growth_background	ENSGALG00010027375	FUT7	0.2412919	0.228556215	1.055722332	0.291095091	0.452485078	positive
PRJNA729271	ed_Lushi_vs_Arbor_Acr	breed_growth_background	ENSGALG00010027376	DSTYK	0.070563583	0.043105835	1.636984479	0.101633713	0.21747574	positive
PRJNA729271	ed_Lushi_vs_Arbor_Acr	breed_growth_background	ENSGALG00010027378	ZBTB47	-0.045888341	0.088062743	-0.521086887	0.602306245	0.734637245	negative
PRJNA729271	ed_Lushi_vs_Arbor_Acr	breed_growth_background	ENSGALG00010027379	OPTC	-0.480402294	0.342968204	-1.400719625	0.161297932	0.300438643	negative
PRJNA729271	ed_Lushi_vs_Arbor_Acr	breed_growth_background	ENSGALG00010027380	GPX1	-0.190300614	0.071115976	-2.675919338	0.007452458	0.033852515	negative
PRJNA729271	ed_Lushi_vs_Arbor_Acr	breed_growth_background	ENSGALG00010027381	KLHL40	-0.376176077	0.105230437	-3.57478394	0.000350517	0.003866627	negative
PRJNA729271	ed_Lushi_vs_Arbor_Acr	breed_growth_background	ENSGALG00010027382	PALM	-0.313470023	0.108696776	-2.883894408	0.003927904	0.021394964	negative
PRJNA729271	ed_Lushi_vs_Arbor_Acr	breed_growth_background	ENSGALG00010027383	ASS1	0.021892484	0.150643494	0.145326451	0.884453132	0.931933811	positive
PRJNA729271	ed_Lushi_vs_Arbor_Acr	breed_growth_background	ENSGALG00010027384	HHATL	0.198526286	0.116804112	1.699651511	0.089196495	0.19900003	positive
PRJNA729271	ed_Lushi_vs_Arbor_Acr	breed_growth_background	ENSGALG00010027385	CNTN2	0.537708448	0.36127415	1.488366791	0.136654189	0.267423043	positive
PRJNA729271	ed_Lushi_vs_Arbor_Acr	breed_growth_background	ENSGALG00010027386	RHOA	-0.119118775	0.035398335	-3.365095381	0.000765172	0.006738256	negative
PRJNA729271	ed_Lushi_vs_Arbor_Acr	breed_growth_background	ENSGALG00010027387	ENSGALG00010027387	-0.026115577	0.149462027	-0.174730516	0.861291388	0.916713909	negative
PRJNA729271	ed_Lushi_vs_Arbor_Acr	breed_growth_background	ENSGALG00010027388	URM1	-0.054831809	0.076994378	-0.712153414	0.476369781	0.629327377	negative
PRJNA729271	ed_Lushi_vs_Arbor_Acr	breed_growth_background	ENSGALG00010027391	TPM3	-0.17875949	0.06145358	-2.908853969	0.003627563	0.020158348	negative
PRJNA729271	ed_Lushi_vs_Arbor_Acr	breed_growth_background	ENSGALG00010027392	RBBP5	0.186734933	0.068289522	2.734459495	0.006248278	0.029861125	positive
PRJNA729271	ed_Lushi_vs_Arbor_Acr	breed_growth_background	ENSGALG00010027393	TEX264	0.194594181	0.101404689	1.918986024	0.054986103	0.141898084	positive
PRJNA729271	ed_Lushi_vs_Arbor_Acr	breed_growth_background	ENSGALG00010027394	SLC27A4	-0.041634845	0.047151544	-0.883000682	0.377235903	0.53967967	negative
PRJNA729271	ed_Lushi_vs_Arbor_Acr	breed_growth_background	ENSGALG00010027395	BCL2L15	0.18816453	0.14637127	1.28552912	0.198607448	0.34722168	positive
PRJNA729271	ed_Lushi_vs_Arbor_Acr	breed_growth_background	ENSGALG00010027397	RFX5	0.083976247	0.067863243	1.237433444	0.215926209	0.36726712	positive
PRJNA729271	ed_Lushi_vs_Arbor_Acr	breed_growth_background	ENSGALG00010027399	MAPKAP1	0.223329051	0.059803309	3.734392887	0.000188169	0.002477296	positive
PRJNA729271	ed_Lushi_vs_Arbor_Acr	breed_growth_background	ENSGALG00010027401	ENSGALG00010027401	0.025777083	0.16794616	0.153484204	0.878016446	0.927680471	positive
PRJNA729271	ed_Lushi_vs_Arbor_Acr	breed_growth_background	ENSGALG00010027402	AP4B1	0.068139322	0.046639912	1.460965921	0.144024794	0.277390299	positive
PRJNA729271	ed_Lushi_vs_Arbor_Acr	breed_growth_background	ENSGALG00010027403	ACKR2	-0.777102635	0.199290433	-3.899347415	9.64523E-05	0.00154914	negative
PRJNA729271	ed_Lushi_vs_Arbor_Acr	breed_growth_background	ENSGALG00010027405	DCLRE1B	-0.284714157	0.097592781	-2.917369041	0.003529978	0.019832185	negative
PRJNA729271	ed_Lushi_vs_Arbor_Acr	breed_growth_background	ENSGALG00010027407	ENSGALG00010027407	-0.483593816	0.536909997	-0.900698102	0.367748858	0.531073155	negative
PRJNA729271	ed_Lushi_vs_Arbor_Acr	breed_growth_background	ENSGALG00010027410	ALAS1	0.027432823	0.0472539	0.580540924	0.561549897	0.702361357	positive
PRJNA729271	ed_Lushi_vs_Arbor_Acr	breed_growth_background	ENSGALG00010027412	ENSGALG00010027412	-0.370230742	0.15060502	-2.458289516	0.013960058	0.052833171	negative
PRJNA729271	ed_Lushi_vs_Arbor_Acr	breed_growth_background	ENSGALG00010027414	PBX3	0.078624496	0.041011752	1.917121101	0.055222548	0.142333389	positive
PRJNA729271	ed_Lushi_vs_Arbor_Acr	breed_growth_background	ENSGALG00010027417	WNT2B	0.200459491	0.198034108	1.012247298	0.311419827	0.474611543	positive
PRJNA729271	ed_Lushi_vs_Arbor_Acr	breed_growth_background	ENSGALG00010027418	ENSGALG00010027418	-0.001744831	0.431773671	-0.004041077	0.996775696	0.998359847	negative
PRJNA729271	ed_Lushi_vs_Arbor_Acr	breed_growth_background	ENSGALG00010027419	PTBP1	-0.102694185	0.045635431	-2.250316981	0.024428831	0.078635554	negative
PRJNA729271	ed_Lushi_vs_Arbor_Acr	breed_growth_background	ENSGALG00010027421	PLPPR3	-0.162100725	0.142065748	-1.141026084	0.253859068	0.410448747	negative
PRJNA729271	ed_Lushi_vs_Arbor_Acr	breed_growth_background	ENSGALG00010027423	PEX11B	0.155234787	0.111525969	1.39191606	0.163947818	0.303731641	positive
PRJNA729271	ed_Lushi_vs_Arbor_Acr	breed_growth_background	ENSGALG00010027425	CFD	0.27183738	0.125901418	2.159128817	0.030840175	0.09286006	positive
PRJNA729271	ed_Lushi_vs_Arbor_Acr	breed_growth_background	ENSGALG00010027427	UBAC1	0.181360573	0.061768711	2.936123647	0.003323418	0.019078395	positive
PRJNA729271	ed_Lushi_vs_Arbor_Acr	breed_growth_background	ENSGALG00010027438	ST7L	0.029832358	0.044986878	0.663134657	0.507244316	0.656708321	positive
PRJNA729271	ed_Lushi_vs_Arbor_Acr	breed_growth_background	ENSGALG00010027440	TWF2	0.38616056	0.101841355	3.791785377	0.000149568	0.002129656	positive
PRJNA729271	ed_Lushi_vs_Arbor_Acr	breed_growth_background	ENSGALG00010027443	MED16	0.210156919	0.084154612	2.49727156	0.012515307	0.048936979	positive
PRJNA729271	ed_Lushi_vs_Arbor_Acr	breed_growth_background	ENSGALG00010027444	SELENBP1	-0.115194056	0.06517402	-1.767484278	0.07714716	0.179350524	negative
PRJNA729271	ed_Lushi_vs_Arbor_Acr	breed_growth_background	ENSGALG00010027445	PPM1M	0.318476761	0.087441443	3.642171842	0.000270347	0.00323324	positive
PRJNA729271	ed_Lushi_vs_Arbor_Acr	breed_growth_background	ENSGALG00010027448	ADAMTSL5	0.520731388	0.164169579	3.171911578	0.001514391	0.010981812	positive
PRJNA729271	ed_Lushi_vs_Arbor_Acr	breed_growth_background	ENSGALG00010027450	CAMSAP1	0.165848283	0.076660034	2.163425648	0.030508457	0.092090812	positive
PRJNA729271	ed_Lushi_vs_Arbor_Acr	breed_growth_background	ENSGALG00010027455	R3HDM4	0.270804297	0.079196325	3.419404857	0.000627583	0.005816376	positive
PRJNA729271	ed_Lushi_vs_Arbor_Acr	breed_growth_background	ENSGALG00010027457	CAPZA1	-0.112401004	0.0686353	-1.637655896	0.101493496	0.217336124	negative
PRJNA729271	ed_Lushi_vs_Arbor_Acr	breed_growth_background	ENSGALG00010027460	WDR82	-0.043658341	0.039790001	-1.097218912	0.272545708	0.43147117	negative
PRJNA729271	ed_Lushi_vs_Arbor_Acr	breed_growth_background	ENSGALG00010027467	GLYCTK	0.094232655	0.073130478	1.288555167	0.197552778	0.346087445	positive
PRJNA729271	ed_Lushi_vs_Arbor_Acr	breed_growth_background	ENSGALG00010027468	BSG	-0.06603224	0.061597623	-1.071993306	0.283723039	0.443881861	negative
PRJNA729271	ed_Lushi_vs_Arbor_Acr	breed_growth_background	ENSGALG00010027471	ENSGALG00010027471	0.217419791	0.069142314	3.144525803	0.001663562	0.011662971	positive
PRJNA729271	ed_Lushi_vs_Arbor_Acr	breed_growth_background	ENSGALG00010027473	DNAH1	0.270073951	0.215990467	1.25039755	0.211154359	0.361255295	positive
PRJNA729271	ed_Lushi_vs_Arbor_Acr	breed_growth_background	ENSGALG00010027475	ARID3A	0.263284973	0.091442735	2.879233359	0.003986432	0.021625501	positive

PRJNA729271	ed_Lushi_vs_Arbor_Acr	breed_growth_background	ENSGALG00010027479	HCN2	-0.070933688	0.151316774	-0.468776107	0.63922968	0.763357572	negative
PRJNA729271	ed_Lushi_vs_Arbor_Acr	breed_growth_background	ENSGALG00010027487	MPZ	0.996332498	0.278234404	3.580910495	0.000342399	0.003799127	positive
PRJNA729271	ed_Lushi_vs_Arbor_Acr	breed_growth_background	ENSGALG00010027490	ATP2B4	-0.150415228	0.036364553	-4.136314532	3.52928E-05	0.000751204	negative
PRJNA729271	ed_Lushi_vs_Arbor_Acr	breed_growth_background	ENSGALG00010027492	PCP4L1	0.692790393	0.257722243	2.688128058	0.007185383	0.03300131	positive
PRJNA729271	ed_Lushi_vs_Arbor_Acr	breed_growth_background	ENSGALG00010027497	KCNT1	0.415567713	0.177557682	2.340465979	0.019259693	0.066595718	positive
PRJNA729271	ed_Lushi_vs_Arbor_Acr	breed_growth_background	ENSGALG00010027499	MOV10	0.001670014	0.215749168	0.007740534	0.993824009	0.996451492	positive
PRJNA729271	ed_Lushi_vs_Arbor_Acr	breed_growth_background	ENSGALG00010027501	BAP1	0.023928692	0.036447173	0.656530813	0.511482649	0.660131867	positive
PRJNA729271	ed_Lushi_vs_Arbor_Acr	breed_growth_background	ENSGALG00010027502	THOP1	-0.049625623	0.0744798	-0.666296408	0.505221661	0.655130385	negative
PRJNA729271	ed_Lushi_vs_Arbor_Acr	breed_growth_background	ENSGALG00010027510	RHOC	0.266768712	0.095371342	2.797158001	0.005155431	0.026137803	positive
PRJNA729271	ed_Lushi_vs_Arbor_Acr	breed_growth_background	ENSGALG00010027511	POLRMT	0.075272621	0.044461767	1.692974111	0.090460395	0.200953782	positive
PRJNA729271	ed_Lushi_vs_Arbor_Acr	breed_growth_background	ENSGALG00010027513	SEMA3G	-0.145244132	0.09711771	-1.495547333	0.134771656	0.264632235	negative
PRJNA729271	ed_Lushi_vs_Arbor_Acr	breed_growth_background	ENSGALG00010027516	PPM1J	0.654155053	0.117628748	5.561183494	2.67951E-08	3.0825E-06	positive
PRJNA729271	ed_Lushi_vs_Arbor_Acr	breed_growth_background	ENSGALG00010027520	FANCE	-0.039917981	0.092771407	-0.430283231	0.666989624	0.784145344	negative
PRJNA729271	ed_Lushi_vs_Arbor_Acr	breed_growth_background	ENSGALG00010027522	PSMD5	0.15568923	0.056243371	2.768134778	0.005637814	0.027729931	positive
PRJNA729271	ed_Lushi_vs_Arbor_Acr	breed_growth_background	ENSGALG00010027524	ENSGALG00010027524	-0.010669621	0.059270128	-0.180016835	0.857139351	0.914151637	negative
PRJNA729271	ed_Lushi_vs_Arbor_Acr	breed_growth_background	ENSGALG00010027525	ENSGALG00010027525	0.269721842	0.135692833	1.987738303	0.04684064	0.12634073	positive
PRJNA729271	ed_Lushi_vs_Arbor_Acr	breed_growth_background	ENSGALG00010027527	ENSGALG00010027527	0.36859225	0.299188477	1.231973419	0.217959014	0.370006418	positive
PRJNA729271	ed_Lushi_vs_Arbor_Acr	breed_growth_background	ENSGALG00010027528	ENSGALG00010027528	-1.761315732	1.244973405	-1.414741653			negative
PRJNA729271	ed_Lushi_vs_Arbor_Acr	breed_growth_background	ENSGALG00010027529	ACY1	0.091182741	0.076456231	1.192613595	0.233020739	0.387505604	positive
PRJNA729271	ed_Lushi_vs_Arbor_Acr	breed_growth_background	ENSGALG00010027530	PHF19	-0.023630064	0.076149495	-0.310311499	0.756324087	0.845997751	negative
PRJNA729271	ed_Lushi_vs_Arbor_Acr	breed_growth_background	ENSGALG00010027531	UFC1	-0.144955644	0.0920465	-1.574808862	0.11530057	0.237904878	negative
PRJNA729271	ed_Lushi_vs_Arbor_Acr	breed_growth_background	ENSGALG00010027533	WDR18	0.096566359	0.053298515	1.811802034	0.070016791	0.167975133	positive
PRJNA729271	ed_Lushi_vs_Arbor_Acr	breed_growth_background	ENSGALG00010027534	KCNN3	-0.072627823	0.101613428	-0.714746311	0.474765816	0.628296403	negative
PRJNA729271	ed_Lushi_vs_Arbor_Acr	breed_growth_background	ENSGALG00010027536	USP21	0.390889984	0.167964703	2.32721504	0.019953823	0.068134432	positive
PRJNA729271	ed_Lushi_vs_Arbor_Acr	breed_growth_background	ENSGALG00010027537	SGTA	0.04818973	0.03674269	1.31154606	0.189673343	0.335978871	positive
PRJNA729271	ed_Lushi_vs_Arbor_Acr	breed_growth_background	ENSGALG00010027538	ABHD14A	-0.091540803	0.072219076	-1.26754326	0.204961106	0.35433657	negative
PRJNA729271	ed_Lushi_vs_Arbor_Acr	breed_growth_background	ENSGALG00010027539	DDR2L	0.027784015	0.05783777	0.480378387	0.63095836	0.756659338	positive
PRJNA729271	ed_Lushi_vs_Arbor_Acr	breed_growth_background	ENSGALG00010027540	PPOX	0.127989518	0.064152581	1.995079806	0.046034176	0.124862535	positive
PRJNA729271	ed_Lushi_vs_Arbor_Acr	breed_growth_background	ENSGALG00010027543	TUBB4B	0.00878401	0.081398806	0.107913249	0.914064507	0.951499307	positive
PRJNA729271	ed_Lushi_vs_Arbor_Acr	breed_growth_background	ENSGALG00010027545	PPARD	0.432384411	0.108307368	3.992197554	6.54638E-05	0.001167156	positive
PRJNA729271	ed_Lushi_vs_Arbor_Acr	breed_growth_background	ENSGALG00010027547	B4GALT3	0.389337777	0.119553414	3.256601064	0.001127548	0.008924626	positive
PRJNA729271	ed_Lushi_vs_Arbor_Acr	breed_growth_background	ENSGALG00010027548	PCBP4	-0.097981217	0.079535431	-1.231919102	0.217979305	0.370006418	negative
PRJNA729271	ed_Lushi_vs_Arbor_Acr	breed_growth_background	ENSGALG00010027553	FGF22	0.082768427	0.166780919	0.496270358	0.619703677	0.747457756	positive
PRJNA729271	ed_Lushi_vs_Arbor_Acr	breed_growth_background	ENSGALG00010027554	ENSGALG00010027554	0.068376419	0.080442771	0.8500008	0.395324642	0.556819617	positive
PRJNA729271	ed_Lushi_vs_Arbor_Acr	breed_growth_background	ENSGALG00010027555	ENSGALG00010027555	0.233921811	0.19526437	1.197974884	0.230926795	0.384877992	positive
PRJNA729271	ed_Lushi_vs_Arbor_Acr	breed_growth_background	ENSGALG00010027557	C6orf132	-0.889256428	0.476762761	-1.865196909	0.062153809	0.154664284	negative
PRJNA729271	ed_Lushi_vs_Arbor_Acr	breed_growth_background	ENSGALG00010027559	ENSGALG00010027559	0.290086124	0.150335622	1.929590068	0.05365765	0.139285036	positive
PRJNA729271	ed_Lushi_vs_Arbor_Acr	breed_growth_background	ENSGALG00010027561	ADAMTS4	-0.330798654	0.182690346	-1.810706814	0.070186247	0.168156968	negative
PRJNA729271	ed_Lushi_vs_Arbor_Acr	breed_growth_background	ENSGALG00010027562	SLC39A3	0.232883968	0.079644919	2.924027917	0.003455338	0.019564499	positive
PRJNA729271	ed_Lushi_vs_Arbor_Acr	breed_growth_background	ENSGALG00010027563	WDR77	-0.045404643	0.066128531	-0.686611997	0.492327266	0.644359704	negative
PRJNA729271	ed_Lushi_vs_Arbor_Acr	breed_growth_background	ENSGALG00010027567	PARP3	-0.284847929	0.068789127	-4.140885956	3.45967E-05	0.000741833	negative
PRJNA729271	ed_Lushi_vs_Arbor_Acr	breed_growth_background	ENSGALG00010027568	NELFB	0.009096285	0.041306467	0.220214559	0.825704059	0.893577746	positive
PRJNA729271	ed_Lushi_vs_Arbor_Acr	breed_growth_background	ENSGALG00010027569	ATP5PB	-0.047751357	0.067004783	-0.712655943	0.476058685	0.629040459	negative
PRJNA729271	ed_Lushi_vs_Arbor_Acr	breed_growth_background	ENSGALG00010027570	NDUFS2	0.12784349	0.073543207	1.738345333	0.082149983	0.187315401	positive
PRJNA729271	ed_Lushi_vs_Arbor_Acr	breed_growth_background	ENSGALG00010027571	RBM10	0.148258874	0.054438207	2.723434173	0.006460709	0.030603914	positive
PRJNA729271	ed_Lushi_vs_Arbor_Acr	breed_growth_background	ENSGALG00010027574	GNG7	-0.078839856	0.051538372	-1.529731192	0.126083279	0.252707015	negative
PRJNA729271	ed_Lushi_vs_Arbor_Acr	breed_growth_background	ENSGALG00010027575	FCER1G	0.320779172	0.180751643	1.774695745	0.075948138	0.177704179	positive
PRJNA729271	ed_Lushi_vs_Arbor_Acr	breed_growth_background	ENSGALG00010027576	GADD45B	0.729624741	0.222659768	3.276859341	0.001049686	0.008457591	positive
PRJNA729271	ed_Lushi_vs_Arbor_Acr	breed_growth_background	ENSGALG00010027578	ENSGALG00010027578	-0.228910754	0.128261122	-1.784724399	0.074306031	0.17518721	negative
PRJNA729271	ed_Lushi_vs_Arbor_Acr	breed_growth_background	ENSGALG00010027579	C26H6ORF125	-0.0425311	0.085204962	-0.499162244	0.617665091	0.746025789	negative
PRJNA729271	ed_Lushi_vs_Arbor_Acr	breed_growth_background	ENSGALG00010027583	GLE1	0.064651937	0.040484758	1.596945141	0.110277936	0.230860584	positive
PRJNA729271	ed_Lushi_vs_Arbor_Acr	breed_growth_background	ENSGALG00010027586	HJV	0.168596925	0.149832933	1.125232761	0.260490414	0.418372138	positive
PRJNA729271	ed_Lushi_vs_Arbor_Acr	breed_growth_background	ENSGALG00010027590	SEMA3F	0.411556118	0.128792199	3.195505019	0.001395864	0.010397247	positive
PRJNA729271	ed_Lushi_vs_Arbor_Acr	breed_growth_background	ENSGALG00010027592	MAP1S	0.243999948	0.090648283	2.691721677	0.007108424	0.032751623	positive
PRJNA729271	ed_Lushi_vs_Arbor_Acr	breed_growth_background	ENSGALG00010027593	ENSGALG00010027593	-0.189093313	0.177452166	-1.065601604	0.286603774	0.447326554	negative
PRJNA729271	ed_Lushi_vs_Arbor_Acr	breed_growth_background	ENSGALG00010027594	ITPR3	-0.099467465	0.0747409	-1.33083045	0.183244806	0.328323049	negative
PRJNA729271	ed_Lushi_vs_Arbor_Acr	breed_growth_background	ENSGALG00010027595	GNAT1	0.002426027	0.243084628	0.009980175	0.992037105	0.99553152	positive
PRJNA729271	ed_Lushi_vs_Arbor_Acr	breed_growth_background	ENSGALG00010027597	EXOSC2	-0.045106078	0.06319877	-0.713717664	0.475401782	0.628793578	negative
PRJNA729271	ed_Lushi_vs_Arbor_Acr	breed_growth_background	ENSGALG00010027600	KIRREL1	-0.074605573	0.075802194	-0.984213892	0.325010357	0.488946163	negative
PRJNA729271	ed_Lushi_vs_Arbor_Acr	breed_growth_background	ENSGALG00010027605	FCHO1	0.342923003	0.176711913	1.940576604	0.052309654	0.136913766	positive
PRJNA729271	ed_Lushi_vs_Arbor_Acr	breed_growth_background	ENSGALG00010027607	ABL1	-0.142896445	0.042696963	-3.346759018	0.000817623	0.007065766	negative
PRJNA729271	ed_Lushi_vs_Arbor_Acr	breed_growth_background	ENSGALG00010027608	GNAI2	-0.141232778	0.077364936	-1.825539913	0.067919594	0.164631189	negative
PRJNA729271	ed_Lushi_vs_Arbor_Acr	breed_growth_background	ENSGALG00010027609	RPL36	-0.1647161	0.099857451	-1.64951236	0.099042713	0.21334944	negative
PRJNA729271	ed_Lushi_vs_Arbor_Acr	breed_growth_background	ENSGALG00010027612	BAK1	-0.107740662	0.088360238	-1.219334224	0.222717364	0.375372259	negative
PRJNA729271	ed_Lushi_vs_Arbor_Acr	breed_growth_background	ENSGALG00010027614	LONP1	0.129546055	0.04298592	3.01368577	0.00258095	0.015933078	positive
PRJNA729271	ed_Lushi_vs_Arbor_Acr	breed_growth_background	ENSGALG00010027616	SLC38A3	0.516759325	0.138116418	3.741476439	0.000182942	0.002433336	positive
PRJNA729271	ed_Lushi_vs_Arbor_Acr	breed_growth_background	ENSGALG00010027618	PPP1R26	0.018231711	0.042497997	0.42900164	0.667922031	0.78486007	positive
PRJNA729271	ed_Lushi_vs_Arbor_Acr	breed_growth_background	ENSGALG00010027619	JAK3	0.125801584	0.186507196	0.674513298	0.499985034	0.651207033	positive
PRJNA729271	ed_Lushi_vs_Arbor_Acr	breed_growth_background	ENSGALG00010027621	TSPO2	-0.230680267	0.225031586	-1.025101725	0.305315192	0.468014351	negative
PRJNA729271	ed_Lushi_vs_Arbor_Acr	breed_growth_background	ENSGALG00010027622	FIBCD1	-0.289610732	0.640164979	-0.45240015	0.650980739	0.772173962	negative
PRJNA729271	ed_Lushi_vs_Arbor_Acr	breed_growth_background	ENSGALG00010027623	APOBEC2	-0.348764342	0.097510153	-3.576697723	0.000347962	0.003841364	negative
PRJNA729271	ed_Lushi_vs_Arbor_Acr	breed_growth_background	ENSGALG00010027624	ENSGALG00010027624	0.118901393	0.21150788	0.562160581	0.574006616	0.711677863	positive
PRJNA729271	ed_Lushi_vs_Arbor_Acr	breed_growth_background	ENSGALG00010027626	SEMA3B	0.506825103	0.113138337	4.479693785	7.47502E-06	0.000233514	positive
PRJNA729271	ed_Lushi_vs_Arbor_Acr	breed_growth_background	ENSGALG00010027627	OARD1	0.101872745	0.049670771	2.050959587	0.040270882	0.112884634	positive
PRJNA729271	ed_Lushi_vs_Arbor_Acr	breed_growth_background	ENSGALG00010027628	PIERCE1	-0.024476376	0.139512496	-0.175442175	0.860732203	0.916307481	negative
PRJNA729271	ed_Lushi_vs_Arbor_Acr	breed_growth_background	ENSGALG00010027632	LAMC3	-0.224059324	0.224067504	-0.99996349	0.317328177	0.480634474	negative
PRJNA729271	ed_Lushi_vs_Arbor_Acr	breed_growth_background	ENSGALG00010027634	OASL	0.31101813	0.379720807	0.819070548	0.412746164	0.573006	positive
PRJNA729271	ed_Lushi_vs_Arbor_Acr	breed_growth_background	ENSGALG00010027635	CD48	0.280514613	0.122584031	2.288345475	0.02211741	0.073378773	positive
PRJNA729271	ed_Lushi_vs_Arbor_Acr	breed_growth_background	ENSGALG00010027636	SLC1A6	-0.130205242	0.321800181	-0.404615193	0.685760389	0.797198186	negative
PRJNA729271	ed_Lushi_vs_Arbor_Acr	breed_growth_background	ENSGALG00010027638	MRPS2	0.17533554	0.075140439	2.333437797	0.019625179	0.067393266	positive
PRJNA729271	ed_Lushi_vs_Arbor_Acr	breed_growth_background	ENSGALG00010027641	HYAL3	-0.312820098	0.088077557	-3.551643656	0.000382833	0.00411659	negative
PRJNA729271	ed_Lushi_vs_Arbor_Acr	breed_growth_background	ENSGALG00010027642	DIPK1B	-0.35185996	0.094540102	-3.721806437	0.000197803	0.002571434	negative
PRJNA729271	ed_Lushi_vs_Arbor_Acr	breed_growth_background	ENSGALG00010027644	NAA80	0.070535675	0.072265226	0.976066624	0.32903145	0.492846773	positive
PRJNA729271	ed_Lushi_vs_Arbor_Acr	breed_growth_background	ENSGALG00010027645	SLAMF1	0.48513394	0.357093239	1.358563778	0.17428486	0.318149661	positive
PRJNA729271	ed_Lushi_vs_Arbor_Acr	breed_growth_background	ENSGALG00010027648	NFYA	0.128439772	0.039486621	3.252741524	0.001142974	0.008994251	positive
PRJNA729271	ed_Lushi_vs_Arbor_Acr	breed_growth_background	ENSGALG00010027649	FBLN2	-0.273742705	0.102489353	-2.67093799	0.007563962	0.034230293	negative
PRJNA729271	ed_Lushi_vs_Arbor_Acr	breed_growth_background	ENSGALG00010027650	CCDC124	0.457735636	0.102428379	4.468836088	7.86464E-06	0.000242034	positive
PRJNA729271	ed_Lushi_vs_Arbor_Acr	breed_growth_background	ENSGALG00010027653	WNT7A	0.153759244	0.331678052	0.463579798	0.642948831	0.766316958	positive
PRJNA729271	ed_Lushi_vs_Arbor_Acr	breed_growth_background	ENSGALG00010027654	AIF1L	-0.02866426	0.048832644	-0.586989721	0.55721061	0.698621901	negative
PRJNA729271	ed_Lushi_vs_Arbor_Acr	breed_growth_background	ENSGALG00010027663	COPA	-0.059283571	0.038003673	-1.559943186	0.118773308	0.242652445	negative
PRJNA729271	ed_Lushi_vs_Arbor_Acr	breed_growth_background	ENSGALG00010027664	TREMB1	-0.182756945	0.170964169	-1.068978057	0.285079557	0.445523254	negative
PRJNA729271	ed_Lushi_vs_Arbor_Acr	breed_growth_background	ENSGALG00010027665	TRAF1	-0.017778244	0.074440449	-0.238825057	0.81124124	0.883798705	negative
PRJNA729271	ed_Lushi_vs_Arbor_Acr	breed_growth_background	ENSGALG00010027667	PTPDC1	0.239974174	0.082326054	2.914923801	0.003557754	0.019934149	positive
PRJNA729271	ed_Lushi_vs_Arbor_Acr	breed_growth_background	ENSGALG00010027668	RANBP3	-0.048367202	0.029322147	-1.649510926	0.099043007	0.21334944	negative
PRJNA729271	ed_Lushi_vs_Arbor_Acr	breed_growth_background	ENSGALG00010027671	BARX1	0.111826714	0.363578707	0.307572232	0.758407853	0.847384708	positive
PRJNA729271	ed_Lushi_vs_Arbor_Acr	breed_growth_background	ENSGALG00010027672	CNTRL	0.08762145	0.093614067	0.935985932	0.349280431	0.513210324	positive
PRJNA729271	ed_Lushi_vs_Arbor_Acr	breed_growth_background	ENSGALG00010027674	TREM2	-0.187961702	0.153618213	-1.223563918	0.221116778	0.373572456	negative
PRJNA729271	ed_Lushi_vs_Arbor_Acr	breed_growth_background	ENSGALG00010027685	ENSGALG00010027685	0.100648101	0.165292255	0.608909962	0.54258412	0.687358576	positive
PRJNA729271	ed_Lushi_vs_Arbor_Acr	breed_growth_background	ENSGALG00010027692	PHF2	0.112304932	0.060362085	1.860521101	0.062811836	0.155793559	positive
PRJNA729271	ed_Lushi_vs_Arbor_Acr	breed_growth_background	ENSGALG00010027693	RAB14	-0.058343599	0.032287572	-1.806998675	0.070762486	0.169090378	negative
PRJNA729271	ed_Lushi_vs_Arbor_Acr	breed_growth_background	ENSGALG00010027695	TREMB2	-0.35443566	0.162651949	-2.17910491	0.029323876	0.08940883	negative
PRJNA729271	ed_Lushi_vs_Arbor_Acr	breed_growth_background	ENSGALG00010027697	CATH2	1.054493289	0.480908572	2.192710526	0.028328242	0.087217049	positive
PRJNA729271	ed_Lushi_vs_Arbor_Acr	breed_growth_background	ENSGALG00010027698	FAM120A	0.014579866	0.035701037	0.408387743	0.682989031	0.795271269	positive
PRJNA729271	ed_Lushi_vs_Arbor_Acr	breed_growth_background	ENSGALG00010027699	VANGL2	-0.260575764	0.069837463	-3.731174557	0.000190589	0.002504614	negative
PRJNA729271	ed_Lushi_vs_Arbor_Acr	breed_growth_background	ENSGALG00010027700	TCF3	-0.103031635	0.051626651	-1.995706352	0.045965895	0.124770721	negative
PRJNA729271	ed_Lushi_vs_Arbor_Acr	breed_growth_background	ENSGALG00010027702	FOXP4	-0.015545429	0.137546863	-0.113019147	0.910015376	0.948901005	negative
PRJNA729271	ed_Lushi_vs_Arbor_Acr	breed_growth_background	ENSGALG00010027703	NCSTN	0.014317885	0.054757485	0.261478133	0.79372381	0.8721876	positive
PRJNA729271	ed_Lushi_vs_Arbor_Acr	breed_growth_background	ENSGALG00010027704	MDFI	0.058141139	0.102899766	0.565026935	0.572055443	0.710413267	positive
PRJNA729271	ed_Lushi_vs_Arbor_Acr	breed_growth_background	ENSGALG00010027705	S100A11	-0.089028443	0.123337155	-0.721829871	0.470399082	0.624684809	negative
PRJNA729271	ed_Lushi_vs_Arbor_Acr	breed_growth_background	ENSGALG00010027706	GSN	-0.273522779	0.091865526	-2.977425713	0.002906801	0.017324867	negative
PRJNA729271	ed_Lushi_vs_Arbor_Acr	breed_growth_background	ENSGALG00010027707	HIGD1C	-0.086995318	0.113534724	-0.766244152	0.443531042	0.600464876	negative
PRJNA729271	ed_Lushi_vs_Arbor_Acr	breed_growth_background	ENSGALG00010027709	PSMA5	0.156494407	0.057077521	2.741787025	0.006110595	0.029416496	positive
PRJNA729271	ed_Lushi_vs_Arbor_Acr	breed_growth_background	ENSGALG00010027710	ENSGALG00010027710	0.609087868	0.145120848	4.197107969	2.70345E-05	0.000606602	positive
PRJNA729271	ed_Lushi_vs_Arbor_Acr	breed_growth_background	ENSGALG00010027711	WNK2	0.424158233	0.099381523	4.267978806	1.97252E-05	0.000476528	positive
PRJNA729271	ed_Lushi_vs_Arbor_Acr	breed_growth_background	ENSGALG00010027712	STOM	-0.326471781	0.083701806	-3.900415026	9.60279E-05	0.001548303	negative
PRJNA729271	ed_Lushi_vs_Arbor_Acr	breed_growth_background	ENSGALG00010027713	ENSGALG00010027713	0.193636943	0.074639343	2.594301275	0.009478339	0.040319652	positive
PRJNA729271	ed_Lushi_vs_Arbor_Acr	breed_growth_background	ENSGALG00010027714	SYPL2	-0.205423661	0.083051794	-2.473440362	0.013381914	0.051301465	negative
PRJNA729271	ed_Lushi_vs_Arbor_Acr	breed_growth_background	ENSGALG00010027715	NINJ1	-0.101986968	0.066611157	-1.531079381	0.125749775	0.252214102	negative
PRJNA729271	ed_Lushi_vs_Arbor_Acr	breed_growth_background	ENSGALG00010027717	ATXN7L2	0.561484946	0.120620405	4.654974809	3.2402E-06	0.000121361	positive
PRJNA729271	ed_Lushi_vs_Arbor_Acr	breed_growth_background	ENSGALG00010027718	ENSGALG00010027718	-0.360519659	0.232918989	-1.547832833	0.121662549	0.246345837	negative
PRJNA729271	ed_Lushi_vs_Arbor_Acr	breed_growth_background	ENSGALG00010027719	GPR61	-0.479478054	0.580183102	-0.826425403	0.408562818	0.569051413	negative
PRJNA729271	ed_Lushi_vs_Arbor_Acr	breed_growth_background	ENSGALG00010027720	DAB2IP	-0.134783463	0.046714312	-2.885271287	0.003910765	0.021350862	negative
PRJNA729271	ed_Lushi_vs_Arbor_Acr	breed_growth_background	ENSGALG00010027721	SUSD3	0.036216584	0.104138384	0.347773637	0.72801019	0.825153912	positive
PRJNA729271	ed_Lushi_vs_Arbor_Acr	breed_growth_background	ENSGALG00010027722	GNAI3	-0.094408669	0.045900028	-2.056832495	0.039702341	0.111854757	negative
PRJNA729271	ed_Lushi_vs_Arbor_Acr	breed_growth_background	ENSGALG00010027723	ENSGALG00010027723	-0.316949994	0.511950379	-0.619102958	0.535848535	0.681087734	negative
PRJNA729271	ed_Lushi_vs_Arbor_Acr	breed_growth_background	ENSGALG00010027724	TTLL11	0.016234352	0.060797777	0.267022129	0.789452123	0.869404993	positive
PRJNA729271	ed_Lushi_vs_Arbor_Acr	breed_growth_background	ENSGALG00010027725	BTN1A1	-1.386840936	0.138507212	-10.01277055	1.33948E-23	3.88315E-20	negative
PRJNA729271	ed_Lushi_vs_Arbor_Acr	breed_growth_background	ENSGALG00010027727	GSTM2	-0.130922012	0.091639972	-1.428656182	0.153103078	0.289565385	negative
PRJNA729271	ed_Lushi_vs_Arbor_Acr	breed_growth_background	ENSGALG00010027728	CRNN	-0.22955415	0.803107487	-0.285832412	0.775006482	0.860293993	negative
PRJNA729271	ed_Lushi_vs_Arbor_Acr	breed_growth_background	ENSGALG00010027729	EPS8L3	-0.080209151	0.133351382	-0.601487216	0.547515524	0.691129279	negative
PRJNA729271	ed_Lushi_vs_Arbor_Acr	breed_growth_background	ENSGALG00010027730	ENSGALG00010027730	0.033297324	0.081240127	0.409863019	0.681906435	0.794479296	positive

PRJNA729271	eed_Lushi_vs_Arbor_Acr	breed_growth_background	ENSGALG00010027731	OLFML2A	0.031482339	0.054116972	0.581746127	0.560737696	0.701587649	positive
PRJNA729271	eed_Lushi_vs_Arbor_Acr	breed_growth_background	ENSGALG00010027732	EEF2	0.062293474	0.062560316	0.995734645	0.319379092	0.482681674	positive
PRJNA729271	eed_Lushi_vs_Arbor_Acr	breed_growth_background	ENSGALG00010027733	RPL35	-0.071845164	0.082359596	-0.872335075	0.383025608	0.544469568	negative
PRJNA729271	eed_Lushi_vs_Arbor_Acr	breed_growth_background	ENSGALG00010027734	CSF1	-0.149263869	0.074420439	-2.005683793	0.044889995	0.122514975	negative
PRJNA729271	eed_Lushi_vs_Arbor_Acr	breed_growth_background	ENSGALG00010027736	ENSGALG00010027736	-0.887547455	0.781016982	-1.136399689	0.255789326	0.412880432	negative
PRJNA729271	eed_Lushi_vs_Arbor_Acr	breed_growth_background	ENSGALG00010027738	ENSGALG00010027738	-0.152788867	0.131597548	-1.161031264	0.245629185	0.401533218	negative
PRJNA729271	eed_Lushi_vs_Arbor_Acr	breed_growth_background	ENSGALG00010027739	FGD3	-0.075829918	0.040873562	-1.855231467	0.06356318	0.157039082	negative
PRJNA729271	eed_Lushi_vs_Arbor_Acr	breed_growth_background	ENSGALG00010027740	MARCHF2	-0.097727274	0.078759705	-1.240828336	0.214669171	0.3654292	negative
PRJNA729271	eed_Lushi_vs_Arbor_Acr	breed_growth_background	ENSGALG00010027741	RAB11B	-0.14294888	0.033779006	-4.231885336	2.31741E-05	0.000536594	negative
PRJNA729271	eed_Lushi_vs_Arbor_Acr	breed_growth_background	ENSGALG00010027743	C1orf74	0.107912028	0.09943419	1.085260794	0.277806165	0.437078081	positive
PRJNA729271	eed_Lushi_vs_Arbor_Acr	breed_growth_background	ENSGALG00010027744	ENSGALG00010027744	-1.054274369	0.51326856	-2.054040423	0.039971779	0.112285067	negative
PRJNA729271	eed_Lushi_vs_Arbor_Acr	breed_growth_background	ENSGALG00010027745	BICD2	-0.272081495	0.074076585	-3.672975684	0.000239742	0.002954987	negative
PRJNA729271	eed_Lushi_vs_Arbor_Acr	breed_growth_background	ENSGALG00010027746	UQCR11	-0.147483052	0.098413707	-1.498602757	0.133976717	0.263857679	negative
PRJNA729271	eed_Lushi_vs_Arbor_Acr	breed_growth_background	ENSGALG00010027748	ENSGALG00010027748	-0.646016187	1.50977047	-0.427890332	0.668730967	0.785324099	negative
PRJNA729271	eed_Lushi_vs_Arbor_Acr	breed_growth_background	ENSGALG00010027751	ANGPTL4	0.003013425	0.113605474	0.026525351	0.978838313	0.987465271	positive
PRJNA729271	eed_Lushi_vs_Arbor_Acr	breed_growth_background	ENSGALG00010027752	SCAI	0.079373267	0.047003114	1.688681033	0.091280572	0.201909339	positive
PRJNA729271	eed_Lushi_vs_Arbor_Acr	breed_growth_background	ENSGALG00010027759	ENSGALG00010027759	-0.832982532	0.477326993	-1.745098315	0.080967757	0.185581907	negative
PRJNA729271	eed_Lushi_vs_Arbor_Acr	breed_growth_background	ENSGALG00010027766	ENSGALG00010027766	0.703580509	0.390728817	1.800687531	0.071752144	0.170583456	positive
PRJNA729271	eed_Lushi_vs_Arbor_Acr	breed_growth_background	ENSGALG00010027767	ENSGALG00010027767	-0.376314184	0.568345903	-0.66212175	0.507893198	0.656958809	negative
PRJNA729271	eed_Lushi_vs_Arbor_Acr	breed_growth_background	ENSGALG00010027772	ENSGALG00010027772	-0.681847609	0.551652515	-1.236009245	0.216455127	0.368037193	negative
PRJNA729271	eed_Lushi_vs_Arbor_Acr	breed_growth_background	ENSGALG00010027775	MVB12B	0.068052323	0.047106654	1.444643546	0.148558035	0.283563468	positive
PRJNA729271	eed_Lushi_vs_Arbor_Acr	breed_growth_background	ENSGALG00010027776	TKT	-0.138810457	0.069904162	-1.985725197	0.047063846	0.126730702	negative
PRJNA729271	eed_Lushi_vs_Arbor_Acr	breed_growth_background	ENSGALG00010027784	PEAR1	-0.168938731	0.102361297	-1.65041608	0.098857864	0.213172381	negative
PRJNA729271	eed_Lushi_vs_Arbor_Acr	breed_growth_background	ENSGALG00010027787	DCP1A	0.216719798	0.074476987	2.909889452	0.003615566	0.020118093	positive
PRJNA729271	eed_Lushi_vs_Arbor_Acr	breed_growth_background	ENSGALG00010027789	UAP1L1	-0.218884351	0.072440534	-3.02157286	0.002514651	0.015653557	negative
PRJNA729271	eed_Lushi_vs_Arbor_Acr	breed_growth_background	ENSGALG00010027801	MBD3	-0.062614543	0.072019418	-0.869411956	0.384621846	0.546417099	negative
PRJNA729271	eed_Lushi_vs_Arbor_Acr	breed_growth_background	ENSGALG00010027803	SAPCD2	0.225985287	0.093868726	2.407460888	0.016063883	0.058489319	positive
PRJNA729271	eed_Lushi_vs_Arbor_Acr	breed_growth_background	ENSGALG00010027811	MEX3D	0.070604795	0.055478055	1.272661688	0.203138118	0.352295648	positive
PRJNA729271	eed_Lushi_vs_Arbor_Acr	breed_growth_background	ENSGALG00010027813	LRRC71	0.03873975	0.173336594	0.223494352	0.823150778	0.891840564	positive
PRJNA729271	eed_Lushi_vs_Arbor_Acr	breed_growth_background	ENSGALG00010027814	ENSGALG00010027814	0.085256762	0.071544377	1.191662657	0.233393546	0.387785356	positive
PRJNA729271	eed_Lushi_vs_Arbor_Acr	breed_growth_background	ENSGALG00010027816	ZMYND19	0.083288596	0.065649379	1.26868824	0.204552278	0.353943569	positive
PRJNA729271	eed_Lushi_vs_Arbor_Acr	breed_growth_background	ENSGALG00010027821	NMRK2	-0.103158049	0.120794252	-0.853997996	0.3931061	0.554556975	negative
PRJNA729271	eed_Lushi_vs_Arbor_Acr	breed_growth_background	ENSGALG00010027822	DPH7	0.069395833	0.048661909	1.426081172	0.153844913	0.290400053	positive
PRJNA729271	eed_Lushi_vs_Arbor_Acr	breed_growth_background	ENSGALG00010027826	ARHGEF11	0.2100798	0.088115039	2.384153737	0.017118449	0.061221792	positive
PRJNA729271	eed_Lushi_vs_Arbor_Acr	breed_growth_background	ENSGALG00010027830	CACNA1D	0.714232149	0.196125509	3.641709601	0.000270833	0.003236382	positive
PRJNA729271	eed_Lushi_vs_Arbor_Acr	breed_growth_background	ENSGALG00010027831	ATCAY	0.368260824	0.119206787	3.089260563	0.002006554	0.01328082	positive
PRJNA729271	eed_Lushi_vs_Arbor_Acr	breed_growth_background	ENSGALG00010027838	ENSGALG00010027838	-0.219314996	0.359999686	-0.609208854	0.542386012	0.687227731	negative
PRJNA729271	eed_Lushi_vs_Arbor_Acr	breed_growth_background	ENSGALG00010027842	SLAMF8	0.356886499	0.119001545	2.999007273	0.002708608	0.016454853	positive
PRJNA729271	eed_Lushi_vs_Arbor_Acr	breed_growth_background	ENSGALG00010027847	NRTN	0.075599926	0.226326669	0.334030126	0.738356825	0.83235979	positive
PRJNA729271	eed_Lushi_vs_Arbor_Acr	breed_growth_background	ENSGALG00010027860	ENSGALG00010027860	0.444090405	0.147707344	3.006556012	0.002642254	0.016173762	positive
PRJNA729271	eed_Lushi_vs_Arbor_Acr	breed_growth_background	ENSGALG00010027861	PNPLA7	0.149629335	0.042870448	3.490267564	0.000482537	0.004844978	positive
PRJNA729271	eed_Lushi_vs_Arbor_Acr	breed_growth_background	ENSGALG00010027863	CHDH	-0.258107679	0.082885172	-3.114039236	0.001845449	0.012546806	negative
PRJNA729271	eed_Lushi_vs_Arbor_Acr	breed_growth_background	ENSGALG00010027864	ACTR8	0.11359354	0.044830455	2.5338476	0.011281779	0.04561489	positive
PRJNA729271	eed_Lushi_vs_Arbor_Acr	breed_growth_background	ENSGALG00010027865	SELENOK	0.111640866	0.066469635	1.67957694	0.093039658	0.204458738	positive
PRJNA729271	eed_Lushi_vs_Arbor_Acr	breed_growth_background	ENSGALG00010027866	KCNA2	-0.100786856	0.075501966	-1.334890481	0.181912194	0.326662196	negative
PRJNA729271	eed_Lushi_vs_Arbor_Acr	breed_growth_background	ENSGALG00010027867	UBAP2L	0.164949065	0.090852201	1.815575873	0.069435457	0.166965321	positive
PRJNA729271	eed_Lushi_vs_Arbor_Acr	breed_growth_background	ENSGALG00010027868	CACNA2D3	-0.07070652	0.170890074	-0.413754401	0.679053982	0.792623996	negative
PRJNA729271	eed_Lushi_vs_Arbor_Acr	breed_growth_background	ENSGALG00010027869	NSMF	0.047837654	0.089423375	0.534956924	0.592679645	0.727240451	positive
PRJNA729271	eed_Lushi_vs_Arbor_Acr	breed_growth_background	ENSGALG00010027870	HAX1	0.074906904	0.064441643	1.162399028	0.245073418	0.400926922	positive
PRJNA729271	eed_Lushi_vs_Arbor_Acr	breed_growth_background	ENSGALG00010027872	KDM5B	0.107078822	0.049206635	2.176105382	0.029547382	0.089957756	positive
PRJNA729271	eed_Lushi_vs_Arbor_Acr	breed_growth_background	ENSGALG00010027873	RFX2	-0.12807091	0.107753938	-1.188549699	0.234616912	0.389059848	negative
PRJNA729271	eed_Lushi_vs_Arbor_Acr	breed_growth_background	ENSGALG00010027874	FRS3	0.039513939	0.072559622	0.544572012	0.586047908	0.72184313	positive
PRJNA729271	eed_Lushi_vs_Arbor_Acr	breed_growth_background	ENSGALG00010027875	WNT5A	-0.050859973	0.090851361	-0.559815199	0.575605495	0.713050308	negative
PRJNA729271	eed_Lushi_vs_Arbor_Acr	breed_growth_background	ENSGALG00010027876	ANAPC2	-0.077904513	0.059066208	-1.318935402	0.187190709	0.332801341	negative
PRJNA729271	eed_Lushi_vs_Arbor_Acr	breed_growth_background	ENSGALG00010027877	OAZ1	-0.150670471	0.061119071	-2.465195703	0.013693844	0.052248555	negative
PRJNA729271	eed_Lushi_vs_Arbor_Acr	breed_growth_background	ENSGALG00010027879	ENSGALG00010027879	0.399229891	0.216219623	1.84640915	0.06483282	0.1590912	positive
PRJNA729271	eed_Lushi_vs_Arbor_Acr	breed_growth_background	ENSGALG00010027880	AMH	0.293648375	0.179627715	1.634760953	0.102099169	0.218310585	positive
PRJNA729271	eed_Lushi_vs_Arbor_Acr	breed_growth_background	ENSGALG00010027881	ENSGALG00010027881	0.291444823	0.168768472	1.726891397	0.08418717	0.190860477	positive
PRJNA729271	eed_Lushi_vs_Arbor_Acr	breed_growth_background	ENSGALG00010027882	CHIA	-1.154128793	0.745648473	-1.547818892	0.121665907	0.246345837	negative
PRJNA729271	eed_Lushi_vs_Arbor_Acr	breed_growth_background	ENSGALG00010027883	ATP8B2	0.008121467	0.062982784	0.128947408	0.897399263	0.940413702	positive
PRJNA729271	eed_Lushi_vs_Arbor_Acr	breed_growth_background	ENSGALG00010027884	SF3A2	0.100653771	0.102674616	0.980317975	0.326929185	0.490613783	positive
PRJNA729271	eed_Lushi_vs_Arbor_Acr	breed_growth_background	ENSGALG00010027885	PLEKHJ1	0.024386739	0.041065868	0.593844472	0.552616141	0.69519148	positive
PRJNA729271	eed_Lushi_vs_Arbor_Acr	breed_growth_background	ENSGALG00010027886	IL6R	0.120307341	0.102111806	1.178192274	0.238719962	0.39410545	positive
PRJNA729271	eed_Lushi_vs_Arbor_Acr	breed_growth_background	ENSGALG00010027887	ERC2	-0.28783658	0.152932832	-1.882111101	0.059820932	0.150532536	negative
PRJNA729271	eed_Lushi_vs_Arbor_Acr	breed_growth_background	ENSGALG00010027888	PFKFB2	-0.060260818	0.07871551	-0.765552019	0.443942903	0.600779795	negative
PRJNA729271	eed_Lushi_vs_Arbor_Acr	breed_growth_background	ENSGALG00010027889	RBM8A	0.104496263	0.089888878	1.16250492	0.245030427	0.400926922	positive
PRJNA729271	eed_Lushi_vs_Arbor_Acr	breed_growth_background	ENSGALG00010027890	ENSGALG00010027890	-0.562548639	0.263727014	-2.133071735	0.032918842	0.097445331	negative
PRJNA729271	eed_Lushi_vs_Arbor_Acr	breed_growth_background	ENSGALG00010027891	CCDC66	0.068881449	0.077920213	0.883999753	0.376696346	0.539173846	positive
PRJNA729271	eed_Lushi_vs_Arbor_Acr	breed_growth_background	ENSGALG00010027892	SHE	-0.208645836	0.089745685	-2.324856451	0.020079637	0.068467265	negative
PRJNA729271	eed_Lushi_vs_Arbor_Acr	breed_growth_background	ENSGALG00010027893	DOT1L	0.107206373	0.084460807	1.269303214	0.20433294	0.353732948	positive
PRJNA729271	eed_Lushi_vs_Arbor_Acr	breed_growth_background	ENSGALG00010027894	RSBN1	0.072160117	0.03745971	1.926339433	0.054062005	0.140033731	positive
PRJNA729271	eed_Lushi_vs_Arbor_Acr	breed_growth_background	ENSGALG00010027895	LIX1L	-0.367021057	0.09620453	-3.81500805	0.000136178	0.001997813	negative
PRJNA729271	eed_Lushi_vs_Arbor_Acr	breed_growth_background	ENSGALG00010027896	TOR4A	-0.146535387	0.084783661	-1.72834465	0.083926452	0.190377767	negative
PRJNA729271	eed_Lushi_vs_Arbor_Acr	breed_growth_background	ENSGALG00010027897	AP3D1	0.184117224	0.05280506	3.48673449	0.000488957	0.00489463	positive
PRJNA729271	eed_Lushi_vs_Arbor_Acr	breed_growth_background	ENSGALG00010027898	TASOR	-0.0447879	0.04978989	-0.899538038	0.368366144	0.531607652	negative
PRJNA729271	eed_Lushi_vs_Arbor_Acr	breed_growth_background	ENSGALG00010027899	PMVK	-0.223322363	0.09777272	-2.284096864	0.022365839	0.073982847	negative
PRJNA729271	eed_Lushi_vs_Arbor_Acr	breed_growth_background	ENSGALG00010027901	MOB3A	-0.072505975	0.053478633	-1.355793343	0.17516493	0.319092077	negative
PRJNA729271	eed_Lushi_vs_Arbor_Acr	breed_growth_background	ENSGALG00010027902	UBE2Q1	0.047786854	0.037773263	1.265097318	0.205836444	0.355190388	positive
PRJNA729271	eed_Lushi_vs_Arbor_Acr	breed_growth_background	ENSGALG00010027903	NDUFA8	0.031475316	0.067223455	0.468219245	0.639627811	0.763702294	positive
PRJNA729271	eed_Lushi_vs_Arbor_Acr	breed_growth_background	ENSGALG00010027904	MORN5	0.106353277	0.332630241	0.319734238	0.749169803	0.840496617	positive
PRJNA729271	eed_Lushi_vs_Arbor_Acr	breed_growth_background	ENSGALG00010027905	SYN2	-0.00981121	0.14700708	-0.06673971	0.946788921	0.970833716	negative
PRJNA729271	eed_Lushi_vs_Arbor_Acr	breed_growth_background	ENSGALG00010027906	LHX6	-0.181169014	0.09135634	-1.983102793	0.047355949	0.127233453	negative
PRJNA729271	eed_Lushi_vs_Arbor_Acr	breed_growth_background	ENSGALG00010027907	MKNK2	0.203707343	0.100583253	2.025261039	0.042840567	0.118384938	positive
PRJNA729271	eed_Lushi_vs_Arbor_Acr	breed_growth_background	ENSGALG00010027908	PBXIP1	-0.121915076	0.056440117	-2.160078361	0.030766604	0.092657786	negative
PRJNA729271	eed_Lushi_vs_Arbor_Acr	breed_growth_background	ENSGALG00010027909	TIMP4	-0.185353733	0.137744832	-1.345631125	0.178421508	0.322632206	negative
PRJNA729271	eed_Lushi_vs_Arbor_Acr	breed_growth_background	ENSGALG00010027910	CHRNB2	0.264347674	0.108410538	2.438394644	0.014752658	0.054858846	positive
PRJNA729271	eed_Lushi_vs_Arbor_Acr	breed_growth_background	ENSGALG00010027911	PTPN22	0.350641779	0.201544732	1.739771497	0.081899149	0.187008218	positive
PRJNA729271	eed_Lushi_vs_Arbor_Acr	breed_growth_background	ENSGALG00010027912	RGS3	-0.245617873	0.070614304	-3.478301986	0.000504601	0.004994066	negative
PRJNA729271	eed_Lushi_vs_Arbor_Acr	breed_growth_background	ENSGALG00010027913	PYGO2	0.174801631	0.123093371	1.420073475	0.155586292	0.292641867	positive
PRJNA729271	eed_Lushi_vs_Arbor_Acr	breed_growth_background	ENSGALG00010027914	BTBD2	0.216137942	0.066999588	3.225959263	0.001255512	0.009628915	positive
PRJNA729271	eed_Lushi_vs_Arbor_Acr	breed_growth_background	ENSGALG00010027915	GSTM3	0.083970757	0.044959013	1.867717991	0.06180139	0.154157829	positive
PRJNA729271	eed_Lushi_vs_Arbor_Acr	breed_growth_background	ENSGALG00010027916	ADAR	-0.04217798	0.069302941	-0.608603029	0.542787596	0.687496173	negative
PRJNA729271	eed_Lushi_vs_Arbor_Acr	breed_growth_background	ENSGALG00010027917	PPARG	-0.516293464	0.242263743	-2.131121467	0.033079137	0.097713103	negative
PRJNA729271	eed_Lushi_vs_Arbor_Acr	breed_growth_background	ENSGALG00010027918	CSNK1G2	0.181350421	0.054146374	3.349262486	0.00081027	0.007024439	positive
PRJNA729271	eed_Lushi_vs_Arbor_Acr	breed_growth_background	ENSGALG00010027919	TSEN2	0.23013801	0.064820598	3.550383924	0.00038467	0.004130213	positive
PRJNA729271	eed_Lushi_vs_Arbor_Acr	breed_growth_background	ENSGALG00010027920	SCAMP4	0.05190006	0.06140624	0.84519195	0.398003676	0.559288733	positive
PRJNA729271	eed_Lushi_vs_Arbor_Acr	breed_growth_background	ENSGALG00010027921	MKRN2OS	-0.190511588	0.124301735	-1.532654293	0.125361057	0.251607382	negative
PRJNA729271	eed_Lushi_vs_Arbor_Acr	breed_growth_background	ENSGALG00010027922	SHC1	0.106482647	0.060907029	1.748281765	0.080415249	0.184637896	positive
PRJNA729271	eed_Lushi_vs_Arbor_Acr	breed_growth_background	ENSGALG00010027923	AMPD2	-0.186266735	0.086121722	-2.162831066	0.030554175	0.092209613	negative
PRJNA729271	eed_Lushi_vs_Arbor_Acr	breed_growth_background	ENSGALG00010027925	MKRN2	0.127514768	0.038108222	3.346122218	0.000819503	0.007066443	positive
PRJNA729271	eed_Lushi_vs_Arbor_Acr	breed_growth_background	ENSGALG00010027926	CKS1B	-0.149587738	0.136087283	-1.099204381	0.271678928	0.430400196	negative
PRJNA729271	eed_Lushi_vs_Arbor_Acr	breed_growth_background	ENSGALG00010027927	ENSGALG00010027927	0.224596645	0.104597642	2.14724386	0.031773873	0.09513784	positive
PRJNA729271	eed_Lushi_vs_Arbor_Acr	breed_growth_background	ENSGALG00010027928	FLAD1	0.068785383	0.0870543	0.790143433	0.429444007	0.588076607	positive
PRJNA729271	eed_Lushi_vs_Arbor_Acr	breed_growth_background	ENSGALG00010027929	CDK5RAP2	0.197754289	0.074022011	2.671560607	0.007549944	0.034209576	positive
PRJNA729271	eed_Lushi_vs_Arbor_Acr	breed_growth_background	ENSGALG00010027930	RAF1	0.0472901	0.04774634	0.990444504	0.321956902	0.485716621	positive
PRJNA729271	eed_Lushi_vs_Arbor_Acr	breed_growth_background	ENSGALG00010027931	ABHD17A	0.238510448	0.092400671	2.581263149	0.009843951	0.041360736	positive
PRJNA729271	eed_Lushi_vs_Arbor_Acr	breed_growth_background	ENSGALG00010027932	POLE3	-0.035874676	0.041901749	-0.856161788	0.391908296	0.553459737	negative
PRJNA729271	eed_Lushi_vs_Arbor_Acr	breed_growth_background	ENSGALG00010027933	ENSGALG00010027933	-0.010808671	0.113325367	-0.095377333	0.92401512	0.957557608	negative
PRJNA729271	eed_Lushi_vs_Arbor_Acr	breed_growth_background	ENSGALG00010027934	CNBP	0.05264886	0.045542185	1.156045967	0.247662355	0.403809431	positive
PRJNA729271	eed_Lushi_vs_Arbor_Acr	breed_growth_background	ENSGALG00010027935	ISY1	0.158183381	0.048075717	3.290296842	0.001000817	0.008154066	positive
PRJNA729271	eed_Lushi_vs_Arbor_Acr	breed_growth_background	ENSGALG00010027936	ENSGALG00010027936	0.078890385	0.046655138	1.690925977	0.090850941	0.201450683	positive
PRJNA729271	eed_Lushi_vs_Arbor_Acr	breed_growth_background	ENSGALG00010027937	HDHD3	0.044772294	0.050244317	0.891091707	0.372879974	0.535403191	positive
PRJNA729271	eed_Lushi_vs_Arbor_Acr	breed_growth_background	ENSGALG00010027938	ENSGALG00010027938	0.022276205	0.062287473	0.357635394	0.720616188	0.821140686	positive
PRJNA729271	eed_Lushi_vs_Arbor_Acr	breed_growth_background	ENSGALG00010027939	BSPRY	0.268553045	0.262677681	1.022367197	0.30660714	0.469250395	positive
PRJNA729271	eed_Lushi_vs_Arbor_Acr	breed_growth_background	ENSGALG00010027940	ZBTB7B	-0.29988764	0.213459899	-1.404889826	0.160054042	0.298882782	negative
PRJNA729271	eed_Lushi_vs_Arbor_Acr	breed_growth_background	ENSGALG00010027941	KLF2	-0.079615424	0.080859993	-0.984608349	0.324816487	0.488864643	negative
PRJNA729271	eed_Lushi_vs_Arbor_Acr	breed_growth_background	ENSGALG00010027942	KHDC4	0.12699345	0.066200352	1.918319876	0.055070464	0.142061999	positive
PRJNA729271	eed_Lushi_vs_Arbor_Acr	breed_growth_background	ENSGALG00010027943	RAB43	-0.025709682	0.051053461	-0.503583517	0.614554078	0.743755645	negative
PRJNA729271	eed_Lushi_vs_Arbor_Acr	breed_growth_background	ENSGALG00010027944	WDR31	0.106249682	0.167433586	0.634578071	0.525703634	0.672164578	positive
PRJNA729271	eed_Lushi_vs_Arbor_Acr	breed_growth_background	ENSGALG00010027946	AP1M1	-0.000755227	0.049919214	-0.015128977	0.987929283	0.992723394	negative
PRJNA729271	eed_Lushi_vs_Arbor_Acr	breed_growth_background	ENSGALG00010027947	GP9	0.068205645	0.190856206	0.357366662	0.720817331	0.82115647	positive
PRJNA729271	eed_Lushi_vs_Arbor_Acr	breed_growth_background	ENSGALG00010027948	PRPF4	0.062859466	0.061389985	1.023936815	0.30586512	0.468339428	positive
PRJNA729271	eed_Lushi_vs_Arbor_Acr	breed_growth_background	ENSGALG00010027950	CDC26	0.037282533	0.068875194	0.541305667	0.588296915	0.723946327	positive
PRJNA729271	eed_Lushi_vs_Arbor_Acr	breed_growth_background	ENSGALG00010027951	FAM32A	0.039260911	0.056056488	0.700381223	0.483689259	0.635867569	positive
PRJNA729271	eed_Lushi_vs_Arbor_Acr	breed_growth_background	ENSGALG00010027952	CIB3	-0.392523797	0.275511572	-1.424708931	0.154241356	0.29073192	negative
PRJNA729271	eed_Lushi_vs_Arbor_Acr	breed_growth_background	ENSGALG00010027953	SLC31A1	-0.111044735	0.065740918	-1.68912663	0.091195166	0.201830019	negative
PRJNA729271	eed_Lushi_vs_Arbor_Acr	breed_growth_background	ENSGALG00010027954	CFAP92	0.155259894	0.125877199	1.233423494	0.217417804	0.369327443	positive
PRJNA729271	eed_Lushi_vs_Arbor_Acr	breed_growth_background	ENSGALG00010027955	ENSGALG00010027955	-0.089420914	0.063327735	-1.412033992	0.157939953	0.295895	negative
PRJNA729271	eed_Lushi_vs_Arbor_Acr	breed_growth_background	ENSGALG00010027956	RAB8A	0.001701785	0.036411643	0.046737393	0.962722528	0.978835813	positive
PRJNA729271	eed_Lushi_vs_Arbor_Acr	breed_growth_background	ENSGALG00010027957	ENTREP3	-0.080779463	0.275282711	-0.293441831	0.769184455	0.856257482	negative
PRJNA729271	eed_Lushi_vs_Arbor_Acr	breed_growth_background	ENSGALG00010027958	ENSGALG00010027958	-0.022483286	0.074389468	-0.302237495	0.762471023	0.850678686	negative

PRJNA729271	ed_Lushi_vs_Arbor_Acr	breed_growth_background	ENSGALG00010027959	TPM4	-0.212344072	0.093307407	-2.275747227	0.022861143	0.075124069	negative
PRJNA729271	ed_Lushi_vs_Arbor_Acr	breed_growth_background	ENSGALG00010027960	PPP6C	0.096781548	0.06484793	1.492438501	0.135584228	0.265769548	positive
PRJNA729271	ed_Lushi_vs_Arbor_Acr	breed_growth_background	ENSGALG00010027961	TINCR	-0.198444524	0.527379675	-0.376283982	0.706705787	0.810740038	negative
PRJNA729271	ed_Lushi_vs_Arbor_Acr	breed_growth_background	ENSGALG00010027962	SCAMP3	0.07534249	0.06881355	1.094878692	0.273569785	0.432619904	positive
PRJNA729271	ed_Lushi_vs_Arbor_Acr	breed_growth_background	ENSGALG00010027964	RABEPK	-0.168955252	0.074383234	-2.27141578	0.02312182	0.075671886	negative
PRJNA729271	ed_Lushi_vs_Arbor_Acr	breed_growth_background	ENSGALG00010027965	ACAD9	-0.0493218	0.05843731	-0.844012157	0.398662612	0.559834777	negative
PRJNA729271	ed_Lushi_vs_Arbor_Acr	breed_growth_background	ENSGALG00010027966	CLK2	0.060472957	0.05405734	1.1186817	0.263275955	0.421398517	positive
PRJNA729271	ed_Lushi_vs_Arbor_Acr	breed_growth_background	ENSGALG00010027968	FKBP15	0.122757915	0.05225653	2.3491402	0.01881682	0.065505157	positive
PRJNA729271	ed_Lushi_vs_Arbor_Acr	breed_growth_background	ENSGALG00010027969	HSPA5	-0.063590746	0.069312113	-0.917455024	0.358904251	0.522397622	negative
PRJNA729271	ed_Lushi_vs_Arbor_Acr	breed_growth_background	ENSGALG00010027970	GRIN3B	0.037438112	0.096600752	0.387555079	0.698345317	0.805308638	positive
PRJNA729271	ed_Lushi_vs_Arbor_Acr	breed_growth_background	ENSGALG00010027971	ENSGALG00010027971	0.55077929	0.230721736	2.387201571	0.01697718	0.060851688	positive
PRJNA729271	ed_Lushi_vs_Arbor_Acr	breed_growth_background	ENSGALG00010027972	TMEM259	0.053148501	0.056936549	0.93346895	0.350577895	0.514490897	positive
PRJNA729271	ed_Lushi_vs_Arbor_Acr	breed_growth_background	ENSGALG00010027973	GAPVD1	0.003776177	0.034608601	0.109110931	0.913114505	0.951037921	positive
PRJNA729271	ed_Lushi_vs_Arbor_Acr	breed_growth_background	ENSGALG00010027974	ENSGALG00010027974	0.022828509	0.169720404	0.134506568	0.893002018	0.937432231	positive
PRJNA729271	ed_Lushi_vs_Arbor_Acr	breed_growth_background	ENSGALG00010027975	IQSEC1	-0.267835982	0.063497212	-4.218074686	2.46397E-05	0.000562193	negative
PRJNA729271	ed_Lushi_vs_Arbor_Acr	breed_growth_background	ENSGALG00010027976	LRSAM1	-0.32057016	0.052835198	-6.067359873	1.3003E-09	2.65463E-07	negative
PRJNA729271	ed_Lushi_vs_Arbor_Acr	breed_growth_background	ENSGALG00010027977	BOLA1	-0.170396665	0.083535967	-2.039799997	0.04137025	0.115186665	negative
PRJNA729271	ed_Lushi_vs_Arbor_Acr	breed_growth_background	ENSGALG00010027979	RPL12	-0.116095517	0.063920681	-1.81624344	0.069333037	0.166802054	negative
PRJNA729271	ed_Lushi_vs_Arbor_Acr	breed_growth_background	ENSGALG00010027980	NUP210	0.017586467	0.075806485	0.231991593	0.816544544	0.886909941	positive
PRJNA729271	ed_Lushi_vs_Arbor_Acr	breed_growth_background	ENSGALG00010027981	CNN2	0.133686738	0.087999209	1.519181134	0.128716909	0.256108661	positive
PRJNA729271	ed_Lushi_vs_Arbor_Acr	breed_growth_background	ENSGALG00010027982	ENTPD2	-0.392775719	0.133029814	-2.952539049	0.003151722	0.018383988	negative
PRJNA729271	ed_Lushi_vs_Arbor_Acr	breed_growth_background	ENSGALG00010027983	POGZ	0.183140101	0.078523723	2.332290082	0.019685436	0.067520207	positive
PRJNA729271	ed_Lushi_vs_Arbor_Acr	breed_growth_background	ENSGALG00010027984	ARHGAP45	0.029731509	0.090147482	0.329809642	0.741543801	0.834913578	positive
PRJNA729271	ed_Lushi_vs_Arbor_Acr	breed_growth_background	ENSGALG00010027985	PSMB4	0.005725059	0.050754776	0.112798434	0.91019036	0.949015197	positive
PRJNA729271	ed_Lushi_vs_Arbor_Acr	breed_growth_background	ENSGALG00010027986	CGN	0.320899387	0.149363306	2.148448605	0.031678135	0.094925253	positive
PRJNA729271	ed_Lushi_vs_Arbor_Acr	breed_growth_background	ENSGALG00010027987	TMEM141	0.07763658	0.074426085	1.04313668	0.296884995	0.45863242	positive
PRJNA729271	ed_Lushi_vs_Arbor_Acr	breed_growth_background	ENSGALG00010027988	HDAC11	0.171762606	0.069126749	2.4847488	0.012964288	0.050205011	positive
PRJNA729271	ed_Lushi_vs_Arbor_Acr	breed_growth_background	ENSGALG00010027989	ENSGALG00010027989	0.011804307	0.075170272	0.157034251	0.875217855	0.926392097	positive
PRJNA729271	ed_Lushi_vs_Arbor_Acr	breed_growth_background	ENSGALG00010027990	ENSGALG00010027990	0.332002621	0.102680831	3.233345661	0.001223494	0.009473584	positive
PRJNA729271	ed_Lushi_vs_Arbor_Acr	breed_growth_background	ENSGALG00010027991	ENSGALG00010027991	-0.180105382	0.078543015	-2.293079538	0.021843426	0.072693436	negative
PRJNA729271	ed_Lushi_vs_Arbor_Acr	breed_growth_background	ENSGALG00010027993	CLIC3	0.192301148	0.089447815	2.149869703	0.031565523	0.094650859	positive
PRJNA729271	ed_Lushi_vs_Arbor_Acr	breed_growth_background	ENSGALG00010027994	TUFT1	0.02118957	0.091114979	0.232558577	0.816104196	0.886680308	positive
PRJNA729271	ed_Lushi_vs_Arbor_Acr	breed_growth_background	ENSGALG00010027995	POLR2E	-0.142295822	0.044369881	-3.207036375	0.0013411	0.010093068	negative
PRJNA729271	ed_Lushi_vs_Arbor_Acr	breed_growth_background	ENSGALG00010027996	GPX4	-0.022780782	0.065232346	-0.349225244	0.726920216	0.824527408	negative
PRJNA729271	ed_Lushi_vs_Arbor_Acr	breed_growth_background	ENSGALG00010027998	SNX27	0.13177218	0.04950226	2.661942693	0.00776911	0.03492967	positive
PRJNA729271	ed_Lushi_vs_Arbor_Acr	breed_growth_background	ENSGALG00010027999	ENSGALG00010027999	0.959762541	0.302846317	3.169140536	0.001528904	0.011031095	positive
PRJNA729271	ed_Lushi_vs_Arbor_Acr	breed_growth_background	ENSGALG00010028000	MRPL9	0.214279315	0.088812688	2.412710607	0.015834386	0.057930194	positive
PRJNA729271	ed_Lushi_vs_Arbor_Acr	breed_growth_background	ENSGALG00010028001	SBNO2	-0.02374274	0.063041383	-0.376621486	0.706454918	0.810580546	negative
PRJNA729271	ed_Lushi_vs_Arbor_Acr	breed_growth_background	ENSGALG00010028002	ABCA2	0.102909646	0.064812595	1.587803194	0.112330849	0.233629223	positive
PRJNA729271	ed_Lushi_vs_Arbor_Acr	breed_growth_background	ENSGALG00010028004	ENSGALG00010028004	0.368042804	0.17028026	2.161394425	0.030664884	0.092462558	positive
PRJNA729271	ed_Lushi_vs_Arbor_Acr	breed_growth_background	ENSGALG00010028005	IER5L	0.196818116	0.076919586	2.558751636	0.010504876	0.043322608	positive
PRJNA729271	ed_Lushi_vs_Arbor_Acr	breed_growth_background	ENSGALG00010028006	ENSGALG00010028006	0.105221432	0.359681712	0.292540401	0.769873475	0.856557109	positive
PRJNA729271	ed_Lushi_vs_Arbor_Acr	breed_growth_background	ENSGALG00010028007	STK11	0.061291232	0.063846876	0.959972286	0.337069163	0.5013666	positive
PRJNA729271	ed_Lushi_vs_Arbor_Acr	breed_growth_background	ENSGALG00010028008	NTMT1	0.0650925	0.060423462	1.077271947	0.281358791	0.441326228	positive
PRJNA729271	ed_Lushi_vs_Arbor_Acr	breed_growth_background	ENSGALG00010028009	RRP9	-0.134308629	0.096636879	-1.389827888	0.164581147	0.304597004	negative
PRJNA729271	ed_Lushi_vs_Arbor_Acr	breed_growth_background	ENSGALG00010028010	ASB6	0.157967517	0.040214033	3.92816899	8.55951E-05	0.001427733	positive
PRJNA729271	ed_Lushi_vs_Arbor_Acr	breed_growth_background	ENSGALG00010028011	ENSGALG00010028011	-0.026263894	0.051712326	-0.50788461	0.611534268	0.741565902	negative
PRJNA729271	ed_Lushi_vs_Arbor_Acr	breed_growth_background	ENSGALG00010028012	PRRX2	-0.068698605	0.138808597	-0.494916071	0.620659366	0.748146154	negative
PRJNA729271	ed_Lushi_vs_Arbor_Acr	breed_growth_background	ENSGALG00010028013	CBARP	0.567062622	0.116113745	4.883682121	1.04123E-06	5.03087E-05	positive
PRJNA729271	ed_Lushi_vs_Arbor_Acr	breed_growth_background	ENSGALG00010028014	ENSGALG00010028014	0.365632366	0.15656184	2.335386239	0.019523253	0.067154615	positive
PRJNA729271	ed_Lushi_vs_Arbor_Acr	breed_growth_background	ENSGALG00010028015	DUSP7	-0.181767697	0.054794371	-3.317269497	0.000909019	0.007589993	negative
PRJNA729271	ed_Lushi_vs_Arbor_Acr	breed_growth_background	ENSGALG00010028016	PRF1	0.663206137	0.621585197	1.066959349	0.285990193	0.446609335	positive
PRJNA729271	ed_Lushi_vs_Arbor_Acr	breed_growth_background	ENSGALG00010028017	ENSGALG00010028017	0.001444412	0.713399616	0.002024688	0.998384534	0.999025066	positive
PRJNA729271	ed_Lushi_vs_Arbor_Acr	breed_growth_background	ENSGALG00010028019	RPL29	-0.176531442	0.121402416	-1.454101562	0.145918151	0.280143522	negative
PRJNA729271	ed_Lushi_vs_Arbor_Acr	breed_growth_background	ENSGALG00010028020	ENSGALG00010028020	-0.354342254	0.080849068	-4.382762359	1.17184E-05	0.00032665	negative
PRJNA729271	ed_Lushi_vs_Arbor_Acr	breed_growth_background	ENSGALG00010028021	ATP5F1D	-0.00316952	0.07755103	-0.040870115	0.967399442	0.981552213	negative
PRJNA729271	ed_Lushi_vs_Arbor_Acr	breed_growth_background	ENSGALG00010028022	ENSGALG00010028022	0.277874465	0.077369252	3.591536137	0.000328735	0.003682386	positive
PRJNA729271	ed_Lushi_vs_Arbor_Acr	breed_growth_background	ENSGALG00010028023	ENSGALG00010028023	0.383793214	0.493111786	0.778308742	0.436387023	0.594045778	positive
PRJNA729271	ed_Lushi_vs_Arbor_Acr	breed_growth_background	ENSGALG00010028024	RBM3	-0.311760947	0.060462089	-5.156304607	2.51871E-07	1.69498E-05	negative
PRJNA729271	ed_Lushi_vs_Arbor_Acr	breed_growth_background	ENSGALG00010028025	TNNC1	-0.159830849	0.126728463	-1.261207197	0.207234206	0.356287489	negative
PRJNA729271	ed_Lushi_vs_Arbor_Acr	breed_growth_background	ENSGALG00010028026	ANKMY1	0.072443868	0.089941955	0.80545134	0.420559276	0.579688731	positive
PRJNA729271	ed_Lushi_vs_Arbor_Acr	breed_growth_background	ENSGALG00010028027	ENSGALG00010028027	0.480916266	0.12310711	3.90648654	9.36479E-05	0.001521778	positive
PRJNA729271	ed_Lushi_vs_Arbor_Acr	breed_growth_background	ENSGALG00010028028	FAM174C	-0.051149254	0.09035373	-0.566100083	0.571325747	0.70962868	negative
PRJNA729271	ed_Lushi_vs_Arbor_Acr	breed_growth_background	ENSGALG00010028029	EFNA2	-0.033759675	0.092971429	-0.363118815	0.71651613	0.818302971	negative
PRJNA729271	ed_Lushi_vs_Arbor_Acr	breed_growth_background	ENSGALG00010028030	PFDN2	0.341670243	0.072521616	4.711288359	2.46156E-06	9.74871E-05	positive
PRJNA729271	ed_Lushi_vs_Arbor_Acr	breed_growth_background	ENSGALG00010028031	NISCH	0.076184609	0.043328455	1.758304299	0.078695747	0.18187099	positive
PRJNA729271	ed_Lushi_vs_Arbor_Acr	breed_growth_background	ENSGALG00010028032	ENSGALG00010028032	-0.267310706	0.145465933	-1.837617246	0.066118826	0.161641952	negative
PRJNA729271	ed_Lushi_vs_Arbor_Acr	breed_growth_background	ENSGALG00010028033	ENSGALG00010028033	0.085617831	0.144137289	0.594001949	0.552510809	0.69519148	positive
PRJNA729271	ed_Lushi_vs_Arbor_Acr	breed_growth_background	ENSGALG00010028034	MUM1L1	0.208655154	0.04223926	4.9398392	7.8187E-07	3.99057E-05	positive
PRJNA729271	ed_Lushi_vs_Arbor_Acr	breed_growth_background	ENSGALG00010028035	ENSGALG00010028035	1.557831204	0.570449822	2.730882091	0.006316507	0.030087993	positive
PRJNA729271	ed_Lushi_vs_Arbor_Acr	breed_growth_background	ENSGALG00010028036	NDUFS7	0.083477333	0.06516146	1.281084461	0.200164001	0.349016865	positive
PRJNA729271	ed_Lushi_vs_Arbor_Acr	breed_growth_background	ENSGALG00010028037	ENSGALG00010028037	0.46214698	0.15364548	3.007878791	0.00263078	0.016117144	positive
PRJNA729271	ed_Lushi_vs_Arbor_Acr	breed_growth_background	ENSGALG00010028039	STAB1	-0.258929748	0.086698788	-2.98654402	0.002821502	0.016969989	negative
PRJNA729271	ed_Lushi_vs_Arbor_Acr	breed_growth_background	ENSGALG00010028042	DPP7	0.491795157	0.102644552	4.79124462	1.6575E-06	7.32232E-05	positive
PRJNA729271	ed_Lushi_vs_Arbor_Acr	breed_growth_background	ENSGALG00010028043	NT5DC2	0.159856931	0.061799947	2.586683926	0.009690444	0.040891698	positive
PRJNA729271	ed_Lushi_vs_Arbor_Acr	breed_growth_background	ENSGALG00010028044	GAMT	-0.004870268	0.081527899	-0.059737445	0.952364748	0.973433394	negative
PRJNA729271	ed_Lushi_vs_Arbor_Acr	breed_growth_background	ENSGALG00010028045	ENSGALG00010028045	0.415225838	0.338826809	1.225481064	0.22039402	0.37294085	positive
PRJNA729271	ed_Lushi_vs_Arbor_Acr	breed_growth_background	ENSGALG00010028046	SMIM4	-0.096787759	0.108352873	-0.89326436	0.371715619	0.534463681	negative
PRJNA729271	ed_Lushi_vs_Arbor_Acr	breed_growth_background	ENSGALG00010028047	ENSGALG00010028047	-0.655962663	0.154179213	-4.254546698	2.09473E-05	0.000496941	negative
PRJNA729271	ed_Lushi_vs_Arbor_Acr	breed_growth_background	ENSGALG00010028048	S100A10	-0.168551095	0.11675848	-1.443587611	0.148855012	0.28378974	negative
PRJNA729271	ed_Lushi_vs_Arbor_Acr	breed_growth_background	ENSGALG00010028049	MAN1B1	-0.089341179	0.032708071	-2.73147199	0.00630521	0.030053936	negative
PRJNA729271	ed_Lushi_vs_Arbor_Acr	breed_growth_background	ENSGALG00010028050	PBRM1	0.128994005	0.047196296	2.733138316	0.006273398	0.029931501	positive
PRJNA729271	ed_Lushi_vs_Arbor_Acr	breed_growth_background	ENSGALG00010028051	GNL3	0.049437753	0.050032123	0.988120215	0.323093777	0.486995449	positive
PRJNA729271	ed_Lushi_vs_Arbor_Acr	breed_growth_background	ENSGALG00010028052	ENSGALG00010028052	-0.198280443	0.118967612	-1.666675833	0.095578881	0.208231947	negative
PRJNA729271	ed_Lushi_vs_Arbor_Acr	breed_growth_background	ENSGALG00010028053	ORM1	-3.544881275	1.172412935	-3.023577418	0.002498051	0.015573871	negative
PRJNA729271	ed_Lushi_vs_Arbor_Acr	breed_growth_background	ENSGALG00010028054	RPS15	-0.098137884	0.075270059	-1.30381037	0.192298246	0.33917779	negative
PRJNA729271	ed_Lushi_vs_Arbor_Acr	breed_growth_background	ENSGALG00010028055	ENSGALG00010028055	-0.156240929	0.063479395	-2.461285742	0.013844006	0.052626718	negative
PRJNA729271	ed_Lushi_vs_Arbor_Acr	breed_growth_background	ENSGALG00010028056	SLC25A25	-0.073121237	0.126995658	-0.57577746	0.564765611	0.704723648	negative
PRJNA729271	ed_Lushi_vs_Arbor_Acr	breed_growth_background	ENSGALG00010028057	APC2	-0.29982252	0.210302965	-1.425669485	0.153963769	0.290408079	negative
PRJNA729271	ed_Lushi_vs_Arbor_Acr	breed_growth_background	ENSGALG00010028058	GLT8D1	-0.025879403	0.062129139	-0.416542126	0.677013348	0.791395845	negative
PRJNA729271	ed_Lushi_vs_Arbor_Acr	breed_growth_background	ENSGALG00010028059	GZMM	1.042409248	0.315437338	3.304647623	0.000950959	0.007860563	positive
PRJNA729271	ed_Lushi_vs_Arbor_Acr	breed_growth_background	ENSGALG00010028060	ENSGALG00010028060	0.175139761	0.0760927	2.301663107	0.021354178	0.071470486	positive
PRJNA729271	ed_Lushi_vs_Arbor_Acr	breed_growth_background	ENSGALG00010028061	NAIF1	0.063588029	0.051539609	1.233770106	0.217288582	0.369237748	positive
PRJNA729271	ed_Lushi_vs_Arbor_Acr	breed_growth_background	ENSGALG00010028062	ENSGALG00010028062	-1.534287139	0.262359442	-5.848034771	4.97415E-09	7.5895E-07	negative
PRJNA729271	ed_Lushi_vs_Arbor_Acr	breed_growth_background	ENSGALG00010028064	SPCS1	0.000520616	0.047279936	0.011011357	0.991214386	0.995105645	positive
PRJNA729271	ed_Lushi_vs_Arbor_Acr	breed_growth_background	ENSGALG00010028065	REEP6	1.471733699	0.396721596	3.70973931	0.000207473	0.002668427	positive
PRJNA729271	ed_Lushi_vs_Arbor_Acr	breed_growth_background	ENSGALG00010028066	ENSGALG00010028066	0.117761203	0.095851699	1.228577106	0.219230404	0.371624188	positive
PRJNA729271	ed_Lushi_vs_Arbor_Acr	breed_growth_background	ENSGALG00010028067	FAM102A	-0.032703311	0.060933999	-0.536700562	0.591474468	0.726253486	negative
PRJNA729271	ed_Lushi_vs_Arbor_Acr	breed_growth_background	ENSGALG00010028068	POLE4	-0.099746334	0.060413059	-1.651072414	0.098723789	0.212946626	negative
PRJNA729271	ed_Lushi_vs_Arbor_Acr	breed_growth_background	ENSGALG00010028069	NEK4	0.065422119	0.06260833	1.044942734	0.296049437	0.457727506	positive
PRJNA729271	ed_Lushi_vs_Arbor_Acr	breed_growth_background	ENSGALG00010028070	MEX3A	-0.043395219	0.08194597	-0.529558913	0.596417788	0.730096768	negative
PRJNA729271	ed_Lushi_vs_Arbor_Acr	breed_growth_background	ENSGALG00010028071	DPM2	-0.017382025	0.062514471	-0.278048021	0.780975496	0.864404385	negative
PRJNA729271	ed_Lushi_vs_Arbor_Acr	breed_growth_background	ENSGALG00010028072	ENSGALG00010028072	-0.344400556	0.437766464	-0.78672211	0.431444558	0.589757532	negative
PRJNA729271	ed_Lushi_vs_Arbor_Acr	breed_growth_background	ENSGALG00010028073	ST6GALNAC4	0.146801884	0.062835437	2.336291299	0.019476065	0.067087823	positive
PRJNA729271	ed_Lushi_vs_Arbor_Acr	breed_growth_background	ENSGALG00010028074	ENSGALG00010028074	-0.166101003	0.242454789	-0.685080315	0.493293242	0.644925186	negative
PRJNA729271	ed_Lushi_vs_Arbor_Acr	breed_growth_background	ENSGALG00010028075	RAB25	0.428868314	0.29149588	1.471267155	0.141218882	0.273841832	positive
PRJNA729271	ed_Lushi_vs_Arbor_Acr	breed_growth_background	ENSGALG00010028076	ST6GALNAC6	0.009687228	0.081195207	0.119307883	0.905031438	0.945468208	positive
PRJNA729271	ed_Lushi_vs_Arbor_Acr	breed_growth_background	ENSGALG00010028078	LAMTOR2	-0.154351276	0.069856664	-2.209542603	0.027136923	0.084573144	negative
PRJNA729271	ed_Lushi_vs_Arbor_Acr	breed_growth_background	ENSGALG00010028079	ITIH3	-1.961075784	0.794525293	-2.468235815	0.013578084	0.051888828	negative
PRJNA729271	ed_Lushi_vs_Arbor_Acr	breed_growth_background	ENSGALG00010028080	MUSTN1	-0.540721863	0.21637843	-2.498963795	0.012455704	0.048822451	negative
PRJNA729271	ed_Lushi_vs_Arbor_Acr	breed_growth_background	ENSGALG00010028081	UBQLN4	0.172205558	0.087087807	1.977378514	0.047998861	0.128621324	positive
PRJNA729271	ed_Lushi_vs_Arbor_Acr	breed_growth_background	ENSGALG00010028082	AK1	0.195191856	0.072867386	2.678727301	0.007390255	0.033675496	positive
PRJNA729271	ed_Lushi_vs_Arbor_Acr	breed_growth_background	ENSGALG00010028083	ELAVL1	0.006651779	0.026680816	0.249309427	0.80312144	0.878475884	positive
PRJNA729271	ed_Lushi_vs_Arbor_Acr	breed_growth_background	ENSGALG00010028084	ENG	-0.027801267	0.103032603	-0.269829803	0.787291193	0.867849953	negative
PRJNA729271	ed_Lushi_vs_Arbor_Acr	breed_growth_background	ENSGALG00010028085	ENSGALG00010028085	0.130302277	0.15654547	0.832360573	0.40520545	0.566061391	positive
PRJNA729271	ed_Lushi_vs_Arbor_Acr	breed_growth_background	ENSGALG00010028086	MATK	-0.421266315	0.150902056	-2.791653909	0.005243941	0.026411024	negative
PRJNA729271	ed_Lushi_vs_Arbor_Acr	breed_growth_background	ENSGALG00010028087	STIMATE	-0.130541521	0.048705947	-2.680196716	0.007357891	0.033549111	negative
PRJNA729271	ed_Lushi_vs_Arbor_Acr	breed_growth_background	ENSGALG00010028088	NAXE	-0.009589649	0.061319883	-0.156387266	0.875727774	0.926613189	negative
PRJNA729271	ed_Lushi_vs_Arbor_Acr	breed_growth_background	ENSGALG00010028091	FPGS	-0.083107237	0.054646724	-1.520809134	0.128307742	0.255504975	negative
PRJNA729271	ed_Lushi_vs_Arbor_Acr	breed_growth_background	ENSGALG00010028093	SFMBT1	-0.087812981	0.078456081	-1.119262904	0.263027997	0.421229946	negative
PRJNA729271	ed_Lushi_vs_Arbor_Acr	breed_growth_background	ENSGALG00010028095	CDK9	0.090840581	0.050983553	1.781762456	0.074787977	0.175925305	positive
PRJNA729271	ed_Lushi_vs_Arbor_Acr	breed_growth_background	ENSGALG00010028096	TOR2A	0.073706111	0.082801523	0.89015405	0.373383174	0.535807257	positive
PRJNA729271	ed_Lushi_vs_Arbor_Acr	breed_growth_background	ENSGALG00010028097	TTC24	-0.086242127	0.115407279	-0.747284984	0.454891555	0.611020944	negative
PRJNA729271	ed_Lushi_vs_Arbor_Acr	breed_growth_background	ENSGALG00010028098	RFT1	-0.111903406	0.065317381	-1.713225555	0.086671052	0.194774713	negative
PRJNA729271	ed_Lushi_vs_Arbor_Acr	breed_growth_background	ENSGALG00010028099	PCASP2	-0.002497703	0.129817262	-0.019240146	0.984649531	0.990870241	negative
PRJNA729271	ed_Lushi_vs_Arbor_Acr	breed_growth_background	ENSGALG00010028100	ENSGALG00010028100	0.280297664	0.237151283	1.181936109	0.237231053	0.39231764	positive
PRJNA729271	ed_Lushi_vs_Arbor_Acr	breed_growth_background	ENSGALG00010028101	MRPL54	-0.006720118	0.068010762	-0.09880963	0.921289422	0.956185749	negative
PRJNA729271	ed_Lushi_vs_Arbor_Acr	breed_growth_background	ENSGALG00010028102	CERCAM	-0.244463966	0.096554144	-2.531884758	0.011345127	0.045756154	negative
PRJNA729271	ed_Lushi_vs_Arbor_Acr	breed_growth_background	ENSGALG00010028103	APBA3	0.179149521	0.070929681	2.525734182	0.01154568	0.046320132	positive
PRJNA729271	ed_Lushi_vs_Arbor_Acr	breed_growth_background	ENSGALG00010028104	GOLGA1	0.151672432	0.061855922	2.452027674	0.014205374	0.053412942	positive
PRJNA729271	ed_Lushi_vs_Arbor_Acr	breed_growth_background	ENSGALG00010028105	PRKCD	-0.159729816	0.077568475	-2.059210488	0.039474079	0.111383448	negative

PRJNA729271	ed_Lushi_vs_Arbor_Acr	breed_growth_background	ENSGALG00010028106	ARPC5L	0.046019574	0.057145297	0.80530815	0.42064188	0.579747462	positive
PRJNA729271	ed_Lushi_vs_Arbor_Acr	breed_growth_background	ENSGALG00010028107	NOTCH1	-0.078676179	0.066725363	-1.179104533	0.238356554	0.393639996	negative
PRJNA729271	ed_Lushi_vs_Arbor_Acr	breed_growth_background	ENSGALG00010028108	IQGAP3	-0.282754775	0.114927456	-2.460289175	0.013882511	0.052704818	negative
PRJNA729271	ed_Lushi_vs_Arbor_Acr	breed_growth_background	ENSGALG00010028109	TJP3	0.362913928	0.119546658	3.035751332	0.002399372	0.015154201	positive
PRJNA729271	ed_Lushi_vs_Arbor_Acr	breed_growth_background	ENSGALG00010028110	COQ4	-0.099176802	0.054612855	-1.815997381	0.069370774	0.166837459	negative
PRJNA729271	ed_Lushi_vs_Arbor_Acr	breed_growth_background	ENSGALG00010028111	TRUB2	-0.045012566	0.049425917	-0.910707766	0.362449371	0.525790996	negative
PRJNA729271	ed_Lushi_vs_Arbor_Acr	breed_growth_background	ENSGALG00010028112	SWI5	-0.030620275	0.044350507	-0.69041543	0.489932976	0.641747559	negative
PRJNA729271	ed_Lushi_vs_Arbor_Acr	breed_growth_background	ENSGALG00010028113	PIP5K1C	-0.113226656	0.053177005	-2.129240953	0.03323433	0.098012537	negative
PRJNA729271	ed_Lushi_vs_Arbor_Acr	breed_growth_background	ENSGALG00010028114	PFKFB4	-0.167673743	0.048909947	-3.428213568	0.000607567	0.005677469	negative
PRJNA729271	ed_Lushi_vs_Arbor_Acr	breed_growth_background	ENSGALG00010028115	CACTIN	0.376436545	0.093816941	4.012458096	6.00897E-05	0.001102533	positive
PRJNA729271	ed_Lushi_vs_Arbor_Acr	breed_growth_background	ENSGALG00010028116	HYAL1	0.034472034	0.078696345	0.438038572	0.661358323	0.780332836	positive
PRJNA729271	ed_Lushi_vs_Arbor_Acr	breed_growth_background	ENSGALG00010028117	ENSGALG00010028117	0.40028632	0.128572967	3.11330079	0.001850074	0.012565983	positive
PRJNA729271	ed_Lushi_vs_Arbor_Acr	breed_growth_background	ENSGALG00010028118	ENSGALG00010028118	0.11960096	0.041581808	2.876280919	0.004023914	0.021723142	positive
PRJNA729271	ed_Lushi_vs_Arbor_Acr	breed_growth_background	ENSGALG00010028119	HYAL2	-0.115740483	0.06852255	-1.68908605	0.091202941	0.201830019	negative
PRJNA729271	ed_Lushi_vs_Arbor_Acr	breed_growth_background	ENSGALG00010028120	TBXA2R	-0.046936751	0.13405646	-0.350126734	0.726243588	0.824474505	negative
PRJNA729271	ed_Lushi_vs_Arbor_Acr	breed_growth_background	ENSGALG00010028121	TUSC2	0.074972544	0.051857619	1.445738267	0.148250627	0.283179482	positive
PRJNA729271	ed_Lushi_vs_Arbor_Acr	breed_growth_background	ENSGALG00010028122	SEC16A	0.084009936	0.047593238	1.765165378	0.077535975	0.179925599	positive
PRJNA729271	ed_Lushi_vs_Arbor_Acr	breed_growth_background	ENSGALG00010028123	RASSF1	-0.214216596	0.057567528	-3.721135901	0.000198329	0.002575962	negative
PRJNA729271	ed_Lushi_vs_Arbor_Acr	breed_growth_background	ENSGALG00010028124	SUGP2	-0.087987047	0.048498731	-1.814213377	0.069644882	0.167385601	negative
PRJNA729271	ed_Lushi_vs_Arbor_Acr	breed_growth_background	ENSGALG00010028125	HMG20B	-0.147637992	0.066735954	-2.212270637	0.026947971	0.08418337	negative
PRJNA729271	ed_Lushi_vs_Arbor_Acr	breed_growth_background	ENSGALG00010028126	ZMYND10	0.054520825	0.044564742	1.223407171	0.221175946	0.373609759	positive
PRJNA729271	ed_Lushi_vs_Arbor_Acr	breed_growth_background	ENSGALG00010028127	MFSD12	-0.071925261	0.095256787	-0.755067045	0.450208755	0.60648475	negative
PRJNA729271	ed_Lushi_vs_Arbor_Acr	breed_growth_background	ENSGALG00010028128	DNM1	-0.272826894	0.077507363	-3.520012597	0.000431526	0.004466547	negative
PRJNA729271	ed_Lushi_vs_Arbor_Acr	breed_growth_background	ENSGALG00010028129	CYB561D2	0.003087772	0.056714322	0.054444312	0.956581176	0.97569799	positive
PRJNA729271	ed_Lushi_vs_Arbor_Acr	breed_growth_background	ENSGALG00010028130	TMEM115	0.195944041	0.067933597	2.88434662	0.003922268	0.021394964	positive
PRJNA729271	ed_Lushi_vs_Arbor_Acr	breed_growth_background	ENSGALG00010028131	TMEM161A	0.004226752	0.108793546	0.038851131	0.969009079	0.982376831	positive
PRJNA729271	ed_Lushi_vs_Arbor_Acr	breed_growth_background	ENSGALG00010028132	CACNA2D2	0.078170449	0.071188862	1.098071348	0.272173335	0.430975803	positive
PRJNA729271	ed_Lushi_vs_Arbor_Acr	breed_growth_background	ENSGALG00010028133	ENSGALG00010028133	0.601438518	0.166879222	3.60403476	0.000313315	0.003564759	positive
PRJNA729271	ed_Lushi_vs_Arbor_Acr	breed_growth_background	ENSGALG00010028134	INPP5E	0.035064905	0.039900897	0.878799934	0.379509762	0.541436417	positive
PRJNA729271	ed_Lushi_vs_Arbor_Acr	breed_growth_background	ENSGALG00010028136	C3orf18	-0.365656134	0.087730119	-4.167965779	3.0733E-05	0.000679078	negative
PRJNA729271	ed_Lushi_vs_Arbor_Acr	breed_growth_background	ENSGALG00010028137	NUP214	-0.038256837	0.036207212	-1.056608198	0.290690437	0.452146999	negative
PRJNA729271	ed_Lushi_vs_Arbor_Acr	breed_growth_background	ENSGALG00010028138	ENSGALG00010028138	0.413898682	0.284781391	1.453390899	0.146115252	0.280410509	positive
PRJNA729271	ed_Lushi_vs_Arbor_Acr	breed_growth_background	ENSGALG00010028139	FZR1	0.018855445	0.047811212	0.394372877	0.693305766	0.802096502	positive
PRJNA729271	ed_Lushi_vs_Arbor_Acr	breed_growth_background	ENSGALG00010028140	HEMK1	-0.243318592	0.061210265	-3.975127219	7.03416E-05	0.001226958	negative
PRJNA729271	ed_Lushi_vs_Arbor_Acr	breed_growth_background	ENSGALG00010028141	CISH	0.161603093	0.099514422	1.623916309	0.104393671	0.221780289	positive
PRJNA729271	ed_Lushi_vs_Arbor_Acr	breed_growth_background	ENSGALG00010028142	PMPCA	0.152070231	0.065418453	2.324576985	0.020094591	0.068502138	positive
PRJNA729271	ed_Lushi_vs_Arbor_Acr	breed_growth_background	ENSGALG00010028144	FAM78A	0.203700647	0.098459925	2.068868595	0.038558419	0.109632069	positive
PRJNA729271	ed_Lushi_vs_Arbor_Acr	breed_growth_background	ENSGALG00010028145	ENSGALG00010028145	0.091569205	0.076399354	1.198559936	0.230699105	0.384542723	positive
PRJNA729271	ed_Lushi_vs_Arbor_Acr	breed_growth_background	ENSGALG00010028146	MAPKAPK3	-0.019239732	0.056210107	-0.342282434	0.732138354	0.828240196	negative
PRJNA729271	ed_Lushi_vs_Arbor_Acr	breed_growth_background	ENSGALG00010028147	SLC25A42	-0.015041439	0.105777671	-0.142198626	0.886923117	0.933484649	negative
PRJNA729271	ed_Lushi_vs_Arbor_Acr	breed_growth_background	ENSGALG00010028148	ENSGALG00010028148	0.597017268	0.11101329	5.377890072	7.53638E-08	6.58071E-06	positive
PRJNA729271	ed_Lushi_vs_Arbor_Acr	breed_growth_background	ENSGALG00010028149	SNAPC4	0.074469144	0.048722022	1.528449361	0.126401007	0.252967135	positive
PRJNA729271	ed_Lushi_vs_Arbor_Acr	breed_growth_background	ENSGALG00010028150	ARMC6	0.074955763	0.044395708	1.688356046	0.091342903	0.201966932	positive
PRJNA729271	ed_Lushi_vs_Arbor_Acr	breed_growth_background	ENSGALG00010028151	DOCK3	0.591896365	0.137220841	4.313458216	1.60721E-05	0.000408413	positive
PRJNA729271	ed_Lushi_vs_Arbor_Acr	breed_growth_background	ENSGALG00010028152	ENSGALG00010028152	0.429975026	0.198172772	2.169697791	0.030029747	0.090910858	positive
PRJNA729271	ed_Lushi_vs_Arbor_Acr	breed_growth_background	ENSGALG00010028153	MANF	-0.050372848	0.079497146	-0.633643483	0.526313517	0.672565849	negative
PRJNA729271	ed_Lushi_vs_Arbor_Acr	breed_growth_background	ENSGALG00010028154	ENSGALG00010028154	-0.008844831	0.073007378	-0.121149828	0.903572363	0.944629023	negative
PRJNA729271	ed_Lushi_vs_Arbor_Acr	breed_growth_background	ENSGALG00010028155	CIZ1	0.033224148	0.042636439	0.779243042	0.43583656	0.59359542	positive
PRJNA729271	ed_Lushi_vs_Arbor_Acr	breed_growth_background	ENSGALG00010028156	DOHH	0.026532625	0.052320652	0.50711571	0.612073633	0.741913407	positive
PRJNA729271	ed_Lushi_vs_Arbor_Acr	breed_growth_background	ENSGALG00010028157	PRRC2B	0.234498137	0.077665901	3.019319096	0.002533435	0.015720097	positive
PRJNA729271	ed_Lushi_vs_Arbor_Acr	breed_growth_background	ENSGALG00010028158	DDX49	0.121069771	0.053697885	2.254646898	0.024155501	0.078060676	positive
PRJNA729271	ed_Lushi_vs_Arbor_Acr	breed_growth_background	ENSGALG00010028159	DCAF1	0.100153082	0.048510815	2.064551629	0.038965444	0.110307844	positive
PRJNA729271	ed_Lushi_vs_Arbor_Acr	breed_growth_background	ENSGALG00010028160	ENSGALG00010028160	-0.260413958	0.282700816	-0.921164505	0.356964547	0.520595745	negative
PRJNA729271	ed_Lushi_vs_Arbor_Acr	breed_growth_background	ENSGALG00010028161	ENSGALG00010028161	-0.571235941	0.31579712	-1.808870015	0.0704712	0.168565914	negative
PRJNA729271	ed_Lushi_vs_Arbor_Acr	breed_growth_background	ENSGALG00010028162	CARD9	-0.112076266	0.094800593	-1.1822317	0.237113778	0.392213193	negative
PRJNA729271	ed_Lushi_vs_Arbor_Acr	breed_growth_background	ENSGALG00010028163	GBP	0.230726171	0.185008838	1.247108913	0.212357565	0.362529725	positive
PRJNA729271	ed_Lushi_vs_Arbor_Acr	breed_growth_background	ENSGALG00010028164	BBLN	-0.02030641	0.076079224	-0.266911376	0.789537398	0.869432848	negative
PRJNA729271	ed_Lushi_vs_Arbor_Acr	breed_growth_background	ENSGALG00010028165	NFIC	0.012730608	0.083470041	0.152517088	0.878779113	0.928012768	positive
PRJNA729271	ed_Lushi_vs_Arbor_Acr	breed_growth_background	ENSGALG00010028168	PTGES2	0.056729983	0.073792946	0.768772434	0.442028416	0.599156055	positive
PRJNA729271	ed_Lushi_vs_Arbor_Acr	breed_growth_background	ENSGALG00010028169	DNLZ	-0.12710583	0.076423018	-1.6631878	0.096274849	0.209189617	negative
PRJNA729271	ed_Lushi_vs_Arbor_Acr	breed_growth_background	ENSGALG00010028170	COPE	0.007287043	0.051188257	0.142357702	0.886797471	0.933420183	positive
PRJNA729271	ed_Lushi_vs_Arbor_Acr	breed_growth_background	ENSGALG00010028171	RNF123	0.201577985	0.084354471	2.389653829	0.01686426	0.060528165	positive
PRJNA729271	ed_Lushi_vs_Arbor_Acr	breed_growth_background	ENSGALG00010028172	CERS1	0.281529549	0.113015403	2.491072375	0.012735817	0.04955857	positive
PRJNA729271	ed_Lushi_vs_Arbor_Acr	breed_growth_background	ENSGALG00010028173	GDF3	0.30006737	0.122310001	2.45333469	0.014153858	0.053274518	positive
PRJNA729271	ed_Lushi_vs_Arbor_Acr	breed_growth_background	ENSGALG00010028174	GMPPB	-0.223972918	0.083713994	-2.675453734	0.007462817	0.033882915	negative
PRJNA729271	ed_Lushi_vs_Arbor_Acr	breed_growth_background	ENSGALG00010028175	POMT1	0.114642597	0.05084182	2.254887771	0.024140374	0.07803629	positive
PRJNA729271	ed_Lushi_vs_Arbor_Acr	breed_growth_background	ENSGALG00010028176	UPF1	0.09792954	0.050884792	1.924534538	0.054287616	0.140513278	positive
PRJNA729271	ed_Lushi_vs_Arbor_Acr	breed_growth_background	ENSGALG00010028177	ENSGALG00010028177	-0.762351737	0.207141147	-3.68034911	0.000232915	0.002890497	negative
PRJNA729271	ed_Lushi_vs_Arbor_Acr	breed_growth_background	ENSGALG00010028178	ENSGALG00010028178	-0.366341483	0.089735552	-4.082456451	4.45622E-05	0.000889709	negative
PRJNA729271	ed_Lushi_vs_Arbor_Acr	breed_growth_background	ENSGALG00010028179	MCM2	-0.259538576	0.077893673	-3.331959646	0.000862368	0.007318512	negative
PRJNA729271	ed_Lushi_vs_Arbor_Acr	breed_growth_background	ENSGALG00010028180	ODF2	-0.121228379	0.057234596	-2.118096187	0.034166922	0.100090853	negative
PRJNA729271	ed_Lushi_vs_Arbor_Acr	breed_growth_background	ENSGALG00010028181	BGLAP	-0.07869505	0.15274168	-0.515216606	0.606401666	0.737646203	negative
PRJNA729271	ed_Lushi_vs_Arbor_Acr	breed_growth_background	ENSGALG00010028182	SMG5	0.248679842	0.078668615	3.16110613	0.001571712	0.011222645	positive
PRJNA729271	ed_Lushi_vs_Arbor_Acr	breed_growth_background	ENSGALG00010028183	CRTC1	0.091214516	0.052850828	1.725886234	0.084367882	0.191050218	positive
PRJNA729271	ed_Lushi_vs_Arbor_Acr	breed_growth_background	ENSGALG00010028184	TMEM79	0.277321275	0.134558749	2.060967986	0.039306094	0.111039142	positive
PRJNA729271	ed_Lushi_vs_Arbor_Acr	breed_growth_background	ENSGALG00010028185	GLMP	0.045660816	0.047944914	0.952359954	0.340914461	0.50482449	positive
PRJNA729271	ed_Lushi_vs_Arbor_Acr	breed_growth_background	ENSGALG00010028186	CCT3	0.054282561	0.070804926	0.766649493	0.44328994	0.600344547	positive
PRJNA729271	ed_Lushi_vs_Arbor_Acr	breed_growth_background	ENSGALG00010028187	TPRA1	-0.008536398	0.037661062	-0.226663759	0.82068521	0.889873737	negative
PRJNA729271	ed_Lushi_vs_Arbor_Acr	breed_growth_background	ENSGALG00010028188	ENSGALG00010028188	0.139638854	0.059028238	2.365628028	0.017999519	0.063541896	positive
PRJNA729271	ed_Lushi_vs_Arbor_Acr	breed_growth_background	ENSGALG00010028189	GPSM1	-0.065771095	0.05733684	-1.147100104	0.251340251	0.407468621	negative
PRJNA729271	ed_Lushi_vs_Arbor_Acr	breed_growth_background	ENSGALG00010028190	ILF2	-0.024609376	0.043618078	-0.564201285	0.572617153	0.710909624	negative
PRJNA729271	ed_Lushi_vs_Arbor_Acr	breed_growth_background	ENSGALG00010028191	KLHL26	-0.066457873	0.053919143	-1.232546916	0.217744852	0.369753003	negative
PRJNA729271	ed_Lushi_vs_Arbor_Acr	breed_growth_background	ENSGALG00010028192	ENSGALG00010028192	-0.283133276	0.163271648	-1.734123952	0.082896092	0.188630904	negative
PRJNA729271	ed_Lushi_vs_Arbor_Acr	breed_growth_background	ENSGALG00010028193	BRINP1	-0.180722415	0.134213896	-1.346525362	0.178133147	0.322392304	negative
PRJNA729271	ed_Lushi_vs_Arbor_Acr	breed_growth_background	ENSGALG00010028194	ENSGALG00010028194	-0.027626404	0.096607998	-0.285963942	0.774905739	0.860248042	negative
PRJNA729271	ed_Lushi_vs_Arbor_Acr	breed_growth_background	ENSGALG00010028195	ENSGALG00010028195	0.495226447	0.156708575	3.160174526	0.001576747	0.01123093	positive
PRJNA729271	ed_Lushi_vs_Arbor_Acr	breed_growth_background	ENSGALG00010028196	ENSGALG00010028196	-0.031726351	0.053507868	-0.592928714	0.553228861	0.695608329	negative
PRJNA729271	ed_Lushi_vs_Arbor_Acr	breed_growth_background	ENSGALG00010028197	INTS3	0.146069667	0.082452629	1.77155864	0.07646785	0.178476796	positive
PRJNA729271	ed_Lushi_vs_Arbor_Acr	breed_growth_background	ENSGALG00010028198	UCK1	-0.038745051	0.050168348	-0.772300713	0.439936343	0.597365554	negative
PRJNA729271	ed_Lushi_vs_Arbor_Acr	breed_growth_background	ENSGALG00010028199	DPP9	0.091413032	0.057520371	1.589228832	0.11200874	0.233304595	positive
PRJNA729271	ed_Lushi_vs_Arbor_Acr	breed_growth_background	ENSGALG00010028201	ENSGALG00010028201	0.067430498	0.085065452	0.79268958	0.427958703	0.58704092	positive
PRJNA729271	ed_Lushi_vs_Arbor_Acr	breed_growth_background	ENSGALG00010028202	QSOX2	0.033758463	0.036567372	0.923185368	0.355910613	0.519666017	positive
PRJNA729271	ed_Lushi_vs_Arbor_Acr	breed_growth_background	ENSGALG00010028205	PRRT1B	-0.330539128	0.257687265	-1.282714253	0.199592205	0.348355088	negative
PRJNA729271	ed_Lushi_vs_Arbor_Acr	breed_growth_background	ENSGALG00010028206	RBM15B	0.08914045	0.05171261	1.723766215	0.084750056	0.191795794	positive
PRJNA729271	ed_Lushi_vs_Arbor_Acr	breed_growth_background	ENSGALG00010028207	ENSGALG00010028207	-0.256304263	0.141483753	-1.811545547	0.070056445	0.16798481	negative
PRJNA729271	ed_Lushi_vs_Arbor_Acr	breed_growth_background	ENSGALG00010028211	GATAD2B	0.193195567	0.079720072	2.423424382	0.015374954	0.056736241	positive
PRJNA729271	ed_Lushi_vs_Arbor_Acr	breed_growth_background	ENSGALG00010028216	NKX2-3	-0.732008841	0.750820738	-0.974944888	0.329587598	0.493530189	negative
PRJNA729271	ed_Lushi_vs_Arbor_Acr	breed_growth_background	ENSGALG00010028221	TNFAIP8L1	-0.238179742	0.103691815	-2.296996562	0.021618966	0.072121268	negative
PRJNA729271	ed_Lushi_vs_Arbor_Acr	breed_growth_background	ENSGALG00010028232	C9orf69	0.193186718	0.058460277	3.304580954	0.000951185	0.007860563	positive
PRJNA729271	ed_Lushi_vs_Arbor_Acr	breed_growth_background	ENSGALG00010028239	EDMPN2	-1.24205067	0.776652762	-1.599235504	0.109768284	0.230101646	negative
PRJNA729271	ed_Lushi_vs_Arbor_Acr	breed_growth_background	ENSGALG00010028242	ENSGALG00010028242	0.034618093	0.149497773	0.231562601	0.816877758	0.887046157	positive
PRJNA729271	ed_Lushi_vs_Arbor_Acr	breed_growth_background	ENSGALG00010028245	JAC1	-0.916870486	0.683283977	-1.341858608	0.179641845	0.323949806	negative
PRJNA729271	ed_Lushi_vs_Arbor_Acr	breed_growth_background	ENSGALG00010028247	MVP	-0.307293797	0.05018979	-6.122635678	9.204E-10	1.96194E-07	negative
PRJNA729271	ed_Lushi_vs_Arbor_Acr	breed_growth_background	ENSGALG00010028262	C1orf43	0.187460683	0.076334993	2.455763395	0.014058568	0.053067435	positive
PRJNA729271	ed_Lushi_vs_Arbor_Acr	breed_growth_background	ENSGALG00010028270	RAPGEF1	0.201066716	0.097233811	2.067868297	0.038652409	0.10976818	positive
PRJNA729271	ed_Lushi_vs_Arbor_Acr	breed_growth_background	ENSGALG00010028279	EDBETA	-0.742849419	0.810854642	-0.916131425	0.359597967	0.523067992	negative
PRJNA729271	ed_Lushi_vs_Arbor_Acr	breed_growth_background	ENSGALG00010028283	ENSGALG00010028283	-0.479942706	0.080876614	-5.934258143	2.95177E-09	5.15493E-07	negative
PRJNA729271	ed_Lushi_vs_Arbor_Acr	breed_growth_background	ENSGALG00010028286	KCTD12A	-0.6471066	0.643106743	-1.006219584	0.314309954	0.477906968	negative
PRJNA729271	ed_Lushi_vs_Arbor_Acr	breed_growth_background	ENSGALG00010028292	ENSGALG00010028292	0.263427754	0.279722166	0.941747869	0.346321741	0.510207707	positive
PRJNA729271	ed_Lushi_vs_Arbor_Acr	breed_growth_background	ENSGALG00010028301	FBN3	-0.300003688	0.09955957	-3.013308401	0.002584162	0.015946114	negative
PRJNA729271	ed_Lushi_vs_Arbor_Acr	breed_growth_background	ENSGALG00010028311	ENSGALG00010028311	0.033185321	0.141981062	0.233730613	0.81519412	0.886306539	positive
PRJNA729271	ed_Lushi_vs_Arbor_Acr	breed_growth_background	ENSGALG00010028314	MEF2D	0.341747811	0.114079559	2.995697155	0.002738182	0.016585855	positive
PRJNA729271	ed_Lushi_vs_Arbor_Acr	breed_growth_background	ENSGALG00010028316	TIMM44	-0.000817328	0.043712168	-0.018697945	0.985082067	0.991099088	negative
PRJNA729271	ed_Lushi_vs_Arbor_Acr	breed_growth_background	ENSGALG00010028318	NACC2	-0.052157162	0.103968272	-0.501664217	0.615903739	0.744456696	negative
PRJNA729271	ed_Lushi_vs_Arbor_Acr	breed_growth_background	ENSGALG00010028321	RHBG	-0.108248756	0.102651622	-1.054525528	0.291642379	0.453044292	negative
PRJNA729271	ed_Lushi_vs_Arbor_Acr	breed_growth_background	ENSGALG00010028322	STRBP	0.08016063	0.060690223	1.320816191	0.18656266	0.332132861	positive
PRJNA729271	ed_Lushi_vs_Arbor_Acr	breed_growth_background	ENSGALG00010028326	CRB2	-0.060732892	0.140994412	-0.430746803	0.666652483	0.783942594	negative
PRJNA729271	ed_Lushi_vs_Arbor_Acr	breed_growth_background	ENSGALG00010028328	HNRNPM	0.200778913	0.088908556	2.258263111	0.023929261	0.077578759	positive
PRJNA729271	ed_Lushi_vs_Arbor_Acr	breed_growth_background	ENSGALG00010028329	HAPLN2	-0.273273432	0.310199641	-0.88095986	0.378339546	0.540448994	negative
PRJNA729271	ed_Lushi_vs_Arbor_Acr	breed_growth_background	ENSGALG00010028337	ENSGALG00010028337	0.148767643	0.099460863	1.495740521	0.134721286	0.26456917	positive
PRJNA729271	ed_Lushi_vs_Arbor_Acr	breed_growth_background	ENSGALG00010028339	BCAN	-0.152066925	0.239668035	-0.634489804	0.525761219	0.672164578	negative
PRJNA729271	ed_Lushi_vs_Arbor_Acr	breed_growth_background	ENSGALG00010028344	LSM7	-0.107457223	0.05244813	-2.048828479	0.04047889	0.113336201	negative
PRJNA729271	ed_Lushi_vs_Arbor_Acr	breed_growth_background	ENSGALG00010028346	DENND1A	-0.150212524	0.039764493	-3.777554161	0.000158376	0.002224478	negative
PRJNA729271	ed_Lushi_vs_Arbor_Acr	breed_growth_background	ENSGALG00010028348	NES	-0.514910854	0.149340553	-3.44789707	0.000564969	0.00539475	negative
PRJNA729271	ed_Lushi_vs_Arbor_Acr	breed_growth_background	ENSGALG00010028353	ENSGALG00010028353	-0.244785392	0.779751802	-0.313927318	0.753576242	0.844264467	negative
PRJNA729271	ed_Lushi_vs_Arbor_Acr	breed_growth_background	ENSGALG00010028355	RBM18	-0.048160156	0.040235577	-1.196954531	0.231324278	0.385274665	negative
PRJNA729271	ed_Lushi_vs_Arbor_Acr	breed_growth_background	ENSGALG00010028357	SPPL2C	0.064637975	0.05039475	1.282633111	0.199620644	0.348362779	positive
PRJNA729271	ed_Lushi_vs_Arbor_Acr	breed_growth_background	ENSGALG00010028360	ENSGALG00010028360	0.260289054	0.399410111	0.651683688	0.514605248	0.662981341	positive
PRJNA729271	ed_Lushi_vs_Arbor_Acr	breed_growth_background	ENSGALG00010028362	TMPRSS9	0.172654166	0.258396664	0.668174903	0.504021953	0.654597749	positive
PRJNA729271	ed_Lushi_vs_Arbor_Acr	breed_growth_background	ENSGALG00010028364	MHM2	0.481653405	0.312155226	1.542993242	0.122832416	0.247715444	positive
PRJNA729271	ed_Lushi_vs_Arbor_Acr	breed_growth_background	ENSGALG00010028366	MRRF	0.182602954	0.04743849	3.849257324	0.000118476	0.001811761	positive

PRJNA729271	:ed_Lushi_vs_Arbor_Acr	breed_growth_background	ENSGALG00010028368	TIMM13	-0.120891647	0.06482055	-1.865020382	0.062178547	0.154699287	negative
PRJNA729271	:ed_Lushi_vs_Arbor_Acr	breed_growth_background	ENSGALG00010028370	S100A12	0.498402961	0.349729119	1.425111416	0.154124997	0.290588168	positive
PRJNA729271	:ed_Lushi_vs_Arbor_Acr	breed_growth_background	ENSGALG00010028372	PTGS1	-0.100551424	0.087484311	-1.149365214	0.250405418	0.406304479	negative
PRJNA729271	:ed_Lushi_vs_Arbor_Acr	breed_growth_background	ENSGALG00010028374	FDPS	-0.027902322	0.071185982	-0.391963717	0.695085023	0.80351363	negative
PRJNA729271	:ed_Lushi_vs_Arbor_Acr	breed_growth_background	ENSGALG00010028375	LMNB2	-0.272670879	0.081748619	-3.335479921	0.000851523	0.007269038	negative
PRJNA729271	:ed_Lushi_vs_Arbor_Acr	breed_growth_background	ENSGALG00010028377	PDCL	-0.116796317	0.03234628	-3.610811435	0.000305241	0.003514075	negative
PRJNA729271	:ed_Lushi_vs_Arbor_Acr	breed_growth_background	ENSGALG00010028380	RUSC1	0.168378734	0.075778371	2.221989373	0.026284024	0.082751287	positive
PRJNA729271	:ed_Lushi_vs_Arbor_Acr	breed_growth_background	ENSGALG00010028384	ENSGALG00010028384	-0.749734289	0.473276739	-1.584135089	0.113162981	0.234707218	negative
PRJNA729271	:ed_Lushi_vs_Arbor_Acr	breed_growth_background	ENSGALG00010028386	NCAN	0.286277985	0.298891315	0.957799608	0.338163817	0.502479193	positive
PRJNA729271	:ed_Lushi_vs_Arbor_Acr	breed_growth_background	ENSGALG00010028391	MED26	0.156625208	0.061217938	2.558485539	0.010512919	0.043328253	positive
PRJNA729271	:ed_Lushi_vs_Arbor_Acr	breed_growth_background	ENSGALG00010028392	ASH1L	0.119809044	0.064591709	1.854867217	0.06361519	0.157140795	positive
PRJNA729271	:ed_Lushi_vs_Arbor_Acr	breed_growth_background	ENSGALG00010028394	ZBTB26	0.08693167	0.05592313	1.554485042	0.120068762	0.244433864	positive
PRJNA729271	:ed_Lushi_vs_Arbor_Acr	breed_growth_background	ENSGALG00010028399	SLC35E1	0.047274335	0.042875298	1.102600739	0.270200589	0.428968544	positive
PRJNA729271	:ed_Lushi_vs_Arbor_Acr	breed_growth_background	ENSGALG00010028405	S100A6	-0.41019635	0.149855265	-2.737283538	0.006194887	0.029669253	negative
PRJNA729271	:ed_Lushi_vs_Arbor_Acr	breed_growth_background	ENSGALG00010028411	LHX2	0.048020952	0.45289584	0.106030896	0.915557839	0.952192002	positive
PRJNA729271	:ed_Lushi_vs_Arbor_Acr	breed_growth_background	ENSGALG00010028412	ENSGALG00010028412	-0.137082626	0.173803436	-0.788722188	0.430274397	0.588497923	negative
PRJNA729271	:ed_Lushi_vs_Arbor_Acr	breed_growth_background	ENSGALG00010028414	KBTBD12	0.310273852	0.169800066	1.827289347	0.067656276	0.164162244	positive
PRJNA729271	:ed_Lushi_vs_Arbor_Acr	breed_growth_background	ENSGALG00010028415	FSD1	0.394727706	0.091054405	4.335075356	1.4571E-05	0.000382283	positive
PRJNA729271	:ed_Lushi_vs_Arbor_Acr	breed_growth_background	ENSGALG00010028418	ENSGALG00010028418	-0.003755669	0.066243437	-0.056694964	0.954788185	0.974885706	negative
PRJNA729271	:ed_Lushi_vs_Arbor_Acr	breed_growth_background	ENSGALG00010028419	ENSGALG00010028419	0.149129866	0.188035437	0.793094471	0.427722784	0.586883922	positive
PRJNA729271	:ed_Lushi_vs_Arbor_Acr	breed_growth_background	ENSGALG00010028420	ENSGALG00010028420	-1.030824757	0.437153161	-2.358040267	0.0183717	0.0644118	negative
PRJNA729271	:ed_Lushi_vs_Arbor_Acr	breed_growth_background	ENSGALG00010028421	ZBTB7A	0.254293117	0.101183043	2.513198957	0.011964184	0.047474588	positive
PRJNA729271	:ed_Lushi_vs_Arbor_Acr	breed_growth_background	ENSGALG00010028422	RXRA	0.407156138	0.111509601	3.65131015	0.000260906	0.003153469	positive
PRJNA729271	:ed_Lushi_vs_Arbor_Acr	breed_growth_background	ENSGALG00010028425	DAP3	0.028968375	0.069274752	0.418166424	0.675825443	0.790569593	positive
PRJNA729271	:ed_Lushi_vs_Arbor_Acr	breed_growth_background	ENSGALG00010028426	GPR21	0.19019611	0.084890065	2.240499036	0.025058543	0.080128742	positive
PRJNA729271	:ed_Lushi_vs_Arbor_Acr	breed_growth_background	ENSGALG00010028427	ENSGALG00010028427	-0.124555408	0.111371289	-1.11837987	0.263404788	0.421466984	negative
PRJNA729271	:ed_Lushi_vs_Arbor_Acr	breed_growth_background	ENSGALG00010028428	RABGAP1	-0.00531481	0.03612651	-0.147116621	0.883039971	0.931087828	negative
PRJNA729271	:ed_Lushi_vs_Arbor_Acr	breed_growth_background	ENSGALG00010028429	REXO1	0.094178607	0.047923331	1.965193252	0.049391875	0.131388369	positive
PRJNA729271	:ed_Lushi_vs_Arbor_Acr	breed_growth_background	ENSGALG00010028430	NR2C2AP	0.043103036	0.062821576	0.686118357	0.492638476	0.644476057	positive
PRJNA729271	:ed_Lushi_vs_Arbor_Acr	breed_growth_background	ENSGALG00010028431	ENSGALG00010028431	-0.263294643	0.146855179	-1.79288633	0.072991102	0.1728768	negative
PRJNA729271	:ed_Lushi_vs_Arbor_Acr	breed_growth_background	ENSGALG00010028432	RFXANK	0.078653709	0.071851873	1.094664703	0.273663558	0.432631565	positive
PRJNA729271	:ed_Lushi_vs_Arbor_Acr	breed_growth_background	ENSGALG00010028433	MSTO1	0.008173717	0.052363847	0.156094655	0.875958411	0.926645502	positive
PRJNA729271	:ed_Lushi_vs_Arbor_Acr	breed_growth_background	ENSGALG00010028434	COL5A1	-0.155441763	0.104721868	-1.484329547	0.137721515	0.268967039	negative
PRJNA729271	:ed_Lushi_vs_Arbor_Acr	breed_growth_background	ENSGALG00010028435	ATP8B3	0.247840347	0.180327829	1.374387687	0.169321357	0.311421529	positive
PRJNA729271	:ed_Lushi_vs_Arbor_Acr	breed_growth_background	ENSGALG00010028436	S100A1	0.481577607	0.096635844	4.983426326	6.24681E-07	3.34124E-05	positive
PRJNA729271	:ed_Lushi_vs_Arbor_Acr	breed_growth_background	ENSGALG00010028437	ENSGALG00010028437	0.08933853	0.03625523	2.464155655	0.013733646	0.052351899	positive
PRJNA729271	:ed_Lushi_vs_Arbor_Acr	breed_growth_background	ENSGALG00010028438	MED27	0.011260344	0.039625947	0.28416592	0.776283231	0.861212243	positive
PRJNA729271	:ed_Lushi_vs_Arbor_Acr	breed_growth_background	ENSGALG00010028439	BORCS8	0.133924464	0.065001312	2.060334798	0.039366545	0.111166584	positive
PRJNA729271	:ed_Lushi_vs_Arbor_Acr	breed_growth_background	ENSGALG00010028440	PSMB7	0.030746979	0.042121346	0.729961928	0.465413456	0.620565546	positive
PRJNA729271	:ed_Lushi_vs_Arbor_Acr	breed_growth_background	ENSGALG00010028442	CHTOP	-0.102818551	0.048875746	-2.103672263	0.035407046	0.102871342	negative
PRJNA729271	:ed_Lushi_vs_Arbor_Acr	breed_growth_background	ENSGALG00010028443	ENSGALG00010028443	-0.174915974	0.183530638	-0.953061436	0.340558944	0.504589787	negative
PRJNA729271	:ed_Lushi_vs_Arbor_Acr	breed_growth_background	ENSGALG00010028444	MEF2B	-0.293548331	0.172237631	-1.704321701	0.088321003	0.197380965	negative
PRJNA729271	:ed_Lushi_vs_Arbor_Acr	breed_growth_background	ENSGALG00010028445	SYT11	-0.601296293	0.109870523	-5.472771734	4.43051E-08	4.55817E-06	negative
PRJNA729271	:ed_Lushi_vs_Arbor_Acr	breed_growth_background	ENSGALG00010028446	ENSGALG00010028446	-0.033189945	0.119655555	-0.277379059	0.78148906	0.864774709	negative
PRJNA729271	:ed_Lushi_vs_Arbor_Acr	breed_growth_background	ENSGALG00010028447	OLFM1	-0.170138696	0.088067319	-1.931916372	0.053369829	0.138886116	negative
PRJNA729271	:ed_Lushi_vs_Arbor_Acr	breed_growth_background	ENSGALG00010028448	RIT1	0.094926916	0.031244241	3.03822116	0.002379792	0.015069939	positive
PRJNA729271	:ed_Lushi_vs_Arbor_Acr	breed_growth_background	ENSGALG00010028449	EFCAB12	-0.122580476	0.183580067	-0.667722146	0.504310971	0.654597749	negative
PRJNA729271	:ed_Lushi_vs_Arbor_Acr	breed_growth_background	ENSGALG00010028450	PTGDS	0.443838208	0.216572333	2.049376308	0.040425332	0.113251873	positive
PRJNA729271	:ed_Lushi_vs_Arbor_Acr	breed_growth_background	ENSGALG00010028451	TMEM59L	-0.562770329	0.181968088	-3.092686937	0.001983533	0.013188298	negative
PRJNA729271	:ed_Lushi_vs_Arbor_Acr	breed_growth_background	ENSGALG00010028452	GOLPH3L	0.335861536	0.392098754	0.856573842	0.391680447	0.553299686	positive
PRJNA729271	:ed_Lushi_vs_Arbor_Acr	breed_growth_background	ENSGALG00010028453	ENSGALG00010028453	0.032970094	0.051058493	0.645731823	0.518453047	0.666614508	positive
PRJNA729271	:ed_Lushi_vs_Arbor_Acr	breed_growth_background	ENSGALG00010028454	C8G	0.269840346	0.119508144	2.257924321	0.023950378	0.077597174	positive
PRJNA729271	:ed_Lushi_vs_Arbor_Acr	breed_growth_background	ENSGALG00010028455	ENSA	0.029077436	0.037258676	0.780420543	0.43514338	0.593247111	positive
PRJNA729271	:ed_Lushi_vs_Arbor_Acr	breed_growth_background	ENSGALG00010028456	FBXW5	0.028900779	0.050163038	0.576136931	0.564522631	0.704620299	positive
PRJNA729271	:ed_Lushi_vs_Arbor_Acr	breed_growth_background	ENSGALG00010028457	KANK3	-0.134230075	0.055833348	-2.404120122	0.016211445	0.058849211	negative
PRJNA729271	:ed_Lushi_vs_Arbor_Acr	breed_growth_background	ENSGALG00010028458	TRAF2	0.129668719	0.058481846	2.217247356	0.026606193	0.083529731	positive
PRJNA729271	:ed_Lushi_vs_Arbor_Acr	breed_growth_background	ENSGALG00010028459	MCL1	0.192253243	0.112176003	1.713853559	0.086555624	0.194666217	positive
PRJNA729271	:ed_Lushi_vs_Arbor_Acr	breed_growth_background	ENSGALG00010028460	CRLF1	-0.557084633	0.135230566	-4.119517127	3.79667E-05	0.000794091	negative
PRJNA729271	:ed_Lushi_vs_Arbor_Acr	breed_growth_background	ENSGALG00010028461	ENSGALG00010028461	0.020047871	0.16959892	0.118207537	0.905903219	0.945955552	positive
PRJNA729271	:ed_Lushi_vs_Arbor_Acr	breed_growth_background	ENSGALG00010028462	EDF1	0.036051869	0.056459702	0.638541608	0.523121178	0.670081431	positive
PRJNA729271	:ed_Lushi_vs_Arbor_Acr	breed_growth_background	ENSGALG00010028463	RPS28	-0.110695588	0.090444827	-1.223901812	0.22098927	0.373424979	negative
PRJNA729271	:ed_Lushi_vs_Arbor_Acr	breed_growth_background	ENSGALG00010028464	ENSGALG00010028464	0.12785646	0.060109413	2.127062208	0.033414915	0.098404955	positive
PRJNA729271	:ed_Lushi_vs_Arbor_Acr	breed_growth_background	ENSGALG00010028465	ENSGALG00010028465	0.057234923	0.073782673	0.775723087	0.437912505	0.595342503	positive
PRJNA729271	:ed_Lushi_vs_Arbor_Acr	breed_growth_background	ENSGALG00010028466	MAMDC4	-0.241144748	0.361903564	-0.66632322	0.505204527	0.655130385	negative
PRJNA729271	:ed_Lushi_vs_Arbor_Acr	breed_growth_background	ENSGALG00010028467	ENSGALG00010028467	-0.024006723	0.135253787	-0.177493903	0.859120454	0.915349107	negative
PRJNA729271	:ed_Lushi_vs_Arbor_Acr	breed_growth_background	ENSGALG00010028468	PHPT1	0.038372991	0.056952343	0.673773702	0.500455196	0.651585204	positive
PRJNA729271	:ed_Lushi_vs_Arbor_Acr	breed_growth_background	ENSGALG00010028470	AJM1	0.04079429	0.232391274	0.1755414	0.860654243	0.916307481	positive
PRJNA729271	:ed_Lushi_vs_Arbor_Acr	breed_growth_background	ENSGALG00010028471	TPGS1	0.020958653	0.091291403	0.2295797	0.818418383	0.888320249	positive
PRJNA729271	:ed_Lushi_vs_Arbor_Acr	breed_growth_background	ENSGALG00010028472	RPRD2	0.132943112	0.074457423	1.785491716	0.074181594	0.174981644	positive
PRJNA729271	:ed_Lushi_vs_Arbor_Acr	breed_growth_background	ENSGALG00010028473	CDC34	0.21382921	0.074293714	2.878160175	0.00400002	0.021666774	positive
PRJNA729271	:ed_Lushi_vs_Arbor_Acr	breed_growth_background	ENSGALG00010028474	PEX19	0.171474842	0.064605485	2.65418397	0.007950047	0.035479043	positive
PRJNA729271	:ed_Lushi_vs_Arbor_Acr	breed_growth_background	ENSGALG00010028475	AMBP	-0.785648538	0.671661375	-1.169709273			negative
PRJNA729271	:ed_Lushi_vs_Arbor_Acr	breed_growth_background	ENSGALG00010028476	ENSGALG00010028476	0.269053487	0.082887676	3.246001107	0.001170384	0.009160213	positive
PRJNA729271	:ed_Lushi_vs_Arbor_Acr	breed_growth_background	ENSGALG00010028477	NRARP	0.236511091	0.116193701	2.035489776	0.041801616	0.116209135	positive
PRJNA729271	:ed_Lushi_vs_Arbor_Acr	breed_growth_background	ENSGALG00010028478	PEA15	-0.182240898	0.086874191	-2.097756485	0.035926662	0.103985018	negative
PRJNA729271	:ed_Lushi_vs_Arbor_Acr	breed_growth_background	ENSGALG00010028479	CASQ1	0.499512343	0.134983073	3.700555422	0.000215128	0.002744966	positive
PRJNA729271	:ed_Lushi_vs_Arbor_Acr	breed_growth_background	ENSGALG00010028480	F11R	-0.225732514	0.117471855	-1.921588068	0.054657611	0.141248364	negative
PRJNA729271	:ed_Lushi_vs_Arbor_Acr	breed_growth_background	ENSGALG00010028481	ENSGALG00010028481	0.206034885	0.07744815	2.660294482	0.007807236	0.035046727	positive
PRJNA729271	:ed_Lushi_vs_Arbor_Acr	breed_growth_background	ENSGALG00010028482	CD320	0.222043064	0.095101215	2.334807853	0.019553461	0.067210674	positive
PRJNA729271	:ed_Lushi_vs_Arbor_Acr	breed_growth_background	ENSGALG00010028483	EXD3	0.032941279	0.049971798	0.659197408	0.509769011	0.658565225	positive
PRJNA729271	:ed_Lushi_vs_Arbor_Acr	breed_growth_background	ENSGALG00010028484	NOXA1	0.311748625	0.289577802	1.076562578	0.28167573	0.441648752	positive
PRJNA729271	:ed_Lushi_vs_Arbor_Acr	breed_growth_background	ENSGALG00010028485	USF1	0.236589312	0.099015408	2.389419162	0.016875037	0.060545461	positive
PRJNA729271	:ed_Lushi_vs_Arbor_Acr	breed_growth_background	ENSGALG00010028486	ARHGAP30	-0.037345948	0.119063504	-0.313664105	0.753776166	0.844357459	negative
PRJNA729271	:ed_Lushi_vs_Arbor_Acr	breed_growth_background	ENSGALG00010028488	PTGES	-0.070219062	0.068693685	-1.022205491	0.306683653	0.469317942	negative
PRJNA729271	:ed_Lushi_vs_Arbor_Acr	breed_growth_background	ENSGALG00010028489	ENSGALG00010028489	0.257870457	0.458289455	0.56268032	0.573652588	0.711482353	positive
PRJNA729271	:ed_Lushi_vs_Arbor_Acr	breed_growth_background	ENSGALG00010028490	NECTIN4	-0.132241361	0.198674167	-0.665619308	0.505654462	0.655293824	negative
PRJNA729271	:ed_Lushi_vs_Arbor_Acr	breed_growth_background	ENSGALG00010028491	TOR1B	-0.162858411	0.053109573	-3.066460576	0.002166094	0.014048111	negative
PRJNA729271	:ed_Lushi_vs_Arbor_Acr	breed_growth_background	ENSGALG00010028492	ENSGALG00010028492	-0.016257952	0.128061092	-0.126954658	0.898976286	0.941398557	negative
PRJNA729271	:ed_Lushi_vs_Arbor_Acr	breed_growth_background	ENSGALG00010028493	TOR1A	0.087597212	0.047189502	1.856285994	0.063412804	0.156873916	positive
PRJNA729271	:ed_Lushi_vs_Arbor_Acr	breed_growth_background	ENSGALG00010028494	ENSGALG00010028494	0.023683684	0.108885604	0.217509778	0.827811088	0.894989313	positive
PRJNA729271	:ed_Lushi_vs_Arbor_Acr	breed_growth_background	ENSGALG00010028495	LINGO3	-0.518114052	0.18777019	-2.759298751	0.005792555	0.028260885	negative
PRJNA729271	:ed_Lushi_vs_Arbor_Acr	breed_growth_background	ENSGALG00010028496	ENSGALG00010028496	0.144582311	0.077314169	1.870062281	0.06147517	0.153541252	positive
PRJNA729271	:ed_Lushi_vs_Arbor_Acr	breed_growth_background	ENSGALG00010028498	C9orf78	0.071167844	0.056360607	1.262723166	0.206688686	0.355721117	positive
PRJNA729271	:ed_Lushi_vs_Arbor_Acr	breed_growth_background	ENSGALG00010028500	VPS45	0.044795601	0.037564406	1.192501225	0.233064771	0.387505604	positive
PRJNA729271	:ed_Lushi_vs_Arbor_Acr	breed_growth_background	ENSGALG00010028501	USP20	0.051080971	0.047135202	1.083711724	0.278492636	0.437835737	positive
PRJNA729271	:ed_Lushi_vs_Arbor_Acr	breed_growth_background	ENSGALG00010028502	PLEKHO1	-0.250527205	0.080936027	-3.09537316	0.001965654	0.013116204	negative
PRJNA729271	:ed_Lushi_vs_Arbor_Acr	breed_growth_background	ENSGALG00010028503	NDOR1	0.135230713	0.068899329	1.962729026	0.049677669	0.131882382	positive
PRJNA729271	:ed_Lushi_vs_Arbor_Acr	breed_growth_background	ENSGALG00010028506	SHD	-0.506091755	0.217783255	-2.323832267	0.020134486	0.068590841	negative
PRJNA729271	:ed_Lushi_vs_Arbor_Acr	breed_growth_background	ENSGALG00010028507	ANP32E	-0.082712729	0.030632053	-2.700201931	0.00692974	0.032145293	negative
PRJNA729271	:ed_Lushi_vs_Arbor_Acr	breed_growth_background	ENSGALG00010028510	CILP2	0.739853641	0.315598188	2.344289892	0.019063348	0.066153514	positive
PRJNA729271	:ed_Lushi_vs_Arbor_Acr	breed_growth_background	ENSGALG00010028511	YJEFN3	0.889252312	0.237036738	3.751537923	0.000175753	0.002369807	positive
PRJNA729271	:ed_Lushi_vs_Arbor_Acr	breed_growth_background	ENSGALG00010028512	NDUFA13	0.00477342	0.06548323	0.072895302	0.941889432	0.96841306	positive
PRJNA729271	:ed_Lushi_vs_Arbor_Acr	breed_growth_background	ENSGALG00010028513	ENSGALG00010028513	-0.269373017	0.613287215	-0.439228163	0.660496221	0.77944254	negative
PRJNA729271	:ed_Lushi_vs_Arbor_Acr	breed_growth_background	ENSGALG00010028514	FNBP1	0.032191635	0.031379967	1.025865823	0.304954834	0.467511404	positive
PRJNA729271	:ed_Lushi_vs_Arbor_Acr	breed_growth_background	ENSGALG00010028515	ENSGALG00010028515	-0.286144598	1.79369841	-0.159527709	0.873253129	0.925210446	negative
PRJNA729271	:ed_Lushi_vs_Arbor_Acr	breed_growth_background	ENSGALG00010028516	GATAD2A	0.004020703	0.034392469	0.116906508	0.906934141	0.94664149	positive
PRJNA729271	:ed_Lushi_vs_Arbor_Acr	breed_growth_background	ENSGALG00010028517	RNF208	0.547415168	0.181714048	3.012508798	0.002590979	0.015967791	positive
PRJNA729271	:ed_Lushi_vs_Arbor_Acr	breed_growth_background	ENSGALG00010028518	MAU2	0.018163488	0.061876118	0.293546012	0.769104835	0.856234608	positive
PRJNA729271	:ed_Lushi_vs_Arbor_Acr	breed_growth_background	ENSGALG00010028519	ENSGALG00010028519	0.104399877	1.062901034	0.098221634	0.921756304	0.956327938	positive
PRJNA729271	:ed_Lushi_vs_Arbor_Acr	breed_growth_background	ENSGALG00010028523	SUGP1	-0.041389976	0.062297417	-0.664393133	0.506438727	0.655899691	negative
PRJNA729271	:ed_Lushi_vs_Arbor_Acr	breed_growth_background	ENSGALG00010028528	UHRF1	-0.301988501	0.099546865	-3.03363145	0.002416294	0.015238839	negative
PRJNA729271	:ed_Lushi_vs_Arbor_Acr	breed_growth_background	ENSGALG00010028534	ENSGALG00010028534	-0.249850823	2.310113534	-0.108155214	0.913872571	0.951485628	negative
PRJNA729271	:ed_Lushi_vs_Arbor_Acr	breed_growth_background	ENSGALG00010028535	TICAM1	-0.114674416	0.057104384	-2.008154347	0.044626893	0.121944524	negative
PRJNA729271	:ed_Lushi_vs_Arbor_Acr	breed_growth_background	ENSGALG00010028536	GPR107	-0.070177093	0.034627763	-2.026613563	0.042701948	0.118123042	negative
PRJNA729271	:ed_Lushi_vs_Arbor_Acr	breed_growth_background	ENSGALG00010028537	ENSGALG00010028537	-0.926074975	1.084306683	-0.854071075	0.393065609	0.554553826	negative
PRJNA729271	:ed_Lushi_vs_Arbor_Acr	breed_growth_background	ENSGALG00010028538	FEM1A	0.116149622	0.062519808	1.857805168	0.063196687	0.156453626	positive
PRJNA729271	:ed_Lushi_vs_Arbor_Acr	breed_growth_background	ENSGALG00010028539	ENSGALG00010028539	-0.391431519	1.771469855	-0.220964256	0.825120264	0.893279426	negative
PRJNA729271	:ed_Lushi_vs_Arbor_Acr	breed_growth_background	ENSGALG00010028541	ENSGALG00010028541	-0.278733853	0.163447254	-1.705344365	0.088130218	0.197094192	negative
PRJNA729271	:ed_Lushi_vs_Arbor_Acr	breed_growth_background	ENSGALG00010028542	ENSGALG00010028542	-0.811519677	0.998983706	-0.812345258	0.416593548	0.576800409	negative
PRJNA729271	:ed_Lushi_vs_Arbor_Acr	breed_growth_background	ENSGALG00010028543	ANP32B	-0.16238108	0.050413397	-3.220990634	0.001277483	0.009743353	negative
PRJNA729271	:ed_Lushi_vs_Arbor_Acr	breed_growth_background	ENSGALG00010028544	ZNF414	0.107338585	0.088041696	1.219178981	0.222776268	0.375372259	positive
PRJNA729271	:ed_Lushi_vs_Arbor_Acr	breed_growth_background	ENSGALG00010028545	ENSGALG00010028545	-1.048991784	0.661474646	-1.585838233	0.11277601	0.234196027	negative
PRJNA729271	:ed_Lushi_vs_Arbor_Acr	breed_growth_background	ENSGALG00010028546	ENSGALG00010028546	-0.765762978	0.834387682	-0.917754414	0.358747453	0.522323865	negative
PRJNA729271	:ed_Lushi_vs_Arbor_Acr	breed_growth_background	ENSGALG00010028547	ZNF618	0.075950288	0.056774127	1.337762312	0.180973931	0.32553915	positive
PRJNA729271	:ed_Lushi_vs_Arbor_Acr	breed_growth_background	ENSGALG00010028548	MYO1F	0.113872171	0.133836627	0.850829652	0.394863988	0.556347402	positive
PRJNA729271	:ed_Lushi_vs_Arbor_Acr	breed_growth_background	ENSGALG00010028549	ADAMTS10	-0.364055203	0.096558437	-3.770309603	0.000163045	0.00226808	negative
PRJNA729271	:ed_Lushi_vs_Arbor_Acr	breed_growth_background	ENSGALG00010028550	ZAP70	0.367420759	0.375885714	0.977479978	0.328331585	0.492157842	positive
PRJNA729271	:ed_Lushi_vs_Arbor_Acr	breed_growth_background	ENSGALG00010028551	NCS1	-0.5740349	0.12916539	-4.444185086	8.82256E-06	0.000264768	negative
PRJNA729271	:ed_Lushi_vs_Arbor_Acr	breed_growth_background	ENSGALG00010028552	ENSGALG00010028552	-0.111704974	0.397653565	-0.280910279	0.778779216	0.862895944	negative
PRJNA729271	:ed_Lushi_vs_Arbor_Acr	breed_growth_background	ENSGALG00010028553	NFILZ	0.409108845	0.366078499	1.117544041	0.26376178	0.421850049	positive
PRJNA729271	:ed_Lushi_vs_Arbor_Acr	breed_growth_background	ENSGALG00010028554	ENSGALG00010028554	-1.032334418	0.494416969	-2.087983387	0.036799333	0.105813596	negative

PRJNA729271	ed_Lushi_vs_Arbor_Acr	breed_growth_background	ENSGALG00010028555	ENSGALG00010028555	-1.119316687	0.645455826	-1.734149174	0.082891618	0.188630904	negative
PRJNA729271	ed_Lushi_vs_Arbor_Acr	breed_growth_background	ENSGALG00010028556	SPTAN1	0.046702508	0.049932269	0.935317153	0.349624878	0.513612305	positive
PRJNA729271	ed_Lushi_vs_Arbor_Acr	breed_growth_background	ENSGALG00010028558	ENSGALG00010028558	-1.88712627	0.717945054	-2.62851072	0.008575965	0.037273948	negative
PRJNA729271	ed_Lushi_vs_Arbor_Acr	breed_growth_background	ENSGALG00010028559	DYNC2I2	-0.070857998	0.069665113	-1.01712313	0.309094858	0.471862029	negative
PRJNA729271	ed_Lushi_vs_Arbor_Acr	breed_growth_background	ENSGALG00010028560	ENSGALG00010028560	-1.183562886	1.841559041	-0.642696139	0.520421287	0.668019523	negative
PRJNA729271	ed_Lushi_vs_Arbor_Acr	breed_growth_background	ENSGALG00010028561	SET	-0.014979292	0.027833057	-0.538183512	0.590450362	0.725549169	negative
PRJNA729271	ed_Lushi_vs_Arbor_Acr	breed_growth_background	ENSGALG00010028562	ACSBG2	-0.140597019	0.106545119	-1.319600758	0.18696835	0.332674092	negative
PRJNA729271	ed_Lushi_vs_Arbor_Acr	breed_growth_background	ENSGALG00010028563	ENSGALG00010028563	-1.081735629	1.177902373	-0.918357628	0.358431666	0.522142208	negative
PRJNA729271	ed_Lushi_vs_Arbor_Acr	breed_growth_background	ENSGALG00010028565	PKN1	-0.177925628	0.065445739	-2.718673989	0.006554417	0.03091646	negative
PRJNA729271	ed_Lushi_vs_Arbor_Acr	breed_growth_background	ENSGALG00010028566	ZDHHC12	0.193279505	0.06743755	2.866051694	0.004156264	0.022222443	positive
PRJNA729271	ed_Lushi_vs_Arbor_Acr	breed_growth_background	ENSGALG00010028567	ENSGALG00010028567	-0.523502102	0.62453791	-0.838223099	0.401905416	0.562916128	negative
PRJNA729271	ed_Lushi_vs_Arbor_Acr	breed_growth_background	ENSGALG00010028568	MLLT1	-0.045973109	0.036424232	-1.262157244	0.20689221	0.355995084	negative
PRJNA729271	ed_Lushi_vs_Arbor_Acr	breed_growth_background	ENSGALG00010028569	ZER1	0.034826392	0.037099412	0.938731629	0.34786855	0.511862211	positive
PRJNA729271	ed_Lushi_vs_Arbor_Acr	breed_growth_background	ENSGALG00010028572	TBC1D13	-0.012489374	0.037235246	-0.335418053	0.737309752	0.83173013	negative
PRJNA729271	ed_Lushi_vs_Arbor_Acr	breed_growth_background	ENSGALG00010028573	KCNN1	0.703832231	0.140797662	4.998891453	5.76608E-07	3.14901E-05	positive
PRJNA729271	ed_Lushi_vs_Arbor_Acr	breed_growth_background	ENSGALG00010028574	ENDOG	0.057771528	0.064673482	0.893279998	0.371707247	0.534463681	positive
PRJNA729271	ed_Lushi_vs_Arbor_Acr	breed_growth_background	ENSGALG00010028575	SETDB1	0.09993131	0.064033571	1.560608118	0.118616242	0.242502458	positive
PRJNA729271	ed_Lushi_vs_Arbor_Acr	breed_growth_background	ENSGALG00010028577	CERS2	0.152139791	0.071129989	2.138897994	0.032443931	0.096622277	positive
PRJNA729271	ed_Lushi_vs_Arbor_Acr	breed_growth_background	ENSGALG00010028578	SPOUT1	0.288076949	0.067447224	4.271146096	1.94471E-05	0.000471381	positive
PRJNA729271	ed_Lushi_vs_Arbor_Acr	breed_growth_background	ENSGALG00010028579	OCLN	0.73279675	0.146246619	5.010691898	5.42347E-07	3.0005E-05	positive
PRJNA729271	ed_Lushi_vs_Arbor_Acr	breed_growth_background	ENSGALG00010028580	PRUNE1	0.086902531	0.08181687	1.062159071	0.288163477	0.449132215	positive
PRJNA729271	ed_Lushi_vs_Arbor_Acr	breed_growth_background	ENSGALG00010028581	NR2F6	0.142452904	0.066551803	2.140481522	0.032315872	0.096322964	positive
PRJNA729271	ed_Lushi_vs_Arbor_Acr	breed_growth_background	ENSGALG00010028582	KYAT1	-0.119822172	0.112341578	-1.066587939	0.286157949	0.44677504	negative
PRJNA729271	ed_Lushi_vs_Arbor_Acr	breed_growth_background	ENSGALG00010028583	USHBP1	-0.422841578	0.104933018	-4.029633238	5.58639E-05	0.001050257	negative
PRJNA729271	ed_Lushi_vs_Arbor_Acr	breed_growth_background	ENSGALG00010028584	LRRC8A	-0.121915063	0.040934886	-2.978268035	0.002898824	0.017291542	negative
PRJNA729271	ed_Lushi_vs_Arbor_Acr	breed_growth_background	ENSGALG00010028585	MINDY1	0.322044061	0.064820366	4.968254321	6.75583E-07	3.58703E-05	positive
PRJNA729271	ed_Lushi_vs_Arbor_Acr	breed_growth_background	ENSGALG00010028586	BABAM1	-0.00027596	0.065328782	-0.004224178	0.996629604	0.998282503	negative
PRJNA729271	ed_Lushi_vs_Arbor_Acr	breed_growth_background	ENSGALG00010028587	ANKLE1	0.036508747	0.108117509	0.337676542	0.735606954	0.830503295	positive
PRJNA729271	ed_Lushi_vs_Arbor_Acr	breed_growth_background	ENSGALG00010028588	ABHD8	-0.92145356	0.41112949	-2.241273326	0.025008376	0.080066796	negative
PRJNA729271	ed_Lushi_vs_Arbor_Acr	breed_growth_background	ENSGALG00010028589	PHYHD1	-0.093015625	0.101611014	-0.915408887	0.359977015	0.523409251	negative
PRJNA729271	ed_Lushi_vs_Arbor_Acr	breed_growth_background	ENSGALG00010028590	ENSGALG00010028590	0.229815183	0.122008748	1.883595947	0.05961965	0.150214989	positive
PRJNA729271	ed_Lushi_vs_Arbor_Acr	breed_growth_background	ENSGALG00010028591	GABPB2	-0.1536673	0.068437845	-2.245355618	0.024745312	0.07946019	negative
PRJNA729271	ed_Lushi_vs_Arbor_Acr	breed_growth_background	ENSGALG00010028592	SEMA6C	-0.186379171	0.07924234	-2.352014985	0.018672023	0.065140594	negative
PRJNA729271	ed_Lushi_vs_Arbor_Acr	breed_growth_background	ENSGALG00010028593	DOLK	0.049878793	0.065936652	0.75646535	0.449370239	0.605692916	positive
PRJNA729271	ed_Lushi_vs_Arbor_Acr	breed_growth_background	ENSGALG00010028594	ENSGALG00010028594	0.167225976	0.076787244	2.177783288	0.029422175	0.08965197	positive
PRJNA729271	ed_Lushi_vs_Arbor_Acr	breed_growth_background	ENSGALG00010028595	SCNM1	0.057317993	0.060041691	0.954636556	0.339761524	0.503820286	positive
PRJNA729271	ed_Lushi_vs_Arbor_Acr	breed_growth_background	ENSGALG00010028596	DDA1	-0.053033155	0.047570209	-1.114839647	0.264919143	0.423187456	negative
PRJNA729271	ed_Lushi_vs_Arbor_Acr	breed_growth_background	ENSGALG00010028597	NUP188	0.027096327	0.031183221	0.868939346	0.384880307	0.54662356	positive
PRJNA729271	ed_Lushi_vs_Arbor_Acr	breed_growth_background	ENSGALG00010028598	TMOD4	0.173195089	0.131432926	1.317745063	0.187589004	0.333386784	positive
PRJNA729271	ed_Lushi_vs_Arbor_Acr	breed_growth_background	ENSGALG00010028599	VPS72	0.113860001	0.063216952	1.801099197	0.071687246	0.1705559	positive
PRJNA729271	ed_Lushi_vs_Arbor_Acr	breed_growth_background	ENSGALG00010028600	PSMD4	0.152041523	0.070787879	2.147846872	0.031725922	0.095033528	positive
PRJNA729271	ed_Lushi_vs_Arbor_Acr	breed_growth_background	ENSGALG00010028601	ANO8	0.357509404	0.118001919	3.029691452	0.002448037	0.015387812	positive
PRJNA729271	ed_Lushi_vs_Arbor_Acr	breed_growth_background	ENSGALG00010028602	ZNF687	0.108424918	0.091394603	1.186338297	0.235488728	0.390193086	positive
PRJNA729271	ed_Lushi_vs_Arbor_Acr	breed_growth_background	ENSGALG00010028603	SH3GLB2	-0.225519937	0.181350157	-1.24356075	0.213661269	0.364183925	negative
PRJNA729271	ed_Lushi_vs_Arbor_Acr	breed_growth_background	ENSGALG00010028604	PI4KB	0.362419056	0.075314091	4.812101553	1.49351E-06	6.74408E-05	positive
PRJNA729271	ed_Lushi_vs_Arbor_Acr	breed_growth_background	ENSGALG00010028605	GTPBP3	0.269972651	0.114503105	2.357775798	0.018384793	0.064431231	positive
PRJNA729271	ed_Lushi_vs_Arbor_Acr	breed_growth_background	ENSGALG00010028606	PRPF3	0.342733952	0.073098352	4.688668669	2.74988E-06	0.000106292	positive
PRJNA729271	ed_Lushi_vs_Arbor_Acr	breed_growth_background	ENSGALG00010028607	ENSGALG00010028607	-0.097875073	0.127737302	-0.766221542	0.443544493	0.600464876	negative
PRJNA729271	ed_Lushi_vs_Arbor_Acr	breed_growth_background	ENSGALG00010028608	MIGA2	-0.063816878	0.085585143	-0.745653695	0.455876638	0.611920636	negative
PRJNA729271	ed_Lushi_vs_Arbor_Acr	breed_growth_background	ENSGALG00010028609	MRPS21	0.168279043	0.077823274	2.162322845	0.0305933	0.092302522	positive
PRJNA729271	ed_Lushi_vs_Arbor_Acr	breed_growth_background	ENSGALG00010028610	ENSGALG00010028610	0.115085813	0.282837514	0.406897272	0.684083439	0.796065306	positive
PRJNA729271	ed_Lushi_vs_Arbor_Acr	breed_growth_background	ENSGALG00010028612	DIRAS1	-0.18000758	0.36898197	-0.487849259	0.625656618	0.752043509	negative
PRJNA729271	ed_Lushi_vs_Arbor_Acr	breed_growth_background	ENSGALG00010028613	ENSGALG00010028613	0.091563204	0.1950716	0.469382544	0.638796223	0.763028534	positive
PRJNA729271	ed_Lushi_vs_Arbor_Acr	breed_growth_background	ENSGALG00010028618	DOLPP1	-0.146830456	0.060832557	-2.413682136	0.015792233	0.057805155	negative
PRJNA729271	ed_Lushi_vs_Arbor_Acr	breed_growth_background	ENSGALG00010028626	CRAT	0.088187704	0.092284111	0.955610197	0.339268844	0.503501781	positive
PRJNA729271	ed_Lushi_vs_Arbor_Acr	breed_growth_background	ENSGALG00010028642	APH1A	0.151339731	0.074883095	2.02101329	0.043278389	0.11917349	positive
PRJNA729271	ed_Lushi_vs_Arbor_Acr	breed_growth_background	ENSGALG00010028649	ENSGALG00010028649	-0.23028623	0.09299392	-2.476357914	0.013273044	0.05099199	negative
PRJNA729271	ed_Lushi_vs_Arbor_Acr	breed_growth_background	ENSGALG00010028658	PTPA	0.018156537	0.044238161	0.410427018	0.68149273	0.794262051	positive
PRJNA729271	ed_Lushi_vs_Arbor_Acr	breed_growth_background	ENSGALG00010028662	FCRL4	-0.193312424	0.123950142	-1.559598237	0.118854853	0.242784823	negative
PRJNA729271	ed_Lushi_vs_Arbor_Acr	breed_growth_background	ENSGALG00010028669	ZBTB34	0.073376525	0.059437875	1.234507881	0.217013712	0.3688139	positive
PRJNA729271	ed_Lushi_vs_Arbor_Acr	breed_growth_background	ENSGALG00010028671	CADM3	-0.193607234	0.113597751	-1.704322771	0.088320803	0.197380965	negative
PRJNA729271	ed_Lushi_vs_Arbor_Acr	breed_growth_background	ENSGALG00010028675	ZBTB43	-0.013657784	0.038554635	-0.354244942	0.723155326	0.822458267	negative
PRJNA729271	ed_Lushi_vs_Arbor_Acr	breed_growth_background	ENSGALG00010028678	ENSGALG00010028678	-0.136932239	0.196419782	-0.697140774	0.485714699	0.63743183	negative
PRJNA729271	ed_Lushi_vs_Arbor_Acr	breed_growth_background	ENSGALG00010028686	TAGLN2	-0.131482478	0.064098599	-2.051253534	0.040242263	0.112857597	negative
PRJNA729271	ed_Lushi_vs_Arbor_Acr	breed_growth_background	ENSGALG00010028689	CFAP45	0.71711793	0.345274198	2.076951984	0.037805991	0.108107682	positive
PRJNA729271	ed_Lushi_vs_Arbor_Acr	breed_growth_background	ENSGALG00010028692	RALGPS1	0.121906296	0.059622311	2.044642265	0.040890139	0.114222889	positive
PRJNA729271	ed_Lushi_vs_Arbor_Acr	breed_growth_background	ENSGALG00010028695	VSIG8	0.240154683	0.460816647	0.52115019	0.602262149	0.734637245	positive
PRJNA729271	ed_Lushi_vs_Arbor_Acr	breed_growth_background	ENSGALG00010028700	ANGPTL2	-0.261495778	0.105527881	-2.477978085	0.013212926	0.05084188	negative
PRJNA729271	ed_Lushi_vs_Arbor_Acr	breed_growth_background	ENSGALG00010028707	ENSGALG00010028707	-0.071684201	0.652527908	-0.109856146	0.912523463	0.950627252	negative
PRJNA729271	ed_Lushi_vs_Arbor_Acr	breed_growth_background	ENSGALG00010028712	FBXW2	-0.076502993	0.045903066	-1.666620529	0.095589884	0.208231947	negative
PRJNA729271	ed_Lushi_vs_Arbor_Acr	breed_growth_background	ENSGALG00010028726	SLC2A8	-0.275390035	0.060745605	-4.533497314	5.8015E-06	0.000193317	negative
PRJNA729271	ed_Lushi_vs_Arbor_Acr	breed_growth_background	ENSGALG00010028727	ENSGALG00010028727	0.053006444	0.098469776	0.538301667	0.5903688	0.72551045	positive
PRJNA729271	ed_Lushi_vs_Arbor_Acr	breed_growth_background	ENSGALG00010028734	ENSGALG00010028734	0.839710958	0.190206862	4.414724846	1.01139E-05	0.000294378	positive
PRJNA729271	ed_Lushi_vs_Arbor_Acr	breed_growth_background	ENSGALG00010028735	ENSGALG00010028735	-0.124687848	0.19287789	-0.646460039	0.517981469	0.666295724	negative
PRJNA729271	ed_Lushi_vs_Arbor_Acr	breed_growth_background	ENSGALG00010028738	MIDN	0.24804286	0.099844122	2.484301062	0.012980601	0.050227928	positive
PRJNA729271	ed_Lushi_vs_Arbor_Acr	breed_growth_background	ENSGALG00010028739	GARNL3	-0.037859717	0.04193776	-0.902759633	0.36665348	0.530160847	negative
PRJNA729271	ed_Lushi_vs_Arbor_Acr	breed_growth_background	ENSGALG00010028740	DAZAP1	0.138640961	0.056905867	2.436321049	0.014837509	0.055117809	positive
PRJNA729271	ed_Lushi_vs_Arbor_Acr	breed_growth_background	ENSGALG00010028742	ENSGALG00010028742	0.304785665	0.079295919	3.843648833	0.000121218	0.001839855	positive
PRJNA729271	ed_Lushi_vs_Arbor_Acr	breed_growth_background	ENSGALG00010028743	ARRDC1	0.253856383	0.099445831	2.552710152	0.010688841	0.043915744	positive
PRJNA729271	ed_Lushi_vs_Arbor_Acr	breed_growth_background	ENSGALG00010028744	HOMER3	0.066107119	0.111541381	0.592669	0.553402691	0.695652763	positive
PRJNA729271	ed_Lushi_vs_Arbor_Acr	breed_growth_background	ENSGALG00010028745	ENSGALG00010028745	0.27482175	0.074546154	3.686598646	0.000227271	0.002839912	positive
PRJNA729271	ed_Lushi_vs_Arbor_Acr	breed_growth_background	ENSGALG00010028746	MYDGF	-0.15920292	0.067580804	-2.355741728	0.018485765	0.064706875	negative
PRJNA729271	ed_Lushi_vs_Arbor_Acr	breed_growth_background	ENSGALG00010028747	ENSGALG00010028747	-0.312322821	0.102786564	-3.038556892	0.002377142	0.015059736	negative
PRJNA729271	ed_Lushi_vs_Arbor_Acr	breed_growth_background	ENSGALG00010028748	PIAS3	0.278124161	0.10685894	2.602722447	0.009248679	0.039580634	positive
PRJNA729271	ed_Lushi_vs_Arbor_Acr	breed_growth_background	ENSGALG00010028750	OTUD7B	0.237343973	0.065067251	3.647671716	0.000264627	0.003183216	positive
PRJNA729271	ed_Lushi_vs_Arbor_Acr	breed_growth_background	ENSGALG00010028751	EHMT1	0.006389836	0.051479707	0.124123397	0.901217573	0.942711173	positive
PRJNA729271	ed_Lushi_vs_Arbor_Acr	breed_growth_background	ENSGALG00010028752	MTMR11	-0.132286986	0.070994416	-1.863343531	0.062413949	0.155072025	negative
PRJNA729271	ed_Lushi_vs_Arbor_Acr	breed_growth_background	ENSGALG00010028753	SV2A	0.020270571	0.335065851	0.060497276	0.951759585	0.973433394	positive
PRJNA729271	ed_Lushi_vs_Arbor_Acr	breed_growth_background	ENSGALG00010028756	ENSGALG00010028756	1.37907912	0.418555987	3.29484982	0.000984743	0.008073444	positive
PRJNA729271	ed_Lushi_vs_Arbor_Acr	breed_growth_background	ENSGALG00010028757	MAT2A	1.001258345	0.730156022	1.371293689	0.170283422	0.312596024	positive
PRJNA729271	ed_Lushi_vs_Arbor_Acr	breed_growth_background	ENSGALG00010028760	SSR2	0.021248658	0.052208572	0.406995572	0.684011241	0.796045197	positive
PRJNA729271	ed_Lushi_vs_Arbor_Acr	breed_growth_background	ENSGALG00010028761	CTSS	-0.049135235	0.12671817	-0.387752089	0.698199504	0.805308638	negative
PRJNA729271	ed_Lushi_vs_Arbor_Acr	breed_growth_background	ENSGALG00010028762	CACNA1B	0.48009144	0.397569625	1.207565692	0.227214362	0.380335933	positive
PRJNA729271	ed_Lushi_vs_Arbor_Acr	breed_growth_background	ENSGALG00010028763	CTSK	-0.217028732	0.116296953	-1.866160086	0.062018972	0.154408278	negative
PRJNA729271	ed_Lushi_vs_Arbor_Acr	breed_growth_background	ENSGALG00010028764	ARNT	0.106399912	0.067126358	1.585069032	0.112950651	0.234424353	positive
PRJNA729271	ed_Lushi_vs_Arbor_Acr	breed_growth_background	ENSGALG00010028768	NTNG2	-0.002149817	0.191005352	-0.01125527	0.991019783	0.995001161	negative
PRJNA729271	ed_Lushi_vs_Arbor_Acr	breed_growth_background	ENSGALG00010028771	SETX	-0.046958586	0.038945621	-1.205747518	0.227914857	0.381128963	negative
PRJNA729271	ed_Lushi_vs_Arbor_Acr	breed_growth_background	ENSGALG00010028772	TTF1	0.018792612	0.039801399	0.472159578	0.636812889	0.761364896	positive
PRJNA729271	ed_Lushi_vs_Arbor_Acr	breed_growth_background	ENSGALG00010028782	MLLT11	-0.007966721	0.137042617	-0.058133162	0.953642559	0.974074966	negative
PRJNA729271	ed_Lushi_vs_Arbor_Acr	breed_growth_background	ENSGALG00010028783	DDX31	0.043869815	0.040846133	1.074026151	0.282810961	0.442789466	positive
PRJNA729271	ed_Lushi_vs_Arbor_Acr	breed_growth_background	ENSGALG00010028786	GTF3C4	-0.021904546	0.037147482	-0.589664351	0.5554157	0.697196692	negative
PRJNA729271	ed_Lushi_vs_Arbor_Acr	breed_growth_background	ENSGALG00010028800	ENSGALG00010028800	0.120956648	0.229311241	0.527478056	0.597861651	0.731123314	positive
PRJNA729271	ed_Lushi_vs_Arbor_Acr	breed_growth_background	ENSGALG00010028806	SPACA9	-0.178940177	0.232507862	-0.769609147	0.441531778	0.598914759	negative
PRJNA729271	ed_Lushi_vs_Arbor_Acr	breed_growth_background	ENSGALG00010028818	TSC1	0.082882148	0.06270951	1.321683866	0.186273443	0.331740209	positive
PRJNA729271	ed_Lushi_vs_Arbor_Acr	breed_growth_background	ENSGALG00010028823	GFI1B	-0.389973863	0.176705903	-2.206909084	0.027320412	0.084944094	negative
PRJNA729271	ed_Lushi_vs_Arbor_Acr	breed_growth_background	ENSGALG00010028826	CEL	-2.579886309	1.45373185	-1.774664502	0.0759533	0.177704179	negative
PRJNA729271	ed_Lushi_vs_Arbor_Acr	breed_growth_background	ENSGALG00010028829	GTF3C5	0.196762264	0.061894712	3.17898344	0.001477925	0.010813997	positive
PRJNA729271	ed_Lushi_vs_Arbor_Acr	breed_growth_background	ENSGALG00010028833	GBGT1	-0.010771853	0.088400428	-0.121852954	0.903015475	0.944114924	negative
PRJNA729271	ed_Lushi_vs_Arbor_Acr	breed_growth_background	ENSGALG00010028849	ENSGALG00010028849	-0.670022757	0.168023961	-3.987661961	6.67276E-05	0.001183864	negative
PRJNA729271	ed_Lushi_vs_Arbor_Acr	breed_growth_background	ENSGALG00010028853	ENSGALG00010028853	-0.77560327	0.654039813	-1.18586553	0.235675408	0.390368533	negative
PRJNA729271	ed_Lushi_vs_Arbor_Acr	breed_growth_background	ENSGALG00010028854	RPL7A	-0.00957905	0.075016277	-0.12769296	0.898391962	0.941162567	negative
PRJNA729271	ed_Lushi_vs_Arbor_Acr	breed_growth_background	ENSGALG00010028855	SURF6	0.251934039	0.09102837	2.767643105	0.005646325	0.027762375	positive
PRJNA729271	ed_Lushi_vs_Arbor_Acr	breed_growth_background	ENSGALG00010028856	ETV3	0.144870342	0.059138771	2.449667768	0.014298808	0.053625155	positive
PRJNA729271	ed_Lushi_vs_Arbor_Acr	breed_growth_background	ENSGALG00010028857	MED22	-0.117395532	0.042508104	-2.761721177	0.005749756	0.028080428	negative
PRJNA729271	ed_Lushi_vs_Arbor_Acr	breed_growth_background	ENSGALG00010028858	ENSGALG00010028858	-1.664115279	0.245812357	-6.769860152	1.28907E-11	4.92795E-09	negative
PRJNA729271	ed_Lushi_vs_Arbor_Acr	breed_growth_background	ENSGALG00010028859	SURF1	0.163265397	0.059001046	2.767161041	0.005654681	0.027794034	positive
PRJNA729271	ed_Lushi_vs_Arbor_Acr	breed_growth_background	ENSGALG00010028860	SURF2	0.080207873	0.092273367	0.86924186	0.384714856	0.546460265	positive
PRJNA729271	ed_Lushi_vs_Arbor_Acr	breed_growth_background	ENSGALG00010028861	SURF4	-0.050306858	0.040090841	-1.254821711	0.209543489	0.359257796	negative
PRJNA729271	ed_Lushi_vs_Arbor_Acr	breed_growth_background	ENSGALG00010028862	ENSGALG00010028862	-1.168394161	0.623428118	-1.87414415	0.06091056	0.152565848	negative
PRJNA729271	ed_Lushi_vs_Arbor_Acr	breed_growth_background	ENSGALG00010028865	STKLD1	0.112612011	0.089990298	1.25137947	0.210796064	0.360955575	positive
PRJNA729271	ed_Lushi_vs_Arbor_Acr	breed_growth_background	ENSGALG00010028866	HORMAD1	0.109700544	0.275358738	0.398391369	0.690341719	0.799752495	positive
PRJNA729271	ed_Lushi_vs_Arbor_Acr	breed_growth_background	ENSGALG00010028867	REXO4	0.081255829	0.064264271	1.264401317	0.206086021	0.355413636	positive
PRJNA729271	ed_Lushi_vs_Arbor_Acr	breed_growth_background	ENSGALG00010028868	ENSGALG00010028868	-0.074835948	2.254040989	-0.033200793	0.973514466	0.984380341	negative
PRJNA729271	ed_Lushi_vs_Arbor_Acr	breed_growth_background	ENSGALG00010028869	ADAMTS13	0.159135762	0.085186018	1.868097204	0.061748523	0.154052469	positive
PRJNA729271	ed_Lushi_vs_Arbor_Acr	breed_growth_background	ENSGALG00010028870	CACFD1	-0.214433771	0.12498034	-1.715740022	0.086209636	0.194129047	negative
PRJNA729271	ed_Lushi_vs_Arbor_Acr	breed_growth_background	ENSGALG00010028871	PIP5K1A	0.016166891	0.05858961	0.275932787	0.782599691	0.865541166	positive
PRJNA729271	ed_Lushi_vs_Arbor_Acr	breed_growth_background	ENSGALG00010028872	SLC2A6	0.107806972	0.180333871	0.597818762	0.549960871	0.69326276	positive
PRJNA729271	ed_Lushi_vs_Arbor_Acr	breed_growth_background	ENSGALG00010028873	TMEM8C	-0.166887344	0.151063845	-1.104747099	0.269269189	0.427872933	negative
PRJNA729271	ed_Lushi_vs_Arbor_Acr	breed_growth_background	ENSGALG00010028874	CRPL1	2.948165292	0.272249395	10.82891404	2.51116E-27	9.09981E-24	positive
PRJNA729271	ed_Lushi_vs_Arbor_Acr	breed_growth_background	ENSGALG00010028875	DUSP23	-0.071131531	0.063844005	-1.11414581	0.265216639	0.423438766	negative
PRJNA729271	ed_Lushi_vs_Arbor_Acr	breed_growth_background	ENSGALG00010028876	ADAMTSL2	0.07460388	0.105472093	0.707332892	0.47935963	0.632182498	positive

PRJNA729271	:ed_Lushi_vs_Arbor_Acr	breed_growth_background	ENSGALG00010028877	FAM163B	0.158897002	0.378815781	0.419457187	0.674882038	0.789732392	positive
PRJNA729271	:ed_Lushi_vs_Arbor_Acr	breed_growth_background	ENSGALG00010028878	IGSF9	0.255093756	0.085202248	2.99397917	0.002753647	0.016651698	positive
PRJNA729271	:ed_Lushi_vs_Arbor_Acr	breed_growth_background	ENSGALG00010028881	SARDH	-0.382759374	0.124220245	-3.081296249	0.002061015	0.013548485	negative
PRJNA729271	:ed_Lushi_vs_Arbor_Acr	breed_growth_background	ENSGALG00010028884	VAV2	-0.098521735	0.070305192	-1.401343661	0.161111331	0.300206806	negative
PRJNA729271	:ed_Lushi_vs_Arbor_Acr	breed_growth_background	ENSGALG00010028885	BRD3	0.299142499	0.076304861	3.920359651	8.84169E-05	0.001463017	positive
PRJNA729271	:ed_Lushi_vs_Arbor_Acr	breed_growth_background	ENSGALG00010028886	ENSGALG00010028886	0.261635887	0.924660557	0.282953441	0.777212527	0.861621077	positive
PRJNA729271	:ed_Lushi_vs_Arbor_Acr	breed_growth_background	ENSGALG00010028887	SF3B4	0.262779671	0.094342615	2.785376156	0.005346566	0.02673283	positive
PRJNA729271	:ed_Lushi_vs_Arbor_Acr	breed_growth_background	ENSGALG00010028888	WDR5B	0.098861839	0.044749858	2.209210137	0.027160028	0.08460877	positive
PRJNA729271	:ed_Lushi_vs_Arbor_Acr	breed_growth_background	ENSGALG00010028889	FUBP3	-0.114525285	0.044982982	-2.545969144	0.010897482	0.044484914	negative
PRJNA729271	:ed_Lushi_vs_Arbor_Acr	breed_growth_background	ENSGALG00010028890	ENSGALG00010028890	0.28037088	0.16542785	1.694822726	0.090109055	0.20041902	positive
PRJNA729271	:ed_Lushi_vs_Arbor_Acr	breed_growth_background	ENSGALG00010028891	SLC31A2	0.326484763	0.119867352	2.723717149	0.006455176	0.030587703	positive
PRJNA729271	:ed_Lushi_vs_Arbor_Acr	breed_growth_background	ENSGALG00010028892	NIBAN2	-0.191282632	0.075513786	-2.533082277	0.011306441	0.045692482	negative
PRJNA729271	:ed_Lushi_vs_Arbor_Acr	breed_growth_background	ENSGALG00010028893	STXBP1	0.221526153	0.106215797	2.085623423	0.037012748	0.106300729	positive
PRJNA729271	:ed_Lushi_vs_Arbor_Acr	breed_growth_background	ENSGALG00010028894	AKNA	-0.039036901	0.087780767	-0.444709043	0.656530033	0.776152258	negative
PRJNA729271	:ed_Lushi_vs_Arbor_Acr	breed_growth_background	ENSGALG00010028895	WHRN	0.08414043	0.40378781	0.208377837	0.83493396	0.899603639	positive
PRJNA729271	:ed_Lushi_vs_Arbor_Acr	breed_growth_background	ENSGALG00010028896	ATP6V1G1	-0.0883297	0.052905805	-1.669565373	0.095005386	0.207457528	negative
PRJNA729271	:ed_Lushi_vs_Arbor_Acr	breed_growth_background	ENSGALG00010028897	TMEM268	-0.334170107	0.152629005	-2.189427276	0.028565799	0.087706261	negative
PRJNA729271	:ed_Lushi_vs_Arbor_Acr	breed_growth_background	ENSGALG00010028898	TNFSF15	0.234809988	0.140053125	1.67657799	0.093625034	0.205244769	positive
PRJNA729271	:ed_Lushi_vs_Arbor_Acr	breed_growth_background	ENSGALG00010028899	TNFSF8	0.667005182	0.323357263	2.062749964	0.03913639	0.110667572	positive
PRJNA729271	:ed_Lushi_vs_Arbor_Acr	breed_growth_background	ENSGALG00010028900	TNC	-0.560687696	0.294200466	-1.905801521	0.056675962	0.144914105	negative
PRJNA729271	:ed_Lushi_vs_Arbor_Acr	breed_growth_background	ENSGALG00010028901	PAPPA	0.154594112	0.063879891	2.420074779	0.015517316	0.057130681	positive
PRJNA729271	:ed_Lushi_vs_Arbor_Acr	breed_growth_background	ENSGALG00010028902	ENSGALG00010028902	0.105180017	0.273875488	0.384043194	0.700946433	0.807058487	positive
PRJNA729271	:ed_Lushi_vs_Arbor_Acr	breed_growth_background	ENSGALG00010028903	ASTN2	0.199725806	0.136766845	1.460337895	0.144197232	0.277501179	positive
PRJNA729271	:ed_Lushi_vs_Arbor_Acr	breed_growth_background	ENSGALG00010028904	TRIM32	0.004176471	0.046254541	0.090293219	0.928054208	0.960044658	positive
PRJNA729271	:ed_Lushi_vs_Arbor_Acr	breed_growth_background	ENSGALG00010028905	COLGALT1	-0.030969346	0.046373016	-0.667831193	0.504241353	0.654597749	negative
PRJNA729271	:ed_Lushi_vs_Arbor_Acr	breed_growth_background	ENSGALG00010028909	ENSGALG00010028909	0.221222663	0.07684361	2.878868683	0.003991045	0.021634328	positive
PRJNA729271	:ed_Lushi_vs_Arbor_Acr	breed_growth_background	ENSGALG00010028911	LMX1B	-1.042318893	0.350443169	-2.974287947	0.002936694	0.017474291	negative
PRJNA729271	:ed_Lushi_vs_Arbor_Acr	breed_growth_background	ENSGALG00010028912	EXFABP	0.583727344	0.266409895	2.191087323	0.028445474	0.087466514	positive
PRJNA729271	:ed_Lushi_vs_Arbor_Acr	breed_growth_background	ENSGALG00010028914	TLR4	-0.009898751	0.12290039	-0.08054288	0.935805494	0.964536447	negative
PRJNA729271	:ed_Lushi_vs_Arbor_Acr	breed_growth_background	ENSGALG00010028915	ADGRD2	-0.263834285	0.258764999	-1.019590308	0.307922803	0.470617992	negative
PRJNA729271	:ed_Lushi_vs_Arbor_Acr	breed_growth_background	ENSGALG00010028917	NR6A1	0.061748757	0.07009431	0.880938223	0.378351258	0.540448994	positive
PRJNA729271	:ed_Lushi_vs_Arbor_Acr	breed_growth_background	ENSGALG00010028918	AGPAT2	0.185240218	0.097909169	1.891959875	0.058496327	0.148027979	positive
PRJNA729271	:ed_Lushi_vs_Arbor_Acr	breed_growth_background	ENSGALG00010028919	EGFL7	-0.481092269	0.090153775	-5.336351885	9.48353E-08	7.94588E-06	negative
PRJNA729271	:ed_Lushi_vs_Arbor_Acr	breed_growth_background	ENSGALG00010028921	MRPL41	0.038200293	0.065834401	0.5802482	0.561747254	0.702486968	positive
PRJNA729271	:ed_Lushi_vs_Arbor_Acr	breed_growth_background	ENSGALG00010028923	ENSGALG00010028923	-0.113873145	0.099093255	-1.149151324	0.250493588	0.40632325	negative
PRJNA729271	:ed_Lushi_vs_Arbor_Acr	breed_growth_background	ENSGALG00010028924	TMEM203	0.226121074	0.064692923	3.495298466	0.000473532	0.004786503	positive
PRJNA729271	:ed_Lushi_vs_Arbor_Acr	breed_growth_background	ENSGALG00010028925	ENSGALG00010028925	-0.072886599	0.175262601	-0.415870805	0.677504544	0.791617779	negative
PRJNA729271	:ed_Lushi_vs_Arbor_Acr	breed_growth_background	ENSGALG00010028926	ENSGALG00010028926	1.341741697	0.351620619	3.815878886	0.000135699	0.001995675	positive
PRJNA729271	:ed_Lushi_vs_Arbor_Acr	breed_growth_background	ENSGALG00010029140	NPDC1	-0.119365238	0.102368947	-1.166029754	0.243602416	0.399210516	negative
PRJNA729271	:ed_Lushi_vs_Arbor_Acr	breed_growth_background	ENSGALG00010029141	C5	-0.704485919	0.308455552	-2.283913887	0.022376592	0.073990888	negative
PRJNA729271	:ed_Lushi_vs_Arbor_Acr	breed_growth_background	ENSGALG00010029142	ENSGALG00010029142	-0.172891634	0.097329863	-1.776347242	0.075675701	0.177253275	negative
PRJNA729271	:ed_Lushi_vs_Arbor_Acr	breed_growth_background	ENSGALG00010029143	SH2D3C	-0.164680383	0.059191019	-2.782185331	0.00539942	0.026913545	negative
PRJNA729271	:ed_Lushi_vs_Arbor_Acr	breed_growth_background	ENSGALG00010029144	ENSGALG00010029144	0.537729535	0.107030366	5.024083852	5.05841E-07	2.84193E-05	positive
PRJNA729271	:ed_Lushi_vs_Arbor_Acr	breed_growth_background	ENSGALG00010029145	RC3H2	0.151224947	0.071513246	2.11464247	0.034460431	0.100779418	positive
PRJNA729271	:ed_Lushi_vs_Arbor_Acr	breed_growth_background	ENSGALG00010029146	NEK6	0.039219591	0.031955415	1.227322237	0.219701501	0.372160016	positive
PRJNA729271	:ed_Lushi_vs_Arbor_Acr	breed_growth_background	ENSGALG00010029147	RABL6	-0.061046353	0.055958319	-1.090925443	0.275305691	0.434225232	negative
PRJNA729271	:ed_Lushi_vs_Arbor_Acr	breed_growth_background	ENSGALG00010029148	RALGDS	-0.165166494	0.063642076	-2.59524051	0.009452475	0.040262599	negative
PRJNA729271	:ed_Lushi_vs_Arbor_Acr	breed_growth_background	ENSGALG00010029149	ENSGALG00010029149	0.244309867	0.125566968	1.94565394	0.051696323	0.135629936	positive
PRJNA729271	:ed_Lushi_vs_Arbor_Acr	breed_growth_background	ENSGALG00010029150	NARF	0.202931919	0.06614172	3.068137905	0.002153972	0.013982008	positive
PRJNA729271	:ed_Lushi_vs_Arbor_Acr	breed_growth_background	ENSGALG00010029151	CYBC1	-0.185346633	0.062354224	-2.972479209	0.002954052	0.017537284	negative
PRJNA729271	:ed_Lushi_vs_Arbor_Acr	breed_growth_background	ENSGALG00010029152	HEXD	0.021027414	0.071517593	0.294017369	0.768744631	0.855899334	positive
PRJNA729271	:ed_Lushi_vs_Arbor_Acr	breed_growth_background	ENSGALG00010029153	OGFOD3	-0.067333079	0.044154211	-1.524952609	0.127270919	0.254208621	negative
PRJNA729271	:ed_Lushi_vs_Arbor_Acr	breed_growth_background	ENSGALG00010029154	UTS2R	-0.099923685	0.077989992	-1.281237276	0.200110337	0.348965271	negative
PRJNA729271	:ed_Lushi_vs_Arbor_Acr	breed_growth_background	ENSGALG00010029155	ENSGALG00010029155	0.45080978	0.311774606	1.445947717	0.148191867	0.283156336	positive
PRJNA729271	:ed_Lushi_vs_Arbor_Acr	breed_growth_background	ENSGALG00010029156	ENSGALG00010029156	0.156258106	0.619593729	0.25219446	0.80089076	0.877102969	positive
PRJNA729271	:ed_Lushi_vs_Arbor_Acr	breed_growth_background	ENSGALG00010029158	CD7	0.301529587	0.236953505	1.272526385	0.203186156	0.352326913	positive
PRJNA729271	:ed_Lushi_vs_Arbor_Acr	breed_growth_background	ENSGALG00010029159	SSC4D	-0.206304733	0.562469428	-0.366783904	0.713780215	0.81615478	negative
PRJNA729271	:ed_Lushi_vs_Arbor_Acr	breed_growth_background	ENSGALG00010029160	DUS1L	0.115697712	0.04943648	2.340330703	0.019266671	0.066603959	positive
PRJNA729271	:ed_Lushi_vs_Arbor_Acr	breed_growth_background	ENSGALG00010029162	ELN	-0.260171899	0.166247494	-1.564967345	0.117590566	0.241085609	negative
PRJNA729271	:ed_Lushi_vs_Arbor_Acr	breed_growth_background	ENSGALG00010029163	ANKFY1	-0.154064026	0.050817921	-3.031686888	0.002431913	0.015306375	negative
PRJNA729271	:ed_Lushi_vs_Arbor_Acr	breed_growth_background	ENSGALG00010029164	CYB5D2	-0.057531237	0.044569876	-1.290809916	0.196769598	0.34517654	negative
PRJNA729271	:ed_Lushi_vs_Arbor_Acr	breed_growth_background	ENSGALG00010029165	RAC3	-0.159182041	0.064787143	-2.45700049	0.01401025	0.052926392	negative
PRJNA729271	:ed_Lushi_vs_Arbor_Acr	breed_growth_background	ENSGALG00010029166	ZZEF1	0.129338784	0.066246637	1.952382624	0.050892794	0.134101264	positive
PRJNA729271	:ed_Lushi_vs_Arbor_Acr	breed_growth_background	ENSGALG00010029167	ENSGALG00010029167	0.137012352	0.067868234	2.018799428	0.043508071	0.119690545	positive
PRJNA729271	:ed_Lushi_vs_Arbor_Acr	breed_growth_background	ENSGALG00010029168	RFNG	0.115294721	0.042968589	2.683232663	0.007291424	0.033344123	positive
PRJNA729271	:ed_Lushi_vs_Arbor_Acr	breed_growth_background	ENSGALG00010029169	SBDS	0.184502972	0.041635505	4.431385468	9.36295E-06	0.000278107	positive
PRJNA729271	:ed_Lushi_vs_Arbor_Acr	breed_growth_background	ENSGALG00010029170	DCXR	-0.419875273	0.076329879	-5.500798378	3.78075E-08	4.05941E-06	negative
PRJNA729271	:ed_Lushi_vs_Arbor_Acr	breed_growth_background	ENSGALG00010029171	ENSGALG00010029171	0.089529743	0.078917732	1.134469289	0.256597749	0.413724624	positive
PRJNA729271	:ed_Lushi_vs_Arbor_Acr	breed_growth_background	ENSGALG00010029172	LRRC45	0.075701416	0.040785839	1.856071054	0.063443431	0.156873916	positive
PRJNA729271	:ed_Lushi_vs_Arbor_Acr	breed_growth_background	ENSGALG00010029173	ZNHIT3	-0.203048265	0.073228999	-2.772784944	0.005557884	0.027457916	negative
PRJNA729271	:ed_Lushi_vs_Arbor_Acr	breed_growth_background	ENSGALG00010029174	CALN1	0.178774706	0.219007609	0.816294497	0.414331721	0.574656807	positive
PRJNA729271	:ed_Lushi_vs_Arbor_Acr	breed_growth_background	ENSGALG00010029175	CENPX	-0.32572154	0.07144488	-4.55906064	5.13829E-06	0.000178181	negative
PRJNA729271	:ed_Lushi_vs_Arbor_Acr	breed_growth_background	ENSGALG00010029176	ENSGALG00010029176	0.033231015	0.270973492	0.122635668	0.902395609	0.94373913	positive
PRJNA729271	:ed_Lushi_vs_Arbor_Acr	breed_growth_background	ENSGALG00010029177	ASPSCR1	-0.045730851	0.043277937	-1.056678158	0.290658497	0.452145837	negative
PRJNA729271	:ed_Lushi_vs_Arbor_Acr	breed_growth_background	ENSGALG00010029178	NOTUM	0.281169364	0.111418917	2.523533442	0.011618201	0.046526765	positive
PRJNA729271	:ed_Lushi_vs_Arbor_Acr	breed_growth_background	ENSGALG00010029179	PIGW	0.133822732	0.081914485	1.633688244	0.102324328	0.218659775	positive
PRJNA729271	:ed_Lushi_vs_Arbor_Acr	breed_growth_background	ENSGALG00010029180	CA4	0.920962358	0.196386417	4.689542043	2.73817E-06	0.000106122	positive
PRJNA729271	:ed_Lushi_vs_Arbor_Acr	breed_growth_background	ENSGALG00010029181	MYO19	0.072583378	0.056818207	1.277466889	0.201437463	0.350352295	positive
PRJNA729271	:ed_Lushi_vs_Arbor_Acr	breed_growth_background	ENSGALG00010029182	HEATR6	0.020513893	0.038044189	0.539212244	0.589740412	0.725230108	positive
PRJNA729271	:ed_Lushi_vs_Arbor_Acr	breed_growth_background	ENSGALG00010029183	ENSGALG00010029183	0.219137641	0.07446658	2.942764954	0.003252953	0.01880046	positive
PRJNA729271	:ed_Lushi_vs_Arbor_Acr	breed_growth_background	ENSGALG00010029184	DYNLL2	0.082239781	0.05140744	1.599764172	0.10965091	0.230079609	positive
PRJNA729271	:ed_Lushi_vs_Arbor_Acr	breed_growth_background	ENSGALG00010029186	ZNF302	0.278555673	0.075463498	3.691263727	0.000223143	0.002812624	positive
PRJNA729271	:ed_Lushi_vs_Arbor_Acr	breed_growth_background	ENSGALG00010029187	SRSF1	-0.069647021	0.028440503	-2.448867399	0.014330619	0.053696092	negative
PRJNA729271	:ed_Lushi_vs_Arbor_Acr	breed_growth_background	ENSGALG00010029188	ENSGALG00010029188	0.105073118	0.135351503	0.776298124	0.43757298	0.595048349	positive
PRJNA729271	:ed_Lushi_vs_Arbor_Acr	breed_growth_background	ENSGALG00010029189	ENSGALG00010029189	0.236696654	0.190012972	1.245686813	0.212879397	0.363192898	positive
PRJNA729271	:ed_Lushi_vs_Arbor_Acr	breed_growth_background	ENSGALG00010029190	DHRS7C	-0.365248321	0.080506388	-4.536886221	5.70909E-06	0.000191558	negative
PRJNA729271	:ed_Lushi_vs_Arbor_Acr	breed_growth_background	ENSGALG00010029192	DTX2	0.136378328	0.054141401	2.518928671	0.01177125	0.046939276	positive
PRJNA729271	:ed_Lushi_vs_Arbor_Acr	breed_growth_background	ENSGALG00010029194	DHRS11	-0.084613625	0.073510487	-1.151041542	0.249715145	0.405696148	negative
PRJNA729271	:ed_Lushi_vs_Arbor_Acr	breed_growth_background	ENSGALG00010029196	MRM1	0.111618027	0.054304366	2.055415338	0.039838905	0.112031338	positive
PRJNA729271	:ed_Lushi_vs_Arbor_Acr	breed_growth_background	ENSGALG00010029197	LHX1	0.238853363	0.273307587	0.873936086	0.382153061	0.543549075	positive
PRJNA729271	:ed_Lushi_vs_Arbor_Acr	breed_growth_background	ENSGALG00010029198	PPM1D	0.151371273	0.093719094	1.615159365	0.106276188	0.224853794	positive
PRJNA729271	:ed_Lushi_vs_Arbor_Acr	breed_growth_background	ENSGALG00010029199	APPBP2	-0.050754181	0.048187683	-1.05326045	0.29222164	0.453652423	negative
PRJNA729271	:ed_Lushi_vs_Arbor_Acr	breed_growth_background	ENSGALG00010029200	GAS7	-0.194654012	0.066528876	-2.925857498	0.003435082	0.01948025	negative
PRJNA729271	:ed_Lushi_vs_Arbor_Acr	breed_growth_background	ENSGALG00010029201	PIGS	0.154275427	0.061251797	2.5187086	0.011778609	0.046955704	positive
PRJNA729271	:ed_Lushi_vs_Arbor_Acr	breed_growth_background	ENSGALG00010029202	PSMD12	0.125590264	0.076612943	1.639282602	0.101154418	0.216866334	positive
PRJNA729271	:ed_Lushi_vs_Arbor_Acr	breed_growth_background	ENSGALG00010029203	ALDOC	-0.190073178	0.092785153	-2.048530096	0.040508086	0.113396043	negative
PRJNA729271	:ed_Lushi_vs_Arbor_Acr	breed_growth_background	ENSGALG00010029204	HELZ	0.060676589	0.05259711	1.153610686	0.248659815	0.404798296	positive
PRJNA729271	:ed_Lushi_vs_Arbor_Acr	breed_growth_background	ENSGALG00010029205	CACNG1	-0.31259639	0.060153057	-5.196683332	2.02876E-07	1.44151E-05	negative
PRJNA729271	:ed_Lushi_vs_Arbor_Acr	breed_growth_background	ENSGALG00010029206	CACNG4	-0.67638853	0.183031745	-3.695471132	0.000219479	0.002788216	negative
PRJNA729271	:ed_Lushi_vs_Arbor_Acr	breed_growth_background	ENSGALG00010029207	SPAG5	-0.36883847	0.120911183	-3.050490954	0.002284676	0.014627373	negative
PRJNA729271	:ed_Lushi_vs_Arbor_Acr	breed_growth_background	ENSGALG00010029208	CACNG5	-0.187807783	0.152013992	-1.23546379	0.216657945	0.368252452	negative
PRJNA729271	:ed_Lushi_vs_Arbor_Acr	breed_growth_background	ENSGALG00010029209	PRKCA	0.083130219	0.051649375	1.609510651	0.107504728	0.226659059	positive
PRJNA729271	:ed_Lushi_vs_Arbor_Acr	breed_growth_background	ENSGALG00010029210	RSKR	0.735927257	0.173202985	4.248929405	2.14795E-05	0.000503781	positive
PRJNA729271	:ed_Lushi_vs_Arbor_Acr	breed_growth_background	ENSGALG00010029211	SDF2	-0.25323573	0.090900212	-2.785865118	0.005338508	0.026701751	negative
PRJNA729271	:ed_Lushi_vs_Arbor_Acr	breed_growth_background	ENSGALG00010029212	SUPT6H	0.114213819	0.060832974	1.877498527	0.060449799	0.151673851	positive
PRJNA729271	:ed_Lushi_vs_Arbor_Acr	breed_growth_background	ENSGALG00010029213	APOH	-1.219538505	0.816785497	-1.4930952	0.135412269	0.265530416	negative
PRJNA729271	:ed_Lushi_vs_Arbor_Acr	breed_growth_background	ENSGALG00010029214	ENSGALG00010029214	0.175947035	0.187453369	0.938617621	0.347927102	0.511896402	positive
PRJNA729271	:ed_Lushi_vs_Arbor_Acr	breed_growth_background	ENSGALG00010029215	ENSGALG00010029215	-0.079897731	0.08459616	-0.944460482	0.344934387	0.508836142	negative
PRJNA729271	:ed_Lushi_vs_Arbor_Acr	breed_growth_background	ENSGALG00010029216	RAB34	0.226661347	0.07322767	3.095296444	0.001966163	0.013116204	positive
PRJNA729271	:ed_Lushi_vs_Arbor_Acr	breed_growth_background	ENSGALG00010029217	CEP112	0.039604127	0.050574483	0.783085153	0.433577109	0.591736607	positive
PRJNA729271	:ed_Lushi_vs_Arbor_Acr	breed_growth_background	ENSGALG00010029218	RPL23A	-0.166625776	0.08029806	-2.075090935	0.037978108	0.108471462	negative
PRJNA729271	:ed_Lushi_vs_Arbor_Acr	breed_growth_background	ENSGALG00010029219	TLCD1	-0.09578428	0.109415415	-0.875418519	0.381346227	0.542827611	negative
PRJNA729271	:ed_Lushi_vs_Arbor_Acr	breed_growth_background	ENSGALG00010029220	NEK8	-0.148711555	0.069646455	-2.135235103	0.032741811	0.097133146	negative
PRJNA729271	:ed_Lushi_vs_Arbor_Acr	breed_growth_background	ENSGALG00010029221	AXIN2	0.230755982	0.069821514	3.304940977	0.000949964	0.007859434	positive
PRJNA729271	:ed_Lushi_vs_Arbor_Acr	breed_growth_background	ENSGALG00010029222	TRAF4	0.297835559	0.126872117	2.347525737	0.018898567	0.065715228	positive
PRJNA729271	:ed_Lushi_vs_Arbor_Acr	breed_growth_background	ENSGALG00010029223	NME2	-0.177771786	0.07797571	-2.279835416	0.022617452	0.074593848	negative
PRJNA729271	:ed_Lushi_vs_Arbor_Acr	breed_growth_background	ENSGALG00010029224	FAM222B	0.184791587	0.087033728	2.123218091	0.033735581	0.099208205	positive
PRJNA729271	:ed_Lushi_vs_Arbor_Acr	breed_growth_background	ENSGALG00010029226	MBTD1	0.064225648	0.041299506	1.55511902	0.119917726	0.244267487	positive
PRJNA729271	:ed_Lushi_vs_Arbor_Acr	breed_growth_background	ENSGALG00010029227	UTP18	0.056093003	0.044327258	1.265429099	0.205717549	0.355069764	positive
PRJNA729271	:ed_Lushi_vs_Arbor_Acr	breed_growth_background	ENSGALG00010029228	ERAL1	0.061198024	0.064114376	0.954513297	0.339823882	0.503861208	positive
PRJNA729271	:ed_Lushi_vs_Arbor_Acr	breed_growth_background	ENSGALG00010029229	CBX4	0.076843422	0.119327999	0.643968075	0.519596136	0.667335282	positive
PRJNA729271	:ed_Lushi_vs_Arbor_Acr	breed_growth_background	ENSGALG00010029230	PPM1E	0.098801645	0.088685791	1.114063976	0.265251742	0.423438766	positive
PRJNA729271	:ed_Lushi_vs_Arbor_Acr	breed_growth_background	ENSGALG00010029231	CBX8	0.112454121	0.061050261	1.84199245	0.065476254	0.160371461	positive
PRJNA729271	:ed_Lushi_vs_Arbor_Acr	breed_growth_background	ENSGALG00010029232	CA10	0.377398755	0.232114835	1.625913976	0.103967957	0.221197054	positive
PRJNA729271	:ed_Lushi_vs_Arbor_Acr	breed_growth_background	ENSGALG00010029233	CBX2	0.005715801	0.09587792	0.059615406	0.952461948	0.973433394	positive
PRJNA729271	:ed_Lushi_vs_Arbor_Acr	breed_growth_background	ENSGALG00010029234	TOM1L1	0.288531353	0.13152736	2.193698346	0.028257103	0.08712757	positive
PRJNA729271	:ed_Lushi_vs_Arbor_Acr	breed_growth_background	ENSGALG00010029235	TRIM37	0.020104063	0.04093417	0.491131577	0.623333389	0.7499973	positive
PRJNA729271	:ed_Lushi_vs_Arbor_Acr	breed_growth_background	ENSGALG00010029237	COX11	0.158774927	0.057864589	2.743904889	0.006071313	0.029276007	positive
PRJNA729271	:ed_Lushi_vs_Arbor_Acr	breed_growth_background	ENSGALG00010029238	CAMKK1	-0.283884696	0.133065437	-2.133421744	0.032890145	0.0974137	negative
PRJNA729271	:ed_Lushi_vs_Arbor_Acr	breed_growth_background	ENSGALG00010029239	RBFOX3	0.050978939	0.25954284	0.196418206	0.844282847	0.905369525	positive
PRJNA729271	:ed_Lushi_vs_Arbor_Acr	breed_growth_background	ENSGALG00010029240	MIS12	-0.315578983	0.139525527	-2.261801047	0.023709698	0.07702198	negative

PRJNA729271	ed_Lushi_vs_Arbor_Acr	breed_growth_background	ENSGALG00010029241	STXBP4	0.145533658	0.053837545	2.703200109	0.006867539	0.031977184	positive
PRJNA729271	ed_Lushi_vs_Arbor_Acr	breed_growth_background	ENSGALG00010029242	DERL2	-0.126398941	0.053547904	-2.360483443	0.018251133	0.064148926	negative
PRJNA729271	ed_Lushi_vs_Arbor_Acr	breed_growth_background	ENSGALG00010029243	NME1	0.070260844	0.19661465	0.357353048	0.720827522	0.82115647	positive
PRJNA729271	ed_Lushi_vs_Arbor_Acr	breed_growth_background	ENSGALG00010029244	HLF	0.040605396	0.097207815	0.417717401	0.676153748	0.790773647	positive
PRJNA729271	ed_Lushi_vs_Arbor_Acr	breed_growth_background	ENSGALG00010029245	SKA2	-0.16875697	0.062322688	-2.70779353	0.006773215	0.03167024	negative
PRJNA729271	ed_Lushi_vs_Arbor_Acr	breed_growth_background	ENSGALG00010029246	DHX33	0.32511827	0.073811006	4.404739731	1.05911E-05	0.000303996	positive
PRJNA729271	ed_Lushi_vs_Arbor_Acr	breed_growth_background	ENSGALG00010029247	CCDC92B	0.528593442	0.189013625	2.796589092	0.005164517	0.026160621	positive
PRJNA729271	ed_Lushi_vs_Arbor_Acr	breed_growth_background	ENSGALG00010029248	C1QBP	-0.018545773	0.043750524	-0.423898311	0.671639976	0.787778446	negative
PRJNA729271	ed_Lushi_vs_Arbor_Acr	breed_growth_background	ENSGALG00010029249	PRR11	-0.281030997	0.156774029	-1.792586432	0.073039078	0.172933915	negative
PRJNA729271	ed_Lushi_vs_Arbor_Acr	breed_growth_background	ENSGALG00010029250	SPAG9	0.091439409	0.04677097	1.955046228	0.050577614	0.133495486	positive
PRJNA729271	ed_Lushi_vs_Arbor_Acr	breed_growth_background	ENSGALG00010029251	ENGASE	-0.299687282	0.111937935	-2.677262922	0.007422636	0.03375937	negative
PRJNA729271	ed_Lushi_vs_Arbor_Acr	breed_growth_background	ENSGALG00010029252	RPAIN	0.12463073	0.074312331	1.677120456	0.093518929	0.205138753	positive
PRJNA729271	ed_Lushi_vs_Arbor_Acr	breed_growth_background	ENSGALG00010029253	NUP88	0.213257768	0.046342829	4.60174259	4.18971E-06	0.00014995	positive
PRJNA729271	ed_Lushi_vs_Arbor_Acr	breed_growth_background	ENSGALG00010029254	MMD	0.038706101	0.063783566	0.606835002	0.543960417	0.688259972	positive
PRJNA729271	ed_Lushi_vs_Arbor_Acr	breed_growth_background	ENSGALG00010029255	RAP1GAP2	0.201841796	0.100699136	2.004404459	0.045026752	0.122775865	positive
PRJNA729271	ed_Lushi_vs_Arbor_Acr	breed_growth_background	ENSGALG00010029256	RABEP1	0.197012714	0.051856556	3.799186248	0.000145172	0.002078902	positive
PRJNA729271	ed_Lushi_vs_Arbor_Acr	breed_growth_background	ENSGALG00010029257	YPEL2	-0.002413728	0.086285131	-0.027973852	0.977683006	0.986943044	negative
PRJNA729271	ed_Lushi_vs_Arbor_Acr	breed_growth_background	ENSGALG00010029258	TMEM100	0.174722393	0.1258397	1.38845208	0.164999427	0.305176304	positive
PRJNA729271	ed_Lushi_vs_Arbor_Acr	breed_growth_background	ENSGALG00010029259	PTRH2	-0.093772485	0.058551491	-1.601538808	0.109257632	0.229420451	negative
PRJNA729271	ed_Lushi_vs_Arbor_Acr	breed_growth_background	ENSGALG00010029260	DHX40	0.009213389	0.05606325	0.164339191	0.869464131	0.922207126	positive
PRJNA729271	ed_Lushi_vs_Arbor_Acr	breed_growth_background	ENSGALG00010029261	IFT22	-0.064085387	0.055089233	-1.163301484	0.244707204	0.400613386	negative
PRJNA729271	ed_Lushi_vs_Arbor_Acr	breed_growth_background	ENSGALG00010029262	CLTC	-0.046256485	0.034979217	-1.322399114	0.186035284	0.331560487	negative
PRJNA729271	ed_Lushi_vs_Arbor_Acr	breed_growth_background	ENSGALG00010029263	TOB1	0.132408524	0.100590169	1.316316742	0.188067754	0.333910104	positive
PRJNA729271	ed_Lushi_vs_Arbor_Acr	breed_growth_background	ENSGALG00010029264	COL26A1	-0.074500432	0.098353	-0.757480016	0.448762334	0.605098608	negative
PRJNA729271	ed_Lushi_vs_Arbor_Acr	breed_growth_background	ENSGALG00010029266	CANT1	0.176627845	0.060382812	2.925134499	0.003443074	0.019503093	positive
PRJNA729271	ed_Lushi_vs_Arbor_Acr	breed_growth_background	ENSGALG00010029267	ENSGALG00010029267	0.307609687	0.135861434	2.264142796	0.023565334	0.076742195	positive
PRJNA729271	ed_Lushi_vs_Arbor_Acr	breed_growth_background	ENSGALG00010029268	PCTP	0.359006858	0.096166092	3.733195871	0.000189065	0.002486845	positive
PRJNA729271	ed_Lushi_vs_Arbor_Acr	breed_growth_background	ENSGALG00010029269	WSCD1	-0.149130322	0.082197502	-1.814292634	0.069632685	0.167384041	negative
PRJNA729271	ed_Lushi_vs_Arbor_Acr	breed_growth_background	ENSGALG00010029270	TIMP2	-0.290886914	0.079892067	-3.640998744	0.000271582	0.003242659	negative
PRJNA729271	ed_Lushi_vs_Arbor_Acr	breed_growth_background	ENSGALG00010029271	MYL10	0.458916005	0.428855928	1.070093651	0.284577157	0.444929984	positive
PRJNA729271	ed_Lushi_vs_Arbor_Acr	breed_growth_background	ENSGALG00010029273	SLC13A5	0.194156743	0.256087531	0.758165548	0.448351883	0.60477939	positive
PRJNA729271	ed_Lushi_vs_Arbor_Acr	breed_growth_background	ENSGALG00010029274	VMP1	-0.134204655	0.079204712	-1.694402411	0.090188842	0.200473434	negative
PRJNA729271	ed_Lushi_vs_Arbor_Acr	breed_growth_background	ENSGALG00010029275	PCYT2	0.08797471	0.038446442	2.288240589	0.022123514	0.073382228	positive
PRJNA729271	ed_Lushi_vs_Arbor_Acr	breed_growth_background	ENSGALG00010029276	TUBD1	-0.143254842	0.093193631	-1.537174174	0.124250663	0.249932467	negative
PRJNA729271	ed_Lushi_vs_Arbor_Acr	breed_growth_background	ENSGALG00010029277	WFIKKN2	-0.837753744	0.392086655	-2.13665457	0.032626098	0.096941996	negative
PRJNA729271	ed_Lushi_vs_Arbor_Acr	breed_growth_background	ENSGALG00010029278	MED31	0.030734463	0.038603531	0.796156788	0.42594091	0.585159084	positive
PRJNA729271	ed_Lushi_vs_Arbor_Acr	breed_growth_background	ENSGALG00010029279	ENSGALG00010029279	-0.17456172	0.084809804	-2.058272883	0.039563946	0.111571866	negative
PRJNA729271	ed_Lushi_vs_Arbor_Acr	breed_growth_background	ENSGALG00010029280	USP36	0.439067225	0.09780406	4.489253575	7.14732E-06	0.00022485	positive
PRJNA729271	ed_Lushi_vs_Arbor_Acr	breed_growth_background	ENSGALG00010029281	KIAA0753	-0.109438769	0.072784173	-1.503606681	0.13268267	0.261842791	negative
PRJNA729271	ed_Lushi_vs_Arbor_Acr	breed_growth_background	ENSGALG00010029282	LUC7L3	0.12710839	0.075676931	1.679618706	0.093031526	0.204458738	positive
PRJNA729271	ed_Lushi_vs_Arbor_Acr	breed_growth_background	ENSGALG00010029283	RPS6KB1	0.02544326	0.041741004	0.609550736	0.542159454	0.687141742	positive
PRJNA729271	ed_Lushi_vs_Arbor_Acr	breed_growth_background	ENSGALG00010029284	CYTH1	-0.066973888	0.050004851	-1.339347802	0.180457474	0.324975908	negative
PRJNA729271	ed_Lushi_vs_Arbor_Acr	breed_growth_background	ENSGALG00010029285	CUX1	-0.103452072	0.03744224	-2.762977649	0.005727669	0.028019764	negative
PRJNA729271	ed_Lushi_vs_Arbor_Acr	breed_growth_background	ENSGALG00010029286	MAFG	0.31398759	0.097627948	3.216165009	0.001299161	0.009842037	positive
PRJNA729271	ed_Lushi_vs_Arbor_Acr	breed_growth_background	ENSGALG00010029287	ANKRD40	0.204919473	0.066866202	3.064619583	0.00217947	0.014109612	positive
PRJNA729271	ed_Lushi_vs_Arbor_Acr	breed_growth_background	ENSGALG00010029288	PYCR1	-0.355637042	0.157520906	-2.257713286	0.02396354	0.077603109	negative
PRJNA729271	ed_Lushi_vs_Arbor_Acr	breed_growth_background	ENSGALG00010029289	PITPNM3	-0.291300224	0.122302514	-2.381800784	0.017228215	0.061417357	negative
PRJNA729271	ed_Lushi_vs_Arbor_Acr	breed_growth_background	ENSGALG00010029290	ENSGALG00010029290	-0.211309434	0.107401928	-1.967464068	0.049129734	0.131027691	negative
PRJNA729271	ed_Lushi_vs_Arbor_Acr	breed_growth_background	ENSGALG00010029291	RNFT1	-0.086165292	0.06217243	-1.385908392	0.165774878	0.306141783	negative
PRJNA729271	ed_Lushi_vs_Arbor_Acr	breed_growth_background	ENSGALG00010029292	ENSGALG00010029292	-0.30400133	0.136107228	-2.233542886	0.025513165	0.081151376	negative
PRJNA729271	ed_Lushi_vs_Arbor_Acr	breed_growth_background	ENSGALG00010029293	DNAH17	-0.269047595	0.079867709	-3.368665474	0.00075533	0.006675923	negative
PRJNA729271	ed_Lushi_vs_Arbor_Acr	breed_growth_background	ENSGALG00010029294	PGS1	0.018274385	0.042164689	0.433404938	0.664720614	0.78255942	positive
PRJNA729271	ed_Lushi_vs_Arbor_Acr	breed_growth_background	ENSGALG00010029295	SOCS3	0.056427574	0.132804352	0.424892507	0.670915037	0.787060008	positive
PRJNA729271	ed_Lushi_vs_Arbor_Acr	breed_growth_background	ENSGALG00010029296	FBXO39	-0.300629939	0.087083333	-3.452209858	0.000556015	0.005351553	negative
PRJNA729271	ed_Lushi_vs_Arbor_Acr	breed_growth_background	ENSGALG00010029297	SH2B2	-0.047131967	0.134035479	-0.351637991	0.72510977	0.824004405	negative
PRJNA729271	ed_Lushi_vs_Arbor_Acr	breed_growth_background	ENSGALG00010029298	ENDOV	-0.077677877	0.079001661	-0.983243601	0.32548756	0.489129961	negative
PRJNA729271	ed_Lushi_vs_Arbor_Acr	breed_growth_background	ENSGALG00010029299	PRKRIP1	0.068504622	0.049305233	1.389398598	0.164711576	0.304714509	positive
PRJNA729271	ed_Lushi_vs_Arbor_Acr	breed_growth_background	ENSGALG00010029300	TEKT1	-0.267707242	0.18297241	-1.463101692	0.143439555	0.2766109	negative
PRJNA729271	ed_Lushi_vs_Arbor_Acr	breed_growth_background	ENSGALG00010029301	RNF213	0.161361369	0.248127953	0.650315159	0.515488667	0.66382447	positive
PRJNA729271	ed_Lushi_vs_Arbor_Acr	breed_growth_background	ENSGALG00010029303	SLC26A11	-0.024346432	0.083506759	-0.291550436	0.770630376	0.857011454	negative
PRJNA729271	ed_Lushi_vs_Arbor_Acr	breed_growth_background	ENSGALG00010029304	ORAI3	-0.19126751	0.063417356	-3.016012054	0.002561231	0.015865402	negative
PRJNA729271	ed_Lushi_vs_Arbor_Acr	breed_growth_background	ENSGALG00010029305	SMTNL2	0.132771899	0.101428899	1.309014493	0.190529441	0.337126159	positive
PRJNA729271	ed_Lushi_vs_Arbor_Acr	breed_growth_background	ENSGALG00010029306	CEP95	-0.16718849	0.087647936	-1.907500592	0.056455794	0.144606245	negative
PRJNA729271	ed_Lushi_vs_Arbor_Acr	breed_growth_background	ENSGALG00010029307	SGSH	-0.144088496	0.090437478	-1.593238764	0.111106638	0.231820931	negative
PRJNA729271	ed_Lushi_vs_Arbor_Acr	breed_growth_background	ENSGALG00010029308	GAA	-0.088695896	0.074567258	-1.18947508	0.234252772	0.388633847	negative
PRJNA729271	ed_Lushi_vs_Arbor_Acr	breed_growth_background	ENSGALG00010029309	ALKBH4	0.300563142	0.088943078	3.379275249	0.000726772	0.006482807	positive
PRJNA729271	ed_Lushi_vs_Arbor_Acr	breed_growth_background	ENSGALG00010029310	LRWD1	-0.071643141	0.049773031	-1.439396783	0.150038135	0.285482118	negative
PRJNA729271	ed_Lushi_vs_Arbor_Acr	breed_growth_background	ENSGALG00010029311	ENSGALG00010029311	-0.16678401	0.089250574	-1.868716397	0.061662282	0.153916785	negative
PRJNA729271	ed_Lushi_vs_Arbor_Acr	breed_growth_background	ENSGALG00010029312	SMURF2	-0.083410823	0.050408668	-1.654692067	0.097986977	0.211764886	negative
PRJNA729271	ed_Lushi_vs_Arbor_Acr	breed_growth_background	ENSGALG00010029313	CCDC40	0.058138508	0.14053725	0.41368753	0.679102961	0.792623996	positive
PRJNA729271	ed_Lushi_vs_Arbor_Acr	breed_growth_background	ENSGALG00010029314	MED13	-0.08510812	0.067461587	-1.261578965	0.207100329	0.356226329	negative
PRJNA729271	ed_Lushi_vs_Arbor_Acr	breed_growth_background	ENSGALG00010029315	TBC1D16	-0.03827442	0.05236464	-0.730921103	0.464827345	0.620069241	negative
PRJNA729271	ed_Lushi_vs_Arbor_Acr	breed_growth_background	ENSGALG00010029316	INTS2	-0.188820663	0.051176671	-3.689584692	0.00022462	0.002821381	negative
PRJNA729271	ed_Lushi_vs_Arbor_Acr	breed_growth_background	ENSGALG00010029317	KPNA2	-0.366377146	0.110411924	-3.318275171	0.000905752	0.007577248	negative
PRJNA729271	ed_Lushi_vs_Arbor_Acr	breed_growth_background	ENSGALG00010029318	C17orf58	-0.189843658	0.106250451	-1.786756251	0.073976893	0.174669338	negative
PRJNA729271	ed_Lushi_vs_Arbor_Acr	breed_growth_background	ENSGALG00010029319	BRIP1	-0.172252365	0.06900063	-2.496388297	0.012546518	0.048992935	negative
PRJNA729271	ed_Lushi_vs_Arbor_Acr	breed_growth_background	ENSGALG00010029320	TBX4	0.741590378	0.426346431	1.739407964	0.081963028	0.187065231	positive
PRJNA729271	ed_Lushi_vs_Arbor_Acr	breed_growth_background	ENSGALG00010029321	TBX2	-0.07531058	0.094696594	-0.795282884	0.426448968	0.585579555	negative
PRJNA729271	ed_Lushi_vs_Arbor_Acr	breed_growth_background	ENSGALG00010029322	BPTF	0.079256651	0.052105148	1.521090607	0.128237101	0.255465599	positive
PRJNA729271	ed_Lushi_vs_Arbor_Acr	breed_growth_background	ENSGALG00010029323	NOL11	0.183935854	0.048950976	3.757552346	0.000171583	0.002326569	positive
PRJNA729271	ed_Lushi_vs_Arbor_Acr	breed_growth_background	ENSGALG00010029324	BCAS3	0.149711291	0.067621035	2.213975155	0.026830488	0.083979254	positive
PRJNA729271	ed_Lushi_vs_Arbor_Acr	breed_growth_background	ENSGALG00010029325	LRRC75A	-0.156264459	0.092617855	-1.687195839	0.091565701	0.202277402	negative
PRJNA729271	ed_Lushi_vs_Arbor_Acr	breed_growth_background	ENSGALG00010029326	NCBP3	0.00962525	0.043796667	0.219771281	0.826049287	0.893684462	positive
PRJNA729271	ed_Lushi_vs_Arbor_Acr	breed_growth_background	ENSGALG00010029327	CRK	-0.024970443	0.029622915	-0.842943473	0.399260059	0.560456571	negative
PRJNA729271	ed_Lushi_vs_Arbor_Acr	breed_growth_background	ENSGALG00010029328	ENSGALG00010029328	0.008274004	0.044344519	0.186584584	0.851986355	0.910666043	positive
PRJNA729271	ed_Lushi_vs_Arbor_Acr	breed_growth_background	ENSGALG00010029329	ENSGALG00010029329	-0.256319	0.095538349	-2.682891252	0.007298872	0.03336397	negative
PRJNA729271	ed_Lushi_vs_Arbor_Acr	breed_growth_background	ENSGALG00010029330	AFMID	0.176214873	0.06964499	2.53018734	0.011400164	0.04585977	positive
PRJNA729271	ed_Lushi_vs_Arbor_Acr	breed_growth_background	ENSGALG00010029331	TK1	-0.450621867	0.149515276	-3.013885124	0.002579254	0.015929397	negative
PRJNA729271	ed_Lushi_vs_Arbor_Acr	breed_growth_background	ENSGALG00010029332	YWHAE	0.045181795	0.079654157	0.567224572	0.570561616	0.709165719	positive
PRJNA729271	ed_Lushi_vs_Arbor_Acr	breed_growth_background	ENSGALG00010029333	ENSGALG00010029333	0.076344502	0.094330491	0.809330056	0.418325322	0.578364015	positive
PRJNA729271	ed_Lushi_vs_Arbor_Acr	breed_growth_background	ENSGALG00010029335	RNF222	-0.062564323	0.765442262	-0.081736176	0.934856511	0.964451953	negative
PRJNA729271	ed_Lushi_vs_Arbor_Acr	breed_growth_background	ENSGALG00010029336	SYNGR2	0.052660087	0.058793039	0.895685736	0.370420631	0.533657803	positive
PRJNA729271	ed_Lushi_vs_Arbor_Acr	breed_growth_background	ENSGALG00010029337	MYO1C	-0.180201165	0.071438602	-2.522462089	0.011653651	0.046585679	negative
PRJNA729271	ed_Lushi_vs_Arbor_Acr	breed_growth_background	ENSGALG00010029338	INPP5K	-0.138519305	0.064648059	-2.14266766	0.032139794	0.09589673	negative
PRJNA729271	ed_Lushi_vs_Arbor_Acr	breed_growth_background	ENSGALG00010029339	PITPNA	0.103416358	0.067958795	1.521750922	0.128071503	0.255315147	positive
PRJNA729271	ed_Lushi_vs_Arbor_Acr	breed_growth_background	ENSGALG00010029340	NDEL1	0.089391246	0.055659074	1.606049807	0.108262967	0.227903096	positive
PRJNA729271	ed_Lushi_vs_Arbor_Acr	breed_growth_background	ENSGALG00010029341	SLC43A2	0.281227447	0.174187888	1.614506327	0.106417646	0.225067635	positive
PRJNA729271	ed_Lushi_vs_Arbor_Acr	breed_growth_background	ENSGALG00010029342	TMC6	0.096945863	0.071408879	1.357616368	0.174585446	0.318436648	positive
PRJNA729271	ed_Lushi_vs_Arbor_Acr	breed_growth_background	ENSGALG00010029343	SCARF1	-0.124923419	0.069317378	-1.802194818	0.07151476	0.170354386	negative
PRJNA729271	ed_Lushi_vs_Arbor_Acr	breed_growth_background	ENSGALG00010029344	ENSGALG00010029344	-0.275823759	0.0878774	-3.138733729	0.001696796	0.011813185	negative
PRJNA729271	ed_Lushi_vs_Arbor_Acr	breed_growth_background	ENSGALG00010029345	RILP	0.166626019	0.129424774	1.287435271	0.197942617	0.346309987	positive
PRJNA729271	ed_Lushi_vs_Arbor_Acr	breed_growth_background	ENSGALG00010029346	ARL16	-0.07643319	0.071327991	-1.071573564	0.283911613	0.444081021	negative
PRJNA729271	ed_Lushi_vs_Arbor_Acr	breed_growth_background	ENSGALG00010029347	PRPF8	0.0329304	0.037517343	0.877738075	0.380085877	0.541972552	positive
PRJNA729271	ed_Lushi_vs_Arbor_Acr	breed_growth_background	ENSGALG00010029348	HGS	0.0412582	0.066281597	0.622468403	0.533633914	0.679106548	positive
PRJNA729271	ed_Lushi_vs_Arbor_Acr	breed_growth_background	ENSGALG00010029349	MYH10	-0.161256284	0.057594938	-2.799834328	0.005112884	0.026014139	negative
PRJNA729271	ed_Lushi_vs_Arbor_Acr	breed_growth_background	ENSGALG00010029350	MRPL12	0.012202597	0.065049941	0.187588131	0.851199531	0.910572119	positive
PRJNA729271	ed_Lushi_vs_Arbor_Acr	breed_growth_background	ENSGALG00010029351	ENSGALG00010029351	-0.072976105	0.11030503	-0.661584561	0.508237505	0.657231031	negative
PRJNA729271	ed_Lushi_vs_Arbor_Acr	breed_growth_background	ENSGALG00010029352	AATF	0.265646063	0.060762804	4.371853243	1.23196E-05	0.000336296	positive
PRJNA729271	ed_Lushi_vs_Arbor_Acr	breed_growth_background	ENSGALG00010029353	GCGR	-0.460901289	0.460045919	-1.001859313	0.316411546	0.479697244	negative
PRJNA729271	ed_Lushi_vs_Arbor_Acr	breed_growth_background	ENSGALG00010029354	TLCD2	-0.110483642	0.156908668	-0.704127081	0.481353637	0.634164332	negative
PRJNA729271	ed_Lushi_vs_Arbor_Acr	breed_growth_background	ENSGALG00010029355	MCRIP1	0.05440037	0.092208665	0.589970254	0.555210592	0.697139426	positive
PRJNA729271	ed_Lushi_vs_Arbor_Acr	breed_growth_background	ENSGALG00010029357	ARHGDIA	-0.115286973	0.052331101	-2.203029766	0.027592651	0.085640864	negative
PRJNA729271	ed_Lushi_vs_Arbor_Acr	breed_growth_background	ENSGALG00010029359	ENSGALG00010029359	-0.437374143	0.28436218	-1.538088302	0.124027026	0.249586525	negative
PRJNA729271	ed_Lushi_vs_Arbor_Acr	breed_growth_background	ENSGALG00010029360	ACACA	-0.063620519	0.046378377	-1.371771126	0.170134699	0.312481305	negative
PRJNA729271	ed_Lushi_vs_Arbor_Acr	breed_growth_background	ENSGALG00010029361	ALYREF	0.081512736	0.073072457	1.115505611	0.264633815	0.422824863	positive
PRJNA729271	ed_Lushi_vs_Arbor_Acr	breed_growth_background	ENSGALG00010029362	TADA2A	0.009317193	0.061524907	0.151437748	0.879630414	0.928505888	positive
PRJNA729271	ed_Lushi_vs_Arbor_Acr	breed_growth_background	ENSGALG00010029363	PIK3R6	-0.033016374	0.101158609	-0.326382243	0.74413517	0.836921112	negative
PRJNA729271	ed_Lushi_vs_Arbor_Acr	breed_growth_background	ENSGALG00010029364	ANAPC11	0.0217356	0.070915061	0.306501881	0.759222548	0.848033508	positive
PRJNA729271	ed_Lushi_vs_Arbor_Acr	breed_growth_background	ENSGALG00010029365	PIK3R5	0.032310219	0.099349349	0.325218222	0.745015918	0.837262036	positive
PRJNA729271	ed_Lushi_vs_Arbor_Acr	breed_growth_background	ENSGALG00010029366	MGAT5B	0.126607732	0.183268298	0.690832694	0.489670687	0.641519939	positive
PRJNA729271	ed_Lushi_vs_Arbor_Acr	breed_growth_background	ENSGALG00010029367	DUSP14	-0.158234001	0.063772141	-2.481240216	0.013092611	0.050486138	negative
PRJNA729271	ed_Lushi_vs_Arbor_Acr	breed_growth_background	ENSGALG00010029368	DDX52	0.052983958	0.050379512	1.051696532	0.292938803	0.454316489	positive
PRJNA729271	ed_Lushi_vs_Arbor_Acr	breed_growth_background	ENSGALG00010029369	MFSD11	-0.03581624	0.045739997	-0.783039853	0.43360371	0.591736607	negative
PRJNA729271	ed_Lushi_vs_Arbor_Acr	breed_growth_background	ENSGALG00010029370	SRSF2	0.125968975	0.068520114	1.8384233	0.066000054	0.161465251	positive
PRJNA729271	ed_Lushi_vs_Arbor_Acr	breed_growth_background	ENSGALG00010029371	AUTS2	0.113949071	0.096180631	1.184740315	0.236120137	0.39088184	positive
PRJNA729271	ed_Lushi_vs_Arbor_Acr	breed_growth_background	ENSGALG00010029372	METTL23	-0.255063905	0.084845454	-3.006217685	0.002645196	0.016184934	negative
PRJNA729271	ed_Lushi_vs_Arbor_Acr	breed_growth_background	ENSGALG00010029373	GATSL2	0.334378312	0.090183713	3.70774611	0.000209112	0.002685348	positive
PRJNA729271	ed_Lushi_vs_Arbor_Acr	breed_growth_background	ENSGALG00010029374	RCC1L	0.182154782	0.064260261	2.834641192	0.004587718	0.023920494	positive
PRJNA729271	ed_Lushi_vs_Arbor_Acr	breed_growth_background	ENSGALG00010029375	JMJD6	0.11262538	0.08621967	1.306260864	0.191463861	0.33832829	positive
PRJNA729271	ed_Lushi_vs_Arbor_Acr	breed_growth_background	ENSGALG00010029376	ENSGALG00010029376	-0.560746435	0.111285651	-5.038802653	4.68453E-07	2.6849E-05	negative
PRJNA729271	ed_Lushi_vs_Arbor_Acr	breed_growth_background	ENSGALG00010029377	NCF1C	-0.38751603	0.156236527	-2.480316454	0.013126583	0.050603674	negative

PRJNA729271	:ed_Lushi_vs_Arbor_Acr	breed_growth_background	ENSGALG00010029378	ST6GALNAC1	0.52334777	0.206409216	2.535486448	0.011229127	0.045465419	positive
PRJNA729271	:ed_Lushi_vs_Arbor_Acr	breed_growth_background	ENSGALG00010029379	GTF2I	0.109983421	0.060423022	1.820223776	0.068724934	0.165862124	positive
PRJNA729271	:ed_Lushi_vs_Arbor_Acr	breed_growth_background	ENSGALG00010029380	RGS9	-0.112653031	0.080432022	-1.400599264	0.161333941	0.300467105	negative
PRJNA729271	:ed_Lushi_vs_Arbor_Acr	breed_growth_background	ENSGALG00010029382	GTF2IRD1	0.569547238	0.110467775	5.15577721	2.52581E-07	1.69498E-05	positive
PRJNA729271	:ed_Lushi_vs_Arbor_Acr	breed_growth_background	ENSGALG00010029383	GNA13	0.004272544	0.042486285	0.100562908	0.919897442	0.955493617	positive
PRJNA729271	:ed_Lushi_vs_Arbor_Acr	breed_growth_background	ENSGALG00010029384	WDR81	-0.043483759	0.0409374	-1.062201295	0.288144312	0.449132215	negative
PRJNA729271	:ed_Lushi_vs_Arbor_Acr	breed_growth_background	ENSGALG00010029385	CLIP2	-0.268231613	0.066077214	-4.059366253	4.92061E-05	0.000961027	negative
PRJNA729271	:ed_Lushi_vs_Arbor_Acr	breed_growth_background	ENSGALG00010029386	SLC16A6	-0.197128063	0.12836927	-1.535632812	0.124628462	0.250502855	negative
PRJNA729271	:ed_Lushi_vs_Arbor_Acr	breed_growth_background	ENSGALG00010029387	SERPINF1	-0.389685634	0.094009075	-4.145191671	3.3953E-05	0.00073019	negative
PRJNA729271	:ed_Lushi_vs_Arbor_Acr	breed_growth_background	ENSGALG00010029388	ST6GALNAC2	-0.326395858	0.173865107	-1.87729363	0.060477861	0.151691745	negative
PRJNA729271	:ed_Lushi_vs_Arbor_Acr	breed_growth_background	ENSGALG00010029389	SMYD4	0.044896344	0.052055873	0.862464521	0.388431948	0.550427323	positive
PRJNA729271	:ed_Lushi_vs_Arbor_Acr	breed_growth_background	ENSGALG00010029390	RPA1	-0.030058468	0.043793191	-0.686373092	0.492477868	0.644383742	negative
PRJNA729271	:ed_Lushi_vs_Arbor_Acr	breed_growth_background	ENSGALG00010029391	ARSG	-0.083561855	0.077300417	-1.081001345	0.279696515	0.439425026	negative
PRJNA729271	:ed_Lushi_vs_Arbor_Acr	breed_growth_background	ENSGALG00010029392	RTN4RL1	0.153398574	0.261726595	0.586102354	0.557806734	0.69912742	positive
PRJNA729271	:ed_Lushi_vs_Arbor_Acr	breed_growth_background	ENSGALG00010029394	CYGB	-0.203264813	0.076589543	-2.653949932	0.007955563	0.035481811	negative
PRJNA729271	:ed_Lushi_vs_Arbor_Acr	breed_growth_background	ENSGALG00010029395	DPH1	0.073261713	0.065667219	1.115651218	0.264571459	0.422771833	positive
PRJNA729271	:ed_Lushi_vs_Arbor_Acr	breed_growth_background	ENSGALG00010029396	WIPI1	-0.06260089	0.082415896	-0.759573007	0.447509859	0.604084132	negative
PRJNA729271	:ed_Lushi_vs_Arbor_Acr	breed_growth_background	ENSGALG00010029397	OVCA2	0.003780644	0.08542758	0.044255541	0.96470071	0.979820371	positive
PRJNA729271	:ed_Lushi_vs_Arbor_Acr	breed_growth_background	ENSGALG00010029398	RHBDF2	0.19458979	0.067588958	2.879017453	0.003989163	0.021632215	positive
PRJNA729271	:ed_Lushi_vs_Arbor_Acr	breed_growth_background	ENSGALG00010029399	PRKAR1A	-0.06200121	0.0289891	-2.138776629	0.032453763	0.096624277	negative
PRJNA729271	:ed_Lushi_vs_Arbor_Acr	breed_growth_background	ENSGALG00010029400	AANAT	-0.135098598	0.346092608	-0.390353897	0.696274874	0.804311787	negative
PRJNA729271	:ed_Lushi_vs_Arbor_Acr	breed_growth_background	ENSGALG00010029401	HIC1	-0.173829966	0.108344163	-1.604423915	0.108620643	0.228413785	negative
PRJNA729271	:ed_Lushi_vs_Arbor_Acr	breed_growth_background	ENSGALG00010029402	SMG6	0.051249564	0.047517005	1.07855206	0.280787462	0.440705816	positive
PRJNA729271	:ed_Lushi_vs_Arbor_Acr	breed_growth_background	ENSGALG00010029403	FAM20A	0.039675612	0.082975944	0.47815801	0.632537747	0.757789574	positive
PRJNA729271	:ed_Lushi_vs_Arbor_Acr	breed_growth_background	ENSGALG00010029404	SRR	-0.112474197	0.100792532	-1.115898111	0.26446575	0.42267421	negative
PRJNA729271	:ed_Lushi_vs_Arbor_Acr	breed_growth_background	ENSGALG00010029405	POLDIP2	-0.059068705	0.042276713	-1.397192459	0.162355704	0.301751865	negative
PRJNA729271	:ed_Lushi_vs_Arbor_Acr	breed_growth_background	ENSGALG00010029406	TMEM199	-0.078554318	0.055888606	-1.405551577	0.159857324	0.298637957	negative
PRJNA729271	:ed_Lushi_vs_Arbor_Acr	breed_growth_background	ENSGALG00010029407	UBE2O	0.154662231	0.101287353	1.526964876	0.126769746	0.253487028	positive
PRJNA729271	:ed_Lushi_vs_Arbor_Acr	breed_growth_background	ENSGALG00010029408	KIF12	0.039053989	0.237162961	0.164671537	0.869202521	0.921997113	positive
PRJNA729271	:ed_Lushi_vs_Arbor_Acr	breed_growth_background	ENSGALG00010029409	ENSGALG00010029409	0.25540069	0.100490219	2.54154776	0.011036287	0.044910437	positive
PRJNA729271	:ed_Lushi_vs_Arbor_Acr	breed_growth_background	ENSGALG00010029410	VTN	-1.037308666	0.469254922	-2.210544029	0.027067428	0.084447347	negative
PRJNA729271	:ed_Lushi_vs_Arbor_Acr	breed_growth_background	ENSGALG00010029411	SARM1	-0.246499976	0.108418733	-2.273592117	0.022990522	0.075429519	negative
PRJNA729271	:ed_Lushi_vs_Arbor_Acr	breed_growth_background	ENSGALG00010029412	SLC46A1	0.002772532	0.059462021	0.046626932	0.962810567	0.978835813	positive
PRJNA729271	:ed_Lushi_vs_Arbor_Acr	breed_growth_background	ENSGALG00010029413	B3GNTL1	0.00223735	0.118858181	0.018823693	0.984981753	0.991099088	positive
PRJNA729271	:ed_Lushi_vs_Arbor_Acr	breed_growth_background	ENSGALG00010029416	UNC119	0.463283103	0.262975251	1.761698493	0.078120253	0.180858179	positive
PRJNA729271	:ed_Lushi_vs_Arbor_Acr	breed_growth_background	ENSGALG00010029417	ENSGALG00010029417	-0.260397605	0.111273994	-2.340147904	0.019276104	0.066620681	negative
PRJNA729271	:ed_Lushi_vs_Arbor_Acr	breed_growth_background	ENSGALG00010029418	ABCA5	0.056254794	0.0710472	0.791794666	0.428480413	0.587534158	positive
PRJNA729271	:ed_Lushi_vs_Arbor_Acr	breed_growth_background	ENSGALG00010029419	RFC2	0.185216816	0.071505529	2.59024466	0.009590774	0.04062486	positive
PRJNA729271	:ed_Lushi_vs_Arbor_Acr	breed_growth_background	ENSGALG00010029420	LAT2	0.402826821	0.170365573	2.364484874	0.018055165	0.063629859	positive
PRJNA729271	:ed_Lushi_vs_Arbor_Acr	breed_growth_background	ENSGALG00010029421	TBCD	-0.011436544	0.05058263	-0.226096268	0.821126547	0.890152517	negative
PRJNA729271	:ed_Lushi_vs_Arbor_Acr	breed_growth_background	ENSGALG00010029422	EIF4H	0.09503041	0.046790708	2.03096755	0.042258286	0.117231361	positive
PRJNA729271	:ed_Lushi_vs_Arbor_Acr	breed_growth_background	ENSGALG00010029423	MYBBP1A	-0.01657807	0.065517888	-0.253031202	0.800244102	0.876495259	negative
PRJNA729271	:ed_Lushi_vs_Arbor_Acr	breed_growth_background	ENSGALG00010029424	MAP2K6	-0.000367093	0.116939013	-0.003139179	0.997495301	0.998883293	negative
PRJNA729271	:ed_Lushi_vs_Arbor_Acr	breed_growth_background	ENSGALG00010029425	SPNS2	-0.06919918	0.087574794	-0.790172338	0.429427128	0.588076607	negative
PRJNA729271	:ed_Lushi_vs_Arbor_Acr	breed_growth_background	ENSGALG00010029426	ZNF750	0.176212543	0.546001695	0.322732593	0.746897766	0.838661641	positive
PRJNA729271	:ed_Lushi_vs_Arbor_Acr	breed_growth_background	ENSGALG00010029427	SPNS3	-0.083413546	0.168826943	-0.494077217	0.621251647	0.7485052	negative
PRJNA729271	:ed_Lushi_vs_Arbor_Acr	breed_growth_background	ENSGALG00010029428	FN3K	0.14228507	0.139954738	1.016650613	0.309319667	0.472134692	positive
PRJNA729271	:ed_Lushi_vs_Arbor_Acr	breed_growth_background	ENSGALG00010029429	UBE2G1	0.030063169	0.047902542	0.627590269	0.530272385	0.676015675	positive
PRJNA729271	:ed_Lushi_vs_Arbor_Acr	breed_growth_background	ENSGALG00010029430	FN3KRP	-0.057375471	0.052958568	-1.083402994	0.278629587	0.437945767	negative
PRJNA729271	:ed_Lushi_vs_Arbor_Acr	breed_growth_background	ENSGALG00010029432	ENSGALG00010029432	-9.922287259	2.915732632	-3.403016844			negative
PRJNA729271	:ed_Lushi_vs_Arbor_Acr	breed_growth_background	ENSGALG00010029436	WDR45B	0.123405474	0.043388325	2.844209223	0.00445218	0.023395951	positive
PRJNA729271	:ed_Lushi_vs_Arbor_Acr	breed_growth_background	ENSGALG00010029437	ENSGALG00010029437	-6.893293263	1.568275443	-4.39546082	1.10538E-05	0.000314784	negative
PRJNA729271	:ed_Lushi_vs_Arbor_Acr	breed_growth_background	ENSGALG00010029438	ENSGALG00010029438	-0.450012113	0.14500416	-3.103442771	0.001912832	0.012860159	negative
PRJNA729271	:ed_Lushi_vs_Arbor_Acr	breed_growth_background	ENSGALG00010029439	ENSGALG00010029439	0.49792262	0.115805511	4.299645298	1.71072E-05	0.000424603	positive
PRJNA729271	:ed_Lushi_vs_Arbor_Acr	breed_growth_background	ENSGALG00010029440	FOXK2	0.239563216	0.097050839	2.468430143	0.013570714	0.051878198	positive
PRJNA729271	:ed_Lushi_vs_Arbor_Acr	breed_growth_background	ENSGALG00010029442	ENSGALG00010029442	0.055757137	0.152350867	0.365978467	0.714381143	0.816572405	positive
PRJNA729271	:ed_Lushi_vs_Arbor_Acr	breed_growth_background	ENSGALG00010029443	ENSGALG00010029443	-0.983030712	0.17065233	-5.76042946	8.39002E-09	1.18071E-06	negative
PRJNA729271	:ed_Lushi_vs_Arbor_Acr	breed_growth_background	ENSGALG00010029445	SPHK1	0.05482879	0.075162419	0.729470804	0.465713721	0.620737506	positive
PRJNA729271	:ed_Lushi_vs_Arbor_Acr	breed_growth_background	ENSGALG00010029446	PRPSAP1	0.123939602	0.043603228	2.842440959	0.004476952	0.023469592	positive
PRJNA729271	:ed_Lushi_vs_Arbor_Acr	breed_growth_background	ENSGALG00010029449	UBALD2	0.310018519	0.096688438	3.206365987	0.001344229	0.01010612	positive
PRJNA729271	:ed_Lushi_vs_Arbor_Acr	breed_growth_background	ENSGALG00010029451	ENSGALG00010029451	0.148027268	0.119670773	1.236954229	0.216104076	0.367526525	positive
PRJNA729271	:ed_Lushi_vs_Arbor_Acr	breed_growth_background	ENSGALG00010029452	ENSGALG00010029452	-2.767711588	0.534281053	-5.180254044	2.21584E-07	1.54416E-05	negative
PRJNA729271	:ed_Lushi_vs_Arbor_Acr	breed_growth_background	ENSGALG00010029453	ENSGALG00010029453	-0.276085687	0.848022736	-0.325564015	0.744754242	0.837162678	negative
PRJNA729271	:ed_Lushi_vs_Arbor_Acr	breed_growth_background	ENSGALG00010029454	ENSGALG00010029454	0.336218894	0.156181211	2.152748668	0.031338436	0.094106202	positive
PRJNA729271	:ed_Lushi_vs_Arbor_Acr	breed_growth_background	ENSGALG00010029455	ENSGALG00010029455	0.080058692	0.138419535	0.578377119	0.563009538	0.703400028	positive
PRJNA729271	:ed_Lushi_vs_Arbor_Acr	breed_growth_background	ENSGALG00010029456	RNF157	0.077282151	0.2958308	0.261237676	0.793909225	0.872325214	positive
PRJNA729271	:ed_Lushi_vs_Arbor_Acr	breed_growth_background	ENSGALG00010029457	ENSGALG00010029457	0.042228823	0.102569603	0.411708944	0.680552769	0.793541921	positive
PRJNA729271	:ed_Lushi_vs_Arbor_Acr	breed_growth_background	ENSGALG00010029460	ENSGALG00010029460	-0.420491997	0.153069197	-2.747071285	0.006013006	0.029081646	negative
PRJNA729271	:ed_Lushi_vs_Arbor_Acr	breed_growth_background	ENSGALG00010029461	ENSGALG00010029461	-0.088291386	0.109170172	-0.808750084	0.418658913	0.578555831	negative
PRJNA729271	:ed_Lushi_vs_Arbor_Acr	breed_growth_background	ENSGALG00010029462	ENSGALG00010029462	-0.686218279	0.968571208	-0.70848511	0.478644056	0.631583576	negative
PRJNA729271	:ed_Lushi_vs_Arbor_Acr	breed_growth_background	ENSGALG00010029463	ENSGALG00010029463	-0.009798946	0.235836568	-0.041549729	0.966857649	0.98113985	negative
PRJNA729271	:ed_Lushi_vs_Arbor_Acr	breed_growth_background	ENSGALG00010029464	ENSGALG00010029464	0.373276326	0.290342702	1.285640464	0.198568568	0.347195584	positive
PRJNA729271	:ed_Lushi_vs_Arbor_Acr	breed_growth_background	ENSGALG00010029465	ENSGALG00010029465	0.368135754	1.295649201	0.284132274	0.776309015	0.861212243	positive
PRJNA729271	:ed_Lushi_vs_Arbor_Acr	breed_growth_background	ENSGALG00010029466	SLC39A11	-0.030716063	0.06725263	-0.45672657	0.647867591	0.770182952	negative
PRJNA729271	:ed_Lushi_vs_Arbor_Acr	breed_growth_background	ENSGALG00010029468	ENSGALG00010029468	0.175081661	0.12990585	1.347758094	0.1777362	0.32191506	positive
PRJNA729271	:ed_Lushi_vs_Arbor_Acr	breed_growth_background	ENSGALG00010029469	ENSGALG00010029469	-0.616466849	0.223159266	-2.762452399	0.005736893	0.028055418	negative
PRJNA729271	:ed_Lushi_vs_Arbor_Acr	breed_growth_background	ENSGALG00010029470	SSTR2	-0.604829433	0.491846708	-1.229711256	0.21880525	0.371055356	negative
PRJNA729271	:ed_Lushi_vs_Arbor_Acr	breed_growth_background	ENSGALG00010029472	ENSGALG00010029472	-0.154365474	0.444531535	-0.347254271	0.728400302	0.825436821	negative
PRJNA729271	:ed_Lushi_vs_Arbor_Acr	breed_growth_background	ENSGALG00010029473	COG1	0.201377008	0.053633972	3.754654013	0.000173581	0.00234926	positive
PRJNA729271	:ed_Lushi_vs_Arbor_Acr	breed_growth_background	ENSGALG00010029474	ENSGALG00010029474	0.261049099	0.575146128	0.453883086	0.649912978	0.771407505	positive
PRJNA729271	:ed_Lushi_vs_Arbor_Acr	breed_growth_background	ENSGALG00010029477	FAM104A	-0.011395637	0.032258418	-0.353260873	0.723892876	0.823095955	negative
PRJNA729271	:ed_Lushi_vs_Arbor_Acr	breed_growth_background	ENSGALG00010029479	ENSGALG00010029479	-0.179367907	0.296471585	-0.605008762	0.545173175	0.689081199	negative
PRJNA729271	:ed_Lushi_vs_Arbor_Acr	breed_growth_background	ENSGALG00010029480	ENSGALG00010029480	0.23314403	0.062258976	3.744745625	0.000180577	0.002421331	positive
PRJNA729271	:ed_Lushi_vs_Arbor_Acr	breed_growth_background	ENSGALG00010029481	ENSGALG00010029481	0.682934166	0.149646627	4.563645567	5.02729E-06	0.00017517	positive
PRJNA729271	:ed_Lushi_vs_Arbor_Acr	breed_growth_background	ENSGALG00010029482	DDX25	-0.276475228	0.101968106	-2.711389268	0.006700192	0.031399702	negative
PRJNA729271	:ed_Lushi_vs_Arbor_Acr	breed_growth_background	ENSGALG00010029484	ENSGALG00010029484	0.723166309	0.341794331	2.115793751	0.034362353	0.100541442	positive
PRJNA729271	:ed_Lushi_vs_Arbor_Acr	breed_growth_background	ENSGALG00010029487	CDC42EP4	-0.115166417	0.079832685	-1.442597323	0.149133938	0.284013715	negative
PRJNA729271	:ed_Lushi_vs_Arbor_Acr	breed_growth_background	ENSGALG00010029489	ENSGALG00010029489	-0.042837674	0.554714046	-0.077224787	0.938444724	0.966310739	negative
PRJNA729271	:ed_Lushi_vs_Arbor_Acr	breed_growth_background	ENSGALG00010029490	ENSGALG00010029490	0.096985812	0.335816746	0.288805764	0.772730021	0.858685608	positive
PRJNA729271	:ed_Lushi_vs_Arbor_Acr	breed_growth_background	ENSGALG00010029491	ENSGALG00010029491	-0.254089485	0.106894979	-2.377001111	0.017454036	0.061985801	negative
PRJNA729271	:ed_Lushi_vs_Arbor_Acr	breed_growth_background	ENSGALG00010029492	SDK2	-0.428064692	0.123676356	-3.461168382	0.000537836	0.005249789	negative
PRJNA729271	:ed_Lushi_vs_Arbor_Acr	breed_growth_background	ENSGALG00010029494	MYH7	-0.08815972	0.294314233	-0.299542835	0.764525894	0.852249699	negative
PRJNA729271	:ed_Lushi_vs_Arbor_Acr	breed_growth_background	ENSGALG00010029495	RPL38	-0.107604338	0.100834312	-1.067140102	0.285908575	0.446529986	negative
PRJNA729271	:ed_Lushi_vs_Arbor_Acr	breed_growth_background	ENSGALG00010029496	TTYH2	-0.097598204	0.079166216	-1.232826431	0.217640528	0.369619151	negative
PRJNA729271	:ed_Lushi_vs_Arbor_Acr	breed_growth_background	ENSGALG00010029497	ENSGALG00010029497	0.135240952	0.321284927	0.420937743	0.673800544	0.789040142	positive
PRJNA729271	:ed_Lushi_vs_Arbor_Acr	breed_growth_background	ENSGALG00010029498	ENSGALG00010029498	0.029134694	0.079887835	0.364695	0.715339091	0.817409549	positive
PRJNA729271	:ed_Lushi_vs_Arbor_Acr	breed_growth_background	ENSGALG00010029501	ENSGALG00010029501	-0.293978811	0.211545308	-1.38967304	0.164628185	0.304645161	negative
PRJNA729271	:ed_Lushi_vs_Arbor_Acr	breed_growth_background	ENSGALG00010029502	BTBD17	0.01725991	0.271629491	0.063542107	0.94933483	0.97207424	positive
PRJNA729271	:ed_Lushi_vs_Arbor_Acr	breed_growth_background	ENSGALG00010029504	GPR142	-0.488916537	0.272848222	-1.791899299	0.073149098	0.173081322	negative
PRJNA729271	:ed_Lushi_vs_Arbor_Acr	breed_growth_background	ENSGALG00010029505	VEZF1	0.1727951	0.059027414	2.927370345	0.003418416	0.019433	positive
PRJNA729271	:ed_Lushi_vs_Arbor_Acr	breed_growth_background	ENSGALG00010029506	OXLD1	-0.090762882	0.089416635	-1.015055884	0.310079189	0.473065767	negative
PRJNA729271	:ed_Lushi_vs_Arbor_Acr	breed_growth_background	ENSGALG00010029507	VPS37D	0.126836122	0.094503689	1.342128795	0.179554239	0.323872411	positive
PRJNA729271	:ed_Lushi_vs_Arbor_Acr	breed_growth_background	ENSGALG00010029508	ENSGALG00010029508	0.153632788	0.081889853	1.876090664	0.060642832	0.151974039	positive
PRJNA729271	:ed_Lushi_vs_Arbor_Acr	breed_growth_background	ENSGALG00010029509	DNAJC30	0.202652259	0.090584445	2.237164004	0.025275623	0.080609495	positive
PRJNA729271	:ed_Lushi_vs_Arbor_Acr	breed_growth_background	ENSGALG00010029510	PDE6G	-0.352192666	0.17382623	-2.026119218	0.042752569	0.118198205	negative
PRJNA729271	:ed_Lushi_vs_Arbor_Acr	breed_growth_background	ENSGALG00010029511	CUEDC1	0.035763319	0.039616807	0.902731002	0.366668678	0.530160847	positive
PRJNA729271	:ed_Lushi_vs_Arbor_Acr	breed_growth_background	ENSGALG00010029512	TSPAN10	0.105362286	0.392122652	0.26869727	0.788162652	0.868446799	positive
PRJNA729271	:ed_Lushi_vs_Arbor_Acr	breed_growth_background	ENSGALG00010029513	WBSCR22	-0.049653434	0.046704718	-1.063135282	0.287720607	0.44873146	negative
PRJNA729271	:ed_Lushi_vs_Arbor_Acr	breed_growth_background	ENSGALG00010029514	MRPS23	0.158031429	0.052669286	3.000447504	0.002695832	0.016390976	positive
PRJNA729271	:ed_Lushi_vs_Arbor_Acr	breed_growth_background	ENSGALG00010029515	NPLOC4	0.193108985	0.081533868	2.368451166	0.017862738	0.063212986	positive
PRJNA729271	:ed_Lushi_vs_Arbor_Acr	breed_growth_background	ENSGALG00010029516	ENSGALG00010029516	-0.160111664	0.115548076	-1.38567139	0.165847268	0.306236452	negative
PRJNA729271	:ed_Lushi_vs_Arbor_Acr	breed_growth_background	ENSGALG00010029517	MSI2	-0.163550407	0.060807951	-2.68962207	0.007153298	0.032899432	negative
PRJNA729271	:ed_Lushi_vs_Arbor_Acr	breed_growth_background	ENSGALG00010029518	FAAP100	-0.266666919	0.100824958	-2.644850275	0.008172707	0.036149951	negative
PRJNA729271	:ed_Lushi_vs_Arbor_Acr	breed_growth_background	ENSGALG00010029519	STX1A	-0.06961818	0.061828992	-1.125979537	0.260174181	0.418049524	negative
PRJNA729271	:ed_Lushi_vs_Arbor_Acr	breed_growth_background	ENSGALG00010029520	MKS1	0.034176473	0.060775136	0.562343006	0.573882342	0.711596991	positive
PRJNA729271	:ed_Lushi_vs_Arbor_Acr	breed_growth_background	ENSGALG00010029521	ACTG1	-0.109427618	0.05260797	-2.080057791	0.037520232	0.107545139	negative
PRJNA729271	:ed_Lushi_vs_Arbor_Acr	breed_growth_background	ENSGALG00010029522	AKAP1	0.003358832	0.059891655	0.056081799	0.955276643	0.975053514	positive
PRJNA729271	:ed_Lushi_vs_Arbor_Acr	breed_growth_background	ENSGALG00010029523	FSCN2	-0.57926267	0.367188862	-1.57756057	0.114666597	0.236866513	negative
PRJNA729271	:ed_Lushi_vs_Arbor_Acr	breed_growth_background	ENSGALG00010029524	ENSGALG00010029524	0.088149735	0.079192492	1.113107236	0.265662376	0.42383473	positive
PRJNA729271	:ed_Lushi_vs_Arbor_Acr	breed_growth_background	ENSGALG00010029525	SLC38A10	-0.153876917	0.07430447	-2.070897167	0.038368407	0.109306222	negative
PRJNA729271	:ed_Lushi_vs_Arbor_Acr	breed_growth_background	ENSGALG00010029526	BCL7B	0.149378283	0.060123618	2.484519211	0.012972651	0.050210567	positive
PRJNA729271	:ed_Lushi_vs_Arbor_Acr	breed_growth_background	ENSGALG00010029527	NDUFAF8	-0.045830514	0.081385457	-0.563129042	0.573347017	0.71134683	negative
PRJNA729271	:ed_Lushi_vs_Arbor_Acr	breed_growth_background	ENSGALG00010029528	TBL2	-0.066593867	0.052622499	-1.265501797	0.205691505	0.355069764	negative
PRJNA729271	:ed_Lushi_vs_Arbor_Acr	breed_growth_background	ENSGALG00010029529	TEPSIN	0.249389777	0.070001423	3.562638696	0.000367146	0.003995329	positive
PRJNA729271	:ed_Lushi_vs_Arbor_Acr	breed_growth_background	ENSGALG00010029530	ENSGALG00010029530	0.040208742	0.189976306	0.21165135	0.832379045	0.898252997	positive
PRJNA729271	:ed_Lushi_vs_Arbor_Acr	breed_growth_background	ENSGALG00010029531	CEP131	0.282983932	0.092338813	3.064626057	0.002179423	0.014109612	positive
PRJNA729271	:ed_Lushi_vs_Arbor_Acr	breed_growth_background	ENSGALG00010029532	EVI2A	0.11415381	0.243911677	0.468012896	0.639775368	0.763757532	positive
PRJNA729271	:ed_Lushi_vs_Arbor_Acr	breed_growth_background	ENSGALG00010029533	MLXIPL	-0.144028939	0.416620277	-0.345707943	0.729562214	0.82630132	negative
PRJNA729271	:ed_Lushi_vs_Arbor_Acr	breed_growth_background	ENSGALG00010029534	NF1	-0.160327515	0.048731833	-3.289995551	0.00100189	0.008154066	negative
PRJNA729271	:ed_Lushi_vs_Arbor_Acr	breed_growth_background	ENSGALG00010029536	BAZ1B	0.016192294	0.041831035	0.387088052	0.698691023	0.805494842	positive
PRJNA729271	:ed_Lushi_vs_Arbor_Acr	breed_growth_background	ENSGALG00010029537	MYH1B	0.113215563	0.110147242	1.027856539	0.304017316	0.46654545	positive

PRJNA729271	ed_Lushi_vs_Arbor_Acr	breed_growth_background	ENSGALG00010029538	FZD9	0.166907254	0.09292971	1.796059124	0.072485114	0.172041294	positive
PRJNA729271	ed_Lushi_vs_Arbor_Acr	breed_growth_background	ENSGALG00010029539	FKBP6	0.158486525	0.226764621	0.698903227	0.484612511	0.636583407	positive
PRJNA729271	ed_Lushi_vs_Arbor_Acr	breed_growth_background	ENSGALG00010029541	ENSGALG00010029541	-0.164591211	0.122297388	-1.345827687	0.178358094	0.322598025	negative
PRJNA729271	ed_Lushi_vs_Arbor_Acr	breed_growth_background	ENSGALG00010029542	AATK	-0.076990276	0.07436617	-1.035286281	0.300535235	0.46298844	negative
PRJNA729271	ed_Lushi_vs_Arbor_Acr	breed_growth_background	ENSGALG00010029543	NSUN5	-0.103482853	0.072885478	-1.419800695	0.155665712	0.292655577	negative
PRJNA729271	ed_Lushi_vs_Arbor_Acr	breed_growth_background	ENSGALG00010029544	ENSGALG00010029544	0.151500489	0.096395295	1.571658539	0.116029762	0.238797587	positive
PRJNA729271	ed_Lushi_vs_Arbor_Acr	breed_growth_background	ENSGALG00010029545	OMG	0.468697526	0.164046722	2.857097787	0.004275341	0.022650244	positive
PRJNA729271	ed_Lushi_vs_Arbor_Acr	breed_growth_background	ENSGALG00010029546	WSB1	-0.109362682	0.088431753	-1.236690201	0.216202118	0.367650129	negative
PRJNA729271	ed_Lushi_vs_Arbor_Acr	breed_growth_background	ENSGALG00010029547	HIP1	0.030709158	0.054147675	0.56713714	0.570621012	0.709178733	positive
PRJNA729271	ed_Lushi_vs_Arbor_Acr	breed_growth_background	ENSGALG00010029548	BAIAP2	-0.160234418	0.119216995	-1.344056841	0.178930007	0.323229119	negative
PRJNA729271	ed_Lushi_vs_Arbor_Acr	breed_growth_background	ENSGALG00010029549	KSR1	-0.100659654	0.066298551	-1.518278354	0.128944242	0.256417194	negative
PRJNA729271	ed_Lushi_vs_Arbor_Acr	breed_growth_background	ENSGALG00010029550	CHMP6	0.37619689	0.128068487	2.9374665	0.003309059	0.01903407	positive
PRJNA729271	ed_Lushi_vs_Arbor_Acr	breed_growth_background	ENSGALG00010029552	NOS2	0.051231957	0.069545426	0.736668968	0.461323683	0.616587071	positive
PRJNA729271	ed_Lushi_vs_Arbor_Acr	breed_growth_background	ENSGALG00010029553	MYH1C	-0.244054616	0.1040949	-2.344539593	0.019050588	0.066125064	negative
PRJNA729271	ed_Lushi_vs_Arbor_Acr	breed_growth_background	ENSGALG00010029554	ENSGALG00010029554	-0.051482681	0.16059452	-0.320575579	0.748532047	0.839976157	negative
PRJNA729271	ed_Lushi_vs_Arbor_Acr	breed_growth_background	ENSGALG00010029555	LYRM9	0.083713123	0.083918207	0.997556146	0.318494635	0.481746816	positive
PRJNA729271	ed_Lushi_vs_Arbor_Acr	breed_growth_background	ENSGALG00010029556	NPTX1	-0.292697117	0.308730986	-0.948065242	0.343096242	0.506950053	negative
PRJNA729271	ed_Lushi_vs_Arbor_Acr	breed_growth_background	ENSGALG00010029557	ENSGALG00010029557	-0.044515855	0.264848506	-0.168080448	0.866519991	0.919959516	negative
PRJNA729271	ed_Lushi_vs_Arbor_Acr	breed_growth_background	ENSGALG00010029558	ENSGALG00010029558	-0.698371699	0.400697654	-1.742889415	0.081352933	0.186171577	negative
PRJNA729271	ed_Lushi_vs_Arbor_Acr	breed_growth_background	ENSGALG00010029560	ENSGALG00010029560	0.294833544	0.079969008	3.686847582	0.000227049	0.002839585	positive
PRJNA729271	ed_Lushi_vs_Arbor_Acr	breed_growth_background	ENSGALG00010029561	SCO1	-0.339310232	0.05614842	-6.043094928	1.51186E-09	3.00197E-07	negative
PRJNA729271	ed_Lushi_vs_Arbor_Acr	breed_growth_background	ENSGALG00010029562	ENSGALG00010029562	0.596882752	0.348149603	1.714443293	0.086447343	0.194513232	positive
PRJNA729271	ed_Lushi_vs_Arbor_Acr	breed_growth_background	ENSGALG00010029563	ADPRM	0.117879289	0.067222118	1.753578919	0.079502686	0.183414202	positive
PRJNA729271	ed_Lushi_vs_Arbor_Acr	breed_growth_background	ENSGALG00010029564	NLK	0.018455241	0.048028884	0.384252961	0.700790968	0.807020345	positive
PRJNA729271	ed_Lushi_vs_Arbor_Acr	breed_growth_background	ENSGALG00010029566	TMEM97	-0.061865068	0.079298833	-0.780151054	0.435301969	0.593295913	negative
PRJNA729271	ed_Lushi_vs_Arbor_Acr	breed_growth_background	ENSGALG00010029567	IFT20	-0.138616969	0.065143066	-2.127885238	0.0333466	0.098283645	negative
PRJNA729271	ed_Lushi_vs_Arbor_Acr	breed_growth_background	ENSGALG00010029568	ENSGALG00010029568	-0.892948471	0.584964827	-1.526499424	0.126885534	0.25368356	negative
PRJNA729271	ed_Lushi_vs_Arbor_Acr	breed_growth_background	ENSGALG00010029569	TMEM220	0.525724624	0.107035278	4.911694859	9.02925E-07	4.4823E-05	positive
PRJNA729271	ed_Lushi_vs_Arbor_Acr	breed_growth_background	ENSGALG00010029570	TNFAIP1	-0.082549608	0.043089007	-1.915792781	0.055391477	0.142636251	negative
PRJNA729271	ed_Lushi_vs_Arbor_Acr	breed_growth_background	ENSGALG00010029571	EPX	0.542896272	0.410481592	1.322583725	0.18597385	0.331491755	positive
PRJNA729271	ed_Lushi_vs_Arbor_Acr	breed_growth_background	ENSGALG00010029572	TSPOAP1	0.308265783	0.064143647	4.805866147	1.54083E-06	6.93613E-05	positive
PRJNA729271	ed_Lushi_vs_Arbor_Acr	breed_growth_background	ENSGALG00010029573	MAP2K4	0.087228418	0.033542053	2.600568886	0.009306933	0.039759504	positive
PRJNA729271	ed_Lushi_vs_Arbor_Acr	breed_growth_background	ENSGALG00010029574	SGSM2	-0.132915895	0.072155612	-1.842072857	0.065464493	0.160369754	negative
PRJNA729271	ed_Lushi_vs_Arbor_Acr	breed_growth_background	ENSGALG00010029575	ANKFN1	0.200786489	0.392971418	0.510944258	0.609390089	0.739977327	positive
PRJNA729271	ed_Lushi_vs_Arbor_Acr	breed_growth_background	ENSGALG00010029576	ATP2A3	-0.072052461	0.069453213	-1.037424443	0.299538092	0.461943254	negative
PRJNA729271	ed_Lushi_vs_Arbor_Acr	breed_growth_background	ENSGALG00010029577	NOG	0.312515592	0.255294329	1.224138401	0.220900022	0.373317689	positive
PRJNA729271	ed_Lushi_vs_Arbor_Acr	breed_growth_background	ENSGALG00010029578	C17orf67	1.716037606	0.353511532	4.854262023	1.20836E-06	5.70526E-05	positive
PRJNA729271	ed_Lushi_vs_Arbor_Acr	breed_growth_background	ENSGALG00010029579	GALNT17	-0.348015883	0.098709663	-3.525651595	0.000422442	0.004405253	negative
PRJNA729271	ed_Lushi_vs_Arbor_Acr	breed_growth_background	ENSGALG00010029580	P2RX1	-0.10538964	0.163996473	-0.642633577	0.52046189	0.668019523	negative
PRJNA729271	ed_Lushi_vs_Arbor_Acr	breed_growth_background	ENSGALG00010029581	DGKE	0.166693504	0.056953551	2.926832485	0.003424333	0.019449726	positive
PRJNA729271	ed_Lushi_vs_Arbor_Acr	breed_growth_background	ENSGALG00010029582	SCPEP1	-0.28400966	0.080517568	-3.527300517	0.00041982	0.004387376	negative
PRJNA729271	ed_Lushi_vs_Arbor_Acr	breed_growth_background	ENSGALG00010029583	LIMK1	-0.309558798	0.115105573	-2.689346753	0.007159201	0.032901908	negative
PRJNA729271	ed_Lushi_vs_Arbor_Acr	breed_growth_background	ENSGALG00010029584	TRIM25	-0.043829949	0.12474574	-0.351354273	0.725322583	0.824053245	negative
PRJNA729271	ed_Lushi_vs_Arbor_Acr	breed_growth_background	ENSGALG00010029585	ENSGALG00010029585	-0.375450798	0.197269476	-1.903238171	0.057009475	0.145495147	negative
PRJNA729271	ed_Lushi_vs_Arbor_Acr	breed_growth_background	ENSGALG00010029586	COIL	0.238021461	0.101786877	2.338429741	0.019364966	0.066816279	positive
PRJNA729271	ed_Lushi_vs_Arbor_Acr	breed_growth_background	ENSGALG00010029588	RAB11FIP4	0.425031879	0.083068007	5.116673632	3.10971E-07	1.96835E-05	positive
PRJNA729271	ed_Lushi_vs_Arbor_Acr	breed_growth_background	ENSGALG00010029590	UTP6	-0.003216965	0.053019934	-0.060674624	0.951618341	0.973375757	negative
PRJNA729271	ed_Lushi_vs_Arbor_Acr	breed_growth_background	ENSGALG00010029591	SUZ12	-0.062455925	0.043821562	-1.425232755	0.154089931	0.290559848	negative
PRJNA729271	ed_Lushi_vs_Arbor_Acr	breed_growth_background	ENSGALG00010029592	MNT	0.147832668	0.089208956	1.657150511	0.097489049	0.211037002	positive
PRJNA729271	ed_Lushi_vs_Arbor_Acr	breed_growth_background	ENSGALG00010029593	PAFAH1B1	0.133053322	0.042232972	3.150460794	0.001630131	0.011498177	positive
PRJNA729271	ed_Lushi_vs_Arbor_Acr	breed_growth_background	ENSGALG00010029594	CRLF3	-0.081128446	0.03711499	-2.185867362	0.028825311	0.088334647	negative
PRJNA729271	ed_Lushi_vs_Arbor_Acr	breed_growth_background	ENSGALG00010029595	CLUH	0.158196868	0.072097949	2.194193731	0.028221485	0.087065688	positive
PRJNA729271	ed_Lushi_vs_Arbor_Acr	breed_growth_background	ENSGALG00010029596	ATAD5	-0.090373604	0.071295354	-1.267594587	0.204942766	0.35433657	negative
PRJNA729271	ed_Lushi_vs_Arbor_Acr	breed_growth_background	ENSGALG00010029597	ADAP2	-0.099114331	0.11042492	-0.897572128	0.369413704	0.532748149	negative
PRJNA729271	ed_Lushi_vs_Arbor_Acr	breed_growth_background	ENSGALG00010029598	SUPT4H1	-0.016434511	0.080208973	-0.204896163	0.837653253	0.901461422	negative
PRJNA729271	ed_Lushi_vs_Arbor_Acr	breed_growth_background	ENSGALG00010029599	RNF43	-0.422259533	0.222585941	-1.897062913	0.057819635	0.146828243	negative
PRJNA729271	ed_Lushi_vs_Arbor_Acr	breed_growth_background	ENSGALG00010029600	RHOT1	0.096784131	0.058157868	1.664162283	0.096080003	0.208975615	positive
PRJNA729271	ed_Lushi_vs_Arbor_Acr	breed_growth_background	ENSGALG00010029601	HSF5	-1.381831852	0.294120063	-4.698189707	2.62478E-06	0.000103106	negative
PRJNA729271	ed_Lushi_vs_Arbor_Acr	breed_growth_background	ENSGALG00010029602	MTMR4	-0.034552419	0.047666584	-0.724877183	0.46852738	0.623398602	negative
PRJNA729271	ed_Lushi_vs_Arbor_Acr	breed_growth_background	ENSGALG00010029603	RHBDL3	-0.446438373	0.243104917	-1.836402069	0.066298214	0.161972076	negative
PRJNA729271	ed_Lushi_vs_Arbor_Acr	breed_growth_background	ENSGALG00010029604	ENSGALG00010029604	-0.031557923	0.065675044	-0.480516213	0.630860377	0.756604432	negative
PRJNA729271	ed_Lushi_vs_Arbor_Acr	breed_growth_background	ENSGALG00010029605	C17orf75	-0.224853386	0.05140841	-4.373863879	1.22067E-05	0.000335701	negative
PRJNA729271	ed_Lushi_vs_Arbor_Acr	breed_growth_background	ENSGALG00010029606	ABHD11	0.252612408	0.111644557	2.262648677	0.023657355	0.076972696	positive
PRJNA729271	ed_Lushi_vs_Arbor_Acr	breed_growth_background	ENSGALG00010029607	ZNF207	0.030018466	0.035167708	0.853580411	0.393337516	0.554829437	positive
PRJNA729271	ed_Lushi_vs_Arbor_Acr	breed_growth_background	ENSGALG00010029608	ENSGALG00010029608	-0.049396594	0.184103939	-0.268308188	0.788462104	0.868633582	negative
PRJNA729271	ed_Lushi_vs_Arbor_Acr	breed_growth_background	ENSGALG00010029609	METTL27	0.126082243	0.081825577	1.540865911	0.123349424	0.248533486	positive
PRJNA729271	ed_Lushi_vs_Arbor_Acr	breed_growth_background	ENSGALG00010029610	ERN1	0.043414489	0.043330819	1.001930966	0.316376936	0.479694946	positive
PRJNA729271	ed_Lushi_vs_Arbor_Acr	breed_growth_background	ENSGALG00010029611	YWHAG	0.027399928	0.069941212	0.391756549	0.695238103	0.803597503	positive
PRJNA729271	ed_Lushi_vs_Arbor_Acr	breed_growth_background	ENSGALG00010029612	HSPB1	0.007438752	0.104025064	0.071509227	0.942992481	0.969134715	positive
PRJNA729271	ed_Lushi_vs_Arbor_Acr	breed_growth_background	ENSGALG00010029614	MDH2	-0.015223456	0.068720053	-0.221528587	0.82468088	0.893003836	negative
PRJNA729271	ed_Lushi_vs_Arbor_Acr	breed_growth_background	ENSGALG00010029615	ENSGALG00010029615	-0.110449133	0.178631159	-0.618308325	0.536372116	0.681394725	negative
PRJNA729271	ed_Lushi_vs_Arbor_Acr	breed_growth_background	ENSGALG00010029616	TMEM120A	0.031949276	0.072189984	0.442572148	0.658075234	0.777493397	positive
PRJNA729271	ed_Lushi_vs_Arbor_Acr	breed_growth_background	ENSGALG00010029617	POR	0.04243758	0.051211389	0.828674651	0.407288533	0.567846171	positive
PRJNA729271	ed_Lushi_vs_Arbor_Acr	breed_growth_background	ENSGALG00010029618	TEX2	-0.099447995	0.060092878	-1.654904847	0.097943801	0.211705248	negative
PRJNA729271	ed_Lushi_vs_Arbor_Acr	breed_growth_background	ENSGALG00010029619	ENSGALG00010029619	0.061600312	0.056574574	1.088833851	0.276227161	0.435255213	positive
PRJNA729271	ed_Lushi_vs_Arbor_Acr	breed_growth_background	ENSGALG00010029620	ENSGALG00010029620	0.350773447	0.21689321	1.617263384	0.10582144	0.22405518	positive
PRJNA729271	ed_Lushi_vs_Arbor_Acr	breed_growth_background	ENSGALG00010029621	TAF15	0.177755546	0.066186223	2.685687998	0.007238064	0.033201184	positive
PRJNA729271	ed_Lushi_vs_Arbor_Acr	breed_growth_background	ENSGALG00010029622	MMP28	-0.351622027	0.085005377	-4.136468065	3.52692E-05	0.000751204	negative
PRJNA729271	ed_Lushi_vs_Arbor_Acr	breed_growth_background	ENSGALG00010029623	ENSGALG00010029623	-0.817509771	0.209329686	-3.905369494	9.40815E-05	0.001523119	negative
PRJNA729271	ed_Lushi_vs_Arbor_Acr	breed_growth_background	ENSGALG00010029624	RASL10B	-0.952489679	0.172242224	-5.529942996	3.20335E-08	3.54447E-06	negative
PRJNA729271	ed_Lushi_vs_Arbor_Acr	breed_growth_background	ENSGALG00010029625	PECAM1	-0.112902576	0.093128154	-1.212335598	0.225383942	0.378468518	negative
PRJNA729271	ed_Lushi_vs_Arbor_Acr	breed_growth_background	ENSGALG00010029626	AP2B1	0.048307956	0.058730598	0.822534712	0.410772639	0.571196221	positive
PRJNA729271	ed_Lushi_vs_Arbor_Acr	breed_growth_background	ENSGALG00010029627	PEX12	0.014914135	0.080415882	0.185462551	0.852866251	0.911136225	positive
PRJNA729271	ed_Lushi_vs_Arbor_Acr	breed_growth_background	ENSGALG00010029628	UNC45B	-0.264097104	0.085783517	-3.078646268	0.002079434	0.013641864	negative
PRJNA729271	ed_Lushi_vs_Arbor_Acr	breed_growth_background	ENSGALG00010029629	ENSGALG00010029629	0.133058937	0.135224685	0.983984074	0.325123344	0.488946163	positive
PRJNA729271	ed_Lushi_vs_Arbor_Acr	breed_growth_background	ENSGALG00010029630	RAD51D	-0.156179367	0.065115507	-2.398497298	0.016462498	0.059500713	negative
PRJNA729271	ed_Lushi_vs_Arbor_Acr	breed_growth_background	ENSGALG00010029631	LIG3	0.049497333	0.047181368	1.04908642	0.294138346	0.455457251	positive
PRJNA729271	ed_Lushi_vs_Arbor_Acr	breed_growth_background	ENSGALG00010029632	TMEM132E	0.108966497	0.120635479	0.903270729	0.366382228	0.529854934	positive
PRJNA729271	ed_Lushi_vs_Arbor_Acr	breed_growth_background	ENSGALG00010029633	POLG2	-0.074898196	0.094890797	-0.78930938	0.429931206	0.588354685	negative
PRJNA729271	ed_Lushi_vs_Arbor_Acr	breed_growth_background	ENSGALG00010029634	DDX5	-0.165881373	0.048811548	-3.398404292	0.000677802	0.006167443	negative
PRJNA729271	ed_Lushi_vs_Arbor_Acr	breed_growth_background	ENSGALG00010029635	ENSGALG00010029635	0.433871345	0.309794506	1.400513364	0.161359644	0.30046714	positive
PRJNA729271	ed_Lushi_vs_Arbor_Acr	breed_growth_background	ENSGALG00010029636	SPATA20	0.010616119	0.040920418	0.259433299	0.79530094	0.873324782	positive
PRJNA729271	ed_Lushi_vs_Arbor_Acr	breed_growth_background	ENSGALG00010029638	EPN3	0.259484716	0.104044841	2.493970038	0.012632321	0.049235141	positive
PRJNA729271	ed_Lushi_vs_Arbor_Acr	breed_growth_background	ENSGALG00010029639	ENSGALG00010029639	0.10723718	0.42004083	0.2553018	0.79849001	0.875112152	positive
PRJNA729271	ed_Lushi_vs_Arbor_Acr	breed_growth_background	ENSGALG00010029641	ENSGALG00010029641	0.365638484	0.392604596	0.931314834	0.351690731	0.515706337	positive
PRJNA729271	ed_Lushi_vs_Arbor_Acr	breed_growth_background	ENSGALG00010029642	NAT9	0.229078815	0.077648296	2.950210455	0.003175575	0.018490252	positive
PRJNA729271	ed_Lushi_vs_Arbor_Acr	breed_growth_background	ENSGALG00010029644	MYCBPAP	-0.238453197	0.089161204	-2.674405291	0.007486192	0.033963177	negative
PRJNA729271	ed_Lushi_vs_Arbor_Acr	breed_growth_background	ENSGALG00010029645	ENSGALG00010029645	0.123225439	0.216482162	0.569217518	0.569208534	0.708151893	positive
PRJNA729271	ed_Lushi_vs_Arbor_Acr	breed_growth_background	ENSGALG00010029646	MRPS17	-0.037416255	0.05829899	-0.64179938	0.521003452	0.668372868	negative
PRJNA729271	ed_Lushi_vs_Arbor_Acr	breed_growth_background	ENSGALG00010029647	RSAD1	-0.08490986	0.082682049	-1.026944319	0.304446684	0.466929921	negative
PRJNA729271	ed_Lushi_vs_Arbor_Acr	breed_growth_background	ENSGALG00010029648	NIPSNAP2	0.040621987	0.065159884	0.623420187	0.533008436	0.678370118	positive
PRJNA729271	ed_Lushi_vs_Arbor_Acr	breed_growth_background	ENSGALG00010029649	PSPH	-0.224111144	0.137018127	-1.635631346	0.101916766	0.217984878	negative
PRJNA729271	ed_Lushi_vs_Arbor_Acr	breed_growth_background	ENSGALG00010029650	ACSF2	-0.07729555	0.073089523	-1.057546235	0.290262365	0.451723533	negative
PRJNA729271	ed_Lushi_vs_Arbor_Acr	breed_growth_background	ENSGALG00010029651	ENSGALG00010029651	-0.288689417	0.149102049	-1.936186794	0.052844827	0.13786641	negative
PRJNA729271	ed_Lushi_vs_Arbor_Acr	breed_growth_background	ENSGALG00010029652	LRRC59	-0.069042901	0.055591344	-1.241972139	0.214246842	0.364881679	negative
PRJNA729271	ed_Lushi_vs_Arbor_Acr	breed_growth_background	ENSGALG00010029653	CCT6A	-0.046148907	0.048376011	-0.95396263	0.340102559	0.504016623	negative
PRJNA729271	ed_Lushi_vs_Arbor_Acr	breed_growth_background	ENSGALG00010029654	EME1	-0.268577122	0.073886584	-3.634991719	0.00027799	0.003305545	negative
PRJNA729271	ed_Lushi_vs_Arbor_Acr	breed_growth_background	ENSGALG00010029655	PHKG1	0.19397012	0.068451499	2.833686954	0.004601438	0.023966169	positive
PRJNA729271	ed_Lushi_vs_Arbor_Acr	breed_growth_background	ENSGALG00010029656	MRPL27	-0.085560498	0.061237616	-1.397188588	0.162356868	0.301751865	negative
PRJNA729271	ed_Lushi_vs_Arbor_Acr	breed_growth_background	ENSGALG00010029657	CHCHD2	-0.034144087	0.078429512	-0.435347443	0.663310253	0.781503479	negative
PRJNA729271	ed_Lushi_vs_Arbor_Acr	breed_growth_background	ENSGALG00010029658	XYLT2	0.157169616	0.067224338	2.337986812	0.019387932	0.066863688	positive
PRJNA729271	ed_Lushi_vs_Arbor_Acr	breed_growth_background	ENSGALG00010029659	VKORC1L1	-0.336947342	0.078762042	-4.278042247	1.88544E-05	0.000459877	negative
PRJNA729271	ed_Lushi_vs_Arbor_Acr	breed_growth_background	ENSGALG00010029660	CD300LG	0.553594518	0.166652099	3.32185746	0.000894204	0.007530254	positive
PRJNA729271	ed_Lushi_vs_Arbor_Acr	breed_growth_background	ENSGALG00010029661	KRABZFP	-0.428343705	0.293471302	-1.459576123	0.144406605	0.277867217	negative
PRJNA729271	ed_Lushi_vs_Arbor_Acr	breed_growth_background	ENSGALG00010029663	GUSB	-0.367157611	0.08265194	-4.442214061	8.90379E-06	0.000266654	negative
PRJNA729271	ed_Lushi_vs_Arbor_Acr	breed_growth_background	ENSGALG00010029664	RAB37	0.185896035	0.202463369	0.9181712	0.358529244	0.522142208	positive
PRJNA729271	ed_Lushi_vs_Arbor_Acr	breed_growth_background	ENSGALG00010029665	ASL1	0.151406672	0.101991582	1.484501664	0.137675882	0.268930298	positive
PRJNA729271	ed_Lushi_vs_Arbor_Acr	breed_growth_background	ENSGALG00010029666	SLC9A3R1	0.028541869	0.1079112	0.264494037	0.791399259	0.870651015	positive
PRJNA729271	ed_Lushi_vs_Arbor_Acr	breed_growth_background	ENSGALG00010029667	TMEM104	-0.010244993	0.050856833	-0.201447718	0.840348506	0.903221978	negative
PRJNA729271	ed_Lushi_vs_Arbor_Acr	breed_growth_background	ENSGALG00010029668	CRCP	-0.13142193	0.097630918	-1.346109745	0.178267126	0.322473729	negative
PRJNA729271	ed_Lushi_vs_Arbor_Acr	breed_growth_background	ENSGALG00010029669	HID1	-0.053529274	0.039890009	-1.341921821	0.179621346	0.323949806	negative
PRJNA729271	ed_Lushi_vs_Arbor_Acr	breed_growth_background	ENSGALG00010029670	TPST1	-0.159412358	0.091153848	-1.748827523	0.080320838	0.184538048	negative
PRJNA729271	ed_Lushi_vs_Arbor_Acr	breed_growth_background	ENSGALG00010029671	GRIN2C	-0.264481231	0.100567699	-2.629882496	0.008541439	0.037146343	negative
PRJNA729271	ed_Lushi_vs_Arbor_Acr	breed_growth_background	ENSGALG00010029672	KCTD7	-0.133754597	0.13236322	-1.010511811	0.312250143	0.475477028	negative
PRJNA729271	ed_Lushi_vs_Arbor_Acr	breed_growth_background	ENSGALG00010029673	TMEM248	-0.149740007	0.048716808	-3.073682625	0.002114342	0.013799882	negative
PRJNA729271	ed_Lushi_vs_Arbor_Acr	breed_growth_background	ENSGALG00010029674	FDXR	-0.09748087	0.062437986	-1.561243026	0.118466421	0.242332877	negative
PRJNA729271	ed_Lushi_vs_Arbor_Acr	breed_growth_background	ENSGALG00010029675	DHRS13	-0.228175159	0.124952476	-1.826095534	0.067835873	0.164455758	negative
PRJNA729271	ed_Lushi_vs_Arbor_Acr	breed_growth_background	ENSGALG00010029676	FADS6	-0.915975394	0.590292873	-1.551730397	0.120726746	0.245294952	negative
PRJNA729271	ed_Lushi_vs_Arbor_Acr	breed_growth_background	ENSGALG00010029677	PHF12	0.072529893	0.053858551	1.346673673	0.178085355	0.322346057	positive
PRJNA729271	ed_Lushi_vs_Arbor_Acr	breed_growth_background	ENSGALG00010029682	CDR2L	-0.123373139	0.074510159	-1.6557895	0.097764455	0.211380635	negative
PRJNA729271	ed_Lushi_vs_Arbor_Acr	breed_growth_background	ENSGALG00010029683	MRPL58	0.095417266	0.059652658	1.599547617	0.109698977	0.230101646	positive

PRJNA729271	ed_Lushi_vs_Arbor_Acr	breed_growth_background	ENSGALG00010029684	ENSGALG00010029684	-0.134769703	0.319752135	-0.421481794	0.673403303	0.788829875	negative
PRJNA729271	ed_Lushi_vs_Arbor_Acr	breed_growth_background	ENSGALG00010029685	ATP5PD	-0.045644395	0.061494725	-0.742248949	0.457936516	0.61376258	negative
PRJNA729271	ed_Lushi_vs_Arbor_Acr	breed_growth_background	ENSGALG00010029686	KCTD2	-0.004891073	0.032210779	-0.151845847	0.879308521	0.92824647	negative
PRJNA729271	ed_Lushi_vs_Arbor_Acr	breed_growth_background	ENSGALG00010029687	SLC16A5	-0.448366316	0.256971845	-1.744807164	0.081018441	0.185581907	negative
PRJNA729271	ed_Lushi_vs_Arbor_Acr	breed_growth_background	ENSGALG00010029689	ENSGALG00010029689	0.263609742	0.093884638	2.807804851	0.004988044	0.025530466	positive
PRJNA729271	ed_Lushi_vs_Arbor_Acr	breed_growth_background	ENSGALG00010029690	ENSGALG00010029690	0.229609887	0.085757958	2.677417854	0.007419204	0.0337578	positive
PRJNA729271	ed_Lushi_vs_Arbor_Acr	breed_growth_background	ENSGALG00010029691	NUFIP2	0.187337926	0.07535168	2.486181162	0.012912222	0.050070265	positive
PRJNA729271	ed_Lushi_vs_Arbor_Acr	breed_growth_background	ENSGALG00010029692	CRYBA1	0.875565123	0.31834609	2.750356139	0.005953052	0.028849714	positive
PRJNA729271	ed_Lushi_vs_Arbor_Acr	breed_growth_background	ENSGALG00010029693	HN1	-0.302307793	0.068067108	-4.441319793	8.94088E-06	0.000267213	negative
PRJNA729271	ed_Lushi_vs_Arbor_Acr	breed_growth_background	ENSGALG00010029694	ARMC7	-0.078272936	0.049419431	-1.583849389	0.113227997	0.234731095	negative
PRJNA729271	ed_Lushi_vs_Arbor_Acr	breed_growth_background	ENSGALG00010029695	TAOK1	0.096441335	0.0463568	2.080414001	0.037487576	0.107472788	positive
PRJNA729271	ed_Lushi_vs_Arbor_Acr	breed_growth_background	ENSGALG00010029696	SUMO2	-0.054451901	0.058302056	-0.93396194	0.350323526	0.514169655	negative
PRJNA729271	ed_Lushi_vs_Arbor_Acr	breed_growth_background	ENSGALG00010029697	GIT1	0.124221084	0.088991882	1.395869848	0.162753692	0.302256857	positive
PRJNA729271	ed_Lushi_vs_Arbor_Acr	breed_growth_background	ENSGALG00010029698	NUP85	-0.147780215	0.046510738	-3.177335384	0.00148635	0.010842803	negative
PRJNA729271	ed_Lushi_vs_Arbor_Acr	breed_growth_background	ENSGALG00010029699	ANKRD13B	-0.16488877	0.097834064	-1.685392226	0.091912922	0.202689459	negative
PRJNA729271	ed_Lushi_vs_Arbor_Acr	breed_growth_background	ENSGALG00010029700	GGA3	0.038586155	0.04320065	0.893184584	0.371758332	0.534463681	positive
PRJNA729271	ed_Lushi_vs_Arbor_Acr	breed_growth_background	ENSGALG00010029701	MRPS7	0.011246284	0.035813666	0.31402215	0.753504216	0.844264467	positive
PRJNA729271	ed_Lushi_vs_Arbor_Acr	breed_growth_background	ENSGALG00010029702	CORO6	-0.201098861	0.089212828	-2.254147363	0.024186899	0.078117001	negative
PRJNA729271	ed_Lushi_vs_Arbor_Acr	breed_growth_background	ENSGALG00010029703	MIF4GD	-0.105808306	0.062053431	-1.705116139	0.088172766	0.197110464	negative
PRJNA729271	ed_Lushi_vs_Arbor_Acr	breed_growth_background	ENSGALG00010029704	SLC25A19	-0.007136207	0.053629088	-0.133065972	0.894141206	0.938112726	negative
PRJNA729271	ed_Lushi_vs_Arbor_Acr	breed_growth_background	ENSGALG00010029705	GRB2	-0.090074679	0.036799529	-2.44771284	0.014376619	0.053777829	negative
PRJNA729271	ed_Lushi_vs_Arbor_Acr	breed_growth_background	ENSGALG00010029706	SSH2	0.22930426	0.115459969	1.986006594	0.047032592	0.126721687	positive
PRJNA729271	ed_Lushi_vs_Arbor_Acr	breed_growth_background	ENSGALG00010029707	NSRP1	0.361450901	0.120350218	3.003325694	0.002670465	0.016298268	positive
PRJNA729271	ed_Lushi_vs_Arbor_Acr	breed_growth_background	ENSGALG00010029708	TMEM94	-0.051591095	0.045647173	-1.130214453	0.258385871	0.415775222	negative
PRJNA729271	ed_Lushi_vs_Arbor_Acr	breed_growth_background	ENSGALG00010029709	SLC6A4	-0.193284954	0.107125295	-1.804288655	0.071186067	0.169822588	negative
PRJNA729271	ed_Lushi_vs_Arbor_Acr	breed_growth_background	ENSGALG00010029710	BLMH	0.030324251	0.044623696	0.679554884	0.496786345	0.648251612	positive
PRJNA729271	ed_Lushi_vs_Arbor_Acr	breed_growth_background	ENSGALG00010029711	CASKIN2	0.121689281	0.059168201	2.056666906	0.039718278	0.111854757	positive
PRJNA729271	ed_Lushi_vs_Arbor_Acr	breed_growth_background	ENSGALG00010029713	CPD	0.138337789	0.062452534	2.215086874	0.026754102	0.083866936	positive
PRJNA729271	ed_Lushi_vs_Arbor_Acr	breed_growth_background	ENSGALG00010029716	GOSR1	0.043246051	0.034204578	1.264335184	0.206109746	0.355413636	positive
PRJNA729271	ed_Lushi_vs_Arbor_Acr	breed_growth_background	ENSGALG00010029717	TRARG1	0.513531933	0.260366348	1.972343729	0.048570381	0.129909179	positive
PRJNA729271	ed_Lushi_vs_Arbor_Acr	breed_growth_background	ENSGALG00010029718	ABR	-0.113191933	0.060283474	-1.877661088	0.060427543	0.151644258	negative
PRJNA729271	ed_Lushi_vs_Arbor_Acr	breed_growth_background	ENSGALG00010029720	TIMM22	0.139338816	0.058557175	2.379534464	0.017334522	0.06164124	positive
PRJNA729271	ed_Lushi_vs_Arbor_Acr	breed_growth_background	ENSGALG00010029721	EXOC7	0.158187913	0.056550518	2.797284947	0.005153406	0.026136677	positive
PRJNA729271	ed_Lushi_vs_Arbor_Acr	breed_growth_background	ENSGALG00010029722	SPECC1	0.068799083	0.075890676	0.906555143	0.364642092	0.528020692	positive
PRJNA729271	ed_Lushi_vs_Arbor_Acr	breed_growth_background	ENSGALG00010029723	ADORA2B	-0.588269052	0.096666506	-6.085552033	1.16091E-09	2.43874E-07	negative
PRJNA729271	ed_Lushi_vs_Arbor_Acr	breed_growth_background	ENSGALG00010029724	CACNA1G	-0.225214777	0.117808632	-1.911700122	0.055914669	0.143549969	negative
PRJNA729271	ed_Lushi_vs_Arbor_Acr	breed_growth_background	ENSGALG00010029725	TSEN54	0.076454386	0.037515554	2.037938349	0.041556099	0.115615288	positive
PRJNA729271	ed_Lushi_vs_Arbor_Acr	breed_growth_background	ENSGALG00010029726	SEPTIN9	-0.233548673	0.067308368	-3.469831172	0.000520786	0.005114308	negative
PRJNA729271	ed_Lushi_vs_Arbor_Acr	breed_growth_background	ENSGALG00010029727	ZSWIM7	0.121743024	0.075707478	1.608071318	0.10781956	0.227190656	positive
PRJNA729271	ed_Lushi_vs_Arbor_Acr	breed_growth_background	ENSGALG00010029728	TNRC6C	0.10952301	0.05553705	1.972071091	0.048601492	0.129953628	positive
PRJNA729271	ed_Lushi_vs_Arbor_Acr	breed_growth_background	ENSGALG00010029729	RASL10A	-0.478677051	0.436682587	-1.096167022	0.273005689	0.431963482	negative
PRJNA729271	ed_Lushi_vs_Arbor_Acr	breed_growth_background	ENSGALG00010029730	TTC19	0.258529408	0.070563745	3.663771065	0.000248529	0.003032346	positive
PRJNA729271	ed_Lushi_vs_Arbor_Acr	breed_growth_background	ENSGALG00010029731	NCOR1	0.13650451	0.098791924	1.381737541	0.167052303	0.307951562	positive
PRJNA729271	ed_Lushi_vs_Arbor_Acr	breed_growth_background	ENSGALG00010029732	PIGL	-0.4071698	0.096540269	-4.217616188	2.46899E-05	0.000562193	negative
PRJNA729271	ed_Lushi_vs_Arbor_Acr	breed_growth_background	ENSGALG00010029733	ENSGALG00010029733	0.17196624	0.087444394	1.966578203	0.049231858	0.131113581	positive
PRJNA729271	ed_Lushi_vs_Arbor_Acr	breed_growth_background	ENSGALG00010029734	SEC14L1	-0.173465122	0.045306953	-3.828664497	0.000128841	0.001913466	negative
PRJNA729271	ed_Lushi_vs_Arbor_Acr	breed_growth_background	ENSGALG00010029735	SRP68	0.139395887	0.061628904	2.26185893	0.023706121	0.07702198	positive
PRJNA729271	ed_Lushi_vs_Arbor_Acr	breed_growth_background	ENSGALG00010029736	ENSGALG00010029736	0.006200359	0.07111835	0.087183668	0.930525521	0.961846069	positive
PRJNA729271	ed_Lushi_vs_Arbor_Acr	breed_growth_background	ENSGALG00010029737	ENSGALG00010029737	0.193322628	0.309124268	0.625388066	0.531716393	0.677499043	positive
PRJNA729271	ed_Lushi_vs_Arbor_Acr	breed_growth_background	ENSGALG00010029738	TRPV2	-0.144907838	0.08923393	-1.623909635	0.104395096	0.221780289	negative
PRJNA729271	ed_Lushi_vs_Arbor_Acr	breed_growth_background	ENSGALG00010029739	P2RX5	-0.214256093	0.108365491	-1.977161657	0.04802336	0.128621324	negative
PRJNA729271	ed_Lushi_vs_Arbor_Acr	breed_growth_background	ENSGALG00010029740	HASPIN	-0.300814335	0.121696743	-2.471835536	0.013442134	0.051464272	negative
PRJNA729271	ed_Lushi_vs_Arbor_Acr	breed_growth_background	ENSGALG00010029741	EMC6	0.06863205	0.087702893	0.782551723	0.4338904	0.591872892	positive
PRJNA729271	ed_Lushi_vs_Arbor_Acr	breed_growth_background	ENSGALG00010029742	TAX1BP3	-0.066210189	0.064010953	-1.034357186	0.300969212	0.463393251	negative
PRJNA729271	ed_Lushi_vs_Arbor_Acr	breed_growth_background	ENSGALG00010029743	MYOCD	0.152602517	0.132867107	1.148534955	0.250747791	0.406690079	positive
PRJNA729271	ed_Lushi_vs_Arbor_Acr	breed_growth_background	ENSGALG00010029744	CTNS	0.101760899	0.048230221	2.109899101	0.034867048	0.101669253	positive
PRJNA729271	ed_Lushi_vs_Arbor_Acr	breed_growth_background	ENSGALG00010029745	SHPK	0.194435149	0.090117475	2.157575021	0.030960889	0.093146135	positive
PRJNA729271	ed_Lushi_vs_Arbor_Acr	breed_growth_background	ENSGALG00010029746	TRPV1	0.343818109	0.178554171	1.925567497	0.0541584	0.140258355	positive
PRJNA729271	ed_Lushi_vs_Arbor_Acr	breed_growth_background	ENSGALG00010029747	TRPV3	0.19820716	0.184030749	1.077032836	0.281465596	0.441445988	positive
PRJNA729271	ed_Lushi_vs_Arbor_Acr	breed_growth_background	ENSGALG00010029749	ASPA	0.360034674	0.154055716	2.337041965	0.019437002	0.066985104	positive
PRJNA729271	ed_Lushi_vs_Arbor_Acr	breed_growth_background	ENSGALG00010029750	TEN1	0.01167202	0.137415003	0.084939923	0.932309152	0.962797176	positive
PRJNA729271	ed_Lushi_vs_Arbor_Acr	breed_growth_background	ENSGALG00010029752	ENSGALG00010029752	-0.165192676	0.091584373	-1.803721204	0.071275024	0.169894996	negative
PRJNA729271	ed_Lushi_vs_Arbor_Acr	breed_growth_background	ENSGALG00010029753	AKAP10	0.046623812	0.040860752	1.141041452	0.253852673	0.410448747	positive
PRJNA729271	ed_Lushi_vs_Arbor_Acr	breed_growth_background	ENSGALG00010029754	NLE1	-0.238642423	0.097600351	-2.445098002	0.014481279	0.05400259	negative
PRJNA729271	ed_Lushi_vs_Arbor_Acr	breed_growth_background	ENSGALG00010029755	ACOX1	-0.01254436	0.064104402	-0.195686402	0.844855628	0.905734415	negative
PRJNA729271	ed_Lushi_vs_Arbor_Acr	breed_growth_background	ENSGALG00010029756	ELAC2	0.097795858	0.045608464	2.144248024	0.032013019	0.095636585	positive
PRJNA729271	ed_Lushi_vs_Arbor_Acr	breed_growth_background	ENSGALG00010029757	ULK2	-0.217329369	0.111911317	-1.941978466	0.052139707	0.136592275	negative
PRJNA729271	ed_Lushi_vs_Arbor_Acr	breed_growth_background	ENSGALG00010029758	DNAH9	0.396711668	0.162094553	2.447409	0.014388746	0.053795426	positive
PRJNA729271	ed_Lushi_vs_Arbor_Acr	breed_growth_background	ENSGALG00010029759	ALDH3A2	0.049338776	0.057711702	0.854918063	0.39259651	0.554036367	positive
PRJNA729271	ed_Lushi_vs_Arbor_Acr	breed_growth_background	ENSGALG00010029760	SHISA6	-0.222243963	0.220908991	-1.006043086	0.314394845	0.477907334	negative
PRJNA729271	ed_Lushi_vs_Arbor_Acr	breed_growth_background	ENSGALG00010029761	MRPL38	0.140227727	0.063838363	2.196605923	0.028048603	0.086650575	positive
PRJNA729271	ed_Lushi_vs_Arbor_Acr	breed_growth_background	ENSGALG00010029762	SLC47A2	-0.109315013	0.219109125	-0.498906711	0.617845106	0.746181038	negative
PRJNA729271	ed_Lushi_vs_Arbor_Acr	breed_growth_background	ENSGALG00010029763	GEMIN4	0.068524578	0.053472248	1.281497996	0.200018803	0.348847618	positive
PRJNA729271	ed_Lushi_vs_Arbor_Acr	breed_growth_background	ENSGALG00010029764	NXN	0.120369769	0.068249303	1.763677632	0.077786269	0.180315364	positive
PRJNA729271	ed_Lushi_vs_Arbor_Acr	breed_growth_background	ENSGALG00010029765	KCNJ16	0.190397005	0.167977317	1.133468547	0.257017543	0.414309306	positive
PRJNA729271	ed_Lushi_vs_Arbor_Acr	breed_growth_background	ENSGALG00010029766	ENSGALG00010029766	0.172439824	0.078863834	2.186551379	0.02877529	0.088200006	positive
PRJNA729271	ed_Lushi_vs_Arbor_Acr	breed_growth_background	ENSGALG00010029767	GLOD4	-0.048087524	0.04721372	-1.018507422	0.308436876	0.471254613	negative
PRJNA729271	ed_Lushi_vs_Arbor_Acr	breed_growth_background	ENSGALG00010029768	KCNJ2	0.194744072	0.093842166	2.075229933	0.03796523	0.108456051	positive
PRJNA729271	ed_Lushi_vs_Arbor_Acr	breed_growth_background	ENSGALG00010029770	TLCD3A	-0.122290225	0.126150817	-0.969397014	0.332347142	0.49673869	negative
PRJNA729271	ed_Lushi_vs_Arbor_Acr	breed_growth_background	ENSGALG00010029771	SOX9	-0.237755203	0.145483889	-1.634237336	0.102209026	0.218513249	negative
PRJNA729271	ed_Lushi_vs_Arbor_Acr	breed_growth_background	ENSGALG00010029772	VPS53	0.031343333	0.037419419	0.837622114	0.402242969	0.563225641	positive
PRJNA729271	ed_Lushi_vs_Arbor_Acr	breed_growth_background	ENSGALG00010029773	TRIM65	-0.013747611	0.071350344	-0.192677572	0.847211489	0.907502995	negative
PRJNA729271	ed_Lushi_vs_Arbor_Acr	breed_growth_background	ENSGALG00010029774	RFLNB	-0.058291988	0.067284505	-0.866350846	0.386297791	0.548260695	negative
PRJNA729271	ed_Lushi_vs_Arbor_Acr	breed_growth_background	ENSGALG00010029775	ENSGALG00010029775	-0.285947695	0.076509725	-3.737403248	0.000185931	0.002456758	negative
PRJNA729271	ed_Lushi_vs_Arbor_Acr	breed_growth_background	ENSGALG00010029776	WBP2	-0.040411619	0.072986855	-0.553683523	0.579795454	0.716587236	negative
PRJNA729271	ed_Lushi_vs_Arbor_Acr	breed_growth_background	ENSGALG00010029777	ENSGALG00010029777	-0.201279981	0.076350079	-2.636277322	0.00838212	0.036751005	negative
PRJNA729271	ed_Lushi_vs_Arbor_Acr	breed_growth_background	ENSGALG00010029778	DOC2B	-0.136127862	0.188578723	-0.721862254	0.47037917	0.624684809	negative
PRJNA729271	ed_Lushi_vs_Arbor_Acr	breed_growth_background	ENSGALG00010029780	TEX14	0.05161232	0.140029131	0.368582737	0.712438765	0.815059187	positive
PRJNA729271	ed_Lushi_vs_Arbor_Acr	breed_growth_background	ENSGALG00010029781	UNC13D	-0.139963154	0.135644141	-1.031840765	0.302146719	0.464582231	negative
PRJNA729271	ed_Lushi_vs_Arbor_Acr	breed_growth_background	ENSGALG00010029782	UNK	0.141470794	0.06584985	2.14838445	0.031683227	0.094925253	positive
PRJNA729271	ed_Lushi_vs_Arbor_Acr	breed_growth_background	ENSGALG00010029783	RAD51C	-0.078950374	0.066135402	-1.193768715	0.232568453	0.386946708	negative
PRJNA729271	ed_Lushi_vs_Arbor_Acr	breed_growth_background	ENSGALG00010029784	CLDN3	-0.511685097	0.424396379	-1.205677341	0.227941925	0.381130258	negative
PRJNA729271	ed_Lushi_vs_Arbor_Acr	breed_growth_background	ENSGALG00010029785	ENSGALG00010029785	-0.074402916	0.125281593	-0.593885453	0.552588729	0.69519148	negative
PRJNA729271	ed_Lushi_vs_Arbor_Acr	breed_growth_background	ENSGALG00010029787	H3-3B	-0.053273654	0.034427135	-1.54743209	0.121759088	0.246450566	negative
PRJNA729271	ed_Lushi_vs_Arbor_Acr	breed_growth_background	ENSGALG00010029791	GALK1	0.175085024	0.061572142	2.843575332	0.004461046	0.02342499	positive
PRJNA729271	ed_Lushi_vs_Arbor_Acr	breed_growth_background	ENSGALG00010029804	ITGB4	-0.01930664	0.122580747	-0.157501406	0.8748497	0.926157347	negative
PRJNA729271	ed_Lushi_vs_Arbor_Acr	breed_growth_background	ENSGALG00010029812	SAP30BP	0.132237164	0.062295414	2.122743172	0.033775379	0.099305096	positive
PRJNA729271	ed_Lushi_vs_Arbor_Acr	breed_growth_background	ENSGALG00010029835	RECQL5	0.067620263	0.060205374	1.123159927	0.261369578	0.419412381	positive
PRJNA729271	ed_Lushi_vs_Arbor_Acr	breed_growth_background	ENSGALG00010029841	SMIM5	0.015175802	0.071179329	0.213205178	0.831166929	0.897317827	positive
PRJNA729271	ed_Lushi_vs_Arbor_Acr	breed_growth_background	ENSGALG00010029859	CSNK1D	0.048011651	0.028806789	1.66667832	0.095578386	0.208231947	positive
PRJNA729271	ed_Lushi_vs_Arbor_Acr	breed_growth_background	ENSGALG00010029864	SLC16A3	0.165387584	0.181756828	0.909938763	0.362854805	0.526135191	positive
PRJNA729271	ed_Lushi_vs_Arbor_Acr	breed_growth_background	ENSGALG00010029879	ENSGALG00010029879	0.079805485	0.182605575	0.437037508	0.662084147	0.780871416	positive
PRJNA729271	ed_Lushi_vs_Arbor_Acr	breed_growth_background	ENSGALG00010029895	FASN	-0.271936577	0.090332104	-3.010408967	0.002608961	0.016030901	negative
PRJNA729271	ed_Lushi_vs_Arbor_Acr	breed_growth_background	ENSGALG00010029907	NTN1	-0.339478418	0.152001913	-2.233382536	0.025523729	0.081151376	negative
PRJNA729271	ed_Lushi_vs_Arbor_Acr	breed_growth_background	ENSGALG00010029921	STX8	0.182501947	0.05660432	3.224169923	0.001263384	0.009679045	positive
PRJNA729271	ed_Lushi_vs_Arbor_Acr	breed_growth_background	ENSGALG00010029925	GGNBP2	0.200727196	0.067137117	2.989809581	0.002791514	0.016845544	positive
PRJNA729271	ed_Lushi_vs_Arbor_Acr	breed_growth_background	ENSGALG00010029926	BLTP2	0.253808626	0.071017953	3.573865703	0.000351749	0.003874973	positive
PRJNA729271	ed_Lushi_vs_Arbor_Acr	breed_growth_background	ENSGALG00010029927	ENSGALG00010029927	0.232659412	0.409084123	0.568732442	0.569537728	0.708439833	positive
PRJNA729271	ed_Lushi_vs_Arbor_Acr	breed_growth_background	ENSGALG00010029928	USP43	-0.159389399	0.073646441	-2.164251204	0.030445075	0.091918635	negative
PRJNA729271	ed_Lushi_vs_Arbor_Acr	breed_growth_background	ENSGALG00010029929	GDPD1	0.113163966	0.050772016	2.22886109	0.025823149	0.081851419	positive
PRJNA729271	ed_Lushi_vs_Arbor_Acr	breed_growth_background	ENSGALG00010029930	ARHGAP44	0.053751801	0.128291292	0.418982458	0.675228953	0.790074562	positive
PRJNA729271	ed_Lushi_vs_Arbor_Acr	breed_growth_background	ENSGALG00010029932	RASA4B	-0.201587091	0.138036124	-1.460393732	0.144181895	0.277501179	negative
PRJNA729271	ed_Lushi_vs_Arbor_Acr	breed_growth_background	ENSGALG00010029933	TVP23B	-0.163815629	0.050716164	-3.230047665	0.001237696	0.009532625	negative
PRJNA729271	ed_Lushi_vs_Arbor_Acr	breed_growth_background	ENSGALG00010029934	TEKT3	-0.305008563	0.229164382	-1.330959727	0.183202263	0.328287403	negative
PRJNA729271	ed_Lushi_vs_Arbor_Acr	breed_growth_background	ENSGALG00010029935	SYNRG	-0.019176698	0.03980701	-0.481741742	0.629989419	0.756060327	negative
PRJNA729271	ed_Lushi_vs_Arbor_Acr	breed_growth_background	ENSGALG00010029936	SERPINF2	-0.539186135	0.267875567	-2.012823123			negative
PRJNA729271	ed_Lushi_vs_Arbor_Acr	breed_growth_background	ENSGALG00010029937	PMP22	0.079728773	0.066011236	1.207806091	0.227121858	0.380329405	positive
PRJNA729271	ed_Lushi_vs_Arbor_Acr	breed_growth_background	ENSGALG00010029938	ENSGALG00010029938	0.125483344	0.171339289	0.732367601	0.463944228	0.619176097	positive
PRJNA729271	ed_Lushi_vs_Arbor_Acr	breed_growth_background	ENSGALG00010029939	ENSGALG00010029939	-3.491196159	0.800967442	-4.358724184	1.30823E-05	0.000352879	negative
PRJNA729271	ed_Lushi_vs_Arbor_Acr	breed_growth_background	ENSGALG00010029941	COX10	0.162202134	0.092352424	1.756338673	0.079030598	0.182518936	positive
PRJNA729271	ed_Lushi_vs_Arbor_Acr	breed_growth_background	ENSGALG00010029942	ENSGALG00010029942	0.36793434	0.84353709	0.436180394	0.662705852	0.78128681	positive
PRJNA729271	ed_Lushi_vs_Arbor_Acr	breed_growth_background	ENSGALG00010029943	HS3ST3A1	0.146720983	0.119110714	1.231803406	0.218022531	0.370007796	positive
PRJNA729271	ed_Lushi_vs_Arbor_Acr	breed_growth_background	ENSGALG00010029945	CCL4	0.334608458	0.36335593	0.920883436	0.357111287	0.52070497	positive
PRJNA729271	ed_Lushi_vs_Arbor_Acr	breed_growth_background	ENSGALG00010029946	METRNL	-0.124582259	0.073476962	-1.695528185	0.089975268	0.200183754	negative
PRJNA729271	ed_Lushi_vs_Arbor_Acr	breed_growth_background	ENSGALG00010029947	CCL5	1.032957428	0.392616065	2.630960674	0.008514389	0.037146343	positive
PRJNA729271	ed_Lushi_vs_Arbor_Acr	breed_growth_background	ENSGALG00010029948	MYADML2	-0.138097227	0.116521246	-1.18516778	0.235951116	0.390735911	negative
PRJNA729271	ed_Lushi_vs_Arbor_Acr	breed_growth_background	ENSGALG00010029950	RFFL	0.045980893	0.056952974	0.807348407	0.419465782	0.579117679	positive
PRJNA729271	ed_Lushi_vs_Arbor_Acr	breed_growth_background	ENSGALG00010029956	CCL15	-0.268374698	0.091842004	-2.922134604	0.003476413	0.019668463	negative
PRJNA729271	ed_Lushi_vs_Arbor_Acr	breed_growth_background	ENSGALG00010029962	ENSGALG00010029962	-0.299297868	0.409422896	-0.731023768	0.464764635	0.62004265	negative
PRJNA729271	ed_Lushi_vs_Arbor_Acr	breed_growth_background	ENSGALG00010029966	RABGEF1	0.079613952	0.043586654	1.826567192	0.06776487	0.164311106	positive
PRJNA729271	ed_Lushi_vs_Arbor_Acr	breed_growth_background	ENSGALG00010030004	FLOT2	-0.018017033	0.037698845	-0.477920023	0.632707129	0.757844698	negative

PRJNA729271	:ed_Lushi_vs_Arbor_Acr	breed_growth_background	ENSGALG00010030014	ENSGALG00010030014	0.33484208	0.165598731	2.022008735	0.043175449	0.11902399	positive
PRJNA729271	:ed_Lushi_vs_Arbor_Acr	breed_growth_background	ENSGALG00010030102	GPS1	-0.012698203	0.044790976	-0.283499133	0.776794246	0.861420901	negative
PRJNA729271	:ed_Lushi_vs_Arbor_Acr	breed_growth_background	ENSGALG00010030103	C1QTNF1	-0.151166484	0.111783039	-1.352320393	0.176272843	0.320224948	negative
PRJNA729271	:ed_Lushi_vs_Arbor_Acr	breed_growth_background	ENSGALG00010030104	P4HB	-0.181174631	0.058967334	-3.072457533	0.00212304	0.013849447	negative
PRJNA729271	:ed_Lushi_vs_Arbor_Acr	breed_growth_background	ENSGALG00010030105	RAB40B	0.084907609	0.105061858	0.808167789	0.418993998	0.57892863	positive
PRJNA729271	:ed_Lushi_vs_Arbor_Acr	breed_growth_background	ENSGALG00010030106	GPRC5C	-0.161474164	0.102152657	-1.580714279	0.113943382	0.235747917	negative
PRJNA729271	:ed_Lushi_vs_Arbor_Acr	breed_growth_background	ENSGALG00010030107	RPTOR	0.156171933	0.074099173	2.107606973	0.035065	0.102061681	positive
PRJNA729271	:ed_Lushi_vs_Arbor_Acr	breed_growth_background	ENSGALG00010030108	LLGL2	-0.025809622	0.064075088	-0.402802751	0.687093337	0.797989263	negative
PRJNA729271	:ed_Lushi_vs_Arbor_Acr	breed_growth_background	ENSGALG00010030109	FBF1	0.084418425	0.048380319	1.744891855	0.081003695	0.185581907	positive
